# Global assessment of organ specific basal gene expression over a diurnal cycle with analyses of gene copies exhibiting cyclic expression patterns

**DOI:** 10.1186/s12864-020-07202-9

**Published:** 2020-11-11

**Authors:** Yuan Lu, Mikki Boswell, William Boswell, Raquel Ybanez Salinas, Markita Savage, Jose Reyes, Sean Walter, Rebecca Marks, Trevor Gonzalez, Geraldo Medrano, Wesley C. Warren, Manfred Schartl, Ronald B. Walter

**Affiliations:** 1 The Xiphophorus Genetic Stock Center, Department of Chemistry and Biochemistry, Texas State University, 419 Centennial Hall, 601 University Drive, San Marcos, TX 78666 USA; 2 The University of Texas MD Anderson Cancer Center, Graduate School of Biomedical Sciences, Houston, TX USA; 3 Bond Life Sciences Center, University of Missouri, Columbia, MO USA; 4 Developmental Biochemistry, Theodor-Boveri-Institute, Biozentrum, University of Würzburg, Würzburg, Germany

**Keywords:** *Xiphophorus*, Organ, Gene expression profiling, Profiling, Light response, Basal level gene expression, Paralog, Genome duplication, Evolution

## Abstract

**Background:**

Studying functional divergences between paralogs that originated from genome duplication is a significant topic in investigating molecular evolution. Genes that exhibit basal level cyclic expression patterns including circadian and light responsive genes are important physiological regulators. Temporal shifts in basal gene expression patterns are important factors to be considered when studying genetic functions. However, adequate efforts have not been applied to studying basal gene expression variation on a global scale to establish transcriptional activity baselines for each organ. Furthermore, the investigation of cyclic expression pattern comparisons between genome duplication created paralogs, and potential functional divergence between them has been neglected. To address these questions, we utilized a teleost fish species, *Xiphophorus maculatus,* and profiled gene expression within 9 organs at 3-h intervals throughout a 24-h diurnal period.

**Results:**

Our results showed 1.3–21.9% of genes in different organs exhibited cyclic expression patterns, with eye showing the highest fraction of cycling genes while gonads yielded the lowest. A majority of the duplicated gene pairs exhibited divergences in their basal level expression patterns wherein only one paralog exhibited an oscillating expression pattern, or both paralogs exhibit oscillating expression patterns, but each gene duplicate showed a different peak expression time, and/or in different organs.

**Conclusions:**

These observations suggest cyclic genes experienced significant sub-, neo-, or non-functionalization following the teleost genome duplication event. In addition, we developed a customized, web-accessible, gene expression browser to facilitate data mining and data visualization for the scientific community.

## Background

There are over 33,000 described fish species, comprising the largest vertebrate group. Teleost fishes make up 96% of all extant fish species and exhibit extreme biodiversity that is thought to be a result of the *teleost specific whole genome duplication* (TGD) event that is estimated to have occurred ≈375 million years ago [1–4]. This duplication, accompanied with further retention of duplicated gene copies (i.e., neo-, sub-, non-functionalization, and chromosomal structural change), shaped the genomes of the teleost common ancestor and allowed each species to tailor its genome to best fit their particular environmental niche [5–8]. Studies of whole genome duplication (WGD) events have provided data to support the ability to track changes in gene function by comparing post-WGD duplicated allele pairs to pre-WGD single genes [9].

It is well known that gene expression does not remain at a steady state over a daily light/dark cycle but is dynamically responsive to a variety of environmental stimuli [10–12]. The dynamic nature of gene expression leads to transcriptional profiles in each organ that reflect complex time-of-day expression patterns that are tuned to properly regulate physiological demands. Genes known to follow cyclic ebbs and flows in their expression patterns serve as examples of the complex homeostatic genetic regulatory networks, yet these cycles only account for one aspect of many potentially differing oscillating gene expression patterns [13]. There are many other instances where basal gene expression at first appear to exhibit random patterns, yet later are shown to actually reflect a dynamic response to an extrinsic temporal stimuli, such as light-dark switching, feeding and other subtle adaptations to environmental conditions [14–19].

Circadian oscillation of gene expression has been shown for all vertebrate organisms thus far studied and is known to affect physiology in different model systems [20]. It is also well known that a group of genes (i.e., circadian genes) have expression patterns that coincide with discrete physiological changes in the diurnal cycle [21–23]. Even Mexican blind cavefish and Antarctic blackfin icefish encode circadian genes in their genomes [24, 25]. Although the blind cavefish do not exhibit *per1* circadian rhythm in the wild, it can be entrained in the lab with light. Although it is observed that different members of the same circadian gene family may exhibit different expression patterns, how gene duplicates, especially those originating from a WGD event (i.e., Ohnologs) behave transcriptionally has not yet been studied [26, 27]. This critical piece of missing information may be useful to infer the molecular evolution of rhythmicity.

The resolution of duplicated genomes in teleost fishes is hypothesized to have taken place soon following the duplication (i.e., 70–80 million years following the duplication), and most genes resolved down to a single copy during that period. However, the genomes of different extant teleost species may contain ≈3–20% of their genes in a duplicated condition when compared to basal actinopterygians (i.e., fishes that did not experience TGD), or tetrapods [8, 9, 28, 29]. Functional analyses showed a majority of the paralogs developed new functions compared to the molecular ancestor. We hypothesize that most paralogs of genes exhibiting cyclic patterns are differentiated into different expression patterns following genome duplication. In this report, we use cyclic expression patterns as a function to compare paralog functions.

It is possible to track WGD event and re-construct the ancestral teleost chromosomes that are hypothesized to share similar structures to those in tetrapods, including human [9, 28]. It has also been shown that teleost genomes represent excellent models to study molecular evolution. We chose *Xiphophorus* as the model for this study for several reasons: 1. *X. maculatus* is a new world, live-bearing vertebrate with an inbred (i.e., 115 generations of sibling matings) pedigree, 2. *Xiphophorus* fishes represent a well-established biomedical model for studies aimed at determining the genetics underlying melanoma development [30], 3. *Xiphophorus* genomes (i.e., *X. maculatus*, *X. couchianus* and *X. hellerii*) represent some of the best-assembled vertebrate genomes in existence, in terms of chromosome contiguity, sequence gaps and unassembled contigs [31, 32]. Also, availability of genomes from three distinct species within the same genus allows inter-species comparative genomic and transcriptomic studies to be performed [17, 33–35]; 4. *Xiphophorus* belongs to teleost fishes that have retained portions of their genomes in duplicated form, enabling the investigation of genomic molecular evolution [4, 29]; 5. A unique feature of *Xiphophorus* fishes is the ability to produce viable interspecies hybrids. This attribute presents researchers with a hybridization model that allows genetic interactions between divergent alleles that are artificially brought together in the interspecies hybrids to be studied [33–35].

Collectively, this study collects a comprehensive list of organ-specific genes exhibiting cyclic expression patterns (i.e., cyclic genes). In addition, comparing the paralog expression patterns forwards our understanding of the molecular evolution of these genes.

## Results

### Gene expression profiling statistics

Gene expression for 9 organs (brain, eye, gills, ovary, testis, heart, liver, muscle, and skin) was profiled every 3 h over a 24-h period from two fish at each time point using RNA-Seq. Overall, 87.9–92.2% of 46.1–51.8 million (M) filtered sequencing reads were mapped to the *X. maculatus* reference genome v5.0, with an average sequencing depth within exonic regions of 98.2–110.3 × in different organs (Table 1). This dataset was further processed as library size-normalized read counts (i.e., cpm). Visualization and data mining of this dataset can be accessed by https://www.xiphophorus.txstate.edu/Gene-Expression-Browser.html.

**Table 1:** RNA-Seq statistics

Organ	Average filtered reads (M)	Average mapped reads (M)	Average mapping rate (%)	Average depth (×)
Brain	46.1	41.9	90.8	98.2
Eye	51.8	46.5	89.8	110.3
Ovary	50.6	46.1	91.2	107.7
Testis	49.6	45.6	92.1	105.6
Heart	49.7	43.6	87.9	105.7
Liver	48.6	43.2	88.8	103.5
Muscle	51.5	45.2	87.9	109.6
Skin	46.8	42.4	90.5	99.7

*M* Million

### Expression patterns of circadian master regulator families

**Fig. 1:**
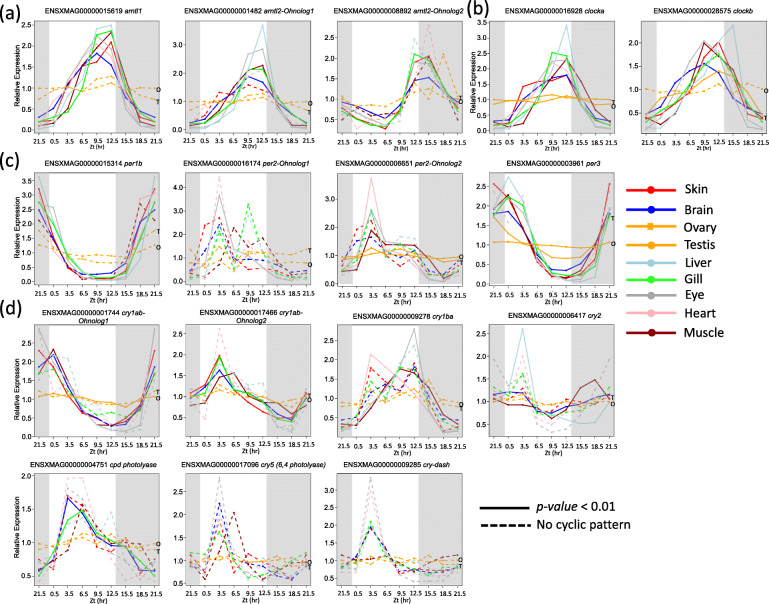
Master circadian regulator gene expression throughout a diurnal cycle As a technical control, each member of known circadian master regulator gene families in the *X. maculatus* genome was tested for a cyclic gene expression pattern. These genes include **a**: The *arntl* gene family, **b**: The *clock* gene family, **c**: The *per* gene family, and **d**: The *cry* gene family. For each gene, relative expression values that were calculated by normalizing average absolute expression to daily expression average are plotted against zeitgeber times to from an organ-specific expression pattern. Expression patterns of 9 organs are plotted, with colors represented organ, and line types represented if a gene exhibit cyclic expression pattern. Gray and white areas represent dark and light phases respectively

The *Xiphophorus* genome encodes 3 *arntl* genes (one *arntl1a*, two *arntl2*), 2 *clock* genes, 4 *per* genes (one *per1b,* one *per2*, one *per3*, and one *per2* paralog named *period circadian regulator 2*) and 7 *cry* genes [three *cry1*, one *cry2*, one *cry-dash, cry5* (*6,4 photolyase*) and CPD photolyase zgc:66475] (Fig. 1). All, except for *cry-dash* and *cry5*, showed circadian expression patterns as expected. These findings serve as a positive control and validate cyclic gene discovery presented in this study: known circadian master regulator genes show cyclic expression patterns and peak expression times at similar zeitgeber time (Zt) in all 9 organs although testis and ovary exhibited smaller amplitude for all circadian master regulator genes (Fig. 1). The *cry-dash* and *cry5* genes do not show cyclic expression pattern, but exhibited peak expression in light-phase. The *cry-dash* gene exhibited statistically different expression between early light and late dark phase in skin, brain and eye; and *cry5* in brain, eye, and muscle (Fig. 1d; Supplement Table 1). Their expression patterns suggest they are light-inducible genes (Fig. 1d).

### Identification of genes exhibiting cyclic expression patterns in 9 *Xiphophorus* organs (skin, brain, liver, gills, testis, ovary, heart, eye and muscle)

Gene expression profiling was performed on 9 organs throughout a 24 h cycle, at a 3 h intervals. Genes exhibiting cyclic expression pattern were subsequently identified (*p*-value < 0.01; normalized gene expression value, cpm, is larger than 1 in at least two time points; skin: 935; brain: 731; ovary: 224; heart: 659; muscle: 2076; eye: 4033; gill: 1740; liver: 1039; testis: 294; Fig. 2; Supplement Tables 2 and 3). There are 34 opsin genes encoded in the *X. maculatus* genome. Except *opn6,* expressed in all tested organs, and universally unexpressed *opn7a*, *opn1sw1*, *tmtops3a* and *parietopsin*, each organ exhibits a different opsin expression profile (Supplement Figure 5a). Although eye expressed most opsin genes (i.e., 28 of the 34) and showed the largest number of cyclic genes (Supplement Figure 5a; Supplement Table 2), the number of opsin genes expressed in any organ is not correlated to the number of cyclic genes. However, *lws2*, *opn6b*, *rrh*, *parapinoopsin* and *lws4* are only expressed in eyes. Considering that cyclic genes are the most abundant in the eyes, it implies that these genes may be related to the higher ocular representation of cyclic genes.

**Fig. 2:**
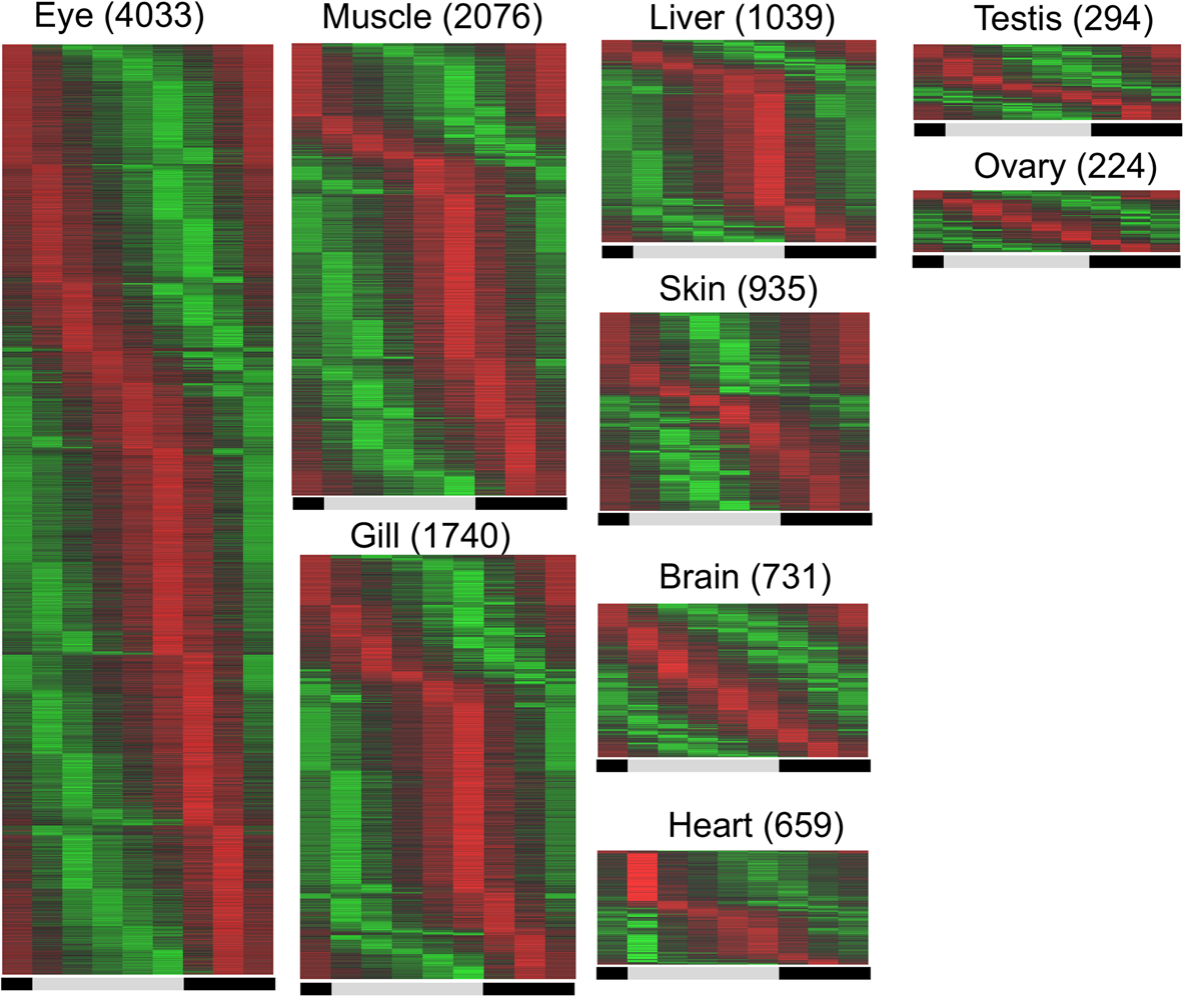
Cyclic genes in each organ: Circadian genes within each organ were identified by rain algorithm (*p-*value < 0.01). Each heatmap represents the circadian gene expression patterns of all genes in that organ over a 24-h period. Lists of circadian gene names and expression levels for each time point are provided in Supplemental Table 3). Gene expression read counts were first normalized to library size to generate CPM values. Mean of CPM values between biological replicates were subsequently calculated to represent expression level of a gene in an organ at a particular Zt, followed by scaling of gene expression at each Zt to the mean expression of a gene throughout a day. The scaled expression levels were represented by color (i.e., red: higher expression; green: lower expression). Circadian genes were ordered by peak expression time and length of time for a gene to reach the minimum expression

Genes showing the cyclic expression patterns were mostly organ specific (Supplement Figure 5b). This remains true even among comparisons that exclude testis and ovary, that exhibited the fewest cyclic genes and therefore showed smallest overlap with the circadian gene sets of other organs.

### Identification of Ohnologs and singletons *Xiphophorus* compared to the spotted gar genome

**Fig. 3:**
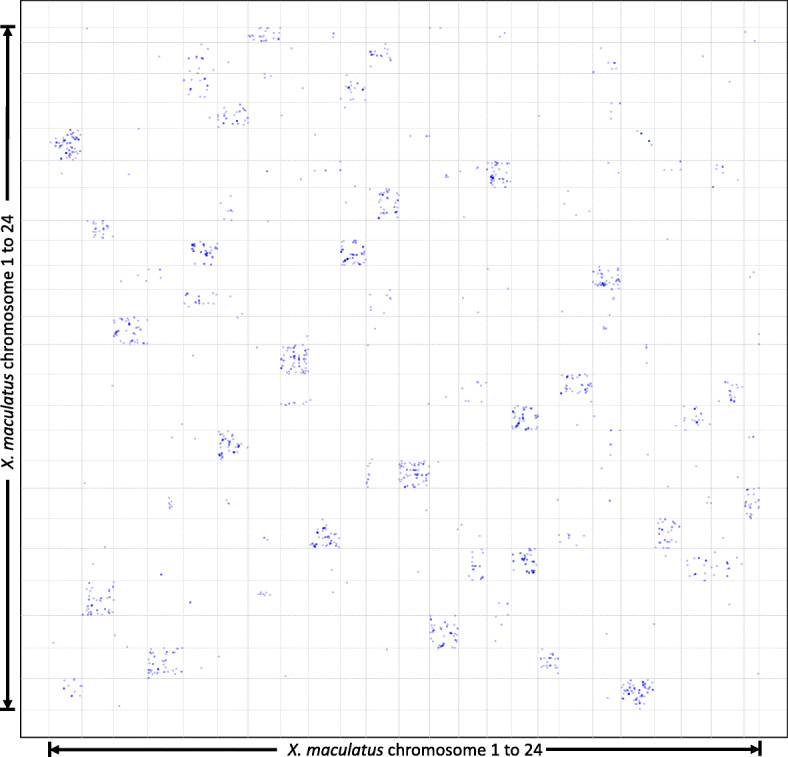
Gene duplication in *X. maculatus* genome compared to gar Chromosome plots showing all genomic locations of 943 Ohnolog pairs. X and Y axis represent chromosome lengths. Each spot on the graph represents one Ohnolog pair that are both orthologs to a single gar gene. The X- and Y- coordinates of each point correspond to the genomic location of each paralog gene on the *Xiphophorus* chromosome

The *X. maculatus* genome (assembly version 5.0; Ensembl release v94) encodes a total of 24,209 genes. We have identified 1886 *Xiphophorus* genes that show 2:1 relationship to gar (i.e., 943 Ohnolog pairs; Fig. 3; Supplement Table 4), and 10,952 *X. maculatus* genes that show 1:1 to gar (i.e., singletons; Supplement Table 5). Therefore, a total of 12,838 *Xiphophorus* genes (53%) can be tracked through the TGD using gar genome as a bridge. For example, among the *Xiphophorus arntl, cry, per* and *clock* gene families, *per2* and period circadian regulator 2, *cry1ab* and crypthochrome-1-like, and two *arntl2* genes are duplication products of unnamed gar genes ENSLOCG00000004441, *cry1ab*, and *arntl2*, respectively (Supplement Figure 6).

An average of 65.2% of the cyclic genes from the different organs can be traced to singletons or Ohnologs (Supplement Table 6). It is hypothesized herein that two Ohnologs preserve the ancestral function, meaning in most cases both Ohnologs should exhibit the same spatial and temporal cyclic based expression pattern. However, it was observed to be very rare for both Ohnologs to exhibit cyclic based expression patterns (i.e., average of only 1.6% of genes; Supplement Table 6) regardless of the expression pattern tested. Therefore, most post-TGD duplicates exhibit neo- (i.e., molecular ancestor exhibit no cyclic cycle, one or both Ohnologs exhibit cyclic cycle) or sub-functionalization (i.e., molecular ancestor exhibit cyclic expression, one Ohnolog lost it, or both Ohnologs exhibit cyclic cycles, but expression patterns do not overlap), instead of superfunctionalization (i.e., both Ohnologs showed the same cyclic expression pattern).

### Functional divergence of cyclic genes in *Xiphophorus*

Although it is impossible to determine if a gene duplicate gained a new function, or lost an ancestral function after a WGD event without knowing the ancestral genetic function, it is important to know how Ohnologs differentiate in function (or not) to study how WGD and the following genome retention patterns have reshaped the cyclic rhythm regulatory circuits. Of the 964 Ohnolog pairs that showed a 2:1 relationship between the *X. maculatus* and gar, 626 of them exhibit cyclic expression patterns for at least one Ohnolog in at least one organ (Supplement Figure 7). Also, 4573 singletons exhibit cyclic based expression patterns among the various different organs (Supplement Figure 8). A majority (i.e., 86.3–100%) of cyclic Ohnologs exhibit intra-organ functional differentiation (i.e., only one of the pair exhibit cyclic gene expression per organ, or both Ohnologs exhibit cyclic expression patterns, but show cyclic pattern different from one another), and only a small fraction (i.e. 2.6%) of these genes exhibit peak expression at the same time in the same organs (i.e., superfunctionalization; Supplement Table 7). Due to the high organ specificity of cyclic gene expression, all of the 626 Ohnolog pairs exhibited inter-organ functional differentiation (i.e., only one of the gene pairs exhibited cyclic gene expression in various organs, or both Ohnologs exhibit cyclic expression patterns, but show peak expression at different times among different organs; Supplement Table 7).

A survey conducted on zebrafish brain circadian genes (http://cgdb.biocuckoo.org/) showed similar observations, that a majority (29 of 32 Ohnolog pairs) of the genes do not share the expression pattern with their Ohnologs (Supplement Table 8). Also, 4 of the 7 genes that show a circadian expression pattern in both brain and pineal gland exhibited different peak expression Ct times (Supplement Table 9). These observations, from a teleost fish model distant from *Xiphophorus*, may suggest that circadian gene sub-functionalization following TGD is a feature shared among fish species.

#### Intra-organ functional divergence

**Fig. 4:**
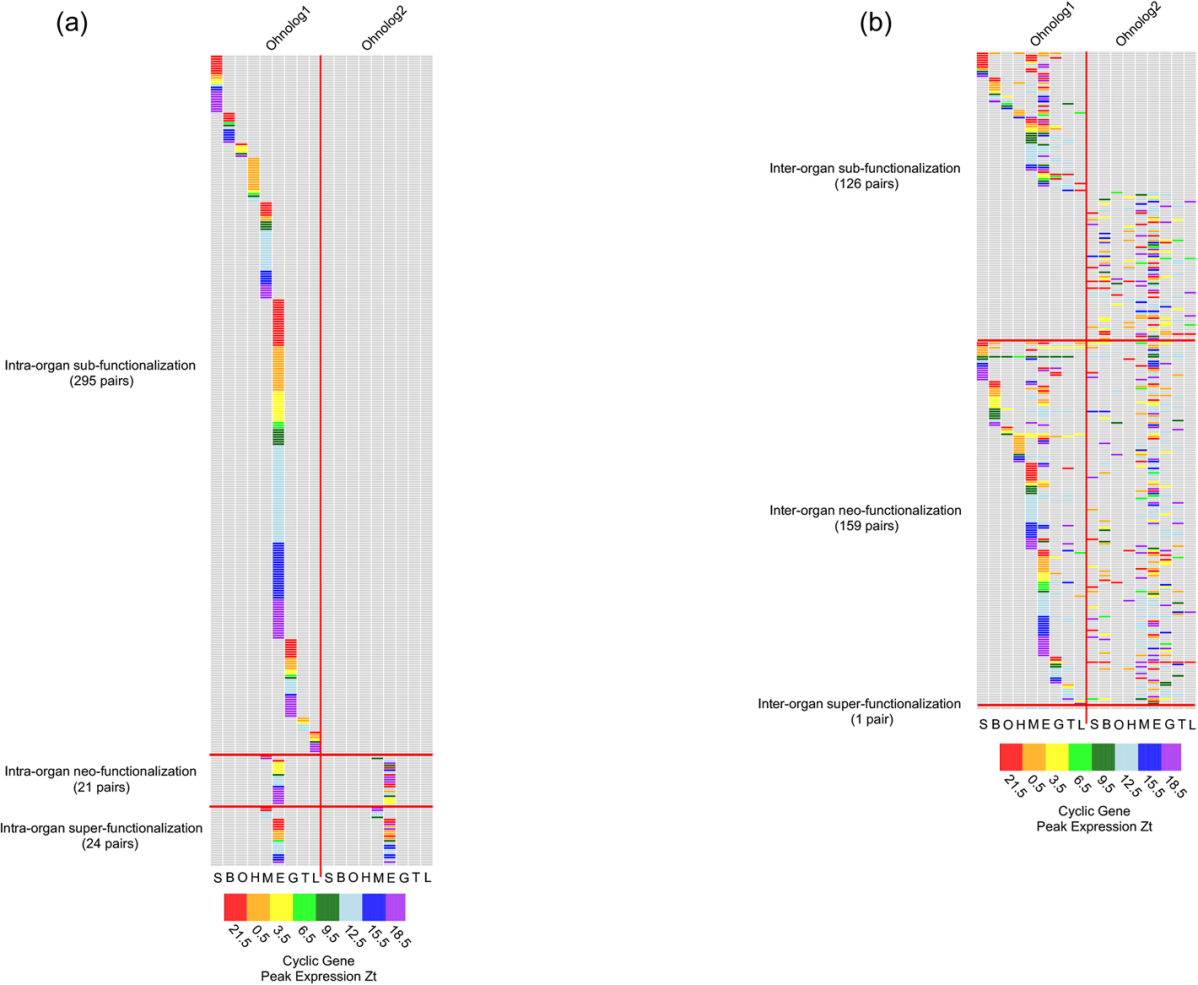
Functional divergence of Ohnologs showing cyclic expression pattern: **a** intra-organ functional divergence, and **b** inter-organ functional divergence. In each case, heatmap was used to describe the organ(s) in which gene showing circadian pattern. Colored blocks represent a particular gene that exhibits circadian expression in a particular organ, with the color of the block represented the peak expression Zt. Light gray means no circadian expression was identified. The center red line dividing each heatmap, splits the left and right half of a heatmap, each half represents Ohnolog1 and Ohnolog2, respectively. A majority of Ohnolog pairs showed Ohnolog-specific circadian expression patterns (i.e., sub- and neo-functionalization). S=Skin; B=Brain; O=Ovary; H=Heart; M = Muscle; E = Eye; G = Gills; L = Liver; T = Testis

Of the 626 Ohnolog pairs that exhibit circadian expression patterns for at least one of the duplicated genes, 340 pairs (54.3%) are also organ specific. Among these are 295 cases where one gene exhibits a cyclic expression pattern, but not the other; 21 cases where both duplicated genes exhibit cyclic expression, but have different expression patterns; and 24 cases where both duplicated genes cycled with the same expression pattern (Fig. 4a; For Ohnologs expression pattern, see Supplement Figure 4).

#### Inter-organ functional divergence

286 of the 626 circadian Ohnolog pairs exhibited circadian expression patterns in multiple organs. These include 126 cases where only one Ohnolog exhibit circadian expression pattern; 159 cases where different Ohnolog exhibit cyclic expression pattern in different organs; and only one case where both Ohnolog show synchronized cyclic cycle in several organs (Fig. 4b; For Ohnologs expression pattern, see Supplement Figure 7). 1427 of the 4573 singleton cyclic genes are present in multiple organs, 423 genes exhibited different expression pattern in different organ and 1004 exhibited synchronized cyclic pattern in different organs (For Ohnologs expression pattern, see Supplement Figure 8).

Taken together, Ohnolog pairs mostly exhibited differentiated expression patterns throughout a 24-h period that contains both dark and light phases. The observed expression pattern differentiation suggested the fate of Ohnologs following WGD is predominantly sub-functionalization.

## Discussion

Gene expression is a dynamic process and under the regulation of constantly changing environmental stimuli [10, 11]. Therefore, researchers should take the complexity of basal gene expression into consideration when investigating biological effects when studying genetic functions for a few simple reasons: (a) Target gene(s) of interest need to be expressed in target organ(s) in order for functional tests, such as knock-down or knock-outs, to be interpreted; (b) Gene(s) that may functionally compensate a target of interest need to be taken into account (e.g., paralogs); (c) Since basal gene expression is often time-dependent and external stimulus related (e.g., circadian genes, light inducible genes), a proper time to apply an experimental intervention needs to be determined. In consideration of these experimental parameters, we assembled a basal gene expression browser to assist researchers in addressing the above concerns. Major genome databases, such as NCBI, ENCODE and Ensembl, carry organ specific gene expression data for a variety of species, but lack temporal expression pattern data along with it. The dataset and expression browser presented in this report therefore serves as a new and supplementary tool to those databases.

We detailed expression patterns of four circadian regulator gene families (i.e., ARNTL, CLOCK, PER and CRY), as examples and technical controls for further investigation, to demonstrate the power of our platform to detect and display gene expression patterns showing temporal shifts during the diurnal period. ARNTL and CLOCK genes form heterodimers to induce transcription of PER and CRY genes, therefore ARNTL and CLOCK expression patterns are expected to be synchronized and diurnally opposite to PER and CRY [For review, see [23]]. This is true for both of the *X. maculatus clock* genes, and two of the *arntl* genes (i.e., *arntl1*, and *anrtl2-*ohnolog1) although in some cases (e.g., heart, testis) a family member shows circadian patterns that are organ-specific. In contrast, the *arntl2-*Ohnolog 2, not only lost cyclic expression pattern in most of the assessed organs, but also shows peak expression that is shifted to the middle of dark phase, instead of at the light-dark phase transition period (Fig. 1). This observation suggests sub-functionalization following the TGD and warrants investigation into the function of the *arntl2* duplicate. In contrast, PER and CRY genes show peak expression ~ 6 h following ARNTL and CLOCK peak expression times, or at the dark-light transition. Both *per1b* and *per3*, as well as *cry1ab* Ohnolog 1, exhibit the anticipated expression patterns (Fig. 1). Both of the *per2* genes are Ohnologs. However, *per2-*Ohnolog1 lost circadian expression in all organs, while *per2-*Ohnolog2 exhibited an unequally shifted expression pattern. This indicates the *per2* genes may have lost tight transcriptional regulation in the *Xiphophorus* lineage and therefore may not serve as sound markers for circadian studies. The *cry* gene family represents the most complex basal gene expression pattern of those discussed herein. The *cry1ab*-Ohnolog2, *cry1ba* and *cry2* all show shifted peak expression towards the light-dark transition, with the *cry1ba* expression pattern resembling that of *arntl* and *clock,* as observed in zebrafish [36]. Three fish specific *cry* genes, *cpd photolyase*, *6,4 photolyase* (*cry5*) and *cry-dash* exhibit high expression only in light phase of a 24-h period (Fig. 1; Supplement Table 1). This observation corresponds to their presumed function in repair of UV induced DNA damage [37–39]. The same observation that *cpd photolyase*, *6,4 photolyase* exhibited up-regulation following 2 h of FL light exposure was made in our previous study [40, 41]. The current study added more dynamic information to the gene expression change. The instantaneous increase of both genes’ transcripts suggests the up-regulation may due to post-transcriptional modulation (e.g., decrease of transcript degradation), rather than enhancement of new transcript synthesis. The molecular evolution of these photolyases, as well as their functional divergence from visible light-inducible DNA repair genes for UV induced damage in fishes, instead of circadian regulators as in mammals, is an interesting research topic. The complex transcriptional behavior of this family of genes supports the importance of studying basal gene expression as a first step in genetic analyses of biomedical research topics.

We found that among all the organs tested eye exhibited the highest percentage of cyclic genes. 18,377 genes are expressed in eye, and 4033 (21.9%) of them showed cyclic expression patterns (Fig. 2; Supplement Table 2 and 3). This is expected considering that eye is the major light-receiving organ, and cyclic gene expression may be light inducible. Although cycling expression patterns of opsins are not the prerequisite for cyclic rhythm, eyes did express most the *Xiphophorus* opsin genes and also exhibited the largest number of opsins exhibiting cyclic expression patterns (Supplement Figure 5). This suggests the cyclic pattern of a large portion of the ocular transcriptome is driven by opsins and their downstream signaling pathways. In contrast, ovary and testis exhibited the least amount of cyclic genes. Transcriptional rhythmicity studies performed in mammalian model systems have shown mixed results regarding gonad circadian gene expression (i.e., cyclic or non-cyclic) [42–48]. This may be due to the different types of molecules measured (i.e., RNA transcripts vs. protein) or sensitivity of the bioassays. We found known circadian regulators did show cyclic expression patterns in gonads, but the amplitudes are very small when compared to other organs, and so exhibited a relatively “muted” pattern of expression. These results support others that indicate gonads have cyclic transcriptional rhythmicity, and also expand potential circadian genes to non-master regulators within gonad, and therefore forward our understanding of transcriptional homeostasis in reproductive organs.

Genomic studies, accompanied with natural biodiversity, enable the study of molecular evolution by comparisons, and translation of discoveries from various aquatic animal models into human biomedical applications [30]. TGD provides the raw genetic material for the formation of the clade comprising the largest numbers of species and also provides a plethora of information for study of molecular evolution and genetic functional adaptation. However, the TGD genome duplication event also confounds identification of orthology between pre- and post-TGD species (e.g., teleost and tetrapods). The recent sequencing of gar genome facilitated amelioration of this confusion because gar has maintained a fish-like lifestyle but did not undergo the teleost genome duplication, and therefore serves as a direct connection between teleost fish and mammals [49, 50]. We were able to trace 12,838 *Xiphophorus* genes to 11,895 gar genes (10,952 singletons and 943 ohnolog pairs; Fig. 3; Supplement Tables 4 and 5). These numbers are similar to what were reported in comparative genomic studies performed between medaka and zebrafish [9]. Because it is impossible to assess gene expression patterns of the common ancestor between *Xiphophorus* and gar, and similar gar basal gene expression data over time is not readily available, we cannot truly access the functional divergence between *Xiphophorus* Ohnologs. However, characterizing how the gene duplicates behave differently is significant to study molecular evolution of particular gene families, and within the scope of the presented study, the fate of cyclic genes following the TGD event. Therefore, we investigated cases where only one gene of the Ohnolog pair exhibits cyclic gene expression, but not the other, or two Ohnologs exhibit different expression patterns within the same organ or among different organs (i.e., subfunctionalization), and both Ohnologs exhibit cyclic expression patterns, but show different cyclic expression peaks (neo-functionalization), under the assumption that molecular ancestors of the *Xiphophorus* cyclic genes are also cyclic. It is intriguing that Ohnologs exhibiting sub- and neo- functionalization account for a majority of the genes showing cyclic expression patterns. This observation suggests that most Ohnologs within a duplication pair have evolved new functions and therefore are not likely to be functionally and temporally redundant to their partners. This raises the question of why only one Ohnolog gene is selected to carry the cycling expression pattern (Fig. 4; Supplement Table 7), or conversely, what are the effects of Ohnolog overexpression. It was suggested that complex genes are preferentially retained as duplicates following TGD [51]. Therefore, we investigated complexity of gene structure (i.e., gene, transcript, CDS, UTR lengths) between the cyclic and non-cyclic gene copies, to test if differential complexity of Ohnologs might differentiate their expression patterns. However, we found no association between these parameters and both expression patterns. Additionally, expressivity of Ohnologs, and the relative expression levels between two Ohnologs in a pair did not correlate to cyclic expression patterns (Supplement Figure 9). Therefore, the sub-functionalization in circadian expression patterns may be rooted in more complex divergence mechanisms within select regulatory regions, or molecular interactions within the context of the whole transcriptome, epigenetic regulation, or DNA/RNA sequences throughout post-TGD evolution.

Master circadian regulator genes were found in a few fish species and their expression patterns appear to be consistent to their mammalian counterparts [52, 53]. Following the teleost genome duplication, most duplicated genes were resolved back to one copy (i.e., singleton) while only ≈5–20% of the ancestral teleost genome remained duplicated [8, 9, 54]. It is important to know whether and how the Ohnologs may differ in terms of cyclic expression pattern, in order to study the molecular evolution of these genes, and deconvolute regulatory mechanisms underlying these expression patterns. Although comparative genomics for known circadian regulators (e.g., per, cry, arntl, and clock genes) have been performed for a few fish species [26], functional comparison of such genes is not studied at transcriptome level. We extended the inter-Ohnologs expression patterns comparison to zebrafish by incorporating reported zebrafish circadian genes in brain and pineal gland, and identified Ohnolog pairs [9, 55]. It is showed that a majority Ohnolog genes do not shared cyclic expression patterns (Supplement Tables 8 and 9). These observations are in accordance to *Xiphophorus* and suggest that cyclic gene sub-functionalization following TGD is a common fate of retained gene copies. Similar observations that sub- and neo-functionalization account for a majority of the extant Ohnologs functions are not unprecedented. Pasquier et al. reported a majority of Ohnologs were found undergone major changes in organ-specific expression patterns in zebrafish and medaka, in comparison to gar [9, 56]. Taken all these evidences together, it suggests neo- and sub-functionalization between Ohnologs is the reason for retention of both gene copies following genome duplication.

## Conclusions

We conclude that despite structural duplication, functional redundancy of expression among gene duplicates is rare in most teleost organs. In addition, we provide an innovative collection of basal transcriptomic data from nine fish organs over a diurnal cycle, and provide a gene expression browser for research community to utilize.

## Methods

### Fish utilized and RNA isolation

*X. maculatus* Jp 163 A in the 116th inbred generation (i.e., sibling mating) were provided by the *Xiphophorus* Genetic Stock Center (http://www.xiphophorus.txstate.edu/). Mature (12 months old) male and female *X. maculatus* (12 months old) were maintained in separate 38-l aquaria filled with filtered aquifer water from San Marcos, TX on a 13 h light/11 h dark cycle under 10,000 K fluorescent light (Coralife T8 Lamp 10,000 K, 32 W). All fish utilized were co-adapted to the same conditions for at least 2 weeks prior to the experiment. Fish were fed with flake food precisely at 8 a.m. and brine shrimp at 3 p.m. during this adaptive synchronizing periods. A total of 24 male and 24 female *Xiphophorus* were used for this study. Two male fish were dissected for skin, brain, liver, heart, gills, muscle, eyes, and testis, two female fish were dissected for ovary at each Zt (Zt 0.5, 3.5, 6.5, 9.5, 12.5, 15.5, 18.5 and 21.5 h; Supplement Figure 1). Heart samples were pooled from 3 males and 3 females at each time point to attain RNA yields required for construction of sequencing libraries. At dissection, fish were euthanized in an ice bath and upon loss of gill movement were sacrificed by cranial resection. All organs, except hearts, were dissected into separate 2.5 ml microcentrifuge tubes filled with RNA*later* (Ambion Inc.) for RNA isolation. A total of 48 animals were utilized (8 time point, 3 fish per sex per time point). All fish were maintained and samples taken in accordance with approved protocols (IACUC# 2015107711).

Total RNA was isolated using a methodology similar to those previously detailed [57]. Briefly, RNA*later* was removed from respective microcentrifuge tubes followed by addition of 750 μL of QIAzol (Qiagen) in 2.0 mL collection tubes designed for automated tissue homogenization and RNA isolation stations. Organs were homogenized using the TisseLyser II (Qiagen) facilitated by stainless beads (Qiagen) for 10 min at 25 Hz. RNA isolation was subsequently performed using a QIAcube HT (Qiagen) automated bio-sample isolation system. The isolation system is equipped with a robotic arm with 8 pipettes. Each pipette is able to pick and eject pipette tips, self-clean, and transfers liquids between well/columns, or between reagent reservoirs and well/columns in standard 96-well plate formats. Each sample was independently maintained throughout the isolation process. Briefly, 150 μL of chloroform was added to each isolation tube and the samples were vigorously shaken for 15 s and then phases partitioned by centrifugation (12,000×g for 15 min at 4 °C). The aqueous phase containing nucleic acids was transferred to a new sample tube by a rack of automated pipettors. After extraction, nucleic acids were precipitated with 500 μL 70% ethanol. RNA was then purified using a Qiagen RNeasy mini RNA kit (96-well plate) and eluted following the manufacturer’s protocol. RNA was quantified with a Qubit 2.0 fluorometer (Life Technologies, Grand Island, NY, USA) and RNA quality was measured on an Agilent 2100 Bioanalyzer (Agilent Technologies, Santa Clara, CA) to confirm RIN scores were above 8.0 prior to sequencing. Concentrations of RNA samples were adjusted to 100 ng/μL with RNase-free water (Qiagen) prior to RNA sequencing.

### RNA-Seq and gene expression profiling

Each RNA sample was used to construct an independent sequencing library using Illumina TruSeq library preparation kit (Illumina, Inc., San Diego, CA, USA). This library strategy led to a total of 136 dual-indexed libraries. Libraries were sequenced as 150 bp paired-end fragments using Illumina HiSeq 2000 system (Illumina, Inc., San Diego, CA, USA). Sequencing files can be accessed in NCBI GEO with accession number GSE158968(“https://www.ncbi.nlm.nih.gov/geo/query/acc.cgi?acc=GSE158968”). Short sequencing reads were filtered using an in-house data processing pipeline [58]. Briefly, sequencing adaptors, if detected, were first removed from sequencing reads. Processed sequencing reads were subsequently trimmed and filtered based on quality scores by using a filtration algorithm that removed low-scoring sections of each read and preserved the longest remaining fragment.

Processed sequencing reads were subsequently mapped to *X. maculatus* genome version 5.0 using Tophat2 [59], and gene expression of gene models annotated by Ensembl database (Release 94) was quantified using FeatureCount [60]. *X. maculatus* genome dataset, including genome sequence, genes, gene chromosomal coordinates are available at “https://uswest.ensembl.org/Xiphophorus_maculatus/Info/Index”.

### Detecting oscillating gene expression patterns

Counts of sequencing reads were normalized to total read counts per sample to eliminate effects of different sequencing depths among the different samples. Normalized read counts were in the format of Count Per Million (cpm) and were calculated as cpm = (read counts / library size) × 10^6^. Averaged cpm values were subsequently calculated using the biological replicates per time points. Relative expression at each time point was further calculated to bring all gene expression to the same scale: Relative expression = (Average cpm of biological replicate) / (Average cpm of all time points) [12].

Expressed genes of each organ were first identified by applying expression criteria to each gene of each organ-specific expression profile. A gene is determined to be expressed if cpm values of at least two of the eight time points are larger than 1. Subsequently, a Rhythmicity Analysis Incorporating Nonparametric algorithm was applied to identify genes exhibiting oscillating expression patterns [61]. The RAIN algorithm is a robust nonparametric method to detect rhythms. It first groups data by measurement time, then test the grouped data against oscillation model consisting of a rising and a falling slope (i.e., monatomic slopes). Slopes are independently tested by summation of Mann-Whitney-U-Tests. A *p*-value cutoff of 0.01 (*p-value <* 0.01) was used to determine genes that showed oscillating expression patterns. Heatmaps, dot plots and line plots were used to represent the expression patterns of these genes over a single diurnal cycle. All plots were created using R plot function and heatmap.2 function of R package gplots.

#### Syntenic group analyses of circadian master regulators

*Xiphophorus* and gar chromosomal coordinates, and gar orthologs of *Xiphophorus* genes were downloaded from Ensembl database (Release 94). *X. maculatus* genome dataset, including genome sequence, genes, gene chromosomal coordinates are available at “https://uswest.ensembl.org/Xiphophorus_maculatus/Info/Index”. Gar genome dataset, including genome sequence, genes, gene chromosomal coordinates, are available at “https://uswest.ensembl.org/Lepisosteus_oculatus/Info/Index”. *Xiphophorus* paralog gene IDs, paralog type, ancestry information, and gar orthologs of *Xiphophorus* genes, and transcript sequences are available at either “http://uswest.ensembl.org/biomart/martview/ae840c9e1d9e5837bec0348203e881ab”, or through R/Bioconductor Biomart at “https://www.bioconductor.org/packages/release/bioc/html/biomaRt.html”. For each *Xiphophorus* gene assigned to a location *i* (gene _*i*_), its upstream (gene_*i + 1*_ … gene_*i + n*_) and downstream neighbor genes (gene_*i-n*_ … gene_*i-1*_) within a window n were acquired. Similarly, the gar ortholog of gene *i* (ortholog _*i*_), along with its chromosome neighbors (ortholog_*i + 1*_ … ortholog_*i + n*_ and ortholog_*i-n*_ … ortholog_*i-1*_) were also retrieved using the gar annotation file. To test the “co-linearization” between the *Xiphophorus* gene and gar orthologs, each gene within the “gene _*i-n*_ *…* gene _*i+n*_” were tested to each gar gene within the “ortholog _*i-n*_ *…* ortholog _*i+n*_” to test if *Xiphophorus* – gar orthology is present for each gene pair. A score is calculated as: *Score = #* orthologs / (window size n) × 2. If a *Xiphophorus* gene appeared orthologous to several gar genes, the gene pair that results in the highest *Score* is assigned as the *Xiphophorus -* gar ortholog.

Circadian regulator gene families, which include any gene that contains “aryl hydrocarbon receptor nuclear translocator”, “cryptochrome circadian”, “period circadian”, and “clock circadian”, as well as paralogs of these genes’ were retrieved from the *Xiphophorus* genome for synteny analyses compared to the gar.

#### Identification of TGD Ohnologs in *X. maculatus* and spotted gar ortholog

We employed the Pasquier et al. methodology (Pasquier et al., 2017) to identify *X. maculatus* Ohnolog pairs. Briefly, *X. maculatus* gene IDs (“ensembl_gene_id”), paralog IDs (“xmaculatus_paralog_ensembl_gene”) and spotted gar ortholog gene IDs (“loculatus_homolog_ensembl_gene”) were first downloaded from Ensembl Release 94 using Bioconductor package “Biomart”. Paralog types and ancestry information (“xmaculatus_paralog_subtype” and “xmaculatus_paralog_orthology_type”, respectively) were also downloaded for paralogous pairs filter. To qualify as an Ohnolog pair, two *X. maculatus* paralogs are expected to be rooted from the TGD, and share one single spotted gar ortholog (i.e., 1:2 gene relationship between gar and *X. maculatus*). First, we removed any paralogous pairs that showed a duplication ancestor as “Clupeocephala”, because this is a duplication point that occurs after the divergence of *Xiphophorus* from spotted gar. Second, paralogous pairs that exhibited more than one spotted gar ortholog were removed. Third, any gene that appeared more than once in all paralogous pairs was removed from the dataset. Additionally, we removed Ohnolog pairs that are not annotated to chromosomes (i.e., un-anchored contigs, and/or mitochondria), and are less than 5 Mbp separated on the same chromosome. This process led to identification of 943 Ohnolog cases (i.e., 943 gar genes, 1886 *Xiphophorus* genes) where two *Xiphophorus* orthologs correspond to one gar gene.

#### Identification of *Xiphophorus* singletons

To identify *Xiphophorus* genes that show 1:1 gene relationship (i.e., singletons, or genes that lost one TGD Ohnolog in *Xiphophorus*) compared to the gar genes, we first retrieved ensembl gene ID of all *Xiphophorus* genes, their paralog gene IDs, paralog type and ancestry information, as well as gar orthologs of *Xiphophorus* genes from Ensembl Release 94 using Biomart. Next, paralogs that had an indication of TGD and post-TGD lineage specific duplication (i.e., “Clupeocephala”, “Euteleosteomorpha”, “Percomorphaceae”, “Ovalentaria”, “Atherinomorphae”, “Cyprinodontlformes”, “Cyprinodontoldel”, “Poecllilnae”, “Xiphophorus”) were removed from the dataset. According to Pasquier et.al [9], genes with duplication ancestor “Neopterygii” because this duplication node can root from Ensembl tree reconstructions artifacts. Third, genes with gene names that suggest gene duplications (i.e., key work “one of many” in gene name) were removed. At last, each singleton is required to have one unique gar ortholog (i.e., *L. oculatus* orthology type = “ortholog_one2one”). This analysis identified 10,952 *Xiphophorus* singletons compared the gar genome.

#### Configuration of user interface and server

The user interface (UI) depicting basal gene expression is designed using the R project Shiny platform (https://cran.r-project.org/web/packages/shiny/index.html). To accurately deliver gene expression patterns, the Ensembl gene ID is required as the input due to ambiguity in common gene names that can confuse the algorithm. The Ensembl gene ID can be found using ensemble tool biomaRt, or the built-in gene ID table [62, 63].

Both UI (“ui” in Shiny is defined as the scripts that describe the user interface) and server (“server” in Shiny is defined as the scripts that process the data in correspondence to the “ui”) were constructed using R language and were assembled as one single project. Once the user inputs a gene ID, the server collects basal expression data of 9 organs from the XGSC data server to calculate cpm and relative expression values resulting in two graphs, as well as a summarizing table: (a) A bar graph showing mean cpm values of a gene in all organs; (b) expression pattern of a gene throughout a 24-h period in all organs; (c) a table showing peak expression time of gene of interest, and if the gene(s) exhibit a circadian expression pattern (RAIN algorithm *p*-value < 0.01). For flowchart, see Supplement Figure 2.

This project can be accessed remotely through the XGSC website (https://www.xiphophorus.txstate.edu), or downloaded from GitHub and launched locally using R command line. For detailed instructions, please see. https://www.xiphophorus.txstate.edu/Gene-Expression-Browser.html.

To utilize the user interface (UI), a query gene ID is required (Supplement Figures 3 and 4). The only input from the user is “Ensembl gene ID”. This input format was selected because the gene ID (e.g., ENSXMAG00000016482) is unique to one single gene model, while the common gene name (e.g., *egfr*) in many cases has multiple gene entries in a given database. Additionally, it is estimated that 5–20% of the teleost genome remains duplicated from the Teleost Genome Duplication [9, 28, 64], rendering duplicate genes to share the same or similar gene names. Using ambiguous common gene names without an in-depth functional interpretation may lead to errors in experimental interpretation. For example, *X. maculatus* encodes two *per2* genes resulting from the TGD. The loci that surround these two genes share orthology, and show synteny with the pre-TGD gar genome. They are annotated as “period circadian clock 2”, and “period circadian regulator 2”. Therefore, it is impossible to clearly distinguish the function of these two genes by these rather arbitrary names. The conversion of common gene name to gene ID can be accomplished using Ensemble Biomart, or by a built-in tool that searches for the Ensemble gene IDs for any gene that contains the user-input key word in a common gene name (Supplement Figure 3). After the gene ID is input into the “Ensembl Gene ID” window, the browser will download gene expression profiles of 9 organs (i.e., skin, brain, ovary, heart, muscle, eye, gill, liver and testis), collected every 3 h throughout a 24-h period and extract cpm values as gene expression values. The cpm values are next used to determine the expressiveness of a gene in different organs, and these data are plotted in a bar graph for visualization (Supplement Figure 4). Relative expression values for the gene-of-interest in each organ, at each time point, is also output as a plot to show basal expression level over the 24 h period (Supplement Figure 4). These two graphs are the main output of the presented browser that can assist users for study design. Along with these graphs is a summary table listing of the gene’s expressivity in each organ, such as peak expression time, amplitude of expression changes over a day, and if it exhibits a circadian expression pattern (Supplement Figure 4).

#### Comparison of circadian genes between *Xiphophorus* and zebrafish

For a parallel comparison of the circadian genes between *Xiphophorus* and zebrafish, Ohnolog pairs, singleton genes and their gar orthologs were downloaded [9]. Known zebrafish circadian gene from brain and pineal gland, and their peak expression time were downloaded from circadian gene database [55] (http://cgdb.biocuckoo.org/). Uniprot IDs were first converted to Ensemble peptide IDs using Uniprot website user interface, and subsequently converted to Ensembl gene IDs using Biomart. Duplicated IDs due to different protein products encoded by the same gene were collapsed. If the same gene were identified to be circadian gene from several studies, only the study using RNA-Seq or microarray was kept. Circadian genes were separated to Ohnologs and singletons to assess sub-functionalization between Ohnologs in circadian expression pattern.

#### Gene expression analysis by NanoString nCounter

A panel of Nanostring nCounter capture and reporter probe set was custom designed to capture and quantify selected *Xiphophorus* transcripts [12]. This NanoString nCounter panel contained 4 *Xiphophorus* genes that exhibited cyclic expression patterns (Supplement Figure 10). Design and production of the nCounter probes, was performed by the Nanostring bioinformatics group (Nanostring, Seattle, WA). Transcript sequences corresponding to each gene target were downloaded from Ensembl database, and used as templates to design probes. Each probe is 100 nt long, with a melting temperature between 73 and 91 °C and will not form secondary structures inhibiting the assay. Probes were tested in silico to avoid cross hybridization to other loci. NanoString hybridization of RNA samples with the target panel was initiated by mixing 500 ng of RNA (100 ng/μL) with the custom designed capture and reporter probe set. Samples were incubated for 12 h at 65 °C and then processed by the NanoString Prep Station (NanoString Technologies, Seattle, WA, USA) and subsequent nCounter analysis to determine gene expression. Built-in positive control probes were used to control the binding efficiency of each sample as read counts generated by these probes are independent of the RNA samples. A scaling factor is calculated based on the mean value of positive control probe generated read counts. Samples with the scaling factor between 0.3 and 3 are qualified for further analyses. Subsequently, the scaled read counts were normalized to the geometrical mean of the housekeeping genes to normalize potentially different total RNA input. Finally, background signal noise was determined by the read counts of negative control probes and was removed from normalized read counts [65–67].

## Supplementary Information

**Additional file 1: Supplement Figure 1**. Sample collection and experimental setup: 9 organs (skin, brain, liver, gill, heart, muscle, testis, ovary and eye) were collected at 5 am, 8 am, 11 am, 2 pm, 5 pm, 8 pm, 11 pm and 2 am. Duplicate samples of total RNA from each organ of individual fish was isolated for gene expression profiling. **Supplement Figure 2**. Flowchart depicting the configuration of the gene expression browser. Establishment of the gene expression browser includes 2 main steps: 1. Sample collection and gene expression profiling, and Gene ID and Gene name conversion: Gene expression data were normalized and scaled and stored in the XGSC website; All *Xiphophorus* Ensembl gene ID and corresponding gene name, as well as other bioinformatics information were downloaded into a custom table and stored in the XGSC website; 2. Configuration of the user interface and server: user interface was designed to convert common gene name to Ensembl gene ID to precisely identify the gene of interest, and to show basal gene expression pattern. These tools are stored in both shiny.io and Github for user to launch the browser remotely or locally. **Supplement Figure 3**. Illustration of the gene ID converter. The user interface (UI) contains a gene ID conversion tool. Users can input a common gene name as a key word to search through the whole genome to identify genes that may be of study interest. An example of “per” for *period* gene family is used in the Fig. 9 lines of data were returned. Among those two are *gper1*, a GPCR gene, and three are *perp,* a TP53 apoptosis effector gene that are all not relevant to the per gene family, and 4 per gene family members: *per1b*, *per2, per3* and a *per2* paralog (ENSMAG00000006651). **Supplement Figure 4**. Illustration of the gene expression browser user interface. Once in the web tool at the *Xiphophorus* Genetic Stock Center web page, an outside user can enter a proper Ensembl gene ID as input in order to generate a bar graph representing the organ-specific average expression levels of the query gene over a 24-h period. This generates an organ-specific expression curve depicting the gene expression at each time point of a day. The example shown is *per1b*. The table underneath the bar graph and expression pattern plot shows basic statics of whether this gene exhibits a cyclic expression pattern, including gene name, description, peak time (in Zt), peak shape (i.e., hours to go from peak expression to the lowest expression level), amplitude (i.e., distance between peak expression and the bottom of a circadian expression pattern). **Supplement Figure 5**. Cyclic genes and opsin expression heatmaps representing (a) circadian genes distribution in each organ and (b) opsin expression in each organ. For each heatmap, each row represents a gene and each column represents an organ. Yellow blocks show a gene (a) exhibiting circadian expression pattern, or (b) expressed. **Supplement Figure 6**. Circadian master regulator gene orthology to gar. Three pairs of circadian master regulators in *Xiphophorus* were found to be Ohnologs. These genes include (a) *arntl2*, (b) *per2*, (c) *cry1ab*. A syntenic block plot is used to show shared synteny of each Ohnolog with a gar ortholog. Red dots (open or closed) represent upstream and downstream neighboring genes of a *Xiphophorus* gene. The larger closed red dot represents a particular circadian gene. Blue dots (open or closed) represent upstream and downstream neighboring genes of gar ortholog of the *Xiphophorus* gene, and the larger closed blue dot represents the gar ortholog. Each *Xiphophorus* gene in the chromosome window is used to scan the gene of gar ortholog neighboring genes for orthology. If orthology exists, the *Xiphophorus* gene and the corresponding gar gene are connected by a gray line, with a closed dot representing *Xiphophorus* gene. **Supplement Figure 7**. Gene expression patterns of cyclic gene Ohnologs. A total of 626 Ohnologs pairs exhibited at least one of the duplicate showing circadian expression cycle in one organ. Per Ohnolog pair, 9 plots representing 9 organs were represent basal gene expression patterns, with solid line and dashed lines represented each gene duplicate. **Supplement Figure 8**. Gene expression patterns of singletons. A total of 4573 singletons exhibited circadian expression cycle in at least one organ. Per gene, 9 plots representing 9 organs were represent basal gene expression patterns, with solid line and dashed lines represented each gene duplicate. **Supplement Figure 9**. Expression analyses of cyclic gene Ohnologs exhibiting functional divergence in expression pattern: (A) intra-organ functional divergence, and (B) inter-organ functional divergence. In each case, two heatmap panels were used to describe (left panel) the expression status (i.e., gray = not expressed, black = expressed) and (right panel) relative expression between the Ohnologs (Ohnolog1 expression / Ohnolog2 expression). For the left panel, the center red line dividing each panel, splits the left and right half that represent Ohnolog1 and Ohnolog2, respectively. Status of expression and relative expression of Ohnologs are not correlated to if one Ohnolog exhibit circadian pattern, but not the other. S=Skin; B=Brain; O=Ovary; H=Heart; M = Muscle; E = Eye; G = Gills; L = Liver; T = Testis. **Supplement Figure 10**. Validating expression patterns of 4 cyclic genes using nanostring nCounter. Expression patterns of 4 cyclic genes identified in *X. maculatus* skin using RNA-Seq (*arntl1, cry1ab, clockb, clocka*) were assessed using nCounter by direct transcript molecule counting.

**Additional file 2: Supplement Table 1**. Differential expression analyses results.

**Additional file 3: Supplement Table 2**. Number of cyclic genes in each organ.

**Additional file 4: Supplement Table 3**. Cyclic genes of all 9 organs.

**Additional file 5: Supplement Table 4**. *Xiphophorus* Ohnologs.

**Additional file 6: Supplement Table 5**. *Xiphophorus* singletons.

**Additional file 7: Supplement Table 6**. Number of cyclic Ohnologs and singletons.

**Additional file 8: Supplement Table 7**. Functional divergence of cyclic gene Ohnologs.

**Additional file 9: Supplement Table 8**. Circadian Ohnologs in zebrafish brain and pineal gland.

**Additional file 10: Supplement Table 9**. Genes showing circadian expression pattern in zebrafish brain and pineal gland.

## Data Availability

*X. maculatus* genome dataset, including genome sequence, genes, gene chromosomal coordinates are available at “https://uswest.ensembl.org/Xiphophorus_maculatus/Info/Index”. This dataset is associated to Supplement Tables 1, 3, 4, 5, and 7. Gar genome dataset, including genome sequence, genes, gene chromosomal coordinates, are available at “https://uswest.ensembl.org/Lepisosteus_oculatus/Info/Index”. The gar genome dataset is associated to Supplement Tables 4, 5, and 7. *Xiphophorus* paralog gene IDs, paralog type, ancestry information, and gar orthologs of *Xiphophorus* genes, and transcript sequences are available at either “http://uswest.ensembl.org/biomart/martview/ae840c9e1d9e5837bec0348203e881ab”, or through R/Bioconductor Biomart at “https://www.bioconductor.org/packages/release/bioc/html/biomaRt.html”. This information is related to Supplement Tables 4, 5, and 7. Sequencing files can be accessed in NCBI GEO with accession number GSE158968 (“https://www.ncbi.nlm.nih.gov/geo/query/acc.cgi?acc=GSE158968”). Zebrafish circadian gene sets were downloaded from circadian gene database at “http://cgdb.biocuckoo.org/”, and are associated to Supplement Tables 8 and 9.

## Abbreviations

Zt: Zeitgeber Time
WGD: whole genome duplication
TGD: Teleost genome duplication
CDS: Coding sequence
UTR: Untranslated region
