# Supplementary material for: Global assessment of organ specific basal gene expression over a diurnal cycle with analyses of gene copies exhibiting cyclic expression patterns
Source: BMC Genomics. 2020 Nov 11;21:787. doi: 10.1186/s12864-020-07202-9 (PMC7659085; doi:10.1186/s12864-020-07202-9)
Supplement: Supplementary file 1 — Additional file 1: Supplement Figure 1. Sample collection and experimental setup: 9 organs (skin, brain, liver, gill, heart, muscle, testis, ovary and eye) were collected at 5 am, 8 am, 11 am, 2 pm, 5 pm, 8 pm, 11 pm and 2 am. Duplicate samples of total RNA from each organ of individual fish was isolated for gene expression profiling. Supplement Figure 2. Flowchart depicting the configuration of the gene expression browser. Establishment of the gene expression browser includes 2 main steps: 1. Sample collection and gene expression profiling, and Gene ID and Gene name conversion: Gene expression data were normalized and scaled and stored in the XGSC website; All Xiphophorus Ensembl gene ID and corresponding gene name, as well as other bioinformatics information were downloaded into a custom table and stored in the XGSC website; 2. Configuration of the user interface and server: user interface was designed to convert common gene name to Ensembl gene ID to precisely identify the gene of interest, and to show basal gene expression pattern. These tools are stored in both shiny.io and Github for user to launch the browser remotely or locally. Supplement Figure 3. Illustration of the gene ID converter. The user interface (UI) contains a gene ID conversion tool. Users can input a common gene name as a key word to search through the whole genome to identify genes that may be of study interest. An example of “per” for period gene family is used in the Fig. 9 lines of data were returned. Among those two are gper1, a GPCR gene, and three are perp, a TP53 apoptosis effector gene that are all not relevant to the per gene family, and 4 per gene family members: per1b, per2, per3 and a per2 paralog (ENSMAG00000006651). Supplement Figure 4. Illustration of the gene expression browser user interface. Once in the web tool at the Xiphophorus Genetic Stock Center web page, an outside user can enter a proper Ensembl gene ID as input in order to generate a bar graph representing the organ- [file 12864_2020_7202_MOESM1_ESM.zip › sup.figures revision.pdf]

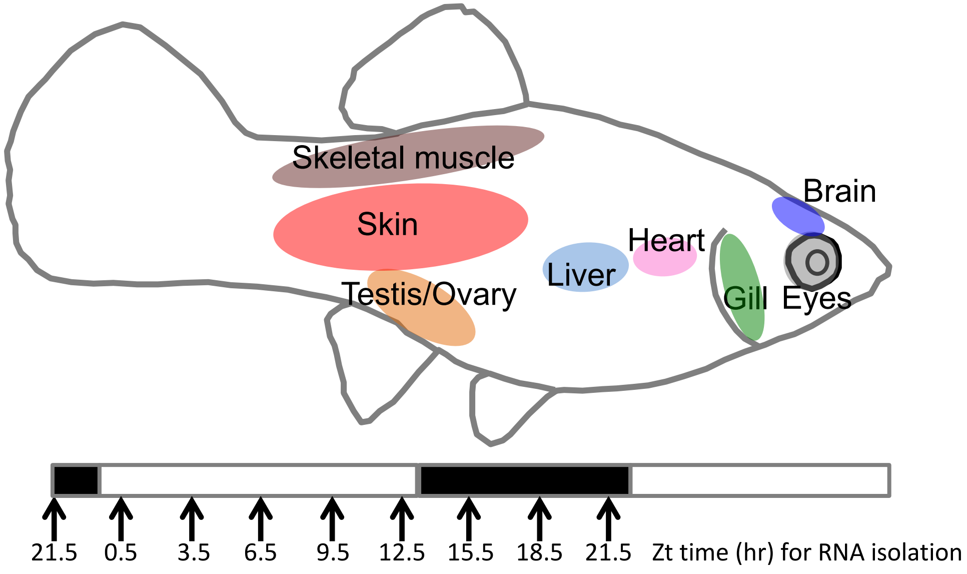

Supplement Figure 1

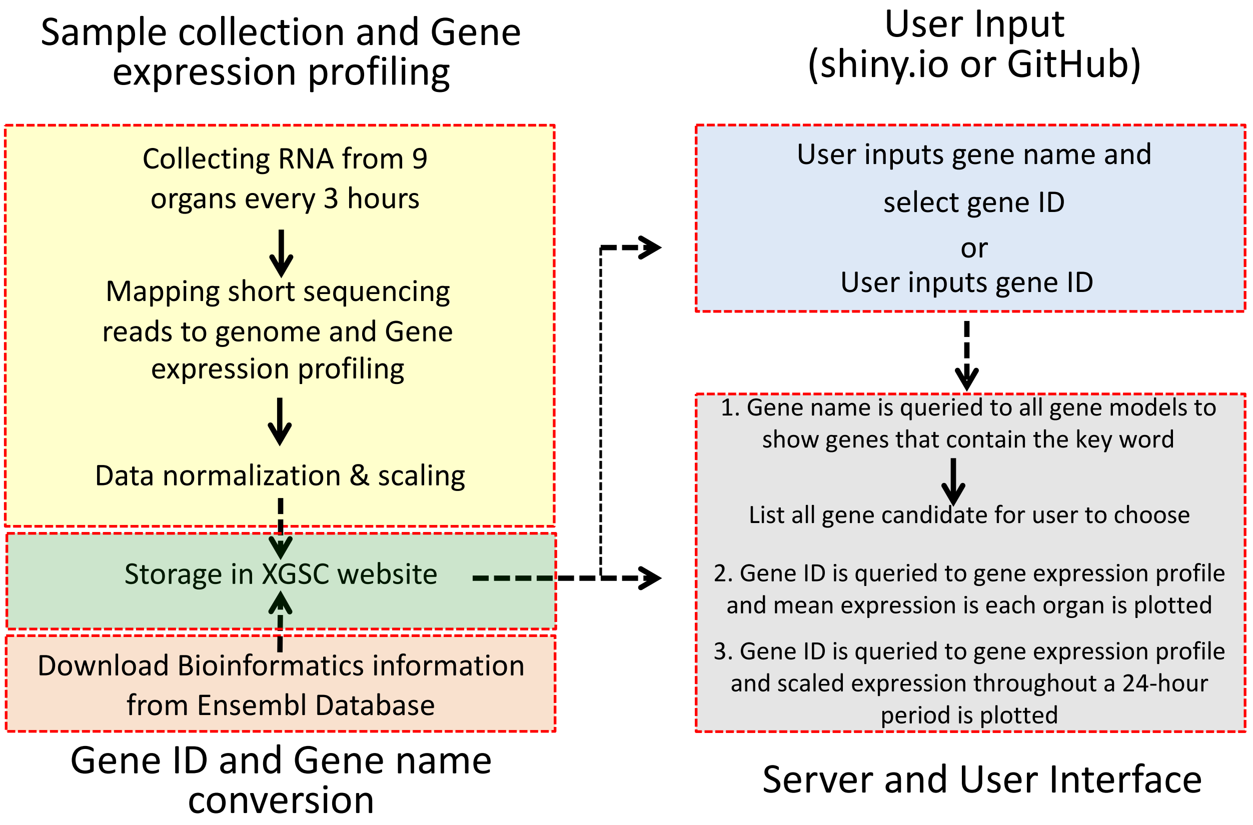

Supplement Figure 2

Gene Name (e.g., per1b)

per

ensembl_gene_id	external_gene_name	description	chromosome_name	start_position	end_position	strand	xmaculatus_paralog_ensembl_gene
ENSXMAG00000003057	scaper	S-phase cyclin A-associated protein in the ER [Source:ZFIN;Acc:ZDB-GENE-060303-5]	2	9649696	9715294	-1	
ENSXMAG00000003961	per3	period circadian clock 3 [Source:ZFIN;Acc:ZDB-GENE-000804-1]	20	22995060	23015472	1	
ENSXMAG00000016174	per2	period circadian clock 2 [Source:ZFIN;Acc:ZDB-GENE-011220-2]	6	14804076	14836161	1	ENSXMAG00000006651
ENSXMAG00000009518	gper1	G protein-coupled estrogen receptor 1 [Source:ZFIN;Acc:ZDB-GENE-090311-1]	16	8891959	8894920	1	ENSXMAG00000019424
ENSXMAG00000009518	gper1	G protein-coupled estrogen receptor 1 [Source:ZFIN;Acc:ZDB-GENE-090311-1]	16	8891959	8894920	1	ENSXMAG00000019535
ENSXMAG00000015314	per1b	period circadian clock 1b [Source:ZFIN;Acc:ZDB-GENE-040419-1]	14	12354593	12377957	1	
ENSXMAG00000015728	perp	PERP, TP53 apoptosis effector [Source:ZFIN;Acc:ZDB-GENE-050104-1]	22	20395989	20408458	-1	ENSXMAG00000008404
ENSXMAG00000015728	perp	PERP, TP53 apoptosis effector [Source:ZFIN;Acc:ZDB-GENE-050104-1]	22	20395989	20408458	-1	ENSXMAG00000023637
ENSXMAG00000015728	perp	PERP, TP53 apoptosis effector [Source:ZFIN;Acc:ZDB-GENE-050104-1]	22	20395989	20408458	-1	ENSXMAG00000027031

Supplement Figure 3

Ensembl Gene ID

ENSXMAG00000015314

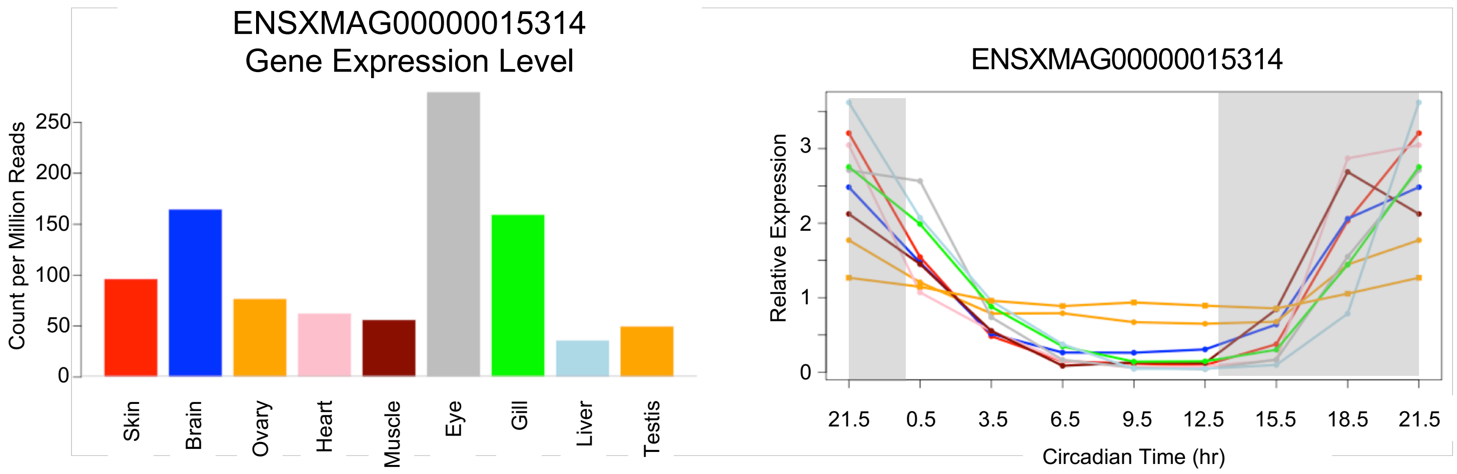

	Gene Name	Peak Time	Peak Shape	Amplitude
Skin	*per1b*	21.5	15	30.9
Brain	*per1b*	21.5	12	9.2
Ovary	*per1b*	21.5	n.a.	n.a.
Heart	*per1b*	0	12	41.9
Muscle	*per1b*	21.5	n.a.	n.a.
Eye	*per1b*	21.5	15	54.5
Gill	*per1b*	21.5	12	18.2
Liver	*per1b*	21.5	12	66.1
Testis	*per1b*	n.a.	n.a.	n.a.

Supplement Figure 4

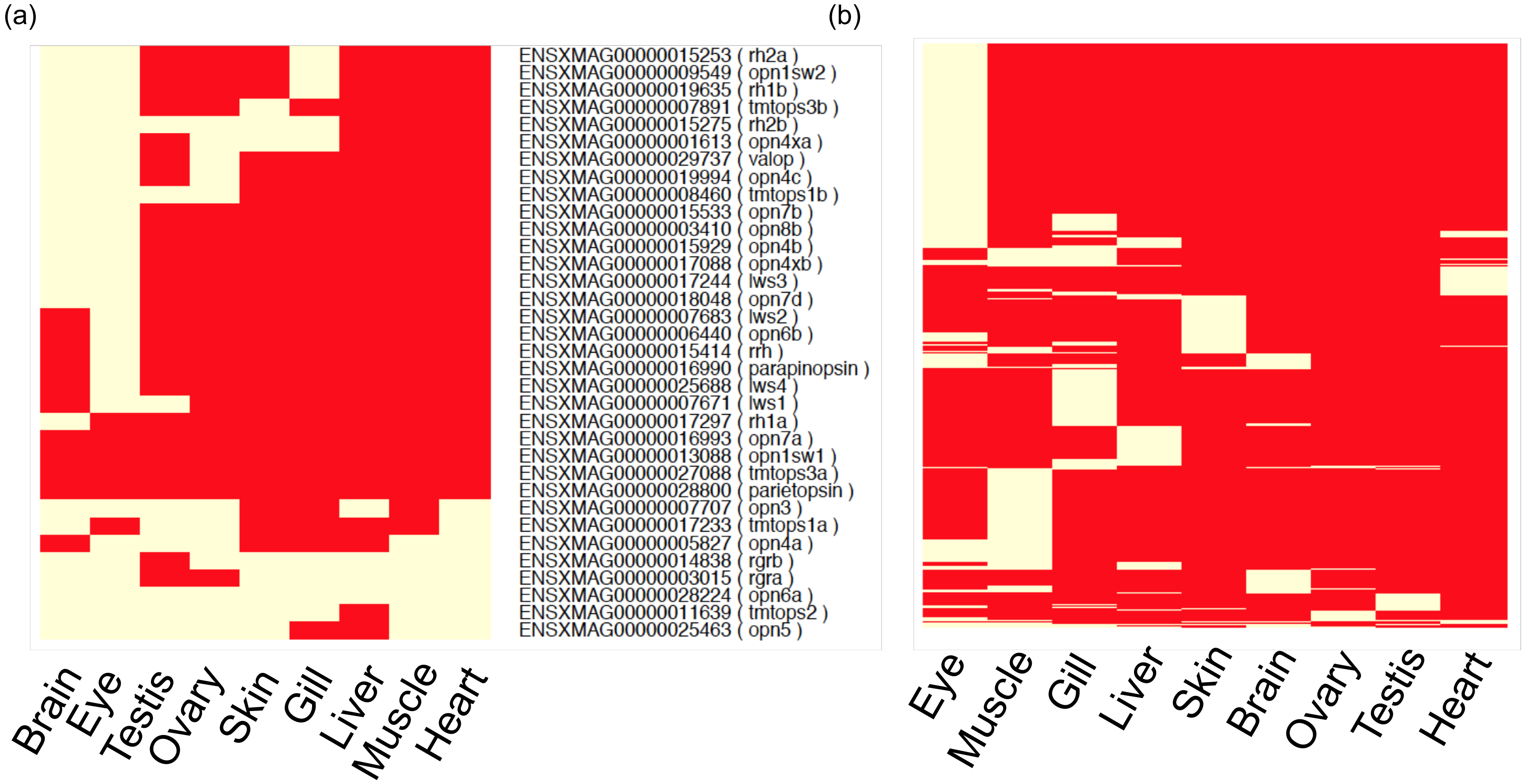

Supplement Figure 5

Supplement Figure 6 (a-c)

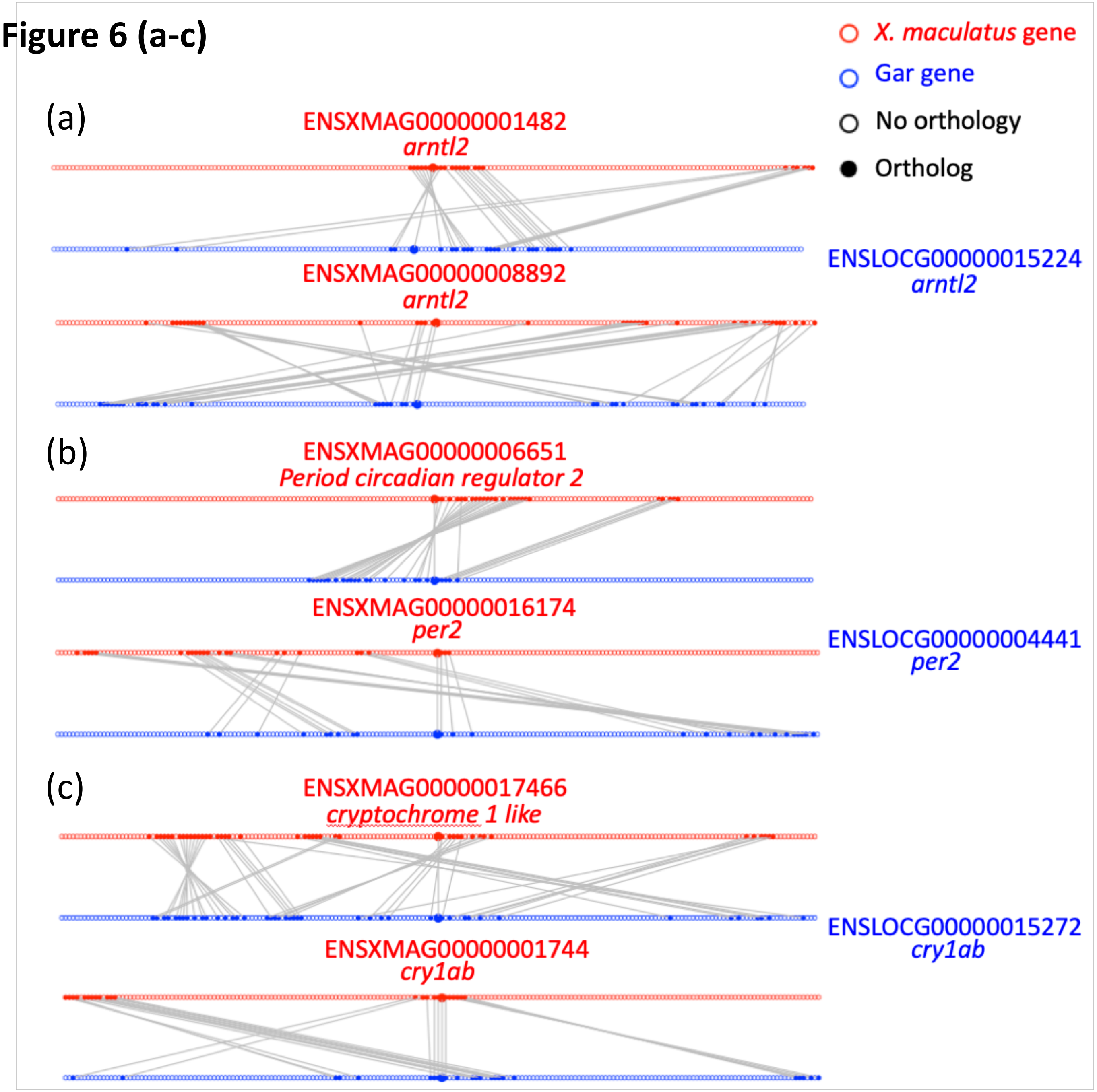

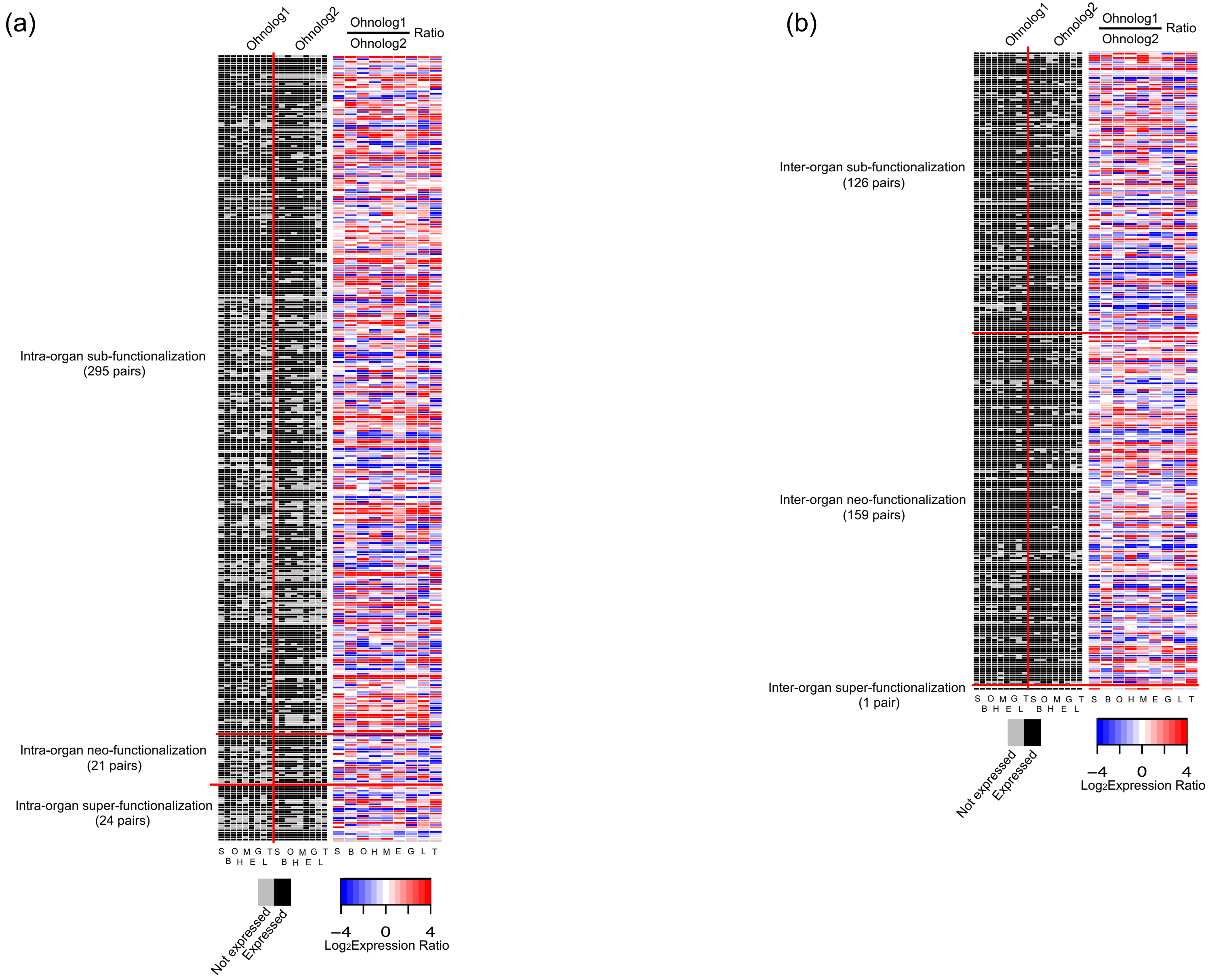

Supplement Figure 9

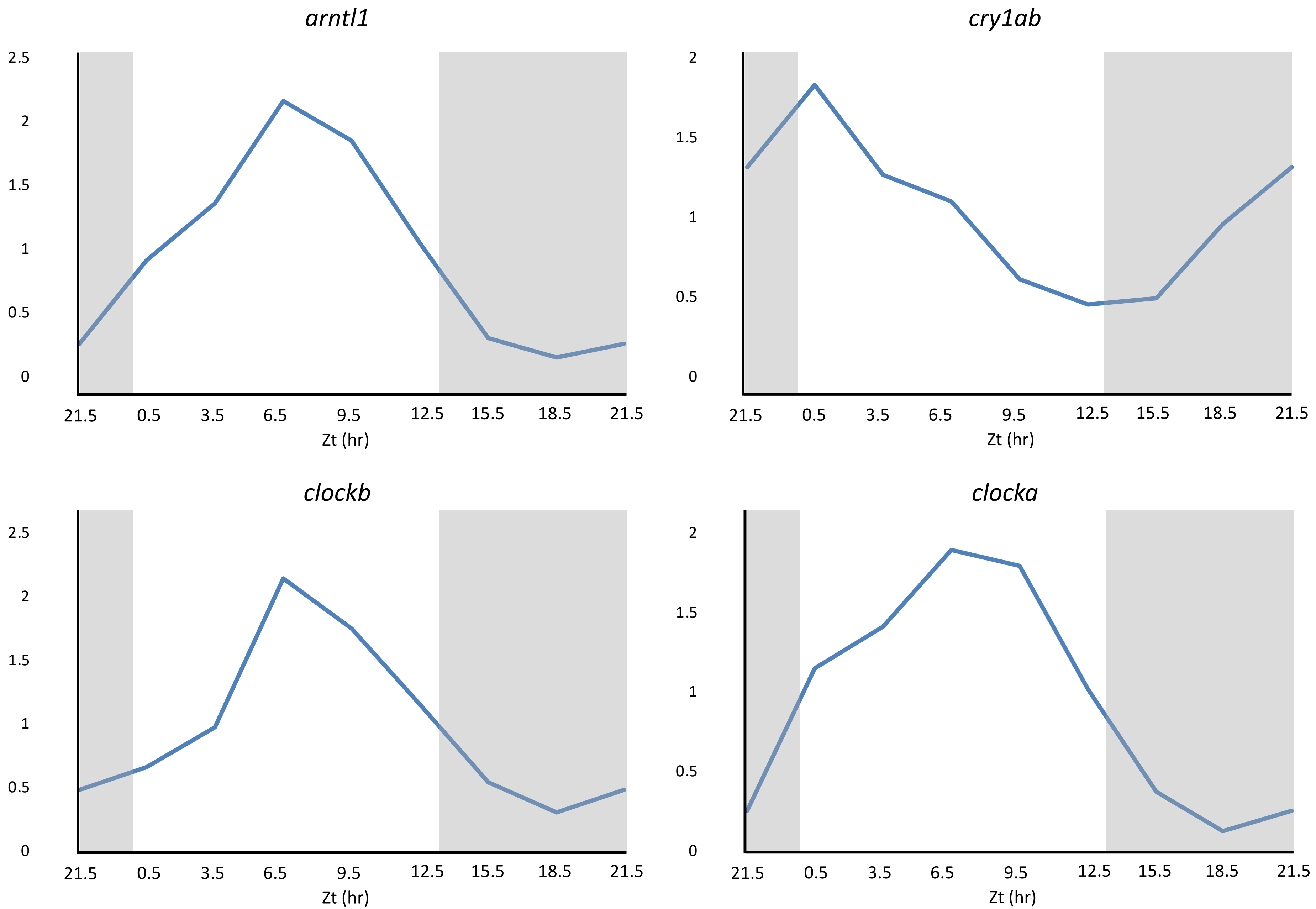

Supplement Figure 10
