# Supplementary material for: Global assessment of organ specific basal gene expression over a diurnal cycle with analyses of gene copies exhibiting cyclic expression patterns
Source: BMC Genomics. 2020 Nov 11;21:787. doi: 10.1186/s12864-020-07202-9 (PMC7659085; doi:10.1186/s12864-020-07202-9)
Supplement: Supplementary file 2 — Additional file 2: Supplement Table 1. Differential expression analyses results. [file 12864_2020_7202_MOESM2_ESM.pdf]

Supplement Table 1. Light responsive genes in all 9 organs

GeneID	logFC	logCPM	PValue	FDR	external_ger	Organ
ENSXMAG00	-1.17	-0.05	0.00	0.03		skin
ENSXMAG00	1.09	3.03	0.00	0.01	slc25a44b	skin
ENSXMAG00	-1.31	0.47	0.00	0.01	chrna5	skin
ENSXMAG00	-2.29	-0.66	0.00	0.02	adamtsl2	skin
ENSXMAG00	-1.01	4.73	0.00	0.01	mrtfbb	skin
ENSXMAG00	-0.79	4.94	0.00	0.00	zgc:154046	skin
ENSXMAG00	-0.78	4.71	0.00	0.01	nprl3	skin
ENSXMAG00	-0.72	3.99	0.00	0.02	si:ch211-235	skin
ENSXMAG00	-0.90	5.16	0.00	0.01		skin
ENSXMAG00	-1.08	2.55	0.00	0.01	raver1	skin
ENSXMAG00	-1.17	0.63	0.00	0.04	grin2aa	skin
ENSXMAG00	0.63	7.06	0.00	0.03	xrn2	skin
ENSXMAG00	1.15	5.96	0.00	0.00	si:dkey-93h2	skin
ENSXMAG00	-1.70	5.29	0.00	0.00	si:ch211-221	skin
ENSXMAG00	1.75	5.58	0.00	0.00	gfm1	skin
ENSXMAG00	-1.39	3.39	0.00	0.00		skin
ENSXMAG00	0.81	4.99	0.00	0.00	GPR37 (1 of	skin
ENSXMAG00	-0.97	2.30	0.00	0.03	b9d1	skin
ENSXMAG00	-0.60	5.30	0.00	0.04	aspscr1	skin
ENSXMAG00	0.76	3.26	0.00	0.05	mars2	skin
ENSXMAG00	-7.63	-1.18	0.00	0.02	adad2	skin
ENSXMAG00	0.85	4.79	0.00	0.01	hgh1	skin
ENSXMAG00	-0.86	6.00	0.00	0.05	COL21A1	skin
ENSXMAG00	0.68	5.86	0.00	0.01	rbm14a	skin
ENSXMAG00	0.77	6.13	0.00	0.02	dpp3	skin
ENSXMAG00	-0.75	3.08	0.00	0.04		skin
ENSXMAG00	0.76	6.65	0.00	0.01	actr1	skin
ENSXMAG00	0.68	6.48	0.00	0.05	thop1	skin
ENSXMAG00	-1.36	6.09	0.00	0.01		skin
ENSXMAG00	1.03	6.16	0.00	0.00	hdac1	skin
ENSXMAG00	0.97	4.88	0.00	0.00	znrd2	skin
ENSXMAG00	1.27	6.98	0.00	0.00	npm1a	skin
ENSXMAG00	0.76	5.61	0.00	0.01	wu:fj64h06	skin
ENSXMAG00	1.19	6.46	0.00	0.00	agps	skin
ENSXMAG00	0.78	5.24	0.00	0.01	anp32a	skin
ENSXMAG00	1.78	5.58	0.00	0.00	prmt5	skin
ENSXMAG00	0.87	7.23	0.00	0.01	acaca	skin
ENSXMAG00	1.00	5.30	0.00	0.00	dapk3	skin
ENSXMAG00	1.59	4.66	0.00	0.00	timm50	skin
ENSXMAG00	1.34	5.36	0.00	0.00	spega	skin
ENSXMAG00	-0.68	4.45	0.00	0.05	rapgef5a	skin

ENSXMAG00	0.62	5.43	0.00	0.04	relch	skin
ENSXMAG00	0.94	5.75	0.00	0.00	ap1m1	skin
ENSXMAG00	1.63	2.09	0.00	0.00	AQP11 (1 of	skin
ENSXMAG00	1.11	5.42	0.00	0.00	rangap1b	skin
ENSXMAG00	-1.02	8.14	0.00	0.01	ccl25a	skin
ENSXMAG00	-1.10	2.43	0.00	0.03		skin
ENSXMAG00	-1.37	4.07	0.00	0.00	znf395a	skin
ENSXMAG00	0.81	4.45	0.00	0.01	snrpb2	skin
ENSXMAG00	0.90	2.96	0.00	0.01	amer2	skin
ENSXMAG00	-0.70	4.16	0.00	0.04	ppp1r9a	skin
ENSXMAG00	-0.89	4.99	0.00	0.02	aspm	skin
ENSXMAG00	0.90	2.13	0.00	0.03		skin
ENSXMAG00	-0.86	5.28	0.00	0.00	pamr1	skin
ENSXMAG00	0.72	3.31	0.00	0.03	ago2	skin
ENSXMAG00	0.86	5.27	0.00	0.03	pdia5	skin
ENSXMAG00	-1.67	-0.11	0.00	0.00	sagb	skin
ENSXMAG00	-0.90	3.91	0.00	0.01	slc15a2	skin
ENSXMAG00	0.96	4.71	0.00	0.00	unc45a	skin
ENSXMAG00	1.04	3.04	0.00	0.01	adcy2a	skin
ENSXMAG00	-1.78	0.62	0.00	0.00	cacng7a	skin
ENSXMAG00	-0.92	5.59	0.00	0.00	PIEZO2	skin
ENSXMAG00	-1.94	6.43	0.00	0.00	CARD10	skin
ENSXMAG00	-0.72	5.90	0.00	0.01	proza	skin
ENSXMAG00	-0.93	3.02	0.00	0.01	gramd1bb	skin
ENSXMAG00	1.14	3.07	0.00	0.01	oxnad1	skin
ENSXMAG00	1.23	3.51	0.00	0.00	pus1	skin
ENSXMAG00	0.81	5.87	0.00	0.03	uqcrc2a	skin
ENSXMAG00	1.32	5.95	0.00	0.00		skin
ENSXMAG00	0.74	5.48	0.00	0.03	snrpd2	skin
ENSXMAG00	0.99	4.26	0.00	0.01	CLTC (1 of m	skin
ENSXMAG00	0.80	6.96	0.00	0.00	chd4a	skin
ENSXMAG00	-0.96	2.08	0.00	0.03		skin
ENSXMAG00	0.82	5.40	0.00	0.00	bysl	skin
ENSXMAG00	0.66	4.27	0.00	0.04		skin
ENSXMAG00	-0.75	5.02	0.00	0.01	ap5z1	skin
ENSXMAG00	0.99	2.82	0.00	0.01	med20	skin
ENSXMAG00	-0.77	4.86	0.00	0.03	acsl2	skin
ENSXMAG00	-0.77	4.32	0.00	0.01	cables2b	skin
ENSXMAG00	1.47	0.73	0.00	0.02	nsmfa	skin
ENSXMAG00	0.60	5.73	0.00	0.03	eif3jb	skin
ENSXMAG00	1.13	5.33	0.00	0.00	tox4b	skin
ENSXMAG00	0.88	6.30	0.00	0.00	SLC12A6	skin
ENSXMAG00	0.77	4.93	0.00	0.01	entpd4	skin

ENSXMAG00	0.68	4.88	0.00	0.01	uhrf1bp1l	skin
ENSXMAG00	0.82	5.59	0.00	0.01	cse1l	skin
ENSXMAG00	1.80	5.54	0.00	0.00		skin
ENSXMAG00	-0.75	5.55	0.00	0.04		skin
ENSXMAG00	0.78	4.86	0.00	0.00	aebp2	skin
ENSXMAG00	-0.63	6.47	0.00	0.04		skin
ENSXMAG00	0.73	4.21	0.00	0.01	taf5	skin
ENSXMAG00	0.82	5.64	0.00	0.03	gdi1	skin
ENSXMAG00	-0.76	4.32	0.00	0.02	nod1	skin
ENSXMAG00	-2.37	-2.63	0.00	0.04	tbpl2	skin
ENSXMAG00	1.20	6.53	0.00	0.01	serpinh1b	skin
ENSXMAG00	1.17	5.60	0.00	0.00	tsr1	skin
ENSXMAG00	0.89	4.73	0.00	0.00	mfsd1	skin
ENSXMAG00	1.38	-0.63	0.00	0.04	camk1gb	skin
ENSXMAG00	-0.99	5.81	0.00	0.02	mical1	skin
ENSXMAG00	0.93	6.61	0.00	0.00	larsb	skin
ENSXMAG00	0.69	5.22	0.00	0.02	snrpa1	skin
ENSXMAG00	1.38	1.78	0.00	0.00	gfer	skin
ENSXMAG00	1.81	5.23	0.00	0.00	arntl2	skin
ENSXMAG00	0.84	4.94	0.00	0.00	dhx36	skin
ENSXMAG00	0.84	5.44	0.00	0.00	slc27a4	skin
ENSXMAG00	-1.77	5.15	0.00	0.00		skin
ENSXMAG00	0.65	4.85	0.00	0.04	polr3b	skin
ENSXMAG00	-1.00	4.98	0.00	0.00	si:dkey-172j4	skin
ENSXMAG00	1.16	3.39	0.00	0.00	ahcyl2	skin
ENSXMAG00	-0.82	6.65	0.00	0.01	stat1a	skin
ENSXMAG00	1.50	5.51	0.00	0.00	hspa4a	skin
ENSXMAG00	1.07	5.57	0.00	0.00	fech	skin
ENSXMAG00	1.61	6.43	0.00	0.00	hyou1	skin
ENSXMAG00	-1.08	6.40	0.00	0.00	calcoco1a	skin
ENSXMAG00	-0.89	4.16	0.00	0.00	plch1	skin
ENSXMAG00	1.27	3.05	0.00	0.00	cyp27c1	skin
ENSXMAG00	0.71	4.26	0.00	0.02	gtf2f2a	skin
ENSXMAG00	-0.99	2.75	0.00	0.03		skin
ENSXMAG00	-1.02	3.58	0.00	0.00	prkab1a	skin
ENSXMAG00	1.41	5.40	0.00	0.00		skin
ENSXMAG00	0.64	7.07	0.00	0.03	prpf4bb	skin
ENSXMAG00	-0.91	6.30	0.00	0.01	itgb6	skin
ENSXMAG00	-1.26	3.51	0.00	0.00		skin
ENSXMAG00	1.28	6.86	0.00	0.00	abce1	skin
ENSXMAG00	0.83	4.85	0.00	0.00	dpysl2b	skin
ENSXMAG00	1.21	4.97	0.00	0.00	umps	skin
ENSXMAG00	0.95	5.53	0.00	0.00	ppp2r2ab	skin

ENSXMAG00	1.31	4.55	0.00	0.00	naprt	skin
ENSXMAG00	0.78	5.55	0.00	0.01	ruvbl1	skin
ENSXMAG00	0.65	5.85	0.00	0.05	atic	skin
ENSXMAG00	0.79	4.76	0.00	0.00	tmem39a	skin
ENSXMAG00	-0.73	5.78	0.00	0.04	prkdc	skin
ENSXMAG00	1.32	4.41	0.00	0.00	pitpnb (1 of r	skin
ENSXMAG00	1.39	6.90	0.00	0.00	abcf2a	skin
ENSXMAG00	-1.35	3.96	0.00	0.00	ulk1a	skin
ENSXMAG00	1.49	5.39	0.00	0.00	sec24d	skin
ENSXMAG00	0.62	7.47	0.00	0.04	snx1a	skin
ENSXMAG00	1.41	5.40	0.00	0.00	e2f4	skin
ENSXMAG00	-1.70	3.15	0.00	0.00	fgf24	skin
ENSXMAG00	0.77	4.71	0.00	0.01	prkag1	skin
ENSXMAG00	-0.88	6.40	0.00	0.00	limch1b	skin
ENSXMAG00	-1.31	4.63	0.00	0.00	bnip3la	skin
ENSXMAG00	-1.43	5.14	0.00	0.00	MX1	skin
ENSXMAG00	0.60	5.65	0.00	0.04	ccnt2a	skin
ENSXMAG00	0.84	6.08	0.00	0.02	phka2	skin
ENSXMAG00	-0.60	4.98	0.00	0.04	vps41	skin
ENSXMAG00	-1.11	6.41	0.00	0.00	stat2	skin
ENSXMAG00	0.76	7.42	0.00	0.01	snrnp200	skin
ENSXMAG00	0.75	3.59	0.00	0.02	fastkd3	skin
ENSXMAG00	-0.95	4.40	0.00	0.00		skin
ENSXMAG00	0.67	3.49	0.00	0.05	si:dkey-229d	skin
ENSXMAG00	-0.87	3.90	0.00	0.01		skin
ENSXMAG00	0.80	6.12	0.00	0.01	larp4ab	skin
ENSXMAG00	-1.22	5.62	0.00	0.00	mst1rb	skin
ENSXMAG00	1.22	4.78	0.00	0.00	ppid	skin
ENSXMAG00	1.15	3.90	0.00	0.00	alg6	skin
ENSXMAG00	1.27	3.91	0.00	0.00	trnt1	skin
ENSXMAG00	-0.74	3.75	0.00	0.02	ak4	skin
ENSXMAG00	-0.82	2.95	0.00	0.02	aga	skin
ENSXMAG00	-0.99	5.36	0.00	0.00	rasgef1bb	skin
ENSXMAG00	-0.93	4.76	0.00	0.00	bloc1s6	skin
ENSXMAG00	-0.70	4.09	0.00	0.05	tnksa	skin
ENSXMAG00	-1.51	0.87	0.00	0.00		skin
ENSXMAG00	0.80	5.34	0.00	0.01	GAR1	skin
ENSXMAG00	1.00	4.07	0.00	0.00	eif4ea	skin
ENSXMAG00	-1.02	3.27	0.00	0.00	sema4c	skin
ENSXMAG00	-1.05	4.14	0.00	0.00	slc39a1	skin
ENSXMAG00	-1.63	7.52	0.00	0.00		skin
ENSXMAG00	0.70	4.51	0.00	0.05	SPRYD3	skin
ENSXMAG00	-0.77	4.11	0.00	0.01	nf2a	skin

ENSXMAG00	0.58	5.68	0.00	0.04	dda1	skin
ENSXMAG00	0.62	6.14	0.00	0.04	dgcr8	skin
ENSXMAG00	0.90	6.11	0.00	0.00	ranbp1	skin
ENSXMAG00	-1.35	5.60	0.00	0.00	ulk1b	skin
ENSXMAG00	0.93	3.25	0.00	0.04	insig1	skin
ENSXMAG00	-4.20	2.47	0.00	0.00	nos1	skin
ENSXMAG00	-1.01	5.47	0.00	0.04	fcho1	skin
ENSXMAG00	0.90	6.96	0.00	0.00	ppp4ca	skin
ENSXMAG00	-2.01	3.44	0.00	0.00	rfx2	skin
ENSXMAG00	0.84	4.59	0.00	0.00	carm1	skin
ENSXMAG00	-1.43	1.07	0.00	0.01	asb14b	skin
ENSXMAG00	-0.78	3.00	0.00	0.02	hpse2	skin
ENSXMAG00	0.97	7.13	0.00	0.01	abat	skin
ENSXMAG00	-3.51	-2.51	0.00	0.01		skin
ENSXMAG00	0.78	3.24	0.00	0.03	mllt1a	skin
ENSXMAG00	0.97	5.36	0.00	0.00	uba5	skin
ENSXMAG00	-0.79	5.84	0.00	0.00	wsb2	skin
ENSXMAG00	-1.37	0.54	0.00	0.01	mpp2a	skin
ENSXMAG00	-4.63	-3.27	0.00	0.04		skin
ENSXMAG00	0.95	7.28	0.00	0.00	stt3b	skin
ENSXMAG00	0.85	3.22	0.00	0.01	coq8b	skin
ENSXMAG00	4.27	-1.70	0.00	0.05		skin
ENSXMAG00	0.93	5.84	0.00	0.00	ap2a1	skin
ENSXMAG00	0.86	3.64	0.00	0.01	TGDS	skin
ENSXMAG00	-0.98	5.01	0.00	0.00	si:ch211-251	skin
ENSXMAG00	2.45	4.55	0.00	0.00	psat1	skin
ENSXMAG00	-1.43	8.80	0.00	0.00		skin
ENSXMAG00	1.30	2.87	0.00	0.00	sfxn3	skin
ENSXMAG00	0.82	6.37	0.00	0.00	xpot	skin
ENSXMAG00	-1.83	4.07	0.00	0.00		skin
ENSXMAG00	0.71	8.08	0.00	0.02	setb	skin
ENSXMAG00	-1.01	1.72	0.00	0.01	zgc:162879	skin
ENSXMAG00	0.88	4.54	0.00	0.00	rtcb	skin
ENSXMAG00	1.31	4.25	0.00	0.00	si:ch211-233	skin
ENSXMAG00	1.10	7.45	0.00	0.00	tars	skin
ENSXMAG00	0.88	3.02	0.00	0.01	ctu1	skin
ENSXMAG00	-0.96	5.55	0.00	0.00	ctsh	skin
ENSXMAG00	-1.33	2.15	0.00	0.00	stxbp5a	skin
ENSXMAG00	0.64	6.29	0.00	0.04	gak	skin
ENSXMAG00	0.85	8.38	0.00	0.00	ywhaqb	skin
ENSXMAG00	-0.75	5.69	0.00	0.01		skin
ENSXMAG00	0.86	4.68	0.00	0.00	snrnp40	skin
ENSXMAG00	1.27	4.65	0.00	0.00	mlec	skin

ENSXMAG00	-0.85	4.99	0.00	0.01	malt3	skin
ENSXMAG00	-0.89	4.84	0.00	0.00	cyba	skin
ENSXMAG00	-0.68	5.06	0.00	0.02	ggcx	skin
ENSXMAG00	0.61	7.05	0.00	0.04	srsf7a	skin
ENSXMAG00	1.54	3.23	0.00	0.00	SCD	skin
ENSXMAG00	1.25	3.52	0.00	0.00	emc8	skin
ENSXMAG00	0.76	9.83	0.00	0.05	ACTN4	skin
ENSXMAG00	3.19	-0.14	0.00	0.01		skin
ENSXMAG00	-0.70	5.84	0.00	0.04	evi5b	skin
ENSXMAG00	1.98	5.98	0.00	0.00	fkbp9	skin
ENSXMAG00	-1.85	-0.68	0.00	0.00	pkd1l2a	skin
ENSXMAG00	-1.00	1.93	0.00	0.02		skin
ENSXMAG00	-0.96	0.86	0.00	0.05	dynlt1	skin
ENSXMAG00	0.91	4.15	0.00	0.04	mxra5a	skin
ENSXMAG00	-1.28	0.80	0.00	0.00	LAPTM4B (1	skin
ENSXMAG00	-0.71	4.65	0.00	0.02	gm2a	skin
ENSXMAG00	-0.78	6.21	0.00	0.02	kat2b	skin
ENSXMAG00	-0.77	2.49	0.00	0.04	cep57l1	skin
ENSXMAG00	1.51	6.41	0.00	0.00	calr	skin
ENSXMAG00	1.64	1.76	0.00	0.00	mmp19	skin
ENSXMAG00	0.71	4.64	0.00	0.02	pmpca	skin
ENSXMAG00	-1.04	3.18	0.00	0.00	inpp5e	skin
ENSXMAG00	1.16	4.27	0.00	0.00	cyb5b	skin
ENSXMAG00	1.63	5.68	0.00	0.00		skin
ENSXMAG00	0.82	3.93	0.00	0.01	cdk2	skin
ENSXMAG00	-1.43	2.12	0.00	0.00		skin
ENSXMAG00	-0.78	3.91	0.00	0.03	atp6v1c1b	skin
ENSXMAG00	0.66	6.38	0.00	0.02	RAB8A (1 of	skin
ENSXMAG00	0.66	6.43	0.00	0.03	plxnb3	skin
ENSXMAG00	-1.92	9.17	0.00	0.01		skin
ENSXMAG00	-1.82	9.10	0.00	0.00	lpl	skin
ENSXMAG00	-1.24	2.28	0.00	0.03		skin
ENSXMAG00	1.16	3.29	0.00	0.00	nudt2	skin
ENSXMAG00	0.72	6.67	0.00	0.02	npepl1	skin
ENSXMAG00	1.37	4.92	0.00	0.00	prep	skin
ENSXMAG00	1.11	1.82	0.00	0.01	wdcp	skin
ENSXMAG00	0.73	6.46	0.00	0.01	si:ch211-193	skin
ENSXMAG00	-1.44	1.34	0.00	0.00	map2k1	skin
ENSXMAG00	0.69	4.75	0.00	0.03	hdgfl2	skin
ENSXMAG00	1.04	7.03	0.00	0.00	si:ch1073-55	skin
ENSXMAG00	0.89	5.09	0.00	0.00	copz2	skin
ENSXMAG00	-0.84	5.36	0.00	0.00		skin
ENSXMAG00	0.78	2.27	0.00	0.04	fam57a	skin

ENSXMAG00	-1.40	5.09	0.00	0.00	trmt1l	skin
ENSXMAG00	-1.10	0.48	0.00	0.03	lgi3	skin
ENSXMAG00	0.86	2.13	0.00	0.03	fam8a1a	skin
ENSXMAG00	-1.09	4.73	0.00	0.00	map3k15	skin
ENSXMAG00	1.15	5.20	0.00	0.00	parp1	skin
ENSXMAG00	0.92	3.81	0.00	0.02		skin
ENSXMAG00	-1.07	5.58	0.00	0.00	scarb2c	skin
ENSXMAG00	1.01	4.71	0.00	0.00	mbtps2	skin
ENSXMAG00	-0.96	5.89	0.00	0.04	cxcr4b	skin
ENSXMAG00	1.05	5.23	0.00	0.00	xpnpep3	skin
ENSXMAG00	0.62	4.63	0.00	0.03	rps6kc1	skin
ENSXMAG00	0.74	5.46	0.00	0.01	rangap1a	skin
ENSXMAG00	1.21	5.37	0.00	0.00	sf3b4	skin
ENSXMAG00	1.19	4.12	0.00	0.00	ABHD1	skin
ENSXMAG00	-1.83	4.83	0.00	0.00	fdft1	skin
ENSXMAG00	1.85	5.23	0.00	0.00	noc2l	skin
ENSXMAG00	-1.65	3.82	0.00	0.00	pnpla2	skin
ENSXMAG00	-0.88	3.45	0.00	0.02	DDA1 (1 of m	skin
ENSXMAG00	0.70	5.37	0.00	0.01	tmem183a	skin
ENSXMAG00	-2.06	3.47	0.00	0.00	pgghg	skin
ENSXMAG00	-1.51	2.57	0.00	0.01		skin
ENSXMAG00	-1.49	4.34	0.00	0.00		skin
ENSXMAG00	1.37	9.49	0.00	0.00	tubb2b	skin
ENSXMAG00	-2.03	1.66	0.00	0.00	pif1	skin
ENSXMAG00	1.62	3.11	0.00	0.00	nsun4	skin
ENSXMAG00	0.79	3.70	0.00	0.01	gtf2h1	skin
ENSXMAG00	0.82	3.50	0.00	0.03	mre11a	skin
ENSXMAG00	1.18	4.72	0.00	0.00	ptcd3	skin
ENSXMAG00	0.87	4.22	0.00	0.00	slc25a32b	skin
ENSXMAG00	-3.20	7.39	0.00	0.00	fosab	skin
ENSXMAG00	1.20	4.91	0.00	0.00	gsk3ba	skin
ENSXMAG00	-0.68	3.47	0.00	0.05	ccdc18	skin
ENSXMAG00	0.79	4.36	0.00	0.01	nudt4b	skin
ENSXMAG00	-0.77	3.90	0.00	0.02	ccdc3b	skin
ENSXMAG00	1.95	0.00	0.00	0.02	adm2b	skin
ENSXMAG00	-0.65	4.94	0.00	0.05	si:dkey-266m	skin
ENSXMAG00	-1.30	7.26	0.00	0.00	prkd2	skin
ENSXMAG00	0.93	7.79	0.00	0.00	hspa9	skin
ENSXMAG00	1.00	6.40	0.00	0.00	dhx57	skin
ENSXMAG00	-0.80	2.77	0.00	0.04	mat2aa	skin
ENSXMAG00	-1.31	2.62	0.00	0.01	strip2	skin
ENSXMAG00	-0.57	5.39	0.00	0.04	man1b1b	skin
ENSXMAG00	1.13	4.14	0.00	0.00	fam136a	skin

ENSXMAG00	1.02	4.71	0.00	0.00	pcyox1	skin
ENSXMAG00	0.86	3.95	0.00	0.01	tmem209	skin
ENSXMAG00	0.76	2.84	0.00	0.04	kif3b	skin
ENSXMAG00	1.90	-0.54	0.00	0.00	fgf23	skin
ENSXMAG00	2.25	1.28	0.00	0.00	tigarb	skin
ENSXMAG00	0.97	5.96	0.00	0.00	clptm1	skin
ENSXMAG00	0.81	4.98	0.00	0.00	stk25b	skin
ENSXMAG00	0.92	7.98	0.00	0.00	kpnb3	skin
ENSXMAG00	0.84	3.88	0.00	0.01	dnajc21	skin
ENSXMAG00	-1.23	2.82	0.00	0.00	ift81	skin
ENSXMAG00	-0.84	2.49	0.00	0.04		skin
ENSXMAG00	-0.72	3.66	0.00	0.03	zgc:110269	skin
ENSXMAG00	-0.82	5.60	0.00	0.00	ppm1f	skin
ENSXMAG00	-0.65	4.59	0.00	0.04	atg13	skin
ENSXMAG00	0.84	2.68	0.00	0.02	tmed4	skin
ENSXMAG00	0.87	5.65	0.00	0.00	lman1	skin
ENSXMAG00	-0.93	5.76	0.00	0.02		skin
ENSXMAG00	-1.28	0.71	0.00	0.04	tlr7	skin
ENSXMAG00	0.72	7.87	0.00	0.01	tmed2	skin
ENSXMAG00	-1.01	5.19	0.00	0.00		skin
ENSXMAG00	1.60	5.19	0.00	0.00	bcl6ab	skin
ENSXMAG00	0.71	6.69	0.00	0.01	sec63	skin
ENSXMAG00	0.79	7.06	0.00	0.01	cmpk	skin
ENSXMAG00	1.34	4.33	0.00	0.00	dcun1d5	skin
ENSXMAG00	0.71	5.13	0.00	0.01	CUL5 (1 of m	skin
ENSXMAG00	-1.17	3.08	0.00	0.04	usp18	skin
ENSXMAG00	0.98	4.96	0.00	0.00	trap1	skin
ENSXMAG00	1.19	4.36	0.00	0.00	pak1	skin
ENSXMAG00	0.90	6.76	0.00	0.00	syncrip	skin
ENSXMAG00	0.95	3.32	0.00	0.00		skin
ENSXMAG00	0.63	7.30	0.00	0.03	rcc2	skin
ENSXMAG00	1.80	3.98	0.00	0.00	creld2	skin
ENSXMAG00	1.01	4.48	0.00	0.00	polr3f	skin
ENSXMAG00	-0.85	3.07	0.00	0.02	arsa	skin
ENSXMAG00	1.98	-1.94	0.00	0.03	vipr2	skin
ENSXMAG00	-0.86	4.50	0.00	0.00	si:ch73-21k1	skin
ENSXMAG00	0.70	6.78	0.00	0.03	pfkpa	skin
ENSXMAG00	0.60	6.00	0.00	0.04	dnaja3a	skin
ENSXMAG00	1.19	5.45	0.00	0.00	sephs1	skin
ENSXMAG00	-0.82	2.93	0.00	0.03	magi2a	skin
ENSXMAG00	0.74	5.55	0.00	0.01	hmox2b	skin
ENSXMAG00	1.69	3.50	0.00	0.00		skin
ENSXMAG00	-1.11	5.00	0.00	0.00	klf6a	skin

ENSXMAG00	1.30	6.11	0.00	0.00	zgc:66475	skin
ENSXMAG00	0.90	5.65	0.00	0.00	nuak1b	skin
ENSXMAG00	-0.85	6.04	0.00	0.01	abca3b	skin
ENSXMAG00	1.14	1.85	0.00	0.01		skin
ENSXMAG00	1.13	2.12	0.00	0.00		skin
ENSXMAG00	0.98	5.66	0.00	0.00	mtrex	skin
ENSXMAG00	0.84	5.94	0.00	0.00	rnps1	skin
ENSXMAG00	-0.71	5.07	0.00	0.02	TMC7	skin
ENSXMAG00	0.83	6.69	0.00	0.00	nomo	skin
ENSXMAG00	-0.68	4.63	0.00	0.05	naga	skin
ENSXMAG00	1.43	4.07	0.00	0.00	dhx29	skin
ENSXMAG00	0.79	7.02	0.00	0.02	fam126a	skin
ENSXMAG00	-2.16	4.47	0.00	0.00		skin
ENSXMAG00	-0.66	5.11	0.00	0.03	phip	skin
ENSXMAG00	1.03	3.72	0.00	0.00	ssb	skin
ENSXMAG00	1.41	4.70	0.00	0.00	imp4	skin
ENSXMAG00	-0.87	4.85	0.00	0.01	pnpla7b	skin
ENSXMAG00	1.08	7.17	0.00	0.00	etf1a	skin
ENSXMAG00	-3.92	-2.76	0.00	0.00	ankrd34bb	skin
ENSXMAG00	-0.91	5.42	0.00	0.01	ugt1ab (1 of	skin
ENSXMAG00	0.74	5.27	0.00	0.04	stoml2	skin
ENSXMAG00	1.17	4.52	0.00	0.00	pigo	skin
ENSXMAG00	0.98	4.89	0.00	0.00	TMEM164	skin
ENSXMAG00	0.90	5.04	0.00	0.00	ppil4	skin
ENSXMAG00	0.68	5.27	0.00	0.02	hspa14	skin
ENSXMAG00	-0.91	1.42	0.00	0.05	si:ch73-139e	skin
ENSXMAG00	0.66	5.01	0.00	0.02	rint1	skin
ENSXMAG00	-1.05	3.64	0.00	0.01	gpr155b	skin
ENSXMAG00	-1.01	4.46	0.00	0.00		skin
ENSXMAG00	-1.09	2.02	0.00	0.02	cdk19	skin
ENSXMAG00	0.73	6.07	0.00	0.01	nup155	skin
ENSXMAG00	0.99	6.19	0.00	0.00	wdr36	skin
ENSXMAG00	0.88	3.93	0.00	0.01	syt11b	skin
ENSXMAG00	0.77	4.85	0.00	0.01		skin
ENSXMAG00	0.86	4.69	0.00	0.00	dus1l	skin
ENSXMAG00	0.81	5.87	0.00	0.00	NUP62	skin
ENSXMAG00	-1.43	1.17	0.00	0.01		skin
ENSXMAG00	0.84	4.46	0.00	0.00	vps51	skin
ENSXMAG00	0.71	3.44	0.00	0.03	angel2	skin
ENSXMAG00	-0.77	3.34	0.00	0.03	stx8	skin
ENSXMAG00	-0.90	4.97	0.00	0.00	galnt14	skin
ENSXMAG00	1.01	1.02	0.00	0.04		skin
ENSXMAG00	1.18	7.50	0.00	0.00	ganab	skin

ENSXMAG00	1.04	5.09	0.00	0.00	dnajc10	skin
ENSXMAG00	1.20	6.53	0.00	0.00	stip1	skin
ENSXMAG00	0.62	7.54	0.00	0.04	copg2	skin
ENSXMAG00	0.82	5.83	0.00	0.03	dab2	skin
ENSXMAG00	-0.70	3.98	0.00	0.02		skin
ENSXMAG00	0.79	4.08	0.00	0.01	nus1	skin
ENSXMAG00	-1.13	4.64	0.00	0.00	smpdl3a	skin
ENSXMAG00	1.05	5.66	0.00	0.00	ddx54	skin
ENSXMAG00	-0.66	5.63	0.00	0.02		skin
ENSXMAG00	0.92	4.66	0.00	0.00	cad	skin
ENSXMAG00	-0.76	4.19	0.00	0.02	ankrd6b	skin
ENSXMAG00	0.79	3.92	0.00	0.02	psmd5	skin
ENSXMAG00	-0.87	2.83	0.00	0.01	ada2a	skin
ENSXMAG00	-1.48	6.65	0.00	0.00	wu:fj29h11	skin
ENSXMAG00	-0.99	5.65	0.00	0.04	ltb4r2b	skin
ENSXMAG00	-0.90	6.45	0.00	0.00	tnpo1	skin
ENSXMAG00	1.01	1.87	0.00	0.01	dnajc5ga	skin
ENSXMAG00	0.87	5.26	0.00	0.00	rtca	skin
ENSXMAG00	0.74	4.75	0.00	0.02	zmynd19	skin
ENSXMAG00	-1.44	-0.20	0.00	0.01	ttll2	skin
ENSXMAG00	0.92	5.82	0.00	0.00	si:ch211-217	skin
ENSXMAG00	1.06	2.33	0.00	0.04	xkr8.3	skin
ENSXMAG00	0.86	6.32	0.00	0.01	ints8	skin
ENSXMAG00	-0.98	1.83	0.00	0.03	wdpcp	skin
ENSXMAG00	-0.68	5.25	0.00	0.03	tesk2	skin
ENSXMAG00	-2.66	-1.64	0.00	0.01		skin
ENSXMAG00	0.80	7.11	0.00	0.01	psmd1	skin
ENSXMAG00	0.68	5.87	0.00	0.01	nup188	skin
ENSXMAG00	-1.30	0.23	0.00	0.01	SRRM3	skin
ENSXMAG00	1.14	6.47	0.00	0.00	c1qbp	skin
ENSXMAG00	0.78	4.80	0.00	0.01	ENTPD7	skin
ENSXMAG00	1.08	6.73	0.00	0.00		skin
ENSXMAG00	-0.88	5.36	0.00	0.01	atg9b	skin
ENSXMAG00	0.63	5.36	0.00	0.04	hells	skin
ENSXMAG00	-0.98	4.94	0.00	0.00		skin
ENSXMAG00	0.76	6.47	0.00	0.01	sympk	skin
ENSXMAG00	0.66	5.53	0.00	0.04	abcc4	skin
ENSXMAG00	0.63	7.59	0.00	0.04	rab1ab	skin
ENSXMAG00	0.81	4.94	0.00	0.00	asdurf	skin
ENSXMAG00	-1.07	4.65	0.00	0.00	snap47	skin
ENSXMAG00	1.11	5.42	0.00	0.00	wdr75	skin
ENSXMAG00	1.14	5.30	0.00	0.01	HIGD1A	skin
ENSXMAG00	-0.80	4.46	0.00	0.01	acer2	skin

ENSXMAG00	-1.64	3.98	0.00	0.05		skin
ENSXMAG00	1.32	0.82	0.00	0.00	surf1	skin
ENSXMAG00	0.69	6.22	0.00	0.02	zzef1	skin
ENSXMAG00	-2.37	-0.88	0.00	0.00	smu1b	skin
ENSXMAG00	0.69	6.64	0.00	0.02		skin
ENSXMAG00	0.55	5.37	0.00	0.05	ankfy1	skin
ENSXMAG00	-1.42	6.55	0.00	0.00	znfx1	skin
ENSXMAG00	1.23	3.35	0.00	0.00	polr2h	skin
ENSXMAG00	1.38	5.48	0.00	0.00	hspe1	skin
ENSXMAG00	1.30	7.56	0.00	0.00	hspd1	skin
ENSXMAG00	0.98	4.82	0.00	0.00	bco2b	skin
ENSXMAG00	1.87	1.47	0.00	0.00	tgm2l	skin
ENSXMAG00	0.64	9.03	0.00	0.04	ncl	skin
ENSXMAG00	1.12	5.22	0.00	0.00	ruvbl2	skin
ENSXMAG00	-1.06	2.03	0.00	0.02	pitx3	skin
ENSXMAG00	-0.96	1.63	0.00	0.03	hspa12a	skin
ENSXMAG00	0.68	4.51	0.00	0.02	naa25	skin
ENSXMAG00	-0.89	2.78	0.00	0.01	st7l	skin
ENSXMAG00	1.03	3.77	0.00	0.00	si:dkey-162b	skin
ENSXMAG00	1.06	4.90	0.00	0.00	dpagt1	skin
ENSXMAG00	1.54	-0.34	0.00	0.00	cisd2	skin
ENSXMAG00	1.34	2.96	0.00	0.00	wnt2ba	skin
ENSXMAG00	0.62	4.61	0.00	0.03		skin
ENSXMAG00	0.73	5.12	0.00	0.01	cept1b	skin
ENSXMAG00	1.09	5.42	0.00	0.00	txndc5	skin
ENSXMAG00	0.79	6.06	0.00	0.01	ipo9	skin
ENSXMAG00	-1.05	2.56	0.00	0.02	kif14	skin
ENSXMAG00	1.45	4.28	0.00	0.00	ppp5c	skin
ENSXMAG00	1.60	5.42	0.00	0.00	ipo4	skin
ENSXMAG00	1.05	6.79	0.00	0.00	KIF5B (1 of n	skin
ENSXMAG00	-0.94	3.39	0.00	0.04	aurka	skin
ENSXMAG00	-0.92	4.75	0.00	0.00		skin
ENSXMAG00	0.80	5.07	0.00	0.03	hmgcs1	skin
ENSXMAG00	0.63	6.79	0.00	0.03	srp68	skin
ENSXMAG00	-2.33	-2.12	0.00	0.03	si:dkey-13p1	skin
ENSXMAG00	-0.94	4.49	0.00	0.00		skin
ENSXMAG00	0.59	7.94	0.00	0.04	DDX5	skin
ENSXMAG00	0.93	7.19	0.00	0.00	slc38a4	skin
ENSXMAG00	1.39	5.39	0.00	0.00	rrbp1a	skin
ENSXMAG00	0.65	6.17	0.00	0.03	snx5	skin
ENSXMAG00	-0.75	6.34	0.00	0.04	dcaf11	skin
ENSXMAG00	0.82	7.14	0.00	0.00	pabpn1	skin
ENSXMAG00	0.79	5.62	0.00	0.01	si:ch211-214	skin

ENSXMAG00	-0.67	4.33	0.00	0.02	nav3	skin
ENSXMAG00	-1.68	2.40	0.00	0.05	irf4b	skin
ENSXMAG00	0.80	3.44	0.00	0.03	nup43	skin
ENSXMAG00	1.09	4.04	0.00	0.00	exosc6	skin
ENSXMAG00	-1.49	0.96	0.00	0.00	phactr1	skin
ENSXMAG00	0.80	5.15	0.00	0.01	magoh	skin
ENSXMAG00	1.01	1.39	0.00	0.02	stard5	skin
ENSXMAG00	-2.83	-1.78	0.00	0.01	uck2a	skin
ENSXMAG00	-1.62	-0.88	0.00	0.02		skin
ENSXMAG00	0.99	4.38	0.00	0.00	setd7 (1 of m	skin
ENSXMAG00	1.14	6.46	0.00	0.00	smarca4a	skin
ENSXMAG00	-0.67	4.66	0.00	0.03	pard3ab	skin
ENSXMAG00	0.76	7.22	0.00	0.01	sf3b3	skin
ENSXMAG00	0.98	3.00	0.00	0.02	mfsd2aa	skin
ENSXMAG00	-0.80	5.50	0.00	0.01	lonp2	skin
ENSXMAG00	-1.38	-0.37	0.00	0.02		skin
ENSXMAG00	1.20	6.54	0.00	0.00	rbbp4	skin
ENSXMAG00	-1.08	2.25	0.00	0.01	srd5a2b	skin
ENSXMAG00	0.74	5.02	0.00	0.03	pitrm1	skin
ENSXMAG00	0.69	4.97	0.00	0.02	dnajc2	skin
ENSXMAG00	0.83	4.40	0.00	0.00	slc25a38b	skin
ENSXMAG00	0.59	7.79	0.00	0.05	khdrbs1a	skin
ENSXMAG00	-1.09	2.55	0.00	0.01	zgc:92873	skin
ENSXMAG00	-1.15	5.81	0.00	0.00	chm	skin
ENSXMAG00	1.13	5.68	0.00	0.00	mthfd1b	skin
ENSXMAG00	0.92	2.79	0.00	0.01	prmt6	skin
ENSXMAG00	0.84	5.81	0.00	0.00	utp20	skin
ENSXMAG00	-2.21	-2.36	0.00	0.04	tppp2	skin
ENSXMAG00	0.84	2.86	0.00	0.04	bcs1l	skin
ENSXMAG00	-0.97	0.39	0.00	0.05	CYP27A1	skin
ENSXMAG00	1.33	2.62	0.00	0.00	b3galnt2	skin
ENSXMAG00	-1.88	0.94	0.00	0.00	apba2a	skin
ENSXMAG00	0.83	2.90	0.00	0.02	fan1	skin
ENSXMAG00	-1.18	2.00	0.00	0.01	trhde.2	skin
ENSXMAG00	-1.75	5.65	0.00	0.00	PIK3R6	skin
ENSXMAG00	-1.07	3.54	0.00	0.00	CPNE8	skin
ENSXMAG00	0.88	6.66	0.00	0.03	nansa	skin
ENSXMAG00	0.65	4.81	0.00	0.02	snupn	skin
ENSXMAG00	1.03	5.65	0.00	0.00	tomm40	skin
ENSXMAG00	0.75	5.02	0.00	0.01	nol11	skin
ENSXMAG00	-0.98	3.23	0.00	0.00	bcl3	skin
ENSXMAG00	-1.42	1.92	0.00	0.00		skin
ENSXMAG00	0.71	5.69	0.00	0.01	ddx31	skin

ENSXMAG00	-1.25	5.71	0.00	0.00	crot	skin
ENSXMAG00	0.74	5.02	0.00	0.01		skin
ENSXMAG00	-1.75	-0.89	0.00	0.02		skin
ENSXMAG00	-1.72	-0.03	0.00	0.00		skin
ENSXMAG00	0.82	3.48	0.00	0.02	mrpl17	skin
ENSXMAG00	0.76	9.35	0.00	0.01		skin
ENSXMAG00	-0.71	4.04	0.00	0.02	psmd10	skin
ENSXMAG00	0.68	5.79	0.00	0.05		skin
ENSXMAG00	0.70	4.50	0.00	0.05		skin
ENSXMAG00	0.64	6.29	0.00	0.02	ncbp3	skin
ENSXMAG00	0.74	6.17	0.00	0.01	sel1l	skin
ENSXMAG00	-0.99	6.05	0.00	0.00	pdcd4b	skin
ENSXMAG00	-1.16	4.14	0.00	0.00	CSDC2 (1 of r	skin
ENSXMAG00	-1.27	4.00	0.00	0.00	PMM1	skin
ENSXMAG00	-0.66	6.44	0.00	0.05	rab18b	skin
ENSXMAG00	-0.62	4.55	0.00	0.05	nbn	skin
ENSXMAG00	-0.69	5.27	0.00	0.03	stard13b	skin
ENSXMAG00	0.85	7.10	0.00	0.00	copb1	skin
ENSXMAG00	1.00	4.20	0.00	0.00		skin
ENSXMAG00	0.87	6.00	0.00	0.00	nhp2	skin
ENSXMAG00	-0.76	4.02	0.00	0.02	si:ch211-79e	skin
ENSXMAG00	-0.74	6.99	0.00	0.03	coq8aa	skin
ENSXMAG00	0.76	6.57	0.00	0.01	slc35a2	skin
ENSXMAG00	-1.22	3.30	0.00	0.04	TRPC4 (1 of r	skin
ENSXMAG00	1.24	5.14	0.00	0.00	WDR77	skin
ENSXMAG00	0.68	7.84	0.00	0.02	fubp1	skin
ENSXMAG00	-1.15	3.43	0.00	0.00	VRK2	skin
ENSXMAG00	0.82	4.44	0.00	0.01	pgm3	skin
ENSXMAG00	0.62	5.04	0.00	0.04	dagla	skin
ENSXMAG00	-0.65	5.60	0.00	0.04	dchs1a	skin
ENSXMAG00	0.80	6.73	0.00	0.01	gspt1l	skin
ENSXMAG00	-0.85	4.81	0.00	0.01	neurl1ab	skin
ENSXMAG00	0.69	4.24	0.00	0.03	usp16	skin
ENSXMAG00	1.28	2.17	0.00	0.00	rwdd2b	skin
ENSXMAG00	0.90	5.69	0.00	0.00	nat10	skin
ENSXMAG00	-1.43	2.47	0.00	0.00	cadm2b	skin
ENSXMAG00	1.48	4.99	0.00	0.00	calub	skin
ENSXMAG00	-0.77	5.33	0.00	0.01	prodhb	skin
ENSXMAG00	0.83	3.94	0.00	0.03	mrps17	skin
ENSXMAG00	-2.19	3.47	0.00	0.00		skin
ENSXMAG00	-1.04	2.93	0.00	0.00		skin
ENSXMAG00	-1.39	-0.17	0.00	0.04	guca1c	skin
ENSXMAG00	-1.22	3.09	0.00	0.01		skin

ENSXMAG00	1.73	2.49	0.00	0.00	DNAJB5 (1 o	skin
ENSXMAG00	-1.60	-0.16	0.00	0.01		skin
ENSXMAG00	0.89	8.16	0.00	0.00	vcp	skin
ENSXMAG00	-0.89	4.49	0.00	0.01	hdac11	skin
ENSXMAG00	0.66	6.83	0.00	0.02	cebpz	skin
ENSXMAG00	-3.92	-2.76	0.00	0.00	STK32C	skin
ENSXMAG00	0.79	3.39	0.00	0.02	xrcc3	skin
ENSXMAG00	-0.72	3.42	0.00	0.04	zgc:158254	skin
ENSXMAG00	-0.85	5.43	0.00	0.01		skin
ENSXMAG00	-1.69	0.25	0.00	0.00	slc22a16	skin
ENSXMAG00	0.96	6.23	0.00	0.04	nt5c2l1	skin
ENSXMAG00	-0.69	5.46	0.00	0.02	SLC8B1	skin
ENSXMAG00	0.83	6.13	0.00	0.01	myo6a	skin
ENSXMAG00	0.77	5.42	0.00	0.01	galk1	skin
ENSXMAG00	0.74	6.88	0.00	0.01	srrt	skin
ENSXMAG00	-0.83	4.93	0.00	0.00	zgc:158619	skin
ENSXMAG00	0.69	3.94	0.00	0.03	cnih1	skin
ENSXMAG00	-1.23	1.78	0.00	0.04	cdkn3	skin
ENSXMAG00	0.93	5.83	0.00	0.00	psmd8	skin
ENSXMAG00	2.06	3.70	0.00	0.00	rwdd	skin
ENSXMAG00	0.86	6.06	0.00	0.00	pes	skin
ENSXMAG00	-0.86	1.76	0.00	0.03	WDR25	skin
ENSXMAG00	0.69	4.93	0.00	0.05		skin
ENSXMAG00	0.66	5.11	0.00	0.03	sirt1	skin
ENSXMAG00	0.69	4.37	0.00	0.01	cacul1	skin
ENSXMAG00	-1.33	1.49	0.00	0.03		skin
ENSXMAG00	0.97	5.44	0.00	0.00	zmpste24	skin
ENSXMAG00	-0.94	7.47	0.00	0.00	susd6	skin
ENSXMAG00	-1.68	5.54	0.00	0.00	znf395b	skin
ENSXMAG00	-0.72	4.96	0.00	0.01	si:ch211-15d	skin
ENSXMAG00	0.99	3.58	0.00	0.01	ccdc43	skin
ENSXMAG00	-0.94	5.66	0.00	0.02		skin
ENSXMAG00	-0.98	6.40	0.00	0.00	acacb	skin
ENSXMAG00	0.90	6.35	0.00	0.01	syncripl	skin
ENSXMAG00	-0.63	6.84	0.00	0.04	eml3	skin
ENSXMAG00	-0.86	5.27	0.00	0.02	SNX14	skin
ENSXMAG00	-2.36	3.97	0.00	0.00	KLF15	skin
ENSXMAG00	0.90	7.45	0.00	0.00	rpn2	skin
ENSXMAG00	0.76	3.07	0.00	0.05	pspc1	skin
ENSXMAG00	0.98	4.49	0.00	0.00	ecsit	skin
ENSXMAG00	1.11	4.29	0.00	0.00		skin
ENSXMAG00	0.92	7.54	0.00	0.00	lmnb2	skin
ENSXMAG00	0.85	6.38	0.00	0.01		skin

ENSXMAG00	0.74	7.15	0.00	0.01	lonp1	skin
ENSXMAG00	-0.90	5.50	0.00	0.03	hsd3b7	skin
ENSXMAG00	-1.07	0.99	0.00	0.04	nrg3b	skin
ENSXMAG00	-1.33	4.67	0.00	0.00	cmpk2	skin
ENSXMAG00	-1.10	4.36	0.00	0.00	rsad2	skin
ENSXMAG00	-2.45	5.50	0.00	0.04		skin
ENSXMAG00	-2.77	5.81	0.00	0.02		skin
ENSXMAG00	0.94	4.86	0.00	0.00	rbm4.2	skin
ENSXMAG00	-0.96	4.96	0.00	0.00	afap1l2	skin
ENSXMAG00	0.80	5.57	0.00	0.01	copz1	skin
ENSXMAG00	0.73	5.36	0.00	0.00	arl8bb	skin
ENSXMAG00	-0.71	4.91	0.00	0.04		skin
ENSXMAG00	-0.63	4.37	0.00	0.04	map3k3	skin
ENSXMAG00	1.17	3.81	0.00	0.05	wnt2bb	skin
ENSXMAG00	0.85	3.18	0.00	0.02	nsd2	skin
ENSXMAG00	1.08	2.22	0.00	0.05	ebp	skin
ENSXMAG00	0.67	7.57	0.00	0.02	EIF4G1	skin
ENSXMAG00	0.94	4.98	0.00	0.00	eed	skin
ENSXMAG00	-1.15	3.46	0.00	0.00	mlh1	skin
ENSXMAG00	1.00	5.58	0.00	0.00	pelp1	skin
ENSXMAG00	0.81	7.30	0.00	0.01	psmd2	skin
ENSXMAG00	-0.88	4.52	0.00	0.02	dact2	skin
ENSXMAG00	-1.00	3.67	0.00	0.00	prss16	skin
ENSXMAG00	-1.22	3.69	0.00	0.00		skin
ENSXMAG00	0.74	5.04	0.00	0.01	senp3b	skin
ENSXMAG00	0.72	5.03	0.00	0.04		skin
ENSXMAG00	-0.69	4.89	0.00	0.02	si:ch73-132f(	skin
ENSXMAG00	1.15	5.35	0.00	0.00	ddx47	skin
ENSXMAG00	-0.78	5.58	0.00	0.00	inpp4ab	skin
ENSXMAG00	-0.75	5.46	0.00	0.00	als2b	skin
ENSXMAG00	0.81	7.87	0.00	0.00	uba1	skin
ENSXMAG00	1.02	7.56	0.00	0.00	sar1b	skin
ENSXMAG00	0.64	6.71	0.00	0.03	sec24a	skin
ENSXMAG00	0.90	5.94	0.00	0.05	hsp90aa1.2	skin
ENSXMAG00	0.86	4.02	0.00	0.04	pdss1	skin
ENSXMAG00	-1.00	5.82	0.00	0.03	kpna2	skin
ENSXMAG00	-0.80	6.43	0.00	0.02	SLC25A36	skin
ENSXMAG00	0.73	6.07	0.00	0.02	yme1l1a	skin
ENSXMAG00	0.70	7.89	0.00	0.01	prpf39	skin
ENSXMAG00	0.65	6.78	0.00	0.03	uspl1	skin
ENSXMAG00	0.96	1.22	0.00	0.04	b3glctb	skin
ENSXMAG00	-1.33	4.40	0.00	0.00	arl5c	skin
ENSXMAG00	-0.67	4.04	0.00	0.04	PACS1	skin

ENSXMAG00	0.96	6.84	0.00	0.00	prpf19	skin
ENSXMAG00	1.14	6.31	0.00	0.00	eftud2	skin
ENSXMAG00	0.74	5.30	0.00	0.01	plaa	skin
ENSXMAG00	0.85	7.07	0.00	0.01	fbl	skin
ENSXMAG00	-0.77	4.50	0.00	0.01	sema4ab	skin
ENSXMAG00	0.91	6.46	0.00	0.00	phb	skin
ENSXMAG00	0.61	4.87	0.00	0.03		skin
ENSXMAG00	-0.76	4.63	0.00	0.02	klhl22	skin
ENSXMAG00	-1.55	0.17	0.00	0.00	chrng	skin
ENSXMAG00	-1.18	4.71	0.00	0.01	ccnb1	skin
ENSXMAG00	1.55	7.36	0.00	0.00	cluha	skin
ENSXMAG00	0.88	4.32	0.00	0.01	capn10	skin
ENSXMAG00	1.26	3.96	0.00	0.00	aldh16a1	skin
ENSXMAG00	0.75	5.32	0.00	0.01	alg5	skin
ENSXMAG00	0.75	2.93	0.00	0.04	lmln	skin
ENSXMAG00	-1.10	4.76	0.00	0.00		skin
ENSXMAG00	-0.77	4.52	0.00	0.01	washc5	skin
ENSXMAG00	-1.97	4.24	0.00	0.00	fbxo32	skin
ENSXMAG00	-0.75	3.40	0.00	0.03	RNF146	skin
ENSXMAG00	1.48	2.30	0.00	0.00	psph	skin
ENSXMAG00	-0.84	4.44	0.00	0.01	c1qtnf5	skin
ENSXMAG00	-1.00	4.53	0.00	0.01	usp28	skin
ENSXMAG00	0.60	4.95	0.00	0.04	slc5a5	skin
ENSXMAG00	0.70	6.93	0.00	0.02	ssr1	skin
ENSXMAG00	0.80	4.36	0.00	0.01	tmem147	skin
ENSXMAG00	2.14	4.37	0.00	0.00	zbtb16a	skin
ENSXMAG00	2.41	1.73	0.00	0.00	slc5a6a	skin
ENSXMAG00	-1.75	0.96	0.00	0.01		skin
ENSXMAG00	-0.94	1.46	0.00	0.05		skin
ENSXMAG00	-1.20	5.24	0.00	0.02	dhx58	skin
ENSXMAG00	0.77	7.33	0.00	0.01	sdha	skin
ENSXMAG00	0.78	6.00	0.00	0.01	rpl7l1	skin
ENSXMAG00	0.76	4.03	0.00	0.01	vezt	skin
ENSXMAG00	1.05	4.73	0.00	0.00	kat2a	skin
ENSXMAG00	0.86	8.19	0.00	0.01	DDX39A	skin
ENSXMAG00	-2.56	4.74	0.00	0.00		skin
ENSXMAG00	0.74	7.37	0.00	0.02	hnrnpaba	skin
ENSXMAG00	-2.14	-1.42	0.00	0.04		skin
ENSXMAG00	0.67	5.25	0.00	0.02	derl1	skin
ENSXMAG00	-1.64	3.56	0.00	0.00	has2	skin
ENSXMAG00	0.64	5.87	0.00	0.02	HOOK3	skin
ENSXMAG00	-1.39	5.04	0.00	0.00	sinhcaf	skin
ENSXMAG00	1.23	5.11	0.00	0.00	abcb7	skin

ENSXMAG00	-0.64	6.23	0.00	0.04	LRRC17 (1 of	skin
ENSXMAG00	-0.93	3.17	0.00	0.01	fbxl13	skin
ENSXMAG00	0.92	5.36	0.00	0.00	atrx	skin
ENSXMAG00	-0.90	4.73	0.00	0.00	pld1a	skin
ENSXMAG00	0.91	4.25	0.00	0.00	erc1a	skin
ENSXMAG00	-3.21	-3.13	0.00	0.05	kctd16a	skin
ENSXMAG00	1.31	7.49	0.00	0.00	hspa4b	skin
ENSXMAG00	-0.92	5.01	0.00	0.01	csrnp1b	skin
ENSXMAG00	-0.71	5.39	0.00	0.01	stag1b	skin
ENSXMAG00	0.88	3.97	0.00	0.01	utp25	skin
ENSXMAG00	2.51	5.53	0.00	0.00	cry3a	skin
ENSXMAG00	0.83	3.95	0.00	0.02	cry-dash	skin
ENSXMAG00	-1.06	1.59	0.00	0.03	tcea2	skin
ENSXMAG00	1.09	3.39	0.00	0.00	cradd	skin
ENSXMAG00	-1.40	2.48	0.00	0.03	mafk	skin
ENSXMAG00	1.13	5.81	0.00	0.00	sarnp	skin
ENSXMAG00	-0.66	5.48	0.00	0.05	yaf2	skin
ENSXMAG00	0.93	2.40	0.00	0.03	rab23	skin
ENSXMAG00	1.38	7.19	0.00	0.00	gars	skin
ENSXMAG00	-0.73	4.70	0.00	0.01	gsap	skin
ENSXMAG00	-2.67	0.59	0.00	0.00	zgc:174917	skin
ENSXMAG00	-0.71	5.67	0.00	0.04	parp12b	skin
ENSXMAG00	0.65	4.50	0.00	0.03	zgc:66447	skin
ENSXMAG00	0.89	4.73	0.00	0.00	tmem41b	skin
ENSXMAG00	0.61	4.60	0.00	0.05	abcc10	skin
ENSXMAG00	0.64	4.94	0.00	0.04	wdr4	skin
ENSXMAG00	-1.92	-0.02	0.00	0.00	zbtb47b	skin
ENSXMAG00	-1.43	1.04	0.00	0.02		skin
ENSXMAG00	0.77	4.10	0.00	0.02	grm8a	skin
ENSXMAG00	-1.01	4.11	0.00	0.00		skin
ENSXMAG00	0.71	4.33	0.00	0.01		skin
ENSXMAG00	-0.71	8.51	0.00	0.02	fgfr2	skin
ENSXMAG00	-0.93	4.62	0.00	0.01	unkl	skin
ENSXMAG00	-1.39	5.49	0.00	0.00	igfbp6b	skin
ENSXMAG00	-0.70	3.31	0.00	0.04	spryd3	skin
ENSXMAG00	-0.64	6.41	0.00	0.05	ing3	skin
ENSXMAG00	0.88	7.60	0.00	0.00		skin
ENSXMAG00	0.81	6.73	0.00	0.00	txnrd3	skin
ENSXMAG00	-0.81	3.83	0.00	0.01	wdr19	skin
ENSXMAG00	-1.00	1.33	0.00	0.04	fut11	skin
ENSXMAG00	-0.84	4.17	0.00	0.01		skin
ENSXMAG00	-0.61	5.18	0.00	0.04	ankrd27	skin
ENSXMAG00	-0.90	4.22	0.00	0.00		skin

ENSXMAG00	0.68	5.90	0.00	0.03	aqr	skin
ENSXMAG00	0.92	5.73	0.00	0.00	top1l	skin
ENSXMAG00	1.13	5.33	0.00	0.00	ugdh	skin
ENSXMAG00	-0.73	4.30	0.00	0.04	znf827	skin
ENSXMAG00	-0.88	2.90	0.00	0.01	map9	skin
ENSXMAG00	0.87	2.94	0.00	0.02	mmaa	skin
ENSXMAG00	-0.94	5.97	0.00	0.05	eda	skin
ENSXMAG00	-0.89	2.70	0.00	0.03		skin
ENSXMAG00	-0.72	4.80	0.00	0.03	mlycd	skin
ENSXMAG00	-0.64	4.58	0.00	0.04	cdkn1bb	skin
ENSXMAG00	0.65	3.81	0.00	0.04	yars2	skin
ENSXMAG00	-0.97	6.54	0.00	0.05	dusp1	skin
ENSXMAG00	1.33	3.26	0.00	0.00	ergic1	skin
ENSXMAG00	1.43	6.50	0.00	0.00	smarca5	skin
ENSXMAG00	-1.48	5.57	0.00	0.00	ypel5	skin
ENSXMAG00	0.74	4.98	0.00	0.02	prelid1a	skin
ENSXMAG00	-0.93	1.56	0.00	0.05		skin
ENSXMAG00	0.83	4.08	0.00	0.01	abcg2a	skin
ENSXMAG00	0.73	6.92	0.00	0.02	phf20a	skin
ENSXMAG00	-1.42	5.55	0.00	0.00	si:ch211-149	skin
ENSXMAG00	0.85	6.13	0.00	0.00	wdr3	skin
ENSXMAG00	0.89	6.35	0.00	0.00	ssrp1a	skin
ENSXMAG00	-0.85	5.81	0.00	0.01	fam13a	skin
ENSXMAG00	0.73	6.62	0.00	0.01	zgc:172295	skin
ENSXMAG00	0.89	6.04	0.00	0.00	uggt1	skin
ENSXMAG00	-0.77	2.93	0.00	0.04	klf11a	skin
ENSXMAG00	-1.83	2.49	0.00	0.00	spsb3b	skin
ENSXMAG00	-0.79	4.26	0.00	0.01		skin
ENSXMAG00	1.26	4.76	0.00	0.00	cdk8	skin
ENSXMAG00	-1.00	0.93	0.00	0.05	doc2d	skin
ENSXMAG00	0.63	4.59	0.00	0.05	ddx51	skin
ENSXMAG00	0.76	7.80	0.00	0.01	g3bp2	skin
ENSXMAG00	-0.90	2.39	0.00	0.03	n4bp3	skin
ENSXMAG00	0.71	5.80	0.00	0.01	uso1	skin
ENSXMAG00	0.71	6.34	0.00	0.02	naa50	skin
ENSXMAG00	-0.76	3.32	0.00	0.04	si:ch211-175	skin
ENSXMAG00	0.88	3.16	0.00	0.01	polr2gl	skin
ENSXMAG00	-0.80	4.34	0.00	0.05	si:dkey-25o1	skin
ENSXMAG00	7.65	1.37	0.00	0.00	gck	skin
ENSXMAG00	-1.89	2.02	0.00	0.02		skin
ENSXMAG00	1.58	5.39	0.00	0.00	adamts12	skin
ENSXMAG00	1.06	3.46	0.00	0.00	snapc3	skin
ENSXMAG00	-0.76	4.30	0.00	0.02	gdpd5b	skin

ENSXMAG00	0.82	6.50	0.00	0.00		skin
ENSXMAG00	1.54	5.90	0.00	0.00	TTC39B	skin
ENSXMAG00	-1.38	7.85	0.00	0.02	mmp9	skin
ENSXMAG00	-1.07	5.70	0.00	0.00	casp6	skin
ENSXMAG00	1.88	-1.45	0.00	0.01		skin
ENSXMAG00	0.71	4.62	0.00	0.02	atp23	skin
ENSXMAG00	-0.80	4.58	0.00	0.02	lef1	skin
ENSXMAG00	-1.47	5.97	0.00	0.00	si:dkey-193c1	skin
ENSXMAG00	1.05	4.68	0.00	0.00		skin
ENSXMAG00	0.80	5.39	0.00	0.00	cpeb4a	skin
ENSXMAG00	1.44	5.42	0.00	0.00	elovl5	skin
ENSXMAG00	-1.12	5.74	0.00	0.00	epgn	skin
ENSXMAG00	1.10	2.44	0.00	0.00	abcb9	skin
ENSXMAG00	-0.64	7.14	0.00	0.05	fbln1	skin
ENSXMAG00	0.65	5.37	0.00	0.02	nup50	skin
ENSXMAG00	0.68	4.98	0.00	0.03	agpat5	skin
ENSXMAG00	1.32	6.34	0.00	0.00	dnajb11	skin
ENSXMAG00	0.93	4.07	0.00	0.01	fam98b	skin
ENSXMAG00	0.68	8.06	0.00	0.04		skin
ENSXMAG00	-0.60	5.46	0.00	0.04		skin
ENSXMAG00	-1.13	7.48	0.00	0.00	adam28	skin
ENSXMAG00	0.88	5.63	0.00	0.00	smarcd1	skin
ENSXMAG00	0.85	7.25	0.00	0.00	csnk2a1	skin
ENSXMAG00	-0.92	3.51	0.00	0.01	ablim3	skin
ENSXMAG00	1.41	6.17	0.00	0.00	adka	skin
ENSXMAG00	-1.72	-1.48	0.00	0.04		skin
ENSXMAG00	2.79	-0.72	0.00	0.00	crispld1a	skin
ENSXMAG00	-0.70	4.42	0.00	0.03	ccndbp1	skin
ENSXMAG00	-0.69	5.06	0.00	0.01		skin
ENSXMAG00	-0.74	4.23	0.00	0.02	rttn	skin
ENSXMAG00	1.34	6.98	0.00	0.00		skin
ENSXMAG00	0.88	5.58	0.00	0.04	lonrf1	skin
ENSXMAG00	1.03	6.42	0.00	0.00	larp4aa	skin
ENSXMAG00	-1.03	1.68	0.00	0.01	lrmda	skin
ENSXMAG00	-1.06	1.44	0.00	0.03	cadm2a	skin
ENSXMAG00	-1.30	0.04	0.00	0.02		skin
ENSXMAG00	0.65	6.32	0.00	0.04	ptges3a	skin
ENSXMAG00	-1.21	0.11	0.00	0.03		skin
ENSXMAG00	-1.22	1.58	0.00	0.00	cacnb3b	skin
ENSXMAG00	1.37	5.48	0.00	0.00	rcc1	skin
ENSXMAG00	0.69	5.19	0.00	0.02	dnajc3b	skin
ENSXMAG00	-0.84	5.13	0.00	0.00	tcea3	skin
ENSXMAG00	0.78	5.82	0.00	0.01	PSMD11	skin

ENSXMAG00	1.00	3.43	0.00	0.00	alg9	skin
ENSXMAG00	0.78	5.03	0.00	0.01	spra	skin
ENSXMAG00	0.71	8.57	0.00	0.02	aldocb	skin
ENSXMAG00	0.59	6.65	0.00	0.05	map1sa	skin
ENSXMAG00	1.02	6.49	0.00	0.00	ssr4	skin
ENSXMAG00	0.73	5.63	0.00	0.01	psme4a	skin
ENSXMAG00	1.13	3.38	0.00	0.02	DYNLL2 (1 of	skin
ENSXMAG00	1.63	4.38	0.00	0.00	heatr6	skin
ENSXMAG00	-1.23	1.89	0.00	0.00	si:dkey-288i2	skin
ENSXMAG00	0.95	7.20	0.00	0.00		skin
ENSXMAG00	1.32	7.70	0.00	0.00	prmt1	skin
ENSXMAG00	0.76	3.45	0.00	0.03	slc35d1a	skin
ENSXMAG00	0.70	7.02	0.00	0.01	baz1b	skin
ENSXMAG00	-1.26	3.69	0.00	0.00	plxna4	skin
ENSXMAG00	0.67	4.29	0.00	0.02	nrf1	skin
ENSXMAG00	-0.98	4.30	0.00	0.00	ypel2a	skin
ENSXMAG00	0.88	2.66	0.00	0.01	gdpd1	skin
ENSXMAG00	-0.91	3.82	0.00	0.00	ino80e	skin
ENSXMAG00	-1.35	5.82	0.00	0.00		skin
ENSXMAG00	0.65	5.06	0.00	0.03	zc3hc1	skin
ENSXMAG00	-0.69	5.10	0.00	0.03	osbpl1a	skin
ENSXMAG00	-0.61	5.17	0.00	0.05	tbc1d4	skin
ENSXMAG00	1.12	7.28	0.00	0.00	tfr1a	skin
ENSXMAG00	0.78	6.98	0.00	0.01	usp5	skin
ENSXMAG00	-0.99	5.71	0.00	0.00	slc31a2	skin
ENSXMAG00	0.88	5.95	0.00	0.01	crym	skin
ENSXMAG00	-1.02	3.98	0.00	0.03	si:ch211-69g	skin
ENSXMAG00	-0.63	5.25	0.00	0.04		skin
ENSXMAG00	0.92	5.56	0.00	0.00	lpcat3	skin
ENSXMAG00	-0.74	4.21	0.00	0.02	mysm1	skin
ENSXMAG00	0.96	6.86	0.00	0.00	pa2g4b	skin
ENSXMAG00	1.28	5.22	0.00	0.00	srm	skin
ENSXMAG00	1.09	5.61	0.00	0.00	kpna3	skin
ENSXMAG00	0.92	4.77	0.00	0.00	itpa	skin
ENSXMAG00	-1.54	4.00	0.00	0.00	RCBTB1	skin
ENSXMAG00	-3.83	2.60	0.00	0.00		skin
ENSXMAG00	-1.36	-0.80	0.00	0.02	uckl1a	skin
ENSXMAG00	0.91	7.77	0.00	0.00	prrc2c	skin
ENSXMAG00	-1.05	1.98	0.00	0.01	lrp8	skin
ENSXMAG00	-1.35	3.25	0.00	0.00	si:dkey-239i2	skin
ENSXMAG00	0.99	4.14	0.00	0.00	plpp6	skin
ENSXMAG00	-0.64	7.03	0.00	0.03	cd302	skin
ENSXMAG00	0.61	5.17	0.00	0.03	fam20b	skin

ENSXMAG00	-1.78	3.03	0.00	0.04		skin
ENSXMAG00	-1.10	0.84	0.00	0.01		skin
ENSXMAG00	-1.15	3.54	0.00	0.00		skin
ENSXMAG00	1.44	3.76	0.00	0.00	coa7	skin
ENSXMAG00	-1.60	4.58	0.00	0.04	nr1d4a	skin
ENSXMAG00	1.10	5.94	0.00	0.00	nop2	skin
ENSXMAG00	-0.90	4.33	0.00	0.01	KLHL12	skin
ENSXMAG00	0.68	4.24	0.00	0.05		skin
ENSXMAG00	0.61	4.51	0.00	0.04		skin
ENSXMAG00	-0.98	3.14	0.00	0.02		skin
ENSXMAG00	-0.81	6.04	0.00	0.01	si:ch73-12o2	skin
ENSXMAG00	-0.67	6.29	0.00	0.02	ifih1	skin
ENSXMAG00	0.72	5.62	0.00	0.02	txndc11	skin
ENSXMAG00	-0.79	6.76	0.00	0.01		skin
ENSXMAG00	-1.20	0.22	0.00	0.03	scn12aa	skin
ENSXMAG00	1.11	6.19	0.00	0.00	kdelr2b	skin
ENSXMAG00	0.75	4.52	0.00	0.01	pym1	skin
ENSXMAG00	-0.70	4.21	0.00	0.04	slc6a16a	skin
ENSXMAG00	1.36	1.82	0.00	0.00	letmd1	skin
ENSXMAG00	-0.86	3.90	0.00	0.00	ephb6	skin
ENSXMAG00	0.67	5.04	0.00	0.01	zgc:55558	skin
ENSXMAG00	0.59	5.89	0.00	0.04		skin
ENSXMAG00	1.02	2.72	0.00	0.02		skin
ENSXMAG00	0.66	3.85	0.00	0.03	pdha1b	skin
ENSXMAG00	-0.81	7.28	0.00	0.03	nfkbiaa	skin
ENSXMAG00	-0.62	4.41	0.00	0.04	cc2d2a	skin
ENSXMAG00	0.65	5.90	0.00	0.05	txnl1	skin
ENSXMAG00	-0.79	4.03	0.00	0.03	ubald1a	skin
ENSXMAG00	0.80	5.36	0.00	0.01	arhgap35b	skin
ENSXMAG00	-0.74	5.00	0.00	0.03	stap2b	skin
ENSXMAG00	-1.32	1.31	0.00	0.00	cep89	skin
ENSXMAG00	-0.99	5.89	0.00	0.04		skin
ENSXMAG00	-3.21	-3.13	0.00	0.05	zgc:165481	skin
ENSXMAG00	-1.42	3.01	0.00	0.03	ucp3	skin
ENSXMAG00	0.81	4.00	0.00	0.01	slc7a10a	skin
ENSXMAG00	-0.75	5.82	0.00	0.01		skin
ENSXMAG00	-1.66	0.27	0.00	0.03	camkk1b	skin
ENSXMAG00	0.73	5.76	0.00	0.02	nudc	skin
ENSXMAG00	-0.83	4.28	0.00	0.01	meak7	skin
ENSXMAG00	-0.75	4.16	0.00	0.02		skin
ENSXMAG00	-0.64	4.99	0.00	0.04	slc27a1b	skin
ENSXMAG00	-0.84	4.24	0.00	0.01	pitpnc1b	skin
ENSXMAG00	1.11	5.84	0.00	0.00	si:ch211-114	skin

ENSXMAG00	-1.07	4.54	0.00	0.00	flcn	skin
ENSXMAG00	-1.11	4.91	0.00	0.03	slc6a4a	skin
ENSXMAG00	-1.87	2.40	0.00	0.00	si:ch211-120	skin
ENSXMAG00	-0.74	5.77	0.00	0.01	syap1	skin
ENSXMAG00	0.79	3.18	0.00	0.02	cdadc1	skin
ENSXMAG00	-0.65	4.20	0.00	0.03	hgsnat	skin
ENSXMAG00	0.92	5.86	0.00	0.00	adpgk	skin
ENSXMAG00	1.49	5.78	0.00	0.00	manf	skin
ENSXMAG00	0.88	4.96	0.00	0.00	cul5b	skin
ENSXMAG00	1.46	4.67	0.00	0.00	blmh	skin
ENSXMAG00	2.39	1.07	0.00	0.00	nptx1l	skin
ENSXMAG00	-0.73	4.51	0.00	0.02	leng1	skin
ENSXMAG00	1.57	4.34	0.00	0.00	dus2	skin
ENSXMAG00	-1.40	0.08	0.00	0.01		skin
ENSXMAG00	-0.83	4.05	0.00	0.01		skin
ENSXMAG00	-1.41	3.84	0.00	0.03		skin
ENSXMAG00	-1.15	2.22	0.00	0.00	stk36	skin
ENSXMAG00	-1.65	4.69	0.00	0.00	atf5a	skin
ENSXMAG00	0.60	6.45	0.00	0.04	MAP4K4 (1 o	skin
ENSXMAG00	0.92	2.71	0.00	0.04	SPHK1 (1 of	skin
ENSXMAG00	-1.11	5.75	0.00	0.00	tbcelb	skin
ENSXMAG00	0.95	6.53	0.00	0.00	dpysl3	skin
ENSXMAG00	1.04	7.16	0.00	0.00	ddost	skin
ENSXMAG00	0.73	6.79	0.00	0.01	zc3h13	skin
ENSXMAG00	0.78	4.58	0.00	0.01	tdg.1	skin
ENSXMAG00	1.00	4.72	0.00	0.00	samm50	skin
ENSXMAG00	0.78	6.94	0.00	0.01	api5	skin
ENSXMAG00	1.53	4.95	0.00	0.00		skin
ENSXMAG00	0.79	3.54	0.00	0.01	mtpap	skin
ENSXMAG00	-2.28	6.16	0.00	0.00	socs3a	skin
ENSXMAG00	1.22	5.61	0.00	0.00	si:dkey-82j4.	skin
ENSXMAG00	-1.64	6.84	0.00	0.00	irs2b	skin
ENSXMAG00	-0.89	3.21	0.00	0.01	zranb1b	skin
ENSXMAG00	0.89	3.24	0.00	0.00	slc25a10	skin
ENSXMAG00	1.18	4.54	0.00	0.00		skin
ENSXMAG00	1.38	2.15	0.00	0.00	adck2	skin
ENSXMAG00	-0.92	4.61	0.00	0.00	hmg20b	skin
ENSXMAG00	-0.89	4.96	0.00	0.00	zgc:163098	skin
ENSXMAG00	-0.79	4.38	0.00	0.00	pqlc2	skin
ENSXMAG00	0.79	5.27	0.00	0.00	pgm2	skin
ENSXMAG00	-0.66	6.29	0.00	0.03	mapk13	skin
ENSXMAG00	-0.99	0.45	0.00	0.04		skin
ENSXMAG00	1.29	3.96	0.00	0.00	knop1	skin

ENSXMAG00	0.66	4.77	0.00	0.04	mthfr	skin
ENSXMAG00	-1.30	4.62	0.00	0.00	bnip3	skin
ENSXMAG00	-0.89	5.32	0.00	0.00	RUSC2	skin
ENSXMAG00	-1.07	4.73	0.00	0.00	cited4a	skin
ENSXMAG00	0.71	6.53	0.00	0.02	ctps1a	skin
ENSXMAG00	-0.79	6.52	0.00	0.01		skin
ENSXMAG00	-1.35	3.02	0.00	0.00		skin
ENSXMAG00	1.98	5.68	0.00	0.00	khsrp	skin
ENSXMAG00	-0.63	4.73	0.00	0.03	fkbp8	skin
ENSXMAG00	0.87	7.71	0.00	0.03	hlfa	skin
ENSXMAG00	-0.91	2.58	0.00	0.01	fn3krp	skin
ENSXMAG00	-0.97	3.99	0.00	0.00	slc26a11	skin
ENSXMAG00	-1.39	4.38	0.00	0.00	sgsh	skin
ENSXMAG00	1.33	5.91	0.00	0.02	pcyt2	skin
ENSXMAG00	1.86	3.79	0.00	0.00	papss2a	skin
ENSXMAG00	-0.87	3.97	0.00	0.02	colgalt2	skin
ENSXMAG00	-0.88	4.68	0.00	0.01	rgl1	skin
ENSXMAG00	0.62	5.16	0.00	0.03	ATP6V1A (1	skin
ENSXMAG00	-1.55	6.05	0.00	0.00	gadd45ba	skin
ENSXMAG00	-0.59	6.59	0.00	0.05	mfge8b	skin
ENSXMAG00	0.70	3.72	0.00	0.03	lipt2	skin
ENSXMAG00	-1.29	0.04	0.00	0.05	map7d2a	skin
ENSXMAG00	0.74	6.18	0.00	0.01	tnpo3	skin
ENSXMAG00	-0.91	1.17	0.00	0.05		skin
ENSXMAG00	-0.98	2.03	0.00	0.01	flr	skin
ENSXMAG00	0.98	5.99	0.00	0.00	dnajb1a	skin
ENSXMAG00	0.83	5.32	0.00	0.04	CKMT1A	skin
ENSXMAG00	1.85	-1.69	0.00	0.03	hhla2a.2	skin
ENSXMAG00	-0.73	5.74	0.00	0.02	si:zfos-80g1	skin
ENSXMAG00	0.81	2.31	0.00	0.04	alg1	skin
ENSXMAG00	0.74	6.40	0.00	0.01	tmem214	skin
ENSXMAG00	-0.97	6.34	0.00	0.02	foxo4	skin
ENSXMAG00	-0.63	4.79	0.00	0.04	tyw1	skin
ENSXMAG00	-1.29	5.21	0.00	0.00	rab40c	skin
ENSXMAG00	-0.83	7.14	0.00	0.01	eml2	skin
ENSXMAG00	-2.21	-0.36	0.00	0.00	slc44a5b	skin
ENSXMAG00	-0.82	2.92	0.00	0.03	tmem67	skin
ENSXMAG00	0.87	4.10	0.00	0.00	tex10	skin
ENSXMAG00	-0.84	3.28	0.00	0.01	stx17	skin
ENSXMAG00	-1.38	1.42	0.00	0.00	adgrl1a	skin
ENSXMAG00	0.81	3.51	0.00	0.01	alg2	skin
ENSXMAG00	0.89	3.39	0.00	0.01	utp11	skin
ENSXMAG00	0.96	2.86	0.00	0.05	olfml2ba	skin

ENSXMAG00	-0.74	3.97	0.00	0.01	rrnad1	skin
ENSXMAG00	0.74	6.67	0.00	0.02	psmc4	skin
ENSXMAG00	0.83	4.22	0.00	0.02	dhdds	skin
ENSXMAG00	-0.77	4.84	0.00	0.01	cpamd8	skin
ENSXMAG00	-1.02	4.58	0.00	0.00	pcmtl	skin
ENSXMAG00	1.04	5.64	0.00	0.00	atp13a1	skin
ENSXMAG00	1.09	6.05	0.00	0.00	gmppb	skin
ENSXMAG00	0.68	6.20	0.00	0.01	zfr	skin
ENSXMAG00	-0.86	5.13	0.00	0.00	tmtc4	skin
ENSXMAG00	0.75	5.16	0.00	0.01	tle2a	skin
ENSXMAG00	-1.03	2.84	0.00	0.00	tle2b	skin
ENSXMAG00	0.92	4.01	0.00	0.00	cox15	skin
ENSXMAG00	0.61	5.79	0.00	0.04	pom121	skin
ENSXMAG00	0.73	4.63	0.00	0.03	tbl2	skin
ENSXMAG00	1.45	5.39	0.00	0.00	pprc1	skin
ENSXMAG00	-1.00	7.09	0.00	0.00	oga	skin
ENSXMAG00	-0.70	5.01	0.00	0.02	GABARAPL2	skin
ENSXMAG00	-1.56	2.93	0.00	0.00	nox1	skin
ENSXMAG00	0.93	6.19	0.00	0.00	rrp12	skin
ENSXMAG00	1.30	3.99	0.00	0.00	znrd1	skin
ENSXMAG00	-0.71	4.65	0.00	0.04	cep290	skin
ENSXMAG00	0.84	6.57	0.00	0.00	eif4g2b	skin
ENSXMAG00	-1.15	0.60	0.00	0.02	capslb	skin
ENSXMAG00	-0.64	4.61	0.00	0.04		skin
ENSXMAG00	-1.23	3.98	0.00	0.05		skin
ENSXMAG00	0.73	5.41	0.00	0.01	nsun2	skin
ENSXMAG00	0.80	4.95	0.00	0.01	ddx24	skin
ENSXMAG00	-0.71	4.81	0.00	0.02	lpcat1	skin
ENSXMAG00	-1.45	-0.88	0.00	0.04	si:dkey-221l4	skin
ENSXMAG00	-0.96	6.01	0.00	0.01	dis3l	skin
ENSXMAG00	-0.86	4.33	0.00	0.00		skin
ENSXMAG00	1.13	6.77	0.00	0.00	nop58	skin
ENSXMAG00	0.72	4.51	0.00	0.02	ZDHHC5 (1 o	skin
ENSXMAG00	1.16	3.86	0.00	0.00	gtf3aa	skin
ENSXMAG00	-0.74	3.94	0.00	0.01	si:dkey-28b4	skin
ENSXMAG00	0.66	6.10	0.00	0.03	slc6a8	skin
ENSXMAG00	0.88	4.27	0.00	0.00	trmt1	skin
ENSXMAG00	-1.28	0.40	0.00	0.04	cobll1a	skin
ENSXMAG00	1.59	1.26	0.00	0.02	fkbp14	skin
ENSXMAG00	0.86	6.89	0.00	0.00	stt3a	skin
ENSXMAG00	-1.45	-0.95	0.00	0.04	crtc1a	skin
ENSXMAG00	0.74	5.88	0.00	0.01	DPP9	skin
ENSXMAG00	0.89	6.46	0.00	0.01	nup93	skin

ENSXMAG00	-0.80	4.91	0.00	0.01	col28a2b	skin
ENSXMAG00	0.63	4.91	0.00	0.04	mpp6a	skin
ENSXMAG00	-1.04	3.03	0.00	0.01		skin
ENSXMAG00	-0.73	4.88	0.00	0.01	dzip1	skin
ENSXMAG00	-0.79	1.93	0.00	0.05	acer3	skin
ENSXMAG00	0.65	5.81	0.00	0.02	arfgap2	skin
ENSXMAG00	1.27	5.41	0.00	0.00	abcf3	skin
ENSXMAG00	-0.97	4.20	0.00	0.01	kdm2bb	skin
ENSXMAG00	1.29	4.35	0.00	0.00	gtpbp1	skin
ENSXMAG00	-1.03	0.38	0.00	0.04	ppfia3	skin
ENSXMAG00	1.18	4.22	0.00	0.00		skin
ENSXMAG00	0.64	6.64	0.00	0.03	luc7l3	skin
ENSXMAG00	-0.98	5.28	0.00	0.00	wfikkn2a	skin
ENSXMAG00	0.68	7.48	0.00	0.02	eif4a3	skin
ENSXMAG00	0.83	5.28	0.00	0.00	nup133	skin
ENSXMAG00	-1.00	5.89	0.00	0.01	klf4	skin
ENSXMAG00	0.98	4.39	0.00	0.00	ogfod1	skin
ENSXMAG00	-0.76	5.89	0.00	0.01	tbc1d17	skin
ENSXMAG00	0.68	5.21	0.00	0.02	nudt21	skin
ENSXMAG00	-0.84	4.32	0.00	0.05		skin
ENSXMAG00	0.78	5.21	0.00	0.01	utp15	skin
ENSXMAG00	1.04	6.45	0.00	0.00	TNPO2 (1 of	skin
ENSXMAG00	-1.34	0.26	0.00	0.05	wt1b	skin
ENSXMAG00	0.98	6.40	0.00	0.00	bms1	skin
ENSXMAG00	-0.99	3.65	0.00	0.01		skin
ENSXMAG00	0.96	5.20	0.00	0.00		skin
ENSXMAG00	-0.85	4.49	0.00	0.01	rfx1a	skin
ENSXMAG00	-1.24	1.79	0.00	0.03		skin
ENSXMAG00	-1.94	5.86	0.00	0.00		skin
ENSXMAG00	1.14	5.40	0.00	0.00	mtr	skin
ENSXMAG00	1.39	3.98	0.00	0.00	sdf2	skin
ENSXMAG00	-1.77	1.14	0.00	0.00	rps6ka2	skin
ENSXMAG00	0.96	5.73	0.00	0.00	rhot1a	skin
ENSXMAG00	0.73	4.36	0.00	0.01	fastkd1	skin
ENSXMAG00	-1.42	0.33	0.00	0.00		skin
ENSXMAG00	0.67	6.57	0.00	0.04		skin
ENSXMAG00	-0.65	6.18	0.00	0.04		skin
ENSXMAG00	0.75	4.72	0.00	0.01	acbd4	skin
ENSXMAG00	0.66	5.18	0.00	0.05	acta2	skin
ENSXMAG00	-2.40	-1.82	0.00	0.04		skin
ENSXMAG00	0.79	6.01	0.00	0.01	atad1b	skin
ENSXMAG00	1.71	2.45	0.00	0.00	foxo6a	skin
ENSXMAG00	-0.80	3.68	0.00	0.01		skin

ENSXMAG00	0.73	3.17	0.00	0.04	ppp1r8b	skin
ENSXMAG00	-2.31	-1.65	0.00	0.02	si:ch211-220	skin
ENSXMAG00	1.12	4.57	0.00	0.00	supv3l1	skin
ENSXMAG00	-1.67	5.10	0.00	0.00	rnf213b	skin
ENSXMAG00	0.69	4.93	0.00	0.02	nup85	skin
ENSXMAG00	0.78	5.98	0.00	0.01	hk1	skin
ENSXMAG00	0.99	3.48	0.00	0.01	fcf1	skin
ENSXMAG00	-1.18	5.84	0.00	0.01	COL4A5	skin
ENSXMAG00	-1.45	4.66	0.00	0.00	plek2	skin
ENSXMAG00	1.03	0.15	0.00	0.04	tacr2	skin
ENSXMAG00	-1.13	2.26	0.00	0.04		skin
ENSXMAG00	-2.53	-1.20	0.00	0.00	si:ch211-10a	skin
ENSXMAG00	0.97	4.14	0.00	0.00	eif3eb	skin
ENSXMAG00	-1.40	1.80	0.00	0.00	OSCP1	skin
ENSXMAG00	0.89	3.95	0.00	0.01	tdrkh	skin
ENSXMAG00	-0.59	6.39	0.00	0.04	acsf2	skin
ENSXMAG00	0.91	6.49	0.00	0.00	srebf1	skin
ENSXMAG00	0.68	4.76	0.00	0.01	parn	skin
ENSXMAG00	-1.63	6.76	0.00	0.02	arg2	skin
ENSXMAG00	-0.82	2.82	0.00	0.04		skin
ENSXMAG00	-0.77	5.32	0.00	0.02	efna1a	skin
ENSXMAG00	0.73	5.17	0.00	0.02	wdr74	skin
ENSXMAG00	-0.80	3.40	0.00	0.04	ift88	skin
ENSXMAG00	-1.48	0.65	0.00	0.00	rgrb	skin
ENSXMAG00	0.74	4.17	0.00	0.01	stx4	skin
ENSXMAG00	-0.78	4.08	0.00	0.02	fam120b	skin
ENSXMAG00	1.07	5.22	0.00	0.00	tbl3	skin
ENSXMAG00	1.11	1.13	0.00	0.02		skin
ENSXMAG00	1.54	2.42	0.00	0.00	mrm1	skin
ENSXMAG00	1.23	6.50	0.00	0.00	ppm1g	skin
ENSXMAG00	0.73	5.84	0.00	0.01	noa1	skin
ENSXMAG00	-1.47	3.12	0.00	0.00	ttll6	skin
ENSXMAG00	0.93	2.74	0.00	0.01	pdcd2	skin
ENSXMAG00	0.90	2.84	0.00	0.03	nudt18	skin
ENSXMAG00	0.64	4.46	0.00	0.03	alg3	skin
ENSXMAG00	1.34	1.09	0.00	0.01	ybey	skin
ENSXMAG00	-1.01	3.25	0.00	0.00	TATDN3	skin
ENSXMAG00	0.72	5.48	0.00	0.01	urb2	skin
ENSXMAG00	-1.54	4.24	0.00	0.00	irf7	skin
ENSXMAG00	0.75	5.38	0.00	0.01	galnt2	skin
ENSXMAG00	-2.08	6.24	0.00	0.00	klhl24b	skin
ENSXMAG00	1.91	2.51	0.00	0.01	kcnab1b	skin
ENSXMAG00	1.22	0.31	0.00	0.05	frmd3	skin

ENSXMAG00	-0.64	4.88	0.00	0.04	slc5a6b	skin
ENSXMAG00	0.94	6.49	0.00	0.01	acsl3b	skin
ENSXMAG00	0.96	3.32	0.00	0.01	afg1la	skin
ENSXMAG00	1.01	4.24	0.00	0.00	dus3l	skin
ENSXMAG00	1.03	4.67	0.00	0.00	rnf130	skin
ENSXMAG00	-0.94	4.26	0.00	0.00		skin
ENSXMAG00	0.82	5.15	0.00	0.00		skin
ENSXMAG00	0.66	7.28	0.00	0.03	tmed7	skin
ENSXMAG00	1.16	4.89	0.00	0.00	eif4eb	skin
ENSXMAG00	-2.11	1.82	0.00	0.00		skin
ENSXMAG00	1.02	6.27	0.00	0.00	prpf40a	skin
ENSXMAG00	-2.16	3.25	0.00	0.01		skin
ENSXMAG00	-1.15	2.40	0.00	0.00		skin
ENSXMAG00	-1.48	2.52	0.00	0.00	ephb1	skin
ENSXMAG00	-1.31	3.20	0.00	0.00	tmem260	skin
ENSXMAG00	-1.35	3.72	0.00	0.00	rbm41	skin
ENSXMAG00	1.06	4.16	0.00	0.00	smarcad1a	skin
ENSXMAG00	0.94	5.28	0.00	0.00		skin
ENSXMAG00	1.63	1.70	0.00	0.00		skin
ENSXMAG00	0.84	1.86	0.00	0.05	vcpkmt	skin
ENSXMAG00	-0.83	5.44	0.00	0.00	spon1b	skin
ENSXMAG00	0.75	6.79	0.00	0.01		skin
ENSXMAG00	-1.27	4.44	0.00	0.00	glb1l	skin
ENSXMAG00	-1.77	4.73	0.00	0.00	ntn4	skin
ENSXMAG00	-0.76	3.49	0.00	0.02		skin
ENSXMAG00	-0.91	1.60	0.00	0.04		skin
ENSXMAG00	1.07	5.86	0.00	0.00	DGKQ	skin
ENSXMAG00	0.94	2.87	0.00	0.01	elac1	skin
ENSXMAG00	0.92	3.50	0.00	0.02		skin
ENSXMAG00	1.48	4.03	0.00	0.00	zgc:158828	skin
ENSXMAG00	0.93	2.86	0.00	0.01	zgc:65997	skin
ENSXMAG00	-1.05	4.10	0.00	0.00	gab2	skin
ENSXMAG00	-1.10	5.29	0.00	0.00	rnf114	skin
ENSXMAG00	-0.84	4.10	0.00	0.01		skin
ENSXMAG00	-0.94	2.75	0.00	0.00	trpm6	skin
ENSXMAG00	0.83	1.92	0.00	0.05	tmem41aa	skin
ENSXMAG00	0.62	4.91	0.00	0.04	EIF4ENIF1	skin
ENSXMAG00	0.98	5.28	0.00	0.00	noc3l	skin
ENSXMAG00	-0.76	6.02	0.00	0.01		skin
ENSXMAG00	-2.18	-1.96	0.00	0.01	nme8	skin
ENSXMAG00	0.93	7.53	0.00	0.00	vclb	skin
ENSXMAG00	-0.77	3.89	0.00	0.01	SMARCAL1	skin
ENSXMAG00	0.77	3.16	0.00	0.05	poglut3	skin

ENSXMAG00	0.99	6.14	0.00	0.00	rars	skin
ENSXMAG00	0.89	5.36	0.00	0.00	tspo (1 of ma	skin
ENSXMAG00	2.12	2.43	0.00	0.00	adck1	skin
ENSXMAG00	0.78	8.49	0.00	0.01	srsf2a	skin
ENSXMAG00	-1.33	4.58	0.00	0.00		skin
ENSXMAG00	1.25	4.17	0.00	0.00	ARL3	skin
ENSXMAG00	-0.91	1.82	0.00	0.02	si:dkey-219e	skin
ENSXMAG00	-1.20	4.62	0.00	0.01		skin
ENSXMAG00	-1.53	1.09	0.00	0.02	oprd1b	skin
ENSXMAG00	1.05	3.86	0.00	0.00	tha1	skin
ENSXMAG00	1.22	2.97	0.00	0.01	YBX2	skin
ENSXMAG00	0.58	5.25	0.00	0.05	polr3e	skin
ENSXMAG00	0.73	2.87	0.00	0.04	ctdnep1b	skin
ENSXMAG00	-0.90	3.35	0.00	0.01		skin
ENSXMAG00	0.69	4.72	0.00	0.04	ipo11	skin
ENSXMAG00	-1.06	1.20	0.00	0.01	arpin	skin
ENSXMAG00	-1.69	-0.54	0.00	0.01		skin
ENSXMAG00	-1.52	-0.58	0.00	0.04	fam169b	skin
ENSXMAG00	-1.08	3.55	0.00	0.00	ldhd	skin
ENSXMAG00	0.72	5.20	0.00	0.01	cacybp	skin
ENSXMAG00	-1.14	5.38	0.00	0.00	elmsan1b	skin
ENSXMAG00	-0.71	7.34	0.00	0.03	amotl2b	skin
ENSXMAG00	-1.10	3.56	0.00	0.00	neil1	skin
ENSXMAG00	0.88	6.63	0.00	0.00	tomm70a	skin
ENSXMAG00	0.95	4.58	0.00	0.03		skin
ENSXMAG00	0.78	4.50	0.00	0.01	slc35f2	skin
ENSXMAG00	-1.83	4.43	0.00	0.00		skin
ENSXMAG00	0.72	4.45	0.00	0.02	polr2c	skin
ENSXMAG00	-1.19	3.56	0.00	0.01	cdc20	skin
ENSXMAG00	-1.00	5.01	0.00	0.02	kdm4aa	skin
ENSXMAG00	-1.32	4.85	0.00	0.03	elovl6l	skin
ENSXMAG00	0.90	6.41	0.00	0.00	lmnb1	skin
ENSXMAG00	1.26	5.12	0.00	0.00	uba2	skin
ENSXMAG00	-1.87	1.40	0.00	0.02		skin
ENSXMAG00	1.30	8.25	0.00	0.00	eif4g1a	skin
ENSXMAG00	-0.78	5.91	0.00	0.01		skin
ENSXMAG00	-0.99	4.59	0.00	0.00	man2b2	skin
ENSXMAG00	-0.87	6.28	0.00	0.05	nt5dc2	skin
ENSXMAG00	0.91	3.26	0.00	0.03	sfxn5b	skin
ENSXMAG00	0.77	5.02	0.00	0.00	sptlc1	skin
ENSXMAG00	0.57	5.15	0.00	0.04	tjap1	skin
ENSXMAG00	0.62	4.91	0.00	0.04	mapkapk5	skin
ENSXMAG00	-0.91	5.96	0.00	0.00	atp8a1	skin

ENSXMAG00	0.99	3.06	0.00	0.02	chac2	skin
ENSXMAG00	-1.02	2.64	0.00	0.03	oca2	skin
ENSXMAG00	0.90	5.03	0.00	0.00	dnajc11a	skin
ENSXMAG00	-0.74	2.78	0.00	0.05	gba	skin
ENSXMAG00	-1.18	3.32	0.00	0.00	xrcc4	skin
ENSXMAG00	-1.07	2.07	0.00	0.04		skin
ENSXMAG00	-0.78	4.70	0.00	0.02	wdr45	skin
ENSXMAG00	0.65	4.13	0.00	0.04	akap17a	skin
ENSXMAG00	-1.04	4.77	0.00	0.00		skin
ENSXMAG00	1.24	3.98	0.00	0.00	exosc4	skin
ENSXMAG00	-1.06	1.16	0.00	0.01	si:ch211-124	skin
ENSXMAG00	-0.80	3.76	0.00	0.02	ube3a	skin
ENSXMAG00	0.86	3.25	0.00	0.01	trit1	skin
ENSXMAG00	1.19	3.83	0.00	0.00	rrp9	skin
ENSXMAG00	0.75	4.40	0.00	0.01	vldlr	skin
ENSXMAG00	1.41	0.74	0.00	0.02		skin
ENSXMAG00	1.85	4.50	0.00	0.00	dhx30	skin
ENSXMAG00	-2.02	4.93	0.00	0.04		skin
ENSXMAG00	1.45	5.40	0.00	0.04	mylk4a	skin
ENSXMAG00	-0.91	3.50	0.00	0.01	bivm	skin
ENSXMAG00	-1.53	-1.54	0.00	0.05	st6galnac5a	skin
ENSXMAG00	0.81	4.52	0.00	0.01	UBA3 (1 of n	skin
ENSXMAG00	-0.73	6.29	0.00	0.02	fgfr1b	skin
ENSXMAG00	0.78	5.28	0.00	0.01	si:ch211-173	skin
ENSXMAG00	-0.77	3.63	0.00	0.03	arl6ip5b	skin
ENSXMAG00	-0.91	1.43	0.00	0.03	AMZ2	skin
ENSXMAG00	0.65	5.28	0.00	0.02	tepsin	skin
ENSXMAG00	-1.01	5.40	0.00	0.00	prkag2b	skin
ENSXMAG00	0.76	6.02	0.00	0.01	heatr3	skin
ENSXMAG00	-0.69	3.22	0.00	0.05	COMMD10	skin
ENSXMAG00	-1.24	5.96	0.00	0.00	hbp1	skin
ENSXMAG00	0.67	4.99	0.00	0.05	bcl6aa	skin
ENSXMAG00	1.41	7.93	0.00	0.00	hsp90b1	skin
ENSXMAG00	0.88	3.56	0.00	0.01	b4galt7	skin
ENSXMAG00	-0.77	6.46	0.00	0.03	egfra	skin
ENSXMAG00	0.77	5.73	0.00	0.01	nt5dc3	skin
ENSXMAG00	-2.04	4.78	0.00	0.00		skin
ENSXMAG00	0.72	6.67	0.00	0.01	myo1b	skin
ENSXMAG00	0.78	4.52	0.00	0.01	ddx10	skin
ENSXMAG00	0.75	4.32	0.00	0.05	hmga1a	skin
ENSXMAG00	-1.87	0.86	0.00	0.00		skin
ENSXMAG00	1.83	5.39	0.00	0.00		skin
ENSXMAG00	2.88	-2.65	0.00	0.01	cacnb2b	skin

ENSXMAG00	0.66	6.78	0.00	0.03	abcf1	skin
ENSXMAG00	-0.86	5.91	0.00	0.00	ccdc136b	skin
ENSXMAG00	1.24	5.00	0.00	0.00	mrpl20	skin
ENSXMAG00	1.51	5.84	0.00	0.01		skin
ENSXMAG00	-1.16	1.01	0.00	0.01	cox4i2	skin
ENSXMAG00	-0.71	4.11	0.00	0.02	ccdc30	skin
ENSXMAG00	-1.60	4.82	0.00	0.00	MFAP2	skin
ENSXMAG00	0.79	5.72	0.00	0.01	nsfl1c	skin
ENSXMAG00	-4.71	-3.23	0.00	0.02	cplane2	skin
ENSXMAG00	0.89	3.36	0.00	0.01	MSH3	skin
ENSXMAG00	-1.78	2.72	0.00	0.00	prdm1b	skin
ENSXMAG00	2.12	3.18	0.00	0.00	slc25a25b	skin
ENSXMAG00	0.62	4.65	0.00	0.05	nckipsd	skin
ENSXMAG00	0.90	5.91	0.00	0.01	dhtkd1	skin
ENSXMAG00	-0.85	4.79	0.00	0.01		skin
ENSXMAG00	1.48	6.30	0.00	0.01	asns	skin
ENSXMAG00	-2.60	2.85	0.00	0.00		skin
ENSXMAG00	0.64	4.80	0.00	0.04	PNPT1	skin
ENSXMAG00	-0.92	3.65	0.00	0.00	fyco1b	skin
ENSXMAG00	-0.80	4.71	0.00	0.01		skin
ENSXMAG00	0.78	7.30	0.00	0.00	top2b	skin
ENSXMAG00	-0.63	6.12	0.00	0.05	CCDC88A	skin
ENSXMAG00	0.73	8.64	0.00	0.02	arf2b	skin
ENSXMAG00	0.92	3.44	0.00	0.01	slc35b4	skin
ENSXMAG00	0.93	4.25	0.00	0.02	golt1bb	skin
ENSXMAG00	-0.86	6.51	0.00	0.01		skin
ENSXMAG00	1.16	4.92	0.00	0.00	tmed3	skin
ENSXMAG00	1.72	2.31	0.00	0.00	si:ch73-52e5	skin
ENSXMAG00	1.36	4.25	0.00	0.00	lrrc59	skin
ENSXMAG00	1.32	3.90	0.00	0.00	riox1	skin
ENSXMAG00	1.29	4.64	0.00	0.00	timm23a	skin
ENSXMAG00	1.48	1.91	0.00	0.00	wdr76	skin
ENSXMAG00	-4.61	-3.28	0.00	0.04	prph2a	skin
ENSXMAG00	-0.99	4.68	0.00	0.00	bmpr1ba	skin
ENSXMAG00	0.66	5.29	0.00	0.04	sec11a	skin
ENSXMAG00	1.08	3.20	0.00	0.00	prmt9	skin
ENSXMAG00	-0.92	2.38	0.00	0.01	spata20	skin
ENSXMAG00	-1.23	3.07	0.00	0.00	miip	skin
ENSXMAG00	0.68	8.91	0.00	0.03	YWHAB	skin
ENSXMAG00	-0.64	4.63	0.00	0.04	bap1	skin
ENSXMAG00	-0.64	5.23	0.00	0.05	map4k3b	skin
ENSXMAG00	0.97	2.53	0.00	0.01	smim4	skin
ENSXMAG00	-1.27	3.15	0.00	0.03	gls2a	skin

ENSXMAG00	-0.72	4.90	0.00	0.02	si:ch211-125	skin
ENSXMAG00	-1.30	1.40	0.00	0.02		skin
ENSXMAG00	-0.90	3.81	0.00	0.05	mindy1	skin
ENSXMAG00	0.73	6.89	0.00	0.04	sacm1la	skin
ENSXMAG00	0.71	4.70	0.00	0.03	cab39l	skin
ENSXMAG00	-1.06	1.79	0.00	0.01		skin
ENSXMAG00	-1.02	3.29	0.00	0.00	IFT122	skin
ENSXMAG00	0.79	5.29	0.00	0.01	rer1	skin
ENSXMAG00	0.98	2.33	0.00	0.03	tamm41	skin
ENSXMAG00	1.20	7.10	0.00	0.00	ptges3b	skin
ENSXMAG00	-0.73	4.31	0.00	0.01	vgll4b	skin
ENSXMAG00	1.35	0.96	0.00	0.00	creld1b	skin
ENSXMAG00	-0.61	4.93	0.00	0.04	mtmr7a	skin
ENSXMAG00	-1.24	3.47	0.00	0.00	trim3b	skin
ENSXMAG00	0.95	4.39	0.00	0.00	ints7	skin
ENSXMAG00	-2.40	5.66	0.00	0.00	lpin1	skin
ENSXMAG00	-1.81	4.52	0.00	0.00		skin
ENSXMAG00	0.68	5.16	0.00	0.03	lpgat1	skin
ENSXMAG00	-0.68	3.36	0.00	0.04	eva1c	skin
ENSXMAG00	0.62	5.31	0.00	0.02	tcea1	skin
ENSXMAG00	-0.67	4.17	0.00	0.04	ephb2b	skin
ENSXMAG00	-1.19	2.60	0.00	0.00	grik1a	skin
ENSXMAG00	0.97	5.69	0.00	0.00		skin
ENSXMAG00	0.90	2.93	0.00	0.01	twnk	skin
ENSXMAG00	0.66	6.08	0.00	0.02	esf1	skin
ENSXMAG00	0.90	3.95	0.00	0.01		skin
ENSXMAG00	-0.73	3.37	0.00	0.05	zmp:000000(	skin
ENSXMAG00	-1.01	2.35	0.00	0.01	kif26ba	skin
ENSXMAG00	0.68	5.14	0.00	0.02	CHORDC1	skin
ENSXMAG00	-0.68	6.36	0.00	0.04	sh2d5	skin
ENSXMAG00	-0.99	1.91	0.00	0.03		skin
ENSXMAG00	1.12	6.08	0.00	0.01	mthfd2	skin
ENSXMAG00	1.16	5.40	0.00	0.00	timm10	skin
ENSXMAG00	-2.34	-1.74	0.00	0.01	SLC43A3 (1 c	skin
ENSXMAG00	-1.17	3.15	0.00	0.02	PLXNA2	skin
ENSXMAG00	1.11	7.10	0.00	0.00	pdia6	skin
ENSXMAG00	0.83	6.42	0.00	0.00	NAA15	skin
ENSXMAG00	-0.86	3.91	0.00	0.01	smpd1	skin
ENSXMAG00	-3.77	8.52	0.00	0.00		skin
ENSXMAG00	0.62	6.82	0.00	0.05	arhgef2	skin
ENSXMAG00	0.72	5.49	0.00	0.01	gpam	skin
ENSXMAG00	0.61	4.41	0.00	0.04	tarbp2	skin
ENSXMAG00	-0.82	4.22	0.00	0.01		skin

ENSXMAG00	0.68	5.73	0.00	0.01	dctn2	skin
ENSXMAG00	-0.97	3.64	0.00	0.00	phc2b	skin
ENSXMAG00	-0.83	5.82	0.00	0.01		skin
ENSXMAG00	2.20	5.62	0.00	0.00	mars	skin
ENSXMAG00	0.62	7.72	0.00	0.04	ewsr1b	skin
ENSXMAG00	0.66	4.88	0.00	0.04	snrpc	skin
ENSXMAG00	-1.66	0.85	0.00	0.00	ENDOV	skin
ENSXMAG00	1.12	5.75	0.00	0.00	psmd11b	skin
ENSXMAG00	-0.99	4.27	0.00	0.00	DYNC2H1	skin
ENSXMAG00	-2.73	3.71	0.00	0.04		skin
ENSXMAG00	0.63	6.45	0.00	0.04	ddx18	skin
ENSXMAG00	-1.36	2.46	0.00	0.00	c18h3orf33	skin
ENSXMAG00	-0.63	5.63	0.00	0.03	trip10a	skin
ENSXMAG00	-2.36	-0.83	0.00	0.02		skin
ENSXMAG00	-1.04	3.44	0.00	0.02	prc1b	skin
ENSXMAG00	-0.75	2.68	0.00	0.04	tctn2	skin
ENSXMAG00	-1.94	5.02	0.00	0.00	pik3ip1	skin
ENSXMAG00	-1.91	-0.80	0.00	0.00	igdcc4	skin
ENSXMAG00	0.86	6.76	0.00	0.00		skin
ENSXMAG00	0.66	4.99	0.00	0.04		skin
ENSXMAG00	-1.89	-0.58	0.00	0.01		skin
ENSXMAG00	0.89	6.97	0.00	0.00	lrpprc	skin
ENSXMAG00	-0.69	3.93	0.00	0.05		skin
ENSXMAG00	-1.58	3.44	0.00	0.02		skin
ENSXMAG00	0.87	6.38	0.00	0.00	ftsj3	skin
ENSXMAG00	0.88	6.20	0.00	0.00	sart3	skin
ENSXMAG00	0.72	3.65	0.00	0.03	ptar1	skin
ENSXMAG00	-1.17	3.45	0.00	0.00	rtkn2a	skin
ENSXMAG00	-1.43	8.41	0.00	0.00	zgc:162730	skin
ENSXMAG00	0.66	3.94	0.00	0.04	vkorc1l1	skin
ENSXMAG00	0.97	4.85	0.00	0.00	actl6a	skin
ENSXMAG00	-0.86	5.16	0.00	0.00	si:dkeyp-97b	skin
ENSXMAG00	0.71	5.39	0.00	0.01	NCBP1	skin
ENSXMAG00	-0.75	4.17	0.00	0.01	mcee	skin
ENSXMAG00	0.64	4.98	0.00	0.04	mphosph10	skin
ENSXMAG00	0.90	3.70	0.00	0.01	dhodh	skin
ENSXMAG00	-1.37	0.30	0.00	0.01		skin
ENSXMAG00	-0.74	3.84	0.00	0.04	loxl2a	skin
ENSXMAG00	-0.62	5.02	0.00	0.04	traf3	skin
ENSXMAG00	1.10	6.02	0.00	0.00	rcor1	skin
ENSXMAG00	-2.30	4.90	0.00	0.00	ankrd9	skin
ENSXMAG00	-1.28	3.30	0.00	0.00	rbks	skin
ENSXMAG00	-0.84	2.88	0.00	0.03	EVC	skin

ENSXMAG00	-1.07	3.12	0.00	0.00	wdr35	skin
ENSXMAG00	1.29	5.14	0.00	0.00	pelo	skin
ENSXMAG00	0.86	4.41	0.00	0.00	dmap1	skin
ENSXMAG00	1.18	4.56	0.00	0.00	nol10	skin
ENSXMAG00	-1.69	5.29	0.00	0.00	apaf1	skin
ENSXMAG00	0.78	4.84	0.00	0.01	dars2	skin
ENSXMAG00	-0.63	4.65	0.00	0.04	fam149b1	skin
ENSXMAG00	1.35	5.08	0.00	0.00	snrpa	skin
ENSXMAG00	1.42	5.74	0.00	0.00	tgm2b	skin
ENSXMAG00	-0.77	5.47	0.00	0.04	tap1	skin
ENSXMAG00	0.67	4.52	0.00	0.03	BRD2 (1 of m	skin
ENSXMAG00	0.68	5.01	0.00	0.01	iars2	skin
ENSXMAG00	-0.77	3.20	0.00	0.04	dpydb	skin
ENSXMAG00	1.22	-0.24	0.00	0.04	espn	skin
ENSXMAG00	-2.60	4.62	0.00	0.02	fosb	skin
ENSXMAG00	-1.55	3.93	0.00	0.00		skin
ENSXMAG00	-0.74	3.14	0.00	0.04	vdrb	skin
ENSXMAG00	0.80	4.81	0.00	0.01	ncln	skin
ENSXMAG00	0.83	5.16	0.00	0.00	ash2l	skin
ENSXMAG00	0.68	6.39	0.00	0.03	cope	skin
ENSXMAG00	-1.99	-1.52	0.00	0.02		skin
ENSXMAG00	-1.23	-0.19	0.00	0.03	DUSP26	skin
ENSXMAG00	1.28	4.69	0.00	0.00	brms1lb	skin
ENSXMAG00	0.62	7.80	0.00	0.03	kpnb1	skin
ENSXMAG00	-0.98	4.43	0.00	0.00	pargl	skin
ENSXMAG00	1.95	4.30	0.00	0.00		skin
ENSXMAG00	-1.36	3.86	0.00	0.00	tmem173	skin
ENSXMAG00	-0.81	4.30	0.00	0.01	wdr41	skin
ENSXMAG00	1.36	5.95	0.00	0.00	ddx19	skin
ENSXMAG00	1.47	7.28	0.00	0.00	pdia4	skin
ENSXMAG00	-2.64	1.12	0.00	0.00	asb10	skin
ENSXMAG00	1.05	7.90	0.00	0.00	rpn1	skin
ENSXMAG00	-1.23	6.39	0.00	0.00	oplah	skin
ENSXMAG00	-0.83	4.31	0.00	0.00	tsr3	skin
ENSXMAG00	0.85	2.47	0.00	0.04	nmnat3	skin
ENSXMAG00	-2.32	4.23	0.00	0.00	nr1d1	skin
ENSXMAG00	0.70	7.51	0.00	0.02	copb2	skin
ENSXMAG00	-1.05	0.44	0.00	0.04		skin
ENSXMAG00	0.95	6.77	0.00	0.00	dync1li2	skin
ENSXMAG00	1.45	1.72	0.00	0.00	zmp:000000(	skin
ENSXMAG00	-1.41	1.37	0.00	0.01		skin
ENSXMAG00	0.73	3.96	0.00	0.04	ankmy2a	skin
ENSXMAG00	0.85	3.53	0.00	0.01	ppie	skin

ENSXMAG00	-0.87	6.47	0.00	0.02	SPIDR	skin
ENSXMAG00	0.82	5.05	0.00	0.00		skin
ENSXMAG00	-0.83	3.45	0.00	0.02		skin
ENSXMAG00	0.65	4.19	0.00	0.05	PRELID3B	skin
ENSXMAG00	-0.63	5.77	0.00	0.03	srgap1a	skin
ENSXMAG00	-1.53	1.99	0.00	0.00		skin
ENSXMAG00	0.76	7.46	0.00	0.03	si:ch1073-44	skin
ENSXMAG00	0.86	6.61	0.00	0.00	sec31a	skin
ENSXMAG00	-0.86	4.82	0.00	0.00	sema6e	skin
ENSXMAG00	-0.86	3.74	0.00	0.00	tshz3a	skin
ENSXMAG00	-0.84	4.89	0.00	0.00	fzr1b	skin
ENSXMAG00	-0.69	5.80	0.00	0.04		skin
ENSXMAG00	0.94	7.84	0.00	0.00	eif2s2	skin
ENSXMAG00	0.93	4.72	0.00	0.00	tpp2	skin
ENSXMAG00	0.81	2.74	0.00	0.02	tfb1m	skin
ENSXMAG00	1.06	4.15	0.00	0.00		skin
ENSXMAG00	1.29	5.48	0.00	0.00	denr	skin
ENSXMAG00	1.64	7.04	0.00	0.00	g3bp1	skin
ENSXMAG00	-0.85	5.78	0.00	0.00		skin
ENSXMAG00	-1.62	1.73	0.00	0.00	tmem17	skin
ENSXMAG00	-1.07	3.04	0.00	0.01	chdh	skin
ENSXMAG00	0.75	4.49	0.00	0.01	plekhj1	skin
ENSXMAG00	0.85	3.47	0.00	0.01	rnf126	skin
ENSXMAG00	1.33	2.95	0.00	0.00	ARMC6	skin
ENSXMAG00	0.92	6.26	0.00	0.00	impdh2	skin
ENSXMAG00	0.87	5.90	0.00	0.00	sec13	skin
ENSXMAG00	1.46	5.12	0.00	0.00	xpc	skin
ENSXMAG00	-0.96	2.68	0.00	0.01	dnajc16	skin
ENSXMAG00	-0.82	3.62	0.00	0.01	casp9	skin
ENSXMAG00	-0.92	8.72	0.00	0.03	aqp3a	skin
ENSXMAG00	0.88	4.77	0.00	0.00	nol6	skin
ENSXMAG00	-1.00	3.34	0.00	0.01	rhobtb4	skin
ENSXMAG00	1.01	1.67	0.00	0.05	smim12	skin
ENSXMAG00	0.93	2.65	0.00	0.01	SLC35A4	skin
ENSXMAG00	-0.67	3.98	0.00	0.03	inhbaa	skin
ENSXMAG00	0.73	5.51	0.00	0.03	prss23	skin
ENSXMAG00	0.94	2.41	0.00	0.04	FASTKD5	skin
ENSXMAG00	-2.21	5.88	0.00	0.00	junba	skin
ENSXMAG00	1.17	0.75	0.00	0.04		skin
ENSXMAG00	0.60	4.87	0.00	0.04	purbb	skin
ENSXMAG00	-1.27	-0.24	0.00	0.04	kcnf1a	skin
ENSXMAG00	1.26	1.07	0.00	0.01		skin
ENSXMAG00	1.14	-0.11	0.00	0.04	TMEM250	skin

ENSXMAG00	-1.72	4.79	0.00	0.00	ier2b	skin
ENSXMAG00	0.61	7.89	0.00	0.04	HNRNPA0 (1	skin
ENSXMAG00	0.95	1.56	0.00	0.03	fahd1	skin
ENSXMAG00	1.34	-0.63	0.00	0.04		skin
ENSXMAG00	0.98	4.13	0.00	0.00	zpr1	skin
ENSXMAG00	-1.14	2.52	0.00	0.03	kcnj2a	skin
ENSXMAG00	0.83	5.79	0.00	0.00	nono	skin
ENSXMAG00	0.82	3.21	0.00	0.01	abhd13	skin
ENSXMAG00	-1.22	2.26	0.00	0.00	trim107	skin
ENSXMAG00	0.98	6.51	0.00	0.02		skin
ENSXMAG00	-2.35	3.08	0.00	0.00	cldni (1 of m	skin
ENSXMAG00	-0.99	3.97	0.00	0.01	nupr1a	skin
ENSXMAG00	-1.21	5.84	0.00	0.00		skin
ENSXMAG00	0.95	1.14	0.00	0.03	ora5	skin
ENSXMAG00	1.31	-0.20	0.00	0.02		skin
ENSXMAG00	0.82	5.55	0.00	0.04		skin
ENSXMAG00	0.82	4.58	0.00	0.01	si:dkey-251i1	skin
ENSXMAG00	-1.14	3.66	0.00	0.00		skin
ENSXMAG00	0.95	3.81	0.00	0.00	timm8b	skin
ENSXMAG00	-1.53	3.37	0.00	0.01		skin
ENSXMAG00	1.21	1.22	0.00	0.01	slitrk5a	skin
ENSXMAG00	1.94	3.44	0.00	0.00	si:ch211-199	skin
ENSXMAG00	0.89	3.78	0.00	0.00	aatf	skin
ENSXMAG00	0.78	5.28	0.00	0.00	nup88	skin
ENSXMAG00	0.73	5.25	0.00	0.02	tpbgb	skin
ENSXMAG00	0.97	3.41	0.00	0.00	dpm3	skin
ENSXMAG00	-1.57	4.93	0.00	0.00	h1f0	skin
ENSXMAG00	-1.10	0.41	0.00	0.05	c2cd4a	skin
ENSXMAG00	0.72	4.30	0.00	0.01	zgc:55781	skin
ENSXMAG00	-2.57	1.83	0.00	0.00		skin
ENSXMAG00	1.22	-0.62	0.00	0.04	SPRY3	skin
ENSXMAG00	-1.03	2.61	0.00	0.04	tlr22	skin
ENSXMAG00	-1.06	4.50	0.00	0.00	zc3h6	skin
ENSXMAG00	0.95	0.88	0.00	0.03	si:dkey-48p1	skin
ENSXMAG00	-0.86	6.26	0.00	0.00	ADAMTS5	skin
ENSXMAG00	-0.91	3.36	0.00	0.01		skin
ENSXMAG00	-0.76	5.86	0.00	0.03		skin
ENSXMAG00	0.80	3.56	0.00	0.05		skin
ENSXMAG00	0.79	5.76	0.00	0.01	lsm12b	skin
ENSXMAG00	0.88	5.94	0.00	0.00	nfe2l3	skin
ENSXMAG00	1.67	2.82	0.00	0.00	aqp11	skin
ENSXMAG00	0.89	2.13	0.00	0.04	PPIG	skin
ENSXMAG00	-0.88	3.28	0.00	0.01	mbd4	skin

ENSXMAG00	-0.65	5.43	0.00	0.02		skin
ENSXMAG00	-2.33	-0.24	0.00	0.00		skin
ENSXMAG00	1.61	-1.14	0.00	0.04	fgf14	skin
ENSXMAG00	4.67	-3.27	0.00	0.04		skin
ENSXMAG00	-1.25	2.05	0.00	0.00		skin
ENSXMAG00	-1.44	3.99	0.00	0.00	parp12a	skin
ENSXMAG00	0.70	2.92	0.00	0.05		skin
ENSXMAG00	0.70	3.65	0.00	0.04	slc30a7	skin
ENSXMAG00	0.90	3.37	0.00	0.00	si:dkey-260j1	skin
ENSXMAG00	1.62	4.53	0.00	0.00	si:ch211-11k	skin
ENSXMAG00	-0.81	5.70	0.00	0.04	txlnbb	skin
ENSXMAG00	-1.23	3.91	0.00	0.00		skin
ENSXMAG00	1.00	6.58	0.00	0.00	SURF4	skin
ENSXMAG00	-0.97	6.19	0.00	0.01		skin
ENSXMAG00	0.62	8.05	0.00	0.03	srcap	skin
ENSXMAG00	-1.36	0.77	0.00	0.00	epb41a	skin
ENSXMAG00	1.80	-1.97	0.00	0.05		skin
ENSXMAG00	0.93	5.61	0.00	0.00	hnrnph1	skin
ENSXMAG00	1.05	5.90	0.00	0.00	mogs	skin
ENSXMAG00	-0.82	3.58	0.00	0.03		skin
ENSXMAG00	-1.65	2.03	0.00	0.00	s1pr4	skin
ENSXMAG00	-2.45	-0.37	0.00	0.03	si:ch211-153	skin
ENSXMAG00	1.07	5.54	0.00	0.00	fpgs	skin
ENSXMAG00	-0.94	7.84	0.00	0.00	pim1	skin
ENSXMAG00	0.92	4.84	0.00	0.01	txn2	skin
ENSXMAG00	-1.13	3.90	0.00	0.01	TNFRSF11B	skin
ENSXMAG00	-0.99	6.01	0.00	0.02	USP53	skin
ENSXMAG00	-0.83	4.16	0.00	0.00	tex264a	skin
ENSXMAG00	-0.78	6.40	0.00	0.00	znf385d	skin
ENSXMAG00	0.68	4.18	0.00	0.05	dcps	skin
ENSXMAG00	1.01	4.06	0.00	0.01	marcksb	skin
ENSXMAG00	1.10	4.65	0.00	0.00	camlg	skin
ENSXMAG00	0.96	0.84	0.00	0.05		skin
ENSXMAG00	0.81	2.97	0.00	0.02	sirt6	skin
ENSXMAG00	-0.73	3.87	0.00	0.03	poglut2	skin
ENSXMAG00	-0.88	2.76	0.00	0.02		skin
ENSXMAG00	-1.23	5.72	0.00	0.04	foxq1a	skin
ENSXMAG00	0.76	6.57	0.00	0.01	papss1	skin
ENSXMAG00	-0.92	5.90	0.00	0.00	apol1	skin
ENSXMAG00	-0.62	4.82	0.00	0.05		skin
ENSXMAG00	-1.35	-0.90	0.00	0.02		skin
ENSXMAG00	-1.26	5.45	0.00	0.00		skin
ENSXMAG00	-0.94	4.51	0.00	0.00	si:ch1073-32	skin

ENSXMAG00	0.70	7.57	0.00	0.01	SRSF6	skin
ENSXMAG00	-1.88	0.57	0.00	0.00		skin
ENSXMAG00	2.41	4.98	0.00	0.00	dhrs3b	skin
ENSXMAG00	0.65	5.00	0.00	0.03	icmt	skin
ENSXMAG00	0.81	3.70	0.00	0.01	pdcd7	skin
ENSXMAG00	0.83	3.13	0.00	0.02	wars2	skin
ENSXMAG00	-5.07	-3.05	0.00	0.01		skin
ENSXMAG00	-1.72	-1.77	0.00	0.03	si:dkeyp-59c1	skin
ENSXMAG00	-0.89	5.78	0.00	0.00	si:ch211-74f1	skin
ENSXMAG00	-0.96	2.34	0.00	0.01		skin
ENSXMAG00	-1.18	3.53	0.00	0.00	ctso	skin
ENSXMAG00	1.56	3.05	0.00	0.01	p3h4	skin
ENSXMAG00	-1.56	1.51	0.00	0.00		skin
ENSXMAG00	-0.77	4.80	0.00	0.04		skin
ENSXMAG00	-0.95	2.97	0.00	0.01		skin
ENSXMAG00	1.20	5.27	0.00	0.00	ahsa1b	skin
ENSXMAG00	1.03	3.12	0.00	0.01	tent5bb	skin
ENSXMAG00	-2.81	4.40	0.00	0.03		skin
ENSXMAG00	1.21	2.63	0.00	0.00	timm8a	skin
ENSXMAG00	-1.80	0.90	0.00	0.00		skin
ENSXMAG00	-0.85	3.48	0.00	0.01	rnf11b	skin
ENSXMAG00	-1.21	0.51	0.00	0.01		skin
ENSXMAG00	0.87	4.49	0.00	0.00	tefm	skin
ENSXMAG00	1.03	3.82	0.00	0.01	si:dkey-122a	skin
ENSXMAG00	1.20	4.32	0.00	0.00	setd7 (1 of m	skin
ENSXMAG00	-1.51	2.97	0.00	0.00		skin
ENSXMAG00	-1.58	0.15	0.00	0.00		skin
ENSXMAG00	-0.90	5.67	0.00	0.00	ZC3H7B (1 of	skin
ENSXMAG00	-1.43	-0.71	0.00	0.03		skin
ENSXMAG00	0.72	7.20	0.00	0.01	nap1l4b	skin
ENSXMAG00	0.72	3.83	0.00	0.03	c1d	skin
ENSXMAG00	0.65	5.12	0.00	0.03	kctd9a	skin
ENSXMAG00	0.65	5.46	0.00	0.01	narf	skin
ENSXMAG00	-0.86	4.51	0.00	0.02	map3k8	skin
ENSXMAG00	0.59	5.89	0.00	0.04	rap2c	skin
ENSXMAG00	-0.84	6.21	0.00	0.00		skin
ENSXMAG00	0.66	5.19	0.00	0.05	pdxdc1	skin
ENSXMAG00	0.84	5.30	0.00	0.01	timm13	skin
ENSXMAG00	-2.05	-1.41	0.00	0.04		skin
ENSXMAG00	-1.12	2.28	0.00	0.03	CDKL5 (1 of r	skin
ENSXMAG00	-1.27	2.49	0.00	0.02		skin
ENSXMAG00	1.67	2.63	0.00	0.00	olfm1b	skin
ENSXMAG00	-1.61	-1.01	0.00	0.03	rab3c	skin

ENSXMAG00	-4.34	0.98	0.00	0.02		skin
ENSXMAG00	-2.65	1.89	0.00	0.00		skin
ENSXMAG00	1.89	-2.01	0.00	0.02	RF00334	skin
ENSXMAG00	-0.63	6.51	0.00	0.03	fuca2	skin
ENSXMAG00	1.01	2.48	0.00	0.03	ccdc58	skin
ENSXMAG00	0.90	5.54	0.00	0.00	gnl3	skin
ENSXMAG00	-0.98	1.37	0.00	0.03		skin
ENSXMAG00	3.39	-0.30	0.00	0.00	nots	skin
ENSXMAG00	-0.70	4.79	0.00	0.02	tspan33a	skin
ENSXMAG00	1.31	2.83	0.00	0.00	si:ch211-231	skin
ENSXMAG00	1.41	-1.05	0.00	0.03		skin
ENSXMAG00	-1.60	1.13	0.00	0.00		skin
ENSXMAG00	1.19	3.45	0.00	0.00	zgc:109986	skin
ENSXMAG00	-0.87	3.15	0.00	0.03		skin
ENSXMAG00	-1.57	0.18	0.00	0.00	tmem151ba	skin
ENSXMAG00	0.63	6.59	0.00	0.03	VPS26B (1 of	skin
ENSXMAG00	-6.25	0.46	0.00	0.01		skin
ENSXMAG00	0.84	4.45	0.00	0.01	dohh	skin
ENSXMAG00	0.88	6.02	0.00	0.00	zgc:153675	skin
ENSXMAG00	-1.70	0.75	0.00	0.00		skin
ENSXMAG00	-1.41	4.79	0.00	0.02		skin
ENSXMAG00	0.83	3.32	0.00	0.04	selenoi	skin
ENSXMAG00	0.61	7.50	0.00	0.04	ppp1caa	skin
ENSXMAG00	0.61	6.08	0.00	0.05	tmed9	skin
ENSXMAG00	-1.34	7.61	0.00	0.00		skin
ENSXMAG00	1.08	4.93	0.00	0.00	KDELR2	skin
ENSXMAG00	1.41	-0.36	0.00	0.01	RF01192	skin
ENSXMAG00	-0.77	4.95	0.00	0.01	ca16b	skin
ENSXMAG00	1.09	5.68	0.00	0.00		skin
ENSXMAG00	-0.83	3.35	0.00	0.01	zgc:162396	skin
ENSXMAG00	1.18	5.70	0.00	0.00	uck2b	skin
ENSXMAG00	-0.82	2.83	0.00	0.03	HOXA4	skin
ENSXMAG00	1.22	0.61	0.00	0.00	rpl12	skin
ENSXMAG00	-1.54	2.04	0.00	0.03		skin
ENSXMAG00	0.98	3.55	0.00	0.00	fbxo45	skin
ENSXMAG00	0.61	5.72	0.00	0.03	necap2	skin
ENSXMAG00	-1.15	3.01	0.00	0.00	si:dkey-78k1	skin
ENSXMAG00	1.84	-0.83	0.00	0.02	mkxa (1 of m	skin
ENSXMAG00	-1.21	1.33	0.00	0.03		skin
ENSXMAG00	0.81	3.97	0.00	0.01	taf5l	skin
ENSXMAG00	-1.93	-0.94	0.00	0.02		skin
ENSXMAG00	-3.03	-2.80	0.00	0.02		skin
ENSXMAG00	-1.10	7.33	0.00	0.00	zfand5a	skin

ENSXMAG00	1.67	-0.39	0.00	0.01		skin
ENSXMAG00	-1.70	-0.90	0.00	0.01	si:dkey-245n	skin
ENSXMAG00	1.05	6.32	0.00	0.00	nifk	skin
ENSXMAG00	-0.73	6.68	0.00	0.02	rin2	skin
ENSXMAG00	0.85	2.21	0.00	0.02	bnip1a	skin
ENSXMAG00	-2.62	1.43	0.00	0.00		skin
ENSXMAG00	1.06	0.91	0.00	0.05		skin
ENSXMAG00	-1.57	0.40	0.00	0.01		skin
ENSXMAG00	-1.51	5.00	0.00	0.00		skin
ENSXMAG00	0.78	5.43	0.00	0.00	chp1	skin
ENSXMAG00	0.86	3.07	0.00	0.01	PSTK	skin
ENSXMAG00	0.86	2.47	0.00	0.03	aqp12	skin
ENSXMAG00	0.91	3.56	0.00	0.02	suox	skin
ENSXMAG00	1.97	4.73	0.00	0.00	eif4e1c	skin
ENSXMAG00	-1.14	0.13	0.00	0.04	zgc:158258	skin
ENSXMAG00	0.69	5.27	0.00	0.01	mettl3	skin
ENSXMAG00	0.93	5.49	0.00	0.00	cdv3	skin
ENSXMAG00	-1.35	4.00	0.00	0.00		skin
ENSXMAG00	-2.77	2.17	0.00	0.00		skin
ENSXMAG00	-1.18	0.93	0.00	0.03		skin
ENSXMAG00	0.98	3.46	0.00	0.00		skin
ENSXMAG00	-1.27	0.38	0.00	0.02		skin
ENSXMAG00	1.13	3.37	0.00	0.00	hmbsa	skin
ENSXMAG00	-2.60	-2.50	0.00	0.02	gpbar1	skin
ENSXMAG00	-2.45	1.06	0.00	0.00		skin
ENSXMAG00	-1.50	4.58	0.00	0.00		skin
ENSXMAG00	-1.15	5.30	0.00	0.00	sp100.1	skin
ENSXMAG00	1.93	-1.28	0.00	0.01		skin
ENSXMAG00	1.10	5.47	0.00	0.00	tecrb	skin
ENSXMAG00	1.04	4.88	0.00	0.00	acp6	skin
ENSXMAG00	-2.57	-2.12	0.00	0.02		skin
ENSXMAG00	0.97	2.06	0.00	0.03		skin
ENSXMAG00	0.85	3.49	0.00	0.01	nepro	skin
ENSXMAG00	0.82	4.47	0.00	0.01	fam207a	skin
ENSXMAG00	0.60	4.26	0.00	0.05		skin
ENSXMAG00	0.98	1.85	0.00	0.01		skin
ENSXMAG00	-1.14	2.13	0.00	0.00	fgf10a	skin
ENSXMAG00	-2.50	3.40	0.00	0.00		skin
ENSXMAG00	-0.76	4.26	0.00	0.01	zgc:110006	skin
ENSXMAG00	1.24	5.41	0.00	0.02	phkg1b	skin
ENSXMAG00	0.69	4.76	0.00	0.02	msra	skin
ENSXMAG00	1.06	3.10	0.00	0.00	ccnyl1	skin
ENSXMAG00	-0.79	2.62	0.00	0.03	rpusd1	skin

ENSXMAG00	-1.93	3.64	0.00	0.00		skin
ENSXMAG00	-2.23	-2.34	0.00	0.04		skin
ENSXMAG00	-1.24	0.37	0.00	0.02		skin
ENSXMAG00	0.61	5.78	0.00	0.04	rab18a	skin
ENSXMAG00	0.77	6.51	0.00	0.02	mybbp1a	skin
ENSXMAG00	0.82	6.37	0.00	0.01		skin
ENSXMAG00	-1.17	2.25	0.00	0.00	CBX2	skin
ENSXMAG00	0.78	2.59	0.00	0.03		skin
ENSXMAG00	-0.74	3.61	0.00	0.05		skin
ENSXMAG00	1.42	4.60	0.00	0.00	hsd11b1la (1	skin
ENSXMAG00	0.90	5.63	0.00	0.00	sec23ip	skin
ENSXMAG00	0.76	7.14	0.00	0.01	akap12b	skin
ENSXMAG00	1.32	1.80	0.00	0.00	ognb	skin
ENSXMAG00	0.73	3.93	0.00	0.02	ubxn2a	skin
ENSXMAG00	-1.35	3.63	0.00	0.00		skin
ENSXMAG00	-1.56	7.28	0.00	0.00	cbx7a	skin
ENSXMAG00	0.91	5.45	0.00	0.00	rps6kb1b	skin
ENSXMAG00	-1.05	4.01	0.00	0.03		skin
ENSXMAG00	0.81	6.16	0.00	0.01	tbca	skin
ENSXMAG00	1.24	-0.43	0.00	0.02	RF00277	skin
ENSXMAG00	0.77	3.91	0.00	0.04		skin
ENSXMAG00	-1.03	2.44	0.00	0.04		skin
ENSXMAG00	-1.45	4.64	0.00	0.00	socs3b	skin
ENSXMAG00	0.78	3.43	0.00	0.03		skin
ENSXMAG00	0.85	2.42	0.00	0.03	adprhl2	skin
ENSXMAG00	1.10	5.32	0.00	0.00	si:dkey-167k	skin
ENSXMAG00	-3.32	-3.08	0.00	0.05		skin
ENSXMAG00	1.57	-1.30	0.00	0.01	cnrip1b	skin
ENSXMAG00	1.01	3.35	0.00	0.01		skin
ENSXMAG00	1.19	2.73	0.00	0.00	sdr42e1	skin
ENSXMAG00	2.73	-0.44	0.00	0.02	lrrtm4l2	skin
ENSXMAG00	-1.74	-0.40	0.00	0.00		skin
ENSXMAG00	1.19	4.54	0.00	0.00	gpatch4	skin
ENSXMAG00	-0.86	2.25	0.00	0.03		skin
ENSXMAG00	0.75	3.07	0.00	0.05	commd7	skin
ENSXMAG00	-2.13	0.34	0.00	0.00		skin
ENSXMAG00	1.21	2.05	0.00	0.02	fuz	skin
ENSXMAG00	1.08	4.52	0.00	0.01	ppic	skin
ENSXMAG00	0.62	4.88	0.00	0.04	gpaa1	skin
ENSXMAG00	-0.96	5.06	0.00	0.00	si:rp71-1g18	skin
ENSXMAG00	-1.97	0.52	0.00	0.00	allc	skin
ENSXMAG00	1.04	1.93	0.00	0.04	MID1	skin
ENSXMAG00	0.67	3.55	0.00	0.04	unc119.1	skin

ENSXMAG00	-0.73	5.79	0.00	0.04	igfbp2a	skin
ENSXMAG00	-1.22	3.38	0.00	0.00		skin
ENSXMAG00	0.70	3.01	0.00	0.05	cnpy2	skin
ENSXMAG00	-1.11	4.92	0.00	0.00	mov10b.1	skin
ENSXMAG00	0.65	4.40	0.00	0.03	mto1	skin
ENSXMAG00	-1.20	0.65	0.00	0.05	si:ch211-197	skin
ENSXMAG00	1.13	5.64	0.00	0.00	slc35e1	skin
ENSXMAG00	0.57	5.22	0.00	0.05	zgc:162634	skin
ENSXMAG00	1.48	4.44	0.00	0.00		skin
ENSXMAG00	0.80	5.79	0.00	0.01	ube2v1	skin
ENSXMAG00	-0.61	4.58	0.00	0.04		skin
ENSXMAG00	0.72	5.98	0.00	0.01	tmx1	skin
ENSXMAG00	-0.97	6.80	0.00	0.04	hivep1	skin
ENSXMAG00	-1.24	-0.36	0.00	0.02	FAM57A	skin
ENSXMAG00	0.94	4.98	0.00	0.00	ssbp1	skin
ENSXMAG00	1.02	2.19	0.00	0.01	nanp	skin
ENSXMAG00	-1.69	0.71	0.00	0.02	noxred1	skin
ENSXMAG00	0.92	2.44	0.00	0.04		skin
ENSXMAG00	1.99	1.50	0.00	0.00	emc9	skin
ENSXMAG00	1.06	5.00	0.00	0.00	rab5if	skin
ENSXMAG00	-1.19	2.51	0.00	0.00		skin
ENSXMAG00	-0.90	1.89	0.00	0.03	si:ch211-63o	skin
ENSXMAG00	0.87	4.95	0.00	0.01	dnajc15	skin
ENSXMAG00	0.72	6.65	0.00	0.01	qsox2	skin
ENSXMAG00	-2.95	-1.72	0.00	0.03		skin
ENSXMAG00	-0.89	4.09	0.00	0.01		skin
ENSXMAG00	-1.18	3.53	0.00	0.01	foxi2	skin
ENSXMAG00	-9.76	0.75	0.00	0.00		skin
ENSXMAG00	0.70	5.30	0.00	0.02		skin
ENSXMAG00	0.67	6.03	0.00	0.03	rsl1d1	skin
ENSXMAG00	0.86	5.59	0.00	0.00	vamp1	skin
ENSXMAG00	-1.40	4.47	0.00	0.00		skin
ENSXMAG00	-0.60	5.61	0.00	0.05	epha7	skin
ENSXMAG00	0.90	8.88	0.00	0.05	pof1b	skin
ENSXMAG00	1.09	2.21	0.00	0.03		skin
ENSXMAG00	-1.42	5.15	0.00	0.01	JDP2 (1 of m	skin
ENSXMAG00	-1.22	3.79	0.00	0.00	gbp	skin
ENSXMAG00	-0.90	6.05	0.00	0.00	badb	skin
ENSXMAG00	1.20	7.34	0.00	0.00	mapre1a	skin
ENSXMAG00	0.77	5.21	0.00	0.01	sec22bb	skin
ENSXMAG00	0.75	2.49	0.00	0.05	MEX3D	skin
ENSXMAG00	-0.97	2.18	0.00	0.04		skin
ENSXMAG00	-0.74	3.99	0.00	0.04	AOPEP	skin

ENSXMAG00	-1.71	0.28	0.00	0.02	dkk1a	skin
ENSXMAG00	-2.56	-2.13	0.00	0.02	slc25a1a	skin
ENSXMAG00	-1.31	1.48	0.00	0.02	zgc:162297	skin
ENSXMAG00	2.32	3.26	0.00	0.00	leap2	skin
ENSXMAG00	-0.78	2.62	0.00	0.04		skin
ENSXMAG00	0.69	7.09	0.00	0.01		skin
ENSXMAG00	-0.87	4.59	0.00	0.04		skin
ENSXMAG00	-0.93	3.71	0.00	0.01	hsdl1	skin
ENSXMAG00	-0.70	4.87	0.00	0.02		skin
ENSXMAG00	-1.77	0.12	0.00	0.00		skin
ENSXMAG00	1.99	0.67	0.00	0.00		skin
ENSXMAG00	-1.85	-1.32	0.00	0.03		skin
ENSXMAG00	-0.74	4.92	0.00	0.03	si:dkey-78k1:	skin
ENSXMAG00	-1.65	2.90	0.00	0.05	si:ch73-361p	skin
ENSXMAG00	-1.54	5.08	0.00	0.00	ccng2	skin
ENSXMAG00	0.84	5.35	0.00	0.00	dnttip2	skin
ENSXMAG00	0.83	4.65	0.00	0.02	tcf19l	skin
ENSXMAG00	0.91	5.21	0.00	0.00		skin
ENSXMAG00	0.73	5.09	0.00	0.00	ccnk	skin
ENSXMAG00	0.83	2.31	0.00	0.05		skin
ENSXMAG00	-1.13	2.96	0.00	0.00	zgc:85936	skin
ENSXMAG00	1.47	3.22	0.00	0.00	qtrt1	skin
ENSXMAG00	0.85	4.51	0.00	0.01	si:dkey-12j5.	skin
ENSXMAG00	0.66	4.35	0.00	0.03	hs2st1b	skin
ENSXMAG00	-1.24	0.33	0.00	0.02	slc10a2	skin
ENSXMAG00	-1.01	4.05	0.00	0.00	rnf44	skin
ENSXMAG00	0.70	6.72	0.00	0.01		skin
ENSXMAG00	-1.34	6.64	0.00	0.00	arrdc2	skin
ENSXMAG00	0.78	6.12	0.00	0.01		skin
ENSXMAG00	-1.11	4.92	0.00	0.00	atn1	skin
ENSXMAG00	1.07	8.58	0.00	0.01	calr3b	skin
ENSXMAG00	-0.64	4.03	0.00	0.03	manbal	skin
ENSXMAG00	1.09	0.39	0.00	0.04	RF00281	skin
ENSXMAG00	0.94	5.63	0.00	0.00	erp44	skin
ENSXMAG00	-0.75	4.65	0.00	0.01	acadl	skin
ENSXMAG00	-1.07	8.61	0.00	0.01	mknk2b	skin
ENSXMAG00	-2.55	2.35	0.00	0.00		skin
ENSXMAG00	-1.24	2.37	0.00	0.01		skin
ENSXMAG00	0.98	3.68	0.00	0.00		skin
ENSXMAG00	1.13	6.06	0.00	0.00	usp10	skin
ENSXMAG00	-1.42	6.12	0.00	0.00		skin
ENSXMAG00	-1.76	3.54	0.00	0.04	ikzf4	skin
ENSXMAG00	-0.91	4.08	0.00	0.01		skin

ENSXMAG00	1.37	0.66	0.00	0.00		skin
ENSXMAG00	1.36	7.91	0.00	0.00		skin
ENSXMAG00	-0.72	5.36	0.00	0.02	TBC1D2	skin
ENSXMAG00	-1.34	-0.80	0.00	0.04	gja3	skin
ENSXMAG00	1.11	2.16	0.00	0.01		skin
ENSXMAG00	0.60	5.33	0.00	0.03	pgap3	skin
ENSXMAG00	0.83	3.88	0.00	0.01	xgb	skin
ENSXMAG00	-1.08	5.78	0.00	0.00	zgc:175214	skin
ENSXMAG00	-1.84	-1.34	0.00	0.01		skin
ENSXMAG00	2.06	-0.38	0.00	0.01		skin
ENSXMAG00	-0.66	3.99	0.00	0.05	sgip1a	skin
ENSXMAG00	-0.94	3.93	0.00	0.00		skin
ENSXMAG00	-1.23	2.39	0.00	0.00	inpp4b	skin
ENSXMAG00	-0.82	4.77	0.00	0.05		skin
ENSXMAG00	0.96	3.70	0.00	0.00	ccdc173	skin
ENSXMAG00	0.84	2.51	0.00	0.03	rpp21	skin
ENSXMAG00	1.08	5.41	0.00	0.00	tomm22	skin
ENSXMAG00	-1.30	2.27	0.00	0.00		skin
ENSXMAG00	1.96	-0.42	0.00	0.05	tbx4	skin
ENSXMAG00	-0.92	4.55	0.00	0.00		skin
ENSXMAG00	1.00	2.01	0.00	0.01		skin
ENSXMAG00	-0.74	4.63	0.00	0.01	idua	skin
ENSXMAG00	-2.32	4.06	0.00	0.02		skin
ENSXMAG00	-0.72	4.89	0.00	0.01	si:dkey-218f9	skin
ENSXMAG00	-1.64	-1.19	0.00	0.05		skin
ENSXMAG00	-0.83	4.45	0.00	0.01	ptprub	skin
ENSXMAG00	0.60	5.30	0.00	0.05	znf346	skin
ENSXMAG00	0.85	4.71	0.00	0.01		skin
ENSXMAG00	-0.73	3.63	0.00	0.03	rab40b	skin
ENSXMAG00	-1.51	1.15	0.00	0.00	ccbe1	skin
ENSXMAG00	1.42	6.39	0.00	0.00	sumo3a	skin
ENSXMAG00	-1.05	5.10	0.00	0.00	rnf13	skin
ENSXMAG00	1.23	6.33	0.00	0.00	tram1	skin
ENSXMAG00	-4.50	-3.33	0.00	0.04		skin
ENSXMAG00	2.06	-2.12	0.00	0.03		skin
ENSXMAG00	1.39	5.70	0.00	0.00	usp14	skin
ENSXMAG00	-1.51	0.08	0.00	0.01		skin
ENSXMAG00	-0.65	3.85	0.00	0.04	borcs5	skin
ENSXMAG00	-1.31	1.57	0.00	0.01		skin
ENSXMAG00	-1.29	7.12	0.00	0.01		skin
ENSXMAG00	-1.51	5.58	0.00	0.00	tp53inp1	skin
ENSXMAG00	-0.98	5.50	0.00	0.00		skin
ENSXMAG00	-1.22	1.44	0.00	0.00		skin

ENSXMAG00	0.72	5.74	0.00	0.04		skin
ENSXMAG00	-0.85	2.38	0.00	0.03		skin
ENSXMAG00	-0.69	6.50	0.00	0.03	mtus1b	skin
ENSXMAG00	-1.02	5.35	0.00	0.01	klf15	skin
ENSXMAG00	-1.33	3.73	0.00	0.00		skin
ENSXMAG00	-0.77	6.16	0.00	0.01	arid5b	skin
ENSXMAG00	-4.94	1.19	0.00	0.01		skin
ENSXMAG00	-0.76	6.24	0.00	0.02	znf532	skin
ENSXMAG00	-1.19	0.45	0.00	0.03	gdnfb	skin
ENSXMAG00	1.03	5.94	0.00	0.00	nolc1	skin
ENSXMAG00	-1.65	3.74	0.00	0.03		skin
ENSXMAG00	-1.09	4.75	0.00	0.02		skin
ENSXMAG00	1.36	-0.37	0.00	0.02	RF00276	skin
ENSXMAG00	-1.36	1.23	0.00	0.01		skin
ENSXMAG00	-0.94	2.60	0.00	0.01	lrch1	skin
ENSXMAG00	-1.03	1.32	0.00	0.04	tbc1d32	skin
ENSXMAG00	-0.89	5.44	0.00	0.01	zgc:100868	skin
ENSXMAG00	-0.70	4.26	0.00	0.05	btbd6b	skin
ENSXMAG00	0.70	3.41	0.00	0.05	h2afx1	skin
ENSXMAG00	-0.99	3.06	0.00	0.00	fam110c	skin
ENSXMAG00	1.05	3.14	0.00	0.00	mri1	skin
ENSXMAG00	-0.86	5.54	0.00	0.00		skin
ENSXMAG00	-0.93	4.15	0.00	0.00	crbn	skin
ENSXMAG00	-1.22	1.78	0.00	0.02		skin
ENSXMAG00	1.30	3.90	0.00	0.01	atp1b1b	skin
ENSXMAG00	-0.71	5.01	0.00	0.01	sftpbb	skin
ENSXMAG00	-1.03	4.62	0.00	0.00		skin
ENSXMAG00	-0.68	4.55	0.00	0.02	gsdmeb	skin
ENSXMAG00	-0.89	3.93	0.00	0.00	tspan4a	skin
ENSXMAG00	1.04	3.71	0.00	0.00	elovl6	skin
ENSXMAG00	0.86	6.99	0.00	0.00	gmfb	skin
ENSXMAG00	1.14	4.50	0.00	0.02	mthfd1l	skin
ENSXMAG00	-4.65	-1.69	0.00	0.04		skin
ENSXMAG00	-3.04	0.65	0.00	0.00		skin
ENSXMAG00	-3.27	3.17	0.00	0.00		skin
ENSXMAG00	0.71	4.99	0.00	0.01	ncdn	skin
ENSXMAG00	0.84	3.99	0.00	0.01	tmem106c	skin
ENSXMAG00	0.87	3.50	0.00	0.01		skin
ENSXMAG00	-1.39	1.44	0.00	0.02		skin
ENSXMAG00	-0.86	2.69	0.00	0.02	ptger1c	skin
ENSXMAG00	-1.11	3.26	0.00	0.01	MAFG (1 of	skin
ENSXMAG00	1.69	3.40	0.00	0.00		skin
ENSXMAG00	-2.01	5.75	0.00	0.01		skin

ENSXMAG00	-0.94	2.28	0.00	0.02		skin
ENSXMAG00	1.07	4.90	0.00	0.00	etnk2	skin
ENSXMAG00	-1.89	2.28	0.00	0.00	agxt2	skin
ENSXMAG00	0.72	5.04	0.00	0.01	kpna1	skin
ENSXMAG00	1.01	3.72	0.00	0.00	endog	skin
ENSXMAG00	1.28	5.06	0.00	0.00	psme3	skin
ENSXMAG00	-2.37	2.34	0.00	0.00	ch25h	skin
ENSXMAG00	0.64	6.45	0.00	0.04	nmt1a	skin
ENSXMAG00	0.76	3.46	0.00	0.05	smx5	skin
ENSXMAG00	-0.98	2.58	0.00	0.01		skin
ENSXMAG00	-1.62	1.44	0.00	0.00	SLC25A45	skin
ENSXMAG00	-0.86	5.57	0.00	0.02	chka	skin
ENSXMAG00	-0.71	4.62	0.00	0.02	zgc:91944	skin
ENSXMAG00	-2.65	0.30	0.00	0.00	il19l	skin
ENSXMAG00	0.90	3.44	0.00	0.01		skin
ENSXMAG00	-0.89	4.51	0.00	0.00		skin
ENSXMAG00	-0.70	4.78	0.00	0.01	snapin	skin
ENSXMAG00	-0.92	5.73	0.00	0.03		skin
ENSXMAG00	-1.90	-2.26	0.00	0.04		skin
ENSXMAG00	-1.10	2.45	0.00	0.04	ect2	skin
ENSXMAG00	0.64	6.04	0.00	0.04	tomm20b	skin
ENSXMAG00	0.85	3.38	0.00	0.01	dnajb9a	skin
ENSXMAG00	0.70	3.82	0.00	0.03	sirt4	skin
ENSXMAG00	-0.89	2.51	0.00	0.03		skin
ENSXMAG00	-3.77	-1.04	0.00	0.05		skin
ENSXMAG00	-0.98	4.21	0.00	0.05	socs2	skin
ENSXMAG00	-1.95	-0.49	0.00	0.00		skin
ENSXMAG00	0.97	5.19	0.00	0.00	krtcap2	skin
ENSXMAG00	0.92	2.43	0.00	0.01	si:ch73-42p1	skin
ENSXMAG00	-0.78	3.45	0.00	0.01		skin
ENSXMAG00	-1.35	0.67	0.00	0.01	si:ch211-13f8	skin
ENSXMAG00	0.96	6.24	0.00	0.00	srpra	skin
ENSXMAG00	-0.99	4.97	0.00	0.00	ctdspla	skin
ENSXMAG00	0.69	5.60	0.00	0.02	abhd16a	skin
ENSXMAG00	0.96	5.26	0.00	0.00	etnppl	skin
ENSXMAG00	-2.15	9.34	0.00	0.00	sesn1	skin
ENSXMAG00	-0.86	2.25	0.00	0.05	elob	skin
ENSXMAG00	0.78	5.20	0.00	0.02		skin
ENSXMAG00	1.21	0.62	0.00	0.05	megf8	skin
ENSXMAG00	0.89	5.95	0.00	0.04	gpt2l	skin
ENSXMAG00	-0.83	3.71	0.00	0.04	bach2b	skin
ENSXMAG00	1.35	4.69	0.00	0.00	setd3	skin
ENSXMAG00	-0.94	2.73	0.00	0.05		skin

ENSXMAG00	1.14	5.66	0.00	0.00	gde1	skin
ENSXMAG00	-1.60	-1.84	0.00	0.04		skin
ENSXMAG00	-1.24	5.73	0.00	0.00	map1lc3b	skin
ENSXMAG00	0.85	5.71	0.00	0.00	ppan	skin
ENSXMAG00	-3.78	-1.11	0.00	0.03		skin
ENSXMAG00	-1.15	0.85	0.00	0.01	A3GALT2	skin
ENSXMAG00	-2.15	5.34	0.00	0.00	nfat5b	skin
ENSXMAG00	-1.63	1.60	0.00	0.01		skin
ENSXMAG00	0.62	5.32	0.00	0.02	UBE2K (1 of	skin
ENSXMAG00	1.26	-0.45	0.00	0.04	RF00571	skin
ENSXMAG00	-0.96	1.61	0.00	0.03	cdkn2c	skin
ENSXMAG00	-0.89	3.62	0.00	0.01	jade2	skin
ENSXMAG00	-1.03	3.22	0.00	0.00	glyctk	skin
ENSXMAG00	1.08	4.25	0.00	0.00	eif1ad	skin
ENSXMAG00	0.74	3.99	0.00	0.01	srd5a1	skin
ENSXMAG00	-1.21	3.28	0.00	0.03		skin
ENSXMAG00	-0.93	6.77	0.00	0.01	jun	skin
ENSXMAG00	0.77	5.88	0.00	0.02	zgc:110843	skin
ENSXMAG00	-0.67	3.97	0.00	0.04	si:dkeyp-74a	skin
ENSXMAG00	-1.26	3.22	0.00	0.00		skin
ENSXMAG00	-1.29	0.08	0.00	0.02	irg1l (1 of ma	skin
ENSXMAG00	1.04	4.30	0.00	0.00	rnf175	skin
ENSXMAG00	0.92	5.50	0.00	0.00	strap	skin
ENSXMAG00	-0.77	5.23	0.00	0.01	stk11ip	skin
ENSXMAG00	0.60	5.30	0.00	0.05	sf3a2	skin
ENSXMAG00	0.62	5.18	0.00	0.04		skin
ENSXMAG00	-2.52	-2.02	0.00	0.02		skin
ENSXMAG00	-0.59	5.09	0.00	0.05	ostm1	skin
ENSXMAG00	-1.43	3.39	0.00	0.00		skin
ENSXMAG00	1.13	7.56	0.00	0.00	HNRNPA0 (1	skin
ENSXMAG00	-1.31	3.85	0.00	0.01		skin
ENSXMAG00	-0.93	3.80	0.00	0.00	wdr92	skin
ENSXMAG00	-0.78	2.66	0.00	0.04	znf512	skin
ENSXMAG00	-5.10	-3.04	0.00	0.02		skin
ENSXMAG00	-0.78	3.65	0.00	0.04		skin
ENSXMAG00	-1.18	1.44	0.00	0.00		skin
ENSXMAG00	0.95	5.56	0.00	0.02	sc5d	skin
ENSXMAG00	1.33	5.67	0.00	0.03	smyhc1	skin
ENSXMAG00	1.51	5.21	0.00	0.00	pgp	skin
ENSXMAG00	-1.59	4.54	0.00	0.00		skin
ENSXMAG00	0.81	4.44	0.00	0.00		skin
ENSXMAG00	1.53	3.65	0.00	0.00	ppil1	skin
ENSXMAG00	-1.38	2.68	0.00	0.00		skin

ENSXMAG00	-0.98	5.03	0.00	0.01	tspan15	skin
ENSXMAG00	-2.15	5.24	0.00	0.00	pcmtd1	skin
ENSXMAG00	1.04	0.58	0.00	0.03	RF00276	skin
ENSXMAG00	-0.75	5.28	0.00	0.01	st6gal2a	skin
ENSXMAG00	0.97	4.63	0.00	0.00	srp9	skin
ENSXMAG00	-1.78	4.60	0.00	0.00	si:ch211-117	skin
ENSXMAG00	-3.11	5.38	0.00	0.00	noxo1b	skin
ENSXMAG00	1.30	0.91	0.00	0.04	si:dkey-121b	skin
ENSXMAG00	-0.91	1.51	0.00	0.04		skin
ENSXMAG00	-0.88	1.70	0.00	0.04	ccdc15	skin
ENSXMAG00	1.34	1.39	0.00	0.00	timm10b	skin
ENSXMAG00	-0.65	4.16	0.00	0.04	atraid	skin
ENSXMAG00	-3.00	2.98	0.00	0.01		skin
ENSXMAG00	-0.80	4.80	0.00	0.01		skin
ENSXMAG00	-1.71	0.24	0.00	0.01		skin
ENSXMAG00	-1.26	7.29	0.00	0.00	dap1b	skin
ENSXMAG00	0.95	4.65	0.00	0.00	ctnnbip1	skin
ENSXMAG00	0.89	4.32	0.00	0.00	vmp1	skin
ENSXMAG00	1.36	1.72	0.00	0.00		skin
ENSXMAG00	0.91	2.63	0.00	0.01	dlx6a	skin
ENSXMAG00	-0.83	3.38	0.00	0.01	tmem192	skin
ENSXMAG00	-0.94	1.86	0.00	0.02	foxp2	skin
ENSXMAG00	-0.68	5.57	0.00	0.01	cep162	skin
ENSXMAG00	-1.58	3.81	0.00	0.00		skin
ENSXMAG00	1.27	1.96	0.00	0.00		skin
ENSXMAG00	-0.73	6.17	0.00	0.01		skin
ENSXMAG00	0.80	5.37	0.00	0.00	prrc1	skin
ENSXMAG00	-0.79	5.76	0.00	0.03	zgc:114045	skin
ENSXMAG00	-1.17	7.13	0.00	0.00	eif4ebp3l	skin
ENSXMAG00	-0.99	1.36	0.00	0.04		skin
ENSXMAG00	-0.85	4.75	0.00	0.01	zgc:153184	skin
ENSXMAG00	1.09	1.17	0.00	0.04	frrs1l	skin
ENSXMAG00	-0.89	3.68	0.00	0.03	spry4	skin
ENSXMAG00	-0.96	5.62	0.00	0.01		skin
ENSXMAG00	-1.19	4.19	0.00	0.04		skin
ENSXMAG00	1.03	5.05	0.00	0.02		skin
ENSXMAG00	-2.57	2.54	0.00	0.00		skin
ENSXMAG00	0.67	5.58	0.00	0.01	impad1	skin
ENSXMAG00	0.81	3.78	0.00	0.01	pitpnb (1 of r	skin
ENSXMAG00	-0.91	3.33	0.00	0.01		skin
ENSXMAG00	-0.82	6.99	0.00	0.01	fbxo33	skin
ENSXMAG00	0.77	3.91	0.00	0.00	si:dkey-93h2	brain

ENSXMAG00	0.48	4.61	0.00	0.00	gfm1	brain
ENSXMAG00	0.56	4.78	0.00	0.00	prmt5	brain
ENSXMAG00	0.33	5.81	0.00	0.03		brain
ENSXMAG00	-0.58	5.79	0.00	0.00	cyp26b1	brain
ENSXMAG00	-0.82	6.52	0.00	0.00		brain
ENSXMAG00	0.42	5.65	0.00	0.03		brain
ENSXMAG00	0.65	3.67	0.00	0.00	rangap1b	brain
ENSXMAG00	-0.63	5.16	0.00	0.00	znf395a	brain
ENSXMAG00	0.57	5.57	0.00	0.00		brain
ENSXMAG00	-0.73	2.38	0.00	0.02	sagb	brain
ENSXMAG00	0.94	2.83	0.00	0.00		brain
ENSXMAG00	0.79	1.57	0.00	0.01	cdr2a	brain
ENSXMAG00	1.03	3.53	0.00	0.00	blvra	brain
ENSXMAG00	0.58	4.48	0.00	0.02	si:dkey-201i6	brain
ENSXMAG00	0.36	5.26	0.00	0.03	tox4b	brain
ENSXMAG00	-1.04	1.01	0.00	0.04	aldh1a3	brain
ENSXMAG00	0.52	4.32	0.00	0.01	prkd1	brain
ENSXMAG00	0.50	6.55	0.00	0.01	prkcda	brain
ENSXMAG00	0.58	3.62	0.00	0.01		brain
ENSXMAG00	0.72	6.30	0.00	0.01	abcf2a	brain
ENSXMAG00	0.66	3.32	0.00	0.00	tmem177	brain
ENSXMAG00	0.39	4.85	0.00	0.04	e2f4	brain
ENSXMAG00	0.44	3.81	0.00	0.04	zgc:91818	brain
ENSXMAG00	-0.55	3.16	0.00	0.04	arl6ip5a	brain
ENSXMAG00	1.54	1.50	0.00	0.00	SHC4	brain
ENSXMAG00	-0.59	3.32	0.00	0.01	zgc:77938	brain
ENSXMAG00	-0.89	6.31	0.00	0.00		brain
ENSXMAG00	0.53	9.40	0.00	0.00	abat	brain
ENSXMAG00	-1.23	2.46	0.00	0.00		brain
ENSXMAG00	0.72	3.94	0.00	0.00	zgc:172302	brain
ENSXMAG00	0.59	6.64	0.00	0.01		brain
ENSXMAG00	0.67	4.49	0.00	0.00	slc25a55a	brain
ENSXMAG00	0.73	4.80	0.00	0.00	SCD	brain
ENSXMAG00	-0.95	0.42	0.00	0.04	clic5b	brain
ENSXMAG00	-0.44	4.53	0.00	0.02	CTSZ	brain
ENSXMAG00	-2.09	-1.35	0.00	0.01		brain
ENSXMAG00	0.41	6.51	0.00	0.01	si:ch1073-55	brain
ENSXMAG00	-0.36	6.12	0.00	0.03	trmt1l	brain
ENSXMAG00	0.49	4.65	0.00	0.05	RFX7 (1 of m	brain
ENSXMAG00	0.42	4.62	0.00	0.02	rai14	brain
ENSXMAG00	1.64	-1.48	0.00	0.03	agxta	brain
ENSXMAG00	0.81	3.11	0.00	0.03		brain
ENSXMAG00	0.39	5.81	0.00	0.03	ABHD1	brain

ENSXMAG00	-0.46	3.95	0.00	0.03	DDA1 (1 of n	brain
ENSXMAG00	0.47	3.74	0.00	0.02		brain
ENSXMAG00	-1.29	4.46	0.00	0.00	fosab	brain
ENSXMAG00	0.43	8.69	0.00	0.04	citb	brain
ENSXMAG00	-0.63	2.94	0.00	0.00	si:dkey-185e	brain
ENSXMAG00	1.34	0.68	0.00	0.00	slc13a5b	brain
ENSXMAG00	-0.54	3.66	0.00	0.00	dpp7	brain
ENSXMAG00	0.55	4.53	0.00	0.00	zbtb7c	brain
ENSXMAG00	-0.57	4.39	0.00	0.00	relb	brain
ENSXMAG00	0.40	5.40	0.00	0.01	plpbp	brain
ENSXMAG00	-0.81	2.72	0.00	0.01		brain
ENSXMAG00	-0.46	5.37	0.00	0.01	pdcd11	brain
ENSXMAG00	0.32	6.00	0.00	0.04		brain
ENSXMAG00	0.53	4.42	0.00	0.00	pigo	brain
ENSXMAG00	0.52	4.91	0.00	0.00	rgl3a	brain
ENSXMAG00	0.47	4.69	0.00	0.01	dnajc10	brain
ENSXMAG00	-0.57	2.55	0.00	0.02	zgc:85789	brain
ENSXMAG00	-0.96	5.39	0.00	0.00	aspa	brain
ENSXMAG00	0.51	5.30	0.00	0.05	cthl	brain
ENSXMAG00	0.49	5.86	0.00	0.00	pde10a	brain
ENSXMAG00	0.52	3.68	0.00	0.01	chrna6	brain
ENSXMAG00	-0.60	4.35	0.00	0.03	ugp2a	brain
ENSXMAG00	0.43	5.09	0.00	0.01	abhd3	brain
ENSXMAG00	0.41	5.47	0.00	0.02	slc38a4	brain
ENSXMAG00	-0.45	4.49	0.00	0.03	utp4	brain
ENSXMAG00	-1.76	0.16	0.00	0.00	tbx19	brain
ENSXMAG00	0.45	5.15	0.00	0.00		brain
ENSXMAG00	0.45	4.54	0.00	0.05	TRIM67	brain
ENSXMAG00	0.46	5.45	0.00	0.00	ttc13	brain
ENSXMAG00	0.41	5.73	0.00	0.00	slc25a38b	brain
ENSXMAG00	0.48	5.00	0.00	0.00		brain
ENSXMAG00	1.53	0.55	0.00	0.00	slc6a11a	brain
ENSXMAG00	0.43	8.38	0.00	0.01		brain
ENSXMAG00	0.39	6.53	0.00	0.01	acsl4a	brain
ENSXMAG00	-0.71	3.23	0.00	0.00	ccn2a	brain
ENSXMAG00	-0.37	4.59	0.00	0.03	brca2	brain
ENSXMAG00	0.92	3.09	0.00	0.00	them4	brain
ENSXMAG00	-0.59	5.97	0.00	0.00	SLC16A6 (1 c	brain
ENSXMAG00	-0.50	3.87	0.00	0.03	urb1	brain
ENSXMAG00	0.43	7.24	0.00	0.01	faxca	brain
ENSXMAG00	1.74	4.18	0.00	0.00	nt5c2l1	brain
ENSXMAG00	-0.49	4.29	0.00	0.01	rbm19	brain
ENSXMAG00	-0.99	5.39	0.00	0.00	KLF15	brain

ENSXMAG00	0.52	4.39	0.00	0.00	crispld2	brain
ENSXMAG00	0.54	4.21	0.00	0.01		brain
ENSXMAG00	-0.38	4.77	0.00	0.03	pwp2h	brain
ENSXMAG00	-0.55	6.95	0.00	0.00	cbsa	brain
ENSXMAG00	-0.35	5.97	0.00	0.02		brain
ENSXMAG00	0.42	4.81	0.00	0.02	ddx47	brain
ENSXMAG00	-0.96	5.62	0.00	0.00		brain
ENSXMAG00	0.33	6.42	0.00	0.05	zgc:158659	brain
ENSXMAG00	-0.39	6.08	0.00	0.01	4-Sep	brain
ENSXMAG00	0.54	3.09	0.00	0.05	adamts9	brain
ENSXMAG00	-0.88	5.80	0.00	0.00	sinhcaf	brain
ENSXMAG00	-2.10	-1.95	0.00	0.02	hsd17b1	brain
ENSXMAG00	-0.62	3.80	0.00	0.03	ramp2	brain
ENSXMAG00	-0.45	5.56	0.00	0.00	atg4db	brain
ENSXMAG00	1.42	6.11	0.00	0.00	cry3a	brain
ENSXMAG00	0.80	4.33	0.00	0.00	cry-dash	brain
ENSXMAG00	0.73	1.97	0.00	0.03	lrrc18b	brain
ENSXMAG00	-0.61	2.41	0.00	0.02		brain
ENSXMAG00	-0.65	2.87	0.00	0.00	smad1	brain
ENSXMAG00	0.35	6.42	0.00	0.04	mboat2b	brain
ENSXMAG00	0.35	8.89	0.00	0.03	TMEM63B	brain
ENSXMAG00	0.34	5.47	0.00	0.04	cdk8	brain
ENSXMAG00	-1.32	1.80	0.00	0.00		brain
ENSXMAG00	0.55	3.51	0.00	0.00	snapc3	brain
ENSXMAG00	0.38	6.34	0.00	0.05		brain
ENSXMAG00	0.77	0.97	0.00	0.04		brain
ENSXMAG00	-1.01	3.06	0.00	0.03	si:dkey-79d1	brain
ENSXMAG00	0.54	3.94	0.00	0.00	heatr6	brain
ENSXMAG00	0.84	5.06	0.00	0.00	cyp46a1.4	brain
ENSXMAG00	0.37	6.39	0.00	0.01	baz1b	brain
ENSXMAG00	0.79	4.41	0.00	0.00	tfr1a	brain
ENSXMAG00	0.46	5.81	0.00	0.00	si:ch211-176	brain
ENSXMAG00	-1.86	-1.33	0.00	0.02		brain
ENSXMAG00	0.43	6.14	0.00	0.02	RASGRP2	brain
ENSXMAG00	1.16	3.87	0.00	0.00	kel	brain
ENSXMAG00	0.77	1.75	0.00	0.02		brain
ENSXMAG00	0.36	5.10	0.00	0.03	stk39	brain
ENSXMAG00	0.39	5.01	0.00	0.01	fhod1	brain
ENSXMAG00	5.36	-0.97	0.00	0.03		brain
ENSXMAG00	1.01	3.49	0.00	0.00	p4ha2	brain
ENSXMAG00	-0.54	2.71	0.00	0.03	stk36	brain
ENSXMAG00	-0.72	5.28	0.00	0.00	atf5a	brain
ENSXMAG00	0.65	5.45	0.00	0.00	crtac1a	brain

ENSXMAG00	0.43	4.32	0.00	0.02	brpf3b	brain
ENSXMAG00	-1.31	-0.37	0.00	0.03	pde6ga	brain
ENSXMAG00	-0.62	2.84	0.00	0.00		brain
ENSXMAG00	-0.39	4.49	0.00	0.02	sgsh	brain
ENSXMAG00	0.58	2.76	0.00	0.04		brain
ENSXMAG00	0.60	4.22	0.00	0.00	dio1	brain
ENSXMAG00	-1.10	4.55	0.00	0.03	si:ch211-153	brain
ENSXMAG00	0.58	5.14	0.00	0.01	gdpd2	brain
ENSXMAG00	-0.37	4.59	0.00	0.04	shdb	brain
ENSXMAG00	-0.50	3.63	0.00	0.04	lmna	brain
ENSXMAG00	-1.86	3.64	0.00	0.00		brain
ENSXMAG00	-0.64	1.73	0.00	0.05	grb10a	brain
ENSXMAG00	0.51	4.33	0.00	0.03	pnpo	brain
ENSXMAG00	0.88	5.97	0.00	0.00		brain
ENSXMAG00	-1.15	2.62	0.00	0.00	ier2a	brain
ENSXMAG00	0.52	5.28	0.00	0.00	abcf3	brain
ENSXMAG00	0.47	4.61	0.00	0.02	si:ch211-106	brain
ENSXMAG00	0.39	4.37	0.00	0.02	HEY1 (1 of m	brain
ENSXMAG00	-0.44	6.79	0.00	0.00		brain
ENSXMAG00	-0.79	1.08	0.00	0.02	arhgap19	brain
ENSXMAG00	-0.48	3.29	0.00	0.05	hsd17b8	brain
ENSXMAG00	-1.18	0.49	0.00	0.01	psmb13a	brain
ENSXMAG00	0.65	2.93	0.00	0.01		brain
ENSXMAG00	-0.65	4.40	0.00	0.01		brain
ENSXMAG00	-0.45	4.91	0.00	0.03	lgals9l3	brain
ENSXMAG00	0.46	4.86	0.00	0.03	slc23a2	brain
ENSXMAG00	0.45	5.72	0.00	0.02	cox17	brain
ENSXMAG00	-1.39	1.88	0.00	0.02	dusp2	brain
ENSXMAG00	-0.66	3.75	0.00	0.02	klhl24b	brain
ENSXMAG00	-0.63	7.06	0.00	0.00	irs2a	brain
ENSXMAG00	-0.42	4.23	0.00	0.03	slc5a6b	brain
ENSXMAG00	-0.40	4.72	0.00	0.05	smc6	brain
ENSXMAG00	0.35	5.43	0.00	0.02		brain
ENSXMAG00	0.44	4.27	0.00	0.04	smarcad1a	brain
ENSXMAG00	0.36	6.71	0.00	0.03	DGKQ	brain
ENSXMAG00	-0.41	5.26	0.00	0.02	rnf114	brain
ENSXMAG00	-0.73	2.80	0.00	0.01	bcl6b	brain
ENSXMAG00	-0.50	4.79	0.00	0.00	pfkla	brain
ENSXMAG00	0.92	3.16	0.00	0.00	zgc:110366	brain
ENSXMAG00	1.56	4.56	0.00	0.00	LONRF3	brain
ENSXMAG00	-0.51	4.67	0.00	0.00	ldhd	brain
ENSXMAG00	0.59	3.40	0.00	0.02		brain
ENSXMAG00	1.40	-0.45	0.00	0.01		brain

ENSXMAG00	0.45	5.07	0.00	0.00	uba2	brain
ENSXMAG00	0.51	7.00	0.00	0.00	eif4g1a	brain
ENSXMAG00	0.38	5.01	0.00	0.02	mctp1b	brain
ENSXMAG00	0.42	5.81	0.00	0.01	mapkapk5	brain
ENSXMAG00	0.80	3.39	0.00	0.01	chac2	brain
ENSXMAG00	0.50	4.94	0.00	0.00	heg1	brain
ENSXMAG00	-0.46	5.07	0.00	0.01	mfsd2ab	brain
ENSXMAG00	-0.59	5.20	0.00	0.00	mcm7	brain
ENSXMAG00	0.46	4.09	0.00	0.02	dhx30	brain
ENSXMAG00	0.48	6.21	0.00	0.02	gpc2	brain
ENSXMAG00	0.45	4.24	0.00	0.04	SLCO1C1	brain
ENSXMAG00	-0.50	4.16	0.00	0.01	prkag2b	brain
ENSXMAG00	-0.44	5.63	0.00	0.01		brain
ENSXMAG00	1.55	-1.06	0.00	0.02	btr12	brain
ENSXMAG00	0.44	7.08	0.00	0.00	RASGRF2 (1	brain
ENSXMAG00	-1.13	1.07	0.00	0.01	FGD6 (1 of m	brain
ENSXMAG00	1.19	2.30	0.00	0.00	myom1a	brain
ENSXMAG00	0.47	5.90	0.00	0.02	slc25a25b	brain
ENSXMAG00	0.44	7.93	0.00	0.01	adcy1b	brain
ENSXMAG00	-0.62	4.10	0.00	0.00		brain
ENSXMAG00	0.64	6.17	0.00	0.02	chchd3b	brain
ENSXMAG00	0.41	5.06	0.00	0.02	si:ch211-277	brain
ENSXMAG00	1.00	1.53	0.00	0.00	wdr76	brain
ENSXMAG00	0.98	3.64	0.00	0.01	cry5	brain
ENSXMAG00	0.63	4.81	0.00	0.00	SHC3	brain
ENSXMAG00	-0.47	3.42	0.00	0.03	itfg2	brain
ENSXMAG00	0.76	4.28	0.00	0.00	pah	brain
ENSXMAG00	1.61	-1.20	0.00	0.04		brain
ENSXMAG00	0.90	4.37	0.00	0.00	si:dkey-5g14.	brain
ENSXMAG00	-0.47	6.28	0.00	0.00		brain
ENSXMAG00	0.71	5.61	0.00	0.00	kctd3	brain
ENSXMAG00	0.36	6.43	0.00	0.02	gpam	brain
ENSXMAG00	1.00	1.31	0.00	0.01		brain
ENSXMAG00	0.36	5.30	0.00	0.03	ppp1r14aa	brain
ENSXMAG00	-0.46	4.64	0.00	0.01	nccrp1	brain
ENSXMAG00	0.59	7.43	0.00	0.01	si:dkey-237i9	brain
ENSXMAG00	-2.07	-1.53	0.00	0.02		brain
ENSXMAG00	0.45	8.30	0.00	0.00	RIMS1 (1 of	brain
ENSXMAG00	-4.16	-0.43	0.00	0.03		brain
ENSXMAG00	-0.55	4.30	0.00	0.02	apaf1	brain
ENSXMAG00	-1.18	5.74	0.00	0.00	tcp11l2	brain
ENSXMAG00	-1.22	7.96	0.00	0.00	nr1d2a	brain
ENSXMAG00	-0.50	3.49	0.00	0.03	ARSB	brain

ENSXMAG00	0.45	6.23	0.00	0.01	bcl7a	brain
ENSXMAG00	0.40	5.73	0.00	0.02	g3bp1	brain
ENSXMAG00	0.52	6.58	0.00	0.04		brain
ENSXMAG00	0.47	3.92	0.00	0.04	nadka	brain
ENSXMAG00	0.84	2.18	0.00	0.04	igfals	brain
ENSXMAG00	-1.16	3.37	0.00	0.00	ier2b	brain
ENSXMAG00	-0.73	2.60	0.00	0.02	phlda2	brain
ENSXMAG00	1.48	2.12	0.00	0.00		brain
ENSXMAG00	-0.53	5.07	0.00	0.00	nupr1a	brain
ENSXMAG00	-0.79	1.94	0.00	0.02	ackr4b	brain
ENSXMAG00	-0.89	4.99	0.00	0.00	h1f0	brain
ENSXMAG00	-0.95	4.36	0.00	0.00	gpr186	brain
ENSXMAG00	0.44	3.77	0.00	0.03	nfe2l3	brain
ENSXMAG00	-1.07	-0.11	0.00	0.03		brain
ENSXMAG00	0.46	6.65	0.00	0.01	RAB37	brain
ENSXMAG00	-0.49	5.12	0.00	0.02	ntd5	brain
ENSXMAG00	-0.49	3.61	0.00	0.03	poglut2	brain
ENSXMAG00	1.43	2.65	0.00	0.00	dhrs3b	brain
ENSXMAG00	-0.78	4.25	0.00	0.00	TOB2	brain
ENSXMAG00	0.92	2.59	0.00	0.03	cilp	brain
ENSXMAG00	-0.53	3.64	0.00	0.01	COMTD1 (1 c	brain
ENSXMAG00	1.50	-1.05	0.00	0.02	si:ch211-243	brain
ENSXMAG00	-0.46	6.45	0.00	0.00		brain
ENSXMAG00	-0.37	6.48	0.00	0.04		brain
ENSXMAG00	-1.21	1.82	0.00	0.00	gpr171	brain
ENSXMAG00	-0.76	4.10	0.00	0.00	gnl3	brain
ENSXMAG00	0.46	9.37	0.00	0.01	syf2	brain
ENSXMAG00	0.70	3.24	0.00	0.02		brain
ENSXMAG00	0.43	4.53	0.00	0.02	uck2b	brain
ENSXMAG00	0.49	5.73	0.00	0.00	hpcal1	brain
ENSXMAG00	0.45	4.44	0.00	0.04	PSTK	brain
ENSXMAG00	-0.43	4.71	0.00	0.02	ccl44	brain
ENSXMAG00	-1.23	-0.13	0.00	0.00	RF00271	brain
ENSXMAG00	-0.66	3.80	0.00	0.00	ifi30	brain
ENSXMAG00	-0.78	4.18	0.00	0.00		brain
ENSXMAG00	0.81	4.66	0.00	0.00	msra	brain
ENSXMAG00	-0.72	2.76	0.00	0.00	hspbap1	brain
ENSXMAG00	-0.42	8.48	0.00	0.02	cbx7a	brain
ENSXMAG00	0.41	5.09	0.00	0.01	rps6kb1b	brain
ENSXMAG00	1.33	3.94	0.00	0.00		brain
ENSXMAG00	0.51	4.73	0.00	0.00	cnrip1b	brain
ENSXMAG00	0.40	4.69	0.00	0.02	MID1	brain
ENSXMAG00	-1.91	0.65	0.00	0.02		brain

ENSXMAG00	0.42	5.34	0.00	0.01	slc35e1	brain
ENSXMAG00	0.73	5.02	0.00	0.00		brain
ENSXMAG00	0.55	5.72	0.00	0.00	dnajc15	brain
ENSXMAG00	4.21	-0.74	0.00	0.03		brain
ENSXMAG00	0.35	5.58	0.00	0.04	mapre1a	brain
ENSXMAG00	0.54	6.05	0.00	0.00	plekho1a	brain
ENSXMAG00	-1.96	2.82	0.00	0.00		brain
ENSXMAG00	-0.70	4.13	0.00	0.00	arrdc2	brain
ENSXMAG00	-0.33	6.87	0.00	0.04	zgc:175214	brain
ENSXMAG00	0.67	4.39	0.00	0.00		brain
ENSXMAG00	-1.55	1.30	0.00	0.00	si:ch211-256	brain
ENSXMAG00	3.04	-2.61	0.00	0.03	RF00134	brain
ENSXMAG00	-0.73	1.54	0.00	0.05	ska3	brain
ENSXMAG00	-3.97	-2.53	0.00	0.00	prr33	brain
ENSXMAG00	0.55	4.34	0.00	0.00		brain
ENSXMAG00	0.59	3.71	0.00	0.02		brain
ENSXMAG00	-3.97	-0.26	0.00	0.00		brain
ENSXMAG00	1.17	4.66	0.00	0.00	dnajc4	brain
ENSXMAG00	1.52	1.31	0.00	0.02	gfi1b	brain
ENSXMAG00	-0.63	5.47	0.00	0.00		brain
ENSXMAG00	0.37	6.13	0.00	0.03	etnk2	brain
ENSXMAG00	-0.69	7.66	0.00	0.00	egr1	brain
ENSXMAG00	0.46	4.47	0.00	0.03	ptgesl	brain
ENSXMAG00	0.66	8.51	0.00	0.00	slc38a3a	brain
ENSXMAG00	-0.93	0.95	0.00	0.01	lysmd1	brain
ENSXMAG00	0.69	3.98	0.00	0.00		brain
ENSXMAG00	-0.42	6.35	0.00	0.01	map1lc3b	brain
ENSXMAG00	0.55	5.09	0.00	0.00	ppm1e	brain
ENSXMAG00	-0.86	2.36	0.00	0.00	nfat5b	brain
ENSXMAG00	0.92	3.48	0.00	0.01		brain
ENSXMAG00	0.52	5.34	0.00	0.01	pgp	brain
ENSXMAG00	-0.72	5.37	0.00	0.00	si:ch211-117	brain
ENSXMAG00	0.42	5.66	0.00	0.03		brain
ENSXMAG00	-0.70	1.88	0.00	0.01	si:dkeyp-26a	brain
ENSXMAG00	0.35	6.36	0.00	0.03	CERK (1 of m	brain
ENSXMAG00	2.36	-1.55	0.00	0.01		brain
ENSXMAG00	-2.52	0.77	0.00	0.02	CAPN14 (1 o	ovary
ENSXMAG00	-1.72	4.38	0.00	0.00	ptgdsb.1	ovary
ENSXMAG00	-2.48	1.09	0.00	0.02		ovary
ENSXMAG00	-1.04	4.77	0.00	0.02		ovary
ENSXMAG00	-1.14	4.76	0.00	0.04	add3a	ovary
ENSXMAG00	-6.50	-0.79	0.00	0.00		ovary

ENSXMAG00	-2.68	-0.05	0.00	0.02	cdh17	ovary
ENSXMAG00	-0.98	7.49	0.00	0.00	atp1a1a.2	ovary
ENSXMAG00	-0.94	6.46	0.00	0.00	per1b	ovary
ENSXMAG00	-1.19	5.20	0.00	0.04	hsd17b3	ovary
ENSXMAG00	-2.07	1.65	0.00	0.01		ovary
ENSXMAG00	-0.95	5.28	0.00	0.05	ghrb	ovary
ENSXMAG00	-1.21	6.61	0.00	0.00	flnca	ovary
ENSXMAG00	-3.37	1.67	0.00	0.04		ovary
ENSXMAG00	-1.05	5.95	0.00	0.03	enpp1	ovary
ENSXMAG00	-1.38	3.58	0.00	0.02	lpin1	ovary
ENSXMAG00	-1.32	4.13	0.00	0.02	angptl1b	ovary
ENSXMAG00	-0.94	6.37	0.00	0.02	pim1	ovary
ENSXMAG00	-3.33	0.54	0.00	0.00	cd59	ovary
ENSXMAG00	-2.85	0.67	0.00	0.01	si:dkey-202l2	ovary
ENSXMAG00	-5.13	2.35	0.00	0.00	leap2	ovary
ENSXMAG00	-4.36	0.24	0.00	0.02		ovary
ENSXMAG00	-1.46	6.58	0.00	0.01	tefb	ovary
ENSXMAG00	-2.64	1.77	0.00	0.02		ovary
ENSXMAG00	-6.71	5.94	0.00	0.03		heart
ENSXMAG00	-6.50	4.87	0.00	0.03	ttc16	heart
ENSXMAG00	-7.80	7.50	0.00	0.03	piwil1	heart
ENSXMAG00	-7.39	4.53	0.00	0.03	lrrc6	heart
ENSXMAG00	-7.12	3.72	0.00	0.03	adad2	heart
ENSXMAG00	-6.61	6.15	0.00	0.03		heart
ENSXMAG00	-5.73	3.92	0.00	0.04		heart
ENSXMAG00	-6.60	4.44	0.00	0.04	armc3	heart
ENSXMAG00	-6.65	1.96	0.00	0.04	henmt1	heart
ENSXMAG00	-5.95	2.58	0.00	0.03		heart
ENSXMAG00	-5.59	3.93	0.00	0.04	whrna	heart
ENSXMAG00	-6.61	4.92	0.00	0.03		heart
ENSXMAG00	-6.89	5.05	0.00	0.03		heart
ENSXMAG00	-5.48	1.87	0.00	0.05	prelid1b	heart
ENSXMAG00	-6.02	5.31	0.00	0.03	depdc1a	heart
ENSXMAG00	-8.22	-0.61	0.00	0.05		heart
ENSXMAG00	-5.51	3.98	0.00	0.04	PTPRM	heart
ENSXMAG00	-7.48	8.01	0.00	0.03	eno4	heart
ENSXMAG00	-5.98	3.73	0.00	0.03	slc1a2b	heart
ENSXMAG00	-7.16	6.76	0.00	0.03		heart
ENSXMAG00	-6.76	3.65	0.00	0.03	fam221a	heart
ENSXMAG00	-7.09	4.22	0.00	0.03		heart
ENSXMAG00	-6.83	3.55	0.00	0.03	si:dkey-83h2.	heart
ENSXMAG00	-7.11	4.85	0.00	0.04	LGALS2	heart

ENSXMAG00	-7.18	7.17	0.00	0.03		heart
ENSXMAG00	-6.84	2.77	0.00	0.04		heart
ENSXMAG00	-7.20	2.74	0.00	0.03	ccnd3	heart
ENSXMAG00	-7.10	7.28	0.00	0.03		heart
ENSXMAG00	-6.91	4.17	0.00	0.03		heart
ENSXMAG00	-5.70	3.39	0.00	0.04	cnga4	heart
ENSXMAG00	-6.99	6.78	0.00	0.03		heart
ENSXMAG00	-6.09	3.79	0.00	0.03		heart
ENSXMAG00	-8.09	4.30	0.00	0.03	nr1h4	heart
ENSXMAG00	-6.20	4.91	0.00	0.04	POLN	heart
ENSXMAG00	-5.59	4.44	0.00	0.04	TTC29	heart
ENSXMAG00	-7.00	5.36	0.00	0.03	stom (1 of m	heart
ENSXMAG00	-6.53	4.55	0.00	0.04		heart
ENSXMAG00	-7.04	5.28	0.00	0.03		heart
ENSXMAG00	-6.29	7.67	0.00	0.03	espl1	heart
ENSXMAG00	-7.03	6.44	0.00	0.03	spata4	heart
ENSXMAG00	-7.61	3.83	0.00	0.03	si:dkey-247m	heart
ENSXMAG00	-6.99	5.43	0.00	0.05	zgc:153317 (	heart
ENSXMAG00	-7.27	6.68	0.00	0.03	tekt1	heart
ENSXMAG00	-5.18	4.62	0.00	0.05	myt1la	heart
ENSXMAG00	-7.23	6.41	0.00	0.03	RGS22	heart
ENSXMAG00	-6.53	4.38	0.00	0.03	pyya	heart
ENSXMAG00	-7.45	6.71	0.00	0.03	cpm	heart
ENSXMAG00	-5.42	3.03	0.00	0.04	st8sia2	heart
ENSXMAG00	-6.57	5.44	0.00	0.03		heart
ENSXMAG00	-4.15	3.59	0.00	0.05	apoa1b	heart
ENSXMAG00	-7.20	6.39	0.00	0.03		heart
ENSXMAG00	-6.05	3.90	0.00	0.03	STAC2 (1 of r	heart
ENSXMAG00	-7.42	7.57	0.00	0.03		heart
ENSXMAG00	-7.33	2.29	0.00	0.03	DIPK1A (1 of	heart
ENSXMAG00	-7.18	7.65	0.00	0.03	ST8SIA3	heart
ENSXMAG00	-6.96	3.46	0.00	0.03		heart
ENSXMAG00	-6.83	3.44	0.00	0.03	ppil6	heart
ENSXMAG00	-6.64	4.66	0.00	0.03	armc2	heart
ENSXMAG00	-5.94	2.44	0.00	0.04		heart
ENSXMAG00	-7.50	6.19	0.00	0.03		heart
ENSXMAG00	-7.40	1.49	0.00	0.03		heart
ENSXMAG00	-5.68	5.23	0.00	0.04	radil	heart
ENSXMAG00	-7.50	3.01	0.00	0.03		heart
ENSXMAG00	-7.21	5.65	0.00	0.03	iqch	heart
ENSXMAG00	-7.56	7.07	0.00	0.03	slc6a14	heart
ENSXMAG00	-6.62	5.54	0.00	0.03		heart
ENSXMAG00	-6.80	5.38	0.00	0.04	cfap161	heart

ENSXMAG00	-5.74	5.14	0.00	0.05	KIF18A	heart
ENSXMAG00	-7.35	5.46	0.00	0.03	ppp1r32	heart
ENSXMAG00	-5.01	3.56	0.00	0.05		heart
ENSXMAG00	-6.85	3.56	0.00	0.03	abhd8a	heart
ENSXMAG00	-8.01	6.44	0.00	0.03	slc2a5	heart
ENSXMAG00	-7.13	5.26	0.00	0.03	maats1	heart
ENSXMAG00	-6.79	5.17	0.00	0.03	slc13a5b	heart
ENSXMAG00	-5.02	4.75	0.00	0.05	nme6	heart
ENSXMAG00	-7.57	0.84	0.00	0.03		heart
ENSXMAG00	-6.03	4.99	0.00	0.03	fbxo36b	heart
ENSXMAG00	-5.10	5.59	0.00	0.05		heart
ENSXMAG00	-6.66	5.68	0.00	0.03		heart
ENSXMAG00	-6.31	1.84	0.00	0.04	wnt2	heart
ENSXMAG00	-6.68	4.25	0.00	0.03	ednrbb	heart
ENSXMAG00	-5.79	6.10	0.00	0.04		heart
ENSXMAG00	-7.06	4.94	0.00	0.03	ccdc88aa	heart
ENSXMAG00	-6.66	4.18	0.00	0.03	DYNLRB1 (1	heart
ENSXMAG00	-5.77	3.43	0.00	0.04		heart
ENSXMAG00	-7.63	5.04	0.00	0.03	zgc:172182	heart
ENSXMAG00	-6.38	7.98	0.00	0.04	kif20ba	heart
ENSXMAG00	-6.29	3.83	0.00	0.03	si:dkey-106n	heart
ENSXMAG00	-5.56	5.62	0.00	0.05	diaph3	heart
ENSXMAG00	-6.92	5.63	0.00	0.03	piwil2	heart
ENSXMAG00	-5.74	1.85	0.00	0.04	panx1b	heart
ENSXMAG00	-7.12	6.60	0.00	0.03		heart
ENSXMAG00	-5.77	0.80	0.00	0.05	zgc:100920	heart
ENSXMAG00	-6.67	6.24	0.00	0.03	RSPH1	heart
ENSXMAG00	-5.73	6.37	0.00	0.04	pcsk1	heart
ENSXMAG00	-4.47	2.86	0.00	0.04	zgc:110789	heart
ENSXMAG00	-6.98	2.48	0.00	0.03	ggt1b	heart
ENSXMAG00	-6.87	3.23	0.00	0.03	slc26a3.2	heart
ENSXMAG00	-6.58	5.01	0.00	0.03	fam161a	heart
ENSXMAG00	-5.74	4.96	0.00	0.05	ccdc13	heart
ENSXMAG00	-6.68	4.96	0.00	0.04		heart
ENSXMAG00	-5.50	3.16	0.00	0.03		heart
ENSXMAG00	-5.80	4.15	0.00	0.03	kif3ca	heart
ENSXMAG00	-5.96	5.43	0.00	0.03	dnaaf4	heart
ENSXMAG00	-6.71	5.65	0.00	0.04	slco1e1	heart
ENSXMAG00	-6.09	3.99	0.00	0.04		heart
ENSXMAG00	-7.13	3.93	0.00	0.03	stk33	heart
ENSXMAG00	-6.18	3.25	0.00	0.03	fkbp6	heart
ENSXMAG00	-6.99	5.42	0.00	0.03		heart
ENSXMAG00	-7.13	3.92	0.00	0.03	PLCZ1	heart

ENSXMAG00	-6.49	7.42	0.00	0.03	dnah7	heart
ENSXMAG00	-8.99	0.10	0.00	0.04		heart
ENSXMAG00	-7.45	4.25	0.00	0.03		heart
ENSXMAG00	-6.37	2.12	0.00	0.03		heart
ENSXMAG00	-6.50	4.68	0.00	0.03		heart
ENSXMAG00	-6.71	4.24	0.00	0.03	dcst2	heart
ENSXMAG00	-6.44	2.67	0.00	0.03		heart
ENSXMAG00	-4.64	4.11	0.00	0.05	knstrn	heart
ENSXMAG00	-6.85	6.59	0.00	0.03	ankef1a	heart
ENSXMAG00	-6.43	2.92	0.00	0.03		heart
ENSXMAG00	-6.29	2.99	0.00	0.03	cadpsa	heart
ENSXMAG00	-7.35	4.56	0.00	0.03	cfap300	heart
ENSXMAG00	-7.10	6.34	0.00	0.03	si:dkey-25g1	heart
ENSXMAG00	-6.73	6.08	0.00	0.03	rsph4a	heart
ENSXMAG00	-6.64	4.64	0.00	0.03	tdrd1	heart
ENSXMAG00	-7.18	4.60	0.00	0.03	rbm46	heart
ENSXMAG00	-6.55	4.44	0.00	0.03	fstl4	heart
ENSXMAG00	-6.31	4.50	0.00	0.03	PAX3	heart
ENSXMAG00	-6.59	5.05	0.00	0.03	gal3st3	heart
ENSXMAG00	-8.02	5.21	0.00	0.03	ccna1	heart
ENSXMAG00	-5.88	5.42	0.00	0.04	sparta	heart
ENSXMAG00	-6.71	3.68	0.00	0.04		heart
ENSXMAG00	-6.74	3.88	0.00	0.03	tmem237a	heart
ENSXMAG00	-4.42	4.14	0.00	0.04		heart
ENSXMAG00	-5.86	6.24	0.00	0.03	MAJIN	heart
ENSXMAG00	-6.53	1.82	0.00	0.05	si:ch211-255	heart
ENSXMAG00	-6.54	4.02	0.00	0.03		heart
ENSXMAG00	-6.15	2.66	0.00	0.03	m1ap	heart
ENSXMAG00	-7.55	5.47	0.00	0.03	ttc12	heart
ENSXMAG00	-6.28	5.22	0.00	0.03		heart
ENSXMAG00	-7.46	5.51	0.00	0.03	chrm2b	heart
ENSXMAG00	-7.37	3.74	0.00	0.03	hormad1	heart
ENSXMAG00	-7.64	5.65	0.00	0.03	DYNLRB2	heart
ENSXMAG00	-6.91	5.22	0.00	0.03	gabrr2a	heart
ENSXMAG00	-6.97	4.75	0.00	0.03	si:dkey-48j7.	heart
ENSXMAG00	-7.09	4.73	0.00	0.03	ampd3a	heart
ENSXMAG00	-7.54	4.95	0.00	0.03	FAM187A	heart
ENSXMAG00	-6.29	3.35	0.00	0.03		heart
ENSXMAG00	-7.02	4.91	0.00	0.03	smc1b	heart
ENSXMAG00	-7.14	4.82	0.00	0.03	lrrc18b	heart
ENSXMAG00	-5.40	2.91	0.00	0.04		heart
ENSXMAG00	-6.42	4.95	0.00	0.03	ppp1r42	heart
ENSXMAG00	-7.21	5.53	0.00	0.03	ddx4	heart

ENSXMAG00	-7.65	4.15	0.00	0.03		heart
ENSXMAG00	-5.68	4.10	0.00	0.04	myo10l1	heart
ENSXMAG00	-8.05	3.97	0.00	0.04		heart
ENSXMAG00	-5.37	4.24	0.00	0.04	otog	heart
ENSXMAG00	-6.20	3.54	0.00	0.03		heart
ENSXMAG00	-6.70	4.89	0.00	0.03		heart
ENSXMAG00	-7.29	5.91	0.00	0.03		heart
ENSXMAG00	-6.13	6.40	0.00	0.03	TACR1	heart
ENSXMAG00	-6.91	5.26	0.00	0.04	sgms2a	heart
ENSXMAG00	-6.45	4.30	0.00	0.03	zgc:112408	heart
ENSXMAG00	-7.64	6.83	0.00	0.03		heart
ENSXMAG00	-5.86	4.60	0.00	0.04	SLC24A1	heart
ENSXMAG00	-7.25	7.79	0.00	0.03	mkrn4	heart
ENSXMAG00	-5.61	2.68	0.00	0.03	TAFA1 (1 of	heart
ENSXMAG00	-7.40	5.45	0.00	0.03		heart
ENSXMAG00	-7.35	3.27	0.00	0.03		heart
ENSXMAG00	-6.52	4.61	0.00	0.03		heart
ENSXMAG00	-5.79	5.19	0.00	0.05		heart
ENSXMAG00	-7.08	4.49	0.00	0.03	daw1	heart
ENSXMAG00	-7.25	5.55	0.00	0.03	fbxo36a	heart
ENSXMAG00	-7.01	4.64	0.00	0.03		heart
ENSXMAG00	-6.83	6.17	0.00	0.03	ribc1	heart
ENSXMAG00	-5.91	4.29	0.00	0.03	tdrd7a	heart
ENSXMAG00	-7.16	4.77	0.00	0.03	styxl1	heart
ENSXMAG00	-5.16	5.19	0.00	0.04	katnal1	heart
ENSXMAG00	-7.78	4.57	0.00	0.03	WDR63	heart
ENSXMAG00	-6.95	1.57	0.00	0.04		heart
ENSXMAG00	-6.12	6.02	0.00	0.04		heart
ENSXMAG00	-6.31	5.47	0.00	0.03	CCDC148	heart
ENSXMAG00	-5.98	5.61	0.00	0.05		heart
ENSXMAG00	-6.26	4.65	0.00	0.03		heart
ENSXMAG00	-6.06	4.41	0.00	0.03	brdt	heart
ENSXMAG00	-7.08	7.07	0.00	0.03	zgc:56699	heart
ENSXMAG00	-6.06	2.44	0.00	0.03	aldoca	heart
ENSXMAG00	-6.63	3.25	0.00	0.03	msrb1b	heart
ENSXMAG00	-6.01	3.80	0.00	0.03	atp1b2b	heart
ENSXMAG00	-6.24	5.48	0.00	0.04	EEF1A1	heart
ENSXMAG00	-7.47	4.60	0.00	0.03	hsd20b2 (1 o	heart
ENSXMAG00	-6.68	4.19	0.00	0.03		heart
ENSXMAG00	-7.22	5.18	0.00	0.03		heart
ENSXMAG00	-6.16	2.77	0.00	0.03	si:ch211-176	heart
ENSXMAG00	-6.50	4.15	0.00	0.04	zgc:193690	heart
ENSXMAG00	-6.19	6.43	0.00	0.03	ttll10	heart

ENSXMAG00	-5.34	3.20	0.00	0.05		heart
ENSXMAG00	-6.85	4.64	0.00	0.03	hsd20b2 (1 o	heart
ENSXMAG00	-6.63	3.85	0.00	0.03	dmc1	heart
ENSXMAG00	-7.14	6.72	0.00	0.03		heart
ENSXMAG00	-7.10	6.28	0.00	0.03		heart
ENSXMAG00	-6.93	2.66	0.00	0.03	CLVS1	heart
ENSXMAG00	-5.87	4.00	0.00	0.05		heart
ENSXMAG00	3.76	4.20	0.00	0.03	si:ch211-244	heart
ENSXMAG00	-5.74	4.33	0.00	0.03	dpysl5b	heart
ENSXMAG00	-5.96	7.60	0.00	0.04		heart
ENSXMAG00	-5.86	6.14	0.00	0.04	slc44a5b	heart
ENSXMAG00	4.72	5.25	0.00	0.03	nr4a3	heart
ENSXMAG00	-5.69	5.00	0.00	0.04		heart
ENSXMAG00	-7.37	5.05	0.00	0.03		heart
ENSXMAG00	-9.51	0.59	0.00	0.05	aipl2	heart
ENSXMAG00	-5.44	3.09	0.00	0.04	drp2	heart
ENSXMAG00	-5.93	0.56	0.00	0.04		heart
ENSXMAG00	-5.44	5.08	0.00	0.04	UNC79	heart
ENSXMAG00	-7.11	3.04	0.00	0.03	fgf11a	heart
ENSXMAG00	-6.15	3.20	0.00	0.03	chrnb1	heart
ENSXMAG00	-6.10	4.77	0.00	0.03	gpr135	heart
ENSXMAG00	-7.47	6.34	0.00	0.03		heart
ENSXMAG00	-6.59	3.94	0.00	0.03		heart
ENSXMAG00	-6.15	2.86	0.00	0.05	cacng3a	heart
ENSXMAG00	-6.55	5.08	0.00	0.03	rad21b	heart
ENSXMAG00	-5.97	5.06	0.00	0.03	slc44a4	heart
ENSXMAG00	-5.39	5.92	0.00	0.04	TPPP	heart
ENSXMAG00	-4.94	4.59	0.00	0.04	ccnb2	heart
ENSXMAG00	-7.28	0.55	0.00	0.04	ociad2	heart
ENSXMAG00	-5.91	4.68	0.00	0.03	si:dkey-83h2.	heart
ENSXMAG00	-7.07	4.90	0.00	0.03	si:dkey-83h2.	heart
ENSXMAG00	-7.17	3.66	0.00	0.03	asz1	heart
ENSXMAG00	-6.11	5.21	0.00	0.03	si:ch211-81a	heart
ENSXMAG00	-6.74	3.73	0.00	0.05	zgc:162324	heart
ENSXMAG00	-5.75	3.23	0.00	0.04	gc2	heart
ENSXMAG00	-7.21	5.48	0.00	0.03	rnf17	heart
ENSXMAG00	-7.00	6.00	0.00	0.03		heart
ENSXMAG00	-6.42	5.64	0.00	0.04	rsph10b	heart
ENSXMAG00	-6.83	4.92	0.00	0.03	dnali1	heart
ENSXMAG00	-5.73	3.44	0.00	0.03	KIF27	heart
ENSXMAG00	-6.40	5.46	0.00	0.03		heart
ENSXMAG00	-4.71	1.98	0.00	0.05	fam161b	heart
ENSXMAG00	-6.74	7.15	0.00	0.03		heart

ENSXMAG00	-7.56	3.07	0.00	0.03		heart
ENSXMAG00	-6.64	3.24	0.00	0.03	dmrt1	heart
ENSXMAG00	-6.58	6.25	0.00	0.03	dnah12	heart
ENSXMAG00	-7.13	7.03	0.00	0.03	ak7b	heart
ENSXMAG00	-6.61	4.54	0.00	0.03	si:dkeyp-93d	heart
ENSXMAG00	-7.24	6.31	0.00	0.03	mapk15	heart
ENSXMAG00	-6.82	4.10	0.00	0.04	nim1k	heart
ENSXMAG00	-4.64	4.56	0.00	0.03	cldn11a	heart
ENSXMAG00	-6.60	6.09	0.00	0.03	spire2	heart
ENSXMAG00	-6.34	2.09	0.00	0.03	apoea	heart
ENSXMAG00	-5.36	1.88	0.00	0.04		heart
ENSXMAG00	-6.88	6.34	0.00	0.03		heart
ENSXMAG00	-6.83	6.89	0.00	0.03	ric8a	heart
ENSXMAG00	-5.32	1.84	0.00	0.05	grm2b	heart
ENSXMAG00	-7.46	4.53	0.00	0.03		heart
ENSXMAG00	-7.05	6.15	0.00	0.03		heart
ENSXMAG00	-6.07	3.49	0.00	0.04	LRRC49	heart
ENSXMAG00	-5.70	9.00	0.00	0.04		heart
ENSXMAG00	-5.11	5.27	0.00	0.04	slco2a1	heart
ENSXMAG00	-6.68	5.36	0.00	0.03	zmynd12	heart
ENSXMAG00	-6.08	3.24	0.00	0.03	lrp2b	heart
ENSXMAG00	-7.01	5.53	0.00	0.03	ttll9	heart
ENSXMAG00	-5.68	4.41	0.00	0.04		heart
ENSXMAG00	-5.93	3.52	0.00	0.03	zmynd10	heart
ENSXMAG00	-6.57	6.06	0.00	0.04	pabpc1l	heart
ENSXMAG00	-6.98	3.69	0.00	0.03	mmel1	heart
ENSXMAG00	-6.40	5.67	0.00	0.03		heart
ENSXMAG00	-6.97	7.45	0.00	0.03	ccdc114	heart
ENSXMAG00	-6.58	2.83	0.00	0.03	tekt3	heart
ENSXMAG00	-7.05	7.46	0.00	0.03		heart
ENSXMAG00	-6.41	1.97	0.00	0.05		heart
ENSXMAG00	-6.91	5.04	0.00	0.03	tcte1	heart
ENSXMAG00	-5.82	3.62	0.00	0.03	atp2b2	heart
ENSXMAG00	-5.80	6.44	0.00	0.04	wdr66	heart
ENSXMAG00	-6.02	5.88	0.00	0.04	nek2	heart
ENSXMAG00	-5.71	5.30	0.00	0.05		heart
ENSXMAG00	-6.83	5.13	0.00	0.03	LRRC43	heart
ENSXMAG00	-6.16	0.78	0.00	0.04	ptpdc1b	heart
ENSXMAG00	-6.55	4.25	0.00	0.03		heart
ENSXMAG00	-6.67	5.61	0.00	0.03	pacrg	heart
ENSXMAG00	-5.88	6.83	0.00	0.05	zmp:000000(	heart
ENSXMAG00	-6.83	4.62	0.00	0.03		heart
ENSXMAG00	-6.72	5.87	0.00	0.03	fam184a	heart

ENSXMAG00	-6.65	2.39	0.00	0.03		heart
ENSXMAG00	-5.59	6.12	0.00	0.04	ccdc40	heart
ENSXMAG00	-6.92	5.70	0.00	0.03		heart
ENSXMAG00	-5.83	6.95	0.00	0.04	prc1b	heart
ENSXMAG00	-7.78	3.30	0.00	0.04		heart
ENSXMAG00	-6.64	4.66	0.00	0.03		heart
ENSXMAG00	-7.12	5.97	0.00	0.03	dnai1.2	heart
ENSXMAG00	-5.64	6.08	0.00	0.04	cita	heart
ENSXMAG00	-7.50	5.03	0.00	0.03	tdrd9	heart
ENSXMAG00	-5.24	6.26	0.00	0.05		heart
ENSXMAG00	-7.50	6.90	0.00	0.03		heart
ENSXMAG00	-6.27	7.33	0.00	0.04	ccdc181	heart
ENSXMAG00	-6.12	4.79	0.00	0.03	zgc:153738	heart
ENSXMAG00	-5.48	6.61	0.00	0.04	wdr35	heart
ENSXMAG00	-5.35	5.45	0.00	0.04	atp2a1l	heart
ENSXMAG00	-6.16	2.54	0.00	0.03		heart
ENSXMAG00	-5.63	6.23	0.00	0.05	acot7	heart
ENSXMAG00	-5.70	3.93	0.00	0.04		heart
ENSXMAG00	-6.82	4.16	0.00	0.03	nek10	heart
ENSXMAG00	-6.35	4.22	0.00	0.03	KLHL10	heart
ENSXMAG00	-6.62	1.92	0.00	0.03	col28a1b	heart
ENSXMAG00	-6.66	4.72	0.00	0.03	si:ch73-81k8.	heart
ENSXMAG00	-6.01	4.91	0.00	0.03	SOWAHC (1 (	heart
ENSXMAG00	-6.49	4.58	0.00	0.03	thsd7ab	heart
ENSXMAG00	-5.63	3.28	0.00	0.04	dgkb	heart
ENSXMAG00	-7.34	3.42	0.00	0.03		heart
ENSXMAG00	-6.93	1.90	0.00	0.04	si:ch211-160	heart
ENSXMAG00	-5.89	7.40	0.00	0.04	ift140	heart
ENSXMAG00	-6.33	5.59	0.00	0.04		heart
ENSXMAG00	-5.48	4.70	0.00	0.03	tmem17	heart
ENSXMAG00	-6.41	4.71	0.00	0.03	nkpd1 (1 of r	heart
ENSXMAG00	-6.44	2.69	0.00	0.03	SLC25A53	heart
ENSXMAG00	-7.37	6.39	0.00	0.03		heart
ENSXMAG00	-6.92	3.72	0.00	0.03	lrrc18a	heart
ENSXMAG00	-5.91	5.45	0.00	0.03		heart
ENSXMAG00	-7.44	7.76	0.00	0.03		heart
ENSXMAG00	-6.59	2.52	0.00	0.03	tdrd12	heart
ENSXMAG00	-6.47	5.71	0.00	0.03		heart
ENSXMAG00	-7.56	4.96	0.00	0.03		heart
ENSXMAG00	-7.05	4.70	0.00	0.03		heart
ENSXMAG00	-7.14	5.19	0.00	0.03		heart
ENSXMAG00	-6.37	6.24	0.00	0.03		heart
ENSXMAG00	-6.06	2.68	0.00	0.04		heart

ENSXMAG00	-6.86	4.55	0.00	0.03		heart
ENSXMAG00	-6.92	6.60	0.00	0.03	xrra1	heart
ENSXMAG00	-6.71	5.42	0.00	0.03	si:ch211-113	heart
ENSXMAG00	-7.59	5.33	0.00	0.03		heart
ENSXMAG00	-7.33	4.21	0.00	0.03		heart
ENSXMAG00	-6.47	3.87	0.00	0.03	gpat2	heart
ENSXMAG00	-6.19	4.60	0.00	0.03	cfap99	heart
ENSXMAG00	-8.61	1.86	0.00	0.04		heart
ENSXMAG00	-6.92	4.38	0.00	0.03	zgc:66426 (1	heart
ENSXMAG00	-6.51	4.96	0.00	0.04		heart
ENSXMAG00	-8.03	2.10	0.00	0.03		heart
ENSXMAG00	-6.19	3.18	0.00	0.04		heart
ENSXMAG00	-6.58	1.85	0.00	0.05	pih1d2	heart
ENSXMAG00	-6.08	8.15	0.00	0.03		heart
ENSXMAG00	-7.49	7.79	0.00	0.03		heart
ENSXMAG00	-6.25	2.19	0.00	0.05		heart
ENSXMAG00	-6.94	0.22	0.00	0.05		heart
ENSXMAG00	-7.12	5.83	0.00	0.03	ropn1l	heart
ENSXMAG00	-6.76	5.09	0.00	0.03	cfap57	heart
ENSXMAG00	-7.53	6.34	0.00	0.03		heart
ENSXMAG00	-7.26	6.77	0.00	0.03		heart
ENSXMAG00	-7.01	3.92	0.00	0.03		heart
ENSXMAG00	-6.90	5.68	0.00	0.03		heart
ENSXMAG00	-8.03	3.93	0.00	0.03		heart
ENSXMAG00	-6.93	5.13	0.00	0.03		heart
ENSXMAG00	-6.41	4.82	0.00	0.03	cbln1	heart
ENSXMAG00	-6.72	3.54	0.00	0.04		heart
ENSXMAG00	-6.96	4.80	0.00	0.03		heart
ENSXMAG00	-6.88	4.34	0.00	0.03		heart
ENSXMAG00	-6.60	5.44	0.00	0.03	rsph9	heart
ENSXMAG00	-6.34	3.25	0.00	0.04	rnf182	heart
ENSXMAG00	-5.55	4.34	0.00	0.04		heart
ENSXMAG00	-5.20	4.05	0.00	0.05		heart
ENSXMAG00	-5.46	1.44	0.00	0.05	FABP7	heart
ENSXMAG00	-6.03	3.77	0.00	0.04	tatdn2	heart
ENSXMAG00	-6.72	4.96	0.00	0.03		heart
ENSXMAG00	-5.86	5.58	0.00	0.05		heart
ENSXMAG00	-6.71	2.81	0.00	0.03		heart
ENSXMAG00	-6.95	4.15	0.00	0.03		heart
ENSXMAG00	-6.62	4.86	0.00	0.03		heart
ENSXMAG00	-7.20	7.92	0.00	0.03		heart
ENSXMAG00	-6.34	4.39	0.00	0.03	si:dkey-70p6.	heart
ENSXMAG00	-7.22	5.59	0.00	0.03		heart

ENSXMAG00	-5.55	4.95	0.00	0.04	ttbk1a	heart
ENSXMAG00	-7.11	5.93	0.00	0.03	cfap206	heart
ENSXMAG00	-7.72	5.61	0.00	0.03		heart
ENSXMAG00	-5.22	3.87	0.00	0.04	LGR5	heart
ENSXMAG00	-7.09	3.18	0.00	0.03		heart
ENSXMAG00	-8.00	2.59	0.00	0.03		heart
ENSXMAG00	-5.27	6.28	0.00	0.05		heart
ENSXMAG00	-7.16	5.76	0.00	0.03	terb2	heart
ENSXMAG00	-6.23	4.49	0.00	0.03	clcf1	heart
ENSXMAG00	-6.89	4.66	0.00	0.03	ubxn11	heart
ENSXMAG00	-5.79	2.71	0.00	0.04	zgc:194246	heart
ENSXMAG00	-6.18	0.83	0.00	0.05		heart
ENSXMAG00	-6.15	2.09	0.00	0.04		heart
ENSXMAG00	-6.64	5.16	0.00	0.03		heart
ENSXMAG00	-6.59	6.10	0.00	0.03		heart
ENSXMAG00	-6.56	6.49	0.00	0.04		heart
ENSXMAG00	-6.66	5.95	0.00	0.03		heart
ENSXMAG00	-4.56	3.92	0.00	0.05		heart
ENSXMAG00	-6.27	7.70	0.00	0.03		heart
ENSXMAG00	-6.83	5.58	0.00	0.03		heart
ENSXMAG00	-5.81	4.55	0.00	0.03		heart
ENSXMAG00	-7.03	5.47	0.00	0.03		heart
ENSXMAG00	-7.02	4.66	0.00	0.03		heart
ENSXMAG00	-5.30	4.60	0.00	0.05		heart
ENSXMAG00	-7.15	5.76	0.00	0.03		heart
ENSXMAG00	-7.03	2.29	0.00	0.03		heart
ENSXMAG00	-7.30	9.90	0.00	0.03		heart
ENSXMAG00	-7.01	1.61	0.00	0.05		heart
ENSXMAG00	-6.73	3.45	0.00	0.03		heart
ENSXMAG00	-5.92	2.73	0.00	0.04		heart
ENSXMAG00	-7.99	5.67	0.00	0.03		heart
ENSXMAG00	-7.12	5.17	0.00	0.03		heart
ENSXMAG00	-6.66	1.96	0.00	0.04		heart
ENSXMAG00	-8.14	3.86	0.00	0.03		heart
ENSXMAG00	-6.93	4.85	0.00	0.04		heart
ENSXMAG00	-6.47	4.75	0.00	0.03		heart
ENSXMAG00	-7.24	6.62	0.00	0.03		heart
ENSXMAG00	-6.82	4.65	0.00	0.03		heart
ENSXMAG00	-6.16	3.95	0.00	0.03	nicn1	heart
ENSXMAG00	-5.66	4.62	0.00	0.04	si:busm1-57f	heart
ENSXMAG00	-6.06	3.64	0.00	0.03		heart
ENSXMAG00	-6.92	4.05	0.00	0.03		heart
ENSXMAG00	-7.21	5.37	0.00	0.03		heart

ENSXMAG00	-7.60	6.75	0.00	0.03		heart
ENSXMAG00	-6.69	5.58	0.00	0.03		heart
ENSXMAG00	-5.98	6.57	0.00	0.04	si:ch211-272	heart
ENSXMAG00	-7.38	5.50	0.00	0.03	tmem136a	heart
ENSXMAG00	-6.47	5.13	0.00	0.03		heart
ENSXMAG00	-7.96	3.68	0.00	0.03		heart
ENSXMAG00	-6.94	4.56	0.00	0.03		heart
ENSXMAG00	-5.84	2.57	0.00	0.04	si:ch1073-75	heart
ENSXMAG00	-5.39	0.72	0.00	0.05		heart
ENSXMAG00	-6.13	4.60	0.00	0.03		heart
ENSXMAG00	-9.78	0.85	0.00	0.03		heart
ENSXMAG00	-6.11	2.60	0.00	0.03		heart
ENSXMAG00	-7.39	6.22	0.00	0.03		heart
ENSXMAG00	-7.35	8.37	0.00	0.03	tdrd6	heart
ENSXMAG00	-7.71	5.22	0.00	0.03		heart
ENSXMAG00	-5.82	4.00	0.00	0.03	grid2	heart
ENSXMAG00	-6.68	4.98	0.00	0.03		heart
ENSXMAG00	-5.96	4.55	0.00	0.04	lrrc34	heart
ENSXMAG00	-7.27	5.46	0.00	0.03		heart
ENSXMAG00	-7.32	3.39	0.00	0.03	slc5a1 (1 of	heart
ENSXMAG00	-6.72	5.03	0.00	0.04		heart
ENSXMAG00	-6.59	5.65	0.00	0.03		heart
ENSXMAG00	-5.76	2.02	0.00	0.05		heart
ENSXMAG00	-7.15	6.67	0.00	0.03	cfap52 (1 of	heart
ENSXMAG00	-9.22	3.78	0.00	0.03	si:dkeyp-7a3	heart
ENSXMAG00	-8.95	2.98	0.00	0.03		heart
ENSXMAG00	-5.96	6.80	0.00	0.04		heart
ENSXMAG00	-6.61	4.29	0.00	0.03		heart
ENSXMAG00	-6.60	3.31	0.00	0.03		heart
ENSXMAG00	-6.85	4.78	0.00	0.03		heart
ENSXMAG00	-5.80	2.18	0.00	0.04	igfbp2b	heart
ENSXMAG00	-6.68	4.61	0.00	0.03		heart
ENSXMAG00	-7.52	7.61	0.00	0.03		heart
ENSXMAG00	-7.51	6.89	0.00	0.03		heart
ENSXMAG00	-4.78	3.94	0.00	0.04	nphs2	heart
ENSXMAG00	-7.23	4.90	0.00	0.03		heart
ENSXMAG00	-7.28	4.92	0.00	0.03		heart
ENSXMAG00	-6.74	4.79	0.00	0.03		heart
ENSXMAG00	-5.61	3.51	0.00	0.03	zgc:109889	heart
ENSXMAG00	-6.80	5.74	0.00	0.03	si:dkeyp-46h	heart
ENSXMAG00	-7.30	5.39	0.00	0.03	ubxn10	heart
ENSXMAG00	-7.17	3.96	0.00	0.03		heart
ENSXMAG00	-6.80	4.15	0.00	0.03		heart

ENSXMAG00	-6.34	5.57	0.00	0.03	TMEM87A	heart
ENSXMAG00	-9.07	4.00	0.00	0.03		heart
ENSXMAG00	-5.53	1.92	0.00	0.03	fbp2	heart
ENSXMAG00	-7.98	2.04	0.00	0.03	tub	heart
ENSXMAG00	-8.28	2.34	0.00	0.04		heart
ENSXMAG00	-5.08	5.63	0.00	0.05	magixb	heart
ENSXMAG00	-6.44	5.48	0.00	0.03	EDNRA (1 of	heart
ENSXMAG00	-6.78	3.76	0.00	0.03		heart
ENSXMAG00	-5.98	4.12	0.00	0.03	spag6 (1 of r	heart
ENSXMAG00	-7.19	2.47	0.00	0.05		heart
ENSXMAG00	-5.71	3.69	0.00	0.04		heart
ENSXMAG00	-5.82	1.77	0.00	0.04	SAMD12	heart
ENSXMAG00	-6.73	6.47	0.00	0.03		heart
ENSXMAG00	-7.11	2.84	0.00	0.03		heart
ENSXMAG00	-8.03	3.92	0.00	0.03		heart
ENSXMAG00	-6.61	3.43	0.00	0.03		heart
ENSXMAG00	-5.99	6.94	0.00	0.04		heart
ENSXMAG00	-7.37	6.75	0.00	0.03		heart
ENSXMAG00	-6.14	3.78	0.00	0.04		heart
ENSXMAG00	-9.13	3.70	0.00	0.03		heart
ENSXMAG00	-7.63	3.17	0.00	0.03	si:ch211-133	heart
ENSXMAG00	-5.78	3.27	0.00	0.03	apc2	heart
ENSXMAG00	-4.99	4.37	0.00	0.04		heart
ENSXMAG00	-6.70	4.83	0.00	0.03	ribc2	heart
ENSXMAG00	-6.17	6.75	0.00	0.03	efcab2	heart
ENSXMAG00	-6.35	4.85	0.00	0.03		heart
ENSXMAG00	-6.75	2.03	0.00	0.03		heart
ENSXMAG00	-10.80	1.85	0.00	0.03		heart
ENSXMAG00	-7.33	6.76	0.00	0.03		heart
ENSXMAG00	-7.26	5.30	0.00	0.03		heart
ENSXMAG00	-7.31	7.08	0.00	0.03	igsf9a	heart
ENSXMAG00	-5.64	3.89	0.00	0.03	jam3a	heart
ENSXMAG00	-6.07	3.87	0.00	0.03		heart
ENSXMAG00	-6.45	6.46	0.00	0.03	A3GALT2	heart
ENSXMAG00	-6.18	3.82	0.00	0.03	dnal1	heart
ENSXMAG00	-9.92	0.99	0.00	0.04		heart
ENSXMAG00	-6.82	5.36	0.00	0.03		heart
ENSXMAG00	-6.63	2.55	0.00	0.03		heart
ENSXMAG00	-9.06	3.11	0.00	0.03		heart
ENSXMAG00	-6.51	6.70	0.00	0.03		heart
ENSXMAG00	-7.07	4.94	0.00	0.03		heart
ENSXMAG00	-7.69	0.93	0.00	0.04		heart
ENSXMAG00	-6.81	6.58	0.00	0.03	ncam1b	heart

ENSXMAG00	-5.80	0.44	0.00	0.04	si:dkey-30c1!	heart
ENSXMAG00	-5.61	0.94	0.00	0.03	tekt2	heart
ENSXMAG00	-8.12	3.39	0.00	0.03		heart
ENSXMAG00	-6.74	5.06	0.00	0.03	ELAVL2	heart
ENSXMAG00	-8.23	2.27	0.00	0.03		heart
ENSXMAG00	-7.41	7.06	0.00	0.03		heart
ENSXMAG00	-7.06	5.80	0.00	0.03		heart
ENSXMAG00	-7.41	4.88	0.00	0.03	ccsapa	heart
ENSXMAG00	-7.46	2.44	0.00	0.05		heart
ENSXMAG00	-7.14	6.99	0.00	0.03		heart
ENSXMAG00	-7.33	5.97	0.00	0.03		heart
ENSXMAG00	-6.73	6.88	0.00	0.03		heart
ENSXMAG00	-6.69	3.75	0.00	0.03		heart
ENSXMAG00	-7.23	6.11	0.00	0.03		heart
ENSXMAG00	-6.31	3.52	0.00	0.05	morn5	heart
ENSXMAG00	-7.20	5.88	0.00	0.03		heart
ENSXMAG00	-7.94	2.02	0.00	0.03		heart
ENSXMAG00	-6.70	5.84	0.00	0.03		heart
ENSXMAG00	1.16	1.74	0.00	0.01	cant1b	muscle
ENSXMAG00	-0.98	2.33	0.00	0.04	slc49a4	muscle
ENSXMAG00	-1.18	0.35	0.00	0.04		muscle
ENSXMAG00	1.78	1.03	0.00	0.03	oxnad1	muscle
ENSXMAG00	0.78	6.15	0.00	0.01	tbc1d16	muscle
ENSXMAG00	0.66	3.49	0.00	0.04	znf598	muscle
ENSXMAG00	-0.88	2.14	0.00	0.02	tbrg1	muscle
ENSXMAG00	1.27	4.66	0.00	0.00	larp4ab	muscle
ENSXMAG00	-0.69	5.67	0.00	0.02	plvapb	muscle
ENSXMAG00	1.33	5.52	0.00	0.00	soul5	muscle
ENSXMAG00	0.78	3.25	0.00	0.01	gak	muscle
ENSXMAG00	1.17	4.17	0.00	0.02	trib1	muscle
ENSXMAG00	0.89	5.99	0.00	0.00	brd2a	muscle
ENSXMAG00	0.95	4.71	0.00	0.03	NR4A1	muscle
ENSXMAG00	1.12	3.55	0.00	0.00	ddb2	muscle
ENSXMAG00	-0.79	2.36	0.00	0.04	pgghg	muscle
ENSXMAG00	0.79	6.22	0.00	0.01	tubb2b	muscle
ENSXMAG00	-1.19	2.11	0.00	0.00	b4galnt3b	muscle
ENSXMAG00	0.96	2.10	0.00	0.04	itsn2a	muscle
ENSXMAG00	0.94	4.16	0.00	0.02	zgc:66475	muscle
ENSXMAG00	1.02	2.70	0.00	0.00	sec23b	muscle
ENSXMAG00	-1.54	7.54	0.00	0.01		muscle
ENSXMAG00	0.64	5.34	0.00	0.02	etf1a	muscle
ENSXMAG00	1.59	-0.08	0.00	0.02		muscle

ENSXMAG00	-0.90	1.40	0.00	0.03	pou6f1	muscle
ENSXMAG00	0.73	2.95	0.00	0.03		muscle
ENSXMAG00	-1.41	1.98	0.00	0.02	aspa	muscle
ENSXMAG00	0.80	4.37	0.00	0.01	arhgap12b	muscle
ENSXMAG00	0.92	2.55	0.00	0.02	zmynd19	muscle
ENSXMAG00	0.77	2.89	0.00	0.05	pi4k2b	muscle
ENSXMAG00	-5.27	-3.05	0.00	0.05		muscle
ENSXMAG00	0.88	1.69	0.00	0.04	ENTPD7	muscle
ENSXMAG00	2.58	4.56	0.00	0.01	prelid3b	muscle
ENSXMAG00	0.78	2.76	0.00	0.03	mycla	muscle
ENSXMAG00	0.77	3.48	0.00	0.01	acsl4a	muscle
ENSXMAG00	1.45	1.58	0.00	0.00	ldlrb	muscle
ENSXMAG00	0.73	4.88	0.00	0.03	she	muscle
ENSXMAG00	-0.78	4.58	0.00	0.02	slc16a9a	muscle
ENSXMAG00	1.60	0.43	0.00	0.01		muscle
ENSXMAG00	0.67	3.66	0.00	0.03	si:ch211-117	muscle
ENSXMAG00	1.81	5.11	0.00	0.00	ppargc1a	muscle
ENSXMAG00	1.07	3.70	0.00	0.00	syncripl	muscle
ENSXMAG00	1.00	4.22	0.00	0.01	hsd3b7	muscle
ENSXMAG00	1.29	2.56	0.00	0.01	pnp5a	muscle
ENSXMAG00	1.18	1.28	0.00	0.01	tlcd2	muscle
ENSXMAG00	-0.99	1.74	0.00	0.04	si:ch211-106	muscle
ENSXMAG00	1.18	2.12	0.00	0.02	adamts9	muscle
ENSXMAG00	-0.89	2.94	0.00	0.01	sinhcaf	muscle
ENSXMAG00	-0.89	4.53	0.00	0.00	fam13a	muscle
ENSXMAG00	1.85	5.77	0.00	0.00	lonrf1l	muscle
ENSXMAG00	-0.81	4.68	0.00	0.02	smg6	muscle
ENSXMAG00	0.86	4.49	0.00	0.00	baz1b	muscle
ENSXMAG00	0.76	5.27	0.00	0.00	acvrl1	muscle
ENSXMAG00	1.03	3.48	0.00	0.00	zgc:55558	muscle
ENSXMAG00	-1.70	-1.01	0.00	0.02		muscle
ENSXMAG00	1.58	5.35	0.00	0.00	si:dkey-190g	muscle
ENSXMAG00	-0.92	3.53	0.00	0.01	si:dkey-17m8	muscle
ENSXMAG00	0.83	5.34	0.00	0.00	slc9a3r1b	muscle
ENSXMAG00	1.83	1.56	0.00	0.00	npas2	muscle
ENSXMAG00	1.64	5.24	0.00	0.00	irs2b	muscle
ENSXMAG00	1.27	5.75	0.00	0.00		muscle
ENSXMAG00	1.05	3.35	0.00	0.00	brpf3b	muscle
ENSXMAG00	1.54	2.60	0.00	0.00	khsrp	muscle
ENSXMAG00	0.77	2.99	0.00	0.02		muscle
ENSXMAG00	4.58	6.88	0.00	0.00	nr4a3	muscle
ENSXMAG00	-0.62	4.71	0.00	0.05	cpamd8	muscle
ENSXMAG00	-0.71	5.21	0.00	0.05	cnksr1	muscle

ENSXMAG00	0.81	3.39	0.00	0.01		muscle
ENSXMAG00	-1.28	1.30	0.00	0.01	hao2	muscle
ENSXMAG00	1.22	1.82	0.00	0.03	camk2n1a	muscle
ENSXMAG00	-1.44	4.28	0.00	0.01	klhl24b	muscle
ENSXMAG00	0.59	5.38	0.00	0.04		muscle
ENSXMAG00	-2.29	5.62	0.00	0.00	per1b	muscle
ENSXMAG00	1.10	6.35	0.00	0.04	actb1	muscle
ENSXMAG00	3.43	4.01	0.00	0.00	LONRF3	muscle
ENSXMAG00	-0.84	4.13	0.00	0.02	elmsan1b	muscle
ENSXMAG00	-1.34	3.90	0.00	0.00	ghra	muscle
ENSXMAG00	1.28	1.26	0.00	0.01	edn1	muscle
ENSXMAG00	2.74	4.45	0.00	0.00	per2	muscle
ENSXMAG00	0.86	5.45	0.00	0.01	heg1	muscle
ENSXMAG00	1.25	8.28	0.00	0.00	mylk4a	muscle
ENSXMAG00	-0.94	3.39	0.00	0.01	CHST15 (1 of	muscle
ENSXMAG00	0.67	5.36	0.00	0.01	amotl2a	muscle
ENSXMAG00	1.93	0.76	0.00	0.02		muscle
ENSXMAG00	-1.39	2.46	0.00	0.00	slc25a34	muscle
ENSXMAG00	1.30	3.09	0.00	0.00	cry5	muscle
ENSXMAG00	-0.82	4.71	0.00	0.00		muscle
ENSXMAG00	1.36	3.33	0.00	0.00	si:dkey-5g14	muscle
ENSXMAG00	-0.70	3.72	0.00	0.03		muscle
ENSXMAG00	1.44	2.34	0.00	0.03	si:ch211-195	muscle
ENSXMAG00	-1.08	4.86	0.00	0.00	tcp11l2	muscle
ENSXMAG00	-1.27	7.30	0.00	0.00	nr1d2a	muscle
ENSXMAG00	1.93	2.99	0.00	0.04	mibp	muscle
ENSXMAG00	1.76	7.19	0.00	0.00	si:ch1073-44	muscle
ENSXMAG00	0.75	3.92	0.00	0.00	selenoo1	muscle
ENSXMAG00	0.86	2.83	0.00	0.02	bcl7a	muscle
ENSXMAG00	1.21	2.24	0.00	0.00	rnf126	muscle
ENSXMAG00	1.62	3.29	0.00	0.01	TMEM100	muscle
ENSXMAG00	2.49	1.56	0.00	0.00		muscle
ENSXMAG00	1.23	4.12	0.00	0.00	otud1	muscle
ENSXMAG00	1.41	1.32	0.00	0.01		muscle
ENSXMAG00	-0.76	4.06	0.00	0.02	h1f0	muscle
ENSXMAG00	-0.79	2.57	0.00	0.04	arl14	muscle
ENSXMAG00	-1.05	5.37	0.00	0.00	mpp7a	muscle
ENSXMAG00	-0.85	4.88	0.00	0.00		muscle
ENSXMAG00	0.89	3.89	0.00	0.02		muscle
ENSXMAG00	0.83	3.21	0.00	0.05	ankrd10b	muscle
ENSXMAG00	0.92	2.53	0.00	0.00	dhrs3b	muscle
ENSXMAG00	2.42	-1.63	0.00	0.02	sh3gl2a	muscle
ENSXMAG00	0.97	5.42	0.00	0.00	cab39l1	muscle

ENSXMAG00	1.71	1.17	0.00	0.00	si:ch211-197	muscle
ENSXMAG00	1.19	2.35	0.00	0.01	cyp1b1	muscle
ENSXMAG00	1.16	6.24	0.00	0.01		muscle
ENSXMAG00	1.45	1.02	0.00	0.01		muscle
ENSXMAG00	1.02	4.07	0.00	0.00	VPS26B (1 of	muscle
ENSXMAG00	0.85	2.37	0.00	0.03	slc30a1a	muscle
ENSXMAG00	1.39	2.96	0.00	0.00	uck2b	muscle
ENSXMAG00	0.88	1.68	0.00	0.05		muscle
ENSXMAG00	-0.66	4.43	0.00	0.04		muscle
ENSXMAG00	0.79	8.23	0.00	0.02	phkg1b	muscle
ENSXMAG00	0.88	4.61	0.00	0.02	akap12b	muscle
ENSXMAG00	-0.91	1.89	0.00	0.03	zgc:113263	muscle
ENSXMAG00	1.95	-0.30	0.00	0.03		muscle
ENSXMAG00	0.70	3.31	0.00	0.02	C18orf32	muscle
ENSXMAG00	0.84	3.18	0.00	0.03	zgc:153115	muscle
ENSXMAG00	-0.88	2.76	0.00	0.01		muscle
ENSXMAG00	2.57	-1.72	0.00	0.02	prrt4	muscle
ENSXMAG00	-1.02	3.37	0.00	0.00	tnrc6c1	muscle
ENSXMAG00	1.47	1.62	0.00	0.02	atf3	muscle
ENSXMAG00	-0.70	5.74	0.00	0.02		muscle
ENSXMAG00	-2.09	1.29	0.00	0.00		muscle
ENSXMAG00	0.96	3.10	0.00	0.00	tram1	muscle
ENSXMAG00	-1.30	4.32	0.00	0.00	FIGNL2	muscle
ENSXMAG00	1.06	2.91	0.00	0.03		muscle
ENSXMAG00	-1.15	6.06	0.00	0.00	dbpa	muscle
ENSXMAG00	0.77	4.25	0.00	0.03	dnajc4	muscle
ENSXMAG00	1.20	2.11	0.00	0.00	etnk2	muscle
ENSXMAG00	1.19	1.59	0.00	0.03	dnajb9a	muscle
ENSXMAG00	2.31	5.41	0.00	0.00	coq10b	muscle
ENSXMAG00	0.73	4.99	0.00	0.01		muscle
ENSXMAG00	-0.72	3.78	0.00	0.01	si:ch211-286	muscle
ENSXMAG00	0.66	5.33	0.00	0.04	stk40	muscle
ENSXMAG00	1.52	2.83	0.00	0.00	nocta	muscle
ENSXMAG00	1.16	2.16	0.00	0.00	chchd4b	muscle
ENSXMAG00	-1.10	1.18	0.00	0.05	ccsapa	muscle
ENSXMAG00	-1.24	3.22	0.00	0.00	si:ch211-117	muscle
ENSXMAG00	0.72	2.77	0.00	0.05	slc52a2	muscle
ENSXMAG00	1.20	2.71	0.00	0.04	zbtb42	muscle
ENSXMAG00	2.05	0.07	0.00	0.02		muscle
ENSXMAG00	-1.07	1.82	0.00	0.04	nwd2	muscle
ENSXMAG00	-2.13	-1.87	0.00	0.01	mymk	eye
ENSXMAG00	-0.38	5.24	0.00	0.02	ascc3	eye

ENSXMAG00	0.90	5.06	0.00	0.00	slc1a8a	eye
ENSXMAG00	-1.48	6.74	0.00	0.00	dyrk4	eye
ENSXMAG00	-1.13	-0.96	0.00	0.05	piwil1	eye
ENSXMAG00	-0.69	2.67	0.00	0.02		eye
ENSXMAG00	-0.50	5.80	0.00	0.01	grin2aa	eye
ENSXMAG00	1.03	3.96	0.00	0.00	si:dkey-93h2	eye
ENSXMAG00	0.92	4.59	0.00	0.00	gfm1	eye
ENSXMAG00	-0.64	1.86	0.00	0.05		eye
ENSXMAG00	0.41	4.48	0.00	0.03	fhl1b	eye
ENSXMAG00	0.59	3.00	0.00	0.05	hgh1	eye
ENSXMAG00	-0.83	0.71	0.00	0.03	npy8ar	eye
ENSXMAG00	0.47	5.68	0.00	0.03	npm1a	eye
ENSXMAG00	-1.03	-0.03	0.00	0.01	fndc5b	eye
ENSXMAG00	0.38	5.15	0.00	0.04	agps	eye
ENSXMAG00	-0.72	5.02	0.00	0.00	ABLIM3 (1 of	eye
ENSXMAG00	1.04	4.67	0.00	0.00	prmt5	eye
ENSXMAG00	-0.89	2.02	0.00	0.00	crebrf	eye
ENSXMAG00	0.50	4.78	0.00	0.01	timm50	eye
ENSXMAG00	0.35	5.97	0.00	0.04		eye
ENSXMAG00	-0.78	6.50	0.00	0.00	ptdss2	eye
ENSXMAG00	-0.41	5.51	0.00	0.04	itgb8	eye
ENSXMAG00	-1.85	2.77	0.00	0.00		eye
ENSXMAG00	0.56	4.02	0.00	0.01	pign	eye
ENSXMAG00	-0.45	5.57	0.00	0.02	klf2b	eye
ENSXMAG00	0.37	5.86	0.00	0.04		eye
ENSXMAG00	-0.65	3.60	0.00	0.00	abca2	eye
ENSXMAG00	-0.65	3.93	0.00	0.00	znf395a	eye
ENSXMAG00	0.47	4.20	0.00	0.03	pdia5	eye
ENSXMAG00	0.75	2.38	0.00	0.00	kcnh8	eye
ENSXMAG00	-1.05	-0.41	0.00	0.05	dnah5	eye
ENSXMAG00	-0.45	3.89	0.00	0.02	polg	eye
ENSXMAG00	-0.87	5.07	0.00	0.00	si:ch211-186	eye
ENSXMAG00	1.52	6.36	0.00	0.00	cdr2a	eye
ENSXMAG00	-0.48	3.73	0.00	0.01	si:dkey-32e2	eye
ENSXMAG00	0.48	2.94	0.00	0.04	oxnad1	eye
ENSXMAG00	-0.56	2.23	0.00	0.03	sdr16c5a	eye
ENSXMAG00	-0.49	3.39	0.00	0.05	bcr	eye
ENSXMAG00	0.58	3.32	0.00	0.01	anks4b	eye
ENSXMAG00	0.48	5.81	0.00	0.02	dhrs12	eye
ENSXMAG00	-0.46	4.24	0.00	0.01	mief1	eye
ENSXMAG00	-1.48	2.80	0.00	0.00	vangl2	eye
ENSXMAG00	-0.39	4.76	0.00	0.05		eye
ENSXMAG00	0.51	10.54	0.00	0.04	gnb3b	eye

ENSXMAG00	-0.71	4.51	0.00	0.00	cnnm4a	eye
ENSXMAG00	-0.65	3.25	0.00	0.01	gdap1l1	eye
ENSXMAG00	0.44	5.81	0.00	0.04		eye
ENSXMAG00	1.11	0.09	0.00	0.04	cnga4	eye
ENSXMAG00	0.52	4.38	0.00	0.00	TMEM184B (	eye
ENSXMAG00	-0.75	1.76	0.00	0.02	lhfpl4a	eye
ENSXMAG00	-1.00	1.60	0.00	0.00		eye
ENSXMAG00	-0.46	4.55	0.00	0.03	tex2	eye
ENSXMAG00	-0.70	1.86	0.00	0.04	kcnj5	eye
ENSXMAG00	-0.88	0.32	0.00	0.04	klf7b	eye
ENSXMAG00	0.40	6.54	0.00	0.03	ubap2a	eye
ENSXMAG00	-0.45	3.97	0.00	0.01	gaa	eye
ENSXMAG00	0.49	4.58	0.00	0.02	tsr1	eye
ENSXMAG00	-0.96	1.18	0.00	0.05		eye
ENSXMAG00	-0.45	4.19	0.00	0.03	sgsm1b	eye
ENSXMAG00	0.88	3.85	0.00	0.00	slc35c1	eye
ENSXMAG00	-0.56	7.01	0.00	0.00	cerkl	eye
ENSXMAG00	0.91	4.27	0.00	0.00	hsd17b12a	eye
ENSXMAG00	0.61	5.40	0.00	0.00	hyou1	eye
ENSXMAG00	0.46	3.72	0.00	0.02	si:dkey-103i1	eye
ENSXMAG00	-0.60	2.36	0.00	0.03	wdsub1	eye
ENSXMAG00	-0.63	7.56	0.00	0.00	ip6k2b	eye
ENSXMAG00	0.48	6.73	0.00	0.01	grik1b	eye
ENSXMAG00	1.27	1.00	0.00	0.00	antxr1a	eye
ENSXMAG00	0.73	3.58	0.00	0.00	naprt	eye
ENSXMAG00	0.58	3.09	0.00	0.01	polr3d	eye
ENSXMAG00	-0.46	5.15	0.00	0.02	prkdc	eye
ENSXMAG00	0.82	2.09	0.00	0.00	pitpnb (1 of r	eye
ENSXMAG00	0.75	4.67	0.00	0.00	e2f4	eye
ENSXMAG00	1.67	-0.98	0.00	0.01	si:dkey-37o8.	eye
ENSXMAG00	-0.38	6.36	0.00	0.02	os9	eye
ENSXMAG00	1.50	-1.32	0.00	0.02	OTOP1	eye
ENSXMAG00	0.40	6.21	0.00	0.02	WDR1	eye
ENSXMAG00	-1.34	-0.60	0.00	0.01	gatm	eye
ENSXMAG00	1.28	4.60	0.00	0.00		eye
ENSXMAG00	-0.54	2.81	0.00	0.03	inhbb	eye
ENSXMAG00	-0.47	4.08	0.00	0.01		eye
ENSXMAG00	0.59	4.01	0.00	0.00	ppid	eye
ENSXMAG00	0.97	5.88	0.00	0.00	etfdh	eye
ENSXMAG00	-0.43	5.60	0.00	0.02		eye
ENSXMAG00	0.67	3.38	0.00	0.00	trnt1	eye
ENSXMAG00	-0.65	1.89	0.00	0.01	enosf1	eye
ENSXMAG00	0.56	2.50	0.00	0.04	inip	eye

ENSXMAG00	-1.65	-1.90	0.00	0.02	tdo2a	eye
ENSXMAG00	-0.42	5.68	0.00	0.03	cacnb2a	eye
ENSXMAG00	-0.83	1.13	0.00	0.02	fgf7	eye
ENSXMAG00	0.62	5.72	0.00	0.00	lrit3a	eye
ENSXMAG00	0.73	5.19	0.00	0.00	arl8	eye
ENSXMAG00	-0.56	1.94	0.00	0.03	adamts3	eye
ENSXMAG00	-0.57	5.08	0.00	0.02	ank1b	eye
ENSXMAG00	-0.77	6.53	0.00	0.00		eye
ENSXMAG00	-0.48	7.01	0.00	0.00	myo1cb	eye
ENSXMAG00	0.45	6.18	0.00	0.01	ppp4ca	eye
ENSXMAG00	0.52	2.82	0.00	0.03	mpv17l2	eye
ENSXMAG00	-0.97	0.80	0.00	0.01	GRHL2 (1 of	eye
ENSXMAG00	-0.70	2.92	0.00	0.00		eye
ENSXMAG00	0.49	4.35	0.00	0.00	dazap1	eye
ENSXMAG00	0.50	3.47	0.00	0.01	zgc:123010	eye
ENSXMAG00	-0.55	3.75	0.00	0.00	GOLGA7B (1	eye
ENSXMAG00	-1.61	2.35	0.00	0.00	cpm	eye
ENSXMAG00	-0.55	6.04	0.00	0.00	ctnnal1	eye
ENSXMAG00	-0.43	4.64	0.00	0.02		eye
ENSXMAG00	0.64	4.85	0.00	0.00	fam174b	eye
ENSXMAG00	0.41	5.34	0.00	0.01	slc25a55a	eye
ENSXMAG00	-0.49	4.49	0.00	0.02	si:ch1073-28	eye
ENSXMAG00	0.46	5.23	0.00	0.02	tars	eye
ENSXMAG00	0.44	3.98	0.00	0.03	mrpl1	eye
ENSXMAG00	0.43	3.43	0.00	0.05	snrnp40	eye
ENSXMAG00	0.52	3.26	0.00	0.02	ccdc88b	eye
ENSXMAG00	-0.63	1.38	0.00	0.03		eye
ENSXMAG00	0.59	3.79	0.00	0.00	emc8	eye
ENSXMAG00	-0.57	3.83	0.00	0.01	flrt1b	eye
ENSXMAG00	0.69	4.32	0.00	0.00	ppfibp2a	eye
ENSXMAG00	-0.53	4.40	0.00	0.01	diaph2	eye
ENSXMAG00	0.99	3.02	0.00	0.00	soat1	eye
ENSXMAG00	-0.43	4.41	0.00	0.01		eye
ENSXMAG00	-1.81	7.13	0.00	0.00	tulp4a	eye
ENSXMAG00	2.39	-1.12	0.00	0.00	mmp19	eye
ENSXMAG00	-0.74	2.25	0.00	0.01	afg1lb	eye
ENSXMAG00	0.50	3.68	0.00	0.02	cyb5b	eye
ENSXMAG00	-0.66	6.96	0.00	0.01	pmela	eye
ENSXMAG00	0.56	4.73	0.00	0.01		eye
ENSXMAG00	-0.55	3.57	0.00	0.03	bend5	eye
ENSXMAG00	0.54	5.74	0.00	0.03	grm6a	eye
ENSXMAG00	0.48	5.01	0.00	0.00	usp3	eye
ENSXMAG00	-0.76	4.37	0.00	0.00		eye

ENSXMAG00	0.99	3.92	0.00	0.00	sqlea	eye
ENSXMAG00	-3.39	0.20	0.00	0.00	abcg2b	eye
ENSXMAG00	0.40	7.78	0.00	0.03		eye
ENSXMAG00	0.41	6.55	0.00	0.02	si:ch1073-55	eye
ENSXMAG00	0.88	1.39	0.00	0.00	iqch	eye
ENSXMAG00	-0.70	2.55	0.00	0.02	NR4A1	eye
ENSXMAG00	-0.57	4.80	0.00	0.00	hey1	eye
ENSXMAG00	0.44	5.48	0.00	0.01	parp1	eye
ENSXMAG00	-0.78	1.33	0.00	0.01		eye
ENSXMAG00	0.46	4.76	0.00	0.01	mbtps2	eye
ENSXMAG00	-0.79	2.74	0.00	0.00	igf1ra	eye
ENSXMAG00	1.77	5.68	0.00	0.00	si:ch211-203	eye
ENSXMAG00	-0.91	6.57	0.00	0.00	dyrk3	eye
ENSXMAG00	0.71	4.57	0.00	0.00	ABHD1	eye
ENSXMAG00	0.75	1.41	0.00	0.04	fads2 (1 of m	eye
ENSXMAG00	-0.66	3.01	0.00	0.00		eye
ENSXMAG00	-0.65	2.12	0.00	0.04	mmp15b	eye
ENSXMAG00	-0.39	4.23	0.00	0.04	DDA1 (1 of m	eye
ENSXMAG00	-0.61	2.80	0.00	0.04		eye
ENSXMAG00	0.72	5.44	0.00	0.00	samd11	eye
ENSXMAG00	-0.92	2.91	0.00	0.00		eye
ENSXMAG00	-0.60	3.98	0.00	0.00	il1rapl2	eye
ENSXMAG00	-0.78	1.87	0.00	0.00	crb2b	eye
ENSXMAG00	-0.72	1.67	0.00	0.02		eye
ENSXMAG00	-0.45	3.65	0.00	0.03	dcc	eye
ENSXMAG00	-0.60	2.73	0.00	0.01	b4galnt3b	eye
ENSXMAG00	-1.68	5.44	0.00	0.01	fosab	eye
ENSXMAG00	0.42	4.23	0.00	0.03	tbc1d10ab	eye
ENSXMAG00	0.49	6.39	0.00	0.00		eye
ENSXMAG00	-0.71	3.81	0.00	0.00	RSBN1L (1 o	eye
ENSXMAG00	-0.48	3.40	0.00	0.03	cux2b	eye
ENSXMAG00	-0.57	2.64	0.00	0.01	rtn4rl1b	eye
ENSXMAG00	0.41	4.76	0.00	0.02	tmem267	eye
ENSXMAG00	-1.11	1.74	0.00	0.00	cdc7	eye
ENSXMAG00	-0.48	4.79	0.00	0.02	zgc:85722	eye
ENSXMAG00	-0.59	4.46	0.00	0.01	strip2	eye
ENSXMAG00	2.18	-2.38	0.00	0.04		eye
ENSXMAG00	0.42	4.28	0.00	0.02	fam136a	eye
ENSXMAG00	0.51	3.66	0.00	0.01	tmem209	eye
ENSXMAG00	1.27	0.07	0.00	0.00	fgf23	eye
ENSXMAG00	-0.43	7.38	0.00	0.04	si:dkey-91m1	eye
ENSXMAG00	0.55	5.07	0.00	0.00	plpbp	eye
ENSXMAG00	-1.03	2.23	0.00	0.00		eye

ENSXMAG00	0.56	4.98	0.00	0.00	dcun1d5	eye
ENSXMAG00	0.65	4.24	0.00	0.00	trap1	eye
ENSXMAG00	0.48	3.56	0.00	0.02		eye
ENSXMAG00	0.90	5.59	0.00	0.00	zgc:77752	eye
ENSXMAG00	-0.67	4.81	0.00	0.00	shank3a	eye
ENSXMAG00	0.47	4.37	0.00	0.03	polr3f	eye
ENSXMAG00	0.67	3.46	0.00	0.00		eye
ENSXMAG00	-0.61	1.35	0.00	0.05		eye
ENSXMAG00	-0.38	5.66	0.00	0.03	adarb2	eye
ENSXMAG00	1.04	3.63	0.00	0.00		eye
ENSXMAG00	0.68	5.08	0.00	0.00	si:dkey-97m3	eye
ENSXMAG00	1.03	5.82	0.00	0.00	esrrgb	eye
ENSXMAG00	-0.96	2.04	0.00	0.00		eye
ENSXMAG00	1.45	5.44	0.00	0.00	zgc:66475	eye
ENSXMAG00	-0.53	5.60	0.00	0.00	abca3b	eye
ENSXMAG00	0.37	5.22	0.00	0.04	sec23b	eye
ENSXMAG00	2.76	-2.63	0.00	0.04	DYNLRB1 (1	eye
ENSXMAG00	-0.86	3.68	0.00	0.00	tmem91	eye
ENSXMAG00	-0.45	3.45	0.00	0.03	rasgrp4	eye
ENSXMAG00	-0.63	3.30	0.00	0.01	ryr1b	eye
ENSXMAG00	-0.51	5.61	0.00	0.00	tnc	eye
ENSXMAG00	-0.51	7.82	0.00	0.00	MPDZ	eye
ENSXMAG00	-0.59	6.23	0.00	0.00	tyrp1a	eye
ENSXMAG00	-0.70	5.76	0.00	0.02	myo3b	eye
ENSXMAG00	1.00	3.36	0.00	0.00	imp4	eye
ENSXMAG00	-0.50	4.34	0.00	0.02	cdh5	eye
ENSXMAG00	0.55	6.28	0.00	0.00	etf1a	eye
ENSXMAG00	0.49	4.54	0.00	0.00	fam151b	eye
ENSXMAG00	-0.70	1.70	0.00	0.03	cdk19	eye
ENSXMAG00	-0.44	4.01	0.00	0.03	kalrna	eye
ENSXMAG00	-0.48	5.13	0.00	0.00	spg11	eye
ENSXMAG00	0.92	-0.15	0.00	0.05		eye
ENSXMAG00	-0.73	0.81	0.00	0.05	cyp27b1	eye
ENSXMAG00	-0.49	4.52	0.00	0.01	frmd8	eye
ENSXMAG00	-0.53	3.21	0.00	0.01	HS3ST5	eye
ENSXMAG00	0.62	4.93	0.00	0.00	dnajc10	eye
ENSXMAG00	0.45	4.34	0.00	0.01	nus1	eye
ENSXMAG00	-0.70	2.57	0.00	0.01	kcng4a	eye
ENSXMAG00	0.41	6.31	0.00	0.02	pik3r3b	eye
ENSXMAG00	-0.48	4.03	0.00	0.01	ada2a	eye
ENSXMAG00	-0.48	4.29	0.00	0.03	wu:fj29h11	eye
ENSXMAG00	0.53	5.86	0.00	0.01	gpr155a	eye
ENSXMAG00	-0.60	6.75	0.00	0.00	slc6a1l	eye

ENSXMAG00	0.45	5.64	0.00	0.01	cthl	eye
ENSXMAG00	-0.47	4.16	0.00	0.01	stmn4l	eye
ENSXMAG00	0.73	6.08	0.00	0.00	adipor2	eye
ENSXMAG00	-0.44	6.91	0.00	0.02	ERC1 (1 of m	eye
ENSXMAG00	0.54	6.50	0.00	0.00	pde10a	eye
ENSXMAG00	-0.64	2.46	0.00	0.04		eye
ENSXMAG00	1.02	2.73	0.00	0.00	myo19	eye
ENSXMAG00	0.52	6.85	0.00	0.00	pcsk1	eye
ENSXMAG00	-0.83	3.26	0.00	0.00	zgc:110789	eye
ENSXMAG00	-0.57	5.57	0.00	0.02	tmtc2b	eye
ENSXMAG00	0.45	5.35	0.00	0.01	CNNM1 (1 of	eye
ENSXMAG00	-0.81	3.19	0.00	0.00	kirrel1b	eye
ENSXMAG00	0.63	5.33	0.00	0.00	c1qbp	eye
ENSXMAG00	-0.89	0.93	0.00	0.01	htr2b	eye
ENSXMAG00	0.41	5.39	0.00	0.04	ENTPD7	eye
ENSXMAG00	0.37	4.92	0.00	0.04	dolpp1	eye
ENSXMAG00	-0.49	4.26	0.00	0.02	hpcal4	eye
ENSXMAG00	-0.56	4.36	0.00	0.02	cntn4	eye
ENSXMAG00	0.60	4.05	0.00	0.00	wdr75	eye
ENSXMAG00	0.48	5.77	0.00	0.00	HIGD1A	eye
ENSXMAG00	0.48	5.26	0.00	0.00	acer2	eye
ENSXMAG00	0.54	6.83	0.00	0.00		eye
ENSXMAG00	0.61	4.46	0.00	0.00	adat3	eye
ENSXMAG00	-0.79	4.73	0.00	0.00	sorl1	eye
ENSXMAG00	0.40	5.73	0.00	0.03	eef1db	eye
ENSXMAG00	-0.79	5.84	0.00	0.00	si:dkey-162b	eye
ENSXMAG00	-0.47	6.16	0.00	0.04	sptbn4b	eye
ENSXMAG00	-0.40	6.16	0.00	0.04	lamc3	eye
ENSXMAG00	-0.61	4.59	0.00	0.00	fibcd1	eye
ENSXMAG00	-0.65	5.24	0.00	0.02	aanat1	eye
ENSXMAG00	0.42	6.45	0.00	0.02	nfs1	eye
ENSXMAG00	0.65	4.55	0.00	0.01	ipo4	eye
ENSXMAG00	-0.60	3.91	0.00	0.00		eye
ENSXMAG00	0.58	5.94	0.00	0.01	hmgcs1	eye
ENSXMAG00	0.55	4.55	0.00	0.04	fam210b	eye
ENSXMAG00	0.96	4.50	0.00	0.01	trib3	eye
ENSXMAG00	-1.15	-1.03	0.00	0.03		eye
ENSXMAG00	-0.77	3.05	0.00	0.00		eye
ENSXMAG00	-0.47	4.34	0.00	0.02	nav3	eye
ENSXMAG00	-0.51	3.06	0.00	0.02	ephx5	eye
ENSXMAG00	0.91	2.80	0.00	0.00	exosc6	eye
ENSXMAG00	-0.99	7.52	0.00	0.00	cngb3.2	eye
ENSXMAG00	0.51	5.77	0.00	0.00	rbbp4	eye

ENSXMAG00	0.65	4.56	0.00	0.00	pitrm1	eye
ENSXMAG00	-0.91	6.67	0.00	0.00	myh9a	eye
ENSXMAG00	0.42	4.68	0.00	0.03	slc26a5	eye
ENSXMAG00	0.56	5.10	0.00	0.00	dpm1	eye
ENSXMAG00	-2.09	-2.09	0.00	0.01	dnaaf4	eye
ENSXMAG00	0.49	5.19	0.00	0.00		eye
ENSXMAG00	0.58	3.16	0.00	0.01	prmt6	eye
ENSXMAG00	1.10	-0.65	0.00	0.04		eye
ENSXMAG00	-0.52	5.93	0.00	0.04	lrmp	eye
ENSXMAG00	-1.16	3.03	0.00	0.00		eye
ENSXMAG00	0.62	3.84	0.00	0.00	fan1	eye
ENSXMAG00	-0.94	3.59	0.00	0.00	slco1e1	eye
ENSXMAG00	-0.48	7.13	0.00	0.00	aqp9b	eye
ENSXMAG00	-0.43	6.36	0.00	0.01	crot	eye
ENSXMAG00	0.69	5.20	0.00	0.00	vps37a	eye
ENSXMAG00	-0.88	3.40	0.00	0.00	iqgap2	eye
ENSXMAG00	-1.51	-1.07	0.00	0.00		eye
ENSXMAG00	0.37	5.17	0.00	0.04	abi1b	eye
ENSXMAG00	0.49	4.84	0.00	0.00	stx7l	eye
ENSXMAG00	4.47	-0.05	0.00	0.04		eye
ENSXMAG00	0.71	2.74	0.00	0.01	them4	eye
ENSXMAG00	-0.65	1.58	0.00	0.05	ano9a	eye
ENSXMAG00	-0.38	4.78	0.00	0.03	usf2	eye
ENSXMAG00	0.39	6.65	0.00	0.04	spartb	eye
ENSXMAG00	-0.54	5.59	0.00	0.02	dgkh	eye
ENSXMAG00	1.94	2.02	0.00	0.00		eye
ENSXMAG00	-0.45	5.01	0.00	0.02	NFIX	eye
ENSXMAG00	1.62	-0.52	0.00	0.00	rskra	eye
ENSXMAG00	-0.60	1.98	0.00	0.05	si:dkey-51a1	eye
ENSXMAG00	0.47	4.46	0.00	0.00	pex13	eye
ENSXMAG00	-0.74	3.37	0.00	0.00	klf8	eye
ENSXMAG00	0.54	5.69	0.00	0.00	sqor	eye
ENSXMAG00	0.52	3.95	0.00	0.04	hebp2	eye
ENSXMAG00	-0.61	5.97	0.00	0.00	tenm4	eye
ENSXMAG00	-0.51	6.11	0.00	0.00	adam15	eye
ENSXMAG00	0.39	4.99	0.00	0.03	H2AFZ	eye
ENSXMAG00	0.79	3.45	0.00	0.00	rwdd	eye
ENSXMAG00	-0.34	5.38	0.00	0.04	ppargc1a	eye
ENSXMAG00	0.81	3.45	0.00	0.00	irx6a	eye
ENSXMAG00	-1.25	6.16	0.00	0.00	znf395b	eye
ENSXMAG00	-0.50	4.91	0.00	0.04	sipa1l1	eye
ENSXMAG00	-0.74	1.79	0.00	0.03	erbb4a	eye
ENSXMAG00	0.63	4.57	0.00	0.00	msmo1	eye

ENSXMAG00	0.61	5.08	0.00	0.00	tpst1l	eye
ENSXMAG00	-0.66	3.75	0.00	0.00		eye
ENSXMAG00	0.86	0.84	0.00	0.05	mybl2b	eye
ENSXMAG00	0.39	6.31	0.00	0.03		eye
ENSXMAG00	0.40	4.87	0.00	0.03	nrg3b	eye
ENSXMAG00	0.51	2.85	0.00	0.04	trpv4	eye
ENSXMAG00	-0.51	7.51	0.00	0.01	1-Mar	eye
ENSXMAG00	-0.86	7.29	0.00	0.00	asap2a	eye
ENSXMAG00	0.51	4.65	0.00	0.00	kcnh6a	eye
ENSXMAG00	1.21	6.73	0.00	0.00	itpr1b	eye
ENSXMAG00	-0.37	5.04	0.00	0.02	cbsa	eye
ENSXMAG00	0.52	4.70	0.00	0.03		eye
ENSXMAG00	0.88	3.03	0.00	0.01	dclre1a	eye
ENSXMAG00	0.57	4.71	0.00	0.00	eed	eye
ENSXMAG00	-0.47	4.26	0.00	0.00	si:ch73-132f(	eye
ENSXMAG00	-0.53	4.48	0.00	0.01	fstl4	eye
ENSXMAG00	0.74	6.56	0.00	0.00		eye
ENSXMAG00	0.64	4.46	0.00	0.00	ddx47	eye
ENSXMAG00	-0.97	5.23	0.00	0.00	inpp4ab	eye
ENSXMAG00	0.40	6.49	0.00	0.04	sar1b	eye
ENSXMAG00	-0.68	3.16	0.00	0.00		eye
ENSXMAG00	-0.97	5.73	0.00	0.00	svilc	eye
ENSXMAG00	0.91	6.46	0.00	0.00	yme1l1a	eye
ENSXMAG00	-0.65	4.22	0.00	0.00	ncam3	eye
ENSXMAG00	0.67	2.52	0.00	0.00	rdh12l	eye
ENSXMAG00	0.52	4.35	0.00	0.00	ccs	eye
ENSXMAG00	-0.75	2.99	0.00	0.00		eye
ENSXMAG00	0.71	2.66	0.00	0.00	b3glctb	eye
ENSXMAG00	-0.59	5.71	0.00	0.00	gpr158a	eye
ENSXMAG00	-0.67	6.69	0.00	0.00		eye
ENSXMAG00	0.38	4.18	0.00	0.04	slc46a1	eye
ENSXMAG00	0.41	5.63	0.00	0.02	eftud2	eye
ENSXMAG00	-0.51	3.83	0.00	0.03	PLCB1	eye
ENSXMAG00	0.93	6.12	0.00	0.00	sema4ab	eye
ENSXMAG00	0.68	4.48	0.00	0.00	klhl22	eye
ENSXMAG00	-1.20	0.41	0.00	0.03	TMC2	eye
ENSXMAG00	-0.38	5.67	0.00	0.03		eye
ENSXMAG00	-0.99	7.15	0.00	0.00	pfkfb3	eye
ENSXMAG00	-0.57	4.96	0.00	0.01	ppp2r5b	eye
ENSXMAG00	0.51	5.78	0.00	0.01	cnot6a	eye
ENSXMAG00	-0.54	6.33	0.00	0.01	trak1a	eye
ENSXMAG00	0.44	7.43	0.00	0.02	lrwd1	eye
ENSXMAG00	-0.50	4.70	0.00	0.01	cbl	eye

ENSXMAG00	-1.29	-0.99	0.00	0.02	si:dkey-183c(	eye
ENSXMAG00	-0.85	4.80	0.00	0.00	ephx1	eye
ENSXMAG00	-1.11	0.64	0.00	0.00	tcf23	eye
ENSXMAG00	-2.23	-1.89	0.00	0.02		eye
ENSXMAG00	-1.84	6.75	0.00	0.00	gas2l3	eye
ENSXMAG00	-4.53	-3.41	0.00	0.04		eye
ENSXMAG00	-0.48	6.09	0.00	0.00	si:ch211-210	eye
ENSXMAG00	0.61	4.88	0.00	0.00	FAM53C	eye
ENSXMAG00	-0.44	6.12	0.00	0.02	magi2b	eye
ENSXMAG00	0.58	4.86	0.00	0.00	dse	eye
ENSXMAG00	0.40	6.82	0.00	0.02	atp6v0a1a	eye
ENSXMAG00	-0.56	3.29	0.00	0.02	afap1	eye
ENSXMAG00	-0.68	4.05	0.00	0.00	TRABD2A	eye
ENSXMAG00	0.46	4.91	0.00	0.00	abcb7	eye
ENSXMAG00	-0.62	2.26	0.00	0.01	LRRC17 (1 of	eye
ENSXMAG00	0.39	4.11	0.00	0.04	gpn3	eye
ENSXMAG00	0.67	3.91	0.00	0.00	fam185a	eye
ENSXMAG00	-0.60	4.01	0.00	0.00	pld1a	eye
ENSXMAG00	-0.56	5.30	0.00	0.00	grip2b	eye
ENSXMAG00	-0.79	6.12	0.00	0.01		eye
ENSXMAG00	-0.88	5.48	0.00	0.00	paplna	eye
ENSXMAG00	-0.79	7.13	0.00	0.00	galnt16	eye
ENSXMAG00	-0.64	4.22	0.00	0.02	csrnp1b	eye
ENSXMAG00	1.60	4.52	0.00	0.00	cry-dash	eye
ENSXMAG00	0.54	2.69	0.00	0.03	cradd	eye
ENSXMAG00	-0.79	3.76	0.00	0.00	pmelb	eye
ENSXMAG00	-0.55	3.77	0.00	0.00	axin1	eye
ENSXMAG00	0.40	5.99	0.00	0.02	gars	eye
ENSXMAG00	0.40	4.40	0.00	0.03	tmem41b	eye
ENSXMAG00	-0.51	5.67	0.00	0.03	bbx	eye
ENSXMAG00	-0.58	3.75	0.00	0.00	hs3st4	eye
ENSXMAG00	-0.64	1.77	0.00	0.03	si:dkey-191m	eye
ENSXMAG00	-0.76	2.59	0.00	0.01		eye
ENSXMAG00	0.54	5.80	0.00	0.00	phactr3b	eye
ENSXMAG00	-1.22	6.06	0.00	0.01	lima1a	eye
ENSXMAG00	0.60	5.83	0.00	0.00	txnrd3	eye
ENSXMAG00	-0.48	4.18	0.00	0.00		eye
ENSXMAG00	0.96	2.11	0.00	0.00	lias	eye
ENSXMAG00	-1.00	1.02	0.00	0.01	cyldb	eye
ENSXMAG00	-0.82	8.09	0.00	0.00	f8	eye
ENSXMAG00	-1.02	4.27	0.00	0.04	lnx2a	eye
ENSXMAG00	0.35	5.91	0.00	0.04	ssrp1a	eye
ENSXMAG00	-0.46	6.71	0.00	0.00	fam13a	eye

ENSXMAG00	-1.09	4.29	0.00	0.03	cntf	eye
ENSXMAG00	0.53	5.16	0.00	0.00	cdk8	eye
ENSXMAG00	-0.80	2.59	0.00	0.00	cdh4	eye
ENSXMAG00	-2.04	0.64	0.00	0.00		eye
ENSXMAG00	-0.81	3.01	0.00	0.00		eye
ENSXMAG00	1.31	7.09	0.00	0.00	lonrf1l	eye
ENSXMAG00	0.42	5.53	0.00	0.01	hbegf	eye
ENSXMAG00	-0.60	5.50	0.00	0.01	si:ch211-253	eye
ENSXMAG00	0.36	6.80	0.00	0.05	dctn4	eye
ENSXMAG00	0.60	3.15	0.00	0.01	snapc3	eye
ENSXMAG00	0.36	6.37	0.00	0.04		eye
ENSXMAG00	2.10	3.27	0.00	0.00	TTC39B	eye
ENSXMAG00	-0.76	2.97	0.00	0.02		eye
ENSXMAG00	-0.56	6.20	0.00	0.04	lipib	eye
ENSXMAG00	0.51	3.35	0.00	0.01	stc2a	eye
ENSXMAG00	-3.73	-3.02	0.00	0.02	lhfpl5b	eye
ENSXMAG00	-0.41	6.30	0.00	0.02	fbln1	eye
ENSXMAG00	-1.70	7.40	0.00	0.00	slkb	eye
ENSXMAG00	0.44	5.34	0.00	0.01	dnajb11	eye
ENSXMAG00	-0.90	3.88	0.00	0.00	sh3pxd2aa	eye
ENSXMAG00	1.15	5.12	0.00	0.00	nab1b	eye
ENSXMAG00	0.62	5.09	0.00	0.00	adka	eye
ENSXMAG00	0.61	7.24	0.00	0.00		eye
ENSXMAG00	-0.62	7.57	0.00	0.02	zgc:195245	eye
ENSXMAG00	0.55	5.49	0.00	0.00	aox1	eye
ENSXMAG00	-0.40	3.83	0.00	0.03	si:dkey-49n2	eye
ENSXMAG00	1.17	2.95	0.00	0.00		eye
ENSXMAG00	1.14	6.74	0.00	0.00	lonrf1	eye
ENSXMAG00	-0.46	5.32	0.00	0.01	pdzd8	eye
ENSXMAG00	-0.55	2.85	0.00	0.02	si:ch211-246	eye
ENSXMAG00	-0.57	3.56	0.00	0.04	cadm2a	eye
ENSXMAG00	0.42	6.90	0.00	0.01	glmnb	eye
ENSXMAG00	0.44	6.81	0.00	0.01	cnga1b	eye
ENSXMAG00	0.43	5.85	0.00	0.02	dnpep	eye
ENSXMAG00	-0.42	7.71	0.00	0.01	si:ch73-389b	eye
ENSXMAG00	0.80	3.69	0.00	0.00	heatr6	eye
ENSXMAG00	-0.68	3.46	0.00	0.01		eye
ENSXMAG00	0.43	5.33	0.00	0.01	cyp46a1.4	eye
ENSXMAG00	-0.80	4.64	0.00	0.00		eye
ENSXMAG00	0.35	5.68	0.00	0.04	hars	eye
ENSXMAG00	0.48	4.14	0.00	0.00	gdpd1	eye
ENSXMAG00	-0.57	3.57	0.00	0.00	ddr1	eye
ENSXMAG00	-0.55	3.69	0.00	0.00	itga9	eye

ENSXMAG00	0.51	6.04	0.00	0.01	tfr1a	eye
ENSXMAG00	0.37	4.53	0.00	0.05	uchl3	eye
ENSXMAG00	-1.55	-1.19	0.00	0.02	cts12	eye
ENSXMAG00	-0.67	1.87	0.00	0.01	elovl8a	eye
ENSXMAG00	0.98	0.35	0.00	0.05	tbxtb	eye
ENSXMAG00	-1.59	6.17	0.00	0.00		eye
ENSXMAG00	0.54	3.72	0.00	0.01	lpcat3	eye
ENSXMAG00	0.52	5.10	0.00	0.00	pa2g4b	eye
ENSXMAG00	-0.46	7.63	0.00	0.00	suco	eye
ENSXMAG00	-0.69	2.89	0.00	0.00	arhgap39	eye
ENSXMAG00	-0.60	5.63	0.00	0.01	sema3aa	eye
ENSXMAG00	0.35	5.15	0.00	0.04	exosc10	eye
ENSXMAG00	0.53	8.23	0.00	0.00	casz1	eye
ENSXMAG00	-0.49	6.03	0.00	0.03	uckl1a	eye
ENSXMAG00	0.36	4.59	0.00	0.05	plpp6	eye
ENSXMAG00	-2.08	-2.20	0.00	0.01	hrh2a	eye
ENSXMAG00	-0.62	2.29	0.00	0.03		eye
ENSXMAG00	0.88	3.06	0.00	0.00	coa7	eye
ENSXMAG00	0.40	4.48	0.00	0.03	FAIM2	eye
ENSXMAG00	0.52	5.11	0.00	0.00	nop2	eye
ENSXMAG00	-0.44	7.54	0.00	0.01	clstn1	eye
ENSXMAG00	0.49	3.86	0.00	0.01	txndc11	eye
ENSXMAG00	0.53	5.61	0.00	0.00	tmem189	eye
ENSXMAG00	0.90	0.81	0.00	0.01	hsd17b7	eye
ENSXMAG00	0.49	5.87	0.00	0.02	zgc:55558	eye
ENSXMAG00	-0.77	2.52	0.00	0.04	igsf10	eye
ENSXMAG00	0.40	5.10	0.00	0.03	psma6b	eye
ENSXMAG00	0.40	5.45	0.00	0.05	meis1b	eye
ENSXMAG00	0.62	5.25	0.00	0.00	stk39	eye
ENSXMAG00	0.46	5.52	0.00	0.03		eye
ENSXMAG00	0.78	4.85	0.00	0.01	si:dkey-190g	eye
ENSXMAG00	-1.92	0.94	0.00	0.00	il11a	eye
ENSXMAG00	-0.36	4.46	0.00	0.04	zdhhc18b	eye
ENSXMAG00	0.39	5.44	0.00	0.02	si:ch211-114	eye
ENSXMAG00	0.50	6.69	0.00	0.03	si:dkey-92j12	eye
ENSXMAG00	-0.96	1.71	0.00	0.00	kcnk9	eye
ENSXMAG00	-0.54	6.17	0.00	0.00	nxnl1 (1 of m	eye
ENSXMAG00	0.61	1.88	0.00	0.03	sat2b	eye
ENSXMAG00	0.55	4.23	0.00	0.00	manf	eye
ENSXMAG00	0.54	4.66	0.00	0.00	blmh	eye
ENSXMAG00	-0.41	7.07	0.00	0.03	myo9ab	eye
ENSXMAG00	0.96	3.64	0.00	0.00	dus2	eye
ENSXMAG00	-1.18	-0.78	0.00	0.02		eye

ENSXMAG00	0.47	4.65	0.00	0.00	radx	eye
ENSXMAG00	-1.12	-0.08	0.00	0.00	tecta	eye
ENSXMAG00	-0.38	5.90	0.00	0.02	agrn	eye
ENSXMAG00	-0.61	2.43	0.00	0.02		eye
ENSXMAG00	0.87	1.29	0.00	0.01	VWA5B1	eye
ENSXMAG00	-0.77	1.20	0.00	0.03	ano11	eye
ENSXMAG00	0.42	5.33	0.00	0.01	ubiad1	eye
ENSXMAG00	-0.57	4.64	0.00	0.02	socs3a	eye
ENSXMAG00	0.54	3.75	0.00	0.02	pdap1a	eye
ENSXMAG00	-0.47	5.22	0.00	0.00	12-Sep	eye
ENSXMAG00	-0.47	3.00	0.00	0.04	unc13d	eye
ENSXMAG00	-0.56	6.25	0.00	0.01	gabra1	eye
ENSXMAG00	0.53	5.13	0.00	0.01	mbnl2	eye
ENSXMAG00	0.62	4.87	0.00	0.00	zbtb7b	eye
ENSXMAG00	-0.70	4.53	0.00	0.00		eye
ENSXMAG00	-1.50	-1.61	0.00	0.02	pax5	eye
ENSXMAG00	0.51	4.25	0.00	0.01	GOLGA7B (1	eye
ENSXMAG00	1.28	2.34	0.00	0.00	wee1	eye
ENSXMAG00	0.76	5.44	0.00	0.00	hs3st3b1a	eye
ENSXMAG00	0.55	3.56	0.00	0.03	bco1	eye
ENSXMAG00	0.42	4.65	0.00	0.02	rerglb	eye
ENSXMAG00	-0.49	2.70	0.00	0.03		eye
ENSXMAG00	-0.46	3.81	0.00	0.01	sgsh	eye
ENSXMAG00	-0.74	1.56	0.00	0.01	sema6bb	eye
ENSXMAG00	-0.50	5.11	0.00	0.00	si:ch73-60h1	eye
ENSXMAG00	0.56	2.46	0.00	0.03	lipt2	eye
ENSXMAG00	1.05	5.17	0.00	0.00		eye
ENSXMAG00	-2.11	-2.55	0.00	0.05		eye
ENSXMAG00	0.71	4.37	0.00	0.01	acot11b	eye
ENSXMAG00	0.72	5.92	0.00	0.00	dnajb1a	eye
ENSXMAG00	0.71	3.66	0.00	0.00	fpgt	eye
ENSXMAG00	-0.73	1.47	0.00	0.02	eef2kmt	eye
ENSXMAG00	-1.74	3.78	0.00	0.01	npas4a	eye
ENSXMAG00	0.52	4.23	0.00	0.03	dmbx1b	eye
ENSXMAG00	-0.77	0.48	0.00	0.04		eye
ENSXMAG00	-2.09	0.93	0.00	0.00		eye
ENSXMAG00	0.92	5.06	0.00	0.01	tle2a	eye
ENSXMAG00	-0.35	5.59	0.00	0.04	cbfa2t2	eye
ENSXMAG00	0.41	7.26	0.00	0.01	map7d3	eye
ENSXMAG00	-1.00	-0.42	0.00	0.04		eye
ENSXMAG00	-0.67	3.13	0.00	0.02		eye
ENSXMAG00	-0.67	2.96	0.00	0.01	drp2	eye
ENSXMAG00	0.79	3.99	0.00	0.00	pprc1	eye

ENSXMAG00	-0.60	2.14	0.00	0.03	cenpi	eye
ENSXMAG00	-0.45	3.36	0.00	0.03	syndig1l	eye
ENSXMAG00	0.55	4.86	0.00	0.01	slc25a47a	eye
ENSXMAG00	-0.81	6.22	0.00	0.00	zfyve28	eye
ENSXMAG00	-0.53	3.65	0.00	0.02	dnase2	eye
ENSXMAG00	3.35	-2.79	0.00	0.03	pigu	eye
ENSXMAG00	0.72	3.39	0.00	0.00	adam8a	eye
ENSXMAG00	0.71	5.12	0.00	0.00	nop58	eye
ENSXMAG00	-0.98	1.85	0.00	0.01	fgf11a	eye
ENSXMAG00	-0.86	5.58	0.00	0.00		eye
ENSXMAG00	0.45	4.33	0.00	0.01	ube2a	eye
ENSXMAG00	-0.74	3.91	0.00	0.01		eye
ENSXMAG00	-0.44	3.96	0.00	0.05	tp53bp2b	eye
ENSXMAG00	-0.59	6.76	0.00	0.00	shank2b	eye
ENSXMAG00	-1.03	5.48	0.00	0.00	akt3a	eye
ENSXMAG00	-0.47	4.48	0.00	0.01	cpvl	eye
ENSXMAG00	-0.86	1.48	0.00	0.00	col28a2b	eye
ENSXMAG00	-0.46	5.20	0.00	0.02	si:rp71-39b2(	eye
ENSXMAG00	0.65	4.83	0.00	0.00	abcf3	eye
ENSXMAG00	0.39	5.76	0.00	0.03	sgsm3	eye
ENSXMAG00	-1.11	4.48	0.00	0.00	si:ch211-257	eye
ENSXMAG00	0.59	6.14	0.00	0.05		eye
ENSXMAG00	-0.72	3.20	0.00	0.00	klf4	eye
ENSXMAG00	-0.54	4.11	0.00	0.00	csmd3a	eye
ENSXMAG00	0.62	4.08	0.00	0.00	ogfod1	eye
ENSXMAG00	-0.41	7.78	0.00	0.03	jmjd1cb	eye
ENSXMAG00	-0.51	3.91	0.00	0.01	clic1	eye
ENSXMAG00	0.37	6.26	0.00	0.03	TNPO2 (1 of	eye
ENSXMAG00	0.67	2.80	0.00	0.01	upp1	eye
ENSXMAG00	-0.55	6.12	0.00	0.00		eye
ENSXMAG00	0.56	6.19	0.00	0.02	thbs1a	eye
ENSXMAG00	0.54	5.98	0.00	0.00		eye
ENSXMAG00	2.12	-1.30	0.00	0.02	GPX2	eye
ENSXMAG00	-0.62	6.74	0.00	0.00	ret	eye
ENSXMAG00	-0.42	4.04	0.00	0.04	hyal4	eye
ENSXMAG00	-0.64	1.69	0.00	0.03	CSMD1	eye
ENSXMAG00	-2.14	1.88	0.00	0.00		eye
ENSXMAG00	-0.89	0.58	0.00	0.04	dla	eye
ENSXMAG00	0.51	4.02	0.00	0.00	supv3l1	eye
ENSXMAG00	-0.50	3.19	0.00	0.03		eye
ENSXMAG00	0.52	4.06	0.00	0.02	2-Mar	eye
ENSXMAG00	0.84	7.11	0.00	0.00	ESRRB	eye
ENSXMAG00	0.49	5.78	0.00	0.00	map4k5	eye

ENSXMAG00	-0.40	5.70	0.00	0.01	CDH8	eye
ENSXMAG00	0.73	2.65	0.00	0.04	ABCA1 (1 of	eye
ENSXMAG00	-1.13	7.44	0.00	0.00	nin	eye
ENSXMAG00	-0.48	6.77	0.00	0.00	arhgap36	eye
ENSXMAG00	-0.58	4.77	0.00	0.01	nhsl2	eye
ENSXMAG00	-0.62	6.44	0.00	0.00	cgnb	eye
ENSXMAG00	-0.77	8.34	0.00	0.00	lrit1b	eye
ENSXMAG00	0.92	3.23	0.00	0.00		eye
ENSXMAG00	0.46	5.23	0.00	0.01	pcna	eye
ENSXMAG00	0.47	4.57	0.00	0.05	hcn5	eye
ENSXMAG00	-0.51	2.74	0.00	0.04	ttll6	eye
ENSXMAG00	0.63	2.72	0.00	0.01	pdcd2	eye
ENSXMAG00	0.51	5.92	0.00	0.01	cox17	eye
ENSXMAG00	-0.48	3.61	0.00	0.02	frmd6	eye
ENSXMAG00	0.96	4.38	0.00	0.00	MSRB1	eye
ENSXMAG00	0.43	4.08	0.00	0.02	tk2	eye
ENSXMAG00	0.41	4.58	0.00	0.04	si:ch211-87m	eye
ENSXMAG00	-0.39	4.52	0.00	0.05	camk2n1a	eye
ENSXMAG00	-0.72	1.59	0.00	0.01	irf7	eye
ENSXMAG00	-0.55	2.06	0.00	0.04	pask	eye
ENSXMAG00	-0.48	5.58	0.00	0.01	coq6	eye
ENSXMAG00	-0.54	7.73	0.00	0.00	irs2a	eye
ENSXMAG00	-0.86	4.58	0.00	0.00	slc5a6b	eye
ENSXMAG00	0.70	6.77	0.00	0.00		eye
ENSXMAG00	-0.51	6.60	0.00	0.01		eye
ENSXMAG00	-0.56	4.24	0.00	0.02		eye
ENSXMAG00	-0.41	3.97	0.00	0.04	smc6	eye
ENSXMAG00	-0.75	2.35	0.00	0.03	slc35f4	eye
ENSXMAG00	0.45	3.73	0.00	0.04	eif4eb	eye
ENSXMAG00	0.51	4.48	0.00	0.00	smarcad1a	eye
ENSXMAG00	0.53	3.40	0.00	0.01	zgc:63863	eye
ENSXMAG00	-0.69	6.81	0.00	0.00		eye
ENSXMAG00	-0.56	3.61	0.00	0.01	glb1l	eye
ENSXMAG00	0.39	4.73	0.00	0.05	GALNT10 (1	eye
ENSXMAG00	0.70	4.09	0.00	0.00	zgc:158828	eye
ENSXMAG00	0.87	2.32	0.00	0.00	zgc:65997	eye
ENSXMAG00	-0.44	3.52	0.00	0.05	nphp1	eye
ENSXMAG00	0.42	6.91	0.00	0.01	ptk2aa	eye
ENSXMAG00	-0.61	4.45	0.00	0.02	slc4a8	eye
ENSXMAG00	-0.72	1.99	0.00	0.00	opn7b	eye
ENSXMAG00	-0.52	6.15	0.00	0.00	pde4d	eye
ENSXMAG00	-0.59	2.72	0.00	0.03	csmd3a	eye
ENSXMAG00	-0.56	6.04	0.00	0.00	rorb	eye

ENSXMAG00	-0.46	3.95	0.00	0.01	sfi1	eye
ENSXMAG00	0.44	4.87	0.00	0.02	rars	eye
ENSXMAG00	-1.05	3.15	0.00	0.00	si:rp71-79p2(	eye
ENSXMAG00	-1.26	1.31	0.00	0.00		eye
ENSXMAG00	3.75	7.05	0.00	0.00	LONRF3	eye
ENSXMAG00	-0.36	9.85	0.00	0.05	slc6a6a	eye
ENSXMAG00	-0.80	3.69	0.00	0.00	mych	eye
ENSXMAG00	-0.44	4.69	0.00	0.03	sptb	eye
ENSXMAG00	-0.36	6.82	0.00	0.04		eye
ENSXMAG00	-0.93	5.15	0.00	0.00	nim1k	eye
ENSXMAG00	0.38	5.48	0.00	0.04	tuba8l3	eye
ENSXMAG00	0.46	4.48	0.00	0.01	polr2c	eye
ENSXMAG00	-0.79	2.05	0.00	0.02	uhmk1	eye
ENSXMAG00	0.54	6.79	0.00	0.00	rcvrna	eye
ENSXMAG00	0.56	4.59	0.00	0.00	uba2	eye
ENSXMAG00	-0.48	4.97	0.00	0.01	zgc:162200	eye
ENSXMAG00	-0.79	5.71	0.00	0.00	mrps27	eye
ENSXMAG00	0.41	7.33	0.00	0.01	eif4g1a	eye
ENSXMAG00	-0.86	5.45	0.00	0.00	nedd9	eye
ENSXMAG00	-1.32	3.84	0.00	0.00	ptger4a	eye
ENSXMAG00	0.58	5.92	0.00	0.00	mctp1b	eye
ENSXMAG00	0.77	3.46	0.00	0.00	chac2	eye
ENSXMAG00	0.38	4.91	0.00	0.03	dnajc11a	eye
ENSXMAG00	0.45	4.98	0.00	0.01	akap17a	eye
ENSXMAG00	0.45	6.01	0.00	0.01	fam49al	eye
ENSXMAG00	-0.46	4.41	0.00	0.01	si:ch1073-51	eye
ENSXMAG00	0.67	2.55	0.00	0.02	rrp9	eye
ENSXMAG00	-1.23	-0.16	0.00	0.01		eye
ENSXMAG00	0.52	4.04	0.00	0.00	dhx30	eye
ENSXMAG00	-0.61	3.74	0.00	0.05	pdzph1	eye
ENSXMAG00	-0.63	3.89	0.00	0.00	SLCO1C1	eye
ENSXMAG00	-0.61	2.07	0.00	0.02	AMZ2	eye
ENSXMAG00	-0.52	4.49	0.00	0.00	prkag2b	eye
ENSXMAG00	-0.59	8.90	0.00	0.00	MACF1	eye
ENSXMAG00	-0.49	4.07	0.00	0.03	ADAM12 (1 c	eye
ENSXMAG00	0.40	5.94	0.00	0.04	si:ch211-79l1	eye
ENSXMAG00	0.53	5.01	0.00	0.01	pcdh1b	eye
ENSXMAG00	-0.57	5.35	0.00	0.02	syt2a	eye
ENSXMAG00	-0.58	3.91	0.00	0.01	mmp16b	eye
ENSXMAG00	0.40	4.79	0.00	0.04	ssu72	eye
ENSXMAG00	-1.69	-1.73	0.00	0.01		eye
ENSXMAG00	0.44	4.49	0.00	0.05		eye
ENSXMAG00	0.43	5.56	0.00	0.05	amotl2a	eye

ENSXMAG00	-0.39	6.70	0.00	0.04	cacnb2b	eye
ENSXMAG00	0.42	4.12	0.00	0.02	mrpl20	eye
ENSXMAG00	-0.71	1.00	0.00	0.04		eye
ENSXMAG00	0.45	10.51	0.00	0.01	pde6gb	eye
ENSXMAG00	-0.57	4.21	0.00	0.00	map2k6	eye
ENSXMAG00	0.39	5.22	0.00	0.02		eye
ENSXMAG00	-0.87	2.27	0.00	0.00	slc24a5	eye
ENSXMAG00	-0.57	2.17	0.00	0.04	slc25a34	eye
ENSXMAG00	1.68	4.12	0.00	0.00	cyp1a	eye
ENSXMAG00	1.84	5.29	0.00	0.01	prdm1b	eye
ENSXMAG00	0.67	4.90	0.00	0.00	slc25a25b	eye
ENSXMAG00	-0.66	4.12	0.00	0.00	cacna2d2a	eye
ENSXMAG00	-0.85	2.26	0.00	0.00	lrp2b	eye
ENSXMAG00	-0.61	5.08	0.00	0.00		eye
ENSXMAG00	-0.66	6.78	0.00	0.00		eye
ENSXMAG00	-0.59	6.71	0.00	0.01	DSCAM (1 of	eye
ENSXMAG00	0.67	3.09	0.00	0.00	TMEM43	eye
ENSXMAG00	-0.40	5.15	0.00	0.02		eye
ENSXMAG00	-0.54	2.21	0.00	0.04	oxsm	eye
ENSXMAG00	-0.51	3.89	0.00	0.02	atm	eye
ENSXMAG00	-0.41	8.86	0.00	0.03	ldhbb	eye
ENSXMAG00	0.59	2.70	0.00	0.02	riox1	eye
ENSXMAG00	0.54	5.03	0.00	0.00	timm23a	eye
ENSXMAG00	0.41	7.53	0.00	0.05	slc12a5b	eye
ENSXMAG00	0.82	0.64	0.00	0.04		eye
ENSXMAG00	1.24	3.63	0.00	0.02	cry5	eye
ENSXMAG00	0.83	6.53	0.00	0.00	rgs9b	eye
ENSXMAG00	-0.77	1.64	0.00	0.02	miip	eye
ENSXMAG00	-0.51	4.65	0.00	0.00	srgap1b	eye
ENSXMAG00	-0.79	6.26	0.00	0.00	kif19	eye
ENSXMAG00	-1.43	1.07	0.00	0.00	gls2a	eye
ENSXMAG00	0.39	5.68	0.00	0.03	si:ch211-30b	eye
ENSXMAG00	-0.49	4.06	0.00	0.05	mindy1	eye
ENSXMAG00	-0.65	4.37	0.00	0.01	cacna2d2b	eye
ENSXMAG00	-1.24	5.13	0.00	0.00	nfatc3a	eye
ENSXMAG00	0.37	4.92	0.00	0.03	rer1	eye
ENSXMAG00	-0.41	5.28	0.00	0.02	abcc9	eye
ENSXMAG00	-0.85	5.61	0.00	0.00	hdac5	eye
ENSXMAG00	0.39	6.69	0.00	0.04	ptges3b	eye
ENSXMAG00	0.55	8.07	0.00	0.03		eye
ENSXMAG00	1.42	2.05	0.00	0.00		eye
ENSXMAG00	0.41	4.26	0.00	0.04	ints7	eye
ENSXMAG00	0.91	5.42	0.00	0.00	si:dkey-5g14	eye

ENSXMAG00	0.38	6.36	0.00	0.03	lpgat1	eye
ENSXMAG00	0.90	1.73	0.00	0.00	greb1	eye
ENSXMAG00	-0.85	0.20	0.00	0.05	si:ch73-352p	eye
ENSXMAG00	-0.66	2.83	0.00	0.00	zgc:171857	eye
ENSXMAG00	-1.06	0.57	0.00	0.03	grm5a	eye
ENSXMAG00	-0.48	5.44	0.00	0.01		eye
ENSXMAG00	0.88	3.92	0.00	0.00	SLC43A3 (1 c	eye
ENSXMAG00	-0.50	4.30	0.00	0.03	ncoa1	eye
ENSXMAG00	1.18	6.71	0.00	0.00	samd7	eye
ENSXMAG00	-0.62	1.20	0.00	0.04	DUSP3	eye
ENSXMAG00	-0.59	2.59	0.00	0.01		eye
ENSXMAG00	0.45	6.32	0.00	0.01	ddit3	eye
ENSXMAG00	-0.61	2.14	0.00	0.02		eye
ENSXMAG00	-0.54	4.05	0.00	0.01	anks1ab	eye
ENSXMAG00	0.36	5.11	0.00	0.03	psmd11b	eye
ENSXMAG00	-0.51	3.01	0.00	0.03	si:ch211-225	eye
ENSXMAG00	0.54	6.38	0.00	0.00		eye
ENSXMAG00	-0.61	7.59	0.00	0.00	ssx2ipb	eye
ENSXMAG00	-0.45	7.23	0.00	0.02	UNC13C	eye
ENSXMAG00	0.43	6.99	0.00	0.01	lrpprc	eye
ENSXMAG00	-2.30	-2.32	0.00	0.03	si:ch211-225	eye
ENSXMAG00	-0.45	6.58	0.00	0.01		eye
ENSXMAG00	0.53	3.81	0.00	0.02	mrps2	eye
ENSXMAG00	-0.54	4.45	0.00	0.00	celf4	eye
ENSXMAG00	-1.13	6.08	0.00	0.00	myorg	eye
ENSXMAG00	-0.64	5.99	0.00	0.00	zgc:162730	eye
ENSXMAG00	-0.35	6.05	0.00	0.04	ssh1a	eye
ENSXMAG00	-0.57	5.54	0.00	0.00	sdccag8	eye
ENSXMAG00	0.47	6.27	0.00	0.00	svopa	eye
ENSXMAG00	-0.74	4.53	0.00	0.00	zbtb18	eye
ENSXMAG00	-0.35	9.29	0.00	0.05	rtn1b	eye
ENSXMAG00	0.45	7.29	0.00	0.04	nexn	eye
ENSXMAG00	0.41	5.28	0.00	0.01	rcor1	eye
ENSXMAG00	-2.19	3.07	0.00	0.00	alkal1	eye
ENSXMAG00	-0.66	3.19	0.00	0.02	bag5	eye
ENSXMAG00	0.55	6.23	0.00	0.02	odc1	eye
ENSXMAG00	0.71	3.69	0.00	0.00	nol10	eye
ENSXMAG00	-0.51	3.73	0.00	0.01	apaf1	eye
ENSXMAG00	-0.62	1.87	0.00	0.05	si:ch211-265	eye
ENSXMAG00	-0.69	1.27	0.00	0.04	ca8	eye
ENSXMAG00	0.51	4.35	0.00	0.01	snrpa	eye
ENSXMAG00	0.48	6.89	0.00	0.02	tgm2b	eye
ENSXMAG00	-0.59	3.59	0.00	0.01	insrb	eye

ENSXMAG00	0.40	5.72	0.00	0.03	map3k10	eye
ENSXMAG00	0.36	6.06	0.00	0.04	tia1	eye
ENSXMAG00	0.35	5.10	0.00	0.04	brms1lb	eye
ENSXMAG00	-0.89	8.12	0.00	0.00	camk1ga	eye
ENSXMAG00	1.98	2.60	0.00	0.00		eye
ENSXMAG00	0.42	5.47	0.00	0.01	ddx19	eye
ENSXMAG00	0.57	5.19	0.00	0.00	mfsd12a	eye
ENSXMAG00	-0.61	4.88	0.00	0.00	spsb4a	eye
ENSXMAG00	-0.71	0.64	0.00	0.04	her12	eye
ENSXMAG00	-0.54	6.44	0.00	0.00	oplah	eye
ENSXMAG00	-0.66	3.44	0.00	0.00	F2RL2 (1 of r	eye
ENSXMAG00	0.59	4.73	0.00	0.00	dgkb	eye
ENSXMAG00	1.64	-0.66	0.00	0.00		eye
ENSXMAG00	0.40	6.22	0.00	0.02	lhx4	eye
ENSXMAG00	-0.86	3.59	0.00	0.00	kif21b	eye
ENSXMAG00	0.37	5.38	0.00	0.03		eye
ENSXMAG00	-0.72	6.48	0.00	0.00	slc6a9	eye
ENSXMAG00	0.92	8.59	0.00	0.00	si:ch1073-44	eye
ENSXMAG00	0.61	2.56	0.00	0.02	mrm3a	eye
ENSXMAG00	0.43	3.78	0.00	0.02	enoph1	eye
ENSXMAG00	-1.43	6.26	0.00	0.00		eye
ENSXMAG00	1.08	5.54	0.00	0.00	zswim6	eye
ENSXMAG00	-0.83	4.60	0.00	0.00	ppargc1b	eye
ENSXMAG00	0.57	5.89	0.00	0.00	selenoo1	eye
ENSXMAG00	1.31	4.08	0.00	0.00		eye
ENSXMAG00	-0.57	3.86	0.00	0.03	glra1	eye
ENSXMAG00	0.55	3.95	0.00	0.00	ifrd2	eye
ENSXMAG00	0.59	3.11	0.00	0.01	ARMC6	eye
ENSXMAG00	1.00	5.03	0.00	0.00	xpc	eye
ENSXMAG00	0.71	7.05	0.00	0.00	itcha	eye
ENSXMAG00	-1.52	1.13	0.00	0.00	tgm5l	eye
ENSXMAG00	-0.39	5.45	0.00	0.02	aqp3a	eye
ENSXMAG00	-1.41	6.29	0.00	0.00	mef2cb	eye
ENSXMAG00	-2.54	-1.60	0.00	0.00		eye
ENSXMAG00	-0.51	2.97	0.00	0.02	KCNA10	eye
ENSXMAG00	0.35	4.86	0.00	0.04		eye
ENSXMAG00	0.55	3.66	0.00	0.01	sco2	eye
ENSXMAG00	-0.77	1.68	0.00	0.00	lrrc4.2	eye
ENSXMAG00	0.41	4.32	0.00	0.04	lrrc8da	eye
ENSXMAG00	-0.50	4.41	0.00	0.00	slc5a3b	eye
ENSXMAG00	-0.59	6.49	0.00	0.00	si:ch73-167i1	eye
ENSXMAG00	-0.93	0.81	0.00	0.02	kcnf1a	eye
ENSXMAG00	-1.26	0.83	0.00	0.01	cxcr1	eye

ENSXMAG00	0.58	5.19	0.00	0.00	s1pr1	eye
ENSXMAG00	0.91	3.70	0.00	0.00		eye
ENSXMAG00	-0.45	3.97	0.00	0.03	lingo2a	eye
ENSXMAG00	0.48	3.71	0.00	0.01	zpr1	eye
ENSXMAG00	0.55	2.22	0.00	0.04	senp8	eye
ENSXMAG00	1.02	0.04	0.00	0.03		eye
ENSXMAG00	-0.41	3.94	0.00	0.05	chst7	eye
ENSXMAG00	-0.61	4.09	0.00	0.00	nupr1a	eye
ENSXMAG00	-0.72	1.80	0.00	0.03		eye
ENSXMAG00	-0.80	0.29	0.00	0.04		eye
ENSXMAG00	-0.96	1.51	0.00	0.01	KCNA1	eye
ENSXMAG00	-0.50	4.28	0.00	0.02	KCNA5	eye
ENSXMAG00	-0.65	3.22	0.00	0.03	si:ch1073-30	eye
ENSXMAG00	-0.60	1.80	0.00	0.05	PTGDR2	eye
ENSXMAG00	-0.45	3.32	0.00	0.04	lpar6a	eye
ENSXMAG00	0.80	2.36	0.00	0.00	hspb11	eye
ENSXMAG00	1.02	5.47	0.00	0.00	six3b	eye
ENSXMAG00	0.51	4.05	0.00	0.01	zgc:55781	eye
ENSXMAG00	-0.83	1.35	0.00	0.01	C2CD4C	eye
ENSXMAG00	1.41	-1.10	0.00	0.01		eye
ENSXMAG00	0.47	3.88	0.00	0.05		eye
ENSXMAG00	-0.44	5.67	0.00	0.01	zc3h6	eye
ENSXMAG00	-0.56	3.31	0.00	0.04		eye
ENSXMAG00	-1.17	2.30	0.00	0.00		eye
ENSXMAG00	0.56	3.93	0.00	0.00	nfe2l3	eye
ENSXMAG00	-0.55	3.62	0.00	0.02	usp54b	eye
ENSXMAG00	0.80	2.86	0.00	0.02		eye
ENSXMAG00	-2.17	-2.26	0.00	0.01		eye
ENSXMAG00	-0.87	1.91	0.00	0.01		eye
ENSXMAG00	1.61	2.03	0.00	0.01	hmox1a	eye
ENSXMAG00	1.03	0.59	0.00	0.03		eye
ENSXMAG00	0.66	2.23	0.00	0.02	slc30a7	eye
ENSXMAG00	-1.55	0.33	0.00	0.00		eye
ENSXMAG00	-0.60	2.04	0.00	0.04		eye
ENSXMAG00	0.34	5.79	0.00	0.04	SURF4	eye
ENSXMAG00	-1.88	-2.20	0.00	0.03		eye
ENSXMAG00	2.79	1.17	0.00	0.04		eye
ENSXMAG00	-1.30	5.02	0.00	0.00		eye
ENSXMAG00	-1.55	1.90	0.00	0.00		eye
ENSXMAG00	0.37	5.11	0.00	0.04	TRAPPC5	eye
ENSXMAG00	-0.87	0.25	0.00	0.03		eye
ENSXMAG00	-1.15	5.55	0.00	0.00	abhd8b	eye
ENSXMAG00	0.73	4.55	0.00	0.00	txn2	eye

ENSXMAG00	-0.44	5.96	0.00	0.03	tet1	eye
ENSXMAG00	-4.94	-3.23	0.00	0.02		eye
ENSXMAG00	0.55	4.03	0.00	0.00	cyb5d2	eye
ENSXMAG00	-0.55	2.86	0.00	0.03	NIPAL2	eye
ENSXMAG00	-0.75	1.53	0.00	0.02		eye
ENSXMAG00	-0.51	2.99	0.00	0.02	ehhadh	eye
ENSXMAG00	5.36	-2.79	0.00	0.00		eye
ENSXMAG00	1.67	-1.39	0.00	0.03	MFAP3	eye
ENSXMAG00	-0.52	4.23	0.00	0.02		eye
ENSXMAG00	1.16	1.56	0.00	0.00	ropn1l	eye
ENSXMAG00	-0.64	3.64	0.00	0.01	slc13a3	eye
ENSXMAG00	0.46	4.99	0.00	0.01	dhrs3b	eye
ENSXMAG00	-0.55	3.63	0.00	0.00	shisa7a	eye
ENSXMAG00	2.55	-2.13	0.00	0.01	cip2a	eye
ENSXMAG00	-0.73	2.28	0.00	0.04	NINJ1 (1 of r	eye
ENSXMAG00	0.67	1.77	0.00	0.04	p3h4	eye
ENSXMAG00	0.45	3.87	0.00	0.02		eye
ENSXMAG00	0.52	3.66	0.00	0.01		eye
ENSXMAG00	-0.78	1.11	0.00	0.01		eye
ENSXMAG00	0.38	6.27	0.00	0.02	cab39l1	eye
ENSXMAG00	-0.80	5.72	0.00	0.00		eye
ENSXMAG00	0.50	4.66	0.00	0.01	tefm	eye
ENSXMAG00	0.35	5.26	0.00	0.04	trabd	eye
ENSXMAG00	-0.66	2.64	0.00	0.01		eye
ENSXMAG00	0.40	5.74	0.00	0.02	narf	eye
ENSXMAG00	-2.44	4.19	0.00	0.00		eye
ENSXMAG00	3.58	-2.63	0.00	0.02		eye
ENSXMAG00	1.04	4.38	0.00	0.00	si:ch211-197	eye
ENSXMAG00	-0.85	0.50	0.00	0.03		eye
ENSXMAG00	-1.30	8.24	0.00	0.00	six7	eye
ENSXMAG00	-2.72	1.54	0.00	0.00		eye
ENSXMAG00	0.36	5.63	0.00	0.04	VPS26B (1 of	eye
ENSXMAG00	-0.51	4.30	0.00	0.00	CBX4 (1 of m	eye
ENSXMAG00	-2.45	0.59	0.00	0.00		eye
ENSXMAG00	0.85	3.65	0.00	0.00		eye
ENSXMAG00	0.64	5.39	0.00	0.00	rnaset2	eye
ENSXMAG00	-0.75	5.22	0.00	0.03		eye
ENSXMAG00	5.81	-1.06	0.00	0.03		eye
ENSXMAG00	0.60	3.31	0.00	0.01		eye
ENSXMAG00	-0.42	5.76	0.00	0.04	si:dkey-70p6	eye
ENSXMAG00	0.76	2.60	0.00	0.00		eye
ENSXMAG00	1.75	0.85	0.00	0.00		eye
ENSXMAG00	1.03	4.59	0.00	0.00	uck2b	eye

ENSXMAG00	0.35	5.55	0.00	0.05	mapk4	eye
ENSXMAG00	-1.27	2.54	0.00	0.00		eye
ENSXMAG00	1.15	1.38	0.00	0.00		eye
ENSXMAG00	-1.73	-0.18	0.00	0.00	si:dkey-245n	eye
ENSXMAG00	0.53	4.75	0.00	0.00	nifk	eye
ENSXMAG00	-0.43	4.89	0.00	0.03		eye
ENSXMAG00	-0.47	7.97	0.00	0.01	sall3a	eye
ENSXMAG00	-0.51	3.69	0.00	0.02		eye
ENSXMAG00	-1.39	-1.23	0.00	0.01		eye
ENSXMAG00	-1.57	0.96	0.00	0.00		eye
ENSXMAG00	-0.92	2.29	0.00	0.04		eye
ENSXMAG00	0.78	4.93	0.00	0.00	eif4e1c	eye
ENSXMAG00	1.04	-0.48	0.00	0.03		eye
ENSXMAG00	-0.54	2.48	0.00	0.04	si:ch211-132	eye
ENSXMAG00	-0.93	0.23	0.00	0.04	avpr1ab	eye
ENSXMAG00	0.46	4.11	0.00	0.02	si:ch73-244f	eye
ENSXMAG00	1.94	-2.32	0.00	0.05		eye
ENSXMAG00	3.12	-2.92	0.00	0.05		eye
ENSXMAG00	8.13	-0.72	0.00	0.01	cbln20 (1 of	eye
ENSXMAG00	0.71	3.39	0.00	0.00		eye
ENSXMAG00	1.24	3.03	0.00	0.00	si:ch211-210	eye
ENSXMAG00	-1.40	-1.02	0.00	0.01		eye
ENSXMAG00	-0.81	3.07	0.00	0.00		eye
ENSXMAG00	0.40	4.50	0.00	0.04	si:cabz01085	eye
ENSXMAG00	0.53	3.25	0.00	0.02	prdm8	eye
ENSXMAG00	0.60	6.32	0.00	0.00	fam89a	eye
ENSXMAG00	0.74	5.18	0.00	0.00	msra	eye
ENSXMAG00	-0.67	3.91	0.00	0.00		eye
ENSXMAG00	-0.54	2.78	0.00	0.02		eye
ENSXMAG00	-1.39	-0.19	0.00	0.00		eye
ENSXMAG00	-0.85	2.45	0.00	0.00	hspbap1	eye
ENSXMAG00	-0.48	4.91	0.00	0.00	kctd7	eye
ENSXMAG00	0.42	4.46	0.00	0.02	MPDU1	eye
ENSXMAG00	5.06	-2.93	0.00	0.02		eye
ENSXMAG00	-0.85	0.14	0.00	0.02		eye
ENSXMAG00	0.48	5.15	0.00	0.00	rps6kb1b	eye
ENSXMAG00	1.01	9.88	0.00	0.00	gngt1	eye
ENSXMAG00	-0.50	5.10	0.00	0.00	zgc:112294	eye
ENSXMAG00	0.63	3.66	0.00	0.00	gpatch4	eye
ENSXMAG00	-0.56	5.40	0.00	0.00		eye
ENSXMAG00	5.29	-2.76	0.00	0.03		eye
ENSXMAG00	-0.82	1.31	0.00	0.00		eye
ENSXMAG00	-1.50	2.96	0.00	0.00	si:ch73-335l	eye

ENSXMAG00	-1.74	-2.18	0.00	0.04		eye
ENSXMAG00	0.57	2.99	0.00	0.04	bdnf	eye
ENSXMAG00	-0.54	2.81	0.00	0.02		eye
ENSXMAG00	-0.65	2.34	0.00	0.05	onecut2	eye
ENSXMAG00	0.41	4.08	0.00	0.03	mto1	eye
ENSXMAG00	0.40	5.33	0.00	0.02	slc35e1	eye
ENSXMAG00	0.43	6.30	0.00	0.01		eye
ENSXMAG00	-0.60	4.80	0.00	0.00		eye
ENSXMAG00	0.45	5.04	0.00	0.02	ssbp1	eye
ENSXMAG00	-0.58	5.05	0.00	0.00		eye
ENSXMAG00	-0.78	4.54	0.00	0.00		eye
ENSXMAG00	-0.71	2.18	0.00	0.01		eye
ENSXMAG00	-0.59	2.95	0.00	0.01		eye
ENSXMAG00	-1.23	-0.29	0.00	0.01	socs1b	eye
ENSXMAG00	0.55	4.27	0.00	0.00	rsl1d1	eye
ENSXMAG00	-0.74	2.43	0.00	0.04		eye
ENSXMAG00	-0.62	2.18	0.00	0.04	dlc	eye
ENSXMAG00	-0.89	3.08	0.00	0.00		eye
ENSXMAG00	0.38	7.92	0.00	0.03	dgcr2	eye
ENSXMAG00	-3.47	-2.74	0.00	0.01		eye
ENSXMAG00	-2.47	4.62	0.00	0.00	prkcha	eye
ENSXMAG00	-0.73	2.63	0.00	0.01	p2ry12	eye
ENSXMAG00	-0.85	5.92	0.00	0.00		eye
ENSXMAG00	-2.56	5.85	0.00	0.00	cipcb	eye
ENSXMAG00	-0.47	7.10	0.00	0.01	nxnl1 (1 of m	eye
ENSXMAG00	-1.43	2.53	0.00	0.00	si:dkey-202l2	eye
ENSXMAG00	0.47	3.89	0.00	0.02	stk17b	eye
ENSXMAG00	-3.00	-2.96	0.00	0.02		eye
ENSXMAG00	-1.16	0.97	0.00	0.02	leap2	eye
ENSXMAG00	-0.60	3.76	0.00	0.04		eye
ENSXMAG00	-0.95	1.01	0.00	0.00		eye
ENSXMAG00	-2.89	-3.01	0.00	0.04		eye
ENSXMAG00	-1.83	4.59	0.00	0.00		eye
ENSXMAG00	-0.83	3.42	0.00	0.00	si:ch211-160	eye
ENSXMAG00	0.47	3.60	0.00	0.02	si:ch211-121	eye
ENSXMAG00	0.51	3.22	0.00	0.03		eye
ENSXMAG00	0.58	3.89	0.00	0.00	scamp2l	eye
ENSXMAG00	0.66	2.47	0.00	0.01	serpine3	eye
ENSXMAG00	0.94	7.80	0.00	0.00	slc20a1b	eye
ENSXMAG00	-0.56	4.79	0.00	0.00	rnf44	eye
ENSXMAG00	-2.18	-2.38	0.00	0.02		eye
ENSXMAG00	-0.75	1.33	0.00	0.03	herc7	eye
ENSXMAG00	-1.78	-1.30	0.00	0.01		eye

ENSXMAG00	0.40	4.18	0.00	0.03	fam49bb	eye
ENSXMAG00	-0.63	5.57	0.00	0.00	celf5a	eye
ENSXMAG00	1.17	5.42	0.00	0.00	camk1db	eye
ENSXMAG00	-0.88	0.24	0.00	0.05	mstna	eye
ENSXMAG00	0.73	2.70	0.00	0.00	ikzf4	eye
ENSXMAG00	0.47	7.33	0.00	0.01		eye
ENSXMAG00	0.76	4.04	0.00	0.00		eye
ENSXMAG00	0.40	4.50	0.00	0.03	tmem178	eye
ENSXMAG00	1.17	4.51	0.00	0.00	abhd4	eye
ENSXMAG00	0.78	2.04	0.00	0.02		eye
ENSXMAG00	-0.65	1.61	0.00	0.02	cdk6	eye
ENSXMAG00	-1.58	-0.89	0.00	0.01		eye
ENSXMAG00	-0.65	2.52	0.00	0.01		eye
ENSXMAG00	0.89	3.38	0.00	0.00	adprm	eye
ENSXMAG00	-0.63	2.43	0.00	0.02	SLC38A2 (1 c	eye
ENSXMAG00	-1.86	-1.85	0.00	0.01		eye
ENSXMAG00	-0.82	1.28	0.00	0.04		eye
ENSXMAG00	-0.59	3.19	0.00	0.01	znf219	eye
ENSXMAG00	-0.55	3.66	0.00	0.00		eye
ENSXMAG00	-0.85	0.25	0.00	0.04	ska3	eye
ENSXMAG00	0.59	4.53	0.00	0.01	klhdc8a	eye
ENSXMAG00	0.61	2.35	0.00	0.01		eye
ENSXMAG00	-0.55	4.19	0.00	0.00		eye
ENSXMAG00	-0.54	5.79	0.00	0.00	ptprub	eye
ENSXMAG00	-0.51	3.78	0.00	0.01	rab40b	eye
ENSXMAG00	-2.28	-2.20	0.00	0.01		eye
ENSXMAG00	0.40	6.16	0.00	0.02	kctd12b	eye
ENSXMAG00	1.24	4.20	0.00	0.04	plcxd2	eye
ENSXMAG00	5.40	-2.68	0.00	0.02	si:dkey-1m11	eye
ENSXMAG00	0.43	4.49	0.00	0.03	tram1	eye
ENSXMAG00	-0.36	5.28	0.00	0.04	FIGNL2	eye
ENSXMAG00	-0.54	5.02	0.00	0.00		eye
ENSXMAG00	0.36	5.13	0.00	0.04	ss18l2	eye
ENSXMAG00	-0.69	2.27	0.00	0.02	dhfr	eye
ENSXMAG00	-0.83	3.09	0.00	0.01		eye
ENSXMAG00	0.90	5.18	0.00	0.00	rfk	eye
ENSXMAG00	1.58	-1.14	0.00	0.03		eye
ENSXMAG00	-1.15	-0.31	0.00	0.01		eye
ENSXMAG00	-0.92	0.62	0.00	0.01		eye
ENSXMAG00	-0.47	5.98	0.00	0.01	cyth1a	eye
ENSXMAG00	-0.50	4.64	0.00	0.01		eye
ENSXMAG00	-0.49	5.30	0.00	0.01	zgc:110591	eye
ENSXMAG00	-0.74	2.92	0.00	0.02		eye

ENSXMAG00	-0.75	1.61	0.00	0.04	P2RY4	eye
ENSXMAG00	0.62	6.22	0.00	0.00	gabrb3	eye
ENSXMAG00	-0.58	4.39	0.00	0.00	zfhx3	eye
ENSXMAG00	0.40	4.83	0.00	0.02	nolc1	eye
ENSXMAG00	-0.65	3.19	0.00	0.00	tbc1d32	eye
ENSXMAG00	0.64	4.69	0.00	0.00	zgc:100868	eye
ENSXMAG00	0.45	3.39	0.00	0.04		eye
ENSXMAG00	-0.56	4.49	0.00	0.00	runx3	eye
ENSXMAG00	-1.22	-0.09	0.00	0.03		eye
ENSXMAG00	0.43	4.49	0.00	0.04	zgc:92335	eye
ENSXMAG00	0.66	4.52	0.00	0.00	inpp5ja	eye
ENSXMAG00	0.53	3.51	0.00	0.03		eye
ENSXMAG00	0.71	5.45	0.00	0.00		eye
ENSXMAG00	0.77	6.21	0.00	0.00	elovl6	eye
ENSXMAG00	-0.60	5.09	0.00	0.04		eye
ENSXMAG00	1.06	-0.11	0.00	0.02		eye
ENSXMAG00	-0.97	0.09	0.00	0.03		eye
ENSXMAG00	0.59	3.02	0.00	0.03	sync	eye
ENSXMAG00	-1.34	-0.56	0.00	0.02		eye
ENSXMAG00	-0.42	6.85	0.00	0.02	plecb	eye
ENSXMAG00	-2.88	-2.74	0.00	0.01	spag8	eye
ENSXMAG00	-0.79	1.51	0.00	0.02	sp5a	eye
ENSXMAG00	-0.97	3.60	0.00	0.00		eye
ENSXMAG00	0.66	2.54	0.00	0.02	cnn1b	eye
ENSXMAG00	-1.25	-0.16	0.00	0.00		eye
ENSXMAG00	-0.64	3.76	0.00	0.01		eye
ENSXMAG00	-0.92	4.17	0.00	0.00		eye
ENSXMAG00	0.68	6.25	0.00	0.00	slc22a23	eye
ENSXMAG00	-1.04	0.05	0.00	0.02		eye
ENSXMAG00	1.06	-0.53	0.00	0.03	erlin1	eye
ENSXMAG00	0.57	4.64	0.00	0.00	psme3	eye
ENSXMAG00	0.93	3.49	0.00	0.00	ypel3	eye
ENSXMAG00	-0.85	2.36	0.00	0.01	ch25h	eye
ENSXMAG00	-1.35	5.16	0.00	0.00	rnf41l	eye
ENSXMAG00	-0.46	7.71	0.00	0.03	chka	eye
ENSXMAG00	-0.65	5.22	0.00	0.00	cgas	eye
ENSXMAG00	-1.41	7.44	0.00	0.00	egr1	eye
ENSXMAG00	-0.39	4.89	0.00	0.02		eye
ENSXMAG00	-1.14	7.06	0.00	0.00	8-Mar	eye
ENSXMAG00	-0.44	4.92	0.00	0.01		eye
ENSXMAG00	0.68	5.47	0.00	0.01		eye
ENSXMAG00	-0.48	2.69	0.00	0.05	ACYP1	eye
ENSXMAG00	-0.42	5.29	0.00	0.05	coq10b	eye

ENSXMAG00	2.45	-2.21	0.00	0.01		eye
ENSXMAG00	-0.88	0.60	0.00	0.03		eye
ENSXMAG00	-0.54	5.23	0.00	0.02		eye
ENSXMAG00	0.65	1.49	0.00	0.04	ndufaf3	eye
ENSXMAG00	-1.47	-0.57	0.00	0.01		eye
ENSXMAG00	0.40	6.15	0.00	0.02	srpra	eye
ENSXMAG00	1.00	1.29	0.00	0.00		eye
ENSXMAG00	0.65	2.80	0.00	0.02	rspo2	eye
ENSXMAG00	0.53	2.20	0.00	0.04		eye
ENSXMAG00	-1.15	0.20	0.00	0.00	RETSAT	eye
ENSXMAG00	-0.42	7.68	0.00	0.01	sesn1	eye
ENSXMAG00	2.10	-2.00	0.00	0.03		eye
ENSXMAG00	0.41	7.36	0.00	0.01	iscub	eye
ENSXMAG00	-0.71	3.30	0.00	0.01	bach2b	eye
ENSXMAG00	-4.92	-3.23	0.00	0.03		eye
ENSXMAG00	-1.15	2.99	0.00	0.00	zgc:113054	eye
ENSXMAG00	-0.80	0.27	0.00	0.04	derl3	eye
ENSXMAG00	-0.87	3.19	0.00	0.00	jade2	eye
ENSXMAG00	0.55	3.73	0.00	0.01	srd5a1	eye
ENSXMAG00	0.81	0.61	0.00	0.03		eye
ENSXMAG00	0.65	1.90	0.00	0.01	RF00411	eye
ENSXMAG00	-0.48	4.77	0.00	0.00	unm_hu7912	eye
ENSXMAG00	0.36	5.56	0.00	0.04	tfr2	eye
ENSXMAG00	-1.15	0.70	0.00	0.00		eye
ENSXMAG00	0.69	4.03	0.00	0.00	rnf175	eye
ENSXMAG00	0.60	4.70	0.00	0.00	cbarpb	eye
ENSXMAG00	0.55	2.64	0.00	0.05	gdf6a	eye
ENSXMAG00	-0.49	4.98	0.00	0.01		eye
ENSXMAG00	-1.06	0.85	0.00	0.00		eye
ENSXMAG00	0.69	2.33	0.00	0.01		eye
ENSXMAG00	-2.03	5.34	0.00	0.00	impg2b	eye
ENSXMAG00	1.31	-0.34	0.00	0.02		eye
ENSXMAG00	-0.45	3.91	0.00	0.03	PHLPP2	eye
ENSXMAG00	0.54	3.00	0.00	0.02		eye
ENSXMAG00	0.60	4.20	0.00	0.00	dhrs11a	eye
ENSXMAG00	0.92	1.11	0.00	0.01	nts	eye
ENSXMAG00	0.54	6.67	0.00	0.01		eye
ENSXMAG00	-0.47	3.88	0.00	0.02	whrnb	eye
ENSXMAG00	-3.32	-2.79	0.00	0.02		eye
ENSXMAG00	-0.88	1.44	0.00	0.01		eye
ENSXMAG00	-1.83	-2.23	0.00	0.02	GAS2 (1 of m	eye
ENSXMAG00	-0.53	3.50	0.00	0.04		eye
ENSXMAG00	-0.84	2.56	0.00	0.00		eye

ENSXMAG00	0.55	3.92	0.00	0.00	vmp1	eye
ENSXMAG00	-0.74	4.79	0.00	0.00		eye
ENSXMAG00	-0.49	4.32	0.00	0.03	tm4sf18	eye
ENSXMAG00	-0.61	2.45	0.00	0.02	tmem26a	eye
ENSXMAG00	0.41	5.53	0.00	0.01		eye
ENSXMAG00	0.53	6.14	0.00	0.01		eye
ENSXMAG00	1.14	4.93	0.00	0.00	nudt6	eye
ENSXMAG00	3.01	6.81	0.00	0.00	NR2F6 (1 of	eye
ENSXMAG00	-0.63	3.03	0.00	0.01	adcy9	eye
ENSXMAG00	0.85	2.41	0.00	0.01		eye
ENSXMAG00	-0.86	1.34	0.00	0.03		eye
ENSXMAG00	-0.73	2.42	0.00	0.01		eye
ENSXMAG00	0.40	5.00	0.00	0.02	ssr2	eye
ENSXMAG00	0.95	2.39	0.00	0.02	slc25a44b	gill
ENSXMAG00	-1.17	6.75	0.00	0.01	CAPN14 (1 o	gill
ENSXMAG00	-0.57	4.36	0.00	0.05	zgc:154046	gill
ENSXMAG00	-0.88	6.32	0.00	0.03	krt98	gill
ENSXMAG00	-0.90	7.71	0.00	0.00	krt15	gill
ENSXMAG00	0.85	3.18	0.00	0.03	dpysl4	gill
ENSXMAG00	0.86	5.31	0.00	0.00	si:dkey-93h2	gill
ENSXMAG00	0.98	4.31	0.00	0.00	prmt5	gill
ENSXMAG00	0.51	5.06	0.00	0.04	dapk3	gill
ENSXMAG00	-2.83	-2.17	0.00	0.04		gill
ENSXMAG00	0.70	6.50	0.00	0.01		gill
ENSXMAG00	0.56	5.19	0.00	0.03	rangap1b	gill
ENSXMAG00	-0.50	5.21	0.00	0.04	tfpi2	gill
ENSXMAG00	-6.63	0.50	0.00	0.02		gill
ENSXMAG00	-3.99	-0.37	0.00	0.02	eno4	gill
ENSXMAG00	0.79	7.55	0.00	0.01	hexb	gill
ENSXMAG00	-0.51	5.46	0.00	0.02	bmp1a	gill
ENSXMAG00	0.51	5.95	0.00	0.03		gill
ENSXMAG00	0.80	3.95	0.00	0.01	blvra	gill
ENSXMAG00	0.66	4.97	0.00	0.00	tox4b	gill
ENSXMAG00	-1.07	2.60	0.00	0.01	cav3	gill
ENSXMAG00	0.80	7.09	0.00	0.00	cyb5a	gill
ENSXMAG00	0.62	5.68	0.00	0.00	hyou1	gill
ENSXMAG00	-0.80	6.68	0.00	0.00	cry1b	gill
ENSXMAG00	0.73	4.27	0.00	0.00		gill
ENSXMAG00	0.57	5.38	0.00	0.03		gill
ENSXMAG00	-1.56	2.34	0.00	0.00	zgc:77112	gill
ENSXMAG00	0.65	5.93	0.00	0.01	abcf2a	gill
ENSXMAG00	4.56	-2.41	0.00	0.03		gill

ENSXMAG00	-1.35	6.71	0.00	0.00		gill
ENSXMAG00	2.09	1.10	0.00	0.00	ptprn2	gill
ENSXMAG00	0.58	5.55	0.00	0.01	acsbg2	gill
ENSXMAG00	-2.07	0.07	0.00	0.00	GOLGA7B (1	gill
ENSXMAG00	-0.66	3.69	0.00	0.04		gill
ENSXMAG00	0.58	4.69	0.00	0.04	vps26c	gill
ENSXMAG00	0.79	4.12	0.00	0.02	gldc	gill
ENSXMAG00	1.76	-0.96	0.00	0.02	zgc:109982	gill
ENSXMAG00	-0.75	5.33	0.00	0.00	fdft1	gill
ENSXMAG00	-0.87	4.58	0.00	0.00		gill
ENSXMAG00	0.55	8.92	0.00	0.01	tubb2b	gill
ENSXMAG00	-0.69	4.52	0.00	0.02	rassf6	gill
ENSXMAG00	0.48	7.11	0.00	0.04	kpnb3	gill
ENSXMAG00	2.79	-1.72	0.00	0.03	glra2	gill
ENSXMAG00	-0.66	5.56	0.00	0.04		gill
ENSXMAG00	-0.70	5.52	0.00	0.01	pim3	gill
ENSXMAG00	-0.73	5.02	0.00	0.03	cpt1b	gill
ENSXMAG00	1.03	3.10	0.00	0.00	cyp26c1	gill
ENSXMAG00	0.54	6.61	0.00	0.02	etf1a	gill
ENSXMAG00	-0.68	3.83	0.00	0.04	galnt14	gill
ENSXMAG00	0.74	2.94	0.00	0.03	pts	gill
ENSXMAG00	-1.24	4.21	0.00	0.00	aspa	gill
ENSXMAG00	-0.93	4.33	0.00	0.00	atp1a2a	gill
ENSXMAG00	0.76	4.73	0.00	0.00	HIGD1A	gill
ENSXMAG00	0.63	3.78	0.00	0.05	ppp5c	gill
ENSXMAG00	-0.62	3.64	0.00	0.05	scara5	gill
ENSXMAG00	-0.87	2.77	0.00	0.03	ttc26	gill
ENSXMAG00	-0.90	3.10	0.00	0.02	si:dkey-24p1	gill
ENSXMAG00	1.40	7.00	0.00	0.00	PER2	gill
ENSXMAG00	0.77	8.40	0.00	0.01	lgmn	gill
ENSXMAG00	0.80	4.73	0.00	0.00		gill
ENSXMAG00	1.15	3.07	0.00	0.00	nox5	gill
ENSXMAG00	-0.66	3.72	0.00	0.05	CSDC2 (1 of	gill
ENSXMAG00	1.70	7.16	0.00	0.00	slc14a2	gill
ENSXMAG00	0.57	5.79	0.00	0.01	myo6a	gill
ENSXMAG00	1.93	-0.91	0.00	0.03		gill
ENSXMAG00	0.70	5.80	0.00	0.01	syncripl	gill
ENSXMAG00	1.64	1.58	0.00	0.05	TMPRSS6	gill
ENSXMAG00	0.73	5.06	0.00	0.00	ddx47	gill
ENSXMAG00	0.54	6.27	0.00	0.03	yme1l1a	gill
ENSXMAG00	0.73	3.92	0.00	0.02	rdh12l	gill
ENSXMAG00	-0.69	4.62	0.00	0.01	fbxo32	gill
ENSXMAG00	-1.14	2.90	0.00	0.00	spon2b	gill

ENSXMAG00	-0.59	4.32	0.00	0.03	phykpl	gill
ENSXMAG00	-0.46	5.67	0.00	0.03	sinhcaf	gill
ENSXMAG00	1.10	3.05	0.00	0.00	fam185a	gill
ENSXMAG00	0.73	6.54	0.00	0.00	hspa4b	gill
ENSXMAG00	5.53	-2.81	0.00	0.02	gdf9	gill
ENSXMAG00	1.64	5.34	0.00	0.00	cry3a	gill
ENSXMAG00	-0.59	5.85	0.00	0.05	igfbp6b	gill
ENSXMAG00	-1.29	4.10	0.00	0.00	gria3b	gill
ENSXMAG00	2.16	6.57	0.00	0.00	cyldb	gill
ENSXMAG00	0.66	5.97	0.00	0.00	smarca5	gill
ENSXMAG00	-0.60	6.34	0.00	0.03	slit2	gill
ENSXMAG00	0.65	5.33	0.00	0.01	ppp1r13ba	gill
ENSXMAG00	0.89	3.28	0.00	0.04	ponzr3	gill
ENSXMAG00	0.76	3.11	0.00	0.02	ISYNA1	gill
ENSXMAG00	-1.35	5.84	0.00	0.02	mmp9	gill
ENSXMAG00	-3.23	-1.90	0.00	0.02		gill
ENSXMAG00	-0.82	5.64	0.00	0.01	sfrp1a	gill
ENSXMAG00	0.89	4.99	0.00	0.01	kirrel3l	gill
ENSXMAG00	-0.75	4.69	0.00	0.01	si:dkey-49n2	gill
ENSXMAG00	1.02	5.98	0.00	0.00	lonrf1	gill
ENSXMAG00	-0.53	6.50	0.00	0.04	fam129ba	gill
ENSXMAG00	-2.86	-0.04	0.00	0.00	prss1	gill
ENSXMAG00	0.69	4.38	0.00	0.00	rcc1	gill
ENSXMAG00	-1.03	1.76	0.00	0.04	angptl5	gill
ENSXMAG00	-0.73	3.77	0.00	0.03		gill
ENSXMAG00	-0.77	4.47	0.00	0.00	p3h3	gill
ENSXMAG00	-0.85	5.76	0.00	0.00	ABI3BP (1 of	gill
ENSXMAG00	-0.48	6.94	0.00	0.03	cd302	gill
ENSXMAG00	0.67	6.71	0.00	0.05		gill
ENSXMAG00	1.77	6.22	0.00	0.00	rbp7a	gill
ENSXMAG00	-1.05	2.75	0.00	0.00	cacng6b	gill
ENSXMAG00	-0.79	5.89	0.00	0.02	si:ch211-120	gill
ENSXMAG00	-0.62	5.15	0.00	0.02	npnta	gill
ENSXMAG00	2.00	2.32	0.00	0.00	npas2	gill
ENSXMAG00	-0.75	5.64	0.00	0.03	atf5a	gill
ENSXMAG00	-0.52	5.88	0.00	0.04	tbcelb	gill
ENSXMAG00	0.48	4.98	0.00	0.05	si:dkey-82j4.	gill
ENSXMAG00	1.50	4.40	0.00	0.00		gill
ENSXMAG00	-0.48	5.01	0.00	0.05	hmg20b	gill
ENSXMAG00	-0.96	4.96	0.00	0.00	cited4a	gill
ENSXMAG00	0.91	4.47	0.00	0.00	khsrp	gill
ENSXMAG00	0.87	4.44	0.00	0.05	pcyt2	gill
ENSXMAG00	-1.05	4.91	0.00	0.00	cpamd8	gill

ENSXMAG00	1.11	3.75	0.00	0.00	pprc1	gill
ENSXMAG00	-1.10	2.61	0.00	0.01	xpnpep2	gill
ENSXMAG00	-0.62	6.00	0.00	0.01	col28a2b	gill
ENSXMAG00	0.72	4.68	0.00	0.00	abcf3	gill
ENSXMAG00	0.69	4.06	0.00	0.03	gtpbp1	gill
ENSXMAG00	0.85	5.26	0.00	0.01	arap3	gill
ENSXMAG00	-0.88	7.11	0.00	0.00	pdk2a	gill
ENSXMAG00	-0.78	6.81	0.00	0.02	irs2a	gill
ENSXMAG00	0.59	5.51	0.00	0.03	acsl3b	gill
ENSXMAG00	0.72	3.39	0.00	0.03	zgc:63863	gill
ENSXMAG00	0.92	2.23	0.00	0.03	zgc:65997	gill
ENSXMAG00	-0.51	6.58	0.00	0.03		gill
ENSXMAG00	1.46	2.24	0.00	0.01	adck1	gill
ENSXMAG00	1.24	5.23	0.00	0.04	LONRF3	gill
ENSXMAG00	0.50	4.71	0.00	0.04	arsh	gill
ENSXMAG00	-5.53	-0.20	0.00	0.00	myoz1b	gill
ENSXMAG00	-0.78	4.96	0.00	0.04	gadd45ga	gill
ENSXMAG00	5.40	-2.88	0.00	0.03	fabp1b.1	gill
ENSXMAG00	0.53	7.34	0.00	0.02	eif4g1a	gill
ENSXMAG00	2.76	5.51	0.00	0.00	per2	gill
ENSXMAG00	2.01	-0.15	0.00	0.03	grm2b	gill
ENSXMAG00	-7.49	3.00	0.00	0.01		gill
ENSXMAG00	0.77	4.28	0.00	0.00		gill
ENSXMAG00	1.57	8.23	0.00	0.01	cyp1a	gill
ENSXMAG00	2.27	-1.32	0.00	0.02		gill
ENSXMAG00	-2.17	-1.16	0.00	0.05		gill
ENSXMAG00	-1.09	5.58	0.00	0.00	tp73	gill
ENSXMAG00	-1.35	0.51	0.00	0.04		gill
ENSXMAG00	-6.10	-2.46	0.00	0.01	LRRC43	gill
ENSXMAG00	-0.53	6.84	0.00	0.01		gill
ENSXMAG00	6.15	1.21	0.00	0.00		gill
ENSXMAG00	1.38	5.08	0.00	0.00	si:ch211-195	gill
ENSXMAG00	-1.81	0.02	0.00	0.03	ccdc181	gill
ENSXMAG00	-2.54	-1.88	0.00	0.03	rnf220b	gill
ENSXMAG00	-0.91	5.16	0.00	0.00	tcp11l2	gill
ENSXMAG00	0.65	7.46	0.00	0.02	tgm2b	gill
ENSXMAG00	-0.92	8.44	0.00	0.03	nr1d2a	gill
ENSXMAG00	-0.59	4.14	0.00	0.03	JMY (1 of ma	gill
ENSXMAG00	1.09	5.29	0.00	0.00		gill
ENSXMAG00	0.53	5.24	0.00	0.01	ddx19	gill
ENSXMAG00	0.80	7.69	0.00	0.01	si:ch1073-44	gill
ENSXMAG00	-0.82	6.07	0.00	0.00	slc51a	gill
ENSXMAG00	0.79	5.67	0.00	0.00	g3bp1	gill

ENSXMAG00	0.81	5.33	0.00	0.02	xpc	gill
ENSXMAG00	-0.94	4.86	0.00	0.01		gill
ENSXMAG00	-0.65	6.11	0.00	0.03		gill
ENSXMAG00	0.82	3.64	0.00	0.01		gill
ENSXMAG00	1.06	2.38	0.00	0.03	hspb11	gill
ENSXMAG00	1.59	1.95	0.00	0.02	scg2b	gill
ENSXMAG00	0.98	2.82	0.00	0.02	zgc:113305	gill
ENSXMAG00	0.82	6.46	0.00	0.00	ADAMTS5	gill
ENSXMAG00	0.70	5.55	0.00	0.00	nfe2l3	gill
ENSXMAG00	1.29	4.93	0.00	0.00	rab27b	gill
ENSXMAG00	-7.43	-1.44	0.00	0.02		gill
ENSXMAG00	0.72	4.04	0.00	0.00	MFAP3	gill
ENSXMAG00	1.59	6.79	0.00	0.00		gill
ENSXMAG00	-0.52	5.43	0.00	0.02	apol1	gill
ENSXMAG00	-5.06	-0.71	0.00	0.00		gill
ENSXMAG00	-1.16	2.02	0.00	0.04	rabgap1l2	gill
ENSXMAG00	-5.96	2.60	0.00	0.00		gill
ENSXMAG00	1.30	0.88	0.00	0.01	si:ch211-197	gill
ENSXMAG00	-0.70	3.98	0.00	0.05	tspan33a	gill
ENSXMAG00	-5.00	-0.23	0.00	0.01		gill
ENSXMAG00	0.75	5.04	0.00	0.00	uck2b	gill
ENSXMAG00	-0.47	7.11	0.00	0.02	zfand5a	gill
ENSXMAG00	-5.99	-1.34	0.00	0.01		gill
ENSXMAG00	1.01	3.57	0.00	0.00	eif4e1c	gill
ENSXMAG00	-0.59	4.83	0.00	0.03		gill
ENSXMAG00	0.83	3.44	0.00	0.05	nkx3-2	gill
ENSXMAG00	5.57	-2.80	0.00	0.03	cth1	gill
ENSXMAG00	-0.72	10.82	0.00	0.00	krt92	gill
ENSXMAG00	7.60	2.85	0.00	0.00		gill
ENSXMAG00	-4.76	-0.89	0.00	0.03		gill
ENSXMAG00	-1.07	4.27	0.00	0.00	itga6b (1 of r	gill
ENSXMAG00	-0.73	3.61	0.00	0.05		gill
ENSXMAG00	-2.73	-0.66	0.00	0.01		gill
ENSXMAG00	-0.63	6.06	0.00	0.01	anxa2a	gill
ENSXMAG00	-2.08	5.87	0.00	0.00	cipcb	gill
ENSXMAG00	-2.97	-1.77	0.00	0.01		gill
ENSXMAG00	-3.77	-0.03	0.00	0.03	tdrd6	gill
ENSXMAG00	-7.58	-1.32	0.00	0.01		gill
ENSXMAG00	-0.67	6.49	0.00	0.04	arrdc2	gill
ENSXMAG00	0.62	7.60	0.00	0.00		gill
ENSXMAG00	-7.30	-1.55	0.00	0.02		gill
ENSXMAG00	-6.02	-2.52	0.00	0.02		gill
ENSXMAG00	-0.80	5.90	0.00	0.00	tp53inp1	gill

ENSXMAG00	0.62	6.93	0.00	0.05	zgc:100868	gill
ENSXMAG00	-1.63	3.57	0.00	0.05		gill
ENSXMAG00	-1.11	2.75	0.00	0.03	fam110c	gill
ENSXMAG00	0.65	6.33	0.00	0.00	mmp11a	gill
ENSXMAG00	0.86	4.98	0.00	0.00		gill
ENSXMAG00	-1.05	5.90	0.00	0.00		gill
ENSXMAG00	0.69	4.12	0.00	0.01	etnk2	gill
ENSXMAG00	-0.55	5.06	0.00	0.04	pcolceb	gill
ENSXMAG00	-0.92	4.56	0.00	0.03	grem1a	gill
ENSXMAG00	1.75	4.63	0.00	0.00		gill
ENSXMAG00	-0.66	7.24	0.00	0.00	aldh1a2	gill
ENSXMAG00	0.79	6.84	0.00	0.01	glulc	gill
ENSXMAG00	-3.40	1.07	0.00	0.00		gill
ENSXMAG00	1.60	2.17	0.00	0.01	nocta	gill
ENSXMAG00	0.45	7.11	0.00	0.04	HNRNPA0 (1	gill
ENSXMAG00	0.83	4.14	0.00	0.01	pgp	gill
ENSXMAG00	-0.52	4.93	0.00	0.03		gill
ENSXMAG00	-0.46	6.17	0.00	0.03	pcmtd1	gill
ENSXMAG00	1.03	1.73	0.00	0.05	slc25a29 (1 c	gill
ENSXMAG00	-0.72	6.83	0.00	0.03		gill
ENSXMAG00	2.87	0.79	0.00	0.02	cyth4a	liver
ENSXMAG00	2.54	1.00	0.00	0.02	si:ch211-241	liver
ENSXMAG00	-6.73	-1.99	0.00	0.01	piwil1	liver
ENSXMAG00	1.09	4.48	0.00	0.00		liver
ENSXMAG00	-1.33	3.92	0.00	0.00	zgc:154046	liver
ENSXMAG00	-1.23	3.36	0.00	0.00	hbba2	liver
ENSXMAG00	-1.17	1.75	0.00	0.04	pde4a	liver
ENSXMAG00	-0.86	3.45	0.00	0.03	enox2	liver
ENSXMAG00	2.08	3.21	0.00	0.00	si:dkey-93h2	liver
ENSXMAG00	1.61	2.71	0.00	0.00	cant1b	liver
ENSXMAG00	-0.96	6.79	0.00	0.01	afmid	liver
ENSXMAG00	2.55	-0.22	0.00	0.01	plekho1b	liver
ENSXMAG00	-0.94	5.23	0.00	0.03		liver
ENSXMAG00	2.05	3.06	0.00	0.00	hdac1	liver
ENSXMAG00	1.14	2.79	0.00	0.03	mfsd4b	liver
ENSXMAG00	1.82	2.55	0.00	0.00	prmt5	liver
ENSXMAG00	-3.02	-2.63	0.00	0.04	itga11a	liver
ENSXMAG00	1.11	2.26	0.00	0.05	dapk3	liver
ENSXMAG00	4.24	2.18	0.00	0.00		liver
ENSXMAG00	-4.84	-3.26	0.00	0.04		liver
ENSXMAG00	-0.93	3.28	0.00	0.01	znf395a	liver
ENSXMAG00	-0.84	4.13	0.00	0.04	si:ch211-198	liver

ENSXMAG00	-1.22	4.99	0.00	0.01	ddc	liver
ENSXMAG00	-1.11	4.33	0.00	0.00	LDLRAD3	liver
ENSXMAG00	-5.75	-1.43	0.00	0.01	eno4	liver
ENSXMAG00	1.36	4.88	0.00	0.00	pdia5	liver
ENSXMAG00	-6.38	-2.24	0.00	0.03		liver
ENSXMAG00	1.72	5.65	0.00	0.00		liver
ENSXMAG00	1.01	3.72	0.00	0.02	ccne1	liver
ENSXMAG00	1.48	1.72	0.00	0.02	blvra	liver
ENSXMAG00	1.43	4.38	0.00	0.00	chd4a	liver
ENSXMAG00	-1.59	-0.96	0.00	0.04	ccnd3	liver
ENSXMAG00	1.94	3.09	0.00	0.00	osbpl3a	liver
ENSXMAG00	1.28	3.07	0.00	0.01		liver
ENSXMAG00	1.88	3.06	0.00	0.00	tox4b	liver
ENSXMAG00	1.21	2.67	0.00	0.03	SLC12A6	liver
ENSXMAG00	1.22	2.69	0.00	0.01	entpd4	liver
ENSXMAG00	-0.98	2.39	0.00	0.04	gaa	liver
ENSXMAG00	4.42	-1.97	0.00	0.03	m17	liver
ENSXMAG00	3.07	1.24	0.00	0.01	ripk3	liver
ENSXMAG00	0.79	5.31	0.00	0.04	larsb	liver
ENSXMAG00	-1.11	2.75	0.00	0.03		liver
ENSXMAG00	-7.03	-1.75	0.00	0.00		liver
ENSXMAG00	2.31	3.05	0.00	0.00	hspa4a	liver
ENSXMAG00	2.01	5.00	0.00	0.00	fech	liver
ENSXMAG00	3.34	0.11	0.00	0.02		liver
ENSXMAG00	3.14	6.43	0.00	0.00	hyou1	liver
ENSXMAG00	3.46	0.46	0.00	0.01	slc7a8a	liver
ENSXMAG00	-1.32	4.30	0.00	0.00	ip6k2b	liver
ENSXMAG00	3.06	6.02	0.00	0.00		liver
ENSXMAG00	4.74	-2.23	0.00	0.01		liver
ENSXMAG00	1.33	5.37	0.00	0.00	abce1	liver
ENSXMAG00	2.02	3.67	0.00	0.00	slc10a7	liver
ENSXMAG00	1.55	5.95	0.00	0.00	gfpt1	liver
ENSXMAG00	-0.85	5.02	0.00	0.04		liver
ENSXMAG00	2.00	3.81	0.00	0.04	fn1a	liver
ENSXMAG00	2.86	-0.58	0.00	0.03	si:ch211-1o7	liver
ENSXMAG00	2.21	1.94	0.00	0.00	pitpnb (1 of r	liver
ENSXMAG00	3.05	4.43	0.00	0.00	abcf2a	liver
ENSXMAG00	2.69	0.31	0.00	0.01	ace2	liver
ENSXMAG00	1.75	6.28	0.00	0.00	sec24d	liver
ENSXMAG00	1.14	2.91	0.00	0.00	myca	liver
ENSXMAG00	1.10	2.45	0.00	0.01	tmem33	liver
ENSXMAG00	1.19	4.76	0.00	0.00	WDR1	liver
ENSXMAG00	1.41	0.74	0.00	0.04	si:ch73-196l6	liver

ENSXMAG00	1.21	4.65	0.00	0.04	larp4ab	liver
ENSXMAG00	3.56	-1.30	0.00	0.02	LPIN2 (1 of n	liver
ENSXMAG00	1.52	3.66	0.00	0.00	arl6ip5a	liver
ENSXMAG00	1.25	1.70	0.00	0.02	inip	liver
ENSXMAG00	1.13	2.75	0.00	0.03		liver
ENSXMAG00	0.97	3.48	0.00	0.01	eif4ea	liver
ENSXMAG00	-1.17	3.25	0.00	0.03	myt1la	liver
ENSXMAG00	1.28	2.68	0.00	0.00	SPRYD3	liver
ENSXMAG00	1.18	3.71	0.00	0.00	dda1	liver
ENSXMAG00	1.98	4.33	0.00	0.03	PTPRC	liver
ENSXMAG00	5.07	3.74	0.00	0.00		liver
ENSXMAG00	0.76	5.43	0.00	0.04		liver
ENSXMAG00	-0.77	8.28	0.00	0.04		liver
ENSXMAG00	2.77	-1.44	0.00	0.04	f3a	liver
ENSXMAG00	1.41	5.21	0.00	0.00	ppp4ca	liver
ENSXMAG00	0.85	4.00	0.00	0.05	mpv17l2	liver
ENSXMAG00	0.92	2.78	0.00	0.02	PGPEP1	liver
ENSXMAG00	1.27	2.29	0.00	0.01		liver
ENSXMAG00	1.65	4.69	0.00	0.00	uba5	liver
ENSXMAG00	1.37	0.57	0.00	0.04	zgc:172302	liver
ENSXMAG00	1.64	5.86	0.00	0.00	atf4b	liver
ENSXMAG00	5.26	1.21	0.00	0.00	zap70	liver
ENSXMAG00	2.62	1.53	0.00	0.04	si:ch211-117	liver
ENSXMAG00	-6.16	-2.45	0.00	0.00		liver
ENSXMAG00	1.63	0.71	0.00	0.01	sfxn3	liver
ENSXMAG00	0.75	4.50	0.00	0.04	xpot	liver
ENSXMAG00	-1.66	2.55	0.00	0.00		liver
ENSXMAG00	1.00	4.63	0.00	0.02	tars	liver
ENSXMAG00	3.05	0.03	0.00	0.01	prkcq	liver
ENSXMAG00	3.75	0.28	0.00	0.03	CD3G	liver
ENSXMAG00	2.95	0.07	0.00	0.02		liver
ENSXMAG00	1.32	3.87	0.00	0.00	ywhaqb	liver
ENSXMAG00	1.29	1.38	0.00	0.03	hps4	liver
ENSXMAG00	-0.96	3.72	0.00	0.01	sfmbt2	liver
ENSXMAG00	2.68	3.18	0.00	0.00	mlec	liver
ENSXMAG00	3.67	6.74	0.00	0.00	aacs	liver
ENSXMAG00	3.63	-0.91	0.00	0.01	ggt5a	liver
ENSXMAG00	1.28	2.84	0.00	0.04	mvda	liver
ENSXMAG00	1.52	2.10	0.00	0.00	emc8	liver
ENSXMAG00	3.85	6.16	0.00	0.00		liver
ENSXMAG00	2.34	4.50	0.00	0.00		liver
ENSXMAG00	1.26	3.17	0.00	0.01	fkbp9	liver
ENSXMAG00	-6.84	-1.89	0.00	0.01	ST8SIA3	liver

ENSXMAG00	-0.98	4.48	0.00	0.01	kat2b	liver
ENSXMAG00	1.61	6.28	0.00	0.00	calr	liver
ENSXMAG00	1.36	3.71	0.00	0.00	rasal2	liver
ENSXMAG00	0.97	4.26	0.00	0.01	RAB8A (1 of	liver
ENSXMAG00	1.17	3.10	0.00	0.00	SRPK3	liver
ENSXMAG00	-0.85	4.94	0.00	0.03	si:dkey-6b12.	liver
ENSXMAG00	-1.83	-0.37	0.00	0.02		liver
ENSXMAG00	1.56	0.20	0.00	0.02	mfsd2b	liver
ENSXMAG00	1.87	2.48	0.00	0.00		liver
ENSXMAG00	1.78	5.81	0.00	0.03	arpc1b	liver
ENSXMAG00	0.98	5.19	0.00	0.00	si:ch1073-55	liver
ENSXMAG00	1.64	4.99	0.00	0.00	copz2	liver
ENSXMAG00	0.86	3.98	0.00	0.03		liver
ENSXMAG00	1.36	3.76	0.00	0.00	si:dkeyp-120l	liver
ENSXMAG00	-1.04	2.31	0.00	0.02	trmt1l	liver
ENSXMAG00	0.92	3.98	0.00	0.01	arfgap1	liver
ENSXMAG00	1.36	2.96	0.00	0.00	parp1	liver
ENSXMAG00	1.03	5.19	0.00	0.00	mtdha	liver
ENSXMAG00	1.05	3.58	0.00	0.00	xpnpep3	liver
ENSXMAG00	1.07	3.93	0.00	0.00	rangap1a	liver
ENSXMAG00	1.98	4.00	0.00	0.00	ABHD1	liver
ENSXMAG00	1.78	2.73	0.00	0.00	noc2l	liver
ENSXMAG00	-1.39	4.23	0.00	0.01	pnpla2	liver
ENSXMAG00	-1.10	5.56	0.00	0.02	rerea	liver
ENSXMAG00	1.28	3.47	0.00	0.00	tmem183a	liver
ENSXMAG00	-1.19	1.95	0.00	0.04	ppfia4	liver
ENSXMAG00	1.90	1.78	0.00	0.01	fam49ba	liver
ENSXMAG00	2.29	1.35	0.00	0.04	dock10	liver
ENSXMAG00	1.30	2.22	0.00	0.01	mbd2	liver
ENSXMAG00	1.76	6.92	0.00	0.01	tubb2b	liver
ENSXMAG00	2.96	-0.06	0.00	0.00	nsun4	liver
ENSXMAG00	3.42	1.87	0.00	0.00	entpd2a.2	liver
ENSXMAG00	3.20	-0.45	0.00	0.04		liver
ENSXMAG00	1.49	2.29	0.00	0.01	il10ra	liver
ENSXMAG00	3.56	-1.53	0.00	0.05	gpr184	liver
ENSXMAG00	-1.32	2.05	0.00	0.01	cux2b	liver
ENSXMAG00	0.82	4.82	0.00	0.02	dhx57	liver
ENSXMAG00	2.01	4.65	0.00	0.02		liver
ENSXMAG00	9.79	0.73	0.00	0.00	mpx (1 of ma	liver
ENSXMAG00	3.46	1.15	0.00	0.00		liver
ENSXMAG00	-0.81	4.65	0.00	0.03	guf1	liver
ENSXMAG00	1.58	2.53	0.00	0.00		liver
ENSXMAG00	2.27	-1.58	0.00	0.04	galnt8b.1	liver

ENSXMAG00	2.48	-2.19	0.00	0.05	fgf23	liver
ENSXMAG00	3.12	-0.61	0.00	0.00	tigarb	liver
ENSXMAG00	1.51	4.32	0.00	0.00	qpctla	liver
ENSXMAG00	1.03	6.61	0.00	0.03	lman1	liver
ENSXMAG00	2.76	0.00	0.00	0.02	TLR8	liver
ENSXMAG00	-1.25	1.83	0.00	0.02		liver
ENSXMAG00	2.15	4.21	0.00	0.00	sdf2l1	liver
ENSXMAG00	-1.19	4.42	0.00	0.00	susd1	liver
ENSXMAG00	1.32	3.89	0.00	0.00	nbas	liver
ENSXMAG00	0.87	4.44	0.00	0.01	syncrip	liver
ENSXMAG00	0.98	4.39	0.00	0.03	ddx1	liver
ENSXMAG00	0.94	5.37	0.00	0.01	arf5	liver
ENSXMAG00	0.86	3.88	0.00	0.03	rcc2	liver
ENSXMAG00	-4.28	-0.95	0.00	0.02		liver
ENSXMAG00	1.22	2.53	0.00	0.01	polr3f	liver
ENSXMAG00	2.12	0.83	0.00	0.05	arhgap25	liver
ENSXMAG00	1.95	0.69	0.00	0.02	si:ch73-21k1	liver
ENSXMAG00	2.16	1.89	0.00	0.00		liver
ENSXMAG00	5.04	5.07	0.00	0.00		liver
ENSXMAG00	4.66	3.66	0.00	0.00		liver
ENSXMAG00	6.12	-2.43	0.00	0.04	tmc5	liver
ENSXMAG00	2.00	2.49	0.00	0.00	dhx29	liver
ENSXMAG00	2.75	0.13	0.00	0.04	rasgrp4	liver
ENSXMAG00	1.11	3.68	0.00	0.00	fam126a	liver
ENSXMAG00	-1.10	5.53	0.00	0.03	abcc6a	liver
ENSXMAG00	1.14	2.11	0.00	0.03	imp4	liver
ENSXMAG00	1.39	6.52	0.00	0.00	etf1a	liver
ENSXMAG00	1.50	2.70	0.00	0.00	pigo	liver
ENSXMAG00	3.34	0.35	0.00	0.02		liver
ENSXMAG00	1.44	2.00	0.00	0.04	slc44a2	liver
ENSXMAG00	5.48	-1.63	0.00	0.03		liver
ENSXMAG00	1.16	2.51	0.00	0.00	vps51	liver
ENSXMAG00	-1.42	2.55	0.00	0.00	rapgef4	liver
ENSXMAG00	0.90	5.98	0.00	0.01	copg2	liver
ENSXMAG00	1.02	3.94	0.00	0.00	nus1	liver
ENSXMAG00	-6.73	0.01	0.00	0.00	camsap1a	liver
ENSXMAG00	-1.34	4.10	0.00	0.00	aspa	liver
ENSXMAG00	1.77	1.00	0.00	0.02		liver
ENSXMAG00	0.85	6.80	0.00	0.03	copa	liver
ENSXMAG00	1.68	2.67	0.00	0.01	zmynd19	liver
ENSXMAG00	1.52	4.32	0.00	0.00	smox	liver
ENSXMAG00	1.28	1.34	0.00	0.02	myo19	liver
ENSXMAG00	-1.04	4.69	0.00	0.01	pcsk1	liver

ENSXMAG00	1.81	1.18	0.00	0.04	ggt1b	liver
ENSXMAG00	2.64	1.48	0.00	0.02		liver
ENSXMAG00	2.07	1.31	0.00	0.03		liver
ENSXMAG00	1.14	4.06	0.00	0.01	ufsp2	liver
ENSXMAG00	-1.21	3.72	0.00	0.01	gucy1b1	liver
ENSXMAG00	1.14	4.90	0.00	0.00	GOT1 (1 of n	liver
ENSXMAG00	0.78	3.75	0.00	0.05	ENTPD7	liver
ENSXMAG00	-1.25	2.27	0.00	0.03		liver
ENSXMAG00	0.76	4.84	0.00	0.04		liver
ENSXMAG00	1.55	3.55	0.00	0.00	dolpp1	liver
ENSXMAG00	0.90	6.51	0.00	0.01	rab1ab	liver
ENSXMAG00	1.65	4.01	0.00	0.00	xpo1a	liver
ENSXMAG00	1.13	3.41	0.00	0.00	zzef1	liver
ENSXMAG00	1.00	5.45	0.00	0.01	gbf1	liver
ENSXMAG00	2.57	1.13	0.00	0.01	tox2	liver
ENSXMAG00	1.84	1.64	0.00	0.00	si:dkey-162b	liver
ENSXMAG00	1.62	5.34	0.00	0.00	dpagt1	liver
ENSXMAG00	-1.25	3.73	0.00	0.00		liver
ENSXMAG00	1.52	2.92	0.00	0.00	ppp5c	liver
ENSXMAG00	1.73	3.66	0.00	0.00	ipo4	liver
ENSXMAG00	2.67	4.26	0.00	0.02	prelid3b	liver
ENSXMAG00	1.48	3.79	0.00	0.00	KIF5B (1 of n	liver
ENSXMAG00	0.93	4.58	0.00	0.03	tm9sf1	liver
ENSXMAG00	1.15	5.84	0.00	0.00	srp68	liver
ENSXMAG00	-1.44	0.58	0.00	0.01		liver
ENSXMAG00	-0.85	4.86	0.00	0.02	dcaf11	liver
ENSXMAG00	1.46	1.48	0.00	0.02	gmds	liver
ENSXMAG00	0.94	3.67	0.00	0.01	si:ch211-214	liver
ENSXMAG00	1.20	4.66	0.00	0.00	tmco1	liver
ENSXMAG00	0.90	2.59	0.00	0.05	setd7 (1 of n	liver
ENSXMAG00	1.13	3.46	0.00	0.00	smarca4a	liver
ENSXMAG00	2.73	4.49	0.00	0.01		liver
ENSXMAG00	2.82	0.29	0.00	0.01	mfsd2aa	liver
ENSXMAG00	0.99	3.40	0.00	0.01	crema	liver
ENSXMAG00	1.25	2.18	0.00	0.02		liver
ENSXMAG00	2.49	2.65	0.00	0.00	rbbp4	liver
ENSXMAG00	1.57	4.13	0.00	0.00	ttc13	liver
ENSXMAG00	-1.13	1.65	0.00	0.04		liver
ENSXMAG00	1.81	1.13	0.00	0.02	prmt6	liver
ENSXMAG00	-4.51	-1.46	0.00	0.04	TPPP2	liver
ENSXMAG00	2.85	0.79	0.00	0.03	jak3	liver
ENSXMAG00	0.99	3.43	0.00	0.02	tomm40	liver
ENSXMAG00	0.76	4.12	0.00	0.04	MARK3 (1 of	liver

ENSXMAG00	1.89	5.58	0.00	0.00	PER2	liver
ENSXMAG00	-0.87	2.82	0.00	0.04	fuk	liver
ENSXMAG00	-2.01	-0.57	0.00	0.01		liver
ENSXMAG00	1.70	5.11	0.00	0.00	acsl4a	liver
ENSXMAG00	2.64	1.57	0.00	0.02	cxcr3.2	liver
ENSXMAG00	1.08	5.77	0.00	0.03	sel1l	liver
ENSXMAG00	1.74	0.03	0.00	0.03	them4	liver
ENSXMAG00	1.17	6.18	0.00	0.00	copb1	liver
ENSXMAG00	2.52	1.98	0.00	0.03		liver
ENSXMAG00	5.36	-1.82	0.00	0.02		liver
ENSXMAG00	-1.07	3.71	0.00	0.01	ANK2	liver
ENSXMAG00	1.10	4.06	0.00	0.00	slc35a2	liver
ENSXMAG00	0.93	3.85	0.00	0.00	calcrla	liver
ENSXMAG00	1.02	4.10	0.00	0.02	AP1M2	liver
ENSXMAG00	1.27	2.74	0.00	0.01	WDR77	liver
ENSXMAG00	2.40	-1.99	0.00	0.04	si:dkeyp-72e	liver
ENSXMAG00	0.90	2.70	0.00	0.04	lipea	liver
ENSXMAG00	1.68	0.67	0.00	0.02	dagla	liver
ENSXMAG00	0.84	6.03	0.00	0.05	gspt1l	liver
ENSXMAG00	1.48	3.20	0.00	0.00	usp16	liver
ENSXMAG00	2.24	4.77	0.00	0.00	calub	liver
ENSXMAG00	1.11	2.59	0.00	0.02		liver
ENSXMAG00	4.05	-1.02	0.00	0.02		liver
ENSXMAG00	-1.28	2.43	0.00	0.01		liver
ENSXMAG00	-6.99	-1.77	0.00	0.01	cfap52 (1 of	liver
ENSXMAG00	1.41	2.52	0.00	0.00	THADA	liver
ENSXMAG00	-0.97	2.83	0.00	0.05	si:dkey-188i1	liver
ENSXMAG00	1.25	4.90	0.00	0.00	acbd3	liver
ENSXMAG00	0.81	2.97	0.00	0.03	capza1b	liver
ENSXMAG00	1.01	3.96	0.00	0.01	H2AFZ	liver
ENSXMAG00	2.04	2.05	0.00	0.00	rwdd	liver
ENSXMAG00	1.24	2.49	0.00	0.02	sirt1	liver
ENSXMAG00	0.95	2.72	0.00	0.02	cacul1	liver
ENSXMAG00	3.02	4.42	0.00	0.01	C1QC	liver
ENSXMAG00	-0.84	3.90	0.00	0.01	si:ch211-15d	liver
ENSXMAG00	3.35	3.25	0.00	0.00	C1QA	liver
ENSXMAG00	1.46	3.88	0.00	0.00	syncripl	liver
ENSXMAG00	-1.14	3.05	0.00	0.03	sorbs1	liver
ENSXMAG00	1.11	4.49	0.00	0.01	lmnb2	liver
ENSXMAG00	3.00	-0.19	0.00	0.03		liver
ENSXMAG00	1.06	5.95	0.00	0.01	tm9sf3	liver
ENSXMAG00	3.01	0.89	0.00	0.00	mvk	liver
ENSXMAG00	0.75	3.42	0.00	0.04	trak2	liver

ENSXMAG00	0.79	3.94	0.00	0.03	mboat2a	liver
ENSXMAG00	-1.81	-0.68	0.00	0.04		liver
ENSXMAG00	3.16	0.41	0.00	0.01	si:ch73-40a2	liver
ENSXMAG00	0.87	4.66	0.00	0.03	copz1	liver
ENSXMAG00	-0.95	6.03	0.00	0.01	slc19a1	liver
ENSXMAG00	0.85	3.58	0.00	0.01	PARD3 (1 of	liver
ENSXMAG00	2.10	1.32	0.00	0.05		liver
ENSXMAG00	-1.00	3.06	0.00	0.04	si:dkey-177p	liver
ENSXMAG00	-1.18	3.01	0.00	0.02		liver
ENSXMAG00	0.75	5.33	0.00	0.04	clint1a	liver
ENSXMAG00	-0.84	3.58	0.00	0.03	zgc:194242	liver
ENSXMAG00	0.82	3.45	0.00	0.03	ddx47	liver
ENSXMAG00	1.19	1.71	0.00	0.03	pdss1	liver
ENSXMAG00	-3.17	-2.54	0.00	0.03	ncam3	liver
ENSXMAG00	1.72	1.78	0.00	0.00	rdh12l	liver
ENSXMAG00	0.91	4.08	0.00	0.02	prpf19	liver
ENSXMAG00	0.87	3.52	0.00	0.02	plaa	liver
ENSXMAG00	1.04	3.82	0.00	0.01		liver
ENSXMAG00	1.95	0.69	0.00	0.00	tlcd2	liver
ENSXMAG00	2.39	3.77	0.00	0.00		liver
ENSXMAG00	1.99	8.12	0.00	0.02	cd74a	liver
ENSXMAG00	2.19	4.15	0.00	0.02	lcp1	liver
ENSXMAG00	3.49	2.93	0.00	0.00	pfkfb3	liver
ENSXMAG00	2.38	0.00	0.00	0.00	psph	liver
ENSXMAG00	-0.95	2.96	0.00	0.03	il17rd	liver
ENSXMAG00	-1.77	-0.35	0.00	0.02		liver
ENSXMAG00	-2.95	-1.24	0.00	0.00		liver
ENSXMAG00	1.88	1.90	0.00	0.05	pkn1b	liver
ENSXMAG00	0.97	5.13	0.00	0.01	hnrnpaba	liver
ENSXMAG00	-0.77	3.53	0.00	0.05	phykpl	liver
ENSXMAG00	1.05	4.78	0.00	0.01	derl1	liver
ENSXMAG00	1.24	2.79	0.00	0.03		liver
ENSXMAG00	0.85	3.41	0.00	0.02	atrx	liver
ENSXMAG00	0.99	4.55	0.00	0.00		liver
ENSXMAG00	1.12	6.30	0.00	0.00	eif4g2a	liver
ENSXMAG00	2.12	4.08	0.00	0.00	cry3a	liver
ENSXMAG00	-0.97	2.83	0.00	0.01	si:dkey-48j7.	liver
ENSXMAG00	1.57	3.14	0.00	0.01		liver
ENSXMAG00	1.43	3.35	0.00	0.00	sarnp	liver
ENSXMAG00	1.14	4.65	0.00	0.00	gars	liver
ENSXMAG00	-1.42	4.21	0.00	0.00	gsap	liver
ENSXMAG00	1.59	3.54	0.00	0.00	tmem41b	liver
ENSXMAG00	0.88	3.13	0.00	0.04	abcc10	liver

ENSXMAG00	2.14	1.47	0.00	0.04	agap2	liver
ENSXMAG00	1.04	2.66	0.00	0.01	fam114a1	liver
ENSXMAG00	-1.17	2.26	0.00	0.05	si:ch73-212j7	liver
ENSXMAG00	1.17	4.12	0.00	0.00	top1l	liver
ENSXMAG00	-1.26	2.27	0.00	0.02	pparab	liver
ENSXMAG00	2.46	1.44	0.00	0.04	tec	liver
ENSXMAG00	1.52	2.93	0.00	0.00	ergic1	liver
ENSXMAG00	1.75	3.21	0.00	0.00	smarca5	liver
ENSXMAG00	-1.23	5.15	0.00	0.00	trim105	liver
ENSXMAG00	2.05	0.80	0.00	0.02	mxd3	liver
ENSXMAG00	-5.13	-3.09	0.00	0.03	otog	liver
ENSXMAG00	0.96	3.76	0.00	0.01	ssrp1a	liver
ENSXMAG00	1.18	2.37	0.00	0.04	cdk8	liver
ENSXMAG00	-0.74	4.11	0.00	0.04	rufy1	liver
ENSXMAG00	-1.33	4.45	0.00	0.00	n4bp3	liver
ENSXMAG00	1.27	4.95	0.00	0.00	uso1	liver
ENSXMAG00	1.42	3.90	0.00	0.00	ATP6V1A (1	liver
ENSXMAG00	2.09	2.97	0.00	0.02	rac2	liver
ENSXMAG00	0.84	4.44	0.00	0.02	ykt6	liver
ENSXMAG00	3.80	7.79	0.00	0.02	gck	liver
ENSXMAG00	2.67	3.06	0.00	0.01		liver
ENSXMAG00	0.97	3.43	0.00	0.03	slc16a4	liver
ENSXMAG00	2.41	1.03	0.00	0.05	samsn1a	liver
ENSXMAG00	1.61	0.95	0.00	0.01	snapc3	liver
ENSXMAG00	1.14	4.41	0.00	0.02		liver
ENSXMAG00	2.02	5.33	0.00	0.00	aclyb	liver
ENSXMAG00	2.69	-2.46	0.00	0.04	si:dkey-202e	liver
ENSXMAG00	1.87	2.40	0.00	0.00		liver
ENSXMAG00	0.86	3.05	0.00	0.03	zdhhc13	liver
ENSXMAG00	2.08	6.95	0.00	0.00	elovl5	liver
ENSXMAG00	2.58	1.01	0.00	0.05	AIF1	liver
ENSXMAG00	0.82	3.62	0.00	0.05	smarcd1	liver
ENSXMAG00	-0.73	4.82	0.00	0.04		liver
ENSXMAG00	1.19	5.00	0.00	0.00	csnk2a1	liver
ENSXMAG00	1.36	1.80	0.00	0.05	nab1b	liver
ENSXMAG00	3.01	1.21	0.00	0.01	plaua	liver
ENSXMAG00	1.00	4.88	0.00	0.01	larp4aa	liver
ENSXMAG00	4.48	-1.81	0.00	0.04		liver
ENSXMAG00	3.23	1.53	0.00	0.03		liver
ENSXMAG00	2.41	2.68	0.00	0.01	hephl1b	liver
ENSXMAG00	2.56	2.41	0.00	0.02	slc43a2b	liver
ENSXMAG00	-4.14	-1.58	0.00	0.01		liver
ENSXMAG00	0.92	2.86	0.00	0.02	man1b1a	liver

ENSXMAG00	2.25	0.66	0.00	0.04	arhgap4a	liver
ENSXMAG00	2.34	1.78	0.00	0.00	heatr6	liver
ENSXMAG00	-4.07	-2.80	0.00	0.03	sphkap	liver
ENSXMAG00	0.89	3.94	0.00	0.01	baz1b	liver
ENSXMAG00	1.41	3.21	0.00	0.03	sil1	liver
ENSXMAG00	4.94	-1.06	0.00	0.04	si:ch211-184	liver
ENSXMAG00	-1.13	5.48	0.00	0.02	itga9	liver
ENSXMAG00	1.40	0.94	0.00	0.02	zgc:77880	liver
ENSXMAG00	-1.78	6.11	0.00	0.00	plpp3	liver
ENSXMAG00	1.53	5.55	0.00	0.00	lpcat3	liver
ENSXMAG00	0.98	3.21	0.00	0.01	cdc42l	liver
ENSXMAG00	1.23	3.34	0.00	0.00	kpna3	liver
ENSXMAG00	2.59	1.89	0.00	0.01	DOCK2	liver
ENSXMAG00	0.90	5.60	0.00	0.01	prrc2c	liver
ENSXMAG00	-1.04	10.02	0.00	0.04	upp2	liver
ENSXMAG00	2.17	3.18	0.00	0.03		liver
ENSXMAG00	0.97	4.35	0.00	0.01	grpel1	liver
ENSXMAG00	0.87	4.78	0.00	0.04	zgc:158564	liver
ENSXMAG00	5.76	0.25	0.00	0.01		liver
ENSXMAG00	1.02	4.20	0.00	0.02	nop2	liver
ENSXMAG00	1.20	4.77	0.00	0.00	gorasp2	liver
ENSXMAG00	1.39	4.95	0.00	0.00	txndc11	liver
ENSXMAG00	1.13	2.94	0.00	0.01	tmem189	liver
ENSXMAG00	1.60	5.45	0.00	0.00	kdelr2b	liver
ENSXMAG00	-0.99	7.45	0.00	0.04	nme4	liver
ENSXMAG00	4.75	-0.84	0.00	0.01	nr4a1	liver
ENSXMAG00	1.26	3.97	0.00	0.00	usp4	liver
ENSXMAG00	-0.75	4.32	0.00	0.05	vps9d1	liver
ENSXMAG00	0.76	3.95	0.00	0.05	psma6b	liver
ENSXMAG00	4.54	-1.85	0.00	0.01	stk39	liver
ENSXMAG00	0.93	2.36	0.00	0.03	me3	liver
ENSXMAG00	-1.01	4.35	0.00	0.01	paxip1	liver
ENSXMAG00	2.12	2.04	0.00	0.05	si:dkey-190g	liver
ENSXMAG00	-0.95	5.77	0.00	0.02	gltpd2	liver
ENSXMAG00	-1.03	2.42	0.00	0.03	pafah1b2	liver
ENSXMAG00	-1.49	4.97	0.00	0.00	mapk8ip3	liver
ENSXMAG00	-1.35	2.93	0.00	0.00	si:ch211-26b	liver
ENSXMAG00	-2.80	-1.91	0.00	0.03	armc4	liver
ENSXMAG00	-1.01	2.88	0.00	0.02	nme3	liver
ENSXMAG00	1.90	3.33	0.00	0.03	arhgap45b	liver
ENSXMAG00	-1.08	3.60	0.00	0.00	slc12a9	liver
ENSXMAG00	-1.18	2.62	0.00	0.01	si:ch211-120	liver
ENSXMAG00	-1.05	4.24	0.00	0.00	plxna2	liver

ENSXMAG00	-1.14	3.14	0.00	0.00	hgsnat	liver
ENSXMAG00	1.25	2.24	0.00	0.00	adpgk	liver
ENSXMAG00	3.69	-0.40	0.00	0.04		liver
ENSXMAG00	1.15	2.52	0.00	0.01	blmh	liver
ENSXMAG00	1.11	4.02	0.00	0.00	ctns	liver
ENSXMAG00	0.84	3.71	0.00	0.02	dag1	liver
ENSXMAG00	1.82	2.11	0.00	0.00	dus2	liver
ENSXMAG00	-1.44	2.14	0.00	0.00	SLC25A42	liver
ENSXMAG00	-0.84	4.48	0.00	0.03	shroom3	liver
ENSXMAG00	2.29	0.94	0.00	0.04	adamts15a	liver
ENSXMAG00	1.47	2.21	0.00	0.01		liver
ENSXMAG00	0.88	6.07	0.00	0.02	arcn1b	liver
ENSXMAG00	-1.19	5.06	0.00	0.01	itgb4	liver
ENSXMAG00	-1.32	3.95	0.00	0.00	raph1b	liver
ENSXMAG00	-2.38	-1.76	0.00	0.04	ttll10	liver
ENSXMAG00	1.28	3.29	0.00	0.03	HSD17B7 (1	liver
ENSXMAG00	1.54	3.68	0.00	0.00	si:dkey-82j4.	liver
ENSXMAG00	0.96	2.56	0.00	0.04	TMEM184B (	liver
ENSXMAG00	-1.03	2.88	0.00	0.01		liver
ENSXMAG00	2.63	-0.28	0.00	0.04	abi3a	liver
ENSXMAG00	-1.15	2.44	0.00	0.00	hmg20b	liver
ENSXMAG00	2.24	1.52	0.00	0.01		liver
ENSXMAG00	1.19	3.28	0.00	0.00	zgc:112148	liver
ENSXMAG00	-0.87	3.47	0.00	0.02	zgc:163098	liver
ENSXMAG00	0.94	2.92	0.00	0.04	brpf3b	liver
ENSXMAG00	-1.13	2.34	0.00	0.02	mgmt	liver
ENSXMAG00	-1.31	1.10	0.00	0.02	ebf3a	liver
ENSXMAG00	1.19	3.40	0.00	0.00		liver
ENSXMAG00	1.29	2.98	0.00	0.00	khsrp	liver
ENSXMAG00	5.54	0.65	0.00	0.00		liver
ENSXMAG00	0.80	3.81	0.00	0.05	ATP6V1A (1	liver
ENSXMAG00	1.33	4.65	0.00	0.00	rorca	liver
ENSXMAG00	1.11	2.26	0.00	0.02	lipt2	liver
ENSXMAG00	1.71	6.77	0.00	0.00	tmem214	liver
ENSXMAG00	-0.73	4.15	0.00	0.04	tyw1	liver
ENSXMAG00	-5.67	-2.72	0.00	0.05	erich3	liver
ENSXMAG00	3.15	0.36	0.00	0.02	nr4a3	liver
ENSXMAG00	-1.19	2.73	0.00	0.01	tex11	liver
ENSXMAG00	1.05	4.61	0.00	0.01	atf6	liver
ENSXMAG00	4.83	-0.78	0.00	0.00	ctsl.1	liver
ENSXMAG00	-3.94	-0.99	0.00	0.01	rhbg	liver
ENSXMAG00	1.97	3.81	0.00	0.00	dhdds	liver
ENSXMAG00	-0.78	5.52	0.00	0.03		liver

ENSXMAG00	-0.98	4.49	0.00	0.02	inppl1b	liver
ENSXMAG00	1.91	-1.06	0.00	0.04		liver
ENSXMAG00	3.92	-2.30	0.00	0.01		liver
ENSXMAG00	1.60	4.93	0.00	0.00	atp13a1	liver
ENSXMAG00	1.98	6.34	0.00	0.00	gmppb	liver
ENSXMAG00	1.22	4.63	0.00	0.01	zfr	liver
ENSXMAG00	2.64	-0.23	0.00	0.04	tnfb	liver
ENSXMAG00	3.37	0.01	0.00	0.02		liver
ENSXMAG00	0.76	3.74	0.00	0.03	pom121	liver
ENSXMAG00	3.57	2.76	0.00	0.00	pprc1	liver
ENSXMAG00	2.55	1.78	0.00	0.00	znrd1	liver
ENSXMAG00	-6.18	-2.38	0.00	0.02	LRRC9	liver
ENSXMAG00	-0.80	3.76	0.00	0.02	vipas39	liver
ENSXMAG00	2.45	1.65	0.00	0.02	sash3	liver
ENSXMAG00	2.02	1.97	0.00	0.03	dnase2	liver
ENSXMAG00	3.41	1.21	0.00	0.01		liver
ENSXMAG00	-0.75	3.79	0.00	0.04	dis3l	liver
ENSXMAG00	2.50	3.20	0.00	0.01	coro1a	liver
ENSXMAG00	0.77	3.05	0.00	0.05	ube2a	liver
ENSXMAG00	1.16	2.67	0.00	0.01	gtf3aa	liver
ENSXMAG00	1.89	2.38	0.00	0.04	fmnl1a	liver
ENSXMAG00	1.22	4.89	0.00	0.01	nacc1a	liver
ENSXMAG00	0.78	4.55	0.00	0.04	arfgap2	liver
ENSXMAG00	-0.91	6.14	0.00	0.05	slc25a39	liver
ENSXMAG00	2.23	4.36	0.00	0.01	grna	liver
ENSXMAG00	2.19	3.97	0.00	0.00	abcf3	liver
ENSXMAG00	-0.85	4.62	0.00	0.04	bcat2	liver
ENSXMAG00	1.03	3.82	0.00	0.01	sgsm3	liver
ENSXMAG00	-1.11	2.71	0.00	0.01	kdm2bb	liver
ENSXMAG00	0.93	2.52	0.00	0.03	ankrd40	liver
ENSXMAG00	-0.84	2.53	0.00	0.05		liver
ENSXMAG00	1.19	4.49	0.00	0.00	TNPO2 (1 of	liver
ENSXMAG00	1.46	1.58	0.00	0.01	upp1	liver
ENSXMAG00	0.90	4.12	0.00	0.01	necap1	liver
ENSXMAG00	5.94	-0.66	0.00	0.00	egr2b	liver
ENSXMAG00	1.14	2.92	0.00	0.01	rcn1	liver
ENSXMAG00	1.40	5.03	0.00	0.00		liver
ENSXMAG00	-1.60	1.35	0.00	0.00	itga8	liver
ENSXMAG00	-0.80	5.34	0.00	0.03		liver
ENSXMAG00	3.51	0.39	0.00	0.02	thbs1a	liver
ENSXMAG00	1.47	4.27	0.00	0.00	sdf2	liver
ENSXMAG00	1.22	5.34	0.00	0.01	OSBPL10	liver
ENSXMAG00	-1.13	2.24	0.00	0.01	rgs6	liver

ENSXMAG00	-0.95	4.14	0.00	0.02	cdan1	liver
ENSXMAG00	3.93	1.11	0.00	0.01	SPAM1	liver
ENSXMAG00	1.31	3.80	0.00	0.00	acbd4	liver
ENSXMAG00	1.21	2.24	0.00	0.04	tes	liver
ENSXMAG00	2.44	1.21	0.00	0.04	PIK3R5 (1 of	liver
ENSXMAG00	3.33	-0.01	0.00	0.03	themis2	liver
ENSXMAG00	4.07	8.90	0.00	0.03	fasn	liver
ENSXMAG00	-3.77	-1.84	0.00	0.03	pyyb	liver
ENSXMAG00	2.37	2.01	0.00	0.04	csf3r	liver
ENSXMAG00	1.20	3.11	0.00	0.01	wdr74	liver
ENSXMAG00	-1.18	1.13	0.00	0.04	ift88	liver
ENSXMAG00	2.73	-0.31	0.00	0.04	rassf2a	liver
ENSXMAG00	0.83	3.13	0.00	0.04	tbl3	liver
ENSXMAG00	3.96	0.32	0.00	0.01		liver
ENSXMAG00	-5.81	-2.66	0.00	0.01	rsph10b	liver
ENSXMAG00	0.94	4.90	0.00	0.01	ppm1g	liver
ENSXMAG00	1.82	2.42	0.00	0.00	clcn5b	liver
ENSXMAG00	1.84	1.24	0.00	0.02	dusp2	liver
ENSXMAG00	-0.95	3.76	0.00	0.00	dedd	liver
ENSXMAG00	2.26	0.53	0.00	0.00	nudt18	liver
ENSXMAG00	0.88	2.48	0.00	0.04	tk2	liver
ENSXMAG00	-0.81	5.21	0.00	0.04	mbnl1	liver
ENSXMAG00	-1.00	4.79	0.00	0.02	slc5a6b	liver
ENSXMAG00	-1.05	3.00	0.00	0.05	pnp6	liver
ENSXMAG00	0.96	3.58	0.00	0.02		liver
ENSXMAG00	0.81	4.57	0.00	0.03		liver
ENSXMAG00	1.75	2.30	0.00	0.00	acsl3b	liver
ENSXMAG00	1.39	3.36	0.00	0.00	rnf130	liver
ENSXMAG00	1.08	5.23	0.00	0.00	tmed7	liver
ENSXMAG00	0.94	3.66	0.00	0.01	rtraf	liver
ENSXMAG00	0.83	4.72	0.00	0.02	ARF5	liver
ENSXMAG00	1.60	1.43	0.00	0.00	zgc:63863	liver
ENSXMAG00	-1.60	6.35	0.00	0.00	aspg	liver
ENSXMAG00	0.78	4.68	0.00	0.02		liver
ENSXMAG00	1.07	2.34	0.00	0.02	zgc:158828	liver
ENSXMAG00	2.46	1.03	0.00	0.04	nphp1	liver
ENSXMAG00	1.40	7.97	0.00	0.01	actb1	liver
ENSXMAG00	0.86	5.12	0.00	0.01	vclb	liver
ENSXMAG00	1.24	4.56	0.00	0.05	zgc:174164	liver
ENSXMAG00	2.15	0.76	0.00	0.00	adck1	liver
ENSXMAG00	1.95	3.08	0.00	0.00	LONRF3	liver
ENSXMAG00	0.82	3.77	0.00	0.02	ssuh2rs1	liver
ENSXMAG00	-0.87	2.58	0.00	0.04	man2c1	liver

ENSXMAG00	1.15	3.69	0.00	0.00	lmnb1	liver
ENSXMAG00	0.80	4.24	0.00	0.04		liver
ENSXMAG00	1.08	6.61	0.00	0.00	eif4g1a	liver
ENSXMAG00	0.90	3.76	0.00	0.01	sptlc1	liver
ENSXMAG00	2.65	1.24	0.00	0.02	myo1g	liver
ENSXMAG00	3.23	6.22	0.00	0.00	per2	liver
ENSXMAG00	0.94	3.17	0.00	0.01	ap1ar	liver
ENSXMAG00	1.35	4.49	0.00	0.01	syvn1	liver
ENSXMAG00	1.46	-0.04	0.00	0.03		liver
ENSXMAG00	2.25	1.45	0.00	0.04		liver
ENSXMAG00	1.75	2.60	0.00	0.00	dhx30	liver
ENSXMAG00	2.13	0.61	0.00	0.05	adcy7	liver
ENSXMAG00	4.69	-0.99	0.00	0.04	spp1	liver
ENSXMAG00	1.20	6.59	0.00	0.01	npc1	liver
ENSXMAG00	1.72	3.04	0.00	0.05	inpp5d	liver
ENSXMAG00	-2.20	0.54	0.00	0.00	si:ch211-22d	liver
ENSXMAG00	2.93	2.76	0.00	0.01	mrc1b (1 of ı	liver
ENSXMAG00	4.47	2.19	0.00	0.00	mrc1b (1 of ı	liver
ENSXMAG00	1.61	4.10	0.00	0.00		liver
ENSXMAG00	1.54	4.70	0.00	0.00	edem1	liver
ENSXMAG00	0.93	3.72	0.00	0.01	mrpl20	liver
ENSXMAG00	-1.02	4.28	0.00	0.01	KAZN (1 of m	liver
ENSXMAG00	-1.17	2.06	0.00	0.05	map2k6	liver
ENSXMAG00	2.25	5.87	0.00	0.00	lpin2	liver
ENSXMAG00	1.33	1.44	0.00	0.03	MSH3	liver
ENSXMAG00	4.92	-1.15	0.00	0.02	xcr1b.1	liver
ENSXMAG00	0.81	4.46	0.00	0.02	top2b	liver
ENSXMAG00	1.52	3.73	0.00	0.00	tmem165	liver
ENSXMAG00	1.54	5.11	0.00	0.00	tmed3	liver
ENSXMAG00	-0.86	5.97	0.00	0.03	zgc:136858	liver
ENSXMAG00	3.56	0.46	0.00	0.00	si:ch73-52e5	liver
ENSXMAG00	1.19	2.25	0.00	0.02	slc33a1	liver
ENSXMAG00	1.63	4.48	0.00	0.00	lrrc59	liver
ENSXMAG00	0.98	3.16	0.00	0.03	timm23a	liver
ENSXMAG00	2.21	-0.31	0.00	0.00	wdr76	liver
ENSXMAG00	3.80	0.41	0.00	0.02		liver
ENSXMAG00	-1.37	0.62	0.00	0.05	znf362a	liver
ENSXMAG00	2.23	-1.76	0.00	0.04	ppp1r9bb	liver
ENSXMAG00	1.01	2.96	0.00	0.00	ints6l	liver
ENSXMAG00	0.92	4.57	0.00	0.01	rer1	liver
ENSXMAG00	2.82	1.79	0.00	0.01	tagapb	liver
ENSXMAG00	0.82	5.01	0.00	0.02		liver
ENSXMAG00	-1.70	0.39	0.00	0.01		liver

ENSXMAG00	-1.31	3.19	0.00	0.00	rhag	liver
ENSXMAG00	-2.08	0.11	0.00	0.00	ush2a	liver
ENSXMAG00	4.11	-0.77	0.00	0.02		liver
ENSXMAG00	-0.77	6.15	0.00	0.05	FBLN2 (1 of	liver
ENSXMAG00	1.15	2.42	0.00	0.04	nup210	liver
ENSXMAG00	3.47	0.84	0.00	0.02	capn3b	liver
ENSXMAG00	1.07	2.65	0.00	0.01	mfsd13a	liver
ENSXMAG00	4.18	-2.60	0.00	0.04		liver
ENSXMAG00	2.56	1.53	0.00	0.02	zgc:172352	liver
ENSXMAG00	3.15	1.04	0.00	0.01		liver
ENSXMAG00	-0.91	6.10	0.00	0.01	unc93b1	liver
ENSXMAG00	1.36	2.39	0.00	0.04	mthfd2	liver
ENSXMAG00	0.77	3.97	0.00	0.04	mob1a	liver
ENSXMAG00	2.03	5.51	0.00	0.00	mat2ab	liver
ENSXMAG00	1.02	5.20	0.00	0.00	arhgef2	liver
ENSXMAG00	4.52	-1.48	0.00	0.02		liver
ENSXMAG00	-1.68	0.23	0.00	0.01		liver
ENSXMAG00	3.55	2.93	0.00	0.00	mars	liver
ENSXMAG00	2.43	1.51	0.00	0.04	rasal3	liver
ENSXMAG00	0.98	3.13	0.00	0.02	si:dkey-237i9	liver
ENSXMAG00	0.81	3.65	0.00	0.04	nsfb	liver
ENSXMAG00	1.10	4.09	0.00	0.00	psmd11b	liver
ENSXMAG00	-0.94	4.05	0.00	0.02	cadm1b	liver
ENSXMAG00	-1.71	-0.41	0.00	0.03		liver
ENSXMAG00	5.57	-2.80	0.00	0.04		liver
ENSXMAG00	2.24	-0.11	0.00	0.02	si:ch211-195	liver
ENSXMAG00	2.27	2.27	0.00	0.02		liver
ENSXMAG00	1.06	2.37	0.00	0.01		liver
ENSXMAG00	5.37	-0.11	0.00	0.00	si:ch211-136	liver
ENSXMAG00	0.74	5.62	0.00	0.05	coro1ca	liver
ENSXMAG00	1.15	3.53	0.00	0.01	trappc6bl	liver
ENSXMAG00	-0.97	2.93	0.00	0.05	map3k21	liver
ENSXMAG00	2.70	1.83	0.00	0.03		liver
ENSXMAG00	2.77	5.28	0.00	0.00	odc1	liver
ENSXMAG00	1.17	1.39	0.00	0.02	dmap1	liver
ENSXMAG00	1.56	2.43	0.00	0.00	nol10	liver
ENSXMAG00	1.09	3.05	0.00	0.01	dars2	liver
ENSXMAG00	0.88	3.57	0.00	0.02	si:dkey-33c1:	liver
ENSXMAG00	-2.82	0.22	0.00	0.04	krt4	liver
ENSXMAG00	1.22	3.51	0.00	0.00	ncln	liver
ENSXMAG00	-1.97	6.96	0.00	0.00	nr1d2a	liver
ENSXMAG00	2.06	2.40	0.00	0.02		liver
ENSXMAG00	1.96	2.38	0.00	0.00	brms1lb	liver

ENSXMAG00	2.97	2.47	0.00	0.00		liver
ENSXMAG00	1.90	3.82	0.00	0.00		liver
ENSXMAG00	1.17	4.11	0.00	0.00	ddx19	liver
ENSXMAG00	3.63	0.50	0.00	0.01	lck	liver
ENSXMAG00	0.85	6.12	0.00	0.02	copb2	liver
ENSXMAG00	1.04	4.90	0.00	0.00	dync1li2	liver
ENSXMAG00	2.38	2.30	0.00	0.02	bpifcl	liver
ENSXMAG00	0.81	3.05	0.00	0.04		liver
ENSXMAG00	0.96	4.59	0.00	0.01	si:ch211-259	liver
ENSXMAG00	1.48	2.60	0.00	0.00	PRELID3B	liver
ENSXMAG00	0.90	5.74	0.00	0.02	si:ch1073-44	liver
ENSXMAG00	1.35	5.99	0.00	0.00	sec31a	liver
ENSXMAG00	-1.06	3.48	0.00	0.01	sema6e	liver
ENSXMAG00	-1.53	-0.03	0.00	0.04		liver
ENSXMAG00	-1.23	4.86	0.00	0.01	mtss1la	liver
ENSXMAG00	-1.39	2.22	0.00	0.05	arhgef37	liver
ENSXMAG00	-1.11	3.84	0.00	0.00	tiam2a	liver
ENSXMAG00	1.10	2.05	0.00	0.01	tpp2	liver
ENSXMAG00	1.51	2.14	0.00	0.00		liver
ENSXMAG00	1.81	3.11	0.00	0.00	g3bp1	liver
ENSXMAG00	1.12	2.90	0.00	0.01	plekhj1	liver
ENSXMAG00	1.09	3.09	0.00	0.01		liver
ENSXMAG00	1.10	5.01	0.00	0.00	sec13	liver
ENSXMAG00	1.04	3.12	0.00	0.04	xpc	liver
ENSXMAG00	1.02	2.30	0.00	0.03	nadka	liver
ENSXMAG00	4.43	1.12	0.00	0.01		liver
ENSXMAG00	2.92	-2.30	0.00	0.03		liver
ENSXMAG00	3.09	-2.19	0.00	0.01		liver
ENSXMAG00	-1.62	1.15	0.00	0.03		liver
ENSXMAG00	1.57	2.93	0.00	0.00	zpr1	liver
ENSXMAG00	2.51	0.40	0.00	0.04	gpr183a	liver
ENSXMAG00	1.43	2.91	0.00	0.04	cyp1c1	liver
ENSXMAG00	2.54	-1.09	0.00	0.04	lpar5b	liver
ENSXMAG00	0.86	3.52	0.00	0.02	nup88	liver
ENSXMAG00	3.03	0.78	0.00	0.04	ccr12a	liver
ENSXMAG00	3.74	-1.71	0.00	0.04	p2ry1	liver
ENSXMAG00	1.35	3.28	0.00	0.00	dpm3	liver
ENSXMAG00	1.38	0.75	0.00	0.03	mfsd6l	liver
ENSXMAG00	1.05	3.19	0.00	0.01	zgc:55781	liver
ENSXMAG00	-6.35	-2.25	0.00	0.03		liver
ENSXMAG00	1.49	1.90	0.00	0.03	ADAMTS5	liver
ENSXMAG00	4.48	2.22	0.00	0.01		liver
ENSXMAG00	3.27	1.59	0.00	0.01		liver

ENSXMAG00	1.02	4.44	0.00	0.01	esrra	liver
ENSXMAG00	1.08	3.12	0.00	0.01	ATE1	liver
ENSXMAG00	6.78	-1.95	0.00	0.00		liver
ENSXMAG00	-1.27	3.47	0.00	0.00	epor	liver
ENSXMAG00	0.98	3.23	0.00	0.01	slc30a7	liver
ENSXMAG00	1.75	2.48	0.00	0.00	si:ch211-11k	liver
ENSXMAG00	1.04	7.07	0.00	0.01	SURF4	liver
ENSXMAG00	-1.27	4.19	0.00	0.04	ddit4	liver
ENSXMAG00	-1.41	7.93	0.00	0.00	ppdpfa	liver
ENSXMAG00	-2.67	-1.95	0.00	0.04		liver
ENSXMAG00	2.05	-1.38	0.00	0.03	RF00421	liver
ENSXMAG00	1.06	3.16	0.00	0.00	hnrnph1	liver
ENSXMAG00	1.53	4.96	0.00	0.00	mogs	liver
ENSXMAG00	3.79	0.91	0.00	0.03	si:ch211-153	liver
ENSXMAG00	3.71	1.67	0.00	0.00		liver
ENSXMAG00	0.91	4.78	0.00	0.02	myd88	liver
ENSXMAG00	-6.23	-2.34	0.00	0.03		liver
ENSXMAG00	1.01	5.52	0.00	0.01		liver
ENSXMAG00	1.56	4.03	0.00	0.01	dnajb9b	liver
ENSXMAG00	1.40	2.56	0.00	0.04		liver
ENSXMAG00	-0.91	2.63	0.00	0.05	poglut2	liver
ENSXMAG00	0.99	4.27	0.00	0.01	papss1	liver
ENSXMAG00	-4.52	-1.96	0.00	0.03		liver
ENSXMAG00	3.77	0.21	0.00	0.01		liver
ENSXMAG00	-5.69	-2.72	0.00	0.04		liver
ENSXMAG00	0.96	5.19	0.00	0.01	agpat2	liver
ENSXMAG00	0.86	3.23	0.00	0.03	icmt	liver
ENSXMAG00	2.56	0.91	0.00	0.04	stat4	liver
ENSXMAG00	1.06	3.34	0.00	0.00	ahsa1b	liver
ENSXMAG00	2.79	1.38	0.00	0.01	cxcr2	liver
ENSXMAG00	1.16	2.31	0.00	0.01	RNF224	liver
ENSXMAG00	0.92	2.79	0.00	0.04	tefm	liver
ENSXMAG00	1.24	5.05	0.00	0.00	trabd	liver
ENSXMAG00	1.41	4.50	0.00	0.00	nxt2	liver
ENSXMAG00	5.97	-2.54	0.00	0.01		liver
ENSXMAG00	2.18	6.29	0.00	0.01		liver
ENSXMAG00	1.23	2.72	0.00	0.00	si:ch211-212	liver
ENSXMAG00	1.06	3.06	0.00	0.00	pdxdc1	liver
ENSXMAG00	4.75	-2.24	0.00	0.01	RF00069	liver
ENSXMAG00	1.16	2.54	0.00	0.04		liver
ENSXMAG00	2.26	4.60	0.00	0.00	si:ch211-197	liver
ENSXMAG00	4.89	-2.13	0.00	0.02		liver
ENSXMAG00	-1.41	0.04	0.00	0.05		liver

ENSXMAG00	1.23	2.33	0.00	0.02	zgc:109986	liver
ENSXMAG00	-0.90	4.36	0.00	0.04	nr1i2	liver
ENSXMAG00	1.32	4.22	0.00	0.00	VPS26B (1 of	liver
ENSXMAG00	-1.80	3.43	0.00	0.00	ciarta	liver
ENSXMAG00	1.47	3.53	0.00	0.00	zgc:153675	liver
ENSXMAG00	1.41	2.30	0.00	0.00		liver
ENSXMAG00	0.90	3.60	0.00	0.01	slc6a16b	liver
ENSXMAG00	2.59	1.39	0.00	0.01	sla1a	liver
ENSXMAG00	1.11	3.52	0.00	0.03	si:ch211-238	liver
ENSXMAG00	2.41	0.84	0.00	0.03	MARCO	liver
ENSXMAG00	0.91	5.19	0.00	0.01	tmed9	liver
ENSXMAG00	-5.83	-1.48	0.00	0.01		liver
ENSXMAG00	1.14	4.14	0.00	0.00	KDELR2	liver
ENSXMAG00	1.70	2.41	0.00	0.03	slc30a1a	liver
ENSXMAG00	2.24	3.81	0.00	0.00	uck2b	liver
ENSXMAG00	1.46	2.59	0.00	0.01	ssh2b	liver
ENSXMAG00	1.22	3.70	0.00	0.00	necap2	liver
ENSXMAG00	-0.95	3.77	0.00	0.05	ctdsp2	liver
ENSXMAG00	1.10	1.72	0.00	0.04	taf5l	liver
ENSXMAG00	0.89	4.78	0.00	0.04	nifk	liver
ENSXMAG00	1.64	1.12	0.00	0.01	bnip1a	liver
ENSXMAG00	-6.23	-2.37	0.00	0.03		liver
ENSXMAG00	1.15	1.87	0.00	0.02	PSTK	liver
ENSXMAG00	2.42	1.06	0.00	0.00	eif4e1c	liver
ENSXMAG00	2.11	1.12	0.00	0.03	zbtb46	liver
ENSXMAG00	0.86	4.37	0.00	0.03	cdv3	liver
ENSXMAG00	-1.14	1.31	0.00	0.04		liver
ENSXMAG00	2.42	0.49	0.00	0.04		liver
ENSXMAG00	0.96	6.63	0.00	0.00	PFN2	liver
ENSXMAG00	1.93	-0.54	0.00	0.01	RF00089	liver
ENSXMAG00	4.50	-1.47	0.00	0.01		liver
ENSXMAG00	-1.01	5.08	0.00	0.02	cbx4	liver
ENSXMAG00	2.32	0.72	0.00	0.04		liver
ENSXMAG00	5.35	0.72	0.00	0.00		liver
ENSXMAG00	1.39	2.03	0.00	0.00	si:ch73-244f	liver
ENSXMAG00	-0.94	2.82	0.00	0.02	si:dkey-178e	liver
ENSXMAG00	3.28	2.92	0.00	0.01		liver
ENSXMAG00	-8.80	-0.20	0.00	0.00		liver
ENSXMAG00	2.01	-0.62	0.00	0.03		liver
ENSXMAG00	5.39	-0.60	0.00	0.02		liver
ENSXMAG00	1.26	0.83	0.00	0.04		liver
ENSXMAG00	2.37	-1.19	0.00	0.04		liver
ENSXMAG00	4.35	-0.47	0.00	0.02	CXCR3 (1 of	liver

ENSXMAG00	-1.07	3.31	0.00	0.02		liver
ENSXMAG00	3.68	1.08	0.00	0.03		liver
ENSXMAG00	0.99	3.22	0.00	0.01	irf9	liver
ENSXMAG00	1.73	1.87	0.00	0.05	si:ch211-137	liver
ENSXMAG00	0.86	4.54	0.00	0.02	sec23ip	liver
ENSXMAG00	1.40	3.29	0.00	0.01	akap12b	liver
ENSXMAG00	1.69	5.44	0.00	0.04		liver
ENSXMAG00	0.95	2.54	0.00	0.02	ubxn2a	liver
ENSXMAG00	-4.45	-1.95	0.00	0.04		liver
ENSXMAG00	3.23	-0.70	0.00	0.01		liver
ENSXMAG00	2.60	-1.02	0.00	0.00	RF00277	liver
ENSXMAG00	2.04	2.54	0.00	0.01		liver
ENSXMAG00	1.58	4.03	0.00	0.00	si:dkey-167k:	liver
ENSXMAG00	1.07	2.60	0.00	0.03	gpatch4	liver
ENSXMAG00	-5.14	-3.11	0.00	0.02		liver
ENSXMAG00	-1.13	3.00	0.00	0.02	gata1a	liver
ENSXMAG00	-1.36	0.50	0.00	0.04	dmtf1	liver
ENSXMAG00	2.22	1.48	0.00	0.03		liver
ENSXMAG00	3.81	1.36	0.00	0.01		liver
ENSXMAG00	0.95	4.48	0.00	0.01	eif4e3	liver
ENSXMAG00	1.41	0.64	0.00	0.03	fuz	liver
ENSXMAG00	1.40	2.16	0.00	0.01	tdp2b	liver
ENSXMAG00	1.65	3.40	0.00	0.00	slc35e1	liver
ENSXMAG00	1.43	2.10	0.00	0.01	ube2v1	liver
ENSXMAG00	1.80	-0.09	0.00	0.04	nanp	liver
ENSXMAG00	1.84	-0.02	0.00	0.03	emc9	liver
ENSXMAG00	1.50	4.73	0.00	0.00	rab5if	liver
ENSXMAG00	2.07	2.90	0.00	0.00	dnajc15	liver
ENSXMAG00	-1.28	4.19	0.00	0.00	arhgdig	liver
ENSXMAG00	2.32	1.04	0.00	0.04		liver
ENSXMAG00	1.62	2.94	0.00	0.00	vamp1	liver
ENSXMAG00	2.03	1.14	0.00	0.00		liver
ENSXMAG00	-2.40	-1.98	0.00	0.03	rpz6	liver
ENSXMAG00	1.19	4.60	0.00	0.00	mapre1a	liver
ENSXMAG00	-1.00	3.34	0.00	0.04	tal1	liver
ENSXMAG00	1.24	4.62	0.00	0.00	sec22bb	liver
ENSXMAG00	4.59	4.15	0.00	0.00		liver
ENSXMAG00	-2.26	3.80	0.00	0.00	cipcb	liver
ENSXMAG00	2.51	-1.86	0.00	0.04	RF00067	liver
ENSXMAG00	2.91	-1.15	0.00	0.03	nrip2	liver
ENSXMAG00	0.85	3.90	0.00	0.01	timm17a	liver
ENSXMAG00	0.75	5.21	0.00	0.05		liver
ENSXMAG00	-1.93	1.85	0.00	0.00	cntfr	liver

ENSXMAG00	1.16	1.40	0.00	0.04	kctd6b	liver
ENSXMAG00	1.01	2.97	0.00	0.01	yipf2	liver
ENSXMAG00	-1.10	1.30	0.00	0.04		liver
ENSXMAG00	5.96	-2.55	0.00	0.03		liver
ENSXMAG00	1.83	1.29	0.00	0.02	si:ch211-121	liver
ENSXMAG00	4.17	0.22	0.00	0.01	si:ch73-361p	liver
ENSXMAG00	-3.11	-1.09	0.00	0.04	tdrd6	liver
ENSXMAG00	-0.98	3.18	0.00	0.01	wnt5b	liver
ENSXMAG00	1.53	1.38	0.00	0.02	qtrt1	liver
ENSXMAG00	0.99	1.97	0.00	0.03	ETHE1 (1 of r	liver
ENSXMAG00	1.19	5.05	0.00	0.00		liver
ENSXMAG00	-0.95	4.50	0.00	0.01	retreg2	liver
ENSXMAG00	5.51	-1.61	0.00	0.03		liver
ENSXMAG00	1.27	2.00	0.00	0.01	ergic3 (1 of r	liver
ENSXMAG00	2.15	-0.94	0.00	0.03	RF00281	liver
ENSXMAG00	3.17	0.27	0.00	0.01	ptger4b	liver
ENSXMAG00	1.52	2.24	0.00	0.02	zgc:174888	liver
ENSXMAG00	1.72	3.70	0.00	0.00	erp44	liver
ENSXMAG00	3.21	-0.39	0.00	0.00		liver
ENSXMAG00	-4.48	-2.49	0.00	0.02		liver
ENSXMAG00	2.32	4.00	0.00	0.00		liver
ENSXMAG00	2.02	5.85	0.00	0.02		liver
ENSXMAG00	-1.45	3.34	0.00	0.00		liver
ENSXMAG00	2.40	-0.91	0.00	0.01	tmem42a	liver
ENSXMAG00	4.74	-0.27	0.00	0.01	ikzf4	liver
ENSXMAG00	3.82	-0.42	0.00	0.04	si:ch211-25d	liver
ENSXMAG00	2.01	1.28	0.00	0.03	si:ch211-219	liver
ENSXMAG00	4.64	0.26	0.00	0.00		liver
ENSXMAG00	0.91	4.29	0.00	0.01	abhd4	liver
ENSXMAG00	0.88	2.94	0.00	0.03	pgap3	liver
ENSXMAG00	1.14	2.58	0.00	0.01	xgb	liver
ENSXMAG00	0.78	3.39	0.00	0.04	bmt2	liver
ENSXMAG00	2.36	1.51	0.00	0.03		liver
ENSXMAG00	1.19	3.10	0.00	0.00	adprm	liver
ENSXMAG00	1.28	4.04	0.00	0.00	tomm22	liver
ENSXMAG00	6.74	-1.98	0.00	0.02		liver
ENSXMAG00	1.55	0.66	0.00	0.03		liver
ENSXMAG00	0.84	7.18	0.00	0.04	pisd	liver
ENSXMAG00	1.69	0.97	0.00	0.00	mfsd9	liver
ENSXMAG00	0.92	4.80	0.00	0.01	sumo3a	liver
ENSXMAG00	1.64	6.58	0.00	0.00	tram1	liver
ENSXMAG00	1.20	4.14	0.00	0.00	usp14	liver
ENSXMAG00	-1.40	5.50	0.00	0.00	tp53inp1	liver

ENSXMAG00	0.90	3.63	0.00	0.03	rab11al	liver
ENSXMAG00	1.84	0.64	0.00	0.04	igflr1	liver
ENSXMAG00	2.09	0.80	0.00	0.00	pole3	liver
ENSXMAG00	-2.34	0.45	0.00	0.00	ERC2 (1 of m	liver
ENSXMAG00	-1.51	1.99	0.00	0.02	reep2	liver
ENSXMAG00	-1.28	1.45	0.00	0.02	btbd6b	liver
ENSXMAG00	5.07	-1.98	0.00	0.01	tnfa	liver
ENSXMAG00	0.84	2.89	0.00	0.03		liver
ENSXMAG00	-0.84	3.39	0.00	0.03	gsdmeb	liver
ENSXMAG00	0.98	6.11	0.00	0.01	tmed10	liver
ENSXMAG00	5.46	-1.16	0.00	0.03		liver
ENSXMAG00	0.96	3.59	0.00	0.04		liver
ENSXMAG00	0.77	3.27	0.00	0.03	RAB41	liver
ENSXMAG00	-2.02	0.67	0.00	0.00		liver
ENSXMAG00	6.09	-2.45	0.00	0.03		liver
ENSXMAG00	2.08	4.75	0.00	0.03		liver
ENSXMAG00	1.66	2.87	0.00	0.00	etnk2	liver
ENSXMAG00	3.62	-0.27	0.00	0.02		liver
ENSXMAG00	5.54	0.76	0.00	0.00	rabepk	liver
ENSXMAG00	1.10	4.27	0.00	0.00	kpna1	liver
ENSXMAG00	0.79	5.02	0.00	0.02		liver
ENSXMAG00	0.83	3.98	0.00	0.02	nmt1a	liver
ENSXMAG00	-1.10	3.84	0.00	0.00	SLC25A45	liver
ENSXMAG00	-1.15	4.94	0.00	0.01	8-Mar	liver
ENSXMAG00	-0.90	3.24	0.00	0.02	zgc:91944	liver
ENSXMAG00	-1.48	2.39	0.00	0.03	alkbh3	liver
ENSXMAG00	1.29	3.23	0.00	0.00	scamp1	liver
ENSXMAG00	1.28	1.19	0.00	0.04	dnajb9a	liver
ENSXMAG00	1.15	2.79	0.00	0.04	opn6a	liver
ENSXMAG00	2.89	0.68	0.00	0.01		liver
ENSXMAG00	2.39	0.29	0.00	0.00	PQLC1	liver
ENSXMAG00	-0.81	3.64	0.00	0.05		liver
ENSXMAG00	1.53	1.74	0.00	0.01	asf1bb	liver
ENSXMAG00	3.78	-0.38	0.00	0.01		liver
ENSXMAG00	1.39	2.39	0.00	0.05		liver
ENSXMAG00	2.32	1.54	0.00	0.04	rgs18	liver
ENSXMAG00	1.43	6.22	0.00	0.00	srpra	liver
ENSXMAG00	4.08	-1.20	0.00	0.04		liver
ENSXMAG00	0.85	4.23	0.00	0.04	ccdc167	liver
ENSXMAG00	2.86	-1.12	0.00	0.00	megf8	liver
ENSXMAG00	-1.03	4.63	0.00	0.02	map1lc3b	liver
ENSXMAG00	3.20	3.29	0.00	0.01	C1QB	liver
ENSXMAG00	3.20	-1.92	0.00	0.00	RF00571	liver

ENSXMAG00	1.39	1.25	0.00	0.03	cnga2b	liver
ENSXMAG00	-0.91	5.90	0.00	0.03	glyctk	liver
ENSXMAG00	3.31	-1.06	0.00	0.03		liver
ENSXMAG00	3.04	0.89	0.00	0.01		liver
ENSXMAG00	-1.23	1.63	0.00	0.05		liver
ENSXMAG00	2.35	1.05	0.00	0.04	irg1l (1 of ma	liver
ENSXMAG00	1.58	1.65	0.00	0.00	rnf175	liver
ENSXMAG00	1.26	4.11	0.00	0.00	strap	liver
ENSXMAG00	-1.43	3.69	0.00	0.01		liver
ENSXMAG00	-0.89	3.74	0.00	0.05	stk11ip	liver
ENSXMAG00	1.91	1.77	0.00	0.02		liver
ENSXMAG00	1.09	2.36	0.00	0.04	WARS	liver
ENSXMAG00	-0.98	4.22	0.00	0.03	ncam1b	liver
ENSXMAG00	3.31	2.85	0.00	0.01	nocta	liver
ENSXMAG00	4.42	1.82	0.00	0.00		liver
ENSXMAG00	1.38	4.75	0.00	0.00	HNRNPA0 (1	liver
ENSXMAG00	2.61	0.19	0.00	0.04		liver
ENSXMAG00	3.13	3.24	0.00	0.00		liver
ENSXMAG00	1.12	2.99	0.00	0.01		liver
ENSXMAG00	-1.27	2.92	0.00	0.01		liver
ENSXMAG00	0.83	3.00	0.00	0.04	RAP1GDS1	liver
ENSXMAG00	2.75	-0.52	0.00	0.01		liver
ENSXMAG00	-3.23	-1.66	0.00	0.01		liver
ENSXMAG00	1.22	2.45	0.00	0.04		liver
ENSXMAG00	3.10	0.18	0.00	0.03	slc2a6	liver
ENSXMAG00	4.89	-1.57	0.00	0.01	CXCR3 (1 of r	liver
ENSXMAG00	-1.58	1.18	0.00	0.01		liver
ENSXMAG00	1.78	-0.64	0.00	0.05		liver
ENSXMAG00	-0.80	3.84	0.00	0.04	cep162	liver
ENSXMAG00	4.48	3.10	0.00	0.00		liver
ENSXMAG00	1.67	-0.47	0.00	0.03		liver
ENSXMAG00	0.83	4.17	0.00	0.01	CERK (1 of m	liver
ENSXMAG00	1.73	4.43	0.00	0.00	prrc1	liver
ENSXMAG00	9.20	0.16	0.00	0.00		liver
ENSXMAG00	-1.12	2.97	0.00	0.01	tmem26a	liver
ENSXMAG00	1.05	3.47	0.00	0.01	nudt6	liver
ENSXMAG00	0.86	3.10	0.00	0.04	nabp1a	liver
ENSXMAG00	4.56	4.81	0.00	0.00		liver
ENSXMAG00	2.67	1.89	0.00	0.01		liver
ENSXMAG00	-3.07	-2.61	0.00	0.04		liver
ENSXMAG00	2.89	1.89	0.00	0.01		liver
ENSXMAG00	1.40	4.23	0.00	0.00	impad1	liver
ENSXMAG00	2.05	1.21	0.00	0.02	si:dkey-32e6.	liver

ENSXMAG00	1.76	0.33	0.00	0.00	nfil3-5	testis
ENSXMAG00	-6.88	-1.79	0.00	0.04		testis
ENSXMAG00	-4.72	0.75	0.00	0.01		testis
ENSXMAG00	-2.69	-2.06	0.00	0.01	chst6 (1 of m	testis
ENSXMAG00	-3.99	-1.51	0.00	0.01		testis
