# Supplementary material for: Global assessment of organ specific basal gene expression over a diurnal cycle with analyses of gene copies exhibiting cyclic expression patterns
Source: BMC Genomics. 2020 Nov 11;21:787. doi: 10.1186/s12864-020-07202-9 (PMC7659085; doi:10.1186/s12864-020-07202-9)
Supplement: Supplementary file 3 — Additional file 3: Supplement Table 2. Number of cyclic genes in each organ. [file 12864_2020_7202_MOESM3_ESM.pdf]

Supplement Table2

	skin	brain	ovary	heart	muscle	eye	gill	liver	testis
Total Gene #	24209	24209	24209	24209	24209	24209	24209	24209	24209
Expressed Gene #	16963	17608	17357	16950	14780	18377	17545	13321	16799
Circadian Gene #	935	731	224	659	2076	4033	1740	1039	294
Ct0	244	111	31	17	336	518	206	61	52
Ct3	101	107	15	272	81	517	129	55	72
Ct6	45	135	45	49	77	297	143	33	31
Ct9	47	46	24	44	34	135	39	27	23
Ct12	84	106	31	65	164	283	89	103	9
Ct15	127	91	36	147	757	895	781	573	24
Ct18	116	65	15	37	273	742	172	111	26
Ct21	171	70	27	28	354	646	181	76	57
% circaidan genes	5.5%	4.2%	1.3%	3.9%	14.0%	21.9%	9.9%	7.8%	1.8%
