# Supplementary material for: Global assessment of organ specific basal gene expression over a diurnal cycle with analyses of gene copies exhibiting cyclic expression patterns
Source: BMC Genomics. 2020 Nov 11;21:787. doi: 10.1186/s12864-020-07202-9 (PMC7659085; doi:10.1186/s12864-020-07202-9)
Supplement: Supplementary file 4 — Additional file 4: Supplement Table 3. Cyclic genes of all 9 organs. [file 12864_2020_7202_MOESM4_ESM.pdf]

Supplement Table 3

Skin circadian genes	GeneID	pVal	phase	peak.shape	external_gene_name	amp	Ct.peak
	ENSXMAG00000000028	0.0016	9	6		2.71	6
	ENSXMAG00000000071	0.0034	21	12	ascc3	1.58	18
	ENSXMAG00000000088	0.0015	9	6	waslb	1.79	6
	ENSXMAG00000000107	0.0029	3	12		3.28	0
	ENSXMAG00000000117	0.0031	24	12	mgrn1b	1.81	21
	ENSXMAG00000000131	0.0026	3	9	slc15a4	2.29	0
	ENSXMAG00000000149	0.0031	6	12	VMP1	3.48	3
	ENSXMAG00000000163	0.0016	15	18	atpaf2	1.87	12
	ENSXMAG00000000220	0.0031	24	12	tyk2	1.94	21
	ENSXMAG00000000221	0.0026	24	9	emp2	1.77	21
	ENSXMAG00000000409	0.0026	12	15	NUP35	1.57	9
	ENSXMAG00000000504	0.0025	3	9	crebrf	6.68	0
	ENSXMAG00000000517	0.0027	18	15	tkfc	5.53	15
	ENSXMAG00000000578	0.0026	6	9	hiat1a	2.22	3
	ENSXMAG00000000652	0.0031	3	12		1.85	0
	ENSXMAG00000000666	0.0024	24	15	fxr1	2.71	21
	ENSXMAG00000000681	0.0025	3	9	abca4b	2.03	0
	ENSXMAG00000000711	0.0015	9	6	fnbp1l	2.59	6
	ENSXMAG00000000716	0.0026	18	15	si:ch211-198n5.11	2.81	15
	ENSXMAG00000000731	0.0029	3	12	si:dkeyp-117h8.2	2.20	0
	ENSXMAG00000000752	0.0026	12	15	sem1	1.83	9
	ENSXMAG00000000786	0.0014	3	6	epb41l3b	1.85	0
	ENSXMAG00000000837	0.0031	24	12	dirc2	4.56	21
	ENSXMAG00000001006	0.0025	3	9	rnf216	1.43	0
	ENSXMAG00000001019	0.0031	12	12	blvra	4.92	9
	ENSXMAG00000001048	0.0030	24	12	wipi2	1.76	21
	ENSXMAG00000001050	0.0031	3	12	bcr	2.02	0
	ENSXMAG00000001054	0.0025	3	9	hoxa5a	2.44	0
	ENSXMAG00000001094	0.0024	24	9	rassf8a	1.58	21
	ENSXMAG00000001133	0.0026	12	15	snrpd3	1.96	9
	ENSXMAG00000001169	0.0025	15	15	tnfrsf19	2.04	12
	ENSXMAG00000001299	0.0026	3	9		5.14	0
	ENSXMAG00000001311	0.0026	9	9	lpcat4	2.76	6
	ENSXMAG00000001324	0.0025	21	15	arfgap3	1.75	18
	ENSXMAG00000001341	0.0031	21	12	atg10	1.41	18
	ENSXMAG00000001367	0.0014	9	6	pacsin2	1.85	6
	ENSXMAG00000001382	0.0026	21	15		3.69	18
	ENSXMAG00000001453	0.0025	24	15		2.78	21
	ENSXMAG00000001521	0.0024	12	15	smn1	1.65	9
	ENSXMAG00000001611	0.0031	24	12	nrxn3b	1.89	21
	ENSXMAG00000001620	0.0026	21	15		2.37	18
	ENSXMAG00000001642	0.0028	6	9	aff4	1.53	3
	ENSXMAG00000001695	0.0031	24	12	slc7a8a	3.75	21
	ENSXMAG00000001712	0.0031	21	12	gpalpp1	1.66	18
	ENSXMAG00000001714	0.0026	3	9	rabgap1	1.96	0
	ENSXMAG00000001721	0.0026	3	9	wdsub1	2.73	0
	ENSXMAG00000001727	0.0071	21	6		2.77	18
	ENSXMAG00000001737	0.0028	3	9	ly75	1.91	0
	ENSXMAG00000001744	0.0025	3	15	cry1ab	7.97	0
	ENSXMAG00000001745	0.0024	3	9	pla2r1	2.41	0
	ENSXMAG00000001747	0.0031	24	12	ip6k2b	4.01	21
	ENSXMAG00000001772	0.0029	15	12	anapc10	1.60	12
	ENSXMAG00000001853	0.0027	15	15		2.28	12
	ENSXMAG00000001882	0.0025	3	9	stom (1 of many)	2.57	0

ENSXMAG00000001912	0.0025	24	15		2.61	21
ENSXMAG00000001928	0.0014	21	18	pir	1.75	18
ENSXMAG00000001961	0.0028	12	15	tmem177	3.30	9
ENSXMAG00000001977	0.0025	24	15	AP3B2	2.43	21
ENSXMAG00000002036	0.0031	24	12	os9	1.73	21
ENSXMAG00000002068	0.0024	24	9	asb1	2.42	21
ENSXMAG00000002194	0.0016	15	18	rnasekb	1.42	12
ENSXMAG00000002248	0.0025	6	15	BHLHE40 (1 of many)	17.60	3
ENSXMAG00000002288	0.0031	24	12	paqr3b	2.31	21
ENSXMAG00000002304	0.0031	24	12	slc30a4	1.43	21
ENSXMAG00000002307	0.0014	12	18	zfand1	1.77	9
ENSXMAG00000002318	0.0025	6	9	rbms2a	2.36	3
ENSXMAG00000002328	0.0027	24	9	INPP5K (1 of many)	2.36	21
ENSXMAG00000002339	0.0029	21	12	nr1d4b	33.59	18
ENSXMAG00000002340	0.0031	21	12	si:ch73-174h16.4	1.46	18
ENSXMAG00000002418	0.0031	3	12	ptgs2b	3.15	0
ENSXMAG00000002452	0.0024	6	9	LRRC75A	2.76	3
ENSXMAG00000002465	0.0071	18	18	ankle2	1.79	15
ENSXMAG00000002511	0.0071	18	18	dlgap1b	3.66	15
ENSXMAG00000002534	0.0026	18	15	hps1	3.23	15
ENSXMAG00000002590	0.0028	18	15	cep76	2.25	15
ENSXMAG00000002594	0.0025	15	15	nosip	1.66	12
ENSXMAG00000002627	0.0016	12	18	arfrp1	1.39	9
ENSXMAG00000002684	0.0031	18	12	si:dkey-174n20.1	1.51	15
ENSXMAG00000002693	0.0026	24	9	wisp1a	2.25	21
ENSXMAG00000002711	0.0014	3	6	si:ch73-61d6.3	2.10	0
ENSXMAG00000002882	0.0024	21	15		3.01	18
ENSXMAG00000002891	0.0031	3	12	fam102aa	2.61	0
ENSXMAG00000002903	0.0024	3	9	zgc:158366	1.99	0
ENSXMAG00000002913	0.0026	18	9	vps26c	1.79	15
ENSXMAG00000002976	0.0071	3	6	tmem110l	1.48	0
ENSXMAG00000002990	0.0024	21	15	cyhr1	3.24	18
ENSXMAG00000003027	0.0031	24	12	def8	2.41	21
ENSXMAG00000003067	0.0027	3	9	mthfsd	3.68	0
ENSXMAG00000003109	0.0026	18	15		2.24	15
ENSXMAG00000003112	0.0031	24	12	fbxl2	3.98	21
ENSXMAG00000003159	0.0029	3	12	tln2b	2.81	0
ENSXMAG00000003173	0.0029	24	12	ATG2A	3.17	21
ENSXMAG00000003220	0.0015	15	18	nudt22	2.42	12
ENSXMAG00000003263	0.0015	6	6	txnipa	2.57	3
ENSXMAG00000003359	0.0026	6	9	azin1b	2.74	3
ENSXMAG00000003386	0.0024	6	9	mycn	5.70	3
ENSXMAG00000003430	0.0026	6	9	csnk1db	2.41	3
ENSXMAG00000003439	0.0024	21	15	mfsd2b	2.17	18
ENSXMAG00000003474	0.0016	12	18	yeats4	1.61	9
ENSXMAG00000003480	0.0031	3	12		2.23	0
ENSXMAG00000003487	0.0026	3	9		2.99	0
ENSXMAG00000003500	0.0027	12	15	mrpl54	2.10	9
ENSXMAG00000003506	0.0031	12	12		1.74	9
ENSXMAG00000003528	0.0031	3	12	fgd1	2.71	0
ENSXMAG00000003577	0.0034	18	12	slc2a15a	2.08	15
ENSXMAG00000003607	0.0031	24	12	znf704	2.18	21
ENSXMAG00000003615	0.0014	9	6	atxn1a	2.59	6
ENSXMAG00000003666	0.0027	3	9	cabin1	1.94	0
ENSXMAG00000003693	0.0024	18	9	rida	2.36	15
ENSXMAG00000003710	0.0026	6	9	synm	2.51	3

ENSXMAG00000003717	0.0025	3	9	igf1ra	3.16	0
ENSXMAG00000003776	0.0016	21	18	farp2	1.76	18
ENSXMAG00000003783	0.0031	24	12	zer1	1.49	21
ENSXMAG00000003795	0.0014	9	6	ccdc6a	1.48	6
ENSXMAG00000003824	0.0024	24	15		1.93	21
ENSXMAG00000003825	0.0014	15	18	dpm2	2.06	12
ENSXMAG00000003840	0.0024	6	15	sik2b	2.51	3
ENSXMAG00000003861	0.0026	3	9		1.82	0
ENSXMAG00000003867	0.0031	24	12	ptprq	2.02	21
ENSXMAG00000003961	0.0025	3	15	per3	17.01	0
ENSXMAG00000003973	0.0024	6	9	pdzd2	2.89	3
ENSXMAG00000004011	0.0027	18	9		3.08	15
ENSXMAG00000004021	0.0026	6	9	ano6	1.79	3
ENSXMAG00000004025	0.0026	18	15	nek4	1.63	15
ENSXMAG00000004042	0.0031	24	12	tbc1d10ab	2.89	21
ENSXMAG00000004060	0.0026	18	15	mtf2	2.52	15
ENSXMAG00000004069	0.0024	9	9		1.85	6
ENSXMAG00000004081	0.0027	21	9		1.72	18
ENSXMAG00000004087	0.0030	3	12	gpr184	2.53	0
ENSXMAG00000004095	0.0031	24	12	RSBN1L (1 of many)	2.32	21
ENSXMAG00000004098	0.0014	18	18	fbxl4	1.76	15
ENSXMAG00000004100	0.0031	3	12	phtf2	1.68	0
ENSXMAG00000004113	0.0025	15	15	rbbp5	1.34	12
ENSXMAG00000004132	0.0014	6	6	slc35e4	3.27	3
ENSXMAG00000004197	0.0024	15	15		1.84	12
ENSXMAG00000004221	0.0031	24	12		1.55	21
ENSXMAG00000004234	0.0024	6	9	tdh	10.22	3
ENSXMAG00000004237	0.0025	3	9	sema3d	1.91	0
ENSXMAG00000004290	0.0031	3	12		1.45	0
ENSXMAG00000004296	0.0016	18	18	PCMTD2	3.12	15
ENSXMAG00000004299	0.0015	6	6	prlra	1.79	3
ENSXMAG00000004333	0.0025	21	15	nedd4l	1.58	18
ENSXMAG00000004336	0.0024	18	15	pparaa	1.73	15
ENSXMAG00000004337	0.0031	3	12	slc2a12	2.02	0
ENSXMAG00000004345	0.0030	3	12	wwp1	1.56	0
ENSXMAG00000004364	0.0026	3	15	ADCY3	3.21	0
ENSXMAG00000004394	0.0026	15	15	ofd1	1.52	12
ENSXMAG00000004422	0.0026	24	9	rasgrp3	2.77	21
ENSXMAG00000004458	0.0016	15	18		2.07	12
ENSXMAG00000004526	0.0026	3	9	pex7	1.84	0
ENSXMAG00000004556	0.0026	6	9	si:dkey-30c15.2	34.05	3
ENSXMAG00000004559	0.0031	3	12		2.27	0
ENSXMAG00000004577	0.0031	3	12	pim3	2.73	0
ENSXMAG00000004631	0.0031	3	12	ch25hl3	3.00	0
ENSXMAG00000004641	0.0028	6	9	itchb	2.08	3
ENSXMAG00000004656	0.0031	3	12	cpt1b	3.29	0
ENSXMAG00000004712	0.0026	18	15	MBNL2 (1 of many)	2.22	15
ENSXMAG00000004737	0.0024	3	9	herpud2	1.48	0
ENSXMAG00000004792	0.0031	3	12	sema3c	3.49	0
ENSXMAG00000004822	0.0026	24	15	vwa1	1.53	21
ENSXMAG00000004842	0.0026	18	15	ap3m2	1.70	15
ENSXMAG00000004877	0.0031	24	12	tmem63a	2.18	21
ENSXMAG00000004919	0.0027	12	9	dkc1	1.73	9
ENSXMAG00000004925	0.0026	21	15	fopnl	1.53	18
ENSXMAG00000004939	0.0034	24	12	tyro3	1.92	21
ENSXMAG00000004948	0.0025	24	15	cdh5	3.01	21

ENSXMAG00000004954	0.0031	3	12		1.62	0
ENSXMAG00000004999	0.0024	6	9	dusp6	3.97	3
ENSXMAG00000005008	0.0031	3	12		2.20	0
ENSXMAG00000005057	0.0026	21	15	C16orf45	1.83	18
ENSXMAG00000005086	0.0034	24	12	mkrn1	1.70	21
ENSXMAG00000005127	0.0025	15	9	nfil3-5	29.36	12
ENSXMAG00000005165	0.0031	24	12	stat5a	2.06	21
ENSXMAG00000005166	0.0031	18	12		2.86	15
ENSXMAG00000005168	0.0031	18	12		2.22	15
ENSXMAG00000005174	0.0025	3	9	UHRF2	2.98	0
ENSXMAG00000005184	0.0031	24	12		2.01	21
ENSXMAG00000005199	0.0025	18	15	HDAC2	1.44	15
ENSXMAG00000005219	0.0025	24	9	mcrs1	1.38	21
ENSXMAG00000005228	0.0026	6	9		2.97	3
ENSXMAG00000005270	0.0071	18	18		1.51	15
ENSXMAG00000005298	0.0026	3	9	cgrrf1	3.21	0
ENSXMAG00000005326	0.0031	3	12	aspa	10.30	0
ENSXMAG00000005331	0.0029	3	12		1.90	0
ENSXMAG00000005337	0.0026	24	15	glis1a	2.71	21
ENSXMAG00000005345	0.0031	24	12	slc4a4b	2.99	21
ENSXMAG00000005355	0.0031	21	12	mpv17	1.74	18
ENSXMAG00000005427	0.0026	18	15	mob1bb	1.44	15
ENSXMAG00000005449	0.0031	3	12	wbp1	2.37	0
ENSXMAG00000005474	0.0071	18	18	rgs12b	1.95	15
ENSXMAG00000005486	0.0016	9	6	ankrd13c	2.25	6
ENSXMAG00000005525	0.0026	6	9	vcam1b	2.63	3
ENSXMAG00000005635	0.0031	3	12		3.29	0
ENSXMAG00000005647	0.0025	18	9	ufsp2	1.58	15
ENSXMAG00000005728	0.0029	3	12	pappaa	8.07	0
ENSXMAG00000005768	0.0026	21	15	pm20d1.2	2.00	18
ENSXMAG00000005775	0.0031	24	12	ulk2	5.30	21
ENSXMAG00000005841	0.0027	6	9	nos1apa	1.77	3
ENSXMAG00000005888	0.0024	3	9		4.58	0
ENSXMAG00000005898	0.0031	3	12	sorl1	2.95	0
ENSXMAG00000005976	0.0031	3	12	abi3bpb	3.72	0
ENSXMAG00000006042	0.0031	3	12	JAM3 (1 of many)	1.97	0
ENSXMAG00000006045	0.0026	6	9	tgm1l1	5.44	3
ENSXMAG00000006087	0.0026	12	15	dad1	1.59	9
ENSXMAG00000006155	0.0026	9	9		2.27	6
ENSXMAG00000006253	0.0031	24	12	tasp1	1.48	21
ENSXMAG00000006278	0.0015	21	18	lats1	1.70	18
ENSXMAG00000006416	0.0029	21	12	spast	1.63	18
ENSXMAG00000006422	0.0031	24	12	zc3h3	1.85	21
ENSXMAG00000006474	0.0026	12	15	cnot9	1.31	9
ENSXMAG00000006478	0.0015	3	6	mcoln1a	1.66	0
ENSXMAG00000006487	0.0031	24	12	abca1a	4.14	21
ENSXMAG00000006569	0.0026	24	15	zfyve19	1.86	21
ENSXMAG00000006597	0.0028	6	9	spint1a	1.60	3
ENSXMAG00000006667	0.0025	18	15	tab1	1.37	15
ENSXMAG00000006670	0.0026	21	15	si:ch211-59o9.10	1.63	18
ENSXMAG00000006681	0.0014	3	6	mgat3b	2.03	0
ENSXMAG00000006723	0.0024	3	9	stag2b	1.34	0
ENSXMAG00000006757	0.0024	3	9	lipeb	2.62	0
ENSXMAG00000006760	0.0025	3	9	INF2	1.77	0
ENSXMAG00000006775	0.0026	18	15	si:ch211-130m23.3	2.58	15
ENSXMAG00000006812	0.0030	24	12	zfyve1	2.74	21

ENSXMAG00000006856	0.0016	3	6		2.13	0
ENSXMAG00000006863	0.0031	3	12	frya	2.99	0
ENSXMAG00000006870	0.0031	3	12	ACO2 (1 of many)	2.85	0
ENSXMAG00000006917	0.0028	15	15	naa20	1.78	12
ENSXMAG00000006919	0.0025	12	15		2.41	9
ENSXMAG00000007020	0.0029	3	12		4.34	0
ENSXMAG00000007067	0.0031	3	12	ANK2	4.83	0
ENSXMAG00000007085	0.0026	12	15	eny2	1.60	9
ENSXMAG00000007093	0.0025	21	15	sergef	1.66	18
ENSXMAG00000007123	0.0026	3	9	smg7	1.42	0
ENSXMAG00000007127	0.0016	21	18	slc35a5	1.70	18
ENSXMAG00000007134	0.0026	21	9	zgc:153521	1.25	18
ENSXMAG00000007150	0.0014	18	18	FAM171B (1 of many)	5.16	15
ENSXMAG00000007172	0.0027	3	9	rgcc	2.04	0
ENSXMAG00000007210	0.0025	3	9	tent2	1.59	0
ENSXMAG00000007213	0.0024	6	9	dgkh	1.93	3
ENSXMAG00000007257	0.0029	3	12	thbs4a	3.48	0
ENSXMAG00000007260	0.0027	15	15	polr2l	1.83	12
ENSXMAG00000007270	0.0031	15	12	sdhaf2	1.90	12
ENSXMAG00000007271	0.0016	18	18	efna1b	1.93	15
ENSXMAG00000007309	0.0014	3	6	bach1a	2.07	0
ENSXMAG00000007445	0.0014	9	6	klf8	5.02	6
ENSXMAG00000007446	0.0031	24	12	ZDHHC14 (1 of many)	1.53	21
ENSXMAG00000007521	0.0024	18	15		2.19	15
ENSXMAG00000007522	0.0025	3	9	ptpdc1a	1.80	0
ENSXMAG00000007551	0.0034	24	12		1.71	21
ENSXMAG00000007572	0.0015	18	18	tpcn1	2.11	15
ENSXMAG00000007583	0.0071	18	18	tmem185	1.75	15
ENSXMAG00000007604	0.0016	18	18		3.46	15
ENSXMAG00000007638	0.0029	21	12		1.94	18
ENSXMAG00000007664	0.0034	15	12	cdc26	2.07	12
ENSXMAG00000007712	0.0031	3	12	rmdn2	1.80	0
ENSXMAG00000007731	0.0031	24	12	fbxo25	15.52	21
ENSXMAG00000007741	0.0026	6	9	ankef1a	2.20	3
ENSXMAG00000007748	0.0034	12	12	ppargc1a	2.39	9
ENSXMAG00000007752	0.0026	24	15	med17	1.71	21
ENSXMAG00000007809	0.0031	15	12	pnocb	1.87	12
ENSXMAG00000007889	0.0026	21	15	dpep2	2.65	18
ENSXMAG00000007976	0.0031	3	12	HDAC4 (1 of many)	2.45	0
ENSXMAG00000008024	0.0026	3	9	atrnl1b	2.23	0
ENSXMAG00000008062	0.0031	3	12	cx30.9 (1 of many)	1.89	0
ENSXMAG00000008087	0.0031	21	12	slc19a1	2.35	18
ENSXMAG00000008099	0.0071	15	18	rps3a	1.44	12
ENSXMAG00000008127	0.0028	12	15	U2AF1L5	1.99	9
ENSXMAG00000008129	0.0015	15	18	fkbpl	2.28	12
ENSXMAG00000008131	0.0025	15	15	ccdc115	1.70	12
ENSXMAG00000008135	0.0026	3	9	PARD3 (1 of many)	1.68	0
ENSXMAG00000008169	0.0026	15	15	rnf113a	1.60	12
ENSXMAG00000008172	0.0031	3	12	si:dkey-177p2.6	7.46	0
ENSXMAG00000008234	0.0025	18	9	lratb.1	4.20	15
ENSXMAG00000008243	0.0025	3	9		2.14	0
ENSXMAG00000008366	0.0031	21	12	NLRX1	1.82	18
ENSXMAG00000008463	0.0031	21	12	slco1c1	1.94	18
ENSXMAG00000008581	0.0026	3	9	ipo8	1.60	0
ENSXMAG00000008651	0.0026	18	15	cdc123	1.26	15
ENSXMAG00000008655	0.0025	15	15	alad	2.50	12

ENSXMAG00000008657	0.0031	24	12	si:ch211-106h11.3	4.11	21
ENSXMAG00000008677	0.0071	15	18	ECHDC1	1.74	12
ENSXMAG00000008742	0.0026	15	15	aars2	1.60	12
ENSXMAG00000008811	0.0026	6	9		1.81	3
ENSXMAG00000008892	0.0024	21	15	ARNTL2 (1 of many)	7.11	18
ENSXMAG00000008961	0.0024	18	15	phykpl	3.96	15
ENSXMAG00000009012	0.0014	6	6	atp6v0a1a	1.28	3
ENSXMAG00000009019	0.0026	6	9		2.45	3
ENSXMAG00000009078	0.0027	6	15		2.12	3
ENSXMAG00000009080	0.0031	3	12	csde1	1.45	0
ENSXMAG00000009103	0.0026	6	9	stk17al	2.62	3
ENSXMAG00000009116	0.0016	12	18	fam185a	2.97	9
ENSXMAG00000009130	0.0025	24	15	EZH1	2.08	21
ENSXMAG00000009152	0.0026	18	15		1.63	15
ENSXMAG00000009184	0.0025	18	9	akr1b1	2.51	15
ENSXMAG00000009196	0.0024	18	9	adrm1	1.80	15
ENSXMAG00000009256	0.0027	18	15	ptrh1	2.14	15
ENSXMAG00000009292	0.0015	12	18	galnt11	1.53	9
ENSXMAG00000009316	0.0026	9	9		4.50	6
ENSXMAG00000009376	0.0031	24	12	pus7l	1.61	21
ENSXMAG00000009432	0.0024	21	15	kdm8	1.92	18
ENSXMAG00000009438	0.0024	24	15	APBA1	2.93	21
ENSXMAG00000009451	0.0027	18	15	nubpl	2.46	15
ENSXMAG00000009483	0.0027	18	15	wrap73	2.56	15
ENSXMAG00000009504	0.0030	24	12	sec22c	1.93	21
ENSXMAG00000009514	0.0031	15	12	phactr3b	4.55	12
ENSXMAG00000009576	0.0024	21	15	tmem184c	1.76	18
ENSXMAG00000009659	0.0014	21	6	cdh26.1	2.08	18
ENSXMAG00000009671	0.0014	6	6	mrtfab	1.56	3
ENSXMAG00000009685	0.0026	24	15	desi1b	1.85	21
ENSXMAG00000009773	0.0016	9	6	PET117	1.94	6
ENSXMAG00000009793	0.0031	24	12	rnf150a	5.18	21
ENSXMAG00000009838	0.0071	18	18		1.96	15
ENSXMAG00000009842	0.0027	18	15	elmod2	1.30	15
ENSXMAG00000009888	0.0025	18	15	ankrd49	1.83	15
ENSXMAG00000009967	0.0024	3	9	rufy1	2.55	0
ENSXMAG00000009974	0.0026	9	15	lonrf1l	2.59	6
ENSXMAG00000009976	0.0034	3	12	TIAM2 (1 of many)	2.27	0
ENSXMAG00000010006	0.0031	6	12	hbegf	2.48	3
ENSXMAG00000010010	0.0026	24	15	rmnd5b	1.70	21
ENSXMAG00000010088	0.0024	3	9	tnip1	2.28	0
ENSXMAG00000010100	0.0026	12	15	agpat9l	2.12	9
ENSXMAG00000010169	0.0024	21	15	shda	1.81	18
ENSXMAG00000010199	0.0015	21	18	arl15a	1.85	18
ENSXMAG00000010239	0.0031	21	12	cyp2u1	1.53	18
ENSXMAG00000010359	0.0031	6	12	lpar6b	4.32	3
ENSXMAG00000010366	0.0026	3	9		1.38	0
ENSXMAG00000010372	0.0025	6	9	trpc2b	2.95	3
ENSXMAG00000010442	0.0025	18	15	fance	2.12	15
ENSXMAG00000010449	0.0014	21	18	ppardb	1.47	18
ENSXMAG00000010499	0.0015	9	6	aplp1	1.94	6
ENSXMAG00000010521	0.0031	3	12	FAM19A1 (1 of many)	2.92	0
ENSXMAG00000010535	0.0015	3	6	nhsa	1.76	0
ENSXMAG00000010573	0.0024	3	9	NDUFS1 (1 of many)	2.46	0
ENSXMAG00000010602	0.0024	6	9	si:dkey-79d12.5	4.88	3
ENSXMAG00000010634	0.0015	15	18	trappc3	1.69	12

ENSXMAG00000010660	0.0016	9	6	nle1	1.87	6
ENSXMAG00000010684	0.0028	3	9	thnsl2	1.78	0
ENSXMAG00000010697	0.0031	24	12		3.82	21
ENSXMAG00000010742	0.0024	15	15	gpn2	1.54	12
ENSXMAG00000010759	0.0071	15	18	fam49a	1.50	12
ENSXMAG00000010822	0.0026	15	15	erlec1	1.49	12
ENSXMAG00000010834	0.0030	3	12	col14a1a	2.47	0
ENSXMAG00000010850	0.0025	3	9	cpt1cb	4.01	0
ENSXMAG00000010911	0.0024	3	9		2.05	0
ENSXMAG00000010934	0.0026	24	15		2.95	21
ENSXMAG00000010972	0.0015	21	18	uvssa	1.54	18
ENSXMAG00000010986	0.0071	9	6	msh6	3.00	6
ENSXMAG00000011014	0.0026	24	15	klf12b	3.10	21
ENSXMAG00000011016	0.0024	3	9	efr3a	1.55	0
ENSXMAG00000011086	0.0015	21	18	fbxl3b	2.02	18
ENSXMAG00000011102	0.0029	21	12	toe1	1.57	18
ENSXMAG00000011133	0.0026	21	15	pex5	1.31	18
ENSXMAG00000011178	0.0025	3	9	erbb3b	1.65	0
ENSXMAG00000011188	0.0025	3	9	lrtm2a	2.50	0
ENSXMAG00000011225	0.0016	3	6	arhgap39	3.32	0
ENSXMAG00000011270	0.0026	3	9		2.39	0
ENSXMAG00000011298	0.0026	24	15	gpsm1b	1.64	21
ENSXMAG00000011304	0.0027	3	9	ACVR1C	4.07	0
ENSXMAG00000011317	0.0025	21	15		1.45	18
ENSXMAG00000011320	0.0029	24	12	rabep1	1.39	21
ENSXMAG00000011321	0.0026	21	15	NACC2	4.86	18
ENSXMAG00000011330	0.0029	6	12	myoc	2.53	3
ENSXMAG00000011345	0.0016	21	18	tardbpl	1.40	18
ENSXMAG00000011358	0.0031	3	12	mpzl1l	1.95	0
ENSXMAG00000011500	0.0028	18	15		2.51	15
ENSXMAG00000011668	0.0031	3	12		1.99	0
ENSXMAG00000011676	0.0029	21	12	ormdl2	1.56	18
ENSXMAG00000011727	0.0027	3	9	vps9d1	2.02	0
ENSXMAG00000011738	0.0027	12	15	psma6b	2.12	9
ENSXMAG00000011795	0.0028	6	9	CDH3 (1 of many)	4.98	3
ENSXMAG00000011819	0.0024	24	15	tmem259	1.41	21
ENSXMAG00000011826	0.0014	21	18	nxpe3 (1 of many)	2.01	18
ENSXMAG00000011832	0.0028	18	15	tada2a	2.08	15
ENSXMAG00000011842	0.0026	3	9	ubxn6	1.58	0
ENSXMAG00000011890	0.0014	3	6	abcc12	2.37	0
ENSXMAG00000011935	0.0025	3	9	mink1	2.05	0
ENSXMAG00000011943	0.0026	12	15	mrps34	2.36	9
ENSXMAG00000011952	0.0029	6	12	serpine1	4.97	3
ENSXMAG00000011967	0.0027	21	15	si:dkey-17m8.1	2.29	18
ENSXMAG00000011971	0.0024	21	15	ccdc106a	1.70	18
ENSXMAG00000011993	0.0028	3	9	FNIP1	1.97	0
ENSXMAG00000012009	0.0031	15	12	tppp3	4.54	12
ENSXMAG00000012029	0.0026	24	15	git1	1.93	21
ENSXMAG00000012054	0.0014	24	18	dbpb	47.58	21
ENSXMAG00000012060	0.0026	18	15	tbck	1.37	15
ENSXMAG00000012065	0.0026	3	9	slc12a9	2.79	0
ENSXMAG00000012105	0.0034	21	12	RRAGB	1.40	18
ENSXMAG00000012112	0.0071	9	6	grwd1	3.53	6
ENSXMAG00000012121	0.0026	12	15	sat2b	3.07	9
ENSXMAG00000012162	0.0031	18	12	phospho2	1.56	15
ENSXMAG00000012187	0.0030	3	12	khk	1.48	0

ENSXMAG00000012189	0.0027	18	9	npas2	7.78	15
ENSXMAG00000012207	0.0026	21	9	rbpja	1.37	18
ENSXMAG00000012234	0.0025	24	9	PPP1R27	6.89	21
ENSXMAG00000012286	0.0071	15	18	SLC25A42	3.48	12
ENSXMAG00000012294	0.0031	24	12	mau2	1.63	21
ENSXMAG00000012303	0.0031	24	12		4.61	21
ENSXMAG00000012305	0.0016	18	18	pycr1a	2.41	15
ENSXMAG00000012410	0.0015	21	18	zmp:0000001088	2.29	18
ENSXMAG00000012425	0.0026	15	15	sap30bp	1.43	12
ENSXMAG00000012494	0.0031	24	12	mdh1b	2.41	21
ENSXMAG00000012517	0.0025	21	9	cby1	1.79	18
ENSXMAG00000012563	0.0028	18	15	helq	1.67	15
ENSXMAG00000012605	0.0027	24	15	klhl21	1.61	21
ENSXMAG00000012620	0.0024	3	9	SRPK2	2.07	0
ENSXMAG00000012674	0.0034	3	12	APP	1.56	0
ENSXMAG00000012704	0.0027	6	15	pde6ga	6.93	3
ENSXMAG00000012734	0.0026	21	15	ampd3b	2.25	18
ENSXMAG00000012762	0.0031	24	12	add3b	2.14	21
ENSXMAG00000012781	0.0024	3	9	si:ch211-114m9.1	2.31	0
ENSXMAG00000012786	0.0026	3	9	ptprua	2.08	0
ENSXMAG00000012866	0.0026	6	9	ndel1b	5.00	3
ENSXMAG00000012936	0.0034	24	12		2.67	21
ENSXMAG00000013014	0.0026	3	9	hdac6	1.30	0
ENSXMAG00000013021	0.0027	15	9	rorca	4.02	12
ENSXMAG00000013057	0.0029	6	12	abhd2b	3.30	3
ENSXMAG00000013059	0.0024	24	9	pld3	1.53	21
ENSXMAG00000013075	0.0025	18	15	elovl8b	2.85	15
ENSXMAG00000013117	0.0015	9	6	irf2bp1 (1 of many)	2.28	6
ENSXMAG00000013149	0.0025	18	9	yif1a	1.31	15
ENSXMAG00000013225	0.0031	24	12	fyco1a	2.82	21
ENSXMAG00000013242	0.0026	21	15		2.13	18
ENSXMAG00000013277	0.0026	21	15	dnajc28	1.78	18
ENSXMAG00000013310	0.0034	9	12	mgat1a	1.80	6
ENSXMAG00000013321	0.0024	3	9	lmbrd1	2.29	0
ENSXMAG00000013325	0.0024	6	9	mcf2a	1.59	3
ENSXMAG00000013336	0.0015	9	6	osgn1	3.71	6
ENSXMAG00000013351	0.0026	3	9	prdm1a	3.31	0
ENSXMAG00000013372	0.0015	21	18	si:ch211-113g11.6	2.53	18
ENSXMAG00000013393	0.0034	3	12	nalcn	2.75	0
ENSXMAG00000013437	0.0026	21	15	CUTC	1.77	18
ENSXMAG00000013469	0.0027	24	15	drp2	2.31	21
ENSXMAG00000013474	0.0029	15	12	trmt5	1.57	12
ENSXMAG00000013507	0.0024	6	9	hivep2a	1.97	3
ENSXMAG00000013544	0.0024	6	9	si:dkey-21a6.5	1.87	3
ENSXMAG00000013580	0.0027	12	15	tent4a	1.94	9
ENSXMAG00000013614	0.0026	6	15	ube2ql1	3.71	3
ENSXMAG00000013651	0.0031	3	12		2.40	0
ENSXMAG00000013667	0.0031	3	12		2.39	0
ENSXMAG00000013680	0.0015	18	18	upf3b	1.49	15
ENSXMAG00000013701	0.0025	3	9		1.80	0
ENSXMAG00000013705	0.0024	24	15	ical1	1.84	21
ENSXMAG00000013710	0.0014	15	18	fuom	2.42	12
ENSXMAG00000013711	0.0031	3	12	cldn7a	4.02	0
ENSXMAG00000013715	0.0026	12	15	cdk5rap3	1.45	9
ENSXMAG00000013725	0.0026	24	15	BMPR2	1.89	21
ENSXMAG00000013738	0.0031	21	12		1.92	18

ENSXMAG00000013778	0.0014	18	18	mapk7	1.58	15
ENSXMAG00000013810	0.0031	24	12	slitrk2	2.44	21
ENSXMAG00000013812	0.0031	3	12	akt3a	2.18	0
ENSXMAG00000013839	0.0029	24	12	hdac9b	1.65	21
ENSXMAG00000013871	0.0031	6	12	umodl1	1.77	3
ENSXMAG00000013896	0.0025	3	9	tax1bp1b	1.43	0
ENSXMAG00000013904	0.0031	24	12	ei24	2.22	21
ENSXMAG00000013912	0.0034	18	12	cbx3b	1.54	15
ENSXMAG00000013946	0.0024	3	9	stox2a	2.52	0
ENSXMAG00000013974	0.0026	18	15	kif7	2.10	15
ENSXMAG00000013989	0.0028	18	15		2.05	15
ENSXMAG00000014007	0.0026	18	15	grinab	1.74	15
ENSXMAG00000014035	0.0026	3	9	usp25	1.34	0
ENSXMAG00000014068	0.0014	15	18	brd9	1.19	12
ENSXMAG00000014077	0.0031	6	12	abcc5	6.80	3
ENSXMAG00000014085	0.0025	18	15	cog4	1.29	15
ENSXMAG00000014132	0.0025	18	9	soul4	3.48	15
ENSXMAG00000014183	0.0026	18	15	ddah2	5.60	15
ENSXMAG00000014231	0.0024	21	9		1.48	18
ENSXMAG00000014297	0.0029	24	12	nf2b	2.06	21
ENSXMAG00000014311	0.0031	3	12	azi2	1.81	0
ENSXMAG00000014330	0.0024	18	15	mettl27	2.35	15
ENSXMAG00000014333	0.0024	15	15	eif4h	1.63	12
ENSXMAG00000014341	0.0031	3	12		2.20	0
ENSXMAG00000014390	0.0027	3	9		2.31	0
ENSXMAG00000014416	0.0026	3	9	cdan1	2.34	0
ENSXMAG00000014436	0.0034	24	12	ZFYVE1 (1 of many)	3.72	21
ENSXMAG00000014459	0.0026	3	9	cadm4	2.02	0
ENSXMAG00000014476	0.0030	3	12	rnf111	1.37	0
ENSXMAG00000014582	0.0034	3	12	rbl1	3.23	0
ENSXMAG00000014607	0.0031	3	12		2.50	0
ENSXMAG00000014675	0.0027	3	9	TC2N	2.19	0
ENSXMAG00000014677	0.0025	24	15	slc44a1b	2.03	21
ENSXMAG00000014803	0.0024	24	9	armc7	1.64	21
ENSXMAG00000014808	0.0031	18	12	igbp1	1.29	15
ENSXMAG00000014902	0.0027	21	15	msl1b	1.60	18
ENSXMAG00000014917	0.0024	24	15		2.19	21
ENSXMAG00000014926	0.0029	18	12	clcn5b	1.39	15
ENSXMAG00000014950	0.0026	12	15	psmc3	1.76	9
ENSXMAG00000014954	0.0026	6	9	pdk2a	5.87	3
ENSXMAG00000014972	0.0024	15	9	ndufv1	1.42	12
ENSXMAG00000014996	0.0026	24	9	ascc1	1.83	21
ENSXMAG00000015001	0.0015	21	18	LRRC2	4.01	18
ENSXMAG00000015002	0.0026	24	15	glyr1	1.79	21
ENSXMAG00000015014	0.0025	21	15	SPOCK2	1.80	18
ENSXMAG00000015032	0.0024	15	15	mul1a	1.52	12
ENSXMAG00000015106	0.0025	18	15	lrrc20	1.75	15
ENSXMAG00000015130	0.0026	3	9	LRRC56	4.67	0
ENSXMAG00000015136	0.0016	9	6	rest	1.58	6
ENSXMAG00000015145	0.0031	24	12	irs2a	5.12	21
ENSXMAG00000015172	0.0014	18	18	gpr137c	9.18	15
ENSXMAG00000015215	0.0026	3	9		1.84	0
ENSXMAG00000015294	0.0071	18	18	gtf2ird1	1.70	15
ENSXMAG00000015314	0.0024	3	15	per1b	30.88	0
ENSXMAG00000015321	0.0031	24	12	peli2	2.70	21
ENSXMAG00000015326	0.0025	3	9	cep120	2.73	0

ENSXMAG00000015335	0.0025	21	15	dlg5b.1	2.51	18
ENSXMAG00000015336	0.0026	3	9	tmem86a	5.21	0
ENSXMAG00000015354	0.0026	3	9	tcp11l1	2.63	0
ENSXMAG00000015455	0.0026	12	15	AMDHD1	3.40	9
ENSXMAG00000015489	0.0031	3	12	mgat4b	1.94	0
ENSXMAG00000015499	0.0029	24	12	fam114a2	1.69	21
ENSXMAG00000015504	0.0029	24	12	ahi1	1.65	21
ENSXMAG00000015528	0.0031	3	12	spata2	1.70	0
ENSXMAG00000015562	0.0016	15	18	commd8	2.74	12
ENSXMAG00000015579	0.0025	3	9	kank1a	1.94	0
ENSXMAG00000015615	0.0024	6	9		2.22	3
ENSXMAG00000015619	0.0026	18	9	arntl1a	10.22	15
ENSXMAG00000015692	0.0024	21	15		2.75	18
ENSXMAG00000015751	0.0024	3	9		2.67	0
ENSXMAG00000015785	0.0026	9	15	LONRF3	6.05	6
ENSXMAG00000015836	0.0031	6	12	DOK7	5.61	3
ENSXMAG00000015843	0.0029	15	12	crip1	1.91	12
ENSXMAG00000015898	0.0026	18	15	chd1l	2.32	15
ENSXMAG00000015937	0.0031	3	12		1.82	0
ENSXMAG00000015950	0.0031	24	12	creb3l3l	1.79	21
ENSXMAG00000015967	0.0015	9	6		2.37	6
ENSXMAG00000016000	0.0025	24	9	ppp1r7	1.47	21
ENSXMAG00000016005	0.0016	24	18	tmem176	1.75	21
ENSXMAG00000016009	0.0031	3	12	vps37c	2.20	0
ENSXMAG00000016011	0.0027	18	15	ubox5	2.18	15
ENSXMAG00000016035	0.0031	3	12	chd3	2.80	0
ENSXMAG00000016063	0.0034	15	12	ltc4s	3.69	12
ENSXMAG00000016065	0.0024	3	15	megf10	1.89	0
ENSXMAG00000016257	0.0031	24	12	si:ch1073-513e17.1	3.25	21
ENSXMAG00000016278	0.0025	6	9	map3k5	1.51	3
ENSXMAG00000016307	0.0024	18	15		1.90	15
ENSXMAG00000016330	0.0026	21	9	pomk	1.83	18
ENSXMAG00000016346	0.0015	12	18	brd7	1.61	9
ENSXMAG00000016362	0.0030	21	12	wrnip1	1.86	18
ENSXMAG00000016387	0.0071	9	6	zgc:172121	1.79	6
ENSXMAG00000016390	0.0026	6	9	ripor1	2.72	3
ENSXMAG00000016403	0.0024	21	15	arsg	2.21	18
ENSXMAG00000016424	0.0031	3	12	SLC16A6	1.78	0
ENSXMAG00000016436	0.0015	15	18	inka1b	2.32	12
ENSXMAG00000016443	0.0031	6	12		2.66	3
ENSXMAG00000016470	0.0027	24	15	c7b	2.42	21
ENSXMAG00000016472	0.0026	24	15	MITF (1 of many)	3.31	21
ENSXMAG00000016506	0.0016	15	18		1.89	12
ENSXMAG00000016511	0.0025	3	9	ghrb	6.75	0
ENSXMAG00000016600	0.0026	18	15	setdb1b	1.48	15
ENSXMAG00000016605	0.0031	3	12	amotl2a	2.04	0
ENSXMAG00000016608	0.0025	21	15	uhrf1bp1	3.71	18
ENSXMAG00000016617	0.0029	24	12	stam	2.08	21
ENSXMAG00000016662	0.0030	24	12		2.44	21
ENSXMAG00000016668	0.0015	21	18		1.70	18
ENSXMAG00000016673	0.0034	24	12	ddi2	1.58	21
ENSXMAG00000016684	0.0034	24	12	FGD6	2.80	21
ENSXMAG00000016741	0.0031	15	12	si:dkey-240h12.4	1.36	12
ENSXMAG00000016801	0.0026	3	9		2.25	0
ENSXMAG00000016822	0.0031	12	12	paics	1.70	9
ENSXMAG00000016828	0.0031	15	12	derl2	1.74	12

ENSXMAG00000016851	0.0024	24	9	igf1rb	1.74	21
ENSXMAG00000016883	0.0031	3	12	RASSF1 (1 of many)	2.33	0
ENSXMAG00000016914	0.0026	21	15	oxsm	2.38	18
ENSXMAG00000016927	0.0024	12	9	chchd3b	1.67	9
ENSXMAG00000016928	0.0026	18	9	clocka	9.91	15
ENSXMAG00000016984	0.0026	3	9	cnot4a	1.54	0
ENSXMAG00000017038	0.0027	21	15	lrsam1	1.60	18
ENSXMAG00000017050	0.0014	18	18	hhat	1.89	15
ENSXMAG00000017069	0.0025	24	9	csf1b	3.16	21
ENSXMAG00000017074	0.0026	15	9	selenbp1	2.34	12
ENSXMAG00000017106	0.0025	6	9	nr1d2b	3.22	3
ENSXMAG00000017127	0.0031	24	12		2.17	21
ENSXMAG00000017128	0.0025	3	9	acot16	1.80	0
ENSXMAG00000017186	0.0026	24	9	CERS5	2.00	21
ENSXMAG00000017196	0.0031	21	12	serpini1	2.14	18
ENSXMAG00000017201	0.0031	3	12	tnk2b	2.63	0
ENSXMAG00000017228	0.0026	3	9	itfg2	2.08	0
ENSXMAG00000017257	0.0027	18	15		1.73	15
ENSXMAG00000017261	0.0034	15	12		2.05	12
ENSXMAG00000017412	0.0024	3	9	hdac5	5.17	0
ENSXMAG00000017430	0.0031	15	12	lcmt1	2.01	12
ENSXMAG00000017438	0.0028	24	15		4.68	21
ENSXMAG00000017466	0.0031	9	12		3.97	6
ENSXMAG00000017513	0.0029	24	12		2.21	21
ENSXMAG00000017517	0.0025	21	15	psmd9	1.53	18
ENSXMAG00000017543	0.0031	3	12		3.00	0
ENSXMAG00000017596	0.0024	21	15	hunk	4.63	18
ENSXMAG00000017597	0.0026	15	9	sod1	1.76	12
ENSXMAG00000017603	0.0024	3	9	ush2a	2.53	0
ENSXMAG00000017604	0.0026	21	15	si:ch73-142c19.1	1.81	18
ENSXMAG00000017646	0.0014	9	6	sema4gb	3.19	6
ENSXMAG00000017665	0.0026	3	9	gas8 (1 of many)	2.12	0
ENSXMAG00000017739	0.0025	3	9	vgll4a	1.75	0
ENSXMAG00000017756	0.0015	18	18	zgc:175264	2.27	15
ENSXMAG00000017778	0.0026	6	9	mat2ab	4.34	3
ENSXMAG00000017782	0.0030	3	12	ncoa1	2.32	0
ENSXMAG00000017789	0.0026	6	9		2.52	3
ENSXMAG00000017791	0.0015	3	6		2.02	0
ENSXMAG00000017858	0.0024	21	15	lrig2	1.76	18
ENSXMAG00000017862	0.0027	18	15	aldh9a1b	3.03	15
ENSXMAG00000017876	0.0025	3	9	nbr1a	1.80	0
ENSXMAG00000017878	0.0028	18	15	kptn	1.75	15
ENSXMAG00000017900	0.0026	24	9		2.29	21
ENSXMAG00000017909	0.0031	6	12	card14	8.03	3
ENSXMAG00000017913	0.0027	3	9	mark4a	2.83	0
ENSXMAG00000018015	0.0031	3	12	phldb1b	2.00	0
ENSXMAG00000018021	0.0015	12	18	anapc11	1.80	9
ENSXMAG00000018080	0.0029	18	12	vps33b	1.77	15
ENSXMAG00000018093	0.0028	12	15	mrpl46	2.55	9
ENSXMAG00000018143	0.0014	18	18	si:ch73-130a3.4	1.70	15
ENSXMAG00000018151	0.0016	9	6		1.57	6
ENSXMAG00000018168	0.0027	21	15		1.48	18
ENSXMAG00000018208	0.0015	12	18	MZT1	1.55	9
ENSXMAG00000018266	0.0025	18	15		1.75	15
ENSXMAG00000018275	0.0025	21	15	ppip5k1a	1.42	18
ENSXMAG00000018334	0.0031	6	12		4.91	3

ENSXMAG00000018336	0.0026	18	15	trim59	2.86	15
ENSXMAG00000018375	0.0031	21	12	r3hcc1	1.55	18
ENSXMAG00000018379	0.0071	9	6	wdr43	2.02	6
ENSXMAG00000018409	0.0027	18	15	supt7l	1.84	15
ENSXMAG00000018424	0.0026	18	15	si:dkey-30h22.11	2.69	15
ENSXMAG00000018468	0.0015	12	18	tsfm	1.83	9
ENSXMAG00000018523	0.0034	24	12	zgc:123305	1.95	21
ENSXMAG00000018543	0.0031	24	12	tcp11l2	7.05	21
ENSXMAG00000018574	0.0031	3	12		1.58	0
ENSXMAG00000018630	0.0026	12	15	lsm7	1.51	9
ENSXMAG00000018641	0.0031	3	12	arhgef18b	2.45	0
ENSXMAG00000018648	0.0025	3	9	insrb	2.69	0
ENSXMAG00000018697	0.0015	15	18		1.39	12
ENSXMAG00000018709	0.0025	6	9	nr1d2a	5.94	3
ENSXMAG00000018710	0.0031	24	12	JMY	6.89	21
ENSXMAG00000018717	0.0026	15	15	prg4a	1.46	12
ENSXMAG00000018811	0.0024	21	15		1.49	18
ENSXMAG00000018827	0.0026	3	9	mios	1.70	0
ENSXMAG00000018879	0.0031	3	12	CAMK2N1	2.78	0
ENSXMAG00000018900	0.0031	24	12	prkx	1.58	21
ENSXMAG00000018903	0.0025	24	15		2.82	21
ENSXMAG00000018925	0.0027	3	9	F2RL2 (1 of many)	2.47	0
ENSXMAG00000018959	0.0024	3	9	rb1cc1	2.10	0
ENSXMAG00000018972	0.0024	21	15	shrprbck1r	1.65	18
ENSXMAG00000018995	0.0025	6	9	cep350	2.30	3
ENSXMAG00000019003	0.0026	18	15	agmo	3.16	15
ENSXMAG00000019028	0.0026	18	15	cwc27	1.65	15
ENSXMAG00000019122	0.0029	24	12	ATG14	1.44	21
ENSXMAG00000019126	0.0031	15	12	dtd2	2.07	12
ENSXMAG00000019131	0.0030	24	12	rxylt1	1.72	21
ENSXMAG00000019169	0.0016	6	6	zswim6	2.94	3
ENSXMAG00000019187	0.0026	12	15	mrpl16	1.76	9
ENSXMAG00000019256	0.0029	24	12	pros1	1.88	21
ENSXMAG00000019284	0.0025	15	15	actr8	1.48	12
ENSXMAG00000019296	0.0025	15	9	rorcb	15.54	12
ENSXMAG00000019336	0.0026	15	15	cirbpa	1.82	12
ENSXMAG00000019370	0.0031	9	12		8.92	6
ENSXMAG00000019465	0.0024	6	9	tob1b	2.51	3
ENSXMAG00000019470	0.0031	24	12		2.61	21
ENSXMAG00000019477	0.0027	18	15	mgat2	1.61	15
ENSXMAG00000019488	0.0031	24	12	adra2da	3.03	21
ENSXMAG00000019515	0.0028	6	9	sox3	2.66	3
ENSXMAG00000019530	0.0025	18	9		10.83	15
ENSXMAG00000019548	0.0031	15	12	MTLN	2.73	12
ENSXMAG00000019564	0.0026	6	9	zgc:154093	2.30	3
ENSXMAG00000019569	0.0025	24	15	ackr3a	3.00	21
ENSXMAG00000019575	0.0024	3	9	ZBTB26	2.31	0
ENSXMAG00000019612	0.0025	24	9		2.36	21
ENSXMAG00000019632	0.0031	3	12		2.20	0
ENSXMAG00000019665	0.0024	6	9	cebpb	7.08	3
ENSXMAG00000019735	0.0014	18	18	TCEANC	1.93	15
ENSXMAG00000019792	0.0031	6	12	tob1a	1.62	3
ENSXMAG00000019818	0.0014	15	18		1.76	12
ENSXMAG00000019819	0.0026	18	15	cyp8b2	2.52	15
ENSXMAG00000019888	0.0024	6	9		2.37	3
ENSXMAG00000019943	0.0025	15	15	nfil3	4.80	12

ENSXMAG00000020012	0.0024	12	15	emc6	1.84	9
ENSXMAG00000020047	0.0071	15	18	brcc3	2.34	12
ENSXMAG00000020048	0.0024	6	9		2.80	3
ENSXMAG00000020084	0.0024	6	9	JUND	14.96	3
ENSXMAG00000020104	0.0034	21	12	tysnd1	2.94	18
ENSXMAG00000020230	0.0031	6	12	lpar6a	2.54	3
ENSXMAG00000020842	0.0026	18	15		1.69	15
ENSXMAG00000020843	0.0024	24	9		2.95	21
ENSXMAG00000020844	0.0026	12	15		1.68	9
ENSXMAG00000020852	0.0026	15	15	asrgl1	3.12	12
ENSXMAG00000020882	0.0024	12	15		1.82	9
ENSXMAG00000020890	0.0031	3	12	ptprsa	1.88	0
ENSXMAG00000020936	0.0026	24	15		3.19	21
ENSXMAG00000020944	0.0026	3	9	ubn2a	2.06	0
ENSXMAG00000020991	0.0031	18	12	spsb3a	2.15	15
ENSXMAG00000021039	0.0031	3	12		1.34	0
ENSXMAG00000021073	0.0031	3	12		2.85	0
ENSXMAG00000021091	0.0031	24	12	scai	1.23	21
ENSXMAG00000021147	0.0029	18	12	PPP1R1A	2.95	15
ENSXMAG00000021149	0.0024	12	15	cdc42ep3	1.58	9
ENSXMAG00000021151	0.0025	6	9	magi3a	2.50	3
ENSXMAG00000021181	0.0025	24	9	rab20	2.07	21
ENSXMAG00000021206	0.0071	15	18	rnf115	1.52	12
ENSXMAG00000021278	0.0026	12	15	polr2i	1.97	9
ENSXMAG00000021307	0.0025	24	15		4.36	21
ENSXMAG00000021333	0.0031	3	12	abhd2a	5.22	0
ENSXMAG00000021341	0.0026	24	9		2.90	21
ENSXMAG00000021396	0.0031	3	12	KCNAB1	2.03	0
ENSXMAG00000021401	0.0031	3	12		1.83	0
ENSXMAG00000021407	0.0014	3	6	slc8a2b	3.28	0
ENSXMAG00000021494	0.0030	3	12	pdgfd	1.92	0
ENSXMAG00000021495	0.0026	15	15	MED9	1.93	12
ENSXMAG00000021528	0.0034	3	12		3.02	0
ENSXMAG00000021559	0.0026	21	15	PNPLA8 (1 of many)	3.01	18
ENSXMAG00000021565	0.0030	3	12	NIPAL2	1.55	0
ENSXMAG00000021579	0.0031	12	12		2.37	9
ENSXMAG00000021638	0.0026	3	15	phyhipla	1.97	0
ENSXMAG00000021677	0.0034	24	12	rgs7bpb	2.86	21
ENSXMAG00000021711	0.0024	21	15		2.22	18
ENSXMAG00000021817	0.0024	24	9	scamp3	2.21	21
ENSXMAG00000021827	0.0024	15	15		1.92	12
ENSXMAG00000021838	0.0071	15	18	IDNK	3.74	12
ENSXMAG00000021842	0.0016	21	18		1.84	18
ENSXMAG00000021855	0.0016	18	18		2.75	15
ENSXMAG00000021879	0.0029	3	12		13.69	0
ENSXMAG00000021882	0.0025	21	15	josd1	1.33	18
ENSXMAG00000021923	0.0027	18	15		1.94	15
ENSXMAG00000021950	0.0014	15	18		2.51	12
ENSXMAG00000021985	0.0031	3	12		1.86	0
ENSXMAG00000021999	0.0030	24	12	TOB2	4.62	21
ENSXMAG00000022038	0.0027	18	15		2.39	15
ENSXMAG00000022122	0.0027	6	9	pacsin3	1.77	3
ENSXMAG00000022179	0.0031	21	12		1.36	18
ENSXMAG00000022200	0.0024	6	15	dgat2	2.76	3
ENSXMAG00000022213	0.0029	3	12		5.75	0
ENSXMAG00000022220	0.0024	6	15	hspb6	10.63	3

ENSXMAG00000022235	0.0031	6	12		2.04	3
ENSXMAG00000022260	0.0014	9	6		3.23	6
ENSXMAG00000022264	0.0029	3	12	C6orf106	2.12	0
ENSXMAG00000022303	0.0014	24	6	CAAP1	1.47	21
ENSXMAG00000022342	0.0015	9	6	cdt1	4.67	6
ENSXMAG00000022378	0.0034	3	12	zgc:154058	3.06	0
ENSXMAG00000022382	0.0031	24	12	prox1b	1.61	21
ENSXMAG00000022413	0.0031	21	12		1.87	18
ENSXMAG00000022456	0.0026	9	9	SLC22A17	3.03	6
ENSXMAG00000022484	0.0025	24	9		2.00	21
ENSXMAG00000022498	0.0031	24	12		2.63	21
ENSXMAG00000022590	0.0031	3	12		3.73	0
ENSXMAG00000022622	0.0025	9	15	KCNK4	1.98	6
ENSXMAG00000022644	0.0026	9	9	RF00045	2.16	6
ENSXMAG00000022713	0.0024	18	15	suds3	1.86	15
ENSXMAG00000022788	0.0031	3	12		1.36	0
ENSXMAG00000022811	0.0029	3	12	ciarta	45.80	0
ENSXMAG00000022833	0.0014	21	18	wdr26b	1.54	18
ENSXMAG00000022886	0.0031	3	12	GPR149	5.95	0
ENSXMAG00000022901	0.0031	15	12	sft2d3	1.80	12
ENSXMAG00000023006	0.0029	21	12		2.47	18
ENSXMAG00000023035	0.0026	18	15	tusc3	1.45	15
ENSXMAG00000023038	0.0031	3	12		2.12	0
ENSXMAG00000023084	0.0024	24	9	asah1b	2.01	21
ENSXMAG00000023114	0.0025	6	15		3.31	3
ENSXMAG00000023165	0.0031	21	12	rnf166	2.34	18
ENSXMAG00000023180	0.0031	21	12	si:ch73-248e21.1	3.79	18
ENSXMAG00000023239	0.0025	3	9		3.08	0
ENSXMAG00000023264	0.0028	18	15	ip6k2a	1.54	15
ENSXMAG00000023278	0.0026	3	9		5.22	0
ENSXMAG00000023285	0.0025	21	15	rab7	1.43	18
ENSXMAG00000023291	0.0031	24	12		2.96	21
ENSXMAG00000023299	0.0016	9	6	slc41a1	2.13	6
ENSXMAG00000023302	0.0028	3	9	ctdsp2	2.73	0
ENSXMAG00000023324	0.0024	12	15	mrps25	1.61	9
ENSXMAG00000023397	0.0031	21	12		2.15	18
ENSXMAG00000023403	0.0030	21	12	ppap2d	2.47	18
ENSXMAG00000023472	0.0031	24	12		2.39	21
ENSXMAG00000023483	0.0031	15	12		1.57	12
ENSXMAG00000023569	0.0030	3	12	scoca	2.27	0
ENSXMAG00000023570	0.0028	3	9	abrab	10.37	0
ENSXMAG00000023579	0.0024	3	9		2.67	0
ENSXMAG00000023739	0.0027	18	15		1.63	15
ENSXMAG00000023781	0.0029	6	12	ugt1a2 (1 of many)	2.36	3
ENSXMAG00000023825	0.0027	6	15		11.42	3
ENSXMAG00000023836	0.0027	21	15	ST3GAL1 (1 of many)	2.88	18
ENSXMAG00000023900	0.0015	9	6	egr3	18.89	6
ENSXMAG00000023946	0.0026	24	15		4.22	21
ENSXMAG00000024008	0.0030	15	12	trappc2	1.62	12
ENSXMAG00000024113	0.0029	6	12	cyp2n13 (1 of many)	5.56	3
ENSXMAG00000024133	0.0031	24	12		1.61	21
ENSXMAG00000024165	0.0024	24	15		2.26	21
ENSXMAG00000024217	0.0031	24	12	hspbap1	3.51	21
ENSXMAG00000024218	0.0031	24	12	WIPI1	5.92	21
ENSXMAG00000024235	0.0026	24	15	gse1	1.71	21
ENSXMAG00000024236	0.0025	18	15		1.87	15

ENSXMAG00000024238	0.0030	3	12	kctd7	2.07	0
ENSXMAG00000024301	0.0015	9	6	ubap1	1.74	6
ENSXMAG00000024362	0.0025	18	15	tmem218	1.99	15
ENSXMAG00000024407	0.0016	18	18	MAST4	1.60	15
ENSXMAG00000024428	0.0027	15	15		1.38	12
ENSXMAG00000024469	0.0025	3	9		2.69	0
ENSXMAG00000024514	0.0024	24	9		8.03	21
ENSXMAG00000024559	0.0028	3	9	dmtf1	2.87	0
ENSXMAG00000024583	0.0027	3	15	ky	4.75	0
ENSXMAG00000024615	0.0014	18	18	SMAD4 (1 of many)	1.76	15
ENSXMAG00000024725	0.0015	12	18		1.73	9
ENSXMAG00000024775	0.0016	21	18		1.98	18
ENSXMAG00000024817	0.0026	6	9	spred3	1.83	3
ENSXMAG00000024840	0.0024	3	9		1.58	0
ENSXMAG00000024956	0.0027	6	9	tnfrsf1a	2.21	3
ENSXMAG00000024959	0.0024	24	15	si:ch211-272n13.3	2.33	21
ENSXMAG00000025000	0.0031	6	12	ppp1r1b	2.12	3
ENSXMAG00000025016	0.0027	21	9	vps36	1.26	18
ENSXMAG00000025030	0.0031	15	12	slc35b1	1.56	12
ENSXMAG00000025060	0.0028	12	15	alg8	1.75	9
ENSXMAG00000025069	0.0026	3	9		1.68	0
ENSXMAG00000025110	0.0027	3	9	zfyve21	1.72	0
ENSXMAG00000025163	0.0031	3	12		3.53	0
ENSXMAG00000025214	0.0026	18	15	cited1	2.49	15
ENSXMAG00000025260	0.0014	9	18	anxa2a	2.34	6
ENSXMAG00000025264	0.0031	24	12	TRAF2	1.91	21
ENSXMAG00000025287	0.0026	3	9	nek7	1.33	0
ENSXMAG00000025334	0.0015	21	18		1.63	18
ENSXMAG00000025368	0.0031	6	12	pdcd6	1.81	3
ENSXMAG00000025371	0.0027	3	15	cipcb	19.93	0
ENSXMAG00000025390	0.0025	18	15		2.34	15
ENSXMAG00000025465	0.0025	6	9	dusp5	6.57	3
ENSXMAG00000025515	0.0025	3	15	bhlhe40	11.93	0
ENSXMAG00000025549	0.0029	24	12	bmp5	1.96	21
ENSXMAG00000025599	0.0030	6	12		3.34	3
ENSXMAG00000025637	0.0015	9	6	zgc:66427	1.93	6
ENSXMAG00000025698	0.0031	18	12	yipf2	1.73	15
ENSXMAG00000025732	0.0031	21	12		1.50	18
ENSXMAG00000025757	0.0034	24	12	prnpb	1.90	21
ENSXMAG00000025805	0.0025	21	15	si:ch73-382f3.1	2.21	18
ENSXMAG00000025830	0.0014	6	6	ptbp3	1.32	3
ENSXMAG00000025856	0.0030	3	12		2.24	0
ENSXMAG00000025924	0.0031	18	12	eif5	1.43	15
ENSXMAG00000025935	0.0031	24	12	retreg2	2.26	21
ENSXMAG00000026011	0.0027	3	9	CAMK2G (1 of many)	2.21	0
ENSXMAG00000026042	0.0027	15	15	fam133b	1.22	12
ENSXMAG00000026077	0.0026	18	15	mecr	1.88	15
ENSXMAG00000026118	0.0024	6	9	atf3	5.43	3
ENSXMAG00000026130	0.0028	18	15	zc4h2	1.68	15
ENSXMAG00000026139	0.0025	24	9	ap5s1	2.18	21
ENSXMAG00000026142	0.0026	6	9		2.10	3
ENSXMAG00000026180	0.0030	6	12	atf7b	1.63	3
ENSXMAG00000026188	0.0026	24	9		11.75	21
ENSXMAG00000026206	0.0025	3	9	tns2a	2.03	0
ENSXMAG00000026286	0.0025	24	15	tbx18	4.07	21
ENSXMAG00000026287	0.0034	3	12		1.62	0

ENSXMAG00000026312	0.0026	24	9	six1b	2.47	21
ENSXMAG00000026333	0.0024	3	15	tefb	24.83	0
ENSXMAG00000026340	0.0025	18	9		2.54	15
ENSXMAG00000026357	0.0014	3	6		5.94	0
ENSXMAG00000026378	0.0031	24	12	vti1a	1.51	21
ENSXMAG00000026397	0.0014	9	6		2.36	6
ENSXMAG00000026411	0.0031	24	12		2.53	21
ENSXMAG00000026457	0.0026	18	15		1.49	15
ENSXMAG00000026503	0.0031	24	12	lamtor1	1.41	21
ENSXMAG00000026570	0.0024	15	15	ciao1	1.69	12
ENSXMAG00000026614	0.0025	24	9	P2RY6	2.41	21
ENSXMAG00000026637	0.0031	21	12	lsm1	1.47	18
ENSXMAG00000026668	0.0071	3	18	myh7	1.86	0
ENSXMAG00000026746	0.0024	21	9	dcun1d2a	1.70	18
ENSXMAG00000026760	0.0029	21	12		1.64	18
ENSXMAG00000026782	0.0026	6	9		1.77	3
ENSXMAG00000026817	0.0029	3	12	CIART	29.13	0
ENSXMAG00000026823	0.0026	6	9	tsc22d2	2.39	3
ENSXMAG00000026826	0.0016	9	6	ormdl3	10.04	6
ENSXMAG00000026911	0.0031	21	12	TMEM179	9.70	18
ENSXMAG00000026936	0.0031	3	12	tnfaip3	2.46	0
ENSXMAG00000026947	0.0026	6	9	fosl2	5.94	3
ENSXMAG00000026971	0.0028	9	9	SRF (1 of many)	1.45	6
ENSXMAG00000026998	0.0030	15	12	cog8	1.45	12
ENSXMAG00000027066	0.0026	6	9		2.10	3
ENSXMAG00000027086	0.0024	3	9	cyth1a	1.53	0
ENSXMAG00000027100	0.0030	3	12		2.41	0
ENSXMAG00000027144	0.0015	15	18	zgc:174906	1.64	12
ENSXMAG00000027224	0.0031	24	12	cmc4	2.34	21
ENSXMAG00000027284	0.0024	24	15		278.94	21
ENSXMAG00000027307	0.0031	21	12	cryzl1	1.80	18
ENSXMAG00000027314	0.0024	21	15		3.14	18
ENSXMAG00000027319	0.0031	3	12	glud1b	1.50	0
ENSXMAG00000027320	0.0026	3	9	tsc1b	2.05	0
ENSXMAG00000027331	0.0026	24	15	akap11	1.63	21
ENSXMAG00000027359	0.0026	3	9		2.26	0
ENSXMAG00000027379	0.0024	3	9	ndrg1a	1.70	0
ENSXMAG00000027468	0.0031	3	12		15.14	0
ENSXMAG00000027540	0.0031	3	12		2.45	0
ENSXMAG00000027609	0.0027	18	15	triqk	2.46	15
ENSXMAG00000027637	0.0016	21	18	RBPMS	1.30	18
ENSXMAG00000027681	0.0026	6	9	SAMD12	2.88	3
ENSXMAG00000027722	0.0071	6	6	si:dkey-160o24.3	5.90	3
ENSXMAG00000027727	0.0026	18	15	UBL5	1.63	15
ENSXMAG00000027821	0.0015	24	18	IL1B	4.86	21
ENSXMAG00000027837	0.0031	15	12	bcap31	1.49	12
ENSXMAG00000027863	0.0029	18	12		2.89	15
ENSXMAG00000027869	0.0024	18	9		1.26	15
ENSXMAG00000027891	0.0034	24	12		2.48	21
ENSXMAG00000028088	0.0031	3	12		7.40	0
ENSXMAG00000028101	0.0028	9	9	GFOD1	2.94	6
ENSXMAG00000028139	0.0024	18	15		28.53	15
ENSXMAG00000028172	0.0027	3	9	khnyn	2.09	0
ENSXMAG00000028318	0.0015	15	18	CDYL	1.33	12
ENSXMAG00000028354	0.0031	24	12	lrrc32	2.25	21
ENSXMAG00000028430	0.0031	6	12	mid1ip1a	3.78	3

ENSXMAG00000028468	0.0026	21	9	cux1b	1.26	18
ENSXMAG00000028471	0.0014	15	18		2.38	12
ENSXMAG00000028474	0.0026	18	15	RAB31	1.42	15
ENSXMAG00000028475	0.0015	24	18		4.76	21
ENSXMAG00000028528	0.0014	15	18	eapp	1.40	12
ENSXMAG00000028532	0.0027	18	15	si:ch211-195e19.1	2.53	15
ENSXMAG00000028554	0.0031	18	12	hoxc10a	1.82	15
ENSXMAG00000028575	0.0025	18	9		5.22	15
ENSXMAG00000028576	0.0026	18	15	mtx2	1.85	15
ENSXMAG00000028604	0.0014	18	18	trim44	1.76	15
ENSXMAG00000028611	0.0031	24	12		1.80	21
ENSXMAG00000028868	0.0071	12	18	zgc:103692	1.39	9
ENSXMAG00000028879	0.0015	15	18		1.44	12
ENSXMAG00000028889	0.0028	21	15	rab5aa	1.37	18
ENSXMAG00000028940	0.0029	6	12		4.11	3
ENSXMAG00000028944	0.0026	3	9	unm_hu7912	4.32	0
ENSXMAG00000028952	0.0026	24	15	jmjd4	1.82	21
ENSXMAG00000029073	0.0031	21	12	fbxl20	1.56	18
ENSXMAG00000029127	0.0014	15	18	nudt5	1.99	12
ENSXMAG00000029131	0.0014	3	6	si:ch211-15b10.6 (1 of many)	3.03	0
ENSXMAG00000029315	0.0027	3	9	PHLPP2	5.23	0
ENSXMAG00000029342	0.0031	3	12	rlim	1.31	0
ENSXMAG00000029485	0.0026	3	15		2.01	0
ENSXMAG00000029494	0.0027	21	15		1.71	18
ENSXMAG00000029561	0.0025	24	15		4.89	21
ENSXMAG00000029568	0.0031	15	12		2.66	12
ENSXMAG00000029585	0.0028	3	15	CCDC137	1.47	0
ENSXMAG00000029631	0.0014	3	6		1.88	0
ENSXMAG00000029652	0.0031	21	12	zgc:77486	1.26	18
ENSXMAG00000029711	0.0026	3	9		2.28	0
ENSXMAG00000029717	0.0071	3	6	epsti1	2.81	0
ENSXMAG00000029794	0.0016	9	6	slc52a2	3.39	6
ENSXMAG00000029811	0.0026	24	15		1.56	21
ENSXMAG00000029843	0.0025	6	9		6.62	3
ENSXMAG00000029874	0.0027	24	15	tmem26a	3.85	21
ENSXMAG00000029891	0.0031	21	12		1.99	18
ENSXMAG00000029932	0.0031	24	12	xk	1.71	21
ENSXMAG00000029954	0.0025	6	9		4.49	3
ENSXMAG00000029994	0.0025	15	15		1.49	12
ENSXMAG00000030038	0.0025	3	9		2.41	0
ENSXMAG00000030052	0.0027	24	15		5.29	21

Brain circadian gene	GeneID	pVal	phase	peak.shape	external_gene_name	amp	Ct.peak
	ENSXMAG00000000054	0.0024	9	9	cnnm2b	1.30	6
	ENSXMAG00000000067	0.0031	15	12	hccsb	1.31	12
	ENSXMAG00000000103	0.0015	3	18	dyrk4	1.59	0
	ENSXMAG00000000124	0.0024	24	9	TMEM132D	1.67	21
	ENSXMAG00000000138	0.0031	24	12	slc39a6	1.22	21
	ENSXMAG00000000230	0.0031	21	12	plcd1b	2.46	18
	ENSXMAG00000000252	0.0027	3	9	si:ch211-221f10.2	2.56	0
	ENSXMAG00000000318	0.0016	3	6	nisch	1.18	0
	ENSXMAG00000000328	0.0016	9	18		1.57	6
	ENSXMAG00000000556	0.0025	3	15	msrb2	1.65	0
	ENSXMAG00000000615	0.0031	9	12	atcayb	1.43	6
	ENSXMAG00000000647	0.0028	9	15	TPM4	1.48	6
	ENSXMAG00000000707	0.0031	21	12		2.06	18

ENSXMAG00000000831	0.0025	3	15		1.22	0
ENSXMAG00000000865	0.0028	21	9	satb1a	1.34	18
ENSXMAG00000000867	0.0025	6	15	NYAP2	1.30	3
ENSXMAG00000000903	0.0024	21	15	mmp14b	2.09	18
ENSXMAG00000000915	0.0031	18	12		1.28	15
ENSXMAG00000000919	0.0030	9	12	gprc5ba	1.28	6
ENSXMAG00000000920	0.0025	3	9	si:dkey-91i10.3	1.20	0
ENSXMAG00000000925	0.0031	6	12		1.27	3
ENSXMAG00000000999	0.0026	21	9	fscn1a	1.24	18
ENSXMAG00000001071	0.0025	9	15	med20	1.32	6
ENSXMAG00000001113	0.0031	3	12	bhlhe41	12.34	0
ENSXMAG00000001119	0.0029	15	12		1.90	12
ENSXMAG00000001156	0.0026	9	9	csnk1e	1.44	6
ENSXMAG00000001172	0.0026	18	15		2.08	15
ENSXMAG00000001198	0.0026	18	9		1.24	15
ENSXMAG00000001208	0.0026	9	9		1.46	6
ENSXMAG00000001247	0.0015	9	18		1.71	6
ENSXMAG00000001251	0.0034	18	12	tmem184bb	1.52	15
ENSXMAG00000001327	0.0026	18	15		1.48	15
ENSXMAG00000001358	0.0024	9	15	angptl7	2.56	6
ENSXMAG00000001387	0.0030	18	12	sestd1	2.22	15
ENSXMAG00000001415	0.0025	9	9		2.23	6
ENSXMAG00000001442	0.0024	15	9	drg2	1.20	12
ENSXMAG00000001458	0.0028	3	15		1.18	0
ENSXMAG00000001476	0.0030	15	12	src	1.39	12
ENSXMAG00000001482	0.0034	15	12	arntl2	7.40	12
ENSXMAG00000001548	0.0024	3	15		2.13	0
ENSXMAG00000001561	0.0029	15	12		1.10	12
ENSXMAG00000001624	0.0015	9	18	fech	4.31	6
ENSXMAG00000001660	0.0024	24	9		1.32	21
ENSXMAG00000001682	0.0024	9	9	tsku	1.54	6
ENSXMAG00000001709	0.0034	15	12	ccni2	2.11	12
ENSXMAG00000001723	0.0031	3	12	baz2ba	1.41	0
ENSXMAG00000001728	0.0025	6	9	hsc70	1.52	3
ENSXMAG00000001732	0.0014	3	6		1.31	0
ENSXMAG00000001744	0.0031	6	12	cry1ab	7.50	3
ENSXMAG00000001762	0.0026	12	9	prpf4bb	1.19	9
ENSXMAG00000001769	0.0026	15	15		1.15	12
ENSXMAG00000001844	0.0024	12	15	naprt	1.57	9
ENSXMAG00000001855	0.0031	21	12	rft1	1.17	18
ENSXMAG00000001915	0.0016	3	6	mn1b	1.14	0
ENSXMAG00000001985	0.0016	6	6	fkbp11	1.57	3
ENSXMAG00000001998	0.0027	12	15	si:dkey-37o8.1	1.44	9
ENSXMAG00000002008	0.0031	21	12	skia	1.41	18
ENSXMAG00000002019	0.0024	9	9	bnip3la	1.45	6
ENSXMAG00000002032	0.0015	9	18	phka2	4.30	6
ENSXMAG00000002084	0.0031	6	12	sox17	1.67	3
ENSXMAG00000002115	0.0026	9	9	ptpra	1.26	6
ENSXMAG00000002148	0.0024	24	9	usp8	1.18	21
ENSXMAG00000002154	0.0025	6	15	mapk6	1.75	3
ENSXMAG00000002165	0.0027	12	15	ppid	1.53	9
ENSXMAG00000002236	0.0031	6	12	tyms	1.51	3
ENSXMAG00000002238	0.0016	3	6	aga	1.38	0
ENSXMAG00000002248	0.0025	6	15	BHLHE40 (1 of many)	10.33	3
ENSXMAG00000002251	0.0026	6	9	slc46a2	1.29	3
ENSXMAG00000002271	0.0024	6	15	rasgef1bb	2.53	3

ENSXMAG00000002274	0.0029	24	12	SLC39A12	2.96	21
ENSXMAG00000002275	0.0029	18	12	asb5a	3.67	15
ENSXMAG00000002313	0.0025	12	15	ivns1abpa	1.22	9
ENSXMAG00000002339	0.0026	24	9	nr1d4b	21.95	21
ENSXMAG00000002356	0.0031	3	12	plbd1	1.46	0
ENSXMAG00000002377	0.0031	9	12	smad3a	1.72	6
ENSXMAG00000002394	0.0014	9	18	spryd3 (1 of many)	1.57	6
ENSXMAG00000002404	0.0027	24	15	arvcfb	1.21	21
ENSXMAG00000002509	0.0024	18	15	fcho1	1.14	15
ENSXMAG00000002550	0.0031	18	12	acsbg2	1.41	15
ENSXMAG00000002645	0.0029	6	12	trpc1	1.44	3
ENSXMAG00000002711	0.0026	21	9	si:ch73-61d6.3	1.29	18
ENSXMAG00000002812	0.0014	3	6		1.20	0
ENSXMAG00000002820	0.0031	15	12		1.80	12
ENSXMAG00000002831	0.0071	6	6		1.45	3
ENSXMAG00000002876	0.0034	6	12	sytl1	1.40	3
ENSXMAG00000002891	0.0027	6	9	fam102aa	1.51	3
ENSXMAG00000002913	0.0027	18	9	vps26c	1.41	15
ENSXMAG00000002915	0.0016	18	18		1.10	15
ENSXMAG00000002955	0.0026	3	9	myofl	1.86	0
ENSXMAG00000002976	0.0026	21	9	tmem110l	1.08	18
ENSXMAG00000003032	0.0025	21	9	gldc	1.48	18
ENSXMAG00000003033	0.0028	3	9	ggcx	1.51	0
ENSXMAG00000003066	0.0024	21	15	DBNDD1	1.33	18
ENSXMAG00000003135	0.0026	6	9	tmem266	1.13	3
ENSXMAG00000003155	0.0025	9	15		1.25	6
ENSXMAG00000003187	0.0027	9	15	soat1	2.72	6
ENSXMAG00000003225	0.0031	15	12	zgc:153018	2.22	12
ENSXMAG00000003252	0.0014	18	18	pcdh12	1.91	15
ENSXMAG00000003295	0.0031	9	12	ttc39a	1.61	6
ENSXMAG00000003340	0.0014	9	6	AGBL4	1.32	6
ENSXMAG00000003373	0.0025	24	15		2.01	21
ENSXMAG00000003430	0.0024	9	9	csnk1db	1.40	6
ENSXMAG00000003439	0.0026	24	9	mfsd2b	1.38	21
ENSXMAG00000003454	0.0014	21	6	si:ch211-193k19.1	1.29	18
ENSXMAG00000003477	0.0015	6	6	malt2	1.45	3
ENSXMAG00000003577	0.0031	18	12	slc2a15a	1.44	15
ENSXMAG00000003595	0.0031	9	12	lgi3	1.35	6
ENSXMAG00000003646	0.0031	6	12	ankrd39	1.43	3
ENSXMAG00000003684	0.0026	6	9	cxcr4b	2.22	3
ENSXMAG00000003694	0.0026	9	15	ddb2	3.48	6
ENSXMAG00000003695	0.0014	3	18	slc5a1 (1 of many)	1.35	0
ENSXMAG00000003725	0.0025	6	15	grhl2b	1.76	3
ENSXMAG00000003731	0.0026	9	9	ttc39c	1.24	6
ENSXMAG00000003761	0.0026	15	15	dyrk3	1.48	12
ENSXMAG00000003855	0.0025	15	15	abhd8a	1.73	12
ENSXMAG00000003890	0.0016	3	18	iqsec3b	1.42	0
ENSXMAG00000003896	0.0031	18	12	dock10	1.59	15
ENSXMAG00000003961	0.0029	6	12	per3	5.24	3
ENSXMAG00000003970	0.0031	6	12	TGFB3 (1 of many)	1.87	3
ENSXMAG00000004040	0.0016	3	6	pwp1	1.40	0
ENSXMAG00000004058	0.0025	15	15	atp6v0a2a	1.23	12
ENSXMAG00000004069	0.0031	15	12		1.29	12
ENSXMAG00000004079	0.0031	3	12	gja5a	1.73	0
ENSXMAG00000004088	0.0025	15	15	fam69aa	1.44	12
ENSXMAG00000004154	0.0024	6	15	ntsr1	1.46	3

ENSXMAG00000004170	0.0027	9	15	si:ch1073-390k14.1	7.29	6
ENSXMAG00000004183	0.0029	6	12		3.26	3
ENSXMAG00000004198	0.0029	24	12		3.40	21
ENSXMAG00000004276	0.0031	24	12		1.34	21
ENSXMAG00000004294	0.0027	18	9	tigarb	1.81	15
ENSXMAG00000004308	0.0025	15	9	tbl1x	1.88	12
ENSXMAG00000004317	0.0026	12	15	dnajc21	1.31	9
ENSXMAG00000004328	0.0014	6	18		1.38	3
ENSXMAG00000004343	0.0015	24	6	CACNA2D1 (1 of many)	1.39	21
ENSXMAG00000004383	0.0031	18	12	specc1la	1.28	15
ENSXMAG00000004460	0.0030	18	12		1.34	15
ENSXMAG00000004555	0.0031	15	12	ddx1	1.16	12
ENSXMAG00000004563	0.0031	18	12	rcc2	1.32	15
ENSXMAG00000004627	0.0026	18	15	dnajc3a	1.27	15
ENSXMAG00000004649	0.0026	12	9	coro7	1.36	9
ENSXMAG00000004689	0.0014	21	18		2.44	18
ENSXMAG00000004691	0.0031	9	12	ppm1j	1.26	6
ENSXMAG00000004714	0.0029	18	12		2.36	15
ENSXMAG00000004751	0.0015	9	18	zgc:66475	2.85	6
ENSXMAG00000004754	0.0025	15	15	nuak1b	1.31	12
ENSXMAG00000004756	0.0015	3	6	abca3b	1.46	0
ENSXMAG00000004769	0.0030	24	12	adgrd2	1.24	21
ENSXMAG00000004839	0.0026	24	9		1.79	21
ENSXMAG00000004886	0.0025	15	9	ppp1r3ca	2.35	12
ENSXMAG00000005023	0.0071	3	6		2.28	0
ENSXMAG00000005039	0.0015	12	6		1.75	9
ENSXMAG00000005067	0.0031	6	12	amd1	1.64	3
ENSXMAG00000005092	0.0026	15	9	wdr36	1.29	12
ENSXMAG00000005101	0.0031	18	12		3.32	15
ENSXMAG00000005127	0.0031	15	12	nfil3-5	20.24	12
ENSXMAG00000005128	0.0034	15	12	si:dkey-119m7.4	1.91	12
ENSXMAG00000005214	0.0029	9	12	fbxl5	1.20	6
ENSXMAG00000005288	0.0025	21	15		1.99	18
ENSXMAG00000005479	0.0024	24	15		1.61	21
ENSXMAG00000005497	0.0026	6	15	cdc14ab	2.24	3
ENSXMAG00000005512	0.0015	12	18		2.52	9
ENSXMAG00000005538	0.0031	3	12	gpr78a	3.49	0
ENSXMAG00000005611	0.0028	24	9	ehbp1	1.18	21
ENSXMAG00000005631	0.0026	21	9	helt	2.77	18
ENSXMAG00000005677	0.0031	9	12	phyhd1	1.78	6
ENSXMAG00000005693	0.0025	15	9	uck1	1.18	12
ENSXMAG00000005791	0.0071	3	6	kyat1	1.29	0
ENSXMAG00000005838	0.0026	15	15		1.31	12
ENSXMAG00000005842	0.0016	24	6	usp20	1.23	21
ENSXMAG00000005844	0.0025	3	9	smu1b	11.59	0
ENSXMAG00000005850	0.0031	15	12	glsb	1.19	12
ENSXMAG00000005855	0.0025	24	9	dnaja1	1.15	21
ENSXMAG00000005965	0.0026	18	15	lamc3	1.42	15
ENSXMAG00000005976	0.0024	3	15	abi3bpb	2.27	0
ENSXMAG00000006010	0.0024	24	9		1.55	21
ENSXMAG00000006030	0.0031	15	12		1.59	12
ENSXMAG00000006052	0.0015	6	18	prelid3b	3.49	3
ENSXMAG00000006107	0.0024	9	15	luzp1	1.51	6
ENSXMAG00000006112	0.0025	9	9	arhgap12a	1.83	6
ENSXMAG00000006134	0.0030	6	12		1.55	3
ENSXMAG00000006246	0.0031	3	12	irf4b	1.97	0

ENSXMAG00000006293	0.0025	24	9	phactr1	1.31	21
ENSXMAG00000006300	0.0024	9	9	OSBPL8	1.40	6
ENSXMAG00000006320	0.0030	3	12	sdk1a	1.37	0
ENSXMAG00000006361	0.0071	6	18	crema	3.33	3
ENSXMAG00000006390	0.0026	9	15	dnajc2	1.55	6
ENSXMAG00000006391	0.0015	12	18	sprtn	1.17	9
ENSXMAG00000006406	0.0026	15	9	psmc2	1.23	12
ENSXMAG00000006417	0.0028	6	9	cry2	1.62	3
ENSXMAG00000006421	0.0015	9	18	pfkpb	1.91	6
ENSXMAG00000006429	0.0014	3	6	pkn1a	1.34	0
ENSXMAG00000006519	0.0025	18	15		2.02	15
ENSXMAG00000006603	0.0027	21	15	slco1e1	1.37	18
ENSXMAG00000006606	0.0034	18	12		1.37	15
ENSXMAG00000006634	0.0031	15	12	snupn	1.31	12
ENSXMAG00000006747	0.0026	18	9	disc1	1.46	15
ENSXMAG00000006817	0.0031	6	12	gchfr	1.49	3
ENSXMAG00000006838	0.0025	18	15	myo10	1.30	15
ENSXMAG00000006854	0.0027	21	15		1.74	18
ENSXMAG00000006870	0.0031	3	12	ACO2 (1 of many)	1.56	0
ENSXMAG00000006918	0.0029	9	12	gstr (1 of many)	3.49	6
ENSXMAG00000006926	0.0025	18	15	PTK7	1.39	15
ENSXMAG00000006999	0.0025	6	15		3.70	3
ENSXMAG00000007002	0.0028	3	9	poc5	1.77	0
ENSXMAG00000007022	0.0026	15	15	syt9a	1.41	12
ENSXMAG00000007048	0.0024	3	9	coq8a	1.20	0
ENSXMAG00000007074	0.0031	3	12	atp8b2	4.41	0
ENSXMAG00000007090	0.0026	21	9	daxx	1.29	18
ENSXMAG00000007094	0.0025	18	15		1.70	15
ENSXMAG00000007144	0.0025	3	9	PLEKHA5	1.23	0
ENSXMAG00000007152	0.0031	3	12		1.46	0
ENSXMAG00000007185	0.0031	18	12	PDE4A (1 of many)	1.32	15
ENSXMAG00000007211	0.0031	6	12	si:dkeyp-72e1.9	1.47	3
ENSXMAG00000007388	0.0026	3	15	cog6	1.07	0
ENSXMAG00000007482	0.0031	21	12	nhsl1b	1.73	18
ENSXMAG00000007532	0.0028	21	15		1.38	18
ENSXMAG00000007534	0.0027	18	9		1.33	15
ENSXMAG00000007552	0.0026	6	9		1.25	3
ENSXMAG00000007562	0.0025	18	9	asb2a.1	1.24	15
ENSXMAG00000007572	0.0015	3	6	tpcn1	1.24	0
ENSXMAG00000007627	0.0024	15	9	srrt	1.23	12
ENSXMAG00000007684	0.0031	21	12	adam19a	1.34	18
ENSXMAG00000007696	0.0028	6	9	scg5	1.15	3
ENSXMAG00000007707	0.0031	15	12	opn3	4.13	12
ENSXMAG00000007731	0.0031	24	12	fbxo25	5.28	21
ENSXMAG00000007745	0.0029	3	12	pole	1.66	0
ENSXMAG00000007776	0.0026	3	9	rlf	1.24	0
ENSXMAG00000007800	0.0026	3	15	znf395b	4.36	0
ENSXMAG00000007821	0.0030	3	12	acacb	1.84	0
ENSXMAG00000007844	0.0025	15	15	csnk2a4	1.18	12
ENSXMAG00000007879	0.0030	15	12	foxn4	4.12	12
ENSXMAG00000008050	0.0026	12	9		1.98	9
ENSXMAG00000008079	0.0026	21	15		1.22	18
ENSXMAG00000008082	0.0031	21	12	zgc:153039	1.42	18
ENSXMAG00000008129	0.0025	15	15	fkbpl	1.24	12
ENSXMAG00000008130	0.0031	18	12	ebp	1.58	15
ENSXMAG00000008152	0.0026	18	9	NAV1 (1 of many)	1.51	15

ENSXMAG00000008172	0.0031	3	12	si:dkey-177p2.6	3.84	0
ENSXMAG00000008234	0.0025	15	9	lratb.1	4.93	12
ENSXMAG00000008270	0.0025	3	9	inpp4ab	1.54	0
ENSXMAG00000008304	0.0029	24	12	pax3b	1.45	21
ENSXMAG00000008354	0.0026	9	15	yme1l1a	1.74	6
ENSXMAG00000008377	0.0016	18	6		1.29	15
ENSXMAG00000008378	0.0031	12	12		1.38	9
ENSXMAG00000008420	0.0031	12	12	b3glctb	1.36	9
ENSXMAG00000008438	0.0027	6	9	zgc:175248	1.69	3
ENSXMAG00000008446	0.0034	18	12	evlb	1.31	15
ENSXMAG00000008453	0.0014	12	18	rflnb	1.84	9
ENSXMAG00000008463	0.0025	18	9	slco1c1	2.08	15
ENSXMAG00000008472	0.0031	15	12	abhd15a	3.65	12
ENSXMAG00000008498	0.0025	18	15	dennd4c	1.49	15
ENSXMAG00000008579	0.0026	6	15	dclk1a	2.53	3
ENSXMAG00000008588	0.0015	21	18	csf1ra	1.41	18
ENSXMAG00000008594	0.0024	24	9	npr2	1.25	21
ENSXMAG00000008595	0.0026	9	15		1.93	6
ENSXMAG00000008604	0.0029	15	12		1.19	12
ENSXMAG00000008689	0.0015	6	18	pfkfb3	2.35	3
ENSXMAG00000008786	0.0014	9	18	lrwd1	2.01	6
ENSXMAG00000008844	0.0025	15	9	SEMA4F	1.33	12
ENSXMAG00000008892	0.0024	21	15	ARNTL2 (1 of many)	2.84	18
ENSXMAG00000008900	0.0026	9	15	sdha	1.82	6
ENSXMAG00000008939	0.0031	12	12	DDX39A	1.36	9
ENSXMAG00000009037	0.0024	9	15		2.19	6
ENSXMAG00000009078	0.0030	18	12		1.72	15
ENSXMAG00000009130	0.0026	3	15	EZH1	1.29	0
ENSXMAG00000009137	0.0031	15	12	adamts1	1.73	12
ENSXMAG00000009312	0.0034	6	12	dlec1	2.39	3
ENSXMAG00000009325	0.0026	6	15	si:ch211-63p21.1	1.94	3
ENSXMAG00000009385	0.0026	21	15	swap70a	1.37	18
ENSXMAG00000009431	0.0025	12	15	zgc:66447	1.25	9
ENSXMAG00000009449	0.0025	6	15	cpa4	1.76	3
ENSXMAG00000009482	0.0024	24	9	grm8a	1.16	21
ENSXMAG00000009576	0.0025	21	15	tmem184c	1.12	18
ENSXMAG00000009638	0.0015	24	18	cacng7b	1.58	21
ENSXMAG00000009720	0.0031	15	12		1.24	12
ENSXMAG00000009739	0.0029	15	12	mxd3	1.90	12
ENSXMAG00000009818	0.0026	3	9	gpr157	1.90	0
ENSXMAG00000009974	0.0025	9	15	lonrf1l	4.35	6
ENSXMAG00000009990	0.0071	3	6	robo1	1.23	0
ENSXMAG00000010019	0.0027	15	15	cdh27	1.38	12
ENSXMAG00000010053	0.0024	3	15	keap1a	1.65	0
ENSXMAG00000010102	0.0026	12	15	dctn4	1.16	9
ENSXMAG00000010108	0.0031	6	12		1.29	3
ENSXMAG00000010130	0.0026	18	15		1.18	15
ENSXMAG00000010202	0.0014	24	18	adora1b	1.18	21
ENSXMAG00000010242	0.0026	18	9		2.12	15
ENSXMAG00000010261	0.0024	9	9	elovl5	2.13	6
ENSXMAG00000010295	0.0027	9	9		2.03	6
ENSXMAG00000010342	0.0025	15	15	dnajb11	1.20	12
ENSXMAG00000010343	0.0034	6	12		1.64	3
ENSXMAG00000010372	0.0026	3	15	trpc2b	2.03	0
ENSXMAG00000010399	0.0024	9	15	SFMBT1	1.65	6
ENSXMAG00000010418	0.0034	15	12	ahr1b	2.53	12

ENSXMAG00000010470	0.0026	24	15	loxl2b	1.89	21
ENSXMAG00000010505	0.0026	9	15	lonrf1	3.62	6
ENSXMAG00000010593	0.0024	9	15	KIAA0895	1.99	6
ENSXMAG00000010643	0.0031	18	12		1.87	15
ENSXMAG00000010644	0.0031	18	12	man1b1a	1.30	15
ENSXMAG00000010656	0.0031	3	12	adcy6b	1.57	0
ENSXMAG00000010657	0.0071	6	18	rcc1	1.20	3
ENSXMAG00000010775	0.0031	15	12	pigs	1.18	12
ENSXMAG00000010825	0.0026	9	15	deptor	1.94	6
ENSXMAG00000010827	0.0031	9	12	dedd1	2.06	6
ENSXMAG00000010850	0.0034	3	12	cpt1cb	2.89	0
ENSXMAG00000010900	0.0031	18	12	nlk1	1.32	15
ENSXMAG00000010902	0.0034	3	12	l1cama	1.43	0
ENSXMAG00000010929	0.0026	9	15	fdxacb1	1.54	6
ENSXMAG00000010930	0.0026	9	15	TOMM40L	1.40	6
ENSXMAG00000010967	0.0031	9	12	porb	1.46	6
ENSXMAG00000010972	0.0034	24	12	uvssa	1.14	21
ENSXMAG00000011018	0.0014	6	6	zgc:103564	2.29	3
ENSXMAG00000011080	0.0014	3	18	slc22a6l	3.78	0
ENSXMAG00000011171	0.0029	15	12	zgc:101559	1.46	12
ENSXMAG00000011192	0.0031	21	12	bcor	1.85	18
ENSXMAG00000011193	0.0024	21	9	myo7ab	2.23	18
ENSXMAG00000011200	0.0029	9	12	oma1	1.46	6
ENSXMAG00000011204	0.0031	12	12	pa2g4b	1.20	9
ENSXMAG00000011257	0.0014	6	18	CYR61	1.96	3
ENSXMAG00000011310	0.0031	18	12	ap1m3	1.22	15
ENSXMAG00000011384	0.0016	24	6	kank4	1.36	21
ENSXMAG00000011390	0.0016	9	18	grpel1	1.52	6
ENSXMAG00000011448	0.0027	9	15	coa7	2.40	6
ENSXMAG00000011464	0.0025	24	9	nr1d4a	20.53	21
ENSXMAG00000011562	0.0031	6	12	slc9a3r1a	1.72	3
ENSXMAG00000011567	0.0031	24	12		1.49	21
ENSXMAG00000011605	0.0031	6	12		1.30	3
ENSXMAG00000011623	0.0030	9	12	HNMT (1 of many)	2.89	6
ENSXMAG00000011681	0.0024	15	9	slc17a7a	1.76	12
ENSXMAG00000011730	0.0028	18	9		1.16	15
ENSXMAG00000011763	0.0031	6	12	nfkbiaa	1.78	3
ENSXMAG00000011870	0.0031	21	12	scamp4	1.46	18
ENSXMAG00000011913	0.0031	24	12	pld1b	1.40	21
ENSXMAG00000011949	0.0029	6	12	ucp2	3.07	3
ENSXMAG00000011967	0.0031	24	12	si:dkey-17m8.1	1.56	21
ENSXMAG00000012037	0.0031	12	12	pipox	5.62	9
ENSXMAG00000012054	0.0025	24	15	dbpb	16.80	21
ENSXMAG00000012059	0.0015	18	18	tjp3	1.61	15
ENSXMAG00000012065	0.0026	24	9	slc12a9	1.67	21
ENSXMAG00000012073	0.0031	18	12	ankrd13b	1.23	15
ENSXMAG00000012134	0.0016	24	6	adgrg1	1.50	21
ENSXMAG00000012189	0.0026	18	9	npas2	4.27	15
ENSXMAG00000012203	0.0031	18	12	abcc3	1.84	15
ENSXMAG00000012248	0.0025	6	15	bco2a	3.27	3
ENSXMAG00000012259	0.0025	3	15		1.53	0
ENSXMAG00000012277	0.0025	9	9	PCYT2 (1 of many)	1.32	6
ENSXMAG00000012278	0.0025	12	15	crmp1	1.36	9
ENSXMAG00000012309	0.0031	18	12		1.27	15
ENSXMAG00000012327	0.0031	3	12		1.21	0
ENSXMAG00000012344	0.0025	15	15		1.31	12

ENSXMAG00000012356	0.0014	9	18	agmat	1.53	6
ENSXMAG00000012364	0.0031	18	12	MAP4K4 (1 of many)	1.42	15
ENSXMAG00000012380	0.0031	21	12	col4a1	1.99	18
ENSXMAG00000012401	0.0027	3	9	usp36	1.43	0
ENSXMAG00000012455	0.0027	12	15	cptp	1.39	9
ENSXMAG00000012479	0.0015	21	6		1.23	18
ENSXMAG00000012512	0.0031	3	12	socs3a	2.42	0
ENSXMAG00000012583	0.0026	6	9		1.73	3
ENSXMAG00000012600	0.0026	9	15		7.23	6
ENSXMAG00000012623	0.0034	9	12	KHDC4	1.36	6
ENSXMAG00000012675	0.0024	3	9		1.45	0
ENSXMAG00000012690	0.0025	9	15		1.73	6
ENSXMAG00000012705	0.0015	6	18	pgam1b	1.35	3
ENSXMAG00000012711	0.0031	3	12	il13ra2	1.38	0
ENSXMAG00000012760	0.0024	12	15	cdc27	1.30	9
ENSXMAG00000012787	0.0030	9	12	rap1gap	1.76	6
ENSXMAG00000012806	0.0026	9	9	fndc4a	1.68	6
ENSXMAG00000012809	0.0029	3	12	ctps1a	3.28	0
ENSXMAG00000012841	0.0031	15	12	oaz1a	1.43	12
ENSXMAG00000012920	0.0024	3	9	slc26a11	1.27	0
ENSXMAG00000012938	0.0025	15	9		3.22	12
ENSXMAG00000013021	0.0024	15	9	rorca	5.89	12
ENSXMAG00000013097	0.0024	12	15	dnajb1a	2.48	9
ENSXMAG00000013258	0.0034	18	12	rhbg	2.35	15
ENSXMAG00000013348	0.0034	15	12	eml1	1.88	12
ENSXMAG00000013381	0.0026	21	9	tle2a	1.27	18
ENSXMAG00000013444	0.0026	9	9	sv2a	1.25	6
ENSXMAG00000013544	0.0025	6	15	si:dkey-21a6.5	1.66	3
ENSXMAG00000013565	0.0014	6	6	plk4	1.97	3
ENSXMAG00000013587	0.0026	18	15	asb2b	1.15	15
ENSXMAG00000013614	0.0031	6	12	ube2ql1	1.31	3
ENSXMAG00000013637	0.0024	6	9	si:dkey-221l4.11	1.27	3
ENSXMAG00000013699	0.0029	15	12	6-Sep	1.15	12
ENSXMAG00000013764	0.0025	6	15	ankrd6a	2.00	3
ENSXMAG00000013834	0.0016	21	6		1.14	18
ENSXMAG00000013871	0.0026	18	9	umodl1	1.45	15
ENSXMAG00000013928	0.0031	6	12	osbpl3b	1.32	3
ENSXMAG00000014010	0.0031	18	12	git2a	2.08	15
ENSXMAG00000014041	0.0031	9	12	ptdss1a	1.17	6
ENSXMAG00000014043	0.0031	6	12	rad21b	1.20	3
ENSXMAG00000014051	0.0025	12	15	dclk2a	1.33	9
ENSXMAG00000014077	0.0031	6	12	abcc5	4.62	3
ENSXMAG00000014084	0.0031	9	12	TPPP	1.56	6
ENSXMAG00000014132	0.0025	18	9	soul4	1.54	15
ENSXMAG00000014155	0.0026	3	15		1.30	0
ENSXMAG00000014170	0.0031	6	12	depdc7a	1.76	3
ENSXMAG00000014302	0.0031	9	12	adap2	2.11	6
ENSXMAG00000014312	0.0028	3	15	rps6ka2	1.64	0
ENSXMAG00000014473	0.0014	6	6	CHMP6 (1 of many)	1.20	3
ENSXMAG00000014545	0.0026	3	9	actn1	1.31	0
ENSXMAG00000014761	0.0024	9	9	acsf2	1.36	6
ENSXMAG00000014792	0.0027	6	15	arg2	1.92	3
ENSXMAG00000014814	0.0025	9	9	pbxip1a	1.43	6
ENSXMAG00000014842	0.0027	24	9	cgnb	1.60	21
ENSXMAG00000014878	0.0027	21	9		1.47	18
ENSXMAG00000014890	0.0015	3	6	mrm1	1.37	0

ENSXMAG00000014898	0.0031	3	12	gnl2	1.41	0
ENSXMAG00000014902	0.0025	24	15	msl1b	1.18	21
ENSXMAG00000014914	0.0026	24	15	tnfaip2b	1.76	21
ENSXMAG00000014920	0.0034	9	12		1.42	6
ENSXMAG00000014926	0.0014	18	6	clcn5b	1.63	15
ENSXMAG00000014943	0.0025	3	15	ppl	1.61	0
ENSXMAG00000014960	0.0024	18	9	dnajb12b	1.48	15
ENSXMAG00000015026	0.0031	15	12	rnls	1.54	12
ENSXMAG00000015089	0.0015	21	18	slc20a1a	1.61	18
ENSXMAG00000015104	0.0031	15	12		1.23	12
ENSXMAG00000015108	0.0025	21	9	prcc	1.13	18
ENSXMAG00000015242	0.0031	6	12	slc35f4	1.64	3
ENSXMAG00000015276	0.0014	3	6	ano10b	1.36	0
ENSXMAG00000015291	0.0014	24	18	GGT7	1.15	21
ENSXMAG00000015314	0.0029	3	12	per1b	9.19	0
ENSXMAG00000015321	0.0024	3	9	peli2	1.67	0
ENSXMAG00000015342	0.0034	15	12	grm1b	1.43	12
ENSXMAG00000015385	0.0024	3	9	aspg	1.77	0
ENSXMAG00000015394	0.0031	24	12		1.30	21
ENSXMAG00000015409	0.0014	9	18	syt9b	1.33	6
ENSXMAG00000015470	0.0024	9	15	zgc:65997	2.45	6
ENSXMAG00000015477	0.0026	3	9	gab2	1.48	0
ENSXMAG00000015558	0.0016	9	6	vclb	1.37	6
ENSXMAG00000015563	0.0026	3	9	VAV1	1.49	0
ENSXMAG00000015612	0.0024	9	15	tspo (1 of many)	2.41	6
ENSXMAG00000015619	0.0031	15	12	arntl1a	5.86	12
ENSXMAG00000015629	0.0024	15	15	si:dkey-38p12.3	1.50	12
ENSXMAG00000015645	0.0015	9	18	adck1	2.11	6
ENSXMAG00000015749	0.0024	6	15	phactr4a	1.45	3
ENSXMAG00000015763	0.0029	12	12	slc25a21	1.52	9
ENSXMAG00000015773	0.0026	9	15	hivep3a	1.50	6
ENSXMAG00000015779	0.0029	18	12	plekhh1	3.13	15
ENSXMAG00000015790	0.0026	9	15	ssuh2rs1	1.38	6
ENSXMAG00000015795	0.0029	6	12	STMN1	1.53	3
ENSXMAG00000015833	0.0031	6	12	crip2	1.79	3
ENSXMAG00000015837	0.0029	3	12	mych	2.99	0
ENSXMAG00000015844	0.0029	12	12	arsh	2.23	9
ENSXMAG00000015871	0.0024	21	9	sptb	1.87	18
ENSXMAG00000015874	0.0034	15	12	msantd1	1.67	12
ENSXMAG00000015904	0.0031	9	12		3.51	6
ENSXMAG00000015924	0.0031	9	12		2.59	6
ENSXMAG00000015928	0.0024	9	15	ogg1	1.69	6
ENSXMAG00000015986	0.0031	3	12	HABP4	1.48	0
ENSXMAG00000015990	0.0031	9	12	ppm1bb	1.15	6
ENSXMAG00000016055	0.0016	3	6	tubgcp5	1.19	0
ENSXMAG00000016068	0.0014	9	18		3.66	6
ENSXMAG00000016104	0.0016	21	6	entpd6	1.41	18
ENSXMAG00000016117	0.0024	9	15	sfxn5b	2.21	6
ENSXMAG00000016147	0.0026	6	9	edn1	3.00	3
ENSXMAG00000016225	0.0027	12	15	akap17a	1.23	9
ENSXMAG00000016260	0.0026	21	9	itga10	1.60	18
ENSXMAG00000016261	0.0027	21	9		1.27	18
ENSXMAG00000016346	0.0026	12	15	brd7	1.13	9
ENSXMAG00000016416	0.0024	18	9	PDE3A	1.57	15
ENSXMAG00000016437	0.0026	15	15	tepsin	1.17	12
ENSXMAG00000016511	0.0031	3	12	ghrb	1.88	0

ENSXMAG00000016524	0.0025	12	15		2.59	9
ENSXMAG00000016554	0.0025	9	9	oat	2.17	6
ENSXMAG00000016582	0.0031	18	12	mrc1b (1 of many)	1.54	15
ENSXMAG00000016644	0.0028	21	9	TMCC3	1.20	18
ENSXMAG00000016663	0.0029	18	12	CDH2	1.19	15
ENSXMAG00000016672	0.0029	9	12		6.38	6
ENSXMAG00000016785	0.0014	3	6	sim1a	1.39	0
ENSXMAG00000016896	0.0025	9	15	slc1a3a	1.35	6
ENSXMAG00000016918	0.0026	9	15	cox11	1.50	6
ENSXMAG00000016928	0.0025	18	9	clocka	5.71	15
ENSXMAG00000016937	0.0071	3	6	CCDC88A	1.20	0
ENSXMAG00000017047	0.0031	15	12		1.10	12
ENSXMAG00000017082	0.0025	24	9	chl1b	1.22	21
ENSXMAG00000017106	0.0025	6	9	nr1d2b	2.45	3
ENSXMAG00000017158	0.0014	21	18		1.30	18
ENSXMAG00000017201	0.0031	3	12	tnk2b	1.38	0
ENSXMAG00000017204	0.0034	15	12	rassf1	3.00	12
ENSXMAG00000017239	0.0025	9	15	gpd1a	1.80	6
ENSXMAG00000017310	0.0027	12	15		1.13	9
ENSXMAG00000017329	0.0025	24	9		1.45	21
ENSXMAG00000017357	0.0015	3	6	IFT122	1.40	0
ENSXMAG00000017367	0.0025	6	9	aida	1.44	3
ENSXMAG00000017449	0.0029	9	12	syt10	1.77	6
ENSXMAG00000017466	0.0024	9	15		3.67	6
ENSXMAG00000017480	0.0029	6	12	slc7a2	2.47	3
ENSXMAG00000017517	0.0029	24	12	psmd9	1.35	21
ENSXMAG00000017520	0.0024	3	15	lpin1	2.49	0
ENSXMAG00000017627	0.0026	12	9	kntc1	1.59	9
ENSXMAG00000017661	0.0016	3	6		1.54	0
ENSXMAG00000017769	0.0027	24	9		1.29	21
ENSXMAG00000017835	0.0026	21	15	prss12	1.29	18
ENSXMAG00000017854	0.0024	9	9		1.89	6
ENSXMAG00000017863	0.0024	21	9	tmem79b	1.52	18
ENSXMAG00000017886	0.0024	21	15		1.27	18
ENSXMAG00000017978	0.0014	9	18	r3hdm2	1.28	6
ENSXMAG00000017993	0.0016	6	18	si:ch211-195b13.1	3.19	3
ENSXMAG00000018003	0.0031	3	12	ddx18	1.59	0
ENSXMAG00000018034	0.0031	15	12	si:ch211-51c14.1	1.59	12
ENSXMAG00000018058	0.0027	18	9	ppp1r12c	1.19	15
ENSXMAG00000018079	0.0026	9	15		1.28	6
ENSXMAG00000018222	0.0024	3	9	gusb	1.39	0
ENSXMAG00000018261	0.0031	3	12	usp13	1.49	0
ENSXMAG00000018334	0.0031	6	12		1.78	3
ENSXMAG00000018356	0.0026	6	15	SPATA5	1.47	3
ENSXMAG00000018457	0.0031	18	12		1.33	15
ENSXMAG00000018476	0.0034	6	12	BRF1 (1 of many)	1.63	3
ENSXMAG00000018570	0.0034	18	12	quo	1.67	15
ENSXMAG00000018577	0.0026	15	9	zgc:153119	1.25	12
ENSXMAG00000018670	0.0031	15	12	si:ch211-160d20.3	1.22	12
ENSXMAG00000018710	0.0031	24	12	JMY	2.73	21
ENSXMAG00000018718	0.0031	9	12		2.21	6
ENSXMAG00000018738	0.0025	21	15	tnfaip8l1	1.52	18
ENSXMAG00000018753	0.0026	15	15	lhfpl2a	1.21	12
ENSXMAG00000018772	0.0015	9	18	brms1lb	1.24	6
ENSXMAG00000018813	0.0031	24	12	pargl	1.75	21
ENSXMAG00000018852	0.0027	6	15	cep70	1.74	3

ENSXMAG00000018879	0.0014	24	18	CAMK2N1	1.33	21
ENSXMAG00000018889	0.0016	21	6	psd2	1.14	18
ENSXMAG00000018934	0.0031	24	12		12.42	21
ENSXMAG00000019074	0.0031	3	12		1.24	0
ENSXMAG00000019139	0.0029	15	12	ppp1r9alb	1.88	12
ENSXMAG00000019146	0.0024	9	15	syt12	1.40	6
ENSXMAG00000019158	0.0026	12	15	mtss1la	1.25	9
ENSXMAG00000019225	0.0026	9	15		5.87	6
ENSXMAG00000019243	0.0031	9	12	abcc8	1.24	6
ENSXMAG00000019263	0.0031	9	12	hdhd5	1.68	6
ENSXMAG00000019296	0.0024	15	9	rorcb	9.08	12
ENSXMAG00000019307	0.0031	15	12		1.39	12
ENSXMAG00000019336	0.0025	18	9	cirbpa	1.54	15
ENSXMAG00000019360	0.0026	9	15	xpc	2.57	6
ENSXMAG00000019366	0.0028	24	9	sema3h	1.30	21
ENSXMAG00000019446	0.0015	21	18	cdca4	1.22	18
ENSXMAG00000019447	0.0026	21	9	SOX1 (1 of many)	1.45	18
ENSXMAG00000019486	0.0026	6	15	TMEM100	2.15	3
ENSXMAG00000019515	0.0071	18	6	sox3	1.47	15
ENSXMAG00000019527	0.0026	6	15	nfil3-6	9.34	3
ENSXMAG00000019530	0.0031	18	12		4.16	15
ENSXMAG00000019538	0.0024	18	9	bola1	1.37	15
ENSXMAG00000019561	0.0025	12	15		1.55	9
ENSXMAG00000019564	0.0016	6	18	zgc:154093	1.54	3
ENSXMAG00000019823	0.0025	15	9	tmem264	1.35	12
ENSXMAG00000019874	0.0031	21	12	arl4d	1.44	18
ENSXMAG00000019888	0.0026	3	9		2.20	0
ENSXMAG00000019943	0.0025	15	15	nfil3	4.70	12
ENSXMAG00000019973	0.0031	12	12		2.14	9
ENSXMAG00000020056	0.0031	12	12	chst2b	1.14	9
ENSXMAG00000020104	0.0016	3	6	tysnd1	1.42	0
ENSXMAG00000020171	0.0024	21	15		1.82	18
ENSXMAG00000020181	0.0031	15	12		1.31	12
ENSXMAG00000020237	0.0031	12	12	NRGN	1.28	9
ENSXMAG00000020294	0.0031	12	12	MRPL21	1.31	9
ENSXMAG00000020345	0.0025	9	9	flrt2	1.35	6
ENSXMAG00000020829	0.0027	12	15	prkar2aa	1.21	9
ENSXMAG00000020886	0.0031	3	12	ADAMTS5	1.88	0
ENSXMAG00000020927	0.0015	6	18		1.52	3
ENSXMAG00000020951	0.0034	15	12		1.18	12
ENSXMAG00000020965	0.0029	18	12	PCDHGB4	8.26	15
ENSXMAG00000020991	0.0024	15	15	spsb3a	1.33	12
ENSXMAG00000021001	0.0034	6	12	lrrc75bb	1.50	3
ENSXMAG00000021055	0.0024	24	15	dsn1	1.34	21
ENSXMAG00000021086	0.0031	15	12	gramd2aa	1.93	12
ENSXMAG00000021148	0.0015	9	18		1.25	6
ENSXMAG00000021188	0.0026	18	15	parp12a (1 of many)	1.83	15
ENSXMAG00000021235	0.0031	12	12	si:ch211-11k18.4	1.55	9
ENSXMAG00000021268	0.0026	18	9	pllp	1.43	15
ENSXMAG00000021313	0.0031	21	12	pdlim2	1.47	18
ENSXMAG00000021333	0.0024	3	15	abhd2a	2.44	0
ENSXMAG00000021334	0.0015	21	6		16.14	18
ENSXMAG00000021370	0.0027	9	15	GPT	2.12	6
ENSXMAG00000021498	0.0025	6	15		1.41	3
ENSXMAG00000021509	0.0031	9	12	sult4a1	1.45	6
ENSXMAG00000021526	0.0026	6	15		1.31	3

ENSXMAG00000021530	0.0029	6	12	USP53	1.81	3
ENSXMAG00000021579	0.0015	9	18		1.94	6
ENSXMAG00000021623	0.0031	15	12	marcksb	1.48	12
ENSXMAG00000021624	0.0016	18	6		1.61	15
ENSXMAG00000021649	0.0025	21	9		1.17	18
ENSXMAG00000021729	0.0026	6	15		1.62	3
ENSXMAG00000021739	0.0026	24	9		3.14	21
ENSXMAG00000021795	0.0025	3	9		1.42	0
ENSXMAG00000021818	0.0015	15	18	smug1	1.50	12
ENSXMAG00000021936	0.0031	15	12		1.35	12
ENSXMAG00000022102	0.0015	12	18	pgbd5	1.23	9
ENSXMAG00000022235	0.0031	9	12		6.74	6
ENSXMAG00000022382	0.0014	3	6	prox1b	1.41	0
ENSXMAG00000022421	0.0016	24	6	si:ch73-22o12.1	1.22	21
ENSXMAG00000022484	0.0031	15	12		1.46	12
ENSXMAG00000022506	0.0024	9	15	SLC35G1	2.22	6
ENSXMAG00000022509	0.0026	24	15		2.24	21
ENSXMAG00000022528	0.0024	9	9	oxsr1b	1.16	6
ENSXMAG00000022593	0.0015	9	18	si:ch211-231f6.6	1.87	6
ENSXMAG00000022604	0.0031	15	12		1.16	12
ENSXMAG00000022647	0.0016	6	18	ahcyl1	1.15	3
ENSXMAG00000022652	0.0026	6	15	RNF208	1.42	3
ENSXMAG00000022745	0.0024	12	9		1.44	9
ENSXMAG00000022784	0.0031	15	12	vps26bl	1.04	12
ENSXMAG00000022811	0.0026	24	15	ciarta	20.12	21
ENSXMAG00000022875	0.0026	15	15	r3hdm4	2.07	12
ENSXMAG00000022940	0.0015	21	6		1.47	18
ENSXMAG00000023022	0.0025	18	15		1.28	15
ENSXMAG00000023082	0.0031	15	12		1.64	12
ENSXMAG00000023379	0.0031	24	12		1.64	21
ENSXMAG00000023401	0.0026	15	9	nifk	1.30	12
ENSXMAG00000023420	0.0028	24	9	cbfa2t3	1.49	21
ENSXMAG00000023558	0.0028	12	15	SUOX	1.45	9
ENSXMAG00000023602	0.0025	6	15		16.22	3
ENSXMAG00000023616	0.0031	6	12	rangrf	1.18	3
ENSXMAG00000023656	0.0031	24	12		1.16	21
ENSXMAG00000023706	0.0030	15	12	PFN2	1.15	12
ENSXMAG00000023716	0.0029	9	12	sybu	2.45	6
ENSXMAG00000023781	0.0024	6	15	ugt1a2 (1 of many)	1.97	3
ENSXMAG00000023825	0.0029	9	12		2.24	6
ENSXMAG00000023919	0.0031	15	12		2.38	12
ENSXMAG00000023965	0.0015	9	18	ccl25b	2.37	6
ENSXMAG00000024024	0.0016	9	18	RERG	1.49	6
ENSXMAG00000024113	0.0029	9	12	cyp2n13 (1 of many)	18.39	6
ENSXMAG00000024139	0.0015	24	6	lrfn4b	1.20	21
ENSXMAG00000024156	0.0027	18	15	slc43a3b (1 of many)	1.39	15
ENSXMAG00000024190	0.0025	9	15	hsd11b1la (1 of many)	2.69	6
ENSXMAG00000024264	0.0031	9	12	ognb	1.72	6
ENSXMAG00000024291	0.0026	3	9	nfkbie	1.34	0
ENSXMAG00000024497	0.0026	9	9	HAAO	1.54	6
ENSXMAG00000024523	0.0031	3	12		1.24	0
ENSXMAG00000024565	0.0031	9	12	myct1b	1.69	6
ENSXMAG00000024586	0.0031	15	12	C8orf82	1.71	12
ENSXMAG00000024644	0.0031	21	12		1.63	18
ENSXMAG00000024672	0.0031	15	12	EIF4E3 (1 of many)	1.65	12
ENSXMAG00000024762	0.0024	15	15		1.79	12

ENSXMAG00000024800	0.0028	9	15	mto1	1.35	6
ENSXMAG00000024924	0.0029	6	12	hivep1	2.26	3
ENSXMAG00000024941	0.0024	3	9	maml3	1.38	0
ENSXMAG00000025029	0.0031	18	12		2.39	15
ENSXMAG00000025059	0.0031	18	12	SHROOM2	1.43	15
ENSXMAG00000025194	0.0031	15	12		1.51	12
ENSXMAG00000025219	0.0015	6	18	JDP2 (1 of many)	2.09	3
ENSXMAG00000025321	0.0027	21	9		1.85	18
ENSXMAG00000025371	0.0024	3	15	cipcb	21.45	0
ENSXMAG00000025404	0.0031	12	12	gstr (1 of many)	1.58	9
ENSXMAG00000025444	0.0025	9	15		1.29	6
ENSXMAG00000025460	0.0031	9	12		1.29	6
ENSXMAG00000025504	0.0014	21	18	zbtb2b	1.35	18
ENSXMAG00000025515	0.0024	3	15	bhlhe40	3.31	0
ENSXMAG00000025607	0.0025	3	15		1.61	0
ENSXMAG00000025699	0.0024	3	15	cobl	1.72	0
ENSXMAG00000025761	0.0025	24	9	cep72	1.39	21
ENSXMAG00000025804	0.0031	15	12	cdc42	1.13	12
ENSXMAG00000025866	0.0025	3	15	ADAM11	1.60	0
ENSXMAG00000025868	0.0025	15	15	syt14a	1.31	12
ENSXMAG00000025869	0.0029	15	12	prrt4	1.81	12
ENSXMAG00000025912	0.0024	9	15		1.31	6
ENSXMAG00000025938	0.0026	24	9		1.20	21
ENSXMAG00000026103	0.0024	9	9		1.67	6
ENSXMAG00000026131	0.0031	15	12	si:dkey-40c11.2	1.41	12
ENSXMAG00000026157	0.0014	9	18	cycsb	2.44	6
ENSXMAG00000026160	0.0031	6	12	sgtb	1.46	3
ENSXMAG00000026166	0.0028	6	9	S1PR3	1.66	3
ENSXMAG00000026180	0.0026	9	9	atf7b	1.32	6
ENSXMAG00000026206	0.0031	6	12	tns2a	1.42	3
ENSXMAG00000026233	0.0030	24	12		2.72	21
ENSXMAG00000026242	0.0027	9	15		1.25	6
ENSXMAG00000026308	0.0015	3	6	C17orf58	1.45	0
ENSXMAG00000026323	0.0024	9	15	abhd4	3.56	6
ENSXMAG00000026332	0.0026	15	9	ubtd2	1.33	12
ENSXMAG00000026333	0.0025	3	15	tefb	8.00	0
ENSXMAG00000026334	0.0024	18	15		1.58	15
ENSXMAG00000026442	0.0031	15	12	gnai2b	1.52	12
ENSXMAG00000026564	0.0027	24	9	rnf26	1.25	21
ENSXMAG00000026649	0.0031	6	12	YWHAG	1.14	3
ENSXMAG00000026666	0.0025	15	15	klhdc8a	1.84	12
ENSXMAG00000026676	0.0027	24	9	gli1	1.48	21
ENSXMAG00000026682	0.0026	18	15	picalmb	1.06	15
ENSXMAG00000026745	0.0025	18	15	GLI2	1.46	15
ENSXMAG00000026760	0.0026	3	9		2.16	0
ENSXMAG00000026801	0.0029	15	12	ppp1r14c	2.44	12
ENSXMAG00000026817	0.0024	3	9	CIART	6.89	0
ENSXMAG00000026878	0.0024	3	9	fam214b	1.28	0
ENSXMAG00000026936	0.0026	3	15	tnfaip3	1.16	0
ENSXMAG00000026943	0.0025	3	9		1.38	0
ENSXMAG00000027050	0.0026	9	15	rfk	1.99	6
ENSXMAG00000027105	0.0026	18	15	kank3	1.54	15
ENSXMAG00000027107	0.0016	6	18	jmjd6	1.51	3
ENSXMAG00000027127	0.0024	3	15	strada	1.10	0
ENSXMAG00000027199	0.0031	15	12	dab1b	1.30	12
ENSXMAG00000027468	0.0030	24	12		5.19	21

ENSXMAG00000027519	0.0026	15	9	mmp11a	3.12	12
ENSXMAG00000027554	0.0031	6	12	sftpbb	1.26	3
ENSXMAG00000027615	0.0014	9	18		3.80	6
ENSXMAG00000027656	0.0025	18	9	cdkn1d	5.33	15
ENSXMAG00000027658	0.0031	6	12		2.21	3
ENSXMAG00000027691	0.0024	21	15	zgc:171704	3.11	18
ENSXMAG00000027721	0.0026	21	15		1.31	18
ENSXMAG00000027761	0.0026	6	9		1.19	3
ENSXMAG00000027929	0.0031	3	12		1.22	0
ENSXMAG00000027948	0.0028	12	15	kpna1	1.23	9
ENSXMAG00000027984	0.0026	15	9	ypel3	1.75	12
ENSXMAG00000027995	0.0025	18	9	shc1	1.90	15
ENSXMAG00000028234	0.0025	21	9	xylt1 (1 of many)	1.28	18
ENSXMAG00000028243	0.0031	12	12		1.33	9
ENSXMAG00000028407	0.0024	18	15		1.32	15
ENSXMAG00000028443	0.0029	18	12		10.99	15
ENSXMAG00000028518	0.0025	24	15	sesn1	1.99	21
ENSXMAG00000028575	0.0029	15	12		3.22	12
ENSXMAG00000028727	0.0014	24	18	CACNA2D3	1.37	21
ENSXMAG00000028787	0.0031	24	12	tgfbr3	1.36	21
ENSXMAG00000028941	0.0026	6	9	jun	1.77	3
ENSXMAG00000029002	0.0016	21	6		1.60	18
ENSXMAG00000029049	0.0015	3	6	stk11ip	1.33	0
ENSXMAG00000029074	0.0031	15	12	ppp2r2ca	1.38	12
ENSXMAG00000029112	0.0024	9	9	ncam1b	1.24	6
ENSXMAG00000029128	0.0024	21	15		2.18	18
ENSXMAG00000029130	0.0031	18	12	TMEM74B	2.58	15
ENSXMAG00000029219	0.0034	9	12		1.64	6
ENSXMAG00000029274	0.0025	6	9	COL26A1	1.41	3
ENSXMAG00000029316	0.0014	9	18	recql	1.77	6
ENSXMAG00000029319	0.0026	6	15	FGF6 (1 of many)	1.25	3
ENSXMAG00000029432	0.0025	12	9	RAP1GDS1	1.17	9
ENSXMAG00000029501	0.0031	6	12	GPATCH2L	2.32	3
ENSXMAG00000029564	0.0024	18	15	angpt1	1.33	15
ENSXMAG00000029616	0.0026	9	15		2.00	6
ENSXMAG00000029740	0.0030	15	12	marcksl1a	1.48	12
ENSXMAG00000029757	0.0024	18	9	scfd1	1.24	15
ENSXMAG00000029758	0.0014	21	18		1.26	18
ENSXMAG00000029860	0.0029	18	12	cspg5a	1.58	15
ENSXMAG00000029886	0.0015	9	18	nudt6	2.30	6
ENSXMAG00000029905	0.0016	6	18		1.22	3
ENSXMAG00000029907	0.0031	15	12	nabp1a	1.24	12
ENSXMAG00000029919	0.0031	18	12		1.19	15
ENSXMAG00000029930	0.0030	24	12		1.21	21
ENSXMAG00000030048	0.0030	24	12	cxadr	1.31	21
ENSXMAG00000030101	0.0025	15	15	raph1a	1.59	12

Ovary circadian gene	GeneID	pVal	phase	peak.shape	external_gene_name	amp	Ct.peak
	ENSXMAG00000000055	0.0014	3	6	adgrd1	5.12	0
	ENSXMAG00000000094	0.0026	24	9	mrtfbb	3.69	21
	ENSXMAG00000000107	0.0031	3	12		2.22	0
	ENSXMAG00000000264	0.0024	6	15	ptprt	1.94	3
	ENSXMAG00000000410	0.0026	6	9	atp5mc3b	1.24	3
	ENSXMAG00000000676	0.0031	15	12	ranbp3b	1.14	12
	ENSXMAG00000000861	0.0026	24	9	slc15a2	3.21	21
	ENSXMAG00000001042	0.0031	12	12	sh3yl1	1.11	9

ENSXMAG00000001191	0.0014	3	18	gnb3b	1.61	0
ENSXMAG00000001428	0.0024	3	9	klf9	5.17	0
ENSXMAG00000001722	0.0026	24	9	GPR21 (1 of many)	6.38	21
ENSXMAG00000001789	0.0024	24	9	disp1	1.58	21
ENSXMAG00000001838	0.0071	12	18		1.41	9
ENSXMAG00000002009	0.0026	12	15	PPP2R2A (1 of many)	1.21	9
ENSXMAG00000002019	0.0071	3	18	bnip3la	2.04	0
ENSXMAG00000002032	0.0015	3	18	phka2	1.27	0
ENSXMAG00000002428	0.0024	18	9	skor1a	1.35	15
ENSXMAG00000002607	0.0034	24	12	cpne4b	2.00	21
ENSXMAG00000002619	0.0026	18	9	arid3a	1.62	15
ENSXMAG00000002628	0.0031	9	12	pcolce2b	7.90	6
ENSXMAG00000002632	0.0031	12	12	pebp1	1.42	9
ENSXMAG00000002704	0.0024	9	15	rgma	1.25	6
ENSXMAG00000003083	0.0024	21	9	scd	1.77	18
ENSXMAG00000003096	0.0015	21	6	prlrb	1.36	18
ENSXMAG00000003253	0.0016	3	18	kat2b	1.87	0
ENSXMAG00000003381	0.0025	15	15	srpk3	1.23	12
ENSXMAG00000003386	0.0031	9	12	mycn	2.91	6
ENSXMAG00000003454	0.0071	18	6	si:ch211-193k19.1	1.23	15
ENSXMAG00000003577	0.0025	3	9	slc2a15a	2.18	0
ENSXMAG00000003736	0.0031	15	12	ZNF706	1.24	12
ENSXMAG00000003923	0.0027	21	9	slc2a5	1.38	18
ENSXMAG00000003961	0.0025	3	15	per3	2.60	0
ENSXMAG00000004008	0.0031	12	12	ptcd3	1.25	9
ENSXMAG00000004088	0.0031	24	12	fam69aa	2.09	21
ENSXMAG00000004200	0.0031	12	12	si:ch1073-111c8.3	1.07	9
ENSXMAG00000004343	0.0031	18	12	CACNA2D1 (1 of many)	1.53	15
ENSXMAG00000004655	0.0014	18	6	pfkpa	1.14	15
ENSXMAG00000004661	0.0031	6	12		1.21	3
ENSXMAG00000004827	0.0015	9	18	RNPS1	1.29	6
ENSXMAG00000004915	0.0026	9	15	imp4	1.34	6
ENSXMAG00000004997	0.0030	6	12	kctd17	1.24	3
ENSXMAG00000005060	0.0026	18	9	adssl	1.17	15
ENSXMAG00000005127	0.0031	15	12	nfil3-5	9.32	12
ENSXMAG00000005231	0.0024	6	15	kyat3	1.91	3
ENSXMAG00000005276	0.0025	15	15	aldh1l2	1.15	12
ENSXMAG00000005325	0.0026	9	9	kcnt1	4.42	6
ENSXMAG00000005650	0.0016	18	6	hoga1	1.24	15
ENSXMAG00000005883	0.0027	15	9	spns3	1.23	12
ENSXMAG00000005992	0.0014	9	18	eef1e1	1.17	6
ENSXMAG00000006108	0.0025	15	15	si:dkey-13p1.4	1.61	12
ENSXMAG00000006318	0.0026	15	9	setd7 (1 of many)	1.17	12
ENSXMAG00000006643	0.0014	15	18	arrb2b	1.13	12
ENSXMAG00000006645	0.0026	6	15	nol11	1.38	3
ENSXMAG00000006651	0.0025	15	9		1.60	12
ENSXMAG00000006665	0.0028	24	9		1.74	21
ENSXMAG00000006758	0.0027	21	9	nox5	1.61	18
ENSXMAG00000006899	0.0025	24	9	slc13a5a	5.32	21
ENSXMAG00000006945	0.0027	9	9	rab18b	1.22	6
ENSXMAG00000007078	0.0028	9	15	ebag9	1.19	6
ENSXMAG00000007139	0.0026	24	9	f5	38.86	21
ENSXMAG00000007367	0.0015	12	18	si:ch211-219a4.3 (1 of many)	1.12	9
ENSXMAG00000007563	0.0026	24	9	mettl24	3.22	21
ENSXMAG00000007882	0.0024	12	15		1.41	9
ENSXMAG00000007930	0.0024	9	9	lgi1b	3.28	6

ENSXMAG00000008113	0.0026	9	9	WNT2B	5.89	6
ENSXMAG00000008243	0.0031	21	12		3.27	18
ENSXMAG00000008446	0.0025	9	9	evlb	3.00	6
ENSXMAG00000008513	0.0031	12	12		1.60	9
ENSXMAG00000008516	0.0031	24	12	cxcl14	2.63	21
ENSXMAG00000008604	0.0028	9	15		1.14	6
ENSXMAG00000008793	0.0015	12	6	pxk	1.12	9
ENSXMAG00000009053	0.0026	3	9	mbpb	1.47	0
ENSXMAG00000009491	0.0026	24	15	ift52	1.36	21
ENSXMAG00000010046	0.0031	3	12	robo2	1.57	0
ENSXMAG00000010058	0.0031	9	12	anxa6	2.83	6
ENSXMAG00000010104	0.0026	9	15	adamts12	1.23	6
ENSXMAG00000010204	0.0026	9	9	aclyb	1.99	6
ENSXMAG00000010322	0.0026	15	9	cers5	1.18	12
ENSXMAG00000010505	0.0027	12	15	lonrf1	1.31	9
ENSXMAG00000010626	0.0034	12	12	stmn1b	1.76	9
ENSXMAG00000010765	0.0024	6	15	trmt13	1.13	3
ENSXMAG00000010829	0.0030	15	12		1.30	12
ENSXMAG00000011486	0.0016	12	18	nlgn3b	1.66	9
ENSXMAG00000011663	0.0016	3	6	slc6a16a	1.31	0
ENSXMAG00000011881	0.0031	9	12	mpzl2b	2.25	6
ENSXMAG00000012054	0.0026	24	15	dbpb	13.40	21
ENSXMAG00000012064	0.0026	24	15	josd2	1.74	21
ENSXMAG00000012113	0.0031	9	12		2.28	6
ENSXMAG00000012118	0.0031	24	12	chmp1b	1.30	21
ENSXMAG00000012259	0.0026	21	9		1.16	18
ENSXMAG00000012287	0.0029	12	12		1.23	9
ENSXMAG00000012356	0.0031	24	12	agmat	1.71	21
ENSXMAG00000012369	0.0026	21	15	SPHK1 (1 of many)	20.17	18
ENSXMAG00000012493	0.0015	3	6		1.45	0
ENSXMAG00000012551	0.0031	3	12	irs2b	2.57	0
ENSXMAG00000012661	0.0029	15	12	crtac1a	1.47	12
ENSXMAG00000012687	0.0016	9	18	brpf3b	1.15	6
ENSXMAG00000012836	0.0028	3	9		1.16	0
ENSXMAG00000013205	0.0034	15	12	faah	1.19	12
ENSXMAG00000013401	0.0024	9	15	zic6	1.40	6
ENSXMAG00000013456	0.0025	21	15		1.44	18
ENSXMAG00000013484	0.0031	3	12	paqr6	2.64	0
ENSXMAG00000013617	0.0026	12	15		1.21	9
ENSXMAG00000013641	0.0025	18	15	cnksr1	1.26	15
ENSXMAG00000013896	0.0071	3	18	tax1bp1b	1.22	0
ENSXMAG00000014132	0.0025	18	15	soul4	1.38	15
ENSXMAG00000014150	0.0030	12	12	ogfod1	1.29	9
ENSXMAG00000014376	0.0016	21	18	serpind1	18.49	18
ENSXMAG00000014539	0.0024	6	15	col4a6	1.96	3
ENSXMAG00000014595	0.0031	6	12	mrpl40	1.12	3
ENSXMAG00000014598	0.0025	18	15	fasn	1.46	15
ENSXMAG00000014667	0.0025	9	9	COL4A5	2.88	6
ENSXMAG00000014672	0.0034	9	12		4.60	6
ENSXMAG00000014714	0.0071	18	6	ABCA1 (1 of many)	1.19	15
ENSXMAG00000014729	0.0024	21	9	gc2	1.45	18
ENSXMAG00000014737	0.0031	21	12	ttll5	1.49	18
ENSXMAG00000014761	0.0031	24	12	acsf2	1.34	21
ENSXMAG00000014783	0.0016	12	6	parn	1.14	9
ENSXMAG00000014799	0.0071	3	18		2.01	0
ENSXMAG00000014865	0.0015	15	18	pygl	1.38	12

ENSXMAG00000014915	0.0071	15	18		1.32	12
ENSXMAG00000015415	0.0031	9	12	NTN4	2.59	6
ENSXMAG00000015416	0.0027	3	9	fkbp5	3.68	0
ENSXMAG00000015499	0.0024	6	9	fam114a2	1.14	3
ENSXMAG00000015573	0.0071	9	18	elovl7a	1.22	6
ENSXMAG00000015596	0.0026	15	15	slc16a13	1.93	12
ENSXMAG00000015722	0.0031	18	12	herpud1	1.31	15
ENSXMAG00000015826	0.0016	18	6	aff2	1.59	15
ENSXMAG00000015860	0.0024	18	9	tmem62	1.18	15
ENSXMAG00000015883	0.0024	12	15	stra6	1.29	9
ENSXMAG00000016127	0.0031	24	12	ptger4a	2.74	21
ENSXMAG00000016181	0.0016	18	6		1.95	15
ENSXMAG00000016565	0.0027	12	15	si:ch211-106h4.4	1.38	9
ENSXMAG00000016726	0.0026	9	15		1.50	6
ENSXMAG00000016914	0.0026	18	15	oxsm	1.22	15
ENSXMAG00000016920	0.0071	18	6	mpp3b	1.87	15
ENSXMAG00000016928	0.0026	15	9	clocka	1.39	12
ENSXMAG00000016948	0.0016	15	18		1.23	12
ENSXMAG00000017142	0.0030	15	12		1.47	12
ENSXMAG00000017193	0.0027	18	9	TMEM266 (1 of many)	2.82	15
ENSXMAG00000017652	0.0026	18	9		1.09	15
ENSXMAG00000017816	0.0016	3	6	aig1	1.46	0
ENSXMAG00000017961	0.0031	6	12	atad1a	1.35	3
ENSXMAG00000018304	0.0026	9	15	TADA1	1.12	6
ENSXMAG00000018758	0.0026	15	9	ythdf2	1.10	12
ENSXMAG00000018992	0.0034	18	12	nlrc3	1.20	15
ENSXMAG00000019107	0.0014	18	6	si:ch1073-440b2.1	1.25	15
ENSXMAG00000019226	0.0024	6	15	TKT (1 of many)	2.00	3
ENSXMAG00000019285	0.0028	3	15	slc41a2a	1.92	0
ENSXMAG00000019296	0.0031	15	12	rorcb	5.40	12
ENSXMAG00000019943	0.0024	18	9	nfil3	1.75	15
ENSXMAG00000020097	0.0031	18	12		1.66	15
ENSXMAG00000020143	0.0031	18	12		1.77	15
ENSXMAG00000020156	0.0026	9	9	slc35g2b	7.28	6
ENSXMAG00000020315	0.0026	3	9	CHST1	1.59	0
ENSXMAG00000020882	0.0026	9	15		1.18	6
ENSXMAG00000020991	0.0025	18	9	spsb3a	1.15	15
ENSXMAG00000021029	0.0014	18	6	e2f3	1.32	15
ENSXMAG00000021164	0.0030	9	12	pid1	1.29	6
ENSXMAG00000021439	0.0071	3	6	gdnfa	6.20	0
ENSXMAG00000021560	0.0015	15	18		1.30	12
ENSXMAG00000021752	0.0026	9	15	pcbp2	1.10	6
ENSXMAG00000022003	0.0029	9	12	pdgfaa	4.12	6
ENSXMAG00000022282	0.0026	21	9		1.24	18
ENSXMAG00000022442	0.0025	24	9		1.55	21
ENSXMAG00000022710	0.0015	9	18	tm2d3	1.20	6
ENSXMAG00000022915	0.0016	6	6	pcdh10a	2.61	3
ENSXMAG00000022920	0.0027	18	9		1.16	15
ENSXMAG00000023092	0.0026	24	15		1.78	21
ENSXMAG00000023103	0.0071	3	18	HIST1H4G	2.26	0
ENSXMAG00000023164	0.0031	24	12	ppm1lb	1.33	21
ENSXMAG00000023180	0.0025	24	15	si:ch73-248e21.1	3.16	21
ENSXMAG00000023654	0.0031	24	12		2.54	21
ENSXMAG00000023883	0.0014	9	18	ppp1r2	1.20	6
ENSXMAG00000024165	0.0026	15	9		1.40	12
ENSXMAG00000024399	0.0026	15	9		1.23	12

	ENSXMAG00000024628	0.0034	6	12	CAMKMT	1.34	3
	ENSXMAG00000024816	0.0027	15	15		1.19	12
	ENSXMAG00000024891	0.0031	9	12	ndp	5.39	6
	ENSXMAG00000025265	0.0016	12	18	GABRR1	1.33	9
	ENSXMAG00000025515	0.0027	3	15	bhlhe40	2.42	0
	ENSXMAG00000025690	0.0031	18	12		1.31	15
	ENSXMAG00000025714	0.0029	9	12	scn4bb	4.08	6
	ENSXMAG00000025803	0.0027	9	15	hprt1	1.28	6
	ENSXMAG00000025834	0.0026	9	15	ccnk	1.18	6
	ENSXMAG00000025839	0.0024	15	9		1.25	12
	ENSXMAG00000025978	0.0034	9	12		2.65	6
	ENSXMAG00000026103	0.0024	18	9		1.23	15
	ENSXMAG00000026145	0.0026	21	15		1.95	18
	ENSXMAG00000026215	0.0024	24	15		2.09	21
	ENSXMAG00000026233	0.0026	24	15		1.98	21
	ENSXMAG00000026259	0.0024	24	9	TMEM229A	3.35	21
	ENSXMAG00000026340	0.0031	15	12		1.20	12
	ENSXMAG00000026522	0.0024	9	15	adprm	1.29	6
	ENSXMAG00000026561	0.0015	18	6		7.72	15
	ENSXMAG00000026755	0.0027	12	9	znf346	1.30	9
	ENSXMAG00000026905	0.0030	6	12	CDH20	2.96	3
	ENSXMAG00000026940	0.0026	3	9		1.76	0
	ENSXMAG00000026979	0.0071	3	18		3.25	0
	ENSXMAG00000027026	0.0031	9	12		2.26	6
	ENSXMAG00000027173	0.0024	9	9	si:ch211-219a15.3	10.32	6
	ENSXMAG00000027263	0.0016	18	6	zmp:0000000760	1.61	15
	ENSXMAG00000027288	0.0027	3	9	zfhx3	1.35	0
	ENSXMAG00000027438	0.0015	9	18		1.37	6
	ENSXMAG00000027468	0.0031	3	12		11.96	0
	ENSXMAG00000027546	0.0026	21	9	atp1b1b	1.19	18
	ENSXMAG00000027592	0.0030	12	12	spag6 (1 of many)	1.64	9
	ENSXMAG00000027656	0.0031	18	12	cdkn1d	6.85	15
	ENSXMAG00000027669	0.0031	15	12	mthfd1l	1.21	12
	ENSXMAG00000027757	0.0025	21	15	gk5	1.67	18
	ENSXMAG00000028033	0.0025	3	9		1.58	0
	ENSXMAG00000028406	0.0024	12	15	b4galnt4a	1.16	9
	ENSXMAG00000028575	0.0024	18	9		2.99	15
	ENSXMAG00000028590	0.0028	9	15	gpt2l	1.22	6
	ENSXMAG00000028781	0.0025	18	9	si:ch211-180a12.2	1.48	15
	ENSXMAG00000028827	0.0025	9	9	wfdc1	2.92	6
	ENSXMAG00000028856	0.0026	18	9		1.10	15
	ENSXMAG00000028971	0.0027	12	15	castor2	1.21	9
	ENSXMAG00000028978	0.0025	15	9		1.20	12
	ENSXMAG00000029488	0.0031	18	12	susd5	4.55	15
	ENSXMAG00000029675	0.0031	3	12		1.13	0
	ENSXMAG00000029888	0.0031	24	12	PRKAB2	1.22	21
	ENSXMAG00000029906	0.0026	15	15		1.25	12
	ENSXMAG00000029946	0.0025	9	9	sirt5	1.24	6

Heart circadian gene	GeneID	pVal	phase	peak.shape	external_gene_name	amp	Ct.peak
	ENSXMAG00000000011	0.0031	6	12	si:ch73-281f12.4	3.48	3
	ENSXMAG00000000113	0.0025	9	9	cdip1	1.90	6
	ENSXMAG00000000148	0.0034	6	12	tex2l	5.33	3
	ENSXMAG00000000211	0.0026	6	15	oxsr1a	38.28	3
	ENSXMAG00000000216	0.0025	15	15	nubp1	2.76	12
	ENSXMAG00000000221	0.0031	18	12	emp2	4.74	15

ENSXMAG00000000222	0.0030	6	12	grin2aa	12.90	3
ENSXMAG00000000252	0.0026	6	15	si:ch211-221f10.2	128.84	3
ENSXMAG00000000294	0.0031	6	12	CPEB4 (1 of many)	29.86	3
ENSXMAG00000000313	0.0031	18	12	hgh1	2.03	15
ENSXMAG00000000322	0.0024	15	15		2.98	12
ENSXMAG00000000384	0.0024	15	15	sssca1	1.51	12
ENSXMAG00000000406	0.0015	12	18	ppp3ccb	2.12	9
ENSXMAG00000000409	0.0026	15	15	NUP35	1.97	12
ENSXMAG00000000442	0.0030	9	12	zgc:110239	2.27	6
ENSXMAG00000000505	0.0071	9	18	timm50	2.10	6
ENSXMAG00000000586	0.0026	6	9		8.64	3
ENSXMAG00000000726	0.0031	18	12		1.89	15
ENSXMAG00000000849	0.0025	15	15	hdac3	1.76	12
ENSXMAG00000000874	0.0026	15	15	unc45a	2.25	12
ENSXMAG00000000946	0.0026	12	15		1.94	9
ENSXMAG00000000960	0.0024	21	15	si:ch211-186j3.6	3.96	18
ENSXMAG00000000983	0.0028	15	15	errfi1a	5.72	12
ENSXMAG00000001095	0.0014	15	6	ltb4r2a	5.89	12
ENSXMAG00000001163	0.0027	6	9	tpi1a	3.57	3
ENSXMAG00000001169	0.0027	21	9	tnfrsf19	4.32	18
ENSXMAG00000001297	0.0015	3	6	gdf3	3.71	0
ENSXMAG00000001387	0.0025	12	15	sestd1	7.04	9
ENSXMAG00000001522	0.0024	6	15	NR3C1	10.28	3
ENSXMAG00000001555	0.0025	6	15		776.54	3
ENSXMAG00000001572	0.0026	6	9	MME (1 of many)	739.82	3
ENSXMAG00000001608	0.0015	6	18	hspa4a	7.83	3
ENSXMAG00000001666	0.0031	18	12	gmpr2	2.62	15
ENSXMAG00000001694	0.0027	3	15		3.84	0
ENSXMAG00000001703	0.0034	6	12		14.42	3
ENSXMAG00000001707	0.0031	6	12	dyrk1ab	7.56	3
ENSXMAG00000001709	0.0016	12	18	ccni2	4.34	9
ENSXMAG00000001778	0.0025	15	9	abce1	2.57	12
ENSXMAG00000001885	0.0030	18	12	dbnlb	1.58	15
ENSXMAG00000001995	0.0026	15	9	psmd6	1.56	12
ENSXMAG00000002028	0.0031	6	12	ccnt2a	3.63	3
ENSXMAG00000002029	0.0030	18	12	b4galnt1b	5.72	15
ENSXMAG00000002092	0.0024	15	15		3.91	12
ENSXMAG00000002171	0.0031	18	12	alg6	3.13	15
ENSXMAG00000002275	0.0026	21	9	asb5a	21.84	18
ENSXMAG00000002300	0.0031	6	12		467.23	3
ENSXMAG00000002339	0.0024	24	9	nr1d4b	104.77	21
ENSXMAG00000002379	0.0071	9	18		2.39	6
ENSXMAG00000002473	0.0029	6	12	chfr	40.58	3
ENSXMAG00000002481	0.0025	21	9		6.67	18
ENSXMAG00000002514	0.0024	21	9		4.29	18
ENSXMAG00000002624	0.0030	18	12	pex11g	3.47	15
ENSXMAG00000002625	0.0016	12	18		96.53	9
ENSXMAG00000002661	0.0015	12	18	stt3b	1.81	9
ENSXMAG00000002739	0.0024	6	15		44.32	3
ENSXMAG00000002759	0.0031	18	12		2.12	15
ENSXMAG00000002760	0.0014	6	18	snrka	59.94	3
ENSXMAG00000002762	0.0024	9	15	si:ch211-251j10.3	1.88	6
ENSXMAG00000002778	0.0031	18	12		31.59	15
ENSXMAG00000002785	0.0016	12	18	prrg2	3.34	9
ENSXMAG00000002827	0.0071	6	18	xpot	29.58	3
ENSXMAG00000002829	0.0030	18	12	ube2z	1.46	15

ENSXMAG00000002891	0.0034	6	12	fam102aa	12.21	3
ENSXMAG00000002959	0.0015	18	6	ywhaqb	2.31	15
ENSXMAG00000002994	0.0031	6	12		38.08	3
ENSXMAG00000003010	0.0026	6	15	abcb6a	47.41	3
ENSXMAG00000003067	0.0015	24	18	mthfsd	3.69	21
ENSXMAG00000003111	0.0025	18	9		2.10	15
ENSXMAG00000003116	0.0031	18	12	scp2b	3.73	15
ENSXMAG00000003160	0.0031	18	12	fkbp9	5.58	15
ENSXMAG00000003170	0.0024	6	15	diaph2	12.47	3
ENSXMAG00000003192	0.0026	6	15		186.28	3
ENSXMAG00000003244	0.0031	18	12	rabl3	2.52	15
ENSXMAG00000003259	0.0027	18	9	calr	3.03	15
ENSXMAG00000003266	0.0031	12	12	rab5c	1.42	9
ENSXMAG00000003361	0.0031	18	12	fbxl22	8.12	15
ENSXMAG00000003396	0.0031	6	12	efhc1	668.52	3
ENSXMAG00000003430	0.0026	9	9	csnk1db	2.06	6
ENSXMAG00000003437	0.0031	6	12		3.39	3
ENSXMAG00000003466	0.0026	12	15	abcd1	2.09	9
ENSXMAG00000003487	0.0029	6	12		5.97	3
ENSXMAG00000003505	0.0029	18	12	hdgfl2	2.13	15
ENSXMAG00000003519	0.0016	6	18		7.68	3
ENSXMAG00000003573	0.0071	6	18	fam57a	28.84	3
ENSXMAG00000003589	0.0029	6	12	chmp7	3.62	3
ENSXMAG00000003692	0.0026	6	9		754.23	3
ENSXMAG00000003820	0.0026	6	15		5.23	3
ENSXMAG00000003898	0.0031	6	12		9.17	3
ENSXMAG00000003909	0.0026	6	15		18.05	3
ENSXMAG00000003975	0.0026	15	15	MTFMT	2.47	12
ENSXMAG00000004014	0.0031	15	12	rab35b	1.52	12
ENSXMAG00000004061	0.0025	21	9	miox	5.24	18
ENSXMAG00000004069	0.0016	6	18		1.75	3
ENSXMAG00000004148	0.0031	15	12		3.52	12
ENSXMAG00000004151	0.0025	6	15	dop1b	7.73	3
ENSXMAG00000004229	0.0026	12	15	tm9sf4	1.37	9
ENSXMAG00000004255	0.0015	21	6		3.43	18
ENSXMAG00000004264	0.0071	15	6		3.74	12
ENSXMAG00000004265	0.0031	6	12	galnt8b.1	7.94	3
ENSXMAG00000004281	0.0024	6	15	lca5	227.49	3
ENSXMAG00000004307	0.0071	6	18	kpnb3	3.55	3
ENSXMAG00000004332	0.0026	6	15	cnot10	2.39	3
ENSXMAG00000004391	0.0031	18	12	cpne3 (1 of many)	2.56	15
ENSXMAG00000004423	0.0029	6	12		3.31	3
ENSXMAG00000004439	0.0031	6	12	WFS1 (1 of many)	5.26	3
ENSXMAG00000004445	0.0031	18	12	tmed2	1.78	15
ENSXMAG00000004576	0.0026	6	9		544.03	3
ENSXMAG00000004589	0.0025	9	15	gcat	2.39	6
ENSXMAG00000004594	0.0016	24	6	idi1	3.45	21
ENSXMAG00000004648	0.0031	6	12	KDM7A	18.82	3
ENSXMAG00000004706	0.0031	9	12		1.69	6
ENSXMAG00000004737	0.0026	6	15	herpud2	6.15	3
ENSXMAG00000004759	0.0031	18	12	zgpat	1.51	15
ENSXMAG00000004827	0.0031	15	12	RNPS1	1.70	12
ENSXMAG00000004838	0.0029	24	12		3.15	21
ENSXMAG00000004839	0.0015	3	6		3.30	0
ENSXMAG00000004842	0.0014	12	18	ap3m2	1.80	9
ENSXMAG00000004875	0.0026	6	15	phip	1.96	3

ENSXMAG00000004902	0.0024	6	15	ubr3	4.01	3
ENSXMAG00000004971	0.0031	6	12		5.99	3
ENSXMAG00000004984	0.0026	21	9		1.95	18
ENSXMAG00000004993	0.0029	9	12	pigo	2.32	6
ENSXMAG00000005041	0.0071	6	18	ugcg	6.54	3
ENSXMAG00000005092	0.0034	15	12	wdr36	1.77	12
ENSXMAG00000005127	0.0024	12	15	nfil3-5	106.64	9
ENSXMAG00000005150	0.0031	18	12	gdi2	2.71	15
ENSXMAG00000005213	0.0031	6	12	ppp1r9ala	9.26	3
ENSXMAG00000005214	0.0025	6	15	fbxl5	15.52	3
ENSXMAG00000005253	0.0071	6	18	dnajc10	10.82	3
ENSXMAG00000005254	0.0031	6	12	dgkg	11.06	3
ENSXMAG00000005317	0.0026	6	9		742.20	3
ENSXMAG00000005401	0.0026	6	15		74.27	3
ENSXMAG00000005409	0.0027	6	15	gpr155a	11.89	3
ENSXMAG00000005437	0.0026	6	9		617.10	3
ENSXMAG00000005462	0.0071	6	18	copa	4.54	3
ENSXMAG00000005479	0.0029	12	12		2.18	9
ENSXMAG00000005497	0.0025	6	15	cdc14ab	22.37	3
ENSXMAG00000005516	0.0024	6	15	igf2r	3.71	3
ENSXMAG00000005570	0.0026	6	15	cldnd1b	1.76	3
ENSXMAG00000005571	0.0027	12	15	mdh1ab	3.38	9
ENSXMAG00000005635	0.0031	6	12		7.48	3
ENSXMAG00000005647	0.0034	18	12	ufsp2	1.88	15
ENSXMAG00000005675	0.0024	6	9	cadm3	4.10	3
ENSXMAG00000005681	0.0031	9	12	SLC25A28 (1 of many)	4.11	6
ENSXMAG00000005775	0.0024	3	15	ulk2	4.11	0
ENSXMAG00000005778	0.0027	21	9	spout1	1.50	18
ENSXMAG00000005799	0.0015	24	6	mpz	4.86	21
ENSXMAG00000005914	0.0026	15	15	ncl	1.42	12
ENSXMAG00000005936	0.0026	18	9		2.64	15
ENSXMAG00000005972	0.0026	6	15		11.06	3
ENSXMAG00000006078	0.0025	6	15	si:zfos-943e10.1	6.27	3
ENSXMAG00000006169	0.0031	6	12	hadhb	1.49	3
ENSXMAG00000006201	0.0031	6	12	pank4	28.85	3
ENSXMAG00000006300	0.0031	9	12	OSBPL8	2.28	6
ENSXMAG00000006360	0.0014	6	18	GLIPR1	7.07	3
ENSXMAG00000006362	0.0025	6	15		2.32	3
ENSXMAG00000006407	0.0015	15	6		5.50	12
ENSXMAG00000006408	0.0029	18	12	zbtb8os	3.06	15
ENSXMAG00000006422	0.0031	6	12	zc3h3	28.99	3
ENSXMAG00000006426	0.0025	6	9	zgc:92873	4.38	3
ENSXMAG00000006433	0.0015	6	18		3.11	3
ENSXMAG00000006493	0.0014	24	18		3.80	21
ENSXMAG00000006505	0.0026	6	9		792.85	3
ENSXMAG00000006539	0.0031	18	12		3.95	15
ENSXMAG00000006646	0.0031	6	12	sycp3	106.07	3
ENSXMAG00000006651	0.0025	9	15		36.30	6
ENSXMAG00000006963	0.0031	6	12	efhc2	505.39	3
ENSXMAG00000007039	0.0026	15	15	psmd4b	1.33	12
ENSXMAG00000007124	0.0027	18	9	eps8l3a	1.92	15
ENSXMAG00000007141	0.0024	9	15	ago3a	4.51	6
ENSXMAG00000007188	0.0031	6	12		10.74	3
ENSXMAG00000007230	0.0034	18	12		6.87	15
ENSXMAG00000007263	0.0031	6	12	slc25a24l	5.61	3
ENSXMAG00000007321	0.0026	6	9	abhd11	3.11	3

ENSXMAG00000007326	0.0026	21	9	cadm2b	11.60	18
ENSXMAG00000007360	0.0030	6	12		140.97	3
ENSXMAG00000007407	0.0014	24	18		2.24	21
ENSXMAG00000007410	0.0026	6	9	cfap52 (1 of many)	729.93	3
ENSXMAG00000007445	0.0015	6	18	klf8	9.61	3
ENSXMAG00000007455	0.0028	15	15	epcam	11.40	12
ENSXMAG00000007470	0.0015	24	18		12.17	21
ENSXMAG00000007613	0.0031	6	12		15.06	3
ENSXMAG00000007880	0.0024	6	15	sgpl1	5.84	3
ENSXMAG00000007950	0.0024	9	15		2.18	6
ENSXMAG00000007959	0.0034	15	12		1.95	12
ENSXMAG00000007961	0.0025	6	15	men1	2.12	3
ENSXMAG00000008035	0.0025	6	15	uchl1	450.29	3
ENSXMAG00000008036	0.0031	6	12		14.87	3
ENSXMAG00000008063	0.0026	6	15	gstt2	3.32	3
ENSXMAG00000008074	0.0031	18	12	arpc2	2.11	15
ENSXMAG00000008085	0.0031	18	12	ttl	3.71	15
ENSXMAG00000008129	0.0025	21	9	fkbpl	3.56	18
ENSXMAG00000008139	0.0025	6	15	cbsa	4.89	3
ENSXMAG00000008149	0.0015	18	6	eed	2.12	15
ENSXMAG00000008165	0.0030	18	12	csrp1a	3.78	15
ENSXMAG00000008320	0.0034	9	12	pdss1	2.33	6
ENSXMAG00000008341	0.0031	6	12	bptf	14.72	3
ENSXMAG00000008356	0.0024	6	9	spock1	5.34	3
ENSXMAG00000008362	0.0031	6	12		4.09	3
ENSXMAG00000008368	0.0026	6	15	snx19b	3.34	3
ENSXMAG00000008370	0.0014	24	6	ncam3	10.32	21
ENSXMAG00000008381	0.0024	6	15	zbtb44	4.36	3
ENSXMAG00000008481	0.0031	6	12	PLCB4	4.22	3
ENSXMAG00000008485	0.0015	18	6	prpf19	2.10	15
ENSXMAG00000008515	0.0031	6	12	znf652	5.13	3
ENSXMAG00000008567	0.0034	6	12	braf	3.21	3
ENSXMAG00000008690	0.0031	6	12	casc1	586.42	3
ENSXMAG00000008797	0.0031	6	12	cbl	11.84	3
ENSXMAG00000008815	0.0025	6	15	orai2	9.55	3
ENSXMAG00000008820	0.0031	6	12		172.19	3
ENSXMAG00000008826	0.0031	15	12	ssr1	1.60	12
ENSXMAG00000008828	0.0024	6	9		571.32	3
ENSXMAG00000008911	0.0026	6	15		3.62	3
ENSXMAG00000008941	0.0024	6	15	negr1	9.40	3
ENSXMAG00000008976	0.0034	18	12	ctsk	19.03	15
ENSXMAG00000009062	0.0031	6	12		3.42	3
ENSXMAG00000009147	0.0026	6	9	nol4lb	39.26	3
ENSXMAG00000009190	0.0031	9	12	dnajc5ab	2.20	6
ENSXMAG00000009207	0.0031	6	12	nfat5a	6.17	3
ENSXMAG00000009278	0.0026	9	15	cry1ba	16.90	6
ENSXMAG00000009312	0.0026	6	15	dlec1	77.12	3
ENSXMAG00000009346	0.0026	15	15		3.87	12
ENSXMAG00000009359	0.0034	18	12	zcrb1	2.42	15
ENSXMAG00000009373	0.0031	18	12	nckap5l	1.58	15
ENSXMAG00000009465	0.0026	9	15	rhogc (1 of many)	2.18	6
ENSXMAG00000009482	0.0027	12	15	grm8a	1.71	9
ENSXMAG00000009503	0.0034	6	12	mapk8b	15.39	3
ENSXMAG00000009527	0.0031	18	12	igfbp6b	6.90	15
ENSXMAG00000009602	0.0031	18	12	ugdh	2.34	15
ENSXMAG00000009617	0.0016	24	6	ACTC1	27.08	21

ENSXMAG00000009657	0.0024	9	9	pparab	6.23	6
ENSXMAG00000009769	0.0027	9	15		5.68	6
ENSXMAG00000009784	0.0026	12	15	cpsf3	1.39	9
ENSXMAG00000009804	0.0026	15	15	aph1b	1.67	12
ENSXMAG00000009877	0.0025	6	15	rbpjb	4.97	3
ENSXMAG00000009898	0.0027	9	9	dido1	1.71	6
ENSXMAG00000009915	0.0031	18	12	tmem134	3.02	15
ENSXMAG00000009971	0.0031	6	12		723.89	3
ENSXMAG00000009974	0.0031	9	12	lonrf1l	12.70	6
ENSXMAG00000009984	0.0025	9	9		2.06	6
ENSXMAG00000010055	0.0071	9	18	ATP6V1A (1 of many)	2.79	6
ENSXMAG00000010056	0.0031	18	12	rac2	5.81	15
ENSXMAG00000010057	0.0031	6	12	wdfy3	12.59	3
ENSXMAG00000010102	0.0071	6	18	dctn4	6.37	3
ENSXMAG00000010118	0.0031	6	12	tspan17	2.10	3
ENSXMAG00000010142	0.0016	18	6	paip1	1.78	15
ENSXMAG00000010152	0.0024	6	15	slc6a5	4.63	3
ENSXMAG00000010172	0.0026	15	15	pla2g12a	1.61	12
ENSXMAG00000010188	0.0015	24	6	fsta	6.07	21
ENSXMAG00000010205	0.0026	24	9	lef1	4.04	21
ENSXMAG00000010227	0.0026	6	15	hadh	3.39	3
ENSXMAG00000010237	0.0025	6	15	e2f8	12.37	3
ENSXMAG00000010261	0.0015	6	18	elovl5	35.02	3
ENSXMAG00000010324	0.0016	18	6	slc22a18	2.23	15
ENSXMAG00000010331	0.0025	6	15	mcph1	28.31	3
ENSXMAG00000010342	0.0027	18	9	dnajb11	2.57	15
ENSXMAG00000010418	0.0031	12	12	ahr1b	4.08	9
ENSXMAG00000010482	0.0027	9	9	shank3b	2.82	6
ENSXMAG00000010534	0.0026	21	9	suclg2	2.44	18
ENSXMAG00000010583	0.0025	6	15	rnf25	7.64	3
ENSXMAG00000010641	0.0031	9	12	pitpnab	5.44	6
ENSXMAG00000010642	0.0026	6	9		445.77	3
ENSXMAG00000010671	0.0014	12	18	dnajc3b	1.99	9
ENSXMAG00000010676	0.0026	6	9		731.61	3
ENSXMAG00000010695	0.0026	15	9	alg9	1.89	12
ENSXMAG00000010743	0.0026	6	15	nadk2	5.48	3
ENSXMAG00000010758	0.0026	9	9	aldocb	1.82	6
ENSXMAG00000010775	0.0027	18	9	pigs	1.92	15
ENSXMAG00000010814	0.0026	6	15	znf526	2.21	3
ENSXMAG00000010816	0.0025	6	15	erfl3	37.04	3
ENSXMAG00000010827	0.0029	6	12	dedd1	9.19	3
ENSXMAG00000010894	0.0014	12	18	cyp46a1.4	2.87	9
ENSXMAG00000010981	0.0025	6	15	pcnx3	64.91	3
ENSXMAG00000011013	0.0030	18	12	cnpy3	3.84	15
ENSXMAG00000011082	0.0034	18	12	crym	2.56	15
ENSXMAG00000011225	0.0029	6	12	arhgap39	9.35	3
ENSXMAG00000011234	0.0031	18	12		5.92	15
ENSXMAG00000011299	0.0025	6	15		546.05	3
ENSXMAG00000011326	0.0034	6	12	uckl1a	37.44	3
ENSXMAG00000011356	0.0071	9	18	pgd	2.96	6
ENSXMAG00000011408	0.0071	6	18	fam20b	2.85	3
ENSXMAG00000011475	0.0031	6	12	mgat4a	2.58	3
ENSXMAG00000011604	0.0031	6	12	THOC2	2.21	3
ENSXMAG00000011609	0.0029	18	12	ecrg4b	3.64	15
ENSXMAG00000011684	0.0030	6	12	letmd1	7.62	3
ENSXMAG00000011810	0.0025	6	15	slc2a3b	265.65	3

ENSXMAG00000011910	0.0031	6	12	armc4	264.33	3
ENSXMAG00000011935	0.0029	6	12	mink1	2.20	3
ENSXMAG00000012040	0.0031	18	12	gnb2	2.68	15
ENSXMAG00000012054	0.0027	24	15	dbpb	106.81	21
ENSXMAG00000012074	0.0030	18	12	sh3bgrl3	3.84	15
ENSXMAG00000012127	0.0034	6	12	brwd3	60.49	3
ENSXMAG00000012204	0.0016	18	6	manf	3.93	15
ENSXMAG00000012210	0.0025	6	15	cep104	2.86	3
ENSXMAG00000012244	0.0031	6	12	nf1a	4.65	3
ENSXMAG00000012252	0.0024	15	15	PMM2	1.67	12
ENSXMAG00000012259	0.0031	6	12		21.47	3
ENSXMAG00000012261	0.0028	3	9	borcs8	1.43	0
ENSXMAG00000012265	0.0026	21	9	tmem161a	2.55	18
ENSXMAG00000012272	0.0031	18	12	prpf31	1.44	15
ENSXMAG00000012411	0.0027	18	9	ddost	2.22	15
ENSXMAG00000012455	0.0014	18	6	cptp	2.37	15
ENSXMAG00000012520	0.0015	18	6	pdap1a	2.18	15
ENSXMAG00000012537	0.0016	18	18	EEF1AKMT2	1.92	15
ENSXMAG00000012542	0.0031	6	12		337.07	3
ENSXMAG00000012575	0.0025	21	9	hsd11b1la (1 of many)	19.25	18
ENSXMAG00000012650	0.0027	9	15	jam2a	1.77	6
ENSXMAG00000012661	0.0014	12	18	crtac1a	6.07	9
ENSXMAG00000012742	0.0029	18	12	hexdc	2.68	15
ENSXMAG00000012854	0.0014	6	18	dot1l	8.58	3
ENSXMAG00000012904	0.0014	6	18		4.90	3
ENSXMAG00000012933	0.0024	6	9	cfap43	179.90	3
ENSXMAG00000013021	0.0026	12	9	rorca	86.46	9
ENSXMAG00000013051	0.0031	6	12	RASSF8 (1 of many)	3.72	3
ENSXMAG00000013069	0.0026	21	9	pdia3	3.59	18
ENSXMAG00000013072	0.0026	6	9		793.16	3
ENSXMAG00000013136	0.0024	6	9	erich3	657.18	3
ENSXMAG00000013137	0.0030	6	12	pigq	3.48	3
ENSXMAG00000013157	0.0024	6	15	fosl1a	10.84	3
ENSXMAG00000013201	0.0025	15	15	cwc22	1.40	12
ENSXMAG00000013220	0.0030	6	12	pim2	3.09	3
ENSXMAG00000013245	0.0026	15	9	psmc4	1.83	12
ENSXMAG00000013246	0.0025	6	15		106.41	3
ENSXMAG00000013283	0.0026	15	15		2.02	12
ENSXMAG00000013314	0.0024	6	9	bbs9	3.46	3
ENSXMAG00000013325	0.0026	6	15	mcf2a	2.91	3
ENSXMAG00000013450	0.0025	15	15		2.10	12
ENSXMAG00000013518	0.0026	6	9	LRRC9	593.74	3
ENSXMAG00000013554	0.0014	24	6	zdhhc9	2.24	21
ENSXMAG00000013556	0.0028	6	15	capslb	12.75	3
ENSXMAG00000013597	0.0027	12	9	nsun2	1.30	9
ENSXMAG00000013614	0.0015	3	18	ube2ql1	10.92	0
ENSXMAG00000013804	0.0034	18	12	rrp36	2.59	15
ENSXMAG00000013812	0.0031	6	12	akt3a	16.15	3
ENSXMAG00000013888	0.0015	24	6	wnt11r	7.73	21
ENSXMAG00000013934	0.0024	24	9	angptl4	5.08	21
ENSXMAG00000014002	0.0024	6	9	acer3	1.78	3
ENSXMAG00000014048	0.0031	18	12		6.28	15
ENSXMAG00000014055	0.0026	6	15	lrch3	16.59	3
ENSXMAG00000014089	0.0031	12	12		3.05	9
ENSXMAG00000014103	0.0024	21	9	nr2f2	4.42	18
ENSXMAG00000014104	0.0031	6	12	spag9a	1.72	3

ENSXMAG00000014106	0.0031	6	12	gnao1b	347.20	3
ENSXMAG00000014130	0.0071	6	18	nup133	3.50	3
ENSXMAG00000014132	0.0031	18	12	soul4	8.60	15
ENSXMAG00000014172	0.0025	18	9	nudt21	1.90	15
ENSXMAG00000014223	0.0031	6	12	zgc:194578	2.25	3
ENSXMAG00000014253	0.0024	18	9		1.98	15
ENSXMAG00000014298	0.0026	6	9	spata18	405.27	3
ENSXMAG00000014310	0.0028	18	15	immp1l	2.27	15
ENSXMAG00000014393	0.0026	21	9	9-Sep	3.21	18
ENSXMAG00000014411	0.0026	6	9	ttbk2a	3.98	3
ENSXMAG00000014563	0.0071	6	18	nploc4	5.95	3
ENSXMAG00000014591	0.0071	6	18	cers2a	3.87	3
ENSXMAG00000014629	0.0030	18	12	inpp5b	2.26	15
ENSXMAG00000014672	0.0026	21	9		12.66	18
ENSXMAG00000014737	0.0034	6	12	ttll5	260.77	3
ENSXMAG00000014776	0.0031	18	12	zdhhc6	1.88	15
ENSXMAG00000014781	0.0026	15	15		3.27	12
ENSXMAG00000014793	0.0015	24	18		1.96	21
ENSXMAG00000014814	0.0031	6	12	pbxip1a	32.07	3
ENSXMAG00000014856	0.0024	6	15	SLC23A2	20.19	3
ENSXMAG00000014926	0.0026	15	15	clcn5b	2.97	12
ENSXMAG00000014941	0.0014	6	18	acadm	1.45	3
ENSXMAG00000014991	0.0031	6	12		1.87	3
ENSXMAG00000015148	0.0026	12	9	card9	4.19	9
ENSXMAG00000015165	0.0034	15	12		1.78	12
ENSXMAG00000015239	0.0031	6	12	pgfb	7.10	3
ENSXMAG00000015304	0.0031	18	12	ZCCHC24 (1 of many)	9.69	15
ENSXMAG00000015306	0.0015	18	6	arf4a	1.57	15
ENSXMAG00000015314	0.0031	3	12	per1b	41.91	0
ENSXMAG00000015322	0.0031	18	12	pggt1b	2.08	15
ENSXMAG00000015468	0.0024	12	15	DHX35	2.10	9
ENSXMAG00000015473	0.0025	6	15	bag4	6.87	3
ENSXMAG00000015491	0.0025	6	15	dapk1	20.36	3
ENSXMAG00000015534	0.0031	6	12	tmem41aa	5.75	3
ENSXMAG00000015619	0.0031	15	12	arntl1a	24.63	12
ENSXMAG00000015644	0.0026	9	9	YTHDF1 (1 of many)	1.73	6
ENSXMAG00000015652	0.0031	6	12	zgc:77151	12.66	3
ENSXMAG00000015712	0.0031	6	12	rps6ka5	18.13	3
ENSXMAG00000015721	0.0031	6	12	dicer1	5.70	3
ENSXMAG00000015785	0.0026	9	15	LONRF3	22.59	6
ENSXMAG00000015925	0.0025	6	15	ptbp1a	3.61	3
ENSXMAG00000015953	0.0031	18	12	tuba8l3	6.83	15
ENSXMAG00000015967	0.0031	6	12		5.11	3
ENSXMAG00000016009	0.0031	6	12	vps37c	14.75	3
ENSXMAG00000016021	0.0026	15	15		2.61	12
ENSXMAG00000016056	0.0027	6	15	uhmk1	6.59	3
ENSXMAG00000016097	0.0031	9	12	zgc:162200	3.48	6
ENSXMAG00000016101	0.0016	6	18		5.58	3
ENSXMAG00000016170	0.0031	18	12	ostf1	2.58	15
ENSXMAG00000016304	0.0031	18	12	crabp2a	15.44	15
ENSXMAG00000016318	0.0026	12	9	nod2	2.74	9
ENSXMAG00000016332	0.0025	9	9	DMXL1	1.92	6
ENSXMAG00000016336	0.0026	9	15		2.27	6
ENSXMAG00000016346	0.0025	15	15	brd7	1.66	12
ENSXMAG00000016375	0.0024	6	15	pigk	2.38	3
ENSXMAG00000016389	0.0027	18	15		1.58	15

ENSXMAG00000016442	0.0025	3	15	lamb2	2.29	0
ENSXMAG00000016630	0.0030	6	12	si:ch211-215a10.4	7.61	3
ENSXMAG00000016750	0.0026	6	15	pvrl2l	2.97	3
ENSXMAG00000016773	0.0015	9	18	slc25a25b	3.37	6
ENSXMAG00000016786	0.0025	3	15	phactr3a	49.50	0
ENSXMAG00000016826	0.0015	18	18	gstt1a	2.88	15
ENSXMAG00000016926	0.0031	6	12	nipbla	4.76	3
ENSXMAG00000016928	0.0031	15	12	clocka	41.69	12
ENSXMAG00000016942	0.0028	12	15	slc35b4	1.71	9
ENSXMAG00000016945	0.0015	18	6	psmc5	1.84	15
ENSXMAG00000017011	0.0014	18	6	lrrc59	3.44	15
ENSXMAG00000017041	0.0014	12	18	naa40	1.40	9
ENSXMAG00000017077	0.0025	6	9	bicral	10.40	3
ENSXMAG00000017241	0.0034	21	12	acy1	3.19	18
ENSXMAG00000017328	0.0031	6	12	hace1	13.59	3
ENSXMAG00000017400	0.0024	6	15	amfra	1.51	3
ENSXMAG00000017424	0.0026	6	9	dnai2b	521.87	3
ENSXMAG00000017586	0.0031	18	12	tcea1	2.18	15
ENSXMAG00000017602	0.0015	24	6	ephb2b	5.96	21
ENSXMAG00000017642	0.0034	6	12	kctd3	8.09	3
ENSXMAG00000017697	0.0031	12	12		10.99	9
ENSXMAG00000017748	0.0025	21	9		58.52	18
ENSXMAG00000017798	0.0026	9	15		11.25	6
ENSXMAG00000017933	0.0024	15	15	ewsr1b	1.53	12
ENSXMAG00000017981	0.0031	6	12		166.22	3
ENSXMAG00000018000	0.0024	18	9	srp72	1.57	15
ENSXMAG00000018028	0.0034	6	12	usp9	22.03	3
ENSXMAG00000018082	0.0024	6	9	ssx2ipb	204.12	3
ENSXMAG00000018125	0.0026	18	15	mfap1	1.28	15
ENSXMAG00000018130	0.0031	18	12		2.61	15
ENSXMAG00000018157	0.0031	18	12		3.14	15
ENSXMAG00000018356	0.0025	6	15	SPATA5	1.87	3
ENSXMAG00000018408	0.0024	15	15	nexn	8.25	12
ENSXMAG00000018476	0.0024	6	9	BRF1 (1 of many)	7.27	3
ENSXMAG00000018580	0.0026	15	9	snrpa	2.93	12
ENSXMAG00000018588	0.0015	21	18	epyc	1.64	18
ENSXMAG00000018673	0.0024	15	15	eif4bb	1.84	12
ENSXMAG00000018717	0.0014	12	18	prg4a	1.88	9
ENSXMAG00000018726	0.0015	9	6	cers1	2.18	6
ENSXMAG00000018729	0.0027	12	15		4.28	9
ENSXMAG00000018734	0.0031	6	12	ubr4	4.11	3
ENSXMAG00000018753	0.0025	15	15	lhfpl2a	5.49	12
ENSXMAG00000018765	0.0025	12	9	kitlga	4.63	9
ENSXMAG00000018827	0.0031	6	12	mios	4.06	3
ENSXMAG00000018871	0.0014	12	18		2.70	9
ENSXMAG00000018874	0.0026	21	9	pdia4	3.60	18
ENSXMAG00000018896	0.0025	18	9	rpn1	1.97	15
ENSXMAG00000018934	0.0025	24	15		13.54	21
ENSXMAG00000018973	0.0031	18	12	mrps24	1.73	15
ENSXMAG00000018995	0.0031	6	12	cep350	19.85	3
ENSXMAG00000019027	0.0024	18	9	ppie	1.80	15
ENSXMAG00000019044	0.0026	6	15	capn15	2.09	3
ENSXMAG00000019093	0.0031	9	12		3.07	6
ENSXMAG00000019094	0.0026	18	15	itga6b (1 of many)	3.96	15
ENSXMAG00000019103	0.0031	6	12	kiz	267.52	3
ENSXMAG00000019136	0.0031	6	12	ptpn23a	3.10	3

ENSXMAG00000019185	0.0026	6	9	PITPNM2	3.53	3
ENSXMAG00000019193	0.0031	18	12	txnl4b	2.83	15
ENSXMAG00000019269	0.0024	6	9	agbl2	590.54	3
ENSXMAG00000019275	0.0026	6	15	washc4	2.35	3
ENSXMAG00000019296	0.0029	12	12	rorcb	32.61	9
ENSXMAG00000019336	0.0029	18	12	cirbpa	2.55	15
ENSXMAG00000019358	0.0031	6	12		338.28	3
ENSXMAG00000019391	0.0031	18	12	gnb1a	1.56	15
ENSXMAG00000019524	0.0025	15	15		10.87	12
ENSXMAG00000019560	0.0024	6	15	b3gnt5b	147.88	3
ENSXMAG00000019566	0.0031	18	12	aplnrb	9.48	15
ENSXMAG00000019607	0.0030	18	12	taf13	1.68	15
ENSXMAG00000019757	0.0025	6	9		1.43	3
ENSXMAG00000019827	0.0025	6	15	socs5b	13.74	3
ENSXMAG00000019923	0.0031	18	12		2.32	15
ENSXMAG00000019936	0.0029	6	12	chst7	7.28	3
ENSXMAG00000019942	0.0031	18	12	si:dkey-237j11.3	4.99	15
ENSXMAG00000019943	0.0025	18	9	nfil3	26.27	15
ENSXMAG00000020053	0.0071	9	18	otud1	11.04	6
ENSXMAG00000020118	0.0034	24	12		1.80	21
ENSXMAG00000020237	0.0026	21	9	NRGN	5.62	18
ENSXMAG00000020271	0.0016	6	18	gpr84	100.40	3
ENSXMAG00000020882	0.0015	18	6		1.95	15
ENSXMAG00000020909	0.0026	6	15		6.63	3
ENSXMAG00000020914	0.0024	6	9	spag6 (1 of many)	696.96	3
ENSXMAG00000020947	0.0025	6	15	dcst1	170.75	3
ENSXMAG00000020970	0.0034	6	12	cacna1db	74.84	3
ENSXMAG00000020997	0.0024	6	15		47.63	3
ENSXMAG00000021012	0.0024	3	9	gtf2a1l	2.32	0
ENSXMAG00000021031	0.0016	12	18		2.48	9
ENSXMAG00000021116	0.0031	15	12	rabggtb	1.52	12
ENSXMAG00000021168	0.0031	18	12		6.48	15
ENSXMAG00000021172	0.0024	6	9		3.93	3
ENSXMAG00000021181	0.0031	18	12	rab20	3.55	15
ENSXMAG00000021232	0.0071	3	18		1.68	0
ENSXMAG00000021269	0.0031	18	12	surf4	2.82	15
ENSXMAG00000021301	0.0026	3	9	epb41a	3.28	0
ENSXMAG00000021333	0.0025	6	15	abhd2a	7.99	3
ENSXMAG00000021343	0.0030	6	12	gdap1	2.01	3
ENSXMAG00000021390	0.0031	12	12		1.74	9
ENSXMAG00000021449	0.0029	24	12	mtnr1ab	37.91	21
ENSXMAG00000021553	0.0026	6	9		753.47	3
ENSXMAG00000021596	0.0031	18	12	si:ch211-1a19.3	5.12	15
ENSXMAG00000021617	0.0015	18	6	comtd1	7.53	15
ENSXMAG00000021656	0.0026	12	9		1.88	9
ENSXMAG00000021691	0.0031	6	12	si:dkey-40m6.8	14.65	3
ENSXMAG00000021736	0.0031	15	12	papss1	1.60	12
ENSXMAG00000021864	0.0031	24	12		2.45	21
ENSXMAG00000021880	0.0026	18	9	ankrd10b	2.31	15
ENSXMAG00000021950	0.0026	15	15		5.32	12
ENSXMAG00000021985	0.0031	6	12		2.78	3
ENSXMAG00000022054	0.0034	18	12		5.43	15
ENSXMAG00000022080	0.0027	9	9	rdh14b	2.15	6
ENSXMAG00000022148	0.0026	6	9		574.95	3
ENSXMAG00000022264	0.0034	6	12	C6orf106	7.06	3
ENSXMAG00000022277	0.0014	18	6	nxt2	2.58	15

ENSXMAG00000022292	0.0026	21	9	cd276	5.29	18
ENSXMAG00000022293	0.0026	21	9		8.27	18
ENSXMAG00000022361	0.0029	18	12	si:ch211-39k3.2	1.60	15
ENSXMAG00000022385	0.0024	15	15	ttc4	2.21	12
ENSXMAG00000022387	0.0026	6	15	si:dkey-110g7.8	3.13	3
ENSXMAG00000022402	0.0029	6	12		5.60	3
ENSXMAG00000022410	0.0031	18	12	arl6ip4	1.81	15
ENSXMAG00000022462	0.0034	6	12	CDKL5 (1 of many)	21.73	3
ENSXMAG00000022531	0.0029	6	12		17.89	3
ENSXMAG00000022609	0.0025	6	15	si:dkey-3h3.3	19.48	3
ENSXMAG00000022646	0.0024	15	15		2.01	12
ENSXMAG00000022778	0.0034	18	12	sypl2a	2.49	15
ENSXMAG00000022816	0.0026	6	9		719.00	3
ENSXMAG00000022854	0.0028	15	15		3.62	12
ENSXMAG00000022904	0.0014	3	18		10.91	0
ENSXMAG00000022942	0.0026	9	9	tacc1	1.87	6
ENSXMAG00000022990	0.0034	6	12	gadd45aa	6.39	3
ENSXMAG00000022994	0.0024	6	15	lrrc61	19.86	3
ENSXMAG00000023067	0.0030	18	12	KDELR2	2.21	15
ENSXMAG00000023272	0.0031	18	12	si:ch211-284b7.3	2.02	15
ENSXMAG00000023383	0.0031	6	12	cdc34a	1.79	3
ENSXMAG00000023428	0.0014	24	6		5.54	21
ENSXMAG00000023460	0.0034	6	12		151.21	3
ENSXMAG00000023499	0.0026	6	15		725.31	3
ENSXMAG00000023539	0.0031	18	12	cbx5	3.04	15
ENSXMAG00000023545	0.0031	6	12		542.12	3
ENSXMAG00000023635	0.0034	6	12		4.93	3
ENSXMAG00000023653	0.0028	21	9	mettl3	4.29	18
ENSXMAG00000023665	0.0029	6	12	adss	2.70	3
ENSXMAG00000023912	0.0030	18	12	acp6	3.27	15
ENSXMAG00000023917	0.0027	15	15	ZBTB14	1.79	12
ENSXMAG00000023931	0.0016	15	18	rapgefl1	4.95	12
ENSXMAG00000023943	0.0026	15	15	si:ch211-210c8.6	4.11	12
ENSXMAG00000023947	0.0026	6	15		4.70	3
ENSXMAG00000023964	0.0030	18	12	fam207a	2.81	15
ENSXMAG00000024057	0.0031	18	12	PHKG1	2.45	15
ENSXMAG00000024058	0.0030	18	12		7.22	15
ENSXMAG00000024147	0.0031	6	12		2.34	3
ENSXMAG00000024224	0.0026	21	9	RMDN1	1.81	18
ENSXMAG00000024306	0.0031	6	12		388.70	3
ENSXMAG00000024404	0.0031	18	12		4.74	15
ENSXMAG00000024410	0.0030	18	12		3.43	15
ENSXMAG00000024417	0.0014	24	6	COQ2	2.23	21
ENSXMAG00000024458	0.0031	18	12	ptrhd1	3.51	15
ENSXMAG00000024473	0.0031	6	12		34.00	3
ENSXMAG00000024531	0.0026	6	15		4.38	3
ENSXMAG00000024576	0.0025	18	9	ppih	3.17	15
ENSXMAG00000024584	0.0034	18	12	commd7	3.31	15
ENSXMAG00000024641	0.0031	3	12		1.28	0
ENSXMAG00000024819	0.0030	18	12	lasp1	3.48	15
ENSXMAG00000024877	0.0027	18	9	ADPRH	1.40	15
ENSXMAG00000024943	0.0016	12	18	gipc1	2.26	9
ENSXMAG00000025067	0.0027	15	15	edaradd	2.80	12
ENSXMAG00000025105	0.0031	18	12	si:ch1073-44g3.1	3.36	15
ENSXMAG00000025111	0.0015	18	6	rsl1d1	1.93	15
ENSXMAG00000025204	0.0027	15	15		1.96	12

ENSXMAG00000025257	0.0031	18	12	sec22bb	1.86	15
ENSXMAG00000025371	0.0016	3	18	cipcb	18.28	0
ENSXMAG00000025373	0.0027	9	15		161.56	6
ENSXMAG00000025472	0.0027	9	9	slc25a19	1.34	6
ENSXMAG00000025492	0.0031	6	12		3.76	3
ENSXMAG00000025577	0.0031	6	12		15.05	3
ENSXMAG00000025579	0.0027	21	9		6.16	18
ENSXMAG00000025583	0.0025	18	9		1.78	15
ENSXMAG00000025600	0.0026	6	15		89.49	3
ENSXMAG00000025616	0.0025	6	15		19.56	3
ENSXMAG00000025719	0.0030	18	12		2.10	15
ENSXMAG00000025720	0.0031	18	12	TMEM208	2.42	15
ENSXMAG00000025726	0.0031	18	12	PUDP	3.14	15
ENSXMAG00000025753	0.0026	21	9	ppp1r14bb	2.51	18
ENSXMAG00000025760	0.0031	6	12		11.46	3
ENSXMAG00000025797	0.0026	6	9		792.55	3
ENSXMAG00000025818	0.0014	12	18		1.66	9
ENSXMAG00000025829	0.0016	12	18	evx2	209.79	9
ENSXMAG00000025871	0.0026	3	15		2.24	0
ENSXMAG00000025881	0.0026	15	15	hs2st1b	1.83	12
ENSXMAG00000025890	0.0071	21	18	slc10a2	4.21	18
ENSXMAG00000025895	0.0031	6	12	neto2b	36.20	3
ENSXMAG00000026103	0.0025	6	15		7.74	3
ENSXMAG00000026218	0.0030	6	12		135.02	3
ENSXMAG00000026280	0.0034	18	12	doc2b	2.84	15
ENSXMAG00000026298	0.0024	18	9	GPR68	1.74	15
ENSXMAG00000026332	0.0031	18	12	ubtd2	2.71	15
ENSXMAG00000026337	0.0031	15	12	psmb6	1.53	12
ENSXMAG00000026375	0.0034	6	12		10.95	3
ENSXMAG00000026547	0.0026	21	9		4.86	18
ENSXMAG00000026570	0.0030	18	12	ciao1	2.09	15
ENSXMAG00000026649	0.0015	12	18	YWHAG	4.42	9
ENSXMAG00000026774	0.0030	18	12		1.45	15
ENSXMAG00000026817	0.0024	6	9	CIART	26.50	3
ENSXMAG00000026885	0.0015	18	6		4.67	15
ENSXMAG00000026911	0.0024	21	9	TMEM179	7.40	18
ENSXMAG00000027001	0.0014	15	18	si:dkeyp-94h10.5	2.00	12
ENSXMAG00000027005	0.0025	21	9	ss18l2	1.86	18
ENSXMAG00000027009	0.0027	21	9		2.94	18
ENSXMAG00000027021	0.0015	24	6	TSHZ1	3.72	21
ENSXMAG00000027252	0.0026	15	9		43.67	12
ENSXMAG00000027269	0.0016	24	6	CTHRC1	13.87	21
ENSXMAG00000027369	0.0031	18	12	SEPT7 (1 of many)	2.43	15
ENSXMAG00000027492	0.0031	6	12	znrf2b	35.46	3
ENSXMAG00000027518	0.0015	6	18	dnajc4	6.26	3
ENSXMAG00000027536	0.0028	12	15		12.04	9
ENSXMAG00000027547	0.0031	18	12	gtf2a2	3.87	15
ENSXMAG00000027573	0.0026	21	9		3.47	18
ENSXMAG00000027624	0.0031	6	12		3.98	3
ENSXMAG00000027653	0.0026	6	15		792.92	3
ENSXMAG00000027708	0.0031	24	12	gpc5a	10.37	21
ENSXMAG00000027742	0.0026	6	15		1.79	3
ENSXMAG00000027768	0.0031	6	12		522.77	3
ENSXMAG00000027779	0.0034	9	12	neu1	1.71	6
ENSXMAG00000027978	0.0031	18	12	dnajc8	2.16	15
ENSXMAG00000028012	0.0025	15	15		2.26	12

	ENSXMAG00000028013	0.0027	18	9		4.85	15
	ENSXMAG00000028142	0.0031	18	12	cmtm3	2.90	15
	ENSXMAG00000028164	0.0015	9	18	scamp1	2.10	6
	ENSXMAG00000028404	0.0027	9	9	znf507	2.72	6
	ENSXMAG00000028406	0.0029	9	12	b4galnt4a	2.97	6
	ENSXMAG00000028479	0.0029	18	12	zgc:64201	1.79	15
	ENSXMAG00000028521	0.0024	6	15	npas1	154.40	3
	ENSXMAG00000028536	0.0026	21	9		6.57	18
	ENSXMAG00000028594	0.0030	18	12		2.02	15
	ENSXMAG00000028597	0.0031	18	12	setd3	2.38	15
	ENSXMAG00000028629	0.0031	18	12	mmd	2.36	15
	ENSXMAG00000028651	0.0026	6	9		788.07	3
	ENSXMAG00000028685	0.0071	3	18		2.35	0
	ENSXMAG00000028814	0.0031	18	12	cryl1	3.73	15
	ENSXMAG00000028862	0.0031	18	12	NAT8	3.15	15
	ENSXMAG00000028864	0.0025	6	15		6.79	3
	ENSXMAG00000028998	0.0026	15	9		1.87	12
	ENSXMAG00000029024	0.0031	18	12		2.96	15
	ENSXMAG00000029174	0.0027	9	9	shisal1b	3.00	6
	ENSXMAG00000029179	0.0031	18	12		3.03	15
	ENSXMAG00000029335	0.0026	15	15	mak16	2.17	12
	ENSXMAG00000029343	0.0026	12	9		12.75	9
	ENSXMAG00000029397	0.0031	15	12		2.08	12
	ENSXMAG00000029457	0.0016	6	6	ero1a	10.53	3
	ENSXMAG00000029462	0.0026	15	15	CMAS	2.10	12
	ENSXMAG00000029512	0.0014	24	6	dia1b	2.18	21
	ENSXMAG00000029648	0.0016	6	18	gdpd4a	9.31	3
	ENSXMAG00000029706	0.0031	6	12	mxd1	6.63	3
	ENSXMAG00000029767	0.0029	6	12		791.37	3
	ENSXMAG00000029853	0.0026	6	9		4.31	3
	ENSXMAG00000029874	0.0025	21	9	tmem26a	6.06	18
	ENSXMAG00000029882	0.0034	9	12	adnp2b	2.01	6
	ENSXMAG00000029893	0.0015	12	18	sptlc3	2.93	9
	ENSXMAG00000029983	0.0025	18	9		1.46	15
	ENSXMAG00000030042	0.0027	6	15		10.72	3
	ENSXMAG00000030044	0.0031	6	12		11.29	3
	ENSXMAG00000030045	0.0025	18	9	insyn1	6.07	15
Muscle circadian ger	GeneID	pVal	phase	peak.shape	external_gene_name	amp	Ct.peak
	ENSXMAG00000000010	0.0024	3	15	bbox1	5.44	0
	ENSXMAG00000000014	0.0025	24	9	nr1h3	1.28	21
	ENSXMAG00000000027	0.0025	18	9	slc25a44b	10.18	15
	ENSXMAG00000000035	0.0027	3	15	nptnb	2.13	0
	ENSXMAG00000000044	0.0026	18	15	hira	1.34	15
	ENSXMAG00000000069	0.0026	24	9	si:ch211-241f5.3	1.57	21
	ENSXMAG00000000085	0.0029	18	12	ints2	2.00	15
	ENSXMAG00000000091	0.0016	3	18		1.43	0
	ENSXMAG00000000103	0.0025	18	15	dyrk4	2.93	15
	ENSXMAG00000000107	0.0024	24	15		1.94	21
	ENSXMAG00000000116	0.0027	3	15	asb15b	2.14	0
	ENSXMAG00000000138	0.0034	24	12	slc39a6	1.96	21
	ENSXMAG00000000145	0.0031	15	12	IER5L	2.14	12
	ENSXMAG00000000147	0.0027	3	15	zgc:154046	2.85	0
	ENSXMAG00000000178	0.0028	15	9	hbba2	2.99	12
	ENSXMAG00000000181	0.0028	15	9		3.78	12
	ENSXMAG00000000204	0.0031	21	12		1.73	18

ENSXMAG00000000212	0.0025	18	15	mfhas1	2.28	15
ENSXMAG00000000242	0.0026	15	15	xrn2	1.38	12
ENSXMAG00000000243	0.0025	18	9		8.73	15
ENSXMAG00000000254	0.0034	21	12		1.79	18
ENSXMAG00000000265	0.0024	18	15	taz	1.73	15
ENSXMAG00000000304	0.0031	15	12	fhl1b	2.15	12
ENSXMAG00000000335	0.0031	21	12	metrnla	2.67	18
ENSXMAG00000000355	0.0031	18	12	actr1	1.34	15
ENSXMAG00000000363	0.0031	6	12	kdm6bb	2.41	3
ENSXMAG00000000379	0.0031	18	12	hdac1	4.87	15
ENSXMAG00000000380	0.0025	3	9		2.00	0
ENSXMAG00000000383	0.0031	21	12	fam89b	1.42	18
ENSXMAG00000000394	0.0026	18	9	erap1b	1.77	15
ENSXMAG00000000397	0.0026	24	9	atf2	1.90	21
ENSXMAG00000000409	0.0025	21	9	NUP35	1.92	18
ENSXMAG00000000415	0.0024	21	9	ap1g2	1.29	18
ENSXMAG00000000440	0.0030	3	12	WDR7	1.47	0
ENSXMAG00000000455	0.0031	21	12	mfsd4b	2.59	18
ENSXMAG00000000473	0.0024	3	15		2.19	0
ENSXMAG00000000484	0.0016	24	18		4.17	21
ENSXMAG00000000490	0.0014	24	18	htatsf1	1.34	21
ENSXMAG00000000503	0.0025	18	9	dapk3	3.98	15
ENSXMAG00000000514	0.0034	18	12		3.54	15
ENSXMAG00000000525	0.0026	6	15	CTDSP1	1.42	3
ENSXMAG00000000532	0.0026	18	15	CLINT1 (1 of many)	2.69	15
ENSXMAG00000000539	0.0029	18	12	tmem168b	1.68	15
ENSXMAG00000000545	0.0026	24	9	ptdss2	1.82	21
ENSXMAG00000000554	0.0026	24	9	cyp26b1	2.74	21
ENSXMAG00000000570	0.0016	3	18	dysf	2.26	0
ENSXMAG00000000608	0.0029	6	12	msi2a	1.59	3
ENSXMAG00000000627	0.0026	18	15	relch	1.98	15
ENSXMAG00000000631	0.0014	24	6	snx13	1.91	21
ENSXMAG00000000634	0.0025	3	9		1.74	0
ENSXMAG00000000642	0.0026	18	15	ap1m1	1.84	15
ENSXMAG00000000647	0.0031	15	12	TPM4	1.86	12
ENSXMAG00000000652	0.0031	3	12		2.29	0
ENSXMAG00000000663	0.0031	18	12	rangap1b	3.64	15
ENSXMAG00000000676	0.0031	18	12	ranbp3b	1.51	15
ENSXMAG00000000699	0.0034	18	12	snrpb2	1.65	15
ENSXMAG00000000731	0.0025	3	15	si:dkeyp-117h8.2	2.45	0
ENSXMAG00000000751	0.0031	21	12		1.40	18
ENSXMAG00000000768	0.0029	21	12		2.05	18
ENSXMAG00000000786	0.0026	3	15	epb41l3b	1.81	0
ENSXMAG00000000791	0.0034	9	12	grb10b	4.66	6
ENSXMAG00000000792	0.0026	18	15	pdia5	2.68	15
ENSXMAG00000000806	0.0026	18	15		1.85	15
ENSXMAG00000000814	0.0026	21	9	ccr8.1	1.88	18
ENSXMAG00000000815	0.0025	18	15	triob	2.20	15
ENSXMAG00000000831	0.0031	18	12		1.62	15
ENSXMAG00000000849	0.0026	18	9	hdac3	1.21	15
ENSXMAG00000000875	0.0031	21	12	mov10a	1.88	18
ENSXMAG00000000891	0.0027	3	9		2.09	0
ENSXMAG00000000901	0.0026	24	9	adam9	1.22	21
ENSXMAG00000000910	0.0031	18	12	im:6912630	1.63	15
ENSXMAG00000000966	0.0026	24	9		1.82	21
ENSXMAG00000000977	0.0026	3	15	gramd1bb	2.17	0

ENSXMAG00000000986	0.0015	15	18	sb:cb649	2.45	12
ENSXMAG00000000990	0.0025	24	15	celsr2	3.06	21
ENSXMAG00000000999	0.0026	18	9	fscn1a	2.46	15
ENSXMAG00000001001	0.0031	3	12	pak1ip1	1.36	0
ENSXMAG00000001006	0.0024	3	15	rnf216	1.54	0
ENSXMAG00000001012	0.0031	21	12	si:dkey-222f8.3	2.05	18
ENSXMAG00000001067	0.0031	18	12		1.93	15
ENSXMAG00000001083	0.0034	18	12	osbpl3a	3.30	15
ENSXMAG00000001096	0.0026	21	15	cdkn1a	19.63	18
ENSXMAG00000001102	0.0015	21	6	STK19	2.29	18
ENSXMAG00000001113	0.0025	24	15	bhlhe41	22.46	21
ENSXMAG00000001125	0.0025	9	15	c1qtnf6a	2.46	6
ENSXMAG00000001138	0.0029	24	12	polm	1.47	21
ENSXMAG00000001141	0.0029	3	12	sgcg	1.25	0
ENSXMAG00000001148	0.0027	12	9	PEBP4	1.50	9
ENSXMAG00000001149	0.0025	24	9	tgfb1a	2.13	21
ENSXMAG00000001153	0.0024	6	15	RHOBTB2	20.34	3
ENSXMAG00000001156	0.0024	9	9	csnk1e	1.63	6
ENSXMAG00000001160	0.0031	21	12	atp5fa1 (1 of many)	1.49	18
ENSXMAG00000001177	0.0027	18	15	adamts17	2.00	15
ENSXMAG00000001182	0.0031	6	12	nsmfa	5.16	3
ENSXMAG00000001200	0.0026	18	9	pdgfbb	1.46	15
ENSXMAG00000001208	0.0014	24	18		1.83	21
ENSXMAG00000001212	0.0031	18	12	tox4b	3.91	15
ENSXMAG00000001219	0.0031	18	12	fitm2	5.03	15
ENSXMAG00000001222	0.0031	18	12	entpd4	1.77	15
ENSXMAG00000001272	0.0034	24	12	lins1	1.67	21
ENSXMAG00000001274	0.0024	18	15		2.77	15
ENSXMAG00000001286	0.0024	3	15	pfkfb1	2.07	0
ENSXMAG00000001289	0.0031	18	12		2.18	15
ENSXMAG00000001302	0.0031	15	12	si:ch211-282j22.3	1.60	12
ENSXMAG00000001317	0.0025	21	15	nop56	1.47	18
ENSXMAG00000001363	0.0026	3	15	gaa	2.50	0
ENSXMAG00000001380	0.0031	3	12	LRP4	2.70	0
ENSXMAG00000001384	0.0026	24	9		2.26	21
ENSXMAG00000001387	0.0025	18	15	sestd1	2.23	15
ENSXMAG00000001398	0.0030	18	12	mfsd1	2.64	15
ENSXMAG00000001406	0.0015	21	6	camk1gb	14.95	18
ENSXMAG00000001424	0.0034	18	12		1.80	15
ENSXMAG00000001427	0.0031	15	12		5.55	12
ENSXMAG00000001429	0.0027	3	15	cbx8a	1.72	0
ENSXMAG00000001444	0.0026	18	15	aadac	3.99	15
ENSXMAG00000001455	0.0031	18	12	snrpa1	2.04	15
ENSXMAG00000001466	0.0031	18	12	slc35c1	2.42	15
ENSXMAG00000001476	0.0026	24	9	src	1.50	21
ENSXMAG00000001482	0.0024	18	9	arntl2	14.93	15
ENSXMAG00000001487	0.0026	3	15	apip	1.86	0
ENSXMAG00000001495	0.0024	18	9	slc27a4	4.14	15
ENSXMAG00000001507	0.0026	12	15		2.50	9
ENSXMAG00000001548	0.0031	6	12		2.94	3
ENSXMAG00000001596	0.0027	24	9	taf2	1.46	21
ENSXMAG00000001624	0.0031	18	12	fech	1.98	15
ENSXMAG00000001626	0.0031	18	12	hyou1	2.26	15
ENSXMAG00000001631	0.0027	3	15	calcoco1a	2.45	0
ENSXMAG00000001648	0.0031	21	12	hoxc11a	2.05	18
ENSXMAG00000001650	0.0026	6	15	plch1	1.48	3

ENSXMAG00000001652	0.0030	24	12	ABHD5	2.43	21
ENSXMAG00000001657	0.0026	18	9	emc3	1.51	15
ENSXMAG00000001694	0.0027	3	15		3.66	0
ENSXMAG00000001700	0.0024	6	9	strbp	2.37	3
ENSXMAG00000001714	0.0027	3	15	rabgap1	2.11	0
ENSXMAG00000001735	0.0014	24	18	rxraa	2.58	21
ENSXMAG00000001736	0.0014	24	18	7-Mar	2.92	21
ENSXMAG00000001744	0.0034	6	12	cry1ab	7.53	3
ENSXMAG00000001751	0.0025	18	9		17.66	15
ENSXMAG00000001758	0.0071	21	6	pxdc1b	1.71	18
ENSXMAG00000001762	0.0025	18	9	prpf4bb	1.83	15
ENSXMAG00000001778	0.0026	18	9	abce1	1.91	15
ENSXMAG00000001787	0.0026	18	15	si:ch73-91k6.2	1.57	15
ENSXMAG00000001794	0.0034	18	12	slc10a7	1.86	15
ENSXMAG00000001802	0.0025	18	9	rbms1a	2.79	15
ENSXMAG00000001814	0.0025	21	9	umps	3.28	18
ENSXMAG00000001821	0.0026	18	9	fn1a	2.08	15
ENSXMAG00000001828	0.0031	18	12	ppp2r2ab	2.13	15
ENSXMAG00000001833	0.0026	6	15	dclk2b	4.47	3
ENSXMAG00000001844	0.0027	18	9	naprt	3.35	15
ENSXMAG00000001845	0.0027	3	9	gba2	1.28	0
ENSXMAG00000001861	0.0014	15	18	prkcda	1.73	12
ENSXMAG00000001862	0.0025	21	9	puf60b	1.63	18
ENSXMAG00000001864	0.0025	18	15	scrib	2.84	15
ENSXMAG00000001878	0.0026	3	15	zgc:77112	4.77	0
ENSXMAG00000001881	0.0031	21	12		1.56	18
ENSXMAG00000001885	0.0025	18	15	dbnlb	1.26	15
ENSXMAG00000001894	0.0025	18	9	slc4a2b	1.42	15
ENSXMAG00000001915	0.0024	18	15	mn1b	2.17	15
ENSXMAG00000001916	0.0031	18	12	PITPNB	4.11	15
ENSXMAG00000001940	0.0026	6	15	SYNPO2 (1 of many)	1.92	3
ENSXMAG00000001944	0.0025	18	9	sec24d	2.78	15
ENSXMAG00000001960	0.0026	24	9	prickle2b	2.75	21
ENSXMAG00000001970	0.0024	24	9	ncoa3	1.70	21
ENSXMAG00000001980	0.0031	21	12	elmo3	1.72	18
ENSXMAG00000001985	0.0026	18	9	fkbp11	2.36	15
ENSXMAG00000001987	0.0024	24	9	ANKS6	1.61	21
ENSXMAG00000002022	0.0029	3	12	ppef1	3.56	0
ENSXMAG00000002031	0.0025	24	9		1.40	21
ENSXMAG00000002033	0.0025	18	9	tmem33	2.04	15
ENSXMAG00000002059	0.0031	21	12	HOMER2	1.70	18
ENSXMAG00000002066	0.0031	3	12	WHAMM	1.88	0
ENSXMAG00000002068	0.0028	3	15	asb1	1.89	0
ENSXMAG00000002082	0.0034	18	12	pde8a	2.48	15
ENSXMAG00000002092	0.0025	18	9		2.46	15
ENSXMAG00000002138	0.0026	24	9	kmt5aa	1.86	21
ENSXMAG00000002141	0.0026	18	15	rapgef2	1.54	15
ENSXMAG00000002154	0.0034	12	12	mapk6	3.21	9
ENSXMAG00000002155	0.0014	21	6	pfdn5	1.36	18
ENSXMAG00000002158	0.0025	24	9	leo1	1.32	21
ENSXMAG00000002221	0.0026	24	9	yes1	2.60	21
ENSXMAG00000002224	0.0027	3	15		1.68	0
ENSXMAG00000002248	0.0031	9	12	BHLHE40 (1 of many)	13.64	6
ENSXMAG00000002249	0.0030	24	12	FBN1	2.62	21
ENSXMAG00000002252	0.0024	24	9		1.86	21
ENSXMAG00000002253	0.0015	15	6		1.56	12

ENSXMAG00000002261	0.0026	15	15	snx18b	1.56	12
ENSXMAG00000002269	0.0026	18	15	pars2	2.42	15
ENSXMAG00000002271	0.0031	6	12	rasgef1bb	4.71	3
ENSXMAG00000002275	0.0026	15	15	asb5a	2.19	12
ENSXMAG00000002306	0.0015	15	18		1.71	12
ENSXMAG00000002313	0.0029	18	12	ivns1abpa	2.67	15
ENSXMAG00000002320	0.0025	18	9		2.56	15
ENSXMAG00000002346	0.0031	21	12	rps7	1.21	18
ENSXMAG00000002351	0.0034	24	12	sema4c	1.55	21
ENSXMAG00000002370	0.0031	21	12	tbx1	2.38	18
ENSXMAG00000002371	0.0027	3	15	bcas3	2.19	0
ENSXMAG00000002394	0.0031	18	12	spryd3 (1 of many)	2.05	15
ENSXMAG00000002402	0.0024	18	15	ano8b	1.97	15
ENSXMAG00000002403	0.0025	18	9	azin1a	1.81	15
ENSXMAG00000002426	0.0030	18	12	dgcr8	1.57	15
ENSXMAG00000002440	0.0031	24	12	dennd1b	3.64	21
ENSXMAG00000002443	0.0031	18	12		1.90	15
ENSXMAG00000002454	0.0027	3	15	poldip2	1.53	0
ENSXMAG00000002501	0.0029	18	12	insig1	4.30	15
ENSXMAG00000002510	0.0026	18	9	ppp4cb	3.13	15
ENSXMAG00000002525	0.0031	18	12	mpv17l2	5.36	15
ENSXMAG00000002532	0.0030	21	12	PGPEP1	1.50	18
ENSXMAG00000002569	0.0027	24	15		20.61	21
ENSXMAG00000002570	0.0030	15	12		3.24	12
ENSXMAG00000002608	0.0026	24	9	u2surp	1.28	21
ENSXMAG00000002613	0.0026	18	15	sars2	2.46	15
ENSXMAG00000002627	0.0031	21	12	arfrp1	1.51	18
ENSXMAG00000002628	0.0029	6	12	pcolce2b	4.27	3
ENSXMAG00000002629	0.0026	18	15	zgc:172302	3.66	15
ENSXMAG00000002648	0.0024	18	9	zdhhc3a	1.95	15
ENSXMAG00000002664	0.0031	15	12	mob3a	1.78	12
ENSXMAG00000002668	0.0028	21	9		1.30	18
ENSXMAG00000002671	0.0016	15	18	coq8b	1.62	12
ENSXMAG00000002681	0.0025	21	9	rexo1	1.81	18
ENSXMAG00000002693	0.0025	9	9	wisp1a	2.81	6
ENSXMAG00000002707	0.0026	24	9	chd2	2.93	21
ENSXMAG00000002738	0.0026	15	15	SMARCB1	2.29	12
ENSXMAG00000002745	0.0031	18	12	ap2a1	2.51	15
ENSXMAG00000002747	0.0024	3	15	tgfbr2b	2.01	0
ENSXMAG00000002749	0.0027	21	15		2.16	18
ENSXMAG00000002750	0.0024	3	15	prpf4	2.10	0
ENSXMAG00000002753	0.0026	18	15	sf3a1	1.80	15
ENSXMAG00000002762	0.0025	18	15	si:ch211-251j10.3	3.89	15
ENSXMAG00000002786	0.0024	18	9	psat1	52.74	15
ENSXMAG00000002805	0.0031	15	12	slc25a55a	2.74	12
ENSXMAG00000002845	0.0034	18	12	seta	1.55	15
ENSXMAG00000002896	0.0026	3	9		1.84	0
ENSXMAG00000002919	0.0025	18	15	arhgef10la	1.89	15
ENSXMAG00000002928	0.0024	18	15	ctu1	2.55	15
ENSXMAG00000002940	0.0028	3	15	ppp6c	1.36	0
ENSXMAG00000002959	0.0025	18	9	ywhaqb	2.16	15
ENSXMAG00000002970	0.0029	18	12	snrnp40	2.73	15
ENSXMAG00000002971	0.0025	18	15	mlec	2.74	15
ENSXMAG00000003001	0.0025	21	9	bbs1	1.64	18
ENSXMAG00000003016	0.0016	24	18	tcf25	1.35	21
ENSXMAG00000003050	0.0015	21	6	srsf7a	1.95	18

ENSXMAG00000003057	0.0026	21	15	scaper	1.74	18
ENSXMAG00000003064	0.0015	3	18	hif1an	1.94	0
ENSXMAG00000003067	0.0014	24	18	mthfsd	3.13	21
ENSXMAG00000003075	0.0030	15	12	mfsd10	2.03	12
ENSXMAG00000003081	0.0024	15	15	armt1	1.46	12
ENSXMAG00000003095	0.0026	3	15	dnajb12a	1.60	0
ENSXMAG00000003101	0.0026	18	15	dr1	2.08	15
ENSXMAG00000003104	0.0031	24	12		2.46	21
ENSXMAG00000003112	0.0024	24	15	fbxl2	3.42	21
ENSXMAG00000003116	0.0024	24	9	scp2b	1.24	21
ENSXMAG00000003118	0.0031	24	12	podn	2.12	21
ENSXMAG00000003129	0.0026	15	15	atmin	1.67	12
ENSXMAG00000003143	0.0030	21	12	rpl5a	1.23	18
ENSXMAG00000003154	0.0025	18	9		1.32	15
ENSXMAG00000003156	0.0031	3	12	evi5b	2.63	0
ENSXMAG00000003160	0.0025	18	9	fkbp9	8.63	15
ENSXMAG00000003179	0.0025	18	15	nfyc	2.47	15
ENSXMAG00000003186	0.0026	9	9	hif1al	2.45	6
ENSXMAG00000003189	0.0024	21	9	myct1a	1.91	18
ENSXMAG00000003200	0.0031	18	12	stx5a	1.57	15
ENSXMAG00000003206	0.0025	18	9	mxra5a	3.54	15
ENSXMAG00000003209	0.0026	12	15	thbs2a	1.75	9
ENSXMAG00000003225	0.0014	18	6	zgc:153018	3.92	15
ENSXMAG00000003232	0.0031	21	12	thoc3	1.94	18
ENSXMAG00000003245	0.0030	24	12	gm2a	2.17	21
ENSXMAG00000003246	0.0024	18	15	ptgs1 (1 of many)	1.79	15
ENSXMAG00000003248	0.0016	15	18	ice2	2.03	12
ENSXMAG00000003253	0.0026	3	15	kat2b	2.55	0
ENSXMAG00000003254	0.0015	24	18	cep57l1	3.61	21
ENSXMAG00000003262	0.0030	15	12	mmp19	12.63	12
ENSXMAG00000003263	0.0024	3	15	txnipa	3.86	0
ENSXMAG00000003269	0.0026	9	15	foxo3	8.56	6
ENSXMAG00000003276	0.0014	24	18	otud6b	1.58	21
ENSXMAG00000003284	0.0025	18	9	cyb5b	4.77	15
ENSXMAG00000003322	0.0014	9	18	fndc1	1.26	6
ENSXMAG00000003328	0.0015	15	18	camsap1b	1.76	12
ENSXMAG00000003362	0.0027	15	15	usp3	1.81	12
ENSXMAG00000003370	0.0026	18	9		3.04	15
ENSXMAG00000003375	0.0024	18	9	smek1	1.76	15
ENSXMAG00000003399	0.0024	18	9	tram2	3.69	15
ENSXMAG00000003409	0.0028	3	15	ube2r2	2.57	0
ENSXMAG00000003419	0.0031	18	12	npepl1	1.65	15
ENSXMAG00000003424	0.0026	18	9	prep	1.81	15
ENSXMAG00000003436	0.0031	24	12	wdcp	2.98	21
ENSXMAG00000003470	0.0025	21	9		1.76	18
ENSXMAG00000003471	0.0026	18	15	arpc1b	1.22	15
ENSXMAG00000003474	0.0014	21	6	yeats4	1.81	18
ENSXMAG00000003514	0.0026	18	15	ap3d1	1.45	15
ENSXMAG00000003535	0.0016	18	18		2.38	15
ENSXMAG00000003623	0.0031	18	12	arfgap1	1.69	15
ENSXMAG00000003630	0.0025	21	15	nfic	1.44	18
ENSXMAG00000003631	0.0024	18	15	nnt	3.20	15
ENSXMAG00000003651	0.0029	9	12	RFX7	1.89	6
ENSXMAG00000003653	0.0029	24	12	bloc1s4	2.48	21
ENSXMAG00000003661	0.0030	18	12	rrm2b	1.46	15
ENSXMAG00000003675	0.0031	18	12	mbtps2	2.94	15

ENSXMAG00000003680	0.0027	3	15	phex	2.63	0
ENSXMAG00000003683	0.0029	18	12	mtdha	2.08	15
ENSXMAG00000003705	0.0026	3	15	ighmbp2	2.58	0
ENSXMAG00000003707	0.0024	21	15	xpnpep3	1.56	18
ENSXMAG00000003712	0.0026	24	9	sppl3	1.49	21
ENSXMAG00000003715	0.0025	15	15	rps6kc1	3.02	12
ENSXMAG00000003717	0.0014	3	18	igf1ra	4.36	0
ENSXMAG00000003725	0.0026	12	15	grhl2b	2.01	9
ENSXMAG00000003742	0.0025	3	15	lgr4	2.33	0
ENSXMAG00000003752	0.0031	21	12	si:ch211-203k16.3	1.68	18
ENSXMAG00000003772	0.0025	18	9	sf3b4	2.64	15
ENSXMAG00000003775	0.0024	21	9	fads2 (1 of many)	2.27	18
ENSXMAG00000003776	0.0034	24	12	farp2	1.41	21
ENSXMAG00000003777	0.0026	21	9	fads2 (1 of many)	6.83	18
ENSXMAG00000003783	0.0024	3	9	zer1	1.91	0
ENSXMAG00000003786	0.0015	24	18	fdft1	4.36	21
ENSXMAG00000003802	0.0031	15	12	vps72a	1.80	12
ENSXMAG00000003822	0.0024	21	15	mettl22	1.79	18
ENSXMAG00000003837	0.0025	9	9	laynb	1.85	6
ENSXMAG00000003840	0.0026	9	15	sik2b	4.08	6
ENSXMAG00000003860	0.0029	18	12	samd11	2.85	15
ENSXMAG00000003868	0.0031	3	12	OXR1	1.21	0
ENSXMAG00000003872	0.0025	3	9	ptpa	1.28	0
ENSXMAG00000003874	0.0030	18	12	tmem183a	2.11	15
ENSXMAG00000003888	0.0031	18	12	fam49ba	2.10	15
ENSXMAG00000003899	0.0027	21	9		1.79	18
ENSXMAG00000003922	0.0026	18	15	ercc2	2.13	15
ENSXMAG00000003958	0.0027	18	15	GPR89B	1.90	15
ENSXMAG00000003961	0.0029	6	12	per3	13.36	3
ENSXMAG00000003973	0.0025	6	9	pdzd2	2.04	3
ENSXMAG00000003986	0.0031	18	12	gtf2h1	2.13	15
ENSXMAG00000004031	0.0025	3	9	pptc7b	2.98	0
ENSXMAG00000004046	0.0030	24	12	klhl32	1.63	21
ENSXMAG00000004063	0.0014	15	18	camk1b	1.58	12
ENSXMAG00000004076	0.0014	24	18	si:dkey-266m15.5	3.33	21
ENSXMAG00000004088	0.0031	15	12	fam69aa	2.61	12
ENSXMAG00000004093	0.0025	18	9	bcl9	2.28	15
ENSXMAG00000004136	0.0031	18	12	dhx57	2.38	15
ENSXMAG00000004140	0.0026	3	9	mat2aa	2.03	0
ENSXMAG00000004148	0.0034	21	12		3.25	18
ENSXMAG00000004170	0.0027	12	15	si:ch1073-390k14.1	35.86	9
ENSXMAG00000004176	0.0031	3	12	strip2	2.37	0
ENSXMAG00000004189	0.0014	24	18	guf1	1.73	21
ENSXMAG00000004202	0.0024	18	9		2.67	15
ENSXMAG00000004210	0.0029	24	12	tmem160	1.49	21
ENSXMAG00000004214	0.0014	24	6	pkn2	1.91	21
ENSXMAG00000004220	0.0025	24	9	snx24	1.73	21
ENSXMAG00000004229	0.0024	18	15	tm9sf4	1.79	15
ENSXMAG00000004249	0.0026	24	9	pex5lb	1.98	21
ENSXMAG00000004254	0.0031	18	12	pcyox1	2.64	15
ENSXMAG00000004256	0.0026	18	9	2-Sep	1.84	15
ENSXMAG00000004259	0.0029	24	12	bmpr1aa	1.36	21
ENSXMAG00000004267	0.0024	18	9	tmem209	3.31	15
ENSXMAG00000004298	0.0030	18	12	clptm1	2.46	15
ENSXMAG00000004311	0.0024	18	9		2.65	15
ENSXMAG00000004337	0.0025	9	15	slc2a12	5.15	6

ENSXMAG00000004350	0.0026	24	9	si:dkey-91m11.5	2.61	21
ENSXMAG00000004358	0.0024	3	15	mospd2	1.99	0
ENSXMAG00000004372	0.0031	18	12		1.56	15
ENSXMAG00000004373	0.0026	3	15	rcan1a	1.40	0
ENSXMAG00000004378	0.0024	3	15	ap4b1	1.68	0
ENSXMAG00000004391	0.0024	24	9	cpne3 (1 of many)	1.42	21
ENSXMAG00000004394	0.0026	24	9	ofd1	1.59	21
ENSXMAG00000004398	0.0027	18	9	lman1	1.64	15
ENSXMAG00000004412	0.0029	6	12		1.29	3
ENSXMAG00000004421	0.0027	3	15	USP15	1.87	0
ENSXMAG00000004422	0.0026	3	9	rasgrp3	1.76	0
ENSXMAG00000004439	0.0030	9	12	WFS1 (1 of many)	2.12	6
ENSXMAG00000004445	0.0029	18	12	tmed2	1.54	15
ENSXMAG00000004460	0.0030	15	12		1.73	12
ENSXMAG00000004462	0.0024	18	15	sec63	1.56	15
ENSXMAG00000004468	0.0014	3	6	EML6	1.50	0
ENSXMAG00000004520	0.0024	21	15	TRAP1	1.53	18
ENSXMAG00000004521	0.0028	24	9	KDM5A	1.41	21
ENSXMAG00000004533	0.0024	3	15	RNF31	1.57	0
ENSXMAG00000004537	0.0025	21	9	clns1a	1.53	18
ENSXMAG00000004553	0.0031	18	12	syncrip	1.67	15
ENSXMAG00000004558	0.0025	18	9	arf5	2.29	15
ENSXMAG00000004563	0.0029	18	12	rcc2	2.47	15
ENSXMAG00000004575	0.0026	3	15		1.71	0
ENSXMAG00000004590	0.0025	3	15	eif4a2	1.65	0
ENSXMAG00000004604	0.0034	18	12	polr3f	2.49	15
ENSXMAG00000004606	0.0026	24	9	tnfsf10	2.79	21
ENSXMAG00000004610	0.0025	21	9	alg12	2.10	18
ENSXMAG00000004618	0.0015	24	18	arsa	2.87	21
ENSXMAG00000004637	0.0025	3	9	mfn2	1.43	0
ENSXMAG00000004641	0.0026	9	9	itchb	2.07	6
ENSXMAG00000004655	0.0025	15	15	pfkpa	1.51	12
ENSXMAG00000004663	0.0026	9	15	si:ch73-21k16.1	3.76	6
ENSXMAG00000004712	0.0015	3	18	MBNL2 (1 of many)	1.56	0
ENSXMAG00000004713	0.0034	21	12	prpf18	1.35	18
ENSXMAG00000004714	0.0031	21	12		3.06	18
ENSXMAG00000004719	0.0031	3	12	VSTM2L	1.37	0
ENSXMAG00000004732	0.0030	18	12	si:ch211-57i17.1	2.57	15
ENSXMAG00000004754	0.0031	18	12	nuak1b	3.71	15
ENSXMAG00000004788	0.0026	24	9	rpl10	1.23	21
ENSXMAG00000004792	0.0031	3	12	sema3c	2.11	0
ENSXMAG00000004807	0.0031	3	12	cdc73	1.44	0
ENSXMAG00000004892	0.0015	24	18	taf1a	1.28	21
ENSXMAG00000004915	0.0026	18	9	imp4	1.70	15
ENSXMAG00000004919	0.0026	21	15	dkc1	1.86	18
ENSXMAG00000004920	0.0016	3	6	PTPRD (1 of many)	1.70	0
ENSXMAG00000004939	0.0015	24	18	tyro3	2.11	21
ENSXMAG00000004941	0.0034	3	12	sgpp1	2.76	0
ENSXMAG00000004967	0.0026	6	15	ugt1a2 (1 of many)	2.90	3
ENSXMAG00000004971	0.0028	3	15		2.54	0
ENSXMAG00000004975	0.0029	18	12	stoml2	2.13	15
ENSXMAG00000004985	0.0024	15	15	grb2b	1.65	12
ENSXMAG00000004993	0.0029	18	12	pigo	3.29	15
ENSXMAG00000004998	0.0025	18	15	ppil4	1.97	15
ENSXMAG00000005014	0.0031	18	12	RINT1	1.58	15
ENSXMAG00000005030	0.0024	18	9	lemd3	1.77	15

ENSXMAG00000005081	0.0031	24	12	kalrna	1.53	21
ENSXMAG00000005085	0.0024	3	15	nudcd1	1.47	0
ENSXMAG00000005086	0.0024	3	15	mkrn1	2.33	0
ENSXMAG00000005127	0.0025	15	9	nfil3-5	98.49	12
ENSXMAG00000005128	0.0031	15	12	si:dkey-119m7.4	9.86	12
ENSXMAG00000005150	0.0031	18	12	gdi2	1.40	15
ENSXMAG00000005153	0.0030	15	12	slc44a2	2.15	12
ENSXMAG00000005163	0.0031	18	12	vps51	2.27	15
ENSXMAG00000005173	0.0030	21	12	kat5b	1.54	18
ENSXMAG00000005240	0.0025	18	9	ganab	3.50	15
ENSXMAG00000005265	0.0031	21	12	exosc2	1.75	18
ENSXMAG00000005273	0.0024	18	9	lrrc8c	1.63	15
ENSXMAG00000005287	0.0024	18	9	dab2	2.04	15
ENSXMAG00000005293	0.0031	18	12	nus1	2.97	15
ENSXMAG00000005298	0.0024	3	15	cgrrf1	2.78	0
ENSXMAG00000005301	0.0026	18	9	ehd1a	1.32	15
ENSXMAG00000005316	0.0026	6	15	scospondin	2.31	3
ENSXMAG00000005320	0.0026	24	9	zgc:85789	2.07	21
ENSXMAG00000005331	0.0026	15	15		1.48	12
ENSXMAG00000005338	0.0031	15	12	slc12a7b	1.71	12
ENSXMAG00000005344	0.0031	18	12		2.79	15
ENSXMAG00000005372	0.0031	15	12	tbx3a	2.74	12
ENSXMAG00000005373	0.0024	3	15	ankrd6b	2.33	0
ENSXMAG00000005379	0.0025	21	9	zswim7	1.69	18
ENSXMAG00000005388	0.0031	18	12	ncor1	1.81	15
ENSXMAG00000005390	0.0028	3	15	ada2a	2.02	0
ENSXMAG00000005419	0.0026	21	15	si:ch211-242b18.1	11.48	18
ENSXMAG00000005436	0.0025	3	9	gpn1	1.37	0
ENSXMAG00000005450	0.0025	21	15	fam222ba	1.92	18
ENSXMAG00000005462	0.0031	18	12	copa	1.58	15
ENSXMAG00000005506	0.0071	21	6	cpz	1.89	18
ENSXMAG00000005510	0.0026	24	9	ERC1 (1 of many)	1.57	21
ENSXMAG00000005523	0.0027	24	9	fbxw5	1.37	21
ENSXMAG00000005535	0.0027	15	9	pde10a	1.84	12
ENSXMAG00000005540	0.0025	24	9	fgd4a	1.92	21
ENSXMAG00000005551	0.0031	15	12	ugp2b	2.44	12
ENSXMAG00000005562	0.0031	18	12		1.43	15
ENSXMAG00000005572	0.0025	18	9	VANGL2	3.09	15
ENSXMAG00000005600	0.0025	18	9		1.90	15
ENSXMAG00000005605	0.0031	24	12	vgll2a	2.26	21
ENSXMAG00000005610	0.0024	3	15	tesk2	2.04	0
ENSXMAG00000005632	0.0031	9	12	RNF43	2.10	6
ENSXMAG00000005640	0.0031	3	12	ttc38	1.67	0
ENSXMAG00000005642	0.0024	18	15	nup188	1.65	15
ENSXMAG00000005651	0.0027	18	9	rgs5a	1.67	15
ENSXMAG00000005698	0.0031	6	12	atp1a2a	1.64	3
ENSXMAG00000005714	0.0024	18	9		1.84	15
ENSXMAG00000005734	0.0025	18	9	dolpp1	6.37	15
ENSXMAG00000005744	0.0014	3	6	camkk1a	2.19	0
ENSXMAG00000005749	0.0026	15	15	ppm1da	1.94	12
ENSXMAG00000005765	0.0024	3	15	ctdp1	2.32	0
ENSXMAG00000005775	0.0031	6	12	ulk2	3.16	3
ENSXMAG00000005786	0.0024	18	15	adck5	1.77	15
ENSXMAG00000005790	0.0024	18	9	xpo1a	3.94	15
ENSXMAG00000005819	0.0015	3	6	USP34	1.71	0
ENSXMAG00000005820	0.0016	24	18	acer2	1.92	21

ENSXMAG00000005842	0.0031	24	12	usp20	1.58	21
ENSXMAG00000005874	0.0024	9	15	greb1l	3.87	6
ENSXMAG00000005891	0.0031	18	12	hspd1	1.52	15
ENSXMAG00000005898	0.0026	3	15	sorl1	3.23	0
ENSXMAG00000005903	0.0031	18	12		2.49	15
ENSXMAG00000005906	0.0014	9	18		6.01	6
ENSXMAG00000005913	0.0031	21	12		1.14	18
ENSXMAG00000005915	0.0031	3	12		1.51	0
ENSXMAG00000005918	0.0026	18	9	ruvbl2	1.87	15
ENSXMAG00000005921	0.0015	24	18	pitx3	1.79	21
ENSXMAG00000005935	0.0026	24	9		1.71	21
ENSXMAG00000005936	0.0025	18	9		3.83	15
ENSXMAG00000005940	0.0031	21	12	ptcd2	1.66	18
ENSXMAG00000005944	0.0026	18	9	alpi.1	5.83	15
ENSXMAG00000005956	0.0026	18	9	map1b	2.17	15
ENSXMAG00000005958	0.0025	24	9		2.55	21
ENSXMAG00000005959	0.0024	18	9	dpagt1	2.73	15
ENSXMAG00000005971	0.0025	18	9	wnt2ba	2.55	15
ENSXMAG00000005983	0.0034	18	12	ap1g1	1.54	15
ENSXMAG00000006013	0.0026	18	9	ipo9	1.73	15
ENSXMAG00000006032	0.0029	18	12	ppp5c	2.64	15
ENSXMAG00000006042	0.0031	3	12	JAM3 (1 of many)	1.64	0
ENSXMAG00000006055	0.0029	18	12	kif5ba	1.66	15
ENSXMAG00000006090	0.0034	18	12	tm9sf1	1.85	15
ENSXMAG00000006101	0.0025	18	15	srp68	1.29	15
ENSXMAG00000006126	0.0031	18	12	lpar1	1.52	15
ENSXMAG00000006131	0.0029	18	12		1.52	15
ENSXMAG00000006136	0.0025	3	9	esr2a	1.91	0
ENSXMAG00000006142	0.0024	18	9	rrbp1a	3.91	15
ENSXMAG00000006148	0.0027	3	15	tango6	2.54	0
ENSXMAG00000006152	0.0034	18	12	poldip3	1.42	15
ENSXMAG00000006160	0.0029	18	12	snx5	2.00	15
ENSXMAG00000006196	0.0027	18	9	pabpn1	1.61	15
ENSXMAG00000006199	0.0014	24	18	osgep	1.36	21
ENSXMAG00000006208	0.0024	3	15	si:dkey-76k16.5	2.77	0
ENSXMAG00000006227	0.0027	3	15		2.08	0
ENSXMAG00000006239	0.0026	21	9	zc3h18	1.31	18
ENSXMAG00000006268	0.0034	21	12	zgc:91860	2.42	18
ENSXMAG00000006275	0.0030	15	12		1.63	12
ENSXMAG00000006283	0.0031	24	12	tbc1d7	1.91	21
ENSXMAG00000006284	0.0026	18	9	ldlra	4.85	15
ENSXMAG00000006293	0.0016	24	18	phactr1	5.11	21
ENSXMAG00000006296	0.0014	21	6	NRP1 (1 of many)	1.74	18
ENSXMAG00000006317	0.0014	3	18	dtnba	2.04	0
ENSXMAG00000006328	0.0026	21	9	med8	1.95	18
ENSXMAG00000006333	0.0031	15	12		2.53	12
ENSXMAG00000006335	0.0027	3	15	TNS3 (1 of many)	1.57	0
ENSXMAG00000006357	0.0027	18	9	mfsd2aa	28.76	15
ENSXMAG00000006362	0.0014	3	18		1.66	0
ENSXMAG00000006381	0.0024	3	15		3.86	0
ENSXMAG00000006417	0.0028	24	15	cry2	2.34	21
ENSXMAG00000006419	0.0015	15	18	khdrbs1a	1.26	12
ENSXMAG00000006421	0.0029	12	12	pfkpb	1.69	9
ENSXMAG00000006429	0.0031	24	12	pkn1a	1.60	21
ENSXMAG00000006439	0.0024	3	15	chm	2.66	0
ENSXMAG00000006441	0.0028	15	9		1.97	12

ENSXMAG00000006454	0.0031	24	12	zgc:136929	2.09	21
ENSXMAG00000006477	0.0031	15	12	mthfd1b	3.48	12
ENSXMAG00000006487	0.0026	3	15	abca1a	3.55	0
ENSXMAG00000006499	0.0024	18	9	cand1	2.20	15
ENSXMAG00000006502	0.0031	21	12		3.11	18
ENSXMAG00000006538	0.0028	3	15	tomm20a	1.57	0
ENSXMAG00000006558	0.0014	18	18	IGFBP5 (1 of many)	1.39	15
ENSXMAG00000006561	0.0030	3	12		2.18	0
ENSXMAG00000006569	0.0026	3	15	zfyve19	2.42	0
ENSXMAG00000006579	0.0025	15	15	fan1	2.20	12
ENSXMAG00000006586	0.0031	3	12		1.80	0
ENSXMAG00000006587	0.0026	3	15	pyroxd1	1.91	0
ENSXMAG00000006596	0.0025	24	9	PIK3R6	2.24	21
ENSXMAG00000006608	0.0031	18	12	slco1d1 (1 of many)	6.18	15
ENSXMAG00000006610	0.0031	18	12		1.89	15
ENSXMAG00000006628	0.0015	15	18	nansa	1.38	12
ENSXMAG00000006635	0.0031	3	12		2.79	0
ENSXMAG00000006651	0.0026	9	15		9.86	6
ENSXMAG00000006670	0.0015	24	18	si:ch211-59o9.10	2.31	21
ENSXMAG00000006672	0.0027	3	15	lyrm5b	3.73	0
ENSXMAG00000006688	0.0027	18	9		1.74	15
ENSXMAG00000006691	0.0024	3	15	crot	5.21	0
ENSXMAG00000006697	0.0025	21	15	trmu	1.16	18
ENSXMAG00000006702	0.0026	15	15		1.54	12
ENSXMAG00000006720	0.0031	6	12		1.97	3
ENSXMAG00000006721	0.0026	24	15	celsr1a	1.66	21
ENSXMAG00000006725	0.0025	18	15	arpp19a	1.60	15
ENSXMAG00000006729	0.0024	3	15	egln1a	3.06	0
ENSXMAG00000006761	0.0031	18	12	ddx3xa	1.65	15
ENSXMAG00000006767	0.0026	3	9	gramd4a	3.29	0
ENSXMAG00000006783	0.0027	3	15	ranbp9	1.43	0
ENSXMAG00000006794	0.0029	24	12	iqgap2	2.78	21
ENSXMAG00000006797	0.0015	21	6	mdkb	2.22	18
ENSXMAG00000006810	0.0014	24	6	PNPLA6	1.51	21
ENSXMAG00000006821	0.0027	9	15		1.70	6
ENSXMAG00000006844	0.0030	21	12	hmgb1b	1.67	18
ENSXMAG00000006872	0.0025	18	9	pcf11	1.86	15
ENSXMAG00000006883	0.0025	18	9		1.44	15
ENSXMAG00000006889	0.0031	18	12		1.95	15
ENSXMAG00000006891	0.0026	24	9	prdm11	1.97	21
ENSXMAG00000006921	0.0031	24	12	polr3h	1.55	21
ENSXMAG00000006943	0.0031	9	12	guca1d	4.40	6
ENSXMAG00000006945	0.0024	3	15	rab18b	2.51	0
ENSXMAG00000006967	0.0031	21	12	mkxb	2.42	18
ENSXMAG00000006994	0.0027	18	9	them4	9.11	15
ENSXMAG00000006996	0.0024	3	15	stard13b	1.55	0
ENSXMAG00000007001	0.0031	18	12	rras2	1.55	15
ENSXMAG00000007010	0.0031	18	12	copb1	1.88	15
ENSXMAG00000007018	0.0026	24	9	polk	2.01	21
ENSXMAG00000007030	0.0024	18	9		2.98	15
ENSXMAG00000007038	0.0025	3	9		2.01	0
ENSXMAG00000007063	0.0026	24	9	gpia	1.52	21
ENSXMAG00000007067	0.0015	3	18	ANK2	1.53	0
ENSXMAG00000007094	0.0026	18	9		2.54	15
ENSXMAG00000007104	0.0029	18	12	KIF2A	2.17	15
ENSXMAG00000007119	0.0026	21	15	nucb2a	1.23	18

ENSXMAG00000007125	0.0016	18	6	calcrla	1.76	15
ENSXMAG00000007148	0.0015	21	6	caprin1a	1.50	18
ENSXMAG00000007167	0.0031	3	12		4.87	0
ENSXMAG00000007169	0.0031	21	12	frem2b	2.99	18
ENSXMAG00000007220	0.0024	21	15		3.08	18
ENSXMAG00000007222	0.0031	15	12	pgm3	1.90	12
ENSXMAG00000007230	0.0031	15	12		2.36	12
ENSXMAG00000007274	0.0031	24	12	armc8	1.50	21
ENSXMAG00000007275	0.0024	18	15	slc50a1	2.25	15
ENSXMAG00000007277	0.0031	21	12	tiam1b	2.28	18
ENSXMAG00000007284	0.0031	18	12	gspt1l	1.85	15
ENSXMAG00000007292	0.0025	24	9	TEAD4	2.39	21
ENSXMAG00000007314	0.0034	18	12	usp16	1.77	15
ENSXMAG00000007316	0.0026	21	15	cdk7	1.32	18
ENSXMAG00000007320	0.0031	18	12	rwdd2b	2.64	15
ENSXMAG00000007326	0.0015	24	6	cadm2b	2.32	21
ENSXMAG00000007329	0.0026	18	9	calub	3.38	15
ENSXMAG00000007333	0.0031	15	12	tprn	2.06	12
ENSXMAG00000007369	0.0014	24	18	sf3b1	1.51	21
ENSXMAG00000007397	0.0031	24	12		1.51	21
ENSXMAG00000007437	0.0024	3	15		2.12	0
ENSXMAG00000007467	0.0016	24	18		2.15	21
ENSXMAG00000007503	0.0030	21	12	thoc5	1.46	18
ENSXMAG00000007515	0.0031	3	12	rhoq	1.86	0
ENSXMAG00000007533	0.0025	3	15		1.46	0
ENSXMAG00000007536	0.0025	18	15	yap1	1.43	15
ENSXMAG00000007567	0.0025	3	15	asb2a.1	1.92	0
ENSXMAG00000007569	0.0024	15	9	nt5c2l1	3.99	12
ENSXMAG00000007571	0.0025	21	15	adam15	1.26	18
ENSXMAG00000007578	0.0025	3	9	sash1a	1.49	0
ENSXMAG00000007594	0.0016	24	18	SLC8B1	2.76	21
ENSXMAG00000007617	0.0014	15	18	galk1	1.95	12
ENSXMAG00000007630	0.0030	18	12	bcl9l	1.75	15
ENSXMAG00000007637	0.0026	15	15	psmb10	1.96	12
ENSXMAG00000007658	0.0029	18	12		2.03	15
ENSXMAG00000007676	0.0026	3	9	slmapa	1.45	0
ENSXMAG00000007684	0.0029	24	12	adam19a	1.72	21
ENSXMAG00000007688	0.0024	24	15	si:dkey-98f17.5	1.41	21
ENSXMAG00000007690	0.0014	21	6		3.23	18
ENSXMAG00000007692	0.0025	21	15	rcan3	1.96	18
ENSXMAG00000007713	0.0029	9	12	soul3	3.35	6
ENSXMAG00000007715	0.0028	3	15		1.17	0
ENSXMAG00000007731	0.0014	24	18	fbxo25	13.47	21
ENSXMAG00000007768	0.0025	18	9	zmpste24	3.04	15
ENSXMAG00000007783	0.0031	18	12	namptb	2.48	15
ENSXMAG00000007798	0.0014	24	18	susd6	1.64	21
ENSXMAG00000007800	0.0014	24	18	znf395b	12.42	21
ENSXMAG00000007822	0.0031	15	12		1.35	12
ENSXMAG00000007827	0.0031	3	12	nkl.1	1.70	0
ENSXMAG00000007831	0.0016	24	18	enpp4	1.64	21
ENSXMAG00000007834	0.0015	15	18	slc6a2	1.75	12
ENSXMAG00000007852	0.0027	3	15	asb14a	2.25	0
ENSXMAG00000007857	0.0028	9	15	cpe	1.67	6
ENSXMAG00000007887	0.0015	3	18		2.03	0
ENSXMAG00000007903	0.0026	24	15	cdk5rap1	1.48	21
ENSXMAG00000007919	0.0024	18	15		2.40	15

ENSXMAG00000007942	0.0015	3	6	cpda	1.36	0
ENSXMAG00000007953	0.0029	18	12	lmnb2	3.21	15
ENSXMAG00000007959	0.0029	18	12		2.27	15
ENSXMAG00000007983	0.0015	24	6	arl5a	1.34	21
ENSXMAG00000008001	0.0031	18	12	tm9sf3	1.91	15
ENSXMAG00000008011	0.0024	21	15	ncf4	1.43	18
ENSXMAG00000008019	0.0025	24	9	fam222a	3.10	21
ENSXMAG00000008036	0.0024	24	15		1.38	21
ENSXMAG00000008057	0.0024	18	15	SYDE1	1.59	15
ENSXMAG00000008059	0.0024	18	9	rbm4.2	2.35	15
ENSXMAG00000008071	0.0026	21	15	gatb	2.32	18
ENSXMAG00000008083	0.0031	15	12	nfe2	2.26	12
ENSXMAG00000008084	0.0024	18	9	copz1	2.77	15
ENSXMAG00000008111	0.0015	24	18	map3k3	1.93	21
ENSXMAG00000008122	0.0025	18	15	peli3	1.77	15
ENSXMAG00000008149	0.0031	18	12	eed	1.97	15
ENSXMAG00000008164	0.0026	18	9	pef1	2.92	15
ENSXMAG00000008172	0.0030	24	12	si:dkey-177p2.6	4.04	21
ENSXMAG00000008191	0.0026	24	9	dact2	2.70	21
ENSXMAG00000008192	0.0031	18	12	fam117aa	3.89	15
ENSXMAG00000008194	0.0014	24	18	prss16	2.03	21
ENSXMAG00000008201	0.0024	18	9	senp3b	2.45	15
ENSXMAG00000008223	0.0026	15	15	clint1a	1.45	12
ENSXMAG00000008227	0.0026	6	15		1.49	3
ENSXMAG00000008232	0.0016	24	18	si:ch73-132f6.5	1.43	21
ENSXMAG00000008233	0.0031	24	12	ehmt2	2.00	21
ENSXMAG00000008266	0.0027	24	15	kcne4	2.65	21
ENSXMAG00000008272	0.0024	3	15	als2b	3.17	0
ENSXMAG00000008279	0.0031	18	12	asb16	1.49	15
ENSXMAG00000008280	0.0031	21	12	farsb	1.35	18
ENSXMAG00000008294	0.0025	18	9	sar1b	3.19	15
ENSXMAG00000008298	0.0026	18	9	sec24a	1.60	15
ENSXMAG00000008318	0.0027	3	15		2.30	0
ENSXMAG00000008340	0.0026	21	15	acbd5a	1.32	18
ENSXMAG00000008350	0.0028	21	9	bgnb	1.46	18
ENSXMAG00000008354	0.0015	12	18	yme1l1a	2.58	9
ENSXMAG00000008372	0.0034	21	12		2.28	18
ENSXMAG00000008377	0.0025	18	9		3.48	15
ENSXMAG00000008387	0.0025	24	9	PLCD3	1.73	21
ENSXMAG00000008398	0.0026	18	9	cdk13	1.97	15
ENSXMAG00000008400	0.0031	21	12	hmgb1a	1.38	18
ENSXMAG00000008424	0.0031	6	12		1.43	3
ENSXMAG00000008469	0.0024	18	9	pak7	5.52	15
ENSXMAG00000008485	0.0025	18	9	prpf19	3.19	15
ENSXMAG00000008496	0.0031	6	12		2.50	3
ENSXMAG00000008512	0.0024	24	15	gkup	2.22	21
ENSXMAG00000008516	0.0026	9	15	cxcl14	2.39	6
ENSXMAG00000008518	0.0028	6	15	fam13b	1.93	3
ENSXMAG00000008538	0.0031	24	12	NFIB	1.64	21
ENSXMAG00000008541	0.0027	18	15		1.52	15
ENSXMAG00000008576	0.0031	21	12	aifm4	2.08	18
ENSXMAG00000008591	0.0030	18	12	cluha	2.79	15
ENSXMAG00000008594	0.0031	21	12	npr2	2.36	18
ENSXMAG00000008617	0.0028	3	15	KAT7	5.21	0
ENSXMAG00000008626	0.0014	18	6	slc3a2a	17.02	15
ENSXMAG00000008643	0.0025	21	9	brms1	2.25	18

ENSXMAG00000008647	0.0014	24	18	fndc7a	3.71	21
ENSXMAG00000008661	0.0016	3	6	ndst1a	1.65	0
ENSXMAG00000008681	0.0026	18	9	rbm22	2.44	15
ENSXMAG00000008710	0.0031	18	12	ctnnb1	1.64	15
ENSXMAG00000008723	0.0028	3	15		1.80	0
ENSXMAG00000008732	0.0071	3	6	mark2b	1.52	0
ENSXMAG00000008807	0.0025	18	15	mapk9	1.58	15
ENSXMAG00000008819	0.0014	15	18	nagk	1.86	12
ENSXMAG00000008856	0.0031	24	12	dnajc7	1.40	21
ENSXMAG00000008870	0.0026	24	9	ephx1	2.05	21
ENSXMAG00000008915	0.0031	18	12	kat2a	2.50	15
ENSXMAG00000008939	0.0025	18	9	DDX39A	1.76	15
ENSXMAG00000008963	0.0031	6	12	gas2l3	1.95	3
ENSXMAG00000009005	0.0029	18	12	dse	1.97	15
ENSXMAG00000009027	0.0024	24	9	smg8	1.58	21
ENSXMAG00000009042	0.0025	18	9		2.83	15
ENSXMAG00000009056	0.0031	3	12	RPP14	1.47	0
ENSXMAG00000009065	0.0024	18	9		1.95	15
ENSXMAG00000009070	0.0031	18	12	abcb7	3.33	15
ENSXMAG00000009077	0.0025	21	9	RBM25	1.53	18
ENSXMAG00000009099	0.0015	18	18	armc10	1.54	15
ENSXMAG00000009110	0.0024	3	15	fbxl13	2.59	0
ENSXMAG00000009130	0.0027	3	15	EZH1	2.01	0
ENSXMAG00000009139	0.0034	18	12		2.31	15
ENSXMAG00000009210	0.0031	21	12		2.21	18
ENSXMAG00000009239	0.0029	21	12		1.54	18
ENSXMAG00000009256	0.0025	18	15	ptrh1	2.01	15
ENSXMAG00000009278	0.0025	15	15	cry1ba	5.68	12
ENSXMAG00000009299	0.0014	21	18	ganc	1.99	18
ENSXMAG00000009324	0.0026	3	9	hltf	1.61	0
ENSXMAG00000009325	0.0031	15	12	si:ch211-63p21.1	1.73	12
ENSXMAG00000009359	0.0031	21	12	zcrb1	1.59	18
ENSXMAG00000009369	0.0024	24	9	prickle1b	1.96	21
ENSXMAG00000009383	0.0029	21	12	si:dkeyp-33b5.4	2.57	18
ENSXMAG00000009398	0.0016	24	18	nfkbiab	2.83	21
ENSXMAG00000009399	0.0031	21	12	zc3h7a	1.30	18
ENSXMAG00000009422	0.0031	24	12	ZNF106	2.26	21
ENSXMAG00000009432	0.0027	3	15	kdm8	1.84	0
ENSXMAG00000009443	0.0015	18	6	klhl40b	2.94	15
ENSXMAG00000009446	0.0015	18	18	abcc10	1.86	15
ENSXMAG00000009450	0.0024	18	15	olfm2b	2.68	15
ENSXMAG00000009482	0.0026	18	9	grm8a	2.48	15
ENSXMAG00000009490	0.0028	3	15		2.69	0
ENSXMAG00000009503	0.0024	3	15	mapk8b	1.83	0
ENSXMAG00000009510	0.0029	21	12	acot8	1.53	18
ENSXMAG00000009514	0.0014	21	6	phactr3b	2.98	18
ENSXMAG00000009517	0.0015	3	18	hipk3b	2.85	0
ENSXMAG00000009538	0.0030	21	12	slc16a1b	3.39	18
ENSXMAG00000009557	0.0031	21	12	meis2a	2.48	18
ENSXMAG00000009578	0.0031	18	12	fem1b	1.74	15
ENSXMAG00000009584	0.0024	18	15	adnpa	1.51	15
ENSXMAG00000009596	0.0025	18	15	aqr	1.78	15
ENSXMAG00000009598	0.0031	18	12	top1l	2.27	15
ENSXMAG00000009600	0.0026	24	9	si:dkey-85a20.4	1.71	21
ENSXMAG00000009602	0.0034	18	12	ugdh	2.40	15
ENSXMAG00000009609	0.0026	24	9	map3k4	1.66	21

ENSXMAG00000009627	0.0029	18	12	hhip	2.51	15
ENSXMAG00000009635	0.0016	18	6	fermt2	1.30	15
ENSXMAG00000009654	0.0031	18	12	get4	1.81	15
ENSXMAG00000009666	0.0031	18	12	ergic1	3.66	15
ENSXMAG00000009667	0.0031	24	12	fgfr1op	1.32	21
ENSXMAG00000009669	0.0025	24	9	n4bp2	1.55	21
ENSXMAG00000009670	0.0031	15	12	pde7a	1.72	12
ENSXMAG00000009671	0.0025	3	9	mrtfab	1.88	0
ENSXMAG00000009678	0.0031	18	12	SMARCA1	6.23	15
ENSXMAG00000009709	0.0025	24	9	manea	1.64	21
ENSXMAG00000009721	0.0029	18	12	USP38	1.84	15
ENSXMAG00000009731	0.0030	18	12	ube3a	1.42	15
ENSXMAG00000009732	0.0031	21	12	ppp1r13ba	1.89	18
ENSXMAG00000009733	0.0025	18	9	prelid1a	3.25	15
ENSXMAG00000009756	0.0026	24	9		1.59	21
ENSXMAG00000009769	0.0024	15	9		1.83	12
ENSXMAG00000009792	0.0026	9	9		9.04	6
ENSXMAG00000009801	0.0026	18	9	atg9a	1.99	15
ENSXMAG00000009820	0.0024	18	15	trim45	2.15	15
ENSXMAG00000009850	0.0034	18	12	ssrp1a	1.92	15
ENSXMAG00000009861	0.0031	18	12	ATP6V0C (1 of many)	1.60	15
ENSXMAG00000009878	0.0025	18	15		2.08	15
ENSXMAG00000009880	0.0025	18	15	uggt1	2.46	15
ENSXMAG00000009883	0.0027	15	9		2.13	12
ENSXMAG00000009886	0.0026	24	9	usp12a	1.48	21
ENSXMAG00000009896	0.0030	21	12	rad9a	2.22	18
ENSXMAG00000009955	0.0024	18	15	FILIP1L	2.08	15
ENSXMAG00000009956	0.0026	3	15	becn1	2.77	0
ENSXMAG00000009959	0.0028	18	9	slc26a10	5.44	15
ENSXMAG00000009967	0.0026	3	15	rufy1	2.26	0
ENSXMAG00000009970	0.0027	3	15	ppp6r2a	2.12	0
ENSXMAG00000009988	0.0015	24	18	ankrd13d	1.50	21
ENSXMAG00000010002	0.0028	3	15	gtpbp1l	2.01	0
ENSXMAG00000010014	0.0031	12	12	rasgef1ba	7.24	9
ENSXMAG00000010051	0.0026	18	9	rae1	1.65	15
ENSXMAG00000010056	0.0029	18	12	rac2	1.87	15
ENSXMAG00000010063	0.0024	18	9	polr2gl	2.34	15
ENSXMAG00000010079	0.0031	18	12	ykt6	1.46	15
ENSXMAG00000010104	0.0025	18	9	adamts12	9.28	15
ENSXMAG00000010114	0.0031	15	12	slc16a4	48.53	12
ENSXMAG00000010133	0.0031	18	12		2.08	15
ENSXMAG00000010134	0.0027	12	15	TTC39B	5.73	9
ENSXMAG00000010143	0.0016	24	18		1.30	21
ENSXMAG00000010189	0.0025	24	9	nav2a	2.05	21
ENSXMAG00000010191	0.0025	3	9	mapkapk3	1.76	0
ENSXMAG00000010221	0.0015	18	6	lipg	11.79	15
ENSXMAG00000010242	0.0031	18	12		4.67	15
ENSXMAG00000010247	0.0031	18	12	cpeb4a	2.89	15
ENSXMAG00000010253	0.0024	15	15	zdhhc13	1.69	12
ENSXMAG00000010281	0.0031	21	12	rpl26	1.25	18
ENSXMAG00000010288	0.0024	3	15	taok1b	2.61	0
ENSXMAG00000010308	0.0029	21	12	abcb9	5.06	18
ENSXMAG00000010317	0.0024	18	9	mapk14a	4.18	15
ENSXMAG00000010322	0.0031	18	12	cers5	2.20	15
ENSXMAG00000010323	0.0029	18	12	SLK (1 of many)	1.35	15
ENSXMAG00000010324	0.0026	18	9	slc22a18	2.89	15

ENSXMAG00000010331	0.0025	9	9	mcph1	3.16	6
ENSXMAG00000010333	0.0029	3	12	RPA1 (1 of many)	1.35	0
ENSXMAG00000010342	0.0031	18	12	dnajb11	1.30	15
ENSXMAG00000010374	0.0071	21	18	stn1	1.67	18
ENSXMAG00000010380	0.0031	21	12	glsa	1.50	18
ENSXMAG00000010389	0.0030	18	12	smarcd1	2.38	15
ENSXMAG00000010440	0.0031	18	12	sh3bp4a	1.38	15
ENSXMAG00000010467	0.0014	21	6	GOLGA7	1.30	18
ENSXMAG00000010468	0.0031	18	12	uba3	1.73	15
ENSXMAG00000010490	0.0029	15	12	hdr	4.21	12
ENSXMAG00000010495	0.0034	24	12		1.74	21
ENSXMAG00000010497	0.0026	18	9		9.21	15
ENSXMAG00000010505	0.0014	12	18	lonrf1	4.09	9
ENSXMAG00000010519	0.0029	18	12	larp4aa	3.14	15
ENSXMAG00000010524	0.0025	18	9	slc38a7	2.34	15
ENSXMAG00000010534	0.0031	21	12	suclg2	1.42	18
ENSXMAG00000010540	0.0026	3	15	scp2a	1.79	0
ENSXMAG00000010546	0.0014	24	6	znf503	3.02	21
ENSXMAG00000010572	0.0031	9	12	hsc70	1.96	6
ENSXMAG00000010581	0.0025	24	9	zeb2b	1.75	21
ENSXMAG00000010601	0.0031	12	12		3.31	9
ENSXMAG00000010628	0.0025	9	9	slc43a2b	5.33	6
ENSXMAG00000010639	0.0026	24	9	znf609b	1.57	21
ENSXMAG00000010644	0.0025	18	15	man1b1a	2.36	15
ENSXMAG00000010649	0.0025	3	9	phactr4b	1.77	0
ENSXMAG00000010652	0.0024	21	9		1.77	18
ENSXMAG00000010657	0.0031	18	12	rcc1	2.71	15
ENSXMAG00000010693	0.0030	18	12		2.49	15
ENSXMAG00000010711	0.0024	18	15	pigv	3.25	15
ENSXMAG00000010747	0.0014	21	6	sf3b6	1.22	18
ENSXMAG00000010755	0.0031	3	12	zgc:158328	1.96	0
ENSXMAG00000010758	0.0034	18	12	aldocb	1.60	15
ENSXMAG00000010769	0.0034	3	12	lrrc39	1.38	0
ENSXMAG00000010771	0.0014	18	6	ehd2a	1.68	15
ENSXMAG00000010775	0.0026	18	15	pigs	2.72	15
ENSXMAG00000010776	0.0026	9	9	cep85	3.74	6
ENSXMAG00000010777	0.0031	18	12	SSR4	1.55	15
ENSXMAG00000010787	0.0024	21	9	mrpl44	1.85	18
ENSXMAG00000010796	0.0026	24	9	cdc42bpab	1.71	21
ENSXMAG00000010797	0.0024	21	9		1.38	18
ENSXMAG00000010798	0.0026	18	9		4.18	15
ENSXMAG00000010799	0.0030	18	12	heatr6	5.78	15
ENSXMAG00000010814	0.0025	18	9	znf526	1.49	15
ENSXMAG00000010818	0.0024	18	15	LIMD1	1.72	15
ENSXMAG00000010827	0.0029	6	12	dedd1	3.66	3
ENSXMAG00000010850	0.0030	24	12	cpt1cb	3.33	21
ENSXMAG00000010853	0.0025	18	9	prmt1	1.67	15
ENSXMAG00000010885	0.0027	18	15		5.78	15
ENSXMAG00000010913	0.0026	15	15		1.81	12
ENSXMAG00000010922	0.0025	18	15	hip1	1.26	15
ENSXMAG00000010957	0.0031	3	12		2.92	0
ENSXMAG00000010963	0.0024	3	15	ino80e	2.91	0
ENSXMAG00000010964	0.0031	6	12		4.52	3
ENSXMAG00000010967	0.0031	12	12	porb	1.61	9
ENSXMAG00000010983	0.0031	18	12	taf15	1.30	15
ENSXMAG00000010985	0.0031	18	12	mmp28	2.40	15

ENSXMAG00000010993	0.0016	24	18	zfat	1.44	21
ENSXMAG00000011002	0.0034	24	12	dachd	2.77	21
ENSXMAG00000011022	0.0027	21	15		1.51	18
ENSXMAG00000011028	0.0027	18	9	tfr1a	5.49	15
ENSXMAG00000011043	0.0031	18	12	zgc:77880	2.87	15
ENSXMAG00000011049	0.0026	18	9	uchl3	1.84	15
ENSXMAG00000011051	0.0024	9	9		1.44	6
ENSXMAG00000011059	0.0026	3	15	nasp	1.96	0
ENSXMAG00000011133	0.0014	24	18	pex5	1.62	21
ENSXMAG00000011158	0.0026	18	9	slain1a	5.35	15
ENSXMAG00000011168	0.0025	21	15		2.14	18
ENSXMAG00000011172	0.0024	3	15	katnal1	1.95	0
ENSXMAG00000011173	0.0024	3	9	prkaa2	1.95	0
ENSXMAG00000011178	0.0024	3	15	erbb3b	1.42	0
ENSXMAG00000011184	0.0025	24	9	dab1a	2.72	21
ENSXMAG00000011186	0.0031	18	12	lpcat3	2.91	15
ENSXMAG00000011188	0.0015	3	18	lrtm2a	2.40	0
ENSXMAG00000011192	0.0025	24	9	bcor	2.99	21
ENSXMAG00000011220	0.0027	21	9	tspan11	1.32	18
ENSXMAG00000011222	0.0029	18	12	tmed5	2.24	15
ENSXMAG00000011234	0.0031	21	12		1.75	18
ENSXMAG00000011242	0.0031	18	12	cdc42l	2.11	15
ENSXMAG00000011265	0.0031	21	12	tie1	1.47	18
ENSXMAG00000011270	0.0027	3	15		3.10	0
ENSXMAG00000011275	0.0034	18	12	srm	1.80	15
ENSXMAG00000011294	0.0024	3	15	pex14	3.08	0
ENSXMAG00000011295	0.0034	18	12	pak2b	1.59	15
ENSXMAG00000011315	0.0025	6	15	DOCK2	1.51	3
ENSXMAG00000011324	0.0031	21	12		1.55	18
ENSXMAG00000011330	0.0025	3	15	myoc	1.64	0
ENSXMAG00000011356	0.0027	15	15	pgd	1.52	12
ENSXMAG00000011366	0.0015	24	18	PLPP7	1.68	21
ENSXMAG00000011374	0.0015	15	18	med24	1.57	12
ENSXMAG00000011383	0.0025	3	15	tanc1b	4.34	0
ENSXMAG00000011384	0.0014	3	6	kank4	1.40	0
ENSXMAG00000011389	0.0031	24	12	atg4c	1.95	21
ENSXMAG00000011408	0.0024	18	15	fam20b	1.94	15
ENSXMAG00000011447	0.0024	15	15	JAKMIP1	1.74	12
ENSXMAG00000011451	0.0031	24	12	zyg11	1.67	21
ENSXMAG00000011456	0.0027	24	9	rev1	2.23	21
ENSXMAG00000011464	0.0029	21	12	nr1d4a	45.52	18
ENSXMAG00000011472	0.0025	3	15	cwc15	1.65	0
ENSXMAG00000011474	0.0029	24	12	RASGRP2	1.79	21
ENSXMAG00000011480	0.0014	24	18	plp1b	3.12	21
ENSXMAG00000011484	0.0014	24	18		2.76	21
ENSXMAG00000011487	0.0026	24	9	cttnbp2	1.67	21
ENSXMAG00000011494	0.0031	24	12		1.98	21
ENSXMAG00000011529	0.0028	3	15	si:ch73-12o23.1	1.95	0
ENSXMAG00000011540	0.0034	18	12	ptpn1	1.92	15
ENSXMAG00000011547	0.0031	24	12	clcn1b	1.66	21
ENSXMAG00000011562	0.0031	9	12	slc9a3r1a	2.06	6
ENSXMAG00000011568	0.0014	3	18	si:ch211-119c20.2	1.75	0
ENSXMAG00000011574	0.0031	21	12	GRB14	4.28	18
ENSXMAG00000011578	0.0030	18	12	slc52a3	4.05	15
ENSXMAG00000011593	0.0025	3	9	si:dkeyp-118h3.6	1.30	0
ENSXMAG00000011594	0.0026	21	9	spopla	2.19	18

ENSXMAG00000011602	0.0024	18	15	kirrel1a	1.57	15
ENSXMAG00000011605	0.0016	18	18		1.94	15
ENSXMAG00000011622	0.0034	21	12	fam173a	1.91	18
ENSXMAG00000011623	0.0026	9	15	HNMT (1 of many)	4.12	6
ENSXMAG00000011639	0.0026	15	15	tmtops2b	2.15	12
ENSXMAG00000011644	0.0034	24	12	foxk2	1.54	21
ENSXMAG00000011645	0.0029	9	12	cyp2n13 (1 of many)	4.42	6
ENSXMAG00000011649	0.0030	18	12	pym1	1.94	15
ENSXMAG00000011669	0.0016	24	18	fxr2	1.99	21
ENSXMAG00000011684	0.0030	18	12	letmd1	3.17	15
ENSXMAG00000011693	0.0034	6	12	ddr2a	1.73	3
ENSXMAG00000011694	0.0026	9	15	nr4a1	14.30	6
ENSXMAG00000011725	0.0027	18	9	dgkaa	4.59	15
ENSXMAG00000011727	0.0014	24	18	vps9d1	2.10	21
ENSXMAG00000011739	0.0024	3	9	meis1b	1.28	0
ENSXMAG00000011744	0.0026	24	9	anapc4	1.50	21
ENSXMAG00000011753	0.0026	18	15		1.13	15
ENSXMAG00000011760	0.0015	21	6	crybb1l3	11.66	18
ENSXMAG00000011828	0.0031	24	12	apoob	1.40	21
ENSXMAG00000011832	0.0031	24	12	tada2a	1.79	21
ENSXMAG00000011842	0.0024	3	15	ubxn6	2.06	0
ENSXMAG00000011868	0.0026	24	9	traf4a	3.46	21
ENSXMAG00000011920	0.0029	18	12	sytl5	3.21	15
ENSXMAG00000011926	0.0031	21	12	rab11bb	1.61	18
ENSXMAG00000011931	0.0028	18	15	ap1s1	1.94	15
ENSXMAG00000011979	0.0024	18	15	u2af2b	1.48	15
ENSXMAG00000012035	0.0031	24	12		2.03	21
ENSXMAG00000012049	0.0026	21	15	rpl22l1	1.33	18
ENSXMAG00000012054	0.0031	24	12	dbpb	33.23	21
ENSXMAG00000012062	0.0031	15	12	pls3	2.06	12
ENSXMAG00000012071	0.0026	18	15		1.94	15
ENSXMAG00000012074	0.0024	15	15	sh3bgrl3	1.72	12
ENSXMAG00000012088	0.0026	15	9	rapgef3	1.70	12
ENSXMAG00000012121	0.0014	21	6	sat2b	2.23	18
ENSXMAG00000012122	0.0025	24	9	xylt2	1.59	21
ENSXMAG00000012125	0.0024	3	15	leng8	1.67	0
ENSXMAG00000012133	0.0015	3	6	recql5	1.78	0
ENSXMAG00000012154	0.0031	18	12	efhd1	1.65	15
ENSXMAG00000012157	0.0026	15	15	ripor3	1.81	12
ENSXMAG00000012179	0.0026	24	15		1.83	21
ENSXMAG00000012180	0.0014	3	18	bean1	3.69	0
ENSXMAG00000012200	0.0025	18	15	adpgk	2.19	15
ENSXMAG00000012216	0.0031	6	12	mcf2la	2.08	3
ENSXMAG00000012222	0.0029	21	12		2.33	18
ENSXMAG00000012228	0.0025	18	15	CUL5	1.85	15
ENSXMAG00000012229	0.0031	24	12	cuedc1b	1.41	21
ENSXMAG00000012234	0.0025	21	9	PPP1R27	2.56	18
ENSXMAG00000012248	0.0024	9	15	bco2a	10.31	6
ENSXMAG00000012250	0.0034	18	12	dag1	1.98	15
ENSXMAG00000012259	0.0024	9	15		1.94	6
ENSXMAG00000012261	0.0026	3	15	borcs8	2.10	0
ENSXMAG00000012264	0.0027	18	9	nptx1l	5.97	15
ENSXMAG00000012266	0.0025	24	9		1.68	21
ENSXMAG00000012268	0.0024	18	9	dus2	1.98	15
ENSXMAG00000012273	0.0026	18	9	cnot3a	1.42	15
ENSXMAG00000012280	0.0026	3	15	sec14l1	1.77	0

ENSXMAG00000012290	0.0034	24	12	shroom3	1.79	21
ENSXMAG00000012295	0.0024	3	15	zgc:136908	1.47	0
ENSXMAG00000012308	0.0025	24	9	wfikkn2b	2.51	21
ENSXMAG00000012317	0.0031	9	12	ptpn11b	2.14	6
ENSXMAG00000012318	0.0029	12	12	usp2b	3.73	9
ENSXMAG00000012321	0.0025	18	9	adamts15a	4.38	15
ENSXMAG00000012323	0.0025	18	9	gatad2ab	1.75	15
ENSXMAG00000012334	0.0014	24	18	plekha7a	1.68	21
ENSXMAG00000012364	0.0027	18	15	MAP4K4 (1 of many)	1.69	15
ENSXMAG00000012376	0.0031	18	12	cant1a	2.34	15
ENSXMAG00000012380	0.0030	24	12	col4a1	2.06	21
ENSXMAG00000012409	0.0030	15	12	dpysl3	1.91	12
ENSXMAG00000012411	0.0031	18	12	ddost	1.87	15
ENSXMAG00000012434	0.0030	18	12	tdg.1	2.08	15
ENSXMAG00000012450	0.0029	18	12	samm50	2.28	15
ENSXMAG00000012472	0.0027	21	9	acap3a	1.72	18
ENSXMAG00000012495	0.0025	18	9		5.78	15
ENSXMAG00000012498	0.0025	9	15	gpat3	3.55	6
ENSXMAG00000012502	0.0024	6	15	jcada	1.65	3
ENSXMAG00000012503	0.0030	21	12	mtpap	2.31	18
ENSXMAG00000012509	0.0031	18	12	MMP23B	2.11	15
ENSXMAG00000012546	0.0025	18	15	tnrc6b	1.48	15
ENSXMAG00000012548	0.0031	18	12	si:dkey-82j4.2	4.02	15
ENSXMAG00000012556	0.0027	3	15	mfsd8	1.93	0
ENSXMAG00000012562	0.0014	24	18	unk	2.32	21
ENSXMAG00000012579	0.0024	12	15	sdf4	1.50	9
ENSXMAG00000012607	0.0029	24	12	b3gntl1	1.71	21
ENSXMAG00000012608	0.0015	3	18	rnf123	1.37	0
ENSXMAG00000012623	0.0030	6	12	KHDC4	1.67	3
ENSXMAG00000012658	0.0031	15	12		2.11	12
ENSXMAG00000012675	0.0014	21	18		2.02	18
ENSXMAG00000012694	0.0025	18	9	pgm2	2.38	15
ENSXMAG00000012696	0.0031	24	12	fdxr	1.91	21
ENSXMAG00000012716	0.0025	24	9	mbd3a	1.79	21
ENSXMAG00000012720	0.0026	18	9	knop1	3.90	15
ENSXMAG00000012736	0.0031	6	12	pkd1a	1.60	3
ENSXMAG00000012749	0.0025	3	15	tsc2	1.50	0
ENSXMAG00000012766	0.0031	18	12	wee1	8.86	15
ENSXMAG00000012770	0.0026	9	9		1.64	6
ENSXMAG00000012780	0.0024	24	15	SCN4A (1 of many)	2.02	21
ENSXMAG00000012781	0.0031	9	12	si:ch211-114m9.1	2.33	6
ENSXMAG00000012801	0.0016	24	18	cited4a	3.66	21
ENSXMAG00000012822	0.0026	18	15		1.30	15
ENSXMAG00000012838	0.0024	9	15		2.56	6
ENSXMAG00000012844	0.0027	18	9	amh	5.01	15
ENSXMAG00000012847	0.0027	6	15	stk35	10.41	3
ENSXMAG00000012863	0.0031	15	12	mak	2.02	12
ENSXMAG00000012883	0.0014	24	18	rmc1	1.84	21
ENSXMAG00000012916	0.0030	15	12	pctp	2.90	12
ENSXMAG00000012951	0.0026	9	15	pcyt2	7.20	6
ENSXMAG00000012952	0.0024	18	15	scamp2	1.52	15
ENSXMAG00000012959	0.0031	15	12	sirt7	2.54	12
ENSXMAG00000012961	0.0031	24	12		1.96	21
ENSXMAG00000012962	0.0025	18	9	papss2a	4.60	15
ENSXMAG00000012963	0.0027	6	15	colgalt2	1.65	3
ENSXMAG00000012975	0.0015	21	6	minpp1a	1.86	18

ENSXMAG00000012982	0.0015	15	18	ATP6V1A (1 of many)	1.49	12
ENSXMAG00000013026	0.0014	18	18	RSPRY1	1.22	15
ENSXMAG00000013051	0.0024	3	15	RASSF8 (1 of many)	1.64	0
ENSXMAG00000013057	0.0030	6	12	abhd2b	2.67	3
ENSXMAG00000013087	0.0025	21	9	hs6st2	2.13	18
ENSXMAG00000013097	0.0015	18	6	dnajb1a	2.92	15
ENSXMAG00000013101	0.0026	21	9		3.61	18
ENSXMAG00000013103	0.0027	3	15	ptpn3	2.02	0
ENSXMAG00000013122	0.0030	18	12	tmem214	2.05	15
ENSXMAG00000013129	0.0025	18	9	cfl1	2.75	15
ENSXMAG00000013130	0.0031	6	12		2.39	3
ENSXMAG00000013138	0.0015	24	18	rab40c	4.59	21
ENSXMAG00000013155	0.0024	18	9	pdp1	8.53	15
ENSXMAG00000013158	0.0014	24	18	si:ch211-262e15.1	2.07	21
ENSXMAG00000013164	0.0025	18	15	dnm2a	1.41	15
ENSXMAG00000013195	0.0029	18	12	alg2	3.55	15
ENSXMAG00000013208	0.0025	18	15	galnt1	2.16	15
ENSXMAG00000013230	0.0031	18	12	akirin1	1.38	15
ENSXMAG00000013231	0.0026	18	9	atf6	1.74	15
ENSXMAG00000013238	0.0031	24	12	slc27a1a	1.48	21
ENSXMAG00000013239	0.0026	24	9	MYOM3	1.95	21
ENSXMAG00000013241	0.0031	24	12	si:dkey-199f5.8	1.21	21
ENSXMAG00000013249	0.0024	18	9	ctsl.1	3.16	15
ENSXMAG00000013253	0.0026	3	9	qkia	2.44	0
ENSXMAG00000013264	0.0026	3	15	scyl3	1.58	0
ENSXMAG00000013272	0.0025	18	9	dhdds	3.68	15
ENSXMAG00000013286	0.0029	18	12	si:zfos-452g4.1	2.36	15
ENSXMAG00000013306	0.0016	24	18	EAF1	2.11	21
ENSXMAG00000013315	0.0016	24	18	GABBR1	1.70	21
ENSXMAG00000013327	0.0028	3	15	pcmtl	2.67	0
ENSXMAG00000013336	0.0026	18	9	osgn1	5.09	15
ENSXMAG00000013346	0.0030	18	12	atp13a1	4.76	15
ENSXMAG00000013356	0.0024	18	15	zfr	1.68	15
ENSXMAG00000013383	0.0025	6	15		3.12	3
ENSXMAG00000013393	0.0015	24	18	nalcn	2.01	21
ENSXMAG00000013428	0.0031	18	12	map7d3	1.71	15
ENSXMAG00000013447	0.0031	18	12		1.83	15
ENSXMAG00000013450	0.0025	18	15		1.98	15
ENSXMAG00000013461	0.0024	24	9	asmtl	1.42	21
ENSXMAG00000013475	0.0028	3	15	GABARAPL2 (1 of many)	1.45	0
ENSXMAG00000013476	0.0014	24	18	mnat1	1.81	21
ENSXMAG00000013477	0.0026	3	9		1.46	0
ENSXMAG00000013479	0.0026	18	15	sufu	1.49	15
ENSXMAG00000013480	0.0026	3	9	six4a	2.66	0
ENSXMAG00000013491	0.0025	18	15	zdhhc16a	2.66	15
ENSXMAG00000013496	0.0025	24	9	gtf2h4	1.93	21
ENSXMAG00000013499	0.0027	3	15	smg5	1.24	0
ENSXMAG00000013517	0.0025	18	9	znrd1	2.18	15
ENSXMAG00000013519	0.0024	3	15	cep290	1.69	0
ENSXMAG00000013520	0.0024	18	15	mgat1b	1.65	15
ENSXMAG00000013536	0.0024	24	9		1.99	21
ENSXMAG00000013546	0.0026	21	9	sytl4	1.52	18
ENSXMAG00000013555	0.0024	18	9	hspa4l	2.01	15
ENSXMAG00000013557	0.0030	15	12		1.87	12
ENSXMAG00000013564	0.0031	21	12	pbx2	1.31	18
ENSXMAG00000013595	0.0028	18	15	snrnp48	1.53	15

ENSXMAG00000013640	0.0031	15	12		3.04	12
ENSXMAG00000013662	0.0030	21	12	adam8a	2.76	18
ENSXMAG00000013666	0.0031	18	12	nop58	3.10	15
ENSXMAG00000013699	0.0024	18	15	6-Sep	1.49	15
ENSXMAG00000013701	0.0016	24	18		1.72	21
ENSXMAG00000013714	0.0034	18	12		3.72	15
ENSXMAG00000013723	0.0034	18	12		2.94	15
ENSXMAG00000013750	0.0026	15	15	si:dkey-28b4.8	1.96	12
ENSXMAG00000013823	0.0026	21	15	trmt1	1.52	18
ENSXMAG00000013839	0.0014	3	18	hdac9b	2.94	0
ENSXMAG00000013844	0.0026	3	15	akap8l	1.46	0
ENSXMAG00000013849	0.0026	24	9	PRDM15	3.03	21
ENSXMAG00000013850	0.0025	18	15	tubg1	2.86	15
ENSXMAG00000013854	0.0034	24	12	PLEKHH3	1.33	21
ENSXMAG00000013878	0.0024	18	9	pde4ca	5.77	15
ENSXMAG00000013887	0.0025	3	9	xirp2a	1.77	0
ENSXMAG00000013892	0.0024	24	9		1.56	21
ENSXMAG00000013902	0.0026	18	9		2.45	15
ENSXMAG00000013912	0.0024	18	15	cbx3b	2.06	15
ENSXMAG00000013937	0.0030	18	12	casc3	1.71	15
ENSXMAG00000013943	0.0025	24	9		2.06	21
ENSXMAG00000013946	0.0031	3	12	stox2a	1.63	0
ENSXMAG00000013967	0.0024	3	15	gtf3c5	1.79	0
ENSXMAG00000013970	0.0026	6	15	klhl41b	1.30	3
ENSXMAG00000014005	0.0031	24	12	cass4	1.78	21
ENSXMAG00000014010	0.0026	24	9	git2a	2.16	21
ENSXMAG00000014016	0.0026	3	9	ext1b	1.91	0
ENSXMAG00000014017	0.0031	6	12	rbm24b	1.74	3
ENSXMAG00000014019	0.0026	3	15	slc25a39	1.94	0
ENSXMAG00000014051	0.0031	21	12	dclk2a	2.24	18
ENSXMAG00000014077	0.0029	6	12	abcc5	5.64	3
ENSXMAG00000014084	0.0031	6	12	TPPP	7.56	3
ENSXMAG00000014096	0.0031	18	12	ankrd40	2.01	15
ENSXMAG00000014097	0.0027	3	15	setd1ba	2.41	0
ENSXMAG00000014098	0.0014	24	18	ccdc12	1.53	21
ENSXMAG00000014132	0.0030	18	12	soul4	2.53	15
ENSXMAG00000014154	0.0026	6	15	jmjd1cb	1.32	3
ENSXMAG00000014172	0.0025	18	15	nudt21	1.78	15
ENSXMAG00000014174	0.0024	6	15		1.29	3
ENSXMAG00000014195	0.0015	3	18		4.53	0
ENSXMAG00000014206	0.0031	24	12	utp15	1.41	21
ENSXMAG00000014213	0.0026	18	9	rbm12b	1.55	15
ENSXMAG00000014217	0.0029	18	12	TNPO2 (1 of many)	2.26	15
ENSXMAG00000014231	0.0031	21	12		1.58	18
ENSXMAG00000014232	0.0025	3	15		1.43	0
ENSXMAG00000014239	0.0024	18	15	rcn1	1.98	15
ENSXMAG00000014250	0.0025	21	9	htra1b	2.42	18
ENSXMAG00000014257	0.0031	24	12	PIGB	1.79	21
ENSXMAG00000014297	0.0071	3	18	nf2b	1.35	0
ENSXMAG00000014302	0.0031	6	12	adap2	2.16	3
ENSXMAG00000014305	0.0025	3	9	atg16l2	1.81	0
ENSXMAG00000014312	0.0026	3	15	rps6ka2	6.96	0
ENSXMAG00000014316	0.0024	18	15	rhot1a	2.40	15
ENSXMAG00000014318	0.0025	21	15	wdr62	1.70	18
ENSXMAG00000014350	0.0025	18	15		3.43	15
ENSXMAG00000014375	0.0014	15	18	mindy3	2.02	12

ENSXMAG00000014387	0.0026	15	9		2.82	12
ENSXMAG00000014422	0.0026	18	9	glipr2l	2.25	15
ENSXMAG00000014437	0.0027	21	9	adam10a	1.31	18
ENSXMAG00000014438	0.0026	18	9	tbrg4	1.72	15
ENSXMAG00000014442	0.0025	18	9	MINPP1	1.84	15
ENSXMAG00000014447	0.0025	18	9	tes	2.31	15
ENSXMAG00000014478	0.0025	18	9	foxo6a	7.72	15
ENSXMAG00000014481	0.0031	18	12	med18	2.18	15
ENSXMAG00000014496	0.0026	3	15	ino80c	1.72	0
ENSXMAG00000014520	0.0031	15	12	map3k9	2.57	12
ENSXMAG00000014527	0.0025	18	15		1.75	15
ENSXMAG00000014533	0.0025	18	9	cavin1b	2.36	15
ENSXMAG00000014534	0.0014	3	18	slc8a3	1.99	0
ENSXMAG00000014558	0.0026	24	9	AREL1	1.48	21
ENSXMAG00000014575	0.0031	24	12	dcaf5	1.62	21
ENSXMAG00000014578	0.0031	3	12	pacs2	1.96	0
ENSXMAG00000014597	0.0031	6	12		1.97	3
ENSXMAG00000014605	0.0027	3	15	angel1	2.20	0
ENSXMAG00000014648	0.0029	18	12		2.42	15
ENSXMAG00000014727	0.0027	3	15		1.27	0
ENSXMAG00000014732	0.0016	24	18	csf3r	2.00	21
ENSXMAG00000014756	0.0025	18	15	tdrkh	1.81	15
ENSXMAG00000014774	0.0025	18	9	srebf1	3.07	15
ENSXMAG00000014782	0.0015	24	18		1.74	21
ENSXMAG00000014783	0.0029	21	12	parn	1.95	18
ENSXMAG00000014785	0.0024	3	9	tecpr1a	3.93	0
ENSXMAG00000014792	0.0027	6	15	arg2	4.77	3
ENSXMAG00000014800	0.0024	24	9		1.57	21
ENSXMAG00000014815	0.0025	24	9	map7d1a	1.84	21
ENSXMAG00000014820	0.0025	18	9	s100u	2.18	15
ENSXMAG00000014850	0.0026	21	15	hdac8	1.70	18
ENSXMAG00000014852	0.0031	18	12	ccz1	1.85	15
ENSXMAG00000014853	0.0031	15	12		1.56	12
ENSXMAG00000014854	0.0024	18	9	stx4	2.21	15
ENSXMAG00000014890	0.0031	18	12	mrm1	5.10	15
ENSXMAG00000014897	0.0031	18	12	ppm1g	2.57	15
ENSXMAG00000014899	0.0031	9	12	EML5	8.17	6
ENSXMAG00000014900	0.0024	3	15		1.39	0
ENSXMAG00000014921	0.0024	24	9	FAHD2A	1.53	21
ENSXMAG00000014926	0.0031	18	12	clcn5b	2.38	15
ENSXMAG00000014929	0.0030	24	12	pdcd2	1.93	21
ENSXMAG00000014939	0.0015	18	18	jmjd7	1.44	15
ENSXMAG00000014967	0.0029	18	12	npc2	1.98	15
ENSXMAG00000014971	0.0025	21	9		1.40	18
ENSXMAG00000014977	0.0031	24	12	plvapa	1.51	21
ENSXMAG00000014983	0.0029	18	12	nr2f6a	1.69	15
ENSXMAG00000015000	0.0029	18	12	nudt18	10.56	15
ENSXMAG00000015029	0.0026	24	9	HAGHL	1.64	21
ENSXMAG00000015037	0.0030	18	12		2.19	15
ENSXMAG00000015101	0.0031	21	12	kcnab1b	7.87	18
ENSXMAG00000015121	0.0016	24	18	rxrbb	1.42	21
ENSXMAG00000015127	0.0026	3	15	krit1	1.85	0
ENSXMAG00000015145	0.0025	3	15	irs2a	12.84	0
ENSXMAG00000015150	0.0014	24	18	cox18	1.45	21
ENSXMAG00000015155	0.0031	9	12	adamts14	1.33	6
ENSXMAG00000015160	0.0014	18	18	selenot2	1.27	15

ENSXMAG00000015174	0.0031	18	12	kdm2ba	1.60	15
ENSXMAG00000015195	0.0024	12	9	ddr2l	2.04	9
ENSXMAG00000015223	0.0026	3	15	tcaim	2.13	0
ENSXMAG00000015227	0.0034	18	12	znf319b	1.93	15
ENSXMAG00000015247	0.0029	21	12	aplnra	7.54	18
ENSXMAG00000015249	0.0029	18	12	afg1la	2.67	15
ENSXMAG00000015250	0.0024	18	15	ZMIZ1 (1 of many)	2.54	15
ENSXMAG00000015252	0.0025	24	9	dus3l	1.75	21
ENSXMAG00000015264	0.0031	18	12	snx2	1.92	15
ENSXMAG00000015265	0.0024	18	15		2.23	15
ENSXMAG00000015283	0.0025	18	9		2.34	15
ENSXMAG00000015287	0.0034	18	12	tmed7	1.76	15
ENSXMAG00000015292	0.0031	18	12	rtraf	1.52	15
ENSXMAG00000015293	0.0014	21	6	eif4eb	1.77	18
ENSXMAG00000015299	0.0031	18	12	prpf40a	2.24	15
ENSXMAG00000015308	0.0031	21	12	rell1	1.69	18
ENSXMAG00000015316	0.0016	24	18	metap1	1.30	21
ENSXMAG00000015326	0.0024	3	15	cep120	2.73	0
ENSXMAG00000015336	0.0014	24	18	tmem86a	5.43	21
ENSXMAG00000015355	0.0024	18	15	zgc:63863	2.52	15
ENSXMAG00000015367	0.0031	18	12		2.47	15
ENSXMAG00000015373	0.0025	18	15	cnpy4	2.39	15
ENSXMAG00000015383	0.0031	24	12	rmnd1	2.31	21
ENSXMAG00000015385	0.0026	6	15	aspg	2.34	3
ENSXMAG00000015394	0.0030	24	12		1.93	21
ENSXMAG00000015434	0.0031	18	12	sap30l	1.46	15
ENSXMAG00000015442	0.0031	24	12	nars2	1.93	21
ENSXMAG00000015468	0.0034	18	12	DHX35	2.61	15
ENSXMAG00000015473	0.0025	6	15	bag4	2.26	3
ENSXMAG00000015491	0.0024	3	15	dapk1	2.93	0
ENSXMAG00000015512	0.0027	3	15	rnf114	2.02	0
ENSXMAG00000015525	0.0025	24	9	trpm6	2.46	21
ENSXMAG00000015527	0.0024	3	15	si:ch73-335l21.1	2.62	0
ENSXMAG00000015541	0.0014	15	18	EIF4ENIF1	1.46	12
ENSXMAG00000015553	0.0029	18	12	clptm1l	3.01	15
ENSXMAG00000015558	0.0071	15	6	vclb	1.57	12
ENSXMAG00000015571	0.0034	24	12	atp10d	1.88	21
ENSXMAG00000015579	0.0031	3	12	kank1a	2.19	0
ENSXMAG00000015583	0.0034	21	12	ercc8	1.59	18
ENSXMAG00000015601	0.0031	21	12	coasy	2.21	18
ENSXMAG00000015605	0.0025	18	15	ZSWIM6	2.11	15
ENSXMAG00000015611	0.0014	24	18	smarca2	1.58	21
ENSXMAG00000015618	0.0025	12	9	uckl1b	1.68	9
ENSXMAG00000015619	0.0025	18	9	arntl1a	22.30	15
ENSXMAG00000015632	0.0028	3	15		6.45	0
ENSXMAG00000015645	0.0026	18	15	adck1	3.31	15
ENSXMAG00000015676	0.0027	18	9	SRSF2	1.47	15
ENSXMAG00000015693	0.0031	18	12	ppm1la	1.76	15
ENSXMAG00000015696	0.0025	18	9	ARL3	2.52	15
ENSXMAG00000015708	0.0031	18	12		1.80	15
ENSXMAG00000015709	0.0014	24	18	pcbp4 (1 of many)	2.41	21
ENSXMAG00000015713	0.0024	3	15	si:dkey-219e21.2	2.50	0
ENSXMAG00000015752	0.0026	9	15	eef2k	3.55	6
ENSXMAG00000015759	0.0030	24	12	eif2b3	1.72	21
ENSXMAG00000015779	0.0031	18	12	plekhh1	3.00	15
ENSXMAG00000015781	0.0034	21	12	DCAF1	1.67	18

ENSXMAG00000015788	0.0025	18	9	ctdnep1b	3.35	15
ENSXMAG00000015790	0.0026	18	9	ssuh2rs1	1.69	15
ENSXMAG00000015803	0.0026	6	15	grip2a	2.16	3
ENSXMAG00000015817	0.0027	18	15	trmt44	1.77	15
ENSXMAG00000015832	0.0029	6	12	slc6a6a	2.32	3
ENSXMAG00000015833	0.0031	9	12	crip2	1.99	6
ENSXMAG00000015840	0.0024	18	9		5.30	15
ENSXMAG00000015841	0.0026	15	9	HGFAC	1.83	12
ENSXMAG00000015843	0.0027	18	9	crip1	1.66	15
ENSXMAG00000015844	0.0031	12	12	arsh	2.97	9
ENSXMAG00000015864	0.0024	24	15	fam20cb	1.80	21
ENSXMAG00000015904	0.0026	6	15		4.49	3
ENSXMAG00000015939	0.0031	15	12	ptprna	3.85	12
ENSXMAG00000015956	0.0014	18	18	pofut2	1.70	15
ENSXMAG00000015984	0.0026	18	9	polr2c	1.61	15
ENSXMAG00000015986	0.0015	18	6	HABP4	2.25	15
ENSXMAG00000015990	0.0025	21	9	ppm1bb	1.20	18
ENSXMAG00000016005	0.0024	6	15	tmem176	1.32	3
ENSXMAG00000016045	0.0016	21	6	gch2	9.13	18
ENSXMAG00000016066	0.0027	24	9		1.32	21
ENSXMAG00000016078	0.0026	21	15	mesd	1.86	18
ENSXMAG00000016094	0.0024	18	15		1.83	15
ENSXMAG00000016101	0.0034	18	12		1.97	15
ENSXMAG00000016104	0.0025	21	15	entpd6	1.30	18
ENSXMAG00000016115	0.0031	24	12	sept5b	3.29	21
ENSXMAG00000016124	0.0016	15	18	nipa2	2.05	12
ENSXMAG00000016125	0.0024	18	9	sptlc1	2.40	15
ENSXMAG00000016133	0.0015	3	6	herc2	1.91	0
ENSXMAG00000016136	0.0030	18	12	tjap1	1.35	15
ENSXMAG00000016139	0.0031	18	12	fdps	1.51	15
ENSXMAG00000016159	0.0031	24	12	atp8a1	1.46	21
ENSXMAG00000016160	0.0024	18	15	pbrm1l	1.50	15
ENSXMAG00000016170	0.0024	15	15	ostf1	1.69	12
ENSXMAG00000016178	0.0034	18	12	dnajc11a	1.90	15
ENSXMAG00000016191	0.0024	24	15		1.43	21
ENSXMAG00000016192	0.0031	24	12	chd9	1.68	21
ENSXMAG00000016215	0.0034	21	12	polr3c	1.73	18
ENSXMAG00000016223	0.0029	18	12		1.84	15
ENSXMAG00000016226	0.0031	18	12		2.74	15
ENSXMAG00000016233	0.0034	21	12		1.71	18
ENSXMAG00000016258	0.0030	21	12	prkacbb	1.49	18
ENSXMAG00000016259	0.0031	18	12	ppp6r3	1.35	15
ENSXMAG00000016261	0.0025	15	15		1.57	12
ENSXMAG00000016312	0.0034	21	12	vldlr	2.65	18
ENSXMAG00000016318	0.0027	6	15	nod2	3.83	3
ENSXMAG00000016330	0.0031	21	12	pomk	1.90	18
ENSXMAG00000016336	0.0034	18	12		4.32	15
ENSXMAG00000016342	0.0026	18	15	ctcf	1.42	15
ENSXMAG00000016346	0.0030	21	12	brd7	1.69	18
ENSXMAG00000016371	0.0025	18	9	lypla2	2.28	15
ENSXMAG00000016454	0.0026	24	9		1.86	21
ENSXMAG00000016460	0.0026	18	9	hsp90b1	2.83	15
ENSXMAG00000016472	0.0029	9	12	MITF (1 of many)	4.96	6
ENSXMAG00000016477	0.0025	18	15	b4galt7	1.59	15
ENSXMAG00000016482	0.0027	15	9	egfra	1.35	12
ENSXMAG00000016508	0.0034	18	12	cers2b	1.68	15

ENSXMAG00000016525	0.0034	24	12	flnca	1.70	21
ENSXMAG00000016530	0.0026	18	9	sema3fa	1.58	15
ENSXMAG00000016561	0.0025	21	9	zgc:91910	1.29	18
ENSXMAG00000016586	0.0030	18	12	ssu72	2.20	15
ENSXMAG00000016594	0.0015	18	6		5.08	15
ENSXMAG00000016601	0.0031	18	12	ATAD3A	6.98	15
ENSXMAG00000016630	0.0030	18	12	si:ch211-215a10.4	2.30	15
ENSXMAG00000016644	0.0016	15	6	TMCC3	1.57	12
ENSXMAG00000016646	0.0031	21	12	taf11	1.80	18
ENSXMAG00000016651	0.0027	24	9	NR2C1	1.45	21
ENSXMAG00000016652	0.0031	18	12		3.36	15
ENSXMAG00000016667	0.0014	18	6		11.15	15
ENSXMAG00000016682	0.0024	24	9		1.51	21
ENSXMAG00000016690	0.0028	15	9		3.28	12
ENSXMAG00000016695	0.0027	15	9		3.33	12
ENSXMAG00000016711	0.0024	18	9	ppp1r10	4.58	15
ENSXMAG00000016718	0.0029	18	12	lpin2	2.84	15
ENSXMAG00000016736	0.0027	18	15	pfkfb2a	2.64	15
ENSXMAG00000016749	0.0025	18	9	uap1l1	1.60	15
ENSXMAG00000016761	0.0026	18	9	cyp1a	24.17	15
ENSXMAG00000016764	0.0025	18	9	prkar2ab	1.68	15
ENSXMAG00000016837	0.0025	18	9	prkaa1	2.07	15
ENSXMAG00000016907	0.0031	21	12	PNPT1	1.36	18
ENSXMAG00000016908	0.0025	18	9		4.05	15
ENSXMAG00000016914	0.0025	3	15	oxsm	2.71	0
ENSXMAG00000016928	0.0031	18	12	clocka	18.46	15
ENSXMAG00000016929	0.0026	24	15	fyco1b	2.79	21
ENSXMAG00000016931	0.0029	18	12	tmcc1b	4.63	15
ENSXMAG00000016936	0.0031	18	12	top2b	2.10	15
ENSXMAG00000016942	0.0031	18	12	slc35b4	1.58	15
ENSXMAG00000016953	0.0031	18	12	golt1bb	4.06	15
ENSXMAG00000016977	0.0026	24	9	brpf3a	1.83	21
ENSXMAG00000016979	0.0026	18	15	pthlhb	1.82	15
ENSXMAG00000016983	0.0024	18	9	tmed3	1.97	15
ENSXMAG00000016994	0.0026	18	9	nt5c1bb	2.77	15
ENSXMAG00000017001	0.0031	18	12	THEM6	8.37	15
ENSXMAG00000017005	0.0014	3	18		1.79	0
ENSXMAG00000017011	0.0031	18	12	lrrc59	2.60	15
ENSXMAG00000017027	0.0031	18	12	timm23a	2.46	15
ENSXMAG00000017032	0.0031	21	12	skib	1.90	18
ENSXMAG00000017048	0.0031	18	12	cpsf2	2.28	15
ENSXMAG00000017055	0.0031	12	12	wdr76	5.43	9
ENSXMAG00000017066	0.0015	15	6	thrb	1.46	12
ENSXMAG00000017101	0.0026	18	9	fzd9b	3.44	15
ENSXMAG00000017133	0.0026	18	15	sec11a	1.59	15
ENSXMAG00000017138	0.0024	18	15	prmt9	2.63	15
ENSXMAG00000017141	0.0024	18	15	ssr3	1.42	15
ENSXMAG00000017148	0.0026	24	9	fut8b	1.32	21
ENSXMAG00000017156	0.0025	18	9		2.38	15
ENSXMAG00000017158	0.0031	21	12		2.27	18
ENSXMAG00000017173	0.0027	3	15	gls2a	8.55	0
ENSXMAG00000017191	0.0024	21	15	TMEM19	1.38	18
ENSXMAG00000017201	0.0015	3	18	tnk2b	2.74	0
ENSXMAG00000017224	0.0025	18	9	HYAL2 (1 of many)	3.97	15
ENSXMAG00000017242	0.0015	3	18	tln2a	2.50	0
ENSXMAG00000017251	0.0026	24	9	slc8a1b	2.36	21

ENSXMAG00000017252	0.0026	3	15		3.41	0
ENSXMAG00000017286	0.0026	6	15	adcy6a	1.93	3
ENSXMAG00000017331	0.0029	18	12	SACM1L	2.43	15
ENSXMAG00000017347	0.0024	21	9	phf6	1.69	18
ENSXMAG00000017376	0.0029	18	12	rer1	1.92	15
ENSXMAG00000017382	0.0024	3	15	tanc2a	2.60	0
ENSXMAG00000017407	0.0025	18	15	tamm41	2.52	15
ENSXMAG00000017411	0.0030	21	12	cyb5r3	1.17	18
ENSXMAG00000017412	0.0014	3	18	hdac5	3.50	0
ENSXMAG00000017416	0.0025	18	9	ptges3b	2.17	15
ENSXMAG00000017432	0.0027	3	15	kbtbd12	2.31	0
ENSXMAG00000017437	0.0030	15	12	ezrb	2.85	12
ENSXMAG00000017448	0.0014	21	6	creld1b	2.97	18
ENSXMAG00000017466	0.0031	12	12		2.61	9
ENSXMAG00000017503	0.0026	18	9	ints7	3.16	15
ENSXMAG00000017520	0.0031	6	12	lpin1	5.28	3
ENSXMAG00000017572	0.0031	18	12	ptprk	2.28	15
ENSXMAG00000017579	0.0026	24	15	SUZ12	1.35	21
ENSXMAG00000017586	0.0024	18	9	tcea1	1.84	15
ENSXMAG00000017597	0.0014	21	6	sod1	1.35	18
ENSXMAG00000017630	0.0015	3	18	grik1a	3.17	0
ENSXMAG00000017655	0.0031	24	12	lzts2b	2.99	21
ENSXMAG00000017656	0.0025	24	15		1.51	21
ENSXMAG00000017670	0.0031	21	12	rab38b	1.96	18
ENSXMAG00000017683	0.0031	18	12	ece1	2.45	15
ENSXMAG00000017688	0.0015	3	18		4.54	0
ENSXMAG00000017703	0.0030	18	12	plekhm1	1.53	15
ENSXMAG00000017707	0.0024	18	15	chordc1b	1.73	15
ENSXMAG00000017736	0.0031	21	12	timm10	1.69	18
ENSXMAG00000017748	0.0031	24	12		31.98	21
ENSXMAG00000017749	0.0027	3	15	rdh8a	2.15	0
ENSXMAG00000017759	0.0026	18	9	myg1	1.76	15
ENSXMAG00000017764	0.0024	24	9	ELF2	1.76	21
ENSXMAG00000017774	0.0031	18	12	NAA15	1.58	15
ENSXMAG00000017782	0.0025	3	15	ncoa1	7.66	0
ENSXMAG00000017805	0.0024	15	15		1.48	12
ENSXMAG00000017814	0.0031	24	12	clgn	2.01	21
ENSXMAG00000017819	0.0029	18	12	gpam	3.32	15
ENSXMAG00000017835	0.0025	24	9	prss12	2.51	21
ENSXMAG00000017846	0.0030	21	12	mettl14	2.10	18
ENSXMAG00000017859	0.0026	24	9	ebf2	1.78	21
ENSXMAG00000017861	0.0025	18	15	proser1	2.22	15
ENSXMAG00000017872	0.0026	18	9		1.28	15
ENSXMAG00000017878	0.0026	24	9	kptn	1.76	21
ENSXMAG00000017888	0.0024	24	9		1.86	21
ENSXMAG00000017892	0.0016	24	18	tmem106a	2.36	21
ENSXMAG00000017899	0.0030	24	12	meox1	1.57	21
ENSXMAG00000017903	0.0014	9	18		5.08	6
ENSXMAG00000017910	0.0025	24	9	si:ch211-102c2.7	3.01	21
ENSXMAG00000017924	0.0026	21	9	ttll7	1.68	18
ENSXMAG00000017935	0.0025	21	9	SNRPC	1.30	18
ENSXMAG00000017938	0.0025	21	9	nsfb	2.05	18
ENSXMAG00000017962	0.0026	18	15	thrap3b	1.10	15
ENSXMAG00000017978	0.0025	18	15	r3hdm2	1.35	15
ENSXMAG00000017996	0.0026	18	9	igfn1.2	2.24	15
ENSXMAG00000018003	0.0026	24	15	ddx18	1.37	21

ENSXMAG00000018008	0.0031	18	12		1.63	15
ENSXMAG00000018071	0.0014	24	18	pik3ip1	5.35	21
ENSXMAG00000018073	0.0025	3	9	igdcc4	3.97	0
ENSXMAG00000018096	0.0031	24	12	plp2 (1 of many)	1.85	21
ENSXMAG00000018113	0.0025	21	15		1.35	18
ENSXMAG00000018125	0.0029	18	12	mfap1	1.24	15
ENSXMAG00000018131	0.0030	18	12		1.53	15
ENSXMAG00000018135	0.0031	3	12		2.17	0
ENSXMAG00000018170	0.0031	24	12	mfn1b	1.63	21
ENSXMAG00000018174	0.0024	24	9	cep170aa	1.87	21
ENSXMAG00000018177	0.0026	18	9	zgc:162344	3.84	15
ENSXMAG00000018190	0.0024	6	15	myorg	1.45	3
ENSXMAG00000018199	0.0024	18	9	coro1ca	2.41	15
ENSXMAG00000018200	0.0025	18	15	timm17b	2.23	15
ENSXMAG00000018203	0.0029	18	12	trappc6bl	1.78	15
ENSXMAG00000018222	0.0034	24	12	gusb	1.33	21
ENSXMAG00000018223	0.0025	18	15	actl6a	2.10	15
ENSXMAG00000018227	0.0031	15	12	bcl2l10	3.37	12
ENSXMAG00000018243	0.0026	3	9		1.95	0
ENSXMAG00000018260	0.0014	18	18	tp53bp1	1.57	15
ENSXMAG00000018262	0.0015	21	6	pxmp2	1.77	18
ENSXMAG00000018304	0.0031	18	12	TADA1	1.85	15
ENSXMAG00000018334	0.0024	6	15		2.56	3
ENSXMAG00000018345	0.0026	18	15	cop1	1.65	15
ENSXMAG00000018348	0.0024	24	9	FAT4	2.60	21
ENSXMAG00000018350	0.0031	15	12	tspy	1.20	12
ENSXMAG00000018370	0.0014	21	6	golga7	1.25	18
ENSXMAG00000018390	0.0031	24	12	erbb2	1.88	21
ENSXMAG00000018398	0.0026	21	15	trmt61b	4.48	18
ENSXMAG00000018422	0.0024	9	9	ankrd9	11.94	6
ENSXMAG00000018424	0.0031	15	12	si:dkey-30h22.11	2.86	12
ENSXMAG00000018442	0.0024	3	15		5.31	0
ENSXMAG00000018474	0.0026	18	9	pelo	2.36	15
ENSXMAG00000018477	0.0026	24	9	PLXNA1	1.48	21
ENSXMAG00000018498	0.0026	18	9	nol10	2.28	15
ENSXMAG00000018511	0.0029	18	12	timm44	1.94	15
ENSXMAG00000018525	0.0025	18	9	MSN	1.76	15
ENSXMAG00000018535	0.0025	21	15	si:dkey-33c12.4	1.39	18
ENSXMAG00000018547	0.0029	18	12	daam2	1.64	15
ENSXMAG00000018549	0.0030	3	12	cryz	1.44	0
ENSXMAG00000018560	0.0015	21	6	tox	2.43	18
ENSXMAG00000018589	0.0024	18	9	tgm2b	4.23	15
ENSXMAG00000018617	0.0031	15	12	zmp:0000000846	1.40	12
ENSXMAG00000018623	0.0015	15	18	capn12	1.74	12
ENSXMAG00000018629	0.0025	15	15	BRD2 (1 of many)	1.86	12
ENSXMAG00000018630	0.0027	18	15	lsm7	1.41	15
ENSXMAG00000018635	0.0014	24	18	enc3	3.33	21
ENSXMAG00000018648	0.0026	3	15	insrb	4.82	0
ENSXMAG00000018662	0.0031	24	12	satb1b	2.28	21
ENSXMAG00000018668	0.0026	3	15	odr4	1.52	0
ENSXMAG00000018691	0.0015	18	6	pxdn	1.70	15
ENSXMAG00000018702	0.0025	9	15	HOMER1 (1 of many)	3.57	6
ENSXMAG00000018707	0.0027	3	15	vdrb	3.22	0
ENSXMAG00000018710	0.0014	24	18	JMY	6.69	21
ENSXMAG00000018717	0.0031	21	12	prg4a	1.76	18
ENSXMAG00000018730	0.0025	18	15	cope	1.28	15

ENSXMAG00000018753	0.0026	18	9	lhfpl2a	2.41	15
ENSXMAG00000018761	0.0027	18	9	ldlrap1b	2.59	15
ENSXMAG00000018773	0.0026	12	15	si:ch1073-358c10.1	2.75	9
ENSXMAG00000018776	0.0028	15	9	phkg2	7.19	12
ENSXMAG00000018780	0.0029	24	12	larp1	2.19	21
ENSXMAG00000018816	0.0031	3	12	lyrm1	1.35	0
ENSXMAG00000018818	0.0026	21	9		1.40	18
ENSXMAG00000018819	0.0027	18	9		13.07	15
ENSXMAG00000018837	0.0026	18	9	ube2ia	1.41	15
ENSXMAG00000018844	0.0028	18	15	rps6ka1	1.62	15
ENSXMAG00000018853	0.0025	18	9	ddx19	2.18	15
ENSXMAG00000018866	0.0025	18	9	slc25a36a (1 of many)	2.51	15
ENSXMAG00000018868	0.0031	18	12	mfsd12a	1.51	15
ENSXMAG00000018883	0.0015	24	18	asb10	9.21	21
ENSXMAG00000018887	0.0025	18	15	spsb4a	5.75	15
ENSXMAG00000018896	0.0024	18	15	rpn1	2.02	15
ENSXMAG00000018900	0.0031	18	12	prkx	1.43	15
ENSXMAG00000018906	0.0031	24	12	zgc:154055	1.51	21
ENSXMAG00000018910	0.0015	24	18	tmem106ba	1.19	21
ENSXMAG00000018925	0.0015	24	18	F2RL2 (1 of many)	3.97	21
ENSXMAG00000018934	0.0029	24	12		23.65	21
ENSXMAG00000018937	0.0026	18	9	copb2	2.01	15
ENSXMAG00000018946	0.0031	18	12	dync1li2	2.52	15
ENSXMAG00000018958	0.0024	3	9	acbd6	1.47	0
ENSXMAG00000018961	0.0026	3	15	med15	2.36	0
ENSXMAG00000018995	0.0024	9	15	cep350	1.86	6
ENSXMAG00000019000	0.0025	24	9	mzt2b	1.46	21
ENSXMAG00000019003	0.0025	21	15	agmo	3.45	18
ENSXMAG00000019005	0.0024	3	15	trim23	2.01	0
ENSXMAG00000019008	0.0025	21	15	cog7	1.41	18
ENSXMAG00000019027	0.0025	18	15	ppie	2.61	15
ENSXMAG00000019046	0.0031	18	12	adamts6	3.21	15
ENSXMAG00000019050	0.0027	18	15	telo2	1.96	15
ENSXMAG00000019054	0.0031	18	12	rrad	5.29	15
ENSXMAG00000019055	0.0034	18	12	actr5	2.41	15
ENSXMAG00000019056	0.0031	18	12	gale	2.32	15
ENSXMAG00000019058	0.0026	18	9	SLC25A12 (1 of many)	1.78	15
ENSXMAG00000019078	0.0025	18	15		6.31	15
ENSXMAG00000019083	0.0024	24	15	PGM1	2.58	21
ENSXMAG00000019084	0.0025	24	9	map4k4	1.76	21
ENSXMAG00000019139	0.0029	21	12	ppp1r9alb	4.24	18
ENSXMAG00000019168	0.0016	24	18	arhgef37	2.93	21
ENSXMAG00000019173	0.0027	18	15		1.25	15
ENSXMAG00000019200	0.0024	18	15	fbxo18	1.67	15
ENSXMAG00000019215	0.0025	24	9	sema5a	6.69	21
ENSXMAG00000019220	0.0024	3	15	nsun5	2.69	0
ENSXMAG00000019224	0.0024	18	15	slc26a2	1.98	15
ENSXMAG00000019225	0.0015	12	18		10.46	9
ENSXMAG00000019228	0.0024	18	9		2.50	15
ENSXMAG00000019259	0.0034	24	12		1.75	21
ENSXMAG00000019296	0.0029	12	12	rorcb	32.83	9
ENSXMAG00000019318	0.0026	18	9	ifrd2	1.60	15
ENSXMAG00000019320	0.0031	18	12	plekhj1	2.98	15
ENSXMAG00000019340	0.0024	18	15	ARMC6	2.64	15
ENSXMAG00000019341	0.0025	3	9		1.39	0
ENSXMAG00000019357	0.0031	15	12	dnase1l4.2	2.35	12

ENSXMAG00000019360	0.0028	12	15	xpc	5.25	9
ENSXMAG00000019370	0.0034	9	12		33.60	6
ENSXMAG00000019391	0.0024	18	9	gnb1a	2.34	15
ENSXMAG00000019414	0.0027	24	9	mef2cb	1.87	21
ENSXMAG00000019415	0.0029	18	12	tmem161b	2.78	15
ENSXMAG00000019427	0.0026	21	9	mterf2	2.98	18
ENSXMAG00000019487	0.0031	18	12	SLC35A4	3.27	15
ENSXMAG00000019499	0.0024	24	9	rab9b	2.68	21
ENSXMAG00000019527	0.0031	9	12	nfil3-6	15.32	6
ENSXMAG00000019531	0.0024	15	9	pnrc2	4.05	12
ENSXMAG00000019533	0.0014	21	6	nog2	2.11	18
ENSXMAG00000019536	0.0024	3	15	rpz	1.47	0
ENSXMAG00000019558	0.0025	15	15	lrrc8da	1.90	12
ENSXMAG00000019575	0.0024	3	15	ZBTB26	2.89	0
ENSXMAG00000019579	0.0025	3	9	socs9	1.58	0
ENSXMAG00000019661	0.0026	15	15	zgc:63470	1.21	12
ENSXMAG00000019746	0.0026	3	15	pop7	2.29	0
ENSXMAG00000019759	0.0025	15	15		1.82	12
ENSXMAG00000019772	0.0015	3	18		1.94	0
ENSXMAG00000019788	0.0029	6	12	NHLRC1	3.44	3
ENSXMAG00000019795	0.0026	24	9	thap11	1.72	21
ENSXMAG00000019818	0.0031	3	12		1.70	0
ENSXMAG00000019861	0.0026	24	9		1.75	21
ENSXMAG00000019865	0.0029	9	12	si:ch73-334d15.1	6.26	6
ENSXMAG00000019885	0.0025	18	15	snrnp35	1.96	15
ENSXMAG00000019893	0.0034	3	12	smim13	1.46	0
ENSXMAG00000019937	0.0029	18	12	abhd13	2.57	15
ENSXMAG00000019943	0.0031	18	12	nfil3	9.91	15
ENSXMAG00000019971	0.0071	3	6	sf3b5	1.21	0
ENSXMAG00000019975	0.0031	12	12		1.53	9
ENSXMAG00000019995	0.0014	24	18	arl4aa	2.41	21
ENSXMAG00000020012	0.0026	21	15	emc6	1.38	18
ENSXMAG00000020022	0.0026	21	9		1.94	18
ENSXMAG00000020062	0.0026	21	15	twist2	2.19	18
ENSXMAG00000020081	0.0024	3	15		2.87	0
ENSXMAG00000020142	0.0031	6	12		2.03	3
ENSXMAG00000020162	0.0029	15	12	col10a1a	2.73	12
ENSXMAG00000020287	0.0029	18	12	dpm3	1.83	15
ENSXMAG00000020395	0.0026	3	15	tlr22 (1 of many)	2.25	0
ENSXMAG00000020519	0.0026	21	15	MIR133B	2.99	18
ENSXMAG00000020673	0.0015	15	6		2.10	12
ENSXMAG00000020852	0.0025	18	15	asrgl1	2.83	15
ENSXMAG00000020870	0.0031	21	12	adprhl1	1.84	18
ENSXMAG00000020882	0.0029	18	12		1.38	15
ENSXMAG00000020886	0.0031	18	12	ADAMTS5	1.57	15
ENSXMAG00000020907	0.0026	18	9		4.41	15
ENSXMAG00000020921	0.0024	18	15		1.62	15
ENSXMAG00000020974	0.0026	21	9		3.84	18
ENSXMAG00000021004	0.0029	9	12		1.85	6
ENSXMAG00000021009	0.0026	24	9		2.54	21
ENSXMAG00000021020	0.0026	18	9	nfe2l3	3.43	15
ENSXMAG00000021031	0.0026	18	15		1.37	15
ENSXMAG00000021037	0.0031	18	12	esrra	5.70	15
ENSXMAG00000021049	0.0028	15	9	si:ch211-103n10.5	4.02	12
ENSXMAG00000021050	0.0031	21	12	CRACR2A (1 of many)	2.62	18
ENSXMAG00000021074	0.0024	18	15	asf1bb	1.62	15

ENSXMAG00000021078	0.0031	18	12		2.16	15
ENSXMAG00000021101	0.0029	18	12	NDE1 (1 of many)	3.87	15
ENSXMAG00000021110	0.0026	24	15	cbll1	1.27	21
ENSXMAG00000021113	0.0014	15	18		2.88	12
ENSXMAG00000021125	0.0026	18	9	smfn	1.96	15
ENSXMAG00000021129	0.0024	3	15	mbd4	3.47	0
ENSXMAG00000021137	0.0014	3	18		1.30	0
ENSXMAG00000021147	0.0024	18	9	PPP1R1A	2.66	15
ENSXMAG00000021149	0.0027	18	9	cdc42ep3	2.68	15
ENSXMAG00000021162	0.0031	15	12		2.09	12
ENSXMAG00000021168	0.0015	21	6		3.20	18
ENSXMAG00000021203	0.0031	18	12	ZHX1 (1 of many)	2.12	15
ENSXMAG00000021224	0.0024	3	15	zgc:101853	1.81	0
ENSXMAG00000021227	0.0026	18	9	slc30a7	1.94	15
ENSXMAG00000021235	0.0025	18	9	si:ch211-11k18.4	4.74	15
ENSXMAG00000021261	0.0028	3	15	stx18	2.20	0
ENSXMAG00000021264	0.0025	18	9	pias4a	2.06	15
ENSXMAG00000021275	0.0026	21	15	mcat	1.45	18
ENSXMAG00000021286	0.0026	3	15	jarid2b	1.78	0
ENSXMAG00000021333	0.0031	6	12	abhd2a	3.48	3
ENSXMAG00000021384	0.0027	24	9	spen	1.83	21
ENSXMAG00000021418	0.0027	15	9		2.37	12
ENSXMAG00000021435	0.0015	3	18		1.93	0
ENSXMAG00000021436	0.0025	3	9	znf1115	2.91	0
ENSXMAG00000021448	0.0024	9	15	keap1b	3.53	6
ENSXMAG00000021546	0.0025	15	15	myd88	1.69	12
ENSXMAG00000021554	0.0031	18	12		1.77	15
ENSXMAG00000021577	0.0027	3	15	rpp25l	1.39	0
ENSXMAG00000021579	0.0029	15	12		1.89	12
ENSXMAG00000021620	0.0030	18	12		2.14	15
ENSXMAG00000021623	0.0025	18	9	marcksb	5.82	15
ENSXMAG00000021634	0.0034	18	12	camlg	2.52	15
ENSXMAG00000021639	0.0027	3	15		2.92	0
ENSXMAG00000021653	0.0025	21	9	mtnr1c	2.32	18
ENSXMAG00000021677	0.0025	3	9	rgs7bpb	1.78	0
ENSXMAG00000021695	0.0025	18	9	zgc:64051	1.33	15
ENSXMAG00000021736	0.0026	18	9	papss1	2.40	15
ENSXMAG00000021795	0.0014	21	18		1.46	18
ENSXMAG00000021830	0.0025	3	15	si:ch1073-322p19.1	1.91	0
ENSXMAG00000021847	0.0026	24	9	p4hb	1.73	21
ENSXMAG00000021850	0.0016	3	18		1.81	0
ENSXMAG00000021901	0.0027	3	15		5.87	0
ENSXMAG00000021908	0.0025	24	9	tbx15	2.86	21
ENSXMAG00000021914	0.0026	18	9	trib2	4.00	15
ENSXMAG00000021924	0.0029	21	12	mprip	1.39	18
ENSXMAG00000021952	0.0031	18	12	pdcd7	1.87	15
ENSXMAG00000021983	0.0025	18	9	CDK2AP1	5.19	15
ENSXMAG00000022009	0.0031	24	12		1.84	21
ENSXMAG00000022013	0.0031	15	12		2.37	12
ENSXMAG00000022075	0.0027	21	9		1.71	18
ENSXMAG00000022076	0.0026	3	15	sharpin	1.38	0
ENSXMAG00000022086	0.0031	18	12	aptx	1.70	15
ENSXMAG00000022104	0.0026	9	9	psenen	1.78	6
ENSXMAG00000022116	0.0030	21	12		1.38	18
ENSXMAG00000022190	0.0025	18	9		2.76	15
ENSXMAG00000022220	0.0025	9	9	hspb6	6.16	6

ENSXMAG00000022235	0.0025	9	15		2.78	6
ENSXMAG00000022242	0.0027	24	9		5.53	21
ENSXMAG00000022277	0.0029	18	12	nxt2	2.12	15
ENSXMAG00000022292	0.0030	21	12	cd276	2.40	18
ENSXMAG00000022293	0.0029	18	12		2.44	15
ENSXMAG00000022324	0.0030	18	12	narf	2.54	15
ENSXMAG00000022328	0.0025	18	9	RHOA	2.16	15
ENSXMAG00000022329	0.0034	18	12	si:ch211-212g7.6	2.24	15
ENSXMAG00000022336	0.0025	9	15		1.98	6
ENSXMAG00000022360	0.0034	18	12	rap2c	1.93	15
ENSXMAG00000022371	0.0016	24	18		1.97	21
ENSXMAG00000022375	0.0031	9	12	rhoub	6.09	6
ENSXMAG00000022380	0.0027	3	15	rnf144b	2.86	0
ENSXMAG00000022390	0.0027	18	15		1.80	15
ENSXMAG00000022556	0.0028	15	9		3.64	12
ENSXMAG00000022566	0.0026	18	9	INHBB (1 of many)	4.23	15
ENSXMAG00000022571	0.0028	15	9	HBE1 (1 of many)	2.98	12
ENSXMAG00000022591	0.0016	24	18		4.34	21
ENSXMAG00000022593	0.0024	18	9	si:ch211-231f6.6	1.80	15
ENSXMAG00000022604	0.0031	21	12		1.88	18
ENSXMAG00000022640	0.0029	18	12		13.45	15
ENSXMAG00000022645	0.0025	18	15	prkacaa	1.81	15
ENSXMAG00000022653	0.0014	15	18		2.28	12
ENSXMAG00000022666	0.0031	18	12		1.67	15
ENSXMAG00000022689	0.0027	18	15	zgc:109986	1.61	15
ENSXMAG00000022701	0.0026	18	15		1.65	15
ENSXMAG00000022719	0.0024	12	15	prelp	1.50	9
ENSXMAG00000022749	0.0025	18	9		1.41	15
ENSXMAG00000022771	0.0026	15	9		1.58	12
ENSXMAG00000022802	0.0014	15	18		1.85	12
ENSXMAG00000022811	0.0025	24	15	ciarta	13.25	21
ENSXMAG00000022828	0.0031	18	12		1.58	15
ENSXMAG00000022862	0.0029	21	12	abhd18	1.58	18
ENSXMAG00000022875	0.0024	15	15	r3hdm4	1.63	12
ENSXMAG00000022904	0.0025	6	15		7.04	3
ENSXMAG00000022939	0.0024	18	9	ppp1caa	2.19	15
ENSXMAG00000022940	0.0026	18	9		25.47	15
ENSXMAG00000022943	0.0025	18	15		1.72	15
ENSXMAG00000023022	0.0026	21	15		1.79	18
ENSXMAG00000023032	0.0014	21	6	rasl11a	2.51	18
ENSXMAG00000023043	0.0014	21	6	opa3	2.29	18
ENSXMAG00000023061	0.0025	9	15		4.95	6
ENSXMAG00000023124	0.0024	18	9	esrrd	2.27	15
ENSXMAG00000023128	0.0026	15	9		4.39	12
ENSXMAG00000023142	0.0031	24	12		1.56	21
ENSXMAG00000023145	0.0026	18	9		2.47	15
ENSXMAG00000023150	0.0025	3	15	HOXA4	3.22	0
ENSXMAG00000023202	0.0026	15	15	gdf10a	2.80	12
ENSXMAG00000023269	0.0029	18	12	necap2	2.83	15
ENSXMAG00000023274	0.0029	18	12	klhdc3	1.63	15
ENSXMAG00000023302	0.0014	24	18	ctdsp2	5.12	21
ENSXMAG00000023303	0.0031	18	12	taf5l	3.25	15
ENSXMAG00000023312	0.0031	24	12	faxdc2	1.62	21
ENSXMAG00000023314	0.0071	21	6		1.20	18
ENSXMAG00000023330	0.0024	21	9	mef2d	1.75	18
ENSXMAG00000023344	0.0026	18	15		2.22	15

ENSXMAG00000023354	0.0015	24	18	zfand5a	1.48	21
ENSXMAG00000023381	0.0024	3	9	thtpa	1.66	0
ENSXMAG00000023412	0.0025	18	9	bnip1a	2.36	15
ENSXMAG00000023420	0.0026	24	9	cbfa2t3	2.04	21
ENSXMAG00000023436	0.0014	24	18		13.32	21
ENSXMAG00000023477	0.0034	18	12		2.21	15
ENSXMAG00000023486	0.0029	18	12	chp1	1.91	15
ENSXMAG00000023487	0.0031	18	12	si:dkey-4e7.3 (1 of many)	2.26	15
ENSXMAG00000023523	0.0024	3	15		2.94	0
ENSXMAG00000023550	0.0031	18	12	aqp12	4.37	15
ENSXMAG00000023558	0.0034	18	12	SUOX	2.81	15
ENSXMAG00000023566	0.0024	3	15	pex2	1.57	0
ENSXMAG00000023567	0.0026	18	9	eif4e1c	3.43	15
ENSXMAG00000023570	0.0027	3	15	abrab	12.66	0
ENSXMAG00000023602	0.0031	9	12		4.71	6
ENSXMAG00000023617	0.0028	3	15	rab12	1.44	0
ENSXMAG00000023624	0.0024	21	15	krr1	1.73	18
ENSXMAG00000023636	0.0025	18	9		2.20	15
ENSXMAG00000023645	0.0027	3	15	kdm4c	2.16	0
ENSXMAG00000023646	0.0026	24	9	sash1b	1.86	21
ENSXMAG00000023653	0.0025	21	9	mettl3	1.87	18
ENSXMAG00000023654	0.0025	6	15		3.35	3
ENSXMAG00000023656	0.0027	3	15		2.15	0
ENSXMAG00000023666	0.0014	24	18		3.97	21
ENSXMAG00000023667	0.0024	21	15	ube2c	1.34	18
ENSXMAG00000023673	0.0031	15	12	slc25a15b	1.62	12
ENSXMAG00000023681	0.0024	9	15		1.55	6
ENSXMAG00000023701	0.0025	21	9	si:ch211-270g19.5	2.24	18
ENSXMAG00000023706	0.0029	18	12	PFN2	1.61	15
ENSXMAG00000023732	0.0034	18	12	rab11a	1.41	15
ENSXMAG00000023736	0.0024	3	15	fam50a	1.41	0
ENSXMAG00000023756	0.0026	18	9	hmbsa	2.36	15
ENSXMAG00000023765	0.0026	18	15		3.14	15
ENSXMAG00000023781	0.0030	6	12	ugt1a2 (1 of many)	3.74	3
ENSXMAG00000023787	0.0034	24	12	avpr1ab	2.55	21
ENSXMAG00000023808	0.0025	12	9	clec11a	2.59	9
ENSXMAG00000023815	0.0029	18	12	ATP6V0C	1.91	15
ENSXMAG00000023819	0.0029	21	12	fth1b	1.34	18
ENSXMAG00000023825	0.0025	6	15		13.43	3
ENSXMAG00000023922	0.0026	24	9	fam83c	2.63	21
ENSXMAG00000023924	0.0027	18	9	mkxa (1 of many)	2.55	15
ENSXMAG00000023965	0.0026	21	9	ccl25b	1.81	18
ENSXMAG00000023987	0.0027	18	15		1.85	15
ENSXMAG00000024036	0.0031	21	12	si:dkey-69o16.5	1.50	18
ENSXMAG00000024071	0.0015	21	18		1.64	18
ENSXMAG00000024078	0.0024	24	9	trim8b	1.89	21
ENSXMAG00000024083	0.0014	18	18	ccnyl1	1.62	15
ENSXMAG00000024091	0.0029	24	12	dachc	4.55	21
ENSXMAG00000024113	0.0031	9	12	cyp2n13 (1 of many)	10.52	6
ENSXMAG00000024152	0.0031	18	12		2.51	15
ENSXMAG00000024190	0.0034	15	12	hsd11b1la (1 of many)	4.55	12
ENSXMAG00000024211	0.0031	3	12	MXRA7	1.30	0
ENSXMAG00000024212	0.0026	24	9	ZHX1 (1 of many)	1.38	21
ENSXMAG00000024227	0.0034	6	12	ncalda	2.11	3
ENSXMAG00000024233	0.0025	18	9		1.90	15
ENSXMAG00000024271	0.0025	18	9	mpdu1b	4.02	15

ENSXMAG00000024272	0.0031	24	12	stc1	2.60	21
ENSXMAG00000024284	0.0026	12	15		1.65	9
ENSXMAG00000024342	0.0024	3	15	nrap	1.51	0
ENSXMAG00000024351	0.0024	3	9	hoxd9a	1.75	0
ENSXMAG00000024355	0.0028	15	9		3.31	12
ENSXMAG00000024399	0.0025	18	9		3.86	15
ENSXMAG00000024404	0.0031	18	12		1.70	15
ENSXMAG00000024444	0.0031	18	12	si:dkey-167k11.5	1.97	15
ENSXMAG00000024489	0.0031	18	12	lmo4a	2.23	15
ENSXMAG00000024497	0.0014	3	18	HAAO	2.25	0
ENSXMAG00000024502	0.0029	18	12	lrrtm4l2	40.22	15
ENSXMAG00000024533	0.0024	15	9	gata1a	2.67	12
ENSXMAG00000024539	0.0031	18	12	cxcl12b	2.18	15
ENSXMAG00000024540	0.0014	3	18	bnip3lb	1.92	0
ENSXMAG00000024576	0.0025	18	9	ppih	3.26	15
ENSXMAG00000024578	0.0031	15	12		3.14	12
ENSXMAG00000024583	0.0026	3	15	ky	2.36	0
ENSXMAG00000024584	0.0025	18	15	commd7	1.56	15
ENSXMAG00000024612	0.0024	18	9		5.77	15
ENSXMAG00000024615	0.0014	24	18	SMAD4 (1 of many)	2.47	21
ENSXMAG00000024654	0.0026	12	9	fam214a	1.51	9
ENSXMAG00000024679	0.0026	18	9	PPIC	3.04	15
ENSXMAG00000024681	0.0014	3	6	wrb	1.70	0
ENSXMAG00000024707	0.0026	6	15	si:ch211-220e11.3	3.59	3
ENSXMAG00000024712	0.0025	18	9	MID1 (1 of many)	3.58	15
ENSXMAG00000024727	0.0026	18	15	unc119.1	2.42	15
ENSXMAG00000024768	0.0025	18	9	cnpy2	3.37	15
ENSXMAG00000024769	0.0034	21	12	rpl21	1.30	18
ENSXMAG00000024777	0.0024	21	9	zdhhc24	1.61	18
ENSXMAG00000024794	0.0031	18	12	acvr1l	1.44	15
ENSXMAG00000024796	0.0027	15	9	HBE1 (1 of many)	3.85	12
ENSXMAG00000024799	0.0027	3	15		1.59	0
ENSXMAG00000024819	0.0026	18	9	lasp1	1.85	15
ENSXMAG00000024820	0.0034	18	12	slc35e1	3.96	15
ENSXMAG00000024836	0.0025	18	9		3.00	15
ENSXMAG00000024876	0.0030	18	12	ube2v1	2.89	15
ENSXMAG00000024898	0.0031	18	12	tmx1	1.94	15
ENSXMAG00000024920	0.0031	6	12	rab11fip5a	2.18	3
ENSXMAG00000024924	0.0025	6	15	hivep1	4.17	3
ENSXMAG00000024927	0.0024	6	15		1.90	3
ENSXMAG00000024941	0.0016	3	18	maml3	1.58	0
ENSXMAG00000024957	0.0031	24	12	srrm2	1.19	21
ENSXMAG00000025000	0.0030	12	12	ppp1r1b	1.73	9
ENSXMAG00000025009	0.0025	24	15		3.25	21
ENSXMAG00000025011	0.0024	6	15	slc48a1a	2.26	3
ENSXMAG00000025017	0.0029	18	12	dnajc15	2.52	15
ENSXMAG00000025022	0.0031	21	12	qsox2	1.35	18
ENSXMAG00000025090	0.0031	18	12	lmo2	1.73	15
ENSXMAG00000025098	0.0026	3	15		1.77	0
ENSXMAG00000025099	0.0014	3	18		5.51	0
ENSXMAG00000025124	0.0034	21	12	ube2s	1.60	18
ENSXMAG00000025135	0.0027	3	15	rnf10	1.55	0
ENSXMAG00000025157	0.0031	18	12	vamp1	2.60	15
ENSXMAG00000025201	0.0024	18	9		5.99	15
ENSXMAG00000025221	0.0031	6	12	gbp	6.73	3
ENSXMAG00000025229	0.0015	24	18	badb	2.07	21

ENSXMAG00000025236	0.0031	18	12	mapre1b	2.79	15
ENSXMAG00000025238	0.0027	15	9	tal1	2.62	12
ENSXMAG00000025255	0.0024	18	15	scdb	1.44	15
ENSXMAG00000025272	0.0025	21	15		1.53	18
ENSXMAG00000025277	0.0031	24	12		1.25	21
ENSXMAG00000025311	0.0026	21	15	METTL18	2.36	18
ENSXMAG00000025316	0.0026	21	9		3.00	18
ENSXMAG00000025368	0.0025	15	9	pdcd6	1.57	12
ENSXMAG00000025371	0.0031	24	12	cipcb	7.46	21
ENSXMAG00000025389	0.0028	18	9	si:dkey-19e4.5	1.26	15
ENSXMAG00000025394	0.0024	9	15		1.99	6
ENSXMAG00000025425	0.0026	3	15	zgc:162297	4.72	0
ENSXMAG00000025471	0.0031	6	12		13.60	3
ENSXMAG00000025483	0.0025	9	15	ccnl1a	1.79	6
ENSXMAG00000025486	0.0025	18	9	zdhhc3b	1.92	15
ENSXMAG00000025502	0.0027	3	15	szrd1	1.47	0
ENSXMAG00000025512	0.0031	15	12	fam3a	3.27	12
ENSXMAG00000025515	0.0015	24	18	bhlhe40	25.19	21
ENSXMAG00000025520	0.0029	18	12		1.62	15
ENSXMAG00000025544	0.0024	18	15	znhit2	1.43	15
ENSXMAG00000025571	0.0031	21	12	RPL19 (1 of many)	1.23	18
ENSXMAG00000025599	0.0014	3	18		6.50	0
ENSXMAG00000025627	0.0014	3	18		5.91	0
ENSXMAG00000025630	0.0031	21	12	fam131bb	2.45	18
ENSXMAG00000025640	0.0026	18	9	kctd6b	1.41	15
ENSXMAG00000025644	0.0016	24	18		12.62	21
ENSXMAG00000025650	0.0024	15	15		2.14	12
ENSXMAG00000025665	0.0028	3	15		2.21	0
ENSXMAG00000025671	0.0026	15	15		1.95	12
ENSXMAG00000025724	0.0027	18	9	SLC2A4	4.29	15
ENSXMAG00000025753	0.0024	18	15	ppp1r14bb	2.46	15
ENSXMAG00000025776	0.0031	18	12	si:ch211-121a2.4	2.98	15
ENSXMAG00000025790	0.0026	18	15	kras	1.48	15
ENSXMAG00000025804	0.0025	18	9	cdc42	2.15	15
ENSXMAG00000025834	0.0024	18	9	ccnk	1.97	15
ENSXMAG00000025836	0.0014	3	18	si:dkey-8e10.3	4.27	0
ENSXMAG00000025867	0.0031	18	12	qtrt1	5.26	15
ENSXMAG00000025918	0.0025	18	9		1.29	15
ENSXMAG00000025954	0.0031	24	12		2.13	21
ENSXMAG00000026002	0.0031	3	12		1.89	0
ENSXMAG00000026003	0.0026	24	9	si:ch211-207k7.4	1.93	21
ENSXMAG00000026038	0.0030	3	12	tmem182b	1.66	0
ENSXMAG00000026052	0.0014	3	6	thumpd1	1.70	0
ENSXMAG00000026069	0.0025	18	9	crkl	1.76	15
ENSXMAG00000026084	0.0015	21	18		1.73	18
ENSXMAG00000026085	0.0031	18	12	erp44	1.91	15
ENSXMAG00000026106	0.0031	21	12	tmem107	1.82	18
ENSXMAG00000026109	0.0027	3	15	rnf24	2.46	0
ENSXMAG00000026112	0.0026	15	9		6.51	12
ENSXMAG00000026129	0.0024	9	9	mknk2b	5.69	6
ENSXMAG00000026139	0.0024	3	9	ap5s1	1.89	0
ENSXMAG00000026149	0.0015	18	6		1.48	15
ENSXMAG00000026179	0.0031	18	12	usp10	2.62	15
ENSXMAG00000026180	0.0016	24	18	atf7b	2.61	21
ENSXMAG00000026210	0.0026	12	9		1.68	9
ENSXMAG00000026229	0.0026	18	15	FKBP14	2.83	15

ENSXMAG00000026233	0.0031	24	12		5.88	21
ENSXMAG00000026246	0.0024	6	15	lin7a	1.82	3
ENSXMAG00000026247	0.0014	3	18		1.33	0
ENSXMAG00000026265	0.0031	18	12		4.75	15
ENSXMAG00000026279	0.0024	18	15		1.87	15
ENSXMAG00000026314	0.0024	3	9		1.71	0
ENSXMAG00000026333	0.0024	24	15	tefb	10.15	21
ENSXMAG00000026443	0.0029	24	12	myod1	1.81	21
ENSXMAG00000026446	0.0031	15	12	gnaia	1.71	12
ENSXMAG00000026448	0.0031	21	12	eaf2	2.05	18
ENSXMAG00000026469	0.0024	18	15		2.08	15
ENSXMAG00000026487	0.0026	18	15	zswim5	1.56	15
ENSXMAG00000026514	0.0016	24	18	mettl9	1.24	21
ENSXMAG00000026532	0.0025	18	9	rnf6	1.52	15
ENSXMAG00000026579	0.0024	21	9		1.76	18
ENSXMAG00000026587	0.0029	9	12	klf13	4.40	6
ENSXMAG00000026639	0.0029	18	12	tomm22	2.01	15
ENSXMAG00000026646	0.0024	21	15		2.08	18
ENSXMAG00000026666	0.0024	18	15	klhdc8a	1.70	15
ENSXMAG00000026675	0.0028	3	15		1.89	0
ENSXMAG00000026682	0.0029	18	12	picalmb	1.41	15
ENSXMAG00000026689	0.0024	24	9	HOXA7	1.52	21
ENSXMAG00000026711	0.0027	24	9	TRAPPC9	1.69	21
ENSXMAG00000026713	0.0030	18	12	srebf2	1.32	15
ENSXMAG00000026720	0.0027	3	15		5.33	0
ENSXMAG00000026724	0.0026	18	9		3.68	15
ENSXMAG00000026750	0.0016	15	6		3.97	12
ENSXMAG00000026751	0.0031	18	12	vegfab	4.14	15
ENSXMAG00000026757	0.0024	18	9	tuba1c	4.10	15
ENSXMAG00000026772	0.0034	15	12	pdyn	2.86	12
ENSXMAG00000026805	0.0027	24	9	rgs3b	1.68	21
ENSXMAG00000026862	0.0025	18	9	mfsd9	3.22	15
ENSXMAG00000026869	0.0025	18	9	sumo3b	4.49	15
ENSXMAG00000026871	0.0028	3	9	ntn5	1.32	0
ENSXMAG00000026888	0.0026	9	9		1.32	6
ENSXMAG00000026911	0.0025	21	9	TMEM179	9.71	18
ENSXMAG00000026912	0.0031	21	12	ppox	1.64	18
ENSXMAG00000026975	0.0026	3	15	borcs5	2.22	0
ENSXMAG00000027021	0.0024	24	9	TSHZ1	3.19	21
ENSXMAG00000027038	0.0026	3	15		1.75	0
ENSXMAG00000027042	0.0025	18	15	mapkapk2a	2.07	15
ENSXMAG00000027043	0.0024	24	9		2.17	21
ENSXMAG00000027054	0.0028	3	15	syngr2b	1.82	0
ENSXMAG00000027058	0.0025	6	15	adamts15b	2.29	3
ENSXMAG00000027072	0.0026	3	15		1.63	0
ENSXMAG00000027082	0.0026	3	15		1.47	0
ENSXMAG00000027087	0.0026	18	9		1.83	15
ENSXMAG00000027105	0.0031	21	12	kank3	1.99	18
ENSXMAG00000027106	0.0016	21	6	ppib	1.73	18
ENSXMAG00000027119	0.0027	3	9	si:dkey-17e16.10	1.22	0
ENSXMAG00000027130	0.0024	18	9		2.71	15
ENSXMAG00000027140	0.0025	24	15	klf15	1.74	21
ENSXMAG00000027151	0.0027	6	15		13.62	3
ENSXMAG00000027171	0.0014	24	18		3.16	21
ENSXMAG00000027215	0.0025	15	15	pole3	1.97	12
ENSXMAG00000027239	0.0025	18	15	si:ch211-261d7.3	1.82	15

ENSXMAG00000027301	0.0028	15	9	cahz	3.33	12
ENSXMAG00000027309	0.0031	21	12		2.28	18
ENSXMAG00000027316	0.0026	15	15		1.95	12
ENSXMAG00000027318	0.0016	24	18	si:ch211-71n6.4	1.42	21
ENSXMAG00000027344	0.0071	3	6	fam219aa	1.65	0
ENSXMAG00000027353	0.0031	24	12	MYNN	1.42	21
ENSXMAG00000027387	0.0031	15	12		1.80	12
ENSXMAG00000027388	0.0031	21	12		1.59	18
ENSXMAG00000027517	0.0014	24	18	crbn	2.23	21
ENSXMAG00000027523	0.0024	18	9		3.79	15
ENSXMAG00000027540	0.0027	3	15		2.30	0
ENSXMAG00000027554	0.0024	3	15	sftpbb	1.62	0
ENSXMAG00000027573	0.0025	24	9		1.49	21
ENSXMAG00000027589	0.0027	15	9	gfi1b	3.96	12
ENSXMAG00000027599	0.0024	18	15		1.98	15
ENSXMAG00000027601	0.0034	21	12	zgc:92140	1.73	18
ENSXMAG00000027615	0.0030	18	12		9.36	15
ENSXMAG00000027618	0.0030	18	12	guk1a	2.10	15
ENSXMAG00000027623	0.0026	18	9	elovl6	2.57	15
ENSXMAG00000027637	0.0024	21	9	RBPMS	1.43	18
ENSXMAG00000027642	0.0024	18	9	gmfb	3.27	15
ENSXMAG00000027672	0.0030	9	12	iqsec2b	1.61	6
ENSXMAG00000027695	0.0030	15	12		2.01	12
ENSXMAG00000027725	0.0029	21	12		9.46	18
ENSXMAG00000027733	0.0034	18	12	tmem106c	2.67	15
ENSXMAG00000027765	0.0025	24	9	tcf7l2	2.32	21
ENSXMAG00000027766	0.0026	24	9	si:ch211-212d10.2	2.22	21
ENSXMAG00000027787	0.0026	15	15	RAB41	1.53	12
ENSXMAG00000027827	0.0014	24	18		2.32	21
ENSXMAG00000027841	0.0026	15	15	stx16	1.53	12
ENSXMAG00000027861	0.0026	21	9		1.78	18
ENSXMAG00000027863	0.0025	21	9		1.93	18
ENSXMAG00000027869	0.0030	15	12		1.84	12
ENSXMAG00000027891	0.0031	3	12		1.70	0
ENSXMAG00000027915	0.0026	21	9	tial1	1.25	18
ENSXMAG00000027923	0.0031	21	12		1.56	18
ENSXMAG00000027968	0.0031	18	12	psme3	2.84	15
ENSXMAG00000027974	0.0031	9	12	fgl2a	2.43	6
ENSXMAG00000027983	0.0026	24	9	ppp2r5cb	1.51	21
ENSXMAG00000027990	0.0027	3	15	timmdc1	2.14	0
ENSXMAG00000028018	0.0026	18	9	smx5	1.70	15
ENSXMAG00000028021	0.0028	6	15		2.28	3
ENSXMAG00000028069	0.0031	21	12	KCNC4	1.71	18
ENSXMAG00000028088	0.0024	3	9		2.40	0
ENSXMAG00000028099	0.0031	18	12	TFAP4	1.61	15
ENSXMAG00000028111	0.0015	3	18	rnf144aa	1.80	0
ENSXMAG00000028116	0.0025	3	9	gramd1a	2.21	0
ENSXMAG00000028133	0.0027	3	15	alkbh3	16.56	0
ENSXMAG00000028164	0.0029	18	12	scamp1	2.07	15
ENSXMAG00000028183	0.0031	15	12	tax1bp3	1.85	12
ENSXMAG00000028189	0.0031	21	12	efnb2a	1.65	18
ENSXMAG00000028212	0.0026	15	9		13.21	12
ENSXMAG00000028246	0.0031	18	12		2.01	15
ENSXMAG00000028260	0.0026	18	9	slc38a3a	2.82	15
ENSXMAG00000028284	0.0026	3	15	si:ch211-215k15.5	1.66	0
ENSXMAG00000028290	0.0031	21	12		2.80	18

ENSXMAG00000028295	0.0024	18	9		2.98	15
ENSXMAG00000028309	0.0024	3	15		4.85	0
ENSXMAG00000028352	0.0026	24	9	atf7ip	1.69	21
ENSXMAG00000028392	0.0034	21	12	zgc:165532	2.33	18
ENSXMAG00000028393	0.0016	24	6		1.80	21
ENSXMAG00000028425	0.0016	3	18		1.47	0
ENSXMAG00000028431	0.0028	3	15		2.11	0
ENSXMAG00000028437	0.0031	18	12		1.70	15
ENSXMAG00000028462	0.0024	3	15	cnstb	2.85	0
ENSXMAG00000028465	0.0024	18	15	abhd16a	1.57	15
ENSXMAG00000028470	0.0031	9	12	egln2	2.88	6
ENSXMAG00000028526	0.0024	18	15	smyd2a	1.99	15
ENSXMAG00000028532	0.0034	21	12	si:ch211-195e19.1	2.14	18
ENSXMAG00000028554	0.0024	21	9	hoxc10a	2.16	18
ENSXMAG00000028573	0.0029	18	12	dhcr7	1.54	15
ENSXMAG00000028575	0.0025	15	15		7.54	12
ENSXMAG00000028577	0.0031	24	12	aasdhppt	1.66	21
ENSXMAG00000028579	0.0014	18	6		1.92	15
ENSXMAG00000028591	0.0031	21	12	gpr161	1.45	18
ENSXMAG00000028599	0.0024	21	15	MGME1	1.94	18
ENSXMAG00000028618	0.0029	18	12	GDE1	3.98	15
ENSXMAG00000028689	0.0026	15	15	abracl	2.57	12
ENSXMAG00000028704	0.0027	3	15	BFSP1	1.90	0
ENSXMAG00000028765	0.0025	24	15	cln5	2.29	21
ENSXMAG00000028849	0.0026	24	9		1.86	21
ENSXMAG00000028853	0.0031	21	12	arxa	2.42	18
ENSXMAG00000028859	0.0014	21	6	eif1ad	1.91	18
ENSXMAG00000028893	0.0027	18	15	tor1 (1 of many)	1.64	15
ENSXMAG00000028932	0.0031	24	12		13.88	21
ENSXMAG00000028969	0.0015	21	6	nln	2.95	18
ENSXMAG00000028971	0.0014	3	18	castor2	1.79	0
ENSXMAG00000028981	0.0031	21	12		1.40	18
ENSXMAG00000028986	0.0031	21	12	timp4.3	3.56	18
ENSXMAG00000028988	0.0025	3	9		2.69	0
ENSXMAG00000028994	0.0025	18	15	selenof	1.54	15
ENSXMAG00000029020	0.0027	3	15		2.66	0
ENSXMAG00000029021	0.0031	15	12	si:ch211-130m23.5	3.81	12
ENSXMAG00000029024	0.0031	18	12		1.97	15
ENSXMAG00000029041	0.0025	18	9		3.21	15
ENSXMAG00000029054	0.0015	3	6		1.37	0
ENSXMAG00000029063	0.0026	18	9		1.91	15
ENSXMAG00000029086	0.0025	21	15	WARS	1.62	18
ENSXMAG00000029095	0.0026	18	9		1.97	15
ENSXMAG00000029096	0.0034	21	12		1.83	18
ENSXMAG00000029107	0.0034	18	12		1.60	15
ENSXMAG00000029109	0.0034	24	12	NFE2L1	1.78	21
ENSXMAG00000029119	0.0031	18	12	tmem150c	1.55	15
ENSXMAG00000029182	0.0028	3	9	PCBD2	1.63	0
ENSXMAG00000029187	0.0030	21	12		2.64	18
ENSXMAG00000029251	0.0027	18	15	tmem117	3.22	15
ENSXMAG00000029258	0.0026	24	9		2.29	21
ENSXMAG00000029269	0.0025	18	15	CCNYL1	2.14	15
ENSXMAG00000029281	0.0026	6	15	lrrc58b	2.32	3
ENSXMAG00000029340	0.0025	18	9	cmbl	4.19	15
ENSXMAG00000029362	0.0025	6	15	si:dkey-238d18.4	1.93	3
ENSXMAG00000029381	0.0026	21	9	dhrs11a	1.97	18

	ENSXMAG00000029397	0.0014	21	6		1.83	18
	ENSXMAG00000029412	0.0029	18	12	cdab	2.01	15
	ENSXMAG00000029418	0.0024	3	15	tspan15	2.32	0
	ENSXMAG00000029424	0.0015	24	18	tprg1l	1.49	21
	ENSXMAG00000029433	0.0014	24	18	pcmtd1	9.47	21
	ENSXMAG00000029462	0.0026	18	15	CMAS	1.42	15
	ENSXMAG00000029515	0.0026	3	15		2.49	0
	ENSXMAG00000029533	0.0025	24	9	efna3b	2.58	21
	ENSXMAG00000029540	0.0026	24	9	inka2	2.36	21
	ENSXMAG00000029545	0.0031	12	12		2.60	9
	ENSXMAG00000029572	0.0031	18	12	timm10b	3.01	15
	ENSXMAG00000029578	0.0031	18	12	caska	1.57	15
	ENSXMAG00000029592	0.0014	12	6		1.74	9
	ENSXMAG00000029616	0.0026	9	15		3.00	6
	ENSXMAG00000029666	0.0026	18	15	ctnnbip1	2.36	15
	ENSXMAG00000029668	0.0031	18	12	vmp1	4.87	15
	ENSXMAG00000029703	0.0026	18	15		5.01	15
	ENSXMAG00000029706	0.0027	6	15	mxd1	5.90	3
	ENSXMAG00000029714	0.0031	9	12	myclb	3.38	6
	ENSXMAG00000029722	0.0015	3	18		2.07	0
	ENSXMAG00000029752	0.0026	24	9		4.12	21
	ENSXMAG00000029762	0.0014	21	6		2.69	18
	ENSXMAG00000029818	0.0026	21	9	foxd3	2.17	18
	ENSXMAG00000029828	0.0030	12	12	TMCC1 (1 of many)	1.43	9
	ENSXMAG00000029834	0.0031	18	12	degs1	1.65	15
	ENSXMAG00000029859	0.0028	18	9	tm4sf18	1.92	15
	ENSXMAG00000029877	0.0027	9	15	pptc7a	8.20	6
	ENSXMAG00000029882	0.0031	18	12	adnp2b	2.41	15
	ENSXMAG00000029896	0.0025	18	9	nck1b	1.63	15
	ENSXMAG00000029905	0.0026	9	15		2.14	6
	ENSXMAG00000029907	0.0026	18	9	nabp1a	2.67	15
	ENSXMAG00000029926	0.0026	24	15		1.53	21
	ENSXMAG00000029936	0.0031	18	12	ca4a	4.10	15
	ENSXMAG00000029954	0.0014	3	18		4.11	0
	ENSXMAG00000029962	0.0026	18	15		1.74	15
	ENSXMAG00000029991	0.0027	18	9	tuba1a	2.96	15
	ENSXMAG00000029999	0.0026	21	9		2.88	18
	ENSXMAG00000030005	0.0030	21	12		1.68	18
	ENSXMAG00000030059	0.0026	18	9		1.62	15
	ENSXMAG00000030062	0.0031	18	12	impad1	1.97	15
	ENSXMAG00000030083	0.0028	3	15	fbxo33	3.74	0
	ENSXMAG00000030095	0.0029	18	12	si:dkey-32e6.3	1.83	15
	ENSXMAG00000030098	0.0071	15	18	ssr2	1.23	12

Eye circadian genes	GeneID	pVal	phase	peak.shape	external_gene_name	amp	Ct.peak
	ENSXMAG00000000010	0.0031	21	12	bbox1	2.26	18
	ENSXMAG00000000011	0.0025	3	15	si:ch73-281f12.4	5.97	0
	ENSXMAG00000000014	0.0071	21	18	nr1h3	1.24	18
	ENSXMAG00000000019	0.0031	21	12	cd151l	1.25	18
	ENSXMAG00000000027	0.0025	15	9	slc25a44b	1.64	12
	ENSXMAG00000000028	0.0031	12	12		1.54	9
	ENSXMAG00000000031	0.0014	21	6	chrnb4	4.08	18
	ENSXMAG00000000039	0.0031	12	12	pkma	1.23	9
	ENSXMAG00000000044	0.0024	18	9	hira	1.47	15
	ENSXMAG00000000045	0.0027	21	9	slc2a8	1.49	18
	ENSXMAG00000000053	0.0030	6	12	ncor2	2.49	3

ENSXMAG00000000054	0.0026	12	15	cnnm2b	1.28	9
ENSXMAG00000000055	0.0031	9	12	adgrd1	2.72	6
ENSXMAG00000000067	0.0028	12	15	hccsb	1.50	9
ENSXMAG00000000068	0.0015	18	6	egln3	1.67	15
ENSXMAG00000000086	0.0031	15	12		1.62	12
ENSXMAG00000000088	0.0025	6	15	waslb	2.50	3
ENSXMAG00000000092	0.0025	6	15	RIMBP2	3.34	3
ENSXMAG00000000095	0.0027	3	15		2.97	0
ENSXMAG00000000107	0.0026	6	15		4.35	3
ENSXMAG00000000116	0.0024	24	9	asb15b	2.44	21
ENSXMAG00000000117	0.0031	24	12	mgrn1b	1.56	21
ENSXMAG00000000122	0.0025	21	15	im:7160594	1.53	18
ENSXMAG00000000124	0.0015	21	6	TMEM132D	6.12	18
ENSXMAG00000000128	0.0025	24	9	aanat2	304.36	21
ENSXMAG00000000129	0.0027	21	9	glt1d1	3.02	18
ENSXMAG00000000133	0.0016	24	18	rhbdf1a	1.35	21
ENSXMAG00000000138	0.0071	21	6	slc39a6	1.43	18
ENSXMAG00000000145	0.0025	15	15	IER5L	1.77	12
ENSXMAG00000000148	0.0031	12	12	tex2l	3.19	9
ENSXMAG00000000151	0.0031	21	12	cavin4a	1.48	18
ENSXMAG00000000155	0.0031	6	12	si:ch211-156l18.7	1.96	3
ENSXMAG00000000160	0.0015	21	6		1.42	18
ENSXMAG00000000170	0.0031	3	12	ncs1a	1.97	0
ENSXMAG00000000174	0.0026	18	9		1.72	15
ENSXMAG00000000175	0.0031	18	12		1.22	15
ENSXMAG00000000180	0.0026	6	15	hmcn2	2.37	3
ENSXMAG00000000194	0.0024	18	9	cacng3b	1.52	15
ENSXMAG00000000204	0.0024	18	9		2.68	15
ENSXMAG00000000209	0.0026	15	9	jupa	1.63	12
ENSXMAG00000000210	0.0026	3	15	slc22a13a (1 of many)	1.41	0
ENSXMAG00000000215	0.0031	24	12	kcnip2	2.23	21
ENSXMAG00000000217	0.0024	21	9	raver1	2.32	18
ENSXMAG00000000223	0.0034	21	12	adgrv1	2.76	18
ENSXMAG00000000226	0.0015	9	18		1.47	6
ENSXMAG00000000234	0.0031	21	12	KAZALD1	1.77	18
ENSXMAG00000000235	0.0026	6	15	cacna1ha	3.32	3
ENSXMAG00000000252	0.0027	3	15	si:ch211-221f10.2	27.65	0
ENSXMAG00000000264	0.0026	24	9	ptprt	3.22	21
ENSXMAG00000000274	0.0027	18	9	med12	1.33	15
ENSXMAG00000000276	0.0015	24	18		2.01	21
ENSXMAG00000000277	0.0014	15	6		1.98	12
ENSXMAG00000000278	0.0014	21	6	TMC6	1.40	18
ENSXMAG00000000281	0.0026	18	9	gpr37a	2.47	15
ENSXMAG00000000282	0.0029	12	12		1.76	9
ENSXMAG00000000291	0.0025	21	9	nsg2	1.75	18
ENSXMAG00000000298	0.0031	3	12	vipr1a	2.32	0
ENSXMAG00000000306	0.0028	3	15	slc9a6b	2.53	0
ENSXMAG00000000328	0.0024	18	9		1.69	15
ENSXMAG00000000335	0.0031	18	12	metrnla	1.65	15
ENSXMAG00000000342	0.0034	18	12	dpp3	1.69	15
ENSXMAG00000000343	0.0027	9	15	zgc:194678	1.47	6
ENSXMAG00000000362	0.0015	18	18	tox4a	1.37	15
ENSXMAG00000000363	0.0030	3	12	kdm6bb	2.43	0
ENSXMAG00000000364	0.0031	12	12		1.82	9
ENSXMAG00000000379	0.0025	18	9	hdac1	2.01	15
ENSXMAG00000000384	0.0024	21	9	sssca1	1.31	18

ENSXMAG00000000385	0.0024	3	15	fgf11b	11.81	0
ENSXMAG00000000386	0.0024	18	15	ptprfb	1.60	15
ENSXMAG00000000387	0.0024	9	9	arr3a	2.58	6
ENSXMAG00000000389	0.0026	9	15		4.62	6
ENSXMAG00000000394	0.0027	24	9	erap1b	1.52	21
ENSXMAG00000000405	0.0015	18	6	kcnip1b	2.27	15
ENSXMAG00000000413	0.0034	24	12	lnpa	1.30	21
ENSXMAG00000000426	0.0026	3	9	si:dkey-166k12.1	1.49	0
ENSXMAG00000000445	0.0014	24	18	AFAP1L1 (1 of many)	3.49	21
ENSXMAG00000000449	0.0028	24	9	hpdl	1.30	21
ENSXMAG00000000452	0.0034	12	12	mturn	1.39	9
ENSXMAG00000000464	0.0031	21	12	lrrc42	1.37	18
ENSXMAG00000000465	0.0028	21	9	anp32a	1.26	18
ENSXMAG00000000471	0.0029	15	12	coro2ba	4.84	12
ENSXMAG00000000476	0.0031	18	12	vav3b	5.74	15
ENSXMAG00000000479	0.0025	3	15	itga11a	1.57	0
ENSXMAG00000000480	0.0026	3	15	rnf145b	1.75	0
ENSXMAG00000000481	0.0024	18	9	prkci	1.38	15
ENSXMAG00000000500	0.0026	21	9	stim2b	1.41	18
ENSXMAG00000000503	0.0026	18	9	dapk3	2.85	15
ENSXMAG00000000514	0.0025	15	15		1.77	12
ENSXMAG00000000516	0.0026	3	15	cp	6.97	0
ENSXMAG00000000519	0.0026	18	9		1.27	15
ENSXMAG00000000521	0.0024	21	15	sppl2	1.32	18
ENSXMAG00000000525	0.0026	3	15	CTDSP1	1.22	0
ENSXMAG00000000530	0.0015	21	6		1.50	18
ENSXMAG00000000548	0.0034	6	12	slc25a24	1.63	3
ENSXMAG00000000553	0.0034	24	12	FAM102B	1.71	21
ENSXMAG00000000554	0.0030	21	12	cyp26b1	1.95	18
ENSXMAG00000000571	0.0026	3	15	sez6a	2.30	0
ENSXMAG00000000576	0.0026	6	15	ttc39b	2.41	3
ENSXMAG00000000577	0.0029	6	12	rapgef5a	1.37	3
ENSXMAG00000000579	0.0031	18	12	myo18aa	5.17	15
ENSXMAG00000000585	0.0031	3	12	rmdn3	1.47	0
ENSXMAG00000000598	0.0014	3	18	ksr1a	1.91	0
ENSXMAG00000000600	0.0025	3	15		1.56	0
ENSXMAG00000000612	0.0031	24	12	si:ch73-290k24.5	1.54	21
ENSXMAG00000000613	0.0031	9	12	nmrk2	2.52	6
ENSXMAG00000000615	0.0025	6	15	atcayb	2.70	3
ENSXMAG00000000626	0.0027	24	15	pip4p1b	1.45	21
ENSXMAG00000000627	0.0026	18	9	relch	1.25	15
ENSXMAG00000000631	0.0026	18	9	snx13	1.92	15
ENSXMAG00000000663	0.0026	18	9	rangap1b	2.12	15
ENSXMAG00000000673	0.0031	18	12		76.43	15
ENSXMAG00000000676	0.0029	18	12	ranbp3b	1.66	15
ENSXMAG00000000677	0.0030	21	12	olfm3b	2.16	18
ENSXMAG00000000679	0.0026	21	9	phf5a	1.75	18
ENSXMAG00000000680	0.0031	15	12	calcr	1.53	12
ENSXMAG00000000684	0.0028	21	9		2.10	18
ENSXMAG00000000699	0.0026	21	9	snrpb2	1.60	18
ENSXMAG00000000702	0.0025	12	9	amer2	1.96	9
ENSXMAG00000000711	0.0026	6	15	fnbp1l	1.86	3
ENSXMAG00000000712	0.0031	18	12	ppp1r9a	1.34	15
ENSXMAG00000000718	0.0025	3	15	pdk4	1.87	0
ENSXMAG00000000733	0.0014	24	18	si:ch211-43f4.1	1.46	21
ENSXMAG00000000737	0.0027	9	15	cmtm8b	1.41	6

ENSXMAG00000000740	0.0024	18	9		1.80	15
ENSXMAG00000000747	0.0031	21	12	PTPRM	2.37	18
ENSXMAG00000000766	0.0031	15	12	bmp4	1.69	12
ENSXMAG00000000768	0.0031	18	12		2.41	15
ENSXMAG00000000769	0.0025	21	9	LDLRAD3	1.75	18
ENSXMAG00000000779	0.0024	6	9		1.76	3
ENSXMAG00000000794	0.0026	15	9	ankrd33ba	2.25	12
ENSXMAG00000000809	0.0031	3	12	sagb	3.99	0
ENSXMAG00000000814	0.0026	18	15	ccr8.1	1.75	15
ENSXMAG00000000815	0.0025	18	9	triob	2.40	15
ENSXMAG00000000822	0.0031	15	12		1.55	12
ENSXMAG00000000823	0.0026	12	15	accs	1.44	9
ENSXMAG00000000842	0.0027	21	9	arhgap32b	2.06	18
ENSXMAG00000000849	0.0015	21	6	hdac3	1.30	18
ENSXMAG00000000851	0.0031	15	12	man2a2	1.45	12
ENSXMAG00000000856	0.0024	18	9	ATP2C1	1.33	15
ENSXMAG00000000861	0.0027	3	15	slc15a2	1.18	0
ENSXMAG00000000867	0.0030	9	12	NYAP2	2.27	6
ENSXMAG00000000877	0.0025	12	15	ogdhb	1.36	9
ENSXMAG00000000886	0.0025	18	9	adcy2a	2.08	15
ENSXMAG00000000888	0.0031	21	12	CIB1	1.48	18
ENSXMAG00000000890	0.0029	9	12	cacng7a	2.22	6
ENSXMAG00000000891	0.0015	3	6		1.78	0
ENSXMAG00000000893	0.0025	12	9		3.75	9
ENSXMAG00000000898	0.0027	6	15	sun1	2.14	3
ENSXMAG00000000919	0.0029	9	12	gprc5ba	1.34	6
ENSXMAG00000000924	0.0029	15	12	slc2a3a	3.01	12
ENSXMAG00000000930	0.0026	18	9	si:dkey-112m2.1	8.04	15
ENSXMAG00000000932	0.0025	18	9	si:dkey-1d7.3	28.04	15
ENSXMAG00000000933	0.0014	3	18		2.19	0
ENSXMAG00000000945	0.0031	9	12	si:ch73-287m6.1	1.65	6
ENSXMAG00000000950	0.0031	6	12		1.60	3
ENSXMAG00000000961	0.0031	6	12	bco1l	6.24	3
ENSXMAG00000000963	0.0031	18	12		1.59	15
ENSXMAG00000000964	0.0030	6	12	noc4l	1.64	3
ENSXMAG00000000966	0.0031	6	12		1.57	3
ENSXMAG00000000977	0.0026	24	15	gramd1bb	2.13	21
ENSXMAG00000000993	0.0031	12	12		2.00	9
ENSXMAG00000000999	0.0025	18	9	fscn1a	3.48	15
ENSXMAG00000001002	0.0025	15	15	eif5b	1.25	12
ENSXMAG00000001016	0.0024	21	9	zgc:56596	1.44	18
ENSXMAG00000001018	0.0026	21	9	snrpd2	1.80	18
ENSXMAG00000001019	0.0029	12	12	blvra	2.65	9
ENSXMAG00000001020	0.0034	18	12	vaspb	2.27	15
ENSXMAG00000001021	0.0016	21	6		1.82	18
ENSXMAG00000001022	0.0015	24	18	cc2d1b	1.93	21
ENSXMAG00000001023	0.0025	12	9	iffo1a	3.81	9
ENSXMAG00000001027	0.0027	21	9	cbx3a	1.61	18
ENSXMAG00000001028	0.0024	18	9		3.35	15
ENSXMAG00000001030	0.0027	6	15	si:dkey-190l8.2	2.98	3
ENSXMAG00000001040	0.0027	18	15	si:ch211-260e23.8	1.84	15
ENSXMAG00000001048	0.0025	21	15	wipi2	1.23	18
ENSXMAG00000001060	0.0027	15	15	bysl	1.50	12
ENSXMAG00000001066	0.0031	9	12		3.34	6
ENSXMAG00000001071	0.0026	18	15	med20	1.95	15
ENSXMAG00000001073	0.0027	24	9	banp	1.31	21

ENSXMAG00000001082	0.0025	21	9	usp49	1.54	18
ENSXMAG00000001089	0.0015	21	6	tmcc2	2.24	18
ENSXMAG00000001096	0.0031	24	12	cdkn1a	31.46	21
ENSXMAG00000001110	0.0028	9	15	eno2	1.40	6
ENSXMAG00000001112	0.0029	3	12	AHCYL1	1.80	0
ENSXMAG00000001113	0.0026	24	15	bhlhe41	75.02	21
ENSXMAG00000001119	0.0025	18	9		2.95	15
ENSXMAG00000001125	0.0014	6	18	c1qtnf6a	3.14	3
ENSXMAG00000001131	0.0026	24	9	plxnb1a	2.60	21
ENSXMAG00000001133	0.0026	21	9	snrpd3	1.58	18
ENSXMAG00000001146	0.0031	18	12		1.73	15
ENSXMAG00000001153	0.0014	3	18	RHOBTB2	9.85	0
ENSXMAG00000001158	0.0025	18	9	KCNV1	4.80	15
ENSXMAG00000001163	0.0016	21	6	tpi1a	1.20	18
ENSXMAG00000001169	0.0024	21	9	tnfrsf19	2.18	18
ENSXMAG00000001172	0.0025	18	9		23.03	15
ENSXMAG00000001178	0.0031	9	12	MIPEP	1.86	6
ENSXMAG00000001180	0.0027	3	15	cables2b	4.87	0
ENSXMAG00000001182	0.0025	6	15	nsmfa	11.58	3
ENSXMAG00000001186	0.0014	3	18		2.24	0
ENSXMAG00000001193	0.0025	6	15	zgc:122979	22.62	3
ENSXMAG00000001198	0.0031	18	12		1.41	15
ENSXMAG00000001212	0.0026	18	9	tox4b	1.75	15
ENSXMAG00000001221	0.0024	9	15	SLC12A6	1.47	6
ENSXMAG00000001241	0.0029	21	12	ttpal	1.90	18
ENSXMAG00000001243	0.0024	3	9	fli1a	1.37	0
ENSXMAG00000001245	0.0025	21	9	pkig	1.59	18
ENSXMAG00000001247	0.0026	6	15		2.95	3
ENSXMAG00000001257	0.0029	15	12	svopb	8.69	12
ENSXMAG00000001262	0.0014	24	18		3.22	21
ENSXMAG00000001276	0.0026	21	15	CPNE9	2.45	18
ENSXMAG00000001279	0.0025	21	9	desi1a	1.30	18
ENSXMAG00000001285	0.0025	6	15	rasgrf2a	3.03	3
ENSXMAG00000001289	0.0024	18	9		3.84	15
ENSXMAG00000001293	0.0024	6	15	pou5f3	2.45	3
ENSXMAG00000001294	0.0025	12	15		1.21	9
ENSXMAG00000001301	0.0031	9	12	npdc1a	1.69	6
ENSXMAG00000001303	0.0014	24	18	aldh1a3	1.56	21
ENSXMAG00000001312	0.0025	9	15	emc4	1.44	6
ENSXMAG00000001319	0.0029	18	12		2.38	15
ENSXMAG00000001324	0.0016	24	18	arfgap3	1.48	21
ENSXMAG00000001325	0.0031	21	12		1.88	18
ENSXMAG00000001331	0.0031	21	12		1.31	18
ENSXMAG00000001340	0.0025	12	9	fam118b	13.64	9
ENSXMAG00000001341	0.0031	21	12	atg10	1.47	18
ENSXMAG00000001346	0.0015	21	6	LLGL1	2.23	18
ENSXMAG00000001358	0.0026	9	15	angptl7	2.97	6
ENSXMAG00000001370	0.0014	3	18	serpinh1b	2.19	0
ENSXMAG00000001380	0.0031	24	12	LRP4	1.41	21
ENSXMAG00000001381	0.0026	24	9	tescb	1.86	21
ENSXMAG00000001386	0.0031	21	12	cdk14	2.57	18
ENSXMAG00000001387	0.0025	18	9	sestd1	15.98	15
ENSXMAG00000001398	0.0028	21	9	mfsd1	1.80	18
ENSXMAG00000001415	0.0031	6	12		4.66	3
ENSXMAG00000001420	0.0024	24	9	klc3	1.36	21
ENSXMAG00000001421	0.0029	18	12	def6c	11.84	15

ENSXMAG00000001427	0.0031	6	12		3.92	3
ENSXMAG00000001444	0.0014	24	18	aadac	1.84	21
ENSXMAG00000001463	0.0029	6	12	slc17a8	4.32	3
ENSXMAG00000001471	0.0016	3	18	slc1a2a	33.94	0
ENSXMAG00000001476	0.0025	18	9	src	4.63	15
ENSXMAG00000001477	0.0026	15	9	ARHGEF26	1.74	12
ENSXMAG00000001482	0.0025	18	9	arntl2	35.34	15
ENSXMAG00000001495	0.0031	18	12	slc27a4	1.92	15
ENSXMAG00000001507	0.0024	3	15		2.78	0
ENSXMAG00000001522	0.0031	6	12	NR3C1	3.32	3
ENSXMAG00000001526	0.0016	15	6	ARHGAP26	1.87	12
ENSXMAG00000001535	0.0026	21	15	cav3	1.61	18
ENSXMAG00000001548	0.0024	3	15		4.22	0
ENSXMAG00000001575	0.0029	9	12	ahcyl2	2.40	6
ENSXMAG00000001579	0.0015	9	18	cyb5a	1.54	6
ENSXMAG00000001584	0.0024	18	9		2.95	15
ENSXMAG00000001593	0.0025	18	15	sh3d21	1.98	15
ENSXMAG00000001597	0.0027	6	15		9.16	3
ENSXMAG00000001611	0.0025	24	9	nrxn3b	1.96	21
ENSXMAG00000001613	0.0029	9	12	opn4xa	3.45	6
ENSXMAG00000001628	0.0031	9	12	pdlim5b	1.60	6
ENSXMAG00000001635	0.0031	21	12	bmpr1bb	2.21	18
ENSXMAG00000001642	0.0031	9	12	aff4	1.40	6
ENSXMAG00000001650	0.0027	6	15	plch1	3.36	3
ENSXMAG00000001667	0.0027	3	15	ches1	2.25	0
ENSXMAG00000001678	0.0027	21	9	dctn5	1.24	18
ENSXMAG00000001682	0.0014	3	18	tsku	2.37	0
ENSXMAG00000001685	0.0026	12	9	gucy2f	1.54	9
ENSXMAG00000001694	0.0030	6	12		2.42	3
ENSXMAG00000001695	0.0025	21	9	slc7a8a	1.97	18
ENSXMAG00000001699	0.0029	18	12	cax2	1.80	15
ENSXMAG00000001701	0.0026	21	15	INKA1	1.31	18
ENSXMAG00000001709	0.0031	15	12	ccni2	7.55	12
ENSXMAG00000001711	0.0028	3	15	GABRG2	1.67	0
ENSXMAG00000001712	0.0030	21	12	gpalpp1	1.51	18
ENSXMAG00000001719	0.0031	6	12	tanc1a	2.77	3
ENSXMAG00000001725	0.0031	6	12	sytl2b	2.35	3
ENSXMAG00000001732	0.0025	24	9		2.12	21
ENSXMAG00000001740	0.0034	21	12		1.78	18
ENSXMAG00000001744	0.0015	3	18	cry1ab	18.15	0
ENSXMAG00000001756	0.0025	18	9	VAV2	2.41	15
ENSXMAG00000001769	0.0027	21	9		1.85	18
ENSXMAG00000001771	0.0024	21	9		1.92	18
ENSXMAG00000001778	0.0031	18	12	abce1	1.62	15
ENSXMAG00000001781	0.0029	9	12	MAPKAPK3	1.85	6
ENSXMAG00000001791	0.0024	24	9	DNAJC13	1.35	21
ENSXMAG00000001799	0.0026	6	15		2.24	3
ENSXMAG00000001812	0.0024	18	9	dpysl2b	1.60	15
ENSXMAG00000001816	0.0034	9	12		1.34	6
ENSXMAG00000001819	0.0026	21	9	psmd14	1.52	18
ENSXMAG00000001828	0.0031	18	12	ppp2r2ab	1.55	15
ENSXMAG00000001845	0.0029	15	12	gba2	1.63	12
ENSXMAG00000001846	0.0024	3	15	AAK1 (1 of many)	2.88	0
ENSXMAG00000001847	0.0026	21	9	ruvbl1	1.47	18
ENSXMAG00000001861	0.0031	15	12	prkcda	1.52	12
ENSXMAG00000001865	0.0027	6	15	sorbs3	1.73	3

ENSXMAG00000001880	0.0015	3	6	stk16	1.22	0
ENSXMAG00000001882	0.0014	24	18	stom (1 of many)	1.37	21
ENSXMAG00000001885	0.0014	24	6	dbnlb	1.20	21
ENSXMAG00000001890	0.0031	18	12		1.73	15
ENSXMAG00000001924	0.0031	18	12	TTC28	2.96	15
ENSXMAG00000001927	0.0031	18	12	abcf2a	1.75	15
ENSXMAG00000001932	0.0029	18	12	ptpn4a	1.47	15
ENSXMAG00000001961	0.0016	9	18	tmem177	2.25	6
ENSXMAG00000001970	0.0025	24	9	ncoa3	2.06	21
ENSXMAG00000001980	0.0024	18	9	elmo3	1.64	15
ENSXMAG00000001981	0.0031	24	12		1.16	21
ENSXMAG00000001986	0.0024	24	9	npm1b	1.38	21
ENSXMAG00000002008	0.0029	21	12	skia	2.46	18
ENSXMAG00000002012	0.0026	21	9		1.61	18
ENSXMAG00000002013	0.0024	6	15	LIMCH1	4.08	3
ENSXMAG00000002016	0.0014	6	18	prkcz	1.65	3
ENSXMAG00000002019	0.0030	6	12	bnip3la	1.77	3
ENSXMAG00000002024	0.0015	24	18	ankrd33ab	52.01	21
ENSXMAG00000002026	0.0014	6	18	arhgef25b	2.09	3
ENSXMAG00000002039	0.0026	21	9	ube2f	1.32	18
ENSXMAG00000002042	0.0026	21	9		1.45	18
ENSXMAG00000002046	0.0024	15	9	es1	1.61	12
ENSXMAG00000002054	0.0025	24	9	apof	4.39	21
ENSXMAG00000002065	0.0034	24	12		1.80	21
ENSXMAG00000002075	0.0030	18	12	prom1a	1.58	15
ENSXMAG00000002082	0.0031	12	12	pde8a	2.59	9
ENSXMAG00000002085	0.0026	24	9	smarcc2	1.33	21
ENSXMAG00000002089	0.0025	18	15	arl13b	2.68	15
ENSXMAG00000002095	0.0026	18	9		2.46	15
ENSXMAG00000002128	0.0015	24	6	cpeb2	1.63	21
ENSXMAG00000002148	0.0016	24	18	usp8	1.26	21
ENSXMAG00000002154	0.0014	3	18	mapk6	3.37	0
ENSXMAG00000002163	0.0027	24	9	mst1rb	2.33	21
ENSXMAG00000002175	0.0031	24	12	pgm1	1.20	21
ENSXMAG00000002184	0.0014	24	6	emilin2b	1.79	21
ENSXMAG00000002194	0.0015	21	6	rnasekb	1.14	18
ENSXMAG00000002195	0.0030	9	12	ireb2	1.25	6
ENSXMAG00000002196	0.0029	18	12	cachd1	1.45	15
ENSXMAG00000002199	0.0016	18	6	fam198b	2.32	15
ENSXMAG00000002203	0.0031	18	12	gldn	2.66	15
ENSXMAG00000002207	0.0024	21	9	wdr61	1.26	18
ENSXMAG00000002209	0.0027	21	9	dmxl2	1.65	18
ENSXMAG00000002212	0.0028	3	15	jak1	1.78	0
ENSXMAG00000002218	0.0026	3	15	atf1	1.67	0
ENSXMAG00000002221	0.0025	3	15	yes1	1.79	0
ENSXMAG00000002223	0.0027	21	9	arl6ip5a	2.06	18
ENSXMAG00000002229	0.0026	3	9	glrbb	1.13	0
ENSXMAG00000002240	0.0024	6	15	dnajc6	2.78	3
ENSXMAG00000002241	0.0025	24	9	si:dkey-237h12.3	1.93	21
ENSXMAG00000002248	0.0031	9	12	BHLHE40 (1 of many)	61.52	6
ENSXMAG00000002251	0.0015	3	6	slc46a2	1.89	0
ENSXMAG00000002257	0.0026	18	9	SHC4	5.02	15
ENSXMAG00000002271	0.0014	6	18	rasgef1bb	3.20	3
ENSXMAG00000002275	0.0031	18	12	asb5a	7.01	15
ENSXMAG00000002281	0.0029	21	12	gpm6aa	1.75	18
ENSXMAG00000002288	0.0024	24	15	paqr3b	1.48	21

ENSXMAG00000002289	0.0024	9	15	NSUN6	2.31	6
ENSXMAG00000002299	0.0025	18	9	zgc:112356	1.63	15
ENSXMAG00000002302	0.0026	3	15	zgc:77938	1.64	0
ENSXMAG00000002307	0.0026	21	9	zfand1	1.86	18
ENSXMAG00000002312	0.0031	6	12	tnksa	1.81	3
ENSXMAG00000002313	0.0014	15	6	ivns1abpa	1.44	12
ENSXMAG00000002317	0.0026	21	9		1.44	18
ENSXMAG00000002327	0.0015	9	18	faim2b	12.34	6
ENSXMAG00000002328	0.0030	3	12	INPP5K (1 of many)	1.72	0
ENSXMAG00000002339	0.0026	24	9	nr1d4b	90.87	21
ENSXMAG00000002351	0.0031	24	12	sema4c	1.73	21
ENSXMAG00000002357	0.0027	21	9	rnaseh1	1.31	18
ENSXMAG00000002360	0.0024	21	9	rargb	1.47	18
ENSXMAG00000002377	0.0031	9	12	smad3a	3.02	6
ENSXMAG00000002378	0.0025	12	9	txnrd2.1	1.39	9
ENSXMAG00000002380	0.0031	12	12	ushbp1	1.72	9
ENSXMAG00000002399	0.0030	21	12		1.64	18
ENSXMAG00000002400	0.0026	3	15	plvapb	1.93	0
ENSXMAG00000002403	0.0016	21	6	azin1a	1.22	18
ENSXMAG00000002404	0.0024	24	9	arvcfb	1.48	21
ENSXMAG00000002407	0.0031	6	12	eif2ak3	2.41	3
ENSXMAG00000002431	0.0030	15	12	camsap3	1.87	12
ENSXMAG00000002436	0.0026	3	15	fbxo21	2.32	0
ENSXMAG00000002444	0.0026	9	9	pdcb	4.40	6
ENSXMAG00000002447	0.0024	3	15	crb1	11.96	0
ENSXMAG00000002459	0.0025	18	9	pgam5	3.81	15
ENSXMAG00000002466	0.0034	21	12	ulk1b	1.42	18
ENSXMAG00000002470	0.0024	3	15	golga3	1.67	0
ENSXMAG00000002475	0.0025	21	15	gfra4a	1.93	18
ENSXMAG00000002480	0.0015	3	18	smtnl	12.95	0
ENSXMAG00000002499	0.0014	21	6	her13	1.57	18
ENSXMAG00000002502	0.0024	6	9	si:dkey-197i20.6	1.43	3
ENSXMAG00000002533	0.0029	6	12	SPAG1	4.04	3
ENSXMAG00000002534	0.0024	21	9	hps1	2.56	18
ENSXMAG00000002549	0.0031	15	12	meis3	2.38	12
ENSXMAG00000002550	0.0025	21	15	acsbg2	2.29	18
ENSXMAG00000002551	0.0031	6	12	ksr2	2.74	3
ENSXMAG00000002556	0.0027	21	9	nucks1a	1.37	18
ENSXMAG00000002557	0.0025	18	15		1.86	15
ENSXMAG00000002559	0.0034	12	12	abat	3.85	9
ENSXMAG00000002566	0.0025	6	15	CNNM1 (1 of many)	5.81	3
ENSXMAG00000002569	0.0031	3	12		5.35	0
ENSXMAG00000002570	0.0029	21	12		1.45	18
ENSXMAG00000002576	0.0029	21	12	acer1	17.93	18
ENSXMAG00000002596	0.0015	6	18	ptpn2a	2.15	3
ENSXMAG00000002601	0.0026	18	15	p3h1	1.73	15
ENSXMAG00000002617	0.0014	21	6	ctdspl3	2.98	18
ENSXMAG00000002624	0.0024	24	9	pex11g	1.63	21
ENSXMAG00000002626	0.0071	21	6	si:ch73-40i7.5	1.41	18
ENSXMAG00000002629	0.0027	3	15	zgc:172302	7.02	0
ENSXMAG00000002631	0.0024	15	9		2.22	12
ENSXMAG00000002632	0.0031	12	12	pebp1	2.36	9
ENSXMAG00000002645	0.0024	6	15	trpc1	2.50	3
ENSXMAG00000002664	0.0031	15	12	mob3a	2.12	12
ENSXMAG00000002665	0.0025	9	9	xbp1	1.38	6
ENSXMAG00000002670	0.0030	18	12	znrf3	3.09	15

ENSXMAG00000002672	0.0014	21	6		1.43	18
ENSXMAG00000002675	0.0026	18	9		1.56	15
ENSXMAG00000002687	0.0031	21	12	glrx2	1.72	18
ENSXMAG00000002688	0.0031	6	12	zgc:101100	5.64	3
ENSXMAG00000002691	0.0025	21	9	uchl5	1.21	18
ENSXMAG00000002705	0.0029	24	12	slc25a16	1.70	21
ENSXMAG00000002707	0.0024	24	15	chd2	1.43	21
ENSXMAG00000002710	0.0014	21	6	dpf1	2.03	18
ENSXMAG00000002714	0.0034	18	12		4.10	15
ENSXMAG00000002743	0.0025	18	9		4.88	15
ENSXMAG00000002747	0.0026	3	15	tgfbr2b	1.48	0
ENSXMAG00000002750	0.0026	21	9	prpf4	1.15	18
ENSXMAG00000002751	0.0016	21	6		1.65	18
ENSXMAG00000002755	0.0028	24	15	zgc:110319	1.46	21
ENSXMAG00000002757	0.0031	9	12		15.30	6
ENSXMAG00000002762	0.0029	21	12	si:ch211-251j10.3	1.48	18
ENSXMAG00000002782	0.0024	9	9	isca1	2.30	6
ENSXMAG00000002786	0.0031	12	12	psat1	2.43	9
ENSXMAG00000002790	0.0024	21	9	VANGL1	2.50	18
ENSXMAG00000002797	0.0031	21	12	si:dkey-184p18.2	12.26	18
ENSXMAG00000002813	0.0026	3	9	aoc2	1.37	0
ENSXMAG00000002816	0.0026	9	9	tspan2a	1.94	6
ENSXMAG00000002827	0.0024	18	9	xpot	1.39	15
ENSXMAG00000002852	0.0025	3	15	pdzd7a	5.35	0
ENSXMAG00000002855	0.0027	21	9	rtcb	1.58	18
ENSXMAG00000002857	0.0027	6	15	lzts2a	3.28	3
ENSXMAG00000002862	0.0030	6	12		2.17	3
ENSXMAG00000002866	0.0024	6	15		39.63	3
ENSXMAG00000002868	0.0025	18	9	si:dkey-84j12.1	1.64	15
ENSXMAG00000002871	0.0027	6	15	idh2	1.76	3
ENSXMAG00000002879	0.0025	3	15	ZDHHC12 (1 of many)	2.99	0
ENSXMAG00000002890	0.0026	15	9	yrk	1.86	12
ENSXMAG00000002891	0.0031	6	12	fam102aa	3.03	3
ENSXMAG00000002892	0.0031	6	12	RASGRF1	2.28	3
ENSXMAG00000002893	0.0025	15	9	sik3	2.24	12
ENSXMAG00000002896	0.0029	15	12		2.77	12
ENSXMAG00000002902	0.0015	21	6	TBC1D30	1.62	18
ENSXMAG00000002912	0.0031	6	12	prkcq	1.91	3
ENSXMAG00000002913	0.0015	21	6	vps26c	2.43	18
ENSXMAG00000002918	0.0014	21	6	kcnv2a	3.93	18
ENSXMAG00000002923	0.0016	24	18		2.70	21
ENSXMAG00000002925	0.0016	9	6	golga1	1.09	6
ENSXMAG00000002928	0.0025	18	9	ctu1	1.39	15
ENSXMAG00000002929	0.0016	3	18	fras1	1.54	0
ENSXMAG00000002941	0.0024	18	9	gap43	1.66	15
ENSXMAG00000002948	0.0027	6	15	stxbp5a	4.12	3
ENSXMAG00000002950	0.0027	15	15	parp12a (1 of many)	1.99	12
ENSXMAG00000002958	0.0026	18	15	gak	3.47	15
ENSXMAG00000002967	0.0031	24	12	sfmbt2	4.19	21
ENSXMAG00000002976	0.0025	24	9	tmem110l	2.15	21
ENSXMAG00000002978	0.0031	6	12	asphd2	1.61	3
ENSXMAG00000002990	0.0034	24	12	cyhr1	1.46	21
ENSXMAG00000003016	0.0029	21	12	tcf25	1.21	18
ENSXMAG00000003019	0.0029	6	12	lrit1a	2.80	3
ENSXMAG00000003021	0.0027	15	9	lrit2	3.36	12
ENSXMAG00000003032	0.0029	15	12	gldc	1.60	12

ENSXMAG00000003034	0.0031	18	12		6.00	15
ENSXMAG00000003040	0.0031	18	12	tnfaip6	1.62	15
ENSXMAG00000003041	0.0024	15	9		4.26	12
ENSXMAG00000003057	0.0031	21	12	scaper	1.80	18
ENSXMAG00000003061	0.0026	21	9	phpt1	1.35	18
ENSXMAG00000003063	0.0027	24	15		1.60	21
ENSXMAG00000003065	0.0026	18	15	entpd2b	1.51	15
ENSXMAG00000003075	0.0031	15	12	mfsd10	1.25	12
ENSXMAG00000003081	0.0024	15	15	armt1	1.12	12
ENSXMAG00000003083	0.0014	18	6	scd	6.63	15
ENSXMAG00000003086	0.0026	18	9	rom1b	1.76	15
ENSXMAG00000003095	0.0024	21	15	dnajb12a	1.21	18
ENSXMAG00000003107	0.0034	6	12	zgc:109982	4.82	3
ENSXMAG00000003122	0.0034	18	12	slc1a7b	5.35	15
ENSXMAG00000003126	0.0025	18	9	ACTN4	1.71	15
ENSXMAG00000003129	0.0025	24	15	atmin	1.95	21
ENSXMAG00000003131	0.0031	18	12	pnpla3	1.70	15
ENSXMAG00000003135	0.0016	3	6	tmem266	1.32	0
ENSXMAG00000003138	0.0027	21	9	lactb	2.04	18
ENSXMAG00000003148	0.0026	15	9	usp47	1.90	12
ENSXMAG00000003157	0.0027	24	15	eif4ebp2	1.45	21
ENSXMAG00000003158	0.0025	15	9		21.43	12
ENSXMAG00000003160	0.0031	18	12	fkbp9	2.58	15
ENSXMAG00000003163	0.0031	18	12	zmiz1a	2.01	15
ENSXMAG00000003167	0.0025	24	15	vat1l	1.43	21
ENSXMAG00000003178	0.0026	24	9		1.15	21
ENSXMAG00000003179	0.0031	3	12	nfyc	1.39	0
ENSXMAG00000003184	0.0031	3	12	KCNQ4	2.67	0
ENSXMAG00000003189	0.0031	21	12	myct1a	1.68	18
ENSXMAG00000003192	0.0026	3	15		1.69	0
ENSXMAG00000003194	0.0024	3	15	TINAGL1	1.93	0
ENSXMAG00000003195	0.0029	15	12	CCNG2 (1 of many)	2.37	12
ENSXMAG00000003205	0.0034	24	12	dynlt1	1.65	21
ENSXMAG00000003209	0.0031	12	12	thbs2a	2.63	9
ENSXMAG00000003225	0.0031	15	12	zgc:153018	5.82	12
ENSXMAG00000003226	0.0026	24	9	kcnma1a	1.53	21
ENSXMAG00000003228	0.0025	6	15	roraa	7.07	3
ENSXMAG00000003234	0.0029	3	12	si:ch211-176g6.2	3.02	0
ENSXMAG00000003236	0.0029	18	12	si:dkey-280e21.3	2.18	15
ENSXMAG00000003252	0.0031	21	12	pcdh12	2.32	18
ENSXMAG00000003253	0.0026	24	9	kat2b	1.42	21
ENSXMAG00000003259	0.0029	21	12	calr	1.55	18
ENSXMAG00000003270	0.0027	3	15	eps15	4.27	0
ENSXMAG00000003285	0.0024	18	15	ilf2	1.35	15
ENSXMAG00000003295	0.0026	12	15	ttc39a	1.30	9
ENSXMAG00000003297	0.0024	18	9	rasal2	3.45	15
ENSXMAG00000003304	0.0026	18	9	prph	3.26	15
ENSXMAG00000003315	0.0031	9	12	trib1	2.09	6
ENSXMAG00000003326	0.0027	18	9	cdk2	1.65	15
ENSXMAG00000003340	0.0025	3	15	AGBL4	1.34	0
ENSXMAG00000003348	0.0026	3	15		1.40	0
ENSXMAG00000003350	0.0025	15	15	dapk2b	2.79	12
ENSXMAG00000003372	0.0031	15	12		1.99	12
ENSXMAG00000003381	0.0024	18	9	srpk3	2.06	15
ENSXMAG00000003385	0.0024	12	15		1.32	9
ENSXMAG00000003393	0.0025	21	9	plxnb3	2.64	18

ENSXMAG00000003404	0.0026	3	9	lpl	2.30	0
ENSXMAG00000003407	0.0014	18	18	dnajc27	1.56	15
ENSXMAG00000003412	0.0025	6	9	brd2a	1.50	3
ENSXMAG00000003413	0.0026	18	9	xkr5a	1.60	15
ENSXMAG00000003424	0.0026	18	9	prep	1.56	15
ENSXMAG00000003430	0.0024	9	9	csnk1db	2.23	6
ENSXMAG00000003436	0.0031	3	12	wdcp	2.29	0
ENSXMAG00000003439	0.0034	3	12	mfsd2b	3.25	0
ENSXMAG00000003442	0.0024	3	15	lrrc8ab	1.76	0
ENSXMAG00000003446	0.0024	18	9	tfap2e	2.98	15
ENSXMAG00000003452	0.0031	18	12		2.45	15
ENSXMAG00000003454	0.0026	18	9	si:ch211-193k19.1	1.79	15
ENSXMAG00000003466	0.0031	12	12	abcd1	1.61	9
ENSXMAG00000003490	0.0031	21	12	MMD2 (1 of many)	1.70	18
ENSXMAG00000003491	0.0026	9	9	NAPEPLD	1.63	6
ENSXMAG00000003494	0.0025	21	9	pip4p2	2.01	18
ENSXMAG00000003497	0.0016	3	6	klhl29	1.75	0
ENSXMAG00000003528	0.0031	18	12	fgd1	3.08	15
ENSXMAG00000003534	0.0014	21	6	map7d1b	1.76	18
ENSXMAG00000003539	0.0031	15	12	ripor2	4.24	12
ENSXMAG00000003548	0.0025	21	9	DGKA	1.51	18
ENSXMAG00000003554	0.0026	6	15	tsc22d1	2.73	3
ENSXMAG00000003563	0.0027	21	15	ntng2a	3.19	18
ENSXMAG00000003573	0.0031	9	12	fam57a	1.99	6
ENSXMAG00000003574	0.0031	9	12	rbp4l	124.78	6
ENSXMAG00000003582	0.0031	12	12	pde6b	1.79	9
ENSXMAG00000003585	0.0025	24	9	dyrk1b	1.59	21
ENSXMAG00000003588	0.0014	21	6	stmn2b	1.79	18
ENSXMAG00000003600	0.0024	18	9	agpat3	2.05	15
ENSXMAG00000003607	0.0026	18	9	znf704	2.61	15
ENSXMAG00000003615	0.0027	6	15	atxn1a	3.67	3
ENSXMAG00000003621	0.0026	24	9	tle3b	2.29	21
ENSXMAG00000003628	0.0014	3	18	mylipa	2.80	0
ENSXMAG00000003631	0.0028	15	9	nnt	1.12	12
ENSXMAG00000003635	0.0025	6	15	dtnbp1b	2.58	3
ENSXMAG00000003652	0.0031	3	12	pard6gb	2.22	0
ENSXMAG00000003654	0.0024	3	15	crocc2	2.32	0
ENSXMAG00000003662	0.0025	18	9	rai14	3.74	15
ENSXMAG00000003669	0.0024	3	15	klhl30	2.17	0
ENSXMAG00000003670	0.0034	21	12	smcr8a	1.40	18
ENSXMAG00000003671	0.0026	18	15	top3a	1.41	15
ENSXMAG00000003673	0.0014	24	18	scarb2c	1.60	21
ENSXMAG00000003680	0.0025	24	15	phex	2.09	21
ENSXMAG00000003682	0.0014	21	6	PTPN14	1.70	18
ENSXMAG00000003690	0.0026	18	9		3.53	15
ENSXMAG00000003695	0.0024	6	15	slc5a1 (1 of many)	3.31	3
ENSXMAG00000003696	0.0026	21	15	kbtbd4	1.45	18
ENSXMAG00000003703	0.0024	24	15	spag1a	2.06	21
ENSXMAG00000003704	0.0014	24	18	ttc23	2.10	21
ENSXMAG00000003710	0.0026	3	15	synm	2.93	0
ENSXMAG00000003718	0.0024	24	9	st13	1.22	21
ENSXMAG00000003720	0.0014	3	18		4.54	0
ENSXMAG00000003721	0.0025	3	15	ncaldb	44.74	0
ENSXMAG00000003725	0.0027	3	15	grhl2b	45.19	0
ENSXMAG00000003726	0.0015	12	18	rangap1a	1.69	9
ENSXMAG00000003728	0.0031	6	12	ptchd1	1.94	3

ENSXMAG00000003731	0.0031	6	12	ttc39c	1.81	3
ENSXMAG00000003737	0.0026	9	15	cracr2b	24.17	6
ENSXMAG00000003753	0.0026	9	15	pabpc1b	1.15	6
ENSXMAG00000003764	0.0014	24	18	ANO2	2.77	21
ENSXMAG00000003769	0.0031	18	12	LIX1L	1.58	15
ENSXMAG00000003772	0.0031	18	12	sf3b4	1.74	15
ENSXMAG00000003777	0.0016	18	6	fads2 (1 of many)	4.04	15
ENSXMAG00000003779	0.0031	18	12	KIF18A	3.62	15
ENSXMAG00000003782	0.0024	3	9	SV2A (1 of many)	1.33	0
ENSXMAG00000003785	0.0025	18	15	srgap2	2.30	15
ENSXMAG00000003798	0.0031	18	12	cd9a	4.29	15
ENSXMAG00000003800	0.0027	3	15	rgs7a	1.67	0
ENSXMAG00000003804	0.0016	24	6	klhl17	1.72	21
ENSXMAG00000003816	0.0014	3	18		4.12	0
ENSXMAG00000003835	0.0030	18	12	noc2l	2.18	15
ENSXMAG00000003836	0.0024	18	9	daam1b	2.73	15
ENSXMAG00000003837	0.0025	6	15	laynb	1.85	3
ENSXMAG00000003840	0.0031	9	12	sik2b	6.87	6
ENSXMAG00000003849	0.0026	3	15	pnpla2	2.60	0
ENSXMAG00000003851	0.0031	18	12		1.81	15
ENSXMAG00000003855	0.0025	18	9	abhd8a	3.52	15
ENSXMAG00000003858	0.0025	15	15	ppp2r1bb	1.09	12
ENSXMAG00000003861	0.0026	24	9		1.53	21
ENSXMAG00000003864	0.0024	6	15	kcnh5a	1.68	3
ENSXMAG00000003872	0.0029	21	12	ptpa	1.39	18
ENSXMAG00000003875	0.0031	15	12	phb2a	1.87	12
ENSXMAG00000003883	0.0025	18	9	ppfia4	1.70	15
ENSXMAG00000003890	0.0029	6	12	iqsec3b	2.91	3
ENSXMAG00000003903	0.0026	6	15	best1	3.86	3
ENSXMAG00000003906	0.0024	18	9	june	2.94	15
ENSXMAG00000003924	0.0031	21	12	RIMS2	2.04	18
ENSXMAG00000003936	0.0031	18	12	tubb2b	1.89	15
ENSXMAG00000003953	0.0026	12	15	gcn1	1.41	9
ENSXMAG00000003960	0.0027	21	9	sae1	1.75	18
ENSXMAG00000003961	0.0031	6	12	per3	22.17	3
ENSXMAG00000003962	0.0025	12	15		1.38	9
ENSXMAG00000003963	0.0034	18	12	fkbp4	1.54	15
ENSXMAG00000003970	0.0026	3	15	TGFB3 (1 of many)	1.44	0
ENSXMAG00000003973	0.0025	3	9	pdzd2	2.19	0
ENSXMAG00000003982	0.0027	24	9		2.47	21
ENSXMAG00000003993	0.0025	24	9	wls	1.53	21
ENSXMAG00000004005	0.0031	3	12		3.50	0
ENSXMAG00000004010	0.0025	21	9	psma5	1.59	18
ENSXMAG00000004014	0.0026	15	15	rab35b	1.61	12
ENSXMAG00000004015	0.0025	24	15		5.13	21
ENSXMAG00000004026	0.0026	18	9	bicdl1	5.19	15
ENSXMAG00000004028	0.0016	21	6		1.97	18
ENSXMAG00000004031	0.0024	24	15	pptc7b	1.94	21
ENSXMAG00000004047	0.0029	21	12	lmf2a	1.46	18
ENSXMAG00000004056	0.0031	24	12	ccdc18	1.76	21
ENSXMAG00000004058	0.0031	15	12	atp6v0a2a	2.46	12
ENSXMAG00000004063	0.0031	15	12	camk1b	1.77	12
ENSXMAG00000004065	0.0025	18	9	slc13a5b	44.61	15
ENSXMAG00000004066	0.0014	24	6	casp3b	1.60	21
ENSXMAG00000004078	0.0031	3	12	adora1a	3.51	0
ENSXMAG00000004088	0.0029	18	12	fam69aa	2.37	15

ENSXMAG00000004093	0.0031	15	12	bcl9	2.75	12
ENSXMAG00000004100	0.0014	18	6	phtf2	2.99	15
ENSXMAG00000004108	0.0031	6	12	chrna2b	5.71	3
ENSXMAG00000004110	0.0031	21	12	morc3a	1.38	18
ENSXMAG00000004120	0.0026	24	9	lrig1	1.81	21
ENSXMAG00000004132	0.0029	6	12	slc35e4	1.70	3
ENSXMAG00000004136	0.0025	12	15	dhx57	1.32	9
ENSXMAG00000004140	0.0024	18	15	mat2aa	1.84	15
ENSXMAG00000004148	0.0029	18	12		1.70	15
ENSXMAG00000004151	0.0031	6	12	dop1b	5.35	3
ENSXMAG00000004154	0.0029	6	12	ntsr1	5.09	3
ENSXMAG00000004156	0.0014	24	18	usp45	1.19	21
ENSXMAG00000004170	0.0026	9	15	si:ch1073-390k14.1	17.84	6
ENSXMAG00000004178	0.0026	3	15	hfm1	2.69	0
ENSXMAG00000004179	0.0034	18	12	slc1a5	2.31	15
ENSXMAG00000004183	0.0025	6	15		13.03	3
ENSXMAG00000004187	0.0027	18	9		1.99	15
ENSXMAG00000004190	0.0031	24	12		1.89	21
ENSXMAG00000004197	0.0024	18	9		2.81	15
ENSXMAG00000004198	0.0030	24	12		11.49	21
ENSXMAG00000004202	0.0028	18	9		2.83	15
ENSXMAG00000004206	0.0016	21	6	gtf2b	1.19	18
ENSXMAG00000004215	0.0030	15	12		2.00	12
ENSXMAG00000004219	0.0031	6	12	si:ch211-278j3.3	2.00	3
ENSXMAG00000004221	0.0026	24	9		1.65	21
ENSXMAG00000004228	0.0015	24	18	dpp7	2.34	21
ENSXMAG00000004234	0.0024	6	15	tdh	4.19	3
ENSXMAG00000004236	0.0016	3	6	ctif	1.74	0
ENSXMAG00000004239	0.0027	15	9	smad7	4.60	12
ENSXMAG00000004240	0.0024	24	9	mtmr9	1.23	21
ENSXMAG00000004256	0.0031	18	12	2-Sep	1.32	15
ENSXMAG00000004258	0.0027	18	9	chac1	1.73	15
ENSXMAG00000004278	0.0024	3	15	fam167ab	6.17	0
ENSXMAG00000004294	0.0031	15	12	tigarb	2.70	12
ENSXMAG00000004296	0.0014	21	6	PCMTD2	1.84	18
ENSXMAG00000004297	0.0026	24	15	relb	2.38	21
ENSXMAG00000004298	0.0031	18	12	clptm1	1.29	15
ENSXMAG00000004299	0.0024	3	15	prlra	33.50	0
ENSXMAG00000004307	0.0024	12	15	kpnb3	1.42	9
ENSXMAG00000004308	0.0031	15	12	tbl1x	2.23	12
ENSXMAG00000004311	0.0026	21	9		2.08	18
ENSXMAG00000004320	0.0028	6	15	tbc1d22b	1.53	3
ENSXMAG00000004324	0.0031	12	12	fam129bb	1.89	9
ENSXMAG00000004328	0.0031	6	12		2.69	3
ENSXMAG00000004335	0.0034	18	12		1.77	15
ENSXMAG00000004336	0.0031	21	12	pparaa	1.73	18
ENSXMAG00000004337	0.0026	3	15	slc2a12	1.71	0
ENSXMAG00000004339	0.0025	6	15	SGK1	3.74	3
ENSXMAG00000004340	0.0026	21	9	zgc:110269	1.51	18
ENSXMAG00000004343	0.0029	21	12	CACNA2D1 (1 of many)	1.50	18
ENSXMAG00000004348	0.0031	21	12	ppm1f	1.67	18
ENSXMAG00000004370	0.0031	21	12	glra2	1.84	18
ENSXMAG00000004371	0.0026	9	15	atp1b3a	1.83	6
ENSXMAG00000004372	0.0031	18	12		1.28	15
ENSXMAG00000004381	0.0030	15	12	GPM6B (1 of many)	1.76	12
ENSXMAG00000004394	0.0030	15	12	ofd1	1.88	12

ENSXMAG00000004418	0.0024	3	15	hcn1	3.97	0
ENSXMAG00000004421	0.0015	18	18	USP15	1.39	15
ENSXMAG00000004422	0.0034	3	12	rasgrp3	1.59	0
ENSXMAG00000004434	0.0025	3	15	tlr7	1.58	0
ENSXMAG00000004435	0.0028	6	15	tlr7	1.96	3
ENSXMAG00000004439	0.0031	6	12	WFS1 (1 of many)	2.49	3
ENSXMAG00000004446	0.0034	15	12	cyfip2	1.65	12
ENSXMAG00000004460	0.0028	18	9		2.76	15
ENSXMAG00000004461	0.0015	12	18	kri1	1.22	9
ENSXMAG00000004462	0.0031	18	12	sec63	1.36	15
ENSXMAG00000004465	0.0034	9	12	esm1	2.13	6
ENSXMAG00000004473	0.0027	21	9	sdf2l1	1.56	18
ENSXMAG00000004476	0.0014	24	6	pla2g3	2.14	21
ENSXMAG00000004480	0.0025	6	15	mapk10	2.51	3
ENSXMAG00000004486	0.0025	12	15		1.50	9
ENSXMAG00000004492	0.0031	18	12	arhgef7b	2.38	15
ENSXMAG00000004494	0.0025	9	15	zgc:56235	1.79	6
ENSXMAG00000004507	0.0031	15	12	DNAAF5	1.44	12
ENSXMAG00000004518	0.0026	6	15	ptpn13	2.03	3
ENSXMAG00000004522	0.0025	18	9	slc35f2l	4.46	15
ENSXMAG00000004537	0.0024	21	9	clns1a	2.09	18
ENSXMAG00000004542	0.0031	18	12	PCDH8 (1 of many)	2.34	15
ENSXMAG00000004547	0.0031	21	12		2.88	18
ENSXMAG00000004548	0.0031	18	12	pak1	2.90	15
ENSXMAG00000004553	0.0025	18	9	syncrip	1.36	15
ENSXMAG00000004577	0.0027	3	15	pim3	1.88	0
ENSXMAG00000004589	0.0031	12	12	gcat	1.81	9
ENSXMAG00000004593	0.0024	18	9	creld2	2.03	15
ENSXMAG00000004603	0.0034	24	12	rtel1	1.48	21
ENSXMAG00000004605	0.0034	15	12		1.79	12
ENSXMAG00000004606	0.0034	24	12	tnfsf10	1.63	21
ENSXMAG00000004613	0.0031	21	12	arhgap25	1.52	18
ENSXMAG00000004615	0.0030	9	12	tbl1xr1b	1.48	6
ENSXMAG00000004623	0.0031	21	12		1.43	18
ENSXMAG00000004627	0.0024	21	9	dnajc3a	1.30	18
ENSXMAG00000004631	0.0026	3	15	ch25hl3	4.22	0
ENSXMAG00000004641	0.0029	6	12	itchb	1.91	3
ENSXMAG00000004653	0.0031	15	12	si:ch73-21k16.5	3.16	12
ENSXMAG00000004655	0.0024	9	15	pfkpa	1.33	6
ENSXMAG00000004673	0.0026	24	9	ephb3a	2.49	21
ENSXMAG00000004675	0.0031	18	12	ccdc88aa	50.81	15
ENSXMAG00000004677	0.0014	21	6	sephs1	1.80	18
ENSXMAG00000004680	0.0029	21	12	hk2	1.56	18
ENSXMAG00000004681	0.0026	18	15	sb:cb81	1.95	15
ENSXMAG00000004691	0.0031	9	12	ppm1j	1.65	6
ENSXMAG00000004692	0.0015	24	6	mmp17b	3.12	21
ENSXMAG00000004700	0.0024	18	9	brat1	1.65	15
ENSXMAG00000004721	0.0025	21	9		1.34	18
ENSXMAG00000004726	0.0026	12	15	atxn7l2a	1.32	9
ENSXMAG00000004727	0.0031	18	12	ppp4r2b	1.32	15
ENSXMAG00000004731	0.0025	21	15	nlgn1	2.60	18
ENSXMAG00000004735	0.0026	6	15	cep170ab	1.79	3
ENSXMAG00000004738	0.0030	6	12	klf6a	1.57	3
ENSXMAG00000004759	0.0031	18	12	zgpat	1.97	15
ENSXMAG00000004765	0.0028	18	9		2.08	15
ENSXMAG00000004767	0.0031	3	12	NAALADL2	2.47	0

ENSXMAG00000004775	0.0031	21	12		2.75	18
ENSXMAG00000004785	0.0025	3	9		32.18	0
ENSXMAG00000004786	0.0031	24	12	exosc5	1.40	21
ENSXMAG00000004796	0.0015	21	6	slc3a2b	1.39	18
ENSXMAG00000004800	0.0027	24	9	ctnna2	1.60	21
ENSXMAG00000004804	0.0014	24	18		3.75	21
ENSXMAG00000004811	0.0031	21	12		2.56	18
ENSXMAG00000004820	0.0024	18	15	BAZ1A	1.58	15
ENSXMAG00000004825	0.0027	3	15	trove2	1.68	0
ENSXMAG00000004832	0.0031	24	12	TMC7	1.38	21
ENSXMAG00000004837	0.0024	18	15		1.54	15
ENSXMAG00000004847	0.0025	15	15	dhx29	1.69	12
ENSXMAG00000004868	0.0029	24	12	ankrd1a	3.31	21
ENSXMAG00000004870	0.0071	21	18		6.42	18
ENSXMAG00000004878	0.0024	9	15	pcgf5a	2.05	6
ENSXMAG00000004882	0.0027	3	9		1.89	0
ENSXMAG00000004883	0.0024	24	15	klhl23	2.35	21
ENSXMAG00000004889	0.0027	15	9	cyp26c1	6.21	12
ENSXMAG00000004893	0.0025	21	9	psma3	1.48	18
ENSXMAG00000004905	0.0024	24	9	traf2a	1.58	21
ENSXMAG00000004908	0.0031	18	12	LURAP1L	3.24	15
ENSXMAG00000004921	0.0031	9	12	mpp1	1.58	6
ENSXMAG00000004936	0.0015	21	6	GORASP2 (1 of many)	1.48	18
ENSXMAG00000004967	0.0071	3	18	ugt1a2 (1 of many)	2.25	0
ENSXMAG00000004973	0.0024	18	9	ppiab	1.33	15
ENSXMAG00000004975	0.0026	12	15	stoml2	1.63	9
ENSXMAG00000004979	0.0014	6	18	si:ch73-139e5.2	14.70	3
ENSXMAG00000004993	0.0025	18	9	pigo	1.88	15
ENSXMAG00000004996	0.0025	6	15	cntnap5a	2.12	3
ENSXMAG00000004997	0.0024	3	15	kctd17	2.41	0
ENSXMAG00000005006	0.0024	6	15	si:ch73-139e5.4	1.77	3
ENSXMAG00000005018	0.0026	3	15	pip5k1ba	7.09	0
ENSXMAG00000005032	0.0014	3	18		3.37	0
ENSXMAG00000005035	0.0025	24	9	panx1b	1.42	21
ENSXMAG00000005055	0.0014	3	6	dera	1.41	0
ENSXMAG00000005060	0.0025	15	9	adssl	1.39	12
ENSXMAG00000005067	0.0014	3	18	amd1	2.94	0
ENSXMAG00000005070	0.0031	21	12	arvcfa	2.04	18
ENSXMAG00000005071	0.0031	12	12	thbs4b	1.56	9
ENSXMAG00000005080	0.0031	15	12	nup155	1.44	12
ENSXMAG00000005092	0.0024	18	9	wdr36	1.66	15
ENSXMAG00000005099	0.0027	18	9	syt11b	1.26	15
ENSXMAG00000005121	0.0014	3	18		2.42	0
ENSXMAG00000005122	0.0031	18	12	itga6a	2.09	15
ENSXMAG00000005123	0.0027	18	9	slc25a46	1.63	15
ENSXMAG00000005127	0.0025	15	9	nfil3-5	153.62	12
ENSXMAG00000005136	0.0027	18	9		1.70	15
ENSXMAG00000005146	0.0030	15	12		4.39	12
ENSXMAG00000005149	0.0026	18	15		2.22	15
ENSXMAG00000005156	0.0025	24	15		2.12	21
ENSXMAG00000005170	0.0025	3	15		1.52	0
ENSXMAG00000005186	0.0034	24	12	cln3	1.41	21
ENSXMAG00000005189	0.0024	3	15	dazap2	1.27	0
ENSXMAG00000005193	0.0025	9	9	chchd3a	1.54	6
ENSXMAG00000005194	0.0031	24	12	LAMA4	1.31	21
ENSXMAG00000005196	0.0015	21	6	ythdc1	1.16	18

ENSXMAG00000005197	0.0029	21	12	MARCKS	1.45	18
ENSXMAG00000005198	0.0026	3	15		1.69	0
ENSXMAG00000005199	0.0025	21	9	HDAC2	1.39	18
ENSXMAG00000005200	0.0025	24	9	vash2	1.70	21
ENSXMAG00000005204	0.0026	18	9	rom1a	1.57	15
ENSXMAG00000005214	0.0024	9	9	fbxl5	2.09	6
ENSXMAG00000005218	0.0030	15	12	angel2	1.66	12
ENSXMAG00000005224	0.0024	6	15		1.99	3
ENSXMAG00000005227	0.0031	21	12		1.72	18
ENSXMAG00000005230	0.0030	6	12	ilf3a	1.08	3
ENSXMAG00000005232	0.0024	3	15	RAPGEF4 (1 of many)	3.82	0
ENSXMAG00000005250	0.0031	21	12	slc41a2b	1.33	18
ENSXMAG00000005251	0.0015	9	6	en1a	1.63	6
ENSXMAG00000005254	0.0025	6	15	dgkg	1.94	3
ENSXMAG00000005255	0.0025	24	9	cep85l	1.45	21
ENSXMAG00000005256	0.0031	9	12	nrsn1	2.01	6
ENSXMAG00000005262	0.0015	21	6	si:dkey-217d24.6	1.29	18
ENSXMAG00000005264	0.0031	24	12		3.02	21
ENSXMAG00000005267	0.0031	18	12	btbd9	1.21	15
ENSXMAG00000005269	0.0026	24	9	cx43	1.33	21
ENSXMAG00000005280	0.0028	3	15	CRIP2	2.69	0
ENSXMAG00000005281	0.0015	21	6	si:ch211-114c17.1	1.49	18
ENSXMAG00000005282	0.0034	18	12	copg2	1.31	15
ENSXMAG00000005286	0.0031	9	12	etv5a	3.89	6
ENSXMAG00000005292	0.0026	21	9		1.73	18
ENSXMAG00000005298	0.0014	24	18	cgrrf1	1.58	21
ENSXMAG00000005316	0.0034	24	12	scospondin	5.64	21
ENSXMAG00000005317	0.0027	3	15		4.34	0
ENSXMAG00000005318	0.0034	15	12	itgb5	1.47	12
ENSXMAG00000005321	0.0026	24	9	mest	1.73	21
ENSXMAG00000005326	0.0014	24	18	aspa	8.12	21
ENSXMAG00000005338	0.0025	6	15	slc12a7b	3.69	3
ENSXMAG00000005342	0.0031	21	12	lhx1a	2.34	18
ENSXMAG00000005353	0.0025	24	15		1.57	21
ENSXMAG00000005355	0.0025	24	9	mpv17	1.57	21
ENSXMAG00000005372	0.0031	21	12	tbx3a	1.88	18
ENSXMAG00000005373	0.0024	6	15	ankrd6b	2.33	3
ENSXMAG00000005374	0.0014	6	18	si:dkey-246g23.4	1.94	3
ENSXMAG00000005379	0.0025	24	15	zswim7	1.76	21
ENSXMAG00000005399	0.0025	21	9	ppa2	1.46	18
ENSXMAG00000005401	0.0026	9	9		3.51	6
ENSXMAG00000005403	0.0027	6	15	bsk146	1.84	3
ENSXMAG00000005407	0.0031	12	12	si:ch211-244a23.1	3.43	9
ENSXMAG00000005419	0.0025	18	9	si:ch211-242b18.1	5.36	15
ENSXMAG00000005432	0.0026	3	9	lman2la	1.30	0
ENSXMAG00000005435	0.0024	21	9	wee2	1.27	18
ENSXMAG00000005436	0.0015	21	6	gpn1	1.49	18
ENSXMAG00000005444	0.0015	21	6	prrx1b	1.56	18
ENSXMAG00000005447	0.0026	6	15	iqsec3a	2.37	3
ENSXMAG00000005450	0.0025	18	9	fam222ba	2.75	15
ENSXMAG00000005480	0.0025	3	15	pdcd4a	3.24	0
ENSXMAG00000005482	0.0015	21	6	rtca	1.40	18
ENSXMAG00000005483	0.0024	3	15		1.57	0
ENSXMAG00000005486	0.0030	9	12	ankrd13c	1.57	6
ENSXMAG00000005488	0.0030	9	12		2.64	6
ENSXMAG00000005497	0.0031	6	12	cdc14ab	8.24	3

ENSXMAG00000005505	0.0027	24	9		1.83	21
ENSXMAG00000005525	0.0031	6	12	vcam1b	6.07	3
ENSXMAG00000005529	0.0015	12	18	si:ch211-217k17.7	1.43	9
ENSXMAG00000005538	0.0024	3	15	gpr78a	9.20	0
ENSXMAG00000005541	0.0030	18	12	fubp3	1.49	15
ENSXMAG00000005554	0.0026	24	15	atp13a3	1.26	21
ENSXMAG00000005558	0.0014	21	18	ASS1	1.17	18
ENSXMAG00000005562	0.0028	18	9		1.30	15
ENSXMAG00000005568	0.0031	15	12	ints8	1.60	12
ENSXMAG00000005585	0.0030	9	12	tacr3l	17.99	6
ENSXMAG00000005597	0.0026	6	15		1.49	3
ENSXMAG00000005602	0.0016	24	6	dnm1a	1.34	21
ENSXMAG00000005604	0.0028	18	9	otx1	2.57	15
ENSXMAG00000005611	0.0031	21	12	ehbp1	1.55	18
ENSXMAG00000005615	0.0024	3	15	rgs16	2.55	0
ENSXMAG00000005616	0.0024	18	9	chrnb3a	1.53	15
ENSXMAG00000005621	0.0025	18	15	pomgnt1	1.15	15
ENSXMAG00000005628	0.0024	24	9	mbd1b	1.30	21
ENSXMAG00000005629	0.0029	18	12	si:ch73-380n15.2	2.36	15
ENSXMAG00000005633	0.0015	21	6	supt4h1	1.38	18
ENSXMAG00000005638	0.0024	21	9	psmd1	1.39	18
ENSXMAG00000005653	0.0026	18	9		6.17	15
ENSXMAG00000005656	0.0031	6	12	gucy1a1	1.66	3
ENSXMAG00000005661	0.0025	6	15	slc24a3	4.54	3
ENSXMAG00000005662	0.0029	18	12	si:ch73-28h20.1	5.76	15
ENSXMAG00000005667	0.0031	18	12	mlxipl	3.07	15
ENSXMAG00000005675	0.0031	24	12	cadm3	2.37	21
ENSXMAG00000005677	0.0029	6	12	phyhd1	2.63	3
ENSXMAG00000005692	0.0029	9	12	usp53b	2.09	6
ENSXMAG00000005693	0.0024	21	9	uck1	1.41	18
ENSXMAG00000005703	0.0015	21	6	rapgef1b	1.54	18
ENSXMAG00000005708	0.0026	3	15		4.49	0
ENSXMAG00000005722	0.0015	24	18	atg9b	1.57	21
ENSXMAG00000005737	0.0014	21	6	sympk	1.51	18
ENSXMAG00000005740	0.0025	9	9	tmem54b	2.22	6
ENSXMAG00000005744	0.0031	3	12	camkk1a	2.16	0
ENSXMAG00000005747	0.0029	24	12	FAM135B	3.88	21
ENSXMAG00000005751	0.0031	6	12	nt5c1aa	3.19	3
ENSXMAG00000005753	0.0026	12	9	abcc4	2.05	9
ENSXMAG00000005761	0.0029	3	12	mttp	2.17	0
ENSXMAG00000005769	0.0031	15	12	tlx1	2.47	12
ENSXMAG00000005783	0.0025	3	15	rubcn	1.25	0
ENSXMAG00000005787	0.0024	12	9	fam161a	3.86	9
ENSXMAG00000005790	0.0031	15	12	xpo1a	1.31	12
ENSXMAG00000005791	0.0027	24	9	kyat1	1.46	21
ENSXMAG00000005800	0.0034	24	12	tspan5b	1.25	21
ENSXMAG00000005815	0.0031	3	12	cx47.1	2.53	0
ENSXMAG00000005827	0.0025	18	9	opn4a	4.05	15
ENSXMAG00000005830	0.0029	6	12	pth1ra	2.41	3
ENSXMAG00000005832	0.0031	6	12	jph3	3.25	3
ENSXMAG00000005838	0.0025	18	9		2.02	15
ENSXMAG00000005841	0.0027	3	9	nos1apa	1.38	0
ENSXMAG00000005844	0.0026	3	15	smu1b	80.20	0
ENSXMAG00000005875	0.0027	9	15	mdh1aa	2.02	6
ENSXMAG00000005876	0.0031	24	12	armc9	1.28	21
ENSXMAG00000005893	0.0025	3	15	ptges	2.00	0

ENSXMAG00000005895	0.0031	9	12	bco2b	3.93	6
ENSXMAG00000005896	0.0031	24	12	cntn3b	1.88	21
ENSXMAG00000005907	0.0031	3	12	SPNS2	2.73	0
ENSXMAG00000005908	0.0034	21	12	necab3	1.59	18
ENSXMAG00000005912	0.0015	18	6	cnga1a	1.83	15
ENSXMAG00000005917	0.0025	9	9	abhd3	1.94	6
ENSXMAG00000005930	0.0024	18	9	naa25	1.27	15
ENSXMAG00000005934	0.0071	21	18	st7l	1.51	18
ENSXMAG00000005950	0.0024	18	9	pdzd3b	3.39	15
ENSXMAG00000005975	0.0024	21	9	pycr3	1.43	18
ENSXMAG00000005976	0.0031	3	12	abi3bpb	2.87	0
ENSXMAG00000005986	0.0031	18	12	rap1ab	1.95	15
ENSXMAG00000005995	0.0025	18	9		4.59	15
ENSXMAG00000005999	0.0034	18	12	txndc5	1.85	15
ENSXMAG00000006017	0.0031	18	12		4.21	15
ENSXMAG00000006023	0.0031	21	12	carmil3	5.24	18
ENSXMAG00000006024	0.0031	21	12	SETD6	1.81	18
ENSXMAG00000006030	0.0025	18	9		16.12	15
ENSXMAG00000006052	0.0031	9	12	prelid3b	4.04	6
ENSXMAG00000006058	0.0025	18	9	wdr47b	2.92	15
ENSXMAG00000006070	0.0024	21	15	vps16	1.26	18
ENSXMAG00000006085	0.0014	3	18		1.27	0
ENSXMAG00000006109	0.0034	18	12	ppp1r16b	1.94	15
ENSXMAG00000006112	0.0029	6	12	arhgap12a	5.55	3
ENSXMAG00000006118	0.0024	18	9		1.77	15
ENSXMAG00000006121	0.0030	15	12	polg2	1.51	12
ENSXMAG00000006131	0.0031	18	12		1.37	15
ENSXMAG00000006133	0.0028	3	15	slc38a4	3.24	0
ENSXMAG00000006134	0.0027	3	15		3.48	0
ENSXMAG00000006136	0.0016	24	6	esr2a	1.79	21
ENSXMAG00000006158	0.0031	9	12		3.52	6
ENSXMAG00000006166	0.0028	9	15	mylk4b	1.91	6
ENSXMAG00000006178	0.0034	18	12	gmds	1.64	15
ENSXMAG00000006180	0.0030	6	12		1.41	3
ENSXMAG00000006183	0.0030	18	12		1.88	15
ENSXMAG00000006216	0.0024	24	9	GAN	1.53	21
ENSXMAG00000006227	0.0029	3	12		1.97	0
ENSXMAG00000006235	0.0024	24	9	draxin	2.70	21
ENSXMAG00000006239	0.0027	18	9	zc3h18	1.19	15
ENSXMAG00000006241	0.0014	3	6		1.34	0
ENSXMAG00000006257	0.0025	9	9	csrp2	2.57	6
ENSXMAG00000006258	0.0029	6	12		1.60	3
ENSXMAG00000006259	0.0026	12	15	prozb	3.01	9
ENSXMAG00000006263	0.0029	15	12		1.77	12
ENSXMAG00000006277	0.0025	18	9		2.60	15
ENSXMAG00000006282	0.0024	18	9	aars	1.46	15
ENSXMAG00000006291	0.0025	21	15	cpt2	1.58	18
ENSXMAG00000006296	0.0031	21	12	NRP1 (1 of many)	1.47	18
ENSXMAG00000006297	0.0024	18	9	magoh	1.58	15
ENSXMAG00000006306	0.0026	9	15		1.32	6
ENSXMAG00000006308	0.0030	21	12	jag1b	1.62	18
ENSXMAG00000006311	0.0024	21	9	tmco1	1.45	18
ENSXMAG00000006320	0.0014	6	6	sdk1a	2.41	3
ENSXMAG00000006329	0.0024	24	9		1.24	21
ENSXMAG00000006338	0.0026	24	9		1.77	21
ENSXMAG00000006346	0.0025	9	15	CNGB3	4.49	6

ENSXMAG00000006352	0.0025	9	15		5.20	6
ENSXMAG00000006355	0.0015	15	6	mycla	5.21	12
ENSXMAG00000006361	0.0031	3	12	crema	4.09	0
ENSXMAG00000006380	0.0026	18	9	TRIM67	3.14	15
ENSXMAG00000006384	0.0027	6	15	KLF6 (1 of many)	1.66	3
ENSXMAG00000006388	0.0031	6	12	srd5a2b	3.99	3
ENSXMAG00000006390	0.0016	9	18	dnajc2	1.66	6
ENSXMAG00000006394	0.0026	24	9		1.32	21
ENSXMAG00000006405	0.0030	18	12	slc25a38b	1.71	15
ENSXMAG00000006418	0.0026	6	15	mapk8ip1a	1.98	3
ENSXMAG00000006419	0.0031	18	12	khdrbs1a	1.28	15
ENSXMAG00000006421	0.0025	9	15	pfkpb	1.92	6
ENSXMAG00000006425	0.0031	21	12	hibch	1.18	18
ENSXMAG00000006426	0.0024	3	15	zgc:92873	1.84	0
ENSXMAG00000006434	0.0024	21	15	si:ch73-111k22.2	1.27	18
ENSXMAG00000006437	0.0071	21	18	RHPN1	3.47	18
ENSXMAG00000006444	0.0024	3	15	mapk1	1.33	0
ENSXMAG00000006445	0.0028	18	9	lss	2.48	15
ENSXMAG00000006471	0.0025	3	15		1.55	0
ENSXMAG00000006487	0.0026	24	15	abca1a	2.33	21
ENSXMAG00000006493	0.0016	24	18		4.54	21
ENSXMAG00000006499	0.0024	18	9	cand1	1.26	15
ENSXMAG00000006524	0.0031	18	12	psmd12	1.32	15
ENSXMAG00000006529	0.0031	6	12	CYP27A1	3.19	3
ENSXMAG00000006538	0.0027	3	15	tomm20a	3.38	0
ENSXMAG00000006540	0.0031	21	12		1.22	18
ENSXMAG00000006547	0.0026	21	9	ddx39aa	1.46	18
ENSXMAG00000006554	0.0024	24	9	pak6a	1.90	21
ENSXMAG00000006557	0.0024	21	9	cacng1a	1.35	18
ENSXMAG00000006562	0.0031	21	12	bub1bb	2.07	18
ENSXMAG00000006572	0.0025	6	15	slc4a3	3.70	3
ENSXMAG00000006578	0.0024	6	15	prkca	2.45	3
ENSXMAG00000006598	0.0014	3	6	si:dkey-24p1.1	2.28	0
ENSXMAG00000006602	0.0029	18	12	SH3GL3	1.67	15
ENSXMAG00000006606	0.0029	18	12		2.85	15
ENSXMAG00000006610	0.0025	18	9		1.73	15
ENSXMAG00000006637	0.0029	18	12		1.85	15
ENSXMAG00000006643	0.0014	21	6	arrb2b	1.29	18
ENSXMAG00000006651	0.0025	9	15		37.65	6
ENSXMAG00000006652	0.0027	24	9	slc37a1	1.23	21
ENSXMAG00000006654	0.0031	12	12	slc6a11a	1.84	9
ENSXMAG00000006663	0.0026	6	15	arhgef9b	2.98	3
ENSXMAG00000006664	0.0025	3	15	slc24a2	4.82	0
ENSXMAG00000006665	0.0030	15	12		52.14	12
ENSXMAG00000006673	0.0031	21	12	akt1	1.36	18
ENSXMAG00000006679	0.0031	15	12	prtgb	3.95	12
ENSXMAG00000006694	0.0031	9	12	ckbb	2.05	6
ENSXMAG00000006699	0.0026	24	9	fuk	1.90	21
ENSXMAG00000006704	0.0014	21	6	pomt1	1.34	18
ENSXMAG00000006708	0.0014	3	18	usp2a	6.19	0
ENSXMAG00000006711	0.0034	21	12	jpt1a	1.22	18
ENSXMAG00000006728	0.0034	24	12	rpgrip1l	1.38	21
ENSXMAG00000006732	0.0015	21	6	ap4m1	1.48	18
ENSXMAG00000006743	0.0031	3	12	ABCB1	1.45	0
ENSXMAG00000006757	0.0025	3	15	lipeb	2.25	0
ENSXMAG00000006760	0.0026	15	9	INF2	1.26	12

ENSXMAG00000006761	0.0024	18	9	ddx3xa	1.72	15
ENSXMAG00000006766	0.0025	12	9	znf207b	1.47	9
ENSXMAG00000006770	0.0034	9	12		1.70	6
ENSXMAG00000006797	0.0031	21	12	mdkb	3.98	18
ENSXMAG00000006803	0.0027	6	15	dgkzb	2.87	3
ENSXMAG00000006813	0.0025	15	9	slco2b1	1.65	12
ENSXMAG00000006816	0.0027	21	9	cdc5l	1.18	18
ENSXMAG00000006817	0.0014	6	18	gchfr	2.04	3
ENSXMAG00000006825	0.0031	15	12	acsl4a	2.70	12
ENSXMAG00000006826	0.0031	15	12	nyx	3.72	12
ENSXMAG00000006829	0.0025	3	15		1.54	0
ENSXMAG00000006830	0.0026	18	9		1.54	15
ENSXMAG00000006835	0.0026	6	15	rap1gap2b	2.87	3
ENSXMAG00000006841	0.0024	9	15		1.42	6
ENSXMAG00000006842	0.0014	15	6		1.68	12
ENSXMAG00000006844	0.0024	21	9	hmgb1b	1.57	18
ENSXMAG00000006856	0.0024	3	15		2.18	0
ENSXMAG00000006863	0.0030	9	12	frya	3.57	6
ENSXMAG00000006865	0.0034	6	12	me1	2.28	3
ENSXMAG00000006869	0.0034	3	12		33.47	0
ENSXMAG00000006872	0.0031	15	12	pcf11	1.50	12
ENSXMAG00000006881	0.0034	3	12	gtpbp10	1.24	0
ENSXMAG00000006882	0.0031	3	12	FMN1 (1 of many)	1.36	0
ENSXMAG00000006891	0.0026	24	9	prdm11	1.66	21
ENSXMAG00000006892	0.0029	6	12	ctgfa	4.42	3
ENSXMAG00000006901	0.0031	15	12	yme1l1b	1.27	12
ENSXMAG00000006902	0.0014	9	18	chga	2.75	6
ENSXMAG00000006903	0.0027	21	9	zgc:158803	1.27	18
ENSXMAG00000006916	0.0027	3	15	ano3	3.25	0
ENSXMAG00000006919	0.0031	12	12		1.57	9
ENSXMAG00000006920	0.0024	21	9	adcy8	1.92	18
ENSXMAG00000006921	0.0015	3	6	polr3h	1.62	0
ENSXMAG00000006923	0.0024	15	15		1.52	12
ENSXMAG00000006926	0.0025	18	15	PTK7	1.39	15
ENSXMAG00000006928	0.0014	24	18	pdcd4b	1.70	21
ENSXMAG00000006940	0.0025	6	15		6.80	3
ENSXMAG00000006943	0.0025	6	15	guca1d	15.40	3
ENSXMAG00000006944	0.0031	9	12	PMM1	2.62	6
ENSXMAG00000006956	0.0026	12	15	FLOT2 (1 of many)	1.22	9
ENSXMAG00000006965	0.0027	24	9	RIC3	1.75	21
ENSXMAG00000006967	0.0025	12	15	mkxb	23.27	9
ENSXMAG00000006968	0.0026	15	15	RBM20	6.62	12
ENSXMAG00000006970	0.0024	18	9	mast1a	1.68	15
ENSXMAG00000006979	0.0025	21	9	mpp7b	11.06	18
ENSXMAG00000006981	0.0031	6	12	trmt9b	14.34	3
ENSXMAG00000006984	0.0031	21	12		1.30	18
ENSXMAG00000006986	0.0031	3	12		1.15	0
ENSXMAG00000006999	0.0031	9	12		81.32	6
ENSXMAG00000007021	0.0031	3	12	ano5a	1.38	0
ENSXMAG00000007029	0.0034	6	12		4.51	3
ENSXMAG00000007032	0.0025	6	15	ibtk	1.30	3
ENSXMAG00000007055	0.0028	6	15		1.66	3
ENSXMAG00000007068	0.0026	18	15	si:dkey-71b5.7	32.03	15
ENSXMAG00000007074	0.0025	3	15	atp8b2	19.75	0
ENSXMAG00000007076	0.0028	24	15	commd3	1.42	21
ENSXMAG00000007077	0.0014	18	18	unc45b	1.97	15

ENSXMAG00000007080	0.0031	24	12	cd81b	1.46	21
ENSXMAG00000007103	0.0031	18	12	tph1a	6.14	15
ENSXMAG00000007105	0.0026	18	9	ldlrb	2.28	15
ENSXMAG00000007106	0.0031	21	12		2.08	18
ENSXMAG00000007119	0.0031	21	12	nucb2a	1.29	18
ENSXMAG00000007120	0.0024	3	15	miga1	2.13	0
ENSXMAG00000007138	0.0014	21	6		1.67	18
ENSXMAG00000007139	0.0031	3	12	f5	2.43	0
ENSXMAG00000007142	0.0031	6	12	rgs9a	2.23	3
ENSXMAG00000007148	0.0024	21	9	caprin1a	1.39	18
ENSXMAG00000007160	0.0024	18	9		1.43	15
ENSXMAG00000007164	0.0025	3	15	cog1	1.93	0
ENSXMAG00000007185	0.0031	15	12	PDE4A (1 of many)	2.02	12
ENSXMAG00000007191	0.0026	15	15	soga3b	1.24	12
ENSXMAG00000007194	0.0014	3	6	ext2	1.31	0
ENSXMAG00000007198	0.0027	15	9	cgna	3.68	12
ENSXMAG00000007201	0.0071	3	18	vrk2	2.47	0
ENSXMAG00000007202	0.0028	15	9	trim3a	1.76	12
ENSXMAG00000007206	0.0026	12	9	zgc:194202 (1 of many)	1.72	9
ENSXMAG00000007210	0.0014	24	18	tent2	1.23	21
ENSXMAG00000007211	0.0027	6	15	si:dkeyp-72e1.9	2.44	3
ENSXMAG00000007222	0.0024	21	9	pgm3	1.54	18
ENSXMAG00000007236	0.0029	9	12	dagla	2.53	6
ENSXMAG00000007242	0.0026	18	9		2.13	15
ENSXMAG00000007246	0.0030	21	12	rims1a	1.45	18
ENSXMAG00000007262	0.0024	6	9	cavin2a	1.55	3
ENSXMAG00000007272	0.0031	6	12	kcnh3	6.55	3
ENSXMAG00000007277	0.0031	15	12	tiam1b	1.50	12
ENSXMAG00000007278	0.0031	6	12	apba2b	2.07	3
ENSXMAG00000007280	0.0029	18	12	tspan18b	2.29	15
ENSXMAG00000007295	0.0031	24	12	sh3pxd2ab	1.23	21
ENSXMAG00000007296	0.0029	9	12	faxca	1.90	6
ENSXMAG00000007297	0.0025	18	9	snx33	2.19	15
ENSXMAG00000007301	0.0025	6	15	neurl1ab	4.46	3
ENSXMAG00000007309	0.0015	24	18	bach1a	1.37	21
ENSXMAG00000007320	0.0031	18	12	rwdd2b	2.07	15
ENSXMAG00000007328	0.0030	21	12	lcmt2	1.93	18
ENSXMAG00000007329	0.0029	18	12	calub	1.84	15
ENSXMAG00000007344	0.0014	24	18		1.53	21
ENSXMAG00000007350	0.0024	21	15	mybpc2b	1.38	18
ENSXMAG00000007351	0.0016	6	6	prodhb	1.53	3
ENSXMAG00000007356	0.0031	24	12	adal	1.36	21
ENSXMAG00000007360	0.0025	18	15		9.20	15
ENSXMAG00000007368	0.0031	21	12	bicc1a	1.42	18
ENSXMAG00000007385	0.0025	18	9	ephb4b	1.56	15
ENSXMAG00000007391	0.0024	18	9		132.65	15
ENSXMAG00000007411	0.0031	6	12	hcn2b	3.69	3
ENSXMAG00000007414	0.0026	18	9	nptna	5.21	15
ENSXMAG00000007415	0.0024	24	9	ANLN	2.37	21
ENSXMAG00000007417	0.0024	18	9		2.48	15
ENSXMAG00000007418	0.0031	12	12	vdac1	1.14	9
ENSXMAG00000007422	0.0024	18	15	THADA	2.04	15
ENSXMAG00000007435	0.0031	6	12	guca1c	14.10	3
ENSXMAG00000007444	0.0031	24	12	scamp5a	1.24	21
ENSXMAG00000007452	0.0025	21	9		10.11	18
ENSXMAG00000007467	0.0025	3	15		1.83	0

ENSXMAG00000007469	0.0025	18	9	dnajb5	1.98	15
ENSXMAG00000007473	0.0031	18	12		15.35	15
ENSXMAG00000007479	0.0027	24	9	ttc7a	2.43	21
ENSXMAG00000007481	0.0031	21	12	prcp	1.86	18
ENSXMAG00000007482	0.0024	18	15	nhsl1b	8.63	15
ENSXMAG00000007485	0.0031	18	12	vcp	1.50	15
ENSXMAG00000007486	0.0031	24	12	cmtr1	1.14	21
ENSXMAG00000007488	0.0014	24	18	iqsec1b	1.92	21
ENSXMAG00000007489	0.0014	24	18	sypb	1.33	21
ENSXMAG00000007515	0.0034	18	12	rhoq	1.65	15
ENSXMAG00000007523	0.0026	9	15		1.74	6
ENSXMAG00000007525	0.0027	3	15		4.57	0
ENSXMAG00000007526	0.0024	18	9	nr5a1b	420.59	15
ENSXMAG00000007534	0.0026	21	9		1.35	18
ENSXMAG00000007535	0.0014	21	6	psmb7	1.20	18
ENSXMAG00000007545	0.0027	24	15	trpc6b	1.42	21
ENSXMAG00000007546	0.0016	24	18	ddhd2	1.63	21
ENSXMAG00000007547	0.0031	3	12		1.24	0
ENSXMAG00000007552	0.0024	3	15		2.03	0
ENSXMAG00000007553	0.0016	3	18	pgr	2.73	0
ENSXMAG00000007555	0.0015	21	6	ppp4r4	1.48	18
ENSXMAG00000007559	0.0014	3	18	TACC1	1.63	0
ENSXMAG00000007570	0.0015	21	6	ubr7	1.53	18
ENSXMAG00000007572	0.0031	24	12	tpcn1	1.71	21
ENSXMAG00000007579	0.0026	24	9	kcnd3	2.19	21
ENSXMAG00000007588	0.0030	6	12	cttnbp2nlb	2.07	3
ENSXMAG00000007593	0.0027	21	9	capza1b	1.32	18
ENSXMAG00000007597	0.0031	21	12	vox	3.69	18
ENSXMAG00000007607	0.0031	21	12		1.52	18
ENSXMAG00000007614	0.0027	3	15		4.27	0
ENSXMAG00000007617	0.0031	18	12	galk1	1.58	15
ENSXMAG00000007649	0.0031	15	12	bckdha	1.42	12
ENSXMAG00000007662	0.0027	24	15	napab	1.65	21
ENSXMAG00000007677	0.0025	6	15		13.63	3
ENSXMAG00000007684	0.0024	24	9	adam19a	1.45	21
ENSXMAG00000007685	0.0031	18	12	psmd8	1.67	15
ENSXMAG00000007692	0.0031	18	12	rcan3	2.33	15
ENSXMAG00000007701	0.0024	18	9	fmn1	62.59	15
ENSXMAG00000007704	0.0024	18	15	st14a	2.59	15
ENSXMAG00000007707	0.0025	18	9	opn3	3.81	15
ENSXMAG00000007713	0.0026	6	15	soul3	3.33	3
ENSXMAG00000007715	0.0025	24	15		1.64	21
ENSXMAG00000007730	0.0030	21	12	DLK1	2.06	18
ENSXMAG00000007731	0.0025	24	15	fbxo25	23.31	21
ENSXMAG00000007735	0.0071	3	18		1.76	0
ENSXMAG00000007739	0.0024	24	15	PYGM	1.16	21
ENSXMAG00000007746	0.0014	21	6	sirt1	1.36	18
ENSXMAG00000007755	0.0030	24	12	stmn4	2.43	21
ENSXMAG00000007759	0.0026	24	15	MYPN	1.63	21
ENSXMAG00000007761	0.0015	24	18	aven	1.30	21
ENSXMAG00000007797	0.0031	15	12	si:dkeyp-94b4.1	1.61	12
ENSXMAG00000007798	0.0027	3	15	susd6	4.89	0
ENSXMAG00000007807	0.0027	21	9	ccdc43	1.59	18
ENSXMAG00000007812	0.0029	9	12		1.92	6
ENSXMAG00000007819	0.0024	21	9	lpcat2	1.47	18
ENSXMAG00000007821	0.0014	24	18	acacb	2.22	21

ENSXMAG00000007822	0.0028	21	9		1.25	18
ENSXMAG00000007827	0.0031	3	12	nkl.1	4.40	0
ENSXMAG00000007844	0.0029	18	12	csnk2a4	1.45	15
ENSXMAG00000007850	0.0026	18	9	mta2	1.64	15
ENSXMAG00000007855	0.0014	21	18	si:ch211-93g23.2	1.59	18
ENSXMAG00000007881	0.0026	18	9	syncripl	1.66	15
ENSXMAG00000007889	0.0031	3	12	dpep2	1.55	0
ENSXMAG00000007895	0.0026	6	15	SCN8A	2.06	3
ENSXMAG00000007908	0.0014	24	18	KLF15	2.42	21
ENSXMAG00000007909	0.0031	15	12	rpp25b	2.04	12
ENSXMAG00000007912	0.0026	6	15		1.80	3
ENSXMAG00000007915	0.0031	18	12	rnd3a	1.55	15
ENSXMAG00000007916	0.0025	18	9	crispld2	2.82	15
ENSXMAG00000007919	0.0024	18	9		1.51	15
ENSXMAG00000007940	0.0026	21	9	prom1b	3.83	18
ENSXMAG00000007942	0.0031	24	12	cpda	1.32	21
ENSXMAG00000007950	0.0024	9	15		2.30	6
ENSXMAG00000007953	0.0034	18	12	lmnb2	1.48	15
ENSXMAG00000007965	0.0015	24	18	pde6c	62.02	21
ENSXMAG00000007976	0.0024	3	15	HDAC4 (1 of many)	2.64	0
ENSXMAG00000007990	0.0024	12	15	LONP1	1.15	9
ENSXMAG00000008001	0.0031	18	12	tm9sf3	1.32	15
ENSXMAG00000008021	0.0025	24	9	setd1a	1.20	21
ENSXMAG00000008030	0.0025	18	9	zgc:110045	3.54	15
ENSXMAG00000008036	0.0024	18	9		2.25	15
ENSXMAG00000008039	0.0025	18	9	atf4a	1.66	15
ENSXMAG00000008068	0.0027	21	9		4.28	18
ENSXMAG00000008069	0.0025	18	9	rbm4.1	1.26	15
ENSXMAG00000008071	0.0014	21	6	gatb	1.74	18
ENSXMAG00000008076	0.0016	3	6		1.42	0
ENSXMAG00000008080	0.0024	18	9	phf19	2.86	15
ENSXMAG00000008089	0.0025	18	9	vwa2	2.72	15
ENSXMAG00000008105	0.0025	3	15		2.28	0
ENSXMAG00000008122	0.0015	21	6	peli3	1.27	18
ENSXMAG00000008126	0.0025	24	9	nhlrc2	1.47	21
ENSXMAG00000008130	0.0031	18	12	ebp	2.63	15
ENSXMAG00000008134	0.0025	21	9	apobec2b	2.95	18
ENSXMAG00000008136	0.0026	9	9	jade1	1.30	6
ENSXMAG00000008152	0.0026	24	9	NAV1 (1 of many)	1.77	21
ENSXMAG00000008158	0.0025	18	9	pelp1	1.53	15
ENSXMAG00000008164	0.0025	18	15	pef1	1.47	15
ENSXMAG00000008172	0.0014	24	18	si:dkey-177p2.6	7.90	21
ENSXMAG00000008179	0.0026	21	9	PSMD2	1.28	18
ENSXMAG00000008189	0.0031	24	12	tead3a	1.51	21
ENSXMAG00000008191	0.0026	3	15	dact2	1.56	0
ENSXMAG00000008192	0.0031	18	12	fam117aa	1.86	15
ENSXMAG00000008194	0.0031	24	12	prss16	1.83	21
ENSXMAG00000008197	0.0016	24	18		1.65	21
ENSXMAG00000008201	0.0026	18	9	senp3b	1.25	15
ENSXMAG00000008211	0.0026	18	9		1.75	15
ENSXMAG00000008217	0.0034	24	12		1.44	21
ENSXMAG00000008231	0.0026	6	15		5.20	3
ENSXMAG00000008233	0.0016	21	6	ehmt2	1.28	18
ENSXMAG00000008234	0.0028	18	9	lratb.1	2.86	15
ENSXMAG00000008252	0.0026	3	15		1.61	0
ENSXMAG00000008262	0.0024	6	15	camkvl	1.37	3

ENSXMAG00000008266	0.0025	18	15	kcne4	2.01	15
ENSXMAG00000008275	0.0024	3	15	myo3a	4.61	0
ENSXMAG00000008283	0.0014	24	6	asic2	1.22	21
ENSXMAG00000008285	0.0015	21	6	si:ch211-222n4.6	1.50	18
ENSXMAG00000008291	0.0024	18	15		3.88	15
ENSXMAG00000008297	0.0025	12	15	arhgef3l	1.63	9
ENSXMAG00000008303	0.0034	3	12	hsp90aa1.2	2.13	0
ENSXMAG00000008306	0.0026	24	9	epha4a	2.04	21
ENSXMAG00000008307	0.0031	9	12	usp21	4.52	6
ENSXMAG00000008312	0.0025	21	9	ddx46	1.14	18
ENSXMAG00000008315	0.0024	18	9	hsp90aa1.1	2.45	15
ENSXMAG00000008317	0.0024	18	9	znf281a	1.92	15
ENSXMAG00000008318	0.0031	9	12		1.47	6
ENSXMAG00000008320	0.0015	9	18	pdss1	1.87	6
ENSXMAG00000008338	0.0014	21	6	pcdh2ab5	2.84	18
ENSXMAG00000008339	0.0028	21	9	si:ch73-379j16.2 (1 of many)	2.35	18
ENSXMAG00000008340	0.0026	21	9	acbd5a	1.60	18
ENSXMAG00000008344	0.0025	18	9	si:ch73-233f7.1	1.95	15
ENSXMAG00000008348	0.0014	21	6	pxylp1	2.29	18
ENSXMAG00000008349	0.0024	21	15	fkbp3	1.33	18
ENSXMAG00000008353	0.0014	24	18	slc25a36a (1 of many)	1.34	21
ENSXMAG00000008356	0.0024	18	9	spock1	1.92	15
ENSXMAG00000008362	0.0025	3	9		2.79	0
ENSXMAG00000008372	0.0026	21	9		1.40	18
ENSXMAG00000008377	0.0025	18	9		2.00	15
ENSXMAG00000008382	0.0031	21	12		1.99	18
ENSXMAG00000008384	0.0029	3	12		1.49	0
ENSXMAG00000008389	0.0025	21	9	ca10b	2.38	18
ENSXMAG00000008391	0.0029	18	12	actr10	1.32	15
ENSXMAG00000008396	0.0026	24	9		1.47	21
ENSXMAG00000008399	0.0025	3	15	ubl3a	2.68	0
ENSXMAG00000008410	0.0031	18	12	znf410	2.02	15
ENSXMAG00000008415	0.0030	24	12	ints10	1.17	21
ENSXMAG00000008426	0.0029	24	12		3.84	21
ENSXMAG00000008433	0.0025	18	9	frmpd1b	2.04	15
ENSXMAG00000008442	0.0031	6	12	arhgap21a	1.90	3
ENSXMAG00000008446	0.0025	21	9	evlb	2.25	18
ENSXMAG00000008449	0.0024	18	9	pacs1a	3.72	15
ENSXMAG00000008461	0.0015	24	6		5.87	21
ENSXMAG00000008463	0.0029	18	12	slco1c1	4.23	15
ENSXMAG00000008464	0.0031	6	12	SLC24A2	2.58	3
ENSXMAG00000008469	0.0026	24	9	pak7	1.31	21
ENSXMAG00000008472	0.0025	18	9	abhd15a	3.12	15
ENSXMAG00000008474	0.0030	9	12	aipl1	1.78	6
ENSXMAG00000008481	0.0026	6	15	PLCB4	1.30	3
ENSXMAG00000008491	0.0031	6	12	plin2	7.58	3
ENSXMAG00000008492	0.0029	24	12	GJC1	2.32	21
ENSXMAG00000008498	0.0031	18	12	dennd4c	2.16	15
ENSXMAG00000008513	0.0031	18	12		5.90	15
ENSXMAG00000008514	0.0034	24	12		10.71	21
ENSXMAG00000008520	0.0029	24	12		1.79	21
ENSXMAG00000008525	0.0027	21	9	ythdf1	1.33	18
ENSXMAG00000008552	0.0025	9	15	zgc:153372	2.48	6
ENSXMAG00000008554	0.0025	21	15	pdgfrb	1.28	18
ENSXMAG00000008555	0.0027	3	15	kcnj10a	3.55	0
ENSXMAG00000008560	0.0031	24	12	PPP3CA	1.42	21

ENSXMAG00000008573	0.0025	9	15	zgc:158659	1.75	6
ENSXMAG00000008574	0.0031	6	12	pcxb	1.38	3
ENSXMAG00000008576	0.0026	18	9	aifm4	1.85	15
ENSXMAG00000008579	0.0031	9	12	dclk1a	3.42	6
ENSXMAG00000008586	0.0031	21	12	rad21a	1.27	18
ENSXMAG00000008595	0.0030	9	12		1.97	6
ENSXMAG00000008597	0.0024	18	9	aldh16a1	1.91	15
ENSXMAG00000008604	0.0031	18	12		1.72	15
ENSXMAG00000008609	0.0026	3	15	camsap2b	2.60	0
ENSXMAG00000008610	0.0034	6	12	gpcpd1	1.82	3
ENSXMAG00000008615	0.0024	18	15	mettl16	2.65	15
ENSXMAG00000008626	0.0024	24	9	slc3a2a	1.49	21
ENSXMAG00000008630	0.0025	24	9		1.56	21
ENSXMAG00000008634	0.0014	21	6		5.21	18
ENSXMAG00000008641	0.0024	18	9	tmem237a	43.41	15
ENSXMAG00000008655	0.0024	18	9	alad	2.32	15
ENSXMAG00000008663	0.0027	24	15	fbxo32	2.26	21
ENSXMAG00000008664	0.0024	6	15	fam102ba	4.83	3
ENSXMAG00000008680	0.0014	3	18		2.05	0
ENSXMAG00000008681	0.0034	18	12	rbm22	1.25	15
ENSXMAG00000008682	0.0026	9	15	tcap	2.53	6
ENSXMAG00000008700	0.0031	12	12	arl3l2	2.08	9
ENSXMAG00000008704	0.0031	24	12	hint3	1.43	21
ENSXMAG00000008708	0.0031	3	12	ITM2B	1.60	0
ENSXMAG00000008710	0.0027	21	9	ctnnb1	2.02	18
ENSXMAG00000008741	0.0026	21	15	rad23aa	1.12	18
ENSXMAG00000008746	0.0026	18	9	adamts9	1.86	15
ENSXMAG00000008754	0.0031	24	12	nfixb	1.92	21
ENSXMAG00000008825	0.0026	9	15	slc5a5	2.35	6
ENSXMAG00000008826	0.0025	18	9	ssr1	1.29	15
ENSXMAG00000008827	0.0031	24	12	plppr2a	2.61	21
ENSXMAG00000008844	0.0024	18	9	SEMA4F	2.74	15
ENSXMAG00000008849	0.0026	12	15		1.41	9
ENSXMAG00000008853	0.0027	6	15	zbtb16a	2.32	3
ENSXMAG00000008855	0.0034	21	12	sox13	1.65	18
ENSXMAG00000008858	0.0025	3	15	slc13a4	1.98	0
ENSXMAG00000008881	0.0026	24	9	slc5a6a	2.80	21
ENSXMAG00000008891	0.0026	3	15		4.00	0
ENSXMAG00000008892	0.0015	18	18	ARNTL2 (1 of many)	5.08	15
ENSXMAG00000008895	0.0024	3	15	pkn1b	1.49	0
ENSXMAG00000008900	0.0024	9	15	sdha	2.62	6
ENSXMAG00000008925	0.0025	6	15	scn3b	3.38	3
ENSXMAG00000008926	0.0025	18	9	ptchd4	3.69	15
ENSXMAG00000008939	0.0025	18	9	DDX39A	1.77	15
ENSXMAG00000008976	0.0025	21	9	ctsk	4.37	18
ENSXMAG00000008989	0.0025	18	9	ephb2a	3.17	15
ENSXMAG00000009020	0.0026	3	15	sfswap	1.23	0
ENSXMAG00000009024	0.0014	15	18	NEMF	1.17	12
ENSXMAG00000009031	0.0024	24	9	ZNF516	1.85	21
ENSXMAG00000009034	0.0015	18	6	ARHGAP6	1.80	15
ENSXMAG00000009037	0.0029	12	12		3.28	9
ENSXMAG00000009054	0.0024	6	15		2.38	3
ENSXMAG00000009073	0.0025	6	15		1.75	3
ENSXMAG00000009078	0.0024	18	9		1.98	15
ENSXMAG00000009103	0.0016	3	18	stk17al	4.84	0
ENSXMAG00000009110	0.0025	6	15	fbxl13	4.07	3

ENSXMAG00000009118	0.0025	3	15	plekhd1	3.25	0
ENSXMAG00000009123	0.0027	21	9	plod2	1.64	18
ENSXMAG00000009136	0.0026	21	9	tomm34	1.46	18
ENSXMAG00000009139	0.0026	18	9		1.95	15
ENSXMAG00000009141	0.0027	18	9		1.15	15
ENSXMAG00000009145	0.0014	24	18	kif3a	1.29	21
ENSXMAG00000009149	0.0027	18	9	cntnap1	2.76	15
ENSXMAG00000009160	0.0024	24	15		2.42	21
ENSXMAG00000009163	0.0025	6	15		4.56	3
ENSXMAG00000009187	0.0014	21	6	psen1	1.22	18
ENSXMAG00000009192	0.0031	21	12	gnpda1	1.86	18
ENSXMAG00000009197	0.0024	21	9		1.46	18
ENSXMAG00000009200	0.0031	18	12	hspa4b	1.70	15
ENSXMAG00000009201	0.0026	6	15	si:ch211-168d23.3	1.68	3
ENSXMAG00000009232	0.0015	3	18	nrcama	1.66	0
ENSXMAG00000009234	0.0027	6	15	gabrr2a	18.87	3
ENSXMAG00000009235	0.0014	24	18	vps39	1.37	21
ENSXMAG00000009241	0.0031	6	12	stag1b	1.52	3
ENSXMAG00000009248	0.0016	24	18	ccng1	1.21	21
ENSXMAG00000009251	0.0024	18	9		6.64	15
ENSXMAG00000009255	0.0026	6	15	CDK18	2.07	3
ENSXMAG00000009256	0.0031	24	12	ptrh1	1.55	21
ENSXMAG00000009263	0.0025	9	9	gabra6a	3.04	6
ENSXMAG00000009271	0.0026	3	9	mctp2a	1.61	0
ENSXMAG00000009278	0.0014	18	6	cry1ba	23.08	15
ENSXMAG00000009280	0.0025	21	9	cntn1b	1.81	18
ENSXMAG00000009304	0.0026	6	15	mrvi1	1.64	3
ENSXMAG00000009306	0.0029	18	12		5.45	15
ENSXMAG00000009312	0.0029	9	12	dlec1	15.85	6
ENSXMAG00000009323	0.0016	24	18	tcea2	1.42	21
ENSXMAG00000009325	0.0031	9	12	si:ch211-63p21.1	6.80	6
ENSXMAG00000009344	0.0016	6	18	ndrg2	1.45	3
ENSXMAG00000009345	0.0025	18	15	fbxl3l	205.45	15
ENSXMAG00000009355	0.0025	24	9	gxylt1b	1.66	21
ENSXMAG00000009356	0.0031	18	12	sarnp	1.81	15
ENSXMAG00000009358	0.0029	24	12		1.85	21
ENSXMAG00000009370	0.0026	3	15	slc1a8b	68.32	0
ENSXMAG00000009373	0.0029	18	12	nckap5l	2.04	15
ENSXMAG00000009391	0.0025	3	15	zgc:92162	2.44	0
ENSXMAG00000009422	0.0024	24	9	ZNF106	1.73	21
ENSXMAG00000009427	0.0026	24	15		2.01	21
ENSXMAG00000009436	0.0016	24	6	tmem206	1.75	21
ENSXMAG00000009448	0.0024	6	15		2.30	3
ENSXMAG00000009450	0.0024	18	9	olfm2b	1.76	15
ENSXMAG00000009467	0.0029	3	12		1.24	0
ENSXMAG00000009473	0.0026	6	15	ogt.1	1.25	3
ENSXMAG00000009500	0.0031	21	12	klhl5	1.74	18
ENSXMAG00000009527	0.0014	18	6	igfbp6b	2.74	15
ENSXMAG00000009532	0.0031	21	12	gemin2	1.35	18
ENSXMAG00000009538	0.0031	6	12	slc16a1b	2.87	3
ENSXMAG00000009541	0.0024	18	9	irbpl	3.51	15
ENSXMAG00000009549	0.0030	15	12	opn1sw2	2.23	12
ENSXMAG00000009551	0.0031	24	12		1.51	21
ENSXMAG00000009563	0.0029	21	12	nlgn3a	1.95	18
ENSXMAG00000009576	0.0015	21	18	tmem184c	1.37	18
ENSXMAG00000009603	0.0026	18	15		1.57	15

ENSXMAG00000009610	0.0016	18	6	pak6b	1.91	15
ENSXMAG00000009616	0.0025	21	9	pds5a	1.22	18
ENSXMAG00000009618	0.0026	24	15	smad1	2.07	21
ENSXMAG00000009638	0.0024	3	15	cacng7b	2.92	0
ENSXMAG00000009641	0.0014	3	6	neurl1b	1.24	0
ENSXMAG00000009644	0.0026	3	15	trpc4apa	1.35	0
ENSXMAG00000009648	0.0026	24	15	cdkn1bb	1.51	21
ENSXMAG00000009662	0.0025	24	9	frem3	1.55	21
ENSXMAG00000009667	0.0031	21	12	fgfr1op	1.37	18
ENSXMAG00000009678	0.0031	18	12	SMARCA1	1.87	15
ENSXMAG00000009691	0.0026	18	9	xrcc6	1.38	15
ENSXMAG00000009709	0.0025	24	9	manea	1.48	21
ENSXMAG00000009711	0.0025	18	9	tagln3a	2.31	15
ENSXMAG00000009720	0.0026	18	9		2.64	15
ENSXMAG00000009734	0.0025	3	15	gab1	1.62	0
ENSXMAG00000009739	0.0031	15	12	mxd3	5.44	12
ENSXMAG00000009742	0.0031	9	12		3.69	6
ENSXMAG00000009749	0.0014	24	18		3.19	21
ENSXMAG00000009757	0.0029	18	12	myo10l1	4.37	15
ENSXMAG00000009759	0.0025	18	9	hs3st3l	6.92	15
ENSXMAG00000009769	0.0031	12	12		1.76	9
ENSXMAG00000009775	0.0025	15	9	TMEM63B	1.66	12
ENSXMAG00000009778	0.0016	9	18	phf20a	1.44	6
ENSXMAG00000009784	0.0024	18	9	cpsf3	1.36	15
ENSXMAG00000009790	0.0029	24	12		1.36	21
ENSXMAG00000009792	0.0031	6	12		7.96	3
ENSXMAG00000009793	0.0024	24	15	rnf150a	2.34	21
ENSXMAG00000009804	0.0026	18	9	aph1b	1.34	15
ENSXMAG00000009805	0.0014	21	6	tbc1d9	1.34	18
ENSXMAG00000009808	0.0015	21	6		1.97	18
ENSXMAG00000009810	0.0027	6	15	adam17b	3.51	3
ENSXMAG00000009827	0.0031	18	12	wdr3	1.85	15
ENSXMAG00000009846	0.0031	12	12	gch1	1.96	9
ENSXMAG00000009858	0.0029	12	12	taf1b	1.74	9
ENSXMAG00000009900	0.0024	18	9	dock3	3.93	15
ENSXMAG00000009912	0.0027	24	9	NDUFS8	1.38	21
ENSXMAG00000009918	0.0034	18	12		1.68	15
ENSXMAG00000009925	0.0031	24	12	asmt2	1.57	21
ENSXMAG00000009937	0.0024	21	9	antxr2a	1.56	18
ENSXMAG00000009945	0.0025	15	9	PPP1R1B (1 of many)	6.60	12
ENSXMAG00000009948	0.0026	24	9	pcdh7b	2.08	21
ENSXMAG00000009950	0.0025	6	15	doc2d	1.69	3
ENSXMAG00000009955	0.0029	18	12	FILIP1L	8.01	15
ENSXMAG00000009964	0.0031	6	12	ampd2b	2.91	3
ENSXMAG00000009970	0.0026	24	9	ppp6r2a	1.23	21
ENSXMAG00000009976	0.0034	24	12	TIAM2 (1 of many)	1.52	21
ENSXMAG00000009990	0.0025	24	9	robo1	2.23	21
ENSXMAG00000010000	0.0031	15	12	prkg2	5.79	12
ENSXMAG00000010022	0.0027	3	15	n4bp3	4.97	0
ENSXMAG00000010029	0.0025	21	9	shisa2b	1.86	18
ENSXMAG00000010032	0.0029	3	12	boc	1.41	0
ENSXMAG00000010034	0.0024	3	15		2.89	0
ENSXMAG00000010048	0.0031	6	12		3.78	3
ENSXMAG00000010051	0.0031	15	12	rae1	1.27	12
ENSXMAG00000010054	0.0025	24	9	sort1b	1.67	21
ENSXMAG00000010075	0.0031	21	12	tada3l	1.32	18

ENSXMAG00000010079	0.0031	18	12	ykt6	1.21	15
ENSXMAG00000010085	0.0025	21	9	zdhhc23b	2.18	18
ENSXMAG00000010088	0.0031	24	12	tnip1	1.28	21
ENSXMAG00000010106	0.0031	15	12	rrn3	1.66	12
ENSXMAG00000010108	0.0031	6	12		2.18	3
ENSXMAG00000010111	0.0034	21	12		1.72	18
ENSXMAG00000010112	0.0027	21	9	PSIP1	1.43	18
ENSXMAG00000010114	0.0025	6	15	slc16a4	4.38	3
ENSXMAG00000010118	0.0024	3	15	tspan17	2.09	0
ENSXMAG00000010123	0.0015	3	18	prex2	1.40	0
ENSXMAG00000010139	0.0031	18	12	ctnna1	1.38	15
ENSXMAG00000010149	0.0025	18	9	VAT1 (1 of many)	1.34	15
ENSXMAG00000010164	0.0027	18	9	ARHGEF25	1.54	15
ENSXMAG00000010166	0.0029	18	12	sncb	1.69	15
ENSXMAG00000010168	0.0026	15	9	SLC16A7	4.40	12
ENSXMAG00000010174	0.0030	18	12	neo1b	1.85	15
ENSXMAG00000010178	0.0029	18	12	lrig3	1.39	15
ENSXMAG00000010194	0.0014	21	18	ndufs4	1.39	18
ENSXMAG00000010204	0.0025	18	9	aclyb	1.85	15
ENSXMAG00000010214	0.0025	6	9	lrrc1	1.44	3
ENSXMAG00000010223	0.0024	15	9	si:dkey-202e22.2	2.00	12
ENSXMAG00000010224	0.0015	24	18	slco5a1a	4.24	21
ENSXMAG00000010226	0.0025	21	15	zgc:110329	1.39	18
ENSXMAG00000010236	0.0031	24	12	si:ch211-48m9.1	10.35	21
ENSXMAG00000010238	0.0025	15	15	SRC	1.36	12
ENSXMAG00000010242	0.0031	15	12		6.24	12
ENSXMAG00000010253	0.0026	21	9	zdhhc13	1.40	18
ENSXMAG00000010258	0.0027	18	9		1.58	15
ENSXMAG00000010261	0.0031	9	12	elovl5	10.70	6
ENSXMAG00000010271	0.0034	21	12	eya1	1.86	18
ENSXMAG00000010278	0.0026	21	15	dtd1	1.25	18
ENSXMAG00000010288	0.0031	6	12	taok1b	2.20	3
ENSXMAG00000010290	0.0031	6	12	srpk1a	1.36	3
ENSXMAG00000010292	0.0031	3	12	STRA6	6.72	0
ENSXMAG00000010295	0.0024	3	15		4.25	0
ENSXMAG00000010304	0.0026	9	9	wnt7bb	2.38	6
ENSXMAG00000010312	0.0027	18	9	nup50	1.38	15
ENSXMAG00000010314	0.0024	3	15		2.04	0
ENSXMAG00000010321	0.0026	3	15	smg6	1.41	0
ENSXMAG00000010331	0.0024	9	9	mcph1	3.05	6
ENSXMAG00000010343	0.0015	3	18		2.27	0
ENSXMAG00000010345	0.0029	18	12		4.21	15
ENSXMAG00000010351	0.0024	24	9		1.51	21
ENSXMAG00000010356	0.0031	18	12	hnrnpua	1.38	15
ENSXMAG00000010362	0.0026	18	9	si:ch211-163l21.8	10.01	15
ENSXMAG00000010371	0.0024	18	9	SLC24A1 (1 of many)	2.30	15
ENSXMAG00000010374	0.0030	21	12	stn1	1.68	18
ENSXMAG00000010375	0.0016	24	18	sh3tc2	1.40	21
ENSXMAG00000010380	0.0031	21	12	glsa	2.46	18
ENSXMAG00000010383	0.0027	9	9	bbs4	1.26	6
ENSXMAG00000010385	0.0031	18	12	rrm1	2.07	15
ENSXMAG00000010396	0.0026	3	15	ablim3	2.51	0
ENSXMAG00000010405	0.0024	18	9	SBSPON	2.56	15
ENSXMAG00000010407	0.0026	21	9	rnf121	1.58	18
ENSXMAG00000010417	0.0016	21	6		1.72	18
ENSXMAG00000010431	0.0026	18	15	ap3m1	1.78	15

ENSXMAG00000010441	0.0031	18	12		1.31	15
ENSXMAG00000010449	0.0026	3	15	ppardb	1.47	0
ENSXMAG00000010458	0.0071	21	18	ccndbp1	1.81	18
ENSXMAG00000010467	0.0015	21	6	GOLGA7	1.47	18
ENSXMAG00000010475	0.0029	15	12	dok6	1.80	12
ENSXMAG00000010492	0.0027	6	15	MYO9A	2.47	3
ENSXMAG00000010499	0.0031	6	12	aplp1	1.25	3
ENSXMAG00000010511	0.0029	6	12	got2b	1.49	3
ENSXMAG00000010512	0.0031	21	12	hmmr	4.29	18
ENSXMAG00000010524	0.0025	6	9	slc38a7	1.40	3
ENSXMAG00000010526	0.0031	21	12	TENM2	3.68	18
ENSXMAG00000010530	0.0031	9	12	si:ch211-113j13.2	3.69	6
ENSXMAG00000010535	0.0025	21	9	nhsa	1.84	18
ENSXMAG00000010562	0.0031	6	12	proser3	1.87	3
ENSXMAG00000010563	0.0024	18	9	slc1a7a	12.37	15
ENSXMAG00000010572	0.0030	9	12	hsc70	1.95	6
ENSXMAG00000010581	0.0031	21	12	zeb2b	2.13	18
ENSXMAG00000010603	0.0027	3	15	hephl1b	2.38	0
ENSXMAG00000010606	0.0031	6	12	ino80da	1.21	3
ENSXMAG00000010628	0.0014	3	18	slc43a2b	2.43	0
ENSXMAG00000010629	0.0027	21	9	zgc:66440	1.42	18
ENSXMAG00000010632	0.0015	18	6	rnd1b	4.39	15
ENSXMAG00000010637	0.0031	21	12		6.83	18
ENSXMAG00000010641	0.0025	12	9	pitpnab	1.58	9
ENSXMAG00000010643	0.0025	18	9		2.58	15
ENSXMAG00000010645	0.0031	24	12	cacnb3b	1.49	21
ENSXMAG00000010646	0.0014	3	18	hivep3b	5.46	0
ENSXMAG00000010653	0.0026	24	15	pcdh15a	1.59	21
ENSXMAG00000010655	0.0031	3	12	inpp5kb	2.17	0
ENSXMAG00000010656	0.0027	3	15	adcy6b	2.84	0
ENSXMAG00000010660	0.0025	3	15	nle1	1.86	0
ENSXMAG00000010671	0.0025	18	9	dnajc3b	1.60	15
ENSXMAG00000010693	0.0031	18	12		1.38	15
ENSXMAG00000010694	0.0031	12	12	tent5ba	26.97	9
ENSXMAG00000010731	0.0028	21	9	pla2g7	1.28	18
ENSXMAG00000010743	0.0031	18	12	nadk2	1.50	15
ENSXMAG00000010750	0.0026	18	9	wdr34	1.98	15
ENSXMAG00000010752	0.0025	9	15	vsnl1a	1.60	6
ENSXMAG00000010758	0.0029	9	12	aldocb	1.48	6
ENSXMAG00000010764	0.0024	21	9	neto1	2.06	18
ENSXMAG00000010771	0.0016	18	18	ehd2a	1.34	15
ENSXMAG00000010776	0.0015	6	18	cep85	1.75	3
ENSXMAG00000010778	0.0024	12	15	dbt	1.50	9
ENSXMAG00000010779	0.0027	6	15	fzd3a	3.73	3
ENSXMAG00000010781	0.0015	12	6	PSME4	2.26	9
ENSXMAG00000010815	0.0014	24	18	sphkap	3.43	21
ENSXMAG00000010816	0.0026	3	15	erfl3	21.29	0
ENSXMAG00000010827	0.0031	6	12	dedd1	5.26	3
ENSXMAG00000010829	0.0027	3	15		3.56	0
ENSXMAG00000010832	0.0025	3	9	rabac1	1.64	0
ENSXMAG00000010834	0.0029	24	12	col14a1a	1.85	21
ENSXMAG00000010835	0.0027	21	9	ccdc25	1.82	18
ENSXMAG00000010837	0.0027	9	15		1.73	6
ENSXMAG00000010838	0.0027	3	9		1.45	0
ENSXMAG00000010841	0.0026	18	9	prl	562.66	15
ENSXMAG00000010848	0.0014	6	18	si:ch211-237i5.4	1.51	3

ENSXMAG00000010850	0.0031	3	12	cpt1cb	6.05	0
ENSXMAG00000010855	0.0031	9	12	RASA2	2.06	6
ENSXMAG00000010862	0.0014	21	6		1.71	18
ENSXMAG00000010863	0.0031	6	12	SLC24A3	6.54	3
ENSXMAG00000010868	0.0034	15	12		2.64	12
ENSXMAG00000010885	0.0026	24	9		1.15	21
ENSXMAG00000010912	0.0026	18	9		1.23	15
ENSXMAG00000010913	0.0030	6	12		1.80	3
ENSXMAG00000010918	0.0031	15	12	naa15a	1.33	12
ENSXMAG00000010922	0.0024	18	9	hip1	2.00	15
ENSXMAG00000010928	0.0016	9	18	snw1	1.15	6
ENSXMAG00000010930	0.0026	21	9	TOMM40L	2.18	18
ENSXMAG00000010942	0.0026	24	9	ppp1r21	1.22	21
ENSXMAG00000010946	0.0031	21	12	nrf1	1.36	18
ENSXMAG00000010954	0.0024	18	9	ypel2a	3.50	15
ENSXMAG00000010960	0.0024	24	9	smc1al	1.34	21
ENSXMAG00000010964	0.0025	3	15		3.95	0
ENSXMAG00000010969	0.0024	18	9		43.41	15
ENSXMAG00000010976	0.0024	18	9	zgc:153426	2.78	15
ENSXMAG00000010979	0.0025	21	9	zc3hc1	1.28	18
ENSXMAG00000010983	0.0031	18	12	taf15	1.82	15
ENSXMAG00000010989	0.0029	6	12	fam124b	4.28	3
ENSXMAG00000010993	0.0025	3	15	zfat	1.58	0
ENSXMAG00000010998	0.0030	9	12	cax1	1.38	6
ENSXMAG00000011002	0.0034	24	12	dachd	1.65	21
ENSXMAG00000011008	0.0031	3	12	sntb1	2.08	0
ENSXMAG00000011013	0.0025	21	9	cnpy3	1.34	18
ENSXMAG00000011015	0.0026	3	15	gnl1	1.38	0
ENSXMAG00000011016	0.0027	24	9	efr3a	1.82	21
ENSXMAG00000011020	0.0030	15	12	tbc1d4	1.92	12
ENSXMAG00000011058	0.0025	6	9	rgs11	1.39	3
ENSXMAG00000011067	0.0034	21	12	gpc1a	2.24	18
ENSXMAG00000011080	0.0031	6	12	slc22a6l	7.37	3
ENSXMAG00000011082	0.0031	18	12	crym	1.48	15
ENSXMAG00000011089	0.0014	3	6	p3h3	1.58	0
ENSXMAG00000011106	0.0031	21	12		1.35	18
ENSXMAG00000011116	0.0026	24	9	cep152	1.72	21
ENSXMAG00000011133	0.0025	12	15	pex5	1.48	9
ENSXMAG00000011149	0.0024	24	9	bahcc1b	3.02	21
ENSXMAG00000011150	0.0016	18	6		2.96	15
ENSXMAG00000011151	0.0031	18	12	dnajc18	1.20	15
ENSXMAG00000011155	0.0030	24	12	clstn3	1.47	21
ENSXMAG00000011158	0.0031	15	12	slain1a	2.38	12
ENSXMAG00000011163	0.0025	15	15	CACNA2D4	3.72	12
ENSXMAG00000011165	0.0031	18	12	plpp3	1.83	15
ENSXMAG00000011168	0.0024	24	9		1.37	21
ENSXMAG00000011172	0.0026	18	9	katnal1	2.07	15
ENSXMAG00000011184	0.0026	15	15	dab1a	2.81	12
ENSXMAG00000011188	0.0031	6	12	lrtm2a	3.01	3
ENSXMAG00000011192	0.0026	24	9	bcor	3.30	21
ENSXMAG00000011193	0.0024	18	9	myo7ab	1.93	15
ENSXMAG00000011194	0.0016	21	6	mettl25	1.37	18
ENSXMAG00000011200	0.0031	6	12	oma1	2.55	3
ENSXMAG00000011203	0.0029	3	12	abi3bpa	3.44	0
ENSXMAG00000011228	0.0014	24	18	tmem30c	1.71	21
ENSXMAG00000011232	0.0026	24	9	PIGG	1.31	21

ENSXMAG00000011234	0.0025	21	9		1.95	18
ENSXMAG00000011236	0.0025	6	15	dlg1	1.68	3
ENSXMAG00000011241	0.0034	21	12	snx7	1.63	18
ENSXMAG00000011253	0.0026	18	9	pafah1b1b	1.33	15
ENSXMAG00000011257	0.0014	6	18	CYR61	3.64	3
ENSXMAG00000011261	0.0025	18	9	NR0B1	2.64	15
ENSXMAG00000011272	0.0025	18	9	mast3a	2.09	15
ENSXMAG00000011274	0.0016	24	18	spryd7b	1.71	21
ENSXMAG00000011277	0.0028	18	9	sept4a	13.89	15
ENSXMAG00000011285	0.0026	21	9	kpna3	1.92	18
ENSXMAG00000011298	0.0025	15	9	gpsm1b	2.57	12
ENSXMAG00000011300	0.0026	21	9	itpa	1.60	18
ENSXMAG00000011303	0.0029	18	12	top2a	1.77	15
ENSXMAG00000011304	0.0024	3	15	ACVR1C	9.09	0
ENSXMAG00000011305	0.0031	15	12	dhx9	1.40	12
ENSXMAG00000011308	0.0024	18	9	LHX3	4.21	15
ENSXMAG00000011310	0.0027	18	9	ap1m3	2.72	15
ENSXMAG00000011311	0.0024	3	15		2.54	0
ENSXMAG00000011324	0.0025	18	9		4.47	15
ENSXMAG00000011339	0.0016	24	18	TMEM125	3.39	21
ENSXMAG00000011345	0.0026	3	15	tardbpl	1.41	0
ENSXMAG00000011356	0.0026	9	15	pgd	2.59	6
ENSXMAG00000011374	0.0014	21	6	med24	1.40	18
ENSXMAG00000011375	0.0025	24	9	dnajc5aa	1.27	21
ENSXMAG00000011379	0.0025	3	15	tpd52l2a	7.72	0
ENSXMAG00000011380	0.0025	18	9		4.79	15
ENSXMAG00000011384	0.0026	24	9	kank4	2.29	21
ENSXMAG00000011388	0.0031	24	12	nol4la	2.85	21
ENSXMAG00000011390	0.0024	9	15	grpel1	1.83	6
ENSXMAG00000011392	0.0027	6	15	atp11a	4.43	3
ENSXMAG00000011406	0.0031	6	12	taf1	1.21	3
ENSXMAG00000011408	0.0027	18	9	fam20b	1.95	15
ENSXMAG00000011415	0.0029	21	12	ralgps2	2.05	18
ENSXMAG00000011422	0.0031	24	12	osbpl2a	1.15	21
ENSXMAG00000011423	0.0024	3	15	iffo1b	3.42	0
ENSXMAG00000011433	0.0014	24	18	CXorf38	3.42	21
ENSXMAG00000011435	0.0029	15	12	slc4a10b	4.04	12
ENSXMAG00000011455	0.0015	3	6	ncapd2	1.28	0
ENSXMAG00000011464	0.0030	24	12	nr1d4a	119.37	21
ENSXMAG00000011470	0.0024	3	15	creg2	4.54	0
ENSXMAG00000011475	0.0034	24	12	mgat4a	1.45	21
ENSXMAG00000011479	0.0027	18	9	MMACHC	1.76	15
ENSXMAG00000011487	0.0029	18	12	cttnbp2	1.77	15
ENSXMAG00000011490	0.0026	18	9		1.45	15
ENSXMAG00000011499	0.0024	24	9		4.47	21
ENSXMAG00000011513	0.0015	24	18	micall2b	1.49	21
ENSXMAG00000011520	0.0025	18	9	gorasp2	1.52	15
ENSXMAG00000011527	0.0027	12	9		1.89	9
ENSXMAG00000011529	0.0014	24	18	si:ch73-12o23.1	1.91	21
ENSXMAG00000011534	0.0031	3	12	lzic	1.38	0
ENSXMAG00000011550	0.0031	24	12		1.55	21
ENSXMAG00000011557	0.0031	6	12		7.27	3
ENSXMAG00000011565	0.0031	6	12	cabp5b	6.70	3
ENSXMAG00000011566	0.0026	18	9	KCNH7	2.85	15
ENSXMAG00000011568	0.0029	24	12	si:ch211-119c20.2	4.83	21
ENSXMAG00000011573	0.0031	3	12	sv2ba	2.93	0

ENSXMAG00000011574	0.0026	21	9	GRB14	3.09	18
ENSXMAG00000011580	0.0025	9	15	stxbp5l	1.41	6
ENSXMAG00000011583	0.0034	24	12	tmem182a	1.34	21
ENSXMAG00000011587	0.0026	18	9	SCN10A	1.52	15
ENSXMAG00000011601	0.0025	12	15	GCDH	1.62	9
ENSXMAG00000011606	0.0014	21	6	kctd13	1.62	18
ENSXMAG00000011607	0.0025	21	9	mrps11	1.36	18
ENSXMAG00000011615	0.0015	3	18		4.92	0
ENSXMAG00000011619	0.0026	24	9	tmem201	1.36	21
ENSXMAG00000011622	0.0026	3	9	fam173a	1.48	0
ENSXMAG00000011627	0.0031	9	12		2.96	6
ENSXMAG00000011635	0.0071	18	18		8.64	15
ENSXMAG00000011639	0.0024	3	15	tmtops2b	2.37	0
ENSXMAG00000011652	0.0031	21	12	zgc:92606	1.48	18
ENSXMAG00000011659	0.0026	18	9		4.85	15
ENSXMAG00000011668	0.0026	3	15		2.13	0
ENSXMAG00000011671	0.0029	6	12		1.77	3
ENSXMAG00000011677	0.0026	21	9	serbp1a	1.14	18
ENSXMAG00000011685	0.0014	21	6	ephb6	3.05	18
ENSXMAG00000011690	0.0027	15	15	eprs	1.54	12
ENSXMAG00000011693	0.0024	3	15	ddr2a	1.61	0
ENSXMAG00000011705	0.0025	15	9	GALNT3	2.44	12
ENSXMAG00000011708	0.0025	18	15	gtse1	2.75	15
ENSXMAG00000011725	0.0025	24	9	dgkaa	1.67	21
ENSXMAG00000011746	0.0034	21	12	atp2b1b	3.47	18
ENSXMAG00000011755	0.0024	18	9	NARS	1.45	15
ENSXMAG00000011808	0.0014	21	6	NYAP1	1.30	18
ENSXMAG00000011810	0.0014	24	18	slc2a3b	2.81	21
ENSXMAG00000011817	0.0025	21	9	SH3BGRL	1.59	18
ENSXMAG00000011825	0.0025	18	9	sat1b	4.03	15
ENSXMAG00000011832	0.0014	21	6	tada2a	1.72	18
ENSXMAG00000011835	0.0016	24	18		1.38	21
ENSXMAG00000011838	0.0027	24	15	rps6kal	2.22	21
ENSXMAG00000011839	0.0025	21	9	mctp2b	2.94	18
ENSXMAG00000011841	0.0014	21	6	nat16	1.83	18
ENSXMAG00000011842	0.0025	3	15	ubxn6	1.72	0
ENSXMAG00000011848	0.0024	21	9	tufm	1.21	18
ENSXMAG00000011867	0.0026	18	15		1.22	15
ENSXMAG00000011870	0.0026	24	9	scamp4	1.30	21
ENSXMAG00000011880	0.0030	9	12	hsd17b12b	1.27	6
ENSXMAG00000011884	0.0031	21	12	jpt2	1.25	18
ENSXMAG00000011886	0.0028	24	15	si:ch211-129c21.1	1.55	21
ENSXMAG00000011888	0.0031	21	12	nat14	1.51	18
ENSXMAG00000011901	0.0071	21	6	hdx	1.49	18
ENSXMAG00000011905	0.0024	3	15		2.06	0
ENSXMAG00000011908	0.0031	6	12	sbk3	3.88	3
ENSXMAG00000011913	0.0031	21	12	pld1b	1.29	18
ENSXMAG00000011915	0.0029	6	12	foxj2	1.37	3
ENSXMAG00000011920	0.0025	18	9	sytl5	1.96	15
ENSXMAG00000011925	0.0027	6	15		2.16	3
ENSXMAG00000011926	0.0015	21	6	rab11bb	1.23	18
ENSXMAG00000011927	0.0026	21	9	nme3	1.18	18
ENSXMAG00000011943	0.0026	15	15	mrps34	1.74	12
ENSXMAG00000011946	0.0031	15	12	rhpn2	3.76	12
ENSXMAG00000011949	0.0025	6	15	ucp2	6.10	3
ENSXMAG00000011954	0.0014	18	6	slc7a10a	2.56	15

ENSXMAG00000011964	0.0026	24	9		1.87	21
ENSXMAG00000011967	0.0026	24	9	si:dkey-17m8.1	2.52	21
ENSXMAG00000011974	0.0024	6	15	camkk1b	13.09	3
ENSXMAG00000011979	0.0030	18	12	u2af2b	1.17	15
ENSXMAG00000011991	0.0025	24	15	cacng6b	2.24	21
ENSXMAG00000011993	0.0027	3	15	FNIP1	1.74	0
ENSXMAG00000011996	0.0024	18	15		6.13	15
ENSXMAG00000012008	0.0027	24	15	ppm1nb	1.64	21
ENSXMAG00000012034	0.0031	12	12	nudt9	1.27	9
ENSXMAG00000012038	0.0027	21	9		2.64	18
ENSXMAG00000012054	0.0025	24	15	dbpb	164.16	21
ENSXMAG00000012059	0.0014	18	6	tjp3	1.29	15
ENSXMAG00000012060	0.0024	24	9	tbck	1.41	21
ENSXMAG00000012069	0.0024	21	9	ttyh1	3.24	18
ENSXMAG00000012070	0.0031	3	12	paqr7a	2.21	0
ENSXMAG00000012073	0.0031	15	12	ankrd13b	2.31	12
ENSXMAG00000012087	0.0024	3	15	slc22a5	2.89	0
ENSXMAG00000012088	0.0026	15	9	rapgef3	2.27	12
ENSXMAG00000012099	0.0026	18	9		1.69	15
ENSXMAG00000012100	0.0031	15	12	grk1b	114.78	12
ENSXMAG00000012105	0.0031	18	12	RRAGB	1.28	15
ENSXMAG00000012106	0.0026	18	9	ssh2a	1.74	15
ENSXMAG00000012109	0.0026	18	9	slc6a4a	6.47	15
ENSXMAG00000012114	0.0014	21	18	sik1	4.53	18
ENSXMAG00000012122	0.0025	18	9	xylt2	2.05	15
ENSXMAG00000012146	0.0026	6	15	cacna1i	1.69	3
ENSXMAG00000012148	0.0027	21	9	TTC39C (1 of many)	1.31	18
ENSXMAG00000012149	0.0025	18	9	naxd	1.61	15
ENSXMAG00000012154	0.0031	21	12	efhd1	1.18	18
ENSXMAG00000012162	0.0031	21	12	phospho2	1.55	18
ENSXMAG00000012164	0.0025	6	15	clmn	2.10	3
ENSXMAG00000012169	0.0025	18	15	dnm2b	1.44	15
ENSXMAG00000012171	0.0027	24	15	nlk2	1.68	21
ENSXMAG00000012174	0.0025	3	15		1.51	0
ENSXMAG00000012179	0.0031	24	12		2.12	21
ENSXMAG00000012189	0.0025	15	9	npas2	14.66	12
ENSXMAG00000012190	0.0014	21	6	mtmr7b	1.56	18
ENSXMAG00000012198	0.0028	21	9	zgc:56106	1.36	18
ENSXMAG00000012200	0.0031	18	12	adpgk	2.02	15
ENSXMAG00000012203	0.0025	18	9	abcc3	3.49	15
ENSXMAG00000012211	0.0031	6	12	kcnk10b	2.30	3
ENSXMAG00000012214	0.0024	18	9	ubash3ba	1.60	15
ENSXMAG00000012216	0.0024	21	15	mcf2la	2.87	18
ENSXMAG00000012224	0.0027	24	9	pltp	1.38	21
ENSXMAG00000012243	0.0024	24	9	hacd2	1.38	21
ENSXMAG00000012248	0.0031	9	12	bco2a	4.50	6
ENSXMAG00000012250	0.0025	18	15	dag1	2.43	15
ENSXMAG00000012254	0.0026	18	9	stim2a	1.94	15
ENSXMAG00000012259	0.0027	3	15		3.12	0
ENSXMAG00000012267	0.0031	3	12	leng1	1.25	0
ENSXMAG00000012277	0.0031	6	12	PCYT2 (1 of many)	2.32	3
ENSXMAG00000012281	0.0030	18	12	hmgb2a	1.53	15
ENSXMAG00000012291	0.0015	24	6	nfatc3b	1.27	21
ENSXMAG00000012295	0.0024	3	15	zgc:136908	1.90	0
ENSXMAG00000012300	0.0024	18	9	zgc:158689	4.08	15
ENSXMAG00000012302	0.0034	6	12	elmod1	1.30	3

ENSXMAG00000012303	0.0025	3	15		3.55	0
ENSXMAG00000012317	0.0031	3	12	ptpn11b	2.23	0
ENSXMAG00000012320	0.0029	9	12		2.60	6
ENSXMAG00000012325	0.0026	18	9	aifm1	1.34	15
ENSXMAG00000012334	0.0024	24	9	plekha7a	2.38	21
ENSXMAG00000012338	0.0025	21	9	samd1b	1.72	18
ENSXMAG00000012351	0.0026	6	15	wfs1a	3.22	3
ENSXMAG00000012352	0.0025	24	15	stk36	1.66	21
ENSXMAG00000012359	0.0014	24	18	atf5a	2.85	21
ENSXMAG00000012364	0.0026	18	9	MAP4K4 (1 of many)	1.78	15
ENSXMAG00000012369	0.0031	18	12	SPHK1 (1 of many)	2.48	15
ENSXMAG00000012372	0.0026	3	15	tbcelb	1.75	0
ENSXMAG00000012374	0.0027	21	9	pik3c2a	1.52	18
ENSXMAG00000012378	0.0031	21	12		1.76	18
ENSXMAG00000012379	0.0026	18	9	glra4b	3.17	15
ENSXMAG00000012380	0.0029	24	12	col4a1	1.88	21
ENSXMAG00000012387	0.0015	12	6		1.41	9
ENSXMAG00000012394	0.0025	3	15	epn2	2.09	0
ENSXMAG00000012409	0.0027	6	15	dpysl3	1.41	3
ENSXMAG00000012411	0.0031	18	12	ddost	1.40	15
ENSXMAG00000012420	0.0031	3	12	midn	1.51	0
ENSXMAG00000012422	0.0016	21	6	polr2j	1.29	18
ENSXMAG00000012425	0.0014	21	6	sap30bp	1.51	18
ENSXMAG00000012435	0.0024	6	15		2.18	3
ENSXMAG00000012451	0.0025	9	15	bsg	1.61	6
ENSXMAG00000012462	0.0031	18	12	api5	1.34	15
ENSXMAG00000012472	0.0029	18	12	acap3a	3.45	15
ENSXMAG00000012476	0.0034	18	12	si:dkey-76b14.2	1.29	15
ENSXMAG00000012494	0.0026	21	15	mdh1b	1.35	18
ENSXMAG00000012495	0.0025	18	9		1.64	15
ENSXMAG00000012498	0.0016	6	18	gpat3	5.01	3
ENSXMAG00000012502	0.0027	3	15	jcada	1.59	0
ENSXMAG00000012511	0.0016	24	18	gucy2d	10.37	21
ENSXMAG00000012515	0.0031	18	12	cdk11b	1.21	15
ENSXMAG00000012519	0.0026	6	15	fbxl16	2.52	3
ENSXMAG00000012532	0.0024	18	9	pik3c2b	1.48	15
ENSXMAG00000012542	0.0025	6	9		4.34	3
ENSXMAG00000012548	0.0025	18	9	si:dkey-82j4.2	1.55	15
ENSXMAG00000012551	0.0031	9	12	irs2b	3.98	6
ENSXMAG00000012554	0.0031	18	12	myo16	2.37	15
ENSXMAG00000012567	0.0029	21	12	LTBP3	1.35	18
ENSXMAG00000012570	0.0014	15	18	slc25a10	1.39	12
ENSXMAG00000012579	0.0026	3	15	sdf4	1.38	0
ENSXMAG00000012583	0.0027	6	15		1.66	3
ENSXMAG00000012585	0.0027	21	9	rgmd	1.55	18
ENSXMAG00000012594	0.0031	24	12		1.70	21
ENSXMAG00000012596	0.0030	18	12	olfml3a	1.55	15
ENSXMAG00000012600	0.0024	9	15		13.78	6
ENSXMAG00000012602	0.0027	18	9	klhl13	1.93	15
ENSXMAG00000012604	0.0026	24	9	pard3ba	1.76	21
ENSXMAG00000012612	0.0016	24	18	otud3	1.53	21
ENSXMAG00000012623	0.0025	6	15	KHDC4	1.77	3
ENSXMAG00000012632	0.0031	21	12	adam12	4.76	18
ENSXMAG00000012645	0.0025	6	9	abi3a	1.39	3
ENSXMAG00000012650	0.0030	24	12	jam2a	3.44	21
ENSXMAG00000012654	0.0014	21	18	trim65	1.29	18

ENSXMAG00000012661	0.0031	12	12	crtac1a	1.71	9
ENSXMAG00000012664	0.0031	24	12	si:dkey-110c1.7	2.05	21
ENSXMAG00000012672	0.0026	3	15		2.09	0
ENSXMAG00000012673	0.0025	18	9	ptpreb	3.04	15
ENSXMAG00000012674	0.0031	21	12	APP	1.75	18
ENSXMAG00000012690	0.0031	6	12		1.65	3
ENSXMAG00000012691	0.0031	24	12	zcchc7	1.23	21
ENSXMAG00000012696	0.0031	21	12	fdxr	2.58	18
ENSXMAG00000012699	0.0031	6	12	mapk13	2.32	3
ENSXMAG00000012704	0.0025	3	9	pde6ga	9.55	0
ENSXMAG00000012705	0.0029	12	12	pgam1b	2.36	9
ENSXMAG00000012710	0.0030	15	12	gnrh3	2.30	12
ENSXMAG00000012716	0.0025	21	9	mbd3a	1.77	18
ENSXMAG00000012717	0.0025	21	15	tbc1d1	1.79	18
ENSXMAG00000012722	0.0024	3	15	gprc5bb	13.32	0
ENSXMAG00000012723	0.0024	9	15	si:dkey-282h22.5	3.23	6
ENSXMAG00000012726	0.0029	21	12	MGAT4C (1 of many)	3.18	18
ENSXMAG00000012734	0.0024	18	9	ampd3b	2.06	15
ENSXMAG00000012739	0.0027	24	15	chmp6a	1.96	21
ENSXMAG00000012742	0.0031	21	12	hexdc	1.77	18
ENSXMAG00000012744	0.0029	18	12	crhr1	2.16	15
ENSXMAG00000012748	0.0031	6	12	slc2a1a	2.39	3
ENSXMAG00000012751	0.0026	18	9	PAK3	2.14	15
ENSXMAG00000012758	0.0030	15	12	MXI1 (1 of many)	2.65	12
ENSXMAG00000012760	0.0027	15	9	cdc27	1.35	12
ENSXMAG00000012774	0.0016	21	6	ccdc22	1.37	18
ENSXMAG00000012788	0.0024	9	15	zc2hc1a	1.30	6
ENSXMAG00000012806	0.0024	9	9	fndc4a	3.17	6
ENSXMAG00000012807	0.0031	12	12	zgc:85843	6.29	9
ENSXMAG00000012809	0.0034	3	12	ctps1a	3.83	0
ENSXMAG00000012814	0.0029	21	12	mxra8b	1.42	18
ENSXMAG00000012822	0.0025	18	15		1.39	15
ENSXMAG00000012823	0.0025	18	9	srsf5a	1.73	15
ENSXMAG00000012825	0.0025	6	15	hectd2	2.32	3
ENSXMAG00000012829	0.0026	18	9		3.91	15
ENSXMAG00000012838	0.0031	9	12		2.46	6
ENSXMAG00000012841	0.0026	12	15	oaz1a	1.38	9
ENSXMAG00000012844	0.0015	18	6	amh	2.81	15
ENSXMAG00000012847	0.0027	6	15	stk35	16.74	3
ENSXMAG00000012851	0.0025	18	9		1.80	15
ENSXMAG00000012854	0.0027	18	9	dot1l	3.41	15
ENSXMAG00000012863	0.0028	3	15	mak	2.83	0
ENSXMAG00000012874	0.0024	24	9	tbcd	1.29	21
ENSXMAG00000012876	0.0031	18	12	khsrp	1.83	15
ENSXMAG00000012893	0.0031	24	12		1.36	21
ENSXMAG00000012903	0.0024	6	15	hlfa	4.28	3
ENSXMAG00000012915	0.0014	3	18		2.59	0
ENSXMAG00000012917	0.0025	18	9	CLVS1	1.75	15
ENSXMAG00000012933	0.0014	15	6	cfap43	1.86	12
ENSXMAG00000012935	0.0031	6	12	si:dkey-120c6.5	5.57	3
ENSXMAG00000012938	0.0030	18	12		8.52	15
ENSXMAG00000012946	0.0024	18	9	mpi	1.23	15
ENSXMAG00000012951	0.0026	9	15	pcyt2	2.63	6
ENSXMAG00000012953	0.0031	24	12	edem3	1.20	21
ENSXMAG00000012961	0.0026	24	9		1.50	21
ENSXMAG00000012964	0.0027	6	15	mafgb	3.77	3

ENSXMAG00000012969	0.0025	24	9	rgl1	2.34	21
ENSXMAG00000012975	0.0014	21	6	minpp1a	1.56	18
ENSXMAG00000012979	0.0027	9	15	suz12a	2.06	6
ENSXMAG00000012988	0.0031	12	12	si:dkeyp-72e1.7	7.79	9
ENSXMAG00000013013	0.0031	21	12	prdx4	1.35	18
ENSXMAG00000013018	0.0015	24	6	ARL2BP	1.17	21
ENSXMAG00000013021	0.0031	12	12	rorca	50.47	9
ENSXMAG00000013027	0.0024	18	9		1.91	15
ENSXMAG00000013033	0.0024	24	15	gadd45bb	2.36	21
ENSXMAG00000013048	0.0025	15	9		2.46	12
ENSXMAG00000013050	0.0025	6	15		4.14	3
ENSXMAG00000013051	0.0026	18	9	RASSF8 (1 of many)	2.96	15
ENSXMAG00000013053	0.0024	24	15		1.15	21
ENSXMAG00000013069	0.0029	18	12	pdia3	1.36	15
ENSXMAG00000013070	0.0025	24	15	saga	1.62	21
ENSXMAG00000013079	0.0028	3	15		14.78	0
ENSXMAG00000013081	0.0025	9	15	dio1	6.85	6
ENSXMAG00000013084	0.0024	3	15		3.40	0
ENSXMAG00000013090	0.0024	18	9	si:ch211-244c8.4	6.74	15
ENSXMAG00000013107	0.0026	21	9	si:zfos-80g12.1	2.06	18
ENSXMAG00000013109	0.0028	21	9	mapre3b	1.39	18
ENSXMAG00000013119	0.0026	18	9	alg1	1.94	15
ENSXMAG00000013124	0.0026	3	15	foxo4	1.90	0
ENSXMAG00000013137	0.0031	21	12	pigq	1.73	18
ENSXMAG00000013138	0.0025	3	15	rab40c	1.85	0
ENSXMAG00000013147	0.0029	21	12	gem	5.24	18
ENSXMAG00000013161	0.0026	12	15	efemp2a	1.42	9
ENSXMAG00000013169	0.0026	3	15		1.83	0
ENSXMAG00000013186	0.0034	24	12	stx17	1.54	21
ENSXMAG00000013193	0.0029	3	12	kdrl	1.39	0
ENSXMAG00000013207	0.0014	21	6	tmem245	1.21	18
ENSXMAG00000013208	0.0031	15	12	galnt1	1.84	12
ENSXMAG00000013228	0.0031	18	12		1.85	15
ENSXMAG00000013245	0.0015	21	6	psmc4	1.39	18
ENSXMAG00000013250	0.0031	21	12	si:dkey-51e6.1	1.61	18
ENSXMAG00000013258	0.0014	18	6	rhbg	3.46	15
ENSXMAG00000013261	0.0024	18	9	scn1ba	2.11	15
ENSXMAG00000013267	0.0026	18	15	grhl1	2.65	15
ENSXMAG00000013279	0.0029	24	12	pgls	1.61	21
ENSXMAG00000013284	0.0026	24	9	colgalt1	2.09	21
ENSXMAG00000013304	0.0024	3	15	atp11c	1.59	0
ENSXMAG00000013310	0.0030	12	12	mgat1a	1.63	9
ENSXMAG00000013321	0.0031	6	12	lmbrd1	1.81	3
ENSXMAG00000013335	0.0026	24	9		1.48	21
ENSXMAG00000013341	0.0031	15	12	pcnx1	1.71	12
ENSXMAG00000013343	0.0025	21	9		2.17	18
ENSXMAG00000013346	0.0031	18	12	atp13a1	1.32	15
ENSXMAG00000013349	0.0031	15	12	gmppb	1.33	12
ENSXMAG00000013357	0.0071	21	6	shdb	1.69	18
ENSXMAG00000013367	0.0034	15	12	tlr18	1.90	12
ENSXMAG00000013383	0.0014	24	18		2.25	21
ENSXMAG00000013407	0.0024	24	9	rbmx	1.26	21
ENSXMAG00000013409	0.0031	24	12	tnni2a.2	1.24	21
ENSXMAG00000013417	0.0031	21	12	tpp1	2.03	18
ENSXMAG00000013436	0.0025	18	9	nr2f5	2.70	15
ENSXMAG00000013437	0.0031	24	12	CUTC	1.44	21

ENSXMAG00000013444	0.0024	6	15	sv2a	1.65	3
ENSXMAG00000013448	0.0030	6	12	hif1aa	2.16	3
ENSXMAG00000013453	0.0026	18	9	mtmr11	2.34	15
ENSXMAG00000013456	0.0031	24	12		13.30	21
ENSXMAG00000013461	0.0030	21	12	asmtl	1.41	18
ENSXMAG00000013464	0.0027	21	9	si:ch211-106m9.1	3.57	18
ENSXMAG00000013465	0.0025	6	15	kcng4b	2.39	3
ENSXMAG00000013483	0.0025	21	9	tmem35	1.56	18
ENSXMAG00000013492	0.0024	3	15	ubtd1a	4.19	0
ENSXMAG00000013503	0.0015	24	18	kita	1.81	21
ENSXMAG00000013507	0.0026	3	15	hivep2a	2.88	0
ENSXMAG00000013517	0.0014	21	6	znrd1	1.96	18
ENSXMAG00000013528	0.0026	6	15		1.78	3
ENSXMAG00000013531	0.0024	18	9		1.49	15
ENSXMAG00000013537	0.0025	18	9	CDC42BPB	1.96	15
ENSXMAG00000013542	0.0024	24	9	antxr1d	1.30	21
ENSXMAG00000013551	0.0026	3	15	reep1	2.58	0
ENSXMAG00000013555	0.0027	6	15	hspa4l	2.40	3
ENSXMAG00000013564	0.0031	21	12	pbx2	1.30	18
ENSXMAG00000013574	0.0025	6	15	itpk1b	1.91	3
ENSXMAG00000013577	0.0031	24	12		5.91	21
ENSXMAG00000013586	0.0026	6	15	PRIMA1	2.53	3
ENSXMAG00000013593	0.0015	24	6	MEGF11 (1 of many)	2.13	21
ENSXMAG00000013600	0.0031	12	12	ddx24	1.48	9
ENSXMAG00000013613	0.0025	21	9	cul4b	1.23	18
ENSXMAG00000013614	0.0026	3	15	ube2ql1	8.99	0
ENSXMAG00000013631	0.0031	6	12	lamp2	1.56	3
ENSXMAG00000013641	0.0025	3	15	cnksr1	3.34	0
ENSXMAG00000013651	0.0030	21	12		1.89	18
ENSXMAG00000013681	0.0031	18	12		1.17	15
ENSXMAG00000013699	0.0031	18	12	6-Sep	1.43	15
ENSXMAG00000013704	0.0025	21	9	syt5b	1.54	18
ENSXMAG00000013706	0.0025	24	15	enpep	3.44	21
ENSXMAG00000013723	0.0014	24	6		1.32	21
ENSXMAG00000013731	0.0016	21	6		2.98	18
ENSXMAG00000013732	0.0027	18	9	zdhhc5a	2.07	15
ENSXMAG00000013733	0.0014	21	6		1.11	18
ENSXMAG00000013741	0.0025	18	9		1.60	15
ENSXMAG00000013742	0.0025	24	9	lrit3b	37.15	21
ENSXMAG00000013750	0.0015	21	6	si:dkey-28b4.8	1.32	18
ENSXMAG00000013762	0.0026	3	15	cx52.7	10.81	0
ENSXMAG00000013763	0.0031	15	12	gtf3aa	1.93	12
ENSXMAG00000013764	0.0027	6	15	ankrd6a	28.94	3
ENSXMAG00000013766	0.0014	9	18	guca1g	9.97	6
ENSXMAG00000013781	0.0034	18	12	slc15a1b	2.05	15
ENSXMAG00000013784	0.0026	3	15	rcor3	1.33	0
ENSXMAG00000013796	0.0030	18	12	eif3c	1.18	15
ENSXMAG00000013814	0.0025	24	9	ndnf	1.81	21
ENSXMAG00000013821	0.0027	6	15		1.82	3
ENSXMAG00000013823	0.0024	18	9	trmt1	1.78	15
ENSXMAG00000013831	0.0025	18	9		7.70	15
ENSXMAG00000013834	0.0031	15	12		1.45	12
ENSXMAG00000013845	0.0030	21	12	GCA	1.25	18
ENSXMAG00000013850	0.0024	18	9	tubg1	1.62	15
ENSXMAG00000013851	0.0024	18	9	cttn	1.84	15
ENSXMAG00000013864	0.0026	6	15	scn1lab	2.05	3

ENSXMAG00000013870	0.0034	9	12	ZBTB21	1.55	6
ENSXMAG00000013878	0.0025	9	15	pde4ca	3.31	6
ENSXMAG00000013882	0.0031	21	12	chn2	2.50	18
ENSXMAG00000013883	0.0025	18	9	PPFIA1	1.79	15
ENSXMAG00000013894	0.0024	18	9	stt3a	1.63	15
ENSXMAG00000013925	0.0024	21	15	serpinh2	1.72	18
ENSXMAG00000013927	0.0015	21	6	nup93	1.78	18
ENSXMAG00000013928	0.0027	9	15	osbpl3b	2.63	6
ENSXMAG00000013935	0.0027	18	9	dscama	5.53	15
ENSXMAG00000013937	0.0024	18	9	casc3	1.51	15
ENSXMAG00000013946	0.0031	9	12	stox2a	2.47	6
ENSXMAG00000013953	0.0026	24	15		1.49	21
ENSXMAG00000013963	0.0026	21	9	psmd3	1.35	18
ENSXMAG00000013977	0.0028	9	15		2.00	6
ENSXMAG00000013979	0.0026	21	9	hs6st3a	2.94	18
ENSXMAG00000013988	0.0024	18	15	frem1b	1.83	15
ENSXMAG00000014005	0.0014	15	18	cass4	2.14	12
ENSXMAG00000014010	0.0024	18	9	git2a	4.80	15
ENSXMAG00000014014	0.0031	21	12		1.85	18
ENSXMAG00000014018	0.0026	18	9	zgc:92275	2.68	15
ENSXMAG00000014034	0.0025	24	9	lrba	1.96	21
ENSXMAG00000014037	0.0031	24	12		1.45	21
ENSXMAG00000014041	0.0025	6	15	ptdss1a	1.33	3
ENSXMAG00000014044	0.0029	6	12		1.69	3
ENSXMAG00000014051	0.0026	21	9	dclk2a	4.12	18
ENSXMAG00000014052	0.0014	3	18	rnf34b	3.12	0
ENSXMAG00000014057	0.0031	6	12	kdm2bb	2.23	3
ENSXMAG00000014061	0.0025	3	15	wscd1b	4.34	0
ENSXMAG00000014065	0.0015	18	6	slc44a4	2.72	15
ENSXMAG00000014069	0.0015	21	6	gtpbp1	1.26	18
ENSXMAG00000014077	0.0024	6	15	abcc5	10.38	3
ENSXMAG00000014084	0.0014	6	18	TPPP	2.33	3
ENSXMAG00000014089	0.0024	18	9		1.47	15
ENSXMAG00000014096	0.0026	18	15	ankrd40	1.23	15
ENSXMAG00000014098	0.0026	21	9	ccdc12	1.41	18
ENSXMAG00000014104	0.0031	24	12	spag9a	1.22	21
ENSXMAG00000014111	0.0027	18	9	eif4a3	1.62	15
ENSXMAG00000014127	0.0031	9	12	si:ch211-106h4.9	3.83	6
ENSXMAG00000014130	0.0014	21	6	nup133	1.38	18
ENSXMAG00000014132	0.0025	18	9	soul4	4.81	15
ENSXMAG00000014155	0.0025	6	15		1.89	3
ENSXMAG00000014176	0.0034	24	12	cnot3b	1.15	21
ENSXMAG00000014178	0.0026	24	9	CDH10	1.73	21
ENSXMAG00000014181	0.0026	24	9	dhps	1.31	21
ENSXMAG00000014187	0.0026	24	9	CTCF	1.26	21
ENSXMAG00000014192	0.0025	18	9	SEMA4D	3.32	15
ENSXMAG00000014199	0.0027	24	15	abcg1	1.58	21
ENSXMAG00000014200	0.0029	6	12	ccdc106b	2.01	3
ENSXMAG00000014211	0.0014	24	18	akt1s1	1.48	21
ENSXMAG00000014220	0.0031	21	12	emc10	1.24	18
ENSXMAG00000014228	0.0025	3	15		1.57	0
ENSXMAG00000014229	0.0030	3	12	necap1	2.17	0
ENSXMAG00000014236	0.0026	12	15	bms1	1.27	9
ENSXMAG00000014239	0.0024	18	15	rcn1	1.49	15
ENSXMAG00000014245	0.0031	6	12	si:ch211-171h4.3	3.11	3
ENSXMAG00000014250	0.0016	6	18	htra1b	3.99	3

ENSXMAG00000014253	0.0071	21	6		1.39	18
ENSXMAG00000014271	0.0025	3	15		1.38	0
ENSXMAG00000014298	0.0025	15	15	spata18	3.49	12
ENSXMAG00000014302	0.0026	3	15	adap2	1.86	0
ENSXMAG00000014314	0.0024	3	15	usp46	2.36	0
ENSXMAG00000014315	0.0025	18	15	CDH18	3.50	15
ENSXMAG00000014318	0.0024	18	9	wdr62	1.59	15
ENSXMAG00000014322	0.0025	18	9	ppm1h	2.62	15
ENSXMAG00000014335	0.0027	9	15	RYR2	3.14	6
ENSXMAG00000014337	0.0026	24	9		3.10	21
ENSXMAG00000014345	0.0028	3	15	si:dkey-83h2.5 (1 of many)	3.67	0
ENSXMAG00000014348	0.0029	18	12	SLC10A1 (1 of many)	4.30	15
ENSXMAG00000014358	0.0025	18	9	RAB15 (1 of many)	6.27	15
ENSXMAG00000014362	0.0014	24	6	gsx2	1.67	21
ENSXMAG00000014377	0.0024	9	15	si:dkey-83h2.5 (1 of many)	4.45	6
ENSXMAG00000014380	0.0031	24	12	dpp6a	1.56	21
ENSXMAG00000014384	0.0034	18	12	erbin	1.53	15
ENSXMAG00000014387	0.0026	24	15		2.42	21
ENSXMAG00000014390	0.0024	6	15		2.50	3
ENSXMAG00000014399	0.0031	9	12	ece2b	1.72	6
ENSXMAG00000014417	0.0031	18	12	hyal6	1.59	15
ENSXMAG00000014419	0.0031	18	12	spam1	2.29	15
ENSXMAG00000014421	0.0031	18	12	acbd4	1.24	15
ENSXMAG00000014427	0.0025	18	9	cx52.6	12.27	15
ENSXMAG00000014436	0.0025	24	9	ZFYVE1 (1 of many)	1.36	21
ENSXMAG00000014437	0.0027	3	15	adam10a	1.53	0
ENSXMAG00000014438	0.0026	9	15	tbrg4	1.65	6
ENSXMAG00000014457	0.0031	18	12	met	1.47	15
ENSXMAG00000014464	0.0027	6	15		2.04	3
ENSXMAG00000014466	0.0026	24	9	sltm	1.36	21
ENSXMAG00000014470	0.0026	24	9	SCMH1	1.30	21
ENSXMAG00000014475	0.0026	24	9	slit1b	1.51	21
ENSXMAG00000014480	0.0031	18	12	hnrnpr	1.20	15
ENSXMAG00000014481	0.0031	18	12	med18	1.50	15
ENSXMAG00000014501	0.0031	24	12		1.33	21
ENSXMAG00000014512	0.0024	18	9		3.69	15
ENSXMAG00000014516	0.0024	21	9	kif1bp	1.25	18
ENSXMAG00000014527	0.0014	21	6		1.27	18
ENSXMAG00000014535	0.0027	24	9		1.33	21
ENSXMAG00000014536	0.0031	21	12	slc39a8	2.87	18
ENSXMAG00000014551	0.0031	18	12	jpt1b	2.00	15
ENSXMAG00000014562	0.0031	15	12	hk1	2.47	12
ENSXMAG00000014567	0.0025	3	15	clk2a	1.43	0
ENSXMAG00000014570	0.0031	18	12	itga3b	1.46	15
ENSXMAG00000014576	0.0025	21	9	fcf1	1.60	18
ENSXMAG00000014582	0.0026	24	9	rbl1	1.58	21
ENSXMAG00000014585	0.0026	24	9		1.46	21
ENSXMAG00000014597	0.0026	24	9		1.63	21
ENSXMAG00000014604	0.0031	24	12	mpp2b	2.39	21
ENSXMAG00000014606	0.0031	15	12	yrdc	1.61	12
ENSXMAG00000014619	0.0027	21	9	celf3a	2.44	18
ENSXMAG00000014624	0.0026	18	9	wdr32	6.52	15
ENSXMAG00000014629	0.0026	21	9	inpp5b	1.65	18
ENSXMAG00000014644	0.0031	21	12	sept5a	1.69	18
ENSXMAG00000014703	0.0031	18	12	tacr2	2.24	15
ENSXMAG00000014708	0.0024	18	9	mbtd1	2.41	15

ENSXMAG00000014725	0.0031	6	12		3.58	3
ENSXMAG00000014726	0.0031	24	12	RNF207	1.95	21
ENSXMAG00000014727	0.0029	15	12		1.44	12
ENSXMAG00000014729	0.0026	18	9	gc2	2.01	15
ENSXMAG00000014733	0.0014	21	6	eif3eb	1.73	18
ENSXMAG00000014734	0.0025	18	15	rac3b	1.53	15
ENSXMAG00000014739	0.0028	3	15		1.58	0
ENSXMAG00000014741	0.0024	24	9	tap2t	1.68	21
ENSXMAG00000014761	0.0031	6	12	acsf2	1.47	3
ENSXMAG00000014773	0.0014	24	18	rai1	1.39	21
ENSXMAG00000014775	0.0030	18	12	HNRNPH3	1.22	15
ENSXMAG00000014783	0.0026	18	9	parn	1.36	15
ENSXMAG00000014791	0.0027	24	9	plpp5	1.41	21
ENSXMAG00000014792	0.0031	6	12	arg2	12.16	3
ENSXMAG00000014799	0.0029	6	12		5.35	3
ENSXMAG00000014802	0.0015	6	18	efna1a	1.62	3
ENSXMAG00000014803	0.0071	21	18	armc7	1.49	18
ENSXMAG00000014806	0.0025	12	15	wdr74	1.86	9
ENSXMAG00000014811	0.0034	24	12		1.61	21
ENSXMAG00000014814	0.0031	6	12	pbxip1a	2.72	3
ENSXMAG00000014820	0.0031	18	12	s100u	1.93	15
ENSXMAG00000014844	0.0029	6	12	rassf2a	3.12	3
ENSXMAG00000014848	0.0014	18	6	LRIT2 (1 of many)	5.20	15
ENSXMAG00000014849	0.0025	24	15	CDHR1 (1 of many)	1.97	21
ENSXMAG00000014851	0.0024	21	9	pvalb9	3.94	18
ENSXMAG00000014855	0.0031	24	12		1.70	21
ENSXMAG00000014856	0.0031	9	12	SLC23A2	2.17	6
ENSXMAG00000014864	0.0016	24	18		1.36	21
ENSXMAG00000014867	0.0025	9	9	ubap2l	1.28	6
ENSXMAG00000014893	0.0025	21	15	chmp2a	1.25	18
ENSXMAG00000014900	0.0024	3	15		1.92	0
ENSXMAG00000014917	0.0015	3	18		1.79	0
ENSXMAG00000014926	0.0029	15	12	clcn5b	3.01	12
ENSXMAG00000014933	0.0015	6	6	dusp2	2.56	3
ENSXMAG00000014950	0.0026	21	9	psmc3	1.32	18
ENSXMAG00000014954	0.0029	6	12	pdk2a	9.72	3
ENSXMAG00000014960	0.0025	18	9	dnajb12b	2.59	15
ENSXMAG00000014962	0.0027	21	9	isca2	1.31	18
ENSXMAG00000014968	0.0025	3	15	hcn3	4.52	0
ENSXMAG00000014977	0.0031	3	12	plvapa	1.40	0
ENSXMAG00000014994	0.0029	21	12		1.73	18
ENSXMAG00000015000	0.0016	21	6	nudt18	1.44	18
ENSXMAG00000015025	0.0031	18	12	ACTA1	1.57	15
ENSXMAG00000015026	0.0031	18	12	rnls	1.57	15
ENSXMAG00000015043	0.0030	21	12	narfl	1.38	18
ENSXMAG00000015052	0.0028	18	15	yeats2	1.23	15
ENSXMAG00000015055	0.0026	21	15	zgc:136564	1.42	18
ENSXMAG00000015060	0.0027	9	9	ppa1a	3.68	6
ENSXMAG00000015071	0.0031	15	12	syn1	1.71	12
ENSXMAG00000015074	0.0031	9	12	gstz1	2.52	6
ENSXMAG00000015080	0.0031	24	12	klhl24b	4.06	21
ENSXMAG00000015092	0.0031	15	12	zswim8	1.82	12
ENSXMAG00000015095	0.0024	6	15	ALDH6A1	2.23	3
ENSXMAG00000015104	0.0031	18	12		2.05	15
ENSXMAG00000015121	0.0024	24	15	rxrbb	1.22	21
ENSXMAG00000015125	0.0016	24	6	cenpf	2.11	21

ENSXMAG00000015128	0.0026	24	9		1.36	21
ENSXMAG00000015131	0.0031	9	12		2.45	6
ENSXMAG00000015140	0.0026	18	9	tsg101a	1.12	15
ENSXMAG00000015146	0.0031	24	12	cenpo	2.08	21
ENSXMAG00000015147	0.0031	9	12	acp2	3.65	6
ENSXMAG00000015149	0.0014	3	6	adpgk2	1.35	0
ENSXMAG00000015165	0.0027	18	9		1.29	15
ENSXMAG00000015184	0.0031	3	12	tbccd1	1.91	0
ENSXMAG00000015191	0.0025	18	9		1.93	15
ENSXMAG00000015195	0.0025	9	15	ddr2l	1.89	6
ENSXMAG00000015207	0.0015	21	18	tmem256	1.36	18
ENSXMAG00000015223	0.0016	24	18	tcaim	1.62	21
ENSXMAG00000015225	0.0014	21	6		1.61	18
ENSXMAG00000015230	0.0031	24	12	cadpsb	1.34	21
ENSXMAG00000015244	0.0024	18	15	sncaip	2.20	15
ENSXMAG00000015247	0.0015	21	6	aplnra	2.98	18
ENSXMAG00000015253	0.0031	15	12		8.86	12
ENSXMAG00000015257	0.0016	21	6	dennd6b	1.26	18
ENSXMAG00000015266	0.0031	21	12	cndp2	1.53	18
ENSXMAG00000015267	0.0025	6	15	slc7a4	2.29	3
ENSXMAG00000015270	0.0031	6	12	pax3a	1.36	3
ENSXMAG00000015275	0.0030	15	12		4.18	12
ENSXMAG00000015282	0.0024	18	9	polr2b	1.39	15
ENSXMAG00000015283	0.0026	15	15		2.13	12
ENSXMAG00000015285	0.0029	21	12	slc6a22.2	1.86	18
ENSXMAG00000015287	0.0025	21	9	tmed7	1.35	18
ENSXMAG00000015288	0.0024	24	15		9.18	21
ENSXMAG00000015290	0.0025	18	9	snrkb	2.99	15
ENSXMAG00000015297	0.0025	15	9	fem1c	1.31	12
ENSXMAG00000015310	0.0031	24	12	ephb1	1.65	21
ENSXMAG00000015314	0.0025	3	15	per1b	54.50	0
ENSXMAG00000015321	0.0025	24	15	peli2	2.94	21
ENSXMAG00000015327	0.0031	6	12	trim36	4.60	3
ENSXMAG00000015329	0.0025	18	9	clip2	1.93	15
ENSXMAG00000015330	0.0030	15	12	ktn1	1.42	12
ENSXMAG00000015335	0.0014	3	18	dlg5b.1	2.00	0
ENSXMAG00000015345	0.0026	21	9	top1	1.50	18
ENSXMAG00000015353	0.0026	9	9		2.19	6
ENSXMAG00000015354	0.0029	6	12	tcp11l1	2.97	3
ENSXMAG00000015357	0.0014	21	6		1.43	18
ENSXMAG00000015367	0.0031	18	12		1.63	15
ENSXMAG00000015371	0.0026	3	15		1.76	0
ENSXMAG00000015378	0.0034	15	12	fbxo30a	1.43	12
ENSXMAG00000015380	0.0024	18	9	prf1.5	2.72	15
ENSXMAG00000015381	0.0015	3	18		1.93	0
ENSXMAG00000015385	0.0029	3	12	aspg	9.84	0
ENSXMAG00000015399	0.0015	24	6	cfap20	1.56	21
ENSXMAG00000015409	0.0029	18	12	syt9b	2.72	15
ENSXMAG00000015412	0.0024	6	15	SLC45A4 (1 of many)	4.58	3
ENSXMAG00000015418	0.0026	24	15	RASSF10	2.59	21
ENSXMAG00000015423	0.0026	24	9	tead1a	1.83	21
ENSXMAG00000015426	0.0030	15	12	nyap2b	3.77	12
ENSXMAG00000015428	0.0025	6	15		15.16	3
ENSXMAG00000015457	0.0016	24	18	zhx3	3.39	21
ENSXMAG00000015472	0.0025	18	9	DENND3	4.14	15
ENSXMAG00000015480	0.0026	24	9	si:ch211-167j6.4	5.12	21

ENSXMAG00000015492	0.0034	21	12	hdhd3	1.54	18
ENSXMAG00000015494	0.0026	15	15		1.23	12
ENSXMAG00000015500	0.0027	15	9	slc2a11a	14.13	12
ENSXMAG00000015506	0.0027	18	9	mical2b	3.07	15
ENSXMAG00000015508	0.0026	6	15	plk2b	1.66	3
ENSXMAG00000015512	0.0024	24	15	rnf114	1.67	21
ENSXMAG00000015523	0.0030	6	12	kat6b	1.32	3
ENSXMAG00000015524	0.0031	6	12	map7a	1.30	3
ENSXMAG00000015527	0.0027	3	15	si:ch73-335l21.1	2.60	0
ENSXMAG00000015534	0.0025	9	9	tmem41aa	1.56	6
ENSXMAG00000015536	0.0034	18	12	acap1	1.43	15
ENSXMAG00000015537	0.0024	6	9	si:dkey-190g11.3	2.20	3
ENSXMAG00000015540	0.0026	21	9	ptcd1	1.35	18
ENSXMAG00000015545	0.0025	18	9	noc3l	1.50	15
ENSXMAG00000015548	0.0025	9	15	far1	1.63	6
ENSXMAG00000015556	0.0029	6	12	nme8	2.19	3
ENSXMAG00000015558	0.0029	12	12	vclb	1.57	9
ENSXMAG00000015576	0.0026	24	9		1.59	21
ENSXMAG00000015577	0.0026	21	9	aar2	1.63	18
ENSXMAG00000015580	0.0026	3	15	si:dkeyp-23e4.3	1.88	0
ENSXMAG00000015581	0.0024	21	9	prpf6	1.33	18
ENSXMAG00000015582	0.0024	24	15		1.62	21
ENSXMAG00000015588	0.0031	18	12	mapre3a	1.91	15
ENSXMAG00000015609	0.0031	24	12	ZNF512B	1.51	21
ENSXMAG00000015610	0.0025	6	15	nrxn3a	1.55	3
ENSXMAG00000015618	0.0030	6	12	uckl1b	1.38	3
ENSXMAG00000015619	0.0031	15	12	arntl1a	24.50	12
ENSXMAG00000015622	0.0026	6	9		1.35	3
ENSXMAG00000015625	0.0025	21	9	xrcc5	1.75	18
ENSXMAG00000015627	0.0029	18	12	cbwd	1.39	15
ENSXMAG00000015629	0.0025	18	9	si:dkey-38p12.3	1.52	15
ENSXMAG00000015642	0.0031	21	12	tmem169b	1.69	18
ENSXMAG00000015645	0.0027	12	15	adck1	2.29	9
ENSXMAG00000015646	0.0027	6	15	pfkla	3.38	3
ENSXMAG00000015651	0.0024	24	9	slc17a9b	1.49	21
ENSXMAG00000015670	0.0025	9	15	zgc:110366	4.10	6
ENSXMAG00000015673	0.0034	6	12	rc3h1b	1.68	3
ENSXMAG00000015687	0.0031	6	12	polr2a	1.45	3
ENSXMAG00000015697	0.0014	21	6	zgc:153615	1.28	18
ENSXMAG00000015699	0.0016	21	6	selenot1a	1.39	18
ENSXMAG00000015701	0.0025	24	9	galcb	1.75	21
ENSXMAG00000015708	0.0024	18	15		1.47	15
ENSXMAG00000015716	0.0031	21	12	tp53	1.51	18
ENSXMAG00000015718	0.0027	6	15	DOCK3 (1 of many)	2.85	3
ENSXMAG00000015732	0.0031	3	12		1.83	0
ENSXMAG00000015749	0.0028	12	9	phactr4a	1.96	9
ENSXMAG00000015752	0.0031	9	12	eef2k	1.87	6
ENSXMAG00000015755	0.0025	18	9	YBX2	5.34	15
ENSXMAG00000015769	0.0026	18	9	slc24a1 (1 of many)	2.49	15
ENSXMAG00000015770	0.0027	6	15	FAM126B	1.71	3
ENSXMAG00000015773	0.0025	6	15	hivep3a	3.94	3
ENSXMAG00000015779	0.0024	18	9	plekhh1	5.95	15
ENSXMAG00000015795	0.0025	6	15	STMN1	1.90	3
ENSXMAG00000015803	0.0026	6	15	grip2a	1.46	3
ENSXMAG00000015820	0.0014	24	18	ldhd	2.41	21
ENSXMAG00000015833	0.0025	6	15	crip2	4.75	3

ENSXMAG00000015836	0.0025	6	15	DOK7	2.48	3
ENSXMAG00000015842	0.0015	21	6	nr2e3	13.59	18
ENSXMAG00000015844	0.0030	12	12	arsh	2.53	9
ENSXMAG00000015850	0.0031	18	12	stoml1	3.08	15
ENSXMAG00000015856	0.0029	24	12	tnr	1.97	21
ENSXMAG00000015860	0.0025	18	9	tmem62	1.59	15
ENSXMAG00000015864	0.0026	18	9	fam20cb	2.47	15
ENSXMAG00000015880	0.0029	6	12	plcxd3	1.58	3
ENSXMAG00000015883	0.0034	3	12	stra6	7.63	0
ENSXMAG00000015884	0.0015	24	18	glrba	1.26	21
ENSXMAG00000015888	0.0031	6	12	bin1a	1.42	3
ENSXMAG00000015892	0.0030	21	12	coro1b	1.89	18
ENSXMAG00000015898	0.0024	24	9	chd1l	1.46	21
ENSXMAG00000015904	0.0027	6	15		4.48	3
ENSXMAG00000015929	0.0026	15	15	opn4b	3.36	12
ENSXMAG00000015938	0.0015	21	6	tbc1d23	1.27	18
ENSXMAG00000015939	0.0031	15	12	ptprna	1.30	12
ENSXMAG00000015941	0.0031	18	12	glud1a	1.69	15
ENSXMAG00000015951	0.0031	21	12		1.31	18
ENSXMAG00000015956	0.0024	21	15	pofut2	1.28	18
ENSXMAG00000015965	0.0027	6	15		2.99	3
ENSXMAG00000015967	0.0025	6	15		3.48	3
ENSXMAG00000015974	0.0015	3	18	cdc14b	1.51	0
ENSXMAG00000015975	0.0030	3	12		5.56	0
ENSXMAG00000015979	0.0031	12	12	bokb	5.74	9
ENSXMAG00000015983	0.0026	15	9	elovl1a	1.97	12
ENSXMAG00000015991	0.0026	24	9	cdc20	2.04	21
ENSXMAG00000015994	0.0025	21	9	mta3	1.29	18
ENSXMAG00000016020	0.0024	6	15	akt3b	1.84	3
ENSXMAG00000016021	0.0029	18	12		4.42	15
ENSXMAG00000016045	0.0030	21	12	gch2	5.19	18
ENSXMAG00000016046	0.0014	21	6	aifm2	1.59	18
ENSXMAG00000016047	0.0026	18	9	slc7a5	2.50	15
ENSXMAG00000016048	0.0031	24	12	smyd1b	1.81	21
ENSXMAG00000016062	0.0031	24	12	kcnn3	1.67	21
ENSXMAG00000016068	0.0025	9	15		3.08	6
ENSXMAG00000016071	0.0031	6	12	gsg1l2a	7.12	3
ENSXMAG00000016078	0.0031	21	12	mesd	1.53	18
ENSXMAG00000016090	0.0026	24	9		1.14	21
ENSXMAG00000016106	0.0026	6	9	tomm40l	2.08	3
ENSXMAG00000016109	0.0024	6	15	nt5dc2	1.60	3
ENSXMAG00000016125	0.0028	21	9	sptlc1	1.23	18
ENSXMAG00000016140	0.0025	3	15	camk1a	1.88	0
ENSXMAG00000016150	0.0031	21	12		3.82	18
ENSXMAG00000016154	0.0024	21	15		4.95	18
ENSXMAG00000016159	0.0024	6	15	atp8a1	2.90	3
ENSXMAG00000016164	0.0029	15	12		97.44	12
ENSXMAG00000016167	0.0025	3	9	camta1a	2.30	0
ENSXMAG00000016171	0.0029	24	12	oca2	2.70	21
ENSXMAG00000016174	0.0025	9	15	per2	85.35	6
ENSXMAG00000016187	0.0025	9	15	heg1	3.01	6
ENSXMAG00000016189	0.0031	21	12	xrcc4	1.90	18
ENSXMAG00000016191	0.0031	15	12		2.12	12
ENSXMAG00000016203	0.0025	18	9	si:ch1073-392o20.1	4.34	15
ENSXMAG00000016211	0.0029	9	12	asmt	1.26	6
ENSXMAG00000016217	0.0025	18	9	SRGAP3 (1 of many)	1.60	15

ENSXMAG00000016224	0.0026	3	15	atp10a	1.49	0
ENSXMAG00000016235	0.0025	15	9		4.16	12
ENSXMAG00000016258	0.0031	21	12	prkacbb	2.39	18
ENSXMAG00000016278	0.0029	9	12	map3k5	1.80	6
ENSXMAG00000016284	0.0025	9	15	ptprea	1.27	6
ENSXMAG00000016293	0.0031	6	12	ric8a	2.40	3
ENSXMAG00000016297	0.0026	24	15		4.71	21
ENSXMAG00000016304	0.0031	18	12	crabp2a	2.07	15
ENSXMAG00000016312	0.0014	21	6	vldlr	1.82	18
ENSXMAG00000016317	0.0031	6	12		2.40	3
ENSXMAG00000016321	0.0030	3	12	prkar1ab	1.27	0
ENSXMAG00000016327	0.0034	24	12	cnga3a	25.62	21
ENSXMAG00000016346	0.0027	18	9	brd7	1.36	15
ENSXMAG00000016365	0.0014	6	18		1.77	3
ENSXMAG00000016369	0.0031	6	12	rbm5	1.18	3
ENSXMAG00000016371	0.0031	18	12	lypla2	1.71	15
ENSXMAG00000016372	0.0024	9	15	mylk4a	8.10	6
ENSXMAG00000016378	0.0029	15	12	bivm	4.17	12
ENSXMAG00000016391	0.0031	21	12	sfpq	1.53	18
ENSXMAG00000016396	0.0031	15	12		2.23	12
ENSXMAG00000016415	0.0015	3	18	dpt	1.49	0
ENSXMAG00000016423	0.0025	3	15	slc38a3b	2.63	0
ENSXMAG00000016424	0.0029	3	12	SLC16A6	1.60	0
ENSXMAG00000016443	0.0025	9	15		1.95	6
ENSXMAG00000016486	0.0015	24	6	cep78	1.52	21
ENSXMAG00000016490	0.0025	21	9	nt5dc3	1.35	18
ENSXMAG00000016496	0.0015	24	18	dhx32a	9.18	21
ENSXMAG00000016505	0.0030	6	12		2.93	3
ENSXMAG00000016513	0.0031	18	12	myo1b	2.10	15
ENSXMAG00000016516	0.0025	24	9	lamb2l	2.51	21
ENSXMAG00000016520	0.0031	21	12	uros	1.53	18
ENSXMAG00000016524	0.0016	18	6		2.24	15
ENSXMAG00000016529	0.0031	9	12		2.30	6
ENSXMAG00000016533	0.0026	15	9	fam53b	2.11	12
ENSXMAG00000016535	0.0015	3	18	si:ch211-22d5.2	117.57	0
ENSXMAG00000016538	0.0014	3	18	lhpp	4.33	0
ENSXMAG00000016543	0.0031	15	12	pea15	2.32	12
ENSXMAG00000016552	0.0031	24	12	pepd	1.46	21
ENSXMAG00000016554	0.0031	9	12	oat	2.63	6
ENSXMAG00000016561	0.0030	18	12	zgc:91910	1.28	15
ENSXMAG00000016568	0.0014	6	18		1.87	3
ENSXMAG00000016576	0.0026	3	15	pacsin1a	2.77	0
ENSXMAG00000016577	0.0029	21	12	ZNF521	1.35	18
ENSXMAG00000016611	0.0025	18	9	gnat1	2.39	15
ENSXMAG00000016612	0.0014	24	6	bub3	1.33	21
ENSXMAG00000016616	0.0025	21	9	psmb2	1.47	18
ENSXMAG00000016617	0.0034	24	12	stam	1.40	21
ENSXMAG00000016618	0.0031	21	12	cep63	1.65	18
ENSXMAG00000016619	0.0026	18	9		1.30	15
ENSXMAG00000016622	0.0024	18	9	arfgef2	1.30	15
ENSXMAG00000016624	0.0026	21	9	psma8	1.47	18
ENSXMAG00000016625	0.0030	15	12		1.60	12
ENSXMAG00000016629	0.0027	6	15	si:dkey-46i9.1	1.57	3
ENSXMAG00000016636	0.0031	18	12	ckmt2b	1.54	15
ENSXMAG00000016637	0.0026	18	9	asah2	5.21	15
ENSXMAG00000016646	0.0025	21	9	taf11	1.33	18

ENSXMAG00000016647	0.0026	9	15	slco2a1	1.95	6
ENSXMAG00000016651	0.0030	21	12	NR2C1	1.24	18
ENSXMAG00000016653	0.0031	21	12	KCTD1	1.62	18
ENSXMAG00000016654	0.0025	24	9	ANKS1A	1.50	21
ENSXMAG00000016658	0.0031	6	12	AQP4	1.71	3
ENSXMAG00000016660	0.0071	9	18	SDHB	1.27	6
ENSXMAG00000016664	0.0031	15	12		5.12	12
ENSXMAG00000016668	0.0026	18	9		2.91	15
ENSXMAG00000016670	0.0025	15	15	zmynd12	1.68	12
ENSXMAG00000016678	0.0026	24	9	cox4i2	2.19	21
ENSXMAG00000016682	0.0016	24	6		2.36	21
ENSXMAG00000016684	0.0026	3	15	FGD6	4.27	0
ENSXMAG00000016688	0.0025	18	9	ext1c	2.76	15
ENSXMAG00000016697	0.0071	21	18		1.98	18
ENSXMAG00000016708	0.0027	18	9	btbd17a	53.18	15
ENSXMAG00000016711	0.0025	21	15	ppp1r10	1.43	18
ENSXMAG00000016720	0.0031	18	12	msh3	1.47	15
ENSXMAG00000016724	0.0024	6	15		8.70	3
ENSXMAG00000016725	0.0014	21	6		2.14	18
ENSXMAG00000016729	0.0031	15	12		1.64	12
ENSXMAG00000016733	0.0031	18	12		4.31	15
ENSXMAG00000016749	0.0027	18	9	uap1l1	1.52	15
ENSXMAG00000016750	0.0025	24	9	pvrl2l	1.76	21
ENSXMAG00000016766	0.0031	24	12	agtpbp1	1.21	21
ENSXMAG00000016767	0.0031	15	12	ELK3	1.36	12
ENSXMAG00000016769	0.0031	18	12	thsd7aa	10.40	15
ENSXMAG00000016777	0.0031	9	12	map3k12	2.37	6
ENSXMAG00000016788	0.0028	24	15	naa35	1.18	21
ENSXMAG00000016806	0.0024	9	15	nckipsd	1.81	6
ENSXMAG00000016817	0.0026	18	15	hs3st1l2	2.69	15
ENSXMAG00000016822	0.0025	21	9	paics	2.10	18
ENSXMAG00000016824	0.0026	3	15	col2a1a	2.29	0
ENSXMAG00000016828	0.0028	21	9	derl2	1.45	18
ENSXMAG00000016840	0.0026	21	9	rpa3	1.67	18
ENSXMAG00000016845	0.0027	18	9	zgc:112334	2.55	15
ENSXMAG00000016846	0.0026	24	9	tapbp.2	1.43	21
ENSXMAG00000016847	0.0031	21	12	TNS3 (1 of many)	1.46	18
ENSXMAG00000016850	0.0026	24	15	rp1	3.21	21
ENSXMAG00000016859	0.0025	18	9		1.82	15
ENSXMAG00000016862	0.0031	18	12		9.15	15
ENSXMAG00000016865	0.0031	18	12	cep135	1.54	15
ENSXMAG00000016868	0.0031	18	12	asns	2.15	15
ENSXMAG00000016873	0.0014	6	18	cfap36	2.74	3
ENSXMAG00000016886	0.0014	24	18		5.44	21
ENSXMAG00000016892	0.0030	18	12	rhoab	1.23	15
ENSXMAG00000016893	0.0029	21	12		1.36	18
ENSXMAG00000016894	0.0026	9	9	PDE1C	5.76	6
ENSXMAG00000016899	0.0026	9	9		8.33	6
ENSXMAG00000016912	0.0026	21	9	slc35a1	1.27	18
ENSXMAG00000016920	0.0025	24	9	mpp3b	1.82	21
ENSXMAG00000016927	0.0025	9	15	chchd3b	2.93	6
ENSXMAG00000016928	0.0025	18	9	clocka	32.38	15
ENSXMAG00000016931	0.0026	18	9	tmcc1b	1.55	15
ENSXMAG00000016936	0.0031	18	12	top2b	1.48	15
ENSXMAG00000016937	0.0031	18	12	CCDC88A	3.21	15
ENSXMAG00000016946	0.0031	18	12	stxbp4	3.43	15

ENSXMAG00000016948	0.0029	6	12		3.03	3
ENSXMAG00000016958	0.0024	15	15	tmem165	1.38	12
ENSXMAG00000016963	0.0024	6	15	fam171a2b	1.61	3
ENSXMAG00000016987	0.0016	3	18	si:ch211-107o10.3	3.94	0
ENSXMAG00000017001	0.0031	18	12	THEM6	2.69	15
ENSXMAG00000017009	0.0027	6	15		2.32	3
ENSXMAG00000017017	0.0014	18	18	extl3	1.22	15
ENSXMAG00000017036	0.0026	18	15	tkta	1.46	15
ENSXMAG00000017041	0.0026	21	9	naa40	1.27	18
ENSXMAG00000017049	0.0024	3	15	ngfrb	2.66	0
ENSXMAG00000017061	0.0030	18	12	EGFLAM	3.56	15
ENSXMAG00000017062	0.0026	24	9		1.39	21
ENSXMAG00000017063	0.0029	6	12	frmd5	1.74	3
ENSXMAG00000017064	0.0025	18	9	bicd2	1.68	15
ENSXMAG00000017074	0.0025	12	15	selenbp1	1.74	9
ENSXMAG00000017079	0.0026	6	15		3.13	3
ENSXMAG00000017082	0.0031	18	12	chl1b	6.23	15
ENSXMAG00000017087	0.0025	18	15		1.91	15
ENSXMAG00000017088	0.0029	15	12	opn4xb	4.95	12
ENSXMAG00000017090	0.0031	18	12	NINJ1 (1 of many)	1.53	15
ENSXMAG00000017100	0.0014	3	18	slc24a4b	2.51	0
ENSXMAG00000017105	0.0025	18	9	fli1b	1.66	15
ENSXMAG00000017106	0.0025	9	9	nr1d2b	6.66	6
ENSXMAG00000017124	0.0031	21	12	bmpr1ba	1.59	18
ENSXMAG00000017125	0.0034	21	12		2.11	18
ENSXMAG00000017134	0.0029	18	12	NRCAM (1 of many)	1.81	15
ENSXMAG00000017140	0.0028	21	9	spata20	1.83	18
ENSXMAG00000017142	0.0031	15	12		4.82	12
ENSXMAG00000017147	0.0031	6	12	tspan3b	1.20	3
ENSXMAG00000017179	0.0029	6	12	gphnb	1.61	3
ENSXMAG00000017180	0.0024	18	9	vip	2.31	15
ENSXMAG00000017184	0.0027	18	9	slmapb	1.70	15
ENSXMAG00000017187	0.0027	18	9	ISL2 (1 of many)	2.77	15
ENSXMAG00000017191	0.0026	21	9	TMEM19	1.36	18
ENSXMAG00000017201	0.0031	6	12	tnk2b	2.96	3
ENSXMAG00000017204	0.0031	15	12	rassf1	3.01	12
ENSXMAG00000017207	0.0026	24	9	asic1a	1.62	21
ENSXMAG00000017208	0.0029	21	12	tpma	1.77	18
ENSXMAG00000017239	0.0029	9	12	gpd1a	2.80	6
ENSXMAG00000017244	0.0025	3	15		4.99	0
ENSXMAG00000017247	0.0015	3	6	mpp5b	2.99	0
ENSXMAG00000017249	0.0027	24	15	thumpd2	1.40	21
ENSXMAG00000017253	0.0026	6	15	kcnab2a	1.95	3
ENSXMAG00000017255	0.0031	21	12	sh3bp5b	1.93	18
ENSXMAG00000017261	0.0024	21	9		2.02	18
ENSXMAG00000017262	0.0071	3	18	rasd1	2.48	0
ENSXMAG00000017280	0.0028	24	15	PI4KB (1 of many)	1.21	21
ENSXMAG00000017282	0.0031	21	12	GXYLT1	2.14	18
ENSXMAG00000017286	0.0027	6	15	adcy6a	2.41	3
ENSXMAG00000017306	0.0025	18	9		4.35	15
ENSXMAG00000017308	0.0029	3	12	reck	1.52	0
ENSXMAG00000017320	0.0034	9	12		1.92	6
ENSXMAG00000017324	0.0024	24	9	ADAMTS20	2.70	21
ENSXMAG00000017334	0.0026	24	15	cab39l	1.23	21
ENSXMAG00000017337	0.0031	6	12	cdc42se1	3.78	3
ENSXMAG00000017351	0.0025	6	15	syn2b	1.30	3

ENSXMAG00000017367	0.0024	3	15	aida	1.44	0
ENSXMAG00000017371	0.0031	15	12	nob1	1.52	12
ENSXMAG00000017374	0.0031	6	12	ints6l	2.12	3
ENSXMAG00000017379	0.0025	21	9	PLPPR2	1.95	18
ENSXMAG00000017382	0.0027	3	9	tanc2a	2.49	0
ENSXMAG00000017397	0.0025	3	15	rab11fip4b	5.41	0
ENSXMAG00000017399	0.0030	18	12	twf1a	1.46	15
ENSXMAG00000017400	0.0025	3	15	amfra	1.88	0
ENSXMAG00000017403	0.0024	18	9		1.23	15
ENSXMAG00000017405	0.0031	6	12	kcnk3a	3.67	3
ENSXMAG00000017432	0.0029	21	12	kbtbd12	2.05	18
ENSXMAG00000017445	0.0031	3	12	eno1b	1.33	0
ENSXMAG00000017447	0.0034	18	12	cnot7	1.50	15
ENSXMAG00000017449	0.0031	9	12	syt10	2.40	6
ENSXMAG00000017450	0.0026	18	9	slc34a2a	113.55	15
ENSXMAG00000017454	0.0031	6	12	slc6a1b	1.21	3
ENSXMAG00000017458	0.0027	24	15	tagapb	3.05	21
ENSXMAG00000017459	0.0025	21	9		1.42	18
ENSXMAG00000017462	0.0016	21	6	ints3	1.35	18
ENSXMAG00000017470	0.0024	24	9	NCOA3	2.88	21
ENSXMAG00000017478	0.0034	6	12	slc6a11b	2.13	3
ENSXMAG00000017480	0.0024	6	15	slc7a2	3.30	3
ENSXMAG00000017487	0.0024	6	15	WNK2	2.45	3
ENSXMAG00000017493	0.0029	18	12	si:dkey-27c15.3	1.45	15
ENSXMAG00000017504	0.0027	24	9	plxnb2b	1.42	21
ENSXMAG00000017520	0.0025	3	15	lpin1	5.15	0
ENSXMAG00000017523	0.0031	9	12		4.89	6
ENSXMAG00000017525	0.0025	18	9	FGD3	2.17	15
ENSXMAG00000017532	0.0031	24	12		1.76	21
ENSXMAG00000017557	0.0016	24	18	mlxip	1.39	21
ENSXMAG00000017562	0.0025	15	15	iars	1.66	12
ENSXMAG00000017581	0.0016	6	18	ptpdc1b	4.65	3
ENSXMAG00000017584	0.0015	6	18	eva1c	5.34	3
ENSXMAG00000017596	0.0031	21	12	hunk	5.00	18
ENSXMAG00000017600	0.0024	24	9	esrrga	1.97	21
ENSXMAG00000017602	0.0031	18	12	ephb2b	1.97	15
ENSXMAG00000017614	0.0026	18	9	strn	2.03	15
ENSXMAG00000017621	0.0025	21	15	nrbp1	1.20	18
ENSXMAG00000017631	0.0025	3	15	POC1A	2.10	0
ENSXMAG00000017634	0.0025	9	9	kitb	3.44	6
ENSXMAG00000017639	0.0027	6	15		2.03	3
ENSXMAG00000017642	0.0028	6	15	kctd3	2.48	3
ENSXMAG00000017644	0.0026	6	15	ITGA7	1.52	3
ENSXMAG00000017647	0.0024	12	15		1.59	9
ENSXMAG00000017653	0.0025	21	15	HIP1R	1.95	18
ENSXMAG00000017655	0.0026	18	15	lzts2b	3.06	15
ENSXMAG00000017668	0.0026	12	9	alpl	1.68	9
ENSXMAG00000017679	0.0024	24	15	scn4aa	3.56	21
ENSXMAG00000017682	0.0025	24	9	pum2	1.25	21
ENSXMAG00000017688	0.0026	6	15		1.66	3
ENSXMAG00000017694	0.0025	6	15		2.40	3
ENSXMAG00000017712	0.0031	3	12	matn3a	3.33	0
ENSXMAG00000017719	0.0025	3	9		1.47	0
ENSXMAG00000017725	0.0026	9	15	syn2a	1.28	6
ENSXMAG00000017745	0.0024	18	9		2.31	15
ENSXMAG00000017749	0.0026	3	15	rdh8a	2.28	0

ENSXMAG00000017754	0.0014	3	18	smtnb	5.13	0
ENSXMAG00000017758	0.0024	6	15	mtnr1bb	4.32	3
ENSXMAG00000017759	0.0024	21	9	myg1	1.85	18
ENSXMAG00000017762	0.0015	21	6	noctb	1.67	18
ENSXMAG00000017771	0.0014	3	6	MAP3K12 (1 of many)	2.71	0
ENSXMAG00000017772	0.0014	3	18	inpp5jb	1.23	0
ENSXMAG00000017773	0.0026	21	9	pdia6	1.39	18
ENSXMAG00000017774	0.0025	18	9	NAA15	1.51	15
ENSXMAG00000017775	0.0034	24	12	smpd1	2.07	21
ENSXMAG00000017793	0.0025	18	15	usp39	1.36	15
ENSXMAG00000017798	0.0031	6	12		2.76	3
ENSXMAG00000017800	0.0025	24	9	fam184a	1.91	21
ENSXMAG00000017816	0.0026	18	9	aig1	1.44	15
ENSXMAG00000017817	0.0014	18	6	bmp7b	2.03	15
ENSXMAG00000017818	0.0031	21	12		1.51	18
ENSXMAG00000017819	0.0014	18	6	gpam	2.53	15
ENSXMAG00000017829	0.0031	15	12	SP1	1.38	12
ENSXMAG00000017842	0.0014	24	18	igsf8	1.55	21
ENSXMAG00000017845	0.0029	6	12	IQSEC1 (1 of many)	1.62	3
ENSXMAG00000017846	0.0025	21	9	mettl14	1.53	18
ENSXMAG00000017850	0.0026	3	15		2.05	0
ENSXMAG00000017854	0.0025	6	15		3.89	3
ENSXMAG00000017874	0.0015	3	18	KIF5A	1.56	0
ENSXMAG00000017879	0.0024	18	9	fnbp1b	1.98	15
ENSXMAG00000017903	0.0024	3	15		2.49	0
ENSXMAG00000017908	0.0031	21	12	adgrl2a	1.96	18
ENSXMAG00000017920	0.0030	18	12	mars	2.03	15
ENSXMAG00000017938	0.0025	21	9	nsfb	1.59	18
ENSXMAG00000017939	0.0031	6	12	ARHGAP9	3.13	3
ENSXMAG00000017962	0.0026	18	9	thrap3b	1.24	15
ENSXMAG00000017964	0.0034	15	12		1.94	12
ENSXMAG00000017978	0.0031	15	12	r3hdm2	1.56	12
ENSXMAG00000017993	0.0026	6	15	si:ch211-195b13.1	6.21	3
ENSXMAG00000018000	0.0031	18	12	srp72	1.42	15
ENSXMAG00000018006	0.0031	24	12	syt5a	1.88	21
ENSXMAG00000018018	0.0024	18	9		1.33	15
ENSXMAG00000018034	0.0029	18	12	si:ch211-51c14.1	2.14	15
ENSXMAG00000018048	0.0034	6	12	opn7d	1.85	3
ENSXMAG00000018058	0.0016	12	6	ppp1r12c	1.48	9
ENSXMAG00000018060	0.0014	21	18	TCTN2	1.37	18
ENSXMAG00000018071	0.0024	3	15	pik3ip1	5.83	0
ENSXMAG00000018076	0.0025	3	15	limk2	1.65	0
ENSXMAG00000018086	0.0026	21	9		1.64	18
ENSXMAG00000018094	0.0029	9	12	prickle3	3.04	6
ENSXMAG00000018095	0.0024	24	9	scrn2	1.51	21
ENSXMAG00000018097	0.0031	18	12		1.90	15
ENSXMAG00000018111	0.0025	3	15	sec14l8	2.57	0
ENSXMAG00000018120	0.0025	21	9	psmb3	1.46	18
ENSXMAG00000018124	0.0025	24	15	PIP4K2B	1.39	21
ENSXMAG00000018135	0.0026	6	15		1.73	3
ENSXMAG00000018160	0.0031	18	12	sart3	1.17	15
ENSXMAG00000018168	0.0025	24	9		1.28	21
ENSXMAG00000018172	0.0026	21	9	tpgs2	1.51	18
ENSXMAG00000018179	0.0025	24	9	SMARCE1	1.43	21
ENSXMAG00000018189	0.0024	15	15	si:ch211-152c8.4	1.50	12
ENSXMAG00000018200	0.0026	3	15	timm17b	1.33	0

ENSXMAG00000018211	0.0024	18	9	RIMS1 (1 of many)	2.76	15
ENSXMAG00000018217	0.0025	6	15	dao.2	1.98	3
ENSXMAG00000018222	0.0026	24	9	gusb	1.94	21
ENSXMAG00000018223	0.0026	18	15	actl6a	1.47	15
ENSXMAG00000018227	0.0025	18	9	bcl2l10	1.94	15
ENSXMAG00000018234	0.0031	24	12		1.38	21
ENSXMAG00000018237	0.0024	24	9		1.58	21
ENSXMAG00000018244	0.0025	15	9	usp30	4.43	12
ENSXMAG00000018261	0.0031	24	12	usp13	1.64	21
ENSXMAG00000018266	0.0015	24	6		1.97	21
ENSXMAG00000018280	0.0016	24	6	lin7c	1.30	21
ENSXMAG00000018281	0.0025	9	9		7.84	6
ENSXMAG00000018286	0.0034	24	12	fggy	1.48	21
ENSXMAG00000018295	0.0026	21	15	mcee	1.52	18
ENSXMAG00000018315	0.0031	21	12	srp54	1.25	18
ENSXMAG00000018319	0.0024	21	9	PORCN	1.89	18
ENSXMAG00000018329	0.0027	21	15	ndufa4	1.31	18
ENSXMAG00000018334	0.0026	6	15		2.88	3
ENSXMAG00000018362	0.0031	21	12	p3h2	1.64	18
ENSXMAG00000018365	0.0031	6	12		2.08	3
ENSXMAG00000018388	0.0031	18	12	mipol1	2.50	15
ENSXMAG00000018394	0.0025	21	9	psmd13	1.48	18
ENSXMAG00000018422	0.0025	6	15	ankrd9	3.05	3
ENSXMAG00000018424	0.0024	18	15	si:dkey-30h22.11	1.46	15
ENSXMAG00000018428	0.0031	3	12	rbks	1.64	0
ENSXMAG00000018437	0.0028	3	15	ckba	1.37	0
ENSXMAG00000018438	0.0031	21	12		1.11	18
ENSXMAG00000018440	0.0026	24	9	babam2	1.39	21
ENSXMAG00000018442	0.0024	6	15		8.76	3
ENSXMAG00000018453	0.0026	24	15	rabgef1l	1.23	21
ENSXMAG00000018455	0.0026	3	15	klc1a	1.35	0
ENSXMAG00000018473	0.0015	21	6	guk1b	5.81	18
ENSXMAG00000018476	0.0031	3	12	BRF1 (1 of many)	2.64	0
ENSXMAG00000018477	0.0031	21	12	PLXNA1	1.52	18
ENSXMAG00000018481	0.0026	9	15	matn3b	5.20	6
ENSXMAG00000018535	0.0026	12	15	si:dkey-33c12.4	1.67	9
ENSXMAG00000018543	0.0014	24	18	tcp11l2	3.32	21
ENSXMAG00000018562	0.0031	18	12		2.76	15
ENSXMAG00000018563	0.0026	18	9	dlgap4b	1.96	15
ENSXMAG00000018577	0.0015	21	6	zgc:153119	1.45	18
ENSXMAG00000018590	0.0031	18	12	chd7	2.25	15
ENSXMAG00000018595	0.0027	6	15	pik3r1	2.43	3
ENSXMAG00000018600	0.0026	6	15	taok2b	1.46	3
ENSXMAG00000018609	0.0015	18	6	osbpl5	1.74	15
ENSXMAG00000018611	0.0016	24	6	kcnk5a	1.45	21
ENSXMAG00000018615	0.0025	9	15		1.77	6
ENSXMAG00000018617	0.0034	21	12	zmp:0000000846	3.04	18
ENSXMAG00000018638	0.0027	6	9	gpr153	1.40	3
ENSXMAG00000018639	0.0029	9	12	cabp5a	31.09	6
ENSXMAG00000018645	0.0025	18	15	dpydb	1.60	15
ENSXMAG00000018650	0.0026	6	15	espn	3.28	3
ENSXMAG00000018656	0.0026	3	15	plcl2	4.91	0
ENSXMAG00000018662	0.0031	21	12	satb1b	1.59	18
ENSXMAG00000018693	0.0025	3	9	tns2b	1.48	0
ENSXMAG00000018709	0.0025	6	9	nr1d2a	13.35	3
ENSXMAG00000018710	0.0031	24	12	JMY	4.56	21

ENSXMAG00000018712	0.0027	24	9	zgc:154075	1.64	21
ENSXMAG00000018715	0.0025	21	9	emc1	1.18	18
ENSXMAG00000018716	0.0024	18	9	trappc12	1.23	15
ENSXMAG00000018718	0.0029	6	12		8.18	3
ENSXMAG00000018724	0.0027	6	15	dmgdh	1.57	3
ENSXMAG00000018726	0.0026	6	15	cers1	3.30	3
ENSXMAG00000018729	0.0016	3	18		3.02	0
ENSXMAG00000018737	0.0026	18	15	sept7b	1.42	15
ENSXMAG00000018743	0.0024	18	9		1.39	15
ENSXMAG00000018748	0.0027	24	15	bsdc1	1.40	21
ENSXMAG00000018753	0.0029	18	12	lhfpl2a	2.26	15
ENSXMAG00000018758	0.0026	21	9	ythdf2	1.14	18
ENSXMAG00000018762	0.0029	3	12		2.75	0
ENSXMAG00000018767	0.0026	9	15	lats2	1.28	6
ENSXMAG00000018773	0.0031	12	12	si:ch1073-358c10.1	3.48	9
ENSXMAG00000018776	0.0027	15	9	phkg2	3.38	12
ENSXMAG00000018780	0.0026	24	15	larp1	1.50	21
ENSXMAG00000018786	0.0027	24	15	maco1b	1.23	21
ENSXMAG00000018789	0.0014	21	6	pou4f2	1.46	18
ENSXMAG00000018792	0.0025	18	9	kpnb1	1.42	15
ENSXMAG00000018793	0.0015	21	6	fat1a	2.21	18
ENSXMAG00000018796	0.0014	24	6	acap3b	1.69	21
ENSXMAG00000018838	0.0026	18	15		1.24	15
ENSXMAG00000018842	0.0024	3	15	pde8b	5.08	0
ENSXMAG00000018844	0.0026	3	15	rps6ka1	2.78	0
ENSXMAG00000018860	0.0031	18	12	ica1	1.41	15
ENSXMAG00000018863	0.0026	18	9	srrm1	1.26	15
ENSXMAG00000018866	0.0031	18	12	slc25a36a (1 of many)	1.81	15
ENSXMAG00000018869	0.0025	21	9	UBE2M	1.30	18
ENSXMAG00000018876	0.0026	24	9	thsd7ab	2.33	21
ENSXMAG00000018882	0.0031	6	12	sorbs2a	2.57	3
ENSXMAG00000018884	0.0015	18	6	btr30	2.24	15
ENSXMAG00000018889	0.0027	21	9	psd2	1.29	18
ENSXMAG00000018890	0.0031	24	12	clstn2	1.72	21
ENSXMAG00000018896	0.0027	18	9	rpn1	1.49	15
ENSXMAG00000018898	0.0025	6	15	agap3	3.35	3
ENSXMAG00000018900	0.0025	21	9	prkx	1.57	18
ENSXMAG00000018902	0.0026	21	9		1.47	18
ENSXMAG00000018905	0.0024	3	15	ppp1r16a	2.95	0
ENSXMAG00000018910	0.0031	18	12	tmem106ba	1.35	15
ENSXMAG00000018922	0.0031	3	12	F2R (1 of many)	1.41	0
ENSXMAG00000018929	0.0016	12	18	NMNAT3	1.25	9
ENSXMAG00000018934	0.0031	24	12		49.78	21
ENSXMAG00000018938	0.0014	21	6	pgk1	1.24	18
ENSXMAG00000018942	0.0029	12	12	ctsf	1.55	9
ENSXMAG00000018945	0.0025	21	15	rprd1b	1.20	18
ENSXMAG00000018946	0.0030	18	12	dync1li2	1.46	15
ENSXMAG00000018950	0.0025	18	9	zmp:0000000624	1.83	15
ENSXMAG00000018959	0.0015	24	18	rb1cc1	1.52	21
ENSXMAG00000018961	0.0025	24	9	med15	1.34	21
ENSXMAG00000018963	0.0031	3	12	COL4A3BP (1 of many)	2.06	0
ENSXMAG00000018967	0.0016	24	6	NPDC1	1.18	21
ENSXMAG00000018976	0.0026	18	9		1.79	15
ENSXMAG00000018988	0.0016	21	18	si:ch211-160o17.6	37.19	18
ENSXMAG00000019000	0.0026	21	9	mzt2b	1.46	18
ENSXMAG00000019003	0.0025	18	15	agmo	3.13	15

ENSXMAG00000019006	0.0025	21	9	sntg1	1.61	18
ENSXMAG00000019010	0.0025	18	9	si:ch211-183d21.3	2.29	15
ENSXMAG00000019015	0.0026	18	9	ispd	1.59	15
ENSXMAG00000019026	0.0029	15	12	ca7	11.36	12
ENSXMAG00000019028	0.0025	24	9	cwc27	1.45	21
ENSXMAG00000019040	0.0026	21	15	cdh16	2.38	18
ENSXMAG00000019046	0.0031	21	12	adamts6	2.02	18
ENSXMAG00000019055	0.0031	18	12	actr5	1.64	15
ENSXMAG00000019056	0.0029	18	12	gale	1.58	15
ENSXMAG00000019059	0.0026	24	9		1.36	21
ENSXMAG00000019068	0.0031	15	12	mapre2	1.53	12
ENSXMAG00000019072	0.0031	3	12	si:ch211-263k4.2	1.81	0
ENSXMAG00000019106	0.0030	6	12		1.89	3
ENSXMAG00000019115	0.0026	15	9	scap	2.41	12
ENSXMAG00000019132	0.0015	15	18	cacna1eb	2.84	12
ENSXMAG00000019146	0.0026	9	15	syt12	2.81	6
ENSXMAG00000019147	0.0031	21	12	uri1	1.51	18
ENSXMAG00000019151	0.0014	24	18	hnrnpd	1.23	21
ENSXMAG00000019158	0.0014	18	6	mtss1la	6.92	15
ENSXMAG00000019164	0.0014	24	18		1.66	21
ENSXMAG00000019165	0.0027	24	15	chmp4bb	1.27	21
ENSXMAG00000019177	0.0024	18	9		7.05	15
ENSXMAG00000019181	0.0027	12	15	pde6a	1.52	9
ENSXMAG00000019191	0.0027	6	15	mgst3a	1.30	3
ENSXMAG00000019198	0.0030	21	12	blzf1	1.30	18
ENSXMAG00000019204	0.0024	9	15	panx2	1.64	6
ENSXMAG00000019215	0.0028	24	9	sema5a	1.58	21
ENSXMAG00000019218	0.0031	6	12	btbd11b	1.99	3
ENSXMAG00000019223	0.0024	3	9		2.30	0
ENSXMAG00000019230	0.0025	24	9	PDE5A	1.51	21
ENSXMAG00000019247	0.0027	15	15	denr	1.43	12
ENSXMAG00000019249	0.0031	18	12	slc36a1	1.71	15
ENSXMAG00000019255	0.0030	18	12	g3bp1	2.32	15
ENSXMAG00000019258	0.0029	15	12	ush1c	1.70	12
ENSXMAG00000019263	0.0025	6	15	hdhd5	2.79	3
ENSXMAG00000019265	0.0031	24	12	grk1a	10.29	21
ENSXMAG00000019267	0.0014	21	6		1.22	18
ENSXMAG00000019274	0.0025	18	15	tmco3	1.55	15
ENSXMAG00000019276	0.0027	18	15	dcun1d2b	1.48	15
ENSXMAG00000019280	0.0024	9	15	GRTP1 (1 of many)	6.18	6
ENSXMAG00000019281	0.0031	24	12	chdh	2.11	21
ENSXMAG00000019282	0.0025	9	15	lamp1a	1.79	6
ENSXMAG00000019288	0.0016	24	18	cul4a	1.30	21
ENSXMAG00000019294	0.0031	21	12	sema3bl	1.61	18
ENSXMAG00000019296	0.0025	15	9	rorcb	61.99	12
ENSXMAG00000019300	0.0015	6	6	sh3gl1a	1.68	3
ENSXMAG00000019303	0.0034	9	12		1.22	6
ENSXMAG00000019309	0.0025	21	15	btbd2b	1.25	18
ENSXMAG00000019310	0.0025	6	15		3.84	3
ENSXMAG00000019322	0.0024	24	15	amt	3.77	21
ENSXMAG00000019336	0.0031	18	12	cirbpa	3.66	15
ENSXMAG00000019339	0.0025	18	9	GATAD2A (1 of many)	1.65	15
ENSXMAG00000019341	0.0015	9	6		1.28	6
ENSXMAG00000019347	0.0030	21	12	si:dkey-43k4.5	1.67	18
ENSXMAG00000019348	0.0025	3	15		1.28	0
ENSXMAG00000019349	0.0026	18	9	dalrd3	1.55	15

ENSXMAG00000019355	0.0031	15	12	arih2	1.70	12
ENSXMAG00000019362	0.0026	24	15	si:dkey-72l14.3	3.85	21
ENSXMAG00000019370	0.0031	9	12		52.92	6
ENSXMAG00000019390	0.0025	9	15	NADK	3.77	6
ENSXMAG00000019391	0.0031	15	12	gnb1a	2.56	12
ENSXMAG00000019394	0.0025	6	15	gabrd	1.73	3
ENSXMAG00000019400	0.0027	18	15	ttc37	1.32	15
ENSXMAG00000019401	0.0026	6	15	mctp1a	2.24	3
ENSXMAG00000019402	0.0031	9	12		2.77	6
ENSXMAG00000019403	0.0025	6	15		2.58	3
ENSXMAG00000019412	0.0027	6	15	rhobtb4	5.42	3
ENSXMAG00000019438	0.0031	21	12		1.45	18
ENSXMAG00000019439	0.0031	21	12	kctd12.2	1.27	18
ENSXMAG00000019441	0.0025	21	9	brinp3b	2.57	18
ENSXMAG00000019465	0.0029	6	12	tob1b	6.36	3
ENSXMAG00000019476	0.0024	18	9	zgc:103697	1.39	15
ENSXMAG00000019488	0.0031	6	12	adra2da	2.63	3
ENSXMAG00000019496	0.0024	24	9	inhbaa	2.67	21
ENSXMAG00000019500	0.0027	6	15		1.57	3
ENSXMAG00000019506	0.0025	18	9		2.65	15
ENSXMAG00000019523	0.0034	24	12	si:ch211-285j22.3	4.04	21
ENSXMAG00000019524	0.0029	15	12		334.37	12
ENSXMAG00000019527	0.0031	9	12	nfil3-6	9.86	6
ENSXMAG00000019530	0.0029	15	12		48.74	12
ENSXMAG00000019541	0.0026	18	9	lingo4b	2.39	15
ENSXMAG00000019561	0.0034	15	12		3.15	12
ENSXMAG00000019564	0.0024	3	15	zgc:154093	3.67	0
ENSXMAG00000019585	0.0016	21	6	triap1	1.43	18
ENSXMAG00000019594	0.0025	18	9	rnf152	3.04	15
ENSXMAG00000019600	0.0014	3	18	si:ch211-213o11.11	14.04	0
ENSXMAG00000019607	0.0025	21	9	taf13	1.40	18
ENSXMAG00000019635	0.0025	12	15	rho	1.48	9
ENSXMAG00000019659	0.0026	15	15	lrrc3ca	2.14	12
ENSXMAG00000019660	0.0026	3	15	kcnj12a	4.48	0
ENSXMAG00000019665	0.0071	3	18	cebpb	3.71	0
ENSXMAG00000019666	0.0024	21	9	si:ch211-210c8.7	1.76	18
ENSXMAG00000019679	0.0031	21	12	flrt2	1.47	18
ENSXMAG00000019691	0.0031	9	12	cebpg	1.77	6
ENSXMAG00000019698	0.0025	9	9		1.19	6
ENSXMAG00000019730	0.0026	3	15		2.68	0
ENSXMAG00000019734	0.0031	24	12	vasna	1.67	21
ENSXMAG00000019735	0.0024	24	15	TCEANC	1.48	21
ENSXMAG00000019737	0.0025	3	9		1.94	0
ENSXMAG00000019739	0.0031	15	12	phospho1	2.12	12
ENSXMAG00000019744	0.0031	6	12		2.39	3
ENSXMAG00000019790	0.0031	24	12	fam43b	2.26	21
ENSXMAG00000019793	0.0031	18	12		1.30	15
ENSXMAG00000019804	0.0026	21	9	HNRNPA0 (1 of many)	1.32	18
ENSXMAG00000019813	0.0031	6	12	b3gnt2a	2.48	3
ENSXMAG00000019815	0.0025	18	9	s1pr5b	2.22	15
ENSXMAG00000019819	0.0031	21	12	cyp8b2	1.64	18
ENSXMAG00000019821	0.0031	15	12	flrt3	1.41	12
ENSXMAG00000019822	0.0028	12	15		1.53	9
ENSXMAG00000019825	0.0016	3	18	sertad2b	2.24	0
ENSXMAG00000019849	0.0026	18	9	fahd1	2.11	15
ENSXMAG00000019863	0.0024	3	15	paqr7b	3.49	0

ENSXMAG00000019865	0.0031	9	12	si:ch73-334d15.1	5.61	6
ENSXMAG00000019866	0.0024	9	15		5.97	6
ENSXMAG00000019871	0.0025	6	15	cdc42ep4b	3.58	3
ENSXMAG00000019874	0.0014	15	18	arl4d	2.70	12
ENSXMAG00000019883	0.0024	18	9	nono	1.53	15
ENSXMAG00000019893	0.0031	15	12	smim13	1.54	12
ENSXMAG00000019917	0.0027	18	9	prr18	2.24	15
ENSXMAG00000019927	0.0014	21	6	kctd12.1	3.05	18
ENSXMAG00000019938	0.0027	18	9	im:7152348	1.92	15
ENSXMAG00000019942	0.0031	21	12	si:dkey-237j11.3	1.50	18
ENSXMAG00000019943	0.0029	15	12	nfil3	36.89	12
ENSXMAG00000019959	0.0024	3	15		1.78	0
ENSXMAG00000019971	0.0027	18	9	sf3b5	1.53	15
ENSXMAG00000019973	0.0026	6	15		3.50	3
ENSXMAG00000019983	0.0029	15	12	GPR35 (1 of many)	5.27	12
ENSXMAG00000019994	0.0025	12	15	opn4.1	3.81	9
ENSXMAG00000019997	0.0025	3	9	s1pr5a	1.32	0
ENSXMAG00000020008	0.0016	21	6	lrrtm1	1.65	18
ENSXMAG00000020010	0.0025	18	9	FLRT1	2.78	15
ENSXMAG00000020020	0.0031	15	12	tmem121b	2.40	12
ENSXMAG00000020021	0.0031	18	12	ora5	2.46	15
ENSXMAG00000020022	0.0016	21	6		1.29	18
ENSXMAG00000020032	0.0026	18	9	lrrc8db	1.36	15
ENSXMAG00000020034	0.0026	12	15	si:dkeyp-66d1.7	1.38	9
ENSXMAG00000020055	0.0030	24	12	ackr4b	2.13	21
ENSXMAG00000020057	0.0027	18	9	thbd	2.03	15
ENSXMAG00000020058	0.0026	18	9		1.79	15
ENSXMAG00000020059	0.0031	6	12	cdk5r2b	21.59	3
ENSXMAG00000020065	0.0024	18	9		5.48	15
ENSXMAG00000020066	0.0028	24	9	lrrn1	1.51	21
ENSXMAG00000020152	0.0031	9	12		1.87	6
ENSXMAG00000020156	0.0031	9	12	slc35g2b	1.49	6
ENSXMAG00000020165	0.0015	21	18		1.85	18
ENSXMAG00000020171	0.0025	18	9		2.31	15
ENSXMAG00000020178	0.0031	15	12	zbtb1	1.42	12
ENSXMAG00000020190	0.0024	6	9	amigo1	2.09	3
ENSXMAG00000020250	0.0024	18	9	fjx1	1.94	15
ENSXMAG00000020272	0.0025	18	9		2.98	15
ENSXMAG00000020293	0.0027	6	15	drd1b	3.56	3
ENSXMAG00000020295	0.0026	18	9	slc10a3	1.45	15
ENSXMAG00000020296	0.0031	6	12	zgc:77929	1.64	3
ENSXMAG00000020299	0.0031	6	12	tpst1	2.15	3
ENSXMAG00000020311	0.0031	15	12	hic2	1.98	12
ENSXMAG00000020314	0.0031	6	12		3.74	3
ENSXMAG00000020315	0.0027	15	9	CHST1	2.76	12
ENSXMAG00000020316	0.0031	6	12		11.39	3
ENSXMAG00000020332	0.0026	18	15	fam84b	4.96	15
ENSXMAG00000020345	0.0027	3	15	flrt2	3.03	0
ENSXMAG00000020348	0.0031	24	12		1.26	21
ENSXMAG00000020359	0.0029	15	12	gas1a	1.51	12
ENSXMAG00000020366	0.0025	9	9		19.94	6
ENSXMAG00000020370	0.0024	3	9	tmem229b	1.95	0
ENSXMAG00000020394	0.0025	6	15	npy8br	3.78	3
ENSXMAG00000020618	0.0025	18	9		3.14	15
ENSXMAG00000020852	0.0031	18	12	asrgl1	2.70	15
ENSXMAG00000020854	0.0026	3	15	si:ch211-258f14.2	5.07	0

ENSXMAG00000020855	0.0026	18	9		2.73	15
ENSXMAG00000020858	0.0016	24	18	cplx4c	3.14	21
ENSXMAG00000020864	0.0024	21	9		2.34	18
ENSXMAG00000020875	0.0026	18	15	cerk	1.84	15
ENSXMAG00000020876	0.0031	15	12	bcar1	1.52	12
ENSXMAG00000020877	0.0031	18	12	syngr3b	1.28	15
ENSXMAG00000020882	0.0024	21	9		1.34	18
ENSXMAG00000020888	0.0025	21	9		1.35	18
ENSXMAG00000020904	0.0026	18	9		2.82	15
ENSXMAG00000020912	0.0031	21	12		1.60	18
ENSXMAG00000020924	0.0031	21	12	cacng8b	1.54	18
ENSXMAG00000020929	0.0015	3	18	si:ch73-25f10.6	1.59	0
ENSXMAG00000020938	0.0031	12	12		1.33	9
ENSXMAG00000020945	0.0026	3	15		1.80	0
ENSXMAG00000020954	0.0025	24	15	parvg	1.52	21
ENSXMAG00000020964	0.0026	24	9		1.59	21
ENSXMAG00000020965	0.0025	18	9	PCDHGB4	16.64	15
ENSXMAG00000020991	0.0024	18	9	spsb3a	2.07	15
ENSXMAG00000021001	0.0031	6	12	lrrc75bb	10.05	3
ENSXMAG00000021008	0.0025	3	15	rgs8	2.90	0
ENSXMAG00000021011	0.0025	6	15		4.03	3
ENSXMAG00000021026	0.0025	18	15	FAM13C	9.13	15
ENSXMAG00000021029	0.0031	18	12	e2f3	1.95	15
ENSXMAG00000021035	0.0027	3	15	tp53i11b	3.18	0
ENSXMAG00000021037	0.0028	18	9	esrra	2.91	15
ENSXMAG00000021039	0.0026	21	9		1.43	18
ENSXMAG00000021051	0.0024	18	9	aqp11	1.63	15
ENSXMAG00000021068	0.0014	3	18	osbpl2b	1.89	0
ENSXMAG00000021080	0.0026	24	9		3.39	21
ENSXMAG00000021085	0.0031	12	12	ndufa4l2a	5.82	9
ENSXMAG00000021091	0.0025	21	9	scai	1.26	18
ENSXMAG00000021101	0.0029	15	12	NDE1 (1 of many)	2.20	12
ENSXMAG00000021108	0.0025	21	9		1.21	18
ENSXMAG00000021112	0.0028	18	9	crx	2.28	15
ENSXMAG00000021123	0.0031	21	12		3.09	18
ENSXMAG00000021130	0.0027	18	9		2.87	15
ENSXMAG00000021136	0.0029	21	12		1.36	18
ENSXMAG00000021137	0.0025	21	9		1.35	18
ENSXMAG00000021141	0.0030	12	12		1.80	9
ENSXMAG00000021146	0.0014	24	18		459.96	21
ENSXMAG00000021149	0.0026	21	9	cdc42ep3	1.31	18
ENSXMAG00000021171	0.0016	3	6		1.46	0
ENSXMAG00000021189	0.0031	21	12	sigmar1	1.38	18
ENSXMAG00000021192	0.0015	21	6	cyth1b	1.61	18
ENSXMAG00000021195	0.0024	21	9		1.26	18
ENSXMAG00000021203	0.0034	18	12	ZHX1 (1 of many)	1.94	15
ENSXMAG00000021215	0.0025	21	9	otulina	1.68	18
ENSXMAG00000021218	0.0025	24	9	vstm4b	2.48	21
ENSXMAG00000021228	0.0031	18	12	EHD4	1.37	15
ENSXMAG00000021229	0.0014	12	18		2.18	9
ENSXMAG00000021244	0.0025	6	15	cables1	1.91	3
ENSXMAG00000021245	0.0028	21	9	ppifb	1.29	18
ENSXMAG00000021252	0.0024	21	9		1.37	18
ENSXMAG00000021255	0.0026	12	15		1.38	9
ENSXMAG00000021284	0.0024	3	15	ddit4	1.52	0
ENSXMAG00000021289	0.0014	21	18	ppdpfa	2.36	18

ENSXMAG00000021299	0.0024	3	15	AATK (1 of many)	2.12	0
ENSXMAG00000021300	0.0014	6	18		1.60	3
ENSXMAG00000021301	0.0034	9	12	epb41a	1.67	6
ENSXMAG00000021303	0.0031	9	12	RAB37	1.67	6
ENSXMAG00000021315	0.0024	12	9		3.81	9
ENSXMAG00000021332	0.0026	21	9	hnrnph1	1.43	18
ENSXMAG00000021333	0.0030	9	12	abhd2a	6.90	6
ENSXMAG00000021353	0.0014	24	18	ppifa	1.85	21
ENSXMAG00000021359	0.0024	3	15	sned1	2.25	0
ENSXMAG00000021370	0.0025	6	15	GPT	5.26	3
ENSXMAG00000021384	0.0025	24	9	spen	1.49	21
ENSXMAG00000021390	0.0025	18	9		1.76	15
ENSXMAG00000021394	0.0026	18	9		3.61	15
ENSXMAG00000021396	0.0014	6	18	KCNAB1	1.41	3
ENSXMAG00000021413	0.0016	24	18	si:ch73-193i2.2	1.57	21
ENSXMAG00000021423	0.0031	3	12		1.49	0
ENSXMAG00000021448	0.0024	9	15	keap1b	3.55	6
ENSXMAG00000021449	0.0014	24	18	mtnr1ab	2.93	21
ENSXMAG00000021465	0.0031	18	12		2.52	15
ENSXMAG00000021468	0.0031	18	12	rhbdl1	1.67	15
ENSXMAG00000021480	0.0027	21	9	pex3	1.36	18
ENSXMAG00000021498	0.0026	6	15		1.71	3
ENSXMAG00000021505	0.0031	9	12	si:dkey-225f23.5	1.85	6
ENSXMAG00000021506	0.0025	15	9	si:dkey-234i14.6	5.14	12
ENSXMAG00000021509	0.0029	9	12	sult4a1	1.85	6
ENSXMAG00000021526	0.0024	9	9		2.09	6
ENSXMAG00000021529	0.0029	15	12	foxq2	10.89	12
ENSXMAG00000021530	0.0029	6	12	USP53	3.11	3
ENSXMAG00000021537	0.0016	18	18		2.89	15
ENSXMAG00000021550	0.0025	18	9		3.24	15
ENSXMAG00000021559	0.0031	15	12	PNPLA8 (1 of many)	2.41	12
ENSXMAG00000021577	0.0024	18	9	rpp25l	2.20	15
ENSXMAG00000021579	0.0014	6	18		3.21	3
ENSXMAG00000021584	0.0029	6	12	tmem241	2.55	3
ENSXMAG00000021595	0.0016	24	18	zbtb49	1.98	21
ENSXMAG00000021613	0.0016	9	6		1.63	6
ENSXMAG00000021623	0.0026	18	9	marcksb	3.03	15
ENSXMAG00000021624	0.0025	18	9		2.13	15
ENSXMAG00000021625	0.0026	6	15		2.34	3
ENSXMAG00000021637	0.0026	18	9		2.82	15
ENSXMAG00000021649	0.0026	21	9		1.50	18
ENSXMAG00000021662	0.0031	15	12		1.64	12
ENSXMAG00000021672	0.0028	24	9	si:dkey-175m17.7	2.01	21
ENSXMAG00000021684	0.0029	24	12	kdelc1	1.66	21
ENSXMAG00000021685	0.0015	24	6	ophn1	1.71	21
ENSXMAG00000021699	0.0015	21	6	PPP1CC	1.47	18
ENSXMAG00000021710	0.0028	18	9		4.67	15
ENSXMAG00000021734	0.0024	6	15	pclob	4.08	3
ENSXMAG00000021736	0.0031	18	12	papss1	1.32	15
ENSXMAG00000021756	0.0026	12	9	si:ch211-195b15.7	2.05	9
ENSXMAG00000021760	0.0026	24	9	synpo2la	1.95	21
ENSXMAG00000021763	0.0016	3	18		2.72	0
ENSXMAG00000021771	0.0025	24	9		1.58	21
ENSXMAG00000021779	0.0026	24	9		1.63	21
ENSXMAG00000021783	0.0031	18	12	rbms3	2.66	15
ENSXMAG00000021787	0.0034	9	12		1.95	6

ENSXMAG00000021801	0.0031	9	12	adcyap1b	2.07	6
ENSXMAG00000021830	0.0034	24	12	si:ch1073-322p19.1	1.74	21
ENSXMAG00000021841	0.0031	12	12		1.74	9
ENSXMAG00000021860	0.0029	21	12	ssbp4	1.95	18
ENSXMAG00000021862	0.0034	3	12	gnas	1.30	0
ENSXMAG00000021867	0.0025	18	9	zgc:109949	4.02	15
ENSXMAG00000021880	0.0030	12	12	ankrd10b	3.23	9
ENSXMAG00000021891	0.0031	21	12	nsmce2	1.65	18
ENSXMAG00000021894	0.0029	15	12		2.07	12
ENSXMAG00000021908	0.0031	24	12	tbx15	1.81	21
ENSXMAG00000021914	0.0031	9	12	trib2	1.58	6
ENSXMAG00000021915	0.0031	24	12	atp6v1h	1.15	21
ENSXMAG00000021918	0.0034	9	12		2.98	6
ENSXMAG00000021924	0.0026	6	15	mprip	2.79	3
ENSXMAG00000021932	0.0031	15	12		4.15	12
ENSXMAG00000021939	0.0025	21	9	COPS6	1.30	18
ENSXMAG00000021941	0.0016	21	18	icmt	1.75	18
ENSXMAG00000021943	0.0026	6	15	sspn	2.40	3
ENSXMAG00000021955	0.0027	21	9	aprt	1.53	18
ENSXMAG00000021983	0.0026	21	9	CDK2AP1	2.02	18
ENSXMAG00000021985	0.0014	24	18		1.65	21
ENSXMAG00000021989	0.0024	18	9	GJD2	3.93	15
ENSXMAG00000021999	0.0031	24	12	TOB2	5.46	21
ENSXMAG00000022010	0.0029	18	12	acbd5b	3.78	15
ENSXMAG00000022028	0.0026	21	9	tor2a	1.43	18
ENSXMAG00000022033	0.0031	21	12	stat4	3.21	18
ENSXMAG00000022041	0.0024	24	9		2.21	21
ENSXMAG00000022048	0.0031	21	12	got1	3.35	18
ENSXMAG00000022051	0.0031	3	12	slco4a1	3.11	0
ENSXMAG00000022054	0.0071	18	18		3.94	15
ENSXMAG00000022055	0.0031	9	12	nrn1lb	3.70	6
ENSXMAG00000022062	0.0025	9	9	paip2b	1.40	6
ENSXMAG00000022072	0.0027	6	15		1.69	3
ENSXMAG00000022074	0.0027	21	9	pfdn6	1.50	18
ENSXMAG00000022076	0.0027	21	9	sharpin	1.33	18
ENSXMAG00000022088	0.0031	3	12		3.08	0
ENSXMAG00000022090	0.0016	21	6		3.33	18
ENSXMAG00000022099	0.0015	3	18	sh3gl2a	4.02	0
ENSXMAG00000022102	0.0024	6	15	pgbd5	1.51	3
ENSXMAG00000022106	0.0025	18	9	si:ch211-204d2.4	1.37	15
ENSXMAG00000022122	0.0025	18	9	pacsin3	2.69	15
ENSXMAG00000022127	0.0031	12	12	guca1b	2.76	9
ENSXMAG00000022145	0.0026	21	15		1.46	18
ENSXMAG00000022151	0.0031	6	12	nrn1la	2.70	3
ENSXMAG00000022155	0.0028	18	9	tent5bb	2.22	15
ENSXMAG00000022156	0.0031	6	12	prr16	1.46	3
ENSXMAG00000022157	0.0025	21	9	psma2	1.44	18
ENSXMAG00000022186	0.0014	21	6	yipf3	1.75	18
ENSXMAG00000022190	0.0030	18	12		2.04	15
ENSXMAG00000022200	0.0015	24	18	dgat2	10.79	21
ENSXMAG00000022204	0.0016	21	6		1.47	18
ENSXMAG00000022209	0.0024	6	15	Xima-DXB	4.09	3
ENSXMAG00000022210	0.0025	9	9		2.38	6
ENSXMAG00000022215	0.0014	3	18	aqp1a.1	1.87	0
ENSXMAG00000022220	0.0024	6	15	hspb6	5.21	3
ENSXMAG00000022222	0.0025	21	9	crcp	1.62	18

ENSXMAG00000022235	0.0026	9	15		3.48	6
ENSXMAG00000022237	0.0030	21	12	lhx9	1.55	18
ENSXMAG00000022255	0.0027	3	15	lrrc38b	2.58	0
ENSXMAG00000022264	0.0024	3	15	C6orf106	2.57	0
ENSXMAG00000022265	0.0030	3	12		2.92	0
ENSXMAG00000022277	0.0026	18	9	nxt2	2.08	15
ENSXMAG00000022293	0.0024	18	9		4.94	15
ENSXMAG00000022295	0.0026	24	9	cbln1	1.71	21
ENSXMAG00000022301	0.0024	3	15		10.05	0
ENSXMAG00000022310	0.0025	21	9		4.16	18
ENSXMAG00000022313	0.0027	6	15		1.80	3
ENSXMAG00000022328	0.0030	18	12	RHOA	2.35	15
ENSXMAG00000022375	0.0026	3	9	rhoub	1.77	0
ENSXMAG00000022378	0.0025	3	15	zgc:154058	2.03	0
ENSXMAG00000022381	0.0025	18	9		2.55	15
ENSXMAG00000022382	0.0026	24	9	prox1b	2.00	21
ENSXMAG00000022387	0.0029	6	12	si:dkey-110g7.8	1.75	3
ENSXMAG00000022388	0.0024	3	15	mpped1	2.81	0
ENSXMAG00000022395	0.0026	6	15		6.59	3
ENSXMAG00000022399	0.0025	18	9	cacng5a	4.70	15
ENSXMAG00000022410	0.0014	21	18	arl6ip4	1.44	18
ENSXMAG00000022412	0.0025	21	9	hmx1	1.76	18
ENSXMAG00000022414	0.0024	6	15		9.08	3
ENSXMAG00000022415	0.0027	18	9	plin6	2.45	15
ENSXMAG00000022418	0.0027	24	15		1.25	21
ENSXMAG00000022428	0.0031	18	12		4.99	15
ENSXMAG00000022434	0.0026	24	15	zbtb41	1.95	21
ENSXMAG00000022438	0.0024	9	15		1.74	6
ENSXMAG00000022447	0.0030	15	12		2.40	12
ENSXMAG00000022450	0.0025	15	15	msx1a	1.51	12
ENSXMAG00000022451	0.0029	18	12	AP1S2 (1 of many)	1.97	15
ENSXMAG00000022464	0.0031	15	12	olfm1b	1.32	12
ENSXMAG00000022470	0.0025	9	9	oaz2a	3.19	6
ENSXMAG00000022482	0.0026	9	15	ponzr1 (1 of many)	1.56	6
ENSXMAG00000022504	0.0026	18	9		10.15	15
ENSXMAG00000022509	0.0025	9	9		18.30	6
ENSXMAG00000022521	0.0071	21	18	shisa3	1.20	18
ENSXMAG00000022524	0.0031	21	12		1.51	18
ENSXMAG00000022548	0.0026	15	15	wnt1	1.35	12
ENSXMAG00000022554	0.0027	21	9	rnaseh2b	1.47	18
ENSXMAG00000022559	0.0026	24	9	fuca2	1.80	21
ENSXMAG00000022561	0.0025	18	15		2.60	15
ENSXMAG00000022593	0.0016	9	18	si:ch211-231f6.6	2.35	6
ENSXMAG00000022604	0.0015	21	6		1.26	18
ENSXMAG00000022625	0.0015	24	6	PHF21B	1.65	21
ENSXMAG00000022628	0.0025	24	9	dffa	1.52	21
ENSXMAG00000022630	0.0024	21	9	smarcd3b	1.49	18
ENSXMAG00000022638	0.0031	3	12	rtn2a	2.99	0
ENSXMAG00000022643	0.0031	15	12	ankrd50l	1.97	12
ENSXMAG00000022647	0.0031	9	12	ahcyl1	1.65	6
ENSXMAG00000022652	0.0031	6	12	RNF208	3.49	3
ENSXMAG00000022653	0.0031	15	12		1.43	12
ENSXMAG00000022657	0.0024	6	15		1.43	3
ENSXMAG00000022678	0.0026	24	9	ntpcr	1.47	21
ENSXMAG00000022692	0.0024	6	15	hpca	2.96	3
ENSXMAG00000022693	0.0031	3	12		1.55	0

ENSXMAG00000022722	0.0034	18	12	FABP7	2.72	15
ENSXMAG00000022723	0.0031	15	12		2.83	12
ENSXMAG00000022753	0.0031	24	12	tmem151ba	2.66	21
ENSXMAG00000022758	0.0025	3	9	slc6a3	1.58	0
ENSXMAG00000022763	0.0014	21	6	pcdh2aa15 (1 of many)	2.00	18
ENSXMAG00000022774	0.0016	21	6	slc39a9	1.22	18
ENSXMAG00000022778	0.0027	18	9	sypl2a	1.35	15
ENSXMAG00000022785	0.0031	6	12	ywhag2	1.90	3
ENSXMAG00000022796	0.0014	21	18		8.40	18
ENSXMAG00000022799	0.0031	15	12		1.66	12
ENSXMAG00000022811	0.0034	3	12	ciarta	37.96	0
ENSXMAG00000022822	0.0026	24	9		1.79	21
ENSXMAG00000022830	0.0031	18	12	dohh	1.95	15
ENSXMAG00000022831	0.0014	21	6	tspan18a	2.36	18
ENSXMAG00000022841	0.0025	15	15	FOXN2	1.66	12
ENSXMAG00000022849	0.0034	21	12		1.21	18
ENSXMAG00000022860	0.0030	21	12		1.79	18
ENSXMAG00000022862	0.0030	21	12	abhd18	1.23	18
ENSXMAG00000022869	0.0024	3	15	si:ch73-62l21.1	1.70	0
ENSXMAG00000022875	0.0024	18	9	r3hdm4	2.38	15
ENSXMAG00000022889	0.0025	3	15		1.46	0
ENSXMAG00000022890	0.0027	24	15	gabarapb	1.29	21
ENSXMAG00000022891	0.0025	21	9		1.55	18
ENSXMAG00000022899	0.0024	24	9	slc6a16b	2.74	21
ENSXMAG00000022904	0.0029	6	12		7.01	3
ENSXMAG00000022919	0.0031	21	12	si:dkey-174m14.3	3.27	18
ENSXMAG00000022940	0.0025	15	15		1.93	12
ENSXMAG00000022942	0.0029	6	12	tacc1	1.92	3
ENSXMAG00000022945	0.0031	18	12		1.49	15
ENSXMAG00000022947	0.0026	24	9		1.92	21
ENSXMAG00000022961	0.0027	18	9		5.90	15
ENSXMAG00000022962	0.0029	15	12	si:dkeyp-113d7.1	1.78	12
ENSXMAG00000022965	0.0026	18	9	adgra1a	2.07	15
ENSXMAG00000022980	0.0026	24	9		2.30	21
ENSXMAG00000022984	0.0034	9	12		4.13	6
ENSXMAG00000022988	0.0014	24	18	ripk2	1.44	21
ENSXMAG00000023004	0.0014	24	18	si:dkeyp-69b9.6	1.54	21
ENSXMAG00000023022	0.0031	21	12		2.05	18
ENSXMAG00000023031	0.0026	21	9	bpgm	1.70	18
ENSXMAG00000023032	0.0026	18	9	rasl11a	2.51	15
ENSXMAG00000023035	0.0027	21	9	tusc3	1.20	18
ENSXMAG00000023037	0.0027	21	9	cdc42ep1b	3.57	18
ENSXMAG00000023038	0.0031	9	12		1.36	6
ENSXMAG00000023045	0.0016	6	6		1.47	3
ENSXMAG00000023048	0.0026	24	9	LEPROT	1.22	21
ENSXMAG00000023054	0.0024	6	15	wdr95	2.72	3
ENSXMAG00000023056	0.0025	18	9	PPP2R2C (1 of many)	5.64	15
ENSXMAG00000023066	0.0025	6	15		8.55	3
ENSXMAG00000023076	0.0031	18	12	fam167b	3.44	15
ENSXMAG00000023087	0.0026	12	15		1.71	9
ENSXMAG00000023108	0.0027	18	9	ccdc85al	1.69	15
ENSXMAG00000023114	0.0027	3	15		1.96	0
ENSXMAG00000023116	0.0015	21	6		1.42	18
ENSXMAG00000023118	0.0028	6	15		1.38	3
ENSXMAG00000023119	0.0027	9	15		2.09	6
ENSXMAG00000023124	0.0030	15	12	esrrd	32.95	12

ENSXMAG00000023151	0.0014	24	18	dusp10	1.52	21
ENSXMAG00000023156	0.0031	9	12	kbtbd11	2.20	6
ENSXMAG00000023165	0.0026	18	9	rnf166	1.93	15
ENSXMAG00000023172	0.0025	18	9	gabrr3a	8.75	15
ENSXMAG00000023173	0.0029	9	12		4.69	6
ENSXMAG00000023186	0.0015	21	6		2.77	18
ENSXMAG00000023187	0.0025	12	9	dnajc22	2.50	9
ENSXMAG00000023189	0.0031	24	12	zgc:113274	2.77	21
ENSXMAG00000023201	0.0026	9	9	mtus2a	1.48	6
ENSXMAG00000023202	0.0030	21	12	gdf10a	1.62	18
ENSXMAG00000023205	0.0024	3	15	tent4b	2.88	0
ENSXMAG00000023220	0.0029	15	12		5.10	12
ENSXMAG00000023230	0.0027	24	9	tshz1	1.86	21
ENSXMAG00000023234	0.0024	18	15		4.06	15
ENSXMAG00000023244	0.0031	6	12	cers3b	1.70	3
ENSXMAG00000023246	0.0016	9	18	cyb561d1	1.89	6
ENSXMAG00000023279	0.0025	9	9		5.66	6
ENSXMAG00000023284	0.0014	21	6	efna2a	1.27	18
ENSXMAG00000023302	0.0031	6	12	ctdsp2	2.61	3
ENSXMAG00000023303	0.0027	18	9	taf5l	1.52	15
ENSXMAG00000023311	0.0016	18	18		37.78	15
ENSXMAG00000023354	0.0071	3	18	zfand5a	2.48	0
ENSXMAG00000023355	0.0024	18	15	KLF7	1.60	15
ENSXMAG00000023365	0.0071	3	6	ptpn11a	1.18	0
ENSXMAG00000023388	0.0028	18	9	znrf1	5.39	15
ENSXMAG00000023396	0.0024	18	9		5.06	15
ENSXMAG00000023399	0.0026	6	15		1.98	3
ENSXMAG00000023403	0.0026	18	15	ppap2d	1.98	15
ENSXMAG00000023407	0.0031	15	12	trim16	2.17	12
ENSXMAG00000023411	0.0028	15	15	tbx21	1.31	12
ENSXMAG00000023420	0.0026	18	15	cbfa2t3	1.95	15
ENSXMAG00000023425	0.0014	12	18	cox5aa	1.33	9
ENSXMAG00000023435	0.0025	12	9	sema7a	4.70	9
ENSXMAG00000023442	0.0031	24	12	AR	1.87	21
ENSXMAG00000023449	0.0030	21	12	loxa	1.54	18
ENSXMAG00000023459	0.0014	24	6		1.45	21
ENSXMAG00000023482	0.0028	24	15		3.38	21
ENSXMAG00000023489	0.0025	6	15	pde6ha	4.11	3
ENSXMAG00000023496	0.0026	21	15	her6	1.80	18
ENSXMAG00000023517	0.0031	18	12	ubl3b	1.51	15
ENSXMAG00000023525	0.0015	21	6	ino80b	1.50	18
ENSXMAG00000023532	0.0025	21	9	rabif	1.39	18
ENSXMAG00000023539	0.0031	18	12	cbx5	1.45	15
ENSXMAG00000023546	0.0014	15	6	si:dkey-85k7.12 (1 of many)	1.99	12
ENSXMAG00000023558	0.0016	9	18	SUOX	2.14	6
ENSXMAG00000023563	0.0024	21	15		4.77	18
ENSXMAG00000023590	0.0015	21	6	barhl2	1.71	18
ENSXMAG00000023595	0.0025	18	9	mllt11	1.88	15
ENSXMAG00000023606	0.0026	24	9		3.61	21
ENSXMAG00000023607	0.0016	15	6	dusp7	1.70	12
ENSXMAG00000023621	0.0029	6	12		35.56	3
ENSXMAG00000023623	0.0031	21	12	tpd52	1.57	18
ENSXMAG00000023636	0.0027	18	9		1.47	15
ENSXMAG00000023639	0.0025	3	15	tmem198a	2.98	0
ENSXMAG00000023643	0.0031	18	12	gal3st4	2.12	15
ENSXMAG00000023651	0.0024	18	9	cnih3	1.67	15

ENSXMAG00000023656	0.0014	24	18		1.43	21
ENSXMAG00000023660	0.0029	15	12	cdv3	1.25	12
ENSXMAG00000023666	0.0031	24	12		1.64	21
ENSXMAG00000023705	0.0014	24	6	efl1	1.31	21
ENSXMAG00000023706	0.0030	18	12	PFN2	1.35	15
ENSXMAG00000023716	0.0031	9	12	sybu	22.11	6
ENSXMAG00000023736	0.0015	24	18	fam50a	1.38	21
ENSXMAG00000023750	0.0025	21	15	cbx4	1.33	18
ENSXMAG00000023760	0.0025	24	9		1.53	21
ENSXMAG00000023765	0.0024	18	15		1.45	15
ENSXMAG00000023769	0.0025	9	15		4.21	6
ENSXMAG00000023780	0.0016	21	6		1.72	18
ENSXMAG00000023781	0.0029	6	12	ugt1a2 (1 of many)	3.21	3
ENSXMAG00000023782	0.0027	18	9	pcdh2ac	2.62	15
ENSXMAG00000023792	0.0024	21	9		2.19	18
ENSXMAG00000023810	0.0031	21	12	znf618	1.56	18
ENSXMAG00000023825	0.0025	6	15		4.37	3
ENSXMAG00000023845	0.0030	9	12		2.81	6
ENSXMAG00000023847	0.0024	3	15	ARPP19 (1 of many)	1.96	0
ENSXMAG00000023853	0.0031	15	12	map1ab	4.26	12
ENSXMAG00000023873	0.0031	3	12	RF00271	7.36	0
ENSXMAG00000023877	0.0025	9	15	stim1a	2.95	6
ENSXMAG00000023878	0.0028	18	9	ankrd33bb	3.28	15
ENSXMAG00000023881	0.0026	24	9	rp9	1.35	21
ENSXMAG00000023882	0.0026	3	15		3.21	0
ENSXMAG00000023905	0.0031	21	12	arrdc1b	1.68	18
ENSXMAG00000023906	0.0025	24	15		1.65	21
ENSXMAG00000023912	0.0025	18	9	acp6	2.27	15
ENSXMAG00000023917	0.0016	21	6	ZBTB14	1.37	18
ENSXMAG00000023919	0.0026	18	9		2.14	15
ENSXMAG00000023926	0.0025	18	9	ndufa4l2b	2.03	15
ENSXMAG00000023938	0.0026	21	9	mmgt1	1.24	18
ENSXMAG00000023939	0.0026	24	9		1.26	21
ENSXMAG00000023953	0.0025	18	9		1.46	15
ENSXMAG00000023958	0.0031	15	12	gngt2b	1.95	12
ENSXMAG00000023979	0.0034	21	12		1.29	18
ENSXMAG00000023988	0.0026	3	15	atcaya	1.66	0
ENSXMAG00000023994	0.0025	3	15	si:ch1073-469d17.2	6.47	0
ENSXMAG00000024003	0.0026	15	15	jam2b	7.93	12
ENSXMAG00000024011	0.0025	21	9	txnl4a	1.45	18
ENSXMAG00000024025	0.0025	3	15	syne3	1.35	0
ENSXMAG00000024036	0.0014	24	6	si:dkey-69o16.5	1.52	21
ENSXMAG00000024037	0.0026	3	15	zgc:110006	2.61	0
ENSXMAG00000024051	0.0031	18	12	si:ch73-345f18.3	3.48	15
ENSXMAG00000024063	0.0027	18	15	fam83fa	2.35	15
ENSXMAG00000024073	0.0031	3	12		2.79	0
ENSXMAG00000024097	0.0029	18	12		68.06	15
ENSXMAG00000024110	0.0031	24	12	foxl1	2.17	21
ENSXMAG00000024113	0.0027	6	15	cyp2n13 (1 of many)	15.43	3
ENSXMAG00000024123	0.0024	3	15	trim62	3.06	0
ENSXMAG00000024140	0.0028	18	9		1.65	15
ENSXMAG00000024158	0.0031	6	12	si:ch211-232m10.6	1.62	3
ENSXMAG00000024169	0.0031	9	12		1.58	6
ENSXMAG00000024170	0.0015	21	18	zgc:65873	1.34	18
ENSXMAG00000024179	0.0014	9	18		12.07	6
ENSXMAG00000024190	0.0025	9	15	hsd11b1la (1 of many)	3.44	6

ENSXMAG00000024208	0.0024	21	9	gng3	1.40	18
ENSXMAG00000024211	0.0029	3	12	MXRA7	8.57	0
ENSXMAG00000024219	0.0024	18	9	sec23ip	1.75	15
ENSXMAG00000024222	0.0027	6	15	zfp36l2	2.00	3
ENSXMAG00000024224	0.0026	21	9	RMDN1	1.28	18
ENSXMAG00000024227	0.0026	3	15	ncalda	10.38	0
ENSXMAG00000024235	0.0015	24	6	gse1	1.48	21
ENSXMAG00000024236	0.0016	24	18		1.34	21
ENSXMAG00000024240	0.0026	24	9	si:ch211-137a8.4	2.22	21
ENSXMAG00000024243	0.0025	24	15	ccdc93	1.15	21
ENSXMAG00000024248	0.0026	6	15	akap12b	1.52	3
ENSXMAG00000024253	0.0024	3	15	mdm4	1.23	0
ENSXMAG00000024258	0.0025	3	15		1.57	0
ENSXMAG00000024267	0.0025	21	9	rab2a	1.21	18
ENSXMAG00000024273	0.0026	3	9	zgc:162331	6.12	0
ENSXMAG00000024275	0.0026	3	15		3.06	0
ENSXMAG00000024287	0.0025	24	15	spina	1.45	21
ENSXMAG00000024290	0.0014	24	18		5.64	21
ENSXMAG00000024292	0.0025	3	15		7.19	0
ENSXMAG00000024300	0.0026	24	15	ankrd33aa	3.24	21
ENSXMAG00000024319	0.0025	3	15		1.42	0
ENSXMAG00000024321	0.0029	18	12		1.85	15
ENSXMAG00000024326	0.0027	18	9	mab21l1	2.27	15
ENSXMAG00000024327	0.0025	3	15	spock3	2.68	0
ENSXMAG00000024328	0.0016	24	18	cbx7a	1.42	21
ENSXMAG00000024329	0.0026	18	9		3.29	15
ENSXMAG00000024331	0.0031	9	12		7.45	6
ENSXMAG00000024333	0.0031	9	12		5.88	6
ENSXMAG00000024342	0.0034	18	12	nrap	2.03	15
ENSXMAG00000024347	0.0030	15	12	tprg1	1.54	12
ENSXMAG00000024357	0.0014	21	6		2.81	18
ENSXMAG00000024399	0.0024	18	9		2.73	15
ENSXMAG00000024435	0.0014	21	6		3.93	18
ENSXMAG00000024455	0.0029	12	12	steap4 (1 of many)	1.65	9
ENSXMAG00000024458	0.0026	21	9	ptrhd1	1.70	18
ENSXMAG00000024463	0.0031	3	12	rsrc2	1.22	0
ENSXMAG00000024465	0.0031	21	12	si:ch211-169p10.1	2.13	18
ENSXMAG00000024476	0.0015	24	18	fam204a	1.44	21
ENSXMAG00000024478	0.0028	24	9	tesca	1.76	21
ENSXMAG00000024484	0.0031	18	12		9.62	15
ENSXMAG00000024487	0.0025	15	15	cnrip1b	3.78	12
ENSXMAG00000024537	0.0034	21	12	nicn1	1.45	18
ENSXMAG00000024539	0.0034	18	12	cxcl12b	1.52	15
ENSXMAG00000024540	0.0031	6	12	bnip3lb	1.39	3
ENSXMAG00000024549	0.0027	21	9	prune	1.62	18
ENSXMAG00000024578	0.0031	15	12		1.64	12
ENSXMAG00000024580	0.0025	24	15		6.29	21
ENSXMAG00000024583	0.0029	6	12	ky	6.49	3
ENSXMAG00000024586	0.0029	18	12	C8orf82	2.01	15
ENSXMAG00000024588	0.0024	3	15	ETHE1 (1 of many)	5.33	0
ENSXMAG00000024590	0.0024	9	15	numb	1.43	6
ENSXMAG00000024591	0.0026	21	9		1.30	18
ENSXMAG00000024597	0.0031	12	12	elk1	1.20	9
ENSXMAG00000024612	0.0031	18	12		2.16	15
ENSXMAG00000024616	0.0031	21	12	mcf2l2	2.48	18
ENSXMAG00000024630	0.0024	24	9	dpy19l3	1.55	21

ENSXMAG00000024636	0.0025	6	15	map1sb	1.51	3
ENSXMAG00000024644	0.0026	18	9		6.61	15
ENSXMAG00000024650	0.0026	18	15	pygo2	1.30	15
ENSXMAG00000024652	0.0026	18	9	pdk3a	1.23	15
ENSXMAG00000024654	0.0014	3	18	fam214a	2.48	0
ENSXMAG00000024658	0.0031	15	12		2.15	12
ENSXMAG00000024669	0.0025	3	15	CNMD	1.57	0
ENSXMAG00000024672	0.0027	12	15	EIF4E3 (1 of many)	2.72	9
ENSXMAG00000024676	0.0015	9	18	fuz	1.88	6
ENSXMAG00000024678	0.0027	24	15	scfd2	2.48	21
ENSXMAG00000024679	0.0025	24	9	PPIC	1.53	21
ENSXMAG00000024687	0.0031	21	12	usb1	1.68	18
ENSXMAG00000024692	0.0014	24	18		1.91	21
ENSXMAG00000024700	0.0024	21	9	ccnh	1.44	18
ENSXMAG00000024712	0.0025	18	9	MID1 (1 of many)	4.31	15
ENSXMAG00000024720	0.0027	3	15	KCNJ10	8.02	0
ENSXMAG00000024734	0.0029	18	12	igfbp2a	2.30	15
ENSXMAG00000024741	0.0029	18	12		2.98	15
ENSXMAG00000024751	0.0031	21	12		13.14	18
ENSXMAG00000024762	0.0025	18	9		2.42	15
ENSXMAG00000024776	0.0031	9	12	si:dkey-205h13.1	2.16	6
ENSXMAG00000024783	0.0026	24	15		58.78	21
ENSXMAG00000024805	0.0031	24	12		1.76	21
ENSXMAG00000024816	0.0025	18	9		1.71	15
ENSXMAG00000024819	0.0029	18	12	lasp1	1.39	15
ENSXMAG00000024840	0.0025	9	9		1.39	6
ENSXMAG00000024853	0.0016	24	18	tmem255a	1.43	21
ENSXMAG00000024854	0.0025	6	15		7.86	3
ENSXMAG00000024855	0.0031	21	12	abhd17b	1.70	18
ENSXMAG00000024858	0.0024	21	9	PRKAR2B	1.45	18
ENSXMAG00000024866	0.0025	18	9		6.79	15
ENSXMAG00000024876	0.0031	18	12	ube2v1	1.35	15
ENSXMAG00000024898	0.0031	18	12	tmx1	1.34	15
ENSXMAG00000024906	0.0014	3	18	faima	1.95	0
ENSXMAG00000024910	0.0031	21	12		1.57	18
ENSXMAG00000024911	0.0027	21	9	samd10a	2.32	18
ENSXMAG00000024919	0.0024	18	15	cotl1	1.67	15
ENSXMAG00000024920	0.0014	3	18	rab11fip5a	4.16	0
ENSXMAG00000024924	0.0024	3	15	hivep1	6.61	0
ENSXMAG00000024925	0.0026	3	15		3.00	0
ENSXMAG00000024934	0.0031	24	12	ppp1r15b	1.24	21
ENSXMAG00000024951	0.0031	24	12	opcml	1.65	21
ENSXMAG00000024969	0.0025	21	15	tmem136a	1.52	18
ENSXMAG00000024975	0.0029	15	12		73.11	12
ENSXMAG00000025000	0.0025	6	15	ppp1r1b	3.66	3
ENSXMAG00000025006	0.0027	21	9	nap1l1	1.23	18
ENSXMAG00000025011	0.0029	9	12	slc48a1a	2.16	6
ENSXMAG00000025017	0.0024	15	9	dnajc15	2.97	12
ENSXMAG00000025031	0.0026	24	9	ldb2a	1.61	21
ENSXMAG00000025038	0.0024	21	15	reep6	1.55	18
ENSXMAG00000025050	0.0026	18	9	mfsd5	1.74	15
ENSXMAG00000025052	0.0015	21	6		1.27	18
ENSXMAG00000025054	0.0025	3	15	ARHGEF17	2.27	0
ENSXMAG00000025056	0.0024	24	9	arhgdig	2.09	21
ENSXMAG00000025058	0.0034	21	12		2.75	18
ENSXMAG00000025064	0.0031	24	12		1.40	21

ENSXMAG00000025066	0.0025	9	9	arr3b	12.80	6
ENSXMAG00000025069	0.0028	3	15		1.60	0
ENSXMAG00000025073	0.0029	6	12	sh3d19	1.42	3
ENSXMAG00000025108	0.0024	18	9		2.23	15
ENSXMAG00000025110	0.0025	3	15	zfyve21	1.76	0
ENSXMAG00000025113	0.0026	24	15	batf	2.13	21
ENSXMAG00000025138	0.0024	18	15	lrrc75ba	3.02	15
ENSXMAG00000025145	0.0026	3	15		135.15	0
ENSXMAG00000025155	0.0014	21	6	retreg3	1.35	18
ENSXMAG00000025156	0.0031	6	12		1.78	3
ENSXMAG00000025157	0.0029	9	12	vamp1	2.51	6
ENSXMAG00000025175	0.0024	6	15		5.16	3
ENSXMAG00000025194	0.0025	18	9		25.82	15
ENSXMAG00000025198	0.0029	24	12	pibf1	1.45	21
ENSXMAG00000025204	0.0031	21	12		1.32	18
ENSXMAG00000025207	0.0015	21	6	retreg1	2.30	18
ENSXMAG00000025218	0.0031	6	12	tmem56b	2.03	3
ENSXMAG00000025219	0.0014	3	18	JDP2 (1 of many)	3.14	0
ENSXMAG00000025221	0.0029	6	12	gbp	3.15	3
ENSXMAG00000025235	0.0026	21	9	rfxank	1.70	18
ENSXMAG00000025240	0.0024	18	9		3.66	15
ENSXMAG00000025257	0.0026	21	9	sec22bb	1.44	18
ENSXMAG00000025259	0.0029	15	12	tgif1	8.84	12
ENSXMAG00000025264	0.0031	6	12	TRAF2	1.50	3
ENSXMAG00000025265	0.0025	18	9	GABRR1	9.94	15
ENSXMAG00000025269	0.0027	24	9	ttc33	1.34	21
ENSXMAG00000025272	0.0026	18	9		3.49	15
ENSXMAG00000025280	0.0016	3	18	HLF (1 of many)	2.55	0
ENSXMAG00000025283	0.0031	18	12	dnlz	1.75	15
ENSXMAG00000025294	0.0025	24	9	foxo6b	2.01	21
ENSXMAG00000025301	0.0025	15	9		2.26	12
ENSXMAG00000025306	0.0025	6	15		2.19	3
ENSXMAG00000025318	0.0031	3	12	trmt12	1.37	0
ENSXMAG00000025320	0.0025	24	15	RAMP3	1.52	21
ENSXMAG00000025324	0.0030	21	12	cldn12	1.40	18
ENSXMAG00000025333	0.0024	6	15		2.12	3
ENSXMAG00000025352	0.0026	18	9	gng13b	2.12	15
ENSXMAG00000025368	0.0025	9	15	pdcd6	1.43	6
ENSXMAG00000025372	0.0014	21	6		1.42	18
ENSXMAG00000025390	0.0031	21	12		1.93	18
ENSXMAG00000025394	0.0026	6	15		2.91	3
ENSXMAG00000025404	0.0025	12	9	gstr (1 of many)	1.81	9
ENSXMAG00000025409	0.0034	18	12	slc25a1a	2.27	15
ENSXMAG00000025417	0.0026	3	15	grk7a	75.13	0
ENSXMAG00000025419	0.0015	24	6		1.68	21
ENSXMAG00000025427	0.0026	24	9	cdh24b	1.70	21
ENSXMAG00000025434	0.0025	6	15	nrip2	1.79	3
ENSXMAG00000025441	0.0024	24	9	hpgd	1.99	21
ENSXMAG00000025444	0.0025	9	15		1.72	6
ENSXMAG00000025446	0.0027	6	15	fryl	1.79	3
ENSXMAG00000025460	0.0024	3	15		1.86	0
ENSXMAG00000025467	0.0026	9	15	slc48a1b	3.81	6
ENSXMAG00000025483	0.0031	9	12	ccnl1a	1.48	6
ENSXMAG00000025488	0.0028	21	9	znf511	1.28	18
ENSXMAG00000025500	0.0014	21	6		2.03	18
ENSXMAG00000025502	0.0024	12	15	szrd1	1.29	9

ENSXMAG00000025505	0.0014	15	18		1.23	12
ENSXMAG00000025507	0.0026	6	15	BTBD11 (1 of many)	1.55	3
ENSXMAG00000025511	0.0015	3	18		1.69	0
ENSXMAG00000025515	0.0015	24	18	bhlhe40	47.07	21
ENSXMAG00000025517	0.0031	24	12		2.62	21
ENSXMAG00000025521	0.0025	18	9	six6a	1.89	15
ENSXMAG00000025536	0.0031	12	12	plekho1a	3.14	9
ENSXMAG00000025555	0.0024	12	15		1.53	9
ENSXMAG00000025576	0.0026	6	15	cntfr	1.99	3
ENSXMAG00000025586	0.0031	21	12		1.34	18
ENSXMAG00000025612	0.0016	24	6	chmp1a	1.37	21
ENSXMAG00000025613	0.0014	3	6		1.49	0
ENSXMAG00000025616	0.0014	3	18		1.36	0
ENSXMAG00000025625	0.0024	24	9	edil3a	1.70	21
ENSXMAG00000025626	0.0025	18	9		3.30	15
ENSXMAG00000025627	0.0031	15	12		1.75	12
ENSXMAG00000025631	0.0025	6	9	oprd1a	1.38	3
ENSXMAG00000025635	0.0029	18	12		1.26	15
ENSXMAG00000025637	0.0014	18	6	zgc:66427	1.92	15
ENSXMAG00000025638	0.0031	18	12	zgc:153115	2.01	15
ENSXMAG00000025640	0.0025	18	9	kctd6b	1.44	15
ENSXMAG00000025647	0.0026	21	9		1.41	18
ENSXMAG00000025648	0.0025	21	9	six2a	2.59	18
ENSXMAG00000025671	0.0025	24	9		1.65	21
ENSXMAG00000025680	0.0025	3	9		1.18	0
ENSXMAG00000025683	0.0034	6	12	elavl4	1.75	3
ENSXMAG00000025690	0.0014	18	6		1.83	15
ENSXMAG00000025698	0.0026	21	9	yipf2	1.49	18
ENSXMAG00000025703	0.0031	9	12		56.46	6
ENSXMAG00000025726	0.0014	21	6	PUDP	1.44	18
ENSXMAG00000025730	0.0025	3	15	sntb2	1.41	0
ENSXMAG00000025736	0.0025	6	15	peak1	1.63	3
ENSXMAG00000025757	0.0015	24	18	prnpb	1.50	21
ENSXMAG00000025761	0.0026	24	9	cep72	1.67	21
ENSXMAG00000025782	0.0025	24	9	ntng1a	1.48	21
ENSXMAG00000025790	0.0027	18	9	kras	1.46	15
ENSXMAG00000025801	0.0031	6	12	st8sia5	1.26	3
ENSXMAG00000025804	0.0024	18	9	cdc42	1.79	15
ENSXMAG00000025812	0.0034	6	12	efna3a	1.84	3
ENSXMAG00000025831	0.0025	18	15	fkbp10b	1.41	15
ENSXMAG00000025837	0.0027	3	9	grid2	1.76	0
ENSXMAG00000025848	0.0034	21	12	rab30	1.29	18
ENSXMAG00000025857	0.0024	12	9		3.67	9
ENSXMAG00000025863	0.0026	18	9	zgc:172067	4.91	15
ENSXMAG00000025865	0.0031	15	12		2.89	12
ENSXMAG00000025867	0.0025	18	9	qtrt1	1.98	15
ENSXMAG00000025868	0.0025	18	9	syt14a	2.61	15
ENSXMAG00000025869	0.0030	15	12	prrt4	6.00	12
ENSXMAG00000025871	0.0025	6	15		3.04	3
ENSXMAG00000025879	0.0026	3	15		7.44	0
ENSXMAG00000025895	0.0027	9	9	neto2b	2.21	6
ENSXMAG00000025903	0.0025	3	15		2.15	0
ENSXMAG00000025909	0.0025	18	9		3.60	15
ENSXMAG00000025910	0.0029	15	12	ETHE1 (1 of many)	2.70	12
ENSXMAG00000025916	0.0025	24	9		1.73	21
ENSXMAG00000025920	0.0031	21	12	si:ch73-379j16.2 (1 of many)	2.32	18

ENSXMAG00000025931	0.0014	3	18	tpd52l1	7.22	0
ENSXMAG00000025944	0.0029	18	12		2.06	15
ENSXMAG00000025950	0.0025	21	9	psmb4	1.42	18
ENSXMAG00000025973	0.0025	18	15		1.83	15
ENSXMAG00000025981	0.0031	21	12		1.67	18
ENSXMAG00000026007	0.0024	18	9	calr3b	1.40	15
ENSXMAG00000026041	0.0031	15	12	si:dkey-175g6.2	3.15	12
ENSXMAG00000026043	0.0029	12	12		4.63	9
ENSXMAG00000026052	0.0016	24	6	thumpd1	1.42	21
ENSXMAG00000026056	0.0025	6	15	gad1a	9.93	3
ENSXMAG00000026062	0.0025	24	9	fgf16	2.13	21
ENSXMAG00000026071	0.0027	6	15		1.91	3
ENSXMAG00000026085	0.0025	15	15	erp44	1.31	12
ENSXMAG00000026090	0.0025	9	15	podxl	1.52	6
ENSXMAG00000026103	0.0031	6	12		2.85	3
ENSXMAG00000026116	0.0034	6	12	acot19	2.15	3
ENSXMAG00000026117	0.0034	18	12	bzw1a	1.37	15
ENSXMAG00000026118	0.0027	3	15	atf3	4.90	0
ENSXMAG00000026119	0.0027	3	15		1.78	0
ENSXMAG00000026127	0.0025	24	9		1.86	21
ENSXMAG00000026129	0.0031	6	12	mknk2b	3.44	3
ENSXMAG00000026130	0.0030	21	12	zc4h2	1.33	18
ENSXMAG00000026131	0.0025	15	9	si:dkey-40c11.2	3.95	12
ENSXMAG00000026139	0.0027	24	9	ap5s1	2.38	21
ENSXMAG00000026143	0.0026	21	9	prpf38a	1.44	18
ENSXMAG00000026154	0.0025	21	9	fam8a1b	1.33	18
ENSXMAG00000026157	0.0031	3	12	cycsb	1.40	0
ENSXMAG00000026168	0.0025	9	15		1.34	6
ENSXMAG00000026180	0.0024	3	15	atf7b	2.42	0
ENSXMAG00000026185	0.0028	21	9	tmem42a	1.40	18
ENSXMAG00000026191	0.0027	21	9		2.61	18
ENSXMAG00000026204	0.0025	18	15		2.30	15
ENSXMAG00000026206	0.0031	6	12	tns2a	2.40	3
ENSXMAG00000026207	0.0031	6	12	rassf3	2.14	3
ENSXMAG00000026216	0.0024	21	9	sulf2a	1.51	18
ENSXMAG00000026233	0.0029	3	12		15.80	0
ENSXMAG00000026242	0.0029	9	12		1.91	6
ENSXMAG00000026246	0.0029	6	12	lin7a	1.94	3
ENSXMAG00000026261	0.0024	18	9		2.16	15
ENSXMAG00000026292	0.0029	3	12	prune2	1.26	0
ENSXMAG00000026307	0.0014	21	6		13.36	18
ENSXMAG00000026320	0.0015	15	6		2.22	12
ENSXMAG00000026327	0.0024	21	9	tmem104	1.31	18
ENSXMAG00000026328	0.0025	6	15	map6a	3.45	3
ENSXMAG00000026332	0.0025	18	9	ubtd2	2.19	15
ENSXMAG00000026333	0.0024	3	15	tefb	14.08	0
ENSXMAG00000026337	0.0014	21	6	psmb6	1.31	18
ENSXMAG00000026338	0.0016	24	6		1.50	21
ENSXMAG00000026340	0.0025	18	9		2.02	15
ENSXMAG00000026347	0.0031	9	12	si:dkey-283b1.7	22.74	6
ENSXMAG00000026349	0.0031	18	12		2.59	15
ENSXMAG00000026357	0.0030	18	12		3.54	15
ENSXMAG00000026365	0.0024	18	9		5.83	15
ENSXMAG00000026379	0.0026	21	9	chst12a	2.10	18
ENSXMAG00000026381	0.0024	24	15		1.75	21
ENSXMAG00000026393	0.0030	21	12	crb3a	2.21	18

ENSXMAG00000026416	0.0031	18	12	srsf5b	1.47	15
ENSXMAG00000026427	0.0034	21	12	zgc:175214	2.60	18
ENSXMAG00000026437	0.0025	3	15	zgc:162144	32.73	0
ENSXMAG00000026438	0.0029	24	12		1.26	21
ENSXMAG00000026442	0.0027	21	9	gnai2b	1.60	18
ENSXMAG00000026450	0.0031	18	12		5.95	15
ENSXMAG00000026459	0.0025	24	9		1.55	21
ENSXMAG00000026469	0.0034	18	12		2.62	15
ENSXMAG00000026476	0.0027	24	9	bmt2	1.25	21
ENSXMAG00000026491	0.0025	6	15	sgip1a	2.23	3
ENSXMAG00000026493	0.0014	21	6	rem2	2.99	18
ENSXMAG00000026500	0.0015	21	6		1.50	18
ENSXMAG00000026506	0.0026	9	15	tmem258	10.73	6
ENSXMAG00000026528	0.0026	21	9	BIN3	1.32	18
ENSXMAG00000026533	0.0029	6	12		1.63	3
ENSXMAG00000026540	0.0029	6	12	hemk1	1.93	3
ENSXMAG00000026550	0.0027	18	9		5.34	15
ENSXMAG00000026570	0.0027	21	9	ciao1	1.44	18
ENSXMAG00000026583	0.0029	9	12	rhbdf1b	2.57	6
ENSXMAG00000026585	0.0014	24	18		1.59	21
ENSXMAG00000026598	0.0031	6	12	cplx3a	1.37	3
ENSXMAG00000026629	0.0026	21	9		1.48	18
ENSXMAG00000026650	0.0027	9	9		1.84	6
ENSXMAG00000026653	0.0024	18	9		1.98	15
ENSXMAG00000026667	0.0034	9	12		2.02	6
ENSXMAG00000026675	0.0014	24	18		1.66	21
ENSXMAG00000026693	0.0031	18	12	znf281b	1.17	15
ENSXMAG00000026701	0.0026	15	15		2.71	12
ENSXMAG00000026707	0.0024	18	9	PM20D2	3.22	15
ENSXMAG00000026723	0.0024	6	15	gsg1l	14.49	3
ENSXMAG00000026739	0.0027	24	15		1.90	21
ENSXMAG00000026746	0.0025	18	15	dcun1d2a	1.32	15
ENSXMAG00000026755	0.0015	24	6	znf346	1.23	21
ENSXMAG00000026760	0.0014	3	6		2.14	0
ENSXMAG00000026761	0.0024	18	9		1.26	15
ENSXMAG00000026765	0.0031	3	12	bcl2l1	1.85	0
ENSXMAG00000026769	0.0029	21	12		1.26	18
ENSXMAG00000026772	0.0026	12	9	pdyn	1.35	9
ENSXMAG00000026779	0.0027	24	15	znf740b	1.65	21
ENSXMAG00000026801	0.0029	18	12	ppp1r14c	4.84	15
ENSXMAG00000026805	0.0026	18	9	rgs3b	2.48	15
ENSXMAG00000026817	0.0026	3	9	CIART	13.96	0
ENSXMAG00000026819	0.0026	24	9		1.26	21
ENSXMAG00000026826	0.0031	6	12	ormdl3	11.72	3
ENSXMAG00000026834	0.0014	3	6	trip10b	1.48	0
ENSXMAG00000026837	0.0029	6	12	rnf150b	2.09	3
ENSXMAG00000026845	0.0027	3	15	ccbe1	1.64	0
ENSXMAG00000026862	0.0027	21	9	mfsd9	1.33	18
ENSXMAG00000026869	0.0031	18	12	sumo3b	1.52	15
ENSXMAG00000026878	0.0029	24	12	fam214b	1.80	21
ENSXMAG00000026885	0.0031	15	12		3.07	12
ENSXMAG00000026888	0.0024	6	15		2.39	3
ENSXMAG00000026895	0.0027	3	15	sh3pxd2b	1.86	0
ENSXMAG00000026897	0.0031	18	12		10.93	15
ENSXMAG00000026899	0.0031	9	12		2.15	6
ENSXMAG00000026909	0.0024	18	9	plppr4b	2.19	15

ENSXMAG00000026921	0.0026	18	9	cspg5b	3.26	15
ENSXMAG00000026922	0.0024	6	15	prrt1	2.26	3
ENSXMAG00000026925	0.0031	24	12	hint2	1.71	21
ENSXMAG00000026934	0.0026	15	15	pcdh18a	1.32	12
ENSXMAG00000026936	0.0015	24	18	tnfaip3	1.78	21
ENSXMAG00000026950	0.0024	18	9	stac	1.98	15
ENSXMAG00000026971	0.0031	15	12	SRF (1 of many)	1.17	12
ENSXMAG00000026972	0.0025	18	9	PCDHAC2 (1 of many)	2.61	15
ENSXMAG00000026977	0.0031	6	12	si:ch73-256g18.2	6.18	3
ENSXMAG00000026997	0.0027	21	9	relt	17.64	18
ENSXMAG00000026998	0.0028	18	9	cog8	1.43	15
ENSXMAG00000027002	0.0026	9	15		1.48	6
ENSXMAG00000027011	0.0016	21	18	tp53inp1	1.85	18
ENSXMAG00000027012	0.0025	15	15		1.42	12
ENSXMAG00000027016	0.0031	21	12	tfap2a	1.71	18
ENSXMAG00000027021	0.0025	24	9	TSHZ1	1.82	21
ENSXMAG00000027026	0.0031	6	12		5.21	3
ENSXMAG00000027040	0.0027	3	15	ABR	3.18	0
ENSXMAG00000027047	0.0015	3	6	si:ch211-153b23.5 (1 of many)	4.24	0
ENSXMAG00000027051	0.0026	21	15	etfb	1.35	18
ENSXMAG00000027054	0.0025	15	15	syngr2b	1.62	12
ENSXMAG00000027058	0.0031	6	12	adamts15b	2.57	3
ENSXMAG00000027074	0.0031	15	12		2.29	12
ENSXMAG00000027084	0.0026	24	9		1.27	21
ENSXMAG00000027091	0.0025	24	9	mycbp	1.51	21
ENSXMAG00000027096	0.0027	24	9	GFRA1	1.97	21
ENSXMAG00000027103	0.0029	15	12	MSI1	2.83	12
ENSXMAG00000027105	0.0025	21	15	kank3	1.39	18
ENSXMAG00000027106	0.0026	21	9	ppib	1.30	18
ENSXMAG00000027108	0.0015	21	6		2.94	18
ENSXMAG00000027112	0.0031	15	12	OPRK1	1.40	12
ENSXMAG00000027122	0.0031	18	12	si:ch73-379j16.2 (1 of many)	2.48	15
ENSXMAG00000027147	0.0024	9	9	srpk1b	1.34	6
ENSXMAG00000027151	0.0024	6	15		11.31	3
ENSXMAG00000027162	0.0024	3	15		8.74	0
ENSXMAG00000027163	0.0071	3	18	si:dkey-178o16.4	2.15	0
ENSXMAG00000027169	0.0024	9	15	GHITM	1.23	6
ENSXMAG00000027172	0.0024	24	9		1.91	21
ENSXMAG00000027181	0.0031	6	12	arid5b	2.02	3
ENSXMAG00000027193	0.0016	21	6	fbxo42	1.20	18
ENSXMAG00000027197	0.0026	9	15	pinx1	1.49	6
ENSXMAG00000027199	0.0024	18	9	dab1b	2.97	15
ENSXMAG00000027208	0.0026	24	9	ppat	1.89	21
ENSXMAG00000027217	0.0031	21	12	snx20	3.35	18
ENSXMAG00000027218	0.0031	6	12	phrf1	1.30	3
ENSXMAG00000027226	0.0025	21	9		1.61	18
ENSXMAG00000027229	0.0026	6	15		1.92	3
ENSXMAG00000027234	0.0025	6	15		5.54	3
ENSXMAG00000027242	0.0015	3	6	znf532	1.47	0
ENSXMAG00000027245	0.0024	18	9	aftpha	1.29	15
ENSXMAG00000027249	0.0015	9	6	zgc:109889	1.22	6
ENSXMAG00000027269	0.0031	18	12	CTHRC1	1.65	15
ENSXMAG00000027295	0.0025	18	15	rflna	1.71	15
ENSXMAG00000027318	0.0027	3	15	si:ch211-71n6.4	1.34	0
ENSXMAG00000027324	0.0071	21	18	c1galt1a	1.58	18
ENSXMAG00000027325	0.0024	15	9	HERPUD2 (1 of many)	2.19	12

ENSXMAG00000027330	0.0024	6	15		3.49	3
ENSXMAG00000027350	0.0026	18	9		3.01	15
ENSXMAG00000027369	0.0029	18	12	SEPT7 (1 of many)	3.69	15
ENSXMAG00000027373	0.0024	6	15		2.13	3
ENSXMAG00000027377	0.0027	3	15		5.57	0
ENSXMAG00000027382	0.0031	6	12	BTBD6	1.96	3
ENSXMAG00000027388	0.0015	24	6		1.19	21
ENSXMAG00000027397	0.0025	24	9	csgalnact2	1.46	21
ENSXMAG00000027398	0.0028	6	15		2.36	3
ENSXMAG00000027430	0.0031	9	12	arrdc3a	1.98	6
ENSXMAG00000027468	0.0031	3	12		19.56	0
ENSXMAG00000027476	0.0026	24	9	wdr37	1.34	21
ENSXMAG00000027480	0.0031	9	12	cyp2ad2	4.77	6
ENSXMAG00000027481	0.0034	24	12	TMEM87A	1.35	21
ENSXMAG00000027485	0.0025	18	9		2.07	15
ENSXMAG00000027487	0.0026	18	9		2.70	15
ENSXMAG00000027488	0.0031	24	12		2.38	21
ENSXMAG00000027492	0.0027	6	15	znrf2b	2.12	3
ENSXMAG00000027507	0.0026	24	9		1.50	21
ENSXMAG00000027513	0.0025	3	15		2.79	0
ENSXMAG00000027516	0.0014	21	6	acads	1.79	18
ENSXMAG00000027518	0.0026	9	15	dnajc4	3.16	6
ENSXMAG00000027525	0.0026	24	9		1.40	21
ENSXMAG00000027531	0.0025	18	15	lcor	2.96	15
ENSXMAG00000027540	0.0031	6	12		2.00	3
ENSXMAG00000027546	0.0025	18	9	atp1b1b	2.21	15
ENSXMAG00000027547	0.0024	18	9	gtf2a2	1.84	15
ENSXMAG00000027581	0.0015	24	6	gsdmeb	1.61	21
ENSXMAG00000027602	0.0025	21	9	VASH1	4.58	18
ENSXMAG00000027603	0.0031	21	12	zmp:0000000521 (1 of many)	1.20	18
ENSXMAG00000027609	0.0031	21	12	triqk	1.88	18
ENSXMAG00000027614	0.0025	12	15	calm1b	1.34	9
ENSXMAG00000027639	0.0031	15	12	PCYOX1L	1.57	12
ENSXMAG00000027646	0.0031	24	12	MMP16	1.78	21
ENSXMAG00000027653	0.0014	9	18		12.61	6
ENSXMAG00000027654	0.0024	6	15		2.69	3
ENSXMAG00000027656	0.0025	18	9	cdkn1d	13.66	15
ENSXMAG00000027657	0.0024	6	15		4.01	3
ENSXMAG00000027658	0.0024	3	15		11.67	0
ENSXMAG00000027671	0.0015	18	18		5.40	15
ENSXMAG00000027680	0.0025	18	9		1.38	15
ENSXMAG00000027687	0.0014	21	6	slc25a11	1.68	18
ENSXMAG00000027691	0.0025	21	9	zgc:171704	13.45	18
ENSXMAG00000027698	0.0027	21	9	ncdn	1.32	18
ENSXMAG00000027733	0.0031	18	12	tmem106c	1.59	15
ENSXMAG00000027735	0.0026	18	15	snai1a	1.22	15
ENSXMAG00000027736	0.0029	9	12	RGS6	2.19	6
ENSXMAG00000027757	0.0031	3	12	gk5	1.31	0
ENSXMAG00000027760	0.0031	18	12		3.83	15
ENSXMAG00000027778	0.0027	3	15		1.93	0
ENSXMAG00000027787	0.0024	9	9	RAB41	4.28	6
ENSXMAG00000027796	0.0025	24	15	ldb1b	1.39	21
ENSXMAG00000027813	0.0024	3	15	ptpn5	1.34	0
ENSXMAG00000027828	0.0030	21	12	lamtor4	1.32	18
ENSXMAG00000027831	0.0025	18	9	ABI2	4.38	15
ENSXMAG00000027832	0.0026	18	15		1.58	15

ENSXMAG00000027837	0.0024	21	9	bcap31	1.40	18
ENSXMAG00000027840	0.0026	24	9	mafga	1.65	21
ENSXMAG00000027862	0.0027	24	9	praf2	1.39	21
ENSXMAG00000027863	0.0025	21	15		2.16	18
ENSXMAG00000027878	0.0024	21	9		6.17	18
ENSXMAG00000027890	0.0025	6	15		1.59	3
ENSXMAG00000027907	0.0024	18	9	si:ch211-212o1.2	1.75	15
ENSXMAG00000027920	0.0030	21	12		1.99	18
ENSXMAG00000027929	0.0025	3	15		4.07	0
ENSXMAG00000027932	0.0026	18	9		1.46	15
ENSXMAG00000027936	0.0016	3	18		1.71	0
ENSXMAG00000027949	0.0027	21	9	endog	2.07	18
ENSXMAG00000027950	0.0028	21	9	psmg1	1.38	18
ENSXMAG00000027955	0.0026	21	15	msrb1b	2.27	18
ENSXMAG00000027971	0.0015	24	6		2.62	21
ENSXMAG00000027974	0.0031	6	12	fgl2a	1.67	3
ENSXMAG00000027975	0.0031	6	12		1.47	3
ENSXMAG00000027976	0.0031	21	12	id4	1.72	18
ENSXMAG00000027981	0.0031	21	12	fam19a5a	1.35	18
ENSXMAG00000027983	0.0029	6	12	ppp2r5cb	2.87	3
ENSXMAG00000027985	0.0026	21	9	fbxl14a	1.20	18
ENSXMAG00000028010	0.0015	21	6	nmt1a	1.37	18
ENSXMAG00000028012	0.0024	9	9		1.97	6
ENSXMAG00000028020	0.0031	9	12	CACNG2	2.25	6
ENSXMAG00000028036	0.0031	9	12		2.40	6
ENSXMAG00000028044	0.0025	12	15	oaz1b	1.28	9
ENSXMAG00000028054	0.0025	6	9	mettl11b	3.21	3
ENSXMAG00000028064	0.0031	12	12	tfr1b	2.19	9
ENSXMAG00000028093	0.0025	18	9	raraa	2.13	15
ENSXMAG00000028101	0.0025	12	15	GFOD1	1.63	9
ENSXMAG00000028106	0.0016	21	18		1.62	18
ENSXMAG00000028116	0.0025	21	9	gramd1a	1.84	18
ENSXMAG00000028136	0.0024	3	15	MMD2 (1 of many)	3.27	0
ENSXMAG00000028153	0.0029	18	12	KLHDC2	1.63	15
ENSXMAG00000028196	0.0031	18	12	MYT1L (1 of many)	1.82	15
ENSXMAG00000028205	0.0027	24	9		1.39	21
ENSXMAG00000028211	0.0014	24	18	TOM1L2	1.77	21
ENSXMAG00000028221	0.0026	9	15	dnajb9a	1.87	6
ENSXMAG00000028233	0.0027	21	9	mydgf	1.56	18
ENSXMAG00000028234	0.0031	18	12	xylt1 (1 of many)	1.89	15
ENSXMAG00000028236	0.0031	21	12	pax7a	1.99	18
ENSXMAG00000028248	0.0026	15	15	b4galt3	1.31	12
ENSXMAG00000028250	0.0014	21	6	pcdh1g32	3.13	18
ENSXMAG00000028251	0.0027	3	15		2.21	0
ENSXMAG00000028252	0.0014	21	6	fgf1a	1.70	18
ENSXMAG00000028254	0.0026	18	15		2.45	15
ENSXMAG00000028259	0.0030	18	12	DPYSL2	1.18	15
ENSXMAG00000028260	0.0025	15	9	slc38a3a	1.90	12
ENSXMAG00000028263	0.0028	6	15	SYDE2	5.55	3
ENSXMAG00000028268	0.0031	18	12		3.03	15
ENSXMAG00000028272	0.0030	6	12	znf385a	1.37	3
ENSXMAG00000028295	0.0016	18	6		2.05	15
ENSXMAG00000028296	0.0031	9	12		2.62	6
ENSXMAG00000028313	0.0026	21	9	krtcap2	1.41	18
ENSXMAG00000028318	0.0015	21	6	CDYL	1.90	18
ENSXMAG00000028331	0.0024	18	15	ADCY9	1.73	15

ENSXMAG00000028338	0.0014	21	6	arl1	1.24	18
ENSXMAG00000028344	0.0029	24	12	RS1	1.78	21
ENSXMAG00000028354	0.0024	3	15	lrrc32	1.75	0
ENSXMAG00000028370	0.0027	3	15		7.81	0
ENSXMAG00000028371	0.0026	18	9	efnb1	2.19	15
ENSXMAG00000028378	0.0016	18	6	guca1a	44.22	15
ENSXMAG00000028381	0.0025	24	9		1.64	21
ENSXMAG00000028390	0.0034	3	12	si:ch211-13f8.1	2.68	0
ENSXMAG00000028396	0.0031	21	12	cers4a	1.77	18
ENSXMAG00000028403	0.0029	21	12		1.36	18
ENSXMAG00000028406	0.0016	24	18	b4galnt4a	1.67	21
ENSXMAG00000028414	0.0031	15	12	fam163b	1.67	12
ENSXMAG00000028416	0.0014	6	18	ctdspla	1.77	3
ENSXMAG00000028435	0.0034	9	12	PTPN20	2.97	6
ENSXMAG00000028437	0.0031	18	12		1.59	15
ENSXMAG00000028443	0.0024	18	9		24.64	15
ENSXMAG00000028449	0.0027	3	15	foxo1a	2.24	0
ENSXMAG00000028452	0.0027	24	15	si:dkey-87o1.2	4.09	21
ENSXMAG00000028457	0.0016	21	6	dph7	1.20	18
ENSXMAG00000028462	0.0024	15	9	cnstb	1.21	12
ENSXMAG00000028476	0.0025	6	15		2.79	3
ENSXMAG00000028477	0.0026	24	9		2.48	21
ENSXMAG00000028479	0.0031	18	12	zgc:64201	2.09	15
ENSXMAG00000028511	0.0025	24	15	adra2c	2.40	21
ENSXMAG00000028526	0.0014	18	6	smyd2a	1.90	15
ENSXMAG00000028529	0.0026	18	15	cap1	1.71	15
ENSXMAG00000028565	0.0031	24	12	elmod3	1.71	21
ENSXMAG00000028572	0.0034	21	12	si:dkey-106g10.7	1.62	18
ENSXMAG00000028573	0.0029	15	12	dhcr7	3.54	12
ENSXMAG00000028575	0.0031	15	12		12.65	12
ENSXMAG00000028582	0.0031	15	12	CSRNP3	1.96	12
ENSXMAG00000028585	0.0031	21	12	ntf3	2.60	18
ENSXMAG00000028590	0.0025	9	15	gpt2l	1.65	6
ENSXMAG00000028599	0.0031	15	12	MGME1	2.28	12
ENSXMAG00000028601	0.0026	21	9		1.37	18
ENSXMAG00000028610	0.0026	3	15	st8sia6 (1 of many)	2.84	0
ENSXMAG00000028614	0.0016	24	18		2.43	21
ENSXMAG00000028618	0.0031	18	12	GDE1	2.22	15
ENSXMAG00000028636	0.0029	12	12		1.38	9
ENSXMAG00000028637	0.0029	21	12	her9	1.82	18
ENSXMAG00000028653	0.0024	21	9	cfl2	1.18	18
ENSXMAG00000028657	0.0028	3	15		4.60	0
ENSXMAG00000028666	0.0031	24	12	map1lc3b	1.65	21
ENSXMAG00000028674	0.0026	6	15	ppm1e	2.14	3
ENSXMAG00000028682	0.0031	18	12	igsf9a	5.88	15
ENSXMAG00000028683	0.0026	24	9		2.84	21
ENSXMAG00000028689	0.0026	21	9	abracl	1.97	18
ENSXMAG00000028698	0.0029	18	12	glulc	4.14	15
ENSXMAG00000028741	0.0014	21	6	si:ch211-128m15.3	1.44	18
ENSXMAG00000028750	0.0016	24	18	nfat5b	2.92	21
ENSXMAG00000028756	0.0016	6	18	cabp1a	1.41	3
ENSXMAG00000028764	0.0030	21	12	RIDA (1 of many)	1.74	18
ENSXMAG00000028766	0.0031	18	12		3.17	15
ENSXMAG00000028769	0.0031	6	12		2.19	3
ENSXMAG00000028770	0.0016	24	6		1.19	21
ENSXMAG00000028774	0.0024	3	15		4.91	0

ENSXMAG00000028777	0.0025	15	9		4.70	12
ENSXMAG00000028778	0.0025	18	9		1.67	15
ENSXMAG00000028787	0.0024	3	15	tgfbr3	1.65	0
ENSXMAG00000028794	0.0028	24	15		2.14	21
ENSXMAG00000028797	0.0014	24	6		1.69	21
ENSXMAG00000028803	0.0029	24	12	gatd1	1.50	21
ENSXMAG00000028828	0.0031	24	12	sept9b	1.58	21
ENSXMAG00000028842	0.0015	21	18		1.26	18
ENSXMAG00000028844	0.0014	21	6	dusp3b	2.22	18
ENSXMAG00000028909	0.0024	3	9	oprk1	3.57	0
ENSXMAG00000028910	0.0031	6	12		4.88	3
ENSXMAG00000028912	0.0031	12	12	rgs9bp	1.97	9
ENSXMAG00000028913	0.0016	24	18	gosr1	1.75	21
ENSXMAG00000028921	0.0015	6	18	slc25a6 (1 of many)	4.04	3
ENSXMAG00000028939	0.0015	3	18	si:ch1073-272o11.3	26.86	0
ENSXMAG00000028941	0.0024	24	15	jun	4.85	21
ENSXMAG00000028951	0.0031	15	12	zgc:110843	1.80	12
ENSXMAG00000028954	0.0028	24	15	trappc13	1.30	21
ENSXMAG00000028956	0.0031	12	12	CALCB	2.50	9
ENSXMAG00000028965	0.0026	6	15		2.28	3
ENSXMAG00000028966	0.0029	15	12	BACE1	1.72	12
ENSXMAG00000028967	0.0030	15	12	FAM124A	4.27	12
ENSXMAG00000028970	0.0031	21	12		3.16	18
ENSXMAG00000028971	0.0024	9	9	castor2	1.58	6
ENSXMAG00000028984	0.0030	3	12		1.60	0
ENSXMAG00000028988	0.0015	18	18		3.02	15
ENSXMAG00000029000	0.0024	24	9	cd82b	1.64	21
ENSXMAG00000029013	0.0025	18	9	ppp2r3a	2.32	15
ENSXMAG00000029041	0.0031	18	12		1.85	15
ENSXMAG00000029056	0.0024	3	15	bbs5	1.33	0
ENSXMAG00000029073	0.0025	18	15	fbxl20	2.58	15
ENSXMAG00000029079	0.0015	24	18		2.05	21
ENSXMAG00000029081	0.0031	9	12	anp32e	1.43	6
ENSXMAG00000029085	0.0031	21	12	ngrn	1.47	18
ENSXMAG00000029088	0.0025	18	9		5.19	15
ENSXMAG00000029092	0.0027	18	9		6.83	15
ENSXMAG00000029096	0.0025	18	15		1.24	15
ENSXMAG00000029098	0.0031	3	12		3.14	0
ENSXMAG00000029099	0.0025	15	15		3.91	12
ENSXMAG00000029112	0.0025	6	15	ncam1b	2.17	3
ENSXMAG00000029115	0.0025	12	15		3.51	9
ENSXMAG00000029129	0.0029	6	12	sat2a	2.04	3
ENSXMAG00000029130	0.0029	18	12	TMEM74B	8.61	15
ENSXMAG00000029136	0.0026	21	9	erh	1.34	18
ENSXMAG00000029137	0.0024	18	15		2.67	15
ENSXMAG00000029143	0.0031	9	12	NOCT	1.92	6
ENSXMAG00000029146	0.0016	6	18		123.08	3
ENSXMAG00000029150	0.0030	18	12	HNRNPA0 (1 of many)	1.60	15
ENSXMAG00000029156	0.0029	3	12	timm9	1.41	0
ENSXMAG00000029157	0.0029	3	12	lrtm1	1.73	0
ENSXMAG00000029182	0.0031	24	12	PCBD2	1.34	21
ENSXMAG00000029185	0.0025	24	15	KCNMB4	2.14	21
ENSXMAG00000029189	0.0027	3	15		1.97	0
ENSXMAG00000029190	0.0025	21	9		144.09	18
ENSXMAG00000029194	0.0026	9	15		20.44	6
ENSXMAG00000029207	0.0015	21	6		1.59	18

ENSXMAG00000029211	0.0028	18	9	elavl2	1.33	15
ENSXMAG00000029229	0.0026	24	9		5.17	21
ENSXMAG00000029251	0.0031	24	12	tmem117	1.69	21
ENSXMAG00000029255	0.0031	18	12		1.18	15
ENSXMAG00000029257	0.0025	6	15	sncgb	2.24	3
ENSXMAG00000029258	0.0026	24	9		1.74	21
ENSXMAG00000029273	0.0026	15	15		1.80	12
ENSXMAG00000029284	0.0025	18	9		2.91	15
ENSXMAG00000029312	0.0029	3	12	nt5c2b	1.61	0
ENSXMAG00000029314	0.0014	12	18	sc5d	1.95	9
ENSXMAG00000029325	0.0031	6	12		2.79	3
ENSXMAG00000029327	0.0031	24	12		1.43	21
ENSXMAG00000029356	0.0031	24	12		7.24	21
ENSXMAG00000029375	0.0025	18	9	pgp	2.66	15
ENSXMAG00000029376	0.0025	15	15		3.68	12
ENSXMAG00000029395	0.0016	21	6		2.05	18
ENSXMAG00000029411	0.0071	3	18	c1qtnf6b	1.74	0
ENSXMAG00000029433	0.0015	24	18	pcmtd1	2.11	21
ENSXMAG00000029448	0.0030	12	12	tagln	1.60	9
ENSXMAG00000029455	0.0026	3	15	si:ch211-197h24.6	1.13	0
ENSXMAG00000029462	0.0027	21	9	CMAS	1.30	18
ENSXMAG00000029469	0.0024	3	15	tpra1	2.61	0
ENSXMAG00000029471	0.0026	24	9	st6gal2a	1.42	21
ENSXMAG00000029474	0.0029	21	12	pip5kl1	1.89	18
ENSXMAG00000029477	0.0025	24	15	rnf165a	1.41	21
ENSXMAG00000029487	0.0031	15	12		4.51	12
ENSXMAG00000029501	0.0024	3	15	GPATCH2L	4.95	0
ENSXMAG00000029509	0.0015	24	18	si:ch211-161c3.5	1.90	21
ENSXMAG00000029515	0.0026	9	9		5.25	6
ENSXMAG00000029516	0.0031	15	12		2.10	12
ENSXMAG00000029530	0.0031	15	12		1.36	12
ENSXMAG00000029533	0.0014	24	6	efna3b	1.59	21
ENSXMAG00000029540	0.0026	18	9	inka2	2.12	15
ENSXMAG00000029542	0.0028	3	15		1.40	0
ENSXMAG00000029543	0.0031	18	12		4.31	15
ENSXMAG00000029546	0.0015	24	18	SLC25A29	2.25	21
ENSXMAG00000029549	0.0026	6	15		1.51	3
ENSXMAG00000029558	0.0029	24	12	ccdc15	1.74	21
ENSXMAG00000029563	0.0027	24	15	ECH1	1.30	21
ENSXMAG00000029569	0.0031	21	12		1.69	18
ENSXMAG00000029585	0.0025	3	15	CCDC137	2.27	0
ENSXMAG00000029591	0.0025	12	15		2.79	9
ENSXMAG00000029601	0.0031	24	12		1.54	21
ENSXMAG00000029616	0.0025	6	15		3.90	3
ENSXMAG00000029620	0.0015	24	18	slc40a1	1.51	21
ENSXMAG00000029631	0.0031	24	12		1.64	21
ENSXMAG00000029648	0.0031	6	12	gdpd4a	5.55	3
ENSXMAG00000029655	0.0031	21	12	dap1b	1.44	18
ENSXMAG00000029656	0.0025	3	15	SYNGR1	1.20	0
ENSXMAG00000029660	0.0031	15	12		15.33	12
ENSXMAG00000029666	0.0031	18	12	ctnnbip1	1.63	15
ENSXMAG00000029670	0.0027	3	9	ahdc1	1.51	0
ENSXMAG00000029677	0.0029	6	12	wasf1	1.66	3
ENSXMAG00000029692	0.0031	9	12	CCKAR	2.69	6
ENSXMAG00000029698	0.0025	18	9		9.20	15
ENSXMAG00000029699	0.0031	18	12	gna11a	1.29	15

	ENSXMAG00000029706	0.0025	6	15	mxd1	2.19	3
	ENSXMAG00000029709	0.0025	3	9	tmem42b	3.35	0
	ENSXMAG00000029740	0.0025	18	9	marcksl1a	3.66	15
	ENSXMAG00000029747	0.0026	15	15	slc25a29 (1 of many)	2.13	12
	ENSXMAG00000029752	0.0031	21	12		1.68	18
	ENSXMAG00000029758	0.0031	3	12		1.53	0
	ENSXMAG00000029766	0.0027	15	9	csrnp1a	4.58	12
	ENSXMAG00000029785	0.0026	12	15		1.59	9
	ENSXMAG00000029787	0.0016	3	18		2.63	0
	ENSXMAG00000029789	0.0024	24	15		2.86	21
	ENSXMAG00000029793	0.0027	6	15	ptn	1.49	3
	ENSXMAG00000029802	0.0031	21	12		1.41	18
	ENSXMAG00000029804	0.0034	6	12		1.52	3
	ENSXMAG00000029821	0.0027	18	9		2.68	15
	ENSXMAG00000029825	0.0030	24	12		1.38	21
	ENSXMAG00000029826	0.0031	21	12		1.49	18
	ENSXMAG00000029844	0.0031	6	12	eif4ebp3l	2.75	3
	ENSXMAG00000029856	0.0031	3	12	si:ch211-207e14.4	2.94	0
	ENSXMAG00000029858	0.0026	9	15	slc32a1	1.46	6
	ENSXMAG00000029860	0.0031	18	12	cspg5a	2.96	15
	ENSXMAG00000029897	0.0029	15	12		2.56	12
	ENSXMAG00000029900	0.0031	3	12	MCU	1.49	0
	ENSXMAG00000029905	0.0026	9	15		3.23	6
	ENSXMAG00000029910	0.0031	3	12	cdo1	1.63	0
	ENSXMAG00000029949	0.0025	24	9	st3gal3b	1.20	21
	ENSXMAG00000029951	0.0030	24	12	immp2l	1.41	21
	ENSXMAG00000029962	0.0025	24	15		1.60	21
	ENSXMAG00000029991	0.0031	18	12	tuba1a	1.94	15
	ENSXMAG00000029993	0.0026	6	15	chrnb2b	1.82	3
	ENSXMAG00000030000	0.0015	24	18	gfap	1.40	21
	ENSXMAG00000030007	0.0026	3	15		1.67	0
	ENSXMAG00000030012	0.0015	18	18		19.67	15
	ENSXMAG00000030028	0.0025	18	9		11.47	15
	ENSXMAG00000030035	0.0025	9	15		1.44	6
	ENSXMAG00000030036	0.0031	9	12		3.87	6
	ENSXMAG00000030049	0.0031	9	12	KCNIP3	3.04	6
	ENSXMAG00000030053	0.0025	18	9	spsb1	1.42	15
	ENSXMAG00000030054	0.0026	3	15		2.27	0
	ENSXMAG00000030057	0.0024	18	9		105.09	15
	ENSXMAG00000030067	0.0031	18	12	pitpnb	1.59	15
	ENSXMAG00000030073	0.0027	24	15		4.12	21
	ENSXMAG00000030083	0.0031	6	12	fbxo33	3.26	3
	ENSXMAG00000030090	0.0030	21	12	CYGB	1.70	18
	ENSXMAG00000030095	0.0031	18	12	si:dkey-32e6.3	1.46	15
	ENSXMAG00000030099	0.0026	18	9	gad1b	1.46	15
	ENSXMAG00000030101	0.0025	18	9	raph1a	3.79	15
Gill circadian genes	GeneID	pVal	phase	peak.shape	external_gene_name	amp	Ct.peak
	ENSXMAG00000000019	0.0026	3	15	cd151l	1.71	0
	ENSXMAG00000000044	0.0026	18	9	hira	1.77	15
	ENSXMAG00000000052	0.0034	18	12	mrps16	1.54	15
	ENSXMAG00000000062	0.0026	3	15	stx2b	3.94	0
	ENSXMAG00000000067	0.0026	18	9	hccsb	1.25	15
	ENSXMAG00000000087	0.0024	3	15	galnt9	1.57	0
	ENSXMAG00000000140	0.0027	18	9	ccdc88c	1.90	15
	ENSXMAG00000000149	0.0024	9	9	VMP1	1.90	6

ENSXMAG00000000155	0.0031	9	12	si:ch211-156l18.7	2.37	6
ENSXMAG00000000174	0.0030	18	12	rtn4a	1.87	15
ENSXMAG00000000175	0.0024	18	9		1.42	15
ENSXMAG00000000189	0.0015	18	6	gnmt	1.88	15
ENSXMAG00000000197	0.0031	24	12	si:dkey-28e7.3	1.31	21
ENSXMAG00000000204	0.0026	21	9	ptp4a2a	1.62	18
ENSXMAG00000000225	0.0034	18	12	capzb	1.26	15
ENSXMAG00000000242	0.0025	18	9	xrn2	1.74	15
ENSXMAG00000000254	0.0024	21	9	ndnl2	1.39	18
ENSXMAG00000000261	0.0024	18	9	gfm1	3.16	15
ENSXMAG00000000276	0.0016	24	18		1.64	21
ENSXMAG00000000285	0.0031	3	12	b9d1	2.41	0
ENSXMAG00000000296	0.0031	18	12	mars2	1.54	15
ENSXMAG00000000304	0.0024	15	9	fhl1b	1.59	12
ENSXMAG00000000313	0.0025	18	9	hgh1	2.02	15
ENSXMAG00000000316	0.0025	18	15	eif2b5	1.32	15
ENSXMAG00000000342	0.0034	18	12	dpp3	2.76	15
ENSXMAG00000000355	0.0031	18	12	actr1	1.83	15
ENSXMAG00000000364	0.0026	3	15		1.69	0
ENSXMAG00000000371	0.0026	9	9		2.11	6
ENSXMAG00000000374	0.0026	21	9		1.32	18
ENSXMAG00000000375	0.0028	18	9	ldah	1.72	15
ENSXMAG00000000384	0.0026	18	9	sssca1	2.11	15
ENSXMAG00000000409	0.0027	18	9	nup35	1.55	15
ENSXMAG00000000493	0.0024	21	9	psmb5	1.42	18
ENSXMAG00000000505	0.0025	18	9	timm50	2.58	15
ENSXMAG00000000510	0.0031	18	12	cln6a	2.17	15
ENSXMAG00000000520	0.0026	3	15		2.38	0
ENSXMAG00000000613	0.0031	12	12	nmrk2	4.45	9
ENSXMAG00000000643	0.0026	3	15	zgc:86896	1.88	0
ENSXMAG00000000678	0.0026	9	9	tefa	1.88	6
ENSXMAG00000000679	0.0031	18	12	phf5a	1.67	15
ENSXMAG00000000699	0.0025	18	9	snrpb2	2.42	15
ENSXMAG00000000708	0.0025	18	9	large2	1.57	15
ENSXMAG00000000712	0.0026	9	9	ppp1r9a	1.58	6
ENSXMAG00000000715	0.0030	9	12	arhgap29b	1.40	6
ENSXMAG00000000718	0.0030	6	12	pdk4	2.90	3
ENSXMAG00000000719	0.0026	24	9	rpe65b	1.40	21
ENSXMAG00000000745	0.0034	18	12	sco1	1.61	15
ENSXMAG00000000752	0.0026	21	9	sem1	1.74	18
ENSXMAG00000000768	0.0029	18	12		2.37	15
ENSXMAG00000000786	0.0026	3	15	epb41l3b	1.90	0
ENSXMAG00000000792	0.0031	18	12	pdia5	1.84	15
ENSXMAG00000000799	0.0026	18	9	clcn4	1.39	15
ENSXMAG00000000829	0.0027	18	9	atg16l1	1.35	15
ENSXMAG00000000837	0.0026	3	15	dirc2	2.17	0
ENSXMAG00000000874	0.0031	18	12	unc45a	1.86	15
ENSXMAG00000000877	0.0026	18	9	OGDH	1.70	15
ENSXMAG00000000925	0.0026	3	15	proza	1.78	0
ENSXMAG00000000933	0.0027	6	15	mppe1	2.83	3
ENSXMAG00000000980	0.0031	18	12	oxnad1	3.94	15
ENSXMAG00000000988	0.0031	18	12	pus1	1.83	15
ENSXMAG00000000993	0.0015	9	18		1.89	6
ENSXMAG00000000996	0.0031	18	12	uqcrc2a	1.91	15
ENSXMAG00000001000	0.0016	24	18	tmem14cb	2.45	21
ENSXMAG00000001016	0.0026	21	9	zgc:56596	1.20	18

ENSXMAG00000001018	0.0031	18	12	snrpd2	2.11	15
ENSXMAG00000001025	0.0026	15	15	lipt1	1.42	12
ENSXMAG00000001041	0.0026	15	9		1.37	12
ENSXMAG00000001060	0.0025	18	9	bysl	1.87	15
ENSXMAG00000001071	0.0024	18	15	med20	2.01	15
ENSXMAG00000001093	0.0031	18	12	slc37a4a	1.73	15
ENSXMAG00000001096	0.0031	24	12	cdkn1a	16.84	21
ENSXMAG00000001098	0.0026	3	15		1.67	0
ENSXMAG00000001107	0.0014	24	6		1.81	21
ENSXMAG00000001113	0.0015	3	18	bhlhe41	39.91	0
ENSXMAG00000001125	0.0026	9	15	c1qtnf6a	5.24	6
ENSXMAG00000001135	0.0029	21	12	ndufa10	1.37	18
ENSXMAG00000001137	0.0015	24	18	ano9b	2.16	21
ENSXMAG00000001141	0.0026	9	9	sgcg	1.69	6
ENSXMAG00000001156	0.0025	9	9		1.74	6
ENSXMAG00000001162	0.0014	21	6	dek	1.23	18
ENSXMAG00000001168	0.0025	18	15		2.18	15
ENSXMAG00000001182	0.0026	9	15	nsmfa	3.63	6
ENSXMAG00000001187	0.0027	3	15		1.94	0
ENSXMAG00000001192	0.0026	18	9	eif3jb	1.49	15
ENSXMAG00000001241	0.0026	15	15	ttpal	1.62	12
ENSXMAG00000001252	0.0026	18	9	anks1b	1.43	15
ENSXMAG00000001311	0.0026	3	15	lpcat4	3.56	0
ENSXMAG00000001317	0.0025	18	9	nop56	1.32	15
ENSXMAG00000001321	0.0027	18	15		1.44	15
ENSXMAG00000001328	0.0031	18	12	aebp2	1.35	15
ENSXMAG00000001358	0.0024	9	15	angptl7	1.99	6
ENSXMAG00000001360	0.0016	24	18	ilvbl	1.37	21
ENSXMAG00000001363	0.0024	3	15	gaa	2.16	0
ENSXMAG00000001365	0.0030	3	12		2.11	0
ENSXMAG00000001372	0.0034	18	12		1.60	15
ENSXMAG00000001387	0.0025	18	9	sestd1	3.63	15
ENSXMAG00000001388	0.0031	18	12	atp6ap1b	1.28	15
ENSXMAG00000001390	0.0031	15	12	tbc1d16	1.31	12
ENSXMAG00000001397	0.0025	18	9	tsr1	2.39	15
ENSXMAG00000001399	0.0026	3	15	si:dkey-224e22.2	2.56	0
ENSXMAG00000001420	0.0026	24	15	klc3	1.61	21
ENSXMAG00000001434	0.0024	6	15		3.97	3
ENSXMAG00000001445	0.0025	9	9	arhgap17b	1.37	6
ENSXMAG00000001454	0.0024	18	9	larsb	2.26	15
ENSXMAG00000001455	0.0034	18	12	snrpa1	1.74	15
ENSXMAG00000001464	0.0031	9	12	GLB1L2	1.89	6
ENSXMAG00000001468	0.0027	18	9	gfer	2.97	15
ENSXMAG00000001478	0.0031	18	12	slc25a17	1.53	15
ENSXMAG00000001480	0.0025	15	15		1.62	12
ENSXMAG00000001482	0.0025	18	9	arntl2	9.47	15
ENSXMAG00000001497	0.0031	9	12	si:ch211-200p22.4	2.50	6
ENSXMAG00000001503	0.0026	9	9	ITPRID2	2.51	6
ENSXMAG00000001528	0.0026	21	9		1.44	18
ENSXMAG00000001548	0.0014	3	18		2.15	0
ENSXMAG00000001568	0.0034	18	12	polr3b	1.45	15
ENSXMAG00000001575	0.0026	18	9	ahcyl2	2.56	15
ENSXMAG00000001584	0.0030	15	12		3.78	12
ENSXMAG00000001628	0.0026	3	15	pdlim5b	2.10	0
ENSXMAG00000001666	0.0026	18	9	gmpr2	1.55	15
ENSXMAG00000001668	0.0025	3	15	ano10a	1.42	0

ENSXMAG00000001677	0.0024	21	9	psmc1a	1.51	18
ENSXMAG00000001683	0.0031	9	12	shroom1	2.23	6
ENSXMAG00000001724	0.0026	24	15	rc3h2	1.51	21
ENSXMAG00000001742	0.0024	24	9		2.00	21
ENSXMAG00000001745	0.0031	6	12	pla2r1	1.89	3
ENSXMAG00000001747	0.0031	3	12	ip6k2b	1.31	0
ENSXMAG00000001753	0.0025	24	15		1.42	21
ENSXMAG00000001756	0.0024	12	9	vav2	1.43	9
ENSXMAG00000001778	0.0025	18	9	abce1	3.48	15
ENSXMAG00000001781	0.0024	9	9	MAPKAPK3	1.96	6
ENSXMAG00000001794	0.0027	18	9	slc10a7	1.67	15
ENSXMAG00000001816	0.0027	9	15		1.52	6
ENSXMAG00000001819	0.0034	18	12	psmd14	1.70	15
ENSXMAG00000001847	0.0025	18	9	ruvbl1	1.54	15
ENSXMAG00000001854	0.0031	6	12	abca4a	3.05	3
ENSXMAG00000001855	0.0016	24	18	rft1	1.46	21
ENSXMAG00000001865	0.0031	6	12	sorbs3	1.91	3
ENSXMAG00000001870	0.0024	18	9	atic	1.55	15
ENSXMAG00000001872	0.0024	18	9	polr3d	1.98	15
ENSXMAG00000001901	0.0024	15	9	tcf7l1b	1.63	12
ENSXMAG00000001902	0.0026	18	9	tmem39a	1.57	15
ENSXMAG00000001944	0.0024	18	9	sec24d	2.57	15
ENSXMAG00000001947	0.0015	21	18	ankrd46b	1.29	18
ENSXMAG00000001950	0.0025	21	9		1.42	18
ENSXMAG00000001974	0.0025	18	9	e2f4	3.28	15
ENSXMAG00000001995	0.0031	18	12	psmd6	1.48	15
ENSXMAG00000002019	0.0030	6	12	bnip3la	2.73	3
ENSXMAG00000002027	0.0026	3	15	ankha	3.04	0
ENSXMAG00000002032	0.0030	9	12	phka2	1.57	6
ENSXMAG00000002043	0.0031	3	12	igsf9b	2.04	0
ENSXMAG00000002087	0.0031	18	12	mrpl15	1.40	15
ENSXMAG00000002092	0.0031	18	12	WDR1	1.31	15
ENSXMAG00000002095	0.0034	18	12		1.49	15
ENSXMAG00000002101	0.0031	18	12	idh3b	1.42	15
ENSXMAG00000002146	0.0016	24	6	espl1	2.06	21
ENSXMAG00000002155	0.0029	18	12	pfdn5	1.47	15
ENSXMAG00000002158	0.0029	18	12	leo1	1.47	15
ENSXMAG00000002165	0.0031	18	12	ppid	3.37	15
ENSXMAG00000002217	0.0026	18	9	CRABP1	2.11	15
ENSXMAG00000002249	0.0031	24	12	FBN1	1.74	21
ENSXMAG00000002290	0.0031	18	12	uqcrc1	1.61	15
ENSXMAG00000002307	0.0029	18	12	zfand1	1.82	15
ENSXMAG00000002309	0.0024	24	9		1.58	21
ENSXMAG00000002316	0.0030	18	12	GAR1	1.91	15
ENSXMAG00000002320	0.0034	18	12		1.87	15
ENSXMAG00000002328	0.0016	24	18	inpp5ka	1.56	21
ENSXMAG00000002339	0.0029	24	12	nr1d4b	51.99	21
ENSXMAG00000002356	0.0016	3	6	plbd1	1.24	0
ENSXMAG00000002377	0.0027	6	15	smad3a	1.78	3
ENSXMAG00000002378	0.0034	18	12	txnrd2.1	1.50	15
ENSXMAG00000002385	0.0025	18	9	atp6v1c1a	1.49	15
ENSXMAG00000002386	0.0015	24	18	CDH3 (1 of many)	2.22	21
ENSXMAG00000002387	0.0030	18	12	brf1b	1.38	15
ENSXMAG00000002399	0.0031	18	12		1.57	15
ENSXMAG00000002410	0.0015	12	18	zmp:0000001048	2.13	9
ENSXMAG00000002413	0.0016	24	18	nf2a	1.75	21

ENSXMAG00000002419	0.0026	9	15	soul5	7.55	6
ENSXMAG00000002450	0.0026	18	9	zdhhc8b	1.83	15
ENSXMAG00000002458	0.0026	18	15	fbxw8	1.54	15
ENSXMAG00000002459	0.0024	15	15	pgam5	1.52	12
ENSXMAG00000002480	0.0031	15	12	smtnl	1.74	12
ENSXMAG00000002483	0.0031	18	12	dhrs13b	1.57	15
ENSXMAG00000002501	0.0031	18	12	insig1	2.55	15
ENSXMAG00000002518	0.0026	15	15		3.35	12
ENSXMAG00000002556	0.0034	18	12	nucks1a	1.42	15
ENSXMAG00000002559	0.0024	9	15	abat	2.98	6
ENSXMAG00000002572	0.0025	18	15	uba5	1.53	15
ENSXMAG00000002601	0.0025	18	15	p3h1	1.44	15
ENSXMAG00000002603	0.0031	18	12	gamt	2.14	15
ENSXMAG00000002605	0.0030	18	12	psma6l	1.47	15
ENSXMAG00000002625	0.0031	12	12	elf3	1.77	9
ENSXMAG00000002629	0.0028	18	9	zgc:172302	2.08	15
ENSXMAG00000002642	0.0034	21	12	smarce1	1.12	18
ENSXMAG00000002648	0.0031	18	12	zdhhc3a	1.23	15
ENSXMAG00000002661	0.0031	18	12	stt3b	1.99	15
ENSXMAG00000002673	0.0026	18	9	GADL1	2.57	15
ENSXMAG00000002712	0.0027	3	15	si:ch211-117l17.5	1.53	0
ENSXMAG00000002730	0.0031	18	12		2.45	15
ENSXMAG00000002731	0.0024	15	15		1.85	12
ENSXMAG00000002753	0.0029	18	12	sf3a1	1.62	15
ENSXMAG00000002775	0.0026	18	9	lrrk2	1.93	15
ENSXMAG00000002786	0.0031	18	12	psat1	11.28	15
ENSXMAG00000002790	0.0027	18	9	VANGL1	1.56	15
ENSXMAG00000002805	0.0025	18	9	slc25a55a	1.69	15
ENSXMAG00000002830	0.0031	18	12		1.46	15
ENSXMAG00000002841	0.0031	9	12		4.41	6
ENSXMAG00000002845	0.0031	18	12		1.60	15
ENSXMAG00000002856	0.0030	9	12	pcsk7	1.20	6
ENSXMAG00000002863	0.0031	9	12	tp53bp2a	1.34	6
ENSXMAG00000002877	0.0025	18	9	gart	1.61	15
ENSXMAG00000002878	0.0031	18	12	tars	3.05	15
ENSXMAG00000002888	0.0015	3	18	EFEMP1	2.05	0
ENSXMAG00000002891	0.0031	6	12	fam102aa	1.72	3
ENSXMAG00000002900	0.0034	6	12	cnot6l	1.72	3
ENSXMAG00000002960	0.0014	24	18		1.45	21
ENSXMAG00000002970	0.0034	18	12	snrnp40	2.37	15
ENSXMAG00000002972	0.0024	12	9	adgb	1.54	9
ENSXMAG00000002975	0.0027	24	15	malt3	1.56	21
ENSXMAG00000002977	0.0026	15	15	rab32a	1.92	12
ENSXMAG00000002994	0.0025	21	9		1.35	18
ENSXMAG00000002998	0.0026	18	15	pdcd5	1.41	15
ENSXMAG00000003001	0.0025	21	9	bbs1	1.48	18
ENSXMAG00000003015	0.0026	9	15	rgra	13.22	6
ENSXMAG00000003050	0.0025	18	9	srsf7a	1.64	15
ENSXMAG00000003067	0.0026	3	15	mthfsd	2.04	0
ENSXMAG00000003116	0.0024	21	9	scp2b	1.47	18
ENSXMAG00000003118	0.0034	21	12	podn	1.89	18
ENSXMAG00000003160	0.0031	18	12	fkbp9	2.63	15
ENSXMAG00000003194	0.0031	6	12	TINAGL1	2.54	3
ENSXMAG00000003229	0.0031	24	12	tulp4a	1.68	21
ENSXMAG00000003232	0.0024	18	9	thoc3	1.67	15
ENSXMAG00000003247	0.0014	21	18		1.67	18

ENSXMAG00000003259	0.0029	18	12	calr	2.41	15
ENSXMAG00000003267	0.0071	21	18	inpp5e	1.37	18
ENSXMAG00000003285	0.0029	18	12	ilf2	1.39	15
ENSXMAG00000003296	0.0024	18	9	hm13	1.59	15
ENSXMAG00000003325	0.0031	18	12	sod2	1.50	15
ENSXMAG00000003345	0.0014	24	18		1.93	21
ENSXMAG00000003381	0.0026	18	9	srpk3	1.93	15
ENSXMAG00000003399	0.0025	21	9	tram2	1.85	18
ENSXMAG00000003418	0.0024	18	9	nudt2	1.87	15
ENSXMAG00000003419	0.0029	18	12	npepl1	1.69	15
ENSXMAG00000003436	0.0015	18	6	wdcp	2.27	15
ENSXMAG00000003443	0.0029	18	12	atp5fa1	1.59	15
ENSXMAG00000003454	0.0027	18	9	si:ch211-193k19.1	2.18	15
ENSXMAG00000003458	0.0071	3	18	safb	1.13	0
ENSXMAG00000003466	0.0026	18	9	abcd1	1.88	15
ENSXMAG00000003474	0.0025	18	15	yeats4	1.51	15
ENSXMAG00000003514	0.0034	15	12	ap3d1	1.55	12
ENSXMAG00000003519	0.0026	18	9	si:ch1073-55a19.2	2.38	15
ENSXMAG00000003534	0.0027	18	9	map7d1b	3.11	15
ENSXMAG00000003564	0.0026	21	9	aagab	1.52	18
ENSXMAG00000003604	0.0031	18	12	morf4l1	1.50	15
ENSXMAG00000003625	0.0015	24	18	gmppaa	1.29	21
ENSXMAG00000003656	0.0025	18	9	pdap1b	1.93	15
ENSXMAG00000003659	0.0031	18	12		2.24	15
ENSXMAG00000003664	0.0031	18	12	bud31	1.45	15
ENSXMAG00000003666	0.0031	6	12	cabin1	1.71	3
ENSXMAG00000003675	0.0024	18	9	mbtps2	2.15	15
ENSXMAG00000003684	0.0016	24	18	cxcr4a	1.46	21
ENSXMAG00000003691	0.0026	9	9	ep300b	1.15	6
ENSXMAG00000003693	0.0031	18	12	rida	2.00	15
ENSXMAG00000003707	0.0031	18	12	xpnpep3	1.86	15
ENSXMAG00000003718	0.0026	18	15	st13	1.36	15
ENSXMAG00000003726	0.0031	18	12	rangap1a	1.81	15
ENSXMAG00000003752	0.0031	21	12	si:ch211-203k16.3	1.35	18
ENSXMAG00000003772	0.0025	18	9	sf3b4	2.50	15
ENSXMAG00000003810	0.0031	18	12	HINT3 (1 of many)	1.94	15
ENSXMAG00000003862	0.0024	18	9	ndor1	1.73	15
ENSXMAG00000003888	0.0027	21	9	fam49ba	1.43	18
ENSXMAG00000003952	0.0025	3	15		2.53	0
ENSXMAG00000003958	0.0030	18	12	si:ch73-390b10.2	1.26	15
ENSXMAG00000003960	0.0029	18	12	sae1	2.11	15
ENSXMAG00000003961	0.0025	6	15	per3	10.02	3
ENSXMAG00000003966	0.0015	12	6	hps5	1.54	9
ENSXMAG00000003973	0.0029	6	12		2.00	3
ENSXMAG00000003975	0.0025	21	9	mtfmt	1.80	18
ENSXMAG00000003976	0.0028	24	15	nfatc2a	1.58	21
ENSXMAG00000004008	0.0031	18	12	ptcd3	1.84	15
ENSXMAG00000004010	0.0029	18	12	psma5	2.24	15
ENSXMAG00000004047	0.0031	18	12	lmf2a	1.58	15
ENSXMAG00000004059	0.0031	18	12		1.69	15
ENSXMAG00000004061	0.0026	21	9	miox	1.62	18
ENSXMAG00000004087	0.0029	3	12	gpr184	1.73	0
ENSXMAG00000004088	0.0030	18	12	fam69aa	1.84	15
ENSXMAG00000004091	0.0031	18	12	RPL5 (1 of many)	1.79	15
ENSXMAG00000004123	0.0025	18	15	glmna	1.77	15
ENSXMAG00000004136	0.0026	18	9	dhx57	2.06	15

ENSXMAG00000004142	0.0031	24	12	nme6	1.64	21
ENSXMAG00000004145	0.0024	21	9	slc25a26	2.02	18
ENSXMAG00000004164	0.0029	6	12	foxp1b	2.16	3
ENSXMAG00000004235	0.0031	18	12	ap2s1	1.75	15
ENSXMAG00000004248	0.0031	18	12	fam136a	1.77	15
ENSXMAG00000004262	0.0025	18	9	pofut1	1.79	15
ENSXMAG00000004267	0.0029	18	12	tmem209	2.07	15
ENSXMAG00000004276	0.0029	18	12	sema5bb	4.41	15
ENSXMAG00000004294	0.0031	18	12	tigarb	4.68	15
ENSXMAG00000004298	0.0031	18	12	clptm1	1.87	15
ENSXMAG00000004304	0.0031	18	12	stk25b	1.58	15
ENSXMAG00000004317	0.0029	18	12	dnajc21	1.55	15
ENSXMAG00000004319	0.0026	18	9	qpctla	1.16	15
ENSXMAG00000004332	0.0034	18	12	cnot10	1.28	15
ENSXMAG00000004336	0.0031	18	12	pparaa	1.49	15
ENSXMAG00000004372	0.0025	18	9		1.41	15
ENSXMAG00000004388	0.0024	3	15	hgfb	1.55	0
ENSXMAG00000004398	0.0031	18	12	lman1	1.75	15
ENSXMAG00000004411	0.0031	3	12	upb1	1.44	0
ENSXMAG00000004434	0.0024	24	15	tlr7	4.40	21
ENSXMAG00000004439	0.0031	6	12	wfs1b	1.92	3
ENSXMAG00000004445	0.0031	18	12	tmed2	1.57	15
ENSXMAG00000004462	0.0030	18	12	sec63	1.55	15
ENSXMAG00000004473	0.0025	21	9	sdf2l1	1.81	18
ENSXMAG00000004494	0.0034	12	12	zgc:56235	2.76	9
ENSXMAG00000004499	0.0025	21	9	COX7A2	1.84	18
ENSXMAG00000004504	0.0031	15	12	tmem30ab	1.18	12
ENSXMAG00000004520	0.0024	18	9	trap1	2.39	15
ENSXMAG00000004537	0.0025	18	9	clns1a	1.48	15
ENSXMAG00000004544	0.0014	24	6	smc2	1.89	21
ENSXMAG00000004548	0.0026	18	9	pak1	2.44	15
ENSXMAG00000004555	0.0031	18	12	ddx1	1.62	15
ENSXMAG00000004556	0.0027	6	15	si:dkey-30c15.2	11.46	3
ENSXMAG00000004561	0.0031	18	12		1.66	15
ENSXMAG00000004563	0.0029	18	12	rcc2	1.75	15
ENSXMAG00000004590	0.0031	3	12	eif4a2	1.37	0
ENSXMAG00000004593	0.0029	18	12	creld2	2.73	15
ENSXMAG00000004594	0.0026	21	9	idi1	1.25	18
ENSXMAG00000004604	0.0025	18	9	polr3f	2.12	15
ENSXMAG00000004605	0.0026	15	15	itsn2a	1.68	12
ENSXMAG00000004610	0.0031	18	12	alg12	1.44	15
ENSXMAG00000004662	0.0015	9	18	si:dkey-97m3.1	1.54	6
ENSXMAG00000004677	0.0031	18	12	sephs1	2.33	15
ENSXMAG00000004678	0.0031	18	12	ctnnbl1	1.49	15
ENSXMAG00000004714	0.0031	21	12		1.78	18
ENSXMAG00000004725	0.0024	18	9	tti1	1.53	15
ENSXMAG00000004751	0.0027	12	15	zgc:66475	2.91	9
ENSXMAG00000004782	0.0024	15	15		2.23	12
ENSXMAG00000004793	0.0027	21	9		2.16	18
ENSXMAG00000004797	0.0027	6	15	adra1d	2.85	3
ENSXMAG00000004819	0.0024	3	15		1.51	0
ENSXMAG00000004822	0.0026	3	15	vwa1	1.90	0
ENSXMAG00000004827	0.0029	18	12	RNPS1	2.09	15
ENSXMAG00000004835	0.0026	18	9	nomo	1.79	15
ENSXMAG00000004840	0.0027	15	9	myo1eb	1.29	12
ENSXMAG00000004842	0.0024	18	9	ap3m2	1.64	15

ENSXMAG00000004847	0.0025	18	9	dhx29	3.15	15
ENSXMAG00000004893	0.0025	21	9	psma3	1.64	18
ENSXMAG00000004915	0.0030	18	12	imp4	2.86	15
ENSXMAG00000004919	0.0025	18	9	dkc1	1.56	15
ENSXMAG00000004999	0.0026	3	15	dusp6	1.83	0
ENSXMAG00000005014	0.0025	18	9	rint1	1.35	15
ENSXMAG00000005017	0.0015	24	6	kif4	1.79	21
ENSXMAG00000005051	0.0031	21	12	smad4a	1.08	18
ENSXMAG00000005060	0.0025	18	9	adssl	1.79	15
ENSXMAG00000005072	0.0027	21	9	arhgef39	1.81	18
ENSXMAG00000005076	0.0025	18	9	slc30a5	1.74	15
ENSXMAG00000005092	0.0025	18	9	wdr36	2.25	15
ENSXMAG00000005103	0.0016	21	6	tdrd3	1.12	18
ENSXMAG00000005116	0.0026	24	15	slc8a4a	11.72	21
ENSXMAG00000005127	0.0031	15	12	nfil3-5	59.37	12
ENSXMAG00000005136	0.0025	18	9		2.09	15
ENSXMAG00000005147	0.0024	18	9	dus1l	2.12	15
ENSXMAG00000005150	0.0029	18	12	gdi2	1.43	15
ENSXMAG00000005189	0.0024	3	15	dazap2	1.44	0
ENSXMAG00000005193	0.0030	21	12	chchd3a	1.28	18
ENSXMAG00000005199	0.0027	21	9	HDAC2	1.28	18
ENSXMAG00000005217	0.0031	3	12	rps6ka4	1.34	0
ENSXMAG00000005240	0.0031	18	12	ganab	2.19	15
ENSXMAG00000005253	0.0026	18	9	dnajc10	1.91	15
ENSXMAG00000005265	0.0031	18	12	exosc2	2.03	15
ENSXMAG00000005275	0.0031	6	12	RSPH1	2.42	3
ENSXMAG00000005281	0.0025	18	9	si:ch211-114c17.1	1.40	15
ENSXMAG00000005282	0.0031	18	12	copg2	1.45	15
ENSXMAG00000005287	0.0025	18	9	dab2	1.60	15
ENSXMAG00000005307	0.0029	18	12	psmc6	1.68	15
ENSXMAG00000005312	0.0031	18	12	ddx54	1.98	15
ENSXMAG00000005313	0.0026	18	15	samm50l	1.57	15
ENSXMAG00000005352	0.0025	21	9	smim8	1.26	18
ENSXMAG00000005434	0.0026	3	15	trpv1 (1 of many)	1.46	0
ENSXMAG00000005461	0.0031	18	12	cthl	2.25	15
ENSXMAG00000005480	0.0025	3	15	pdcd4a	2.20	0
ENSXMAG00000005492	0.0025	6	9	tmem141	1.85	3
ENSXMAG00000005505	0.0031	15	12	smox	1.89	12
ENSXMAG00000005525	0.0025	6	15	vcam1b	2.02	3
ENSXMAG00000005551	0.0030	15	12	ugp2b	1.48	12
ENSXMAG00000005568	0.0024	18	9	ints8	2.60	15
ENSXMAG00000005638	0.0031	18	12	psmd1	2.08	15
ENSXMAG00000005651	0.0015	24	18	rgs5a	1.32	21
ENSXMAG00000005652	0.0025	21	9	ciao2b	1.50	18
ENSXMAG00000005660	0.0014	18	6		2.27	15
ENSXMAG00000005671	0.0034	18	12	c1qbp	2.53	15
ENSXMAG00000005677	0.0026	9	15	phyhd1	1.83	6
ENSXMAG00000005678	0.0026	24	15	acap2	1.27	21
ENSXMAG00000005736	0.0028	24	9		2.04	21
ENSXMAG00000005737	0.0025	18	9	sympk	2.23	15
ENSXMAG00000005757	0.0031	18	12	rab1ab	1.60	15
ENSXMAG00000005775	0.0025	3	15	ulk2	3.40	0
ENSXMAG00000005798	0.0034	15	12	cpsf1	1.43	12
ENSXMAG00000005811	0.0025	18	9	wdr75	2.57	15
ENSXMAG00000005859	0.0024	24	15	znfx1	1.90	21
ENSXMAG00000005881	0.0031	18	12	fkbp7	1.60	15

ENSXMAG00000005889	0.0027	18	9	hspe1	2.20	15
ENSXMAG00000005891	0.0025	18	9	hspd1	2.88	15
ENSXMAG00000005906	0.0024	18	9	tgm2l	1.33	15
ENSXMAG00000005918	0.0029	18	12	ruvbl2	2.32	15
ENSXMAG00000005934	0.0026	24	15	st7l	1.37	21
ENSXMAG00000005937	0.0029	6	12	si:ch73-209e20.5	1.28	3
ENSXMAG00000005970	0.0031	12	12		1.42	9
ENSXMAG00000005976	0.0031	6	12	abi3bpb	2.22	3
ENSXMAG00000005999	0.0029	18	12	txndc5	1.98	15
ENSXMAG00000006020	0.0026	3	9	zgc:101569	1.31	0
ENSXMAG00000006121	0.0030	18	12	polg2	1.53	15
ENSXMAG00000006123	0.0024	18	9	shmt2	1.89	15
ENSXMAG00000006131	0.0034	18	12	DDX5	1.52	15
ENSXMAG00000006166	0.0026	6	15	mylk4b	6.58	3
ENSXMAG00000006198	0.0016	21	6		2.15	18
ENSXMAG00000006241	0.0031	24	12		1.45	21
ENSXMAG00000006260	0.0015	3	18	GPRC5D	1.66	0
ENSXMAG00000006262	0.0026	18	9	nup43	1.98	15
ENSXMAG00000006297	0.0031	18	12	magoh	1.93	15
ENSXMAG00000006300	0.0031	9	12	OSBPL8	1.70	6
ENSXMAG00000006311	0.0031	18	12	tmco1	1.92	15
ENSXMAG00000006318	0.0030	18	12	setd7 (1 of many)	3.73	15
ENSXMAG00000006321	0.0024	18	9	smarca4a	2.77	15
ENSXMAG00000006328	0.0026	18	9	med8	1.41	15
ENSXMAG00000006331	0.0026	24	9	igfbp3	1.26	21
ENSXMAG00000006345	0.0026	21	9	pbk	2.47	18
ENSXMAG00000006383	0.0031	18	12	rbbp4	2.91	15
ENSXMAG00000006395	0.0026	21	9	fam168b	1.19	18
ENSXMAG00000006406	0.0034	18	12	psmc2	1.73	15
ENSXMAG00000006419	0.0034	18	12	khdrbs1a	1.51	15
ENSXMAG00000006426	0.0015	24	18	zgc:92873	2.17	21
ENSXMAG00000006446	0.0031	24	12	si:ch1073-291c23.1	1.60	21
ENSXMAG00000006474	0.0026	21	9	cnot9	1.28	18
ENSXMAG00000006524	0.0034	18	12	psmd12	1.64	15
ENSXMAG00000006532	0.0026	3	15	lrmp	1.64	0
ENSXMAG00000006547	0.0026	18	9	ddx39aa	1.77	15
ENSXMAG00000006551	0.0026	24	15	kdm4b	1.42	21
ENSXMAG00000006563	0.0026	18	15	b3galnt2	2.87	15
ENSXMAG00000006578	0.0014	3	18	prkcaa	1.85	0
ENSXMAG00000006600	0.0027	15	15	apbb3	2.27	12
ENSXMAG00000006602	0.0034	18	12		1.58	15
ENSXMAG00000006627	0.0027	24	15	PLD4	2.18	21
ENSXMAG00000006634	0.0025	18	9	snupn	1.78	15
ENSXMAG00000006645	0.0025	18	9	nol11	1.75	15
ENSXMAG00000006678	0.0027	18	9	ddx31	1.82	15
ENSXMAG00000006697	0.0031	15	12	trmu	1.36	12
ENSXMAG00000006702	0.0029	18	12		1.63	15
ENSXMAG00000006725	0.0029	18	12	ARPP19 (1 of many)	1.70	15
ENSXMAG00000006741	0.0031	18	12	mrpl17	1.60	15
ENSXMAG00000006786	0.0016	21	18	si:dkey-3k24.5	1.33	18
ENSXMAG00000006794	0.0025	24	9	iqgap2	1.75	21
ENSXMAG00000006797	0.0031	21	12	mdkb	2.02	18
ENSXMAG00000006801	0.0031	21	12	pigh	1.27	18
ENSXMAG00000006867	0.0025	21	9	serhl	1.48	18
ENSXMAG00000006874	0.0025	3	15	si:dkey-222n6.2	4.20	0
ENSXMAG00000006892	0.0024	6	15	ctgfa	3.10	3

ENSXMAG00000006915	0.0034	9	12	mao	1.99	6
ENSXMAG00000006933	0.0016	9	18		1.42	6
ENSXMAG00000006994	0.0031	18	12	them4	2.84	15
ENSXMAG00000006999	0.0030	12	12		1.81	9
ENSXMAG00000007024	0.0024	18	9	ube3d	1.79	15
ENSXMAG00000007026	0.0031	21	12	cdca8	1.92	18
ENSXMAG00000007031	0.0025	18	9	nhp2	1.82	15
ENSXMAG00000007045	0.0028	24	15	itpkb	1.65	21
ENSXMAG00000007075	0.0027	24	9	ttk	1.86	21
ENSXMAG00000007085	0.0029	18	12	eny2	1.39	15
ENSXMAG00000007110	0.0025	12	15	ccdc80	1.52	9
ENSXMAG00000007111	0.0031	9	12		1.87	6
ENSXMAG00000007118	0.0015	3	6		1.61	0
ENSXMAG00000007120	0.0025	18	9	miga1	1.71	15
ENSXMAG00000007134	0.0031	18	12	zgc:153521	1.28	15
ENSXMAG00000007144	0.0031	6	12	PLEKHA5	1.80	3
ENSXMAG00000007148	0.0031	18	12	caprin1a	1.43	15
ENSXMAG00000007160	0.0031	18	12	WDR77	2.24	15
ENSXMAG00000007182	0.0031	9	12	tuft1a	2.16	6
ENSXMAG00000007257	0.0026	9	15	thbs4a	3.59	6
ENSXMAG00000007262	0.0027	3	15	cavin2a	1.53	0
ENSXMAG00000007299	0.0031	18	12	slc35b3	1.36	15
ENSXMAG00000007316	0.0026	18	9	cdk7	1.34	15
ENSXMAG00000007325	0.0025	18	9	nat10	1.87	15
ENSXMAG00000007329	0.0034	18	12	calub	2.73	15
ENSXMAG00000007351	0.0025	3	15	prodhb	2.75	0
ENSXMAG00000007357	0.0028	24	9		1.32	21
ENSXMAG00000007362	0.0015	24	6	erap2	1.58	21
ENSXMAG00000007389	0.0031	6	12	arglu1a	1.46	3
ENSXMAG00000007415	0.0025	24	9	anln	2.10	21
ENSXMAG00000007457	0.0031	15	12	si:ch211-117c9.1	2.28	12
ENSXMAG00000007467	0.0034	6	12		1.62	3
ENSXMAG00000007483	0.0026	9	9	sqor	1.23	6
ENSXMAG00000007484	0.0034	9	12	hebp2	2.26	6
ENSXMAG00000007485	0.0034	18	12	vcp	2.09	15
ENSXMAG00000007490	0.0025	6	9	tenm4	1.58	3
ENSXMAG00000007491	0.0025	3	15	hdac11	1.54	0
ENSXMAG00000007499	0.0024	18	9	cebpz	1.86	15
ENSXMAG00000007505	0.0025	21	9		2.83	18
ENSXMAG00000007512	0.0014	9	18		1.50	6
ENSXMAG00000007535	0.0024	21	9	psmb7	1.54	18
ENSXMAG00000007538	0.0026	18	9	xrcc3	1.95	15
ENSXMAG00000007552	0.0026	21	9		1.18	18
ENSXMAG00000007559	0.0024	3	15		1.86	0
ENSXMAG00000007584	0.0015	9	18	snx14	1.23	6
ENSXMAG00000007586	0.0025	9	9		2.38	6
ENSXMAG00000007594	0.0027	3	15	SLC8B1	1.36	0
ENSXMAG00000007617	0.0029	18	12	galk1	1.64	15
ENSXMAG00000007621	0.0031	18	12		1.45	15
ENSXMAG00000007637	0.0026	21	9	psmb10	1.69	18
ENSXMAG00000007649	0.0025	15	15	bckdha	1.49	12
ENSXMAG00000007658	0.0029	18	12	H2AFZ	1.88	15
ENSXMAG00000007685	0.0026	18	9	psmd8	2.32	15
ENSXMAG00000007692	0.0024	18	9	rcan3	1.84	15
ENSXMAG00000007701	0.0024	3	15	fmn1	1.61	0
ENSXMAG00000007705	0.0026	18	9	rwdd	3.85	15

ENSXMAG00000007708	0.0031	18	12	pes	1.78	15
ENSXMAG00000007731	0.0024	24	15	fbxo25	9.08	21
ENSXMAG00000007768	0.0031	18	12	zmpste24	2.60	15
ENSXMAG00000007770	0.0025	15	15	katnbl1	1.31	12
ENSXMAG00000007775	0.0026	9	9	thbs1b	1.73	6
ENSXMAG00000007785	0.0034	18	12	znf593	1.55	15
ENSXMAG00000007800	0.0030	6	12	znf395b	5.11	3
ENSXMAG00000007807	0.0031	18	12	ccdc43	2.73	15
ENSXMAG00000007815	0.0025	3	9		1.70	0
ENSXMAG00000007835	0.0024	21	9	taf6l	1.62	18
ENSXMAG00000007846	0.0024	9	9	mgat5	1.39	6
ENSXMAG00000007910	0.0026	18	15	lap3	1.36	15
ENSXMAG00000007919	0.0029	18	12	rpn2	1.95	15
ENSXMAG00000007931	0.0029	18	12	ecsit	2.04	15
ENSXMAG00000007959	0.0031	18	12	actr3	1.68	15
ENSXMAG00000007970	0.0030	18	12	fam45a	1.37	15
ENSXMAG00000007990	0.0025	18	9	lonp1	1.63	15
ENSXMAG00000008009	0.0014	3	6	cep55l	2.50	0
ENSXMAG00000008025	0.0031	18	12	aamp	1.32	15
ENSXMAG00000008040	0.0026	3	15		1.84	0
ENSXMAG00000008045	0.0025	3	15		3.96	0
ENSXMAG00000008049	0.0026	3	15	cpo	2.07	0
ENSXMAG00000008050	0.0027	3	15		4.19	0
ENSXMAG00000008059	0.0026	18	9		2.18	15
ENSXMAG00000008078	0.0030	6	12	afap1l2	1.70	3
ENSXMAG00000008079	0.0027	18	9		1.84	15
ENSXMAG00000008084	0.0031	18	12	copz1	1.69	15
ENSXMAG00000008122	0.0029	18	12	peli3	1.53	15
ENSXMAG00000008130	0.0029	18	12	ebp	8.43	15
ENSXMAG00000008156	0.0026	3	15	mlh1	1.93	0
ENSXMAG00000008158	0.0026	18	9	pelp1	2.22	15
ENSXMAG00000008179	0.0034	18	12	psmd2	1.94	15
ENSXMAG00000008184	0.0027	3	15	PKP1 (1 of many)	1.88	0
ENSXMAG00000008199	0.0031	6	12	ddx21	1.73	3
ENSXMAG00000008210	0.0026	9	15	sox19b	3.85	6
ENSXMAG00000008234	0.0026	18	9	lratb.1	2.88	15
ENSXMAG00000008273	0.0030	21	12	skp1	1.22	18
ENSXMAG00000008290	0.0026	21	9		2.71	18
ENSXMAG00000008294	0.0031	18	12	sar1b	1.69	15
ENSXMAG00000008315	0.0031	15	12	hsp90aa1.1	3.05	12
ENSXMAG00000008320	0.0031	18	12	pdss1	2.18	15
ENSXMAG00000008323	0.0024	21	15	kpna2	1.98	18
ENSXMAG00000008389	0.0030	24	12	ca10b	2.19	21
ENSXMAG00000008421	0.0031	24	12	gpr158a	2.82	21
ENSXMAG00000008431	0.0031	18	12	vps53	1.44	15
ENSXMAG00000008454	0.0026	18	9	dus4l	2.18	15
ENSXMAG00000008455	0.0026	9	15	apodb	2.71	6
ENSXMAG00000008485	0.0029	18	12	prpf19	1.95	15
ENSXMAG00000008494	0.0024	18	9	eftud2	2.25	15
ENSXMAG00000008496	0.0026	6	15		1.61	3
ENSXMAG00000008512	0.0031	21	12	gkup	1.47	18
ENSXMAG00000008516	0.0031	12	12	cxcl14	2.93	9
ENSXMAG00000008518	0.0024	9	15	fam13b	1.35	6
ENSXMAG00000008522	0.0031	18	12	fbl	1.78	15
ENSXMAG00000008553	0.0027	6	15	tmc2a	1.96	3
ENSXMAG00000008554	0.0015	24	18	pdgfrb	1.43	21

ENSXMAG00000008570	0.0026	3	15	sparta	1.90	0
ENSXMAG00000008579	0.0031	9	12	dclk1a	5.85	6
ENSXMAG00000008592	0.0034	18	12	trmt6	1.43	15
ENSXMAG00000008607	0.0029	18	12	alg5	1.56	15
ENSXMAG00000008643	0.0024	21	9	brms1	1.56	18
ENSXMAG00000008651	0.0015	21	6	cdc123	1.23	18
ENSXMAG00000008697	0.0031	18	12	nipsnap2	1.37	15
ENSXMAG00000008746	0.0026	18	9	adamts9	4.20	15
ENSXMAG00000008807	0.0024	18	9	mapk9	1.27	15
ENSXMAG00000008822	0.0025	18	9	pdhb	1.42	15
ENSXMAG00000008825	0.0030	18	12	slc5a5	1.49	15
ENSXMAG00000008826	0.0026	18	9	ssr1	1.67	15
ENSXMAG00000008830	0.0029	18	12	tmem147	1.78	15
ENSXMAG00000008841	0.0026	12	9		1.44	9
ENSXMAG00000008844	0.0031	3	12	SEMA4F	1.53	0
ENSXMAG00000008849	0.0029	12	12	gapdhs	1.39	9
ENSXMAG00000008878	0.0024	6	15	clk4a	1.40	3
ENSXMAG00000008891	0.0026	3	15		2.65	0
ENSXMAG00000008892	0.0014	18	18	ARNTL2 (1 of many)	6.14	15
ENSXMAG00000008900	0.0026	9	15	sdha	1.80	6
ENSXMAG00000008904	0.0026	18	9	vezt	1.62	15
ENSXMAG00000008936	0.0024	18	15	eri1	1.32	15
ENSXMAG00000008939	0.0031	18	12	DDX39A	2.01	15
ENSXMAG00000008963	0.0031	6	12	gas2l3	2.18	3
ENSXMAG00000008965	0.0024	24	9		2.23	21
ENSXMAG00000009027	0.0026	18	9	smg8	1.46	15
ENSXMAG00000009067	0.0029	24	12	DEPDC1B	2.10	21
ENSXMAG00000009070	0.0024	18	9	abcb7	2.30	15
ENSXMAG00000009077	0.0031	18	12	RBM25	1.48	15
ENSXMAG00000009101	0.0034	18	12	klhl36	1.53	15
ENSXMAG00000009131	0.0024	6	15	smoc1	1.77	3
ENSXMAG00000009141	0.0031	18	12		1.38	15
ENSXMAG00000009151	0.0034	18	12	lsm3	1.42	15
ENSXMAG00000009155	0.0026	18	9	erc1a	2.12	15
ENSXMAG00000009179	0.0026	15	15	psmd7	1.69	12
ENSXMAG00000009186	0.0031	6	12	paplna	1.99	3
ENSXMAG00000009196	0.0015	21	6	adrm1	1.63	18
ENSXMAG00000009241	0.0031	6	12	stag1a	1.73	3
ENSXMAG00000009292	0.0028	18	9	galnt11	1.62	15
ENSXMAG00000009304	0.0034	6	12	mrvi1	1.77	3
ENSXMAG00000009312	0.0024	9	9	dlec1	1.82	6
ENSXMAG00000009316	0.0015	6	18		6.22	3
ENSXMAG00000009322	0.0031	9	12	atp10b	1.59	6
ENSXMAG00000009329	0.0025	18	9	cradd	2.68	15
ENSXMAG00000009332	0.0031	18	12		1.62	15
ENSXMAG00000009356	0.0029	18	12	sarnp	2.90	15
ENSXMAG00000009372	0.0029	18	12	ftsj1	1.31	15
ENSXMAG00000009373	0.0025	24	9	nckap5l	1.20	21
ENSXMAG00000009385	0.0028	24	15	swap70a	2.46	21
ENSXMAG00000009402	0.0031	18	12	gars	2.80	15
ENSXMAG00000009419	0.0025	18	9	frmpd3	3.07	15
ENSXMAG00000009432	0.0027	24	15	kdm8	1.40	21
ENSXMAG00000009470	0.0025	24	9		1.91	21
ENSXMAG00000009501	0.0030	18	12		1.60	15
ENSXMAG00000009534	0.0031	18	12	nsdhl	2.93	15
ENSXMAG00000009538	0.0031	15	12	slc16a1b	1.75	12

ENSXMAG00000009587	0.0015	15	18	si:dkeyp-97e7.9	1.93	12
ENSXMAG00000009592	0.0024	3	15	ednraa	1.72	0
ENSXMAG00000009594	0.0027	18	15	lias	1.52	15
ENSXMAG00000009600	0.0034	18	12	si:dkey-85a20.4	1.95	15
ENSXMAG00000009612	0.0024	18	9	vps35l	1.43	15
ENSXMAG00000009615	0.0031	18	12	mmaa	1.85	15
ENSXMAG00000009645	0.0031	6	12	mlycd	1.97	3
ENSXMAG00000009666	0.0034	18	12	ergic1	2.54	15
ENSXMAG00000009671	0.0031	6	12	mrtfab	1.84	3
ENSXMAG00000009691	0.0025	21	9	xrcc6	1.54	18
ENSXMAG00000009769	0.0026	18	9	abcg2a	2.36	15
ENSXMAG00000009784	0.0014	21	6	cpsf3	1.53	18
ENSXMAG00000009796	0.0014	24	6	zgc:92107	1.34	21
ENSXMAG00000009810	0.0031	15	12	adam17b	1.42	12
ENSXMAG00000009815	0.0015	3	18	zmynd8	1.36	0
ENSXMAG00000009822	0.0031	15	12		1.41	12
ENSXMAG00000009858	0.0026	18	9	taf1b	2.10	15
ENSXMAG00000009861	0.0030	18	12	ATP6V0C	1.76	15
ENSXMAG00000009893	0.0034	18	12	EME2	2.19	15
ENSXMAG00000009913	0.0031	9	12	bmp2k	1.48	6
ENSXMAG00000009973	0.0026	3	9		3.64	0
ENSXMAG00000010009	0.0024	18	9	ddx51	1.58	15
ENSXMAG00000010014	0.0031	6	12	RASGEF1B	2.24	3
ENSXMAG00000010019	0.0031	9	12	cdh27	1.70	6
ENSXMAG00000010051	0.0025	18	9	rae1	1.30	15
ENSXMAG00000010052	0.0030	18	12	naa50	1.75	15
ENSXMAG00000010055	0.0031	18	12	ATP6V1A (1 of many)	2.19	15
ENSXMAG00000010063	0.0031	18	12	polr2gl	2.16	15
ENSXMAG00000010101	0.0031	18	12	fkbp2	1.70	15
ENSXMAG00000010105	0.0029	3	12	cntln	1.28	0
ENSXMAG00000010106	0.0031	18	12	rrn3	1.35	15
ENSXMAG00000010108	0.0026	9	9	pfkfb4a	1.83	6
ENSXMAG00000010120	0.0031	18	12	snapc3	2.19	15
ENSXMAG00000010188	0.0026	3	15	fsta	1.28	0
ENSXMAG00000010196	0.0025	15	15	atp23	1.46	12
ENSXMAG00000010205	0.0026	24	9	lef1	1.50	21
ENSXMAG00000010245	0.0014	3	6	SEC61A1 (1 of many)	1.24	0
ENSXMAG00000010255	0.0026	21	9	aimp1	1.48	18
ENSXMAG00000010288	0.0031	9	12		1.75	6
ENSXMAG00000010309	0.0026	3	15	FBLN1	2.27	0
ENSXMAG00000010310	0.0024	21	9		1.51	18
ENSXMAG00000010342	0.0029	18	12	dnajb11	2.35	15
ENSXMAG00000010360	0.0024	15	9	angpt2a	1.90	12
ENSXMAG00000010369	0.0026	3	15	kcnk5b	1.67	0
ENSXMAG00000010378	0.0027	3	15		1.62	0
ENSXMAG00000010385	0.0016	24	6	rrm1	1.91	21
ENSXMAG00000010437	0.0027	3	15	ube2w	1.81	0
ENSXMAG00000010475	0.0027	24	15	dok6	2.41	21
ENSXMAG00000010480	0.0024	21	9	si:ch211-156b7.4	2.69	18
ENSXMAG00000010488	0.0027	3	15	ECM2	2.34	0
ENSXMAG00000010496	0.0014	24	18	tmf1	1.21	21
ENSXMAG00000010497	0.0031	18	12		3.90	15
ENSXMAG00000010531	0.0026	3	15		1.58	0
ENSXMAG00000010534	0.0031	18	12	suclg2	1.39	15
ENSXMAG00000010575	0.0024	18	9	ttc27	1.78	15
ENSXMAG00000010583	0.0014	6	18	rnf25	1.32	3

ENSXMAG00000010602	0.0034	24	12	si:dkey-79d12.5	1.35	21
ENSXMAG00000010615	0.0028	21	9	orc4	1.34	18
ENSXMAG00000010628	0.0024	9	9	slc43a2b	3.22	6
ENSXMAG00000010644	0.0031	18	12	man1b1a	1.73	15
ENSXMAG00000010671	0.0031	15	12	dnajc3b	1.60	12
ENSXMAG00000010691	0.0029	9	12	slc12a7a	1.25	6
ENSXMAG00000010700	0.0030	21	12	nhej1	2.18	18
ENSXMAG00000010711	0.0026	18	9	pigv	1.62	15
ENSXMAG00000010735	0.0031	21	12		2.31	18
ENSXMAG00000010740	0.0028	18	9	med16	1.58	15
ENSXMAG00000010755	0.0024	24	15	zgc:158328	1.58	21
ENSXMAG00000010777	0.0029	18	12	ssr4	2.10	15
ENSXMAG00000010798	0.0031	18	12		3.23	15
ENSXMAG00000010825	0.0030	9	12	deptor	1.78	6
ENSXMAG00000010827	0.0027	9	9	dedd1	3.64	6
ENSXMAG00000010838	0.0014	24	18		1.74	21
ENSXMAG00000010850	0.0024	3	15	cpt1cb	3.61	0
ENSXMAG00000010853	0.0029	18	12	prmt1	2.64	15
ENSXMAG00000010883	0.0028	18	9	cdipt	1.62	15
ENSXMAG00000010901	0.0028	6	9	amigo3	1.82	3
ENSXMAG00000010906	0.0015	21	6	si:dkeyp-69c1.6	1.29	18
ENSXMAG00000010926	0.0071	18	6	spns1	1.40	15
ENSXMAG00000010938	0.0024	18	9	hars	1.50	15
ENSXMAG00000010946	0.0025	21	9	nrf1	1.51	18
ENSXMAG00000010964	0.0024	6	15		2.24	3
ENSXMAG00000010983	0.0034	18	12	taf15	1.64	15
ENSXMAG00000011005	0.0031	3	12	smo	1.31	0
ENSXMAG00000011012	0.0030	15	12		1.82	12
ENSXMAG00000011051	0.0029	9	12	lmo7a	1.54	6
ENSXMAG00000011082	0.0029	18	12	crym	1.77	15
ENSXMAG00000011091	0.0024	21	9	PSMA4	1.95	18
ENSXMAG00000011130	0.0024	24	15	si:ch211-69g19.2	2.28	21
ENSXMAG00000011161	0.0026	3	15		1.43	0
ENSXMAG00000011185	0.0030	15	12		1.40	12
ENSXMAG00000011196	0.0024	18	9	tpi1	1.48	15
ENSXMAG00000011204	0.0030	18	12	pa2g4b	2.67	15
ENSXMAG00000011212	0.0015	24	18	suco	1.25	21
ENSXMAG00000011227	0.0024	21	9	mlf2	1.26	18
ENSXMAG00000011229	0.0024	15	15	pigc	1.40	12
ENSXMAG00000011234	0.0025	21	9		1.45	18
ENSXMAG00000011241	0.0029	18	12	snx7	1.68	15
ENSXMAG00000011243	0.0031	18	12	cmss1	1.66	15
ENSXMAG00000011257	0.0015	6	18	cyr61	1.83	3
ENSXMAG00000011288	0.0029	18	12	mettl13	1.96	15
ENSXMAG00000011302	0.0029	18	12	foxred1	1.51	15
ENSXMAG00000011305	0.0025	18	9	dhx9	1.96	15
ENSXMAG00000011317	0.0025	3	15		1.57	0
ENSXMAG00000011328	0.0031	18	12		5.25	15
ENSXMAG00000011363	0.0029	6	12	PKP4	1.31	3
ENSXMAG00000011397	0.0025	21	9	prdx6	1.59	18
ENSXMAG00000011405	0.0030	9	12	RBMS1 (1 of many)	1.39	6
ENSXMAG00000011408	0.0031	18	12	fam20b	1.37	15
ENSXMAG00000011433	0.0024	3	15		2.66	0
ENSXMAG00000011455	0.0014	24	6	ncapd2	1.66	21
ENSXMAG00000011464	0.0025	24	15	nr1d4a	11.69	21
ENSXMAG00000011470	0.0026	3	15	creg2	3.28	0

ENSXMAG00000011483	0.0031	18	12	nop2	1.87	15
ENSXMAG00000011488	0.0031	12	12	vegfc	2.08	9
ENSXMAG00000011490	0.0025	18	9		1.58	15
ENSXMAG00000011529	0.0025	3	15	si:ch73-12o23.1	2.18	0
ENSXMAG00000011613	0.0027	24	15	gstk4	1.66	21
ENSXMAG00000011615	0.0031	6	12	scn1laa	2.22	3
ENSXMAG00000011620	0.0014	18	6	fer	1.47	15
ENSXMAG00000011645	0.0025	6	15	cyp2n13 (1 of many)	3.48	3
ENSXMAG00000011666	0.0024	6	15	col6a1	1.21	3
ENSXMAG00000011720	0.0031	6	12	igsf10	2.92	3
ENSXMAG00000011738	0.0029	18	12	psma6b	2.14	15
ENSXMAG00000011755	0.0029	18	12	nars	2.76	15
ENSXMAG00000011762	0.0014	15	18	zgc:162816	1.70	12
ENSXMAG00000011817	0.0024	21	9	sh3bgrl	1.54	18
ENSXMAG00000011826	0.0024	24	15	nxpe3 (1 of many)	1.77	21
ENSXMAG00000011839	0.0031	3	12	mctp2b	1.30	0
ENSXMAG00000011867	0.0031	15	12		1.45	12
ENSXMAG00000011885	0.0024	3	15	pafah1b2	1.51	0
ENSXMAG00000011888	0.0024	24	9	nat14	1.81	21
ENSXMAG00000011901	0.0025	12	15	hdx	1.26	9
ENSXMAG00000011913	0.0031	3	12	pld1b	1.44	0
ENSXMAG00000011915	0.0031	6	12	foxj2	1.29	3
ENSXMAG00000011952	0.0031	6	12	serpine1	5.96	3
ENSXMAG00000011978	0.0014	24	6	ppme1	1.13	21
ENSXMAG00000011981	0.0026	21	9		1.34	18
ENSXMAG00000011986	0.0031	6	12		1.92	3
ENSXMAG00000012011	0.0031	18	12	nudc	1.63	15
ENSXMAG00000012014	0.0026	3	15	meak7	1.43	0
ENSXMAG00000012027	0.0026	9	9	atp11a	1.93	6
ENSXMAG00000012038	0.0031	18	12		1.89	15
ENSXMAG00000012054	0.0024	3	15	dbpb	26.06	0
ENSXMAG00000012064	0.0024	24	15	josd2	1.71	21
ENSXMAG00000012066	0.0027	15	9		3.21	12
ENSXMAG00000012132	0.0025	18	9	taf6	1.24	15
ENSXMAG00000012149	0.0024	18	9	naxd	1.31	15
ENSXMAG00000012179	0.0025	3	15		1.64	0
ENSXMAG00000012180	0.0014	6	18		2.91	3
ENSXMAG00000012204	0.0029	18	12	manf	2.92	15
ENSXMAG00000012218	0.0024	21	9	alyref	1.23	18
ENSXMAG00000012228	0.0030	18	12	cul5b	1.99	15
ENSXMAG00000012240	0.0030	18	12	blmh	2.27	15
ENSXMAG00000012248	0.0031	9	12	bco2a	14.73	6
ENSXMAG00000012252	0.0024	18	9	pmm2	1.31	15
ENSXMAG00000012268	0.0031	18	12	dus2	3.31	15
ENSXMAG00000012276	0.0024	3	15	rap1gap2a	1.68	0
ENSXMAG00000012303	0.0031	6	12		4.39	3
ENSXMAG00000012321	0.0031	15	12	adamts15a	4.13	12
ENSXMAG00000012334	0.0016	9	18	plekha7a	1.35	6
ENSXMAG00000012337	0.0024	9	15	si:ch211-132b12.2	7.22	6
ENSXMAG00000012344	0.0029	18	12		1.98	15
ENSXMAG00000012347	0.0031	18	12	ctsa	1.62	15
ENSXMAG00000012361	0.0031	6	12	phldb1a	1.76	3
ENSXMAG00000012378	0.0028	18	9		1.82	15
ENSXMAG00000012411	0.0029	18	12	ddost	2.10	15
ENSXMAG00000012434	0.0029	18	12	tdg.1	2.05	15
ENSXMAG00000012443	0.0025	18	9		2.15	15

ENSXMAG00000012463	0.0028	18	9		1.54	15
ENSXMAG00000012479	0.0031	15	12	tsen2	1.43	12
ENSXMAG00000012498	0.0024	9	15	gpat3	2.55	6
ENSXMAG00000012502	0.0034	6	12	jcada	1.69	3
ENSXMAG00000012503	0.0026	18	9	mtpap	1.79	15
ENSXMAG00000012520	0.0029	18	12	pdap1a	1.76	15
ENSXMAG00000012532	0.0034	15	12	pik3c2b	1.84	12
ENSXMAG00000012536	0.0031	18	12	HSD17B7 (1 of many)	1.79	15
ENSXMAG00000012564	0.0026	6	9	HID1 (1 of many)	1.39	3
ENSXMAG00000012643	0.0014	21	6	mrpl39	1.24	18
ENSXMAG00000012653	0.0027	24	9	melk	2.24	21
ENSXMAG00000012679	0.0031	18	12	pfdn4	1.54	15
ENSXMAG00000012699	0.0024	3	15	mapk13	2.16	0
ENSXMAG00000012716	0.0029	18	12		1.22	15
ENSXMAG00000012720	0.0031	18	12	knop1	2.76	15
ENSXMAG00000012723	0.0025	9	15	si:dkey-282h22.5	9.58	6
ENSXMAG00000012760	0.0027	18	9	cdc27	1.52	15
ENSXMAG00000012766	0.0034	18	12	wee1	1.71	15
ENSXMAG00000012822	0.0024	18	9	ubtfl	1.33	15
ENSXMAG00000012834	0.0031	18	12	immt	1.48	15
ENSXMAG00000012841	0.0029	18	12	oaz1a	1.29	15
ENSXMAG00000012852	0.0024	21	9	si:ch73-302a13.2	1.36	18
ENSXMAG00000012865	0.0026	18	9	slc4a7	1.41	15
ENSXMAG00000012938	0.0024	18	15	si:ch211-157p22.10	1.83	15
ENSXMAG00000012946	0.0030	18	12	mpi	2.89	15
ENSXMAG00000012959	0.0034	18	12	sirt7	1.42	15
ENSXMAG00000013006	0.0031	18	12		1.41	15
ENSXMAG00000013021	0.0031	12	12	rorca	4.95	9
ENSXMAG00000013030	0.0026	18	9	lrrc29	2.05	15
ENSXMAG00000013062	0.0026	18	9	tnpo3	2.18	15
ENSXMAG00000013069	0.0029	18	12	pdia3	1.64	15
ENSXMAG00000013081	0.0030	9	12	dio1	8.47	6
ENSXMAG00000013103	0.0026	3	15	ptpn3	1.91	0
ENSXMAG00000013119	0.0024	18	9	alg1	1.92	15
ENSXMAG00000013137	0.0034	21	12	pigq	1.30	18
ENSXMAG00000013185	0.0031	18	12	mknk1	1.73	15
ENSXMAG00000013205	0.0031	18	12	faah	1.80	15
ENSXMAG00000013216	0.0025	21	9	uqcrh	1.73	18
ENSXMAG00000013219	0.0031	18	12	utp11l	2.00	15
ENSXMAG00000013245	0.0025	18	9	psmc4	2.17	15
ENSXMAG00000013250	0.0025	18	15	si:dkey-51e6.1	1.70	15
ENSXMAG00000013256	0.0025	3	9	si:ch73-373m9.1	1.40	0
ENSXMAG00000013279	0.0027	21	9	pgls	1.76	18
ENSXMAG00000013297	0.0026	9	9	si:dkeyp-97a10.2	2.01	6
ENSXMAG00000013327	0.0016	24	18	pcmtl	2.06	21
ENSXMAG00000013349	0.0029	18	12	gmppb	1.88	15
ENSXMAG00000013357	0.0026	24	9	shdb	1.68	21
ENSXMAG00000013380	0.0031	12	12	tspan13a	1.78	9
ENSXMAG00000013381	0.0026	18	9	tle2a	1.66	15
ENSXMAG00000013383	0.0031	6	12		1.28	3
ENSXMAG00000013407	0.0025	18	9	rbmx	1.46	15
ENSXMAG00000013450	0.0031	18	12	tbl2	2.07	15
ENSXMAG00000013453	0.0016	21	6	mtmr11	1.40	18
ENSXMAG00000013474	0.0031	18	12	trmt5	1.31	15
ENSXMAG00000013483	0.0024	18	9	tmem35	2.39	15
ENSXMAG00000013484	0.0029	3	12	paqr6	2.48	0

ENSXMAG00000013494	0.0031	9	12	epha3	1.40	6
ENSXMAG00000013521	0.0026	15	15	slc25a47a	1.61	12
ENSXMAG00000013537	0.0014	18	6	cdc42bpb	1.98	15
ENSXMAG00000013544	0.0026	3	15	si:dkey-21a6.5	2.34	0
ENSXMAG00000013575	0.0026	18	15	ndufaf6	1.40	15
ENSXMAG00000013596	0.0034	6	12	OTUB2	2.03	3
ENSXMAG00000013597	0.0024	18	9	nsun2	1.73	15
ENSXMAG00000013600	0.0031	18	12	ddx24	1.59	15
ENSXMAG00000013666	0.0026	18	9	nop58	2.22	15
ENSXMAG00000013686	0.0030	18	12	rexo4	1.38	15
ENSXMAG00000013701	0.0024	3	15		2.16	0
ENSXMAG00000013714	0.0030	18	12	si:ch211-113e8.3	2.45	15
ENSXMAG00000013715	0.0030	21	12	cdk5rap3	1.32	18
ENSXMAG00000013731	0.0015	18	6		1.63	15
ENSXMAG00000013765	0.0031	18	12	wdr83	1.39	15
ENSXMAG00000013803	0.0030	21	12	nif3l1	1.52	18
ENSXMAG00000013810	0.0024	3	15	slitrk2	1.53	0
ENSXMAG00000013821	0.0026	3	9	dpp4	1.84	0
ENSXMAG00000013823	0.0025	18	9	trmt1	2.27	15
ENSXMAG00000013831	0.0014	21	6		2.78	18
ENSXMAG00000013845	0.0024	21	9	zgc:92027	1.28	18
ENSXMAG00000013894	0.0025	18	9	stt3a	1.70	15
ENSXMAG00000013899	0.0026	21	9	hibadhb	1.40	18
ENSXMAG00000013918	0.0025	18	15	atp5f1b	1.25	15
ENSXMAG00000013927	0.0024	18	9	nup93	2.60	15
ENSXMAG00000014009	0.0025	18	9	phf2	1.23	15
ENSXMAG00000014012	0.0027	18	9	arfgap2	1.78	15
ENSXMAG00000014022	0.0029	6	12	gdpd5a	2.61	3
ENSXMAG00000014046	0.0025	18	9	bcat2	1.79	15
ENSXMAG00000014057	0.0026	9	9	kdm2bb	2.20	6
ENSXMAG00000014059	0.0031	6	12	RNF5	1.59	3
ENSXMAG00000014061	0.0026	12	15	wscd1b	2.09	9
ENSXMAG00000014063	0.0031	6	12	hsd17b14	2.84	3
ENSXMAG00000014077	0.0025	6	15	abcc5	4.94	3
ENSXMAG00000014084	0.0031	6	12	TPPP	2.21	3
ENSXMAG00000014089	0.0025	18	9		3.94	15
ENSXMAG00000014094	0.0016	21	18	rhof	1.38	18
ENSXMAG00000014110	0.0034	18	12	zgc:158403	1.34	15
ENSXMAG00000014111	0.0027	18	9	eif4a3	1.92	15
ENSXMAG00000014115	0.0015	18	18		1.29	15
ENSXMAG00000014129	0.0025	9	9	sh3rf1	1.52	6
ENSXMAG00000014132	0.0031	18	12	soul4	3.34	15
ENSXMAG00000014141	0.0025	18	9	sos1	2.09	15
ENSXMAG00000014150	0.0025	18	9	ogfod1	2.01	15
ENSXMAG00000014167	0.0031	18	12		5.76	15
ENSXMAG00000014172	0.0034	18	12	nudt21	1.58	15
ENSXMAG00000014191	0.0015	18	6	gemin6	1.79	15
ENSXMAG00000014236	0.0030	18	12	bms1	1.97	15
ENSXMAG00000014237	0.0025	18	9	spcs2	1.67	15
ENSXMAG00000014239	0.0031	18	12	rcn1	1.57	15
ENSXMAG00000014250	0.0015	18	6	HTRA1	2.82	15
ENSXMAG00000014251	0.0024	24	9		1.60	21
ENSXMAG00000014253	0.0029	18	12		1.69	15
ENSXMAG00000014277	0.0031	6	12		3.19	3
ENSXMAG00000014278	0.0024	18	9	mtr	2.71	15
ENSXMAG00000014282	0.0031	18	12	sdf2	2.41	15

ENSXMAG00000014290	0.0071	3	6		2.49	0
ENSXMAG00000014297	0.0015	24	18	nf2b	1.46	21
ENSXMAG00000014302	0.0029	9	12	adap2	2.47	6
ENSXMAG00000014318	0.0024	18	9	wdr62	1.53	15
ENSXMAG00000014336	0.0034	18	12	fastkd1	1.58	15
ENSXMAG00000014355	0.0024	6	9	MAX	1.45	3
ENSXMAG00000014366	0.0029	18	12	phgdh	3.70	15
ENSXMAG00000014370	0.0026	3	15	SIPA1L1 (1 of many)	1.39	0
ENSXMAG00000014398	0.0026	9	9	ret	1.56	6
ENSXMAG00000014407	0.0031	15	12	cltcl1	1.65	12
ENSXMAG00000014414	0.0027	3	15		1.53	0
ENSXMAG00000014421	0.0031	18	12	acbd4	1.65	15
ENSXMAG00000014422	0.0026	24	9	glipr2l	1.72	21
ENSXMAG00000014437	0.0024	24	15	adam10a	1.56	21
ENSXMAG00000014442	0.0031	18	12	MINPP1	1.29	15
ENSXMAG00000014445	0.0029	18	12	atad1b	1.71	15
ENSXMAG00000014459	0.0024	3	15	cadm4	1.52	0
ENSXMAG00000014472	0.0027	24	9	arhgap19	3.36	21
ENSXMAG00000014474	0.0024	12	9	gpr20	1.79	9
ENSXMAG00000014477	0.0014	24	18	dgat1b	1.79	21
ENSXMAG00000014515	0.0025	24	9	pomt2	1.30	21
ENSXMAG00000014516	0.0031	18	12	kif1bp	1.20	15
ENSXMAG00000014522	0.0024	18	9	aldh5a1	1.48	15
ENSXMAG00000014544	0.0029	18	12	cyc1	1.82	15
ENSXMAG00000014546	0.0026	12	15		1.63	9
ENSXMAG00000014551	0.0031	21	12	jpt1b	1.68	18
ENSXMAG00000014561	0.0030	18	12	hkdc1	2.34	15
ENSXMAG00000014576	0.0029	18	12	fcf1	2.46	15
ENSXMAG00000014605	0.0071	21	18	angel1	1.71	18
ENSXMAG00000014606	0.0025	18	9	yrdc	2.17	15
ENSXMAG00000014627	0.0015	24	6		2.05	21
ENSXMAG00000014629	0.0025	15	15	inpp5b	1.30	12
ENSXMAG00000014651	0.0016	21	18	l2hgdh	1.44	18
ENSXMAG00000014704	0.0027	18	9	ncoa5	1.30	15
ENSXMAG00000014712	0.0015	24	18	lrrc45	1.40	21
ENSXMAG00000014734	0.0026	18	9	rac3b	2.28	15
ENSXMAG00000014792	0.0031	9	12	arg2	4.72	6
ENSXMAG00000014814	0.0026	9	15	pbxip1a	1.34	6
ENSXMAG00000014854	0.0031	18	12	stx4	1.56	15
ENSXMAG00000014866	0.0025	18	9	tbl3	1.89	15
ENSXMAG00000014869	0.0031	18	12	pcca	1.28	15
ENSXMAG00000014894	0.0024	18	9	mis12	2.05	15
ENSXMAG00000014897	0.0031	18	12	ppm1g	2.21	15
ENSXMAG00000014921	0.0031	21	12	FAHD2A	1.47	18
ENSXMAG00000014925	0.0025	24	9	exoc3l4	1.47	21
ENSXMAG00000014929	0.0026	18	9	pdcd2	2.53	15
ENSXMAG00000014939	0.0031	18	12	jmjd7	2.04	15
ENSXMAG00000014950	0.0031	18	12	psmc3	1.65	15
ENSXMAG00000015000	0.0029	18	12	nudt18	2.67	15
ENSXMAG00000015011	0.0031	18	12	hectd3	1.51	15
ENSXMAG00000015012	0.0031	18	12	ybey	2.90	15
ENSXMAG00000015027	0.0025	18	9	si:ch211-87m7.2	1.87	15
ENSXMAG00000015073	0.0031	18	12	galnt2	1.37	15
ENSXMAG00000015076	0.0025	18	9	polr1c	1.67	15
ENSXMAG00000015115	0.0025	24	9	entpd5a	4.21	21
ENSXMAG00000015125	0.0031	24	12	cenpf	2.00	21

ENSXMAG00000015130	0.0031	6	12	si:ch211-103e16.5	3.10	3
ENSXMAG00000015160	0.0025	18	9	selenot2	1.85	15
ENSXMAG00000015165	0.0016	21	6	rnf4	1.42	18
ENSXMAG00000015249	0.0024	18	9	afg1la	1.88	15
ENSXMAG00000015266	0.0026	21	9	cndp2	1.64	18
ENSXMAG00000015287	0.0034	18	12	tmed7	1.67	15
ENSXMAG00000015292	0.0029	18	12	rtraf	1.42	15
ENSXMAG00000015293	0.0030	18	12	eif4eb	2.31	15
ENSXMAG00000015299	0.0031	18	12	prpf40a	1.89	15
ENSXMAG00000015314	0.0031	3	12	per1b	18.24	0
ENSXMAG00000015329	0.0031	15	12	clip2	2.33	12
ENSXMAG00000015357	0.0034	18	12		1.44	15
ENSXMAG00000015367	0.0031	18	12		2.25	15
ENSXMAG00000015371	0.0031	9	12		1.38	6
ENSXMAG00000015373	0.0024	18	15	cnpy4	2.23	15
ENSXMAG00000015385	0.0029	3	12	aspg	1.52	0
ENSXMAG00000015395	0.0016	24	18	cpt1ab	2.15	21
ENSXMAG00000015401	0.0031	9	12	grk5l	1.57	6
ENSXMAG00000015415	0.0031	6	12	ntn4	2.16	3
ENSXMAG00000015445	0.0031	18	12	lipf	1.41	15
ENSXMAG00000015446	0.0030	18	12	DGKQ	3.34	15
ENSXMAG00000015454	0.0025	18	9		2.09	15
ENSXMAG00000015468	0.0030	18	12	zgc:158828	2.93	15
ENSXMAG00000015492	0.0026	24	9	hdhd3	1.52	21
ENSXMAG00000015521	0.0026	21	9	cnot8	1.29	18
ENSXMAG00000015538	0.0024	18	9	gemin5	1.49	15
ENSXMAG00000015548	0.0034	9	12	far1	1.64	6
ENSXMAG00000015559	0.0014	9	6	trps1	1.51	6
ENSXMAG00000015564	0.0025	21	9	mrpl22	1.38	18
ENSXMAG00000015567	0.0031	9	12	rorb	4.52	6
ENSXMAG00000015578	0.0029	18	12	kdelc2	1.89	15
ENSXMAG00000015595	0.0029	18	12	rars	2.94	15
ENSXMAG00000015619	0.0025	18	9	arntl1a	11.29	15
ENSXMAG00000015642	0.0025	21	9	tmem169b	1.72	18
ENSXMAG00000015696	0.0029	18	12	arl3	1.97	15
ENSXMAG00000015700	0.0026	18	9	sfxn2	1.43	15
ENSXMAG00000015704	0.0026	21	9	kif2c	2.23	18
ENSXMAG00000015733	0.0024	15	15	slc8a1a	1.40	12
ENSXMAG00000015748	0.0026	24	9	vps28	1.19	21
ENSXMAG00000015755	0.0030	18	12	YBX2	4.04	15
ENSXMAG00000015770	0.0014	3	18	zgc:158316	1.55	0
ENSXMAG00000015773	0.0028	9	15	hivep3a	9.01	6
ENSXMAG00000015778	0.0031	18	12	slc24a1 (1 of many)	1.78	15
ENSXMAG00000015819	0.0025	24	15	arih1	1.22	21
ENSXMAG00000015822	0.0026	18	9	cacybp	1.45	15
ENSXMAG00000015857	0.0027	18	9	smpd4	1.41	15
ENSXMAG00000015874	0.0031	18	12	msantd1	2.01	15
ENSXMAG00000015877	0.0029	18	12		1.61	15
ENSXMAG00000015904	0.0031	9	12		6.40	6
ENSXMAG00000015937	0.0031	6	12		2.11	3
ENSXMAG00000015941	0.0031	18	12		2.50	15
ENSXMAG00000015955	0.0026	3	15		1.86	0
ENSXMAG00000015965	0.0034	12	12		1.67	9
ENSXMAG00000015972	0.0031	21	12	nit2	1.61	18
ENSXMAG00000015974	0.0025	3	15	cdc14b	1.38	0
ENSXMAG00000015975	0.0025	6	9		3.72	3

ENSXMAG00000015984	0.0031	18	12	polr2c	1.52	15
ENSXMAG00000015995	0.0031	6	12	rp2	1.35	3
ENSXMAG00000015996	0.0029	18	12	spcs3	1.76	15
ENSXMAG00000016021	0.0026	18	15		3.73	15
ENSXMAG00000016060	0.0026	24	15	ttc14	1.26	21
ENSXMAG00000016068	0.0024	12	9		1.87	9
ENSXMAG00000016073	0.0031	18	12	uba2	2.29	15
ENSXMAG00000016078	0.0025	21	9	mesd	1.62	18
ENSXMAG00000016098	0.0034	18	12	mrps27	1.76	15
ENSXMAG00000016115	0.0031	18	12	sept5b	4.40	15
ENSXMAG00000016138	0.0024	18	9	sars	1.67	15
ENSXMAG00000016175	0.0031	18	12	stau1	1.72	15
ENSXMAG00000016178	0.0031	18	12	dnajc11a	1.77	15
ENSXMAG00000016187	0.0031	9	12	heg1	2.24	6
ENSXMAG00000016233	0.0026	24	9		1.39	21
ENSXMAG00000016253	0.0026	24	15	dip2a	1.26	21
ENSXMAG00000016257	0.0015	24	18	si:ch1073-513e17.1	2.31	21
ENSXMAG00000016308	0.0031	18	12	rrp9	2.57	15
ENSXMAG00000016318	0.0028	6	15	nod2	1.76	3
ENSXMAG00000016322	0.0031	18	12		2.21	15
ENSXMAG00000016348	0.0029	18	12	yars	2.03	15
ENSXMAG00000016365	0.0015	9	18	PTPRB	1.89	6
ENSXMAG00000016391	0.0014	18	18	sfpq	1.30	15
ENSXMAG00000016437	0.0024	18	9	tepsin	1.53	15
ENSXMAG00000016447	0.0031	3	12	prkag2b	1.79	0
ENSXMAG00000016460	0.0029	18	12	hsp90b1	2.28	15
ENSXMAG00000016464	0.0031	6	12		1.75	3
ENSXMAG00000016477	0.0029	18	12	b4galt7	1.83	15
ENSXMAG00000016507	0.0014	21	6		1.55	18
ENSXMAG00000016561	0.0025	18	9	zgc:91910	1.41	15
ENSXMAG00000016574	0.0026	3	15	CPXM2	1.76	0
ENSXMAG00000016576	0.0025	6	15	pacsin1a	3.49	3
ENSXMAG00000016602	0.0029	3	12	LRRC49	2.28	0
ENSXMAG00000016616	0.0025	21	9	psmb2	1.71	18
ENSXMAG00000016624	0.0031	18	12	psma8	1.84	15
ENSXMAG00000016643	0.0014	24	18	ccdc136b	2.14	21
ENSXMAG00000016653	0.0027	3	15	kctd1	1.45	0
ENSXMAG00000016660	0.0029	18	12	si:dkey-31b16.7	1.54	15
ENSXMAG00000016712	0.0026	3	15	dsc2l	1.58	0
ENSXMAG00000016739	0.0031	9	12		2.68	6
ENSXMAG00000016745	0.0026	6	15		1.63	3
ENSXMAG00000016771	0.0014	21	6		1.36	18
ENSXMAG00000016773	0.0025	18	9	slc25a25b	6.83	15
ENSXMAG00000016787	0.0015	24	18	znf384l	1.50	21
ENSXMAG00000016827	0.0025	6	15	ttll9	3.09	3
ENSXMAG00000016828	0.0026	21	9	derl2	1.44	18
ENSXMAG00000016862	0.0014	3	6		2.85	0
ENSXMAG00000016868	0.0024	18	9	asns	6.67	15
ENSXMAG00000016907	0.0025	18	9	PNPT1	1.52	15
ENSXMAG00000016927	0.0030	12	12	chchd3b	1.66	9
ENSXMAG00000016928	0.0029	15	12	clocka	14.55	12
ENSXMAG00000016935	0.0014	24	18		1.96	21
ENSXMAG00000016936	0.0029	18	12	top2b	1.71	15
ENSXMAG00000016937	0.0031	21	12	CCDC88A	1.54	18
ENSXMAG00000016945	0.0031	18	12	psmc5	1.68	15
ENSXMAG00000016973	0.0031	3	12	tubd1	1.21	0

ENSXMAG00000016974	0.0030	18	12	mon1bb	1.21	15
ENSXMAG00000016987	0.0031	9	12	si:ch211-107o10.3	3.52	6
ENSXMAG00000017011	0.0031	18	12	lrrc59	2.53	15
ENSXMAG00000017026	0.0031	18	12	riox1	2.40	15
ENSXMAG00000017047	0.0029	18	12	dnm1l	1.35	15
ENSXMAG00000017048	0.0031	18	12	cpsf2	1.62	15
ENSXMAG00000017060	0.0025	21	9	arl2	1.57	18
ENSXMAG00000017066	0.0030	9	12	thrb	1.72	6
ENSXMAG00000017074	0.0026	12	15	selenbp1	1.93	9
ENSXMAG00000017106	0.0024	9	9	nr1d2b	3.89	6
ENSXMAG00000017107	0.0026	9	9	fam83e	1.63	6
ENSXMAG00000017125	0.0026	21	9		1.77	18
ENSXMAG00000017128	0.0031	6	12	acot16	1.50	3
ENSXMAG00000017138	0.0031	18	12	prmt9	2.10	15
ENSXMAG00000017144	0.0031	15	12		1.72	12
ENSXMAG00000017150	0.0026	3	15	miip	2.33	0
ENSXMAG00000017173	0.0024	3	15	gls2a	3.11	0
ENSXMAG00000017185	0.0026	3	15	ptx3b	1.71	0
ENSXMAG00000017189	0.0031	6	12		1.97	3
ENSXMAG00000017203	0.0028	3	15	slc16a12a	2.59	0
ENSXMAG00000017251	0.0026	18	9	slc8a1b	3.58	15
ENSXMAG00000017272	0.0034	18	12	lmf1	1.25	15
ENSXMAG00000017330	0.0024	3	15	PPARG	1.55	0
ENSXMAG00000017348	0.0031	3	12	nfatc3a	1.21	0
ENSXMAG00000017361	0.0031	18	12	trnau1apa	1.81	15
ENSXMAG00000017403	0.0031	18	12	idh3a	1.55	15
ENSXMAG00000017416	0.0029	18	12	ptges3b	2.09	15
ENSXMAG00000017430	0.0025	18	9	lcmt1	1.81	15
ENSXMAG00000017443	0.0028	24	9		1.28	21
ENSXMAG00000017456	0.0015	24	18	mtmr7a	1.60	21
ENSXMAG00000017466	0.0025	9	15		4.76	6
ENSXMAG00000017493	0.0024	18	9	si:dkey-27c15.3	1.71	15
ENSXMAG00000017501	0.0027	15	15	zgc:100906	1.39	12
ENSXMAG00000017503	0.0025	18	9	ints7	2.12	15
ENSXMAG00000017520	0.0025	3	15	lpin1	5.22	0
ENSXMAG00000017532	0.0026	3	15		3.84	0
ENSXMAG00000017560	0.0026	21	9	nek2	3.04	18
ENSXMAG00000017591	0.0026	18	9	glra3	1.89	15
ENSXMAG00000017601	0.0026	21	9	glmp	1.30	18
ENSXMAG00000017613	0.0025	3	15		1.56	0
ENSXMAG00000017614	0.0026	18	9	strn	2.00	15
ENSXMAG00000017631	0.0015	24	18	POC1A	1.79	21
ENSXMAG00000017647	0.0026	18	9		2.49	15
ENSXMAG00000017658	0.0026	3	15	zmp:0000000711	1.85	0
ENSXMAG00000017711	0.0025	18	15	plxdc1	1.66	15
ENSXMAG00000017736	0.0034	18	12	timm10	2.34	15
ENSXMAG00000017749	0.0031	6	12	rdh8a	1.87	3
ENSXMAG00000017759	0.0029	18	12	myg1	2.20	15
ENSXMAG00000017773	0.0029	18	12	pdia6	1.91	15
ENSXMAG00000017806	0.0026	21	9	si:dkey-12e7.4	1.46	18
ENSXMAG00000017818	0.0025	18	9	ARHGEF2	1.49	15
ENSXMAG00000017846	0.0029	18	12	mettl14	1.57	15
ENSXMAG00000017867	0.0026	3	15	stoml3b	1.56	0
ENSXMAG00000017896	0.0029	15	12	sost	1.60	12
ENSXMAG00000017919	0.0026	3	15		1.74	0
ENSXMAG00000017920	0.0030	18	12	mars	7.96	15

ENSXMAG00000017932	0.0025	24	15		1.49	21
ENSXMAG00000017933	0.0029	18	12	ewsr1b	1.71	15
ENSXMAG00000017935	0.0031	18	12	snrpc	1.76	15
ENSXMAG00000017969	0.0031	18	12	rpf1	1.38	15
ENSXMAG00000017972	0.0026	18	9	eya3	1.28	15
ENSXMAG00000018034	0.0031	18	12	si:ch211-51c14.1	1.75	15
ENSXMAG00000018075	0.0025	18	9	ascc2	1.68	15
ENSXMAG00000018080	0.0026	21	9	vps33b	1.29	18
ENSXMAG00000018082	0.0024	3	9	ssx2ipb	2.88	0
ENSXMAG00000018090	0.0026	6	15	UNC13C	1.64	3
ENSXMAG00000018096	0.0026	3	9	plp2 (1 of many)	1.67	0
ENSXMAG00000018097	0.0031	18	12	IDH2	1.57	15
ENSXMAG00000018118	0.0031	15	12	znf710b	1.38	12
ENSXMAG00000018120	0.0029	18	12	psmb3	1.81	15
ENSXMAG00000018125	0.0031	18	12	mfap1	1.38	15
ENSXMAG00000018129	0.0031	3	12		2.21	0
ENSXMAG00000018139	0.0025	18	9	ftsj3	1.65	15
ENSXMAG00000018142	0.0015	24	6		1.54	21
ENSXMAG00000018149	0.0025	21	15	PCGF2	1.18	18
ENSXMAG00000018196	0.0026	24	15	rtkn2a	2.25	21
ENSXMAG00000018203	0.0031	18	12	trappc6bl	1.44	15
ENSXMAG00000018217	0.0031	12	12	dao.2	2.76	9
ENSXMAG00000018219	0.0028	21	9	vkorc1l1	1.37	18
ENSXMAG00000018223	0.0029	18	12	actl6a	2.13	15
ENSXMAG00000018224	0.0026	3	15	sdccag8	1.31	0
ENSXMAG00000018240	0.0024	18	9	tubgcp4	1.94	15
ENSXMAG00000018286	0.0025	24	9	fggy	1.83	21
ENSXMAG00000018315	0.0025	21	9	srp54	1.23	18
ENSXMAG00000018333	0.0029	18	12	htra1a	2.66	15
ENSXMAG00000018355	0.0014	18	6		3.74	15
ENSXMAG00000018356	0.0026	18	9	SPATA5	1.61	15
ENSXMAG00000018393	0.0031	18	12	cpox (1 of many)	1.32	15
ENSXMAG00000018394	0.0031	18	12	psmd13	1.90	15
ENSXMAG00000018432	0.0027	9	9	klhl20	1.12	6
ENSXMAG00000018454	0.0031	18	12	ELOB	1.28	15
ENSXMAG00000018474	0.0031	18	12	pelo	3.32	15
ENSXMAG00000018492	0.0029	18	12	dmap1	2.13	15
ENSXMAG00000018498	0.0030	18	12	nol10	2.62	15
ENSXMAG00000018500	0.0024	18	15	bud23	1.40	15
ENSXMAG00000018502	0.0031	18	12	hnrnpm	1.25	15
ENSXMAG00000018516	0.0026	3	15	PAOX (1 of many)	1.70	0
ENSXMAG00000018525	0.0016	24	6	msna	1.44	21
ENSXMAG00000018529	0.0031	18	12	dars2	2.54	15
ENSXMAG00000018535	0.0031	18	12	si:dkey-33c12.4	1.57	15
ENSXMAG00000018575	0.0026	9	9	itpkcb	1.36	6
ENSXMAG00000018580	0.0029	18	12	snrpa	3.62	15
ENSXMAG00000018601	0.0028	6	15	apoeb	1.72	3
ENSXMAG00000018630	0.0024	21	9	lsm7	1.53	18
ENSXMAG00000018643	0.0031	6	12	fam234a	1.29	3
ENSXMAG00000018667	0.0031	3	12	scarb1	1.70	0
ENSXMAG00000018673	0.0025	18	9	eif4bb	1.42	15
ENSXMAG00000018696	0.0031	18	12	dtwd2	1.71	15
ENSXMAG00000018701	0.0031	3	12	micu2	1.30	0
ENSXMAG00000018707	0.0027	6	9	vdrb	2.18	3
ENSXMAG00000018718	0.0026	9	15		5.17	6
ENSXMAG00000018720	0.0025	6	15	tmem41ab	2.09	3

ENSXMAG00000018730	0.0031	18	12	cope	1.54	15
ENSXMAG00000018739	0.0025	15	15	kifap3b	1.64	12
ENSXMAG00000018772	0.0031	18	12	brms1lb	2.88	15
ENSXMAG00000018773	0.0026	12	15	si:ch1073-358c10.1	4.41	9
ENSXMAG00000018814	0.0029	18	12	mrpl37	1.65	15
ENSXMAG00000018874	0.0034	18	12	pdia4	2.32	15
ENSXMAG00000018887	0.0025	24	15	spsb4a	1.70	21
ENSXMAG00000018896	0.0031	18	12	rpn1	1.98	15
ENSXMAG00000018902	0.0031	18	12		1.57	15
ENSXMAG00000018928	0.0031	18	12	gnptg	1.48	15
ENSXMAG00000018934	0.0025	3	9	nr1d1	6.69	0
ENSXMAG00000018936	0.0031	18	12	lck	1.52	15
ENSXMAG00000018938	0.0025	18	15	pgk1	1.54	15
ENSXMAG00000018959	0.0026	24	15	rb1cc1	1.53	21
ENSXMAG00000018965	0.0026	18	9	plrg1	1.23	15
ENSXMAG00000018970	0.0025	18	9	wdr24	1.47	15
ENSXMAG00000018973	0.0025	21	9	mrps24	1.65	18
ENSXMAG00000019001	0.0025	18	9	ap2m1a	1.68	15
ENSXMAG00000019003	0.0024	21	9	agmo	2.70	18
ENSXMAG00000019024	0.0029	18	12	ankmy2a	1.75	15
ENSXMAG00000019055	0.0031	18	12	actr5	2.22	15
ENSXMAG00000019061	0.0024	3	9	ift140	1.46	0
ENSXMAG00000019070	0.0024	18	9		1.65	15
ENSXMAG00000019078	0.0034	18	12	IL1R2	1.67	15
ENSXMAG00000019080	0.0024	24	15	metap1d	1.56	21
ENSXMAG00000019157	0.0030	9	12		1.75	6
ENSXMAG00000019165	0.0025	3	15	chmp4bb	1.38	0
ENSXMAG00000019172	0.0029	18	12	eif2s2	2.02	15
ENSXMAG00000019187	0.0026	18	15	mrpl16	1.56	15
ENSXMAG00000019200	0.0029	18	12	fbxo18	1.81	15
ENSXMAG00000019211	0.0030	6	12	mlc1	2.77	3
ENSXMAG00000019227	0.0024	3	15	filip1a	1.82	0
ENSXMAG00000019247	0.0029	18	12	denr	2.15	15
ENSXMAG00000019273	0.0026	9	9	ptprsa	1.39	6
ENSXMAG00000019274	0.0024	3	15	tmco3	1.42	0
ENSXMAG00000019296	0.0030	15	12	rorcb	13.67	12
ENSXMAG00000019300	0.0031	9	12	sh3gl1a	1.47	6
ENSXMAG00000019302	0.0015	3	18	sema4e	1.70	0
ENSXMAG00000019318	0.0031	18	12	ifrd2	1.47	15
ENSXMAG00000019320	0.0026	18	9	plekhj1	1.55	15
ENSXMAG00000019336	0.0031	21	12	cirbpa	1.64	18
ENSXMAG00000019340	0.0024	18	9	ARMC6	2.28	15
ENSXMAG00000019354	0.0024	18	9	impdh2	1.92	15
ENSXMAG00000019389	0.0071	21	18	SLC35E2A	1.44	18
ENSXMAG00000019403	0.0014	24	18		1.61	21
ENSXMAG00000019404	0.0031	24	12	fam172a	1.40	21
ENSXMAG00000019408	0.0031	18	12	nol6	1.91	15
ENSXMAG00000019455	0.0027	3	15		7.42	0
ENSXMAG00000019465	0.0029	9	12		1.58	6
ENSXMAG00000019476	0.0015	18	6	zgc:103697	1.55	15
ENSXMAG00000019487	0.0024	15	15	SLC35A4	1.71	12
ENSXMAG00000019530	0.0025	18	9		15.37	15
ENSXMAG00000019564	0.0025	9	9	zgc:154093	2.44	6
ENSXMAG00000019572	0.0031	18	12	FASTKD5	1.37	15
ENSXMAG00000019607	0.0029	21	12	taf13	1.42	18
ENSXMAG00000019661	0.0016	18	18	zgc:63470	1.20	15

ENSXMAG00000019675	0.0024	18	9	dusp14	4.37	15
ENSXMAG00000019701	0.0024	12	15	tmem203	1.21	9
ENSXMAG00000019792	0.0024	6	15	tob1a	1.84	3
ENSXMAG00000019863	0.0031	9	12	paqr7b	2.89	6
ENSXMAG00000019865	0.0031	9	12	si:ch73-334d15.1	8.39	6
ENSXMAG00000019942	0.0034	21	12	si:dkey-237j11.3	1.42	18
ENSXMAG00000019943	0.0030	15	12	nfil3	5.21	12
ENSXMAG00000019958	0.0031	18	12	aldh7a1	1.41	15
ENSXMAG00000019975	0.0024	9	9		2.33	6
ENSXMAG00000019996	0.0024	24	9	vasnb	1.37	21
ENSXMAG00000020001	0.0034	18	12	adob	1.71	15
ENSXMAG00000020012	0.0029	18	12	emc6	1.56	15
ENSXMAG00000020013	0.0029	18	12	pigw	1.39	15
ENSXMAG00000020015	0.0030	6	12	KCNS2	1.54	3
ENSXMAG00000020092	0.0026	18	9	timm8b	1.99	15
ENSXMAG00000020094	0.0031	18	12	ptrh2	1.94	15
ENSXMAG00000020149	0.0031	15	12	slitrk5a	1.54	12
ENSXMAG00000020299	0.0026	9	15	tpst1	1.88	6
ENSXMAG00000020382	0.0031	18	12	FH	1.66	15
ENSXMAG00000020394	0.0031	9	12	npy8br	3.94	6
ENSXMAG00000020786	0.0031	18	12	ND1	1.41	15
ENSXMAG00000020818	0.0031	15	12	rdh12	2.21	12
ENSXMAG00000020822	0.0031	6	12	zc3h6	2.10	3
ENSXMAG00000020840	0.0014	18	6		5.80	15
ENSXMAG00000020878	0.0028	18	9	nol7	1.48	15
ENSXMAG00000020890	0.0027	3	15	ptprsa	1.57	0
ENSXMAG00000020901	0.0026	18	9		1.27	15
ENSXMAG00000020966	0.0025	9	9	slc38a2 (1 of many)	1.42	6
ENSXMAG00000021003	0.0025	12	15	rbp4	2.47	9
ENSXMAG00000021035	0.0025	3	15	tp53i11a	1.62	0
ENSXMAG00000021046	0.0027	3	15		1.69	0
ENSXMAG00000021068	0.0025	9	9	osbpl2b	1.54	6
ENSXMAG00000021085	0.0031	15	12	ndufa4l2a	4.99	12
ENSXMAG00000021116	0.0031	18	12	rabggtb	1.36	15
ENSXMAG00000021118	0.0025	9	9	sipa1l3	1.24	6
ENSXMAG00000021162	0.0015	24	6		1.52	21
ENSXMAG00000021171	0.0026	3	15		1.83	0
ENSXMAG00000021188	0.0015	24	18	parp12a	2.12	21
ENSXMAG00000021244	0.0015	9	6	cables1	1.59	6
ENSXMAG00000021252	0.0014	21	6		1.68	18
ENSXMAG00000021253	0.0026	3	15		2.81	0
ENSXMAG00000021269	0.0029	18	12	SURF4	1.66	15
ENSXMAG00000021301	0.0014	24	18	epb41a	2.46	21
ENSXMAG00000021325	0.0026	3	15		1.44	0
ENSXMAG00000021337	0.0034	18	12	mogs	2.18	15
ENSXMAG00000021359	0.0015	12	6	sned1	1.35	9
ENSXMAG00000021390	0.0026	18	9	fpgs	2.64	15
ENSXMAG00000021439	0.0028	6	15	gdnfa	1.48	3
ENSXMAG00000021451	0.0034	21	12	eci2	1.30	18
ENSXMAG00000021487	0.0031	3	12	tet1	1.84	0
ENSXMAG00000021496	0.0034	18	12		1.56	15
ENSXMAG00000021506	0.0014	9	18	si:dkey-234i14.6	5.20	6
ENSXMAG00000021531	0.0014	21	6	gpr78b	6.46	18
ENSXMAG00000021541	0.0015	21	18	cyb5d2	1.39	18
ENSXMAG00000021572	0.0031	18	12	lrrc14b	1.82	15
ENSXMAG00000021577	0.0028	18	9	rpp25l	1.78	15

ENSXMAG00000021579	0.0025	9	15	si:ch211-140m22.7	3.27	6
ENSXMAG00000021584	0.0031	6	12	tmem241	2.41	3
ENSXMAG00000021590	0.0034	18	12	tomm5	1.80	15
ENSXMAG00000021614	0.0030	18	12	dcps	2.13	15
ENSXMAG00000021623	0.0034	18	12	marcksb	2.02	15
ENSXMAG00000021625	0.0028	6	15		3.48	3
ENSXMAG00000021649	0.0024	18	9	C11orf98	1.50	15
ENSXMAG00000021718	0.0025	24	15		1.61	21
ENSXMAG00000021736	0.0031	18	12	papss1	1.82	15
ENSXMAG00000021740	0.0071	21	18	pick1	1.25	18
ENSXMAG00000021778	0.0026	21	15		1.61	18
ENSXMAG00000021847	0.0029	18	12	p4hb	1.32	15
ENSXMAG00000021848	0.0025	18	15		1.39	15
ENSXMAG00000021856	0.0034	18	12	clta	1.25	15
ENSXMAG00000021880	0.0026	15	15	ankrd10b	1.89	12
ENSXMAG00000021891	0.0031	21	12	nsmce2	1.64	18
ENSXMAG00000021912	0.0031	15	12	dhrs3b	2.19	12
ENSXMAG00000021939	0.0031	18	12	COPS6	1.58	15
ENSXMAG00000021955	0.0031	18	12	aprt	1.80	15
ENSXMAG00000021972	0.0028	24	15		1.81	21
ENSXMAG00000021983	0.0024	21	9	CDK2AP1	1.90	18
ENSXMAG00000022015	0.0024	15	9	pkn3	1.63	12
ENSXMAG00000022050	0.0025	18	9		1.42	15
ENSXMAG00000022075	0.0026	18	9		1.44	15
ENSXMAG00000022108	0.0031	6	12		1.38	3
ENSXMAG00000022123	0.0026	24	9	zgc:162898	1.58	21
ENSXMAG00000022157	0.0031	18	12	psma2	1.90	15
ENSXMAG00000022186	0.0028	18	9	yipf3	1.60	15
ENSXMAG00000022203	0.0031	6	12	AHNAK	1.32	3
ENSXMAG00000022217	0.0031	18	12	tefm	1.86	15
ENSXMAG00000022219	0.0031	18	12	si:dkey-122a22.2	3.11	15
ENSXMAG00000022224	0.0031	18	12	setd7 (1 of many)	4.13	15
ENSXMAG00000022258	0.0026	3	15	ZC3H7B (1 of many)	1.79	0
ENSXMAG00000022273	0.0031	18	12	c1d	1.64	15
ENSXMAG00000022277	0.0025	15	15	nxt2	1.70	12
ENSXMAG00000022312	0.0027	18	9		1.85	15
ENSXMAG00000022314	0.0026	15	15	kctd9a	1.42	12
ENSXMAG00000022322	0.0030	9	12		1.54	6
ENSXMAG00000022353	0.0026	15	9		1.66	12
ENSXMAG00000022370	0.0030	24	12	COL8A1	1.60	21
ENSXMAG00000022378	0.0026	9	9	zgc:154058	1.96	6
ENSXMAG00000022390	0.0031	18	12		1.29	15
ENSXMAG00000022402	0.0026	3	15		1.53	0
ENSXMAG00000022411	0.0031	18	12	timm13	1.80	15
ENSXMAG00000022428	0.0025	21	9	snx22	1.47	18
ENSXMAG00000022429	0.0029	18	12	cdca5	1.88	15
ENSXMAG00000022547	0.0024	24	15	srxn1	1.86	21
ENSXMAG00000022563	0.0026	21	9	ccdc58	1.55	18
ENSXMAG00000022572	0.0024	18	9	si:ch211-195o20.7	3.71	15
ENSXMAG00000022578	0.0034	15	12	TMEM60	2.09	12
ENSXMAG00000022607	0.0026	3	15		1.55	0
ENSXMAG00000022669	0.0031	24	12		2.35	21
ENSXMAG00000022678	0.0071	21	18	ntpcr	1.61	18
ENSXMAG00000022683	0.0030	12	12	ugt5d1 (1 of many)	2.24	9
ENSXMAG00000022686	0.0026	9	9	PELI1 (1 of many)	1.75	6
ENSXMAG00000022698	0.0025	9	15	syf2	1.39	6

ENSXMAG00000022708	0.0015	18	6	tm2d1	1.47	15
ENSXMAG00000022730	0.0025	3	15	zgc:100829	1.83	0
ENSXMAG00000022781	0.0031	18	12		1.58	15
ENSXMAG00000022794	0.0031	18	12	mrps14	1.66	15
ENSXMAG00000022799	0.0031	3	12		1.58	0
ENSXMAG00000022811	0.0030	3	12	ciarta	12.43	0
ENSXMAG00000022822	0.0031	18	12		1.93	15
ENSXMAG00000022824	0.0015	24	18	rad1	1.97	21
ENSXMAG00000022828	0.0071	3	6		1.33	0
ENSXMAG00000022830	0.0031	18	12	dohh	2.21	15
ENSXMAG00000022845	0.0026	18	9		1.64	15
ENSXMAG00000022849	0.0024	18	9		1.41	15
ENSXMAG00000022856	0.0025	6	15	gpr142	3.49	3
ENSXMAG00000022858	0.0030	9	12	cdc42ep5	1.28	6
ENSXMAG00000022862	0.0031	18	12	abhd18	1.53	15
ENSXMAG00000022869	0.0015	24	18	si:ch73-62l21.1	1.93	21
ENSXMAG00000022882	0.0026	24	15		1.68	21
ENSXMAG00000022904	0.0024	6	15		4.42	3
ENSXMAG00000022915	0.0026	18	9	pcdh10a	2.57	15
ENSXMAG00000022920	0.0025	18	9		1.42	15
ENSXMAG00000023067	0.0029	18	12	KDELR2	2.07	15
ENSXMAG00000023088	0.0031	9	12		9.93	6
ENSXMAG00000023102	0.0025	9	15	ca16b	1.54	6
ENSXMAG00000023115	0.0031	9	12	TNS4	8.29	6
ENSXMAG00000023123	0.0031	18	12		1.91	15
ENSXMAG00000023124	0.0014	21	6	esrrd	1.43	18
ENSXMAG00000023139	0.0031	9	12		1.43	6
ENSXMAG00000023163	0.0025	12	9	cd81a	1.34	9
ENSXMAG00000023193	0.0026	21	9	cldn5a	1.96	18
ENSXMAG00000023230	0.0026	24	9		1.41	21
ENSXMAG00000023248	0.0015	24	18	cttnbp2nla	1.33	21
ENSXMAG00000023259	0.0026	3	15		1.52	0
ENSXMAG00000023265	0.0031	3	12	pla2g15	1.19	0
ENSXMAG00000023269	0.0030	18	12	necap2	1.79	15
ENSXMAG00000023291	0.0016	24	18		1.88	21
ENSXMAG00000023326	0.0031	18	12		2.81	15
ENSXMAG00000023328	0.0025	9	15		9.72	6
ENSXMAG00000023334	0.0031	9	12		4.11	6
ENSXMAG00000023368	0.0029	21	12	mrpl11	1.58	18
ENSXMAG00000023371	0.0026	15	15	amacr	1.25	12
ENSXMAG00000023373	0.0026	3	15	krt98 (1 of many)	3.78	0
ENSXMAG00000023378	0.0031	12	12	npr3	2.60	9
ENSXMAG00000023380	0.0025	3	15	si:dkey-245n4.2	2.72	0
ENSXMAG00000023382	0.0034	6	12	exosc1	1.52	3
ENSXMAG00000023393	0.0029	3	12		1.37	0
ENSXMAG00000023425	0.0034	18	12	cox5aa (1 of many)	1.74	15
ENSXMAG00000023436	0.0014	24	18		4.75	21
ENSXMAG00000023470	0.0026	3	15		2.43	0
ENSXMAG00000023525	0.0024	21	9	ino80b	1.32	18
ENSXMAG00000023528	0.0014	3	6		1.07	0
ENSXMAG00000023539	0.0031	18	12	cbx5	1.53	15
ENSXMAG00000023561	0.0029	15	12	anxa13	1.57	12
ENSXMAG00000023595	0.0025	21	9	mllt11	1.58	18
ENSXMAG00000023602	0.0024	9	9		5.18	6
ENSXMAG00000023626	0.0031	3	12	dcxr	1.34	0
ENSXMAG00000023645	0.0030	3	12	kdm4c	1.73	0

ENSXMAG00000023653	0.0024	18	9	mettl3	1.76	15
ENSXMAG00000023706	0.0031	18	12	PFN2	1.63	15
ENSXMAG00000023720	0.0025	18	9		2.09	15
ENSXMAG00000023756	0.0024	18	9	hmbsa	2.35	15
ENSXMAG00000023761	0.0014	24	18		1.81	21
ENSXMAG00000023776	0.0029	18	12	med28	1.39	15
ENSXMAG00000023781	0.0031	9	12	ugt1a7 (1 of many)	5.87	6
ENSXMAG00000023803	0.0031	18	12	PCBP2	1.31	15
ENSXMAG00000023808	0.0031	6	12	clec11a	2.08	3
ENSXMAG00000023819	0.0015	24	6	fth1b	1.52	21
ENSXMAG00000023825	0.0014	6	18		11.09	3
ENSXMAG00000023836	0.0025	3	15	ST3GAL1 (1 of many)	2.71	0
ENSXMAG00000023842	0.0029	18	12	si:ch73-244f7.3	2.11	15
ENSXMAG00000023861	0.0014	3	6		1.34	0
ENSXMAG00000023884	0.0026	3	9	si:ch211-150o23.2	1.21	0
ENSXMAG00000023901	0.0029	18	12	tecrb	2.86	15
ENSXMAG00000023912	0.0031	18	12	acp6	2.44	15
ENSXMAG00000023930	0.0024	9	15		1.48	6
ENSXMAG00000023955	0.0026	15	15	rab27a	1.81	12
ENSXMAG00000023964	0.0031	18	12	fam207a	1.92	15
ENSXMAG00000023968	0.0025	18	9		1.72	15
ENSXMAG00000023987	0.0014	18	6		1.68	15
ENSXMAG00000023990	0.0029	6	12		2.34	3
ENSXMAG00000024011	0.0031	18	12	txnl4a	1.59	15
ENSXMAG00000024012	0.0026	18	9	ABHD17A	1.38	15
ENSXMAG00000024057	0.0024	15	9	phkg1a	1.57	12
ENSXMAG00000024113	0.0031	9	12	cyp2n13 (1 of many)	10.97	6
ENSXMAG00000024119	0.0031	21	12	zgc:112185	1.43	18
ENSXMAG00000024131	0.0026	18	9	mybbp1a	1.61	15
ENSXMAG00000024152	0.0031	18	12		2.12	15
ENSXMAG00000024168	0.0025	6	15		1.75	3
ENSXMAG00000024190	0.0031	12	12	hsd11b1la (1 of many)	3.15	9
ENSXMAG00000024192	0.0026	18	9	RF00443	1.40	15
ENSXMAG00000024193	0.0031	15	12	crls1	1.35	12
ENSXMAG00000024196	0.0026	21	9	prdx1	1.54	18
ENSXMAG00000024208	0.0029	18	12	gng3	2.28	15
ENSXMAG00000024219	0.0025	18	9	sec23ip	2.14	15
ENSXMAG00000024246	0.0034	15	12	zmp:0000000936 (1 of many)	1.47	12
ENSXMAG00000024257	0.0031	12	12	cldnf	1.52	9
ENSXMAG00000024259	0.0031	21	12		1.38	18
ENSXMAG00000024261	0.0029	18	12	mrpl27	1.41	15
ENSXMAG00000024266	0.0014	21	6	tsen34	1.57	18
ENSXMAG00000024271	0.0034	18	12	mpdu1b	1.79	15
ENSXMAG00000024283	0.0024	15	9	slc38a2 (1 of many)	1.65	12
ENSXMAG00000024303	0.0031	18	12	zgc:114119	2.10	15
ENSXMAG00000024338	0.0026	18	15		1.96	15
ENSXMAG00000024347	0.0031	18	12	tprg1	1.27	15
ENSXMAG00000024381	0.0031	18	12	tbca	1.62	15
ENSXMAG00000024411	0.0031	18	12	adprhl2	1.63	15
ENSXMAG00000024425	0.0024	3	15		2.26	0
ENSXMAG00000024443	0.0027	24	9	mvb12ba	1.14	21
ENSXMAG00000024444	0.0030	18	12	si:dkey-167k11.5	1.99	15
ENSXMAG00000024448	0.0025	24	15		1.58	21
ENSXMAG00000024482	0.0025	24	15		1.34	21
ENSXMAG00000024489	0.0031	18	12		2.34	15
ENSXMAG00000024494	0.0025	18	9	sdr42e1	1.81	15

ENSXMAG00000024497	0.0031	6	12	HAAO	1.64	3
ENSXMAG00000024507	0.0027	3	15	rnf167	1.38	0
ENSXMAG00000024531	0.0025	9	9	mfge8a	2.54	6
ENSXMAG00000024543	0.0031	24	12		1.28	21
ENSXMAG00000024562	0.0029	21	12	mif	1.44	18
ENSXMAG00000024588	0.0025	3	15	ethe1	2.40	0
ENSXMAG00000024590	0.0027	15	9	numb	1.62	12
ENSXMAG00000024605	0.0027	24	15	nit1	1.77	21
ENSXMAG00000024615	0.0015	24	18		1.35	21
ENSXMAG00000024624	0.0024	24	9	spag7	1.22	21
ENSXMAG00000024627	0.0027	3	15	si:busm1-57f23.1 (1 of many)	1.77	0
ENSXMAG00000024654	0.0024	6	9	fam214a	1.46	3
ENSXMAG00000024676	0.0034	15	12	fuz	3.16	12
ENSXMAG00000024682	0.0031	18	12	gpaa1	1.39	15
ENSXMAG00000024692	0.0026	3	15	si:rp71-1g18.13	2.19	0
ENSXMAG00000024800	0.0031	15	12	mto1	1.25	12
ENSXMAG00000024807	0.0016	24	18	C3 (1 of many)	2.02	21
ENSXMAG00000024825	0.0026	21	9	kif11	1.92	18
ENSXMAG00000024833	0.0031	18	12	zgc:162634	1.54	15
ENSXMAG00000024846	0.0026	18	9		2.24	15
ENSXMAG00000024847	0.0031	18	12	btf3l4 (1 of many)	1.89	15
ENSXMAG00000024876	0.0029	18	12	ube2v1	1.85	15
ENSXMAG00000024877	0.0029	18	12	ADPRH	1.40	15
ENSXMAG00000024938	0.0026	18	9	cnbpa	1.36	15
ENSXMAG00000024964	0.0015	24	18	noxred1	2.65	21
ENSXMAG00000024976	0.0024	24	15	ATP9A	1.37	21
ENSXMAG00000024977	0.0031	18	12	ube2d2	1.25	15
ENSXMAG00000024981	0.0029	18	12	emc9	4.26	15
ENSXMAG00000025010	0.0031	9	12		1.69	6
ENSXMAG00000025017	0.0025	18	9	dnajc15	2.66	15
ENSXMAG00000025024	0.0014	3	18	paqr5a	6.23	0
ENSXMAG00000025030	0.0029	18	12	slc35b1	1.47	15
ENSXMAG00000025042	0.0016	3	6		1.32	0
ENSXMAG00000025043	0.0024	15	9	si:dkey-206d17.12	1.67	12
ENSXMAG00000025060	0.0028	18	9	alg8	2.10	15
ENSXMAG00000025110	0.0034	6	12	zfyve21	2.09	3
ENSXMAG00000025111	0.0025	18	9	rsl1d1	2.17	15
ENSXMAG00000025151	0.0031	18	12		1.29	15
ENSXMAG00000025157	0.0031	18	12	vamp1	1.78	15
ENSXMAG00000025161	0.0027	12	9		1.29	9
ENSXMAG00000025172	0.0031	3	12	slitrk6	1.46	0
ENSXMAG00000025204	0.0031	9	12		1.21	6
ENSXMAG00000025219	0.0024	9	9		2.28	6
ENSXMAG00000025277	0.0034	18	12		1.48	15
ENSXMAG00000025289	0.0028	6	9	runx2b	1.48	3
ENSXMAG00000025297	0.0034	18	12	rps19bp1	1.42	15
ENSXMAG00000025322	0.0025	3	15	krt98 (1 of many)	2.44	0
ENSXMAG00000025341	0.0031	3	12		1.59	0
ENSXMAG00000025389	0.0024	18	9	si:dkey-19e4.5	1.33	15
ENSXMAG00000025443	0.0026	21	9		2.27	18
ENSXMAG00000025496	0.0015	24	18		2.24	21
ENSXMAG00000025515	0.0024	3	15	bhlhe40	10.80	0
ENSXMAG00000025555	0.0026	9	15		1.63	6
ENSXMAG00000025607	0.0031	6	12		1.40	3
ENSXMAG00000025613	0.0031	24	12		1.22	21
ENSXMAG00000025644	0.0030	3	12		2.52	0

ENSXMAG00000025645	0.0034	18	12	glrx5	1.39	15
ENSXMAG00000025656	0.0029	6	12		1.66	3
ENSXMAG00000025690	0.0025	18	9		1.51	15
ENSXMAG00000025714	0.0016	21	6	scn4bb	3.75	18
ENSXMAG00000025719	0.0026	21	9		1.31	18
ENSXMAG00000025725	0.0027	24	15	BLNK	1.70	21
ENSXMAG00000025726	0.0029	18	12	PUDP	1.89	15
ENSXMAG00000025739	0.0014	24	18		2.16	21
ENSXMAG00000025753	0.0026	24	9	ppp1r14bb	1.60	21
ENSXMAG00000025761	0.0014	24	18	cep72	1.49	21
ENSXMAG00000025780	0.0025	9	9		1.49	6
ENSXMAG00000025818	0.0025	18	9		2.10	15
ENSXMAG00000025831	0.0031	21	12	fkbp10b	1.50	18
ENSXMAG00000025839	0.0026	18	9		2.49	15
ENSXMAG00000025849	0.0014	24	18		1.46	21
ENSXMAG00000025860	0.0031	18	12	dnph1	1.45	15
ENSXMAG00000025863	0.0015	18	6	zgc:172067	3.35	15
ENSXMAG00000025867	0.0031	18	12	qtrt1	2.33	15
ENSXMAG00000025868	0.0031	18	12	syt14a	1.93	15
ENSXMAG00000025910	0.0031	18	12	ETHE1 (1 of many)	1.76	15
ENSXMAG00000025918	0.0025	18	9		1.50	15
ENSXMAG00000025950	0.0031	18	12	psmb4	1.88	15
ENSXMAG00000025967	0.0015	3	6		1.46	0
ENSXMAG00000025973	0.0026	18	9		1.81	15
ENSXMAG00000025977	0.0025	21	9	psma1	1.55	18
ENSXMAG00000025992	0.0025	18	15		3.51	15
ENSXMAG00000025997	0.0014	21	6	si:dkey-19b23.7	1.88	18
ENSXMAG00000026007	0.0031	18	12	calr3b	1.98	15
ENSXMAG00000026049	0.0026	18	9		1.90	15
ENSXMAG00000026085	0.0031	18	12	erp44	1.69	15
ENSXMAG00000026106	0.0031	18	12	tmem107	1.61	15
ENSXMAG00000026117	0.0029	18	12	bzw1a	1.39	15
ENSXMAG00000026133	0.0031	12	12		1.92	9
ENSXMAG00000026143	0.0024	18	9	prpf38a	1.47	15
ENSXMAG00000026177	0.0034	18	12	DPF3	1.72	15
ENSXMAG00000026180	0.0026	3	15		1.71	0
ENSXMAG00000026205	0.0026	21	9		2.46	18
ENSXMAG00000026233	0.0031	3	12		4.38	0
ENSXMAG00000026284	0.0034	21	12		1.93	18
ENSXMAG00000026300	0.0024	24	9	hmgn3	1.43	21
ENSXMAG00000026333	0.0027	3	15	tefb	15.01	0
ENSXMAG00000026337	0.0026	21	9	psmb6	1.54	18
ENSXMAG00000026340	0.0031	18	12		1.79	15
ENSXMAG00000026379	0.0026	18	15	chst12a	1.70	15
ENSXMAG00000026415	0.0015	24	18		1.74	21
ENSXMAG00000026424	0.0026	18	9		1.75	15
ENSXMAG00000026468	0.0031	18	12		7.57	15
ENSXMAG00000026491	0.0025	9	9	sgip1a	2.04	6
ENSXMAG00000026565	0.0026	24	9		1.87	21
ENSXMAG00000026570	0.0026	21	9	ciao1	1.44	18
ENSXMAG00000026573	0.0026	9	9	dennd2c	1.71	6
ENSXMAG00000026586	0.0031	18	12	ccdc173	1.91	15
ENSXMAG00000026600	0.0015	24	6		1.69	21
ENSXMAG00000026607	0.0029	9	12		1.50	6
ENSXMAG00000026608	0.0014	21	6	dph5	1.56	18
ENSXMAG00000026637	0.0024	24	9	lsm1	1.47	21

ENSXMAG00000026676	0.0025	3	15	gli1	1.63	0
ENSXMAG00000026720	0.0029	6	12		2.10	3
ENSXMAG00000026738	0.0024	24	9	COQ4	1.63	21
ENSXMAG00000026739	0.0014	24	18		1.62	21
ENSXMAG00000026745	0.0025	18	15	gli2a	1.46	15
ENSXMAG00000026774	0.0027	18	9		1.67	15
ENSXMAG00000026799	0.0031	15	12	pisd	1.88	12
ENSXMAG00000026816	0.0024	24	15		1.37	21
ENSXMAG00000026817	0.0030	3	12	CIART	8.91	0
ENSXMAG00000026829	0.0026	3	15		1.54	0
ENSXMAG00000026839	0.0026	21	9	dut	1.85	18
ENSXMAG00000026845	0.0071	21	18	ccbe1	1.62	18
ENSXMAG00000026878	0.0024	24	15	fam214b	1.69	21
ENSXMAG00000026911	0.0014	24	6	TMEM179	3.32	21
ENSXMAG00000026959	0.0034	18	12	usp14	2.62	15
ENSXMAG00000026979	0.0014	24	18		2.32	21
ENSXMAG00000027026	0.0031	6	12		1.87	3
ENSXMAG00000027033	0.0031	18	12	si:dkey-78l4.14	1.52	15
ENSXMAG00000027050	0.0030	18	12	rfk	1.57	15
ENSXMAG00000027051	0.0026	21	9	etfb	1.36	18
ENSXMAG00000027062	0.0027	3	15		2.56	0
ENSXMAG00000027106	0.0026	21	9	ppib	1.44	18
ENSXMAG00000027108	0.0024	3	15	mtus1b	1.50	0
ENSXMAG00000027109	0.0014	24	6		2.29	21
ENSXMAG00000027135	0.0015	9	18		1.46	6
ENSXMAG00000027208	0.0031	18	12	ppat	1.97	15
ENSXMAG00000027219	0.0026	3	15	ccdc3a	2.41	0
ENSXMAG00000027265	0.0031	9	12	RASGEF1C	1.63	6
ENSXMAG00000027303	0.0031	21	12		1.47	18
ENSXMAG00000027347	0.0029	18	12		1.65	15
ENSXMAG00000027348	0.0031	24	12		1.23	21
ENSXMAG00000027364	0.0025	15	15		1.33	12
ENSXMAG00000027407	0.0014	18	6	h2afx1	1.46	15
ENSXMAG00000027468	0.0025	3	15		28.60	0
ENSXMAG00000027480	0.0026	9	15	cyp2ad2	4.82	6
ENSXMAG00000027505	0.0026	18	9	MRI1	2.19	15
ENSXMAG00000027506	0.0031	18	12	trmt112	1.40	15
ENSXMAG00000027516	0.0014	21	6	acads	2.92	18
ENSXMAG00000027517	0.0016	24	18	crbn	1.71	21
ENSXMAG00000027547	0.0031	18	12	gtf2a2	1.92	15
ENSXMAG00000027560	0.0026	21	15	tnip2	1.45	18
ENSXMAG00000027572	0.0015	3	6		1.55	0
ENSXMAG00000027608	0.0031	3	12	rgmb	1.44	0
ENSXMAG00000027614	0.0015	18	6	calm1b	1.41	15
ENSXMAG00000027672	0.0029	9	12	iqsec2b	1.91	6
ENSXMAG00000027681	0.0024	9	15	SAMD12	1.70	6
ENSXMAG00000027683	0.0031	18	12		1.33	15
ENSXMAG00000027693	0.0015	24	6		3.01	21
ENSXMAG00000027789	0.0031	3	12	anapc15	1.54	0
ENSXMAG00000027837	0.0031	18	12	bcap31	1.51	15
ENSXMAG00000027856	0.0025	9	9	si:dkey-85k7.12 (1 of many)	2.24	6
ENSXMAG00000027863	0.0031	3	12		1.64	0
ENSXMAG00000027888	0.0025	21	9	commd1	1.56	18
ENSXMAG00000027932	0.0031	18	12	si:dkey-181m9.8	1.41	15
ENSXMAG00000027934	0.0027	3	15	agxt2	3.14	0
ENSXMAG00000027946	0.0025	21	9	fkbp1b	1.55	18

ENSXMAG00000027949	0.0034	18	12	endog	2.07	15
ENSXMAG00000027965	0.0024	18	9		1.91	15
ENSXMAG00000027969	0.0026	3	15		2.62	0
ENSXMAG00000027970	0.0031	6	12	tmtc1	1.51	3
ENSXMAG00000027972	0.0027	18	9	eif2b1	1.61	15
ENSXMAG00000027974	0.0031	6	12	fgl2a	1.82	3
ENSXMAG00000027978	0.0031	18	12	dnajc8	1.30	15
ENSXMAG00000028010	0.0034	18	12	nmt1a	1.62	15
ENSXMAG00000028021	0.0024	9	15		1.56	6
ENSXMAG00000028036	0.0026	9	9		1.32	6
ENSXMAG00000028080	0.0014	24	18	8-Mar	1.56	21
ENSXMAG00000028131	0.0031	6	12	FUNDC1	1.50	3
ENSXMAG00000028132	0.0015	18	6		1.55	15
ENSXMAG00000028146	0.0024	21	15	ppp1r27b	26.99	18
ENSXMAG00000028206	0.0029	18	12	tomm20a	1.53	15
ENSXMAG00000028226	0.0034	15	12	zgc:77158	1.66	12
ENSXMAG00000028233	0.0034	18	12	mydgf	1.56	15
ENSXMAG00000028249	0.0014	3	18	unc119b	1.49	0
ENSXMAG00000028272	0.0027	3	15	znf385a	2.19	0
ENSXMAG00000028299	0.0015	18	6	unm_sa1506	4.80	15
ENSXMAG00000028311	0.0016	24	18	OTOR	2.31	21
ENSXMAG00000028313	0.0029	18	12	krtcap2	2.20	15
ENSXMAG00000028340	0.0031	6	12		2.41	3
ENSXMAG00000028402	0.0014	24	6	psme2	1.46	21
ENSXMAG00000028406	0.0024	6	15	b4galnt4a	1.41	3
ENSXMAG00000028408	0.0031	18	12	mettl26	1.53	15
ENSXMAG00000028416	0.0027	9	9	ctdspla	2.06	6
ENSXMAG00000028472	0.0027	24	15		1.76	21
ENSXMAG00000028474	0.0027	24	15	RAB31	1.38	21
ENSXMAG00000028476	0.0025	6	15		2.46	3
ENSXMAG00000028478	0.0031	6	12		1.44	3
ENSXMAG00000028482	0.0025	6	9	lysmd1	1.89	3
ENSXMAG00000028514	0.0027	24	15		1.34	21
ENSXMAG00000028535	0.0016	9	18		1.68	6
ENSXMAG00000028549	0.0016	24	6	tmem222a	1.18	21
ENSXMAG00000028575	0.0025	18	9	clockb	5.49	15
ENSXMAG00000028589	0.0029	18	12	megf8	4.01	15
ENSXMAG00000028590	0.0030	18	12	gpt2l	3.07	15
ENSXMAG00000028597	0.0031	18	12	setd3	2.80	15
ENSXMAG00000028608	0.0026	21	15	nedd8	1.37	18
ENSXMAG00000028625	0.0026	18	9		1.95	15
ENSXMAG00000028708	0.0031	18	12	ppan	1.78	15
ENSXMAG00000028713	0.0027	18	9		3.91	15
ENSXMAG00000028747	0.0025	18	9		1.95	15
ENSXMAG00000028755	0.0030	18	12	zgc:56622	2.00	15
ENSXMAG00000028817	0.0026	3	15	rspo1	1.43	0
ENSXMAG00000028873	0.0025	21	15	lin52	1.73	18
ENSXMAG00000028874	0.0029	18	12	mrps15	1.79	15
ENSXMAG00000028878	0.0026	3	15	si:ch211-51e12.7	1.53	0
ENSXMAG00000028922	0.0031	18	12	cdc23	1.91	15
ENSXMAG00000028940	0.0031	9	12		3.71	6
ENSXMAG00000028951	0.0031	18	12	zgc:110843	1.92	15
ENSXMAG00000028984	0.0031	6	12		1.54	3
ENSXMAG00000028988	0.0026	3	15		1.42	0
ENSXMAG00000028994	0.0025	21	9	selenof	1.55	18
ENSXMAG00000028998	0.0027	21	9	si:ch211-282b22.1	1.44	18

	ENSXMAG00000028999	0.0028	6	15	sb:cb1058	4.53	3
	ENSXMAG00000029002	0.0025	18	9	rnf175	2.04	15
	ENSXMAG00000029016	0.0031	18	12	strap	1.78	15
	ENSXMAG00000029020	0.0028	24	15		3.05	21
	ENSXMAG00000029040	0.0071	21	6	slc39a10	1.40	18
	ENSXMAG00000029068	0.0031	18	12	sf3a2	1.68	15
	ENSXMAG00000029086	0.0031	18	12	WARS	2.71	15
	ENSXMAG00000029102	0.0031	18	12	aurkaip1	1.84	15
	ENSXMAG00000029107	0.0034	18	12		1.51	15
	ENSXMAG00000029183	0.0029	18	12	batf3	1.72	15
	ENSXMAG00000029198	0.0026	18	15	cox10	1.38	15
	ENSXMAG00000029199	0.0031	18	12	RSRC1	1.50	15
	ENSXMAG00000029235	0.0029	6	12		1.25	3
	ENSXMAG00000029269	0.0031	15	12	CCNYL1	1.34	12
	ENSXMAG00000029301	0.0029	21	12		3.29	18
	ENSXMAG00000029306	0.0025	18	9		3.24	15
	ENSXMAG00000029313	0.0031	15	12		1.35	12
	ENSXMAG00000029314	0.0025	15	15	sc5d	2.22	12
	ENSXMAG00000029335	0.0025	18	9	mak16	1.42	15
	ENSXMAG00000029336	0.0028	24	15		4.95	21
	ENSXMAG00000029340	0.0024	12	15	cmbl	2.06	9
	ENSXMAG00000029352	0.0015	3	18	pam16	1.48	0
	ENSXMAG00000029409	0.0029	18	12	ppil1	4.34	15
	ENSXMAG00000029412	0.0031	18	12	cdab	1.42	15
	ENSXMAG00000029414	0.0024	3	15		2.91	0
	ENSXMAG00000029448	0.0027	15	9	tagln	1.94	12
	ENSXMAG00000029462	0.0029	18	12	CMAS	1.41	15
	ENSXMAG00000029533	0.0034	24	12	efna3b	2.21	21
	ENSXMAG00000029534	0.0024	3	15		2.24	0
	ENSXMAG00000029558	0.0030	21	12	ccdc15	1.57	18
	ENSXMAG00000029603	0.0034	18	12	mtch2	1.43	15
	ENSXMAG00000029620	0.0024	6	9	slc40a1	1.35	3
	ENSXMAG00000029652	0.0015	24	18	zgc:77486	1.29	21
	ENSXMAG00000029680	0.0028	12	15		1.80	9
	ENSXMAG00000029684	0.0025	24	15	pbx1a	1.43	21
	ENSXMAG00000029686	0.0016	24	18		1.71	21
	ENSXMAG00000029706	0.0031	6	12	mxd1	2.08	3
	ENSXMAG00000029757	0.0027	18	9	scfd1	1.50	15
	ENSXMAG00000029763	0.0031	6	12	timp2b	1.56	3
	ENSXMAG00000029788	0.0026	18	15	srp19	1.26	15
	ENSXMAG00000029833	0.0030	6	12	zgc:114045	2.11	3
	ENSXMAG00000029843	0.0026	3	15		5.60	0
	ENSXMAG00000029845	0.0016	3	6	zgc:103678	1.37	0
	ENSXMAG00000029855	0.0026	15	15		1.34	12
	ENSXMAG00000029856	0.0034	3	12	si:ch211-207e14.4	1.63	0
	ENSXMAG00000029865	0.0027	24	15		1.38	21
	ENSXMAG00000029935	0.0030	24	12	zgc:113531	1.40	21
	ENSXMAG00000029964	0.0027	6	15	spry4	2.47	3
	ENSXMAG00000029983	0.0029	18	12	sephs2	1.51	15
	ENSXMAG00000030083	0.0031	6	12	fbxo33	2.31	3
	ENSXMAG00000030086	0.0029	15	12	chst14	2.43	12
	ENSXMAG00000030098	0.0034	18	12	ssr2	1.48	15

Liver circadian genes	GeneID	pVal	phase	peak.shape	external_gene_name	amp	Ct.peak
	ENSXMAG00000000045	0.0026	9	15	slc2a8	2.16	6
	ENSXMAG00000000054	0.0028	15	9	cnnm2b	13.78	12

ENSXMAG00000000094	0.0016	21	18	mrtfbb	2.63	18
ENSXMAG00000000102	0.0031	18	12	slc1a8a	1.66	15
ENSXMAG00000000127	0.0031	18	12	tbc1d24	2.72	15
ENSXMAG00000000160	0.0026	18	9	MPP5 (1 of many)	3.01	15
ENSXMAG00000000163	0.0030	24	12	atpaf2	1.78	21
ENSXMAG00000000175	0.0024	18	9		1.82	15
ENSXMAG00000000204	0.0031	24	12	ptp4a2a	1.71	21
ENSXMAG00000000221	0.0024	18	9	emp2	1.71	15
ENSXMAG00000000227	0.0031	3	12	si:dkey-96f10.1	1.76	0
ENSXMAG00000000251	0.0030	18	12	CDC37	1.73	15
ENSXMAG00000000313	0.0015	18	6	hgh1	5.34	15
ENSXMAG00000000317	0.0030	18	12	snx12	1.64	15
ENSXMAG00000000319	0.0015	18	6	slc7a3a	8.13	15
ENSXMAG00000000415	0.0031	18	12	ap1g2	5.28	15
ENSXMAG00000000417	0.0026	3	15	ldlrap1a	2.64	0
ENSXMAG00000000424	0.0031	6	12	f9b	1.34	3
ENSXMAG00000000464	0.0025	21	9	lrrc42	1.72	18
ENSXMAG00000000465	0.0030	18	12	anp32a	4.25	15
ENSXMAG00000000496	0.0024	15	9	tsc1a	1.48	12
ENSXMAG00000000505	0.0026	18	9	timm50	11.67	15
ENSXMAG00000000597	0.0026	21	9	tpk1	1.91	18
ENSXMAG00000000598	0.0027	18	9	ksr1a	2.18	15
ENSXMAG00000000609	0.0031	6	12		1.39	3
ENSXMAG00000000642	0.0024	18	9	ap1m1	4.70	15
ENSXMAG00000000663	0.0014	18	6	rangap1b	41.60	15
ENSXMAG00000000679	0.0031	18	12	phf5a	2.34	15
ENSXMAG00000000699	0.0031	18	12	snrpb2	2.04	15
ENSXMAG00000000777	0.0030	6	12	masp1	2.04	3
ENSXMAG00000000782	0.0026	18	15	shtn1	2.78	15
ENSXMAG00000000829	0.0030	18	12	atg16l1	1.53	15
ENSXMAG00000000980	0.0026	18	9	oxnad1	9.90	15
ENSXMAG00000001002	0.0031	18	12	eif5b	2.11	15
ENSXMAG00000001018	0.0031	18	12	snrpd2	2.84	15
ENSXMAG00000001030	0.0025	15	9	si:dkey-190l8.2	3.61	12
ENSXMAG00000001040	0.0026	18	9	si:ch211-260e23.8	4.44	15
ENSXMAG00000001073	0.0026	18	9	banp	1.48	15
ENSXMAG00000001097	0.0025	18	9	srsf3b	2.08	15
ENSXMAG00000001102	0.0030	18	12	stk19	2.03	15
ENSXMAG00000001113	0.0025	3	15	bhlhe41	39.60	0
ENSXMAG00000001125	0.0031	12	12	c1qtnf6a	4.67	9
ENSXMAG00000001236	0.0031	18	12	ano5b	3.48	15
ENSXMAG00000001241	0.0031	18	12	ttpal	5.12	15
ENSXMAG00000001254	0.0029	15	12	ckmt2a	4.48	12
ENSXMAG00000001279	0.0031	18	12	desi1a	1.53	15
ENSXMAG00000001302	0.0024	15	9	si:ch211-282j22.3	2.76	12
ENSXMAG00000001306	0.0014	18	6	cse1l	7.58	15
ENSXMAG00000001321	0.0027	3	9		1.42	0
ENSXMAG00000001345	0.0024	18	9	plekhg7	4.03	15
ENSXMAG00000001349	0.0026	9	15	lmbrd2b	1.91	6
ENSXMAG00000001360	0.0031	21	12	ilvbl	1.58	18
ENSXMAG00000001372	0.0029	18	12		1.98	15
ENSXMAG00000001387	0.0031	18	12	sestd1	6.40	15
ENSXMAG00000001390	0.0031	18	12	tbc1d16	1.99	15
ENSXMAG00000001421	0.0026	21	9	def6c	2.63	18
ENSXMAG00000001446	0.0031	18	12	selenos	2.10	15
ENSXMAG00000001468	0.0030	18	12	gfer	5.43	15

ENSXMAG00000001470	0.0014	18	6	gnptab	6.21	15
ENSXMAG00000001481	0.0014	18	6	chpt1	1.68	15
ENSXMAG00000001482	0.0025	18	9	arntl2	91.49	15
ENSXMAG00000001507	0.0015	6	18		46.09	3
ENSXMAG00000001514	0.0027	12	15	yipf5	1.72	9
ENSXMAG00000001520	0.0025	21	15	cerkl	11.97	18
ENSXMAG00000001575	0.0014	18	6	ahcyl2	5.33	15
ENSXMAG00000001628	0.0025	3	15	pdlim5b	3.40	0
ENSXMAG00000001633	0.0030	18	12	dnajc25	1.62	15
ENSXMAG00000001657	0.0030	18	12	emc3	1.85	15
ENSXMAG00000001666	0.0031	18	12	gmpr2	1.99	15
ENSXMAG00000001680	0.0014	18	6	urm1	2.20	15
ENSXMAG00000001769	0.0031	18	12		3.16	15
ENSXMAG00000001787	0.0028	15	9	si:ch73-91k6.2	1.70	12
ENSXMAG00000001839	0.0025	15	15	eefsec	2.14	12
ENSXMAG00000001844	0.0031	18	12	naprt	2.45	15
ENSXMAG00000001845	0.0014	21	6	gba2	3.52	18
ENSXMAG00000001890	0.0031	18	12		2.76	15
ENSXMAG00000001913	0.0025	18	9	pa2g4a	2.13	15
ENSXMAG00000001986	0.0025	18	15	npm1b	2.23	15
ENSXMAG00000001995	0.0031	18	12	psmd6	1.57	15
ENSXMAG00000002018	0.0034	21	12	acmsd	1.46	18
ENSXMAG00000002047	0.0030	12	12	plod3	3.73	9
ENSXMAG00000002135	0.0014	21	18		2.56	18
ENSXMAG00000002145	0.0031	18	12	si:dkey-229d2.4	2.60	15
ENSXMAG00000002171	0.0025	18	9	alg6	3.58	15
ENSXMAG00000002227	0.0016	18	6	trnt1	3.63	15
ENSXMAG00000002268	0.0031	6	12	tdo2a	4.83	3
ENSXMAG00000002281	0.0014	21	6	gpm6aa	2.62	18
ENSXMAG00000002310	0.0027	21	9	bloc1s6	1.89	18
ENSXMAG00000002313	0.0026	18	9	ivns1abpa	5.12	15
ENSXMAG00000002339	0.0030	24	12	nr1d4b	114.22	21
ENSXMAG00000002357	0.0029	18	12	rnaseh1	2.04	15
ENSXMAG00000002387	0.0026	18	9	brf1b	1.64	15
ENSXMAG00000002442	0.0025	18	9	cdk4	2.53	15
ENSXMAG00000002451	0.0031	18	12	nom1	2.34	15
ENSXMAG00000002539	0.0015	3	6	clpp	1.25	0
ENSXMAG00000002550	0.0016	18	6	acsbg2	41.11	15
ENSXMAG00000002570	0.0031	18	12		3.94	15
ENSXMAG00000002579	0.0025	18	9	rcn3	1.29	15
ENSXMAG00000002591	0.0030	21	12	acad11	1.60	18
ENSXMAG00000002592	0.0014	6	6		1.40	3
ENSXMAG00000002604	0.0030	18	12	tspan4b	1.59	15
ENSXMAG00000002661	0.0031	15	12	stt3b	1.46	12
ENSXMAG00000002691	0.0031	18	12	uchl5	2.25	15
ENSXMAG00000002738	0.0031	18	12	smarcb1a	2.04	15
ENSXMAG00000002791	0.0030	18	12	pnkp	1.88	15
ENSXMAG00000002810	0.0025	18	15	poglut1	2.51	15
ENSXMAG00000002875	0.0016	18	6	n6amt1	1.79	15
ENSXMAG00000002901	0.0031	18	12	insig2	1.54	15
ENSXMAG00000002913	0.0025	18	9	vps26c	1.51	15
ENSXMAG00000002920	0.0031	18	12	mrpl1	1.23	15
ENSXMAG00000002945	0.0031	3	12	pum1	1.51	0
ENSXMAG00000002958	0.0016	18	6	gak	7.48	15
ENSXMAG00000002990	0.0016	21	18	cyhr1	3.65	18
ENSXMAG00000003002	0.0025	6	15	chata	21.46	3

ENSXMAG00000003030	0.0025	21	15	rcn2	3.35	18
ENSXMAG00000003032	0.0014	18	6	gldc	4.80	15
ENSXMAG00000003102	0.0026	21	15	etfa	1.21	18
ENSXMAG00000003109	0.0031	18	12	spock2	1.92	15
ENSXMAG00000003136	0.0026	18	15	ppa1b	1.64	15
ENSXMAG00000003167	0.0026	18	9	vat1l	3.25	15
ENSXMAG00000003169	0.0026	18	9	scarb2a	50.08	15
ENSXMAG00000003193	0.0026	21	9	zgc:152863	5.13	18
ENSXMAG00000003195	0.0024	18	9	CCNG2 (1 of many)	8.81	15
ENSXMAG00000003237	0.0025	18	9	mrps10	1.82	15
ENSXMAG00000003265	0.0016	18	6	pmpca	3.68	15
ENSXMAG00000003284	0.0016	18	6	cyb5b	54.60	15
ENSXMAG00000003323	0.0014	18	6		54.94	15
ENSXMAG00000003325	0.0025	18	15	sod2	1.65	15
ENSXMAG00000003401	0.0031	6	12		5.17	3
ENSXMAG00000003424	0.0014	18	6	prep	6.30	15
ENSXMAG00000003454	0.0031	18	12	si:ch211-193k19.1	3.06	15
ENSXMAG00000003479	0.0016	18	6	atad2b	2.31	15
ENSXMAG00000003500	0.0031	18	12	mrpl54	2.91	15
ENSXMAG00000003501	0.0026	3	9	fam32a	1.48	0
ENSXMAG00000003529	0.0031	24	12	acot13	2.14	21
ENSXMAG00000003552	0.0029	18	12		1.91	15
ENSXMAG00000003600	0.0024	18	9	agpat3	2.30	15
ENSXMAG00000003615	0.0016	18	6	atxn1a	2.21	15
ENSXMAG00000003644	0.0026	3	15	shmt1	1.45	0
ENSXMAG00000003659	0.0024	15	15		2.59	12
ENSXMAG00000003725	0.0031	18	12	grhl2b	8.32	15
ENSXMAG00000003731	0.0029	24	12	TTC39C (1 of many)	1.68	21
ENSXMAG00000003740	0.0026	24	9	zgc:103559	1.93	21
ENSXMAG00000003752	0.0031	18	12	si:ch211-203k16.3	4.50	15
ENSXMAG00000003762	0.0026	3	15	atg4b	2.99	0
ENSXMAG00000003775	0.0031	18	12	fads2 (1 of many)	2.99	15
ENSXMAG00000003803	0.0024	21	9	plekhn1	1.87	18
ENSXMAG00000003953	0.0015	18	6	gcn1	1.80	15
ENSXMAG00000003955	0.0024	9	15	FLVCR2 (1 of many)	4.65	6
ENSXMAG00000003960	0.0029	18	12	sae1	2.04	15
ENSXMAG00000003961	0.0025	6	15	per3	40.30	3
ENSXMAG00000003973	0.0031	3	12		2.38	0
ENSXMAG00000003975	0.0031	18	12	mtfmt	2.22	15
ENSXMAG00000003988	0.0014	18	6	ylpm1	2.36	15
ENSXMAG00000004003	0.0030	18	12	mre11a	6.09	15
ENSXMAG00000004037	0.0016	18	6	ACSL1 (1 of many)	3.14	15
ENSXMAG00000004040	0.0024	3	9	pwp1	1.56	0
ENSXMAG00000004061	0.0024	18	9	miox	2.76	15
ENSXMAG00000004091	0.0030	18	12	RPL5 (1 of many)	3.73	15
ENSXMAG00000004093	0.0026	21	9	bcl9	3.75	18
ENSXMAG00000004098	0.0026	21	9	fbxl4	2.17	18
ENSXMAG00000004114	0.0030	18	12	hspa9	3.12	15
ENSXMAG00000004163	0.0016	21	6	mpx (1 of many)	3.34	18
ENSXMAG00000004235	0.0031	18	12	ap2s1	2.01	15
ENSXMAG00000004254	0.0025	18	9	pcyox1	1.93	15
ENSXMAG00000004262	0.0014	15	18	pofut1	1.41	12
ENSXMAG00000004263	0.0026	21	15		1.54	18
ENSXMAG00000004285	0.0024	3	15		1.43	0
ENSXMAG00000004298	0.0014	18	6	clptm1	4.29	15
ENSXMAG00000004307	0.0026	18	9	kpnb3	3.24	15

ENSXMAG00000004309	0.0030	18	12	tbcb	2.85	15
ENSXMAG00000004312	0.0024	18	9	zufsp	1.79	15
ENSXMAG00000004317	0.0014	18	6	dnajc21	4.92	15
ENSXMAG00000004328	0.0024	9	9		3.08	6
ENSXMAG00000004337	0.0024	6	15	slc2a12	9.62	3
ENSXMAG00000004346	0.0034	15	12		13.87	12
ENSXMAG00000004354	0.0016	18	6	plpbp	2.34	15
ENSXMAG00000004420	0.0026	18	9	chchd7	1.61	15
ENSXMAG00000004439	0.0026	9	15	wfs1b	5.25	6
ENSXMAG00000004470	0.0031	15	12	cmpk	3.86	12
ENSXMAG00000004471	0.0015	18	6	dcun1d5	11.23	15
ENSXMAG00000004496	0.0024	15	9	usp18	3.34	12
ENSXMAG00000004561	0.0015	18	6		3.84	15
ENSXMAG00000004593	0.0027	15	15	creld2	7.44	12
ENSXMAG00000004603	0.0029	18	12	rtel1	4.33	15
ENSXMAG00000004661	0.0034	15	12		3.65	12
ENSXMAG00000004677	0.0025	18	9	sephs1	6.46	15
ENSXMAG00000004679	0.0015	18	6	si:ch73-21k16.4	1.53	15
ENSXMAG00000004697	0.0025	6	9	ACADL (1 of many)	1.50	3
ENSXMAG00000004780	0.0029	18	12	plpp1a	1.71	15
ENSXMAG00000004807	0.0026	24	15	cdc73	1.31	21
ENSXMAG00000004812	0.0015	24	18		2.54	21
ENSXMAG00000004837	0.0026	15	15		2.56	12
ENSXMAG00000004872	0.0025	18	9	pex10	1.64	15
ENSXMAG00000004888	0.0025	18	9	ssb	3.01	15
ENSXMAG00000004931	0.0025	15	15	cinp	1.79	12
ENSXMAG00000004938	0.0014	18	6		2.01	15
ENSXMAG00000004967	0.0028	9	15	ugt1a7 (1 of many)	4.28	6
ENSXMAG00000004998	0.0031	18	12	ppil4	2.50	15
ENSXMAG00000005127	0.0024	15	9	nfil3-5	308.96	12
ENSXMAG00000005150	0.0031	18	12	gdi2	1.63	15
ENSXMAG00000005234	0.0031	18	12	nfybb	1.89	15
ENSXMAG00000005289	0.0031	18	12	ubxn1	1.64	15
ENSXMAG00000005305	0.0026	9	15	GLP2R	3.04	6
ENSXMAG00000005307	0.0031	18	12	psmc6	2.21	15
ENSXMAG00000005313	0.0025	24	9	samm50l	1.50	21
ENSXMAG00000005374	0.0024	15	15	si:dkey-246g23.4	2.07	12
ENSXMAG00000005428	0.0027	15	9	shpk	1.66	12
ENSXMAG00000005432	0.0026	18	15	lman2la	3.68	15
ENSXMAG00000005452	0.0031	6	12	kifap3a	2.94	3
ENSXMAG00000005457	0.0034	24	12	ndufb6	1.39	21
ENSXMAG00000005460	0.0025	18	9	dnajc5ga	7.54	15
ENSXMAG00000005461	0.0031	12	12	cthl	2.15	9
ENSXMAG00000005480	0.0026	3	15	pdcd4a	2.05	0
ENSXMAG00000005529	0.0015	18	6	si:ch211-217k17.7	5.17	15
ENSXMAG00000005562	0.0026	18	9		2.85	15
ENSXMAG00000005593	0.0014	18	6	opa1	1.73	15
ENSXMAG00000005671	0.0030	18	12	c1qbp	4.99	15
ENSXMAG00000005681	0.0015	18	6		5.02	15
ENSXMAG00000005694	0.0014	24	18	rgs4	5.02	21
ENSXMAG00000005735	0.0024	15	9	edc4	2.55	12
ENSXMAG00000005768	0.0030	6	12	pm20d1.2	1.60	3
ENSXMAG00000005770	0.0026	18	9	nutf2	1.82	15
ENSXMAG00000005775	0.0031	6	12	ulk2	10.16	3
ENSXMAG00000005786	0.0026	9	15	adck5	3.25	6
ENSXMAG00000005796	0.0026	3	15		12.03	0

ENSXMAG00000005800	0.0031	6	12		3.85	3
ENSXMAG00000005817	0.0014	18	6	HIGD1A	24.14	15
ENSXMAG00000005835	0.0026	15	9	hykk.1	3.13	12
ENSXMAG00000005861	0.0031	18	12	polr2h	4.76	15
ENSXMAG00000005878	0.0031	21	12	capn1a	2.12	18
ENSXMAG00000005889	0.0031	18	12	hspe1	2.31	15
ENSXMAG00000005914	0.0031	18	12	ncl	2.45	15
ENSXMAG00000005929	0.0031	15	12		2.86	12
ENSXMAG00000005940	0.0025	18	9	ptcd2	3.15	15
ENSXMAG00000005943	0.0026	18	9	hinfp	2.12	15
ENSXMAG00000005945	0.0024	9	15		1.93	6
ENSXMAG00000006028	0.0014	18	6	cpne1	2.04	15
ENSXMAG00000006057	0.0015	21	6	blvrb	2.47	18
ENSXMAG00000006087	0.0031	18	12	dad1	1.64	15
ENSXMAG00000006091	0.0014	18	6	hmgcs1	129.61	15
ENSXMAG00000006208	0.0014	12	18	si:dkey-76k16.5	2.91	9
ENSXMAG00000006227	0.0016	18	18	slc23a1	1.77	15
ENSXMAG00000006261	0.0026	3	15	ces3	1.23	0
ENSXMAG00000006262	0.0026	18	9	nup43	3.01	15
ENSXMAG00000006282	0.0024	15	9	aars	1.41	12
ENSXMAG00000006335	0.0026	3	15	TNS3 (1 of many)	2.52	0
ENSXMAG00000006356	0.0025	18	9	gnpat	3.71	15
ENSXMAG00000006417	0.0031	9	12	cry2	4.99	6
ENSXMAG00000006423	0.0029	6	12	slc26a5	1.97	3
ENSXMAG00000006424	0.0031	18	12	atpaf1	1.69	15
ENSXMAG00000006474	0.0025	18	9	cnot9	1.58	15
ENSXMAG00000006477	0.0025	18	9	mthfd1b	3.14	15
ENSXMAG00000006499	0.0014	18	6	cand1	2.07	15
ENSXMAG00000006502	0.0071	15	18		2.55	12
ENSXMAG00000006522	0.0025	18	9	xrcc1	2.84	15
ENSXMAG00000006563	0.0030	18	12	b3galnt2	5.79	15
ENSXMAG00000006612	0.0015	21	18	lsr	2.14	18
ENSXMAG00000006626	0.0071	21	18	commd9	2.12	18
ENSXMAG00000006628	0.0031	15	12	nansa	2.03	12
ENSXMAG00000006644	0.0026	21	9	cep170b	1.86	18
ENSXMAG00000006652	0.0014	18	6	slc37a1	2.04	15
ENSXMAG00000006674	0.0034	15	12	nedd1	2.06	12
ENSXMAG00000006678	0.0015	18	6	ddx31	2.91	15
ENSXMAG00000006741	0.0031	18	12	mrpl17	3.10	15
ENSXMAG00000006747	0.0031	18	12	disc1	45.59	15
ENSXMAG00000006761	0.0014	18	6		4.42	15
ENSXMAG00000006763	0.0016	18	6		2.36	15
ENSXMAG00000006794	0.0031	18	12	iqgap2	1.97	15
ENSXMAG00000006798	0.0031	24	12	atp5pd	1.35	21
ENSXMAG00000006832	0.0015	18	6	EIF2AK4	1.32	15
ENSXMAG00000006879	0.0025	21	9	mrpl2	1.44	18
ENSXMAG00000006883	0.0026	18	9		2.05	15
ENSXMAG00000006964	0.0027	18	9	SRF	6.39	15
ENSXMAG00000007001	0.0031	18	12	rras2	2.33	15
ENSXMAG00000007078	0.0026	18	9	ebag9	1.76	15
ENSXMAG00000007104	0.0024	15	9	zgc:103670	3.29	12
ENSXMAG00000007134	0.0014	18	6	zgc:153521	2.35	15
ENSXMAG00000007222	0.0014	18	6	pgm3	3.34	15
ENSXMAG00000007271	0.0024	24	15	efna1b	4.39	21
ENSXMAG00000007311	0.0031	21	12	dbr1	1.57	18
ENSXMAG00000007357	0.0024	18	9		2.07	15

ENSXMAG00000007369	0.0024	6	9	sf3b1	1.39	3
ENSXMAG00000007434	0.0027	18	9	tnnt3a	9.56	15
ENSXMAG00000007452	0.0026	9	15		6.55	6
ENSXMAG00000007467	0.0029	6	12		2.53	3
ENSXMAG00000007469	0.0031	15	12	DNAJB5 (1 of many)	5.69	12
ENSXMAG00000007474	0.0031	21	12	akr1a1a	1.58	18
ENSXMAG00000007529	0.0029	18	12	FIG4	2.04	15
ENSXMAG00000007570	0.0024	18	9	ubr7	1.71	15
ENSXMAG00000007584	0.0024	15	15	snx14	1.26	12
ENSXMAG00000007602	0.0071	18	6	mfsd11	1.43	15
ENSXMAG00000007607	0.0024	21	9	apeh	3.22	18
ENSXMAG00000007617	0.0031	18	12	galk1	1.99	15
ENSXMAG00000007619	0.0031	18	12	mogat3b	2.11	15
ENSXMAG00000007627	0.0031	18	12	srrt	2.32	15
ENSXMAG00000007667	0.0015	24	18	heca	2.41	21
ENSXMAG00000007702	0.0071	15	6		2.19	12
ENSXMAG00000007712	0.0031	3	12	rmdn2	2.78	0
ENSXMAG00000007713	0.0014	18	6	soul3	8.50	15
ENSXMAG00000007731	0.0027	24	15	fbxo25	35.65	21
ENSXMAG00000007748	0.0015	18	6	ppargc1a	3.73	15
ENSXMAG00000007800	0.0029	3	12	znf395b	5.08	0
ENSXMAG00000007822	0.0031	18	12	sf1	2.14	15
ENSXMAG00000007850	0.0015	18	6		2.33	15
ENSXMAG00000007882	0.0026	15	15		2.94	12
ENSXMAG00000007888	0.0029	6	12	myo1ha	73.12	3
ENSXMAG00000007902	0.0028	3	9	lancl1	1.89	0
ENSXMAG00000007937	0.0031	18	12	zgc:171480	2.12	15
ENSXMAG00000007949	0.0031	18	12	gpd1c	1.57	15
ENSXMAG00000007954	0.0030	18	12	marveld3	3.54	15
ENSXMAG00000007990	0.0031	18	12	lonp1	2.56	15
ENSXMAG00000008006	0.0015	3	6	colec11	1.40	0
ENSXMAG00000008048	0.0014	6	6	TMPRSS6	1.70	3
ENSXMAG00000008071	0.0031	18	12	gatb	1.75	15
ENSXMAG00000008126	0.0025	18	15	nhlrc2	2.13	15
ENSXMAG00000008130	0.0031	18	12	ebp	9.73	15
ENSXMAG00000008187	0.0031	18	12	ccny	3.26	15
ENSXMAG00000008215	0.0031	18	12	mblac1	2.22	15
ENSXMAG00000008240	0.0031	18	12	thg1l	3.27	15
ENSXMAG00000008262	0.0025	3	15	camkvl	3.65	0
ENSXMAG00000008278	0.0031	9	12		2.76	6
ENSXMAG00000008303	0.0015	18	6	hsp90aa1.2	32.14	15
ENSXMAG00000008340	0.0031	24	12	acbd5a	1.72	21
ENSXMAG00000008372	0.0029	18	12		1.76	15
ENSXMAG00000008378	0.0024	18	15		2.60	15
ENSXMAG00000008416	0.0034	3	12	fbxl7	1.53	0
ENSXMAG00000008458	0.0024	18	15	bcap29	1.40	15
ENSXMAG00000008494	0.0031	18	12	eftud2	3.97	15
ENSXMAG00000008537	0.0031	18	12	phb	2.20	15
ENSXMAG00000008538	0.0024	3	9	NFIB	2.75	0
ENSXMAG00000008587	0.0031	18	12	eif4e2rs1	3.63	15
ENSXMAG00000008607	0.0024	15	15	alg5	3.55	12
ENSXMAG00000008655	0.0025	24	9	alad	1.76	21
ENSXMAG00000008666	0.0026	15	15	plcb3	3.38	12
ENSXMAG00000008671	0.0026	18	9	cnot11	1.47	15
ENSXMAG00000008699	0.0030	21	12	zgc:101765	1.81	18
ENSXMAG00000008704	0.0024	18	9	hint3	1.96	15

ENSXMAG00000008725	0.0024	3	9	ulk4	2.69	0
ENSXMAG00000008826	0.0026	15	9	ssr1	2.28	12
ENSXMAG00000008830	0.0031	18	12	tmem147	2.65	15
ENSXMAG00000008845	0.0024	18	9		4.09	15
ENSXMAG00000008850	0.0031	18	12	snrpe	1.77	15
ENSXMAG00000008857	0.0014	18	18	coa6	1.56	15
ENSXMAG00000008881	0.0015	18	18	slc5a6a	1.67	15
ENSXMAG00000008936	0.0024	18	9	eri1	2.27	15
ENSXMAG00000008975	0.0026	18	9	FAM53C	5.36	15
ENSXMAG00000008985	0.0031	24	12	spon2a	3.23	21
ENSXMAG00000009007	0.0031	18	12	slu7	1.59	15
ENSXMAG00000009026	0.0024	15	15	si:ch211-225b10.3	1.43	12
ENSXMAG00000009050	0.0016	18	6	EML4	2.12	15
ENSXMAG00000009064	0.0025	18	9	acss2	2.07	15
ENSXMAG00000009083	0.0031	12	12	tmem168a	1.59	9
ENSXMAG00000009093	0.0071	6	18	slc25a38a	6.93	3
ENSXMAG00000009097	0.0031	18	12	rnf170	1.60	15
ENSXMAG00000009098	0.0026	15	9		10.09	12
ENSXMAG00000009115	0.0028	21	9	dnmt3bb.1	4.42	18
ENSXMAG00000009116	0.0030	18	12	fam185a	6.48	15
ENSXMAG00000009128	0.0025	18	9	ramp2	2.26	15
ENSXMAG00000009155	0.0026	18	9	erc1a	4.14	15
ENSXMAG00000009179	0.0031	18	12	psmd7	2.92	15
ENSXMAG00000009200	0.0014	18	6	hspa4b	10.27	15
ENSXMAG00000009314	0.0026	15	15	mrm2	2.31	12
ENSXMAG00000009353	0.0014	15	6	vill	2.20	12
ENSXMAG00000009355	0.0014	18	6	gxylt1b	2.96	15
ENSXMAG00000009375	0.0024	15	9	znf451	1.90	12
ENSXMAG00000009398	0.0026	24	15	nfkbiab	2.61	21
ENSXMAG00000009431	0.0014	18	6	zgc:66447	2.95	15
ENSXMAG00000009451	0.0031	21	12	nubpl	1.69	18
ENSXMAG00000009460	0.0015	24	18	dennd5a	2.23	21
ENSXMAG00000009513	0.0025	21	15	vipr1b	2.43	18
ENSXMAG00000009531	0.0027	18	15	spryd3	2.83	15
ENSXMAG00000009534	0.0024	18	9	nsdhl	16.76	15
ENSXMAG00000009544	0.0014	18	6	txnrd3	6.30	15
ENSXMAG00000009571	0.0025	21	15		2.93	18
ENSXMAG00000009615	0.0014	18	6	mmaa	2.44	15
ENSXMAG00000009654	0.0031	18	12	get4	4.86	15
ENSXMAG00000009672	0.0026	18	9	ankrd10a	7.17	15
ENSXMAG00000009705	0.0026	24	9	tagln3b	2.33	21
ENSXMAG00000009729	0.0027	6	9	spata13	1.67	3
ENSXMAG00000009743	0.0025	18	9		21.28	15
ENSXMAG00000009804	0.0031	18	12	aph1b	2.08	15
ENSXMAG00000009853	0.0024	18	9	mtrf1	2.62	15
ENSXMAG00000009861	0.0031	18	12	ATP6V0C	2.44	15
ENSXMAG00000009862	0.0031	9	12	fam13a	6.35	6
ENSXMAG00000009878	0.0031	18	12	zgc:172295	2.95	15
ENSXMAG00000009889	0.0024	21	9	LGALS8	1.56	18
ENSXMAG00000009896	0.0024	15	15	rad9a	2.07	12
ENSXMAG00000009918	0.0026	18	9	ANP32B (1 of many)	3.60	15
ENSXMAG00000009925	0.0024	3	15	asmt2	2.30	0
ENSXMAG00000009972	0.0016	21	18		3.35	18
ENSXMAG00000010011	0.0014	18	6	g3bp2	2.32	15
ENSXMAG00000010027	0.0034	6	12		2.51	3
ENSXMAG00000010052	0.0026	18	9	naa50	3.59	15

ENSXMAG00000010174	0.0024	21	15	neo1b	1.77	18
ENSXMAG00000010241	0.0031	21	12	pin4	1.48	18
ENSXMAG00000010245	0.0025	12	15	SEC61A1 (1 of many)	2.09	9
ENSXMAG00000010310	0.0026	15	15		1.99	12
ENSXMAG00000010314	0.0025	21	15		1.32	18
ENSXMAG00000010325	0.0024	24	9		3.26	21
ENSXMAG00000010334	0.0024	3	15	trpa1b	6.77	0
ENSXMAG00000010342	0.0025	15	15	dnajb11	4.50	12
ENSXMAG00000010413	0.0015	18	6	adka	3.17	15
ENSXMAG00000010427	0.0025	18	9	zgc:195245	10.03	15
ENSXMAG00000010431	0.0031	18	12	ap3m1	2.92	15
ENSXMAG00000010441	0.0025	18	9	vcla	1.83	15
ENSXMAG00000010442	0.0071	21	18	fance	3.75	18
ENSXMAG00000010446	0.0031	21	12	aox1	2.51	18
ENSXMAG00000010497	0.0034	18	12		6.21	15
ENSXMAG00000010501	0.0025	21	15	eogt	1.72	18
ENSXMAG00000010559	0.0034	3	12	si:dkey-27m7.4	3.33	0
ENSXMAG00000010574	0.0014	18	6	si:dkey-175g6.5	2.01	15
ENSXMAG00000010616	0.0030	18	12	ptges3a	2.88	15
ENSXMAG00000010671	0.0027	15	9	dnajc3b	8.50	12
ENSXMAG00000010693	0.0026	18	9	PSMD11	4.71	15
ENSXMAG00000010727	0.0031	15	12	slc1a3a	1.74	12
ENSXMAG00000010738	0.0026	21	15		1.72	18
ENSXMAG00000010760	0.0015	18	6	map1sa	4.26	15
ENSXMAG00000010763	0.0014	18	6	wdtc1	1.99	15
ENSXMAG00000010774	0.0034	18	12	pafah1b3	1.84	15
ENSXMAG00000010777	0.0025	15	15	ssr4	3.17	12
ENSXMAG00000010798	0.0031	18	12		2.54	15
ENSXMAG00000010822	0.0030	12	12	erlec1	3.17	9
ENSXMAG00000010828	0.0014	18	6	huwe1	2.53	15
ENSXMAG00000010835	0.0031	18	12	ccdc25	2.96	15
ENSXMAG00000010913	0.0026	18	9		3.76	15
ENSXMAG00000011013	0.0025	15	15	cnpy3	2.67	12
ENSXMAG00000011048	0.0028	21	9	commd6	2.15	18
ENSXMAG00000011069	0.0016	24	18	stard10	1.43	21
ENSXMAG00000011122	0.0025	6	15	gnb3a	2.91	3
ENSXMAG00000011127	0.0030	18	12	cxxc5a	3.44	15
ENSXMAG00000011168	0.0027	24	9	si:ch211-59d17.3	2.89	21
ENSXMAG00000011222	0.0026	18	9	tmed5	3.82	15
ENSXMAG00000011236	0.0014	18	6	dlg1	1.84	15
ENSXMAG00000011274	0.0016	12	6	sprydd7b	1.40	9
ENSXMAG00000011275	0.0015	18	6	srm	3.44	15
ENSXMAG00000011288	0.0026	18	9	mettl13	4.08	15
ENSXMAG00000011324	0.0025	21	9		3.00	18
ENSXMAG00000011365	0.0031	21	12	si:dkey-239i20.2	2.20	18
ENSXMAG00000011377	0.0031	18	12	edf1	1.35	15
ENSXMAG00000011403	0.0071	21	18	si:dkey-86e18.1	4.08	18
ENSXMAG00000011458	0.0029	15	12	si:cabz01078036.1	3.14	12
ENSXMAG00000011464	0.0025	24	15	nr1d4a	132.40	21
ENSXMAG00000011470	0.0031	6	12	creg2	4.53	3
ENSXMAG00000011477	0.0025	21	9	rad51d	2.29	18
ENSXMAG00000011479	0.0034	21	12	mmachc	2.51	18
ENSXMAG00000011516	0.0029	18	12	spg21	1.74	15
ENSXMAG00000011577	0.0030	3	12	BFAR	4.62	0
ENSXMAG00000011601	0.0031	18	12	GCDH	1.28	15
ENSXMAG00000011606	0.0027	18	9	kctd13	2.70	15

ENSXMAG00000011620	0.0014	18	6	fer	2.40	15
ENSXMAG00000011686	0.0024	18	9	hsd17b7	7.26	15
ENSXMAG00000011691	0.0026	18	9	zgc:55558	3.11	15
ENSXMAG00000011735	0.0025	15	9	uap1	6.34	12
ENSXMAG00000011744	0.0029	18	12	anapc4	1.94	15
ENSXMAG00000011851	0.0031	24	12	chrne	2.24	21
ENSXMAG00000011874	0.0014	18	6	cd248a	2.53	15
ENSXMAG00000011943	0.0031	18	12	mrps34	2.50	15
ENSXMAG00000012013	0.0027	18	9	c2cd3	2.34	15
ENSXMAG00000012036	0.0027	18	9	p4ha3	12.70	15
ENSXMAG00000012054	0.0027	3	15	dbpb	56.57	0
ENSXMAG00000012060	0.0031	18	12	tbck	1.62	15
ENSXMAG00000012074	0.0031	18	12	sh3bgrl3	2.22	15
ENSXMAG00000012087	0.0031	6	12	slc22a5	1.85	3
ENSXMAG00000012129	0.0026	18	9	ube2al	18.85	15
ENSXMAG00000012148	0.0026	15	15	TTC39C (1 of many)	4.41	12
ENSXMAG00000012149	0.0031	18	12	naxd	2.31	15
ENSXMAG00000012189	0.0029	15	12	npas2	39.16	12
ENSXMAG00000012204	0.0025	15	15	manf	4.38	12
ENSXMAG00000012243	0.0031	18	12	hacd2	1.73	15
ENSXMAG00000012280	0.0026	9	15		3.94	6
ENSXMAG00000012356	0.0026	21	15	agmat	1.42	18
ENSXMAG00000012376	0.0031	9	12	cant1a	5.58	6
ENSXMAG00000012451	0.0026	21	9	bsg	1.47	18
ENSXMAG00000012456	0.0031	18	12	ints11	1.72	15
ENSXMAG00000012520	0.0030	18	12	pdap1a	3.42	15
ENSXMAG00000012557	0.0024	3	15	zranb1b	1.61	0
ENSXMAG00000012579	0.0026	9	15	sdf4	2.83	6
ENSXMAG00000012607	0.0024	18	9	b3gntl1	2.51	15
ENSXMAG00000012617	0.0025	18	9	zbtb48	3.54	15
ENSXMAG00000012634	0.0027	18	9	trim33	3.60	15
ENSXMAG00000012637	0.0026	21	9		4.95	18
ENSXMAG00000012658	0.0027	18	15		1.58	15
ENSXMAG00000012670	0.0029	24	12	atp5pf	1.42	21
ENSXMAG00000012679	0.0026	18	9	pfdn4	1.79	15
ENSXMAG00000012750	0.0025	15	15	zgc:195081	3.24	12
ENSXMAG00000012766	0.0031	18	12	wee1	39.17	15
ENSXMAG00000012801	0.0029	24	12	cited4a	6.85	21
ENSXMAG00000012823	0.0025	18	15	srsf5a	1.52	15
ENSXMAG00000012852	0.0031	18	12	si:ch73-302a13.2	2.00	15
ENSXMAG00000012874	0.0031	18	12	tbcd	3.03	15
ENSXMAG00000012952	0.0027	18	9	scamp2	1.46	15
ENSXMAG00000013026	0.0014	18	6	rspry1	1.87	15
ENSXMAG00000013030	0.0015	18	6	lrrc29	4.45	15
ENSXMAG00000013062	0.0014	18	6	tnpo3	3.81	15
ENSXMAG00000013065	0.0031	6	12	pcyt1bb	6.41	3
ENSXMAG00000013080	0.0025	21	15	l3mbtl1b	1.99	18
ENSXMAG00000013097	0.0014	18	6	dnajb1a	13.10	15
ENSXMAG00000013137	0.0027	18	9	pigq	2.86	15
ENSXMAG00000013158	0.0026	6	9	si:ch211-262e15.1	3.21	3
ENSXMAG00000013165	0.0029	24	12	btf3l4 (1 of many)	1.59	21
ENSXMAG00000013185	0.0031	12	12	mknk1	2.10	9
ENSXMAG00000013219	0.0028	18	9	utp11l	3.77	15
ENSXMAG00000013310	0.0034	21	12	mgat1a	2.31	18
ENSXMAG00000013343	0.0025	18	9		6.05	15
ENSXMAG00000013355	0.0029	18	12	sugt1	2.41	15

ENSXMAG00000013370	0.0026	18	9	pde12	2.94	15
ENSXMAG00000013387	0.0025	3	15	f9a	1.80	0
ENSXMAG00000013407	0.0031	18	12	rbmx	1.87	15
ENSXMAG00000013436	0.0031	18	12	nr2f5	5.49	15
ENSXMAG00000013448	0.0026	9	15	hif1aa	2.38	6
ENSXMAG00000013450	0.0025	15	15	tbl2	1.78	12
ENSXMAG00000013499	0.0014	18	6	smg5	2.31	15
ENSXMAG00000013548	0.0030	18	12	mrpl35	2.38	15
ENSXMAG00000013596	0.0025	15	9	OTUB2	2.28	12
ENSXMAG00000013703	0.0026	21	9	pnpo	1.85	18
ENSXMAG00000013710	0.0024	21	15	fuom	1.64	18
ENSXMAG00000013715	0.0031	12	12	cdk5rap3	2.91	9
ENSXMAG00000013738	0.0034	18	12	PAOX (1 of many)	1.88	15
ENSXMAG00000013784	0.0071	6	6	rcor3	2.63	3
ENSXMAG00000013860	0.0025	6	15	tpcn2	2.34	3
ENSXMAG00000013883	0.0027	9	15	PPFIA1	1.49	6
ENSXMAG00000013887	0.0026	3	15	xirp2a	3.01	0
ENSXMAG00000013922	0.0031	18	12		2.49	15
ENSXMAG00000013925	0.0014	3	6	serpinh2	4.09	0
ENSXMAG00000014069	0.0014	18	6	gtpbp1	7.39	15
ENSXMAG00000014070	0.0026	21	9	mad1l1	1.33	18
ENSXMAG00000014077	0.0031	9	12	abcc5	6.62	6
ENSXMAG00000014092	0.0025	18	9	acvr2ba	3.39	15
ENSXMAG00000014111	0.0025	18	9	eif4a3	2.50	15
ENSXMAG00000014117	0.0026	21	15		1.54	18
ENSXMAG00000014132	0.0029	18	12	soul4	5.03	15
ENSXMAG00000014141	0.0014	18	6	sos1	2.19	15
ENSXMAG00000014150	0.0026	18	9	ogfod1	3.96	15
ENSXMAG00000014237	0.0025	15	15	spcs2	2.45	12
ENSXMAG00000014261	0.0014	18	6	PI4KA	2.40	15
ENSXMAG00000014336	0.0026	18	9	fastkd1	3.10	15
ENSXMAG00000014347	0.0031	12	12	L3MBTL4	1.68	9
ENSXMAG00000014360	0.0026	18	9		3.66	15
ENSXMAG00000014375	0.0026	18	9	mindy3	2.20	15
ENSXMAG00000014482	0.0025	18	15	cyp4t8	3.86	15
ENSXMAG00000014496	0.0026	18	9	ino80c	1.48	15
ENSXMAG00000014572	0.0026	18	9	riok1	2.32	15
ENSXMAG00000014576	0.0030	18	12	fcf1	3.08	15
ENSXMAG00000014578	0.0027	21	9	pacs2	2.75	18
ENSXMAG00000014696	0.0031	18	12	eif2d	2.01	15
ENSXMAG00000014723	0.0014	18	6	zfyve26	2.66	15
ENSXMAG00000014754	0.0029	21	12	pbld	3.80	18
ENSXMAG00000014772	0.0026	15	15	ttf2	2.32	12
ENSXMAG00000014781	0.0026	18	9		4.52	15
ENSXMAG00000014783	0.0026	18	9	parn	3.02	15
ENSXMAG00000014794	0.0024	24	15	enpp6	1.79	21
ENSXMAG00000014808	0.0026	21	9	igbp1	1.52	18
ENSXMAG00000014814	0.0031	12	12	pbxip1a	1.75	9
ENSXMAG00000014852	0.0030	18	12	ccz1	2.37	15
ENSXMAG00000014865	0.0031	21	12	pygl	2.11	18
ENSXMAG00000014891	0.0031	15	12	pcna	3.60	12
ENSXMAG00000014906	0.0015	18	6	eif2ak1	4.36	15
ENSXMAG00000014967	0.0031	18	12	npc2	4.68	15
ENSXMAG00000015003	0.0025	18	9	alg3	2.15	15
ENSXMAG00000015008	0.0031	3	12	kmt2ba	1.87	0
ENSXMAG00000015011	0.0014	18	6	hectd3	2.01	15

ENSXMAG00000015016	0.0015	24	18	cyp21a2	2.57	21
ENSXMAG00000015025	0.0025	18	9	ACTA1	6.62	15
ENSXMAG00000015031	0.0031	18	12		3.24	15
ENSXMAG00000015055	0.0025	24	9	zgc:136564	2.14	21
ENSXMAG00000015074	0.0026	24	9	gstz1	1.37	21
ENSXMAG00000015160	0.0014	18	6	selenot2	3.91	15
ENSXMAG00000015172	0.0031	18	12	gpr137c	5.37	15
ENSXMAG00000015181	0.0026	18	9	orai1a	2.23	15
ENSXMAG00000015249	0.0014	18	6	afg1la	4.35	15
ENSXMAG00000015266	0.0025	18	15	cndp2	1.43	15
ENSXMAG00000015274	0.0031	18	12	zgc:172341	3.11	15
ENSXMAG00000015314	0.0030	3	12	per1b	66.15	0
ENSXMAG00000015366	0.0025	18	9	dgat1a	5.99	15
ENSXMAG00000015372	0.0026	21	9		2.67	18
ENSXMAG00000015455	0.0026	3	15	si:ch73-71d17.1	2.14	0
ENSXMAG00000015461	0.0031	18	12	parvaa	1.73	15
ENSXMAG00000015492	0.0026	18	15	hdhd3	1.67	15
ENSXMAG00000015550	0.0024	3	9		1.72	0
ENSXMAG00000015557	0.0030	18	12	pin1	2.77	15
ENSXMAG00000015561	0.0026	18	15	cyb5r2	4.09	15
ENSXMAG00000015564	0.0034	18	12	mrpl22	1.43	15
ENSXMAG00000015578	0.0031	15	12	kdelc2	2.62	12
ENSXMAG00000015595	0.0031	18	12	rars	1.79	15
ENSXMAG00000015619	0.0025	18	9	arntl1a	68.05	15
ENSXMAG00000015627	0.0031	21	12	cbwd	1.94	18
ENSXMAG00000015699	0.0031	18	12	selenot1a	2.51	15
ENSXMAG00000015745	0.0014	18	6	tha1	2.22	15
ENSXMAG00000015755	0.0024	18	9	YBX2	6.09	15
ENSXMAG00000015763	0.0026	21	15	slc25a21	2.11	18
ENSXMAG00000015778	0.0024	18	9	slc24a1 (1 of many)	2.45	15
ENSXMAG00000015797	0.0034	18	12	hacd3	7.91	15
ENSXMAG00000015820	0.0030	6	12	ldhd	2.02	3
ENSXMAG00000015858	0.0031	18	12	zgc:162858	1.77	15
ENSXMAG00000015904	0.0025	15	9		1.92	12
ENSXMAG00000015930	0.0014	18	6	tomm70a	2.61	15
ENSXMAG00000015952	0.0014	24	18	gadd45ga	5.03	21
ENSXMAG00000015975	0.0031	6	12		5.71	3
ENSXMAG00000015984	0.0031	18	12	polr2c	2.17	15
ENSXMAG00000015996	0.0015	24	6	spcs3	1.78	21
ENSXMAG00000016036	0.0027	24	9	pigf	1.83	21
ENSXMAG00000016067	0.0014	18	6	canx	2.47	15
ENSXMAG00000016077	0.0025	18	9	abhd12	1.75	15
ENSXMAG00000016133	0.0015	18	6	herc2	1.89	15
ENSXMAG00000016139	0.0015	18	6	fdps	69.61	15
ENSXMAG00000016159	0.0031	18	12	atp8a1	3.12	15
ENSXMAG00000016161	0.0026	18	9	chac2	9.43	15
ENSXMAG00000016253	0.0024	18	15	dip2a	7.41	15
ENSXMAG00000016277	0.0025	18	9		3.15	15
ENSXMAG00000016399	0.0014	18	6	UBA3 (1 of many)	3.06	15
ENSXMAG00000016418	0.0014	18	6	zbtb11	2.22	15
ENSXMAG00000016443	0.0027	9	15		6.37	6
ENSXMAG00000016447	0.0027	24	15	prkag2b	2.76	21
ENSXMAG00000016460	0.0025	15	15	hsp90b1	13.77	12
ENSXMAG00000016477	0.0026	12	9	b4galt7	2.60	9
ENSXMAG00000016490	0.0014	18	6	nt5dc3	5.40	15
ENSXMAG00000016641	0.0031	18	12	abcf1	2.07	15

ENSXMAG00000016663	0.0026	21	15	CDH2	1.48	18
ENSXMAG00000016672	0.0025	9	15		3.60	6
ENSXMAG00000016686	0.0031	18	12	myef2	2.10	15
ENSXMAG00000016773	0.0031	18	12	slc25a25b	28.08	15
ENSXMAG00000016828	0.0026	15	15	derl2	1.63	12
ENSXMAG00000016861	0.0026	15	15	si:ch211-117n7.8	2.52	12
ENSXMAG00000016897	0.0026	21	9	bpnt1	1.83	18
ENSXMAG00000016913	0.0026	21	9	si:ch211-106j24.1	2.50	18
ENSXMAG00000016915	0.0030	18	12	ngly1	2.71	15
ENSXMAG00000016921	0.0025	21	9	rars2	1.63	18
ENSXMAG00000016928	0.0030	18	12	clocka	64.45	15
ENSXMAG00000017050	0.0034	24	12	hhat	2.62	21
ENSXMAG00000017066	0.0014	18	6	thrb	1.89	15
ENSXMAG00000017106	0.0034	9	12	nr1d2b	9.91	6
ENSXMAG00000017125	0.0025	21	15		1.96	18
ENSXMAG00000017162	0.0026	21	9	slc17a5	1.56	18
ENSXMAG00000017240	0.0029	18	12		3.04	15
ENSXMAG00000017243	0.0024	15	9	slc35e3	2.07	12
ENSXMAG00000017256	0.0029	18	12	g6pd	3.87	15
ENSXMAG00000017270	0.0027	18	9	megf6b	2.53	15
ENSXMAG00000017293	0.0015	24	18	th2	5.94	21
ENSXMAG00000017365	0.0014	18	6	fam122b	2.73	15
ENSXMAG00000017366	0.0027	21	15	esrp2	1.41	18
ENSXMAG00000017375	0.0024	24	15	pld6	4.76	21
ENSXMAG00000017379	0.0031	24	12		2.11	21
ENSXMAG00000017399	0.0026	18	9	twf1a	3.68	15
ENSXMAG00000017400	0.0031	6	12	amfra	1.83	3
ENSXMAG00000017408	0.0028	21	15	enpp1	2.61	18
ENSXMAG00000017416	0.0026	18	9	ptges3b	7.08	15
ENSXMAG00000017429	0.0024	15	15	atg7	1.48	12
ENSXMAG00000017435	0.0026	9	15		3.43	6
ENSXMAG00000017448	0.0031	15	12	creld1b	6.60	12
ENSXMAG00000017480	0.0015	6	18	slc7a2	2.77	3
ENSXMAG00000017502	0.0024	6	15	pdgfrl	3.35	3
ENSXMAG00000017510	0.0031	21	12	zgc:101679	1.24	18
ENSXMAG00000017520	0.0025	6	9	lpin1	12.17	3
ENSXMAG00000017549	0.0026	18	9	lpgat1	2.27	15
ENSXMAG00000017571	0.0031	21	12	cfap298	1.84	18
ENSXMAG00000017591	0.0024	15	15	glra3	2.01	12
ENSXMAG00000017595	0.0014	18	6	g6pc3	4.65	15
ENSXMAG00000017637	0.0026	18	9	btbd3a	1.91	15
ENSXMAG00000017647	0.0016	18	6		5.94	15
ENSXMAG00000017715	0.0027	18	15	hp1bp3	2.42	15
ENSXMAG00000017736	0.0030	18	12	timm10	5.66	15
ENSXMAG00000017746	0.0014	24	18	pkdccb	3.57	21
ENSXMAG00000017759	0.0030	18	12	myg1	3.00	15
ENSXMAG00000017770	0.0031	18	12	selenom	1.91	15
ENSXMAG00000017773	0.0030	12	12	pdia6	16.93	9
ENSXMAG00000017793	0.0030	18	12	usp39	2.16	15
ENSXMAG00000017798	0.0016	15	6		4.30	12
ENSXMAG00000017836	0.0025	18	9	tarbp2	4.51	15
ENSXMAG00000017878	0.0016	15	18	kptn	1.53	12
ENSXMAG00000017883	0.0027	18	9	si:dkey-166d12.2	2.85	15
ENSXMAG00000017927	0.0024	18	9	ckma	18.98	15
ENSXMAG00000018001	0.0027	18	9	ckmb	12.17	15
ENSXMAG00000018079	0.0014	18	6		2.47	15

ENSXMAG00000018130	0.0031	18	12		2.12	15
ENSXMAG00000018138	0.0015	18	18		2.26	15
ENSXMAG00000018150	0.0026	18	9	ttll11	8.92	15
ENSXMAG00000018157	0.0031	18	12		4.15	15
ENSXMAG00000018169	0.0026	21	15	lyrm7	2.50	18
ENSXMAG00000018170	0.0027	18	9	mfn1b	2.72	15
ENSXMAG00000018249	0.0031	18	12	mrpl47	1.87	15
ENSXMAG00000018254	0.0029	15	12	unga	6.87	12
ENSXMAG00000018257	0.0031	24	12	ndufb5	1.38	21
ENSXMAG00000018262	0.0031	21	12	pxmp2	1.30	18
ENSXMAG00000018298	0.0031	18	12	mphosph10	3.51	15
ENSXMAG00000018304	0.0026	15	15	TADA1	2.15	12
ENSXMAG00000018315	0.0024	21	9	srp54	1.41	18
ENSXMAG00000018360	0.0071	15	6	pxna	3.74	12
ENSXMAG00000018402	0.0026	18	9	tab3	2.43	15
ENSXMAG00000018422	0.0027	6	15	ankrd9	26.22	3
ENSXMAG00000018428	0.0025	3	15	rbks	2.04	0
ENSXMAG00000018454	0.0025	18	9	ELOB	1.64	15
ENSXMAG00000018474	0.0029	18	12	pelo	3.70	15
ENSXMAG00000018487	0.0034	15	12	atp2a1l	28.96	12
ENSXMAG00000018508	0.0031	24	12	chchd6b	1.89	21
ENSXMAG00000018557	0.0031	21	12		1.82	18
ENSXMAG00000018569	0.0026	18	9	ecd	1.36	15
ENSXMAG00000018613	0.0014	18	6		1.99	15
ENSXMAG00000018630	0.0025	18	9	lsm7	1.58	15
ENSXMAG00000018636	0.0031	18	12	iars2	3.05	15
ENSXMAG00000018652	0.0031	18	12	impdh1b	4.88	15
ENSXMAG00000018726	0.0015	18	6	cers1	15.68	15
ENSXMAG00000018730	0.0030	12	12	cope	3.84	9
ENSXMAG00000018770	0.0024	18	9	atp6v0d1	1.78	15
ENSXMAG00000018792	0.0026	18	9	kpnb1	2.84	15
ENSXMAG00000018796	0.0031	24	12	acap3b	7.31	21
ENSXMAG00000018846	0.0014	18	18	pusl1	2.27	15
ENSXMAG00000018874	0.0025	15	15	pdia4	8.05	12
ENSXMAG00000018882	0.0027	3	15	sorbs2a	2.25	0
ENSXMAG00000018896	0.0025	15	15	rpn1	2.10	12
ENSXMAG00000018921	0.0015	24	18	tsr3	2.16	21
ENSXMAG00000018928	0.0030	18	12	gnptg	2.99	15
ENSXMAG00000018934	0.0015	3	18	nr1d1	9.53	0
ENSXMAG00000018966	0.0034	18	12	nudcd3	1.67	15
ENSXMAG00000018977	0.0031	18	12	gnsb	2.18	15
ENSXMAG00000018978	0.0024	3	15	qsox1	2.72	0
ENSXMAG00000019038	0.0031	18	12	ube2v2	2.41	15
ENSXMAG00000019055	0.0014	18	6	actr5	3.68	15
ENSXMAG00000019056	0.0030	18	12	gale	3.41	15
ENSXMAG00000019062	0.0025	24	9		1.42	21
ENSXMAG00000019110	0.0031	18	12	elp6	2.48	15
ENSXMAG00000019116	0.0031	21	12		2.39	18
ENSXMAG00000019126	0.0029	18	12	dtd2	1.66	15
ENSXMAG00000019183	0.0026	18	9	cct5	3.74	15
ENSXMAG00000019247	0.0030	18	12	denr	6.06	15
ENSXMAG00000019263	0.0027	15	9	hdhd5	2.29	12
ENSXMAG00000019296	0.0025	15	9	rorcb	55.53	12
ENSXMAG00000019315	0.0026	18	15	zgc:136971	2.03	15
ENSXMAG00000019336	0.0015	21	6	cirbpa	1.51	18
ENSXMAG00000019340	0.0031	18	12	ARMC6	5.27	15

ENSXMAG00000019400	0.0015	18	6	ttc37	1.60	15
ENSXMAG00000019418	0.0027	24	15	cox7c	1.34	21
ENSXMAG00000019438	0.0031	24	12		1.76	21
ENSXMAG00000019530	0.0031	18	12		107.53	15
ENSXMAG00000019534	0.0014	18	6	glrx3	2.75	15
ENSXMAG00000019568	0.0071	15	6	slc5a3b	12.06	12
ENSXMAG00000019575	0.0024	6	9	si:ch211-236k19.4	2.86	3
ENSXMAG00000019860	0.0026	18	15		2.40	15
ENSXMAG00000019902	0.0014	24	18		4.17	21
ENSXMAG00000019943	0.0027	18	9	nfil3	18.91	15
ENSXMAG00000019975	0.0029	9	12		4.19	6
ENSXMAG00000020009	0.0031	24	12	mthfs	1.41	21
ENSXMAG00000020102	0.0031	6	12	slc18a3a	12.53	3
ENSXMAG00000020150	0.0031	24	12	HTD2	1.80	21
ENSXMAG00000020317	0.0031	15	12	adra2b	9.14	12
ENSXMAG00000020322	0.0031	18	12	smim15	2.36	15
ENSXMAG00000020356	0.0071	15	6	arl14	2.29	12
ENSXMAG00000020786	0.0031	21	12	ND1	1.42	18
ENSXMAG00000020821	0.0027	15	9	pogzb	2.79	12
ENSXMAG00000020844	0.0029	18	12		1.95	15
ENSXMAG00000020852	0.0026	21	15	asrgl1	1.90	18
ENSXMAG00000020879	0.0031	15	12		2.05	12
ENSXMAG00000020894	0.0028	21	9		1.99	18
ENSXMAG00000020901	0.0030	12	12		2.61	9
ENSXMAG00000020918	0.0031	18	12	fxyd1	2.89	15
ENSXMAG00000020921	0.0031	18	12	SYNGR2 (1 of many)	2.02	15
ENSXMAG00000021003	0.0029	12	12	rbp4	2.18	9
ENSXMAG00000021019	0.0026	24	9	uraha	1.88	21
ENSXMAG00000021026	0.0031	21	12	FAM13C	3.55	18
ENSXMAG00000021116	0.0024	15	15	rabggtb	1.54	12
ENSXMAG00000021189	0.0027	18	15	sigmar1	2.51	15
ENSXMAG00000021202	0.0025	18	9	ARL6	4.08	15
ENSXMAG00000021245	0.0024	21	9	ppifb	2.06	18
ENSXMAG00000021252	0.0031	18	12		2.32	15
ENSXMAG00000021268	0.0014	18	6	pllp	2.45	15
ENSXMAG00000021278	0.0031	18	12	polr2i	2.05	15
ENSXMAG00000021312	0.0031	15	12		1.95	12
ENSXMAG00000021324	0.0025	18	9	dnmt3ab	9.55	15
ENSXMAG00000021333	0.0026	9	15	abhd2a	11.66	6
ENSXMAG00000021410	0.0031	18	12	si:ch211-168f7.5	2.21	15
ENSXMAG00000021447	0.0026	9	15		9.80	6
ENSXMAG00000021536	0.0025	18	9		5.87	15
ENSXMAG00000021541	0.0024	3	15	cyb5d2	3.26	0
ENSXMAG00000021590	0.0029	18	12	tomm5	2.51	15
ENSXMAG00000021594	0.0031	18	12	EIF1AY	2.04	15
ENSXMAG00000021634	0.0014	18	6	camlg	4.59	15
ENSXMAG00000021654	0.0025	18	15	ttll12	1.57	15
ENSXMAG00000021670	0.0026	3	15		3.45	0
ENSXMAG00000021725	0.0025	18	9	MRPL34	2.90	15
ENSXMAG00000021830	0.0071	21	18	si:ch1073-322p19.1	2.08	18
ENSXMAG00000021881	0.0015	18	6		3.51	15
ENSXMAG00000021893	0.0029	18	12	C5orf15	2.01	15
ENSXMAG00000021931	0.0026	21	15	si:dkey-30j16.3	3.31	18
ENSXMAG00000021939	0.0031	18	12	COPS6	1.54	15
ENSXMAG00000022006	0.0026	9	9	zbtb16b	13.68	6
ENSXMAG00000022027	0.0034	24	12	tctex1d2	3.07	21

ENSXMAG00000022050	0.0031	18	12		2.09	15
ENSXMAG00000022052	0.0025	3	9	dpy30	1.48	0
ENSXMAG00000022075	0.0030	18	12		4.34	15
ENSXMAG00000022106	0.0031	18	12	si:ch211-204d2.4	2.56	15
ENSXMAG00000022128	0.0025	6	15		2.11	3
ENSXMAG00000022168	0.0031	18	12	timm8a	5.05	15
ENSXMAG00000022222	0.0031	18	12	crcp	1.83	15
ENSXMAG00000022224	0.0030	18	12	setd7 (1 of many)	3.48	15
ENSXMAG00000022235	0.0026	9	9		2.30	6
ENSXMAG00000022292	0.0026	21	9	CD276	2.24	18
ENSXMAG00000022324	0.0014	18	6	narf	2.37	15
ENSXMAG00000022332	0.0024	3	15	map3k8	8.12	0
ENSXMAG00000022408	0.0029	18	12		1.88	15
ENSXMAG00000022522	0.0031	15	12	lygl2	2.76	12
ENSXMAG00000022577	0.0029	18	12		2.33	15
ENSXMAG00000022593	0.0031	18	12	si:ch211-231f6.6	3.32	15
ENSXMAG00000022599	0.0026	15	9	rev3l	1.86	12
ENSXMAG00000022639	0.0031	6	12	micu1	1.28	3
ENSXMAG00000022664	0.0030	12	12	GNG12 (1 of many)	3.99	9
ENSXMAG00000022665	0.0031	18	12	MTFP1	4.75	15
ENSXMAG00000022756	0.0025	24	15	gpc4	2.36	21
ENSXMAG00000022781	0.0015	21	6		2.13	18
ENSXMAG00000022841	0.0025	24	15	FOXN2	2.21	21
ENSXMAG00000022846	0.0031	24	12		1.36	21
ENSXMAG00000022885	0.0025	3	15		2.57	0
ENSXMAG00000022964	0.0031	18	12	zgc:154077	2.24	15
ENSXMAG00000023049	0.0025	18	9	imp3	2.22	15
ENSXMAG00000023082	0.0026	18	15		65.63	15
ENSXMAG00000023128	0.0031	6	12		2.71	3
ENSXMAG00000023164	0.0029	18	12		3.63	15
ENSXMAG00000023178	0.0030	12	12		4.26	9
ENSXMAG00000023187	0.0031	3	12	dnajc22	1.40	0
ENSXMAG00000023264	0.0031	6	12	ip6k2a	5.15	3
ENSXMAG00000023272	0.0030	24	12	si:ch211-284b7.3	1.40	21
ENSXMAG00000023274	0.0026	21	9	klhdc3	2.29	18
ENSXMAG00000023293	0.0030	18	12	ralba	2.25	15
ENSXMAG00000023298	0.0031	18	12	rd3	2.16	15
ENSXMAG00000023324	0.0031	18	12	mrps25	1.78	15
ENSXMAG00000023339	0.0034	21	12	mcts1	1.40	18
ENSXMAG00000023341	0.0025	18	9		2.72	15
ENSXMAG00000023379	0.0014	18	6		2.29	15
ENSXMAG00000023425	0.0026	21	9	cox5aa (1 of many)	1.36	18
ENSXMAG00000023487	0.0026	18	9	si:dkey-4e7.3 (1 of many)	3.36	15
ENSXMAG00000023532	0.0029	18	12	rabif	2.03	15
ENSXMAG00000023539	0.0029	18	12	cbx5	1.75	15
ENSXMAG00000023561	0.0024	18	9	anxa13	1.50	15
ENSXMAG00000023603	0.0014	18	6	aco1	2.06	15
ENSXMAG00000023607	0.0026	18	9	dusp7	4.07	15
ENSXMAG00000023611	0.0031	12	12		1.73	9
ENSXMAG00000023620	0.0024	24	15		2.02	21
ENSXMAG00000023716	0.0025	3	15	sybu	9.79	0
ENSXMAG00000023742	0.0025	15	9	GOLT1B (1 of many)	2.29	12
ENSXMAG00000023756	0.0015	18	6	hmbsa	5.11	15
ENSXMAG00000023781	0.0026	6	15	ugt1a7 (1 of many)	3.59	3
ENSXMAG00000023782	0.0031	18	12	pcdh2ac	2.40	15
ENSXMAG00000023847	0.0026	18	9	arpp19a	1.92	15

ENSXMAG00000023857	0.0031	18	12	tfam	2.25	15
ENSXMAG00000023901	0.0030	18	12	tecrb	7.38	15
ENSXMAG00000023909	0.0025	21	9	gstr (1 of many)	1.40	18
ENSXMAG00000023943	0.0025	18	15	si:ch211-210c8.6	1.42	15
ENSXMAG00000023993	0.0026	18	9		4.32	15
ENSXMAG00000024082	0.0014	24	18		12.23	21
ENSXMAG00000024117	0.0071	15	6	gnpnat1	11.57	12
ENSXMAG00000024152	0.0031	18	12		3.81	15
ENSXMAG00000024156	0.0030	18	12	slc43a3b (1 of many)	4.42	15
ENSXMAG00000024190	0.0014	18	6	hsd11b1la (1 of many)	5.58	15
ENSXMAG00000024193	0.0025	18	9	crls1	4.52	15
ENSXMAG00000024208	0.0031	18	12	gng3	3.10	15
ENSXMAG00000024211	0.0025	18	9	MXRA7	1.54	15
ENSXMAG00000024229	0.0024	18	15	mrpl14	1.36	15
ENSXMAG00000024264	0.0026	18	9	ognb	3.39	15
ENSXMAG00000024303	0.0034	18	12	zgc:114119	3.78	15
ENSXMAG00000024324	0.0031	6	12		1.30	3
ENSXMAG00000024334	0.0031	6	12	GYS2	1.43	3
ENSXMAG00000024339	0.0029	18	12	PRPSAP1	2.54	15
ENSXMAG00000024381	0.0029	18	12	tbca	1.62	15
ENSXMAG00000024398	0.0029	15	12	socs3b	4.46	12
ENSXMAG00000024428	0.0026	15	15		2.69	12
ENSXMAG00000024451	0.0031	24	12		1.40	21
ENSXMAG00000024476	0.0024	24	9	fam204a	1.43	21
ENSXMAG00000024615	0.0071	21	18		4.27	18
ENSXMAG00000024679	0.0031	18	12	PPIC	4.61	15
ENSXMAG00000024712	0.0030	18	12	MID1	6.36	15
ENSXMAG00000024722	0.0024	18	9	pvalb4	10.35	15
ENSXMAG00000024759	0.0024	24	9		1.40	21
ENSXMAG00000024791	0.0031	18	12		1.88	15
ENSXMAG00000024799	0.0026	18	9		2.37	15
ENSXMAG00000024827	0.0026	18	9	tsr2	3.08	15
ENSXMAG00000024833	0.0025	18	9	zgc:162634	2.11	15
ENSXMAG00000024847	0.0031	18	12	btf3l4 (1 of many)	2.15	15
ENSXMAG00000024884	0.0026	9	15		1.42	6
ENSXMAG00000024897	0.0031	18	12	sec62	1.82	15
ENSXMAG00000024912	0.0030	21	12		2.37	18
ENSXMAG00000024940	0.0027	18	9	rpp40	2.72	15
ENSXMAG00000024949	0.0014	18	6	ssbp1	4.69	15
ENSXMAG00000024962	0.0024	18	9	mylpfa	9.38	15
ENSXMAG00000025000	0.0024	6	15	ppp1r1b	2.00	3
ENSXMAG00000025030	0.0025	18	9	slc35b1	2.44	15
ENSXMAG00000025060	0.0031	15	12	alg8	1.90	12
ENSXMAG00000025182	0.0031	18	12		3.88	15
ENSXMAG00000025269	0.0031	18	12	ttc33	1.41	15
ENSXMAG00000025272	0.0024	15	9		3.01	12
ENSXMAG00000025294	0.0025	24	9	foxo6b	3.74	21
ENSXMAG00000025303	0.0014	18	6	C18orf32	3.34	15
ENSXMAG00000025324	0.0016	24	18	cldn12	1.60	21
ENSXMAG00000025396	0.0024	21	9		1.57	18
ENSXMAG00000025436	0.0024	15	15	tmed1b	1.57	12
ENSXMAG00000025485	0.0030	18	12		1.35	15
ENSXMAG00000025488	0.0024	18	15	znf511	1.73	15
ENSXMAG00000025493	0.0014	18	6	stk17b	3.10	15
ENSXMAG00000025526	0.0030	24	12	ndufs5	1.40	21
ENSXMAG00000025552	0.0024	3	15	pgpep1l	2.59	0

ENSXMAG00000025556	0.0031	21	12	fam69c	1.26	18
ENSXMAG00000025560	0.0026	24	9	cbx1b	1.73	21
ENSXMAG00000025586	0.0031	18	12		1.31	15
ENSXMAG00000025654	0.0024	18	9	polr3k	2.68	15
ENSXMAG00000025658	0.0014	18	6		3.23	15
ENSXMAG00000025685	0.0030	12	12	ufl1	2.74	9
ENSXMAG00000025696	0.0027	24	15	ing4	2.14	21
ENSXMAG00000025720	0.0024	15	15	zgc:77041	2.31	12
ENSXMAG00000025729	0.0027	18	9	zcchc10	2.85	15
ENSXMAG00000025748	0.0024	18	9	tnnc2	21.36	15
ENSXMAG00000025750	0.0024	18	9	si:ch1073-314i13.4	1.70	15
ENSXMAG00000025753	0.0031	18	12	ppp1r14bb	3.05	15
ENSXMAG00000025757	0.0031	3	12	prnpb	1.62	0
ENSXMAG00000025780	0.0031	3	12		3.01	0
ENSXMAG00000025850	0.0031	18	12		3.18	15
ENSXMAG00000025859	0.0014	18	6	cul1b	2.40	15
ENSXMAG00000025881	0.0031	18	12	hs2st1b	1.57	15
ENSXMAG00000025933	0.0026	18	9	ublcp1	5.93	15
ENSXMAG00000025967	0.0026	18	9		2.17	15
ENSXMAG00000025973	0.0027	18	9		1.79	15
ENSXMAG00000026007	0.0025	15	15	calr3b	5.85	12
ENSXMAG00000026080	0.0030	12	12	ergic3 (1 of many)	7.37	9
ENSXMAG00000026091	0.0026	18	9		1.84	15
ENSXMAG00000026109	0.0015	24	18	rnf24	2.48	21
ENSXMAG00000026116	0.0024	9	15	acot19	1.94	6
ENSXMAG00000026139	0.0028	18	15	ap5s1	1.58	15
ENSXMAG00000026144	0.0026	18	9		3.29	15
ENSXMAG00000026161	0.0026	18	9	fam49bb	6.93	15
ENSXMAG00000026179	0.0026	18	9	usp10	5.47	15
ENSXMAG00000026233	0.0029	6	12		12.84	3
ENSXMAG00000026309	0.0031	18	12	flvcr2a	12.23	15
ENSXMAG00000026333	0.0014	3	18	tefb	28.64	0
ENSXMAG00000026346	0.0014	18	6	mboat7	2.79	15
ENSXMAG00000026350	0.0031	15	12		1.58	12
ENSXMAG00000026416	0.0031	21	12	srsf5b	1.77	18
ENSXMAG00000026480	0.0031	18	12	rnf7	2.26	15
ENSXMAG00000026517	0.0027	18	9	c1ql3b	2.62	15
ENSXMAG00000026608	0.0015	18	6	dph5	1.90	15
ENSXMAG00000026619	0.0031	18	12	rpp21	3.76	15
ENSXMAG00000026665	0.0031	24	12		1.92	21
ENSXMAG00000026707	0.0034	18	12	PM20D2	2.09	15
ENSXMAG00000026714	0.0026	21	15	dnal4a	1.53	18
ENSXMAG00000026720	0.0026	9	9		3.09	6
ENSXMAG00000026769	0.0025	18	15		1.78	15
ENSXMAG00000026802	0.0014	24	18	rab40b	6.34	21
ENSXMAG00000026908	0.0025	12	15		2.37	9
ENSXMAG00000026974	0.0024	21	9	slc35f6	1.77	18
ENSXMAG00000027001	0.0024	21	9	si:dkeyp-94h10.5	1.46	18
ENSXMAG00000027063	0.0027	18	9	gdf11	8.55	15
ENSXMAG00000027132	0.0024	18	9		2.06	15
ENSXMAG00000027145	0.0031	3	12		2.09	0
ENSXMAG00000027150	0.0031	21	12	sypl2b	1.50	18
ENSXMAG00000027213	0.0014	21	6	znf706	1.43	18
ENSXMAG00000027231	0.0031	15	12		2.39	12
ENSXMAG00000027359	0.0026	6	15		2.67	3
ENSXMAG00000027379	0.0014	18	6	ndrg1a	8.07	15

ENSXMAG00000027417	0.0025	21	15	tspan36	1.90	18
ENSXMAG00000027468	0.0014	3	18		12.11	0
ENSXMAG00000027480	0.0016	6	18	cyp2ad2	1.94	3
ENSXMAG00000027615	0.0014	18	6		35.39	15
ENSXMAG00000027646	0.0031	18	12	MMP16 (1 of many)	3.26	15
ENSXMAG00000027656	0.0014	21	6	cdkn1d	23.31	18
ENSXMAG00000027716	0.0026	6	15	hibadha	1.77	3
ENSXMAG00000027733	0.0031	18	12	tmem106c	13.00	15
ENSXMAG00000027774	0.0026	18	9		7.46	15
ENSXMAG00000027840	0.0014	24	18	mafga	5.81	21
ENSXMAG00000027907	0.0030	18	12	si:ch211-212o1.2	1.73	15
ENSXMAG00000027928	0.0025	3	9		1.46	0
ENSXMAG00000027949	0.0030	18	12	endog	4.46	15
ENSXMAG00000027968	0.0014	18	6	psme3	8.29	15
ENSXMAG00000027969	0.0031	9	12		6.39	6
ENSXMAG00000027978	0.0031	18	12	dnajc8	2.14	15
ENSXMAG00000028047	0.0031	15	12	laptm4a	1.66	12
ENSXMAG00000028103	0.0031	6	12	prg4b	2.48	3
ENSXMAG00000028105	0.0026	18	9		3.95	15
ENSXMAG00000028160	0.0031	18	12	ptgesl	2.59	15
ENSXMAG00000028172	0.0031	3	12	khnyn	4.20	0
ENSXMAG00000028202	0.0031	12	12		2.95	9
ENSXMAG00000028233	0.0025	15	15	mydgf	4.15	12
ENSXMAG00000028308	0.0031	18	12	sap18	1.75	15
ENSXMAG00000028356	0.0024	21	9	nsd3	1.47	18
ENSXMAG00000028392	0.0026	18	9	zgc:165532	1.69	15
ENSXMAG00000028408	0.0031	18	12	mettl26	2.19	15
ENSXMAG00000028425	0.0024	3	9	ablim2	2.67	0
ENSXMAG00000028444	0.0031	18	12	mrpl12	3.48	15
ENSXMAG00000028470	0.0071	18	18	egln2	3.12	15
ENSXMAG00000028517	0.0026	15	15	chchd6a	1.70	12
ENSXMAG00000028526	0.0031	18	12	smyd2a	4.45	15
ENSXMAG00000028532	0.0025	21	9	si:ch211-195e19.1	3.99	18
ENSXMAG00000028549	0.0028	18	9	tmem222a	1.95	15
ENSXMAG00000028556	0.0026	18	9	yipf6	2.18	15
ENSXMAG00000028575	0.0026	21	9	clockb	12.74	18
ENSXMAG00000028577	0.0014	18	6	aasdhppt	3.14	15
ENSXMAG00000028597	0.0014	18	6	setd3	10.01	15
ENSXMAG00000028608	0.0034	18	12	nedd8	1.82	15
ENSXMAG00000028629	0.0030	18	12	mmd	1.97	15
ENSXMAG00000028723	0.0025	6	15	slc25a33	8.47	3
ENSXMAG00000028792	0.0026	21	9	ADI1	1.72	18
ENSXMAG00000028827	0.0031	18	12	wfdc1	5.97	15
ENSXMAG00000028868	0.0031	18	12	zgc:103692	1.97	15
ENSXMAG00000028882	0.0014	18	6	srd5a1	4.34	15
ENSXMAG00000028901	0.0026	18	9		4.81	15
ENSXMAG00000028963	0.0024	18	9	tmem135	2.92	15
ENSXMAG00000028967	0.0071	15	6	si:dkeyp-74a11.1	1.84	12
ENSXMAG00000028998	0.0025	18	15	si:ch211-282b22.1	1.62	15
ENSXMAG00000029095	0.0026	18	9		4.10	15
ENSXMAG00000029102	0.0031	18	12	aurkaip1	3.26	15
ENSXMAG00000029119	0.0031	18	12	tmem150c	3.38	15
ENSXMAG00000029126	0.0027	18	9		58.27	15
ENSXMAG00000029144	0.0014	18	6		1.92	15
ENSXMAG00000029182	0.0024	18	15	PCBD2	1.71	15
ENSXMAG00000029216	0.0024	15	9		1.88	12

ENSXMAG00000029275	0.0025	18	9	zgc:171489	5.80	15
ENSXMAG00000029312	0.0031	18	12	nt5c2b	4.33	15
ENSXMAG00000029314	0.0029	18	12	sc5d	17.08	15
ENSXMAG00000029375	0.0026	18	9	pgp	4.84	15
ENSXMAG00000029408	0.0031	24	12		1.32	21
ENSXMAG00000029411	0.0027	24	15	c1qtnf6b	2.00	21
ENSXMAG00000029444	0.0031	18	12	eef1akmt1	2.17	15
ENSXMAG00000029463	0.0029	15	12	irak1bp1	9.14	12
ENSXMAG00000029501	0.0014	6	18	GPATCH2L	4.33	3
ENSXMAG00000029542	0.0025	18	15		2.32	15
ENSXMAG00000029555	0.0031	24	12	atp5f1e	1.50	21
ENSXMAG00000029562	0.0026	18	9	pdrg1	1.30	15
ENSXMAG00000029603	0.0034	18	12	mtch2	1.49	15
ENSXMAG00000029666	0.0031	18	12	ctnnbip1	2.83	15
ENSXMAG00000029694	0.0014	18	6		4.98	15
ENSXMAG00000029757	0.0030	12	12	scfd1	2.68	9
ENSXMAG00000029765	0.0029	18	12	bola3	2.45	15
ENSXMAG00000029788	0.0030	18	12	srp19	2.03	15
ENSXMAG00000029801	0.0024	9	9	ACSS1	6.25	6
ENSXMAG00000029813	0.0028	3	15		2.32	0
ENSXMAG00000029834	0.0026	18	9	degs1	3.53	15
ENSXMAG00000029843	0.0027	3	15		34.88	0
ENSXMAG00000029844	0.0031	6	12	eif4ebp3l	8.86	3
ENSXMAG00000029900	0.0026	18	15	mcu	1.76	15
ENSXMAG00000029918	0.0015	24	18	trnau1apb	2.00	21
ENSXMAG00000029978	0.0027	18	9		2.12	15
ENSXMAG00000029979	0.0034	3	12	SLC39A11	1.35	0
ENSXMAG00000029983	0.0031	18	12	sephs2	2.62	15
ENSXMAG00000029989	0.0026	12	9	nwd2	5.74	9

Testis circadian gene

GeneID	pVal	phase	peak.shape	external_gene_name	amp	Ct.peak
ENSXMAG00000000336	0.0029	6	12	magi1b	1.38	3
ENSXMAG00000000389	0.0034	6	12		1.69	3
ENSXMAG00000000471	0.0026	18	9	coro2bb	1.29	15
ENSXMAG00000000538	0.0026	24	15	tm4sf4	1.27	21
ENSXMAG00000000631	0.0026	12	9	snx13	1.08	9
ENSXMAG00000000645	0.0026	21	9	grk3	1.08	18
ENSXMAG00000000726	0.0024	18	9		1.17	15
ENSXMAG00000000900	0.0014	24	18	bmp1a	1.26	21
ENSXMAG00000000903	0.0024	6	9	mmp14b	1.51	3
ENSXMAG00000001020	0.0025	24	15	vaspb	1.18	21
ENSXMAG00000001096	0.0025	24	15	cdkn1a	1.84	21
ENSXMAG00000001358	0.0028	6	9	angptl7	1.36	3
ENSXMAG00000001418	0.0071	3	18	ripk3	1.76	0
ENSXMAG00000001522	0.0016	12	6	NR3C1 (1 of many)	1.05	9
ENSXMAG00000001671	0.0026	21	9	PCDH9	1.43	18
ENSXMAG00000001744	0.0024	6	15	cry1ab	1.45	3
ENSXMAG00000001821	0.0031	24	12	fn1a	1.71	21
ENSXMAG00000001866	0.0031	6	12		1.69	3
ENSXMAG00000001944	0.0025	6	15	sec24d	1.11	3
ENSXMAG00000001997	0.0034	6	12		1.67	3
ENSXMAG00000002074	0.0028	18	9	myl6 (1 of many)	1.09	15
ENSXMAG00000002205	0.0025	21	9	hdhd2	1.17	18
ENSXMAG00000002246	0.0026	6	15	lepr	2.06	3
ENSXMAG00000002249	0.0031	24	12	FBN1	2.08	21
ENSXMAG00000002363	0.0026	24	15	esyt2b	1.68	21

ENSXMAG00000002400	0.0031	12	12	plvapb	1.86	9
ENSXMAG00000002481	0.0026	6	15		1.28	3
ENSXMAG00000002499	0.0024	6	15	her13	1.41	3
ENSXMAG00000002545	0.0025	18	9	rnf19a	1.12	15
ENSXMAG00000002827	0.0029	18	12	xpot	1.04	15
ENSXMAG00000002856	0.0034	6	12	pcsk7	1.14	3
ENSXMAG00000002863	0.0031	3	12	tp53bp2a	1.12	0
ENSXMAG00000003013	0.0027	3	9	cyba	1.45	0
ENSXMAG00000003163	0.0016	3	18	zmiz1a	1.35	0
ENSXMAG00000003194	0.0026	3	15	TINAGL1	1.08	0
ENSXMAG00000003209	0.0031	9	12	thbs2a	1.64	6
ENSXMAG00000003252	0.0014	24	6	pcdh12	2.01	21
ENSXMAG00000003494	0.0027	6	9	pip4p2	1.77	3
ENSXMAG00000003539	0.0026	21	9	ripor2	1.09	18
ENSXMAG00000003639	0.0024	15	15		1.11	12
ENSXMAG00000003727	0.0025	12	15	zgc:113307	1.27	9
ENSXMAG00000003908	0.0031	18	12	lhx2b	1.55	15
ENSXMAG00000003909	0.0015	21	6		1.08	18
ENSXMAG00000003934	0.0024	6	9		1.54	3
ENSXMAG00000003961	0.0028	6	15	per3	1.17	3
ENSXMAG00000004069	0.0031	9	12		1.12	6
ENSXMAG00000004192	0.0027	24	9		1.34	21
ENSXMAG00000004209	0.0026	24	15	man1b1b	1.25	21
ENSXMAG00000004228	0.0026	24	15	dpp7	1.24	21
ENSXMAG00000004311	0.0028	3	9		1.31	0
ENSXMAG00000004437	0.0026	12	15	si:rp71-84d19.3	1.12	9
ENSXMAG00000004446	0.0024	3	15	cyfip2	1.24	0
ENSXMAG00000004555	0.0031	3	12	ddx1	1.10	0
ENSXMAG00000004564	0.0031	24	12	znf703	1.64	21
ENSXMAG00000004652	0.0029	15	12		1.12	12
ENSXMAG00000004772	0.0030	9	12	chrna4b	1.30	6
ENSXMAG00000004892	0.0016	15	6	taf1a	1.02	12
ENSXMAG00000004967	0.0025	9	9	ugt1a7 (1 of many)	2.31	6
ENSXMAG00000004975	0.0026	15	15	stoml2	1.16	12
ENSXMAG00000005326	0.0027	6	15	aspa	1.76	3
ENSXMAG00000005396	0.0030	24	12	MED13L	1.34	21
ENSXMAG00000005417	0.0034	21	12	auts2a	1.15	18
ENSXMAG00000005592	0.0015	24	18	comtb	1.52	21
ENSXMAG00000005861	0.0027	18	9	polr2h	1.23	15
ENSXMAG00000005954	0.0024	24	9	slc29a2	1.24	21
ENSXMAG00000006152	0.0014	21	6	poldip3	1.09	18
ENSXMAG00000006235	0.0031	6	12	draxin	2.21	3
ENSXMAG00000006473	0.0031	6	12	si:ch211-214c7.5	1.08	3
ENSXMAG00000006616	0.0031	9	12	aqp9b	2.31	6
ENSXMAG00000006651	0.0031	9	12	PER2	1.42	6
ENSXMAG00000006780	0.0016	24	18	epdr1	1.37	21
ENSXMAG00000006995	0.0015	21	6		1.14	18
ENSXMAG00000007056	0.0034	24	12	mxi1	1.06	21
ENSXMAG00000007076	0.0031	6	12	commd3	1.55	3
ENSXMAG00000007288	0.0026	24	15	ogfrl1	1.84	21
ENSXMAG00000007347	0.0029	12	12	pcgf6	1.08	9
ENSXMAG00000007351	0.0026	3	15	prodhb	1.24	0
ENSXMAG00000007776	0.0015	3	18	rlf	1.17	0
ENSXMAG00000007800	0.0025	3	15	znf395b	2.53	0
ENSXMAG00000007836	0.0024	24	15	vtnb	8.71	21
ENSXMAG00000008116	0.0016	18	18	nsd2	1.14	15

ENSXMAG00000008165	0.0026	24	15	csrp1a	1.28	21
ENSXMAG00000008190	0.0031	9	12	gpd2	1.15	6
ENSXMAG00000008243	0.0024	24	15	fga	1.36	21
ENSXMAG00000008274	0.0014	3	18	uba1	1.08	0
ENSXMAG00000008424	0.0026	24	15	arhgap23a	1.30	21
ENSXMAG00000008529	0.0031	15	12	gria4b	1.13	12
ENSXMAG00000008804	0.0016	6	6		1.60	3
ENSXMAG00000008815	0.0031	3	12	orai2	1.10	0
ENSXMAG00000008976	0.0025	21	9	ctsk	4.56	18
ENSXMAG00000009137	0.0031	6	12	adamts1	2.12	3
ENSXMAG00000009479	0.0025	3	9	nktr	1.10	0
ENSXMAG00000009551	0.0015	3	18		1.06	0
ENSXMAG00000009563	0.0025	3	9	nlgn3a	1.14	0
ENSXMAG00000009571	0.0031	3	12		1.24	0
ENSXMAG00000009783	0.0028	18	9	clp1	1.24	15
ENSXMAG00000009878	0.0014	21	6	zgc:172295	1.09	18
ENSXMAG00000010154	0.0027	9	9	prmt3	1.11	6
ENSXMAG00000010214	0.0034	6	12	lrrc1	1.32	3
ENSXMAG00000010380	0.0027	3	15	glsa	1.10	0
ENSXMAG00000010620	0.0034	9	12	pcsk2	1.71	6
ENSXMAG00000010823	0.0027	24	15	pou2f2a	1.63	21
ENSXMAG00000010838	0.0014	24	18		1.50	21
ENSXMAG00000010842	0.0025	3	9	trpc6a	1.86	0
ENSXMAG00000011464	0.0027	24	15	nr1d4a	21.58	21
ENSXMAG00000011787	0.0030	6	12	gys1	1.12	3
ENSXMAG00000011799	0.0025	3	15	slain2	1.10	0
ENSXMAG00000011910	0.0027	3	15	armc4	1.14	0
ENSXMAG00000011919	0.0014	9	18	agtr2	1.87	6
ENSXMAG00000012054	0.0031	24	12	dbpb	1.60	21
ENSXMAG00000012065	0.0016	24	18	slc12a9	1.42	21
ENSXMAG00000012125	0.0026	6	15	leng8	1.13	3
ENSXMAG00000012177	0.0027	24	15	hgsnat	1.20	21
ENSXMAG00000012248	0.0024	9	15	bco2a	4.93	6
ENSXMAG00000012249	0.0016	21	18	epha2b	1.43	18
ENSXMAG00000012272	0.0031	18	12	prpf31	1.07	15
ENSXMAG00000012373	0.0026	18	9	pink1	1.06	15
ENSXMAG00000012579	0.0026	9	9	sdf4	1.12	6
ENSXMAG00000012587	0.0025	3	9	c1qtnf12	1.33	0
ENSXMAG00000012673	0.0016	6	18	ptpreb	1.59	3
ENSXMAG00000012727	0.0031	24	12	aatka	1.69	21
ENSXMAG00000012750	0.0025	12	15	zgc:195081	1.15	9
ENSXMAG00000012814	0.0028	24	15	mxra8b	1.39	21
ENSXMAG00000012914	0.0024	3	15		1.21	0
ENSXMAG00000013205	0.0026	18	15	faah	1.23	15
ENSXMAG00000013221	0.0026	3	15	si:dkey-235d18.5	1.65	0
ENSXMAG00000013365	0.0071	6	6		1.88	3
ENSXMAG00000013432	0.0016	24	18		2.79	21
ENSXMAG00000013476	0.0071	3	6	mnat1	1.09	0
ENSXMAG00000013484	0.0024	6	9	paqr6	2.15	3
ENSXMAG00000013541	0.0031	9	12	kdr	1.67	6
ENSXMAG00000014001	0.0028	6	9	rpl3	1.11	3
ENSXMAG00000014027	0.0016	12	6	mmp23bb	1.07	9
ENSXMAG00000014055	0.0031	18	12	lrch3	1.17	15
ENSXMAG00000014077	0.0031	9	12	abcc5	1.37	6
ENSXMAG00000014224	0.0016	3	18	cratb	1.13	0
ENSXMAG00000014241	0.0026	6	15	HEY1 (1 of many)	2.68	3

ENSXMAG00000014297	0.0016	3	6	nf2b	1.10	0
ENSXMAG00000014445	0.0034	15	12	atad1b	1.08	12
ENSXMAG00000014579	0.0031	6	12	acaa2	1.25	3
ENSXMAG00000014591	0.0024	6	15	cers2a	1.12	3
ENSXMAG00000014758	0.0031	18	12	cenpj	1.18	15
ENSXMAG00000014925	0.0024	6	9	exoc3l4	1.89	3
ENSXMAG00000014934	0.0028	6	9	pla1a	1.83	3
ENSXMAG00000015347	0.0031	6	12	si:dkey-19b23.12	1.20	3
ENSXMAG00000015372	0.0026	24	15		1.31	21
ENSXMAG00000015412	0.0016	6	6	SLC45A4 (1 of many)	1.30	3
ENSXMAG00000015504	0.0071	12	6	ahi1	1.05	9
ENSXMAG00000015580	0.0034	6	12	si:dkeyp-23e4.3	1.68	3
ENSXMAG00000015600	0.0025	21	9	dmrt3a	1.42	18
ENSXMAG00000015613	0.0025	21	15	bcl6b	1.18	18
ENSXMAG00000015803	0.0025	24	15	grip2a	2.11	21
ENSXMAG00000015832	0.0030	6	12	slc6a6a	1.58	3
ENSXMAG00000015904	0.0031	9	12		2.13	6
ENSXMAG00000016055	0.0031	24	12	tubgcp5	1.08	21
ENSXMAG00000016068	0.0031	12	12		4.83	9
ENSXMAG00000016371	0.0015	21	6	lypla2	1.10	18
ENSXMAG00000016651	0.0026	15	15	nr2c1	1.06	12
ENSXMAG00000016652	0.0029	12	12	mrpl20	1.11	9
ENSXMAG00000016684	0.0024	18	9	FGD6 (1 of many)	1.19	15
ENSXMAG00000016697	0.0026	6	9		1.22	3
ENSXMAG00000016836	0.0024	6	15	p2rx7	1.38	3
ENSXMAG00000016897	0.0025	18	15	bpnt1	1.06	15
ENSXMAG00000016928	0.0014	18	18	clocka	1.24	15
ENSXMAG00000016967	0.0015	6	6	rngtt	1.11	3
ENSXMAG00000016987	0.0025	12	9	si:ch211-107o10.3	1.99	9
ENSXMAG00000017049	0.0025	24	15	ngfrb	1.23	21
ENSXMAG00000017106	0.0014	9	6	nr1d2b	1.83	6
ENSXMAG00000017348	0.0031	6	12	nfatc3a	1.08	3
ENSXMAG00000017456	0.0031	3	12	mtmr7a	1.19	0
ENSXMAG00000017520	0.0026	3	15	lpin1	1.98	0
ENSXMAG00000017543	0.0025	6	9		1.56	3
ENSXMAG00000017677	0.0034	3	12	elmsan1a	1.30	0
ENSXMAG00000017724	0.0027	3	15	gucy2g	1.54	0
ENSXMAG00000017753	0.0016	12	6	col5a3a	1.25	9
ENSXMAG00000017803	0.0030	24	12		2.14	21
ENSXMAG00000017831	0.0024	6	9	thsd1	1.60	3
ENSXMAG00000017877	0.0031	24	12	AXIN2	1.43	21
ENSXMAG00000017900	0.0016	3	18	tcn2	1.19	0
ENSXMAG00000017913	0.0031	3	12	mark4a	1.37	0
ENSXMAG00000017919	0.0026	12	9		1.06	9
ENSXMAG00000018411	0.0031	21	12	gpbp1l1	1.11	18
ENSXMAG00000018738	0.0026	21	15	tnfaip8l1	1.49	18
ENSXMAG00000018868	0.0031	9	12	mfsd12a	1.22	6
ENSXMAG00000018880	0.0014	24	18	galns	1.31	21
ENSXMAG00000018915	0.0030	6	12	oplah	1.88	3
ENSXMAG00000019183	0.0031	12	12	cct5	1.08	9
ENSXMAG00000019195	0.0016	21	6		1.07	18
ENSXMAG00000019227	0.0031	6	12	filip1a	1.77	3
ENSXMAG00000019296	0.0025	12	15	rorcb	2.60	9
ENSXMAG00000019322	0.0031	21	12	amt	1.16	18
ENSXMAG00000019511	0.0031	15	12	med7	1.12	12
ENSXMAG00000019531	0.0015	3	18	pnrc2	1.19	0

ENSXMAG00000019535	0.0028	18	9	gpr182	2.11	15
ENSXMAG00000019603	0.0031	24	12	ddx28	1.17	21
ENSXMAG00000019865	0.0031	9	12	si:ch73-334d15.1	3.80	6
ENSXMAG00000019917	0.0027	6	9	prr18	1.93	3
ENSXMAG00000019975	0.0024	6	15		2.39	3
ENSXMAG00000020010	0.0016	6	6	FLRT1	1.21	3
ENSXMAG00000020055	0.0031	6	12	ackr4b	4.16	3
ENSXMAG00000020125	0.0014	24	18		1.29	21
ENSXMAG00000020131	0.0031	3	12	lingo1a	1.84	0
ENSXMAG00000020208	0.0031	9	12	cyp1c1	2.64	6
ENSXMAG00000020273	0.0015	24	18		1.52	21
ENSXMAG00000020950	0.0026	18	9	lsm12b	1.13	15
ENSXMAG00000021060	0.0031	6	12	TIMP3	1.43	3
ENSXMAG00000021093	0.0016	9	6		1.38	6
ENSXMAG00000021299	0.0026	6	15	aatkb	1.76	3
ENSXMAG00000021366	0.0031	6	12		1.50	3
ENSXMAG00000021369	0.0031	6	12		1.59	3
ENSXMAG00000021622	0.0031	9	12		1.94	6
ENSXMAG00000021627	0.0024	6	9		1.15	3
ENSXMAG00000021672	0.0031	24	12	si:dkey-175m17.7	1.99	21
ENSXMAG00000021796	0.0031	6	12	slc16a10	1.31	3
ENSXMAG00000021848	0.0024	6	9		1.10	3
ENSXMAG00000021975	0.0026	18	9	shisa7a	1.40	15
ENSXMAG00000021994	0.0014	21	6	si:ch211-74f19.2	1.11	18
ENSXMAG00000022027	0.0026	12	15	tctex1d2	1.19	9
ENSXMAG00000022235	0.0024	6	15		2.57	3
ENSXMAG00000022298	0.0027	12	15		1.29	9
ENSXMAG00000022434	0.0026	21	15	zbtb41	1.37	18
ENSXMAG00000022643	0.0034	6	12	ankrd50l	1.31	3
ENSXMAG00000022895	0.0024	18	15		1.06	15
ENSXMAG00000022947	0.0025	24	15		1.53	21
ENSXMAG00000022969	0.0028	6	9		1.35	3
ENSXMAG00000023420	0.0014	24	18	cbfa2t3	1.86	21
ENSXMAG00000023447	0.0031	6	12	sall3a	2.08	3
ENSXMAG00000023462	0.0024	24	15	clic2	1.38	21
ENSXMAG00000023506	0.0026	3	15		1.81	0
ENSXMAG00000023781	0.0029	9	12	ugt1a7 (1 of many)	2.68	6
ENSXMAG00000023820	0.0034	6	12	krt5	1.25	3
ENSXMAG00000023838	0.0015	9	6	creg1	1.41	6
ENSXMAG00000023871	0.0026	9	9		1.57	6
ENSXMAG00000023883	0.0029	18	12	ppp1r2	1.08	15
ENSXMAG00000023915	0.0015	12	6	ifi30	1.21	9
ENSXMAG00000024113	0.0029	9	12	cyp2n13 (1 of many)	7.68	6
ENSXMAG00000024119	0.0015	24	6	zgc:112185	1.19	21
ENSXMAG00000024417	0.0026	9	9	COQ2	1.56	6
ENSXMAG00000024642	0.0024	3	15		1.41	0
ENSXMAG00000024755	0.0026	21	15		1.83	18
ENSXMAG00000024769	0.0026	9	9	rpl21	1.18	6
ENSXMAG00000024881	0.0028	6	9	rps19	1.15	3
ENSXMAG00000025156	0.0031	24	12		1.24	21
ENSXMAG00000025261	0.0031	24	12	ehd3	1.49	21
ENSXMAG00000025311	0.0031	9	12	si:dkey-15f17.8	1.18	6
ENSXMAG00000025433	0.0025	6	9	YAF2 (1 of many)	1.11	3
ENSXMAG00000025515	0.0031	3	12	bhlhe40	3.14	0
ENSXMAG00000025624	0.0026	6	15		1.54	3
ENSXMAG00000025637	0.0025	24	15	zgc:66427	1.23	21

ENSXMAG00000025639	0.0031	21	12	emx2	2.01	18
ENSXMAG00000025902	0.0024	12	9	mmp25b	5.17	9
ENSXMAG00000025965	0.0026	21	9	LACTB2	1.18	18
ENSXMAG00000026080	0.0025	12	15	ergic3 (1 of many)	1.20	9
ENSXMAG00000026087	0.0030	3	12		1.33	0
ENSXMAG00000026161	0.0031	12	12	fam49bb	1.23	9
ENSXMAG00000026202	0.0024	3	9	rab3il1	1.57	0
ENSXMAG00000026316	0.0016	24	18	f2rl2	1.67	21
ENSXMAG00000026331	0.0025	21	15	smim20	1.46	18
ENSXMAG00000026333	0.0014	3	18	tefb	3.08	0
ENSXMAG00000026342	0.0025	24	15	olfml2a	1.60	21
ENSXMAG00000026493	0.0025	3	15	rem2	1.29	0
ENSXMAG00000026646	0.0031	18	12		1.55	15
ENSXMAG00000026676	0.0025	6	9	gli1	1.54	3
ENSXMAG00000026740	0.0024	6	9	wsb1	1.23	3
ENSXMAG00000026936	0.0031	3	12	tnfaip3	1.74	0
ENSXMAG00000027105	0.0016	21	18	kank3	1.27	18
ENSXMAG00000027138	0.0071	6	6		1.22	3
ENSXMAG00000027290	0.0071	3	6	vtna	1.59	0
ENSXMAG00000027317	0.0024	21	15	samd10b	1.32	18
ENSXMAG00000027517	0.0030	3	12	crbn	1.15	0
ENSXMAG00000027759	0.0031	12	12	znf407	1.31	9
ENSXMAG00000028080	0.0031	24	12	8-Mar	1.55	21
ENSXMAG00000028108	0.0031	3	12	zzz3	1.22	0
ENSXMAG00000028333	0.0014	3	18		1.56	0
ENSXMAG00000028352	0.0024	24	9	atf7ip	1.11	21
ENSXMAG00000028489	0.0031	3	12	RETSAT	1.71	0
ENSXMAG00000028502	0.0026	24	15	prex1	1.38	21
ENSXMAG00000028538	0.0034	3	12		1.51	0
ENSXMAG00000028560	0.0014	6	6	mala	1.35	3
ENSXMAG00000028601	0.0026	6	9		1.39	3
ENSXMAG00000028679	0.0027	18	9		1.51	15
ENSXMAG00000028760	0.0025	9	9	rps18	1.15	6
ENSXMAG00000028872	0.0028	6	9	rps10	1.14	3
ENSXMAG00000028875	0.0024	3	15		1.52	0
ENSXMAG00000028896	0.0026	3	9		1.29	0
ENSXMAG00000029265	0.0034	24	12	tfap2b	1.19	21
ENSXMAG00000029275	0.0024	6	9	zgc:171489	1.35	3
ENSXMAG00000029359	0.0026	15	15		1.58	12
ENSXMAG00000029501	0.0031	6	12	GPATCH2L	1.76	3
ENSXMAG00000029620	0.0031	24	12	slc40a1	1.19	21
ENSXMAG00000029781	0.0015	9	6	rpl24	1.14	6
ENSXMAG00000029796	0.0026	24	15	rtf2	1.11	21
ENSXMAG00000029801	0.0027	3	15	ACSS1	1.77	0
ENSXMAG00000029843	0.0030	9	12		1.32	6
