# Supplementary material for: Global assessment of organ specific basal gene expression over a diurnal cycle with analyses of gene copies exhibiting cyclic expression patterns
Source: BMC Genomics. 2020 Nov 11;21:787. doi: 10.1186/s12864-020-07202-9 (PMC7659085; doi:10.1186/s12864-020-07202-9)
Supplement: Supplementary file 5 — Additional file 5: Supplement Table 4. Xiphophorus Ohnologs. [file 12864_2020_7202_MOESM5_ESM.pdf]

Supplement Table 4

ensembl_gene_id	xmaculatus_paralog_ensembl_gene	loculatus_homolog_ensembl_gene	Chr.1	location.1	Chr.2	location.2
ENSXMAG00000022293	ENSXMAG00000024612	ENSLOCG00000000072	3	2149759	13	12350662
ENSXMAG00000024612	ENSXMAG00000022293	ENSLOCG00000000072	13	12350662	3	2149759
ENSXMAG00000005497	ENSXMAG00000014128	ENSLOCG00000000103	6	1400887	9	30017113
ENSXMAG00000014128	ENSXMAG00000005497	ENSLOCG00000000103	9	30017113	6	1400887
ENSXMAG00000026493	ENSXMAG00000027141	ENSLOCG00000000150	9	22669695	6	7864484
ENSXMAG00000027141	ENSXMAG00000026493	ENSLOCG00000000150	6	7864484	9	22669695
ENSXMAG00000020917	ENSXMAG00000028386	ENSLOCG00000000151	18	3252534	14	5169091
ENSXMAG00000028386	ENSXMAG00000020917	ENSLOCG00000000151	14	5169091	18	3252534
ENSXMAG00000006023	ENSXMAG00000016807	ENSLOCG00000000168	9	22604568	6	7949964
ENSXMAG00000016807	ENSXMAG00000006023	ENSLOCG00000000168	6	7949964	9	22604568
ENSXMAG00000011992	ENSXMAG00000029414	ENSLOCG00000000180	6	19746230	13	27624537
ENSXMAG00000029414	ENSXMAG00000011992	ENSLOCG00000000180	13	27624537	6	19746230
ENSXMAG00000011663	ENSXMAG00000022899	ENSLOCG00000000202	10	1591983	16	16140769
ENSXMAG00000022899	ENSXMAG00000011663	ENSLOCG00000000202	16	16140769	10	1591983
ENSXMAG00000006078	ENSXMAG00000019289	ENSLOCG00000000216	6	11553879	9	10518890
ENSXMAG00000019289	ENSXMAG00000006078	ENSLOCG00000000216	9	10518890	6	11553879
ENSXMAG00000022622	ENSXMAG00000029196	ENSLOCG00000000224	23	13583016	11	24100606
ENSXMAG00000029196	ENSXMAG00000022622	ENSLOCG00000000224	11	24100606	23	13583016
ENSXMAG00000012023	ENSXMAG00000014145	ENSLOCG00000000249	13	11257834	3	19343638
ENSXMAG00000014145	ENSXMAG00000012023	ENSLOCG00000000249	3	19343638	13	11257834
ENSXMAG00000009362	ENSXMAG00000030088	ENSLOCG00000000284	9	31521760	19	18013764
ENSXMAG00000030088	ENSXMAG00000009362	ENSLOCG00000000284	19	18013764	9	31521760
ENSXMAG00000018005	ENSXMAG00000023350	ENSLOCG00000000341	10	3034932	16	3904996
ENSXMAG00000023350	ENSXMAG00000018005	ENSLOCG00000000341	16	3904996	10	3034932
ENSXMAG00000022464	ENSXMAG00000026065	ENSLOCG00000000392	12	1043869	8	20786119
ENSXMAG00000026065	ENSXMAG00000022464	ENSLOCG00000000392	8	20786119	12	1043869
ENSXMAG00000004738	ENSXMAG00000006384	ENSLOCG00000000413	6	26746117	21	18770069
ENSXMAG00000006384	ENSXMAG00000004738	ENSLOCG00000000413	21	18770069	6	26746117
ENSXMAG00000006847	ENSXMAG00000008420	ENSLOCG00000000422	11	30370074	18	8895131
ENSXMAG00000008420	ENSXMAG00000006847	ENSLOCG00000000422	18	8895131	11	30370074
ENSXMAG00000009695	ENSXMAG00000022498	ENSLOCG00000000482	15	6770464	19	16591109
ENSXMAG00000022498	ENSXMAG00000009695	ENSLOCG00000000482	19	16591109	15	6770464
ENSXMAG00000016655	ENSXMAG00000029158	ENSLOCG00000000510	20	15046616	1	11123679
ENSXMAG00000029158	ENSXMAG00000016655	ENSLOCG00000000510	1	11123679	20	15046616
ENSXMAG00000011681	ENSXMAG00000018700	ENSLOCG00000000530	10	1608542	16	16159091
ENSXMAG00000018700	ENSXMAG00000011681	ENSLOCG00000000530	16	16159091	10	1608542
ENSXMAG00000015131	ENSXMAG00000021485	ENSLOCG00000000545	15	11788851	4	12742038
ENSXMAG00000021485	ENSXMAG00000015131	ENSLOCG00000000545	4	12742038	15	11788851
ENSXMAG00000016750	ENSXMAG00000022421	ENSLOCG00000000554	3	2281239	13	12480200
ENSXMAG00000022421	ENSXMAG00000016750	ENSLOCG00000000554	13	12480200	3	2281239
ENSXMAG00000011629	ENSXMAG00000026955	ENSLOCG00000000592	13	11066677	3	1242439
ENSXMAG00000026955	ENSXMAG00000011629	ENSLOCG00000000592	3	1242439	13	11066677
ENSXMAG00000000886	ENSXMAG00000013559	ENSLOCG00000000614	13	17297155	3	22482956
ENSXMAG00000013559	ENSXMAG00000000886	ENSLOCG00000000614	3	22482956	13	17297155
ENSXMAG00000007816	ENSXMAG00000015923	ENSLOCG00000000749	14	13347209	5	29679029
ENSXMAG00000015923	ENSXMAG00000007816	ENSLOCG00000000749	5	29679029	14	13347209
ENSXMAG00000013347	ENSXMAG00000015186	ENSLOCG00000000765	2	15743078	4	1650123
ENSXMAG00000015186	ENSXMAG00000013347	ENSLOCG00000000765	4	1650123	2	15743078
ENSXMAG00000007350	ENSXMAG00000017975	ENSLOCG00000000775	16	3827854	10	3071805
ENSXMAG00000017975	ENSXMAG00000007350	ENSLOCG00000000775	10	3071805	16	3827854
ENSXMAG00000006296	ENSXMAG00000008055	ENSLOCG00000000790	21	19864811	6	25962631
ENSXMAG00000008055	ENSXMAG00000006296	ENSLOCG00000000790	6	25962631	21	19864811
ENSXMAG00000005570	ENSXMAG00000028728	ENSLOCG00000000825	14	13374776	5	15549238
ENSXMAG00000028728	ENSXMAG00000005570	ENSLOCG00000000825	5	15549238	14	13374776
ENSXMAG00000002328	ENSXMAG00000010655	ENSLOCG00000000846	11	17596124	18	11023480
ENSXMAG00000010655	ENSXMAG00000002328	ENSLOCG00000000846	18	11023480	11	17596124
ENSXMAG00000010760	ENSXMAG00000024636	ENSLOCG00000000850	19	17888585	9	31395759
ENSXMAG00000024636	ENSXMAG00000010760	ENSLOCG00000000850	9	31395759	19	17888585
ENSXMAG00000009380	ENSXMAG00000014736	ENSLOCG00000000877	5	28304337	16	8370801
ENSXMAG00000014736	ENSXMAG00000009380	ENSLOCG00000000877	16	8370801	5	28304337
ENSXMAG00000016079	ENSXMAG00000025385	ENSLOCG00000000878	19	21817565	23	10992176
ENSXMAG00000025385	ENSXMAG00000016079	ENSLOCG00000000878	23	10992176	19	21817565

ENSXMAG00000010119	ENSXMAG00000029062	ENSLOCG00000000927	11	27983084	18	13479203
ENSXMAG00000029062	ENSXMAG00000010119	ENSLOCG00000000927	18	13479203	11	27983084
ENSXMAG00000011022	ENSXMAG00000017047	ENSLOCG00000000949	2	21194877	17	13025650
ENSXMAG00000017047	ENSXMAG00000011022	ENSLOCG00000000949	17	13025650	2	21194877
ENSXMAG00000010132	ENSXMAG00000014022	ENSLOCG00000000967	11	27959905	18	13504337
ENSXMAG00000014022	ENSXMAG00000010132	ENSLOCG00000000967	18	13504337	11	27959905
ENSXMAG00000002475	ENSXMAG00000016091	ENSLOCG00000000979	23	11030106	19	21422651
ENSXMAG00000016091	ENSXMAG00000002475	ENSLOCG00000000979	19	21422651	23	11030106
ENSXMAG00000011569	ENSXMAG00000026944	ENSLOCG00000001007	6	24385743	14	2791372
ENSXMAG00000026944	ENSXMAG00000011569	ENSLOCG00000001007	14	2791372	6	24385743
ENSXMAG00000021574	ENSXMAG00000028221	ENSLOCG00000001014	2	21209340	17	11324123
ENSXMAG00000028221	ENSXMAG00000021574	ENSLOCG00000001014	17	11324123	2	21209340
ENSXMAG00000003086	ENSXMAG00000005204	ENSLOCG00000001099	11	24283124	23	13971854
ENSXMAG00000005204	ENSXMAG00000003086	ENSLOCG00000001099	23	13971854	11	24283124
ENSXMAG00000000586	ENSXMAG00000004639	ENSLOCG00000001119	6	3687271	9	1866275
ENSXMAG00000004639	ENSXMAG00000000586	ENSLOCG00000001119	9	1866275	6	3687271
ENSXMAG00000003979	ENSXMAG00000026119	ENSLOCG00000001132	24	1298109	7	13532030
ENSXMAG00000026119	ENSXMAG00000003979	ENSLOCG00000001132	7	13532030	24	1298109
ENSXMAG00000009232	ENSXMAG00000017134	ENSLOCG00000001143	2	21275826	17	11350888
ENSXMAG00000017134	ENSXMAG00000009232	ENSLOCG00000001143	17	11350888	2	21275826
ENSXMAG00000001590	ENSXMAG00000011880	ENSLOCG00000001197	4	5362417	2	26668030
ENSXMAG00000011880	ENSXMAG00000001590	ENSLOCG00000001197	2	26668030	4	5362417
ENSXMAG00000008139	ENSXMAG00000012095	ENSLOCG00000001205	7	12883167	24	1347317
ENSXMAG00000012095	ENSXMAG00000008139	ENSLOCG00000001205	24	1347317	7	12883167
ENSXMAG00000010713	ENSXMAG00000016048	ENSLOCG00000001222	12	14482293	8	1193228
ENSXMAG00000016048	ENSXMAG00000010713	ENSLOCG00000001222	8	1193228	12	14482293
ENSXMAG00000016762	ENSXMAG00000018563	ENSLOCG00000001238	20	24187170	1	22593214
ENSXMAG00000018563	ENSXMAG00000016762	ENSLOCG00000001238	1	22593214	20	24187170
ENSXMAG00000021249	ENSXMAG00000021613	ENSLOCG00000001251	15	4796340	19	24781564
ENSXMAG00000021613	ENSXMAG00000021249	ENSLOCG00000001251	19	24781564	15	4796340
ENSXMAG00000009280	ENSXMAG00000017213	ENSLOCG00000001255	2	21350612	17	11410983
ENSXMAG00000017213	ENSXMAG00000009280	ENSLOCG00000001255	17	11410983	2	21350612
ENSXMAG00000016683	ENSXMAG00000017122	ENSLOCG00000001262	20	14786877	1	11505546
ENSXMAG00000017122	ENSXMAG00000016683	ENSLOCG00000001262	1	11505546	20	14786877
ENSXMAG00000009225	ENSXMAG00000024443	ENSLOCG00000001270	8	27547138	12	25178720
ENSXMAG00000024443	ENSXMAG00000009225	ENSLOCG00000001270	12	25178720	8	27547138
ENSXMAG00000000398	ENSXMAG00000022838	ENSLOCG00000001314	3	24304215	13	17756492
ENSXMAG00000022838	ENSXMAG00000000398	ENSLOCG00000001314	13	17756492	3	24304215
ENSXMAG00000004249	ENSXMAG00000018305	ENSLOCG00000001351	9	13806603	6	29839868
ENSXMAG00000018305	ENSXMAG00000004249	ENSLOCG00000001351	6	29839868	9	13806603
ENSXMAG00000000796	ENSXMAG00000001471	ENSLOCG00000001388	4	5166606	2	15242002
ENSXMAG00000001471	ENSXMAG00000000796	ENSLOCG00000001388	2	15242002	4	5166606
ENSXMAG00000004905	ENSXMAG00000025264	ENSLOCG00000001420	8	22762531	11	1917405
ENSXMAG00000025264	ENSXMAG00000004905	ENSLOCG00000001420	11	1917405	8	22762531
ENSXMAG00000000759	ENSXMAG00000002607	ENSLOCG00000001474	13	22409122	3	556215
ENSXMAG00000002607	ENSXMAG00000000759	ENSLOCG00000001474	3	556215	13	22409122
ENSXMAG00000005074	ENSXMAG00000010415	ENSLOCG00000001506	4	3521565	2	25809662
ENSXMAG00000010415	ENSXMAG00000005074	ENSLOCG00000001506	2	25809662	4	3521565
ENSXMAG00000001726	ENSXMAG00000026219	ENSLOCG00000001533	12	27102149	8	8301782
ENSXMAG00000026219	ENSXMAG00000001726	ENSLOCG00000001533	8	8301782	12	27102149
ENSXMAG00000016148	ENSXMAG00000019401	ENSLOCG00000001557	12	21506236	8	25861995
ENSXMAG00000019401	ENSXMAG00000016148	ENSLOCG00000001557	8	25861995	12	21506236
ENSXMAG00000004457	ENSXMAG00000022003	ENSLOCG00000001558	5	26345696	16	12769142
ENSXMAG00000022003	ENSXMAG00000004457	ENSLOCG00000001558	16	12769142	5	26345696
ENSXMAG00000012214	ENSXMAG00000012415	ENSLOCG00000001560	18	30088829	11	20285375
ENSXMAG00000012415	ENSXMAG00000012214	ENSLOCG00000001560	11	20285375	18	30088829
ENSXMAG00000019818	ENSXMAG00000027512	ENSLOCG00000001561	24	9754514	7	30863998
ENSXMAG00000027512	ENSXMAG00000019818	ENSLOCG00000001561	7	30863998	24	9754514
ENSXMAG00000004029	ENSXMAG00000007358	ENSLOCG00000001584	8	4777444	11	821780
ENSXMAG00000007358	ENSXMAG00000004029	ENSLOCG00000001584	11	821780	8	4777444
ENSXMAG00000001929	ENSXMAG00000023871	ENSLOCG00000001609	11	4618879	14	9591838
ENSXMAG00000023871	ENSXMAG00000001929	ENSLOCG00000001609	14	9591838	11	4618879
ENSXMAG00000006737	ENSXMAG00000012424	ENSLOCG00000001610	18	30006433	18	9086397
ENSXMAG00000012424	ENSXMAG00000006737	ENSLOCG00000001610	18	9086397	18	30006433

ENSXMAG00000002915	ENSXMAG00000018360	ENSLOCG00000001696	20	25033671	12	27229964
ENSXMAG00000018360	ENSXMAG00000002915	ENSLOCG00000001696	12	27229964	20	25033671
ENSXMAG00000014607	ENSXMAG00000019113	ENSLOCG00000001718	23	5356462	19	23018547
ENSXMAG00000019113	ENSXMAG00000014607	ENSLOCG00000001718	19	23018547	23	5356462
ENSXMAG00000002220	ENSXMAG00000027722	ENSLOCG00000001735	23	25984513	8	11220557
ENSXMAG00000027722	ENSXMAG00000002220	ENSLOCG00000001735	8	11220557	23	25984513
ENSXMAG00000008912	ENSXMAG00000018541	ENSLOCG00000001745	19	20952121	14	19112016
ENSXMAG00000018541	ENSXMAG00000008912	ENSLOCG00000001745	14	19112016	19	20952121
ENSXMAG00000002028	ENSXMAG00000007871	ENSLOCG00000001764	24	9543819	7	25269781
ENSXMAG00000007871	ENSXMAG00000002028	ENSLOCG00000001764	7	25269781	24	9543819
ENSXMAG00000004268	ENSXMAG00000010597	ENSLOCG00000001798	8	6150842	11	670875
ENSXMAG00000010597	ENSXMAG00000004268	ENSLOCG00000001798	11	670875	8	6150842
ENSXMAG00000008907	ENSXMAG00000025606	ENSLOCG00000001799	14	4286255	14	19107943
ENSXMAG00000025606	ENSXMAG00000008907	ENSLOCG00000001799	14	19107943	14	4286255
ENSXMAG00000004796	ENSXMAG00000008626	ENSLOCG00000001843	14	17974605	19	20017602
ENSXMAG00000008626	ENSXMAG00000004796	ENSLOCG00000001843	19	20017602	14	17974605
ENSXMAG00000024090	ENSXMAG00000024525	ENSLOCG00000001847	9	31457428	19	26741027
ENSXMAG00000024525	ENSXMAG00000024090	ENSLOCG00000001847	19	26741027	9	31457428
ENSXMAG00000005452	ENSXMAG00000018739	ENSLOCG00000001850	6	10496558	9	30358605
ENSXMAG00000018739	ENSXMAG00000005452	ENSLOCG00000001850	9	30358605	6	10496558
ENSXMAG00000017262	ENSXMAG00000021886	ENSLOCG00000001864	16	12095793	10	20959111
ENSXMAG00000021886	ENSXMAG00000017262	ENSLOCG00000001864	10	20959111	16	12095793
ENSXMAG00000011954	ENSXMAG00000012176	ENSLOCG00000001884	2	30624886	4	29848237
ENSXMAG00000012176	ENSXMAG00000011954	ENSLOCG00000001884	4	29848237	2	30624886
ENSXMAG00000001301	ENSXMAG00000018967	ENSLOCG00000001892	12	352894	8	8911264
ENSXMAG00000018967	ENSXMAG00000001301	ENSLOCG00000001892	8	8911264	12	352894
ENSXMAG00000000091	ENSXMAG00000025944	ENSLOCG00000001927	16	16246345	10	2322126
ENSXMAG00000025944	ENSXMAG00000000091	ENSLOCG00000001927	10	2322126	16	16246345
ENSXMAG00000023647	ENSXMAG00000028167	ENSLOCG00000001958	6	1772498	9	6620505
ENSXMAG00000028167	ENSXMAG00000023647	ENSLOCG00000001958	9	6620505	6	1772498
ENSXMAG00000001213	ENSXMAG00000004935	ENSLOCG00000001986	11	1825020	8	8968647
ENSXMAG00000004935	ENSXMAG00000001213	ENSLOCG00000001986	8	8968647	11	1825020
ENSXMAG00000012120	ENSXMAG00000026761	ENSLOCG00000001994	16	12057198	10	20862670
ENSXMAG00000026761	ENSXMAG00000012120	ENSLOCG00000001994	10	20862670	16	12057198
ENSXMAG00000005116	ENSXMAG00000008678	ENSLOCG00000001997	14	18178306	11	24182726
ENSXMAG00000008678	ENSXMAG00000005116	ENSLOCG00000001997	11	24182726	14	18178306
ENSXMAG00000008541	ENSXMAG00000017331	ENSLOCG00000002009	13	25100637	3	33078839
ENSXMAG00000017331	ENSXMAG00000008541	ENSLOCG00000002009	3	33078839	13	25100637
ENSXMAG00000006830	ENSXMAG00000029578	ENSLOCG00000002012	7	4106510	24	7374017
ENSXMAG00000029578	ENSXMAG00000006830	ENSLOCG00000002012	24	7374017	7	4106510
ENSXMAG00000002125	ENSXMAG00000022566	ENSLOCG00000002019	24	14588250	7	31667129
ENSXMAG00000022566	ENSXMAG00000002125	ENSLOCG00000002019	7	31667129	24	14588250
ENSXMAG00000016189	ENSXMAG00000025960	ENSLOCG00000002026	12	19676264	8	26050554
ENSXMAG00000025960	ENSXMAG00000016189	ENSLOCG00000002026	8	26050554	12	19676264
ENSXMAG00000001182	ENSXMAG00000004853	ENSLOCG00000002067	11	2142203	8	9016014
ENSXMAG00000004853	ENSXMAG00000001182	ENSLOCG00000002067	8	9016014	11	2142203
ENSXMAG00000001703	ENSXMAG00000002540	ENSLOCG00000002072	9	6676690	6	1730864
ENSXMAG00000002540	ENSXMAG00000001703	ENSLOCG00000002072	6	1730864	9	6676690
ENSXMAG00000007063	ENSXMAG00000016488	ENSLOCG00000002085	4	9009679	2	5383906
ENSXMAG00000016488	ENSXMAG00000007063	ENSLOCG00000002085	2	5383906	4	9009679
ENSXMAG00000002567	ENSXMAG00000008513	ENSLOCG00000002136	6	1698470	9	6716092
ENSXMAG00000008513	ENSXMAG00000002567	ENSLOCG00000002136	9	6716092	6	1698470
ENSXMAG00000007434	ENSXMAG00000027991	ENSLOCG00000002181	4	3204824	2	30264591
ENSXMAG00000027991	ENSXMAG00000007434	ENSLOCG00000002181	2	30264591	4	3204824
ENSXMAG00000006381	ENSXMAG00000027712	ENSLOCG00000002200	13	18110111	19	10842059
ENSXMAG00000027712	ENSXMAG00000006381	ENSLOCG00000002200	19	10842059	13	18110111
ENSXMAG00000012472	ENSXMAG00000018796	ENSLOCG00000002244	20	23593821	1	27238320
ENSXMAG00000018796	ENSXMAG00000012472	ENSLOCG00000002244	1	27238320	20	23593821
ENSXMAG00000004923	ENSXMAG00000014985	ENSLOCG00000002262	12	11380295	8	18461836
ENSXMAG00000014985	ENSXMAG00000004923	ENSLOCG00000002262	8	18461836	12	11380295
ENSXMAG00000005713	ENSXMAG00000006419	ENSLOCG00000002273	19	10827959	13	16029485
ENSXMAG00000006419	ENSXMAG00000005713	ENSLOCG00000002273	13	16029485	19	10827959
ENSXMAG00000005370	ENSXMAG00000013465	ENSLOCG00000002291	4	916948	2	1154364
ENSXMAG00000013465	ENSXMAG00000005370	ENSLOCG00000002291	2	1154364	4	916948

ENSXMAG00000004917	ENSXMAG00000016744	ENSLOCG00000002294	12	15598058	8	18438796
ENSXMAG00000016744	ENSXMAG00000004917	ENSLOCG00000002294	8	18438796	12	15598058
ENSXMAG00000003976	ENSXMAG00000010231	ENSLOCG00000002372	20	7077747	1	28135549
ENSXMAG00000010231	ENSXMAG00000003976	ENSLOCG00000002372	1	28135549	20	7077747
ENSXMAG00000000528	ENSXMAG00000014284	ENSLOCG00000002398	3	23163229	13	4586415
ENSXMAG00000014284	ENSXMAG00000000528	ENSLOCG00000002398	13	4586415	3	23163229
ENSXMAG00000002404	ENSXMAG00000005070	ENSLOCG00000002429	8	14456730	12	28507710
ENSXMAG00000005070	ENSXMAG00000002404	ENSLOCG00000002429	12	28507710	8	14456730
ENSXMAG00000004671	ENSXMAG00000007950	ENSLOCG00000002434	5	26319092	16	12372728
ENSXMAG00000007950	ENSXMAG00000004671	ENSLOCG00000002434	16	12372728	5	26319092
ENSXMAG00000009697	ENSXMAG00000011416	ENSLOCG00000002537	14	1511613	19	16615776
ENSXMAG00000011416	ENSXMAG00000009697	ENSLOCG00000002537	19	16615776	14	1511613
ENSXMAG00000000379	ENSXMAG00000025106	ENSLOCG00000002539	19	12571634	13	16442432
ENSXMAG00000025106	ENSXMAG00000000379	ENSLOCG00000002539	13	16442432	19	12571634
ENSXMAG00000000412	ENSXMAG00000029740	ENSLOCG00000002564	19	13141987	13	16452341
ENSXMAG00000029740	ENSXMAG00000000412	ENSLOCG00000002564	13	16452341	19	13141987
ENSXMAG00000027478	ENSXMAG00000028099	ENSLOCG00000002626	5	23014492	16	12533224
ENSXMAG00000028099	ENSXMAG00000027478	ENSLOCG00000002626	16	12533224	5	23014492
ENSXMAG00000004625	ENSXMAG00000027598	ENSLOCG00000002644	5	23034374	16	12545315
ENSXMAG00000027598	ENSXMAG00000004625	ENSLOCG00000002644	16	12545315	5	23034374
ENSXMAG00000028414	ENSXMAG00000028795	ENSLOCG00000002663	12	2813133	8	8723169
ENSXMAG00000028795	ENSXMAG00000028414	ENSLOCG00000002663	8	8723169	12	2813133
ENSXMAG00000028331	ENSXMAG00000029963	ENSLOCG00000002667	5	23055085	16	12569119
ENSXMAG00000029963	ENSXMAG00000028331	ENSLOCG00000002667	16	12569119	5	23055085
ENSXMAG00000004557	ENSXMAG00000011929	ENSLOCG00000002701	5	23101680	16	12607901
ENSXMAG00000011929	ENSXMAG00000004557	ENSLOCG00000002701	16	12607901	5	23101680
ENSXMAG00000016178	ENSXMAG00000018728	ENSLOCG00000002720	20	23771771	1	23731599
ENSXMAG00000018728	ENSXMAG00000016178	ENSLOCG00000002720	1	23731599	20	23771771
ENSXMAG00000009251	ENSXMAG00000029000	ENSLOCG00000002767	2	5934115	4	851642
ENSXMAG00000029000	ENSXMAG00000009251	ENSLOCG00000002767	4	851642	2	5934115
ENSXMAG00000007192	ENSXMAG00000025062	ENSLOCG00000002770	16	12652294	5	23161688
ENSXMAG00000025062	ENSXMAG00000007192	ENSLOCG00000002770	5	23161688	16	12652294
ENSXMAG00000003140	ENSXMAG00000015869	ENSLOCG00000002790	7	19752356	17	19520485
ENSXMAG00000015869	ENSXMAG00000003140	ENSLOCG00000002790	17	19520485	7	19752356
ENSXMAG00000007280	ENSXMAG00000022831	ENSLOCG00000002791	4	819710	2	6018644
ENSXMAG00000022831	ENSXMAG00000007280	ENSLOCG00000002791	2	6018644	4	819710
ENSXMAG00000014983	ENSXMAG00000029958	ENSLOCG00000002897	9	5650669	6	2285800
ENSXMAG00000029958	ENSXMAG00000014983	ENSLOCG00000002897	6	2285800	9	5650669
ENSXMAG00000003614	ENSXMAG00000026154	ENSLOCG00000002898	3	15859087	13	671900
ENSXMAG00000026154	ENSXMAG00000003614	ENSLOCG00000002898	13	671900	3	15859087
ENSXMAG00000014044	ENSXMAG00000028756	ENSLOCG00000002914	12	27040380	8	13397545
ENSXMAG00000028756	ENSXMAG00000014044	ENSLOCG00000002914	8	13397545	12	27040380
ENSXMAG00000004007	ENSXMAG00000012814	ENSLOCG00000002916	1	24204736	20	23015870
ENSXMAG00000012814	ENSXMAG00000004007	ENSLOCG00000002916	20	23015870	1	24204736
ENSXMAG00000003855	ENSXMAG00000021422	ENSLOCG00000002983	9	5789508	6	2386699
ENSXMAG00000021422	ENSXMAG00000003855	ENSLOCG00000002983	6	2386699	9	5789508
ENSXMAG00000002938	ENSXMAG00000018271	ENSLOCG00000002987	20	25124874	12	27215100
ENSXMAG00000018271	ENSXMAG00000002938	ENSLOCG00000002987	12	27215100	20	25124874
ENSXMAG00000002414	ENSXMAG00000003852	ENSLOCG00000003013	6	2380591	9	5783611
ENSXMAG00000003852	ENSXMAG00000002414	ENSLOCG00000003013	9	5783611	6	2380591
ENSXMAG00000002402	ENSXMAG00000021728	ENSLOCG00000003024	6	2356422	9	5739581
ENSXMAG00000021728	ENSXMAG00000002402	ENSLOCG00000003024	9	5739581	6	2356422
ENSXMAG00000014478	ENSXMAG00000025294	ENSLOCG00000003028	13	26627758	3	6171145
ENSXMAG00000025294	ENSXMAG00000014478	ENSLOCG00000003028	3	6171145	13	26627758
ENSXMAG00000002063	ENSXMAG00000008002	ENSLOCG00000003034	5	29415011	23	3585211
ENSXMAG00000008002	ENSXMAG00000002063	ENSLOCG00000003034	23	3585211	5	29415011
ENSXMAG00000013475	ENSXMAG00000016058	ENSLOCG00000003061	2	19829102	4	924496
ENSXMAG00000016058	ENSXMAG00000013475	ENSLOCG00000003061	4	924496	2	19829102
ENSXMAG00000002400	ENSXMAG00000014977	ENSLOCG00000003074	6	2320154	9	5706463
ENSXMAG00000014977	ENSXMAG00000002400	ENSLOCG00000003074	9	5706463	6	2320154
ENSXMAG00000000786	ENSXMAG00000014334	ENSLOCG00000003075	21	24459219	6	22526694
ENSXMAG00000014334	ENSXMAG00000000786	ENSLOCG00000003075	6	22526694	21	24459219
ENSXMAG00000009036	ENSXMAG00000017576	ENSLOCG00000003096	20	9666180	1	30436787
ENSXMAG00000017576	ENSXMAG00000009036	ENSLOCG00000003096	1	30436787	20	9666180

ENSXMAG00000001106	ENSXMAG00000002511	ENSLOCG00000003107	21	8854562	6	735821
ENSXMAG00000002511	ENSXMAG00000001106	ENSLOCG00000003107	6	735821	21	8854562
ENSXMAG00000014733	ENSXMAG00000024808	ENSLOCG00000003110	3	26504352	13	15105619
ENSXMAG00000024808	ENSXMAG00000014733	ENSLOCG00000003110	13	15105619	3	26504352
ENSXMAG00000007066	ENSXMAG00000028956	ENSLOCG00000003120	2	27365255	4	18792682
ENSXMAG00000028956	ENSXMAG00000007066	ENSLOCG00000003120	4	18792682	2	27365255
ENSXMAG00000018743	ENSXMAG00000024528	ENSLOCG00000003238	16	754869	10	1739649
ENSXMAG00000024528	ENSXMAG00000018743	ENSLOCG00000003238	10	1739649	16	754869
ENSXMAG00000006965	ENSXMAG00000029930	ENSLOCG00000003266	2	27401325	4	18570735
ENSXMAG00000029930	ENSXMAG00000006965	ENSLOCG00000003266	4	18570735	2	27401325
ENSXMAG00000003342	ENSXMAG00000005325	ENSLOCG00000003283	8	18913966	12	7784680
ENSXMAG00000005325	ENSXMAG00000003342	ENSLOCG00000003283	12	7784680	8	18913966
ENSXMAG00000009514	ENSXMAG00000016786	ENSLOCG00000003291	1	11754629	20	2300954
ENSXMAG00000016786	ENSXMAG00000009514	ENSLOCG00000003291	20	2300954	1	11754629
ENSXMAG00000008442	ENSXMAG00000016331	ENSLOCG00000003296	6	15071364	21	4715241
ENSXMAG00000016331	ENSXMAG00000008442	ENSLOCG00000003296	21	4715241	6	15071364
ENSXMAG00000014146	ENSXMAG00000015566	ENSLOCG00000003327	13	1769396	3	23242749
ENSXMAG00000015566	ENSXMAG00000014146	ENSLOCG00000003327	3	23242749	13	1769396
ENSXMAG00000008989	ENSXMAG00000017602	ENSLOCG00000003331	20	9432703	1	30510146
ENSXMAG00000017602	ENSXMAG00000008989	ENSLOCG00000003331	1	30510146	20	9432703
ENSXMAG00000000673	ENSXMAG00000028000	ENSLOCG00000003359	9	16163537	6	21087335
ENSXMAG00000028000	ENSXMAG00000000673	ENSLOCG00000003359	6	21087335	9	16163537
ENSXMAG00000008432	ENSXMAG00000016301	ENSLOCG00000003364	6	15060373	21	4809951
ENSXMAG00000016301	ENSXMAG00000008432	ENSLOCG00000003364	21	4809951	6	15060373
ENSXMAG00000000675	ENSXMAG00000023965	ENSLOCG00000003372	9	16150300	6	21095819
ENSXMAG00000023965	ENSXMAG00000000675	ENSLOCG00000003372	6	21095819	9	16150300
ENSXMAG00000008782	ENSXMAG00000025064	ENSLOCG00000003395	11	14202548	18	20904864
ENSXMAG00000025064	ENSXMAG00000008782	ENSLOCG00000003395	18	20904864	11	14202548
ENSXMAG00000003277	ENSXMAG00000005227	ENSLOCG00000003419	8	18864813	12	7923823
ENSXMAG00000005227	ENSXMAG00000003277	ENSLOCG00000003419	12	7923823	8	18864813
ENSXMAG00000006556	ENSXMAG00000007298	ENSLOCG00000003459	21	12900629	17	1210425
ENSXMAG00000007298	ENSXMAG00000006556	ENSLOCG00000003459	17	1210425	21	12900629
ENSXMAG00000003370	ENSXMAG00000029254	ENSLOCG00000003485	6	21047918	9	16227156
ENSXMAG00000029254	ENSXMAG00000003370	ENSLOCG00000003485	9	16227156	6	21047918
ENSXMAG00000003211	ENSXMAG00000021119	ENSLOCG00000003498	8	2131504	12	8290426
ENSXMAG00000021119	ENSXMAG00000003211	ENSLOCG00000003498	12	8290426	8	2131504
ENSXMAG00000020107	ENSXMAG00000028666	ENSLOCG00000003518	2	2647533	8	5236463
ENSXMAG00000028666	ENSXMAG00000020107	ENSLOCG00000003518	8	5236463	2	2647533
ENSXMAG00000005286	ENSXMAG00000026320	ENSLOCG00000003519	24	5646505	7	2329855
ENSXMAG00000026320	ENSXMAG00000005286	ENSLOCG00000003519	7	2329855	24	5646505
ENSXMAG00000002095	ENSXMAG00000010868	ENSLOCG00000003520	7	17265542	24	11318213
ENSXMAG00000010868	ENSXMAG00000002095	ENSLOCG00000003520	24	11318213	7	17265542
ENSXMAG00000008830	ENSXMAG00000008883	ENSLOCG00000003521	20	32645811	3	24392446
ENSXMAG00000008883	ENSXMAG00000008830	ENSLOCG00000003521	3	24392446	20	32645811
ENSXMAG00000012786	ENSXMAG00000026721	ENSLOCG00000003524	3	13334204	13	26852241
ENSXMAG00000026721	ENSXMAG00000012786	ENSLOCG00000003524	13	26852241	3	13334204
ENSXMAG00000013162	ENSXMAG00000016398	ENSLOCG00000003568	6	23648382	9	17624639
ENSXMAG00000016398	ENSXMAG00000013162	ENSLOCG00000003568	9	17624639	6	23648382
ENSXMAG00000010457	ENSXMAG00000010748	ENSLOCG00000003582	6	6740282	21	12534647
ENSXMAG00000010748	ENSXMAG00000010457	ENSLOCG00000003582	21	12534647	6	6740282
ENSXMAG00000013101	ENSXMAG00000027431	ENSLOCG00000003615	7	15486399	24	6819902
ENSXMAG00000027431	ENSXMAG00000013101	ENSLOCG00000003615	24	6819902	7	15486399
ENSXMAG00000010649	ENSXMAG00000015749	ENSLOCG00000003653	13	27401558	3	19846313
ENSXMAG00000015749	ENSXMAG00000010649	ENSLOCG00000003653	3	19846313	13	27401558
ENSXMAG00000001396	ENSXMAG00000002409	ENSLOCG00000003661	12	7136456	8	13843281
ENSXMAG00000002409	ENSXMAG00000001396	ENSLOCG00000003661	8	13843281	12	7136456
ENSXMAG00000002295	ENSXMAG00000011221	ENSLOCG00000003733	1	15568234	20	21065303
ENSXMAG00000011221	ENSXMAG00000002295	ENSLOCG00000003733	20	21065303	1	15568234
ENSXMAG00000012787	ENSXMAG00000017883	ENSLOCG00000003744	20	8572858	1	24224630
ENSXMAG00000017883	ENSXMAG00000012787	ENSLOCG00000003744	1	24224630	20	8572858
ENSXMAG00000010013	ENSXMAG00000015481	ENSLOCG00000003746	1	21455204	20	27276925
ENSXMAG00000015481	ENSXMAG00000010013	ENSLOCG00000003746	20	27276925	1	21455204
ENSXMAG00000003195	ENSXMAG00000025807	ENSLOCG00000003764	8	2481622	12	8318653
ENSXMAG00000025807	ENSXMAG00000003195	ENSLOCG00000003764	12	8318653	8	2481622

ENSXMAG00000003609	ENSXMAG00000003658	ENSLOCG00000003790	22	26930519	7	22460871
ENSXMAG00000003658	ENSXMAG00000003609	ENSLOCG00000003790	7	22460871	22	26930519
ENSXMAG00000003484	ENSXMAG00000009400	ENSLOCG00000003816	1	29107186	20	21950444
ENSXMAG00000009400	ENSXMAG00000003484	ENSLOCG00000003816	20	21950444	1	29107186
ENSXMAG00000026705	ENSXMAG00000028774	ENSLOCG00000003817	6	644875	21	12144035
ENSXMAG00000028774	ENSXMAG00000026705	ENSLOCG00000003817	21	12144035	6	644875
ENSXMAG00000013492	ENSXMAG00000022287	ENSLOCG00000003833	10	22119220	22	13238901
ENSXMAG00000022287	ENSXMAG00000013492	ENSLOCG00000003833	22	13238901	10	22119220
ENSXMAG00000012884	ENSXMAG00000028236	ENSLOCG00000003864	20	14456905	1	6488114
ENSXMAG00000028236	ENSXMAG00000012884	ENSLOCG00000003864	1	6488114	20	14456905
ENSXMAG00000018858	ENSXMAG00000021214	ENSLOCG00000003889	6	20841163	9	14977208
ENSXMAG00000021214	ENSXMAG00000018858	ENSLOCG00000003889	9	14977208	6	20841163
ENSXMAG00000005867	ENSXMAG00000005902	ENSLOCG00000003898	11	19965359	18	32168048
ENSXMAG00000005902	ENSXMAG00000005867	ENSLOCG00000003898	18	32168048	11	19965359
ENSXMAG00000009265	ENSXMAG00000013534	ENSLOCG00000003911	2	25167715	4	17949870
ENSXMAG00000013534	ENSXMAG00000009265	ENSLOCG00000003911	4	17949870	2	25167715
ENSXMAG00000003053	ENSXMAG00000015650	ENSLOCG00000003930	2	19032724	4	17803438
ENSXMAG00000015650	ENSXMAG00000003053	ENSLOCG00000003930	4	17803438	2	19032724
ENSXMAG00000023202	ENSXMAG00000028699	ENSLOCG00000003931	10	22018149	22	13177965
ENSXMAG00000028699	ENSXMAG00000023202	ENSLOCG00000003931	22	13177965	10	22018149
ENSXMAG00000027879	ENSXMAG00000028549	ENSLOCG00000003943	3	20059008	13	27820176
ENSXMAG00000028549	ENSXMAG00000027879	ENSLOCG00000003943	13	27820176	3	20059008
ENSXMAG00000009815	ENSXMAG00000010040	ENSLOCG00000003951	1	22036422	20	26697688
ENSXMAG00000010040	ENSXMAG00000009815	ENSLOCG00000003951	20	26697688	1	22036422
ENSXMAG00000000615	ENSXMAG00000023988	ENSLOCG00000003955	9	14915518	6	20808318
ENSXMAG00000023988	ENSXMAG00000000615	ENSLOCG00000003955	6	20808318	9	14915518
ENSXMAG00000000983	ENSXMAG00000023654	ENSLOCG00000003964	1	10296462	20	14549659
ENSXMAG00000023654	ENSXMAG00000000983	ENSLOCG00000003964	20	14549659	1	10296462
ENSXMAG00000000613	ENSXMAG00000018750	ENSLOCG00000003973	9	14910451	6	20805954
ENSXMAG00000018750	ENSXMAG00000000613	ENSLOCG00000003973	6	20805954	9	14910451
ENSXMAG00000002405	ENSXMAG00000009508	ENSLOCG00000003996	1	28998362	20	21489978
ENSXMAG00000009508	ENSXMAG00000002405	ENSLOCG00000003996	20	21489978	1	28998362
ENSXMAG00000002401	ENSXMAG00000009527	ENSLOCG00000004011	1	28992046	20	21478280
ENSXMAG00000009527	ENSXMAG00000002401	ENSLOCG00000004011	20	21478280	1	28992046
ENSXMAG00000014288	ENSXMAG00000014354	ENSLOCG00000004017	18	3139833	11	31029626
ENSXMAG00000014354	ENSXMAG00000014288	ENSLOCG00000004017	11	31029626	18	3139833
ENSXMAG00000004183	ENSXMAG00000029606	ENSLOCG00000004029	4	17480823	2	11642977
ENSXMAG00000029606	ENSXMAG00000004183	ENSLOCG00000004029	2	11642977	4	17480823
ENSXMAG00000002394	ENSXMAG00000009531	ENSLOCG00000004042	1	28939196	20	21453947
ENSXMAG00000009531	ENSXMAG00000002394	ENSLOCG00000004042	20	21453947	1	28939196
ENSXMAG00000009486	ENSXMAG00000019543	ENSLOCG00000004077	22	13045908	10	21872214
ENSXMAG00000019543	ENSXMAG00000009486	ENSLOCG00000004077	10	21872214	22	13045908
ENSXMAG00000001453	ENSXMAG00000013962	ENSLOCG00000004081	12	18106257	8	13898594
ENSXMAG00000013962	ENSXMAG00000001453	ENSLOCG00000004081	8	13898594	12	18106257
ENSXMAG00000013584	ENSXMAG00000021218	ENSLOCG00000004090	10	21851284	22	13011605
ENSXMAG00000021218	ENSXMAG00000013584	ENSLOCG00000004090	22	13011605	10	21851284
ENSXMAG00000008269	ENSXMAG00000015222	ENSLOCG00000004093	6	13179843	18	10233841
ENSXMAG00000015222	ENSXMAG00000008269	ENSLOCG00000004093	18	10233841	6	13179843
ENSXMAG00000003128	ENSXMAG00000015506	ENSLOCG00000004102	2	18768430	4	17607618
ENSXMAG00000015506	ENSXMAG00000003128	ENSLOCG00000004102	4	17607618	2	18768430
ENSXMAG00000003962	ENSXMAG00000013216	ENSLOCG00000004105	9	17481118	6	2608040
ENSXMAG00000013216	ENSXMAG00000003962	ENSLOCG00000004105	6	2608040	9	17481118
ENSXMAG00000010314	ENSXMAG00000021301	ENSLOCG00000004107	13	25017750	3	14567701
ENSXMAG00000021301	ENSXMAG00000010314	ENSLOCG00000004107	3	14567701	13	25017750
ENSXMAG00000000591	ENSXMAG00000018704	ENSLOCG00000004111	9	14659035	6	20688692
ENSXMAG00000018704	ENSXMAG00000000591	ENSLOCG00000004111	6	20688692	9	14659035
ENSXMAG00000026288	ENSXMAG00000028807	ENSLOCG00000004117	22	12994371	10	21827789
ENSXMAG00000028807	ENSXMAG00000026288	ENSLOCG00000004117	10	21827789	22	12994371
ENSXMAG00000001494	ENSXMAG00000014010	ENSLOCG00000004167	12	18197563	8	13914501
ENSXMAG00000014010	ENSXMAG00000001494	ENSLOCG00000004167	8	13914501	12	18197563
ENSXMAG00000009778	ENSXMAG00000023796	ENSLOCG00000004172	1	30936282	20	26397930
ENSXMAG00000023796	ENSXMAG00000009778	ENSLOCG00000004172	20	26397930	1	30936282
ENSXMAG00000000547	ENSXMAG00000018648	ENSLOCG00000004179	9	14558452	6	20593427
ENSXMAG00000018648	ENSXMAG00000000547	ENSLOCG00000004179	6	20593427	9	14558452

ENSXMAG00000013763	ENSXMAG00000029776	ENSLOCG00000004190	21	11057434	5	25749808
ENSXMAG00000029776	ENSXMAG00000013763	ENSLOCG00000004190	5	25749808	21	11057434
ENSXMAG00000024042	ENSXMAG00000024539	ENSLOCG00000004193	5	23224325	22	12923894
ENSXMAG00000024539	ENSXMAG00000024042	ENSLOCG00000004193	22	12923894	5	23224325
ENSXMAG00000003262	ENSXMAG00000011637	ENSLOCG00000004229	1	25666450	20	21664366
ENSXMAG00000011637	ENSXMAG00000003262	ENSLOCG00000004229	20	21664366	1	25666450
ENSXMAG00000008353	ENSXMAG00000018866	ENSLOCG00000004239	6	10564824	18	9756904
ENSXMAG00000018866	ENSXMAG00000008353	ENSLOCG00000004239	18	9756904	6	10564824
ENSXMAG00000000544	ENSXMAG00000018641	ENSLOCG00000004244	9	14484591	6	20569995
ENSXMAG00000018641	ENSXMAG00000000544	ENSLOCG00000004244	6	20569995	9	14484591
ENSXMAG00000004516	ENSXMAG00000005135	ENSLOCG00000004274	12	680632	8	24791429
ENSXMAG00000005135	ENSXMAG00000004516	ENSLOCG00000004274	8	24791429	12	680632
ENSXMAG00000015051	ENSXMAG00000024881	ENSLOCG00000004313	3	6683468	13	3370872
ENSXMAG00000024881	ENSXMAG00000015051	ENSLOCG00000004313	13	3370872	3	6683468
ENSXMAG00000007022	ENSXMAG00000015409	ENSLOCG00000004325	2	18525530	4	14536716
ENSXMAG00000015409	ENSXMAG00000007022	ENSLOCG00000004325	4	14536716	2	18525530
ENSXMAG00000002037	ENSXMAG00000028360	ENSLOCG00000004383	24	4118779	7	25299609
ENSXMAG00000028360	ENSXMAG00000002037	ENSLOCG00000004383	7	25299609	24	4118779
ENSXMAG00000008630	ENSXMAG00000011277	ENSLOCG00000004398	11	31323795	18	20512768
ENSXMAG00000011277	ENSXMAG00000008630	ENSLOCG00000004398	18	20512768	11	31323795
ENSXMAG00000001259	ENSXMAG00000007888	ENSLOCG00000004399	12	18418969	8	13731128
ENSXMAG00000007888	ENSXMAG00000001259	ENSLOCG00000004399	8	13731128	12	18418969
ENSXMAG00000027838	ENSXMAG00000028861	ENSLOCG00000004411	11	31338313	18	20495271
ENSXMAG00000028861	ENSXMAG00000027838	ENSLOCG00000004411	18	20495271	11	31338313
ENSXMAG00000006651	ENSXMAG00000016174	ENSLOCG00000004441	6	14804076	18	9184728
ENSXMAG00000016174	ENSXMAG00000006651	ENSLOCG00000004441	18	9184728	6	14804076
ENSXMAG00000018511	ENSXMAG00000018819	ENSLOCG00000004442	6	10533365	9	14265784
ENSXMAG00000018819	ENSXMAG00000018511	ENSLOCG00000004442	9	14265784	6	10533365
ENSXMAG00000008604	ENSXMAG00000011253	ENSLOCG00000004448	11	31362140	18	20438931
ENSXMAG00000011253	ENSXMAG00000008604	ENSLOCG00000004448	18	20438931	11	31362140
ENSXMAG00000003074	ENSXMAG00000016276	ENSLOCG00000004472	6	14939370	9	27443221
ENSXMAG00000016276	ENSXMAG00000003074	ENSLOCG00000004472	9	27443221	6	14939370
ENSXMAG00000018861	ENSXMAG00000026178	ENSLOCG00000004473	9	20696667	6	10552794
ENSXMAG00000026178	ENSXMAG00000018861	ENSLOCG00000004473	6	10552794	9	20696667
ENSXMAG00000003490	ENSXMAG00000028136	ENSLOCG00000004492	16	18869454	5	31473698
ENSXMAG00000028136	ENSXMAG00000003490	ENSLOCG00000004492	5	31473698	16	18869454
ENSXMAG00000003548	ENSXMAG00000011725	ENSLOCG00000004530	1	25845926	20	22004384
ENSXMAG00000011725	ENSXMAG00000003548	ENSLOCG00000004530	20	22004384	1	25845926
ENSXMAG00000009598	ENSXMAG00000015345	ENSLOCG00000004545	1	17961661	20	26236023
ENSXMAG00000015345	ENSXMAG00000009598	ENSLOCG00000004545	20	26236023	1	17961661
ENSXMAG00000010823	ENSXMAG00000025547	ENSLOCG00000004622	13	12970507	3	2513183
ENSXMAG00000025547	ENSXMAG00000010823	ENSLOCG00000004622	3	2513183	13	12970507
ENSXMAG00000010816	ENSXMAG00000029798	ENSLOCG00000004635	13	13030680	3	2606196
ENSXMAG00000029798	ENSXMAG00000010816	ENSLOCG00000004635	3	2606196	13	13030680
ENSXMAG00000027403	ENSXMAG00000028190	ENSLOCG00000004648	3	2686367	13	13063935
ENSXMAG00000028190	ENSXMAG00000027403	ENSLOCG00000004648	13	13063935	3	2686367
ENSXMAG00000008861	ENSXMAG00000029112	ENSLOCG00000004658	18	26725356	11	13791588
ENSXMAG00000029112	ENSXMAG00000008861	ENSLOCG00000004658	11	13791588	18	26725356
ENSXMAG00000010792	ENSXMAG00000017195	ENSLOCG00000004659	13	9441458	3	2886437
ENSXMAG00000017195	ENSXMAG00000010792	ENSLOCG00000004659	3	2886437	13	9441458
ENSXMAG00000019914	ENSXMAG00000020173	ENSLOCG00000004673	20	26164069	1	18316616
ENSXMAG00000020173	ENSXMAG00000019914	ENSLOCG00000004673	1	18316616	20	26164069
ENSXMAG00000003143	ENSXMAG00000004091	ENSLOCG00000004677	6	2845574	9	27300281
ENSXMAG00000004091	ENSXMAG00000003143	ENSLOCG00000004677	9	27300281	6	2845574
ENSXMAG00000018187	ENSXMAG00000028586	ENSLOCG00000004690	8	13656213	12	17975714
ENSXMAG00000028586	ENSXMAG00000018187	ENSLOCG00000004690	12	17975714	8	13656213
ENSXMAG00000012398	ENSXMAG00000017979	ENSLOCG00000004714	18	26404252	11	13098862
ENSXMAG00000017979	ENSXMAG00000012398	ENSLOCG00000004714	11	13098862	18	26404252
ENSXMAG00000012163	ENSXMAG00000022489	ENSLOCG00000004727	6	12364055	9	20288748
ENSXMAG00000022489	ENSXMAG00000012163	ENSLOCG00000004727	9	20288748	6	12364055
ENSXMAG00000004123	ENSXMAG00000010662	ENSLOCG00000004740	9	27220443	6	2887633
ENSXMAG00000010662	ENSXMAG00000004123	ENSLOCG00000004740	6	2887633	9	27220443
ENSXMAG00000003226	ENSXMAG00000015365	ENSLOCG00000004752	10	8636232	22	15688889
ENSXMAG00000015365	ENSXMAG00000003226	ENSLOCG00000004752	22	15688889	10	8636232

ENSXMAG00000012702	ENSXMAG00000018190	ENSLOCG00000004772	20	25282691	12	11318450
ENSXMAG00000018190	ENSXMAG00000012702	ENSLOCG00000004772	12	11318450	20	25282691
ENSXMAG00000009661	ENSXMAG00000013827	ENSLOCG00000004781	6	29920382	21	10639762
ENSXMAG00000013827	ENSXMAG00000009661	ENSLOCG00000004781	21	10639762	6	29920382
ENSXMAG00000023613	ENSXMAG00000028127	ENSLOCG00000004835	12	1632184	8	15413240
ENSXMAG00000028127	ENSXMAG00000023613	ENSLOCG00000004835	8	15413240	12	1632184
ENSXMAG00000000579	ENSXMAG00000012050	ENSLOCG00000004837	18	30662592	11	22358674
ENSXMAG00000012050	ENSXMAG00000000579	ENSLOCG00000004837	11	22358674	18	30662592
ENSXMAG00000009571	ENSXMAG00000012016	ENSLOCG00000004844	1	26149308	20	2445733
ENSXMAG00000012016	ENSXMAG00000009571	ENSLOCG00000004844	20	2445733	1	26149308
ENSXMAG00000006054	ENSXMAG00000017997	ENSLOCG00000004847	18	26253924	11	16506300
ENSXMAG00000017997	ENSXMAG00000006054	ENSLOCG00000004847	11	16506300	18	26253924
ENSXMAG00000003180	ENSXMAG00000015304	ENSLOCG00000004863	10	8568817	22	15579158
ENSXMAG00000015304	ENSXMAG00000003180	ENSLOCG00000004863	22	15579158	10	8568817
ENSXMAG00000000530	ENSXMAG00000024206	ENSLOCG00000004869	21	10699544	6	29826434
ENSXMAG00000024206	ENSXMAG00000000530	ENSLOCG00000004869	6	29826434	21	10699544
ENSXMAG00000006042	ENSXMAG00000028684	ENSLOCG00000004891	18	26225186	11	16654401
ENSXMAG00000028684	ENSXMAG00000006042	ENSLOCG00000004891	11	16654401	18	26225186
ENSXMAG00000002832	ENSXMAG00000005753	ENSLOCG00000004910	24	1390800	24	10533426
ENSXMAG00000005753	ENSXMAG00000002832	ENSLOCG00000004910	24	10533426	24	1390800
ENSXMAG00000005622	ENSXMAG00000014976	ENSLOCG00000005005	12	26951901	8	13444112
ENSXMAG00000014976	ENSXMAG00000005622	ENSLOCG00000005005	8	13444112	12	26951901
ENSXMAG00000002940	ENSXMAG00000013741	ENSLOCG00000005012	8	1099131	11	1710006
ENSXMAG00000013741	ENSXMAG00000002940	ENSLOCG00000005012	11	1710006	8	1099131
ENSXMAG00000021001	ENSXMAG00000025138	ENSLOCG00000005021	12	26966349	8	13464662
ENSXMAG00000025138	ENSXMAG00000021001	ENSLOCG00000005021	8	13464662	12	26966349
ENSXMAG00000014992	ENSXMAG00000029541	ENSLOCG00000005049	10	911423	16	13863798
ENSXMAG00000029541	ENSXMAG00000014992	ENSLOCG00000005049	16	13863798	10	911423
ENSXMAG00000023059	ENSXMAG00000027553	ENSLOCG00000005090	1	19015697	1	25051882
ENSXMAG00000027553	ENSXMAG00000023059	ENSLOCG00000005090	1	25051882	1	19015697
ENSXMAG00000004449	ENSXMAG00000018355	ENSLOCG00000005111	9	13963259	6	3327961
ENSXMAG00000018355	ENSXMAG00000004449	ENSLOCG00000005111	6	3327961	9	13963259
ENSXMAG00000016824	ENSXMAG00000017719	ENSLOCG00000005115	20	13229992	1	22511770
ENSXMAG00000017719	ENSXMAG00000016824	ENSLOCG00000005115	1	22511770	20	13229992
ENSXMAG00000006099	ENSXMAG00000018057	ENSLOCG00000005121	18	26127948	11	16402893
ENSXMAG00000018057	ENSXMAG00000006099	ENSLOCG00000005121	11	16402893	18	26127948
ENSXMAG00000000484	ENSXMAG00000005749	ENSLOCG00000005124	18	31720854	11	22709681
ENSXMAG00000005749	ENSXMAG00000000484	ENSLOCG00000005124	11	22709681	18	31720854
ENSXMAG00000000754	ENSXMAG00000016443	ENSLOCG00000005155	2	31444185	4	33876221
ENSXMAG00000016443	ENSXMAG00000000754	ENSLOCG00000005155	4	33876221	2	31444185
ENSXMAG00000012441	ENSXMAG00000019329	ENSLOCG00000005159	6	12150866	9	19713668
ENSXMAG00000019329	ENSXMAG00000012441	ENSLOCG00000005159	9	19713668	6	12150866
ENSXMAG00000001732	ENSXMAG00000006106	ENSLOCG00000005170	11	16156931	18	25984050
ENSXMAG00000006106	ENSXMAG00000001732	ENSLOCG00000005170	18	25984050	11	16156931
ENSXMAG00000009269	ENSXMAG00000024926	ENSLOCG00000005187	21	16992389	19	18120094
ENSXMAG00000024926	ENSXMAG00000009269	ENSLOCG00000005187	19	18120094	21	16992389
ENSXMAG00000025011	ENSXMAG00000025467	ENSLOCG00000005231	20	23796385	1	26234910
ENSXMAG00000025467	ENSXMAG00000025011	ENSLOCG00000005231	1	26234910	20	23796385
ENSXMAG00000004547	ENSXMAG00000028214	ENSLOCG00000005237	4	22015092	7	14932196
ENSXMAG00000028214	ENSXMAG00000004547	ENSLOCG00000005237	7	14932196	4	22015092
ENSXMAG00000001718	ENSXMAG00000011531	ENSLOCG00000005256	11	32007152	18	25880011
ENSXMAG00000011531	ENSXMAG00000001718	ENSLOCG00000005256	18	25880011	11	32007152
ENSXMAG00000015893	ENSXMAG00000016605	ENSLOCG00000005262	9	25628770	6	14524110
ENSXMAG00000016605	ENSXMAG00000015893	ENSLOCG00000005262	6	14524110	9	25628770
ENSXMAG00000001285	ENSXMAG00000016680	ENSLOCG00000005270	12	15475040	8	25017132
ENSXMAG00000016680	ENSXMAG00000001285	ENSLOCG00000005270	8	25017132	12	15475040
ENSXMAG00000011605	ENSXMAG00000016485	ENSLOCG00000005313	9	25565513	6	6196649
ENSXMAG00000016485	ENSXMAG00000011605	ENSLOCG00000005313	6	6196649	9	25565513
ENSXMAG00000003095	ENSXMAG00000014960	ENSLOCG00000005316	10	8127133	22	15329611
ENSXMAG00000014960	ENSXMAG00000003095	ENSLOCG00000005316	22	15329611	10	8127133
ENSXMAG00000007098	ENSXMAG00000025613	ENSLOCG00000005321	3	3890565	13	14184115
ENSXMAG00000025613	ENSXMAG00000007098	ENSLOCG00000005321	13	14184115	3	3890565
ENSXMAG00000005806	ENSXMAG00000007838	ENSLOCG00000005354	11	3966645	18	31911941
ENSXMAG00000007838	ENSXMAG00000005806	ENSLOCG00000005354	18	31911941	11	3966645

ENSXMAG00000000134	ENSXMAG00000001445	ENSLOCG00000005365	16	13762201	10	816055
ENSXMAG00000001445	ENSXMAG00000000134	ENSLOCG00000005365	10	816055	16	13762201
ENSXMAG00000006482	ENSXMAG00000018982	ENSLOCG00000005433	8	23553508	12	625832
ENSXMAG00000018982	ENSXMAG00000006482	ENSLOCG00000005433	12	625832	8	23553508
ENSXMAG00000000201	ENSXMAG00000005339	ENSLOCG00000005443	5	32941500	16	17774113
ENSXMAG00000005339	ENSXMAG00000000201	ENSLOCG00000005443	16	17774113	5	32941500
ENSXMAG00000007034	ENSXMAG00000018963	ENSLOCG00000005451	8	23532669	12	19385127
ENSXMAG00000018963	ENSXMAG00000007034	ENSLOCG00000005451	12	19385127	8	23532669
ENSXMAG00000007558	ENSXMAG00000010856	ENSLOCG00000005493	11	17830013	18	6582154
ENSXMAG00000010856	ENSXMAG00000007558	ENSLOCG00000005493	18	6582154	11	17830013
ENSXMAG00000003022	ENSXMAG00000014849	ENSLOCG00000005567	10	7834389	22	15047006
ENSXMAG00000014849	ENSXMAG00000003022	ENSLOCG00000005567	22	15047006	10	7834389
ENSXMAG00000013257	ENSXMAG00000016199	ENSLOCG00000005573	13	13737556	3	4413947
ENSXMAG00000016199	ENSXMAG00000013257	ENSLOCG00000005573	3	4413947	13	13737556
ENSXMAG00000016777	ENSXMAG00000017771	ENSLOCG00000005581	20	13161278	1	22551197
ENSXMAG00000017771	ENSXMAG00000016777	ENSLOCG00000005581	1	22551197	20	13161278
ENSXMAG00000006118	ENSXMAG00000015332	ENSLOCG00000005582	7	18773737	24	14093537
ENSXMAG00000015332	ENSXMAG00000006118	ENSLOCG00000005582	24	14093537	7	18773737
ENSXMAG00000021752	ENSXMAG00000023803	ENSLOCG00000005598	20	13145817	1	22571806
ENSXMAG00000023803	ENSXMAG00000021752	ENSLOCG00000005598	1	22571806	20	13145817
ENSXMAG00000021441	ENSXMAG00000027283	ENSLOCG00000005607	12	1457743	8	6748189
ENSXMAG00000027283	ENSXMAG00000021441	ENSLOCG00000005607	8	6748189	12	1457743
ENSXMAG00000003019	ENSXMAG00000014845	ENSLOCG00000005616	10	7825819	22	15033967
ENSXMAG00000014845	ENSXMAG00000003019	ENSLOCG00000005616	22	15033967	10	7825819
ENSXMAG00000004722	ENSXMAG00000025668	ENSLOCG00000005668	3	4779421	13	13731252
ENSXMAG00000025668	ENSXMAG00000004722	ENSLOCG00000005668	13	13731252	3	4779421
ENSXMAG00000016619	ENSXMAG00000018907	ENSLOCG00000005670	8	23386210	12	15447440
ENSXMAG00000018907	ENSXMAG00000016619	ENSLOCG00000005670	12	15447440	8	23386210
ENSXMAG00000001997	ENSXMAG00000021403	ENSLOCG00000005680	20	15308616	1	6190894
ENSXMAG00000021403	ENSXMAG00000001997	ENSLOCG00000005680	1	6190894	20	15308616
ENSXMAG00000002955	ENSXMAG00000016283	ENSLOCG00000005726	10	23542664	22	14941167
ENSXMAG00000016283	ENSXMAG00000002955	ENSLOCG00000005726	22	14941167	10	23542664
ENSXMAG00000023708	ENSXMAG00000029627	ENSLOCG00000005731	9	16942200	19	17453835
ENSXMAG00000029627	ENSXMAG00000023708	ENSLOCG00000005731	19	17453835	9	16942200
ENSXMAG00000008707	ENSXMAG00000027891	ENSLOCG00000005732	5	33093901	16	22525488
ENSXMAG00000027891	ENSXMAG00000008707	ENSLOCG00000005732	16	22525488	5	33093901
ENSXMAG00000027112	ENSXMAG00000028909	ENSLOCG00000005766	6	19305916	21	7615995
ENSXMAG00000028909	ENSXMAG00000027112	ENSLOCG00000005766	21	7615995	6	19305916
ENSXMAG00000017874	ENSXMAG00000018983	ENSLOCG00000005807	1	10133069	20	12929899
ENSXMAG00000018983	ENSXMAG00000017874	ENSLOCG00000005807	20	12929899	1	10133069
ENSXMAG00000024922	ENSXMAG00000028164	ENSLOCG00000005808	8	23137248	12	15346725
ENSXMAG00000028164	ENSXMAG00000024922	ENSLOCG00000005808	12	15346725	8	23137248
ENSXMAG00000022252	ENSXMAG00000024728	ENSLOCG00000005817	13	288311	24	14797233
ENSXMAG00000024728	ENSXMAG00000022252	ENSLOCG00000005817	24	14797233	13	288311
ENSXMAG00000012486	ENSXMAG00000021557	ENSLOCG00000005822	16	17010168	5	33276486
ENSXMAG00000021557	ENSXMAG00000012486	ENSLOCG00000005822	5	33276486	16	17010168
ENSXMAG00000014885	ENSXMAG00000023091	ENSLOCG00000005868	1	4257010	3	5283457
ENSXMAG00000023091	ENSXMAG00000014885	ENSLOCG00000005868	3	5283457	1	4257010
ENSXMAG00000002830	ENSXMAG00000008364	ENSLOCG00000005870	8	1129718	12	5917500
ENSXMAG00000008364	ENSXMAG00000002830	ENSLOCG00000005870	12	5917500	8	1129718
ENSXMAG00000006895	ENSXMAG00000023562	ENSLOCG00000005926	24	14135630	2	5131873
ENSXMAG00000023562	ENSXMAG00000006895	ENSLOCG00000005926	2	5131873	24	14135630
ENSXMAG00000005071	ENSXMAG00000007257	ENSLOCG00000006036	12	19187278	8	18655632
ENSXMAG00000007257	ENSXMAG00000005071	ENSLOCG00000006036	8	18655632	12	19187278
ENSXMAG00000007337	ENSXMAG00000021211	ENSLOCG00000006050	11	17728142	18	28256355
ENSXMAG00000021211	ENSXMAG00000007337	ENSLOCG00000006050	18	28256355	11	17728142
ENSXMAG00000002879	ENSXMAG00000016743	ENSLOCG00000006071	8	1052379	12	5877886
ENSXMAG00000016743	ENSXMAG00000002879	ENSLOCG00000006071	12	5877886	8	1052379
ENSXMAG00000007125	ENSXMAG00000007989	ENSLOCG00000006075	24	3088907	7	29944311
ENSXMAG00000007989	ENSXMAG00000007125	ENSLOCG00000006075	7	29944311	24	3088907
ENSXMAG00000013868	ENSXMAG00000026168	ENSLOCG00000006099	13	20857791	3	15074881
ENSXMAG00000026168	ENSXMAG00000013868	ENSLOCG00000006099	3	15074881	13	20857791
ENSXMAG00000013027	ENSXMAG00000024751	ENSLOCG00000006113	2	468523	4	635503
ENSXMAG00000024751	ENSXMAG00000013027	ENSLOCG00000006113	4	635503	2	468523

ENSXMAG00000011950	ENSXMAG00000011986	ENSLOCG00000006121	18	28780517	11	21337009
ENSXMAG00000011986	ENSXMAG00000011950	ENSLOCG00000006121	11	21337009	18	28780517
ENSXMAG00000019295	ENSXMAG00000027507	ENSLOCG00000006132	7	10649295	4	21718219
ENSXMAG00000027507	ENSXMAG00000019295	ENSLOCG00000006132	4	21718219	7	10649295
ENSXMAG00000013879	ENSXMAG00000026229	ENSLOCG00000006139	13	20871816	3	15094358
ENSXMAG00000026229	ENSXMAG00000013879	ENSLOCG00000006139	3	15094358	13	20871816
ENSXMAG00000002891	ENSXMAG00000028388	ENSLOCG00000006144	8	1316412	12	5835976
ENSXMAG00000028388	ENSXMAG00000002891	ENSLOCG00000006144	12	5835976	8	1316412
ENSXMAG00000012793	ENSXMAG00000014217	ENSLOCG00000006178	16	14970637	5	21849065
ENSXMAG00000014217	ENSXMAG00000012793	ENSLOCG00000006178	5	21849065	16	14970637
ENSXMAG00000007999	ENSXMAG00000028362	ENSLOCG00000006192	7	30160386	24	3067004
ENSXMAG00000028362	ENSXMAG00000007999	ENSLOCG00000006192	24	3067004	7	30160386
ENSXMAG00000003122	ENSXMAG00000010563	ENSLOCG00000006199	6	4843400	9	27698402
ENSXMAG00000010563	ENSXMAG00000003122	ENSLOCG00000006199	9	27698402	6	4843400
ENSXMAG00000000222	ENSXMAG00000000907	ENSLOCG00000006209	5	31245677	16	17284807
ENSXMAG00000000907	ENSXMAG00000000222	ENSLOCG00000006209	16	17284807	5	31245677
ENSXMAG00000012615	ENSXMAG00000023280	ENSLOCG00000006221	6	11750829	9	19145266
ENSXMAG00000023280	ENSXMAG00000012615	ENSLOCG00000006221	9	19145266	6	11750829
ENSXMAG00000003416	ENSXMAG00000025018	ENSLOCG00000006228	16	17991699	5	31427015
ENSXMAG00000025018	ENSXMAG00000003416	ENSLOCG00000006228	5	31427015	16	17991699
ENSXMAG00000010169	ENSXMAG00000013357	ENSLOCG00000006271	9	16879235	19	17874919
ENSXMAG00000013357	ENSXMAG00000010169	ENSLOCG00000006271	19	17874919	9	16879235
ENSXMAG00000004092	ENSXMAG00000023494	ENSLOCG00000006353	11	14950780	18	27879580
ENSXMAG00000023494	ENSXMAG00000004092	ENSLOCG00000006353	18	27879580	11	14950780
ENSXMAG00000007837	ENSXMAG00000010653	ENSLOCG00000006360	22	16332212	10	5428135
ENSXMAG00000010653	ENSXMAG00000007837	ENSLOCG00000006360	10	5428135	22	16332212
ENSXMAG00000004102	ENSXMAG00000010333	ENSLOCG00000006397	11	30601957	18	27850858
ENSXMAG00000010333	ENSXMAG00000004102	ENSLOCG00000006397	18	27850858	11	30601957
ENSXMAG00000014642	ENSXMAG00000016304	ENSLOCG00000006421	3	18333462	13	4186084
ENSXMAG00000016304	ENSXMAG00000014642	ENSLOCG00000006421	13	4186084	3	18333462
ENSXMAG00000019305	ENSXMAG00000023383	ENSLOCG00000006431	9	16420562	6	11711561
ENSXMAG00000023383	ENSXMAG00000019305	ENSLOCG00000006431	6	11711561	9	16420562
ENSXMAG00000011017	ENSXMAG00000012174	ENSLOCG00000006462	6	15497641	9	22411400
ENSXMAG00000012174	ENSXMAG00000011017	ENSLOCG00000006462	9	22411400	6	15497641
ENSXMAG00000007014	ENSXMAG00000028499	ENSLOCG00000006488	11	12482146	18	2221872
ENSXMAG00000028499	ENSXMAG00000007014	ENSLOCG00000006488	18	2221872	11	12482146
ENSXMAG00000014619	ENSXMAG00000016475	ENSLOCG00000006518	3	18517333	13	4242935
ENSXMAG00000016475	ENSXMAG00000014619	ENSLOCG00000006518	13	4242935	3	18517333
ENSXMAG00000006561	ENSXMAG00000008657	ENSLOCG00000006523	16	21333029	5	8915082
ENSXMAG00000008657	ENSXMAG00000006561	ENSLOCG00000006523	5	8915082	16	21333029
ENSXMAG00000006547	ENSXMAG00000008939	ENSLOCG00000006534	16	21343271	5	8905077
ENSXMAG00000008939	ENSXMAG00000006547	ENSLOCG00000006534	5	8905077	16	21343271
ENSXMAG00000003686	ENSXMAG00000015917	ENSLOCG00000006554	6	14573947	9	25989055
ENSXMAG00000015917	ENSXMAG00000003686	ENSLOCG00000006554	9	25989055	6	14573947
ENSXMAG00000001298	ENSXMAG00000006985	ENSLOCG00000006582	2	7167520	4	19934428
ENSXMAG00000006985	ENSXMAG00000001298	ENSLOCG00000006582	4	19934428	2	7167520
ENSXMAG00000011255	ENSXMAG00000029648	ENSLOCG00000006596	18	2400454	11	12521830
ENSXMAG00000029648	ENSXMAG00000011255	ENSLOCG00000006596	11	12521830	18	2400454
ENSXMAG00000011345	ENSXMAG00000016713	ENSLOCG00000006604	20	15926268	1	12635017
ENSXMAG00000016713	ENSXMAG00000011345	ENSLOCG00000006604	1	12635017	20	15926268
ENSXMAG00000006429	ENSXMAG00000008895	ENSLOCG00000006607	16	21395422	5	8700699
ENSXMAG00000008895	ENSXMAG00000006429	ENSLOCG00000006607	5	8700699	16	21395422
ENSXMAG00000000947	ENSXMAG00000010042	ENSLOCG00000006610	9	3794928	6	4618866
ENSXMAG00000010042	ENSXMAG00000000947	ENSLOCG00000006610	6	4618866	9	3794928
ENSXMAG00000012106	ENSXMAG00000023256	ENSLOCG00000006621	11	30959560	18	13129035
ENSXMAG00000023256	ENSXMAG00000012106	ENSLOCG00000006621	18	13129035	11	30959560
ENSXMAG00000004628	ENSXMAG00000011193	ENSLOCG00000006622	11	12549691	18	2417977
ENSXMAG00000011193	ENSXMAG00000004628	ENSLOCG00000006622	18	2417977	11	12549691
ENSXMAG00000013007	ENSXMAG00000014067	ENSLOCG00000006623	4	15835476	2	17750455
ENSXMAG00000014067	ENSXMAG00000013007	ENSLOCG00000006623	2	17750455	4	15835476
ENSXMAG00000001137	ENSXMAG00000007047	ENSLOCG00000006642	2	7209668	4	33275922
ENSXMAG00000007047	ENSXMAG00000001137	ENSLOCG00000006642	4	33275922	2	7209668
ENSXMAG00000002112	ENSXMAG00000016054	ENSLOCG00000006670	1	15050077	20	20595399
ENSXMAG00000016054	ENSXMAG00000002112	ENSLOCG00000006670	20	20595399	1	15050077

ENSXMAG00000004039	ENSXMAG00000011865	ENSLOCG00000006671	11	21153986	18	22341412
ENSXMAG00000011865	ENSXMAG00000004039	ENSLOCG00000006671	18	22341412	11	21153986
ENSXMAG00000002738	ENSXMAG00000016803	ENSLOCG00000006685	8	1034268	12	6020668
ENSXMAG00000016803	ENSXMAG00000002738	ENSLOCG00000006685	12	6020668	8	1034268
ENSXMAG00000007080	ENSXMAG00000023163	ENSLOCG00000006694	4	33206433	2	7261924
ENSXMAG00000023163	ENSXMAG00000007080	ENSLOCG00000006694	2	7261924	4	33206433
ENSXMAG00000010614	ENSXMAG00000017461	ENSLOCG00000006699	1	22193341	1	14532143
ENSXMAG00000017461	ENSXMAG00000010614	ENSLOCG00000006699	1	14532143	1	22193341
ENSXMAG00000006509	ENSXMAG00000008873	ENSLOCG00000006717	16	21511959	5	8839323
ENSXMAG00000008873	ENSXMAG00000006509	ENSLOCG00000006717	5	8839323	16	21511959
ENSXMAG00000013002	ENSXMAG00000019292	ENSLOCG00000006724	6	11585732	9	17941279
ENSXMAG00000019292	ENSXMAG00000013002	ENSLOCG00000006724	9	17941279	6	11585732
ENSXMAG00000004486	ENSXMAG00000012228	ENSLOCG00000006732	11	12697485	18	2275165
ENSXMAG00000012228	ENSXMAG00000004486	ENSLOCG00000006732	18	2275165	11	12697485
ENSXMAG00000005262	ENSXMAG00000008866	ENSLOCG00000006765	16	21519249	5	19779877
ENSXMAG00000008866	ENSXMAG00000005262	ENSLOCG00000006765	5	19779877	16	21519249
ENSXMAG00000004522	ENSXMAG00000015958	ENSLOCG00000006766	11	12714437	18	2295910
ENSXMAG00000015958	ENSXMAG00000004522	ENSLOCG00000006766	18	2295910	11	12714437
ENSXMAG00000002228	ENSXMAG00000007895	ENSLOCG00000006769	1	14363155	20	20784877
ENSXMAG00000007895	ENSXMAG00000002228	ENSLOCG00000006769	20	20784877	1	14363155
ENSXMAG00000006835	ENSXMAG00000012276	ENSLOCG00000006811	18	13217568	11	14598970
ENSXMAG00000012276	ENSXMAG00000006835	ENSLOCG00000006811	11	14598970	18	13217568
ENSXMAG00000017106	ENSXMAG00000018709	ENSLOCG00000006818	3	7742934	13	26471631
ENSXMAG00000018709	ENSXMAG00000017106	ENSLOCG00000006818	13	26471631	3	7742934
ENSXMAG00000001583	ENSXMAG00000002823	ENSLOCG00000006820	16	25026832	10	3213011
ENSXMAG00000002823	ENSXMAG00000001583	ENSLOCG00000006820	10	3213011	16	25026832
ENSXMAG00000010183	ENSXMAG00000017075	ENSLOCG00000006842	1	20250529	20	7169716
ENSXMAG00000017075	ENSXMAG00000010183	ENSLOCG00000006842	20	7169716	1	20250529
ENSXMAG00000004660	ENSXMAG00000013138	ENSLOCG00000006888	16	1562796	5	20811626
ENSXMAG00000013138	ENSXMAG00000004660	ENSLOCG00000006888	5	20811626	16	1562796
ENSXMAG00000014187	ENSXMAG00000016342	ENSLOCG00000006895	2	31301064	4	15948682
ENSXMAG00000016342	ENSXMAG00000014187	ENSLOCG00000006895	4	15948682	2	31301064
ENSXMAG00000001184	ENSXMAG00000017442	ENSLOCG00000006899	13	8457533	3	213034
ENSXMAG00000017442	ENSXMAG00000001184	ENSLOCG00000006899	3	213034	13	8457533
ENSXMAG00000003808	ENSXMAG00000017468	ENSLOCG00000006906	17	9061173	2	11259050
ENSXMAG00000017468	ENSXMAG00000003808	ENSLOCG00000006906	2	11259050	17	9061173
ENSXMAG00000001007	ENSXMAG00000011432	ENSLOCG00000006948	3	1451024	13	10965979
ENSXMAG00000011432	ENSXMAG00000001007	ENSLOCG00000006948	13	10965979	3	1451024
ENSXMAG00000018365	ENSXMAG00000026491	ENSLOCG00000006955	9	4613515	6	5060410
ENSXMAG00000026491	ENSXMAG00000018365	ENSLOCG00000006955	6	5060410	9	4613515
ENSXMAG00000008529	ENSXMAG00000023735	ENSLOCG00000006993	18	18694557	11	12785681
ENSXMAG00000023735	ENSXMAG00000008529	ENSLOCG00000006993	11	12785681	18	18694557
ENSXMAG00000004881	ENSXMAG00000028234	ENSLOCG00000006998	16	1522285	5	20216055
ENSXMAG00000028234	ENSXMAG00000004881	ENSLOCG00000006998	5	20216055	16	1522285
ENSXMAG00000002015	ENSXMAG00000014114	ENSLOCG00000007005	6	1412730	2	22662148
ENSXMAG00000014114	ENSXMAG00000002015	ENSLOCG00000007005	2	22662148	6	1412730
ENSXMAG00000007882	ENSXMAG00000028239	ENSLOCG00000007012	14	22589281	11	3996459
ENSXMAG00000028239	ENSXMAG00000007882	ENSLOCG00000007012	11	3996459	14	22589281
ENSXMAG00000008459	ENSXMAG00000026844	ENSLOCG00000007053	6	20368164	21	4682273
ENSXMAG00000026844	ENSXMAG00000008459	ENSLOCG00000007053	21	4682273	6	20368164
ENSXMAG00000007231	ENSXMAG00000014814	ENSLOCG00000007064	13	3691666	3	17630289
ENSXMAG00000014814	ENSXMAG00000007231	ENSLOCG00000007064	3	17630289	13	3691666
ENSXMAG00000011686	ENSXMAG00000012536	ENSLOCG00000007122	6	4426208	9	20886997
ENSXMAG00000012536	ENSXMAG00000011686	ENSLOCG00000007122	9	20886997	6	4426208
ENSXMAG00000015876	ENSXMAG00000028593	ENSLOCG00000007126	5	957832	14	13313502
ENSXMAG00000028593	ENSXMAG00000015876	ENSLOCG00000007126	14	13313502	5	957832
ENSXMAG00000002271	ENSXMAG00000010014	ENSLOCG00000007128	12	4842207	8	10695061
ENSXMAG00000010014	ENSXMAG00000002271	ENSLOCG00000007128	8	10695061	12	4842207
ENSXMAG00000006236	ENSXMAG00000014323	ENSLOCG00000007160	21	3727247	6	25893198
ENSXMAG00000014323	ENSXMAG00000006236	ENSLOCG00000007160	6	25893198	21	3727247
ENSXMAG00000011209	ENSXMAG00000017698	ENSLOCG00000007176	11	29018212	18	18208691
ENSXMAG00000017698	ENSXMAG00000011209	ENSLOCG00000007176	18	18208691	11	29018212
ENSXMAG00000007142	ENSXMAG00000017103	ENSLOCG00000007185	10	12601731	16	23764794
ENSXMAG00000017103	ENSXMAG00000007142	ENSLOCG00000007185	16	23764794	10	12601731

ENSXMAG00000000413	ENSXMAG00000026819	ENSLOCG00000007190	22	25478763	7	16485471
ENSXMAG00000026819	ENSXMAG00000000413	ENSLOCG00000007190	7	16485471	22	25478763
ENSXMAG00000000410	ENSXMAG00000019062	ENSLOCG00000007211	4	20923708	7	16366092
ENSXMAG00000019062	ENSXMAG00000000410	ENSLOCG00000007211	7	16366092	4	20923708
ENSXMAG00000005615	ENSXMAG00000019234	ENSLOCG00000007246	6	4354315	9	8213749
ENSXMAG00000019234	ENSXMAG00000005615	ENSLOCG00000007246	9	8213749	6	4354315
ENSXMAG00000002114	ENSXMAG00000010493	ENSLOCG00000007256	1	14236364	20	20653929
ENSXMAG00000010493	ENSXMAG00000002114	ENSLOCG00000007256	20	20653929	1	14236364
ENSXMAG00000019441	ENSXMAG00000022365	ENSLOCG00000007272	9	7972276	6	4255739
ENSXMAG00000022365	ENSXMAG00000019441	ENSLOCG00000007272	6	4255739	9	7972276
ENSXMAG00000015971	ENSXMAG00000021629	ENSLOCG00000007277	21	25681766	19	26923258
ENSXMAG00000021629	ENSXMAG00000015971	ENSLOCG00000007277	19	26923258	21	25681766
ENSXMAG00000005488	ENSXMAG00000005951	ENSLOCG00000007278	6	25190449	21	20837870
ENSXMAG00000005951	ENSXMAG00000005488	ENSLOCG00000007278	21	20837870	6	25190449
ENSXMAG00000012058	ENSXMAG00000015976	ENSLOCG00000007288	19	26936183	21	25701672
ENSXMAG00000015976	ENSXMAG00000012058	ENSLOCG00000007288	21	25701672	19	26936183
ENSXMAG00000010616	ENSXMAG00000017416	ENSLOCG00000007299	1	22229542	1	14538833
ENSXMAG00000017416	ENSXMAG00000010616	ENSLOCG00000007299	1	14538833	1	22229542
ENSXMAG00000007574	ENSXMAG00000022965	ENSLOCG00000007306	10	176995	22	3988324
ENSXMAG00000022965	ENSXMAG00000007574	ENSLOCG00000007306	22	3988324	10	176995
ENSXMAG00000002288	ENSXMAG00000009926	ENSLOCG00000007313	12	5394143	8	10668656
ENSXMAG00000009926	ENSXMAG00000002288	ENSLOCG00000007313	8	10668656	12	5394143
ENSXMAG00000004031	ENSXMAG00000029877	ENSLOCG00000007322	11	3162677	8	13206861
ENSXMAG00000029877	ENSXMAG00000004031	ENSLOCG00000007322	8	13206861	11	3162677
ENSXMAG00000005021	ENSXMAG00000005409	ENSLOCG00000007323	7	16283587	22	29192811
ENSXMAG00000005409	ENSXMAG00000005021	ENSLOCG00000007323	22	29192811	7	16283587
ENSXMAG00000002712	ENSXMAG00000024646	ENSLOCG00000007328	6	4082047	9	7513684
ENSXMAG00000024646	ENSXMAG00000002712	ENSLOCG00000007328	9	7513684	6	4082047
ENSXMAG00000012988	ENSXMAG00000029897	ENSLOCG00000007339	5	20009357	16	1737326
ENSXMAG00000029897	ENSXMAG00000012988	ENSLOCG00000007339	16	1737326	5	20009357
ENSXMAG00000002706	ENSXMAG00000025681	ENSLOCG00000007350	6	4066176	9	7502984
ENSXMAG00000025681	ENSXMAG00000002706	ENSLOCG00000007350	9	7502984	6	4066176
ENSXMAG00000010632	ENSXMAG00000017409	ENSLOCG00000007372	1	22259957	1	14580585
ENSXMAG00000017409	ENSXMAG00000010632	ENSLOCG00000007372	1	14580585	1	22259957
ENSXMAG00000007023	ENSXMAG00000015212	ENSLOCG00000007396	13	3471211	3	17396198
ENSXMAG00000015212	ENSXMAG00000007023	ENSLOCG00000007396	3	17396198	13	3471211
ENSXMAG00000009132	ENSXMAG00000011913	ENSLOCG00000007400	21	25782323	19	18450047
ENSXMAG00000011913	ENSXMAG00000009132	ENSLOCG00000007400	19	18450047	21	25782323
ENSXMAG00000010656	ENSXMAG00000017286	ENSLOCG00000007430	1	22320851	1	14632971
ENSXMAG00000017286	ENSXMAG00000010656	ENSLOCG00000007430	1	14632971	1	22320851
ENSXMAG00000007284	ENSXMAG00000022050	ENSLOCG00000007431	10	1309140	16	24017853
ENSXMAG00000022050	ENSXMAG00000007284	ENSLOCG00000007431	16	24017853	10	1309140
ENSXMAG00000017827	ENSXMAG00000024968	ENSLOCG00000007453	5	17565437	2	11152371
ENSXMAG00000024968	ENSXMAG00000017827	ENSLOCG00000007453	2	11152371	5	17565437
ENSXMAG00000005291	ENSXMAG00000022912	ENSLOCG00000007479	6	11024864	7	16105466
ENSXMAG00000022912	ENSXMAG00000005291	ENSLOCG00000007479	7	16105466	6	11024864
ENSXMAG00000002447	ENSXMAG00000004785	ENSLOCG00000007531	6	3970874	9	6162196
ENSXMAG00000004785	ENSXMAG00000002447	ENSLOCG00000007531	9	6162196	6	3970874
ENSXMAG00000000208	ENSXMAG00000007185	ENSLOCG00000007575	5	28495839	16	9189409
ENSXMAG00000007185	ENSXMAG00000000208	ENSLOCG00000007575	16	9189409	5	28495839
ENSXMAG00000002002	ENSXMAG00000010544	ENSLOCG00000007586	9	24178834	4	23779196
ENSXMAG00000010544	ENSXMAG00000002002	ENSLOCG00000007586	4	23779196	9	24178834
ENSXMAG00000008340	ENSXMAG00000022010	ENSLOCG00000007590	6	5948518	21	5433863
ENSXMAG00000022010	ENSXMAG00000008340	ENSLOCG00000007590	21	5433863	6	5948518
ENSXMAG00000005348	ENSXMAG00000005990	ENSLOCG00000007616	12	18683896	8	14767058
ENSXMAG00000005990	ENSXMAG00000005348	ENSLOCG00000007616	8	14767058	12	18683896
ENSXMAG00000004822	ENSXMAG00000026357	ENSLOCG00000007622	20	18534514	1	1308686
ENSXMAG00000026357	ENSXMAG00000004822	ENSLOCG00000007622	1	1308686	20	18534514
ENSXMAG00000001967	ENSXMAG00000004476	ENSLOCG00000007625	8	24551208	12	23956309
ENSXMAG00000004476	ENSXMAG00000001967	ENSLOCG00000007625	12	23956309	8	24551208
ENSXMAG00000012673	ENSXMAG00000016284	ENSLOCG00000007653	10	10715842	22	17541888
ENSXMAG00000016284	ENSXMAG00000012673	ENSLOCG00000007653	22	17541888	10	10715842
ENSXMAG00000006861	ENSXMAG00000008314	ENSLOCG00000007664	21	5529382	6	5993259
ENSXMAG00000008314	ENSXMAG00000006861	ENSLOCG00000007664	6	5993259	21	5529382

ENSXMAG00000014446	ENSXMAG00000015472	ENSLOCG00000007668	13	2681769	3	16211497
ENSXMAG00000015472	ENSXMAG00000014446	ENSLOCG00000007668	3	16211497	13	2681769
ENSXMAG00000009188	ENSXMAG00000009319	ENSLOCG00000007672	19	18065734	21	9842557
ENSXMAG00000009319	ENSXMAG00000009188	ENSLOCG00000007672	21	9842557	19	18065734
ENSXMAG00000019058	ENSXMAG00000028633	ENSLOCG00000007673	7	15900167	6	10903821
ENSXMAG00000028633	ENSXMAG00000019058	ENSLOCG00000007673	6	10903821	7	15900167
ENSXMAG00000010911	ENSXMAG00000015476	ENSLOCG00000007702	13	2608773	3	20532429
ENSXMAG00000015476	ENSXMAG00000010911	ENSLOCG00000007702	3	20532429	13	2608773
ENSXMAG00000004860	ENSXMAG00000017327	ENSLOCG00000007704	13	8819990	3	155631
ENSXMAG00000017327	ENSXMAG00000004860	ENSLOCG00000007704	3	155631	13	8819990
ENSXMAG00000026235	ENSXMAG00000027871	ENSLOCG00000007735	20	20327527	1	15482236
ENSXMAG00000027871	ENSXMAG00000026235	ENSLOCG00000007735	1	15482236	20	20327527
ENSXMAG00000012600	ENSXMAG00000016660	ENSLOCG00000007761	20	18494824	1	7030832
ENSXMAG00000016660	ENSXMAG00000012600	ENSLOCG00000007761	1	7030832	20	18494824
ENSXMAG00000008421	ENSXMAG00000011728	ENSLOCG00000007776	6	6091224	21	4890197
ENSXMAG00000011728	ENSXMAG00000008421	ENSLOCG00000007776	21	4890197	6	6091224
ENSXMAG00000015605	ENSXMAG00000019169	ENSLOCG00000007783	8	24047076	12	12859818
ENSXMAG00000019169	ENSXMAG00000015605	ENSLOCG00000007783	12	12859818	8	24047076
ENSXMAG00000001916	ENSXMAG00000030067	ENSLOCG00000007813	8	24682437	12	23658164
ENSXMAG00000030067	ENSXMAG00000001916	ENSLOCG00000007813	12	23658164	8	24682437
ENSXMAG00000006321	ENSXMAG00000007030	ENSLOCG00000007820	5	10216111	16	21002276
ENSXMAG00000007030	ENSXMAG00000006321	ENSLOCG00000007820	16	21002276	5	10216111
ENSXMAG00000000740	ENSXMAG00000007104	ENSLOCG00000007823	12	19296461	8	24130339
ENSXMAG00000007104	ENSXMAG00000000740	ENSLOCG00000007823	8	24130339	12	19296461
ENSXMAG00000016721	ENSXMAG00000025502	ENSLOCG00000007847	1	7060293	20	18434923
ENSXMAG00000025502	ENSXMAG00000016721	ENSLOCG00000007847	20	18434923	1	7060293
ENSXMAG00000007825	ENSXMAG00000011147	ENSLOCG00000007850	18	18133321	10	5357529
ENSXMAG00000011147	ENSXMAG00000007825	ENSLOCG00000007850	10	5357529	18	18133321
ENSXMAG00000006970	ENSXMAG00000009142	ENSLOCG00000007853	16	21046047	5	10167748
ENSXMAG00000009142	ENSXMAG00000006970	ENSLOCG00000007853	5	10167748	16	21046047
ENSXMAG00000017772	ENSXMAG00000027563	ENSLOCG00000007855	8	13102533	12	17261542
ENSXMAG00000027563	ENSXMAG00000017772	ENSLOCG00000007855	12	17261542	8	13102533
ENSXMAG00000001163	ENSXMAG00000011196	ENSLOCG00000007859	3	1747801	13	10862614
ENSXMAG00000011196	ENSXMAG00000001163	ENSLOCG00000007859	13	10862614	3	1747801
ENSXMAG00000006403	ENSXMAG00000011955	ENSLOCG00000007862	7	12714656	24	14808005
ENSXMAG00000011955	ENSXMAG00000006403	ENSLOCG00000007862	24	14808005	7	12714656
ENSXMAG00000006158	ENSXMAG00000030090	ENSLOCG00000007868	16	16949330	5	8467381
ENSXMAG00000030090	ENSXMAG00000006158	ENSLOCG00000007868	5	8467381	16	16949330
ENSXMAG00000008061	ENSXMAG00000016753	ENSLOCG00000007873	20	18411429	1	7517726
ENSXMAG00000016753	ENSXMAG00000008061	ENSLOCG00000007873	1	7517726	20	18411429
ENSXMAG00000000761	ENSXMAG00000009087	ENSLOCG00000007875	3	26091013	13	15266338
ENSXMAG00000009087	ENSXMAG00000000761	ENSLOCG00000007875	13	15266338	3	26091013
ENSXMAG00000017728	ENSXMAG00000026412	ENSLOCG00000007888	23	15877851	5	17918234
ENSXMAG00000026412	ENSXMAG00000017728	ENSLOCG00000007888	5	17918234	23	15877851
ENSXMAG00000021384	ENSXMAG00000029887	ENSLOCG00000007896	20	18382203	1	7482836
ENSXMAG00000029887	ENSXMAG00000021384	ENSLOCG00000007896	1	7482836	20	18382203
ENSXMAG00000000320	ENSXMAG00000006441	ENSLOCG00000007922	21	17007474	6	8652781
ENSXMAG00000006441	ENSXMAG00000000320	ENSLOCG00000007922	6	8652781	21	17007474
ENSXMAG00000003793	ENSXMAG00000008983	ENSLOCG00000007929	3	9732606	13	1416018
ENSXMAG00000008983	ENSXMAG00000003793	ENSLOCG00000007929	13	1416018	3	9732606
ENSXMAG00000000476	ENSXMAG00000006442	ENSLOCG00000007934	21	17044074	6	21743608
ENSXMAG00000006442	ENSXMAG00000000476	ENSLOCG00000007934	6	21743608	21	17044074
ENSXMAG00000004335	ENSXMAG00000010385	ENSLOCG00000007941	11	20758841	18	23364030
ENSXMAG00000010385	ENSXMAG00000004335	ENSLOCG00000007941	18	23364030	11	20758841
ENSXMAG00000009745	ENSXMAG00000017738	ENSLOCG00000007942	5	17865369	23	15843205
ENSXMAG00000017738	ENSXMAG00000009745	ENSLOCG00000007942	23	15843205	5	17865369
ENSXMAG00000015852	ENSXMAG00000016470	ENSLOCG00000007953	8	3337827	12	13565186
ENSXMAG00000016470	ENSXMAG00000015852	ENSLOCG00000007953	12	13565186	8	3337827
ENSXMAG00000003851	ENSXMAG00000009717	ENSLOCG00000007961	23	15888614	5	17996071
ENSXMAG00000009717	ENSXMAG00000003851	ENSLOCG00000007961	5	17996071	23	15888614
ENSXMAG00000011034	ENSXMAG00000016787	ENSLOCG00000007968	13	12325749	3	2005764
ENSXMAG00000016787	ENSXMAG00000011034	ENSLOCG00000007968	3	2005764	13	12325749
ENSXMAG00000000677	ENSXMAG00000025233	ENSLOCG00000008015	21	17712865	6	22101377
ENSXMAG00000025233	ENSXMAG00000000677	ENSLOCG00000008015	6	22101377	21	17712865

ENSXMAG00000010903	ENSXMAG00000025417	ENSLOCG00000008038	6	3205378	18	17418832
ENSXMAG00000025417	ENSXMAG00000010903	ENSLOCG00000008038	18	17418832	6	3205378
ENSXMAG00000013970	ENSXMAG00000014325	ENSLOCG00000008040	7	15605973	24	1499159
ENSXMAG00000014325	ENSXMAG00000013970	ENSLOCG00000008040	24	1499159	7	15605973
ENSXMAG00000009803	ENSXMAG00000013723	ENSLOCG00000008046	5	5073422	23	15692410
ENSXMAG00000013723	ENSXMAG00000009803	ENSLOCG00000008046	23	15692410	5	5073422
ENSXMAG00000009791	ENSXMAG00000013732	ENSLOCG00000008054	5	5053013	23	15698467
ENSXMAG00000013732	ENSXMAG00000009791	ENSLOCG00000008054	23	15698467	5	5053013
ENSXMAG00000008768	ENSXMAG00000024059	ENSLOCG00000008073	5	10596140	16	22057993
ENSXMAG00000024059	ENSXMAG00000008768	ENSLOCG00000008073	16	22057993	5	10596140
ENSXMAG00000011911	ENSXMAG00000014223	ENSLOCG00000008077	13	11677040	3	19589366
ENSXMAG00000014223	ENSXMAG00000011911	ENSLOCG00000008077	3	19589366	13	11677040
ENSXMAG00000017368	ENSXMAG00000025391	ENSLOCG00000008126	16	22017452	5	10605608
ENSXMAG00000025391	ENSXMAG00000017368	ENSLOCG00000008126	5	10605608	16	22017452
ENSXMAG00000002229	ENSXMAG00000015884	ENSLOCG00000008187	5	6057137	23	2558093
ENSXMAG00000015884	ENSXMAG00000002229	ENSLOCG00000008187	23	2558093	5	6057137
ENSXMAG00000003263	ENSXMAG00000004870	ENSLOCG00000008221	3	151291	13	24813290
ENSXMAG00000004870	ENSXMAG00000003263	ENSLOCG00000008221	13	24813290	3	151291
ENSXMAG00000005130	ENSXMAG00000011502	ENSLOCG00000008281	16	21690026	5	19889623
ENSXMAG00000011502	ENSXMAG00000005130	ENSLOCG00000008281	5	19889623	16	21690026
ENSXMAG00000010370	ENSXMAG00000026909	ENSLOCG00000008301	21	1386901	6	1240820
ENSXMAG00000026909	ENSXMAG00000010370	ENSLOCG00000008301	6	1240820	21	1386901
ENSXMAG00000004187	ENSXMAG00000010347	ENSLOCG00000008316	9	24788821	24	9109629
ENSXMAG00000010347	ENSXMAG00000004187	ENSLOCG00000008316	24	9109629	9	24788821
ENSXMAG00000003635	ENSXMAG00000015449	ENSLOCG00000008322	3	32835581	13	853328
ENSXMAG00000015449	ENSXMAG00000003635	ENSLOCG00000008322	13	853328	3	32835581
ENSXMAG00000004208	ENSXMAG00000016186	ENSLOCG00000008325	9	24834081	24	9087494
ENSXMAG00000016186	ENSXMAG00000004208	ENSLOCG00000008325	24	9087494	9	24834081
ENSXMAG00000005153	ENSXMAG00000011550	ENSLOCG00000008330	16	21662400	5	19852303
ENSXMAG00000011550	ENSXMAG00000005153	ENSLOCG00000008330	5	19852303	16	21662400
ENSXMAG00000014180	ENSXMAG00000018354	ENSLOCG00000008348	22	19624397	10	16735247
ENSXMAG00000018354	ENSXMAG00000014180	ENSLOCG00000008348	10	16735247	22	19624397
ENSXMAG00000016959	ENSXMAG00000018363	ENSLOCG00000008385	22	19306141	10	17069261
ENSXMAG00000018363	ENSXMAG00000016959	ENSLOCG00000008385	10	17069261	22	19306141
ENSXMAG00000007084	ENSXMAG00000008579	ENSLOCG00000008398	11	29814251	18	8457943
ENSXMAG00000008579	ENSXMAG00000007084	ENSLOCG00000008398	18	8457943	11	29814251
ENSXMAG00000001119	ENSXMAG00000014597	ENSLOCG00000008410	10	23087169	16	13234349
ENSXMAG00000014597	ENSXMAG00000001119	ENSLOCG00000008410	16	13234349	10	23087169
ENSXMAG00000007440	ENSXMAG00000010744	ENSLOCG00000008420	12	2652325	8	27701093
ENSXMAG00000010744	ENSXMAG00000007440	ENSLOCG00000008420	8	27701093	12	2652325
ENSXMAG00000007108	ENSXMAG00000008570	ENSLOCG00000008434	11	29870282	18	8447371
ENSXMAG00000008570	ENSXMAG00000007108	ENSLOCG00000008434	18	8447371	11	29870282
ENSXMAG00000000205	ENSXMAG00000008277	ENSLOCG00000008498	14	22612295	5	32400732
ENSXMAG00000008277	ENSXMAG00000000205	ENSLOCG00000008498	5	32400732	14	22612295
ENSXMAG00000010707	ENSXMAG00000012164	ENSLOCG00000008535	19	6611092	15	19869734
ENSXMAG00000012164	ENSXMAG00000010707	ENSLOCG00000008535	15	19869734	19	6611092
ENSXMAG00000011435	ENSXMAG00000013798	ENSLOCG00000008549	24	2160698	7	17899313
ENSXMAG00000013798	ENSXMAG00000011435	ENSLOCG00000008549	7	17899313	24	2160698
ENSXMAG00000014657	ENSXMAG00000014851	ENSLOCG00000008552	10	23815217	16	14402020
ENSXMAG00000014851	ENSXMAG00000014657	ENSLOCG00000008552	16	14402020	10	23815217
ENSXMAG00000012028	ENSXMAG00000013238	ENSLOCG00000008584	16	9925395	5	22946194
ENSXMAG00000013238	ENSXMAG00000012028	ENSLOCG00000008584	5	22946194	16	9925395
ENSXMAG00000010736	ENSXMAG00000029258	ENSLOCG00000008589	8	22776659	12	2636211
ENSXMAG00000029258	ENSXMAG00000010736	ENSLOCG00000008589	12	2636211	8	22776659
ENSXMAG00000007449	ENSXMAG00000025073	ENSLOCG00000008605	5	29011952	14	23852218
ENSXMAG00000025073	ENSXMAG00000007449	ENSLOCG00000008605	14	23852218	5	29011952
ENSXMAG00000001802	ENSXMAG00000011405	ENSLOCG00000008622	7	17978572	4	23410865
ENSXMAG00000011405	ENSXMAG00000001802	ENSLOCG00000008622	4	23410865	7	17978572
ENSXMAG00000007202	ENSXMAG00000017483	ENSLOCG00000008640	11	29492955	18	8311197
ENSXMAG00000017483	ENSXMAG00000007202	ENSLOCG00000008640	18	8311197	11	29492955
ENSXMAG00000002961	ENSXMAG00000029837	ENSLOCG00000008654	7	8856546	12	27091555
ENSXMAG00000029837	ENSXMAG00000002961	ENSLOCG00000008654	12	27091555	7	8856546
ENSXMAG00000007243	ENSXMAG00000017516	ENSLOCG00000008672	11	29336772	18	8171795
ENSXMAG00000017516	ENSXMAG00000007243	ENSLOCG00000008672	18	8171795	11	29336772

ENSXMAG00000005304	ENSXMAG00000018383	ENSLOCG00000008725	10	4321653	21	21403104
ENSXMAG00000018383	ENSXMAG00000005304	ENSLOCG00000008725	21	21403104	10	4321653
ENSXMAG00000000364	ENSXMAG00000010908	ENSLOCG00000008777	15	18068460	19	496621
ENSXMAG00000010908	ENSXMAG00000000364	ENSLOCG00000008777	19	496621	15	18068460
ENSXMAG00000001820	ENSXMAG00000009576	ENSLOCG00000008784	5	4540250	14	23746066
ENSXMAG00000009576	ENSXMAG00000001820	ENSLOCG00000008784	14	23746066	5	4540250
ENSXMAG00000007371	ENSXMAG00000023673	ENSLOCG00000008787	11	20425431	18	7596418
ENSXMAG00000023673	ENSXMAG00000007371	ENSLOCG00000008787	18	7596418	11	20425431
ENSXMAG00000011165	ENSXMAG00000018151	ENSLOCG00000008788	6	5439088	9	22101653
ENSXMAG00000018151	ENSXMAG00000011165	ENSLOCG00000008788	9	22101653	6	5439088
ENSXMAG00000010910	ENSXMAG00000015361	ENSLOCG00000008789	19	759189	15	18086156
ENSXMAG00000015361	ENSXMAG00000010910	ENSLOCG00000008789	15	18086156	19	759189
ENSXMAG00000022020	ENSXMAG00000028449	ENSLOCG00000008807	18	7559383	11	20338234
ENSXMAG00000028449	ENSXMAG00000022020	ENSLOCG00000008807	11	20338234	18	7559383
ENSXMAG00000000156	ENSXMAG00000029202	ENSLOCG00000008829	16	13943058	10	768781
ENSXMAG00000029202	ENSXMAG00000000156	ENSLOCG00000008829	10	768781	16	13943058
ENSXMAG00000029417	ENSXMAG00000029525	ENSLOCG00000008840	10	781207	16	13938309
ENSXMAG00000029525	ENSXMAG00000029417	ENSLOCG00000008840	16	13938309	10	781207
ENSXMAG00000011184	ENSXMAG00000027199	ENSLOCG00000008845	6	5369285	9	21822948
ENSXMAG00000027199	ENSXMAG00000011184	ENSLOCG00000008845	9	21822948	6	5369285
ENSXMAG00000007079	ENSXMAG00000013395	ENSLOCG00000008848	3	12124218	13	14198694
ENSXMAG00000013395	ENSXMAG00000007079	ENSLOCG00000008848	13	14198694	3	12124218
ENSXMAG00000003233	ENSXMAG00000028745	ENSLOCG00000008881	3	12075959	13	23936371
ENSXMAG00000028745	ENSXMAG00000003233	ENSLOCG00000008881	13	23936371	3	12075959
ENSXMAG00000018972	ENSXMAG00000022076	ENSLOCG00000008910	6	16716091	21	13643422
ENSXMAG00000022076	ENSXMAG00000018972	ENSLOCG00000008910	21	13643422	6	16716091
ENSXMAG00000012169	ENSXMAG00000013164	ENSLOCG00000008920	16	10227687	5	22774486
ENSXMAG00000013164	ENSXMAG00000012169	ENSLOCG00000008920	5	22774486	16	10227687
ENSXMAG00000000148	ENSXMAG00000014947	ENSLOCG00000008925	16	13902517	10	792765
ENSXMAG00000014947	ENSXMAG00000000148	ENSLOCG00000008925	10	792765	16	13902517
ENSXMAG00000005256	ENSXMAG00000029349	ENSLOCG00000008928	13	16997165	3	21720287
ENSXMAG00000029349	ENSXMAG00000005256	ENSLOCG00000008928	3	21720287	13	16997165
ENSXMAG00000014989	ENSXMAG00000027955	ENSLOCG00000008938	10	812492	16	13899250
ENSXMAG00000027955	ENSXMAG00000014989	ENSLOCG00000008938	16	13899250	10	812492
ENSXMAG00000007339	ENSXMAG00000016068	ENSLOCG00000008941	11	16956428	18	7642290
ENSXMAG00000016068	ENSXMAG00000007339	ENSLOCG00000008941	18	7642290	11	16956428
ENSXMAG00000009861	ENSXMAG00000023815	ENSLOCG00000008943	16	16347652	10	2468085
ENSXMAG00000023815	ENSXMAG00000009861	ENSLOCG00000008943	10	2468085	16	16347652
ENSXMAG00000007326	ENSXMAG00000010551	ENSLOCG00000008983	11	17224797	18	7803316
ENSXMAG00000010551	ENSXMAG00000007326	ENSLOCG00000008983	18	7803316	11	17224797
ENSXMAG00000009701	ENSXMAG00000028955	ENSLOCG00000008994	13	10648559	3	21007742
ENSXMAG00000028955	ENSXMAG00000009701	ENSLOCG00000008994	3	21007742	13	10648559
ENSXMAG00000002580	ENSXMAG00000005681	ENSLOCG00000009024	5	7107926	22	13926527
ENSXMAG00000005681	ENSXMAG00000002580	ENSLOCG00000009024	22	13926527	5	7107926
ENSXMAG00000009808	ENSXMAG00000026556	ENSLOCG00000009040	5	3501679	14	23344572
ENSXMAG00000026556	ENSXMAG00000009808	ENSLOCG00000009040	14	23344572	5	3501679
ENSXMAG00000000604	ENSXMAG00000009343	ENSLOCG00000009065	19	15405713	19	25483096
ENSXMAG00000009343	ENSXMAG00000000604	ENSLOCG00000009065	19	25483096	19	15405713
ENSXMAG00000002566	ENSXMAG00000005657	ENSLOCG00000009070	5	7013860	22	13864243
ENSXMAG00000005657	ENSXMAG00000002566	ENSLOCG00000009070	22	13864243	5	7013860
ENSXMAG00000009793	ENSXMAG00000026837	ENSLOCG00000009076	14	23406233	5	3461504
ENSXMAG00000026837	ENSXMAG00000009793	ENSLOCG00000009076	5	3461504	14	23406233
ENSXMAG00000007309	ENSXMAG00000026281	ENSLOCG00000009077	11	29062380	18	8003026
ENSXMAG00000026281	ENSXMAG00000007309	ENSLOCG00000009077	18	8003026	11	29062380
ENSXMAG00000002457	ENSXMAG00000026439	ENSLOCG00000009090	19	17473929	9	6195916
ENSXMAG00000026439	ENSXMAG00000002457	ENSLOCG00000009090	9	6195916	19	17473929
ENSXMAG00000011392	ENSXMAG00000012027	ENSLOCG00000009109	24	6019018	7	11719061
ENSXMAG00000012027	ENSXMAG00000011392	ENSLOCG00000009109	7	11719061	24	6019018
ENSXMAG00000000294	ENSXMAG00000010247	ENSLOCG00000009131	23	22174099	11	4821287
ENSXMAG00000010247	ENSXMAG00000000294	ENSLOCG00000009131	11	4821287	23	22174099
ENSXMAG00000007277	ENSXMAG00000017609	ENSLOCG00000009135	11	29122490	18	8056498
ENSXMAG00000017609	ENSXMAG00000007277	ENSLOCG00000009135	18	8056498	11	29122490
ENSXMAG00000009398	ENSXMAG00000011763	ENSLOCG00000009153	15	19123094	19	15456023
ENSXMAG00000011763	ENSXMAG00000009398	ENSLOCG00000009153	19	15456023	15	19123094

ENSXMAG00000015959	ENSXMAG00000025663	ENSLOCG00000009155	8	3613288	12	13941994
ENSXMAG00000025663	ENSXMAG00000015959	ENSLOCG00000009155	12	13941994	8	3613288
ENSXMAG00000000497	ENSXMAG00000023412	ENSLOCG00000009167	23	22254779	11	4117888
ENSXMAG00000023412	ENSXMAG00000000497	ENSLOCG00000009167	11	4117888	23	22254779
ENSXMAG00000018473	ENSXMAG00000027618	ENSLOCG00000009170	6	17925779	9	14178644
ENSXMAG00000027618	ENSXMAG00000018473	ENSLOCG00000009170	9	14178644	6	17925779
ENSXMAG00000011306	ENSXMAG00000013757	ENSLOCG00000009231	24	6183163	7	11650580
ENSXMAG00000013757	ENSXMAG00000011306	ENSLOCG00000009231	7	11650580	24	6183163
ENSXMAG00000024734	ENSXMAG00000026555	ENSLOCG00000009232	7	30218436	24	4391718
ENSXMAG00000026555	ENSXMAG00000024734	ENSLOCG00000009232	24	4391718	7	30218436
ENSXMAG00000006943	ENSXMAG00000007435	ENSLOCG00000009269	18	7251565	11	14560130
ENSXMAG00000007435	ENSXMAG00000006943	ENSLOCG00000009269	11	14560130	18	7251565
ENSXMAG00000000532	ENSXMAG00000008223	ENSLOCG00000009271	23	22380951	11	4038800
ENSXMAG00000008223	ENSXMAG00000000532	ENSLOCG00000009271	11	4038800	23	22380951
ENSXMAG00000021113	ENSXMAG00000024920	ENSLOCG00000009311	19	21129237	23	11133693
ENSXMAG00000024920	ENSXMAG00000021113	ENSLOCG00000009311	23	11133693	19	21129237
ENSXMAG00000000470	ENSXMAG00000019510	ENSLOCG00000009352	23	22577324	11	4383709
ENSXMAG00000019510	ENSXMAG00000000470	ENSLOCG00000009352	11	4383709	23	22577324
ENSXMAG00000005008	ENSXMAG00000022318	ENSLOCG00000009367	11	21380807	18	28894034
ENSXMAG00000022318	ENSXMAG00000005008	ENSLOCG00000009367	18	28894034	11	21380807
ENSXMAG00000008175	ENSXMAG00000028711	ENSLOCG00000009369	7	29834216	24	2980714
ENSXMAG00000028711	ENSXMAG00000008175	ENSLOCG00000009369	24	2980714	7	29834216
ENSXMAG00000014755	ENSXMAG00000015648	ENSLOCG00000009371	6	13872457	18	73969
ENSXMAG00000015648	ENSXMAG00000014755	ENSLOCG00000009371	18	73969	6	13872457
ENSXMAG00000005018	ENSXMAG00000007055	ENSLOCG00000009412	12	19341617	8	3801289
ENSXMAG00000007055	ENSXMAG00000005018	ENSLOCG00000009412	8	3801289	12	19341617
ENSXMAG00000009754	ENSXMAG00000015370	ENSLOCG00000009417	7	18844997	19	16851079
ENSXMAG00000015370	ENSXMAG00000009754	ENSLOCG00000009417	19	16851079	7	18844997
ENSXMAG00000009757	ENSXMAG00000015372	ENSLOCG00000009424	7	18850787	19	16862821
ENSXMAG00000015372	ENSXMAG00000009757	ENSLOCG00000009424	19	16862821	7	18850787
ENSXMAG00000007041	ENSXMAG00000009000	ENSLOCG00000009467	23	18948238	11	14332906
ENSXMAG00000009000	ENSXMAG00000007041	ENSLOCG00000009467	11	14332906	23	18948238
ENSXMAG00000013714	ENSXMAG00000024096	ENSLOCG00000009499	14	10107752	5	5081959
ENSXMAG00000024096	ENSXMAG00000013714	ENSLOCG00000009499	5	5081959	14	10107752
ENSXMAG00000006002	ENSXMAG00000025103	ENSLOCG00000009501	7	11080934	24	14200873
ENSXMAG00000025103	ENSXMAG00000006002	ENSLOCG00000009501	24	14200873	7	11080934
ENSXMAG00000006344	ENSXMAG00000016932	ENSLOCG00000009545	6	19656775	9	9352364
ENSXMAG00000016932	ENSXMAG00000006344	ENSLOCG00000009545	9	9352364	6	19656775
ENSXMAG00000012915	ENSXMAG00000026622	ENSLOCG00000009594	23	30441703	11	19048768
ENSXMAG00000026622	ENSXMAG00000012915	ENSLOCG00000009594	11	19048768	23	30441703
ENSXMAG00000002596	ENSXMAG00000021556	ENSLOCG00000009609	13	1252764	3	29694266
ENSXMAG00000021556	ENSXMAG00000002596	ENSLOCG00000009609	3	29694266	13	1252764
ENSXMAG00000002574	ENSXMAG00000003725	ENSLOCG00000009655	13	1301901	3	29388645
ENSXMAG00000003725	ENSXMAG00000002574	ENSLOCG00000009655	3	29388645	13	1301901
ENSXMAG00000000293	ENSXMAG00000009443	ENSLOCG00000009659	21	10295098	6	8711078
ENSXMAG00000009443	ENSXMAG00000000293	ENSLOCG00000009659	6	8711078	21	10295098
ENSXMAG00000000405	ENSXMAG00000021925	ENSLOCG00000009667	23	16050845	11	23464263
ENSXMAG00000021925	ENSXMAG00000000405	ENSLOCG00000009667	11	23464263	23	16050845
ENSXMAG00000001917	ENSXMAG00000011769	ENSLOCG00000009679	7	12549054	4	23633380
ENSXMAG00000011769	ENSXMAG00000001917	ENSLOCG00000009679	4	23633380	7	12549054
ENSXMAG00000011687	ENSXMAG00000015599	ENSLOCG00000009684	7	19209506	6	8978830
ENSXMAG00000015599	ENSXMAG00000011687	ENSLOCG00000009684	6	8978830	7	19209506
ENSXMAG00000000230	ENSXMAG00000009326	ENSLOCG00000009737	21	10204190	6	8750277
ENSXMAG00000009326	ENSXMAG00000000230	ENSLOCG00000009737	6	8750277	21	10204190
ENSXMAG00000008152	ENSXMAG00000018024	ENSLOCG00000009769	20	7925659	1	16510121
ENSXMAG00000018024	ENSXMAG00000008152	ENSLOCG00000009769	1	16510121	20	7925659
ENSXMAG00000008957	ENSXMAG00000015407	ENSLOCG00000009773	11	10795325	23	18636970
ENSXMAG00000015407	ENSXMAG00000008957	ENSLOCG00000009773	23	18636970	11	10795325
ENSXMAG00000004957	ENSXMAG00000017401	ENSLOCG00000009798	12	16764288	8	18533390
ENSXMAG00000017401	ENSXMAG00000004957	ENSLOCG00000009798	8	18533390	12	16764288
ENSXMAG00000010477	ENSXMAG00000014975	ENSLOCG00000009807	11	8752625	23	22900005
ENSXMAG00000014975	ENSXMAG00000010477	ENSLOCG00000009807	23	22900005	11	8752625
ENSXMAG00000005772	ENSXMAG00000016876	ENSLOCG00000009813	1	11902917	20	1706999
ENSXMAG00000016876	ENSXMAG00000005772	ENSLOCG00000009813	20	1706999	1	11902917

ENSXMAG00000008184	ENSXMAG00000018083	ENSLOCG00000009825	20	7868326	1	16638588
ENSXMAG00000018083	ENSXMAG00000008184	ENSLOCG00000009825	1	16638588	20	7868326
ENSXMAG00000003693	ENSXMAG00000028764	ENSLOCG00000009827	3	28821827	13	1196092
ENSXMAG00000028764	ENSXMAG00000003693	ENSLOCG00000009827	13	1196092	3	28821827
ENSXMAG00000004944	ENSXMAG00000017274	ENSLOCG00000009852	12	16601727	8	18496226
ENSXMAG00000017274	ENSXMAG00000004944	ENSLOCG00000009852	8	18496226	12	16601727
ENSXMAG00000011144	ENSXMAG00000017082	ENSLOCG00000009860	20	1326015	1	12039881
ENSXMAG00000017082	ENSXMAG00000011144	ENSLOCG00000009860	1	12039881	20	1326015
ENSXMAG00000011659	ENSXMAG00000019265	ENSLOCG00000009923	4	21644841	7	12463486
ENSXMAG00000019265	ENSXMAG00000011659	ENSLOCG00000009923	7	12463486	4	21644841
ENSXMAG00000014092	ENSXMAG00000026459	ENSLOCG00000009939	6	22219452	21	24528600
ENSXMAG00000026459	ENSXMAG00000014092	ENSLOCG00000009939	21	24528600	6	22219452
ENSXMAG00000004727	ENSXMAG00000009582	ENSLOCG00000009942	1	23378613	20	820137
ENSXMAG00000009582	ENSXMAG00000004727	ENSLOCG00000009942	20	820137	1	23378613
ENSXMAG00000017940	ENSXMAG00000022264	ENSLOCG00000009988	1	22948427	20	8369045
ENSXMAG00000022264	ENSXMAG00000017940	ENSLOCG00000009988	20	8369045	1	22948427
ENSXMAG00000021074	ENSXMAG00000028295	ENSLOCG00000009997	5	22648146	16	10369456
ENSXMAG00000028295	ENSXMAG00000021074	ENSLOCG00000009997	16	10369456	5	22648146
ENSXMAG00000011334	ENSXMAG00000028424	ENSLOCG00000010030	18	1320910	6	6622327
ENSXMAG00000028424	ENSXMAG00000011334	ENSLOCG00000010030	6	6622327	18	1320910
ENSXMAG00000006498	ENSXMAG00000008738	ENSLOCG00000010111	23	18178481	11	15242939
ENSXMAG00000008738	ENSXMAG00000006498	ENSLOCG00000010111	11	15242939	23	18178481
ENSXMAG00000010468	ENSXMAG00000016399	ENSLOCG00000010113	1	23493541	20	27973376
ENSXMAG00000016399	ENSXMAG00000010468	ENSLOCG00000010113	20	27973376	1	23493541
ENSXMAG00000004439	ENSXMAG00000012351	ENSLOCG00000010122	5	12409514	23	15984968
ENSXMAG00000012351	ENSXMAG00000004439	ENSLOCG00000010122	23	15984968	5	12409514
ENSXMAG00000003378	ENSXMAG00000011463	ENSLOCG00000010123	1	25365772	20	21892848
ENSXMAG00000011463	ENSXMAG00000003378	ENSLOCG00000010123	20	21892848	1	25365772
ENSXMAG00000003004	ENSXMAG00000003465	ENSLOCG00000010137	8	12203540	12	8608250
ENSXMAG00000003465	ENSXMAG00000003004	ENSLOCG00000010137	12	8608250	8	12203540
ENSXMAG00000017760	ENSXMAG00000019976	ENSLOCG00000010167	23	4994631	19	19059209
ENSXMAG00000019976	ENSXMAG00000017760	ENSLOCG00000010167	19	19059209	23	4994631
ENSXMAG00000006854	ENSXMAG00000016385	ENSLOCG00000010177	1	23594396	20	28558960
ENSXMAG00000016385	ENSXMAG00000006854	ENSLOCG00000010177	20	28558960	1	23594396
ENSXMAG00000003470	ENSXMAG00000004780	ENSLOCG00000010196	8	18281861	12	14623244
ENSXMAG00000004780	ENSXMAG00000003470	ENSLOCG00000010196	12	14623244	8	18281861
ENSXMAG00000004941	ENSXMAG00000022329	ENSLOCG00000010232	15	12044472	19	18986342
ENSXMAG00000022329	ENSXMAG00000004941	ENSLOCG00000010232	19	18986342	15	12044472
ENSXMAG00000004514	ENSXMAG00000025257	ENSLOCG00000010249	9	18285347	6	3419010
ENSXMAG00000025257	ENSXMAG00000004514	ENSLOCG00000010249	6	3419010	9	18285347
ENSXMAG00000006136	ENSXMAG00000023811	ENSLOCG00000010280	19	18702779	15	193846
ENSXMAG00000023811	ENSXMAG00000006136	ENSLOCG00000010280	15	193846	19	18702779
ENSXMAG00000000875	ENSXMAG00000024782	ENSLOCG00000010292	1	16399502	20	7328887
ENSXMAG00000024782	ENSXMAG00000000875	ENSLOCG00000010292	20	7328887	1	16399502
ENSXMAG00000008285	ENSXMAG00000009800	ENSLOCG00000010293	7	5559392	24	2762363
ENSXMAG00000009800	ENSXMAG00000008285	ENSLOCG00000010293	24	2762363	7	5559392
ENSXMAG00000000634	ENSXMAG00000025537	ENSLOCG00000010321	15	79726	19	18843915
ENSXMAG00000025537	ENSXMAG00000000634	ENSLOCG00000010321	19	18843915	15	79726
ENSXMAG00000011393	ENSXMAG00000018278	ENSLOCG00000010338	6	5203725	9	21501984
ENSXMAG00000018278	ENSXMAG00000011393	ENSLOCG00000010338	9	21501984	6	5203725
ENSXMAG00000012467	ENSXMAG00000030101	ENSLOCG00000010348	7	1968826	24	6374734
ENSXMAG00000030101	ENSXMAG00000012467	ENSLOCG00000010348	24	6374734	7	1968826
ENSXMAG00000007579	ENSXMAG00000019946	ENSLOCG00000010353	20	12014677	1	17593151
ENSXMAG00000019946	ENSXMAG00000007579	ENSLOCG00000010353	1	17593151	20	12014677
ENSXMAG00000002387	ENSXMAG00000018476	ENSLOCG00000010360	19	20703141	15	5199451
ENSXMAG00000018476	ENSXMAG00000002387	ENSLOCG00000010360	15	5199451	19	20703141
ENSXMAG00000005986	ENSXMAG00000025746	ENSLOCG00000010384	1	25346309	20	12153948
ENSXMAG00000025746	ENSXMAG00000005986	ENSLOCG00000010384	20	12153948	1	25346309
ENSXMAG00000008695	ENSXMAG00000012465	ENSLOCG00000010386	24	6368481	7	5121586
ENSXMAG00000012465	ENSXMAG00000008695	ENSLOCG00000010386	7	5121586	24	6368481
ENSXMAG00000016541	ENSXMAG00000027646	ENSLOCG00000010400	21	16706438	6	18742337
ENSXMAG00000027646	ENSXMAG00000016541	ENSLOCG00000010400	6	18742337	21	16706438
ENSXMAG00000011330	ENSXMAG00000028903	ENSLOCG00000010402	6	5209941	9	21632377
ENSXMAG00000028903	ENSXMAG00000011330	ENSLOCG00000010402	9	21632377	6	5209941

ENSXMAG00000019066	ENSXMAG00000023223	ENSLOCG00000010430	7	21661652	4	20955388
ENSXMAG00000023223	ENSXMAG00000019066	ENSLOCG00000010430	4	20955388	7	21661652
ENSXMAG00000001512	ENSXMAG00000009166	ENSLOCG00000010491	23	19354294	11	5277581
ENSXMAG00000009166	ENSXMAG00000001512	ENSLOCG00000010491	11	5277581	23	19354294
ENSXMAG00000015437	ENSXMAG00000023569	ENSLOCG00000010495	5	17600726	23	30013310
ENSXMAG00000023569	ENSXMAG00000015437	ENSLOCG00000010495	23	30013310	5	17600726
ENSXMAG00000003333	ENSXMAG00000012596	ENSLOCG00000010503	1	27377668	20	21741033
ENSXMAG00000012596	ENSXMAG00000003333	ENSLOCG00000010503	20	21741033	1	27377668
ENSXMAG00000001522	ENSXMAG00000009167	ENSLOCG00000010508	23	19376506	11	5408451
ENSXMAG00000009167	ENSXMAG00000001522	ENSLOCG00000010508	11	5408451	23	19376506
ENSXMAG00000015104	ENSXMAG00000018595	ENSLOCG00000010510	8	22642505	12	2054592
ENSXMAG00000018595	ENSXMAG00000015104	ENSLOCG00000010510	12	2054592	8	22642505
ENSXMAG00000012561	ENSXMAG00000018751	ENSLOCG00000010511	20	23669573	1	27302262
ENSXMAG00000018751	ENSXMAG00000012561	ENSLOCG00000010511	1	27302262	20	23669573
ENSXMAG00000014805	ENSXMAG00000025219	ENSLOCG00000010526	15	12327498	19	8846594
ENSXMAG00000025219	ENSXMAG00000014805	ENSLOCG00000010526	19	8846594	15	12327498
ENSXMAG00000026160	ENSXMAG00000028476	ENSLOCG00000010567	22	14698586	8	22326528
ENSXMAG00000028476	ENSXMAG00000026160	ENSLOCG00000010567	8	22326528	22	14698586
ENSXMAG00000001585	ENSXMAG00000002519	ENSLOCG00000010578	9	1911061	11	5671854
ENSXMAG00000002519	ENSXMAG00000001585	ENSLOCG00000010578	11	5671854	9	1911061
ENSXMAG00000005338	ENSXMAG00000010691	ENSLOCG00000010585	6	6700027	21	21197128
ENSXMAG00000010691	ENSXMAG00000005338	ENSLOCG00000010585	21	21197128	6	6700027
ENSXMAG00000000059	ENSXMAG00000025480	ENSLOCG00000010587	7	19861156	24	14734732
ENSXMAG00000025480	ENSXMAG00000000059	ENSLOCG00000010587	24	14734732	7	19861156
ENSXMAG00000003970	ENSXMAG00000014713	ENSLOCG00000010588	19	8945238	15	12159416
ENSXMAG00000014713	ENSXMAG00000003970	ENSLOCG00000010588	15	12159416	19	8945238
ENSXMAG00000001608	ENSXMAG00000009200	ENSLOCG00000010589	23	19420439	11	5678641
ENSXMAG00000009200	ENSXMAG00000001608	ENSLOCG00000010589	11	5678641	23	19420439
ENSXMAG00000002816	ENSXMAG00000026349	ENSLOCG00000010592	1	16922524	20	7646659
ENSXMAG00000026349	ENSXMAG00000002816	ENSLOCG00000010592	20	7646659	1	16922524
ENSXMAG00000002805	ENSXMAG00000008253	ENSLOCG00000010612	1	16939906	20	7640874
ENSXMAG00000008253	ENSXMAG00000002805	ENSLOCG00000010612	20	7640874	1	16939906
ENSXMAG00000001689	ENSXMAG00000009231	ENSLOCG00000010651	23	19443032	11	5773461
ENSXMAG00000009231	ENSXMAG00000001689	ENSLOCG00000010651	11	5773461	23	19443032
ENSXMAG00000009263	ENSXMAG00000012573	ENSLOCG00000010706	11	6173871	23	19462639
ENSXMAG00000012573	ENSXMAG00000009263	ENSLOCG00000010706	23	19462639	11	6173871
ENSXMAG00000012670	ENSXMAG00000021579	ENSLOCG00000010745	7	592170	24	6859362
ENSXMAG00000021579	ENSXMAG00000012670	ENSLOCG00000010745	24	6859362	7	592170
ENSXMAG00000022061	ENSXMAG00000023589	ENSLOCG00000010752	20	9173111	1	22841245
ENSXMAG00000023589	ENSXMAG00000022061	ENSLOCG00000010752	1	22841245	20	9173111
ENSXMAG00000003039	ENSXMAG00000014761	ENSLOCG00000010783	10	15907157	16	10642198
ENSXMAG00000014761	ENSXMAG00000003039	ENSLOCG00000010783	16	10642198	10	15907157
ENSXMAG00000000760	ENSXMAG00000005349	ENSLOCG00000010828	10	19389218	16	1117980
ENSXMAG00000005349	ENSXMAG00000000760	ENSLOCG00000010828	16	1117980	10	19389218
ENSXMAG00000027508	ENSXMAG00000028007	ENSLOCG00000010905	23	18330789	11	10863673
ENSXMAG00000028007	ENSXMAG00000027508	ENSLOCG00000010905	11	10863673	23	18330789
ENSXMAG00000000011	ENSXMAG00000003234	ENSLOCG00000010906	10	19546153	16	1022467
ENSXMAG00000003234	ENSXMAG00000000011	ENSLOCG00000010906	16	1022467	10	19546153
ENSXMAG00000008426	ENSXMAG00000015095	ENSLOCG00000010934	19	8684421	15	16106837
ENSXMAG00000015095	ENSXMAG00000008426	ENSLOCG00000010934	15	16106837	19	8684421
ENSXMAG00000015114	ENSXMAG00000018152	ENSLOCG00000010949	8	20400272	19	8674749
ENSXMAG00000018152	ENSXMAG00000015114	ENSLOCG00000010949	19	8674749	8	20400272
ENSXMAG00000024169	ENSXMAG00000028632	ENSLOCG00000010954	20	8966901	1	26637431
ENSXMAG00000028632	ENSXMAG00000024169	ENSLOCG00000010954	1	26637431	20	8966901
ENSXMAG00000008092	ENSXMAG00000016669	ENSLOCG00000010957	20	16036996	20	30253814
ENSXMAG00000016669	ENSXMAG00000008092	ENSLOCG00000010957	20	30253814	20	16036996
ENSXMAG00000021981	ENSXMAG00000028316	ENSLOCG00000011037	23	17849730	22	21192562
ENSXMAG00000028316	ENSXMAG00000021981	ENSLOCG00000011037	22	21192562	23	17849730
ENSXMAG00000015236	ENSXMAG00000025371	ENSLOCG00000011077	15	16074674	19	8560202
ENSXMAG00000025371	ENSXMAG00000015236	ENSLOCG00000011077	19	8560202	15	16074674
ENSXMAG00000002157	ENSXMAG00000016718	ENSLOCG00000011093	6	19182326	21	7680807
ENSXMAG00000016718	ENSXMAG00000002157	ENSLOCG00000011093	21	7680807	6	19182326
ENSXMAG00000006394	ENSXMAG00000008554	ENSLOCG00000011097	23	17800895	11	15301682
ENSXMAG00000008554	ENSXMAG00000006394	ENSLOCG00000011097	11	15301682	23	17800895

ENSXMAG00000002124	ENSXMAG00000016754	ENSLOCG00000011117	6	19268196	21	7648234
ENSXMAG00000016754	ENSXMAG00000002124	ENSLOCG00000011117	21	7648234	6	19268196
ENSXMAG00000012966	ENSXMAG00000017142	ENSLOCG00000011140	10	12651593	16	1896604
ENSXMAG00000017142	ENSXMAG00000012966	ENSLOCG00000011140	16	1896604	10	12651593
ENSXMAG00000001881	ENSXMAG00000022847	ENSLOCG00000011142	20	27903599	20	20155528
ENSXMAG00000022847	ENSXMAG00000001881	ENSLOCG00000011142	20	20155528	20	27903599
ENSXMAG00000001160	ENSXMAG00000003443	ENSLOCG00000011157	12	14613238	8	18695965
ENSXMAG00000003443	ENSXMAG00000001160	ENSLOCG00000011157	8	18695965	12	14613238
ENSXMAG00000016769	ENSXMAG00000018876	ENSLOCG00000011174	3	8065436	13	25455089
ENSXMAG00000018876	ENSXMAG00000016769	ENSLOCG00000011174	13	25455089	3	8065436
ENSXMAG00000002110	ENSXMAG00000016811	ENSLOCG00000011176	6	19298610	21	7626427
ENSXMAG00000016811	ENSXMAG00000002110	ENSLOCG00000011176	21	7626427	6	19298610
ENSXMAG00000016760	ENSXMAG00000018910	ENSLOCG00000011187	3	8308535	13	25410036
ENSXMAG00000018910	ENSXMAG00000016760	ENSLOCG00000011187	13	25410036	3	8308535
ENSXMAG00000005112	ENSXMAG00000016129	ENSLOCG00000011190	12	14558713	8	18739686
ENSXMAG00000016129	ENSXMAG00000005112	ENSLOCG00000011190	8	18739686	12	14558713
ENSXMAG00000004086	ENSXMAG00000023235	ENSLOCG00000011204	1	13982968	20	23236745
ENSXMAG00000023235	ENSXMAG00000004086	ENSLOCG00000011204	20	23236745	1	13982968
ENSXMAG00000009997	ENSXMAG00000014053	ENSLOCG00000011273	16	15547777	5	27524467
ENSXMAG00000014053	ENSXMAG00000009997	ENSLOCG00000011273	5	27524467	16	15547777
ENSXMAG00000001406	ENSXMAG00000018817	ENSLOCG00000011356	20	30889690	1	28284098
ENSXMAG00000018817	ENSXMAG00000001406	ENSLOCG00000011356	1	28284098	20	30889690
ENSXMAG00000000015	ENSXMAG00000010043	ENSLOCG00000011363	5	27110496	16	974892
ENSXMAG00000010043	ENSXMAG00000000015	ENSLOCG00000011363	16	974892	5	27110496
ENSXMAG00000022583	ENSXMAG00000023809	ENSLOCG00000011379	5	27038056	16	948756
ENSXMAG00000023809	ENSXMAG00000022583	ENSLOCG00000011379	16	948756	5	27038056
ENSXMAG00000008551	ENSXMAG00000010149	ENSLOCG00000011410	10	6802252	16	6317629
ENSXMAG00000010149	ENSXMAG00000008551	ENSLOCG00000011410	16	6317629	10	6802252
ENSXMAG00000008197	ENSXMAG00000023930	ENSLOCG00000011420	10	14410759	19	1535315
ENSXMAG00000023930	ENSXMAG00000008197	ENSLOCG00000011420	19	1535315	10	14410759
ENSXMAG00000001200	ENSXMAG00000008034	ENSLOCG00000011442	5	26592848	16	18644092
ENSXMAG00000008034	ENSXMAG00000001200	ENSLOCG00000011442	16	18644092	5	26592848
ENSXMAG00000002178	ENSXMAG00000025866	ENSLOCG00000011485	10	6889120	16	6719381
ENSXMAG00000025866	ENSXMAG00000002178	ENSLOCG00000011485	16	6719381	10	6889120
ENSXMAG00000008387	ENSXMAG00000014404	ENSLOCG00000011522	10	24774484	16	5886548
ENSXMAG00000014404	ENSXMAG00000008387	ENSLOCG00000011522	16	5886548	10	24774484
ENSXMAG00000001279	ENSXMAG00000009685	ENSLOCG00000011526	10	22296191	16	18263511
ENSXMAG00000009685	ENSXMAG00000001279	ENSLOCG00000011526	16	18263511	10	22296191
ENSXMAG00000014677	ENSXMAG00000016107	ENSLOCG00000011528	5	13991725	23	393911
ENSXMAG00000016107	ENSXMAG00000014677	ENSLOCG00000011528	23	393911	5	13991725
ENSXMAG00000005345	ENSXMAG00000006322	ENSLOCG00000011541	12	4555822	8	21663653
ENSXMAG00000006322	ENSXMAG00000005345	ENSLOCG00000011541	8	21663653	12	4555822
ENSXMAG00000000577	ENSXMAG00000015176	ENSLOCG00000011542	3	14807538	13	22232758
ENSXMAG00000015176	ENSXMAG00000000577	ENSLOCG00000011542	13	22232758	3	14807538
ENSXMAG00000005844	ENSXMAG00000019096	ENSLOCG00000011577	23	5347786	5	15212725
ENSXMAG00000019096	ENSXMAG00000005844	ENSLOCG00000011577	5	15212725	23	5347786
ENSXMAG00000000663	ENSXMAG00000003726	ENSLOCG00000011582	16	19260728	10	22391864
ENSXMAG00000003726	ENSXMAG00000000663	ENSLOCG00000011582	10	22391864	16	19260728
ENSXMAG00000008317	ENSXMAG00000026693	ENSLOCG00000011584	10	5089656	16	5693733
ENSXMAG00000026693	ENSXMAG00000008317	ENSLOCG00000011584	16	5693733	10	5089656
ENSXMAG00000000678	ENSXMAG00000026333	ENSLOCG00000011595	16	19290930	10	22418036
ENSXMAG00000026333	ENSXMAG00000000678	ENSLOCG00000011595	10	22418036	16	19290930
ENSXMAG00000000683	ENSXMAG00000006870	ENSLOCG00000011607	16	19321290	10	22434319
ENSXMAG00000006870	ENSXMAG00000000683	ENSLOCG00000011607	10	22434319	16	19321290
ENSXMAG00000008446	ENSXMAG00000024161	ENSLOCG00000011645	19	5228151	15	16168497
ENSXMAG00000024161	ENSXMAG00000008446	ENSLOCG00000011645	15	16168497	19	5228151
ENSXMAG00000000838	ENSXMAG00000013995	ENSLOCG00000011653	3	15432894	13	21232909
ENSXMAG00000013995	ENSXMAG00000000838	ENSLOCG00000011653	13	21232909	3	15432894
ENSXMAG00000008287	ENSXMAG00000016920	ENSLOCG00000011654	10	4816808	16	5638252
ENSXMAG00000016920	ENSXMAG00000008287	ENSLOCG00000011654	16	5638252	10	4816808
ENSXMAG00000008204	ENSXMAG00000017049	ENSLOCG00000011662	10	4694776	16	5122991
ENSXMAG00000017049	ENSXMAG00000008204	ENSLOCG00000011662	16	5122991	10	4694776
ENSXMAG00000000115	ENSXMAG00000003577	ENSLOCG00000011671	22	24334870	5	21064604
ENSXMAG00000003577	ENSXMAG00000000115	ENSLOCG00000011671	5	21064604	22	24334870

ENSXMAG00000021661	ENSXMAG00000025032	ENSLOCG00000011679	22	24370603	5	21022169
ENSXMAG00000025032	ENSXMAG00000021661	ENSLOCG00000011679	5	21022169	22	24370603
ENSXMAG00000001083	ENSXMAG00000013928	ENSLOCG00000011683	3	15359175	13	20404848
ENSXMAG00000013928	ENSXMAG00000001083	ENSLOCG00000011683	13	20404848	3	15359175
ENSXMAG00000001077	ENSXMAG00000012546	ENSLOCG00000011695	10	1369668	16	19506242
ENSXMAG00000012546	ENSXMAG00000001077	ENSLOCG00000011695	16	19506242	10	1369668
ENSXMAG00000018329	ENSXMAG00000022120	ENSLOCG00000011696	16	4742768	10	4400570
ENSXMAG00000022120	ENSXMAG00000018329	ENSLOCG00000011696	10	4400570	16	4742768
ENSXMAG00000018332	ENSXMAG00000020987	ENSLOCG00000011714	16	4695882	10	4371527
ENSXMAG00000020987	ENSXMAG00000018332	ENSLOCG00000011714	10	4371527	16	4695882
ENSXMAG00000005784	ENSXMAG00000029312	ENSLOCG00000011770	5	20901346	22	24438366
ENSXMAG00000029312	ENSXMAG00000005784	ENSLOCG00000011770	22	24438366	5	20901346
ENSXMAG00000001035	ENSXMAG00000022732	ENSLOCG00000011778	3	15306783	13	20760326
ENSXMAG00000022732	ENSXMAG00000001035	ENSLOCG00000011778	13	20760326	3	15306783
ENSXMAG00000001318	ENSXMAG00000005095	ENSLOCG00000011794	5	19978828	16	19564506
ENSXMAG00000005095	ENSXMAG00000001318	ENSLOCG00000011794	16	19564506	5	19978828
ENSXMAG00000028422	ENSXMAG00000029054	ENSLOCG00000011830	13	20607372	3	15266881
ENSXMAG00000029054	ENSXMAG00000028422	ENSLOCG00000011830	3	15266881	13	20607372
ENSXMAG00000007352	ENSXMAG00000026427	ENSLOCG00000011833	5	20836281	22	5700423
ENSXMAG00000026427	ENSXMAG00000007352	ENSLOCG00000011833	22	5700423	5	20836281
ENSXMAG00000001603	ENSXMAG00000021473	ENSLOCG00000011834	5	10116420	16	19593432
ENSXMAG00000021473	ENSXMAG00000001603	ENSLOCG00000011834	16	19593432	5	10116420
ENSXMAG00000001062	ENSXMAG00000026913	ENSLOCG00000011842	3	15254579	13	20586618
ENSXMAG00000026913	ENSXMAG00000001062	ENSLOCG00000011842	13	20586618	3	15254579
ENSXMAG00000013824	ENSXMAG00000016886	ENSLOCG00000011862	10	12068932	16	7604824
ENSXMAG00000016886	ENSXMAG00000013824	ENSLOCG00000011862	16	7604824	10	12068932
ENSXMAG00000007789	ENSXMAG00000015589	ENSLOCG00000011894	10	9337725	16	7302050
ENSXMAG00000015589	ENSXMAG00000007789	ENSLOCG00000011894	16	7302050	10	9337725
ENSXMAG00000022941	ENSXMAG00000028587	ENSLOCG00000011934	16	19817789	5	10061961
ENSXMAG00000028587	ENSXMAG00000022941	ENSLOCG00000011934	5	10061961	16	19817789
ENSXMAG00000017518	ENSXMAG00000022792	ENSLOCG00000011960	23	17633859	4	11258383
ENSXMAG00000022792	ENSXMAG00000017518	ENSLOCG00000011960	4	11258383	23	17633859
ENSXMAG00000006209	ENSXMAG00000027718	ENSLOCG00000011974	5	11137065	16	19867593
ENSXMAG00000027718	ENSXMAG00000006209	ENSLOCG00000011974	16	19867593	5	11137065
ENSXMAG00000005912	ENSXMAG00000010681	ENSLOCG00000011990	14	20759915	5	15095270
ENSXMAG00000010681	ENSXMAG00000005912	ENSLOCG00000011990	5	15095270	14	20759915
ENSXMAG00000022007	ENSXMAG00000022735	ENSLOCG00000012003	14	20745457	6	12775940
ENSXMAG00000022735	ENSXMAG00000022007	ENSLOCG00000012003	6	12775940	14	20745457
ENSXMAG00000006407	ENSXMAG00000008955	ENSLOCG00000012010	16	21263343	5	8636108
ENSXMAG00000008955	ENSXMAG00000006407	ENSLOCG00000012010	5	8636108	16	21263343
ENSXMAG00000006405	ENSXMAG00000009093	ENSLOCG00000012029	16	21254559	5	8623859
ENSXMAG00000009093	ENSXMAG00000006405	ENSLOCG00000012029	5	8623859	16	21254559
ENSXMAG00000002099	ENSXMAG00000005936	ENSLOCG00000012034	5	15009509	14	20710722
ENSXMAG00000005936	ENSXMAG00000002099	ENSLOCG00000012034	14	20710722	5	15009509
ENSXMAG00000009106	ENSXMAG00000020944	ENSLOCG00000012045	5	8575792	16	21236499
ENSXMAG00000020944	ENSXMAG00000009106	ENSLOCG00000012045	16	21236499	5	8575792
ENSXMAG00000004607	ENSXMAG00000005288	ENSLOCG00000012051	5	19698970	16	20812618
ENSXMAG00000005288	ENSXMAG00000004607	ENSLOCG00000012051	16	20812618	5	19698970
ENSXMAG00000008209	ENSXMAG00000017002	ENSLOCG00000012065	10	4756351	16	5205886
ENSXMAG00000017002	ENSXMAG00000008209	ENSLOCG00000012065	16	5205886	10	4756351
ENSXMAG00000000750	ENSXMAG00000005241	ENSLOCG00000012127	10	19069303	16	1125346
ENSXMAG00000005241	ENSXMAG00000000750	ENSLOCG00000012127	16	1125346	10	19069303
ENSXMAG00000013166	ENSXMAG00000027975	ENSLOCG00000012185	10	18632346	16	1313550
ENSXMAG00000027975	ENSXMAG00000013166	ENSLOCG00000012185	16	1313550	10	18632346
ENSXMAG00000008244	ENSXMAG00000014532	ENSLOCG00000012189	6	30108615	21	5826931
ENSXMAG00000014532	ENSXMAG00000008244	ENSLOCG00000012189	21	5826931	6	30108615
ENSXMAG00000019835	ENSXMAG00000028405	ENSLOCG00000012193	19	680437	23	1434403
ENSXMAG00000028405	ENSXMAG00000019835	ENSLOCG00000012193	23	1434403	19	680437
ENSXMAG00000002337	ENSXMAG00000015293	ENSLOCG00000012198	19	722988	23	1847590
ENSXMAG00000015293	ENSXMAG00000002337	ENSLOCG00000012198	23	1847590	19	722988
ENSXMAG00000011644	ENSXMAG00000013158	ENSLOCG00000012201	16	1413494	10	18505003
ENSXMAG00000013158	ENSXMAG00000011644	ENSLOCG00000012201	10	18505003	16	1413494
ENSXMAG00000012903	ENSXMAG00000025280	ENSLOCG00000012233	10	17530704	16	2709533
ENSXMAG00000025280	ENSXMAG00000012903	ENSLOCG00000012233	16	2709533	10	17530704

ENSXMAG00000004645	ENSXMAG00000006770	ENSLOCG00000012249	6	27744600	21	18317154
ENSXMAG00000006770	ENSXMAG00000004645	ENSLOCG00000012249	21	18317154	6	27744600
ENSXMAG00000009223	ENSXMAG00000016947	ENSLOCG00000012256	10	12229980	16	7801574
ENSXMAG00000016947	ENSXMAG00000009223	ENSLOCG00000012256	16	7801574	10	12229980
ENSXMAG00000005745	ENSXMAG00000008444	ENSLOCG00000012324	14	16547549	5	15302364
ENSXMAG00000008444	ENSXMAG00000005745	ENSLOCG00000012324	5	15302364	14	16547549
ENSXMAG00000005739	ENSXMAG00000008441	ENSLOCG00000012328	14	16522301	5	15309759
ENSXMAG00000008441	ENSXMAG00000005739	ENSLOCG00000012328	5	15309759	14	16522301
ENSXMAG00000006812	ENSXMAG00000014436	ENSLOCG00000012337	19	9579169	15	14958345
ENSXMAG00000014436	ENSXMAG00000006812	ENSLOCG00000012337	15	14958345	19	9579169
ENSXMAG00000017382	ENSXMAG00000017606	ENSLOCG00000012344	10	13955994	16	25539871
ENSXMAG00000017606	ENSXMAG00000017382	ENSLOCG00000012344	16	25539871	10	13955994
ENSXMAG00000007056	ENSXMAG00000012758	ENSLOCG00000012358	22	17266239	5	23490031
ENSXMAG00000012758	ENSXMAG00000007056	ENSLOCG00000012358	5	23490031	22	17266239
ENSXMAG00000014401	ENSXMAG00000027736	ENSLOCG00000012364	19	9617720	15	6270984
ENSXMAG00000027736	ENSXMAG00000014401	ENSLOCG00000012364	15	6270984	19	9617720
ENSXMAG00000024355	ENSXMAG00000029848	ENSLOCG00000012370	10	14125934	16	3233509
ENSXMAG00000029848	ENSXMAG00000024355	ENSLOCG00000012370	16	3233509	10	14125934
ENSXMAG00000002363	ENSXMAG00000003829	ENSLOCG00000012376	6	27181308	21	8233656
ENSXMAG00000003829	ENSXMAG00000002363	ENSLOCG00000012376	21	8233656	6	27181308
ENSXMAG00000007059	ENSXMAG00000012762	ENSLOCG00000012377	22	17241514	5	23346610
ENSXMAG00000012762	ENSXMAG00000007059	ENSLOCG00000012377	5	23346610	22	17241514
ENSXMAG00000015502	ENSXMAG00000025635	ENSLOCG00000012384	23	26179222	19	7882473
ENSXMAG00000025635	ENSXMAG00000015502	ENSLOCG00000012384	19	7882473	23	26179222
ENSXMAG00000007856	ENSXMAG00000014370	ENSLOCG00000012388	19	9669317	15	6138422
ENSXMAG00000014370	ENSXMAG00000007856	ENSLOCG00000012388	15	6138422	19	9669317
ENSXMAG00000007826	ENSXMAG00000014355	ENSLOCG00000012423	19	9720860	15	6075070
ENSXMAG00000014355	ENSXMAG00000007826	ENSLOCG00000012423	15	6075070	19	9720860
ENSXMAG00000016197	ENSXMAG00000021816	ENSLOCG00000012429	19	15336583	23	11283925
ENSXMAG00000021816	ENSXMAG00000016197	ENSLOCG00000012429	23	11283925	19	15336583
ENSXMAG00000007798	ENSXMAG00000014341	ENSLOCG00000012443	19	9736276	15	6020446
ENSXMAG00000014341	ENSXMAG00000007798	ENSLOCG00000012443	15	6020446	19	9736276
ENSXMAG00000017092	ENSXMAG00000029593	ENSLOCG00000012460	16	23782940	10	12582158
ENSXMAG00000029593	ENSXMAG00000017092	ENSLOCG00000012460	10	12582158	16	23782940
ENSXMAG00000012137	ENSXMAG00000024797	ENSLOCG00000012498	10	21523198	16	11872939
ENSXMAG00000024797	ENSXMAG00000012137	ENSLOCG00000012498	16	11872939	10	21523198
ENSXMAG00000007770	ENSXMAG00000025396	ENSLOCG00000012512	19	9863979	15	5797035
ENSXMAG00000025396	ENSXMAG00000007770	ENSLOCG00000012512	15	5797035	19	9863979
ENSXMAG00000015921	ENSXMAG00000021023	ENSLOCG00000012516	10	10014803	22	2909078
ENSXMAG00000021023	ENSXMAG00000015921	ENSLOCG00000012516	22	2909078	10	10014803
ENSXMAG00000002918	ENSXMAG00000015430	ENSLOCG00000012521	12	27271184	8	2936719
ENSXMAG00000015430	ENSXMAG00000002918	ENSLOCG00000012521	8	2936719	12	27271184
ENSXMAG00000021695	ENSXMAG00000026852	ENSLOCG00000012534	12	29395703	11	1852519
ENSXMAG00000026852	ENSXMAG00000021695	ENSLOCG00000012534	11	1852519	12	29395703
ENSXMAG00000012255	ENSXMAG00000023416	ENSLOCG00000012550	5	21672915	16	11698678
ENSXMAG00000023416	ENSXMAG00000012255	ENSLOCG00000012550	16	11698678	5	21672915
ENSXMAG00000008959	ENSXMAG00000026045	ENSLOCG00000012553	10	7111127	16	6994324
ENSXMAG00000026045	ENSXMAG00000008959	ENSLOCG00000012553	16	6994324	10	7111127
ENSXMAG00000005692	ENSXMAG00000021530	ENSLOCG00000012556	14	15725766	5	15380105
ENSXMAG00000021530	ENSXMAG00000005692	ENSLOCG00000012556	5	15380105	14	15725766
ENSXMAG00000012277	ENSXMAG00000012951	ENSLOCG00000012558	5	21662114	16	11687086
ENSXMAG00000012951	ENSXMAG00000012277	ENSLOCG00000012558	16	11687086	5	21662114
ENSXMAG00000005682	ENSXMAG00000027561	ENSLOCG00000012560	14	13870664	5	15401213
ENSXMAG00000027561	ENSXMAG00000005682	ENSLOCG00000012560	5	15401213	14	13870664
ENSXMAG00000008886	ENSXMAG00000010258	ENSLOCG00000012589	10	6995144	16	6818140
ENSXMAG00000010258	ENSXMAG00000008886	ENSLOCG00000012589	16	6818140	10	6995144
ENSXMAG00000012330	ENSXMAG00000023668	ENSLOCG00000012591	5	21503842	16	11646868
ENSXMAG00000023668	ENSXMAG00000012330	ENSLOCG00000012591	16	11646868	5	21503842
ENSXMAG00000022730	ENSXMAG00000027090	ENSLOCG00000012612	23	25823175	11	18450104
ENSXMAG00000027090	ENSXMAG00000022730	ENSLOCG00000012612	11	18450104	23	25823175
ENSXMAG00000008856	ENSXMAG00000010242	ENSLOCG00000012614	10	6981830	16	6801307
ENSXMAG00000010242	ENSXMAG00000008856	ENSLOCG00000012614	16	6801307	10	6981830
ENSXMAG00000007693	ENSXMAG00000030056	ENSLOCG00000012618	19	10101800	15	5567595
ENSXMAG00000030056	ENSXMAG00000007693	ENSLOCG00000012618	15	5567595	19	10101800

ENSXMAG00000003899	ENSXMAG00000016494	ENSLOCG00000012627	12	15259809	8	1445183
ENSXMAG00000016494	ENSXMAG00000003899	ENSLOCG00000012627	8	1445183	12	15259809
ENSXMAG00000002862	ENSXMAG00000017646	ENSLOCG00000012636	10	14251691	22	14749341
ENSXMAG00000017646	ENSXMAG00000002862	ENSLOCG00000012636	22	14749341	10	14251691
ENSXMAG00000008800	ENSXMAG00000010204	ENSLOCG00000012637	10	6953162	16	6768833
ENSXMAG00000010204	ENSXMAG00000008800	ENSLOCG00000012637	16	6768833	10	6953162
ENSXMAG00000010246	ENSXMAG00000025617	ENSLOCG00000012660	14	21932014	5	2195137
ENSXMAG00000025617	ENSXMAG00000010246	ENSLOCG00000012660	5	2195137	14	21932014
ENSXMAG00000008750	ENSXMAG00000025831	ENSLOCG00000012674	10	6932522	16	6753018
ENSXMAG00000025831	ENSXMAG00000008750	ENSLOCG00000012674	16	6753018	10	6932522
ENSXMAG00000010255	ENSXMAG00000021224	ENSLOCG00000012682	14	21941332	5	2072272
ENSXMAG00000021224	ENSXMAG00000010255	ENSLOCG00000012682	5	2072272	14	21941332
ENSXMAG00000013477	ENSXMAG00000024078	ENSLOCG00000012685	10	9560482	22	13280335
ENSXMAG00000024078	ENSXMAG00000013477	ENSLOCG00000012685	22	13280335	10	9560482
ENSXMAG00000000246	ENSXMAG00000012376	ENSLOCG00000012695	16	11534763	5	21421836
ENSXMAG00000012376	ENSXMAG00000000246	ENSLOCG00000012695	5	21421836	16	11534763
ENSXMAG00000011299	ENSXMAG00000018369	ENSLOCG00000012708	19	14651043	1	12333785
ENSXMAG00000018369	ENSXMAG00000011299	ENSLOCG00000012708	1	12333785	19	14651043
ENSXMAG00000021192	ENSXMAG00000027086	ENSLOCG00000012720	16	11449689	10	9642306
ENSXMAG00000027086	ENSXMAG00000021192	ENSLOCG00000012720	10	9642306	16	11449689
ENSXMAG00000009945	ENSXMAG00000025000	ENSLOCG00000012752	16	6553216	10	7333878
ENSXMAG00000025000	ENSXMAG00000009945	ENSLOCG00000012752	10	7333878	16	6553216
ENSXMAG00000001349	ENSXMAG00000016875	ENSLOCG00000012758	12	15811184	8	915880
ENSXMAG00000016875	ENSXMAG00000001349	ENSLOCG00000012758	8	915880	12	15811184
ENSXMAG00000011141	ENSXMAG00000020865	ENSLOCG00000012771	16	11360600	10	23197639
ENSXMAG00000020865	ENSXMAG00000011141	ENSLOCG00000012771	10	23197639	16	11360600
ENSXMAG00000007614	ENSXMAG00000008396	ENSLOCG00000012787	21	5192406	6	20277590
ENSXMAG00000008396	ENSXMAG00000007614	ENSLOCG00000012787	6	20277590	21	5192406
ENSXMAG00000013379	ENSXMAG00000014472	ENSLOCG00000012802	5	6728407	22	13610453
ENSXMAG00000014472	ENSXMAG00000013379	ENSLOCG00000012802	22	13610453	5	6728407
ENSXMAG00000008471	ENSXMAG00000025571	ENSLOCG00000012805	16	6115832	10	7211847
ENSXMAG00000025571	ENSXMAG00000008471	ENSLOCG00000012805	10	7211847	16	6115832
ENSXMAG00000006610	ENSXMAG00000009599	ENSLOCG00000012808	15	4375937	19	10574142
ENSXMAG00000009599	ENSXMAG00000006610	ENSLOCG00000012808	19	10574142	15	4375937
ENSXMAG00000005595	ENSXMAG00000010024	ENSLOCG00000012810	23	15397750	5	19160155
ENSXMAG00000010024	ENSXMAG00000005595	ENSLOCG00000012810	5	19160155	23	15397750
ENSXMAG00000010008	ENSXMAG00000028542	ENSLOCG00000012813	5	19210986	23	15454594
ENSXMAG00000028542	ENSXMAG00000010008	ENSLOCG00000012813	23	15454594	5	19210986
ENSXMAG00000006597	ENSXMAG00000009603	ENSLOCG00000012816	15	4365681	19	10614455
ENSXMAG00000009603	ENSXMAG00000006597	ENSLOCG00000012816	19	10614455	15	4365681
ENSXMAG00000017225	ENSXMAG00000025717	ENSLOCG00000012818	16	10786220	10	13319361
ENSXMAG00000025717	ENSXMAG00000017225	ENSLOCG00000012818	10	13319361	16	10786220
ENSXMAG00000005648	ENSXMAG00000027912	ENSLOCG00000012827	22	13767408	5	6941945
ENSXMAG00000027912	ENSXMAG00000005648	ENSLOCG00000012827	5	6941945	22	13767408
ENSXMAG00000006554	ENSXMAG00000009610	ENSLOCG00000012854	15	4345105	19	10659109
ENSXMAG00000009610	ENSXMAG00000006554	ENSLOCG00000012854	19	10659109	15	4345105
ENSXMAG00000012510	ENSXMAG00000026217	ENSLOCG00000012855	23	17548139	5	12632255
ENSXMAG00000026217	ENSXMAG00000012510	ENSLOCG00000012855	5	12632255	23	17548139
ENSXMAG00000006477	ENSXMAG00000009631	ENSLOCG00000012891	15	4300882	19	10759898
ENSXMAG00000009631	ENSXMAG00000006477	ENSLOCG00000012891	19	10759898	15	4300882
ENSXMAG00000012739	ENSXMAG00000014473	ENSLOCG00000012913	10	16427916	16	10979007
ENSXMAG00000014473	ENSXMAG00000012739	ENSLOCG00000012913	16	10979007	10	16427916
ENSXMAG00000015398	ENSXMAG00000025590	ENSLOCG00000012919	15	4228923	19	8148789
ENSXMAG00000025590	ENSXMAG00000015398	ENSLOCG00000012919	19	8148789	15	4228923
ENSXMAG00000001628	ENSXMAG00000021636	ENSLOCG00000012933	12	15964856	8	14083048
ENSXMAG00000021636	ENSXMAG00000001628	ENSLOCG00000012933	8	14083048	12	15964856
ENSXMAG00000006717	ENSXMAG00000015406	ENSLOCG00000012939	19	7954661	15	4499076
ENSXMAG00000015406	ENSXMAG00000006717	ENSLOCG00000012939	15	4499076	19	7954661
ENSXMAG00000001635	ENSXMAG00000017124	ENSLOCG00000012942	12	16020706	8	14125607
ENSXMAG00000017124	ENSXMAG00000001635	ENSLOCG00000012942	8	14125607	12	16020706
ENSXMAG00000013225	ENSXMAG00000016929	ENSLOCG00000012949	6	19609904	9	17407530
ENSXMAG00000016929	ENSXMAG00000013225	ENSLOCG00000012949	9	17407530	6	19609904
ENSXMAG00000023445	ENSXMAG00000027096	ENSLOCG00000012958	22	27957993	10	6190155
ENSXMAG00000027096	ENSXMAG00000023445	ENSLOCG00000012958	10	6190155	22	27957993

ENSXMAG00000010812	ENSXMAG00000017938	ENSLOCG00000012959	10	15218032	16	23283969
ENSXMAG00000017938	ENSXMAG00000010812	ENSLOCG00000012959	16	23283969	10	15218032
ENSXMAG00000008384	ENSXMAG00000009679	ENSLOCG00000012969	5	13216080	23	17466352
ENSXMAG00000009679	ENSXMAG00000008384	ENSLOCG00000012969	23	17466352	5	13216080
ENSXMAG00000006166	ENSXMAG00000016372	ENSLOCG00000012976	6	16671818	9	8498255
ENSXMAG00000016372	ENSXMAG00000006166	ENSLOCG00000012976	9	8498255	6	16671818
ENSXMAG00000007544	ENSXMAG00000013610	ENSLOCG00000012977	19	6164299	15	5220946
ENSXMAG00000013610	ENSXMAG00000007544	ENSLOCG00000012977	15	5220946	19	6164299
ENSXMAG00000010829	ENSXMAG00000017922	ENSLOCG00000013012	10	14996395	16	23210255
ENSXMAG00000017922	ENSXMAG00000010829	ENSLOCG00000013012	16	23210255	10	14996395
ENSXMAG00000006258	ENSXMAG00000023204	ENSLOCG00000013015	6	16462085	9	8862808
ENSXMAG00000023204	ENSXMAG00000006258	ENSLOCG00000013015	9	8862808	6	16462085
ENSXMAG00000016931	ENSXMAG00000029828	ENSLOCG00000013017	1	20404220	20	18243940
ENSXMAG00000029828	ENSXMAG00000016931	ENSLOCG00000013017	20	18243940	1	20404220
ENSXMAG00000016028	ENSXMAG00000017341	ENSLOCG00000013046	10	13874712	16	25315693
ENSXMAG00000017341	ENSXMAG00000016028	ENSLOCG00000013046	16	25315693	10	13874712
ENSXMAG00000016816	ENSXMAG00000028548	ENSLOCG00000013051	9	8957883	6	16180087
ENSXMAG00000028548	ENSXMAG00000016816	ENSLOCG00000013051	6	16180087	9	8957883
ENSXMAG00000016710	ENSXMAG00000023975	ENSLOCG00000013055	16	11136678	10	11799707
ENSXMAG00000023975	ENSXMAG00000016710	ENSLOCG00000013055	10	11799707	16	11136678
ENSXMAG00000006335	ENSXMAG00000016847	ENSLOCG00000013065	6	16200473	9	9185645
ENSXMAG00000016847	ENSXMAG00000006335	ENSLOCG00000013065	9	9185645	6	16200473
ENSXMAG00000019029	ENSXMAG00000022228	ENSLOCG00000013069	23	11654020	6	10894018
ENSXMAG00000022228	ENSXMAG00000019029	ENSLOCG00000013069	6	10894018	23	11654020
ENSXMAG00000007470	ENSXMAG00000017432	ENSLOCG00000013077	20	17545653	1	17809327
ENSXMAG00000017432	ENSXMAG00000007470	ENSLOCG00000013077	1	17809327	20	17545653
ENSXMAG00000006331	ENSXMAG00000028732	ENSLOCG00000013080	6	16250517	9	9120641
ENSXMAG00000028732	ENSXMAG00000006331	ENSLOCG00000013080	9	9120641	6	16250517
ENSXMAG00000006325	ENSXMAG00000016888	ENSLOCG00000013086	6	16258828	9	9114930
ENSXMAG00000016888	ENSXMAG00000006325	ENSLOCG00000013086	9	9114930	6	16258828
ENSXMAG00000006304	ENSXMAG00000016898	ENSLOCG00000013092	6	16264690	9	9053634
ENSXMAG00000016898	ENSXMAG00000006304	ENSLOCG00000013092	9	9053634	6	16264690
ENSXMAG00000012564	ENSXMAG00000016830	ENSLOCG00000013094	10	12000174	16	11200162
ENSXMAG00000016830	ENSXMAG00000012564	ENSLOCG00000013094	16	11200162	10	12000174
ENSXMAG00000006293	ENSXMAG00000016948	ENSLOCG00000013116	6	16329799	9	9002833
ENSXMAG00000016948	ENSXMAG00000006293	ENSLOCG00000013116	9	9002833	6	16329799
ENSXMAG00000012603	ENSXMAG00000023407	ENSLOCG00000013122	10	11976218	16	11187671
ENSXMAG00000023407	ENSXMAG00000012603	ENSLOCG00000013122	16	11187671	10	11976218
ENSXMAG00000012649	ENSXMAG00000016778	ENSLOCG00000013126	10	11968756	16	11171037
ENSXMAG00000016778	ENSXMAG00000012649	ENSLOCG00000013126	16	11171037	10	11968756
ENSXMAG00000012781	ENSXMAG00000014836	ENSLOCG00000013174	10	15817220	16	10666440
ENSXMAG00000014836	ENSXMAG00000012781	ENSLOCG00000013174	16	10666440	10	15817220
ENSXMAG00000025314	ENSXMAG00000025640	ENSLOCG00000013186	1	20331051	20	3175552
ENSXMAG00000025640	ENSXMAG00000025314	ENSLOCG00000013186	20	3175552	1	20331051
ENSXMAG00000012493	ENSXMAG00000023015	ENSLOCG00000013206	5	12064443	14	13805092
ENSXMAG00000023015	ENSXMAG00000012493	ENSLOCG00000013206	14	13805092	5	12064443
ENSXMAG00000004882	ENSXMAG00000012102	ENSLOCG00000013236	5	11830342	14	14602048
ENSXMAG00000012102	ENSXMAG00000004882	ENSLOCG00000013236	14	14602048	5	11830342
ENSXMAG00000004907	ENSXMAG00000012077	ENSLOCG00000013247	5	11765554	14	14901931
ENSXMAG00000012077	ENSXMAG00000004907	ENSLOCG00000013247	14	14901931	5	11765554
ENSXMAG00000013776	ENSXMAG00000025029	ENSLOCG00000013280	20	2699898	1	17126054
ENSXMAG00000025029	ENSXMAG00000013776	ENSLOCG00000013280	1	17126054	20	2699898
ENSXMAG00000007410	ENSXMAG00000026073	ENSLOCG00000013290	5	9419813	12	18909485
ENSXMAG00000026073	ENSXMAG00000007410	ENSLOCG00000013290	12	18909485	5	9419813
ENSXMAG00000012675	ENSXMAG00000014570	ENSLOCG00000013315	10	23027631	16	2964155
ENSXMAG00000014570	ENSXMAG00000012675	ENSLOCG00000013315	16	2964155	10	23027631
ENSXMAG00000003371	ENSXMAG00000009186	ENSLOCG00000013346	19	23497762	15	9261680
ENSXMAG00000009186	ENSXMAG00000003371	ENSLOCG00000013346	15	9261680	19	23497762
ENSXMAG00000000572	ENSXMAG00000000942	ENSLOCG00000013353	6	433169	21	13935201
ENSXMAG00000000942	ENSXMAG00000000572	ENSLOCG00000013353	21	13935201	6	433169
ENSXMAG00000013669	ENSXMAG00000017472	ENSLOCG00000013354	16	25403838	10	15535029
ENSXMAG00000017472	ENSXMAG00000013669	ENSLOCG00000013354	10	15535029	16	25403838
ENSXMAG00000006711	ENSXMAG00000014551	ENSLOCG00000013364	16	14587709	5	9620416
ENSXMAG00000014551	ENSXMAG00000006711	ENSLOCG00000013364	5	9620416	16	14587709

ENSXMAG00000009197	ENSXMAG00000029136	ENSLOCG00000013365	15	9257345	19	23471833
ENSXMAG00000029136	ENSXMAG00000009197	ENSLOCG00000013365	19	23471833	15	9257345
ENSXMAG00000014393	ENSXMAG00000028828	ENSLOCG00000013371	5	9676838	16	14626359
ENSXMAG00000028828	ENSXMAG00000014393	ENSLOCG00000013371	16	14626359	5	9676838
ENSXMAG00000012250	ENSXMAG00000015708	ENSLOCG00000013399	1	19529448	20	5385126
ENSXMAG00000015708	ENSXMAG00000012250	ENSLOCG00000013399	20	5385126	1	19529448
ENSXMAG00000000363	ENSXMAG00000008979	ENSLOCG00000013420	14	19171836	11	23675605
ENSXMAG00000008979	ENSXMAG00000000363	ENSLOCG00000013420	11	23675605	14	19171836
ENSXMAG00000009792	ENSXMAG00000009932	ENSLOCG00000013435	5	3239924	14	23434001
ENSXMAG00000009932	ENSXMAG00000009792	ENSLOCG00000013435	14	23434001	5	3239924
ENSXMAG00000005972	ENSXMAG00000015781	ENSLOCG00000013471	1	19679006	20	10533345
ENSXMAG00000015781	ENSXMAG00000005972	ENSLOCG00000013471	20	10533345	1	19679006
ENSXMAG00000012369	ENSXMAG00000025799	ENSLOCG00000013477	5	8101772	16	16980880
ENSXMAG00000025799	ENSXMAG00000012369	ENSLOCG00000013477	16	16980880	5	8101772
ENSXMAG00000006679	ENSXMAG00000014280	ENSLOCG00000013480	4	16046519	2	6883257
ENSXMAG00000014280	ENSXMAG00000006679	ENSLOCG00000013480	2	6883257	4	16046519
ENSXMAG00000000824	ENSXMAG00000011244	ENSLOCG00000013495	6	6813907	21	15091978
ENSXMAG00000011244	ENSXMAG00000000824	ENSLOCG00000013495	21	15091978	6	6813907
ENSXMAG00000015790	ENSXMAG00000024535	ENSLOCG00000013502	20	10366931	1	19694664
ENSXMAG00000024535	ENSXMAG00000015790	ENSLOCG00000013502	1	19694664	20	10366931
ENSXMAG00000012511	ENSXMAG00000013383	ENSLOCG00000013521	2	30073461	11	20450088
ENSXMAG00000013383	ENSXMAG00000012511	ENSLOCG00000013521	11	20450088	2	30073461
ENSXMAG00000011271	ENSXMAG00000019153	ENSLOCG00000013527	21	13319071	6	6777793
ENSXMAG00000019153	ENSXMAG00000011271	ENSLOCG00000013527	6	6777793	21	13319071
ENSXMAG00000014411	ENSXMAG00000017673	ENSLOCG00000013533	15	8319523	19	26371061
ENSXMAG00000017673	ENSXMAG00000014411	ENSLOCG00000013533	19	26371061	15	8319523
ENSXMAG00000006131	ENSXMAG00000008305	ENSLOCG00000013534	16	20424237	5	8357393
ENSXMAG00000008305	ENSXMAG00000006131	ENSLOCG00000013534	5	8357393	16	20424237
ENSXMAG00000009165	ENSXMAG00000015803	ENSLOCG00000013537	1	19719563	20	4147259
ENSXMAG00000015803	ENSXMAG00000009165	ENSLOCG00000013537	20	4147259	1	19719563
ENSXMAG00000011847	ENSXMAG00000022641	ENSLOCG00000013543	14	21853260	11	20594109
ENSXMAG00000022641	ENSXMAG00000011847	ENSLOCG00000013543	11	20594109	14	21853260
ENSXMAG00000015849	ENSXMAG00000017677	ENSLOCG00000013555	15	8326925	19	26313374
ENSXMAG00000017677	ENSXMAG00000015849	ENSLOCG00000013555	19	26313374	15	8326925
ENSXMAG00000002642	ENSXMAG00000018179	ENSLOCG00000013577	5	16480495	16	24226091
ENSXMAG00000018179	ENSXMAG00000002642	ENSLOCG00000013577	16	24226091	5	16480495
ENSXMAG00000006616	ENSXMAG00000029098	ENSLOCG00000013583	2	6803310	4	16455771
ENSXMAG00000029098	ENSXMAG00000006616	ENSLOCG00000013583	4	16455771	2	6803310
ENSXMAG00000001186	ENSXMAG00000006578	ENSLOCG00000013639	5	25192040	16	13289438
ENSXMAG00000006578	ENSXMAG00000001186	ENSLOCG00000013639	16	13289438	5	25192040
ENSXMAG00000001253	ENSXMAG00000017877	ENSLOCG00000013680	5	17343853	16	13511687
ENSXMAG00000017877	ENSXMAG00000001253	ENSLOCG00000013680	16	13511687	5	17343853
ENSXMAG00000001283	ENSXMAG00000017886	ENSLOCG00000013688	5	17246802	16	13578496
ENSXMAG00000017886	ENSXMAG00000001283	ENSLOCG00000013688	16	13578496	5	17246802
ENSXMAG00000006814	ENSXMAG00000014316	ENSLOCG00000013741	16	14883711	5	9993225
ENSXMAG00000014316	ENSXMAG00000006814	ENSLOCG00000013741	5	9993225	16	14883711
ENSXMAG00000000385	ENSXMAG00000013668	ENSLOCG00000013755	14	27067132	11	23517410
ENSXMAG00000013668	ENSXMAG00000000385	ENSLOCG00000013755	11	23517410	14	27067132
ENSXMAG00000006859	ENSXMAG00000014302	ENSLOCG00000013764	16	14903213	5	10012299
ENSXMAG00000014302	ENSXMAG00000006859	ENSLOCG00000013764	5	10012299	16	14903213
ENSXMAG00000012979	ENSXMAG00000017579	ENSLOCG00000013792	5	11294444	16	1761004
ENSXMAG00000017579	ENSXMAG00000012979	ENSLOCG00000013792	16	1761004	5	11294444
ENSXMAG00000017789	ENSXMAG00000023607	ENSLOCG00000013799	20	13572715	1	21248020
ENSXMAG00000023607	ENSXMAG00000017789	ENSLOCG00000013799	1	21248020	20	13572715
ENSXMAG00000001429	ENSXMAG00000021089	ENSLOCG00000013847	5	17113315	16	13739728
ENSXMAG00000021089	ENSXMAG00000001429	ENSLOCG00000013847	16	13739728	5	17113315
ENSXMAG00000010483	ENSXMAG00000024264	ENSLOCG00000013860	1	20321995	20	6514470
ENSXMAG00000024264	ENSXMAG00000010483	ENSLOCG00000013860	20	6514470	1	20321995
ENSXMAG00000002372	ENSXMAG00000003550	ENSLOCG00000013892	2	8259691	4	25022587
ENSXMAG00000003550	ENSXMAG00000002372	ENSLOCG00000013892	4	25022587	2	8259691
ENSXMAG00000012523	ENSXMAG00000026810	ENSLOCG00000013910	16	10402381	5	22477702
ENSXMAG00000026810	ENSXMAG00000012523	ENSLOCG00000013910	5	22477702	16	10402381
ENSXMAG00000017419	ENSXMAG00000024245	ENSLOCG00000013962	5	24006744	23	25257849
ENSXMAG00000024245	ENSXMAG00000017419	ENSLOCG00000013962	23	25257849	5	24006744

ENSXMAG00000012593	ENSXMAG00000012922	ENSLOCG00000013963	16	10517903	5	22371986
ENSXMAG00000012922	ENSXMAG00000012593	ENSLOCG00000013963	5	22371986	16	10517903
ENSXMAG00000006485	ENSXMAG00000012490	ENSLOCG00000013976	14	13775239	5	24357431
ENSXMAG00000012490	ENSXMAG00000006485	ENSLOCG00000013976	5	24357431	14	13775239
ENSXMAG00000012443	ENSXMAG00000017656	ENSLOCG00000013994	1	21132507	20	5799935
ENSXMAG00000017656	ENSXMAG00000012443	ENSLOCG00000013994	20	5799935	1	21132507
ENSXMAG00000007500	ENSXMAG00000017618	ENSLOCG00000014039	1	20915989	20	31353418
ENSXMAG00000017618	ENSXMAG00000007500	ENSLOCG00000014039	20	31353418	1	20915989
ENSXMAG00000007513	ENSXMAG00000017615	ENSLOCG00000014046	1	20865002	20	31313141
ENSXMAG00000017615	ENSXMAG00000007513	ENSLOCG00000014046	20	31313141	1	20865002
ENSXMAG00000007522	ENSXMAG00000017581	ENSLOCG00000014058	1	20849029	20	31301991
ENSXMAG00000017581	ENSXMAG00000007522	ENSLOCG00000014058	20	31301991	1	20849029
ENSXMAG00000025916	ENSXMAG00000026041	ENSLOCG00000014060	11	17632498	14	3501319
ENSXMAG00000026041	ENSXMAG00000025916	ENSLOCG00000014060	14	3501319	11	17632498
ENSXMAG00000017090	ENSXMAG00000022037	ENSLOCG00000014087	1	20701191	20	18012288
ENSXMAG00000022037	ENSXMAG00000017090	ENSLOCG00000014087	20	18012288	1	20701191
ENSXMAG00000027075	ENSXMAG00000029244	ENSLOCG00000014090	20	18022926	1	20710858
ENSXMAG00000029244	ENSXMAG00000027075	ENSLOCG00000014090	1	20710858	20	18022926
ENSXMAG00000016999	ENSXMAG00000019252	ENSLOCG00000014111	1	9787543	20	18062837
ENSXMAG00000019252	ENSXMAG00000016999	ENSLOCG00000014111	20	18062837	1	9787543
ENSXMAG00000006869	ENSXMAG00000018049	ENSLOCG00000014132	4	2946020	2	7041586
ENSXMAG00000018049	ENSXMAG00000006869	ENSLOCG00000014132	2	7041586	4	2946020
ENSXMAG00000000160	ENSXMAG00000017247	ENSLOCG00000014151	15	7611592	19	329751
ENSXMAG00000017247	ENSXMAG00000000160	ENSLOCG00000014151	19	329751	15	7611592
ENSXMAG00000013503	ENSXMAG00000017634	ENSLOCG00000014166	5	11354950	9	22894785
ENSXMAG00000017634	ENSXMAG00000013503	ENSLOCG00000014166	9	22894785	5	11354950
ENSXMAG00000010771	ENSXMAG00000029134	ENSLOCG00000014200	18	5340967	18	17036944
ENSXMAG00000029134	ENSXMAG00000010771	ENSLOCG00000014200	18	17036944	18	5340967
ENSXMAG00000012976	ENSXMAG00000018275	ENSLOCG00000014203	4	2482535	2	26926625
ENSXMAG00000018275	ENSXMAG00000012976	ENSLOCG00000014203	2	26926625	4	2482535
ENSXMAG00000012577	ENSXMAG00000018328	ENSLOCG00000014218	4	2459267	2	26892948
ENSXMAG00000018328	ENSXMAG00000012577	ENSLOCG00000014218	2	26892948	4	2459267
ENSXMAG00000013036	ENSXMAG00000024531	ENSLOCG00000014224	4	24953037	2	26848289
ENSXMAG00000024531	ENSXMAG00000013036	ENSLOCG00000014224	2	26848289	4	24953037
ENSXMAG00000002347	ENSXMAG00000013063	ENSLOCG00000014238	4	24981918	2	26823640
ENSXMAG00000013063	ENSXMAG00000002347	ENSLOCG00000014238	2	26823640	4	24981918
ENSXMAG00000002892	ENSXMAG00000017009	ENSLOCG00000014310	4	10144195	2	9893279
ENSXMAG00000017009	ENSXMAG00000002892	ENSLOCG00000014310	2	9893279	4	10144195
ENSXMAG00000002886	ENSXMAG00000016987	ENSLOCG00000014321	4	10123042	2	9956073
ENSXMAG00000016987	ENSXMAG00000002886	ENSLOCG00000014321	2	9956073	4	10123042
ENSXMAG00000007547	ENSXMAG00000017369	ENSLOCG00000014345	20	17561406	1	17762099
ENSXMAG00000017369	ENSXMAG00000007547	ENSLOCG00000014345	1	17762099	20	17561406
ENSXMAG00000002826	ENSXMAG00000024050	ENSLOCG00000014355	4	28244033	2	10138298
ENSXMAG00000024050	ENSXMAG00000002826	ENSLOCG00000014355	2	10138298	4	28244033
ENSXMAG00000001747	ENSXMAG00000023264	ENSLOCG00000014413	20	27522724	20	19880703
ENSXMAG00000023264	ENSXMAG00000001747	ENSLOCG00000014413	20	19880703	20	27522724
ENSXMAG00000003824	ENSXMAG00000012880	ENSLOCG00000014427	1	6515084	20	22732615
ENSXMAG00000012880	ENSXMAG00000003824	ENSLOCG00000014427	20	22732615	1	6515084
ENSXMAG00000001861	ENSXMAG00000010429	ENSLOCG00000014468	20	27875194	20	20129027
ENSXMAG00000010429	ENSXMAG00000001861	ENSLOCG00000014468	20	20129027	20	27875194
ENSXMAG00000016085	ENSXMAG00000018069	ENSLOCG00000014521	4	28614132	2	30923762
ENSXMAG00000018069	ENSXMAG00000016085	ENSLOCG00000014521	2	30923762	4	28614132
ENSXMAG00000009589	ENSXMAG00000011526	ENSLOCG00000014528	11	20950517	23	20429779
ENSXMAG00000011526	ENSXMAG00000009589	ENSLOCG00000014528	23	20429779	11	20950517
ENSXMAG00000000035	ENSXMAG00000007414	ENSLOCG00000014545	2	6366759	4	6814266
ENSXMAG00000007414	ENSXMAG00000000035	ENSLOCG00000014545	4	6814266	2	6366759
ENSXMAG00000023285	ENSXMAG00000024665	ENSLOCG00000014547	20	19976984	20	27654964
ENSXMAG00000024665	ENSXMAG00000023285	ENSLOCG00000014547	20	27654964	20	19976984
ENSXMAG00000002966	ENSXMAG00000017063	ENSLOCG00000014643	4	10184484	2	9811454
ENSXMAG00000017063	ENSXMAG00000002966	ENSLOCG00000014643	2	9811454	4	10184484
ENSXMAG00000008911	ENSXMAG00000027672	ENSLOCG00000014645	1	24600868	1	30770609
ENSXMAG00000027672	ENSXMAG00000008911	ENSLOCG00000014645	1	30770609	1	24600868
ENSXMAG00000017127	ENSXMAG00000026513	ENSLOCG00000014657	2	9783603	4	10259265
ENSXMAG00000026513	ENSXMAG00000017127	ENSLOCG00000014657	4	10259265	2	9783603

ENSXMAG00000006418	ENSXMAG00000027053	ENSLOCG00000014667	4	4887189	2	4507747
ENSXMAG00000027053	ENSXMAG00000006418	ENSLOCG00000014667	2	4507747	4	4887189
ENSXMAG00000003020	ENSXMAG00000023194	ENSLOCG00000014687	4	10304442	2	9721223
ENSXMAG00000023194	ENSXMAG00000003020	ENSLOCG00000014687	2	9721223	4	10304442
ENSXMAG00000003135	ENSXMAG00000017193	ENSLOCG00000014719	4	10424166	2	9615567
ENSXMAG00000017193	ENSXMAG00000003135	ENSLOCG00000014719	2	9615567	4	10424166
ENSXMAG00000003228	ENSXMAG00000018442	ENSLOCG00000014779	4	2211566	2	9173740
ENSXMAG00000018442	ENSXMAG00000003228	ENSLOCG00000014779	2	9173740	4	2211566
ENSXMAG00000021321	ENSXMAG00000027551	ENSLOCG00000014780	1	24655195	1	31896980
ENSXMAG00000027551	ENSXMAG00000021321	ENSLOCG00000014780	1	31896980	1	24655195
ENSXMAG00000013331	ENSXMAG00000025260	ENSLOCG00000014787	2	9141555	4	670733
ENSXMAG00000025260	ENSXMAG00000013331	ENSLOCG00000014787	4	670733	2	9141555
ENSXMAG00000013562	ENSXMAG00000021311	ENSLOCG00000014836	2	8879777	4	1058462
ENSXMAG00000021311	ENSXMAG00000013562	ENSLOCG00000014836	4	1058462	2	8879777
ENSXMAG00000000226	ENSXMAG00000010902	ENSLOCG00000014844	1	14916973	1	30352112
ENSXMAG00000010902	ENSXMAG00000000226	ENSLOCG00000014844	1	30352112	1	14916973
ENSXMAG00000001773	ENSXMAG00000012008	ENSLOCG00000014876	11	21431732	18	22955898
ENSXMAG00000012008	ENSXMAG00000001773	ENSLOCG00000014876	18	22955898	11	21431732
ENSXMAG00000000900	ENSXMAG00000003972	ENSLOCG00000014917	8	13249503	12	22662538
ENSXMAG00000003972	ENSXMAG00000000900	ENSLOCG00000014917	12	22662538	8	13249503
ENSXMAG00000000325	ENSXMAG00000001480	ENSLOCG00000014927	14	6825063	11	4671648
ENSXMAG00000001480	ENSXMAG00000000325	ENSLOCG00000014927	11	4671648	14	6825063
ENSXMAG00000012952	ENSXMAG00000025884	ENSLOCG00000014941	4	26516169	2	27058742
ENSXMAG00000025884	ENSXMAG00000012952	ENSLOCG00000014941	2	27058742	4	26516169
ENSXMAG00000001846	ENSXMAG00000006860	ENSLOCG00000014966	8	13289895	12	22786397
ENSXMAG00000006860	ENSXMAG00000001846	ENSLOCG00000014966	12	22786397	8	13289895
ENSXMAG00000010311	ENSXMAG00000015842	ENSLOCG00000014973	2	8621904	4	3694536
ENSXMAG00000015842	ENSXMAG00000010311	ENSLOCG00000014973	4	3694536	2	8621904
ENSXMAG00000001785	ENSXMAG00000003926	ENSLOCG00000014974	8	13268236	12	22727041
ENSXMAG00000003926	ENSXMAG00000001785	ENSLOCG00000014974	12	22727041	8	13268236
ENSXMAG00000006593	ENSXMAG00000023737	ENSLOCG00000014995	4	26939378	2	4696715
ENSXMAG00000023737	ENSXMAG00000006593	ENSLOCG00000014995	2	4696715	4	26939378
ENSXMAG00000004824	ENSXMAG00000012123	ENSLOCG00000014996	11	21519939	23	24897133
ENSXMAG00000012123	ENSXMAG00000004824	ENSLOCG00000014996	23	24897133	11	21519939
ENSXMAG00000016650	ENSXMAG00000022379	ENSLOCG00000015037	20	15225005	1	11115223
ENSXMAG00000022379	ENSXMAG00000016650	ENSLOCG00000015037	1	11115223	20	15225005
ENSXMAG00000000027	ENSXMAG00000002204	ENSLOCG00000015045	2	22763194	4	6551916
ENSXMAG00000002204	ENSXMAG00000000027	ENSLOCG00000015045	4	6551916	2	22763194
ENSXMAG00000002217	ENSXMAG00000027139	ENSLOCG00000015050	2	22779267	4	6538315
ENSXMAG00000027139	ENSXMAG00000002217	ENSLOCG00000015050	4	6538315	2	22779267
ENSXMAG00000017435	ENSXMAG00000023145	ENSLOCG00000015078	8	26819441	13	8531315
ENSXMAG00000023145	ENSXMAG00000017435	ENSLOCG00000015078	13	8531315	8	26819441
ENSXMAG00000000832	ENSXMAG00000014039	ENSLOCG00000015082	4	15638912	2	14484280
ENSXMAG00000014039	ENSXMAG00000000832	ENSLOCG00000015082	2	14484280	4	15638912
ENSXMAG00000018766	ENSXMAG00000022958	ENSLOCG00000015143	12	24375932	8	25428715
ENSXMAG00000022958	ENSXMAG00000018766	ENSLOCG00000015143	8	25428715	12	24375932
ENSXMAG00000023425	ENSXMAG00000029675	ENSLOCG00000015147	4	9948038	2	6295846
ENSXMAG00000029675	ENSXMAG00000023425	ENSLOCG00000015147	2	6295846	4	9948038
ENSXMAG00000000404	ENSXMAG00000013325	ENSLOCG00000015164	23	7423167	11	4515508
ENSXMAG00000013325	ENSXMAG00000000404	ENSLOCG00000015164	11	4515508	23	7423167
ENSXMAG00000001482	ENSXMAG00000008892	ENSLOCG00000015224	17	16999971	2	13768437
ENSXMAG00000008892	ENSXMAG00000001482	ENSLOCG00000015224	2	13768437	17	16999971
ENSXMAG00000002364	ENSXMAG00000017872	ENSLOCG00000015227	12	17446807	8	10382140
ENSXMAG00000017872	ENSXMAG00000002364	ENSLOCG00000015227	8	10382140	12	17446807
ENSXMAG00000001195	ENSXMAG00000016797	ENSLOCG00000015250	12	3265929	8	25136790
ENSXMAG00000016797	ENSXMAG00000001195	ENSLOCG00000015250	8	25136790	12	3265929
ENSXMAG00000005432	ENSXMAG00000024814	ENSLOCG00000015252	12	29410529	8	15143840
ENSXMAG00000024814	ENSXMAG00000005432	ENSLOCG00000015252	8	15143840	12	29410529
ENSXMAG00000001744	ENSXMAG00000017466	ENSLOCG00000015272	17	9176816	2	17084201
ENSXMAG00000017466	ENSXMAG00000001744	ENSLOCG00000015272	2	17084201	17	9176816
ENSXMAG00000001409	ENSXMAG00000004062	ENSLOCG00000015286	17	12669622	2	13854858
ENSXMAG00000004062	ENSXMAG00000001409	ENSLOCG00000015286	2	13854858	17	12669622
ENSXMAG00000002689	ENSXMAG00000025444	ENSLOCG00000015299	8	25278344	12	6129687
ENSXMAG00000025444	ENSXMAG00000002689	ENSLOCG00000015299	12	6129687	8	25278344

ENSXMAG00000005068	ENSXMAG00000028275	ENSLOCG00000015342	11	2588303	8	14452489
ENSXMAG00000028275	ENSXMAG00000005068	ENSLOCG00000015342	8	14452489	11	2588303
ENSXMAG00000010236	ENSXMAG00000018615	ENSLOCG00000015371	8	25553336	11	3764948
ENSXMAG00000018615	ENSXMAG00000010236	ENSLOCG00000015371	11	3764948	8	25553336
ENSXMAG00000001294	ENSXMAG00000016966	ENSLOCG00000015385	2	21005600	17	19714282
ENSXMAG00000016966	ENSXMAG00000001294	ENSLOCG00000015385	17	19714282	2	21005600
ENSXMAG00000001328	ENSXMAG00000026296	ENSLOCG00000015421	2	20124426	17	19897071
ENSXMAG00000026296	ENSXMAG00000001328	ENSLOCG00000015421	17	19897071	2	20124426
ENSXMAG00000001309	ENSXMAG00000007144	ENSLOCG00000015422	2	5429125	17	19728158
ENSXMAG00000007144	ENSXMAG00000001309	ENSLOCG00000015422	17	19728158	2	5429125
ENSXMAG00000010470	ENSXMAG00000018377	ENSLOCG00000015445	20	24970142	12	29883726
ENSXMAG00000018377	ENSXMAG00000010470	ENSLOCG00000015445	12	29883726	20	24970142
ENSXMAG00000010490	ENSXMAG00000022145	ENSLOCG00000015460	8	22265330	12	29862978
ENSXMAG00000022145	ENSXMAG00000010490	ENSLOCG00000015460	12	29862978	8	22265330
ENSXMAG00000015021	ENSXMAG00000029289	ENSLOCG00000015472	5	6324778	4	12952316
ENSXMAG00000029289	ENSXMAG00000015021	ENSLOCG00000015472	4	12952316	5	6324778
ENSXMAG00000014461	ENSXMAG00000014878	ENSLOCG00000015493	4	13082481	5	6694888
ENSXMAG00000014878	ENSXMAG00000014461	ENSLOCG00000015493	5	6694888	4	13082481
ENSXMAG00000004343	ENSXMAG00000011342	ENSLOCG00000015521	17	3641501	2	12027734
ENSXMAG00000011342	ENSXMAG00000004343	ENSLOCG00000015521	2	12027734	17	3641501
ENSXMAG00000004015	ENSXMAG00000030049	ENSLOCG00000015636	12	11023079	8	13224161
ENSXMAG00000030049	ENSXMAG00000004015	ENSLOCG00000015636	8	13224161	12	11023079
ENSXMAG00000002416	ENSXMAG00000003685	ENSLOCG00000015672	12	26628840	8	10323155
ENSXMAG00000003685	ENSXMAG00000002416	ENSLOCG00000015672	8	10323155	12	26628840
ENSXMAG00000016859	ENSXMAG00000024223	ENSLOCG00000015690	17	2570712	2	20856193
ENSXMAG00000024223	ENSXMAG00000016859	ENSLOCG00000015690	2	20856193	17	2570712
ENSXMAG00000015733	ENSXMAG00000017251	ENSLOCG00000015740	22	22281658	5	384922
ENSXMAG00000017251	ENSXMAG00000015733	ENSLOCG00000015740	5	384922	22	22281658
ENSXMAG00000003397	ENSXMAG00000007839	ENSLOCG00000015818	24	9472567	15	17469769
ENSXMAG00000007839	ENSXMAG00000003397	ENSLOCG00000015818	15	17469769	24	9472567
ENSXMAG00000007586	ENSXMAG00000016455	ENSLOCG00000015822	2	20375098	17	17172160
ENSXMAG00000016455	ENSXMAG00000007586	ENSLOCG00000015822	17	17172160	2	20375098
ENSXMAG00000008881	ENSXMAG00000015153	ENSLOCG00000015895	19	24880251	15	21742123
ENSXMAG00000015153	ENSXMAG00000008881	ENSLOCG00000015895	15	21742123	19	24880251
ENSXMAG00000021335	ENSXMAG00000026835	ENSLOCG00000015906	5	4973688	22	8179460
ENSXMAG00000026835	ENSXMAG00000021335	ENSLOCG00000015906	22	8179460	5	4973688
ENSXMAG00000000690	ENSXMAG00000007800	ENSLOCG00000015964	15	20535340	4	736729
ENSXMAG00000007800	ENSXMAG00000000690	ENSLOCG00000015964	4	736729	15	20535340
ENSXMAG00000009104	ENSXMAG00000009384	ENSLOCG00000015990	17	11705360	2	13366764
ENSXMAG00000009384	ENSXMAG00000009104	ENSLOCG00000015990	2	13366764	17	11705360
ENSXMAG00000027974	ENSXMAG00000028171	ENSLOCG00000015996	2	13343390	17	11745857
ENSXMAG00000028171	ENSXMAG00000027974	ENSLOCG00000015996	17	11745857	2	13343390
ENSXMAG00000027245	ENSXMAG00000029191	ENSLOCG00000016046	22	23387020	5	421039
ENSXMAG00000029191	ENSXMAG00000027245	ENSLOCG00000016046	5	421039	22	23387020
ENSXMAG00000005970	ENSXMAG00000011845	ENSLOCG00000016049	22	23412248	5	7830094
ENSXMAG00000011845	ENSXMAG00000005970	ENSLOCG00000016049	5	7830094	22	23412248
ENSXMAG00000020875	ENSXMAG00000029787	ENSLOCG00000016052	2	25918975	17	14425491
ENSXMAG00000029787	ENSXMAG00000020875	ENSLOCG00000016052	17	14425491	2	25918975
ENSXMAG00000017888	ENSXMAG00000025049	ENSLOCG00000016072	8	10433116	12	17496697
ENSXMAG00000025049	ENSXMAG00000017888	ENSLOCG00000016072	12	17496697	8	10433116
ENSXMAG00000002728	ENSXMAG00000004964	ENSLOCG00000016087	17	14788123	2	24550044
ENSXMAG00000004964	ENSXMAG00000002728	ENSLOCG00000016087	2	24550044	17	14788123
ENSXMAG00000002043	ENSXMAG00000028682	ENSLOCG00000016092	8	6953556	12	24067649
ENSXMAG00000028682	ENSXMAG00000002043	ENSLOCG00000016092	12	24067649	8	6953556
ENSXMAG00000002761	ENSXMAG00000006611	ENSLOCG00000016098	17	14846325	2	24607158
ENSXMAG00000006611	ENSXMAG00000002761	ENSLOCG00000016098	2	24607158	17	14846325
ENSXMAG00000001792	ENSXMAG00000003750	ENSLOCG00000016114	12	26731222	8	7309402
ENSXMAG00000003750	ENSXMAG00000001792	ENSLOCG00000016114	8	7309402	12	26731222
ENSXMAG00000007578	ENSXMAG00000023646	ENSLOCG00000016121	19	25024336	15	10694705
ENSXMAG00000023646	ENSXMAG00000007578	ENSLOCG00000016121	15	10694705	19	25024336
ENSXMAG00000005658	ENSXMAG00000023108	ENSLOCG00000016142	5	20482236	22	24081830
ENSXMAG00000023108	ENSXMAG00000005658	ENSLOCG00000016142	22	24081830	5	20482236
ENSXMAG00000012035	ENSXMAG00000024491	ENSLOCG00000016173	22	22581203	5	16232946
ENSXMAG00000024491	ENSXMAG00000012035	ENSLOCG00000016173	5	16232946	22	22581203

ENSXMAG00000007246	ENSXMAG00000018211	ENSLOCG00000016181	5	16345305	22	5994963
ENSXMAG00000018211	ENSXMAG00000007246	ENSLOCG00000016181	22	5994963	5	16345305
ENSXMAG00000016365	ENSXMAG00000028202	ENSLOCG00000016219	17	3082636	2	20090132
ENSXMAG00000028202	ENSXMAG00000016365	ENSLOCG00000016219	2	20090132	17	3082636
ENSXMAG00000003229	ENSXMAG00000015466	ENSLOCG00000016263	19	25379733	15	9836527
ENSXMAG00000015466	ENSXMAG00000003229	ENSLOCG00000016263	15	9836527	19	25379733
ENSXMAG00000003189	ENSXMAG00000024565	ENSLOCG00000016271	19	25374456	15	9875904
ENSXMAG00000024565	ENSXMAG00000003189	ENSLOCG00000016271	15	9875904	19	25374456
ENSXMAG00000010885	ENSXMAG00000016119	ENSLOCG00000016276	10	10487198	22	10646257
ENSXMAG00000016119	ENSXMAG00000010885	ENSLOCG00000016276	22	10646257	10	10487198
ENSXMAG00000015620	ENSXMAG00000021324	ENSLOCG00000016317	19	25506239	15	865100
ENSXMAG00000021324	ENSXMAG00000015620	ENSLOCG00000016317	15	865100	19	25506239
ENSXMAG00000026395	ENSXMAG00000028289	ENSLOCG00000016339	3	13027776	3	1113125
ENSXMAG00000028289	ENSXMAG00000026395	ENSLOCG00000016339	3	1113125	3	13027776
ENSXMAG00000018655	ENSXMAG00000029551	ENSLOCG00000016342	15	8257990	3	7042096
ENSXMAG00000029551	ENSXMAG00000018655	ENSLOCG00000016342	3	7042096	15	8257990
ENSXMAG00000003995	ENSXMAG00000018765	ENSLOCG00000016368	2	25408908	17	12583597
ENSXMAG00000018765	ENSXMAG00000003995	ENSLOCG00000016368	17	12583597	2	25408908
ENSXMAG00000019592	ENSXMAG00000020326	ENSLOCG00000016373	10	10265871	22	21007402
ENSXMAG00000020326	ENSXMAG00000019592	ENSLOCG00000016373	22	21007402	10	10265871
ENSXMAG00000009952	ENSXMAG00000017405	ENSLOCG00000016383	15	7902024	19	22932752
ENSXMAG00000017405	ENSXMAG00000009952	ENSLOCG00000016383	19	22932752	15	7902024
ENSXMAG00000016497	ENSXMAG00000017072	ENSLOCG00000016479	10	12541935	22	3319318
ENSXMAG00000017072	ENSXMAG00000016497	ENSLOCG00000016479	22	3319318	10	12541935
ENSXMAG00000010752	ENSXMAG00000015237	ENSLOCG00000016480	19	24938319	15	19934056
ENSXMAG00000015237	ENSXMAG00000010752	ENSLOCG00000016480	15	19934056	19	24938319
ENSXMAG00000023610	ENSXMAG00000025787	ENSLOCG00000016482	15	19914503	19	24913656
ENSXMAG00000025787	ENSXMAG00000023610	ENSLOCG00000016482	19	24913656	15	19914503
ENSXMAG00000021354	ENSXMAG00000025978	ENSLOCG00000016514	15	14278127	3	23864733
ENSXMAG00000025978	ENSXMAG00000021354	ENSLOCG00000016514	3	23864733	15	14278127
ENSXMAG00000016785	ENSXMAG00000019075	ENSLOCG00000016561	15	8701171	3	31816416
ENSXMAG00000019075	ENSXMAG00000016785	ENSLOCG00000016561	3	31816416	15	8701171
ENSXMAG00000013677	ENSXMAG00000024056	ENSLOCG00000016575	19	13519774	15	8616166
ENSXMAG00000024056	ENSXMAG00000013677	ENSLOCG00000016575	15	8616166	19	13519774
ENSXMAG00000011188	ENSXMAG00000025768	ENSLOCG00000016641	2	30366840	17	4123135
ENSXMAG00000025768	ENSXMAG00000011188	ENSLOCG00000016641	17	4123135	2	30366840
ENSXMAG00000023214	ENSXMAG00000028462	ENSLOCG00000016645	15	20275540	4	12127027
ENSXMAG00000028462	ENSXMAG00000023214	ENSLOCG00000016645	4	12127027	15	20275540
ENSXMAG00000010348	ENSXMAG00000017700	ENSLOCG00000016650	4	11939781	15	20232370
ENSXMAG00000017700	ENSXMAG00000010348	ENSLOCG00000016650	15	20232370	4	11939781
ENSXMAG00000024752	ENSXMAG00000029613	ENSLOCG00000016684	13	10704783	24	14687600
ENSXMAG00000029613	ENSXMAG00000024752	ENSLOCG00000016684	24	14687600	13	10704783
ENSXMAG00000007048	ENSXMAG00000015084	ENSLOCG00000016780	19	24833936	15	14645153
ENSXMAG00000015084	ENSXMAG00000007048	ENSLOCG00000016780	15	14645153	19	24833936
ENSXMAG00000004075	ENSXMAG00000005131	ENSLOCG00000016819	17	19023427	2	11504530
ENSXMAG00000005131	ENSXMAG00000004075	ENSLOCG00000016819	2	11504530	17	19023427
ENSXMAG00000002960	ENSXMAG00000009810	ENSLOCG00000016828	15	3973070	19	4791970
ENSXMAG00000009810	ENSXMAG00000002960	ENSLOCG00000016828	19	4791970	15	3973070
ENSXMAG00000014385	ENSXMAG00000014415	ENSLOCG00000016832	5	6599740	4	14008202
ENSXMAG00000014415	ENSXMAG00000014385	ENSLOCG00000016832	4	14008202	5	6599740
ENSXMAG00000009885	ENSXMAG00000010947	ENSLOCG00000016837	19	4755498	15	3944541
ENSXMAG00000010947	ENSXMAG00000009885	ENSLOCG00000016837	15	3944541	19	4755498
ENSXMAG00000007446	ENSXMAG00000009927	ENSLOCG00000016842	15	3916281	19	10968831
ENSXMAG00000009927	ENSXMAG00000007446	ENSLOCG00000016842	19	10968831	15	3916281
ENSXMAG00000006338	ENSXMAG00000009940	ENSLOCG00000016845	15	3888502	19	11020119
ENSXMAG00000009940	ENSXMAG00000006338	ENSLOCG00000016845	19	11020119	15	3888502
ENSXMAG00000009976	ENSXMAG00000019210	ENSLOCG00000016852	19	11768034	15	3823872
ENSXMAG00000019210	ENSXMAG00000009976	ENSLOCG00000016852	15	3823872	19	11768034
ENSXMAG00000007492	ENSXMAG00000025237	ENSLOCG00000016873	19	12235949	15	5086701
ENSXMAG00000025237	ENSXMAG00000007492	ENSLOCG00000016873	15	5086701	19	12235949
ENSXMAG00000000284	ENSXMAG00000027111	ENSLOCG00000016895	15	4924397	19	11626953
ENSXMAG00000027111	ENSXMAG00000000284	ENSLOCG00000016895	19	11626953	15	4924397
ENSXMAG00000010212	ENSXMAG00000014414	ENSLOCG00000016902	5	6581071	22	12355493
ENSXMAG00000014414	ENSXMAG00000010212	ENSLOCG00000016902	22	12355493	5	6581071

ENSXMAG00000007610	ENSXMAG00000007951	ENSLOCG00000016906	19	2763858	15	5301101
ENSXMAG00000007951	ENSXMAG00000007610	ENSLOCG00000016906	15	5301101	19	2763858
ENSXMAG00000009234	ENSXMAG00000027658	ENSLOCG00000016939	15	23020080	19	6384563
ENSXMAG00000027658	ENSXMAG00000009234	ENSLOCG00000016939	19	6384563	15	23020080
ENSXMAG00000003764	ENSXMAG00000008729	ENSLOCG00000017053	17	17201062	2	11195090
ENSXMAG00000008729	ENSXMAG00000003764	ENSLOCG00000017053	2	11195090	17	17201062
ENSXMAG00000010369	ENSXMAG00000018611	ENSLOCG00000017178	19	13638818	3	14552167
ENSXMAG00000018611	ENSXMAG00000010369	ENSLOCG00000017178	3	14552167	19	13638818
ENSXMAG00000021472	ENSXMAG00000022080	ENSLOCG00000017188	15	6961381	19	7446439
ENSXMAG00000022080	ENSXMAG00000021472	ENSLOCG00000017188	19	7446439	15	6961381
ENSXMAG00000002367	ENSXMAG00000028196	ENSLOCG00000017216	19	13326865	15	5164766
ENSXMAG00000028196	ENSXMAG00000002367	ENSLOCG00000017216	15	5164766	19	13326865
ENSXMAG00000000268	ENSXMAG00000017553	ENSLOCG00000017224	15	6634459	19	11333316
ENSXMAG00000017553	ENSXMAG00000000268	ENSLOCG00000017224	19	11333316	15	6634459
ENSXMAG00000004048	ENSXMAG00000004406	ENSLOCG00000017241	24	5820312	15	12484845
ENSXMAG00000004406	ENSXMAG00000004048	ENSLOCG00000017241	15	12484845	24	5820312
ENSXMAG00000021883	ENSXMAG00000024962	ENSLOCG00000017299	16	4466036	10	4257728
ENSXMAG00000024962	ENSXMAG00000021883	ENSLOCG00000017299	10	4257728	16	4466036
ENSXMAG00000019680	ENSXMAG00000020251	ENSLOCG00000017316	2	14885983	4	5291027
ENSXMAG00000020251	ENSXMAG00000019680	ENSLOCG00000017316	4	5291027	2	14885983
ENSXMAG00000020277	ENSXMAG00000020353	ENSLOCG00000017378	6	13086141	18	24879563
ENSXMAG00000020353	ENSXMAG00000020277	ENSLOCG00000017378	18	24879563	6	13086141
ENSXMAG00000021789	ENSXMAG00000028778	ENSLOCG00000017388	6	13196963	18	10203641
ENSXMAG00000028778	ENSXMAG00000021789	ENSLOCG00000017388	18	10203641	6	13196963
ENSXMAG00000019016	ENSXMAG00000029906	ENSLOCG00000017443	8	1517053	21	13494327
ENSXMAG00000029906	ENSXMAG00000019016	ENSLOCG00000017443	21	13494327	8	1517053
ENSXMAG00000020084	ENSXMAG00000025875	ENSLOCG00000017628	20	26455092	9	6643332
ENSXMAG00000025875	ENSXMAG00000020084	ENSLOCG00000017628	9	6643332	20	26455092
ENSXMAG00000019316	ENSXMAG00000019590	ENSLOCG00000017635	9	18773063	6	11951624
ENSXMAG00000019590	ENSXMAG00000019316	ENSLOCG00000017635	6	11951624	9	18773063
ENSXMAG00000019271	ENSXMAG00000020383	ENSLOCG00000017638	9	16446673	6	11440740
ENSXMAG00000020383	ENSXMAG00000019271	ENSLOCG00000017638	6	11440740	9	16446673
ENSXMAG00000019679	ENSXMAG00000020345	ENSLOCG00000017699	19	6828805	15	19450732
ENSXMAG00000020345	ENSXMAG00000019679	ENSLOCG00000017699	15	19450732	19	6828805
ENSXMAG00000014252	ENSXMAG00000019711	ENSLOCG00000017709	15	5785205	19	9870563
ENSXMAG00000019711	ENSXMAG00000014252	ENSLOCG00000017709	19	9870563	15	5785205
ENSXMAG00000011339	ENSXMAG00000019647	ENSLOCG00000017739	6	4758318	9	3365783
ENSXMAG00000019647	ENSXMAG00000011339	ENSLOCG00000017739	9	3365783	6	4758318
ENSXMAG00000019972	ENSXMAG00000028941	ENSLOCG00000017745	9	21399819	6	5171273
ENSXMAG00000028941	ENSXMAG00000019972	ENSLOCG00000017745	6	5171273	9	21399819
ENSXMAG00000019454	ENSXMAG00000019871	ENSLOCG00000017759	10	13452599	16	10764791
ENSXMAG00000019871	ENSXMAG00000019454	ENSLOCG00000017759	16	10764791	10	13452599
ENSXMAG00000021609	ENSXMAG00000022071	ENSLOCG00000017761	16	16928061	5	9183216
ENSXMAG00000022071	ENSXMAG00000021609	ENSLOCG00000017761	5	9183216	16	16928061
ENSXMAG00000019457	ENSXMAG00000019687	ENSLOCG00000017767	5	22396290	16	10502083
ENSXMAG00000019687	ENSXMAG00000019457	ENSLOCG00000017767	16	10502083	5	22396290
ENSXMAG00000019934	ENSXMAG00000020169	ENSLOCG00000017791	24	5082370	9	24339880
ENSXMAG00000020169	ENSXMAG00000019934	ENSLOCG00000017791	9	24339880	24	5082370
ENSXMAG00000019657	ENSXMAG00000019775	ENSLOCG00000017831	24	1606200	7	17425554
ENSXMAG00000019775	ENSXMAG00000019657	ENSLOCG00000017831	7	17425554	24	1606200
ENSXMAG00000019897	ENSXMAG00000020119	ENSLOCG00000017909	17	6097318	2	21089940
ENSXMAG00000020119	ENSXMAG00000019897	ENSLOCG00000017909	2	21089940	17	6097318
ENSXMAG00000008984	ENSXMAG00000017689	ENSLOCG00000017918	17	8421808	2	13571403
ENSXMAG00000017689	ENSXMAG00000008984	ENSLOCG00000017918	2	13571403	17	8421808
ENSXMAG00000027985	ENSXMAG00000029210	ENSLOCG00000017919	17	5582064	2	13233437
ENSXMAG00000029210	ENSXMAG00000027985	ENSLOCG00000017919	2	13233437	17	5582064
ENSXMAG00000019464	ENSXMAG00000019922	ENSLOCG00000017921	20	7714834	1	16849849
ENSXMAG00000019922	ENSXMAG00000019464	ENSLOCG00000017921	1	16849849	20	7714834
ENSXMAG00000009062	ENSXMAG00000020192	ENSLOCG00000017940	1	13834004	20	18946555
ENSXMAG00000020192	ENSXMAG00000009062	ENSLOCG00000017940	20	18946555	1	13834004
ENSXMAG00000019727	ENSXMAG00000020023	ENSLOCG00000017947	4	33574538	10	6322984
ENSXMAG00000020023	ENSXMAG00000019727	ENSLOCG00000017947	10	6322984	4	33574538
ENSXMAG00000001788	ENSXMAG00000006310	ENSLOCG00000018000	15	812526	15	20782870
ENSXMAG00000006310	ENSXMAG00000001788	ENSLOCG00000018000	15	20782870	15	812526

ENSXMAG00000019909	ENSXMAG00000028511	ENSLOCG00000018038	5	19412810	14	13173548
ENSXMAG00000028511	ENSXMAG00000019909	ENSLOCG00000018038	14	13173548	5	19412810
ENSXMAG00000010891	ENSXMAG00000019458	ENSLOCG00000018067	1	16805819	20	7748335
ENSXMAG00000019458	ENSXMAG00000010891	ENSLOCG00000018067	20	7748335	1	16805819
ENSXMAG00000014093	ENSXMAG00000019743	ENSLOCG00000018089	16	8031143	5	28600671
ENSXMAG00000019743	ENSXMAG00000014093	ENSLOCG00000018089	5	28600671	16	8031143
ENSXMAG00000012731	ENSXMAG00000014160	ENSLOCG00000018093	16	15067401	5	21996004
ENSXMAG00000014160	ENSXMAG00000012731	ENSLOCG00000018093	5	21996004	16	15067401
ENSXMAG00000010941	ENSXMAG00000018562	ENSLOCG00000018113	10	3977555	16	489520
ENSXMAG00000018562	ENSXMAG00000010941	ENSLOCG00000018113	16	489520	10	3977555
ENSXMAG00000023632	ENSXMAG00000026647	ENSLOCG00000018130	11	8904136	13	16204719
ENSXMAG00000026647	ENSXMAG00000023632	ENSLOCG00000018130	13	16204719	11	8904136
ENSXMAG00000016113	ENSXMAG00000023193	ENSLOCG00000018139	12	10816794	8	25830987
ENSXMAG00000023193	ENSXMAG00000016113	ENSLOCG00000018139	8	25830987	12	10816794
ENSXMAG00000020272	ENSXMAG00000027447	ENSLOCG00000018210	1	15071987	20	20543008
ENSXMAG00000027447	ENSXMAG00000020272	ENSLOCG00000018210	20	20543008	1	15071987
ENSXMAG00000019856	ENSXMAG00000020102	ENSLOCG00000018229	22	15013517	10	7803750
ENSXMAG00000020102	ENSXMAG00000019856	ENSLOCG00000018229	10	7803750	22	15013517
ENSXMAG00000014234	ENSXMAG00000020001	ENSLOCG00000018234	22	2477938	10	24541322
ENSXMAG00000020001	ENSXMAG00000014234	ENSLOCG00000018234	10	24541322	22	2477938
ENSXMAG00000019483	ENSXMAG00000020215	ENSLOCG00000018254	5	23733864	22	17280975
ENSXMAG00000020215	ENSXMAG00000019483	ENSLOCG00000018254	22	17280975	5	23733864
ENSXMAG00000005127	ENSXMAG00000019530	ENSLOCG00000018299	16	21736972	5	19923372
ENSXMAG00000019530	ENSXMAG00000005127	ENSLOCG00000018299	5	19923372	16	21736972
ENSXMAG00000025105	ENSXMAG00000025956	ENSLOCG00000018304	11	7929753	23	19591834
ENSXMAG00000025956	ENSXMAG00000025105	ENSLOCG00000018304	23	19591834	11	7929753
ENSXMAG00000008654	ENSXMAG00000020332	ENSLOCG00000018342	13	24389572	3	23734877
ENSXMAG00000020332	ENSXMAG00000008654	ENSLOCG00000018342	3	23734877	13	24389572
ENSXMAG00000002730	ENSXMAG00000019568	ENSLOCG00000018353	7	14000120	24	5375803
ENSXMAG00000019568	ENSXMAG00000002730	ENSLOCG00000018353	24	5375803	7	14000120
ENSXMAG00000020168	ENSXMAG00000023700	ENSLOCG00000018359	1	11809217	20	2354788
ENSXMAG00000023700	ENSXMAG00000020168	ENSLOCG00000018359	20	2354788	1	11809217
