# Supplementary material for: Global assessment of organ specific basal gene expression over a diurnal cycle with analyses of gene copies exhibiting cyclic expression patterns
Source: BMC Genomics. 2020 Nov 11;21:787. doi: 10.1186/s12864-020-07202-9 (PMC7659085; doi:10.1186/s12864-020-07202-9)
Supplement: Supplementary file 6 — Additional file 6: Supplement Table 5. Xiphophorus singletons. [file 12864_2020_7202_MOESM6_ESM.pdf]

Supplement Table 5

ensembl_gene_id	external_gene_name	loculatus_homolog_ensembl_gene	loculatus_homolog_associated_gene_name
ENSXMAG00000020786	ND1	ENSLOCG00000020971	ND1
ENSXMAG00000020790	ND2	ENSLOCG00000020975	ND2
ENSXMAG00000020796	COX1	ENSLOCG00000020981	COX1
ENSXMAG00000020799	COX2	ENSLOCG00000020984	COX2
ENSXMAG00000020802	ATP6	ENSLOCG00000020987	ATP6
ENSXMAG00000020803	COX3	ENSLOCG00000020988	COX3
ENSXMAG00000020805	ND3	ENSLOCG00000020990	ND3
ENSXMAG00000020807	ND4L	ENSLOCG00000020992	ND4L
ENSXMAG00000020808	ND4	ENSLOCG00000020993	ND4
ENSXMAG00000020812	ND5	ENSLOCG00000020997	ND5
ENSXMAG00000020813	ND6	ENSLOCG00000020998	ND6
ENSXMAG00000020815	CYTB	ENSLOCG00000021000	CYTB
ENSXMAG00000023113	fam19a5l	ENSLOCG00000011050	fam19a5l
ENSXMAG00000012381	agrn	ENSLOCG00000007329	agrn
ENSXMAG00000012317	ptpn11b	ENSLOCG00000007344	ptpn11b
ENSXMAG00000026573	dennd2c	ENSLOCG00000010452	dennd2c
ENSXMAG00000009029	AMPD1	ENSLOCG00000010444	ampd1
ENSXMAG00000009080	csde1	ENSLOCG00000010413	csde1
ENSXMAG00000009079	si:ch211-261n11.3	ENSLOCG00000010433	
ENSXMAG00000000910	im:6912630	ENSLOCG00000010407	im:6912630
ENSXMAG00000006941		ENSLOCG00000007593	tmco4
ENSXMAG00000004783	ralgapb	ENSLOCG00000000654	ralgapb
ENSXMAG00000028502	prex1	ENSLOCG00000001552	prex1
ENSXMAG00000004772	chrna4b	ENSLOCG00000001635	chrna4a
ENSXMAG00000004759	zgpat	ENSLOCG00000001659	zgpat
ENSXMAG00000004725	tti1	ENSLOCG00000001130	tti1
ENSXMAG00000004678	ctnnbl1	ENSLOCG00000001069	ctnnbl1
ENSXMAG00000004669	e2f1	ENSLOCG00000001016	e2f1
ENSXMAG00000014133	znf341	ENSLOCG00000000947	znf341
ENSXMAG00000004623		ENSLOCG00000005174	
ENSXMAG00000001327		ENSLOCG00000013731	SLC41A3
ENSXMAG00000001273	lhfpl4a	ENSLOCG00000013665	lhfpl4a
ENSXMAG00000001270		ENSLOCG00000013656	
ENSXMAG00000001076	zgc:171775	ENSLOCG00000013616	zgc:171775
ENSXMAG00000027733	tmem106c	ENSLOCG00000005136	tmem106c
ENSXMAG00000016305		ENSLOCG00000005157	
ENSXMAG00000016138	sars	ENSLOCG00000012557	sars
ENSXMAG00000029856	si:ch211-207e14.4	ENSLOCG00000014378	si:ch211-207e14.4
ENSXMAG00000023736	fam50a	ENSLOCG00000014548	fam50a
ENSXMAG00000016356	gss	ENSLOCG00000004359	GSS
ENSXMAG00000021393	pim1	ENSLOCG00000011041	pim1
ENSXMAG00000025836	si:dkey-8e10.3	ENSLOCG00000014538	si:dkey-8e10.3
ENSXMAG00000020962	klhdc8b	ENSLOCG00000013109	klhdc8b
ENSXMAG00000030095	si:dkey-32e6.3	ENSLOCG00000013114	si:dkey-32e6.3
ENSXMAG00000016414	usp19	ENSLOCG00000013120	usp19
ENSXMAG00000016442	lamb2	ENSLOCG00000013129	lamb2
ENSXMAG00000016516	lamb2l	ENSLOCG00000013159	lamb2l
ENSXMAG00000016526	si:rp71-17i16.5	ENSLOCG00000013171	si:rp71-17i16.5
ENSXMAG00000028434	si:rp71-17i16.4	ENSLOCG00000000401	
ENSXMAG00000019607	taf13	ENSLOCG00000002878	taf13
ENSXMAG00000025204		ENSLOCG00000002883	
ENSXMAG00000006459	dpm1	ENSLOCG00000002964	dpm1
ENSXMAG00000016610	pard6b	ENSLOCG00000003029	pard6b
ENSXMAG00000016678	cox4i2	ENSLOCG00000006091	cox4i2

ENSXMAG00000016691	nsfl1c	ENSLOCG00000006055	nsfl1c
ENSXMAG00000016714	fkbp1ab	ENSLOCG00000006019	fkbp1ab
ENSXMAG00000004154	ntsr1	ENSLOCG00000005946	ntsr1
ENSXMAG00000022051	slco4a1	ENSLOCG00000005933	slco4a1
ENSXMAG00000020423	mir1-1	ENSLOCG00000019404	mir1-1
ENSXMAG00000004186	gata5	ENSLOCG00000005910	gata5
ENSXMAG00000028090		ENSLOCG00000005890	
ENSXMAG00000004194	hck	ENSLOCG00000005873	hck
ENSXMAG00000004229	tm9sf4	ENSLOCG00000005859	tm9sf4
ENSXMAG00000004262	pofut1	ENSLOCG00000005824	pofut1
ENSXMAG00000004280	kif3b	ENSLOCG00000005800	kif3b
ENSXMAG00000004296	PCMTD2	ENSLOCG00000005219	pcmtd2
ENSXMAG00000027801	myt1b	ENSLOCG00000005236	myt1b
ENSXMAG00000004320	tbc1d22b	ENSLOCG00000011057	tbc1d22b
ENSXMAG00000004353	snrpg	ENSLOCG00000015662	snrpg
ENSXMAG00000004354	plpbp	ENSLOCG00000015656	plpbp
ENSXMAG00000004360	tmed4	ENSLOCG00000015187	TMED4
ENSXMAG00000004368	ikbkg	ENSLOCG00000014262	ikbkg
ENSXMAG00000029983		ENSLOCG00000014256	sephs2
ENSXMAG00000004378	ap4b1	ENSLOCG00000010255	ap4b1
ENSXMAG00000004404	ccnq	ENSLOCG00000015536	ccnq
ENSXMAG00000004407	atp2b3b	ENSLOCG00000015538	atp2b3b
ENSXMAG00000013649	crebzf	ENSLOCG00000005355	
ENSXMAG00000023987		ENSLOCG00000014635	las1l
ENSXMAG00000004475	tpx2	ENSLOCG00000006128	tpx2
ENSXMAG00000004495		ENSLOCG00000005408	plod1a
ENSXMAG00000004560	si:dkeyp-104h9.5	ENSLOCG00000005478	si:dkeyp-104h9.5
ENSXMAG00000004561	phc2a	ENSLOCG00000003682	
ENSXMAG00000027747	si:ch211-215j19.12	ENSLOCG00000005508	
ENSXMAG00000004563	rcc2	ENSLOCG00000003000	rcc2
ENSXMAG00000021941	icmt	ENSLOCG00000004867	icmt
ENSXMAG00000004603	rtel1	ENSLOCG00000000543	
ENSXMAG00000004637	mfn2	ENSLOCG00000005375	mfn2
ENSXMAG00000004661		ENSLOCG00000015550	haus7
ENSXMAG00000004664		ENSLOCG00000015557	
ENSXMAG00000004665	flna	ENSLOCG00000015505	flna
ENSXMAG00000004788	rpl10	ENSLOCG00000015500	rpl10
ENSXMAG00000004821	renbp	ENSLOCG00000014488	renbp
ENSXMAG00000004839		ENSLOCG00000011476	dennd2db
ENSXMAG00000004852	stk38a	ENSLOCG00000011519	stk38a
ENSXMAG00000004872	pex10	ENSLOCG00000005635	pex10
ENSXMAG00000002008	skia	ENSLOCG00000005728	skia
ENSXMAG00000002016	prkcz	ENSLOCG00000005744	prkcz
ENSXMAG00000012872	suv39h1a	ENSLOCG00000014420	suv39h1b
ENSXMAG00000012852		ENSLOCG00000001741	GMEB2
ENSXMAG00000026772	pdyn	ENSLOCG00000001827	pdyn
ENSXMAG00000012847	stk35	ENSLOCG00000001817	stk35
ENSXMAG00000012838		ENSLOCG00000011538	
ENSXMAG00000012836		ENSLOCG00000014298	ndufb11
ENSXMAG00000027860	tbc1d25	ENSLOCG00000014341	tbc1d25
ENSXMAG00000012774	ccdc22	ENSLOCG00000014464	ccdc22
ENSXMAG00000012737	mthfr	ENSLOCG00000005606	mthfr
ENSXMAG00000012699	mapk13	ENSLOCG00000011102	mapk13
ENSXMAG00000012669	pqlc2	ENSLOCG00000007530	pqlc2
ENSXMAG00000012634	trim33	ENSLOCG00000010479	trim33
ENSXMAG00000012622	bcas2	ENSLOCG00000010471	bcas2

ENSXMAG00000012612	otud3	ENSLOCG00000007718	otud3
ENSXMAG00000012610	tmem240a	ENSLOCG00000007658	tmem240b
ENSXMAG00000012558	atp13a2	ENSLOCG00000007777	atp13a2
ENSXMAG00000012515	cdk11b	ENSLOCG00000006226	cdk11b
ENSXMAG00000012509	MMP23B	ENSLOCG00000006275	MMP23B
ENSXMAG00000012507	ubiad1	ENSLOCG00000006329	ubiad1
ENSXMAG00000012469	mib2	ENSLOCG00000006296	mib2
ENSXMAG00000012468	ttll10	ENSLOCG00000007378	
ENSXMAG00000001754	arhgef19	ENSLOCG00000007424	arhgef19
ENSXMAG00000012445	ano11	ENSLOCG00000007442	ano11
ENSXMAG00000012429	plekhm2	ENSLOCG00000008039	plekhm2
ENSXMAG00000012411	ddost	ENSLOCG00000008002	ddost
ENSXMAG00000012388	zbtb17	ENSLOCG00000007923	zbtb17
ENSXMAG00000012383	hspb7	ENSLOCG00000007940	hspb7
ENSXMAG00000012341	clcnk	ENSLOCG00000007948	clcnk
ENSXMAG00000022368	fam131c	ENSLOCG00000007966	fam131c
ENSXMAG00000012210	cep104	ENSLOCG00000005920	cep104
ENSXMAG00000020025	b3gnt7l	ENSLOCG00000018085	b3gnt7l
ENSXMAG00000005169	dffb	ENSLOCG00000005950	dffb
ENSXMAG00000005185		ENSLOCG00000005969	
ENSXMAG00000026634	ajap1	ENSLOCG00000006021	ajap1
ENSXMAG00000019394	gabrd	ENSLOCG00000006079	GABRD
ENSXMAG00000020970	cacna1db	ENSLOCG00000006094	cacna1db
ENSXMAG00000019390	NADK	ENSLOCG00000006173	nadka
ENSXMAG00000027232		ENSLOCG00000006194	
ENSXMAG00000019389	SLC35E2A	ENSLOCG00000006207	SLC35E2A
ENSXMAG00000019387	mad2l2	ENSLOCG00000005872	mad2l2
ENSXMAG00000019386	lrrc47	ENSLOCG00000005902	lrrc47
ENSXMAG00000019384	casp9	ENSLOCG00000001860	CASP9
ENSXMAG00000019369	tmem115	ENSLOCG00000014244	tmem115
ENSXMAG00000022328	RHOA	ENSLOCG00000014438	
ENSXMAG00000019366	sema3h	ENSLOCG00000014437	sema3h
ENSXMAG00000025285	si:dkey-72l14.4	ENSLOCG00000014431	si:dkey-72l14.4
ENSXMAG00000019362	si:dkey-72l14.3	ENSLOCG00000014421	si:dkey-72l14.3
ENSXMAG00000019360	xpc	ENSLOCG00000013060	XPC
ENSXMAG00000019355	arih2	ENSLOCG00000014400	arih2
ENSXMAG00000019354	impdh2	ENSLOCG00000014390	impdh2
ENSXMAG00000019349	dalrd3	ENSLOCG00000014389	dalrd3
ENSXMAG00000019342	celsr3	ENSLOCG00000014385	celsr3
ENSXMAG00000025403	wdr6	ENSLOCG00000014377	wdr6
ENSXMAG00000019330	slc26a6	ENSLOCG00000000029	
ENSXMAG00000009052	mgll	ENSLOCG00000013071	mgll
ENSXMAG00000025817	hyal3	ENSLOCG00000014281	hyal3
ENSXMAG00000019318	ifrd2	ENSLOCG00000014287	ifrd2
ENSXMAG00000019315	zgc:136971	ENSLOCG00000014295	zgc:136971
ENSXMAG00000019303		ENSLOCG00000014325	sema3b
ENSXMAG00000019294	sema3bl	ENSLOCG00000014307	sema3bl
ENSXMAG00000019284	actr8	ENSLOCG00000014161	actr8
ENSXMAG00000019293	selenok	ENSLOCG00000014164	selenok
ENSXMAG00000019281	chdh	ENSLOCG00000014155	chdh
ENSXMAG00000028395	dcp1a	ENSLOCG00000014108	dcp1a
ENSXMAG00000019220	nsun5	ENSLOCG00000001105	nsun5
ENSXMAG00000019197	sgk2a	ENSLOCG00000001397	sgk2a
ENSXMAG00000019188	l3mbtl1a	ENSLOCG00000001414	l3mbtl1a
ENSXMAG00000021849	SRSF6	ENSLOCG00000004202	
ENSXMAG00000019172	eif2s2	ENSLOCG00000007205	eif2s2

ENSXMAG00000019165	chmp4bb	ENSLOCG00000007168	chmp4ba
ENSXMAG00000029555	atp5f1e	ENSLOCG00000006308	atp5f1e
ENSXMAG00000019093		ENSLOCG00000006326	
ENSXMAG00000019060	bpifcl	ENSLOCG00000006358	
ENSXMAG00000019055	actr5	ENSLOCG00000006403	ACTR5
ENSXMAG00000019019	stac3	ENSLOCG00000005998	stac3
ENSXMAG00000018945	rprd1b	ENSLOCG00000001144	rprd1b
ENSXMAG00000018734	ubr4	ENSLOCG00000004534	ubr4
ENSXMAG00000018715	emc1	ENSLOCG00000004489	emc1
ENSXMAG00000018712	zgc:154075	ENSLOCG00000004036	zgc:154075
ENSXMAG00000018697		ENSLOCG00000002478	taf10
ENSXMAG00000024576	ppih	ENSLOCG00000002496	PPIH
ENSXMAG00000018674	ybx1	ENSLOCG00000002530	ybx1
ENSXMAG00000018659	arhgef16	ENSLOCG00000005013	arhgef16
ENSXMAG00000018650	espn	ENSLOCG00000005036	espn
ENSXMAG00000018646	acot7	ENSLOCG00000005099	acot7
ENSXMAG00000018638	gpr153	ENSLOCG00000005146	gpr153
ENSXMAG00000028058	her3	ENSLOCG00000005161	HES3
ENSXMAG00000017360	tp73	ENSLOCG00000005255	tp73
ENSXMAG00000009483	wrap73	ENSLOCG00000005279	wrap73
ENSXMAG00000029424	tprg1l	ENSLOCG00000005319	tprg1l
ENSXMAG00000019072	si:ch211-263k4.2	ENSLOCG00000005336	si:ch211-263k4.2
ENSXMAG00000016665	rimkla	ENSLOCG00000001562	rimkla
ENSXMAG00000016670	zmynd12	ENSLOCG00000001538	zmynd12
ENSXMAG00000016679	ccdc30	ENSLOCG00000001300	
ENSXMAG00000023747	si:ch211-112c15.8	ENSLOCG00000004304	
ENSXMAG00000022230	ttc34	ENSLOCG00000004222	TTC34
ENSXMAG00000016690		ENSLOCG00000014541	alas2
ENSXMAG00000025632	RF00009	ENSLOCG00000019720	RF00009
ENSXMAG00000028148	RF00030	ENSLOCG00000019585	RF00030
ENSXMAG00000016733		ENSLOCG00000000750	
ENSXMAG00000016768	pigt	ENSLOCG00000003262	pigt
ENSXMAG00000016827	ttll9	ENSLOCG00000003352	ttll9
ENSXMAG00000011246	exosc10	ENSLOCG00000006443	exosc10
ENSXMAG00000011275	srm	ENSLOCG00000006482	srm
ENSXMAG00000011283	cort	ENSLOCG00000006494	cort
ENSXMAG00000011286	masp2	ENSLOCG00000006509	masp2
ENSXMAG00000022628	dffa	ENSLOCG00000006536	dffa
ENSXMAG00000011294	pex14	ENSLOCG00000006553	pex14
ENSXMAG00000011322	casz1	ENSLOCG00000006571	casz1
ENSXMAG00000011356	pgd	ENSLOCG00000006643	pgd
ENSXMAG00000011378	kif1b	ENSLOCG00000006679	kif1b
ENSXMAG00000011476	ube4b	ENSLOCG00000006740	ube4b
ENSXMAG00000011530	nmnat1	ENSLOCG00000006792	nmnat1
ENSXMAG00000011534	lzic	ENSLOCG00000006822	lzic
ENSXMAG00000011543	clstn1	ENSLOCG00000006860	clstn1
ENSXMAG00000029666	ctnnbip1	ENSLOCG00000006849	ctnnbip1
ENSXMAG00000011579	pik3cd	ENSLOCG00000006919	pik3cd
ENSXMAG00000011619	tmem201	ENSLOCG00000006950	tmem201
ENSXMAG00000029241	zgc:194189	ENSLOCG00000006935	zgc:194189
ENSXMAG00000028637	her9	ENSLOCG00000007298	her9
ENSXMAG00000023655		ENSLOCG00000007290	si:dkey-98j1.5
ENSXMAG00000003803	plekhn1	ENSLOCG00000007279	plekhn1
ENSXMAG00000003804	klhl17	ENSLOCG00000007252	klhl17
ENSXMAG00000003835	noc2l	ENSLOCG00000007227	noc2l
ENSXMAG00000003860	samd11	ENSLOCG00000007210	samd11

ENSXMAG00000021776	ca6	ENSLOCG00000007130	ca6
ENSXMAG00000003923	slc2a5	ENSLOCG00000007109	slc2a5
ENSXMAG00000009818	gpr157	ENSLOCG00000007093	gpr157
ENSXMAG00000030053	spsb1	ENSLOCG00000007002	spsb1
ENSXMAG00000028723	slc25a33	ENSLOCG00000006983	slc25a33
ENSXMAG00000023884	si:ch211-150o23.2	ENSLOCG00000015513	si:ch211-150o23.2
ENSXMAG00000009859	dnase1l1l	ENSLOCG00000015497	dnase1l1l
ENSXMAG00000009883		ENSLOCG00000011279	TRAF3IP3
ENSXMAG00000028165	atp5pb	ENSLOCG00000011269	atp5pb
ENSXMAG00000009964	ampd2b	ENSLOCG00000012353	ampd2b
ENSXMAG00000020846	gnat2	ENSLOCG00000012347	gnat2
ENSXMAG00000029085	ngrn	ENSLOCG00000012302	ngrn
ENSXMAG00000028069	KCNC4	ENSLOCG00000011699	KCNC4
ENSXMAG00000010113	ALX3	ENSLOCG00000011685	ALX3
ENSXMAG00000010114	slc16a4	ENSLOCG00000011704	slc16a4
ENSXMAG00000027457	lamtor5	ENSLOCG00000011713	lamtor5
ENSXMAG00000024068	prok1	ENSLOCG00000011719	prok1
ENSXMAG00000010129	ZNF335	ENSLOCG00000006887	ZNF335
ENSXMAG00000010153	mmp9	ENSLOCG00000006863	mmp9
ENSXMAG00000010206	dclre1b	ENSLOCG00000010747	dclre1b
ENSXMAG00000010362	si:ch211-163l21.8	ENSLOCG00000010201	KIAA1324
ENSXMAG00000010404	smc5	ENSLOCG00000009494	smc5
ENSXMAG00000010442	fance	ENSLOCG00000000495	fance
ENSXMAG00000010448	mkrn4	ENSLOCG00000000455	mkrn4
ENSXMAG00000010449	ppardb	ENSLOCG00000011628	ppardb
ENSXMAG00000010474	DEF6	ENSLOCG00000011619	def6a
ENSXMAG00000010519	larp4aa	ENSLOCG00000007113	larp4aa
ENSXMAG00000010537	atf7a	ENSLOCG00000006882	atf7a
ENSXMAG00000026987	FIGNL2	ENSLOCG00000018208	si:dkey-157g16.6
ENSXMAG00000010611	stat6	ENSLOCG00000006721	stat6
ENSXMAG00000010629	zgc:66440	ENSLOCG00000007354	zgc:66440
ENSXMAG00000028926		ENSLOCG00000007362	
ENSXMAG00000010739	zgc:158263	ENSLOCG00000014277	zgc:158263
ENSXMAG00000016782	naa10	ENSLOCG00000014858	naa10
ENSXMAG00000010777	SSR4	ENSLOCG00000014259	ssr4
ENSXMAG00000010795	idh3g	ENSLOCG00000014632	
ENSXMAG00000021004		ENSLOCG00000014785	WNK3
ENSXMAG00000010813	phf8	ENSLOCG00000014794	
ENSXMAG00000010828	huwe1	ENSLOCG00000014797	huwe1
ENSXMAG00000020586	MIRLET7F2	ENSLOCG00000018716	MIRLET7F2
ENSXMAG00000010988	cacna1sb	ENSLOCG00000011555	cacna1sb
ENSXMAG00000011047		ENSLOCG00000011609	tmem9
ENSXMAG00000023757	birc5b	ENSLOCG00000016573	
ENSXMAG00000027324	c1galt1a	ENSLOCG00000007520	c1galt1a
ENSXMAG00000009959	slc26a10	ENSLOCG00000007565	slc26a10
ENSXMAG00000010161	sp5l	ENSLOCG00000007577	sp5l
ENSXMAG00000010164	ARHGEF25	ENSLOCG00000007588	ARHGEF25
ENSXMAG00000011072	acvrl1	ENSLOCG00000007620	acvrl1
ENSXMAG00000025100	acvr1ba	ENSLOCG00000007634	acvr1bb
ENSXMAG00000011132	dhh	ENSLOCG00000007693	dhh
ENSXMAG00000011135	lmbr1l	ENSLOCG00000007708	lmbr1l
ENSXMAG00000023187	dnajc22	ENSLOCG00000007723	dnajc22
ENSXMAG00000022548	wnt1	ENSLOCG00000007786	wnt1
ENSXMAG00000011174	wnt10b	ENSLOCG00000007804	wnt10b
ENSXMAG00000011178	erbb3b	ENSLOCG00000003677	erbb3b
ENSXMAG00000011245	fmnl3	ENSLOCG00000004076	fmnl3

ENSXMAG00000028151		ENSLOCG00000001123	lrp1ba
ENSXMAG00000011425		ENSLOCG00000006126	
ENSXMAG00000011443	si:dkey-21c19.3	ENSLOCG00000006182	si:dkey-21c19.3
ENSXMAG00000025050	mfsd5	ENSLOCG00000018206	mfsd5
ENSXMAG00000001641	hoxc13a	ENSLOCG00000006295	hoxc13a
ENSXMAG00000001643	hoxc12a	ENSLOCG00000006304	hoxc12b
ENSXMAG00000001648	hoxc11a	ENSLOCG00000006316	hoxc11a
ENSXMAG00000001655	hoxc9a	ENSLOCG00000007106	
ENSXMAG00000001663	hoxc6a	ENSLOCG00000006348	hoxc6a
ENSXMAG00000025781	hoxc5a	ENSLOCG00000006365	hoxc5a
ENSXMAG00000023829	hoxc4a	ENSLOCG00000006379	hoxc4a
ENSXMAG00000023539	cbx5	ENSLOCG00000006460	cbx5
ENSXMAG00000008083	nfe2	ENSLOCG00000006498	nfe2
ENSXMAG00000008084	copz1	ENSLOCG00000006517	copz1
ENSXMAG00000026779	znf740b	ENSLOCG00000006558	znf740b
ENSXMAG00000008102	csad	ENSLOCG00000006576	csad
ENSXMAG00000027144	zgc:174906	ENSLOCG00000006593	zgc:174906
ENSXMAG00000008129	fkbpl	ENSLOCG00000014344	
ENSXMAG00000008130	ebp	ENSLOCG00000014346	ebp
ENSXMAG00000008131	ccdc115	ENSLOCG00000014417	ccdc115
ENSXMAG00000008134	apobec2b	ENSLOCG00000012415	apobec2b
ENSXMAG00000019456	phlda3	ENSLOCG00000018057	phlda3
ENSXMAG00000026538	LGR6	ENSLOCG00000009662	
ENSXMAG00000008235	ube2t	ENSLOCG00000009649	UBE2T
ENSXMAG00000008241	etv7	ENSLOCG00000010545	etv7
ENSXMAG00000008274	uba1	ENSLOCG00000014330	uba1
ENSXMAG00000008297	arhgef3l	ENSLOCG00000014322	arhgef3l
ENSXMAG00000008307	usp21	ENSLOCG00000014293	usp21
ENSXMAG00000024965	lrrc23	ENSLOCG00000014290	
ENSXMAG00000008325	tktb	ENSLOCG00000015562	tktb
ENSXMAG00000008350	bgnb	ENSLOCG00000015519	bgnb
ENSXMAG00000028248	b4galt3	ENSLOCG00000000001	b4galt3
ENSXMAG00000029157	lrtm1	ENSLOCG00000014190	lrtm1
ENSXMAG00000002614	wnt5a	ENSLOCG00000014191	wnt5a
ENSXMAG00000007629	tlr9	ENSLOCG00000014202	tlr9
ENSXMAG00000007607		ENSLOCG00000014206	apeh
ENSXMAG00000007593	capza1b	ENSLOCG00000010303	capza1b
ENSXMAG00000007573	ddx20	ENSLOCG00000010372	ddx20
ENSXMAG00000007548		ENSLOCG00000014350	
ENSXMAG00000007523		ENSLOCG00000014354	si:ch211-117c9.5
ENSXMAG00000007463		ENSLOCG00000005620	AMHR2
ENSXMAG00000007439	samhd1	ENSLOCG00000004382	
ENSXMAG00000023061		ENSLOCG00000004455	tp53inp2
ENSXMAG00000015291	GGT7	ENSLOCG00000004507	GGT7
ENSXMAG00000023426		ENSLOCG00000004487	ncoa6
ENSXMAG00000015379	plcg1	ENSLOCG00000004569	plcg1
ENSXMAG00000026603		ENSLOCG00000014935	
ENSXMAG00000015457	zhx3	ENSLOCG00000004596	zhx3
ENSXMAG00000024990	rab5if	ENSLOCG00000004604	rab5if
ENSXMAG00000015468	DHX35	ENSLOCG00000004639	DHX35
ENSXMAG00000003696	kbtbd4	ENSLOCG00000006020	kbtbd4
ENSXMAG00000015512	rnf114	ENSLOCG00000004685	rnf114
ENSXMAG00000015528	spata2	ENSLOCG00000004705	spata2
ENSXMAG00000003694	ddb2	ENSLOCG00000005486	ddb2
ENSXMAG00000015549	slc9a8	ENSLOCG00000004737	slc9a8
ENSXMAG00000015569	b4galt5	ENSLOCG00000004750	b4galt5

ENSXMAG00000015577	aar2	ENSLOCG00000004809	aar2
ENSXMAG00000015581	prpf6	ENSLOCG00000004827	prpf6
ENSXMAG00000015609	ZNF512B	ENSLOCG00000004884	ZNF512B
ENSXMAG00000022123	zgc:162898	ENSLOCG00000013920	zgc:162898
ENSXMAG00000003568	iqch	ENSLOCG00000013914	iqch
ENSXMAG00000020213	irgq2	ENSLOCG00000017672	irgq2
ENSXMAG00000015618	uckl1b	ENSLOCG00000004906	uckl1b
ENSXMAG00000010306	lipca	ENSLOCG00000013598	lipca
ENSXMAG00000003564	aagab	ENSLOCG00000013907	aagab
ENSXMAG00000023594	zbtb46	ENSLOCG00000005130	zbtb46
ENSXMAG00000027023		ENSLOCG00000011228	IFT27
ENSXMAG00000028201		ENSLOCG00000005741	
ENSXMAG00000003549	snapc5	ENSLOCG00000013891	snapc5
ENSXMAG00000006656		ENSLOCG00000011404	
ENSXMAG00000014456	mindy2	ENSLOCG00000013626	mindy2
ENSXMAG00000015632		ENSLOCG00000005112	si:rp71-79p20.2
ENSXMAG00000015634	nkain4	ENSLOCG00000005053	nkain4
ENSXMAG00000014466	sltm	ENSLOCG00000013629	sltm
ENSXMAG00000006548	drd2l	ENSLOCG00000005675	drd2l
ENSXMAG00000020742	MIR153-1	ENSLOCG00000020785	
ENSXMAG00000026292	prune2	ENSLOCG00000009830	
ENSXMAG00000015942		ENSLOCG00000012427	DNAJB6
ENSXMAG00000006522	xrcc1	ENSLOCG00000005639	xrcc1
ENSXMAG00000003532	zwilch	ENSLOCG00000013873	zwilch
ENSXMAG00000019916	bhlhe23	ENSLOCG00000018362	bhlhe23
ENSXMAG00000014476	rnf111	ENSLOCG00000013642	RNF111
ENSXMAG00000029529	rpl4	ENSLOCG00000013867	rpl4
ENSXMAG00000009869	nubp2	ENSLOCG00000002301	NUBP2
ENSXMAG00000015956	pofut2	ENSLOCG00000004410	
ENSXMAG00000027547	gtf2a2	ENSLOCG00000014796	gtf2a2
ENSXMAG00000004965		ENSLOCG00000004466	UNC13B
ENSXMAG00000015970	abca12	ENSLOCG00000004451	abca12
ENSXMAG00000003499	map2k1	ENSLOCG00000013858	map2k1
ENSXMAG00000000127	tbc1d24	ENSLOCG00000008951	
ENSXMAG00000004975	stoml2	ENSLOCG00000004431	stoml2
ENSXMAG00000014524	cep131	ENSLOCG00000012955	cep131
ENSXMAG00000015788	ctdnep1b	ENSLOCG00000014490	ctdnep1b
ENSXMAG00000000130		ENSLOCG00000008962	
ENSXMAG00000006657	myo1ea	ENSLOCG00000013657	myo1ea
ENSXMAG00000026071		ENSLOCG00000001008	
ENSXMAG00000004993	pigo	ENSLOCG00000004414	pigo
ENSXMAG00000028910		ENSLOCG00000001062	
ENSXMAG00000024303	zgc:114119	ENSLOCG00000004930	zgc:114119
ENSXMAG00000015792	elp5	ENSLOCG00000014484	elp5
ENSXMAG00000018060	TCTN2	ENSLOCG00000005931	TCTN2
ENSXMAG00000015798	phf23b	ENSLOCG00000014481	phf23b
ENSXMAG00000023971	bard1	ENSLOCG00000004470	bard1
ENSXMAG00000005471	il17a/f1	ENSLOCG00000015957	il17a/f1
ENSXMAG00000016688	ext1c	ENSLOCG00000004913	ext1c
ENSXMAG00000014866	tbl3	ENSLOCG00000008853	tbl3
ENSXMAG00000015666	dnah12	ENSLOCG00000013287	dnah12
ENSXMAG00000008784	ntn2	ENSLOCG00000008971	ntn2
ENSXMAG00000015802	plscr3b	ENSLOCG00000014476	plscr3b
ENSXMAG00000023254	vwc2l	ENSLOCG00000004485	vwc2l
ENSXMAG00000010808	rpain	ENSLOCG00000002804	rpain
ENSXMAG00000005436	gpn1	ENSLOCG00000015949	gpn1

ENSXMAG00000019533	nog2	ENSLOCG00000018109	nog2
ENSXMAG00000026156	ikzf2	ENSLOCG00000004501	ikzf2
ENSXMAG00000018064	si:ch211-225b11.4	ENSLOCG00000004874	si:ch211-225b11.4
ENSXMAG00000015814	tnk1	ENSLOCG00000014472	tnk1
ENSXMAG00000006736	mtmr10	ENSLOCG00000013672	mtmr10
ENSXMAG00000016012	erbb4b	ENSLOCG00000004529	erbb4b
ENSXMAG00000010016		ENSLOCG00000011320	
ENSXMAG00000008777		ENSLOCG00000008981	
ENSXMAG00000006278	lats1	ENSLOCG00000017006	lats1
ENSXMAG00000001847	ruvbl1	ENSLOCG00000014532	ruvbl1
ENSXMAG00000025943	ptgir	ENSLOCG00000014873	ptgir
ENSXMAG00000021228	EHD4	ENSLOCG00000009774	ehd4
ENSXMAG00000022611	atxn1l	ENSLOCG00000004385	atxn1l
ENSXMAG00000015817	trmt44	ENSLOCG00000010316	trmt44
ENSXMAG00000016657	ago4	ENSLOCG00000000584	ago4
ENSXMAG00000026279		ENSLOCG00000004371	
ENSXMAG00000014501		ENSLOCG00000012947	
ENSXMAG00000019556	gemin7	ENSLOCG00000017726	gemin7
ENSXMAG00000026621	bmp16	ENSLOCG00000014862	bmp16
ENSXMAG00000023858	rab10	ENSLOCG00000016323	rab10
ENSXMAG00000012503	mtpap	ENSLOCG00000007357	mtpap
ENSXMAG00000016110	cps1	ENSLOCG00000004580	cps1
ENSXMAG00000016634	CLSPN	ENSLOCG00000000631	CLSPN
ENSXMAG00000016010	fam98a	ENSLOCG00000016699	fam98a
ENSXMAG00000019557		ENSLOCG00000017804	
ENSXMAG00000018071	pik3ip1	ENSLOCG00000004841	pik3ip1
ENSXMAG00000005983	ap1g1	ENSLOCG00000004347	ap1g1
ENSXMAG00000022332	map3k8	ENSLOCG00000007364	map3k8
ENSXMAG00000020834	ppp1r37	ENSLOCG00000014857	ppp1r37
ENSXMAG00000004820	BAZ1A	ENSLOCG00000009448	BAZ1A
ENSXMAG00000001855	rft1	ENSLOCG00000014475	rft1
ENSXMAG00000028852	kmt5b	ENSLOCG00000002639	kmt5b
ENSXMAG00000019044	capn15	ENSLOCG00000009003	capn15
ENSXMAG00000002553	fam208ab	ENSLOCG00000013340	FAM208A
ENSXMAG00000028838		ENSLOCG00000010357	
ENSXMAG00000004293	mrpl28	ENSLOCG00000014850	mrpl28
ENSXMAG00000018076	limk2	ENSLOCG00000004821	limk2
ENSXMAG00000027215	pole3	ENSLOCG00000002521	pole3
ENSXMAG00000013316	nags	ENSLOCG00000013066	nags
ENSXMAG00000028211	TOM1L2	ENSLOCG00000008795	TOM1L2
ENSXMAG00000016616	psmb2	ENSLOCG00000000700	psmb2
ENSXMAG00000003464	dennd4a	ENSLOCG00000013827	dennd4a
ENSXMAG00000015836	DOK7	ENSLOCG00000010376	dok7
ENSXMAG00000004297	relb	ENSLOCG00000014849	relb
ENSXMAG00000003446	tfap2e	ENSLOCG00000000725	tfap2e
ENSXMAG00000028653	cfl2	ENSLOCG00000009463	cfl2
ENSXMAG00000018114	si:ch211-225b11.1	ENSLOCG00000004784	si:ch211-225b11.1
ENSXMAG00000027698	ncdn	ENSLOCG00000000757	ncdn
ENSXMAG00000001627		ENSLOCG00000007282	CHRNA1
ENSXMAG00000004298	clptm1	ENSLOCG00000014846	clptm1
ENSXMAG00000019435		ENSLOCG00000009022	
ENSXMAG00000029315	PHLPP2	ENSLOCG00000004332	PHLPP2
ENSXMAG00000004865	snx6	ENSLOCG00000009469	snx6
ENSXMAG00000007211	si:dkeyp-72e1.9	ENSLOCG00000001005	si:dkeyp-72e1.9
ENSXMAG00000020212		ENSLOCG00000005391	
ENSXMAG00000018145	wscd2	ENSLOCG00000004759	wscd2

ENSXMAG00000019008	cog7	ENSLOCG00000003874	cog7
ENSXMAG00000003454	si:ch211-193k19.1	ENSLOCG00000000785	
ENSXMAG00000021928	si:dkey-121h17.7	ENSLOCG00000006577	
ENSXMAG00000023962		ENSLOCG00000002794	
ENSXMAG00000005485	znf438	ENSLOCG00000007254	znf438
ENSXMAG00000001660		ENSLOCG00000005226	cdh11
ENSXMAG00000016184	kansl1l	ENSLOCG00000004691	KANSL1L
ENSXMAG00000004309	tbcb	ENSLOCG00000014843	tbcb
ENSXMAG00000018159	ficd	ENSLOCG00000004731	ficd
ENSXMAG00000001888	ccdc174	ENSLOCG00000011131	ccdc174
ENSXMAG00000022070		ENSLOCG00000002428	
ENSXMAG00000028529	cap1	ENSLOCG00000000820	cap1
ENSXMAG00000007954	marveld3	ENSLOCG00000004317	
ENSXMAG00000014774	srebf1	ENSLOCG00000008766	srebf1
ENSXMAG00000004893	psma3	ENSLOCG00000009368	psma3
ENSXMAG00000025473	six5	ENSLOCG00000014840	six5
ENSXMAG00000014943	ppl	ENSLOCG00000001033	ppl
ENSXMAG00000015780	ints14	ENSLOCG00000013812	ints14
ENSXMAG00000016187	heg1	ENSLOCG00000002732	heg1
ENSXMAG00000018160	sart3	ENSLOCG00000004710	sart3
ENSXMAG00000005105	zgc:77838	ENSLOCG00000011177	zgc:77838
ENSXMAG00000022737	tatdn2	ENSLOCG00000011120	tatdn2
ENSXMAG00000027051	etfb	ENSLOCG00000005340	etfb
ENSXMAG00000000735	rheb	ENSLOCG00000010004	rheb
ENSXMAG00000026179	usp10	ENSLOCG00000004297	usp10
ENSXMAG00000016190	slc12a8	ENSLOCG00000002752	slc12a8
ENSXMAG00000027460	vhl	ENSLOCG00000011111	vhl
ENSXMAG00000004319	qpctla	ENSLOCG00000014833	qpctlb
ENSXMAG00000004904	arid4a	ENSLOCG00000009355	arid4a
ENSXMAG00000003488	ppt1	ENSLOCG00000000848	ppt1
ENSXMAG00000014773	rai1	ENSLOCG00000008754	rai1
ENSXMAG00000015002	glyr1	ENSLOCG00000001106	glyr1
ENSXMAG00000009987		ENSLOCG00000011263	mfng
ENSXMAG00000005063	MAMDC2	ENSLOCG00000009479	mamdc2a
ENSXMAG00000027517	crbn	ENSLOCG00000011083	crbn
ENSXMAG00000016196	znf148	ENSLOCG00000002769	znf148
ENSXMAG00000014286		ENSLOCG00000016449	mcm8
ENSXMAG00000015812	ppcdc	ENSLOCG00000014263	ppcdc
ENSXMAG00000010788		ENSLOCG00000016777	si:ch211-245h14.1
ENSXMAG00000005472	arhgap12b	ENSLOCG00000007199	arhgap12b
ENSXMAG00000007200	rogdi	ENSLOCG00000001146	rogdi
ENSXMAG00000029741	si:ch211-191d15.2	ENSLOCG00000018146	si:ch211-191d15.2
ENSXMAG00000007916	crispld2	ENSLOCG00000004276	crispld2
ENSXMAG00000021118	sipa1l3	ENSLOCG00000014617	sipa1l3
ENSXMAG00000022800		ENSLOCG00000014260	si:dkey-73n10.1
ENSXMAG00000000351	man2b1	ENSLOCG00000006499	man2b1
ENSXMAG00000029156	timm9	ENSLOCG00000009345	timm9
ENSXMAG00000005076	slc30a5	ENSLOCG00000010499	slc30a5
ENSXMAG00000016211	asmt	ENSLOCG00000010259	asmt
ENSXMAG00000001913	pa2g4a	ENSLOCG00000003714	pa2g4a
ENSXMAG00000021671	rpl32	ENSLOCG00000013512	rpl32
ENSXMAG00000015819	arih1	ENSLOCG00000014970	arih1
ENSXMAG00000016225	akap17a	ENSLOCG00000010269	akap17a
ENSXMAG00000004916		ENSLOCG00000009320	si:ch211-185a18.2
ENSXMAG00000008543	gpr108	ENSLOCG00000007111	
ENSXMAG00000007164	cog1	ENSLOCG00000012473	cog1

ENSXMAG00000000747	PTPRM		ENSLOCG00000002692	PTPRM	
ENSXMAG00000016235			ENSLOCG00000010278	sowahcb	
ENSXMAG00000015874	msantd1		ENSLOCG00000010411	msantd1	
ENSXMAG00000013303			ENSLOCG00000005297		
ENSXMAG00000007901			ENSLOCG00000004238		
ENSXMAG00000024193	crls1		ENSLOCG00000016448	crls1	
ENSXMAG00000016237		10-Sep	ENSLOCG00000010284		10-Sep
ENSXMAG00000018686	map3k10		ENSLOCG00000014624	map3k10	
ENSXMAG00000027275	RF00548		ENSLOCG00000019391	RF00548	
ENSXMAG00000013122	tmem214		ENSLOCG00000016307	tmem214	
ENSXMAG00000025801	st8sia5		ENSLOCG00000011209	st8sia5	
ENSXMAG00000018213	ssh1a		ENSLOCG00000004601	ssh1b	
ENSXMAG00000029324	ttc9b		ENSLOCG00000014626	ttc9b	
ENSXMAG00000003525	meaf6		ENSLOCG00000005842	meaf6	
ENSXMAG00000027288	zfhx3		ENSLOCG00000004206	zfhx3	
ENSXMAG00000023166	ubn1		ENSLOCG00000001085	UBN1	
ENSXMAG00000018670	si:ch211-160d20.3		ENSLOCG00000014629	si:ch211-160d20.3	
ENSXMAG00000013297	si:dkeyp-97a10.2		ENSLOCG00000004674		
ENSXMAG00000010835	ccdc25		ENSLOCG00000016773	ccdc25	
ENSXMAG00000015892	coro1b		ENSLOCG00000007079		
ENSXMAG00000015862	abtb1		ENSLOCG00000013573	abtb1	
ENSXMAG00000028285	hexa		ENSLOCG00000014972	hexa	
ENSXMAG00000018663	fosb		ENSLOCG00000014630	fosb	
ENSXMAG00000018217	dao.2		ENSLOCG00000004565	dao.2	
ENSXMAG00000003292	dhx38		ENSLOCG00000004169	dhx38	
ENSXMAG00000001985	fkbp11		ENSLOCG00000007767	fkbp11	
ENSXMAG00000005141	rpl37		ENSLOCG00000011220	rpl37	
ENSXMAG00000014669	lmtk2		ENSLOCG00000008620	lmtk2	
ENSXMAG00000010833	esco2		ENSLOCG00000015834	esco2	
ENSXMAG00000004918	DACT1		ENSLOCG00000009312	dact1	
ENSXMAG00000030011	RF00577		ENSLOCG00000019430	RF00577	
ENSXMAG00000023732	rab11a		ENSLOCG00000013835	rab11a	
ENSXMAG00000018623	capn12		ENSLOCG00000014633	capn12	
ENSXMAG00000016298	edar		ENSLOCG00000010312	edar	
ENSXMAG00000004919	dkc1		ENSLOCG00000013713	dkc1	
ENSXMAG00000003284	cyb5b		ENSLOCG00000004148	cyb5b	
ENSXMAG00000006345	pbk		ENSLOCG00000015839	PBK	
ENSXMAG00000003282	zgc:162592		ENSLOCG00000004138	zgc:162592	
ENSXMAG00000005181	pdcl		ENSLOCG00000003058	pdcl	
ENSXMAG00000013272	dhdds		ENSLOCG00000005552	dhdds	
ENSXMAG00000006816	cdc5l		ENSLOCG00000015811	cdc5l	
ENSXMAG00000006351	SCARA5		ENSLOCG00000015840	scara5	
ENSXMAG00000010106	rrn3		ENSLOCG00000007390	rrn3	
ENSXMAG00000018599	clasrp		ENSLOCG00000014647	clasrp	
ENSXMAG00000005190	rpl28		ENSLOCG00000003073	rpl28	
ENSXMAG00000018226	svopa		ENSLOCG00000004555	svopa	
ENSXMAG00000029022	C1orf232		ENSLOCG00000000174		
ENSXMAG00000029944	rps26		ENSLOCG00000007743	rps26	
ENSXMAG00000016314	vgll3		ENSLOCG00000010554	VGLL3	
ENSXMAG00000021863			ENSLOCG00000003085		
ENSXMAG00000015881	rbsn		ENSLOCG00000011180	rbsn	
ENSXMAG00000006354			ENSLOCG00000015843		
ENSXMAG00000004931	cinp		ENSLOCG00000009791	CINP	
ENSXMAG00000003227	cpne7		ENSLOCG00000004083	cpne7	
ENSXMAG00000008780	chmp2ba		ENSLOCG00000010544	chmp2bb	
ENSXMAG00000023324	mrps25		ENSLOCG00000011170	mrps25	

ENSXMAG00000019963	oscp1a	ENSLOCG00000018277	oscp1a
ENSXMAG00000010131	hao1	ENSLOCG00000016440	hao1
ENSXMAG00000015850	stoml1	ENSLOCG00000014929	stoml1
ENSXMAG00000014649	aimp2	ENSLOCG00000008482	aimp2
ENSXMAG00000008771	pou1f1	ENSLOCG00000010523	pou1f1
ENSXMAG00000013661	dis3l	ENSLOCG00000013846	dis3l
ENSXMAG00000015890	nr2c2	ENSLOCG00000011160	nr2c2
ENSXMAG00000018244	usp30	ENSLOCG00000004518	usp30
ENSXMAG00000005198		ENSLOCG00000003461	SEC16A
ENSXMAG00000006720		ENSLOCG00000015813	supt3h
ENSXMAG00000015863	cyp11a2	ENSLOCG00000014925	cyp11a2
ENSXMAG00000014648		ENSLOCG00000013038	zgc:161969
ENSXMAG00000029804		ENSLOCG00000003032	TMEM200C
ENSXMAG00000013256		ENSLOCG00000005609	
ENSXMAG00000029925	wdr46	ENSLOCG00000014416	wdr46
ENSXMAG00000002003	rhebl1	ENSLOCG00000007682	rhebl1
ENSXMAG00000018580	snrpa	ENSLOCG00000014661	snrpa
ENSXMAG00000008584	ccnb1	ENSLOCG00000010488	ccnb1
ENSXMAG00000007344		ENSLOCG00000000866	
ENSXMAG00000010144	smg1	ENSLOCG00000007061	smg1
ENSXMAG00000017972	eya3	ENSLOCG00000004228	eya3
ENSXMAG00000018577	zgc:153119	ENSLOCG00000014662	zgc:153119
ENSXMAG00000018252	alkbh2	ENSLOCG00000004502	alkbh2
ENSXMAG00000002012		ENSLOCG00000007652	PRKAG1
ENSXMAG00000022558	tipin	ENSLOCG00000013854	tipin
ENSXMAG00000006356	gnpat	ENSLOCG00000015770	gnpat
ENSXMAG00000003221	rpl13	ENSLOCG00000004063	rpl13
ENSXMAG00000011563	txndc11	ENSLOCG00000000563	txndc11
ENSXMAG00000027730	spag8	ENSLOCG00000010480	spag8
ENSXMAG00000021719	zgc:152951	ENSLOCG00000010509	zgc:152951
ENSXMAG00000018575	itpkcb	ENSLOCG00000014664	itpkca
ENSXMAG00000021993	RF00324	ENSLOCG00000019701	RF00324
ENSXMAG00000026302	rbm45	ENSLOCG00000006862	rbm45
ENSXMAG00000018254	unga	ENSLOCG00000004484	unga
ENSXMAG00000015928	ogg1	ENSLOCG00000014515	ogg1
ENSXMAG00000011577		ENSLOCG00000000688	BFAR
ENSXMAG00000028433	zgc:194224	ENSLOCG00000015768	zgc:194224
ENSXMAG00000013749	crybgx	ENSLOCG00000013888	crybgx
ENSXMAG00000023953		ENSLOCG00000004145	litaf
ENSXMAG00000012556	mfsd8	ENSLOCG00000010695	mfsd8
ENSXMAG00000003191	kars	ENSLOCG00000004002	kars
ENSXMAG00000027605	si:ch211-261p9.4	ENSLOCG00000010497	C3orf38
ENSXMAG00000015931	qars	ENSLOCG00000014502	qars
ENSXMAG00000012519	fbxl16	ENSLOCG00000006759	fbxl16
ENSXMAG00000018262	pxmp2	ENSLOCG00000004024	pxmp2
ENSXMAG00000019893	smim13	ENSLOCG00000007898	smim13
ENSXMAG00000006380	TRIM67	ENSLOCG00000015766	TRIM67
ENSXMAG00000000261	GFM1	ENSLOCG00000003778	gfm1
ENSXMAG00000008594	npr2	ENSLOCG00000002488	si:dkey-37g12.1
ENSXMAG00000000989	zc3h15	ENSLOCG00000006786	zc3h15
ENSXMAG00000015911	commd4	ENSLOCG00000014904	commd4
ENSXMAG00000014857	zgc:153247	ENSLOCG00000004065	EARS2
ENSXMAG00000006385	exoc8	ENSLOCG00000017989	exoc8
ENSXMAG00000013494	epha3	ENSLOCG00000010477	epha3
ENSXMAG00000025522	lxn	ENSLOCG00000003797	lxn
ENSXMAG00000018267	gatc	ENSLOCG00000004056	gatc

ENSXMAG00000015912	neil1	ENSLOCG00000014898	neil1
ENSXMAG00000006391	sprtn	ENSLOCG00000015764	sprtn
ENSXMAG00000005543	xkr8.3	ENSLOCG00000004211	xkr8.3
ENSXMAG00000019531	pnrc2	ENSLOCG00000018289	pnrc2
ENSXMAG00000014846		ENSLOCG00000004053	GGA2
ENSXMAG00000012528	12-Sep	ENSLOCG00000006723	12-Sep
ENSXMAG00000000259	neu4	ENSLOCG00000009785	neu4
ENSXMAG00000015918	man2c1	ENSLOCG00000014890	man2c1
ENSXMAG00000027637	RBPMS	ENSLOCG00000013199	RBPMS
ENSXMAG00000025517		ENSLOCG00000006742	znf804a
ENSXMAG00000024065	fam89a	ENSLOCG00000015762	fam89a
ENSXMAG00000022535	wdr53	ENSLOCG00000009777	wdr53
ENSXMAG00000006397	arv1	ENSLOCG00000015761	arv1
ENSXMAG00000014783	parn	ENSLOCG00000000715	parn
ENSXMAG00000013230	akirin1	ENSLOCG00000005708	akirin1
ENSXMAG00000002036	os9	ENSLOCG00000007533	os9
ENSXMAG00000008655	alad	ENSLOCG00000000657	alad
ENSXMAG00000028725	srprb	ENSLOCG00000009740	srprb
ENSXMAG00000005548	msto1	ENSLOCG00000007178	
ENSXMAG00000002343	rhbdl2	ENSLOCG00000005736	rhbdl2
ENSXMAG00000014299		ENSLOCG00000012878	ABCA5
ENSXMAG00000021391		ENSLOCG00000017417	STX19
ENSXMAG00000012522	DMRTA1	ENSLOCG00000012567	DMRTA1
ENSXMAG00000003380	ca12	ENSLOCG00000014880	ca12
ENSXMAG00000006400	ttc13	ENSLOCG00000015756	ttc13
ENSXMAG00000019635	rho	ENSLOCG00000018246	rho
ENSXMAG00000028101	GFOD1	ENSLOCG00000010603	GFOD1
ENSXMAG00000005562		ENSLOCG00000005956	DPY19L4
ENSXMAG00000029274	COL26A1	ENSLOCG00000001578	COL26A1
ENSXMAG00000026290	rragca	ENSLOCG00000005778	rragca
ENSXMAG00000021716		ENSLOCG00000012571	
ENSXMAG00000019529	pou3f1	ENSLOCG00000018292	pou3f1
ENSXMAG00000014292	asb3	ENSLOCG00000016273	asb3
ENSXMAG00000013219	utp11l	ENSLOCG00000005795	utp11l
ENSXMAG00000005568	ints8	ENSLOCG00000006404	ints8
ENSXMAG00000014289	si:rp71-19m20.1	ENSLOCG00000008269	C10orf88
ENSXMAG00000003362	usp3	ENSLOCG00000014869	usp3
ENSXMAG00000014766		ENSLOCG00000000776	
ENSXMAG00000013948	lrrc57	ENSLOCG00000011675	lrrc57
ENSXMAG00000026351	APCDD1	ENSLOCG00000002365	APCDD1
ENSXMAG00000002056	stat2	ENSLOCG00000007484	stat2
ENSXMAG00000020110	FUT4	ENSLOCG00000018021	FUT4
ENSXMAG00000013512	nckap1	ENSLOCG00000006648	nckap1
ENSXMAG00000022386	pdxdc1	ENSLOCG00000007352	pdxdc1
ENSXMAG00000026052	thumpd1	ENSLOCG00000000854	thumpd1
ENSXMAG00000029211	elavl2	ENSLOCG00000012575	elavl2
ENSXMAG00000027421		ENSLOCG00000011669	si:dkey-170l10.1
ENSXMAG00000000805	vapal	ENSLOCG00000002404	vapal
ENSXMAG00000028817	rspo1	ENSLOCG00000005961	rspo1
ENSXMAG00000025472	slc25a19	ENSLOCG00000013316	slc25a19
ENSXMAG00000013966	slc6a4b	ENSLOCG00000004125	slc6a4b
ENSXMAG00000003455		ENSLOCG00000010408	
ENSXMAG00000027303		ENSLOCG00000008287	
ENSXMAG00000023808	clec11a	ENSLOCG00000015704	
ENSXMAG00000022303	CAAP1	ENSLOCG00000012584	CAAP1
ENSXMAG00000003361	fbxl22	ENSLOCG00000014866	fbxl22

ENSXMAG00000028474	RAB31	ENSLOCG00000002436	RAB31
ENSXMAG00000023257	MRPS7	ENSLOCG00000013326	mrps7
ENSXMAG00000026233		ENSLOCG00000014863	
ENSXMAG00000018315	srp54	ENSLOCG00000009440	srp54
ENSXMAG00000006434	si:ch73-111k22.2	ENSLOCG00000015705	si:ch73-111k22.2
ENSXMAG00000025723		ENSLOCG00000005294	
ENSXMAG00000018520		ENSLOCG00000001531	cux1a
ENSXMAG00000024329		ENSLOCG00000017416	CLDN14
ENSXMAG00000003350	dapk2b	ENSLOCG00000014848	dapk2b
ENSXMAG00000000825	ralbp1	ENSLOCG00000002522	ralbp1
ENSXMAG00000014594	MIF4GD	ENSLOCG00000013322	mif4gdb
ENSXMAG00000020271	gpr84	ENSLOCG00000007191	gpr84
ENSXMAG00000028124	SH2B2	ENSLOCG00000001510	SH2B2
ENSXMAG00000013686		ENSLOCG00000003577	REXO4
ENSXMAG00000014886	dnali1	ENSLOCG00000005894	dnali1
ENSXMAG00000027847	twsg1a	ENSLOCG00000002541	twsg1a
ENSXMAG00000024647	pigp	ENSLOCG00000004992	pigp
ENSXMAG00000003338	tbc1d2b	ENSLOCG00000014842	tbc1d2b
ENSXMAG00000005407	si:ch211-244a23.1	ENSLOCG00000003007	si:ch211-244a23.1
ENSXMAG00000018467		ENSLOCG00000007236	
ENSXMAG00000003483		ENSLOCG00000010377	
ENSXMAG00000025533	hsdl1	ENSLOCG00000017252	hsdl1
ENSXMAG00000018500	bud23	ENSLOCG00000001485	bud23
ENSXMAG00000014853		ENSLOCG00000002899	si:ch211-1i11.3
ENSXMAG00000026996		ENSLOCG00000002556	ankrd12
ENSXMAG00000014557	nup85	ENSLOCG00000013342	nup85
ENSXMAG00000013990	ankrd13a	ENSLOCG00000004140	ankrd13a
ENSXMAG00000018352	sec23a	ENSLOCG00000009415	sec23a
ENSXMAG00000018468	tsfm	ENSLOCG00000006608	tsfm
ENSXMAG00000014815	map7d1a	ENSLOCG00000002929	map7d1a
ENSXMAG00000013545	ppp1r1c	ENSLOCG00000006587	ppp1r1c
ENSXMAG00000016982	magi1a	ENSLOCG00000010248	magi1a
ENSXMAG00000027349	tbc1d32	ENSLOCG00000017251	tbc1d32
ENSXMAG00000023041	cib2	ENSLOCG00000014834	cib2
ENSXMAG00000002074	MYL6	ENSLOCG00000006627	MYL6
ENSXMAG00000004781		ENSLOCG00000013213	
ENSXMAG00000005375	psmd5	ENSLOCG00000003231	psmd5
ENSXMAG00000014693	GTF3C1	ENSLOCG00000000909	GTF3C1
ENSXMAG00000000845	ndufv2	ENSLOCG00000002593	ndufv2
ENSXMAG00000022994	lrrc61	ENSLOCG00000014831	lrrc61
ENSXMAG00000003677		ENSLOCG00000006289	plekha3
ENSXMAG00000005391	B3GNT10	ENSLOCG00000017340	B3GNT10
ENSXMAG00000024022	atoh1c	ENSLOCG00000018237	atoh1c
ENSXMAG00000018485		ENSLOCG00000001466	
ENSXMAG00000001593	sh3d21	ENSLOCG00000002978	sh3d21
ENSXMAG00000003323	DNAJA4	ENSLOCG00000014817	DNAJA4
ENSXMAG00000013801	si:ch1073-450f2.1	ENSLOCG00000005253	shank2
ENSXMAG00000005328	aoc1	ENSLOCG00000008713	aoc1
ENSXMAG00000001589	eva1ba	ENSLOCG00000003102	eva1ba
ENSXMAG00000003822	mettl22	ENSLOCG00000006624	mettl22
ENSXMAG00000000852	acss2l	ENSLOCG00000002610	acss2l
ENSXMAG00000029028	stk40	ENSLOCG00000003116	stk40
ENSXMAG00000018388	mipol1	ENSLOCG00000009377	mipol1
ENSXMAG00000025698	yipf2	ENSLOCG00000006061	yipf2
ENSXMAG00000019701	tmem203	ENSLOCG00000017333	tmem203
ENSXMAG00000017782	ncoa1	ENSLOCG00000015891	ncoa1

ENSXMAG00000002675		ENSLOCG00000006044	timm29
ENSXMAG00000005085	nudcd1	ENSLOCG00000003063	nudcd1
ENSXMAG00000007333	tprn	ENSLOCG00000000692	tprn
ENSXMAG00000005618	zgc:55582	ENSLOCG00000003100	
ENSXMAG00000013851	cttn	ENSLOCG00000005239	cttn
ENSXMAG00000014752	OSCP1	ENSLOCG00000003127	OSCP1
ENSXMAG00000007336	ssna1	ENSLOCG00000000723	ssna1
ENSXMAG00000003167	vat1l	ENSLOCG00000003861	vat1l
ENSXMAG00000005316	scospondin	ENSLOCG00000008668	scospondin
ENSXMAG00000013547	itsn1	ENSLOCG00000006667	itsn1
ENSXMAG00000018391	traf3	ENSLOCG00000009826	traf3
ENSXMAG00000003293	OTUD7A	ENSLOCG00000014801	OTUD7A
ENSXMAG00000024458	ptrhd1	ENSLOCG00000015890	ptrhd1
ENSXMAG00000024500		ENSLOCG00000004533	
ENSXMAG00000017042	ren	ENSLOCG00000012053	ren
ENSXMAG00000028874	mrps15	ENSLOCG00000003165	MRPS15
ENSXMAG00000018414	rcor1	ENSLOCG00000009821	rcor1
ENSXMAG00000017069	csf1b	ENSLOCG00000012067	
ENSXMAG00000020327	gcnt3	ENSLOCG00000017958	gcnt3
ENSXMAG00000014732	csf3r	ENSLOCG00000003184	csf3r
ENSXMAG00000027307	cryzl1	ENSLOCG00000006728	cryzl1
ENSXMAG00000021544	MTCL1	ENSLOCG00000002658	MTCL1
ENSXMAG00000018422	ankrd9	ENSLOCG00000009816	ankrd9
ENSXMAG00000014696	eif2d	ENSLOCG00000012230	eif2d
ENSXMAG00000003275	bnip2	ENSLOCG00000014789	bnip2
ENSXMAG00000003519		ENSLOCG00000010333	RANBP2
ENSXMAG00000003148	usp47	ENSLOCG00000004131	usp47
ENSXMAG00000018423	tecpr2	ENSLOCG00000009804	tecpr2
ENSXMAG00000013588	setd4	ENSLOCG00000006764	setd4
ENSXMAG00000002146	espl1	ENSLOCG00000007163	espl1
ENSXMAG00000005642	nup188	ENSLOCG00000004351	nup188
ENSXMAG00000014647	GRIK3	ENSLOCG00000003209	GRIK3
ENSXMAG00000003274	foxb1a	ENSLOCG00000017957	foxb1a
ENSXMAG00000023617	rab12	ENSLOCG00000002680	rab12
ENSXMAG00000029096		ENSLOCG00000009783	
ENSXMAG00000018451		ENSLOCG00000001375	
ENSXMAG00000021281	si:ch211-175f12.2	ENSLOCG00000003245	si:ch211-175f12.2
ENSXMAG00000002155	pfdn5	ENSLOCG00000007134	pfdn5
ENSXMAG00000013883	PPFIA1	ENSLOCG00000005197	PPFIA1
ENSXMAG00000000881	napgb	ENSLOCG00000002353	napga
ENSXMAG00000022815	sptssb	ENSLOCG00000017385	sptssb
ENSXMAG00000005677	phyhd1	ENSLOCG00000004384	phyhd1
ENSXMAG00000014629	inpp5b	ENSLOCG00000003290	inpp5b
ENSXMAG00000019587	dolk	ENSLOCG00000017343	dolk
ENSXMAG00000025971	TNRC6C	ENSLOCG00000013377	tnrc6c1
ENSXMAG00000022721	gltpa	ENSLOCG00000004248	gltpa
ENSXMAG00000003248	ice2	ENSLOCG00000014783	ice2
ENSXMAG00000018406	nmd3	ENSLOCG00000003561	nmd3
ENSXMAG00000025283	dnlz	ENSLOCG00000004465	DNLZ
ENSXMAG00000014614	mtf1	ENSLOCG00000003310	mtf1
ENSXMAG00000013739	si:dkey-16p21.7	ENSLOCG00000017118	si:ch211-255p10.4
ENSXMAG00000018447	trmt61a	ENSLOCG00000009920	trmt61a
ENSXMAG00000005693	uck1	ENSLOCG00000006214	UCK1
ENSXMAG00000008020	trpv4	ENSLOCG00000004262	trpv4
ENSXMAG00000014606	yrdc	ENSLOCG00000003321	yrdc
ENSXMAG00000014070	mad1l1	ENSLOCG00000001393	mad1l1

ENSXMAG00000028654	tpt1	ENSLOCG00000005488	tpt1
ENSXMAG00000010956	hvcn1	ENSLOCG00000007346	hvcn1
ENSXMAG00000018450	bag5	ENSLOCG00000009928	bag5
ENSXMAG00000005607	AFDN	ENSLOCG00000015746	AFDN
ENSXMAG00000010953	zgc:153146	ENSLOCG00000013800	zgc:153146
ENSXMAG00000026077	mecr	ENSLOCG00000003375	mecr
ENSXMAG00000008019	fam222a	ENSLOCG00000004291	fam222a
ENSXMAG00000017099	rbm39b	ENSLOCG00000006562	rbm39a
ENSXMAG00000017784	dnd1	ENSLOCG00000011212	
ENSXMAG00000026250	smpdl3b	ENSLOCG00000003393	smpdl3b
ENSXMAG00000000891		ENSLOCG00000002260	piezo2a.2
ENSXMAG00000015744	si:dkeyp-93d12.1	ENSLOCG00000017206	si:dkeyp-93d12.1
ENSXMAG00000008007	mvk	ENSLOCG00000004323	mvk
ENSXMAG00000002604	tspan4b	ENSLOCG00000017164	tspan4b
ENSXMAG00000018455	klc1a	ENSLOCG00000009937	klc1a
ENSXMAG00000018357	kpna4	ENSLOCG00000003608	kpna4
ENSXMAG00000017135	pabpc1l	ENSLOCG00000007519	pabpc1l
ENSXMAG00000010938	hars	ENSLOCG00000011198	hars
ENSXMAG00000015726	rfx6	ENSLOCG00000017133	rfx6
ENSXMAG00000013755	irf3	ENSLOCG00000017144	irf3
ENSXMAG00000027496	EPPK1	ENSLOCG00000008673	
ENSXMAG00000023180	si:ch73-248e21.1	ENSLOCG00000017166	si:ch73-248e21.1
ENSXMAG00000014373		ENSLOCG00000013721	
ENSXMAG00000014548	themis2	ENSLOCG00000003069	themis2
ENSXMAG00000008005	mmab	ENSLOCG00000004341	mmab
ENSXMAG00000020324	c2cd4a	ENSLOCG00000017956	c2cd4a
ENSXMAG00000005703	rapgef1b	ENSLOCG00000004322	rapgef1a
ENSXMAG00000002149		ENSLOCG00000008682	
ENSXMAG00000005605	vgll2a	ENSLOCG00000017136	vgll2a
ENSXMAG00000002218	atf1	ENSLOCG00000006826	atf1
ENSXMAG00000014368	zgc:153240	ENSLOCG00000013733	zgc:153240
ENSXMAG00000002594	nosip	ENSLOCG00000017172	nosip
ENSXMAG00000014527		ENSLOCG00000003050	
ENSXMAG00000007995	aldh3b2	ENSLOCG00000000404	aldh3b2
ENSXMAG00000010936	zmp:0000000758	ENSLOCG00000010540	zmp:0000000758
ENSXMAG00000020389	kctd4	ENSLOCG00000017785	kctd4
ENSXMAG00000005574	plg	ENSLOCG00000015732	plg
ENSXMAG00000025110	zfyve21	ENSLOCG00000009956	zfyve21
ENSXMAG00000016946	stxbp4	ENSLOCG00000012226	stxbp4
ENSXMAG00000017172	smim4	ENSLOCG00000010575	smim4
ENSXMAG00000023759	RF00553	ENSLOCG00000020536	RF00553
ENSXMAG00000002579	rcn3	ENSLOCG00000017170	rcn3
ENSXMAG00000003554	tsc22d1	ENSLOCG00000005602	tsc22d1
ENSXMAG00000002150	zgc:109744	ENSLOCG00000008699	zgc:109744
ENSXMAG00000017177	abhd6b	ENSLOCG00000013212	abhd6b
ENSXMAG00000018289	smc4	ENSLOCG00000003657	smc4
ENSXMAG00000007971	ube3b	ENSLOCG00000004355	ube3b
ENSXMAG00000029794	slc52a2	ENSLOCG00000008703	slc52a2
ENSXMAG00000020546		ENSLOCG00000020433	MIR190A
ENSXMAG00000014481	med18	ENSLOCG00000003042	med18
ENSXMAG00000003088	tead1b	ENSLOCG00000004054	tead1b
ENSXMAG00000026887	kctd10	ENSLOCG00000004391	kctd10
ENSXMAG00000016934	ANKFN1	ENSLOCG00000012248	ANKFN1
ENSXMAG00000002559	abat	ENSLOCG00000006598	abat
ENSXMAG00000005268		ENSLOCG00000003135	ift74
ENSXMAG00000005516	igf2r	ENSLOCG00000015707	igf2r

ENSXMAG00000003144	TPM1	ENSLOCG00000014736	tpma
ENSXMAG00000014034	lrba	ENSLOCG00000008628	lrba
ENSXMAG00000017210	trh	ENSLOCG00000011194	
ENSXMAG00000014470	SCMH1	ENSLOCG00000003009	SCMH1
ENSXMAG00000028478		ENSLOCG00000004219	
ENSXMAG00000001249	ada	ENSLOCG00000001870	ada
ENSXMAG00000025706		ENSLOCG00000003161	LRRC19
ENSXMAG00000000933		ENSLOCG00000002161	MPPE1
ENSXMAG00000001245	pkig	ENSLOCG00000001911	pkig
ENSXMAG00000003138	lactb	ENSLOCG00000014732	lactb
ENSXMAG00000010874	slc7a11	ENSLOCG00000010595	slc7a11
ENSXMAG00000014468	cited4b	ENSLOCG00000018276	cited4b
ENSXMAG00000026224		ENSLOCG00000010044	zcchc2
ENSXMAG00000003070	btbd10b	ENSLOCG00000003960	btbd10b
ENSXMAG00000000763	atp9b	ENSLOCG00000007857	atp9b
ENSXMAG00000014012	arfgap2	ENSLOCG00000005421	arfgap2
ENSXMAG00000005514	ttll2	ENSLOCG00000015741	ttll2
ENSXMAG00000029317	rab42b	ENSLOCG00000003467	si:dkey-34d22.5
ENSXMAG00000002542	carm1	ENSLOCG00000006077	carm1
ENSXMAG00000001241	ttpal	ENSLOCG00000001940	ttpal
ENSXMAG00000017238	PLXND1	ENSLOCG00000013025	PLXND1
ENSXMAG00000021586		ENSLOCG00000017386	
ENSXMAG00000022985		ENSLOCG00000002166	
ENSXMAG00000003600	agpat3	ENSLOCG00000003758	agpat3
ENSXMAG00000003102	etfa	ENSLOCG00000014711	etfa
ENSXMAG00000024081	msra	ENSLOCG00000015881	msra
ENSXMAG00000014465	taf12	ENSLOCG00000003480	taf12
ENSXMAG00000018493	pank2	ENSLOCG00000002151	pank2
ENSXMAG00000001220	hnf4a	ENSLOCG00000001957	hnf4a
ENSXMAG00000018251	si:dkeyp-97b10.3	ENSLOCG00000003695	si:dkeyp-97b10.3
ENSXMAG00000014054	pex16	ENSLOCG00000005389	pex16
ENSXMAG00000001219	fitm2	ENSLOCG00000001999	fitm2
ENSXMAG00000003616	chpfa	ENSLOCG00000005334	chpfa
ENSXMAG00000002539	clpp	ENSLOCG00000006106	clpp
ENSXMAG00000007821	acacb	ENSLOCG00000004439	acacb
ENSXMAG00000001199	gdap1l1	ENSLOCG00000002008	gdap1l1
ENSXMAG00000001000	tmem14cb	ENSLOCG00000013157	tmem14cb
ENSXMAG00000001331		ENSLOCG00000011027	slbp
ENSXMAG00000003624	asic4a	ENSLOCG00000000689	asic4a
ENSXMAG00000027198	mab21l2	ENSLOCG00000018024	mab21l2
ENSXMAG00000014293	atad5a	ENSLOCG00000013779	atad5a
ENSXMAG00000024075	aldh3b1	ENSLOCG00000000747	
ENSXMAG00000017297	exorh	ENSLOCG00000013037	exorh
ENSXMAG00000012834	immt	ENSLOCG00000010858	immt
ENSXMAG00000005470	ift172	ENSLOCG00000015878	ift172
ENSXMAG00000008099	rps3a	ENSLOCG00000008617	rps3a
ENSXMAG00000001001	pak1ip1	ENSLOCG00000013164	pak1ip1
ENSXMAG00000001194	jph2	ENSLOCG00000002027	jph2
ENSXMAG00000018497	loxl3b	ENSLOCG00000002120	loxl3b
ENSXMAG00000004908	LURAP1L	ENSLOCG00000013262	LURAP1L
ENSXMAG00000009282	ctr9	ENSLOCG00000003894	ctr9
ENSXMAG00000023036	si:ch211-79k12.1	ENSLOCG00000004280	si:ch211-79k12.1
ENSXMAG00000014222	ccnb2	ENSLOCG00000013651	ccnb2
ENSXMAG00000001760	prkcea	ENSLOCG00000016351	prkceb
ENSXMAG00000017357	IFT122	ENSLOCG00000013048	IFT122
ENSXMAG00000003625	gmppaa	ENSLOCG00000000759	gmppaa

ENSXMAG00000027084		ENSLOCG00000011017	ccdc142
ENSXMAG00000005734	dolpp1	ENSLOCG00000004245	dolpp1
ENSXMAG00000014459	cadm4	ENSLOCG00000004299	cadm4
ENSXMAG00000017418	nell2a	ENSLOCG00000016436	nell2a
ENSXMAG00000001022	cc2d1b	ENSLOCG00000003904	cc2d1b
ENSXMAG00000026802	rab40b	ENSLOCG00000012215	rab40b
ENSXMAG00000007745	pole	ENSLOCG00000003978	pole
ENSXMAG00000026945	schip1	ENSLOCG00000003742	schip1
ENSXMAG00000003057	scaper	ENSLOCG00000014697	scaper
ENSXMAG00000005746	miga2	ENSLOCG00000004191	miga2
ENSXMAG00000022862	abhd18	ENSLOCG00000010714	abhd18
ENSXMAG00000009304	mrvi1	ENSLOCG00000003841	mrvi1
ENSXMAG00000020826		ENSLOCG00000017168	pax10
ENSXMAG00000025028	mrps26	ENSLOCG00000002051	mrps26
ENSXMAG00000018528	si:dkey-33c12.3	ENSLOCG00000002004	si:dkey-33c12.3
ENSXMAG00000008071	gatb	ENSLOCG00000008564	gatb
ENSXMAG00000005147	dus1l	ENSLOCG00000012342	dus1l
ENSXMAG00000014808	igbp1	ENSLOCG00000014729	igbp1
ENSXMAG00000021129	mbd4	ENSLOCG00000013054	MBD4
ENSXMAG00000005758	st6galnac4	ENSLOCG00000004230	st6galnac4
ENSXMAG00000009316		ENSLOCG00000003812	LYVE1
ENSXMAG00000014794	enpp6	ENSLOCG00000013730	enpp6
ENSXMAG00000005798	cpsf1	ENSLOCG00000008484	cpsf1
ENSXMAG00000025466	txndc12	ENSLOCG00000003852	txndc12
ENSXMAG00000023588	st6galnac6	ENSLOCG00000004212	st6galnac6
ENSXMAG00000018535	si:dkey-33c12.4	ENSLOCG00000001861	
ENSXMAG00000004689		ENSLOCG00000016431	
ENSXMAG00000005460	dnajc5ga	ENSLOCG00000015869	dnajc5ga
ENSXMAG00000001081	gipc2	ENSLOCG00000003819	gipc2
ENSXMAG00000014119	lhb	ENSLOCG00000015412	lhb
ENSXMAG00000003657	dars	ENSLOCG00000000902	dars
ENSXMAG00000007717	p2rx2	ENSLOCG00000003961	p2rx2
ENSXMAG00000004694	szl	ENSLOCG00000015495	szl
ENSXMAG00000016918	cox11	ENSLOCG00000012222	cox11
ENSXMAG00000001398	mfsd1	ENSLOCG00000003756	mfsd1
ENSXMAG00000027168	dnajb4	ENSLOCG00000003800	dnajb4
ENSXMAG00000028098	csnk1a1	ENSLOCG00000012101	csnk1a1
ENSXMAG00000023181	slc16a3	ENSLOCG00000012307	si:ch211-234h8.7
ENSXMAG00000027318	si:ch211-71n6.4	ENSLOCG00000005410	si:ch211-71n6.4
ENSXMAG00000005369	cad	ENSLOCG00000015871	cad
ENSXMAG00000019725	dctpp1	ENSLOCG00000002747	DCTPP1
ENSXMAG00000013778	mapk7	ENSLOCG00000000046	mapk7
ENSXMAG00000005778	spout1	ENSLOCG00000004143	spout1
ENSXMAG00000009559	fam120c	ENSLOCG00000014788	fam120c
ENSXMAG00000015102	ndst2a	ENSLOCG00000012849	ndst2a
ENSXMAG00000005786	adck5	ENSLOCG00000008470	adck5
ENSXMAG00000018579		ENSLOCG00000004417	
ENSXMAG00000003430	csnk1db	ENSLOCG00000012299	csnk1db
ENSXMAG00000020030	uts1	ENSLOCG00000017992	uts1
ENSXMAG00000025141	vkorc1	ENSLOCG00000000048	
ENSXMAG00000002286	SUMF1	ENSLOCG00000011045	
ENSXMAG00000007708	pes	ENSLOCG00000003935	pes
ENSXMAG00000023997	fbxw7	ENSLOCG00000008551	fbxw7
ENSXMAG00000005791	kyat1	ENSLOCG00000004106	kyat1
ENSXMAG00000028393		ENSLOCG00000014522	
ENSXMAG00000024593	fbxw9	ENSLOCG00000006165	fbxw9

ENSXMAG00000026688	VSTM2A	ENSLOCG00000000372	VSTM2A	
ENSXMAG00000005355	mpv17	ENSLOCG00000015864	mpv17	
ENSXMAG00000011636	thsd7ba	ENSLOCG00000000959	thsd7bb	
ENSXMAG00000007168	fubp1	ENSLOCG00000003745	fubp1	
ENSXMAG00000003449	engase	ENSLOCG00000012284	ENGASE	
ENSXMAG00000004754	nuak1b	ENSLOCG00000015451	nuak1b	
ENSXMAG00000010642		ENSLOCG00000001841		
ENSXMAG00000000708	large2	ENSLOCG00000004996	large2	
ENSXMAG00000029407	si:dkey-117i10.1	ENSLOCG00000006143	si:dkey-117i10.1	
ENSXMAG00000001426	p2ry13	ENSLOCG00000017375	p2ry13	
ENSXMAG00000008051	tma16	ENSLOCG00000007945	tma16	
ENSXMAG00000024608	surf6	ENSLOCG00000004072	surf6	
ENSXMAG00000024226	arg1	ENSLOCG00000016630	arg1	
ENSXMAG00000014181	dhps	ENSLOCG00000006119	dhps	
ENSXMAG00000016231	fbxo38	ENSLOCG00000012089	fbxo38	
ENSXMAG00000009340	sbf2	ENSLOCG00000003691	sbf2	
ENSXMAG00000022403	si:dkey-28n18.9	ENSLOCG00000011067		
ENSXMAG00000003463	scpep1	ENSLOCG00000013821	scpep1	
ENSXMAG00000017474	brpf1	ENSLOCG00000014525	brpf1	
ENSXMAG00000007698	lztr1	ENSLOCG00000003881	lztr1	
ENSXMAG00000025279	p2ry12	ENSLOCG00000001975	p2ry12	
ENSXMAG00000007120	miga1	ENSLOCG00000003715	miga1	
ENSXMAG00000005824	surf1	ENSLOCG00000004058	surf1	
ENSXMAG00000008047	1-Mar	ENSLOCG00000007938		1-Mar
ENSXMAG00000005319	gtf3c2	ENSLOCG00000015848	gtf3c2	
ENSXMAG00000017453	appl2	ENSLOCG00000015456	appl2	
ENSXMAG00000011594	spopla	ENSLOCG00000001012	spopla	
ENSXMAG00000015092	zswim8	ENSLOCG00000012853	zswim8	
ENSXMAG00000011720	igsf10	ENSLOCG00000001996	igsf10	
ENSXMAG00000009536		ENSLOCG00000003551	lima1a	
ENSXMAG00000026861		ENSLOCG00000013816	coil	
ENSXMAG00000005765	ctdp1	ENSLOCG00000008457	ctdp1	
ENSXMAG00000021562		ENSLOCG00000000538		
ENSXMAG00000019671	nxph2a	ENSLOCG00000001038	nxph2a	
ENSXMAG00000001444	aadac	ENSLOCG00000002005	aadac	
ENSXMAG00000016309	usp33	ENSLOCG00000003680	usp33	
ENSXMAG00000027279	RF00049	ENSLOCG00000020187	RF00049	
ENSXMAG00000020240	si:dkey-98f17.3	ENSLOCG00000018143	si:dkey-98f17.3	
ENSXMAG00000019071	tmem129	ENSLOCG00000011038	tmem129	
ENSXMAG00000020274	SUCNR1	ENSLOCG00000002017	SUCNR1	
ENSXMAG00000029232	znf512	ENSLOCG00000015845	znf512	
ENSXMAG00000026813	RF00049	ENSLOCG00000020269	RF00049	
ENSXMAG00000029736	tmem192	ENSLOCG00000007925	tmem192	
ENSXMAG00000026138	RF00069	ENSLOCG00000018891	RF00069	
ENSXMAG00000019616	kcng2	ENSLOCG00000008447	kcng2	
ENSXMAG00000019079		ENSLOCG00000010919	atoh8	
ENSXMAG00000007689	aifm3	ENSLOCG00000003842	aifm3	
ENSXMAG00000003030	rcn2	ENSLOCG00000014693	rcn2	
ENSXMAG00000012043	rnf103	ENSLOCG00000010801	rnf103	
ENSXMAG00000018653		ENSLOCG00000006002		
ENSXMAG00000017489	appl2	ENSLOCG00000015459	appl2	
ENSXMAG00000018435		ENSLOCG00000003252		
ENSXMAG00000028108	zzz3	ENSLOCG00000003656	zzz3	
ENSXMAG00000028261	PQLC1	ENSLOCG00000008439	PQLC1	
ENSXMAG00000011057	slc31a2	ENSLOCG00000001771	slc31a2	
ENSXMAG00000005842	usp20	ENSLOCG00000004021	usp20	

ENSXMAG00000018419	csnk2a2a	ENSLOCG00000003699	csnk2a2b
ENSXMAG00000023905	arrdc1b	ENSLOCG00000001694	arrdc1b
ENSXMAG00000016357	ak5	ENSLOCG00000003639	AK5
ENSXMAG00000019535	gpr182	ENSLOCG00000018184	gpr182
ENSXMAG00000022759		ENSLOCG00000013686	
ENSXMAG00000007688	si:dkey-98f17.5	ENSLOCG00000003824	si:dkey-98f17.5
ENSXMAG00000005756	hpcal4	ENSLOCG00000002031	hpcal4
ENSXMAG00000003513	igf2bp1	ENSLOCG00000013497	igf2bp1
ENSXMAG00000003014	si:dkey-24l11.2	ENSLOCG00000014681	si:dkey-24l11.2
ENSXMAG00000018394	psmd13	ENSLOCG00000002439	psmd13
ENSXMAG00000016375	pigk	ENSLOCG00000003605	pigk
ENSXMAG00000012021		ENSLOCG00000013463	
ENSXMAG00000015076	polr1c	ENSLOCG00000016710	polr1c
ENSXMAG00000000183	clvs2	ENSLOCG00000017259	clvs2
ENSXMAG00000001944	sec24d	ENSLOCG00000012522	sec24d
ENSXMAG00000026718	kti12	ENSLOCG00000014679	kti12
ENSXMAG00000005893	ptges	ENSLOCG00000004008	ptges
ENSXMAG00000018384		ENSLOCG00000002426	SIRT3
ENSXMAG00000005295	smpdl3a	ENSLOCG00000017258	smpdl3a
ENSXMAG00000020275	p2ry1	ENSLOCG00000017377	p2ry1
ENSXMAG00000022692	hpca	ENSLOCG00000001992	hpca
ENSXMAG00000020321	ch25hl1.1	ENSLOCG00000017955	ch25hl1.1
ENSXMAG00000009410	ipo7	ENSLOCG00000003606	ipo7
ENSXMAG00000003541	copz2	ENSLOCG00000013403	COPZ2
ENSXMAG00000015070	fbxo28	ENSLOCG00000016709	fbxo28
ENSXMAG00000018656	plcl2	ENSLOCG00000006475	plcl2
ENSXMAG00000016401	st6galnac3	ENSLOCG00000003555	st6galnac3
ENSXMAG00000003005	znf592	ENSLOCG00000014672	znf592
ENSXMAG00000018374	drd4b	ENSLOCG00000002345	drd4a
ENSXMAG00000013788	aldoaa	ENSLOCG00000000155	
ENSXMAG00000029834	degs1	ENSLOCG00000016708	degs1
ENSXMAG00000002988	slc28a1	ENSLOCG00000014663	slc28a1
ENSXMAG00000021116	rabggtb	ENSLOCG00000003528	rabggtb
ENSXMAG00000024675		ENSLOCG00000016937	
ENSXMAG00000001979		ENSLOCG00000012515	si:ch211-145b13.5
ENSXMAG00000009442	tspan31	ENSLOCG00000003959	tspan31
ENSXMAG00000018351	ctsd	ENSLOCG00000002297	ctsd
ENSXMAG00000008035	uchl1	ENSLOCG00000012930	uchl1
ENSXMAG00000005901	si:ch211-182d3.1	ENSLOCG00000003976	si:ch211-182d3.1
ENSXMAG00000009444	tmem41b	ENSLOCG00000003585	tmem41b
ENSXMAG00000015015	nvl	ENSLOCG00000016706	nvl
ENSXMAG00000004413	gtf3c3	ENSLOCG00000005238	gtf3c3
ENSXMAG00000014941	acadm	ENSLOCG00000003500	acadm
ENSXMAG00000020883	emx1	ENSLOCG00000009348	emx1
ENSXMAG00000001477	ARHGEF26	ENSLOCG00000002086	ARHGEF26
ENSXMAG00000018662	satb1b	ENSLOCG00000006524	satb1b
ENSXMAG00000005267	btbd9	ENSLOCG00000017047	btbd9
ENSXMAG00000016703	a1cf	ENSLOCG00000006302	a1cf
ENSXMAG00000017525	FGD3	ENSLOCG00000014094	FGD3
ENSXMAG00000005732	noto	ENSLOCG00000009299	noto
ENSXMAG00000014999	rps8a	ENSLOCG00000003039	rps8a
ENSXMAG00000028102	zgc:91944	ENSLOCG00000001916	zgc:91944
ENSXMAG00000004408		ENSLOCG00000011005	
ENSXMAG00000009460	dennd5a	ENSLOCG00000003559	dennd5a
ENSXMAG00000003244	rabl3	ENSLOCG00000006265	rabl3
ENSXMAG00000002975	malt3	ENSLOCG00000014651	malt3

ENSXMAG00000027690		ENSLOCG00000005140	
ENSXMAG00000007641	rbm19	ENSLOCG00000008341	rbm19
ENSXMAG00000017565		ENSLOCG00000014097	BICD2
ENSXMAG00000005736	CDC25B	ENSLOCG00000001061	CDC25B
ENSXMAG00000004396	runx1	ENSLOCG00000010990	runx1
ENSXMAG00000001490	DHX36	ENSLOCG00000002125	dhx36
ENSXMAG00000015013		ENSLOCG00000005122	tk2
ENSXMAG00000005224		ENSLOCG00000016787	galnt14
ENSXMAG00000023302	ctdsp2	ENSLOCG00000000635	
ENSXMAG00000028175		ENSLOCG00000010988	clic6
ENSXMAG00000023934	sinhcafl	ENSLOCG00000003540	sinhcafl
ENSXMAG00000007771	adgra3	ENSLOCG00000012367	adgra3
ENSXMAG00000011584	gtf2e1	ENSLOCG00000006285	gtf2e1
ENSXMAG00000030079	hoxb2a	ENSLOCG00000007028	
ENSXMAG00000017604	si:ch73-142c19.1	ENSLOCG00000014050	si:ch73-142c19.1
ENSXMAG00000004373	rcan1a	ENSLOCG00000010699	rcan1a
ENSXMAG00000021955	aprt	ENSLOCG00000004763	aprt
ENSXMAG00000016637	asah2	ENSLOCG00000006273	asah2
ENSXMAG00000026139	ap5s1	ENSLOCG00000001046	ap5s1
ENSXMAG00000009377	tegt	ENSLOCG00000004059	tegt
ENSXMAG00000022886	GPR149	ENSLOCG00000002178	GPR149
ENSXMAG00000022738	hoxb3a	ENSLOCG00000013436	hoxb3a
ENSXMAG00000000350	kpna6	ENSLOCG00000002327	kpna6
ENSXMAG00000002951	blm	ENSLOCG00000014302	blm
ENSXMAG00000024779	RF00218	ENSLOCG00000019259	RF00218
ENSXMAG00000009507	scube2	ENSLOCG00000003510	scube2
ENSXMAG00000005218	angel2	ENSLOCG00000016793	angel2
ENSXMAG00000007640	zgc:158619	ENSLOCG00000008313	PLBD2
ENSXMAG00000015011	hectd3	ENSLOCG00000003011	hectd3
ENSXMAG00000023964	fam207a	ENSLOCG00000009039	FAM207A
ENSXMAG00000022342	cdt1	ENSLOCG00000004785	cdt1
ENSXMAG00000007766	gba3	ENSLOCG00000012360	gba3
ENSXMAG00000009373	nckap5l	ENSLOCG00000003584	nckap5l
ENSXMAG00000025641		ENSLOCG00000003292	
ENSXMAG00000007748	ppargc1a	ENSLOCG00000012350	ppargc1a
ENSXMAG00000029970	txlna	ENSLOCG00000002359	txlna
ENSXMAG00000028291	si:dkey-34e4.1	ENSLOCG00000003958	si:dkey-34e4.1
ENSXMAG00000002937	ctsh	ENSLOCG00000014308	ctsh
ENSXMAG00000015069	best4	ENSLOCG00000003046	best4
ENSXMAG00000029174	shisal1b	ENSLOCG00000001158	shisal1a
ENSXMAG00000005200	vash2	ENSLOCG00000000914	vash2
ENSXMAG00000014905	piezo1	ENSLOCG00000004799	piezo1
ENSXMAG00000026905	CDH20	ENSLOCG00000012876	CDH20
ENSXMAG00000018852	cep70	ENSLOCG00000003377	cep70
ENSXMAG00000005937	CACFD1	ENSLOCG00000003536	CACFD1
ENSXMAG00000027358	hoxb8a	ENSLOCG00000013451	hoxb8a
ENSXMAG00000002433	atrn	ENSLOCG00000000928	atrn
ENSXMAG00000009365	kansl2	ENSLOCG00000003604	kansl2
ENSXMAG00000022320		ENSLOCG00000003062	
ENSXMAG00000001566	olfcs1	ENSLOCG00000002830	
ENSXMAG00000027632	EIF3I	ENSLOCG00000002433	
ENSXMAG00000020954	parvg	ENSLOCG00000015368	parvg
ENSXMAG00000005940	ptcd2	ENSLOCG00000011122	ptcd2
ENSXMAG00000009524	tmem9b	ENSLOCG00000003446	tmem9b
ENSXMAG00000008425	dhx15	ENSLOCG00000012339	dhx15
ENSXMAG00000025578	RF00410	ENSLOCG00000020303	RF00410

ENSXMAG00000014096	ankrd40	ENSLOCG00000010960	ankrd40
ENSXMAG00000019594	rnf152	ENSLOCG00000018162	rnf152
ENSXMAG00000007611	morc2	ENSLOCG00000008283	morc2
ENSXMAG00000002838	hoxb9a	ENSLOCG00000013453	hoxb9a
ENSXMAG00000009357	ccnt1	ENSLOCG00000003660	ccnt1
ENSXMAG00000002835	hoxb13a	ENSLOCG00000013457	hoxb13a
ENSXMAG00000009533	st5	ENSLOCG00000003366	st5
ENSXMAG00000018870	cd99	ENSLOCG00000010250	cd99
ENSXMAG00000014869	pcca	ENSLOCG00000003005	pcca
ENSXMAG00000005956	map1b	ENSLOCG00000011141	map1b
ENSXMAG00000014091	luc7l3	ENSLOCG00000010953	luc7l3
ENSXMAG00000017574	parvb	ENSLOCG00000015365	parvb
ENSXMAG00000018872	gyg2	ENSLOCG00000010234	gyg2
ENSXMAG00000009356	sarnp	ENSLOCG00000003621	sarnp
ENSXMAG00000004485	kbtbd2	ENSLOCG00000012888	kbtbd2
ENSXMAG00000020319		ENSLOCG00000017949	
ENSXMAG00000020860	clrn2	ENSLOCG00000003202	clrn2
ENSXMAG00000002829	ube2z	ENSLOCG00000013488	ube2z
ENSXMAG00000011580	stxbp5l	ENSLOCG00000006301	stxbp5l
ENSXMAG00000016837	prkaa1	ENSLOCG00000011222	prkaa1
ENSXMAG00000003206	mxra5a	ENSLOCG00000010205	mxra5b
ENSXMAG00000007910	lap3	ENSLOCG00000003221	LAP3
ENSXMAG00000027254	trim66	ENSLOCG00000003340	trim66
ENSXMAG00000029846	avl9	ENSLOCG00000012894	avl9
ENSXMAG00000020619	RF00001	ENSLOCG00000020430	RF00001
ENSXMAG00000026757	tuba1c	ENSLOCG00000004153	TUBA1C
ENSXMAG00000021930	aif1l	ENSLOCG00000006343	aif1l
ENSXMAG00000006951	stk33	ENSLOCG00000003301	stk33
ENSXMAG00000002813	aoc2	ENSLOCG00000012190	aoc2
ENSXMAG00000003061	phpt1	ENSLOCG00000001151	PHPT1
ENSXMAG00000011548	eme1	ENSLOCG00000011088	eme1
ENSXMAG00000023776	med28	ENSLOCG00000003244	med28
ENSXMAG00000018900	prkx	ENSLOCG00000010188	prkx
ENSXMAG00000007874	klhl2	ENSLOCG00000007894	klhl2
ENSXMAG00000024261	mrpl27	ENSLOCG00000011095	mrpl27
ENSXMAG00000005965	lamc3	ENSLOCG00000006369	lamc3
ENSXMAG00000007604		ENSLOCG00000008423	
ENSXMAG00000025568	lmo1	ENSLOCG00000003286	lmo1
ENSXMAG00000002861	sema4ba	ENSLOCG00000014333	sema4ba
ENSXMAG00000009341	ccm2l	ENSLOCG00000005436	ccm2l
ENSXMAG00000017843	gp9	ENSLOCG00000018261	gp9
ENSXMAG00000014834	vps8	ENSLOCG00000009601	vps8
ENSXMAG00000018908	nlgn4xa	ENSLOCG00000010176	nlgn4xb
ENSXMAG00000002407	eif2ak3	ENSLOCG00000009370	eif2ak3
ENSXMAG00000025726	PUDP	ENSLOCG00000010173	pudp
ENSXMAG00000007869	msmo1	ENSLOCG00000007882	msmo1
ENSXMAG00000026251		ENSLOCG00000008772	SRFBP1
ENSXMAG00000007594	SLC8B1	ENSLOCG00000008436	SLC8B1
ENSXMAG00000024938	cnbpa	ENSLOCG00000013985	cnbpb
ENSXMAG00000027543	tub	ENSLOCG00000003233	tub
ENSXMAG00000007857	cpe	ENSLOCG00000007871	cpe
ENSXMAG00000029988	lsm5	ENSLOCG00000012903	lsm5
ENSXMAG00000002846	zgc:162879	ENSLOCG00000014347	zgc:162879
ENSXMAG00000007001	rras2	ENSLOCG00000003220	rras2
ENSXMAG00000017691	raf1a	ENSLOCG00000013974	raf1b
ENSXMAG00000005973	fibcd1	ENSLOCG00000006394	fibcd1

ENSXMAG00000025637	zgc:66427	ENSLOCG00000000804	
ENSXMAG00000003092	grin1a	ENSLOCG00000000420	grin1a
ENSXMAG00000002395	rpia	ENSLOCG00000009361	rpia
ENSXMAG00000018718		ENSLOCG00000006890	RARB
ENSXMAG00000009323	tcea2	ENSLOCG00000005363	tcea2
ENSXMAG00000018954		ENSLOCG00000001109	
ENSXMAG00000001977	AP3B2	ENSLOCG00000015010	AP3B2
ENSXMAG00000007829	tll1	ENSLOCG00000003509	tll1
ENSXMAG00000005394	si:ch211-284o19.8	ENSLOCG00000000259	
ENSXMAG00000026315	gemin8	ENSLOCG00000010057	GEMIN8
ENSXMAG00000007572	tpcn1	ENSLOCG00000008448	tpcn1
ENSXMAG00000007010	copb1	ENSLOCG00000003198	copb1
ENSXMAG00000011568	si:ch211-119c20.2	ENSLOCG00000011372	si:ch211-119c20.2
ENSXMAG00000018731	nek10	ENSLOCG00000006970	NEK10
ENSXMAG00000018150		ENSLOCG00000003942	ttll11
ENSXMAG00000024197	irf9	ENSLOCG00000001187	irf9
ENSXMAG00000017725	syn2a	ENSLOCG00000013941	syn2a
ENSXMAG00000002837	fkbp16	ENSLOCG00000014352	fkbp16
ENSXMAG00000019735	TCEANC	ENSLOCG00000010028	TCEANC
ENSXMAG00000018737	sept7b	ENSLOCG00000006999	sept7b
ENSXMAG00000005402	parp2	ENSLOCG00000000267	parp2
ENSXMAG00000025570	rgs19	ENSLOCG00000005333	rgs19
ENSXMAG00000024101	mrpl36	ENSLOCG00000008869	mrpl36
ENSXMAG00000007969	PRPS2	ENSLOCG00000009964	PRPS2
ENSXMAG00000009318		ENSLOCG00000012335	
ENSXMAG00000024981	emc9	ENSLOCG00000001201	
ENSXMAG00000002819	pex11a	ENSLOCG00000014367	pex11a
ENSXMAG00000025929	ostc	ENSLOCG00000011223	ostc
ENSXMAG00000014830	chchd1	ENSLOCG00000012875	chchd1
ENSXMAG00000007043	PDE3B	ENSLOCG00000003151	PDE3B
ENSXMAG00000001972	sv2	ENSLOCG00000015007	sv2
ENSXMAG00000002815		ENSLOCG00000014360	gnrhr4
ENSXMAG00000018794	si:dkey-256h2.1	ENSLOCG00000011030	si:dkey-256h2.1
ENSXMAG00000009310	oprl1	ENSLOCG00000005285	oprl1
ENSXMAG00000027214	olfch1	ENSLOCG00000002816	
ENSXMAG00000021988	si:dkeyp-59c12.1	ENSLOCG00000014372	si:dkeyp-59c12.1
ENSXMAG00000014799		ENSLOCG00000012880	si:dkey-191g9.5
ENSXMAG00000010849	pex12	ENSLOCG00000001797	pex12
ENSXMAG00000021120	TNNC1	ENSLOCG00000013793	tnnc1a
ENSXMAG00000028512	etnppl	ENSLOCG00000011208	etnppl
ENSXMAG00000026514	mettl9	ENSLOCG00000008339	mettl9
ENSXMAG00000025792	MED10	ENSLOCG00000008783	med10
ENSXMAG00000009307		ENSLOCG00000011378	si:dkey-48j7.3
ENSXMAG00000014782		ENSLOCG00000015782	galm
ENSXMAG00000002780	st8sia2	ENSLOCG00000014381	st8sia2
ENSXMAG00000011618	kdelr2b	ENSLOCG00000008372	kdelr2b
ENSXMAG00000007057	cyp2r1	ENSLOCG00000003133	cyp2r1
ENSXMAG00000013614	ube2ql1	ENSLOCG00000008774	ube2ql1
ENSXMAG00000017799		ENSLOCG00000003490	spo11
ENSXMAG00000029823		ENSLOCG00000011406	RAB29
ENSXMAG00000013953		ENSLOCG00000013742	casp3a
ENSXMAG00000008479	larp7	ENSLOCG00000011608	
ENSXMAG00000002779	fam174b	ENSLOCG00000014386	fam174b
ENSXMAG00000020034	si:dkeyp-66d1.7	ENSLOCG00000018221	si:dkeyp-66d1.7
ENSXMAG00000014018	zgc:92275	ENSLOCG00000000468	zgc:92275
ENSXMAG00000013597	nsun2	ENSLOCG00000008755	nsun2

ENSXMAG00000002195	ireb2	ENSLOCG00000015029	ireb2
ENSXMAG00000005441	pex19	ENSLOCG00000001928	
ENSXMAG00000008487		ENSLOCG00000005442	
ENSXMAG00000017817	bmp7b	ENSLOCG00000003514	bmp7b
ENSXMAG00000018827	mios	ENSLOCG00000011101	mios
ENSXMAG00000008025	aamp	ENSLOCG00000009130	aamp
ENSXMAG00000009278	cry1ba	ENSLOCG00000011417	cry1ba
ENSXMAG00000027380	wdr45b	ENSLOCG00000012208	wdr45b
ENSXMAG00000005462	copa	ENSLOCG00000001891	copa
ENSXMAG00000028915	RF00415	ENSLOCG00000020020	RF00415
ENSXMAG00000018260	tp53bp1	ENSLOCG00000014193	tp53bp1
ENSXMAG00000000349	fam69b	ENSLOCG00000003692	fam69b
ENSXMAG00000017848	irf10	ENSLOCG00000003532	irf10
ENSXMAG00000008488		ENSLOCG00000011591	ADAMTSL1
ENSXMAG00000023806	gpbar1	ENSLOCG00000009139	gpbar1
ENSXMAG00000028882	srd5a1	ENSLOCG00000008745	srd5a1
ENSXMAG00000024583	ky	ENSLOCG00000000153	ky
ENSXMAG00000018132	mrps2	ENSLOCG00000003705	mrps2
ENSXMAG00000002707	chd2	ENSLOCG00000014387	chd2
ENSXMAG00000008050		ENSLOCG00000009145	
ENSXMAG00000028516	sox6	ENSLOCG00000003065	sox6
ENSXMAG00000022106	si:ch211-204d2.4	ENSLOCG00000015100	PLEKHA2
ENSXMAG00000025164	rps21	ENSLOCG00000005469	rps21
ENSXMAG00000018131		ENSLOCG00000003728	
ENSXMAG00000013967	gtf3c5	ENSLOCG00000005767	gtf3c5
ENSXMAG00000014740	cdk1	ENSLOCG00000006690	cdk1
ENSXMAG00000021684	kdelc1	ENSLOCG00000008683	kdelc1
ENSXMAG00000028965		ENSLOCG00000003546	
ENSXMAG00000018240	tubgcp4	ENSLOCG00000014183	tubgcp4
ENSXMAG00000002207	wdr61	ENSLOCG00000015047	wdr61
ENSXMAG00000027589	gfi1b	ENSLOCG00000005750	gfi1b
ENSXMAG00000025582		ENSLOCG00000017751	
ENSXMAG00000008074	arpc2	ENSLOCG00000009169	arpc2
ENSXMAG00000025744		ENSLOCG00000005484	lama5
ENSXMAG00000018860	ica1	ENSLOCG00000011135	ica1
ENSXMAG00000007136	si:ch211-194c3.5	ENSLOCG00000000908	C20orf194
ENSXMAG00000005522	NCSTN	ENSLOCG00000001868	NCSTN
ENSXMAG00000023956	nepro	ENSLOCG00000008667	nepro
ENSXMAG00000014730	ctu2	ENSLOCG00000004836	ctu2
ENSXMAG00000027320	tsc1b	ENSLOCG00000005714	tsc1a
ENSXMAG00000009799		ENSLOCG00000018059	
ENSXMAG00000008087	slc19a1	ENSLOCG00000009360	slc19a1
ENSXMAG00000018239	cdkn2aip	ENSLOCG00000014181	cdkn2aip
ENSXMAG00000023293	ralba	ENSLOCG00000002006	RALB
ENSXMAG00000024800	mto1	ENSLOCG00000000453	
ENSXMAG00000023165	rnf166	ENSLOCG00000004854	rnf166
ENSXMAG00000001895	gtf3c4	ENSLOCG00000005643	gtf3c4
ENSXMAG00000017898	col5a1	ENSLOCG00000004625	col5a1
ENSXMAG00000007097	plekha7b	ENSLOCG00000003006	plekha7b
ENSXMAG00000007796	pcm1	ENSLOCG00000013882	pcm1
ENSXMAG00000008100	CYP4F8	ENSLOCG00000008791	CYP4F8
ENSXMAG00000018181	iqgap1	ENSLOCG00000014170	iqgap1
ENSXMAG00000026352		ENSLOCG00000012568	kmt2ca
ENSXMAG00000024536		ENSLOCG00000007291	wipf1a
ENSXMAG00000014056	sgsm3	ENSLOCG00000011278	sgsm3
ENSXMAG00000005610	tesk2	ENSLOCG00000002838	tesk2

ENSXMAG00000011226	ptpn9a	ENSLOCG00000015058	ptpn9a
ENSXMAG00000007546	ddhd2	ENSLOCG00000015094	ddhd2
ENSXMAG00000002704	rgma	ENSLOCG00000014392	rgma
ENSXMAG00000008498	dennd4c	ENSLOCG00000011126	DENND4C
ENSXMAG00000025063	foxi2	ENSLOCG00000009392	foxi2
ENSXMAG00000002703	lysmd4	ENSLOCG00000014395	lysmd4
ENSXMAG00000018931	vwde	ENSLOCG00000011205	vwde
ENSXMAG00000018164	crtc3	ENSLOCG00000014162	crtc3
ENSXMAG00000009182	trim101	ENSLOCG00000004940	trim101
ENSXMAG00000004701		ENSLOCG00000001723	
ENSXMAG00000006634	snupn	ENSLOCG00000015064	snupn
ENSXMAG00000005031	XRCC2	ENSLOCG00000012562	XRCC2
ENSXMAG00000011648		ENSLOCG00000012163	
ENSXMAG00000018720		ENSLOCG00000009578	tmem41ab
ENSXMAG00000021818	smug1	ENSLOCG00000006445	smug1
ENSXMAG00000007532		ENSLOCG00000008657	
ENSXMAG00000012180	bean1	ENSLOCG00000005177	bean1
ENSXMAG00000007297	snx33	ENSLOCG00000014253	snx33
ENSXMAG00000004948	cdh5	ENSLOCG00000005195	cdh5
ENSXMAG00000007305	cspg4	ENSLOCG00000014254	cspg4
ENSXMAG00000009161	ptk6b	ENSLOCG00000005191	ptk6a
ENSXMAG00000007119	nucb2a	ENSLOCG00000002925	nucb2a
ENSXMAG00000019995	arl4aa	ENSLOCG00000018349	arl4aa
ENSXMAG00000001828	ppp2r2ab	ENSLOCG00000016105	ppp2r2ab
ENSXMAG00000018161	adamtsl5	ENSLOCG00000014158	adamtsl5
ENSXMAG00000026115	fgfr3	ENSLOCG00000007042	fgfr3
ENSXMAG00000022940		ENSLOCG00000016751	si:ch73-340m8.2
ENSXMAG00000007531	srrd	ENSLOCG00000008675	SRRD
ENSXMAG00000029609	mef2aa	ENSLOCG00000014408	mef2aa
ENSXMAG00000000587	hmg20a	ENSLOCG00000014255	hmg20a
ENSXMAG00000009147	nol4lb	ENSLOCG00000005753	nol4lb
ENSXMAG00000018148	serinc4	ENSLOCG00000014156	serinc4
ENSXMAG00000018944	etv1	ENSLOCG00000011232	etv1
ENSXMAG00000007524	tfip11	ENSLOCG00000008684	tfip11
ENSXMAG00000028576	mtx2	ENSLOCG00000007000	mtx2
ENSXMAG00000016513	myo1b	ENSLOCG00000004425	myo1b
ENSXMAG00000024584	commd7	ENSLOCG00000005720	commd7
ENSXMAG00000025736	peak1	ENSLOCG00000014257	peak1
ENSXMAG00000029941		ENSLOCG00000011640	haus6
ENSXMAG00000022232	RF00286	ENSLOCG00000018522	RF00286
ENSXMAG00000018140	hypk	ENSLOCG00000014153	hypk
ENSXMAG00000015450	mtg2	ENSLOCG00000003710	MTG2
ENSXMAG00000019146	syt12	ENSLOCG00000001528	
ENSXMAG00000009115	dnmt3bb.1	ENSLOCG00000005701	dnmt3bb.1
ENSXMAG00000022687		ENSLOCG00000003723	SS18L1
ENSXMAG00000018947	dgkb	ENSLOCG00000011246	dgkb
ENSXMAG00000018125	mfap1	ENSLOCG00000014150	mfap1
ENSXMAG00000023084	asah1b	ENSLOCG00000013874	asah1b
ENSXMAG00000008506	plaa	ENSLOCG00000011346	plaa
ENSXMAG00000007503	thoc5	ENSLOCG00000008401	thoc5
ENSXMAG00000018121	hddc3	ENSLOCG00000014146	hddc3
ENSXMAG00000005228		ENSLOCG00000012138	MYOCD
ENSXMAG00000007194	ext2	ENSLOCG00000002809	ext2
ENSXMAG00000025087	v2ra18	ENSLOCG00000002475	v2ra18
ENSXMAG00000025437	cat	ENSLOCG00000001015	cat
ENSXMAG00000014047	gtf2f1	ENSLOCG00000006340	gtf2f1

ENSXMAG00000003704	ttc23	ENSLOCG00000014422	ttc23
ENSXMAG00000005675	cadm3	ENSLOCG00000001559	CADM3
ENSXMAG00000007751	frg1	ENSLOCG00000013868	frg1
ENSXMAG00000008107	letm1	ENSLOCG00000007015	letm1
ENSXMAG00000018080	vps33b	ENSLOCG00000014138	vps33b
ENSXMAG00000003710	synm	ENSLOCG00000014429	synm
ENSXMAG00000022284	nmbr	ENSLOCG00000000275	nmbr
ENSXMAG00000008523	zdhhc21	ENSLOCG00000011340	zdhhc21
ENSXMAG00000000361	rpl14	ENSLOCG00000001044	RPL14
ENSXMAG00000025552	pgpep1l	ENSLOCG00000014432	pgpep1l
ENSXMAG00000021134	cx23	ENSLOCG00000000290	cx23
ENSXMAG00000025236	mapre1b	ENSLOCG00000005626	mapre1b
ENSXMAG00000002726	gcm2	ENSLOCG00000013145	gcm2
ENSXMAG00000026522	adprm	ENSLOCG00000012117	adprm
ENSXMAG00000026783	alkbh7	ENSLOCG00000006378	alkbh7
ENSXMAG00000024708	wnt10a	ENSLOCG00000005361	wnt10a
ENSXMAG00000005275		ENSLOCG00000004034	RSPH1
ENSXMAG00000005698	atp1a2a	ENSLOCG00000001762	
ENSXMAG00000021844	vta1	ENSLOCG00000000349	vta1
ENSXMAG00000020059	cdk5r2b	ENSLOCG00000017827	cdk5r2b
ENSXMAG00000009064	acss2	ENSLOCG00000005576	acss2
ENSXMAG00000008538	NFIB	ENSLOCG00000011325	NFIB
ENSXMAG00000001807	cryba2b	ENSLOCG00000005419	cryba2b
ENSXMAG00000018793	fat1a	ENSLOCG00000013845	fat1a
ENSXMAG00000018033		ENSLOCG00000006031	ndufs3
ENSXMAG00000019003	agmo	ENSLOCG00000011262	agmo
ENSXMAG00000027488		ENSLOCG00000001212	
ENSXMAG00000005292		ENSLOCG00000011847	
ENSXMAG00000001814	umps	ENSLOCG00000005441	umps
ENSXMAG00000001639	v2rx4	ENSLOCG00000002892	v2rx4
ENSXMAG00000023917	ZBTB14	ENSLOCG00000017810	zbtb14
ENSXMAG00000029268	c1qtnf4	ENSLOCG00000017323	c1qtnf4
ENSXMAG00000000392	trim71	ENSLOCG00000001260	trim71
ENSXMAG00000005299	pts	ENSLOCG00000011839	pts
ENSXMAG00000005799	mpz	ENSLOCG00000001603	
ENSXMAG00000019012	meox2a	ENSLOCG00000011275	meox2a
ENSXMAG00000025275		ENSLOCG00000002932	v2rx1
ENSXMAG00000028024		ENSLOCG00000003908	TCFL5
ENSXMAG00000021933	agpat2	ENSLOCG00000003674	agpat2
ENSXMAG00000019015	ispd	ENSLOCG00000011281	ispd
ENSXMAG00000008734	alg13	ENSLOCG00000014291	alg13
ENSXMAG00000008136	jade1	ENSLOCG00000010648	jade1
ENSXMAG00000005305		ENSLOCG00000011822	GLP2R
ENSXMAG00000014359	ARHGAP28	ENSLOCG00000002975	arhgap28
ENSXMAG00000005711	egfl7	ENSLOCG00000003643	egfl7
ENSXMAG00000005804	lig1	ENSLOCG00000001777	lig1
ENSXMAG00000003746		ENSLOCG00000012830	cfap161
ENSXMAG00000019021	sostdc1a	ENSLOCG00000011289	sostdc1a
ENSXMAG00000008559		ENSLOCG00000011321	BANK1
ENSXMAG00000007502		ENSLOCG00000004660	
ENSXMAG00000014101	PAIP2	ENSLOCG00000011563	PAIP2
ENSXMAG00000019024	ankmy2a	ENSLOCG00000011307	ankmy2a
ENSXMAG00000009767	gsg1l2b	ENSLOCG00000011813	gsg1l2b
ENSXMAG00000001796	si:ch211-127i16.2	ENSLOCG00000016112	si:ch211-127i16.2
ENSXMAG00000026396	pgap3	ENSLOCG00000012716	pgap3
ENSXMAG00000009759	hs3st3l	ENSLOCG00000010810	hs3st3l

ENSXMAG00000029026	si:busm1-52i16.2	ENSLOCG00000005008	si:busm1-52i16.2
ENSXMAG00000026272	tbc1d2	ENSLOCG00000011539	tbc1d2
ENSXMAG00000018390	erbb2	ENSLOCG00000012694	erbb2
ENSXMAG00000000762	nsa2	ENSLOCG00000005380	nsa2
ENSXMAG00000014347	L3MBTL4	ENSLOCG00000003008	L3MBTL4
ENSXMAG00000019045		ENSLOCG00000002508	dpy19l1l
ENSXMAG00000027222	pih1d3	ENSLOCG00000014965	
ENSXMAG00000009992	adgrg6	ENSLOCG00000000429	adgrg6
ENSXMAG00000008560	PPP3CA	ENSLOCG00000011303	PPP3CA
ENSXMAG00000009925	asmt2	ENSLOCG00000010666	asmt2
ENSXMAG00000013980	dzip1	ENSLOCG00000005084	dzip1
ENSXMAG00000008742	aars2	ENSLOCG00000015678	aars2
ENSXMAG00000024749	EIF3F	ENSLOCG00000000938	eif3f
ENSXMAG00000021924	mprip	ENSLOCG00000010854	mprip
ENSXMAG00000000772	gfm2	ENSLOCG00000005368	GFM2
ENSXMAG00000002430	aass	ENSLOCG00000015888	aass
ENSXMAG00000024645	pgrmc2	ENSLOCG00000010679	pgrmc2
ENSXMAG00000004704	tbx20	ENSLOCG00000002483	tbx20
ENSXMAG00000000442	zgc:110239	ENSLOCG00000004812	zgc:103438
ENSXMAG00000003784	vwf	ENSLOCG00000017042	vwf
ENSXMAG00000013060	msh5	ENSLOCG00000000809	
ENSXMAG00000018848	cyp4v8	ENSLOCG00000013836	cyp4v8
ENSXMAG00000023406	kcnj9	ENSLOCG00000000922	kcnj9
ENSXMAG00000029575	taco1	ENSLOCG00000011738	taco1
ENSXMAG00000008150	larp1b	ENSLOCG00000010684	larp1b
ENSXMAG00000010064	zgc:113278	ENSLOCG00000003967	zgc:113278
ENSXMAG00000025154	pth1rb	ENSLOCG00000011754	
ENSXMAG00000018871		ENSLOCG00000013826	tlr3
ENSXMAG00000001756	VAV2	ENSLOCG00000004506	vav2
ENSXMAG00000000795	hexb	ENSLOCG00000011014	hexb
ENSXMAG00000005400		ENSLOCG00000010874	
ENSXMAG00000028642	oxgr1a.2	ENSLOCG00000017784	oxgr1a.2
ENSXMAG00000022743	DDIT4L	ENSLOCG00000011286	DDIT4L
ENSXMAG00000005403	bsk146	ENSLOCG00000010878	bsk146
ENSXMAG00000005888		ENSLOCG00000017610	tmem151a
ENSXMAG00000000265	taz	ENSLOCG00000015485	taz
ENSXMAG00000023069	enc1	ENSLOCG00000017543	enc1
ENSXMAG00000002442	cdk4	ENSLOCG00000003932	cdk4
ENSXMAG00000007501	fancg	ENSLOCG00000006614	fancg
ENSXMAG00000000452	mturn	ENSLOCG00000001882	mturn
ENSXMAG00000000254		ENSLOCG00000015466	ndnl2
ENSXMAG00000005408	wu:fj29h11	ENSLOCG00000010884	wu:fj29h11
ENSXMAG00000021451		ENSLOCG00000012125	eci2
ENSXMAG00000024011	txnl4a	ENSLOCG00000008419	txnl4a
ENSXMAG00000002453	fezf1	ENSLOCG00000015885	FEZF1
ENSXMAG00000018882	sorbs2a	ENSLOCG00000013818	sorbs2a
ENSXMAG00000028027		ENSLOCG00000016274	BBS10
ENSXMAG00000005656	gucy1a1	ENSLOCG00000008256	gucy1a1
ENSXMAG00000029038	METTL1	ENSLOCG00000003897	mettl1
ENSXMAG00000027780		ENSLOCG00000001446	
ENSXMAG00000008574	pcxb	ENSLOCG00000001936	pcxb
ENSXMAG00000028197	nipsnap3a	ENSLOCG00000011488	nipsnap3a
ENSXMAG00000016252	fam49al	ENSLOCG00000002041	fam49al
ENSXMAG00000007485	vcp	ENSLOCG00000006589	VCP
ENSXMAG00000008774	mrps28	ENSLOCG00000017359	mrps28
ENSXMAG00000013041	fam171a1	ENSLOCG00000010436	fam171a1

ENSXMAG00000005176	cyp27b1	ENSLOCG00000003882	cyp27b1
ENSXMAG00000029816		ENSLOCG00000006761	
ENSXMAG00000030010		ENSLOCG00000016290	otogl
ENSXMAG00000022268		ENSLOCG00000013136	
ENSXMAG00000023662	rbm38	ENSLOCG00000003456	rbm38
ENSXMAG00000005647	ufsp2	ENSLOCG00000013795	ufsp2
ENSXMAG00000022589	sde2	ENSLOCG00000015633	sde2
ENSXMAG00000014164	nub1	ENSLOCG00000009978	nub1
ENSXMAG00000017460		ENSLOCG00000006686	
ENSXMAG00000005413	sult2st2	ENSLOCG00000010892	sult2st2
ENSXMAG00000004447	mtif2	ENSLOCG00000015555	mtif2
ENSXMAG00000018312	SPOP	ENSLOCG00000011688	spop
ENSXMAG00000016302	trit1	ENSLOCG00000002057	trit1
ENSXMAG00000002455	CADPS2	ENSLOCG00000015875	CADPS2
ENSXMAG00000014136	xylb	ENSLOCG00000009962	xylb
ENSXMAG00000005219	mcrs1	ENSLOCG00000004250	
ENSXMAG00000014913	noa1	ENSLOCG00000011439	noa1
ENSXMAG00000007474	akr1a1a	ENSLOCG00000006557	akr1a1a
ENSXMAG00000003971	nmt2	ENSLOCG00000010423	nmt2
ENSXMAG00000019446	cdca4	ENSLOCG00000017716	cdca4
ENSXMAG00000012911	slc25a4	ENSLOCG00000013773	slc25a4
ENSXMAG00000008172	si:dkey-177p2.6	ENSLOCG00000013156	si:dkey-177p2.6
ENSXMAG00000024139	lrfn4b	ENSLOCG00000001963	lrfn4b
ENSXMAG00000007286	sord	ENSLOCG00000013722	sord
ENSXMAG00000028846		ENSLOCG00000018127	
ENSXMAG00000005631	helt	ENSLOCG00000013768	helt
ENSXMAG00000024007		ENSLOCG00000013163	
ENSXMAG00000007469	dnajb5	ENSLOCG00000006541	dnajb5
ENSXMAG00000000316	eif2b5	ENSLOCG00000004645	eif2b5
ENSXMAG00000006046	pold3	ENSLOCG00000002298	pold3
ENSXMAG00000009076	ankra2	ENSLOCG00000011062	ankra2
ENSXMAG00000004468	EML6	ENSLOCG00000015539	EML6
ENSXMAG00000029159		ENSLOCG00000014209	
ENSXMAG00000023558	SUOX	ENSLOCG00000018190	suox
ENSXMAG00000009089	btf3	ENSLOCG00000011068	btf3
ENSXMAG00000027898		ENSLOCG00000006481	epg5
ENSXMAG00000023171		ENSLOCG00000011972	sugct
ENSXMAG00000019974	ighv1-4	ENSLOCG00000001310	ighv1-1
ENSXMAG00000021806		ENSLOCG00000004541	BRD3
ENSXMAG00000019612		ENSLOCG00000002888	inip
ENSXMAG00000009102	foxd1	ENSLOCG00000017544	foxd1
ENSXMAG00000023786		ENSLOCG00000007330	naca
ENSXMAG00000026388		ENSLOCG00000006461	lix1
ENSXMAG00000015034	si:dkeyp-110e4.11	ENSLOCG00000011527	si:dkeyp-110e4.11
ENSXMAG00000024151	tmem174	ENSLOCG00000011080	tmem174
ENSXMAG00000016348	yars	ENSLOCG00000002165	yars
ENSXMAG00000013890		ENSLOCG00000008192	abcb11b
ENSXMAG00000001746	wdr5	ENSLOCG00000004568	wdr5
ENSXMAG00000023402	terb2	ENSLOCG00000013718	terb2
ENSXMAG00000007425	riok2	ENSLOCG00000006432	riok2
ENSXMAG00000025556	fam69c	ENSLOCG00000012097	fam69c
ENSXMAG00000012203	abcc3	ENSLOCG00000010918	abcc3
ENSXMAG00000019067		ENSLOCG00000006655	
ENSXMAG00000026422	snx15	ENSLOCG00000002188	
ENSXMAG00000018913	pdlim3a	ENSLOCG00000013811	pdlim3b
ENSXMAG00000005603	primpol	ENSLOCG00000013749	primpol

ENSXMAG00000024591		ENSLOCG00000007274	vps29
ENSXMAG00000015053	tgfbi	ENSLOCG00000011520	tgfbi
ENSXMAG00000017428	prim1	ENSLOCG00000007341	prim1
ENSXMAG00000027061	si:ch211-194e15.5	ENSLOCG00000002185	si:ch211-194e15.5
ENSXMAG00000027799		ENSLOCG00000000527	si:ch211-202p1.5
ENSXMAG00000022382	prox1b	ENSLOCG00000002197	
ENSXMAG00000001582	C18orf63	ENSLOCG00000012092	C18orf63
ENSXMAG00000003280	timeless	ENSLOCG00000004180	timeless
ENSXMAG00000027608	rgmb	ENSLOCG00000006395	rgmb
ENSXMAG00000005481	fcho2	ENSLOCG00000011085	fcho2
ENSXMAG00000025924	eif5	ENSLOCG00000009881	eif5
ENSXMAG00000018949	fgg	ENSLOCG00000008358	FGG
ENSXMAG00000001735	rxraa	ENSLOCG00000004579	rxraa
ENSXMAG00000007362	erap2	ENSLOCG00000006368	erap2
ENSXMAG00000005585	tacr3l	ENSLOCG00000012019	tacr3a
ENSXMAG00000027159	zgc:110591	ENSLOCG00000002224	zgc:110591
ENSXMAG00000018965	plrg1	ENSLOCG00000008405	plrg1
ENSXMAG00000017387	ddx23	ENSLOCG00000007385	ddx23
ENSXMAG00000019523	si:ch211-285j22.3	ENSLOCG00000017611	si:dkey-17e16.15
ENSXMAG00000009055	zgc:195023	ENSLOCG00000005514	zgc:195023
ENSXMAG00000016384	tert	ENSLOCG00000003516	tert
ENSXMAG00000007345	lnpep	ENSLOCG00000006318	lnpep
ENSXMAG00000027565	nppb	ENSLOCG00000005558	nppb
ENSXMAG00000001579	cyb5a	ENSLOCG00000012082	cyb5a
ENSXMAG00000019110	elp6	ENSLOCG00000004361	ELP6
ENSXMAG00000017350	cacnb3a	ENSLOCG00000007413	cacnb3a
ENSXMAG00000025753	ppp1r14bb	ENSLOCG00000017608	ppp1r14ba
ENSXMAG00000009372	ftsj1	ENSLOCG00000015688	ftsj1
ENSXMAG00000006691	crot	ENSLOCG00000010406	crot
ENSXMAG00000005532	glb1	ENSLOCG00000000812	glb1
ENSXMAG00000013864	scn1lab	ENSLOCG00000008347	scn1lab
ENSXMAG00000003304	prph	ENSLOCG00000004165	prph
ENSXMAG00000028334		ENSLOCG00000008415	
ENSXMAG00000016410	slc6a18	ENSLOCG00000003533	slc6a18
ENSXMAG00000018189	si:ch211-152c8.4	ENSLOCG00000011663	si:ch211-152c8.4
ENSXMAG00000019115	scap	ENSLOCG00000004346	
ENSXMAG00000008666	plcb3	ENSLOCG00000001978	
ENSXMAG00000007334	slc14a2	ENSLOCG00000006284	slc14a2
ENSXMAG00000027185	fars2	ENSLOCG00000011667	fars2
ENSXMAG00000001724	rc3h2	ENSLOCG00000002894	rc3h2
ENSXMAG00000021363	sfrp2	ENSLOCG00000008437	sfrp2
ENSXMAG00000015111	exosc3	ENSLOCG00000011471	exosc3
ENSXMAG00000027560	tnip2	ENSLOCG00000013545	tnip2
ENSXMAG00000014792	arg2	ENSLOCG00000014189	arg2
ENSXMAG00000026109	rnf24	ENSLOCG00000002454	rnf24
ENSXMAG00000003326	cdk2	ENSLOCG00000004109	CDK2
ENSXMAG00000026975	borcs5	ENSLOCG00000016492	borcs5
ENSXMAG00000009013	vps13d	ENSLOCG00000003214	vps13d
ENSXMAG00000008106	fam193a	ENSLOCG00000013548	fam193a
ENSXMAG00000005505		ENSLOCG00000002465	smox
ENSXMAG00000014780	vti1b	ENSLOCG00000014187	vti1b
ENSXMAG00000020818	rdh12	ENSLOCG00000014184	rdh12
ENSXMAG00000004604	polr3f	ENSLOCG00000015701	polr3f
ENSXMAG00000003916	wash1	ENSLOCG00000016868	wash1
ENSXMAG00000028665	setbp1	ENSLOCG00000006153	setbp1
ENSXMAG00000001714	rabgap1	ENSLOCG00000002828	rabgap1

ENSXMAG00000009397	smc1b	ENSLOCG00000017088	
ENSXMAG00000005499	fbxo41	ENSLOCG00000002435	fbxo41
ENSXMAG00000012873	rreb1a	ENSLOCG00000011702	rreb1b
ENSXMAG00000009755	dzank1	ENSLOCG00000015699	dzank1
ENSXMAG00000007316	cdk7	ENSLOCG00000006117	cdk7
ENSXMAG00000014723	zfyve26	ENSLOCG00000014174	zfyve26
ENSXMAG00000003929	ddx11	ENSLOCG00000016875	ddx11
ENSXMAG00000005440	tnpo1	ENSLOCG00000011092	tnpo1
ENSXMAG00000013845	GCA	ENSLOCG00000008441	GCA
ENSXMAG00000008121		ENSLOCG00000008525	arfip1
ENSXMAG00000019136	ptpn23a	ENSLOCG00000004316	ptpn23a
ENSXMAG00000009770	kat14	ENSLOCG00000015696	kat14
ENSXMAG00000021337	mogs	ENSLOCG00000002414	mogs
ENSXMAG00000003963	fkbp4	ENSLOCG00000016880	fkbp4
ENSXMAG00000009938	tmem242	ENSLOCG00000016844	tmem242
ENSXMAG00000012883	rmc1	ENSLOCG00000001613	rmc1
ENSXMAG00000005480	pdcd4a	ENSLOCG00000002395	
ENSXMAG00000009773	PET117	ENSLOCG00000015693	PET117
ENSXMAG00000007303	serinc5	ENSLOCG00000006068	serinc5
ENSXMAG00000012881	fhdc1	ENSLOCG00000008511	fhdc1
ENSXMAG00000015136	rest	ENSLOCG00000011457	rest
ENSXMAG00000014969	tnnc1b	ENSLOCG00000015523	tnnc1b
ENSXMAG00000027063	gdf11	ENSLOCG00000003644	gdf11
ENSXMAG00000009814	vtcn1	ENSLOCG00000011115	vtcn1
ENSXMAG00000003989	b4galnt3b	ENSLOCG00000016886	b4galnt3b
ENSXMAG00000014721	si:ch211-10a23.2	ENSLOCG00000014173	si:ch211-10a23.2
ENSXMAG00000027619	gpr25	ENSLOCG00000018061	gpr25
ENSXMAG00000009907	rrm2	ENSLOCG00000016838	rrm2
ENSXMAG00000005473	mxd4	ENSLOCG00000002303	mxd4
ENSXMAG00000009449	cpa4	ENSLOCG00000015944	cpa4
ENSXMAG00000009820	trim45	ENSLOCG00000011099	trim45
ENSXMAG00000014990	dedd	ENSLOCG00000015531	DEDD
ENSXMAG00000021258	spryd4	ENSLOCG00000004897	spryd4
ENSXMAG00000006834	rundc3b	ENSLOCG00000010363	rundc3b
ENSXMAG00000014691	plek2	ENSLOCG00000014169	plek2
ENSXMAG00000005457	ndufb6	ENSLOCG00000013412	ndufb6
ENSXMAG00000012902		ENSLOCG00000001652	riok3
ENSXMAG00000014993	gorasp1a	ENSLOCG00000009900	gorasp1a
ENSXMAG00000005427	mob1bb	ENSLOCG00000011573	mob1bb
ENSXMAG00000005446	mtap	ENSLOCG00000013426	mtap
ENSXMAG00000012858		ENSLOCG00000001483	iqce
ENSXMAG00000012391	daglb	ENSLOCG00000008384	daglb
ENSXMAG00000009827	wdr3	ENSLOCG00000011052	wdr3
ENSXMAG00000011491	wdr48b	ENSLOCG00000009906	wdr48b
ENSXMAG00000003364	adipor1a	ENSLOCG00000010160	adipor1a
ENSXMAG00000005412	dck	ENSLOCG00000011567	dck
ENSXMAG00000005443	cdkn2a/b	ENSLOCG00000013433	cdkn2a/b
ENSXMAG00000012414	MRPL58	ENSLOCG00000011474	MRPL58
ENSXMAG00000012917	CLVS1	ENSLOCG00000005043	CLVS1
ENSXMAG00000006851	slc25a40	ENSLOCG00000010352	slc25a40
ENSXMAG00000007621		ENSLOCG00000013697	FAM189A1
ENSXMAG00000024047	cxxc4	ENSLOCG00000012594	cxxc4
ENSXMAG00000016961	smarcd2	ENSLOCG00000012004	smarcd2
ENSXMAG00000008709	gpha2	ENSLOCG00000002035	GPHA2
ENSXMAG00000004004	c2cd5	ENSLOCG00000016894	c2cd5
ENSXMAG00000001700	strbp	ENSLOCG00000002814	strbp

ENSXMAG00000023532	rabif	ENSLOCG00000010143	rabif
ENSXMAG00000021720	tnfaip8l2b	ENSLOCG00000007775	tnfaip8l2b
ENSXMAG00000005438	tet2	ENSLOCG00000012598	tet2
ENSXMAG00000022052	dpy30	ENSLOCG00000015945	dpy30
ENSXMAG00000028482	lysmd1	ENSLOCG00000007790	lysmd1
ENSXMAG00000016945	psmc5	ENSLOCG00000011995	psmc5
ENSXMAG00000012832	PLK1	ENSLOCG00000005649	PLK1
ENSXMAG00000012425	sap30bp	ENSLOCG00000011509	sap30bp
ENSXMAG00000007237	mtx3	ENSLOCG00000006008	mtx3
ENSXMAG00000005399	ppa2	ENSLOCG00000012607	ppa2
ENSXMAG00000006880	dbf4	ENSLOCG00000010346	dbf4
ENSXMAG00000019171	scnm1	ENSLOCG00000007801	scnm1
ENSXMAG00000009831	psen2	ENSLOCG00000015948	psen2
ENSXMAG00000009858	taf1b	ENSLOCG00000016834	taf1b
ENSXMAG00000014675	TC2N	ENSLOCG00000013220	TC2N
ENSXMAG00000007605	casc4	ENSLOCG00000013752	casc4
ENSXMAG00000009875	gdap2	ENSLOCG00000011071	gdap2
ENSXMAG00000005387	arhgef38	ENSLOCG00000012616	arhgef38
ENSXMAG00000019176	tmod4	ENSLOCG00000007811	tmod4
ENSXMAG00000007224	cmya5	ENSLOCG00000005987	cmya5
ENSXMAG00000012831		ENSLOCG00000005592	
ENSXMAG00000029835	ints12	ENSLOCG00000012622	ints12
ENSXMAG00000012151	gstcd	ENSLOCG00000012628	gstcd
ENSXMAG00000017060	arl2	ENSLOCG00000002173	arl2
ENSXMAG00000007210	tent2	ENSLOCG00000005954	tent2
ENSXMAG00000017484	gps1	ENSLOCG00000012349	gps1
ENSXMAG00000028458	mcl1b	ENSLOCG00000007846	mcl1b
ENSXMAG00000015141	igfbp7	ENSLOCG00000011413	igfbp7
ENSXMAG00000026146		ENSLOCG00000009071	
ENSXMAG00000025519	adam22	ENSLOCG00000010320	adam22
ENSXMAG00000027443	RF00099	ENSLOCG00000020047	RF00099
ENSXMAG00000012430	itgb4	ENSLOCG00000011516	itgb4
ENSXMAG00000001681	sh2d3ca	ENSLOCG00000002795	sh2d3ca
ENSXMAG00000012135	npnt	ENSLOCG00000012639	npnt
ENSXMAG00000012956	HUS1	ENSLOCG00000013303	
ENSXMAG00000011666	col6a1	ENSLOCG00000009665	col6a1
ENSXMAG00000022992	si:ch211-150j10.4	ENSLOCG00000013767	
ENSXMAG00000008997	ensab	ENSLOCG00000007864	ensab
ENSXMAG00000015150	cox18	ENSLOCG00000011403	cox18
ENSXMAG00000004047	lmf2a	ENSLOCG00000016821	lmf2a
ENSXMAG00000026766	NPHS2	ENSLOCG00000009057	nphs2
ENSXMAG00000001192	eif3jb	ENSLOCG00000013769	eif3ja
ENSXMAG00000028842		ENSLOCG00000011089	
ENSXMAG00000015078	man1a2	ENSLOCG00000011087	man1a2
ENSXMAG00000017981		ENSLOCG00000009045	axdnd1
ENSXMAG00000015160	selenot2	ENSLOCG00000015360	selenot2
ENSXMAG00000004061	miox	ENSLOCG00000016820	miox
ENSXMAG00000022017	cilp	ENSLOCG00000013772	cilp
ENSXMAG00000024545	onecutl	ENSLOCG00000007915	onecutl
ENSXMAG00000024168		ENSLOCG00000007052	si:dkey-118k5.3
ENSXMAG00000007167		ENSLOCG00000007511	
ENSXMAG00000015165		ENSLOCG00000006957	RNF4
ENSXMAG00000005310	fbp1b	ENSLOCG00000011449	fbp1b
ENSXMAG00000001680	urm1	ENSLOCG00000002768	urm1
ENSXMAG00000015098		ENSLOCG00000017806	tent5c
ENSXMAG00000015604	SH2D3A	ENSLOCG00000007275	SH2D3A

ENSXMAG00000002461	RABGAP1L	ENSLOCG00000009098	RABGAP1L
ENSXMAG00000003398	nelfcd	ENSLOCG00000006254	nelfcd
ENSXMAG00000001217	igdcc3	ENSLOCG00000013782	igdcc3
ENSXMAG00000008976	ctsk	ENSLOCG00000007964	ctsk
ENSXMAG00000007137	dimt1l	ENSLOCG00000007837	dimt1l
ENSXMAG00000021908	tbx15	ENSLOCG00000011023	tbx15
ENSXMAG00000014661	fbln5	ENSLOCG00000013226	fbln5
ENSXMAG00000017982		ENSLOCG00000001695	
ENSXMAG00000001646	cercam	ENSLOCG00000002746	cercam
ENSXMAG00000006950	heyl	ENSLOCG00000001853	heyl
ENSXMAG00000012060	tbck	ENSLOCG00000012644	tbck
ENSXMAG00000017041	naa40	ENSLOCG00000001830	naa40
ENSXMAG00000005297	c9	ENSLOCG00000011377	c9
ENSXMAG00000004076	si:dkey-266m15.5	ENSLOCG00000016806	si:dkey-266m15.5
ENSXMAG00000023813		ENSLOCG00000011473	tbc1d9
ENSXMAG00000008145	zbtb8b	ENSLOCG00000000323	zbtb8b
ENSXMAG00000013372	si:ch211-113g11.6	ENSLOCG00000007977	si:ch211-113g11.6
ENSXMAG00000027999	spred2a	ENSLOCG00000016028	spred2b
ENSXMAG00000015563	VAV1	ENSLOCG00000007259	VAV1
ENSXMAG00000003406	ctsz	ENSLOCG00000006242	ctsz
ENSXMAG00000008148	fuca1.2	ENSLOCG00000001807	fuca1.2
ENSXMAG00000001650	plch1	ENSLOCG00000003130	plch1
ENSXMAG00000014645	trip11	ENSLOCG00000013235	trip11
ENSXMAG00000013367	tlr18	ENSLOCG00000007992	tlr18
ENSXMAG00000027364		ENSLOCG00000016025	si:dkey-78p8.1
ENSXMAG00000021964	wars2	ENSLOCG00000011009	wars2
ENSXMAG00000015180		ENSLOCG00000010608	
ENSXMAG00000008724	rcor2	ENSLOCG00000001803	rcor2
ENSXMAG00000001573	sardh	ENSLOCG00000002697	sardh
ENSXMAG00000005287	dab2	ENSLOCG00000011371	si:ch211-204c21.1
ENSXMAG00000029193	zgc:171734	ENSLOCG00000003199	zgc:171734
ENSXMAG00000025324	cldn12	ENSLOCG00000018346	cldn12
ENSXMAG00000026821	FBXO48	ENSLOCG00000016009	FBXO48
ENSXMAG00000003419	npepl1	ENSLOCG00000006225	npepl1
ENSXMAG00000008151	dlgap3	ENSLOCG00000001754	dlgap3
ENSXMAG00000015265		ENSLOCG00000011483	rnf130
ENSXMAG00000011762	zgc:162816	ENSLOCG00000009716	zgc:162816
ENSXMAG00000023522	zgc:165656	ENSLOCG00000016006	APLF
ENSXMAG00000013351	prdm1a	ENSLOCG00000016531	prdm1a
ENSXMAG00000006881	gtpbp10	ENSLOCG00000010313	gtpbp10
ENSXMAG00000021414		ENSLOCG00000011364	
ENSXMAG00000015278	F2RL3	ENSLOCG00000011493	F2RL3
ENSXMAG00000001914	epb41l5	ENSLOCG00000001937	epb41l5
ENSXMAG00000000699	snrpb2	ENSLOCG00000015992	snrpb2
ENSXMAG00000013338	atg5	ENSLOCG00000016528	atg5
ENSXMAG00000001554	dbh	ENSLOCG00000002677	dbh
ENSXMAG00000015033	hao2	ENSLOCG00000010993	hao2
ENSXMAG00000015279		ENSLOCG00000011419	zgc:123244
ENSXMAG00000011765	orc2	ENSLOCG00000009832	orc2
ENSXMAG00000015557	PIN1	ENSLOCG00000007240	pin1
ENSXMAG00000026145		ENSLOCG00000015566	
ENSXMAG00000008163	cx39.4	ENSLOCG00000001736	cx39.4
ENSXMAG00000026466	osgin2	ENSLOCG00000010290	osgin2
ENSXMAG00000019467	smim12	ENSLOCG00000018272	smim12
ENSXMAG00000015282	polr2b	ENSLOCG00000011424	polr2b
ENSXMAG00000002775	lrrk2	ENSLOCG00000016102	lrrk2

ENSXMAG00000017402	cyb561	ENSLOCG00000012338	cyb561	
ENSXMAG00000025325		ENSLOCG00000004522	dctn3	
ENSXMAG00000024310		ENSLOCG00000005551		
ENSXMAG00000008826	ssr1	ENSLOCG00000011718	SSR1	
ENSXMAG00000008164	pef1	ENSLOCG00000001726	pef1	
ENSXMAG00000008732	mark2b	ENSLOCG00000001763	mark2b	
ENSXMAG00000018017	c18h3orf33	ENSLOCG00000003149	c18h3orf33	
ENSXMAG00000022437	cfap410	ENSLOCG00000009819	cfap410	
ENSXMAG00000006954	nbn	ENSLOCG00000010276	nbn	
ENSXMAG00000001932	ptpn4a	ENSLOCG00000001897	ptpn4a	
ENSXMAG00000027105	kank3	ENSLOCG00000002646	kank3	
ENSXMAG00000022077	zgc:171772	ENSLOCG00000009762	RPL37A	
ENSXMAG00000006361	crema	ENSLOCG00000000580	CREM	
ENSXMAG00000025993		ENSLOCG00000011477		
ENSXMAG00000024709	allc	ENSLOCG00000017209	allc	
ENSXMAG00000011806	pecr	ENSLOCG00000009750	pecr	
ENSXMAG00000014633		ENSLOCG00000016389		2-Mar
ENSXMAG00000015544	fbxl18	ENSLOCG00000004725	FBXL18	
ENSXMAG00000022136	si:rp71-68n21.9	ENSLOCG00000010981	si:rp71-68n21.9	
ENSXMAG00000015357		ENSLOCG00000012054	UBE2D3	
ENSXMAG00000011937		ENSLOCG00000005151		
ENSXMAG00000015540	ptcd1	ENSLOCG00000004833	ATP5MF-PTCD1	
ENSXMAG00000013314	bbs9	ENSLOCG00000002553	bbs9	
ENSXMAG00000012547	si:ch211-250n8.1	ENSLOCG00000011566	si:ch211-250n8.1	
ENSXMAG00000014626	hhipl2	ENSLOCG00000016424	HHIPL2	
ENSXMAG00000017654	ndufaf5	ENSLOCG00000015981	ndufaf5	
ENSXMAG00000022357	si:ch211-150g13.3	ENSLOCG00000006219	si:ch211-150g13.3	
ENSXMAG00000000694	ndufb3	ENSLOCG00000009855	ndufb3	
ENSXMAG00000009782	otog	ENSLOCG00000002110	otog	
ENSXMAG00000014624	wdr32	ENSLOCG00000012118	wdr32	
ENSXMAG00000001961	tmem177	ENSLOCG00000017826	tmem177	
ENSXMAG00000022781		ENSLOCG00000015548		
ENSXMAG00000022863		ENSLOCG00000004911		
ENSXMAG00000015369		ENSLOCG00000012080	NFKB1	
ENSXMAG00000023572	slc39a13	ENSLOCG00000006236	slc39a13	
ENSXMAG00000014959	hsd3b1	ENSLOCG00000010975	hsd3b1	
ENSXMAG00000001962	MRAS	ENSLOCG00000001886	mras	
ENSXMAG00000007077	unc45b	ENSLOCG00000009386	unc45b	
ENSXMAG00000012562	unk	ENSLOCG00000011578	unk	
ENSXMAG00000011589	hook1	ENSLOCG00000008956	hook1	
ENSXMAG00000017651	esf1	ENSLOCG00000015979	esf1	
ENSXMAG00000010387	csmd2	ENSLOCG00000001676	csmd2	
ENSXMAG00000001976	PTH2R	ENSLOCG00000001855	pth2r	
ENSXMAG00000021165		ENSLOCG00000011737	NEBL	
ENSXMAG00000006994	them4	ENSLOCG00000007440		
ENSXMAG00000024768	cnpy2	ENSLOCG00000004671		
ENSXMAG00000015514	alkbh5	ENSLOCG00000005006	ALKBH5	
ENSXMAG00000015446	DGKQ	ENSLOCG00000012108	DGKQ	
ENSXMAG00000004170	si:ch1073-390k14.1	ENSLOCG00000004051	si:ch1073-390k14.1	
ENSXMAG00000017640	acaa1	ENSLOCG00000009768	acaa1	
ENSXMAG00000008225	mybpc3	ENSLOCG00000006281	mybpc3	
ENSXMAG00000012584	unc13d	ENSLOCG00000011594	UNC13D	
ENSXMAG00000009693	kidins220b	ENSLOCG00000016815	kidins220b	
ENSXMAG00000001473	ambp	ENSLOCG00000002312	ambp	
ENSXMAG00000009804	aph1b	ENSLOCG00000014889	aph1b	
ENSXMAG00000007797	si:dkeyp-94b4.1	ENSLOCG00000011559		

ENSXMAG00000003535		ENSLOCG00000003869	EEF1AKMT3
ENSXMAG00000006389	pitrm1	ENSLOCG00000010785	pitrm1
ENSXMAG00000001698	zgc:112083	ENSLOCG00000004586	zgc:112083
ENSXMAG00000015489	mgat4b	ENSLOCG00000011450	mgat4b
ENSXMAG00000007867	tmem163a	ENSLOCG00000001812	tmem163a
ENSXMAG00000027841	stx16	ENSLOCG00000006206	stx16
ENSXMAG00000021482	wbp2	ENSLOCG00000011614	wbp2
ENSXMAG00000014934	popdc2	ENSLOCG00000002084	popdc2
ENSXMAG00000013307		ENSLOCG00000002531	bmper
ENSXMAG00000028954	trappc13	ENSLOCG00000010577	trappc13
ENSXMAG00000002018	acmsd	ENSLOCG00000001787	acmsd
ENSXMAG00000014931	cox17	ENSLOCG00000002098	cox17
ENSXMAG00000012654	trim65	ENSLOCG00000011623	trim65
ENSXMAG00000007019		ENSLOCG00000007284	
ENSXMAG00000011585	si:dkey-183n20.15	ENSLOCG00000008930	si:dkey-183n20.15
ENSXMAG00000020266	nrarpa	ENSLOCG00000017338	nrarpa
ENSXMAG00000014924	si:rp71-68n21.12	ENSLOCG00000010952	si:rp71-68n21.12
ENSXMAG00000003742	lgr4	ENSLOCG00000006401	lgr4
ENSXMAG00000008760	cct7	ENSLOCG00000000826	
ENSXMAG00000012684	mrpl38	ENSLOCG00000011636	mrpl38
ENSXMAG00000013306	EAF1	ENSLOCG00000001869	eaf1
ENSXMAG00000001467	exd3	ENSLOCG00000002330	exd3
ENSXMAG00000004218	grm3	ENSLOCG00000016703	grm3
ENSXMAG00000014586	zmp:0000000662	ENSLOCG00000001669	zmp:0000000662
ENSXMAG00000016067	canx	ENSLOCG00000011437	canx
ENSXMAG00000001707	dyrk1ab	ENSLOCG00000004953	dyrk1ab
ENSXMAG00000010460	angpt2b	ENSLOCG00000001647	angpt2b
ENSXMAG00000024824	mettl15	ENSLOCG00000006371	mettl15
ENSXMAG00000013302	METTL6	ENSLOCG00000001835	mettl6
ENSXMAG00000008789	hspa12b	ENSLOCG00000001328	
ENSXMAG00000003779	KIF18A	ENSLOCG00000006384	KIF18A
ENSXMAG00000023227		ENSLOCG00000004440	
ENSXMAG00000011539	pfas	ENSLOCG00000010645	pfas
ENSXMAG00000002525	mpv17l2	ENSLOCG00000001941	mpv17l2
ENSXMAG00000010479	col9a2	ENSLOCG00000001441	col9a2
ENSXMAG00000018299	mcm6	ENSLOCG00000000872	mcm6
ENSXMAG00000004237	sema3d	ENSLOCG00000016404	sema3d
ENSXMAG00000014542	manba	ENSLOCG00000012066	manba
ENSXMAG00000001715	alcamb	ENSLOCG00000005203	alcama
ENSXMAG00000011622	fam173a	ENSLOCG00000003152	fam173a
ENSXMAG00000003591	nab2	ENSLOCG00000004409	nab2
ENSXMAG00000024748	bdnf	ENSLOCG00000017324	bdnf
ENSXMAG00000001459	cdc37l1	ENSLOCG00000011249	
ENSXMAG00000018767	lats2	ENSLOCG00000010924	lats2
ENSXMAG00000000781	map1lc3c	ENSLOCG00000015907	map1lc3c
ENSXMAG00000018280	lin7c	ENSLOCG00000000316	lin7c
ENSXMAG00000022062	paip2b	ENSLOCG00000001347	paip2b
ENSXMAG00000014536	slc39a8	ENSLOCG00000012098	slc39a8
ENSXMAG00000001440	ak3	ENSLOCG00000011239	ak3
ENSXMAG00000017258	capn7	ENSLOCG00000001789	capn7
ENSXMAG00000012696	fdxr	ENSLOCG00000011730	
ENSXMAG00000026672	lsm4	ENSLOCG00000002025	LSM4
ENSXMAG00000003592	dnajc14	ENSLOCG00000004370	dnajc14
ENSXMAG00000008819	nagk	ENSLOCG00000001362	nagk
ENSXMAG00000024495	maml1	ENSLOCG00000011427	maml1
ENSXMAG00000004274	sema3ab	ENSLOCG00000016397	sema3ab

ENSXMAG00000018320	xrn1	ENSLOCG00000007944	xrn1
ENSXMAG00000008952	clcn6	ENSLOCG00000005577	clcn6
ENSXMAG00000014884	xpo4	ENSLOCG00000010910	xpo4
ENSXMAG00000023115	TNS4	ENSLOCG00000013572	TNS4
ENSXMAG00000022366	RCL1	ENSLOCG00000010148	rcl1
ENSXMAG00000011630	xpo6	ENSLOCG00000003304	xpo6
ENSXMAG00000020189	socs4	ENSLOCG00000017708	socs4
ENSXMAG00000016063	ltc4s	ENSLOCG00000011416	ltc4s
ENSXMAG00000002532	PGPEP1	ENSLOCG00000002042	PGPEP1
ENSXMAG00000003597	si:ch211-160o17.2	ENSLOCG00000004294	INPP1
ENSXMAG00000007068	si:dkey-71b5.7	ENSLOCG00000007463	si:dkey-71b5.7
ENSXMAG00000024285		ENSLOCG00000001750	map3k19
ENSXMAG00000008833	dok1b	ENSLOCG00000001428	dok1b
ENSXMAG00000022324	narf	ENSLOCG00000011760	narf
ENSXMAG00000018295	mcee	ENSLOCG00000013690	mcee
ENSXMAG00000018191	CCR7	ENSLOCG00000017973	ccr7
ENSXMAG00000008836	m1ap	ENSLOCG00000001444	m1ap
ENSXMAG00000009476	nenf	ENSLOCG00000016364	nenf
ENSXMAG00000009667	fgfr1op	ENSLOCG00000017219	FGFR1OP
ENSXMAG00000003423	abcg2b	ENSLOCG00000011349	
ENSXMAG00000018298	mphosph10	ENSLOCG00000013681	mphosph10
ENSXMAG00000029444	eef1akmt1	ENSLOCG00000010895	eef1akmt1
ENSXMAG00000012738	cybc1	ENSLOCG00000011775	CYBC1
ENSXMAG00000007018	polk	ENSLOCG00000005477	polk
ENSXMAG00000018307	ist1	ENSLOCG00000004525	ist1
ENSXMAG00000009823	wdhd1	ENSLOCG00000012086	wdhd1
ENSXMAG00000009436	tmem206	ENSLOCG00000016363	tmem206
ENSXMAG00000015981		ENSLOCG00000012214	slit3
ENSXMAG00000014880	il17d	ENSLOCG00000010904	il17d
ENSXMAG00000029480		ENSLOCG00000005918	plce1
ENSXMAG00000002814	slc6a15	ENSLOCG00000016334	slc6a15
ENSXMAG00000018345	cop1	ENSLOCG00000002311	cop1
ENSXMAG00000018176	IGFBP4	ENSLOCG00000013567	IGFBP4
ENSXMAG00000022506	SLC35G1	ENSLOCG00000005895	SLC35G1
ENSXMAG00000018327	dhodh	ENSLOCG00000004457	dhodh
ENSXMAG00000000643	zgc:86896	ENSLOCG00000012417	zgc:86896
ENSXMAG00000011485	IPO13	ENSLOCG00000010620	ipo13
ENSXMAG00000012742	hexdc	ENSLOCG00000011783	hexdc
ENSXMAG00000002039	ube2f	ENSLOCG00000004366	ube2f
ENSXMAG00000008844	SEMA4F	ENSLOCG00000001476	SEMA4F
ENSXMAG00000004318	sema3e	ENSLOCG00000016395	sema3e
ENSXMAG00000003602		ENSLOCG00000004277	
ENSXMAG00000011650	nme4	ENSLOCG00000003288	NME4
ENSXMAG00000017591	glra3	ENSLOCG00000013603	glra3
ENSXMAG00000007009	ankdd1b	ENSLOCG00000005494	ankdd1b
ENSXMAG00000003448	arpc1a	ENSLOCG00000004549	arpc1a
ENSXMAG00000018380	pappa2	ENSLOCG00000002271	pappa2
ENSXMAG00000002554	ddx49	ENSLOCG00000002119	ddx49
ENSXMAG00000007002	poc5	ENSLOCG00000005510	poc5
ENSXMAG00000018158	MLLT6	ENSLOCG00000013676	MLLT6
ENSXMAG00000007074	atp8b2	ENSLOCG00000007522	atp8b2
ENSXMAG00000001302	si:ch211-282j22.3	ENSLOCG00000001748	si:ch211-282j22.3
ENSXMAG00000025441	hpgd	ENSLOCG00000013597	hpgd
ENSXMAG00000012750	zgc:195081	ENSLOCG00000011795	zgc:195081
ENSXMAG00000018343	sult5a1	ENSLOCG00000004402	sult5a1
ENSXMAG00000026682	picalmb	ENSLOCG00000005274	picalma

ENSXMAG00000003471	arpc1b	ENSLOCG00000004536	arpc1b
ENSXMAG00000009846	gch1	ENSLOCG00000012096	gch1
ENSXMAG00000003611	sys1	ENSLOCG00000002789	sys1
ENSXMAG00000014828	ift88	ENSLOCG00000010875	ift88
ENSXMAG00000007956	fra10ac1	ENSLOCG00000005865	fra10ac1
ENSXMAG00000026261		ENSLOCG00000013329	ezh2
ENSXMAG00000012752		ENSLOCG00000017606	sstr1b
ENSXMAG00000002850		ENSLOCG00000016340	lrriq1
ENSXMAG00000021734	pclob	ENSLOCG00000015517	
ENSXMAG00000010598	adgrb2	ENSLOCG00000001563	adgrb2
ENSXMAG00000008847	syt4	ENSLOCG00000009420	syt4
ENSXMAG00000028525		ENSLOCG00000010616	
ENSXMAG00000025859	cul1b	ENSLOCG00000013339	cul1b
ENSXMAG00000009864	samd4a	ENSLOCG00000012106	samd4a
ENSXMAG00000011479	MMACHC	ENSLOCG00000010596	MMACHC
ENSXMAG00000007965	pde6c	ENSLOCG00000005849	pde6c
ENSXMAG00000018124	PIP4K2B	ENSLOCG00000013700	PIP4K2B
ENSXMAG00000024946	slc22a31	ENSLOCG00000004592	slc22a31
ENSXMAG00000025965	LACTB2	ENSLOCG00000004014	LACTB2
ENSXMAG00000012281	hmgb2a	ENSLOCG00000013578	hmgb2a
ENSXMAG00000027755		ENSLOCG00000007258	PHC3
ENSXMAG00000021510	alx1	ENSLOCG00000016343	alx1
ENSXMAG00000009299	ganc	ENSLOCG00000013291	GANC
ENSXMAG00000018120	psmb3	ENSLOCG00000013692	PSMB3
ENSXMAG00000018404	astn1	ENSLOCG00000002232	astn1
ENSXMAG00000008748	si:ch211-255i20.3	ENSLOCG00000010948	si:ch211-255i20.3
ENSXMAG00000026404	echdc2	ENSLOCG00000010557	echdc2
ENSXMAG00000002858	rassf9	ENSLOCG00000016344	rassf9
ENSXMAG00000028237	ankrd11	ENSLOCG00000004570	ankrd11
ENSXMAG00000008875	si:dkey-183c6.8	ENSLOCG00000001521	si:dkey-183c6.8
ENSXMAG00000012252	PMM2	ENSLOCG00000006565	pmm2
ENSXMAG00000026604		ENSLOCG00000002800	
ENSXMAG00000018113		ENSLOCG00000013707	CWC25
ENSXMAG00000029384	nts	ENSLOCG00000016345	nts
ENSXMAG00000001293	pou5f3	ENSLOCG00000001900	pou5f3
ENSXMAG00000017091		ENSLOCG00000010538	
ENSXMAG00000008749	spon2b	ENSLOCG00000010961	spon2b
ENSXMAG00000018112	sp6	ENSLOCG00000013714	sp6
ENSXMAG00000021003	rbp4	ENSLOCG00000005811	rbp4
ENSXMAG00000024852	cacna1g	ENSLOCG00000010668	cacna1g
ENSXMAG00000006794	iqgap2	ENSLOCG00000005540	iqgap2
ENSXMAG00000003913	uba52	ENSLOCG00000002162	uba52
ENSXMAG00000027481	TMEM87A	ENSLOCG00000013311	TMEM87A
ENSXMAG00000018095	scrn2	ENSLOCG00000013717	scrn2
ENSXMAG00000013022	svopl	ENSLOCG00000016932	SVOPL
ENSXMAG00000013258	rhbg	ENSLOCG00000008132	rhbg
ENSXMAG00000003492	radil	ENSLOCG00000004464	radil
ENSXMAG00000029457	ero1a	ENSLOCG00000012049	ero1a
ENSXMAG00000001265	clic3	ENSLOCG00000001932	clic3
ENSXMAG00000006454	zgc:136929	ENSLOCG00000010970	zgc:136929
ENSXMAG00000025940	arrdc2	ENSLOCG00000002205	arrdc2
ENSXMAG00000011451	zyg11	ENSLOCG00000010546	zyg11
ENSXMAG00000011702	pold1	ENSLOCG00000000769	
ENSXMAG00000018086		ENSLOCG00000013879	alyref
ENSXMAG00000018392	spg7	ENSLOCG00000004540	spg7
ENSXMAG00000012049	rpl22l1	ENSLOCG00000007304	rpl22l1

ENSXMAG00000003623	arfgap1	ENSLOCG00000002853	arfgap1
ENSXMAG00000009235	vps39	ENSLOCG00000013325	vps39
ENSXMAG00000022122	pacsin3	ENSLOCG00000005453	pacsin3
ENSXMAG00000013252	hapln2	ENSLOCG00000008116	hapln2
ENSXMAG00000005520	maea	ENSLOCG00000010983	maea
ENSXMAG00000013834		ENSLOCG00000000717	PATL1
ENSXMAG00000025244	brinp2	ENSLOCG00000002216	brinp2
ENSXMAG00000009348		ENSLOCG00000014394	
ENSXMAG00000013246		ENSLOCG00000008083	ISG20L2
ENSXMAG00000013244	rrnad1	ENSLOCG00000008069	rrnad1
ENSXMAG00000028814	cryl1	ENSLOCG00000010863	cryl1
ENSXMAG00000021102		ENSLOCG00000007578	si:dkey-92i15.4
ENSXMAG00000009203	galnt16	ENSLOCG00000013336	galnt16
ENSXMAG00000018052		ENSLOCG00000002763	QPRT
ENSXMAG00000024188	RF01291	ENSLOCG00000018826	RF01291
ENSXMAG00000027665	zbtb25	ENSLOCG00000012884	zbtb25
ENSXMAG00000023682	eif5a	ENSLOCG00000007318	eif5a2
ENSXMAG00000011448	coa7	ENSLOCG00000010537	coa7
ENSXMAG00000018416	cdh15	ENSLOCG00000004612	cdh15
ENSXMAG00000018022		ENSLOCG00000003502	
ENSXMAG00000020178	zbtb1	ENSLOCG00000017713	zbtb1
ENSXMAG00000007906		ENSLOCG00000000480	
ENSXMAG00000004415	rbm28	ENSLOCG00000015541	rbm28
ENSXMAG00000018432		ENSLOCG00000009186	klhl20
ENSXMAG00000003634	elmo2	ENSLOCG00000002700	elmo2
ENSXMAG00000024590	numb	ENSLOCG00000013341	numb
ENSXMAG00000008532	klhl3	ENSLOCG00000012130	klhl3
ENSXMAG00000020892	zgc:103499	ENSLOCG00000016350	zgc:103499
ENSXMAG00000002593	dazap1	ENSLOCG00000002243	dazap1
ENSXMAG00000008909	zgc:86609	ENSLOCG00000014569	zgc:86609
ENSXMAG00000011415	ralgps2	ENSLOCG00000010515	ralgps2
ENSXMAG00000011373	ACSF3	ENSLOCG00000004650	ACSF3
ENSXMAG00000018461		ENSLOCG00000003447	lhx8a
ENSXMAG00000014819	zmym2	ENSLOCG00000010842	zmym2
ENSXMAG00000008869	usp11	ENSLOCG00000014568	
ENSXMAG00000002603	gamt	ENSLOCG00000002281	gamt
ENSXMAG00000025470	traf3ip1	ENSLOCG00000009074	traf3ip1
ENSXMAG00000013595	snrnp48	ENSLOCG00000004187	snrnp48
ENSXMAG00000008862	rfc2	ENSLOCG00000000891	
ENSXMAG00000021847	p4hb	ENSLOCG00000013887	p4hb
ENSXMAG00000003565	exd2	ENSLOCG00000013376	exd2
ENSXMAG00000004706		ENSLOCG00000007090	
ENSXMAG00000002609	ndufs7	ENSLOCG00000002302	ndufs7
ENSXMAG00000013519	cep290	ENSLOCG00000016353	cep290
ENSXMAG00000023420	cbfa2t3	ENSLOCG00000004667	cbfa2t3
ENSXMAG00000008518	fam13b	ENSLOCG00000012259	fam13b
ENSXMAG00000027190		ENSLOCG00000002336	
ENSXMAG00000009201	si:ch211-168d23.3	ENSLOCG00000013370	si:ch211-168d23.3
ENSXMAG00000006775	si:ch211-130m23.3	ENSLOCG00000002081	si:ch211-130m23.3
ENSXMAG00000013048		ENSLOCG00000015570	
ENSXMAG00000003660		ENSLOCG00000017941	LRRN2
ENSXMAG00000013590		ENSLOCG00000004141	
ENSXMAG00000002617	ctdspl3	ENSLOCG00000002346	ctdspl3
ENSXMAG00000020843		ENSLOCG00000008299	
ENSXMAG00000029004		ENSLOCG00000017846	
ENSXMAG00000011408	fam20b	ENSLOCG00000010505	fam20b

ENSXMAG00000013578		ENSLOCG00000002160	wdr73
ENSXMAG00000017140	spata20	ENSLOCG00000010715	spata20
ENSXMAG00000008078	afap1l2	ENSLOCG00000013005	afap1l2
ENSXMAG00000009187	psen1	ENSLOCG00000013357	PSEN1
ENSXMAG00000029894	fmoda	ENSLOCG00000012131	fmodb
ENSXMAG00000001133	snrpd3	ENSLOCG00000006978	snrpd3
ENSXMAG00000027914	faslg	ENSLOCG00000010484	faslg
ENSXMAG00000019540	plekhf2	ENSLOCG00000018337	plekhf2
ENSXMAG00000013056	irf5	ENSLOCG00000015563	irf5
ENSXMAG00000017200	asb12a	ENSLOCG00000014613	asb12a
ENSXMAG00000013506	tmtc3	ENSLOCG00000016362	tmtc3
ENSXMAG00000003727	zgc:113307	ENSLOCG00000012134	zgc:113307
ENSXMAG00000011403	si:dkey-86e18.1	ENSLOCG00000008902	si:dkey-86e18.1
ENSXMAG00000026046		ENSLOCG00000008972	GPANK1
ENSXMAG00000002619	arid3a	ENSLOCG00000002384	arid3a
ENSXMAG00000029979	SLC39A11	ENSLOCG00000012489	SLC39A11
ENSXMAG00000028788	ptp4a2b	ENSLOCG00000001611	ptp4a2b
ENSXMAG00000013062	tnpo3	ENSLOCG00000015556	tnpo3
ENSXMAG00000027995	shc1	ENSLOCG00000007649	shc1
ENSXMAG00000018878	trappc2l	ENSLOCG00000004728	trappc2l
ENSXMAG00000008089	vwa2	ENSLOCG00000012022	vwa2
ENSXMAG00000010459	SORCS1	ENSLOCG00000011997	SORCS1
ENSXMAG00000022719	prelp	ENSLOCG00000012141	prelp
ENSXMAG00000013575	ndufaf6	ENSLOCG00000008276	ndufaf6
ENSXMAG00000024820	slc35e1	ENSLOCG00000001576	slc35e1
ENSXMAG00000029783	col8a2	ENSLOCG00000001624	col8a2
ENSXMAG00000007249	flad1	ENSLOCG00000007025	flad1
ENSXMAG00000028964	natd1	ENSLOCG00000002050	natd1
ENSXMAG00000028104	il19l	ENSLOCG00000012268	il19l
ENSXMAG00000017216		ENSLOCG00000017756	
ENSXMAG00000013096	calua	ENSLOCG00000015549	calua
ENSXMAG00000013573	nagpa	ENSLOCG00000006341	nagpa
ENSXMAG00000011861	tmem11	ENSLOCG00000002065	tmem11
ENSXMAG00000010634	trappc3	ENSLOCG00000001636	trappc3
ENSXMAG00000004994	si:dkey-106n21.1	ENSLOCG00000016369	si:dkey-106n21.1
ENSXMAG00000014758	cenpj	ENSLOCG00000010780	cenpj
ENSXMAG00000011863	DHRS7B	ENSLOCG00000002078	dhrs7b
ENSXMAG00000018880	galns	ENSLOCG00000004743	galns
ENSXMAG00000008104	tdrd1	ENSLOCG00000012030	tdrd1
ENSXMAG00000004460		ENSLOCG00000016730	FRMD4A
ENSXMAG00000003745	il10	ENSLOCG00000012252	il10
ENSXMAG00000027011	tp53inp1	ENSLOCG00000006359	tp53inp1
ENSXMAG00000001731	lim2.3	ENSLOCG00000005467	lim2.1
ENSXMAG00000008516	cxcl14	ENSLOCG00000012251	cxcl14
ENSXMAG00000002624	pex11g	ENSLOCG00000002580	pex11g
ENSXMAG00000002068	asb1	ENSLOCG00000009088	asb1
ENSXMAG00000004999	dusp6	ENSLOCG00000016377	dusp6
ENSXMAG00000006568	zmynd11	ENSLOCG00000012334	zmynd11
ENSXMAG00000027042	mapkapk2a	ENSLOCG00000012245	mapkapk2a
ENSXMAG00000018679	si:ch211-127d4.3	ENSLOCG00000015328	si:ch211-127d4.3
ENSXMAG00000010647	rims3	ENSLOCG00000001399	rims3
ENSXMAG00000019502	sco2	ENSLOCG00000017903	sco2
ENSXMAG00000026399	ccne2	ENSLOCG00000006388	ccne2
ENSXMAG00000027897	si:dkey-34f9.3	ENSLOCG00000009094	si:dkey-34f9.3
ENSXMAG00000006320	sdk1a	ENSLOCG00000004340	sdk1a
ENSXMAG00000002626	si:ch73-40i7.5	ENSLOCG00000002548	si:ch73-40i7.5

ENSXMAG00000011872	cramp1	ENSLOCG00000002093	cramp1
ENSXMAG00000018918	CKLF	ENSLOCG00000005103	CKLF
ENSXMAG00000029021	si:ch211-130m23.5	ENSLOCG00000002054	si:ch211-130m23.5
ENSXMAG00000002491	cops3	ENSLOCG00000001909	cops3
ENSXMAG00000013569	si:dkey-22o22.2	ENSLOCG00000005972	si:dkey-22o22.2
ENSXMAG00000017957	rptor	ENSLOCG00000013425	rptor
ENSXMAG00000003761	dyrk3	ENSLOCG00000012243	dyrk3
ENSXMAG00000029533	efna3b	ENSLOCG00000006835	EFNA3
ENSXMAG00000027342	tmem167a	ENSLOCG00000002040	tmem167a
ENSXMAG00000028142	cmtm3	ENSLOCG00000005088	cmtm3
ENSXMAG00000000236	tspan12	ENSLOCG00000015791	tspan12
ENSXMAG00000005016	cep41	ENSLOCG00000015609	cep41
ENSXMAG00000014747	rnf17	ENSLOCG00000010768	rnf17
ENSXMAG00000018940	cmtm4	ENSLOCG00000005075	cmtm4
ENSXMAG00000002512		ENSLOCG00000001889	NT5M
ENSXMAG00000008110	ccdc186	ENSLOCG00000012041	ccdc186
ENSXMAG00000006694	ckbb	ENSLOCG00000009908	ckbb
ENSXMAG00000024018	RASSF5	ENSLOCG00000012225	RASSF5
ENSXMAG00000009535	ing3	ENSLOCG00000015792	ing3
ENSXMAG00000002629	zgc:172302	ENSLOCG00000002469	zgc:172302
ENSXMAG00000018946	dync1li2	ENSLOCG00000005055	dync1li2
ENSXMAG00000018696		ENSLOCG00000008859	dtwd2
ENSXMAG00000009523	wnt16	ENSLOCG00000015799	wnt16
ENSXMAG00000010410		ENSLOCG00000011988	SORCS3
ENSXMAG00000000988	pus1	ENSLOCG00000006127	pus1
ENSXMAG00000011884	jpt2	ENSLOCG00000002135	jpt2
ENSXMAG00000025874	lrrc34	ENSLOCG00000007136	lrrc34
ENSXMAG00000019728	adrb1	ENSLOCG00000018253	adrb1
ENSXMAG00000002316		ENSLOCG00000011711	GAR1
ENSXMAG00000007290	tmem107l	ENSLOCG00000013332	tmem107l
ENSXMAG00000003768	ikbke	ENSLOCG00000012207	ikbke
ENSXMAG00000000964	noc4l	ENSLOCG00000006060	noc4l
ENSXMAG00000025580	arl8a	ENSLOCG00000012467	arl8a
ENSXMAG00000028313	krtcap2	ENSLOCG00000006824	krtcap2
ENSXMAG00000018710	JMY	ENSLOCG00000005899	JMY
ENSXMAG00000017263	pemt	ENSLOCG00000001840	pemt
ENSXMAG00000013564	pbx2	ENSLOCG00000000428	pbx2
ENSXMAG00000008126	nhlrc2	ENSLOCG00000012050	nhlrc2
ENSXMAG00000011895	lrrc31	ENSLOCG00000007167	lrrc31
ENSXMAG00000025542	s100a10b	ENSLOCG00000007141	s100a10b
ENSXMAG00000013537	CDC42BPB	ENSLOCG00000009847	cdc42bpb
ENSXMAG00000020863	acad8	ENSLOCG00000005041	acad8
ENSXMAG00000010324	slc22a18	ENSLOCG00000001662	slc22a18
ENSXMAG00000017266		ENSLOCG00000001791	RAB26
ENSXMAG00000018987	nae1	ENSLOCG00000005017	nae1
ENSXMAG00000009421	stab2	ENSLOCG00000015003	stab2
ENSXMAG00000022875	r3hdm4	ENSLOCG00000002423	r3hdm4
ENSXMAG00000010401	neurl1aa	ENSLOCG00000011969	neurl1aa
ENSXMAG00000011891	mapk8ip3	ENSLOCG00000002193	mapk8ip3
ENSXMAG00000008958	tdrd7b	ENSLOCG00000011373	tdrd7b
ENSXMAG00000018037	thyn1	ENSLOCG00000005023	thyn1
ENSXMAG00000011878	eif2s3	ENSLOCG00000003369	eif2s3
ENSXMAG00000008142	dclre1a	ENSLOCG00000012058	dclre1a
ENSXMAG00000018724	dmgdh	ENSLOCG00000005858	dmgdh
ENSXMAG00000013373	itpr3	ENSLOCG00000012422	itpr3
ENSXMAG00000024236		ENSLOCG00000002283	

ENSXMAG00000026926	zgc:158404	ENSLOCG00000012069	zgc:158404
ENSXMAG00000003785	srgap2	ENSLOCG00000012180	srgap2
ENSXMAG00000027623	elovl6	ENSLOCG00000011759	elovl6
ENSXMAG00000017272	lmf1	ENSLOCG00000001804	lmf1
ENSXMAG00000018745	ARSB	ENSLOCG00000005844	ARSB
ENSXMAG00000010273	mpped2a	ENSLOCG00000004698	mpped2a
ENSXMAG00000002287	egf	ENSLOCG00000011771	egf
ENSXMAG00000025625	edil3a	ENSLOCG00000001965	edil3a
ENSXMAG00000023286	klhl15	ENSLOCG00000003383	klhl15
ENSXMAG00000029392	casp7	ENSLOCG00000012087	casp7
ENSXMAG00000018753	lhfpl2a	ENSLOCG00000005821	lhfpl2a
ENSXMAG00000010253	zdhhc13	ENSLOCG00000004798	zdhhc13
ENSXMAG00000027329		ENSLOCG00000004315	myh7ba
ENSXMAG00000013526	vipas39	ENSLOCG00000013144	
ENSXMAG00000002285	lrit3a	ENSLOCG00000011791	lrit3a
ENSXMAG00000019418	cox7c	ENSLOCG00000001956	cox7c
ENSXMAG00000006377		ENSLOCG00000004292	gna12a
ENSXMAG00000010374	stn1	ENSLOCG00000011951	stn1
ENSXMAG00000019026	ca7	ENSLOCG00000005000	ca7
ENSXMAG00000027973	csrp3	ENSLOCG00000004817	csrp3
ENSXMAG00000003257	SULF2	ENSLOCG00000004015	sulf2b
ENSXMAG00000013539	phf1	ENSLOCG00000000218	
ENSXMAG00000011927	nme3	ENSLOCG00000002228	nme3
ENSXMAG00000029162	si:dkey-30c15.13	ENSLOCG00000002682	
ENSXMAG00000025979	FAM72B	ENSLOCG00000012177	FAM72B
ENSXMAG00000002278	spata4	ENSLOCG00000013625	spata4
ENSXMAG00000019040	cdh16	ENSLOCG00000004983	cdh16
ENSXMAG00000011943	mrps34	ENSLOCG00000002240	mrps34
ENSXMAG00000018805	ap3b1a	ENSLOCG00000005786	ap3b1a
ENSXMAG00000020991	spsb3a	ENSLOCG00000002279	spsb3a
ENSXMAG00000009417	parp12b	ENSLOCG00000016961	parp12b
ENSXMAG00000003291	mrgbp	ENSLOCG00000004057	mrgbp
ENSXMAG00000010237	e2f8	ENSLOCG00000004831	e2f8
ENSXMAG00000026325	cox19	ENSLOCG00000001744	cox19
ENSXMAG00000019054	rrad	ENSLOCG00000004955	rrad
ENSXMAG00000019416	rasa1a	ENSLOCG00000001929	rasa1b
ENSXMAG00000004525	pdp2	ENSLOCG00000017591	pdp2
ENSXMAG00000009144		ENSLOCG00000013204	
ENSXMAG00000006362		ENSLOCG00000005667	
ENSXMAG00000003296	hm13	ENSLOCG00000004095	hm13
ENSXMAG00000004541		ENSLOCG00000005037	TERB1
ENSXMAG00000016123	si:dkey-7f3.9	ENSLOCG00000006299	si:dkey-7f3.9
ENSXMAG00000009131	smoc1	ENSLOCG00000014213	smoc1
ENSXMAG00000017948	ENDOV	ENSLOCG00000013414	ENDOV
ENSXMAG00000022021	ctso	ENSLOCG00000008229	CTSO
ENSXMAG00000010278	dtd1	ENSLOCG00000016152	dtd1
ENSXMAG00000019063	nsun3	ENSLOCG00000010460	nsun3
ENSXMAG00000013499	smg5	ENSLOCG00000008380	smg5
ENSXMAG00000011825	sat1b	ENSLOCG00000003427	sat1a.2
ENSXMAG00000024381	tbca	ENSLOCG00000005775	tbca
ENSXMAG00000024342	nrap	ENSLOCG00000012095	nrap
ENSXMAG00000027458	arpc3	ENSLOCG00000007235	arpc3
ENSXMAG00000005100		ENSLOCG00000014054	ciao2a
ENSXMAG00000019645		ENSLOCG00000018092	
ENSXMAG00000009121		ENSLOCG00000014211	
ENSXMAG00000022808		ENSLOCG00000012155	MDFI

ENSXMAG00000002268	tdo2a	ENSLOCG00000008247	tdo2b
ENSXMAG00000004556	si:dkey-30c15.2	ENSLOCG00000016779	si:dkey-30c15.2
ENSXMAG00000017937	SEC14L1	ENSLOCG00000013406	SEC14L1
ENSXMAG00000028306	socs2	ENSLOCG00000015773	socs2
ENSXMAG00000003305		ENSLOCG00000015492	dnase1l1
ENSXMAG00000025996	tfeb	ENSLOCG00000012145	tfeb
ENSXMAG00000011796	acot9.2	ENSLOCG00000003460	acot9.2
ENSXMAG00000021842		ENSLOCG00000006319	rusc1
ENSXMAG00000005107	spg11	ENSLOCG00000014059	spg11
ENSXMAG00000018834	wdr41	ENSLOCG00000005746	wdr41
ENSXMAG00000009118	plekhd1	ENSLOCG00000014201	plekhd1
ENSXMAG00000009108	gpn3	ENSLOCG00000007250	gpn3
ENSXMAG00000003313		ENSLOCG00000003229	snx21
ENSXMAG00000009329	cradd	ENSLOCG00000015772	cradd
ENSXMAG00000013484	paqr6	ENSLOCG00000008357	paqr6
ENSXMAG00000010189	nav2a	ENSLOCG00000004849	nav2a
ENSXMAG00000004562	zgc:77752	ENSLOCG00000016774	zgc:77752
ENSXMAG00000018842	pde8b	ENSLOCG00000005705	pde8b
ENSXMAG00000028620	evx1	ENSLOCG00000011857	evx1
ENSXMAG00000013468	ntrk1	ENSLOCG00000000408	ntrk1
ENSXMAG00000022774	slc39a9	ENSLOCG00000014198	slc39a9
ENSXMAG00000021202	arl6	ENSLOCG00000010419	arl6
ENSXMAG00000003317		ENSLOCG00000012391	
ENSXMAG00000009097	rnf170	ENSLOCG00000003248	rnf170
ENSXMAG00000017931	rasal3	ENSLOCG00000006735	rasal3
ENSXMAG00000009466		ENSLOCG00000004150	
ENSXMAG00000004569	rabl2	ENSLOCG00000016767	rabl2
ENSXMAG00000020089	b3galt2	ENSLOCG00000017742	b3galt2
ENSXMAG00000027852	hoxa11a	ENSLOCG00000011836	hoxa11a
ENSXMAG00000024700	ccnh	ENSLOCG00000001902	ccnh
ENSXMAG00000029629		ENSLOCG00000004263	edem2
ENSXMAG00000002687	glrx2	ENSLOCG00000007393	glrx2
ENSXMAG00000013467	afp4	ENSLOCG00000000481	afp4
ENSXMAG00000016165	dap3	ENSLOCG00000006373	dap3
ENSXMAG00000003874	tmem183a	ENSLOCG00000010682	tmem183a
ENSXMAG00000009065		ENSLOCG00000003264	HOOK3
ENSXMAG00000002691	uchl5	ENSLOCG00000007370	uchl5
ENSXMAG00000013463	rbm8a	ENSLOCG00000009082	rbm8a
ENSXMAG00000017816	aig1	ENSLOCG00000000675	aig1
ENSXMAG00000009306		ENSLOCG00000016467	ptpro
ENSXMAG00000019078		ENSLOCG00000010694	
ENSXMAG00000013925	serpinh2	ENSLOCG00000013424	serpinh2
ENSXMAG00000019415	tmem161b	ENSLOCG00000001873	tmem161b
ENSXMAG00000006719	gtpbp4	ENSLOCG00000012279	gtpbp4
ENSXMAG00000009059		ENSLOCG00000003298	
ENSXMAG00000001054	hoxa5a	ENSLOCG00000011819	hoxa5a
ENSXMAG00000029527	efnb3b	ENSLOCG00000013430	efnb3b
ENSXMAG00000004582	shank3a	ENSLOCG00000016766	shank3a
ENSXMAG00000016183	gba	ENSLOCG00000006385	
ENSXMAG00000013458	otud7b	ENSLOCG00000009073	otud7b
ENSXMAG00000022404		ENSLOCG00000015928	znf277
ENSXMAG00000017923	dgke	ENSLOCG00000013841	dgke
ENSXMAG00000021235	si:ch211-11k18.4	ENSLOCG00000000324	
ENSXMAG00000013589	wrap53	ENSLOCG00000013434	wrap53
ENSXMAG00000009020	sfswap	ENSLOCG00000006202	sfswap
ENSXMAG00000018916	s100z	ENSLOCG00000005654	s100z

ENSXMAG00000020580	MIR9-2	ENSLOCG00000019063	MIR9-2
ENSXMAG00000021736	papss1	ENSLOCG00000012667	papss1
ENSXMAG00000021480	pex3	ENSLOCG00000000733	pex3
ENSXMAG00000027630	tmem131l	ENSLOCG00000008465	tmem131l
ENSXMAG00000019143	lmo7b	ENSLOCG00000011412	LMO7
ENSXMAG00000009489		ENSLOCG00000003986	arhgap22
ENSXMAG00000019422	ch25hl2	ENSLOCG00000017529	ch25hl2
ENSXMAG00000019414	mef2cb	ENSLOCG00000001833	mef2cb
ENSXMAG00000013591	gigyf1a	ENSLOCG00000013446	gigyf1b
ENSXMAG00000013720	fam117ba	ENSLOCG00000010182	fam117bb
ENSXMAG00000028550	hoxa3a	ENSLOCG00000011801	hoxa3a
ENSXMAG00000003883	ppfia4	ENSLOCG00000010689	ppfia4
ENSXMAG00000006733	golga5	ENSLOCG00000013242	golga5
ENSXMAG00000009503	mapk8b	ENSLOCG00000003968	mapk8b
ENSXMAG00000013453	mtmr11	ENSLOCG00000009056	mtmr11
ENSXMAG00000019107	si:ch1073-440b2.1	ENSLOCG00000010632	LONRF2
ENSXMAG00000002716	brinp3a.2	ENSLOCG00000007286	brinp3a.2
ENSXMAG00000008159	dnmbp	ENSLOCG00000012128	dnmbp
ENSXMAG00000001039	hoxa1a	ENSLOCG00000011798	hoxa1a
ENSXMAG00000016215	polr3c	ENSLOCG00000006400	polr3c
ENSXMAG00000006716	rtf1	ENSLOCG00000013279	rtf1
ENSXMAG00000026118	atf3	ENSLOCG00000000814	atf3
ENSXMAG00000021219	si:dkey-85k7.11	ENSLOCG00000013221	si:dkey-85k7.11
ENSXMAG00000026131	si:dkey-40c11.2	ENSLOCG00000005134	si:dkey-40c11.2
ENSXMAG00000017909	card14	ENSLOCG00000013861	card14
ENSXMAG00000002466	ulk1b	ENSLOCG00000006141	ulk1b
ENSXMAG00000003320	cdk16	ENSLOCG00000014555	
ENSXMAG00000000488	pdia2	ENSLOCG00000006290	
ENSXMAG00000029183	batf3	ENSLOCG00000000836	batf3
ENSXMAG00000010239	cyp2u1	ENSLOCG00000012294	cyp2u1
ENSXMAG00000013705	ical1	ENSLOCG00000010193	ical1
ENSXMAG00000004618	arsa	ENSLOCG00000016758	arsa
ENSXMAG00000021657	dclre1c	ENSLOCG00000016717	dclre1c
ENSXMAG00000010227	hadh	ENSLOCG00000012288	hadh
ENSXMAG00000004629	mapk8ip2	ENSLOCG00000016754	mapk8ip2
ENSXMAG00000024770		ENSLOCG00000010617	PDCL3
ENSXMAG00000019412	rhobtb4	ENSLOCG00000005114	rhobtb4
ENSXMAG00000016260	itga10	ENSLOCG00000006355	
ENSXMAG00000013685	wdr12	ENSLOCG00000010208	wdr12
ENSXMAG00000022959	cpn1	ENSLOCG00000012139	cpn1
ENSXMAG00000000498		ENSLOCG00000006270	zgc:136472
ENSXMAG00000012409	dpysl3	ENSLOCG00000010797	dpysl3
ENSXMAG00000001027	cbx3a	ENSLOCG00000011747	cbx3a
ENSXMAG00000024873	TMEM243	ENSLOCG00000016714	tmem243a
ENSXMAG00000019137		ENSLOCG00000010590	TBC1D8
ENSXMAG00000017901	ccdc40	ENSLOCG00000013876	ccdc40
ENSXMAG00000006709	ndufaf1	ENSLOCG00000013276	ndufaf1
ENSXMAG00000019545	ufsp1	ENSLOCG00000017554	ufsp1
ENSXMAG00000013666	nop58	ENSLOCG00000010225	nop58
ENSXMAG00000026069	crkl	ENSLOCG00000006537	crkl
ENSXMAG00000010205	lef1	ENSLOCG00000012276	lef1
ENSXMAG00000021020	nfe2l3	ENSLOCG00000011733	nfe2l3
ENSXMAG00000019538	bola1	ENSLOCG00000017692	bola1
ENSXMAG00000004650	chkb	ENSLOCG00000016752	chkb
ENSXMAG00000026910	itgb3b	ENSLOCG00000011968	itgb3b
ENSXMAG00000019746	pop7	ENSLOCG00000017553	pop7

ENSXMAG00000009477	tmprss5	ENSLOCG00000004438	TMPRSS5
ENSXMAG00000024559	dmtf1	ENSLOCG00000016713	dmtf1
ENSXMAG00000013436	nr2f5	ENSLOCG00000009006	nr2f5
ENSXMAG00000006695	nusap1	ENSLOCG00000013267	nusap1
ENSXMAG00000009540	gdf2	ENSLOCG00000003917	gdf2
ENSXMAG00000005651	rgs5a	ENSLOCG00000007218	rgs5a
ENSXMAG00000021721	pih1d2	ENSLOCG00000001526	pih1d2
ENSXMAG00000029768		ENSLOCG00000013444	EPO
ENSXMAG00000017884		ENSLOCG00000017763	
ENSXMAG00000009283	cwf19l1	ENSLOCG00000017035	cwf19l1
ENSXMAG00000005694	rgs4	ENSLOCG00000007206	rgs4
ENSXMAG00000019408		ENSLOCG00000004991	nol6
ENSXMAG00000028076		ENSLOCG00000005952	cacna1hb
ENSXMAG00000010197	MCUB	ENSLOCG00000012272	MCUB
ENSXMAG00000019942	si:dkey-237j11.3	ENSLOCG00000001365	
ENSXMAG00000030066		ENSLOCG00000005561	NUFIP1
ENSXMAG00000021679	fetub	ENSLOCG00000001616	FETUB
ENSXMAG00000004656	cpt1b	ENSLOCG00000016744	cpt1b
ENSXMAG00000003341	abt1	ENSLOCG00000014561	abt1
ENSXMAG00000010181	casp6l1	ENSLOCG00000012266	casp6l2
ENSXMAG00000021805	pgam2	ENSLOCG00000015670	pgam2
ENSXMAG00000016324		ENSLOCG00000006442	
ENSXMAG00000003344	rad51c	ENSLOCG00000001305	rad51c
ENSXMAG00000023369	rps6ka3b	ENSLOCG00000003638	rps6ka3a
ENSXMAG00000000345	jakmip2	ENSLOCG00000010819	jakmip2
ENSXMAG00000022428		ENSLOCG00000015001	snx22
ENSXMAG00000004591	tatdn3	ENSLOCG00000000884	tatdn3
ENSXMAG00000027708	gpc5a	ENSLOCG00000004783	GPC5
ENSXMAG00000012200	adpgk	ENSLOCG00000014950	adpgk
ENSXMAG00000010172	pla2g12a	ENSLOCG00000012254	PLA2G12A
ENSXMAG00000021846		ENSLOCG00000001540	zgc:162339
ENSXMAG00000006662	lgmn	ENSLOCG00000013252	lgmn
ENSXMAG00000004677	sephs1	ENSLOCG00000016742	sephs1
ENSXMAG00000009505	LRFN1	ENSLOCG00000014614	lrfn1
ENSXMAG00000005841	nos1apa	ENSLOCG00000007192	nos1apa
ENSXMAG00000010157	dnajb14	ENSLOCG00000012236	dnajb14
ENSXMAG00000022771		ENSLOCG00000011121	RAB37
ENSXMAG00000027106	ppib	ENSLOCG00000015000	ppib
ENSXMAG00000009570	mms19	ENSLOCG00000003810	mms19
ENSXMAG00000023542	si:dkeyp-87d8.8	ENSLOCG00000017524	gcnt4a
ENSXMAG00000009956	becn1	ENSLOCG00000011368	becn1
ENSXMAG00000022470	oaz2a	ENSLOCG00000015021	oaz2b
ENSXMAG00000003931	myog	ENSLOCG00000010722	myog
ENSXMAG00000025364	lhx5	ENSLOCG00000008328	lhx5
ENSXMAG00000000986	sb:cb649	ENSLOCG00000006162	WIPF3
ENSXMAG00000003381	srpk3	ENSLOCG00000014658	SRPK3
ENSXMAG00000004699	si:ch211-220f12.4	ENSLOCG00000016737	si:ch211-220f12.4
ENSXMAG00000004587	flvcr1	ENSLOCG00000000898	flvcr1
ENSXMAG00000019407	aqp3a	ENSLOCG00000005015	aqp3a
ENSXMAG00000008318		ENSLOCG00000010164	DMD
ENSXMAG00000019000	mzt2b	ENSLOCG00000008364	mzt2b
ENSXMAG00000012122	xylt2	ENSLOCG00000011114	xylt2
ENSXMAG00000019159	neb	ENSLOCG00000002637	neb
ENSXMAG00000028948	lamtor2	ENSLOCG00000008891	lamtor2
ENSXMAG00000023380		ENSLOCG00000005052	si:dkey-245n4.2
ENSXMAG00000020143		ENSLOCG00000018331	si:dkeyp-86h10.3

ENSXMAG00000002238	aga	ENSLOCG00000013645	aga
ENSXMAG00000019005	trim23	ENSLOCG00000010591	trim23
ENSXMAG00000005913		ENSLOCG00000008527	eef1db
ENSXMAG00000004713	prpf18	ENSLOCG00000016736	prpf18
ENSXMAG00000000980	oxnad1	ENSLOCG00000006425	oxnad1
ENSXMAG00000027071	rab11al	ENSLOCG00000008865	rab11al
ENSXMAG00000009949		ENSLOCG00000011391	
ENSXMAG00000013412		ENSLOCG00000008857	
ENSXMAG00000004575		ENSLOCG00000016904	SENP6
ENSXMAG00000021348		ENSLOCG00000017581	
ENSXMAG00000013341	pcnx1	ENSLOCG00000014027	pcnx1
ENSXMAG00000009612	vps35l	ENSLOCG00000002839	vps35l
ENSXMAG00000024253	mdm4	ENSLOCG00000012014	mdm4
ENSXMAG00000019013	ppwd1	ENSLOCG00000010598	ppwd1
ENSXMAG00000012133	recql5	ENSLOCG00000011489	recql5
ENSXMAG00000019579	socs9	ENSLOCG00000014611	socs9
ENSXMAG00000019186		ENSLOCG00000010976	
ENSXMAG00000028638	neil3	ENSLOCG00000013638	neil3
ENSXMAG00000019406	nr2f1a	ENSLOCG00000001663	nr2f1a
ENSXMAG00000005975	pycr3	ENSLOCG00000008510	pycr3
ENSXMAG00000010120	SNAPC3	ENSLOCG00000011852	SNAPC3
ENSXMAG00000023083	arhgap15	ENSLOCG00000001273	arhgap15
ENSXMAG00000019187	mrpl16	ENSLOCG00000010979	
ENSXMAG00000003956	VAMP3	ENSLOCG00000002645	vamp3
ENSXMAG00000003393	plxnb3	ENSLOCG00000014660	plxnb3
ENSXMAG00000004200	si:ch1073-111c8.3	ENSLOCG00000014606	si:ch1073-111c8.3
ENSXMAG00000019028	cwc27	ENSLOCG00000010631	cwc27
ENSXMAG00000012283	p4ha2	ENSLOCG00000010375	P4HA2
ENSXMAG00000022913	wwox	ENSLOCG00000000486	wwox
ENSXMAG00000019193	txnl4b	ENSLOCG00000009526	txnl4b
ENSXMAG00000028194	bmb	ENSLOCG00000008496	bmb
ENSXMAG00000021594	EIF1AY	ENSLOCG00000003622	eif1axb
ENSXMAG00000019404	fam172a	ENSLOCG00000001629	fam172a
ENSXMAG00000010112	PSIP1	ENSLOCG00000011863	psip1a
ENSXMAG00000010179	htatip2	ENSLOCG00000004900	htatip2
ENSXMAG00000019198	blzf1	ENSLOCG00000010075	blzf1
ENSXMAG00000000965	dazl	ENSLOCG00000006452	dazl
ENSXMAG00000002233	pdgfc	ENSLOCG00000008204	pdgfc
ENSXMAG00000019205	trmt10c	ENSLOCG00000017802	trmt10c
ENSXMAG00000003961	per3	ENSLOCG00000002607	per3
ENSXMAG00000006026	tsta3	ENSLOCG00000008481	tsta3
ENSXMAG00000019207	ercc5	ENSLOCG00000010061	ercc5
ENSXMAG00000010154	prmt3	ENSLOCG00000004922	prmt3
ENSXMAG00000019046	adamts6	ENSLOCG00000010611	adamts6
ENSXMAG00000024970		ENSLOCG00000016701	BUB1
ENSXMAG00000013394	pear1	ENSLOCG00000008831	pear1
ENSXMAG00000004192		ENSLOCG00000014604	
ENSXMAG00000006132	serpinb1	ENSLOCG00000012967	serpinb1
ENSXMAG00000019209	zgc:92518	ENSLOCG00000010052	zgc:92518
ENSXMAG00000021630		ENSLOCG00000017268	hey2
ENSXMAG00000009654	get4	ENSLOCG00000001666	get4
ENSXMAG00000004165	PAF1	ENSLOCG00000014601	paf1
ENSXMAG00000019403		ENSLOCG00000001617	
ENSXMAG00000021018	plac8.1	ENSLOCG00000010660	plac8.1
ENSXMAG00000024270	bnc2	ENSLOCG00000011896	bnc2
ENSXMAG00000008300	cyp20a1	ENSLOCG00000010325	cyp20a1

ENSXMAG00000003466	abcd1	ENSLOCG00000014676	abcd1
ENSXMAG00000019092	cops4	ENSLOCG00000010665	cops4
ENSXMAG00000021198	pdlim4	ENSLOCG00000010368	pdlim4
ENSXMAG00000011106		ENSLOCG00000014505	hmgb3a
ENSXMAG00000010152	slc6a5	ENSLOCG00000004950	slc6a5
ENSXMAG00000024520	gpatch4	ENSLOCG00000008820	gpatch4
ENSXMAG00000020077	cldn23a	ENSLOCG00000017550	cldn23a
ENSXMAG00000009189		ENSLOCG00000003883	
ENSXMAG00000024655		ENSLOCG00000011478	
ENSXMAG00000019214	tpp2	ENSLOCG00000010014	tpp2
ENSXMAG00000009671	mrtfab	ENSLOCG00000011497	
ENSXMAG00000016545		ENSLOCG00000006585	ARNT
ENSXMAG00000019104	lin54	ENSLOCG00000010680	lin54
ENSXMAG00000010256	si:ch73-264p11.1	ENSLOCG00000003966	si:ch73-264p11.1
ENSXMAG00000007983	arl5a	ENSLOCG00000002608	arl5a
ENSXMAG00000025070	SLC35D3	ENSLOCG00000017271	SLC35D3
ENSXMAG00000001374	NELL1	ENSLOCG00000007691	si:ch211-37e10.2
ENSXMAG00000017823	exosc9	ENSLOCG00000002999	exosc9
ENSXMAG00000009176	si:ch211-147k9.8	ENSLOCG00000000574	si:ch211-147k9.8
ENSXMAG00000003645	stard3nl	ENSLOCG00000011426	stard3nl
ENSXMAG00000004010	psma5	ENSLOCG00000012319	psma5
ENSXMAG00000009179	psmd7	ENSLOCG00000003971	psmd7
ENSXMAG00000016600	setdb1b	ENSLOCG00000006560	
ENSXMAG00000001397	tsr1	ENSLOCG00000006491	TSR1
ENSXMAG00000003480		ENSLOCG00000012309	sort1a
ENSXMAG00000003511	irak1	ENSLOCG00000014730	irak1
ENSXMAG00000024812	med6	ENSLOCG00000014035	med6
ENSXMAG00000017814	clgn	ENSLOCG00000010483	clgn
ENSXMAG00000006780	epdr1	ENSLOCG00000011436	epdr1
ENSXMAG00000004499	COX7A2	ENSLOCG00000016899	cox7a2a
ENSXMAG00000006178	gmds	ENSLOCG00000012983	gmds
ENSXMAG00000021429		ENSLOCG00000014725	MECP2
ENSXMAG00000019118	sec31a	ENSLOCG00000010692	sec31a
ENSXMAG00000020878	nol7	ENSLOCG00000011505	nol7
ENSXMAG00000023343	hic1	ENSLOCG00000006478	hic1
ENSXMAG00000010951	TTC9	ENSLOCG00000014042	TTC9
ENSXMAG00000020632		ENSLOCG00000020145	
ENSXMAG00000002199	fam198b	ENSLOCG00000008146	fam198b
ENSXMAG00000001380	LRP4	ENSLOCG00000014674	LRP4
ENSXMAG00000004025	nek4	ENSLOCG00000010516	nek4
ENSXMAG00000002131	gtdc1	ENSLOCG00000001286	gtdc1
ENSXMAG00000020182	foxe1	ENSLOCG00000018054	foxe1
ENSXMAG00000021873	dusp4	ENSLOCG00000012199	dusp4
ENSXMAG00000006783	ranbp9	ENSLOCG00000011518	ranbp9
ENSXMAG00000002191	tmem144b	ENSLOCG00000008135	tmem144b
ENSXMAG00000019239	rasa3	ENSLOCG00000009993	rasa3
ENSXMAG00000011336	prrc2c	ENSLOCG00000010383	prrc2c
ENSXMAG00000004147	dph1	ENSLOCG00000006434	dph1
ENSXMAG00000024941	maml3	ENSLOCG00000010506	maml3
ENSXMAG00000019444		ENSLOCG00000017859	gig2p
ENSXMAG00000009691	xrcc6	ENSLOCG00000011533	xrcc6
ENSXMAG00000014520	map3k9	ENSLOCG00000014044	map3k9
ENSXMAG00000004036	spcs1	ENSLOCG00000010522	spcs1
ENSXMAG00000000878	tbc1d5	ENSLOCG00000006501	tbc1d5
ENSXMAG00000006203	foxf2b	ENSLOCG00000012987	foxf2b
ENSXMAG00000022096	trmo	ENSLOCG00000011933	trmo

ENSXMAG00000012271	stam2	ENSLOCG00000002547	stam2	
ENSXMAG00000004038	glt8d1	ENSLOCG00000010530	glt8d1	
ENSXMAG00000019810	foxq1a	ENSLOCG00000012990	foxq1a	
ENSXMAG00000016700	pklr	ENSLOCG00000006611	PKLR	
ENSXMAG00000021882	josd1	ENSLOCG00000011543	josd1	
ENSXMAG00000002186	rxfp1	ENSLOCG00000008114	rxfp1	
ENSXMAG00000019400	ttc37	ENSLOCG00000001504	ttc37	
ENSXMAG00000012487	SLC5A10	ENSLOCG00000005804	SLC5A10	
ENSXMAG00000019256	pros1	ENSLOCG00000009972	pros1	
ENSXMAG00000012198	zgc:56106	ENSLOCG00000003054	zgc:56106	
ENSXMAG00000025207	retreg1	ENSLOCG00000012630		
ENSXMAG00000022716	anp32b	ENSLOCG00000011941	anp32b	
ENSXMAG00000003528	fgd1	ENSLOCG00000014698	fgd1	
ENSXMAG00000006917	naa20	ENSLOCG00000016890	naa20	
ENSXMAG00000022565	gnl3	ENSLOCG00000010542	gnl3	
ENSXMAG00000010062	coro2a	ENSLOCG00000011950	coro2a	
ENSXMAG00000019259		ENSLOCG00000009953		
ENSXMAG00000002301	si:dkey-247m21.3	ENSLOCG00000012209	si:dkey-247m21.3	
ENSXMAG00000014534	slc8a3	ENSLOCG00000014049	slc8a3	
ENSXMAG00000006205	exoc2	ENSLOCG00000012994	exoc2	
ENSXMAG00000012243	hacd2	ENSLOCG00000002527	hacd2	
ENSXMAG00000019262		ENSLOCG00000009934		
ENSXMAG00000006896	crnkl1	ENSLOCG00000016889	crnkl1	
ENSXMAG00000006827	znf622	ENSLOCG00000012640	znf622	
ENSXMAG00000002291	slc26a1	ENSLOCG00000012231	slc26a1	
ENSXMAG00000010026	uso1	ENSLOCG00000013485	uso1	
ENSXMAG00000022163	11-Mar	ENSLOCG00000012646		11-Mar
ENSXMAG00000017764	ELF2	ENSLOCG00000010581	elf2b	
ENSXMAG00000029119	tmem150c	ENSLOCG00000010721	tmem150c	
ENSXMAG00000002176	etfdh	ENSLOCG00000008102	etfdh	
ENSXMAG00000004074	ccdc3b	ENSLOCG00000010493	ccdc3b	
ENSXMAG00000001456		ENSLOCG00000005304		
ENSXMAG00000019151	hnrnpd	ENSLOCG00000010743	hnrnpd	
ENSXMAG00000000770	adcy5	ENSLOCG00000002507	adcy5	
ENSXMAG00000006838	myo10	ENSLOCG00000000037		
ENSXMAG00000000864	kcnh8	ENSLOCG00000006545	kcnh8	
ENSXMAG00000012623	KHDC4	ENSLOCG00000006286	KHDC4	
ENSXMAG00000023616	rangrf	ENSLOCG00000013898	rangrf	
ENSXMAG00000004700	brat1	ENSLOCG00000004235	brat1	
ENSXMAG00000026641	cox16	ENSLOCG00000014052	cox16	
ENSXMAG00000020315	CHST1	ENSLOCG00000017322	CHST1	
ENSXMAG00000012344		ENSLOCG00000013483	FBF1	
ENSXMAG00000010011	g3bp2	ENSLOCG00000013470	g3bp2	
ENSXMAG00000001466	slc35c1	ENSLOCG00000014653	slc35c1	
ENSXMAG00000019534	glrx3	ENSLOCG00000007580	glrx3	
ENSXMAG00000021186	zgc:113363	ENSLOCG00000014689	zgc:113363	
ENSXMAG00000011288	mettl13	ENSLOCG00000010429	mettl13	
ENSXMAG00000002165	ppid	ENSLOCG00000008085	ppid	
ENSXMAG00000017762	noctb	ENSLOCG00000010593	noctb	
ENSXMAG00000013043	cxxc1a	ENSLOCG00000014684	cxxc1a	
ENSXMAG00000024714	wdr81	ENSLOCG00000006334	wdr81	
ENSXMAG00000006876	si:zfos-223e1.2	ENSLOCG00000016887	CFAP61	
ENSXMAG00000019149	enoph1	ENSLOCG00000010725	enoph1	
ENSXMAG00000019399	arsk	ENSLOCG00000001492	arsk	
ENSXMAG00000000862	gpnmb	ENSLOCG00000011583	GPNMB	
ENSXMAG00000001487	apip	ENSLOCG00000001341	apip	

ENSXMAG00000021022	si:ch73-234b20.5	ENSLOCG00000010753	si:ch73-234b20.5
ENSXMAG00000011300	itpa	ENSLOCG00000010409	itpa
ENSXMAG00000004705	eif3bb	ENSLOCG00000004215	eif3bb
ENSXMAG00000014545	actn1	ENSLOCG00000014057	actn1
ENSXMAG00000000784	sec22a	ENSLOCG00000002494	sec22a
ENSXMAG00000019163	mat2al	ENSLOCG00000010761	mat2al
ENSXMAG00000013014	hdac6	ENSLOCG00000014762	
ENSXMAG00000028290		ENSLOCG00000017415	ZBED1
ENSXMAG00000009758	l3mbtl2	ENSLOCG00000011574	
ENSXMAG00000009982	pkd2	ENSLOCG00000013447	pkd2
ENSXMAG00000000240		ENSLOCG00000016882	
ENSXMAG00000000792	pdia5	ENSLOCG00000002474	pdia5
ENSXMAG00000000885		ENSLOCG00000001809	rnf2
ENSXMAG00000011885	pafah1b2	ENSLOCG00000001838	pafah1b2
ENSXMAG00000012038		ENSLOCG00000010317	
ENSXMAG00000006270	bphl	ENSLOCG00000013027	bphl
ENSXMAG00000000897	aglb	ENSLOCG00000001098	aglb
ENSXMAG00000027959	igf2bp3	ENSLOCG00000011599	igf2bp3
ENSXMAG00000029918	trnau1apb	ENSLOCG00000013175	trnau1apb
ENSXMAG00000021513		ENSLOCG00000013039	
ENSXMAG00000000242	xrn2	ENSLOCG00000016874	xrn2
ENSXMAG00000027430	arrdc3a	ENSLOCG00000001698	arrdc3a
ENSXMAG00000006655	lhfpl4b	ENSLOCG00000014763	lhfpl4b
ENSXMAG00000006920	adcy8	ENSLOCG00000012663	adcy8
ENSXMAG00000012399	si:ch211-120k19.1	ENSLOCG00000000016	si:ch211-120k19.1
ENSXMAG00000018512		ENSLOCG00000009179	
ENSXMAG00000011837	ttc17	ENSLOCG00000001159	ttc17
ENSXMAG00000016820	aaas	ENSLOCG00000005559	aaas
ENSXMAG00000009134	rad52	ENSLOCG00000017010	
ENSXMAG00000019274	tmco3	ENSLOCG00000009887	tmco3
ENSXMAG00000012396	rpl18	ENSLOCG00000000017	rpl18
ENSXMAG00000011903	rab6a	ENSLOCG00000006205	rab6a
ENSXMAG00000014575	dcaf5	ENSLOCG00000014066	dcaf5
ENSXMAG00000018529		ENSLOCG00000009206	dars2
ENSXMAG00000000223	adgrv1	ENSLOCG00000001716	adgrv1
ENSXMAG00000006464	tyw3	ENSLOCG00000003437	tyw3
ENSXMAG00000000841	ccdc126	ENSLOCG00000011624	ccdc126
ENSXMAG00000020399		ENSLOCG00000019151	MIR214
ENSXMAG00000026436	snx30	ENSLOCG00000002869	snx30
ENSXMAG00000002141	rapgef2	ENSLOCG00000008011	rapgef2
ENSXMAG00000011229	pigc	ENSLOCG00000010464	pigc
ENSXMAG00000011921	dnajb13	ENSLOCG00000006157	dnajb13
ENSXMAG00000000839	fam221a	ENSLOCG00000011634	fam221a
ENSXMAG00000017190	si:ch211-125a15.1	ENSLOCG00000016974	si:ch211-125a15.1
ENSXMAG00000011222	tmed5	ENSLOCG00000010470	tmed5
ENSXMAG00000011827	traf6	ENSLOCG00000001118	traf6
ENSXMAG00000020870	adprhl1	ENSLOCG00000009871	adprhl1
ENSXMAG00000012394	epn2	ENSLOCG00000005885	epn2
ENSXMAG00000002251	slc46a2	ENSLOCG00000002855	slc46a2
ENSXMAG00000006283	tbc1d7	ENSLOCG00000013127	tbc1d7
ENSXMAG00000026306	ghdc	ENSLOCG00000012540	ghdc
ENSXMAG00000018549	cryz	ENSLOCG00000003420	cryz
ENSXMAG00000021066	tmem68	ENSLOCG00000005519	tmem68
ENSXMAG00000011016	efr3a	ENSLOCG00000012671	efr3a
ENSXMAG00000011993	FNIP1	ENSLOCG00000010308	fnip1
ENSXMAG00000009116	fam185a	ENSLOCG00000015991	fam185a

ENSXMAG00000011949	ucp2	ENSLOCG00000006138	ucp2
ENSXMAG00000007499	cebpz	ENSLOCG00000016867	CEBPZ
ENSXMAG00000008190	gpd2	ENSLOCG00000005757	gpd2
ENSXMAG00000017191	TMEM19	ENSLOCG00000016245	TMEM19
ENSXMAG00000011779	trpm5	ENSLOCG00000000857	trpm5
ENSXMAG00000026502	zc3h7bb	ENSLOCG00000011588	zc3h7bb
ENSXMAG00000014578	pacs2	ENSLOCG00000014081	pacs2
ENSXMAG00000017105	fli1b	ENSLOCG00000004852	fli1b
ENSXMAG00000024775		ENSLOCG00000003326	
ENSXMAG00000000285	b9d1	ENSLOCG00000005936	b9d1
ENSXMAG00000009110	fbxl13	ENSLOCG00000015989	FBXL13
ENSXMAG00000004002	pitpnm3	ENSLOCG00000006245	pitpnm3
ENSXMAG00000028702		ENSLOCG00000007932	
ENSXMAG00000000931	XKR4	ENSLOCG00000005543	XKR4
ENSXMAG00000018560	tox	ENSLOCG00000005171	tox
ENSXMAG00000017736	timm10	ENSLOCG00000007919	timm10
ENSXMAG00000017104	etv2	ENSLOCG00000004877	etv2
ENSXMAG00000017819	gpam	ENSLOCG00000012286	gpam
ENSXMAG00000012300	zgc:158689	ENSLOCG00000000011	
ENSXMAG00000017731	unc93b1	ENSLOCG00000007905	unc93b1
ENSXMAG00000011978	ppme1	ENSLOCG00000006074	ppme1
ENSXMAG00000018565	ca8	ENSLOCG00000005132	ca8
ENSXMAG00000000822		ENSLOCG00000011661	
ENSXMAG00000011212	suco	ENSLOCG00000010475	
ENSXMAG00000002242	plppr1	ENSLOCG00000002781	plppr1
ENSXMAG00000019288	cul4a	ENSLOCG00000009814	cul4a
ENSXMAG00000023141	lysmd3	ENSLOCG00000001782	lysmd3
ENSXMAG00000017202		ENSLOCG00000016204	rab3ip
ENSXMAG00000024267	rab2a	ENSLOCG00000005108	rab2a
ENSXMAG00000009099	armc10	ENSLOCG00000015988	armc10
ENSXMAG00000007008		ENSLOCG00000012689	OC90
ENSXMAG00000001385	cdk20	ENSLOCG00000014752	cdk20
ENSXMAG00000000787	ago2	ENSLOCG00000007766	ago2
ENSXMAG00000022608	tfpt	ENSLOCG00000000009	
ENSXMAG00000001019	blvra	ENSLOCG00000010720	blvra
ENSXMAG00000025139	luzp2	ENSLOCG00000006529	luzp2
ENSXMAG00000003994	si:dkey-243i1.1	ENSLOCG00000006260	si:dkey-243i1.1
ENSXMAG00000028050	mblac2	ENSLOCG00000001811	mblac2
ENSXMAG00000007011		ENSLOCG00000012698	
ENSXMAG00000000837	dirc2	ENSLOCG00000002401	dirc2
ENSXMAG00000027147	srpk1b	ENSLOCG00000011166	srpk1b
ENSXMAG00000002239	GRIN3A	ENSLOCG00000002735	
ENSXMAG00000024934	ppp1r15b	ENSLOCG00000002993	ppp1r15b
ENSXMAG00000019189	MPHOSPH9	ENSLOCG00000005656	MPHOSPH9
ENSXMAG00000001372		ENSLOCG00000014570	uxt
ENSXMAG00000019660	kcnj12a	ENSLOCG00000018090	kcnj12a
ENSXMAG00000021539	rtn2b	ENSLOCG00000014882	
ENSXMAG00000012823	srsf5a	ENSLOCG00000014075	srsf5a
ENSXMAG00000019291	tgfbrap1	ENSLOCG00000008737	tgfbrap1
ENSXMAG00000001029		ENSLOCG00000005341	penkb
ENSXMAG00000012360	rps13	ENSLOCG00000002984	rps13
ENSXMAG00000024597	elk1	ENSLOCG00000014572	elk1
ENSXMAG00000007012	kcnq3	ENSLOCG00000012702	kcnq3
ENSXMAG00000024217	hspbap1	ENSLOCG00000002380	hspbap1
ENSXMAG00000001544	si:dkey-40c11.1	ENSLOCG00000005167	si:dkey-40c11.1
ENSXMAG00000001031	sdr16c5a	ENSLOCG00000005373	sdr16c5b

ENSXMAG00000023346	dlx5a	ENSLOCG00000011012	dlx5a
ENSXMAG00000011374	med24	ENSLOCG00000012864	med24
ENSXMAG00000012840		ENSLOCG00000014071	
ENSXMAG00000008157	atp7b	ENSLOCG00000010371	atp7b
ENSXMAG00000022648	med1	ENSLOCG00000012767	med1
ENSXMAG00000012272	prpf31	ENSLOCG00000000007	prpf31
ENSXMAG00000011201	mysm1	ENSLOCG00000008884	mysm1
ENSXMAG00000008227		ENSLOCG00000010294	rapgef6
ENSXMAG00000012022	ccdc61	ENSLOCG00000014713	ccdc61
ENSXMAG00000025196	txndc17	ENSLOCG00000006280	TXNDC17
ENSXMAG00000029724	dlx6a	ENSLOCG00000011008	dlx6a
ENSXMAG00000018642		ENSLOCG00000008224	ptbp2a
ENSXMAG00000000010	bbox1	ENSLOCG00000006463	BBOX1
ENSXMAG00000027500		ENSLOCG00000015697	
ENSXMAG00000003982		ENSLOCG00000006137	
ENSXMAG00000001053	exoc3	ENSLOCG00000013261	exoc3
ENSXMAG00000012856	lgals3a	ENSLOCG00000011781	lgals3a
ENSXMAG00000000165	lrrc6	ENSLOCG00000012709	lrrc6
ENSXMAG00000012374	pik3c2a	ENSLOCG00000002963	pik3c2a
ENSXMAG00000008149	eed	ENSLOCG00000009588	eed
ENSXMAG00000002140	sbno1	ENSLOCG00000005725	sbno1
ENSXMAG00000013277	dnajc28	ENSLOCG00000017420	dnajc28
ENSXMAG00000011200	oma1	ENSLOCG00000008868	oma1
ENSXMAG00000018645	dpydb	ENSLOCG00000008253	dpydb
ENSXMAG00000001521	smn1	ENSLOCG00000011339	smn1
ENSXMAG00000016699	SCUBE3	ENSLOCG00000010047	scube3
ENSXMAG00000008124	pwp2h	ENSLOCG00000003663	pwp2h
ENSXMAG00000026673	si:ch211-225p5.8	ENSLOCG00000004921	si:ch211-225p5.8
ENSXMAG00000019111	glod4	ENSLOCG00000006096	glod4
ENSXMAG00000000861	slc15a2	ENSLOCG00000002320	slc15a2
ENSXMAG00000027642	gmfb	ENSLOCG00000011787	gmfb
ENSXMAG00000000161	si:ch211-235o23.1	ENSLOCG00000012712	si:ch211-235o23.1
ENSXMAG00000000012	madd	ENSLOCG00000006317	madd
ENSXMAG00000017209	UPF2	ENSLOCG00000015698	UPF2
ENSXMAG00000001849	si:ch211-191a24.4	ENSLOCG00000010018	si:ch211-191a24.4
ENSXMAG00000029777	zgc:158640	ENSLOCG00000011348	zgc:158640
ENSXMAG00000007530		ENSLOCG00000016865	ndufaf7
ENSXMAG00000009868	herc3	ENSLOCG00000009178	herc3
ENSXMAG00000028116	gramd1a	ENSLOCG00000004963	GRAMD1A
ENSXMAG00000012062	pls3	ENSLOCG00000013613	pls3
ENSXMAG00000024586	C8orf82	ENSLOCG00000008450	C8orf82
ENSXMAG00000008123	pjvk	ENSLOCG00000006261	pjvk
ENSXMAG00000002346	rps7	ENSLOCG00000017211	rps7
ENSXMAG00000019085	si:ch211-259g3.4	ENSLOCG00000006063	si:ch211-259g3.4
ENSXMAG00000007643	cnih1	ENSLOCG00000011793	cnih1
ENSXMAG00000018989	tmem167b	ENSLOCG00000010229	tmem167b
ENSXMAG00000000153	ift57	ENSLOCG00000012726	ift57
ENSXMAG00000001513	lzts1	ENSLOCG00000014910	lzts1
ENSXMAG00000018668	odr4	ENSLOCG00000001553	odr4
ENSXMAG00000014702	crygmxl2	ENSLOCG00000012191	crygmxl2
ENSXMAG00000000752	sem1	ENSLOCG00000011004	sem1
ENSXMAG00000007646		ENSLOCG00000011806	cdkn3
ENSXMAG00000019957	b3galt6	ENSLOCG00000018082	b3galt6
ENSXMAG00000028832	cnga2b	ENSLOCG00000014964	cnga2a
ENSXMAG00000026346	mboat7	ENSLOCG00000007984	MBOAT7
ENSXMAG00000015183	kcnd1	ENSLOCG00000014523	kcnd1

ENSXMAG00000002127	FSTL5	ENSLOCG00000007998	FSTL5
ENSXMAG00000002357	rnaseh1	ENSLOCG00000017212	rnaseh1
ENSXMAG00000000722	DYNC1I1	ENSLOCG00000010982	DYNC1I1
ENSXMAG00000012105	RRAGB	ENSLOCG00000014979	rraga
ENSXMAG00000000138	slc39a6	ENSLOCG00000012781	
ENSXMAG00000012175	rps9	ENSLOCG00000008007	rps9
ENSXMAG00000015151	ptf1a	ENSLOCG00000003186	ptf1a
ENSXMAG00000029412	cdab	ENSLOCG00000001971	cdab
ENSXMAG00000000221	emp2	ENSLOCG00000006189	emp2
ENSXMAG00000014615	sos2	ENSLOCG00000011825	sos2
ENSXMAG00000001816		ENSLOCG00000008541	
ENSXMAG00000026867	zgc:153395	ENSLOCG00000012403	zgc:153395
ENSXMAG00000027765	tcf7l2	ENSLOCG00000012211	tcf7l2
ENSXMAG00000015220	gripap1	ENSLOCG00000014510	gripap1
ENSXMAG00000000216	nubp1	ENSLOCG00000006142	nubp1
ENSXMAG00000027800	qdpra	ENSLOCG00000003181	zgc:171517
ENSXMAG00000005027	diaph3	ENSLOCG00000004676	diaph3
ENSXMAG00000009862	fam13a	ENSLOCG00000009161	fam13a
ENSXMAG00000017243	slc35e3	ENSLOCG00000016184	slc35e3
ENSXMAG00000024598	zswim2	ENSLOCG00000008690	zswim2
ENSXMAG00000000718	pdk4	ENSLOCG00000010966	pdk4
ENSXMAG00000001441	ela3l	ENSLOCG00000004644	
ENSXMAG00000000014	nr1h3	ENSLOCG00000006349	nr1h3
ENSXMAG00000028559	zgc:77739	ENSLOCG00000010633	zgc:77739
ENSXMAG00000013245	psmc4	ENSLOCG00000004980	psmc4
ENSXMAG00000018722	dusp12	ENSLOCG00000001308	dusp12
ENSXMAG00000030016		ENSLOCG00000009321	
ENSXMAG00000002205	hdhd2	ENSLOCG00000012392	hdhd2
ENSXMAG00000025031	ldb2a	ENSLOCG00000003158	ldb2a
ENSXMAG00000012127	brwd3	ENSLOCG00000014998	brwd3
ENSXMAG00000000920	si:dkey-91i10.3	ENSLOCG00000002266	si:dkey-91i10.3
ENSXMAG00000000714	asb4	ENSLOCG00000010959	asb4
ENSXMAG00000014651	l2hgdh	ENSLOCG00000011835	l2hgdh
ENSXMAG00000005636	sclt1	ENSLOCG00000010642	sclt1
ENSXMAG00000011766	srsf1a	ENSLOCG00000005994	srsf1a
ENSXMAG00000000016	arl14ep	ENSLOCG00000006362	arl14ep
ENSXMAG00000013241	si:dkey-199f5.8	ENSLOCG00000004999	si:dkey-199f5.8
ENSXMAG00000026448	eaf2	ENSLOCG00000002255	eaf2
ENSXMAG00000020103	dexi	ENSLOCG00000018106	dexi
ENSXMAG00000002201	katnal2	ENSLOCG00000012385	katnal2
ENSXMAG00000022134	atp5s	ENSLOCG00000011844	atp5s
ENSXMAG00000018760	ptpn18	ENSLOCG00000014499	ptpn18
ENSXMAG00000019200	fbxo18	ENSLOCG00000015655	fbxo18
ENSXMAG00000001341	atg10	ENSLOCG00000002071	atg10
ENSXMAG00000018846	pusl1	ENSLOCG00000002210	PUSL1
ENSXMAG00000008251	FSTL4	ENSLOCG00000010287	fstl4
ENSXMAG00000011303	top2a	ENSLOCG00000012897	top2a
ENSXMAG00000000712	ppp1r9a	ENSLOCG00000010917	ppp1r9a
ENSXMAG00000014662	cdkl1	ENSLOCG00000011854	cdkl1
ENSXMAG00000007974	mrps9	ENSLOCG00000008722	mrps9
ENSXMAG00000000967	si:dkey-91i10.2	ENSLOCG00000002209	si:dkey-91i10.2
ENSXMAG00000000213	clec16a	ENSLOCG00000006114	clec16a
ENSXMAG00000001428	klf9	ENSLOCG00000009508	klf9
ENSXMAG00000028797		ENSLOCG00000012499	cnpy1
ENSXMAG00000022538	tsn	ENSLOCG00000002191	tsn
ENSXMAG00000025735		ENSLOCG00000009248	

ENSXMAG00000017276	mdm2	ENSLOCG00000016186	mdm2
ENSXMAG00000011301		ENSLOCG00000017520	GJD3
ENSXMAG00000021189	sigmar1	ENSLOCG00000012381	sigmar1
ENSXMAG00000005103	tdrd3	ENSLOCG00000004652	tdrd3
ENSXMAG00000021922	pou3f3b	ENSLOCG00000008712	pou3f3b
ENSXMAG00000014674	map4k5	ENSLOCG00000011867	map4k5
ENSXMAG00000028967	FAM124A	ENSLOCG00000010298	FAM124A
ENSXMAG00000021893	C5orf15	ENSLOCG00000010282	C5orf15
ENSXMAG00000027837	bcap31	ENSLOCG00000014585	bcap31
ENSXMAG00000011824	paxip1	ENSLOCG00000012517	paxip1
ENSXMAG00000028768		ENSLOCG00000009211	
ENSXMAG00000002181	galt	ENSLOCG00000012369	galt
ENSXMAG00000007418	vdac1	ENSLOCG00000010266	vdac1
ENSXMAG00000023792		ENSLOCG00000009689	
ENSXMAG00000000021		ENSLOCG00000002464	cfap70
ENSXMAG00000013815	SLC6A8	ENSLOCG00000014590	slc6a8
ENSXMAG00000012189	npas2	ENSLOCG00000014750	npas2
ENSXMAG00000019863	paqr7b	ENSLOCG00000018278	paqr7b
ENSXMAG00000001333	trpm3	ENSLOCG00000009513	trpm3
ENSXMAG00000012069	ttyh1	ENSLOCG00000001424	
ENSXMAG00000025897	serpine3	ENSLOCG00000010310	serpine3
ENSXMAG00000013111	dusp28	ENSLOCG00000009143	dusp28
ENSXMAG00000026378	vti1a	ENSLOCG00000012227	vti1a
ENSXMAG00000028075		ENSLOCG00000005794	
ENSXMAG00000014627		ENSLOCG00000007643	
ENSXMAG00000005117	PCDH20	ENSLOCG00000017781	PCDH20
ENSXMAG00000001017	ints6	ENSLOCG00000010329	ints6
ENSXMAG00000020911	vcpip1	ENSLOCG00000004697	vcpip1
ENSXMAG00000000709	sgce	ENSLOCG00000010901	sgce
ENSXMAG00000025576	cntfr	ENSLOCG00000012362	cntfr
ENSXMAG00000007115	slc35a2	ENSLOCG00000014583	slc35a2
ENSXMAG00000020181		ENSLOCG00000009144	
ENSXMAG00000022063	ube2j2	ENSLOCG00000002308	ube2j2
ENSXMAG00000011173	prkaa2	ENSLOCG00000008799	prkaa2
ENSXMAG00000014776	zdhhc6	ENSLOCG00000012241	zdhhc6
ENSXMAG00000001671	pcdh9	ENSLOCG00000004624	pcdh9
ENSXMAG00000029064	tcf7	ENSLOCG00000010252	tcf7
ENSXMAG00000000022	ddb1	ENSLOCG00000000012	DDB1
ENSXMAG00000016646	taf11	ENSLOCG00000010021	taf11
ENSXMAG00000009842	elmod2	ENSLOCG00000009137	elmod2
ENSXMAG00000007538	xrcc3	ENSLOCG00000009948	xrcc3
ENSXMAG00000025737	pou4f1	ENSLOCG00000004613	pou4f1
ENSXMAG00000014757	atl1	ENSLOCG00000011885	atl1
ENSXMAG00000027272	tut1	ENSLOCG00000013105	tut1
ENSXMAG00000016608	uhrf1bp1	ENSLOCG00000010005	uhrf1bp1
ENSXMAG00000001676	RNF219	ENSLOCG00000004597	RNF219
ENSXMAG00000012287		ENSLOCG00000014778	si:ch211-26b3.2
ENSXMAG00000013220	pim2	ENSLOCG00000014577	pim2
ENSXMAG00000011807	synrg	ENSLOCG00000005903	synrg
ENSXMAG00000004144	tmem267	ENSLOCG00000012351	tmem267
ENSXMAG00000009829	ucp1	ENSLOCG00000009127	ucp1
ENSXMAG00000001056	wdfy2	ENSLOCG00000010340	wdfy2
ENSXMAG00000012449	si:dkey-42p14.3	ENSLOCG00000015844	si:dkey-42p14.3
ENSXMAG00000013211	otud5a	ENSLOCG00000014573	otud5a
ENSXMAG00000007700	kmo	ENSLOCG00000016577	kmo
ENSXMAG00000001679	rbm26	ENSLOCG00000004557	rbm26

ENSXMAG00000021349	ntd5	ENSLOCG00000007480	
ENSXMAG00000025612	chmp1a	ENSLOCG00000003012	chmp1a
ENSXMAG00000005577	zgc:195282	ENSLOCG00000012820	zgc:195282
ENSXMAG00000007555	ppp4r4	ENSLOCG00000012982	ppp4r4
ENSXMAG00000011157	bloc1s2	ENSLOCG00000008776	bloc1s2
ENSXMAG00000009805	tbc1d9	ENSLOCG00000009091	tbc1d9
ENSXMAG00000020295	slc10a3	ENSLOCG00000017590	slc10a3
ENSXMAG00000014788	sav1	ENSLOCG00000011893	sav1
ENSXMAG00000005559	pi4k2b	ENSLOCG00000013173	pi4k2b
ENSXMAG00000000024	taldo1	ENSLOCG00000000006	taldo1
ENSXMAG00000004159	rassf6	ENSLOCG00000002613	rassf6
ENSXMAG00000023726	TBC1D8B	ENSLOCG00000014769	TBC1D8B
ENSXMAG00000000704	casd1	ENSLOCG00000010891	casd1
ENSXMAG00000001088	dhrs12	ENSLOCG00000010359	dhrs12
ENSXMAG00000023511	htra3a	ENSLOCG00000010288	htra3a
ENSXMAG00000002903	zgc:158366	ENSLOCG00000002928	ZNF276
ENSXMAG00000014801	nin	ENSLOCG00000011900	nin
ENSXMAG00000007707	opn3	ENSLOCG00000016574	opn3
ENSXMAG00000012462	api5	ENSLOCG00000001136	api5
ENSXMAG00000008273	skp1	ENSLOCG00000010241	skp1
ENSXMAG00000021149	cdc42ep3	ENSLOCG00000016572	cdc42ep3
ENSXMAG00000007480	mylka	ENSLOCG00000005547	mylka
ENSXMAG00000016573	smim29	ENSLOCG00000009940	smim29
ENSXMAG00000010350	ttpa	ENSLOCG00000004948	ttpa
ENSXMAG00000007712	rmdn2	ENSLOCG00000016567	
ENSXMAG00000011727	vps9d1	ENSLOCG00000002943	vps9d1
ENSXMAG00000007949	gpd1c	ENSLOCG00000002845	
ENSXMAG00000016571	hmga1a	ENSLOCG00000009932	hmga1a
ENSXMAG00000001116	olig2	ENSLOCG00000017506	olig2
ENSXMAG00000030008	znf330	ENSLOCG00000009067	znf330
ENSXMAG00000001300	cemip2	ENSLOCG00000009537	cemip2
ENSXMAG00000022694	olig1	ENSLOCG00000017507	olig1
ENSXMAG00000022520	cyp1b1	ENSLOCG00000016566	cyp1b1
ENSXMAG00000014511	ace	ENSLOCG00000012317	ace
ENSXMAG00000004125	MUSK	ENSLOCG00000002642	MUSK
ENSXMAG00000007933		ENSLOCG00000002826	osbpl11
ENSXMAG00000000698	col1a2	ENSLOCG00000010835	col1a2
ENSXMAG00000005493	lrpap1	ENSLOCG00000010362	lrpap1
ENSXMAG00000010364		ENSLOCG00000005010	
ENSXMAG00000024950	nanp	ENSLOCG00000016565	nanp
ENSXMAG00000007468	ube2g2	ENSLOCG00000009402	UBE2G2
ENSXMAG00000027189	ube2b	ENSLOCG00000010219	ube2b
ENSXMAG00000024855	abhd17b	ENSLOCG00000009555	abhd17b
ENSXMAG00000025117		ENSLOCG00000004735	unc80
ENSXMAG00000007723	polr1b	ENSLOCG00000016558	polr1b
ENSXMAG00000000028		ENSLOCG00000014280	
ENSXMAG00000003641	ercc3	ENSLOCG00000010907	ercc3
ENSXMAG00000008286	cdkn2aipnl	ENSLOCG00000010215	cdkn2aipnl
ENSXMAG00000001287	zgc:101016	ENSLOCG00000009561	zgc:101016
ENSXMAG00000022643	ankrd50l	ENSLOCG00000006484	ankrd50l
ENSXMAG00000011110	pcsk9	ENSLOCG00000008732	pcsk9
ENSXMAG00000006126	lpar1	ENSLOCG00000002675	lpar1
ENSXMAG00000014865	pygl	ENSLOCG00000011949	pygl
ENSXMAG00000005474	rgs12b	ENSLOCG00000010388	rgs12b
ENSXMAG00000028835	jade2	ENSLOCG00000010206	jade2
ENSXMAG00000027132		ENSLOCG00000002985	

ENSXMAG00000001250	gda	ENSLOCG00000009573	gda
ENSXMAG00000001697	tnrc6a	ENSLOCG00000005517	tnrc6a
ENSXMAG00000001204	fam160a2	ENSLOCG00000006531	fam160a2
ENSXMAG00000006379	ghrhrb	ENSLOCG00000009570	
ENSXMAG00000023904	pdf	ENSLOCG00000002834	pdf
ENSXMAG00000020940	si:ch211-214j8.12	ENSLOCG00000004846	si:ch211-214j8.12
ENSXMAG00000008294	sar1b	ENSLOCG00000010197	sar1b
ENSXMAG00000001242	cnga4	ENSLOCG00000006552	cnga4
ENSXMAG00000001712	gpalpp1	ENSLOCG00000005528	gpalpp1
ENSXMAG00000023712	zgc:85777	ENSLOCG00000010811	zgc:85777
ENSXMAG00000013123	alkbh3	ENSLOCG00000001237	alkbh3
ENSXMAG00000009040	tat	ENSLOCG00000002775	tat
ENSXMAG00000011090	dhcr24	ENSLOCG00000008711	dhcr24
ENSXMAG00000007742	dnajc12	ENSLOCG00000011266	dnajc12
ENSXMAG00000010623	si:dkey-34d22.1	ENSLOCG00000003494	si:dkey-34d22.1
ENSXMAG00000007570	ubr7	ENSLOCG00000013081	ubr7
ENSXMAG00000025009		ENSLOCG00000006381	ccdc141
ENSXMAG00000027392		ENSLOCG00000017481	
ENSXMAG00000000695	bet1	ENSLOCG00000010821	bet1
ENSXMAG00000001261	trappc10	ENSLOCG00000003708	trappc10
ENSXMAG00000007746	sirt1	ENSLOCG00000011271	SIRT1
ENSXMAG00000028755	zgc:56622	ENSLOCG00000002811	zgc:56622
ENSXMAG00000001222	entpd4	ENSLOCG00000015008	entpd4
ENSXMAG00000024374	gngt1	ENSLOCG00000010805	gngt1
ENSXMAG00000008298	sec24a	ENSLOCG00000010187	sec24a
ENSXMAG00000027795	RF00322	ENSLOCG00000019019	RF00322
ENSXMAG00000019709	tmem251	ENSLOCG00000017714	tmem251
ENSXMAG00000000682	tfpi2	ENSLOCG00000010795	tfpi2
ENSXMAG00000024898	tmx1	ENSLOCG00000011971	tmx1
ENSXMAG00000000032	chrna3	ENSLOCG00000014294	chrna3
ENSXMAG00000009101	klhl36	ENSLOCG00000002761	klhl36
ENSXMAG00000019675	dusp14	ENSLOCG00000017439	dusp14
ENSXMAG00000005378	htt	ENSLOCG00000010421	htt
ENSXMAG00000001675	si:dkey-103i16.6	ENSLOCG00000015265	si:dkey-103i16.6
ENSXMAG00000007753	cacul1	ENSLOCG00000009720	CACUL1
ENSXMAG00000007577	nt5e	ENSLOCG00000016916	nt5e
ENSXMAG00000021685	ophn1	ENSLOCG00000014675	ophn1
ENSXMAG00000023363	ndufab1b	ENSLOCG00000005580	ndufab1b
ENSXMAG00000028133	alkbh3	ENSLOCG00000017314	alkbh3
ENSXMAG00000024919	cotl1	ENSLOCG00000002734	cotl1
ENSXMAG00000001661	ric8b	ENSLOCG00000015262	ric8b
ENSXMAG00000006420	cyldl	ENSLOCG00000008578	cyldl
ENSXMAG00000027169	GHITM	ENSLOCG00000005554	GHITM
ENSXMAG00000000033	CHRNA5	ENSLOCG00000014297	CHRNA5
ENSXMAG00000013108		ENSLOCG00000015077	TICRR
ENSXMAG00000012014	tldc1	ENSLOCG00000002721	tldc1
ENSXMAG00000014965	frmd6	ENSLOCG00000011982	frmd6
ENSXMAG00000017404	abcc9	ENSLOCG00000015376	abcc9
ENSXMAG00000001678	dctn5	ENSLOCG00000005632	dctn5
ENSXMAG00000011832	tada2a	ENSLOCG00000005887	tada2a
ENSXMAG00000025776	si:ch211-121a2.4	ENSLOCG00000008653	si:ch211-121a2.4
ENSXMAG00000000680	calcr	ENSLOCG00000010787	calcr
ENSXMAG00000001638	rfx4	ENSLOCG00000015258	rfx4
ENSXMAG00000009734	gab1	ENSLOCG00000008996	gab1
ENSXMAG00000001218	slc25a37	ENSLOCG00000015013	slc25a37
ENSXMAG00000007584		ENSLOCG00000016917	snx14

ENSXMAG00000001297	gdf3		ENSLOCG00000016443	gdf3	
ENSXMAG00000010878	slc35d1a		ENSLOCG00000007054	slc35d1a	
ENSXMAG00000011966	plcg2		ENSLOCG00000002618	plcg2	
ENSXMAG00000006422	zc3h3		ENSLOCG00000008621	ZC3H3	
ENSXMAG00000020262	nkx3-1		ENSLOCG00000015015	nkx3-1	
ENSXMAG00000019423	HCAR1		ENSLOCG00000018151		
ENSXMAG00000018933	rbp1		ENSLOCG00000004318	rbp1	
ENSXMAG00000007759	MYPN		ENSLOCG00000011326	MYPN	
ENSXMAG00000019247	denr		ENSLOCG00000005501	denr	
ENSXMAG00000011979	u2af2b		ENSLOCG00000008050	u2af2a	
ENSXMAG00000023550	aqp12		ENSLOCG00000005627	aqp12	
ENSXMAG00000019811	mafaa		ENSLOCG00000018124	mafaa	
ENSXMAG00000013099	ckmt1		ENSLOCG00000015070	ckmt1	
ENSXMAG00000024302	RF00582		ENSLOCG00000020639	RF00582	
ENSXMAG00000011081			ENSLOCG00000008397		
ENSXMAG00000019251	bcl7a		ENSLOCG00000005295	bcl7a	
ENSXMAG00000026619	rpp21		ENSLOCG00000005595	rpp21	
ENSXMAG00000010612	prss1		ENSLOCG00000004195	prss1	
ENSXMAG00000001723	baz2ba		ENSLOCG00000006024	baz2ba	
ENSXMAG00000011868	traf4a		ENSLOCG00000005871	traf4a	
ENSXMAG00000006424	atpaf1		ENSLOCG00000009121	atpaf1	
ENSXMAG00000018937	copb2		ENSLOCG00000004368	copb2	
ENSXMAG00000001181	ido1		ENSLOCG00000015024	ido1	
ENSXMAG00000015006	gng2		ENSLOCG00000011994	gng2	
ENSXMAG00000001568	polr3b		ENSLOCG00000015249	polr3b	
ENSXMAG00000021268	pllp		ENSLOCG00000007690	pllp	
ENSXMAG00000021656			ENSLOCG00000001990	znf1168	
ENSXMAG00000007902	lancl1		ENSLOCG00000004634	lancl1	
ENSXMAG00000011082	crym		ENSLOCG00000008130	CRYM	
ENSXMAG00000020069	fgfbp2a		ENSLOCG00000018182	fgfbp2b	
ENSXMAG00000022127	guca1b		ENSLOCG00000011305	guca1b	
ENSXMAG00000005315	grk4		ENSLOCG00000010446	grk4	
ENSXMAG00000018525	MSN		ENSLOCG00000014640	msna	
ENSXMAG00000013018	ARL2BP		ENSLOCG00000007707	ARL2BP	
ENSXMAG00000027972	eif2b1		ENSLOCG00000005846	EIF2B1	
ENSXMAG00000006437	RHPN1		ENSLOCG00000008592	RHPN1	
ENSXMAG00000020068	fgfbp1b		ENSLOCG00000018180	fgfbp1b	
ENSXMAG00000001153	RHOBTB2		ENSLOCG00000014937	rhobtb2b	
ENSXMAG00000014889	FBXL15		ENSLOCG00000005464	FBXL15	
ENSXMAG00000003875	phb2a		ENSLOCG00000001389	phb2b	
ENSXMAG00000011117	uqcrc2b		ENSLOCG00000008162	uqcrc2b	
ENSXMAG00000008312	ddx46		ENSLOCG00000010169	ddx46	
ENSXMAG00000013069	pdia3		ENSLOCG00000014242	pdia3	
ENSXMAG00000013026	RSPRY1		ENSLOCG00000007716	rspry1	
ENSXMAG00000011731	ddx55		ENSLOCG00000005828	ddx55	
ENSXMAG00000018548	zc3h12b		ENSLOCG00000014631	zc3h12b	
ENSXMAG00000017536	usp6nl		ENSLOCG00000015695	usp6nl	
ENSXMAG00000014109			ENSLOCG00000003329	fam151a	
ENSXMAG00000015009	dusp23a		ENSLOCG00000017207	dusp23b	
ENSXMAG00000021367	amer1		ENSLOCG00000014609	amer1	
ENSXMAG00000001736		7-Mar	ENSLOCG00000006058		7-Mar
ENSXMAG00000026879	phf24		ENSLOCG00000004626	phf24	
ENSXMAG00000022369	pigx		ENSLOCG00000005556		
ENSXMAG00000018981	dvl3a		ENSLOCG00000008386	dvl3a	
ENSXMAG00000002057	ccna2		ENSLOCG00000002979	ccna2	
ENSXMAG00000019855	fpr1		ENSLOCG00000018169	fpr1	

ENSXMAG00000027930	tlcd1	ENSLOCG00000005834	tlcd1
ENSXMAG00000012324	GAB3	ENSLOCG00000014605	GAB3
ENSXMAG00000011142	birc5a	ENSLOCG00000012776	birc5a
ENSXMAG00000011279	cep19	ENSLOCG00000006635	cep19
ENSXMAG00000001612	usp7	ENSLOCG00000006243	usp7
ENSXMAG00000019829	atoh7	ENSLOCG00000018251	atoh7
ENSXMAG00000001737	ly75	ENSLOCG00000008671	ly75
ENSXMAG00000023900	egr3	ENSLOCG00000014936	egr3
ENSXMAG00000010604	has1	ENSLOCG00000004185	has1
ENSXMAG00000011967	si:dkey-17m8.1	ENSLOCG00000008068	
ENSXMAG00000025919	lsm8	ENSLOCG00000015580	lsm8
ENSXMAG00000026496	rab34a	ENSLOCG00000005802	rab34a
ENSXMAG00000022426		ENSLOCG00000017945	
ENSXMAG00000000669	vps50	ENSLOCG00000010759	vps50
ENSXMAG00000013066	isg20	ENSLOCG00000014240	isg20
ENSXMAG00000014892	si:ch73-111m19.2	ENSLOCG00000005449	
ENSXMAG00000005285	asna1	ENSLOCG00000007957	asna1
ENSXMAG00000011143	FAAP100	ENSLOCG00000012780	si:dkey-57h18.1
ENSXMAG00000011282	ing5a	ENSLOCG00000006651	ing5a
ENSXMAG00000026528	BIN3	ENSLOCG00000014932	bin3
ENSXMAG00000007774	pkd2l1	ENSLOCG00000011334	pkd2l1
ENSXMAG00000028231	anapc13	ENSLOCG00000004767	anapc13
ENSXMAG00000012325	aifm1	ENSLOCG00000015353	aifm1
ENSXMAG00000020509		ENSLOCG00000019335	MIRLET7C
ENSXMAG00000023887	zgc:56556	ENSLOCG00000012861	zgc:56556
ENSXMAG00000024023	mei4	ENSLOCG00000016914	mei4
ENSXMAG00000003457		ENSLOCG00000017870	
ENSXMAG00000019001	ap2m1a	ENSLOCG00000008365	ap2m1a
ENSXMAG00000002045	bbs7	ENSLOCG00000002959	bbs7
ENSXMAG00000009721	USP38	ENSLOCG00000009016	usp38
ENSXMAG00000008328	txndc15	ENSLOCG00000010163	txndc15
ENSXMAG00000024173	kcnn4	ENSLOCG00000004166	kcnn4
ENSXMAG00000025069		ENSLOCG00000004251	plekhm3
ENSXMAG00000007657		ENSLOCG00000012692	
ENSXMAG00000016489	eevs	ENSLOCG00000010050	eevs
ENSXMAG00000022559	fuca2	ENSLOCG00000000762	FUCA2
ENSXMAG00000019476	zgc:103697	ENSLOCG00000014705	zgc:103697
ENSXMAG00000028046	zgc:174895	ENSLOCG00000005790	zgc:174895
ENSXMAG00000011256	plekhb2	ENSLOCG00000006720	plekhb2
ENSXMAG00000005274	best2	ENSLOCG00000007943	best2
ENSXMAG00000018673	eif4bb	ENSLOCG00000005500	eif4ba
ENSXMAG00000001745	pla2r1	ENSLOCG00000008647	pla2r1
ENSXMAG00000018812		ENSLOCG00000003665	tmem70
ENSXMAG00000001075	acsl2	ENSLOCG00000015588	acsl2
ENSXMAG00000015058	TATDN3	ENSLOCG00000000863	TATDN3
ENSXMAG00000025835		ENSLOCG00000012703	
ENSXMAG00000019582	junba	ENSLOCG00000018297	junba
ENSXMAG00000010591	smg9	ENSLOCG00000004127	smg9
ENSXMAG00000027915	tial1	ENSLOCG00000011405	tial1
ENSXMAG00000013927	nup93	ENSLOCG00000007863	nup93
ENSXMAG00000024070	proca1	ENSLOCG00000005777	proca1
ENSXMAG00000018814		ENSLOCG00000003277	mrpl37
ENSXMAG00000009678	SMARCA1	ENSLOCG00000014591	smarca1
ENSXMAG00000011936	atg12	ENSLOCG00000008119	atg12
ENSXMAG00000019014	pcyt1aa	ENSLOCG00000008590	pcyt1aa
ENSXMAG00000008331	pitx1	ENSLOCG00000010144	pitx1

ENSXMAG00000029323	klhl26	ENSLOCG00000001457	klhl26
ENSXMAG00000025293	rln1	ENSLOCG00000010131	rln1
ENSXMAG00000011233	ankrd29	ENSLOCG00000001523	ankrd29
ENSXMAG00000006468	grip1	ENSLOCG00000016167	grip1
ENSXMAG00000007666		ENSLOCG00000012688	
ENSXMAG00000003524	plgrkt	ENSLOCG00000010124	plgrkt
ENSXMAG00000022616	bag3	ENSLOCG00000011418	bag3
ENSXMAG00000004445	tmed2	ENSLOCG00000005792	tmed2
ENSXMAG00000000608	msi2a	ENSLOCG00000004675	msi2b
ENSXMAG00000002033	tmem33	ENSLOCG00000012912	tmem33
ENSXMAG00000001770	itgb6	ENSLOCG00000008636	itgb6
ENSXMAG00000011915	foxj2	ENSLOCG00000008171	
ENSXMAG00000018868	mfsd12a	ENSLOCG00000001259	mfsd12a
ENSXMAG00000020154	mtif3	ENSLOCG00000004205	mtif3
ENSXMAG00000011068	cts12	ENSLOCG00000001478	cts12
ENSXMAG00000023365	ptpn11a	ENSLOCG00000007663	ptpn11a
ENSXMAG00000027056	si:ch211-157c3.4	ENSLOCG00000006257	
ENSXMAG00000001028		ENSLOCG00000015583	
ENSXMAG00000007667	heca	ENSLOCG00000001223	heca
ENSXMAG00000015128		ENSLOCG00000015886	TP53I3
ENSXMAG00000000667	hepacam2	ENSLOCG00000010740	hepacam2
ENSXMAG00000009662	frem3	ENSLOCG00000008948	frem3
ENSXMAG00000007830	inpp5f	ENSLOCG00000011429	inpp5f
ENSXMAG00000022004	phox2bb	ENSLOCG00000012918	phox2bb
ENSXMAG00000023463	mrrf	ENSLOCG00000003021	mrrf
ENSXMAG00000029637	SLC2A6	ENSLOCG00000003548	
ENSXMAG00000024555	cx36.7	ENSLOCG00000017944	cx36.7
ENSXMAG00000005225	acp5a	ENSLOCG00000008404	acp5a
ENSXMAG00000030033	hapln3	ENSLOCG00000014221	hapln3
ENSXMAG00000028689	abracl	ENSLOCG00000001211	abracl
ENSXMAG00000016444	slc38a10	ENSLOCG00000012804	slc38a10
ENSXMAG00000026356	cdk6	ENSLOCG00000010732	cdk6
ENSXMAG00000015146	cenpo	ENSLOCG00000015889	CENPO
ENSXMAG00000001016	zgc:56596	ENSLOCG00000015578	zgc:56596
ENSXMAG00000015263		ENSLOCG00000006263	
ENSXMAG00000014895	ndufb8	ENSLOCG00000005420	ndufb8
ENSXMAG00000000041	parp6a	ENSLOCG00000015133	parp6a
ENSXMAG00000007848	CREB1	ENSLOCG00000004320	creb1b
ENSXMAG00000001819	psmd14	ENSLOCG00000008596	psmd14
ENSXMAG00000001505	clcn7	ENSLOCG00000006424	clcn7
ENSXMAG00000003538	rbm18	ENSLOCG00000003002	rbm18
ENSXMAG00000023312	faxdc2	ENSLOCG00000012471	faxdc2
ENSXMAG00000018589	tgm2b	ENSLOCG00000001167	tgm2a
ENSXMAG00000015264	snx2	ENSLOCG00000008709	snx2
ENSXMAG00000026042	fam133b	ENSLOCG00000010716	fam133b
ENSXMAG00000000991	si:dkey-32e23.4	ENSLOCG00000015567	si:dkey-32e23.4
ENSXMAG00000013030	lrrc29	ENSLOCG00000006224	lrrc29
ENSXMAG00000003545	lhx6	ENSLOCG00000002986	lhx6
ENSXMAG00000026231	mettl21a	ENSLOCG00000004303	mettl21a
ENSXMAG00000014901	sec31b	ENSLOCG00000005377	sec31b
ENSXMAG00000009627	hhip	ENSLOCG00000008934	hhip
ENSXMAG00000007670	reps1	ENSLOCG00000001188	reps1
ENSXMAG00000018780	larp1	ENSLOCG00000012457	larp1
ENSXMAG00000029911	morn5	ENSLOCG00000002968	morn5
ENSXMAG00000018570	quo	ENSLOCG00000001193	si:dkey-65j6.2
ENSXMAG00000027074		ENSLOCG00000011440	mcmbp

ENSXMAG00000001980	elmo3	ENSLOCG00000006255	elmo3
ENSXMAG00000016243	rab36	ENSLOCG00000002825	RAB36
ENSXMAG00000003556	ndufa8	ENSLOCG00000002944	ndufa8
ENSXMAG00000000598	ksr1a	ENSLOCG00000004773	ksr1b
ENSXMAG00000024679	PPIC	ENSLOCG00000008677	PPIC
ENSXMAG00000016437	tepsin	ENSLOCG00000012812	tepsin
ENSXMAG00000011889	aicda	ENSLOCG00000008158	aicda
ENSXMAG00000024219	sec23ip	ENSLOCG00000011455	sec23ip
ENSXMAG00000025056	arhgdig	ENSLOCG00000006313	arhgdig
ENSXMAG00000018808		ENSLOCG00000012451	
ENSXMAG00000025017	dnajc15	ENSLOCG00000005690	dnajc15
ENSXMAG00000019080	metap1d	ENSLOCG00000007621	METAP1D
ENSXMAG00000024084	rpusd1	ENSLOCG00000005682	rpusd1
ENSXMAG00000029393	PRDM6	ENSLOCG00000008663	PRDM6
ENSXMAG00000015399	cfap20	ENSLOCG00000007614	cfap20
ENSXMAG00000001478		ENSLOCG00000011492	slc25a17
ENSXMAG00000027397	csgalnact2	ENSLOCG00000011491	csgalnact2
ENSXMAG00000026755	znf346	ENSLOCG00000012305	znf346
ENSXMAG00000016432	AMZ2	ENSLOCG00000012454	AMZ2
ENSXMAG00000001387	sestd1	ENSLOCG00000006398	sestd1
ENSXMAG00000027734	ANKRD66	ENSLOCG00000016487	ANKRD66
ENSXMAG00000001463	slc17a8	ENSLOCG00000015210	slc17a8
ENSXMAG00000027197	pinx1	ENSLOCG00000016761	pinx1
ENSXMAG00000000975		ENSLOCG00000007482	DISP3
ENSXMAG00000009615	mmaa	ENSLOCG00000008866	mmaa
ENSXMAG00000001469	znf598	ENSLOCG00000006011	znf598
ENSXMAG00000018769		ENSLOCG00000015141	
ENSXMAG00000015326	cep120	ENSLOCG00000008635	cep120
ENSXMAG00000014398	ret	ENSLOCG00000011498	ret
ENSXMAG00000029876		ENSLOCG00000011234	
ENSXMAG00000012946	mpi	ENSLOCG00000014939	mpi
ENSXMAG00000022964	zgc:154077	ENSLOCG00000016667	zgc:154077
ENSXMAG00000011398	TBC1D14	ENSLOCG00000010162	tbc1d14
ENSXMAG00000003582	pde6b	ENSLOCG00000012238	pde6b
ENSXMAG00000001433	nr1h4	ENSLOCG00000015206	nr1h4
ENSXMAG00000018788	fgfr4	ENSLOCG00000012293	fgfr4
ENSXMAG00000016403	arsg	ENSLOCG00000012439	arsg
ENSXMAG00000004234	tdh	ENSLOCG00000016762	tdh
ENSXMAG00000011857	rasip1	ENSLOCG00000008104	
ENSXMAG00000001442	drg2	ENSLOCG00000006082	drg2
ENSXMAG00000029733		ENSLOCG00000001261	
ENSXMAG00000010905	ZNF608	ENSLOCG00000008603	ZNF608
ENSXMAG00000015355	zgc:63863	ENSLOCG00000007587	zgc:63863
ENSXMAG00000016383	si:ch73-390p7.2	ENSLOCG00000010198	si:ch73-390p7.2
ENSXMAG00000018556	myl9a	ENSLOCG00000001277	myl9a
ENSXMAG00000027372	si:dkey-192p21.6	ENSLOCG00000011511	
ENSXMAG00000028226	zgc:77158	ENSLOCG00000016668	zgc:77158
ENSXMAG00000021356	vezf1a	ENSLOCG00000004823	vezf1a
ENSXMAG00000009608	znf827	ENSLOCG00000008858	znf827
ENSXMAG00000009447	NMNAT2	ENSLOCG00000002037	nmnat2
ENSXMAG00000004240	mtmr9	ENSLOCG00000016765	mtmr9
ENSXMAG00000007673	clu	ENSLOCG00000016768	clu
ENSXMAG00000027173	si:ch211-219a15.3	ENSLOCG00000017898	si:ch211-219a15.3
ENSXMAG00000007889	dpep2	ENSLOCG00000006974	dpep2
ENSXMAG00000029049	stk11ip	ENSLOCG00000009252	stk11ip
ENSXMAG00000026833		ENSLOCG00000017437	

ENSXMAG00000001468	gfer	ENSLOCG00000006067	gfer
ENSXMAG00000011007	impact	ENSLOCG00000006429	impact
ENSXMAG00000014988	anapc16	ENSLOCG00000005282	anapc16
ENSXMAG00000011810	slc2a3b	ENSLOCG00000008181	slc2a3b
ENSXMAG00000011409	zgc:158564	ENSLOCG00000010153	zgc:158564
ENSXMAG00000006500	ndufa7	ENSLOCG00000002666	ndufa7
ENSXMAG00000016321	prkar1ab	ENSLOCG00000012407	prkar1ab
ENSXMAG00000018523	zgc:123305	ENSLOCG00000001295	zgc:123305
ENSXMAG00000014996	ascc1	ENSLOCG00000005266	ascc1
ENSXMAG00000007865	slc12a4	ENSLOCG00000006994	slc12a4
ENSXMAG00000007877	pcbd1	ENSLOCG00000011517	pcbd1
ENSXMAG00000015339		ENSLOCG00000007544	
ENSXMAG00000007680	scara3	ENSLOCG00000016771	scara3
ENSXMAG00000011414	S100P	ENSLOCG00000010147	S100P
ENSXMAG00000016300	fam20a	ENSLOCG00000012394	fam20a
ENSXMAG00000023003	ccm2	ENSLOCG00000015826	ccm2
ENSXMAG00000000511	inha	ENSLOCG00000009276	inha
ENSXMAG00000015014	SPOCK2	ENSLOCG00000005246	spock2
ENSXMAG00000004984		ENSLOCG00000002549	SLC29A2
ENSXMAG00000015315	recql4	ENSLOCG00000007478	recql4
ENSXMAG00000001366	eea1	ENSLOCG00000015288	eea1
ENSXMAG00000005930	naa25	ENSLOCG00000007981	naa25
ENSXMAG00000007880	sgpl1	ENSLOCG00000011521	sgpl1
ENSXMAG00000011305	dhx9	ENSLOCG00000002158	dhx9
ENSXMAG00000023765		ENSLOCG00000016605	FOXO3
ENSXMAG00000000631	snx13	ENSLOCG00000011366	snx13
ENSXMAG00000006506	jak3	ENSLOCG00000002715	jak3
ENSXMAG00000010572	hsc70	ENSLOCG00000004098	hsc70
ENSXMAG00000018789	pou4f2	ENSLOCG00000008825	pou4f2
ENSXMAG00000000042		ENSLOCG00000009146	NUDT12
ENSXMAG00000012892		ENSLOCG00000003091	
ENSXMAG00000018893	rab24	ENSLOCG00000012274	rab24
ENSXMAG00000029469	tpra1	ENSLOCG00000013785	tpra1
ENSXMAG00000007834	slc6a2	ENSLOCG00000007036	slc6a2
ENSXMAG00000022695	cdh13	ENSLOCG00000002199	cdh13
ENSXMAG00000024788	nanos1	ENSLOCG00000018172	nanos1
ENSXMAG00000004278	fam167ab	ENSLOCG00000015823	fam167aa
ENSXMAG00000003631	nnt	ENSLOCG00000012282	nnt
ENSXMAG00000001492	znf385b	ENSLOCG00000006420	znf385b
ENSXMAG00000001779	kansl3	ENSLOCG00000014926	kansl3
ENSXMAG00000005125		ENSLOCG00000011812	C22orf23
ENSXMAG00000015272	zbtb24	ENSLOCG00000017157	zbtb24
ENSXMAG00000003303	lamc1	ENSLOCG00000002091	lamc1
ENSXMAG00000020141	ferd3l	ENSLOCG00000018351	ferd3l
ENSXMAG00000005121		ENSLOCG00000011805	MICALL1
ENSXMAG00000004281	lca5	ENSLOCG00000016242	lca5
ENSXMAG00000023338	lbx2	ENSLOCG00000001890	lbx2
ENSXMAG00000000044	hira	ENSLOCG00000001895	hira
ENSXMAG00000013201	cwc22	ENSLOCG00000006456	cwc22
ENSXMAG00000000629	TMEM196	ENSLOCG00000011414	TMEM196
ENSXMAG00000009645	mlycd	ENSLOCG00000002214	mlycd
ENSXMAG00000023519	neurog3	ENSLOCG00000011531	neurog3
ENSXMAG00000010570	zgc:171592	ENSLOCG00000004085	ctrl
ENSXMAG00000015266	cndp2	ENSLOCG00000004123	cndp2
ENSXMAG00000001994	pcgf1	ENSLOCG00000001907	pcgf1
ENSXMAG00000004283	sh3bgrl2	ENSLOCG00000016240	sh3bgrl2

ENSXMAG00000007696	scg5	ENSLOCG00000012605	scg5
ENSXMAG00000001345	plekhg7	ENSLOCG00000015295	plekhg7
ENSXMAG00000007819	lpcat2	ENSLOCG00000007060	lpcat2
ENSXMAG00000007899	pdlim1	ENSLOCG00000011548	pdlim1
ENSXMAG00000005335	zgc:173742	ENSLOCG00000002229	zgc:173742
ENSXMAG00000000593	itgb8	ENSLOCG00000011431	itgb8
ENSXMAG00000007399	TMEM50B	ENSLOCG00000004113	TMEM50B
ENSXMAG00000016266	aarsd1	ENSLOCG00000012212	aarsd1
ENSXMAG00000017138	prmt9	ENSLOCG00000008764	prmt9
ENSXMAG00000012891	INSC	ENSLOCG00000003105	INSC
ENSXMAG00000010657	rcc1	ENSLOCG00000003672	rcc1
ENSXMAG00000005915		ENSLOCG00000007908	brap
ENSXMAG00000000045	slc2a8	ENSLOCG00000002280	slc2a8
ENSXMAG00000001503	itprid2	ENSLOCG00000006573	itprid2
ENSXMAG00000005768	pm20d1.2	ENSLOCG00000011875	pm20d1.1
ENSXMAG00000015106	lrrc20	ENSLOCG00000005145	lrrc20
ENSXMAG00000015252	dus3l	ENSLOCG00000010531	dus3l
ENSXMAG00000024094	si:dkey-42l23.2	ENSLOCG00000010437	
ENSXMAG00000005364	hsbp1b	ENSLOCG00000002268	hsbp1b
ENSXMAG00000003297	rasal2	ENSLOCG00000009149	rasal2
ENSXMAG00000007912		ENSLOCG00000011554	
ENSXMAG00000006542		ENSLOCG00000016177	mdm1
ENSXMAG00000021304	si:dkey-246g23.2	ENSLOCG00000002278	si:dkey-246g23.2
ENSXMAG00000015109	ndr2	ENSLOCG00000005102	ndr2
ENSXMAG00000000588	sp4	ENSLOCG00000011495	sp4
ENSXMAG00000003662	rai14	ENSLOCG00000012300	rai14
ENSXMAG00000029849	zgc:92287	ENSLOCG00000011858	zgc:92287
ENSXMAG00000001346	LLGL1	ENSLOCG00000004976	LLGL1
ENSXMAG00000008825	slc5a5	ENSLOCG00000002786	slc5a5
ENSXMAG00000013826	arhgap10	ENSLOCG00000008721	arhgap10
ENSXMAG00000010566	lin37	ENSLOCG00000004067	lin37
ENSXMAG00000000046	ttc16	ENSLOCG00000002597	
ENSXMAG00000007397		ENSLOCG00000004158	
ENSXMAG00000001517	neurod1	ENSLOCG00000017829	neurod1
ENSXMAG00000016256	mfsd4ab	ENSLOCG00000011823	mfsd4ab
ENSXMAG00000001130	itpr2	ENSLOCG00000015302	itpr2
ENSXMAG00000000584	cdca7b	ENSLOCG00000011532	CDCA7L
ENSXMAG00000015118	pald1a	ENSLOCG00000005068	pald1b
ENSXMAG00000028106		ENSLOCG00000004177	IL10RB
ENSXMAG00000007921		ENSLOCG00000011598	
ENSXMAG00000005374	si:dkey-246g23.4	ENSLOCG00000002305	si:dkey-246g23.4
ENSXMAG00000008801	cluap1	ENSLOCG00000003361	cluap1
ENSXMAG00000007790	mmp2	ENSLOCG00000007073	mmp2
ENSXMAG00000025977	psma1	ENSLOCG00000003163	psma1
ENSXMAG00000004491	tor4aa	ENSLOCG00000017339	tor4aa
ENSXMAG00000001520	cerkl	ENSLOCG00000006533	cerkl
ENSXMAG00000019208	dnah9l	ENSLOCG00000007428	
ENSXMAG00000028827	wfdc1	ENSLOCG00000002319	wfdc1
ENSXMAG00000001991	fgf24	ENSLOCG00000001921	fgf24
ENSXMAG00000000047	mymk	ENSLOCG00000002633	mymk
ENSXMAG00000022646		ENSLOCG00000007379	NTAN1
ENSXMAG00000021488	ccdc124	ENSLOCG00000002810	ccdc124
ENSXMAG00000024476	fam204a	ENSLOCG00000009714	fam204a
ENSXMAG00000018924	pgrmc1	ENSLOCG00000013885	pgrmc1
ENSXMAG00000013809	nr3c2	ENSLOCG00000008694	nr3c2
ENSXMAG00000000048	adamtsl2	ENSLOCG00000002647	adamtsl2

ENSXMAG00000000103	dyrk4	ENSLOCG00000016661	dyrk4
ENSXMAG00000024451		ENSLOCG00000013880	COX7B
ENSXMAG00000007932	rab11fip2	ENSLOCG00000009709	rab11fip2
ENSXMAG00000027762		ENSLOCG00000016239	
ENSXMAG00000001986	npm1b	ENSLOCG00000001942	npm1b
ENSXMAG00000011272	mast3a	ENSLOCG00000001498	mast3a
ENSXMAG00000011458		ENSLOCG00000002503	MAP3K11
ENSXMAG00000006546	DYRK2	ENSLOCG00000016174	dyrk2
ENSXMAG00000005057	C16orf45	ENSLOCG00000007324	C16orf45
ENSXMAG00000015155	adamts14	ENSLOCG00000005042	adamts14
ENSXMAG00000003673	scarb2c	ENSLOCG00000003913	SCARB2
ENSXMAG00000007786	irx6a	ENSLOCG00000007092	irx6a
ENSXMAG00000029852	rbm34	ENSLOCG00000015902	rbm34
ENSXMAG00000007761	aven	ENSLOCG00000012537	aven
ENSXMAG00000025048	rps12	ENSLOCG00000016236	rps12
ENSXMAG00000025639	emx2	ENSLOCG00000009698	emx2
ENSXMAG00000015173	cyp51	ENSLOCG00000010601	cyp51
ENSXMAG00000021943	sspn	ENSLOCG00000015325	sspn
ENSXMAG00000018938	pgk1	ENSLOCG00000013865	pgk1
ENSXMAG00000007123	smg7	ENSLOCG00000002010	smg7
ENSXMAG00000030014		ENSLOCG00000004031	kmt2bb
ENSXMAG00000007390		ENSLOCG00000004223	
ENSXMAG00000001281	flii	ENSLOCG00000004957	flii
ENSXMAG00000014915		ENSLOCG00000008435	usp42
ENSXMAG00000008753	uhrf1	ENSLOCG00000000097	uhrf1
ENSXMAG00000027134	igflr1	ENSLOCG00000004010	igflr1
ENSXMAG00000012811		ENSLOCG00000003313	rerglb
ENSXMAG00000029170	irx5a	ENSLOCG00000007105	irx5a
ENSXMAG00000013806	tbce	ENSLOCG00000015896	tbce
ENSXMAG00000010685	tcea3	ENSLOCG00000003716	tcea3
ENSXMAG00000007944	eif3s10	ENSLOCG00000009729	eif3s10
ENSXMAG00000027406	RF00186	ENSLOCG00000020509	RF00186
ENSXMAG00000001982	tlx2	ENSLOCG00000001968	tlx2
ENSXMAG00000005046	MARF1	ENSLOCG00000007292	MARF1
ENSXMAG00000019500		ENSLOCG00000017776	C21orf62
ENSXMAG00000007762	emc7	ENSLOCG00000012520	emc7
ENSXMAG00000024238	kctd7	ENSLOCG00000001422	kctd7
ENSXMAG00000000111	ndufa9a	ENSLOCG00000016659	ndufa9a
ENSXMAG00000025171	irx3a	ENSLOCG00000007127	irx3b
ENSXMAG00000010530	si:ch211-113j13.2	ENSLOCG00000004000	si:ch211-113j13.2
ENSXMAG00000015127	krit1	ENSLOCG00000010622	krit1
ENSXMAG00000014906	eif2ak1	ENSLOCG00000008458	eif2ak1
ENSXMAG00000001113	bhlhe41	ENSLOCG00000015327	bhlhe41
ENSXMAG00000019733		ENSLOCG00000004938	KCNJ6
ENSXMAG00000007389	arglu1a	ENSLOCG00000011090	arglu1a
ENSXMAG00000001973	adad1	ENSLOCG00000002873	adad1
ENSXMAG00000022095	fto	ENSLOCG00000007139	
ENSXMAG00000005178		ENSLOCG00000003356	RPL27A
ENSXMAG00000004289	eya4	ENSLOCG00000016233	eya4
ENSXMAG00000022652	RNF208	ENSLOCG00000017332	RNF208
ENSXMAG00000022028	tor2a	ENSLOCG00000002778	tor2a
ENSXMAG00000006632	rabgef1	ENSLOCG00000001391	rabgef1
ENSXMAG00000018457		ENSLOCG00000010056	EVC
ENSXMAG00000001540	itga4	ENSLOCG00000006512	itga4
ENSXMAG00000016185	aktip	ENSLOCG00000007183	aktip
ENSXMAG00000005177	akip1	ENSLOCG00000003390	AKIP1

ENSXMAG00000006551	kdm4b	ENSLOCG00000000105	kdm4b
ENSXMAG00000028189	efnb2a	ENSLOCG00000011084	efnb2a
ENSXMAG00000025591		ENSLOCG00000003984	
ENSXMAG00000014911	ANKRD61	ENSLOCG00000008471	ANKRD61
ENSXMAG00000004879	TOMM7	ENSLOCG00000011551	TOMM7
ENSXMAG00000002913	vps26c	ENSLOCG00000004966	vps26c
ENSXMAG00000007970	fam45a	ENSLOCG00000009752	fam45a
ENSXMAG00000013897		ENSLOCG00000001470	
ENSXMAG00000015175		ENSLOCG00000005011	
ENSXMAG00000014877	pms2	ENSLOCG00000008495	pms2
ENSXMAG00000004866	fam126a	ENSLOCG00000011562	fam126a
ENSXMAG00000024440	cetn4	ENSLOCG00000002846	cetn4
ENSXMAG00000009041	KCNJ15	ENSLOCG00000018193	KCNJ15
ENSXMAG00000028531		ENSLOCG00000016576	ribc2
ENSXMAG00000006565		ENSLOCG00000016231	TSPAN8
ENSXMAG00000010499	aplp1	ENSLOCG00000003972	aplp1
ENSXMAG00000005152	znf143b	ENSLOCG00000003619	znf143b
ENSXMAG00000015185	QRFPR	ENSLOCG00000004968	qrfpra
ENSXMAG00000019978	bbs12	ENSLOCG00000002831	bbs12
ENSXMAG00000009027	smg8	ENSLOCG00000001234	smg8
ENSXMAG00000017781	rpl8	ENSLOCG00000002206	rpl8
ENSXMAG00000024887	fgf2	ENSLOCG00000002819	fgf2
ENSXMAG00000004437		ENSLOCG00000008641	GTPBP8
ENSXMAG00000007978	sfxn4	ENSLOCG00000009759	sfxn4
ENSXMAG00000019223		ENSLOCG00000006817	epha4b
ENSXMAG00000025890	slc10a2	ENSLOCG00000008651	slc10a2
ENSXMAG00000028674	ppm1e	ENSLOCG00000001318	ppm1e
ENSXMAG00000005401		ENSLOCG00000016580	
ENSXMAG00000004816	klhl7	ENSLOCG00000011576	klhl7
ENSXMAG00000029886	nudt6	ENSLOCG00000002784	nudt6
ENSXMAG00000015108	prcc	ENSLOCG00000008014	PRCC
ENSXMAG00000014870	dlgap5	ENSLOCG00000011388	
ENSXMAG00000025790	kras	ENSLOCG00000015334	kras
ENSXMAG00000016192	chd9	ENSLOCG00000007225	CHD9
ENSXMAG00000013902		ENSLOCG00000002533	
ENSXMAG00000021014	GNAZ	ENSLOCG00000002815	GNAZ
ENSXMAG00000007795	gpr176	ENSLOCG00000012456	gpr176
ENSXMAG00000016822	paics	ENSLOCG00000013998	paics
ENSXMAG00000015363	shprh	ENSLOCG00000016153	shprh
ENSXMAG00000016167	camta1a	ENSLOCG00000002655	camta1b
ENSXMAG00000025538		ENSLOCG00000008030	HDGF
ENSXMAG00000018356	SPATA5	ENSLOCG00000002749	SPATA5
ENSXMAG00000015213	polr3a	ENSLOCG00000004942	polr3a
ENSXMAG00000022118	cxcr2	ENSLOCG00000017836	cxcr2
ENSXMAG00000007981	prdx3	ENSLOCG00000009763	prdx3
ENSXMAG00000018028	usp9	ENSLOCG00000002227	usp9
ENSXMAG00000014852	ccz1	ENSLOCG00000008536	ccz1
ENSXMAG00000027737		ENSLOCG00000017589	
ENSXMAG00000015099	mrpl24	ENSLOCG00000008058	mrpl24
ENSXMAG00000018445	EVC2	ENSLOCG00000010037	EVC2
ENSXMAG00000010711	pigv	ENSLOCG00000003786	pigv
ENSXMAG00000003712	sppl3	ENSLOCG00000002863	sppl3
ENSXMAG00000013910	rps15	ENSLOCG00000002230	rps15
ENSXMAG00000012766	wee1	ENSLOCG00000003652	wee1
ENSXMAG00000026271		ENSLOCG00000009869	NIPAL4
ENSXMAG00000005404	slc25a18	ENSLOCG00000016583	slc25a18

ENSXMAG00000010464	kirrel3l	ENSLOCG00000003936	kirrel3l
ENSXMAG00000029496	tcf21	ENSLOCG00000016232	tcf21
ENSXMAG00000004690	smarcc1b	ENSLOCG00000004394	smarcc1a
ENSXMAG00000024906	faima	ENSLOCG00000003397	faima
ENSXMAG00000001268	ccdc97	ENSLOCG00000005995	ccdc97
ENSXMAG00000001073	banp	ENSLOCG00000002495	banp
ENSXMAG00000027208	ppat	ENSLOCG00000014005	ppat
ENSXMAG00000004323	tbpl1	ENSLOCG00000016230	tbpl1
ENSXMAG00000026416	srsf5b	ENSLOCG00000012438	srsf5b
ENSXMAG00000019977	spry1	ENSLOCG00000018178	spry1
ENSXMAG00000008001	tm9sf3	ENSLOCG00000011244	tm9sf3
ENSXMAG00000001813	gfpt1	ENSLOCG00000014971	gfpt1
ENSXMAG00000018353	ANKRD50	ENSLOCG00000002723	ANKRD50
ENSXMAG00000006591	trhde.2	ENSLOCG00000016252	trhde.2
ENSXMAG00000023862	sp100.3	ENSLOCG00000011736	
ENSXMAG00000020646		ENSLOCG00000020779	
ENSXMAG00000016836	p2rx7	ENSLOCG00000006925	p2rx7
ENSXMAG00000004337	slc2a12	ENSLOCG00000016228	slc2a12
ENSXMAG00000007569	nt5c2l1	ENSLOCG00000015189	
ENSXMAG00000001063	fbxo31	ENSLOCG00000003496	fbxo31
ENSXMAG00000010451	nphs1	ENSLOCG00000003902	nphs1
ENSXMAG00000016826	gstt1a	ENSLOCG00000006891	gstt1a
ENSXMAG00000004339	SGK1	ENSLOCG00000016226	SGK1
ENSXMAG00000005431	bcl2l13	ENSLOCG00000016587	bcl2l13
ENSXMAG00000018348	FAT4	ENSLOCG00000002707	FAT4
ENSXMAG00000007581	slc2a11l	ENSLOCG00000006758	slc2a11l
ENSXMAG00000022993	CYTL1	ENSLOCG00000010022	CYTL1
ENSXMAG00000001824		ENSLOCG00000014968	nfu1
ENSXMAG00000004925	fopnl	ENSLOCG00000007248	fopnl
ENSXMAG00000016856	aasdh	ENSLOCG00000014012	aasdh
ENSXMAG00000005435	wee2	ENSLOCG00000016591	wee2
ENSXMAG00000015375	si:dkeyp-110a12.4	ENSLOCG00000016156	si:dkeyp-110a12.4
ENSXMAG00000019625	purbb	ENSLOCG00000015273	purbb
ENSXMAG00000007833	fntb	ENSLOCG00000012412	fntb
ENSXMAG00000021261	stx18	ENSLOCG00000009998	stx18
ENSXMAG00000010742	gpn2	ENSLOCG00000003822	gpn2
ENSXMAG00000004906	abcc6a	ENSLOCG00000007152	abcc6b.1
ENSXMAG00000007615	KCNN2	ENSLOCG00000008989	KCNN2
ENSXMAG00000000571	sez6a	ENSLOCG00000004892	sez6a
ENSXMAG00000018007	med14	ENSLOCG00000002286	med14
ENSXMAG00000015378	fbxo30a	ENSLOCG00000016157	fbxo30a
ENSXMAG00000010749	gpatch3	ENSLOCG00000003858	gpatch3
ENSXMAG00000016812	ckap2l	ENSLOCG00000015267	
ENSXMAG00000016152	tnfrsf9a	ENSLOCG00000002559	tnfrsf9b
ENSXMAG00000004351	aldh8a1	ENSLOCG00000016225	aldh8a1
ENSXMAG00000022936		ENSLOCG00000001713	
ENSXMAG00000002586	mrpl3	ENSLOCG00000001514	mrpl3
ENSXMAG00000011532	ALPK1	ENSLOCG00000006584	
ENSXMAG00000027865	NSG1	ENSLOCG00000009986	NSG1
ENSXMAG00000029604	med22	ENSLOCG00000000579	med22
ENSXMAG00000010556	nr0b2a	ENSLOCG00000003893	nr0b2a
ENSXMAG00000013950	rps28	ENSLOCG00000002654	rps28
ENSXMAG00000004359	hbs1l	ENSLOCG00000016221	hbs1l
ENSXMAG00000026232	si:dkey-262k9.4	ENSLOCG00000003866	
ENSXMAG00000002702		ENSLOCG00000016187	CPM
ENSXMAG00000016128	dvl1a	ENSLOCG00000002858	dvl1a

ENSXMAG00000007359	esyt3	ENSLOCG00000003337	
ENSXMAG00000000731	si:dkeyp-117h8.2	ENSLOCG00000014016	si:dkeyp-117h8.2
ENSXMAG00000013952	si:rp71-39b20.4	ENSLOCG00000002693	si:rp71-39b20.4
ENSXMAG00000014001	rpl3	ENSLOCG00000011210	rpl3
ENSXMAG00000004295	stard7	ENSLOCG00000015208	stard7
ENSXMAG00000030086	chst14	ENSLOCG00000014019	chst14
ENSXMAG00000004901	coq7	ENSLOCG00000007132	coq7
ENSXMAG00000015383	rmnd1	ENSLOCG00000016293	rmnd1
ENSXMAG00000010763	wdtc1	ENSLOCG00000003956	wdtc1
ENSXMAG00000004897	notum2	ENSLOCG00000007115	notum2
ENSXMAG00000014756	tdrkh	ENSLOCG00000011381	
ENSXMAG00000028646	RF00430	ENSLOCG00000020142	RF00430
ENSXMAG00000018000	srp72	ENSLOCG00000002628	srp72
ENSXMAG00000014979	ncf1	ENSLOCG00000001899	ncf1
ENSXMAG00000004392	myb	ENSLOCG00000016218	myb
ENSXMAG00000000936	fanci	ENSLOCG00000014566	fanci
ENSXMAG00000007276	dnah7	ENSLOCG00000005048	dnah7
ENSXMAG00000004894	rps15a	ENSLOCG00000007017	rps15a
ENSXMAG00000002630	dync1li1	ENSLOCG00000001443	dync1li1
ENSXMAG00000005469	agk	ENSLOCG00000016598	agk
ENSXMAG00000010439	FCGBP	ENSLOCG00000000311	
ENSXMAG00000013964	kcnn1a	ENSLOCG00000002827	kcnn1a
ENSXMAG00000016242	atp8b5a	ENSLOCG00000004524	atp8b5a
ENSXMAG00000016268	tox3	ENSLOCG00000007257	tox3
ENSXMAG00000000487	acaca	ENSLOCG00000005003	acaca
ENSXMAG00000015022	st6gal1	ENSLOCG00000014041	
ENSXMAG00000029102	aurkaip1	ENSLOCG00000002935	aurkaip1
ENSXMAG00000026375		ENSLOCG00000016474	EPS8
ENSXMAG00000010776	cep85	ENSLOCG00000003980	CEP85
ENSXMAG00000004254	pcyox1	ENSLOCG00000015660	pcyox1
ENSXMAG00000014019	slc25a39	ENSLOCG00000013283	slc25a39
ENSXMAG00000012730	rnf141	ENSLOCG00000003803	rnf141
ENSXMAG00000004423		ENSLOCG00000016213	PDE7B
ENSXMAG00000002727	dusp16	ENSLOCG00000016491	dusp16
ENSXMAG00000004248	fam136a	ENSLOCG00000015664	fam136a
ENSXMAG00000022750	ccdc28a	ENSLOCG00000015788	
ENSXMAG00000011582	ndufs2	ENSLOCG00000000980	ndufs2
ENSXMAG00000013982	si:dkey-208k22.6	ENSLOCG00000002847	si:dkey-208k22.6
ENSXMAG00000016273	sall1a	ENSLOCG00000007283	sall1b
ENSXMAG00000007674	bicra	ENSLOCG00000014205	bicra
ENSXMAG00000000917	polg	ENSLOCG00000014557	
ENSXMAG00000026585		ENSLOCG00000002670	
ENSXMAG00000018418	aggf1	ENSLOCG00000005688	aggf1
ENSXMAG00000023555		ENSLOCG00000004023	UBXN11
ENSXMAG00000008027		ENSLOCG00000005087	MOCOS
ENSXMAG00000025655	crebl2	ENSLOCG00000016490	crebl2
ENSXMAG00000004223	gmcl1	ENSLOCG00000015668	gmcl1
ENSXMAG00000002657	adsl	ENSLOCG00000011409	
ENSXMAG00000009965		ENSLOCG00000015786	ccdc28a
ENSXMAG00000015041	cx28.8	ENSLOCG00000018012	cx28.8
ENSXMAG00000021245	ppifb	ENSLOCG00000004875	ppifb
ENSXMAG00000029971		ENSLOCG00000007068	tacc3
ENSXMAG00000020338	gpr19	ENSLOCG00000017916	gpr19
ENSXMAG00000019267		ENSLOCG00000004616	gpx4b
ENSXMAG00000007662	napab	ENSLOCG00000014212	napab
ENSXMAG00000016901	exoc1	ENSLOCG00000014025	exoc1

ENSXMAG00000018339	arl9	ENSLOCG00000002598	ARL9
ENSXMAG00000004835	nomo	ENSLOCG00000006964	NOMO1
ENSXMAG00000004136	dhx57	ENSLOCG00000015774	dhx57
ENSXMAG00000016279	cylda	ENSLOCG00000007312	cylda
ENSXMAG00000019542	adrb3a	ENSLOCG00000014961	adrb3a
ENSXMAG00000002736	tbk1	ENSLOCG00000016486	tbk1
ENSXMAG00000014670	TMEM94	ENSLOCG00000011408	tmem94
ENSXMAG00000015491	dapk1	ENSLOCG00000011423	dapk1
ENSXMAG00000012709	hdac10	ENSLOCG00000015364	hdac10
ENSXMAG00000004279		ENSLOCG00000004895	cyp7b1
ENSXMAG00000029040	slc39a10	ENSLOCG00000005081	slc39a10
ENSXMAG00000015045		ENSLOCG00000012311	
ENSXMAG00000023530	tbxa2r	ENSLOCG00000000178	tbxa2r
ENSXMAG00000020234	b3gnt2l	ENSLOCG00000017562	b3gnt2l
ENSXMAG00000008075	vwc2	ENSLOCG00000005007	vwc2
ENSXMAG00000003033	ggcx	ENSLOCG00000015209	ggcx
ENSXMAG00000009485	mtfr2	ENSLOCG00000016211	mtfr2
ENSXMAG00000006082	gipc3	ENSLOCG00000000197	gipc3
ENSXMAG00000001856		ENSLOCG00000014952	
ENSXMAG00000011718	si:ch211-218d20.15	ENSLOCG00000002373	si:ch211-218d20.15
ENSXMAG00000018296	sec24b	ENSLOCG00000009547	
ENSXMAG00000007649	bckdha	ENSLOCG00000014217	bckdha
ENSXMAG00000018375	r3hcc1	ENSLOCG00000015442	r3hcc1
ENSXMAG00000023437		ENSLOCG00000004989	SPATA48
ENSXMAG00000009933	nfkbiz	ENSLOCG00000009576	nfkbiz
ENSXMAG00000000888	CIB1	ENSLOCG00000015112	cib1
ENSXMAG00000013540	zfyve28	ENSLOCG00000011975	
ENSXMAG00000007265	tmeff2a	ENSLOCG00000005097	tmeff2b
ENSXMAG00000022005	TMEM158	ENSLOCG00000018323	TMEM158
ENSXMAG00000001857	adgra2	ENSLOCG00000014947	adgra2
ENSXMAG00000002781		ENSLOCG00000016494	lrp6
ENSXMAG00000012668	pus7	ENSLOCG00000015489	pus7
ENSXMAG00000021071	zfyve16	ENSLOCG00000005189	zfyve16
ENSXMAG00000001606	si:ch211-269e2.1	ENSLOCG00000002393	si:ch211-269e2.1
ENSXMAG00000002693	wisp1a	ENSLOCG00000007334	wisp1a
ENSXMAG00000001151	mief1	ENSLOCG00000011112	mief1
ENSXMAG00000019982	FEM1A	ENSLOCG00000017624	FEM1A
ENSXMAG00000022901	sft2d3	ENSLOCG00000017411	sft2d3
ENSXMAG00000024633	ikzf1	ENSLOCG00000004967	ikzf1
ENSXMAG00000011628	dock11	ENSLOCG00000014215	dock11
ENSXMAG00000029907	nabp1a	ENSLOCG00000005133	nabp1b
ENSXMAG00000014598	fasn	ENSLOCG00000012320	fasn
ENSXMAG00000004220	snx24	ENSLOCG00000008691	SNX24
ENSXMAG00000010803	lars2	ENSLOCG00000002048	lars2
ENSXMAG00000022907	sla1	ENSLOCG00000007319	sla1
ENSXMAG00000018367	rplp0	ENSLOCG00000001683	rplp0
ENSXMAG00000018284		ENSLOCG00000009569	TSTD2
ENSXMAG00000004832	TMC7	ENSLOCG00000006941	TMC7
ENSXMAG00000016109	nt5dc2	ENSLOCG00000014477	nt5dc2
ENSXMAG00000025217	RF00092	ENSLOCG00000019246	RF00092
ENSXMAG00000029696	RUNDC1	ENSLOCG00000012232	RUNDC1
ENSXMAG00000022490	si:dkey-13i19.8	ENSLOCG00000017103	si:dkey-13i19.8
ENSXMAG00000020253	GDPGP1	ENSLOCG00000017961	GDPGP1
ENSXMAG00000021725	MRPL34	ENSLOCG00000002997	mrpl34
ENSXMAG00000007635	cdon	ENSLOCG00000000869	cdon
ENSXMAG00000004188	WDR33	ENSLOCG00000009122	WDR33

ENSXMAG00000019831	fignl1	ENSLOCG00000018328	fignl1
ENSXMAG00000018253	NCBP1	ENSLOCG00000009579	NCBP1
ENSXMAG00000021313	pdlim2	ENSLOCG00000014946	pdlim2
ENSXMAG00000004810		ENSLOCG00000006928	tmc5
ENSXMAG00000001694		ENSLOCG00000002466	PPP2R3B
ENSXMAG00000029290	morn2	ENSLOCG00000015771	morn2
ENSXMAG00000029091	rpl27	ENSLOCG00000012239	rpl27
ENSXMAG00000024830	fam206a	ENSLOCG00000007233	fam206a
ENSXMAG00000023022		ENSLOCG00000016500	
ENSXMAG00000010458	ccndbp1	ENSLOCG00000007104	ccndbp1
ENSXMAG00000008085	ttl	ENSLOCG00000016555	ttl
ENSXMAG00000007630	bcl9l	ENSLOCG00000000815	bcl9l
ENSXMAG00000006072	lim2.2	ENSLOCG00000008990	lim2.2
ENSXMAG00000012013	c2cd3	ENSLOCG00000006088	c2cd3
ENSXMAG00000004176	strip2	ENSLOCG00000015952	strip2
ENSXMAG00000000887	rccd1	ENSLOCG00000015109	rccd1
ENSXMAG00000021649		ENSLOCG00000016502	
ENSXMAG00000022083	cxcr5	ENSLOCG00000000794	cxcr5
ENSXMAG00000009487	armc1l	ENSLOCG00000016210	armc1l
ENSXMAG00000013544	si:dkey-21a6.5	ENSLOCG00000006968	
ENSXMAG00000001176		ENSLOCG00000015245	CNNM3
ENSXMAG00000016338	nkd1	ENSLOCG00000007336	nkd1
ENSXMAG00000014042	ifi35	ENSLOCG00000012246	ifi35
ENSXMAG00000027172		ENSLOCG00000000981	PALMD
ENSXMAG00000002715	ctnnal1	ENSLOCG00000007216	ctnnal1
ENSXMAG00000014945	hax1	ENSLOCG00000007563	hax1
ENSXMAG00000001865	sorbs3	ENSLOCG00000014940	sorbs3
ENSXMAG00000025711	cox5b2	ENSLOCG00000011288	cox5b2
ENSXMAG00000011656		ENSLOCG00000004964	haus4
ENSXMAG00000015122	si:ch211-286f9.2	ENSLOCG00000016202	si:ch211-286f9.2
ENSXMAG00000007620	ddx6	ENSLOCG00000000770	ddx6
ENSXMAG00000025740	apex2	ENSLOCG00000014467	apex2
ENSXMAG00000004757	sec23b	ENSLOCG00000016145	sec23b
ENSXMAG00000027530	fbp2	ENSLOCG00000011443	fbp2
ENSXMAG00000000874	unc45a	ENSLOCG00000015105	unc45a
ENSXMAG00000002821	rpl18a	ENSLOCG00000016509	rpl18a
ENSXMAG00000016922	nmu	ENSLOCG00000014037	nmu
ENSXMAG00000013548	mrpl35	ENSLOCG00000010848	mrpl35
ENSXMAG00000001125	c1qtnf6a	ENSLOCG00000011192	c1qtnf6a
ENSXMAG00000005878	capn1a	ENSLOCG00000015637	capn1a
ENSXMAG00000014571	rab3gap1	ENSLOCG00000001701	rab3gap1
ENSXMAG00000026379	chst12a	ENSLOCG00000018101	chst12a
ENSXMAG00000001716	shox	ENSLOCG00000002524	shox
ENSXMAG00000026801	ppp1r14c	ENSLOCG00000016203	ppp1r14c
ENSXMAG00000010445	pck1	ENSLOCG00000007137	pck1
ENSXMAG00000014867	ubap2l	ENSLOCG00000007592	ubap2l
ENSXMAG00000016346	brd7	ENSLOCG00000007355	brd7
ENSXMAG00000002741	elp2	ENSLOCG00000007202	elp2
ENSXMAG00000018421	slc38a5a	ENSLOCG00000014448	slc38a5a
ENSXMAG00000010818	LIMD1	ENSLOCG00000002038	LIMD1
ENSXMAG00000011669	fxr2	ENSLOCG00000013240	fxr2
ENSXMAG00000018242	SLC49A3	ENSLOCG00000009600	SLC49A3
ENSXMAG00000007963		ENSLOCG00000017282	
ENSXMAG00000009853	mtrf1	ENSLOCG00000005937	MTRF1
ENSXMAG00000013551	reep1	ENSLOCG00000010840	reep1
ENSXMAG00000012036	p4ha3	ENSLOCG00000006051	P4HA3

ENSXMAG00000017308	reck	ENSLOCG00000010656	reck
ENSXMAG00000010432		ENSLOCG00000003787	
ENSXMAG00000008095	herc4	ENSLOCG00000011296	herc4
ENSXMAG00000007609	treh	ENSLOCG00000000719	treh
ENSXMAG00000002827	xpot	ENSLOCG00000016512	xpot
ENSXMAG00000001085	card11	ENSLOCG00000004308	card11
ENSXMAG00000024229	MRPL14	ENSLOCG00000015632	mrpl14
ENSXMAG00000024383	chmp3	ENSLOCG00000010813	chmp3
ENSXMAG00000029739		ENSLOCG00000005967	WBP4
ENSXMAG00000004462	sec63	ENSLOCG00000016596	sec63
ENSXMAG00000001138	polm	ENSLOCG00000015276	polm
ENSXMAG00000000851	man2a2	ENSLOCG00000015097	man2a2
ENSXMAG00000010430	hcst	ENSLOCG00000003804	
ENSXMAG00000001575	ahcyl2	ENSLOCG00000015950	ahcyl2
ENSXMAG00000013461	asmtl	ENSLOCG00000002631	asmtl
ENSXMAG00000018412	wasa	ENSLOCG00000014446	wasb
ENSXMAG00000018235	PCGF3	ENSLOCG00000009614	PCGF3
ENSXMAG00000002752	gabbr2	ENSLOCG00000007187	gabbr2
ENSXMAG00000010825	deptor	ENSLOCG00000009183	deptor
ENSXMAG00000017278	nkx6.3	ENSLOCG00000015030	nkx6.3
ENSXMAG00000012620	SRPK2	ENSLOCG00000015487	SRPK2
ENSXMAG00000026264		ENSLOCG00000003818	
ENSXMAG00000029949	st3gal3b	ENSLOCG00000004247	st3gal3b
ENSXMAG00000009852		ENSLOCG00000005651	
ENSXMAG00000013555	hspa4l	ENSLOCG00000010767	hspa4l
ENSXMAG00000004749	bcl11ab	ENSLOCG00000016135	bcl11aa
ENSXMAG00000018977	gnsb	ENSLOCG00000015279	gnsb
ENSXMAG00000028515	adhfe1	ENSLOCG00000004744	adhfe1
ENSXMAG00000015579	kank1a	ENSLOCG00000011458	kank1a
ENSXMAG00000018358	sema3ga	ENSLOCG00000014436	sema3ga
ENSXMAG00000017264	inpp5l	ENSLOCG00000015035	inpp5l
ENSXMAG00000007608	nectin1b	ENSLOCG00000005852	nectin1b
ENSXMAG00000015048		ENSLOCG00000012316	
ENSXMAG00000001068	ap5z1	ENSLOCG00000004430	ap5z1
ENSXMAG00000016360	adcy7	ENSLOCG00000007376	adcy7
ENSXMAG00000021855		ENSLOCG00000015622	
ENSXMAG00000010395	csnk2a1	ENSLOCG00000007377	csnk2a1
ENSXMAG00000018230	cplx2	ENSLOCG00000009620	cplx2
ENSXMAG00000028840	p2ry8	ENSLOCG00000017771	p2ry8
ENSXMAG00000013565	plk4	ENSLOCG00000010729	plk4
ENSXMAG00000004267	tmem209	ENSLOCG00000015946	tmem209
ENSXMAG00000003046	slc1a9	ENSLOCG00000006878	
ENSXMAG00000023857	tfam	ENSLOCG00000006454	tfam
ENSXMAG00000002855	rtcb	ENSLOCG00000016515	rtcb
ENSXMAG00000000566	tgfbr1b	ENSLOCG00000010673	tgfbr1a
ENSXMAG00000018973	mrps24	ENSLOCG00000015305	mrps24
ENSXMAG00000007606	ttc36	ENSLOCG00000005813	ttc36
ENSXMAG00000009847		ENSLOCG00000013317	HESX1
ENSXMAG00000002771	galnt12	ENSLOCG00000007174	galnt12
ENSXMAG00000013452	upf3a	ENSLOCG00000002665	upf3a
ENSXMAG00000014555		ENSLOCG00000006467	zgc:171971
ENSXMAG00000029108	SAMD8	ENSLOCG00000004590	SAMD8
ENSXMAG00000018966	nudcd3	ENSLOCG00000015304	nudcd3
ENSXMAG00000007757	DNAJC5B	ENSLOCG00000000217	dnajc5b
ENSXMAG00000006040	ddx59	ENSLOCG00000007710	ddx59
ENSXMAG00000007575	cntn5	ENSLOCG00000005476	cntn5

ENSXMAG00000018350	tspy	ENSLOCG00000014443	tspy
ENSXMAG00000014549		ENSLOCG00000006391	ipmkb
ENSXMAG00000025355		ENSLOCG00000006896	NTF4
ENSXMAG00000002784	poc1bl	ENSLOCG00000007151	poc1bl
ENSXMAG00000004286	prdm4	ENSLOCG00000017092	prdm4
ENSXMAG00000015585		ENSLOCG00000002725	cfap157
ENSXMAG00000021637		ENSLOCG00000015935	
ENSXMAG00000005673	nsmce4a	ENSLOCG00000008786	nsmce4a
ENSXMAG00000017123	lancl2	ENSLOCG00000010686	lancl2
ENSXMAG00000023205	tent4b	ENSLOCG00000007410	tent4b
ENSXMAG00000014537	NAGLU	ENSLOCG00000012481	naglu
ENSXMAG00000003941		ENSLOCG00000004232	ARTN
ENSXMAG00000018346	GPR173	ENSLOCG00000017977	gpr173
ENSXMAG00000013574	itpk1b	ENSLOCG00000013095	itpk1b
ENSXMAG00000005552	bicd1a	ENSLOCG00000016614	bicd1a
ENSXMAG00000001048	wipi2	ENSLOCG00000004511	wipi2
ENSXMAG00000009824	cars2	ENSLOCG00000009049	cars2
ENSXMAG00000008153		ENSLOCG00000007936	ADTRP
ENSXMAG00000026789	ptger4c	ENSLOCG00000004217	ptger4c
ENSXMAG00000018325	wdr13	ENSLOCG00000014362	wdr13
ENSXMAG00000015591	dmrt1	ENSLOCG00000011472	dmrt1
ENSXMAG00000006025	kif14	ENSLOCG00000007678	kif14
ENSXMAG00000013442	cdc16	ENSLOCG00000002708	cdc16
ENSXMAG00000022632		ENSLOCG00000005307	
ENSXMAG00000001872	polr3d	ENSLOCG00000016080	polr3d
ENSXMAG00000002797	si:dkey-184p18.2	ENSLOCG00000007138	si:dkey-184p18.2
ENSXMAG00000016449	heatr3	ENSLOCG00000007426	heatr3
ENSXMAG00000002884	lta4h	ENSLOCG00000015731	lta4h
ENSXMAG00000016958	tmem165	ENSLOCG00000014048	tmem165
ENSXMAG00000023881	rp9	ENSLOCG00000007119	rp9
ENSXMAG00000003946	pif1	ENSLOCG00000004184	pif1
ENSXMAG00000015487	slc18b1	ENSLOCG00000016237	slc18b1
ENSXMAG00000019549	fam84a	ENSLOCG00000018002	fam84a
ENSXMAG00000029421		ENSLOCG00000016083	
ENSXMAG00000015600	dmrt3a	ENSLOCG00000011480	dmrt3a
ENSXMAG00000022662		ENSLOCG00000001252	DDRGK1
ENSXMAG00000024650	pygo2	ENSLOCG00000007047	pygo2
ENSXMAG00000015603	dmrt2a	ENSLOCG00000011487	dmrt2a
ENSXMAG00000016481	cnep1r1	ENSLOCG00000007445	cnep1r1
ENSXMAG00000001878	zgc:77112	ENSLOCG00000016085	zgc:77112
ENSXMAG00000024865	ASCL4	ENSLOCG00000017091	
ENSXMAG00000027893	vopp1	ENSLOCG00000010696	vopp1
ENSXMAG00000021890	srd5a3	ENSLOCG00000014051	srd5a3
ENSXMAG00000013579	btbd7	ENSLOCG00000013073	btbd7
ENSXMAG00000000820	rag1	ENSLOCG00000001283	rag1
ENSXMAG00000001013	SMURF1	ENSLOCG00000004637	smurf1
ENSXMAG00000012609	adck2	ENSLOCG00000016681	adck2
ENSXMAG00000005584		ENSLOCG00000016954	
ENSXMAG00000009806	atg3	ENSLOCG00000001724	atg3
ENSXMAG00000001879	gins4	ENSLOCG00000016089	gins4
ENSXMAG00000010389	smarcd1	ENSLOCG00000005337	smarcd1
ENSXMAG00000003968	nsun4	ENSLOCG00000004075	nsun4
ENSXMAG00000028988		ENSLOCG00000008802	
ENSXMAG00000020252	rag2	ENSLOCG00000017315	rag2
ENSXMAG00000018319	PORCN	ENSLOCG00000014348	porcn
ENSXMAG00000008156	mlh1	ENSLOCG00000004851	mlh1

ENSXMAG00000015611	smarca2	ENSLOCG00000011499	smarca2
ENSXMAG00000002809	elmo1	ENSLOCG00000007091	elmo1
ENSXMAG00000024494	sdr42e1	ENSLOCG00000002140	sdr42e1
ENSXMAG00000002902	TBC1D30	ENSLOCG00000017068	tbc1d30
ENSXMAG00000004303		ENSLOCG00000017083	
ENSXMAG00000021156	fgf14	ENSLOCG00000002765	fgf14
ENSXMAG00000017572	ptprk	ENSLOCG00000017283	ptprk
ENSXMAG00000013541	kdr	ENSLOCG00000014177	kdr
ENSXMAG00000016487	wtip	ENSLOCG00000002111	wtip
ENSXMAG00000000396	npm1a	ENSLOCG00000009683	npm1a
ENSXMAG00000023185	si:dkey-85n7.8	ENSLOCG00000013068	si:dkey-85n7.8
ENSXMAG00000024124		ENSLOCG00000016206	
ENSXMAG00000004501	nbas	ENSLOCG00000016470	nbas
ENSXMAG00000021987	ehf	ENSLOCG00000001316	ehf
ENSXMAG00000013426	ITGBL1	ENSLOCG00000002782	ITGBL1
ENSXMAG00000000808		ENSLOCG00000016770	ODF3
ENSXMAG00000018813	pargl	ENSLOCG00000015289	pargl
ENSXMAG00000014816		ENSLOCG00000007082	s100a11
ENSXMAG00000010846	mrpl13	ENSLOCG00000009210	mrpl13
ENSXMAG00000005044	apbb1	ENSLOCG00000009556	apbb1
ENSXMAG00000000802	pdhx	ENSLOCG00000001356	pdhx
ENSXMAG00000009795	slc19a2	ENSLOCG00000001660	slc19a2
ENSXMAG00000028408	mettl26	ENSLOCG00000006910	mettl26
ENSXMAG00000001885	dbnlb	ENSLOCG00000015057	dbnlb
ENSXMAG00000004135	smtna	ENSLOCG00000008548	
ENSXMAG00000013583	UNC79	ENSLOCG00000013044	UNC79
ENSXMAG00000024184		ENSLOCG00000001378	
ENSXMAG00000028105		ENSLOCG00000005665	arl6ip6
ENSXMAG00000021913	nr5a2	ENSLOCG00000007670	nr5a2
ENSXMAG00000010847	dscc1	ENSLOCG00000009176	dscc1
ENSXMAG00000000369	nlgn2b	ENSLOCG00000013735	nlgn2b
ENSXMAG00000018310	PDZD4	ENSLOCG00000014784	
ENSXMAG00000004132	slc35e4	ENSLOCG00000008006	slc35e4
ENSXMAG00000019873	cox14	ENSLOCG00000018200	cox14
ENSXMAG00000014122	nucb1	ENSLOCG00000000841	
ENSXMAG00000004331		ENSLOCG00000016653	CDPF1
ENSXMAG00000007292	TEAD4	ENSLOCG00000016688	
ENSXMAG00000029177	bcdin3d	ENSLOCG00000005406	bcdin3d
ENSXMAG00000001006	rnf216	ENSLOCG00000004666	rnf216
ENSXMAG00000003993	wls	ENSLOCG00000004552	wls
ENSXMAG00000004666	wfikkn1	ENSLOCG00000006900	wfikkn1
ENSXMAG00000010296		ENSLOCG00000007045	
ENSXMAG00000010857		ENSLOCG00000003378	CPQ
ENSXMAG00000013393	nalcn	ENSLOCG00000002812	nalcn
ENSXMAG00000026070		ENSLOCG00000016690	MANSC4
ENSXMAG00000008181	lrrfip2	ENSLOCG00000004883	lrrfip2
ENSXMAG00000002912	prkcq	ENSLOCG00000015666	prkcq
ENSXMAG00000000999	fscn1a	ENSLOCG00000004693	fscn1a
ENSXMAG00000009990	robo1	ENSLOCG00000009515	robo1
ENSXMAG00000007553	pgr	ENSLOCG00000005538	pgr
ENSXMAG00000000785	pamr1	ENSLOCG00000001426	si:ch211-102l7.3
ENSXMAG00000020287	dpm3	ENSLOCG00000017679	dpm3
ENSXMAG00000023627		ENSLOCG00000016692	
ENSXMAG00000014157	tbc1d17	ENSLOCG00000000798	tbc1d17
ENSXMAG00000002348	adamts3	ENSLOCG00000011512	adamts3
ENSXMAG00000022167		ENSLOCG00000003410	sdc2

ENSXMAG00000028928	si:dkeyp-80c12.5	ENSLOCG00000011849	calhm1
ENSXMAG00000018246	kdm5c	ENSLOCG00000014644	
ENSXMAG00000016500	si:ch211-79l17.1	ENSLOCG00000002069	si:ch211-79l17.1
ENSXMAG00000019128	elp1	ENSLOCG00000011549	elp1
ENSXMAG00000018233		ENSLOCG00000001525	TMEM233
ENSXMAG00000021095	rab22a	ENSLOCG00000007029	rab22a
ENSXMAG00000010869		ENSLOCG00000003470	mrs2
ENSXMAG00000018749	ddx56	ENSLOCG00000015185	ddx56
ENSXMAG00000020415		ENSLOCG00000020326	
ENSXMAG00000015523	kat6b	ENSLOCG00000004535	kat6b
ENSXMAG00000009731	ube3a	ENSLOCG00000008388	ube3a
ENSXMAG00000025843		ENSLOCG00000002965	
ENSXMAG00000004056	ccdc18	ENSLOCG00000004628	ccdc18
ENSXMAG00000018221	dnai1.2	ENSLOCG00000004708	dnai1.2
ENSXMAG00000002844	pex1	ENSLOCG00000010675	pex1
ENSXMAG00000000969	tnrc18	ENSLOCG00000004756	tnrc18
ENSXMAG00000023098	ube2d4	ENSLOCG00000015062	ube2d4
ENSXMAG00000014365	pdgfra	ENSLOCG00000014152	pdgfra
ENSXMAG00000005957	alpi.2	ENSLOCG00000004769	alpi.2
ENSXMAG00000027005	ss18l2	ENSLOCG00000002055	ss18l2
ENSXMAG00000004638	pdcd11	ENSLOCG00000011818	pdcd11
ENSXMAG00000005624	unc5b	ENSLOCG00000008838	unc5b
ENSXMAG00000018732	aebp1	ENSLOCG00000015167	aebp1
ENSXMAG00000004352	atxn10	ENSLOCG00000017022	atxn10
ENSXMAG00000029644	rfng	ENSLOCG00000012361	rfng
ENSXMAG00000004060	mtf2	ENSLOCG00000004649	mtf2
ENSXMAG00000002967	sfmbt2	ENSLOCG00000015669	sfmbt2
ENSXMAG00000002869	rbm48	ENSLOCG00000010688	rbm48
ENSXMAG00000023671		ENSLOCG00000005619	
ENSXMAG00000005944	alpi.1	ENSLOCG00000004789	alpi.1
ENSXMAG00000023626	dcxr	ENSLOCG00000012368	dcxr
ENSXMAG00000015588	mapre3a	ENSLOCG00000016310	mapre3a
ENSXMAG00000020250	fjx1	ENSLOCG00000017317	fjx1
ENSXMAG00000025766	slc29a4	ENSLOCG00000004787	slc29a4
ENSXMAG00000005939		ENSLOCG00000010643	srek1ip1
ENSXMAG00000009677		ENSLOCG00000010887	ddx4
ENSXMAG00000007536	yap1	ENSLOCG00000005660	yap1
ENSXMAG00000002876	sytl1	ENSLOCG00000004774	sytl1
ENSXMAG00000018194	zgc:109965	ENSLOCG00000004742	zgc:109965
ENSXMAG00000001343	taf5	ENSLOCG00000011799	taf5
ENSXMAG00000018711	ash2l	ENSLOCG00000015161	ash2l
ENSXMAG00000015558	vclb	ENSLOCG00000004474	vcla
ENSXMAG00000014734	rac3b	ENSLOCG00000012372	rac3a
ENSXMAG00000005627	tspan33b	ENSLOCG00000016839	tspan33b
ENSXMAG00000014362	gsx2	ENSLOCG00000014145	gsx2
ENSXMAG00000005914	ncl	ENSLOCG00000004828	ncl
ENSXMAG00000009648	cdkn1bb	ENSLOCG00000016694	cdkn1ba
ENSXMAG00000000769	LDLRAD3	ENSLOCG00000001462	LDLRAD3
ENSXMAG00000005640	ttc38	ENSLOCG00000016836	ttc38
ENSXMAG00000027346	RF00068	ENSLOCG00000020177	RF00068
ENSXMAG00000007534		ENSLOCG00000005696	
ENSXMAG00000009650	yars2	ENSLOCG00000016695	yars2
ENSXMAG00000024670	cpsf4	ENSLOCG00000004814	cpsf4
ENSXMAG00000002976	tmem110l	ENSLOCG00000015671	tmem110l
ENSXMAG00000008191	dact2	ENSLOCG00000016207	dact2
ENSXMAG00000019890	ascl1b	ENSLOCG00000017318	ascl1b

ENSXMAG00000004636	atp5md	ENSLOCG00000011809	atp5md
ENSXMAG00000014349	lnx1	ENSLOCG00000014140	lnx1
ENSXMAG00000014712	lrrc45	ENSLOCG00000012375	lrrc45
ENSXMAG00000002885	wasf2	ENSLOCG00000004668	wasf2
ENSXMAG00000004572	smoc2	ENSLOCG00000016200	smoc2
ENSXMAG00000004921	mpp1	ENSLOCG00000013734	mpp1
ENSXMAG00000016550	CHST8	ENSLOCG00000001953	si:ch211-269c21.2
ENSXMAG00000022540	atp5mf	ENSLOCG00000004800	atp5mf
ENSXMAG00000004104	rpap2	ENSLOCG00000004726	rpap2
ENSXMAG00000025137	cactin	ENSLOCG00000006566	cactin
ENSXMAG00000012784	rusc2	ENSLOCG00000004546	rusc2
ENSXMAG00000002985	itih5	ENSLOCG00000015674	itih5
ENSXMAG00000013596	OTUB2	ENSLOCG00000013011	OTUB2
ENSXMAG00000015063	irf7	ENSLOCG00000000378	irf7
ENSXMAG00000002889		ENSLOCG00000004653	
ENSXMAG00000005619	slc29a3	ENSLOCG00000008830	slc29a3
ENSXMAG00000027714	apcdd1l	ENSLOCG00000007006	apcdd1l
ENSXMAG00000019802	b3gnt7	ENSLOCG00000004855	b3gnt7
ENSXMAG00000015477	gab2	ENSLOCG00000005755	gab2
ENSXMAG00000016552	pepd	ENSLOCG00000001905	pepd
ENSXMAG00000024678	scfd2	ENSLOCG00000014121	scfd2
ENSXMAG00000029670	ahdc1	ENSLOCG00000004640	ahdc1
ENSXMAG00000013600	ddx24	ENSLOCG00000012997	ddx24
ENSXMAG00000003644	shmt1	ENSLOCG00000004890	shmt1
ENSXMAG00000013374	tmtc4	ENSLOCG00000002966	tmtc4
ENSXMAG00000015582		ENSLOCG00000011955	C1QL1
ENSXMAG00000023611		ENSLOCG00000015074	
ENSXMAG00000026790	cryaa	ENSLOCG00000001257	cryaa
ENSXMAG00000019168	arhgef37	ENSLOCG00000012204	arhgef37
ENSXMAG00000004578	eif3s6ip	ENSLOCG00000016195	eif3s6ip
ENSXMAG00000014701	trub1	ENSLOCG00000012979	trub1
ENSXMAG00000010304	wnt7bb	ENSLOCG00000017025	wnt7bb
ENSXMAG00000018178	celf4	ENSLOCG00000004792	celf4
ENSXMAG00000004928	tmlhe	ENSLOCG00000013710	tmlhe
ENSXMAG00000015584	dcakd	ENSLOCG00000011948	dcakd
ENSXMAG00000014332	rasl11b	ENSLOCG00000014119	rasl11b
ENSXMAG00000020058		ENSLOCG00000018156	zgc:153044
ENSXMAG00000016588	THSD4	ENSLOCG00000014576	THSD4
ENSXMAG00000003008	itih2	ENSLOCG00000015677	itih2
ENSXMAG00000004158	cdc7	ENSLOCG00000004901	CDC7
ENSXMAG00000029937	hsf2bp	ENSLOCG00000001309	hsf2bp
ENSXMAG00000018667	scarb1	ENSLOCG00000001734	scarb1
ENSXMAG00000018216	gpkow	ENSLOCG00000014776	gpkow
ENSXMAG00000004555	ddx1	ENSLOCG00000016473	ddx1
ENSXMAG00000016602	LRRC49	ENSLOCG00000014584	LRRC49
ENSXMAG00000004933		ENSLOCG00000013757	gabrb4
ENSXMAG00000014314	usp46	ENSLOCG00000014113	usp46
ENSXMAG00000001915	mn1b	ENSLOCG00000007833	mn1a
ENSXMAG00000003656	pdap1b	ENSLOCG00000004873	pdap1b
ENSXMAG00000004178	hfm1	ENSLOCG00000004931	
ENSXMAG00000015442	nars2	ENSLOCG00000005772	nars2
ENSXMAG00000027218	phrf1	ENSLOCG00000000493	phrf1
ENSXMAG00000010046	robo2	ENSLOCG00000009474	robo2
ENSXMAG00000005590	psap	ENSLOCG00000008645	psap
ENSXMAG00000010309		ENSLOCG00000017021	
ENSXMAG00000018212	pqbp1	ENSLOCG00000014775	pqbp1

ENSXMAG00000015647	dbn1	ENSLOCG00000012321	dbn1
ENSXMAG00000021956	nfkbil1	ENSLOCG00000005912	nfkbil1
ENSXMAG00000019179	ppargc1b	ENSLOCG00000012195	ppargc1b
ENSXMAG00000021015	larp6a	ENSLOCG00000014592	larp6a
ENSXMAG00000010995	zic2a	ENSLOCG00000003077	zic2a
ENSXMAG00000009655		ENSLOCG00000005536	chrm4a
ENSXMAG00000002916	sesn2	ENSLOCG00000005106	SESN2
ENSXMAG00000018200	timm17b	ENSLOCG00000014774	timm17b
ENSXMAG00000003664	bud31	ENSLOCG00000004858	bud31
ENSXMAG00000022710	tm2d3	ENSLOCG00000014600	tm2d3
ENSXMAG00000003029	kin	ENSLOCG00000015679	kin
ENSXMAG00000019308	eef2b	ENSLOCG00000006185	
ENSXMAG00000007102	gulp1a	ENSLOCG00000006118	gulp1a
ENSXMAG00000019807	mrps6	ENSLOCG00000018238	mrps6
ENSXMAG00000022319	clybl	ENSLOCG00000003101	clybl
ENSXMAG00000014142	exoc6	ENSLOCG00000007011	exoc6
ENSXMAG00000014298	spata18	ENSLOCG00000014109	spata18
ENSXMAG00000004609	cdh23	ENSLOCG00000008670	cdh23
ENSXMAG00000026032	manbal	ENSLOCG00000002342	manbal
ENSXMAG00000005876	armc9	ENSLOCG00000004868	armc9
ENSXMAG00000003670	smcr8a	ENSLOCG00000004927	smcr8a
ENSXMAG00000004953	gabra3	ENSLOCG00000013763	gabra3
ENSXMAG00000025239		ENSLOCG00000011021	
ENSXMAG00000016645	tarsl2	ENSLOCG00000014607	tarsl2
ENSXMAG00000018111	sec14l8	ENSLOCG00000007503	sec14l8
ENSXMAG00000027289	nolc1	ENSLOCG00000008634	nolc1
ENSXMAG00000005670	hipk2	ENSLOCG00000016911	hipk2
ENSXMAG00000000054	cnnm2b	ENSLOCG00000011756	cnnm2b
ENSXMAG00000004203	znf644a	ENSLOCG00000004952	znf644b
ENSXMAG00000015628	grk6	ENSLOCG00000012330	grk6
ENSXMAG00000002933	matn1	ENSLOCG00000005128	matn1
ENSXMAG00000004421	USP15	ENSLOCG00000016990	USP15
ENSXMAG00000011000	si:ch211-184m13.4	ENSLOCG00000017773	si:ch211-184m13.4
ENSXMAG00000003671	top3a	ENSLOCG00000004935	top3a
ENSXMAG00000019181	pde6a	ENSLOCG00000012187	pde6a
ENSXMAG00000019925	gpr183a	ENSLOCG00000017774	gpr183b
ENSXMAG00000003042	atp5f1c	ENSLOCG00000015681	atp5f1c
ENSXMAG00000010218	cyp24a1	ENSLOCG00000002513	cyp24a1
ENSXMAG00000000056	as3mt	ENSLOCG00000011748	as3mt
ENSXMAG00000014291	sgcb	ENSLOCG00000014106	sgcb
ENSXMAG00000011001	gpr18	ENSLOCG00000017775	gpr18
ENSXMAG00000015130	LRRC56	ENSLOCG00000000649	LRRC56
ENSXMAG00000018177	zgc:162344	ENSLOCG00000004807	
ENSXMAG00000019309	btbd2b	ENSLOCG00000006170	btbd2a
ENSXMAG00000018104	rnf215	ENSLOCG00000007489	RNF215
ENSXMAG00000019558	lrrc8da	ENSLOCG00000017738	lrrc8da
ENSXMAG00000022639	micu1	ENSLOCG00000008610	micu1
ENSXMAG00000000063	wbp1lb	ENSLOCG00000011721	wbp1la
ENSXMAG00000004206	gtf2b	ENSLOCG00000005057	gtf2b
ENSXMAG00000027994		ENSLOCG00000005164	
ENSXMAG00000004995	TMEM164	ENSLOCG00000013802	TMEM164
ENSXMAG00000005699	tbxas1	ENSLOCG00000016908	tbxas1
ENSXMAG00000018172	tpgs2	ENSLOCG00000004820	tpgs2
ENSXMAG00000015641	dtnbb	ENSLOCG00000016318	dtnbb
ENSXMAG00000016706	ulk3	ENSLOCG00000014610	ulk3
ENSXMAG00000029113	nop16	ENSLOCG00000012348	nop16

ENSXMAG00000015132	hrasb	ENSLOCG00000000684	hrasb
ENSXMAG00000018075	ascc2	ENSLOCG00000007474	ascc2
ENSXMAG00000000075	poll	ENSLOCG00000011706	poll
ENSXMAG00000026670	ammecr1	ENSLOCG00000013808	ammecr1
ENSXMAG00000004653	si:ch73-21k16.5	ENSLOCG00000001114	
ENSXMAG00000015622		ENSLOCG00000012356	ARL10
ENSXMAG00000007490	tenm4	ENSLOCG00000005799	tenm4
ENSXMAG00000011759	cd99l2	ENSLOCG00000014495	cd99l2
ENSXMAG00000014276	dcun1d4	ENSLOCG00000014103	dcun1d4
ENSXMAG00000015617	wwc1	ENSLOCG00000012357	wwc1
ENSXMAG00000004214	pkn2	ENSLOCG00000005077	PKN2
ENSXMAG00000026201	gata3	ENSLOCG00000015684	gata3
ENSXMAG00000022016	atxn3	ENSLOCG00000012965	atxn3
ENSXMAG00000002945	pum1	ENSLOCG00000005210	pum1
ENSXMAG00000018067	slc39a14	ENSLOCG00000015416	slc39a14
ENSXMAG00000015140	tsg101a	ENSLOCG00000000710	tsg101b
ENSXMAG00000011014	klf12b	ENSLOCG00000011354	klf12b
ENSXMAG00000004819	vsir	ENSLOCG00000008708	vsir
ENSXMAG00000018281		ENSLOCG00000007367	si:ch211-251p5.5
ENSXMAG00000016729		ENSLOCG00000017953	
ENSXMAG00000019224	slc26a2	ENSLOCG00000012182	slc26a2
ENSXMAG00000011764	mtmr1a	ENSLOCG00000014489	mtmr1b
ENSXMAG00000029007	trim69	ENSLOCG00000008317	trim69
ENSXMAG00000026372	zgc:64106	ENSLOCG00000004832	zgc:64106
ENSXMAG00000005735	edc4	ENSLOCG00000006715	edc4
ENSXMAG00000026598	cplx3a	ENSLOCG00000014618	cplx3a
ENSXMAG00000005017	kif4	ENSLOCG00000013822	kif4
ENSXMAG00000014144	cyp26a1	ENSLOCG00000006966	cyp26a1
ENSXMAG00000000098	dpcd	ENSLOCG00000011698	dpcd
ENSXMAG00000001924	TTC28	ENSLOCG00000007797	TTC28
ENSXMAG00000029900	MCU	ENSLOCG00000008593	MCU
ENSXMAG00000010993	zfat	ENSLOCG00000003701	zfat
ENSXMAG00000004823	plpp4	ENSLOCG00000008720	plpp4
ENSXMAG00000015595	rars	ENSLOCG00000012373	rars
ENSXMAG00000009988	ankrd13d	ENSLOCG00000008259	ankrd13d
ENSXMAG00000015495	stambpb	ENSLOCG00000015080	stambpa
ENSXMAG00000016897	bpnt1	ENSLOCG00000000317	bpnt1
ENSXMAG00000022304	ldha	ENSLOCG00000000737	ldha
ENSXMAG00000015679	asxl2	ENSLOCG00000016319	asxl2
ENSXMAG00000005861	polr2h	ENSLOCG00000008946	polr2h
ENSXMAG00000011794	mtm1	ENSLOCG00000014471	mtm1
ENSXMAG00000006572	slc4a3	ENSLOCG00000009239	slc4a3
ENSXMAG00000019312		ENSLOCG00000006043	MBD3
ENSXMAG00000015081	si:dkey-8k3.2	ENSLOCG00000008608	c4
ENSXMAG00000010999	mtbp	ENSLOCG00000009214	mtbp
ENSXMAG00000005563	oit3	ENSLOCG00000008577	oit3
ENSXMAG00000004679	si:ch73-21k16.4	ENSLOCG00000016058	si:ch73-21k16.4
ENSXMAG00000018587	sez6l	ENSLOCG00000008591	sez6l
ENSXMAG00000026669	RF00406	ENSLOCG00000020478	RF00406
ENSXMAG00000019228		ENSLOCG00000012176	htr4
ENSXMAG00000015681	kif3cb	ENSLOCG00000016321	kif3cb
ENSXMAG00000011020	tbc1d4	ENSLOCG00000011360	tbc1d4
ENSXMAG00000005858		ENSLOCG00000008655	
ENSXMAG00000006458	ppil2	ENSLOCG00000001974	ppil2
ENSXMAG00000010406	mon2	ENSLOCG00000016986	mon2
ENSXMAG00000030013		ENSLOCG00000011906	

ENSXMAG00000023327		ENSLOCG00000000395		
ENSXMAG00000015580	si:dkeyp-23e4.3	ENSLOCG00000012379	si:dkeyp-23e4.3	
ENSXMAG00000018072	zgc:171501	ENSLOCG00000006039	zgc:171501	
ENSXMAG00000020986		ENSLOCG00000007784		
ENSXMAG00000011008	sntb1	ENSLOCG00000009223	sntb1	
ENSXMAG00000014154	jmjd1cb	ENSLOCG00000006879	jmjd1cb	
ENSXMAG00000002851	ero1b	ENSLOCG00000015824	ero1b	
ENSXMAG00000001933	SDS	ENSLOCG00000007734	SDS	
ENSXMAG00000015694		ENSLOCG00000016329	hadhab	
ENSXMAG00000011841	nat16	ENSLOCG00000014063	nat16	
ENSXMAG00000005770	nutf2	ENSLOCG00000006733	nutf2	
ENSXMAG00000015601	coasy	ENSLOCG00000012461	coasy	
ENSXMAG00000001870	atic	ENSLOCG00000010002	atic	
ENSXMAG00000000139	cenpk	ENSLOCG00000017098		
ENSXMAG00000002965	nkain1	ENSLOCG00000005227	nkain1	
ENSXMAG00000004148		ENSLOCG00000011186	PTGFRN	
ENSXMAG00000000628	MYO18B	ENSLOCG00000008574	MYO18B	
ENSXMAG00000005535	pde10a	ENSLOCG00000016520	pde10a	
ENSXMAG00000005785	ranbp10	ENSLOCG00000006779	ranbp10	
ENSXMAG00000011846	acox3	ENSLOCG00000010299	acox3	
ENSXMAG00000028994	selenof	ENSLOCG00000005156	selenof	
ENSXMAG00000015615		ENSLOCG00000008238		
ENSXMAG00000014147	CDH6	ENSLOCG00000012841	cdh6	
ENSXMAG00000003707	xpnpep3	ENSLOCG00000011565	xpnpep3	
ENSXMAG00000001877	mnx2b	ENSLOCG00000010942	mnx2b	
ENSXMAG00000016912	slc35a1	ENSLOCG00000016927	slc35a1	
ENSXMAG00000006444	mapk1	ENSLOCG00000002024	mapk1	
ENSXMAG00000005060	adssl	ENSLOCG00000001306	adssl	
ENSXMAG00000002970	snrnp40	ENSLOCG00000005258	snrnp40	
ENSXMAG00000018051		ENSLOCG00000005965	rad21l1	
ENSXMAG00000014704		ENSLOCG00000006815	ncoa5	
ENSXMAG00000000155	si:ch211-156l18.7	ENSLOCG00000013607	si:ch211-156l18.7	
ENSXMAG00000015256	abtb2b	ENSLOCG00000001988	abtb2a	
ENSXMAG00000027998	sgcd	ENSLOCG00000012386	sgcd	
ENSXMAG00000011812	zar1	ENSLOCG00000014077	zar1	
ENSXMAG00000019231	lman2	ENSLOCG00000012170	lman2	
ENSXMAG00000001880	stk16	ENSLOCG00000010936	stk16	
ENSXMAG00000005810	det1	ENSLOCG00000014536	det1	
ENSXMAG00000004256	2-Sep	ENSLOCG00000006303		2-Sep
ENSXMAG00000027667	NOV	ENSLOCG00000009114	NOV	
ENSXMAG00000014178	CDH10	ENSLOCG00000012835	cdh10a	
ENSXMAG00000002980	zcchc17	ENSLOCG00000005280	zcchc17	
ENSXMAG00000016921	rars2	ENSLOCG00000016928	rars2	
ENSXMAG00000001393	dcaf12	ENSLOCG00000004915	dcaf12	
ENSXMAG00000026931	znf16l	ENSLOCG00000006000		
ENSXMAG00000013805	slc22a7a	ENSLOCG00000015596	slc22a7a	
ENSXMAG00000015712	rps6ka5	ENSLOCG00000014006	rps6ka5	
ENSXMAG00000022833	wdr26b	ENSLOCG00000015931	wdr26a	
ENSXMAG00000012541	si:dkey-24p1.6	ENSLOCG00000007930	si:dkey-24p1.6	
ENSXMAG00000002986	fabp3	ENSLOCG00000005299	fabp3	
ENSXMAG00000001886	pcnp	ENSLOCG00000009530	pcnp	
ENSXMAG00000006886	anxa4	ENSLOCG00000014962	anxa4	
ENSXMAG00000004304	stk25b	ENSLOCG00000006383	stk25a	
ENSXMAG00000000645	grk3	ENSLOCG00000003030	grk3	
ENSXMAG00000001891		ENSLOCG00000008704	IL18RAP	
ENSXMAG00000019238	fat2	ENSLOCG00000011694	fat2	

ENSXMAG00000020009	mthfs	ENSLOCG00000012766	mthfs
ENSXMAG00000011048	commd6	ENSLOCG00000011386	commd6
ENSXMAG00000028406	b4galnt4a	ENSLOCG00000001959	b4galnt4b
ENSXMAG00000024301	ubap1	ENSLOCG00000004937	ubap1
ENSXMAG00000002989	dclk3	ENSLOCG00000004794	DCLK3
ENSXMAG00000015564	mrpl22	ENSLOCG00000012398	mrpl22
ENSXMAG00000029068	sf3a2	ENSLOCG00000005835	sf3a2
ENSXMAG00000011809	slc10a4	ENSLOCG00000014074	slc10a4
ENSXMAG00000023877	stim1a	ENSLOCG00000007988	STIM1
ENSXMAG00000013804	rrp36	ENSLOCG00000015862	RRP36
ENSXMAG00000011049	uchl3	ENSLOCG00000011397	uchl3
ENSXMAG00000015742	mbip	ENSLOCG00000014003	mbip
ENSXMAG00000009922	ran	ENSLOCG00000008279	
ENSXMAG00000004691	ppm1j	ENSLOCG00000010272	ppm1j
ENSXMAG00000015671	RNF157	ENSLOCG00000011217	RNF157
ENSXMAG00000006401	rufy3	ENSLOCG00000011601	rufy3
ENSXMAG00000015336	tmem86a	ENSLOCG00000001930	tmem86a
ENSXMAG00000011123	tbxtb	ENSLOCG00000016526	tbxtb
ENSXMAG00000005837	klhdc4	ENSLOCG00000003558	klhdc4
ENSXMAG00000016070	cryba4	ENSLOCG00000008625	cryba4
ENSXMAG00000011887	alox12	ENSLOCG00000014089	alox12
ENSXMAG00000029778	nkx2.1	ENSLOCG00000014000	nkx2.1
ENSXMAG00000019321		ENSLOCG00000001367	fzr1a
ENSXMAG00000002990	cyhr1	ENSLOCG00000005765	cyhr1
ENSXMAG00000014205	reep3b	ENSLOCG00000006912	reep3b
ENSXMAG00000016967	rngtt	ENSLOCG00000016935	rngtt
ENSXMAG00000004846	naga	ENSLOCG00000011942	NAGA
ENSXMAG00000028981		ENSLOCG00000017803	PRSS23
ENSXMAG00000015760	NKX2-8	ENSLOCG00000013997	NKX2-8
ENSXMAG00000010312	nup50	ENSLOCG00000016533	nup50
ENSXMAG00000019917	prr18	ENSLOCG00000017366	prr18
ENSXMAG00000014315	CDH18	ENSLOCG00000012821	CDH18
ENSXMAG00000027471	si:dkey-238f9.1	ENSLOCG00000010264	si:dkey-238f9.1
ENSXMAG00000015538	gemin5	ENSLOCG00000012402	gemin5
ENSXMAG00000015340	spty2d1	ENSLOCG00000001901	spty2d1
ENSXMAG00000019248	mtnr1al	ENSLOCG00000011690	mtnr1al
ENSXMAG00000027450	sft2d1	ENSLOCG00000016529	sft2d1
ENSXMAG00000023586	tbc1d10c	ENSLOCG00000008291	
ENSXMAG00000001902	tmem39a	ENSLOCG00000009633	tmem39a
ENSXMAG00000015762	pax9	ENSLOCG00000013992	pax9
ENSXMAG00000003418	nudt2	ENSLOCG00000004965	nudt2
ENSXMAG00000011799	slain2	ENSLOCG00000014068	slain2
ENSXMAG00000005835	hykk.2	ENSLOCG00000008619	hykk.1
ENSXMAG00000019249	slc36a1	ENSLOCG00000011680	slc36a1
ENSXMAG00000016047	slc7a5	ENSLOCG00000003570	slc7a5
ENSXMAG00000004340	zgc:110269	ENSLOCG00000004298	
ENSXMAG00000010316	zgc:172139	ENSLOCG00000016527	zgc:172139
ENSXMAG00000024578		ENSLOCG00000004766	
ENSXMAG00000014226	nrbf2b	ENSLOCG00000006865	nrbf2b
ENSXMAG00000016088	crybb1	ENSLOCG00000008637	CRYBB1
ENSXMAG00000009896	rad9a	ENSLOCG00000008300	rad9a
ENSXMAG00000014231		ENSLOCG00000008843	NHLRC3
ENSXMAG00000011907	ache	ENSLOCG00000014072	ache
ENSXMAG00000020233	FAM181B	ENSLOCG00000018197	FAM181B
ENSXMAG00000003422	si:dkey-6b12.5	ENSLOCG00000001453	si:dkey-6b12.5
ENSXMAG00000019326		ENSLOCG00000005192	HCN2

ENSXMAG00000019253	atox1	ENSLOCG00000011673	atox1
ENSXMAG00000026683	si:dkeyp-19e1.3	ENSLOCG00000001862	si:dkeyp-19e1.3
ENSXMAG00000022027	tctex1d2	ENSLOCG00000008576	tctex1d2
ENSXMAG00000015763	slc25a21	ENSLOCG00000013986	slc25a21
ENSXMAG00000026751	vegfab	ENSLOCG00000016005	vegfab
ENSXMAG00000016978		ENSLOCG00000010902	
ENSXMAG00000016049	ca5a	ENSLOCG00000003596	ca5a
ENSXMAG00000010329		ENSLOCG00000016522	fbxo7
ENSXMAG00000007481	prcp	ENSLOCG00000005836	prcp
ENSXMAG00000005968		ENSLOCG00000009466	ADGRG7
ENSXMAG00000024959	si:ch211-272n13.3	ENSLOCG00000015575	
ENSXMAG00000021999	TOB2	ENSLOCG00000011604	TOB2
ENSXMAG00000002742	sycp1	ENSLOCG00000010662	
ENSXMAG00000019254	sparc	ENSLOCG00000011666	sparc
ENSXMAG00000004521	KDM5A	ENSLOCG00000017001	
ENSXMAG00000016761	cyp1a	ENSLOCG00000014628	cyp1a
ENSXMAG00000021872	htr7a	ENSLOCG00000000025	htr7a
ENSXMAG00000014236	bms1	ENSLOCG00000012914	bms1
ENSXMAG00000005547	ell2	ENSLOCG00000001432	ell2
ENSXMAG00000022474	rsph9	ENSLOCG00000016010	rsph9
ENSXMAG00000016073	uba2	ENSLOCG00000003617	uba2
ENSXMAG00000015676	SRSF2	ENSLOCG00000011252	srsf2a
ENSXMAG00000001988	vps33a	ENSLOCG00000005360	vps33a
ENSXMAG00000019255	g3bp1	ENSLOCG00000011657	g3bp1
ENSXMAG00000015521	cnot8	ENSLOCG00000012411	cnot8
ENSXMAG00000005927	ftcd	ENSLOCG00000008855	ftcd
ENSXMAG00000025848	rab30	ENSLOCG00000005884	rab30
ENSXMAG00000027757	gk5	ENSLOCG00000007996	gk5
ENSXMAG00000029178	ggh	ENSLOCG00000004962	ggh
ENSXMAG00000020521	MIR128-2	ENSLOCG00000019560	MIR128-2
ENSXMAG00000015499	fam114a2	ENSLOCG00000012416	fam114a2
ENSXMAG00000004864	kif20ba	ENSLOCG00000000026	kif20bb
ENSXMAG00000019331	SUGP1	ENSLOCG00000005094	
ENSXMAG00000015395	cpt1ab	ENSLOCG00000001740	cpt1aa
ENSXMAG00000016994	nt5c1bb	ENSLOCG00000017187	nt5c1ba
ENSXMAG00000009850	ssrp1a	ENSLOCG00000008342	ssrp1a
ENSXMAG00000005137	usf1	ENSLOCG00000000641	usf1
ENSXMAG00000021253		ENSLOCG00000001415	
ENSXMAG00000003000	pdcd6ip	ENSLOCG00000004712	pdcd6ip
ENSXMAG00000019257	glra1	ENSLOCG00000011646	glra1
ENSXMAG00000014278	mtr	ENSLOCG00000016612	mtr
ENSXMAG00000028264		ENSLOCG00000013034	tubb2
ENSXMAG00000005846		ENSLOCG00000002698	
ENSXMAG00000018137	cd2bp2	ENSLOCG00000000081	cd2bp2
ENSXMAG00000005561	pcsk1	ENSLOCG00000001386	pcsk1
ENSXMAG00000011092	lhcgr	ENSLOCG00000016530	lhcgr
ENSXMAG00000015779	plekhh1	ENSLOCG00000013964	plekhh1
ENSXMAG00000021665	MFAP3	ENSLOCG00000012425	MFAP3
ENSXMAG00000004867	mrps18a	ENSLOCG00000016008	mrps18a
ENSXMAG00000020232	thrsp	ENSLOCG00000018201	thrsp
ENSXMAG00000021766		ENSLOCG00000004003	BOLL
ENSXMAG00000017017	extl3	ENSLOCG00000016002	extl3
ENSXMAG00000011744	anapc4	ENSLOCG00000014055	anapc4
ENSXMAG00000003023	ubp1	ENSLOCG00000002718	ubp1
ENSXMAG00000019332	stk11	ENSLOCG00000005072	stk11
ENSXMAG00000028496	slc25a48	ENSLOCG00000011632	slc25a48

ENSXMAG00000006921	polr3h	ENSLOCG00000011621	polr3h
ENSXMAG00000002764	slc5a8l	ENSLOCG00000010641	slc5a8l
ENSXMAG00000004877	tmem63a	ENSLOCG00000016012	tmem63a
ENSXMAG00000005847		ENSLOCG00000007780	
ENSXMAG00000005797		ENSLOCG00000009459	
ENSXMAG00000027683		ENSLOCG00000014065	snapc2
ENSXMAG00000005592	comtb	ENSLOCG00000014559	comtb
ENSXMAG00000017026	riox1	ENSLOCG00000015694	riox1
ENSXMAG00000023504	clcf1	ENSLOCG00000008329	clcf1
ENSXMAG00000001925	vegfd	ENSLOCG00000007773	vegfd
ENSXMAG00000013768	p2rx3b	ENSLOCG00000008356	
ENSXMAG00000004868	ankrd1a	ENSLOCG00000000035	ankrd1a
ENSXMAG00000027516	acads	ENSLOCG00000002843	ACADS
ENSXMAG00000029895	zgc:153184	ENSLOCG00000009297	
ENSXMAG00000017046	NDUFB1	ENSLOCG00000014100	NDUFB1
ENSXMAG00000011931	ap1s1	ENSLOCG00000014056	ap1s1
ENSXMAG00000026476	bmt2	ENSLOCG00000015934	bmt2
ENSXMAG00000006514	bcs1l	ENSLOCG00000008964	bcs1l
ENSXMAG00000019927	kctd12.1	ENSLOCG00000017786	kctd12.1
ENSXMAG00000009825	slc43a1a	ENSLOCG00000008020	SLC43A1
ENSXMAG00000003043	clasp2	ENSLOCG00000002740	clasp2
ENSXMAG00000015725		ENSLOCG00000016040	
ENSXMAG00000008003	tbc1d10b	ENSLOCG00000003422	
ENSXMAG00000024208	gng3	ENSLOCG00000001294	gng3
ENSXMAG00000004878	pcgf5a	ENSLOCG00000000040	pcgf5b
ENSXMAG00000017048	cpsf2	ENSLOCG00000014102	cpsf2
ENSXMAG00000028025	mlnr	ENSLOCG00000009308	mlnr
ENSXMAG00000011952	serpine1	ENSLOCG00000014047	serpine1
ENSXMAG00000011074	acod1	ENSLOCG00000006179	acod1
ENSXMAG00000001928	pir	ENSLOCG00000007757	pir
ENSXMAG00000024729	znf142	ENSLOCG00000008959	znf142
ENSXMAG00000021580		ENSLOCG00000003565	
ENSXMAG00000008008	hsd3b7	ENSLOCG00000003439	hsd3b7
ENSXMAG00000027462	tshba	ENSLOCG00000010628	tshba
ENSXMAG00000013089	mbnl3	ENSLOCG00000015065	mbnl3
ENSXMAG00000007281	ccnc	ENSLOCG00000017086	ccnc
ENSXMAG00000015700	sfxn2	ENSLOCG00000000674	sfxn2
ENSXMAG00000021843	zgc:162944	ENSLOCG00000006169	zgc:162944
ENSXMAG00000004577	pim3	ENSLOCG00000016042	pim3
ENSXMAG00000008021	setd1a	ENSLOCG00000003450	
ENSXMAG00000011391	dnmt3ba	ENSLOCG00000005655	dnmt3ba
ENSXMAG00000022234	zfp36l1a	ENSLOCG00000013957	zfp36l1a
ENSXMAG00000006944	PMM1	ENSLOCG00000011637	PMM1
ENSXMAG00000004593	creld2	ENSLOCG00000016043	creld2
ENSXMAG00000028765	cln5	ENSLOCG00000006151	cln5
ENSXMAG00000000545	ptdss2	ENSLOCG00000000829	ptdss2
ENSXMAG00000014335	RYR2	ENSLOCG00000016594	RYR2
ENSXMAG00000029263	med19a	ENSLOCG00000008028	med19a
ENSXMAG00000008231		ENSLOCG00000013041	
ENSXMAG00000001938	zgc:113276	ENSLOCG00000007736	zgc:113276
ENSXMAG00000020370	tmem229b	ENSLOCG00000017474	tmem229b
ENSXMAG00000005783	rubcn	ENSLOCG00000008501	rubcn
ENSXMAG00000004610	alg12	ENSLOCG00000016045	alg12
ENSXMAG00000021133	adra1aa	ENSLOCG00000016099	adra1aa
ENSXMAG00000028035		ENSLOCG00000005596	
ENSXMAG00000009812	si:dkey-6i22.5	ENSLOCG00000008782	

ENSXMAG00000015493	si:ch211-236l14.4	ENSLOCG00000001786	si:ch211-236l14.4
ENSXMAG00000004470	cmpk	ENSLOCG00000010183	cmpk
ENSXMAG00000008033	ccdc47	ENSLOCG00000011851	ccdc47
ENSXMAG00000000550	cdkn1cb	ENSLOCG00000000889	cdkn1cb
ENSXMAG00000007420	fndc3a	ENSLOCG00000009316	fndc3a
ENSXMAG00000019631	tmem121ab	ENSLOCG00000017477	tmem121ab
ENSXMAG00000027978	dnajc8	ENSLOCG00000004544	
ENSXMAG00000012825	hectd2	ENSLOCG00000000043	hectd2
ENSXMAG00000019337	NDUFA13	ENSLOCG00000004936	NDUFA13
ENSXMAG00000022360	rap2c	ENSLOCG00000015061	rap2c
ENSXMAG00000003461	xdh	ENSLOCG00000015803	
ENSXMAG00000006474	cnot9	ENSLOCG00000008931	cnot9
ENSXMAG00000017148	fut8b	ENSLOCG00000014133	fut8a
ENSXMAG00000019580	zbed4	ENSLOCG00000017908	zbed4
ENSXMAG00000028138		ENSLOCG00000000936	
ENSXMAG00000015811	mta1	ENSLOCG00000013945	mta1
ENSXMAG00000013102	zgc:92907	ENSLOCG00000014897	zgc:92907
ENSXMAG00000001941	ace2	ENSLOCG00000007694	ace2
ENSXMAG00000003112	fbxl2	ENSLOCG00000002704	fbxl2
ENSXMAG00000015166	si:ch211-254p10.2	ENSLOCG00000011557	si:ch211-254p10.2
ENSXMAG00000011027	rplp2	ENSLOCG00000010566	rplp2
ENSXMAG00000013107	si:zfos-80g12.1	ENSLOCG00000014911	si:zfos-80g12.1
ENSXMAG00000014163	palld	ENSLOCG00000013061	palld
ENSXMAG00000008052	DCAF7	ENSLOCG00000011848	dcaf7
ENSXMAG00000022480	ADORA3	ENSLOCG00000009694	
ENSXMAG00000005853	cpne2	ENSLOCG00000007746	cpne2
ENSXMAG00000015734	pgs1	ENSLOCG00000012756	pgs1
ENSXMAG00000013124	foxo4	ENSLOCG00000014915	foxo4
ENSXMAG00000015434	sap30l	ENSLOCG00000012440	sap30l
ENSXMAG00000014480	hnrnpr	ENSLOCG00000004400	hnrnpr
ENSXMAG00000028775	ifitm5	ENSLOCG00000000971	ifitm5
ENSXMAG00000014971		ENSLOCG00000008918	usp37
ENSXMAG00000028619	CNIH2	ENSLOCG00000002236	cnih2
ENSXMAG00000007222	pgm3	ENSLOCG00000017072	pgm3
ENSXMAG00000019340	ARMC6	ENSLOCG00000004866	ARMC6
ENSXMAG00000021500	TNFRSF11B	ENSLOCG00000009086	TNFRSF11B
ENSXMAG00000003728	ptchd1	ENSLOCG00000003811	ptchd1
ENSXMAG00000026823	tsc22d2	ENSLOCG00000013526	
ENSXMAG00000023658	nhsb	ENSLOCG00000007630	nhsb
ENSXMAG00000004886	ppp1r3ca	ENSLOCG00000006006	ppp1r3ca
ENSXMAG00000009783	clp1	ENSLOCG00000008074	clp1
ENSXMAG00000008073	kcnh6a	ENSLOCG00000011829	kcnh6a
ENSXMAG00000029488	susd5	ENSLOCG00000002694	susd5
ENSXMAG00000023049	imp3	ENSLOCG00000010237	
ENSXMAG00000003680	phex	ENSLOCG00000003780	phex
ENSXMAG00000009740	COLEC10	ENSLOCG00000009099	COLEC10
ENSXMAG00000015496	mrpl23	ENSLOCG00000001825	mrpl23
ENSXMAG00000015745	tha1	ENSLOCG00000012764	tha1
ENSXMAG00000024013	HAND1	ENSLOCG00000012444	HAND1
ENSXMAG00000018117	tulp1b	ENSLOCG00000009723	tulp1a
ENSXMAG00000026147	tm7sf2	ENSLOCG00000014353	
ENSXMAG00000023756	hmbsa	ENSLOCG00000003711	hmbsa
ENSXMAG00000015012	ybey	ENSLOCG00000008907	ybey
ENSXMAG00000019512	prss23	ENSLOCG00000017484	prss23
ENSXMAG00000009738	mal2	ENSLOCG00000009108	mal2
ENSXMAG00000013706	enpep	ENSLOCG00000011743	enpep

ENSXMAG00000026239	igf2b	ENSLOCG00000001806	igf2b
ENSXMAG00000003153	crtap	ENSLOCG00000002668	crtap
ENSXMAG00000004887	zgc:165461	ENSLOCG00000018231	zgc:165461
ENSXMAG00000008215	mblac1	ENSLOCG00000014024	mblac1
ENSXMAG00000026884	rnf13	ENSLOCG00000001774	rnf13
ENSXMAG00000005906		ENSLOCG00000007654	tgm2l
ENSXMAG00000003420	si:ch211-132f19.7	ENSLOCG00000016676	si:ch211-132f19.7
ENSXMAG00000024271	mpdu1b	ENSLOCG00000014460	mpdu1b
ENSXMAG00000025487	th	ENSLOCG00000001834	th
ENSXMAG00000005163	vps51	ENSLOCG00000002267	vps51
ENSXMAG00000018099	tead3b	ENSLOCG00000009736	tead3b
ENSXMAG00000030000	gfap	ENSLOCG00000011811	gfap
ENSXMAG00000017179	gphnb	ENSLOCG00000014136	gphna
ENSXMAG00000011150		ENSLOCG00000006032	
ENSXMAG00000002046	es1	ENSLOCG00000016090	es1
ENSXMAG00000021205	FAM241A	ENSLOCG00000018052	fam241a
ENSXMAG00000014074	morn3	ENSLOCG00000005137	morn3
ENSXMAG00000023883	ppp1r2	ENSLOCG00000004753	ppp1r2
ENSXMAG00000015028	mcm3ap	ENSLOCG00000008893	mcm3ap
ENSXMAG00000003407	dnajc27	ENSLOCG00000016679	dnajc27
ENSXMAG00000004530	tsen15	ENSLOCG00000010289	TSEN15
ENSXMAG00000004889	cyp26c1	ENSLOCG00000006984	cyp26c1
ENSXMAG00000010057	wdfy3	ENSLOCG00000007056	wdfy3
ENSXMAG00000008210	sox19b	ENSLOCG00000014463	sox19a
ENSXMAG00000013131	atl3	ENSLOCG00000000849	atl3
ENSXMAG00000013744	jkamp	ENSLOCG00000009267	jkamp
ENSXMAG00000015843	crip1	ENSLOCG00000013566	crip1
ENSXMAG00000003160	fkbp9	ENSLOCG00000002630	fkbp9
ENSXMAG00000026743	tmem127	ENSLOCG00000015207	tmem127
ENSXMAG00000003675	mbtps2	ENSLOCG00000003747	mbtps2
ENSXMAG00000014080	tmem120b	ENSLOCG00000005150	tmem120b
ENSXMAG00000021225	tbc1d22a	ENSLOCG00000016050	tbc1d22a
ENSXMAG00000015752	eef2k	ENSLOCG00000008265	eef2k
ENSXMAG00000008111	map3k3	ENSLOCG00000011723	map3k3
ENSXMAG00000007665	gnl3l	ENSLOCG00000014702	gnl3l
ENSXMAG00000004307	kpnb3	ENSLOCG00000003556	kpnb3
ENSXMAG00000026570	ciao1	ENSLOCG00000015205	ciao1
ENSXMAG00000004538	asip2b	ENSLOCG00000010318	
ENSXMAG00000000799	clcn4	ENSLOCG00000007307	CLCN4
ENSXMAG00000013746	gpr135	ENSLOCG00000017700	gpr135
ENSXMAG00000022270		ENSLOCG00000001679	nap1l4b
ENSXMAG00000015386	slc25a14	ENSLOCG00000014944	slc25a14
ENSXMAG00000022988	ripk2	ENSLOCG00000010422	ripk2
ENSXMAG00000006445	lss	ENSLOCG00000008883	lss
ENSXMAG00000015191		ENSLOCG00000005974	tbc1d12b
ENSXMAG00000008167	eif4a1b	ENSLOCG00000014465	eif4a1a
ENSXMAG00000014153	cbr4	ENSLOCG00000013078	cbr4
ENSXMAG00000021942	si:ch211-145o7.3	ENSLOCG00000000880	si:ch211-145o7.3
ENSXMAG00000003672	smpx	ENSLOCG00000003737	smpx
ENSXMAG00000028736	DNAL1	ENSLOCG00000014125	dnal1
ENSXMAG00000014094	rhof	ENSLOCG00000005166	rhof
ENSXMAG00000000882		ENSLOCG00000009141	
ENSXMAG00000018088	smpd2b	ENSLOCG00000009741	smpd2b
ENSXMAG00000004550		ENSLOCG00000010435	
ENSXMAG00000026188		ENSLOCG00000003725	KLHL34
ENSXMAG00000013149	yif1a	ENSLOCG00000000900	yif1a

ENSXMAG00000019767	phlda2	ENSLOCG00000017319	phlda2
ENSXMAG00000007413	rcbtb2	ENSLOCG00000008911	rcbtb2
ENSXMAG00000015851	ptgr2	ENSLOCG00000013550	ptgr2
ENSXMAG00000015382	GPR119	ENSLOCG00000017485	GPR119
ENSXMAG00000014129	sh3rf1	ENSLOCG00000010301	sh3rf1
ENSXMAG00000005678	acap2	ENSLOCG00000004722	acap2
ENSXMAG00000024164	RF00618	ENSLOCG00000019475	RF00618
ENSXMAG00000023618	zgc:110712	ENSLOCG00000013640	zgc:110712
ENSXMAG00000006425	hibch	ENSLOCG00000008833	hibch
ENSXMAG00000014097	setd1ba	ENSLOCG00000005190	setd1ba
ENSXMAG00000027261	RF01290	ENSLOCG00000019026	RF01290
ENSXMAG00000002058	snrnp200	ENSLOCG00000015198	snrnp200
ENSXMAG00000013157	fosl1a	ENSLOCG00000000771	fosl1a
ENSXMAG00000013701		ENSLOCG00000015533	
ENSXMAG00000003172	rprd1a	ENSLOCG00000003632	rprd1a
ENSXMAG00000006658	slc22a15	ENSLOCG00000006947	slc22a15
ENSXMAG00000014400	chrm3b	ENSLOCG00000017368	chrm3b
ENSXMAG00000018609	osbpl5	ENSLOCG00000001627	osbpl5
ENSXMAG00000028013		ENSLOCG00000013149	med11
ENSXMAG00000008138	ddx42	ENSLOCG00000011764	ddx42
ENSXMAG00000019513	ccdc85b	ENSLOCG00000017605	ccdc85b
ENSXMAG00000025800	hapln1b	ENSLOCG00000001981	hapln1b
ENSXMAG00000029954		ENSLOCG00000001920	
ENSXMAG00000014326	zfpl1	ENSLOCG00000002285	
ENSXMAG00000026653		ENSLOCG00000017834	
ENSXMAG00000009549	opn1sw2	ENSLOCG00000014721	opn1sw2
ENSXMAG00000013152	fibpa	ENSLOCG00000000738	fibpb
ENSXMAG00000015860	tmem62	ENSLOCG00000013540	tmem62
ENSXMAG00000004349	atg13	ENSLOCG00000005677	atg13
ENSXMAG00000004576		ENSLOCG00000000553	
ENSXMAG00000005904	pms1	ENSLOCG00000008805	pms1
ENSXMAG00000007407		ENSLOCG00000008861	
ENSXMAG00000006721	celsr1a	ENSLOCG00000016061	celsr1a
ENSXMAG00000018596		ENSLOCG00000001607	ptprja
ENSXMAG00000005948	hsf4	ENSLOCG00000007976	hsf4
ENSXMAG00000025461	lyrm5a	ENSLOCG00000015336	lyrm5a
ENSXMAG00000014121	car15	ENSLOCG00000004958	car15
ENSXMAG00000010049	cds1	ENSLOCG00000007080	cds1
ENSXMAG00000005214	fbxl5	ENSLOCG00000009829	fbxl5
ENSXMAG00000008158	pelp1	ENSLOCG00000014096	pelp1
ENSXMAG00000029185	KCNMB4	ENSLOCG00000016217	KCNMB4
ENSXMAG00000007405	LHFPL6	ENSLOCG00000008837	lhfpl6
ENSXMAG00000015362	si:ch211-247j9.1	ENSLOCG00000010063	si:ch211-247j9.1
ENSXMAG00000000197	si:dkey-28e7.3	ENSLOCG00000013653	si:dkey-28e7.3
ENSXMAG00000013764	ankrd6a	ENSLOCG00000016943	ankrd6a
ENSXMAG00000008690	casc1	ENSLOCG00000015337	casc1
ENSXMAG00000013161	efemp2a	ENSLOCG00000000683	efemp2a
ENSXMAG00000005217	rps6ka4	ENSLOCG00000001071	rps6ka4
ENSXMAG00000005897	adat3	ENSLOCG00000017833	adat3
ENSXMAG00000011065	mertka	ENSLOCG00000016537	mertka
ENSXMAG00000005900	ormdl1	ENSLOCG00000008794	ormdl1
ENSXMAG00000003179	nfyc	ENSLOCG00000001376	nfyc
ENSXMAG00000010037	tmem175	ENSLOCG00000007095	tmem175
ENSXMAG00000003638		ENSLOCG00000003389	
ENSXMAG00000013170	npas4a	ENSLOCG00000000650	npas4a
ENSXMAG00000007388	cog6	ENSLOCG00000008824	cog6

ENSXMAG00000027127	strada	ENSLOCG00000011776	strada
ENSXMAG00000025200	pof1b	ENSLOCG00000014948	pof1b
ENSXMAG00000028344	RS1	ENSLOCG00000004091	
ENSXMAG00000024144	zcchc9	ENSLOCG00000005331	zcchc9
ENSXMAG00000029888	PRKAB2	ENSLOCG00000001948	PRKAB2
ENSXMAG00000011784	cc2d2a	ENSLOCG00000009843	cc2d2a
ENSXMAG00000001129	slc13a1	ENSLOCG00000015873	slc13a1
ENSXMAG00000021749	si:dkey-13a21.4	ENSLOCG00000014749	si:dkey-13a21.4
ENSXMAG00000025202	dgcr2	ENSLOCG00000004943	dgcr2
ENSXMAG00000015782	polr3e	ENSLOCG00000008290	polr3e
ENSXMAG00000022299	rab1ba	ENSLOCG00000001709	rab1ba
ENSXMAG00000011053	TMEM87B	ENSLOCG00000016544	TMEM87B
ENSXMAG00000002022	ppef1	ENSLOCG00000007507	ppef1
ENSXMAG00000011494		ENSLOCG00000007989	NAF1
ENSXMAG00000024533	gata1a	ENSLOCG00000014759	gata1a
ENSXMAG00000011170	kbtbd7	ENSLOCG00000005953	kbtbd7
ENSXMAG00000014416	cdan1	ENSLOCG00000013516	cdan1
ENSXMAG00000015351	zgc:158291	ENSLOCG00000014949	zgc:158291
ENSXMAG00000024171	harbi1	ENSLOCG00000005661	harbi1
ENSXMAG00000011171	zgc:101559	ENSLOCG00000005904	zgc:101559
ENSXMAG00000005881	fkbp7	ENSLOCG00000006276	fkbp7
ENSXMAG00000011488		ENSLOCG00000013634	vegfc
ENSXMAG00000023568	si:dkey-35m8.1	ENSLOCG00000003659	si:dkey-35m8.1
ENSXMAG00000002091	TMC1	ENSLOCG00000009593	TMC1
ENSXMAG00000022475	cbfb	ENSLOCG00000007913	cbfb
ENSXMAG00000023368	mrpl11	ENSLOCG00000000617	mrpl11
ENSXMAG00000027454	klf5l	ENSLOCG00000014951	klf5l
ENSXMAG00000011172	katnal1	ENSLOCG00000000149	katnal1
ENSXMAG00000009682	mboat1	ENSLOCG00000008952	MBOAT1
ENSXMAG00000025931	tpd52l1	ENSLOCG00000017266	tpd52l1
ENSXMAG00000016208	wdr45	ENSLOCG00000015571	wdr45
ENSXMAG00000004363		ENSLOCG00000005600	creb3l1
ENSXMAG00000008169	rnf113a	ENSLOCG00000011785	rnf113a
ENSXMAG00000010421	RAB21	ENSLOCG00000016246	RAB21
ENSXMAG00000011046	fbln7	ENSLOCG00000016546	fbln7
ENSXMAG00000009587	si:dkeyp-97e7.9	ENSLOCG00000002918	
ENSXMAG00000010000	prkg2	ENSLOCG00000007145	prkg2
ENSXMAG00000014151	ess2	ENSLOCG00000004923	ess2
ENSXMAG00000027813	ptpn5	ENSLOCG00000001577	ptpn5
ENSXMAG00000004899	slc30a6	ENSLOCG00000015520	slc30a6
ENSXMAG00000022516	tmem265	ENSLOCG00000000575	tmem265
ENSXMAG00000003618	map3k15	ENSLOCG00000003527	map3k15
ENSXMAG00000004365		ENSLOCG00000005631	zgc:110699
ENSXMAG00000005302	akap10	ENSLOCG00000003685	akap10
ENSXMAG00000010676		ENSLOCG00000015872	iqub
ENSXMAG00000007445	klf8	ENSLOCG00000014956	klf8
ENSXMAG00000002032	phka2	ENSLOCG00000007449	phka2
ENSXMAG00000005967	si:dkey-102f14.5	ENSLOCG00000007895	si:dkey-102f14.5
ENSXMAG00000024025	syne3	ENSLOCG00000008520	syne3
ENSXMAG00000006697	trmu	ENSLOCG00000016066	trmu
ENSXMAG00000003184	KCNQ4	ENSLOCG00000001349	KCNQ4
ENSXMAG00000023222	LGR5	ENSLOCG00000016234	LGR5
ENSXMAG00000016229	fbxw2	ENSLOCG00000002095	fbxw2
ENSXMAG00000026559	c1qtnf7	ENSLOCG00000009865	c1qtnf7
ENSXMAG00000004579	dcun1d1	ENSLOCG00000000735	DCUN1D1
ENSXMAG00000020822	zc3h6	ENSLOCG00000016548	zc3h6

ENSXMAG00000011484		ENSLOCG00000010150	KLHL12
ENSXMAG00000005320	zgc:85789	ENSLOCG00000003948	zgc:85789
ENSXMAG00000009771	smtnl1	ENSLOCG00000006926	
ENSXMAG00000009595	fam83d	ENSLOCG00000004621	fam83d
ENSXMAG00000021029	e2f3	ENSLOCG00000008942	e2f3
ENSXMAG00000023694	tprkb	ENSLOCG00000016078	tprkb
ENSXMAG00000015338	rbm41	ENSLOCG00000014958	rbm41
ENSXMAG00000029104	dusp8a	ENSLOCG00000001567	dusp8a
ENSXMAG00000029374	hddc2	ENSLOCG00000017267	hddc2
ENSXMAG00000011189		ENSLOCG00000007951	si:dkey-100n23.5
ENSXMAG00000023631	sp8b	ENSLOCG00000011486	sp8b
ENSXMAG00000024391	cldn2	ENSLOCG00000017486	cldn2
ENSXMAG00000019559	setd9	ENSLOCG00000007551	setd9
ENSXMAG00000029116		ENSLOCG00000016552	
ENSXMAG00000006692	si:ch73-263o4.3	ENSLOCG00000016071	si:ch73-263o4.3
ENSXMAG00000022623	mob2a	ENSLOCG00000001539	
ENSXMAG00000015871	sptb	ENSLOCG00000013491	sptb
ENSXMAG00000010861	macc1	ENSLOCG00000011422	macc1
ENSXMAG00000016267		ENSLOCG00000017043	
ENSXMAG00000004590	eif4a2	ENSLOCG00000000847	eif4a2
ENSXMAG00000014417	hyal6	ENSLOCG00000015861	hyal6
ENSXMAG00000014175	rnf185	ENSLOCG00000004810	rnf185
ENSXMAG00000006413	agap1	ENSLOCG00000004775	agap1
ENSXMAG00000011540	ptpn1	ENSLOCG00000002289	ptpn1
ENSXMAG00000015821	si:ch73-127m5.1	ENSLOCG00000012866	
ENSXMAG00000007159	rnf217	ENSLOCG00000017264	rnf217
ENSXMAG00000004903	sult6b1	ENSLOCG00000015518	sult6b1
ENSXMAG00000002128	cpeb2	ENSLOCG00000009876	cpeb2
ENSXMAG00000000209	jupa	ENSLOCG00000013664	jupb
ENSXMAG00000013181	slc25a43	ENSLOCG00000015228	slc25a43
ENSXMAG00000005638	psmd1	ENSLOCG00000004682	psmd1
ENSXMAG00000026196	atp6v1d	ENSLOCG00000014159	atp6v1d
ENSXMAG00000005991	b3gnt9	ENSLOCG00000017599	b3gnt9
ENSXMAG00000006682	alg10	ENSLOCG00000016079	alg10
ENSXMAG00000009998	bmp3	ENSLOCG00000007173	bmp3
ENSXMAG00000025030	slc35b1	ENSLOCG00000011700	slc35b1
ENSXMAG00000014418	hyal4	ENSLOCG00000015859	hyal4
ENSXMAG00000019665	cebpb	ENSLOCG00000018358	cebpb
ENSXMAG00000025600		ENSLOCG00000016212	myrfl
ENSXMAG00000005326	aspa	ENSLOCG00000003642	aspa
ENSXMAG00000014421	acbd4	ENSLOCG00000011535	acbd4
ENSXMAG00000026465	kcnj11	ENSLOCG00000002016	kcnj11
ENSXMAG00000015329	clip2	ENSLOCG00000002159	clip2
ENSXMAG00000009663	CDKAL1	ENSLOCG00000008933	cdkal1
ENSXMAG00000023851	RF01299	ENSLOCG00000019102	RF01299
ENSXMAG00000023340		ENSLOCG00000002982	
ENSXMAG00000007157	nkain2	ENSLOCG00000017263	nkain2
ENSXMAG00000015258	zrsr2	ENSLOCG00000007427	zrsr2
ENSXMAG00000007924	cfap300	ENSLOCG00000005645	cfap300
ENSXMAG00000013187	lnx2b	ENSLOCG00000015226	lnx2b
ENSXMAG00000014419	spam1	ENSLOCG00000015858	spam1
ENSXMAG00000006003	bbs2	ENSLOCG00000007883	bbs2
ENSXMAG00000017281	eif2s1b	ENSLOCG00000014165	eif2s1b
ENSXMAG00000006615	CPNE8	ENSLOCG00000016081	CPNE8
ENSXMAG00000002103	cyp1d1	ENSLOCG00000009638	cyp1d1
ENSXMAG00000003194	TINAGL1	ENSLOCG00000001320	TINAGL1

ENSXMAG00000019243	abcc8	ENSLOCG00000002032	abcc8
ENSXMAG00000015231	TM6SF1	ENSLOCG00000012911	TM6SF1
ENSXMAG00000016290		ENSLOCG00000009107	pam
ENSXMAG00000023688		ENSLOCG00000007805	
ENSXMAG00000015211	BTBD1	ENSLOCG00000012920	BTBD1
ENSXMAG00000022690	fgf5	ENSLOCG00000007208	fgf5
ENSXMAG00000015765		ENSLOCG00000013649	TENM3
ENSXMAG00000015721	dicer1	ENSLOCG00000008544	dicer1
ENSXMAG00000009633	cyldb	ENSLOCG00000004523	cyldb
ENSXMAG00000021288	CCT2	ENSLOCG00000016196	cct2
ENSXMAG00000013190	chic1	ENSLOCG00000015222	chic1
ENSXMAG00000015208	RAMAC	ENSLOCG00000012944	RAMAC
ENSXMAG00000021552	prdm8b	ENSLOCG00000007226	prdm8b
ENSXMAG00000026014	tmem119b	ENSLOCG00000004681	TMEM119
ENSXMAG00000014445	atad1b	ENSLOCG00000006195	atad1b
ENSXMAG00000014447	tes	ENSLOCG00000015603	tes
ENSXMAG00000023458	unc50	ENSLOCG00000001604	unc50
ENSXMAG00000013843	ankrd28b	ENSLOCG00000006030	ankrd28b
ENSXMAG00000013191	cdx4	ENSLOCG00000015221	cdx4
ENSXMAG00000006024	SETD6	ENSLOCG00000006305	setd6
ENSXMAG00000003208	ubqln4	ENSLOCG00000008897	ubqln4
ENSXMAG00000013814	ndnf	ENSLOCG00000009792	ndnf
ENSXMAG00000022618	gbx2	ENSLOCG00000004803	gbx2
ENSXMAG00000002059	HOMER2	ENSLOCG00000012948	homer2
ENSXMAG00000005676	htr2b	ENSLOCG00000004704	htr2b
ENSXMAG00000015906	snap23.1	ENSLOCG00000013482	snap23.1
ENSXMAG00000029118		ENSLOCG00000004647	selplg
ENSXMAG00000008203	nxph3	ENSLOCG00000011674	nxph3
ENSXMAG00000026123	golga4	ENSLOCG00000004917	golga4
ENSXMAG00000003596	sh3kbp1	ENSLOCG00000003567	sh3kbp1
ENSXMAG00000011578	slc52a3	ENSLOCG00000002259	slc52a3
ENSXMAG00000019258	ush1c	ENSLOCG00000002073	ush1c
ENSXMAG00000011198	tfg	ENSLOCG00000009472	tfg
ENSXMAG00000016330	pomk	ENSLOCG00000012140	pomk
ENSXMAG00000023496	her6	ENSLOCG00000004988	her6
ENSXMAG00000028567	scrt2	ENSLOCG00000002246	scrt2
ENSXMAG00000000219	armh3	ENSLOCG00000012746	armh3
ENSXMAG00000022547	srxn1	ENSLOCG00000002239	srxn1
ENSXMAG00000005414	gsc	ENSLOCG00000008557	gsc
ENSXMAG00000013193	kdrl	ENSLOCG00000015218	kdrl
ENSXMAG00000006031	cnot1	ENSLOCG00000006324	cnot1
ENSXMAG00000009644	trpc4apa	ENSLOCG00000004284	trpc4apa
ENSXMAG00000011591	tcf15	ENSLOCG00000002219	tcf15
ENSXMAG00000013825	prdm5	ENSLOCG00000009778	prdm5
ENSXMAG00000017313	hsp90ab1	ENSLOCG00000016396	hsp90ab1
ENSXMAG00000028418	cav2	ENSLOCG00000015600	cav2
ENSXMAG00000002066	WHAMM	ENSLOCG00000012953	WHAMM
ENSXMAG00000015294	gtf2ird1	ENSLOCG00000002184	gtf2ird1
ENSXMAG00000003247		ENSLOCG00000006661	
ENSXMAG00000007112	med23	ENSLOCG00000017274	med23
ENSXMAG00000023073	cav1	ENSLOCG00000015597	cav1
ENSXMAG00000015719	mgst1.1	ENSLOCG00000016144	mgst1.1
ENSXMAG00000027040	ABR	ENSLOCG00000003587	abr
ENSXMAG00000015026	rnls	ENSLOCG00000006122	rnls
ENSXMAG00000021887	slc13a3	ENSLOCG00000002096	SLC13A3
ENSXMAG00000002076	FSD2	ENSLOCG00000012962	FSD2

ENSXMAG00000015925	ptbp1a	ENSLOCG00000001702	ptbp1a
ENSXMAG00000022573	PGA3	ENSLOCG00000016198	nots
ENSXMAG00000026965	gbe1b	ENSLOCG00000008974	gbe1b
ENSXMAG00000010991	KCNK12	ENSLOCG00000016299	KCNK12
ENSXMAG00000015445	lipf	ENSLOCG00000006105	lipf
ENSXMAG00000014457	met	ENSLOCG00000015595	met
ENSXMAG00000009656		ENSLOCG00000018365	sox12
ENSXMAG00000003253	kat2b	ENSLOCG00000006617	kat2b
ENSXMAG00000002145	si:dkey-229d2.4	ENSLOCG00000009703	si:dkey-229d2.4
ENSXMAG00000026313	slc35b2	ENSLOCG00000016398	slc35b2
ENSXMAG00000005593	opa1	ENSLOCG00000005004	opa1
ENSXMAG00000005811	wdr75	ENSLOCG00000008719	wdr75
ENSXMAG00000010986	msh6	ENSLOCG00000016298	msh6
ENSXMAG00000004672	si:ch211-38m6.7	ENSLOCG00000001177	si:ch211-38m6.7
ENSXMAG00000019260	ccdc77	ENSLOCG00000017079	
ENSXMAG00000015701	galcb	ENSLOCG00000008580	galcb
ENSXMAG00000014471	CAPZA2	ENSLOCG00000015593	CAPZA2
ENSXMAG00000011632	ocstamp	ENSLOCG00000002083	
ENSXMAG00000015255	dtx2	ENSLOCG00000002231	dtx2
ENSXMAG00000012859		ENSLOCG00000010098	
ENSXMAG00000028942	ACYP2	ENSLOCG00000015524	
ENSXMAG00000013857	hacl1	ENSLOCG00000006085	hacl1
ENSXMAG00000024291	nfkbie	ENSLOCG00000016399	nfkbie
ENSXMAG00000005009	ABCD2	ENSLOCG00000016096	abcd2
ENSXMAG00000029620	slc40a1	ENSLOCG00000008735	slc40a1
ENSXMAG00000009913	bmp2k	ENSLOCG00000007340	bmp2k
ENSXMAG00000026267	si:ch211-25d12.7	ENSLOCG00000004198	
ENSXMAG00000008609	camsap2b	ENSLOCG00000007726	camsap2b
ENSXMAG00000005263	DCDC2	ENSLOCG00000008919	DCDC2
ENSXMAG00000011634		ENSLOCG00000002043	
ENSXMAG00000015485	stambpl1	ENSLOCG00000006073	stambpl1
ENSXMAG00000004417	st7	ENSLOCG00000015591	st7
ENSXMAG00000002082	pde8a	ENSLOCG00000012971	pde8a
ENSXMAG00000019263	hdhd5	ENSLOCG00000017080	
ENSXMAG00000016377	reep5	ENSLOCG00000009034	reep5
ENSXMAG00000008457	cops9	ENSLOCG00000007062	cops9
ENSXMAG00000022447		ENSLOCG00000006834	znf365
ENSXMAG00000028869	tmem132e	ENSLOCG00000002145	tmem132e
ENSXMAG00000013218	tex11	ENSLOCG00000015211	tex11
ENSXMAG00000015567	rorb	ENSLOCG00000009712	rorb
ENSXMAG00000016389		ENSLOCG00000015581	
ENSXMAG00000025261	ehd3	ENSLOCG00000016786	ehd3
ENSXMAG00000022758	slc6a3	ENSLOCG00000008899	slc6a3
ENSXMAG00000024562	mif	ENSLOCG00000006798	mif
ENSXMAG00000014319	OSBPL10	ENSLOCG00000002233	osbpl10b
ENSXMAG00000008456	otos	ENSLOCG00000017403	otos
ENSXMAG00000004673	ephb3a	ENSLOCG00000008630	EPHB3
ENSXMAG00000004456	wnt2	ENSLOCG00000015590	wnt2
ENSXMAG00000005802	asdurf	ENSLOCG00000008747	asdurf
ENSXMAG00000023469	si:ch211-216l23.2	ENSLOCG00000007455	si:ch211-216l23.2
ENSXMAG00000017355	tcte1	ENSLOCG00000016401	tcte1
ENSXMAG00000003276	otud6b	ENSLOCG00000010181	otud6b
ENSXMAG00000010734	dnpep	ENSLOCG00000010615	dnpep
ENSXMAG00000017358	rnf8	ENSLOCG00000016402	rnf8
ENSXMAG00000021236	zgc:153293	ENSLOCG00000006433	CCDC34
ENSXMAG00000011651		ENSLOCG00000004233	

ENSXMAG00000011477	rad51d	ENSLOCG00000002045	rad51d
ENSXMAG00000014458	asz1	ENSLOCG00000015587	asz1
ENSXMAG00000005737	sympk	ENSLOCG00000014229	
ENSXMAG00000002105	gatm	ENSLOCG00000012986	gatm
ENSXMAG00000028003	ch25h	ENSLOCG00000018232	ch25h
ENSXMAG00000003286		ENSLOCG00000018325	
ENSXMAG00000017367	aida	ENSLOCG00000016416	aida
ENSXMAG00000019266	SLC15A5	ENSLOCG00000016146	SLC15A5
ENSXMAG00000015522		ENSLOCG00000000031	rpp30
ENSXMAG00000021240	irx2a	ENSLOCG00000008842	irx2a
ENSXMAG00000007081	stum	ENSLOCG00000016785	stum
ENSXMAG00000008248	ubtf	ENSLOCG00000012057	ubtf
ENSXMAG00000029124	runx1t1	ENSLOCG00000010158	runx1t1
ENSXMAG00000022002	irx1a	ENSLOCG00000008834	irx1a
ENSXMAG00000001559	POLR2F	ENSLOCG00000011827	POLR2F
ENSXMAG00000025395		ENSLOCG00000008532	NUDT13
ENSXMAG00000011661	pan2	ENSLOCG00000004333	pan2
ENSXMAG00000011457	lig3	ENSLOCG00000002087	lig3
ENSXMAG00000015525	trpm6	ENSLOCG00000009721	trpm6
ENSXMAG00000006113	ZNF423	ENSLOCG00000007466	ZNF423
ENSXMAG00000004751	zgc:66475	ENSLOCG00000008545	zgc:66475
ENSXMAG00000006603	slco1e1	ENSLOCG00000015401	slco1e1
ENSXMAG00000004259	bmpr1aa	ENSLOCG00000012466	bmpr1aa
ENSXMAG00000015879	sec24c	ENSLOCG00000012547	sec24c
ENSXMAG00000019268	tmem17	ENSLOCG00000007806	tmem17
ENSXMAG00000010472	BEST3	ENSLOCG00000016201	BEST3
ENSXMAG00000006463	cftr	ENSLOCG00000015585	cftr
ENSXMAG00000025918		ENSLOCG00000016418	mia3
ENSXMAG00000025486	zdhhc3b	ENSLOCG00000002134	zdhhc3b
ENSXMAG00000022841	FOXN2	ENSLOCG00000016292	foxn2b
ENSXMAG00000013874	plekha8	ENSLOCG00000006111	plekha8
ENSXMAG00000027609	triqk	ENSLOCG00000010149	triqk
ENSXMAG00000007045	itpkb	ENSLOCG00000016782	itpkb
ENSXMAG00000005718	SLC35F5	ENSLOCG00000002955	SLC35F5
ENSXMAG00000022282		ENSLOCG00000000094	ticam1
ENSXMAG00000024575	hdc	ENSLOCG00000013010	hdc
ENSXMAG00000010480	si:ch211-156b7.4	ENSLOCG00000016753	si:ch211-156b7.4
ENSXMAG00000019269	agbl2	ENSLOCG00000006167	agbl2
ENSXMAG00000013842	adamts16	ENSLOCG00000008808	adamts16
ENSXMAG00000004764		ENSLOCG00000008490	FYTTD1
ENSXMAG00000003312	fam92a1	ENSLOCG00000010142	fam92a1
ENSXMAG00000007320	rwdd2b	ENSLOCG00000009019	rwdd2b
ENSXMAG00000011214		ENSLOCG00000007993	atp6ap2
ENSXMAG00000014296	exosc7	ENSLOCG00000002116	exosc7
ENSXMAG00000013224	dlg3	ENSLOCG00000015203	dlg3
ENSXMAG00000011447	JAKMIP1	ENSLOCG00000010109	jakmip1
ENSXMAG00000015545	noc3l	ENSLOCG00000005949	noc3l
ENSXMAG00000015688	si:dkey-288a3.2	ENSLOCG00000008644	si:dkey-288a3.2
ENSXMAG00000007314	usp16	ENSLOCG00000009029	USP16
ENSXMAG00000010942	ppp1r21	ENSLOCG00000016286	PPP1R21
ENSXMAG00000002148	usp8	ENSLOCG00000013028	usp8
ENSXMAG00000008593	capn10	ENSLOCG00000005781	capn10
ENSXMAG00000008271	atxn7l3	ENSLOCG00000012046	atxn7l3
ENSXMAG00000010726		ENSLOCG00000010664	
ENSXMAG00000011676	ormdl2	ENSLOCG00000004357	ormdl2
ENSXMAG00000025382	tmem248	ENSLOCG00000002034	tmem248

ENSXMAG00000000249	afmid	ENSLOCG00000012683	afmid
ENSXMAG00000003315	trib1	ENSLOCG00000010029	trib1
ENSXMAG00000015675	vrk1	ENSLOCG00000008664	vrk1
ENSXMAG00000003047		ENSLOCG00000011873	
ENSXMAG00000011679	nemp1	ENSLOCG00000004393	nemp1
ENSXMAG00000019272	fnbp4	ENSLOCG00000006124	FNBP4
ENSXMAG00000011218	dcbld2	ENSLOCG00000009438	dcbld2
ENSXMAG00000000355	actr1	ENSLOCG00000014980	actr1
ENSXMAG00000004776		ENSLOCG00000001810	TSC22D2
ENSXMAG00000001371	hspb15	ENSLOCG00000003196	hspb15
ENSXMAG00000004892	taf1a	ENSLOCG00000016421	taf1a
ENSXMAG00000019979	gpr39	ENSLOCG00000002937	gpr39
ENSXMAG00000026366	tubgcp3	ENSLOCG00000009147	tubgcp3
ENSXMAG00000002152	USP50	ENSLOCG00000013040	
ENSXMAG00000007004	EPB41L2	ENSLOCG00000017276	EPB41L2
ENSXMAG00000008587	eif4e2rs1	ENSLOCG00000005798	eif4e2rs1
ENSXMAG00000026420	IAPP	ENSLOCG00000015398	IAPP
ENSXMAG00000011682	cd63	ENSLOCG00000004424	cd63
ENSXMAG00000003318	fam91a1	ENSLOCG00000010016	fam91a1
ENSXMAG00000000253	tk1	ENSLOCG00000012676	tk1
ENSXMAG00000020344	BCL11B	ENSLOCG00000008679	BCL11B
ENSXMAG00000023151	dusp10	ENSLOCG00000016394	dusp10
ENSXMAG00000026280	doc2b	ENSLOCG00000002007	doc2b
ENSXMAG00000006587	pyroxd1	ENSLOCG00000015395	pyroxd1
ENSXMAG00000023993		ENSLOCG00000005816	
ENSXMAG00000002154	mapk6	ENSLOCG00000013351	mapk6
ENSXMAG00000024444	si:dkey-167k11.5	ENSLOCG00000011860	si:dkey-167k11.5
ENSXMAG00000010700	nhej1	ENSLOCG00000010861	nhej1
ENSXMAG00000019279	cnot2	ENSLOCG00000016216	cnot2
ENSXMAG00000011684	letmd1	ENSLOCG00000004454	letmd1
ENSXMAG00000015516	carnmt1	ENSLOCG00000009746	carnmt1
ENSXMAG00000005554	atp13a3	ENSLOCG00000004949	atp13a3
ENSXMAG00000008565	chrng	ENSLOCG00000005837	chrng
ENSXMAG00000008276	tmub2	ENSLOCG00000012040	tmub2
ENSXMAG00000028597	setd3	ENSLOCG00000008698	setd3
ENSXMAG00000010933	gtf2a1l	ENSLOCG00000016284	STON1-GTF2A1L
ENSXMAG00000000266	tmc8	ENSLOCG00000012647	tmc8
ENSXMAG00000007980	map4k2	ENSLOCG00000002101	
ENSXMAG00000017389	hlx1	ENSLOCG00000016392	hlx1
ENSXMAG00000013833		ENSLOCG00000008797	ice1
ENSXMAG00000011692	zgc:56699	ENSLOCG00000004469	zgc:56699
ENSXMAG00000013882	chn2	ENSLOCG00000006208	chn2
ENSXMAG00000009796	zgc:92107	ENSLOCG00000004130	
ENSXMAG00000011228	tmem30c	ENSLOCG00000009411	tmem30c
ENSXMAG00000027393		ENSLOCG00000001600	AHSG
ENSXMAG00000004945	arhgap5	ENSLOCG00000009544	arhgap5
ENSXMAG00000008279	asb16	ENSLOCG00000012033	asb16
ENSXMAG00000029658		ENSLOCG00000005875	prss56
ENSXMAG00000002168	tmod2	ENSLOCG00000013334	tmod2
ENSXMAG00000015565	llgl2	ENSLOCG00000011447	llgl2
ENSXMAG00000028971	castor2	ENSLOCG00000001947	castor2
ENSXMAG00000021830	si:ch1073-322p19.1	ENSLOCG00000000550	
ENSXMAG00000000374		ENSLOCG00000015054	
ENSXMAG00000014287		ENSLOCG00000002088	cdcp1a
ENSXMAG00000011243	cmss1	ENSLOCG00000009391	cmss1
ENSXMAG00000028217	etnk1	ENSLOCG00000015350	etnk1

ENSXMAG00000019275	washc4	ENSLOCG00000015056	washc4
ENSXMAG00000015667		ENSLOCG00000012414	YIPF7
ENSXMAG00000011711	grasp	ENSLOCG00000004513	grasp
ENSXMAG00000011487	cttnbp2	ENSLOCG00000015582	cttnbp2
ENSXMAG00000026974	slc35f6	ENSLOCG00000016385	slc35f6
ENSXMAG00000001402	zmat5	ENSLOCG00000003675	zmat5
ENSXMAG00000028562	zgc:153901	ENSLOCG00000005144	zgc:153901
ENSXMAG00000009451	nubpl	ENSLOCG00000009552	nubpl
ENSXMAG00000010925		ENSLOCG00000016282	
ENSXMAG00000027907	si:ch211-212o1.2	ENSLOCG00000007493	si:ch211-212o1.2
ENSXMAG00000012166	cdadc1	ENSLOCG00000009213	cdadc1
ENSXMAG00000008282		ENSLOCG00000012024	si:dkey-260c8.8
ENSXMAG00000013886	cpvl	ENSLOCG00000004266	cpvl
ENSXMAG00000003339	spire1a	ENSLOCG00000009960	spire1a
ENSXMAG00000026713	srebf2	ENSLOCG00000011887	srebf2
ENSXMAG00000007961	men1	ENSLOCG00000002090	men1
ENSXMAG00000027022	lysmd2	ENSLOCG00000013330	lysmd2
ENSXMAG00000017966	nudt3b	ENSLOCG00000009949	nudt3b
ENSXMAG00000011421		ENSLOCG00000009674	TLX3
ENSXMAG00000006550	sox5	ENSLOCG00000015347	sox5
ENSXMAG00000028556	yipf6	ENSLOCG00000014686	yipf6
ENSXMAG00000011469	snd1	ENSLOCG00000015537	snd1
ENSXMAG00000007134	zgc:153521	ENSLOCG00000005160	zgc:153521
ENSXMAG00000002929	fras1	ENSLOCG00000007363	fras1
ENSXMAG00000002179	scg3	ENSLOCG00000013323	scg3
ENSXMAG00000013255	gdpd2	ENSLOCG00000015200	gdpd2
ENSXMAG00000022295	cbln1	ENSLOCG00000007512	cbln1
ENSXMAG00000017408	enpp1	ENSLOCG00000016378	enpp1
ENSXMAG00000028872	rps10	ENSLOCG00000009959	rps10
ENSXMAG00000017334	cab39l	ENSLOCG00000009202	cab39l
ENSXMAG00000025493	stk17b	ENSLOCG00000005185	stk17b
ENSXMAG00000006371	cybb	ENSLOCG00000007849	cybb
ENSXMAG00000025147	zgc:113279	ENSLOCG00000003506	zgc:113279
ENSXMAG00000015656	alkbh1	ENSLOCG00000008800	alkbh1
ENSXMAG00000016783		ENSLOCG00000014634	
ENSXMAG00000006141	n4bp1	ENSLOCG00000007525	n4bp1
ENSXMAG00000007306	zgc:110333	ENSLOCG00000009128	zgc:110333
ENSXMAG00000008429	THNSL1	ENSLOCG00000018121	THNSL1
ENSXMAG00000021452		ENSLOCG00000009686	
ENSXMAG00000013891	creb5b	ENSLOCG00000011905	creb5b
ENSXMAG00000017529	si:dkey-194e6.1	ENSLOCG00000007683	si:dkey-194e6.1
ENSXMAG00000019126	dtd2	ENSLOCG00000009563	dtd2
ENSXMAG00000007617	galk1	ENSLOCG00000011468	galk1
ENSXMAG00000016809		ENSLOCG00000015150	fam219b
ENSXMAG00000011797	rnf41	ENSLOCG00000004550	rnf41
ENSXMAG00000015645	adck1	ENSLOCG00000008823	adck1
ENSXMAG00000017540	reps2	ENSLOCG00000007659	reps2
ENSXMAG00000001438	srsf9	ENSLOCG00000004044	srsf9
ENSXMAG00000002189	tnfaip8l3	ENSLOCG00000013314	tnfaip8l3
ENSXMAG00000014958	vwa5b2	ENSLOCG00000005907	vwa5b2
ENSXMAG00000012244	nf1a	ENSLOCG00000000318	
ENSXMAG00000006366	dusp27	ENSLOCG00000009695	dusp27
ENSXMAG00000007931	ecsit	ENSLOCG00000007679	
ENSXMAG00000025765	pdzd11	ENSLOCG00000015196	pdzd11
ENSXMAG00000003001	bbs1	ENSLOCG00000002571	
ENSXMAG00000013895	jazf1b	ENSLOCG00000011888	jazf1b

ENSXMAG00000011467	pax4	ENSLOCG00000015535	pax4
ENSXMAG00000019122	ATG14	ENSLOCG00000011741	ATG14
ENSXMAG00000017942	SPDEF	ENSLOCG00000009979	SPDEF
ENSXMAG00000000292	aspscr1	ENSLOCG00000012603	aspscr1
ENSXMAG00000000390	hpse	ENSLOCG00000012076	hpse
ENSXMAG00000011261	NR0B1	ENSLOCG00000009335	nr0b1
ENSXMAG00000017437	ezrb	ENSLOCG00000016372	ezra
ENSXMAG00000006148	tango6	ENSLOCG00000002394	tango6
ENSXMAG00000007189	PGAP1	ENSLOCG00000005267	PGAP1
ENSXMAG00000003973	pdzd2	ENSLOCG00000006774	pdzd2
ENSXMAG00000011315	DOCK2	ENSLOCG00000009726	DOCK2
ENSXMAG00000015003	alg3	ENSLOCG00000005922	alg3
ENSXMAG00000015932	ldb3b	ENSLOCG00000001141	ldb3b
ENSXMAG00000014201	rps5	ENSLOCG00000000731	rps5
ENSXMAG00000002203	gldn	ENSLOCG00000013299	gldn
ENSXMAG00000020176	RAI2	ENSLOCG00000017793	RAI2
ENSXMAG00000016831	lrrc28	ENSLOCG00000014419	lrrc28
ENSXMAG00000007896	eml3	ENSLOCG00000002062	
ENSXMAG00000029524	CXorf21	ENSLOCG00000017801	CXorf21
ENSXMAG00000006159	has3	ENSLOCG00000002379	has3
ENSXMAG00000008416	fbxl7	ENSLOCG00000011912	fbxl7
ENSXMAG00000006329		ENSLOCG00000016711	
ENSXMAG00000015610	nrxn3a	ENSLOCG00000008835	nrxn3a
ENSXMAG00000000406	ppp3ccb	ENSLOCG00000015419	ppp3ccb
ENSXMAG00000006534	bcat1	ENSLOCG00000015344	bcat1
ENSXMAG00000015032	mul1a	ENSLOCG00000005991	mul1a
ENSXMAG00000022554	rnaseh2b	ENSLOCG00000009296	rnaseh2b
ENSXMAG00000007203	maip1	ENSLOCG00000005288	maip1
ENSXMAG00000006168	chtf8	ENSLOCG00000002367	chtf8
ENSXMAG00000013003	etfbkmt	ENSLOCG00000015095	etfbkmt
ENSXMAG00000003967	golph3	ENSLOCG00000006756	golph3
ENSXMAG00000005351	specc1	ENSLOCG00000003564	specc1
ENSXMAG00000015044	camk2n1a	ENSLOCG00000005980	camk2n1a
ENSXMAG00000007207	tyw5	ENSLOCG00000005300	tyw5
ENSXMAG00000014183	ddah2	ENSLOCG00000000751	ddah2
ENSXMAG00000008412	zgc:111976	ENSLOCG00000011924	zgc:111976
ENSXMAG00000019286	si:ch73-62b13.1	ENSLOCG00000015269	si:ch73-62b13.1
ENSXMAG00000006364	zgc:194275	ENSLOCG00000016725	zgc:194275
ENSXMAG00000019518	trim13	ENSLOCG00000009265	trim13
ENSXMAG00000022609	si:dkey-3h3.3	ENSLOCG00000006799	si:dkey-3h3.3
ENSXMAG00000006171	utp4	ENSLOCG00000002332	utp4
ENSXMAG00000015473	bag4	ENSLOCG00000015148	bag4
ENSXMAG00000017455	rsph3	ENSLOCG00000016370	rsph3
ENSXMAG00000013899	hibadhb	ENSLOCG00000011866	hibadhb
ENSXMAG00000026314		ENSLOCG00000012576	
ENSXMAG00000000884	dnah5	ENSLOCG00000000680	dnah5
ENSXMAG00000011274	sprydd7b	ENSLOCG00000009258	spryd7b
ENSXMAG00000026519	isl1	ENSLOCG00000006816	isl1
ENSXMAG00000023135	adora2b	ENSLOCG00000003553	adora2b
ENSXMAG00000002209	dmxl2	ENSLOCG00000013273	dmxl2
ENSXMAG00000012959	sirt7	ENSLOCG00000012572	sirt7
ENSXMAG00000019287	zgc:153031	ENSLOCG00000015270	zgc:153031
ENSXMAG00000026637	lsm1	ENSLOCG00000015149	lsm1
ENSXMAG00000006312	map3k2	ENSLOCG00000010929	map3k2
ENSXMAG00000004807	cdc73	ENSLOCG00000007403	cdc73
ENSXMAG00000005536	lsg1	ENSLOCG00000004918	lsg1

ENSXMAG00000006143	ndufa6	ENSLOCG00000011929	ndufa6
ENSXMAG00000017458	tagapb	ENSLOCG00000016367	tagapb
ENSXMAG00000015855	RS1	ENSLOCG00000007527	RS1
ENSXMAG00000011285	kpna3	ENSLOCG00000009241	kpna3
ENSXMAG00000005379	zswim7	ENSLOCG00000003544	zswim7
ENSXMAG00000015049	ece2a	ENSLOCG00000005939	ece2a
ENSXMAG00000011708	gtse1	ENSLOCG00000016073	GTSE1
ENSXMAG00000004357	top3b	ENSLOCG00000002058	top3b
ENSXMAG00000029718	dmtn	ENSLOCG00000014865	DMTN
ENSXMAG00000022627	dio2	ENSLOCG00000008880	dio2
ENSXMAG00000026299	ralaa	ENSLOCG00000011940	ralaa
ENSXMAG00000006193	cmip	ENSLOCG00000002603	cmip
ENSXMAG00000017463	ppp2r5a	ENSLOCG00000016361	ppp2r5a
ENSXMAG00000002920	mrpl1	ENSLOCG00000007408	mrpl1
ENSXMAG00000006306		ENSLOCG00000010236	sumo1
ENSXMAG00000029122	map6d1	ENSLOCG00000006098	map6d1
ENSXMAG00000029882	adnp2b	ENSLOCG00000008400	adnp2b
ENSXMAG00000012229	cuedc1b	ENSLOCG00000004806	cuedc1b
ENSXMAG00000006532	lrmp	ENSLOCG00000015340	lrmp
ENSXMAG00000017919		ENSLOCG00000010042	TCP11X2
ENSXMAG00000013906	hoxa9b	ENSLOCG00000011824	
ENSXMAG00000028247	dapp1	ENSLOCG00000011693	dapp1
ENSXMAG00000025033	pbdc1	ENSLOCG00000014881	pbdc1
ENSXMAG00000015390	star	ENSLOCG00000015158	star
ENSXMAG00000005388	ncor1	ENSLOCG00000003513	ncor1
ENSXMAG00000000846	tmem200a	ENSLOCG00000017883	tmem200a
ENSXMAG00000008398	cdk13	ENSLOCG00000011954	cdk13
ENSXMAG00000017491	dtl	ENSLOCG00000016359	dtl
ENSXMAG00000027482	id1	ENSLOCG00000004086	id1
ENSXMAG00000011840	si:dkey-190g6.2	ENSLOCG00000004633	si:dkey-190g6.2
ENSXMAG00000012222		ENSLOCG00000004703	akap1b
ENSXMAG00000015594	lamtor3	ENSLOCG00000011703	lamtor3
ENSXMAG00000003367		ENSLOCG00000001935	ggcta
ENSXMAG00000002900	cnot6l	ENSLOCG00000007420	cnot6l
ENSXMAG00000006152	poldip3	ENSLOCG00000011953	poldip3
ENSXMAG00000023885	cth1	ENSLOCG00000002047	cth1
ENSXMAG00000015052	yeats2	ENSLOCG00000006062	yeats2
ENSXMAG00000013908	skap2	ENSLOCG00000011789	skap2
ENSXMAG00000013266	stard14	ENSLOCG00000015192	stard14
ENSXMAG00000028565	elmod3	ENSLOCG00000015165	elmod3
ENSXMAG00000004356		ENSLOCG00000002076	
ENSXMAG00000004825	trove2	ENSLOCG00000007384	trove2
ENSXMAG00000026722	cs	ENSLOCG00000004655	cs
ENSXMAG00000007835	taf6l	ENSLOCG00000002156	taf6l
ENSXMAG00000005534	fam43a	ENSLOCG00000017393	fam43a
ENSXMAG00000015859		ENSLOCG00000013678	dctd
ENSXMAG00000003368	snrpd1	ENSLOCG00000002003	snrpd1
ENSXMAG00000027626	ap3s2	ENSLOCG00000014474	ap3s2
ENSXMAG00000023928	BET1L	ENSLOCG00000002376	BET1L
ENSXMAG00000000433	xpo7	ENSLOCG00000014884	xpo7
ENSXMAG00000004348	ppm1f	ENSLOCG00000002046	ppm1f
ENSXMAG00000017503	ints7	ENSLOCG00000016358	ints7
ENSXMAG00000012171	nlk2	ENSLOCG00000005662	nlk2
ENSXMAG00000005526	xxylt1	ENSLOCG00000004904	xxylt1
ENSXMAG00000013352	deaf1	ENSLOCG00000002360	deaf1
ENSXMAG00000015571	atp10d	ENSLOCG00000011676	atp10d

ENSXMAG00000025059	SHROOM2	ENSLOCG00000007347	SHROOM2
ENSXMAG00000017542	cep44	ENSLOCG00000013590	cep44
ENSXMAG00000014152	csnk2b	ENSLOCG00000000693	csnk2b
ENSXMAG00000028479	zgc:64201	ENSLOCG00000015559	zgc:64201
ENSXMAG00000008316		ENSLOCG00000011590	
ENSXMAG00000011867		ENSLOCG00000004688	
ENSXMAG00000006216	GAN	ENSLOCG00000002584	GAN
ENSXMAG00000007822		ENSLOCG00000002130	sf1
ENSXMAG00000006512	slc5a8	ENSLOCG00000015201	slc5a8
ENSXMAG00000011362	emsy	ENSLOCG00000004825	emsy
ENSXMAG00000006983		ENSLOCG00000000955	fkbp6
ENSXMAG00000028712	gphb5	ENSLOCG00000010213	GPHB5
ENSXMAG00000011329		ENSLOCG00000009220	
ENSXMAG00000012930	sgsh	ENSLOCG00000012535	sgsh
ENSXMAG00000019211	mlc1	ENSLOCG00000015509	mlc1
ENSXMAG00000015080	klhl24b	ENSLOCG00000006049	klhl24b
ENSXMAG00000017544	fbxo8	ENSLOCG00000013587	fbxo8
ENSXMAG00000013913	pde11al	ENSLOCG00000011716	pde11al
ENSXMAG00000019933	CLDN34	ENSLOCG00000017792	CLDN34
ENSXMAG00000005418	pigl	ENSLOCG00000003499	pigl
ENSXMAG00000000171		ENSLOCG00000015551	rps27a
ENSXMAG00000006231		ENSLOCG00000006856	
ENSXMAG00000017549	lpgat1	ENSLOCG00000016357	lpgat1
ENSXMAG00000015352	pold2	ENSLOCG00000015171	pold2
ENSXMAG00000023432	hand2	ENSLOCG00000013581	hand2
ENSXMAG00000004848	rgs1	ENSLOCG00000007316	rgs1
ENSXMAG00000008366	NLRX1	ENSLOCG00000003928	nlrx1
ENSXMAG00000006948		ENSLOCG00000017279	arhgap18
ENSXMAG00000006232	fam192a	ENSLOCG00000007733	fam192a
ENSXMAG00000028354	lrrc32	ENSLOCG00000004838	lrrc32
ENSXMAG00000005423	cenpv	ENSLOCG00000003481	cenpv
ENSXMAG00000012920	slc26a11	ENSLOCG00000012525	slc26a11
ENSXMAG00000015873	wwc3	ENSLOCG00000007321	wwc3
ENSXMAG00000028801	zgc:113054	ENSLOCG00000010041	zgc:113054
ENSXMAG00000026428	cops8	ENSLOCG00000004879	cops8
ENSXMAG00000005486	ankrd13c	ENSLOCG00000010043	ankrd13c
ENSXMAG00000015872	zgc:171887	ENSLOCG00000000556	zgc:171887
ENSXMAG00000006503	utp20	ENSLOCG00000015197	utp20
ENSXMAG00000002234	sema6dl	ENSLOCG00000013259	sema6dl
ENSXMAG00000017560	nek2	ENSLOCG00000016356	nek2
ENSXMAG00000001567		ENSLOCG00000012656	WDR70
ENSXMAG00000025973		ENSLOCG00000017355	
ENSXMAG00000002883		ENSLOCG00000006247	slc25a23b
ENSXMAG00000026479	trpc7b	ENSLOCG00000011343	TRPC7
ENSXMAG00000028350	rgs18	ENSLOCG00000007296	rgs18
ENSXMAG00000015990	ppm1bb	ENSLOCG00000016386	ppm1bb
ENSXMAG00000019883	nono	ENSLOCG00000014739	nono
ENSXMAG00000011358	mpzl1l	ENSLOCG00000004876	mpzl1l
ENSXMAG00000019204	panx2	ENSLOCG00000015503	panx2
ENSXMAG00000027001	si:dkeyp-94h10.5	ENSLOCG00000006029	si:dkeyp-94h10.5
ENSXMAG00000012552	p2rx5	ENSLOCG00000003384	p2rx8
ENSXMAG00000012154	efhd1	ENSLOCG00000009199	efhd1
ENSXMAG00000023943	si:ch211-210c8.6	ENSLOCG00000004747	si:ch211-210c8.6
ENSXMAG00000008324	tmem98	ENSLOCG00000011552	tmem98
ENSXMAG00000013397	dldh	ENSLOCG00000015897	dldh
ENSXMAG00000015318	NPC1L1	ENSLOCG00000015181	NPC1L1

ENSXMAG00000015895	traf3ip2l	ENSLOCG00000010562	traf3ip2l
ENSXMAG00000006239	zc3h18	ENSLOCG00000002443	zc3h18
ENSXMAG00000013289	inppl1b	ENSLOCG00000015182	inppl1b
ENSXMAG00000012143	atp6v1b2	ENSLOCG00000014912	atp6v1b2
ENSXMAG00000012145	kcnj13	ENSLOCG00000009192	kcnj13
ENSXMAG00000015093	pask	ENSLOCG00000006009	pask
ENSXMAG00000006913	sel1l	ENSLOCG00000013123	sel1l
ENSXMAG00000013920		ENSLOCG00000011709	C7orf31
ENSXMAG00000022221	trabd	ENSLOCG00000015501	trabd
ENSXMAG00000007253	urb1	ENSLOCG00000009190	urb1
ENSXMAG00000011320	rabep1	ENSLOCG00000002386	rabep1
ENSXMAG00000023136	slc30a1a	ENSLOCG00000016355	slc30a1a
ENSXMAG00000000174		ENSLOCG00000015545	rtn4a
ENSXMAG00000015994	mta3	ENSLOCG00000016454	mta3
ENSXMAG00000016983	tmed3	ENSLOCG00000014324	tmed3
ENSXMAG00000020012	emc6	ENSLOCG00000017425	emc6
ENSXMAG00000015898	chd1l	ENSLOCG00000010574	chd1l
ENSXMAG00000005428	shpk	ENSLOCG00000003362	shpk
ENSXMAG00000017079		ENSLOCG00000003049	
ENSXMAG00000029661	noxa1	ENSLOCG00000001867	NOXA1
ENSXMAG00000023298	rd3	ENSLOCG00000016354	rd3
ENSXMAG00000007722	zgc:112271	ENSLOCG00000013282	zgc:112271
ENSXMAG00000028643		ENSLOCG00000015075	
ENSXMAG00000015562	commd8	ENSLOCG00000011660	commd8
ENSXMAG00000013405	SYT8	ENSLOCG00000002263	SYT8
ENSXMAG00000005461	cthl	ENSLOCG00000010025	cthl
ENSXMAG00000017585	rhag	ENSLOCG00000016348	rhag
ENSXMAG00000012904		ENSLOCG00000006267	CC2D1A
ENSXMAG00000011239	DIAPH1	ENSLOCG00000011329	DIAPH1
ENSXMAG00000004228	dpp7	ENSLOCG00000001486	dpp7
ENSXMAG00000015277	rictora	ENSLOCG00000012599	rictora
ENSXMAG00000004164	foxp1b	ENSLOCG00000010009	foxp1b
ENSXMAG00000007720	slx1b	ENSLOCG00000013274	slx1b
ENSXMAG00000026078	RF00096	ENSLOCG00000020598	RF00096
ENSXMAG00000002845	seta	ENSLOCG00000006027	seta
ENSXMAG00000027188	P2RY4	ENSLOCG00000017494	P2RY4
ENSXMAG00000003385		ENSLOCG00000008582	
ENSXMAG00000008337	cdk21	ENSLOCG00000013828	cdk21
ENSXMAG00000014138		ENSLOCG00000000793	
ENSXMAG00000019666	si:ch211-210c8.7	ENSLOCG00000018196	si:ch211-210c8.7
ENSXMAG00000010822	erlec1	ENSLOCG00000016265	erlec1
ENSXMAG00000024829	ptger3	ENSLOCG00000010015	ptger3
ENSXMAG00000015772	mapk15	ENSLOCG00000008598	mapk15
ENSXMAG00000012563	helq	ENSLOCG00000012099	helq
ENSXMAG00000006255	trhr2	ENSLOCG00000002461	trhr2
ENSXMAG00000028338	arl1	ENSLOCG00000015195	arl1
ENSXMAG00000021072	si:ch211-51h9.7	ENSLOCG00000006014	si:ch211-51h9.7
ENSXMAG00000005189	dazap2	ENSLOCG00000004791	dazap2
ENSXMAG00000011318	si:ch211-284f22.3	ENSLOCG00000002350	si:ch211-284f22.3
ENSXMAG00000005445	zranb2	ENSLOCG00000010010	zranb2
ENSXMAG00000006905	si:dkey-177p2.18	ENSLOCG00000013113	si:dkey-177p2.18
ENSXMAG00000017055	wdr76	ENSLOCG00000014639	wdr76
ENSXMAG00000016036	pigf	ENSLOCG00000016331	pigf
ENSXMAG00000000185	si:ch211-134m17.9	ENSLOCG00000012430	si:ch211-134m17.9
ENSXMAG00000015467	GABRG1	ENSLOCG00000012390	gabrg1
ENSXMAG00000005195	pou6f1	ENSLOCG00000004801	pou6f1

ENSXMAG00000023833	cript	ENSLOCG00000016330	cript
ENSXMAG00000026922	prrt1	ENSLOCG00000000716	
ENSXMAG00000011220	tspan11	ENSLOCG00000016843	tspan11
ENSXMAG00000008347	MASTL	ENSLOCG00000007561	mastl
ENSXMAG00000015920	tmem45a	ENSLOCG00000010607	tmem45a
ENSXMAG00000020048		ENSLOCG00000014777	rnf128a
ENSXMAG00000007705	rwdd	ENSLOCG00000013699	rwdd
ENSXMAG00000005455	eral1	ENSLOCG00000003302	eral1
ENSXMAG00000015748	vps28	ENSLOCG00000008615	vps28
ENSXMAG00000019900	gpr75	ENSLOCG00000017362	gpr75
ENSXMAG00000016045	gch2	ENSLOCG00000016326	gch2
ENSXMAG00000020236	gpr27	ENSLOCG00000018245	gpr27
ENSXMAG00000009855	zfpm1	ENSLOCG00000002480	zfpm1
ENSXMAG00000015441	gnpda2	ENSLOCG00000012405	gnpda2
ENSXMAG00000014079	skiv2l	ENSLOCG00000000691	
ENSXMAG00000016727	slc24a5	ENSLOCG00000013255	slc24a5
ENSXMAG00000027089	rybpb	ENSLOCG00000009980	rybpa
ENSXMAG00000006902	chga	ENSLOCG00000013102	chga
ENSXMAG00000028250	pcdh1g32	ENSLOCG00000011316	pcdh1g32
ENSXMAG00000028772	chst10	ENSLOCG00000010623	chst10
ENSXMAG00000008941	negr1	ENSLOCG00000008597	negr1
ENSXMAG00000015930	tomm70a	ENSLOCG00000010625	tomm70a
ENSXMAG00000002812		ENSLOCG00000005909	gle1
ENSXMAG00000016686	myef2	ENSLOCG00000013241	myef2
ENSXMAG00000027120	zgc:136864	ENSLOCG00000006293	zgc:136864
ENSXMAG00000015941	glud1a	ENSLOCG00000012436	glud1a
ENSXMAG00000006892	ctgfa	ENSLOCG00000017020	ctgfa
ENSXMAG00000004758	gxylt2	ENSLOCG00000009958	gxylt2
ENSXMAG00000005466	flot2a	ENSLOCG00000003267	flot2b
ENSXMAG00000007684	adam19a	ENSLOCG00000001814	
ENSXMAG00000012527	serbp1b	ENSLOCG00000007107	serbp1b
ENSXMAG00000006878	stx7l	ENSLOCG00000017026	stx7l
ENSXMAG00000012423	zc3h13	ENSLOCG00000010561	zc3h13
ENSXMAG00000018018		ENSLOCG00000002976	actr3
ENSXMAG00000012555	mrps18c	ENSLOCG00000012115	mrps18c
ENSXMAG00000005202	tfcp2	ENSLOCG00000004816	tfcp2
ENSXMAG00000015273		ENSLOCG00000012552	zgc:153352
ENSXMAG00000000193	nfkb2	ENSLOCG00000012413	nfkb2
ENSXMAG00000015414	rrh	ENSLOCG00000011717	rrh
ENSXMAG00000014078	nelfe	ENSLOCG00000000208	nelfe
ENSXMAG00000017865	padi2	ENSLOCG00000002953	padi2
ENSXMAG00000016567	slc12a1	ENSLOCG00000013230	slc12a1
ENSXMAG00000005478	si:ch211-283g2.1	ENSLOCG00000003247	si:ch211-283g2.1
ENSXMAG00000004695	pdzrn3b	ENSLOCG00000009917	pdzrn3b
ENSXMAG00000017870	vtg3	ENSLOCG00000008476	
ENSXMAG00000015938	tbc1d23	ENSLOCG00000010638	tbc1d23
ENSXMAG00000017858	lrig2	ENSLOCG00000010986	lrig2
ENSXMAG00000015267	slc7a4	ENSLOCG00000008226	slc7a4
ENSXMAG00000019201	mier1b	ENSLOCG00000007038	mier1b
ENSXMAG00000026911	TMEM179	ENSLOCG00000012925	tmem179
ENSXMAG00000029141	leg1.1	ENSLOCG00000017285	leg1.1
ENSXMAG00000008423	hexim1	ENSLOCG00000017511	hexim1
ENSXMAG00000024134		ENSLOCG00000011592	si:zfos-1192g2.3
ENSXMAG00000012438	cpb2	ENSLOCG00000010552	CPB2
ENSXMAG00000012012	csrnp2	ENSLOCG00000004834	csrnp2
ENSXMAG00000018003	ddx18	ENSLOCG00000003108	ddx18

ENSXMAG00000020939	nkx6.1	ENSLOCG00000012142	nkx6.1
ENSXMAG00000015957	GALNT7	ENSLOCG00000013565	galnt7
ENSXMAG00000019515	sox3	ENSLOCG00000017493	sox3
ENSXMAG00000022585	si:dkey-12h9.6	ENSLOCG00000016341	si:dkey-12h9.6
ENSXMAG00000026868	prr33	ENSLOCG00000017320	prr33
ENSXMAG00000007661	lrch4	ENSLOCG00000013372	lrch4
ENSXMAG00000015251		ENSLOCG00000016179	drc1
ENSXMAG00000012876	khsrp	ENSLOCG00000006229	khsrp
ENSXMAG00000029381	dhrs11a	ENSLOCG00000003224	dhrs11a
ENSXMAG00000011199	ccdc59	ENSLOCG00000016324	ccdc59
ENSXMAG00000013998	smpd5	ENSLOCG00000008553	smpd5
ENSXMAG00000010767		ENSLOCG00000016248	
ENSXMAG00000019147	uri1	ENSLOCG00000005700	uri1
ENSXMAG00000005896	cntn3b	ENSLOCG00000009897	cntn3b
ENSXMAG00000012219	rpl17	ENSLOCG00000012148	rpl17
ENSXMAG00000015159	cul3a	ENSLOCG00000003963	cul3b
ENSXMAG00000019563		ENSLOCG00000001708	
ENSXMAG00000026215		ENSLOCG00000006973	tctex1d1
ENSXMAG00000023863		ENSLOCG00000012100	
ENSXMAG00000028160	ptgesl	ENSLOCG00000005855	ptgesl
ENSXMAG00000003997	nelfb	ENSLOCG00000001721	nelfb
ENSXMAG00000011194	mettl25	ENSLOCG00000016327	mettl25
ENSXMAG00000019191	mgst3a	ENSLOCG00000010594	mgst3a
ENSXMAG00000017133	sec11a	ENSLOCG00000014673	sec11a
ENSXMAG00000005013	glis3	ENSLOCG00000012972	glis3
ENSXMAG00000026839	dut	ENSLOCG00000013224	dut
ENSXMAG00000005524	ggnbp2	ENSLOCG00000003189	ggnbp2
ENSXMAG00000012868	dcaf15	ENSLOCG00000006211	dcaf15
ENSXMAG00000007658		ENSLOCG00000011277	H2AFZ
ENSXMAG00000008320	pdss1	ENSLOCG00000007637	pdss1
ENSXMAG00000002753	sf3a1	ENSLOCG00000008251	sf3a1
ENSXMAG00000000212	mfhas1	ENSLOCG00000012154	mfhas1
ENSXMAG00000019131	rxylt1	ENSLOCG00000016980	rxylt1
ENSXMAG00000002762	si:ch211-251j10.3	ENSLOCG00000005803	si:ch211-251j10.3
ENSXMAG00000015385	aspg	ENSLOCG00000012905	aspg
ENSXMAG00000016086	fshr	ENSLOCG00000016280	fshr
ENSXMAG00000020013	pigw	ENSLOCG00000017424	pigw
ENSXMAG00000016046	aifm2	ENSLOCG00000012383	aifm2
ENSXMAG00000005015	rfx3	ENSLOCG00000012975	rfx3
ENSXMAG00000014065	slc44a4	ENSLOCG00000000132	
ENSXMAG00000014021	kif13a	ENSLOCG00000008249	kif13a
ENSXMAG00000021305		ENSLOCG00000010824	nrg1
ENSXMAG00000005550	myo19	ENSLOCG00000003147	myo19
ENSXMAG00000007644	MLLT3	ENSLOCG00000011259	mllt3
ENSXMAG00000015975		ENSLOCG00000013627	ASB5
ENSXMAG00000019162	fzr1b	ENSLOCG00000010634	fzr1b
ENSXMAG00000003974	fgf17	ENSLOCG00000014874	
ENSXMAG00000012088	rapgef3	ENSLOCG00000005254	rapgef3
ENSXMAG00000015972	nit2	ENSLOCG00000010659	NIT2
ENSXMAG00000015996	spcs3	ENSLOCG00000013631	spcs3
ENSXMAG00000007655	slc38a9	ENSLOCG00000010879	slc38a9
ENSXMAG00000027038		ENSLOCG00000002242	HEPACAM
ENSXMAG00000027487		ENSLOCG00000016750	NINL
ENSXMAG00000010650		ENSLOCG00000001544	si:dkey-61l1.4
ENSXMAG00000006846	dpf3	ENSLOCG00000012352	dpf3
ENSXMAG00000012805	si:dkey-219c10.4	ENSLOCG00000002172	si:dkey-219c10.4

ENSXMAG00000003209	thbs2a	ENSLOCG00000016241	thbs2a
ENSXMAG00000017894	adgrl4	ENSLOCG00000008464	adgrl4
ENSXMAG00000019097	srgap1a	ENSLOCG00000016975	srgap1a
ENSXMAG00000003947	si:ch73-199g24.2	ENSLOCG00000002569	
ENSXMAG00000015247	aplnra	ENSLOCG00000018158	aplnra
ENSXMAG00000028009	gins1	ENSLOCG00000016757	gins1
ENSXMAG00000011154		ENSLOCG00000001692	styxl1
ENSXMAG00000006268	zgc:91860	ENSLOCG00000002529	zgc:91860
ENSXMAG00000019788	NHLRC1	ENSLOCG00000018335	NHLRC1
ENSXMAG00000008296	gad2	ENSLOCG00000007705	gad2
ENSXMAG00000023401	nifk	ENSLOCG00000002155	nifk
ENSXMAG00000015980	gtpbp6	ENSLOCG00000008009	gtpbp6
ENSXMAG00000007654	purg	ENSLOCG00000017542	purg
ENSXMAG00000016077	abhd12	ENSLOCG00000016763	abhd12
ENSXMAG00000006423	SLC26A5	ENSLOCG00000015975	slc26a5
ENSXMAG00000015216	hpdb	ENSLOCG00000005223	hpdb
ENSXMAG00000022820	arf6b	ENSLOCG00000017705	arf6b
ENSXMAG00000007632	FOCAD	ENSLOCG00000011240	focad
ENSXMAG00000013304	atp11c	ENSLOCG00000015169	atp11c
ENSXMAG00000007595	si:dkey-66a8.7	ENSLOCG00000008026	si:dkey-66a8.7
ENSXMAG00000005575	znhit3	ENSLOCG00000003139	znhit3
ENSXMAG00000006828	wdr21	ENSLOCG00000012343	wdr21
ENSXMAG00000016102		ENSLOCG00000016781	
ENSXMAG00000023048	LEPROT	ENSLOCG00000006886	LEPROT
ENSXMAG00000014058	utp23	ENSLOCG00000003289	utp23
ENSXMAG00000015982	plcxd1	ENSLOCG00000008059	zgc:64065
ENSXMAG00000006406	psmc2	ENSLOCG00000015980	psmc2
ENSXMAG00000024110	foxl1	ENSLOCG00000002568	foxl1
ENSXMAG00000030093	fbxo22	ENSLOCG00000014728	fbxo22
ENSXMAG00000019083	PGM1	ENSLOCG00000006665	PGM1
ENSXMAG00000006280	exosc6	ENSLOCG00000017597	exosc6
ENSXMAG00000010051	rae1	ENSLOCG00000003469	rae1
ENSXMAG00000012778	nthl1	ENSLOCG00000003090	nthl1
ENSXMAG00000015377	vcpkmt	ENSLOCG00000011242	vcpkmt
ENSXMAG00000028818	derl3	ENSLOCG00000006673	derl3
ENSXMAG00000012482	fastkd2	ENSLOCG00000010389	
ENSXMAG00000006390	dnajc2	ENSLOCG00000015983	dnajc2
ENSXMAG00000023788		ENSLOCG00000003688	ccdc113
ENSXMAG00000006282	aars	ENSLOCG00000007641	
ENSXMAG00000015987	jade3	ENSLOCG00000008100	jade3
ENSXMAG00000016104	entpd6	ENSLOCG00000016784	entpd6
ENSXMAG00000028216	cacnb1	ENSLOCG00000012811	cacnb1
ENSXMAG00000002257	SHC4	ENSLOCG00000013189	SHC4
ENSXMAG00000012749	tsc2	ENSLOCG00000003041	tsc2
ENSXMAG00000026740	wsb1	ENSLOCG00000004758	wsb1
ENSXMAG00000012494	mdh1b	ENSLOCG00000010397	
ENSXMAG00000015359	msh4	ENSLOCG00000011230	msh4
ENSXMAG00000026749	PMPCB	ENSLOCG00000015984	pmpcb
ENSXMAG00000011153	dcp1b	ENSLOCG00000016629	dcp1b
ENSXMAG00000016135	vsx1	ENSLOCG00000016788	vsx1
ENSXMAG00000008275	myo3a	ENSLOCG00000007720	myo3a
ENSXMAG00000003934		ENSLOCG00000002409	
ENSXMAG00000020014	nupr1a	ENSLOCG00000017423	nupr1a
ENSXMAG00000017636	capn3b	ENSLOCG00000013428	CAPN3
ENSXMAG00000015174	kdm2ba	ENSLOCG00000005104	kdm2ba
ENSXMAG00000007623	dcp2	ENSLOCG00000009013	dcp2

ENSXMAG00000014032	slc30a8	ENSLOCG00000003250	slc30a8
ENSXMAG00000014049		ENSLOCG00000008155	
ENSXMAG00000007229	fhdc4	ENSLOCG00000008650	fhdc4
ENSXMAG00000002266	SECISBP2L	ENSLOCG00000013180	SECISBP2L
ENSXMAG00000020204	phax	ENSLOCG00000008559	phax
ENSXMAG00000018219	vkorc1l1	ENSLOCG00000003060	vkorc1l1
ENSXMAG00000010075	tada3l	ENSLOCG00000014552	tada3l
ENSXMAG00000020021	ora5	ENSLOCG00000017551	
ENSXMAG00000006324	st3gal2	ENSLOCG00000007629	st3gal2
ENSXMAG00000003917		ENSLOCG00000005298	crb2b
ENSXMAG00000006801	pigh	ENSLOCG00000012333	pigh
ENSXMAG00000014029	med30	ENSLOCG00000003235	med30
ENSXMAG00000016005	tmem176	ENSLOCG00000008186	
ENSXMAG00000006387	kcnc2	ENSLOCG00000016255	kcnc2
ENSXMAG00000018222	gusb	ENSLOCG00000003067	gusb
ENSXMAG00000016136	tjap1	ENSLOCG00000016719	tjap1
ENSXMAG00000011036	cacna1c	ENSLOCG00000016617	cacna1c
ENSXMAG00000016009	vps37c	ENSLOCG00000008197	
ENSXMAG00000021774	rhov	ENSLOCG00000012329	rhov
ENSXMAG00000005080	nup155	ENSLOCG00000012677	nup155
ENSXMAG00000025935	retreg2	ENSLOCG00000010995	retreg2
ENSXMAG00000009628	agpat4	ENSLOCG00000016235	agpat4
ENSXMAG00000024822	dlk2	ENSLOCG00000016721	dlk2
ENSXMAG00000006768	vps18	ENSLOCG00000012325	vps18
ENSXMAG00000015995	rp2	ENSLOCG00000008112	rp2
ENSXMAG00000007618		ENSLOCG00000009001	YTHDC2
ENSXMAG00000006334	sf3b3	ENSLOCG00000007566	sf3b3
ENSXMAG00000002906	HACD4	ENSLOCG00000011238	HACD4
ENSXMAG00000027681	SAMD12	ENSLOCG00000003206	SAMD12
ENSXMAG00000000273		ENSLOCG00000015853	pot1
ENSXMAG00000029484	lrrc73	ENSLOCG00000016718	lrrc73
ENSXMAG00000017908	adgrl2a	ENSLOCG00000008424	adgrl2a
ENSXMAG00000015288		ENSLOCG00000005742	
ENSXMAG00000030063	aff3	ENSLOCG00000001338	
ENSXMAG00000015161	dgcr6	ENSLOCG00000008215	DGCR6L
ENSXMAG00000016015	capn9	ENSLOCG00000015444	capn9
ENSXMAG00000015998	slc9a7	ENSLOCG00000008129	slc9a7
ENSXMAG00000006742	ino80	ENSLOCG00000012304	ino80
ENSXMAG00000003908	lhx2b	ENSLOCG00000005257	lhx2b
ENSXMAG00000003024	cnppd1	ENSLOCG00000011010	cnppd1
ENSXMAG00000016153	mea1	ENSLOCG00000016738	mea1
ENSXMAG00000012302	elmod1	ENSLOCG00000006784	elmod1
ENSXMAG00000012037	pipox	ENSLOCG00000004870	pipox
ENSXMAG00000022944	trmt10a	ENSLOCG00000012298	trmt10a
ENSXMAG00000002692		ENSLOCG00000015308	
ENSXMAG00000015330	ktn1	ENSLOCG00000011215	ktn1
ENSXMAG00000015120	RASAL1	ENSLOCG00000008183	RASAL1
ENSXMAG00000004769	adgrd2	ENSLOCG00000005172	adgrd2
ENSXMAG00000005583		ENSLOCG00000003001	
ENSXMAG00000002272	cops2	ENSLOCG00000013176	cops2
ENSXMAG00000025402	si:cabz01093077.1	ENSLOCG00000000892	si:cabz01093077.1
ENSXMAG00000012001	phf12b	ENSLOCG00000004908	phf12b
ENSXMAG00000003010	abcb6a	ENSLOCG00000000956	abcb6a
ENSXMAG00000019936	chst7	ENSLOCG00000017795	chst7
ENSXMAG00000015100	DTX1	ENSLOCG00000008172	DTX1
ENSXMAG00000016055	tubgcp5	ENSLOCG00000008164	tubgcp5

ENSXMAG00000023303	taf5l	ENSLOCG00000015464	taf5l
ENSXMAG00000024774		ENSLOCG00000011033	
ENSXMAG00000025422	tada2b	ENSLOCG00000010175	tada2b
ENSXMAG00000019603	ddx28	ENSLOCG00000017604	ddx28
ENSXMAG00000027751		ENSLOCG00000011020	ptpn22
ENSXMAG00000011456	rev1	ENSLOCG00000001353	rev1
ENSXMAG00000011390	grpel1	ENSLOCG00000010180	grpel1
ENSXMAG00000025699	cobl	ENSLOCG00000000944	cobl
ENSXMAG00000009061	nr6a1a	ENSLOCG00000005138	nr6a1a
ENSXMAG00000022586	tspan33a	ENSLOCG00000015974	tspan33a
ENSXMAG00000012291	nfatc3b	ENSLOCG00000008087	nfatc3b
ENSXMAG00000014130	nup133	ENSLOCG00000015468	nup133
ENSXMAG00000006091	hmgcs1	ENSLOCG00000010958	hmgcs1
ENSXMAG00000012327		ENSLOCG00000011013	
ENSXMAG00000011005	smo	ENSLOCG00000015973	smo
ENSXMAG00000008494	eftud2	ENSLOCG00000011460	eftud2
ENSXMAG00000026342	olfml2a	ENSLOCG00000005109	olfml2a
ENSXMAG00000014060	nkd2a	ENSLOCG00000008140	nkd2a
ENSXMAG00000023624	krr1	ENSLOCG00000016262	krr1
ENSXMAG00000011355	sorcs2	ENSLOCG00000010190	sorcs2
ENSXMAG00000002280	fgf7	ENSLOCG00000013162	fgf7
ENSXMAG00000019948		ENSLOCG00000017935	
ENSXMAG00000016084	cyfip1	ENSLOCG00000008180	cyfip1
ENSXMAG00000012268	dus2	ENSLOCG00000008097	dus2
ENSXMAG00000025010		ENSLOCG00000001454	EPB41L1
ENSXMAG00000005170		ENSLOCG00000010062	KIAA2026
ENSXMAG00000023272	si:ch211-284b7.3	ENSLOCG00000006123	si:ch211-284b7.3
ENSXMAG00000009607	kcnk1b	ENSLOCG00000016222	kcnk1a
ENSXMAG00000002282	dtwd1	ENSLOCG00000013150	dtwd1
ENSXMAG00000029217	ora1	ENSLOCG00000017925	ora1
ENSXMAG00000021151	magi3a	ENSLOCG00000010996	magi3a
ENSXMAG00000017366	esrp2	ENSLOCG00000008062	esrp2
ENSXMAG00000014062	trip13	ENSLOCG00000008122	trip13
ENSXMAG00000024725		ENSLOCG00000001452	mrpl30
ENSXMAG00000022681		ENSLOCG00000007759	
ENSXMAG00000002921	RPL35	ENSLOCG00000005076	RPL35
ENSXMAG00000017756	zgc:175264	ENSLOCG00000010802	zgc:175264
ENSXMAG00000002284	nnt2	ENSLOCG00000013143	nnt2
ENSXMAG00000026339		ENSLOCG00000016264	
ENSXMAG00000011465	txndc9	ENSLOCG00000001434	txndc9
ENSXMAG00000023144	uck2b	ENSLOCG00000006084	uck2b
ENSXMAG00000009605	slc35f3b	ENSLOCG00000016220	slc35f3a
ENSXMAG00000013515	vrtn	ENSLOCG00000017704	vrtn
ENSXMAG00000005174	UHRF2	ENSLOCG00000010051	
ENSXMAG00000029387		ENSLOCG00000016733	ZNF318
ENSXMAG00000011466	mitd1	ENSLOCG00000001479	mitd1
ENSXMAG00000002947	pskh1	ENSLOCG00000006672	pskh1
ENSXMAG00000005613	hpda	ENSLOCG00000003037	hpda
ENSXMAG00000015087	vgll4l	ENSLOCG00000014414	vgll4l
ENSXMAG00000024776	si:dkey-205h13.1	ENSLOCG00000001653	si:dkey-205h13.1
ENSXMAG00000021011		ENSLOCG00000018058	
ENSXMAG00000002371	bcas3	ENSLOCG00000005098	bcas3
ENSXMAG00000015321	peli2	ENSLOCG00000011202	peli2
ENSXMAG00000002925	golga1	ENSLOCG00000005040	golga1
ENSXMAG00000002907	mbtps1	ENSLOCG00000006637	mbtps1
ENSXMAG00000017729	pfkfb2b	ENSLOCG00000010773	PFKFB2

ENSXMAG00000014068	brd9	ENSLOCG00000008095	brd9
ENSXMAG00000014169		ENSLOCG00000013535	cdc6
ENSXMAG00000002298	spata5l1	ENSLOCG00000013121	spata5l1
ENSXMAG00000008515	znf652	ENSLOCG00000011452	znf652
ENSXMAG00000016124	nipa2	ENSLOCG00000008207	nipa2
ENSXMAG00000003573	fam57a	ENSLOCG00000006852	fam57a
ENSXMAG00000009600	si:dkey-85a20.4	ENSLOCG00000016214	TARBP1
ENSXMAG00000006374	lonp2	ENSLOCG00000007553	lonp2
ENSXMAG00000013521	slc25a47a	ENSLOCG00000011606	slc25a47a
ENSXMAG00000015312	tmem260	ENSLOCG00000011193	tmem260
ENSXMAG00000006341	si:ch73-89b15.3	ENSLOCG00000016266	si:dkey-30c15.17
ENSXMAG00000015071	syn1	ENSLOCG00000014401	syn1
ENSXMAG00000012720	knop1	ENSLOCG00000002872	
ENSXMAG00000008537	phb	ENSLOCG00000011441	phb
ENSXMAG00000012335	hspg2	ENSLOCG00000001778	hspg2
ENSXMAG00000003285	ilf2	ENSLOCG00000006883	ilf2
ENSXMAG00000011470	creg2	ENSLOCG00000001535	creg2
ENSXMAG00000024585	faap24	ENSLOCG00000001761	faap24
ENSXMAG00000028044	oaz1b	ENSLOCG00000010027	oaz1b
ENSXMAG00000016132	nipa1	ENSLOCG00000008231	nipa1
ENSXMAG00000023382	exosc1	ENSLOCG00000003776	exosc1
ENSXMAG00000002304	slc30a4	ENSLOCG00000013108	slc30a4
ENSXMAG00000028085	pex11b	ENSLOCG00000006969	pex11b
ENSXMAG00000012336	tecta	ENSLOCG00000001765	tecta
ENSXMAG00000020908	tdrd12	ENSLOCG00000001690	tdrd12
ENSXMAG00000020294	MRPL21	ENSLOCG00000001501	
ENSXMAG00000025339		ENSLOCG00000011421	
ENSXMAG00000002310	bloc1s6	ENSLOCG00000013103	bloc1s6
ENSXMAG00000010979	zc3hc1	ENSLOCG00000015963	zc3hc1
ENSXMAG00000025684	zte38	ENSLOCG00000006033	
ENSXMAG00000011475	mgat4a	ENSLOCG00000001549	mgat4a
ENSXMAG00000005632	RNF43	ENSLOCG00000003019	RNF43
ENSXMAG00000028123	snapin	ENSLOCG00000006741	snapin
ENSXMAG00000015268	exoc5	ENSLOCG00000011161	exoc5
ENSXMAG00000005226	ERMP1	ENSLOCG00000010085	ERMP1
ENSXMAG00000017400	amfra	ENSLOCG00000006944	amfra
ENSXMAG00000017727	yod1	ENSLOCG00000010760	yod1
ENSXMAG00000002314	mibp2	ENSLOCG00000013090	mibp2
ENSXMAG00000020296	zgc:77929	ENSLOCG00000017353	zgc:77929
ENSXMAG00000027594	pi4k2a	ENSLOCG00000003739	pi4k2a
ENSXMAG00000026254	ube2h	ENSLOCG00000015962	ube2h
ENSXMAG00000020951		ENSLOCG00000004778	
ENSXMAG00000024196	prdx1	ENSLOCG00000006005	prdx1
ENSXMAG00000023362	siah1	ENSLOCG00000007536	siah1
ENSXMAG00000000755	ddc	ENSLOCG00000000998	ddc
ENSXMAG00000027768		ENSLOCG00000002988	HSF5
ENSXMAG00000016133	herc2	ENSLOCG00000008243	herc2
ENSXMAG00000025006	nap1l1	ENSLOCG00000016269	nap1l1
ENSXMAG00000014082	zdhhc11	ENSLOCG00000008072	zdhhc11
ENSXMAG00000007560	tpm2	ENSLOCG00000011000	tpm2
ENSXMAG00000014198		ENSLOCG00000013761	npepps
ENSXMAG00000024117	gnpnat1	ENSLOCG00000012081	gnpnat1
ENSXMAG00000016161	chac2	ENSLOCG00000016270	chac2
ENSXMAG00000005633	supt4h1	ENSLOCG00000002970	supt4h1
ENSXMAG00000020559	MIR96	ENSLOCG00000020463	MIR96
ENSXMAG00000013000	necab2	ENSLOCG00000006599	necab2

ENSXMAG00000009561	ankrd27	ENSLOCG00000001654	ankrd27
ENSXMAG00000020495		ENSLOCG00000019144	
ENSXMAG00000023176		ENSLOCG00000016803	SYNDIG1
ENSXMAG00000017723		ENSLOCG00000003655	
ENSXMAG00000011505	coa5	ENSLOCG00000001639	coa5
ENSXMAG00000024138	styx	ENSLOCG00000012074	styx
ENSXMAG00000015246	ap5m1	ENSLOCG00000011145	ap5m1
ENSXMAG00000009591	fam135a	ENSLOCG00000017093	fam135a
ENSXMAG00000014084	TPPP	ENSLOCG00000008061	TPPP
ENSXMAG00000006417	cry2	ENSLOCG00000014655	cry2
ENSXMAG00000022653		ENSLOCG00000013288	
ENSXMAG00000010946	nrf1	ENSLOCG00000015960	nrf1
ENSXMAG00000000746	entpd3	ENSLOCG00000001020	entpd3
ENSXMAG00000005243	RIC1	ENSLOCG00000010095	RIC1
ENSXMAG00000012387		ENSLOCG00000006222	PLEKHB1
ENSXMAG00000025761	cep72	ENSLOCG00000008048	cep72
ENSXMAG00000022835	FGF13	ENSLOCG00000015157	FGF13
ENSXMAG00000010412	gatad2b	ENSLOCG00000008705	gatad2b
ENSXMAG00000012689	zfyve27	ENSLOCG00000008885	zfyve27
ENSXMAG00000023669		ENSLOCG00000006542	hydin
ENSXMAG00000008580	mrc2	ENSLOCG00000011341	mrc2
ENSXMAG00000028457	dph7	ENSLOCG00000001966	dph7
ENSXMAG00000017441	mdga1	ENSLOCG00000017050	mdga1
ENSXMAG00000008303	hsp90aa1.2	ENSLOCG00000000071	
ENSXMAG00000026662	tbx4	ENSLOCG00000005064	tbx4
ENSXMAG00000029314	sc5d	ENSLOCG00000004754	sc5d
ENSXMAG00000007556	dennd1a	ENSLOCG00000005271	dennd1a
ENSXMAG00000015242	slc35f4	ENSLOCG00000011125	slc35f4
ENSXMAG00000014088	si:ch211-257p13.3	ENSLOCG00000004279	si:ch211-257p13.3
ENSXMAG00000019576	mrpl41	ENSLOCG00000017334	mrpl41
ENSXMAG00000002684	si:dkey-174n20.1	ENSLOCG00000003066	
ENSXMAG00000018411	gpbp1l1	ENSLOCG00000005900	gpbp1l1
ENSXMAG00000021783	rbms3	ENSLOCG00000002328	rbms3
ENSXMAG00000008315	hsp90aa1.1	ENSLOCG00000011504	hsp90aa1.1
ENSXMAG00000015020	gapvd1	ENSLOCG00000001381	gapvd1
ENSXMAG00000025720	TMEM208	ENSLOCG00000006204	TMEM208
ENSXMAG00000005635		ENSLOCG00000002948	
ENSXMAG00000009569	TMED6	ENSLOCG00000001361	tmed6
ENSXMAG00000017924	ttll7	ENSLOCG00000008403	ttll7
ENSXMAG00000015178	pnp6	ENSLOCG00000006511	pnp6
ENSXMAG00000007549	nek6	ENSLOCG00000005235	nek6
ENSXMAG00000018395	IPP	ENSLOCG00000005843	IPP
ENSXMAG00000005742	brip1	ENSLOCG00000005046	brip1
ENSXMAG00000014301	mrpl45	ENSLOCG00000013748	mrpl45
ENSXMAG00000027207	col19a1	ENSLOCG00000008161	
ENSXMAG00000015235	zdhhc22	ENSLOCG00000011073	zdhhc22
ENSXMAG00000012356	agmat	ENSLOCG00000001924	agmat
ENSXMAG00000014098	ccdc12	ENSLOCG00000005683	ccdc12
ENSXMAG00000010939	plxna4	ENSLOCG00000015635	plxna4
ENSXMAG00000013401	zic6	ENSLOCG00000015156	zic6
ENSXMAG00000012643	mrpl39	ENSLOCG00000010708	MRPL39
ENSXMAG00000002678	kremen1	ENSLOCG00000003115	kremen1
ENSXMAG00000013404	zic3	ENSLOCG00000015155	zic3
ENSXMAG00000006426	zgc:92873	ENSLOCG00000014671	zgc:92873
ENSXMAG00000006111		ENSLOCG00000001534	
ENSXMAG00000019477	mgat2	ENSLOCG00000017706	mgat2

ENSXMAG00000017822	calb2a	ENSLOCG00000006495	calb2a
ENSXMAG00000015171	tmem63c	ENSLOCG00000011047	tmem63c
ENSXMAG00000014957	stx12	ENSLOCG00000004069	stx12
ENSXMAG00000023266	gpr101	ENSLOCG00000017492	gpr101
ENSXMAG00000008326	RPS29	ENSLOCG00000011385	RPS29
ENSXMAG00000003058		ENSLOCG00000012173	
ENSXMAG00000014112	nbeal2	ENSLOCG00000005650	nbeal2
ENSXMAG00000012373	pink1	ENSLOCG00000001952	pink1
ENSXMAG00000007535	psmb7	ENSLOCG00000005208	psmb7
ENSXMAG00000017467	slc7a9	ENSLOCG00000001714	slc7a9
ENSXMAG00000026745	GLI2	ENSLOCG00000002028	gli2a
ENSXMAG00000009578	fem1b	ENSLOCG00000013948	fem1b
ENSXMAG00000014311	azi2	ENSLOCG00000002349	azi2
ENSXMAG00000008640	mettl2a	ENSLOCG00000011314	mettl2a
ENSXMAG00000015664	extl2	ENSLOCG00000000145	EXTL2
ENSXMAG00000022613	si:ch211-156l18.8	ENSLOCG00000013743	si:ch211-156l18.8
ENSXMAG00000006428	ckap5	ENSLOCG00000014683	ckap5
ENSXMAG00000014309	cmc1	ENSLOCG00000002385	cmc1
ENSXMAG00000013321	lmbrd1	ENSLOCG00000017055	lmbrd1
ENSXMAG00000017701	eif4g3a	ENSLOCG00000003549	eif4g3b
ENSXMAG00000015670	zgc:110366	ENSLOCG00000000209	zgc:110366
ENSXMAG00000002396	map2k5	ENSLOCG00000013923	map2k5
ENSXMAG00000002670	znrf3	ENSLOCG00000003143	znrf3
ENSXMAG00000023105	nudt19	ENSLOCG00000001704	nudt19
ENSXMAG00000004922	araf	ENSLOCG00000014384	araf
ENSXMAG00000005752	appbp2	ENSLOCG00000005143	appbp2
ENSXMAG00000011549	slc9a2	ENSLOCG00000008661	slc9a2
ENSXMAG00000019606	RGS9BP	ENSLOCG00000017584	RGS9BP
ENSXMAG00000020256	dsela	ENSLOCG00000018159	dsela
ENSXMAG00000004985	grb2b	ENSLOCG00000013310	grb2b
ENSXMAG00000012655	r3hcc1l	ENSLOCG00000008944	r3hcc1l
ENSXMAG00000029922	ngb	ENSLOCG00000011029	ngb
ENSXMAG00000013407	rbmx	ENSLOCG00000015142	rbmx
ENSXMAG00000006300	OSBPL8	ENSLOCG00000016275	OSBPL8
ENSXMAG00000015162	fam161b	ENSLOCG00000011006	fam161b
ENSXMAG00000027567	abl1	ENSLOCG00000005460	abl1
ENSXMAG00000014263	ankib1b	ENSLOCG00000010636	ankib1b
ENSXMAG00000001749	TMX3	ENSLOCG00000012164	tmx3a
ENSXMAG00000017490	zdhhc7	ENSLOCG00000001307	zdhhc7
ENSXMAG00000015673	rc3h1b	ENSLOCG00000000279	rc3h1a
ENSXMAG00000008358	usp43b	ENSLOCG00000013295	usp43b
ENSXMAG00000002665	xbp1	ENSLOCG00000003156	xbp1
ENSXMAG00000014321	sp2	ENSLOCG00000013729	sp2
ENSXMAG00000010280	cidec	ENSLOCG00000014579	cidec
ENSXMAG00000023089	cyyr1	ENSLOCG00000010775	cyyr1
ENSXMAG00000023265	pla2g15	ENSLOCG00000000419	pla2g15
ENSXMAG00000004915	imp4	ENSLOCG00000014398	
ENSXMAG00000015139	coq6	ENSLOCG00000010998	coq6
ENSXMAG00000026862	mfsd9	ENSLOCG00000008676	mfsd9
ENSXMAG00000008349	fkbp3	ENSLOCG00000011306	fkbp3
ENSXMAG00000014538	chd1	ENSLOCG00000009047	chd1
ENSXMAG00000019740	rprml	ENSLOCG00000017512	rprml
ENSXMAG00000021413	si:ch73-193i2.2	ENSLOCG00000016499	si:ch73-193i2.2
ENSXMAG00000029012	nradd	ENSLOCG00000005615	nradd
ENSXMAG00000008682	tcap	ENSLOCG00000012731	tcap
ENSXMAG00000017495		ENSLOCG00000001317	TERF2

ENSXMAG00000002660	hspb8	ENSLOCG00000003205	hspb8
ENSXMAG00000016171	oca2	ENSLOCG00000008282	oca2
ENSXMAG00000024687	usb1	ENSLOCG00000000465	usb1
ENSXMAG00000007919		ENSLOCG00000001480	RPN2
ENSXMAG00000014525	ntn1b	ENSLOCG00000013269	ntn1b
ENSXMAG00000006476	dnaaf4	ENSLOCG00000013474	dnaaf4
ENSXMAG00000008686	zgc:91968	ENSLOCG00000012737	zgc:91968
ENSXMAG00000016337	clrn3	ENSLOCG00000007677	
ENSXMAG00000007497	mapkap1	ENSLOCG00000001342	mapkap1
ENSXMAG00000021611	churc1	ENSLOCG00000013510	churc1
ENSXMAG00000002290		ENSLOCG00000014560	uqcrc1
ENSXMAG00000008360		ENSLOCG00000011282	prpf39
ENSXMAG00000017932		ENSLOCG00000008370	
ENSXMAG00000002658	srrm4	ENSLOCG00000003219	srrm4
ENSXMAG00000001758	pxdc1b	ENSLOCG00000012158	pxdc1b
ENSXMAG00000016341	foxi1	ENSLOCG00000007685	foxi1
ENSXMAG00000009446	abcc10	ENSLOCG00000016723	abcc10
ENSXMAG00000019935	GPR45	ENSLOCG00000017796	GPR45
ENSXMAG00000025276	nip7	ENSLOCG00000001379	nip7
ENSXMAG00000028192	si:ch211-204a13.2	ENSLOCG00000018280	si:ch211-204a13.2
ENSXMAG00000004911	rabl6b	ENSLOCG00000001402	rabl6b
ENSXMAG00000017552	pias1a	ENSLOCG00000013933	pias1b
ENSXMAG00000005294	rab14	ENSLOCG00000005509	rab14
ENSXMAG00000011593	si:dkeyp-118h3.6	ENSLOCG00000008750	si:dkeyp-118h3.6
ENSXMAG00000024326	mab21l1	ENSLOCG00000018039	mab21l1
ENSXMAG00000017361	trnau1apa	ENSLOCG00000004041	trnau1apa
ENSXMAG00000008687	stard3	ENSLOCG00000012741	stard3
ENSXMAG00000009430	adgrb3	ENSLOCG00000017045	adgrb3
ENSXMAG00000009437	usp32	ENSLOCG00000005178	usp32
ENSXMAG00000001762	prpf4bb	ENSLOCG00000012136	prpf4ba
ENSXMAG00000022776	cdc42se2	ENSLOCG00000013085	
ENSXMAG00000005205	exoc4	ENSLOCG00000015631	exoc4
ENSXMAG00000028390	si:ch211-13f8.1	ENSLOCG00000009981	si:dkey-121a11.3
ENSXMAG00000002300		ENSLOCG00000014558	ARPC4-TTLL3
ENSXMAG00000025414	uox	ENSLOCG00000008337	uox
ENSXMAG00000022713	suds3	ENSLOCG00000003254	suds3
ENSXMAG00000026565		ENSLOCG00000012846	ska1
ENSXMAG00000022396	nck2a	ENSLOCG00000008780	nck2a
ENSXMAG00000025238	tal1	ENSLOCG00000009966	tal1
ENSXMAG00000005312	ddx54	ENSLOCG00000008485	ddx54
ENSXMAG00000008853	zbtb16a	ENSLOCG00000004636	zbtb16a
ENSXMAG00000007493	KIAA1024L	ENSLOCG00000008296	KIAA1024L
ENSXMAG00000017683	ece1	ENSLOCG00000003479	ece1
ENSXMAG00000011609	ecrg4b	ENSLOCG00000008792	ecrg4a
ENSXMAG00000009137	adamts1	ENSLOCG00000010782	adamts1
ENSXMAG00000002305	hmces	ENSLOCG00000014549	hmces
ENSXMAG00000003878	prkn	ENSLOCG00000016503	prkn
ENSXMAG00000017944	samd13	ENSLOCG00000008363	samd13
ENSXMAG00000020358	mfsd6l	ENSLOCG00000013244	mfsd6l
ENSXMAG00000006274	zdhhc17	ENSLOCG00000016277	zdhhc17
ENSXMAG00000027136	naa30	ENSLOCG00000011139	naa30
ENSXMAG00000015074	gstz1	ENSLOCG00000010781	gstz1
ENSXMAG00000008835	htr3a	ENSLOCG00000004605	htr3a
ENSXMAG00000017960	dnase2b	ENSLOCG00000008324	dnase2b
ENSXMAG00000007939	mybl2b	ENSLOCG00000001337	mybl2b
ENSXMAG00000008388		ENSLOCG00000011110	

ENSXMAG00000016344	dock1	ENSLOCG00000007697	dock1
ENSXMAG00000011616	uxs1	ENSLOCG00000008816	uxs1
ENSXMAG00000014994		ENSLOCG00000001302	
ENSXMAG00000023544		ENSLOCG00000016638	
ENSXMAG00000018896	rpn1	ENSLOCG00000014543	rpn1
ENSXMAG00000015262	fa2h	ENSLOCG00000003741	fa2h
ENSXMAG00000008391	actr10	ENSLOCG00000011094	actr10
ENSXMAG00000020886	ADAMTS5	ENSLOCG00000010800	ADAMTS5
ENSXMAG00000028628		ENSLOCG00000015628	lrguk
ENSXMAG00000005653		ENSLOCG00000002933	
ENSXMAG00000006982	hdac12	ENSLOCG00000008308	hdac12
ENSXMAG00000027600	syk	ENSLOCG00000008182	syk
ENSXMAG00000017969	rpf1	ENSLOCG00000010053	rpf1
ENSXMAG00000015067	zgc:163014	ENSLOCG00000010791	zgc:163014
ENSXMAG00000006257	csrp2	ENSLOCG00000016278	csrp2
ENSXMAG00000008834	htr3b	ENSLOCG00000004587	htr3b
ENSXMAG00000009491	ift52	ENSLOCG00000001372	ift52
ENSXMAG00000001782	lztfl1	ENSLOCG00000012008	lztfl1
ENSXMAG00000000373	uacab	ENSLOCG00000014022	uacab
ENSXMAG00000018304	TADA1	ENSLOCG00000010492	TADA1
ENSXMAG00000008716	cdk12	ENSLOCG00000012760	cdk12
ENSXMAG00000015276	ano10b	ENSLOCG00000003269	ano10b
ENSXMAG00000006981	trmt9b	ENSLOCG00000008334	trmt9b
ENSXMAG00000005372	tbx3a	ENSLOCG00000007598	tbx3b
ENSXMAG00000006586		ENSLOCG00000012937	
ENSXMAG00000014219	upp1	ENSLOCG00000010218	UPP1
ENSXMAG00000022192	hspb1	ENSLOCG00000002907	hspb1
ENSXMAG00000028204	gata2a	ENSLOCG00000014539	gata2a
ENSXMAG00000006252	e2f7	ENSLOCG00000016279	e2f7
ENSXMAG00000017668	alpl	ENSLOCG00000003451	alpl
ENSXMAG00000021655		ENSLOCG00000014180	cart3
ENSXMAG00000016111		ENSLOCG00000006839	PARD6A
ENSXMAG00000017984	SPATA1	ENSLOCG00000010073	SPATA1
ENSXMAG00000029073	fbxl20	ENSLOCG00000012778	FBXL20
ENSXMAG00000006974	brca2	ENSLOCG00000008205	
ENSXMAG00000009510	acot8	ENSLOCG00000003241	acot8
ENSXMAG00000008410	znf410	ENSLOCG00000011015	znf410
ENSXMAG00000005396		ENSLOCG00000007571	
ENSXMAG00000018286	fggy	ENSLOCG00000008915	fggy
ENSXMAG00000005663	SRRM3	ENSLOCG00000002879	SRRM3
ENSXMAG00000008818	usp28	ENSLOCG00000004503	usp28
ENSXMAG00000011639	tmtops2b	ENSLOCG00000008828	tmtops2a
ENSXMAG00000010303		ENSLOCG00000014482	ITIH3
ENSXMAG00000026822	ctbs	ENSLOCG00000010081	ctbs
ENSXMAG00000015290	snrkb	ENSLOCG00000003299	snrkb
ENSXMAG00000020028	ucn3l	ENSLOCG00000017902	ucn3l
ENSXMAG00000002456	ncoa4	ENSLOCG00000012898	ncoa4
ENSXMAG00000030102	mrpl4	ENSLOCG00000016632	MRPL4
ENSXMAG00000017664	nmur3	ENSLOCG00000003425	nmur3
ENSXMAG00000029471	st6gal2a	ENSLOCG00000008836	st6gal2b
ENSXMAG00000016121	galn	ENSLOCG00000002486	galn
ENSXMAG00000008815	orai2	ENSLOCG00000001815	orai2
ENSXMAG00000017682	pum2	ENSLOCG00000017196	pum2
ENSXMAG00000006626	commd9	ENSLOCG00000001074	commd9
ENSXMAG00000005667	mlxipl	ENSLOCG00000002824	mlxipl
ENSXMAG00000005282	copg2	ENSLOCG00000015612	copg2

ENSXMAG00000009474	tmem97	ENSLOCG00000005215	tmem97
ENSXMAG00000022665		ENSLOCG00000007521	MTFP1
ENSXMAG00000008345	fgf18a	ENSLOCG00000009693	fgf18b
ENSXMAG00000013408	arhgef6	ENSLOCG00000015127	arhgef6
ENSXMAG00000008026	cherp	ENSLOCG00000003654	cherp
ENSXMAG00000016130	auh	ENSLOCG00000008202	auh
ENSXMAG00000017663	lactbl1b	ENSLOCG00000003404	lactbl1b
ENSXMAG00000006646	sycp3	ENSLOCG00000011501	
ENSXMAG00000004869	zgc:113162	ENSLOCG00000001461	
ENSXMAG00000008786	lrwd1	ENSLOCG00000001842	lrwd1
ENSXMAG00000019943	nfil3	ENSLOCG00000008217	nfil3
ENSXMAG00000005671		ENSLOCG00000002779	c1qbp
ENSXMAG00000006670	si:ch211-59o9.10	ENSLOCG00000015335	
ENSXMAG00000014356	si:dkey-23f9.4	ENSLOCG00000016635	si:dkey-23f9.4
ENSXMAG00000000203	angptl2b	ENSLOCG00000001221	angptl2b
ENSXMAG00000022830	dohh	ENSLOCG00000001335	dohh
ENSXMAG00000005775	ulk2	ENSLOCG00000005372	ulk2
ENSXMAG00000002180	si:dkey-208k4.2	ENSLOCG00000012792	si:dkey-208k4.2
ENSXMAG00000021582	gskip	ENSLOCG00000010833	gskip
ENSXMAG00000005679	dhx33	ENSLOCG00000002764	dhx33
ENSXMAG00000012607	b3gntl1	ENSLOCG00000013977	b3gntl1
ENSXMAG00000009544	txnrd3	ENSLOCG00000013740	txnrd3
ENSXMAG00000027240	gabrb3	ENSLOCG00000008344	gabrb3
ENSXMAG00000022186	yipf3	ENSLOCG00000016712	yipf3
ENSXMAG00000006674	nedd1	ENSLOCG00000015354	nedd1
ENSXMAG00000002632	pebp1	ENSLOCG00000003317	pebp1
ENSXMAG00000014967	npc2	ENSLOCG00000010850	npc2
ENSXMAG00000008018	rsph4a	ENSLOCG00000014226	rsph4a
ENSXMAG00000014962	isca2	ENSLOCG00000010860	isca2
ENSXMAG00000002588	VSIG10	ENSLOCG00000003360	
ENSXMAG00000016125	sptlc1	ENSLOCG00000008246	sptlc1
ENSXMAG00000025860	dnph1	ENSLOCG00000016740	dnph1
ENSXMAG00000007990	LONP1	ENSLOCG00000003119	lonp1
ENSXMAG00000022255	lrrc38b	ENSLOCG00000003320	lrrc38b
ENSXMAG00000008785		ENSLOCG00000001872	alkbh4
ENSXMAG00000002575	wsb2	ENSLOCG00000003373	wsb2
ENSXMAG00000020876	bcar1	ENSLOCG00000008018	bcar1
ENSXMAG00000021912	dhrs3b	ENSLOCG00000003190	dhrs3a
ENSXMAG00000015699	selenot1a	ENSLOCG00000001846	selenot1a
ENSXMAG00000005449	wbp1	ENSLOCG00000015254	wbp1
ENSXMAG00000006699	fuk	ENSLOCG00000007604	fuk
ENSXMAG00000014405	ptp4a1	ENSLOCG00000017023	ptp4a1
ENSXMAG00000012595	mycbpap	ENSLOCG00000013960	MYCBPAP
ENSXMAG00000005701	p2rx1	ENSLOCG00000002716	p2rx1
ENSXMAG00000009084	apmap	ENSLOCG00000016798	apmap
ENSXMAG00000004842	ap3m2	ENSLOCG00000015606	ap3m2
ENSXMAG00000028047	laptm4a	ENSLOCG00000017194	laptm4a
ENSXMAG00000002561	rfc5	ENSLOCG00000003332	rfc5
ENSXMAG00000004958	etf1a	ENSLOCG00000009715	etf1b
ENSXMAG00000016072	lmnb1	ENSLOCG00000008506	lmnb1
ENSXMAG00000005321	mest	ENSLOCG00000015610	mest
ENSXMAG00000009424	phf3	ENSLOCG00000017027	phf3
ENSXMAG00000016224	atp10a	ENSLOCG00000008355	atp10a
ENSXMAG00000023754	klhdc7a	ENSLOCG00000003121	klhdc7a
ENSXMAG00000017566	greb1	ENSLOCG00000016669	greb1
ENSXMAG00000014899	EML5	ENSLOCG00000010403	EML5

ENSXMAG00000014412	lgsn	ENSLOCG00000017019	lgsn
ENSXMAG00000009082		ENSLOCG00000016800	CST7
ENSXMAG00000026566	ttc32	ENSLOCG00000017191	ttc32
ENSXMAG00000022394	tet3	ENSLOCG00000016077	tet3
ENSXMAG00000015354	tcp11l1	ENSLOCG00000004434	tcp11l1
ENSXMAG00000028131	FUNDC1	ENSLOCG00000001923	fundc1
ENSXMAG00000015704		ENSLOCG00000002551	kif2c
ENSXMAG00000020547		ENSLOCG00000018418	MIR199A2
ENSXMAG00000023054	wdr95	ENSLOCG00000000294	wdr95
ENSXMAG00000016065	megf10	ENSLOCG00000008463	megf10
ENSXMAG00000005467	pax8	ENSLOCG00000015261	PAX8
ENSXMAG00000004830	npffr1l2	ENSLOCG00000016076	npffr1l3
ENSXMAG00000002551	ksr2	ENSLOCG00000003407	ksr2
ENSXMAG00000016473	INSYN2	ENSLOCG00000007774	INSYN2
ENSXMAG00000012570	slc25a10	ENSLOCG00000013922	si:ch1073-100f3.2
ENSXMAG00000001139	cenpl	ENSLOCG00000009201	cenpl
ENSXMAG00000009071	tspan14	ENSLOCG00000012923	tspan14
ENSXMAG00000004680	hk2	ENSLOCG00000015312	hk2
ENSXMAG00000017600	esrrga	ENSLOCG00000016054	esrrga
ENSXMAG00000006213	nav3	ENSLOCG00000016281	nav3
ENSXMAG00000005331		ENSLOCG00000016380	
ENSXMAG00000011667	tfdp1a	ENSLOCG00000009902	tfdp1a
ENSXMAG00000008787	NT5C3B	ENSLOCG00000012665	NT5C3B
ENSXMAG00000009420	si:dkey-103j14.5	ENSLOCG00000017017	si:dkey-103j14.5
ENSXMAG00000005479		ENSLOCG00000015264	
ENSXMAG00000020299	tpst1	ENSLOCG00000005284	tpst1
ENSXMAG00000009050		ENSLOCG00000016471	EML4
ENSXMAG00000009415	khdrbs2	ENSLOCG00000017016	khdrbs2
ENSXMAG00000029802		ENSLOCG00000008454	prrc1
ENSXMAG00000015720	ptch2	ENSLOCG00000002509	ptch2
ENSXMAG00000016162		ENSLOCG00000002586	lrp5
ENSXMAG00000005755	atp2a3	ENSLOCG00000002683	atp2a3
ENSXMAG00000009646	svbp	ENSLOCG00000001111	svbp
ENSXMAG00000008569	eif3ha	ENSLOCG00000003303	eif3ha
ENSXMAG00000009452	actr6	ENSLOCG00000015163	actr6
ENSXMAG00000009396	prim2	ENSLOCG00000017003	prim2
ENSXMAG00000023703		ENSLOCG00000010816	
ENSXMAG00000007984		ENSLOCG00000000467	sass6
ENSXMAG00000002919	arhgef10la	ENSLOCG00000003027	arhgef10lb
ENSXMAG00000025024	paqr5a	ENSLOCG00000013987	paqr5a
ENSXMAG00000013336	osgn1	ENSLOCG00000007297	osgn1
ENSXMAG00000006199	osgep	ENSLOCG00000015512	osgep
ENSXMAG00000005829	sgsm2	ENSLOCG00000005260	sgsm2
ENSXMAG00000026799		ENSLOCG00000001477	pisd
ENSXMAG00000024396	glcea	ENSLOCG00000013983	glcea
ENSXMAG00000016339	tmem131	ENSLOCG00000008449	tmem131
ENSXMAG00000029181	bccip	ENSLOCG00000007872	bccip
ENSXMAG00000018193	c8b	ENSLOCG00000008822	C8B
ENSXMAG00000009387	rab23	ENSLOCG00000016999	rab23
ENSXMAG00000002504	nos1	ENSLOCG00000003433	nos1
ENSXMAG00000022194	RNF224	ENSLOCG00000000622	si:ch211-202f5.2
ENSXMAG00000017603	ush2a	ENSLOCG00000016044	ush2a
ENSXMAG00000004674	RBL2	ENSLOCG00000007200	rbl2
ENSXMAG00000009382	bag2	ENSLOCG00000016997	bag2
ENSXMAG00000008845		ENSLOCG00000012633	cnp
ENSXMAG00000018166	C8A	ENSLOCG00000008810	C8A

ENSXMAG00000011620	fer	ENSLOCG00000010449	fer
ENSXMAG00000028307		ENSLOCG00000007671	sdc4
ENSXMAG00000014274	itga8	ENSLOCG00000010450	itga8
ENSXMAG00000013349	gmppb	ENSLOCG00000013363	gmppb
ENSXMAG00000016520	uros	ENSLOCG00000007886	uros
ENSXMAG00000004676	figla	ENSLOCG00000015315	figla
ENSXMAG00000014881	ttc8	ENSLOCG00000010427	TTC8
ENSXMAG00000012476	si:dkey-76b14.2	ENSLOCG00000009044	si:dkey-76b14.2
ENSXMAG00000006195	apex1	ENSLOCG00000015516	apex1
ENSXMAG00000008851	ttc25	ENSLOCG00000012625	ttc25
ENSXMAG00000025191	oser1	ENSLOCG00000007656	oser1
ENSXMAG00000015759	eif2b3	ENSLOCG00000002492	eif2b3
ENSXMAG00000000162	adamts13	ENSLOCG00000002168	adamts13
ENSXMAG00000011697	pou2f1b	ENSLOCG00000009711	pou2f1b
ENSXMAG00000016233		ENSLOCG00000002620	
ENSXMAG00000016521	mmp21	ENSLOCG00000007899	mmp21
ENSXMAG00000012204	manf	ENSLOCG00000013413	manf
ENSXMAG00000003630	nfic	ENSLOCG00000001284	nfic
ENSXMAG00000019790	fam43b	ENSLOCG00000018080	fam43b
ENSXMAG00000007675	5-Mar	ENSLOCG00000007112	5-Mar
ENSXMAG00000013370	pde12	ENSLOCG00000013277	pde12
ENSXMAG00000007664	cdc26	ENSLOCG00000000546	cdc26
ENSXMAG00000004651	add2	ENSLOCG00000015316	add2
ENSXMAG00000008620	washc5	ENSLOCG00000010046	washc5
ENSXMAG00000014375	mindy3	ENSLOCG00000010478	mindy3
ENSXMAG00000018102	lpar3	ENSLOCG00000005571	lpar3
ENSXMAG00000012421	mul1b	ENSLOCG00000002052	mul1b
ENSXMAG00000006728	rpgrip1l	ENSLOCG00000007156	rpgrip1l
ENSXMAG00000023629	agr2	ENSLOCG00000011333	agr2
ENSXMAG00000025951		ENSLOCG00000000910	
ENSXMAG00000026093	tmem170a	ENSLOCG00000008045	tmem170a
ENSXMAG00000016370	si:ch211-201h21.5	ENSLOCG00000008477	si:ch211-201h21.5
ENSXMAG00000004517	foxred2	ENSLOCG00000012061	foxred2
ENSXMAG00000002750	prpf4	ENSLOCG00000000581	prpf4
ENSXMAG00000012428	VWA5B1	ENSLOCG00000002077	VWA5B1
ENSXMAG00000018106	mcoln2	ENSLOCG00000005533	mcoln2
ENSXMAG00000005834	zzef1	ENSLOCG00000002640	zzef1
ENSXMAG00000006840		ENSLOCG00000018029	
ENSXMAG00000007982	lrrc40	ENSLOCG00000009608	lrrc40
ENSXMAG00000028341	si:ch211-266k8.4	ENSLOCG00000004039	si:ch211-266k8.4
ENSXMAG00000016378	bivm	ENSLOCG00000008489	bivm
ENSXMAG00000012197	rbm15b	ENSLOCG00000013409	rbm15b
ENSXMAG00000015786	plk3	ENSLOCG00000002457	plk3
ENSXMAG00000008879	nkiras2	ENSLOCG00000012608	nkiras2
ENSXMAG00000016741	si:dkey-240h12.4	ENSLOCG00000007353	si:dkey-240h12.4
ENSXMAG00000000465	anp32a	ENSLOCG00000013972	anp32a
ENSXMAG00000002756		ENSLOCG00000002572	
ENSXMAG00000004633	ch25hl3	ENSLOCG00000015341	ch25hl3
ENSXMAG00000007031	nhp2	ENSLOCG00000009630	nhp2
ENSXMAG00000012440	DDX19B	ENSLOCG00000002118	DDX19A
ENSXMAG00000028125	tmem8a	ENSLOCG00000006386	
ENSXMAG00000005848	tnfaip1	ENSLOCG00000005243	tnfaip1
ENSXMAG00000030098	ssr2	ENSLOCG00000007294	ssr2
ENSXMAG00000021604	rps23	ENSLOCG00000013284	rps23
ENSXMAG00000015418	RASSF10	ENSLOCG00000017321	RASSF10
ENSXMAG00000016387	zgc:172121	ENSLOCG00000008512	zgc:172121

ENSXMAG00000013428	map7d3	ENSLOCG00000015111	map7d3
ENSXMAG00000004508	ankrd54	ENSLOCG00000012068	ankrd54
ENSXMAG00000005196	ythdc1	ENSLOCG00000003045	ythdc1
ENSXMAG00000006758	nox5	ENSLOCG00000013975	nox5
ENSXMAG00000002490	nipsnap1	ENSLOCG00000003492	nipsnap1
ENSXMAG00000021697	pde6d	ENSLOCG00000001371	pde6d
ENSXMAG00000016259	ppp6r3	ENSLOCG00000002546	ppp6r3
ENSXMAG00000008899	dhx58	ENSLOCG00000012582	dhx58
ENSXMAG00000007652	cpeb3	ENSLOCG00000007131	cpeb3
ENSXMAG00000014847	NEK9	ENSLOCG00000010439	NEK9
ENSXMAG00000011058	rgs11	ENSLOCG00000006354	rgs11
ENSXMAG00000005860	ift20	ENSLOCG00000005230	ift20
ENSXMAG00000029172	fktn	ENSLOCG00000003089	fktn
ENSXMAG00000025877	si:dkey-12j5.1	ENSLOCG00000010489	si:dkey-12j5.1
ENSXMAG00000007975	rpl36	ENSLOCG00000003106	rpl36
ENSXMAG00000027375	ubxn10	ENSLOCG00000002149	ubxn10
ENSXMAG00000025210	asb6	ENSLOCG00000002248	asb6
ENSXMAG00000004613	arhgap25	ENSLOCG00000015343	arhgap25
ENSXMAG00000008915	kat2a	ENSLOCG00000012570	kat2a
ENSXMAG00000025912		ENSLOCG00000003511	
ENSXMAG00000029681	lyrm9	ENSLOCG00000005647	lyrm9
ENSXMAG00000005492	tmem141	ENSLOCG00000000999	tmem141
ENSXMAG00000026512		ENSLOCG00000013595	ccdc51
ENSXMAG00000016394	nme7	ENSLOCG00000008530	nme7
ENSXMAG00000024441	zmat2	ENSLOCG00000011219	zmat2
ENSXMAG00000024199		ENSLOCG00000010314	apc
ENSXMAG00000015822	cacybp	ENSLOCG00000002438	cacybp
ENSXMAG00000012455	cptp	ENSLOCG00000002163	cptp
ENSXMAG00000015461	parvaa	ENSLOCG00000004074	parvab
ENSXMAG00000011052	zgc:56409	ENSLOCG00000006510	zgc:56409
ENSXMAG00000008663	fbxo32	ENSLOCG00000009995	fbxo32
ENSXMAG00000001791	DNAJC13	ENSLOCG00000012015	DNAJC13
ENSXMAG00000014821	MLH3	ENSLOCG00000010467	mlh3
ENSXMAG00000007953	lmnb2	ENSLOCG00000003086	lmnb2
ENSXMAG00000021541	cyb5d2	ENSLOCG00000002621	cyb5d2
ENSXMAG00000005494	zmynd19	ENSLOCG00000000957	zmynd19
ENSXMAG00000005459	slc6a1l	ENSLOCG00000016858	slc6a1l
ENSXMAG00000006779	ambra1b	ENSLOCG00000005523	ambra1a
ENSXMAG00000004583	cds2	ENSLOCG00000015345	cds2
ENSXMAG00000026284		ENSLOCG00000010504	ZNF804B
ENSXMAG00000012456	ints11	ENSLOCG00000002177	ints11
ENSXMAG00000027415	zgc:110063	ENSLOCG00000003526	zgc:110063
ENSXMAG00000010496	tmf1	ENSLOCG00000010119	tmf1
ENSXMAG00000005142		ENSLOCG00000003114	FSD1L
ENSXMAG00000005852	ankfy1	ENSLOCG00000002602	ankfy1
ENSXMAG00000000131	slc15a4	ENSLOCG00000006344	slc15a4
ENSXMAG00000027993	atp6v1g1	ENSLOCG00000000772	atp6v1g1
ENSXMAG00000008356	spock1	ENSLOCG00000011355	spock1
ENSXMAG00000002464	rnft2	ENSLOCG00000003534	rnft2
ENSXMAG00000021788	prelid3a	ENSLOCG00000009987	prelid3a
ENSXMAG00000026738	COQ4	ENSLOCG00000000864	coq4
ENSXMAG00000011790	me3	ENSLOCG00000009615	me3
ENSXMAG00000000449	hpdl	ENSLOCG00000008001	hpdl
ENSXMAG00000016415	dpt	ENSLOCG00000008571	dpt
ENSXMAG00000008469	pak7	ENSLOCG00000016429	pak7
ENSXMAG00000020905	traf2b	ENSLOCG00000000840	traf2b

ENSXMAG00000012471		ENSLOCG00000018081	
ENSXMAG00000000129	glt1d1	ENSLOCG00000006330	glt1d1
ENSXMAG00000002458	fbxw8	ENSLOCG00000003552	fbxw8
ENSXMAG00000006797	mdkb	ENSLOCG00000005557	mdkb
ENSXMAG00000005123	slc25a46	ENSLOCG00000010395	slc25a46
ENSXMAG00000007938	ndufa11	ENSLOCG00000003164	ndufa11
ENSXMAG00000018139	ftsj3	ENSLOCG00000008979	ftsj3
ENSXMAG00000021404	ube2g1a	ENSLOCG00000002581	ube2g1a
ENSXMAG00000016418	zbtb11	ENSLOCG00000009538	zbtb11
ENSXMAG00000017201	tnk2b	ENSLOCG00000006982	tnk2b
ENSXMAG00000005092	wdr36	ENSLOCG00000010374	wdr36
ENSXMAG00000014769	ERG28	ENSLOCG00000010553	ERG28
ENSXMAG00000016538	lhpp	ENSLOCG00000008067	lhpp
ENSXMAG00000024392		ENSLOCG00000016855	
ENSXMAG00000012245		ENSLOCG00000003402	ctns
ENSXMAG00000009066	MSL3	ENSLOCG00000009931	MSL3
ENSXMAG00000010501	eogt	ENSLOCG00000010125	eogt
ENSXMAG00000022794	mrps14	ENSLOCG00000002424	mrps14
ENSXMAG00000018167	HRASLS	ENSLOCG00000008958	hrasls
ENSXMAG00000002436	fbxo21	ENSLOCG00000003589	fbxo21
ENSXMAG00000000124	TMEM132D	ENSLOCG00000008220	
ENSXMAG00000005883	spns3	ENSLOCG00000002570	
ENSXMAG00000027534		ENSLOCG00000009509	timd4
ENSXMAG00000016554	oat	ENSLOCG00000008099	oat
ENSXMAG00000013618		ENSLOCG00000011365	bicc2
ENSXMAG00000016425	tp63	ENSLOCG00000005148	tp63
ENSXMAG00000016293	ric8a	ENSLOCG00000002406	ric8a
ENSXMAG00000008969	si:ch211-210g13.5	ENSLOCG00000012546	si:ch211-210g13.5
ENSXMAG00000005898	sorl1	ENSLOCG00000006168	sorl1
ENSXMAG00000008478	LAMP5	ENSLOCG00000016433	LAMP5
ENSXMAG00000014737	ttll5	ENSLOCG00000010563	ttll5
ENSXMAG00000005062		ENSLOCG00000010432	man2a1
ENSXMAG00000018503		ENSLOCG00000012192	trappc8
ENSXMAG00000002413	nf2a	ENSLOCG00000003628	nf2a
ENSXMAG00000007817	C1QA	ENSLOCG00000005764	c1qa
ENSXMAG00000005523	fbxw5	ENSLOCG00000000818	fbxw5
ENSXMAG00000016569	CHST15	ENSLOCG00000008128	chst15
ENSXMAG00000006803	dgkzb	ENSLOCG00000005570	DGKZ
ENSXMAG00000014422	glipr2l	ENSLOCG00000005303	glipr2l
ENSXMAG00000020088	fzd10	ENSLOCG00000018155	fzd10
ENSXMAG00000011833	CEP97	ENSLOCG00000009560	cep97
ENSXMAG00000014726	RNF207	ENSLOCG00000004882	rnf207b
ENSXMAG00000013617		ENSLOCG00000011390	
ENSXMAG00000027509		ENSLOCG00000016434	
ENSXMAG00000013459	taf7	ENSLOCG00000015317	taf7
ENSXMAG00000007788	C1QC	ENSLOCG00000005782	c1qc
ENSXMAG00000005907	SPNS2	ENSLOCG00000002511	SPNS2
ENSXMAG00000004479	spc24	ENSLOCG00000007731	spc24
ENSXMAG00000016574	CPXM2	ENSLOCG00000008139	CPXM2
ENSXMAG00000021824		ENSLOCG00000005734	nacad
ENSXMAG00000016328	carmil2	ENSLOCG00000006875	carmil2
ENSXMAG00000029781	rpl24	ENSLOCG00000009548	rpl24
ENSXMAG00000024347	tprg1	ENSLOCG00000005183	tprg1
ENSXMAG00000008481	PLCB4	ENSLOCG00000016435	PLCB4
ENSXMAG00000000110	piwil1	ENSLOCG00000006283	piwil1
ENSXMAG00000004461	kri1	ENSLOCG00000007754	kri1

ENSXMAG00000006969	rhogb	ENSLOCG00000018023	rhoga
ENSXMAG00000028696	C1QB	ENSLOCG00000005797	C1QB
ENSXMAG00000025694	nxph1	ENSLOCG00000011148	nxph1
ENSXMAG00000014438	tbrg4	ENSLOCG00000005721	TBRG4
ENSXMAG00000029845	mpc2	ENSLOCG00000008587	zgc:103678
ENSXMAG00000016612	bub3	ENSLOCG00000008177	bub3
ENSXMAG00000013464	si:ch211-106m9.1	ENSLOCG00000015314	si:ch211-106m9.1
ENSXMAG00000005487		ENSLOCG00000016643	adipor2
ENSXMAG00000024414	zgc:112294	ENSLOCG00000003686	zgc:112294
ENSXMAG00000018207	GNB4	ENSLOCG00000001216	GNB4
ENSXMAG00000024805		ENSLOCG00000006920	mocs2
ENSXMAG00000005918	ruvbl2	ENSLOCG00000002468	ruvbl2
ENSXMAG00000010534	suclg2	ENSLOCG00000010157	suclg2
ENSXMAG00000025861	wnt5b	ENSLOCG00000016647	wnt5b
ENSXMAG00000001905		ENSLOCG00000007411	
ENSXMAG00000019854	zpr1	ENSLOCG00000005578	zpr1
ENSXMAG00000025129		ENSLOCG00000006931	
ENSXMAG00000008972	stat3	ENSLOCG00000012514	stat3
ENSXMAG00000012579	sdf4	ENSLOCG00000002339	SDF4
ENSXMAG00000016800	phf14	ENSLOCG00000011164	phf14
ENSXMAG00000016640	acadsb	ENSLOCG00000008211	acadsb
ENSXMAG00000029470	agtrap	ENSLOCG00000004850	agtrap
ENSXMAG00000013469	drp2	ENSLOCG00000015313	drp2
ENSXMAG00000005041	ugcg	ENSLOCG00000002974	ugcg
ENSXMAG00000011126		ENSLOCG00000009664	slc5a9
ENSXMAG00000008502	PLCB1	ENSLOCG00000016437	PLCB1
ENSXMAG00000015548	far1	ENSLOCG00000004234	far1
ENSXMAG00000014700	IFT43	ENSLOCG00000010599	IFT43
ENSXMAG00000030012		ENSLOCG00000015476	rhoua
ENSXMAG00000012587	c1qtnf12	ENSLOCG00000002323	
ENSXMAG00000006235	draxin	ENSLOCG00000004818	draxin
ENSXMAG00000011866	dcaf6	ENSLOCG00000008600	dcaf6
ENSXMAG00000026661	lpp	ENSLOCG00000005199	lpp
ENSXMAG00000025830	ptbp3	ENSLOCG00000002942	ptbp3
ENSXMAG00000007625	ccnj	ENSLOCG00000007212	ccnj
ENSXMAG00000025060	alg8	ENSLOCG00000005958	alg8
ENSXMAG00000005946	ftr82	ENSLOCG00000002432	ftr82
ENSXMAG00000023748	kbtbd8	ENSLOCG00000010195	kbtbd8
ENSXMAG00000012599	syt6a	ENSLOCG00000010494	syt6b
ENSXMAG00000004987	hsdl2	ENSLOCG00000002917	hsdl2
ENSXMAG00000019958	aldh7a1	ENSLOCG00000008570	aldh7a1
ENSXMAG00000005949	atp5l	ENSLOCG00000004026	atp5l
ENSXMAG00000006134		ENSLOCG00000015202	ANO4
ENSXMAG00000008401	klb	ENSLOCG00000013013	klb
ENSXMAG00000013478	cenpi	ENSLOCG00000015310	cenpi
ENSXMAG00000004120	lrig1	ENSLOCG00000010211	lrig1
ENSXMAG00000004574	zgc:65851	ENSLOCG00000015323	zgc:65851
ENSXMAG00000025172	slitrk6	ENSLOCG00000017777	slitrk6
ENSXMAG00000008563	bmp2b	ENSLOCG00000016442	bmp2b
ENSXMAG00000006890	ccdc90b	ENSLOCG00000005941	ccdc90b
ENSXMAG00000019723	SLITRK1	ENSLOCG00000017778	SLITRK1
ENSXMAG00000012605	klhl21	ENSLOCG00000002774	klhl21
ENSXMAG00000018223	actl6a	ENSLOCG00000001256	actl6a
ENSXMAG00000006201	pank4	ENSLOCG00000004790	pank4
ENSXMAG00000026877	ovch2	ENSLOCG00000004255	
ENSXMAG00000008568	fermt1	ENSLOCG00000016445	fermt1

ENSXMAG00000021458	spry2	ENSLOCG00000017779	spry2
ENSXMAG00000013483	tmem35	ENSLOCG00000015309	tmem35
ENSXMAG00000029501	GPATCH2L	ENSLOCG00000010621	GPATCH2L
ENSXMAG00000008415	ints10	ENSLOCG00000012873	ints10
ENSXMAG00000002529	glcci1a	ENSLOCG00000011123	glcci1a
ENSXMAG00000003460	rtn4r	ENSLOCG00000003749	rtn4r
ENSXMAG00000021238		ENSLOCG00000012395	zgc:92749
ENSXMAG00000029014	NDFIP2	ENSLOCG00000004539	ndfip2
ENSXMAG00000013556	capslb	ENSLOCG00000012779	capslb
ENSXMAG00000007676	slmapa	ENSLOCG00000013251	slmapa
ENSXMAG00000004145	slc25a26	ENSLOCG00000010233	slc25a26
ENSXMAG00000006872	pcf11	ENSLOCG00000005901	pcf11
ENSXMAG00000026003	si:ch211-207k7.4	ENSLOCG00000003759	si:ch211-207k7.4
ENSXMAG00000005558	ASS1	ENSLOCG00000005378	ASS1
ENSXMAG00000000471	coro2ba	ENSLOCG00000013967	coro2bb
ENSXMAG00000013485	arl13a	ENSLOCG00000015307	arl13a
ENSXMAG00000018249	mrpl47	ENSLOCG00000001289	mrpl47
ENSXMAG00000014672		ENSLOCG00000010630	
ENSXMAG00000003442	lrrc8ab	ENSLOCG00000004397	lrrc8ab
ENSXMAG00000012629	nol9	ENSLOCG00000002798	nol9
ENSXMAG00000016840	rpa3	ENSLOCG00000011113	rpa3
ENSXMAG00000015561	cyb5r2	ENSLOCG00000004270	cyb5r2
ENSXMAG00000008592	trmt6	ENSLOCG00000016451	trmt6
ENSXMAG00000018257	ndufb5	ENSLOCG00000001304	ndufb5
ENSXMAG00000003440	zgc:101858	ENSLOCG00000003168	zgc:101858
ENSXMAG00000015578	kdelc2	ENSLOCG00000002033	kdelc2
ENSXMAG00000006849	ncbp3	ENSLOCG00000006706	ncbp3
ENSXMAG00000017642	kctd3	ENSLOCG00000016039	kctd3
ENSXMAG00000026827	xkrx	ENSLOCG00000015306	xkrx
ENSXMAG00000006871	phf21ab	ENSLOCG00000005618	phf21ab
ENSXMAG00000016456	bcl6a	ENSLOCG00000005212	bcl6a
ENSXMAG00000014663	prox2	ENSLOCG00000010701	prox2
ENSXMAG00000012638	plekhg5b	ENSLOCG00000002820	plekhg5b
ENSXMAG00000011225	arhgap39	ENSLOCG00000008461	arhgap39
ENSXMAG00000013488	nox1	ENSLOCG00000015303	nox1
ENSXMAG00000021269	surf4	ENSLOCG00000005358	
ENSXMAG00000006116	cfap53	ENSLOCG00000012829	cfap53
ENSXMAG00000028591	gpr161	ENSLOCG00000008616	si:ch211-237c6.4
ENSXMAG00000008610	gpcpd1	ENSLOCG00000016457	gpcpd1
ENSXMAG00000018261	usp13	ENSLOCG00000001321	usp13
ENSXMAG00000008414	tlr1	ENSLOCG00000012910	tlr1
ENSXMAG00000005594	surf2	ENSLOCG00000005343	
ENSXMAG00000021814		ENSLOCG00000004116	si:ch1073-342h5.2
ENSXMAG00000016693	pde6gb	ENSLOCG00000013026	pde6gb
ENSXMAG00000025586		ENSLOCG00000004654	dnajc24
ENSXMAG00000007634	dennd6aa	ENSLOCG00000013265	dennd6aa
ENSXMAG00000019482	slc25a51b	ENSLOCG00000017922	slc25a51a
ENSXMAG00000006594	pik3cb	ENSLOCG00000004421	pik3cb
ENSXMAG00000016697		ENSLOCG00000013029	OXLD1
ENSXMAG00000007601	DNTT	ENSLOCG00000007264	DNTT
ENSXMAG00000006083	rad54l	ENSLOCG00000004144	rad54l
ENSXMAG00000011883	otc	ENSLOCG00000007928	otc
ENSXMAG00000008433	frmpd1b	ENSLOCG00000012868	FRMPD1
ENSXMAG00000027531	lcor	ENSLOCG00000012823	lcor
ENSXMAG00000022856	gpr142	ENSLOCG00000013050	gpr142
ENSXMAG00000006177		ENSLOCG00000013825	atrip

ENSXMAG00000016708	btbd17a	ENSLOCG00000013052	btbd17a
ENSXMAG00000027885	scrt1b	ENSLOCG00000007573	scrt1b
ENSXMAG00000028018	smx5	ENSLOCG00000005228	smx5
ENSXMAG00000024801	ppp1r3b	ENSLOCG00000017547	ppp1r3b
ENSXMAG00000014631	rps6kl1	ENSLOCG00000010678	rps6kl1
ENSXMAG00000008381	zbtb44	ENSLOCG00000004162	zbtb44
ENSXMAG00000012658		ENSLOCG00000006904	
ENSXMAG00000012295	zgc:136908	ENSLOCG00000009961	zgc:136908
ENSXMAG00000006175	trib3	ENSLOCG00000006629	trib3
ENSXMAG00000014496	ino80c	ENSLOCG00000005268	ino80c
ENSXMAG00000005599	gb:bc139872	ENSLOCG00000004822	gb:bc139872
ENSXMAG00000005650	hoga1	ENSLOCG00000012833	hoga1
ENSXMAG00000006133	slc38a4	ENSLOCG00000016411	slc38a4
ENSXMAG00000013524	cstf2	ENSLOCG00000015300	cstf2
ENSXMAG00000019828		ENSLOCG00000018236	
ENSXMAG00000015856	tnr	ENSLOCG00000002355	tnr
ENSXMAG00000012663	irx7	ENSLOCG00000006946	irx7
ENSXMAG00000019862	kcnj2a	ENSLOCG00000017760	kcnj2a
ENSXMAG00000027058	adamts15b	ENSLOCG00000004181	adamts15b
ENSXMAG00000016471	inpp5d	ENSLOCG00000005346	inpp5d
ENSXMAG00000006157	pnp4a	ENSLOCG00000006609	pnp4a
ENSXMAG00000016747	galr2a	ENSLOCG00000013067	galr2a
ENSXMAG00000007597	vox	ENSLOCG00000007276	vox
ENSXMAG00000006806	zgc:152891	ENSLOCG00000009102	
ENSXMAG00000008437	GRHPR	ENSLOCG00000012851	GRHPR
ENSXMAG00000006891	prdm11	ENSLOCG00000005840	prdm11
ENSXMAG00000011909	srpx	ENSLOCG00000007901	srpx
ENSXMAG00000017681	KRTCAP3	ENSLOCG00000016033	
ENSXMAG00000021226		ENSLOCG00000003755	eps15l1a
ENSXMAG00000026579		ENSLOCG00000002594	
ENSXMAG00000025731	soga3a	ENSLOCG00000017056	soga3a
ENSXMAG00000016866	sdhaf3	ENSLOCG00000011022	sdhaf3
ENSXMAG00000021867	zgc:109949	ENSLOCG00000007879	zgc:109949
ENSXMAG00000014608	lrrc74a	ENSLOCG00000010657	lrrc74a
ENSXMAG00000013208	galnt1	ENSLOCG00000005248	galnt1
ENSXMAG00000013531		ENSLOCG00000015296	bcorl1
ENSXMAG00000020219	foxl2b	ENSLOCG00000017389	foxl2b
ENSXMAG00000020094	ptrh2	ENSLOCG00000017426	ptrh2
ENSXMAG00000016867	tac1	ENSLOCG00000011048	tac1
ENSXMAG00000020115	gpr17	ENSLOCG00000017397	gpr17
ENSXMAG00000007589	KNDC1	ENSLOCG00000007289	KNDC1
ENSXMAG00000012671	cbln4	ENSLOCG00000002576	cbln4
ENSXMAG00000006786	si:dkey-3k24.5	ENSLOCG00000013030	si:dkey-3k24.5
ENSXMAG00000028985		ENSLOCG00000013561	tfr2
ENSXMAG00000006897	kcnj11l	ENSLOCG00000017162	kcnj11l
ENSXMAG00000017688		ENSLOCG00000016027	fzd3b
ENSXMAG00000006580	mrps22	ENSLOCG00000004403	mrps22
ENSXMAG00000016515		ENSLOCG00000002074	ddx10
ENSXMAG00000014605	angel1	ENSLOCG00000010649	angel1
ENSXMAG00000021914	trib2	ENSLOCG00000016468	trib2
ENSXMAG00000013532	tspan7	ENSLOCG00000015294	tspan7
ENSXMAG00000016868	asns	ENSLOCG00000011056	asns
ENSXMAG00000012293	ndufb7	ENSLOCG00000009951	ndufb7
ENSXMAG00000016828	derl2	ENSLOCG00000002403	derl2
ENSXMAG00000008677	ECHDC1	ENSLOCG00000016511	ECHDC1
ENSXMAG00000011920	sytl5	ENSLOCG00000007874	sytl5

ENSXMAG00000012679	pfdn4	ENSLOCG00000002535	pfdn4
ENSXMAG00000027263	zmp:0000000760	ENSLOCG00000016918	zmp:0000000760
ENSXMAG00000008445	si:ch211-203d1.3	ENSLOCG00000008248	si:ch211-203d1.3
ENSXMAG00000016748	exoc7	ENSLOCG00000013076	exoc7
ENSXMAG00000009113	hook2	ENSLOCG00000007916	hook2
ENSXMAG00000006130	st3gal8	ENSLOCG00000006466	st3gal8
ENSXMAG00000013538	srpx2	ENSLOCG00000015293	srpx2
ENSXMAG00000027602	VASH1	ENSLOCG00000010644	VASH1
ENSXMAG00000008746	adamts9	ENSLOCG00000010273	adamts9
ENSXMAG00000008684	rnf146	ENSLOCG00000016513	rnf146
ENSXMAG00000009103	stk17al	ENSLOCG00000012486	stk17al
ENSXMAG00000012683	znf217	ENSLOCG00000002484	znf217
ENSXMAG00000003497	klhl29	ENSLOCG00000016613	zmp:0000000619
ENSXMAG00000013207	tmem245	ENSLOCG00000005216	tmem245
ENSXMAG00000029171		ENSLOCG00000017394	GP5
ENSXMAG00000005683	ENTPD7	ENSLOCG00000009002	
ENSXMAG00000006098	unm_sa1614	ENSLOCG00000006485	unm_sa1614
ENSXMAG00000019526	mdh2	ENSLOCG00000001699	mdh2
ENSXMAG00000029234		ENSLOCG00000016294	fbxo11a
ENSXMAG00000014576	fcf1	ENSLOCG00000010728	fcf1
ENSXMAG00000020092	timm8b	ENSLOCG00000006037	timm8b
ENSXMAG00000009111	trir	ENSLOCG00000007974	trir
ENSXMAG00000013546	sytl4	ENSLOCG00000015291	sytl4
ENSXMAG00000011518	MRPL48	ENSLOCG00000006190	mrpl48
ENSXMAG00000006544	rbp2a	ENSLOCG00000004353	rbp2b
ENSXMAG00000007415	ANLN	ENSLOCG00000007059	ANLN
ENSXMAG00000012685	tshz2	ENSLOCG00000002451	tshz2
ENSXMAG00000022449	sdhdb	ENSLOCG00000006052	sdhda
ENSXMAG00000000494		ENSLOCG00000014032	
ENSXMAG00000009114	hsd17b1	ENSLOCG00000012469	hsd17b1
ENSXMAG00000012686	zfp64	ENSLOCG00000002444	zfp64
ENSXMAG00000021823	uts2d	ENSLOCG00000005045	uts2d
ENSXMAG00000029792	MRPL18	ENSLOCG00000008109	
ENSXMAG00000029932	xk	ENSLOCG00000007838	xk
ENSXMAG00000014558	AREL1	ENSLOCG00000010734	AREL1
ENSXMAG00000017068		ENSLOCG00000007338	gabrz
ENSXMAG00000023224		ENSLOCG00000006507	fer1l4
ENSXMAG00000027250	LANCL3	ENSLOCG00000007822	LANCL3
ENSXMAG00000015619	arntl1a	ENSLOCG00000003999	arntl1a
ENSXMAG00000008688	trmt11	ENSLOCG00000016519	trmt11
ENSXMAG00000016749	uap1l1	ENSLOCG00000006083	uap1l1
ENSXMAG00000007624	hesx1	ENSLOCG00000013306	hesx1
ENSXMAG00000016543	pea15	ENSLOCG00000002124	pea15
ENSXMAG00000021763		ENSLOCG00000008992	
ENSXMAG00000018340	ccdc50	ENSLOCG00000005056	ccdc50
ENSXMAG00000026883		ENSLOCG00000016940	
ENSXMAG00000025318	trmt12	ENSLOCG00000015290	trmt12
ENSXMAG00000005546	si:rp71-84d9.1	ENSLOCG00000016637	si:rp71-84d9.1
ENSXMAG00000007566		ENSLOCG00000007359	CFAP46
ENSXMAG00000008450	tcirg1b	ENSLOCG00000006458	tcirg1a
ENSXMAG00000012692	sall4	ENSLOCG00000002427	sall4
ENSXMAG00000006028	cpne1	ENSLOCG00000006532	cpne1
ENSXMAG00000025549	bmp5	ENSLOCG00000016915	bmp5
ENSXMAG00000003479	atad2b	ENSLOCG00000016615	atad2b
ENSXMAG00000027815	atpv0e2	ENSLOCG00000008982	atpv0e2
ENSXMAG00000005519	ahctf1	ENSLOCG00000016634	ahctf1

ENSXMAG00000013554	zdhhc9	ENSLOCG00000015287	zdhhc9
ENSXMAG00000029956	frrs1l	ENSLOCG00000005200	frrs1l
ENSXMAG00000017818		ENSLOCG00000007365	arhgef2
ENSXMAG00000028931	rnft1	ENSLOCG00000003795	rnft1
ENSXMAG00000000507		ENSLOCG00000013940	calml4b
ENSXMAG00000029737		ENSLOCG00000007383	valopb
ENSXMAG00000011743	anxa13l	ENSLOCG00000007737	
ENSXMAG00000006933		ENSLOCG00000016238	ZFC3H1
ENSXMAG00000018362	p3h2	ENSLOCG00000005129	p3h2
ENSXMAG00000000723	aspm	ENSLOCG00000007494	aspm
ENSXMAG00000010176	hmgcll1	ENSLOCG00000016910	hmgcll1
ENSXMAG00000000510		ENSLOCG00000013946	cln6a
ENSXMAG00000010593	KIAA0895	ENSLOCG00000007046	KIAA0895
ENSXMAG00000024976	ATP9A	ENSLOCG00000002392	ATP9A
ENSXMAG00000007554	nkx6.2	ENSLOCG00000007395	nkx6.2
ENSXMAG00000008778	il17rd	ENSLOCG00000013320	il17rd
ENSXMAG00000005782		ENSLOCG00000012224	
ENSXMAG00000006016	nfs1	ENSLOCG00000006546	nfs1
ENSXMAG00000004127	steap3	ENSLOCG00000003240	steap3
ENSXMAG00000013558	sash3	ENSLOCG00000015281	sash3
ENSXMAG00000024833		ENSLOCG00000003946	PYURF
ENSXMAG00000029984	si:dkey-29b11.3	ENSLOCG00000016521	si:dkey-29b11.3
ENSXMAG00000022253	c1ql2	ENSLOCG00000003228	c1ql2
ENSXMAG00000000055	adgrd1	ENSLOCG00000006216	adgrd1
ENSXMAG00000007372	trappc1	ENSLOCG00000013695	trappc1
ENSXMAG00000016556	sap130a	ENSLOCG00000005291	sap130a
ENSXMAG00000000515	CHRNA7	ENSLOCG00000014571	
ENSXMAG00000018387	ccdc181	ENSLOCG00000010088	ccdc181
ENSXMAG00000025773	zgc:162255	ENSLOCG00000012396	zgc:162255
ENSXMAG00000013561	xpnpep2	ENSLOCG00000015278	xpnpep2
ENSXMAG00000010192	hcrtr2	ENSLOCG00000016905	hcrtr2
ENSXMAG00000008485	prpf19	ENSLOCG00000008123	prpf19
ENSXMAG00000008348	pxylp1	ENSLOCG00000004225	pxylp1
ENSXMAG00000007542	INPP5A	ENSLOCG00000007409	INPP5A
ENSXMAG00000026337	psmb6	ENSLOCG00000013698	psmb6
ENSXMAG00000024294	ubxn2a	ENSLOCG00000016618	ubxn2a
ENSXMAG00000029822	ltv1	ENSLOCG00000015440	ltv1
ENSXMAG00000016869	eepd1	ENSLOCG00000007030	eepd1
ENSXMAG00000013195	alg2	ENSLOCG00000005323	alg2
ENSXMAG00000000720	depdc1a	ENSLOCG00000009559	depdc1a
ENSXMAG00000006008	dram2b	ENSLOCG00000011490	dram2a
ENSXMAG00000011688		ENSLOCG00000004804	dis3l2
ENSXMAG00000003439	mfsd2b	ENSLOCG00000016620	mfsd2b
ENSXMAG00000008793	pxk	ENSLOCG00000013200	pxk
ENSXMAG00000013581	apln	ENSLOCG00000015277	apln
ENSXMAG00000003487		ENSLOCG00000016941	wnk1a
ENSXMAG00000007812		ENSLOCG00000016756	rp1l1a
ENSXMAG00000006966	tbc1d15	ENSLOCG00000016247	TBC1D15
ENSXMAG00000028520	sec61b	ENSLOCG00000005335	sec61b
ENSXMAG00000000526	trip4	ENSLOCG00000014993	trip4
ENSXMAG00000008306	epha4a	ENSLOCG00000004201	epha4l
ENSXMAG00000005988	cept1b	ENSLOCG00000011510	cept1b
ENSXMAG00000022880		ENSLOCG00000016900	
ENSXMAG00000003436	wdcp	ENSLOCG00000016622	wdcp
ENSXMAG00000008822	pdhb	ENSLOCG00000013193	pdhb
ENSXMAG00000007385	ephb4b	ENSLOCG00000013666	ephb4b

ENSXMAG00000013188	nr4a3	ENSLOCG00000005350	nr4a3
ENSXMAG00000016813	FADS6	ENSLOCG00000013119	FADS6
ENSXMAG00000024088	piga	ENSLOCG00000007789	PIGA
ENSXMAG00000017705	smyd3	ENSLOCG00000016648	smyd3
ENSXMAG00000020573	MIR199A1	ENSLOCG00000018412	MIR199A1
ENSXMAG00000000326	decr1	ENSLOCG00000010263	decr1
ENSXMAG00000026324	tph2	ENSLOCG00000016249	TPH2
ENSXMAG00000018396	prrg1	ENSLOCG00000010126	prrg1
ENSXMAG00000007540	pwwp2b	ENSLOCG00000007432	pwwp2b
ENSXMAG00000015658	slc12a3	ENSLOCG00000007841	slc12a3
ENSXMAG00000000737	cmtm8b	ENSLOCG00000001400	cmtm8b
ENSXMAG00000027946	fkbp1b	ENSLOCG00000016624	fkbp1b
ENSXMAG00000002194	rnasekb	ENSLOCG00000014085	rnaseka
ENSXMAG00000010214	lrrc1	ENSLOCG00000016898	lrrc1
ENSXMAG00000021086	gramd2aa	ENSLOCG00000014988	gramd2aa
ENSXMAG00000007815		ENSLOCG00000016748	
ENSXMAG00000028891		ENSLOCG00000007441	LRRC27
ENSXMAG00000006997	zgc:103508	ENSLOCG00000014080	zgc:103508
ENSXMAG00000021822		ENSLOCG00000016420	scaf11
ENSXMAG00000013186	stx17	ENSLOCG00000005385	stx17
ENSXMAG00000025423	pnp4b	ENSLOCG00000010235	pnp4b
ENSXMAG00000001965	slc2a9l1	ENSLOCG00000010306	slc2a9l1
ENSXMAG00000020114	si:dkeyp-87e7.4	ENSLOCG00000013182	si:dkeyp-87e7.4
ENSXMAG00000008710	ctnnb1	ENSLOCG00000001383	ctnnb1
ENSXMAG00000013582	ocrl	ENSLOCG00000015266	ocrl
ENSXMAG00000018402	tab3	ENSLOCG00000010262	tab3
ENSXMAG00000019864	senp8	ENSLOCG00000017960	senp8
ENSXMAG00000008302	SGPP2	ENSLOCG00000004157	SGPP2
ENSXMAG00000000716	si:ch211-198n5.11	ENSLOCG00000009522	si:ch211-198n5.11
ENSXMAG00000006991		ENSLOCG00000017557	
ENSXMAG00000027217	snx20	ENSLOCG00000007326	snx20
ENSXMAG00000026085	erp44	ENSLOCG00000005398	erp44
ENSXMAG00000024902	kcng1	ENSLOCG00000002356	kcng1
ENSXMAG00000006636	arid2	ENSLOCG00000016423	arid2
ENSXMAG00000019621	lrrc3b	ENSLOCG00000018332	lrrc3b
ENSXMAG00000007528	STK32C	ENSLOCG00000007451	STK32C
ENSXMAG00000027957	ANKRD34A	ENSLOCG00000017681	ANKRD34A
ENSXMAG00000017710	TFB2M	ENSLOCG00000016646	tfb2m
ENSXMAG00000027153	mphosph6	ENSLOCG00000002187	mphosph6
ENSXMAG00000016914	oxsm	ENSLOCG00000006960	oxsm
ENSXMAG00000008500	rnf20	ENSLOCG00000008174	rnf20
ENSXMAG00000019938	im:7152348	ENSLOCG00000005370	im:7152348
ENSXMAG00000005654	golga2	ENSLOCG00000004929	golga2
ENSXMAG00000006984		ENSLOCG00000014030	zgc:77439
ENSXMAG00000007831	enpp4	ENSLOCG00000015820	enpp4
ENSXMAG00000005729	pus10	ENSLOCG00000016123	pus10
ENSXMAG00000016915	ngly1	ENSLOCG00000006940	ngly1
ENSXMAG00000012068	gabpa	ENSLOCG00000010754	gabpa
ENSXMAG00000022533	eloal	ENSLOCG00000016897	eloal
ENSXMAG00000013178	invs	ENSLOCG00000005427	invs
ENSXMAG00000003328	camsap1b	ENSLOCG00000003342	camsap1b
ENSXMAG00000008963	gas2l3	ENSLOCG00000015204	gas2l3
ENSXMAG00000021345	sccpdha	ENSLOCG00000016642	sccpdha
ENSXMAG00000008280	farsb	ENSLOCG00000004136	farsb
ENSXMAG00000001995	psmd6	ENSLOCG00000010311	psmd6
ENSXMAG00000007525		ENSLOCG00000012908	

ENSXMAG00000015900	aire	ENSLOCG00000005382	aire
ENSXMAG00000013731		ENSLOCG00000012750	skp2
ENSXMAG00000008953	tm7sf3	ENSLOCG00000015214	tm7sf3
ENSXMAG00000018408	nexn	ENSLOCG00000003732	NEXN
ENSXMAG00000009095	rpsa	ENSLOCG00000012032	rpsa
ENSXMAG00000016647	slco2a1	ENSLOCG00000005400	slco2a1
ENSXMAG00000005750	cct4	ENSLOCG00000016088	cct4
ENSXMAG00000000711	fnbp1l	ENSLOCG00000009505	fnbp1l
ENSXMAG00000007846	mgat5	ENSLOCG00000001826	mgat5
ENSXMAG00000002005	atxn7	ENSLOCG00000010319	atxn7
ENSXMAG00000016936	top2b	ENSLOCG00000006909	top2b
ENSXMAG00000001958	ep400	ENSLOCG00000006092	ep400
ENSXMAG00000000085	ints2	ENSLOCG00000003709	ints2
ENSXMAG00000015722	herpud1	ENSLOCG00000007829	herpud1
ENSXMAG00000003414	mut	ENSLOCG00000016626	mut
ENSXMAG00000008947	fgfr1op2	ENSLOCG00000015212	fgfr1op2
ENSXMAG00000007807	ccdc43	ENSLOCG00000011939	ccdc43
ENSXMAG00000010230	gclc	ENSLOCG00000016888	gclc
ENSXMAG00000008511	aldob	ENSLOCG00000008151	aldob
ENSXMAG00000016880		ENSLOCG00000012107	
ENSXMAG00000017733		ENSLOCG00000001009	GCFC2
ENSXMAG00000013171	tex10	ENSLOCG00000005439	tex10
ENSXMAG00000021784	stab1	ENSLOCG00000010584	stab1
ENSXMAG00000008725	ulk4	ENSLOCG00000001346	ulk4
ENSXMAG00000011994	adgre5b.3	ENSLOCG00000008623	adgre5b.3
ENSXMAG00000001437	mical1	ENSLOCG00000011641	mical1
ENSXMAG00000022772	ryk	ENSLOCG00000005432	ryk
ENSXMAG00000007515	rhoq	ENSLOCG00000016335	rhoq
ENSXMAG00000019865	si:ch73-334d15.1	ENSLOCG00000018102	
ENSXMAG00000005787	fam161a	ENSLOCG00000016091	fam161a
ENSXMAG00000023326		ENSLOCG00000012389	
ENSXMAG00000021091	scai	ENSLOCG00000005027	scai
ENSXMAG00000029455	si:ch211-197h24.6	ENSLOCG00000005455	
ENSXMAG00000002020		ENSLOCG00000006389	
ENSXMAG00000025251	stk38l	ENSLOCG00000015219	stk38l
ENSXMAG00000016885	ccr10	ENSLOCG00000017517	ccr10
ENSXMAG00000003410	opn8b	ENSLOCG00000016633	opn8b
ENSXMAG00000012114	sik1	ENSLOCG00000001279	sik1
ENSXMAG00000007514		ENSLOCG00000016325	
ENSXMAG00000026516	ubac1	ENSLOCG00000003381	ubac1
ENSXMAG00000010383	bbs4	ENSLOCG00000014945	bbs4
ENSXMAG00000009128	ramp2	ENSLOCG00000012382	ramp2
ENSXMAG00000007510	mcfd2	ENSLOCG00000016322	mcfd2
ENSXMAG00000010261	elovl5	ENSLOCG00000016885	elovl5
ENSXMAG00000000189	gnmt	ENSLOCG00000017179	gnmt
ENSXMAG00000008539	tmco6	ENSLOCG00000011172	tmco6
ENSXMAG00000013602	si:ch73-335m24.2	ENSLOCG00000015263	si:ch73-335m24.2
ENSXMAG00000000707		ENSLOCG00000009489	bcar3
ENSXMAG00000006884	serpinf1	ENSLOCG00000006372	serpinf1
ENSXMAG00000018424	si:dkey-30h22.11	ENSLOCG00000009519	si:dkey-30h22.11
ENSXMAG00000028053	chka	ENSLOCG00000002673	chka
ENSXMAG00000011981		ENSLOCG00000006825	
ENSXMAG00000002014	thoc7	ENSLOCG00000010342	thoc7
ENSXMAG00000025868	syt14a	ENSLOCG00000017181	syt14a
ENSXMAG00000013160	tmem67	ENSLOCG00000010134	tmem67
ENSXMAG00000007479	ttc7a	ENSLOCG00000016316	ttc7a

ENSXMAG00000007875	zranb3	ENSLOCG00000001673	zranb3
ENSXMAG00000010262	fbxo9	ENSLOCG00000016879	fbxo9
ENSXMAG00000012139	ing1	ENSLOCG00000009063	ing1
ENSXMAG00000021931	SYNPR	ENSLOCG00000010367	SYNPR
ENSXMAG00000018426	alkal1	ENSLOCG00000005827	alkal1
ENSXMAG00000029101	sdsl	ENSLOCG00000007758	SDSL
ENSXMAG00000006813	slco2b1	ENSLOCG00000010831	
ENSXMAG00000012149	naxd	ENSLOCG00000009033	naxd
ENSXMAG00000001389		ENSLOCG00000012681	
ENSXMAG00000018427	zgc:153738	ENSLOCG00000009323	zgc:153738
ENSXMAG00000021595	zbtb49	ENSLOCG00000009974	zbtb49
ENSXMAG00000022218	si:dkey-12e7.1	ENSLOCG00000017950	si:dkey-12e7.1
ENSXMAG00000021359	sned1	ENSLOCG00000005458	sned1
ENSXMAG00000009130	EZH1	ENSLOCG00000012393	EZH1
ENSXMAG00000013155	pdp1	ENSLOCG00000018343	pdp1
ENSXMAG00000001391		ENSLOCG00000012673	taf8
ENSXMAG00000008858	slc13a4	ENSLOCG00000017106	slc13a4
ENSXMAG00000025543		ENSLOCG00000013591	
ENSXMAG00000024651	arsia	ENSLOCG00000011152	ARSI
ENSXMAG00000010174	neo1b	ENSLOCG00000014953	neo1b
ENSXMAG00000023462	clic2	ENSLOCG00000015260	clic2
ENSXMAG00000021181	rab20	ENSLOCG00000009026	rab20
ENSXMAG00000012803	ttc19	ENSLOCG00000010277	ttc19
ENSXMAG00000007885	r3hdm1	ENSLOCG00000001641	r3hdm1
ENSXMAG00000005417	auts2a	ENSLOCG00000003979	auts2a
ENSXMAG00000014515	pomt2	ENSLOCG00000010763	pomt2
ENSXMAG00000000688	hps3	ENSLOCG00000001628	hps3
ENSXMAG00000008857	coa6	ENSLOCG00000017104	coa6
ENSXMAG00000017066	thrb	ENSLOCG00000006846	thrb
ENSXMAG00000013150	cdh17	ENSLOCG00000010121	cdh17
ENSXMAG00000025258	urp1	ENSLOCG00000015259	urp1
ENSXMAG00000005672	hspa5	ENSLOCG00000004979	hspa5
ENSXMAG00000001355	gdi1	ENSLOCG00000015477	gdi1
ENSXMAG00000009149	cntnap1	ENSLOCG00000012408	cntnap1
ENSXMAG00000015978	thap4	ENSLOCG00000006455	THAP4
ENSXMAG00000023867	ENPP5	ENSLOCG00000015821	enpp5
ENSXMAG00000028873	lin52	ENSLOCG00000010928	lin52
ENSXMAG00000002067	lyar	ENSLOCG00000009963	lyar
ENSXMAG00000026413	tmub1	ENSLOCG00000009394	tmub1
ENSXMAG00000003459	cpsf6	ENSLOCG00000016190	cpsf6
ENSXMAG00000010285	ICK	ENSLOCG00000016878	ICK
ENSXMAG00000004972		ENSLOCG00000008760	gnrhr1
ENSXMAG00000019525	c1galt1c1	ENSLOCG00000017496	c1galt1c1
ENSXMAG00000013147	gem	ENSLOCG00000010118	gem
ENSXMAG00000015804	arpin	ENSLOCG00000014461	arpin
ENSXMAG00000003267	inpp5e	ENSLOCG00000003484	inpp5e
ENSXMAG00000027947	rabepk	ENSLOCG00000004994	RABEPK
ENSXMAG00000008246	flj13639	ENSLOCG00000004049	flj13639
ENSXMAG00000026106	tmem107	ENSLOCG00000009955	tmem107
ENSXMAG00000024543		ENSLOCG00000001583	
ENSXMAG00000008737	trak1a	ENSLOCG00000001329	trak1a
ENSXMAG00000017312		ENSLOCG00000008768	si:ch211-57n23.4
ENSXMAG00000004875	phip	ENSLOCG00000016244	phip
ENSXMAG00000013854	PLEKHH3	ENSLOCG00000012420	PLEKHH3
ENSXMAG00000013141	rad54b	ENSLOCG00000010100	rad54b
ENSXMAG00000024093	TMEM14A	ENSLOCG00000016876	TMEM14A

ENSXMAG00000003474	yeats4	ENSLOCG00000016192	yeats4
ENSXMAG00000012822		ENSLOCG00000010224	
ENSXMAG00000023339	mcts1	ENSLOCG00000015255	mcts1
ENSXMAG00000015818	fam169b	ENSLOCG00000014459	fam169b
ENSXMAG00000007915	rnd3a	ENSLOCG00000001573	rnd3b
ENSXMAG00000002077	OTOP1	ENSLOCG00000009946	OTOP1
ENSXMAG00000016627	btr12	ENSLOCG00000002362	btr12
ENSXMAG00000003265		ENSLOCG00000003505	pmpca
ENSXMAG00000029611	RF00100	ENSLOCG00000018951	RF00100
ENSXMAG00000003669	klhl30	ENSLOCG00000005505	klhl30
ENSXMAG00000007455	epcam	ENSLOCG00000016306	epcam
ENSXMAG00000008220	wdfy1	ENSLOCG00000004030	wdfy1
ENSXMAG00000015820	ldhd	ENSLOCG00000003318	ldhd
ENSXMAG00000003491	NAPEPLD	ENSLOCG00000015986	NAPEPLD
ENSXMAG00000029706	mxd1	ENSLOCG00000015320	mxd1
ENSXMAG00000014464		ENSLOCG00000012264	RASGRP1
ENSXMAG00000020065		ENSLOCG00000018034	
ENSXMAG00000023146	RF00091	ENSLOCG00000020417	RF00091
ENSXMAG00000013850	tubg1	ENSLOCG00000012432	tubg1
ENSXMAG00000010743	nadk2	ENSLOCG00000012738	nadk2
ENSXMAG00000026805	rgs3b	ENSLOCG00000004760	
ENSXMAG00000008823	ptprz1b	ENSLOCG00000015893	ptprz1b
ENSXMAG00000013613	cul4b	ENSLOCG00000015253	cul4b
ENSXMAG00000016060	ttc14	ENSLOCG00000000432	ttc14
ENSXMAG00000017136	rpl15	ENSLOCG00000006808	rpl15
ENSXMAG00000015991	cdc20	ENSLOCG00000003921	cdc20
ENSXMAG00000010319	agpat5	ENSLOCG00000016869	agpat5
ENSXMAG00000028014	rnf41l	ENSLOCG00000010092	rnf41l
ENSXMAG00000007431	plekhh2	ENSLOCG00000016422	plekhh2
ENSXMAG00000023388	znrf1	ENSLOCG00000003334	znrf1
ENSXMAG00000028207	lypd6	ENSLOCG00000001546	lypd6
ENSXMAG00000027366	snrnp27	ENSLOCG00000015319	snrnp27
ENSXMAG00000025155	retreg3	ENSLOCG00000012437	retreg3
ENSXMAG00000026605	znf219	ENSLOCG00000002482	
ENSXMAG00000017139	nkiras1	ENSLOCG00000006787	nkiras1
ENSXMAG00000002081	SLC2A9	ENSLOCG00000009930	SLC2A9
ENSXMAG00000011434	SLC25A30	ENSLOCG00000005454	SLC25A30
ENSXMAG00000015831	gins3	ENSLOCG00000003348	gins3
ENSXMAG00000014462		ENSLOCG00000012278	
ENSXMAG00000008196	serpine2	ENSLOCG00000004009	serpine2
ENSXMAG00000003678	scly	ENSLOCG00000005534	scly
ENSXMAG00000015215		ENSLOCG00000017070	gnsa
ENSXMAG00000016633	arhgef40	ENSLOCG00000002497	
ENSXMAG00000021371	fastk	ENSLOCG00000010214	fastk
ENSXMAG00000000666	fxr1	ENSLOCG00000000589	fxr1
ENSXMAG00000030048	cxadr	ENSLOCG00000004512	cxadr
ENSXMAG00000024087	mlx	ENSLOCG00000012453	mlx
ENSXMAG00000008194	prss16	ENSLOCG00000003995	prss16
ENSXMAG00000016000	ppp1r7	ENSLOCG00000008999	ppp1r7
ENSXMAG00000010331	mcph1	ENSLOCG00000016859	mcph1
ENSXMAG00000007929	pspc1	ENSLOCG00000010832	pspc1
ENSXMAG00000013134	virma	ENSLOCG00000010076	virma
ENSXMAG00000012861	afg3l2	ENSLOCG00000012103	afg3l2
ENSXMAG00000001510	btg3	ENSLOCG00000004531	btg3
ENSXMAG00000014449		ENSLOCG00000012287	DLL4
ENSXMAG00000008772	mroh1	ENSLOCG00000007509	mroh1

ENSXMAG00000027138		ENSLOCG00000005353	ELF1
ENSXMAG00000024037	zgc:110006	ENSLOCG00000004543	zgc:110006
ENSXMAG00000018492	dmap1	ENSLOCG00000002793	dmap1
ENSXMAG00000020363	tal2	ENSLOCG00000017967	tal2
ENSXMAG00000022991	ubxn7	ENSLOCG00000009799	ubxn7
ENSXMAG00000029463	irak1bp1	ENSLOCG00000016253	irak1bp1
ENSXMAG00000001277	wisp2	ENSLOCG00000001790	wisp2
ENSXMAG00000007422	THADA	ENSLOCG00000016430	THADA
ENSXMAG00000017165	sf3a3	ENSLOCG00000003273	sf3a3
ENSXMAG00000028824	rchy1	ENSLOCG00000012485	rchy1
ENSXMAG00000000660	dnajc19	ENSLOCG00000000624	dnajc19
ENSXMAG00000029450	ripk4	ENSLOCG00000004558	ripk4
ENSXMAG00000012380	col4a1	ENSLOCG00000008957	col4a1
ENSXMAG00000029842	psmc3ip	ENSLOCG00000012441	psmc3ip
ENSXMAG00000017639		ENSLOCG00000009442	lrp12
ENSXMAG00000002092		ENSLOCG00000009921	WDR1
ENSXMAG00000015838	ndrg4	ENSLOCG00000003358	ndrg4
ENSXMAG00000010824	si:dkey-261l7.2	ENSLOCG00000016251	si:dkey-261l7.2
ENSXMAG00000023810	znf618	ENSLOCG00000004746	znf618
ENSXMAG00000026774		ENSLOCG00000010750	FDX1
ENSXMAG00000013355	SUGT1	ENSLOCG00000005330	sugt1
ENSXMAG00000015300	gcc1	ENSLOCG00000016778	gcc1
ENSXMAG00000013631	lamp2	ENSLOCG00000015251	lamp2
ENSXMAG00000010360	angpt2a	ENSLOCG00000016861	angpt2a
ENSXMAG00000008179	PSMD2	ENSLOCG00000008710	psmd2
ENSXMAG00000013849	PRDM15	ENSLOCG00000004600	PRDM15
ENSXMAG00000019487	SLC35A4	ENSLOCG00000018306	SLC35A4
ENSXMAG00000000318	nisch	ENSLOCG00000010609	nisch
ENSXMAG00000020128	sox2	ENSLOCG00000017346	sox2
ENSXMAG00000010509	klhl18	ENSLOCG00000010189	klhl18
ENSXMAG00000004512		ENSLOCG00000012428	
ENSXMAG00000016919	grb7	ENSLOCG00000012291	grb7
ENSXMAG00000026286	tbx18	ENSLOCG00000016254	tbx18
ENSXMAG00000016030	tfa	ENSLOCG00000009747	
ENSXMAG00000024669	CNMD	ENSLOCG00000005306	cnmd
ENSXMAG00000015257	dennd6b	ENSLOCG00000016783	dennd6b
ENSXMAG00000016938	arf2b	ENSLOCG00000012283	arf2b
ENSXMAG00000002101	idh3b	ENSLOCG00000001083	idh3b
ENSXMAG00000024806	si:ch211-197l9.5	ENSLOCG00000016257	
ENSXMAG00000023453		ENSLOCG00000013869	
ENSXMAG00000011433	CXorf38	ENSLOCG00000002335	CXorf38
ENSXMAG00000026218		ENSLOCG00000003363	pdik1l
ENSXMAG00000004505	smad2	ENSLOCG00000012409	smad2
ENSXMAG00000021471	txn2	ENSLOCG00000012007	
ENSXMAG00000018502	hnrnpm	ENSLOCG00000004456	hnrnpm
ENSXMAG00000005728	pappaa	ENSLOCG00000004730	pappaa
ENSXMAG00000000313	hgh1	ENSLOCG00000007462	hgh1
ENSXMAG00000013863	C2CD2	ENSLOCG00000004620	c2cd2
ENSXMAG00000008646	cd74a	ENSLOCG00000011007	cd74a
ENSXMAG00000027643		ENSLOCG00000002504	
ENSXMAG00000025506	atp1b4	ENSLOCG00000015247	atp1b4
ENSXMAG00000007992		ENSLOCG00000010719	STRADB
ENSXMAG00000002115	ptpra	ENSLOCG00000001040	ptpra
ENSXMAG00000028095	jtb	ENSLOCG00000008648	jtb
ENSXMAG00000010533	bfsp2	ENSLOCG00000012071	bfsp2
ENSXMAG00000007186	PLCZ1	ENSLOCG00000015427	PLCZ1

ENSXMAG00000022932	selenoi	ENSLOCG00000016178	selenoi
ENSXMAG00000021402		ENSLOCG00000004551	
ENSXMAG00000017601		ENSLOCG00000007215	glmp
ENSXMAG00000027828	lamtor4	ENSLOCG00000016775	lamtor4
ENSXMAG00000008010	trak2	ENSLOCG00000010723	trak2
ENSXMAG00000022860		ENSLOCG00000011432	zgc:153675
ENSXMAG00000000140	ccdc88c	ENSLOCG00000013183	ccdc88c
ENSXMAG00000011430		ENSLOCG00000000788	catip
ENSXMAG00000008813	sult2st3	ENSLOCG00000003588	sult2st3
ENSXMAG00000008792	pik3cg	ENSLOCG00000015829	pik3cg
ENSXMAG00000015320	atp6v1f	ENSLOCG00000015574	atp6v1f
ENSXMAG00000013867	atg101	ENSLOCG00000018192	atg101
ENSXMAG00000016963	fam171a2b	ENSLOCG00000013275	fam171a2b
ENSXMAG00000008661	ndst1a	ENSLOCG00000010994	ndst1a
ENSXMAG00000019994	opn4.1	ENSLOCG00000018136	opn4.1
ENSXMAG00000021416	epb41l4b	ENSLOCG00000005168	epb41l4b
ENSXMAG00000003740	zgc:103559	ENSLOCG00000006539	zgc:103559
ENSXMAG00000024853	tmem255a	ENSLOCG00000015244	tmem255a
ENSXMAG00000012554	myo16	ENSLOCG00000008929	myo16
ENSXMAG00000020121	KCNA5	ENSLOCG00000017910	
ENSXMAG00000017547	cct3	ENSLOCG00000007229	cct3
ENSXMAG00000013870	ZBTB21	ENSLOCG00000004657	ZBTB21
ENSXMAG00000000614	ATP11B	ENSLOCG00000000659	ATP11B
ENSXMAG00000010851	agbl5	ENSLOCG00000016175	agbl5
ENSXMAG00000025917		ENSLOCG00000013328	arhgef3
ENSXMAG00000019826	hcar1-3	ENSLOCG00000017364	hcar1-3
ENSXMAG00000021662		ENSLOCG00000012150	si:dkey-154p10.3
ENSXMAG00000003601	rbpjl	ENSLOCG00000007645	rbpjl
ENSXMAG00000012809	ctps1a	ENSLOCG00000003415	ctps1a
ENSXMAG00000004493	susd1	ENSLOCG00000002960	susd1
ENSXMAG00000026298	GPR68	ENSLOCG00000017717	GPR68
ENSXMAG00000013871	umodl1	ENSLOCG00000004670	
ENSXMAG00000024497	HAAO	ENSLOCG00000016447	haao
ENSXMAG00000010066	csnk1g1	ENSLOCG00000014997	csnk1g1
ENSXMAG00000008147	EIF4G1	ENSLOCG00000008729	EIF4G1
ENSXMAG00000008681	rbm22	ENSLOCG00000010989	rbm22
ENSXMAG00000015152	ctsba	ENSLOCG00000000768	ctsba
ENSXMAG00000028004	tmem186	ENSLOCG00000006581	tmem186
ENSXMAG00000013673	zbtb33	ENSLOCG00000015243	zbtb33
ENSXMAG00000003756	dtymk	ENSLOCG00000006505	dtymk
ENSXMAG00000008049		ENSLOCG00000011079	
ENSXMAG00000008849		ENSLOCG00000003537	gapdhs
ENSXMAG00000028318	CDYL	ENSLOCG00000011639	CDYL
ENSXMAG00000002136		ENSLOCG00000001311	cd8b
ENSXMAG00000028115	myoz3a	ENSLOCG00000010984	myoz3a
ENSXMAG00000014948	eno3	ENSLOCG00000013637	eno3
ENSXMAG00000019908	ost4	ENSLOCG00000017994	ost4
ENSXMAG00000000309	lmcd1	ENSLOCG00000013380	
ENSXMAG00000024940		ENSLOCG00000011650	rpp40
ENSXMAG00000005754	ASTN2	ENSLOCG00000004702	
ENSXMAG00000015149	adpgk2	ENSLOCG00000016655	adpgk2
ENSXMAG00000013103	ptpn3	ENSLOCG00000005125	ptpn3
ENSXMAG00000025834	ccnk	ENSLOCG00000008714	ccnk
ENSXMAG00000008700	arl3l2	ENSLOCG00000010965	arl3l2
ENSXMAG00000025946	RF00405	ENSLOCG00000020071	RF00405
ENSXMAG00000019575	ZBTB26	ENSLOCG00000002911	ZBTB26

ENSXMAG00000003762	atg4b	ENSLOCG00000006470	atg4b
ENSXMAG00000023316	scxb	ENSLOCG00000007444	scxa
ENSXMAG00000015899	smpd3	ENSLOCG00000003455	SMPD3
ENSXMAG00000000302	mcm2	ENSLOCG00000013582	mcm2
ENSXMAG00000003580	matn4	ENSLOCG00000007617	matn4
ENSXMAG00000014572		ENSLOCG00000011728	riok1
ENSXMAG00000004452	p2rx4b	ENSLOCG00000006936	p2rx4b
ENSXMAG00000013680	upf3b	ENSLOCG00000015238	upf3b
ENSXMAG00000027452		ENSLOCG00000010624	col15a1b
ENSXMAG00000022922	RF00544	ENSLOCG00000018520	RF00544
ENSXMAG00000016995	kansl1a	ENSLOCG00000013228	kansl1b
ENSXMAG00000010431	ap3m1	ENSLOCG00000004509	ap3m1
ENSXMAG00000028544	efcab2	ENSLOCG00000016651	efcab2
ENSXMAG00000014369	GPX2	ENSLOCG00000012401	GPX2
ENSXMAG00000013872	mogat2	ENSLOCG00000004715	mogat2
ENSXMAG00000008715	rgs14a	ENSLOCG00000010943	rgs14a
ENSXMAG00000029815	RF00190	ENSLOCG00000020740	RF00190
ENSXMAG00000007113	xpnpep1	ENSLOCG00000012012	xpnpep1
ENSXMAG00000028376	rab42a	ENSLOCG00000003453	rab42a
ENSXMAG00000015144	cgref1	ENSLOCG00000000653	cgref1
ENSXMAG00000014946	zgc:85858	ENSLOCG00000013662	zgc:85858
ENSXMAG00000013690	rpl39	ENSLOCG00000015236	rpl39
ENSXMAG00000004438	gucd1	ENSLOCG00000006997	gucd1
ENSXMAG00000022895	siah2l	ENSLOCG00000014942	siah2l
ENSXMAG00000029047	RF00265	ENSLOCG00000020941	RF00265
ENSXMAG00000022200	dgat2	ENSLOCG00000004729	dgat2
ENSXMAG00000003773	boka	ENSLOCG00000006419	boka
ENSXMAG00000021779		ENSLOCG00000013579	PODXL2
ENSXMAG00000004411	upb1	ENSLOCG00000007024	upb1
ENSXMAG00000001955		ENSLOCG00000013712	CAMTA2
ENSXMAG00000007383	papolg	ENSLOCG00000016130	papolg
ENSXMAG00000013692	sowahd	ENSLOCG00000017495	sowahd
ENSXMAG00000018626		ENSLOCG00000004375	
ENSXMAG00000017493	si:dkey-27c15.3	ENSLOCG00000007268	gon4l
ENSXMAG00000007226	RELN	ENSLOCG00000015474	RELN
ENSXMAG00000027833	cpeb1b	ENSLOCG00000015009	cpeb1b
ENSXMAG00000019488	adra2da	ENSLOCG00000010920	adra2da
ENSXMAG00000019463	blcap	ENSLOCG00000018367	blcap
ENSXMAG00000010894	cyp46a1.4	ENSLOCG00000008757	cyp46a1.4
ENSXMAG00000018630	lsm7	ENSLOCG00000004356	lsm7
ENSXMAG00000015125	cenpf	ENSLOCG00000016707	cenpf
ENSXMAG00000013881	uvrag	ENSLOCG00000004748	uvrag
ENSXMAG00000012582	tnfsf13b	ENSLOCG00000008908	TNFSF13B
ENSXMAG00000025079	15-Sep	ENSLOCG00000003765	
ENSXMAG00000005801	trim32	ENSLOCG00000017344	trim32
ENSXMAG00000012157	ripor3	ENSLOCG00000007402	ripor3
ENSXMAG00000008874		ENSLOCG00000003452	HPN
ENSXMAG00000019634	kcnf1a	ENSLOCG00000018004	kcnf1b
ENSXMAG00000006732	ap4m1	ENSLOCG00000013087	ap4m1
ENSXMAG00000000375		ENSLOCG00000017202	ldah
ENSXMAG00000003776	farp2	ENSLOCG00000006339	farp2
ENSXMAG00000019937	abhd13	ENSLOCG00000017797	abhd13
ENSXMAG00000008730	cnot6b	ENSLOCG00000010102	cnot6b
ENSXMAG00000010724	sprb	ENSLOCG00000001239	spra
ENSXMAG00000019800	MYL4	ENSLOCG00000017341	
ENSXMAG00000017462	ints3	ENSLOCG00000006927	ints3

ENSXMAG00000026483		ENSLOCG00000003194		
ENSXMAG00000000368		ENSLOCG00000014116	rin3	
ENSXMAG00000013699	6-Sep	ENSLOCG00000015233		6-Sep
ENSXMAG00000004383	specc1la	ENSLOCG00000007071	specc1la	
ENSXMAG00000015142	lig4	ENSLOCG00000008901	lig4	
ENSXMAG00000011281	brinp1	ENSLOCG00000003763	brinp1	
ENSXMAG00000025274		ENSLOCG00000008767	alp3	
ENSXMAG00000018631	tmprss9	ENSLOCG00000004321	tmprss9	
ENSXMAG00000013888	wnt11r	ENSLOCG00000004765	wnt11r	
ENSXMAG00000003219	poli	ENSLOCG00000003386	POLI	
ENSXMAG00000001210	rbpms2b	ENSLOCG00000015018	rbpms2b	
ENSXMAG00000003944	pdzk1	ENSLOCG00000008862	pdzk1	
ENSXMAG00000010692	dqx1	ENSLOCG00000001140	dqx1	
ENSXMAG00000020891		ENSLOCG00000014124	pth2	
ENSXMAG00000025101		ENSLOCG00000003783		
ENSXMAG00000018632	si:dkey-159a18.1	ENSLOCG00000015801	si:dkey-159a18.1	
ENSXMAG00000008115	zgc:165508	ENSLOCG00000008790	zgc:165508	
ENSXMAG00000013892		ENSLOCG00000004777		
ENSXMAG00000015172	gpr137c	ENSLOCG00000012038	gpr137c	
ENSXMAG00000007244	si:dkey-29p10.4	ENSLOCG00000006435	si:dkey-29p10.4	
ENSXMAG00000003958	GPR89B	ENSLOCG00000008847	GPR89B	
ENSXMAG00000008114		ENSLOCG00000008803		
ENSXMAG00000020957		ENSLOCG00000007386	chd6	
ENSXMAG00000020918	fxyd1	ENSLOCG00000003478		
ENSXMAG00000013716	nkrf	ENSLOCG00000015232	nkrf	
ENSXMAG00000011091	PSMA4	ENSLOCG00000015026	psma4	
ENSXMAG00000006643	arrb2b	ENSLOCG00000013132	arrb2a	
ENSXMAG00000021035	tp53i11b	ENSLOCG00000002759	tp53i11b	
ENSXMAG00000023968		ENSLOCG00000011779	dnajc1	
ENSXMAG00000010684	thnsl2	ENSLOCG00000001186		
ENSXMAG00000028526	smyd2a	ENSLOCG00000016700	smyd2a	
ENSXMAG00000014337		ENSLOCG00000012463		
ENSXMAG00000020365	zgc:55781	ENSLOCG00000011919	zgc:55781	
ENSXMAG00000013717	ube2a	ENSLOCG00000015231	ube2a	
ENSXMAG00000011293	ak1	ENSLOCG00000003793	ak1	
ENSXMAG00000011100	ubl7b	ENSLOCG00000014272	ubl7a	
ENSXMAG00000000286	rspo4	ENSLOCG00000007448	rspo4	
ENSXMAG00000027932		ENSLOCG00000012202	si:dkey-181m9.8	
ENSXMAG00000024429		ENSLOCG00000003802		
ENSXMAG00000001968	cyb5d1	ENSLOCG00000013978	cyb5d1	
ENSXMAG00000023348	hpcal1	ENSLOCG00000016657	HPCAL1	
ENSXMAG00000015187	txndc16	ENSLOCG00000012026	txndc16	
ENSXMAG00000027180	elf1	ENSLOCG00000015349	elf1	
ENSXMAG00000003778		ENSLOCG00000005818	TBC1D13	
ENSXMAG00000009325	si:ch211-63p21.1	ENSLOCG00000002544		
ENSXMAG00000022411	timm13	ENSLOCG00000004301	timm13	
ENSXMAG00000029346	si:ch211-266i6.3	ENSLOCG00000008856		
ENSXMAG00000015210		ENSLOCG00000016428	TPO	
ENSXMAG00000000611	mccc1	ENSLOCG00000000778	mccc1	
ENSXMAG00000017773	pdia6	ENSLOCG00000016663	pdia6	
ENSXMAG00000018624	fam3c	ENSLOCG00000015800	fam3c	
ENSXMAG00000027956	tnmd	ENSLOCG00000015348	tnmd	
ENSXMAG00000003787	hdlbpa	ENSLOCG00000006278	hdlbpa	
ENSXMAG00000022292	cd276	ENSLOCG00000014271	cd276	
ENSXMAG00000025469	amph	ENSLOCG00000011353	amph	
ENSXMAG00000015073	galnt2	ENSLOCG00000015454	galnt2	

ENSXMAG00000018635	enc3	ENSLOCG00000017631	enc3
ENSXMAG00000008900	sdha	ENSLOCG00000009351	sdha
ENSXMAG00000018526	pcdh19	ENSLOCG00000015346	pcdh19
ENSXMAG00000005819	USP34	ENSLOCG00000016101	USP34
ENSXMAG00000003783	zer1	ENSLOCG00000005833	zer1
ENSXMAG00000007379	REL	ENSLOCG00000016127	rel
ENSXMAG00000015934	exoc3l1	ENSLOCG00000003829	
ENSXMAG00000010473	plaua	ENSLOCG00000004467	
ENSXMAG00000011298	gpsm1b	ENSLOCG00000003828	gpsm1b
ENSXMAG00000030045	insyn1	ENSLOCG00000014270	insyn1
ENSXMAG00000018622	btg1	ENSLOCG00000015002	btg1
ENSXMAG00000015059	urb2	ENSLOCG00000015462	urb2
ENSXMAG00000010928	snw1	ENSLOCG00000008813	snw1
ENSXMAG00000000264	ptprt	ENSLOCG00000007327	ptprt
ENSXMAG00000008775	gfpt2	ENSLOCG00000010084	gfpt2
ENSXMAG00000004329	ift81	ENSLOCG00000007148	ift81
ENSXMAG00000014258	acp7	ENSLOCG00000012497	acp7
ENSXMAG00000025090	lmo2	ENSLOCG00000002844	lmo2
ENSXMAG00000009206	gosr2	ENSLOCG00000012271	gosr2
ENSXMAG00000013894	stt3a	ENSLOCG00000002777	stt3a
ENSXMAG00000006623	taf9	ENSLOCG00000013708	taf9
ENSXMAG00000025381	loxl1	ENSLOCG00000014268	loxl1
ENSXMAG00000025465	dusp5	ENSLOCG00000012340	dusp5
ENSXMAG00000028648		ENSLOCG00000016393	wu:fc23c09
ENSXMAG00000006652	slc37a1	ENSLOCG00000003987	slc37a1
ENSXMAG00000007376	pex13	ENSLOCG00000016122	pex13
ENSXMAG00000003985	maats1	ENSLOCG00000004498	maats1
ENSXMAG00000008091	chrd	ENSLOCG00000008850	chrd
ENSXMAG00000002079	ZNHIT1	ENSLOCG00000013646	ZNHIT1
ENSXMAG00000006978	smc3	ENSLOCG00000012326	smc3
ENSXMAG00000015946	prmt7	ENSLOCG00000003153	prmt7
ENSXMAG00000027036	dcn	ENSLOCG00000016391	dcn
ENSXMAG00000019624	sox14	ENSLOCG00000017402	sox14
ENSXMAG00000024948	ptger2a	ENSLOCG00000012035	ptger2a
ENSXMAG00000000609		ENSLOCG00000000828	
ENSXMAG00000021467	zmp:0000000930	ENSLOCG00000009365	zmp:0000000930
ENSXMAG00000011308	LHX3	ENSLOCG00000003880	LHX3
ENSXMAG00000009217	gpatch8	ENSLOCG00000012265	gpatch8
ENSXMAG00000015039	abcb10	ENSLOCG00000015465	abcb10
ENSXMAG00000022773	nr1i2	ENSLOCG00000004476	nr1i2
ENSXMAG00000015225		ENSLOCG00000016425	sntg2
ENSXMAG00000017424	dnai2b	ENSLOCG00000012856	dnai2b
ENSXMAG00000007370	si:ch211-189k9.2	ENSLOCG00000016119	si:ch211-189k9.2
ENSXMAG00000008930		ENSLOCG00000009372	
ENSXMAG00000018604	lum	ENSLOCG00000016388	lum
ENSXMAG00000018073	igdcc4	ENSLOCG00000013791	igdcc4
ENSXMAG00000027963	zgc:194887	ENSLOCG00000012532	zgc:194887
ENSXMAG00000013904	ei24	ENSLOCG00000002857	ei24
ENSXMAG00000014955	lmo4b	ENSLOCG00000005105	lmo4b
ENSXMAG00000004019	GSK3B	ENSLOCG00000007035	gsk3b
ENSXMAG00000002153	traip	ENSLOCG00000010841	traip
ENSXMAG00000019658	TMEM250	ENSLOCG00000017342	TMEM250
ENSXMAG00000018602	kera	ENSLOCG00000016387	kera
ENSXMAG00000024432	fam173b	ENSLOCG00000013585	fam173b
ENSXMAG00000015966	ciapin1	ENSLOCG00000003170	ciapin1
ENSXMAG00000008807	mapk9	ENSLOCG00000010067	mapk9

ENSXMAG00000029474	pip5kl1	ENSLOCG00000006176	pip5kl1
ENSXMAG00000003170	diaph2	ENSLOCG00000015338	diaph2
ENSXMAG00000006688		ENSLOCG00000001598	NMI
ENSXMAG00000022157	psma2	ENSLOCG00000009306	si:rp71-45k5.4
ENSXMAG00000007325	nat10	ENSLOCG00000002896	nat10
ENSXMAG00000015977	coq9	ENSLOCG00000003188	coq9
ENSXMAG00000018588	epyc	ENSLOCG00000016384	epyc
ENSXMAG00000027962	erlin1	ENSLOCG00000004418	erlin1
ENSXMAG00000006968	RBM20	ENSLOCG00000012315	RBM20
ENSXMAG00000026193	rab4a	ENSLOCG00000015475	rab4a
ENSXMAG00000003155		ENSLOCG00000004147	
ENSXMAG00000003825	dpm2	ENSLOCG00000006158	dpm2
ENSXMAG00000004049	gpr156	ENSLOCG00000007020	gpr156
ENSXMAG00000005231	kyat3	ENSLOCG00000005032	kyat3
ENSXMAG00000005191	UBXN4	ENSLOCG00000001630	UBXN4
ENSXMAG00000002162	mon1a	ENSLOCG00000010888	mon1a
ENSXMAG00000000053	ncor2	ENSLOCG00000006023	ncor2
ENSXMAG00000003134		ENSLOCG00000011989	
ENSXMAG00000006600		ENSLOCG00000011042	apbb3
ENSXMAG00000025122		ENSLOCG00000012006	nid2b
ENSXMAG00000014974	phf10	ENSLOCG00000015494	phf10
ENSXMAG00000018090	UNC13C	ENSLOCG00000013431	UNC13C
ENSXMAG00000015984	polr2c	ENSLOCG00000003215	polr2c
ENSXMAG00000029708	naif1	ENSLOCG00000006120	naif1
ENSXMAG00000024209	ecel1	ENSLOCG00000008900	ecel1
ENSXMAG00000026925	hint2	ENSLOCG00000011981	hint2
ENSXMAG00000006613		ENSLOCG00000011051	SRA1
ENSXMAG00000015292	rtraf	ENSLOCG00000012000	rtraf
ENSXMAG00000029281	lrrc58b	ENSLOCG00000007004	lrrc58a
ENSXMAG00000018584	si:ch211-244b2.4	ENSLOCG00000015170	si:ch211-244b2.4
ENSXMAG00000027265	RASGEF1C	ENSLOCG00000010054	RASGEF1C
ENSXMAG00000021390		ENSLOCG00000005327	fpgs
ENSXMAG00000015188		ENSLOCG00000001108	zmat3
ENSXMAG00000005273	lrrc8c	ENSLOCG00000005018	lrrc8c
ENSXMAG00000005209		ENSLOCG00000009021	itgb2
ENSXMAG00000003123	mrps30	ENSLOCG00000011976	mrps30
ENSXMAG00000014952	psmb1	ENSLOCG00000015486	psmb1
ENSXMAG00000028585	ntf3	ENSLOCG00000017067	ntf3
ENSXMAG00000019492	tmem126a	ENSLOCG00000005364	tmem126a
ENSXMAG00000006928	pdcd4b	ENSLOCG00000012312	pdcd4b
ENSXMAG00000021526		ENSLOCG00000013564	zgc:171566
ENSXMAG00000004055	fstl1b	ENSLOCG00000006981	fstl1a
ENSXMAG00000023590	barhl2	ENSLOCG00000004982	barhl2
ENSXMAG00000014950	psmc3	ENSLOCG00000006198	psmc3
ENSXMAG00000009240	si:dkey-159f12.2	ENSLOCG00000006848	rps11
ENSXMAG00000022116		ENSLOCG00000003268	dbi
ENSXMAG00000003838	cdk9	ENSLOCG00000005311	cdk9
ENSXMAG00000008878	clk4a	ENSLOCG00000010039	clk4a
ENSXMAG00000007356	adal	ENSLOCG00000014596	adal
ENSXMAG00000006555		ENSLOCG00000011137	camk2a
ENSXMAG00000028308	sap18	ENSLOCG00000010931	sap18
ENSXMAG00000014937	tbp	ENSLOCG00000015481	tbp
ENSXMAG00000004226	anapc7	ENSLOCG00000007213	anapc7
ENSXMAG00000027497	zgc:101851	ENSLOCG00000003255	zgc:101851
ENSXMAG00000002107	corin	ENSLOCG00000012013	corin
ENSXMAG00000028787	tgfbr3	ENSLOCG00000004872	tgfbr3

ENSXMAG00000022427	RF00211	ENSLOCG00000018587	RF00211
ENSXMAG00000022971		ENSLOCG00000003213	
ENSXMAG00000017011	lrrc59	ENSLOCG00000011171	lrrc59
ENSXMAG00000021372	TRAPPC5	ENSLOCG00000006678	trappc5
ENSXMAG00000024489	lmo4a	ENSLOCG00000002487	lmo4a
ENSXMAG00000014929	pdcd2	ENSLOCG00000015478	pdcd2
ENSXMAG00000006927	bbip1	ENSLOCG00000012310	bbip1
ENSXMAG00000008940	rack1	ENSLOCG00000009758	rack1
ENSXMAG00000003121	emb	ENSLOCG00000011959	EMB
ENSXMAG00000008971	zgc:101716	ENSLOCG00000009268	zgc:101716
ENSXMAG00000029121	lfng	ENSLOCG00000006097	lfng
ENSXMAG00000004959	zgc:100846	ENSLOCG00000012179	zgc:100846
ENSXMAG00000017038	lrsam1	ENSLOCG00000002536	lrsam1
ENSXMAG00000006907	shoc2	ENSLOCG00000012301	shoc2
ENSXMAG00000017020	nat9	ENSLOCG00000011162	nat9
ENSXMAG00000004205		ENSLOCG00000007310	RAD9B
ENSXMAG00000006553	rps14	ENSLOCG00000011001	rps14
ENSXMAG00000002323		ENSLOCG00000006789	XAF1
ENSXMAG00000025323		ENSLOCG00000009259	FAM83A
ENSXMAG00000020483	mir140	ENSLOCG00000019891	mir140
ENSXMAG00000014897	ppm1g	ENSLOCG00000015498	ppm1g
ENSXMAG00000018567	SCYL2	ENSLOCG00000015168	SCYL2
ENSXMAG00000016043	adat1	ENSLOCG00000003068	
ENSXMAG00000000582		ENSLOCG00000005693	
ENSXMAG00000002901	insig2	ENSLOCG00000003172	insig2
ENSXMAG00000002324		ENSLOCG00000006751	tekt1
ENSXMAG00000004073	gja8a	ENSLOCG00000017790	gja8a
ENSXMAG00000003106	malt1	ENSLOCG00000011876	malt1
ENSXMAG00000003862	ndor1	ENSLOCG00000002261	ndor1
ENSXMAG00000008951	nme5	ENSLOCG00000009764	nme5
ENSXMAG00000007377	edc3	ENSLOCG00000014625	edc3
ENSXMAG00000001619	CEP128	ENSLOCG00000008895	CEP128
ENSXMAG00000015316	metap1	ENSLOCG00000012206	metap1
ENSXMAG00000008980	tbc1d31	ENSLOCG00000009250	tbc1d31
ENSXMAG00000020072	UTP14C	ENSLOCG00000015298	si:dkey-251i10.3
ENSXMAG00000023912	acp6	ENSLOCG00000006932	acp6
ENSXMAG00000011367	PRRC2B	ENSLOCG00000006253	PRRC2B
ENSXMAG00000025488	znf511	ENSLOCG00000004344	znf511
ENSXMAG00000014894		ENSLOCG00000017421	mis12
ENSXMAG00000010782	ephx4	ENSLOCG00000004839	ephx4
ENSXMAG00000026764	ntmt1	ENSLOCG00000002241	ntmt1
ENSXMAG00000025631	oprd1a	ENSLOCG00000003690	oprd1a
ENSXMAG00000017022	syt15	ENSLOCG00000012890	syt15
ENSXMAG00000000568	stxbp3	ENSLOCG00000007816	stxbp3
ENSXMAG00000006496	ndufa2	ENSLOCG00000011183	ndufa2
ENSXMAG00000007347		ENSLOCG00000011792	pcgf6
ENSXMAG00000021699	PPP1CC	ENSLOCG00000007358	PPP1CC
ENSXMAG00000017391	vps45	ENSLOCG00000008558	vps45
ENSXMAG00000024243	ccdc93	ENSLOCG00000003144	ccdc93
ENSXMAG00000003872	ptpa	ENSLOCG00000002220	ptpa
ENSXMAG00000013710	fuom	ENSLOCG00000004282	fuom
ENSXMAG00000004093	bcl9	ENSLOCG00000006913	bcl9
ENSXMAG00000003699	rapsn	ENSLOCG00000005948	rapsn
ENSXMAG00000002778		ENSLOCG00000006464	
ENSXMAG00000006470		ENSLOCG00000011188	ik
ENSXMAG00000008961	phykpl	ENSLOCG00000009784	

ENSXMAG00000011377	edf1	ENSLOCG00000004805	edf1
ENSXMAG00000026611	glra4a	ENSLOCG00000015333	glra4a
ENSXMAG00000006810	PNPLA6	ENSLOCG00000008681	pnpla6
ENSXMAG00000013719	echs1	ENSLOCG00000004265	echs1
ENSXMAG00000020348		ENSLOCG00000017787	nhlh2
ENSXMAG00000019825	sertad2b	ENSLOCG00000017361	sertad2b
ENSXMAG00000001629	tshr	ENSLOCG00000008909	tshr
ENSXMAG00000029160	zgc:112285	ENSLOCG00000012892	zgc:112285
ENSXMAG00000011386	PRAG1	ENSLOCG00000010023	PRAG1
ENSXMAG00000010761	C1orf146	ENSLOCG00000004762	C1orf146
ENSXMAG00000007315	slc1a4	ENSLOCG00000016041	slc1a4
ENSXMAG00000016083	spire2	ENSLOCG00000002891	spire2
ENSXMAG00000024549	prune	ENSLOCG00000007674	prune
ENSXMAG00000018558	si:ch211-265o23.1	ENSLOCG00000015166	si:ch211-265o23.1
ENSXMAG00000017027	timm23a	ENSLOCG00000012900	timm23b
ENSXMAG00000011387	TMEM38B	ENSLOCG00000009996	TMEM38B
ENSXMAG00000000307	gla	ENSLOCG00000015326	gla
ENSXMAG00000006439	chm	ENSLOCG00000014266	chm
ENSXMAG00000014864		ENSLOCG00000015490	fam120b
ENSXMAG00000023727	onecut1	ENSLOCG00000013415	onecut1
ENSXMAG00000003090	secisbp2	ENSLOCG00000011742	secisbp2
ENSXMAG00000002365	SLC43A2	ENSLOCG00000007037	slc43a2a
ENSXMAG00000009300	snx8a	ENSLOCG00000001459	snx8b
ENSXMAG00000022582	use1	ENSLOCG00000004161	use1
ENSXMAG00000019347	si:dkey-43k4.5	ENSLOCG00000007582	si:dkey-43k4.5
ENSXMAG00000008689	pfkfb3	ENSLOCG00000015663	pfkfb3
ENSXMAG00000004101	chaf1b	ENSLOCG00000006847	chaf1b
ENSXMAG00000018543	tcp11l2	ENSLOCG00000015162	tcp11l2
ENSXMAG00000013734	mtg1	ENSLOCG00000004254	mtg1
ENSXMAG00000025730	sntb2	ENSLOCG00000002866	sntb2
ENSXMAG00000024654	fam214a	ENSLOCG00000013407	fam214a
ENSXMAG00000001644	gtf2a1	ENSLOCG00000008922	gtf2a1
ENSXMAG00000008991	derl1	ENSLOCG00000009243	derl1
ENSXMAG00000017056	qki2	ENSLOCG00000016506	qki2
ENSXMAG00000014102	ZNF462	ENSLOCG00000009983	ZNF462
ENSXMAG00000000305	rpl36a	ENSLOCG00000015330	rpl36a
ENSXMAG00000004840	myo1eb	ENSLOCG00000008426	myo1eb
ENSXMAG00000009311	nudt1	ENSLOCG00000001447	nudt1
ENSXMAG00000008978	ctbp1	ENSLOCG00000009571	ctbp1
ENSXMAG00000007289	b3gat2	ENSLOCG00000017110	b3gat2
ENSXMAG00000003075	mfsd10	ENSLOCG00000011631	mfsd10
ENSXMAG00000001818	topbp1	ENSLOCG00000012044	topbp1
ENSXMAG00000016114	vps4a	ENSLOCG00000002848	vps4a
ENSXMAG00000008854	ilf3b	ENSLOCG00000008430	ilf3b
ENSXMAG00000009314	mrm2	ENSLOCG00000001431	mrm2
ENSXMAG00000007288	ogfrl1	ENSLOCG00000017116	ogfrl1
ENSXMAG00000006829		ENSLOCG00000012655	
ENSXMAG00000018504	apaf1	ENSLOCG00000015153	apaf1
ENSXMAG00000016214	GPR55	ENSLOCG00000004542	GPR55
ENSXMAG00000004090	mtmr3	ENSLOCG00000007446	mtmr3
ENSXMAG00000004105	KIF1C	ENSLOCG00000013701	KIF1C
ENSXMAG00000006436		ENSLOCG00000015199	
ENSXMAG00000014116	rad23b	ENSLOCG00000009976	rad23b
ENSXMAG00000014817	rufy2	ENSLOCG00000009235	rufy2
ENSXMAG00000009328	psmg3	ENSLOCG00000001382	psmg3
ENSXMAG00000003906	june	ENSLOCG00000017968	june

ENSXMAG00000023595	mllt11	ENSLOCG00000017680	mllt11
ENSXMAG00000019868	tbcc	ENSLOCG00000017365	tbcc
ENSXMAG00000002810	poglut1	ENSLOCG00000006595	POGLUT1
ENSXMAG00000008985	spon2a	ENSLOCG00000009562	spon2a
ENSXMAG00000016145	cdk10	ENSLOCG00000002991	cdk10
ENSXMAG00000020852	asrgl1	ENSLOCG00000016189	asrgl1
ENSXMAG00000021919	THAP1	ENSLOCG00000003237	THAP1
ENSXMAG00000028970		ENSLOCG00000012686	
ENSXMAG00000000555	henmt1	ENSLOCG00000007885	henmt1
ENSXMAG00000009315	tmem184a	ENSLOCG00000001359	tmem184a
ENSXMAG00000014131	klf4	ENSLOCG00000009967	klf4
ENSXMAG00000001672	kcnk13b	ENSLOCG00000009008	kcnk13a
ENSXMAG00000017077	bicral	ENSLOCG00000016477	bicral
ENSXMAG00000008674	rbm17	ENSLOCG00000015659	rbm17
ENSXMAG00000013942		ENSLOCG00000001392	tmprss2
ENSXMAG00000027990	timmdc1	ENSLOCG00000006630	
ENSXMAG00000011176	lyn	ENSLOCG00000005468	lyn
ENSXMAG00000009011	has2	ENSLOCG00000009236	has2
ENSXMAG00000010523	gfi1ab	ENSLOCG00000004711	gfi1ab
ENSXMAG00000016216	gigyf2	ENSLOCG00000009165	gigyf2
ENSXMAG00000002223	arl6ip5a	ENSLOCG00000010101	arl6ip5a
ENSXMAG00000003909		ENSLOCG00000003472	wdr91
ENSXMAG00000008990	kdm3b	ENSLOCG00000009551	kdm3b
ENSXMAG00000009331	mafk	ENSLOCG00000001343	mafk
ENSXMAG00000001677	psmc1a	ENSLOCG00000009015	psmc1a
ENSXMAG00000021103	tmprss3a	ENSLOCG00000003832	tmprss3a
ENSXMAG00000019350	cox6c	ENSLOCG00000007557	cox6c
ENSXMAG00000027759	znf407	ENSLOCG00000004046	znf407
ENSXMAG00000021378	s1pr4	ENSLOCG00000017630	s1pr4
ENSXMAG00000028831		ENSLOCG00000012814	
ENSXMAG00000008832	farsa	ENSLOCG00000008389	farsa
ENSXMAG00000024417	COQ2	ENSLOCG00000010011	
ENSXMAG00000028398	tmem108	ENSLOCG00000012078	tmem108
ENSXMAG00000019351		ENSLOCG00000007459	angpt4
ENSXMAG00000012011	nudc	ENSLOCG00000003879	nudc
ENSXMAG00000004151	dop1b	ENSLOCG00000006795	dop1b
ENSXMAG00000011160	urod	ENSLOCG00000002977	urod
ENSXMAG00000017295	rfx5	ENSLOCG00000008459	rfx5
ENSXMAG00000006798	atp5pd	ENSLOCG00000010744	atp5pd
ENSXMAG00000002227	trnt1	ENSLOCG00000011105	trnt1
ENSXMAG00000023660	cdv3	ENSLOCG00000012062	cdv3
ENSXMAG00000009023	ZADH2	ENSLOCG00000004028	ZADH2
ENSXMAG00000014137	NOP14	ENSLOCG00000011611	NOP14
ENSXMAG00000003932	mbd2	ENSLOCG00000003370	mbd2
ENSXMAG00000002875	n6amt1	ENSLOCG00000010815	n6amt1
ENSXMAG00000020208	cyp1c1	ENSLOCG00000017710	cyp1c2
ENSXMAG00000026487	zswim5	ENSLOCG00000002938	zswim5
ENSXMAG00000001840	nrbp2b	ENSLOCG00000010069	nrbp2a
ENSXMAG00000000300	btk	ENSLOCG00000015322	btk
ENSXMAG00000009336	ints1	ENSLOCG00000001281	ints1
ENSXMAG00000012033	tbxta	ENSLOCG00000003838	tbxta
ENSXMAG00000028002	pknox1.1	ENSLOCG00000003857	pknox1.2
ENSXMAG00000023017	caly	ENSLOCG00000004319	caly
ENSXMAG00000003032	gldc	ENSLOCG00000010035	gldc
ENSXMAG00000002379		ENSLOCG00000007063	myo1cb
ENSXMAG00000014775	HNRNPH3	ENSLOCG00000009247	hnrnph3

ENSXMAG00000027340	reep2	ENSLOCG00000009527	reep2
ENSXMAG00000011601	GCDH	ENSLOCG00000008352	gcdhb
ENSXMAG00000029127	nudt5	ENSLOCG00000015711	nudt5
ENSXMAG00000018708	ncln	ENSLOCG00000004061	ncln
ENSXMAG00000009151	lsm3	ENSLOCG00000013560	lsm3
ENSXMAG00000027822	ripply2	ENSLOCG00000014954	
ENSXMAG00000009031	ZNF516	ENSLOCG00000003974	ZNF516
ENSXMAG00000002877	gart	ENSLOCG00000010823	gart
ENSXMAG00000028879		ENSLOCG00000004001	nsmce1
ENSXMAG00000022193	RF00409	ENSLOCG00000019633	RF00409
ENSXMAG00000019353	cand2	ENSLOCG00000013520	cand2
ENSXMAG00000028061	egr1	ENSLOCG00000009516	egr1
ENSXMAG00000006817	gchfr	ENSLOCG00000012719	gchfr
ENSXMAG00000004026	bicdl1	ENSLOCG00000001618	bicdl1
ENSXMAG00000021535	fabp6	ENSLOCG00000009437	fabp6
ENSXMAG00000014754	pbld	ENSLOCG00000009266	pbld
ENSXMAG00000009150		ENSLOCG00000007472	abraa
ENSXMAG00000020066	lrrn1	ENSLOCG00000018250	lrrn1
ENSXMAG00000001292	CREB3L4	ENSLOCG00000008658	CREB3L4
ENSXMAG00000020844		ENSLOCG00000003890	
ENSXMAG00000006791	dnajc17	ENSLOCG00000012721	dnajc17
ENSXMAG00000006749	vps37a	ENSLOCG00000013932	vps37a
ENSXMAG00000001862	puf60b	ENSLOCG00000010106	puf60b
ENSXMAG00000004014	rab35b	ENSLOCG00000001637	rab35b
ENSXMAG00000029083	setmar	ENSLOCG00000011063	setmar
ENSXMAG00000003156	evi5b	ENSLOCG00000004695	evi5b
ENSXMAG00000002335		ENSLOCG00000013654	
ENSXMAG00000011120	elovl8a	ENSLOCG00000002926	elovl8a
ENSXMAG00000009148	rims4	ENSLOCG00000007486	rims4
ENSXMAG00000020207		ENSLOCG00000017711	
ENSXMAG00000017267	mindy1	ENSLOCG00000008413	mindy1
ENSXMAG00000026111	znf236	ENSLOCG00000003926	znf236
ENSXMAG00000008651	cdc123	ENSLOCG00000015714	cdc123
ENSXMAG00000014738		ENSLOCG00000009273	DNA2
ENSXMAG00000000538	tm4sf4	ENSLOCG00000001729	tm4sf4
ENSXMAG00000011119	selenop2	ENSLOCG00000002913	
ENSXMAG00000013745	rassf4	ENSLOCG00000004218	rassf4
ENSXMAG00000003959	DCC	ENSLOCG00000003319	dcc
ENSXMAG00000018180		ENSLOCG00000013042	trpm7
ENSXMAG00000007454	parp16	ENSLOCG00000013778	parp16
ENSXMAG00000029018	uncx	ENSLOCG00000001224	uncx
ENSXMAG00000001252	anks1b	ENSLOCG00000015145	anks1b
ENSXMAG00000000527		ENSLOCG00000001711	
ENSXMAG00000025805	si:ch73-382f3.1	ENSLOCG00000002876	si:ch73-382f3.1
ENSXMAG00000014168	add1	ENSLOCG00000011644	add1
ENSXMAG00000008118	itpr1b	ENSLOCG00000010973	itpr1b
ENSXMAG00000009136	tomm34	ENSLOCG00000007539	tomm34
ENSXMAG00000007465	si:ch73-330k17.3	ENSLOCG00000013774	si:ch73-330k17.3
ENSXMAG00000009462	WDR4	ENSLOCG00000003903	wdr4
ENSXMAG00000011102	toe1	ENSLOCG00000002852	toe1
ENSXMAG00000029859	tm4sf18	ENSLOCG00000001686	tm4sf18
ENSXMAG00000027295	rflna	ENSLOCG00000006003	rflna
ENSXMAG00000022259	zfand2a	ENSLOCG00000001194	zfand2a
ENSXMAG00000018726	cers1	ENSLOCG00000004047	cers1
ENSXMAG00000025667	tmem53	ENSLOCG00000002808	tmem53
ENSXMAG00000000516	cp	ENSLOCG00000001648	cp

ENSXMAG00000019356	sec13	ENSLOCG00000013529	sec13
ENSXMAG00000029418	tspan15	ENSLOCG00000009224	tspan15
ENSXMAG00000009002	ccnjl	ENSLOCG00000009433	ccnjl
ENSXMAG00000012070	paqr7a	ENSLOCG00000018279	paqr7a
ENSXMAG00000006713	adssl1	ENSLOCG00000012740	adssl1
ENSXMAG00000018168		ENSLOCG00000013070	SPPL2A
ENSXMAG00000018730	cope	ENSLOCG00000004027	cope
ENSXMAG00000012074	sh3bgrl3	ENSLOCG00000004007	sh3bgrl3
ENSXMAG00000009006	c1qtnf2	ENSLOCG00000009423	c1qtnf2
ENSXMAG00000010698	serpina1l	ENSLOCG00000008567	serpina1l
ENSXMAG00000004245	rpe	ENSLOCG00000004757	rpe
ENSXMAG00000017161	kif19	ENSLOCG00000012867	kif19
ENSXMAG00000003450	LIN28B	ENSLOCG00000016539	lin28b
ENSXMAG00000002144	ercc6	ENSLOCG00000004168	ercc6
ENSXMAG00000006707	siva1	ENSLOCG00000012749	siva1
ENSXMAG00000007483	sqor	ENSLOCG00000013097	sqor
ENSXMAG00000009053	mbpb	ENSLOCG00000003900	
ENSXMAG00000020364	arl4cb	ENSLOCG00000017842	arl4cb
ENSXMAG00000006333		ENSLOCG00000009457	ttc1
ENSXMAG00000029814	zbtb42	ENSLOCG00000012759	zbtb42
ENSXMAG00000027219	ccdc3a	ENSLOCG00000015717	ccdc3a
ENSXMAG00000014703	tacr2	ENSLOCG00000009218	tacr2
ENSXMAG00000002438	ywhae1	ENSLOCG00000007116	ywhae1
ENSXMAG00000018738	tnfaip8l1	ENSLOCG00000004018	tnfaip8l1
ENSXMAG00000009007	slu7	ENSLOCG00000009410	slu7
ENSXMAG00000026711	TRAPPC9	ENSLOCG00000003865	trappc9
ENSXMAG00000002065		ENSLOCG00000011130	NCAM2
ENSXMAG00000028791	RF00231	ENSLOCG00000020803	RF00231
ENSXMAG00000006673	akt1	ENSLOCG00000012762	akt1
ENSXMAG00000010096	ncbp2	ENSLOCG00000001518	ncbp2
ENSXMAG00000004255		ENSLOCG00000011156	IGSF3
ENSXMAG00000003424	prep	ENSLOCG00000016532	prep
ENSXMAG00000028233	mydgf	ENSLOCG00000004004	mydgf
ENSXMAG00000019896		ENSLOCG00000002331	prkrip1
ENSXMAG00000024736	pfdn1	ENSLOCG00000012528	pfdn1
ENSXMAG00000008624	optn	ENSLOCG00000015719	optn
ENSXMAG00000023123	rrp1	ENSLOCG00000004064	
ENSXMAG00000003595	lgi3	ENSLOCG00000014999	lgi3
ENSXMAG00000001864	scrib	ENSLOCG00000010133	scrib
ENSXMAG00000006686	kpna7	ENSLOCG00000004619	kpna7
ENSXMAG00000011137	atp8b4	ENSLOCG00000013128	ATP8B4
ENSXMAG00000029684	pbx1a	ENSLOCG00000004452	
ENSXMAG00000025645	glrx5	ENSLOCG00000008507	glrx5
ENSXMAG00000002981	aacs	ENSLOCG00000001938	aacs
ENSXMAG00000006316	zgc:110540	ENSLOCG00000012501	zgc:110540
ENSXMAG00000010170	ncaph	ENSLOCG00000015362	ncaph
ENSXMAG00000020195	si:ch211-162e15.3	ENSLOCG00000017372	si:ch211-162e15.3
ENSXMAG00000022168	timm8a	ENSLOCG00000015321	timm8a
ENSXMAG00000006644	cep170b	ENSLOCG00000012777	cep170b
ENSXMAG00000004264		ENSLOCG00000011168	
ENSXMAG00000009067		ENSLOCG00000007713	DEPDC1B
ENSXMAG00000008619	mcm10	ENSLOCG00000015721	mcm10
ENSXMAG00000002452	LRRC75A	ENSLOCG00000007159	LRRC75A
ENSXMAG00000023628		ENSLOCG00000009592	XPA
ENSXMAG00000009391	zgc:92162	ENSLOCG00000000651	zgc:92162
ENSXMAG00000010103	MELTF	ENSLOCG00000001493	meltf

ENSXMAG00000007242		ENSLOCG00000014646	
ENSXMAG00000006254		ENSLOCG00000002079	
ENSXMAG00000010715	ints9	ENSLOCG00000015999	ints9
ENSXMAG00000021220	zgc:101583	ENSLOCG00000015318	zgc:101583
ENSXMAG00000025457	drgx	ENSLOCG00000004137	drgx
ENSXMAG00000009051	glod5	ENSLOCG00000014868	glod5
ENSXMAG00000022267	galk2	ENSLOCG00000013168	galk2
ENSXMAG00000009399	zc3h7a	ENSLOCG00000000609	zc3h7a
ENSXMAG00000006627		ENSLOCG00000012785	
ENSXMAG00000020981		ENSLOCG00000013917	
ENSXMAG00000002454	poldip2	ENSLOCG00000000789	poldip2
ENSXMAG00000006251	PWWP2A	ENSLOCG00000009452	pwwp2a
ENSXMAG00000004996	cntnap5a	ENSLOCG00000010405	cntnap5b
ENSXMAG00000000296	mars2	ENSLOCG00000015357	mars2
ENSXMAG00000012082	kcnk9	ENSLOCG00000003851	kcnk9
ENSXMAG00000017217	rpl38	ENSLOCG00000012842	rpl38
ENSXMAG00000003413	xkr5a	ENSLOCG00000000839	xkr5a
ENSXMAG00000023447	sall3a	ENSLOCG00000007831	sall3a
ENSXMAG00000006227		ENSLOCG00000012533	
ENSXMAG00000026425	xgb	ENSLOCG00000012798	xgb
ENSXMAG00000029342	rlim	ENSLOCG00000013643	rlim
ENSXMAG00000011116	cep152	ENSLOCG00000000298	cep152
ENSXMAG00000008603	phyh	ENSLOCG00000015723	phyh
ENSXMAG00000006628	nansa	ENSLOCG00000011173	nansb
ENSXMAG00000002089	arl13b	ENSLOCG00000010465	arl13b
ENSXMAG00000010873	klf17	ENSLOCG00000002742	klf17
ENSXMAG00000027281	nexmifb	ENSLOCG00000013644	nexmifb
ENSXMAG00000002469	tmem199	ENSLOCG00000000808	tmem199
ENSXMAG00000022314	kctd9a	ENSLOCG00000014981	kctd9b
ENSXMAG00000006625	srp14	ENSLOCG00000012793	srp14
ENSXMAG00000001162	dek	ENSLOCG00000005923	dek
ENSXMAG00000004290		ENSLOCG00000011214	
ENSXMAG00000009428	rmi2	ENSLOCG00000004133	rmi2
ENSXMAG00000007223	fancl	ENSLOCG00000016137	fancl
ENSXMAG00000010226	zgc:110329	ENSLOCG00000015369	zgc:110329
ENSXMAG00000010731	pla2g7	ENSLOCG00000016485	pla2g7
ENSXMAG00000001135	ndufa10	ENSLOCG00000010079	ndufa10
ENSXMAG00000000274	med12	ENSLOCG00000015382	med12
ENSXMAG00000012260		ENSLOCG00000015617	col7a1l
ENSXMAG00000006214		ENSLOCG00000012478	mat2b
ENSXMAG00000018784	zfr2	ENSLOCG00000003939	zfr2
ENSXMAG00000023902	ubfd1	ENSLOCG00000004087	ubfd1
ENSXMAG00000009070	abcb7	ENSLOCG00000013647	abcb7
ENSXMAG00000019099	slc6a9	ENSLOCG00000002674	slc6a9
ENSXMAG00000003399	tram2	ENSLOCG00000000868	tram2
ENSXMAG00000001158	KCNV1	ENSLOCG00000005940	KCNV1
ENSXMAG00000025218	tmem56b	ENSLOCG00000008184	tmem56b
ENSXMAG00000003820		ENSLOCG00000003538	TCF4
ENSXMAG00000014192	SEMA4D	ENSLOCG00000011753	SEMA4D
ENSXMAG00000021856	clta	ENSLOCG00000011155	clta
ENSXMAG00000009063	atp2b2	ENSLOCG00000012023	atp2b2
ENSXMAG00000025221	gbp	ENSLOCG00000018330	gbp
ENSXMAG00000002958	gak	ENSLOCG00000011267	gak
ENSXMAG00000014561	hkdc1	ENSLOCG00000009177	hkdc1
ENSXMAG00000010747	sf3b6	ENSLOCG00000016481	sf3b6
ENSXMAG00000027794	cnn1b	ENSLOCG00000008170	cnn1b

ENSXMAG00000005747	FAM135B	ENSLOCG00000003757	FAM135B
ENSXMAG00000009085	uprt	ENSLOCG00000013655	uprt
ENSXMAG00000019367	itcha	ENSLOCG00000000888	itcha
ENSXMAG00000007201	vrk2	ENSLOCG00000016138	vrk2
ENSXMAG00000009432	kdm8	ENSLOCG00000003965	kdm8
ENSXMAG00000024401		ENSLOCG00000016194	frs2a
ENSXMAG00000018839	matk	ENSLOCG00000003919	matk
ENSXMAG00000000346		ENSLOCG00000006749	si:dkey-222b8.1
ENSXMAG00000003396	efhc1	ENSLOCG00000000899	efhc1
ENSXMAG00000027455	tmem205	ENSLOCG00000008148	tmem205
ENSXMAG00000009092	zdhhc15b	ENSLOCG00000013659	zdhhc15a
ENSXMAG00000008550	KLHL22	ENSLOCG00000006522	klhl22
ENSXMAG00000026916	si:ch211-223a10.1	ENSLOCG00000004022	si:ch211-223a10.1
ENSXMAG00000024951	opcml	ENSLOCG00000005387	opcml
ENSXMAG00000010759	fam49a	ENSLOCG00000016478	fam49a
ENSXMAG00000006581	gne	ENSLOCG00000011146	gne
ENSXMAG00000005081	kalrna	ENSLOCG00000005462	kalrna
ENSXMAG00000005362	KHDRBS3	ENSLOCG00000003734	KHDRBS3
ENSXMAG00000000394	erap1b	ENSLOCG00000004362	erap1b
ENSXMAG00000004308	tbl1x	ENSLOCG00000010116	tbl1x
ENSXMAG00000009469	hs3st4	ENSLOCG00000003950	hs3st4
ENSXMAG00000006087	dad1	ENSLOCG00000017661	dad1
ENSXMAG00000026062	fgf16	ENSLOCG00000013663	fgf16
ENSXMAG00000021208	epor	ENSLOCG00000008110	epor
ENSXMAG00000014547	supv3l1	ENSLOCG00000009159	supv3l1
ENSXMAG00000028534	hs3st2	ENSLOCG00000003905	hs3st2
ENSXMAG00000019371		ENSLOCG00000000996	PXMP4
ENSXMAG00000002118	chn1	ENSLOCG00000007269	chn1
ENSXMAG00000002485	gemin4	ENSLOCG00000006840	gemin4
ENSXMAG00000003107	zgc:109982	ENSLOCG00000004583	zgc:109982
ENSXMAG00000000252	si:ch211-221f10.2	ENSLOCG00000015366	si:ch211-221f10.2
ENSXMAG00000001296	uhrf1bp1l	ENSLOCG00000015138	uhrf1bp1l
ENSXMAG00000018862	haus5	ENSLOCG00000003630	haus5
ENSXMAG00000029432	RAP1GDS1	ENSLOCG00000012715	RAP1GDS1
ENSXMAG00000019372	tgm5l	ENSLOCG00000001036	tgm5l
ENSXMAG00000008790	swsap1	ENSLOCG00000008092	swsap1
ENSXMAG00000027761		ENSLOCG00000003529	
ENSXMAG00000017097	SHC3	ENSLOCG00000011720	SHC3
ENSXMAG00000006184	panx3	ENSLOCG00000005345	panx3
ENSXMAG00000009112		ENSLOCG00000013667	atrx
ENSXMAG00000020150	HTD2	ENSLOCG00000013205	HTD2
ENSXMAG00000003101	dr1	ENSLOCG00000004566	dr1
ENSXMAG00000008545	cep83	ENSLOCG00000015765	CEP83
ENSXMAG00000003388	mrpl19	ENSLOCG00000000969	mrpl19
ENSXMAG00000003289	usp31	ENSLOCG00000003884	usp31
ENSXMAG00000006569	zfyve19	ENSLOCG00000012832	zfyve19
ENSXMAG00000017277	tekt3	ENSLOCG00000013131	tekt3
ENSXMAG00000009056	RPP14	ENSLOCG00000013207	rpp14
ENSXMAG00000008783	znf653	ENSLOCG00000008078	znf653
ENSXMAG00000010768	fbxo16	ENSLOCG00000016029	fbxo16
ENSXMAG00000003386	mycn	ENSLOCG00000016476	mycn
ENSXMAG00000007153	wdr11	ENSLOCG00000008740	wdr11
ENSXMAG00000014529	vps26a	ENSLOCG00000009150	vps26a
ENSXMAG00000004334	gpr143	ENSLOCG00000010107	gpr143
ENSXMAG00000019373	eya2	ENSLOCG00000006186	eya2
ENSXMAG00000006176	pknox2	ENSLOCG00000005322	pknox2

ENSXMAG00000003375	smek1	ENSLOCG00000013202	smek1
ENSXMAG00000009145	kif3a	ENSLOCG00000010399	kif3a
ENSXMAG00000018864	SIN3B	ENSLOCG00000000085	sin3b
ENSXMAG00000001319		ENSLOCG00000015137	CKAP4
ENSXMAG00000000646		ENSLOCG00000013396	
ENSXMAG00000029198	cox10	ENSLOCG00000013147	cox10
ENSXMAG00000014516	kif1bp	ENSLOCG00000009142	kif1bp
ENSXMAG00000003089	sec16b	ENSLOCG00000004491	sec16b
ENSXMAG00000010796	cdc42bpab	ENSLOCG00000016021	cdc42bpab
ENSXMAG00000005104	si:ch211-67e16.11	ENSLOCG00000011002	si:ch211-67e16.11
ENSXMAG00000020933		ENSLOCG00000015767	plxnc1
ENSXMAG00000008054	si:ch211-216b21.2	ENSLOCG00000013160	si:ch211-216b21.2
ENSXMAG00000002506	her8.2	ENSLOCG00000004481	her8.2
ENSXMAG00000025833	ccdc84	ENSLOCG00000003234	ccdc84
ENSXMAG00000006038		ENSLOCG00000001219	
ENSXMAG00000001321		ENSLOCG00000015136	
ENSXMAG00000000331	si:dkey-23o4.6	ENSLOCG00000008528	si:dkey-23o4.6
ENSXMAG00000006094	tmod1	ENSLOCG00000011358	
ENSXMAG00000020298		ENSLOCG00000015920	RRP15
ENSXMAG00000009471	baiap3	ENSLOCG00000003707	baiap3
ENSXMAG00000009163		ENSLOCG00000010451	
ENSXMAG00000005106	ttll4	ENSLOCG00000011018	ttll4
ENSXMAG00000011789	txnl1	ENSLOCG00000003581	txnl1
ENSXMAG00000024410		ENSLOCG00000011059	
ENSXMAG00000006531	PLCB2	ENSLOCG00000012860	PLCB2
ENSXMAG00000026878	fam214b	ENSLOCG00000004450	fam214b
ENSXMAG00000028435	PTPN20	ENSLOCG00000003949	
ENSXMAG00000020377	znf648	ENSLOCG00000017736	znf648
ENSXMAG00000029796	rtf2	ENSLOCG00000006976	rtf2
ENSXMAG00000028249	unc119b	ENSLOCG00000005507	unc119b
ENSXMAG00000001324	arfgap3	ENSLOCG00000015134	arfgap3
ENSXMAG00000001651	lrfn5a	ENSLOCG00000009242	lrfn5a
ENSXMAG00000024362	tmem218	ENSLOCG00000005308	tmem218
ENSXMAG00000002508	cab39	ENSLOCG00000004495	cab39
ENSXMAG00000004358	mospd2	ENSLOCG00000010087	mospd2
ENSXMAG00000004232	zbtb7c	ENSLOCG00000011712	zbtb7c
ENSXMAG00000002911	si:ch211-214j8.1	ENSLOCG00000004396	si:ch211-214j8.1
ENSXMAG00000011755	NARS	ENSLOCG00000003598	nars
ENSXMAG00000003666	cabin1	ENSLOCG00000006568	cabin1
ENSXMAG00000023209		ENSLOCG00000016811	mpc1
ENSXMAG00000014683	lft1	ENSLOCG00000016018	lft2
ENSXMAG00000026338		ENSLOCG00000015810	
ENSXMAG00000017074	selenbp1	ENSLOCG00000008479	selenbp1
ENSXMAG00000015784	frem1a	ENSLOCG00000011064	frem1a
ENSXMAG00000009518	gper1	ENSLOCG00000018100	gper1
ENSXMAG00000005115	prkag3b	ENSLOCG00000011025	prkag3b
ENSXMAG00000024060	prdm8	ENSLOCG00000003895	prdm8
ENSXMAG00000023679	INAFM2	ENSLOCG00000017712	INAFM2
ENSXMAG00000028411	rspo2	ENSLOCG00000003131	rspo2
ENSXMAG00000009520	unkl	ENSLOCG00000003648	unkl
ENSXMAG00000001367	pacsin2	ENSLOCG00000015131	pacsin2
ENSXMAG00000004369	fancb	ENSLOCG00000010078	fancb
ENSXMAG00000029010	si:ch73-290k24.6	ENSLOCG00000002324	si:ch73-290k24.6
ENSXMAG00000006530		ENSLOCG00000012881	ccdc9b
ENSXMAG00000004236	ctif	ENSLOCG00000011697	ctif
ENSXMAG00000026994	gcnt7	ENSLOCG00000006989	

ENSXMAG00000004787	ippk	ENSLOCG00000013831	ippk
ENSXMAG00000002142		ENSLOCG00000009336	
ENSXMAG00000000622	cyp17a2	ENSLOCG00000002364	cyp17a2
ENSXMAG00000013542	antxr1d	ENSLOCG00000003873	antxr1d
ENSXMAG00000009192	gnpda1	ENSLOCG00000010571	gnpda1
ENSXMAG00000002537		ENSLOCG00000014309	
ENSXMAG00000005129		ENSLOCG00000010977	fer1l6
ENSXMAG00000025227	CRTC2	ENSLOCG00000008695	CRTC2
ENSXMAG00000003360	PLEKHG1	ENSLOCG00000016199	PLEKHG1
ENSXMAG00000019745	igfals	ENSLOCG00000003531	igfals
ENSXMAG00000014002	acer3	ENSLOCG00000001064	acer3
ENSXMAG00000010009		ENSLOCG00000006858	ddx51
ENSXMAG00000010356	hnrnpua	ENSLOCG00000016652	hnrnpua
ENSXMAG00000005803	tfap2c	ENSLOCG00000006963	tfap2c
ENSXMAG00000023480	zmp:0000001301	ENSLOCG00000010231	zmp:0000001301
ENSXMAG00000027638	ttll1	ENSLOCG00000015129	ttll1
ENSXMAG00000018958	acbd6	ENSLOCG00000009237	acbd6
ENSXMAG00000002549	meis3	ENSLOCG00000014306	meis3
ENSXMAG00000030083	fbxo33	ENSLOCG00000009198	fbxo33
ENSXMAG00000000239	enox2	ENSLOCG00000015363	enox2
ENSXMAG00000023868		ENSLOCG00000011091	
ENSXMAG00000002878	tars	ENSLOCG00000011402	tars
ENSXMAG00000004370	glra2	ENSLOCG00000010066	glra2
ENSXMAG00000011044	stra6l	ENSLOCG00000011382	stra6l
ENSXMAG00000012211	kcnk10b	ENSLOCG00000008480	kcnk10a
ENSXMAG00000009199	emc2	ENSLOCG00000003094	emc2
ENSXMAG00000023410		ENSLOCG00000013367	PAQR9
ENSXMAG00000021275	mcat	ENSLOCG00000015128	mcat
ENSXMAG00000017322	sgca	ENSLOCG00000013033	sgca
ENSXMAG00000027769	map1lc3a	ENSLOCG00000006440	map1lc3a
ENSXMAG00000018968	lhx4	ENSLOCG00000009245	lhx4
ENSXMAG00000003689	dock5	ENSLOCG00000015676	dock5
ENSXMAG00000028152	IGKV4-1	ENSLOCG00000000243	
ENSXMAG00000024024	RERG	ENSLOCG00000016464	RERG
ENSXMAG00000002034	dennd4b	ENSLOCG00000008793	DENND4B
ENSXMAG00000023603	aco1	ENSLOCG00000011082	aco1
ENSXMAG00000000112	snrnp25	ENSLOCG00000007572	snrnp25
ENSXMAG00000002565		ENSLOCG00000014300	
ENSXMAG00000009512	fgfr2	ENSLOCG00000008752	fgfr2
ENSXMAG00000004761	eif6	ENSLOCG00000007763	eif6
ENSXMAG00000009224	uqcrq	ENSLOCG00000010627	uqcrq
ENSXMAG00000005816	cdh22	ENSLOCG00000002614	cdh22
ENSXMAG00000019516	TMEM74	ENSLOCG00000018324	TMEM74
ENSXMAG00000018978	qsox1	ENSLOCG00000009251	qsox1
ENSXMAG00000025654	polr3k	ENSLOCG00000007584	polr3k
ENSXMAG00000003351	tcp1	ENSLOCG00000016197	tcp1
ENSXMAG00000006475	C14orf28	ENSLOCG00000011251	C14orf28
ENSXMAG00000009227	gdf9	ENSLOCG00000010635	gdf9
ENSXMAG00000022471	SYN3	ENSLOCG00000016458	SYN3
ENSXMAG00000002868	si:dkey-84j12.1	ENSLOCG00000011389	si:dkey-84j12.1
ENSXMAG00000006328	med8	ENSLOCG00000004404	med8
ENSXMAG00000001421	def6c	ENSLOCG00000015122	
ENSXMAG00000006105	MSANTD2	ENSLOCG00000005139	MSANTD2
ENSXMAG00000000113	cdip1	ENSLOCG00000007597	cdip1
ENSXMAG00000020824	sirt2	ENSLOCG00000014292	sirt2
ENSXMAG00000013498	rrp12	ENSLOCG00000003846	rrp12

ENSXMAG00000004241	dym	ENSLOCG00000011668	dym
ENSXMAG00000006465	KLHL28	ENSLOCG00000011256	KLHL28
ENSXMAG00000004394	ofd1	ENSLOCG00000010040	ofd1
ENSXMAG00000024917	ASL	ENSLOCG00000005320	asl
ENSXMAG00000021060	TIMP3	ENSLOCG00000016462	
ENSXMAG00000004709	mmp24	ENSLOCG00000007747	mmp24
ENSXMAG00000006313	aldh9a1a.1	ENSLOCG00000006045	aldh9a1a.1
ENSXMAG00000012209	gpr65	ENSLOCG00000008491	GPR65
ENSXMAG00000027750		ENSLOCG00000011272	TOGARAM1
ENSXMAG00000022956	si:ch73-215d9.1	ENSLOCG00000005830	si:ch73-215d9.1
ENSXMAG00000001926	si:ch73-173p19.1	ENSLOCG00000010267	si:ch73-173p19.1
ENSXMAG00000023716	sybu	ENSLOCG00000002949	sybu
ENSXMAG00000000117	mgrn1b	ENSLOCG00000007626	mgrn1a
ENSXMAG00000029107		ENSLOCG00000005876	si:dkey-276j7.1
ENSXMAG00000009021	afap1	ENSLOCG00000010207	afap1
ENSXMAG00000006545	duox2	ENSLOCG00000013747	duox2
ENSXMAG00000002853	mccc2	ENSLOCG00000011352	mccc2
ENSXMAG00000003334	acat2	ENSLOCG00000016193	acat2
ENSXMAG00000029231	gdf6a	ENSLOCG00000002936	gdf6a
ENSXMAG00000011038	lrrc7	ENSLOCG00000009583	lrrc7
ENSXMAG00000007741	ankef1a	ENSLOCG00000015942	ankef1b
ENSXMAG00000016447	prkag2b	ENSLOCG00000012585	prkag2a
ENSXMAG00000022459		ENSLOCG00000017987	
ENSXMAG00000026800	ccdc28b	ENSLOCG00000002375	ccdc28b
ENSXMAG00000006079	fez1	ENSLOCG00000005073	fez1
ENSXMAG00000022651	si:ch1073-228b5.2	ENSLOCG00000014282	si:ch1073-228b5.2
ENSXMAG00000006311	tmco1	ENSLOCG00000006070	tmco1
ENSXMAG00000023922	fam83c	ENSLOCG00000007724	fam83c
ENSXMAG00000024405	si:ch73-252i11.1	ENSLOCG00000016450	si:ch73-252i11.1
ENSXMAG00000005859	znfx1	ENSLOCG00000002641	znfx1
ENSXMAG00000007085	eny2	ENSLOCG00000003051	eny2
ENSXMAG00000003331	wtap	ENSLOCG00000016191	wtap
ENSXMAG00000014522	aldh5a1	ENSLOCG00000005880	aldh5a1
ENSXMAG00000006431	tmem39b	ENSLOCG00000002293	tmem39b
ENSXMAG00000028626	nrip1b	ENSLOCG00000000906	nrip1b
ENSXMAG00000006062	cfap58	ENSLOCG00000011978	cfap58
ENSXMAG00000014110	zgc:158403	ENSLOCG00000008037	zgc:158403
ENSXMAG00000007078	ebag9	ENSLOCG00000002958	ebag9
ENSXMAG00000012231	uqcc1	ENSLOCG00000007712	uqcc1
ENSXMAG00000022824	rad1	ENSLOCG00000011768	rad1
ENSXMAG00000001927	abcf2a	ENSLOCG00000009041	abcf2b
ENSXMAG00000007755	stmn4	ENSLOCG00000015955	stmn4
ENSXMAG00000020205	aatf	ENSLOCG00000004972	aatf
ENSXMAG00000002589	nop53	ENSLOCG00000014178	nop53
ENSXMAG00000003325	sod2	ENSLOCG00000016188	sod2
ENSXMAG00000004416	egfl6	ENSLOCG00000010006	egfl6
ENSXMAG00000002803	TLE4	ENSLOCG00000009894	TLE4
ENSXMAG00000005880	tbc1d20	ENSLOCG00000006680	TBC1D20
ENSXMAG00000006579	fan1	ENSLOCG00000013674	FAN1
ENSXMAG00000000128	aanat2	ENSLOCG00000007662	aanat2
ENSXMAG00000006297	magoh	ENSLOCG00000006104	magoh
ENSXMAG00000008199	DDX21	ENSLOCG00000009123	ddx21
ENSXMAG00000022222	crcp	ENSLOCG00000005302	crcp
ENSXMAG00000025983	C14orf132	ENSLOCG00000017698	C14orf132
ENSXMAG00000000469	prmt5	ENSLOCG00000000583	
ENSXMAG00000009248	ccng1	ENSLOCG00000010677	ccng1

ENSXMAG00000004433	TLR8	ENSLOCG00000009990	TLR8 (1 of many)
ENSXMAG00000006408	zbtb8os	ENSLOCG00000002238	zbtb8os
ENSXMAG00000025303	C18orf32	ENSLOCG00000011664	C18orf32
ENSXMAG00000001356	anapc5	ENSLOCG00000005044	anapc5
ENSXMAG00000029748		ENSLOCG00000017900	
ENSXMAG00000012227	gdf5	ENSLOCG00000007687	gdf5
ENSXMAG00000007764	mcm3	ENSLOCG00000015961	mcm3
ENSXMAG00000003322	fndc1	ENSLOCG00000016185	fndc1
ENSXMAG00000004434	tlr7	ENSLOCG00000009977	tlr7
ENSXMAG00000027884		ENSLOCG00000008689	SLC39A1
ENSXMAG00000001943	chpf2	ENSLOCG00000009059	chpf2
ENSXMAG00000004284		ENSLOCG00000011774	BRIX1
ENSXMAG00000019892	gpr22b	ENSLOCG00000017904	gpr22b
ENSXMAG00000006383	RBBP4	ENSLOCG00000002213	rbbp4
ENSXMAG00000021575	bcl2b	ENSLOCG00000012609	bcl2a
ENSXMAG00000005890		ENSLOCG00000006659	
ENSXMAG00000023533	gadd45gip1	ENSLOCG00000008024	gadd45gip1
ENSXMAG00000007075	ttk	ENSLOCG00000017061	ttk
ENSXMAG00000012290	shroom3	ENSLOCG00000003809	shroom3
ENSXMAG00000009257	nudcd2	ENSLOCG00000010683	nudcd2
ENSXMAG00000006291	cpt2	ENSLOCG00000006146	cpt2
ENSXMAG00000016480	kdsr	ENSLOCG00000012611	kdsr
ENSXMAG00000014035	usp25	ENSLOCG00000000838	usp25
ENSXMAG00000022361	si:ch211-39k3.2	ENSLOCG00000014349	si:ch211-39k3.2
ENSXMAG00000027513		ENSLOCG00000016444	
ENSXMAG00000008186		ENSLOCG00000011434	
ENSXMAG00000025311	METTL18	ENSLOCG00000018228	METTL18
ENSXMAG00000000150	mpg	ENSLOCG00000007699	mpg
ENSXMAG00000028425		ENSLOCG00000010240	ablim2
ENSXMAG00000022630	smarcd3b	ENSLOCG00000009069	smarcd3b
ENSXMAG00000002605		ENSLOCG00000014186	psma6l
ENSXMAG00000019722	paqr8	ENSLOCG00000017993	paqr8
ENSXMAG00000000856	ATP2C1	ENSLOCG00000001574	ATP2C1
ENSXMAG00000027777		ENSLOCG00000010139	FAM19A4
ENSXMAG00000000154	nprl3	ENSLOCG00000007714	nprl3
ENSXMAG00000016491	vps4b	ENSLOCG00000012619	vps4b
ENSXMAG00000003116	scp2b	ENSLOCG00000006164	SCP2
ENSXMAG00000007052	bckdhb	ENSLOCG00000017063	bckdhb
ENSXMAG00000021121	ATE1	ENSLOCG00000008771	ATE1
ENSXMAG00000028153	KLHDC2	ENSLOCG00000011444	KLHDC2
ENSXMAG00000002786	psat1	ENSLOCG00000009885	PSAT1
ENSXMAG00000024537	nicn1	ENSLOCG00000013395	nicn1
ENSXMAG00000025307		ENSLOCG00000003796	
ENSXMAG00000027934	agxt2	ENSLOCG00000011796	agxt2
ENSXMAG00000024858	PRKAR2B	ENSLOCG00000015827	PRKAR2B
ENSXMAG00000003118	podn	ENSLOCG00000006181	podn
ENSXMAG00000000589		ENSLOCG00000012242	CFI
ENSXMAG00000013414	cbfa2t2	ENSLOCG00000000816	cbfa2t2
ENSXMAG00000025014	si:ch211-63o20.7	ENSLOCG00000015970	si:ch211-63o20.7
ENSXMAG00000010633	MBD5	ENSLOCG00000001417	mbd5
ENSXMAG00000023035	tusc3	ENSLOCG00000013971	tusc3
ENSXMAG00000002613	sars2	ENSLOCG00000014192	SARS2
ENSXMAG00000028077	ccni	ENSLOCG00000003779	ccni
ENSXMAG00000001963	si:dkey-10f23.2	ENSLOCG00000011211	
ENSXMAG00000015698	GRM7	ENSLOCG00000013382	GRM7
ENSXMAG00000026624	smyd5	ENSLOCG00000009281	smyd5

ENSXMAG00000003300	otofa	ENSLOCG00000016183	otofa
ENSXMAG00000002782	isca1	ENSLOCG00000002455	isca1
ENSXMAG00000001825	elp3	ENSLOCG00000015972	elp3
ENSXMAG00000004317	dnajc21	ENSLOCG00000011784	dnajc21
ENSXMAG00000009024	NEMF	ENSLOCG00000011454	NEMF
ENSXMAG00000007032	ibtk	ENSLOCG00000017066	ibtk
ENSXMAG00000021209		ENSLOCG00000001396	LAMA3
ENSXMAG00000013479	sufu	ENSLOCG00000012672	sufu
ENSXMAG00000016460	hsp90b1	ENSLOCG00000015012	hsp90b1
ENSXMAG00000010615	orc4	ENSLOCG00000001369	ORC4
ENSXMAG00000003187	soat1	ENSLOCG00000009767	soat1
ENSXMAG00000016117	sfxn5b	ENSLOCG00000009330	sfxn5b
ENSXMAG00000000437	wu:fj64h06	ENSLOCG00000003731	wu:fj64h06
ENSXMAG00000023475		ENSLOCG00000014148	
ENSXMAG00000028015		ENSLOCG00000003875	pikfyve
ENSXMAG00000023046	SNTA1	ENSLOCG00000000786	SNTA1
ENSXMAG00000009322	atp10b	ENSLOCG00000010731	atp10b
ENSXMAG00000020245	tpbgb	ENSLOCG00000017873	tpbgb
ENSXMAG00000000828	pik3r4	ENSLOCG00000001622	pik3r4
ENSXMAG00000010168	SLC16A7	ENSLOCG00000016547	SLC16A7
ENSXMAG00000029145	ostm1	ENSLOCG00000016601	ostm1
ENSXMAG00000016490	nt5dc3	ENSLOCG00000015006	nt5dc3
ENSXMAG00000002639	slc5a2	ENSLOCG00000001542	
ENSXMAG00000002769	CCNO	ENSLOCG00000002485	CCNO
ENSXMAG00000012608	rnf123	ENSLOCG00000013373	rnf123
ENSXMAG00000002766	gpx8	ENSLOCG00000002554	gpx8
ENSXMAG00000010178	lrig3	ENSLOCG00000016545	lrig3
ENSXMAG00000007024	ube3d	ENSLOCG00000017069	ube3d
ENSXMAG00000027738	si:dkey-57a22.13	ENSLOCG00000003713	si:dkey-57a22.13
ENSXMAG00000001448	washc3	ENSLOCG00000015173	washc3
ENSXMAG00000026225	rsl24d1	ENSLOCG00000013445	rsl24d1
ENSXMAG00000008180		ENSLOCG00000011479	MOK
ENSXMAG00000016024		ENSLOCG00000003684	C19orf44
ENSXMAG00000016156	BEND4	ENSLOCG00000012472	BEND4
ENSXMAG00000018093	mrpl46	ENSLOCG00000014530	
ENSXMAG00000027823	FBXO46	ENSLOCG00000017565	FBXO46
ENSXMAG00000001975	asap1b	ENSLOCG00000011231	asap1b
ENSXMAG00000022568		ENSLOCG00000011882	ccdc171
ENSXMAG00000003283	nr2e1	ENSLOCG00000016602	nr2e1
ENSXMAG00000000440	WDR7	ENSLOCG00000003671	WDR7
ENSXMAG00000003197	abl2	ENSLOCG00000009782	abl2
ENSXMAG00000004333	nedd4l	ENSLOCG00000011831	nedd4l
ENSXMAG00000001465	dram1	ENSLOCG00000015183	dram1
ENSXMAG00000013473	npm3	ENSLOCG00000000705	
ENSXMAG00000009345	fbxl3l	ENSLOCG00000010751	fbxl3l
ENSXMAG00000004482	cog3	ENSLOCG00000005428	cog3
ENSXMAG00000022521	shisa3	ENSLOCG00000012459	shisa3
ENSXMAG00000025118	tmem101	ENSLOCG00000013062	tmem101
ENSXMAG00000010196	atp23	ENSLOCG00000016543	atp23
ENSXMAG00000002671	coq8b	ENSLOCG00000014507	coq8b
ENSXMAG00000026933	parietopsin	ENSLOCG00000015794	parietopsin
ENSXMAG00000016033	akr1a1b	ENSLOCG00000005983	akr1a1b
ENSXMAG00000021469	si:dkey-57a22.15	ENSLOCG00000003782	si:dkey-57a22.15
ENSXMAG00000027888	commd1	ENSLOCG00000016086	commd1
ENSXMAG00000023958	gngt2b	ENSLOCG00000013057	gngt2a
ENSXMAG00000009349	atp7a	ENSLOCG00000013870	atp7a

ENSXMAG00000010207	rpap3	ENSLOCG00000016541	rpap3
ENSXMAG00000014061	wscd1b	ENSLOCG00000005722	wscd1b
ENSXMAG00000010901	amigo3	ENSLOCG00000018259	amigo3
ENSXMAG00000003281	snx3	ENSLOCG00000016603	snx3
ENSXMAG00000013471	oga	ENSLOCG00000012705	oga
ENSXMAG00000006960	dop1a	ENSLOCG00000017071	dop1a
ENSXMAG00000005934	st7l	ENSLOCG00000010322	st7l
ENSXMAG00000020950	lsm12b	ENSLOCG00000013079	lsm12b
ENSXMAG00000001470	gnptab	ENSLOCG00000015184	gnptab
ENSXMAG00000005863		ENSLOCG00000016082	
ENSXMAG00000016056	uhmk1	ENSLOCG00000007180	uhmk1
ENSXMAG00000026692	bhlha9	ENSLOCG00000005703	bhlha9
ENSXMAG00000017595	g6pc3	ENSLOCG00000013084	g6pc3
ENSXMAG00000007904	si:dkey-156n14.3	ENSLOCG00000013702	si:dkey-156n14.3
ENSXMAG00000010565	kynu	ENSLOCG00000001250	kynu
ENSXMAG00000006735	myo5c	ENSLOCG00000013362	myo5c
ENSXMAG00000014430		ENSLOCG00000016854	CSMD1
ENSXMAG00000019824		ENSLOCG00000012083	si:ch211-28p3.4
ENSXMAG00000021185	sfxn1	ENSLOCG00000010830	sfxn1
ENSXMAG00000002971	mlec	ENSLOCG00000002903	mlec
ENSXMAG00000024225	numbl	ENSLOCG00000014504	
ENSXMAG00000004509	ednrbb	ENSLOCG00000005403	ednrbb
ENSXMAG00000021621		ENSLOCG00000001438	
ENSXMAG00000008741	rad23aa	ENSLOCG00000007980	rad23aa
ENSXMAG00000000842	arhgap32b	ENSLOCG00000004776	arhgap32b
ENSXMAG00000024316	ASCL5	ENSLOCG00000017939	ASCL5
ENSXMAG00000016159	atp8a1	ENSLOCG00000012435	ATP8A1
ENSXMAG00000005971	wnt2ba	ENSLOCG00000010337	wnt2ba
ENSXMAG00000001789	disp1	ENSLOCG00000016410	disp1
ENSXMAG00000007029		ENSLOCG00000012991	ablim1b
ENSXMAG00000010259	mthfd2l	ENSLOCG00000012052	
ENSXMAG00000002923		ENSLOCG00000013604	
ENSXMAG00000003268	armc2	ENSLOCG00000016606	armc2
ENSXMAG00000009386		ENSLOCG00000010230	PPP2CA
ENSXMAG00000027639	PCYOX1L	ENSLOCG00000009203	pcyox1l
ENSXMAG00000013470	pprc1	ENSLOCG00000012714	pprc1
ENSXMAG00000010550	si:ch211-246m6.5	ENSLOCG00000001087	si:ch211-246m6.5
ENSXMAG00000001481	chpt1	ENSLOCG00000015186	chpt1
ENSXMAG00000003213	osbpl9	ENSLOCG00000009802	osbpl9
ENSXMAG00000012532	pik3c2b	ENSLOCG00000012102	pik3c2b
ENSXMAG00000006524	psmd12	ENSLOCG00000013614	psmd12
ENSXMAG00000000504	crebrf	ENSLOCG00000009174	crebrf
ENSXMAG00000004545	pcdh8	ENSLOCG00000005273	pcdh8
ENSXMAG00000023104	si:ch211-262h13.5	ENSLOCG00000001586	si:ch211-262h13.5
ENSXMAG00000020057	thbd	ENSLOCG00000017998	
ENSXMAG00000024785	onecut2	ENSLOCG00000003635	onecut2
ENSXMAG00000025416	atp5po	ENSLOCG00000006652	atp5po
ENSXMAG00000010244	slc20a2	ENSLOCG00000011322	SLC20A2
ENSXMAG00000018701	micu2	ENSLOCG00000001747	micu2
ENSXMAG00000008132	tmem30b	ENSLOCG00000004514	tmem30b
ENSXMAG00000001787	si:ch73-91k6.2	ENSLOCG00000017996	si:ch73-91k6.2
ENSXMAG00000008722	traf7	ENSLOCG00000005718	traf7
ENSXMAG00000028518	sesn1	ENSLOCG00000016607	sesn1
ENSXMAG00000001624	fech	ENSLOCG00000003614	fech
ENSXMAG00000001776	brox	ENSLOCG00000016414	brox
ENSXMAG00000029540	inka2	ENSLOCG00000018063	inka2

ENSXMAG00000001998	si:dkey-37o8.1	ENSLOCG00000011245	si:dkey-37o8.1
ENSXMAG00000002697	si:ch211-11n16.2	ENSLOCG00000014497	si:ch211-11n16.2
ENSXMAG00000022079	mad2l1	ENSLOCG00000010803	mad2l1
ENSXMAG00000005706	slc26a3.1	ENSLOCG00000015805	slc26a3.1
ENSXMAG00000029640	KCNMB1	ENSLOCG00000001089	kcnmb2
ENSXMAG00000016651	NR2C1	ENSLOCG00000015757	nr2c1
ENSXMAG00000006535	helz	ENSLOCG00000013617	helz
ENSXMAG00000019572	FASTKD5	ENSLOCG00000001287	
ENSXMAG00000001633	dnajc25	ENSLOCG00000003035	dnajc25
ENSXMAG00000008128	kdm1a	ENSLOCG00000004475	kdm1a
ENSXMAG00000001498	mybpc1	ENSLOCG00000015188	mybpc1
ENSXMAG00000012529	FAM107A	ENSLOCG00000010486	si:ch211-236d3.4
ENSXMAG00000027710		ENSLOCG00000010291	tdgf1
ENSXMAG00000002710	dpf1	ENSLOCG00000014388	dpf1
ENSXMAG00000003254	cep57l1	ENSLOCG00000016609	cep57l1
ENSXMAG00000022469	bod1	ENSLOCG00000009148	bod1
ENSXMAG00000021110	cbll1	ENSLOCG00000015804	cbll1
ENSXMAG00000013456		ENSLOCG00000012727	prom2
ENSXMAG00000000807	COL6A6	ENSLOCG00000001732	
ENSXMAG00000006980	fam160b1	ENSLOCG00000012984	fam160b1
ENSXMAG00000004376	oacyl	ENSLOCG00000011895	oacyl
ENSXMAG00000025518	timm17a	ENSLOCG00000010082	timm17a
ENSXMAG00000029902	si:ch211-246m6.4	ENSLOCG00000001058	si:ch211-246m6.4
ENSXMAG00000002025	myca	ENSLOCG00000011270	myca
ENSXMAG00000020047	brcc3	ENSLOCG00000013781	brcc3
ENSXMAG00000001768		ENSLOCG00000016488	mep1a.1
ENSXMAG00000000490	htatsf1	ENSLOCG00000015118	htatsf1
ENSXMAG00000006007	lmod1b	ENSLOCG00000010089	lmod1b
ENSXMAG00000029318	si:ch211-153b23.3	ENSLOCG00000015063	si:ch211-153b23.3
ENSXMAG00000027391	si:dkey-243k1.3	ENSLOCG00000017139	
ENSXMAG00000006865	me1	ENSLOCG00000017074	me1
ENSXMAG00000008232	si:ch73-132f6.5	ENSLOCG00000008333	si:ch73-132f6.5
ENSXMAG00000001763	mep1a.2	ENSLOCG00000016489	mep1a.2
ENSXMAG00000002027	ankha	ENSLOCG00000011283	ankhb
ENSXMAG00000013200	arsj	ENSLOCG00000013135	arsj
ENSXMAG00000010221	lipg	ENSLOCG00000011301	
ENSXMAG00000003243	ppil6	ENSLOCG00000017158	ppil6
ENSXMAG00000007872	mtmr14	ENSLOCG00000013669	mtmr14
ENSXMAG00000013454	HPS6	ENSLOCG00000012743	HPS6
ENSXMAG00000006012	shisa4	ENSLOCG00000010094	shisa4
ENSXMAG00000016577	ZNF521	ENSLOCG00000006396	ZNF521
ENSXMAG00000012508	mkrn2	ENSLOCG00000013970	mkrn2
ENSXMAG00000027242	znf532	ENSLOCG00000011902	znf532
ENSXMAG00000005909	VPS54	ENSLOCG00000016056	vps54
ENSXMAG00000006841		ENSLOCG00000014110	clpxa
ENSXMAG00000004570	dct	ENSLOCG00000004857	dct
ENSXMAG00000012504	mkrn2os.2	ENSLOCG00000013968	mkrn2os.1
ENSXMAG00000013440	cox15	ENSLOCG00000012765	
ENSXMAG00000002031		ENSLOCG00000011294	otulinb
ENSXMAG00000010220	si:dkey-193c22.1	ENSLOCG00000007041	si:dkey-193c22.1
ENSXMAG00000024566		ENSLOCG00000017237	GEN1
ENSXMAG00000006107	luzp1	ENSLOCG00000004433	luzp1
ENSXMAG00000000489	EBF1	ENSLOCG00000009282	ebf1b
ENSXMAG00000019573	sox21b	ENSLOCG00000017783	sox21b
ENSXMAG00000006013	ipo9	ENSLOCG00000010108	ipo9
ENSXMAG00000010446	aox1	ENSLOCG00000010455	aox6

ENSXMAG00000023156	kbtbd11	ENSLOCG00000017221	kbtbd11
ENSXMAG00000004387	rx3	ENSLOCG00000011917	rx3
ENSXMAG00000009414	zgc:174917	ENSLOCG00000014875	zgc:174917
ENSXMAG00000012479		ENSLOCG00000013958	tsen2
ENSXMAG00000021215	otulina	ENSLOCG00000011304	otulina
ENSXMAG00000013437	CUTC	ENSLOCG00000012769	cutc
ENSXMAG00000011607	mrps11	ENSLOCG00000014531	mrps11
ENSXMAG00000002791	pnkp	ENSLOCG00000006597	pnkp
ENSXMAG00000005343	scube1	ENSLOCG00000015066	scube1
ENSXMAG00000029481	cplx4a	ENSLOCG00000011926	cplx4a
ENSXMAG00000016590	ss18	ENSLOCG00000006375	ss18
ENSXMAG00000024135	pou6f2	ENSLOCG00000011317	pou6f2
ENSXMAG00000011614		ENSLOCG00000001551	PDPR
ENSXMAG00000017936	nccrp1	ENSLOCG00000014359	
ENSXMAG00000000262	arhgef10	ENSLOCG00000017222	arhgef10
ENSXMAG00000011208	WDR63	ENSLOCG00000005471	WDR63
ENSXMAG00000022893	rnaset2	ENSLOCG00000016824	rnaset2
ENSXMAG00000022283	RF00152	ENSLOCG00000020115	RF00152
ENSXMAG00000006900		ENSLOCG00000006249	spi1b
ENSXMAG00000028878	si:ch211-51e12.7	ENSLOCG00000017238	si:ch211-51e12.7
ENSXMAG00000029662	prdm13	ENSLOCG00000017084	prdm13
ENSXMAG00000004767	NAALADL2	ENSLOCG00000001022	NAALADL2
ENSXMAG00000004291	fgf23	ENSLOCG00000016671	fgf23
ENSXMAG00000004398	lman1	ENSLOCG00000011935	lman1
ENSXMAG00000006108	si:dkey-13p1.4	ENSLOCG00000014704	si:dkey-13p1.4
ENSXMAG00000008234	lratb.1	ENSLOCG00000008349	lratb.1
ENSXMAG00000003472	hnf1a	ENSLOCG00000002874	hnf1a
ENSXMAG00000003237	mrps10	ENSLOCG00000017239	mrps10
ENSXMAG00000016198	slc30a9	ENSLOCG00000012479	SLC30A9
ENSXMAG00000010199	arl15a	ENSLOCG00000006988	arl15b
ENSXMAG00000028263	SYDE2	ENSLOCG00000005452	SYDE2
ENSXMAG00000007862	setd5	ENSLOCG00000013641	setd5
ENSXMAG00000026055	RF00281	ENSLOCG00000020096	RF00281
ENSXMAG00000016767	ELK3	ENSLOCG00000015730	elk3
ENSXMAG00000002050	vps41	ENSLOCG00000011337	vps41
ENSXMAG00000008237	rbm46	ENSLOCG00000008298	rbm46
ENSXMAG00000024571	tor3a	ENSLOCG00000009789	tor3a
ENSXMAG00000024170	zgc:65873	ENSLOCG00000005440	zgc:65873
ENSXMAG00000000480	rnf145b	ENSLOCG00000009293	rnf145b
ENSXMAG00000006926	PTK7	ENSLOCG00000015880	ptk7a
ENSXMAG00000024016		ENSLOCG00000012799	
ENSXMAG00000016037	PAXBP1	ENSLOCG00000008965	
ENSXMAG00000014312	rps6ka2	ENSLOCG00000016813	rps6ka2
ENSXMAG00000002926	pum3	ENSLOCG00000012529	pum3
ENSXMAG00000012458		ENSLOCG00000018364	si:ch211-15p9.2
ENSXMAG00000010194	ndufs4	ENSLOCG00000006961	ndufs4
ENSXMAG00000006645	nol11	ENSLOCG00000013594	nol11
ENSXMAG00000027398		ENSLOCG00000005315	
ENSXMAG00000026845	ccbe1	ENSLOCG00000011947	ccbe1
ENSXMAG00000004620	cldn10l2	ENSLOCG00000005024	cldn10l2
ENSXMAG00000006916	ano3	ENSLOCG00000006513	ano3
ENSXMAG00000016624	psma8	ENSLOCG00000006356	psma8
ENSXMAG00000011641	kif13ba	ENSLOCG00000015976	kif13ba
ENSXMAG00000017913	mark4a	ENSLOCG00000014735	mark4a
ENSXMAG00000003259	calr	ENSLOCG00000009468	calr
ENSXMAG00000003212	tmem181	ENSLOCG00000016260	tmem181

ENSXMAG00000025933	ublcp1	ENSLOCG00000009305	ublcp1
ENSXMAG00000004731	nlgn1	ENSLOCG00000000991	nlgn1
ENSXMAG00000002831		ENSLOCG00000006477	
ENSXMAG00000011254	znhit6	ENSLOCG00000005376	znhit6
ENSXMAG00000027556	cln8	ENSLOCG00000017223	cln8
ENSXMAG00000012452	isy1	ENSLOCG00000013988	isy1
ENSXMAG00000009419	frmpd3	ENSLOCG00000014883	frmpd3
ENSXMAG00000027835	si:dkey-165n16.1	ENSLOCG00000017087	si:dkey-165n16.1
ENSXMAG00000025389	si:dkey-19e4.5	ENSLOCG00000013652	si:dkey-19e4.5
ENSXMAG00000010188	fsta	ENSLOCG00000006942	fsta
ENSXMAG00000003508		ENSLOCG00000002924	pop5
ENSXMAG00000026207	rassf3	ENSLOCG00000017077	rassf3
ENSXMAG00000016653	KCTD1	ENSLOCG00000006322	KCTD1
ENSXMAG00000010438	s100b	ENSLOCG00000010431	s100b
ENSXMAG00000026750		ENSLOCG00000006292	tubb1
ENSXMAG00000004418	hcn1	ENSLOCG00000011966	hcn1
ENSXMAG00000003205	dynlt1	ENSLOCG00000016258	dynlt1
ENSXMAG00000004627	dnajc3a	ENSLOCG00000005118	dnajc3a
ENSXMAG00000016221	gsr	ENSLOCG00000012126	gsr
ENSXMAG00000004156	usp45	ENSLOCG00000017089	usp45
ENSXMAG00000026212	camk1db	ENSLOCG00000015716	CAMK1D
ENSXMAG00000000806		ENSLOCG00000001429	cmtm6
ENSXMAG00000020052		ENSLOCG00000001896	ESCO1
ENSXMAG00000003202	gtf2h5	ENSLOCG00000016267	gtf2h5
ENSXMAG00000017623		ENSLOCG00000012359	
ENSXMAG00000025135	rnf10	ENSLOCG00000002934	rnf10
ENSXMAG00000029385		ENSLOCG00000007952	nudt8
ENSXMAG00000011260	DDAH1	ENSLOCG00000005404	DDAH1
ENSXMAG00000007844	csnk2a4	ENSLOCG00000002198	csnk2a4
ENSXMAG00000006889		ENSLOCG00000015877	
ENSXMAG00000019814	tmem246	ENSLOCG00000017357	tmem246
ENSXMAG00000021519	prpsap2	ENSLOCG00000005788	prpsap2
ENSXMAG00000004330		ENSLOCG00000017111	
ENSXMAG00000026332	ubtd2	ENSLOCG00000009340	ubtd2
ENSXMAG00000010158	itga2.2	ENSLOCG00000006872	itga2.2
ENSXMAG00000016271		ENSLOCG00000009912	
ENSXMAG00000008250	fgb	ENSLOCG00000008395	FGB
ENSXMAG00000006069	aurka	ENSLOCG00000006337	aurka
ENSXMAG00000004128		ENSLOCG00000017090	
ENSXMAG00000016835	dhtkd1	ENSLOCG00000015702	dhtkd1
ENSXMAG00000003190	serac1	ENSLOCG00000016268	serac1
ENSXMAG00000004647	uggt2	ENSLOCG00000005154	uggt2
ENSXMAG00000026035	HS3ST1	ENSLOCG00000009907	HS3ST1
ENSXMAG00000014310	immp1l	ENSLOCG00000004632	immp1l
ENSXMAG00000002084	sox17	ENSLOCG00000005646	sox17
ENSXMAG00000011264	mpl	ENSLOCG00000006315	mpl
ENSXMAG00000017905		ENSLOCG00000014731	exoc3l2b
ENSXMAG00000028172	khnyn	ENSLOCG00000009322	
ENSXMAG00000001597		ENSLOCG00000013542	CEP95
ENSXMAG00000012427	pcif1	ENSLOCG00000006757	pcif1
ENSXMAG00000021829		ENSLOCG00000002967	
ENSXMAG00000006879	mrpl2	ENSLOCG00000015874	MRPL2
ENSXMAG00000013385	tpcn3	ENSLOCG00000015605	tpcn3
ENSXMAG00000028187	ect2	ENSLOCG00000000941	ect2
ENSXMAG00000004124	coq3	ENSLOCG00000017096	coq3
ENSXMAG00000011680	tdp1	ENSLOCG00000008997	tdp1

ENSXMAG00000004377	creb3l2	ENSLOCG00000017109	
ENSXMAG00000008120	itgb1bp1	ENSLOCG00000016822	itgb1bp1
ENSXMAG00000002848	cbln18	ENSLOCG00000006332	cbln18
ENSXMAG00000016658	AQP4	ENSLOCG00000006310	aqp4
ENSXMAG00000003295	ttc39a	ENSLOCG00000009436	ttc39a
ENSXMAG00000011265	tie1	ENSLOCG00000006350	tie1
ENSXMAG00000004465	esm1	ENSLOCG00000012017	esm1
ENSXMAG00000023245		ENSLOCG00000001410	cmtm7
ENSXMAG00000008676	carhsp1	ENSLOCG00000006547	carhsp1
ENSXMAG00000010574	LTN1	ENSLOCG00000008998	LTN1
ENSXMAG00000029448	tagln	ENSLOCG00000006298	tagln
ENSXMAG00000002087	mrpl15	ENSLOCG00000005663	mrpl15
ENSXMAG00000014304	elp4	ENSLOCG00000004617	elp4
ENSXMAG00000006086		ENSLOCG00000006390	
ENSXMAG00000002930	crybb2	ENSLOCG00000003022	crybb2
ENSXMAG00000005276	aldh1l2	ENSLOCG00000015053	aldh1l2
ENSXMAG00000024949	ssbp1	ENSLOCG00000017107	ssbp1
ENSXMAG00000017632	vps25	ENSLOCG00000012366	vps25
ENSXMAG00000004469	arhgap24	ENSLOCG00000012018	arhgap24
ENSXMAG00000000276		ENSLOCG00000017225	
ENSXMAG00000009431	zgc:66447	ENSLOCG00000014887	zgc:66447
ENSXMAG00000023793	nkx3.2	ENSLOCG00000009892	nkx3.2
ENSXMAG00000018856	usp48	ENSLOCG00000001685	usp48
ENSXMAG00000006842		ENSLOCG00000015867	si:ch211-126j24.1
ENSXMAG00000010142	paip1	ENSLOCG00000012270	paip1
ENSXMAG00000008066	asap2a	ENSLOCG00000016818	asap2a
ENSXMAG00000024107		ENSLOCG00000013187	
ENSXMAG00000002928	ctu1	ENSLOCG00000001719	ctu1
ENSXMAG00000016291		ENSLOCG00000009884	
ENSXMAG00000028330	nat15	ENSLOCG00000006527	nat15
ENSXMAG00000004098	fbxl4	ENSLOCG00000017099	fbxl4
ENSXMAG00000029002		ENSLOCG00000008446	RNF175
ENSXMAG00000002856	pcsk7	ENSLOCG00000006279	pcsk7
ENSXMAG00000028963	tmem135	ENSLOCG00000007117	tmem135
ENSXMAG00000002093	lypla1	ENSLOCG00000005686	lypla1
ENSXMAG00000022564	znf711	ENSLOCG00000014891	znf711
ENSXMAG00000023537	pou3f2b	ENSLOCG00000017875	pou3f2b
ENSXMAG00000010104	adamts12	ENSLOCG00000012331	ADAMTS12
ENSXMAG00000014260	pax6a	ENSLOCG00000004578	pax6a
ENSXMAG00000004480	mapk10	ENSLOCG00000012028	mapk10
ENSXMAG00000000279	CD109	ENSLOCG00000016893	CD109
ENSXMAG00000011310	ap1m3	ENSLOCG00000006392	ap1m3
ENSXMAG00000008652	dnase1	ENSLOCG00000006492	dnase1
ENSXMAG00000004070	mms22l	ENSLOCG00000017105	mms22l
ENSXMAG00000010603	hephl1b	ENSLOCG00000007464	
ENSXMAG00000026859	kctd12b	ENSLOCG00000014860	kctd12b
ENSXMAG00000019823	tmem264	ENSLOCG00000018111	tmem264
ENSXMAG00000023118		ENSLOCG00000015601	ttbk1b
ENSXMAG00000026308	C17orf58	ENSLOCG00000013588	C17orf58
ENSXMAG00000020873	si:dkey-48p11.3	ENSLOCG00000015052	si:dkey-48p11.3
ENSXMAG00000016292	aurkb	ENSLOCG00000013333	
ENSXMAG00000004474		ENSLOCG00000017075	zgc:194209
ENSXMAG00000021260	eci1	ENSLOCG00000006473	eci1
ENSXMAG00000006839		ENSLOCG00000015860	
ENSXMAG00000017641	mfsd13a	ENSLOCG00000012657	mfsd13a
ENSXMAG00000016881	taf3	ENSLOCG00000015683	

ENSXMAG00000028966	BACE1	ENSLOCG00000006246	bace1
ENSXMAG00000024667	fam183a	ENSLOCG00000006457	fam183a
ENSXMAG00000017878	kptn	ENSLOCG00000014286	kptn
ENSXMAG00000005250	slc41a2b	ENSLOCG00000015043	slc41a2b
ENSXMAG00000001472		ENSLOCG00000001322	
ENSXMAG00000008636		ENSLOCG00000000032	
ENSXMAG00000004046	klhl32	ENSLOCG00000017108	klhl32
ENSXMAG00000008929	TNFRSF21	ENSLOCG00000016498	tnfrsf21
ENSXMAG00000023274	klhdc3	ENSLOCG00000015854	klhdc3
ENSXMAG00000012927	zgc:153896	ENSLOCG00000006693	zgc:153896
ENSXMAG00000002108	FASTKD3	ENSLOCG00000011153	fastkd3
ENSXMAG00000011341	ebna1bp2	ENSLOCG00000006438	ebna1bp2
ENSXMAG00000021939	COPS6	ENSLOCG00000013063	COPS6
ENSXMAG00000016312	vldlr	ENSLOCG00000012505	vldlr
ENSXMAG00000002874	apoa1b	ENSLOCG00000006231	apoa1a
ENSXMAG00000028048	myl7	ENSLOCG00000015174	myl7
ENSXMAG00000027119	si:dkey-17e16.10	ENSLOCG00000000535	
ENSXMAG00000028823	cdkn2c	ENSLOCG00000009408	cdkn2c
ENSXMAG00000029795	cd2ap	ENSLOCG00000016501	cd2ap
ENSXMAG00000020900		ENSLOCG00000006210	
ENSXMAG00000026792	fam208b	ENSLOCG00000015650	
ENSXMAG00000004524		ENSLOCG00000016568	pex26
ENSXMAG00000014239	rcn1	ENSLOCG00000004562	rcn1
ENSXMAG00000019069	tmem150aa	ENSLOCG00000015355	tmem150aa
ENSXMAG00000006119	fam210b	ENSLOCG00000006416	fam210b
ENSXMAG00000010091	gck	ENSLOCG00000015175	gck
ENSXMAG00000023984	chst11	ENSLOCG00000015041	chst11
ENSXMAG00000028060		ENSLOCG00000013301	si:ch211-131k2.2
ENSXMAG00000022040	dmrt2b	ENSLOCG00000006480	dmrt2b
ENSXMAG00000000424	f9b	ENSLOCG00000015159	f9b
ENSXMAG00000005984		ENSLOCG00000015586	pex6
ENSXMAG00000017648	mrpl43	ENSLOCG00000012629	mrpl43
ENSXMAG00000016334	MCM7	ENSLOCG00000013075	MCM7
ENSXMAG00000017649	twnk	ENSLOCG00000012623	twnk
ENSXMAG00000004536	llph	ENSLOCG00000015432	llph
ENSXMAG00000005234	nfybb	ENSLOCG00000015038	nfyba
ENSXMAG00000010641	pitpnab	ENSLOCG00000007016	pitpnaa
ENSXMAG00000012938		ENSLOCG00000007971	TMEM116
ENSXMAG00000004034	fzd6	ENSLOCG00000009524	fzd6
ENSXMAG00000012422	polr2j	ENSLOCG00000001885	polr2j
ENSXMAG00000009468		ENSLOCG00000014845	
ENSXMAG00000014190	eif3m	ENSLOCG00000004520	eif3m
ENSXMAG00000017655	lzts2b	ENSLOCG00000012615	lzts2b
ENSXMAG00000004571	golgb1	ENSLOCG00000015469	golgb1
ENSXMAG00000022678	ntpcr	ENSLOCG00000015850	ntpcr
ENSXMAG00000023597		ENSLOCG00000001757	ldlrad2
ENSXMAG00000026090	podxl	ENSLOCG00000015641	
ENSXMAG00000012406	ube2c	ENSLOCG00000006709	ube2c
ENSXMAG00000010079	ykt6	ENSLOCG00000015179	ykt6
ENSXMAG00000025123		ENSLOCG00000013098	
ENSXMAG00000001458		ENSLOCG00000009194	col14a1b
ENSXMAG00000002882		ENSLOCG00000001852	sidt2
ENSXMAG00000012945		ENSLOCG00000007253	
ENSXMAG00000014189	prrg4	ENSLOCG00000004488	prrg4
ENSXMAG00000004518	ptpn13	ENSLOCG00000012036	ptpn13
ENSXMAG00000017861	proser1	ENSLOCG00000008707	proser1

ENSXMAG00000027269	CTHRC1	ENSLOCG00000009514	CTHRC1
ENSXMAG00000002526	st3gal7	ENSLOCG00000009100	st3gal7
ENSXMAG00000028378	guca1a	ENSLOCG00000015471	guca1a
ENSXMAG00000019822		ENSLOCG00000015849	
ENSXMAG00000010077		ENSLOCG00000012617	osmr
ENSXMAG00000016349	sh3bp2	ENSLOCG00000012502	sh3bp2
ENSXMAG00000005201	mkln1	ENSLOCG00000015642	mkln1
ENSXMAG00000016419	gpc2	ENSLOCG00000013124	gpc2
ENSXMAG00000026675		ENSLOCG00000012221	idua
ENSXMAG00000011753		ENSLOCG00000017046	GLO1
ENSXMAG00000012402	zgc:109913	ENSLOCG00000006691	zgc:109913
ENSXMAG00000009473	ogt.1	ENSLOCG00000014832	ogt.1
ENSXMAG00000007783	namptb	ENSLOCG00000010385	namptb
ENSXMAG00000014179	qser1	ENSLOCG00000004471	qser1
ENSXMAG00000004586	orc5	ENSLOCG00000015480	orc5
ENSXMAG00000019222	tfb1m	ENSLOCG00000016850	tfb1m
ENSXMAG00000022432	sigirr	ENSLOCG00000006662	SIGIRR
ENSXMAG00000002534	hps1	ENSLOCG00000009087	hps1
ENSXMAG00000021148		ENSLOCG00000018366	
ENSXMAG00000010660	nle1	ENSLOCG00000000833	nle1
ENSXMAG00000025016	vps36	ENSLOCG00000008718	vps36
ENSXMAG00000004016	slc25a32b	ENSLOCG00000009507	slc25a32b
ENSXMAG00000021577	rpp25l	ENSLOCG00000018327	rpp25l
ENSXMAG00000004854	zgc:172182	ENSLOCG00000008105	zgc:172182
ENSXMAG00000003084	esr1	ENSLOCG00000016285	esr1
ENSXMAG00000024616	mcf2l2	ENSLOCG00000000896	mcf2l2
ENSXMAG00000016380	spp1	ENSLOCG00000012495	
ENSXMAG00000028767	snrpb	ENSLOCG00000002687	snrpb
ENSXMAG00000010414	mfsd6a	ENSLOCG00000009915	mfsd6a
ENSXMAG00000009545	cstf3	ENSLOCG00000004415	cstf3
ENSXMAG00000003310	faf1	ENSLOCG00000009388	faf1
ENSXMAG00000025409	slc25a1a	ENSLOCG00000006737	slc25a1a
ENSXMAG00000002558	hpse2	ENSLOCG00000009078	hpse2
ENSXMAG00000026809	dcaf13	ENSLOCG00000009498	dcaf13
ENSXMAG00000027849	slc22a23	ENSLOCG00000013043	slc22a23
ENSXMAG00000017831	thsd1	ENSLOCG00000008731	thsd1
ENSXMAG00000012347	ctsa	ENSLOCG00000006788	ctsa
ENSXMAG00000006750	sipa1l2	ENSLOCG00000015837	sipa1l2
ENSXMAG00000025504	zbtb2b	ENSLOCG00000016295	zbtb2a
ENSXMAG00000009522	gcna	ENSLOCG00000014829	gcna
ENSXMAG00000010400	nab1a	ENSLOCG00000009947	nab1a
ENSXMAG00000007088	kcnc1b	ENSLOCG00000006708	kcnc1b
ENSXMAG00000010703		ENSLOCG00000006772	FBXO39
ENSXMAG00000024248	akap12b	ENSLOCG00000016296	akap12b
ENSXMAG00000022505	usp1	ENSLOCG00000006551	usp1
ENSXMAG00000017825	si:ch211-287a12.9	ENSLOCG00000008761	si:ch211-287a12.9
ENSXMAG00000029056	bbs5	ENSLOCG00000008052	bbs5
ENSXMAG00000004619	kmt2e	ENSLOCG00000015484	kmt2e
ENSXMAG00000016942	slc35b4	ENSLOCG00000015627	slc35b4
ENSXMAG00000007093	sergef	ENSLOCG00000006722	sergef
ENSXMAG00000028041	camk2d1	ENSLOCG00000013115	camk2d1
ENSXMAG00000003327	dmrta2	ENSLOCG00000009379	dmrta2
ENSXMAG00000009517	hipk3b	ENSLOCG00000004380	hipk3a
ENSXMAG00000009534	nsdhl	ENSLOCG00000014825	nsdhl
ENSXMAG00000027669	mthfd1l	ENSLOCG00000016297	mthfd1l
ENSXMAG00000005180	asb13a.1	ENSLOCG00000015647	asb13a.1

ENSXMAG00000016406	c6	ENSLOCG00000007935	c6
ENSXMAG00000014336	fastkd1	ENSLOCG00000008010	fastkd1
ENSXMAG00000003924	RIMS2	ENSLOCG00000009471	RIMS2
ENSXMAG00000017783	tpte	ENSLOCG00000008770	tpte
ENSXMAG00000006001	UBE2O	ENSLOCG00000013468	UBE2O
ENSXMAG00000027102		ENSLOCG00000014821	
ENSXMAG00000014206	utp15	ENSLOCG00000011049	utp15
ENSXMAG00000005150	gdi2	ENSLOCG00000015651	gdi2
ENSXMAG00000007130	saal1	ENSLOCG00000006776	saal1
ENSXMAG00000025683	elavl4	ENSLOCG00000009363	elavl4
ENSXMAG00000027829	nkx2.3	ENSLOCG00000009050	
ENSXMAG00000023050	nkx3.3	ENSLOCG00000009031	nkx3.3
ENSXMAG00000002893	sik3	ENSLOCG00000001801	sik3
ENSXMAG00000025463	opn5	ENSLOCG00000016508	opn5
ENSXMAG00000001553	ITGB1BP2	ENSLOCG00000014938	
ENSXMAG00000008926	ptchd4	ENSLOCG00000016510	ptchd4
ENSXMAG00000021900	fezf2	ENSLOCG00000010412	fezf2
ENSXMAG00000005145	ankrd16	ENSLOCG00000015654	ankrd16
ENSXMAG00000021053	PPIG	ENSLOCG00000007999	ppig
ENSXMAG00000016904		ENSLOCG00000000164	
ENSXMAG00000004646	ndufb2	ENSLOCG00000016677	ndufb2
ENSXMAG00000005132	glt8d2	ENSLOCG00000015027	GLT8D2
ENSXMAG00000029562	pdrg1	ENSLOCG00000003371	pdrg1
ENSXMAG00000023651	cnih3	ENSLOCG00000015918	cnih3
ENSXMAG00000010732	tmigd1	ENSLOCG00000006218	tmigd1
ENSXMAG00000014244		ENSLOCG00000001125	
ENSXMAG00000007571	adam15	ENSLOCG00000007839	
ENSXMAG00000020311	hic2	ENSLOCG00000002169	hic2
ENSXMAG00000010735		ENSLOCG00000006235	
ENSXMAG00000001570	si:dkey-172j4.3	ENSLOCG00000014305	si:dkey-172j4.3
ENSXMAG00000009550	fut11	ENSLOCG00000000619	fut11
ENSXMAG00000017775	smpd1	ENSLOCG00000009565	smpd1
ENSXMAG00000021793		ENSLOCG00000015917	si:dkey-1j5.4
ENSXMAG00000019192	SCAF8	ENSLOCG00000016856	SCAF8
ENSXMAG00000005034	piwil2	ENSLOCG00000015407	piwil2
ENSXMAG00000011389	atg4c	ENSLOCG00000006628	atg4c
ENSXMAG00000016984	cnot4a	ENSLOCG00000016126	cnot4a
ENSXMAG00000011890	abcc12	ENSLOCG00000005287	abcc12
ENSXMAG00000029015		ENSLOCG00000018360	
ENSXMAG00000005098	sbf1	ENSLOCG00000016809	sbf1
ENSXMAG00000002917		ENSLOCG00000006367	
ENSXMAG00000006973	spon1a	ENSLOCG00000004343	spon1a
ENSXMAG00000010737	cct8	ENSLOCG00000009042	cct8
ENSXMAG00000029818	foxd3	ENSLOCG00000017740	foxd3
ENSXMAG00000013986	cstf1	ENSLOCG00000003418	cstf1
ENSXMAG00000002924	CD3G	ENSLOCG00000006406	
ENSXMAG00000006018	RHBDF2	ENSLOCG00000013458	RHBDF2
ENSXMAG00000028111	rnf144aa	ENSLOCG00000016812	rnf144aa
ENSXMAG00000019164		ENSLOCG00000016857	
ENSXMAG00000004652		ENSLOCG00000015116	ergic2
ENSXMAG00000002171	alg6	ENSLOCG00000006645	alg6
ENSXMAG00000020947	dcst1	ENSLOCG00000007858	
ENSXMAG00000023740	INHBE	ENSLOCG00000005963	INHBC
ENSXMAG00000019690	gpr185b	ENSLOCG00000017472	gpr185b
ENSXMAG00000016998	zgc:136858	ENSLOCG00000016131	zgc:136858
ENSXMAG00000026586	ccdc173	ENSLOCG00000007987	ccdc173

ENSXMAG00000008017	rsad2	ENSLOCG00000016810	rsad2
ENSXMAG00000003343	bend5	ENSLOCG00000009332	bend5
ENSXMAG00000014005	cass4	ENSLOCG00000003431	cass4
ENSXMAG00000004662	si:dkey-97m3.1	ENSLOCG00000015119	si:dkey-97m3.1
ENSXMAG00000027249	zgc:109889	ENSLOCG00000013694	si:ch73-362m14.4
ENSXMAG00000017959	htr7c	ENSLOCG00000015406	htr7b
ENSXMAG00000002598	zgc:123010	ENSLOCG00000008967	zgc:123010
ENSXMAG00000017703	plekhm1	ENSLOCG00000013146	plekhm1
ENSXMAG00000003920	DCSTAMP	ENSLOCG00000009465	DCSTAMP
ENSXMAG00000014366	phgdh	ENSLOCG00000007973	phgdh
ENSXMAG00000017758	mtnr1bb	ENSLOCG00000007314	mtnr1bb
ENSXMAG00000017961	atad1a	ENSLOCG00000015404	atad1a
ENSXMAG00000003345		ENSLOCG00000009309	spata6
ENSXMAG00000007582	dcst2	ENSLOCG00000005838	DCST2
ENSXMAG00000005086	mkrn1	ENSLOCG00000016132	mkrn1
ENSXMAG00000004554	sdad1	ENSLOCG00000004170	sdad1
ENSXMAG00000019148	mocs3	ENSLOCG00000016860	MOCS3
ENSXMAG00000008016	cmpk2	ENSLOCG00000016807	cmpk2
ENSXMAG00000003904	dpys	ENSLOCG00000009447	dpys
ENSXMAG00000010758	aldocb	ENSLOCG00000006034	aldocb
ENSXMAG00000002213	mettl4	ENSLOCG00000011054	mettl4
ENSXMAG00000015068	htr2cl1	ENSLOCG00000013705	htr2cl2
ENSXMAG00000006747	disc1	ENSLOCG00000015836	disc1
ENSXMAG00000002185	ror1	ENSLOCG00000006711	ror1
ENSXMAG00000002941	gap43	ENSLOCG00000002709	GAP43
ENSXMAG00000014376	serpind1	ENSLOCG00000002284	serpind1
ENSXMAG00000010775	pigs	ENSLOCG00000006015	pigs
ENSXMAG00000000087	galnt9	ENSLOCG00000003048	galnt9
ENSXMAG00000002216	adcyap1a	ENSLOCG00000011028	adcyap1a
ENSXMAG00000025809		ENSLOCG00000009635	dnttip2
ENSXMAG00000012284	ddx27	ENSLOCG00000002659	ddx27
ENSXMAG00000004883	klhl23	ENSLOCG00000007956	klhl23
ENSXMAG00000002944	LSAMP	ENSLOCG00000002678	LSAMP
ENSXMAG00000006738	tsnax	ENSLOCG00000015831	tsnax
ENSXMAG00000017955		ENSLOCG00000005919	
ENSXMAG00000006043	acox1	ENSLOCG00000013494	acox1
ENSXMAG00000004888	ssb	ENSLOCG00000007933	ssb
ENSXMAG00000003349	gclm	ENSLOCG00000009650	gclm
ENSXMAG00000011941	zgc:165481	ENSLOCG00000017585	zgc:165481
ENSXMAG00000002221	yes1	ENSLOCG00000011016	yes1
ENSXMAG00000020020	tmem121b	ENSLOCG00000017912	tmem121b
ENSXMAG00000008006	colec11	ENSLOCG00000017210	colec11
ENSXMAG00000007527	barx1	ENSLOCG00000014062	barx1
ENSXMAG00000004597	klhl8	ENSLOCG00000004119	klhl8
ENSXMAG00000027361		ENSLOCG00000012815	
ENSXMAG00000002196	cachd1	ENSLOCG00000006750	cachd1
ENSXMAG00000011946	rhpn2	ENSLOCG00000001772	rhpn2
ENSXMAG00000014386	snap29	ENSLOCG00000002299	snap29
ENSXMAG00000022809	mettl5	ENSLOCG00000007918	mettl5
ENSXMAG00000006729	egln1a	ENSLOCG00000015828	egln1b
ENSXMAG00000007006	rfc4	ENSLOCG00000000876	rfc4
ENSXMAG00000012224	pltp	ENSLOCG00000006771	pltp
ENSXMAG00000003403	zfpm2a	ENSLOCG00000009425	zfpm2a
ENSXMAG00000011998	igsf11	ENSLOCG00000002662	igsf11
ENSXMAG00000017722	tectb	ENSLOCG00000012269	tectb
ENSXMAG00000027691	zgc:171704	ENSLOCG00000007010	zgc:171704

ENSXMAG00000003356	pole4	ENSLOCG00000003351	pole4
ENSXMAG00000006706	gpr137ba	ENSLOCG00000015819	gpr137bb
ENSXMAG00000017939	ARHGAP9	ENSLOCG00000005905	ARHGAP9
ENSXMAG00000026447	si:dkey-178e17.3	ENSLOCG00000002314	si:dkey-178e17.3
ENSXMAG00000017724	gucy2g	ENSLOCG00000012255	gucy2g
ENSXMAG00000012187	khk	ENSLOCG00000015943	khk
ENSXMAG00000001052		ENSLOCG00000005908	fbxo3
ENSXMAG00000023031	bpgm	ENSLOCG00000000860	bpgm
ENSXMAG00000016960	phlpp1	ENSLOCG00000010026	phlpp1
ENSXMAG00000005072	arhgef39	ENSLOCG00000016136	arhgef39
ENSXMAG00000022219	si:dkey-122a22.2	ENSLOCG00000009416	si:dkey-122a22.2
ENSXMAG00000010799	heatr6	ENSLOCG00000005957	heatr6
ENSXMAG00000002232	enosf1	ENSLOCG00000011003	enosf1
ENSXMAG00000012029	git1	ENSLOCG00000006544	git1
ENSXMAG00000022481	fbrsl1	ENSLOCG00000002713	fbrsl1
ENSXMAG00000002638	sfrp5	ENSLOCG00000008904	sfrp5
ENSXMAG00000016270	foxa2	ENSLOCG00000016870	foxa2
ENSXMAG00000012000	lrp3	ENSLOCG00000001859	
ENSXMAG00000008901	rpl7l1	ENSLOCG00000015894	rpl7l1
ENSXMAG00000005099	syt11b	ENSLOCG00000006943	syt11b
ENSXMAG00000002641	zgc:171482	ENSLOCG00000008863	zgc:171482
ENSXMAG00000005065	lmo3	ENSLOCG00000016141	lmo3
ENSXMAG00000003358	slc1a6	ENSLOCG00000003270	slc1a6
ENSXMAG00000019114	pax1a	ENSLOCG00000016871	pax1a
ENSXMAG00000002206	raver2	ENSLOCG00000006773	raver2
ENSXMAG00000003139		ENSLOCG00000000608	
ENSXMAG00000006650	nid1a	ENSLOCG00000015814	nid1b
ENSXMAG00000017750	acsl5	ENSLOCG00000012247	acsl5
ENSXMAG00000020097		ENSLOCG00000003729	
ENSXMAG00000014407		ENSLOCG00000002354	
ENSXMAG00000005055	dera	ENSLOCG00000016147	dera
ENSXMAG00000020099	marveld1	ENSLOCG00000003718	marveld1
ENSXMAG00000019112	nkx2.2a	ENSLOCG00000016872	nkx2.2a
ENSXMAG00000002236	tyms	ENSLOCG00000010997	tyms
ENSXMAG00000028753	cmc2	ENSLOCG00000000647	cmc2
ENSXMAG00000028183	tax1bp3	ENSLOCG00000003428	
ENSXMAG00000006101	srp68	ENSLOCG00000013514	srp68
ENSXMAG00000009563	nlgn3a	ENSLOCG00000014807	nlgn3a
ENSXMAG00000003132	cenpn	ENSLOCG00000000681	cenpn
ENSXMAG00000007491	hdac11	ENSLOCG00000014033	hdac11
ENSXMAG00000007941	impg1b	ENSLOCG00000016912	impg1b
ENSXMAG00000002212	jak1	ENSLOCG00000006806	jak1
ENSXMAG00000019103	kiz	ENSLOCG00000016881	kiz
ENSXMAG00000003369	hsh2d	ENSLOCG00000003473	
ENSXMAG00000025268	mtx1a	ENSLOCG00000008303	mtx1b
ENSXMAG00000001042	sh3yl1	ENSLOCG00000017230	sh3yl1
ENSXMAG00000017920	mars	ENSLOCG00000000371	
ENSXMAG00000003868	OXR1	ENSLOCG00000009399	OXR1
ENSXMAG00000003129	atmin	ENSLOCG00000000709	atmin
ENSXMAG00000023170		ENSLOCG00000016150	msrb3
ENSXMAG00000010805	ddx52	ENSLOCG00000005928	ddx52
ENSXMAG00000027834		ENSLOCG00000017583	
ENSXMAG00000017730	si:ch211-214k5.6	ENSLOCG00000007262	si:ch211-214k5.6
ENSXMAG00000012073	ankrd13b	ENSLOCG00000006564	ankrd13b
ENSXMAG00000002647		ENSLOCG00000012649	
ENSXMAG00000001260	rnf139	ENSLOCG00000009007	rnf139

ENSXMAG00000006121	polg2	ENSLOCG00000013528	polg2
ENSXMAG00000001030	si:dkey-190l8.2	ENSLOCG00000017229	si:dkey-190l8.2
ENSXMAG00000016993	opn7a	ENSLOCG00000012203	opn7a
ENSXMAG00000021282		ENSLOCG00000001213	
ENSXMAG00000012067	itfg1	ENSLOCG00000005347	itfg1
ENSXMAG00000005030	lemd3	ENSLOCG00000016151	lemd3
ENSXMAG00000005029	thbs3a	ENSLOCG00000008271	THBS3
ENSXMAG00000010817		ENSLOCG00000005681	birc2
ENSXMAG00000006576		ENSLOCG00000015802	lyst
ENSXMAG00000001248	tatdn1	ENSLOCG00000009012	tatdn1
ENSXMAG00000008870	ephx1	ENSLOCG00000015901	ephx1
ENSXMAG00000002473	chfr	ENSLOCG00000002699	chfr
ENSXMAG00000003376		ENSLOCG00000003523	
ENSXMAG00000020705	MIR101-1	ENSLOCG00000020087	MIR101-1
ENSXMAG00000005014	RINT1	ENSLOCG00000015491	rint1
ENSXMAG00000010836	rpl23a	ENSLOCG00000005815	rpl23a
ENSXMAG00000002237	ak4	ENSLOCG00000006837	ak4
ENSXMAG00000001025	lipt1	ENSLOCG00000017769	lipt1
ENSXMAG00000000724		ENSLOCG00000001580	
ENSXMAG00000029514	srp9	ENSLOCG00000015903	srp9
ENSXMAG00000001240	ndufb9	ENSLOCG00000009028	ndufb9
ENSXMAG00000022740		ENSLOCG00000001592	
ENSXMAG00000017918	ddit3	ENSLOCG00000005864	ddit3
ENSXMAG00000016997	ripk1l	ENSLOCG00000013018	ripk1l
ENSXMAG00000019065	ralgapa2	ENSLOCG00000016884	ralgapa2
ENSXMAG00000006563	b3galnt2	ENSLOCG00000015892	b3galnt2
ENSXMAG00000002244	hacd1	ENSLOCG00000010955	hacd1
ENSXMAG00000009253	prl2	ENSLOCG00000016432	prl2
ENSXMAG00000002470	golga3	ENSLOCG00000002643	golga3
ENSXMAG00000002240	dnajc6	ENSLOCG00000006853	dnajc6
ENSXMAG00000008843	enah	ENSLOCG00000015905	enah
ENSXMAG00000000726		ENSLOCG00000001605	
ENSXMAG00000006559	ggps1	ENSLOCG00000015898	ggps1
ENSXMAG00000017906	dctn2	ENSLOCG00000005825	dctn2
ENSXMAG00000005002	hspa14	ENSLOCG00000016720	hspa14
ENSXMAG00000003006	epm2a	ENSLOCG00000016158	epm2a
ENSXMAG00000010840	angptl5	ENSLOCG00000005594	angptl5
ENSXMAG00000007881	syncripl	ENSLOCG00000016919	syncripl
ENSXMAG00000012109	slc6a4a	ENSLOCG00000006668	
ENSXMAG00000017015	irf4a	ENSLOCG00000013008	irf4a
ENSXMAG00000015047	ccnb3	ENSLOCG00000014317	ccnb3
ENSXMAG00000002252		ENSLOCG00000010898	
ENSXMAG00000020849		ENSLOCG00000004068	
ENSXMAG00000009620	eda	ENSLOCG00000014715	eda
ENSXMAG00000011685	ephb6	ENSLOCG00000008258	
ENSXMAG00000007460	acad9	ENSLOCG00000014020	acad9
ENSXMAG00000029130	TMEM74B	ENSLOCG00000007423	tmem74b
ENSXMAG00000003986	gtf2h1	ENSLOCG00000006819	
ENSXMAG00000003390	si:ch1073-396h14.1	ENSLOCG00000003603	si:ch1073-396h14.1
ENSXMAG00000012078	phkb	ENSLOCG00000005324	phkb
ENSXMAG00000002465	ankle2	ENSLOCG00000002623	ankle2
ENSXMAG00000009623		ENSLOCG00000014717	
ENSXMAG00000004763	cd36	ENSLOCG00000016019	cd36
ENSXMAG00000000961	bco1l	ENSLOCG00000003923	bco1l
ENSXMAG00000003411	sqlea	ENSLOCG00000009390	sqlea
ENSXMAG00000029557	tmem38a	ENSLOCG00000001550	tmem38a

ENSXMAG00000014441	plat	ENSLOCG00000015608	plat
ENSXMAG00000003966	hps5	ENSLOCG00000006802	hps5
ENSXMAG00000013660	c3b.2	ENSLOCG00000000125	c3b.2
ENSXMAG00000007878	cga	ENSLOCG00000016920	
ENSXMAG00000004913	myo3b	ENSLOCG00000007869	myo3b
ENSXMAG00000012240	blmh	ENSLOCG00000006688	blmh
ENSXMAG00000002246	lepr	ENSLOCG00000006903	lepr
ENSXMAG00000014582	rbl1	ENSLOCG00000007368	rbl1
ENSXMAG00000002274	SLC39A12	ENSLOCG00000010889	SLC39A12
ENSXMAG00000002459	pgam5	ENSLOCG00000002606	pgam5
ENSXMAG00000006538	tomm20a	ENSLOCG00000015899	tomm20a
ENSXMAG00000003401		ENSLOCG00000003571	
ENSXMAG00000015036	tnfsf10l	ENSLOCG00000014326	tnfsf10l
ENSXMAG00000011664	trpv6	ENSLOCG00000008244	trpv6
ENSXMAG00000022388	mpped1	ENSLOCG00000015072	mpped1
ENSXMAG00000022380	rnf144b	ENSLOCG00000009375	rnf144b
ENSXMAG00000000342	dpp3	ENSLOCG00000001626	
ENSXMAG00000002450	ZDHHC8	ENSLOCG00000002575	zdhhc8b
ENSXMAG00000000733	si:ch211-43f4.1	ENSLOCG00000001385	si:ch211-43f4.1
ENSXMAG00000003943	epx	ENSLOCG00000007014	
ENSXMAG00000002256	ttc22	ENSLOCG00000008687	ttc22
ENSXMAG00000019885	snrnp35	ENSLOCG00000018154	snrnp35
ENSXMAG00000004657		ENSLOCG00000004025	AFF1
ENSXMAG00000012282	lbr	ENSLOCG00000015911	lbr
ENSXMAG00000010906	si:dkeyp-69c1.6	ENSLOCG00000005915	si:dkeyp-69c1.6
ENSXMAG00000002443		ENSLOCG00000002540	ranbp1
ENSXMAG00000014460	ikbkb	ENSLOCG00000015613	ikbkb
ENSXMAG00000026202	rab3il1	ENSLOCG00000007007	
ENSXMAG00000004792	sema3c	ENSLOCG00000016023	sema3c
ENSXMAG00000003404	lpl	ENSLOCG00000003542	lpl
ENSXMAG00000005254	dgkg	ENSLOCG00000003539	dgkg
ENSXMAG00000009632		ENSLOCG00000014747	vma21
ENSXMAG00000003119	gins2	ENSLOCG00000000923	gins2
ENSXMAG00000021010	efcc1	ENSLOCG00000014014	efcc1
ENSXMAG00000007863	orc3	ENSLOCG00000016930	ORC3
ENSXMAG00000021509	sult4a1	ENSLOCG00000015081	sult4a1
ENSXMAG00000024353	ccdc130	ENSLOCG00000006327	ccdc130
ENSXMAG00000003903	best1	ENSLOCG00000006995	
ENSXMAG00000021653	mtnr1c	ENSLOCG00000014745	mtnr1c
ENSXMAG00000014926	clcn5b	ENSLOCG00000014329	clcn5b
ENSXMAG00000022970		ENSLOCG00000002221	IQCB1
ENSXMAG00000003110	emc8	ENSLOCG00000000943	emc8
ENSXMAG00000010912		ENSLOCG00000005867	
ENSXMAG00000002434	trmt2a	ENSLOCG00000002517	trmt2a
ENSXMAG00000009641	neurl1b	ENSLOCG00000012037	neurl1b
ENSXMAG00000016362	wrnip1	ENSLOCG00000012970	wrnip1
ENSXMAG00000027772	sp5a	ENSLOCG00000007856	sp5a
ENSXMAG00000003458	safb	ENSLOCG00000003613	safb
ENSXMAG00000003100	cox4i1	ENSLOCG00000000961	cox4i1
ENSXMAG00000003887		ENSLOCG00000006979	
ENSXMAG00000027335		ENSLOCG00000006364	PSPN
ENSXMAG00000021895	cfap57	ENSLOCG00000006408	cfap57
ENSXMAG00000025988		ENSLOCG00000004013	
ENSXMAG00000025179	dusp22a	ENSLOCG00000000986	dusp22a
ENSXMAG00000009944		ENSLOCG00000011923	
ENSXMAG00000012246	KATNA1	ENSLOCG00000017005	katna1

ENSXMAG00000013765	wdr83	ENSLOCG00000006399	wdr83
ENSXMAG00000023554		ENSLOCG00000000845	
ENSXMAG00000009651	dusp1	ENSLOCG00000012031	dusp1
ENSXMAG00000023336		ENSLOCG00000012200	g6pca.2
ENSXMAG00000016336		ENSLOCG00000010283	
ENSXMAG00000007854	AKIRIN2	ENSLOCG00000016934	AKIRIN2
ENSXMAG00000028206	tomm20a	ENSLOCG00000015904	tomm20b
ENSXMAG00000017849	soat2	ENSLOCG00000005783	soat2
ENSXMAG00000011652	zgc:92606	ENSLOCG00000008340	zgc:92606
ENSXMAG00000003082	irf8	ENSLOCG00000001013	irf8
ENSXMAG00000004863	mtpn	ENSLOCG00000016957	mtpn
ENSXMAG00000003477	malt2	ENSLOCG00000003877	malt2
ENSXMAG00000002426	dgcr8	ENSLOCG00000002491	dgcr8
ENSXMAG00000027101	g6pca.1	ENSLOCG00000012196	g6pca.1
ENSXMAG00000030006		ENSLOCG00000000245	
ENSXMAG00000014544	cyc1	ENSLOCG00000007232	cyc1
ENSXMAG00000013770	wdr83os	ENSLOCG00000006415	wdr83os
ENSXMAG00000003079	foxf1	ENSLOCG00000001042	foxf1
ENSXMAG00000027339	GINM1	ENSLOCG00000017004	GINM1
ENSXMAG00000024815	RF00397	ENSLOCG00000018997	RF00397
ENSXMAG00000014531		ENSLOCG00000015619	
ENSXMAG00000009666	ergic1	ENSLOCG00000012027	ergic1
ENSXMAG00000003849	pnpla2	ENSLOCG00000000228	pnpla2
ENSXMAG00000010921	CHEK1	ENSLOCG00000000934	chek1
ENSXMAG00000021654	ttll12	ENSLOCG00000015088	ttll12
ENSXMAG00000029589	ap5b1	ENSLOCG00000001028	ap5b1
ENSXMAG00000026707	PM20D2	ENSLOCG00000016938	PM20D2
ENSXMAG00000021853		ENSLOCG00000008323	
ENSXMAG00000003067	mthfsd	ENSLOCG00000001066	mthfsd
ENSXMAG00000027968	psme3	ENSLOCG00000012181	psme3
ENSXMAG00000003500	mrpl54	ENSLOCG00000003860	mrpl54
ENSXMAG00000004684		ENSLOCG00000003957	NUP54
ENSXMAG00000027433		ENSLOCG00000007193	ASIP
ENSXMAG00000004998	ppil4	ENSLOCG00000017002	ppil4
ENSXMAG00000002385	atp6v1c1a	ENSLOCG00000009938	atp6v1c1b
ENSXMAG00000002289	NSUN6	ENSLOCG00000010862	nsun6
ENSXMAG00000014540	polb	ENSLOCG00000015620	polb
ENSXMAG00000003501	fam32a	ENSLOCG00000003843	fam32a
ENSXMAG00000003066	DBNDD1	ENSLOCG00000001107	si:ch1073-174d20.1
ENSXMAG00000022385	ttc4	ENSLOCG00000008662	ttc4
ENSXMAG00000025038	reep6	ENSLOCG00000000590	reep6
ENSXMAG00000017842	igsf8	ENSLOCG00000005749	igsf8
ENSXMAG00000020382		ENSLOCG00000016581	FH
ENSXMAG00000009676	flt4	ENSLOCG00000012009	flt4
ENSXMAG00000023691	slc7a6	ENSLOCG00000005997	slc7a6
ENSXMAG00000029793	ptn	ENSLOCG00000016549	ptn
ENSXMAG00000017876	nbr1a	ENSLOCG00000012171	nbr1b
ENSXMAG00000028345		ENSLOCG00000014331	SHROOM4
ENSXMAG00000003041		ENSLOCG00000001134	
ENSXMAG00000002269	pars2	ENSLOCG00000008678	pars2
ENSXMAG00000021884	ahcy	ENSLOCG00000007175	ahcy
ENSXMAG00000006376	ttc26	ENSLOCG00000011992	
ENSXMAG00000017840		ENSLOCG00000005679	sp7
ENSXMAG00000003505	hdgfl2	ENSLOCG00000006682	hdgfl2
ENSXMAG00000023663	M6PR	ENSLOCG00000008310	m6pr
ENSXMAG00000003828	slc25a22a	ENSLOCG00000000003	slc25a22a

ENSXMAG00000017836	tarbp2	ENSLOCG00000005711	tarbp2
ENSXMAG00000021798	insm1b	ENSLOCG00000018009	insm1b
ENSXMAG00000000319	slc7a3a	ENSLOCG00000014920	slc7a3a
ENSXMAG00000002411	tango2	ENSLOCG00000002476	tango2
ENSXMAG00000002296	arl8	ENSLOCG00000010851	arl8
ENSXMAG00000001594	stat1a	ENSLOCG00000009965	stat1a
ENSXMAG00000024656	bmp15	ENSLOCG00000014334	
ENSXMAG00000023137		ENSLOCG00000000669	
ENSXMAG00000007828	tonsl	ENSLOCG00000005779	tonsl
ENSXMAG00000000789	si:ch73-238c9.1	ENSLOCG00000000630	si:ch73-238c9.1
ENSXMAG00000024334	gys2	ENSLOCG00000015387	gys2
ENSXMAG00000002299	zgc:112356	ENSLOCG00000008827	zgc:112356
ENSXMAG00000005023		ENSLOCG00000017000	
ENSXMAG00000012134	adgrg1	ENSLOCG00000005960	
ENSXMAG00000000317	snx12	ENSLOCG00000014918	snx12
ENSXMAG00000028667		ENSLOCG00000005735	
ENSXMAG00000023371	amacr	ENSLOCG00000012318	amacr
ENSXMAG00000003027	def8	ENSLOCG00000001166	def8
ENSXMAG00000017892	tmem106a	ENSLOCG00000012162	tmem106a
ENSXMAG00000019056	gale	ENSLOCG00000001779	gale
ENSXMAG00000028417	apc2	ENSLOCG00000000661	apc2
ENSXMAG00000017707	chordc1b	ENSLOCG00000007242	chordc1a
ENSXMAG00000011631	PHC1	ENSLOCG00000008278	PHC1
ENSXMAG00000019501	npy7r	ENSLOCG00000018314	npy7r
ENSXMAG00000002307	zfand1	ENSLOCG00000011892	zfand1
ENSXMAG00000013643	slc45a2	ENSLOCG00000012323	slc45a2
ENSXMAG00000026838	rpl6	ENSLOCG00000007680	rpl6
ENSXMAG00000009733	prelid1a	ENSLOCG00000011987	prelid1a
ENSXMAG00000022584		ENSLOCG00000016551	
ENSXMAG00000029016	strap	ENSLOCG00000015352	strap
ENSXMAG00000002311	chmp4c	ENSLOCG00000011903	chmp4c
ENSXMAG00000003026	mc1r	ENSLOCG00000001220	mc1r
ENSXMAG00000005024	tab2	ENSLOCG00000016998	tab2
ENSXMAG00000027359		ENSLOCG00000011732	CCDC69
ENSXMAG00000023246	cyb561d1	ENSLOCG00000012336	cyb561d1
ENSXMAG00000017829	SP1	ENSLOCG00000005658	sp1
ENSXMAG00000024358	dynll1	ENSLOCG00000008706	
ENSXMAG00000019874	arl4d	ENSLOCG00000017515	arl4d
ENSXMAG00000009739	mxd3	ENSLOCG00000011977	mxd3
ENSXMAG00000019765	RXFP3	ENSLOCG00000017549	RXFP3
ENSXMAG00000017658	zmp:0000000711	ENSLOCG00000000367	
ENSXMAG00000012138		ENSLOCG00000007380	syap1
ENSXMAG00000013033	gadd45bb	ENSLOCG00000000752	gadd45ba
ENSXMAG00000014579	acaa2	ENSLOCG00000011285	
ENSXMAG00000005022	mettl8	ENSLOCG00000007749	mettl8
ENSXMAG00000002429	ubr5	ENSLOCG00000009882	ubr5
ENSXMAG00000003016	tcf25	ENSLOCG00000001236	tcf25
ENSXMAG00000009756		ENSLOCG00000011965	FAM193B
ENSXMAG00000006508	gpatch11	ENSLOCG00000015919	gpatch11
ENSXMAG00000002321		ENSLOCG00000010799	plxdc2
ENSXMAG00000013640		ENSLOCG00000012070	btc
ENSXMAG00000013823	trmt1	ENSLOCG00000006479	trmt1
ENSXMAG00000019047	hmgcl	ENSLOCG00000001792	HMGCL
ENSXMAG00000004726	atxn7l2a	ENSLOCG00000012332	atxn7l2a
ENSXMAG00000005042	dcaf17	ENSLOCG00000007739	dcaf17
ENSXMAG00000016477	b4galt7	ENSLOCG00000012160	b4galt7

ENSXMAG00000012162	phospho2	ENSLOCG00000017830	phospho2
ENSXMAG00000005037	cdk19	ENSLOCG00000016995	CDK19
ENSXMAG00000014595	mrpl40	ENSLOCG00000001879	MRPL40
ENSXMAG00000014862	hdac8	ENSLOCG00000014449	hdac8
ENSXMAG00000021277	SLC25A3	ENSLOCG00000015447	slc25a3b
ENSXMAG00000017896	sost	ENSLOCG00000012122	sost
ENSXMAG00000009768	DDX41	ENSLOCG00000011956	DDX41
ENSXMAG00000014600	si:ch211-51h9.6	ENSLOCG00000001866	C22orf39
ENSXMAG00000029688	foxm1	ENSLOCG00000016556	foxm1
ENSXMAG00000013638	gng10	ENSLOCG00000003023	gng10
ENSXMAG00000019989	cx35.4	ENSLOCG00000018274	cx35.4
ENSXMAG00000002333	malrd1	ENSLOCG00000010809	malrd1
ENSXMAG00000005318	itgb5	ENSLOCG00000002706	itgb5
ENSXMAG00000017899	meox1	ENSLOCG00000012129	meox1
ENSXMAG00000005050	cybrd1	ENSLOCG00000007725	cybrd1
ENSXMAG00000023981	mtss1	ENSLOCG00000007280	mtss1
ENSXMAG00000014684		ENSLOCG00000005249	CDH8
ENSXMAG00000013635	nxnl2	ENSLOCG00000010217	nxnl2
ENSXMAG00000017826		ENSLOCG00000005638	
ENSXMAG00000013029	si:ch73-60h1.1	ENSLOCG00000001661	si:ch73-60h1.1
ENSXMAG00000005067	amd1	ENSLOCG00000016993	amd1
ENSXMAG00000003013	cyba	ENSLOCG00000001258	cyba
ENSXMAG00000014601	ufd1l	ENSLOCG00000001849	ufd1l
ENSXMAG00000011599		ENSLOCG00000008359	si:ch1073-90m23.1
ENSXMAG00000021568	etv4	ENSLOCG00000012135	etv4
ENSXMAG00000026950	stac	ENSLOCG00000004780	stac
ENSXMAG00000017594	si:ch73-352p4.8	ENSLOCG00000016571	si:ch73-352p4.8
ENSXMAG00000006412	map2k7	ENSLOCG00000006590	map2k7
ENSXMAG00000022560		ENSLOCG00000006515	cfb
ENSXMAG00000006472	heatr5b	ENSLOCG00000015922	heatr5b
ENSXMAG00000003003	mvda	ENSLOCG00000001275	mvda
ENSXMAG00000003040	tnfaip6	ENSLOCG00000002695	tnfaip6
ENSXMAG00000004752	zc3h11a	ENSLOCG00000011932	zc3h11a
ENSXMAG00000002677	zfand4	ENSLOCG00000009414	zfand4
ENSXMAG00000003514	ap3d1	ENSLOCG00000006451	ap3d1
ENSXMAG00000021706	RPF2	ENSLOCG00000016992	RPF2
ENSXMAG00000016498	TMEM216	ENSLOCG00000000388	
ENSXMAG00000026908		ENSLOCG00000001689	si:ch211-262i1.6
ENSXMAG00000002340	si:ch73-174h16.4	ENSLOCG00000008819	si:ch73-174h16.4
ENSXMAG00000017907	dhx8	ENSLOCG00000012147	dhx8
ENSXMAG00000019007	pabpc4	ENSLOCG00000001877	pabpc4
ENSXMAG00000021796	slc16a10	ENSLOCG00000016991	slc16a10
ENSXMAG00000014796		ENSLOCG00000005272	adamts18
ENSXMAG00000014623	dhx37	ENSLOCG00000001768	dhx37
ENSXMAG00000002998	pdcd5	ENSLOCG00000001645	pdcd5
ENSXMAG00000029720		ENSLOCG00000008809	si:ch73-174h16.5
ENSXMAG00000006323		ENSLOCG00000009739	
ENSXMAG00000009786	atoh1b	ENSLOCG00000011944	atoh1b
ENSXMAG00000000506	rif1	ENSLOCG00000002657	rif1
ENSXMAG00000002134	mtfr1l	ENSLOCG00000004287	mtfr1l
ENSXMAG00000015853	romo1	ENSLOCG00000007270	romo1
ENSXMAG00000011588	casp2	ENSLOCG00000008375	casp2
ENSXMAG00000012328	vwa7	ENSLOCG00000014690	vwa7
ENSXMAG00000002350	wdr60	ENSLOCG00000012365	wdr60
ENSXMAG00000022599	rev3l	ENSLOCG00000016989	rev3l
ENSXMAG00000002378	txnrd2.1	ENSLOCG00000002405	txnrd2.1

ENSXMAG00000012337	si:ch211-132b12.2	ENSLOCG00000014677	si:ch211-132b12.2	
ENSXMAG00000023848	CRYGS	ENSLOCG00000004099	crygs2	
ENSXMAG00000028080	8-Mar	ENSLOCG00000009384		8-Mar
ENSXMAG00000009787	unc5a	ENSLOCG00000011930	unc5a	
ENSXMAG00000020946		ENSLOCG00000009068	map3k7cl	
ENSXMAG00000015184	tbccd1	ENSLOCG00000004084	tbccd1	
ENSXMAG00000015857	smpd4	ENSLOCG00000006412	smpd4	
ENSXMAG00000005091		ENSLOCG00000016988		
ENSXMAG00000026606		ENSLOCG00000001590		
ENSXMAG00000023838	creg1	ENSLOCG00000009735	creg1	
ENSXMAG00000010342	dnajb11	ENSLOCG00000004052	dnajb11	
ENSXMAG00000012439	si:ch211-132b12.8	ENSLOCG00000014668	si:ch211-132b12.8	
ENSXMAG00000020312	gp1bb	ENSLOCG00000018138	gp1bb	
ENSXMAG00000023818		ENSLOCG00000005086		
ENSXMAG00000026523		ENSLOCG00000006041		
ENSXMAG00000002309		ENSLOCG00000001554	anos1b	
ENSXMAG00000001329	ndufa5	ENSLOCG00000015870	ndufa5	
ENSXMAG00000003255	MATN2	ENSLOCG00000009845	MATN2	
ENSXMAG00000010695	alg9	ENSLOCG00000001352	alg9	
ENSXMAG00000009769		ENSLOCG00000013438	abcg2a	
ENSXMAG00000004799	mtrex	ENSLOCG00000010186	mtrex	
ENSXMAG00000026826	ormdl3	ENSLOCG00000012999	ormdl3	
ENSXMAG00000003252	pcdh12	ENSLOCG00000011407	pcdh12	
ENSXMAG00000018936	lck	ENSLOCG00000002518	lck	
ENSXMAG00000012453	akt2l	ENSLOCG00000014670	akt2	
ENSXMAG00000005096	hat1	ENSLOCG00000007651	hat1	
ENSXMAG00000003245	gm2a	ENSLOCG00000011710	gm2a	
ENSXMAG00000028209		ENSLOCG00000006054		
ENSXMAG00000023933	wnt3	ENSLOCG00000012996	wnt3	
ENSXMAG00000001337	COG5	ENSLOCG00000015817	COG5	
ENSXMAG00000003748	myrf	ENSLOCG00000000407	myrf	
ENSXMAG00000017759	myg1	ENSLOCG00000005545	C12orf10	
ENSXMAG00000023472		ENSLOCG00000001749	swt1	
ENSXMAG00000002374	gnb1l	ENSLOCG00000002390	gnb1l	
ENSXMAG00000021807		ENSLOCG00000008609	stil	
ENSXMAG00000013974	kif7	ENSLOCG00000014540	kif7	
ENSXMAG00000007412	satb2	ENSLOCG00000004032	satb2	
ENSXMAG00000027756	ppfia2	ENSLOCG00000016312	ppfia2	
ENSXMAG00000003236	si:dkey-280e21.3	ENSLOCG00000011605	si:dkey-280e21.3	
ENSXMAG00000023076	fam167b	ENSLOCG00000002498	fam167b	
ENSXMAG00000012466	dhx40	ENSLOCG00000003916	dhx40	
ENSXMAG00000006314	f7	ENSLOCG00000009765	f7	
ENSXMAG00000017757		ENSLOCG00000018202		
ENSXMAG00000018906	zgc:154055	ENSLOCG00000002462	zgc:154055	
ENSXMAG00000002370	tbx1	ENSLOCG00000002370	TBX1	
ENSXMAG00000005566	serpinc1	ENSLOCG00000000247	serpinc1	
ENSXMAG00000022208		ENSLOCG00000008986	col4a2	
ENSXMAG00000009841	pdlim7	ENSLOCG00000011920	pdlim7	
ENSXMAG00000014718	zgc:63587	ENSLOCG00000001793	zgc:63587	
ENSXMAG00000003232	thoc3	ENSLOCG00000011610	thoc3	
ENSXMAG00000007793	selenon	ENSLOCG00000004324	selenon	
ENSXMAG00000026317	dlx1a	ENSLOCG00000007608	dlx1a	
ENSXMAG00000002683	alox5a	ENSLOCG00000009354	alox5a	
ENSXMAG00000000966		ENSLOCG00000013348	CCDC66	
ENSXMAG00000027662	wisp3	ENSLOCG00000016984	wisp3	
ENSXMAG00000005565	zbtb37	ENSLOCG00000000232	zbtb37	

ENSXMAG00000017282	GXYLT1	ENSLOCG00000001360	GXYLT1
ENSXMAG00000018894	tdh2	ENSLOCG00000002446	tdh2
ENSXMAG00000002313	ivns1abpa	ENSLOCG00000001731	ivns1abpb
ENSXMAG00000002351	sema4c	ENSLOCG00000015594	sema4c
ENSXMAG00000005118	dlx2a	ENSLOCG00000007591	dlx2a
ENSXMAG00000028508	CPLX2	ENSLOCG00000011618	cplx2
ENSXMAG00000019798	s1pr1	ENSLOCG00000017643	s1pr1
ENSXMAG00000007406	plcl1	ENSLOCG00000004019	plcl1
ENSXMAG00000027970	TMTC1	ENSLOCG00000015106	tmtc1
ENSXMAG00000005172	tube1	ENSLOCG00000016981	tube1
ENSXMAG00000007785	znf593	ENSLOCG00000004338	znf593
ENSXMAG00000026608	dph5	ENSLOCG00000000186	dph5
ENSXMAG00000010930	TOMM40L	ENSLOCG00000001939	
ENSXMAG00000022670		ENSLOCG00000015599	
ENSXMAG00000002472	POP1	ENSLOCG00000009817	pop1
ENSXMAG00000006473	si:ch211-214c7.5	ENSLOCG00000006625	si:ch211-214c7.5
ENSXMAG00000014750	cdc45	ENSLOCG00000001829	cdc45
ENSXMAG00000005122	itga6a	ENSLOCG00000007555	itga6a
ENSXMAG00000003225	zgc:153018	ENSLOCG00000000978	zgc:153018
ENSXMAG00000026278	tmem187	ENSLOCG00000017979	tmem187
ENSXMAG00000017949	psmd11b	ENSLOCG00000012951	psmd11b
ENSXMAG00000007776	rlf	ENSLOCG00000004348	rlf
ENSXMAG00000014011	fes	ENSLOCG00000015089	fes
ENSXMAG00000021227	slc30a7	ENSLOCG00000000160	slc30a7
ENSXMAG00000025433	yaf2	ENSLOCG00000001370	yaf2
ENSXMAG00000025214	cited1	ENSLOCG00000017480	cited1
ENSXMAG00000011533	zyx	ENSLOCG00000008407	zyx
ENSXMAG00000018849	clic4	ENSLOCG00000002762	clic4
ENSXMAG00000005194	LAMA4	ENSLOCG00000016979	LAMA4
ENSXMAG00000021565	NIPAL2	ENSLOCG00000009809	NIPAL2
ENSXMAG00000010122	hspa13	ENSLOCG00000000950	hspa13
ENSXMAG00000009870	cltb	ENSLOCG00000011899	cltb
ENSXMAG00000003224		ENSLOCG00000001003	HNRNPUL2
ENSXMAG00000007403	rftn2	ENSLOCG00000003988	rftn2
ENSXMAG00000021487	tet1	ENSLOCG00000009344	tet1
ENSXMAG00000007768	zmpste24	ENSLOCG00000004363	zmpste24
ENSXMAG00000010515	kat6a	ENSLOCG00000015602	kat6a
ENSXMAG00000004113	rbbp5	ENSLOCG00000011387	rbbp5
ENSXMAG00000030031		ENSLOCG00000007407	cdc42l
ENSXMAG00000004847	dhx29	ENSLOCG00000010159	dhx29
ENSXMAG00000025104	higd2a	ENSLOCG00000011907	higd2a
ENSXMAG00000014827	rps4x	ENSLOCG00000014445	rps4x
ENSXMAG00000017963	cdk5r1b	ENSLOCG00000017971	cdk5r1b
ENSXMAG00000010929	fdxacb1	ENSLOCG00000001403	fdxacb1
ENSXMAG00000027409	runx3	ENSLOCG00000002744	runx3
ENSXMAG00000008581	ipo8	ENSLOCG00000015102	ipo8
ENSXMAG00000007395	mob4	ENSLOCG00000003970	mob4
ENSXMAG00000023567	eif4e1c	ENSLOCG00000009334	eif4e1c
ENSXMAG00000006446	VIT	ENSLOCG00000015932	VIT
ENSXMAG00000009665	TEC	ENSLOCG00000011964	tec
ENSXMAG00000010937	bicdl2	ENSLOCG00000001903	bicdl2
ENSXMAG00000002977	rab32a	ENSLOCG00000016148	rab32a
ENSXMAG00000002326	hmcn1	ENSLOCG00000001656	hmcn1
ENSXMAG00000020015	KCNS2	ENSLOCG00000018341	KCNS2
ENSXMAG00000005154	NUP62	ENSLOCG00000014967	
ENSXMAG00000027604	rpl13a	ENSLOCG00000004388	rpl13a

ENSXMAG00000002381	ncapg2	ENSLOCG00000012399	ncapg2
ENSXMAG00000005525	vcam1b	ENSLOCG00000000130	vcam1b
ENSXMAG00000021405	si:dkey-228d14.5	ENSLOCG00000009329	si:dkey-228d14.5
ENSXMAG00000009881	ankhd1	ENSLOCG00000012482	ankhd1
ENSXMAG00000003220	nudt22	ENSLOCG00000001142	nudt22
ENSXMAG00000028356	nsd3	ENSLOCG00000015087	nsd3
ENSXMAG00000017964		ENSLOCG00000012936	MYO1D
ENSXMAG00000002498	osr2	ENSLOCG00000009790	osr2
ENSXMAG00000028208	mia	ENSLOCG00000001418	mia
ENSXMAG00000029738	RF00133	ENSLOCG00000020948	RF00133
ENSXMAG00000007966	pla2g6	ENSLOCG00000011469	
ENSXMAG00000005197	MARCKS	ENSLOCG00000016978	MARCKS
ENSXMAG00000028573	dhcr7	ENSLOCG00000005014	dhcr7
ENSXMAG00000022698	syf2	ENSLOCG00000002728	syf2
ENSXMAG00000010944	cryaba	ENSLOCG00000001451	cryabb
ENSXMAG00000017597	sod1	ENSLOCG00000009152	sod1
ENSXMAG00000005162	pdk1	ENSLOCG00000007542	pdk1
ENSXMAG00000011508	emg1	ENSLOCG00000008451	emg1
ENSXMAG00000027444	tmem178b	ENSLOCG00000016125	zgc:194665
ENSXMAG00000005889	hspe1	ENSLOCG00000003954	hspe1
ENSXMAG00000004155		ENSLOCG00000013492	
ENSXMAG00000006230	pcid2	ENSLOCG00000009805	pcid2
ENSXMAG00000005199	HDAC2	ENSLOCG00000016977	HDAC2
ENSXMAG00000028382	si:ch211-133n4.10	ENSLOCG00000000488	
ENSXMAG00000005891	hspd1	ENSLOCG00000003937	hspd1
ENSXMAG00000002972	adgb	ENSLOCG00000016140	adgb
ENSXMAG00000026240	mgst3b	ENSLOCG00000002714	mgst3b
ENSXMAG00000008517	mrps33	ENSLOCG00000016124	mrps33
ENSXMAG00000014823	nhsl2	ENSLOCG00000014439	nhsl2
ENSXMAG00000028803	gatd1	ENSLOCG00000000004	gatd1
ENSXMAG00000010495		ENSLOCG00000013481	si:dkey-49n23.1
ENSXMAG00000021332	hnrnph1	ENSLOCG00000012510	hnrnph1
ENSXMAG00000017324	ADAMTS20	ENSLOCG00000001507	ADAMTS20
ENSXMAG00000024682	gpaa1	ENSLOCG00000008890	gpaa1
ENSXMAG00000023433	tmem50a	ENSLOCG00000002702	tmem50a
ENSXMAG00000006226	tbx19	ENSLOCG00000010586	tbx19
ENSXMAG00000006528		ENSLOCG00000006572	
ENSXMAG00000004871	pstpip2	ENSLOCG00000011149	PSTPIP2
ENSXMAG00000003200	stx5a	ENSLOCG00000001157	stx5a
ENSXMAG00000028301	coq10b	ENSLOCG00000003924	coq10b
ENSXMAG00000014954	pdk2a	ENSLOCG00000013349	pdk2a
ENSXMAG00000018815	rhd	ENSLOCG00000002671	rhd
ENSXMAG00000011483	nop2	ENSLOCG00000007050	nop2
ENSXMAG00000005236	HS3ST5	ENSLOCG00000016976	HS3ST5
ENSXMAG00000003714	chid1	ENSLOCG00000000010	CHID1
ENSXMAG00000009967	rufy1	ENSLOCG00000012518	rufy1
ENSXMAG00000002765	ccar1	ENSLOCG00000009307	ccar1
ENSXMAG00000016287	exosc4	ENSLOCG00000008878	exosc4
ENSXMAG00000005213	ppp1r9ala	ENSLOCG00000007524	ppp1r9ala
ENSXMAG00000023674	letm2	ENSLOCG00000015084	letm2
ENSXMAG00000005239	frk	ENSLOCG00000016973	frk
ENSXMAG00000003705	ighmbp2	ENSLOCG00000001512	ighmbp2
ENSXMAG00000013648	tbkbp1	ENSLOCG00000013343	tbkbp1
ENSXMAG00000025696	ing4	ENSLOCG00000007026	ing4
ENSXMAG00000008567	braf	ENSLOCG00000016118	braf
ENSXMAG00000029698		ENSLOCG00000017707	

ENSXMAG00000010488	ECM2	ENSLOCG00000013843	ECM2
ENSXMAG00000002652	cntn2	ENSLOCG00000011962	cntn2
ENSXMAG00000010513	tm2d2	ENSLOCG00000015103	tm2d2
ENSXMAG00000021508		ENSLOCG00000017246	mcm9
ENSXMAG00000007730	DLK1	ENSLOCG00000011579	DLK1
ENSXMAG00000001685	gucy2f	ENSLOCG00000001011	gucy2f
ENSXMAG00000005482	rtca	ENSLOCG00000000093	rtca
ENSXMAG00000023411	tbx21	ENSLOCG00000013335	tbx21
ENSXMAG00000013441	gna15.4	ENSLOCG00000006414	gna15.4
ENSXMAG00000007369	sf3b1	ENSLOCG00000003899	sf3b1
ENSXMAG00000017590		ENSLOCG00000011169	haus1
ENSXMAG00000010485	aspn	ENSLOCG00000013849	aspn
ENSXMAG00000004890	itga1	ENSLOCG00000006845	itga1
ENSXMAG00000021383		ENSLOCG00000013786	si:dkey-11m19.5
ENSXMAG00000010564	strc	ENSLOCG00000015068	strc
ENSXMAG00000027384		ENSLOCG00000012544	
ENSXMAG00000002500	VPS13B	ENSLOCG00000009760	VPS13B
ENSXMAG00000000303	adgrg4b	ENSLOCG00000015115	
ENSXMAG00000029699	gna11a	ENSLOCG00000006393	gna11a
ENSXMAG00000010958	dlat	ENSLOCG00000001499	dlat
ENSXMAG00000011455	ncapd2	ENSLOCG00000006987	ncapd2
ENSXMAG00000003188	tgfb2l	ENSLOCG00000001175	tgfb2l
ENSXMAG00000005255	cep85l	ENSLOCG00000017245	cep85l
ENSXMAG00000002424	ube3c	ENSLOCG00000012433	ube3c
ENSXMAG00000028973	IRAK4	ENSLOCG00000001620	irak4
ENSXMAG00000025802	large1	ENSLOCG00000016113	large1
ENSXMAG00000014818	arhgap36	ENSLOCG00000014433	arhgap36
ENSXMAG00000010010	rmnd5b	ENSLOCG00000012143	rmnd5b
ENSXMAG00000006566		ENSLOCG00000013393	
ENSXMAG00000013015		ENSLOCG00000006282	orc1
ENSXMAG00000001772	anapc10	ENSLOCG00000008926	anapc10
ENSXMAG00000027506	trmt112	ENSLOCG00000001190	trmt112
ENSXMAG00000014844	rassf2a	ENSLOCG00000015629	rassf2b
ENSXMAG00000017571	cfap298	ENSLOCG00000009219	cfap298
ENSXMAG00000021858	kcmf1	ENSLOCG00000011313	kcmf1
ENSXMAG00000002397	pla2g4ab	ENSLOCG00000001619	pla2g4aa
ENSXMAG00000007725	WDR25	ENSLOCG00000011593	WDR25
ENSXMAG00000010494	unc5db	ENSLOCG00000015107	unc5db
ENSXMAG00000010022	n4bp3	ENSLOCG00000012151	n4bp3
ENSXMAG00000006416	spast	ENSLOCG00000015939	spast
ENSXMAG00000017399	twf1a	ENSLOCG00000001638	twf1b
ENSXMAG00000026824	mnx1	ENSLOCG00000012445	mnx1
ENSXMAG00000024561	olig4	ENSLOCG00000018275	olig4
ENSXMAG00000021387	stox1	ENSLOCG00000009290	stox1
ENSXMAG00000017687	NCKAP1L	ENSLOCG00000005047	NCKAP1L
ENSXMAG00000029086	WARS	ENSLOCG00000011600	WARS
ENSXMAG00000017548	acss3	ENSLOCG00000016308	acss3
ENSXMAG00000008011	ncf4	ENSLOCG00000011446	ncf4
ENSXMAG00000018748	bsdc1	ENSLOCG00000002577	bsdc1
ENSXMAG00000001682	tsku	ENSLOCG00000001049	tsku
ENSXMAG00000002451	nom1	ENSLOCG00000012452	nom1
ENSXMAG00000001778	abce1	ENSLOCG00000008914	abce1
ENSXMAG00000010027		ENSLOCG00000011745	
ENSXMAG00000014856	SLC23A2	ENSLOCG00000015630	slc23a2
ENSXMAG00000001621		ENSLOCG00000001094	
ENSXMAG00000029316	recql	ENSLOCG00000015393	recql

ENSXMAG00000005257	gopc	ENSLOCG00000017146	gopc
ENSXMAG00000024630	dpy19l3	ENSLOCG00000005805	dpy19l3
ENSXMAG00000022473	zgc:165604	ENSLOCG00000002821	zgc:165604
ENSXMAG00000002460	lmbr1	ENSLOCG00000012464	lmbr1
ENSXMAG00000010542		ENSLOCG00000006351	si:ch211-122f10.4
ENSXMAG00000002418	ptgs2b	ENSLOCG00000001597	ptgs2b
ENSXMAG00000029120		ENSLOCG00000006949	
ENSXMAG00000013700	mrpl10	ENSLOCG00000013385	mrpl10
ENSXMAG00000026126	usp12b	ENSLOCG00000014426	usp12b
ENSXMAG00000025420		ENSLOCG00000001235	mmp20b
ENSXMAG00000028792	ADI1	ENSLOCG00000017213	adi1
ENSXMAG00000017524	synj1	ENSLOCG00000009230	synj1
ENSXMAG00000007341	ankrd44	ENSLOCG00000003887	ankrd44
ENSXMAG00000002627	arfrp1	ENSLOCG00000001677	arfrp1
ENSXMAG00000010524	slc38a7	ENSLOCG00000006410	slc38a7
ENSXMAG00000007702		ENSLOCG00000011318	FANCM
ENSXMAG00000013703	pnpo	ENSLOCG00000013387	pnpo
ENSXMAG00000005269	cx43	ENSLOCG00000017880	cx43
ENSXMAG00000021150		ENSLOCG00000008896	
ENSXMAG00000028484		ENSLOCG00000012477	rnf32
ENSXMAG00000002625		ENSLOCG00000012450	elf3
ENSXMAG00000018716	trappc12	ENSLOCG00000017214	trappc12
ENSXMAG00000005270		ENSLOCG00000017256	si:ch73-267c23.10
ENSXMAG00000006596	PIK3R6	ENSLOCG00000013248	pik3r6b
ENSXMAG00000023032	rasl11a	ENSLOCG00000014424	rasl11a
ENSXMAG00000002811	mat1a	ENSLOCG00000012578	mat1a
ENSXMAG00000006396	memo1	ENSLOCG00000015951	memo1
ENSXMAG00000010511	got2b	ENSLOCG00000006474	got2a
ENSXMAG00000000291	nsg2	ENSLOCG00000010853	nsg2
ENSXMAG00000026246	lin7a	ENSLOCG00000016303	lin7a
ENSXMAG00000007713	soul3	ENSLOCG00000011338	soul3
ENSXMAG00000013381	tle2a	ENSLOCG00000006361	tle2a
ENSXMAG00000024356	shha	ENSLOCG00000012487	shha
ENSXMAG00000005293	nus1	ENSLOCG00000017148	nus1
ENSXMAG00000002601	p3h1	ENSLOCG00000000935	p3h1
ENSXMAG00000013715	cdk5rap3	ENSLOCG00000013394	cdk5rap3
ENSXMAG00000018706	eipr1	ENSLOCG00000017215	eipr1
ENSXMAG00000005298	cgrrf1	ENSLOCG00000012123	cgrrf1
ENSXMAG00000014724	cldn15lb	ENSLOCG00000009302	cldn15lb
ENSXMAG00000014891	pcna	ENSLOCG00000015638	pcna
ENSXMAG00000001558	mmp20a	ENSLOCG00000001242	mmp20a
ENSXMAG00000020293	drd1b	ENSLOCG00000010837	drd1b
ENSXMAG00000010058	anxa6	ENSLOCG00000011765	anxa6
ENSXMAG00000028404	znf507	ENSLOCG00000005787	znf507
ENSXMAG00000014753	MB21D2	ENSLOCG00000009364	MB21D2
ENSXMAG00000002488	rbm33a	ENSLOCG00000012490	rbm33a
ENSXMAG00000005307	psmc6	ENSLOCG00000012064	psmc6
ENSXMAG00000017492	myf6	ENSLOCG00000016302	myf6
ENSXMAG00000010981	pcnx3	ENSLOCG00000002523	PCNX3
ENSXMAG00000007328	lcmt2	ENSLOCG00000004108	lcmt2
ENSXMAG00000014904		ENSLOCG00000013519	mns1
ENSXMAG00000027274		ENSLOCG00000002415	tmem234
ENSXMAG00000006388	srd5a2b	ENSLOCG00000015953	srd5a2b
ENSXMAG00000028770		ENSLOCG00000008566	EFCAB14
ENSXMAG00000024845	REELD1	ENSLOCG00000008841	
ENSXMAG00000008040		ENSLOCG00000012980	

ENSXMAG00000002600	cldn19	ENSLOCG00000000886	cldn19
ENSXMAG00000030077	rps16	ENSLOCG00000016289	rps16
ENSXMAG00000010489	cyb561a3a	ENSLOCG00000015453	cyb561a3b
ENSXMAG00000022199	rab5b	ENSLOCG00000004128	rab5b
ENSXMAG00000026360	tshz3b	ENSLOCG00000005747	tshz3b
ENSXMAG00000020352	ZNF830	ENSLOCG00000005038	ZNF830
ENSXMAG00000001353	rad50	ENSLOCG00000010392	rad50
ENSXMAG00000014066	cmtr2	ENSLOCG00000017595	cmtr2
ENSXMAG00000029109	NFE2L1	ENSLOCG00000013411	NFE2L1
ENSXMAG00000025297	rps19bp1	ENSLOCG00000011136	
ENSXMAG00000014729	gc2	ENSLOCG00000014403	gc2
ENSXMAG00000005352	smim8	ENSLOCG00000016923	smim8
ENSXMAG00000002501	insig1	ENSLOCG00000012507	insig1
ENSXMAG00000014781		ENSLOCG00000009378	IL1RAP
ENSXMAG00000007692	rcan3	ENSLOCG00000002797	rcan3
ENSXMAG00000014909	zgc:152830	ENSLOCG00000015639	zgc:152830
ENSXMAG00000024956	tnfrsf1a	ENSLOCG00000006877	
ENSXMAG00000002825	sfxn3	ENSLOCG00000012590	sfxn3
ENSXMAG00000007311	dbr1	ENSLOCG00000004160	dbr1
ENSXMAG00000025560	cbx1b	ENSLOCG00000013417	cbx1b
ENSXMAG00000001794	slc10a7	ENSLOCG00000008832	slc10a7
ENSXMAG00000022057	htr5ab	ENSLOCG00000012511	htr5ab
ENSXMAG00000007686	nipal3	ENSLOCG00000002817	nipal3
ENSXMAG00000011412		ENSLOCG00000006859	
ENSXMAG00000006382	mkks	ENSLOCG00000015954	mkks
ENSXMAG00000021147	PPP1R1A	ENSLOCG00000005001	PPP1R1A
ENSXMAG00000002557		ENSLOCG00000002688	
ENSXMAG00000023168	cfap206	ENSLOCG00000016926	cfap206
ENSXMAG00000021330	gmnc	ENSLOCG00000009396	gmnc
ENSXMAG00000029233		ENSLOCG00000013423	snx11
ENSXMAG00000026044	slx4ip	ENSLOCG00000015956	SLX4IP
ENSXMAG00000017521	si:dkey-5g14.1	ENSLOCG00000009246	si:dkey-5g14.1
ENSXMAG00000007274	armc8	ENSLOCG00000004183	armc8
ENSXMAG00000017674		ENSLOCG00000004987	
ENSXMAG00000014786		ENSLOCG00000009046	
ENSXMAG00000013634	grhl3	ENSLOCG00000002832	grhl3
ENSXMAG00000010088	tnip1	ENSLOCG00000011786	tnip1
ENSXMAG00000011411		ENSLOCG00000007125	
ENSXMAG00000005222	stx8	ENSLOCG00000013278	stx8
ENSXMAG00000019795	thap11	ENSLOCG00000017596	thap11
ENSXMAG00000028387	si:ch211-241j12.3	ENSLOCG00000016925	
ENSXMAG00000019945	s1pr3a	ENSLOCG00000017637	s1pr3a
ENSXMAG00000008048		ENSLOCG00000011175	
ENSXMAG00000001810	TTC29	ENSLOCG00000008818	TTC29
ENSXMAG00000023804	gfod2	ENSLOCG00000006794	gfod2
ENSXMAG00000005311	cdca7a	ENSLOCG00000007465	cdca7a
ENSXMAG00000013637	si:dkey-221l4.11	ENSLOCG00000018286	si:dkey-221l4.11
ENSXMAG00000001551	tbrg1	ENSLOCG00000003093	tbrg1
ENSXMAG00000013728	skap1	ENSLOCG00000013427	skap1
ENSXMAG00000001005	ccne1	ENSLOCG00000005671	ccne1
ENSXMAG00000005229		ENSLOCG00000013286	
ENSXMAG00000010097	gpx3	ENSLOCG00000011804	gpx3
ENSXMAG00000011410		ENSLOCG00000007108	MRPL51
ENSXMAG00000013641	cnksr1	ENSLOCG00000005495	cnksr1
ENSXMAG00000010467	GOLGA7	ENSLOCG00000015441	GOLGA7
ENSXMAG00000001413	rbm27	ENSLOCG00000010426	rbm27

ENSXMAG00000017644	ITGA7	ENSLOCG00000004959	ITGA7
ENSXMAG00000025425	zgc:162297	ENSLOCG00000005653	zgc:162297
ENSXMAG00000021290	gpat2	ENSLOCG00000015643	gpat2
ENSXMAG00000018691	pxdn	ENSLOCG00000017217	pxdn
ENSXMAG00000000585	rmdn3	ENSLOCG00000012713	RMDN3
ENSXMAG00000002556	nucks1a	ENSLOCG00000011797	nucks1a
ENSXMAG00000025259	tgif1	ENSLOCG00000003162	tgif1
ENSXMAG00000025601	hoxb1b	ENSLOCG00000007019	
ENSXMAG00000027644	si:ch211-260e23.9	ENSLOCG00000005633	si:ch211-260e23.9
ENSXMAG00000001534	siae	ENSLOCG00000003053	siae
ENSXMAG00000006681	mgat3b	ENSLOCG00000011196	mgat3b
ENSXMAG00000014085	cog4	ENSLOCG00000007581	cog4
ENSXMAG00000010463	sfrp1a	ENSLOCG00000015437	sfrp1a
ENSXMAG00000000077	hoxb5b	ENSLOCG00000013440	hoxb5a
ENSXMAG00000010102	dctn4	ENSLOCG00000011815	dctn4
ENSXMAG00000000592	RAD51	ENSLOCG00000012707	rad51
ENSXMAG00000020317	adra2b	ENSLOCG00000015645	adra2b
ENSXMAG00000020053	otud1	ENSLOCG00000003243	otud1
ENSXMAG00000021318	hoxb6a	ENSLOCG00000013443	hoxb6a
ENSXMAG00000007256	idh1	ENSLOCG00000004227	idh1
ENSXMAG00000011401	tapbpl	ENSLOCG00000006844	
ENSXMAG00000010461	zmat4a	ENSLOCG00000015435	zmat4a
ENSXMAG00000017534	ppp1r12a	ENSLOCG00000016288	ppp1r12a
ENSXMAG00000014933	dusp2	ENSLOCG00000015652	dusp2
ENSXMAG00000013329	ankrd24	ENSLOCG00000006239	ANKRD24
ENSXMAG00000000600		ENSLOCG00000016942	ube2j1
ENSXMAG00000001043		ENSLOCG00000006959	AP2A2
ENSXMAG00000029523	si:ch211-117k10.3	ENSLOCG00000011807	si:ch211-117k10.3
ENSXMAG00000014927	ttll6	ENSLOCG00000015123	
ENSXMAG00000029717	epsti1	ENSLOCG00000005706	epsti1
ENSXMAG00000001454	larsb	ENSLOCG00000010442	larsb
ENSXMAG00000014106	gnao1b	ENSLOCG00000007927	gnao1a
ENSXMAG00000002535	KIAA1217	ENSLOCG00000003260	si:ch211-285f17.1
ENSXMAG00000018844	rps6ka1	ENSLOCG00000005457	rps6ka1
ENSXMAG00000003154		ENSLOCG00000001254	nxf1
ENSXMAG00000021775	btbd3b	ENSLOCG00000015968	btbd3b
ENSXMAG00000000329	tnfsf11	ENSLOCG00000005745	tnfsf11
ENSXMAG00000022306	elk4	ENSLOCG00000011817	elk4
ENSXMAG00000027331	akap11	ENSLOCG00000005768	akap11
ENSXMAG00000029893	sptlc3	ENSLOCG00000015971	sptlc3
ENSXMAG00000024308		ENSLOCG00000018310	
ENSXMAG00000014149	tradd	ENSLOCG00000007950	tradd
ENSXMAG00000017643		ENSLOCG00000013817	si:dkey-42i9.6
ENSXMAG00000014917		ENSLOCG00000013473	
ENSXMAG00000002509	fcho1	ENSLOCG00000000887	fcho1
ENSXMAG00000029548	shc2	ENSLOCG00000005770	shc2
ENSXMAG00000002513	RGS22	ENSLOCG00000009743	RGS22
ENSXMAG00000014840	zgc:162872	ENSLOCG00000009061	zgc:162872
ENSXMAG00000014961	rmi1	ENSLOCG00000017966	rmi1
ENSXMAG00000014229	necap1	ENSLOCG00000008144	
ENSXMAG00000027555	irs4a	ENSLOCG00000017479	irs4a
ENSXMAG00000024439	CIR1	ENSLOCG00000007371	CIR1
ENSXMAG00000006273	ism1	ENSLOCG00000015977	ism1
ENSXMAG00000014150	ogfod1	ENSLOCG00000007991	ogfod1
ENSXMAG00000009255	CDK18	ENSLOCG00000011838	CDK18
ENSXMAG00000024409	ccr6b	ENSLOCG00000017877	

ENSXMAG00000027097	snf8	ENSLOCG00000013493	snf8
ENSXMAG00000010118	tspan17	ENSLOCG00000011826	tspan17
ENSXMAG00000006253	tasp1	ENSLOCG00000015978	tasp1
ENSXMAG00000014172	nudt21	ENSLOCG00000008005	nudt21
ENSXMAG00000020054	si:dkey-96n2.3	ENSLOCG00000011630	si:dkey-96n2.3
ENSXMAG00000026143	prpf38a	ENSLOCG00000006262	prpf38a
ENSXMAG00000014667	COL4A5	ENSLOCG00000014368	COL4A5
ENSXMAG00000026252	sp9	ENSLOCG00000007391	sp9
ENSXMAG00000013318	cks2	ENSLOCG00000005754	cks2
ENSXMAG00000005397	scrn3	ENSLOCG00000007349	scrn3
ENSXMAG00000017631	POC1A	ENSLOCG00000013801	poc1a
ENSXMAG00000029089	mchr2	ENSLOCG00000011843	mchr2
ENSXMAG00000021827		ENSLOCG00000009095	ilkap
ENSXMAG00000002552	nphp3	ENSLOCG00000011613	nphp3
ENSXMAG00000014902	msl1b	ENSLOCG00000013507	msl1b
ENSXMAG00000029248	FBXO43	ENSLOCG00000009732	FBXO43
ENSXMAG00000028460	plac8l1	ENSLOCG00000010461	plac8l1
ENSXMAG00000015055	zgc:136564	ENSLOCG00000002249	zgc:136564
ENSXMAG00000001360	ilvbl	ENSLOCG00000002885	ilvbl
ENSXMAG00000026666	klhdc8a	ENSLOCG00000011868	klhdc8a
ENSXMAG00000006115	rgn	ENSLOCG00000008081	rgn
ENSXMAG00000029786		ENSLOCG00000015985	macrod2
ENSXMAG00000010139	ctnna1	ENSLOCG00000011845	ctnna1
ENSXMAG00000007213	dgkh	ENSLOCG00000005796	dgkh
ENSXMAG00000029473		ENSLOCG00000017329	
ENSXMAG00000013288	cpamd8	ENSLOCG00000005716	cpamd8
ENSXMAG00000015065	KIF27	ENSLOCG00000002225	KIF27
ENSXMAG00000014890	mrm1	ENSLOCG00000004945	mrm1
ENSXMAG00000001340	fam118b	ENSLOCG00000002758	fam118b
ENSXMAG00000014900		ENSLOCG00000014544	
ENSXMAG00000018795	asap3	ENSLOCG00000005435	asap3
ENSXMAG00000011370		ENSLOCG00000007609	
ENSXMAG00000001509	GRXCR2	ENSLOCG00000010466	GRXCR2
ENSXMAG00000019821	flrt3	ENSLOCG00000017360	flrt3
ENSXMAG00000003149		ENSLOCG00000001280	bscl2
ENSXMAG00000002572	uba5	ENSLOCG00000011596	uba5
ENSXMAG00000006070	vps16	ENSLOCG00000005310	vps16
ENSXMAG00000007067	ANK2	ENSLOCG00000013099	ANK2
ENSXMAG00000006243	mad2l1bp	ENSLOCG00000015718	MAD2L1BP
ENSXMAG00000014888	fbrs	ENSLOCG00000003354	fbrs
ENSXMAG00000002545	rnf19a	ENSLOCG00000009704	rnf19a
ENSXMAG00000017628	pacrg	ENSLOCG00000004588	pacrg
ENSXMAG00000011354	rbp5	ENSLOCG00000007648	rbp5
ENSXMAG00000027527		ENSLOCG00000013368	
ENSXMAG00000020948	rpl29	ENSLOCG00000013798	rpl29
ENSXMAG00000007576	ust	ENSLOCG00000016120	ust
ENSXMAG00000027634	lrrtm2	ENSLOCG00000018312	lrrtm2
ENSXMAG00000023299	slc41a1	ENSLOCG00000011890	slc41a1
ENSXMAG00000002530	rfx2	ENSLOCG00000000921	rfx2
ENSXMAG00000006228		ENSLOCG00000015715	
ENSXMAG00000014232		ENSLOCG00000006929	
ENSXMAG00000027093		ENSLOCG00000001327	
ENSXMAG00000011352	ptpn6	ENSLOCG00000007660	ptpn6
ENSXMAG00000010308	abcb9	ENSLOCG00000005562	abcb9
ENSXMAG00000000397	atf2	ENSLOCG00000007222	atf2
ENSXMAG00000015077	gkap1	ENSLOCG00000002195	gkap1

ENSXMAG00000010166	sncb	ENSLOCG00000011871	sncb
ENSXMAG00000019119	klhl14	ENSLOCG00000000466	klhl14
ENSXMAG00000007565	slc22a16	ENSLOCG00000017150	slc22a16
ENSXMAG00000001514	yipf5	ENSLOCG00000010498	yipf5
ENSXMAG00000002591	acad11	ENSLOCG00000011586	acad11
ENSXMAG00000018783	id3	ENSLOCG00000005395	id3
ENSXMAG00000023188		ENSLOCG00000017800	arl11
ENSXMAG00000021295	srcap	ENSLOCG00000003374	srcap
ENSXMAG00000010305	zgc:113436	ENSLOCG00000005585	zgc:113436
ENSXMAG00000003575	trmt1l	ENSLOCG00000001781	trmt1l
ENSXMAG00000023955	rab27a	ENSLOCG00000013450	rab27a
ENSXMAG00000026128	si:dkeyp-7a3.1	ENSLOCG00000000425	si:dkeyp-7a3.1
ENSXMAG00000030040	tirap	ENSLOCG00000002669	tirap
ENSXMAG00000007563	mettl24	ENSLOCG00000017151	METTL24
ENSXMAG00000010173		ENSLOCG00000011878	
ENSXMAG00000003142	TRPT1	ENSLOCG00000001345	TRPT1
ENSXMAG00000026686	UBQLN1	ENSLOCG00000002171	UBQLN1
ENSXMAG00000018661	qrsl1	ENSLOCG00000016582	qrsl1
ENSXMAG00000014257	PIGB	ENSLOCG00000013454	pigb
ENSXMAG00000013286	si:zfos-452g4.1	ENSLOCG00000005697	si:zfos-452g4.1
ENSXMAG00000023124	esrrd	ENSLOCG00000014553	esrrd
ENSXMAG00000021951	st3gal4	ENSLOCG00000002625	st3gal4
ENSXMAG00000019076	dtna	ENSLOCG00000006152	dtna
ENSXMAG00000010301	ogfod2	ENSLOCG00000005604	ogfod2
ENSXMAG00000021838	IDNK	ENSLOCG00000002152	IDNK
ENSXMAG00000017562	iars	ENSLOCG00000013871	iars
ENSXMAG00000001177	adamts17	ENSLOCG00000012754	adamts17
ENSXMAG00000002550	acsbg2	ENSLOCG00000000968	acsbg2
ENSXMAG00000014964	SHKBP1	ENSLOCG00000014556	SHKBP1
ENSXMAG00000007550	cdc40	ENSLOCG00000017152	cdc40
ENSXMAG00000003120	fermt3b	ENSLOCG00000001364	fermt3b
ENSXMAG00000001290	kcnj5	ENSLOCG00000002615	kcnj5
ENSXMAG00000002870	XPO5	ENSLOCG00000015733	XPO5
ENSXMAG00000013281		ENSLOCG00000005676	
ENSXMAG00000014854	stx4	ENSLOCG00000002833	stx4
ENSXMAG00000019068	mapre2	ENSLOCG00000006134	mapre2
ENSXMAG00000019505		ENSLOCG00000018313	
ENSXMAG00000020979		ENSLOCG00000010790	dytn
ENSXMAG00000014277		ENSLOCG00000013465	
ENSXMAG00000018758	ythdf2	ENSLOCG00000005362	ythdf2
ENSXMAG00000014426		ENSLOCG00000016949	casp8ap2
ENSXMAG00000018636	iars2	ENSLOCG00000017033	iars2
ENSXMAG00000025911	rnf44	ENSLOCG00000011884	rnf44
ENSXMAG00000013278	foxe3	ENSLOCG00000017748	foxe3
ENSXMAG00000015107	frmd3	ENSLOCG00000002132	frmd3
ENSXMAG00000011080	slc22a6l	ENSLOCG00000001481	
ENSXMAG00000005465	fam129aa	ENSLOCG00000001823	fam129aa
ENSXMAG00000011250	wnt4b	ENSLOCG00000007422	wnt4b
ENSXMAG00000001243	fli1a	ENSLOCG00000002563	fli1a
ENSXMAG00000019057		ENSLOCG00000006093	SPIDR
ENSXMAG00000027316		ENSLOCG00000017747	FOXD2
ENSXMAG00000003736	ZNF706	ENSLOCG00000009661	ZNF706
ENSXMAG00000002564	mllt1b	ENSLOCG00000001004	mllt1b
ENSXMAG00000021564	rnmt	ENSLOCG00000009540	rnmt
ENSXMAG00000025829	evx2	ENSLOCG00000007179	evx2
ENSXMAG00000022972	macrod1	ENSLOCG00000001373	MACROD1

ENSXMAG00000020621	MIR216A	ENSLOCG00000019062	MIR216A
ENSXMAG00000013243	iah1	ENSLOCG00000016826	iah1
ENSXMAG00000001526	ARHGAP26	ENSLOCG00000010520	ARHGAP26
ENSXMAG00000019437	cebpd	ENSLOCG00000017816	cebpd
ENSXMAG00000006208	si:dkey-76k16.5	ENSLOCG00000015708	si:dkey-76k16.5
ENSXMAG00000000425	hoxd12a	ENSLOCG00000007146	hoxd12a
ENSXMAG00000015037		ENSLOCG00000014720	
ENSXMAG00000001947	ankrd46b	ENSLOCG00000014469	ankrd46a
ENSXMAG00000023570	abrab	ENSLOCG00000003192	abrab
ENSXMAG00000028595	bach2b	ENSLOCG00000016952	bach2b
ENSXMAG00000010225	faf2	ENSLOCG00000011889	faf2
ENSXMAG00000000427	hoxd11a	ENSLOCG00000007133	hoxd11a
ENSXMAG00000029677	wasf1	ENSLOCG00000017153	wasf1
ENSXMAG00000002576	acer1	ENSLOCG00000001026	acer1
ENSXMAG00000022043	ets1	ENSLOCG00000002526	ets1
ENSXMAG00000002608	u2surp	ENSLOCG00000007828	u2surp
ENSXMAG00000002888		ENSLOCG00000015373	si:ch73-173h19.3
ENSXMAG00000013269	lpxn	ENSLOCG00000010161	lpxn
ENSXMAG00000028599	MGME1	ENSLOCG00000015691	mgme1
ENSXMAG00000000431	hoxd10a	ENSLOCG00000007118	hoxd10a
ENSXMAG00000019042	mcm4	ENSLOCG00000006026	mcm4
ENSXMAG00000024227	ncalda	ENSLOCG00000009644	ncalda
ENSXMAG00000015129	rasef	ENSLOCG00000002094	rasef
ENSXMAG00000019585	triap1	ENSLOCG00000004071	triap1
ENSXMAG00000001272	lins1	ENSLOCG00000012748	lins1
ENSXMAG00000028054	mettl11b	ENSLOCG00000001865	mettl11b
ENSXMAG00000004986	map3k7	ENSLOCG00000016953	map3k7
ENSXMAG00000011227	mlf2	ENSLOCG00000007392	mlf2
ENSXMAG00000013264	scyl3	ENSLOCG00000001482	scyl3
ENSXMAG00000018616	rab3gap2	ENSLOCG00000017036	
ENSXMAG00000001203	smtla	ENSLOCG00000002505	smtla
ENSXMAG00000010241		ENSLOCG00000014511	pin4
ENSXMAG00000004169	supt5h	ENSLOCG00000000237	
ENSXMAG00000006160	snx5	ENSLOCG00000015685	snx5
ENSXMAG00000005448	gorab	ENSLOCG00000001880	gorab
ENSXMAG00000000435	hoxd3a	ENSLOCG00000007043	hoxd3a
ENSXMAG00000027976	id4	ENSLOCG00000009639	id4
ENSXMAG00000007176	vwa8	ENSLOCG00000005831	vwa8
ENSXMAG00000002577	myo1f	ENSLOCG00000001068	myo1f
ENSXMAG00000013253	qkia	ENSLOCG00000004615	qkia
ENSXMAG00000001275	asb7	ENSLOCG00000012745	asb7
ENSXMAG00000005444	prrx1b	ENSLOCG00000001893	prrx1
ENSXMAG00000019038	ube2v2	ENSLOCG00000006012	ube2v2
ENSXMAG00000013251	si:dkey-97o5.1	ENSLOCG00000001436	si:dkey-97o5.1
ENSXMAG00000002590	cep76	ENSLOCG00000009631	cep76
ENSXMAG00000021057	RF01684	ENSLOCG00000019946	RF01684
ENSXMAG00000001303	aldh1a3	ENSLOCG00000012733	aldh1a3
ENSXMAG00000022728	hoxd4a	ENSLOCG00000007074	hoxd4a
ENSXMAG00000000958	KIRREL3	ENSLOCG00000002470	kirrel3a
ENSXMAG00000013259	ptafr	ENSLOCG00000018282	ptafr
ENSXMAG00000015157		ENSLOCG00000009924	TLE1
ENSXMAG00000000439	hnrnpa3	ENSLOCG00000006986	hnrnpa3
ENSXMAG00000019887	puraa	ENSLOCG00000018317	puraa
ENSXMAG00000003069		ENSLOCG00000002300	SCYL1
ENSXMAG00000022496	kcnk17	ENSLOCG00000017177	kcnk17
ENSXMAG00000019006	sntg1	ENSLOCG00000005945	sntg1

ENSXMAG00000002628	pcolce2b	ENSLOCG00000007866	pcolce2a
ENSXMAG00000029295	nfe2l2a	ENSLOCG00000006972	nfe2l2a
ENSXMAG00000013250	si:dkey-51e6.1	ENSLOCG00000001423	si:dkey-51e6.1
ENSXMAG00000022172	ufm1	ENSLOCG00000008568	ufm1
ENSXMAG00000018605	KCNK16	ENSLOCG00000017176	KCNK16
ENSXMAG00000011127	cxxc5a	ENSLOCG00000012610	cxxc5b
ENSXMAG00000007172	rgcc	ENSLOCG00000005886	rgcc
ENSXMAG00000006142	rrbp1a	ENSLOCG00000015682	rrbp1a
ENSXMAG00000029809	rbm24a	ENSLOCG00000006244	rbm24a
ENSXMAG00000026000	atn1	ENSLOCG00000007356	
ENSXMAG00000027899	etnk2	ENSLOCG00000011908	etnk2
ENSXMAG00000006766	znf207b	ENSLOCG00000013726	znf207a
ENSXMAG00000023477		ENSLOCG00000012483	
ENSXMAG00000018578	kif6	ENSLOCG00000017175	kif6
ENSXMAG00000002645	trpc1	ENSLOCG00000007889	trpc1
ENSXMAG00000007529	FIG4	ENSLOCG00000017155	FIG4
ENSXMAG00000002610	seh1l	ENSLOCG00000009595	seh1l
ENSXMAG00000021042	barx2	ENSLOCG00000002440	barx2
ENSXMAG00000017449	syt10	ENSLOCG00000015235	syt10
ENSXMAG00000000454	agps	ENSLOCG00000006956	agps
ENSXMAG00000014803	armc7	ENSLOCG00000010777	armc7
ENSXMAG00000028432	rnaset2l	ENSLOCG00000014694	rnaset2l
ENSXMAG00000029433	pcmtd1	ENSLOCG00000005913	pcmtd1
ENSXMAG00000000970	rbm7	ENSLOCG00000002408	rbm7
ENSXMAG00000020056	chst2b	ENSLOCG00000017407	chst2b
ENSXMAG00000025370	ppp3ca	ENSLOCG00000012574	ppp3ca
ENSXMAG00000003437		ENSLOCG00000008267	nup153
ENSXMAG00000002633	adamts10	ENSLOCG00000001103	adamts10
ENSXMAG00000002897	MARK1	ENSLOCG00000015568	MARK1
ENSXMAG00000006787	rhbdl3	ENSLOCG00000013739	rhbdl3
ENSXMAG00000002611	CEP192	ENSLOCG00000009581	si:rp71-15i12.1
ENSXMAG00000007139	f5	ENSLOCG00000001675	f5
ENSXMAG00000024431		ENSLOCG00000002369	
ENSXMAG00000029512	dia1b	ENSLOCG00000007771	dia1a
ENSXMAG00000010266	nkx2.5	ENSLOCG00000009163	nkx2.5
ENSXMAG00000008855	sox13	ENSLOCG00000011918	sox13
ENSXMAG00000018975	ST18	ENSLOCG00000005847	ST18
ENSXMAG00000018547	daam2	ENSLOCG00000017173	daam2
ENSXMAG00000014798	chad	ENSLOCG00000010806	chad
ENSXMAG00000021512	chp2	ENSLOCG00000014696	chp2
ENSXMAG00000015283		ENSLOCG00000012858	mex3b
ENSXMAG00000007583	tmem185	ENSLOCG00000014486	tmem185
ENSXMAG00000015287	tmed7	ENSLOCG00000008936	tmed7
ENSXMAG00000004762	nt5dc1	ENSLOCG00000016969	nt5dc1
ENSXMAG00000021044	atp6v0e1	ENSLOCG00000009182	ATP6V0E1
ENSXMAG00000017498	atp2b2	ENSLOCG00000013890	atp2b2
ENSXMAG00000023835	pde11a	ENSLOCG00000006915	pde11a
ENSXMAG00000014735	slc16a5a	ENSLOCG00000010765	slc16a5b
ENSXMAG00000026441		ENSLOCG00000017156	ak9
ENSXMAG00000005240	ganab	ENSLOCG00000001117	zgc:171967
ENSXMAG00000015297	fem1c	ENSLOCG00000008945	fem1c
ENSXMAG00000011151	dnajc18	ENSLOCG00000012551	dnajc18
ENSXMAG00000002656	zap70	ENSLOCG00000001129	zap70
ENSXMAG00000011186	lpcat3	ENSLOCG00000007684	lpcat3
ENSXMAG00000009123	plod2	ENSLOCG00000007750	plod2
ENSXMAG00000010281	rpl26	ENSLOCG00000009189	rpl26

ENSXMAG00000014722	kctd2	ENSLOCG00000010755	kctd2
ENSXMAG00000008850	snrpe	ENSLOCG00000011927	snrpe
ENSXMAG00000007508	si:dkey-119f1.1	ENSLOCG00000017236	
ENSXMAG00000001569	endou2	ENSLOCG00000010565	endou2
ENSXMAG00000005398	mast2	ENSLOCG00000005784	mast2
ENSXMAG00000002640	ldlrad4b	ENSLOCG00000009567	ldlrad4a
ENSXMAG00000009005	dse	ENSLOCG00000016968	dse
ENSXMAG00000018959	rb1cc1	ENSLOCG00000005780	rb1cc1
ENSXMAG00000003462	mterf3	ENSLOCG00000008210	mterf3
ENSXMAG00000025804	cdc42	ENSLOCG00000001073	cdc42
ENSXMAG00000014666	tubgcp2	ENSLOCG00000004354	tubgcp2
ENSXMAG00000015322	pggt1b	ENSLOCG00000008973	pggt1b
ENSXMAG00000007127	slc35a5	ENSLOCG00000001739	slc35a5
ENSXMAG00000000505	timm50	ENSLOCG00000014699	timm50
ENSXMAG00000011155	clstn3	ENSLOCG00000007696	clstn3
ENSXMAG00000029074	ppp2r2ca	ENSLOCG00000009212	ppp2r2bb
ENSXMAG00000022217	tefm	ENSLOCG00000013775	TEFM
ENSXMAG00000018544	mocs1	ENSLOCG00000017171	mocs1
ENSXMAG00000002664	mob3a	ENSLOCG00000001154	mob3a
ENSXMAG00000018550	gpr63	ENSLOCG00000016966	gpr63
ENSXMAG00000001439	chsy1	ENSLOCG00000014073	chsy1
ENSXMAG00000008829		ENSLOCG00000001126	wnt4a
ENSXMAG00000014307	col11a2	ENSLOCG00000000702	
ENSXMAG00000006867		ENSLOCG00000011963	
ENSXMAG00000003679	znf280d	ENSLOCG00000013531	znf280d
ENSXMAG00000018259		ENSLOCG00000001325	rxrgb
ENSXMAG00000021572	lrrc14b	ENSLOCG00000008169	lrrc14b
ENSXMAG00000026129	mknk2b	ENSLOCG00000001172	mknk2a
ENSXMAG00000007506	tmem18	ENSLOCG00000017233	tmem18
ENSXMAG00000013813	fam210ab	ENSLOCG00000009549	fam210aa
ENSXMAG00000001446	selenos	ENSLOCG00000014078	selenos
ENSXMAG00000000977	gramd1bb	ENSLOCG00000002340	gramd1bb
ENSXMAG00000007110	ccdc80	ENSLOCG00000001769	ccdc80l1
ENSXMAG00000025685	ufl1	ENSLOCG00000016960	ufl1
ENSXMAG00000001455	snrpa1	ENSLOCG00000014084	snrpa1
ENSXMAG00000010498	osbpl6	ENSLOCG00000006831	osbpl6
ENSXMAG00000025729	zcchc10	ENSLOCG00000010604	zcchc10
ENSXMAG00000017411	cyb5r3	ENSLOCG00000015246	cyb5r3
ENSXMAG00000010315	ADAM19	ENSLOCG00000009244	adam19b
ENSXMAG00000009159	si:ch73-206p6.1	ENSLOCG00000007741	si:ch73-206p6.1
ENSXMAG00000018514	atp6v1c2	ENSLOCG00000016662	atp6v1c2
ENSXMAG00000003473	uqcrb	ENSLOCG00000008199	uqcrb
ENSXMAG00000026536	gng8	ENSLOCG00000014913	gng8
ENSXMAG00000002681	rexo1	ENSLOCG00000001203	rexo1
ENSXMAG00000023014	chrac1	ENSLOCG00000007800	chrac1
ENSXMAG00000013231	atf6	ENSLOCG00000001278	atf6
ENSXMAG00000006903	zgc:158803	ENSLOCG00000011945	
ENSXMAG00000005620	tspan1	ENSLOCG00000005739	tspan1
ENSXMAG00000011175	tmem45b	ENSLOCG00000001184	tmem45b
ENSXMAG00000009162	zic4	ENSLOCG00000007717	zic4
ENSXMAG00000000560	zgc:101731	ENSLOCG00000014754	zgc:101731
ENSXMAG00000007740		ENSLOCG00000017231	
ENSXMAG00000002643	mc5rb	ENSLOCG00000018340	mc5ra
ENSXMAG00000015327	trim36	ENSLOCG00000008980	trim36
ENSXMAG00000008240	thg1l	ENSLOCG00000009255	thg1l
ENSXMAG00000007003	kdm6a	ENSLOCG00000001802	kdm6a

ENSXMAG00000018898	agap3	ENSLOCG00000009358	agap3
ENSXMAG00000001642	aff4	ENSLOCG00000010610	aff4
ENSXMAG00000014602	chuk	ENSLOCG00000004432	chuk
ENSXMAG00000003494	pip4p2	ENSLOCG00000010191	pip4p2
ENSXMAG00000013619	ARHGEF4	ENSLOCG00000009733	ARHGEF4
ENSXMAG00000029482	zic1	ENSLOCG00000007706	zic1
ENSXMAG00000005301	ehd1a	ENSLOCG00000000924	ehd1b
ENSXMAG00000011133	pex5	ENSLOCG00000007738	pex5
ENSXMAG00000008238	lsm11	ENSLOCG00000009264	lsm11
ENSXMAG00000002646	mc2r	ENSLOCG00000009532	mc2r
ENSXMAG00000020155	agtr1b	ENSLOCG00000017406	agtr1b
ENSXMAG00000005621	pomgnt1	ENSLOCG00000005707	pomgnt1
ENSXMAG00000018498	nol10	ENSLOCG00000016660	nol10
ENSXMAG00000009168	cpb1	ENSLOCG00000007688	cpb1
ENSXMAG00000011179	nfrkb	ENSLOCG00000001152	nfrkb
ENSXMAG00000029657	abcc1	ENSLOCG00000007196	abcc1
ENSXMAG00000027732	gbx1	ENSLOCG00000009350	gbx1
ENSXMAG00000027764	prkra	ENSLOCG00000006829	prkra
ENSXMAG00000007731	fbxo25	ENSLOCG00000017227	fbxo25
ENSXMAG00000004420	chchd7	ENSLOCG00000005393	chchd7
ENSXMAG00000018883	asb10	ENSLOCG00000009338	asb10
ENSXMAG00000002711	si:ch73-61d6.3	ENSLOCG00000002860	si:ch73-61d6.3
ENSXMAG00000003504	necab1	ENSLOCG00000010199	necab1
ENSXMAG00000024389		ENSLOCG00000000057	
ENSXMAG00000009195	bdh1	ENSLOCG00000007627	bdh1
ENSXMAG00000013267	grhl1	ENSLOCG00000016835	grhl1
ENSXMAG00000017832	plag1	ENSLOCG00000005402	plag1
ENSXMAG00000009817	ttc9c	ENSLOCG00000000958	ttc9c
ENSXMAG00000015341	ccser1	ENSLOCG00000012882	ccser1
ENSXMAG00000006963	efhc2	ENSLOCG00000001949	efhc2
ENSXMAG00000029896	nck1b	ENSLOCG00000007610	nck1b
ENSXMAG00000018490	odc1	ENSLOCG00000016658	odc1
ENSXMAG00000002380	ushbp1	ENSLOCG00000002908	ushbp1
ENSXMAG00000002659	zgc:77056	ENSLOCG00000002203	zgc:77056
ENSXMAG00000000978	steap2	ENSLOCG00000010448	steap2
ENSXMAG00000020156	slc35g2b	ENSLOCG00000017405	slc35g2a
ENSXMAG00000010882	tollip	ENSLOCG00000006828	tollip
ENSXMAG00000014352	cgnl1	ENSLOCG00000013547	cgnl1
ENSXMAG00000024891	ndp	ENSLOCG00000001980	ndp
ENSXMAG00000028008	tmem64	ENSLOCG00000010212	tmem64
ENSXMAG00000010949		ENSLOCG00000011401	dnaaf2
ENSXMAG00000001683	shroom1	ENSLOCG00000010639	
ENSXMAG00000025837	grid2	ENSLOCG00000012893	grid2
ENSXMAG00000015221	zgc:162698	ENSLOCG00000014761	zgc:162698
ENSXMAG00000018482		ENSLOCG00000017131	
ENSXMAG00000000985	mos	ENSLOCG00000017815	mos
ENSXMAG00000010950	zgc:77287	ENSLOCG00000011393	zgc:77287
ENSXMAG00000010538	FAM237A	ENSLOCG00000010804	FAM237A
ENSXMAG00000026575		ENSLOCG00000001052	frrs1b
ENSXMAG00000007486	cmtr1	ENSLOCG00000017052	cmtr1
ENSXMAG00000011516	spg21	ENSLOCG00000014105	spg21
ENSXMAG00000002383	babam1	ENSLOCG00000002941	babam1
ENSXMAG00000009212		ENSLOCG00000009870	
ENSXMAG00000003478	ALG14	ENSLOCG00000008176	alg14
ENSXMAG00000006915	mao	ENSLOCG00000001995	mao
ENSXMAG00000017478	slc6a11b	ENSLOCG00000013904	slc6a11b

ENSXMAG00000002959	ywhaqb	ENSLOCG00000016833	ywhaqb
ENSXMAG00000003529	acot13	ENSLOCG00000004572	acot13
ENSXMAG00000017081	cnn3a	ENSLOCG00000008165	cnn3a
ENSXMAG00000011323	sms	ENSLOCG00000003761	sms
ENSXMAG00000021952	pdcd7	ENSLOCG00000014112	PDCD7
ENSXMAG00000014372	myzap	ENSLOCG00000013563	myzap
ENSXMAG00000028624	igf1	ENSLOCG00000015180	igf1
ENSXMAG00000024240	si:ch211-137a8.4	ENSLOCG00000017722	
ENSXMAG00000009790		ENSLOCG00000014107	SLC24A4
ENSXMAG00000018465	wdr35	ENSLOCG00000017192	wdr35
ENSXMAG00000002392	gtpbp3	ENSLOCG00000003052	gtpbp3
ENSXMAG00000010539	ADAM23	ENSLOCG00000010807	adam23a
ENSXMAG00000011089	p3h3	ENSLOCG00000007785	p3h3
ENSXMAG00000013222		ENSLOCG00000009533	GHRHR
ENSXMAG00000028162	rasl12	ENSLOCG00000014117	rasl12
ENSXMAG00000013005	clip3	ENSLOCG00000014773	clip3
ENSXMAG00000024777	zdhhc24	ENSLOCG00000001541	zdhhc24
ENSXMAG00000017346	parpbp	ENSLOCG00000015177	parpbp
ENSXMAG00000010375	sh3tc2	ENSLOCG00000009362	sh3tc2
ENSXMAG00000001709	ccni2	ENSLOCG00000010667	ccni2
ENSXMAG00000009764	pola2	ENSLOCG00000002801	
ENSXMAG00000000343	zgc:194678	ENSLOCG00000005584	zgc:194678
ENSXMAG00000015349	atoh1a	ENSLOCG00000017552	atoh1a
ENSXMAG00000028442	aldh1a2	ENSLOCG00000013574	aldh1a2
ENSXMAG00000013221		ENSLOCG00000009009	
ENSXMAG00000003531	zgc:111986	ENSLOCG00000004584	zgc:111986
ENSXMAG00000018810	cdk5	ENSLOCG00000009292	cdk5
ENSXMAG00000001008	dact3a	ENSLOCG00000014520	dact3a
ENSXMAG00000015350	smarcad1a	ENSLOCG00000012909	smarcad1a
ENSXMAG00000007484	hebp2	ENSLOCG00000015783	hebp2
ENSXMAG00000001483	ccnd1	ENSLOCG00000005067	ccnd1
ENSXMAG00000020263	C5AR1	ENSLOCG00000017566	
ENSXMAG00000006953	rnaseh2a	ENSLOCG00000007890	rnaseh2a
ENSXMAG00000017326	nup37	ENSLOCG00000015176	nup37
ENSXMAG00000015201	abcd3b	ENSLOCG00000008096	abcd3a
ENSXMAG00000011056	usp5	ENSLOCG00000007807	usp5
ENSXMAG00000003536	gmnn	ENSLOCG00000004598	gmnn
ENSXMAG00000013276	rpap1	ENSLOCG00000014706	rpap1
ENSXMAG00000029743	HRH1	ENSLOCG00000013921	HRH1
ENSXMAG00000006347	abcb8	ENSLOCG00000009275	abcb8
ENSXMAG00000018463	kcns3a	ENSLOCG00000017189	kcns3a
ENSXMAG00000022903	lto1	ENSLOCG00000005078	lto1
ENSXMAG00000001711	GABRG2	ENSLOCG00000010690	gabrg2
ENSXMAG00000021278	polr2i	ENSLOCG00000014818	polr2i
ENSXMAG00000013217		ENSLOCG00000011119	
ENSXMAG00000026947	fosl2	ENSLOCG00000017169	fosl2
ENSXMAG00000001504	fgf3	ENSLOCG00000005127	fgf3
ENSXMAG00000007482	nhsl1b	ENSLOCG00000015784	nhsl1b
ENSXMAG00000029057	pglyrp5	ENSLOCG00000014823	pglyrp5
ENSXMAG00000015389	mpeg1.2	ENSLOCG00000007854	mpeg1.2
ENSXMAG00000017429	atg7	ENSLOCG00000013924	atg7
ENSXMAG00000003015	rgra	ENSLOCG00000005641	rgra
ENSXMAG00000003539	ripor2	ENSLOCG00000004646	ripor2
ENSXMAG00000005722	atg9b	ENSLOCG00000009263	atg9b
ENSXMAG00000001018	snrpd2	ENSLOCG00000014824	snrpd2
ENSXMAG00000001523	fgf19	ENSLOCG00000005100	fgf19

ENSXMAG00000027417	tspan36	ENSLOCG00000012836	tspan36
ENSXMAG00000012611	gabra1	ENSLOCG00000010698	gabra1
ENSXMAG00000018440	babam2	ENSLOCG00000017167	babam2
ENSXMAG00000014563	nploc4	ENSLOCG00000013007	nploc4
ENSXMAG00000024925		ENSLOCG00000010462	
ENSXMAG00000010137	zmp:0000001073	ENSLOCG00000014588	zmp:0000001073
ENSXMAG00000013205	faah	ENSLOCG00000004038	faah
ENSXMAG00000008573	zgc:158659	ENSLOCG00000013570	zgc:158659
ENSXMAG00000023198	mapk4	ENSLOCG00000012850	MAPK4
ENSXMAG00000002905	eif1b	ENSLOCG00000013648	eif1b
ENSXMAG00000001529	zgc:153993	ENSLOCG00000005142	zgc:153993
ENSXMAG00000007475	acbd3	ENSLOCG00000015790	acbd3
ENSXMAG00000007602	mfsd11	ENSLOCG00000011241	mfsd11
ENSXMAG00000008631	supt20	ENSLOCG00000008519	supt20
ENSXMAG00000007016	atg4da	ENSLOCG00000007842	atg4da
ENSXMAG00000013305	ltk	ENSLOCG00000014716	ltk
ENSXMAG00000015408	me2	ENSLOCG00000012857	me2
ENSXMAG00000013206		ENSLOCG00000000830	
ENSXMAG00000027922	gpr1	ENSLOCG00000017843	gpr1
ENSXMAG00000018428	rbks	ENSLOCG00000017165	rbks
ENSXMAG00000017271	pah	ENSLOCG00000015172	pah
ENSXMAG00000025361	ubald2	ENSLOCG00000011227	ubald2
ENSXMAG00000009742		ENSLOCG00000013636	SLC16A2
ENSXMAG00000028932		ENSLOCG00000007155	si:ch73-86n18.1
ENSXMAG00000021546	myd88	ENSLOCG00000009811	myd88
ENSXMAG00000007451	lin9	ENSLOCG00000015793	lin9
ENSXMAG00000000190	kank2	ENSLOCG00000007602	kank2
ENSXMAG00000014435	sst1.1	ENSLOCG00000009439	sst1.1
ENSXMAG00000013185	mknk1	ENSLOCG00000003998	mknk1
ENSXMAG00000002758	fam198a	ENSLOCG00000001055	fam198a
ENSXMAG00000015453	elac1	ENSLOCG00000012870	elac1
ENSXMAG00000003559	si:dkeyp-120h9.1	ENSLOCG00000004664	si:dkeyp-120h9.1
ENSXMAG00000002421	PTPRC	ENSLOCG00000007650	PTPRC
ENSXMAG00000011042	cops7a	ENSLOCG00000007876	cops7a
ENSXMAG00000026855		ENSLOCG00000009140	
ENSXMAG00000009285	cry-dash	ENSLOCG00000009796	cry-dash
ENSXMAG00000017407	tamm41	ENSLOCG00000013931	tamm41
ENSXMAG00000002768	pomgnt2	ENSLOCG00000018322	pomgnt2
ENSXMAG00000014399	ece2b	ENSLOCG00000008680	ece2b
ENSXMAG00000028876	mier3b	ENSLOCG00000007562	mier3b
ENSXMAG00000018425	si:dkey-16j16.4	ENSLOCG00000017163	si:dkey-16j16.4
ENSXMAG00000017228	itfg2	ENSLOCG00000016560	itfg2
ENSXMAG00000028563		ENSLOCG00000007900	
ENSXMAG00000000119	amdhd2	ENSLOCG00000008993	amdhd2
ENSXMAG00000008621	exosc8	ENSLOCG00000008503	exosc8
ENSXMAG00000000198	dock6	ENSLOCG00000007613	dock6
ENSXMAG00000013182	mob3c	ENSLOCG00000003969	mob3c
ENSXMAG00000002760	snrka	ENSLOCG00000001112	snrka
ENSXMAG00000026636	itpka	ENSLOCG00000014738	itpka
ENSXMAG00000003632	parp1	ENSLOCG00000015798	parp1
ENSXMAG00000011041	si:ch211-154o6.3	ENSLOCG00000007920	si:ch211-154o6.3
ENSXMAG00000010606	ino80da	ENSLOCG00000010852	ino80db
ENSXMAG00000025434	nrip2	ENSLOCG00000016559	nrip2
ENSXMAG00000018415	slc4a1ap	ENSLOCG00000017161	slc4a1ap
ENSXMAG00000013313	ivd	ENSLOCG00000014741	ivd
ENSXMAG00000005776	zgc:63882	ENSLOCG00000009188	zgc:63882

ENSXMAG00000003606	klhl42	ENSLOCG00000017034	klhl42
ENSXMAG00000009286	map3k20	ENSLOCG00000009780	
ENSXMAG00000015508	plk2b	ENSLOCG00000007594	plk2b
ENSXMAG00000002734	cubn	ENSLOCG00000010501	cubn
ENSXMAG00000028119		ENSLOCG00000017328	
ENSXMAG00000029207		ENSLOCG00000009043	
ENSXMAG00000026895	sh3pxd2b	ENSLOCG00000010766	sh3pxd2b
ENSXMAG00000008607	alg5	ENSLOCG00000008488	alg5
ENSXMAG00000003598	zbtb10	ENSLOCG00000002796	zbtb10
ENSXMAG00000003572		ENSLOCG00000016704	si:dkey-153k10.9
ENSXMAG00000019547		ENSLOCG00000017990	
ENSXMAG00000005789	wnt3a	ENSLOCG00000009208	wnt3a
ENSXMAG00000015537	si:dkey-190g11.3	ENSLOCG00000007623	si:dkey-190g11.3
ENSXMAG00000028986	timp4.3	ENSLOCG00000013951	timp4.2
ENSXMAG00000000120	zgc:113223	ENSLOCG00000000559	zgc:113223
ENSXMAG00000000803	fadd	ENSLOCG00000005182	fadd
ENSXMAG00000006826	nyx	ENSLOCG00000002113	nyx
ENSXMAG00000001668	ano10a	ENSLOCG00000001145	ano10a
ENSXMAG00000018287	elavl3	ENSLOCG00000007647	elavl3
ENSXMAG00000005793	wnt9a	ENSLOCG00000009215	wnt9a
ENSXMAG00000025287	nek7	ENSLOCG00000007624	nek7
ENSXMAG00000009722	zgc:152774	ENSLOCG00000014959	zgc:152774
ENSXMAG00000023623	tpd52	ENSLOCG00000002803	tpd52
ENSXMAG00000018409	supt7l	ENSLOCG00000017160	supt7l
ENSXMAG00000022499	rab3c	ENSLOCG00000007639	rab3c
ENSXMAG00000003056	cuedc2	ENSLOCG00000005475	cuedc2
ENSXMAG00000005805	snap47	ENSLOCG00000009233	snap47
ENSXMAG00000013324	ccdc32	ENSLOCG00000014751	ccdc32
ENSXMAG00000001093	slc37a4a	ENSLOCG00000003300	SLC37A4
ENSXMAG00000018292	prkcsh	ENSLOCG00000007655	prkcsh
ENSXMAG00000017330	PPARG	ENSLOCG00000013954	PPARG
ENSXMAG00000028952	jmjd4	ENSLOCG00000009240	jmjd4
ENSXMAG00000003607	znf704	ENSLOCG00000002837	znf704
ENSXMAG00000013327	pcmtl	ENSLOCG00000014753	pcmtl
ENSXMAG00000010643		ENSLOCG00000003216	DOCK9
ENSXMAG00000025348		ENSLOCG00000002703	
ENSXMAG00000001123	trappc4	ENSLOCG00000003274	trappc4
ENSXMAG00000008595		ENSLOCG00000008478	
ENSXMAG00000014333	eif4h	ENSLOCG00000000875	eif4h
ENSXMAG00000008475	zgc:55262	ENSLOCG00000001490	zgc:55262
ENSXMAG00000022237	lhx9	ENSLOCG00000007595	lhx9
ENSXMAG00000003064	hif1an	ENSLOCG00000005437	hif1an
ENSXMAG00000018405	LRFN2	ENSLOCG00000016804	lrfn2b
ENSXMAG00000029078	iba57	ENSLOCG00000009395	iba57
ENSXMAG00000015455	AMDHD1	ENSLOCG00000015737	AMDHD1
ENSXMAG00000007151		ENSLOCG00000007782	ap1m2
ENSXMAG00000015552	pde4d	ENSLOCG00000007665	pde4d
ENSXMAG00000029603	mtch2	ENSLOCG00000006180	mtch2
ENSXMAG00000001136	rps25	ENSLOCG00000003259	rps25
ENSXMAG00000012460	stk10	ENSLOCG00000010779	stk10
ENSXMAG00000027744	pag1	ENSLOCG00000002856	pag1
ENSXMAG00000000409	NUP35	ENSLOCG00000006726	nup35
ENSXMAG00000005815	cx47.1	ENSLOCG00000018128	cx47.1
ENSXMAG00000001141	sgcg	ENSLOCG00000001705	sgcg
ENSXMAG00000026577	si:dkey-121a9.3	ENSLOCG00000007585	si:dkey-121a9.3
ENSXMAG00000021821	rpusd2	ENSLOCG00000014755	rpusd2

ENSXMAG00000018401	spdya	ENSLOCG00000000572	
ENSXMAG00000018764	rffl	ENSLOCG00000000911	zgc:171740
ENSXMAG00000024348	mrpl53	ENSLOCG00000002871	mrpl53
ENSXMAG00000029072	rfxap	ENSLOCG00000008468	rfxap
ENSXMAG00000013163	ptgfr	ENSLOCG00000003835	ptgfr
ENSXMAG00000001622		ENSLOCG00000004094	
ENSXMAG00000015448	snrpf	ENSLOCG00000015744	snrpf
ENSXMAG00000010994	ddr1	ENSLOCG00000000077	
ENSXMAG00000005253	dnajc10	ENSLOCG00000006697	dnajc10
ENSXMAG00000001161	sacs	ENSLOCG00000001682	sacs
ENSXMAG00000017329		ENSLOCG00000013378	TERF2IP
ENSXMAG00000028481	wnt8b	ENSLOCG00000005351	wnt8b
ENSXMAG00000005817	HIGD1A	ENSLOCG00000009418	HIGD1A
ENSXMAG00000022149	STK32A	ENSLOCG00000010793	STK32A
ENSXMAG00000000217	raver1	ENSLOCG00000007500	raver1
ENSXMAG00000026849		ENSLOCG00000007689	
ENSXMAG00000013340	disp2	ENSLOCG00000014758	disp2
ENSXMAG00000002440	dennd1b	ENSLOCG00000007556	dennd1b
ENSXMAG00000001169	tnfrsf19	ENSLOCG00000001649	tnfrsf19
ENSXMAG00000014318	wdr62	ENSLOCG00000014830	
ENSXMAG00000007179	si:zfos-323e3.4	ENSLOCG00000007742	si:zfos-323e3.4
ENSXMAG00000013143	slc44a5b	ENSLOCG00000003464	slc44a5a
ENSXMAG00000005821	ccdc13	ENSLOCG00000009427	ccdc13
ENSXMAG00000017316	iffo2b	ENSLOCG00000004638	
ENSXMAG00000015573	elovl7a	ENSLOCG00000007727	elovl7a
ENSXMAG00000021448	keap1b	ENSLOCG00000007721	keap1b
ENSXMAG00000017746	pkdccb	ENSLOCG00000014760	pkdccb
ENSXMAG00000022296	mid2	ENSLOCG00000013934	mid2
ENSXMAG00000015583	ercc8	ENSLOCG00000007753	ercc8
ENSXMAG00000001178	MIPEP	ENSLOCG00000001633	si:ch73-1a9.4
ENSXMAG00000005826	tmie	ENSLOCG00000009462	tmie
ENSXMAG00000000831		ENSLOCG00000014101	plekho2
ENSXMAG00000000220	tyk2	ENSLOCG00000007532	tyk2
ENSXMAG00000005511	fdx2	ENSLOCG00000007698	fdx2
ENSXMAG00000006740	dusp19a	ENSLOCG00000006612	dusp19a
ENSXMAG00000013055		ENSLOCG00000009441	
ENSXMAG00000001596	taf2	ENSLOCG00000009156	taf2
ENSXMAG00000015598	ndufaf2	ENSLOCG00000007769	ndufaf2
ENSXMAG00000005830	pth1ra	ENSLOCG00000009481	pth1ra
ENSXMAG00000000834		ENSLOCG00000008213	RWDD3
ENSXMAG00000014305	atg16l2	ENSLOCG00000002627	
ENSXMAG00000020994		ENSLOCG00000007686	
ENSXMAG00000006701	pde1a	ENSLOCG00000006602	pde1a
ENSXMAG00000008578	ccdc169	ENSLOCG00000008425	
ENSXMAG00000017270	megf6b	ENSLOCG00000004327	megf6b
ENSXMAG00000003615	atxn1a	ENSLOCG00000002910	atxn1a
ENSXMAG00000008449	pacs1a	ENSLOCG00000001515	pacs1a
ENSXMAG00000010887	rdh1	ENSLOCG00000008152	rdh1
ENSXMAG00000026478		ENSLOCG00000001608	BRWD1
ENSXMAG00000029406		ENSLOCG00000012902	BNC1
ENSXMAG00000020104	tysnd1	ENSLOCG00000005229	tysnd1
ENSXMAG00000009140		ENSLOCG00000016799	
ENSXMAG00000015410	usp44	ENSLOCG00000015748	usp44
ENSXMAG00000003617	gmpr	ENSLOCG00000002927	gmpr
ENSXMAG00000022724	myl13	ENSLOCG00000009496	myl13
ENSXMAG00000027376		ENSLOCG00000007669	

ENSXMAG00000014246	arrb1	ENSLOCG00000002578	arrb1
ENSXMAG00000003648	EFR3B	ENSLOCG00000016682	EFR3B
ENSXMAG00000013136	erich3	ENSLOCG00000003403	
ENSXMAG00000013846	HDGFL3	ENSLOCG00000012906	HDGFL3
ENSXMAG00000021184	sh3bgr	ENSLOCG00000001571	sh3bgr
ENSXMAG00000005843		ENSLOCG00000009503	
ENSXMAG00000013047		ENSLOCG00000003609	map7d2b
ENSXMAG00000003628	mylipa	ENSLOCG00000008309	mylipb
ENSXMAG00000013348	eml1	ENSLOCG00000014764	eml1
ENSXMAG00000023518	PCP4	ENSLOCG00000001548	PCP4
ENSXMAG00000013847	rbb4l	ENSLOCG00000007412	rbb4l
ENSXMAG00000015616	pgm5	ENSLOCG00000009373	pgm5
ENSXMAG00000024707	si:ch211-220e11.3	ENSLOCG00000013918	si:ch211-220e11.3
ENSXMAG00000015392	metap2b	ENSLOCG00000015749	METAP2
ENSXMAG00000009152		ENSLOCG00000016797	
ENSXMAG00000002672		ENSLOCG00000001503	
ENSXMAG00000009353	vill	ENSLOCG00000009718	vill
ENSXMAG00000022215	aqp1a.1	ENSLOCG00000009523	aqp1a.1
ENSXMAG00000013855		ENSLOCG00000007397	txlng
ENSXMAG00000008564	ccna1	ENSLOCG00000008456	ccna1
ENSXMAG00000026890	foxd5	ENSLOCG00000017537	foxd5
ENSXMAG00000021286	jarid2b	ENSLOCG00000008335	jarid2a
ENSXMAG00000016834	dscamb	ENSLOCG00000001471	dscamb
ENSXMAG00000003157	eif4ebp2	ENSLOCG00000005123	eif4ebp2
ENSXMAG00000013115	tnni3k	ENSLOCG00000003355	tnni3k
ENSXMAG00000002353	thoc1	ENSLOCG00000002097	thoc1
ENSXMAG00000008904	vezt	ENSLOCG00000015752	vezt
ENSXMAG00000015627	cbwd	ENSLOCG00000009357	cbwd
ENSXMAG00000013856	ccdc102a	ENSLOCG00000005943	ccdc102a
ENSXMAG00000003162	rps24	ENSLOCG00000004926	rps24
ENSXMAG00000008438	zgc:175248	ENSLOCG00000001570	zgc:175248
ENSXMAG00000003652	pard6gb	ENSLOCG00000008371	pard6gb
ENSXMAG00000009402	gars	ENSLOCG00000009688	gars
ENSXMAG00000013013	prdx4	ENSLOCG00000003488	prdx4
ENSXMAG00000002794	rsu1	ENSLOCG00000010490	rsu1
ENSXMAG00000023042	dok4	ENSLOCG00000005930	dok4
ENSXMAG00000022276	akr7a3	ENSLOCG00000004453	akr7a3
ENSXMAG00000027557	SERTM1	ENSLOCG00000018041	SERTM1
ENSXMAG00000019951	s1pr2	ENSLOCG00000018294	s1pr2
ENSXMAG00000008908	fgd6	ENSLOCG00000015755	fgd6
ENSXMAG00000003653	bloc1s4	ENSLOCG00000008379	bloc1s4
ENSXMAG00000022550	nadsyn1	ENSLOCG00000005029	nadsyn1
ENSXMAG00000030030	RF01229	ENSLOCG00000020652	RF01229
ENSXMAG00000018063	dnmt1	ENSLOCG00000007421	dnmt1
ENSXMAG00000026364	pcdh11	ENSLOCG00000013908	pcdh11
ENSXMAG00000002807	trdmt1	ENSLOCG00000010560	trdmt1
ENSXMAG00000012994		ENSLOCG00000003347	zfx
ENSXMAG00000026959	usp14	ENSLOCG00000002049	usp14
ENSXMAG00000008510	nbeaa	ENSLOCG00000008354	nbeaa
ENSXMAG00000016905	bace2	ENSLOCG00000001455	bace2
ENSXMAG00000007349	RUBCNL	ENSLOCG00000010487	rubcnl
ENSXMAG00000008394	actn3b	ENSLOCG00000001588	actn3a
ENSXMAG00000003661	rrm2b	ENSLOCG00000009872	rrm2b
ENSXMAG00000010476	tmsb2	ENSLOCG00000009797	tmsb2
ENSXMAG00000003682	PTPN14	ENSLOCG00000016705	PTPN14
ENSXMAG00000009226	sobpa	ENSLOCG00000016593	sobpa

ENSXMAG00000013860	tpcn2	ENSLOCG00000005051	tpcn2
ENSXMAG00000000067	hccsb	ENSLOCG00000008198	hccsb
ENSXMAG00000016925	atm	ENSLOCG00000001332	atm
ENSXMAG00000009652	klhl4	ENSLOCG00000013889	klhl4
ENSXMAG00000003683	mtdha	ENSLOCG00000009864	mtdha
ENSXMAG00000027328	lrch1	ENSLOCG00000010453	lrch1
ENSXMAG00000023195	G6PC2	ENSLOCG00000008214	G6PC2
ENSXMAG00000022672	pdss2	ENSLOCG00000016588	pdss2
ENSXMAG00000008936	ERI1	ENSLOCG00000012161	eri1
ENSXMAG00000015659	dock8	ENSLOCG00000009324	dock8
ENSXMAG00000018061	eif3g	ENSLOCG00000007404	eif3g
ENSXMAG00000021087	ssbp3b	ENSLOCG00000003285	ssbp3b
ENSXMAG00000002398	rock1	ENSLOCG00000001989	rock1
ENSXMAG00000011823	nostrin	ENSLOCG00000008240	nostrin
ENSXMAG00000013046	pgm2l1	ENSLOCG00000002326	pgm2l1
ENSXMAG00000029879	si:ch211-206k20.5	ENSLOCG00000016585	si:ch211-206k20.5
ENSXMAG00000023687	park7	ENSLOCG00000003827	park7
ENSXMAG00000001228		ENSLOCG00000011260	mettl23
ENSXMAG00000022203	AHNAK	ENSLOCG00000001394	
ENSXMAG00000009242	bend3	ENSLOCG00000016586	bend3
ENSXMAG00000013862	pop4	ENSLOCG00000005597	pop4
ENSXMAG00000009244	tmem151bb	ENSLOCG00000016400	tmem151bb
ENSXMAG00000027107	jmjd6	ENSLOCG00000011268	jmjd6
ENSXMAG00000011241	snx7	ENSLOCG00000008275	snx7
ENSXMAG00000028708	ppan	ENSLOCG00000007381	ppan
ENSXMAG00000011853	spc25	ENSLOCG00000008228	spc25
ENSXMAG00000022466		ENSLOCG00000001312	
ENSXMAG00000010963	ino80e	ENSLOCG00000011107	ino80e
ENSXMAG00000013042	lipt2	ENSLOCG00000002316	lipt2
ENSXMAG00000012978	arhgap31	ENSLOCG00000002771	arhgap31
ENSXMAG00000013081	dio1	ENSLOCG00000003136	dio1
ENSXMAG00000009245	utp25	ENSLOCG00000017182	utp25
ENSXMAG00000013865	arnt2	ENSLOCG00000012783	arnt2
ENSXMAG00000029230	degs2	ENSLOCG00000011633	degs2
ENSXMAG00000025369	C9orf3	ENSLOCG00000009303	C9orf3
ENSXMAG00000010070	eef1g	ENSLOCG00000014527	
ENSXMAG00000011800	cers6	ENSLOCG00000008257	cers6
ENSXMAG00000009279	esd	ENSLOCG00000010443	esd
ENSXMAG00000003687	rpl30	ENSLOCG00000009836	rpl30
ENSXMAG00000012973	b4galt4	ENSLOCG00000002862	b4galt4
ENSXMAG00000014237	spcs2	ENSLOCG00000002208	spcs2
ENSXMAG00000001743	fah	ENSLOCG00000012774	fah
ENSXMAG00000016973	tubd1	ENSLOCG00000003847	tubd1
ENSXMAG00000018050		ENSLOCG00000007369	
ENSXMAG00000011767	stk39	ENSLOCG00000008262	stk39
ENSXMAG00000010505	lonrf1	ENSLOCG00000009823	lonrf1
ENSXMAG00000020454	MIR27B	ENSLOCG00000018576	MIR27B
ENSXMAG00000003715	rps6kc1	ENSLOCG00000016691	rps6kc1
ENSXMAG00000022592	six7	ENSLOCG00000001646	six7
ENSXMAG00000008962	CALD1	ENSLOCG00000000883	cald1b
ENSXMAG00000023507		ENSLOCG00000017292	
ENSXMAG00000014199	abcg1	ENSLOCG00000002133	abcg1
ENSXMAG00000010927	vars	ENSLOCG00000004240	vars
ENSXMAG00000018441	zgc:92818	ENSLOCG00000005445	zgc:92818
ENSXMAG00000017375	PLD6	ENSLOCG00000008138	PLD6
ENSXMAG00000000052	mrps16	ENSLOCG00000003927	mrps16

ENSXMAG00000015706	fancc	ENSLOCG00000009272	fancc
ENSXMAG00000025534	plppr5b	ENSLOCG00000008289	plppr5a
ENSXMAG00000014705	b4gat1	ENSLOCG00000001655	b4gat1
ENSXMAG00000018792	kpnb1	ENSLOCG00000013753	kpnb1
ENSXMAG00000024411	adprhl2	ENSLOCG00000000800	adprhl2
ENSXMAG00000009479	nktr	ENSLOCG00000009632	nktr
ENSXMAG00000013417	tpp1	ENSLOCG00000011342	
ENSXMAG00000012971	casr	ENSLOCG00000002893	casr
ENSXMAG00000019537	lrrn3b	ENSLOCG00000017906	lrrn3b
ENSXMAG00000003722	moxd1l	ENSLOCG00000016792	moxd1l
ENSXMAG00000018438		ENSLOCG00000005504	vps35
ENSXMAG00000009303	htr2aa	ENSLOCG00000010438	htr2aa
ENSXMAG00000024605	nit1	ENSLOCG00000001670	nit1
ENSXMAG00000018681	tas1r3	ENSLOCG00000003982	tas1r3
ENSXMAG00000015711	PTCH1	ENSLOCG00000009249	ptch1
ENSXMAG00000023479	stk3	ENSLOCG00000009795	stk3
ENSXMAG00000028959	rfc3	ENSLOCG00000008321	rfc3
ENSXMAG00000013438	zgc:174193	ENSLOCG00000002104	zgc:174193
ENSXMAG00000017234	ccdc114	ENSLOCG00000004006	ccdc114
ENSXMAG00000013011	itk	ENSLOCG00000009833	itk
ENSXMAG00000008974	dock4b	ENSLOCG00000015916	dock4b
ENSXMAG00000009320	nrde2	ENSLOCG00000009037	nrde2
ENSXMAG00000008503	kl	ENSLOCG00000008273	kl
ENSXMAG00000021764		ENSLOCG00000008208	
ENSXMAG00000001910	clcn3	ENSLOCG00000013089	clcn3
ENSXMAG00000005874	greb1l	ENSLOCG00000001913	greb1l
ENSXMAG00000013448	hif1aa	ENSLOCG00000010146	hif1aa
ENSXMAG00000003732	snx9b	ENSLOCG00000016687	snx9a
ENSXMAG00000001359	nod1	ENSLOCG00000001910	nod1
ENSXMAG00000018884	btr30	ENSLOCG00000003109	btr30
ENSXMAG00000023695	ercc6l2	ENSLOCG00000009238	ercc6l2
ENSXMAG00000003975	MTFMT	ENSLOCG00000014123	MTFMT
ENSXMAG00000017212	slc45a1	ENSLOCG00000004082	slc45a1
ENSXMAG00000014126	rnpepl1	ENSLOCG00000008399	rnpepl1
ENSXMAG00000013067		ENSLOCG00000002889	MUTYH
ENSXMAG00000017004	slc33a1	ENSLOCG00000003159	slc33a1
ENSXMAG00000008480	pds5b	ENSLOCG00000008252	pds5b
ENSXMAG00000008721	sucla2	ENSLOCG00000010416	sucla2
ENSXMAG00000015794	hsd17b3	ENSLOCG00000009228	hsd17b3
ENSXMAG00000029339	CELF1	ENSLOCG00000005966	celf1
ENSXMAG00000004446	cyfip2	ENSLOCG00000009856	cyfip2
ENSXMAG00000014077	abcc5	ENSLOCG00000008431	abcc5
ENSXMAG00000018902		ENSLOCG00000011061	UTP18
ENSXMAG00000010061	si:ch211-175m2.5	ENSLOCG00000012491	
ENSXMAG00000001909	mfap3l	ENSLOCG00000012768	mfap3l
ENSXMAG00000009043	ifrd1	ENSLOCG00000015930	ifrd1
ENSXMAG00000017018	gmps	ENSLOCG00000003174	gmps
ENSXMAG00000025270	prkcha	ENSLOCG00000010128	prkcha
ENSXMAG00000013061	mpnd	ENSLOCG00000006613	mpnd
ENSXMAG00000007090	daxx	ENSLOCG00000000498	
ENSXMAG00000008718	med4	ENSLOCG00000000694	MED4
ENSXMAG00000001887	aadat	ENSLOCG00000012761	aadat
ENSXMAG00000027492	znrf2b	ENSLOCG00000001894	znrf2b
ENSXMAG00000028582	CSRNP3	ENSLOCG00000008295	CSRNP3
ENSXMAG00000008708	ITM2B	ENSLOCG00000010398	itm2bb
ENSXMAG00000017197	fam213b	ENSLOCG00000004121	fam213b

ENSXMAG00000014075	rpl35a	ENSLOCG00000008453	rpl35a
ENSXMAG00000015801	ipo11	ENSLOCG00000007848	ipo11
ENSXMAG00000028919		ENSLOCG00000010570	fzd1
ENSXMAG00000017414	utp6	ENSLOCG00000013797	utp6
ENSXMAG00000023158		ENSLOCG00000013506	
ENSXMAG00000011705	GALNT3	ENSLOCG00000008304	GALNT3
ENSXMAG00000023525	ino80b	ENSLOCG00000012755	ino80b
ENSXMAG00000008702		ENSLOCG00000010393	NEK5
ENSXMAG00000010512	hmmr	ENSLOCG00000009883	hmmr
ENSXMAG00000008476	zar1l	ENSLOCG00000008190	zar1l
ENSXMAG00000017051	v2rl1	ENSLOCG00000003230	v2rl1
ENSXMAG00000005350	psmg2	ENSLOCG00000009625	psmg2
ENSXMAG00000014055	lrch3	ENSLOCG00000008472	lrch3
ENSXMAG00000009504	sec22c	ENSLOCG00000009619	sec22c
ENSXMAG00000028253	cemip	ENSLOCG00000012817	cemip
ENSXMAG00000005917	abhd3	ENSLOCG00000001874	abhd3
ENSXMAG00000013474	trmt5	ENSLOCG00000010120	trmt5
ENSXMAG00000001874	intu	ENSLOCG00000012742	intu
ENSXMAG00000019539	gpr85	ENSLOCG00000017907	gpr85
ENSXMAG00000017153	mmel1	ENSLOCG00000004155	mmel1
ENSXMAG00000001386	cdk14	ENSLOCG00000010548	cdk14
ENSXMAG00000011678	ttc21b	ENSLOCG00000008327	ttc21b
ENSXMAG00000018961	med15	ENSLOCG00000006469	med15
ENSXMAG00000009513	vipr1b	ENSLOCG00000009610	vipr1b
ENSXMAG00000003720		ENSLOCG00000008362	zgc:136493
ENSXMAG00000013476	mnat1	ENSLOCG00000010103	mnat1
ENSXMAG00000005931	mib1	ENSLOCG00000001783	mib1
ENSXMAG00000018980		ENSLOCG00000000135	thoc6
ENSXMAG00000015846	RNF180	ENSLOCG00000007893	
ENSXMAG00000012990	zgc:77486	ENSLOCG00000006705	zgc:77486
ENSXMAG00000008673	lcp1	ENSLOCG00000010513	lcp1
ENSXMAG00000010832	rabac1	ENSLOCG00000004589	rabac1
ENSXMAG00000029750	foxp2	ENSLOCG00000015936	foxp2
ENSXMAG00000017150	miip	ENSLOCG00000004256	miip
ENSXMAG00000021396	KCNAB1	ENSLOCG00000003246	KCNAB1
ENSXMAG00000014038	abcf3	ENSLOCG00000013815	abcf3
ENSXMAG00000001869		ENSLOCG00000012735	trmt10b
ENSXMAG00000024204	si:dkey-121j17.5	ENSLOCG00000000687	si:dkey-121j17.5
ENSXMAG00000026312	six1b	ENSLOCG00000010086	six1b
ENSXMAG00000010827	dedd1	ENSLOCG00000004607	dedd1
ENSXMAG00000009552	si:ch211-161h7.5	ENSLOCG00000011881	si:dkey-29d8.3
ENSXMAG00000025521	six6a	ENSLOCG00000010080	six6b
ENSXMAG00000008671	cnot11	ENSLOCG00000010579	cnot11
ENSXMAG00000017144		ENSLOCG00000004286	tnfrsf1b
ENSXMAG00000018999	stx1b	ENSLOCG00000002864	
ENSXMAG00000017141	ssr3	ENSLOCG00000003280	ssr3
ENSXMAG00000002362	notch3	ENSLOCG00000007129	notch3
ENSXMAG00000009371	ppp2r3c	ENSLOCG00000009180	ppp2r3c
ENSXMAG00000029243	MDFIC	ENSLOCG00000015940	MDFIC
ENSXMAG00000015880	plcxd3	ENSLOCG00000007985	plcxd3
ENSXMAG00000029286		ENSLOCG00000008331	
ENSXMAG00000003774		ENSLOCG00000000740	
ENSXMAG00000013482	ppm1aa	ENSLOCG00000010070	ppm1aa
ENSXMAG00000008427	rxfp2a	ENSLOCG00000008133	rxfp2a
ENSXMAG00000022705	TIPARP	ENSLOCG00000003293	TIPARP
ENSXMAG00000025406	gata6	ENSLOCG00000001775	gata6

ENSXMAG00000017479		ENSLOCG00000012093	
ENSXMAG00000002936		ENSLOCG00000010541	
ENSXMAG00000024123	trim62	ENSLOCG00000001163	trim62
ENSXMAG00000009383	si:dkeyp-33b5.4	ENSLOCG00000009172	si:dkeyp-33b5.4
ENSXMAG00000027948	kpna1	ENSLOCG00000003272	kpna1
ENSXMAG00000012969	rgl1	ENSLOCG00000010305	rgl1
ENSXMAG00000025856		ENSLOCG00000002996	
ENSXMAG00000013493	dhrs7	ENSLOCG00000010060	dhrs7
ENSXMAG00000015885	oxct1a	ENSLOCG00000007994	oxct1a
ENSXMAG00000014037		ENSLOCG00000009112	si:ch1073-184j22.2
ENSXMAG00000010526	TENM2	ENSLOCG00000010864	si:ch211-12m10.1
ENSXMAG00000002410	zmp:0000001048	ENSLOCG00000007096	zmp:0000001048
ENSXMAG00000021244	cables1	ENSLOCG00000001715	cables1
ENSXMAG00000025483	ccnl1a	ENSLOCG00000003314	ccnl1a
ENSXMAG00000002934	si:ch211-208k15.1	ENSLOCG00000004538	si:ch211-208k15.1
ENSXMAG00000003786	fdft1	ENSLOCG00000000791	fdft1
ENSXMAG00000014031	polr2d	ENSLOCG00000009106	polr2d
ENSXMAG00000023033	tfec	ENSLOCG00000015941	tfec
ENSXMAG00000013505	pcnx4	ENSLOCG00000010048	pcnx4
ENSXMAG00000014027	mmp23bb	ENSLOCG00000008353	
ENSXMAG00000009574	fam162a	ENSLOCG00000003258	fam162a
ENSXMAG00000008627	ENOX1	ENSLOCG00000005664	enox1
ENSXMAG00000015907	si:dkey-46a10.3	ENSLOCG00000008022	si:dkey-46a10.3
ENSXMAG00000017171	veph1	ENSLOCG00000003346	veph1
ENSXMAG00000009238	gata4	ENSLOCG00000000817	gata4
ENSXMAG00000015910	fbxo4	ENSLOCG00000008043	fbxo4
ENSXMAG00000022563	ccdc58	ENSLOCG00000003236	ccdc58
ENSXMAG00000028726		ENSLOCG00000001290	
ENSXMAG00000008622	LACC1	ENSLOCG00000005637	lacc1
ENSXMAG00000016078	mesd	ENSLOCG00000012824	mesd
ENSXMAG00000016426	npc1	ENSLOCG00000001532	npc1
ENSXMAG00000008413	alox5ap	ENSLOCG00000000235	alox5ap
ENSXMAG00000009411	ralgapa1	ENSLOCG00000009103	ralgapa1
ENSXMAG00000014008		ENSLOCG00000001831	eif2a
ENSXMAG00000008611	lmln	ENSLOCG00000002877	lmln
ENSXMAG00000015937		ENSLOCG00000008094	SELENOP
ENSXMAG00000019479	tlnrd1	ENSLOCG00000012828	tlnrd1
ENSXMAG00000017185	ptx3b	ENSLOCG00000003357	ptx3b
ENSXMAG00000008419		ENSLOCG00000000255	
ENSXMAG00000002420	nr5a5	ENSLOCG00000007039	nr5a5
ENSXMAG00000013518	LRRC9	ENSLOCG00000010036	LRRC9
ENSXMAG00000012963	colgalt2	ENSLOCG00000010295	si:ch211-13f8.2
ENSXMAG00000023680		ENSLOCG00000012837	il16
ENSXMAG00000024036	si:dkey-69o16.5	ENSLOCG00000010697	si:dkey-69o16.5
ENSXMAG00000011612	slc38a11	ENSLOCG00000008368	slc38a11
ENSXMAG00000008411	uspl1	ENSLOCG00000000198	USPL1
ENSXMAG00000017189		ENSLOCG00000003367	
ENSXMAG00000012940	kif20a	ENSLOCG00000009613	
ENSXMAG00000006301	stard5	ENSLOCG00000012843	stard5
ENSXMAG00000015940	znf131	ENSLOCG00000008117	znf131
ENSXMAG00000008521	itgav	ENSLOCG00000006805	itgav
ENSXMAG00000024266	tsen34	ENSLOCG00000001887	tsen34
ENSXMAG00000017071	utp3	ENSLOCG00000001449	
ENSXMAG00000003769	LIX1L	ENSLOCG00000009089	LIX1L
ENSXMAG00000011610	COBLL1	ENSLOCG00000008378	COBLL1
ENSXMAG00000016482	egfra	ENSLOCG00000011537	

ENSXMAG00000012953	edem3	ENSLOCG00000010265	edem3
ENSXMAG00000012791	adgrb1a	ENSLOCG00000004554	adgrb1a
ENSXMAG00000003772	sf3b4	ENSLOCG00000009048	sf3b4
ENSXMAG00000014003	mindy4b	ENSLOCG00000001918	mindy4b
ENSXMAG00000000954	eif3d	ENSLOCG00000011396	
ENSXMAG00000000356	thop1	ENSLOCG00000000074	thop1
ENSXMAG00000028922	cdc23	ENSLOCG00000009602	cdc23
ENSXMAG00000017194		ENSLOCG00000003382	
ENSXMAG00000008399	ubl3a	ENSLOCG00000000124	UBL3
ENSXMAG00000009216		ENSLOCG00000001060	
ENSXMAG00000017196	serpini1	ENSLOCG00000003396	serpini1
ENSXMAG00000013999	clrn1	ENSLOCG00000001934	clrn1
ENSXMAG00000026200	avpr2l	ENSLOCG00000001076	
ENSXMAG00000006287	si:dkey-15b23.3	ENSLOCG00000012848	si:dkey-15b23.3
ENSXMAG00000022039	si:ch211-243a20.3	ENSLOCG00000011980	
ENSXMAG00000025672	ppcs	ENSLOCG00000001524	ppcs
ENSXMAG00000009585	arfgef1	ENSLOCG00000004389	arfgef1
ENSXMAG00000015948	nim1k	ENSLOCG00000008131	nim1k
ENSXMAG00000001858	tex261	ENSLOCG00000001643	tex261
ENSXMAG00000000777	masp1	ENSLOCG00000009432	masp1
ENSXMAG00000011574	GRB14	ENSLOCG00000008406	GRB14
ENSXMAG00000018283	rtn1b	ENSLOCG00000010017	rtn1b
ENSXMAG00000009501		ENSLOCG00000009207	mia2
ENSXMAG00000015950	creb3l3l	ENSLOCG00000008143	creb3l3l
ENSXMAG00000012932	npl	ENSLOCG00000010258	npl
ENSXMAG00000017223		ENSLOCG00000003462	
ENSXMAG00000028352	atf7ip	ENSLOCG00000011986	atf7ip
ENSXMAG00000000779		ENSLOCG00000009023	
ENSXMAG00000015952	gadd45ga	ENSLOCG00000008173	GADD45G (1 of many)
ENSXMAG00000001850	vax2	ENSLOCG00000001602	vax2
ENSXMAG00000012908	zbtb20	ENSLOCG00000002726	zbtb20
ENSXMAG00000030022	prdm2b	ENSLOCG00000001614	prdm2b
ENSXMAG00000000797	DGKD	ENSLOCG00000009226	DGKD
ENSXMAG00000018300	tdrd9	ENSLOCG00000009992	tdrd9
ENSXMAG00000009532	gemin2	ENSLOCG00000009221	gemin2
ENSXMAG00000012925	notch2	ENSLOCG00000010216	notch2
ENSXMAG00000017559	3-Sep	ENSLOCG00000011996	3-Sep
ENSXMAG00000011566	KCNH7	ENSLOCG00000008418	KCNH7
ENSXMAG00000009547	pnn	ENSLOCG00000009234	pnn
ENSXMAG00000023705	efl1	ENSLOCG00000012862	efl1
ENSXMAG00000003802	vps72a	ENSLOCG00000007832	vps72a
ENSXMAG00000003836	daam1b	ENSLOCG00000009284	daam1a
ENSXMAG00000000013	grap2a	ENSLOCG00000011347	grap2b
ENSXMAG00000007417		ENSLOCG00000009985	RD3L
ENSXMAG00000029844	eif4ebp3l	ENSLOCG00000012500	eif4ebp3
ENSXMAG00000011536	ifih1	ENSLOCG00000008460	ifih1
ENSXMAG00000010706	patj	ENSLOCG00000009943	patj
ENSXMAG00000013525	kif15	ENSLOCG00000002182	kif15
ENSXMAG00000001845	gba2	ENSLOCG00000009428	gba2
ENSXMAG00000024352		ENSLOCG00000004516	cspp1a
ENSXMAG00000025539		ENSLOCG00000003493	
ENSXMAG00000006825	acsl4a	ENSLOCG00000014514	acsl4a
ENSXMAG00000010368	ptpn21	ENSLOCG00000008432	ptpn21
ENSXMAG00000026462	bmp8a	ENSLOCG00000001276	bmp8a
ENSXMAG00000012913	slc35a3a	ENSLOCG00000010209	slc35a3a
ENSXMAG00000015964	aaed1	ENSLOCG00000009173	aaed1

ENSXMAG00000000768		ENSLOCG00000010843	
ENSXMAG00000012882	dlg4a	ENSLOCG00000013673	dlg4a
ENSXMAG00000019627	slitrk3a	ENSLOCG00000017384	slitrk3a
ENSXMAG00000010344	fam98b	ENSLOCG00000012257	fam98b
ENSXMAG00000009626	cops5	ENSLOCG00000004575	cops5
ENSXMAG00000000829	atg16l1	ENSLOCG00000009283	atg16l1
ENSXMAG00000024002	rex1bd	ENSLOCG00000005666	
ENSXMAG00000022277	nxt2	ENSLOCG00000014519	nxt2
ENSXMAG00000024522	spred1	ENSLOCG00000012250	spred1
ENSXMAG00000009649	ppp1r42	ENSLOCG00000004595	ppp1r42
ENSXMAG00000022708	tm2d1	ENSLOCG00000009936	tm2d1
ENSXMAG00000006808	psmd10	ENSLOCG00000014526	psmd10
ENSXMAG00000001832	rgp1	ENSLOCG00000009443	rgp1
ENSXMAG00000012900	tmem59l	ENSLOCG00000000452	tmem59l
ENSXMAG00000020937	chic2	ENSLOCG00000014142	chic2
ENSXMAG00000011482	fap	ENSLOCG00000008483	fap
ENSXMAG00000024821	TCF24	ENSLOCG00000004611	TCF24
ENSXMAG00000029759	cep162	ENSLOCG00000016570	cep162
ENSXMAG00000006799	xiap	ENSLOCG00000014562	xiap
ENSXMAG00000015639		ENSLOCG00000017682	
ENSXMAG00000017620		ENSLOCG00000014127	fip1l1b
ENSXMAG00000001580	MCMDC2	ENSLOCG00000004629	MCMDC2
ENSXMAG00000009557	meis2a	ENSLOCG00000012229	meis2a
ENSXMAG00000014228		ENSLOCG00000010256	cdk15
ENSXMAG00000016452		ENSLOCG00000001182	MACF1
ENSXMAG00000000348	nfia	ENSLOCG00000009919	nfia
ENSXMAG00000012879	fkbp8	ENSLOCG00000005546	fkbp8
ENSXMAG00000000749	tmem220	ENSLOCG00000012113	tmem220
ENSXMAG00000000745	SCO1	ENSLOCG00000012104	SCO1
ENSXMAG00000001600	sgk3	ENSLOCG00000004656	sgk3
ENSXMAG00000001808	pik3c3	ENSLOCG00000009397	pik3c3
ENSXMAG00000012869	ell	ENSLOCG00000005568	ell
ENSXMAG00000003859	l3hypdh	ENSLOCG00000009274	l3hypdh
ENSXMAG00000013514		ENSLOCG00000012091	
ENSXMAG00000009579	zgc:154061	ENSLOCG00000012223	zgc:154061
ENSXMAG00000006723	stag2b	ENSLOCG00000014565	stag2b
ENSXMAG00000003864	kcnh5a	ENSLOCG00000010194	kcnh5a
ENSXMAG00000000863		ENSLOCG00000009786	TM4SF19-TCTEX1D2
ENSXMAG00000015986	HABP4	ENSLOCG00000009195	zgc:103482
ENSXMAG00000012854	dot1l	ENSLOCG00000005809	dot1l
ENSXMAG00000023404		ENSLOCG00000006785	scn2b
ENSXMAG00000016617	stam	ENSLOCG00000010930	stam
ENSXMAG00000001658	MYBL1	ENSLOCG00000004709	mybl1
ENSXMAG00000016974	mon1bb	ENSLOCG00000014503	mon1bb
ENSXMAG00000015989	znf367	ENSLOCG00000009209	znf367
ENSXMAG00000009590	dph6	ENSLOCG00000012210	dph6
ENSXMAG00000013988	frem1b	ENSLOCG00000009896	frem1b
ENSXMAG00000027872	slc35d2	ENSLOCG00000009217	slc35d2
ENSXMAG00000016972	tcta	ENSLOCG00000014440	tcta
ENSXMAG00000014504	cyb5r4	ENSLOCG00000016563	CYB5R4
ENSXMAG00000014141	sos1	ENSLOCG00000015763	sos1
ENSXMAG00000010745	mecom	ENSLOCG00000009557	mecom
ENSXMAG00000009596	aqr	ENSLOCG00000012184	aqr
ENSXMAG00000020158	crhb	ENSLOCG00000017813	crha
ENSXMAG00000003885	abcd4	ENSLOCG00000010882	abcd4
ENSXMAG00000001774	faah2b	ENSLOCG00000009502	faah2a

ENSXMAG00000022359	si:dkey-245p14.4	ENSLOCG00000014034	si:dkey-245p14.4
ENSXMAG00000021443	lzts3b	ENSLOCG00000001268	lzts3b
ENSXMAG00000025526	ndufs5	ENSLOCG00000001164	ndufs5
ENSXMAG00000029367	anapc2	ENSLOCG00000000748	anapc2
ENSXMAG00000001766	nelfa	ENSLOCG00000002190	nelfa
ENSXMAG00000009670	pde7a	ENSLOCG00000004815	pde7a
ENSXMAG00000026540	hemk1	ENSLOCG00000014444	hemk1
ENSXMAG00000004544	smc2	ENSLOCG00000013338	smc2
ENSXMAG00000009617	ACTC1	ENSLOCG00000012178	ACTC1
ENSXMAG00000028371	efnb1	ENSLOCG00000014708	efnb1
ENSXMAG00000011427	tbr1b	ENSLOCG00000008583	tbr1b
ENSXMAG00000016438	rnf19b	ENSLOCG00000001101	rnf19b
ENSXMAG00000010755	zgc:158328	ENSLOCG00000009575	zgc:158328
ENSXMAG00000029801	ACSS1	ENSLOCG00000010909	
ENSXMAG00000021989	GJD2	ENSLOCG00000012174	gjd2b
ENSXMAG00000001765	si:dkeyp-75h12.5	ENSLOCG00000002204	si:dkeyp-75h12.5
ENSXMAG00000009622	STXBP6	ENSLOCG00000012166	stxbp6
ENSXMAG00000021412	ak2	ENSLOCG00000001124	ak2
ENSXMAG00000010794	rab3b	ENSLOCG00000009849	rab3b
ENSXMAG00000027861		ENSLOCG00000013691	KCNAB3
ENSXMAG00000001761	nat8l	ENSLOCG00000002217	nat8l
ENSXMAG00000013146	arl6ip1	ENSLOCG00000007049	arl6ip1
ENSXMAG00000009687	mtfr1	ENSLOCG00000004859	mtfr1
ENSXMAG00000025754	tank	ENSLOCG00000008612	tank
ENSXMAG00000016082	fabp1a	ENSLOCG00000001202	fabp1a
ENSXMAG00000012844	amh	ENSLOCG00000005854	
ENSXMAG00000016917	nprl2	ENSLOCG00000014239	nprl2
ENSXMAG00000001752	POLN	ENSLOCG00000002235	poln
ENSXMAG00000010778	dbt	ENSLOCG00000000299	dbt
ENSXMAG00000014191	gemin6	ENSLOCG00000015777	gemin6
ENSXMAG00000026276		ENSLOCG00000013324	ecpas
ENSXMAG00000025113	batf	ENSLOCG00000010533	batf
ENSXMAG00000020159	bhlhe22	ENSLOCG00000017814	bhlhe22
ENSXMAG00000026322	tmem178	ENSLOCG00000015750	tmem178
ENSXMAG00000012775	neu3.1	ENSLOCG00000002179	neu3.1
ENSXMAG00000009635	fermt2	ENSLOCG00000012153	fermt2
ENSXMAG00000016095	znf366	ENSLOCG00000011104	ZNF366
ENSXMAG00000000071	ascc3	ENSLOCG00000016550	ascc3
ENSXMAG00000014132	soul4	ENSLOCG00000015879	soul4
ENSXMAG00000023645	kdm4c	ENSLOCG00000013308	
ENSXMAG00000001748	haus3	ENSLOCG00000002277	haus3
ENSXMAG00000016098	mrps27	ENSLOCG00000011134	MRPS27
ENSXMAG00000010785		ENSLOCG00000006656	DNAAF1
ENSXMAG00000019878	or108-3	ENSLOCG00000010866	or108-3
ENSXMAG00000016903	ZMYND10	ENSLOCG00000014237	zmynd10
ENSXMAG00000014111	eif4a3	ENSLOCG00000015796	eif4a3
ENSXMAG00000009698	ythdf3	ENSLOCG00000004932	ythdf3
ENSXMAG00000010787	mrpl44	ENSLOCG00000009636	mrpl44
ENSXMAG00000029614		ENSLOCG00000012194	
ENSXMAG00000010769	lrrc39	ENSLOCG00000000384	lrrc39
ENSXMAG00000021167	xrra1	ENSLOCG00000002223	xrra1
ENSXMAG00000010381	smyd4	ENSLOCG00000006387	SMYD4
ENSXMAG00000010765	trmt13	ENSLOCG00000000418	trmt13
ENSXMAG00000009705	tagln3b	ENSLOCG00000004461	tagln3b
ENSXMAG00000010801	slc19a3a	ENSLOCG00000009677	slc19a3a
ENSXMAG00000010770	cnfn	ENSLOCG00000003611	CNFN

ENSXMAG00000026130	zc4h2	ENSLOCG00000014627	zc4h2
ENSXMAG00000009711	tagln3a	ENSLOCG00000004445	tagln3a
ENSXMAG00000021000	slc19a3b	ENSLOCG00000008297	slc19a3b
ENSXMAG00000020167	CXorf40B	ENSLOCG00000014485	CXorf40A
ENSXMAG00000009725	c1qtnf9	ENSLOCG00000004497	c1qtnf9
ENSXMAG00000029664		ENSLOCG00000002265	chrdl2
ENSXMAG00000010806	daw1	ENSLOCG00000008284	daw1
ENSXMAG00000009680	ddhd1a	ENSLOCG00000012137	ddhd1a
ENSXMAG00000024624	spag7	ENSLOCG00000013728	spag7
ENSXMAG00000013137	pigq	ENSLOCG00000006867	pigq
ENSXMAG00000009729	spata13	ENSLOCG00000004477	SPATA13
ENSXMAG00000004464		ENSLOCG00000007487	panx1a
ENSXMAG00000010545	lrmda	ENSLOCG00000004734	lrmda
ENSXMAG00000010321	smg6	ENSLOCG00000006448	smg6
ENSXMAG00000003988	ylpm1	ENSLOCG00000010710	ylpm1
ENSXMAG00000013132	eef2kmt	ENSLOCG00000006854	eef2kmt
ENSXMAG00000004471	dcun1d5	ENSLOCG00000006719	dcun1d5
ENSXMAG00000024991		ENSLOCG00000004426	TMPRSS7
ENSXMAG00000013119	alg1	ENSLOCG00000006836	alg1
ENSXMAG00000026961		ENSLOCG00000004407	
ENSXMAG00000010815	sphkap	ENSLOCG00000008263	sphkap
ENSXMAG00000023440	si:ch211-76l23.7	ENSLOCG00000006823	si:ch211-76l23.7
ENSXMAG00000011383	tanc1b	ENSLOCG00000005973	tanc1b
ENSXMAG00000006663	arhgef9b	ENSLOCG00000014608	arhgef9b
ENSXMAG00000012841	oaz1a	ENSLOCG00000005874	oaz1a
ENSXMAG00000000766	bmp4	ENSLOCG00000012132	bmp4
ENSXMAG00000027456	mepce	ENSLOCG00000013586	MEPCE
ENSXMAG00000021418		ENSLOCG00000017627	
ENSXMAG00000001729	svep1	ENSLOCG00000013366	svep1
ENSXMAG00000010559	si:dkey-27m7.4	ENSLOCG00000004642	
ENSXMAG00000010288	taok1b	ENSLOCG00000006508	taok1b
ENSXMAG00000006811	megf8	ENSLOCG00000004499	
ENSXMAG00000003481	atxn2	ENSLOCG00000007880	atxn2
ENSXMAG00000030019	org	ENSLOCG00000005893	org
ENSXMAG00000013083	flr	ENSLOCG00000006800	flr
ENSXMAG00000010740	med16	ENSLOCG00000001808	med16
ENSXMAG00000020845	lclat1	ENSLOCG00000006486	lclat1
ENSXMAG00000009774	flt1	ENSLOCG00000004379	flt1
ENSXMAG00000010569	vdac2	ENSLOCG00000004602	vdac2
ENSXMAG00000004537	clns1a	ENSLOCG00000006519	clns1a
ENSXMAG00000012751	PAK3	ENSLOCG00000013833	PAK3
ENSXMAG00000015559		ENSLOCG00000003316	trps1
ENSXMAG00000025153	si:ch211-76l23.4	ENSLOCG00000006791	si:ch211-76l23.4
ENSXMAG00000021164	pid1	ENSLOCG00000008242	pid1
ENSXMAG00000012771	atp8b3	ENSLOCG00000005917	
ENSXMAG00000009359	zcrb1	ENSLOCG00000001409	zcrb1
ENSXMAG00000001695	slc7a8a	ENSLOCG00000001344	
ENSXMAG00000011819	tmem259	ENSLOCG00000001828	tmem259
ENSXMAG00000021937		ENSLOCG00000006528	ABHD15
ENSXMAG00000010820	dner	ENSLOCG00000008223	dner
ENSXMAG00000024662	erfl1	ENSLOCG00000004449	
ENSXMAG00000013072		ENSLOCG00000001229	si:dkey-27p23.3
ENSXMAG00000017328	hace1	ENSLOCG00000016540	hace1
ENSXMAG00000010580	polh	ENSLOCG00000015724	polh
ENSXMAG00000004548	pak1	ENSLOCG00000006555	pak1
ENSXMAG00000010830	trip12	ENSLOCG00000008188	trip12

ENSXMAG00000009709	manea	ENSLOCG00000016959	manea
ENSXMAG00000012769	onecut3a	ENSLOCG00000005935	onecut3a
ENSXMAG00000016170	ostf1	ENSLOCG00000009781	ostf1
ENSXMAG00000016391	sfpq	ENSLOCG00000001067	sfpq
ENSXMAG00000004017	fosab	ENSLOCG00000010507	fosab
ENSXMAG00000021529	foxq2	ENSLOCG00000005947	foxq2
ENSXMAG00000009821	pan3	ENSLOCG00000004336	pan3
ENSXMAG00000025228	wdr18	ENSLOCG00000001858	wdr18
ENSXMAG00000027668	tmed10	ENSLOCG00000010496	tmed10
ENSXMAG00000021264	pias4a	ENSLOCG00000005962	pias4a
ENSXMAG00000016201		ENSLOCG00000015140	
ENSXMAG00000004027	eif2b2	ENSLOCG00000010473	eif2b2
ENSXMAG00000016371	lypla2	ENSLOCG00000001047	lypla2
ENSXMAG00000011363	PKP4	ENSLOCG00000005927	PKP4
ENSXMAG00000028311	OTOR	ENSLOCG00000015995	OTOR
ENSXMAG00000006776	arhgef1b	ENSLOCG00000004462	arhgef1b
ENSXMAG00000026956	ZBTB38	ENSLOCG00000017408	ZBTB38
ENSXMAG00000015532	rplp1	ENSLOCG00000007729	rplp1
ENSXMAG00000021375	map2k2a	ENSLOCG00000005968	map2k2a
ENSXMAG00000016239		ENSLOCG00000009973	stx6
ENSXMAG00000021700	BVES	ENSLOCG00000016536	bves
ENSXMAG00000027176	pithd1	ENSLOCG00000001023	pithd1
ENSXMAG00000010855	RASA2	ENSLOCG00000008121	RASA2
ENSXMAG00000010620	pcsk2	ENSLOCG00000015998	pcsk2
ENSXMAG00000000710	fhl5	ENSLOCG00000016962	fhl5
ENSXMAG00000011925		ENSLOCG00000014581	tenm1
ENSXMAG00000016203	si:ch1073-392o20.1	ENSLOCG00000015139	si:ch1073-392o20.1
ENSXMAG00000028540	dhrs4	ENSLOCG00000000499	dhrs4
ENSXMAG00000016354	eloa	ENSLOCG00000000997	eloa
ENSXMAG00000012740	zbtb7a	ENSLOCG00000005993	zbtb7a
ENSXMAG00000028704	BFSP1	ENSLOCG00000016001	BFSP1
ENSXMAG00000012887	urad	ENSLOCG00000004296	urad
ENSXMAG00000004041	zc3h14	ENSLOCG00000010387	zc3h14
ENSXMAG00000012986	crlf3	ENSLOCG00000013787	crlf3
ENSXMAG00000025974	popdc3	ENSLOCG00000016535	popdc3
ENSXMAG00000021062	bag1	ENSLOCG00000011163	bag1
ENSXMAG00000016340	klhl43	ENSLOCG00000000972	klhl43
ENSXMAG00000019562	pdx1	ENSLOCG00000004260	pdx1
ENSXMAG00000010640	plek	ENSLOCG00000016011	plek
ENSXMAG00000010897	si:dkey-283j8.1	ENSLOCG00000008084	si:dkey-283j8.1
ENSXMAG00000012886	si:ch211-140b10.6	ENSLOCG00000004237	
ENSXMAG00000012721		ENSLOCG00000013852	
ENSXMAG00000009376	pus7l	ENSLOCG00000001587	pus7l
ENSXMAG00000012718	tcf3b	ENSLOCG00000006013	tcf3b
ENSXMAG00000020971	sh2b3	ENSLOCG00000007870	sh2b3
ENSXMAG00000016325	rpl11	ENSLOCG00000000953	rpl11
ENSXMAG00000026480	rnf7	ENSLOCG00000008065	rnf7
ENSXMAG00000016227	si:ch211-193c2.2	ENSLOCG00000018157	si:ch211-193c2.2
ENSXMAG00000027031		ENSLOCG00000013859	
ENSXMAG00000021985		ENSLOCG00000012839	arhgap23b
ENSXMAG00000009830	lnx2a	ENSLOCG00000004220	lnx2a
ENSXMAG00000016322		ENSLOCG00000000926	
ENSXMAG00000022156	prr16	ENSLOCG00000008796	prr16
ENSXMAG00000024027		ENSLOCG00000007931	aldh2.2
ENSXMAG00000021915	atp6v1h	ENSLOCG00000005751	atp6v1h
ENSXMAG00000012141	gtf2e2	ENSLOCG00000013191	gtf2e2

ENSXMAG00000012711	il13ra2	ENSLOCG00000013632	il13ra2
ENSXMAG00000016234	HSD17B4	ENSLOCG00000008806	hsd17b4
ENSXMAG00000004681	sb:cb81	ENSLOCG00000014587	sb:cb81
ENSXMAG00000018818		ENSLOCG00000011191	tsen54
ENSXMAG00000016289	EEF1A2	ENSLOCG00000000907	
ENSXMAG00000029464	nsrp1	ENSLOCG00000006654	NSRP1
ENSXMAG00000017101	fzd9b	ENSLOCG00000017869	fzd9b
ENSXMAG00000006774	cd79a	ENSLOCG00000004482	cd79a
ENSXMAG00000004683		ENSLOCG00000014692	
ENSXMAG00000012688	lrch2	ENSLOCG00000013621	lrch2
ENSXMAG00000009388	ccdc146	ENSLOCG00000015993	ccdc146
ENSXMAG00000011360		ENSLOCG00000005892	CCDC148
ENSXMAG00000012827	rbm42	ENSLOCG00000003368	rbm42
ENSXMAG00000024769	rpl21	ENSLOCG00000004171	rpl21
ENSXMAG00000012177	hgsnat	ENSLOCG00000013178	hgsnat
ENSXMAG00000026327	tmem104	ENSLOCG00000011150	tmem104
ENSXMAG00000021430		ENSLOCG00000006065	mbd3b
ENSXMAG00000004703	rps3	ENSLOCG00000006405	rps3
ENSXMAG00000010907	baz1b	ENSLOCG00000000983	baz1b
ENSXMAG00000016257	si:ch1073-513e17.1	ENSLOCG00000000882	si:ch1073-513e17.1
ENSXMAG00000024192	RF00443	ENSLOCG00000018489	RF00443
ENSXMAG00000024905	sh2d1ab	ENSLOCG00000014578	sh2d1ab
ENSXMAG00000009886	usp12a	ENSLOCG00000004146	usp12a
ENSXMAG00000012662	zgc:163098	ENSLOCG00000013596	zgc:163098
ENSXMAG00000026912	ppox	ENSLOCG00000004755	ppox
ENSXMAG00000011919	agtr2	ENSLOCG00000017471	agtr2
ENSXMAG00000017586	tcea1	ENSLOCG00000005699	tcea1
ENSXMAG00000016332	DMXL1	ENSLOCG00000008849	DMXL1
ENSXMAG00000011348	upp2	ENSLOCG00000005911	upp2
ENSXMAG00000020160	gpr12	ENSLOCG00000018189	gpr12
ENSXMAG00000011914		ENSLOCG00000014895	
ENSXMAG00000027590	kctd14	ENSLOCG00000006426	kctd14
ENSXMAG00000016245	cx52.9	ENSLOCG00000018291	cx52.9
ENSXMAG00000009903	wasf3b	ENSLOCG00000004135	wasf3b
ENSXMAG00000003585	dyrk1b	ENSLOCG00000004700	dyrk1b
ENSXMAG00000016740	mst1	ENSLOCG00000014267	mst1
ENSXMAG00000010917	bcl7bb	ENSLOCG00000001059	bcl7bb
ENSXMAG00000012621	wdr44	ENSLOCG00000015409	wdr44
ENSXMAG00000012939	rbfox3b	ENSLOCG00000012267	rbfox3a
ENSXMAG00000029337	SRSF10	ENSLOCG00000005685	srsf10b
ENSXMAG00000004108	chrna2b	ENSLOCG00000017242	chrna2b
ENSXMAG00000011905		ENSLOCG00000015048	
ENSXMAG00000009404	gsap	ENSLOCG00000015997	gsap
ENSXMAG00000009914	cdk8	ENSLOCG00000004124	cdk8
ENSXMAG00000004746	ints4	ENSLOCG00000006446	ints4
ENSXMAG00000016739		ENSLOCG00000014273	
ENSXMAG00000023612	baalcb	ENSLOCG00000016722	baalcb
ENSXMAG00000008522		ENSLOCG00000004721	fbl
ENSXMAG00000029509	si:ch211-161c3.5	ENSLOCG00000014275	si:ch211-161c3.5
ENSXMAG00000012278	crmp1	ENSLOCG00000010077	crmp1
ENSXMAG00000021461	SEMA6A	ENSLOCG00000008872	sema6a
ENSXMAG00000012602	klhl13	ENSLOCG00000015414	klhl13
ENSXMAG00000010922	hip1	ENSLOCG00000001155	hip1
ENSXMAG00000006741	mrpl17	ENSLOCG00000004435	mrpl17
ENSXMAG00000014282	sdf2	ENSLOCG00000005733	SDF2
ENSXMAG00000016894	PDE1C	ENSLOCG00000012915	PDE1C

ENSXMAG00000016725		ENSLOCG00000006154	GNB1
ENSXMAG00000004784	aamdc	ENSLOCG00000006465	aamdc
ENSXMAG00000020519	MIR133B	ENSLOCG00000019262	MIR133B
ENSXMAG00000021314	RF00421	ENSLOCG00000019407	RF00421
ENSXMAG00000011901	hdx	ENSLOCG00000015046	hdx
ENSXMAG00000012928	ENPP7	ENSLOCG00000012260	enpp7.2
ENSXMAG00000029279	RF00393	ENSLOCG00000018528	RF00393
ENSXMAG00000029347	RF00425	ENSLOCG00000018472	RF00425
ENSXMAG00000018270	JAKMIP3	ENSLOCG00000007499	jakmip3
ENSXMAG00000011304	ACVR1C	ENSLOCG00000005848	ACVR1C
ENSXMAG00000004149	eif2b4	ENSLOCG00000016732	EIF2B4
ENSXMAG00000011838	rps6kal	ENSLOCG00000015032	rps6kal
ENSXMAG00000021155	si:ch1073-224n8.1	ENSLOCG00000002836	
ENSXMAG00000004786	exosc5	ENSLOCG00000014220	exosc5
ENSXMAG00000027069	si:dkey-145c18.2	ENSLOCG00000015835	si:dkey-145c18.3
ENSXMAG00000011296	cytip	ENSLOCG00000005812	cytip
ENSXMAG00000016453	COMMD10	ENSLOCG00000008888	COMMD10
ENSXMAG00000014297	nf2b	ENSLOCG00000004088	nf2b
ENSXMAG00000004802	tmem91	ENSLOCG00000014223	tmem91
ENSXMAG00000025671		ENSLOCG00000001970	SLC6A20
ENSXMAG00000009957	atp8a2	ENSLOCG00000004080	atp8a2
ENSXMAG00000029854	POU3F4	ENSLOCG00000017491	POU3F4
ENSXMAG00000012916	pctp	ENSLOCG00000012244	pctp
ENSXMAG00000023638	neurod6b	ENSLOCG00000012935	neurod6a
ENSXMAG00000021183		ENSLOCG00000018163	
ENSXMAG00000026091		ENSLOCG00000006618	CENPS
ENSXMAG00000016466	AP3S1	ENSLOCG00000008916	AP3S1
ENSXMAG00000009433	sypl1	ENSLOCG00000015838	sypl1
ENSXMAG00000029577	atraid	ENSLOCG00000016731	
ENSXMAG00000010954	ypel2a	ENSLOCG00000001200	ypel2a
ENSXMAG00000028629	mmd	ENSLOCG00000012237	mmd
ENSXMAG00000023043	opa3	ENSLOCG00000014233	opa3
ENSXMAG00000025798	GGCT	ENSLOCG00000001951	ggctb
ENSXMAG00000011821		ENSLOCG00000015022	
ENSXMAG00000016195		ENSLOCG00000010300	CFAP69
ENSXMAG00000004815	HNRNPUL1	ENSLOCG00000014236	HNRNPUL1
ENSXMAG00000016704	rbp7b	ENSLOCG00000006778	rbp7b
ENSXMAG00000009458	ATXN7L1	ENSLOCG00000015842	ATXN7L1
ENSXMAG00000011292	GALNT5	ENSLOCG00000005773	GALNT5
ENSXMAG00000029910	cdo1	ENSLOCG00000008927	cdo1
ENSXMAG00000000241		ENSLOCG00000007471	dpysl4
ENSXMAG00000004181	snx17	ENSLOCG00000016735	snx17
ENSXMAG00000015223	tcaim	ENSLOCG00000001243	tcaim
ENSXMAG00000024194	h6pd	ENSLOCG00000007009	h6pd
ENSXMAG00000010955	gdpd1	ENSLOCG00000001218	gdpd1
ENSXMAG00000004851	rasgrp4	ENSLOCG00000014243	rasgrp4
ENSXMAG00000014330	mettl27	ENSLOCG00000004329	mettl27
ENSXMAG00000016486	cep78	ENSLOCG00000009875	cep78
ENSXMAG00000010583	rnf25	ENSLOCG00000000126	
ENSXMAG00000012400	hpf1	ENSLOCG00000013083	hpf1
ENSXMAG00000023527	cblc	ENSLOCG00000004326	cblc
ENSXMAG00000027262		ENSLOCG00000015846	znf800a
ENSXMAG00000016188	KDM1B	ENSLOCG00000005898	KDM1B
ENSXMAG00000004332	cnot10	ENSLOCG00000001271	cnot10
ENSXMAG00000011817	SH3BGRL	ENSLOCG00000015016	sh3bgrl
ENSXMAG00000000282		ENSLOCG00000000718	si:ch1073-416j23.1

ENSXMAG00000006668	bcl3	ENSLOCG00000004342	
ENSXMAG00000022249	znf513a	ENSLOCG00000016739	znf513b
ENSXMAG00000011280	nr4a2a	ENSLOCG00000005738	nr4a2a
ENSXMAG00000016495		ENSLOCG00000012789	spef2
ENSXMAG00000010967	porb	ENSLOCG00000001640	porb
ENSXMAG00000009482	grm8a	ENSLOCG00000015847	grm8a
ENSXMAG00000016692	cplane2	ENSLOCG00000007458	cplane2
ENSXMAG00000010029	shisa2b	ENSLOCG00000004070	shisa2b
ENSXMAG00000016546	pdxp	ENSLOCG00000007538	pdxp
ENSXMAG00000004326	calhm5.2	ENSLOCG00000017113	calhm5.2
ENSXMAG00000004219	si:ch211-278j3.3	ENSLOCG00000016741	si:ch211-278j3.3
ENSXMAG00000011278	kcnj3a	ENSLOCG00000005729	kcnj3a
ENSXMAG00000010030	spice1	ENSLOCG00000002962	spice1
ENSXMAG00000029830		ENSLOCG00000007468	si:ch73-15b2.5
ENSXMAG00000016150		ENSLOCG00000005748	MYO1G
ENSXMAG00000016548	mrps36	ENSLOCG00000007526	mrps36
ENSXMAG00000008057	SYDE1	ENSLOCG00000008804	SYDE1
ENSXMAG00000004325	calhm6	ENSLOCG00000017112	calhm6
ENSXMAG00000025068	si:dkey-71h2.2	ENSLOCG00000016746	si:dkey-71h2.2
ENSXMAG00000024394		ENSLOCG00000000730	
ENSXMAG00000025380	dkk1a	ENSLOCG00000006347	dkk1b
ENSXMAG00000010983	taf15	ENSLOCG00000001615	taf15
ENSXMAG00000009748	usp40	ENSLOCG00000010114	usp40
ENSXMAG00000004313	rwdd1	ENSLOCG00000017114	rwdd1
ENSXMAG00000027427	tm4sf5	ENSLOCG00000014141	tm4sf5
ENSXMAG00000026259	TMEM229A	ENSLOCG00000017905	TMEM229A
ENSXMAG00000010032	boc	ENSLOCG00000003031	boc
ENSXMAG00000025496	LGALS4	ENSLOCG00000014276	
ENSXMAG00000010985	mmp28	ENSLOCG00000001596	mmp28
ENSXMAG00000019599	si:dkeyp-92c9.2	ENSLOCG00000018329	si:dkeyp-92c9.2
ENSXMAG00000010661	prkg1a	ENSLOCG00000006321	prkg1b
ENSXMAG00000016673	ddi2	ENSLOCG00000008021	ddi2
ENSXMAG00000011252	galnt13	ENSLOCG00000005678	galnt13
ENSXMAG00000016147	edn1	ENSLOCG00000007983	edn1
ENSXMAG00000010989	fam124b	ENSLOCG00000003983	fam124b
ENSXMAG00000012817	blk	ENSLOCG00000016755	blk
ENSXMAG00000024924	hivep1	ENSLOCG00000007963	hivep1
ENSXMAG00000025779	si:ch73-361p23.3	ENSLOCG00000007415	
ENSXMAG00000022745		ENSLOCG00000000852	
ENSXMAG00000007319	cldnh	ENSLOCG00000017427	cldnh
ENSXMAG00000023372	TBX22	ENSLOCG00000014991	TBX22
ENSXMAG00000029935	zgc:113531	ENSLOCG00000002650	zgc:113531
ENSXMAG00000026685		ENSLOCG00000007401	
ENSXMAG00000010050	usf3	ENSLOCG00000003056	usf3
ENSXMAG00000021733	tmem170b	ENSLOCG00000007926	tmem170b
ENSXMAG00000006612		ENSLOCG00000005186	
ENSXMAG00000030069	ITM2A	ENSLOCG00000014989	ITM2A
ENSXMAG00000028907	zgc:91976	ENSLOCG00000006259	zgc:91976
ENSXMAG00000005926		ENSLOCG00000014171	
ENSXMAG00000017019	b4galt2	ENSLOCG00000002636	b4galt2
ENSXMAG00000004225	sox7	ENSLOCG00000016760	sox7
ENSXMAG00000027221	cnih4	ENSLOCG00000015807	cnih4
ENSXMAG00000007321	abhd11	ENSLOCG00000004295	abhd11
ENSXMAG00000012435		ENSLOCG00000002679	si:ch73-335m24.5
ENSXMAG00000009750	TSPEAR	ENSLOCG00000009387	tspearb
ENSXMAG00000016120	nedd9	ENSLOCG00000007912	nedd9

ENSXMAG00000021395	gpr174	ENSLOCG00000017489	gpr174
ENSXMAG00000010052	naa50	ENSLOCG00000003064	naa50
ENSXMAG00000003842		ENSLOCG00000008693	
ENSXMAG00000017029	atp6v0b	ENSLOCG00000002617	atp6v0b
ENSXMAG00000018321	p2ry10	ENSLOCG00000014987	p2ry10
ENSXMAG00000022783	preb	ENSLOCG00000015926	preb
ENSXMAG00000018318	lpar4	ENSLOCG00000017488	lpar4
ENSXMAG00000024710	kiss1ra	ENSLOCG00000006937	KISS1R
ENSXMAG00000019655	rprmb	ENSLOCG00000017828	rprmb
ENSXMAG00000007335	cct6a	ENSLOCG00000004174	cct6a
ENSXMAG00000016642	edem1	ENSLOCG00000010944	edem1
ENSXMAG00000011011	PDE2A	ENSLOCG00000007646	PDE2A
ENSXMAG00000006601	mag	ENSLOCG00000005232	MAG
ENSXMAG00000000109	lmod2b	ENSLOCG00000015865	lmod2b
ENSXMAG00000017054	IFT22	ENSLOCG00000002416	ift22
ENSXMAG00000015299	prpf40a	ENSLOCG00000005629	prpf40a
ENSXMAG00000010671	dnajc3b	ENSLOCG00000013984	dnajc3b
ENSXMAG00000000116	asb15b	ENSLOCG00000015866	asb15b
ENSXMAG00000004364	ADCY3	ENSLOCG00000015927	adcy3a
ENSXMAG00000012962	papss2a	ENSLOCG00000006215	papss2b
ENSXMAG00000017543		ENSLOCG00000014978	
ENSXMAG00000016611	gnat1	ENSLOCG00000010938	gnat1
ENSXMAG00000017062		ENSLOCG00000005418	bcl10
ENSXMAG00000012418	zmp:0000001168	ENSLOCG00000002884	
ENSXMAG00000017538		ENSLOCG00000014976	
ENSXMAG00000028369		ENSLOCG00000013981	naa38
ENSXMAG00000007366		ENSLOCG00000004118	mrps17
ENSXMAG00000016090		ENSLOCG00000004352	ddx61
ENSXMAG00000010068	gramd1c	ENSLOCG00000003088	gramd1c
ENSXMAG00000017522	sybl1	ENSLOCG00000014975	sybl1
ENSXMAG00000009801	atg9a	ENSLOCG00000009485	atg9a
ENSXMAG00000015864	fam20cb	ENSLOCG00000008723	fam20cb
ENSXMAG00000016035	chd3	ENSLOCG00000013965	chd3
ENSXMAG00000012957		ENSLOCG00000006145	
ENSXMAG00000010089	ccdc191	ENSLOCG00000003111	ccdc191
ENSXMAG00000011055	ARAP1	ENSLOCG00000007666	ARAP1
ENSXMAG00000001149	tgfb1a	ENSLOCG00000014160	tgfb1a
ENSXMAG00000010094	qtrt2	ENSLOCG00000003146	qtrt2
ENSXMAG00000012473	fbxo10	ENSLOCG00000012865	fbxo10
ENSXMAG00000000163	atpaf2	ENSLOCG00000008821	atpaf2
ENSXMAG00000010098	drd3	ENSLOCG00000003166	drd3
ENSXMAG00000012647	hmg20b	ENSLOCG00000006147	hmg20b
ENSXMAG00000009826		ENSLOCG00000009517	pcnt
ENSXMAG00000027083	olah	ENSLOCG00000010414	olah
ENSXMAG00000021079		ENSLOCG00000006042	
ENSXMAG00000012484	polr1e	ENSLOCG00000012859	polr1e
ENSXMAG00000026597	tnfsf12	ENSLOCG00000013961	tnfsf12
ENSXMAG00000003999	ints13	ENSLOCG00000015297	ints13
ENSXMAG00000023162	rbm6	ENSLOCG00000010900	rbm6
ENSXMAG00000024676	fuz	ENSLOCG00000018125	fuz
ENSXMAG00000029129	sat2a	ENSLOCG00000014002	sat2a
ENSXMAG00000028590	gpt2l	ENSLOCG00000008969	gpt2l
ENSXMAG00000012518	grhprb	ENSLOCG00000012844	
ENSXMAG00000010115	cpa6	ENSLOCG00000004376	cpa6
ENSXMAG00000025997	si:dkey-19b23.7	ENSLOCG00000013959	si:dkey-19b23.7
ENSXMAG00000005941	si:ch211-117m20.4	ENSLOCG00000003836	si:ch211-117m20.4

ENSXMAG00000000146	neurl2	ENSLOCG00000006803	neurl2
ENSXMAG00000014120	pter	ENSLOCG00000010468	pter
ENSXMAG00000012627	rxfp3.2b	ENSLOCG00000017636	rxfp3.2b
ENSXMAG00000021651	FFAR1	ENSLOCG00000017673	FFAR1
ENSXMAG00000009934	agrp	ENSLOCG00000008154	agrp
ENSXMAG00000005943	hinfp	ENSLOCG00000003801	hinfp
ENSXMAG00000026025		ENSLOCG00000017429	
ENSXMAG00000016108	man2b2	ENSLOCG00000010140	man2b2
ENSXMAG00000029176	tekt2	ENSLOCG00000000469	tekt2
ENSXMAG00000012933	cfap43	ENSLOCG00000011880	cfap43
ENSXMAG00000004021	ano6	ENSLOCG00000016426	ANO6
ENSXMAG00000009832	senp2	ENSLOCG00000009546	senp2
ENSXMAG00000017502	pdgfrl	ENSLOCG00000013903	pdgfrl
ENSXMAG00000005959	dpagt1	ENSLOCG00000003785	dpagt1
ENSXMAG00000012548	si:dkey-82j4.2	ENSLOCG00000012494	si:dkey-82j4.2
ENSXMAG00000011069	stard10	ENSLOCG00000007700	stard10
ENSXMAG00000026192	celf5a	ENSLOCG00000005481	celf5a
ENSXMAG00000010123	prex2	ENSLOCG00000004272	prex2
ENSXMAG00000012931	sfr1	ENSLOCG00000011891	sfr1
ENSXMAG00000026331	smim20	ENSLOCG00000009742	smim20
ENSXMAG00000009963	galr1b	ENSLOCG00000008111	galr1b
ENSXMAG00000011076	CLPB	ENSLOCG00000007715	CLPB
ENSXMAG00000016439	uba7	ENSLOCG00000010829	uba7
ENSXMAG00000004040	pwp1	ENSLOCG00000015280	pwp1
ENSXMAG00000012403	c2cd2l	ENSLOCG00000003760	c2cd2l
ENSXMAG00000009877	rbpjb	ENSLOCG00000009731	rbpjb
ENSXMAG00000012585	rgmd	ENSLOCG00000005465	rgmd
ENSXMAG00000009840	ola1	ENSLOCG00000007399	ola1
ENSXMAG00000024115	phox2a	ENSLOCG00000007728	phox2a
ENSXMAG00000027475	GH1	ENSLOCG00000013185	gh1
ENSXMAG00000007622	lrrcc1	ENSLOCG00000012790	lrrcc1
ENSXMAG00000017456	mtmr7a	ENSLOCG00000013919	mtmr7a
ENSXMAG00000012760	cdc27	ENSLOCG00000013232	cdc27
ENSXMAG00000029975	APOA1BP	ENSLOCG00000008812	naxe
ENSXMAG00000012597	polq	ENSLOCG00000005709	polq
ENSXMAG00000029692	CCKAR	ENSLOCG00000009722	CCKAR
ENSXMAG00000011097	inppl1a	ENSLOCG00000007744	inppl1a
ENSXMAG00000004059	ube2nb	ENSLOCG00000015285	ube2na
ENSXMAG00000027033	si:dkey-78l4.14	ENSLOCG00000005430	si:dkey-78l4.14
ENSXMAG00000009880	uggt1	ENSLOCG00000006770	uggt1
ENSXMAG00000009908	tbc1d19	ENSLOCG00000009710	tbc1d19
ENSXMAG00000010687	zgc:162928	ENSLOCG00000004246	zgc:162928
ENSXMAG00000012492		ENSLOCG00000005390	
ENSXMAG00000017447	cnot7	ENSLOCG00000013938	cnot7
ENSXMAG00000012626	ptpn9b	ENSLOCG00000012772	ptpn9b
ENSXMAG00000023592	cldnj	ENSLOCG00000017435	cldnj
ENSXMAG00000012009	tppp3	ENSLOCG00000008212	tppp3
ENSXMAG00000009977	hs6st1b	ENSLOCG00000006754	hs6st1b
ENSXMAG00000027341	trim37	ENSLOCG00000001333	trim37
ENSXMAG00000011128	FOLR2	ENSLOCG00000007764	FOLR2
ENSXMAG00000012004	zdhhc1	ENSLOCG00000008200	zdhhc1
ENSXMAG00000021005	zdhhc2	ENSLOCG00000013942	zdhhc2
ENSXMAG00000004067		ENSLOCG00000015230	
ENSXMAG00000027789	anapc15	ENSLOCG00000007778	anapc15
ENSXMAG00000016369	rbm5	ENSLOCG00000010774	rbm5
ENSXMAG00000009978	otomp	ENSLOCG00000006736	otomp

ENSXMAG00000012653	melk	ENSLOCG00000012822	melk
ENSXMAG00000010180	sulf1	ENSLOCG00000004178	sulf1
ENSXMAG00000006065	sbds	ENSLOCG00000004073	sbds
ENSXMAG00000012475	fgf22	ENSLOCG00000005240	fgf22
ENSXMAG00000019380	pou2f3	ENSLOCG00000002109	pou2f3
ENSXMAG00000012352	stk36	ENSLOCG00000003525	stk36
ENSXMAG00000017807	samd7	ENSLOCG00000007177	samd7
ENSXMAG00000011134	tomt	ENSLOCG00000007792	tomt
ENSXMAG00000029564	angpt1	ENSLOCG00000003157	angpt1
ENSXMAG00000015413	glb1l	ENSLOCG00000009458	glb1l
ENSXMAG00000027839	zdhhc4	ENSLOCG00000005276	zdhhc4
ENSXMAG00000029375	pgp	ENSLOCG00000005241	pgp
ENSXMAG00000021046	sfrp2l	ENSLOCG00000007815	sfrp2l
ENSXMAG00000009494	slc37a2	ENSLOCG00000002183	slc37a2
ENSXMAG00000009948	pcdh7b	ENSLOCG00000009687	pcdh7b
ENSXMAG00000007036	tpmt.2	ENSLOCG00000003449	tpmt.1
ENSXMAG00000024582	bricd5	ENSLOCG00000005225	bricd5
ENSXMAG00000012464	polrmt	ENSLOCG00000005222	polrmt
ENSXMAG00000026503	lamtor1	ENSLOCG00000007824	lamtor1
ENSXMAG00000016766	agtpbp1	ENSLOCG00000002341	agtpbp1
ENSXMAG00000012714	itga2b	ENSLOCG00000013257	itga2b
ENSXMAG00000012680	pax5	ENSLOCG00000012825	pax5
ENSXMAG00000013125	tyw1	ENSLOCG00000004035	tyw1
ENSXMAG00000029495	TEX264	ENSLOCG00000010705	tex264a
ENSXMAG00000024897	sec62	ENSLOCG00000007194	sec62
ENSXMAG00000019696		ENSLOCG00000018287	
ENSXMAG00000025970	mlst8	ENSLOCG00000005213	mlst8
ENSXMAG00000015465	ankzf1	ENSLOCG00000009473	ankzf1
ENSXMAG00000029558	ccdc15	ENSLOCG00000002226	ccdc15
ENSXMAG00000007033	c19h1orf109	ENSLOCG00000005970	c19h1orf109
ENSXMAG00000000472	nadkb	ENSLOCG00000007207	nadkb
ENSXMAG00000011777	msrb1b	ENSLOCG00000005071	msrb1b
ENSXMAG00000027950	psmg1	ENSLOCG00000004889	psmg1
ENSXMAG00000011018	zgc:103564	ENSLOCG00000013179	zgc:103564
ENSXMAG00000012691	zcchc7	ENSLOCG00000012834	zcchc7
ENSXMAG00000024794	acvr1l	ENSLOCG00000006702	acvr1l
ENSXMAG00000007026	cdca8	ENSLOCG00000005985	cdca8
ENSXMAG00000016308	rrp9	ENSLOCG00000010672	rrp9
ENSXMAG00000029186		ENSLOCG00000013610	
ENSXMAG00000000477	skila	ENSLOCG00000007221	skila
ENSXMAG00000024131	mybbp1a	ENSLOCG00000006996	mybbp1a
ENSXMAG00000010249	prdm14	ENSLOCG00000004139	prdm14
ENSXMAG00000010020		ENSLOCG00000017634	
ENSXMAG00000025128	her5	ENSLOCG00000013605	her5
ENSXMAG00000012694	pgm2	ENSLOCG00000012883	pgm2
ENSXMAG00000016808		ENSLOCG00000006007	
ENSXMAG00000019600	si:ch211-213o11.11	ENSLOCG00000018247	gpr61l
ENSXMAG00000017430	lcmt1	ENSLOCG00000005381	lcmt1
ENSXMAG00000000481	prkci	ENSLOCG00000007237	prkci
ENSXMAG00000012451	bsg	ENSLOCG00000005175	bsg
ENSXMAG00000022074	pfdn6	ENSLOCG00000000454	pfdn6
ENSXMAG00000015483	si:ch211-167j6.5	ENSLOCG00000009506	si:ch211-167j6.5
ENSXMAG00000011164	naalad2	ENSLOCG00000007231	naalad2
ENSXMAG00000013450		ENSLOCG00000001082	tbl2
ENSXMAG00000023938	mmgt1	ENSLOCG00000011507	mmgt1
ENSXMAG00000026595	aqp8a.2	ENSLOCG00000005407	aqp8a.2

ENSXMAG00000016788	naa35	ENSLOCG00000002381	naa35
ENSXMAG00000017374	ints6l	ENSLOCG00000015093	ints6l
ENSXMAG00000013447		ENSLOCG00000001122	pom121
ENSXMAG00000012717	tbc1d1	ENSLOCG00000012895	tbc1d1
ENSXMAG00000016771		ENSLOCG00000004946	
ENSXMAG00000010053	keap1a	ENSLOCG00000005625	keap1a
ENSXMAG00000026920	tram1	ENSLOCG00000004050	tram1
ENSXMAG00000000503	dapk3	ENSLOCG00000001875	dapk3
ENSXMAG00000027887	mospd1	ENSLOCG00000015092	mospd1
ENSXMAG00000013415	zw10	ENSLOCG00000004478	zw10
ENSXMAG00000028161	xkr9	ENSLOCG00000003990	xkr9
ENSXMAG00000017403		ENSLOCG00000014826	idh3a
ENSXMAG00000016765	prdm1b	ENSLOCG00000004978	prdm1b
ENSXMAG00000015507	igf2bp2b	ENSLOCG00000009564	igf2bp2b
ENSXMAG00000017365	fam122b	ENSLOCG00000015090	fam122b
ENSXMAG00000000521	sppl2	ENSLOCG00000004334	sppl2
ENSXMAG00000016871	GOLM1	ENSLOCG00000002400	GOLM1
ENSXMAG00000012436	fstl3	ENSLOCG00000005141	fstl3
ENSXMAG00000013400	mks1	ENSLOCG00000001032	mks1
ENSXMAG00000029989	nwd2	ENSLOCG00000009653	nwd2
ENSXMAG00000012645	abi3a	ENSLOCG00000013134	abi3a
ENSXMAG00000025803	hprt1	ENSLOCG00000015083	hprt1
ENSXMAG00000029070	cbarpb	ENSLOCG00000005061	cbarpb
ENSXMAG00000016751		ENSLOCG00000005016	
ENSXMAG00000006449	ebi3	ENSLOCG00000006256	EBI3
ENSXMAG00000022567	klf3	ENSLOCG00000012901	KLF3
ENSXMAG00000025998	atp5f1d	ENSLOCG00000000356	atp5f1d
ENSXMAG00000012196	f11r.1	ENSLOCG00000000677	
ENSXMAG00000017347	phf6	ENSLOCG00000015079	phf6
ENSXMAG00000009492	fam114a1	ENSLOCG00000013058	fam114a1
ENSXMAG00000021635	ehhadh	ENSLOCG00000009587	ehhadh
ENSXMAG00000010271	eya1	ENSLOCG00000003941	eya1
ENSXMAG00000012420	midn	ENSLOCG00000005039	midn
ENSXMAG00000016175	stau1	ENSLOCG00000003134	stau1
ENSXMAG00000015308	rell1	ENSLOCG00000009640	RELL1
ENSXMAG00000022603	zgc:162707	ENSLOCG00000009596	zgc:162707
ENSXMAG00000013363	ap2b1	ENSLOCG00000001756	ap2b1
ENSXMAG00000026998	cog8	ENSLOCG00000001398	cog8
ENSXMAG00000009500	klhl5	ENSLOCG00000013045	klhl5
ENSXMAG00000022176	cab39l1	ENSLOCG00000015073	cab39l1
ENSXMAG00000027463	ctsc	ENSLOCG00000007149	ctsc
ENSXMAG00000017371	nob1	ENSLOCG00000001411	nob1
ENSXMAG00000015556	nme8	ENSLOCG00000009626	nme8
ENSXMAG00000013214	galnt17	ENSLOCG00000003993	galnt17
ENSXMAG00000015314	per1b	ENSLOCG00000013344	per1a
ENSXMAG00000023931	rapgefl1	ENSLOCG00000013525	rapgefl1
ENSXMAG00000027766	si:ch211-212d10.2	ENSLOCG00000004977	si:ch211-212d10.2
ENSXMAG00000017332	gpc3	ENSLOCG00000015071	gpc3
ENSXMAG00000012165		ENSLOCG00000008798	MRPS31
ENSXMAG00000009548	wdr19	ENSLOCG00000013035	wdr19
ENSXMAG00000012353	cilp2	ENSLOCG00000004969	cilp2
ENSXMAG00000017352	wdr59	ENSLOCG00000001430	wdr59
ENSXMAG00000013189	rhbdd1	ENSLOCG00000009504	rhbdd1
ENSXMAG00000000578	hiat1a	ENSLOCG00000004101	hiat1a
ENSXMAG00000022124	fzd4	ENSLOCG00000007103	fzd4
ENSXMAG00000012132	taf6	ENSLOCG00000014015	taf6

ENSXMAG00000028715	yjefn3	ENSLOCG00000004954	yjefn3
ENSXMAG00000022756	gpc4	ENSLOCG00000015069	gpc4
ENSXMAG00000004100	phtf2	ENSLOCG00000016007	phtf2
ENSXMAG00000015575		ENSLOCG00000009637	smarcal1
ENSXMAG00000017031		ENSLOCG00000006780	ndc80
ENSXMAG00000012755	smndc1	ENSLOCG00000012355	smndc1
ENSXMAG00000011269		ENSLOCG00000007086	
ENSXMAG00000013142	eml2	ENSLOCG00000014791	eml2
ENSXMAG00000013937	casc3	ENSLOCG00000013518	casc3
ENSXMAG00000017342		ENSLOCG00000000240	
ENSXMAG00000012753	bnip4	ENSLOCG00000007528	bnip3
ENSXMAG00000018824		ENSLOCG00000012559	
ENSXMAG00000009566	rfc1	ENSLOCG00000013023	rfc1
ENSXMAG00000018182		ENSLOCG00000001800	gpatch1
ENSXMAG00000021507	tiprl	ENSLOCG00000005101	tiprl
ENSXMAG00000012294	mau2	ENSLOCG00000004881	mau2
ENSXMAG00000018826	tmem173	ENSLOCG00000012569	tmem173
ENSXMAG00000019692	cebpa	ENSLOCG00000017586	cebpa
ENSXMAG00000010332	msc	ENSLOCG00000003912	msc
ENSXMAG00000012743	ppp2r2d	ENSLOCG00000007541	ppp2r2d
ENSXMAG00000017973	DYNC2H1	ENSLOCG00000007034	DYNC2H1
ENSXMAG00000015348	si:dkey-19b23.13	ENSLOCG00000014011	si:dkey-19b23.13
ENSXMAG00000019691	cebpg	ENSLOCG00000017587	cebpg
ENSXMAG00000010334	trpa1b	ENSLOCG00000003830	trpa1a
ENSXMAG00000026967		ENSLOCG00000017723	
ENSXMAG00000000605	loxl5b	ENSLOCG00000003994	loxl5b
ENSXMAG00000017061	EGFLAM	ENSLOCG00000012642	EGFLAM
ENSXMAG00000012100	grk1b	ENSLOCG00000013881	grk1b
ENSXMAG00000011977	fhod1	ENSLOCG00000008274	fhod1
ENSXMAG00000012712	MGMT	ENSLOCG00000007632	MGMT
ENSXMAG00000015625	xrcc5	ENSLOCG00000009725	xrcc5
ENSXMAG00000009586	rpl9	ENSLOCG00000013006	rpl9
ENSXMAG00000012090	slc25a35	ENSLOCG00000013902	slc25a35
ENSXMAG00000010977	trpc5a	ENSLOCG00000014299	trpc5a
ENSXMAG00000020978	terf1	ENSLOCG00000003784	terf1
ENSXMAG00000015373	cnpy4	ENSLOCG00000014021	cnpy4
ENSXMAG00000011507	amot	ENSLOCG00000013862	amot
ENSXMAG00000013963	psmd3	ENSLOCG00000012886	psmd3
ENSXMAG00000009594	lias	ENSLOCG00000013001	lias
ENSXMAG00000010405	SBSPON	ENSLOCG00000003770	SBSPON
ENSXMAG00000013127		ENSLOCG00000014803	cd3eap
ENSXMAG00000015642	tmem169b	ENSLOCG00000009744	tmem169b
ENSXMAG00000012265	tmem161a	ENSLOCG00000004829	tmem161a
ENSXMAG00000026871	ntn5	ENSLOCG00000014453	ntn5
ENSXMAG00000020858	cplx4c	ENSLOCG00000003907	cplx4c
ENSXMAG00000012715	ebf3a	ENSLOCG00000007615	ebf3a
ENSXMAG00000010409		ENSLOCG00000003754	rpl7
ENSXMAG00000027028		ENSLOCG00000010476	FAM3D
ENSXMAG00000010125	pdcd10b	ENSLOCG00000003413	pdcd10b
ENSXMAG00000028583	foxa3	ENSLOCG00000014809	foxa3
ENSXMAG00000015646	pfkla	ENSLOCG00000009776	pfklb
ENSXMAG00000011013	cnpy3	ENSLOCG00000014285	cnpy3
ENSXMAG00000021494	pdgfd	ENSLOCG00000007018	pdgfd
ENSXMAG00000015396	si:dkey-33c9.6	ENSLOCG00000014441	si:dkey-33c9.6
ENSXMAG00000028530	RF00581	ENSLOCG00000020859	RF00581
ENSXMAG00000009602	ugdh	ENSLOCG00000012995	ugdh

ENSXMAG00000026007	calr3b	ENSLOCG00000003722	CALR3
ENSXMAG00000021241	mef2b	ENSLOCG00000004811	mef2b
ENSXMAG00000013059	pld3	ENSLOCG00000014810	pld3
ENSXMAG00000024509	rdh10a	ENSLOCG00000003738	rdh10b
ENSXMAG00000010138	ISYNA1	ENSLOCG00000005587	ISYNA1
ENSXMAG00000021052	dachb	ENSLOCG00000014279	dachb
ENSXMAG00000012262	si:ch211-125m10.6	ENSLOCG00000004796	si:ch211-125m10.6
ENSXMAG00000017087		ENSLOCG00000015239	si:dkey-251i10.2
ENSXMAG00000014709	zgc:162324	ENSLOCG00000010469	zgc:162324
ENSXMAG00000012261	borcs8	ENSLOCG00000004779	borcs8
ENSXMAG00000009448		ENSLOCG00000005438	dlg2
ENSXMAG00000009611	map9	ENSLOCG00000008285	map9
ENSXMAG00000011951	slc9a5	ENSLOCG00000008264	slc9a5
ENSXMAG00000011030	si:ch211-243g6.3	ENSLOCG00000014264	si:ch211-243g6.3
ENSXMAG00000029144		ENSLOCG00000001357	
ENSXMAG00000025235	rfxank	ENSLOCG00000004761	rfxank
ENSXMAG00000028475		ENSLOCG00000014524	
ENSXMAG00000010426	stau2	ENSLOCG00000003717	stau2
ENSXMAG00000015421	zgc:194312	ENSLOCG00000014411	zgc:194312
ENSXMAG00000024977	ube2d2	ENSLOCG00000012597	ube2d2
ENSXMAG00000024620	smim14	ENSLOCG00000000136	
ENSXMAG00000015692		ENSLOCG00000009793	MAP3K13
ENSXMAG00000012225	nr2c2ap	ENSLOCG00000004745	nr2c2ap
ENSXMAG00000029960	kcnk6	ENSLOCG00000014534	kcnk6
ENSXMAG00000015425	acadvl	ENSLOCG00000014404	acadvl
ENSXMAG00000028951	zgc:110843	ENSLOCG00000012606	zgc:110843
ENSXMAG00000023102	ca16b	ENSLOCG00000010428	ptprga
ENSXMAG00000028577	aasdhppt	ENSLOCG00000006918	aasdhppt
ENSXMAG00000009457	BBX	ENSLOCG00000005165	bbx
ENSXMAG00000011031		ENSLOCG00000014261	stk26
ENSXMAG00000015501	dvl2	ENSLOCG00000014396	dvl2
ENSXMAG00000017354	zgc:163057	ENSLOCG00000007770	zgc:163057
ENSXMAG00000009616	pds5a	ENSLOCG00000012981	pds5a
ENSXMAG00000010437	ube2w	ENSLOCG00000003697	ube2w
ENSXMAG00000018889	psd2	ENSLOCG00000012621	psd2
ENSXMAG00000027383	hapln4	ENSLOCG00000004701	hapln4
ENSXMAG00000010875	cwf19l2	ENSLOCG00000006855	cwf19l2
ENSXMAG00000025914	elocb	ENSLOCG00000003689	eloca
ENSXMAG00000009406		ENSLOCG00000001487	
ENSXMAG00000011043	zgc:77880	ENSLOCG00000000963	zgc:77880
ENSXMAG00000024596	si:ch73-335l21.4	ENSLOCG00000013165	si:ch73-335l21.4
ENSXMAG00000000640	klf2b	ENSLOCG00000003791	klf2a
ENSXMAG00000021262	foxj1b	ENSLOCG00000007642	foxj1b
ENSXMAG00000010444	jph1a	ENSLOCG00000003641	jph1a
ENSXMAG00000011792	ubald1b	ENSLOCG00000007611	ubald1b
ENSXMAG00000006057	blvrb	ENSLOCG00000014574	blvrb
ENSXMAG00000009669	n4bp2	ENSLOCG00000008241	
ENSXMAG00000000642	ap1m1	ENSLOCG00000003821	ap1m1
ENSXMAG00000028762		ENSLOCG00000005059	SIM2
ENSXMAG00000021343	gdap1	ENSLOCG00000003626	gdap1
ENSXMAG00000009467		ENSLOCG00000005030	HLCS
ENSXMAG00000020170	rhoh	ENSLOCG00000017924	rhoh
ENSXMAG00000010455	pi15a	ENSLOCG00000003602	pi15a
ENSXMAG00000010665	mrtfba	ENSLOCG00000007529	mrtfba
ENSXMAG00000021125	smfn	ENSLOCG00000002389	smfn
ENSXMAG00000028859	eif1ad	ENSLOCG00000001883	eif1ad

ENSXMAG00000010877	alkbh8	ENSLOCG00000006833	alkbh8
ENSXMAG00000012091	si:ch73-63e15.2	ENSLOCG00000004643	si:ch73-63e15.2
ENSXMAG00000007920	ercc4	ENSLOCG00000007513	ercc4
ENSXMAG00000027532	kbtbd3	ENSLOCG00000006804	kbtbd3
ENSXMAG00000012065	slc12a9	ENSLOCG00000013452	slc12a9
ENSXMAG00000018942	ctsf	ENSLOCG00000001898	
ENSXMAG00000021682	bri3bp	ENSLOCG00000001962	bri3bp
ENSXMAG00000002696	gng7	ENSLOCG00000000727	gng7
ENSXMAG00000022405		ENSLOCG00000014822	zgc:171929
ENSXMAG00000017218	si:ch73-266o15.4	ENSLOCG00000002358	si:ch73-266o15.4
ENSXMAG00000018078	fam168a	ENSLOCG00000006237	fam168a
ENSXMAG00000015536	acap1	ENSLOCG00000013309	acap1
ENSXMAG00000012639	cpped1	ENSLOCG00000007483	cpped1
ENSXMAG00000000665	cib3	ENSLOCG00000003454	cib3
ENSXMAG00000017236		ENSLOCG00000009866	
ENSXMAG00000023281		ENSLOCG00000006166	katnb1
ENSXMAG00000017073	vps11	ENSLOCG00000005692	vps11
ENSXMAG00000026614	P2RY6	ENSLOCG00000017975	P2RY6
ENSXMAG00000010475	dok6	ENSLOCG00000003566	dok6
ENSXMAG00000009689	itln3	ENSLOCG00000002870	itln3
ENSXMAG00000018953	aup1	ENSLOCG00000002068	aup1
ENSXMAG00000000671	admp	ENSLOCG00000003305	admp
ENSXMAG00000012040	gnb2	ENSLOCG00000013499	gnb2
ENSXMAG00000015596	slc16a13	ENSLOCG00000013302	slc16a13
ENSXMAG00000025054	ARHGEF17	ENSLOCG00000006306	ARHGEF17
ENSXMAG00000010484	cd226	ENSLOCG00000003554	cd226
ENSXMAG00000014566	KIFC3	ENSLOCG00000006130	kifc3
ENSXMAG00000000672	im:7138239	ENSLOCG00000003323	
ENSXMAG00000015613	bcl6b	ENSLOCG00000013298	bcl6b
ENSXMAG00000012628	snx29	ENSLOCG00000007467	snx29
ENSXMAG00000018969	fnta	ENSLOCG00000012159	fnta
ENSXMAG00000023066		ENSLOCG00000007788	
ENSXMAG00000001626	hyou1	ENSLOCG00000005640	hyou1
ENSXMAG00000026997	relt	ENSLOCG00000006269	relt
ENSXMAG00000020934		ENSLOCG00000013293	
ENSXMAG00000009716	slit2	ENSLOCG00000003341	slit2
ENSXMAG00000011232	PIGG	ENSLOCG00000012169	si:ch73-49o8.1
ENSXMAG00000025111	rsl1d1	ENSLOCG00000007416	rsl1d1
ENSXMAG00000018109	p2ry2.2	ENSLOCG00000018217	p2ry2.1
ENSXMAG00000010486	rttn	ENSLOCG00000003504	rttn
ENSXMAG00000017381	foxb2	ENSLOCG00000017539	foxb2
ENSXMAG00000000570	dysf	ENSLOCG00000001150	dysf
ENSXMAG00000018119	FCHSD2	ENSLOCG00000006328	FCHSD2
ENSXMAG00000000676	ranbp3b	ENSLOCG00000003225	
ENSXMAG00000011935	mink1	ENSLOCG00000013479	mink1
ENSXMAG00000029917		ENSLOCG00000009837	
ENSXMAG00000015770	FAM126B	ENSLOCG00000009846	zgc:158316
ENSXMAG00000004189	guf1	ENSLOCG00000012397	guf1
ENSXMAG00000015629	si:dkey-38p12.3	ENSLOCG00000013928	si:dkey-38p12.3
ENSXMAG00000012059	tjp3	ENSLOCG00000004553	tjp3
ENSXMAG00000026639	tomm22	ENSLOCG00000011773	tomm22
ENSXMAG00000000684		ENSLOCG00000003179	nrtn
ENSXMAG00000015789		ENSLOCG00000009863	cflara
ENSXMAG00000015633	hdlbpb	ENSLOCG00000013909	hdlbpb
ENSXMAG00000000554	cyp26b1	ENSLOCG00000001249	cyp26b1
ENSXMAG00000018136		ENSLOCG00000006376	

ENSXMAG00000027050	rfk	ENSLOCG00000009824	rfk
ENSXMAG00000024251		ENSLOCG00000017721	
ENSXMAG00000004117	gabra4	ENSLOCG00000012374	gabra4
ENSXMAG00000009370	slc1a8b	ENSLOCG00000000305	slc1a8b
ENSXMAG00000012589	edrf1	ENSLOCG00000007911	edrf1
ENSXMAG00000016253	dip2a	ENSLOCG00000009880	dip2a
ENSXMAG00000001595		ENSLOCG00000005483	bsx
ENSXMAG00000020161	socs6a	ENSLOCG00000017811	socs6b
ENSXMAG00000018175	yif1b	ENSLOCG00000014575	yif1b
ENSXMAG00000025350		ENSLOCG00000004249	zgc:86811
ENSXMAG00000027663	tspan37	ENSLOCG00000000268	
ENSXMAG00000016247		ENSLOCG00000013709	cfap100
ENSXMAG00000000535	exoc6b	ENSLOCG00000001267	exoc6b
ENSXMAG00000009809	lcorl	ENSLOCG00000003324	lcorl
ENSXMAG00000021787		ENSLOCG00000017567	
ENSXMAG00000012031	hmha1b	ENSLOCG00000004582	hmha1a
ENSXMAG00000030085		ENSLOCG00000004231	rad17
ENSXMAG00000020853	cbln2b	ENSLOCG00000003482	cbln2a
ENSXMAG00000015663	neurl4	ENSLOCG00000013886	neurl4
ENSXMAG00000018184	si:ch211-136a13.1	ENSLOCG00000014537	si:ch211-136a13.1
ENSXMAG00000009813	ncapg	ENSLOCG00000003287	ncapg
ENSXMAG00000026495	polr2eb	ENSLOCG00000004599	polr2eb
ENSXMAG00000011897	pld2	ENSLOCG00000013467	pld2
ENSXMAG00000000386	ptprfb	ENSLOCG00000004311	ptprfb
ENSXMAG00000029408		ENSLOCG00000002117	ube2l3a
ENSXMAG00000028975	bloc1s3	ENSLOCG00000017570	bloc1s3
ENSXMAG00000009837	fam184b	ENSLOCG00000003271	fam184b
ENSXMAG00000016264	ap1ar	ENSLOCG00000011808	ap1ar
ENSXMAG00000018203	trappc6bl	ENSLOCG00000014595	TRAPPC6A
ENSXMAG00000015416	fkbp5	ENSLOCG00000012508	fkbp5
ENSXMAG00000018536		ENSLOCG00000013619	
ENSXMAG00000000517	tkfc	ENSLOCG00000009511	
ENSXMAG00000011851	chrne	ENSLOCG00000013546	chrne
ENSXMAG00000018206	zgc:162730	ENSLOCG00000014597	zgc:162730
ENSXMAG00000025215	smad5	ENSLOCG00000011514	smad5
ENSXMAG00000016351		ENSLOCG00000010580	
ENSXMAG00000026791	si:dkey-118j18.2	ENSLOCG00000013593	si:dkey-118j18.2
ENSXMAG00000029731	si:dkeyp-26a9.2	ENSLOCG00000003251	HASPIN
ENSXMAG00000026350		ENSLOCG00000006647	pias2
ENSXMAG00000012539	si:ch211-141o9.10	ENSLOCG00000011691	si:ch211-141o9.10
ENSXMAG00000008422		ENSLOCG00000014616	zgc:172282
ENSXMAG00000029595	si:dkey-9k7.3	ENSLOCG00000009525	si:dkey-9k7.3
ENSXMAG00000016296	syvn1	ENSLOCG00000001464	syvn1
ENSXMAG00000015793	prmt2	ENSLOCG00000010568	prmt2
ENSXMAG00000021013	zbtb4	ENSLOCG00000013872	zbtb4
ENSXMAG00000009324	hltf	ENSLOCG00000007640	hltf
ENSXMAG00000012525	sh3bp1	ENSLOCG00000011670	sh3bp1
ENSXMAG00000015478	trub2	ENSLOCG00000000885	
ENSXMAG00000001420	klc3	ENSLOCG00000014748	klc3
ENSXMAG00000022468		ENSLOCG00000004951	
ENSXMAG00000003796	paxx	ENSLOCG00000001950	paxx
ENSXMAG00000015806	ercc1	ENSLOCG00000006213	ercc1
ENSXMAG00000012520	pdap1a	ENSLOCG00000000519	pdap1a
ENSXMAG00000015497	tgfa	ENSLOCG00000015665	TGFA
ENSXMAG00000019215	sema5a	ENSLOCG00000013571	sema5a
ENSXMAG00000013416	rab11ba	ENSLOCG00000004490	rab11ba

ENSXMAG00000011808	NYAP1	ENSLOCG00000013530	
ENSXMAG00000015687	polr2a	ENSLOCG00000013863	polr2a
ENSXMAG00000012557	zranb1b	ENSLOCG00000007990	zranb1b
ENSXMAG00000009297	pls1	ENSLOCG00000007909	pls1
ENSXMAG00000017500	gtf2h3	ENSLOCG00000005881	gtf2h3
ENSXMAG00000015500	slc2a11a	ENSLOCG00000006718	slc2a11a
ENSXMAG00000007619	mogat3b	ENSLOCG00000013592	mogat3b
ENSXMAG00000015740	si:dkeyp-110e4.6	ENSLOCG00000001445	
ENSXMAG00000009863		ENSLOCG00000012956	NSUN7
ENSXMAG00000025278	smim19	ENSLOCG00000012509	smim19
ENSXMAG00000021481	abraxas2	ENSLOCG00000008016	abraxas2
ENSXMAG00000017517	psmd9	ENSLOCG00000005247	psmd9
ENSXMAG00000009281	atr	ENSLOCG00000007924	atr
ENSXMAG00000008431	vps53	ENSLOCG00000004547	vps53
ENSXMAG00000003922	ercc2	ENSLOCG00000014383	ercc2
ENSXMAG00000017545		ENSLOCG00000008272	
ENSXMAG00000007627	srrt	ENSLOCG00000013658	srrt
ENSXMAG00000015756	fam199x	ENSLOCG00000014795	fam199x
ENSXMAG00000012537	EEF1AKMT2	ENSLOCG00000008027	eef1akmt2
ENSXMAG00000015844	arsh	ENSLOCG00000010227	arsh
ENSXMAG00000017551	wdr66	ENSLOCG00000005265	wdr66
ENSXMAG00000015541	EIF4ENIF1	ENSLOCG00000001513	EIF4ENIF1
ENSXMAG00000011902	fsd1	ENSLOCG00000006601	fsd1
ENSXMAG00000015707	capgb	ENSLOCG00000013857	capgb
ENSXMAG00000019481	nkx1.2la	ENSLOCG00000008089	nkx1.2la
ENSXMAG00000008453	rflnb	ENSLOCG00000004567	rflnb
ENSXMAG00000009874	rbm47	ENSLOCG00000012966	rbm47
ENSXMAG00000015774	COMMD5	ENSLOCG00000014798	commd5
ENSXMAG00000017557	mlxip	ENSLOCG00000005328	mlxip
ENSXMAG00000008454	dus4l	ENSLOCG00000015815	dus4l
ENSXMAG00000015570	sfi1	ENSLOCG00000001495	sfi1
ENSXMAG00000019564	zgc:154093	ENSLOCG00000017564	zgc:154093
ENSXMAG00000019194	mtrr	ENSLOCG00000013559	mtrr
ENSXMAG00000024150		ENSLOCG00000013851	
ENSXMAG00000012792	ydjc	ENSLOCG00000002105	ydjc
ENSXMAG00000029247		ENSLOCG00000013842	
ENSXMAG00000022619	hmx2	ENSLOCG00000008191	hmx2
ENSXMAG00000009241	stag1b	ENSLOCG00000007568	stag1a
ENSXMAG00000015785	LONRF3	ENSLOCG00000014802	LONRF3
ENSXMAG00000017563	LRRC43	ENSLOCG00000005344	LRRC43
ENSXMAG00000003957	zc3h4	ENSLOCG00000014380	zc3h4
ENSXMAG00000027461	HMX3	ENSLOCG00000008201	hmx3a
ENSXMAG00000011870	scamp4	ENSLOCG00000006526	scamp4
ENSXMAG00000019183	cct5	ENSLOCG00000013549	cct5
ENSXMAG00000018311	ikzf5	ENSLOCG00000008225	ikzf5
ENSXMAG00000015574	zgc:172271	ENSLOCG00000001465	
ENSXMAG00000023890		ENSLOCG00000006663	
ENSXMAG00000018816	lyrm1	ENSLOCG00000007969	lyrm1
ENSXMAG00000024507		ENSLOCG00000013630	
ENSXMAG00000029340	cmbl	ENSLOCG00000013544	cmbl
ENSXMAG00000024167	dcun1d3	ENSLOCG00000007958	dcun1d3
ENSXMAG00000018186	ptar1	ENSLOCG00000009461	ptar1
ENSXMAG00000008472	abhd15a	ENSLOCG00000004594	abhd15a
ENSXMAG00000022840	si:ch211-113e8.10	ENSLOCG00000007033	
ENSXMAG00000019907	zgc:112163	ENSLOCG00000017936	zgc:112163
ENSXMAG00000021380		ENSLOCG00000017456	

ENSXMAG00000018633	si:dkey-161j23.7	ENSLOCG00000007921	
ENSXMAG00000003960	sae1	ENSLOCG00000014375	sae1
ENSXMAG00000025686	agt	ENSLOCG00000015448	agt
ENSXMAG00000023040	uqcc2	ENSLOCG00000012635	uqcc2
ENSXMAG00000009213	pccb	ENSLOCG00000007550	pccb
ENSXMAG00000008474	aipl1	ENSLOCG00000004610	aipl1
ENSXMAG00000025724	SLC2A4	ENSLOCG00000013823	SLC2A4
ENSXMAG00000011864	chaf1a	ENSLOCG00000006653	chaf1a
ENSXMAG00000008483	trarg1a	ENSLOCG00000004627	trarg1a
ENSXMAG00000026768	FXN	ENSLOCG00000009422	fxn
ENSXMAG00000009888	ankrd49	ENSLOCG00000007534	ankrd49
ENSXMAG00000010423	cnksr2b	ENSLOCG00000003669	cnksr2b
ENSXMAG00000018640	cox6b1	ENSLOCG00000007947	cox6b1
ENSXMAG00000018269	cog2	ENSLOCG00000015450	cog2
ENSXMAG00000028913	gosr1	ENSLOCG00000004641	gosr1
ENSXMAG00000028552	GPR83	ENSLOCG00000007502	GPR83
ENSXMAG00000015755	YBX2	ENSLOCG00000013819	YBX2
ENSXMAG00000023699	frs3	ENSLOCG00000012711	frs3
ENSXMAG00000011842	ubxn6	ENSLOCG00000006666	ubxn6
ENSXMAG00000021614		ENSLOCG00000002653	dcps
ENSXMAG00000003196	pak4	ENSLOCG00000014365	pak4
ENSXMAG00000000457	tceanc2	ENSLOCG00000003227	tceanc2
ENSXMAG00000015768	EIF5AL1	ENSLOCG00000013814	EIF5AL1
ENSXMAG00000019170	si:rp71-1f1.4	ENSLOCG00000018167	si:rp71-1f1.4
ENSXMAG00000021976	aip	ENSLOCG00000006893	aip
ENSXMAG00000000462	tmem59	ENSLOCG00000003207	tmem59
ENSXMAG00000007942	cpda	ENSLOCG00000005601	
ENSXMAG00000029013	ppp2r3a	ENSLOCG00000007518	ppp2r3a
ENSXMAG00000000464	lrrc42	ENSLOCG00000003176	lrrc42
ENSXMAG00000003193	zgc:152863	ENSLOCG00000014363	zgc:152863
ENSXMAG00000015808		ENSLOCG00000015405	ids
ENSXMAG00000010407	rnf121	ENSLOCG00000007891	rnf121
ENSXMAG00000022102	pgbd5	ENSLOCG00000015452	pgbd5
ENSXMAG00000000466	yipf1	ENSLOCG00000003122	yipf1
ENSXMAG00000018643	fam234a	ENSLOCG00000007887	fam234a
ENSXMAG00000015777	gps2	ENSLOCG00000013806	gps2
ENSXMAG00000017608	zcchc8	ENSLOCG00000005425	zcchc8
ENSXMAG00000009930	serping1	ENSLOCG00000006939	
ENSXMAG00000004473	sdf2l1	ENSLOCG00000002092	sdf2l1
ENSXMAG00000012889	ndc1	ENSLOCG00000003080	ndc1
ENSXMAG00000024463	rsrc2	ENSLOCG00000005444	rsrc2
ENSXMAG00000009950	doc2d	ENSLOCG00000006958	doc2d
ENSXMAG00000018644	luc7l	ENSLOCG00000007877	luc7l
ENSXMAG00000018232	pibf1	ENSLOCG00000011336	pibf1
ENSXMAG00000011723	cryba1a	ENSLOCG00000005548	cryba1a
ENSXMAG00000009157	fndc3ba	ENSLOCG00000007456	fndc3ba
ENSXMAG00000011735	uap1	ENSLOCG00000007170	uap1
ENSXMAG00000015826	aff2	ENSLOCG00000015403	aff2
ENSXMAG00000011760	crybb1l3	ENSLOCG00000005525	crybb1l3
ENSXMAG00000000473		ENSLOCG00000002418	
ENSXMAG00000009974	lonrf1l	ENSLOCG00000013993	lonrf1l
ENSXMAG00000003186	hif1al	ENSLOCG00000014356	hif1al
ENSXMAG00000022890	gabarapb	ENSLOCG00000014491	gabarapb
ENSXMAG00000024652	pdk3a	ENSLOCG00000001784	pdk3a
ENSXMAG00000028193	gtf2h2	ENSLOCG00000004339	gtf2h2
ENSXMAG00000010372	trpc2b	ENSLOCG00000007903	trpc2b

ENSXMAG00000028254		ENSLOCG00000000824	ovol1a
ENSXMAG00000017627	kntc1	ENSLOCG00000005474	kntc1
ENSXMAG00000011786	foxn1	ENSLOCG00000005491	foxn1
ENSXMAG00000018215	dis3	ENSLOCG00000011319	dis3
ENSXMAG00000029409	ppil1	ENSLOCG00000012595	ppil1
ENSXMAG00000003182	DMWD	ENSLOCG00000014351	DMWD
ENSXMAG00000009873	mus81	ENSLOCG00000000795	mus81
ENSXMAG00000000827	fmr1	ENSLOCG00000015397	fmr1
ENSXMAG00000027010	dhfr	ENSLOCG00000005217	dhfr
ENSXMAG00000008489	slc46a1	ENSLOCG00000005466	slc46a1
ENSXMAG00000003164	MAP4K1	ENSLOCG00000014339	MAP4K1
ENSXMAG00000009985		ENSLOCG00000013982	dlc1
ENSXMAG00000009156	ghsrb	ENSLOCG00000007476	ghsrb
ENSXMAG00000008493	sarm1	ENSLOCG00000005448	sarm1
ENSXMAG00000000843	rab33a	ENSLOCG00000015358	rab33a
ENSXMAG00000028958	si:dkey-165a24.9	ENSLOCG00000013250	si:dkey-165a24.9
ENSXMAG00000018210	bora	ENSLOCG00000011308	bora
ENSXMAG00000004966	fam151b	ENSLOCG00000005204	fam151b
ENSXMAG00000018208	MZT1	ENSLOCG00000011295	MZT1
ENSXMAG00000000847	si:ch211-159i8.4	ENSLOCG00000015394	si:ch211-159i8.4
ENSXMAG00000019037	pola1	ENSLOCG00000001836	pola1
ENSXMAG00000013356	zfr	ENSLOCG00000006727	zfr
ENSXMAG00000011673		ENSLOCG00000007069	
ENSXMAG00000013711	cldn7a	ENSLOCG00000013784	cldn7a
ENSXMAG00000017434	si:ch211-157b11.12	ENSLOCG00000007360	
ENSXMAG00000018198	ubac2	ENSLOCG00000003185	UBAC2
ENSXMAG00000017261		ENSLOCG00000001324	
ENSXMAG00000015729	rb1	ENSLOCG00000008882	rb1
ENSXMAG00000024327	spock3	ENSLOCG00000003489	spock3
ENSXMAG00000003111		ENSLOCG00000014328	zgc:85932
ENSXMAG00000023378	npr3	ENSLOCG00000006676	npr3
ENSXMAG00000028853	arxa	ENSLOCG00000001912	arxb
ENSXMAG00000023571	ugt8	ENSLOCG00000003443	ugt8
ENSXMAG00000019018	hgd	ENSLOCG00000001925	hgd
ENSXMAG00000003098	eif3k	ENSLOCG00000014336	eif3k
ENSXMAG00000008505	prpf8	ENSLOCG00000005409	prpf8
ENSXMAG00000013663	TMEM102	ENSLOCG00000013750	TMEM102
ENSXMAG00000027528		ENSLOCG00000003719	si:ch211-248e11.2
ENSXMAG00000017808	ndst3	ENSLOCG00000003401	ndst3
ENSXMAG00000003795	ccdc6a	ENSLOCG00000006632	ccdc6b
ENSXMAG00000015207	tmem256	ENSLOCG00000013745	tmem256
ENSXMAG00000017835	prss12	ENSLOCG00000003365	prss12
ENSXMAG00000007355	slc16a9a	ENSLOCG00000006615	slc16a9a
ENSXMAG00000004424	znf668	ENSLOCG00000003222	
ENSXMAG00000017846	mettl14	ENSLOCG00000003385	mettl14
ENSXMAG00000021741	rilpl2	ENSLOCG00000005762	rilpl2
ENSXMAG00000008552	zgc:153372	ENSLOCG00000000881	zgc:153372
ENSXMAG00000020007		ENSLOCG00000018174	
ENSXMAG00000018747	rnf40	ENSLOCG00000000446	
ENSXMAG00000004797	adra1d	ENSLOCG00000002516	adra1d
ENSXMAG00000003861		ENSLOCG00000014782	si:zfos-2326c3.2
ENSXMAG00000018984	prlh2	ENSLOCG00000011568	prlh2
ENSXMAG00000003566	med27	ENSLOCG00000004302	med27
ENSXMAG00000008576	aifm4	ENSLOCG00000004526	aifm4
ENSXMAG00000008589	tlcd2	ENSLOCG00000004500	tlcd2
ENSXMAG00000003050	srsf7a	ENSLOCG00000014314	

ENSXMAG00000007368	bicc1a	ENSLOCG00000006500	bicc1a
ENSXMAG00000018915	oplah	ENSLOCG00000008846	oplah
ENSXMAG00000027949	endog	ENSLOCG00000004163	endog
ENSXMAG00000028447	si:ch73-376l24.4	ENSLOCG00000005572	si:ch73-376l24.4
ENSXMAG00000018776	phkg2	ENSLOCG00000003344	phkg2
ENSXMAG00000020008	lrrtm1	ENSLOCG00000018176	lrrtm1
ENSXMAG00000003797	ube2d1a	ENSLOCG00000006441	ube2d1b
ENSXMAG00000008591	cluha	ENSLOCG00000004458	cluha
ENSXMAG00000027146	foxh1	ENSLOCG00000008937	foxh1
ENSXMAG00000018831		ENSLOCG00000003315	si:ch73-81k8.2
ENSXMAG00000018905	ppp1r16a	ENSLOCG00000008960	ppp1r16a
ENSXMAG00000019924		ENSLOCG00000018045	
ENSXMAG00000005966	suclg1	ENSLOCG00000002579	suclg1
ENSXMAG00000007752	med17	ENSLOCG00000007406	med17
ENSXMAG00000003800	rgs7a	ENSLOCG00000016589	rgs7a
ENSXMAG00000011380		ENSLOCG00000007970	slc6a7
ENSXMAG00000018837	ube2ia	ENSLOCG00000003753	ube2ia
ENSXMAG00000005961	cisd2	ENSLOCG00000012043	cisd2
ENSXMAG00000003815	grem2a	ENSLOCG00000017367	grem2a
ENSXMAG00000006125	nudt14	ENSLOCG00000010351	nudt14
ENSXMAG00000009046	slc2a2	ENSLOCG00000007335	slc2a2
ENSXMAG00000018874	pdia4	ENSLOCG00000013319	pdia4
ENSXMAG00000026424		ENSLOCG00000007382	
ENSXMAG00000003816		ENSLOCG00000016592	fmn2a
ENSXMAG00000029782	kctd5a	ENSLOCG00000003769	kctd5a
ENSXMAG00000008615	mettl16	ENSLOCG00000004429	mettl16
ENSXMAG00000011406	taf1	ENSLOCG00000014847	taf1
ENSXMAG00000027211	syne2b	ENSLOCG00000010242	SYNE2
ENSXMAG00000022410	arl6ip4	ENSLOCG00000005617	ARL6IP4
ENSXMAG00000025338	bdh2	ENSLOCG00000012025	bdh2
ENSXMAG00000018875	pdpk1b	ENSLOCG00000003794	pdpk1b
ENSXMAG00000018830		ENSLOCG00000013313	
ENSXMAG00000001229		ENSLOCG00000003771	chek2
ENSXMAG00000017735	mthfd2	ENSLOCG00000015377	mthfd2
ENSXMAG00000003898		ENSLOCG00000014790	il1rapl2
ENSXMAG00000018921	tsr3	ENSLOCG00000003693	TSR3
ENSXMAG00000022001	RF00493	ENSLOCG00000019702	RF00493
ENSXMAG00000008063	gstt2	ENSLOCG00000003799	gstt2
ENSXMAG00000000540	SKIDA1	ENSLOCG00000011750	SKIDA1
ENSXMAG00000018928	gnptg	ENSLOCG00000003683	GNPTG
ENSXMAG00000017752	trafd1	ENSLOCG00000008523	trafd1
ENSXMAG00000011459		ENSLOCG00000007628	MTMR2
ENSXMAG00000018950	zmp:0000000624	ENSLOCG00000003601	C7orf50
ENSXMAG00000008665	limk1a	ENSLOCG00000004378	limk1a
ENSXMAG00000000542	mllt10	ENSLOCG00000011763	mllt10
ENSXMAG00000029415		ENSLOCG00000003612	
ENSXMAG00000005905		ENSLOCG00000013517	
ENSXMAG00000011472	cwc15	ENSLOCG00000007570	cwc15
ENSXMAG00000003919	snx25	ENSLOCG00000013780	snx25
ENSXMAG00000017241	acy1	ENSLOCG00000014246	acy1
ENSXMAG00000022593	si:ch211-231f6.6	ENSLOCG00000003623	si:ch211-231f6.6
ENSXMAG00000005879	sparcl1	ENSLOCG00000013522	sparcl1
ENSXMAG00000024119	zgc:112185	ENSLOCG00000003583	
ENSXMAG00000022086	aptx	ENSLOCG00000011556	aptx
ENSXMAG00000007076	commd3	ENSLOCG00000011800	commd3
ENSXMAG00000021713	PPP2R5E	ENSLOCG00000010222	ppp2r5eb

ENSXMAG00000003939		ENSLOCG00000013893	
ENSXMAG00000007733	prdm10	ENSLOCG00000000989	prdm10
ENSXMAG00000018960	anks3	ENSLOCG00000003543	anks3
ENSXMAG00000003848	actn2b	ENSLOCG00000016619	actn2b
ENSXMAG00000025330	abhd14a	ENSLOCG00000014245	abhd14a
ENSXMAG00000008711	psph	ENSLOCG00000004152	psph
ENSXMAG00000007060	bmi1a	ENSLOCG00000011803	bmi1a
ENSXMAG00000005855	dnaja1	ENSLOCG00000011564	dnaja1
ENSXMAG00000024057	PHKG1	ENSLOCG00000004210	phkg1b
ENSXMAG00000017770	selenom	ENSLOCG00000008562	selenom
ENSXMAG00000018970	wdr24	ENSLOCG00000003515	wdr24
ENSXMAG00000026527	chchd2	ENSLOCG00000004252	CHCHD2
ENSXMAG00000017748		ENSLOCG00000009529	akap6
ENSXMAG00000003889	p4ha1b	ENSLOCG00000008546	p4ha1b
ENSXMAG00000007715		ENSLOCG00000001018	aplp2
ENSXMAG00000006169	hadhb	ENSLOCG00000016333	hadhb
ENSXMAG00000005833	rps6	ENSLOCG00000011108	rps6
ENSXMAG00000018971	zgc:112496	ENSLOCG00000003501	zgc:112496
ENSXMAG00000026153	cyb561d2	ENSLOCG00000014241	cyb561d2
ENSXMAG00000017793	usp39	ENSLOCG00000015215	usp39
ENSXMAG00000008726	si:ch211-235m3.5	ENSLOCG00000004273	
ENSXMAG00000005820	acer2	ENSLOCG00000011100	acer2
ENSXMAG00000024340	si:ch211-248a14.8	ENSLOCG00000008508	si:ch211-248a14.8
ENSXMAG00000000549	pip4k2aa	ENSLOCG00000011828	pip4k2ab
ENSXMAG00000021468	rhbdl1	ENSLOCG00000003471	rhbdl1
ENSXMAG00000008728	ube4a	ENSLOCG00000003975	ube4a
ENSXMAG00000020306	h1f0	ENSLOCG00000018098	h1f0
ENSXMAG00000000074	NPAS3	ENSLOCG00000009512	NPAS3
ENSXMAG00000021464	tfap2d	ENSLOCG00000016336	tfap2d
ENSXMAG00000000552	armc3	ENSLOCG00000011846	armc3
ENSXMAG00000018569	ecd	ENSLOCG00000008493	ecd
ENSXMAG00000005761	mttp	ENSLOCG00000012306	mttp
ENSXMAG00000000068	egln3	ENSLOCG00000009500	egln3
ENSXMAG00000000556	msrb2	ENSLOCG00000011855	msrb2
ENSXMAG00000029265	tfap2b	ENSLOCG00000016338	tfap2b
ENSXMAG00000001357	rhot2	ENSLOCG00000003444	rhot2
ENSXMAG00000029361	c8g	ENSLOCG00000003306	c8g
ENSXMAG00000004008	ptcd3	ENSLOCG00000010871	ptcd3
ENSXMAG00000017859	ebf2	ENSLOCG00000015225	ebf2
ENSXMAG00000028528	eapp	ENSLOCG00000009484	eapp
ENSXMAG00000017166	bap1	ENSLOCG00000014219	bap1
ENSXMAG00000007699	dhx34	ENSLOCG00000014928	dhx34
ENSXMAG00000016278	map3k5	ENSLOCG00000017015	map3k5
ENSXMAG00000018564	fam149b1	ENSLOCG00000008469	fam149b1
ENSXMAG00000004032	polr1a	ENSLOCG00000010881	polr1a
ENSXMAG00000011604	THOC2	ENSLOCG00000014546	thoc2
ENSXMAG00000029871		ENSLOCG00000003416	wdr90
ENSXMAG00000001499	prkd1	ENSLOCG00000009700	prkd1
ENSXMAG00000007685	psmd8	ENSLOCG00000014197	
ENSXMAG00000008776	c1qtnf5	ENSLOCG00000003445	c1qtnf5
ENSXMAG00000020235	nup88	ENSLOCG00000002368	nup88
ENSXMAG00000026564	rnf26	ENSLOCG00000003440	rnf26
ENSXMAG00000005743	sod3b	ENSLOCG00000018062	sod3b
ENSXMAG00000018992	nlrc3	ENSLOCG00000003349	nlrc3
ENSXMAG00000017897		ENSLOCG00000016065	DGUOK
ENSXMAG00000008080	phf19	ENSLOCG00000006428	phf19

ENSXMAG00000024501	foxg1a	ENSLOCG00000017702	foxg1a
ENSXMAG00000017900		ENSLOCG00000008019	tcn2
ENSXMAG00000014939	jmjd7	ENSLOCG00000011726	jmjd7
ENSXMAG00000018555	dnajc9	ENSLOCG00000008452	dnajc9
ENSXMAG00000019009	si:ch211-183d21.1	ENSLOCG00000003333	si:ch211-183d21.1
ENSXMAG00000004033	st3gal5	ENSLOCG00000010912	st3gal5
ENSXMAG00000025098		ENSLOCG00000011757	fbxo34
ENSXMAG00000017910	si:ch211-102c2.7	ENSLOCG00000008033	si:ch211-102c2.7
ENSXMAG00000022746	ndnfl	ENSLOCG00000008443	ndnfl
ENSXMAG00000019010	si:ch211-183d21.3	ENSLOCG00000003322	si:ch211-183d21.3
ENSXMAG00000021431	lrp11	ENSLOCG00000017013	lrp11
ENSXMAG00000019011	RDH13	ENSLOCG00000015760	
ENSXMAG00000006249	pcmt	ENSLOCG00000017009	pcmt
ENSXMAG00000009354	hectd1	ENSLOCG00000009590	hectd1
ENSXMAG00000017911	si:ch211-106a19.1	ENSLOCG00000008057	si:ch211-106a19.1
ENSXMAG00000020869	decr2	ENSLOCG00000003276	decr2
ENSXMAG00000017131	erc2	ENSLOCG00000014194	erc2
ENSXMAG00000004064		ENSLOCG00000013756	si:dkey-185e18.7
ENSXMAG00000020825	rab11fip3	ENSLOCG00000003257	RAB11FIP3
ENSXMAG00000000597	tpk1	ENSLOCG00000013400	tpk1
ENSXMAG00000024600	ap4s1	ENSLOCG00000009611	ap4s1
ENSXMAG00000009308	strn3	ENSLOCG00000009618	strn3
ENSXMAG00000004071	irf2	ENSLOCG00000013738	irf2
ENSXMAG00000008816	grik4	ENSLOCG00000002001	grik4
ENSXMAG00000029757	scfd1	ENSLOCG00000009663	scfd1
ENSXMAG00000012797		ENSLOCG00000015384	fam160b2
ENSXMAG00000019050	telo2	ENSLOCG00000003226	telo2
ENSXMAG00000017916	ap1b1	ENSLOCG00000008079	ap1b1
ENSXMAG00000021954	sec61g	ENSLOCG00000013405	sec61g
ENSXMAG00000019653	slitrk4	ENSLOCG00000017498	slitrk4
ENSXMAG00000021971	gas2l1	ENSLOCG00000008106	gas2l1
ENSXMAG00000013810	slitrk2	ENSLOCG00000017499	slitrk2
ENSXMAG00000017933	ewsr1b	ENSLOCG00000008118	ewsr1b
ENSXMAG00000006704	pomt1	ENSLOCG00000006233	pomt1
ENSXMAG00000008860	arhgef12a	ENSLOCG00000002060	arhgef12b
ENSXMAG00000020885	PLEKHA1	ENSLOCG00000008307	plekha1a
ENSXMAG00000000627	relch	ENSLOCG00000013429	relch
ENSXMAG00000004114		ENSLOCG00000012077	hspa9
ENSXMAG00000008884	oafa	ENSLOCG00000002126	oafb
ENSXMAG00000008885	pde9al	ENSLOCG00000002144	pde9al
ENSXMAG00000025022	qsox2	ENSLOCG00000003859	qsox2
ENSXMAG00000024384	zgc:113425	ENSLOCG00000010524	zgc:113425
ENSXMAG00000019061	ift140	ENSLOCG00000003182	ift140
ENSXMAG00000021916		ENSLOCG00000002254	
ENSXMAG00000018331	phactr2	ENSLOCG00000015443	phactr2
ENSXMAG00000021266	mgst2	ENSLOCG00000010514	mgst2
ENSXMAG00000008925	scn3b	ENSLOCG00000002321	scn3b
ENSXMAG00000006678	ddx31	ENSLOCG00000005630	ddx31
ENSXMAG00000000636	pign	ENSLOCG00000013442	pign
ENSXMAG00000008934	drd2a	ENSLOCG00000004412	drd2a
ENSXMAG00000019971	sf3b5	ENSLOCG00000017352	sf3b5
ENSXMAG00000020133	si:dkey-94e7.1	ENSLOCG00000018166	
ENSXMAG00000018317	oprm1	ENSLOCG00000015439	oprm1
ENSXMAG00000022197	hnf4g	ENSLOCG00000013459	hnf4g
ENSXMAG00000019095	tmem204	ENSLOCG00000003210	tmem204
ENSXMAG00000003037	rgs17	ENSLOCG00000015438	rgs17

ENSXMAG00000023566	pex2	ENSLOCG00000013475	pex2
ENSXMAG00000029036	metrn	ENSLOCG00000003142	metrn
ENSXMAG00000027645		ENSLOCG00000015434	pcnx2
ENSXMAG00000000076	stub1	ENSLOCG00000009032	stub1
ENSXMAG00000018313	map3k21	ENSLOCG00000015431	map3k21
ENSXMAG00000000693	zfhx4	ENSLOCG00000013472	zfhx4
ENSXMAG00000024931	ankk1	ENSLOCG00000004369	ankk1
ENSXMAG00000024415		ENSLOCG00000003113	crp1
ENSXMAG00000008956	ttc12	ENSLOCG00000004328	ttc12
ENSXMAG00000025899		ENSLOCG00000015429	IL22RA2
ENSXMAG00000000090	si:ch211-248l17.3	ENSLOCG00000003132	si:ch211-248l17.3
ENSXMAG00000016367	dok2	ENSLOCG00000014900	dok2
ENSXMAG00000022084	si:dkey-103g5.3	ENSLOCG00000001204	si:dkey-103g5.3
ENSXMAG00000018245	OLIG3	ENSLOCG00000017350	olig3
ENSXMAG00000018291		ENSLOCG00000015787	ATL2
ENSXMAG00000000703	mtmr6	ENSLOCG00000004016	mtmr6
ENSXMAG00000003840	sik2b	ENSLOCG00000001288	sik2b
ENSXMAG00000000234	KAZALD1	ENSLOCG00000014896	kazald3
ENSXMAG00000000717	nup58	ENSLOCG00000004040	nup58
ENSXMAG00000003858	ppp2r1bb	ENSLOCG00000001319	ppp2r1bb
ENSXMAG00000023634	hyi	ENSLOCG00000004345	hyi
ENSXMAG00000027582	MLF1	ENSLOCG00000003813	MLF1
ENSXMAG00000003879	med29	ENSLOCG00000014598	med29
ENSXMAG00000021825	ropn1l	ENSLOCG00000013523	ropn1l
ENSXMAG00000029199	RSRC1	ENSLOCG00000003826	RSRC1
ENSXMAG00000028685		ENSLOCG00000010472	
ENSXMAG00000000794	ankrd33ba	ENSLOCG00000013515	ankrd33ba
ENSXMAG00000023351	rpusd4	ENSLOCG00000003124	
ENSXMAG00000020823	shox2	ENSLOCG00000003840	shox2
ENSXMAG00000000816	dap	ENSLOCG00000013575	
ENSXMAG00000004692	mmp17b	ENSLOCG00000000182	
ENSXMAG00000018241	zbtb18	ENSLOCG00000017349	zbtb18
ENSXMAG00000011012		ENSLOCG00000012072	FCHSD1
ENSXMAG00000003896	dock10	ENSLOCG00000003862	dock10
ENSXMAG00000013812	akt3a	ENSLOCG00000015396	akt3a
ENSXMAG00000023092		ENSLOCG00000012060	
ENSXMAG00000021319		ENSLOCG00000005479	c5
ENSXMAG00000010987	trappc11	ENSLOCG00000013704	trappc11
ENSXMAG00000018224	sdccag8	ENSLOCG00000015392	sdccag8
ENSXMAG00000000122	im:7160594	ENSLOCG00000002174	
ENSXMAG00000003992		ENSLOCG00000007612	cep57
ENSXMAG00000016507		ENSLOCG00000015132	tti2
ENSXMAG00000004003	mre11a	ENSLOCG00000007516	mre11a
ENSXMAG00000009893		ENSLOCG00000002257	EME2
ENSXMAG00000012788	zc2hc1a	ENSLOCG00000013490	zc2hc1a
ENSXMAG00000026936	tnfaip3	ENSLOCG00000015425	tnfaip3
ENSXMAG00000004981		ENSLOCG00000015125	SLC18A1
ENSXMAG00000016879	TMEM43	ENSLOCG00000013056	TMEM43
ENSXMAG00000015728	perp	ENSLOCG00000015423	perp
ENSXMAG00000012808	bco1	ENSLOCG00000003896	bco1
ENSXMAG00000029874	tmem26a	ENSLOCG00000006731	tmem26a
ENSXMAG00000010982		ENSLOCG00000012111	spdl1
ENSXMAG00000016845	zgc:112334	ENSLOCG00000014406	zgc:112334
ENSXMAG00000004973	ppiab	ENSLOCG00000000251	ppiab
ENSXMAG00000027181	arid5b	ENSLOCG00000006809	arid5b
ENSXMAG00000004024	TMPRSS13	ENSLOCG00000006710	tmprss13b

ENSXMAG00000012833	tmem56a	ENSLOCG00000008185	tmem56a
ENSXMAG00000004962	zmiz2	ENSLOCG00000015282	zmiz2
ENSXMAG00000028361	ndufaf3	ENSLOCG00000014399	NDUFAF3
ENSXMAG00000004035	fxyd6	ENSLOCG00000006698	fxyd6
ENSXMAG00000018196	rtkn2a	ENSLOCG00000006821	rtkn2a
ENSXMAG00000010975		ENSLOCG00000012109	sil1
ENSXMAG00000016806	nckipsd	ENSLOCG00000014379	nckipsd
ENSXMAG00000010972	uvssa	ENSLOCG00000010991	uvssa
ENSXMAG00000000877	ogdhb	ENSLOCG00000015292	OGDH
ENSXMAG00000016779	slc25a20	ENSLOCG00000014407	slc25a20
ENSXMAG00000018174	cep170aa	ENSLOCG00000015386	cep170aa
ENSXMAG00000004081		ENSLOCG00000017728	
ENSXMAG00000004087	gpr184	ENSLOCG00000017729	gpr184
ENSXMAG00000016755	fblim1	ENSLOCG00000007884	fblim1
ENSXMAG00000004089	PRKD2	ENSLOCG00000014916	PRKD2
ENSXMAG00000017541		ENSLOCG00000010999	nkx1.2lb
ENSXMAG00000026903	plcxd2	ENSLOCG00000003981	plcxd2
ENSXMAG00000018169	lyrm7	ENSLOCG00000008270	lyrm7
ENSXMAG00000010962		ENSLOCG00000007337	tctn1
ENSXMAG00000021360	DYNC2LI1	ENSLOCG00000016417	DYNC2LI1
ENSXMAG00000000901	adam9	ENSLOCG00000015391	adam9
ENSXMAG00000027193	fbxo42	ENSLOCG00000007861	fbxo42
ENSXMAG00000004130	strn4	ENSLOCG00000014921	strn4
ENSXMAG00000018141	abcg5	ENSLOCG00000016413	abcg5
ENSXMAG00000012863	mak	ENSLOCG00000013148	
ENSXMAG00000000948		ENSLOCG00000013925	ercc6l
ENSXMAG00000018127	abcg8	ENSLOCG00000016405	abcg8
ENSXMAG00000023269	necap2	ENSLOCG00000007836	necap2
ENSXMAG00000014998	si:ch211-191j22.3	ENSLOCG00000014856	si:ch211-191j22.3
ENSXMAG00000004177	fkrp	ENSLOCG00000017730	fkrp
ENSXMAG00000019886	sdhaf1	ENSLOCG00000017725	sdhaf1
ENSXMAG00000004179	slc1a5	ENSLOCG00000014907	slc1a5
ENSXMAG00000015000	nudt18	ENSLOCG00000015389	nudt18
ENSXMAG00000028470	egln2	ENSLOCG00000014905	egln2
ENSXMAG00000018108	lrpprc	ENSLOCG00000016390	lrpprc
ENSXMAG00000004210	tmem160	ENSLOCG00000014903	tmem160
ENSXMAG00000015001	LRRC2	ENSLOCG00000010286	
ENSXMAG00000028521	npas1	ENSLOCG00000014899	npas1
ENSXMAG00000027016	tfap2a	ENSLOCG00000013190	tfap2a
ENSXMAG00000018089	slc3a1	ENSLOCG00000016381	slc3a1
ENSXMAG00000004235	ap2s1	ENSLOCG00000014892	ap2s1
ENSXMAG00000029335	mak16	ENSLOCG00000015135	mak16
ENSXMAG00000016681	MFAP2	ENSLOCG00000007802	MFAP2
ENSXMAG00000018077	prepl	ENSLOCG00000016376	prepl
ENSXMAG00000007299	slc35b3	ENSLOCG00000013210	slc35b3
ENSXMAG00000004238	rab4b	ENSLOCG00000014888	rab4b
ENSXMAG00000022375	rhoub	ENSLOCG00000014885	rhoub
ENSXMAG00000024628	CAMKMT	ENSLOCG00000016374	camkmt
ENSXMAG00000005992	eef1e1	ENSLOCG00000013217	eef1e1
ENSXMAG00000016601	ATAD3A	ENSLOCG00000007636	ATAD3A
ENSXMAG00000000652		ENSLOCG00000014859	
ENSXMAG00000024758	RF02252	ENSLOCG00000020618	RF02252
ENSXMAG00000027299	bloc1s5	ENSLOCG00000013223	bloc1s5
ENSXMAG00000025648	six2a	ENSLOCG00000016371	six2a
ENSXMAG00000016586	ssu72	ENSLOCG00000007667	ssu72
ENSXMAG00000005999	txndc5	ENSLOCG00000013233	txndc5

ENSXMAG00000006011	bmp6	ENSLOCG00000013238	bmp6
ENSXMAG00000016861	SRBD1	ENSLOCG00000016360	SRBD1
ENSXMAG00000025608		ENSLOCG00000002788	rsph14
ENSXMAG00000006020	zgc:101569	ENSLOCG00000013243	zgc:101569
ENSXMAG00000029788		ENSLOCG00000010302	SRP19
ENSXMAG00000025368	pdcd6	ENSLOCG00000013249	pdcd6
ENSXMAG00000006041	ipo4	ENSLOCG00000001149	ipo4
ENSXMAG00000013455	stxbp2	ENSLOCG00000006977	stxbp2
ENSXMAG00000016873	cfap36	ENSLOCG00000015379	cfap36
ENSXMAG00000000233	NBL1	ENSLOCG00000007543	NBL1
ENSXMAG00000023961	pet100	ENSLOCG00000006954	pet100
ENSXMAG00000021944		ENSLOCG00000004493	ccdc180
ENSXMAG00000016887	ppp4r3b	ENSLOCG00000015378	PPP4R3B
ENSXMAG00000006090	tm9sf1	ENSLOCG00000001034	tm9sf1
ENSXMAG00000000225	capzb	ENSLOCG00000007567	capzb
ENSXMAG00000016907	PNPT1	ENSLOCG00000015375	PNPT1
ENSXMAG00000015148	card9	ENSLOCG00000004437	card9
ENSXMAG00000006127		ENSLOCG00000017984	
ENSXMAG00000000990	celsr2	ENSLOCG00000012563	celsr2
ENSXMAG00000022144	psme1	ENSLOCG00000001050	psme1
ENSXMAG00000024706	pbx4	ENSLOCG00000006841	pbx4
ENSXMAG00000001003	dstyk	ENSLOCG00000012699	dstyk
ENSXMAG00000023381	thtpa	ENSLOCG00000001092	
ENSXMAG00000025530	nuak2	ENSLOCG00000012687	nuak2
ENSXMAG00000013346	atp13a1	ENSLOCG00000006801	atp13a1
ENSXMAG00000001044	ccnd3	ENSLOCG00000012668	ccnd3
ENSXMAG00000001060	bysl	ENSLOCG00000012661	bysl
ENSXMAG00000013334	lmx1al	ENSLOCG00000001366	
ENSXMAG00000001071	med20	ENSLOCG00000012654	med20
ENSXMAG00000013308	gmip	ENSLOCG00000008518	gmip
ENSXMAG00000006196	pabpn1	ENSLOCG00000000796	pabpn1
ENSXMAG00000001082	usp49	ENSLOCG00000012648	usp49
ENSXMAG00000006206	ngdn	ENSLOCG00000000774	
ENSXMAG00000001089	tmcc2	ENSLOCG00000012691	TMCC2
ENSXMAG00000001096	cdkn1a	ENSLOCG00000012523	
ENSXMAG00000013279	pgls	ENSLOCG00000008572	pgls
ENSXMAG00000001097	SRSF3	ENSLOCG00000012519	srsf3a
ENSXMAG00000022456	SLC22A17	ENSLOCG00000001006	
ENSXMAG00000015195	ddr2l	ENSLOCG00000002383	ddr2l
ENSXMAG00000001170	strip1	ENSLOCG00000011672	strip1
ENSXMAG00000025320	RAMP3	ENSLOCG00000013297	
ENSXMAG00000024622		ENSLOCG00000005906	vac14
ENSXMAG00000006295	pomp	ENSLOCG00000001993	pomp
ENSXMAG00000015244	sncaip	ENSLOCG00000008733	sncaip
ENSXMAG00000027818	ptger1c	ENSLOCG00000008602	ptger1c
ENSXMAG00000006359		ENSLOCG00000005188	NSMAF
ENSXMAG00000013192	adgrl1a	ENSLOCG00000008817	adgrl1a
ENSXMAG00000007143	irf6	ENSLOCG00000011297	irf6
ENSXMAG00000007160		ENSLOCG00000011331	WDR77
ENSXMAG00000006393		ENSLOCG00000001733	RBBP8
ENSXMAG00000006395	fam168b	ENSLOCG00000009724	fam168b
ENSXMAG00000001271	pbrm1	ENSLOCG00000010556	pbrm1
ENSXMAG00000023237	fbxo45	ENSLOCG00000009707	fbxo45
ENSXMAG00000017050	hhat	ENSLOCG00000015584	hhat
ENSXMAG00000006402	nrros	ENSLOCG00000009699	nrros
ENSXMAG00000017065	KCNH1	ENSLOCG00000015579	kcnh1b

ENSXMAG00000001306	cse1l	ENSLOCG00000003099	cse1l
ENSXMAG00000001423	THUMPD3	ENSLOCG00000009800	thumpd3
ENSXMAG00000006494	prmt6	ENSLOCG00000017743	prmt6
ENSXMAG00000006495	rnpc3	ENSLOCG00000007986	rnpc3
ENSXMAG00000025867	qtrt1	ENSLOCG00000008949	qtrt1
ENSXMAG00000024233		ENSLOCG00000015410	desi2
ENSXMAG00000001516	rad18	ENSLOCG00000010629	rad18
ENSXMAG00000013133		ENSLOCG00000008961	
ENSXMAG00000017158		ENSLOCG00000015502	mtrf1l
ENSXMAG00000006525	col11a1a	ENSLOCG00000008000	col11a1a
ENSXMAG00000029459	fbxo5	ENSLOCG00000015506	fbxo5
ENSXMAG00000017180	vip	ENSLOCG00000015508	vip
ENSXMAG00000001535	cav3	ENSLOCG00000010650	cav3
ENSXMAG00000024943	gipc1	ENSLOCG00000009925	gipc1
ENSXMAG00000021153	parp3	ENSLOCG00000010661	parp3
ENSXMAG00000001841	tecrl2a	ENSLOCG00000008031	tecrl2a
ENSXMAG00000022645	prkacaa	ENSLOCG00000009984	prkacaa
ENSXMAG00000001577	fancd2	ENSLOCG00000010711	fancd2
ENSXMAG00000017203	slc16a12a	ENSLOCG00000000022	slc16a12b
ENSXMAG00000012974	zgc:103625	ENSLOCG00000013936	zgc:103625
ENSXMAG00000012955	hgs	ENSLOCG00000013943	hgs
ENSXMAG00000011719	usp4	ENSLOCG00000010739	usp4
ENSXMAG00000015452	pank1a	ENSLOCG00000000023	pank1b
ENSXMAG00000012923	rsad1	ENSLOCG00000013969	rsad1
ENSXMAG00000015470	zgc:65997	ENSLOCG00000000024	zgc:65997
ENSXMAG00000001657	emc3	ENSLOCG00000010724	emc3
ENSXMAG00000012919	epn3a	ENSLOCG00000013956	epn3a
ENSXMAG00000015471	nphp1	ENSLOCG00000015611	nphp1
ENSXMAG00000012910	fn3krp	ENSLOCG00000014013	fn3krp
ENSXMAG00000012874	tbcd	ENSLOCG00000013980	tbcd
ENSXMAG00000012909	znf750	ENSLOCG00000014010	
ENSXMAG00000028833	mrps5	ENSLOCG00000015618	mrps5
ENSXMAG00000017249	thumpd2	ENSLOCG00000015747	THUMPD2
ENSXMAG00000017170	map4k3b	ENSLOCG00000015753	map4k3b
ENSXMAG00000017168	arhgef33	ENSLOCG00000015769	arhgef33
ENSXMAG00000001803		ENSLOCG00000014458	pfkfb4a
ENSXMAG00000017162		ENSLOCG00000016166	slc17a5
ENSXMAG00000012733	baiap2a	ENSLOCG00000012924	baiap2a
ENSXMAG00000028055	mb21d1	ENSLOCG00000016169	mb21d1
ENSXMAG00000017126	ddx43	ENSLOCG00000016170	ddx43
ENSXMAG00000022836	ppp3r1a	ENSLOCG00000016015	ppp3r1a
ENSXMAG00000021459	pno1	ENSLOCG00000016017	pno1
ENSXMAG00000029168	wdr92	ENSLOCG00000016020	wdr92
ENSXMAG00000001839	eefsec	ENSLOCG00000014535	eefsec
ENSXMAG00000021376	tspan10	ENSLOCG00000013019	tspan10
ENSXMAG00000022273	c1d	ENSLOCG00000016024	c1d
ENSXMAG00000001907	prkdc	ENSLOCG00000006046	prkdc
ENSXMAG00000011739	meis1b	ENSLOCG00000016026	meis1b
ENSXMAG00000011813	actr2a	ENSLOCG00000016032	actr2a
ENSXMAG00000022686	peli1b	ENSLOCG00000016053	peli1b
ENSXMAG00000029585	CCDC137	ENSLOCG00000013031	CCDC137
ENSXMAG00000027229		ENSLOCG00000016051	abch1
ENSXMAG00000005591	wdpcp	ENSLOCG00000016068	wdpcp
ENSXMAG00000024045		ENSLOCG00000013072	
ENSXMAG00000005604	otx1	ENSLOCG00000016074	otx1
ENSXMAG00000005611	ehbp1	ENSLOCG00000016075	ehbp1

ENSXMAG00000019450	amn	ENSLOCG00000009838	AMN
ENSXMAG00000005652	ciao2b	ENSLOCG00000004939	ciao2b
ENSXMAG00000022977	si:ch211-250c4.4	ENSLOCG00000009491	si:ch211-250c4.4
ENSXMAG00000005697	ncapd3	ENSLOCG00000004914	ncapd3
ENSXMAG00000005726	calhm3	ENSLOCG00000011856	calhm3
ENSXMAG00000005731		ENSLOCG00000009470	hells
ENSXMAG00000012401	usp36	ENSLOCG00000012710	usp36
ENSXMAG00000021554		ENSLOCG00000016038	CEP68
ENSXMAG00000021357	sdhaf4	ENSLOCG00000017100	sdhaf4
ENSXMAG00000005769	tlx1	ENSLOCG00000011642	tlx1
ENSXMAG00000027566	fbxw4	ENSLOCG00000011687	fbxw4
ENSXMAG00000025827	borcs7	ENSLOCG00000011739	borcs7
ENSXMAG00000005813	cyp17a1	ENSLOCG00000011729	cyp17a1
ENSXMAG00000005864	gbf1	ENSLOCG00000009553	gbf1
ENSXMAG00000005921	pitx3	ENSLOCG00000009566	pitx3
ENSXMAG00000005923	hspa12a	ENSLOCG00000009586	hspa12a
ENSXMAG00000012345		ENSLOCG00000012620	
ENSXMAG00000000773	ENO4	ENSLOCG00000009597	eno4
ENSXMAG00000012305	pycr1a	ENSLOCG00000012583	pycr1a
ENSXMAG00000000782	shtn1	ENSLOCG00000009606	shtn1
ENSXMAG00000003759	atp6v1ba	ENSLOCG00000009629	atp6v1ba
ENSXMAG00000025346	vax1	ENSLOCG00000009641	vax1
ENSXMAG00000003754		ENSLOCG00000009651	
ENSXMAG00000012142	rnf213a	ENSLOCG00000012503	rnf213a
ENSXMAG00000003738	slc18a2	ENSLOCG00000009666	slc18a2
ENSXMAG00000010508	pdzd8	ENSLOCG00000009680	pdzd8
ENSXMAG00000025067	edaradd	ENSLOCG00000016636	edaradd
ENSXMAG00000010514	exo1	ENSLOCG00000016639	exo1
ENSXMAG00000010517	yipf4	ENSLOCG00000016640	yipf4
ENSXMAG00000010525	birc6	ENSLOCG00000016644	birc6
ENSXMAG00000026097	garem	ENSLOCG00000000552	garem
ENSXMAG00000001959	ncf2	ENSLOCG00000001984	ncf2
ENSXMAG00000021495	MED9	ENSLOCG00000001878	MED9
ENSXMAG00000001969	dph2	ENSLOCG00000002599	
ENSXMAG00000028392	zgc:165532	ENSLOCG00000011525	zgc:165532
ENSXMAG00000028912	rgs9bp	ENSLOCG00000011529	rgs9bp
ENSXMAG00000010575	ttc27	ENSLOCG00000016680	ttc27
ENSXMAG00000029370		ENSLOCG00000005434	zgc:56231
ENSXMAG00000024498	RPS20	ENSLOCG00000005417	RPS20
ENSXMAG00000026539	ltbp1	ENSLOCG00000016686	ltbp1
ENSXMAG00000001987	ANKS6	ENSLOCG00000013281	ANKS6
ENSXMAG00000025864		ENSLOCG00000013271	AHRR
ENSXMAG00000004827	RNPS1	ENSLOCG00000001758	
ENSXMAG00000004422	rasgrp3	ENSLOCG00000016689	rasgrp3
ENSXMAG00000001999	pigm	ENSLOCG00000013256	pigm
ENSXMAG00000000061	snx16	ENSLOCG00000010309	snx16
ENSXMAG00000004345	wwp1	ENSLOCG00000010335	wwp1
ENSXMAG00000024224	RMDN1	ENSLOCG00000010345	RMDN1
ENSXMAG00000004594	idi1	ENSLOCG00000012273	idi1
ENSXMAG00000019589	fzd8a	ENSLOCG00000000457	fzd8a
ENSXMAG00000029425	GJD4	ENSLOCG00000000502	GJD4
ENSXMAG00000008171	cul2	ENSLOCG00000000655	cul2
ENSXMAG00000011009	itgb1a	ENSLOCG00000000819	itgb1a
ENSXMAG00000004756	abca3b	ENSLOCG00000002542	abca3b
ENSXMAG00000024122	rab18a	ENSLOCG00000007491	rab18a
ENSXMAG00000004711	ccnf	ENSLOCG00000002525	

ENSXMAG00000025815		ENSLOCG00000002506	
ENSXMAG00000021302	mpp7a	ENSLOCG00000007418	mpp7a
ENSXMAG00000004649	coro7	ENSLOCG00000002377	coro7
ENSXMAG00000000741	waca	ENSLOCG00000007394	waca
ENSXMAG00000025513	bambia	ENSLOCG00000007374	bambia
ENSXMAG00000004642	glis2b	ENSLOCG00000002612	glis2a
ENSXMAG00000004520	TRAP1	ENSLOCG00000002737	trap1
ENSXMAG00000004507	DNAAF5	ENSLOCG00000001606	DNAAF5
ENSXMAG00000027445	prkar1b	ENSLOCG00000001572	prkar1b
ENSXMAG00000023393		ENSLOCG00000002807	CCP110
ENSXMAG00000000935		ENSLOCG00000002954	GPR139
ENSXMAG00000029275	zgc:171489	ENSLOCG00000008315	zgc:171489
ENSXMAG00000000979	sdr42e2	ENSLOCG00000008254	sdr42e2
ENSXMAG00000020995	MOSMO	ENSLOCG00000008203	mosmob
ENSXMAG00000001021		ENSLOCG00000008136	si:ch211-256e16.3
ENSXMAG00000001065	anks4b	ENSLOCG00000008115	anks4b
ENSXMAG00000001067		ENSLOCG00000008103	
ENSXMAG00000001092	DXO	ENSLOCG00000008086	DXO
ENSXMAG00000001102	STK19	ENSLOCG00000008071	
ENSXMAG00000001226	cep112	ENSLOCG00000013670	cep112
ENSXMAG00000001361	zmp:0000000735	ENSLOCG00000017764	zmp:0000000735
ENSXMAG00000001363	gaa	ENSLOCG00000013866	gaa
ENSXMAG00000001390	tbc1d16	ENSLOCG00000013855	tbc1d16
ENSXMAG00000023750	cbx4	ENSLOCG00000013850	cbx4
ENSXMAG00000001479	slc5a11	ENSLOCG00000005342	slc5a11
ENSXMAG00000015082	e4f1	ENSLOCG00000005261	e4f1
ENSXMAG00000015043	narfl	ENSLOCG00000005181	narfl
ENSXMAG00000015029	HAGHL	ENSLOCG00000005131	HAGHL
ENSXMAG00000014995	hagh	ENSLOCG00000005110	hagh
ENSXMAG00000019849	fahd1	ENSLOCG00000005085	fahd1
ENSXMAG00000022110	RPL3L	ENSLOCG00000008905	RPL3L
ENSXMAG00000014932	ndufb10	ENSLOCG00000008898	ndufb10
ENSXMAG00000014912	rps2	ENSLOCG00000008889	rps2
ENSXMAG00000024160		ENSLOCG00000008879	rnf151
