# Supplementary material for: Global assessment of organ specific basal gene expression over a diurnal cycle with analyses of gene copies exhibiting cyclic expression patterns
Source: BMC Genomics. 2020 Nov 11;21:787. doi: 10.1186/s12864-020-07202-9 (PMC7659085; doi:10.1186/s12864-020-07202-9)
Supplement: Supplementary file 7 — Additional file 7: Supplement Table 6. Number of cyclic Ohnologs and singletons. [file 12864_2020_7202_MOESM7_ESM.pdf]

Supplment Table 6

	skin	brain	ovary	heart	muscle	eye	gill	liver	testis
Circadian.genes	935	731	224	659	2076	4033	1740	1039	294
tractable.cir.gene	609	470	144	432	1385	2517	1197	720	178
Singletons	529	373	117	376	1179	2030	1072	649	151
Ohnologs	80	97	27	56	206	487	125	71	27
Paired Ohnologs	12	8	0	0	36	174	12	4	0
Ohnolog pairs	6	4	0	0	18	87	6	2	0
rate.of.orthology	65.1%	64.3%	64.3%	65.6%	66.7%	62.4%	68.8%	69.3%	60.5%
rate.of.both.Ohnolog	2.0%	1.7%	0.0%	0.0%	2.6%	6.9%	1.0%	0.6%	0.0%
