# Supplementary material for: Global assessment of organ specific basal gene expression over a diurnal cycle with analyses of gene copies exhibiting cyclic expression patterns
Source: BMC Genomics. 2020 Nov 11;21:787. doi: 10.1186/s12864-020-07202-9 (PMC7659085; doi:10.1186/s12864-020-07202-9)
Supplement: Supplementary file 8 — Additional file 8: Supplement Table 7. Functional divergence of cyclic gene Ohnologs. [file 12864_2020_7202_MOESM8_ESM.pdf]

Supplement Table 7

id	skin	brain	gonad.F	heart	muscle	eye	gill	liver	gonad.M	gar.ortho	external_gene_name	Intra-organ peak time difference Skin	Intra-organ peak time difference Brain	Intra-organ peak time difference Ovary	Intra-organ peak time difference Heart	Intra-organ peak time difference Muscle	Intra-organ peak time difference Eye	Intra-organ peak time difference Gill	Intra-organ peak time difference Liver	Intra-organ peak time difference Testis	Inter-organ peak time difference
ENSXMAG00000022293	no	no	no	18	15	15	no	no	no	ENSLOCG00000000072.ENSXMAG00000022293.ENSXMAG00000024612		none	none	none	sub	no diff	no diff	none	none	none	sub
ENSXMAG00000024612	no	no	no	no	15	15	no	no	no	ENSLOCG00000000072.ENSXMAG00000024612.ENSXMAG00000022293		none	none	none	sub	no diff	no diff	none	none	none	sub
ENSXMAG00000005497	no	3	no	3	no	3	no	no	no	ENSLOCG00000000103.ENSXMAG00000005497.ENSXMAG00000014128	cdc14ab	none	sub	none	sub	none	sub	none	none	none	sub
ENSXMAG00000014128	no	no	no	no	no	no	no	no	no	ENSLOCG00000000103.ENSXMAG00000014128.ENSXMAG00000005497		none	sub	none	sub	none	sub	none	none	none	sub
ENSXMAG00000026493	no	no	no	no	no	18	no	no	0	ENSLOCG00000000150.ENSXMAG00000026493.ENSXMAG00000027141	rem2	none	none	none	none	none	sub	none	none	sub	sub
ENSXMAG00000027141	no	no	no	no	no	no	no	no	no	ENSLOCG00000000150.ENSXMAG00000027141.ENSXMAG00000026493		none	none	none	none	none	sub	none	none	sub	sub
ENSXMAG00000006023	no	no	no	no	no	18	no	no	no	ENSLOCG00000000168.ENSXMAG00000006023.ENSXMAG00000016807	carmil3	none	none	none	none	none	sub	none	none	none	sub
ENSXMAG00000016807	no	no	no	no	no	no	no	no	no	ENSLOCG00000000168.ENSXMAG00000016807.ENSXMAG00000006023	CARMIL3	none	none	none	none	none	sub	none	none	none	sub
ENSXMAG00000011992	no	no	no	no	no	no	no	no	no	ENSLOCG00000000180.ENSXMAG00000011992.ENSXMAG00000029414		none	none	none	none	none	none	sub	none	none	sub
ENSXMAG00000029414	no	no	no	no	no	no	0	no	no	ENSLOCG00000000180.ENSXMAG00000029414.ENSXMAG00000011992		none	none	none	none	none	none	sub	none	none	sub
ENSXMAG00000011663	no	no	0	no	no	no	no	no	no	ENSLOCG00000000202.ENSXMAG00000011663.ENSXMAG00000022899	slc6a16a	none	none	sub	none	none	sub	none	none	none	sub
ENSXMAG00000022899	no	no	no	no	no	21	no	no	no	ENSLOCG00000000202.ENSXMAG00000022899.ENSXMAG00000011663	slc6a16b	none	none	sub	none	none	sub	none	none	none	sub
ENSXMAG00000006078	no	no	no	3	no	no	no	no	no	ENSLOCG00000000216.ENSXMAG00000006078.ENSXMAG00000019289	si:zfos-943e10.1	none	none	none	sub	none	none	none	none	none	sub
ENSXMAG00000019289	no	no	no	no	no	no	no	no	no	ENSLOCG00000000216.ENSXMAG00000019289.ENSXMAG00000006078		none	none	none	sub	none	none	none	none	none	sub
ENSXMAG00000022622	6	no	no	no	no	no	no	no	no	ENSLOCG00000000224.ENSXMAG00000022622.ENSXMAG00000029196	KCNK4	sub	none	none	none	none	none	none	none	none	sub
ENSXMAG00000029196	no	no	no	no	no	no	no	no	no	ENSLOCG00000000224.ENSXMAG00000029196.ENSXMAG00000022622	kcnk4a	sub	none	none	none	none	none	none	none	none	sub
ENSXMAG00000022464	no	no	no	no	no	12	no	no	no	ENSLOCG00000000392.ENSXMAG00000022464.ENSXMAG00000026065	olfm1b	none	none	none	none	none	sub	none	none	none	sub
ENSXMAG00000026065	no	no	no	no	no	no	no	no	no	ENSLOCG00000000392.ENSXMAG00000026065.ENSXMAG00000022464		none	none	none	none	none	sub	none	none	none	sub
ENSXMAG00000004738	no	no	no	no	no	3	no	no	no	ENSLOCG00000000413.ENSXMAG00000004738.ENSXMAG00000006384	klf6a	none	none	none	none	none	no diff	none	none	none	sub
ENSXMAG00000006384	no	no	no	no	no	3	no	no	no	ENSLOCG00000000413.ENSXMAG00000006384.ENSXMAG00000004738	KLF6 (1 of many)	none	none	none	none	none	no diff	none	none	none	sub
ENSXMAG00000006847	no	no	no	no	no	no	no	no	no	ENSLOCG00000000422.ENSXMAG00000006847.ENSXMAG00000008420	b3glcta	none	sub	none	none	none	none	none	none	none	sub
ENSXMAG00000008420	no	9	no	no	no	no	no	no	no	ENSLOCG00000000422.ENSXMAG00000008420.ENSXMAG00000006847	b3glctb	none	sub	none	none	none	none	none	none	none	sub
ENSXMAG00000009695	no	no	no	no	no	no	no	no	no	ENSLOCG00000000482.ENSXMAG00000009695.ENSXMAG00000022498	ypel5	sub	none	none	none	none	none	none	none	none	sub
ENSXMAG00000022498	21	no	no	no	no	no	no	no	no	ENSLOCG00000000482.ENSXMAG00000022498.ENSXMAG00000009695		sub	none	none	none	none	none	none	none	none	sub
ENSXMAG00000011681	no	12	no	no	no	no	no	no	no	ENSLOCG00000000530.ENSXMAG00000011681.ENSXMAG00000018700	slc17a7a	none	sub	none	none	none	none	none	none	none	sub
ENSXMAG00000018700	no	no	no	no	no	no	no	no	no	ENSLOCG00000000530.ENSXMAG00000018700.ENSXMAG00000011681	slc17a7b	none	sub	none	none	none	none	none	none	none	sub
ENSXMAG00000015131	no	no	no	no	no	6	no	no	no	ENSLOCG00000000545.ENSXMAG00000015131.ENSXMAG00000021485		none	none	none	none	none	sub	none	none	none	sub
ENSXMAG00000021485	no	no	no	no	no	no	no	no	no	ENSLOCG00000000545.ENSXMAG00000021485.ENSXMAG00000015131	KCNK2	none	none	none	none	none	sub	none	none	none	sub
ENSXMAG00000016750	no	no	no	3	no	21	no	no	no	ENSLOCG00000000554.ENSXMAG00000016750.ENSXMAG00000022421	pvrl2l	none	sub	none	sub	none	sub	none	none	none	sub
ENSXMAG00000022421	no	21	no	no	no	no	no	no	no	ENSLOCG00000000554.ENSXMAG00000022421.ENSXMAG00000016750	si:ch73-22o12.1	none	sub	none	sub	none	sub	none	none	none	sub
ENSXMAG00000000886	no	no	no	no	no	15	no	no	no	ENSLOCG00000000614.ENSXMAG00000000886.ENSXMAG00000013559	adcy2a	none	none	none	none	none	sub	none	none	none	sub
ENSXMAG00000013559	no	no	no	no	no	no	no	no	no	ENSLOCG00000000614.ENSXMAG00000013559.ENSXMAG00000000886	adcy2b	none	none	none	none	none	sub	none	none	none	sub
ENSXMAG00000007350	no	no	no	no	no	18	no	no	no	ENSLOCG00000000775.ENSXMAG00000007350.ENSXMAG00000017975	mybpc2b	none	none	none	none	none	sub	none	none	none	sub
ENSXMAG00000017975	no	no	no	no	no	no	no	no	no	ENSLOCG00000000775.ENSXMAG00000017975.ENSXMAG00000007350	mybpc2a	none	none	none	none	none	sub	none	none	none	sub
ENSXMAG00000006296	no	no	no	no	18	18	no	no	no	ENSLOCG00000000790.ENSXMAG00000006296.ENSXMAG00000008055	NRP1 (1 of many)	none	none	none	none	sub	sub	none	none	none	sub
ENSXMAG00000008055	no	no	no	no	no	no	no	no	no	ENSLOCG00000000790.ENSXMAG00000008055.ENSXMAG00000006296	nrp1a	none	none	none	none	sub	sub	none	none	none	sub
ENSXMAG00000005570	no	no	no	3	no	no	no	no	no	ENSLOCG00000000825.ENSXMAG00000005570.ENSXMAG00000028728	cldnd1b	none	none	none	sub	none	none	none	none	none	sub
ENSXMAG00000028728	no	no	no	no	no	no	no	no	no	ENSLOCG00000000825.ENSXMAG00000028728.ENSXMAG00000005570	cldnd1a	none	none	none	sub	none	none	none	none	none	sub
ENSXMAG00000002328	21	no	no	no	no	0	21	no	no	ENSLOCG00000000846.ENSXMAG00000002328.ENSXMAG00000010655	INPP5K (1 of many)	sub	none	none	none	none	no diff	sub	none	none	sub
ENSXMAG00000010655	no	no	no	no	no	0	no	no	no	ENSLOCG00000000846.ENSXMAG00000010655.ENSXMAG00000002328	inpp5kb	sub	none	none	none	none	no diff	sub	none	none	sub
ENSXMAG00000010760	no	no	no	no	no	no	no	15	no	ENSLOCG00000000850.ENSXMAG00000010760.ENSXMAG00000024636		none	none	none	none	none	sub	none	sub	none	sub
ENSXMAG00000024636	no	no	no	no	no	3	no	no	no	ENSLOCG00000000850.ENSXMAG00000024636.ENSXMAG00000010760	map1sb	none	none	none	none	none	sub	none	sub	none	sub
ENSXMAG00000011022	no	no	no	no	18	no	no	no	no	ENSLOCG00000000949.ENSXMAG00000011022.ENSXMAG00000017047		none	sub	none	none	sub	none	sub	none	none	sub
ENSXMAG00000017047	no	12	no	no	no	no	15	no	no	ENSLOCG00000000949.ENSXMAG00000017047.ENSXMAG00000011022		none	sub	none	none	sub	none	sub	none	none	sub
ENSXMAG00000010132	no	no	no	no	no	no	no	no	no	ENSLOCG00000000967.ENSXMAG00000010132.ENSXMAG00000014022	gdpd5b	none	none	none	none	none	none	sub	none	none	sub
ENSXMAG00000014022	no	no	no	no	no	no	3	no	no	ENSLOCG00000000967.ENSXMAG00000014022.ENSXMAG00000010132	gdpd5a	none	none	none	none	none	none	sub	none	none	sub
ENSXMAG00000002475	no	no	no	no	no	18	no	no	no	ENSLOCG00000000979.ENSXMAG00000002475.ENSXMAG00000016091	gfra4a	none	none	none	none	none	sub	none	none	none	sub
ENSXMAG00000016091	no	no	no	no	no	no	no	no	no	ENSLOCG00000000979.ENSXMAG00000016091.ENSXMAG00000002475	gfra4b	none	none	none	none	none	sub	none	none	none	sub
ENSXMAG00000021574	no	no	no	no	no	no	no	no	no	ENSLOCG00000001014.ENSXMAG00000021574.ENSXMAG00000028221	dnajb9b	none	none	none	none	none	sub	none	none	none	sub
ENSXMAG00000028221	no	no	no	no	no	6	no	no	no	ENSLOCG00000001014.ENSXMAG00000028221.ENSXMAG00000021574	dnajb9a	none	none	none	none	none	sub	none	none	none	sub
ENSXMAG00000003086	no	no	no	no	no	15	no	no	no	ENSLOCG00000001099.ENSXMAG00000003086.ENSXMAG00000005204	rom1b	none	none	none	none	none	no diff	none	none	none	sub
ENSXMAG00000005204	no	no	no	no	no	15	no	no	no	ENSLOCG00000001099.ENSXMAG00000005204.ENSXMAG00000003086	rom1a	none	none	none	none	none	no diff	none	none	none	sub
ENSXMAG00000000586	no	no	no	3	no	no	no	no	no	ENSLOCG00000001119.ENSXMAG00000000586.ENSXMAG00000004639		none	none	none	sub	none	none	none	none	none	sub
ENSXMAG00000004639	no	no	no	no	no	no	no	no	no	ENSLOCG00000001119.ENSXMAG00000004639.ENSXMAG00000000586	PIK3CA	none	none	none	sub	none	none	none	none	none	sub
ENSXMAG00000003979	no	no	no	no	no	no	no	no	no	ENSLOCG00000001132.ENSXMAG00000003979.ENSXMAG00000026119	zgc:113337	none	none	none	none	none	sub	none	none	none	sub
ENSXMAG00000026119	no	no	no	no	no	0	no	no	no	ENSLOCG00000001132.ENSXMAG00000026119.ENSXMAG00000003979		none	none	none	none	none	sub	none	none	none	sub
ENSXMAG00000009232	no	no	no	no	no	0	no	no	no	ENSLOCG00000001143.ENSXMAG00000009232.ENSXMAG00000017134	nrcama	none	none	none	none	none	neo	none	none	none	sub
ENSXMAG00000017134	no	no	no	no	no	15	no	no	no	ENSLOCG00000001143.ENSXMAG00000017134.ENSXMAG00000009232	NRCAM (1 of many)	none	none	none	none	none	neo	none	none	none	sub
ENSXMAG00000001590	no	no	no	no	no	no	no	no	no	ENSLOCG00000001197.ENSXMAG00000001590.ENSXMAG00000011880	hsd17b12a	none	none	none	none	none	sub	none	none	none	sub
ENSXMAG00000011880	no	no	no	no	no	6	no	no	no	ENSLOCG00000001197.ENSXMAG00000011880.ENSXMAG00000001590	hsd17b12b	none	none	none	none	none	sub	none	none	none	sub
ENSXMAG00000008139	no	no	no	3	no	no	no	no	no	ENSLOCG00000001205.ENSXMAG00000008139.ENSXMAG00000012095	cbsa	none	none	none	sub	none	none	none	none	none	sub
ENSXMAG00000012095	no	no	no	no	no	no	no	no	no	ENSLOCG00000001205.ENSXMAG00000012095.ENSXMAG00000008139		none	none	none	sub	none	none	none	none	none	sub
ENSXMAG00000010713	no	no	no	no	no	no	no	no	no	ENSLOCG00000001222.ENSXMAG00000010713.ENSXMAG00000016048	smyd1a	none	none	none	none	none	sub	none	none	none	sub
ENSXMAG00000016048	no	no	no	no	no	21	no	no	no	ENSLOCG00000001222.ENSXMAG00000016048.ENSXMAG00000010713	smyd1b	none	none	none	none	none	sub	none	none	none	sub
ENSXMAG00000016762	no	no	no	no	no	no	no	no	no	ENSLOCG00000001238.ENSXMAG00000016762.ENSXMAG00000018563	DLGAP4	none	none	none	none	none	sub	none	none	none	sub
ENSXMAG00000018563	no	no	no	no	no	15	no	no	no	ENSLOCG00000001238.ENSXMAG00000018563.ENSXMAG00000016762	dlgap4b	none	none	none	none	none	sub	none	none	none	sub
ENSXMAG00000021249	no	no	no	no	no	no	no	no	no	ENSLOCG00000001251.ENSXMAG00000021249.ENSXMAG00000021613	txlnbb	none	none	none	none	none	sub	none	none	none	sub
ENSXMAG00000021613	no	no	no	no	no	6	no	no	no	ENSLOCG00000001251.ENSXMAG00000021613.ENSXMAG00000021249		none	none	none	none	none	sub	none	none	none	sub
ENSXMAG00000009280	no	no	no	no	no	18	no	no	no	ENSLOCG00000001255.ENSXMAG00000009280.ENSXMAG00000017213	cntn1b	none	none	none	none	none	sub	none	none	none	sub
ENSXMAG00000017213	no	no	no	no	no	no	no	no	no	ENSLOCG00000001255.ENSXMAG00000017213.ENSXMAG00000009280	CNTN1	none	none	none	none	none	sub	none	none	none	sub
ENSXMAG00000009225	no	no	no	no	no	no	no	no	no	ENSLOCG00000001270.ENSXMAG00000009225.ENSXMAG00000024443	mvb12bb	none	none	none	none	none	none	sub	none	none	sub
ENSXMAG00000024443	no	no	no	no	no	no	21	no	no	ENSLOCG00000001270.ENSXMAG00000024443.ENSXMAG00000009225	mvb12ba	none	none	none	none	none	none	sub	none	none	sub
ENSXMAG00000004249	no	no	no	no	21	no	no	no	no	ENSLOCG00000001351.ENSXMAG00000004249.ENSXMAG00000018305	pex5lb	none	none	none	none	sub	none	none	none	none	sub
ENSXMAG00000018305	no	no	no	no	no	no	no	no	no	ENSLOCG00000001351.ENSXMAG00000018305.ENSXMAG00000004249	pex5la	none	none	none	none	sub	none	none	none	none	sub
ENSXMAG00000000796	no	no	no	no	no	no	no	no	no	ENSLOCG00000001388.ENSXMAG00000000796.ENSXMAG00000001471	slc1a2b	none	none	none	none	none	sub	none	none	none	sub
ENSXMAG00000001471	no	no	no	no	no	0	no	no	no	ENSLOCG00000001388.ENSXMAG00000001471.ENSXMAG00000000796	slc1a2a	none	none	none	none	none	sub	none	none	none	sub
ENSXMAG00000004905	no	no	no	no	no	21	no	no	no	ENSLOCG00000001420.ENSXMAG00000004905.ENSXMAG00000025264	traf2a	sub	none	none	none	none	neo	none	none	none	sub
ENSXMAG00000025264	21	no	no	no	no	3	no	no	no	ENSLOCG00000001420.ENSXMAG00000025264.ENSXMAG00000004905	TRAF2	sub	none	none	none	none	neo	none	none	none	sub
ENSXMAG00000000759	no	no	no	no	no	no	no	no	no	ENSLOCG00000001474.ENSXMAG00000000759.ENSXMAG00000002607		none	none	sub	none	none	none	none	none	none	sub
ENSXMAG00000002607	no	no	21	no	no	no	no	no	no	ENSLOCG00000001474.ENSXMAG00000002607.ENSXMAG00000000759	cpne4b	none	none	sub	sub	none	none	none	none	none	sub
ENSXMAG00000016148	no	no	no	no	no	no	no	no	no	ENSLOCG00000001557.ENSXMAG00000016148.ENSXMAG00000019401	mctp1b	none	none	none	none	none	sub	none	none	none	sub
ENSXMAG00000019401	no	no	no	no	no	3	no	no	no	ENSLOCG00000001557.ENSXMAG00000019401.ENSXMAG00000016148	mctp1a	none	none	none	none	none	sub	none	none	none	sub
ENSXMAG00000004457	no	no	no	no	no	no	no	no	no	ENSLOCG00000001558.ENSXMAG00000004457.ENSXMAG00000022003	PDGFA	none	none	sub	none	none	none	none	none	none	sub
ENSXMAG00000022003	no	no	6	no	no	no	no	no	no	ENSLOCG00000001558.ENSXMAG00000022003.ENSXMAG00000004457	pdgfaa	none	none	sub	none	none	none	none	none	none	sub
ENSXMAG00000012214	no	no	no	no	no	15	no	no	no	ENSLOCG00000001560.ENSXMAG00000012214.ENSXMAG00000012415	ubash3ba	none	none	none	none	none	sub	none	none	none	sub
ENSXMAG00000012415	no	no	no	no	no	no	no	no	no	ENSLOCG00000001560.ENSXMAG00000012415.ENSXMAG00000012214	ubash3bb	none	none	none	none	none	sub	none	none	none	sub
ENSXMAG00000019818	12	no	no	no	0	no	no	no	no	ENSLOCG00000001561.ENSXMAG00000019818.ENSXMAG00000027512		sub	none	none	none	sub	none	none	none	none	sub
ENSXMAG00000027512	no	no	no	no	no	no	no	no	no	ENSLOCG00000001561.ENSXMAG00000027512.ENSXMAG00000019818	zgc:92335	sub	none	none	none	sub	none	none	none	none	sub
ENSXMAG00000001929	no	no	no	no	no	no	no	no	no	ENSLOCG00000001609.ENSXMAG00000001929.ENSXMAG00000023871	si:ch211-168d1.3	none	none	none	none	none	none	none	none	sub	sub
ENSXMAG00000023871	no	no	no	no	no	no	no	no	6	ENSLOCG00000001609.ENSXMAG00000023871.ENSXMAG00000001929		none	none	none	none	none	none	none	none	sub	sub
ENSXMAG00000002915	no	15	no	no	no	no	no	no	no	ENSLOCG00000001696.ENSXMAG00000002915.ENSXMAG00000018360		none	sub	none	none	none	none	none	sub	none	sub
ENSXMAG00000018360	no	no	no	no	no	no	no	12	no	ENSLOCG00000001696.ENSXMAG00000018360.ENSXMAG00000002915	pxna	none	sub	none	none	none	none	none	sub	none	sub
ENSXMAG00000014607	0	no	no	no	no	no	no	no	no	ENSLOCG00000001718.ENSXMAG00000014607.ENSXMAG00000019113		sub	none	none	none	none	none	none	none	none	sub
ENSXMAG00000019113	no	no	no	no	no	no	no	no	no	ENSLOCG00000001718.ENSXMAG00000019113.ENSXMAG00000014607	cpxm1b	sub	none	none	none	none	none	none	none	none	sub
ENSXMAG00000002220	no	no	no	no	no	no	no	no	no	ENSLOCG00000001735.ENSXMAG00000002220.ENSXMAG00000027722	si:dkey-175m17.6	sub	none	none	none	none	none	none	none	none	sub
ENSXMAG00000027722	3	no	no	no	no	no	no	no	no	ENSLOCG00000001735.ENSXMAG00000027722.ENSXMAG00000002220	si:dkey-160o24.3	sub	none	none	none	none	none	none	none	none	sub
ENSXMAG00000002028	no	no	no	3	no	no	no	no	no	ENSLOCG00000001764.ENSXMAG00000002028.ENSXMAG00000007871	ccnt2a	none	none	none	sub	none	none	none	none	none	sub
ENSXMAG00000007871	no	no	no	no	no	no	no	no	no	ENSLOCG00000001764.ENSXMAG00000007871.ENSXMAG00000002028		none	none	none	sub	none	none	none	none	none	sub
ENSXMAG00000004796	no	no	no	no	no	18	no	no	no	ENSLOCG00000001843.ENSXMAG00000004796.ENSXMAG00000008626	slc3a2b	none	none	none	none	sub	no diff	none	none	none	sub
ENSXMAG00000008626	no	no	no	no	15	21	no	no	no	ENSLOCG00000001843.ENSXMAG00000008626.ENSXMAG00000004796	slc3a2a	none	none	none	none	sub	no diff	none	none	none	sub
ENSXMAG00000005452	no	no	no	no	no	no	no	3	no	ENSLOCG00000001850.ENSXMAG00000005452.ENSXMAG00000018739	kifap3a	none	none	none	none	none	none	sub	sub	none	sub
ENSXMAG00000018739	no	no	no	no	no	no	12	no	no	ENSLOCG00000001850.ENSXMAG00000018739.ENSXMAG00000005452		none	none	none	none	none	none	sub	sub	none	sub
ENSXMAG00000017262	no	no	no	no	no	0	no	no	no	ENSLOCG00000001864.ENSXMAG00000017262.ENSXMAG00000021886	rasd1	none	none	none	none	none	sub	none	none	none	sub
ENSXMAG00000021886	no	no	no	no	no	no	no	no	no	ENSLOCG00000001864.ENSXMAG00000021886.ENSXMAG00000017262		none	none	none	none	none	sub	none	none	none	sub
ENSXMAG00000011954	no	no	no	no	no	15	no	no	no	ENSLOCG00000001884.ENSXMAG00000011954.ENSXMAG00000012176	slc7a10a	none	none	none	none	none	sub	none	none	none	sub
ENSXMAG00000012176	no	no	no	no	no	no	no	no	no	ENSLOCG00000001884.ENSXMAG00000012176.ENSXMAG00000011954	slc7a10b	none	none	none	none	none	sub	none	none	none	sub
ENSXMAG00000001301	no	no	no	no	no	6	no	no	no	ENSLOCG00000001892.ENSXMAG00000001301.ENSXMAG00000018967	npdc1a	none	none	none	none	none	neo	none	none	none	sub
ENSXMAG00000018967	no	no	no	no	no	21	no	no	no	ENSLOCG00000001892.ENSXMAG00000018967.ENSXMAG00000001301	NPDC1	none	none	none	none	none	neo	none	none	none	sub
ENSXMAG00000000091	no	no	no	no	0	no	no	no	no	ENSLOCG00000001927.ENSXMAG00000000091.ENSXMAG00000025944		none	none	none	none	sub	sub	none	none	none	sub
ENSXMAG00000025944	no	no	no	no	no	15	no	no	no	ENSLOCG00000001927.ENSXMAG00000025944.ENSXMAG00000000091		none	none	none	none	sub	sub	none	none	none	sub
ENSXMAG00000012120	no	no	no	no	no	no	no	no	no	ENSLOCG00000001994.ENSXMAG00000012120.ENSXMAG00000026761	usp22	none	none	none	none	none	sub	none	none	none	sub
ENSXMAG00000026761	no	no	no	no	no	15	no	no	no	ENSLOCG00000001994.ENSXMAG00000026761.ENSXMAG00000012120		none	none	none	none	none	sub	none	none	none	sub
ENSXMAG00000005116	no	no	no	no	no	no	21	no	no	ENSLOCG00000001997.ENSXMAG00000005116.ENSXMAG00000008678	slc8a4a	none	none	none	none	none	none	sub	none	none	sub
ENSXMAG00000008678	no	no	no	no	no	no	no	no	no	ENSLOCG00000001997.ENSXMAG00000008678.ENSXMAG00000005116	slc8a4b	none	none	none	none	none	none	sub	none	none	sub
ENSXMAG00000008541	no	no	no	no	15	no	no	no	no	ENSLOCG00000002009.ENSXMAG00000008541.ENSXMAG00000017331		none	none	none	none	no diff	none	none	none	none	sub
ENSXMAG00000017331	no	no	no	no	15	no	no	no	no	ENSLOCG00000002009.ENSXMAG00000017331.ENSXMAG00000008541	SACM1L	none	none	none	none	no diff	none	none	none	none	sub
ENSXMAG00000006830	no	no	no	no	15	no	no	no	no	ENSLOCG00000002012.ENSXMAG00000006830.ENSXMAG00000029578		none	none	none	none	sub	sub	none	none	none	sub
ENSXMAG00000029578	no	no	no	no	15	no	no	no	no	ENSLOCG00000002012.ENSXMAG00000029578.ENSXMAG00000006830	caska	none	none	none	none	sub	sub	none	none	none	sub
ENSXMAG00000002125	no	no	no	no	no	no	no	no	no	ENSLOCG00000002019.ENSXMAG00000002125.ENSXMAG00000022566	inhbb	none	none	none	none	sub	none	none	none	none	sub
ENSXMAG00000022566	no	no	no	no	15	no	no	no	no	ENSLOCG00000002019.ENSXMAG00000022566.ENSXMAG00000002125	INHBB (1 of many)	none	none	none	none	sub	none	none	none	none	sub
ENSXMAG00000016189	no	no	no	no	no	18	no	no	no	ENSLOCG00000002026.ENSXMAG00000016189.ENSXMAG00000025960	xrcc4	none	none	none	none	none	sub	none	none	none	sub
ENSXMAG00000025960	no	no	no	no	no	no	no	no	no	ENSLOCG00000002026.ENSXMAG00000025960.ENSXMAG00000016189		none	none	none	none	none	sub	none	none	none	sub
ENSXMAG00000001182	no	no	no	no	3	3	6	no	no	ENSLOCG00000002067.ENSXMAG00000001182.ENSXMAG00000004853	nsmfa	none	none	none	none	sub	sub	sub	none	none	sub
ENSXMAG00000004853	no	no	no	no	no	no	no	no	no	ENSLOCG00000002067.ENSXMAG00000004853.ENSXMAG00000001182	nsmfb	none	none	none	none	sub	sub	sub	none	none	sub
ENSXMAG00000001703	no	no	no	3	no	no	no	no	no	ENSLOCG00000002072.ENSXMAG00000001703.ENSXMAG00000002540		none	none	none	sub	none	none	none	none	none	sub
ENSXMAG00000002540	no	no	no	no	no	no	no	no	no	ENSLOCG00000002072.ENSXMAG00000002540.ENSXMAG00000001703		none	none	none	sub	none	none	none	none	none	sub
ENSXMAG00000007063	no	no	no	no	21	no	no	no	no	ENSLOCG00000002085.ENSXMAG00000007063.ENSXMAG00000016488	gpia	none	none	none	none	sub	none	none	none	none	sub
ENSXMAG00000016488	no	no	no	no	no	no	no	no	no	ENSLOCG00000002085.ENSXMAG00000016488.ENSXMAG00000007063	gpib	none	none	none	none	sub	none	none	none	none	sub
ENSXMAG00000002567	no	no	no	no	no	no	no	no	no	ENSLOCG00000002136.ENSXMAG00000002567.ENSXMAG00000008513	homer3b	none	none	sub	none	none	sub	none	none	none	sub
ENSXMAG00000008513	no	no	9	no	no	15	no	no	no	ENSLOCG00000002136.ENSXMAG00000008513.ENSXMAG00000002567		none	none	sub	none	none	sub	none	none	none	sub
ENSXMAG00000007434	no	no	no	no	no	no	no	15	no	ENSLOCG00000002181.ENSXMAG00000007434.ENSXMAG00000027991	tnnt3a	none	none	none	none	none	none	none	sub	none	sub
ENSXMAG00000027991	no	no	no	no	no	no	no	no	no	ENSLOCG00000002181.ENSXMAG00000027991.ENSXMAG00000007434		none	none	none	none	none	none	none	sub	none	sub
ENSXMAG00000006381	no	no	no	no	0	no	no	no	no	ENSLOCG00000002200.ENSXMAG00000006381.ENSXMAG00000027712		none	none	none	none	sub	none	none	none	none	sub
ENSXMAG00000027712	no	no	no	no	no	no	no	no	no	ENSLOCG00000002200.ENSXMAG00000027712.ENSXMAG00000006381	sync	none	none	none	none	sub	none	none	none	none	sub
ENSXMAG00000012472	no	no	no	no	18	15	no	no	no	ENSLOCG00000002244.ENSXMAG00000012472.ENSXMAG00000018796	acap3a	none	none	none	none	sub	neo	none	sub	none	sub
ENSXMAG00000018796	no	no	no	no	no	21	no	21	no	ENSLOCG00000002244.ENSXMAG00000018796.ENSXMAG00000012472	acap3b	none	none	none	none	sub	neo	none	sub	none	sub
ENSXMAG00000005713	no	no	no	no	no	no	no	no	no	ENSLOCG00000002273.ENSXMAG00000005713.ENSXMAG00000006419	khdrbs1b	none	none	none	none	sub	sub	sub	none	none	sub
ENSXMAG00000006419	no	no	no	no	12	15	15	no	no	ENSLOCG00000002273.ENSXMAG00000006419.ENSXMAG00000005713	khdrbs1a	none	none	none	none	sub	sub	sub	none	none	sub
ENSXMAG00000005370	no	no	no	no	no	no	no	no	no	ENSLOCG00000002291.ENSXMAG00000005370.ENSXMAG00000013465	kcng4a	none	none	none	none	none	sub	none	none	none	sub
ENSXMAG00000013465	no	no	no	no	no	3	no	no	no	ENSLOCG00000002291.ENSXMAG00000013465.ENSXMAG00000005370	kcng4b	none	none	none	none	none	sub	none	none	none	sub
ENSXMAG00000003976	no	no	no	no	no	no	21	no	no	ENSLOCG00000002372.ENSXMAG00000003976.ENSXMAG00000010231	nfatc2a	none	none	none	none	none	none	sub	none	none	sub
ENSXMAG00000010231	no	no	no	no	no	no	no	no	no	ENSLOCG00000002372.ENSXMAG00000010231.ENSXMAG00000003976		none	none	none	none	none	none	sub	none	none	sub
ENSXMAG00000002404	no	21	no	no	no	21	no	no	no	ENSLOCG00000002429.ENSXMAG00000002404.ENSXMAG00000005070	arvcfb	none	sub	none	none	none	no diff	none	none	none	sub
ENSXMAG00000005070	no	no	no	no	no	18	no	no	no	ENSLOCG00000002429.ENSXMAG00000005070.ENSXMAG00000002404	arvcfa	none	sub	none	none	none	no diff	none	none	none	sub
ENSXMAG00000004671	no	no	no	no	no	no	no	no	no	ENSLOCG00000002434.ENSXMAG00000004671.ENSXMAG00000007950	dnaja3a	none	none	none	sub	none	sub	none	none	none	sub
ENSXMAG00000007950	no	no	no	6	no	6	no	no	no	ENSLOCG00000002434.ENSXMAG00000007950.ENSXMAG00000004671		none	none	none	sub	none	sub	none	none	none	sub
ENSXMAG00000000379	no	no	no	no	15	15	no	no	no	ENSLOCG00000002539.ENSXMAG00000000379.ENSXMAG00000025106	hdac1	none	none	none	none	sub	sub	none	none	none	sub
ENSXMAG00000025106	no	no	no	no	no	no	no	no	no	ENSLOCG00000002539.ENSXMAG00000025106.ENSXMAG00000000379		none	none	none	none	sub	sub	none	none	none	sub
ENSXMAG00000000412	no	no	no	no	no	no	no	no	no	ENSLOCG00000002564.ENSXMAG00000000412.ENSXMAG00000029740	marcksl1b	none	sub	none	none	none	sub	none	none	none	sub
ENSXMAG00000029740	no	12	no	no	no	15	no	no	no	ENSLOCG00000002564.ENSXMAG00000029740.ENSXMAG00000000412	marcksl1a	none	sub	none	none	none	sub	none	none	none	sub
ENSXMAG00000027478	no	no	no	no	no	no	no	no	no	ENSLOCG00000002626.ENSXMAG00000027478.ENSXMAG00000028099	tfap4	none	none	none	none	sub	none	none	none	none	sub
ENSXMAG00000028099	no	no	no	no	15	no	no	no	no	ENSLOCG00000002626.ENSXMAG00000028099.ENSXMAG00000027478	TFAP4	none	none	none	none	sub	none	none	none	none	sub
ENSXMAG00000028414	no	no	no	no	no	12	no	no	no	ENSLOCG00000002663.ENSXMAG00000028414.ENSXMAG00000028795	fam163b	none	none	none	none	none	sub	none	none	none	sub
ENSXMAG00000028795	no	no	no	no	no	no	no	no	no	ENSLOCG00000002663.ENSXMAG00000028795.ENSXMAG00000028414	FAM163B	none	none	none	none	none	sub	none	none	none	sub
ENSXMAG00000028331	no	no	no	no	no	15	no	no	no	ENSLOCG00000002667.ENSXMAG00000028331.ENSXMAG00000029963	ADCY9	none	none	none	none	none	sub	none	none	none	sub
ENSXMAG00000029963	no	no	no	no	no	no	no	no	no	ENSLOCG00000002667.ENSXMAG00000029963.ENSXMAG00000028331	adcy9	none	none	none	none	none	sub	none	none	none	sub
ENSXMAG00000016178	no	no	no	no	15	no	15	no	no	ENSLOCG00000002720.ENSXMAG00000016178.ENSXMAG00000018728	dnajc11a	none	none	none	none	sub	none	sub	none	none	sub
ENSXMAG00000018728	no	no	no	no	no	no	no	no	no	ENSLOCG00000002720.ENSXMAG00000018728.ENSXMAG00000016178	dnajc11b	none	none	none	none	sub	none	sub	none	none	sub
ENSXMAG00000009251	no	no	no	no	no	15	no	no	no	ENSLOCG00000002767.ENSXMAG00000009251.ENSXMAG00000029000		none	none	none	none	none	neo	none	none	none	sub
ENSXMAG00000029000	no	no	no	no	no	21	no	no	no	ENSLOCG00000002767.ENSXMAG00000029000.ENSXMAG00000009251	cd82b	none	none	none	none	none	neo	none	none	none	sub
ENSXMAG00000007280	no	no	no	no	no	15	no	no	no	ENSLOCG00000002791.ENSXMAG00000007280.ENSXMAG00000022831	tspan18b	none	none	none	none	none	no diff	none	none	none	sub
ENSXMAG00000022831	no	no	no	no	no	18	no	no	no	ENSLOCG00000002791.ENSXMAG00000022831.ENSXMAG00000007280	tspan18a	none	none	none	none	none	no diff	none	none	none	sub
ENSXMAG00000014983	no	no	no	no	15	no	no	no	no	ENSLOCG00000002897.ENSXMAG00000014983.ENSXMAG00000029958	nr2f6a	none	none	none	none	sub	none	none	none	none	sub
ENSXMAG00000029958	no	no	no	no	no	no	no	no	no	ENSLOCG00000002897.ENSXMAG00000029958.ENSXMAG00000014983	nr2f6b	none	none	none	none	sub	none	none	none	none	sub
ENSXMAG00000003614	no	no	no	no	no	no	no	no	no	ENSLOCG00000002898.ENSXMAG00000003614.ENSXMAG00000026154	fam8a1a	none	none	none	none	none	sub	none	none	none	sub
ENSXMAG00000026154	no	no	no	no	no	18	no	no	no	ENSLOCG00000002898.ENSXMAG00000026154.ENSXMAG00000003614	fam8a1b	none	none	none	none	none	sub	none	none	none	sub
ENSXMAG00000014044	no	no	no	no	no	3	no	no	no	ENSLOCG00000002914.ENSXMAG00000014044.ENSXMAG00000028756		none	none	none	none	none	no diff	none	none	none	sub
ENSXMAG00000028756	no	no	no	no	no	3	no	no	no	ENSLOCG00000002914.ENSXMAG00000028756.ENSXMAG00000014044	cabp1a	none	none	none	none	none	no diff	none	none	none	sub
ENSXMAG00000004007	no	no	no	no	no	no	no	no	no	ENSLOCG00000002916.ENSXMAG00000004007.ENSXMAG00000012814		none	none	none	none	none	sub	none	none	sub	sub
ENSXMAG00000012814	no	no	no	no	no	18	no	no	21	ENSLOCG00000002916.ENSXMAG00000012814.ENSXMAG00000004007	mxra8b	none	none	none	none	none	sub	none	none	sub	sub
ENSXMAG00000003855	no	12	no	no	no	15	no	no	no	ENSLOCG00000002983.ENSXMAG00000003855.ENSXMAG00000021422	abhd8a	none	sub	none	none	none	sub	none	none	none	sub
ENSXMAG00000021422	no	no	no	no	no	no	no	no	no	ENSLOCG00000002983.ENSXMAG00000021422.ENSXMAG00000003855	abhd8b	none	sub	none	none	none	sub	none	none	none	sub
ENSXMAG00000002402	no	no	no	no	15	no	no	no	no	ENSLOCG00000003024.ENSXMAG00000002402.ENSXMAG00000021728	ano8b	none	none	none	none	sub	none	none	none	none	sub
ENSXMAG00000021728	no	no	no	no	no	no	no	no	no	ENSLOCG00000003024.ENSXMAG00000021728.ENSXMAG00000002402	ano8a	none	none	none	none	sub	none	none	none	none	sub
ENSXMAG00000014478	no	no	no	no	15	no	no	no	no	ENSLOCG00000003028.ENSXMAG00000014478.ENSXMAG00000025294	foxo6a	none	none	none	none	sub	sub	none	sub	none	sub
ENSXMAG00000025294	no	no	no	no	no	21	no	21	no	ENSLOCG00000003028.ENSXMAG00000025294.ENSXMAG00000014478	foxo6b	none	none	none	none	sub	sub	none	sub	none	sub
ENSXMAG00000013475	no	no	no	no	no	0	no	no	no	ENSLOCG00000003061.ENSXMAG00000013475.ENSXMAG00000016058	GABARAPL2 (1 of many)	none	none	none	none	sub	none	none	none	none	sub
ENSXMAG00000016058	no	no	no	no	no	no	no	no	no	ENSLOCG00000003061.ENSXMAG00000016058.ENSXMAG00000013475	gabarapl2	none	none	none	none	sub	none	none	none	none	sub
ENSXMAG00000002400	no	no	no	no	no	0	no	no	9	ENSLOCG00000003074.ENSXMAG00000002400.ENSXMAG00000014977	plvapb	none	none	none	none	sub	no diff	none	none	sub	sub
ENSXMAG00000014977	no	no	no	no	21	0	no	no	no	ENSLOCG00000003074.ENSXMAG00000014977.ENSXMAG00000002400	plvapa	none	none	none	none	sub	no diff	none	none	sub	sub
ENSXMAG00000000786	0	no	no	no	0	no	0	no	no	ENSLOCG00000003075.ENSXMAG00000000786.ENSXMAG00000014334	epb41l3b	sub	none	none	none	sub	none	sub	none	none	sub
ENSXMAG00000014334	no	no	no	no	no	no	no	no	no	ENSLOCG00000003075.ENSXMAG00000014334.ENSXMAG00000000786	EPB41L3	sub	none	none	none	sub	none	sub	none	none	sub
ENSXMAG00000001106	no	no	no	no	no	no	no	no	no	ENSLOCG00000003107.ENSXMAG00000001106.ENSXMAG00000002511	dlgap1a	sub	none	none	none	none	none	none	none	none	sub

ENSXMAG00000002511	15	no	no	no	no	no	no	no	no	ENSLOCG00000003107.ENSXMAG00000002511.ENSXMAG00000001106	dlgap1b	sub	none	none	none	none	none	none	none	none	sub
ENSXMAG00000014733	no	no	no	no	no	18	no	no	no	ENSLOCG00000003110.ENSXMAG00000014733.ENSXMAG00000024808	eif3eb	none	none	none	none	none	sub	none	none	none	sub
ENSXMAG00000024808	no	no	no	no	no	no	no	no	no	ENSLOCG00000003110.ENSXMAG00000024808.ENSXMAG00000014733	EIF3E	none	none	none	none	none	sub	none	none	none	sub
ENSXMAG00000007066	no	no	no	no	no	no	no	no	no	ENSLOCG00000003120.ENSXMAG00000007066.ENSXMAG00000028956	calca (1 of many)	none	none	none	none	none	sub	none	none	none	sub
ENSXMAG00000028956	no	no	no	no	no	9	no	no	no	ENSLOCG00000003120.ENSXMAG00000028956.ENSXMAG00000007066	CALCB	none	none	none	none	none	sub	none	none	none	sub
ENSXMAG00000018743	no	no	no	no	no	15	no	no	no	ENSLOCG00000003238.ENSXMAG00000018743.ENSXMAG00000024528		none	none	none	none	none	sub	none	none	none	sub
ENSXMAG00000024528	no	no	no	no	no	no	no	no	no	ENSLOCG00000003238.ENSXMAG00000024528.ENSXMAG00000018743	si:dkey-89b17.4	none	none	none	none	none	sub	none	none	none	sub
ENSXMAG00000006965	no	no	no	no	no	21	no	no	no	ENSLOCG00000003266.ENSXMAG00000006965.ENSXMAG00000029930	RIC3	none	sub	none	none	none	sub	none	none	none	sub
ENSXMAG00000029930	no	21	no	no	no	no	no	no	no	ENSLOCG00000003266.ENSXMAG00000029930.ENSXMAG00000006965		none	sub	none	none	none	sub	none	none	none	sub
ENSXMAG00000003342	no	no	no	no	no	no	no	no	no	ENSLOCG00000003283.ENSXMAG00000003342.ENSXMAG00000005325	si:dkey-21e5.1	none	none	sub	none	none	none	none	none	none	sub
ENSXMAG00000005325	no	no	6	no	no	no	no	no	no	ENSLOCG00000003283.ENSXMAG00000005325.ENSXMAG00000003342	kcnt1	none	none	sub	none	none	none	none	none	none	sub
ENSXMAG00000009514	12	no	no	no	18	no	no	no	no	ENSLOCG00000003291.ENSXMAG00000009514.ENSXMAG00000016786	phactr3b	sub	none	none	sub	sub	none	none	none	none	sub
ENSXMAG00000016786	no	no	no	0	no	no	no	no	no	ENSLOCG00000003291.ENSXMAG00000016786.ENSXMAG00000009514	phactr3a	sub	none	none	sub	sub	none	none	none	none	sub
ENSXMAG00000008442	no	no	no	no	no	3	no	no	no	ENSLOCG00000003296.ENSXMAG00000008442.ENSXMAG00000016331	arhgap21a	none	none	none	none	none	sub	none	none	none	sub
ENSXMAG00000016331	no	no	no	no	no	no	no	no	no	ENSLOCG00000003296.ENSXMAG00000016331.ENSXMAG00000008442	arhgap21b	none	none	none	none	none	sub	none	none	none	sub
ENSXMAG00000008989	no	no	no	no	no	15	no	no	no	ENSLOCG00000003331.ENSXMAG00000008989.ENSXMAG00000017602	ephb2a	none	none	none	sub	none	no diff	none	none	none	sub
ENSXMAG00000017602	no	no	no	21	no	15	no	no	no	ENSLOCG00000003331.ENSXMAG00000017602.ENSXMAG00000008989	ephb2b	none	none	none	sub	none	no diff	none	none	none	sub
ENSXMAG00000000673	no	no	no	no	no	15	no	no	no	ENSLOCG00000003359.ENSXMAG00000000673.ENSXMAG00000028000		none	none	none	none	none	sub	none	none	none	sub
ENSXMAG00000028000	no	no	no	no	no	no	no	no	no	ENSLOCG00000003359.ENSXMAG00000028000.ENSXMAG00000000673		none	none	none	none	none	sub	none	none	none	sub
ENSXMAG00000000675	no	no	no	no	no	no	no	no	no	ENSLOCG00000003372.ENSXMAG00000000675.ENSXMAG00000023965	ccl25a	none	sub	none	none	sub	none	none	none	none	sub
ENSXMAG00000023965	no	6	no	no	18	no	no	no	no	ENSLOCG00000003372.ENSXMAG00000023965.ENSXMAG00000000675	ccl25b	none	sub	none	none	sub	none	none	none	none	sub
ENSXMAG00000008782	no	no	no	no	no	no	no	no	no	ENSLOCG00000003395.ENSXMAG00000008782.ENSXMAG00000025064	mcamb	none	none	none	none	none	sub	none	none	none	sub
ENSXMAG00000025064	no	no	no	no	no	21	no	no	no	ENSLOCG00000003395.ENSXMAG00000025064.ENSXMAG00000008782		none	none	none	none	none	sub	none	none	none	sub
ENSXMAG00000003277	no	no	no	no	no	no	no	no	no	ENSLOCG00000003419.ENSXMAG00000003277.ENSXMAG00000005227	notch1b	none	none	none	none	none	sub	none	none	none	sub
ENSXMAG00000005227	no	no	no	no	no	18	no	no	no	ENSLOCG00000003419.ENSXMAG00000005227.ENSXMAG00000003277		none	none	none	none	none	sub	none	none	none	sub
ENSXMAG00000003370	no	no	no	no	15	no	no	no	no	ENSLOCG00000003485.ENSXMAG00000003370.ENSXMAG00000029254		none	none	none	none	sub	none	none	none	none	sub
ENSXMAG00000029254	no	no	no	no	no	no	no	no	no	ENSLOCG00000003485.ENSXMAG00000029254.ENSXMAG00000003370	rab8a	none	none	none	none	sub	none	none	none	none	sub
ENSXMAG00000020107	no	no	no	no	no	no	no	no	no	ENSLOCG00000003518.ENSXMAG00000020107.ENSXMAG00000028666		none	none	none	none	none	sub	none	none	none	sub
ENSXMAG00000028666	no	no	no	no	no	21	no	no	no	ENSLOCG00000003518.ENSXMAG00000028666.ENSXMAG00000020107	map1lc3b	none	none	none	none	none	sub	none	none	none	sub
ENSXMAG00000005286	no	no	no	no	no	6	no	no	no	ENSLOCG00000003519.ENSXMAG00000005286.ENSXMAG00000026320	etv5a	none	none	none	none	none	neo	none	none	none	sub
ENSXMAG00000026320	no	no	no	no	no	12	no	no	no	ENSLOCG00000003519.ENSXMAG00000026320.ENSXMAG00000005286		none	none	none	none	none	neo	none	none	none	sub
ENSXMAG00000002095	no	no	no	no	no	15	15	no	no	ENSLOCG00000003520.ENSXMAG00000002095.ENSXMAG00000010868		none	none	none	none	none	no diff	sub	none	none	sub
ENSXMAG00000010868	no	no	no	no	no	12	no	no	no	ENSLOCG00000003520.ENSXMAG00000010868.ENSXMAG00000002095		none	none	none	none	none	no diff	sub	none	none	sub
ENSXMAG00000008830	no	no	no	no	no	no	15	15	no	ENSLOCG00000003521.ENSXMAG00000008830.ENSXMAG00000008883	tmem147	none	none	none	none	none	none	sub	sub	none	sub
ENSXMAG00000008883	no	no	no	no	no	no	no	no	no	ENSLOCG00000003521.ENSXMAG00000008883.ENSXMAG00000008830		none	none	none	none	none	none	sub	sub	none	sub
ENSXMAG00000012786	0	no	no	no	no	no	no	no	no	ENSLOCG00000003524.ENSXMAG00000012786.ENSXMAG00000026721	ptprua	sub	none	none	none	none	none	none	none	none	sub
ENSXMAG00000026721	no	no	no	no	no	no	no	no	no	ENSLOCG00000003524.ENSXMAG00000026721.ENSXMAG00000012786	ptprub	sub	none	none	none	none	none	none	none	none	sub
ENSXMAG00000013101	no	no	no	no	18	no	no	no	no	ENSLOCG00000003615.ENSXMAG00000013101.ENSXMAG00000027431		none	none	none	none	sub	none	none	none	none	sub
ENSXMAG00000027431	no	no	no	no	no	no	no	no	no	ENSLOCG00000003615.ENSXMAG00000027431.ENSXMAG00000013101	RAP2A	none	none	none	none	sub	none	none	none	none	sub
ENSXMAG00000010649	no	no	no	no	0	no	no	no	no	ENSLOCG00000003653.ENSXMAG00000010649.ENSXMAG00000015749	phactr4b	none	sub	none	none	sub	sub	none	none	none	sub
ENSXMAG00000015749	no	3	no	no	no	9	no	no	no	ENSLOCG00000003653.ENSXMAG00000015749.ENSXMAG00000010649	phactr4a	none	sub	none	none	sub	sub	none	none	none	sub
ENSXMAG00000012787	no	6	no	no	no	no	no	no	no	ENSLOCG00000003744.ENSXMAG00000012787.ENSXMAG00000017883	rap1gap	none	sub	none	none	none	none	none	sub	none	sub
ENSXMAG00000017883	no	no	no	no	no	no	no	15	no	ENSLOCG00000003744.ENSXMAG00000017883.ENSXMAG00000012787	si:dkey-166d12.2	none	sub	none	none	none	none	none	sub	none	sub
ENSXMAG00000003195	no	no	no	no	no	12	no	15	no	ENSLOCG00000003764.ENSXMAG00000003195.ENSXMAG00000025807	CCNG2 (1 of many)	none	none	none	none	none	sub	none	sub	none	sub
ENSXMAG00000025807	no	no	no	no	no	no	no	no	no	ENSLOCG00000003764.ENSXMAG00000025807.ENSXMAG00000003195	ccng2	none	none	none	none	none	sub	none	sub	none	sub
ENSXMAG00000026705	no	no	no	no	no	no	no	no	no	ENSLOCG00000003817.ENSXMAG00000026705.ENSXMAG00000028774	KCNB2	none	none	none	none	none	sub	none	none	none	sub
ENSXMAG00000028774	no	no	no	no	no	0	no	no	no	ENSLOCG00000003817.ENSXMAG00000028774.ENSXMAG00000026705		none	none	none	none	none	sub	none	none	none	sub
ENSXMAG00000013492	no	no	no	no	no	0	no	no	no	ENSLOCG00000003833.ENSXMAG00000013492.ENSXMAG00000022287	ubtd1a	none	none	none	none	none	sub	none	none	none	sub
ENSXMAG00000022287	no	no	no	no	no	no	no	no	no	ENSLOCG00000003833.ENSXMAG00000022287.ENSXMAG00000013492	UBTD1	none	none	none	none	none	sub	none	none	none	sub
ENSXMAG00000012884	no	no	no	no	no	no	no	no	no	ENSLOCG00000003864.ENSXMAG00000012884.ENSXMAG00000028236		none	none	none	none	none	sub	none	none	none	sub
ENSXMAG00000028236	no	no	no	no	no	18	no	no	no	ENSLOCG00000003864.ENSXMAG00000028236.ENSXMAG00000012884	pax7a	none	none	none	none	none	sub	none	none	none	sub
ENSXMAG00000023202	no	no	no	no	12	18	no	no	no	ENSLOCG00000003931.ENSXMAG00000023202.ENSXMAG00000028699	gdf10a	none	none	none	none	sub	sub	none	none	none	sub
ENSXMAG00000028699	no	no	no	no	no	no	no	no	no	ENSLOCG00000003931.ENSXMAG00000028699.ENSXMAG00000023202	gdf10b	none	none	none	none	sub	sub	none	none	none	sub
ENSXMAG00000027879	no	no	no	no	no	no	no	no	no	ENSLOCG00000003943.ENSXMAG00000027879.ENSXMAG00000028549	tmem222b	none	none	none	none	none	none	sub	sub	none	sub
ENSXMAG00000028549	no	no	no	no	no	no	21	15	no	ENSLOCG00000003943.ENSXMAG00000028549.ENSXMAG00000027879	tmem222a	none	none	none	none	none	none	sub	sub	none	sub
ENSXMAG00000009815	no	no	no	no	no	no	0	no	no	ENSLOCG00000003951.ENSXMAG00000009815.ENSXMAG00000010040	zmynd8	none	none	none	none	none	none	sub	none	none	sub
ENSXMAG00000010040	no	no	no	no	no	no	no	no	no	ENSLOCG00000003951.ENSXMAG00000010040.ENSXMAG00000009815	prkcbp1l	none	none	none	none	none	none	sub	none	none	sub
ENSXMAG00000000615	no	6	no	no	no	3	no	no	no	ENSLOCG00000003955.ENSXMAG00000000615.ENSXMAG00000023988	atcayb	none	sub	none	none	none	no diff	none	none	none	sub
ENSXMAG00000023988	no	no	no	no	no	0	no	no	no	ENSLOCG00000003955.ENSXMAG00000023988.ENSXMAG00000000615	atcaya	none	sub	none	none	none	no diff	none	none	none	sub
ENSXMAG00000000983	no	no	no	12	no	no	no	no	no	ENSLOCG00000003964.ENSXMAG00000000983.ENSXMAG00000023654	errfi1a	none	none	sub	sub	sub	none	none	none	none	sub
ENSXMAG00000023654	no	no	21	no	3	no	no	no	no	ENSLOCG00000003964.ENSXMAG00000023654.ENSXMAG00000000983		none	none	sub	sub	sub	none	none	none	none	sub
ENSXMAG00000000613	no	no	no	no	no	6	9	no	no	ENSLOCG00000003973.ENSXMAG00000000613.ENSXMAG00000018750	nmrk2	none	none	none	none	none	sub	sub	none	none	sub
ENSXMAG00000018750	no	no	no	no	no	no	no	no	no	ENSLOCG00000003973.ENSXMAG00000018750.ENSXMAG00000000613	mibp	none	none	none	none	none	sub	sub	none	none	sub
ENSXMAG00000002401	no	no	no	no	no	no	no	no	no	ENSLOCG00000004011.ENSXMAG00000002401.ENSXMAG00000009527		none	none	none	sub	none	sub	none	none	none	sub
ENSXMAG00000009527	no	no	no	15	no	15	no	no	no	ENSLOCG00000004011.ENSXMAG00000009527.ENSXMAG00000002401	igfbp6b	none	none	none	sub	none	sub	none	none	none	sub
ENSXMAG00000004183	no	3	no	no	no	3	no	no	no	ENSLOCG00000004029.ENSXMAG00000004183.ENSXMAG00000029606		none	sub	none	none	none	sub	none	none	none	sub
ENSXMAG00000029606	no	no	no	no	no	no	no	no	no	ENSLOCG00000004029.ENSXMAG00000029606.ENSXMAG00000004183	gcshb	none	sub	none	none	none	sub	none	none	none	sub
ENSXMAG00000002394	no	6	no	no	15	no	no	no	no	ENSLOCG00000004042.ENSXMAG00000002394.ENSXMAG00000009531	spryd3 (1 of many)	none	sub	none	none	sub	none	none	sub	none	sub
ENSXMAG00000009531	no	no	no	no	no	no	no	15	no	ENSLOCG00000004042.ENSXMAG00000009531.ENSXMAG00000002394	spryd3 (1 of many)	none	sub	none	none	sub	none	none	sub	none	sub
ENSXMAG00000001453	21	no	no	no	no	no	no	no	no	ENSLOCG00000004081.ENSXMAG00000001453.ENSXMAG00000013962		sub	none	none	none	none	none	none	none	none	sub
ENSXMAG00000013962	no	no	no	no	no	no	no	no	no	ENSLOCG00000004081.ENSXMAG00000013962.ENSXMAG00000001453	sgsm1a	sub	none	none	none	none	none	none	none	none	sub
ENSXMAG00000013584	no	no	no	no	no	no	no	no	no	ENSLOCG00000004090.ENSXMAG00000013584.ENSXMAG00000021218	vstm4a	none	none	none	none	none	sub	none	none	none	sub
ENSXMAG00000021218	no	no	no	no	no	21	no	no	no	ENSLOCG00000004090.ENSXMAG00000021218.ENSXMAG00000013584	vstm4b	none	none	none	none	none	sub	none	none	none	sub
ENSXMAG00000003128	no	no	no	no	no	no	no	no	no	ENSLOCG00000004102.ENSXMAG00000003128.ENSXMAG00000015506	mical2a	none	none	none	none	none	sub	none	none	none	sub
ENSXMAG00000015506	no	no	no	no	no	15	no	no	no	ENSLOCG00000004102.ENSXMAG00000015506.ENSXMAG00000003128	mical2b	none	none	none	none	none	sub	none	none	none	sub
ENSXMAG00000003962	no	no	no	no	no	9	no	no	no	ENSLOCG00000004105.ENSXMAG00000003962.ENSXMAG00000013216		none	none	none	none	none	sub	sub	none	none	sub
ENSXMAG00000013216	no	no	no	no	no	no	18	no	no	ENSLOCG00000004105.ENSXMAG00000013216.ENSXMAG00000003962	uqcrh	none	none	none	none	none	sub	sub	none	none	sub
ENSXMAG00000010314	no	no	no	no	no	0	no	18	no	ENSLOCG00000004107.ENSXMAG00000010314.ENSXMAG00000021301		none	none	none	sub	none	neo	sub	sub	none	sub
ENSXMAG00000021301	no	no	no	0	no	6	21	no	no	ENSLOCG00000004107.ENSXMAG00000021301.ENSXMAG00000010314	epb41a	none	none	none	sub	none	neo	sub	sub	none	sub
ENSXMAG00000001494	no	no	no	no	no	no	no	no	no	ENSLOCG00000004167.ENSXMAG00000001494.ENSXMAG00000014010	git2b	none	sub	none	none	sub	sub	none	none	none	sub
ENSXMAG00000014010	no	15	no	no	21	15	no	no	no	ENSLOCG00000004167.ENSXMAG00000014010.ENSXMAG00000001494	git2a	none	sub	none	none	sub	sub	none	none	none	sub
ENSXMAG00000009778	no	no	no	no	no	6	no	no	no	ENSLOCG00000004172.ENSXMAG00000009778.ENSXMAG00000023796	phf20a	none	none	none	none	none	sub	none	none	none	sub
ENSXMAG00000023796	no	no	no	no	no	no	no	no	no	ENSLOCG00000004172.ENSXMAG00000023796.ENSXMAG00000009778		none	none	none	none	none	sub	none	none	none	sub
ENSXMAG00000000547	no	no	no	no	no	no	no	no	no	ENSLOCG00000004179.ENSXMAG00000000547.ENSXMAG00000018648	insra	sub	none	none	none	sub	none	none	none	none	sub
ENSXMAG00000018648	0	no	no	no	0	no	no	no	no	ENSLOCG00000004179.ENSXMAG00000018648.ENSXMAG00000000547	insrb	sub	none	none	none	sub	none	none	none	none	sub
ENSXMAG00000013763	no	no	no	no	no	12	no	no	no	ENSLOCG00000004190.ENSXMAG00000013763.ENSXMAG00000029776	gtf3aa	none	none	none	none	none	sub	none	none	none	sub
ENSXMAG00000029776	no	no	no	no	no	no	no	no	no	ENSLOCG00000004190.ENSXMAG00000029776.ENSXMAG00000013763	gtf3ab	none	none	none	none	none	sub	none	none	none	sub
ENSXMAG00000024042	no	no	no	no	no	no	no	no	no	ENSLOCG00000004193.ENSXMAG00000024042.ENSXMAG00000024539	cxcl12a	none	none	none	none	sub	sub	none	none	none	sub
ENSXMAG00000024539	no	no	no	15	no	15	no	no	no	ENSLOCG00000004193.ENSXMAG00000024539.ENSXMAG00000024042	cxcl12b	none	none	none	none	sub	sub	none	none	none	sub
ENSXMAG00000003262	no	no	no	no	12	no	no	no	no	ENSLOCG00000004229.ENSXMAG00000003262.ENSXMAG00000011637	mmp19	none	none	none	none	sub	none	none	none	none	sub
ENSXMAG00000011637	no	no	no	no	no	no	no	no	no	ENSLOCG00000004229.ENSXMAG00000011637.ENSXMAG00000003262		none	none	none	none	sub	none	none	none	none	sub
ENSXMAG00000008353	no	no	no	no	no	21	no	no	no	ENSLOCG00000004239.ENSXMAG00000008353.ENSXMAG00000018866	slc25a36a (1 of many)	none	none	none	none	sub	neo	none	none	none	sub
ENSXMAG00000018866	no	no	no	no	15	15	no	no	no	ENSLOCG00000004239.ENSXMAG00000018866.ENSXMAG00000008353	slc25a36a (1 of many)	none	none	none	none	sub	neo	none	none	none	sub
ENSXMAG00000000544	no	no	no	no	no	no	no	no	no	ENSLOCG00000004244.ENSXMAG00000000544.ENSXMAG00000018641	arhgef18a	sub	none	none	none	none	none	none	none	none	sub
ENSXMAG00000018641	0	no	no	no	no	no	no	no	no	ENSLOCG00000004244.ENSXMAG00000018641.ENSXMAG00000000544	arhgef18b	sub	none	none	none	none	none	none	none	none	sub
ENSXMAG00000015051	no	no	no	no	no	no	no	no	no	ENSLOCG00000004313.ENSXMAG00000015051.ENSXMAG00000024881		none	none	none	none	none	none	none	none	sub	sub
ENSXMAG00000024881	no	no	no	no	no	no	no	no	3	ENSLOCG00000004313.ENSXMAG00000024881.ENSXMAG00000015051	rps19	none	none	none	none	none	none	none	none	sub	sub
ENSXMAG00000007022	no	12	no	no	no	no	no	no	no	ENSLOCG00000004325.ENSXMAG00000007022.ENSXMAG00000015409	syt9a	none	neo	none	none	none	sub	none	none	none	sub
ENSXMAG00000015409	no	6	no	no	no	15	no	no	no	ENSLOCG00000004325.ENSXMAG00000015409.ENSXMAG00000007022	syt9b	none	neo	none	none	none	sub	none	none	none	sub
ENSXMAG00000008630	no	no	no	no	no	21	no	no	no	ENSLOCG00000004398.ENSXMAG00000008630.ENSXMAG00000011277		none	none	none	none	none	neo	none	none	none	sub
ENSXMAG00000011277	no	no	no	no	no	15	no	no	no	ENSLOCG00000004398.ENSXMAG00000011277.ENSXMAG00000008630	sept4a	none	none	none	none	none	neo	none	none	none	sub
ENSXMAG00000001259	no	no	no	no	no	no	no	no	no	ENSLOCG00000004399.ENSXMAG00000001259.ENSXMAG00000007888	myo1hb	none	none	none	none	none	none	none	sub	none	sub
ENSXMAG00000007888	no	no	no	no	no	no	no	3	no	ENSLOCG00000004399.ENSXMAG00000007888.ENSXMAG00000001259	myo1ha	none	none	none	none	none	none	none	sub	none	sub
ENSXMAG00000006651	no	no	12	6	6	6	no	no	6	ENSLOCG00000004441.ENSXMAG00000006651.ENSXMAG00000016174		none	none	sub	sub	sub	no diff	none	none	sub	sub
ENSXMAG00000016174	no	no	no	no	no	6	no	no	no	ENSLOCG00000004441.ENSXMAG00000016174.ENSXMAG00000006651	per2	none	none	sub	sub	sub	no diff	none	none	sub	sub
ENSXMAG00000018511	no	no	no	no	15	no	no	no	no	ENSLOCG00000004442.ENSXMAG00000018511.ENSXMAG00000018819	timm44	none	none	none	none	no diff	none	none	none	none	sub
ENSXMAG00000018819	no	no	no	no	15	no	no	no	no	ENSLOCG00000004442.ENSXMAG00000018819.ENSXMAG00000018511		none	none	none	none	no diff	none	none	none	none	sub
ENSXMAG00000008604	no	12	6	no	no	15	no	no	no	ENSLOCG00000004448.ENSXMAG00000008604.ENSXMAG00000011253		none	sub	sub	none	none	no diff	none	none	none	sub
ENSXMAG00000011253	no	no	no	no	no	15	no	no	no	ENSLOCG00000004448.ENSXMAG00000011253.ENSXMAG00000008604	pafah1b1b	none	sub	sub	none	none	no diff	none	none	none	sub
ENSXMAG00000003490	no	no	no	no	no	18	no	no	no	ENSLOCG00000004492.ENSXMAG00000003490.ENSXMAG00000028136	MMD2 (1 of many)	none	none	none	none	none	neo	none	none	none	sub
ENSXMAG00000028136	no	no	no	no	no	0	no	no	no	ENSLOCG00000004492.ENSXMAG00000028136.ENSXMAG00000003490	MMD2 (1 of many)	none	none	none	none	none	neo	none	none	none	sub
ENSXMAG00000003548	no	no	no	no	no	18	no	no	no	ENSLOCG00000004530.ENSXMAG00000003548.ENSXMAG00000011725	DGKA	none	none	none	none	sub	no diff	none	none	none	sub
ENSXMAG00000011725	no	no	no	no	15	21	no	no	no	ENSLOCG00000004530.ENSXMAG00000011725.ENSXMAG00000003548	dgkaa	none	none	none	none	sub	no diff	none	none	none	sub
ENSXMAG00000009598	no	no	no	no	15	no	no	no	no	ENSLOCG00000004545.ENSXMAG00000009598.ENSXMAG00000015345	top1l	none	none	none	none	sub	sub	none	none	none	sub
ENSXMAG00000015345	no	no	no	no	no	18	no	no	no	ENSLOCG00000004545.ENSXMAG00000015345.ENSXMAG00000009598	top1	none	none	none	none	sub	sub	none	none	none	sub
ENSXMAG00000010823	no	no	no	no	no	no	no	no	21	ENSLOCG00000004622.ENSXMAG00000010823.ENSXMAG00000025547	pou2f2a	none	none	none	none	none	none	none	none	sub	sub
ENSXMAG00000025547	no	no	no	no	no	no	no	no	no	ENSLOCG00000004622.ENSXMAG00000025547.ENSXMAG00000010823		none	none	none	none	none	none	none	none	sub	sub
ENSXMAG00000010816	no	no	no	3	no	0	no	no	no	ENSLOCG00000004635.ENSXMAG00000010816.ENSXMAG00000029798	erfl3	none	none	none	sub	none	sub	none	none	none	sub
ENSXMAG00000029798	no	no	no	no	no	no	no	no	no	ENSLOCG00000004635.ENSXMAG00000029798.ENSXMAG00000010816	erf	none	none	none	sub	none	sub	none	none	none	sub
ENSXMAG00000008861	no	no	no	no	no	no	no	no	no	ENSLOCG00000004658.ENSXMAG00000008861.ENSXMAG00000029112	ncam1a	none	sub	none	none	none	sub	none	none	none	sub
ENSXMAG00000029112	no	6	no	no	no	3	no	no	no	ENSLOCG00000004658.ENSXMAG00000029112.ENSXMAG00000008861	ncam1b	none	sub	none	none	none	sub	none	none	none	sub
ENSXMAG00000003143	no	no	no	no	18	no	no	no	no	ENSLOCG00000004677.ENSXMAG00000003143.ENSXMAG00000004091	rpl5a	none	none	none	none	sub	none	sub	sub	none	sub
ENSXMAG00000004091	no	no	no	no	no	no	15	15	no	ENSLOCG00000004677.ENSXMAG00000004091.ENSXMAG00000003143		none	none	none	none	sub	none	sub	sub	none	sub
ENSXMAG00000004123	no	no	no	no	no	no	15	no	no	ENSLOCG00000004740.ENSXMAG00000004123.ENSXMAG00000010662	glmna	none	none	none	none	none	none	sub	none	none	sub
ENSXMAG00000010662	no	no	no	no	no	no	no	no	no	ENSLOCG00000004740.ENSXMAG00000010662.ENSXMAG00000004123	glmnb	none	none	none	none	none	none	sub	none	none	sub
ENSXMAG00000003226	no	no	no	no	no	21	no	no	no	ENSLOCG00000004752.ENSXMAG00000003226.ENSXMAG00000015365	kcnma1a	none	none	none	none	none	sub	none	none	none	sub
ENSXMAG00000015365	no	no	no	no	no	no	no	no	no	ENSLOCG00000004752.ENSXMAG00000015365.ENSXMAG00000003226	KCNMA1	none	none	none	none	none	sub	none	none	none	sub
ENSXMAG00000012702	no	no	no	no	no	no	no	no	no	ENSLOCG00000004772.ENSXMAG00000012702.ENSXMAG00000018190		none	none	none	none	sub	none	none	none	none	sub
ENSXMAG00000018190	no	no	no	no	3	no	no	no	no	ENSLOCG00000004772.ENSXMAG00000018190.ENSXMAG00000012702	myorg	none	none	none	none	sub	none	none	none	none	sub
ENSXMAG00000000579	no	no	no	no	no	15	no	no	no	ENSLOCG00000004837.ENSXMAG00000000579.ENSXMAG00000012050	myo18aa	none	none	none	none	none	sub	none	none	none	sub
ENSXMAG00000012050	no	no	no	no	no	no	no	no	no	ENSLOCG00000004837.ENSXMAG00000012050.ENSXMAG00000000579	myo18ab	none	none	none	none	none	sub	none	none	none	sub
ENSXMAG00000009571	no	no	no	no	no	no	no	18	0	ENSLOCG00000004844.ENSXMAG00000009571.ENSXMAG00000012016		none	none	none	none	none	none	none	sub	sub	sub
ENSXMAG00000012016	no	no	no	no	no	no	no	no	no	ENSLOCG00000004844.ENSXMAG00000012016.ENSXMAG00000009571	itga5	none	none	none	none	none	none	none	sub	sub	sub
ENSXMAG00000003180	no	no	no	no	no	no	no	no	no	ENSLOCG00000004863.ENSXMAG00000003180.ENSXMAG00000015304	zcchc24	none	none	none	sub	none	none	none	none	none	sub
ENSXMAG00000015304	no	no	no	15	no	no	no	no	no	ENSLOCG00000004863.ENSXMAG00000015304.ENSXMAG00000003180	ZCCHC24 (1 of many)	none	none	none	sub	none	none	none	none	none	sub
ENSXMAG00000000530	no	no	no	no	no	18	no	no	no	ENSLOCG00000004869.ENSXMAG00000000530.ENSXMAG00000024206		none	none	none	none	none	sub	none	none	none	sub
ENSXMAG00000024206	no	no	no	no	no	no	no	no	no	ENSLOCG00000004869.ENSXMAG00000024206.ENSXMAG00000000530	armc1	none	none	none	none	none	sub	none	none	none	sub
ENSXMAG00000006042	0	no	no	no	0	no	no	no	no	ENSLOCG00000004891.ENSXMAG00000006042.ENSXMAG00000028684	JAM3 (1 of many)	sub	none	none	none	sub	none	none	none	none	sub
ENSXMAG00000028684	no	no	no	no	no	no	no	no	no	ENSLOCG00000004891.ENSXMAG00000028684.ENSXMAG00000006042	jam3a	sub	none	none	none	sub	none	none	none	none	sub
ENSXMAG00000002832	no	no	no	no	no	no	no	no	no	ENSLOCG00000004910.ENSXMAG00000002832.ENSXMAG00000005753		none	none	none	none	none	sub	none	none	none	sub
ENSXMAG00000005753	no	no	no	no	no	9	no	no	no	ENSLOCG00000004910.ENSXMAG00000005753.ENSXMAG00000002832	abcc4	none	none	none	none	none	sub	none	none	none	sub
ENSXMAG00000002940	no	no	no	no	0	no	no	no	no	ENSLOCG00000005012.ENSXMAG00000002940.ENSXMAG00000013741	ppp6c	none	none	none	none	sub	sub	none	none	none	sub
ENSXMAG00000013741	no	no	no	no	no	15	no	no	no	ENSLOCG00000005012.ENSXMAG00000013741.ENSXMAG00000002940		none	none	none	none	sub	sub	none	none	none	sub
ENSXMAG00000021001	no	3	no	no	no	3	no	no	no	ENSLOCG00000005021.ENSXMAG00000021001.ENSXMAG00000025138	lrrc75bb	none	sub	none	none	none	neo	none	none	none	sub
ENSXMAG00000025138	no	no	no	no	no	15	no	no	no	ENSLOCG00000005021.ENSXMAG00000025138.ENSXMAG00000021001	lrrc75ba	none	sub	none	none	none	neo	none	none	none	sub
ENSXMAG00000004449	no	no	no	no	no	no	no	no	no	ENSLOCG00000005111.ENSXMAG00000004449.ENSXMAG00000018355		none	none	none	none	none	none	sub	none	none	sub
ENSXMAG00000018355	no	no	no	no	no	no	15	no	no	ENSLOCG00000005111.ENSXMAG00000018355.ENSXMAG00000004449		none	none	none	none	none	none	sub	none	none	sub
ENSXMAG00000016824	no	no	no	no	no	0	no	no	no	ENSLOCG00000005115.ENSXMAG00000016824.ENSXMAG00000017719	col2a1a	none	none	none	none	none	no diff	none	none	none	sub
ENSXMAG00000017719	no	no	no	no	no	0	no	no	no	ENSLOCG00000005115.ENSXMAG00000017719.ENSXMAG00000016824		none	none	none	none	none	no diff	none	none	none	sub
ENSXMAG00000000484	no	no	no	no	21	no	no	no	no	ENSLOCG00000005124.ENSXMAG00000000484.ENSXMAG00000005749		none	none	none	none	neo	none	none	none	none	sub
ENSXMAG00000005749	no	no	no	no	12	no	no	no	no	ENSLOCG00000005124.ENSXMAG00000005749.ENSXMAG00000000484	ppm1da	none	none	none	none	neo	none	none	none	none	sub
ENSXMAG00000000754	no	no	no	no	no	no	no	no	no	ENSLOCG00000005155.ENSXMAG00000000754.ENSXMAG00000016443	ano1	sub	none	none	none	none	sub	none	sub	none	sub
ENSXMAG00000016443	3	no	no	no	no	6	no	6	no	ENSLOCG00000005155.ENSXMAG00000016443.ENSXMAG00000000754		sub	none	none	none	none	sub	none	sub	none	sub
ENSXMAG00000001732	no	0	no	no	no	21	no	no	no	ENSLOCG00000005170.ENSXMAG00000001732.ENSXMAG00000006106		none	sub	none	none	none	sub	none	none	none	sub
ENSXMAG00000006106	no	no	no	no	no	no	no	no	no	ENSLOCG00000005170.ENSXMAG00000006106.ENSXMAG00000001732	robo3	none	sub	none	none	none	sub	none	none	none	sub
ENSXMAG00000025011	no	no	no	no	3	6	no	no	no	ENSLOCG00000005231.ENSXMAG00000025011.ENSXMAG00000025467	slc48a1a	none	none	none	none	sub	no diff	none	none	none	sub
ENSXMAG00000025467	no	no	no	no	no	6	no	no	no	ENSLOCG00000005231.ENSXMAG00000025467.ENSXMAG00000025011	slc48a1b	none	none	none	none	sub	no diff	none	none	none	sub
ENSXMAG00000004547	no	no	no	no	no	18	no	no	no	ENSLOCG00000005237.ENSXMAG00000004547.ENSXMAG00000028214		none	none	none	none	none	sub	none	none	none	sub
ENSXMAG00000028214	no	no	no	no	no	no	no	no	no	ENSLOCG00000005237.ENSXMAG00000028214.ENSXMAG00000004547	pcdh17	none	none	none	none	none	sub	none	none	none	sub
ENSXMAG00000015893	no	no	no	no	no	no	no	no	no	ENSLOCG00000005262.ENSXMAG00000015893.ENSXMAG00000016605	amotl2b	sub	none	none	none	none	none	none	none	none	sub
ENSXMAG00000016605	0	no	no	no	no	no	no	no	no	ENSLOCG00000005262.ENSXMAG00000016605.ENSXMAG00000015893	amotl2a	sub	none	none	none	none	none	none	none	none	sub
ENSXMAG00000001285	no	no	no	no	no	3	no	no	no	ENSLOCG00000005270.ENSXMAG00000001285.ENSXMAG00000016680	rasgrf2a	none	none	none	none	none	sub	none	none	none	sub
ENSXMAG00000016680	no	no	no	no	no	no	no	no	no	ENSLOCG00000005270.ENSXMAG00000016680.ENSXMAG00000001285	rasgrf2b	none	none	none	none	none	sub	none	none	none	sub
ENSXMAG00000011605	no	3	no	no	15	no	no	no	no	ENSLOCG00000005313.ENSXMAG00000011605.ENSXMAG00000016485		none	sub	none	none	sub	none	none	none	none	sub
ENSXMAG00000016485	no	no	no	no	no	no	no	no	no	ENSLOCG00000005313.ENSXMAG00000016485.ENSXMAG00000011605	si:ch73-194h10.2	none	sub	none	none	sub	none	none	none	none	sub
ENSXMAG00000003095	no	no	no	no	0	18	no	no	no	ENSLOCG00000005316.ENSXMAG00000003095.ENSXMAG00000014960	dnajb12a	none	sub	none	none	sub	no diff	none	none	none	sub
ENSXMAG00000014960	no	15	no	no	no	15	no	no	no	ENSLOCG00000005316.ENSXMAG00000014960.ENSXMAG00000003095	dnajb12b	none	sub	none	none	sub	no diff	none	none	none	sub
ENSXMAG00000007098	no	no	no	no	no	no	no	no	no	ENSLOCG00000005321.ENSXMAG00000007098.ENSXMAG00000025613	usf2	none	none	none	none	none	sub	sub	none	none	sub
ENSXMAG00000025613	no	no	no	no	no	0	21	no	no	ENSLOCG00000005321.ENSXMAG00000025613.ENSXMAG00000007098		none	none	none	none	none	sub	sub	none	none	sub
ENSXMAG00000000134	no	no	no	no	no	no	no	no	no	ENSLOCG00000005365.ENSXMAG00000000134.ENSXMAG00000001445		none	none	none	none	none	none	sub	none	none	sub
ENSXMAG00000001445	no	no	no	no	no	no	6	no	no	ENSLOCG00000005365.ENSXMAG00000001445.ENSXMAG00000000134	arhgap17b	none	none	none	none	none	none	sub	none	none	sub
ENSXMAG00000007034	no	no	no	no	no	no	no	no	no	ENSLOCG00000005451.ENSXMAG00000007034.ENSXMAG00000018963	COL4A3BP (1 of many)	none	none	none	none	none	sub	none	none	none	sub
ENSXMAG00000018963	no	no	no	no	no	0	no	no	no	ENSLOCG00000005451.ENSXMAG00000018963.ENSXMAG00000007034	COL4A3BP (1 of many)	none	none	none	none	none	sub	none	none	none	sub
ENSXMAG00000003022	no	no	no	no	no	no	no	no	no	ENSLOCG00000005567.ENSXMAG00000003022.ENSXMAG00000014849	cdhr1a	none	none	none	none	none	sub	none	none	none	sub
ENSXMAG00000014849	no	no	no	no	no	21	no	no	no	ENSLOCG00000005567.ENSXMAG00000014849.ENSXMAG00000003022	CDHR1 (1 of many)	none	none	none	none	none	sub	none	none	none	sub
ENSXMAG00000016777	no	no	no	no	no	6	no	no	no	ENSLOCG00000005581.ENSXMAG00000016777.ENSXMAG00000017771	map3k12	none	none	none	none	none	neo	none	none	none	sub
ENSXMAG00000017771	no	no	no	no	no	0	no	no	no	ENSLOCG00000005581.ENSXMAG00000017771.ENSXMAG00000016777	MAP3K12 (1 of many)	none	none	none	none	none	neo	none	none	none	sub
ENSXMAG00000006118	no	no	no	no	no	15	no	no	no	ENSLOCG00000005582.ENSXMAG00000006118.ENSXMAG00000015332		none	none	none	none	none	sub	none	none	none	sub
ENSXMAG00000015332	no	no	no	no	no	no	no	no	no	ENSLOCG00000005582.ENSXMAG00000015332.ENSXMAG00000006118	FMNL2	none	none	none	none	none	sub	none	none	none	sub
ENSXMAG00000021752	no	no	6	no	no	no	no	no	no	ENSLOCG00000005598.ENSXMAG00000021752.ENSXMAG00000023803	pcbp2	none	none	sub	none	none	none	sub	none	none	sub
ENSXMAG00000023803	no	no	no	no	no	no	15	no	no	ENSLOCG00000005598.ENSXMAG00000023803.ENSXMAG00000021752	PCBP2	none	none	sub	none	none	none	sub	none	none	sub
ENSXMAG00000003019	no	no	no	no	no	3	no	no	no	ENSLOCG00000005616.ENSXMAG00000003019.ENSXMAG00000014845	lrit1a	none	none	none	none	none	sub	none	none	none	sub
ENSXMAG00000014845	no	no	no	no	no	no	no	no	no	ENSLOCG00000005616.ENSXMAG00000014845.ENSXMAG00000003019	lrit1b	none	none	none	none	none	sub	none	none	none	sub
ENSXMAG00000016619	no	no	no	no	no	15	no	no	no	ENSLOCG00000005670.ENSXMAG00000016619.ENSXMAG00000018907		none	none	none	none	none	sub	none	none	none	sub
ENSXMAG00000018907	no	no	no	no	no	no	no	no	no	ENSLOCG00000005670.ENSXMAG00000018907.ENSXMAG00000016619	crhbp	none	none	none	none	none	sub	none	none	none	sub
ENSXMAG00000001997	no	no	no	no	no	no	no	no	3	ENSLOCG00000005680.ENSXMAG00000001997.ENSXMAG00000021403	si:ch211-251b21.1	none	none	none	none	none	none	none	none	sub	sub
ENSXMAG00000021403	no	no	no	no	no	no	no	no	no	ENSLOCG00000005680.ENSXMAG00000021403.ENSXMAG00000001997	si:dkey-183j2.10	none	none	none	none	none	none	none	none	sub	sub

ENSXMAG00000002955	no	0	no	no	no	no	no	no	no	ENSLOCG00000005726.ENSXMAG00000002955.ENSXMAG00000016283	myofl	none	sub	none	none	none	none	none	none	none	sub
ENSXMAG00000016283	no	no	no	no	no	no	no	no	no	ENSLOCG00000005726.ENSXMAG00000016283.ENSXMAG00000002955	MYOF	none	sub	none	none	none	none	none	none	none	sub
ENSXMAG00000008707	no	no	no	no	no	no	no	no	no	ENSLOCG00000005732.ENSXMAG00000008707.ENSXMAG00000027891	caskin1	sub	none	none	none	sub	none	none	none	none	sub
ENSXMAG00000027891	21	no	no	no	0	no	no	no	no	ENSLOCG00000005732.ENSXMAG00000027891.ENSXMAG00000008707		sub	none	none	none	sub	none	none	none	none	sub
ENSXMAG00000027112	no	no	no	no	no	12	no	no	no	ENSLOCG00000005766.ENSXMAG00000027112.ENSXMAG00000028909	OPRK1	none	none	none	none	none	neo	none	none	none	sub
ENSXMAG00000028909	no	no	no	no	no	0	no	no	no	ENSLOCG00000005766.ENSXMAG00000028909.ENSXMAG00000027112	oprk1	none	none	none	none	none	neo	none	none	none	sub
ENSXMAG00000017874	no	no	no	no	no	0	no	no	no	ENSLOCG00000005807.ENSXMAG00000017874.ENSXMAG00000018983	KIF5A	none	none	none	none	none	sub	none	none	none	sub
ENSXMAG00000018983	no	no	no	no	no	no	no	no	no	ENSLOCG00000005807.ENSXMAG00000018983.ENSXMAG00000017874	kif5aa	none	none	none	none	none	sub	none	none	none	sub
ENSXMAG00000024922	no	no	no	no	no	no	no	no	no	ENSLOCG00000005808.ENSXMAG00000024922.ENSXMAG00000028164	SCAMP1	none	none	none	sub	sub	none	none	none	none	sub
ENSXMAG00000028164	no	no	no	6	15	no	no	no	no	ENSLOCG00000005808.ENSXMAG00000028164.ENSXMAG00000024922	scamp1	none	none	none	sub	sub	none	none	none	none	sub
ENSXMAG00000002830	no	no	no	no	no	no	15	no	no	ENSLOCG00000005870.ENSXMAG00000002830.ENSXMAG00000008364		none	none	none	none	none	none	sub	none	none	sub
ENSXMAG00000008364	no	no	no	no	no	no	no	no	no	ENSLOCG00000005870.ENSXMAG00000008364.ENSXMAG00000002830	odf2a	none	none	none	none	none	none	sub	none	none	sub
ENSXMAG00000005071	no	no	no	no	no	9	no	no	no	ENSLOCG00000006036.ENSXMAG00000005071.ENSXMAG00000007257	thbs4b	sub	none	none	none	none	sub	sub	none	none	sub
ENSXMAG00000007257	0	no	no	no	no	no	6	no	no	ENSLOCG00000006036.ENSXMAG00000007257.ENSXMAG00000005071	thbs4a	sub	none	none	none	none	sub	sub	none	none	sub
ENSXMAG00000002879	no	no	no	no	no	0	no	no	no	ENSLOCG00000006071.ENSXMAG00000002879.ENSXMAG00000016743	ZDHHC12 (1 of many)	none	none	none	none	none	sub	none	none	none	sub
ENSXMAG00000016743	no	no	no	no	no	no	no	no	no	ENSLOCG00000006071.ENSXMAG00000016743.ENSXMAG00000002879	zdhhc12b	none	none	none	none	none	sub	none	none	none	sub
ENSXMAG00000007125	no	no	no	no	15	no	no	no	no	ENSLOCG00000006075.ENSXMAG00000007125.ENSXMAG00000007989	calcrla	none	none	none	none	sub	none	none	none	none	sub
ENSXMAG00000007989	no	no	no	no	no	no	no	no	no	ENSLOCG00000006075.ENSXMAG00000007989.ENSXMAG00000007125		none	none	none	none	sub	none	none	none	none	sub
ENSXMAG00000013868	no	no	no	no	no	no	no	no	no	ENSLOCG00000006099.ENSXMAG00000013868.ENSXMAG00000026168	colq	none	none	none	none	none	sub	none	none	none	sub
ENSXMAG00000026168	no	no	no	no	no	6	no	no	no	ENSLOCG00000006099.ENSXMAG00000026168.ENSXMAG00000013868		none	none	none	none	none	sub	none	none	none	sub
ENSXMAG00000013027	no	no	no	no	no	15	no	no	no	ENSLOCG00000006113.ENSXMAG00000013027.ENSXMAG00000024751		none	none	none	none	none	no diff	none	none	none	sub
ENSXMAG00000024751	no	no	no	no	no	18	no	no	no	ENSLOCG00000006113.ENSXMAG00000024751.ENSXMAG00000013027		none	none	none	none	none	no diff	none	none	none	sub
ENSXMAG00000011950	no	no	no	no	no	no	no	no	no	ENSLOCG00000006121.ENSXMAG00000011950.ENSXMAG00000011986	ucp3	none	none	none	none	none	none	sub	none	none	sub
ENSXMAG00000011986	no	no	no	no	no	no	3	no	no	ENSLOCG00000006121.ENSXMAG00000011986.ENSXMAG00000011950		none	none	none	none	none	none	sub	none	none	sub
ENSXMAG00000019295	no	no	no	no	no	no	no	no	no	ENSLOCG00000006132.ENSXMAG00000019295.ENSXMAG00000027507		none	none	none	none	none	sub	none	none	none	sub
ENSXMAG00000027507	no	no	no	no	no	21	no	no	no	ENSLOCG00000006132.ENSXMAG00000027507.ENSXMAG00000019295		none	none	none	none	none	sub	none	none	none	sub
ENSXMAG00000013879	no	no	no	no	no	no	no	no	no	ENSLOCG00000006139.ENSXMAG00000013879.ENSXMAG00000026229	fkbp14	none	none	none	none	sub	none	none	none	none	sub
ENSXMAG00000026229	no	no	no	no	15	no	no	no	no	ENSLOCG00000006139.ENSXMAG00000026229.ENSXMAG00000013879	FKBP14	none	none	none	none	sub	none	none	none	none	sub
ENSXMAG00000002891	0	3	no	3	no	3	3	no	no	ENSLOCG00000006144.ENSXMAG00000002891.ENSXMAG00000028388	fam102aa	sub	sub	none	sub	none	sub	sub	none	none	sub
ENSXMAG00000028388	no	no	no	no	no	no	no	no	no	ENSLOCG00000006144.ENSXMAG00000028388.ENSXMAG00000002891	fam102ab	sub	sub	none	sub	none	sub	sub	none	none	sub
ENSXMAG00000012793	no	no	no	no	no	no	no	no	no	ENSLOCG00000006178.ENSXMAG00000012793.ENSXMAG00000014217	TNPO2 (1 of many)	none	none	none	none	sub	none	none	none	none	sub
ENSXMAG00000014217	no	no	no	no	no	15	no	no	no	ENSLOCG00000006178.ENSXMAG00000014217.ENSXMAG00000012793	TNPO2 (1 of many)	none	none	none	none	sub	none	none	none	none	sub
ENSXMAG00000003122	no	no	no	no	no	15	no	no	no	ENSLOCG00000006199.ENSXMAG00000003122.ENSXMAG00000010563	slc1a7b	none	none	none	none	none	no diff	none	none	none	sub
ENSXMAG00000010563	no	no	no	no	no	15	no	no	no	ENSLOCG00000006199.ENSXMAG00000010563.ENSXMAG00000003122	slc1a7a	none	none	none	none	none	no diff	none	none	none	sub
ENSXMAG00000000222	no	no	no	3	no	no	no	no	no	ENSLOCG00000006209.ENSXMAG00000000222.ENSXMAG00000000907	grin2aa	none	none	none	sub	none	none	none	none	none	sub
ENSXMAG00000000907	no	no	no	no	no	no	no	no	no	ENSLOCG00000006209.ENSXMAG00000000907.ENSXMAG00000000222	grin2ab	none	none	none	sub	none	none	none	none	none	sub
ENSXMAG00000010169	18	no	no	no	no	no	no	no	no	ENSLOCG00000006271.ENSXMAG00000010169.ENSXMAG00000013357	shda	sub	none	none	none	none	sub	sub	none	none	sub
ENSXMAG00000013357	no	no	no	no	no	18	21	no	no	ENSLOCG00000006271.ENSXMAG00000013357.ENSXMAG00000010169	shdb	sub	none	none	none	none	sub	sub	none	none	sub
ENSXMAG00000007837	no	no	no	no	no	no	no	no	no	ENSLOCG00000006360.ENSXMAG00000007837.ENSXMAG00000010653		none	none	none	none	none	sub	none	none	none	sub
ENSXMAG00000010653	no	no	no	no	no	21	no	no	no	ENSLOCG00000006360.ENSXMAG00000010653.ENSXMAG00000007837	pcdh15a	none	none	none	none	none	sub	none	none	none	sub
ENSXMAG00000004102	no	no	no	no	no	no	no	no	no	ENSLOCG00000006397.ENSXMAG00000004102.ENSXMAG00000010333	rpa1	none	none	none	none	sub	none	none	none	none	sub
ENSXMAG00000010333	no	no	no	no	0	no	no	no	no	ENSLOCG00000006397.ENSXMAG00000010333.ENSXMAG00000004102	RPA1 (1 of many)	none	none	none	none	sub	none	none	none	none	sub
ENSXMAG00000014642	no	no	no	no	no	no	no	no	no	ENSLOCG00000006421.ENSXMAG00000014642.ENSXMAG00000016304	crabp2b	none	none	none	sub	none	sub	none	none	none	sub
ENSXMAG00000016304	no	no	no	15	no	15	no	no	no	ENSLOCG00000006421.ENSXMAG00000016304.ENSXMAG00000014642	crabp2a	none	none	none	sub	none	sub	none	none	none	sub
ENSXMAG00000019305	no	no	no	no	no	no	no	no	no	ENSLOCG00000006431.ENSXMAG00000019305.ENSXMAG00000023383	cdc34b	none	none	none	sub	none	none	none	none	none	sub
ENSXMAG00000023383	no	no	no	3	no	no	no	no	no	ENSLOCG00000006431.ENSXMAG00000023383.ENSXMAG00000019305	cdc34a	none	none	none	sub	none	none	none	none	none	sub
ENSXMAG00000011017	no	no	no	no	no	no	no	no	no	ENSLOCG00000006462.ENSXMAG00000011017.ENSXMAG00000012174	osbpl1a	none	none	none	none	none	sub	none	none	none	sub
ENSXMAG00000012174	no	no	no	no	no	0	no	no	no	ENSLOCG00000006462.ENSXMAG00000012174.ENSXMAG00000011017		none	none	none	none	none	sub	none	none	none	sub
ENSXMAG00000014619	no	no	no	no	no	18	no	no	no	ENSLOCG00000006518.ENSXMAG00000014619.ENSXMAG00000016475	celf3a	none	none	none	none	none	sub	none	none	none	sub
ENSXMAG00000016475	no	no	no	no	no	no	no	no	no	ENSLOCG00000006518.ENSXMAG00000016475.ENSXMAG00000014619	celf3b	none	none	none	none	none	sub	none	none	none	sub
ENSXMAG00000006561	no	no	no	no	0	no	no	no	no	ENSLOCG00000006523.ENSXMAG00000006561.ENSXMAG00000008657		sub	none	none	none	sub	none	none	none	none	sub
ENSXMAG00000008657	21	no	no	no	no	no	no	no	no	ENSLOCG00000006523.ENSXMAG00000008657.ENSXMAG00000006561	si:ch211-106h11.3	sub	none	none	none	sub	none	none	none	none	sub
ENSXMAG00000006547	no	no	no	no	no	18	15	no	no	ENSLOCG00000006534.ENSXMAG00000006547.ENSXMAG00000008939	ddx39aa	none	sub	none	none	sub	no diff	no diff	none	none	sub
ENSXMAG00000008939	no	9	no	no	15	15	15	no	no	ENSLOCG00000006534.ENSXMAG00000008939.ENSXMAG00000006547	DDX39A	none	sub	none	none	sub	no diff	no diff	none	none	sub
ENSXMAG00000011255	no	no	no	no	no	no	no	no	no	ENSLOCG00000006596.ENSXMAG00000011255.ENSXMAG00000029648	gdpd4b	none	none	none	sub	none	sub	none	none	none	sub
ENSXMAG00000029648	no	no	no	3	no	3	no	no	no	ENSLOCG00000006596.ENSXMAG00000029648.ENSXMAG00000011255	gdpd4a	none	none	none	sub	none	sub	none	none	none	sub
ENSXMAG00000011345	18	no	no	no	no	0	no	no	no	ENSLOCG00000006604.ENSXMAG00000011345.ENSXMAG00000016713	tardbpl	sub	none	none	none	none	sub	none	none	none	sub
ENSXMAG00000016713	no	no	no	no	no	no	no	no	no	ENSLOCG00000006604.ENSXMAG00000016713.ENSXMAG00000011345	tardbp	sub	none	none	none	none	sub	none	none	none	sub
ENSXMAG00000006429	no	0	no	no	21	no	no	no	no	ENSLOCG00000006607.ENSXMAG00000006429.ENSXMAG00000008895	pkn1a	none	sub	none	none	sub	sub	none	none	none	sub
ENSXMAG00000008895	no	no	no	no	no	0	no	no	no	ENSLOCG00000006607.ENSXMAG00000008895.ENSXMAG00000006429	pkn1b	none	sub	none	none	sub	sub	none	none	none	sub
ENSXMAG00000012106	no	no	no	no	no	15	no	no	no	ENSLOCG00000006621.ENSXMAG00000012106.ENSXMAG00000023256	ssh2a	none	none	none	none	none	sub	none	none	none	sub
ENSXMAG00000023256	no	no	no	no	no	no	no	no	no	ENSLOCG00000006621.ENSXMAG00000023256.ENSXMAG00000012106	ssh2b	none	none	none	none	none	sub	none	none	none	sub
ENSXMAG00000004628	no	no	no	no	no	no	no	no	no	ENSLOCG00000006622.ENSXMAG00000004628.ENSXMAG00000011193	myo7aa	none	sub	none	none	none	sub	none	none	none	sub
ENSXMAG00000011193	no	18	no	no	no	15	no	no	no	ENSLOCG00000006622.ENSXMAG00000011193.ENSXMAG00000004628	myo7ab	none	sub	none	none	none	sub	none	none	none	sub
ENSXMAG00000001137	no	no	no	no	no	no	21	no	no	ENSLOCG00000006642.ENSXMAG00000001137.ENSXMAG00000007047	ano9b	none	none	none	none	none	none	sub	none	none	sub
ENSXMAG00000007047	no	no	no	no	no	no	no	no	no	ENSLOCG00000006642.ENSXMAG00000007047.ENSXMAG00000001137	ano9a	none	none	none	none	none	none	sub	none	none	sub
ENSXMAG00000002738	no	no	no	no	12	no	no	15	no	ENSLOCG00000006685.ENSXMAG00000002738.ENSXMAG00000016803	SMARCB1	none	none	none	none	sub	none	none	sub	none	sub
ENSXMAG00000016803	no	no	no	no	no	no	no	no	no	ENSLOCG00000006685.ENSXMAG00000016803.ENSXMAG00000002738	smarcb1b	none	none	none	none	sub	none	none	sub	none	sub
ENSXMAG00000007080	no	no	no	no	no	21	no	no	no	ENSLOCG00000006694.ENSXMAG00000007080.ENSXMAG00000023163	cd81b	none	none	none	none	none	sub	sub	none	none	sub
ENSXMAG00000023163	no	no	no	no	no	no	9	no	no	ENSLOCG00000006694.ENSXMAG00000023163.ENSXMAG00000007080	cd81a	none	none	none	none	none	sub	sub	none	none	sub
ENSXMAG00000004486	no	no	no	no	no	9	no	no	no	ENSLOCG00000006732.ENSXMAG00000004486.ENSXMAG00000012228		none	none	none	none	sub	sub	sub	none	none	sub
ENSXMAG00000012228	no	no	no	no	15	no	15	no	no	ENSLOCG00000006732.ENSXMAG00000012228.ENSXMAG00000004486	CUL5	none	none	none	none	sub	sub	sub	none	none	sub
ENSXMAG00000005262	no	no	no	no	no	18	no	no	no	ENSLOCG00000006765.ENSXMAG00000005262.ENSXMAG00000008866	si:dkey-217d24.6	none	none	none	none	none	sub	none	none	none	sub
ENSXMAG00000008866	no	no	no	no	no	no	no	no	no	ENSLOCG00000006765.ENSXMAG00000008866.ENSXMAG00000005262	si:ch211-194b1.1	none	none	none	none	none	sub	none	none	none	sub
ENSXMAG00000004522	no	no	no	no	no	15	no	no	no	ENSLOCG00000006766.ENSXMAG00000004522.ENSXMAG00000015958	slc35f2l	none	none	none	none	none	sub	none	none	none	sub
ENSXMAG00000015958	no	no	no	no	no	no	no	no	no	ENSLOCG00000006766.ENSXMAG00000015958.ENSXMAG00000004522	slc35f2	none	none	none	none	none	sub	none	none	none	sub
ENSXMAG00000002228	no	no	no	no	no	no	no	no	no	ENSLOCG00000006769.ENSXMAG00000002228.ENSXMAG00000007895		none	none	none	none	none	sub	none	none	none	sub
ENSXMAG00000007895	no	no	no	no	no	3	no	no	no	ENSLOCG00000006769.ENSXMAG00000007895.ENSXMAG00000002228	SCN8A	none	none	none	none	none	sub	none	none	none	sub
ENSXMAG00000006835	no	no	no	no	no	3	no	no	no	ENSLOCG00000006811.ENSXMAG00000006835.ENSXMAG00000012276	rap1gap2b	none	none	none	none	none	sub	sub	none	none	sub
ENSXMAG00000012276	no	no	no	no	no	no	0	no	no	ENSLOCG00000006811.ENSXMAG00000012276.ENSXMAG00000006835		none	none	none	none	none	sub	sub	none	none	sub
ENSXMAG00000017106	3	3	no	no	no	6	6	6	6	ENSLOCG00000006818.ENSXMAG00000017106.ENSXMAG00000018709	nr1d2b	no diff	sub	none	none	none	no diff	sub	sub	sub	sub
ENSXMAG00000018709	3	no	no	no	no	3	no	no	no	ENSLOCG00000006818.ENSXMAG00000018709.ENSXMAG00000017106	nr1d2a	no diff	sub	none	none	none	no diff	sub	sub	sub	sub
ENSXMAG00000004660	no	no	no	no	no	no	no	no	no	ENSLOCG00000006888.ENSXMAG00000004660.ENSXMAG00000013138	RAB40C	none	none	none	none	sub	sub	none	none	none	sub
ENSXMAG00000013138	no	no	no	no	21	0	no	no	no	ENSLOCG00000006888.ENSXMAG00000013138.ENSXMAG00000004660	rab40c	none	none	none	none	sub	sub	none	none	none	sub
ENSXMAG00000014187	no	no	no	no	no	21	no	no	no	ENSLOCG00000006895.ENSXMAG00000014187.ENSXMAG00000016342	CTCF	none	none	none	none	sub	sub	none	none	none	sub
ENSXMAG00000016342	no	no	no	no	15	no	no	no	no	ENSLOCG00000006895.ENSXMAG00000016342.ENSXMAG00000014187	ctcf	none	none	none	none	sub	sub	none	none	none	sub
ENSXMAG00000018365	no	no	no	no	no	3	no	no	no	ENSLOCG00000006955.ENSXMAG00000018365.ENSXMAG00000026491		none	none	none	none	none	no diff	sub	none	none	sub
ENSXMAG00000026491	no	no	no	no	no	3	6	no	no	ENSLOCG00000006955.ENSXMAG00000026491.ENSXMAG00000018365	sgip1a	none	none	none	none	none	no diff	sub	none	none	sub
ENSXMAG00000008529	no	no	no	no	no	no	no	no	12	ENSLOCG00000006993.ENSXMAG00000008529.ENSXMAG00000023735	gria4b	none	none	none	none	none	none	none	none	sub	sub
ENSXMAG00000023735	no	no	no	no	no	no	no	no	no	ENSLOCG00000006993.ENSXMAG00000023735.ENSXMAG00000008529	gria4a	none	none	none	none	none	none	none	none	sub	sub
ENSXMAG00000004881	no	no	no	no	no	no	no	no	no	ENSLOCG00000006998.ENSXMAG00000004881.ENSXMAG00000028234	xylt1 (1 of many)	none	sub	none	none	none	sub	none	none	none	sub
ENSXMAG00000028234	no	18	no	no	no	15	no	no	no	ENSLOCG00000006998.ENSXMAG00000028234.ENSXMAG00000004881	xylt1 (1 of many)	none	sub	none	none	none	sub	none	none	none	sub
ENSXMAG00000007882	no	no	9	no	no	no	no	12	no	ENSLOCG00000007012.ENSXMAG00000007882.ENSXMAG00000028239		none	none	sub	none	none	none	none	sub	none	sub
ENSXMAG00000028239	no	no	no	no	no	no	no	no	no	ENSLOCG00000007012.ENSXMAG00000028239.ENSXMAG00000007882		none	none	sub	none	none	none	none	sub	none	sub
ENSXMAG00000007231	no	no	no	no	no	no	no	no	no	ENSLOCG00000007064.ENSXMAG00000007231.ENSXMAG00000014814		none	sub	none	sub	none	sub	sub	sub	none	sub
ENSXMAG00000014814	no	6	no	3	no	3	6	9	no	ENSLOCG00000007064.ENSXMAG00000014814.ENSXMAG00000007231	pbxip1a	none	sub	none	sub	none	sub	sub	sub	none	sub
ENSXMAG00000011686	no	no	no	no	no	no	no	15	no	ENSLOCG00000007122.ENSXMAG00000011686.ENSXMAG00000012536	hsd17b7	none	none	none	none	none	none	sub	sub	none	sub
ENSXMAG00000012536	no	no	no	no	no	no	no	15	no	ENSLOCG00000007122.ENSXMAG00000012536.ENSXMAG00000011686		none	none	none	none	none	none	sub	sub	none	sub
ENSXMAG00000002271	no	3	no	no	3	3	no	no	no	ENSLOCG00000007128.ENSXMAG00000002271.ENSXMAG00000010014	rasgef1bb	none	sub	none	none	neo	sub	sub	none	none	sub
ENSXMAG00000010014	no	no	no	no	9	no	3	no	no	ENSLOCG00000007128.ENSXMAG00000010014.ENSXMAG00000002271	rasgef1ba	none	sub	none	none	neo	sub	sub	none	none	sub
ENSXMAG00000007142	no	no	no	no	no	3	no	no	no	ENSLOCG00000007185.ENSXMAG00000007142.ENSXMAG00000017103	rgs9a	none	none	none	none	none	sub	none	none	none	sub
ENSXMAG00000017103	no	no	no	no	no	no	no	no	no	ENSLOCG00000007185.ENSXMAG00000017103.ENSXMAG00000007142	rgs9b	none	none	none	none	none	sub	none	none	none	sub
ENSXMAG00000000413	no	no	no	no	no	21	no	no	no	ENSLOCG00000007190.ENSXMAG00000000413.ENSXMAG00000026819	lnpa	none	none	none	none	none	no diff	none	none	none	sub
ENSXMAG00000026819	no	no	no	no	no	21	no	no	no	ENSLOCG00000007190.ENSXMAG00000026819.ENSXMAG00000000413		none	none	none	none	none	no diff	none	none	none	sub
ENSXMAG00000000410	no	no	3	no	no	no	no	no	no	ENSLOCG00000007211.ENSXMAG00000000410.ENSXMAG00000019062	atp5mc3b	none	none	sub	none	none	none	none	sub	none	sub
ENSXMAG00000019062	no	no	no	no	no	no	no	21	no	ENSLOCG00000007211.ENSXMAG00000019062.ENSXMAG00000000410		none	none	sub	none	none	none	none	sub	none	sub
ENSXMAG00000005615	no	no	no	no	no	0	no	no	no	ENSLOCG00000007246.ENSXMAG00000005615.ENSXMAG00000019234	rgs16	none	none	none	none	none	sub	none	none	none	sub
ENSXMAG00000019234	no	no	no	no	no	no	no	no	no	ENSLOCG00000007246.ENSXMAG00000019234.ENSXMAG00000005615	rgs5b	none	none	none	none	none	sub	none	none	none	sub
ENSXMAG00000019441	no	no	no	no	no	18	no	no	no	ENSLOCG00000007272.ENSXMAG00000019441.ENSXMAG00000022365	brinp3b	none	none	none	none	none	sub	none	none	none	sub
ENSXMAG00000022365	no	no	no	no	no	no	no	no	no	ENSLOCG00000007272.ENSXMAG00000022365.ENSXMAG00000019441	brinp3a.1	none	none	none	none	none	sub	none	none	none	sub
ENSXMAG00000005488	no	no	no	no	no	6	no	no	no	ENSLOCG00000007278.ENSXMAG00000005488.ENSXMAG00000005951		none	none	none	none	none	sub	none	none	none	sub
ENSXMAG00000005951	no	no	no	no	no	no	no	no	no	ENSLOCG00000007278.ENSXMAG00000005951.ENSXMAG00000005488		none	none	none	none	none	sub	none	none	none	sub
ENSXMAG00000010616	no	no	no	no	no	no	no	15	no	ENSLOCG00000007299.ENSXMAG00000010616.ENSXMAG00000017416	ptges3a	none	none	none	none	sub	none	sub	no diff	none	sub
ENSXMAG00000017416	no	no	no	no	15	no	15	15	no	ENSLOCG00000007299.ENSXMAG00000017416.ENSXMAG00000010616	ptges3b	none	none	none	none	sub	none	sub	no diff	none	sub
ENSXMAG00000007574	no	no	no	no	no	no	no	no	no	ENSLOCG00000007306.ENSXMAG00000007574.ENSXMAG00000022965		none	none	none	none	none	sub	none	none	none	sub
ENSXMAG00000022965	no	no	no	no	no	15	no	no	no	ENSLOCG00000007306.ENSXMAG00000022965.ENSXMAG00000007574	adgra1a	none	none	none	none	none	sub	none	none	none	sub
ENSXMAG00000002288	21	no	no	no	no	21	no	no	no	ENSLOCG00000007313.ENSXMAG00000002288.ENSXMAG00000009926	paqr3b	sub	none	none	none	none	sub	none	none	none	sub
ENSXMAG00000009926	no	no	no	no	no	no	no	no	no	ENSLOCG00000007313.ENSXMAG00000009926.ENSXMAG00000002288	paqr3a	sub	none	none	none	none	sub	none	none	none	sub
ENSXMAG00000004031	no	no	no	no	0	21	no	no	no	ENSLOCG00000007322.ENSXMAG00000004031.ENSXMAG00000029877	pptc7b	none	none	none	none	neo	sub	none	none	none	sub
ENSXMAG00000029877	no	no	no	no	6	no	no	no	no	ENSLOCG00000007322.ENSXMAG00000029877.ENSXMAG00000004031	pptc7a	none	none	none	none	neo	sub	none	none	none	sub
ENSXMAG00000005021	no	no	no	no	no	no	no	no	no	ENSLOCG00000007323.ENSXMAG00000005021.ENSXMAG00000005409	gpr155b	none	none	none	sub	none	none	none	none	none	sub
ENSXMAG00000005409	no	no	no	3	no	no	no	no	no	ENSLOCG00000007323.ENSXMAG00000005409.ENSXMAG00000005021	gpr155a	none	none	none	sub	none	none	none	none	none	sub
ENSXMAG00000002712	no	no	no	no	no	no	0	no	no	ENSLOCG00000007328.ENSXMAG00000002712.ENSXMAG00000024646	RGS13 (1 of many)	none	none	none	none	none	none	sub	none	none	sub
ENSXMAG00000024646	no	no	no	no	no	no	no	no	no	ENSLOCG00000007328.ENSXMAG00000024646.ENSXMAG00000002712	rgs13	none	none	none	none	none	none	sub	none	none	sub
ENSXMAG00000012988	no	no	no	no	no	9	no	no	no	ENSLOCG00000007339.ENSXMAG00000012988.ENSXMAG00000029897	si:dkeyp-72e1.7	none	none	none	none	none	no diff	none	none	none	sub
ENSXMAG00000029897	no	no	no	no	no	12	no	no	no	ENSLOCG00000007339.ENSXMAG00000029897.ENSXMAG00000012988		none	none	none	none	none	no diff	none	none	none	sub
ENSXMAG00000010632	no	no	no	no	no	15	no	no	no	ENSLOCG00000007372.ENSXMAG00000010632.ENSXMAG00000017409	rnd1b	none	none	none	none	none	sub	none	none	none	sub
ENSXMAG00000017409	no	no	no	no	no	no	no	no	no	ENSLOCG00000007372.ENSXMAG00000017409.ENSXMAG00000010632	rnd1a	none	none	none	none	none	sub	none	none	none	sub
ENSXMAG00000009132	no	no	no	no	no	no	no	no	no	ENSLOCG00000007400.ENSXMAG00000009132.ENSXMAG00000011913	pld1a	none	sub	none	none	none	sub	sub	none	none	sub
ENSXMAG00000011913	no	21	no	no	no	18	0	no	no	ENSLOCG00000007400.ENSXMAG00000011913.ENSXMAG00000009132	pld1b	none	sub	none	none	none	sub	sub	none	none	sub
ENSXMAG00000010656	no	0	no	no	no	0	no	no	no	ENSLOCG00000007430.ENSXMAG00000010656.ENSXMAG00000017286	adcy6b	none	sub	none	none	sub	no diff	none	none	none	sub
ENSXMAG00000017286	no	no	no	no	3	3	no	no	no	ENSLOCG00000007430.ENSXMAG00000017286.ENSXMAG00000010656	adcy6a	none	sub	none	none	sub	no diff	none	none	none	sub
ENSXMAG00000007284	no	no	no	no	15	no	no	no	no	ENSLOCG00000007431.ENSXMAG00000007284.ENSXMAG00000022050	gspt1l	none	none	none	none	sub	none	sub	sub	none	sub
ENSXMAG00000022050	no	no	no	no	no	no	15	15	no	ENSLOCG00000007431.ENSXMAG00000022050.ENSXMAG00000007284	GSPT1	none	none	none	none	sub	none	sub	sub	none	sub
ENSXMAG00000002447	no	no	no	no	no	0	no	no	no	ENSLOCG00000007531.ENSXMAG00000002447.ENSXMAG00000004785	crb1	none	none	none	none	none	no diff	none	none	none	sub
ENSXMAG00000004785	no	no	no	no	no	0	no	no	no	ENSLOCG00000007531.ENSXMAG00000004785.ENSXMAG00000002447		none	none	none	none	none	no diff	none	none	none	sub
ENSXMAG00000000208	no	no	no	no	no	no	no	no	no	ENSLOCG00000007575.ENSXMAG00000000208.ENSXMAG00000007185	pde4a	none	sub	none	none	none	sub	none	none	none	sub
ENSXMAG00000007185	no	15	no	no	no	12	no	no	no	ENSLOCG00000007575.ENSXMAG00000007185.ENSXMAG00000000208	PDE4A (1 of many)	none	sub	none	none	none	sub	none	none	none	sub
ENSXMAG00000008340	no	no	no	no	18	18	no	21	no	ENSLOCG00000007590.ENSXMAG00000008340.ENSXMAG00000022010	acbd5a	none	none	none	none	sub	no diff	none	sub	none	sub
ENSXMAG00000022010	no	no	no	no	no	15	no	no	no	ENSLOCG00000007590.ENSXMAG00000022010.ENSXMAG00000008340	acbd5b	none	none	none	none	sub	no diff	none	sub	none	sub
ENSXMAG00000004822	21	no	no	no	no	no	0	no	no	ENSLOCG00000007622.ENSXMAG00000004822.ENSXMAG00000026357	vwa1	no diff	none	none	none	none	sub	sub	none	none	sub
ENSXMAG00000026357	0	no	no	no	no	15	no	no	no	ENSLOCG00000007622.ENSXMAG00000026357.ENSXMAG00000004822		no diff	none	none	none	none	sub	sub	none	none	sub
ENSXMAG00000001967	no	no	no	no	no	no	no	no	no	ENSLOCG00000007625.ENSXMAG00000001967.ENSXMAG00000004476		none	none	none	none	none	sub	none	none	none	sub
ENSXMAG00000004476	no	no	no	no	no	21	no	no	no	ENSLOCG00000007625.ENSXMAG00000004476.ENSXMAG00000001967	pla2g3	none	none	none	none	none	sub	none	none	none	sub
ENSXMAG00000012673	no	no	no	no	no	15	no	no	3	ENSLOCG00000007653.ENSXMAG00000012673.ENSXMAG00000016284	ptpreb	none	none	none	none	none	neo	none	none	sub	sub
ENSXMAG00000016284	no	no	no	no	no	6	no	no	no	ENSLOCG00000007653.ENSXMAG00000016284.ENSXMAG00000012673	ptprea	none	none	none	none	none	neo	none	none	sub	sub
ENSXMAG00000014446	no	no	no	no	no	no	no	no	no	ENSLOCG00000007668.ENSXMAG00000014446.ENSXMAG00000015472		none	none	none	none	none	sub	none	none	none	sub
ENSXMAG00000015472	no	no	no	no	no	15	no	no	no	ENSLOCG00000007668.ENSXMAG00000015472.ENSXMAG00000014446	DENND3	none	none	none	none	none	sub	none	none	none	sub
ENSXMAG00000019058	no	no	no	no	15	no	no	no	no	ENSLOCG00000007673.ENSXMAG00000019058.ENSXMAG00000028633	SLC25A12 (1 of many)	none	none	none	none	sub	none	none	none	none	sub
ENSXMAG00000028633	no	no	no	no	no	no	no	no	no	ENSLOCG00000007673.ENSXMAG00000028633.ENSXMAG00000019058	SLC25A12 (1 of many)	none	none	none	none	sub	none	none	none	none	sub
ENSXMAG00000010911	0	no	no	no	no	no	no	no	no	ENSLOCG00000007702.ENSXMAG00000010911.ENSXMAG00000015476		sub	none	none	none	none	none	none	none	none	sub
ENSXMAG00000015476	no	no	no	no	no	no	no	no	no	ENSLOCG00000007702.ENSXMAG00000015476.ENSXMAG00000010911	ptk2aa	sub	none	none	none	none	none	none	none	none	sub
ENSXMAG00000012600	no	6	no	no	no	6	no	no	no	ENSLOCG00000007761.ENSXMAG00000012600.ENSXMAG00000016660		none	sub	none	none	none	no diff	sub	none	none	sub
ENSXMAG00000016660	no	no	no	no	no	6	15	no	no	ENSLOCG00000007761.ENSXMAG00000016660.ENSXMAG00000012600	SDHB	none	sub	none	none	none	no diff	sub	none	none	sub
ENSXMAG00000008421	no	no	no	no	no	no	21	no	no	ENSLOCG00000007776.ENSXMAG00000008421.ENSXMAG00000011728	gpr158a	none	none	none	none	none	none	sub	none	none	sub
ENSXMAG00000011728	no	no	no	no	no	no	no	no	no	ENSLOCG00000007776.ENSXMAG00000011728.ENSXMAG00000008421	gpr158b	none	none	none	none	none	none	sub	none	none	sub
ENSXMAG00000015605	no	no	no	no	15	no	no	no	no	ENSLOCG00000007783.ENSXMAG00000015605.ENSXMAG00000019169	ZSWIM6	sub	none	none	none	sub	none	none	none	none	sub
ENSXMAG00000019169	3	no	no	no	no	no	no	no	no	ENSLOCG00000007783.ENSXMAG00000019169.ENSXMAG00000015605	zswim6	sub	none	none	none	sub	none	none	none	none	sub
ENSXMAG00000001916	no	no	no	no	15	no	no	no	no	ENSLOCG00000007813.ENSXMAG00000001916.ENSXMAG00000030067	PITPNB	none	none	none	none	sub	sub	none	none	none	sub
ENSXMAG00000030067	no	no	no	no	no	15	no	no	no	ENSLOCG00000007813.ENSXMAG00000030067.ENSXMAG00000001916	pitpnb	none	none	none	none	sub	sub	none	none	none	sub
ENSXMAG00000006321	no	no	no	no	no	no	15	no	no	ENSLOCG00000007820.ENSXMAG00000006321.ENSXMAG00000007030	smarca4a	none	none	none	none	sub	none	sub	none	none	sub
ENSXMAG00000007030	no	no	no	no	15	no	no	no	no	ENSLOCG00000007820.ENSXMAG00000007030.ENSXMAG00000006321		none	none	none	none	sub	none	sub	none	none	sub
ENSXMAG00000000740	no	no	no	no	no	15	no	no	no	ENSLOCG00000007823.ENSXMAG00000000740.ENSXMAG00000007104		none	none	none	none	sub	sub	none	sub	none	sub
ENSXMAG00000007104	no	no	no	no	15	no	no	12	no	ENSLOCG00000007823.ENSXMAG00000007104.ENSXMAG00000000740	KIF2A	none	none	none	none	sub	sub	none	sub	none	sub
ENSXMAG00000016721	no	no	no	no	no	no	no	no	no	ENSLOCG00000007847.ENSXMAG00000016721.ENSXMAG00000025502	SZRD1	none	none	none	none	sub	sub	none	none	none	sub
ENSXMAG00000025502	no	no	no	no	0	9	no	no	no	ENSLOCG00000007847.ENSXMAG00000025502.ENSXMAG00000016721	szrd1	none	none	none	none	sub	sub	none	none	none	sub
ENSXMAG00000006970	no	no	no	no	no	15	no	no	no	ENSLOCG00000007853.ENSXMAG00000006970.ENSXMAG00000009142	mast1a	none	none	none	none	none	sub	none	none	none	sub
ENSXMAG00000009142	no	no	no	no	no	no	no	no	no	ENSLOCG00000007853.ENSXMAG00000009142.ENSXMAG00000006970	mast1b	none	none	none	none	none	sub	none	none	none	sub
ENSXMAG00000017772	no	no	no	no	no	0	no	no	no	ENSLOCG00000007855.ENSXMAG00000017772.ENSXMAG00000027563	inpp5jb	none	none	none	none	none	sub	none	none	none	sub
ENSXMAG00000027563	no	no	no	no	no	no	no	no	no	ENSLOCG00000007855.ENSXMAG00000027563.ENSXMAG00000017772	inpp5ja	none	none	none	none	none	sub	none	none	none	sub
ENSXMAG00000001163	no	no	no	3	no	18	no	no	no	ENSLOCG00000007859.ENSXMAG00000001163.ENSXMAG00000011196	tpi1a	none	none	none	sub	none	sub	sub	none	none	sub
ENSXMAG00000011196	no	no	no	no	no	no	15	no	no	ENSLOCG00000007859.ENSXMAG00000011196.ENSXMAG00000001163	tpi1b	none	none	none	sub	none	sub	sub	none	none	sub
ENSXMAG00000006158	no	no	no	no	no	6	no	no	no	ENSLOCG00000007868.ENSXMAG00000006158.ENSXMAG00000030090		none	none	none	none	none	neo	none	none	none	sub
ENSXMAG00000030090	no	no	no	no	no	18	no	no	no	ENSLOCG00000007868.ENSXMAG00000030090.ENSXMAG00000006158	CYGB	none	none	none	none	none	neo	none	none	none	sub
ENSXMAG00000021384	no	no	no	no	21	21	no	no	no	ENSLOCG00000007896.ENSXMAG00000021384.ENSXMAG00000029887	spen	none	none	none	none	sub	sub	none	none	none	sub
ENSXMAG00000029887	no	no	no	no	no	no	no	no	no	ENSLOCG00000007896.ENSXMAG00000029887.ENSXMAG00000021384	si:ch1073-335m2.2	none	none	none	none	sub	sub	none	none	none	sub
ENSXMAG00000000320	no	no	no	no	no	no	no	no	no	ENSLOCG00000007922.ENSXMAG00000000320.ENSXMAG00000006441	selenoj	none	none	none	none	sub	none	none	none	none	sub
ENSXMAG00000006441	no	no	no	no	12	no	no	no	no	ENSLOCG00000007922.ENSXMAG00000006441.ENSXMAG00000000320		none	none	none	none	sub	none	none	none	none	sub
ENSXMAG00000000476	no	no	no	no	no	15	no	no	no	ENSLOCG00000007934.ENSXMAG00000000476.ENSXMAG00000006442	vav3b	none	none	none	none	none	sub	none	none	none	sub
ENSXMAG00000006442	no	no	no	no	no	no	no	no	no	ENSLOCG00000007934.ENSXMAG00000006442.ENSXMAG00000000476	VAV3 (1 of many)	none	none	none	none	none	sub	none	none	none	sub
ENSXMAG00000004335	no	no	no	no	no	15	no	no	no	ENSLOCG00000007941.ENSXMAG00000004335.ENSXMAG00000010385		none	none	none	none	none	no diff	sub	none	none	sub
ENSXMAG00000010385	no	no	no	no	no	15	21	no	no	ENSLOCG00000007941.ENSXMAG00000010385.ENSXMAG00000004335	rrm1	none	none	none	none	none	no diff	sub	none	none	sub
ENSXMAG00000015852	no	no	no	no	no	no	no	no	no	ENSLOCG00000007953.ENSXMAG00000015852.ENSXMAG00000016470	c7a	sub	none	none	none	none	none	none	none	none	sub
ENSXMAG00000016470	21	no	no	no	no	no	no	no	no	ENSLOCG00000007953.ENSXMAG00000016470.ENSXMAG00000015852	c7b	sub	none	none	none	none	none	none	none	none	sub
ENSXMAG00000003851	no	no	no	no	no	15	no	no	no	ENSLOCG00000007961.ENSXMAG00000003851.ENSXMAG00000009717		none	none	none	none	none	sub	none	none	none	sub
ENSXMAG00000009717	no	no	no	no	no	no	no	no	no	ENSLOCG00000007961.ENSXMAG00000009717.ENSXMAG00000003851	cabp2b	none	none	none	none	none	sub	none	none	none	sub
ENSXMAG00000011034	no	no	no	no	no	no	no	no	no	ENSLOCG00000007968.ENSXMAG00000011034.ENSXMAG00000016787	si:ch211-154o6.2	none	none	none	none	none	none	sub	none	none	sub

ENSXMAG00000016787	no	no	no	no	no	no	21	no	no	ENSLOCG00000007968.ENSXMAG00000016787.ENSXMAG00000011034	znf384l	none	none	none	none	none	none	sub	none	none	sub
ENSXMAG00000000677	no	no	no	no	no	18	no	no	no	ENSLOCG00000008015.ENSXMAG00000000677.ENSXMAG00000025233	olfm3b	none	none	none	none	none	sub	none	none	none	sub
ENSXMAG00000025233	no	no	no	no	no	no	no	no	no	ENSLOCG00000008015.ENSXMAG00000025233.ENSXMAG00000000677	olfm3a	none	none	none	none	none	sub	none	none	none	sub
ENSXMAG00000010903	no	no	no	no	no	no	no	no	no	ENSLOCG00000008038.ENSXMAG00000010903.ENSXMAG00000025417	grk7b	none	none	none	none	none	sub	none	none	none	sub
ENSXMAG00000025417	no	no	no	no	no	0	no	no	no	ENSLOCG00000008038.ENSXMAG00000025417.ENSXMAG00000010903	grk7a	none	none	none	none	none	sub	none	none	none	sub
ENSXMAG00000013970	no	no	no	no	3	no	no	no	no	ENSLOCG00000008040.ENSXMAG00000013970.ENSXMAG00000014325	klhl41b	none	none	none	none	sub	none	none	none	none	sub
ENSXMAG00000014325	no	no	no	no	no	no	no	no	no	ENSLOCG00000008040.ENSXMAG00000014325.ENSXMAG00000013970	klhl41a	none	none	none	none	sub	none	none	none	none	sub
ENSXMAG00000009803	no	no	no	no	no	no	no	no	no	ENSLOCG00000008046.ENSXMAG00000009803.ENSXMAG00000013723	tmx2a	none	none	none	none	sub	sub	none	none	none	sub
ENSXMAG00000013723	no	no	no	no	15	21	no	no	no	ENSLOCG00000008046.ENSXMAG00000013723.ENSXMAG00000009803		none	none	none	none	sub	sub	none	none	none	sub
ENSXMAG00000009791	no	no	no	no	no	no	no	no	no	ENSLOCG00000008054.ENSXMAG00000009791.ENSXMAG00000013732	zdhhc5b	none	none	none	none	none	sub	none	none	none	sub
ENSXMAG00000013732	no	no	no	no	no	15	no	no	no	ENSLOCG00000008054.ENSXMAG00000013732.ENSXMAG00000009791	zdhhc5a	none	none	none	none	none	sub	none	none	none	sub
ENSXMAG00000011911	no	no	no	no	no	no	no	no	no	ENSLOCG00000008077.ENSXMAG00000011911.ENSXMAG00000014223	epn1	none	none	none	sub	none	none	none	none	none	sub
ENSXMAG00000014223	no	no	no	3	no	no	no	no	no	ENSLOCG00000008077.ENSXMAG00000014223.ENSXMAG00000011911	zgc:194578	none	none	none	sub	none	none	none	none	none	sub
ENSXMAG00000002229	no	no	no	no	no	0	no	no	no	ENSLOCG00000008187.ENSXMAG00000002229.ENSXMAG00000015884	glrbb	none	none	none	none	none	none	no diff	none	none	sub
ENSXMAG00000015884	no	no	no	no	no	21	no	no	no	ENSLOCG00000008187.ENSXMAG00000015884.ENSXMAG00000002229	glrba	none	none	none	none	none	none	no diff	none	none	sub
ENSXMAG00000003263	3	no	no	no	0	no	no	no	no	ENSLOCG00000008221.ENSXMAG00000003263.ENSXMAG00000004870	txnipa	sub	none	none	none	sub	sub	none	none	none	sub
ENSXMAG00000004870	no	no	no	no	no	18	no	no	no	ENSLOCG00000008221.ENSXMAG00000004870.ENSXMAG00000003263		sub	none	none	none	sub	sub	none	none	none	sub
ENSXMAG00000010370	no	no	no	no	no	no	no	no	no	ENSLOCG00000008301.ENSXMAG00000010370.ENSXMAG00000026909	PLPPR4 (1 of many)	none	none	none	none	none	sub	none	none	none	sub
ENSXMAG00000026909	no	no	no	no	no	15	no	no	no	ENSLOCG00000008301.ENSXMAG00000026909.ENSXMAG00000010370	plppr4b	none	none	none	none	none	sub	none	none	none	sub
ENSXMAG00000004187	no	no	no	no	no	15	no	no	no	ENSLOCG00000008316.ENSXMAG00000004187.ENSXMAG00000010347		none	none	none	none	none	sub	none	none	none	sub
ENSXMAG00000010347	no	no	no	no	no	no	no	no	no	ENSLOCG00000008316.ENSXMAG00000010347.ENSXMAG00000004187	GABRG3	none	none	none	none	none	sub	none	none	none	sub
ENSXMAG00000003635	no	no	no	no	no	3	no	no	no	ENSLOCG00000008322.ENSXMAG00000003635.ENSXMAG00000015449	dtnbp1b	none	none	none	none	none	sub	none	none	none	sub
ENSXMAG00000015449	no	no	no	no	no	no	no	no	no	ENSLOCG00000008322.ENSXMAG00000015449.ENSXMAG00000003635		none	none	none	none	none	sub	none	none	none	sub
ENSXMAG00000005153	no	no	no	no	12	no	no	no	no	ENSLOCG00000008330.ENSXMAG00000005153.ENSXMAG00000011550	slc44a2	none	none	none	none	sub	sub	none	none	none	sub
ENSXMAG00000011550	no	no	no	no	no	21	no	no	no	ENSLOCG00000008330.ENSXMAG00000011550.ENSXMAG00000005153		none	none	none	none	sub	sub	none	none	none	sub
ENSXMAG00000007084	no	no	no	no	no	no	no	no	no	ENSLOCG00000008398.ENSXMAG00000007084.ENSXMAG00000008579	dclk1b	none	sub	none	none	none	sub	sub	none	none	sub
ENSXMAG00000008579	no	3	no	no	no	6	6	no	no	ENSLOCG00000008398.ENSXMAG00000008579.ENSXMAG00000007084	dclk1a	none	sub	none	none	none	sub	sub	none	none	sub
ENSXMAG00000001119	no	12	no	no	no	15	no	no	no	ENSLOCG00000008410.ENSXMAG00000001119.ENSXMAG00000014597		none	sub	none	none	sub	neo	none	none	none	sub
ENSXMAG00000014597	no	no	no	no	3	21	no	no	no	ENSLOCG00000008410.ENSXMAG00000014597.ENSXMAG00000001119		none	sub	none	none	sub	neo	none	none	none	sub
ENSXMAG00000007108	no	no	no	no	no	no	no	no	no	ENSLOCG00000008434.ENSXMAG00000007108.ENSXMAG00000008570	spartb	none	none	none	none	none	none	sub	none	none	sub
ENSXMAG00000008570	no	no	no	no	no	no	0	no	no	ENSLOCG00000008434.ENSXMAG00000008570.ENSXMAG00000007108	sparta	none	none	none	none	none	none	sub	none	none	sub
ENSXMAG00000010707	no	no	no	no	no	no	no	no	no	ENSLOCG00000008535.ENSXMAG00000010707.ENSXMAG00000012164	zgc:100997	none	none	none	none	none	sub	none	none	none	sub
ENSXMAG00000012164	no	no	no	no	no	3	no	no	no	ENSLOCG00000008535.ENSXMAG00000012164.ENSXMAG00000010707	clmn	none	none	none	none	none	sub	none	none	none	sub
ENSXMAG00000011435	no	no	no	no	no	12	no	no	no	ENSLOCG00000008549.ENSXMAG00000011435.ENSXMAG00000013798	slc4a10b	none	none	none	none	none	sub	none	none	none	sub
ENSXMAG00000013798	no	no	no	no	no	no	no	no	no	ENSLOCG00000008549.ENSXMAG00000013798.ENSXMAG00000011435		none	none	none	none	none	sub	none	none	none	sub
ENSXMAG00000014657	no	no	no	no	no	no	no	no	no	ENSLOCG00000008552.ENSXMAG00000014657.ENSXMAG00000014851	pvalb8	none	none	none	none	none	sub	none	none	none	sub
ENSXMAG00000014851	no	no	no	no	no	18	no	no	no	ENSLOCG00000008552.ENSXMAG00000014851.ENSXMAG00000014657	pvalb9	none	none	none	none	none	sub	none	none	none	sub
ENSXMAG00000012028	no	no	no	no	no	no	no	no	no	ENSLOCG00000008584.ENSXMAG00000012028.ENSXMAG00000013238	slc27a1b	none	none	none	none	sub	none	none	none	none	sub
ENSXMAG00000013238	no	no	no	no	21	no	no	no	no	ENSLOCG00000008584.ENSXMAG00000013238.ENSXMAG00000012028	slc27a1a	none	none	none	none	sub	none	none	none	none	sub
ENSXMAG00000010736	no	no	no	no	no	no	no	no	no	ENSLOCG00000008589.ENSXMAG00000010736.ENSXMAG00000029258	GRAMD2B	none	none	none	none	sub	sub	none	none	none	sub
ENSXMAG00000029258	no	no	no	no	21	21	no	no	no	ENSLOCG00000008589.ENSXMAG00000029258.ENSXMAG00000010736		none	none	none	none	sub	sub	none	none	none	sub
ENSXMAG00000007449	no	no	no	no	no	no	no	no	no	ENSLOCG00000008605.ENSXMAG00000007449.ENSXMAG00000025073		none	none	none	none	none	sub	none	none	none	sub
ENSXMAG00000025073	no	no	no	no	3	no	no	no	no	ENSLOCG00000008605.ENSXMAG00000025073.ENSXMAG00000007449	sh3d19	none	none	none	none	none	sub	none	none	none	sub
ENSXMAG00000001802	no	no	no	no	15	no	no	no	no	ENSLOCG00000008622.ENSXMAG00000001802.ENSXMAG00000011405	rbms1a	none	none	none	none	sub	none	sub	none	none	sub
ENSXMAG00000011405	no	no	no	no	no	no	6	no	no	ENSLOCG00000008622.ENSXMAG00000011405.ENSXMAG00000001802	RBMS1 (1 of many)	none	none	none	none	sub	none	sub	none	none	sub
ENSXMAG00000007202	no	no	no	no	no	12	no	no	no	ENSLOCG00000008640.ENSXMAG00000007202.ENSXMAG00000017483	trim3a	none	none	none	none	none	sub	none	none	none	sub
ENSXMAG00000017483	no	no	no	no	no	no	no	no	no	ENSLOCG00000008640.ENSXMAG00000017483.ENSXMAG00000007202	trim3b	none	none	none	none	none	sub	none	none	none	sub
ENSXMAG00000000364	no	no	no	no	no	9	0	no	no	ENSLOCG00000008777.ENSXMAG00000000364.ENSXMAG00000010908		none	none	none	none	none	sub	sub	none	none	sub
ENSXMAG00000010908	no	no	no	no	no	no	no	no	no	ENSLOCG00000008777.ENSXMAG00000010908.ENSXMAG00000000364	ism2b	none	none	none	none	none	sub	sub	none	none	sub
ENSXMAG00000001820	no	no	no	no	no	no	no	no	no	ENSLOCG00000008784.ENSXMAG00000001820.ENSXMAG00000009576	TMEM184C (1 of many)	sub	sub	none	none	none	sub	none	none	none	sub
ENSXMAG00000009576	18	18	no	no	no	18	no	no	no	ENSLOCG00000008784.ENSXMAG00000009576.ENSXMAG00000001820	tmem184c	sub	sub	none	none	none	sub	none	none	none	sub
ENSXMAG00000007371	no	no	no	no	no	no	no	no	no	ENSLOCG00000008787.ENSXMAG00000007371.ENSXMAG00000023673	slc25a15a	none	none	none	none	sub	none	none	none	none	sub
ENSXMAG00000023673	no	no	no	no	12	no	no	no	no	ENSLOCG00000008787.ENSXMAG00000023673.ENSXMAG00000007371	slc25a15b	none	none	none	none	sub	none	none	none	none	sub
ENSXMAG00000011165	no	no	no	no	no	15	no	no	no	ENSLOCG00000008788.ENSXMAG00000011165.ENSXMAG00000018151	plpp3	sub	none	none	none	none	sub	none	none	none	sub
ENSXMAG00000018151	6	no	no	no	no	no	no	no	no	ENSLOCG00000008788.ENSXMAG00000018151.ENSXMAG00000011165		sub	none	none	none	none	sub	none	none	none	sub
ENSXMAG00000022020	no	no	no	no	no	no	no	no	no	ENSLOCG00000008807.ENSXMAG00000022020.ENSXMAG00000028449		none	none	none	none	none	sub	none	none	none	sub
ENSXMAG00000028449	no	no	no	no	no	0	no	no	no	ENSLOCG00000008807.ENSXMAG00000028449.ENSXMAG00000022020	foxo1a	none	none	none	none	none	sub	none	none	none	sub
ENSXMAG00000011184	no	no	no	no	21	12	no	no	no	ENSLOCG00000008845.ENSXMAG00000011184.ENSXMAG00000027199	dab1a	none	sub	none	none	sub	no diff	none	none	none	sub
ENSXMAG00000027199	no	12	no	no	no	15	no	no	no	ENSLOCG00000008845.ENSXMAG00000027199.ENSXMAG00000011184	dab1b	none	sub	none	none	sub	no diff	none	none	none	sub
ENSXMAG00000018972	18	no	no	no	no	no	no	no	no	ENSLOCG00000008910.ENSXMAG00000018972.ENSXMAG00000022076	shrprbck1r	sub	none	none	none	sub	sub	none	none	none	sub
ENSXMAG00000022076	no	no	no	no	0	18	no	no	no	ENSLOCG00000008910.ENSXMAG00000022076.ENSXMAG00000018972	sharpin	sub	none	none	none	sub	sub	none	none	none	sub
ENSXMAG00000012169	no	no	no	no	no	15	no	no	no	ENSLOCG00000008920.ENSXMAG00000012169.ENSXMAG00000013164	dnm2b	none	none	none	none	sub	sub	none	none	none	sub
ENSXMAG00000013164	no	no	no	no	15	no	no	no	no	ENSLOCG00000008920.ENSXMAG00000013164.ENSXMAG00000012169	dnm2a	none	none	none	none	sub	sub	none	none	none	sub
ENSXMAG00000000148	no	no	no	3	no	9	no	no	no	ENSLOCG00000008925.ENSXMAG00000000148.ENSXMAG00000014947	tex2l	none	none	none	sub	none	sub	none	none	none	sub
ENSXMAG00000014947	no	no	no	no	no	no	no	no	no	ENSLOCG00000008925.ENSXMAG00000014947.ENSXMAG00000000148		none	none	none	sub	none	sub	none	none	none	sub
ENSXMAG00000005256	no	no	no	no	no	6	no	no	no	ENSLOCG00000008928.ENSXMAG00000005256.ENSXMAG00000029349	nrsn1	none	none	none	none	none	sub	none	none	none	sub
ENSXMAG00000029349	no	no	no	no	no	no	no	no	no	ENSLOCG00000008928.ENSXMAG00000029349.ENSXMAG00000005256		none	none	none	none	none	sub	none	none	none	sub
ENSXMAG00000014989	no	no	no	no	no	no	no	no	no	ENSLOCG00000008938.ENSXMAG00000014989.ENSXMAG00000027955	MSRB1 (1 of many)	none	none	none	none	none	sub	none	none	none	sub
ENSXMAG00000027955	no	no	no	no	no	18	no	no	no	ENSLOCG00000008938.ENSXMAG00000027955.ENSXMAG00000014989	msrb1b	none	none	none	none	none	sub	none	none	none	sub
ENSXMAG00000007339	no	no	no	no	no	no	no	no	no	ENSLOCG00000008941.ENSXMAG00000007339.ENSXMAG00000016068	SLC7A1	none	sub	none	none	none	sub	sub	none	sub	sub
ENSXMAG00000016068	no	6	no	no	no	6	9	no	9	ENSLOCG00000008941.ENSXMAG00000016068.ENSXMAG00000007339		none	sub	none	none	none	sub	sub	none	sub	sub
ENSXMAG00000009861	no	no	no	no	15	no	15	15	no	ENSLOCG00000008943.ENSXMAG00000009861.ENSXMAG00000023815	ATP6V0C (1 of many)	none	none	none	none	no diff	none	sub	sub	none	sub
ENSXMAG00000023815	no	no	no	no	15	no	no	no	no	ENSLOCG00000008943.ENSXMAG00000023815.ENSXMAG00000009861	ATP6V0C	none	none	none	none	no diff	none	sub	sub	none	sub
ENSXMAG00000007326	no	no	no	18	21	no	no	no	no	ENSLOCG00000008983.ENSXMAG00000007326.ENSXMAG00000010551	cadm2b	none	none	none	sub	sub	none	none	none	none	sub
ENSXMAG00000010551	no	no	no	no	no	no	no	no	no	ENSLOCG00000008983.ENSXMAG00000010551.ENSXMAG00000007326		none	none	none	sub	sub	none	none	none	none	sub
ENSXMAG00000002580	no	no	no	no	no	no	no	no	no	ENSLOCG00000009024.ENSXMAG00000002580.ENSXMAG00000005681	slc25a28	none	none	none	sub	none	none	none	sub	none	sub
ENSXMAG00000005681	no	no	no	6	no	no	no	15	no	ENSLOCG00000009024.ENSXMAG00000005681.ENSXMAG00000002580	SLC25A28 (1 of many)	none	none	none	sub	none	none	none	sub	none	sub
ENSXMAG00000009808	no	no	no	no	no	18	no	no	no	ENSLOCG00000009040.ENSXMAG00000009808.ENSXMAG00000026556		none	none	none	none	none	sub	none	none	none	sub
ENSXMAG00000026556	no	no	no	no	no	no	no	no	no	ENSLOCG00000009040.ENSXMAG00000026556.ENSXMAG00000009808	inpp4b	none	none	none	none	none	sub	none	none	none	sub
ENSXMAG00000002566	no	no	no	no	no	3	no	no	no	ENSLOCG00000009070.ENSXMAG00000002566.ENSXMAG00000005657	CNNM1 (1 of many)	none	none	none	none	none	sub	none	none	none	sub
ENSXMAG00000005657	no	no	no	no	no	no	no	no	no	ENSLOCG00000009070.ENSXMAG00000005657.ENSXMAG00000002566	CNNM1 (1 of many)	none	none	none	none	none	sub	none	none	none	sub
ENSXMAG00000009793	21	no	no	no	no	21	no	no	no	ENSLOCG00000009076.ENSXMAG00000009793.ENSXMAG00000026837	rnf150a	sub	none	none	none	none	neo	none	none	none	sub
ENSXMAG00000026837	no	no	no	no	no	3	no	no	no	ENSLOCG00000009076.ENSXMAG00000026837.ENSXMAG00000009793	rnf150b	sub	none	none	none	none	neo	none	none	none	sub
ENSXMAG00000007309	0	no	no	no	no	21	no	no	no	ENSLOCG00000009077.ENSXMAG00000007309.ENSXMAG00000026281	bach1a	sub	none	none	none	none	sub	none	none	none	sub
ENSXMAG00000026281	no	no	no	no	no	no	no	no	no	ENSLOCG00000009077.ENSXMAG00000026281.ENSXMAG00000007309		sub	none	none	none	none	sub	none	none	none	sub
ENSXMAG00000011392	no	no	no	no	no	3	no	no	no	ENSLOCG00000009109.ENSXMAG00000011392.ENSXMAG00000012027	atp11a	none	none	none	none	none	sub	sub	none	none	sub
ENSXMAG00000012027	no	no	no	no	no	no	6	no	no	ENSLOCG00000009109.ENSXMAG00000012027.ENSXMAG00000011392		none	none	none	none	none	sub	sub	none	none	sub
ENSXMAG00000000294	no	no	no	3	no	no	no	no	no	ENSLOCG00000009131.ENSXMAG00000000294.ENSXMAG00000010247	CPEB4 (1 of many)	none	none	none	sub	sub	none	none	none	none	sub
ENSXMAG00000010247	no	no	no	no	15	no	no	no	no	ENSLOCG00000009131.ENSXMAG00000010247.ENSXMAG00000000294	cpeb4a	none	none	none	sub	sub	none	none	none	none	sub
ENSXMAG00000007277	no	no	no	no	18	12	no	no	no	ENSLOCG00000009135.ENSXMAG00000007277.ENSXMAG00000017609	tiam1b	none	none	none	none	sub	sub	none	none	none	sub
ENSXMAG00000017609	no	no	no	no	no	no	no	no	no	ENSLOCG00000009135.ENSXMAG00000017609.ENSXMAG00000007277	tiam1a	none	none	none	none	sub	sub	none	none	none	sub
ENSXMAG00000009398	no	no	no	no	21	no	no	21	no	ENSLOCG00000009153.ENSXMAG00000009398.ENSXMAG00000011763	nfkbiab	none	sub	none	none	sub	none	none	sub	none	sub
ENSXMAG00000011763	no	3	no	no	no	no	no	no	no	ENSLOCG00000009153.ENSXMAG00000011763.ENSXMAG00000009398	nfkbiaa	none	sub	none	none	sub	none	none	sub	none	sub
ENSXMAG00000000497	no	no	no	no	no	no	no	no	no	ENSLOCG00000009167.ENSXMAG00000000497.ENSXMAG00000023412	bnip1b	none	none	none	none	sub	none	none	none	none	sub
ENSXMAG00000023412	no	no	no	no	15	no	no	no	no	ENSLOCG00000009167.ENSXMAG00000023412.ENSXMAG00000000497	bnip1a	none	none	none	none	sub	none	none	none	none	sub
ENSXMAG00000018473	no	no	no	no	no	18	no	no	no	ENSLOCG00000009170.ENSXMAG00000018473.ENSXMAG00000027618	guk1b	none	none	none	none	sub	sub	none	none	none	sub
ENSXMAG00000027618	no	no	no	no	15	no	no	no	no	ENSLOCG00000009170.ENSXMAG00000027618.ENSXMAG00000018473	guk1a	none	none	none	none	sub	sub	none	none	none	sub
ENSXMAG00000024734	no	no	no	no	no	15	no	no	no	ENSLOCG00000009232.ENSXMAG00000024734.ENSXMAG00000026555	igfbp2a	none	none	none	none	none	sub	none	none	none	sub
ENSXMAG00000026555	no	no	no	no	no	no	no	no	no	ENSLOCG00000009232.ENSXMAG00000026555.ENSXMAG00000024734	igfbp2b	none	none	none	none	none	sub	none	none	none	sub
ENSXMAG00000006943	no	no	no	no	6	3	no	no	no	ENSLOCG00000009269.ENSXMAG00000006943.ENSXMAG00000007435	guca1d	none	none	none	none	sub	no diff	none	none	none	sub
ENSXMAG00000007435	no	no	no	no	no	3	no	no	no	ENSLOCG00000009269.ENSXMAG00000007435.ENSXMAG00000006943	guca1c	none	none	none	none	sub	no diff	none	none	none	sub
ENSXMAG00000000532	no	no	no	no	15	no	no	no	no	ENSLOCG00000009271.ENSXMAG00000000532.ENSXMAG00000008223	CLINT1 (1 of many)	none	none	none	none	no diff	none	none	none	none	sub
ENSXMAG00000008223	no	no	no	no	12	no	no	no	no	ENSLOCG00000009271.ENSXMAG00000008223.ENSXMAG00000000532	clint1a	none	none	none	none	no diff	none	none	none	none	sub
ENSXMAG00000021113	no	no	no	no	12	no	no	no	no	ENSLOCG00000009311.ENSXMAG00000021113.ENSXMAG00000024920		none	none	none	none	neo	sub	none	none	none	sub
ENSXMAG00000024920	no	no	no	no	3	0	no	no	no	ENSLOCG00000009311.ENSXMAG00000024920.ENSXMAG00000021113	rab11fip5a	none	none	none	none	neo	sub	none	none	none	sub
ENSXMAG00000005008	0	no	no	no	no	no	no	no	no	ENSLOCG00000009367.ENSXMAG00000005008.ENSXMAG00000022318		sub	none	none	none	none	none	none	none	none	sub
ENSXMAG00000022318	no	no	no	no	no	no	no	no	no	ENSLOCG00000009367.ENSXMAG00000022318.ENSXMAG00000005008	nectin3b	sub	none	none	none	none	none	none	none	none	sub
ENSXMAG00000005018	no	no	no	no	no	0	no	no	no	ENSLOCG00000009412.ENSXMAG00000005018.ENSXMAG00000007055	pip5k1ba	none	none	none	none	none	no diff	none	none	none	sub
ENSXMAG00000007055	no	no	no	no	no	3	no	no	no	ENSLOCG00000009412.ENSXMAG00000007055.ENSXMAG00000005018		none	none	none	none	none	no diff	none	none	none	sub
ENSXMAG00000009757	no	no	no	no	no	15	no	no	no	ENSLOCG00000009424.ENSXMAG00000009757.ENSXMAG00000015372	myo10l1	none	none	none	none	none	sub	none	sub	sub	sub
ENSXMAG00000015372	no	no	no	no	no	no	no	18	21	ENSLOCG00000009424.ENSXMAG00000015372.ENSXMAG00000009757		none	none	none	none	none	sub	none	sub	sub	sub
ENSXMAG00000013714	no	no	no	no	15	no	15	no	no	ENSLOCG00000009499.ENSXMAG00000013714.ENSXMAG00000024096		none	none	none	none	sub	none	sub	none	none	sub
ENSXMAG00000024096	no	no	no	no	no	no	no	no	no	ENSLOCG00000009499.ENSXMAG00000024096.ENSXMAG00000013714	si:dkeyp-75h12.2	none	none	none	none	sub	none	sub	none	none	sub
ENSXMAG00000012915	no	no	no	no	no	0	no	no	no	ENSLOCG00000009594.ENSXMAG00000012915.ENSXMAG00000026622		none	none	none	none	none	sub	none	none	none	sub
ENSXMAG00000026622	no	no	no	no	no	no	no	no	no	ENSLOCG00000009594.ENSXMAG00000026622.ENSXMAG00000012915	gfra3	none	none	none	none	none	sub	none	none	none	sub
ENSXMAG00000002596	no	no	no	no	no	3	no	no	no	ENSLOCG00000009609.ENSXMAG00000002596.ENSXMAG00000021556	ptpn2a	none	none	none	none	none	sub	none	none	none	sub
ENSXMAG00000021556	no	no	no	no	no	no	no	no	no	ENSLOCG00000009609.ENSXMAG00000021556.ENSXMAG00000002596	ptpn2b	none	none	none	none	none	sub	none	none	none	sub
ENSXMAG00000002574	no	no	no	no	no	no	no	no	no	ENSLOCG00000009655.ENSXMAG00000002574.ENSXMAG00000003725	grhl2a	none	sub	none	none	sub	sub	none	sub	none	sub
ENSXMAG00000003725	no	3	no	no	9	0	no	15	no	ENSLOCG00000009655.ENSXMAG00000003725.ENSXMAG00000002574	grhl2b	none	sub	none	none	sub	sub	none	sub	none	sub
ENSXMAG00000000293	no	no	no	no	no	no	no	no	no	ENSLOCG00000009659.ENSXMAG00000000293.ENSXMAG00000009443	klhl40a	none	none	none	none	sub	none	none	none	none	sub
ENSXMAG00000009443	no	no	no	no	15	no	no	no	no	ENSLOCG00000009659.ENSXMAG00000009443.ENSXMAG00000000293	klhl40b	none	none	none	none	sub	none	none	none	none	sub
ENSXMAG00000000405	no	no	no	no	no	15	no	no	no	ENSLOCG00000009667.ENSXMAG00000000405.ENSXMAG00000021925	kcnip1b	none	none	none	none	none	sub	none	none	none	sub
ENSXMAG00000021925	no	no	no	no	no	no	no	no	no	ENSLOCG00000009667.ENSXMAG00000021925.ENSXMAG00000000405	KCNIP1	none	none	none	none	none	sub	none	none	none	sub
ENSXMAG00000000230	no	18	no	no	no	no	no	no	no	ENSLOCG00000009737.ENSXMAG00000000230.ENSXMAG00000009326	plcd1b	none	sub	none	none	none	none	none	none	none	sub
ENSXMAG00000009326	no	no	no	no	no	no	no	no	no	ENSLOCG00000009737.ENSXMAG00000009326.ENSXMAG00000000230	plcd1a	none	sub	none	none	none	none	none	none	none	sub
ENSXMAG00000008152	no	15	no	no	no	21	no	no	no	ENSLOCG00000009769.ENSXMAG00000008152.ENSXMAG00000018024	NAV1 (1 of many)	none	sub	none	none	none	sub	none	none	none	sub
ENSXMAG00000018024	no	no	no	no	no	no	no	no	no	ENSLOCG00000009769.ENSXMAG00000018024.ENSXMAG00000008152	nav1b	none	sub	none	none	none	sub	none	none	none	sub
ENSXMAG00000008184	no	no	no	no	no	no	0	no	no	ENSLOCG00000009825.ENSXMAG00000008184.ENSXMAG00000018083	PKP1 (1 of many)	none	none	none	none	none	none	sub	none	none	sub
ENSXMAG00000018083	no	no	no	no	no	no	no	no	no	ENSLOCG00000009825.ENSXMAG00000018083.ENSXMAG00000008184	pkp1b	none	none	none	none	none	none	sub	none	none	sub
ENSXMAG00000003693	15	no	no	no	no	no	15	no	no	ENSLOCG00000009827.ENSXMAG00000003693.ENSXMAG00000028764	rida	sub	none	none	none	none	sub	sub	none	none	sub
ENSXMAG00000028764	no	no	no	no	no	18	no	no	no	ENSLOCG00000009827.ENSXMAG00000028764.ENSXMAG00000003693	RIDA (1 of many)	sub	none	none	none	none	sub	sub	none	none	sub
ENSXMAG00000011144	no	no	no	no	no	no	no	no	no	ENSLOCG00000009860.ENSXMAG00000011144.ENSXMAG00000017082		none	sub	none	none	none	sub	none	none	none	sub
ENSXMAG00000017082	no	21	no	no	no	15	no	no	no	ENSLOCG00000009860.ENSXMAG00000017082.ENSXMAG00000011144	chl1b	none	sub	none	none	none	sub	none	none	none	sub
ENSXMAG00000011659	no	no	no	no	no	15	no	no	no	ENSLOCG00000009923.ENSXMAG00000011659.ENSXMAG00000019265		none	none	none	none	none	neo	none	none	none	sub
ENSXMAG00000019265	no	no	no	no	no	21	no	no	no	ENSLOCG00000009923.ENSXMAG00000019265.ENSXMAG00000011659	grk1a	none	none	none	none	none	neo	none	none	none	sub
ENSXMAG00000014092	no	no	no	no	no	no	no	15	no	ENSLOCG00000009939.ENSXMAG00000014092.ENSXMAG00000026459	acvr2ba	none	none	none	none	none	sub	none	sub	none	sub
ENSXMAG00000026459	no	no	no	no	no	21	no	no	no	ENSLOCG00000009939.ENSXMAG00000026459.ENSXMAG00000014092		none	none	none	none	none	sub	none	sub	none	sub
ENSXMAG00000004727	no	no	no	no	no	15	no	no	no	ENSLOCG00000009942.ENSXMAG00000004727.ENSXMAG00000009582	ppp4r2b	none	none	none	none	none	sub	none	none	none	sub
ENSXMAG00000009582	no	no	no	no	no	no	no	no	no	ENSLOCG00000009942.ENSXMAG00000009582.ENSXMAG00000004727		none	none	none	none	none	sub	none	none	none	sub
ENSXMAG00000017940	no	no	no	no	no	no	no	no	no	ENSLOCG00000009988.ENSXMAG00000017940.ENSXMAG00000022264	zgc:101577	sub	none	none	sub	none	sub	none	none	none	sub
ENSXMAG00000022264	0	no	no	3	no	0	no	no	no	ENSLOCG00000009988.ENSXMAG00000022264.ENSXMAG00000017940	C6orf106	sub	none	none	sub	none	sub	none	none	none	sub
ENSXMAG00000021074	no	no	no	no	15	no	no	no	no	ENSLOCG00000009997.ENSXMAG00000021074.ENSXMAG00000028295	asf1bb	none	none	none	none	no diff	sub	none	none	none	sub
ENSXMAG00000028295	no	no	no	no	15	15	no	no	no	ENSLOCG00000009997.ENSXMAG00000028295.ENSXMAG00000021074		none	none	none	none	no diff	sub	none	none	none	sub
ENSXMAG00000010468	no	no	no	no	15	no	no	no	no	ENSLOCG00000010113.ENSXMAG00000010468.ENSXMAG00000016399	uba3	none	none	none	none	sub	none	none	sub	none	sub
ENSXMAG00000016399	no	no	no	no	no	no	no	15	no	ENSLOCG00000010113.ENSXMAG00000016399.ENSXMAG00000010468	UBA3 (1 of many)	none	none	none	none	sub	none	none	sub	none	sub
ENSXMAG00000004439	no	no	no	3	6	3	3	6	no	ENSLOCG00000010122.ENSXMAG00000004439.ENSXMAG00000012351	WFS1 (1 of many)	none	none	none	sub	sub	no diff	sub	sub	none	sub
ENSXMAG00000012351	no	no	no	no	no	3	no	no	no	ENSLOCG00000010122.ENSXMAG00000012351.ENSXMAG00000004439	wfs1a	none	none	none	sub	sub	no diff	sub	sub	none	sub
ENSXMAG00000006854	no	18	no	no	no	no	no	no	no	ENSLOCG00000010177.ENSXMAG00000006854.ENSXMAG00000016385		none	sub	none	none	none	none	none	none	none	sub
ENSXMAG00000016385	no	no	no	no	no	no	no	no	no	ENSLOCG00000010177.ENSXMAG00000016385.ENSXMAG00000006854	slc16a7	none	sub	none	none	none	none	none	none	none	sub
ENSXMAG00000003470	no	no	no	no	18	no	no	no	no	ENSLOCG00000010196.ENSXMAG00000003470.ENSXMAG00000004780		none	none	none	none	sub	none	none	sub	none	sub
ENSXMAG00000004780	no	no	no	no	no	no	no	15	no	ENSLOCG00000010196.ENSXMAG00000004780.ENSXMAG00000003470	plpp1a	none	none	none	none	sub	none	none	sub	none	sub
ENSXMAG00000004941	no	no	no	no	0	no	no	no	no	ENSLOCG00000010232.ENSXMAG00000004941.ENSXMAG00000022329	sgpp1	none	none	none	none	neo	none	none	none	none	sub
ENSXMAG00000022329	no	no	no	no	15	no	no	no	no	ENSLOCG00000010232.ENSXMAG00000022329.ENSXMAG00000004941	si:ch211-212g7.6	none	none	none	none	neo	none	none	none	none	sub
ENSXMAG00000004514	no	no	no	no	no	no	no	no	no	ENSLOCG00000010249.ENSXMAG00000004514.ENSXMAG00000025257	sec22ba	none	none	none	sub	none	sub	none	none	none	sub
ENSXMAG00000025257	no	no	no	15	no	18	no	no	no	ENSLOCG00000010249.ENSXMAG00000025257.ENSXMAG00000004514	sec22bb	none	none	none	sub	none	sub	none	none	none	sub
ENSXMAG00000006136	no	no	no	no	0	21	no	no	no	ENSLOCG00000010280.ENSXMAG00000006136.ENSXMAG00000023811	esr2a	none	none	none	none	sub	sub	none	none	none	sub
ENSXMAG00000023811	no	no	no	no	no	no	no	no	no	ENSLOCG00000010280.ENSXMAG00000023811.ENSXMAG00000006136	esr2b	none	none	none	none	sub	sub	none	none	none	sub
ENSXMAG00000000875	no	no	no	no	18	no	no	no	no	ENSLOCG00000010292.ENSXMAG00000000875.ENSXMAG00000024782	mov10a	none	none	none	none	sub	none	none	none	none	sub
ENSXMAG00000024782	no	no	no	no	no	no	no	no	no	ENSLOCG00000010292.ENSXMAG00000024782.ENSXMAG00000000875	mov10b.1	none	none	none	none	sub	none	none	none	none	sub
ENSXMAG00000008285	no	no	no	no	no	18	no	no	no	ENSLOCG00000010293.ENSXMAG00000008285.ENSXMAG00000009800	si:ch211-222n4.6	none	none	none	none	none	sub	none	none	none	sub
ENSXMAG00000009800	no	no	no	no	no	no	no	no	no	ENSLOCG00000010293.ENSXMAG00000009800.ENSXMAG00000008285	mpp4a	none	none	none	none	none	sub	none	none	none	sub
ENSXMAG00000000634	no	no	no	no	0	no	no	no	no	ENSLOCG00000010321.ENSXMAG00000000634.ENSXMAG00000025537		none	none	none	none	sub	none	none	none	none	sub
ENSXMAG00000025537	no	no	no	no	no	no	no	no	no	ENSLOCG00000010321.ENSXMAG00000025537.ENSXMAG00000000634	jag2b	none	none	none	none	sub	none	none	none	none	sub
ENSXMAG00000012467	no	no	no	no	no	no	no	no	no	ENSLOCG00000010348.ENSXMAG00000012467.ENSXMAG00000030101	raph1b	none	sub	none	none	none	sub	none	none	none	sub
ENSXMAG00000030101	no	12	no	no	no	15	no	no	no	ENSLOCG00000010348.ENSXMAG00000030101.ENSXMAG00000012467	raph1a	none	sub	none	none	none	sub	none	none	none	sub
ENSXMAG00000007579	no	no	no	no	no	21	no	no	no	ENSLOCG00000010353.ENSXMAG00000007579.ENSXMAG00000019946	kcnd3	none	none	none	none	none	sub	none	none	none	sub
ENSXMAG00000019946	no	no	no	no	no	no	no	no	no	ENSLOCG00000010353.ENSXMAG00000019946.ENSXMAG00000007579	KCND3 (1 of many)	none	none	none	none	none	sub	none	none	none	sub
ENSXMAG00000002387	no	no	no	no	no	no	15	15	no	ENSLOCG00000010360.ENSXMAG00000002387.ENSXMAG00000018476	brf1b	none	sub	none	sub	none	sub	sub	sub	none	sub
ENSXMAG00000018476	no	3	no	3	no	0	no	no	no	ENSLOCG00000010360.ENSXMAG00000018476.ENSXMAG00000002387	BRF1 (1 of many)	none	sub	none	sub	none	sub	sub	sub	none	sub
ENSXMAG00000005986	no	no	no	no	no	15	no	no	no	ENSLOCG00000010384.ENSXMAG00000005986.ENSXMAG00000025746	rap1ab	none	none	none	none	none	sub	none	none	none	sub
ENSXMAG00000025746	no	no	no	no	no	no	no	no	no	ENSLOCG00000010384.ENSXMAG00000025746.ENSXMAG00000005986		none	none	none	none	none	sub	none	none	none	sub
ENSXMAG00000016541	no	no	no	no	no	no	no	no	no	ENSLOCG00000010400.ENSXMAG00000016541.ENSXMAG00000027646	mmp16b	none	none	none	none	none	sub	none	sub	none	sub
ENSXMAG00000027646	no	no	no	no	no	21	no	15	no	ENSLOCG00000010400.ENSXMAG00000027646.ENSXMAG00000016541	MMP16	none	none	none	none	none	sub	none	sub	none	sub
ENSXMAG00000011330	3	no	no	no	0	no	no	no	no	ENSLOCG00000010402.ENSXMAG00000011330.ENSXMAG00000028903	myoc	sub	none	none	none	sub	none	none	none	none	sub
ENSXMAG00000028903	no	no	no	no	no	no	no	no	no	ENSLOCG00000010402.ENSXMAG00000028903.ENSXMAG00000011330		sub	none	none	none	sub	none	none	none	none	sub
ENSXMAG00000015437	no	no	no	no	no	no	no	no	no	ENSLOCG00000010495.ENSXMAG00000015437.ENSXMAG00000023569		sub	none	none	none	none	none	none	none	none	sub
ENSXMAG00000023569	0	no	no	no	no	no	no	no	no	ENSLOCG00000010495.ENSXMAG00000023569.ENSXMAG00000015437	scoca	sub	none	none	none	none	none	none	none	none	sub
ENSXMAG00000003333	no	no	no	no	no	no	no	no	no	ENSLOCG00000010503.ENSXMAG00000003333.ENSXMAG00000012596	olfml3b	none	none	none	none	none	sub	none	none	none	sub
ENSXMAG00000012596	no	no	no	no	no	15	no	no	no	ENSLOCG00000010503.ENSXMAG00000012596.ENSXMAG00000003333	olfml3a	none	none	none	none	none	sub	none	none	none	sub
ENSXMAG00000001522	no	no	no	3	no	3	no	no	9	ENSLOCG00000010508.ENSXMAG00000001522.ENSXMAG00000009167	NR3C1	none	none	none	sub	none	sub	none	none	sub	sub
ENSXMAG00000009167	no	no	no	no	no	no	no	no	no	ENSLOCG00000010508.ENSXMAG00000009167.ENSXMAG00000001522	nr3c1	none	none	none	sub	none	sub	none	none	sub	sub
ENSXMAG00000015104	no	12	no	no	no	15	no	no	no	ENSLOCG00000010510.ENSXMAG00000015104.ENSXMAG00000018595		none	sub	none	none	none	neo	none	none	none	sub
ENSXMAG00000018595	no	no	no	no	no	3	no	no	no	ENSLOCG00000010510.ENSXMAG00000018595.ENSXMAG00000015104	pik3r1	none	sub	none	none	none	neo	none	none	none	sub
ENSXMAG00000014805	no	no	no	no	no	no	no	no	no	ENSLOCG00000010526.ENSXMAG00000014805.ENSXMAG00000025219	jdp2a	none	sub	none	none	none	sub	sub	none	none	sub
ENSXMAG00000025219	no	3	no	no	no	0	6	no	no	ENSLOCG00000010526.ENSXMAG00000025219.ENSXMAG00000014805	JDP2 (1 of many)	none	sub	none	none	none	sub	sub	none	none	sub
ENSXMAG00000026160	no	3	no	no	no	no	no	no	no	ENSLOCG00000010567.ENSXMAG00000026160.ENSXMAG00000028476	sgtb	none	sub	none	none	none	sub	sub	none	none	sub
ENSXMAG00000028476	no	no	no	no	no	3	3	no	no	ENSLOCG00000010567.ENSXMAG00000028476.ENSXMAG00000026160		none	sub	none	none	none	sub	sub	none	none	sub

ENSXMAG00000005338	no	no	no	no	12	3	no	no	no	ENSLOCG00000010585.ENSXMAG00000005338.ENSXMAG00000010691	slc12a7b	none	none	none	none	sub	sub	sub	none	none	sub
ENSXMAG00000010691	no	no	no	no	no	no	6	no	no	ENSLOCG00000010585.ENSXMAG00000010691.ENSXMAG00000005338		none	none	none	none	sub	sub	sub	none	none	sub
ENSXMAG00000003970	no	3	no	no	no	0	no	no	no	ENSLOCG00000010588.ENSXMAG00000003970.ENSXMAG00000014713	TGFB3 (1 of many)	none	sub	none	none	none	sub	none	none	none	sub
ENSXMAG00000014713	no	no	no	no	no	no	no	no	no	ENSLOCG00000010588.ENSXMAG00000014713.ENSXMAG00000003970	tgfb3	none	sub	none	none	none	sub	none	none	none	sub
ENSXMAG00000001608	no	no	no	3	no	no	no	no	no	ENSLOCG00000010589.ENSXMAG00000001608.ENSXMAG00000009200	hspa4a	none	none	none	sub	none	sub	none	sub	none	sub
ENSXMAG00000009200	no	no	no	no	no	15	no	15	no	ENSLOCG00000010589.ENSXMAG00000009200.ENSXMAG00000001608	hspa4b	none	none	none	sub	none	sub	none	sub	none	sub
ENSXMAG00000002816	no	no	no	no	no	6	no	no	no	ENSLOCG00000010592.ENSXMAG00000002816.ENSXMAG00000026349	tspan2a	none	none	none	none	none	neo	none	none	none	sub
ENSXMAG00000026349	no	no	no	no	no	15	no	no	no	ENSLOCG00000010592.ENSXMAG00000026349.ENSXMAG00000002816		none	none	none	none	none	neo	none	none	none	sub
ENSXMAG00000002805	no	no	no	no	12	no	15	no	no	ENSLOCG00000010612.ENSXMAG00000002805.ENSXMAG00000008253	slc25a55a	none	none	none	none	sub	none	sub	none	none	sub
ENSXMAG00000008253	no	no	no	no	no	no	no	no	no	ENSLOCG00000010612.ENSXMAG00000008253.ENSXMAG00000002805		none	none	none	none	sub	none	sub	none	none	sub
ENSXMAG00000009263	no	no	no	no	no	6	no	no	no	ENSLOCG00000010706.ENSXMAG00000009263.ENSXMAG00000012573	gabra6a	none	none	none	none	none	sub	none	none	none	sub
ENSXMAG00000012573	no	no	no	no	no	no	no	no	no	ENSLOCG00000010706.ENSXMAG00000012573.ENSXMAG00000009263	gabra6b	none	none	none	none	none	sub	none	none	none	sub
ENSXMAG00000012670	no	no	no	no	no	no	no	21	no	ENSLOCG00000010745.ENSXMAG00000012670.ENSXMAG00000021579	atp5pf	sub	sub	none	none	sub	sub	sub	sub	none	sub
ENSXMAG00000021579	9	6	no	no	12	3	6	no	no	ENSLOCG00000010745.ENSXMAG00000021579.ENSXMAG00000012670		sub	sub	none	none	sub	sub	sub	sub	none	sub
ENSXMAG00000003039	no	no	no	no	no	no	no	no	no	ENSLOCG00000010783.ENSXMAG00000003039.ENSXMAG00000014761		none	sub	sub	none	none	sub	none	none	none	sub
ENSXMAG00000014761	no	6	21	no	no	3	no	no	no	ENSLOCG00000010783.ENSXMAG00000014761.ENSXMAG00000003039	acsf2	none	sub	sub	none	none	sub	none	none	none	sub
ENSXMAG00000000011	no	no	no	3	no	0	no	no	no	ENSLOCG00000010906.ENSXMAG00000000011.ENSXMAG00000003234	si:ch73-281f12.4	none	none	none	sub	none	no diff	none	none	none	sub
ENSXMAG00000003234	no	no	no	no	no	0	no	no	no	ENSLOCG00000010906.ENSXMAG00000003234.ENSXMAG00000000011	si:ch211-176g6.2	none	none	none	sub	none	no diff	none	none	none	sub
ENSXMAG00000008426	no	no	no	no	no	21	no	no	no	ENSLOCG00000010934.ENSXMAG00000008426.ENSXMAG00000015095		none	none	none	none	none	neo	none	none	none	sub
ENSXMAG00000015095	no	no	no	no	no	3	no	no	no	ENSLOCG00000010934.ENSXMAG00000015095.ENSXMAG00000008426	ALDH6A1	none	none	none	none	none	neo	none	none	none	sub
ENSXMAG00000024169	no	no	no	no	no	6	no	no	no	ENSLOCG00000010954.ENSXMAG00000024169.ENSXMAG00000028632		none	none	none	none	none	sub	none	none	none	sub
ENSXMAG00000028632	no	no	no	no	no	no	no	no	no	ENSLOCG00000010954.ENSXMAG00000028632.ENSXMAG00000024169	si:ch211-286o17.1	none	none	none	none	none	sub	none	none	none	sub
ENSXMAG00000015236	no	no	no	no	no	no	no	no	no	ENSLOCG00000011077.ENSXMAG00000015236.ENSXMAG00000025371	cipca	sub	sub	none	sub	sub	none	none	none	none	sub
ENSXMAG00000025371	0	0	no	0	21	no	no	no	no	ENSLOCG00000011077.ENSXMAG00000025371.ENSXMAG00000015236	cipcb	sub	sub	none	sub	sub	none	none	none	none	sub
ENSXMAG00000002157	no	no	no	no	no	no	no	no	no	ENSLOCG00000011093.ENSXMAG00000002157.ENSXMAG00000016718	LPIN2 (1 of many)	none	none	none	none	sub	none	none	none	none	sub
ENSXMAG00000016718	no	no	no	no	15	no	no	no	no	ENSLOCG00000011093.ENSXMAG00000016718.ENSXMAG00000002157	lpin2	none	none	none	none	sub	none	none	none	none	sub
ENSXMAG00000006394	no	no	no	no	no	21	no	no	no	ENSLOCG00000011097.ENSXMAG00000006394.ENSXMAG00000008554		none	none	none	none	none	no diff	sub	none	none	sub
ENSXMAG00000008554	no	no	no	no	no	18	21	no	no	ENSLOCG00000011097.ENSXMAG00000008554.ENSXMAG00000006394	pdgfrb	none	none	none	none	none	no diff	sub	none	none	sub
ENSXMAG00000012966	no	no	no	no	no	no	no	no	no	ENSLOCG00000011140.ENSXMAG00000012966.ENSXMAG00000017142	grin2ca	none	none	sub	none	none	sub	none	none	none	sub
ENSXMAG00000017142	no	no	12	no	no	12	no	no	no	ENSLOCG00000011140.ENSXMAG00000017142.ENSXMAG00000012966		none	none	sub	none	none	sub	none	none	none	sub
ENSXMAG00000001881	no	no	no	no	18	no	no	no	no	ENSLOCG00000011142.ENSXMAG00000001881.ENSXMAG00000022847		none	none	none	none	sub	none	none	none	none	sub
ENSXMAG00000022847	no	no	no	no	no	no	no	no	no	ENSLOCG00000011142.ENSXMAG00000022847.ENSXMAG00000001881	fgd5a	none	none	none	none	sub	sub	none	none	none	sub
ENSXMAG00000001160	no	no	no	no	18	no	no	no	no	ENSLOCG00000011157.ENSXMAG00000001160.ENSXMAG00000003443	atp5fa1 (1 of many)	none	none	none	none	sub	none	sub	none	none	sub
ENSXMAG00000003443	no	no	no	no	no	no	15	no	no	ENSLOCG00000011157.ENSXMAG00000003443.ENSXMAG00000001160	atp5fa1 (1 of many)	none	none	none	none	sub	none	sub	none	none	sub
ENSXMAG00000016769	no	no	no	no	no	15	no	no	no	ENSLOCG00000011174.ENSXMAG00000016769.ENSXMAG00000018876	thsd7aa	none	none	none	none	none	neo	none	none	none	sub
ENSXMAG00000018876	no	no	no	no	no	21	no	no	no	ENSLOCG00000011174.ENSXMAG00000018876.ENSXMAG00000016769	thsd7ab	none	none	none	none	none	neo	none	none	none	sub
ENSXMAG00000016760	no	no	no	no	no	no	no	no	no	ENSLOCG00000011187.ENSXMAG00000016760.ENSXMAG00000018910	tmem106bb	none	none	none	none	sub	sub	none	none	none	sub
ENSXMAG00000018910	no	no	no	no	21	15	no	no	no	ENSLOCG00000011187.ENSXMAG00000018910.ENSXMAG00000016760	tmem106ba	none	none	none	none	sub	sub	none	none	none	sub
ENSXMAG00000001406	no	no	no	no	18	no	no	no	no	ENSLOCG00000011356.ENSXMAG00000001406.ENSXMAG00000018817	camk1gb	none	none	none	none	sub	none	none	none	none	sub
ENSXMAG00000018817	no	no	no	no	no	no	no	no	no	ENSLOCG00000011356.ENSXMAG00000018817.ENSXMAG00000001406	camk1ga	none	none	none	none	sub	none	none	none	none	sub
ENSXMAG00000008551	no	no	no	no	no	no	no	no	no	ENSLOCG00000011410.ENSXMAG00000008551.ENSXMAG00000010149	vat1	none	none	none	none	none	sub	none	none	none	sub
ENSXMAG00000010149	no	no	no	no	no	15	no	no	no	ENSLOCG00000011410.ENSXMAG00000010149.ENSXMAG00000008551	VAT1 (1 of many)	none	none	none	none	none	sub	none	none	none	sub
ENSXMAG00000008197	no	no	no	no	no	21	no	no	no	ENSLOCG00000011420.ENSXMAG00000008197.ENSXMAG00000023930		none	none	none	none	none	sub	sub	none	none	sub
ENSXMAG00000023930	no	no	no	no	no	no	6	no	no	ENSLOCG00000011420.ENSXMAG00000023930.ENSXMAG00000008197		none	none	none	none	none	sub	sub	none	none	sub
ENSXMAG00000001200	no	no	no	no	15	no	no	no	no	ENSLOCG00000011442.ENSXMAG00000001200.ENSXMAG00000008034	pdgfbb	none	none	none	none	sub	none	none	none	none	sub
ENSXMAG00000008034	no	no	no	no	no	no	no	no	no	ENSLOCG00000011442.ENSXMAG00000008034.ENSXMAG00000001200		none	none	none	none	sub	none	none	none	none	sub
ENSXMAG00000002178	no	no	no	no	no	no	no	no	no	ENSLOCG00000011485.ENSXMAG00000002178.ENSXMAG00000025866		none	sub	none	none	none	none	none	none	none	sub
ENSXMAG00000025866	no	0	no	no	no	no	no	no	no	ENSLOCG00000011485.ENSXMAG00000025866.ENSXMAG00000002178	ADAM11	none	sub	none	none	none	none	none	none	none	sub
ENSXMAG00000008387	no	no	no	no	21	no	no	no	no	ENSLOCG00000011522.ENSXMAG00000008387.ENSXMAG00000014404	PLCD3	none	none	none	none	sub	none	none	none	none	sub
ENSXMAG00000014404	no	no	no	no	no	no	no	no	no	ENSLOCG00000011522.ENSXMAG00000014404.ENSXMAG00000008387	plcd3b	none	none	none	none	sub	none	none	none	none	sub
ENSXMAG00000001279	no	no	no	no	no	18	no	15	no	ENSLOCG00000011526.ENSXMAG00000001279.ENSXMAG00000009685	desi1a	sub	none	none	none	none	sub	none	sub	none	sub
ENSXMAG00000009685	21	no	no	no	no	no	no	no	no	ENSLOCG00000011526.ENSXMAG00000009685.ENSXMAG00000001279	desi1b	sub	none	none	none	none	sub	none	sub	none	sub
ENSXMAG00000014677	21	no	no	no	no	no	no	no	no	ENSLOCG00000011528.ENSXMAG00000014677.ENSXMAG00000016107	slc44a1b	sub	none	none	none	none	none	none	none	none	sub
ENSXMAG00000016107	no	no	no	no	no	no	no	no	no	ENSLOCG00000011528.ENSXMAG00000016107.ENSXMAG00000014677		sub	none	none	none	none	none	none	none	none	sub
ENSXMAG00000005345	21	no	no	no	no	no	no	no	no	ENSLOCG00000011541.ENSXMAG00000005345.ENSXMAG00000006322	slc4a4b	sub	none	none	none	none	none	none	none	none	sub
ENSXMAG00000006322	no	no	no	no	no	no	no	no	no	ENSLOCG00000011541.ENSXMAG00000006322.ENSXMAG00000005345		sub	none	none	none	none	none	none	none	none	sub
ENSXMAG00000000577	no	no	no	no	no	3	no	no	no	ENSLOCG00000011542.ENSXMAG00000000577.ENSXMAG00000015176	rapgef5a	none	none	none	none	none	sub	none	none	none	sub
ENSXMAG00000015176	no	no	no	no	no	no	no	no	no	ENSLOCG00000011542.ENSXMAG00000015176.ENSXMAG00000000577	RAPGEF5	none	none	none	none	none	sub	none	none	none	sub
ENSXMAG00000005844	no	0	no	no	no	0	no	no	no	ENSLOCG00000011577.ENSXMAG00000005844.ENSXMAG00000019096	smu1b	none	sub	none	none	none	sub	none	none	none	sub
ENSXMAG00000019096	no	no	no	no	no	no	no	no	no	ENSLOCG00000011577.ENSXMAG00000019096.ENSXMAG00000005844	smu1a	none	sub	none	none	none	sub	none	none	none	sub
ENSXMAG00000000663	no	no	no	no	15	15	no	15	no	ENSLOCG00000011582.ENSXMAG00000000663.ENSXMAG00000003726	rangap1b	none	none	none	none	sub	neo	sub	sub	none	sub
ENSXMAG00000003726	no	no	no	no	no	9	15	no	no	ENSLOCG00000011582.ENSXMAG00000003726.ENSXMAG00000000663	rangap1a	none	none	none	none	sub	neo	sub	sub	none	sub
ENSXMAG00000008317	no	no	no	no	no	15	no	no	no	ENSLOCG00000011584.ENSXMAG00000008317.ENSXMAG00000026693	znf281a	none	none	none	none	none	no diff	none	none	none	sub
ENSXMAG00000026693	no	no	no	no	no	15	no	no	no	ENSLOCG00000011584.ENSXMAG00000026693.ENSXMAG00000008317	znf281b	none	none	none	none	none	no diff	none	none	none	sub
ENSXMAG00000000678	no	no	no	no	no	no	6	no	no	ENSLOCG00000011595.ENSXMAG00000000678.ENSXMAG00000026333	tefa	sub	sub	none	none	sub	sub	neo	sub	sub	sub
ENSXMAG00000026333	0	0	no	no	21	0	0	0	0	ENSLOCG00000011595.ENSXMAG00000026333.ENSXMAG00000000678	tefb	sub	sub	none	none	sub	sub	neo	sub	sub	sub
ENSXMAG00000000683	no	no	no	no	no	no	no	no	no	ENSLOCG00000011607.ENSXMAG00000000683.ENSXMAG00000006870	aco2	sub	sub	none	none	none	none	none	none	none	sub
ENSXMAG00000006870	0	0	no	no	no	no	no	no	no	ENSLOCG00000011607.ENSXMAG00000006870.ENSXMAG00000000683	ACO2 (1 of many)	sub	sub	none	none	none	none	none	none	none	sub
ENSXMAG00000008446	no	15	6	no	no	18	no	no	no	ENSLOCG00000011645.ENSXMAG00000008446.ENSXMAG00000024161	evlb	none	sub	sub	none	none	sub	none	none	none	sub
ENSXMAG00000024161	no	no	no	no	no	no	no	no	no	ENSLOCG00000011645.ENSXMAG00000024161.ENSXMAG00000008446	evla	none	sub	sub	none	none	sub	none	none	none	sub
ENSXMAG00000008287	no	no	no	no	no	no	no	no	no	ENSLOCG00000011654.ENSXMAG00000008287.ENSXMAG00000016920	mpp3a	none	none	sub	none	none	sub	none	none	none	sub
ENSXMAG00000016920	no	no	15	no	no	21	no	no	no	ENSLOCG00000011654.ENSXMAG00000016920.ENSXMAG00000008287	mpp3b	none	none	sub	none	none	sub	none	none	none	sub
ENSXMAG00000008204	no	no	no	no	no	no	no	no	no	ENSLOCG00000011662.ENSXMAG00000008204.ENSXMAG00000017049	ngfra	none	none	none	none	none	sub	none	none	sub	sub
ENSXMAG00000017049	no	no	no	no	no	0	no	no	21	ENSLOCG00000011662.ENSXMAG00000017049.ENSXMAG00000008204	ngfrb	none	none	none	none	none	sub	none	none	sub	sub
ENSXMAG00000000115	no	no	no	no	no	no	no	no	no	ENSLOCG00000011671.ENSXMAG00000000115.ENSXMAG00000003577	slc2a15b	sub	sub	sub	none	none	none	none	none	none	sub
ENSXMAG00000003577	15	15	0	no	no	no	no	no	no	ENSLOCG00000011671.ENSXMAG00000003577.ENSXMAG00000000115	slc2a15a	sub	sub	sub	none	none	none	none	none	none	sub
ENSXMAG00000001083	no	no	no	no	15	no	no	no	no	ENSLOCG00000011683.ENSXMAG00000001083.ENSXMAG00000013928	osbpl3a	none	sub	none	none	sub	sub	none	none	none	sub
ENSXMAG00000013928	no	3	no	no	no	6	no	no	no	ENSLOCG00000011683.ENSXMAG00000013928.ENSXMAG00000001083	osbpl3b	none	sub	none	none	sub	sub	none	none	none	sub
ENSXMAG00000001077	no	no	no	no	no	no	no	no	no	ENSLOCG00000011695.ENSXMAG00000001077.ENSXMAG00000012546	si:dkey-46g23.1	none	none	none	none	sub	none	none	none	none	sub
ENSXMAG00000012546	no	no	no	no	15	no	no	no	no	ENSLOCG00000011695.ENSXMAG00000012546.ENSXMAG00000001077	tnrc6b	none	none	none	none	sub	none	none	none	none	sub
ENSXMAG00000018329	no	no	no	no	no	18	no	no	no	ENSLOCG00000011696.ENSXMAG00000018329.ENSXMAG00000022120	ndufa4	none	none	none	none	none	sub	none	none	none	sub
ENSXMAG00000022120	no	no	no	no	no	no	no	no	no	ENSLOCG00000011696.ENSXMAG00000022120.ENSXMAG00000018329		none	none	none	none	none	sub	none	none	none	sub
ENSXMAG00000005784	no	no	no	no	no	no	no	no	no	ENSLOCG00000011770.ENSXMAG00000005784.ENSXMAG00000029312	nt5c2a	none	none	none	none	none	sub	none	sub	none	sub
ENSXMAG00000029312	no	no	no	no	no	0	no	15	no	ENSLOCG00000011770.ENSXMAG00000029312.ENSXMAG00000005784	nt5c2b	none	none	none	none	none	sub	none	sub	none	sub
ENSXMAG00000028422	no	no	no	no	no	no	no	no	no	ENSLOCG00000011830.ENSXMAG00000028422.ENSXMAG00000029054	hoxa10b	none	none	none	none	sub	none	none	none	none	sub
ENSXMAG00000029054	no	no	no	no	0	no	no	no	no	ENSLOCG00000011830.ENSXMAG00000029054.ENSXMAG00000028422		none	none	none	none	sub	none	none	none	none	sub
ENSXMAG00000007352	no	no	no	no	no	no	no	no	no	ENSLOCG00000011833.ENSXMAG00000007352.ENSXMAG00000026427	si:dkey-51a16.9	none	none	none	none	none	sub	none	none	none	sub
ENSXMAG00000026427	no	no	no	no	no	18	no	no	no	ENSLOCG00000011833.ENSXMAG00000026427.ENSXMAG00000007352	zgc:175214	none	none	none	none	none	sub	none	none	none	sub
ENSXMAG00000013824	no	no	no	no	no	no	no	no	no	ENSLOCG00000011862.ENSXMAG00000013824.ENSXMAG00000016886	fmnl1a	none	none	none	none	none	sub	none	none	none	sub
ENSXMAG00000016886	no	no	no	no	no	21	no	no	no	ENSLOCG00000011862.ENSXMAG00000016886.ENSXMAG00000013824		none	none	none	none	none	sub	none	none	none	sub
ENSXMAG00000005912	no	no	no	no	no	15	no	no	no	ENSLOCG00000011990.ENSXMAG00000005912.ENSXMAG00000010681	cnga1a	none	none	none	none	none	sub	none	none	none	sub
ENSXMAG00000010681	no	no	no	no	no	no	no	no	no	ENSLOCG00000011990.ENSXMAG00000010681.ENSXMAG00000005912	cnga1b	none	none	none	none	none	sub	none	none	none	sub
ENSXMAG00000006407	no	no	no	12	no	no	no	no	no	ENSLOCG00000012010.ENSXMAG00000006407.ENSXMAG00000008955		none	none	none	sub	none	none	none	none	none	sub
ENSXMAG00000008955	no	no	no	no	no	no	no	no	no	ENSLOCG00000012010.ENSXMAG00000008955.ENSXMAG00000006407	myh9b	none	none	none	sub	none	none	none	none	none	sub
ENSXMAG00000006405	no	no	no	no	no	15	no	no	no	ENSLOCG00000012029.ENSXMAG00000006405.ENSXMAG00000009093	slc25a38b	none	none	none	none	none	sub	none	sub	none	sub
ENSXMAG00000009093	no	no	no	no	no	no	no	3	no	ENSLOCG00000012029.ENSXMAG00000009093.ENSXMAG00000006405	SLC25A38	none	none	none	none	none	sub	none	sub	none	sub
ENSXMAG00000002099	no	no	no	no	no	no	no	no	no	ENSLOCG00000012034.ENSXMAG00000002099.ENSXMAG00000005936		none	none	none	sub	sub	none	none	none	none	sub
ENSXMAG00000005936	no	no	no	15	15	no	no	no	no	ENSLOCG00000012034.ENSXMAG00000005936.ENSXMAG00000002099		none	none	none	sub	sub	none	none	none	none	sub
ENSXMAG00000009106	no	no	no	no	no	no	no	no	no	ENSLOCG00000012045.ENSXMAG00000009106.ENSXMAG00000020944	ubn2b	sub	none	none	none	none	none	none	none	none	sub
ENSXMAG00000020944	0	no	no	no	no	no	no	no	no	ENSLOCG00000012045.ENSXMAG00000020944.ENSXMAG00000009106	ubn2a	sub	none	none	none	none	none	none	none	none	sub
ENSXMAG00000004607	no	no	no	no	no	no	no	no	no	ENSLOCG00000012051.ENSXMAG00000004607.ENSXMAG00000005288		none	sub	none	none	none	none	none	none	none	sub
ENSXMAG00000005288	no	18	no	no	no	no	no	no	no	ENSLOCG00000012051.ENSXMAG00000005288.ENSXMAG00000004607		none	sub	none	none	none	none	none	none	none	sub
ENSXMAG00000013166	no	no	no	no	no	no	no	no	no	ENSLOCG00000012185.ENSXMAG00000013166.ENSXMAG00000027975	SHISA6	none	none	none	none	none	sub	none	none	none	sub
ENSXMAG00000027975	no	no	no	no	no	3	no	no	no	ENSLOCG00000012185.ENSXMAG00000027975.ENSXMAG00000013166		none	none	none	none	none	sub	none	none	none	sub
ENSXMAG00000002337	no	no	no	no	no	no	no	no	no	ENSLOCG00000012198.ENSXMAG00000002337.ENSXMAG00000015293		none	none	none	none	sub	none	sub	none	none	sub
ENSXMAG00000015293	no	no	no	no	18	no	15	no	no	ENSLOCG00000012198.ENSXMAG00000015293.ENSXMAG00000002337	eif4eb	none	none	none	none	sub	none	sub	none	none	sub
ENSXMAG00000011644	no	no	no	no	21	no	no	no	no	ENSLOCG00000012201.ENSXMAG00000011644.ENSXMAG00000013158	foxk2	none	none	none	none	no diff	none	none	sub	none	sub
ENSXMAG00000013158	no	no	no	no	21	no	no	3	no	ENSLOCG00000012201.ENSXMAG00000013158.ENSXMAG00000011644	si:ch211-262e15.1	none	none	none	none	no diff	none	none	sub	none	sub
ENSXMAG00000012903	no	no	no	no	no	3	no	no	no	ENSLOCG00000012233.ENSXMAG00000012903.ENSXMAG00000025280	hlfa	none	none	none	none	none	no diff	none	none	none	sub
ENSXMAG00000025280	no	no	no	no	no	0	no	no	no	ENSLOCG00000012233.ENSXMAG00000025280.ENSXMAG00000012903	HLF (1 of many)	none	none	none	none	none	no diff	none	none	none	sub
ENSXMAG00000004645	no	no	no	no	no	no	no	no	no	ENSLOCG00000012249.ENSXMAG00000004645.ENSXMAG00000006770	adarb2	none	none	none	none	none	sub	none	none	none	sub
ENSXMAG00000006770	no	no	no	no	no	6	no	no	no	ENSLOCG00000012249.ENSXMAG00000006770.ENSXMAG00000004645		none	none	none	none	none	sub	none	none	none	sub
ENSXMAG00000006812	21	no	no	no	no	no	no	no	no	ENSLOCG00000012337.ENSXMAG00000006812.ENSXMAG00000014436	zfyve1	no diff	none	none	none	none	sub	none	none	none	sub
ENSXMAG00000014436	21	no	no	no	no	21	no	no	no	ENSLOCG00000012337.ENSXMAG00000014436.ENSXMAG00000006812	ZFYVE1 (1 of many)	no diff	none	none	none	none	sub	none	none	none	sub
ENSXMAG00000017382	no	no	no	no	0	0	no	no	no	ENSLOCG00000012344.ENSXMAG00000017382.ENSXMAG00000017606	tanc2a	none	none	none	none	sub	sub	none	none	none	sub
ENSXMAG00000017606	no	no	no	no	no	no	no	no	no	ENSLOCG00000012344.ENSXMAG00000017606.ENSXMAG00000017382	TANC2	none	none	none	none	sub	sub	none	none	none	sub
ENSXMAG00000007056	no	no	no	no	no	no	no	no	21	ENSLOCG00000012358.ENSXMAG00000007056.ENSXMAG00000012758	mxi1	none	none	none	none	none	sub	none	none	sub	sub
ENSXMAG00000012758	no	no	no	no	no	12	no	no	no	ENSLOCG00000012358.ENSXMAG00000012758.ENSXMAG00000007056	MXI1 (1 of many)	none	none	none	none	none	sub	none	none	sub	sub
ENSXMAG00000014401	no	no	no	no	no	no	no	no	no	ENSLOCG00000012364.ENSXMAG00000014401.ENSXMAG00000027736	rgs6	none	none	none	none	none	sub	none	none	none	sub
ENSXMAG00000027736	no	no	no	no	no	6	no	no	no	ENSLOCG00000012364.ENSXMAG00000027736.ENSXMAG00000014401	RGS6	none	none	none	none	none	sub	none	none	none	sub
ENSXMAG00000024355	no	no	no	no	12	no	no	no	no	ENSLOCG00000012370.ENSXMAG00000024355.ENSXMAG00000029848		none	none	none	none	sub	none	none	none	none	sub
ENSXMAG00000029848	no	no	no	no	no	no	no	no	no	ENSLOCG00000012370.ENSXMAG00000029848.ENSXMAG00000024355	WNK4	none	none	none	none	sub	none	none	none	none	sub
ENSXMAG00000002363	no	no	no	no	no	no	no	no	21	ENSLOCG00000012376.ENSXMAG00000002363.ENSXMAG00000003829	esyt2b	none	none	none	none	none	none	none	none	sub	sub
ENSXMAG00000003829	no	no	no	no	no	no	no	no	no	ENSLOCG00000012376.ENSXMAG00000003829.ENSXMAG00000002363	esyt2a	none	none	none	none	none	none	none	none	sub	sub
ENSXMAG00000007059	no	no	no	no	no	no	no	no	no	ENSLOCG00000012377.ENSXMAG00000007059.ENSXMAG00000012762	add3a	sub	none	none	none	none	none	none	none	none	sub
ENSXMAG00000012762	21	no	no	no	no	no	no	no	no	ENSLOCG00000012377.ENSXMAG00000012762.ENSXMAG00000007059	add3b	sub	none	none	none	none	none	none	none	none	sub
ENSXMAG00000015502	no	no	no	no	no	no	no	no	no	ENSLOCG00000012384.ENSXMAG00000015502.ENSXMAG00000025635	GABRA2	none	none	none	none	none	sub	none	none	none	sub
ENSXMAG00000025635	no	no	no	no	no	15	no	no	no	ENSLOCG00000012384.ENSXMAG00000025635.ENSXMAG00000015502		none	none	none	none	none	sub	none	none	none	sub
ENSXMAG00000007856	no	no	no	no	no	no	no	no	no	ENSLOCG00000012388.ENSXMAG00000007856.ENSXMAG00000014370	sipa1l1	none	none	none	none	none	none	sub	none	none	sub
ENSXMAG00000014370	no	no	no	no	no	no	0	no	no	ENSLOCG00000012388.ENSXMAG00000014370.ENSXMAG00000007856	SIPA1L1 (1 of many)	none	none	none	none	none	none	sub	none	none	sub
ENSXMAG00000007826	no	no	no	no	no	no	no	no	no	ENSLOCG00000012423.ENSXMAG00000007826.ENSXMAG00000014355	max	none	none	none	none	none	none	sub	none	none	sub
ENSXMAG00000014355	no	no	no	no	no	no	3	no	no	ENSLOCG00000012423.ENSXMAG00000014355.ENSXMAG00000007826	MAX (1 of many)	none	none	none	none	none	none	sub	none	none	sub
ENSXMAG00000007798	no	no	no	no	21	0	no	no	no	ENSLOCG00000012443.ENSXMAG00000007798.ENSXMAG00000014341	susd6	sub	none	none	none	sub	sub	none	none	none	sub
ENSXMAG00000014341	0	no	no	no	no	no	no	no	no	ENSLOCG00000012443.ENSXMAG00000014341.ENSXMAG00000007798		sub	none	none	none	sub	sub	none	none	none	sub
ENSXMAG00000007770	no	no	no	no	no	no	12	no	no	ENSLOCG00000012512.ENSXMAG00000007770.ENSXMAG00000025396	katnbl1	none	none	none	none	none	none	sub	sub	none	sub
ENSXMAG00000025396	no	no	no	no	no	no	no	18	no	ENSLOCG00000012512.ENSXMAG00000025396.ENSXMAG00000007770		none	none	none	none	none	none	sub	sub	none	sub
ENSXMAG00000002918	no	no	no	no	no	18	no	no	no	ENSLOCG00000012521.ENSXMAG00000002918.ENSXMAG00000015430	kcnv2a	none	none	none	none	none	sub	none	none	none	sub
ENSXMAG00000015430	no	no	no	no	no	no	no	no	no	ENSLOCG00000012521.ENSXMAG00000015430.ENSXMAG00000002918	kcnv2b	none	none	none	none	none	sub	none	none	none	sub
ENSXMAG00000021695	no	no	no	no	15	no	no	no	no	ENSLOCG00000012534.ENSXMAG00000021695.ENSXMAG00000026852	zgc:64051	none	none	none	none	sub	none	none	none	none	sub
ENSXMAG00000026852	no	no	no	no	no	no	no	no	no	ENSLOCG00000012534.ENSXMAG00000026852.ENSXMAG00000021695		none	none	none	none	sub	none	none	none	none	sub
ENSXMAG00000005692	no	no	no	no	no	6	no	no	no	ENSLOCG00000012556.ENSXMAG00000005692.ENSXMAG00000021530	usp53b	none	sub	none	none	none	no diff	none	none	none	sub
ENSXMAG00000021530	no	3	no	no	no	3	no	no	no	ENSLOCG00000012556.ENSXMAG00000021530.ENSXMAG00000005692	USP53	none	sub	none	none	none	no diff	none	none	none	sub
ENSXMAG00000012277	no	6	no	no	no	3	no	no	no	ENSLOCG00000012558.ENSXMAG00000012277.ENSXMAG00000012951	PCYT2 (1 of many)	none	sub	none	none	sub	no diff	none	none	none	sub
ENSXMAG00000012951	no	no	no	no	6	6	no	no	no	ENSLOCG00000012558.ENSXMAG00000012951.ENSXMAG00000012277	pcyt2	none	sub	none	none	sub	no diff	none	none	none	sub
ENSXMAG00000008886	no	no	no	no	no	no	no	no	no	ENSLOCG00000012589.ENSXMAG00000008886.ENSXMAG00000010258	znf385c	none	none	none	none	none	sub	none	none	none	sub
ENSXMAG00000010258	no	no	no	no	no	15	no	no	no	ENSLOCG00000012589.ENSXMAG00000010258.ENSXMAG00000008886		none	none	none	none	none	sub	none	none	none	sub
ENSXMAG00000022730	no	no	no	no	no	no	0	no	no	ENSLOCG00000012612.ENSXMAG00000022730.ENSXMAG00000027090	zgc:100829	none	none	none	none	none	none	sub	none	none	sub
ENSXMAG00000027090	no	no	no	no	no	no	no	no	no	ENSLOCG00000012612.ENSXMAG00000027090.ENSXMAG00000022730		none	none	none	none	none	none	sub	none	none	sub
ENSXMAG00000008856	no	no	no	no	21	no	no	no	no	ENSLOCG00000012614.ENSXMAG00000008856.ENSXMAG00000010242	dnajc7	none	sub	none	none	neo	sub	none	none	none	sub
ENSXMAG00000010242	no	15	no	no	15	12	no	no	no	ENSLOCG00000012614.ENSXMAG00000010242.ENSXMAG00000008856		none	sub	none	none	neo	sub	none	none	none	sub
ENSXMAG00000003899	no	no	no	no	18	no	no	no	no	ENSLOCG00000012627.ENSXMAG00000003899.ENSXMAG00000016494		none	none	none	none	sub	none	none	none	none	sub
ENSXMAG00000016494	no	no	no	no	no	no	no	no	no	ENSLOCG00000012627.ENSXMAG00000016494.ENSXMAG00000003899		none	none	none	none	sub	none	none	none	none	sub
ENSXMAG00000002862	no	no	no	no	no	3	no	no	no	ENSLOCG00000012636.ENSXMAG00000002862.ENSXMAG00000017646		sub	none	none	none	none	sub	none	none	none	sub
ENSXMAG00000017646	6	no	no	no	no	no	no	no	no	ENSLOCG00000012636.ENSXMAG00000017646.ENSXMAG00000002862	sema4gb	sub	none	none	none	none	sub	none	none	none	sub
ENSXMAG00000008800	no	no	no	no	no	no	no	no	no	ENSLOCG00000012637.ENSXMAG00000008800.ENSXMAG00000010204	aclya	none	none	sub	none	none	sub	none	none	none	sub
ENSXMAG00000010204	no	no	6	no	no	15	no	no	no	ENSLOCG00000012637.ENSXMAG00000010204.ENSXMAG00000008800	aclyb	none	none	sub	none	none	sub	none	none	none	sub
ENSXMAG00000008750	no	no	no	no	no	no	no	no	no	ENSLOCG00000012674.ENSXMAG00000008750.ENSXMAG00000025831	fkbp10a	none	none	none	none	none	sub	sub	none	none	sub
ENSXMAG00000025831	no	no	no	no	no	15	18	no	no	ENSLOCG00000012674.ENSXMAG00000025831.ENSXMAG00000008750	fkbp10b	none	none	none	none	none	sub	sub	none	none	sub
ENSXMAG00000010255	no	no	no	no	no	no	18	no	no	ENSLOCG00000012682.ENSXMAG00000010255.ENSXMAG00000021224	aimp1	none	none	none	none	sub	none	sub	none	none	sub
ENSXMAG00000021224	no	no	no	no	0	no	no	no	no	ENSLOCG00000012682.ENSXMAG00000021224.ENSXMAG00000010255	zgc:101853	none	none	none	none	sub	none	sub	none	none	sub
ENSXMAG00000013477	no	no	no	no	0	no	no	no	no	ENSLOCG00000012685.ENSXMAG00000013477.ENSXMAG00000024078		none	none	none	none	no diff	none	none	none	none	sub
ENSXMAG00000024078	no	no	no	no	21	no	no	no	no	ENSLOCG00000012685.ENSXMAG00000024078.ENSXMAG00000013477	trim8b	none	none	none	none	no diff	none	none	none	none	sub
ENSXMAG00000000246	no	no	no	no	no	no	no	no	no	ENSLOCG00000012695.ENSXMAG00000000246.ENSXMAG00000012376	cant1b	none	none	none	none	sub	none	none	sub	none	sub
ENSXMAG00000012376	no	no	no	no	15	no	no	6	no	ENSLOCG00000012695.ENSXMAG00000012376.ENSXMAG00000000246	cant1a	none	none	none	none	sub	none	none	sub	none	sub
ENSXMAG00000011299	no	no	no	3	no	no	no	no	no	ENSLOCG00000012708.ENSXMAG00000011299.ENSXMAG00000018369		none	none	none	sub	none	none	none	none	none	sub
ENSXMAG00000018369	no	no	no	no	no	no	no	no	no	ENSLOCG00000012708.ENSXMAG00000018369.ENSXMAG00000011299		none	none	none	sub	none	none	none	none	none	sub
ENSXMAG00000021192	no	no	no	no	no	18	no	no	no	ENSLOCG00000012720.ENSXMAG00000021192.ENSXMAG00000027086	cyth1b	sub	none	none	none	none	sub	none	none	none	sub
ENSXMAG00000027086	0	no	no	no	no	no	no	no	no	ENSLOCG00000012720.ENSXMAG00000027086.ENSXMAG00000021192	cyth1a	sub	none	none	none	none	sub	none	none	none	sub
ENSXMAG00000009945	no	no	no	no	no	12	no	no	no	ENSLOCG00000012752.ENSXMAG00000009945.ENSXMAG00000025000	PPP1R1B (1 of many)	sub	none	none	none	sub	neo	none	sub	none	sub
ENSXMAG00000025000	3	no	no	no	9	3	no	3	no	ENSLOCG00000012752.ENSXMAG00000025000.ENSXMAG00000009945	ppp1r1b	sub	none	none	none	sub	neo	none	sub	none	sub
ENSXMAG00000001349	no	no	no	no	no	no	no	6	no	ENSLOCG00000012758.ENSXMAG00000001349.ENSXMAG00000016875		none	none	none	none	none	none	none	sub	none	sub
ENSXMAG00000016875	no	no	no	no	no	no	no	no	no	ENSLOCG00000012758.ENSXMAG00000016875.ENSXMAG00000001349	lmbrd2a	none	none	none	none	none	none	none	sub	none	sub
ENSXMAG00000007614	no	no	no	no	no	0	no	no	no	ENSLOCG00000012787.ENSXMAG00000007614.ENSXMAG00000008396		none	none	none	none	none	no diff	none	none	none	sub
ENSXMAG00000008396	no	no	no	no	no	21	no	no	no	ENSLOCG00000012787.ENSXMAG00000008396.ENSXMAG00000007614		none	none	none	none	none	no diff	none	none	none	sub
ENSXMAG00000013379	no	no	no	no	no	no	no	no	no	ENSLOCG00000012802.ENSXMAG00000013379.ENSXMAG00000014472		none	none	none	none	none	none	sub	none	none	sub
ENSXMAG00000014472	no	no	no	no	no	no	21	no	no	ENSLOCG00000012802.ENSXMAG00000014472.ENSXMAG00000013379	arhgap19	none	none	none	none	none	none	sub	none	none	sub
ENSXMAG00000008471	no	no	no	no	no	no	no	no	no	ENSLOCG00000012805.ENSXMAG00000008471.ENSXMAG00000025571	rpl19	none	none	none	none	sub	none	none	none	none	sub
ENSXMAG00000025571	no	no	no	no	18	no	no	no	no	ENSLOCG00000012805.ENSXMAG00000025571.ENSXMAG00000008471	RPL19 (1 of many)	none	none	none	none	sub	none	none	none	none	sub
ENSXMAG00000006610	no	no	no	no	15	15	no	no	no	ENSLOCG00000012808.ENSXMAG00000006610.ENSXMAG00000009599		none	none	none	none	sub	sub	none	none	none	sub
ENSXMAG00000009599	no	no	no	no	no	no	no	no	no	ENSLOCG00000012808.ENSXMAG00000009599.ENSXMAG00000006610		none	none	none	none	sub	sub	none	none	none	sub
ENSXMAG00000006597	3	no	no	no	no	no	no	no	no	ENSLOCG00000012816.ENSXMAG00000006597.ENSXMAG00000009603	spint1a	sub	none	none	none	none	sub	none	none	none	sub
ENSXMAG00000009603	no	no	no	no	no	15	no	no	no	ENSLOCG00000012816.ENSXMAG00000009603.ENSXMAG00000006597		sub	none	none	none	none	sub	none	none	none	sub
ENSXMAG00000006554	no	no	no	no	no	21	no	no	no	ENSLOCG00000012854.ENSXMAG00000006554.ENSXMAG00000009610	pak6a	none	none	none	none	none	neo	none	none	none	sub
ENSXMAG00000009610	no	no	no	no	no	15	no	no	no	ENSLOCG00000012854.ENSXMAG00000009610.ENSXMAG00000006554	pak6b	none	none	none	none	none	neo	none	none	none	sub
ENSXMAG00000006477	no	no	no	no	12	no	no	15	no	ENSLOCG00000012891.ENSXMAG00000006477.ENSXMAG00000009631	mthfd1b	none	none	none	none	sub	none	none	sub	none	sub
ENSXMAG00000009631	no	no	no	no	no	no	no	no	no	ENSLOCG00000012891.ENSXMAG00000009631.ENSXMAG00000006477		none	none	none	none	sub	none	none	sub	none	sub
ENSXMAG00000012739	no	no	no	no	no	21	no	no	no	ENSLOCG00000012913.ENSXMAG00000012739.ENSXMAG00000014473	chmp6a	none	sub	none	none	none	sub	none	none	none	sub

ENSXMAG00000014473	no	3	no	no	no	no	no	no	no	ENSLOCG00000012913.ENSXMAG00000014473.ENSXMAG00000012739	CHMP6 (1 of many)	none	sub	none	none	none	sub	none	none	none	sub
ENSXMAG00000001628	no	no	no	no	no	6	0	0	no	ENSLOCG00000012933.ENSXMAG00000001628.ENSXMAG00000021636	pdlim5b	none	none	none	none	none	sub	sub	sub	none	sub
ENSXMAG00000021636	no	no	no	no	no	no	no	no	no	ENSLOCG00000012933.ENSXMAG00000021636.ENSXMAG00000001628	pdlim5a	none	none	none	none	none	sub	sub	sub	none	sub
ENSXMAG00000001635	no	no	no	no	no	18	no	no	no	ENSLOCG00000012942.ENSXMAG00000001635.ENSXMAG00000017124	bmpr1bb	none	none	none	none	none	no diff	none	none	none	sub
ENSXMAG00000017124	no	no	no	no	no	18	no	no	no	ENSLOCG00000012942.ENSXMAG00000017124.ENSXMAG00000001635	bmpr1ba	none	none	none	none	none	no diff	none	none	none	sub
ENSXMAG00000013225	21	no	no	no	no	no	no	no	no	ENSLOCG00000012949.ENSXMAG00000013225.ENSXMAG00000016929	fyco1a	sub	none	none	none	sub	none	none	none	none	sub
ENSXMAG00000016929	no	no	no	no	21	no	no	no	no	ENSLOCG00000012949.ENSXMAG00000016929.ENSXMAG00000013225	fyco1b	sub	none	none	none	sub	none	none	none	none	sub
ENSXMAG00000023445	no	no	no	no	no	no	no	no	no	ENSLOCG00000012958.ENSXMAG00000023445.ENSXMAG00000027096	gfra1b	none	none	none	none	none	sub	none	none	none	sub
ENSXMAG00000027096	no	no	no	no	no	21	no	no	no	ENSLOCG00000012958.ENSXMAG00000027096.ENSXMAG00000023445	GFRA1	none	none	none	none	none	sub	none	none	none	sub
ENSXMAG00000010812	no	no	no	no	no	no	no	no	no	ENSLOCG00000012959.ENSXMAG00000010812.ENSXMAG00000017938	nsfa	none	none	none	none	sub	sub	none	none	none	sub
ENSXMAG00000017938	no	no	no	no	18	18	no	no	no	ENSLOCG00000012959.ENSXMAG00000017938.ENSXMAG00000010812	nsfb	none	none	none	none	sub	sub	none	none	none	sub
ENSXMAG00000008384	no	no	no	no	no	0	no	no	no	ENSLOCG00000012969.ENSXMAG00000008384.ENSXMAG00000009679		none	none	none	none	none	sub	none	none	none	sub
ENSXMAG00000009679	no	no	no	no	no	no	no	no	no	ENSLOCG00000012969.ENSXMAG00000009679.ENSXMAG00000008384	chrna9	none	none	none	none	none	sub	none	none	none	sub
ENSXMAG00000006166	no	no	no	no	no	6	3	no	no	ENSLOCG00000012976.ENSXMAG00000006166.ENSXMAG00000016372	mylk4b	none	none	none	none	none	no diff	sub	none	none	sub
ENSXMAG00000016372	no	no	no	no	no	6	no	no	no	ENSLOCG00000012976.ENSXMAG00000016372.ENSXMAG00000006166	mylk4a	none	none	none	none	none	no diff	sub	none	none	sub
ENSXMAG00000010829	no	no	12	no	no	0	no	no	no	ENSLOCG00000013012.ENSXMAG00000010829.ENSXMAG00000017922		none	none	sub	none	none	sub	none	none	none	sub
ENSXMAG00000017922	no	no	no	no	no	no	no	no	no	ENSLOCG00000013012.ENSXMAG00000017922.ENSXMAG00000010829		none	none	sub	none	none	sub	none	none	none	sub
ENSXMAG00000006258	no	no	no	no	no	3	no	no	no	ENSLOCG00000013015.ENSXMAG00000006258.ENSXMAG00000023204		none	none	none	none	none	sub	none	none	none	sub
ENSXMAG00000023204	no	no	no	no	no	no	no	no	no	ENSLOCG00000013015.ENSXMAG00000023204.ENSXMAG00000006258	dusp22b	none	none	none	none	none	sub	none	none	none	sub
ENSXMAG00000016931	no	no	no	no	15	15	no	no	no	ENSLOCG00000013017.ENSXMAG00000016931.ENSXMAG00000029828	tmcc1b	none	none	none	none	neo	sub	none	none	none	sub
ENSXMAG00000029828	no	no	no	no	9	no	no	no	no	ENSLOCG00000013017.ENSXMAG00000029828.ENSXMAG00000016931	TMCC1 (1 of many)	none	none	none	none	neo	sub	none	none	none	sub
ENSXMAG00000006335	no	no	no	no	0	no	no	0	no	ENSLOCG00000013065.ENSXMAG00000006335.ENSXMAG00000016847	TNS3 (1 of many)	none	none	none	none	sub	sub	none	sub	none	sub
ENSXMAG00000016847	no	no	no	no	no	18	no	no	no	ENSLOCG00000013065.ENSXMAG00000016847.ENSXMAG00000006335	TNS3 (1 of many)	none	none	none	none	sub	sub	none	sub	none	sub
ENSXMAG00000007470	no	no	no	21	no	no	no	no	no	ENSLOCG00000013077.ENSXMAG00000007470.ENSXMAG00000017432		none	none	none	sub	sub	sub	none	none	none	sub
ENSXMAG00000017432	no	no	no	no	0	18	no	no	no	ENSLOCG00000013077.ENSXMAG00000017432.ENSXMAG00000007470	kbtbd12	none	none	none	sub	sub	sub	none	none	none	sub
ENSXMAG00000006331	no	no	no	no	no	no	21	no	no	ENSLOCG00000013080.ENSXMAG00000006331.ENSXMAG00000028732	igfbp3	none	none	none	none	none	none	sub	none	none	sub
ENSXMAG00000028732	no	no	no	no	no	no	no	no	no	ENSLOCG00000013080.ENSXMAG00000028732.ENSXMAG00000006331	IGFBP3	none	none	none	none	none	none	sub	none	none	sub
ENSXMAG00000012564	no	no	no	no	no	no	3	no	no	ENSLOCG00000013094.ENSXMAG00000012564.ENSXMAG00000016830	HID1 (1 of many)	none	none	none	none	none	none	sub	none	none	sub
ENSXMAG00000016830	no	no	no	no	no	no	no	no	no	ENSLOCG00000013094.ENSXMAG00000016830.ENSXMAG00000012564	HID1 (1 of many)	none	none	none	none	none	none	sub	none	none	sub
ENSXMAG00000006293	no	21	no	no	21	no	no	no	no	ENSLOCG00000013116.ENSXMAG00000006293.ENSXMAG00000016948	phactr1	none	sub	sub	none	sub	sub	none	none	none	sub
ENSXMAG00000016948	no	no	12	no	no	3	no	no	no	ENSLOCG00000013116.ENSXMAG00000016948.ENSXMAG00000006293		none	sub	sub	none	sub	sub	none	none	none	sub
ENSXMAG00000012603	no	no	no	no	no	no	no	no	no	ENSLOCG00000013122.ENSXMAG00000012603.ENSXMAG00000023407		none	none	none	none	none	sub	none	none	none	sub
ENSXMAG00000023407	no	no	no	no	no	12	no	no	no	ENSLOCG00000013122.ENSXMAG00000023407.ENSXMAG00000012603	trim16	none	none	none	none	none	sub	none	none	none	sub
ENSXMAG00000012781	0	no	no	no	6	no	no	no	no	ENSLOCG00000013174.ENSXMAG00000012781.ENSXMAG00000014836	si:ch211-114m9.1	sub	none	none	none	sub	none	none	none	none	sub
ENSXMAG00000014836	no	no	no	no	no	no	no	no	no	ENSLOCG00000013174.ENSXMAG00000014836.ENSXMAG00000012781		sub	none	none	none	sub	none	none	none	none	sub
ENSXMAG00000025314	no	no	no	no	no	no	no	no	no	ENSLOCG00000013186.ENSXMAG00000025314.ENSXMAG00000025640	kctd6a	none	none	none	none	sub	sub	none	none	none	sub
ENSXMAG00000025640	no	no	no	no	15	15	no	no	no	ENSLOCG00000013186.ENSXMAG00000025640.ENSXMAG00000025314	kctd6b	none	none	none	none	sub	sub	none	none	none	sub
ENSXMAG00000012493	no	no	0	no	no	no	no	no	no	ENSLOCG00000013206.ENSXMAG00000012493.ENSXMAG00000023015		none	none	sub	none	none	none	none	none	none	sub
ENSXMAG00000023015	no	no	no	no	no	no	no	no	no	ENSLOCG00000013206.ENSXMAG00000023015.ENSXMAG00000012493	DCTN6	none	none	sub	none	none	none	none	none	none	sub
ENSXMAG00000004882	no	no	no	no	no	0	no	no	no	ENSLOCG00000013236.ENSXMAG00000004882.ENSXMAG00000012102		none	none	none	none	none	sub	none	none	none	sub
ENSXMAG00000012102	no	no	no	no	no	no	no	no	no	ENSLOCG00000013236.ENSXMAG00000012102.ENSXMAG00000004882	adgrl3.1	none	none	none	none	none	sub	none	none	none	sub
ENSXMAG00000013776	no	no	no	no	no	no	no	no	no	ENSLOCG00000013280.ENSXMAG00000013776.ENSXMAG00000025029	atp6ap1lb	none	sub	none	none	none	none	none	none	none	sub
ENSXMAG00000025029	no	15	no	no	no	no	no	no	no	ENSLOCG00000013280.ENSXMAG00000025029.ENSXMAG00000013776		none	sub	none	none	none	none	none	none	none	sub
ENSXMAG00000007410	no	no	no	3	no	no	no	no	no	ENSLOCG00000013290.ENSXMAG00000007410.ENSXMAG00000026073	cfap52 (1 of many)	none	none	none	sub	none	none	none	none	none	sub
ENSXMAG00000026073	no	no	no	no	no	no	no	no	no	ENSLOCG00000013290.ENSXMAG00000026073.ENSXMAG00000007410	cfap52 (1 of many)	none	none	none	sub	none	none	none	none	none	sub
ENSXMAG00000012675	no	0	no	no	18	no	no	no	no	ENSLOCG00000013315.ENSXMAG00000012675.ENSXMAG00000014570		none	sub	none	none	sub	sub	none	none	none	sub
ENSXMAG00000014570	no	no	no	no	no	15	no	no	no	ENSLOCG00000013315.ENSXMAG00000014570.ENSXMAG00000012675	itga3b	none	sub	none	none	sub	sub	none	none	none	sub
ENSXMAG00000003371	no	no	no	no	no	no	no	no	no	ENSLOCG00000013346.ENSXMAG00000003371.ENSXMAG00000009186	paplnb	none	none	none	none	none	none	sub	none	none	sub
ENSXMAG00000009186	no	no	no	no	no	no	3	no	no	ENSLOCG00000013346.ENSXMAG00000009186.ENSXMAG00000003371	paplna	none	none	none	none	none	none	sub	none	none	sub
ENSXMAG00000006711	no	no	no	no	no	18	no	no	no	ENSLOCG00000013364.ENSXMAG00000006711.ENSXMAG00000014551	jpt1a	none	none	none	none	none	no diff	sub	none	none	sub
ENSXMAG00000014551	no	no	no	no	no	15	18	no	no	ENSLOCG00000013364.ENSXMAG00000014551.ENSXMAG00000006711	jpt1b	none	none	none	none	none	no diff	sub	none	none	sub
ENSXMAG00000009197	no	no	no	no	no	18	no	no	no	ENSLOCG00000013365.ENSXMAG00000009197.ENSXMAG00000029136		none	none	none	none	none	no diff	none	none	none	sub
ENSXMAG00000029136	no	no	no	no	no	18	no	no	no	ENSLOCG00000013365.ENSXMAG00000029136.ENSXMAG00000009197	erh	none	none	none	none	none	no diff	none	none	none	sub
ENSXMAG00000014393	no	no	no	18	no	no	no	no	no	ENSLOCG00000013371.ENSXMAG00000014393.ENSXMAG00000028828	9-Sep	none	none	none	sub	none	sub	none	none	none	sub
ENSXMAG00000028828	no	no	no	no	no	21	no	no	no	ENSLOCG00000013371.ENSXMAG00000028828.ENSXMAG00000014393	sept9b	none	none	none	sub	none	sub	none	none	none	sub
ENSXMAG00000012250	no	no	no	no	15	15	no	no	no	ENSLOCG00000013399.ENSXMAG00000012250.ENSXMAG00000015708	dag1	none	none	none	none	no diff	no diff	none	none	none	sub
ENSXMAG00000015708	no	no	no	no	15	15	no	no	no	ENSLOCG00000013399.ENSXMAG00000015708.ENSXMAG00000012250		none	none	none	none	no diff	no diff	none	none	none	sub
ENSXMAG00000000363	no	no	no	no	3	0	no	no	no	ENSLOCG00000013420.ENSXMAG00000000363.ENSXMAG00000008979	kdm6bb	none	none	none	none	sub	sub	none	none	none	sub
ENSXMAG00000008979	no	no	no	no	no	no	no	no	no	ENSLOCG00000013420.ENSXMAG00000008979.ENSXMAG00000000363	kdm6ba	none	none	none	none	sub	sub	none	none	none	sub
ENSXMAG00000009792	no	no	no	no	6	3	no	no	no	ENSLOCG00000013435.ENSXMAG00000009792.ENSXMAG00000009932		none	none	none	none	sub	sub	none	none	none	sub
ENSXMAG00000009932	no	no	no	no	no	no	no	no	no	ENSLOCG00000013435.ENSXMAG00000009932.ENSXMAG00000009792	ppm1k	none	none	none	none	sub	sub	none	none	none	sub
ENSXMAG00000005972	no	no	no	3	no	no	no	no	no	ENSLOCG00000013471.ENSXMAG00000005972.ENSXMAG00000015781		none	none	none	sub	sub	none	none	none	none	sub
ENSXMAG00000015781	no	no	no	no	18	no	no	no	no	ENSLOCG00000013471.ENSXMAG00000015781.ENSXMAG00000005972	DCAF1	none	none	none	sub	sub	none	none	none	none	sub
ENSXMAG00000012369	no	no	18	no	no	15	no	no	no	ENSLOCG00000013477.ENSXMAG00000012369.ENSXMAG00000025799	SPHK1 (1 of many)	none	none	sub	none	none	sub	none	none	none	sub
ENSXMAG00000025799	no	no	no	no	no	no	no	no	no	ENSLOCG00000013477.ENSXMAG00000025799.ENSXMAG00000012369	SPHK1 (1 of many)	none	none	sub	none	none	sub	none	none	none	sub
ENSXMAG00000006679	no	no	no	no	no	12	no	no	no	ENSLOCG00000013480.ENSXMAG00000006679.ENSXMAG00000014280	prtgb	none	none	none	none	none	sub	none	none	none	sub
ENSXMAG00000014280	no	no	no	no	no	no	no	no	no	ENSLOCG00000013480.ENSXMAG00000014280.ENSXMAG00000006679	PRTG	none	none	none	none	none	sub	none	none	none	sub
ENSXMAG00000015790	no	6	no	no	15	no	no	no	no	ENSLOCG00000013502.ENSXMAG00000015790.ENSXMAG00000024535	ssuh2rs1	none	sub	none	none	sub	none	none	none	none	sub
ENSXMAG00000024535	no	no	no	no	no	no	no	no	no	ENSLOCG00000013502.ENSXMAG00000024535.ENSXMAG00000015790		none	sub	none	none	sub	none	none	none	none	sub
ENSXMAG00000012511	no	no	no	no	no	21	no	no	no	ENSLOCG00000013521.ENSXMAG00000012511.ENSXMAG00000013383	gucy2d	none	none	none	none	sub	no diff	sub	none	none	sub
ENSXMAG00000013383	no	no	no	no	3	21	3	no	no	ENSLOCG00000013521.ENSXMAG00000013383.ENSXMAG00000012511		none	none	none	none	sub	no diff	sub	none	none	sub
ENSXMAG00000014411	no	no	no	3	no	no	no	no	no	ENSLOCG00000013533.ENSXMAG00000014411.ENSXMAG00000017673	ttbk2a	none	none	none	sub	none	none	none	none	none	sub
ENSXMAG00000017673	no	no	no	no	no	no	no	no	no	ENSLOCG00000013533.ENSXMAG00000017673.ENSXMAG00000014411	ttbk2b	none	none	none	sub	none	none	none	none	none	sub
ENSXMAG00000006131	no	no	no	no	15	15	15	no	no	ENSLOCG00000013534.ENSXMAG00000006131.ENSXMAG00000008305		none	none	none	none	sub	sub	sub	none	none	sub
ENSXMAG00000008305	no	no	no	no	no	no	no	no	no	ENSLOCG00000013534.ENSXMAG00000008305.ENSXMAG00000006131	ddx5	none	none	none	none	sub	sub	sub	none	none	sub
ENSXMAG00000009165	no	no	no	no	no	no	no	no	no	ENSLOCG00000013537.ENSXMAG00000009165.ENSXMAG00000015803	grip2b	none	none	none	none	sub	sub	none	none	sub	sub
ENSXMAG00000015803	no	no	no	no	3	3	no	no	21	ENSLOCG00000013537.ENSXMAG00000015803.ENSXMAG00000009165	grip2a	none	none	none	none	sub	sub	none	none	sub	sub
ENSXMAG00000015849	no	no	no	no	no	no	no	no	no	ENSLOCG00000013555.ENSXMAG00000015849.ENSXMAG00000017677	elmsan1b	none	none	none	none	none	none	none	none	sub	sub
ENSXMAG00000017677	no	no	no	no	no	no	no	no	0	ENSLOCG00000013555.ENSXMAG00000017677.ENSXMAG00000015849	elmsan1a	none	none	none	none	none	none	none	none	sub	sub
ENSXMAG00000002642	no	no	no	no	no	no	18	no	no	ENSLOCG00000013577.ENSXMAG00000002642.ENSXMAG00000018179	smarce1	none	none	none	none	none	sub	sub	none	none	sub
ENSXMAG00000018179	no	no	no	no	no	21	no	no	no	ENSLOCG00000013577.ENSXMAG00000018179.ENSXMAG00000002642	SMARCE1	none	none	none	none	none	sub	sub	none	none	sub
ENSXMAG00000006616	no	no	no	no	no	no	no	no	6	ENSLOCG00000013583.ENSXMAG00000006616.ENSXMAG00000029098	aqp9b	none	none	none	none	none	sub	none	none	sub	sub
ENSXMAG00000029098	no	no	no	no	no	0	no	no	no	ENSLOCG00000013583.ENSXMAG00000029098.ENSXMAG00000006616		none	none	none	none	none	sub	none	none	sub	sub
ENSXMAG00000001186	no	no	no	no	no	0	no	no	no	ENSLOCG00000013639.ENSXMAG00000001186.ENSXMAG00000006578		none	none	none	none	none	no diff	sub	none	none	sub
ENSXMAG00000006578	no	no	no	no	no	3	0	no	no	ENSLOCG00000013639.ENSXMAG00000006578.ENSXMAG00000001186	prkca	none	none	none	none	none	no diff	sub	none	none	sub
ENSXMAG00000001253	no	no	no	no	no	no	no	no	no	ENSLOCG00000013680.ENSXMAG00000001253.ENSXMAG00000017877	axin2	none	none	none	none	none	none	none	none	sub	sub
ENSXMAG00000017877	no	no	no	no	no	no	no	no	21	ENSLOCG00000013680.ENSXMAG00000017877.ENSXMAG00000001253	AXIN2	none	none	none	none	none	none	none	none	sub	sub
ENSXMAG00000001283	no	no	no	no	no	no	no	no	no	ENSLOCG00000013688.ENSXMAG00000001283.ENSXMAG00000017886	tex2	none	sub	none	none	none	none	none	none	none	sub
ENSXMAG00000017886	no	18	no	no	no	no	no	no	no	ENSLOCG00000013688.ENSXMAG00000017886.ENSXMAG00000001283		none	sub	none	none	none	none	none	none	none	sub
ENSXMAG00000006814	no	no	no	no	no	no	no	no	no	ENSLOCG00000013741.ENSXMAG00000006814.ENSXMAG00000014316	rhot1b	none	none	none	none	sub	none	none	none	none	sub
ENSXMAG00000014316	no	no	no	no	15	no	no	no	no	ENSLOCG00000013741.ENSXMAG00000014316.ENSXMAG00000006814	rhot1a	none	none	none	none	sub	none	none	none	none	sub
ENSXMAG00000000385	no	no	no	no	no	0	no	no	no	ENSLOCG00000013755.ENSXMAG00000000385.ENSXMAG00000013668	fgf11b	none	none	none	none	none	sub	none	none	none	sub
ENSXMAG00000013668	no	no	no	no	no	no	no	no	no	ENSLOCG00000013755.ENSXMAG00000013668.ENSXMAG00000000385	fgf11a	none	none	none	none	none	sub	none	none	none	sub
ENSXMAG00000006859	no	no	no	no	no	no	no	no	no	ENSLOCG00000013764.ENSXMAG00000006859.ENSXMAG00000014302		none	sub	none	none	sub	sub	sub	none	none	sub
ENSXMAG00000014302	no	6	no	no	3	0	6	no	no	ENSLOCG00000013764.ENSXMAG00000014302.ENSXMAG00000006859	adap2	none	sub	none	none	sub	sub	sub	none	none	sub
ENSXMAG00000012979	no	no	no	no	no	6	no	no	no	ENSLOCG00000013792.ENSXMAG00000012979.ENSXMAG00000017579	suz12a	none	none	none	none	sub	sub	none	none	none	sub
ENSXMAG00000017579	no	no	no	no	21	no	no	no	no	ENSLOCG00000013792.ENSXMAG00000017579.ENSXMAG00000012979	SUZ12	none	none	none	none	sub	sub	none	none	none	sub
ENSXMAG00000017789	3	no	no	no	no	no	no	no	no	ENSLOCG00000013799.ENSXMAG00000017789.ENSXMAG00000023607		sub	none	none	none	none	sub	none	sub	none	sub
ENSXMAG00000023607	no	no	no	no	no	12	no	15	no	ENSLOCG00000013799.ENSXMAG00000023607.ENSXMAG00000017789	dusp7	sub	none	none	none	none	sub	none	sub	none	sub
ENSXMAG00000001429	no	no	no	no	0	no	no	no	no	ENSLOCG00000013847.ENSXMAG00000001429.ENSXMAG00000021089	cbx8a	none	none	none	none	sub	none	none	none	none	sub
ENSXMAG00000021089	no	no	no	no	no	no	no	no	no	ENSLOCG00000013847.ENSXMAG00000021089.ENSXMAG00000001429	cbx8b	none	none	none	none	sub	none	none	none	none	sub
ENSXMAG00000010483	no	no	no	no	no	no	no	no	no	ENSLOCG00000013860.ENSXMAG00000010483.ENSXMAG00000024264	ogna	none	sub	none	none	none	none	none	sub	none	sub
ENSXMAG00000024264	no	6	no	no	no	no	no	15	no	ENSLOCG00000013860.ENSXMAG00000024264.ENSXMAG00000010483	ognb	none	sub	none	none	none	none	none	sub	none	sub
ENSXMAG00000012443	no	no	no	no	no	no	15	no	no	ENSLOCG00000013994.ENSXMAG00000012443.ENSXMAG00000017656		none	none	none	none	sub	none	sub	none	none	sub
ENSXMAG00000017656	no	no	no	no	21	no	no	no	no	ENSLOCG00000013994.ENSXMAG00000017656.ENSXMAG00000012443		none	none	none	none	sub	none	sub	none	none	sub
ENSXMAG00000007522	0	no	no	no	no	no	no	no	no	ENSLOCG00000014058.ENSXMAG00000007522.ENSXMAG00000017581	ptpdc1a	sub	none	none	none	none	sub	none	none	none	sub
ENSXMAG00000017581	no	no	no	no	no	3	no	no	no	ENSLOCG00000014058.ENSXMAG00000017581.ENSXMAG00000007522	ptpdc1b	sub	none	none	none	none	sub	none	none	none	sub
ENSXMAG00000025916	no	no	no	no	no	21	no	no	no	ENSLOCG00000014060.ENSXMAG00000025916.ENSXMAG00000026041		none	none	none	none	none	neo	none	none	none	sub
ENSXMAG00000026041	no	no	no	no	no	12	no	no	no	ENSLOCG00000014060.ENSXMAG00000026041.ENSXMAG00000025916	si:dkey-175g6.2	none	none	none	none	none	neo	none	none	none	sub
ENSXMAG00000017090	no	no	no	no	no	15	no	no	no	ENSLOCG00000014087.ENSXMAG00000017090.ENSXMAG00000022037	NINJ1 (1 of many)	none	none	none	none	none	sub	none	none	none	sub
ENSXMAG00000022037	no	no	no	no	no	no	no	no	no	ENSLOCG00000014087.ENSXMAG00000022037.ENSXMAG00000017090	NINJ1 (1 of many)	none	none	none	none	none	sub	none	none	none	sub
ENSXMAG00000006869	no	no	no	no	no	0	no	no	no	ENSLOCG00000014132.ENSXMAG00000006869.ENSXMAG00000018049		none	none	none	none	none	sub	none	none	none	sub
ENSXMAG00000018049	no	no	no	no	no	no	no	no	no	ENSLOCG00000014132.ENSXMAG00000018049.ENSXMAG00000006869	prc1b	none	none	none	none	none	sub	none	none	none	sub
ENSXMAG00000000160	no	no	no	no	no	18	no	15	no	ENSLOCG00000014151.ENSXMAG00000000160.ENSXMAG00000017247		none	none	none	none	none	neo	none	sub	none	sub
ENSXMAG00000017247	no	no	no	no	no	0	no	no	no	ENSLOCG00000014151.ENSXMAG00000017247.ENSXMAG00000000160	mpp5b	none	none	none	none	none	neo	none	sub	none	sub
ENSXMAG00000013503	no	no	no	no	no	21	no	no	no	ENSLOCG00000014166.ENSXMAG00000013503.ENSXMAG00000017634	kita	none	none	none	none	none	neo	none	none	none	sub
ENSXMAG00000017634	no	no	no	no	no	6	no	no	no	ENSLOCG00000014166.ENSXMAG00000017634.ENSXMAG00000013503	kitb	none	none	none	none	none	neo	none	none	none	sub
ENSXMAG00000010771	no	no	no	no	15	15	no	no	no	ENSLOCG00000014200.ENSXMAG00000010771.ENSXMAG00000029134	ehd2a	none	none	none	none	sub	sub	none	none	none	sub
ENSXMAG00000029134	no	no	no	no	no	no	no	no	no	ENSLOCG00000014200.ENSXMAG00000029134.ENSXMAG00000010771	ehd2b	none	none	none	none	sub	sub	none	none	none	sub
ENSXMAG00000012976	no	no	no	no	no	no	no	no	no	ENSLOCG00000014203.ENSXMAG00000012976.ENSXMAG00000018275	ppip5k1b	sub	none	none	none	none	none	none	none	none	sub
ENSXMAG00000018275	18	no	no	no	no	no	no	no	no	ENSLOCG00000014203.ENSXMAG00000018275.ENSXMAG00000012976	ppip5k1a	sub	none	none	none	none	none	none	none	none	sub
ENSXMAG00000013036	no	no	no	no	no	no	no	no	no	ENSLOCG00000014224.ENSXMAG00000013036.ENSXMAG00000024531	mfge8b	none	none	none	sub	none	none	sub	none	none	sub
ENSXMAG00000024531	no	no	no	3	no	no	6	no	no	ENSLOCG00000014224.ENSXMAG00000024531.ENSXMAG00000013036		none	none	none	sub	none	none	sub	none	none	sub
ENSXMAG00000002892	no	no	no	no	no	3	no	no	no	ENSLOCG00000014310.ENSXMAG00000002892.ENSXMAG00000017009	RASGRF1	none	none	none	none	none	no diff	none	none	none	sub
ENSXMAG00000017009	no	no	no	no	no	3	no	no	no	ENSLOCG00000014310.ENSXMAG00000017009.ENSXMAG00000002892		none	none	none	none	none	no diff	none	none	none	sub
ENSXMAG00000002886	no	no	no	no	no	no	no	no	no	ENSLOCG00000014321.ENSXMAG00000002886.ENSXMAG00000016987		none	none	none	none	none	sub	sub	none	sub	sub
ENSXMAG00000016987	no	no	no	no	no	0	6	no	9	ENSLOCG00000014321.ENSXMAG00000016987.ENSXMAG00000002886	si:ch211-107o10.3	none	none	none	none	none	sub	sub	none	sub	sub
ENSXMAG00000007547	no	no	no	no	no	0	no	no	no	ENSLOCG00000014345.ENSXMAG00000007547.ENSXMAG00000017369		none	none	none	none	none	sub	none	none	none	sub
ENSXMAG00000017369	no	no	no	no	no	no	no	no	no	ENSLOCG00000014345.ENSXMAG00000017369.ENSXMAG00000007547	p4htm	none	none	none	none	none	sub	none	none	none	sub
ENSXMAG00000001747	21	no	no	no	no	no	0	no	no	ENSLOCG00000014413.ENSXMAG00000001747.ENSXMAG00000023264	ip6k2b	neo	none	none	none	none	none	sub	sub	none	sub
ENSXMAG00000023264	15	no	no	no	no	no	no	3	no	ENSLOCG00000014413.ENSXMAG00000023264.ENSXMAG00000001747	ip6k2a	neo	none	none	none	none	none	sub	sub	none	sub
ENSXMAG00000003824	21	no	no	no	no	no	no	no	no	ENSLOCG00000014427.ENSXMAG00000003824.ENSXMAG00000012880		sub	none	none	none	none	none	none	none	none	sub
ENSXMAG00000012880	no	no	no	no	no	no	no	no	no	ENSLOCG00000014427.ENSXMAG00000012880.ENSXMAG00000003824		sub	none	none	none	none	none	none	none	none	sub
ENSXMAG00000001861	no	no	no	no	12	12	no	no	no	ENSLOCG00000014468.ENSXMAG00000001861.ENSXMAG00000010429	prkcda	none	none	none	none	sub	sub	none	none	none	sub
ENSXMAG00000010429	no	no	no	no	no	no	no	no	no	ENSLOCG00000014468.ENSXMAG00000010429.ENSXMAG00000001861	prkcdb	none	none	none	none	sub	sub	none	none	none	sub
ENSXMAG00000000035	no	no	no	no	0	no	no	no	no	ENSLOCG00000014545.ENSXMAG00000000035.ENSXMAG00000007414	nptnb	none	none	none	none	sub	sub	none	none	none	sub
ENSXMAG00000007414	no	no	no	no	no	15	no	no	no	ENSLOCG00000014545.ENSXMAG00000007414.ENSXMAG00000000035	nptna	none	none	none	none	sub	sub	none	none	none	sub
ENSXMAG00000023285	18	no	no	no	no	no	no	no	no	ENSLOCG00000014547.ENSXMAG00000023285.ENSXMAG00000024665	rab7	sub	none	none	none	none	none	none	none	none	sub
ENSXMAG00000024665	no	no	no	no	no	no	no	no	no	ENSLOCG00000014547.ENSXMAG00000024665.ENSXMAG00000023285	RAB7A	sub	none	none	none	none	none	none	none	none	sub
ENSXMAG00000002966	no	no	no	no	no	no	no	no	no	ENSLOCG00000014643.ENSXMAG00000002966.ENSXMAG00000017063		none	none	none	none	none	sub	none	none	none	sub
ENSXMAG00000017063	no	no	no	no	no	3	no	no	no	ENSLOCG00000014643.ENSXMAG00000017063.ENSXMAG00000002966	frmd5	none	none	none	none	none	sub	none	none	none	sub
ENSXMAG00000008911	no	no	no	3	no	no	no	no	no	ENSLOCG00000014645.ENSXMAG00000008911.ENSXMAG00000027672		none	none	none	sub	sub	none	sub	none	none	sub
ENSXMAG00000027672	no	no	no	no	6	no	6	no	no	ENSLOCG00000014645.ENSXMAG00000027672.ENSXMAG00000008911	iqsec2b	none	none	none	sub	sub	none	sub	none	none	sub
ENSXMAG00000017127	21	no	no	no	no	no	no	no	no	ENSLOCG00000014657.ENSXMAG00000017127.ENSXMAG00000026513		sub	none	none	none	none	none	none	none	none	sub
ENSXMAG00000026513	no	no	no	no	no	no	no	no	no	ENSLOCG00000014657.ENSXMAG00000026513.ENSXMAG00000017127		sub	none	none	none	none	none	none	none	none	sub
ENSXMAG00000006418	no	no	no	no	no	3	no	no	no	ENSLOCG00000014667.ENSXMAG00000006418.ENSXMAG00000027053	mapk8ip1a	none	none	none	none	none	sub	none	none	none	sub
ENSXMAG00000027053	no	no	no	no	no	no	no	no	no	ENSLOCG00000014667.ENSXMAG00000027053.ENSXMAG00000006418		none	none	none	none	none	sub	none	none	none	sub
ENSXMAG00000003135	no	3	no	no	no	0	no	no	no	ENSLOCG00000014719.ENSXMAG00000003135.ENSXMAG00000017193	tmem266	none	sub	sub	none	none	sub	none	none	none	sub
ENSXMAG00000017193	no	no	15	no	no	no	no	no	no	ENSLOCG00000014719.ENSXMAG00000017193.ENSXMAG00000003135	TMEM266 (1 of many)	none	sub	sub	none	none	sub	none	none	none	sub
ENSXMAG00000003228	no	no	no	no	no	3	no	no	no	ENSLOCG00000014779.ENSXMAG00000003228.ENSXMAG00000018442	roraa	none	none	none	none	sub	no diff	none	none	none	sub
ENSXMAG00000018442	no	no	no	no	0	3	no	no	no	ENSLOCG00000014779.ENSXMAG00000018442.ENSXMAG00000003228		none	none	none	none	sub	no diff	none	none	none	sub
ENSXMAG00000013331	no	no	no	no	no	no	no	no	no	ENSLOCG00000014787.ENSXMAG00000013331.ENSXMAG00000025260	anxa2b	sub	none	none	none	none	none	none	none	none	sub
ENSXMAG00000025260	6	no	no	no	no	no	no	no	no	ENSLOCG00000014787.ENSXMAG00000025260.ENSXMAG00000013331	anxa2a	sub	none	none	none	none	none	none	none	none	sub
ENSXMAG00000000226	no	no	no	no	no	6	no	no	no	ENSLOCG00000014844.ENSXMAG00000000226.ENSXMAG00000010902		none	sub	none	none	none	sub	none	none	none	sub
ENSXMAG00000010902	no	0	no	no	no	no	no	no	no	ENSLOCG00000014844.ENSXMAG00000010902.ENSXMAG00000000226	l1cama	none	sub	none	none	none	sub	none	none	none	sub
ENSXMAG00000001773	no	no	no	no	no	no	no	no	no	ENSLOCG00000014876.ENSXMAG00000001773.ENSXMAG00000012008	ppm1na	none	none	none	none	none	sub	none	none	none	sub
ENSXMAG00000012008	no	no	no	no	no	21	no	no	no	ENSLOCG00000014876.ENSXMAG00000012008.ENSXMAG00000001773	ppm1nb	none	none	none	none	none	sub	none	none	none	sub
ENSXMAG00000000900	no	no	no	no	no	no	no	no	21	ENSLOCG00000014917.ENSXMAG00000000900.ENSXMAG00000003972	bmp1a	none	none	none	none	none	none	none	none	sub	sub
ENSXMAG00000003972	no	no	no	no	no	no	no	no	no	ENSLOCG00000014917.ENSXMAG00000003972.ENSXMAG00000000900	bmp1b	none	none	none	none	none	none	none	none	sub	sub
ENSXMAG00000000325	no	no	no	no	no	no	no	no	no	ENSLOCG00000014927.ENSXMAG00000000325.ENSXMAG00000001480	RAB39B	none	none	none	none	none	none	sub	none	none	sub
ENSXMAG00000001480	no	no	no	no	no	no	12	no	no	ENSLOCG00000014927.ENSXMAG00000001480.ENSXMAG00000000325		none	none	none	none	none	none	sub	none	none	sub
ENSXMAG00000012952	no	no	no	no	15	no	no	15	no	ENSLOCG00000014941.ENSXMAG00000012952.ENSXMAG00000025884	scamp2	none	none	none	none	sub	none	none	sub	none	sub
ENSXMAG00000025884	no	no	no	no	no	no	no	no	no	ENSLOCG00000014941.ENSXMAG00000025884.ENSXMAG00000012952	SCAMP2	none	none	none	none	sub	none	none	sub	none	sub
ENSXMAG00000001846	no	no	no	no	no	0	no	no	no	ENSLOCG00000014966.ENSXMAG00000001846.ENSXMAG00000006860	AAK1 (1 of many)	none	none	none	none	none	sub	none	none	none	sub
ENSXMAG00000006860	no	no	no	no	no	no	no	no	no	ENSLOCG00000014966.ENSXMAG00000006860.ENSXMAG00000001846	aak1b	none	none	none	none	none	sub	none	none	none	sub
ENSXMAG00000010311	no	no	no	no	no	no	no	no	no	ENSLOCG00000014973.ENSXMAG00000010311.ENSXMAG00000015842		none	none	none	none	none	sub	none	none	none	sub
ENSXMAG00000015842	no	no	no	no	no	18	no	no	no	ENSLOCG00000014973.ENSXMAG00000015842.ENSXMAG00000010311	nr2e3	none	none	none	none	none	sub	none	none	none	sub
ENSXMAG00000000027	no	no	no	no	15	12	no	no	no	ENSLOCG00000015045.ENSXMAG00000000027.ENSXMAG00000002204	slc25a44b	none	none	none	none	sub	sub	none	none	none	sub
ENSXMAG00000002204	no	no	no	no	no	no	no	no	no	ENSLOCG00000015045.ENSXMAG00000002204.ENSXMAG00000000027	slc25a44a	none	none	none	none	sub	sub	none	none	none	sub
ENSXMAG00000002217	no	no	no	no	no	no	15	no	no	ENSLOCG00000015050.ENSXMAG00000002217.ENSXMAG00000027139	crabp1b	none	none	none	none	none	none	sub	none	none	sub
ENSXMAG00000027139	no	no	no	no	no	no	no	no	no	ENSLOCG00000015050.ENSXMAG00000027139.ENSXMAG00000002217		none	none	none	none	none	none	sub	none	none	sub
ENSXMAG00000017435	no	no	no	no	no	no	no	6	no	ENSLOCG00000015078.ENSXMAG00000017435.ENSXMAG00000023145		none	none	none	none	sub	none	none	sub	none	sub
ENSXMAG00000023145	no	no	no	no	15	no	no	no	no	ENSLOCG00000015078.ENSXMAG00000023145.ENSXMAG00000017435		none	none	none	none	sub	none	none	sub	none	sub
ENSXMAG00000023425	no	no	no	no	no	9	15	18	no	ENSLOCG00000015147.ENSXMAG00000023425.ENSXMAG00000029675	cox5aa	none	none	sub	none	none	sub	sub	sub	none	sub
ENSXMAG00000029675	no	no	0	no	no	no	no	no	no	ENSLOCG00000015147.ENSXMAG00000029675.ENSXMAG00000023425		none	none	sub	none	none	sub	sub	sub	none	sub
ENSXMAG00000000404	no	no	no	no	no	no	no	no	no	ENSLOCG00000015164.ENSXMAG00000000404.ENSXMAG00000013325	mcf2b	sub	none	none	sub	none	none	none	none	none	sub
ENSXMAG00000013325	3	no	no	3	no	no	no	no	no	ENSLOCG00000015164.ENSXMAG00000013325.ENSXMAG00000000404	mcf2a	sub	none	none	sub	none	none	none	none	none	sub
ENSXMAG00000001482	no	12	no	no	15	15	15	15	no	ENSLOCG00000015224.ENSXMAG00000001482.ENSXMAG00000008892	arntl2	sub	neo	none	none	sub	no diff	no diff	sub	none	sub
ENSXMAG00000008892	18	18	no	no	no	15	15	no	no	ENSLOCG00000015224.ENSXMAG00000008892.ENSXMAG00000001482	ARNTL2 (1 of many)	sub	neo	none	none	sub	no diff	no diff	sub	none	sub
ENSXMAG00000002364	no	no	no	no	no	no	no	no	no	ENSLOCG00000015227.ENSXMAG00000002364.ENSXMAG00000017872	ank1b	none	none	none	none	sub	none	none	none	none	sub
ENSXMAG00000017872	no	no	no	no	15	no	no	no	no	ENSLOCG00000015227.ENSXMAG00000017872.ENSXMAG00000002364		none	none	none	none	sub	none	none	none	none	sub
ENSXMAG00000005432	no	no	no	no	no	0	no	15	no	ENSLOCG00000015252.ENSXMAG00000005432.ENSXMAG00000024814	lman2la	none	none	none	none	none	sub	none	sub	none	sub
ENSXMAG00000024814	no	no	no	no	no	no	no	no	no	ENSLOCG00000015252.ENSXMAG00000024814.ENSXMAG00000005432	lman2lb	none	none	none	none	none	sub	none	sub	none	sub
ENSXMAG00000001744	0	3	no	no	3	0	no	no	3	ENSLOCG00000015272.ENSXMAG00000001744.ENSXMAG00000017466	cry1ab	neo	no diff	none	none	neo	sub	sub	none	sub	sub
ENSXMAG00000017466	6	6	no	no	9	no	6	no	no	ENSLOCG00000015272.ENSXMAG00000017466.ENSXMAG00000001744		neo	no diff	none	none	neo	sub	sub	none	sub	sub
ENSXMAG00000002689	no	no	no	no	no	no	no	no	no	ENSLOCG00000015299.ENSXMAG00000002689.ENSXMAG00000025444		none	sub	none	none	none	sub	none	none	none	sub
ENSXMAG00000025444	no	6	no	no	no	6	no	no	no	ENSLOCG00000015299.ENSXMAG00000025444.ENSXMAG00000002689		none	sub	none	none	none	sub	none	none	none	sub

ENSXMAG00000010236	no	no	no	no	no	21	no	no	no	ENSLOCG00000015371.ENSXMAG00000010236.ENSXMAG00000018615	si:ch211-48m9.1	none	none	none	none	none	neo	none	none	none	sub
ENSXMAG00000018615	no	no	no	no	no	6	no	no	no	ENSLOCG00000015371.ENSXMAG00000018615.ENSXMAG00000010236		none	none	none	none	none	neo	none	none	none	sub
ENSXMAG00000001294	no	no	no	no	no	9	no	no	no	ENSLOCG00000015385.ENSXMAG00000001294.ENSXMAG00000016966		none	none	none	none	none	sub	none	none	none	sub
ENSXMAG00000016966	no	no	no	no	no	no	no	no	no	ENSLOCG00000015385.ENSXMAG00000016966.ENSXMAG00000001294	ldhbb	none	none	none	none	none	sub	none	none	none	sub
ENSXMAG00000001328	no	no	no	no	no	no	15	no	no	ENSLOCG00000015421.ENSXMAG00000001328.ENSXMAG00000026296	aebp2	none	none	none	none	none	none	sub	none	none	sub
ENSXMAG00000026296	no	no	no	no	no	no	no	no	no	ENSLOCG00000015421.ENSXMAG00000026296.ENSXMAG00000001328	AEBP2	none	none	none	none	none	none	sub	none	none	sub
ENSXMAG00000001309	no	no	no	no	no	no	no	no	no	ENSLOCG00000015422.ENSXMAG00000001309.ENSXMAG00000007144		none	sub	none	none	none	none	sub	none	none	sub
ENSXMAG00000007144	no	0	no	no	no	no	3	no	no	ENSLOCG00000015422.ENSXMAG00000007144.ENSXMAG00000001309	PLEKHA5	none	sub	none	none	none	none	sub	none	none	sub
ENSXMAG00000010470	no	21	no	no	no	no	no	no	no	ENSLOCG00000015445.ENSXMAG00000010470.ENSXMAG00000018377	loxl2b	none	sub	none	none	none	none	none	none	none	sub
ENSXMAG00000018377	no	no	no	no	no	no	no	no	no	ENSLOCG00000015445.ENSXMAG00000018377.ENSXMAG00000010470	loxl2a	none	sub	none	none	none	none	none	none	none	sub
ENSXMAG00000010490	no	no	no	no	12	no	no	no	no	ENSLOCG00000015460.ENSXMAG00000010490.ENSXMAG00000022145	hdr	none	none	none	none	sub	sub	none	none	none	sub
ENSXMAG00000022145	no	no	no	no	no	18	no	no	no	ENSLOCG00000015460.ENSXMAG00000022145.ENSXMAG00000010490		none	none	none	none	sub	sub	none	none	none	sub
ENSXMAG00000014461	no	no	no	no	no	no	no	no	no	ENSLOCG00000015493.ENSXMAG00000014461.ENSXMAG00000014878	dla	none	sub	none	none	none	none	none	none	none	sub
ENSXMAG00000014878	no	18	no	no	no	no	no	no	no	ENSLOCG00000015493.ENSXMAG00000014878.ENSXMAG00000014461		none	sub	none	none	none	none	none	none	none	sub
ENSXMAG00000004343	no	21	15	no	no	18	no	no	no	ENSLOCG00000015521.ENSXMAG00000004343.ENSXMAG00000011342	CACNA2D1 (1 of many)	none	sub	sub	none	none	sub	none	none	none	sub
ENSXMAG00000011342	no	no	no	no	no	no	no	no	no	ENSLOCG00000015521.ENSXMAG00000011342.ENSXMAG00000004343	cacna2d1a	none	sub	sub	none	none	sub	none	none	none	sub
ENSXMAG00000004015	no	no	no	no	no	21	no	no	no	ENSLOCG00000015636.ENSXMAG00000004015.ENSXMAG00000030049		none	none	none	none	none	neo	none	none	none	sub
ENSXMAG00000030049	no	no	no	no	no	6	no	no	no	ENSLOCG00000015636.ENSXMAG00000030049.ENSXMAG00000004015	KCNIP3	none	none	none	none	none	neo	none	none	none	sub
ENSXMAG00000016859	no	no	no	no	no	15	no	no	no	ENSLOCG00000015690.ENSXMAG00000016859.ENSXMAG00000024223		none	none	none	none	none	sub	none	none	none	sub
ENSXMAG00000024223	no	no	no	no	no	no	no	no	no	ENSLOCG00000015690.ENSXMAG00000024223.ENSXMAG00000016859	celf2	none	none	none	none	none	sub	none	none	none	sub
ENSXMAG00000015733	no	no	no	no	no	no	12	no	no	ENSLOCG00000015740.ENSXMAG00000015733.ENSXMAG00000017251	slc8a1a	none	none	none	none	sub	none	no diff	none	none	sub
ENSXMAG00000017251	no	no	no	no	21	no	15	no	no	ENSLOCG00000015740.ENSXMAG00000017251.ENSXMAG00000015733	slc8a1b	none	none	none	none	sub	none	no diff	none	none	sub
ENSXMAG00000007586	no	no	no	no	no	no	6	no	no	ENSLOCG00000015822.ENSXMAG00000007586.ENSXMAG00000016455		none	none	none	none	none	none	sub	none	none	sub
ENSXMAG00000016455	no	no	no	no	no	no	no	no	no	ENSLOCG00000015822.ENSXMAG00000016455.ENSXMAG00000007586	hbp1	none	none	none	none	none	none	sub	none	none	sub
ENSXMAG00000008881	no	no	no	no	no	21	no	15	no	ENSLOCG00000015895.ENSXMAG00000008881.ENSXMAG00000015153	slc5a6a	none	none	none	none	none	sub	none	sub	none	sub
ENSXMAG00000015153	no	no	no	no	no	no	no	no	no	ENSLOCG00000015895.ENSXMAG00000015153.ENSXMAG00000008881	slc5a6b	none	none	none	none	none	sub	none	sub	none	sub
ENSXMAG00000000690	no	no	no	no	no	no	no	no	no	ENSLOCG00000015964.ENSXMAG00000000690.ENSXMAG00000007800		none	sub	none	none	sub	none	sub	sub	sub	sub
ENSXMAG00000007800	no	0	no	no	21	no	3	0	0	ENSLOCG00000015964.ENSXMAG00000007800.ENSXMAG00000000690	znf395b	none	sub	none	none	sub	none	sub	sub	sub	sub
ENSXMAG00000027974	no	no	no	no	6	3	3	no	no	ENSLOCG00000015996.ENSXMAG00000027974.ENSXMAG00000028171	fgl2a	none	none	none	none	sub	sub	sub	none	none	sub
ENSXMAG00000028171	no	no	no	no	no	no	no	no	no	ENSLOCG00000015996.ENSXMAG00000028171.ENSXMAG00000027974		none	none	none	none	sub	sub	sub	none	none	sub
ENSXMAG00000027245	no	no	no	no	no	15	no	no	no	ENSLOCG00000016046.ENSXMAG00000027245.ENSXMAG00000029191	aftpha	none	none	none	none	none	sub	none	none	none	sub
ENSXMAG00000029191	no	no	no	no	no	no	no	no	no	ENSLOCG00000016046.ENSXMAG00000029191.ENSXMAG00000027245		none	none	none	none	none	sub	none	none	none	sub
ENSXMAG00000005970	no	no	no	no	no	no	9	no	no	ENSLOCG00000016049.ENSXMAG00000005970.ENSXMAG00000011845	lgalsla	none	none	none	none	none	none	sub	none	none	sub
ENSXMAG00000011845	no	no	no	no	no	no	no	no	no	ENSLOCG00000016049.ENSXMAG00000011845.ENSXMAG00000005970	lgalslb	none	none	none	none	none	none	sub	none	none	sub
ENSXMAG00000020875	no	no	no	no	no	15	no	no	no	ENSLOCG00000016052.ENSXMAG00000020875.ENSXMAG00000029787	cerk	none	none	none	none	none	neo	none	none	none	sub
ENSXMAG00000029787	no	no	no	no	no	0	no	no	no	ENSLOCG00000016052.ENSXMAG00000029787.ENSXMAG00000020875		none	none	none	none	none	neo	none	none	none	sub
ENSXMAG00000017888	no	no	no	no	21	no	no	no	no	ENSLOCG00000016072.ENSXMAG00000017888.ENSXMAG00000025049		none	none	none	none	sub	none	none	none	none	sub
ENSXMAG00000025049	no	no	no	no	no	no	no	no	no	ENSLOCG00000016072.ENSXMAG00000025049.ENSXMAG00000017888	eif4ebp1	none	none	none	none	sub	none	none	none	none	sub
ENSXMAG00000002043	no	no	no	no	no	no	0	no	no	ENSLOCG00000016092.ENSXMAG00000002043.ENSXMAG00000028682	igsf9b	none	none	none	none	none	sub	sub	none	none	sub
ENSXMAG00000028682	no	no	no	no	no	15	no	no	no	ENSLOCG00000016092.ENSXMAG00000028682.ENSXMAG00000002043	igsf9a	none	none	none	none	none	sub	sub	none	none	sub
ENSXMAG00000007578	no	no	no	no	no	0	no	no	no	ENSLOCG00000016121.ENSXMAG00000007578.ENSXMAG00000023646	sash1a	none	none	none	none	no diff	none	none	none	none	sub
ENSXMAG00000023646	no	no	no	no	21	no	no	no	no	ENSLOCG00000016121.ENSXMAG00000023646.ENSXMAG00000007578	sash1b	none	none	none	none	no diff	none	none	none	none	sub
ENSXMAG00000005658	no	no	no	no	no	no	no	no	no	ENSLOCG00000016142.ENSXMAG00000005658.ENSXMAG00000023108	ccdc85a	none	none	none	none	none	sub	none	none	none	sub
ENSXMAG00000023108	no	no	no	no	no	15	no	no	no	ENSLOCG00000016142.ENSXMAG00000023108.ENSXMAG00000005658	ccdc85al	none	none	none	none	none	sub	none	none	none	sub
ENSXMAG00000012035	no	no	no	no	21	no	no	no	no	ENSLOCG00000016173.ENSXMAG00000012035.ENSXMAG00000024491		none	none	none	none	sub	none	none	none	none	sub
ENSXMAG00000024491	no	no	no	no	no	no	no	no	no	ENSLOCG00000016173.ENSXMAG00000024491.ENSXMAG00000012035	kcnq5a	none	none	none	none	sub	none	none	none	none	sub
ENSXMAG00000007246	no	no	no	no	no	18	no	no	no	ENSLOCG00000016181.ENSXMAG00000007246.ENSXMAG00000018211	rims1a	none	none	none	none	none	no diff	none	none	none	sub
ENSXMAG00000018211	no	no	no	no	no	15	no	no	no	ENSLOCG00000016181.ENSXMAG00000018211.ENSXMAG00000007246	RIMS1 (1 of many)	none	none	none	none	none	no diff	none	none	none	sub
ENSXMAG00000016365	no	no	no	no	no	3	6	no	no	ENSLOCG00000016219.ENSXMAG00000016365.ENSXMAG00000028202		none	none	none	none	none	sub	sub	sub	none	sub
ENSXMAG00000028202	no	no	no	no	no	no	no	9	no	ENSLOCG00000016219.ENSXMAG00000028202.ENSXMAG00000016365		none	none	none	none	none	sub	sub	sub	none	sub
ENSXMAG00000003229	no	no	no	no	no	no	21	no	no	ENSLOCG00000016263.ENSXMAG00000003229.ENSXMAG00000015466	tulp4a	none	none	none	none	none	none	sub	none	none	sub
ENSXMAG00000015466	no	no	no	no	no	no	no	no	no	ENSLOCG00000016263.ENSXMAG00000015466.ENSXMAG00000003229	tulp4b	none	none	none	none	none	none	sub	none	none	sub
ENSXMAG00000003189	no	no	no	no	18	18	no	no	no	ENSLOCG00000016271.ENSXMAG00000003189.ENSXMAG00000024565	myct1a	none	sub	none	none	sub	sub	none	none	none	sub
ENSXMAG00000024565	no	6	no	no	no	no	no	no	no	ENSLOCG00000016271.ENSXMAG00000024565.ENSXMAG00000003189	myct1b	none	sub	none	none	sub	sub	none	none	none	sub
ENSXMAG00000010885	no	no	no	no	15	21	no	no	no	ENSLOCG00000016276.ENSXMAG00000010885.ENSXMAG00000016119		none	none	none	none	sub	sub	none	none	none	sub
ENSXMAG00000016119	no	no	no	no	no	no	no	no	no	ENSLOCG00000016276.ENSXMAG00000016119.ENSXMAG00000010885	NRXN1	none	none	none	none	sub	sub	none	none	none	sub
ENSXMAG00000015620	no	no	no	no	no	no	no	no	no	ENSLOCG00000016317.ENSXMAG00000015620.ENSXMAG00000021324		none	none	none	none	none	none	none	sub	none	sub
ENSXMAG00000021324	no	no	no	no	no	no	no	15	no	ENSLOCG00000016317.ENSXMAG00000021324.ENSXMAG00000015620	dnmt3ab	none	none	none	none	none	none	none	sub	none	sub
ENSXMAG00000003995	no	no	no	no	no	no	no	no	no	ENSLOCG00000016368.ENSXMAG00000003995.ENSXMAG00000018765		none	none	none	sub	none	none	none	none	none	sub
ENSXMAG00000018765	no	no	no	9	no	no	no	no	no	ENSLOCG00000016368.ENSXMAG00000018765.ENSXMAG00000003995	kitlga	none	none	none	sub	none	none	none	none	none	sub
ENSXMAG00000009952	no	no	no	no	no	no	no	no	no	ENSLOCG00000016383.ENSXMAG00000009952.ENSXMAG00000017405		none	none	none	none	none	sub	none	none	none	sub
ENSXMAG00000017405	no	no	no	no	no	3	no	no	no	ENSLOCG00000016383.ENSXMAG00000017405.ENSXMAG00000009952	kcnk3a	none	none	none	none	none	sub	none	none	none	sub
ENSXMAG00000010752	no	no	no	no	no	6	no	no	no	ENSLOCG00000016480.ENSXMAG00000010752.ENSXMAG00000015237	vsnl1a	none	none	none	none	none	sub	none	none	none	sub
ENSXMAG00000015237	no	no	no	no	no	no	no	no	no	ENSLOCG00000016480.ENSXMAG00000015237.ENSXMAG00000010752		none	none	none	none	none	sub	none	none	none	sub
ENSXMAG00000021354	no	no	no	no	no	no	no	no	no	ENSLOCG00000016514.ENSXMAG00000021354.ENSXMAG00000025978	rspo3	none	none	sub	none	none	none	none	none	none	sub
ENSXMAG00000025978	no	no	6	no	no	no	no	no	no	ENSLOCG00000016514.ENSXMAG00000025978.ENSXMAG00000021354		none	none	sub	none	none	none	none	none	none	sub
ENSXMAG00000016785	no	0	no	no	no	no	no	no	no	ENSLOCG00000016561.ENSXMAG00000016785.ENSXMAG00000019075	sim1a	none	sub	none	none	none	none	none	none	none	sub
ENSXMAG00000019075	no	no	no	no	no	no	no	no	no	ENSLOCG00000016561.ENSXMAG00000019075.ENSXMAG00000016785		none	sub	none	none	none	none	none	none	none	sub
ENSXMAG00000011188	0	no	no	no	0	3	no	no	no	ENSLOCG00000016641.ENSXMAG00000011188.ENSXMAG00000025768	lrtm2a	sub	none	none	none	sub	sub	none	none	none	sub
ENSXMAG00000025768	no	no	no	no	no	no	no	no	no	ENSLOCG00000016641.ENSXMAG00000025768.ENSXMAG00000011188		sub	none	none	none	sub	sub	none	none	none	sub
ENSXMAG00000023214	no	no	no	no	no	no	no	no	no	ENSLOCG00000016645.ENSXMAG00000023214.ENSXMAG00000028462		none	none	none	none	sub	sub	none	none	none	sub
ENSXMAG00000028462	no	no	no	no	0	12	no	no	no	ENSLOCG00000016645.ENSXMAG00000028462.ENSXMAG00000023214	cnstb	none	none	none	none	sub	sub	none	none	none	sub
ENSXMAG00000007048	no	0	no	no	no	no	no	no	no	ENSLOCG00000016780.ENSXMAG00000007048.ENSXMAG00000015084	coq8a	none	sub	none	none	none	none	none	none	none	sub
ENSXMAG00000015084	no	no	no	no	no	no	no	no	no	ENSLOCG00000016780.ENSXMAG00000015084.ENSXMAG00000007048		none	sub	none	none	none	none	none	none	none	sub
ENSXMAG00000002960	no	no	no	no	no	no	21	no	no	ENSLOCG00000016828.ENSXMAG00000002960.ENSXMAG00000009810		none	none	none	none	none	sub	neo	none	none	sub
ENSXMAG00000009810	no	no	no	no	no	3	12	no	no	ENSLOCG00000016828.ENSXMAG00000009810.ENSXMAG00000002960	adam17b	none	none	none	none	none	sub	neo	none	none	sub
ENSXMAG00000007446	21	no	no	no	no	no	no	no	no	ENSLOCG00000016842.ENSXMAG00000007446.ENSXMAG00000009927	ZDHHC14 (1 of many)	sub	none	none	none	none	none	none	none	none	sub
ENSXMAG00000009927	no	no	no	no	no	no	no	no	no	ENSLOCG00000016842.ENSXMAG00000009927.ENSXMAG00000007446	zdhhc14	sub	none	none	none	none	none	none	none	none	sub
ENSXMAG00000006338	no	no	no	no	no	21	no	no	no	ENSLOCG00000016845.ENSXMAG00000006338.ENSXMAG00000009940		none	none	none	none	none	sub	none	none	none	sub
ENSXMAG00000009940	no	no	no	no	no	no	no	no	no	ENSLOCG00000016845.ENSXMAG00000009940.ENSXMAG00000006338	arid1b	none	none	none	none	none	sub	none	none	none	sub
ENSXMAG00000009976	0	no	no	no	no	21	no	no	no	ENSLOCG00000016852.ENSXMAG00000009976.ENSXMAG00000019210	TIAM2 (1 of many)	sub	none	none	none	none	sub	none	none	none	sub
ENSXMAG00000019210	no	no	no	no	no	no	no	no	no	ENSLOCG00000016852.ENSXMAG00000019210.ENSXMAG00000009976	tiam2a	sub	none	none	none	none	sub	none	none	none	sub
ENSXMAG00000010212	no	no	no	no	no	no	no	no	no	ENSLOCG00000016902.ENSXMAG00000010212.ENSXMAG00000014414	fam83b	none	none	none	none	none	none	sub	none	none	sub
ENSXMAG00000014414	no	no	no	no	no	no	0	no	no	ENSLOCG00000016902.ENSXMAG00000014414.ENSXMAG00000010212		none	none	none	none	none	none	sub	none	none	sub
ENSXMAG00000009234	no	no	no	no	no	3	no	no	no	ENSLOCG00000016939.ENSXMAG00000009234.ENSXMAG00000027658	gabrr2a	none	sub	none	none	none	no diff	none	none	none	sub
ENSXMAG00000027658	no	3	no	no	no	0	no	no	no	ENSLOCG00000016939.ENSXMAG00000027658.ENSXMAG00000009234		none	sub	none	none	none	no diff	none	none	none	sub
ENSXMAG00000003764	no	no	no	no	no	21	no	no	no	ENSLOCG00000017053.ENSXMAG00000003764.ENSXMAG00000008729	ANO2	none	none	none	none	none	sub	none	none	none	sub
ENSXMAG00000008729	no	no	no	no	no	no	no	no	no	ENSLOCG00000017053.ENSXMAG00000008729.ENSXMAG00000003764		none	none	none	none	none	sub	none	none	none	sub
ENSXMAG00000010369	no	no	no	no	no	no	0	no	no	ENSLOCG00000017178.ENSXMAG00000010369.ENSXMAG00000018611	kcnk5b	none	none	none	none	none	sub	sub	none	none	sub
ENSXMAG00000018611	no	no	no	no	no	21	no	no	no	ENSLOCG00000017178.ENSXMAG00000018611.ENSXMAG00000010369	kcnk5a	none	none	none	none	none	sub	sub	none	none	sub
ENSXMAG00000021472	no	no	no	no	no	no	no	no	no	ENSLOCG00000017188.ENSXMAG00000021472.ENSXMAG00000022080	RDH14 (1 of many)	none	none	none	sub	none	none	none	none	none	sub
ENSXMAG00000022080	no	no	no	6	no	no	no	no	no	ENSLOCG00000017188.ENSXMAG00000022080.ENSXMAG00000021472	rdh14b	none	none	none	sub	none	none	none	none	none	sub
ENSXMAG00000002367	no	no	no	no	no	no	no	no	no	ENSLOCG00000017216.ENSXMAG00000002367.ENSXMAG00000028196	myt1la	none	none	none	none	none	sub	none	none	none	sub
ENSXMAG00000028196	no	no	no	no	no	15	no	no	no	ENSLOCG00000017216.ENSXMAG00000028196.ENSXMAG00000002367	MYT1L (1 of many)	none	none	none	none	none	sub	none	none	none	sub
ENSXMAG00000021883	no	no	no	no	no	no	no	no	no	ENSLOCG00000017299.ENSXMAG00000021883.ENSXMAG00000024962		none	none	none	none	none	none	none	sub	none	sub
ENSXMAG00000024962	no	no	no	no	no	no	no	15	no	ENSLOCG00000017299.ENSXMAG00000024962.ENSXMAG00000021883	mylpfa	none	none	none	none	none	none	none	sub	none	sub
ENSXMAG00000021789	no	no	no	no	no	no	no	no	no	ENSLOCG00000017388.ENSXMAG00000021789.ENSXMAG00000028778		none	none	none	none	none	sub	none	none	none	sub
ENSXMAG00000028778	no	no	no	no	no	15	no	no	no	ENSLOCG00000017388.ENSXMAG00000028778.ENSXMAG00000021789		none	none	none	none	none	sub	none	none	none	sub
ENSXMAG00000019016	no	no	no	no	no	no	no	no	no	ENSLOCG00000017443.ENSXMAG00000019016.ENSXMAG00000029906		none	none	sub	none	none	none	none	none	none	sub
ENSXMAG00000029906	no	no	12	no	no	no	no	no	no	ENSLOCG00000017443.ENSXMAG00000029906.ENSXMAG00000019016		none	none	sub	none	none	none	none	none	none	sub
ENSXMAG00000020084	3	no	no	no	no	no	no	no	no	ENSLOCG00000017628.ENSXMAG00000020084.ENSXMAG00000025875	JUND	sub	none	none	none	none	none	none	none	none	sub
ENSXMAG00000025875	no	no	no	no	no	no	no	no	no	ENSLOCG00000017628.ENSXMAG00000025875.ENSXMAG00000020084		sub	none	none	none	none	none	none	none	none	sub
ENSXMAG00000019679	no	no	no	no	no	18	no	no	no	ENSLOCG00000017699.ENSXMAG00000019679.ENSXMAG00000020345	flrt2	none	sub	none	none	none	neo	none	none	none	sub
ENSXMAG00000020345	no	6	no	no	no	0	no	no	no	ENSLOCG00000017699.ENSXMAG00000020345.ENSXMAG00000019679	flrt2	none	sub	none	none	none	neo	none	none	none	sub
ENSXMAG00000011339	no	no	no	no	no	21	no	no	no	ENSLOCG00000017739.ENSXMAG00000011339.ENSXMAG00000019647	TMEM125	none	none	none	none	none	sub	none	none	none	sub
ENSXMAG00000019647	no	no	no	no	no	no	no	no	no	ENSLOCG00000017739.ENSXMAG00000019647.ENSXMAG00000011339	tmem125b	none	none	none	none	none	sub	none	none	none	sub
ENSXMAG00000019972	no	no	no	no	no	no	no	no	no	ENSLOCG00000017745.ENSXMAG00000019972.ENSXMAG00000028941		none	sub	none	none	none	sub	none	none	none	sub
ENSXMAG00000028941	no	3	no	no	no	21	no	no	no	ENSLOCG00000017745.ENSXMAG00000028941.ENSXMAG00000019972	jun	none	sub	none	none	none	sub	none	none	none	sub
ENSXMAG00000019454	no	no	no	no	no	no	no	no	no	ENSLOCG00000017759.ENSXMAG00000019454.ENSXMAG00000019871	cdc42ep4a	none	none	none	none	none	sub	none	none	none	sub
ENSXMAG00000019871	no	no	no	no	no	3	no	no	no	ENSLOCG00000017759.ENSXMAG00000019871.ENSXMAG00000019454	cdc42ep4b	none	none	none	none	none	sub	none	none	none	sub
ENSXMAG00000027985	no	no	no	no	no	18	no	no	no	ENSLOCG00000017919.ENSXMAG00000027985.ENSXMAG00000029210	fbxl14a	none	none	none	none	none	sub	none	none	none	sub
ENSXMAG00000029210	no	no	no	no	no	no	no	no	no	ENSLOCG00000017919.ENSXMAG00000029210.ENSXMAG00000027985	fbxl14b	none	none	none	none	none	sub	none	none	none	sub
ENSXMAG00000009062	no	no	no	3	no	no	no	no	no	ENSLOCG00000017940.ENSXMAG00000009062.ENSXMAG00000020192		none	none	none	sub	none	none	none	none	none	sub
ENSXMAG00000020192	no	no	no	no	no	no	no	no	no	ENSLOCG00000017940.ENSXMAG00000020192.ENSXMAG00000009062	rbm15	none	none	none	sub	none	none	none	none	none	sub
ENSXMAG00000019909	no	no	no	no	no	no	no	no	no	ENSLOCG00000018038.ENSXMAG00000019909.ENSXMAG00000028511	ADRA2C	none	none	none	none	none	sub	none	none	none	sub
ENSXMAG00000028511	no	no	no	no	no	21	no	no	no	ENSLOCG00000018038.ENSXMAG00000028511.ENSXMAG00000019909	adra2c	none	none	none	none	none	sub	none	none	none	sub
ENSXMAG00000010941	no	no	no	no	no	no	no	no	no	ENSLOCG00000018113.ENSXMAG00000010941.ENSXMAG00000018562		none	none	none	none	none	sub	none	none	none	sub
ENSXMAG00000018562	no	no	no	no	no	15	no	no	no	ENSLOCG00000018113.ENSXMAG00000018562.ENSXMAG00000010941		none	none	none	none	none	sub	none	none	none	sub
ENSXMAG00000016113	no	no	no	no	no	no	no	no	no	ENSLOCG00000018139.ENSXMAG00000016113.ENSXMAG00000023193	cldn5b	none	none	none	none	none	none	sub	none	none	sub
ENSXMAG00000023193	no	no	no	no	no	no	18	no	no	ENSLOCG00000018139.ENSXMAG00000023193.ENSXMAG00000016113	cldn5a	none	none	none	none	none	none	sub	none	none	sub
ENSXMAG00000020272	no	no	no	no	no	15	no	no	no	ENSLOCG00000018210.ENSXMAG00000020272.ENSXMAG00000027447		none	none	none	none	none	sub	none	none	none	sub
ENSXMAG00000027447	no	no	no	no	no	no	no	no	no	ENSLOCG00000018210.ENSXMAG00000027447.ENSXMAG00000020272	neurod4	none	none	none	none	none	sub	none	none	none	sub
ENSXMAG00000019856	no	no	no	no	no	no	no	no	no	ENSLOCG00000018229.ENSXMAG00000019856.ENSXMAG00000020102	SLC18A3	none	none	none	none	none	none	none	sub	none	sub
ENSXMAG00000020102	no	no	no	no	no	no	no	3	no	ENSLOCG00000018229.ENSXMAG00000020102.ENSXMAG00000019856	slc18a3a	none	none	none	none	none	none	none	sub	none	sub
ENSXMAG00000014234	no	no	no	no	no	no	no	no	no	ENSLOCG00000018234.ENSXMAG00000014234.ENSXMAG00000020001	adoa	none	none	none	none	none	none	sub	none	none	sub
ENSXMAG00000020001	no	no	no	no	no	no	15	no	no	ENSLOCG00000018234.ENSXMAG00000020001.ENSXMAG00000014234	adob	none	none	none	none	none	none	sub	none	none	sub
ENSXMAG00000005127	12	12	12	9	12	12	12	12	no	ENSLOCG00000018299.ENSXMAG00000005127.ENSXMAG00000019530	nfil3-5	no diff	no diff	sub	sub	sub	no diff	no diff	no diff	none	sub
ENSXMAG00000019530	15	15	no	no	no	12	15	15	no	ENSLOCG00000018299.ENSXMAG00000019530.ENSXMAG00000005127		no diff	no diff	sub	sub	sub	no diff	no diff	no diff	none	sub
ENSXMAG00000025105	no	no	no	15	no	no	no	no	no	ENSLOCG00000018304.ENSXMAG00000025105.ENSXMAG00000025956	si:ch1073-44g3.1	none	none	none	sub	none	none	none	none	none	sub
ENSXMAG00000025956	no	no	no	no	no	no	no	no	no	ENSLOCG00000018304.ENSXMAG00000025956.ENSXMAG00000025105	zgc:77058	none	none	none	sub	none	none	none	none	none	sub
ENSXMAG00000008654	no	no	no	no	no	no	no	no	no	ENSLOCG00000018342.ENSXMAG00000008654.ENSXMAG00000020332	zgc:110182	none	none	none	none	none	sub	none	none	none	sub
ENSXMAG00000020332	no	no	no	no	no	15	no	no	no	ENSLOCG00000018342.ENSXMAG00000020332.ENSXMAG00000008654	fam84b	none	none	none	none	none	sub	none	none	none	sub
ENSXMAG00000002730	no	no	no	no	no	no	15	no	no	ENSLOCG00000018353.ENSXMAG00000002730.ENSXMAG00000019568		none	none	none	none	none	none	sub	sub	none	sub
ENSXMAG00000019568	no	no	no	no	no	no	no	12	no	ENSLOCG00000018353.ENSXMAG00000019568.ENSXMAG00000002730	slc5a3b	none	none	none	none	none	none	sub	sub	none	sub
										**Summary Table:**	**not circadian**	**1104**	**1066**	**1198**	**1140**	**876**	**452**	**1014**	**1114**	**1198**	
											**no diff (gene)**	**8**	**4**	**0**	**0**	**20**	**110**	**8**	**4**	**0**	
											**sub (gene)**	**136**	**178**	**54**	**112**	**340**	**626**	**226**	**134**	**54**	
											**neo (gene)**	**4**	**4**	**0**	**0**	**16**	**64**	**4**	**0**	**0**	
											**sub%neo.ratio**	**94.6%**	**97.8%**	**100.0%**	**100.0%**	**94.7%**	**86.3%**	**96.6%**	**97.1%**	**100.0%**	
