# Supplementary material for: Global assessment of organ specific basal gene expression over a diurnal cycle with analyses of gene copies exhibiting cyclic expression patterns
Source: BMC Genomics. 2020 Nov 11;21:787. doi: 10.1186/s12864-020-07202-9 (PMC7659085; doi:10.1186/s12864-020-07202-9)
Supplement: Supplementary file 9 — Additional file 9: Supplement Table 8. Circadian Ohnologs in zebrafish brain and pineal gland. [file 12864_2020_7202_MOESM9_ESM.pdf]

Supplement Table 8

Brain

CGDB.ID	Protein.Nam	Organism	Phase	Peak	Trough	Amplitude.Fc	ensembl_gene_id	Gar Gene ID	functional divergence
CGD-DaR-006813	Uncharacteri	Danio rerio	ZT 12 16	12	16	1.17	ENSDARG00000068894	ENSLOCG00000000906	subfunctionalization
CGD-DaR-006829	Uncharacteri	Danio rerio	ZT 0 20	0	20	1.32	ENSDARG00000019949	ENSLOCG00000000987	subfunctionalization
CGD-DaR-006849	Uncharacteri	Danio rerio	ZT 0 20	0	20	1.06	ENSDARG00000012005	ENSLOCG00000001539	subfunctionalization
CGD-DaR-006866	Ras-like prot	Danio rerio	ZT 0 20	0	20	1.04	ENSDARG00000007396	ENSLOCG00000001573	subfunctionalization
CGD-DaR-006815	Influenza viru	Danio rerio	ZT 0 8	0	8	1.12	ENSDARG00000013946	ENSLOCG00000001731	subfunctionalization
CGD-DaR-006799	Uncharacteri	Danio rerio	ZT 4 20	4	20	1.14	ENSDARG00000044511	ENSLOCG00000003519	subfunctionalization
CGD-DaR-006968	Bmal1	Danio rerio	ZT 12 0	12	0	1.26	ENSDARG00000006791	ENSLOCG00000003999	superfunctionalization
CGD-DaR-006772	Uncharacteri	Danio rerio	ZT 12 0	12	0	1.62	ENSDARG00000035732	ENSLOCG00000003999	superfunctionalization
CGD-DaR-006834	Uncharacteri	Danio rerio	ZT 12 0	12	0	1.05	ENSDARG00000068716	ENSLOCG00000004806	subfunctionalization
CGD-DaR-006816	Uridine kinas	Danio rerio	ZT 4 16	4	16	1.06	ENSDARG00000078973	ENSLOCG00000004906	subfunctionalization
CGD-DaR-006916	Pancreatic pr	Danio rerio	ZT 20 4	20	4	1.03	ENSDARG00000031317	ENSLOCG00000005173	subfunctionalization
CGD-DaR-006814	Uncharacteri	Danio rerio	ZT 0 12	0	12	1.2	ENSDARG00000059719	ENSLOCG00000005396	subfunctionalization
CGD-DaR-038822	ppp1r3cb	Danio rerio	ZT 8 20	8	20	1.07	ENSDARG00000014554	ENSLOCG00000006006	subfunctionalization
CGD-DaR-006901	Ras-GEF don	Danio rerio	ZT 0 4	0	4	1.04	ENSDARG00000033614	ENSLOCG00000007128	subfunctionalization
CGD-DaR-006781	Uncharacteri	Danio rerio	ZT 0 16	0	16	1.09	ENSDARG00000074287	ENSLOCG00000008789	subfunctionalization
CGD-DaR-006743	Myosin, heav	Danio rerio	ZT 4 20	4	20	1.06	ENSDARG00000009782	ENSLOCG00000009053	subfunctionalization
CGD-DaR-006889	Uncharacteri	Danio rerio	ZT 0 16	0	16	1.08	ENSDARG00000006508	ENSLOCG00000009412	superfunctionalization
CGD-DaR-006887	Phosphatidyl	Danio rerio	ZT 0 12	0	12	1.05	ENSDARG00000044295	ENSLOCG00000009412	superfunctionalization
CGD-DaR-006917	Uncharacteri	Danio rerio	ZT 0 8	0	8	1.16	ENSDARG00000018898	ENSLOCG00000009585	subfunctionalization
CGD-DaR-006828	Uncharacteri	Danio rerio	ZT 12 0	12	0	1.08	ENSDARG00000014180	ENSLOCG00000009660	subfunctionalization
CGD-DaR-006761	Uncharacteri	Danio rerio	ZT 4 16	4	16	1.15	ENSDARG00000041394	ENSLOCG00000009935	subfunctionalization
CGD-DaR-006945	Heat shock p	Danio rerio	ZT 4 20	4	20	1.14	ENSDARG00000004754	ENSLOCG00000010589	subfunctionalization
CGD-DaR-044024	mycb	Danio rerio	ZT 8 20	8	20	1.04	ENSDARG00000007241	ENSLOCG00000011270	subfunctionalization
CGD-DaR-052456	tefa	Danio rerio	ZT 4 16	4	16	1.13	ENSDARG00000039117	ENSLOCG00000011595	subfunctionalization
CGD-DaR-072764		Danio rerio	ZT 0 12	0	12	1.25	ENSDARG00000012499	ENSLOCG00000013344	superfunctionalization
CGD-DaR-006939	Uncharacteri	Danio rerio	ZT 0 12	0	12	1.11	ENSDARG00000056885	ENSLOCG00000013344	superfunctionalization
CGD-DaR-006951	Monoacylglyc	Danio rerio	ZT 4 12	4	12	1.1	ENSDARG00000025797	ENSLOCG00000014235	subfunctionalization
CGD-DaR-006806	Tetraspanin	Danio rerio	ZT 12 0	12	0	1.06	ENSDARG00000053559	ENSLOCG00000014685	subfunctionalization
CGD-DaR-006811	Uncharacteri	Danio rerio	ZT 12 0	12	0	1.46	ENSDARG00000058103	ENSLOCG00000015333	subfunctionalization
CGD-DaR-006805	Delta-like pr	Danio rerio	ZT 12 16	12	16	1.28	ENSDARG00000030289	ENSLOCG00000015958	subfunctionalization
CGD-DaR-006876	Uncharacteri	Danio rerio	ZT 0 8	0	8	1.18	ENSDARG00000024195	ENSLOCG00000015964	subfunctionalization
CGD-DaR-006872	Uncharacteri	Danio rerio	ZT 0 20	0	20	1.04	ENSDARG00000002656	ENSLOCG00000016129	subfunctionalization
CGD-DaR-006764	Uncharacteri	Danio rerio	ZT 0 20	0	20	1.16	ENSDARG00000078992	ENSLOCG00000016941	subfunctionalization
CGD-DaR-006904	Tetraspanin	Danio rerio	ZT 12 0	12	0	1.03	ENSDARG00000005842	ENSLOCG00000017037	subfunctionalization
CGD-DaR-006800	Uncharacteri	Danio rerio	ZT 12 0	12	0	1.11	ENSDARG00000077513	ENSLOCG00000018359	subfunctionalization

Pineal gland

CGDB.ID	Protein.Nam	Organism	Phase	Peak	Trough	Amplitude.Fc	ensembl_gene_id	Gar Gene ID	
CGD-DaR-031579	arntl1a	Danio rerio	CT 6 18	6	18	18.89	ENSDARG00000006791	ENSLOCG00000003999	subfunctionalzation
CGD-DaR-006830	Uncharacteri	Danio rerio	CT 10 22	10	22	18.67	ENSDARG00000010420	ENSLOCG00000007343	subfunctionalzation
CGD-DaR-006846	Progressive a	Danio rerio	CT 10 22	10	22	2.05	ENSDARG00000014969	ENSLOCG00000011283	subfunctionalzation
CGD-DaR-006936	Transducer o	Danio rerio	CT 18 6	18	6	2.86	ENSDARG00000021372	ENSLOCG00000010978	subfunctionalzation
CGD-DaR-006954	SH3-domain	Danio rerio	CT 22 10	22	10	5.46	ENSDARG00000023600	ENSLOCG00000011923	subfunctionalzation
