# Supplementary material for: Global assessment of organ specific basal gene expression over a diurnal cycle with analyses of gene copies exhibiting cyclic expression patterns
Source: BMC Genomics. 2020 Nov 11;21:787. doi: 10.1186/s12864-020-07202-9 (PMC7659085; doi:10.1186/s12864-020-07202-9)
Supplement: Supplementary file 10 — Additional file 10: Supplement Table 9. Genes showing circadian expression pattern in zebrafish brain and pineal gland. [file 12864_2020_7202_MOESM10_ESM.pdf]

Supplement Table 9

Brain and Pineal gland shared circadain genes

CGDB.ID	Protein.Name	Organism	Phase	Peak	Trough	Amplitude.F(	Tissue.Cell	ensembl_gene_id
CGD-DaR-072766		Danio rerio	ZT 0 16	0	16	1.22	brain	ENSDARG00000004060
CGD-DaR-006968	Bmal1	Danio rerio	ZT 12 0	12	0	1.26	brain	ENSDARG00000006791
CGD-DaR-006820	Mid1-interacting protein 1-like	Danio rerio	ZT 4 16	4	16	1.05	brain	ENSDARG00000018145
CGD-DaR-006874	Uncharacterized protein	Danio rerio	ZT 0 8	0	8	1.11	brain	ENSDARG00000027316
CGD-DaR-006864	Cry2a protein	Danio rerio	ZT 9 21	9	21	12	brain	ENSDARG00000069074
CGD-DaR-006927	F-box only protein 25	Danio rerio	ZT 20 8	20	8	1.18	brain	ENSDARG00000075172
CGD-DaR-006972	Arylalkylamine N-acetyltransferase	Danio rerio	ZT 15 3	15	3	2	brain	ENSDARG00000079802
CGD-DaR-072766		Danio rerio	CT 18 6	18	6	37.6	pineal gland	ENSDARG00000004060
CGD-DaR-031579	arntl1a	Danio rerio	CT 6 18	6	18	18.89	pineal gland	ENSDARG00000006791
CGD-DaR-006820	Mid1-interacting protein 1-like	Danio rerio	CT 18 6	18	6	4.3	pineal gland	ENSDARG00000018145
CGD-DaR-006874	Uncharacterized protein	Danio rerio	CT 14 2	14	2	2.15	pineal gland	ENSDARG00000027316
CGD-DaR-006736	Uncharacterized protein	Danio rerio	CT 10 22	10	22	7.17	pineal gland	ENSDARG00000069074
CGD-DaR-006927	F-box only protein 25	Danio rerio	CT 18 6	18	6	2.97	pineal gland	ENSDARG00000075172
CGD-DaR-006972	Arylalkylamine N-acetyltransferase	Danio rerio	CT 14 2	14	2	5.78	pineal gland	ENSDARG00000079802
