# Supplementary material for: Global assessment of organ specific basal gene expression over a diurnal cycle with analyses of gene copies exhibiting cyclic expression patterns
Source: BMC Genomics. 2020 Nov 11;21:787. doi: 10.1186/s12864-020-07202-9 (PMC7659085; doi:10.1186/s12864-020-07202-9)

Ohnolog 1: ENSXMAG00000022293
Ohnolog 2: ENSXMAG00000024612

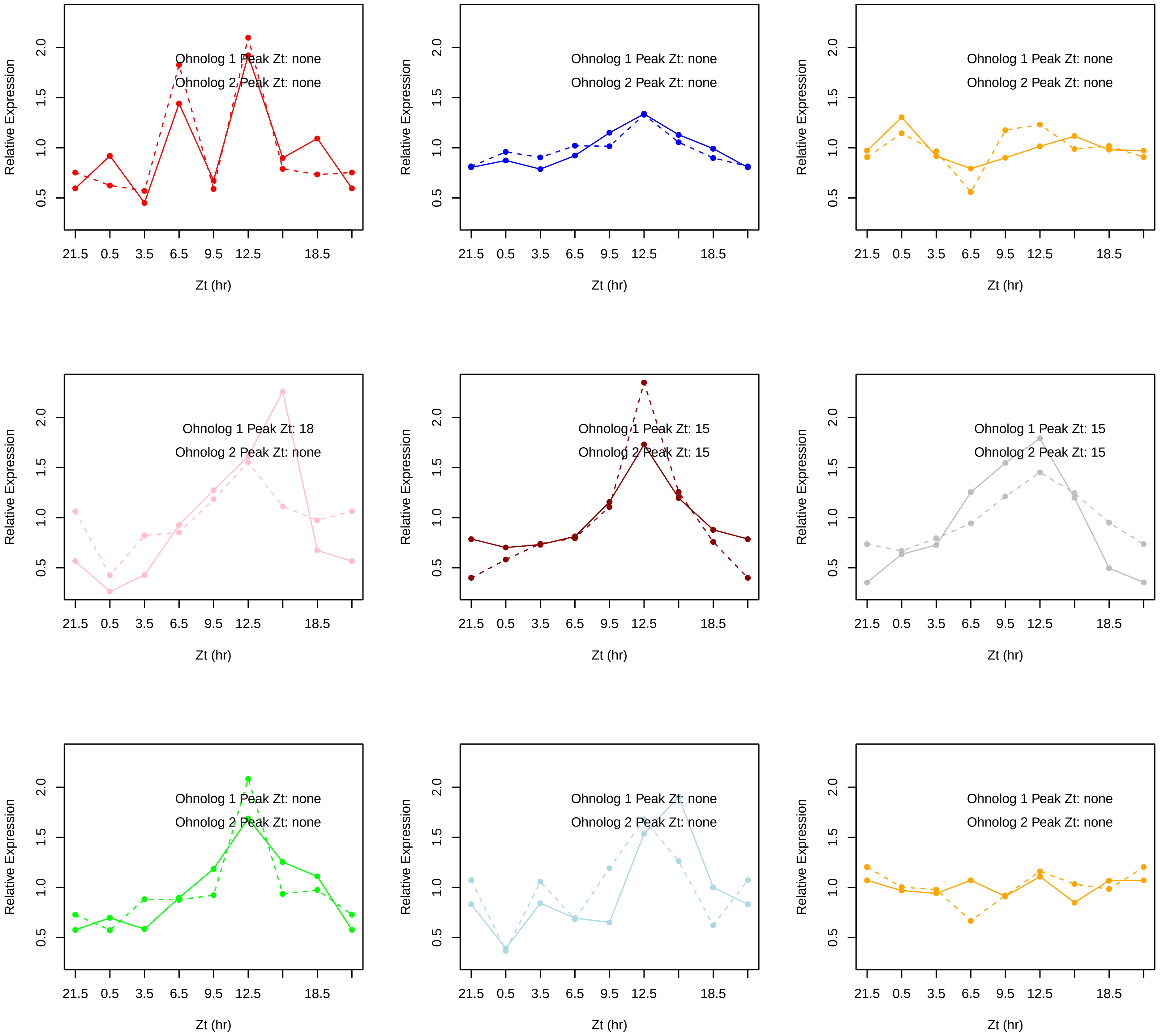

Ohnolog 1: ENSXMAG00000005497 cdc14ab
Ohnolog 2: ENSXMAG00000014128

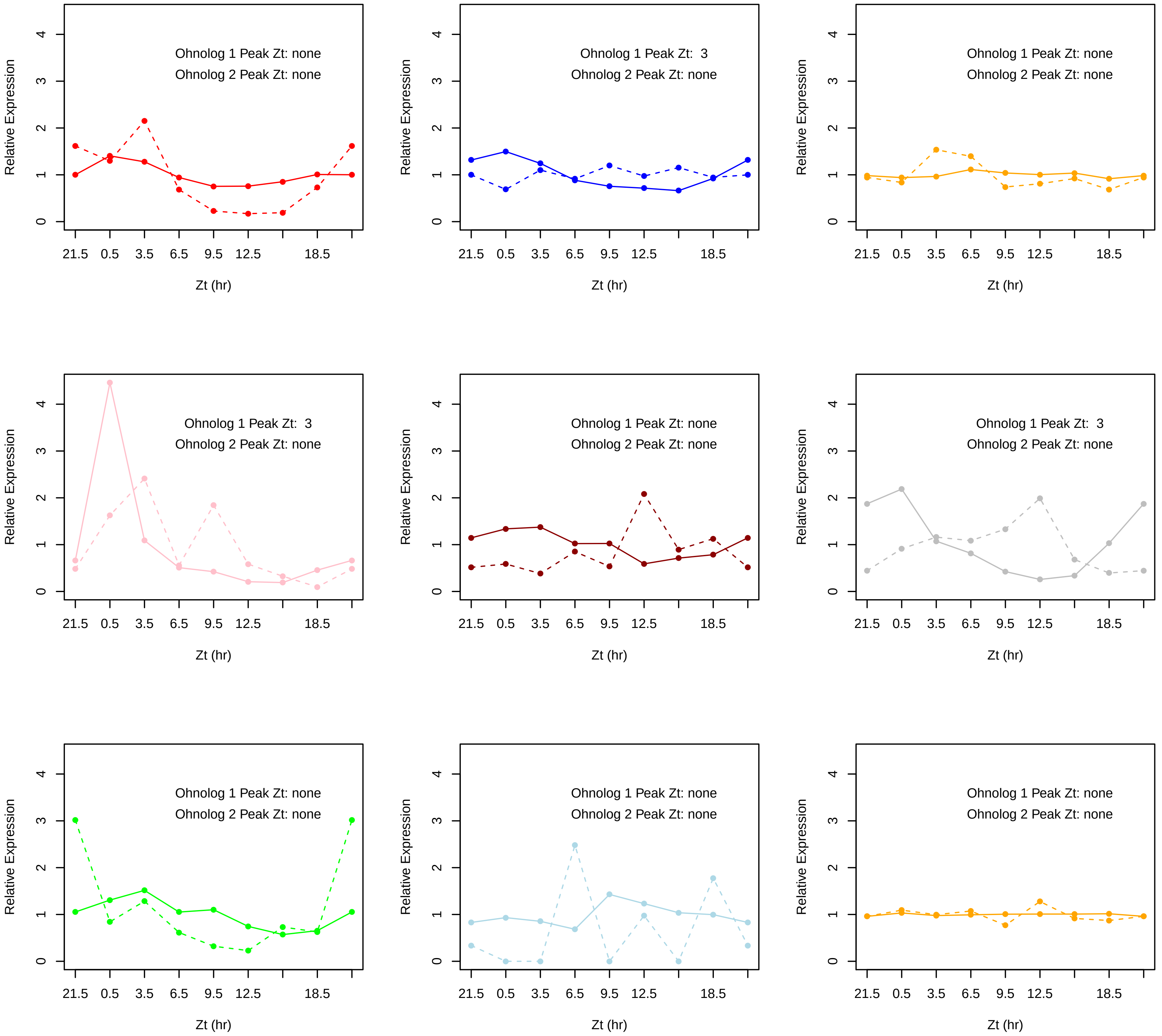

Ohnolog 1: ENSXMAG00000026493 rem2
Ohnolog 2: ENSXMAG00000027141

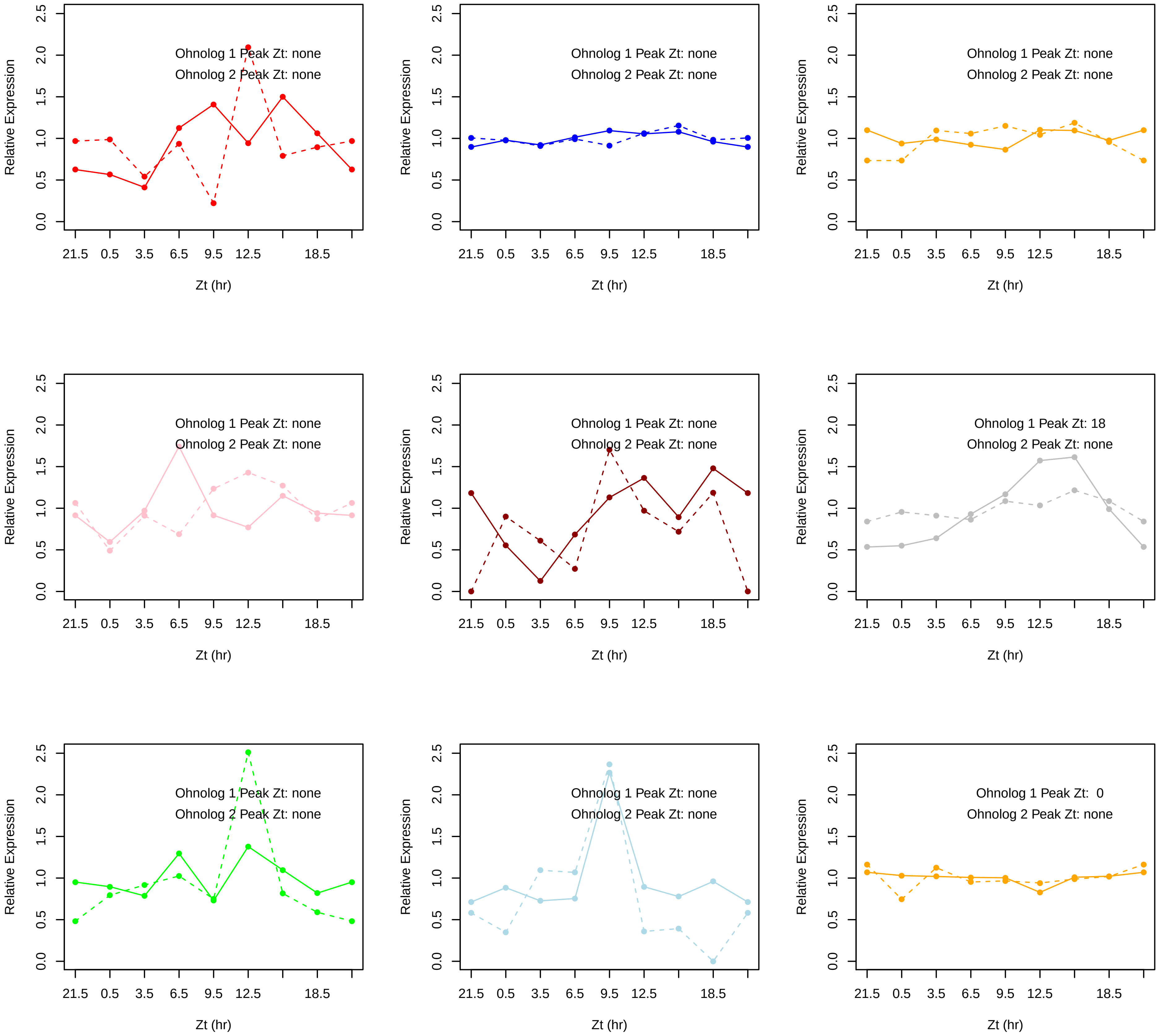

Ohnolog 1: ENSXMAG00000006023 carmil3
Ohnolog 2: ENSXMAG00000016807 CARMIL3

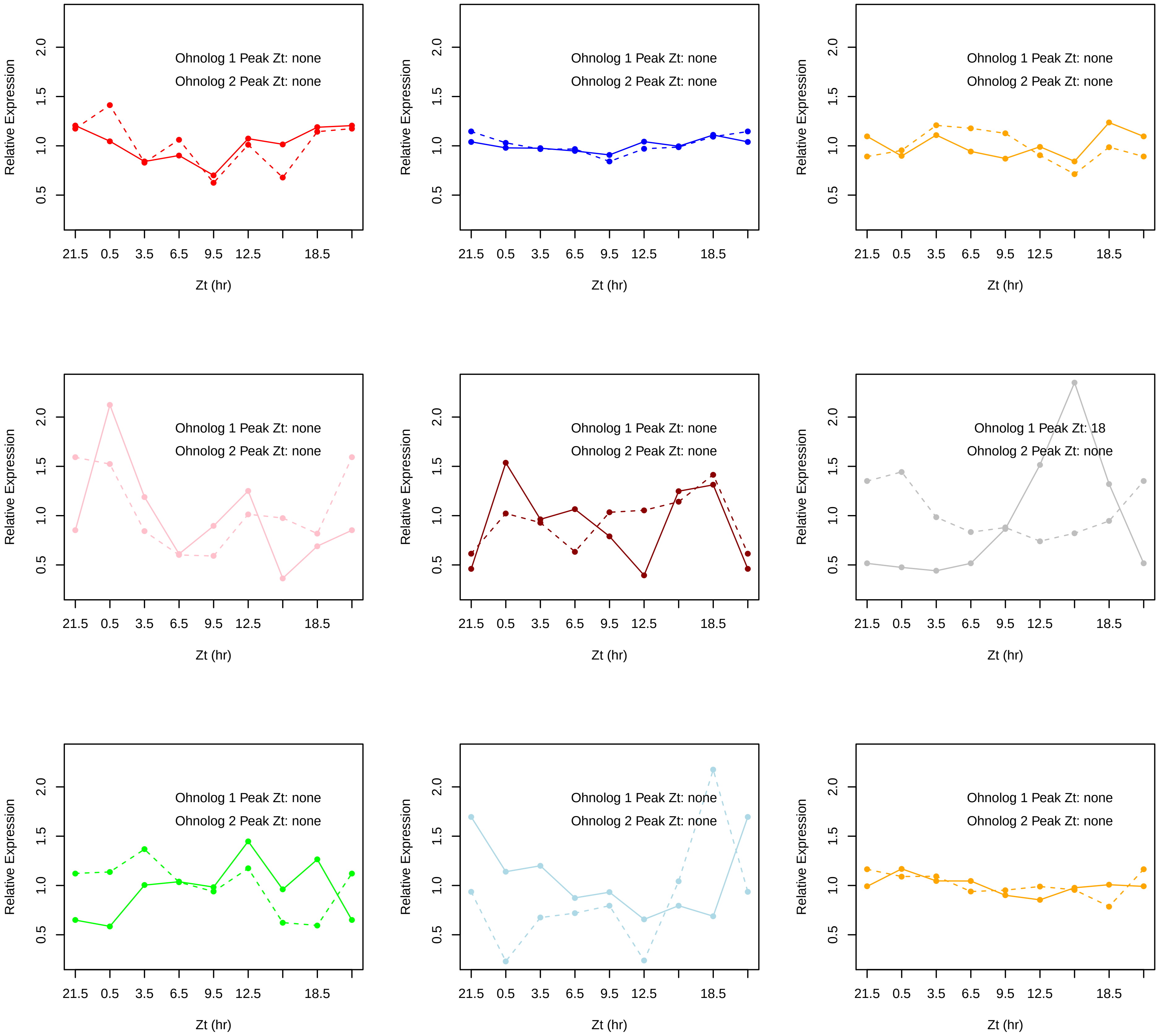

Ohnolog 1: ENSXMAG00000011992
Ohnolog 2: ENSXMAG00000029414

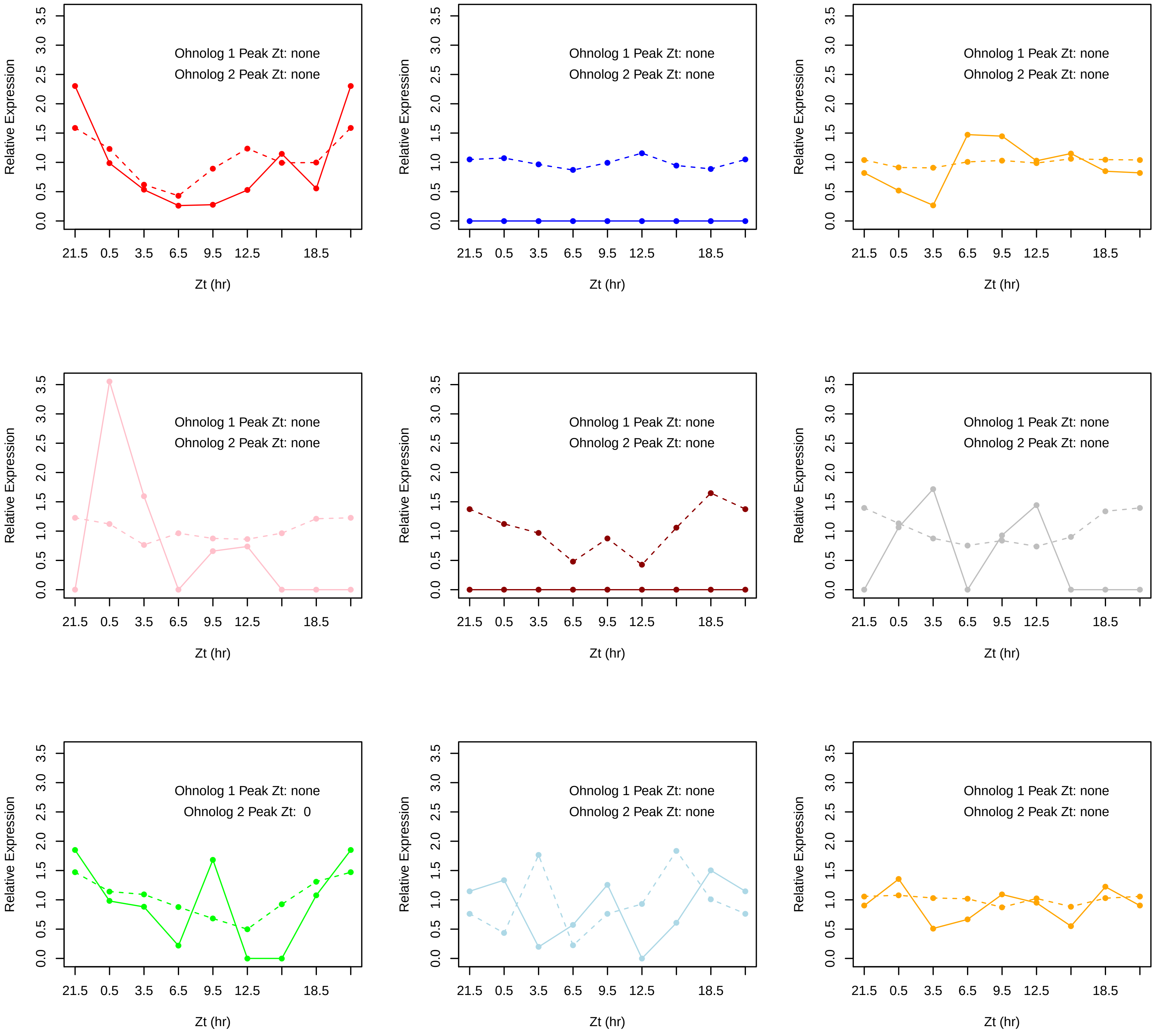

Ohnolog 1: ENSXMAG00000011663 slc6a16a
Ohnolog 2: ENSXMAG00000022899 slc6a16b

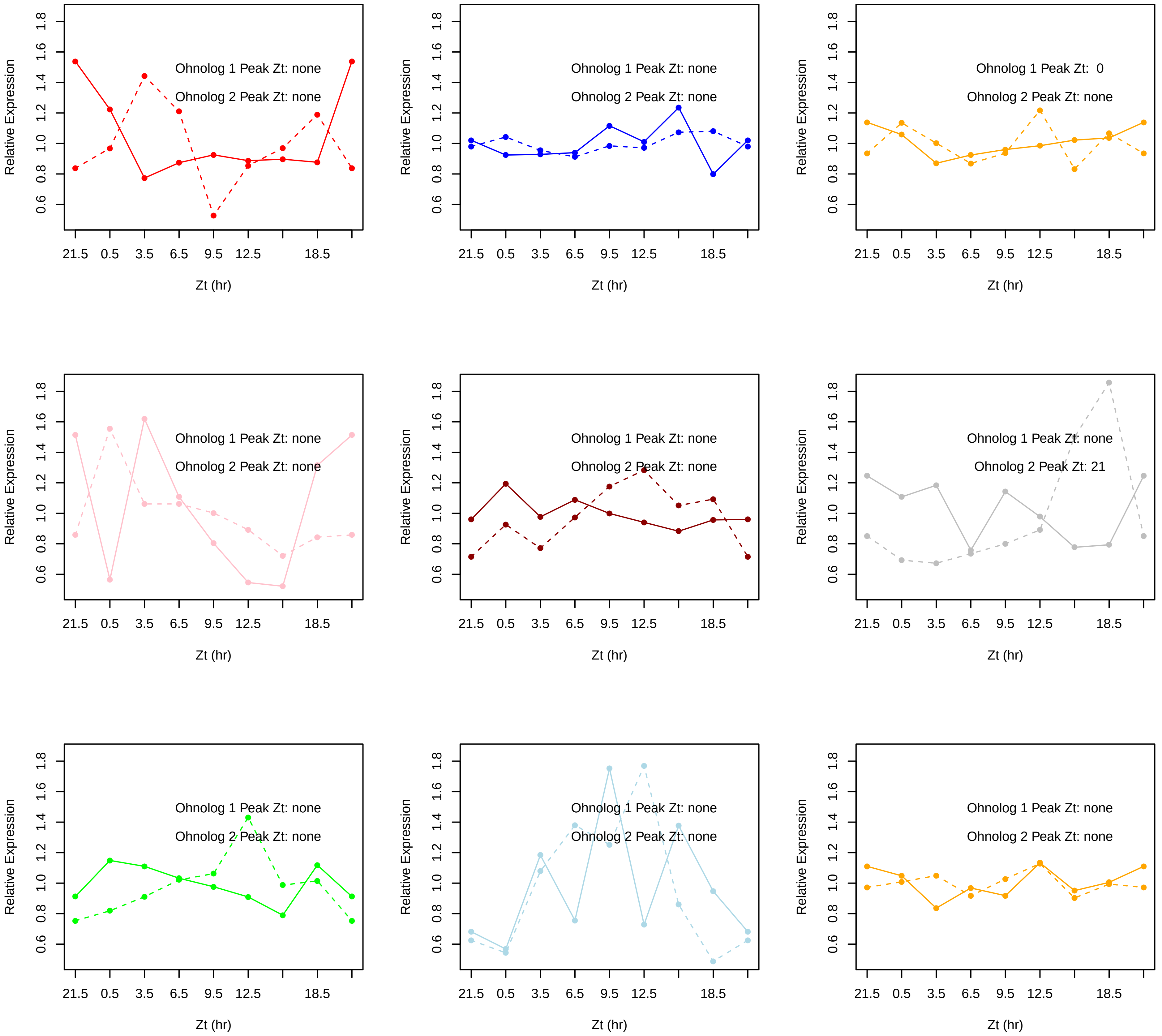

Ohnolog 1: ENSXMAG00000006078 si:zfos−943e10.1
Ohnolog 2: ENSXMAG00000019289

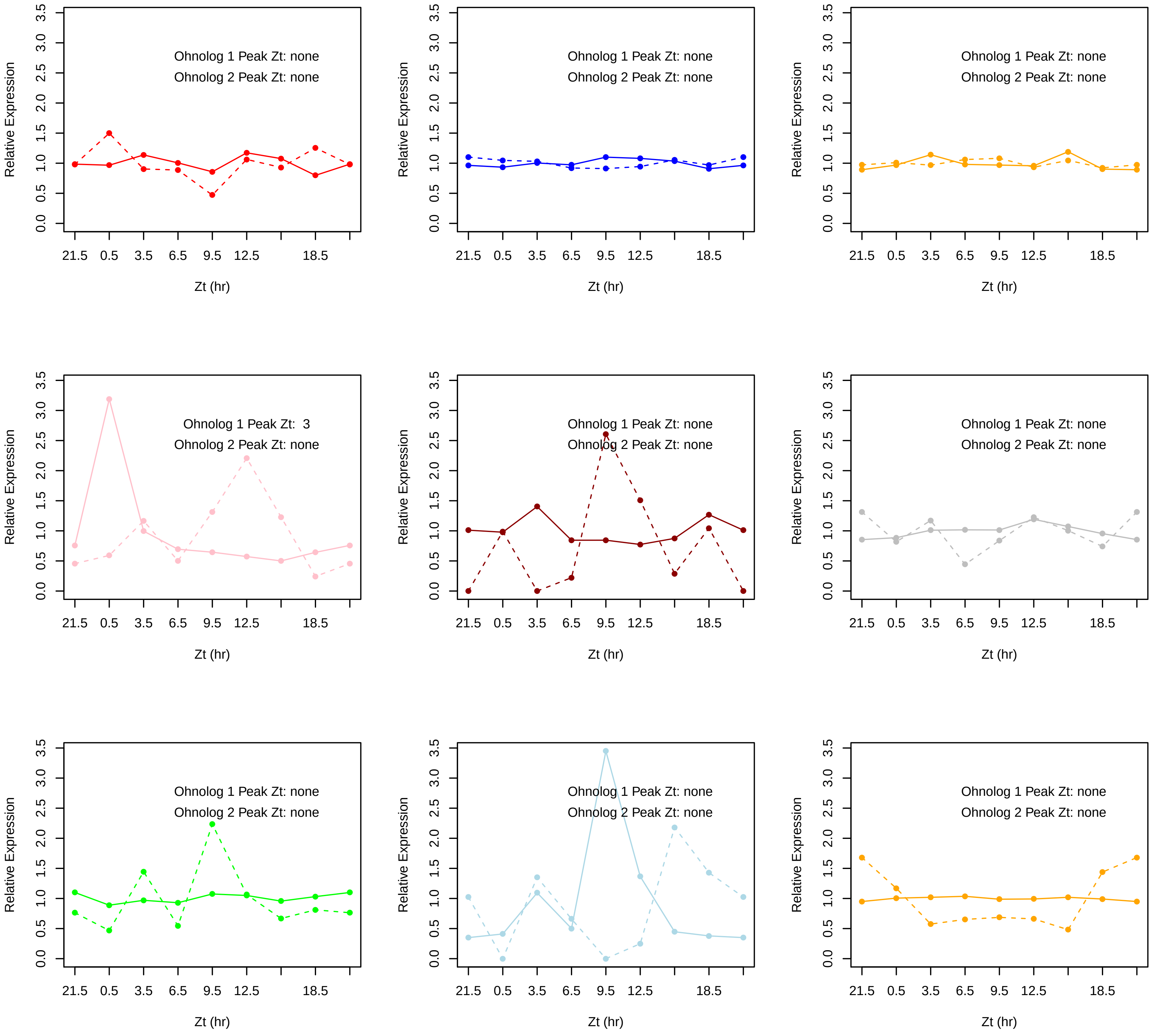

Ohnolog 1: ENSXMAG00000022622 KCNK4
Ohnolog 2: ENSXMAG00000029196 kcnk4a

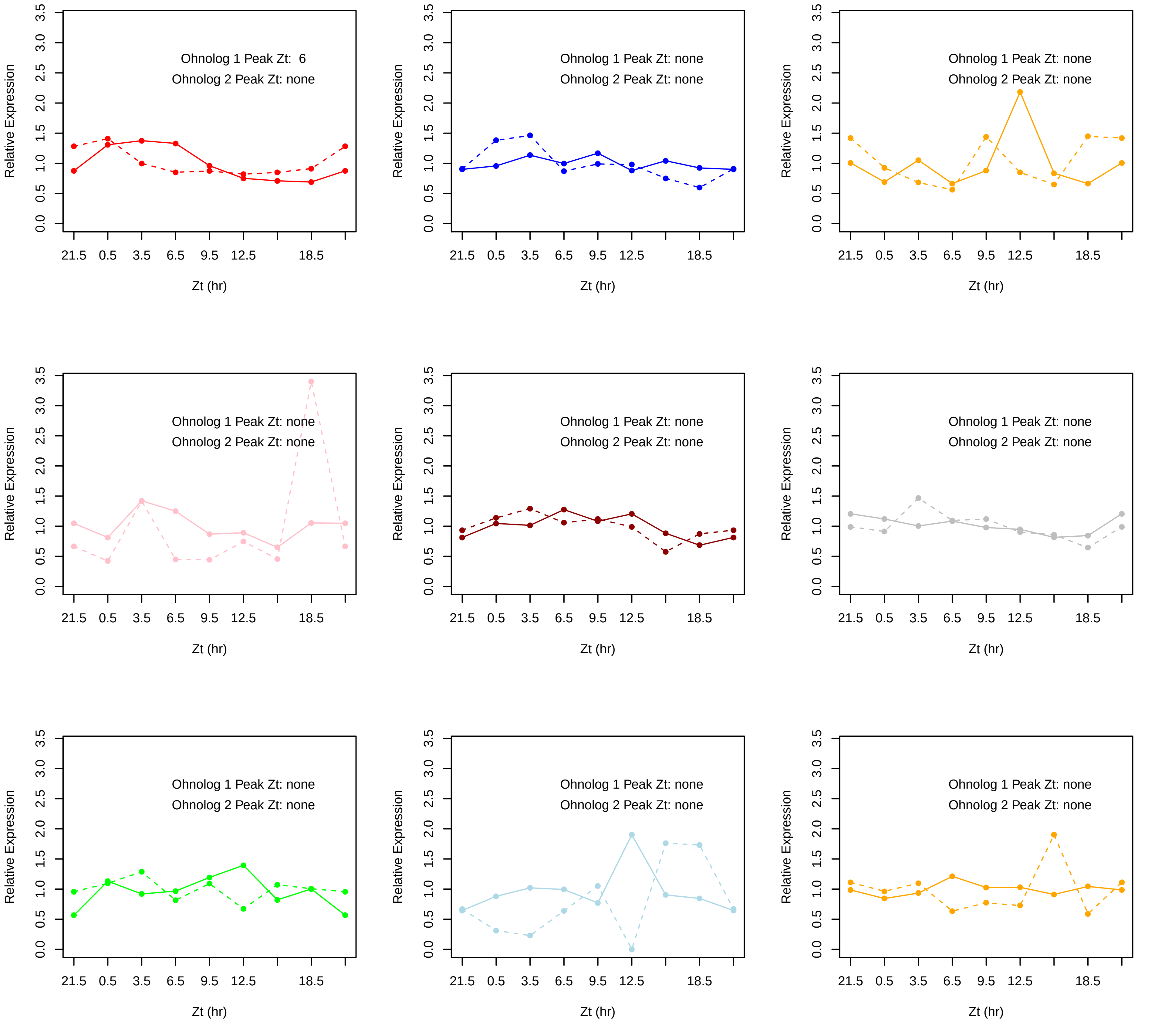

Ohnolog 1: ENSXMAG00000022464 olfm1b
Ohnolog 2: ENSXMAG00000026065

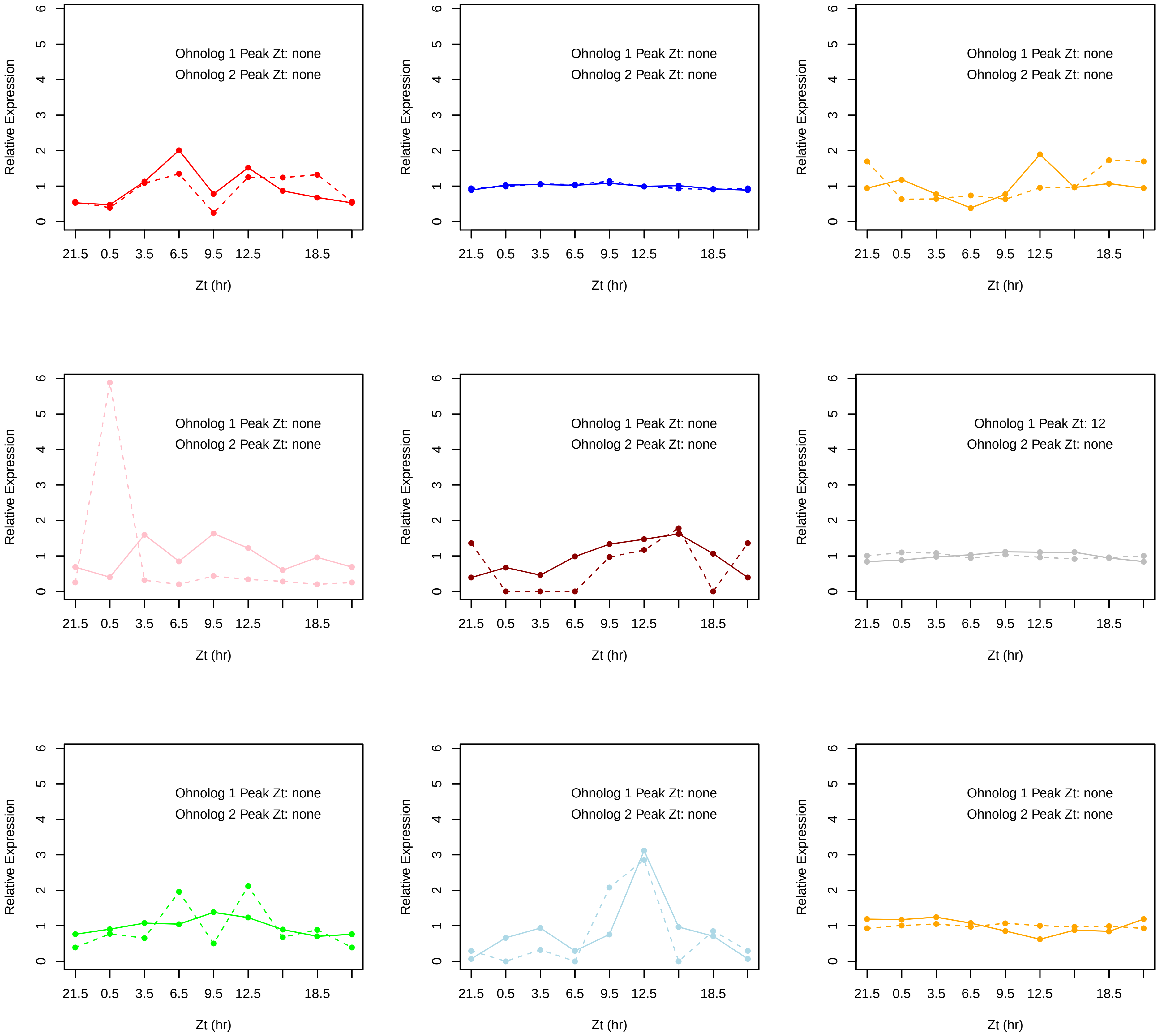

Ohnolog 1: ENSXMAG00000004738 klf6a
Ohnolog 2: ENSXMAG00000006384 KLF6 (1 of many)

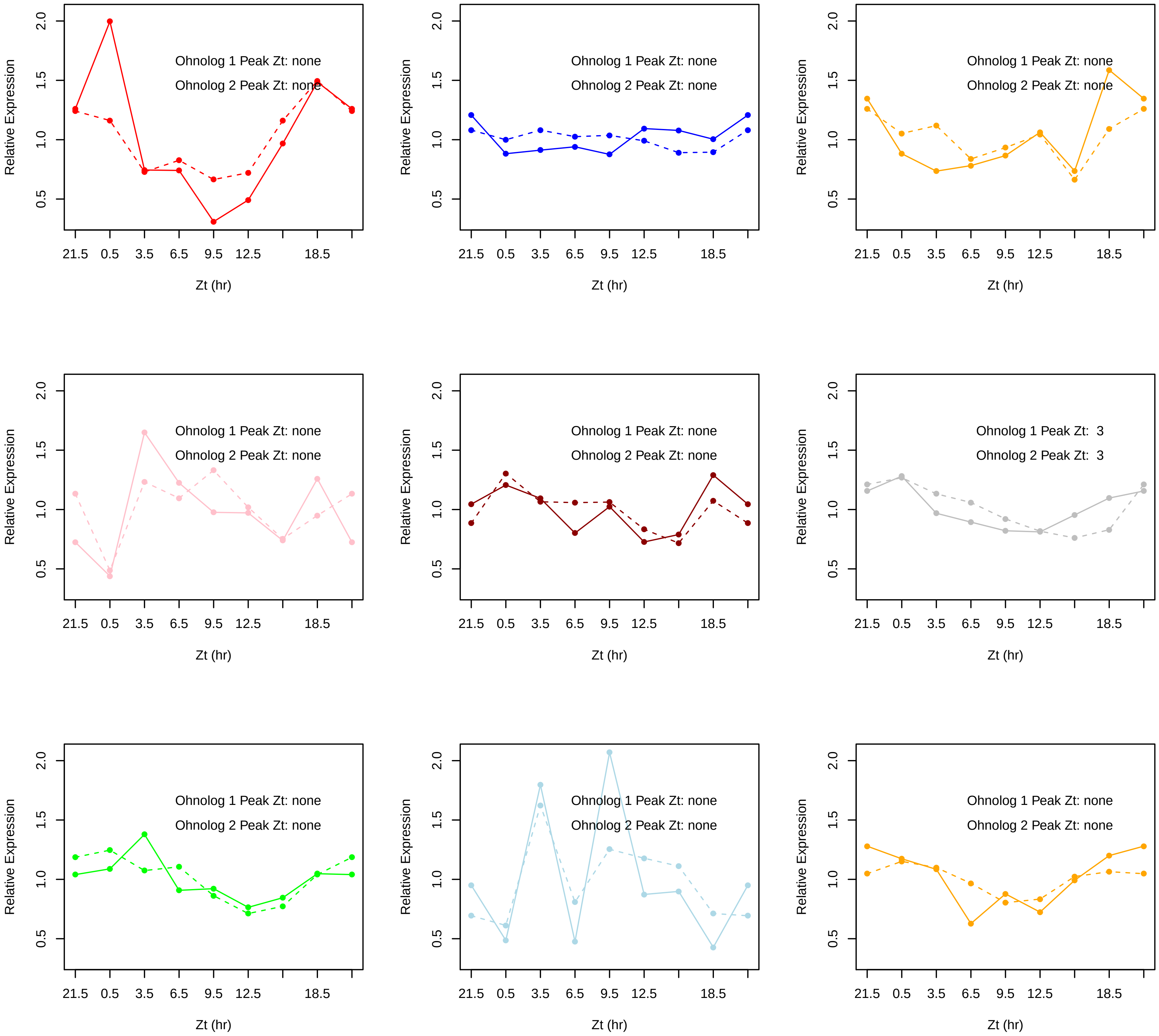

Ohnolog 1: ENSXMAG00000006847 b3glcta
Ohnolog 2: ENSXMAG00000008420 b3glctb

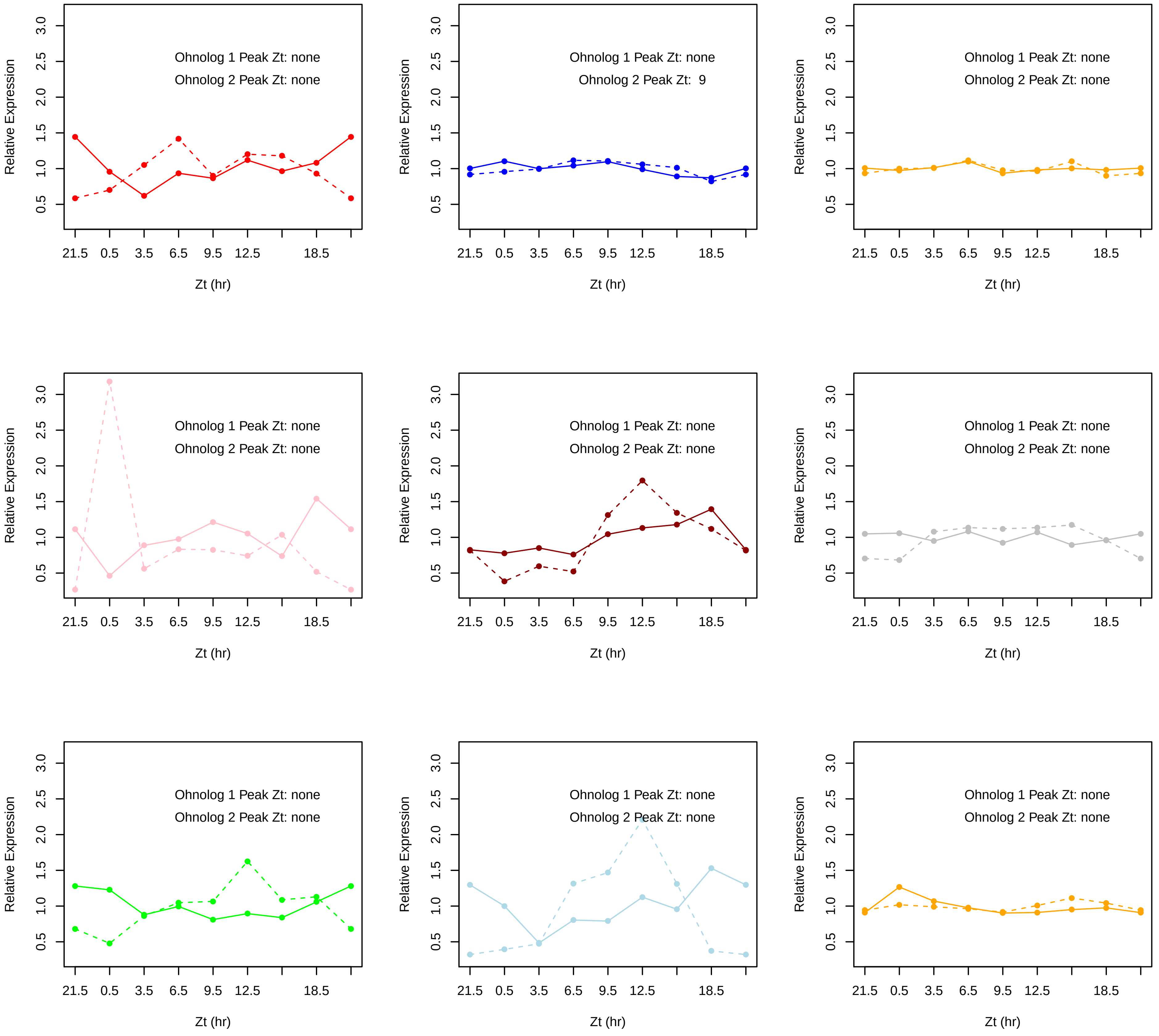

Ohnolog 1: ENSXMAG00000009695 ypel5
Ohnolog 2: ENSXMAG00000022498

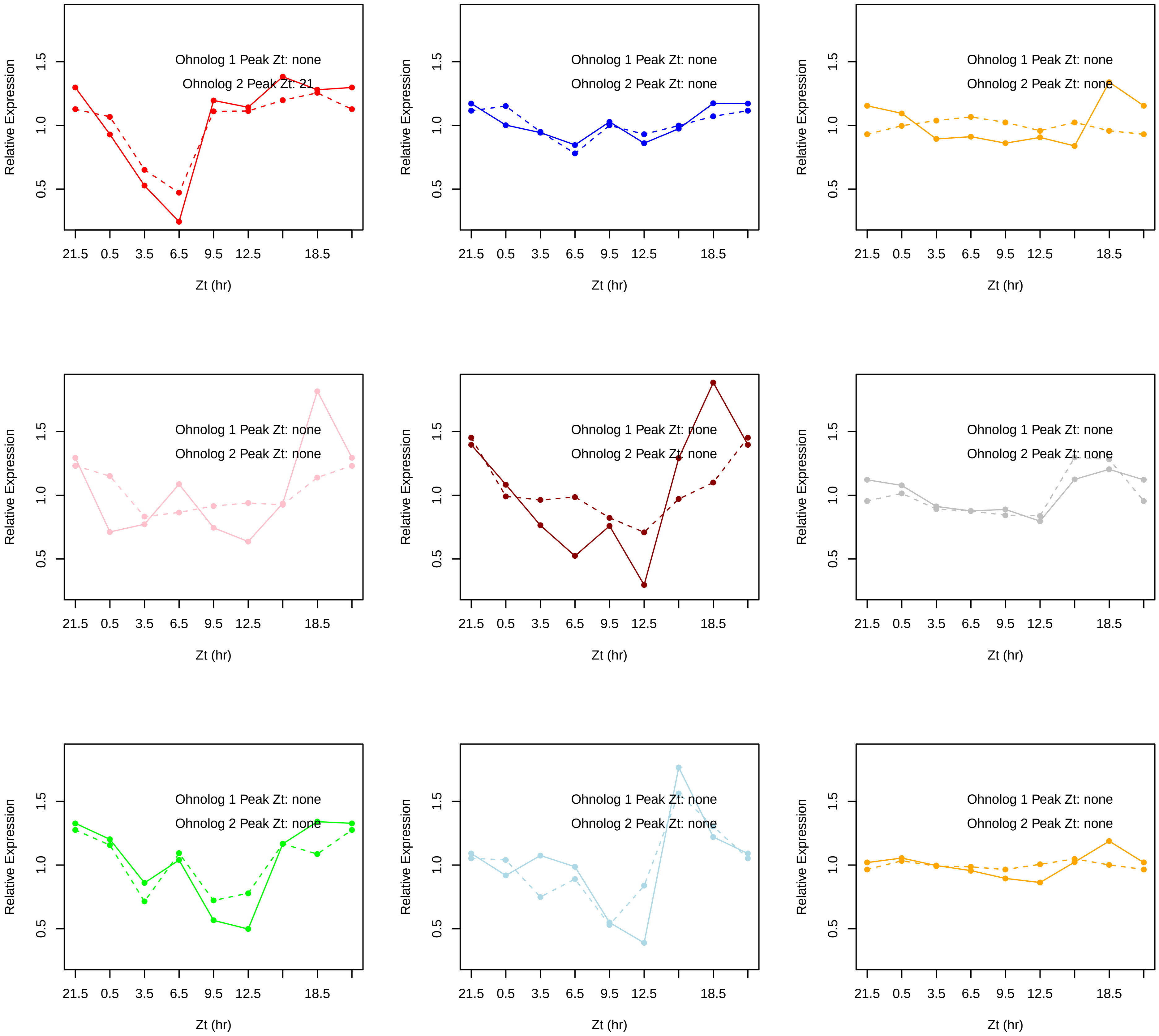

Ohnolog 1: ENSXMAG00000011681 slc17a7a
Ohnolog 2: ENSXMAG00000018700 slc17a7b

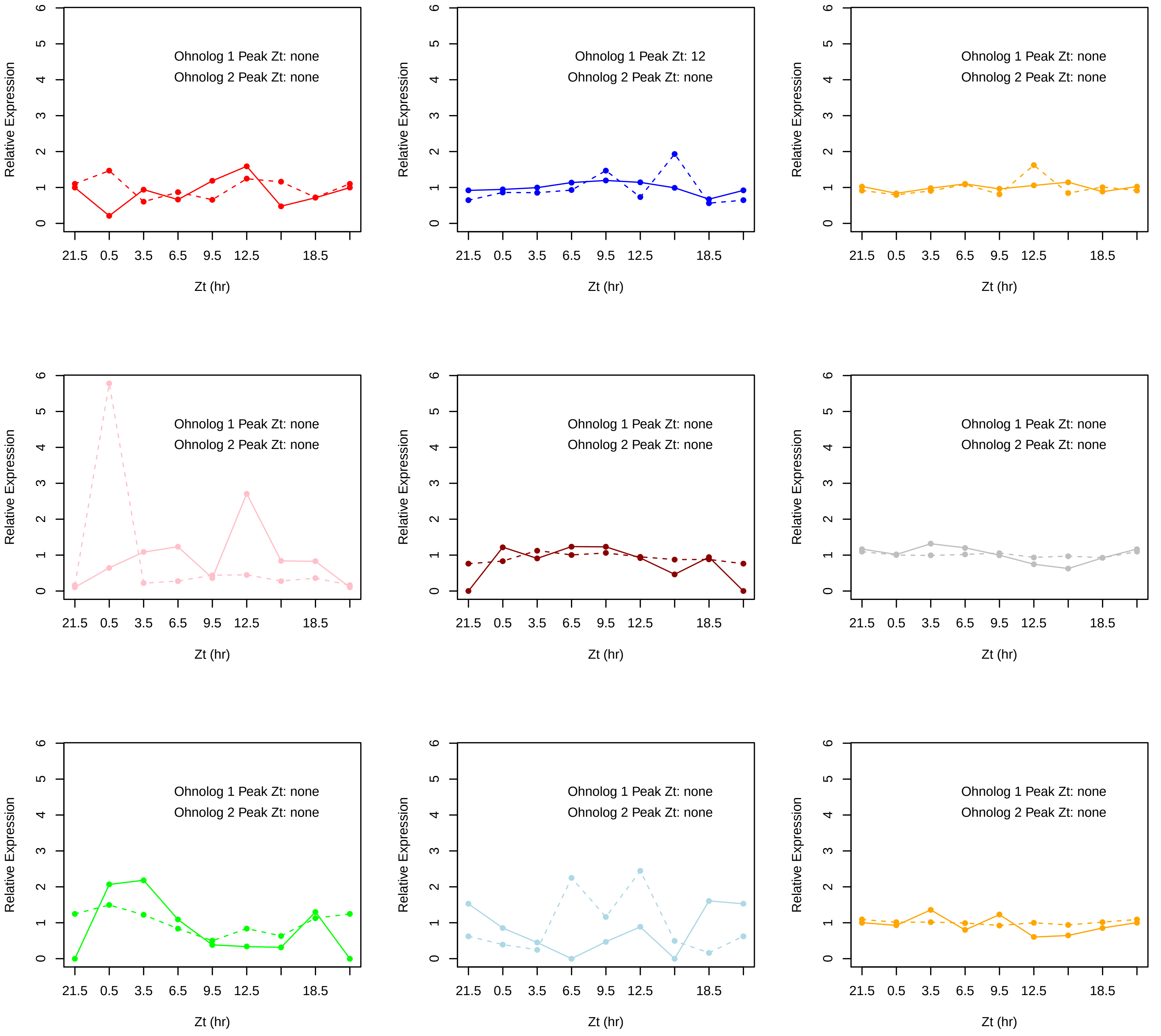

Ohnolog 1: ENSXMAG00000015131
Ohnolog 2: ENSXMAG00000021485 KCNK2

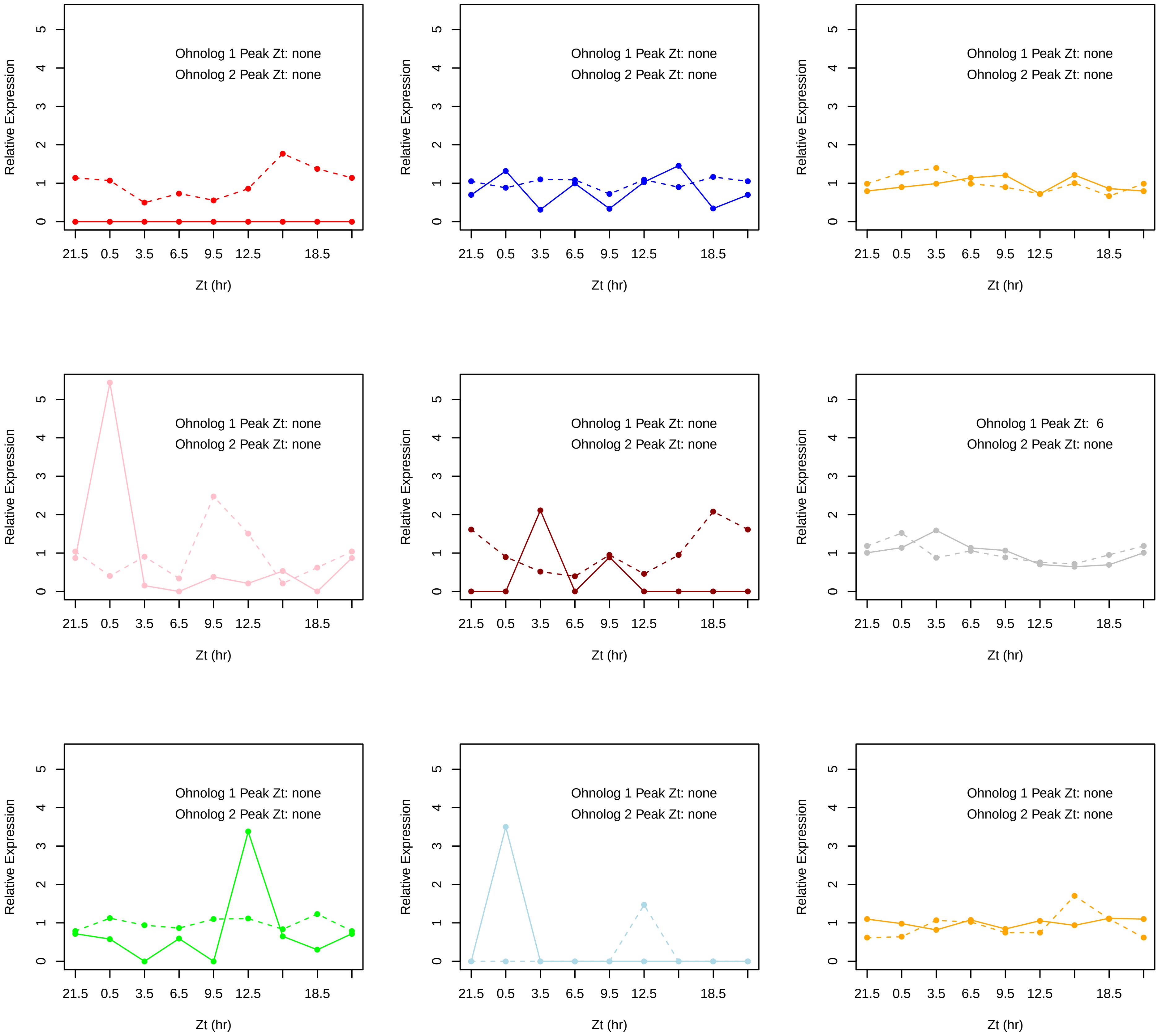

Ohnolog 1: ENSXMAG00000016750 pvrl2l
Ohnolog 2: ENSXMAG00000022421 si:ch73−22o12.1

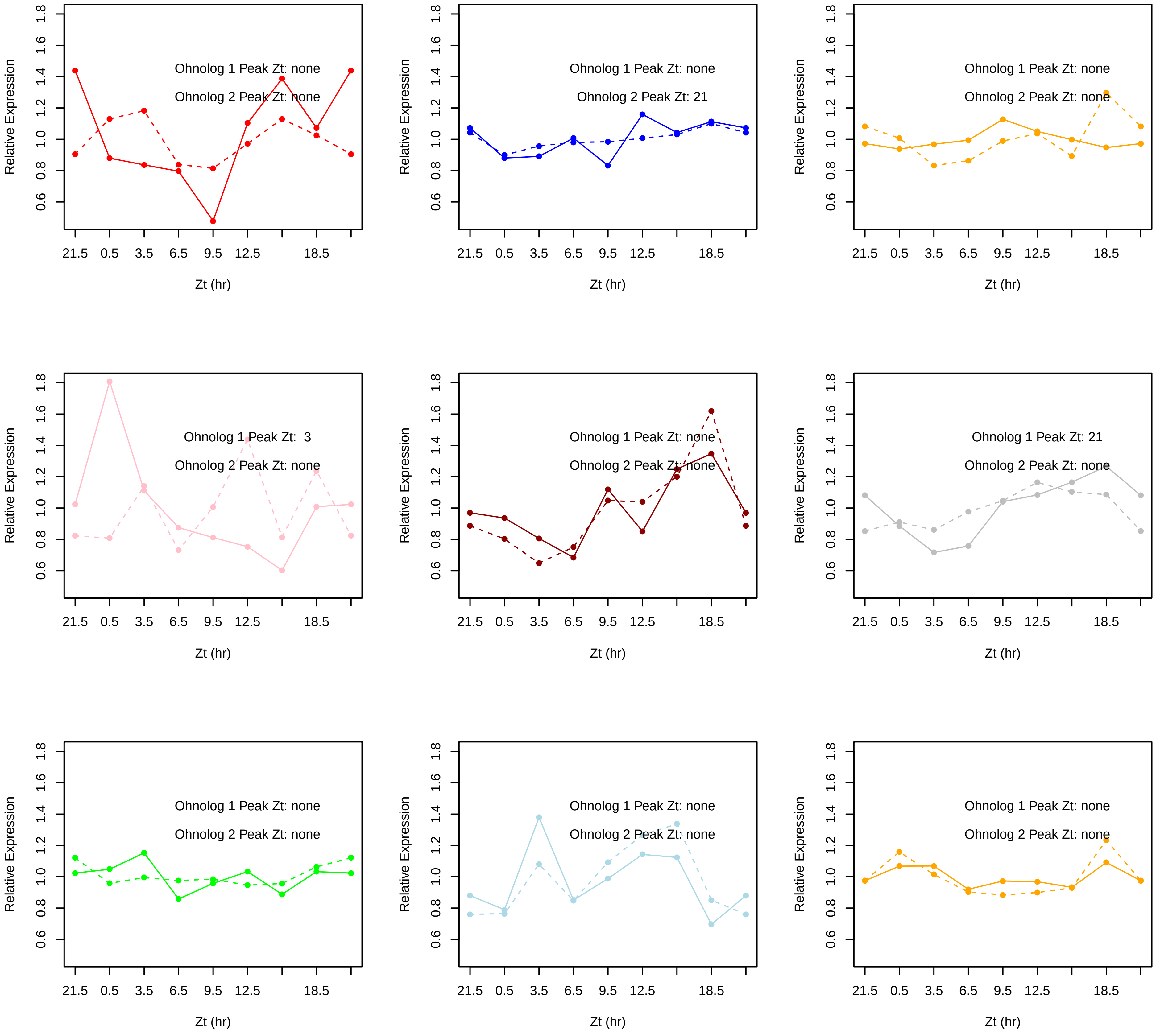

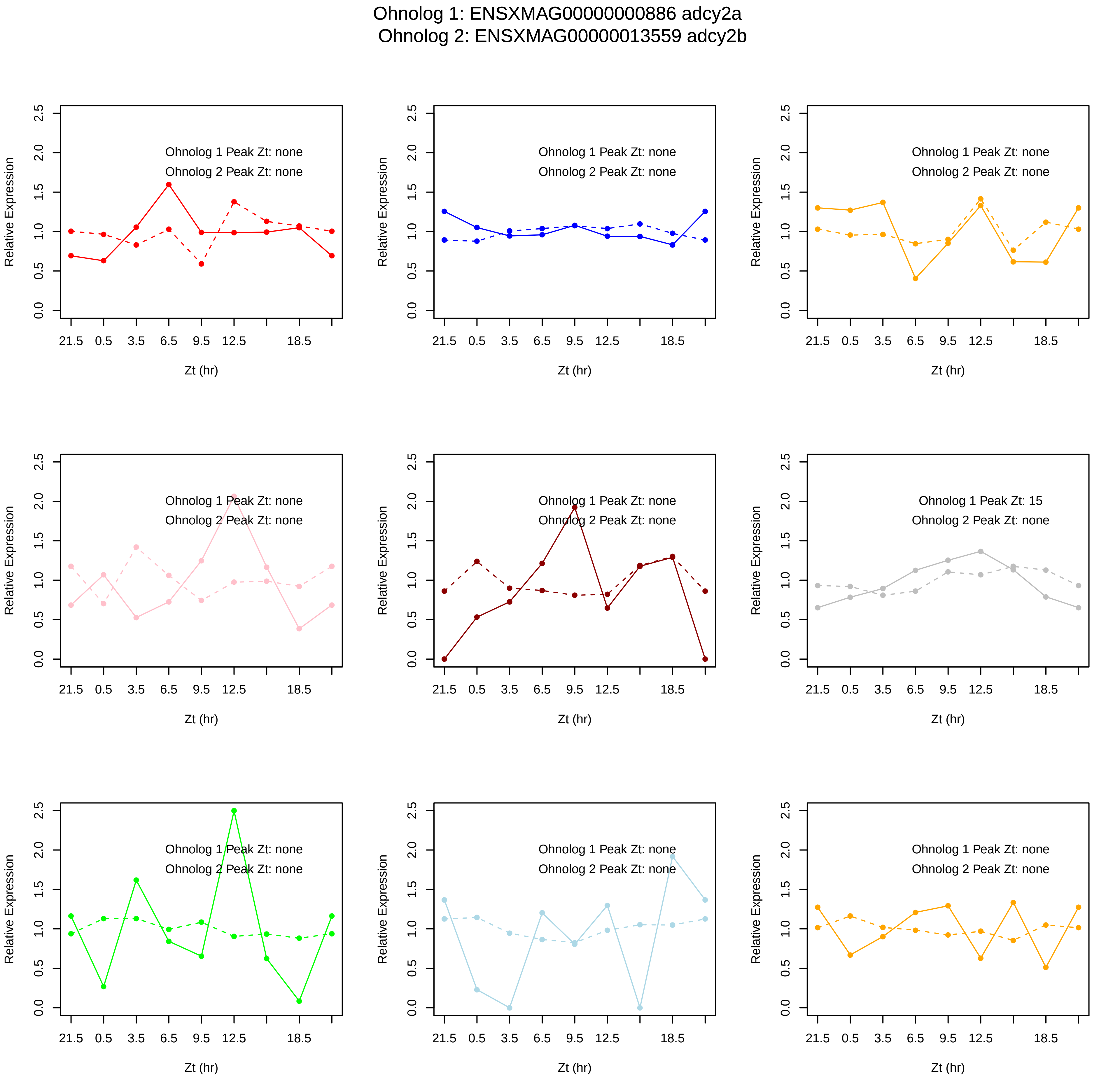

Ohnolog 1: ENSXMAG00000000886 adcy2a
Ohnolog 2: ENSXMAG00000013559 adcy2b
Ohnolog 1 Peak Zt: none
Ohnolog 2 Peak Zt: none
Ohnolog 1 Peak Zt: none
Ohnolog 2 Peak Zt: none
Ohnolog 1 Peak Zt: none
Ohnolog 2 Peak Zt: none
Ohnolog 1 Peak Zt: none
Ohnolog 2 Peak Zt: none
Ohnolog 1 Peak Zt: none
Ohnolog 2 Peak Zt: none
Ohnolog 1 Peak Zt: 15
Ohnolog 2 Peak Zt: none
Ohnolog 1 Peak Zt: none
Ohnolog 2 Peak Zt: none
Ohnolog 1 Peak Zt: none
Ohnolog 2 Peak Zt: none
Ohnolog 1 Peak Zt: none
Ohnolog 2 Peak Zt: none
Relative Expression
Zt (hr)
21.5
0.5
3.5
6.5
9.5
12.5
18.5
0.0
0.5
1.0
1.5
2.0
2.5

Ohnolog 1: ENSXMAG00000007350 mybpc2b
Ohnolog 2: ENSXMAG00000017975 mybpc2a

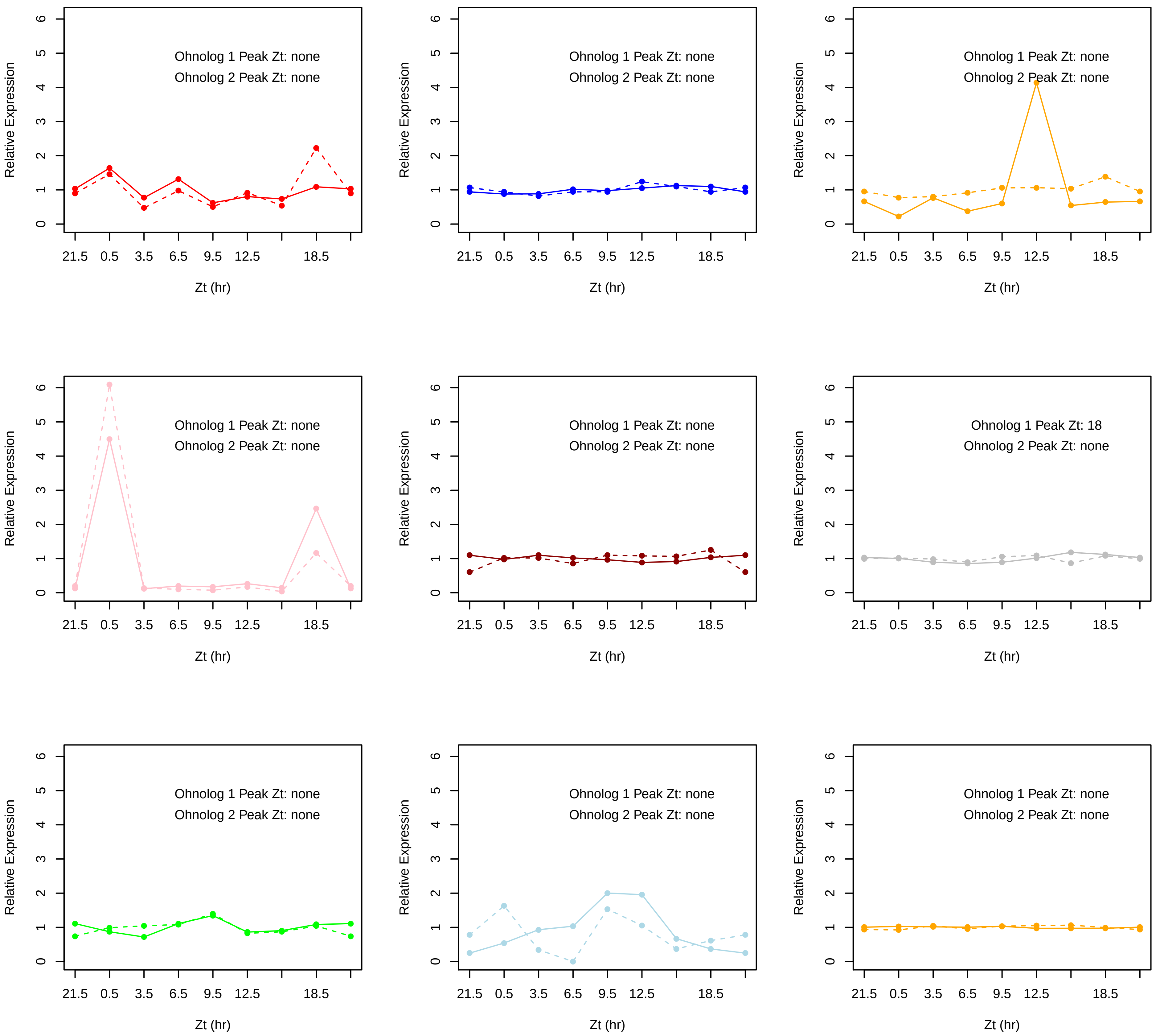

Ohnolog 1: ENSXMAG00000006296 NRP1 (1 of many)
Ohnolog 2: ENSXMAG00000008055 nrp1a

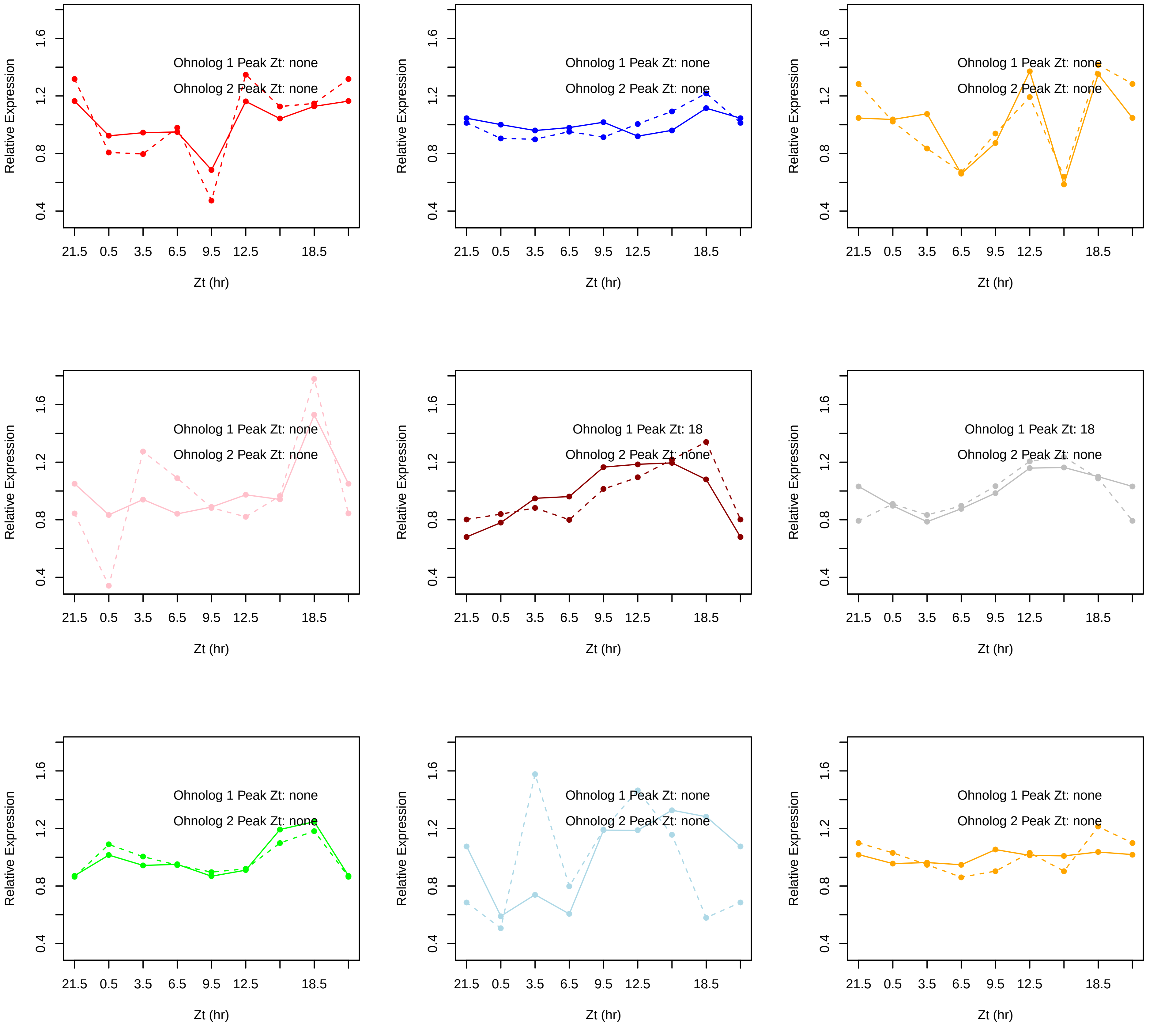

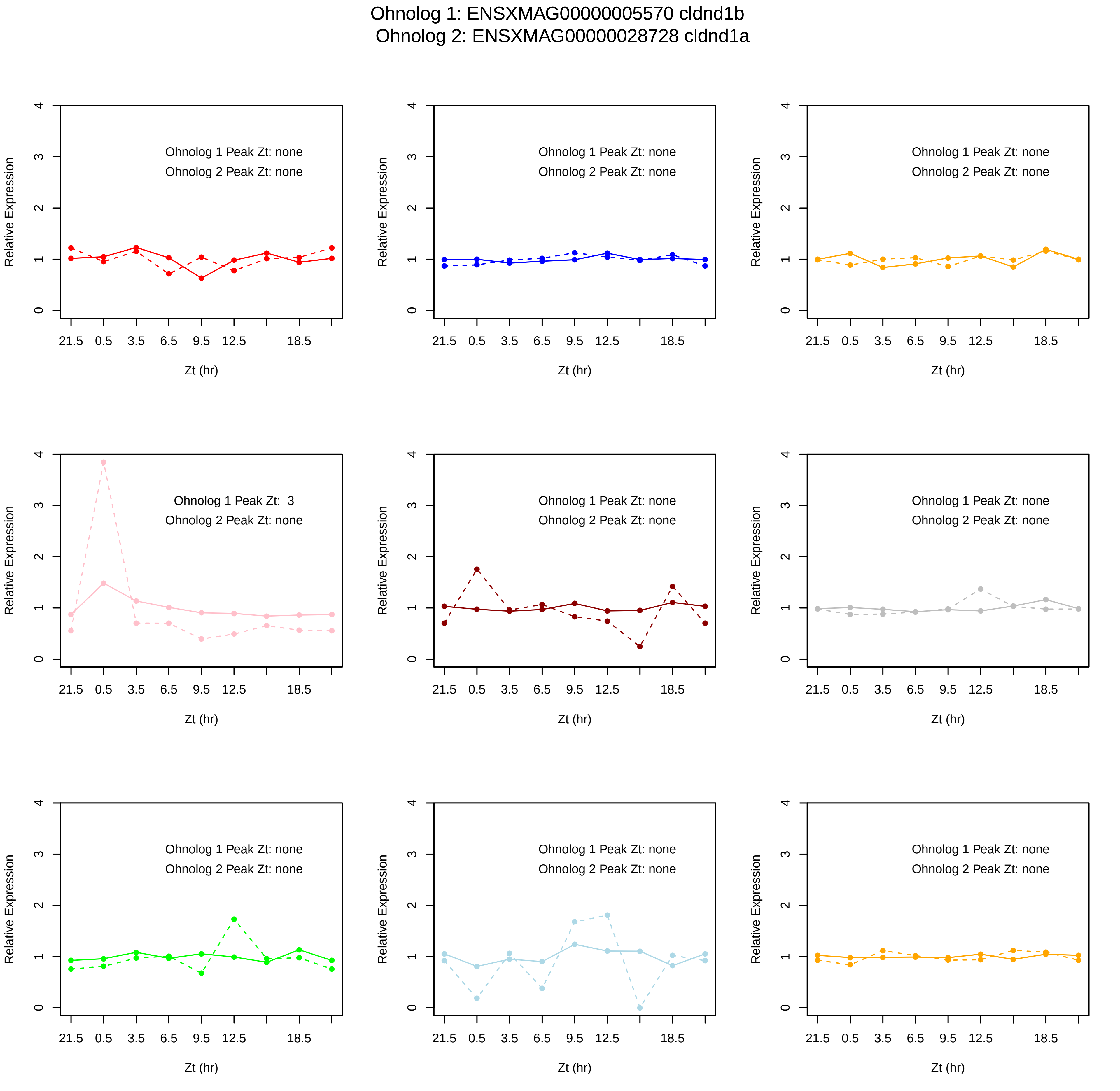
Ohnolog 1: ENSXMAG00000005570 cldnd1b
Ohnolog 2: ENSXMAG00000028728 cldnd1a
Ohnolog 1 Peak Zt: none
Ohnolog 2 Peak Zt: none
Ohnolog 1 Peak Zt: 3
Ohnolog 2 Peak Zt: none
Relative Expression
Zt (hr)
21.5
0.5
3.5
6.5
9.5
12.5
18.5
0
1
2
3
4

Ohnolog 1: ENSXMAG00000002328 INPP5K (1 of many)
Ohnolog 2: ENSXMAG00000010655 inpp5kb

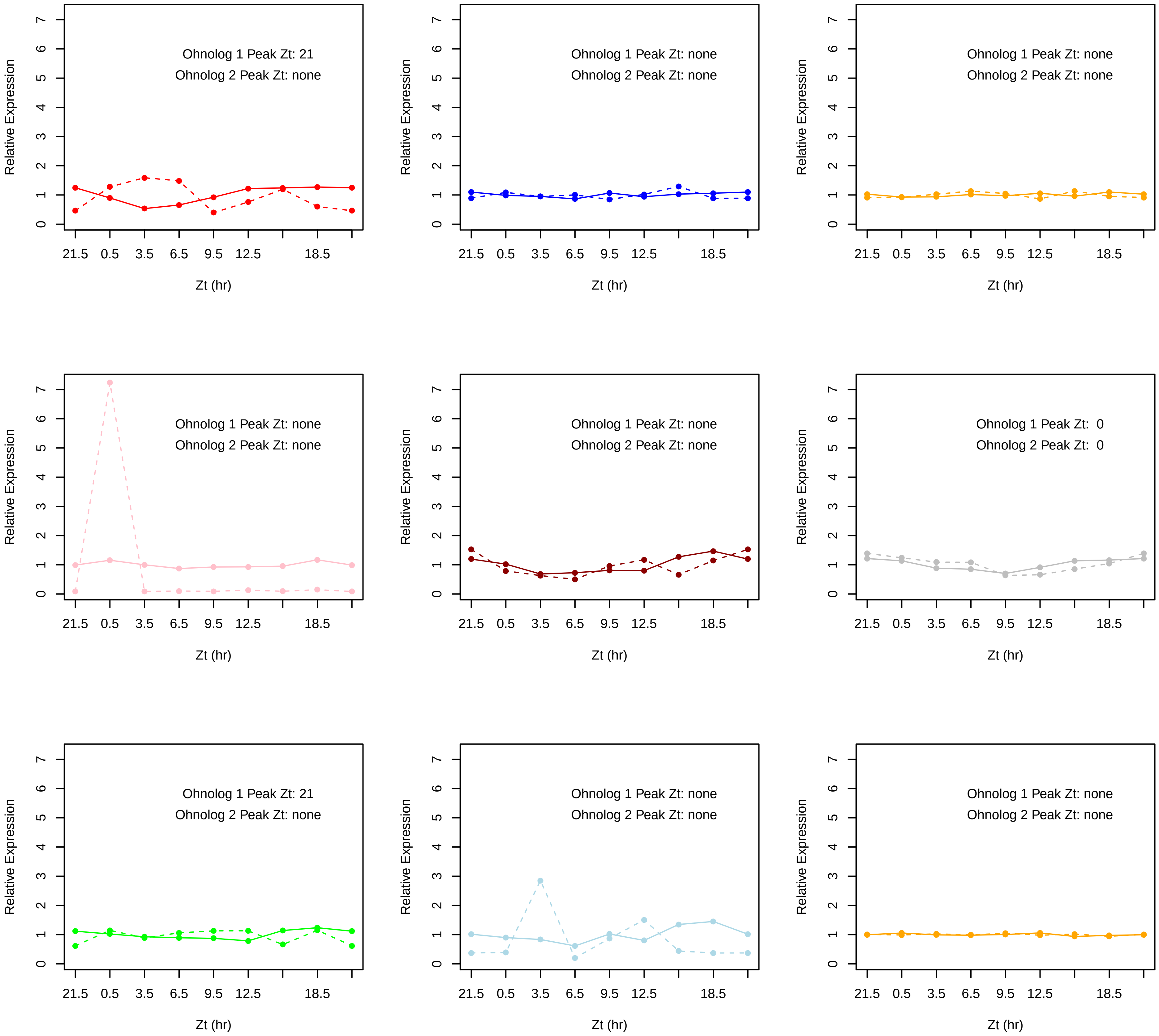

Ohnolog 1: ENSXMAG00000010760
Ohnolog 2: ENSXMAG00000024636 map1sb

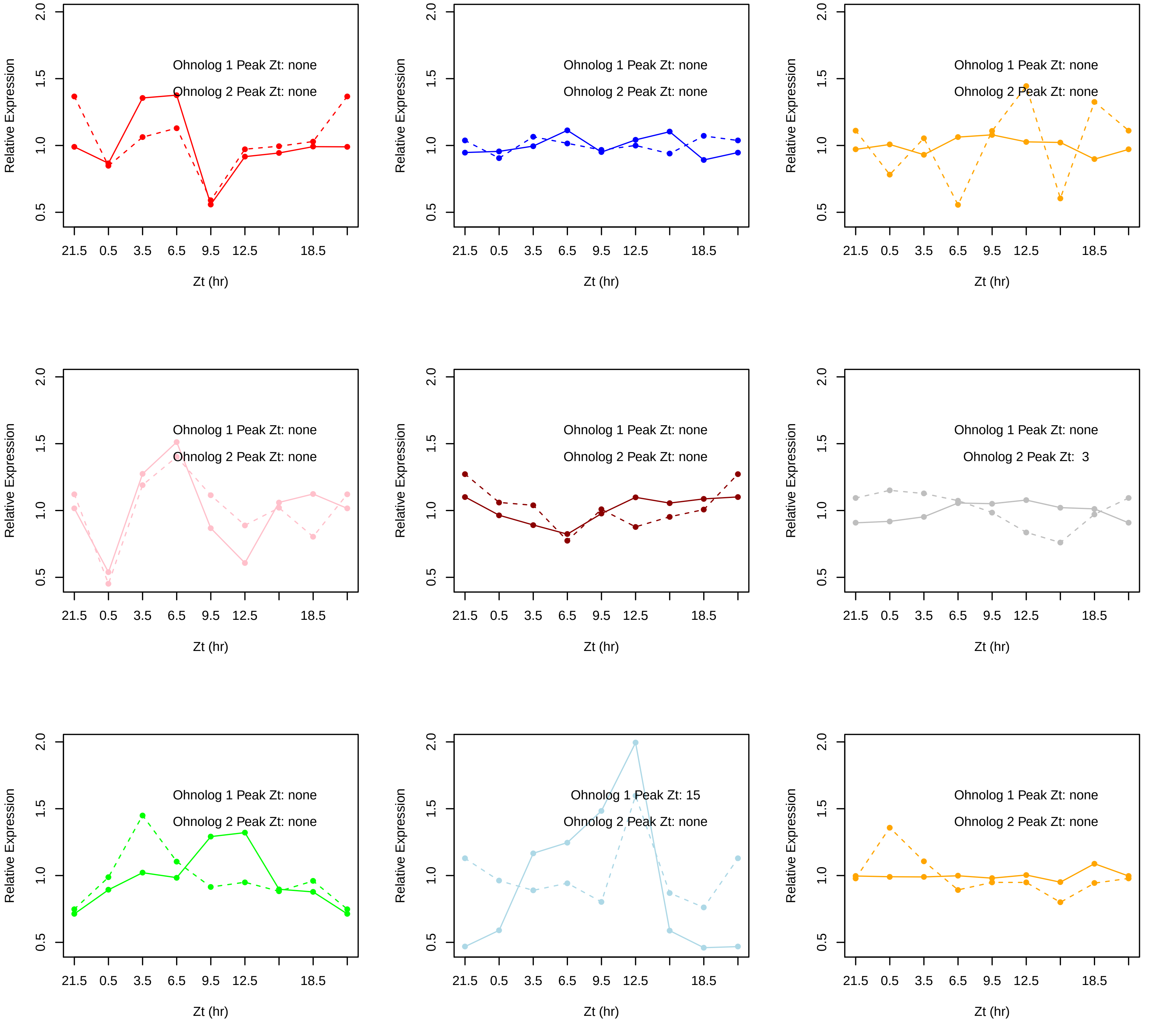

Ohnolog 1: ENSXMAG00000011022
Ohnolog 2: ENSXMAG00000017047

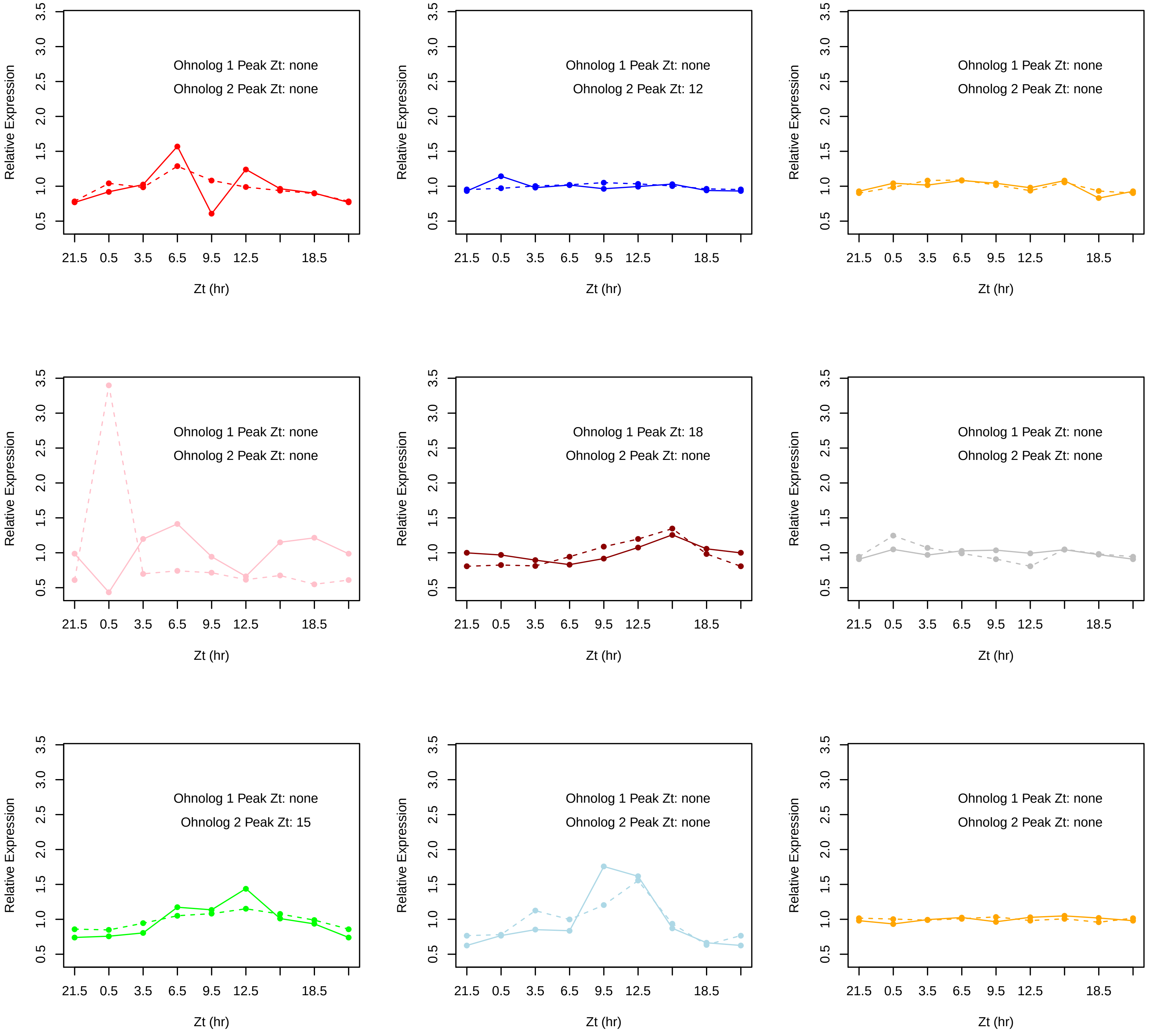

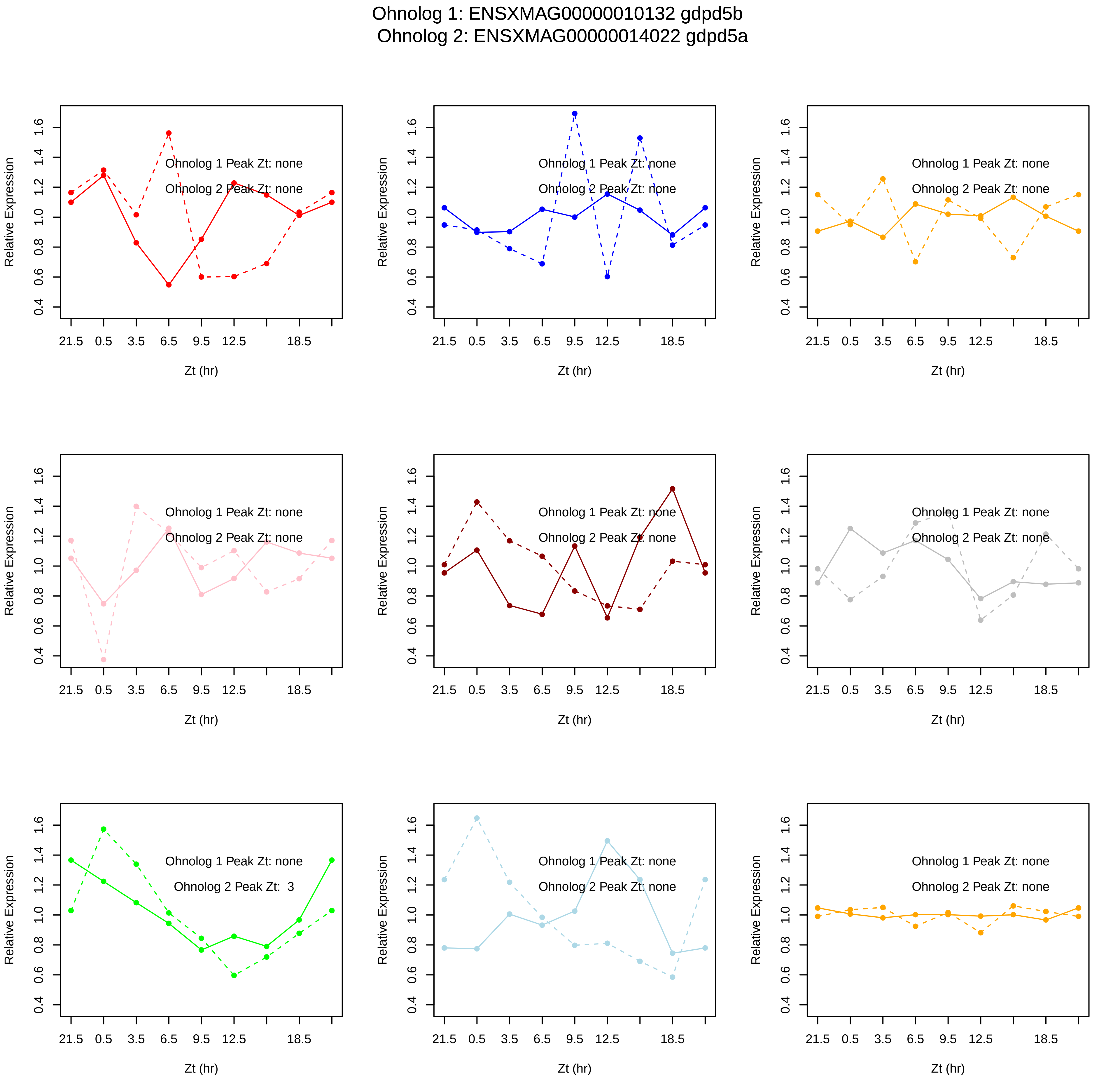

Ohnolog 1: ENSXMAG00000010132 gdpd5b
Ohnolog 2: ENSXMAG00000014022 gdpd5a
Ohnolog 1 Peak Zt: none
Ohnolog 2 Peak Zt: none
Ohnolog 2 Peak Zt: 3
Relative Expression
Zt (hr)
21.5 0.5 3.5 6.5 9.5 12.5 18.5
0.4 0.6 0.8 1.0 1.2 1.4 1.6

Ohnolog 1: ENSXMAG00000002475 gfra4a
Ohnolog 2: ENSXMAG00000016091 gfra4b

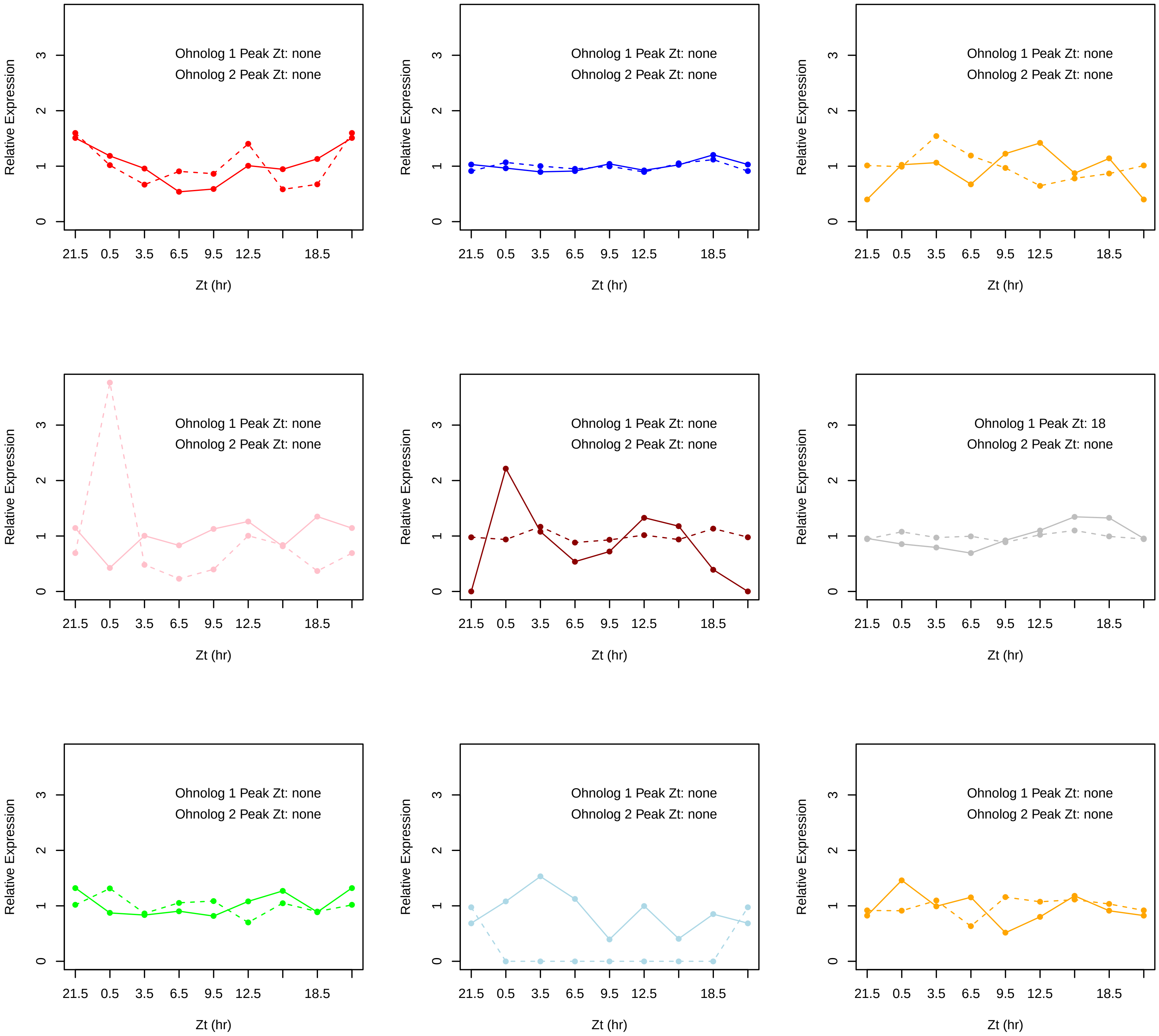

Ohnolog 1: ENSXMAG00000021574 dnajb9b
Ohnolog 2: ENSXMAG00000028221 dnajb9a

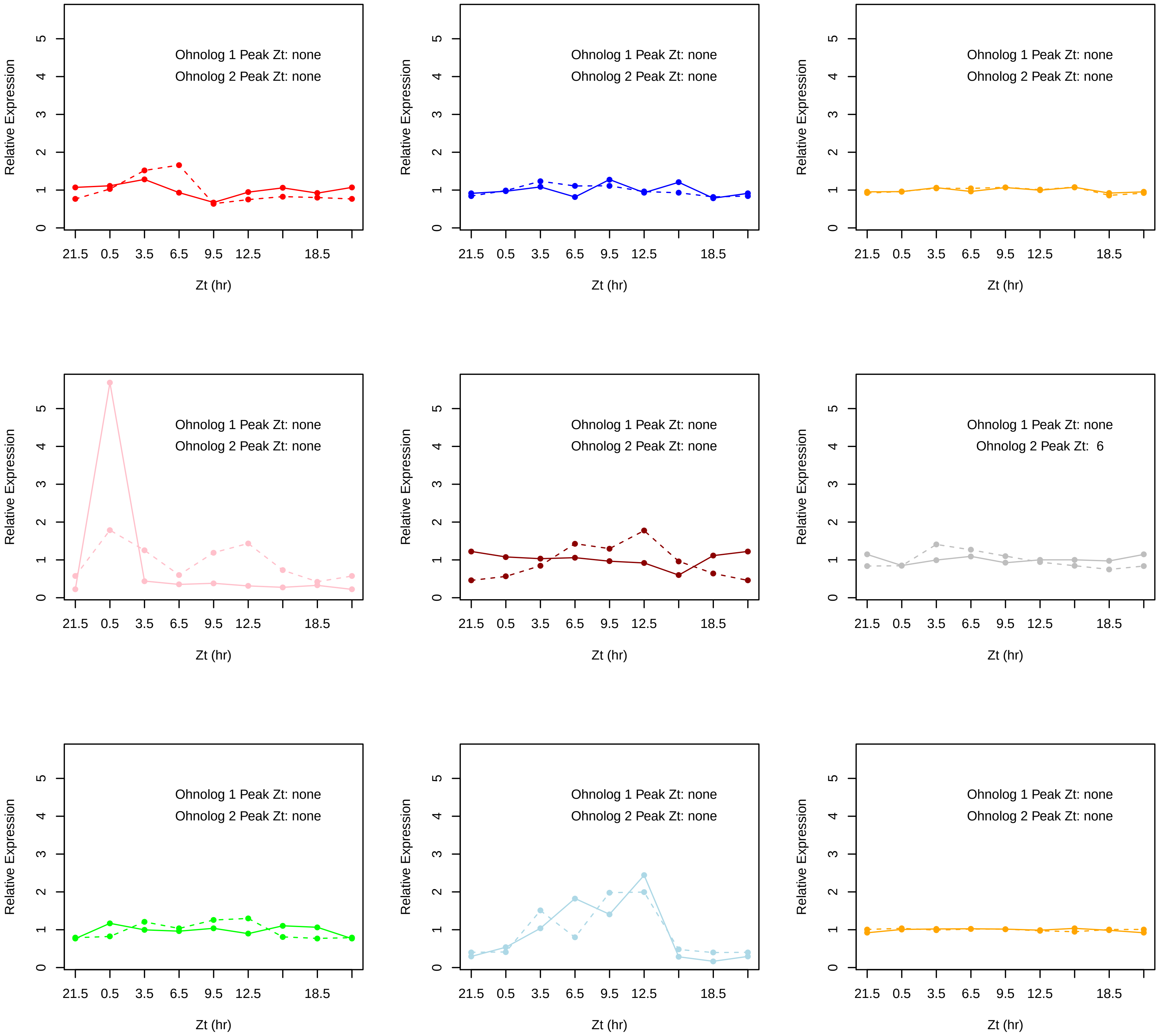

Ohnolog 1: ENSXMAG00000003086 rom1b
Ohnolog 2: ENSXMAG00000005204 rom1a

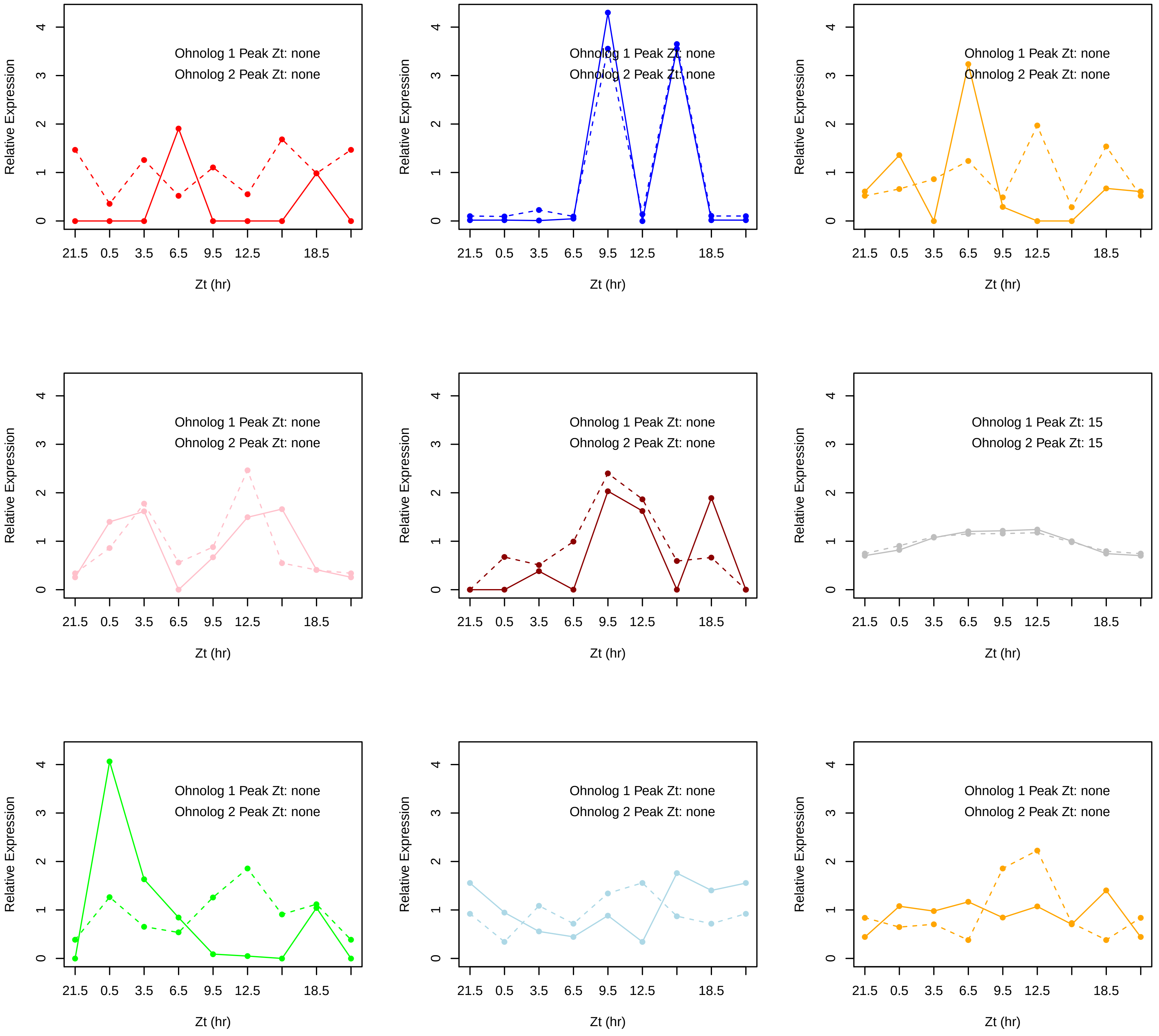

Ohnolog 1: ENSXMAG00000000586
Ohnolog 2: ENSXMAG00000004639 PIK3CA

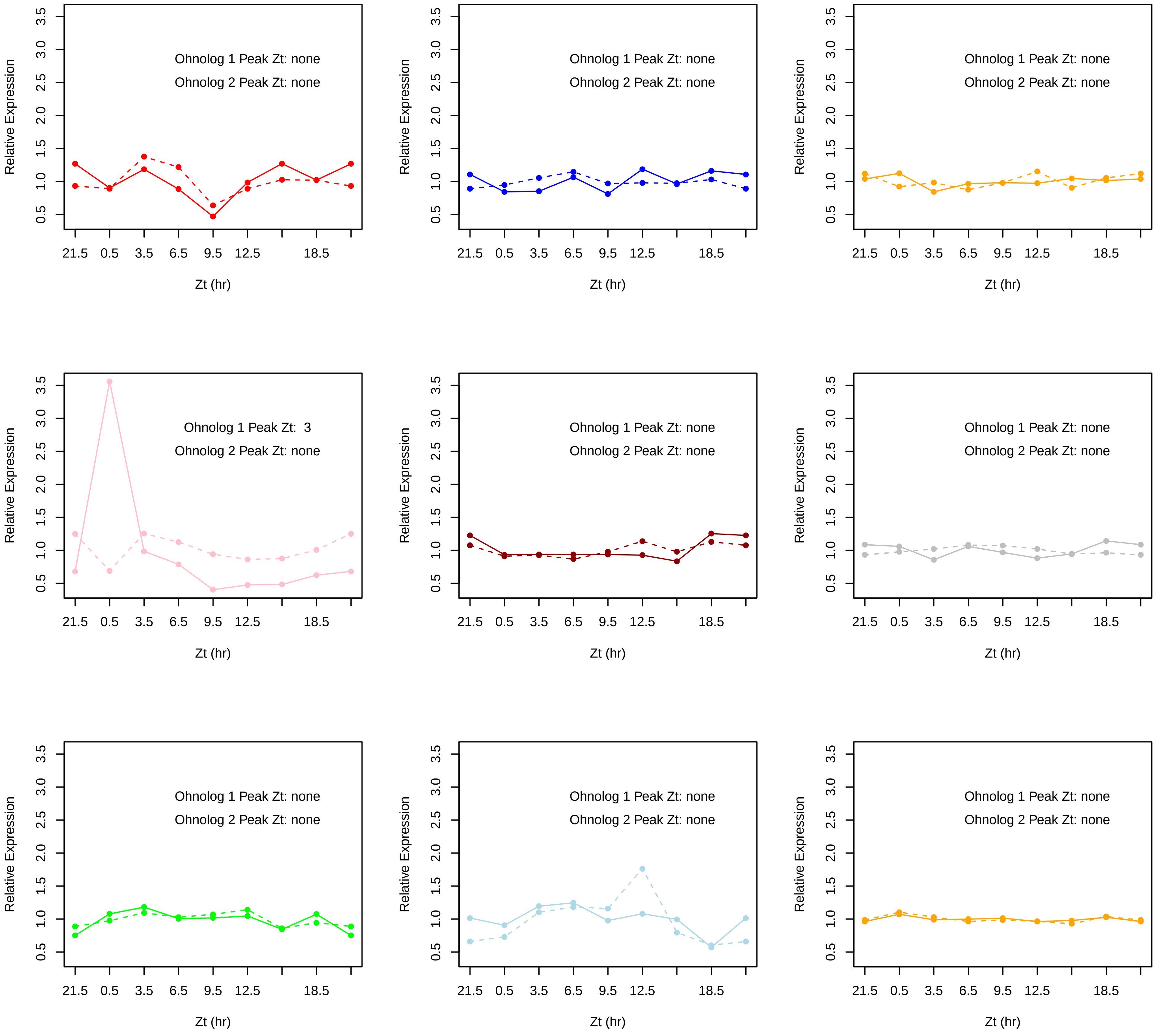

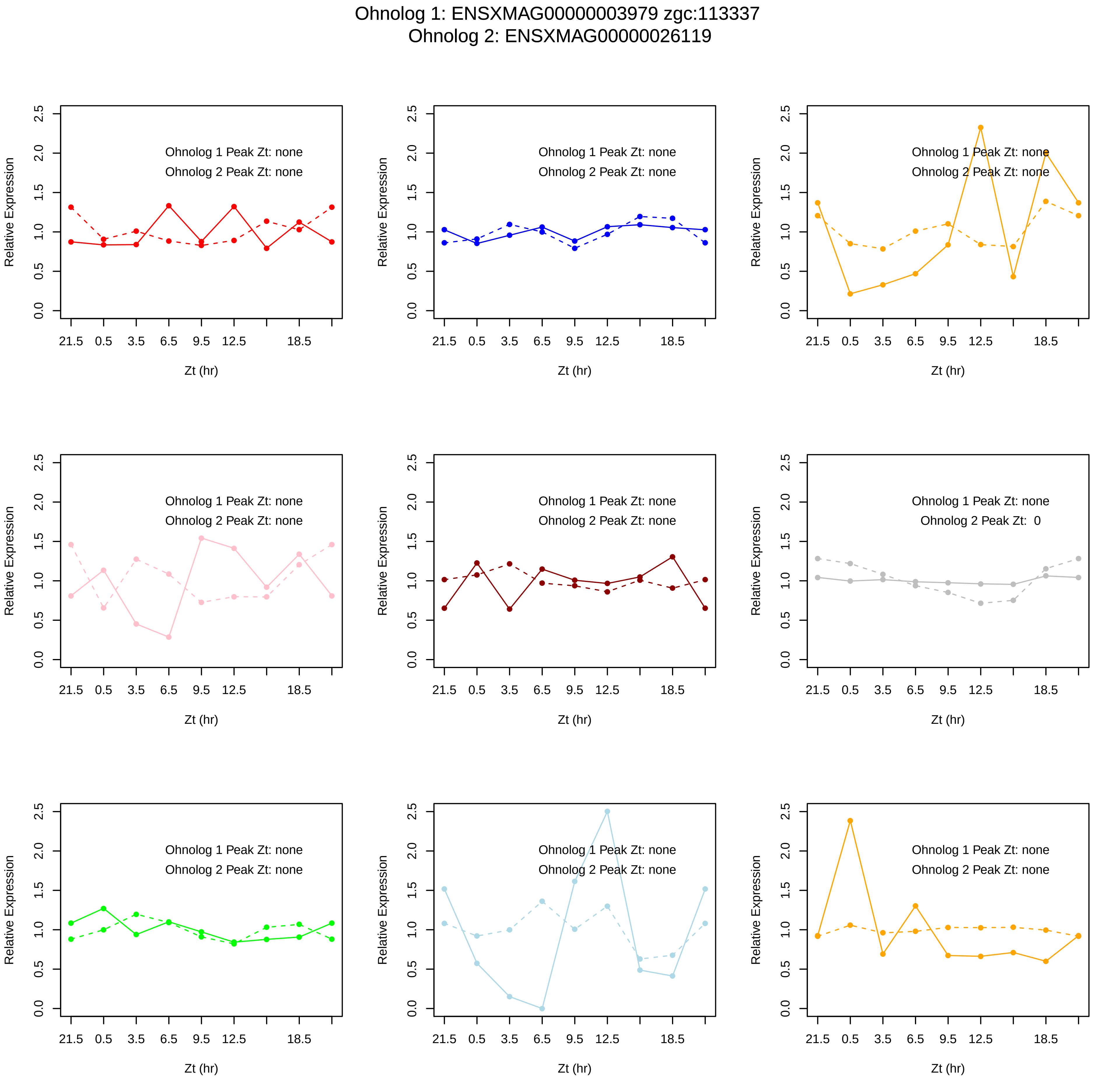

Ohnolog 1: ENSXMAG00000003979 zgc:113337
Ohnolog 2: ENSXMAG00000026119
Ohnolog 1 Peak Zt: none
Ohnolog 2 Peak Zt: none
Ohnolog 1 Peak Zt: none
Ohnolog 2 Peak Zt: none
Ohnolog 1 Peak Zt: none
Ohnolog 2 Peak Zt: none
Ohnolog 1 Peak Zt: none
Ohnolog 2 Peak Zt: none
Ohnolog 1 Peak Zt: none
Ohnolog 2 Peak Zt: none
Ohnolog 1 Peak Zt: none
Ohnolog 2 Peak Zt: 0
Ohnolog 1 Peak Zt: none
Ohnolog 2 Peak Zt: none
Ohnolog 1 Peak Zt: none
Ohnolog 2 Peak Zt: none
Ohnolog 1 Peak Zt: none
Ohnolog 2 Peak Zt: none
Relative Expression
0.0 0.5 1.0 1.5 2.0 2.5
21.5 0.5 3.5 6.5 9.5 12.5 18.5
Zt (hr)

Ohnolog 1: ENSXMAG00000009232 nrcama
Ohnolog 2: ENSXMAG00000017134 NRCAM (1 of many)

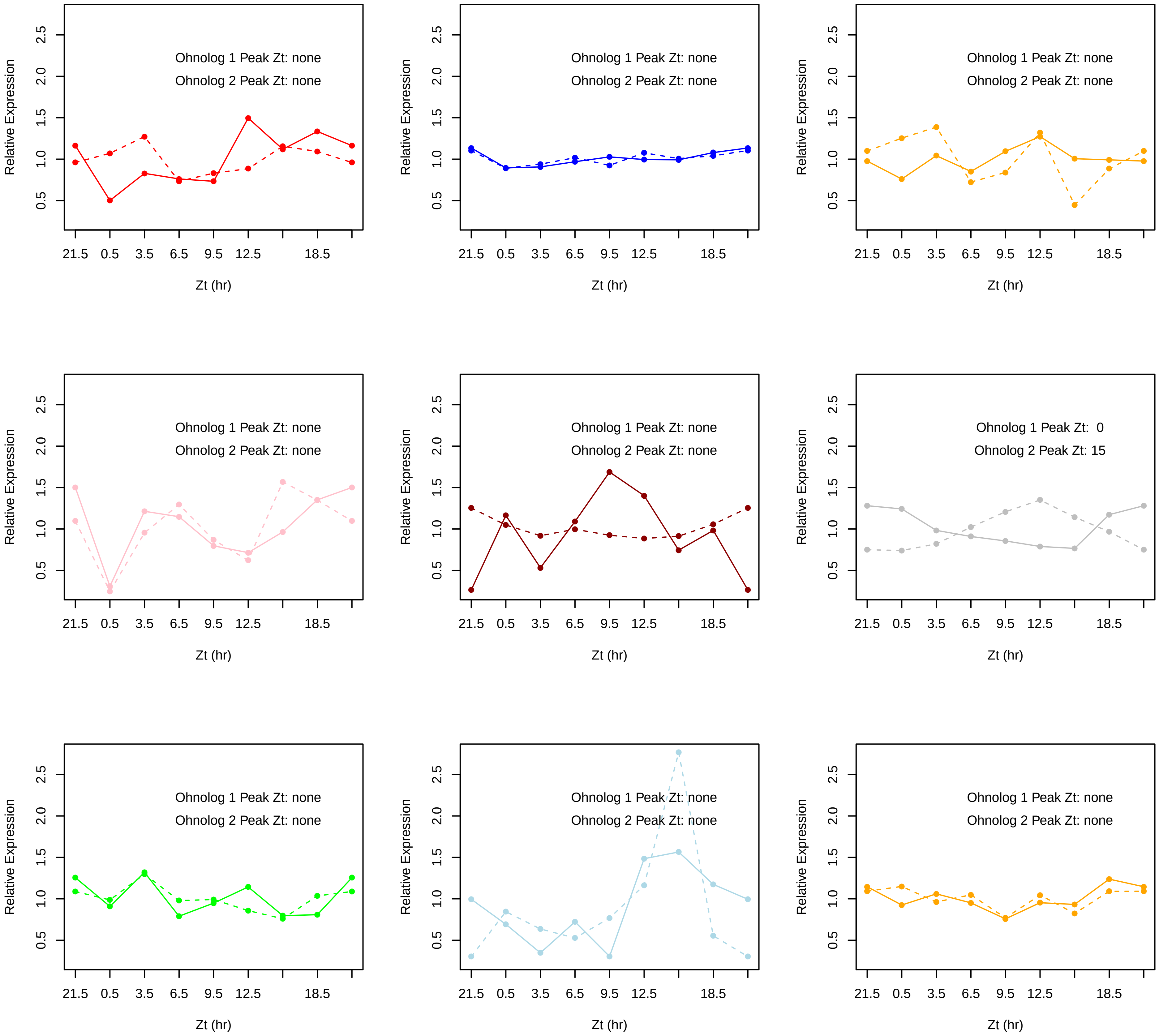

Ohnolog 1: ENSXMAG00000001590 hsd17b12a
Ohnolog 2: ENSXMAG00000011880 hsd17b12b

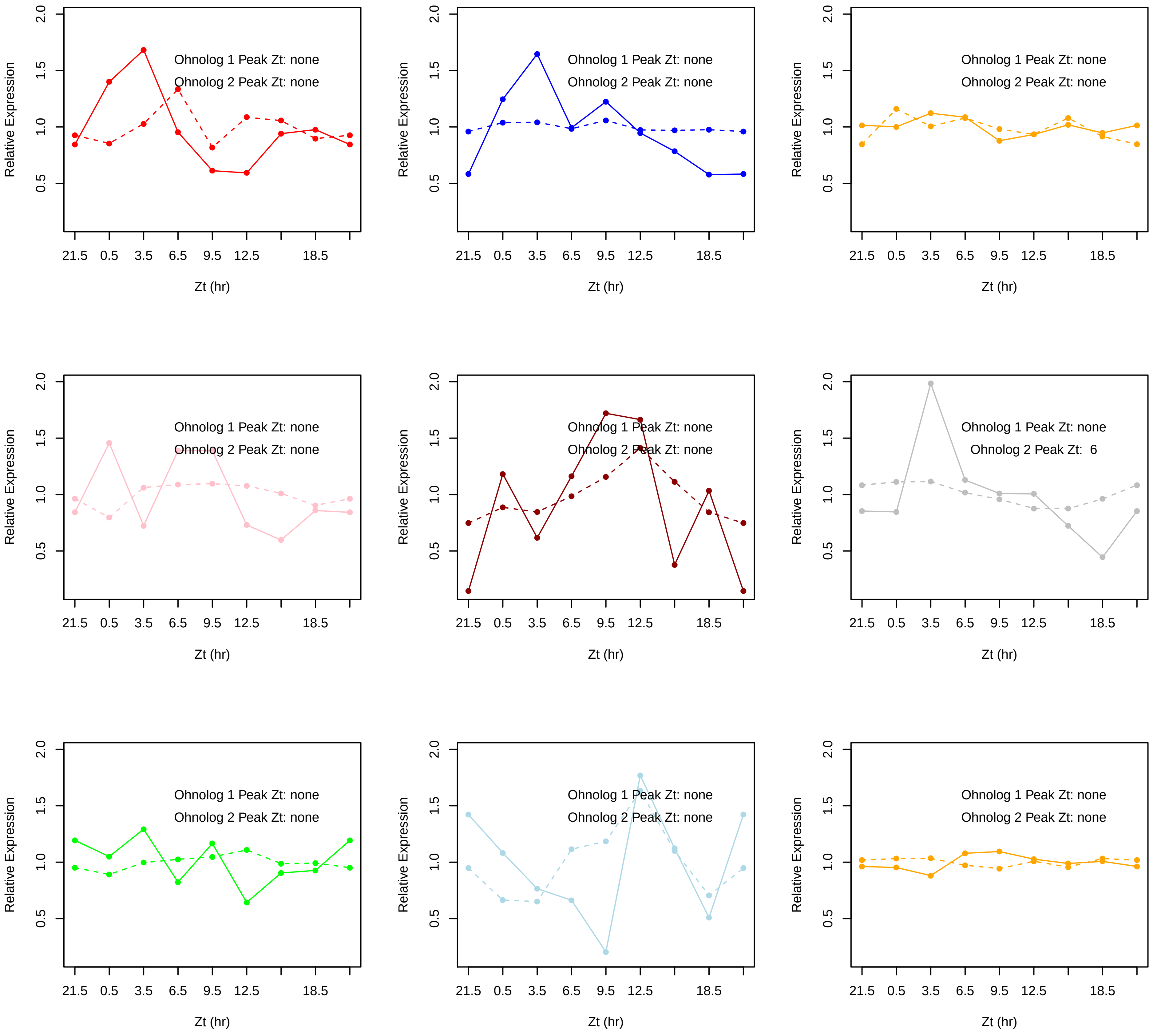

Ohnolog 1: ENSXMAG00000008139 cbsa
Ohnolog 2: ENSXMAG00000012095

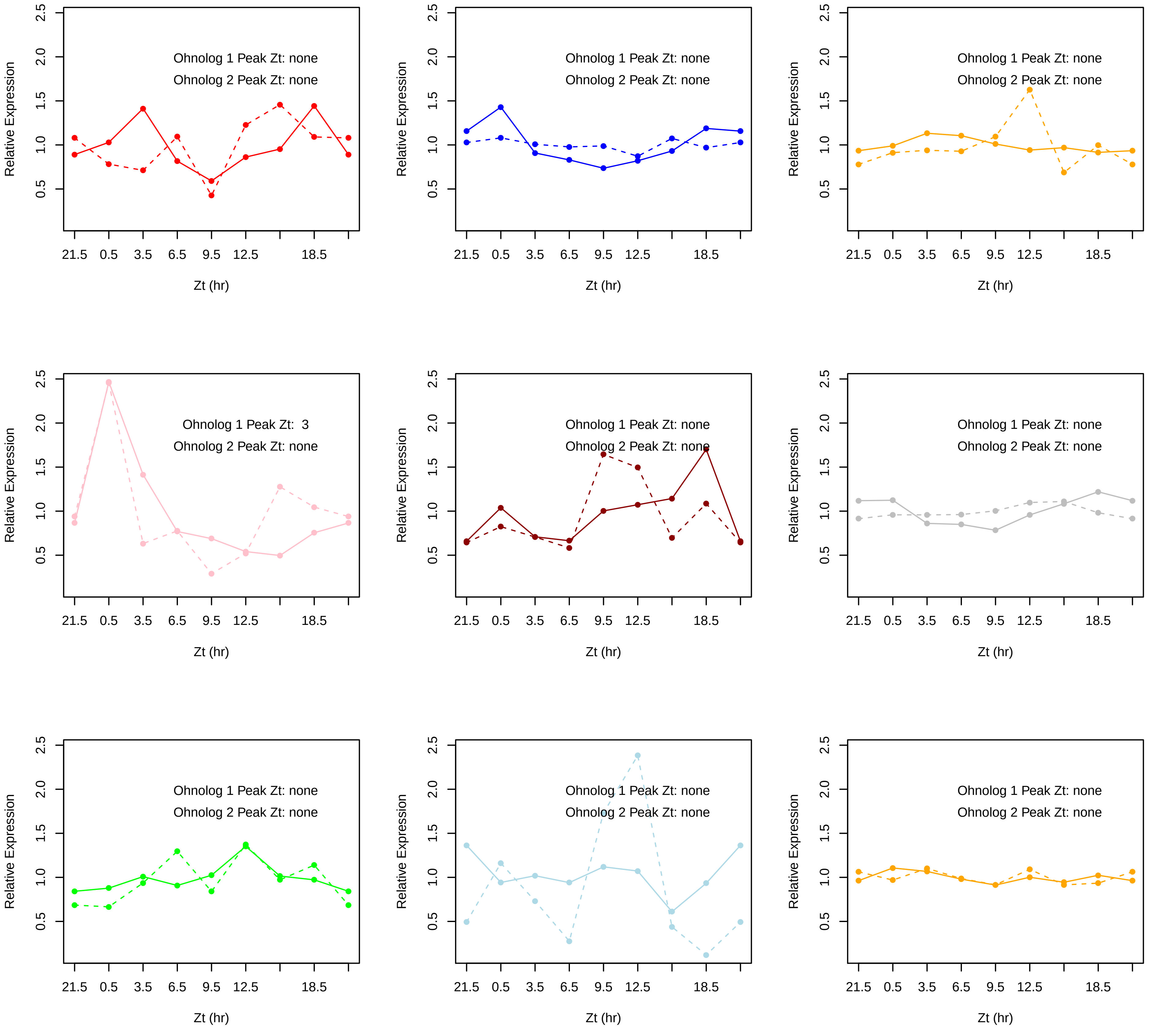

Ohnolog 1: ENSXMAG00000010713 smyd1a
Ohnolog 2: ENSXMAG00000016048 smyd1b

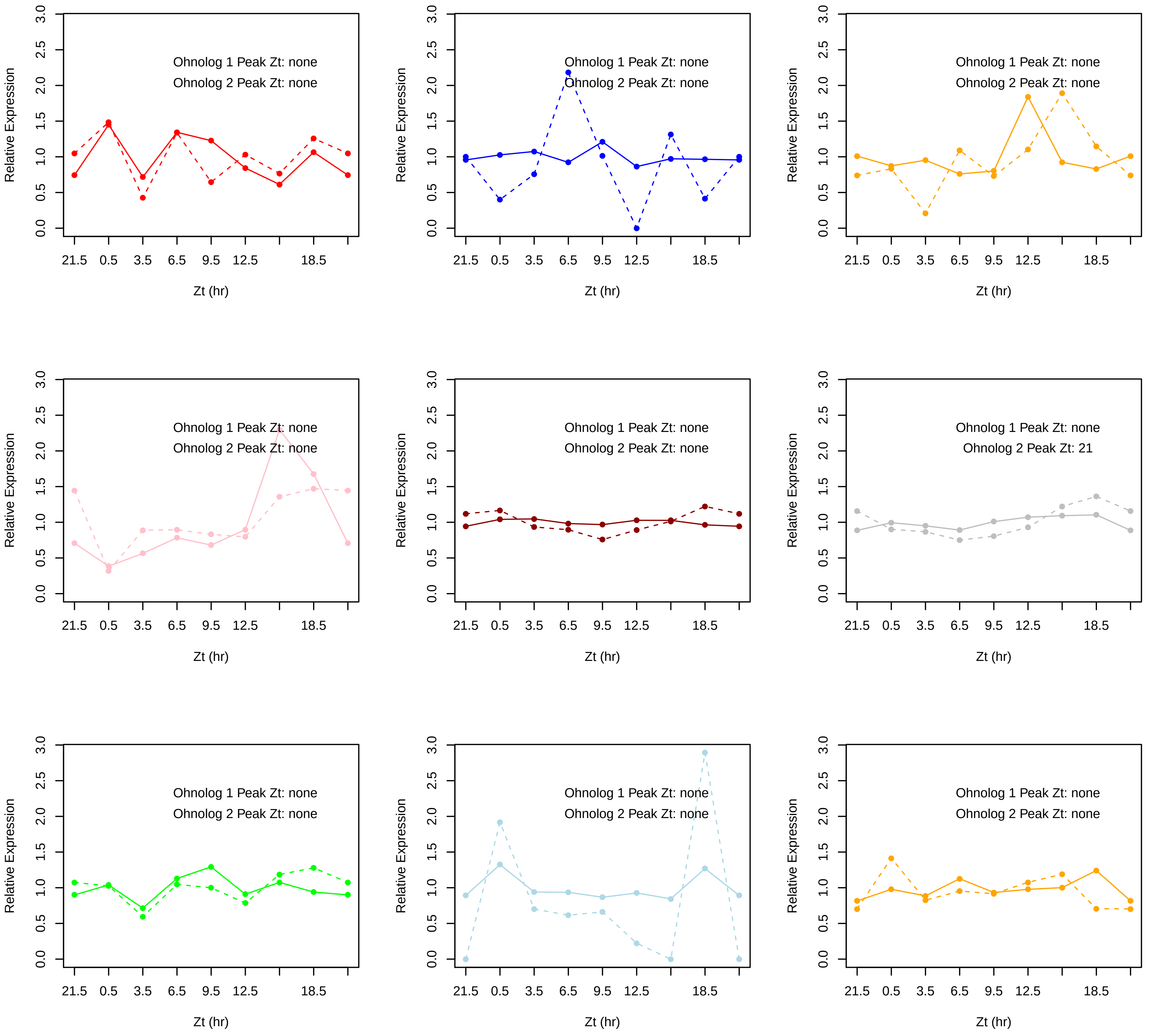

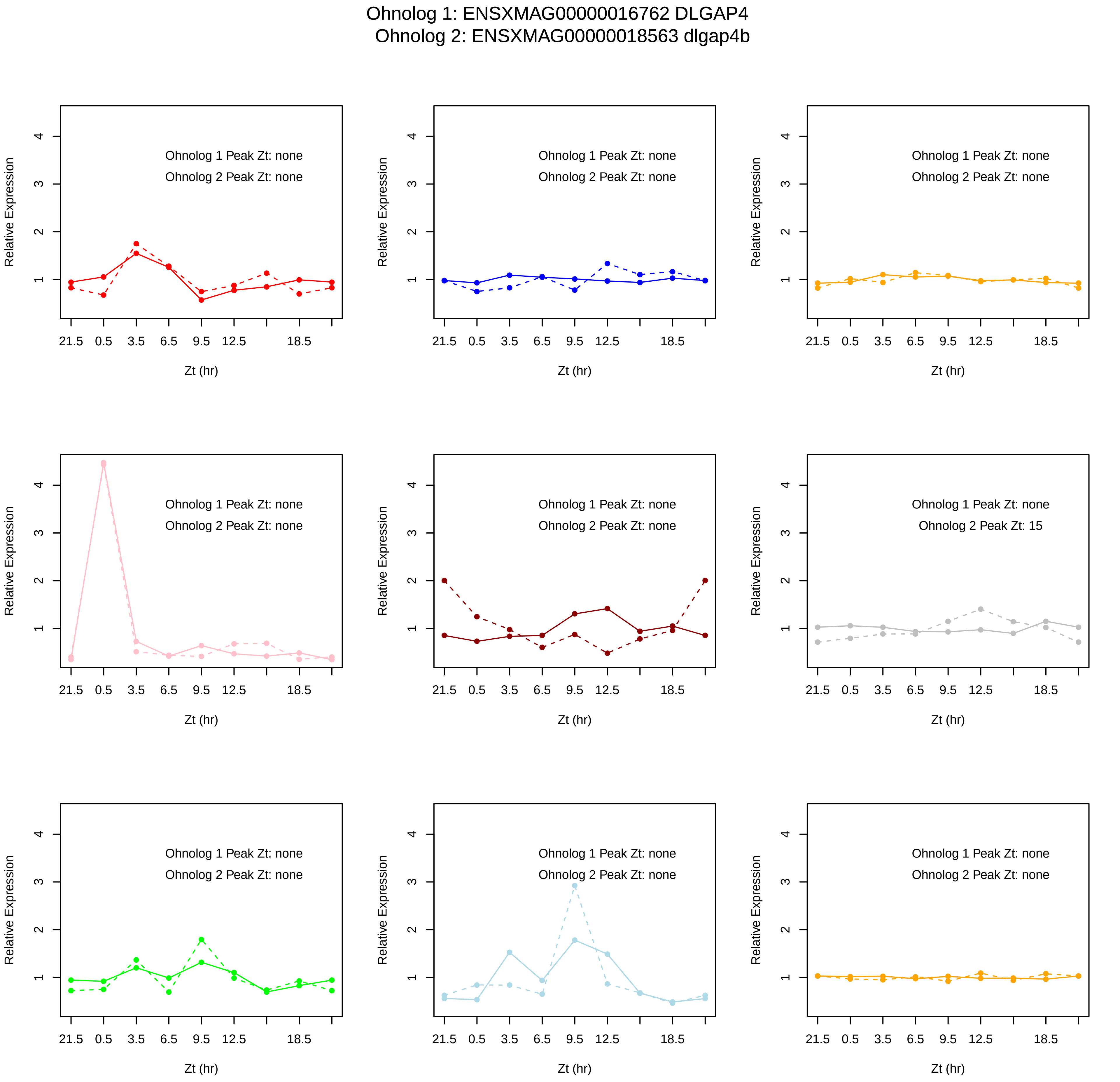

Ohnolog 1: ENSXMAG00000016762 DLGAP4
Ohnolog 2: ENSXMAG00000018563 dlgap4b
Ohnolog 1 Peak Zt: none
Ohnolog 2 Peak Zt: none
Ohnolog 1 Peak Zt: none
Ohnolog 2 Peak Zt: none
Ohnolog 1 Peak Zt: none
Ohnolog 2 Peak Zt: none
Ohnolog 1 Peak Zt: none
Ohnolog 2 Peak Zt: none
Ohnolog 1 Peak Zt: none
Ohnolog 2 Peak Zt: none
Ohnolog 1 Peak Zt: none
Ohnolog 2 Peak Zt: 15
Ohnolog 1 Peak Zt: none
Ohnolog 2 Peak Zt: none
Ohnolog 1 Peak Zt: none
Ohnolog 2 Peak Zt: none
Ohnolog 1 Peak Zt: none
Ohnolog 2 Peak Zt: none
Relative Expression
Zt (hr)
21.5
0.5
3.5
6.5
9.5
12.5
18.5
1
2
3
4

Ohnolog 1: ENSXMAG00000021249 txlnbb
Ohnolog 2: ENSXMAG00000021613

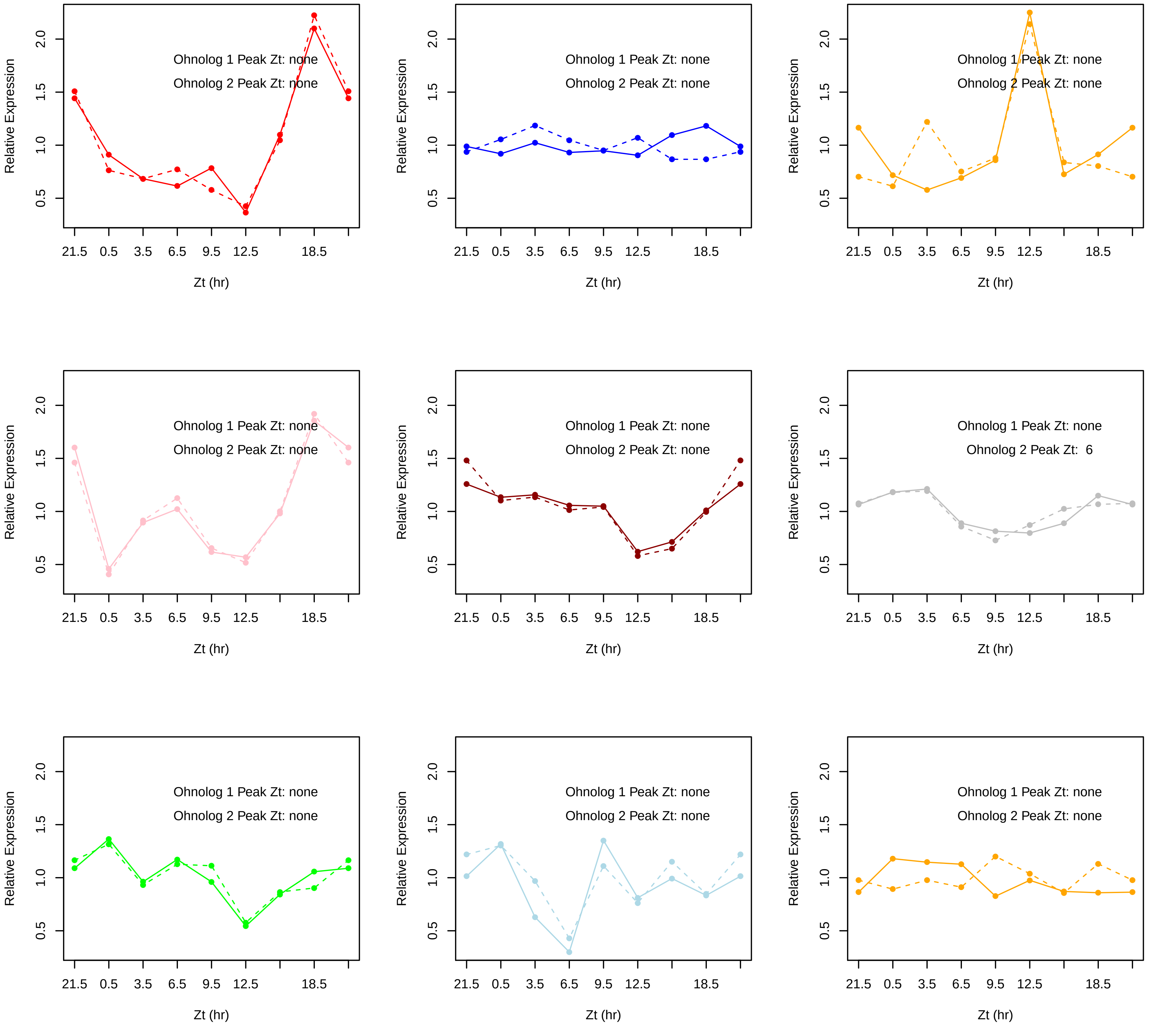

Ohnolog 1: ENSXMAG00000009280 cntn1b
Ohnolog 2: ENSXMAG00000017213 CNTN1

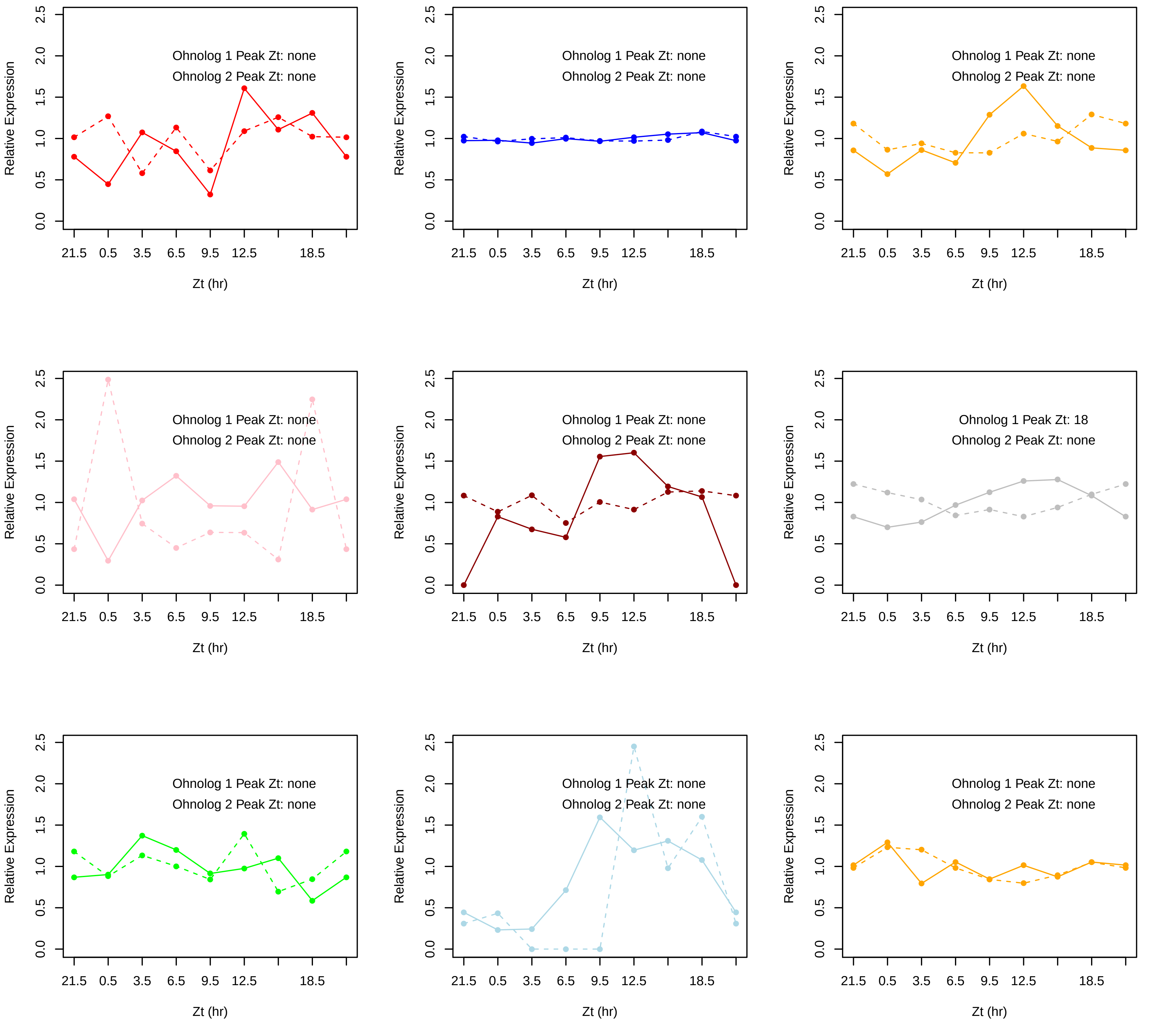

Ohnolog 1: ENSXMAG00000009225 mvb12bb
Ohnolog 2: ENSXMAG00000024443 mvb12ba

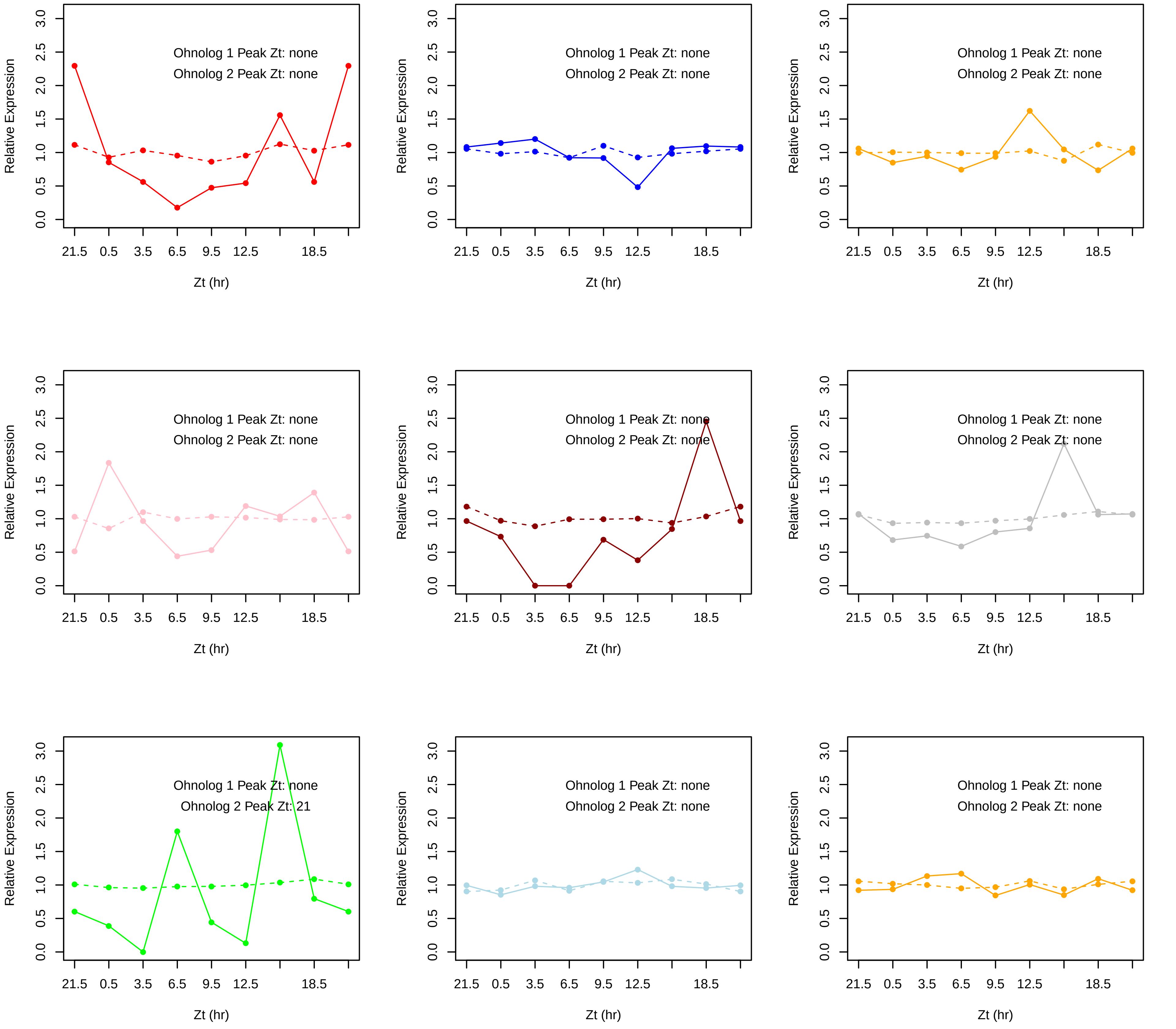

Ohnolog 1: ENSXMAG00000004249 pex5lb
Ohnolog 2: ENSXMAG00000018305 pex5la

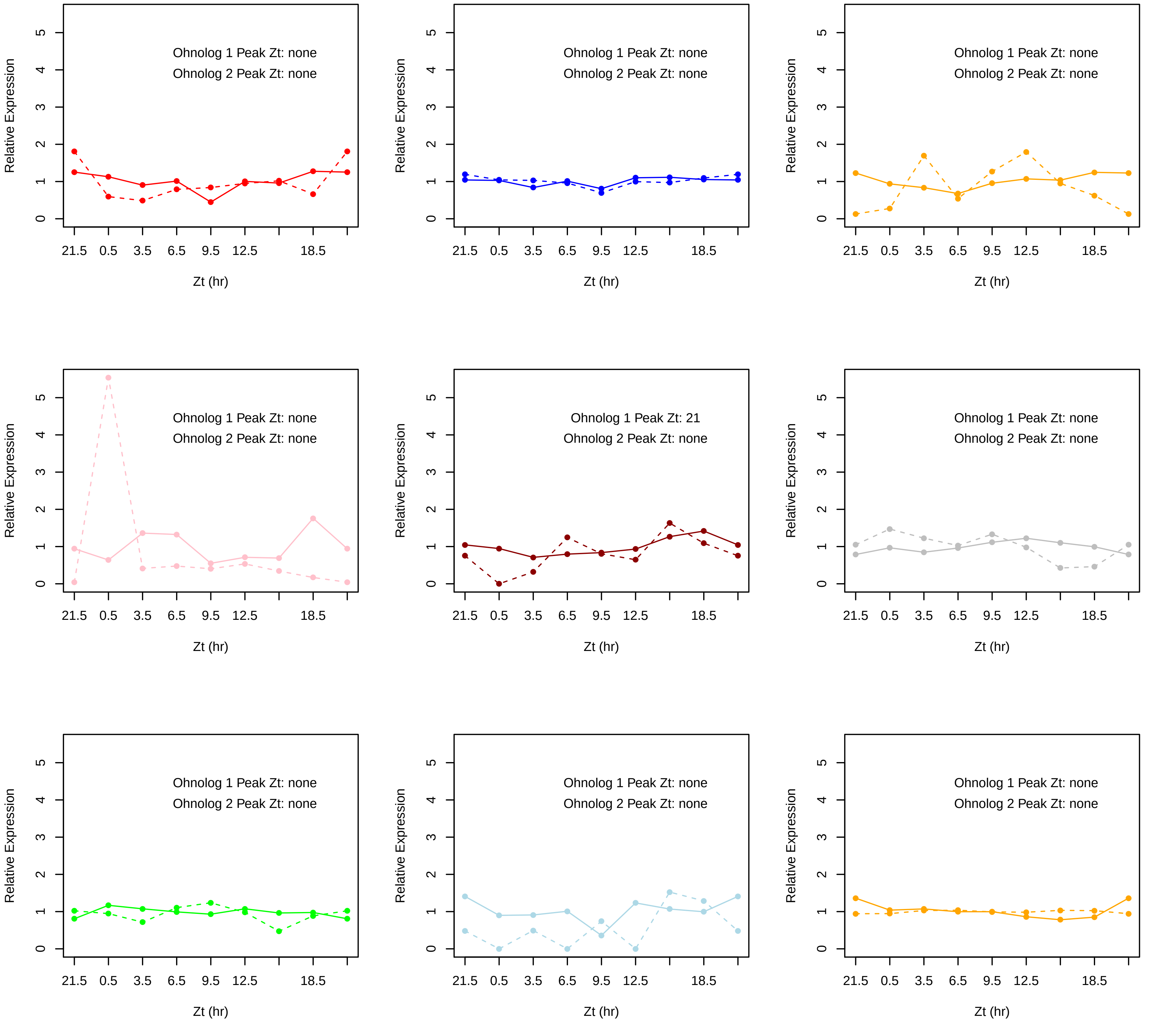

Ohnolog 1: ENSXMAG00000000796 slc1a2b
Ohnolog 2: ENSXMAG00000001471 slc1a2a

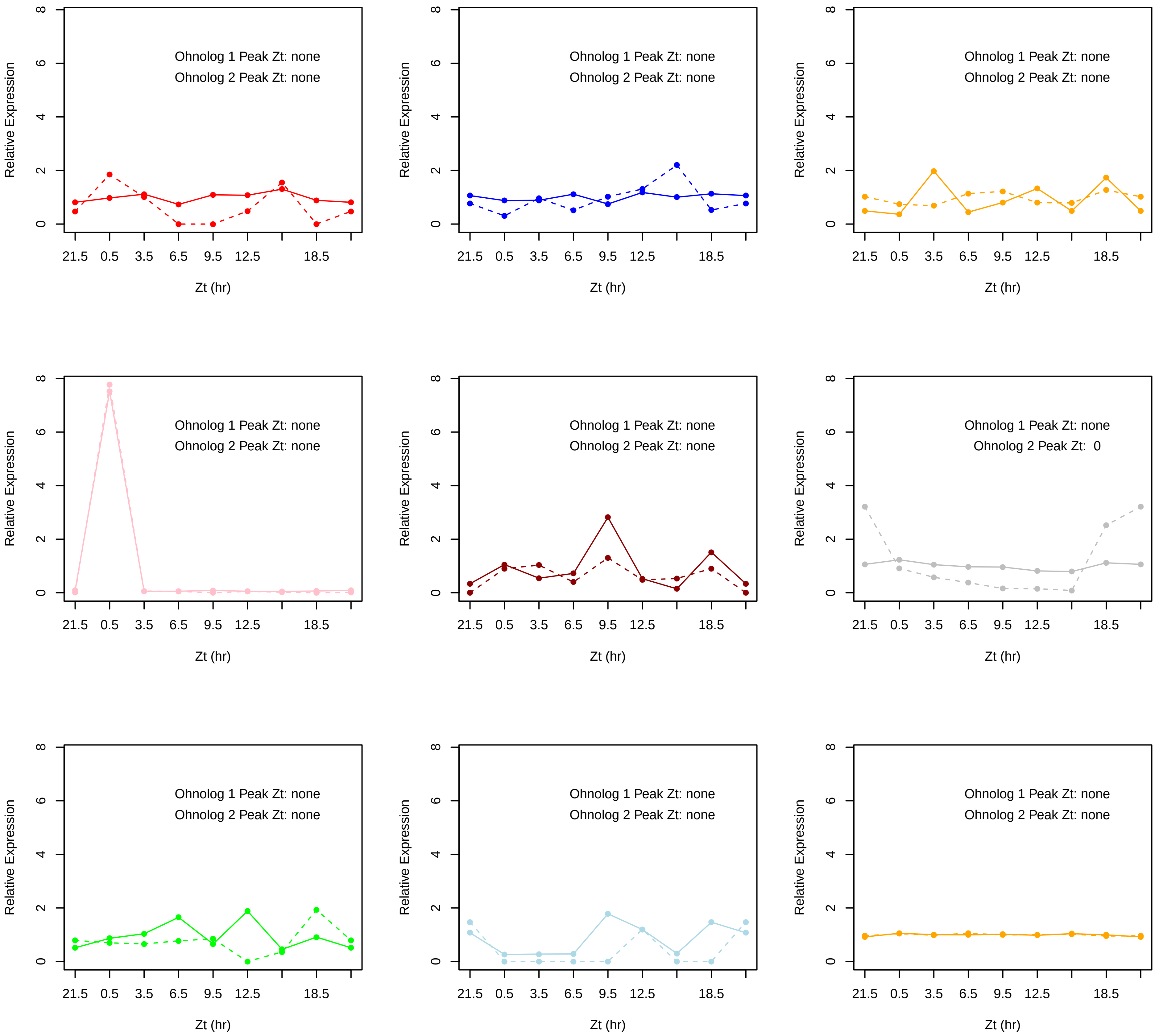

Ohnolog 1: ENSXMAG00000004905 traf2a
Ohnolog 2: ENSXMAG00000025264 TRAF2

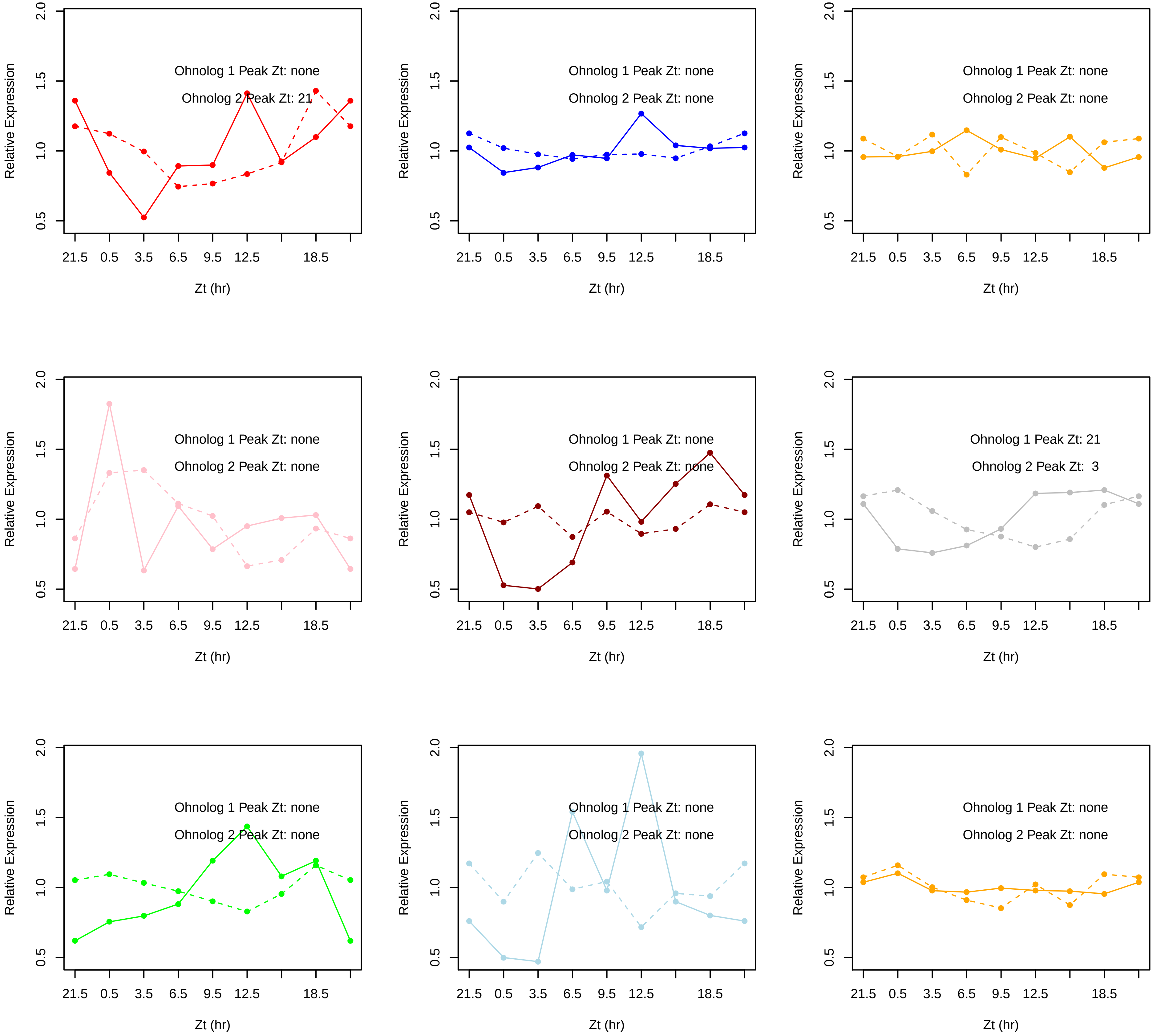

Ohnolog 1: ENSXMAG00000000759
Ohnolog 2: ENSXMAG00000002607 cpne4b

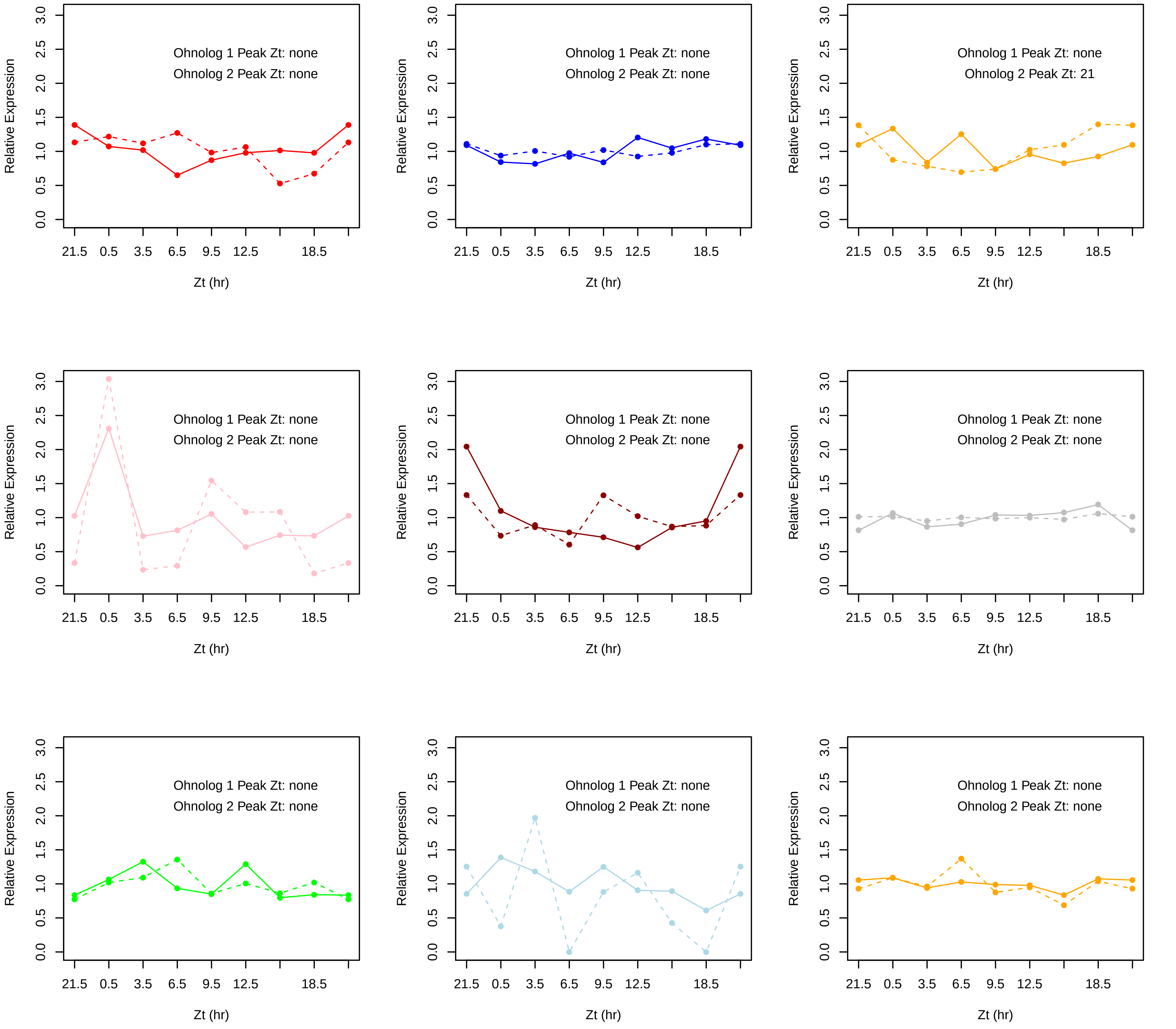

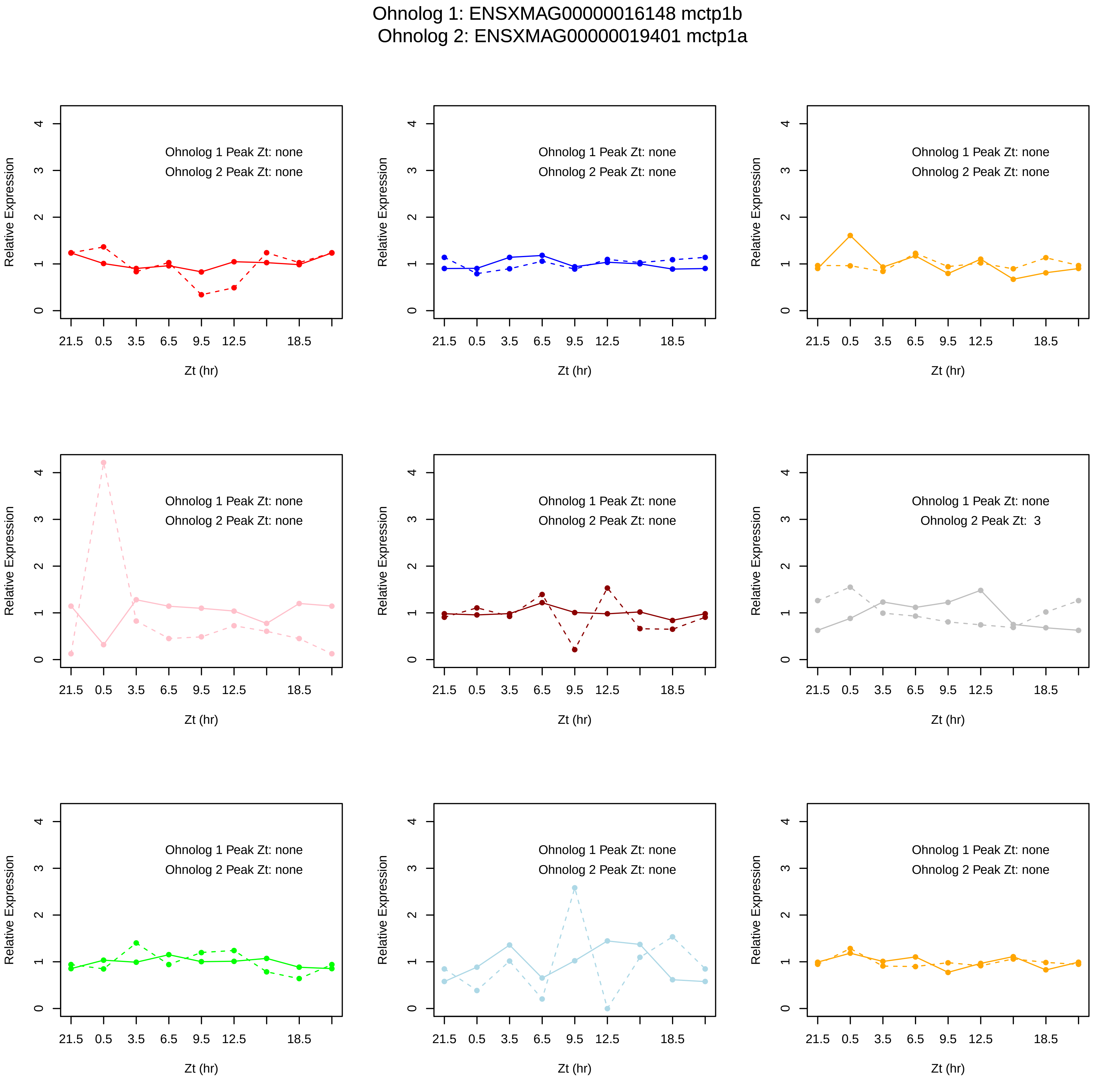
Ohnolog 1: ENSXMAG00000016148 mctp1b
Ohnolog 2: ENSXMAG00000019401 mctp1a
Ohnolog 1 Peak Zt: none
Ohnolog 2 Peak Zt: none
Ohnolog 1 Peak Zt: none
Ohnolog 2 Peak Zt: none
Ohnolog 1 Peak Zt: none
Ohnolog 2 Peak Zt: none
Ohnolog 1 Peak Zt: none
Ohnolog 2 Peak Zt: none
Ohnolog 1 Peak Zt: none
Ohnolog 2 Peak Zt: none
Ohnolog 1 Peak Zt: none
Ohnolog 2 Peak Zt: 3
Ohnolog 1 Peak Zt: none
Ohnolog 2 Peak Zt: none
Ohnolog 1 Peak Zt: none
Ohnolog 2 Peak Zt: none
Ohnolog 1 Peak Zt: none
Ohnolog 2 Peak Zt: none
Relative Expression
Zt (hr)
21.5 0.5 3.5 6.5 9.5 12.5 18.5
0 1 2 3 4

Ohnolog 1: ENSXMAG00000004457 PDGFA
Ohnolog 2: ENSXMAG00000022003 pdgfaa

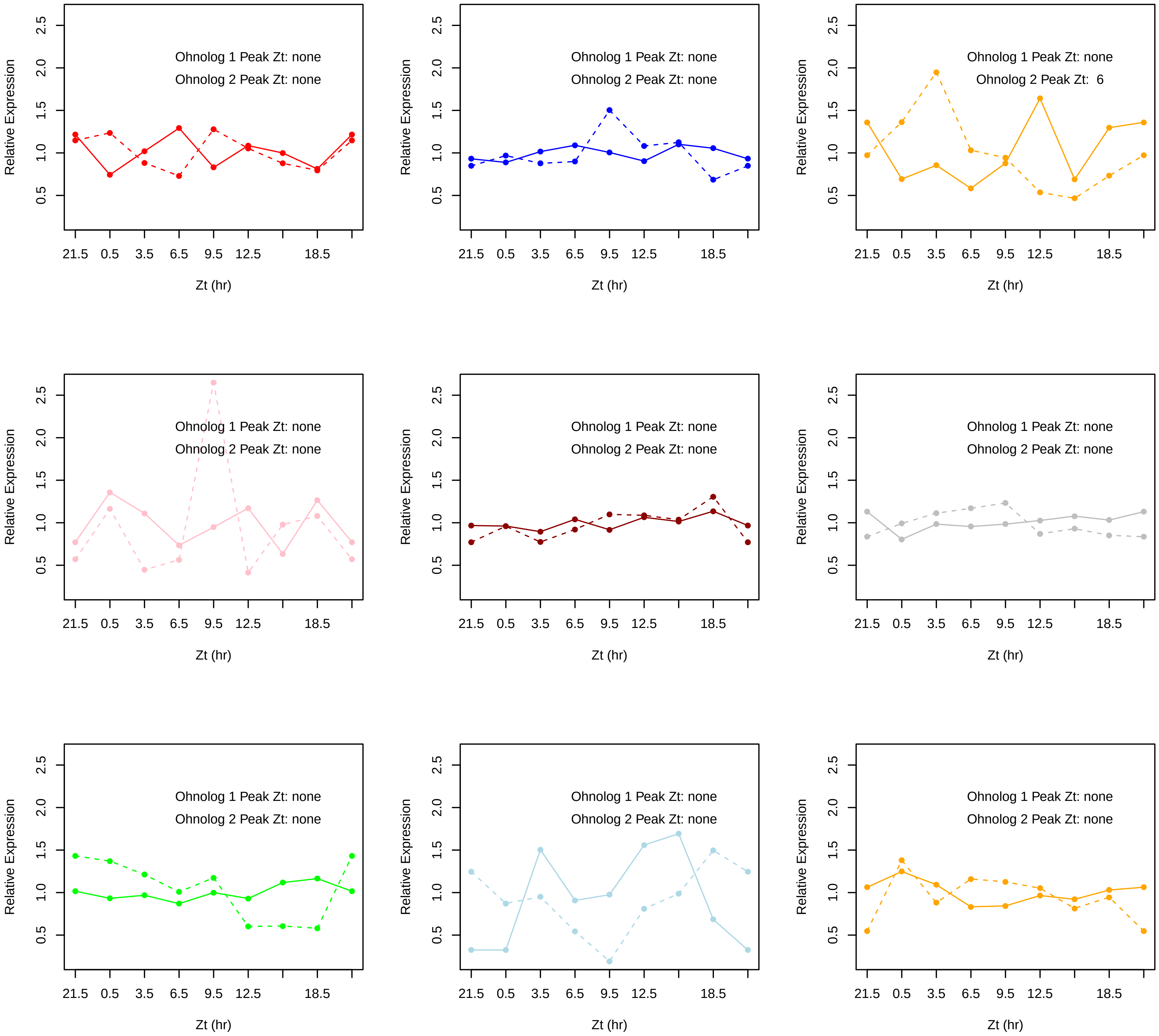

Ohnolog 1: ENSXMAG00000012214 ubash3ba
Ohnolog 2: ENSXMAG00000012415 ubash3bb

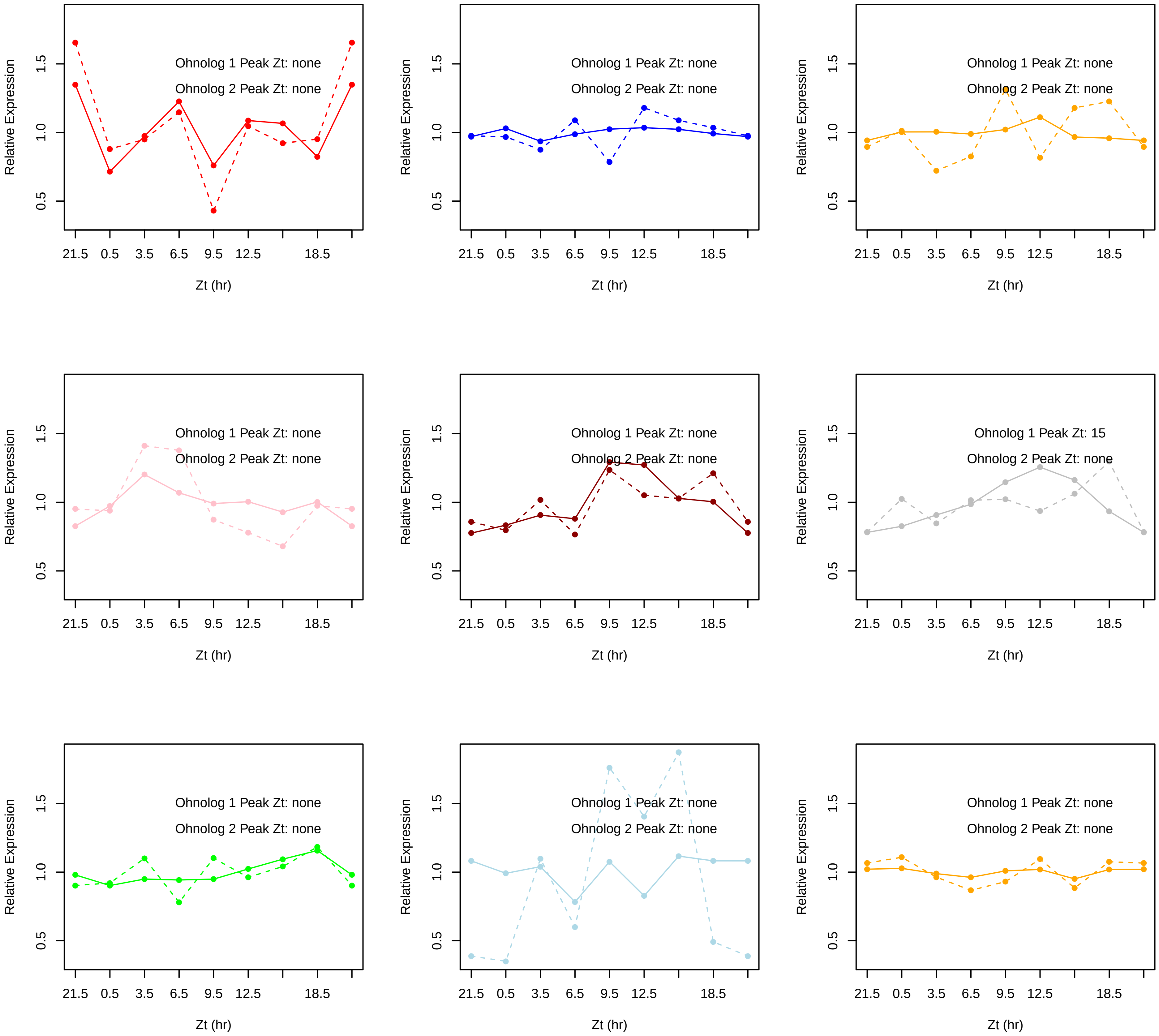

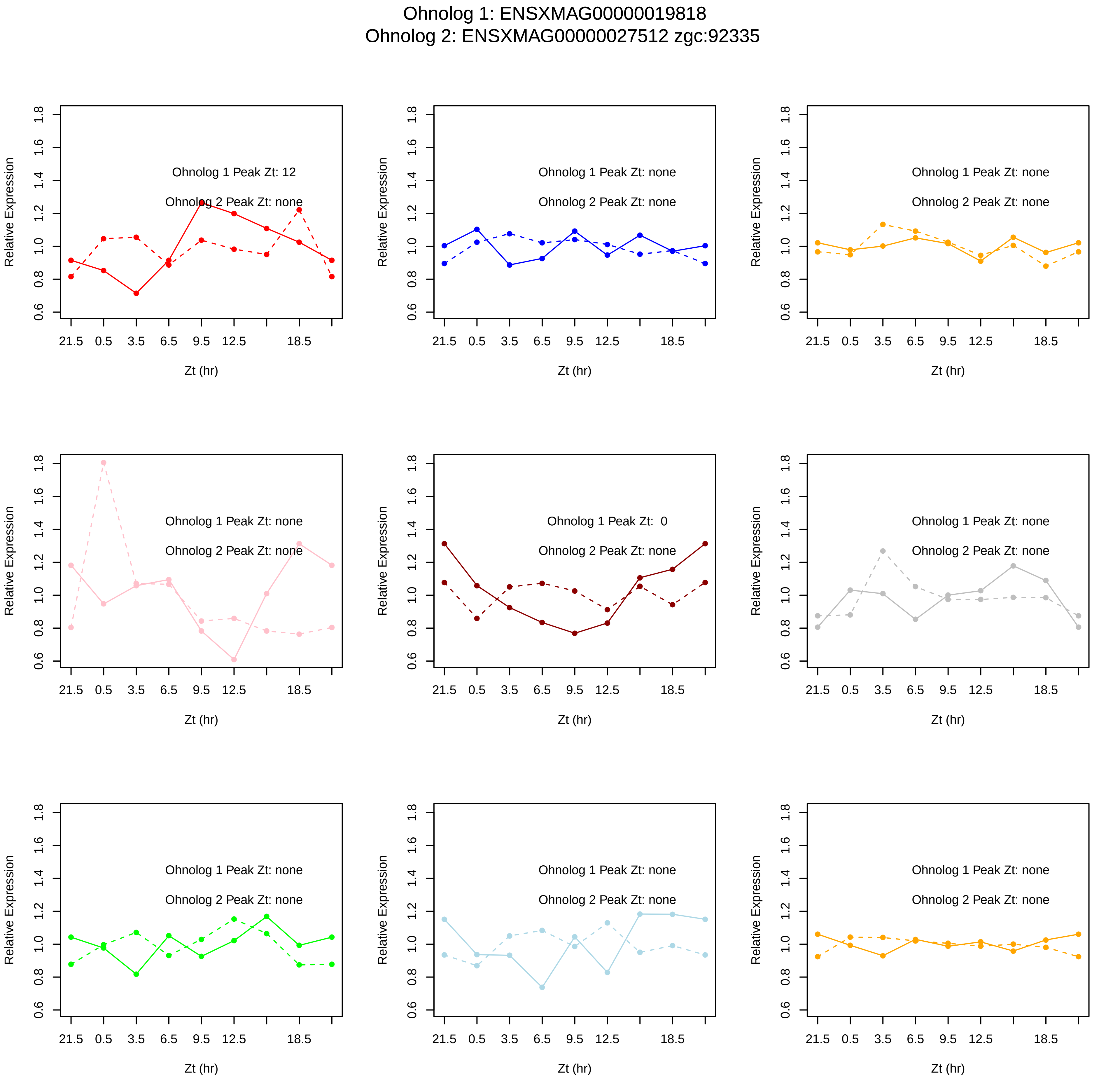
Ohnolog 1: ENSXMAG00000019818
Ohnolog 2: ENSXMAG00000027512 zgc:92335
Ohnolog 1 Peak Zt: 12
Ohnolog 2 Peak Zt: none
Ohnolog 1 Peak Zt: none
Ohnolog 2 Peak Zt: none
Ohnolog 1 Peak Zt: none
Ohnolog 2 Peak Zt: none
Ohnolog 1 Peak Zt: none
Ohnolog 2 Peak Zt: none
Ohnolog 1 Peak Zt: 0
Ohnolog 2 Peak Zt: none
Ohnolog 1 Peak Zt: none
Ohnolog 2 Peak Zt: none
Ohnolog 1 Peak Zt: none
Ohnolog 2 Peak Zt: none
Ohnolog 1 Peak Zt: none
Ohnolog 2 Peak Zt: none
Ohnolog 1 Peak Zt: none
Ohnolog 2 Peak Zt: none
Relative Expression
Zt (hr)
21.5
0.5
3.5
6.5
9.5
12.5
18.5
0.6
0.8
1.0
1.2
1.4
1.6
1.8

Ohnolog 1: ENSXMAG00000001929 si:ch211−168d1.3
Ohnolog 2: ENSXMAG00000023871

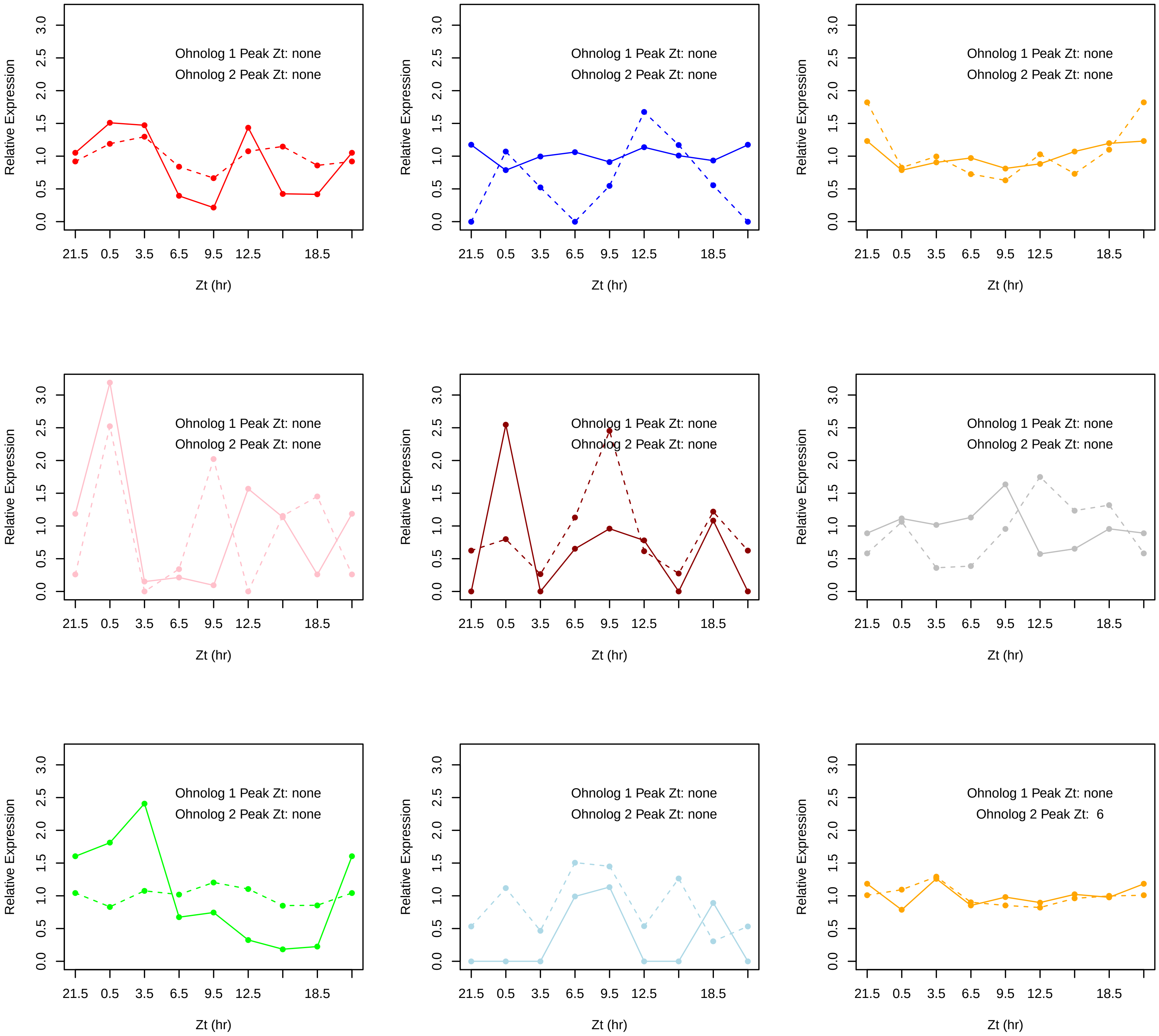

Ohnolog 1: ENSXMAG00000002915
Ohnolog 2: ENSXMAG00000018360 pxna

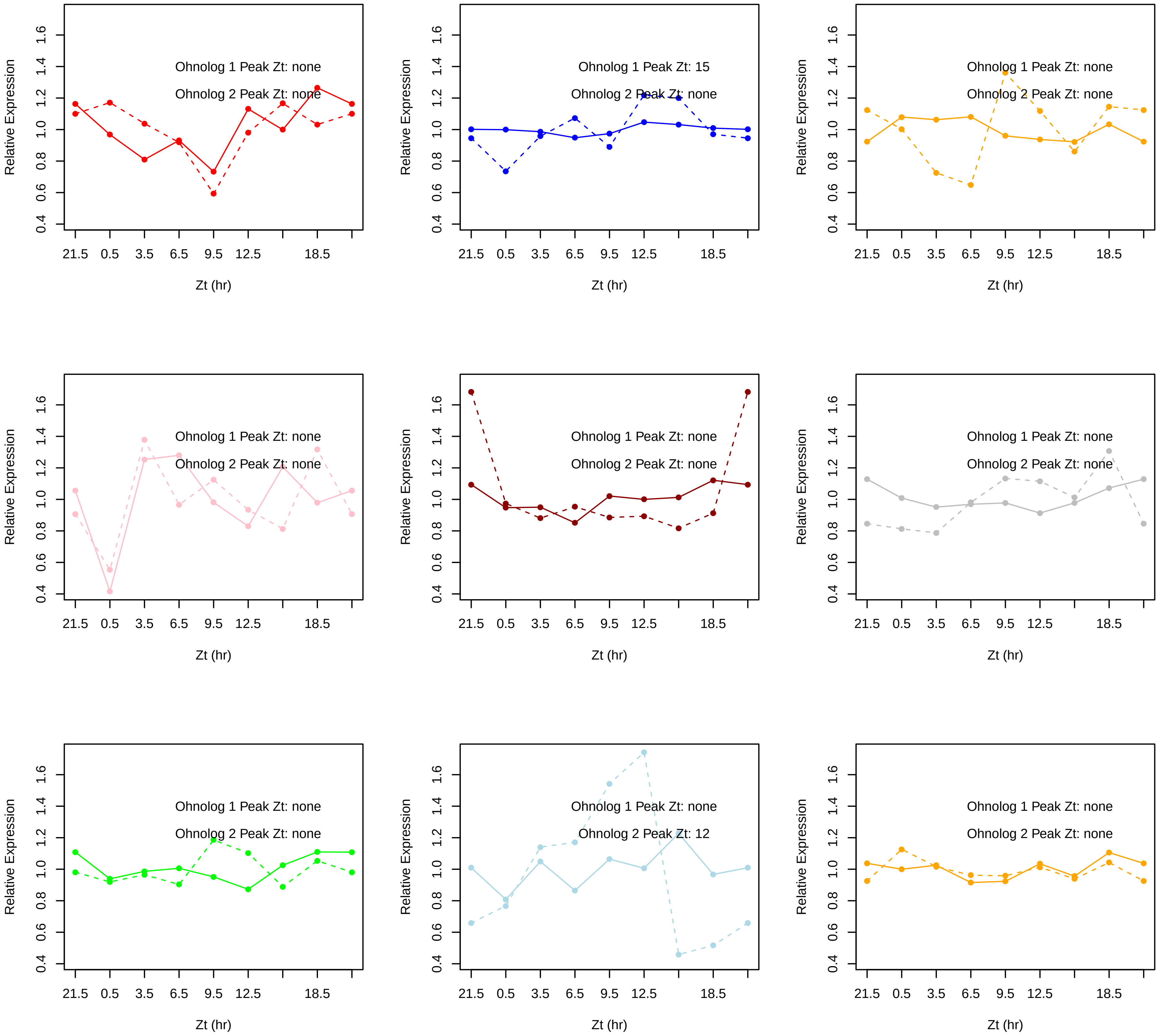

Ohnolog 1: ENSXMAG00000014607
Ohnolog 2: ENSXMAG00000019113 cpxm1b

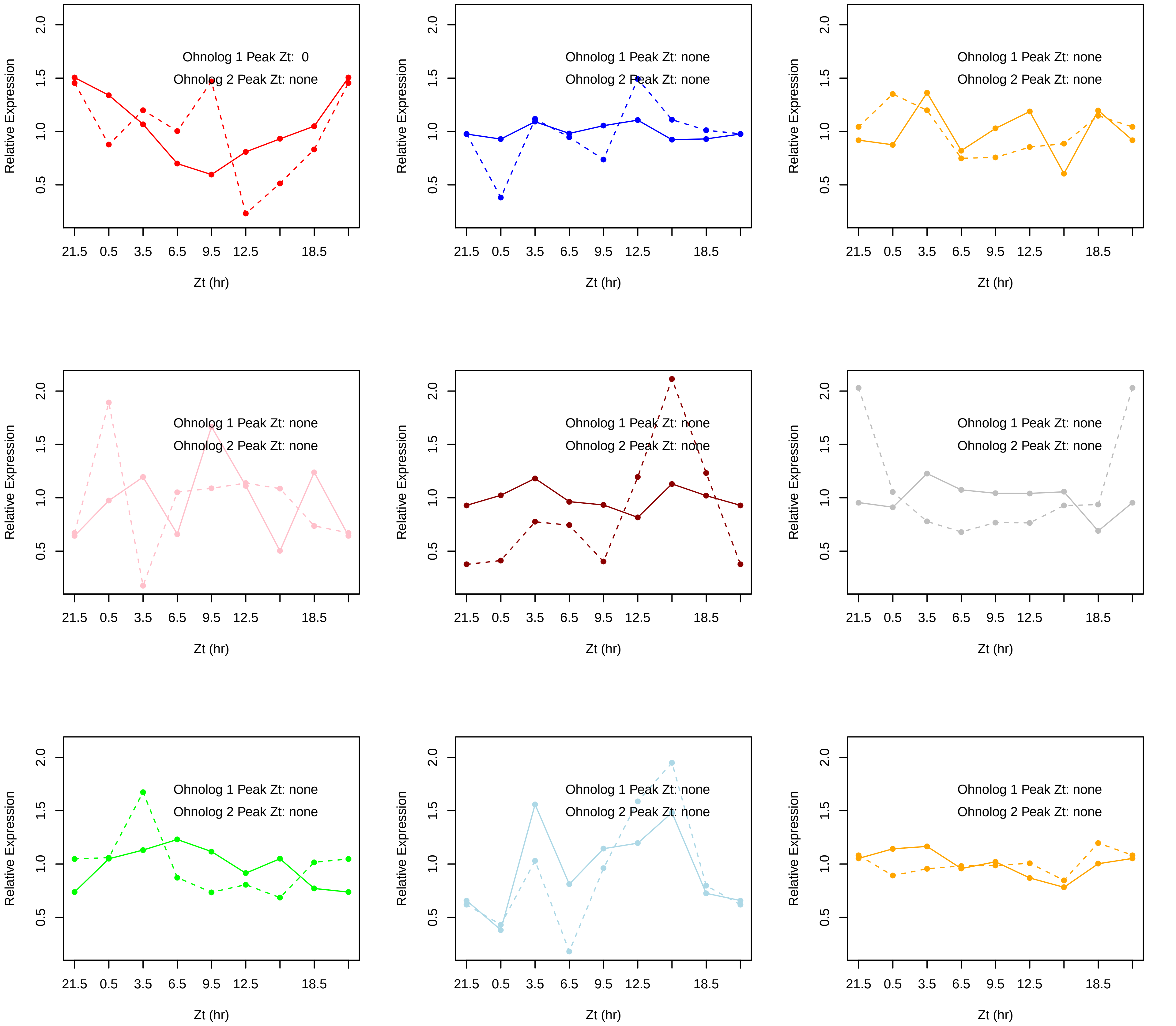

Ohnolog 1: ENSXMAG00000002220 si:dkey−175m17.6
Ohnolog 2: ENSXMAG00000027722 si:dkey−160o24.3

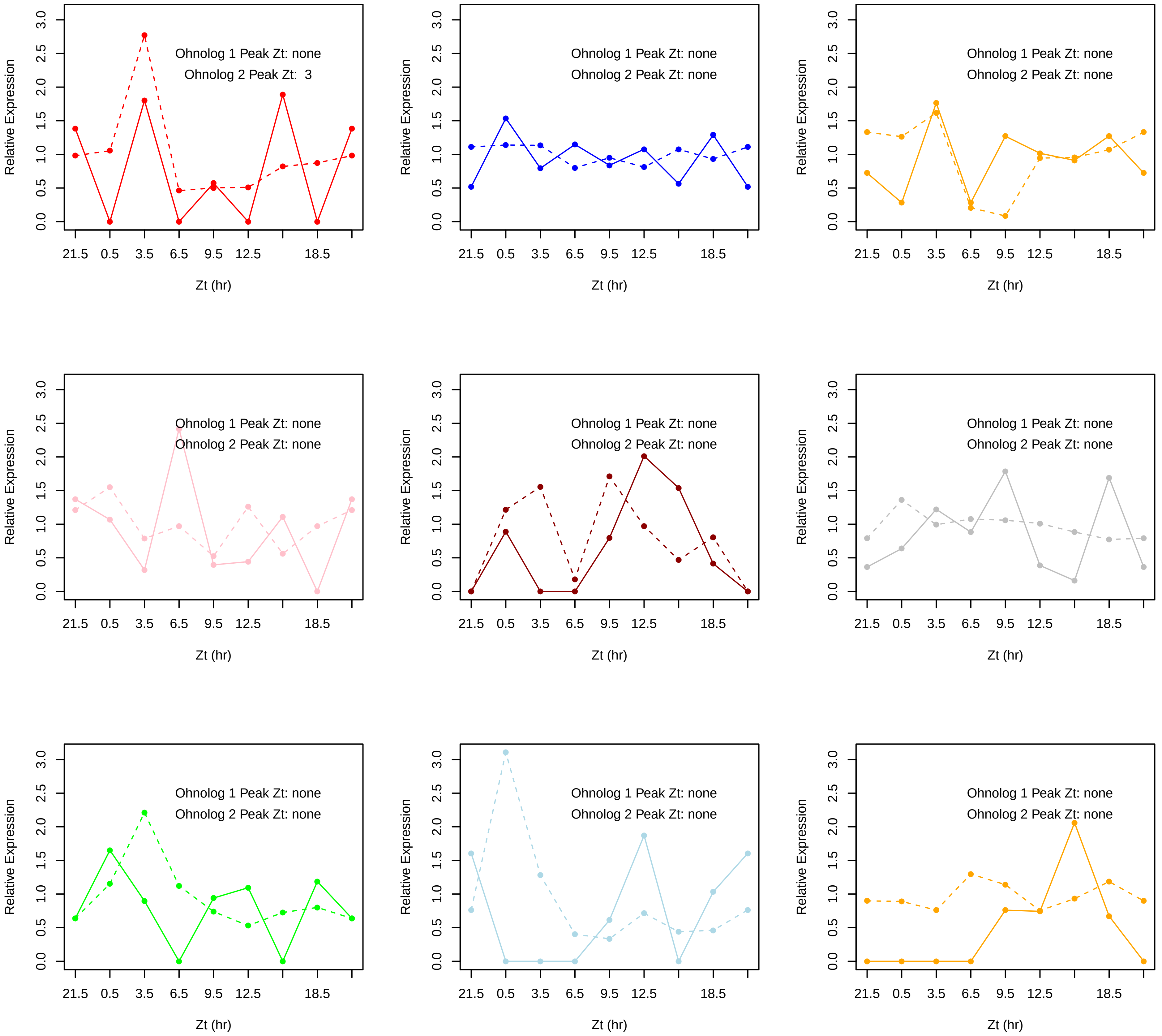

Ohnolog 1: ENSXMAG00000002028 ccnt2a
Ohnolog 2: ENSXMAG00000007871

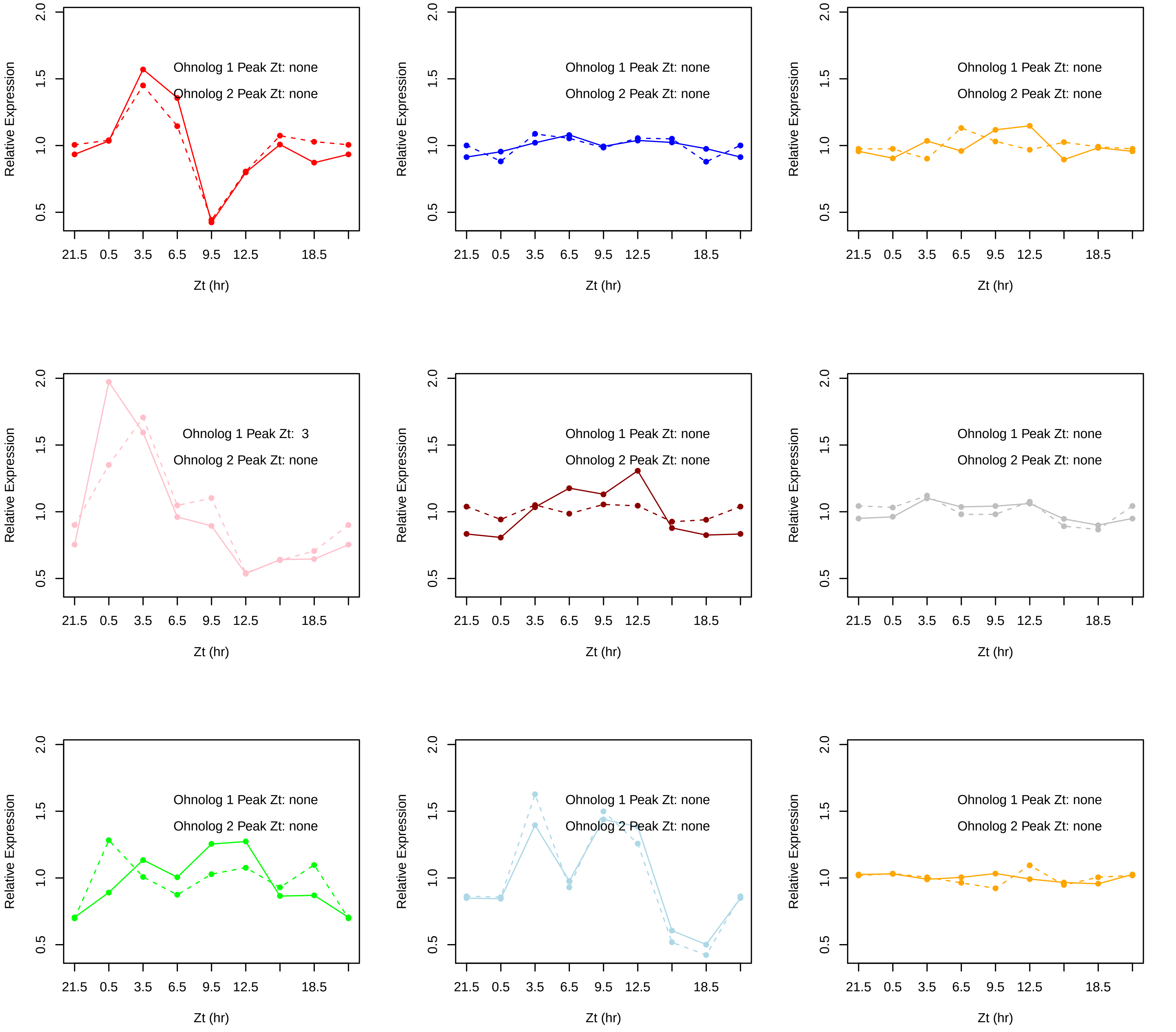

Ohnolog 1: ENSXMAG00000004796 slc3a2b
Ohnolog 2: ENSXMAG00000008626 slc3a2a

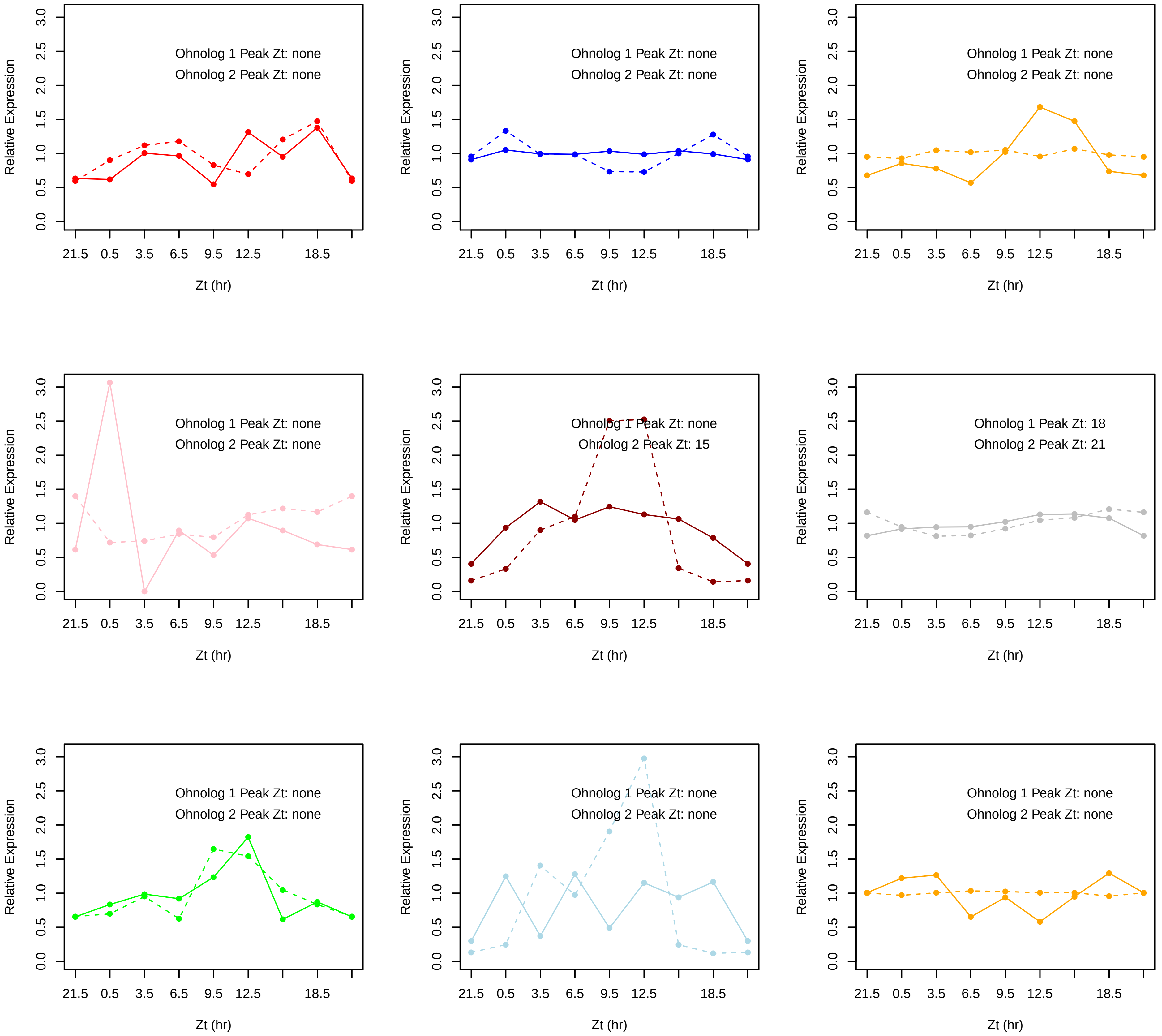

Ohnolog 1: ENSXMAG00000005452 kifap3a
Ohnolog 2: ENSXMAG00000018739

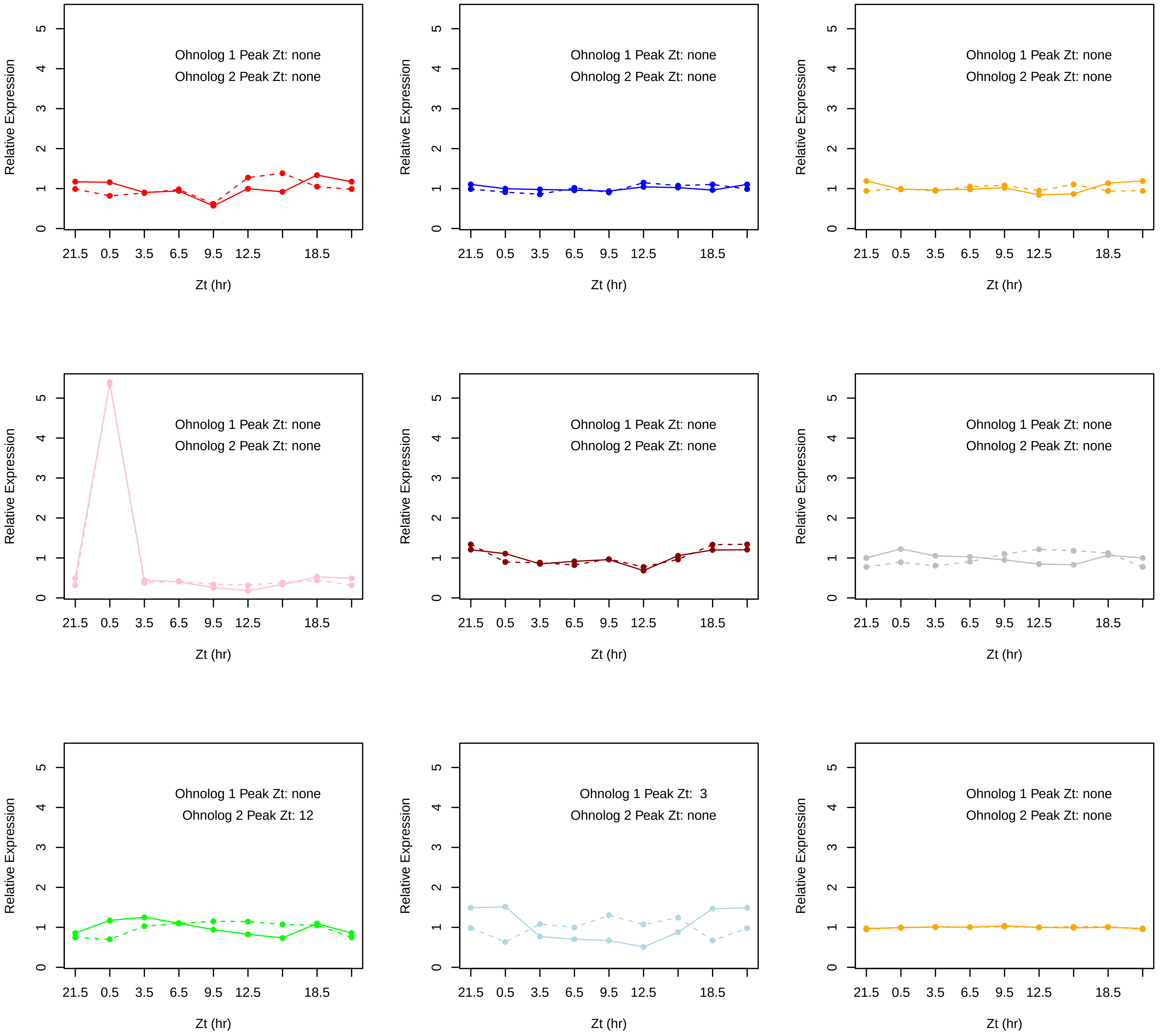

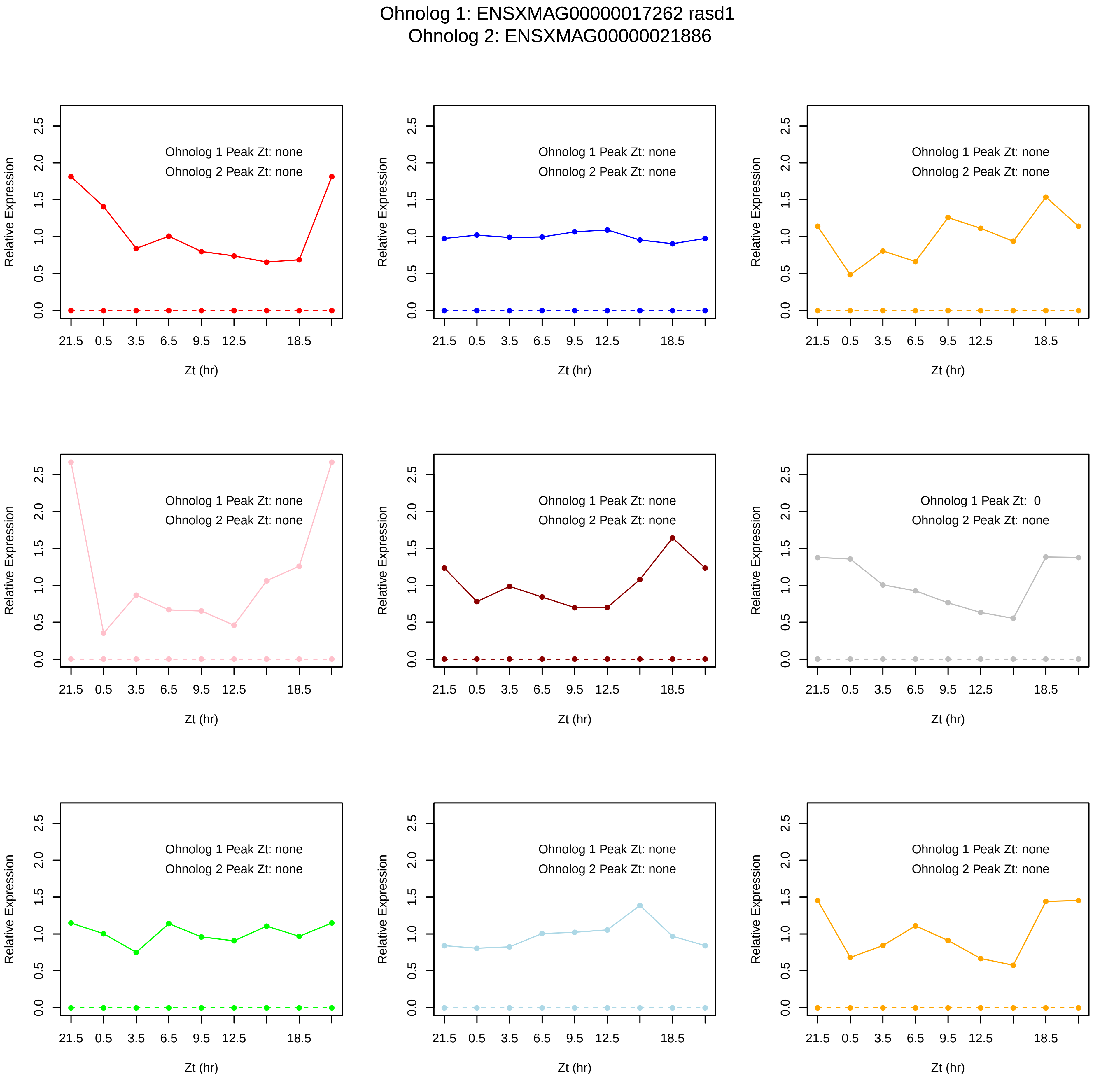

Ohnolog 1: ENSXMAG00000017262 rasd1
Ohnolog 2: ENSXMAG00000021886
Ohnolog 1 Peak Zt: none
Ohnolog 2 Peak Zt: none
Ohnolog 1 Peak Zt: 0
Relative Expression
Zt (hr)
21.5
0.5
3.5
6.5
9.5
12.5
18.5
0.0
0.5
1.0
1.5
2.0
2.5

Ohnolog 1: ENSXMAG00000011954 slc7a10a
Ohnolog 2: ENSXMAG00000012176 slc7a10b

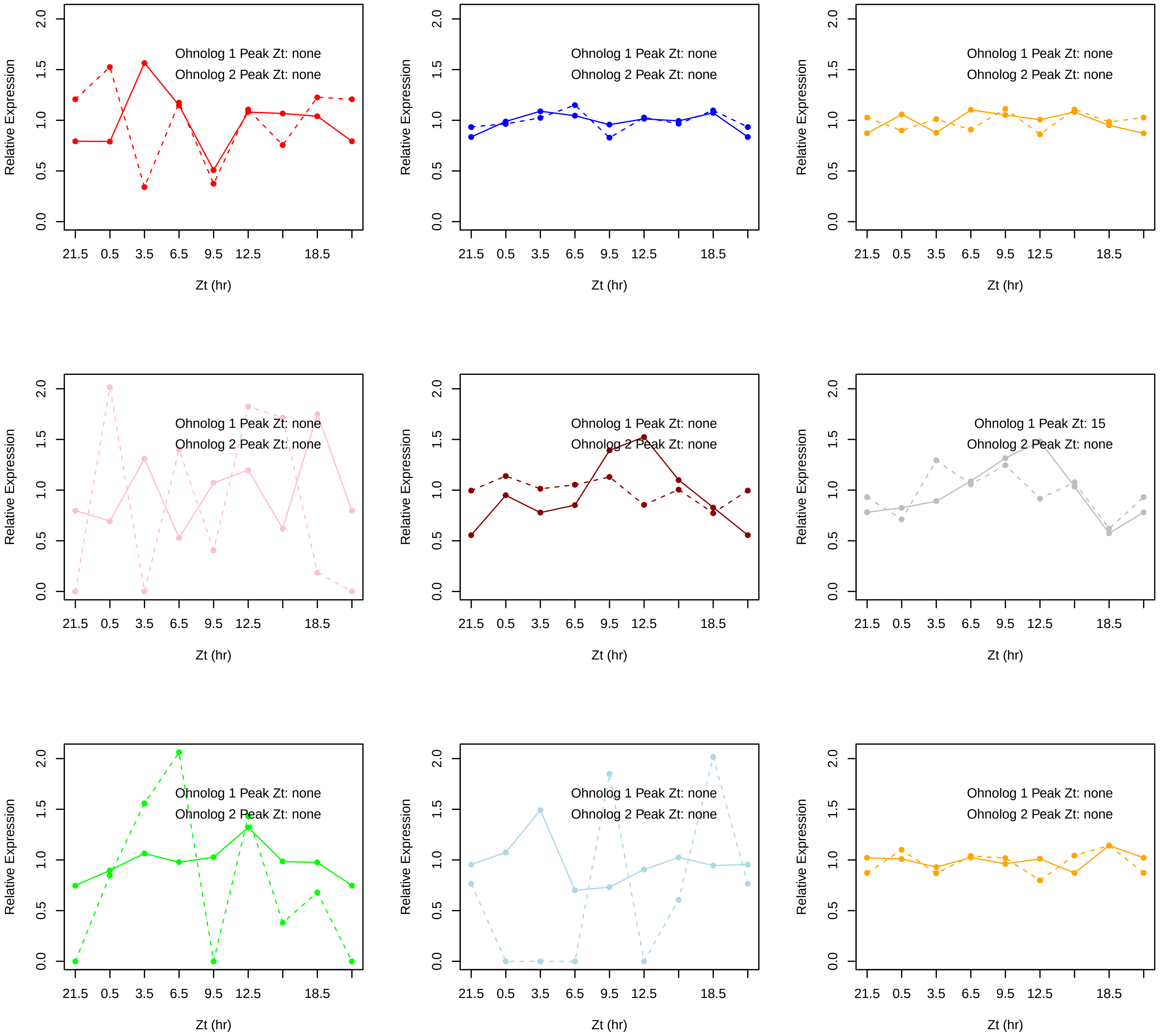

Ohnolog 1: ENSXMAG00000001301 npdc1a
Ohnolog 2: ENSXMAG00000018967 NPDC1

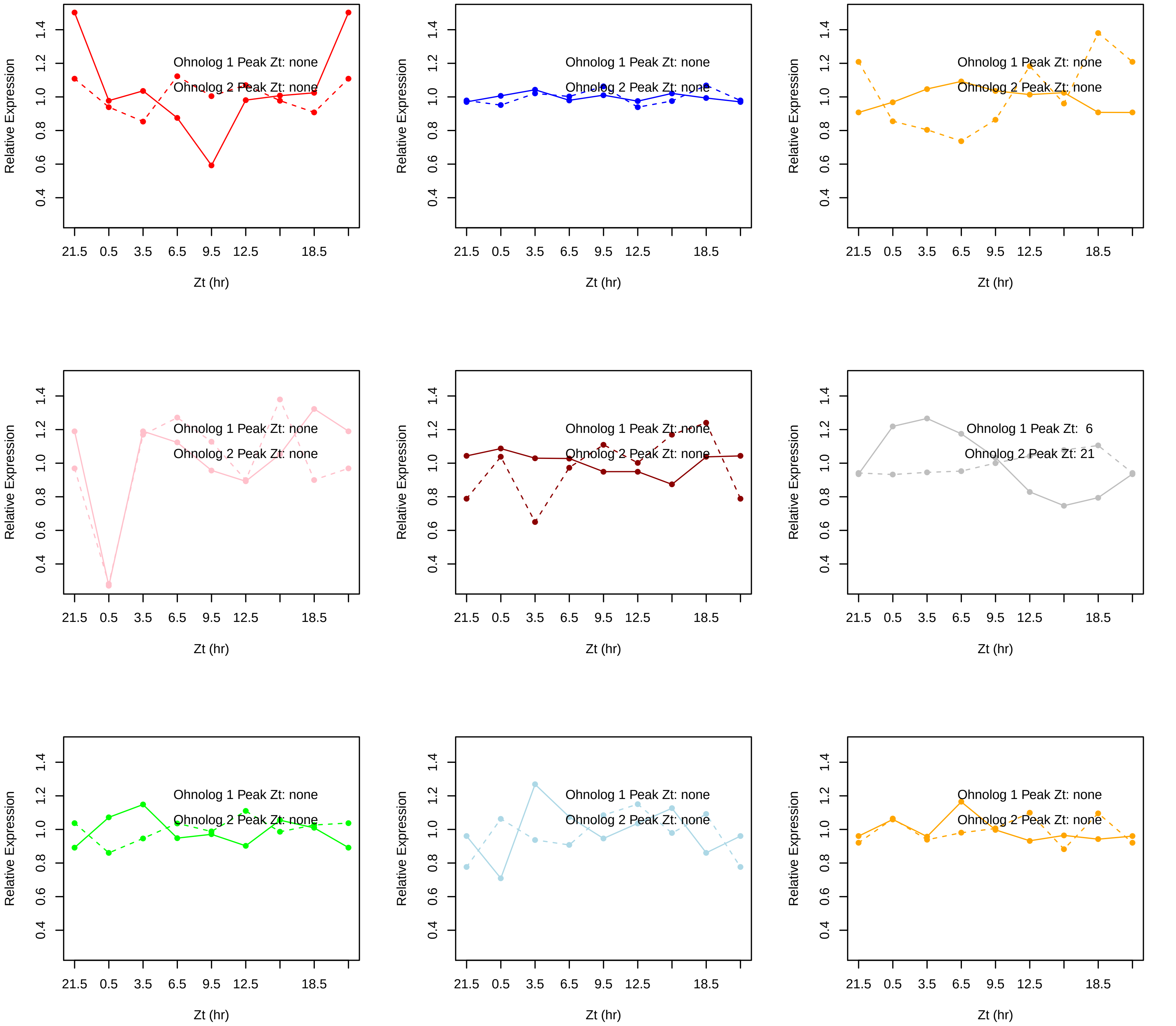

Ohnolog 1: ENSXMAG00000000091
Ohnolog 2: ENSXMAG00000025944

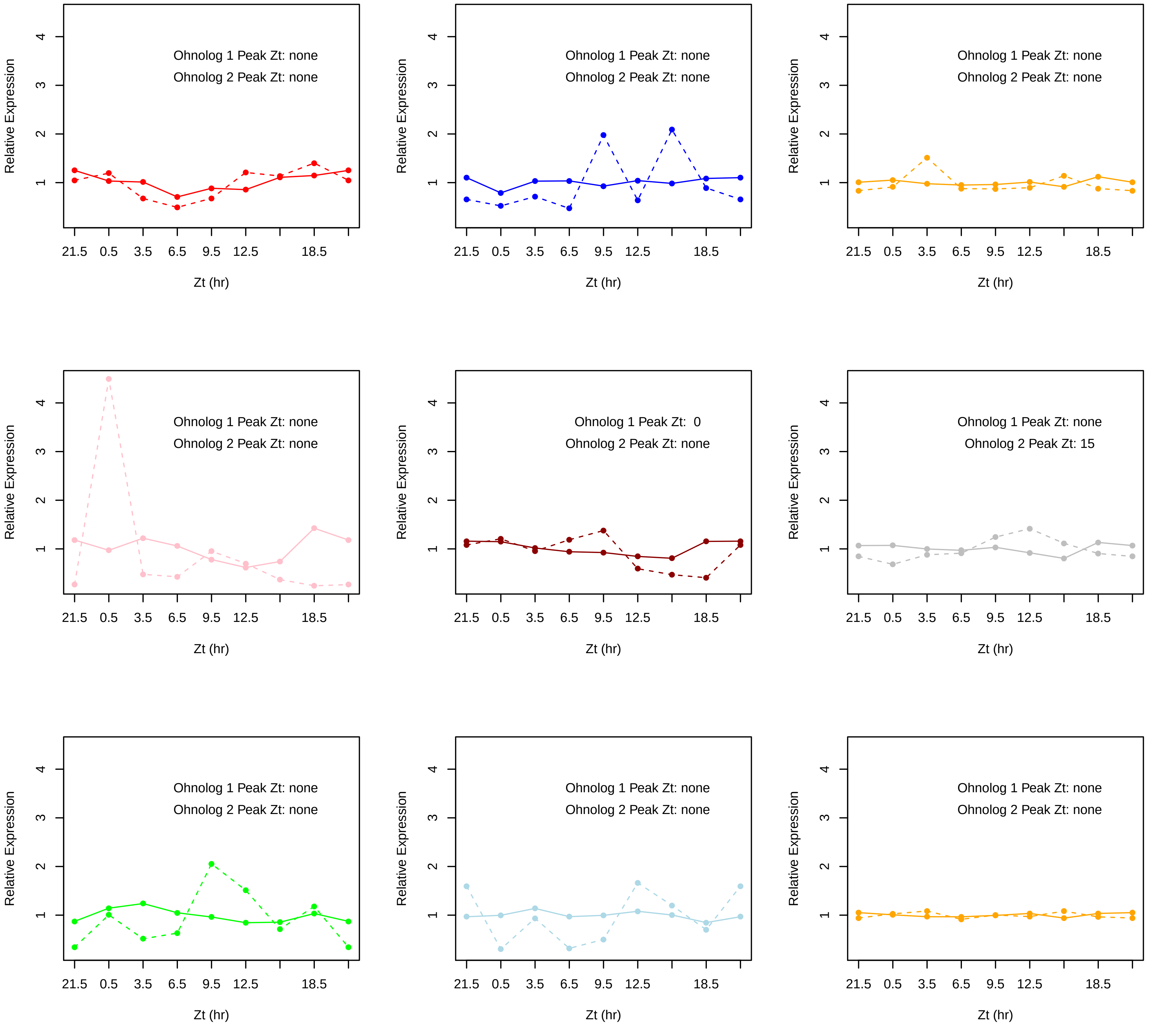

Ohnolog 1: ENSXMAG00000012120 usp22
Ohnolog 2: ENSXMAG00000026761

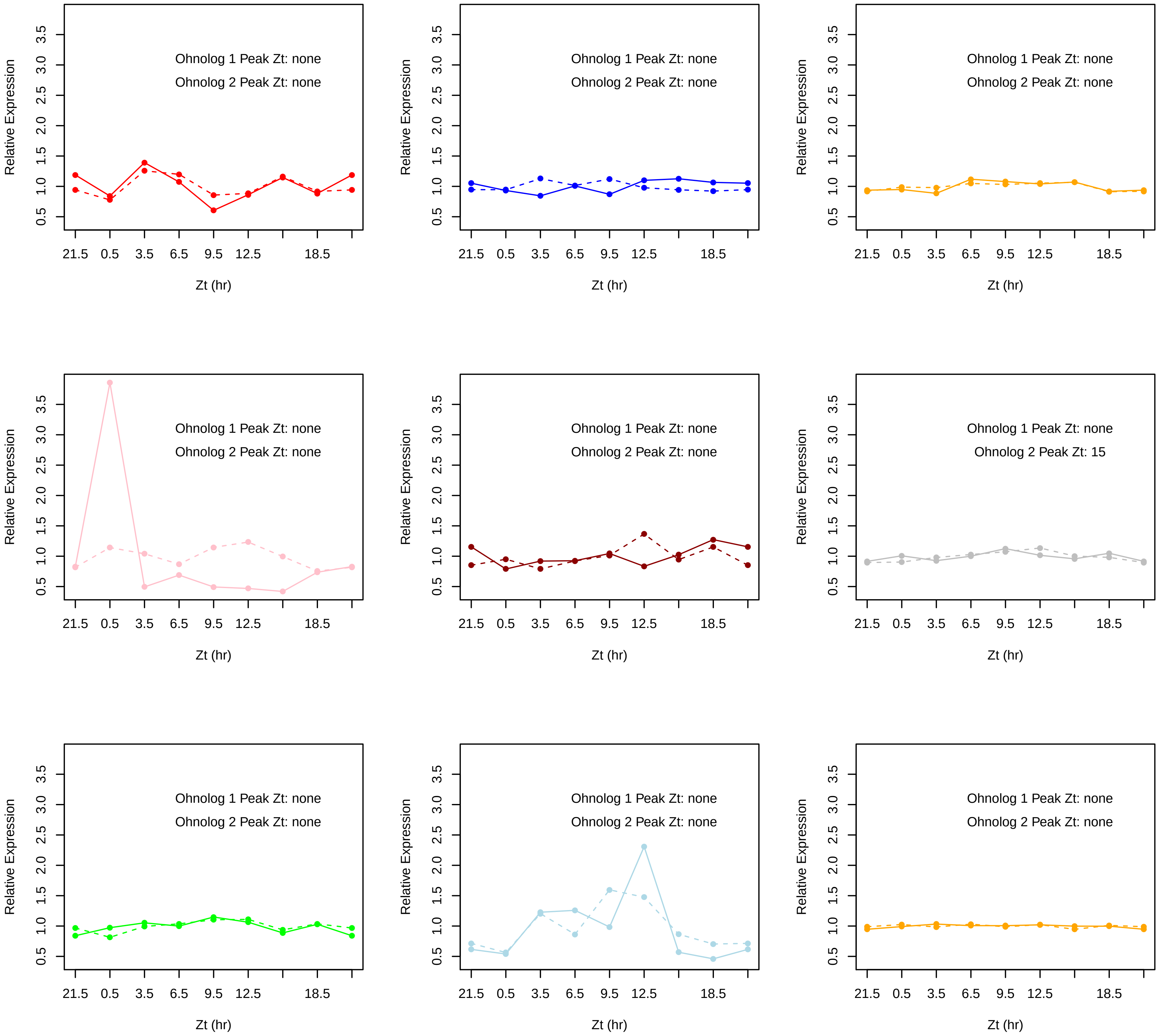

Ohnolog 1: ENSXMAG00000005116 slc8a4a
Ohnolog 2: ENSXMAG00000008678 slc8a4b

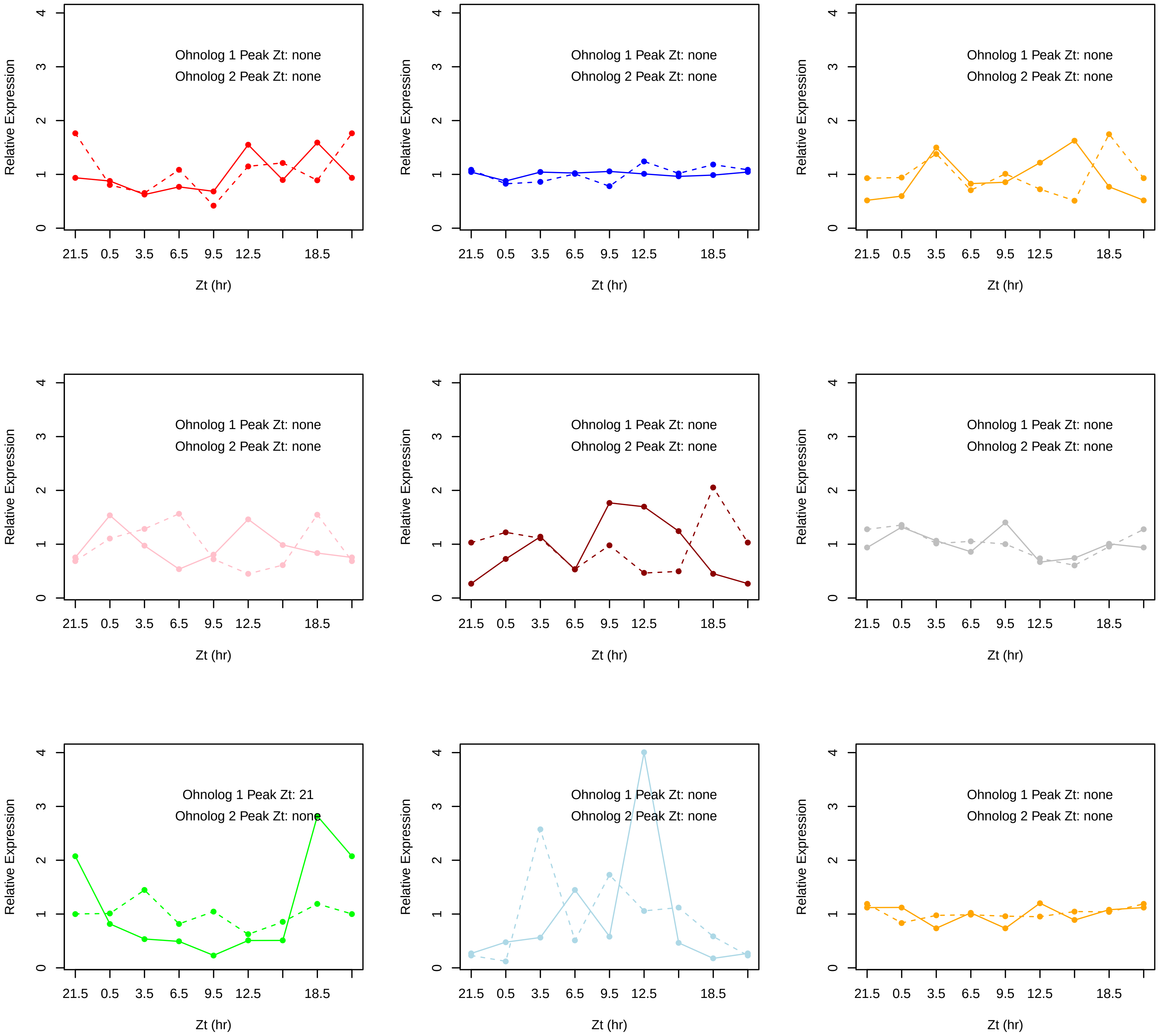

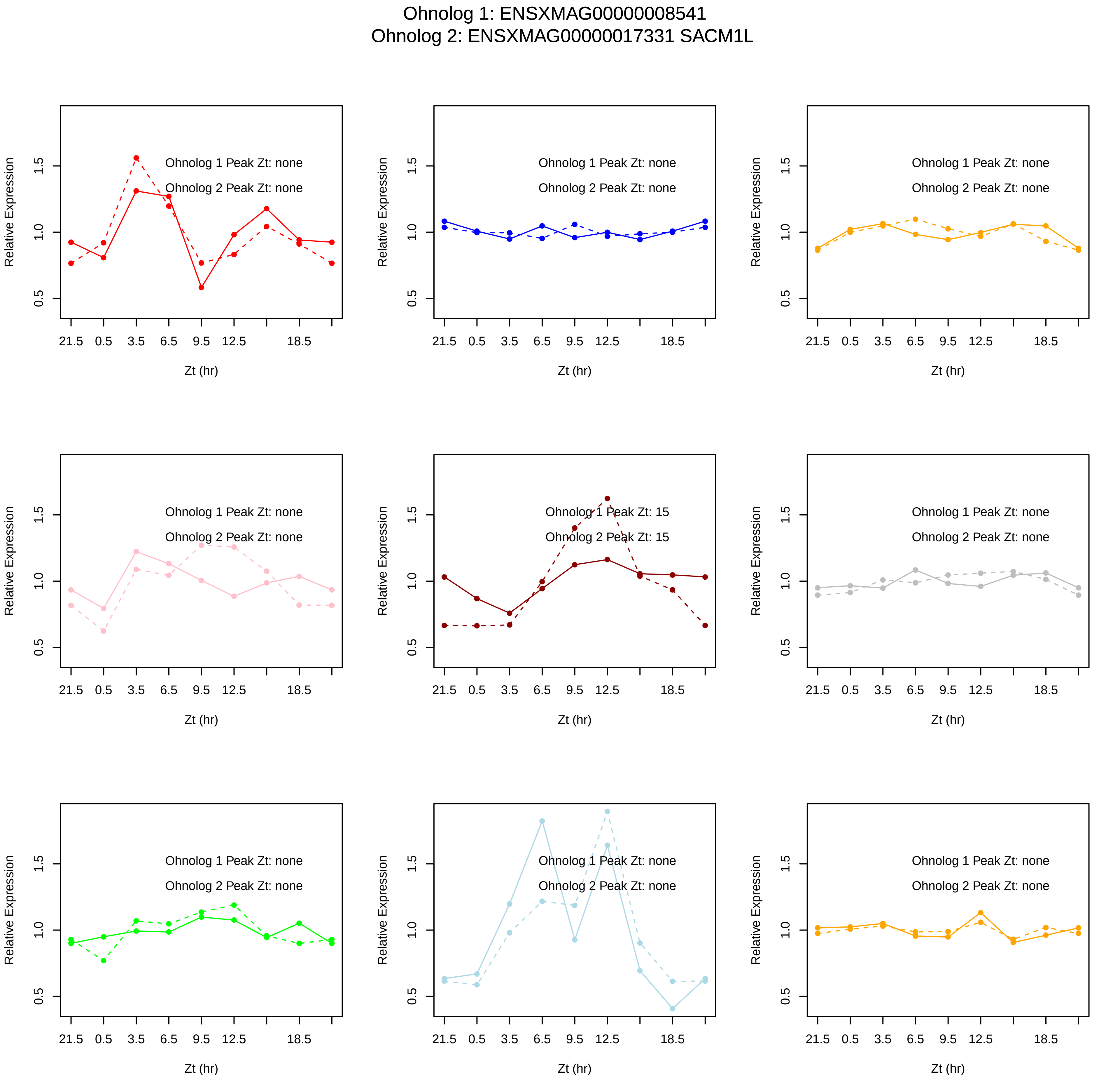
Ohnolog 1: ENSXMAG00000008541
Ohnolog 2: ENSXMAG00000017331 SACM1L
Ohnolog 1 Peak Zt: none
Ohnolog 2 Peak Zt: none
Ohnolog 1 Peak Zt: 15
Ohnolog 2 Peak Zt: 15
Relative Expression
Zt (hr)
21.5
0.5
3.5
6.5
9.5
12.5
18.5
0.5
1.0
1.5

Ohnolog 1: ENSXMAG00000006830
Ohnolog 2: ENSXMAG00000029578 caska

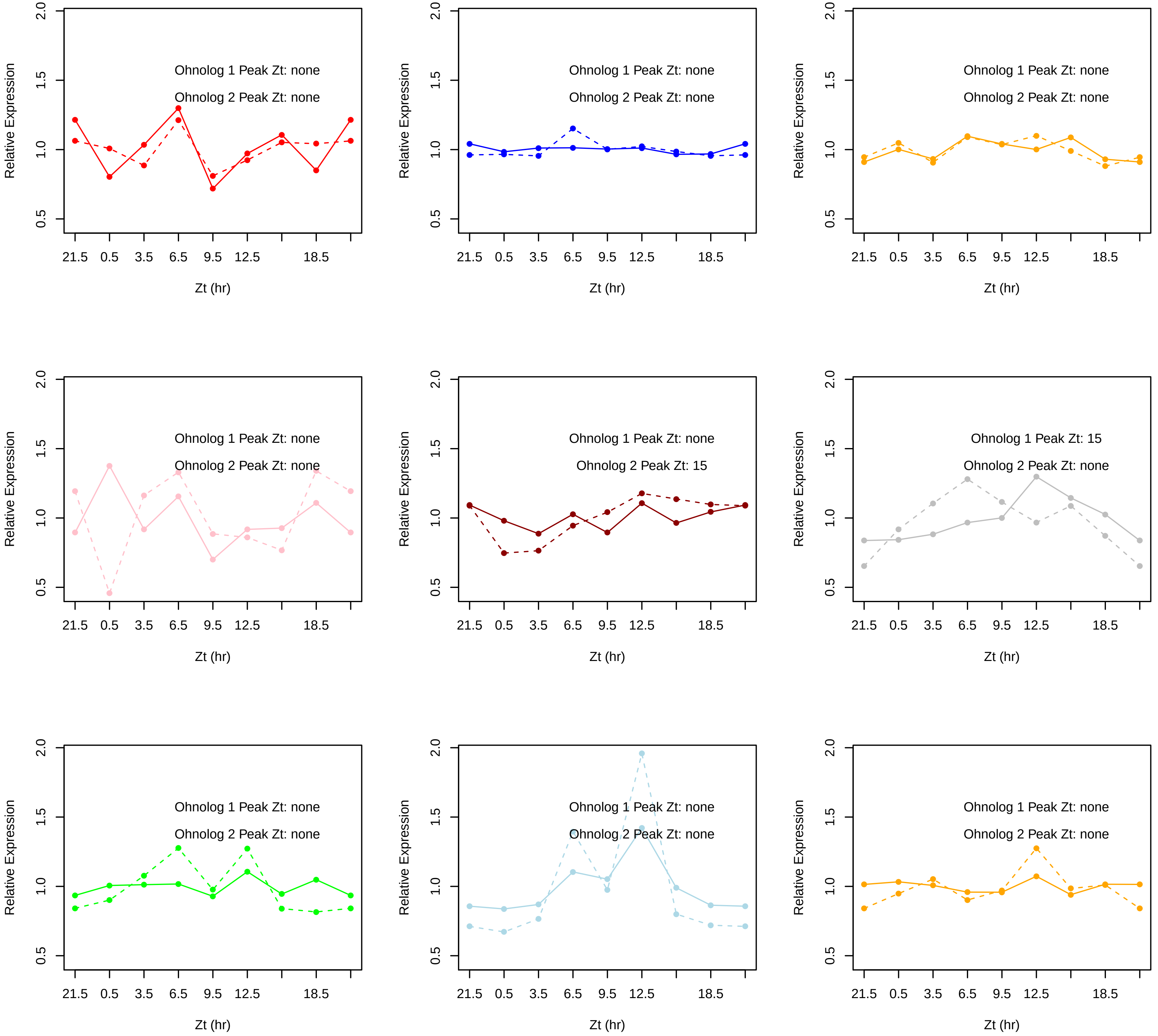

Ohnolog 1: ENSXMAG00000002125 inhbb
Ohnolog 2: ENSXMAG00000022566 INHBB (1 of many)

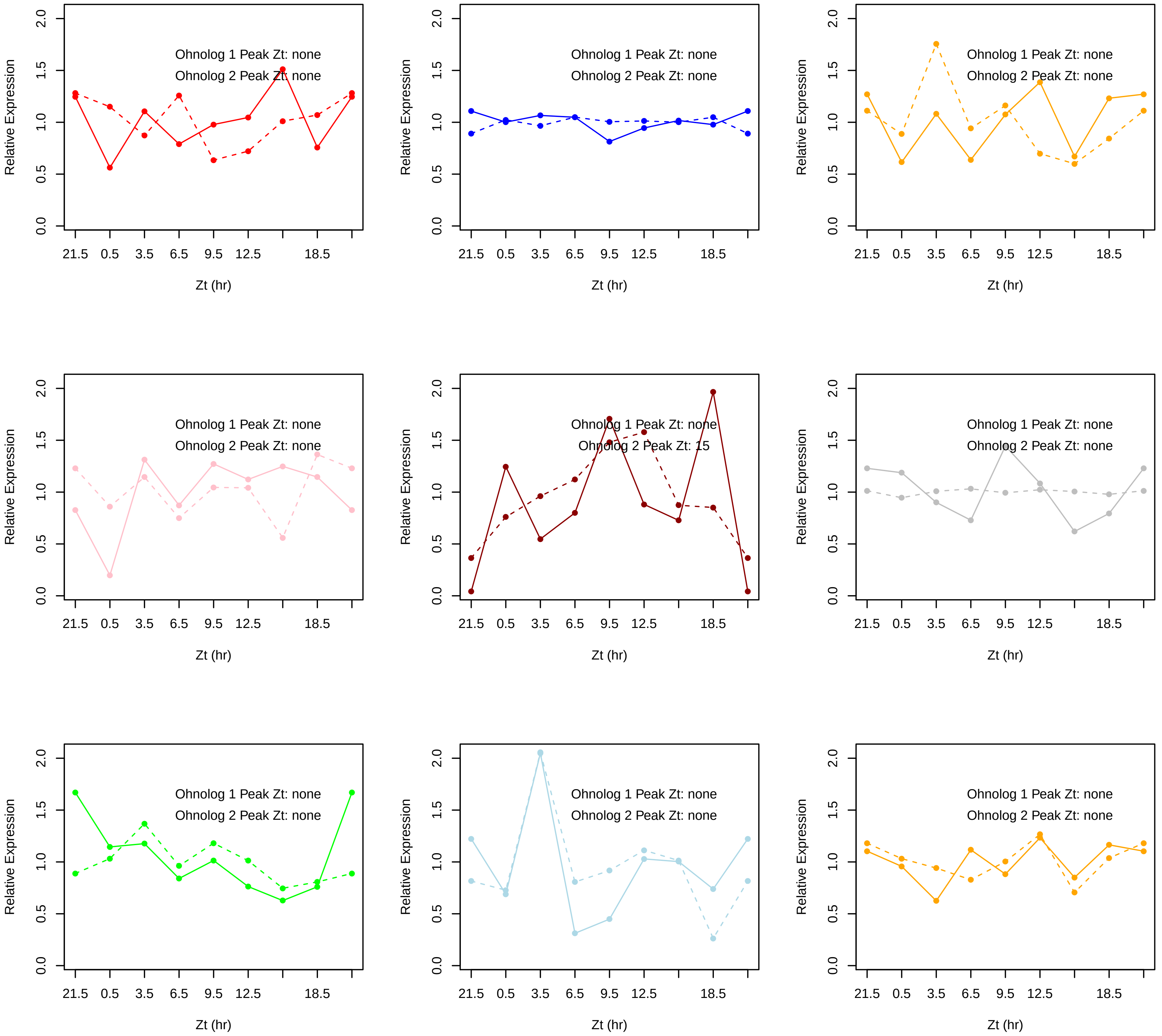

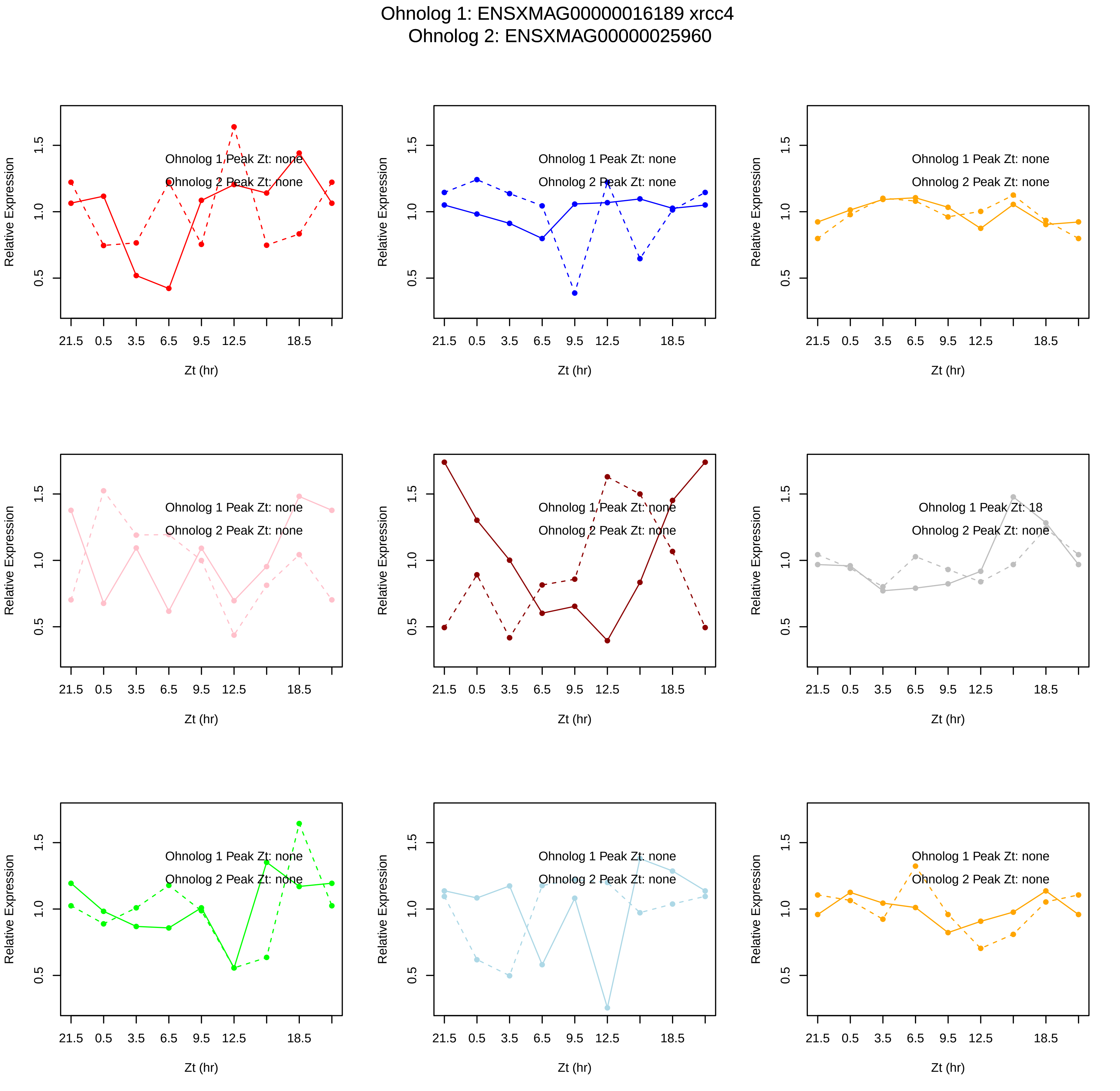
Ohnolog 1: ENSXMAG00000016189 xrcc4
Ohnolog 2: ENSXMAG00000025960
Relative Expression
Zt (hr)
21.5 0.5 3.5 6.5 9.5 12.5 18.5
0.5 1.0 1.5
Ohnolog 1 Peak Zt: none
Ohnolog 2 Peak Zt: none
Ohnolog 1 Peak Zt: none
Ohnolog 2 Peak Zt: none
Ohnolog 1 Peak Zt: none
Ohnolog 2 Peak Zt: none
Ohnolog 1 Peak Zt: none
Ohnolog 2 Peak Zt: none
Ohnolog 1 Peak Zt: none
Ohnolog 2 Peak Zt: none
Ohnolog 1 Peak Zt: 18
Ohnolog 2 Peak Zt: none
Ohnolog 1 Peak Zt: none
Ohnolog 2 Peak Zt: none
Ohnolog 1 Peak Zt: none
Ohnolog 2 Peak Zt: none
Ohnolog 1 Peak Zt: none
Ohnolog 2 Peak Zt: none

Ohnolog 1: ENSXMAG00000001182 nsmfa
Ohnolog 2: ENSXMAG00000004853 nsmfb

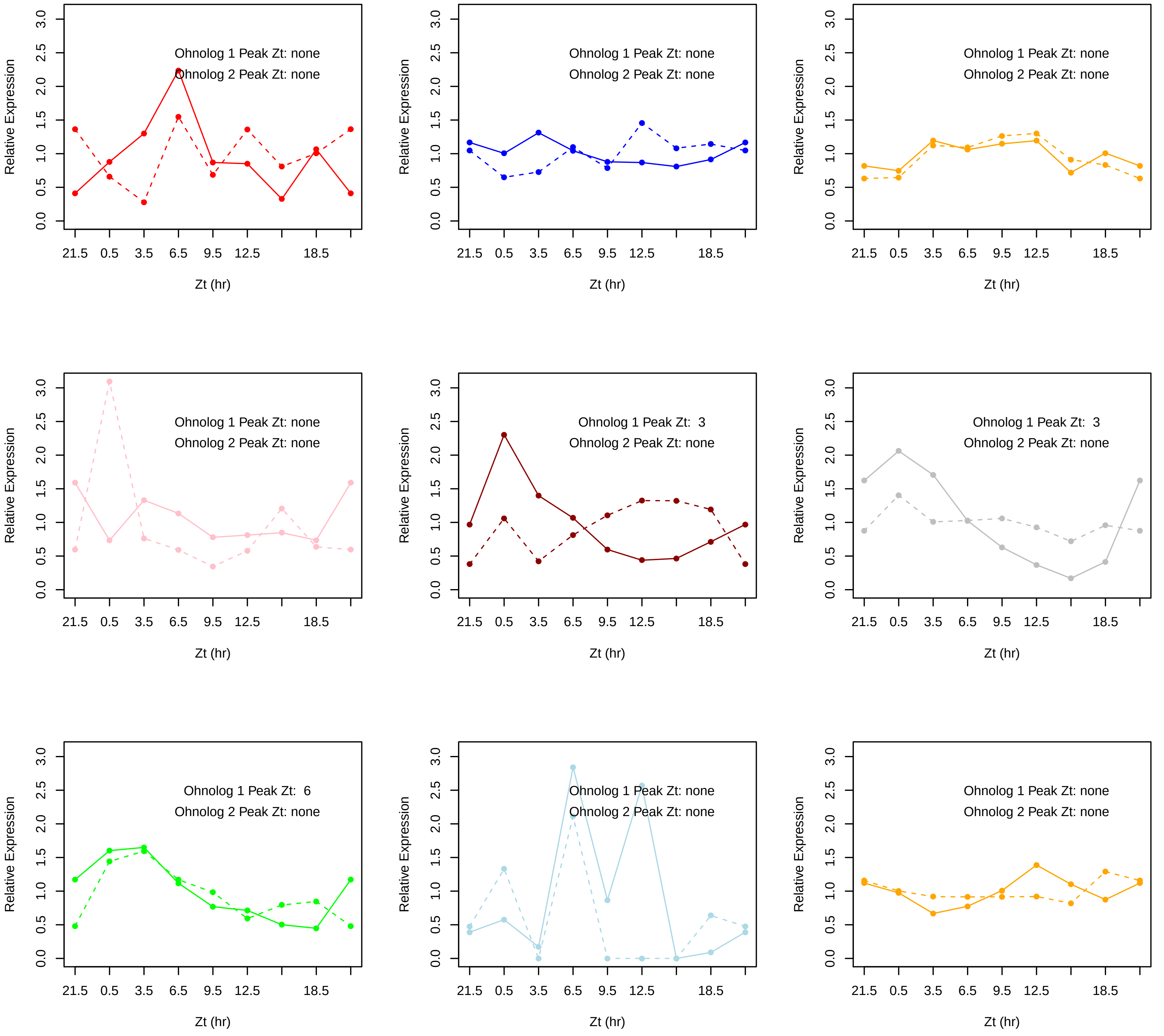

Ohnolog 1: ENSXMAG00000001703
Ohnolog 2: ENSXMAG00000002540

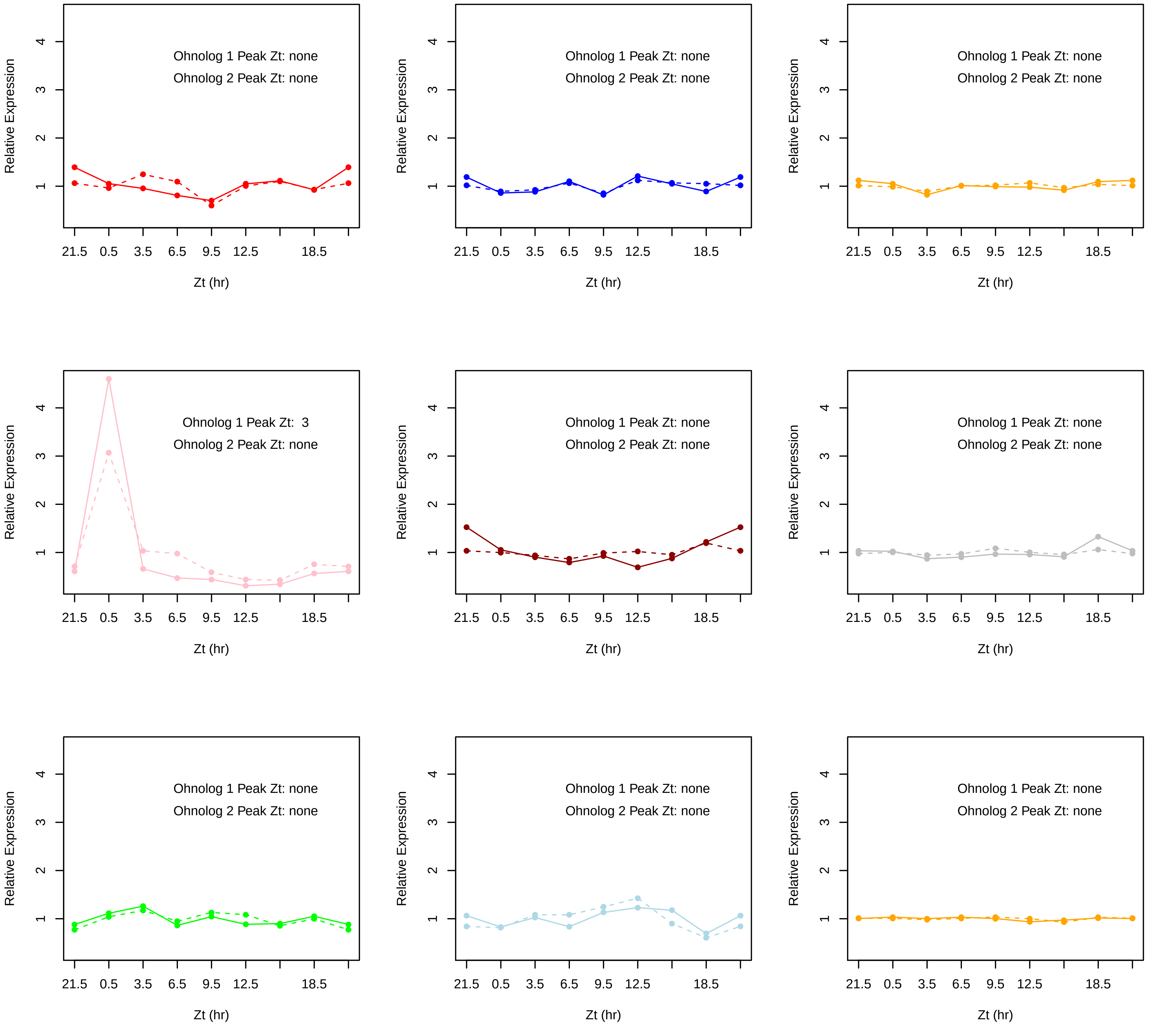

Ohnolog 1: ENSXMAG00000007063 gpia
Ohnolog 2: ENSXMAG00000016488 gpib

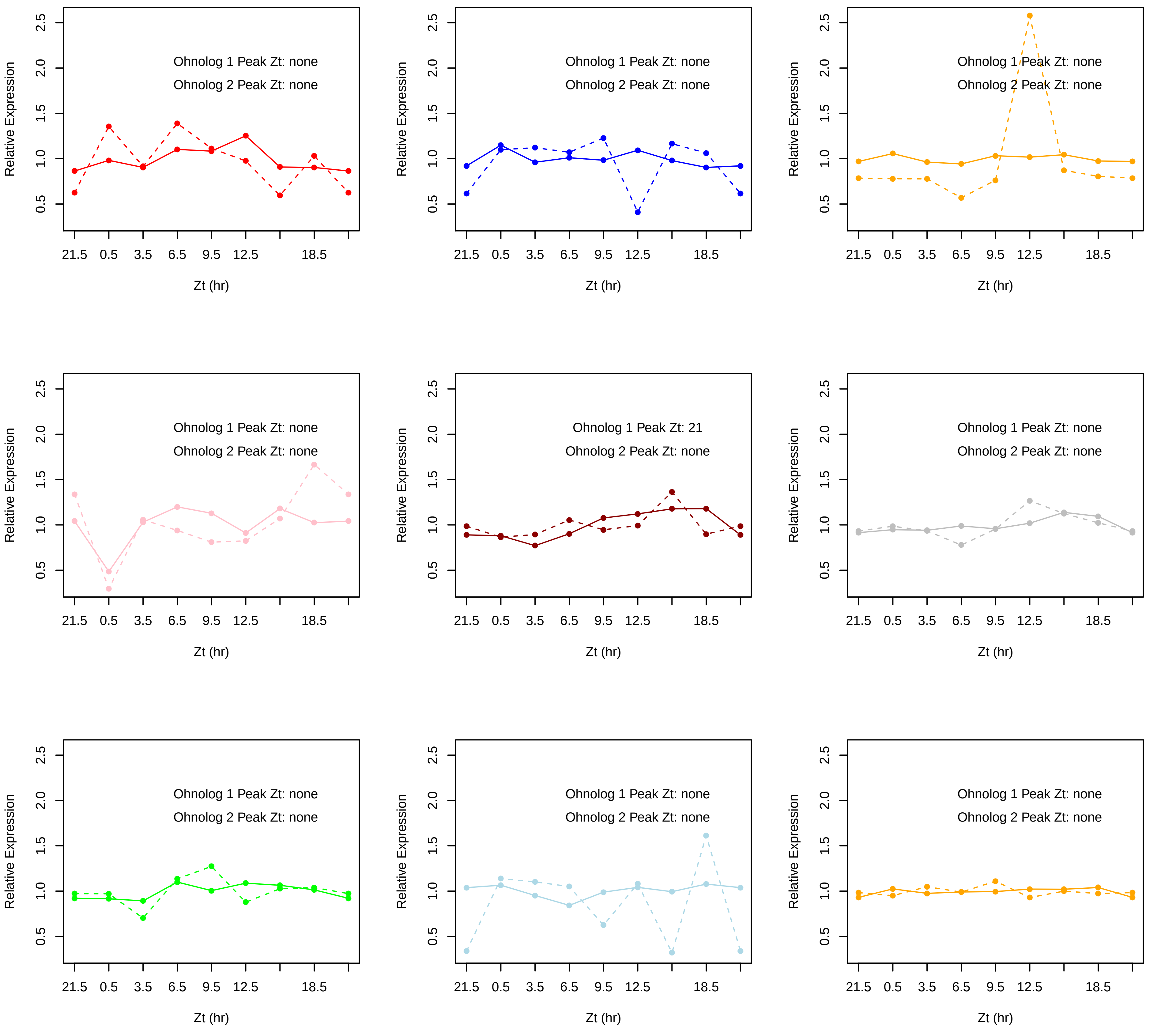

Ohnolog 1: ENSXMAG00000002567 homer3b
Ohnolog 2: ENSXMAG00000008513

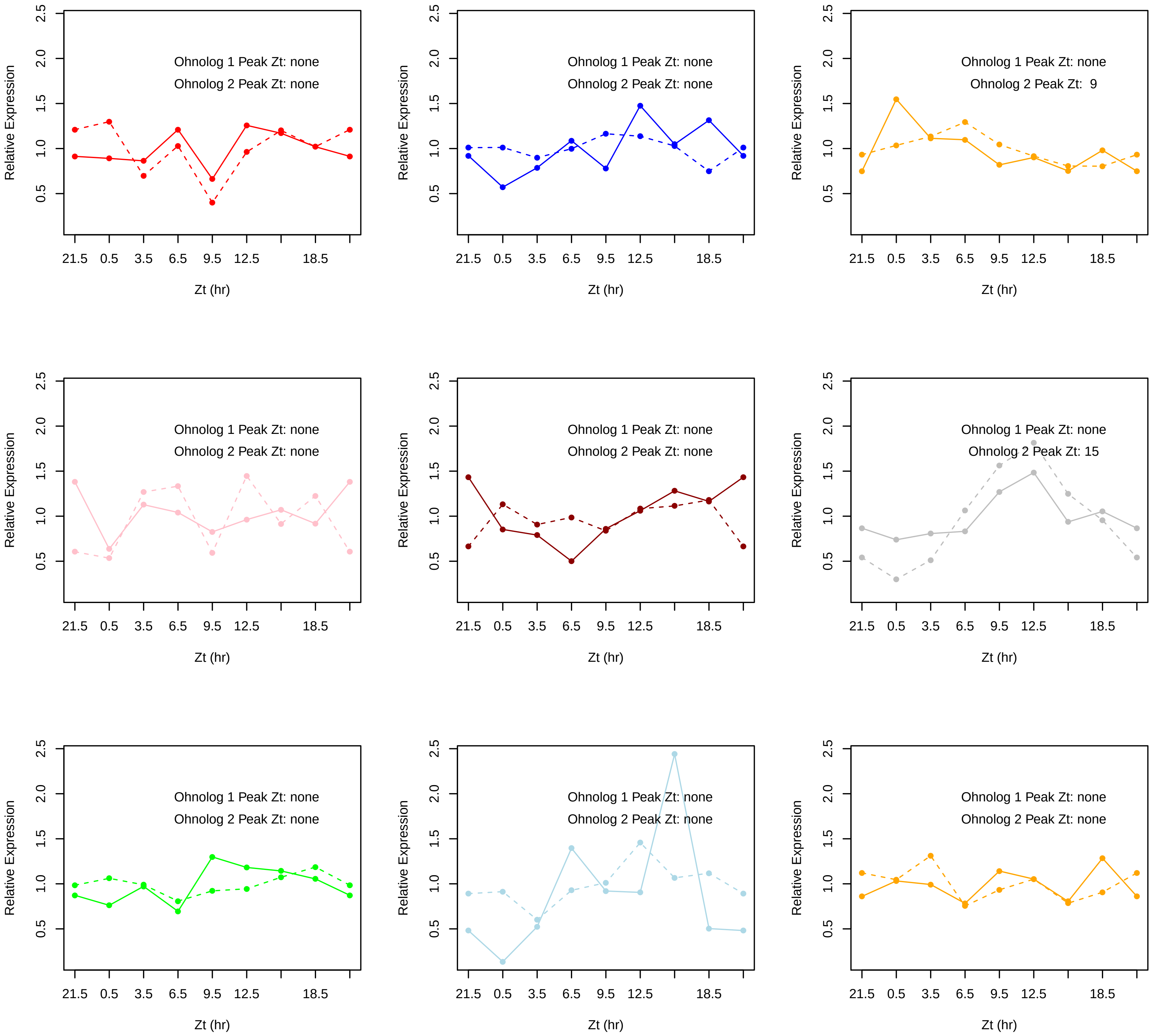

Ohnolog 1: ENSXMAG00000007434 tnnt3a
Ohnolog 2: ENSXMAG00000027991

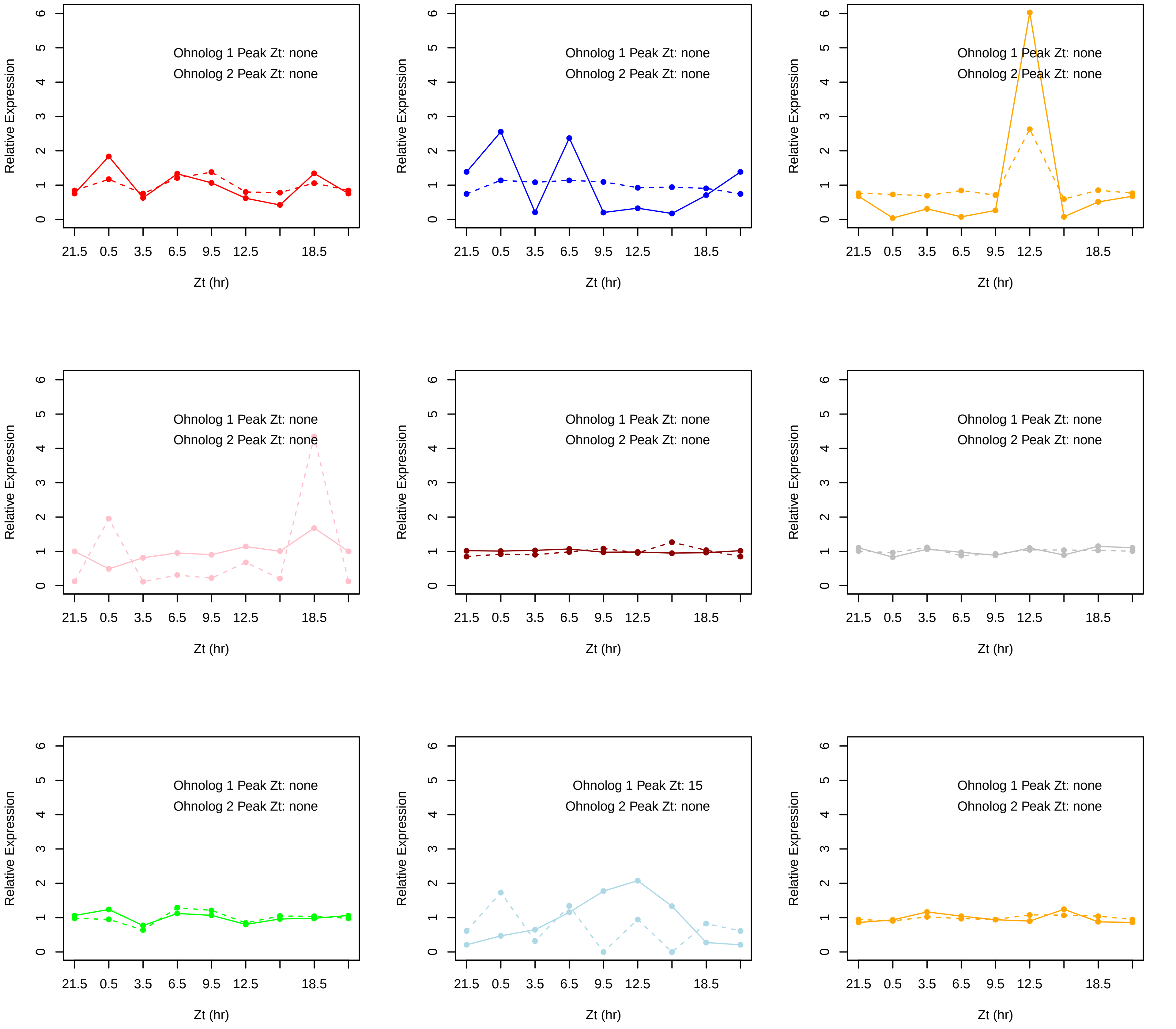

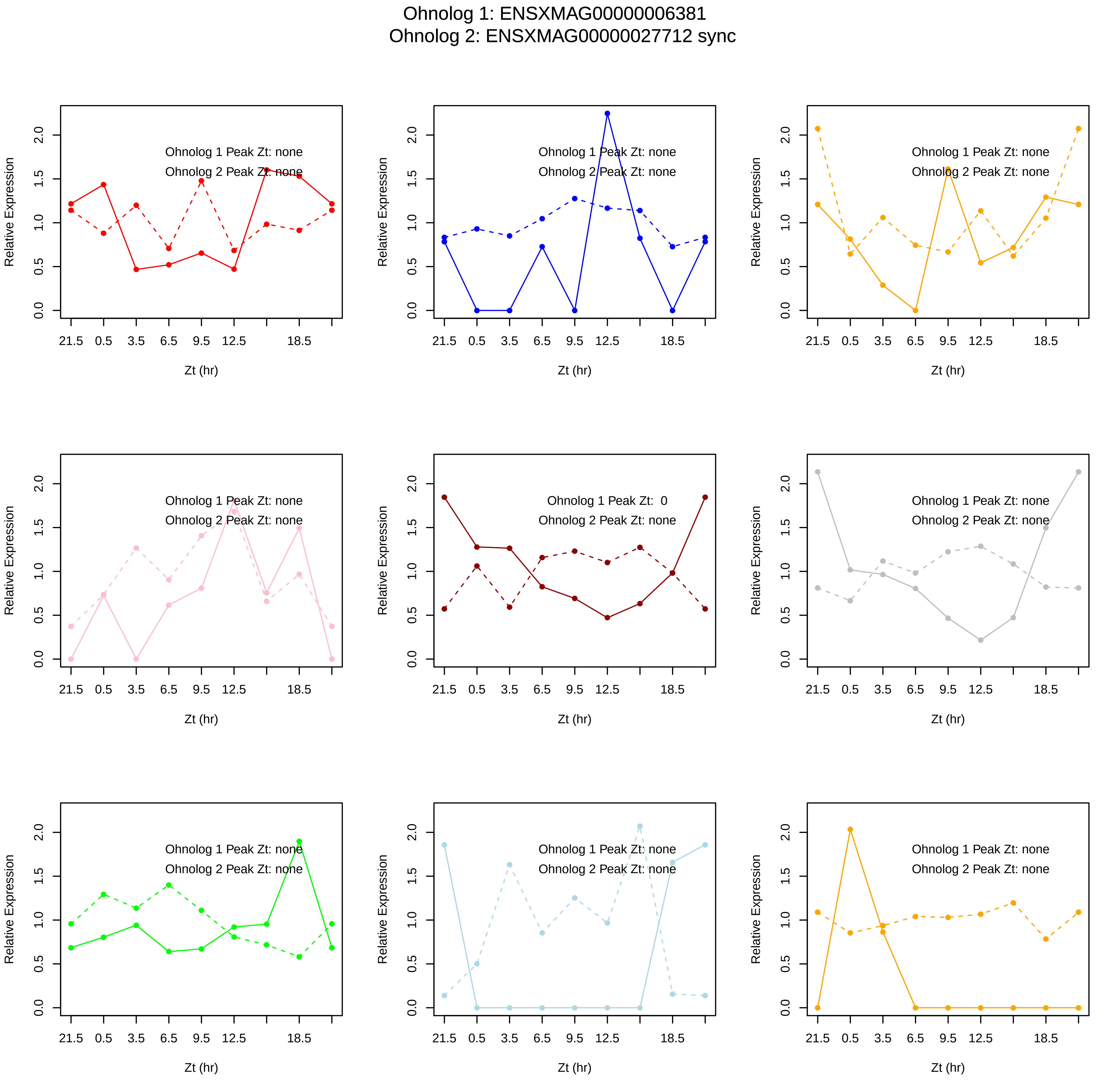
Ohnolog 1: ENSXMAG00000006381
Ohnolog 2: ENSXMAG00000027712 sync
Ohnolog 1 Peak Zt: none
Ohnolog 2 Peak Zt: none
Ohnolog 1 Peak Zt: 0
Relative Expression
Zt (hr)
21.5 0.5 3.5 6.5 9.5 12.5 18.5
0.0 0.5 1.0 1.5 2.0

Ohnolog 1: ENSXMAG00000012472 acap3a
Ohnolog 2: ENSXMAG00000018796 acap3b

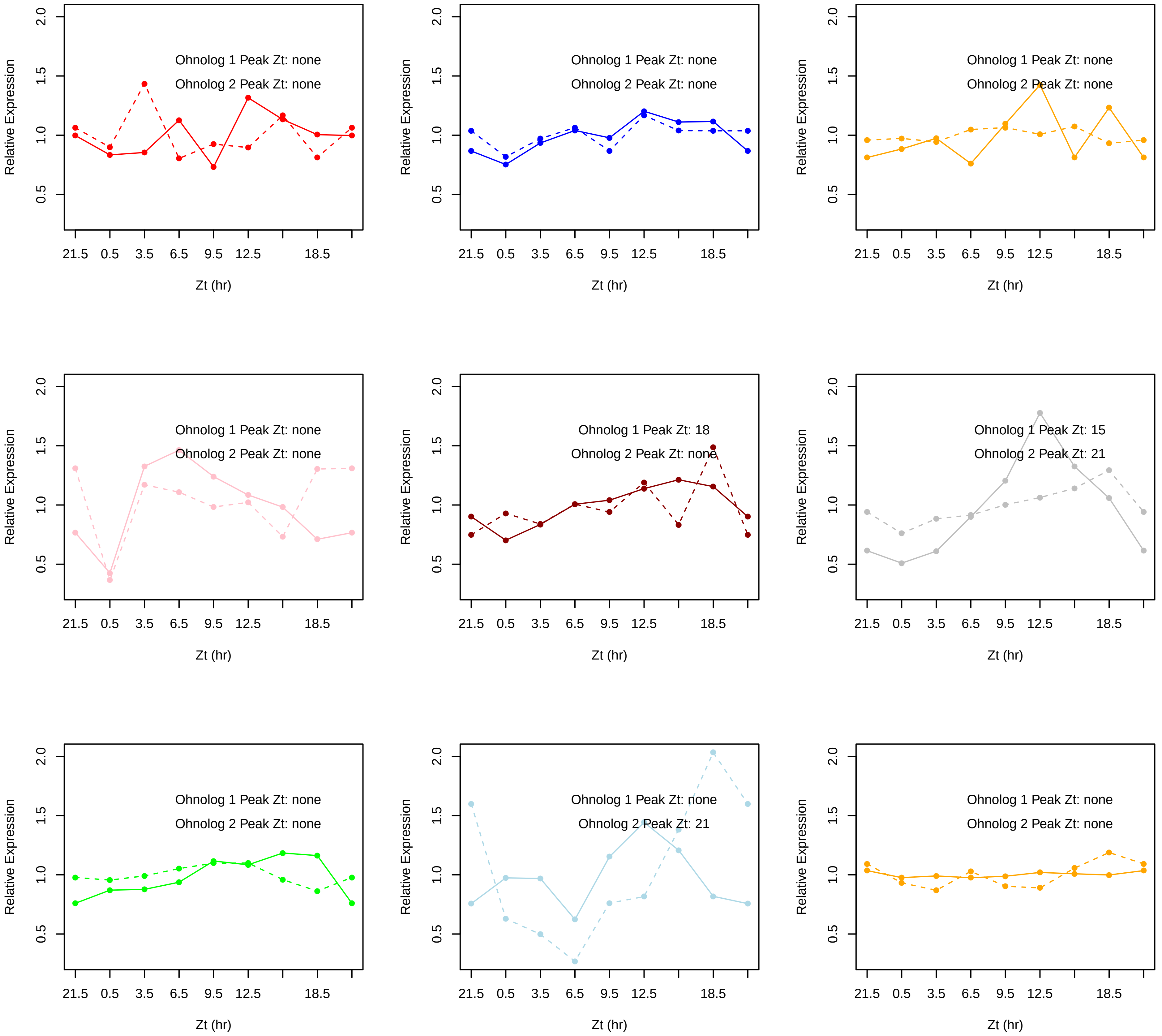

Ohnolog 1: ENSXMAG00000005713 khdrbs1b
Ohnolog 2: ENSXMAG00000006419 khdrbs1a

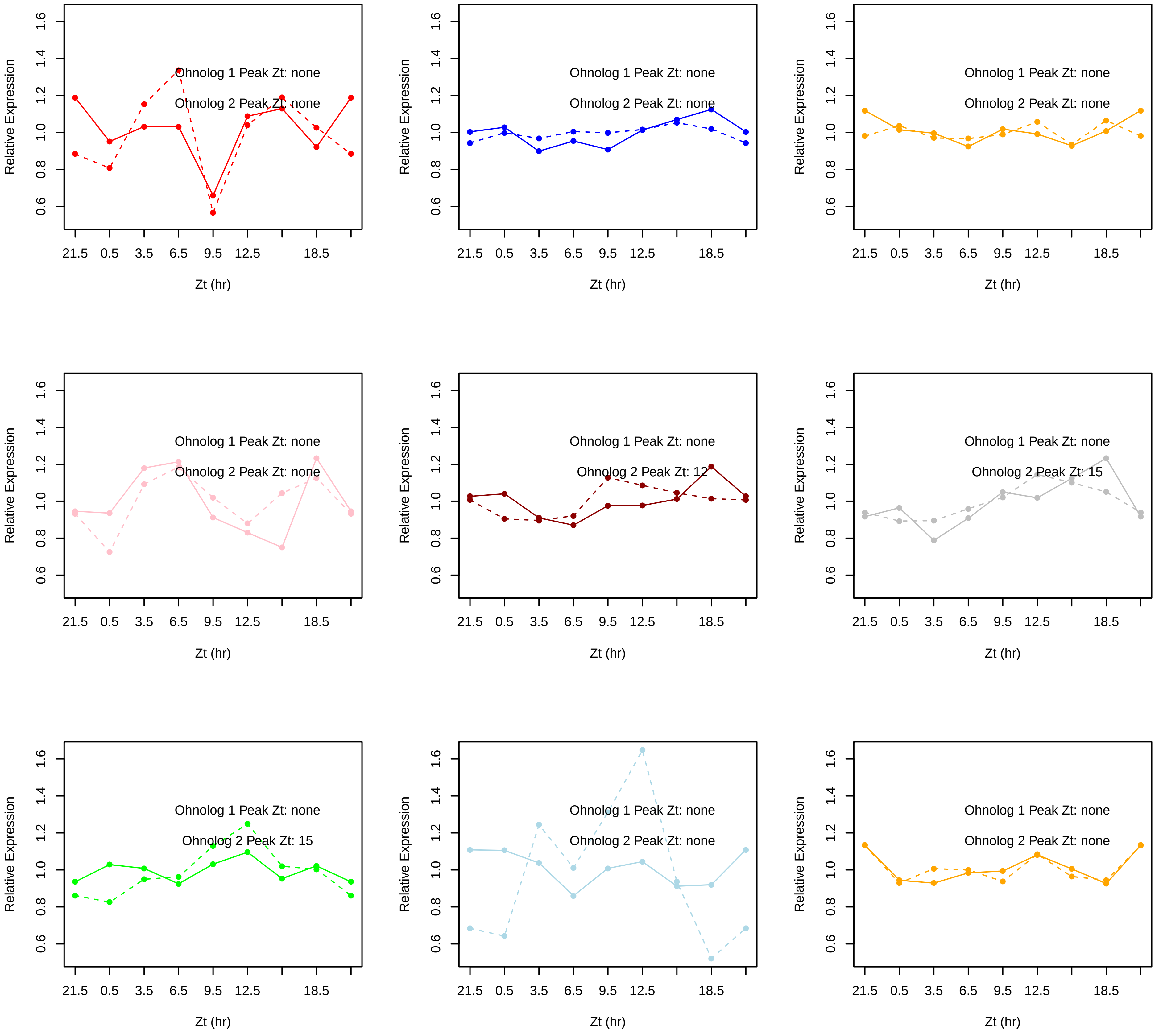

Ohnolog 1: ENSXMAG00000005370 kcng4a
Ohnolog 2: ENSXMAG00000013465 kcng4b

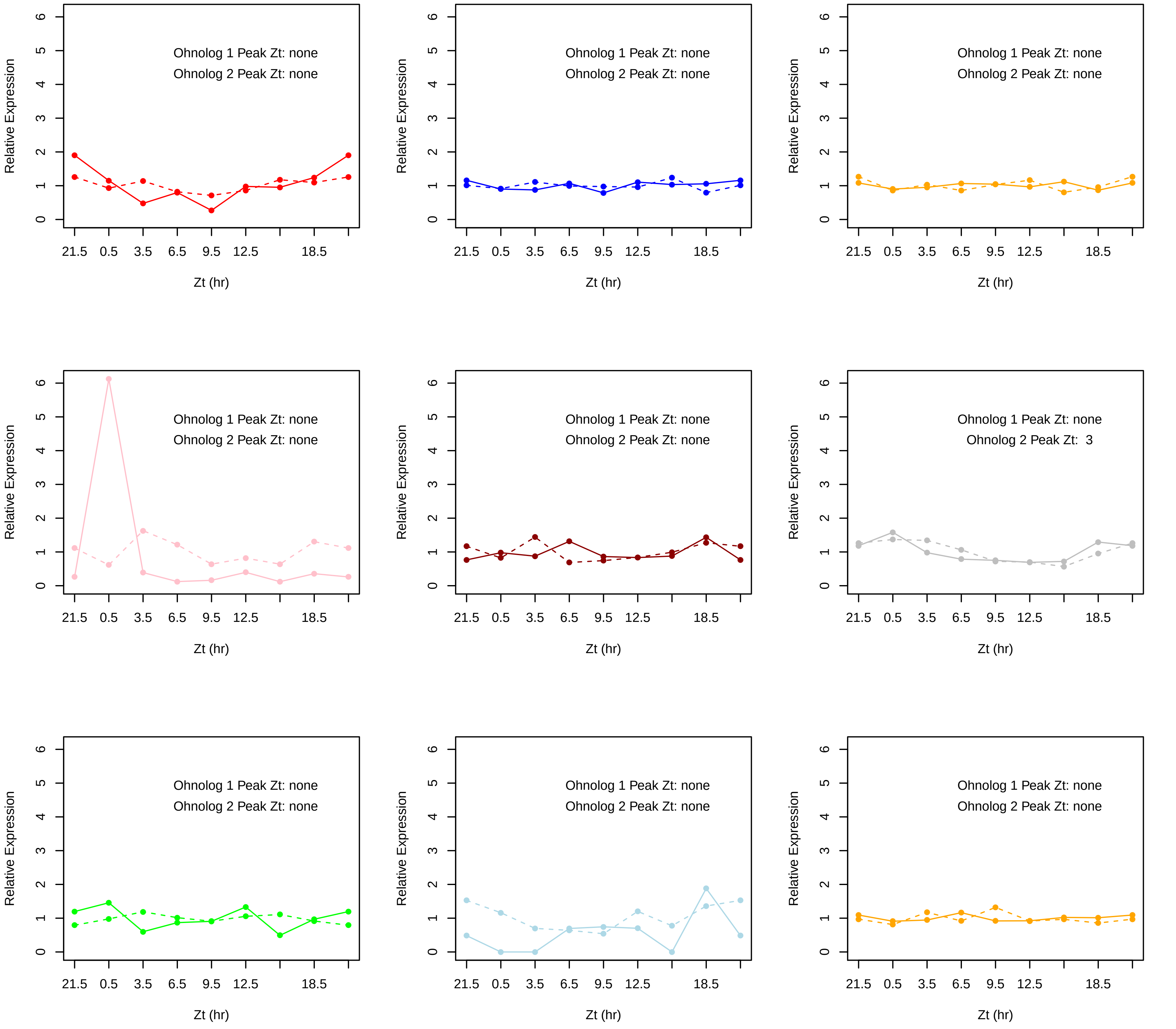

Ohnolog 1: ENSXMAG00000003976 nfatc2a
Ohnolog 2: ENSXMAG00000010231

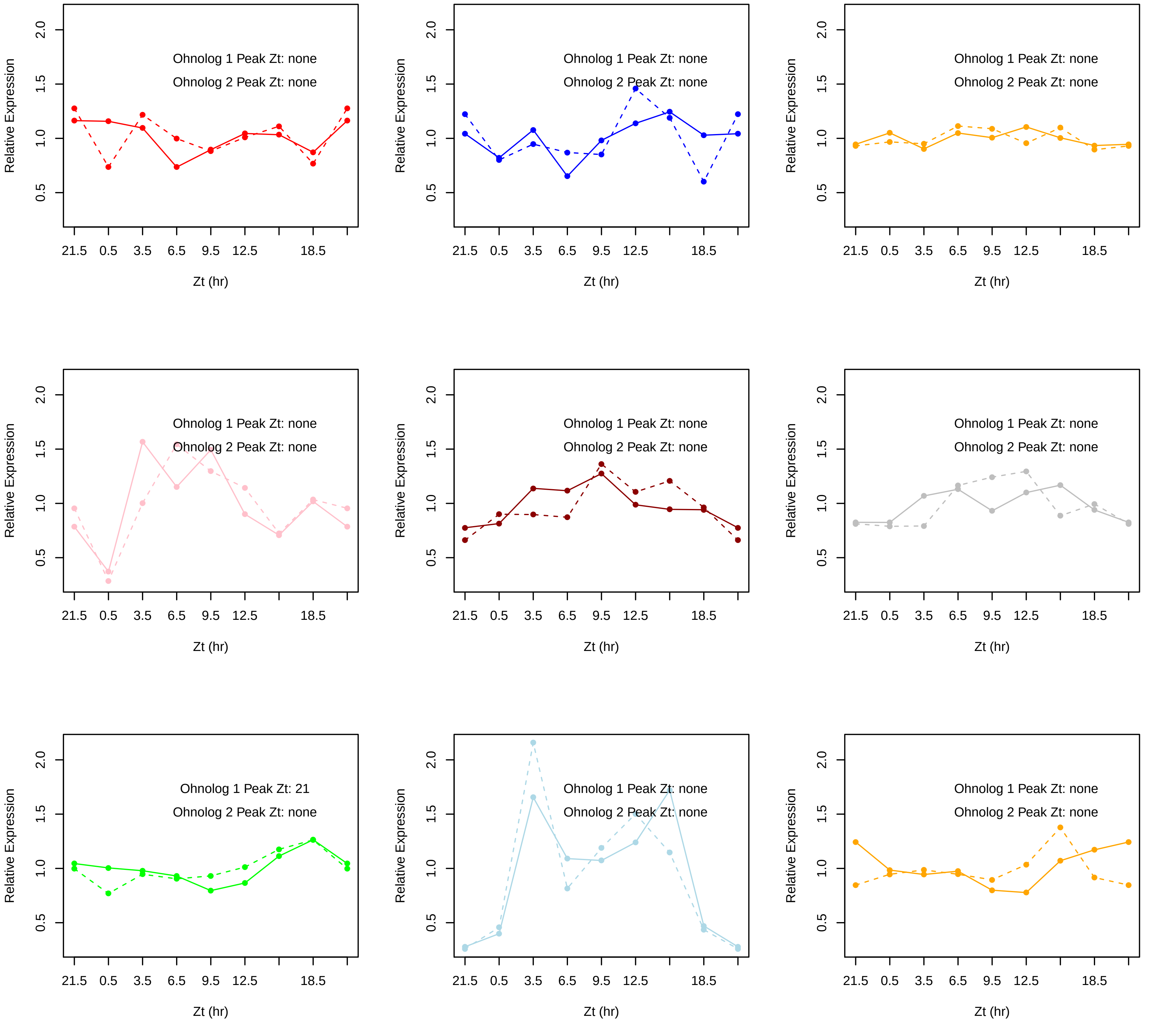

Ohnolog 1: ENSXMAG00000002404 arvcfb
Ohnolog 2: ENSXMAG00000005070 arvcfa

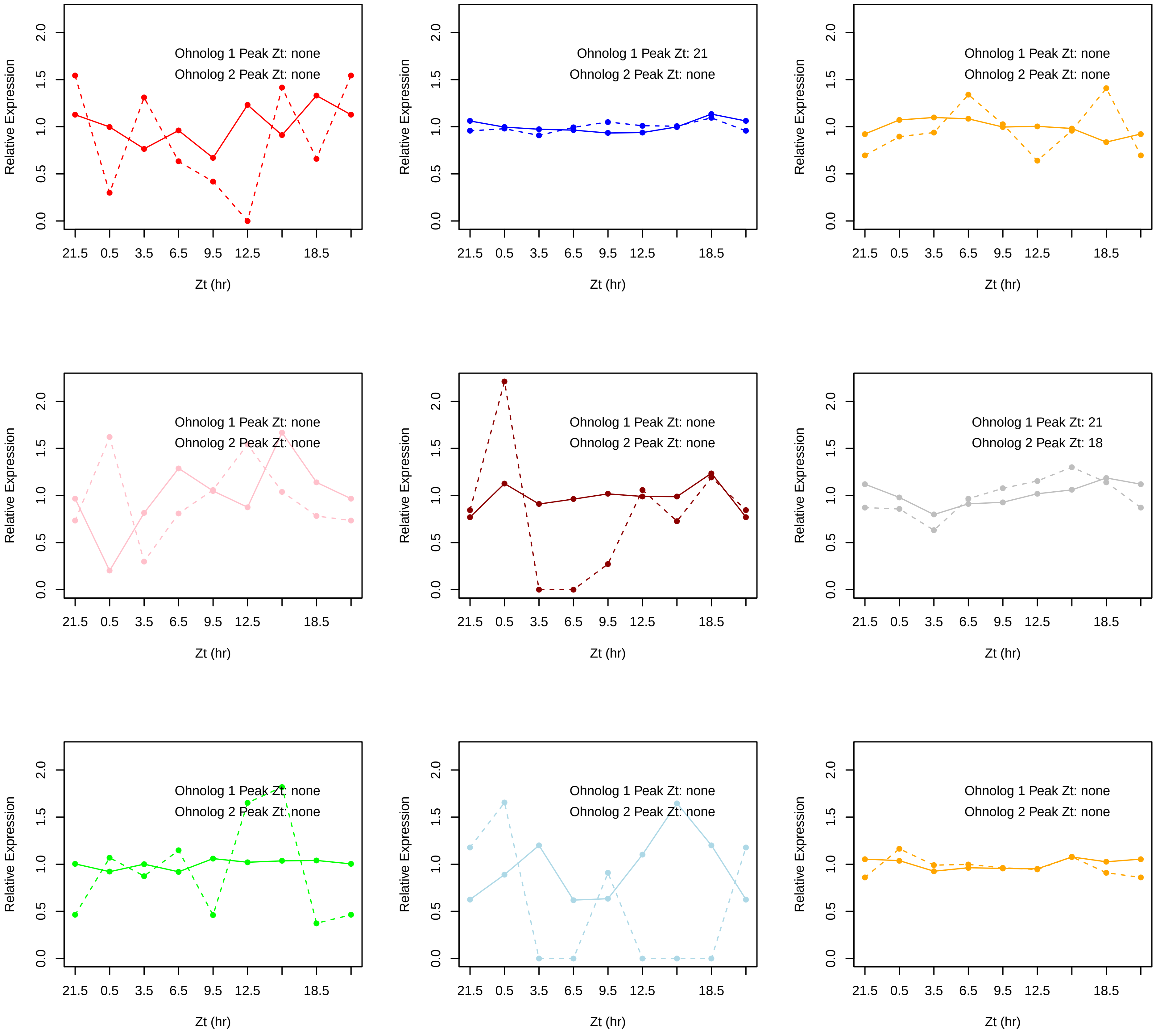

Ohnolog 1: ENSXMAG00000004671 dnaja3a
Ohnolog 2: ENSXMAG00000007950

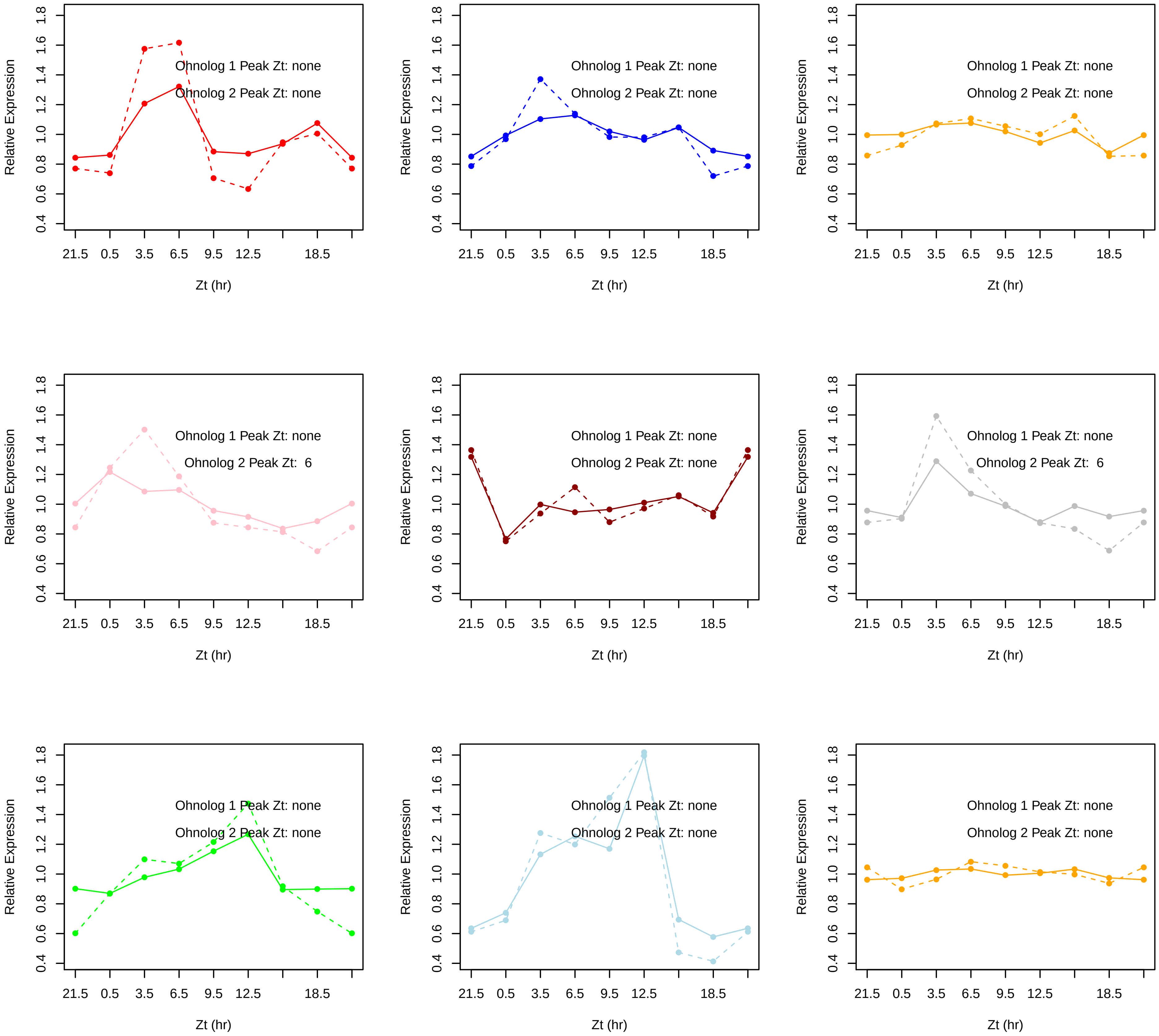

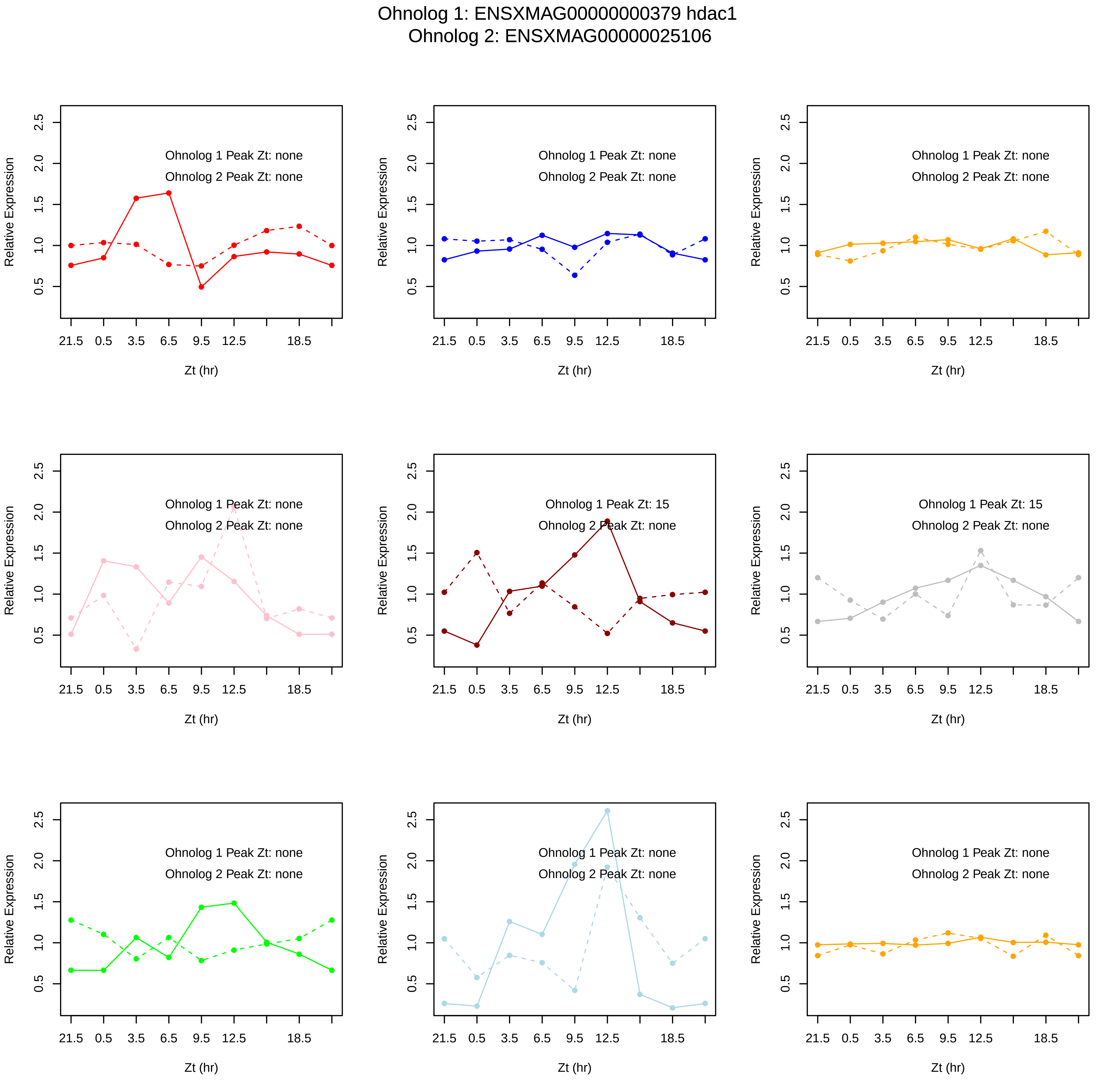
Ohnolog 1: ENSXMAG00000000379 hdac1
Ohnolog 2: ENSXMAG00000025106
Ohnolog 1 Peak Zt: none
Ohnolog 2 Peak Zt: none
Ohnolog 1 Peak Zt: 15
Ohnolog 2 Peak Zt: none
Relative Expression
Zt (hr)
21.5
0.5
3.5
6.5
9.5
12.5
18.5

Ohnolog 1: ENSXMAG00000000412 marcksl1b
Ohnolog 2: ENSXMAG00000029740 marcksl1a

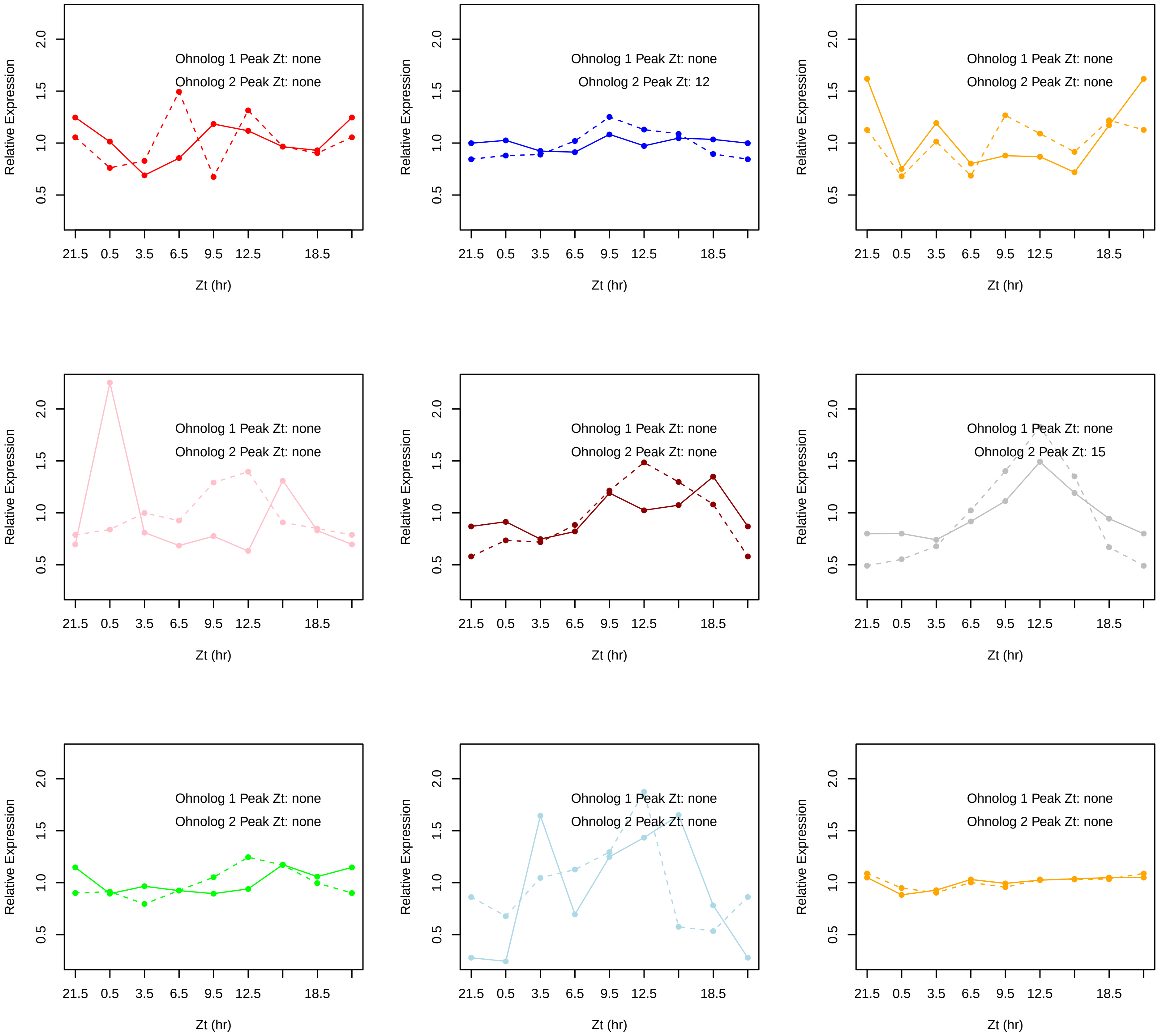

Ohnolog 1: ENSXMAG00000027478 tfap4
Ohnolog 2: ENSXMAG00000028099 TFAP4

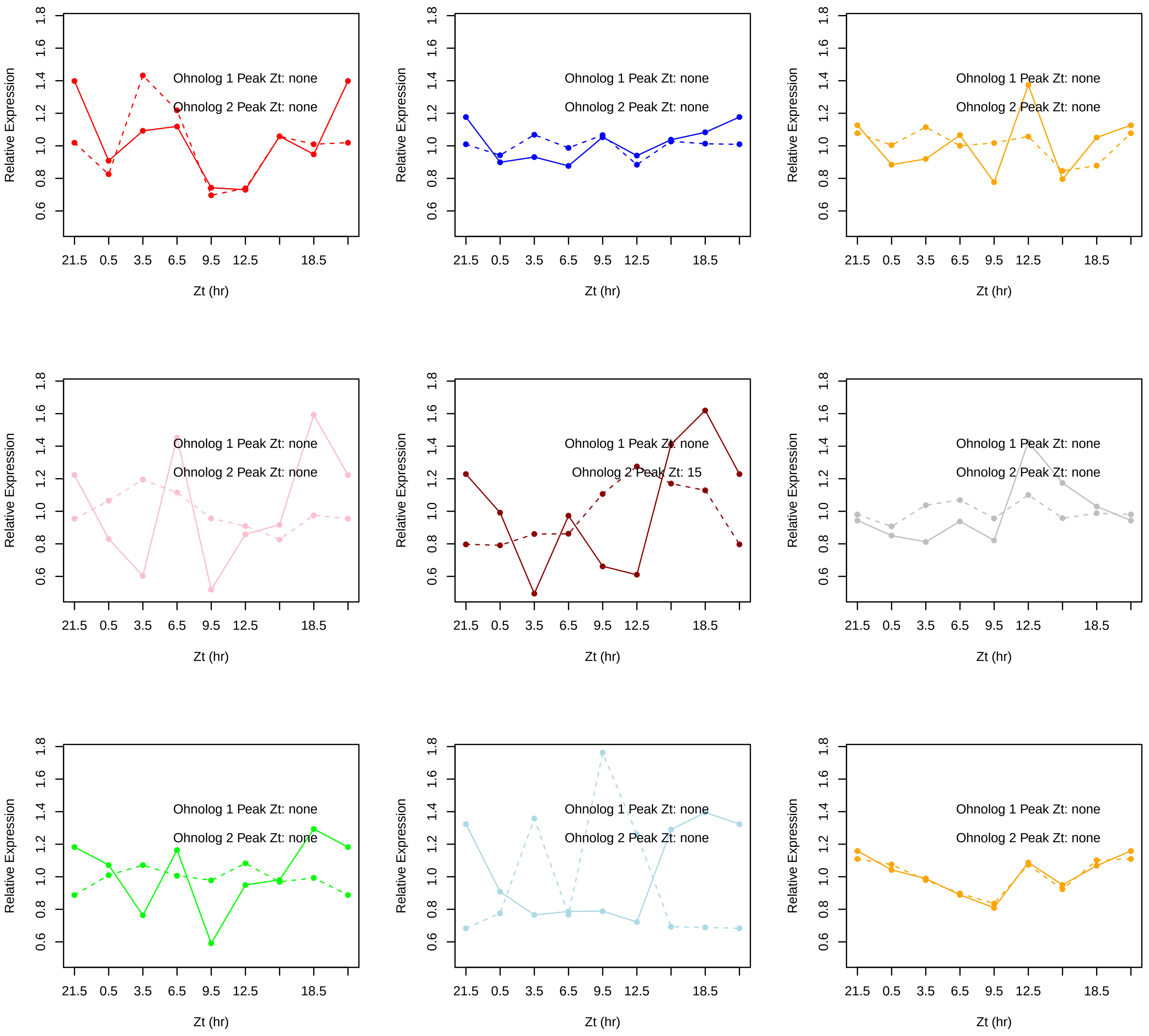

Ohnolog 1: ENSXMAG00000028414 fam163b
Ohnolog 2: ENSXMAG00000028795 FAM163B

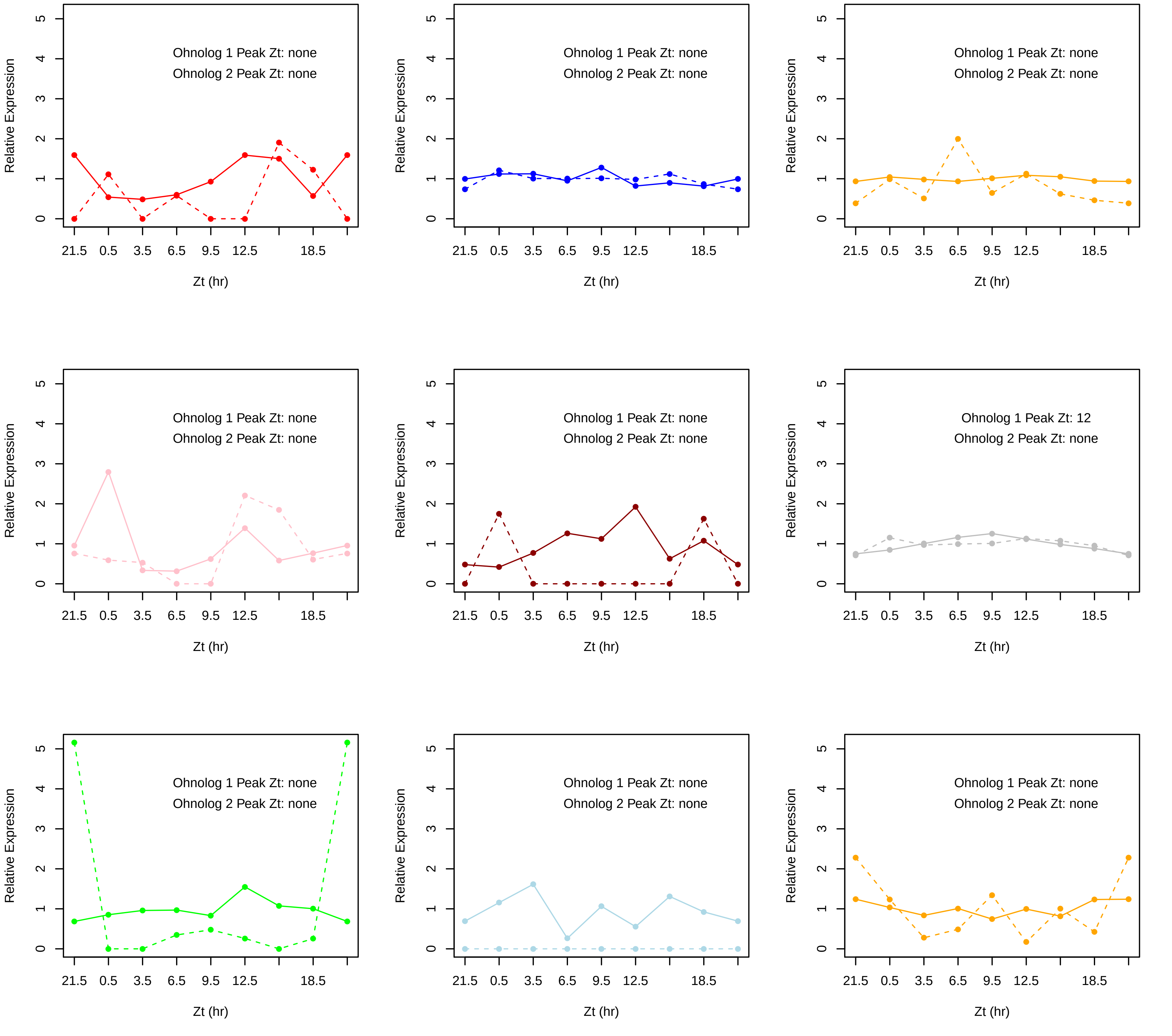

Ohnolog 1: ENSXMAG00000028331 ADCY9
Ohnolog 2: ENSXMAG00000029963 adcy9

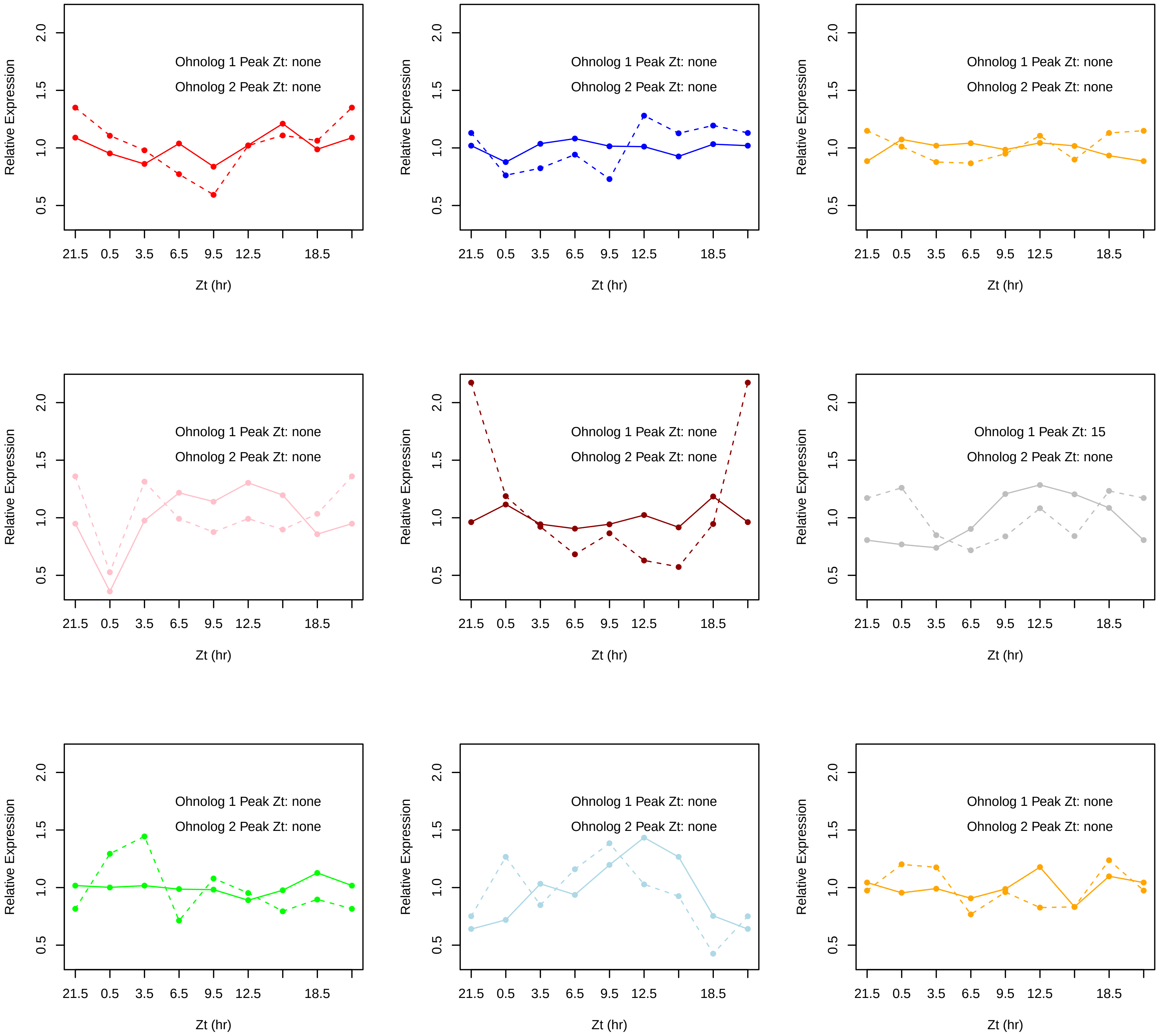

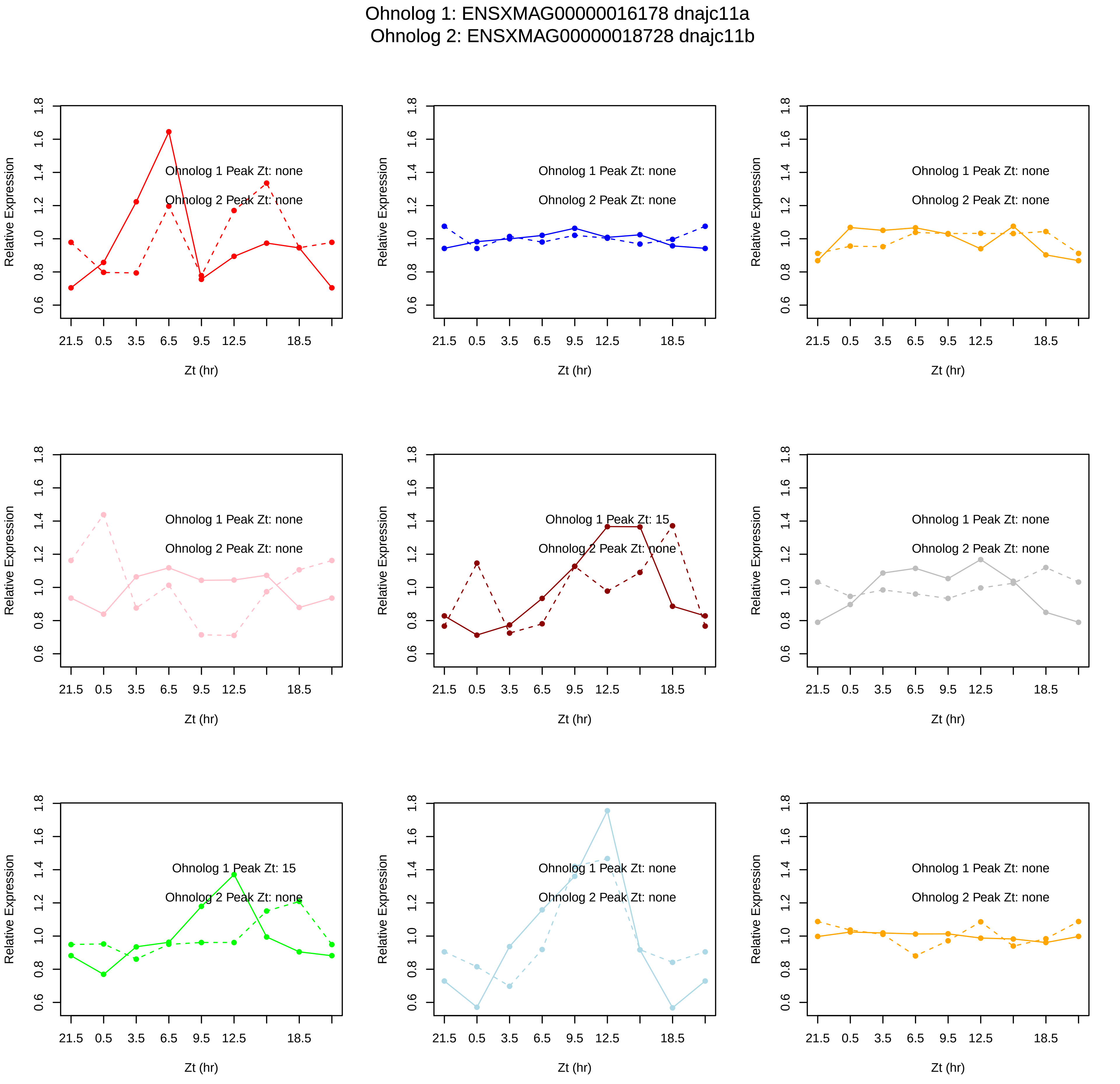
Ohnolog 1: ENSXMAG00000016178 dnajc11a
Ohnolog 2: ENSXMAG00000018728 dnajc11b
Ohnolog 1 Peak Zt: none
Ohnolog 2 Peak Zt: none
Ohnolog 1 Peak Zt: none
Ohnolog 2 Peak Zt: none
Ohnolog 1 Peak Zt: none
Ohnolog 2 Peak Zt: none
Ohnolog 1 Peak Zt: none
Ohnolog 2 Peak Zt: none
Ohnolog 1 Peak Zt: 15
Ohnolog 2 Peak Zt: none
Ohnolog 1 Peak Zt: none
Ohnolog 2 Peak Zt: none
Ohnolog 1 Peak Zt: 15
Ohnolog 2 Peak Zt: none
Ohnolog 1 Peak Zt: none
Ohnolog 2 Peak Zt: none
Ohnolog 1 Peak Zt: none
Ohnolog 2 Peak Zt: none
Relative Expression
Zt (hr)
21.5
0.5
3.5
6.5
9.5
12.5
18.5
0.6
0.8
1.0
1.2
1.4
1.6
1.8

Ohnolog 1: ENSXMAG00000009251
Ohnolog 2: ENSXMAG00000029000 cd82b

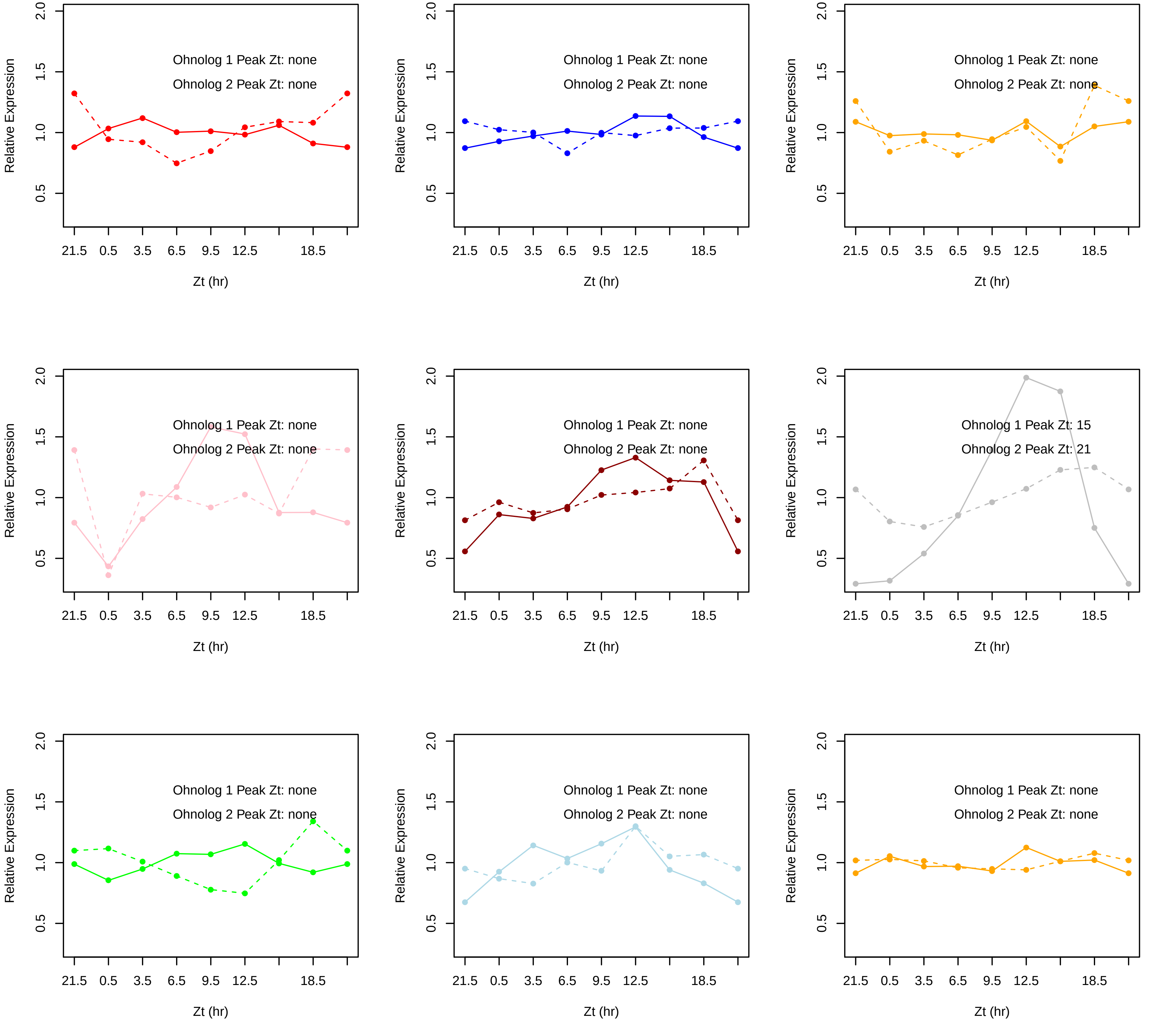

Ohnolog 1: ENSXMAG00000007280 tspan18b
Ohnolog 2: ENSXMAG00000022831 tspan18a

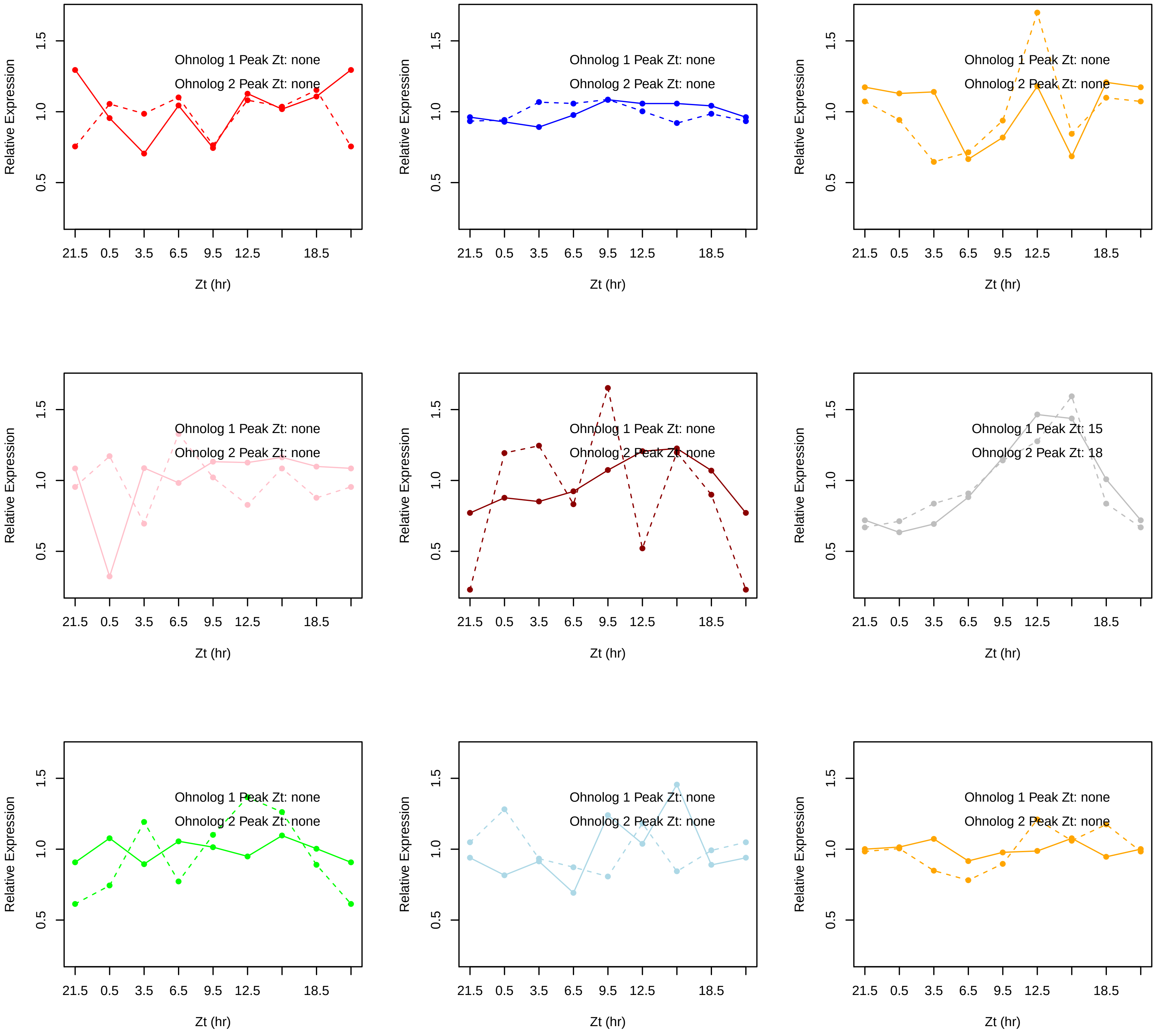

Ohnolog 1: ENSXMAG00000014983 nr2f6a
Ohnolog 2: ENSXMAG00000029958 nr2f6b

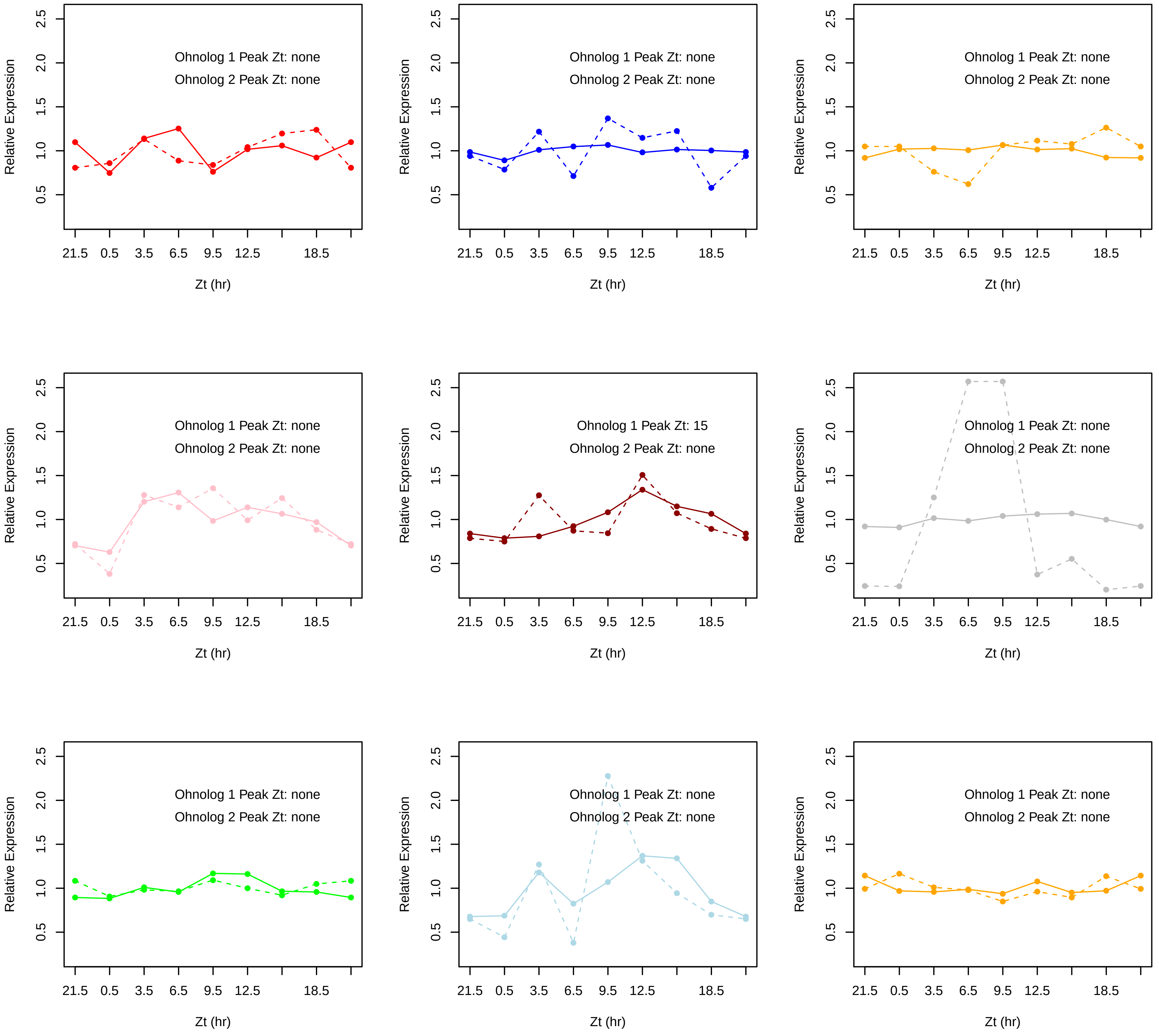

Ohnolog 1: ENSXMAG00000003614 fam8a1a
Ohnolog 2: ENSXMAG00000026154 fam8a1b

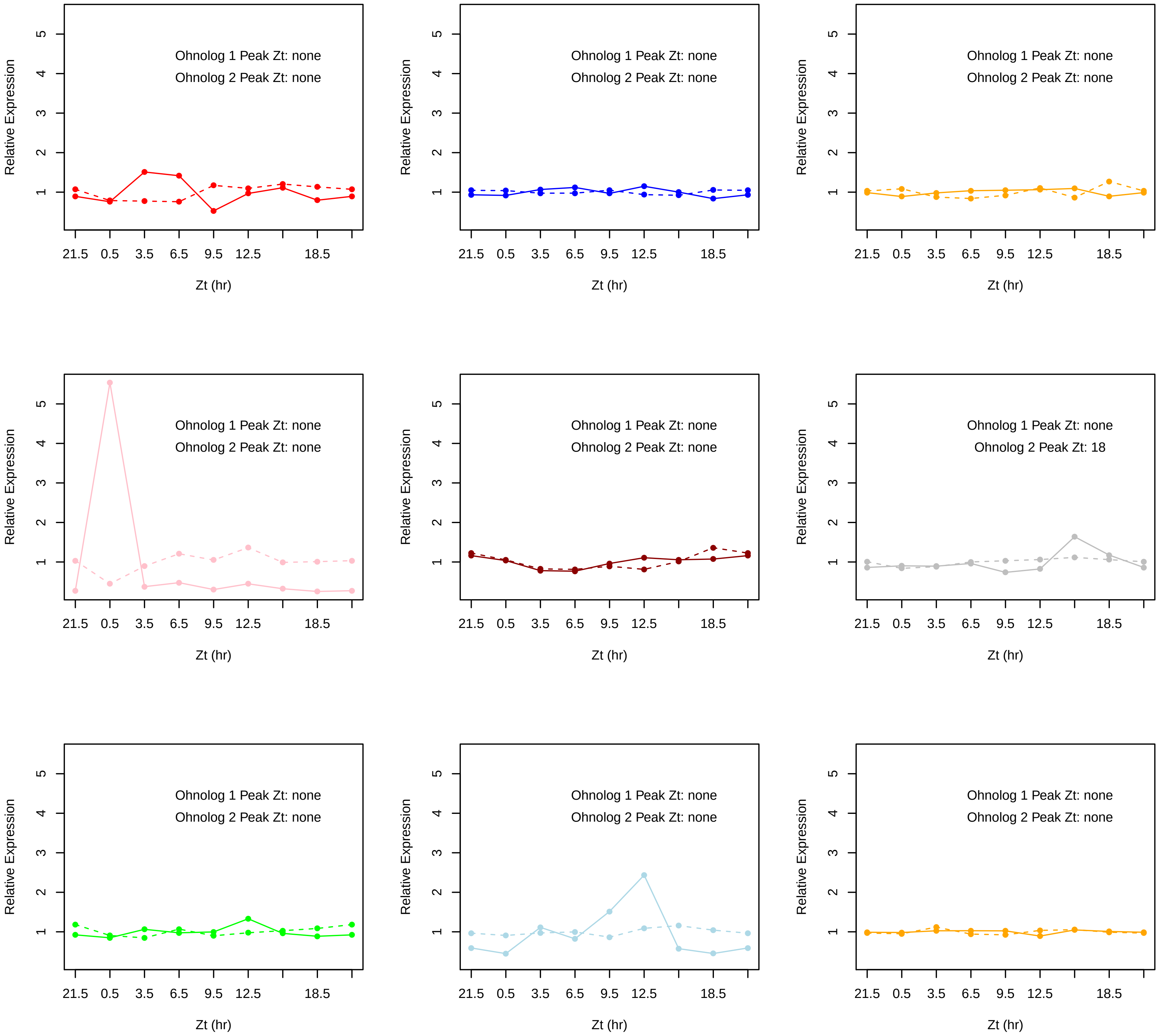

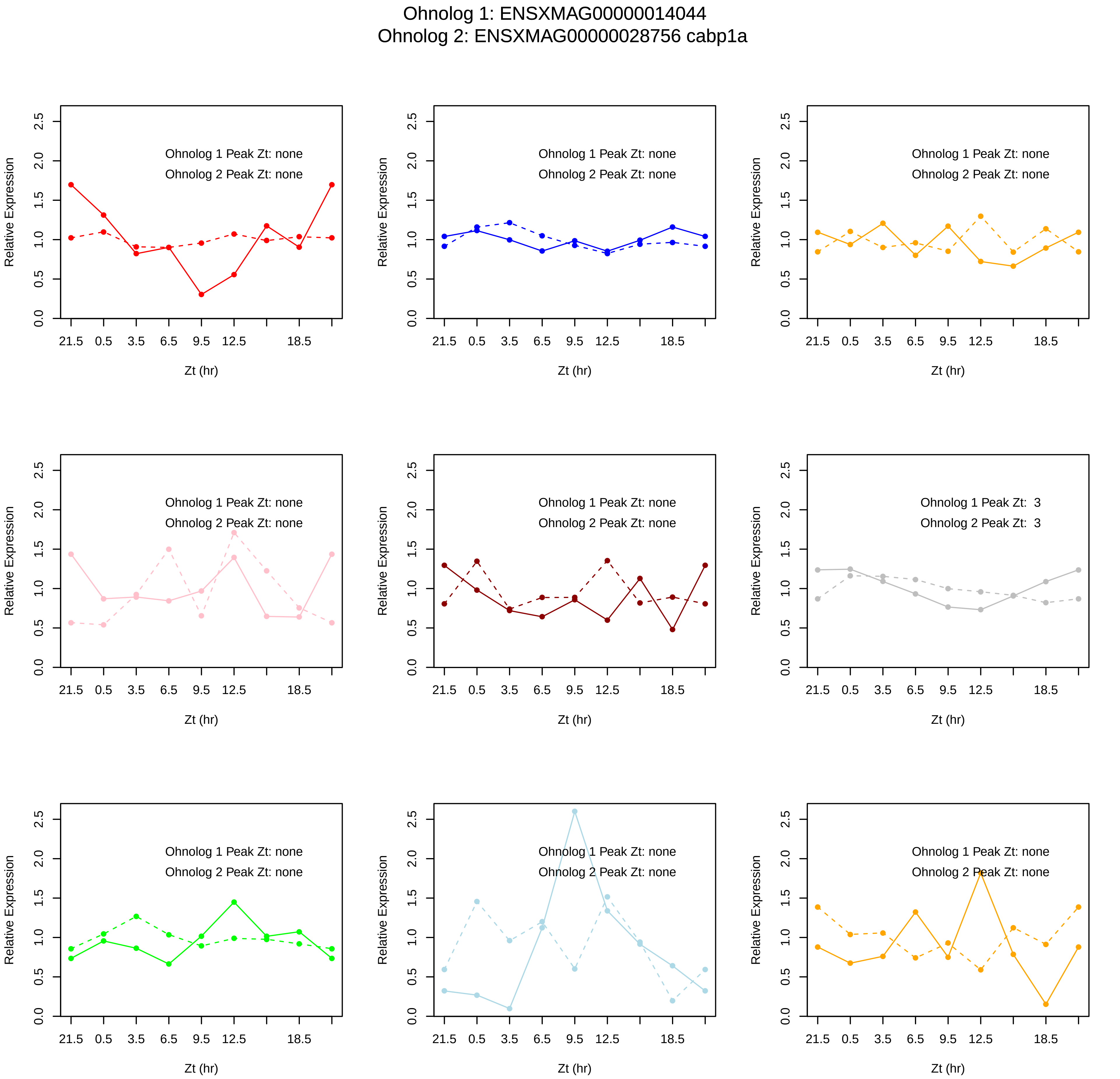
Ohnolog 1: ENSXMAG00000014044
Ohnolog 2: ENSXMAG00000028756 cabp1a
Ohnolog 1 Peak Zt: none
Ohnolog 2 Peak Zt: none
Ohnolog 1 Peak Zt: 3
Ohnolog 2 Peak Zt: 3
Relative Expression
Zt (hr)
21.5 0.5 3.5 6.5 9.5 12.5 18.5
0.0 0.5 1.0 1.5 2.0 2.5

Ohnolog 1: ENSXMAG00000004007
Ohnolog 2: ENSXMAG00000012814 mxra8b

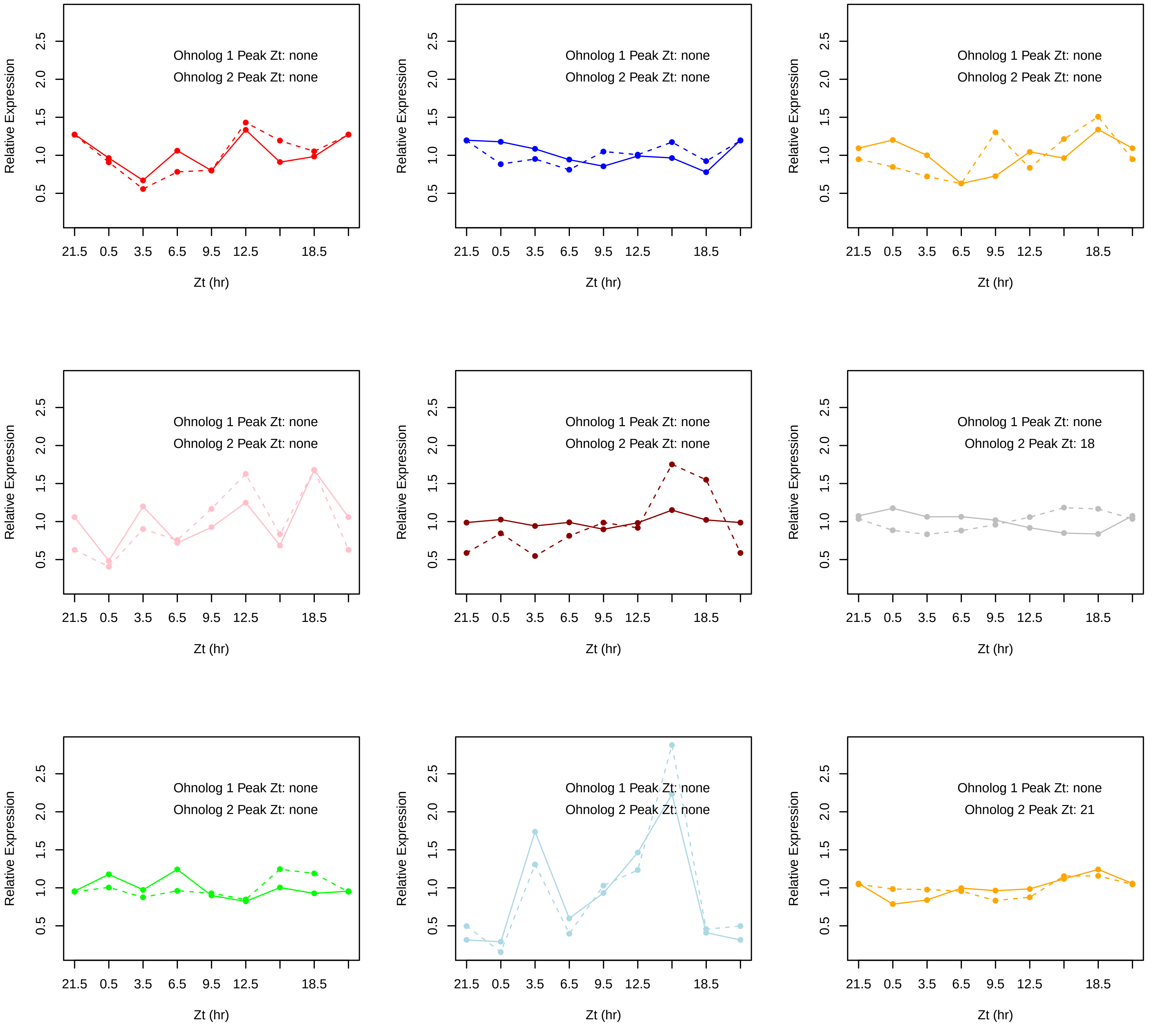

Ohnolog 1: ENSXMAG00000003855 abhd8a
Ohnolog 2: ENSXMAG00000021422 abhd8b

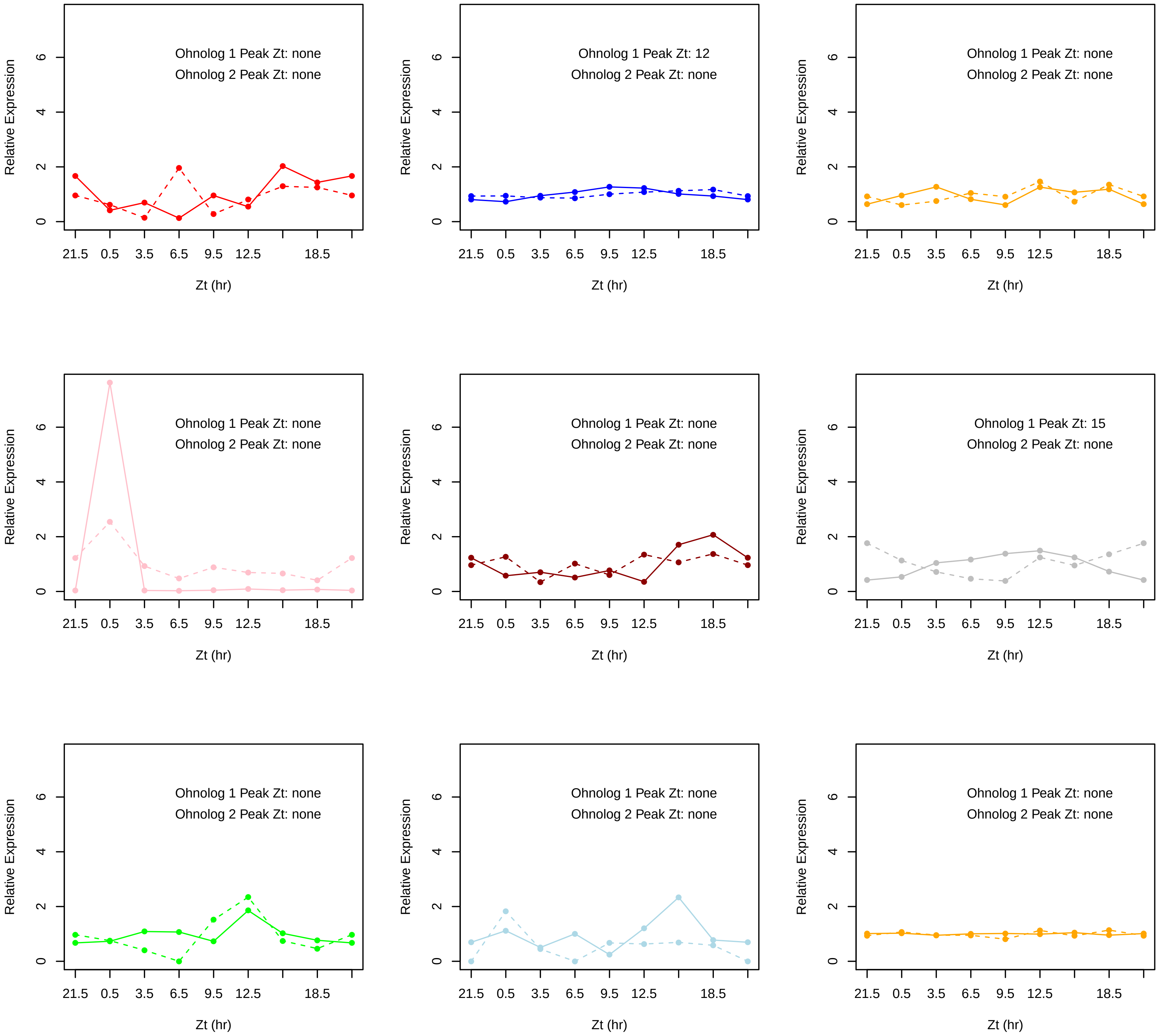

Ohnolog 1: ENSXMAG00000002402 ano8b
Ohnolog 2: ENSXMAG00000021728 ano8a

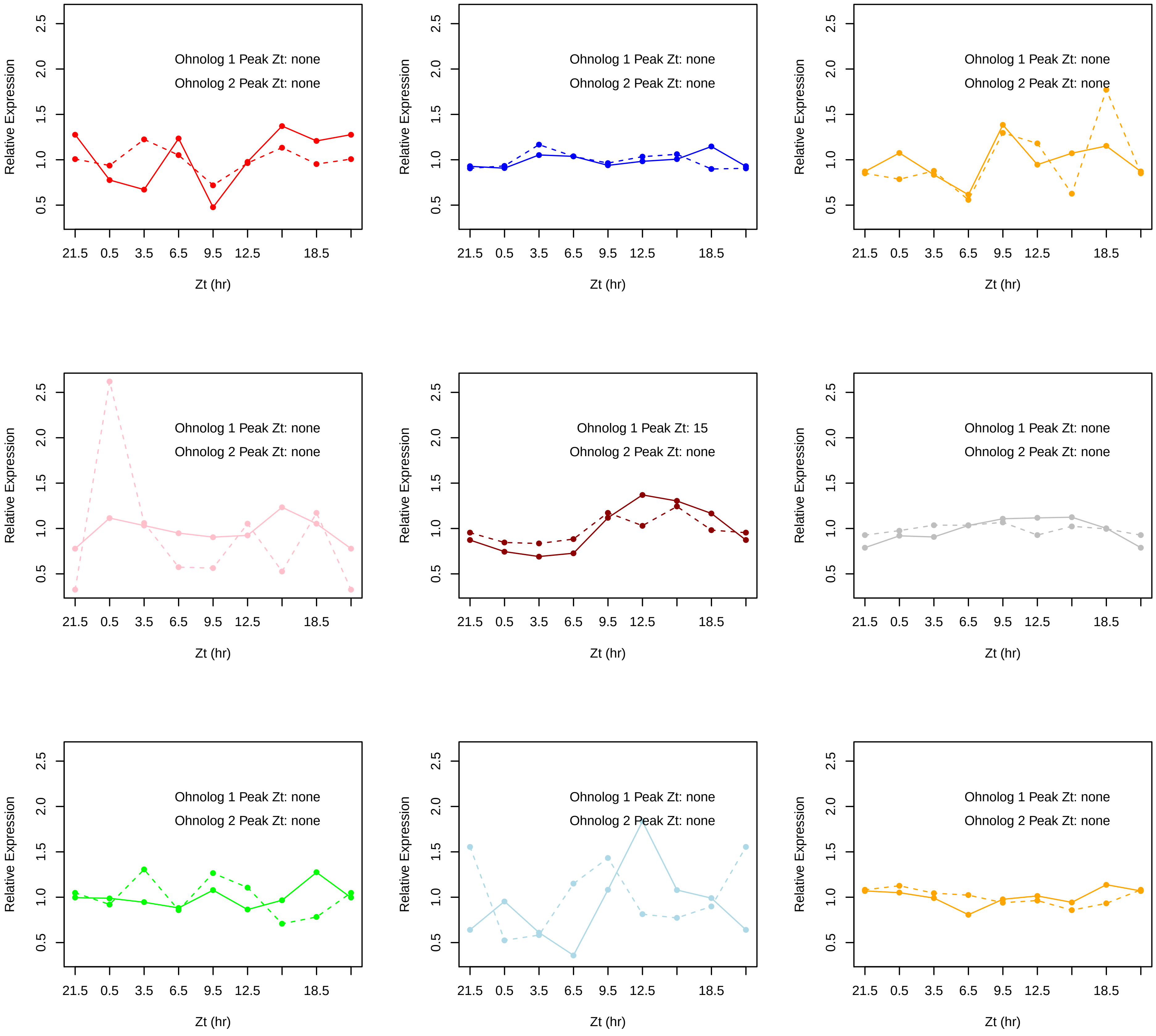

Ohnolog 1: ENSXMAG00000014478 foxo6a
Ohnolog 2: ENSXMAG00000025294 foxo6b

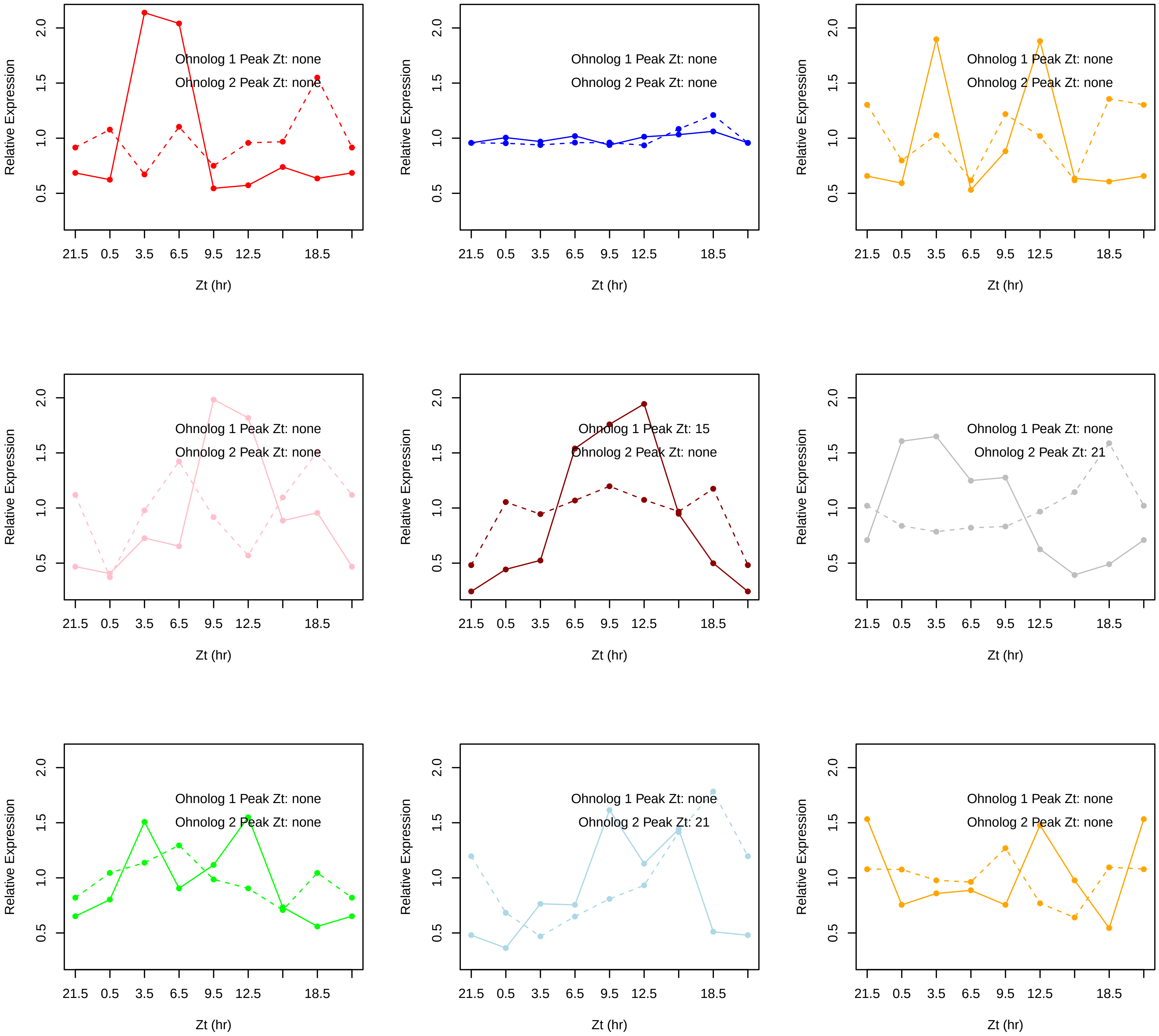

Ohnolog 1: ENSXMAG00000013475 GABARAPL2 (1 of many)
Ohnolog 2: ENSXMAG00000016058 gabarapl2

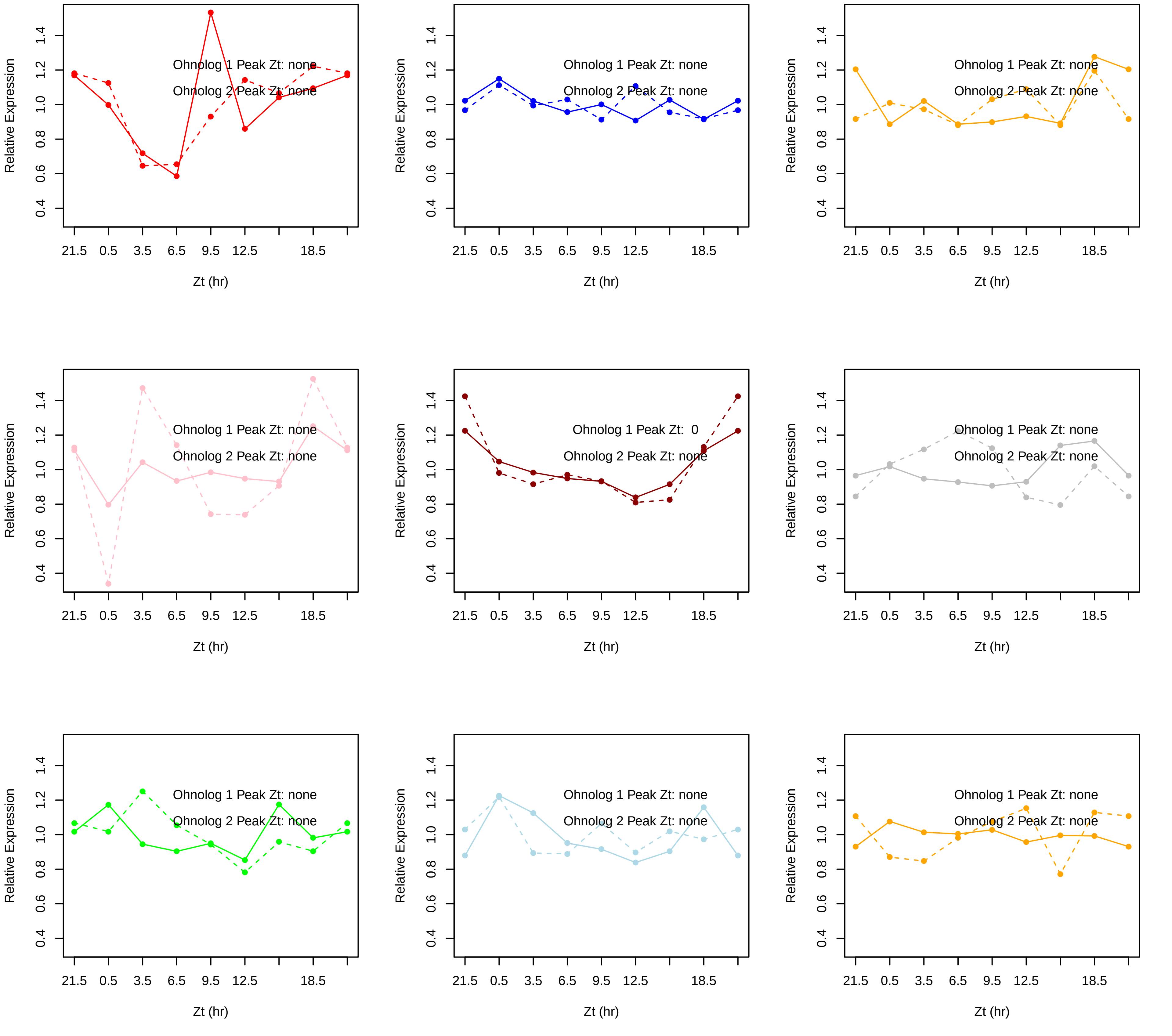

Ohnolog 1: ENSXMAG00000002400 plvapb
Ohnolog 2: ENSXMAG00000014977 plvapa

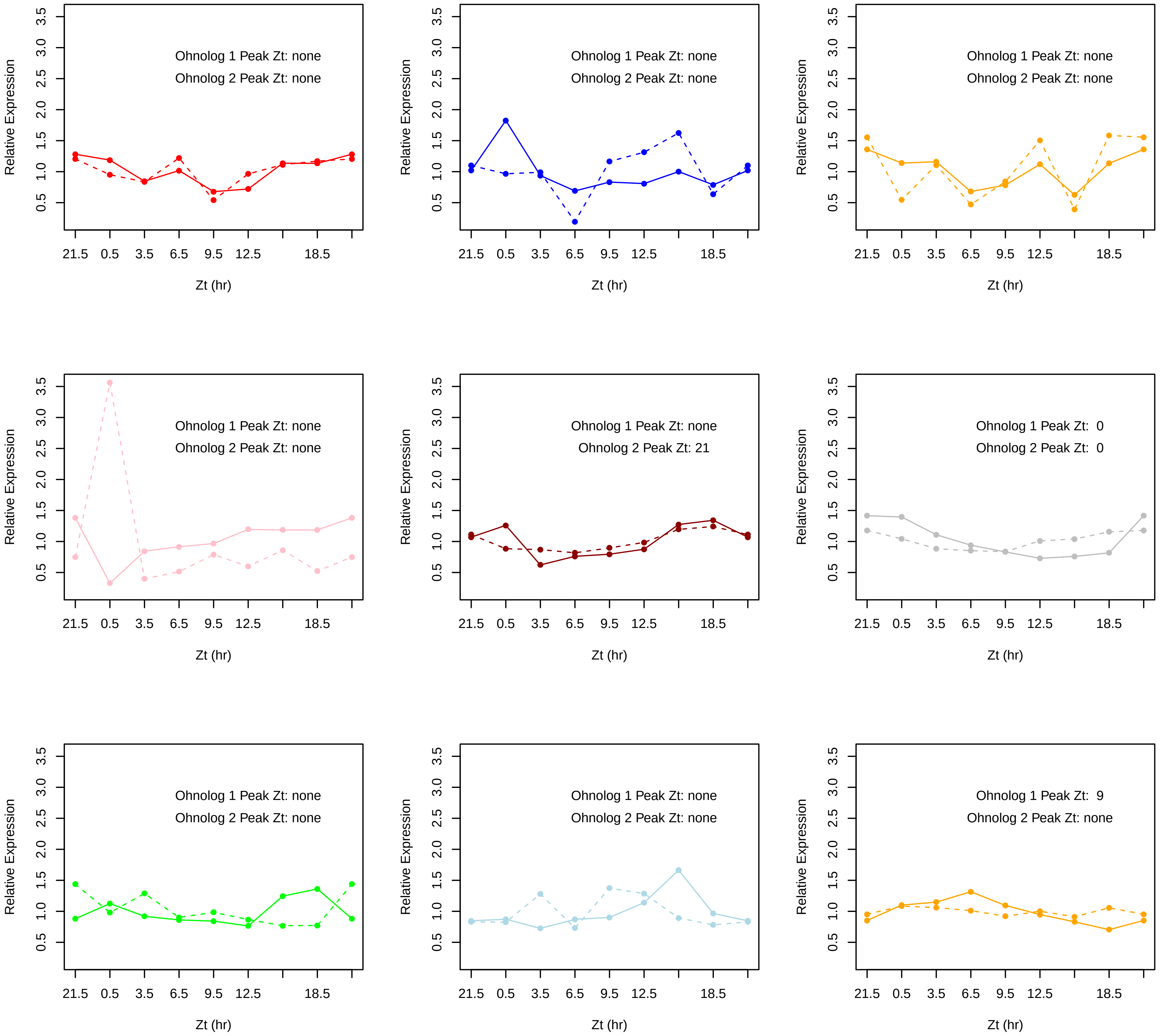

Ohnolog 1: ENSXMAG00000000786 epb41l3b
Ohnolog 2: ENSXMAG00000014334 EPB41L3

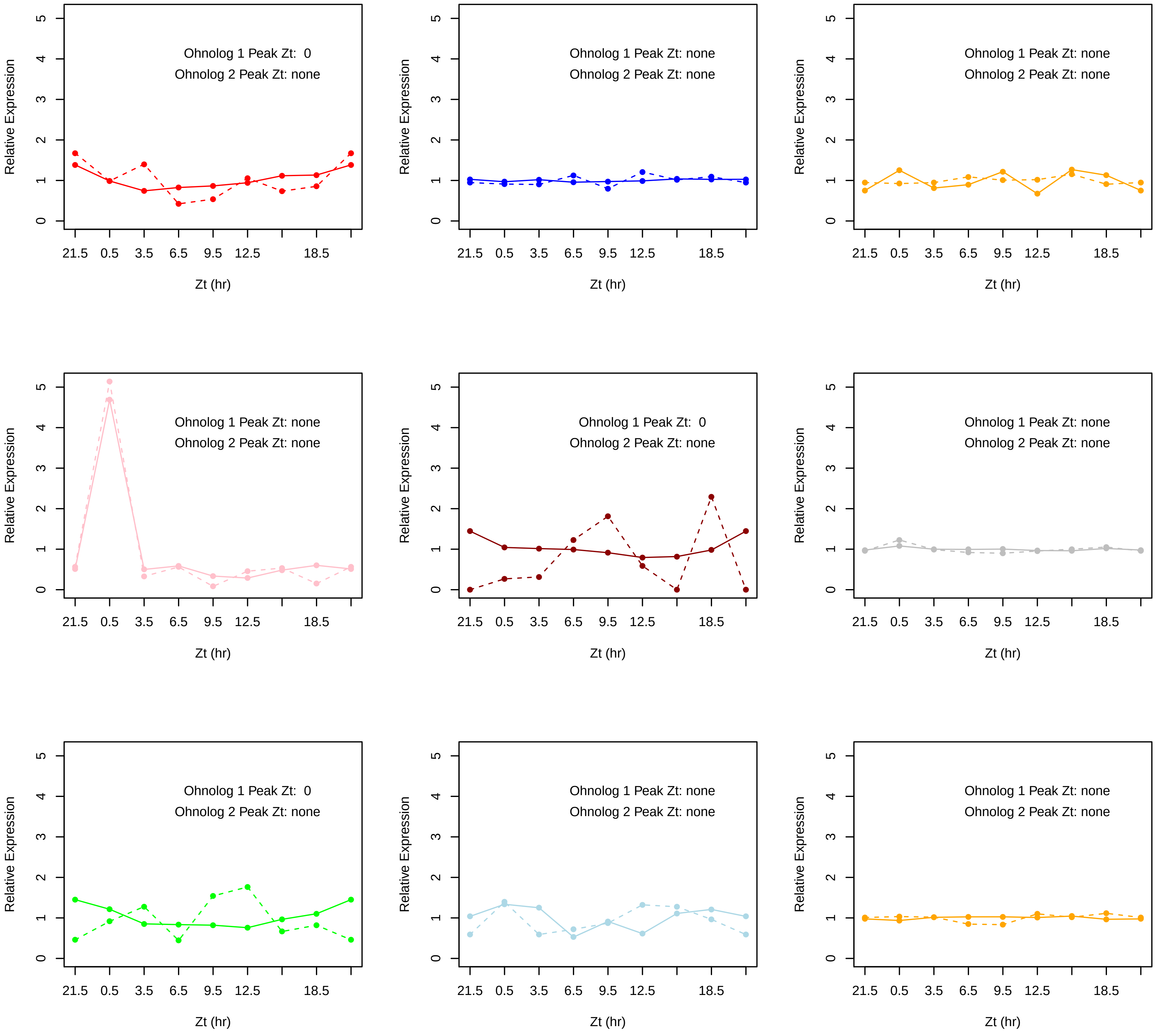

Ohnolog 1: ENSXMAG00000001106 dlgap1a
Ohnolog 2: ENSXMAG00000002511 dlgap1b

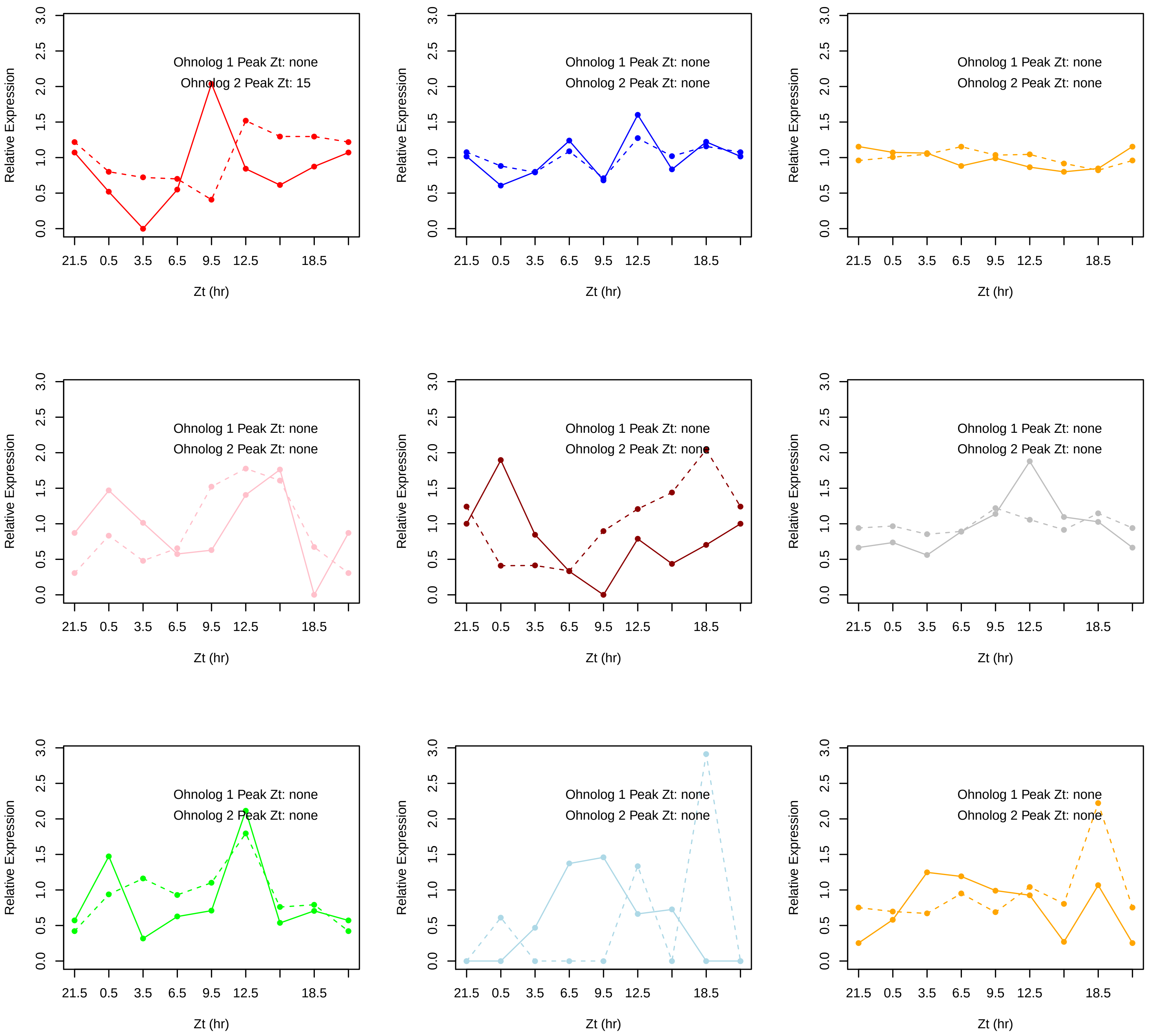

Ohnolog 1: ENSXMAG00000014733 eif3eb
Ohnolog 2: ENSXMAG00000024808 EIF3E

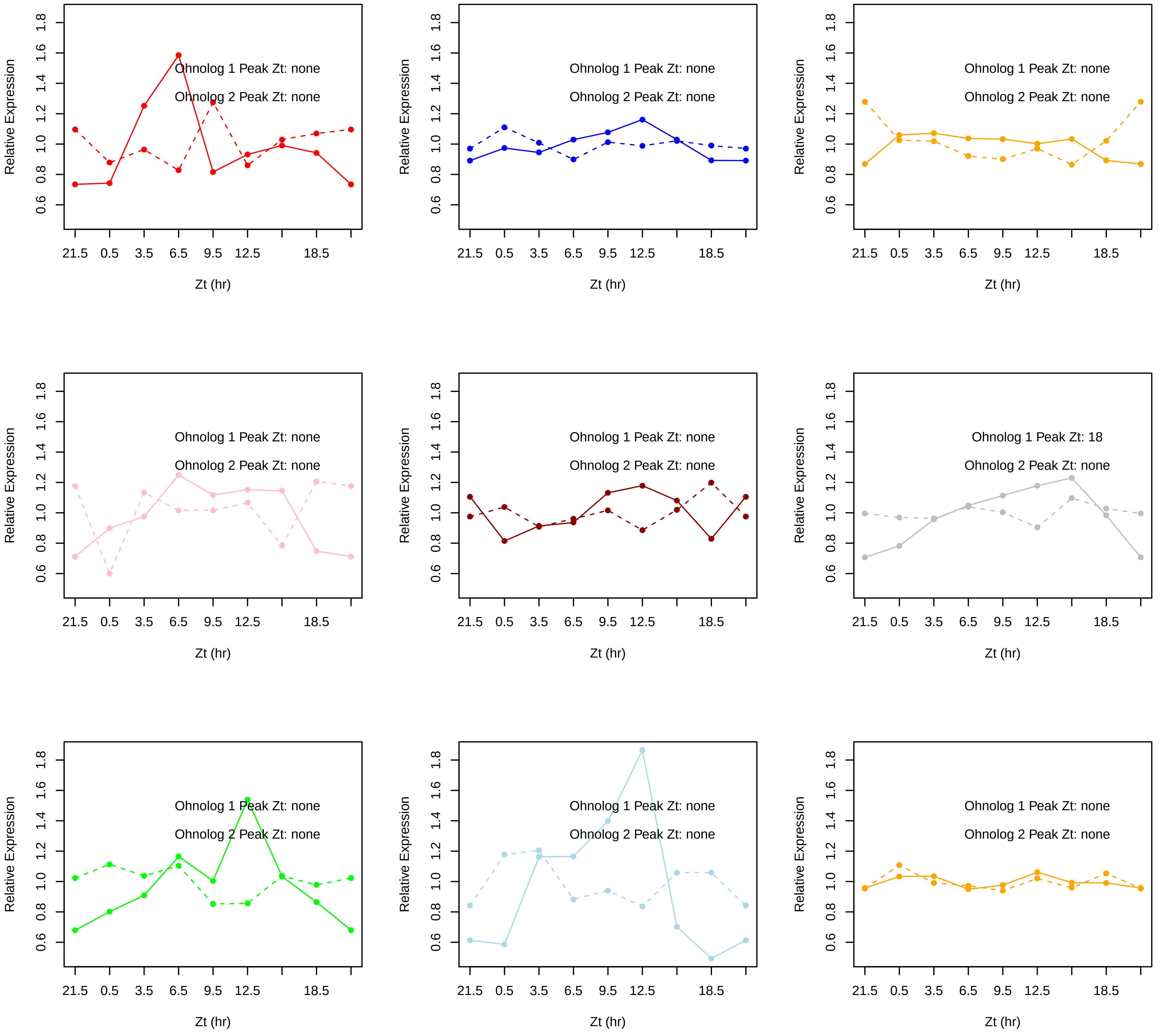

Ohnolog 1: ENSXMAG00000007066 calca (1 of many)
Ohnolog 2: ENSXMAG00000028956 CALCB

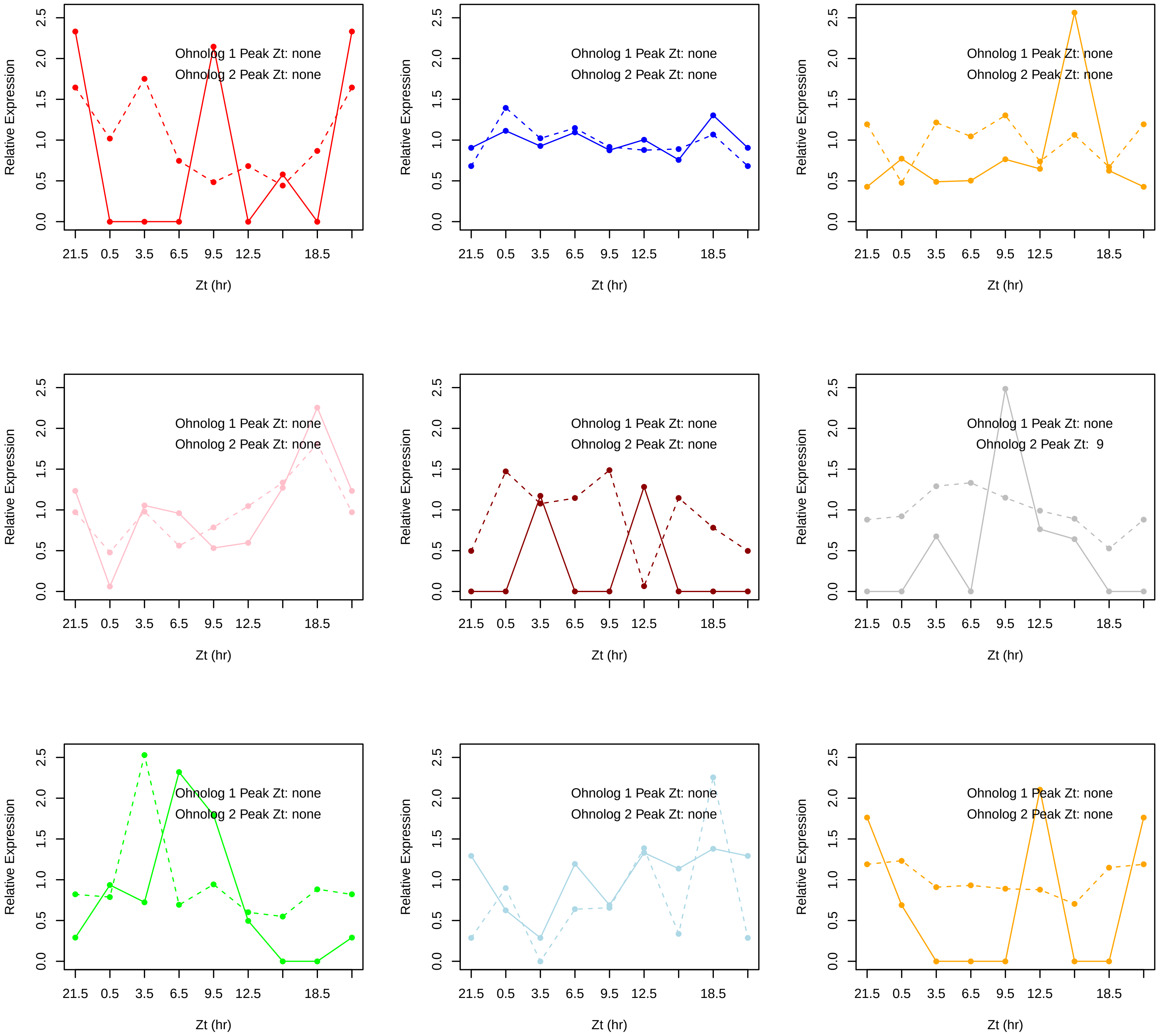

Ohnolog 1: ENSXMAG00000018743
Ohnolog 2: ENSXMAG00000024528 si:dkey−89b17.4

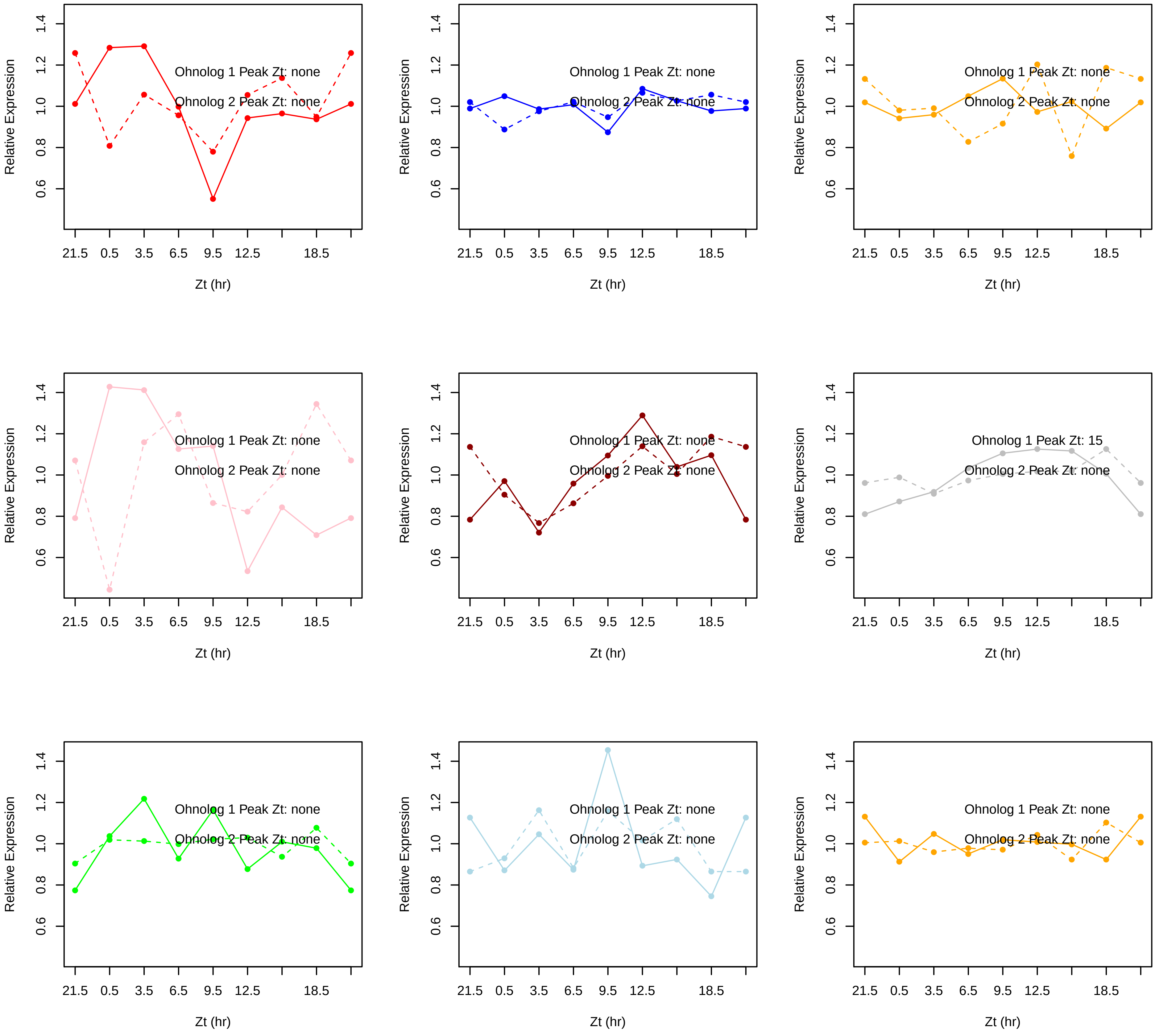

Ohnolog 1: ENSXMAG00000006965 RIC3
Ohnolog 2: ENSXMAG00000029930

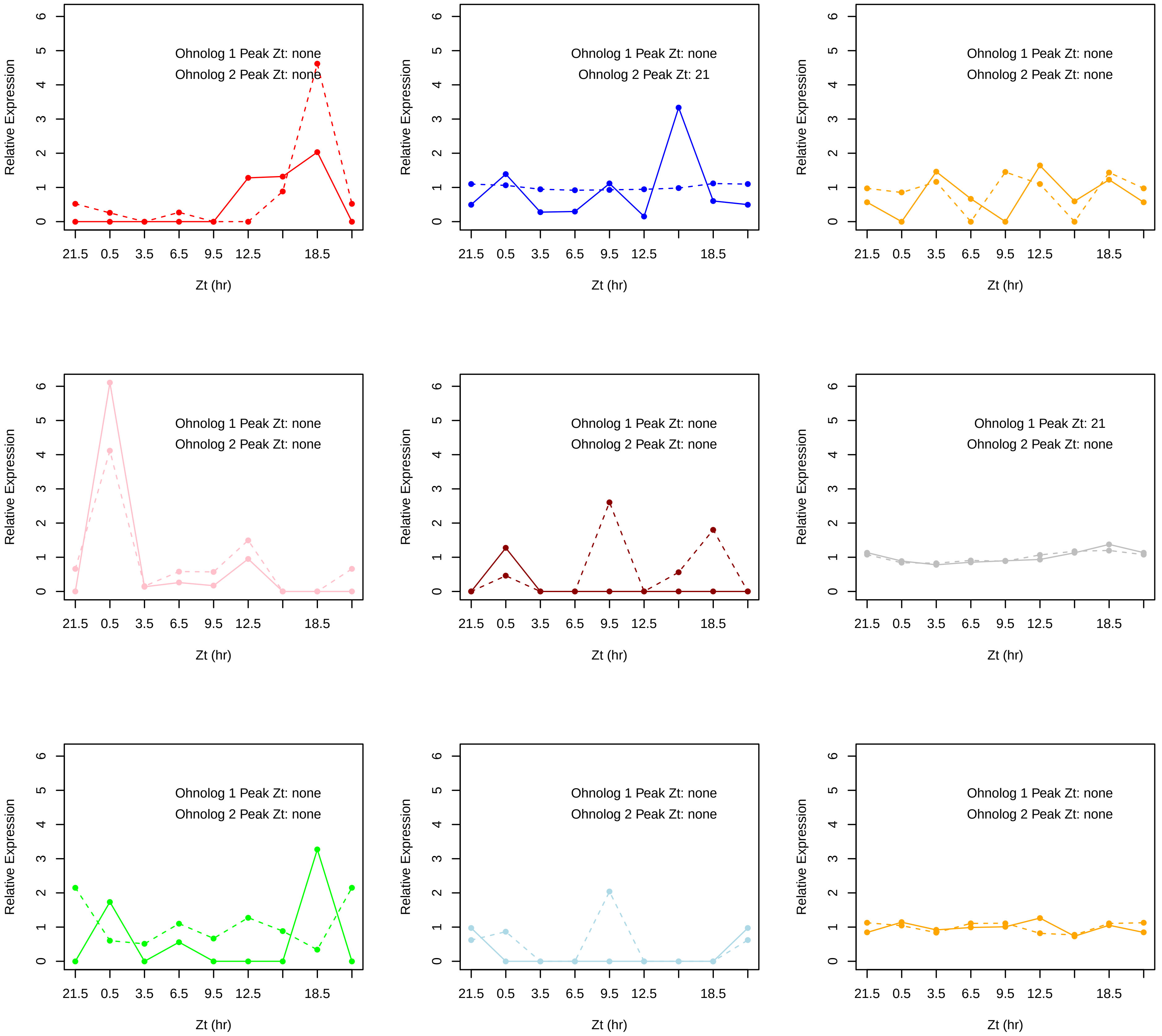

Ohnolog 1: ENSXMAG00000003342 si:dkey−21e5.1
Ohnolog 2: ENSXMAG00000005325 kcnt1

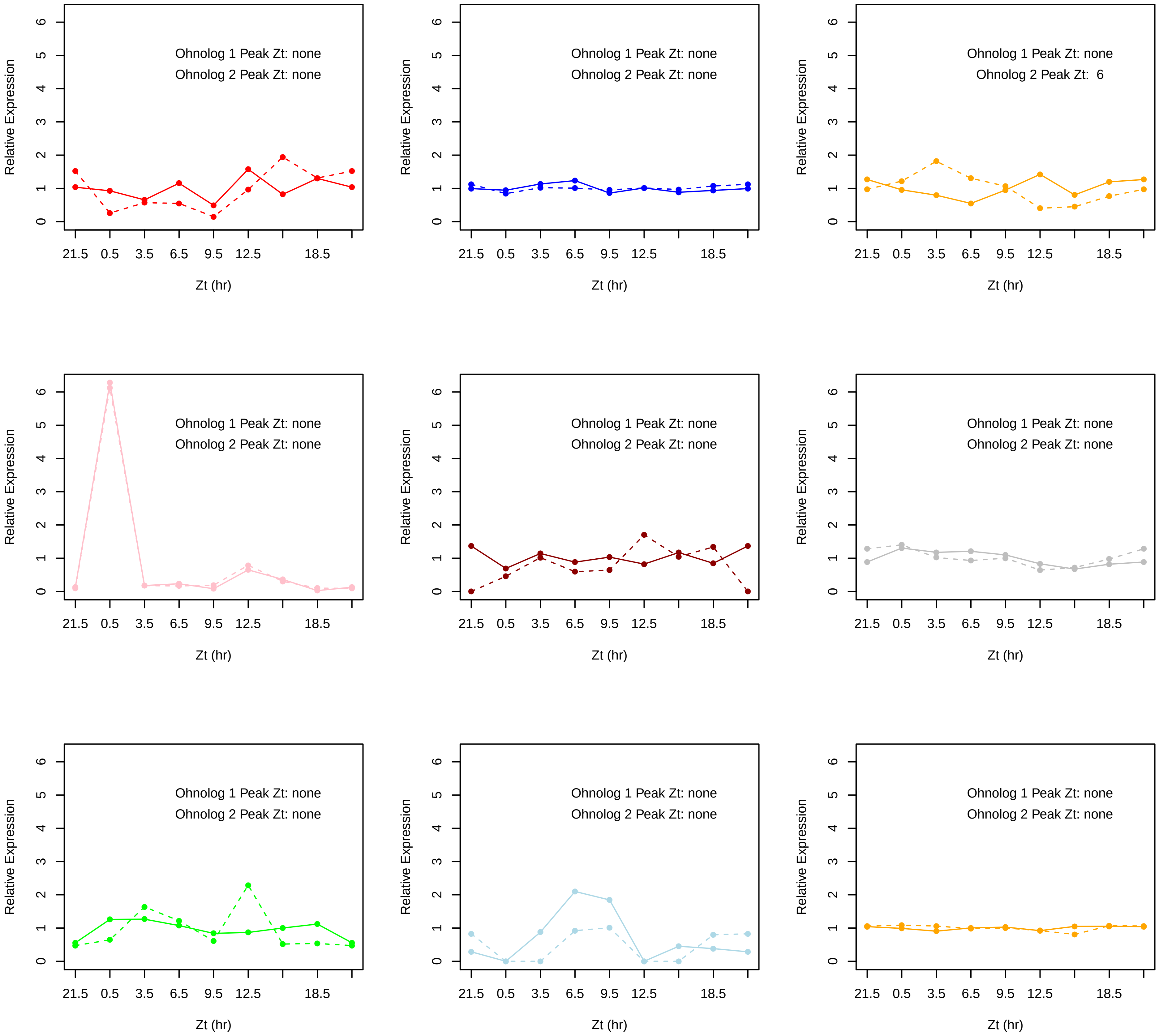

Ohnolog 1: ENSXMAG00000009514 phactr3b
Ohnolog 2: ENSXMAG00000016786 phactr3a

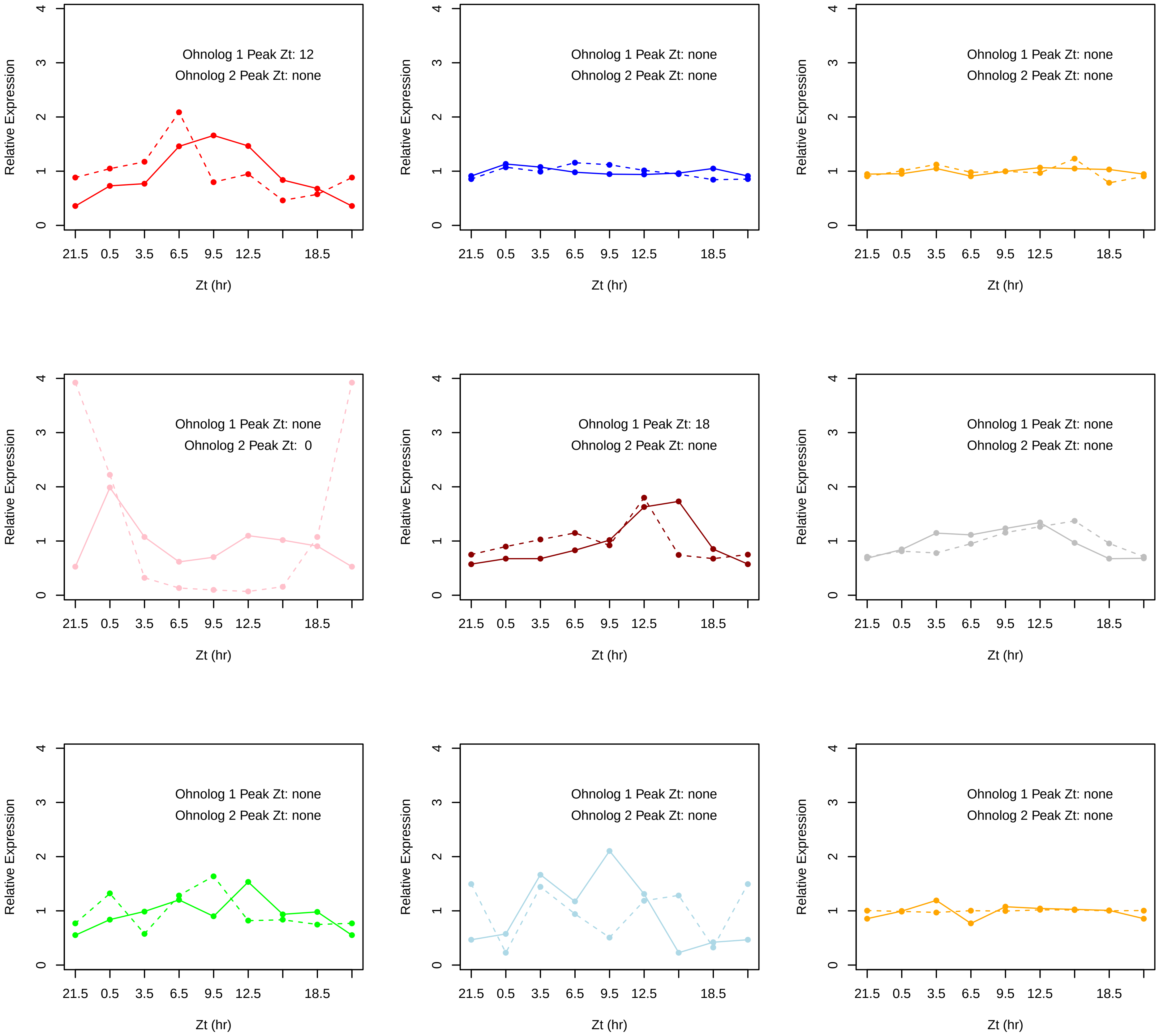

Ohnolog 1: ENSXMAG00000008442 arhgap21a
Ohnolog 2: ENSXMAG00000016331 arhgap21b

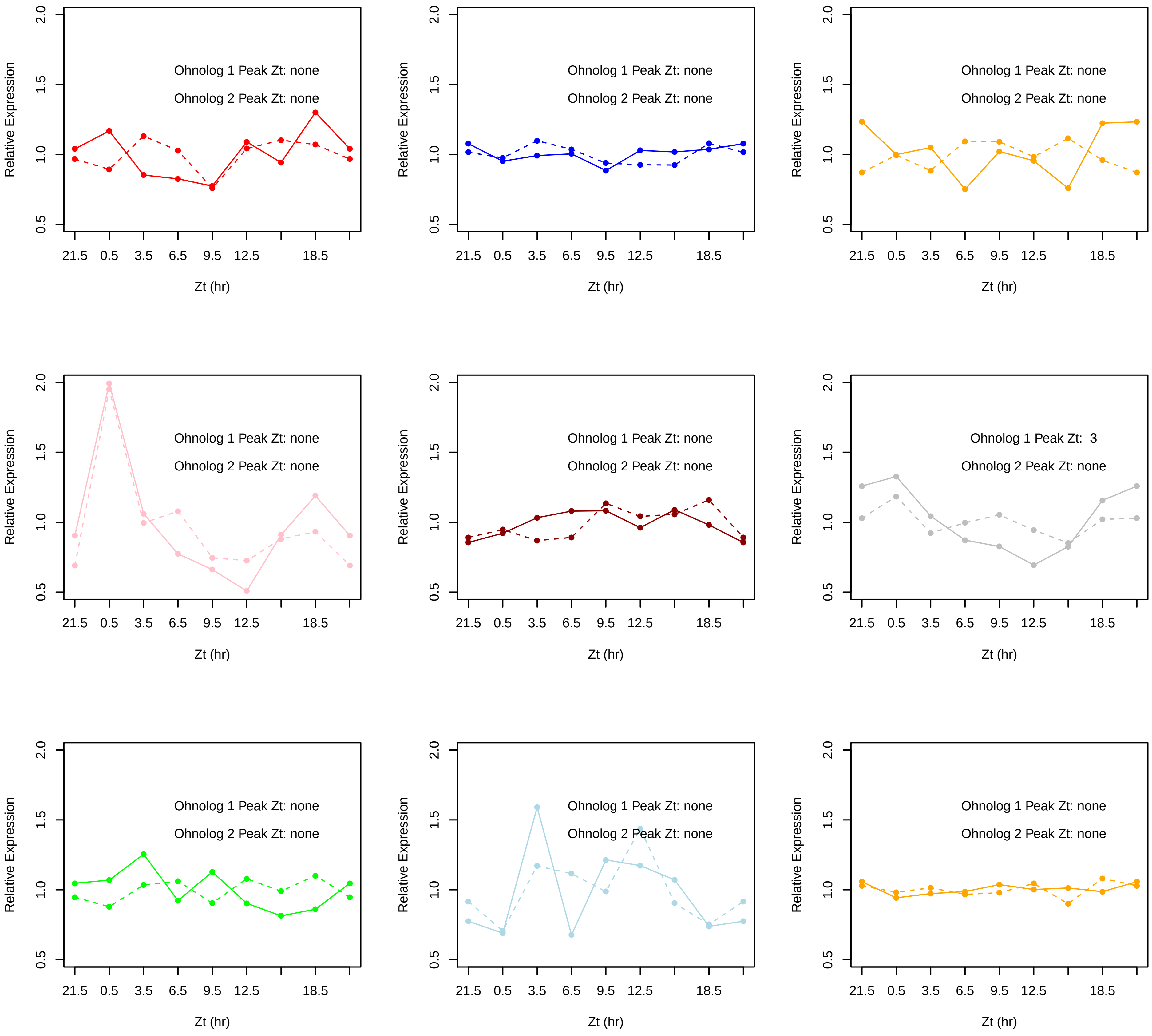

Ohnolog 1: ENSXMAG00000008989 ephb2a
Ohnolog 2: ENSXMAG00000017602 ephb2b

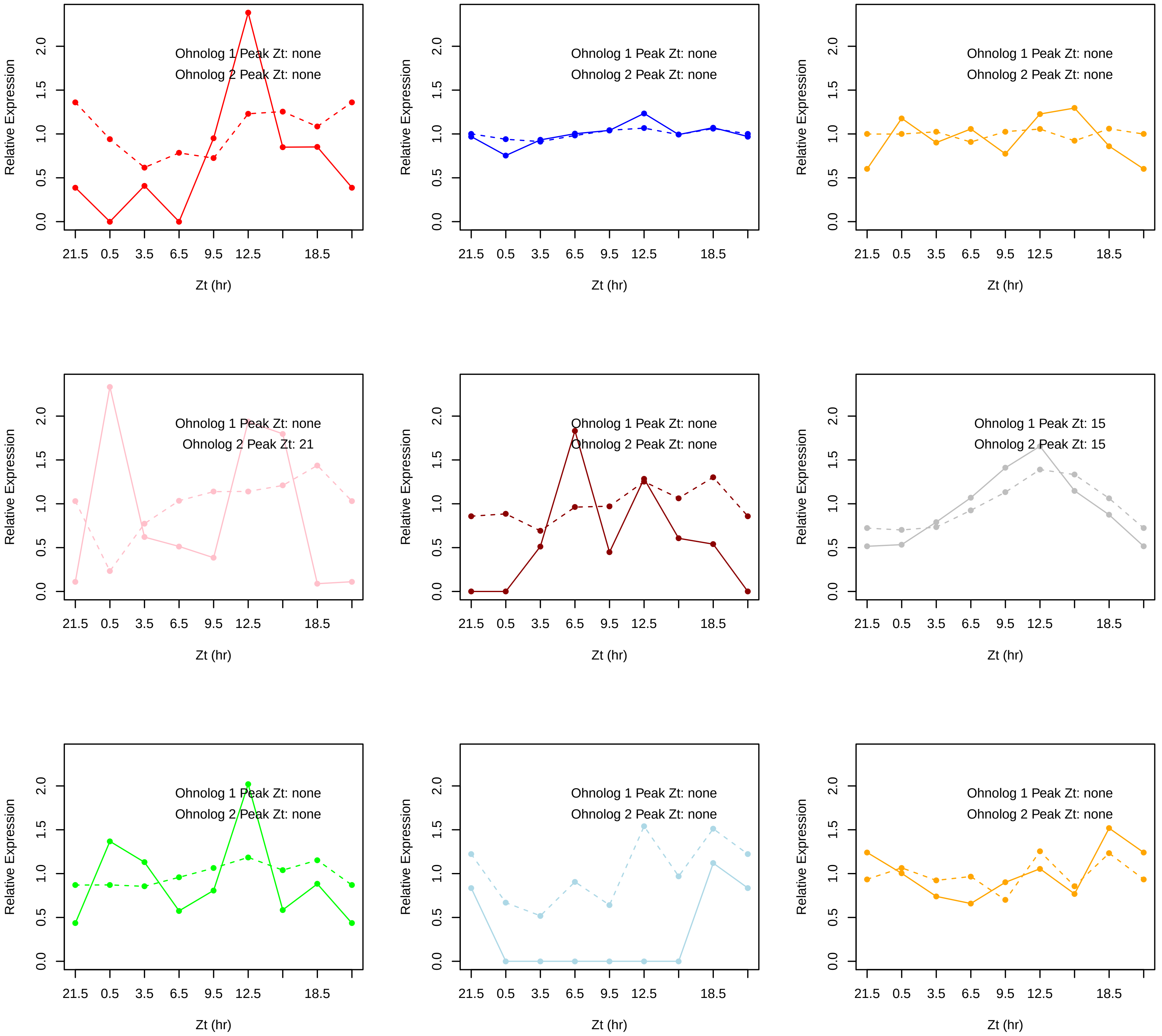

Ohnolog 1: ENSXMAG00000000673
Ohnolog 2: ENSXMAG00000028000

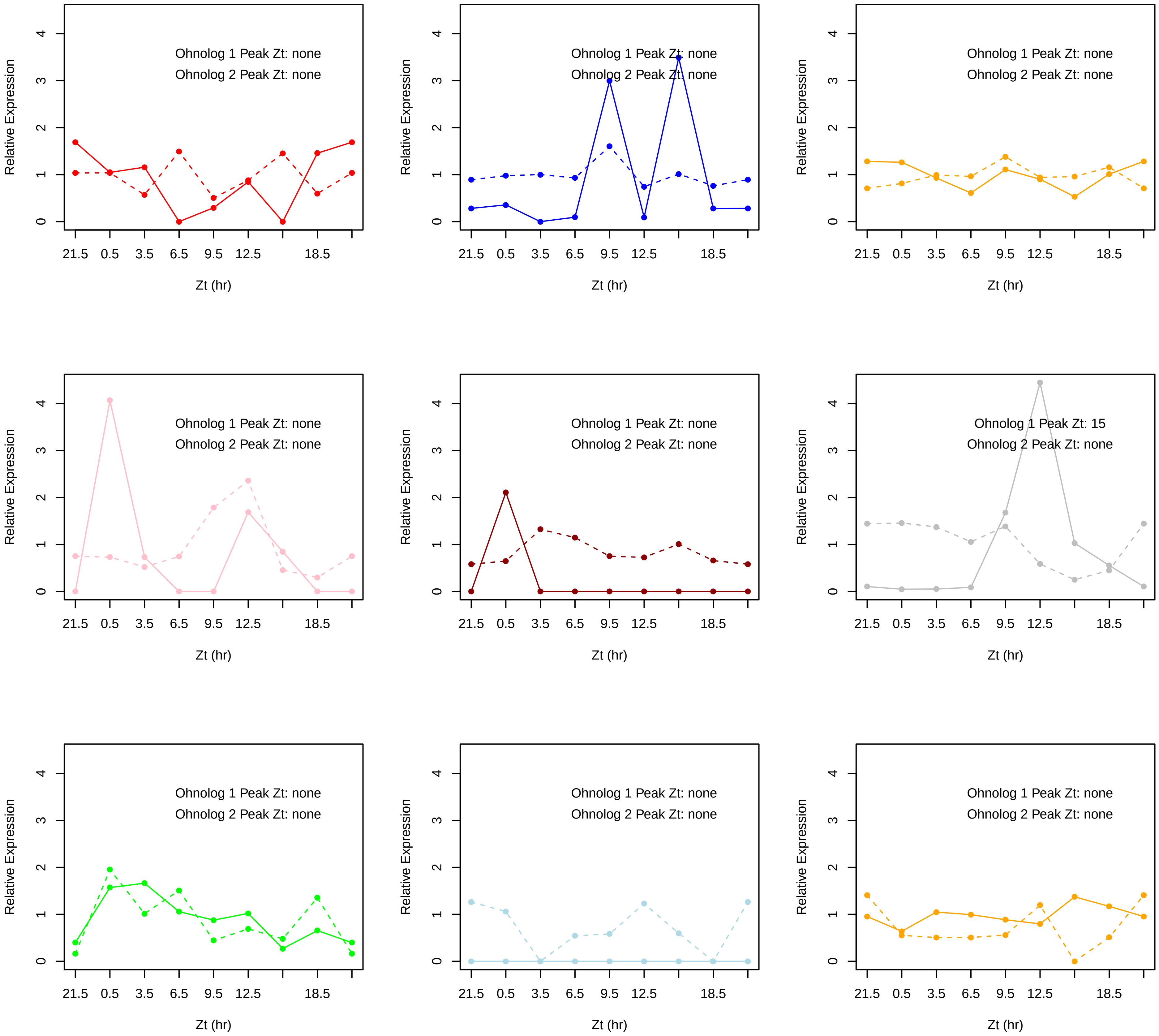

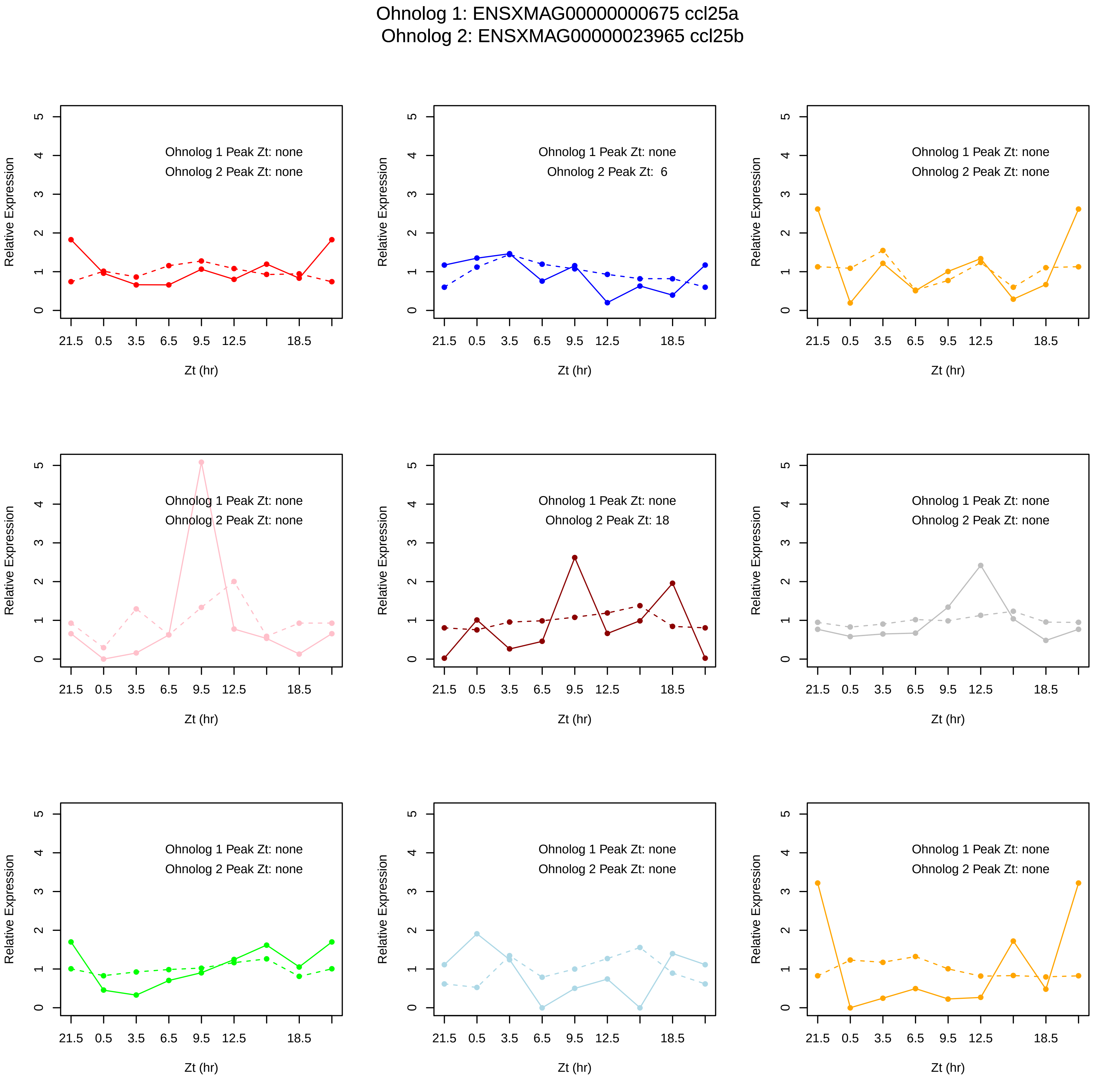
Ohnolog 1: ENSXMAG00000000675 ccl25a
Ohnolog 2: ENSXMAG00000023965 ccl25b
Ohnolog 1 Peak Zt: none
Ohnolog 2 Peak Zt: none
Ohnolog 1 Peak Zt: none
Ohnolog 2 Peak Zt: 6
Ohnolog 1 Peak Zt: none
Ohnolog 2 Peak Zt: none
Ohnolog 1 Peak Zt: none
Ohnolog 2 Peak Zt: none
Ohnolog 1 Peak Zt: none
Ohnolog 2 Peak Zt: 18
Ohnolog 1 Peak Zt: none
Ohnolog 2 Peak Zt: none
Ohnolog 1 Peak Zt: none
Ohnolog 2 Peak Zt: none
Ohnolog 1 Peak Zt: none
Ohnolog 2 Peak Zt: none
Ohnolog 1 Peak Zt: none
Ohnolog 2 Peak Zt: none
Relative Expression
Zt (hr)
21.5
0.5
3.5
6.5
9.5
12.5
18.5

Ohnolog 1: ENSXMAG00000008782 mcamb
Ohnolog 2: ENSXMAG00000025064

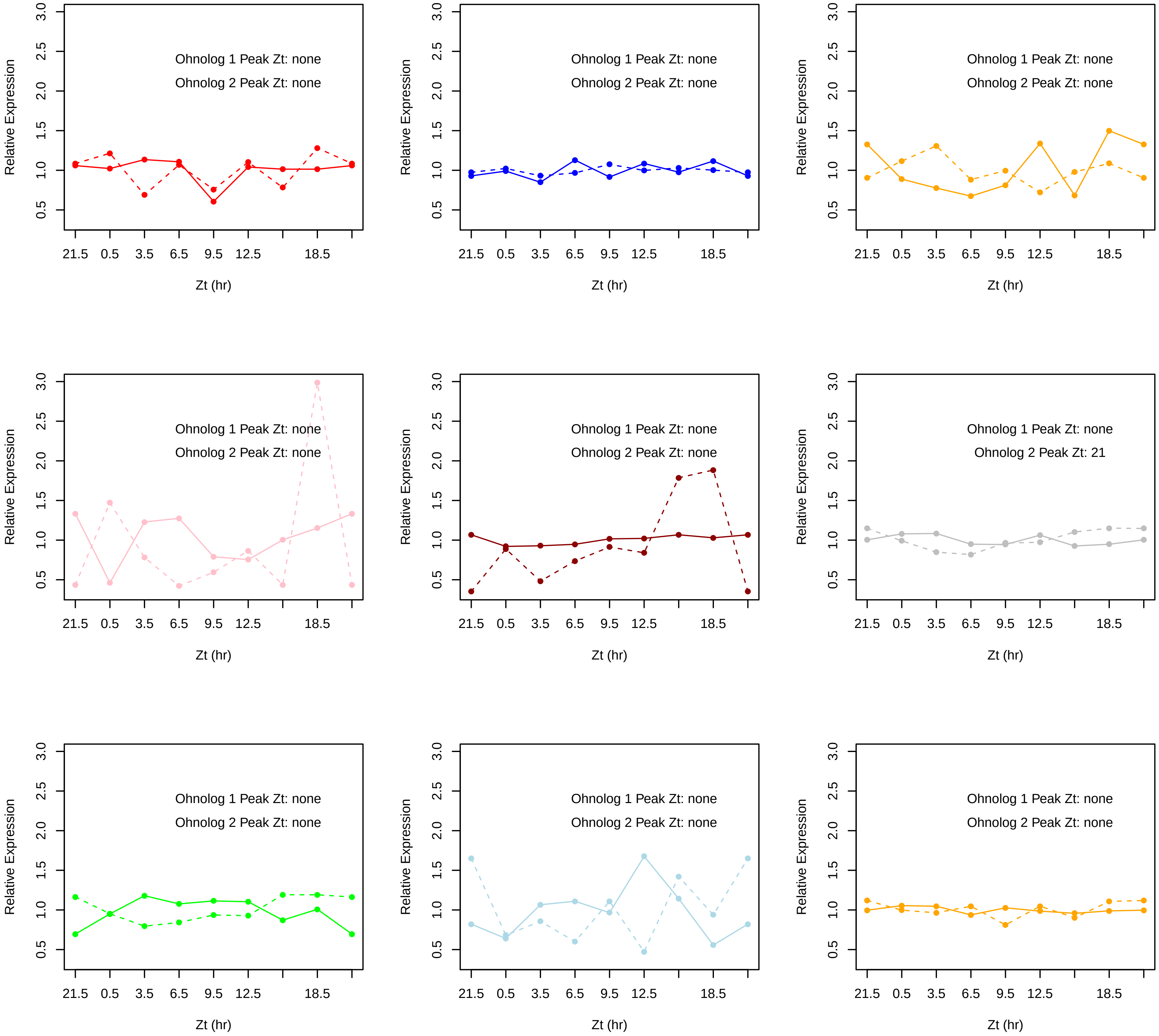

Ohnolog 1: ENSXMAG00000003277 notch1b
Ohnolog 2: ENSXMAG00000005227

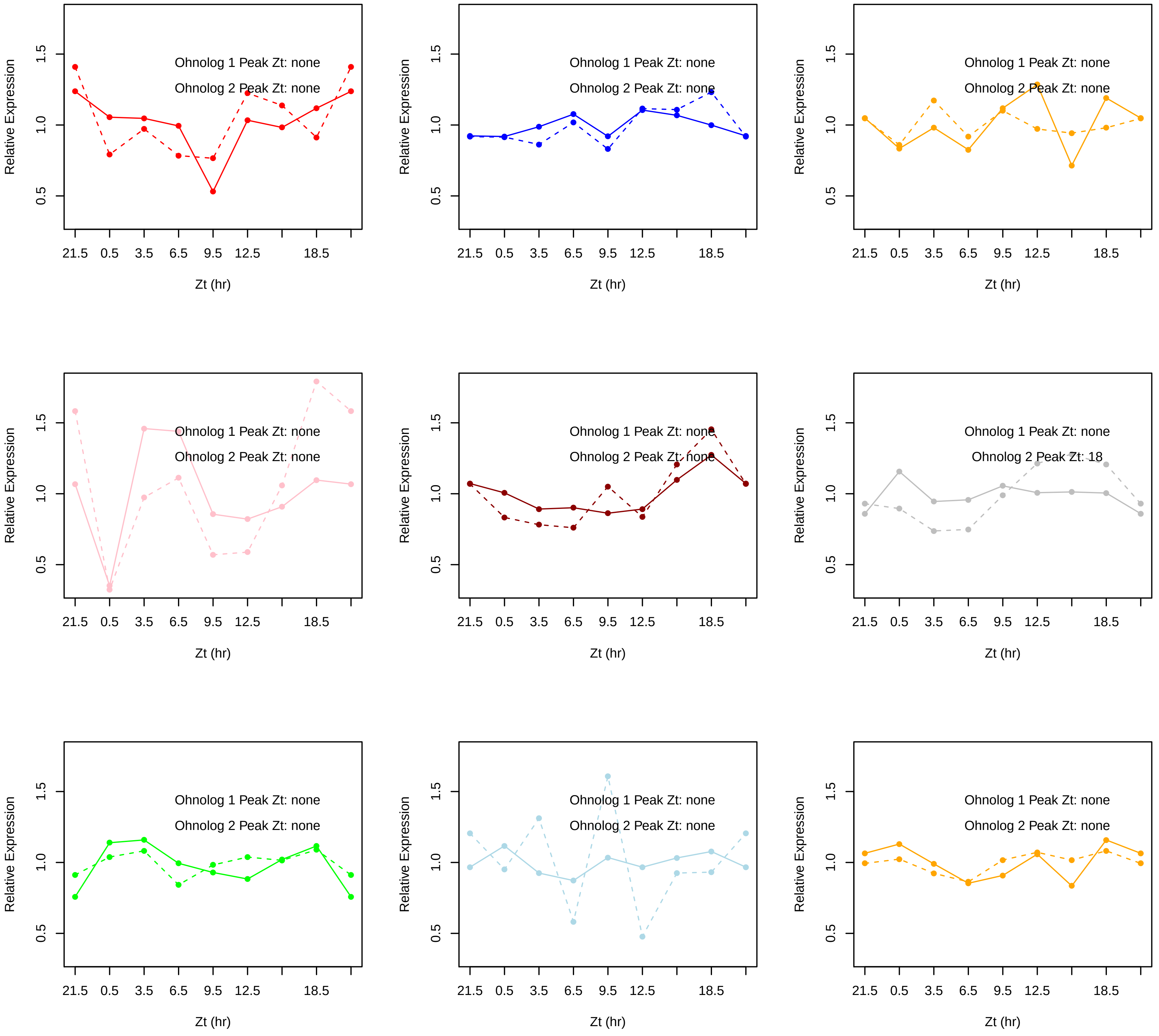

Ohnolog 1: ENSXMAG00000003370
Ohnolog 2: ENSXMAG00000029254 rab8a

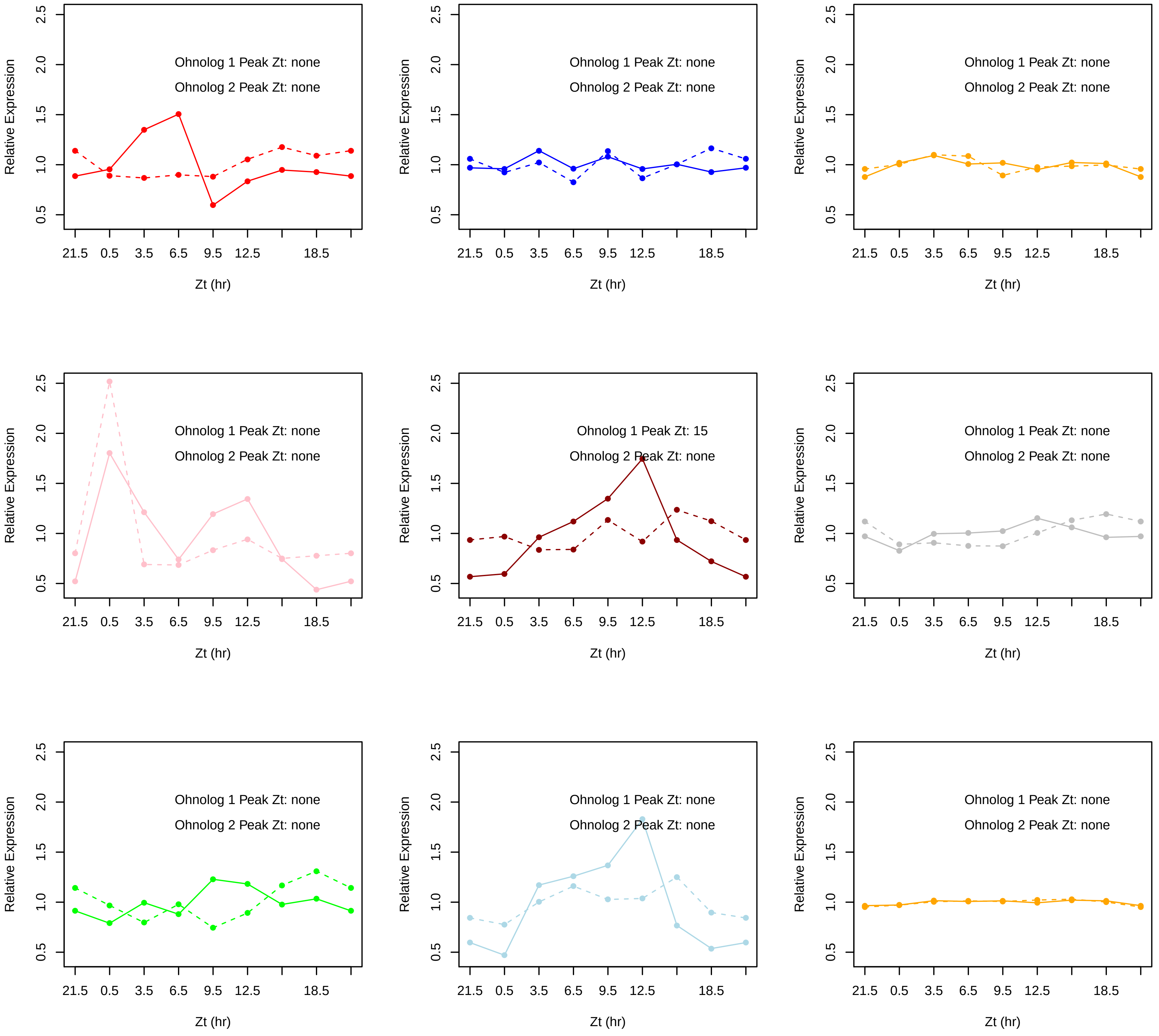

Ohnolog 1: ENSXMAG00000020107
Ohnolog 2: ENSXMAG00000028666 map1lc3b

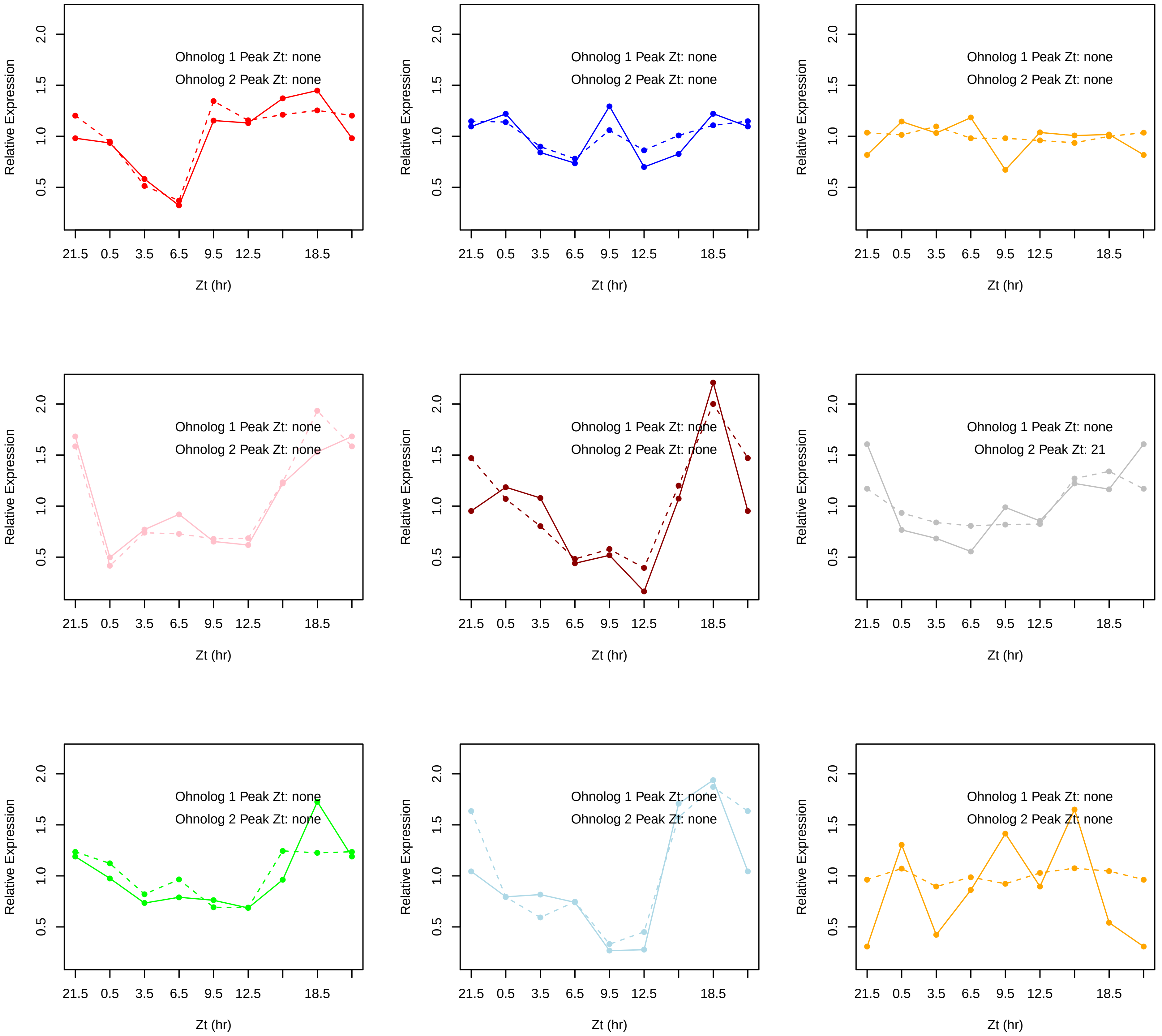

Ohnolog 1: ENSXMAG00000005286 etv5a
Ohnolog 2: ENSXMAG00000026320

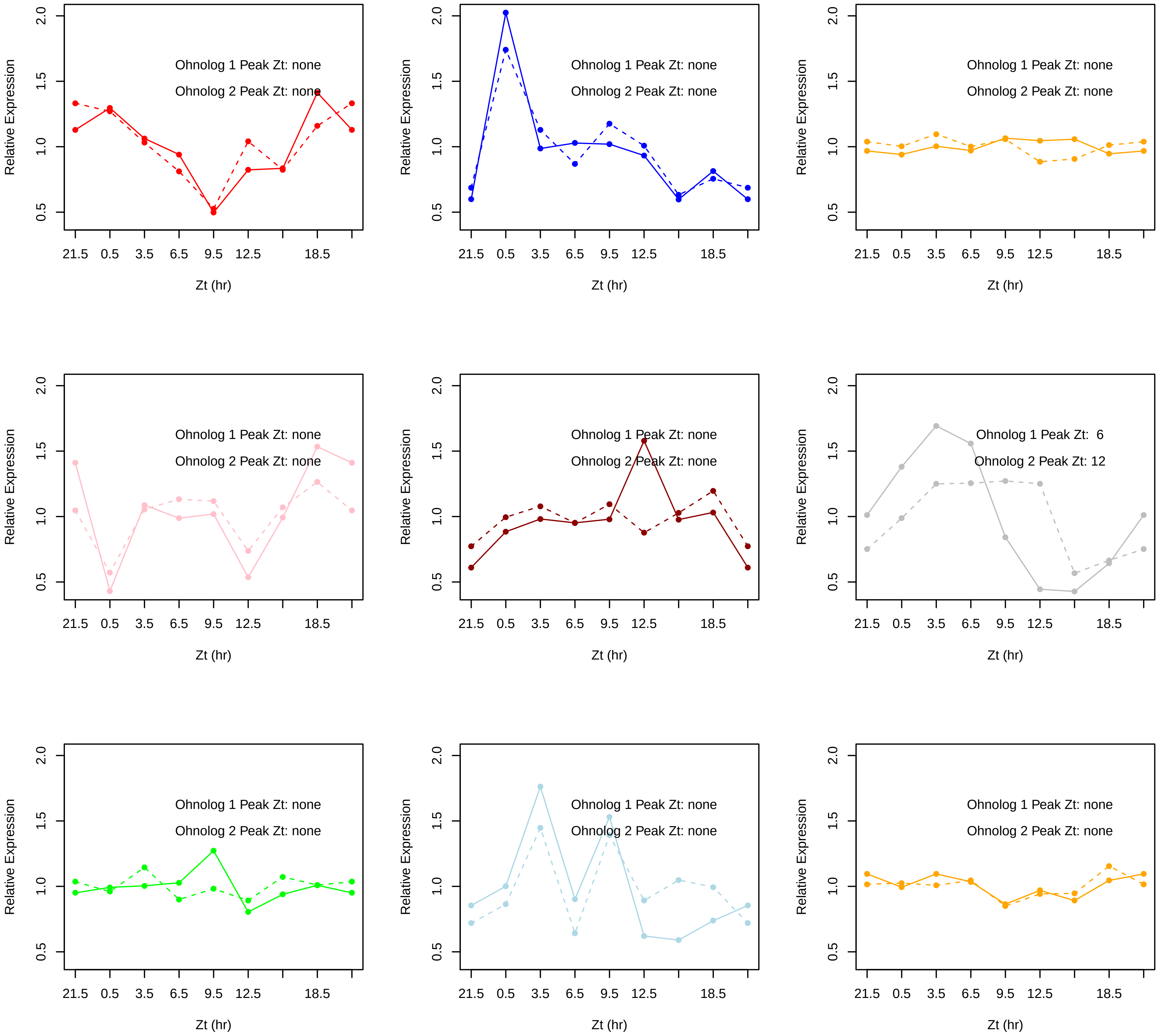

Ohnolog 1: ENSXMAG00000002095
Ohnolog 2: ENSXMAG00000010868

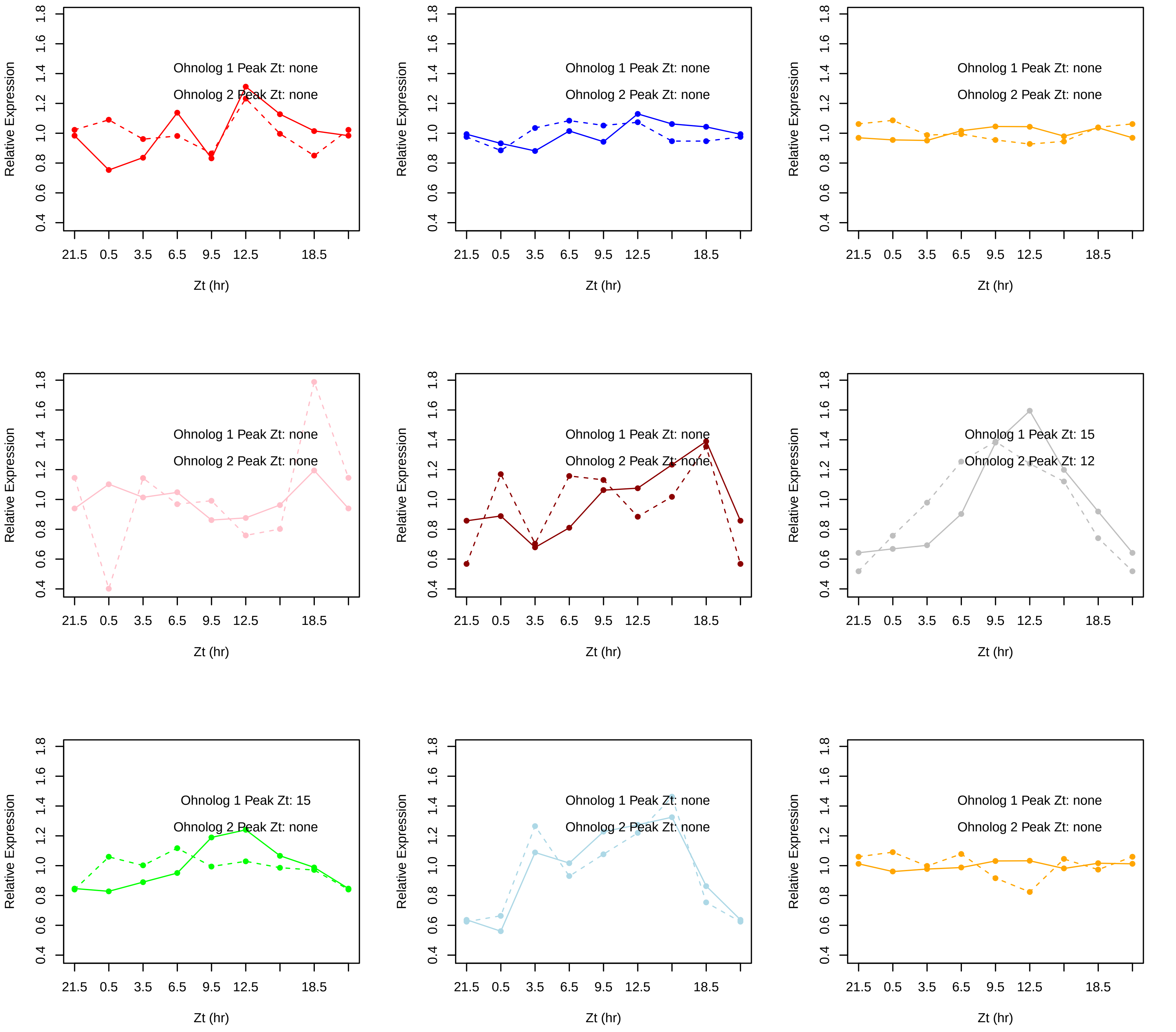

Ohnolog 1: ENSXMAG00000008830 tmem147
Ohnolog 2: ENSXMAG00000008883

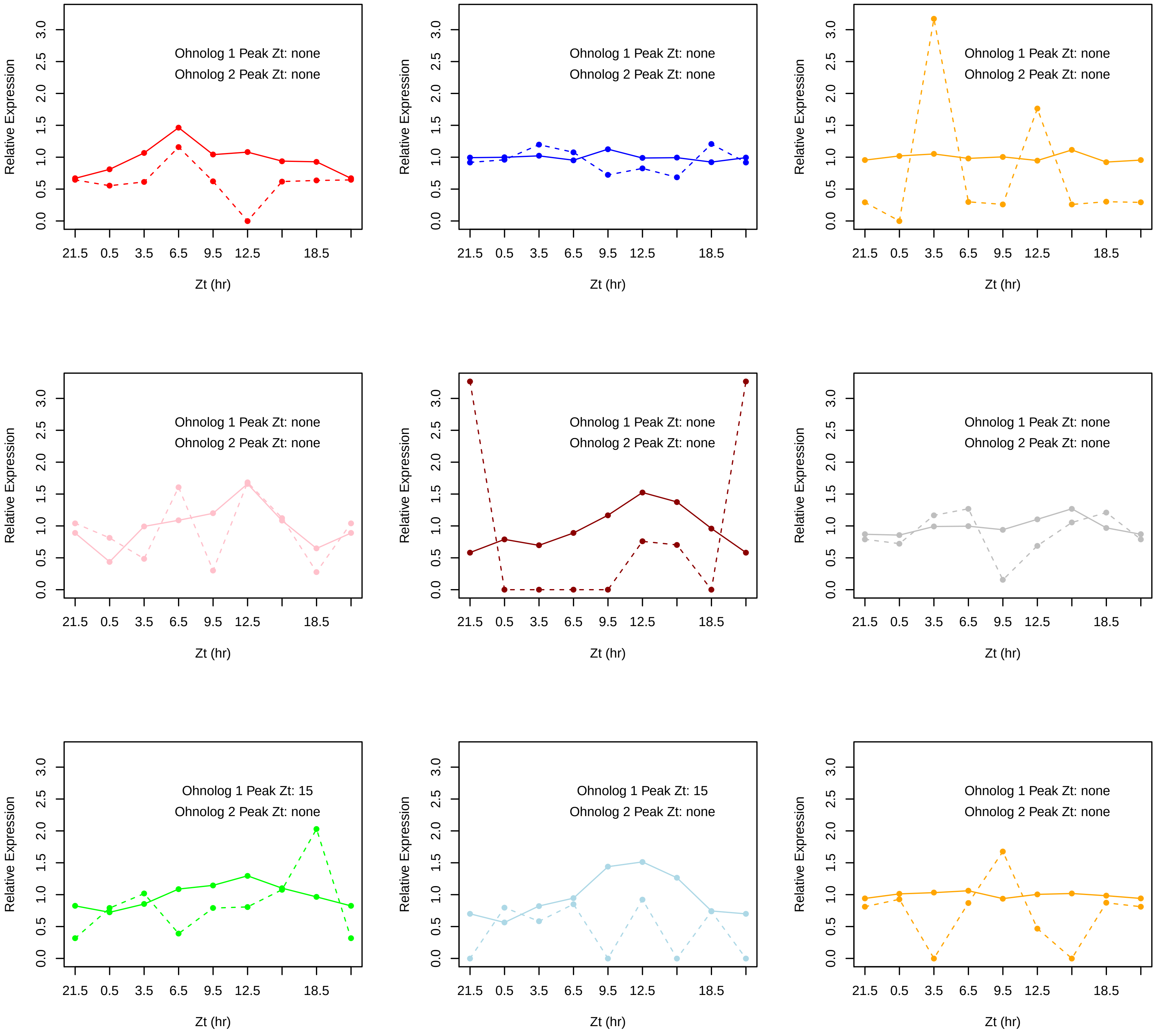

Ohnolog 1: ENSXMAG00000012786 ptprua
Ohnolog 2: ENSXMAG00000026721 ptprub

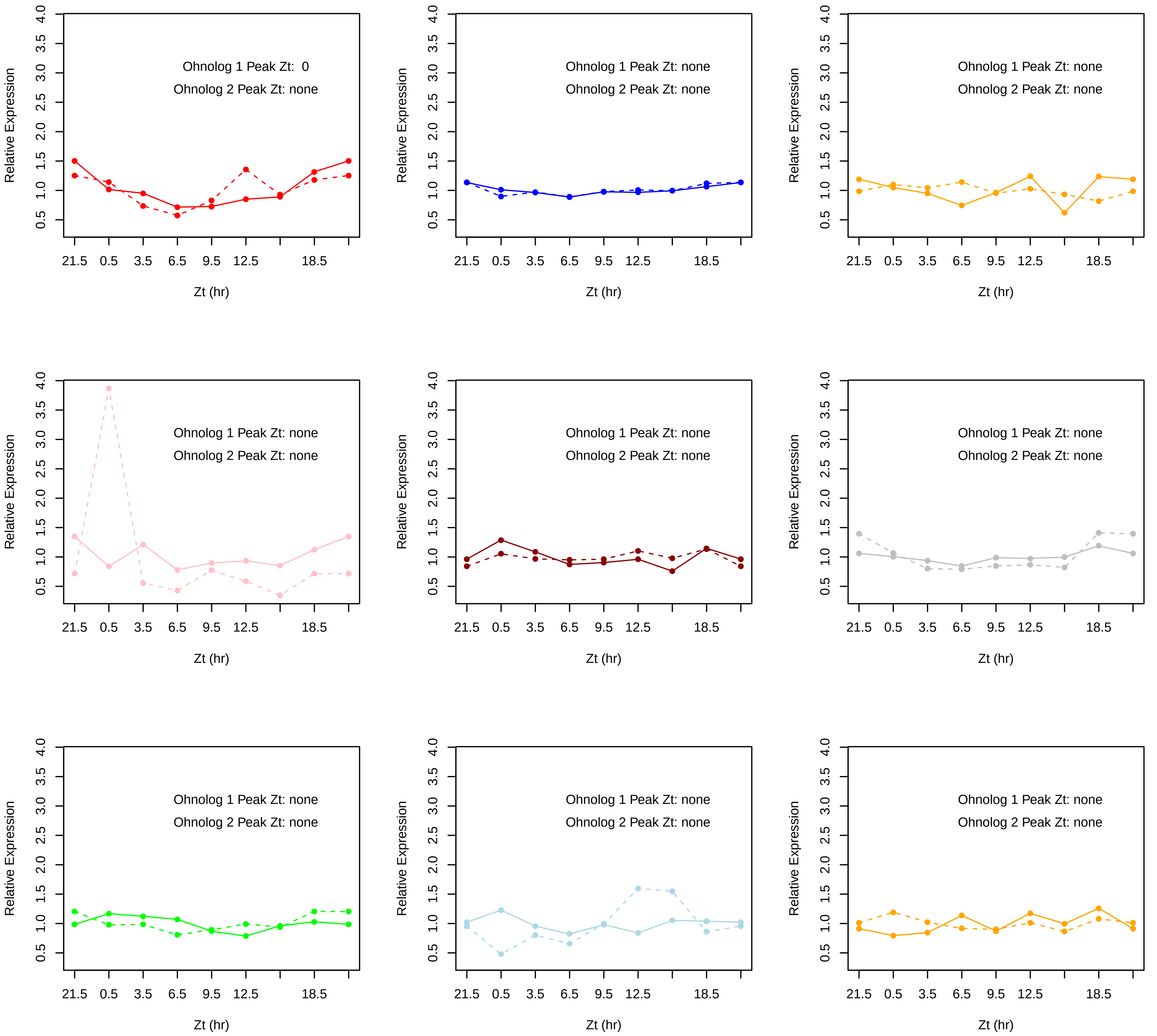

Ohnolog 1: ENSXMAG00000013101
Ohnolog 2: ENSXMAG00000027431 RAP2A

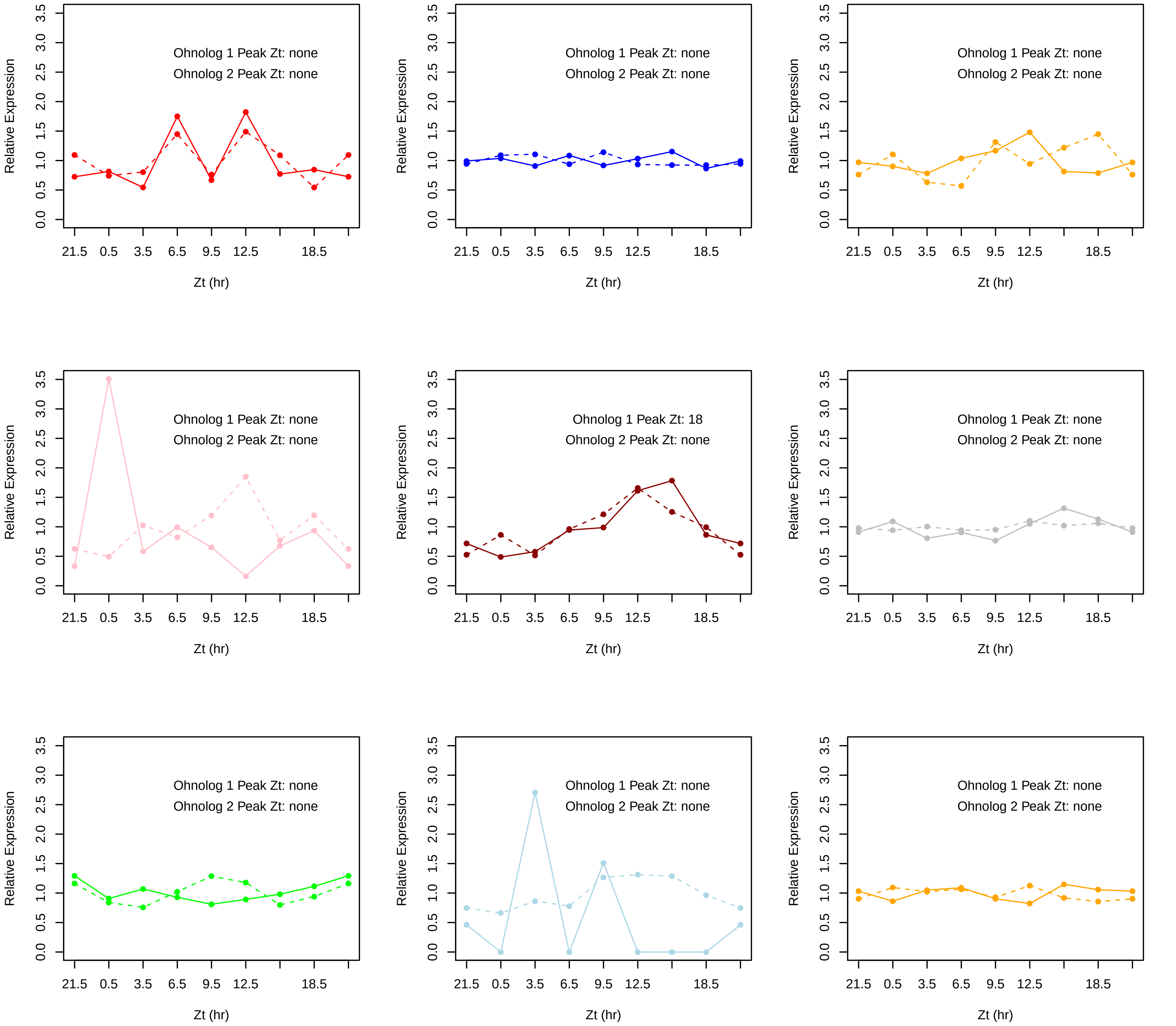

Ohnolog 1: ENSXMAG00000010649 phactr4b
Ohnolog 2: ENSXMAG00000015749 phactr4a

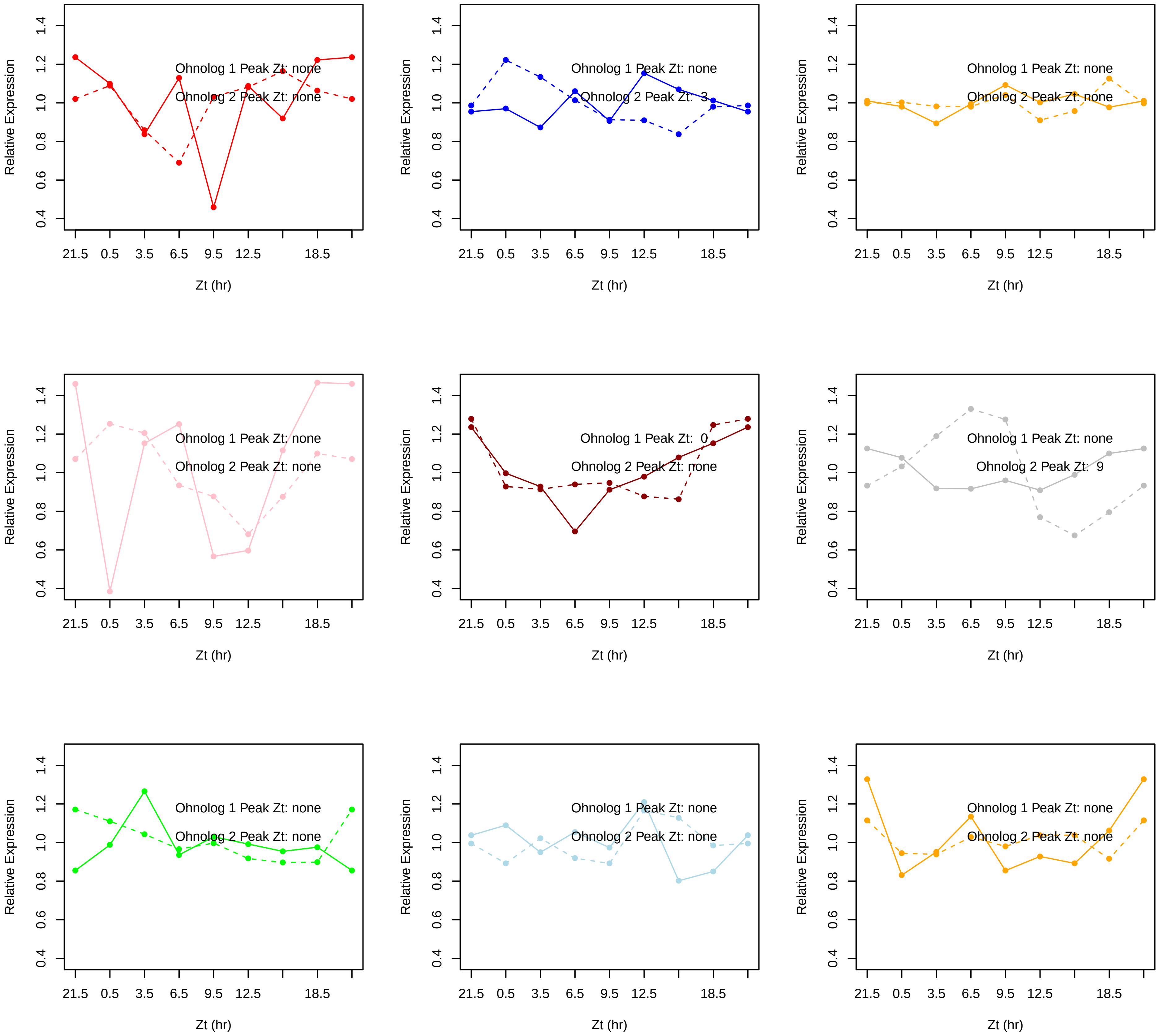

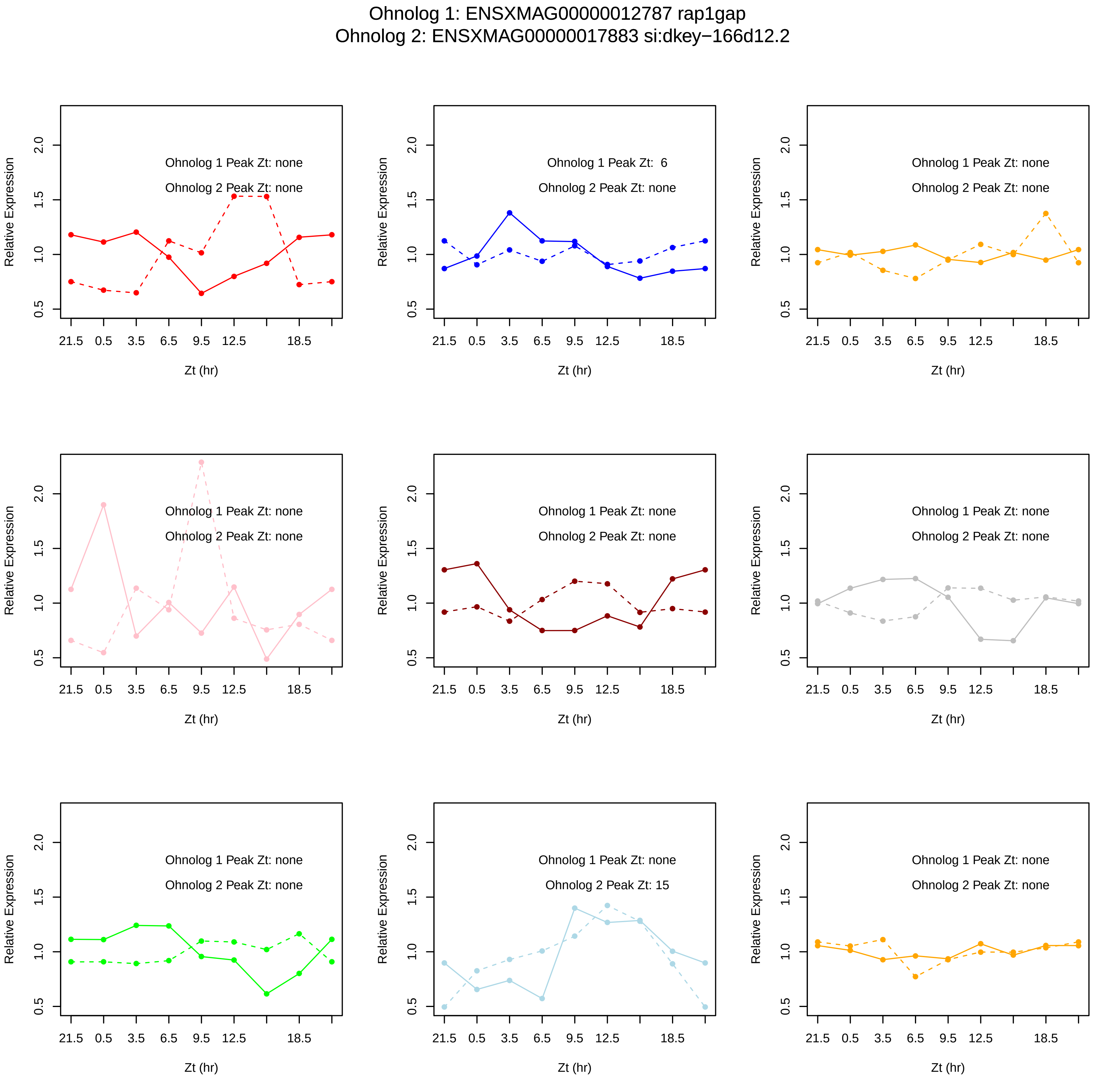
Ohnolog 1: ENSXMAG00000012787 rap1gap
Ohnolog 2: ENSXMAG00000017883 si:dkey−166d12.2
Ohnolog 1 Peak Zt: none
Ohnolog 2 Peak Zt: none
Ohnolog 1 Peak Zt: 6
Ohnolog 2 Peak Zt: none
Ohnolog 1 Peak Zt: none
Ohnolog 2 Peak Zt: none
Ohnolog 1 Peak Zt: none
Ohnolog 2 Peak Zt: none
Ohnolog 1 Peak Zt: none
Ohnolog 2 Peak Zt: none
Ohnolog 1 Peak Zt: none
Ohnolog 2 Peak Zt: none
Ohnolog 1 Peak Zt: none
Ohnolog 2 Peak Zt: none
Ohnolog 1 Peak Zt: none
Ohnolog 2 Peak Zt: 15
Ohnolog 1 Peak Zt: none
Ohnolog 2 Peak Zt: none
Relative Expression
Zt (hr)
21.5 0.5 3.5 6.5 9.5 12.5 18.5
0.5 1.0 1.5 2.0

Ohnolog 1: ENSXMAG00000003195 CCNG2 (1 of many)
Ohnolog 2: ENSXMAG00000025807 ccng2

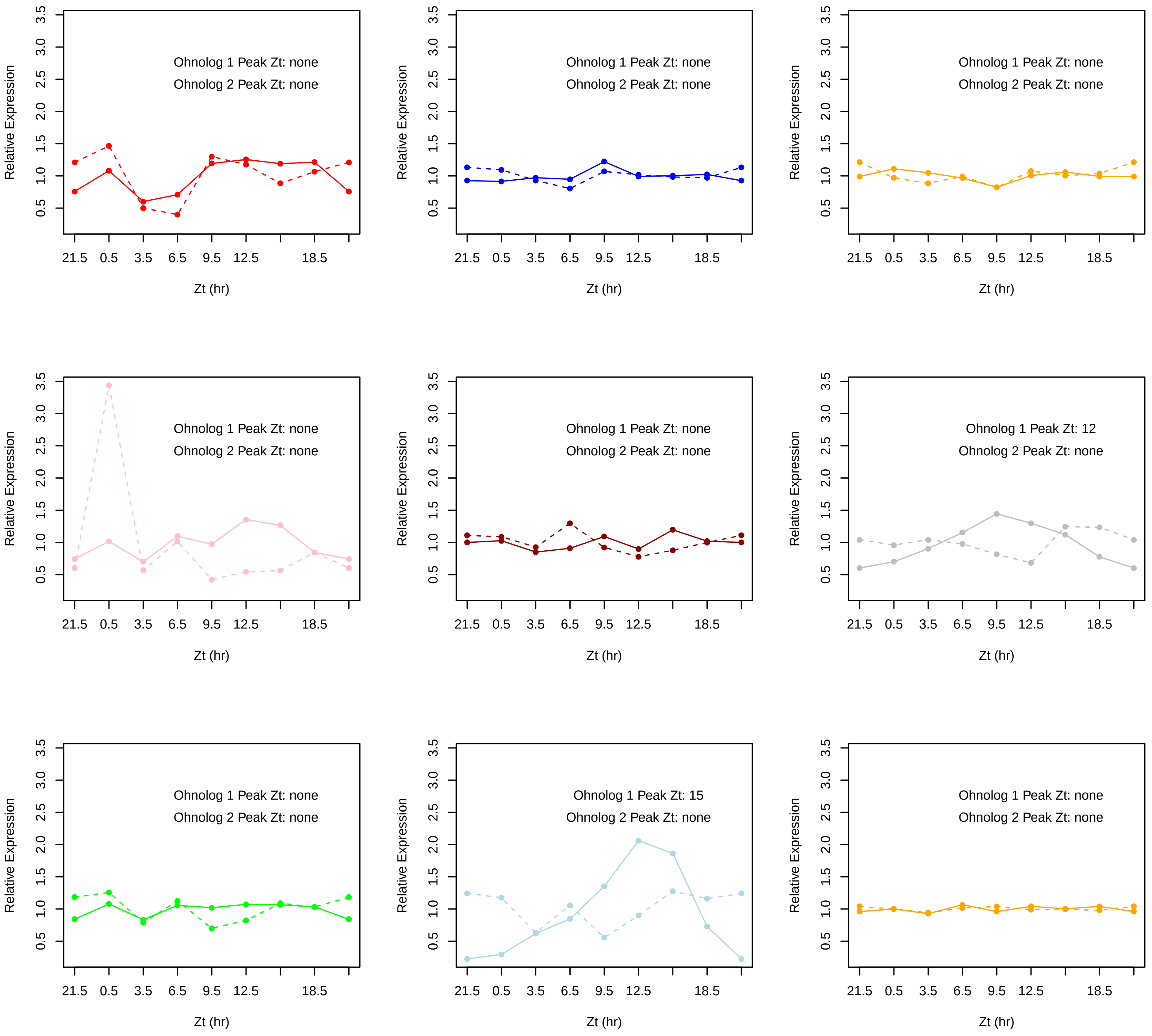

Ohnolog 1: ENSXMAG00000026705 KCNB2
Ohnolog 2: ENSXMAG00000028774

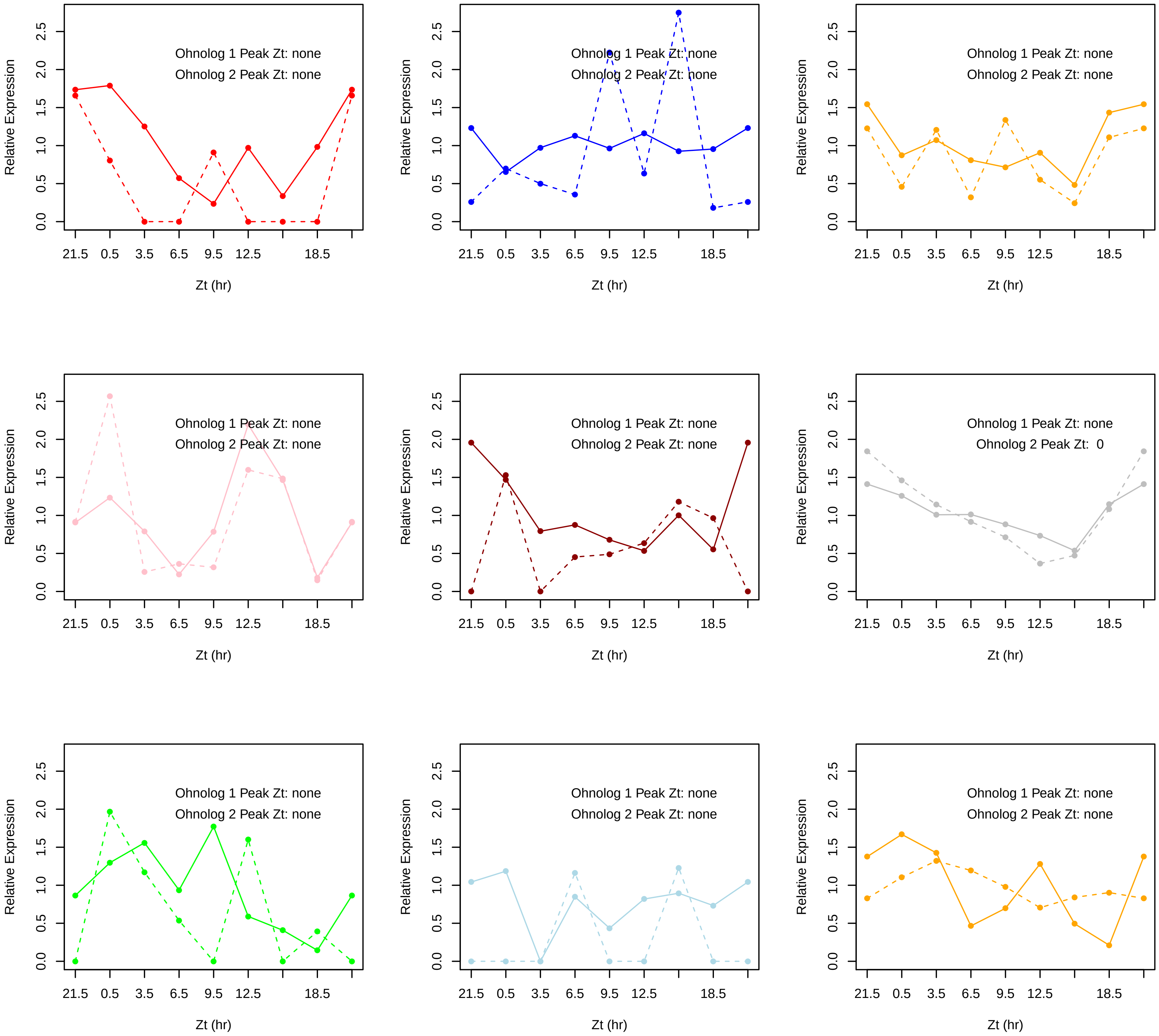

Ohnolog 1: ENSXMAG00000013492 ubtd1a
Ohnolog 2: ENSXMAG00000022287 UBTD1

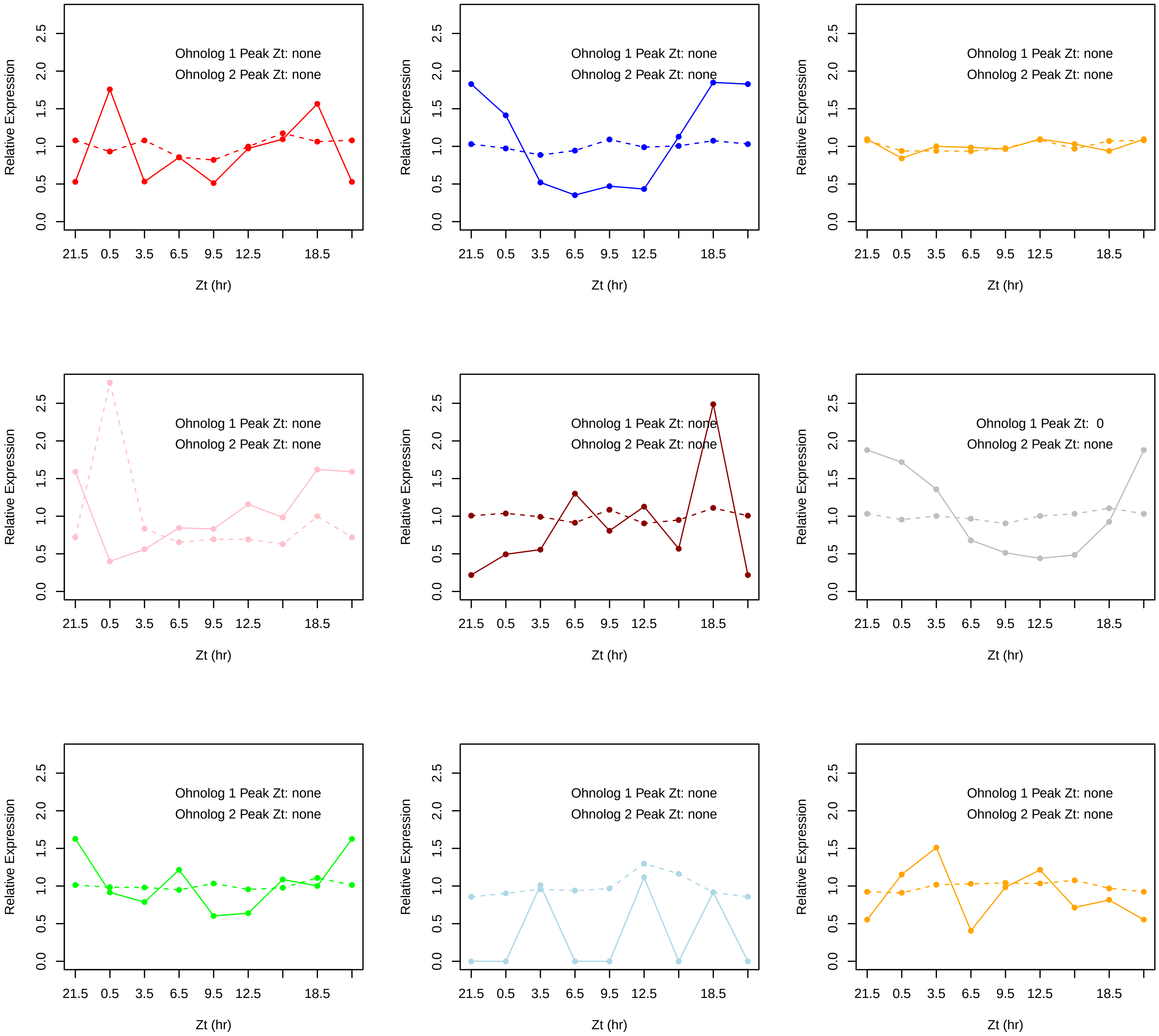

Ohnolog 1: ENSXMAG00000012884
Ohnolog 2: ENSXMAG00000028236 pax7a

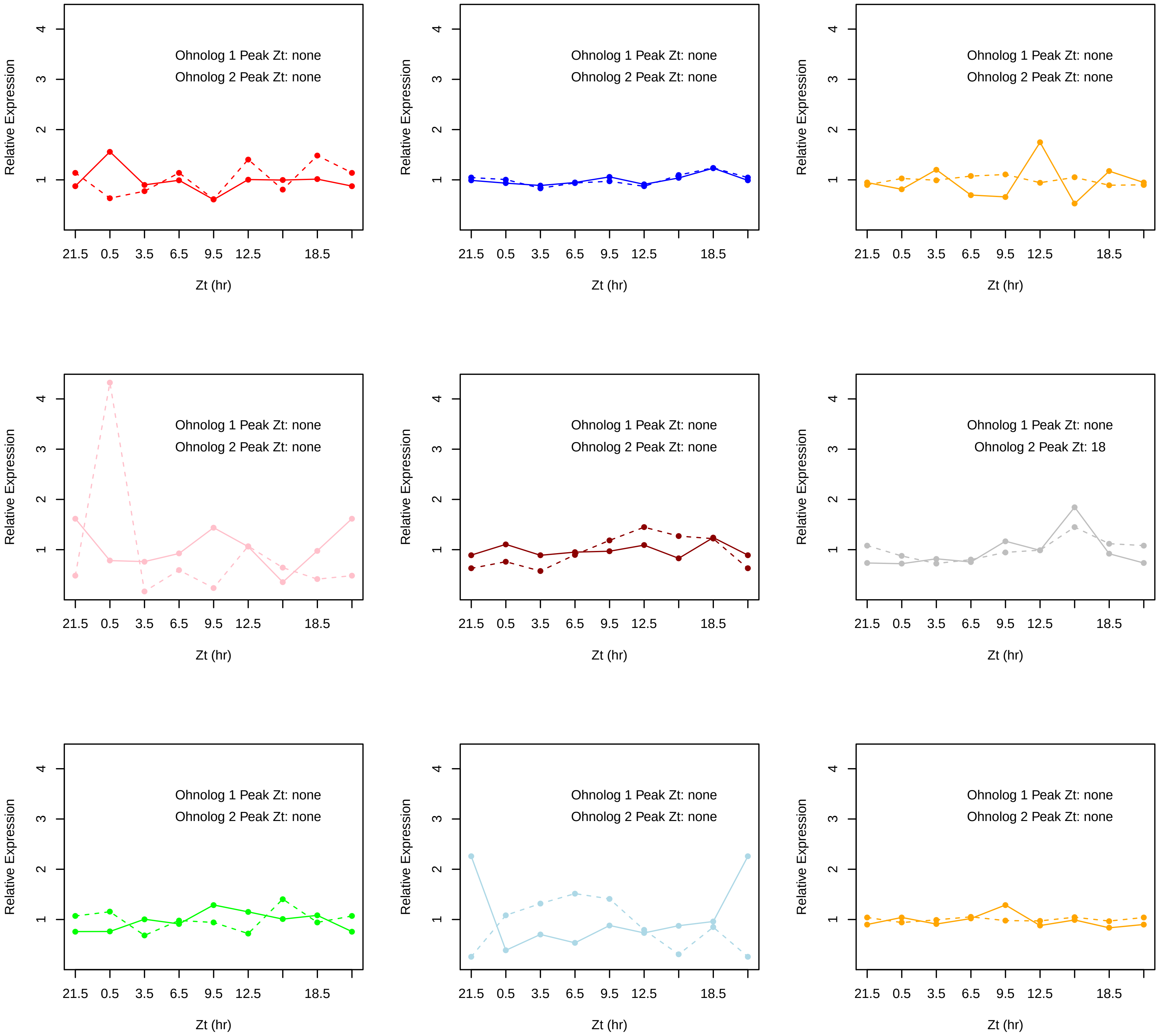

Ohnolog 1: ENSXMAG00000023202 gdf10a
Ohnolog 2: ENSXMAG00000028699 gdf10b

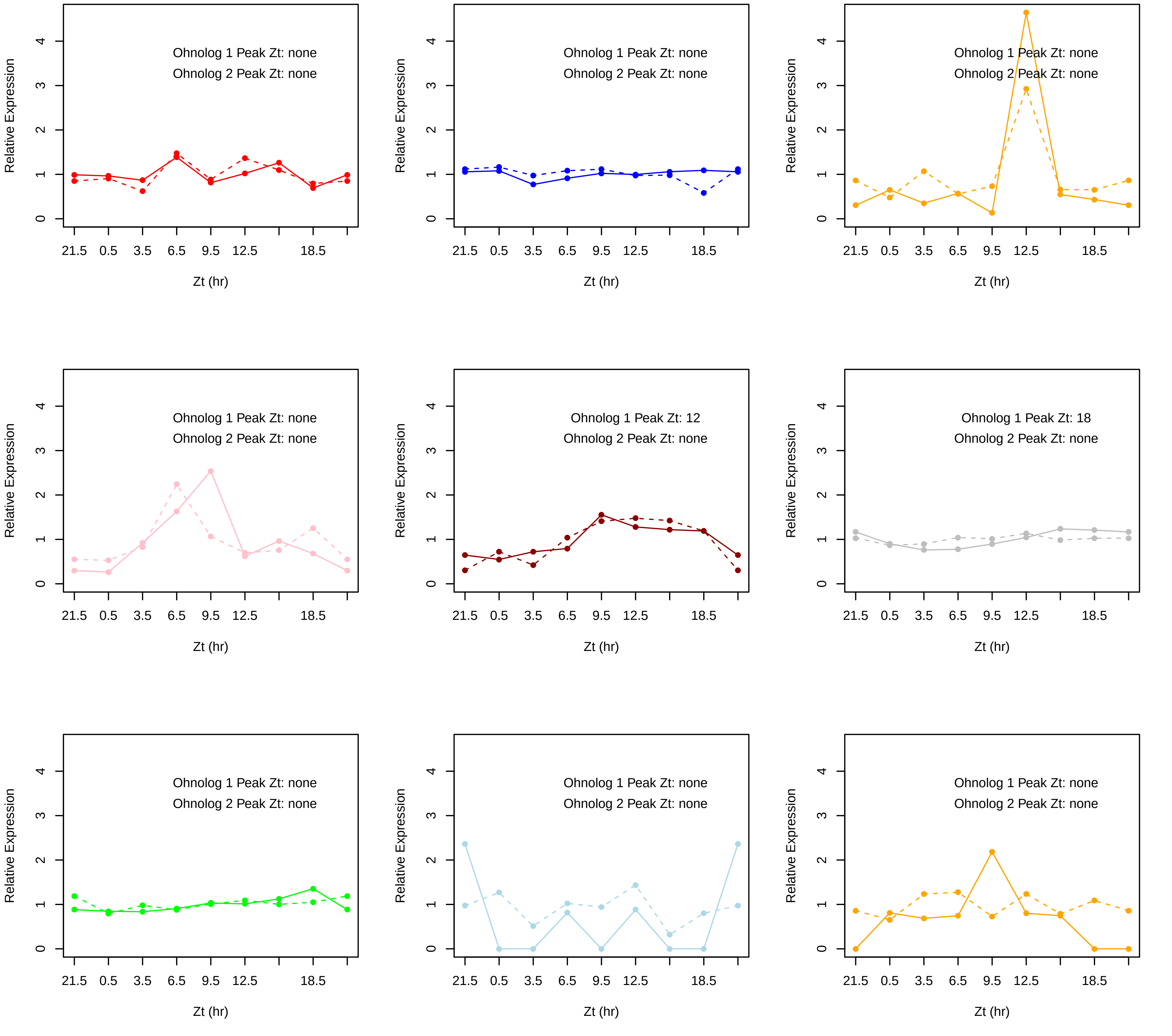

Ohnolog 1: ENSXMAG00000027879 tmem222b
Ohnolog 2: ENSXMAG00000028549 tmem222a

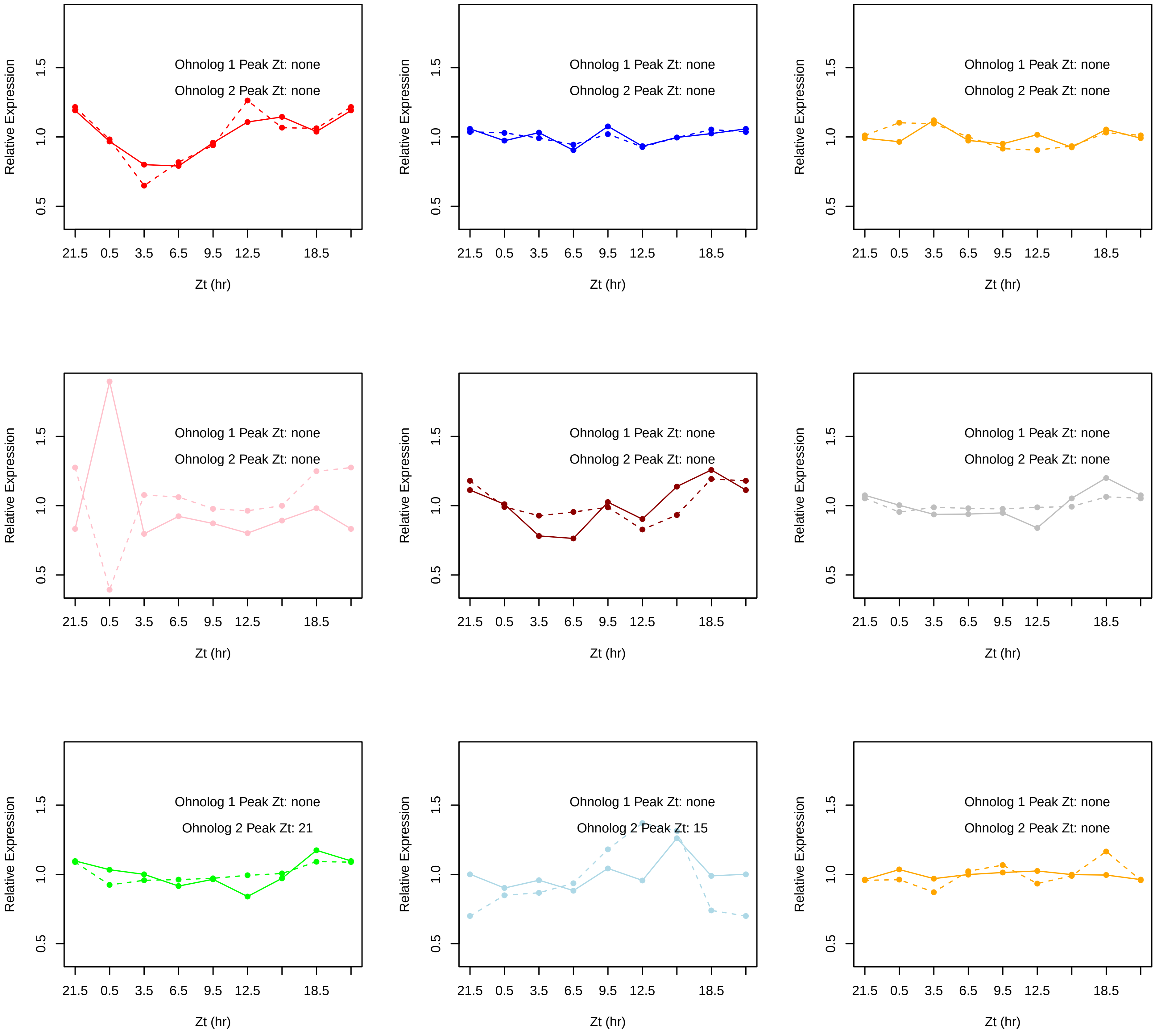

Ohnolog 1: ENSXMAG00000009815 zmynd8
Ohnolog 2: ENSXMAG00000010040 prkcbp1l

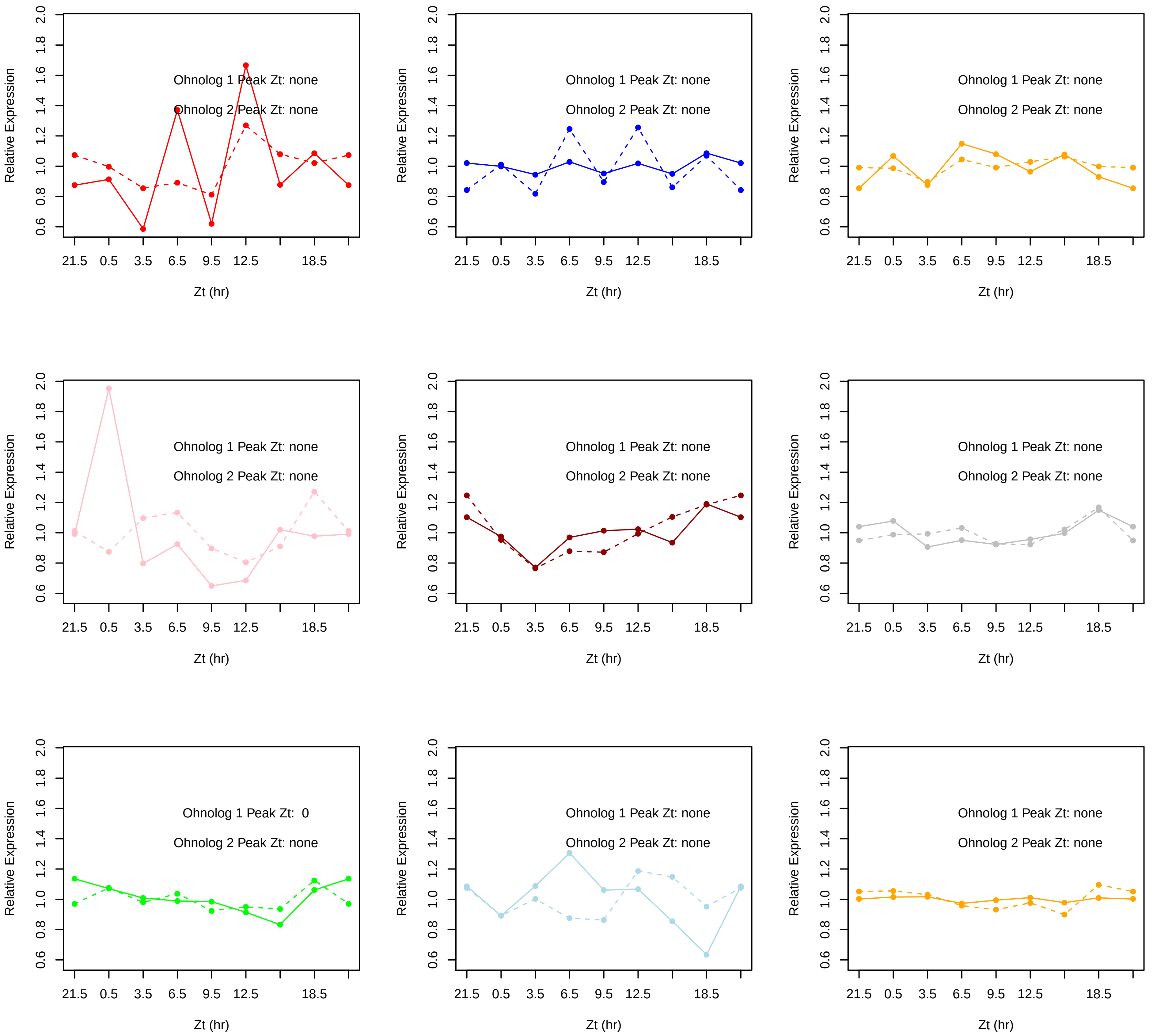

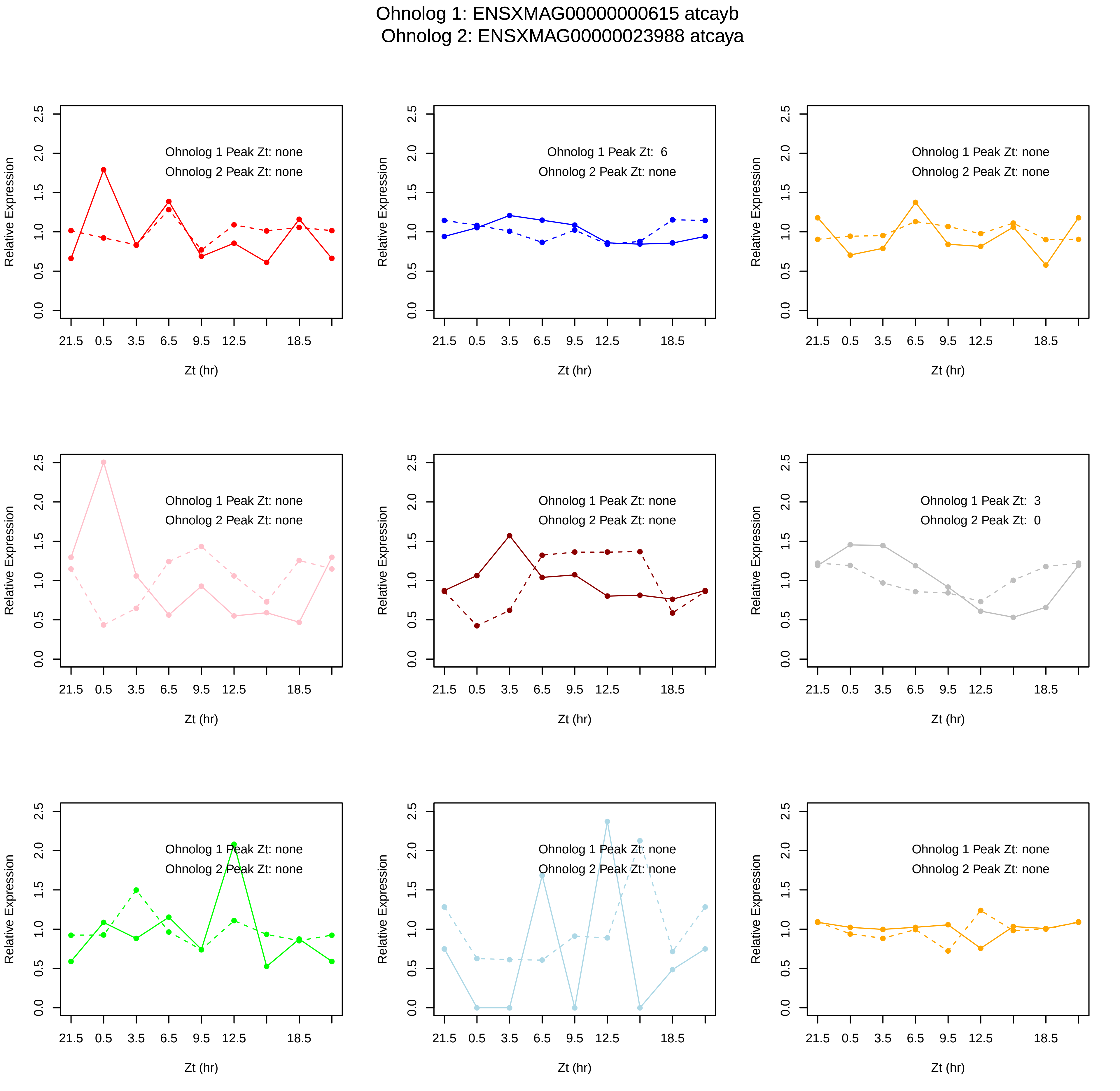
Ohnolog 1: ENSXMAG00000000615 atcayb
Ohnolog 2: ENSXMAG00000023988 atcaya
Ohnolog 1 Peak Zt: none
Ohnolog 2 Peak Zt: none
Ohnolog 1 Peak Zt: 6
Ohnolog 2 Peak Zt: none
Ohnolog 1 Peak Zt: none
Ohnolog 2 Peak Zt: none
Ohnolog 1 Peak Zt: none
Ohnolog 2 Peak Zt: none
Ohnolog 1 Peak Zt: none
Ohnolog 2 Peak Zt: none
Ohnolog 1 Peak Zt: 3
Ohnolog 2 Peak Zt: 0
Ohnolog 1 Peak Zt: none
Ohnolog 2 Peak Zt: none
Ohnolog 1 Peak Zt: none
Ohnolog 2 Peak Zt: none
Ohnolog 1 Peak Zt: none
Ohnolog 2 Peak Zt: none
Relative Expression
Zt (hr)

Ohnolog 1: ENSXMAG00000000983 errfi1a
Ohnolog 2: ENSXMAG00000023654

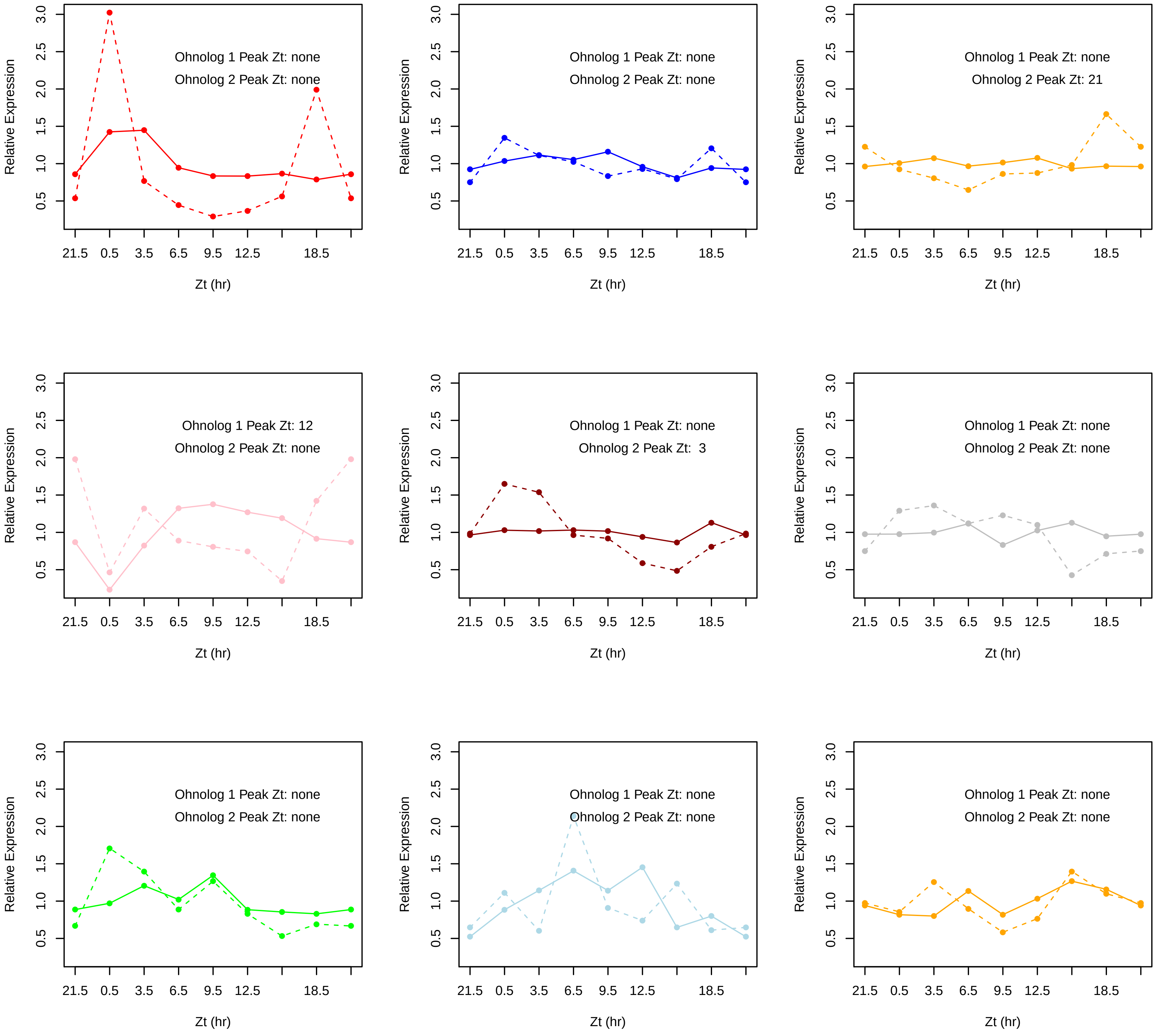

Ohnolog 1: ENSXMAG00000000613 nmrk2
Ohnolog 2: ENSXMAG00000018750 mibp

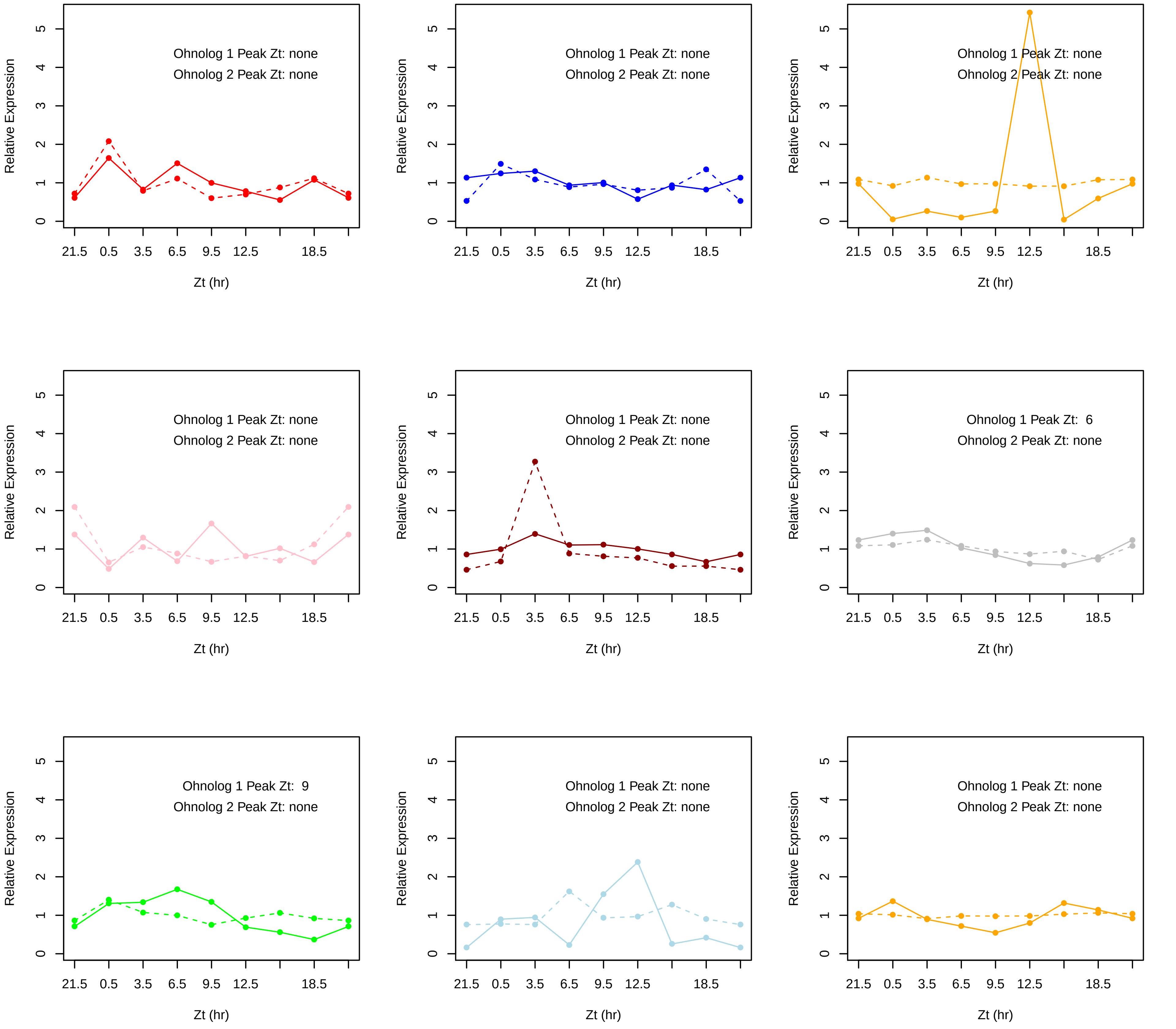

Ohnolog 1: ENSXMAG00000002401
Ohnolog 2: ENSXMAG00000009527 igfbp6b

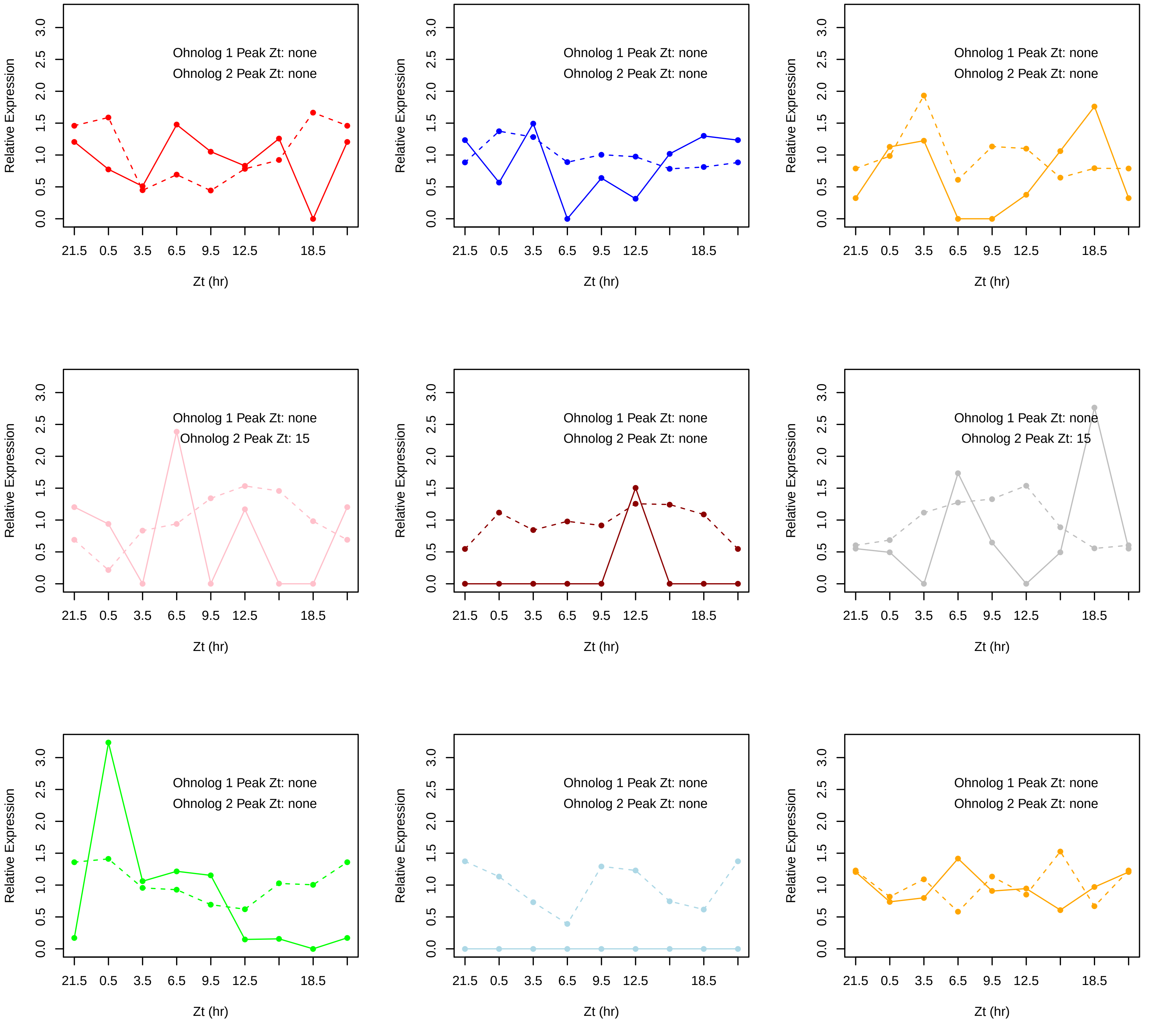

Ohnolog 1: ENSXMAG00000004183
Ohnolog 2: ENSXMAG00000029606 gcshb

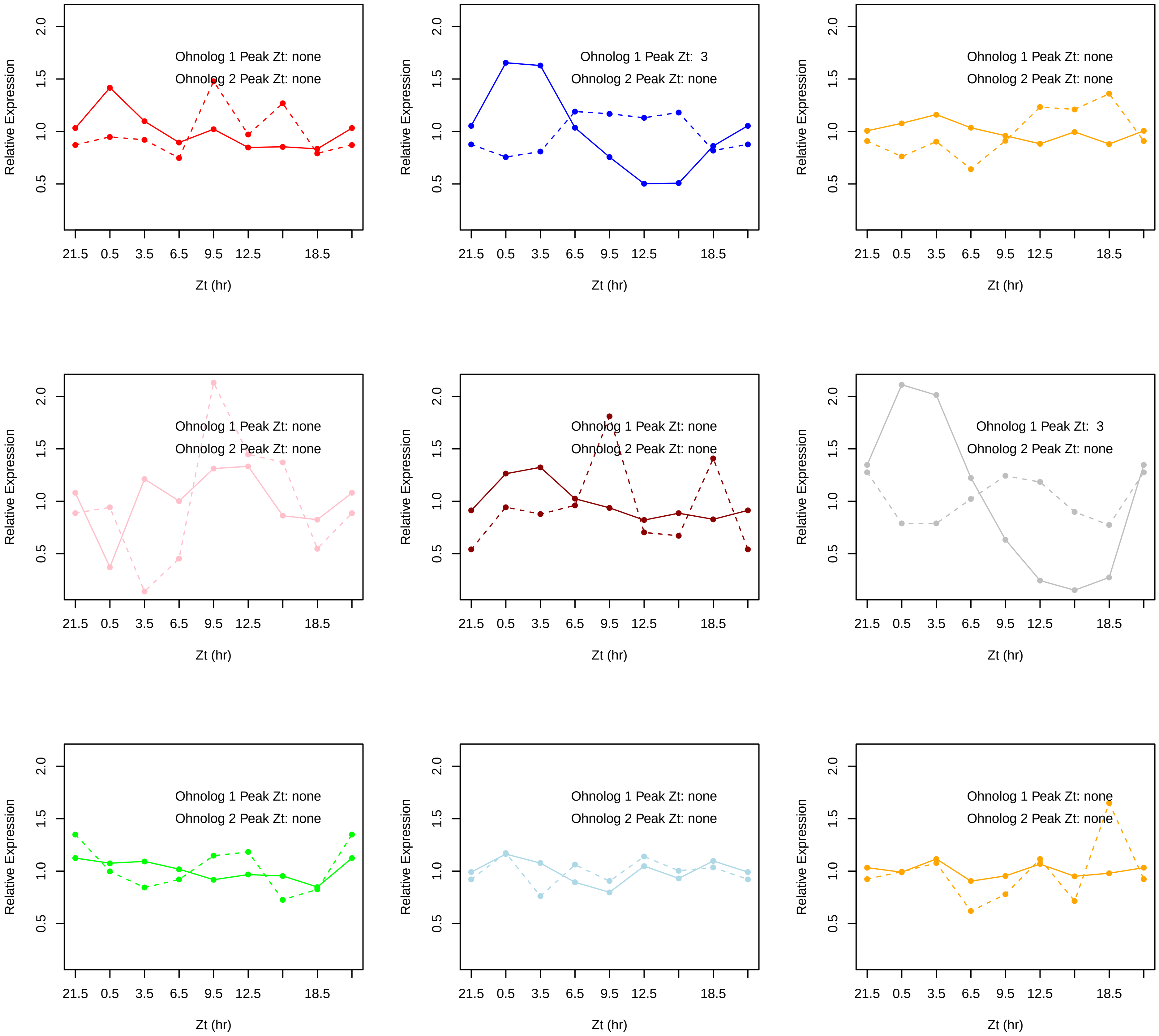

Ohnolog 1: ENSXMAG00000002394 spryd3 (1 of many)
Ohnolog 2: ENSXMAG00000009531 spryd3 (1 of many)

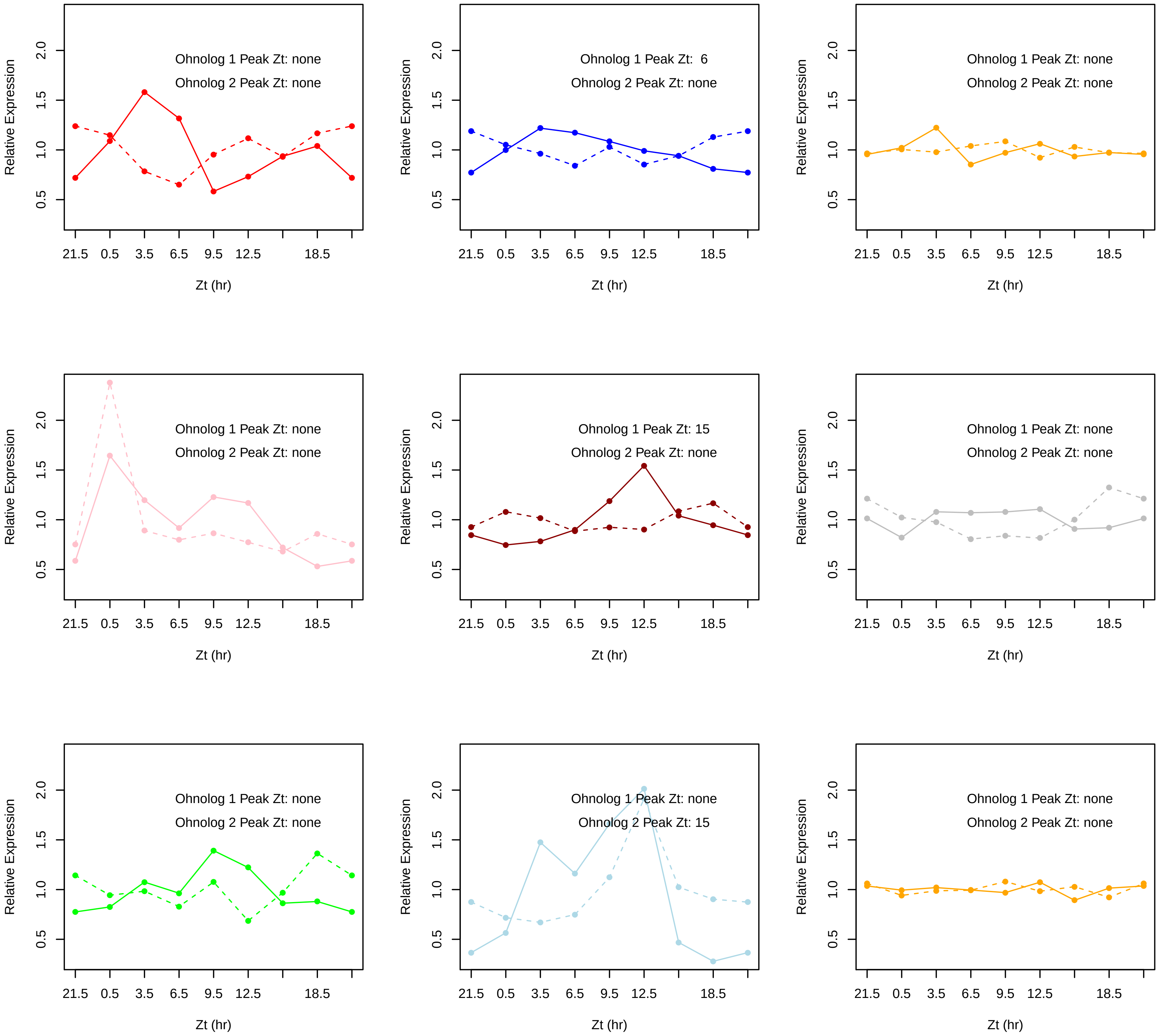

Ohnolog 1: ENSXMAG00000001453
Ohnolog 2: ENSXMAG00000013962 sgsm1a

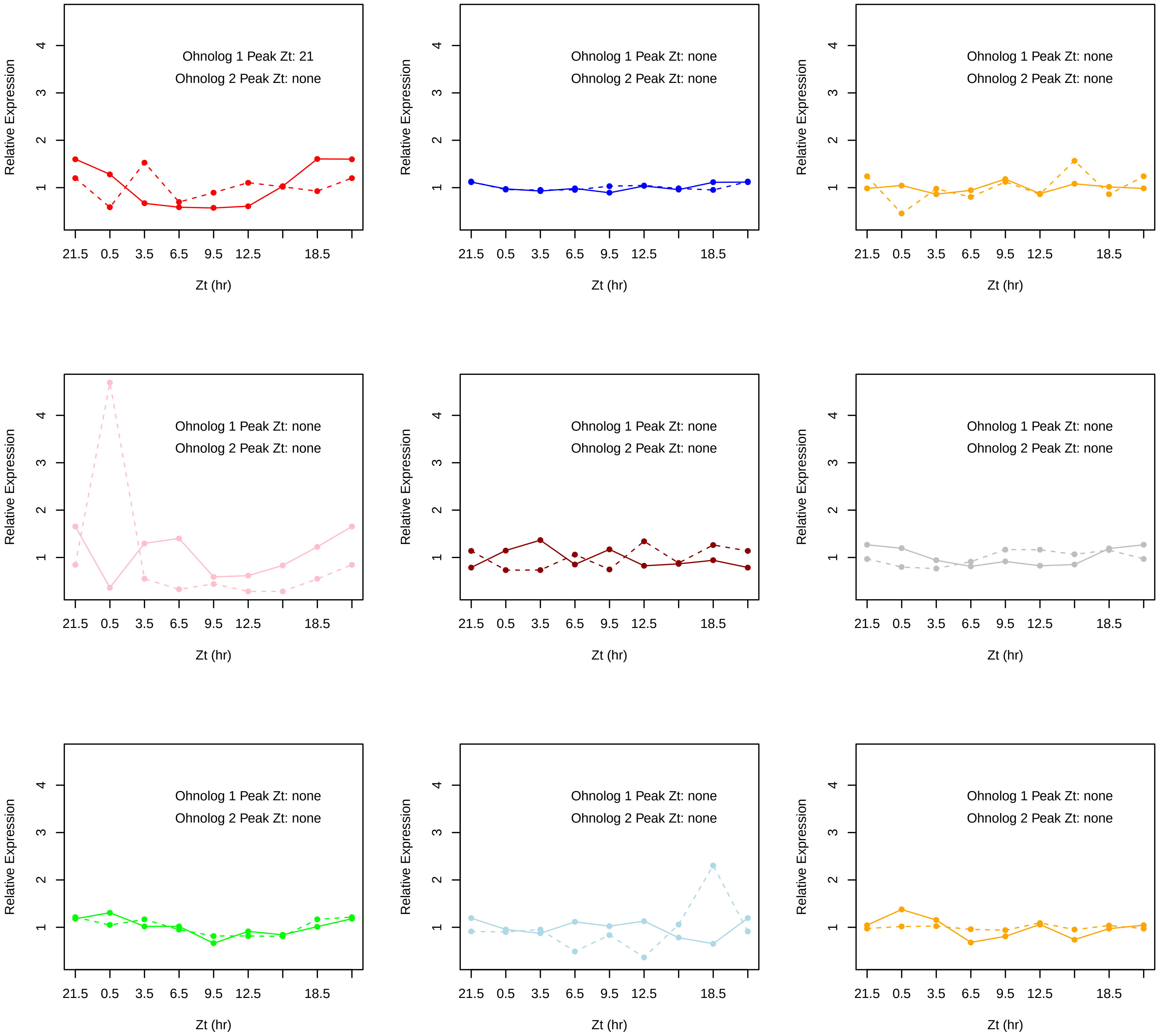

Ohnolog 1: ENSXMAG00000013584 vstm4a
Ohnolog 2: ENSXMAG00000021218 vstm4b

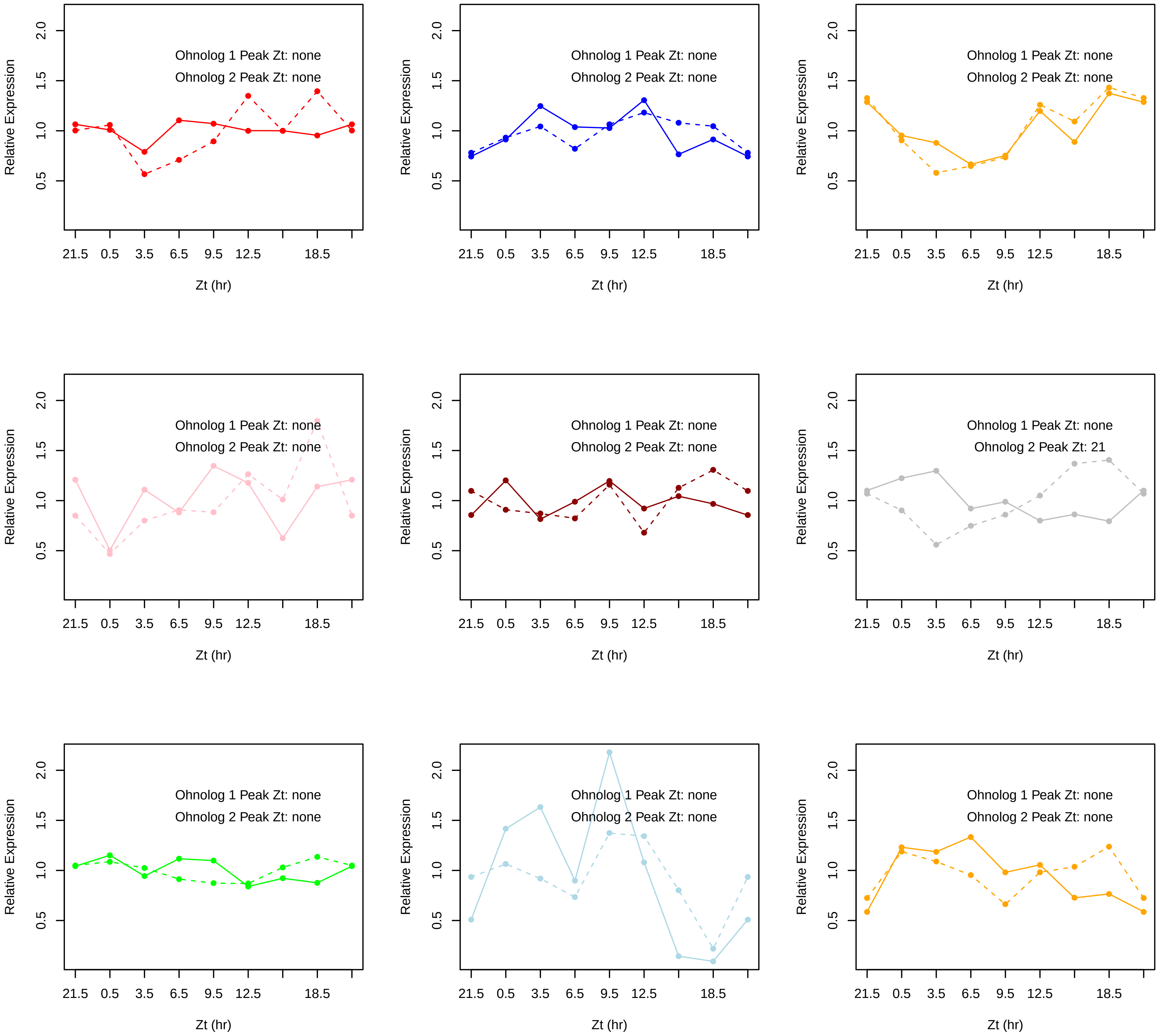

Ohnolog 1: ENSXMAG00000003128 mical2a
Ohnolog 2: ENSXMAG00000015506 mical2b

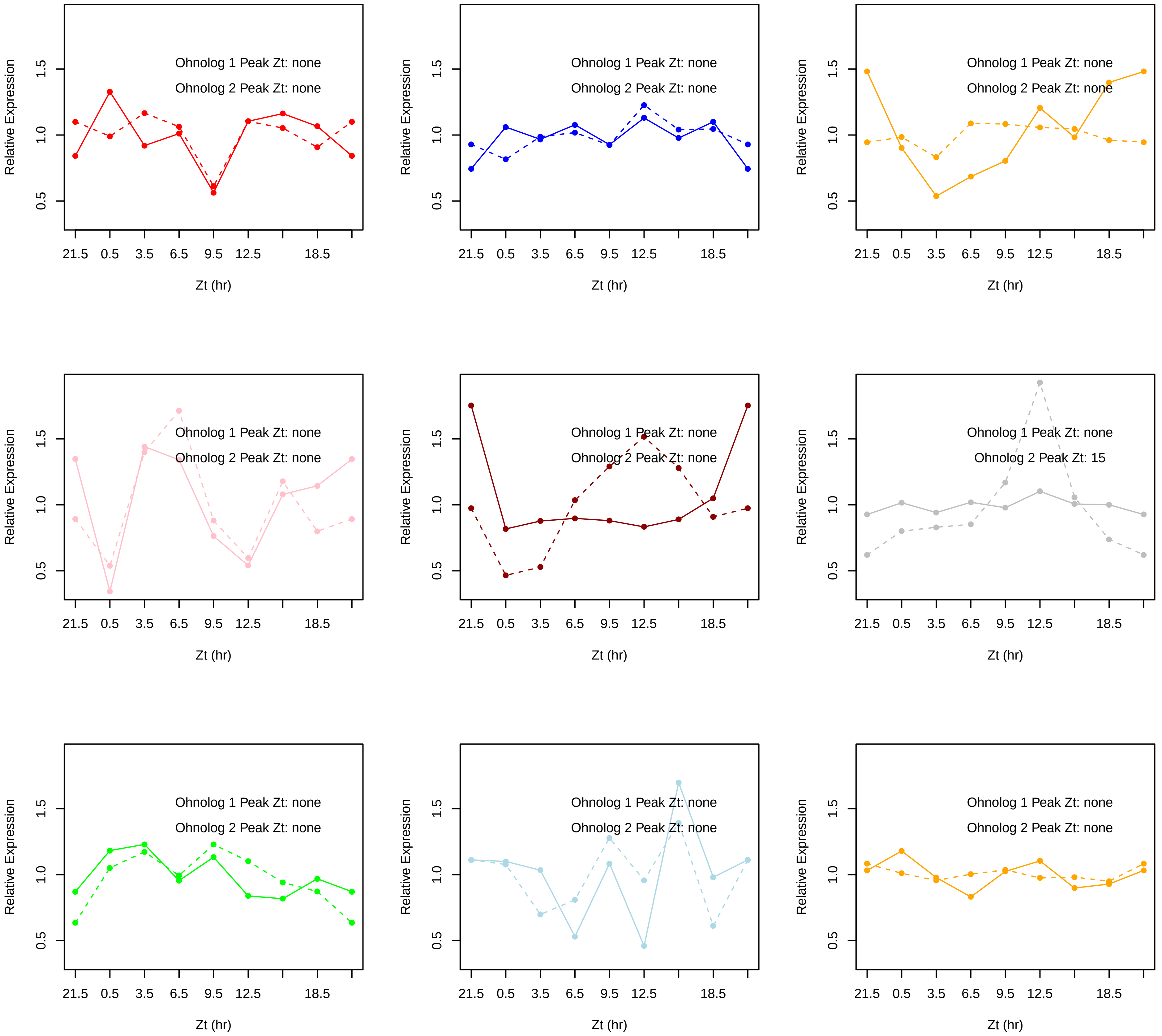

Ohnolog 1: ENSXMAG00000003962
Ohnolog 2: ENSXMAG00000013216 uqcrh

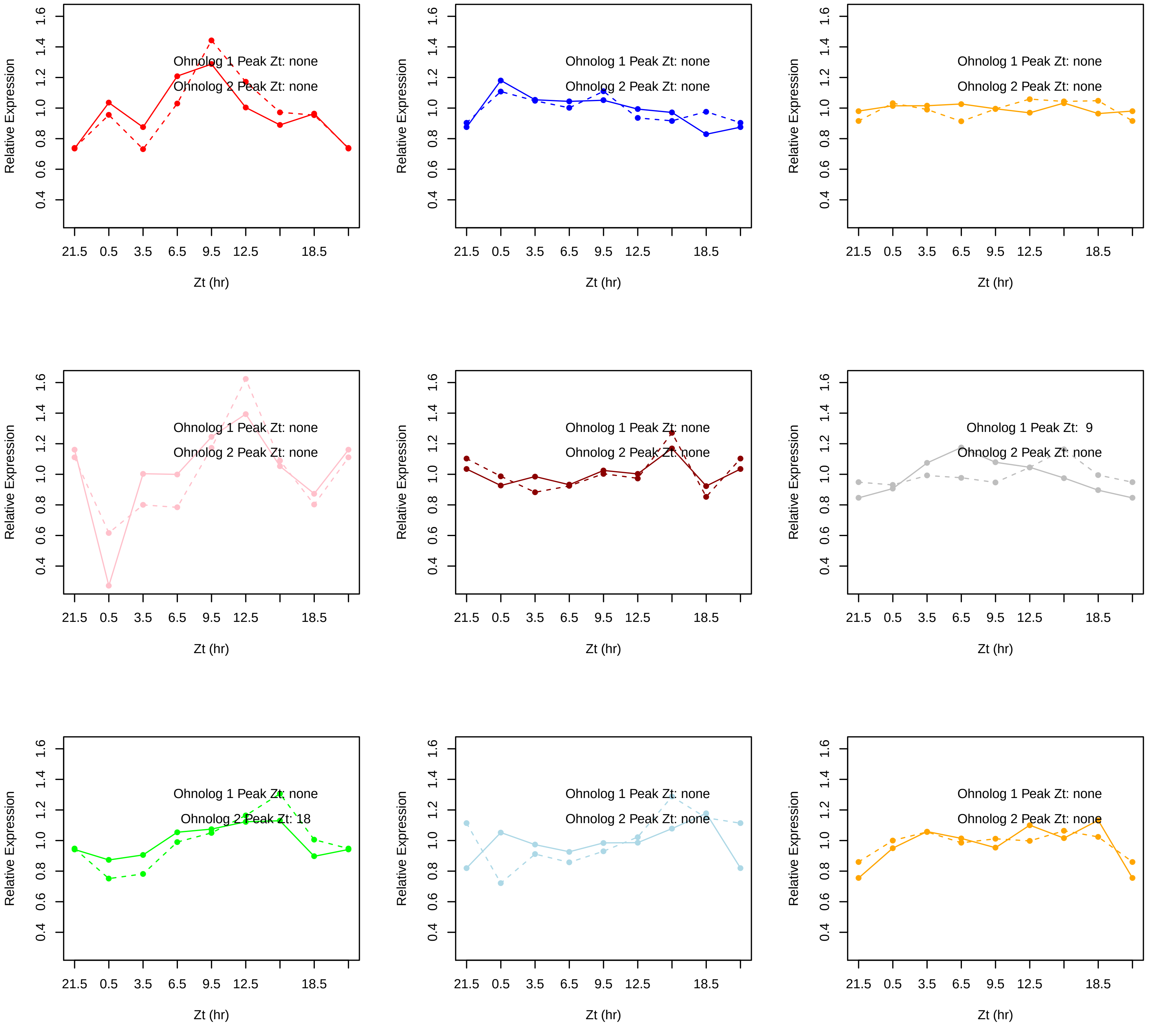

Ohnolog 1: ENSXMAG00000010314
Ohnolog 2: ENSXMAG00000021301 epb41a

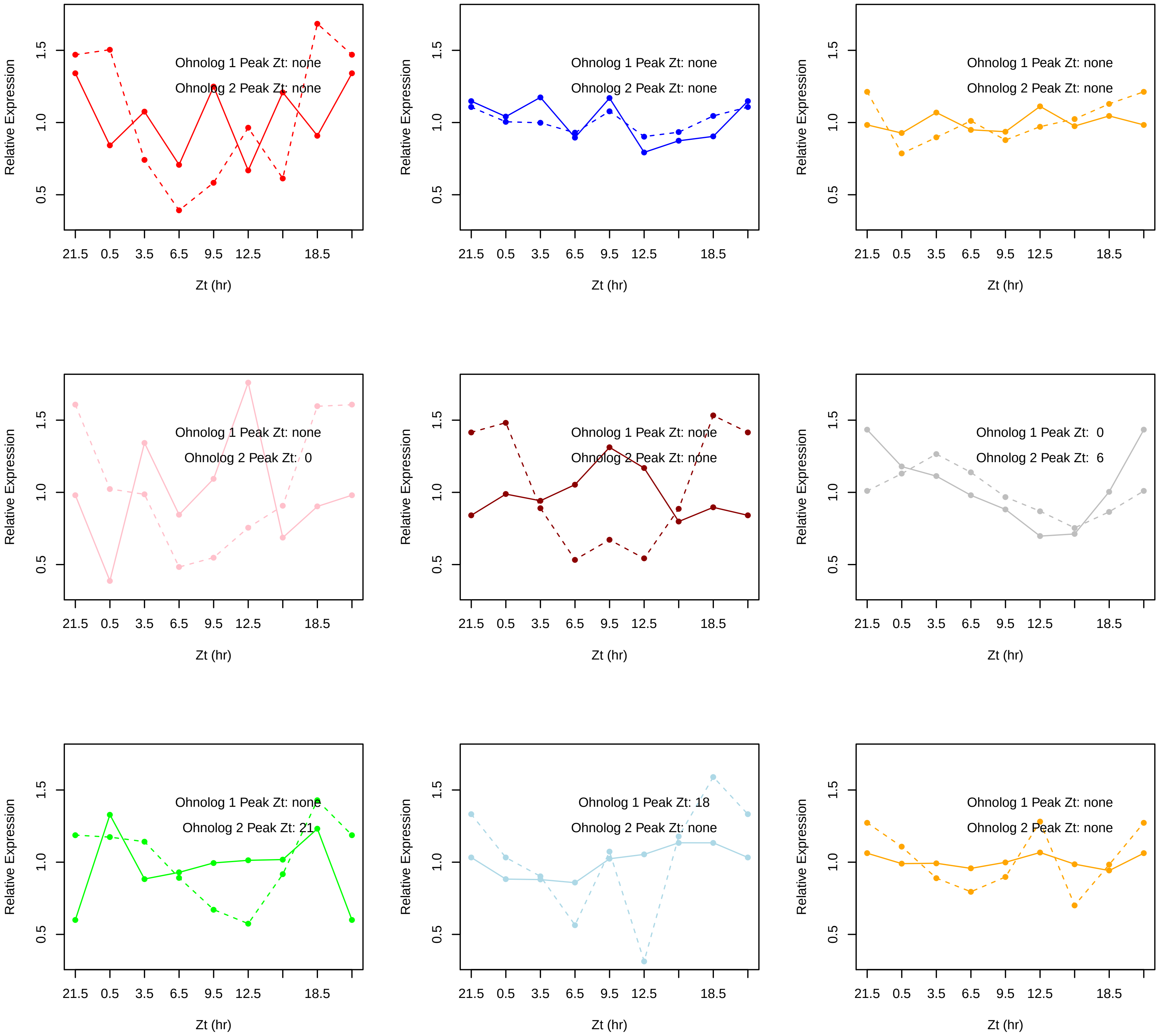

Ohnolog 1: ENSXMAG00000001494 git2b
Ohnolog 2: ENSXMAG00000014010 git2a

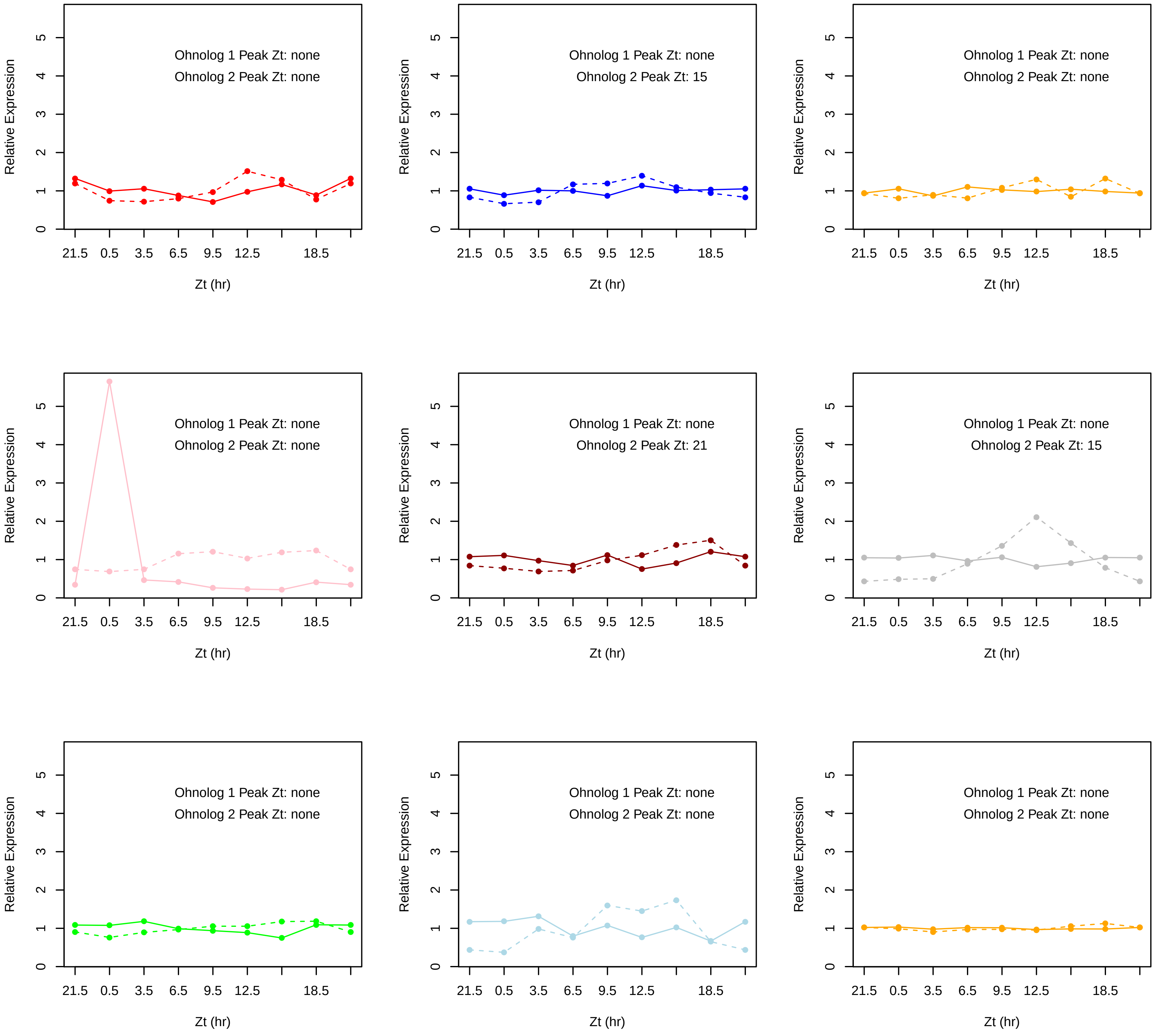

Ohnolog 1: ENSXMAG00000009778 phf20a
Ohnolog 2: ENSXMAG00000023796

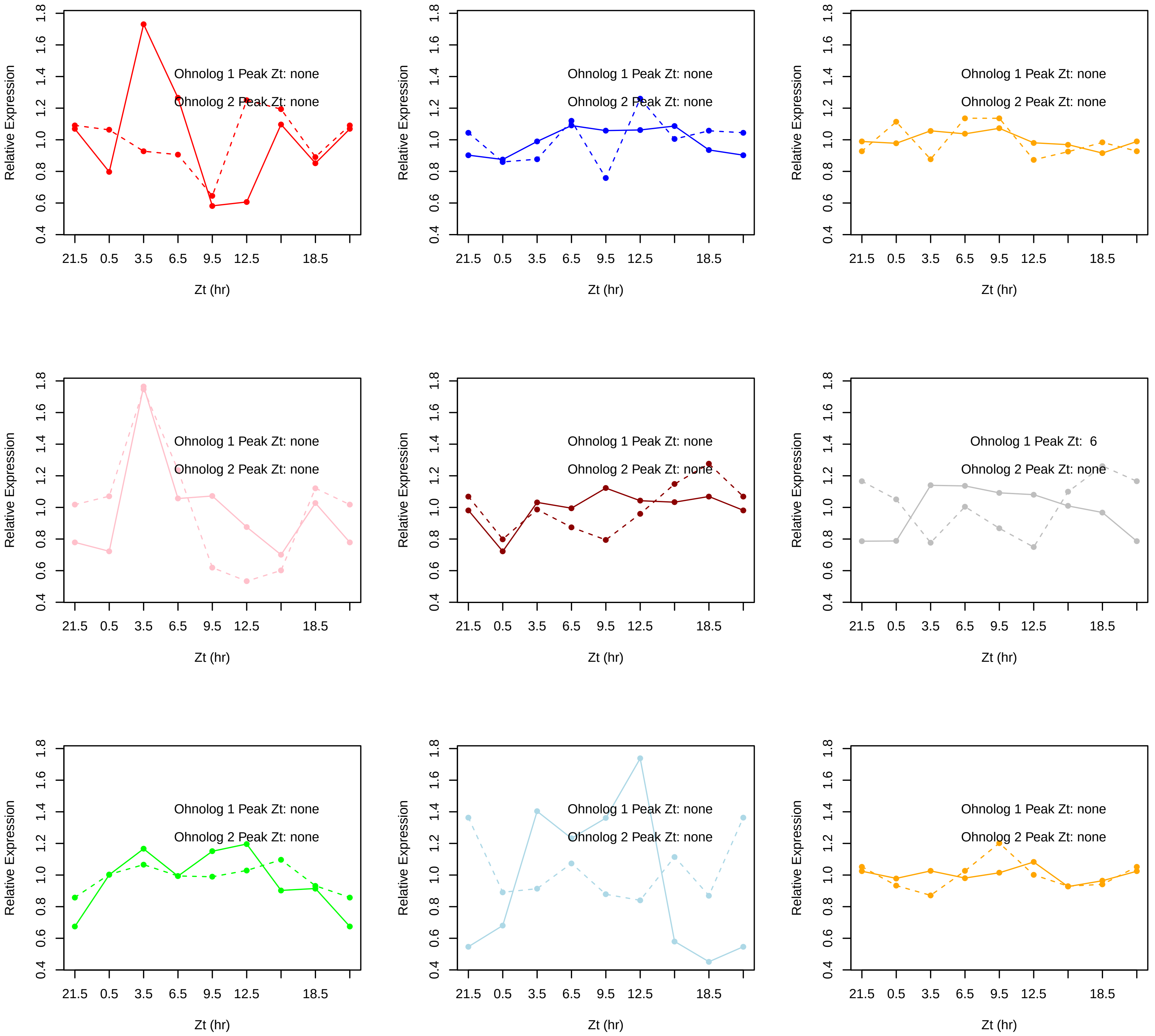

Ohnolog 1: ENSXMAG00000000547 insra
Ohnolog 2: ENSXMAG00000018648 insrb

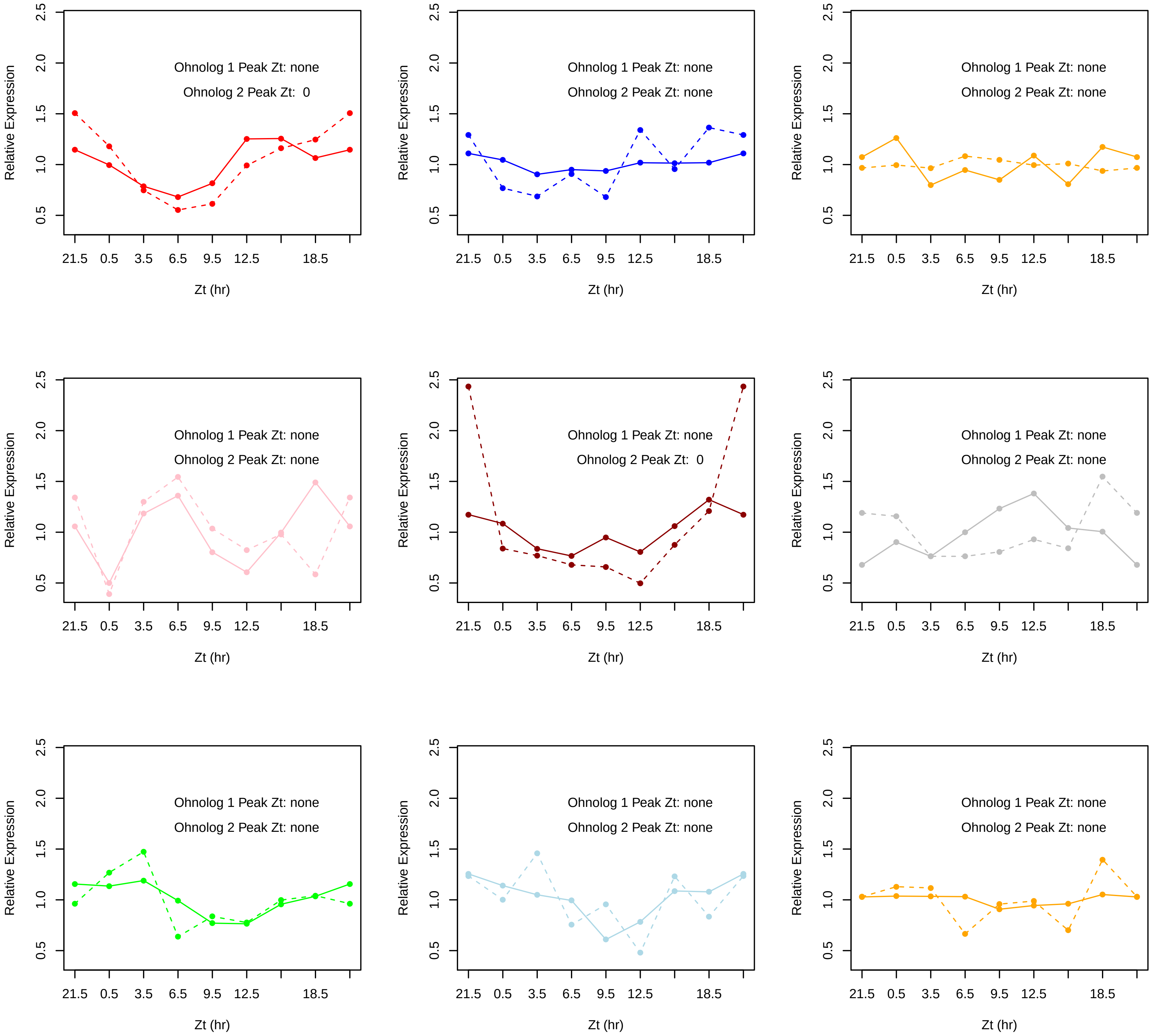

Ohnolog 1: ENSXMAG00000013763 gtf3aa
Ohnolog 2: ENSXMAG00000029776 gtf3ab

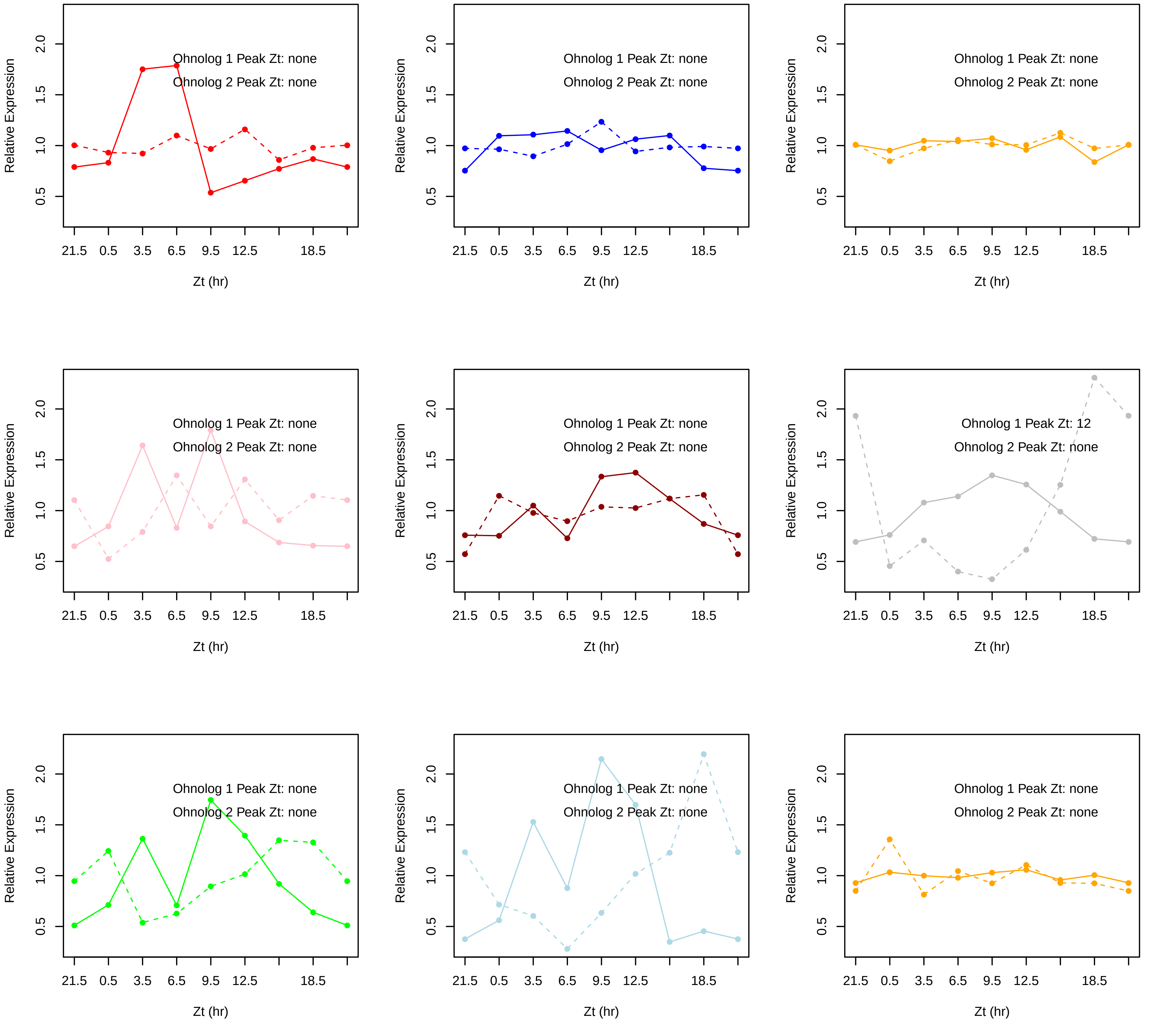

Ohnolog 1: ENSXMAG00000024042 cxcl12a
Ohnolog 2: ENSXMAG00000024539 cxcl12b

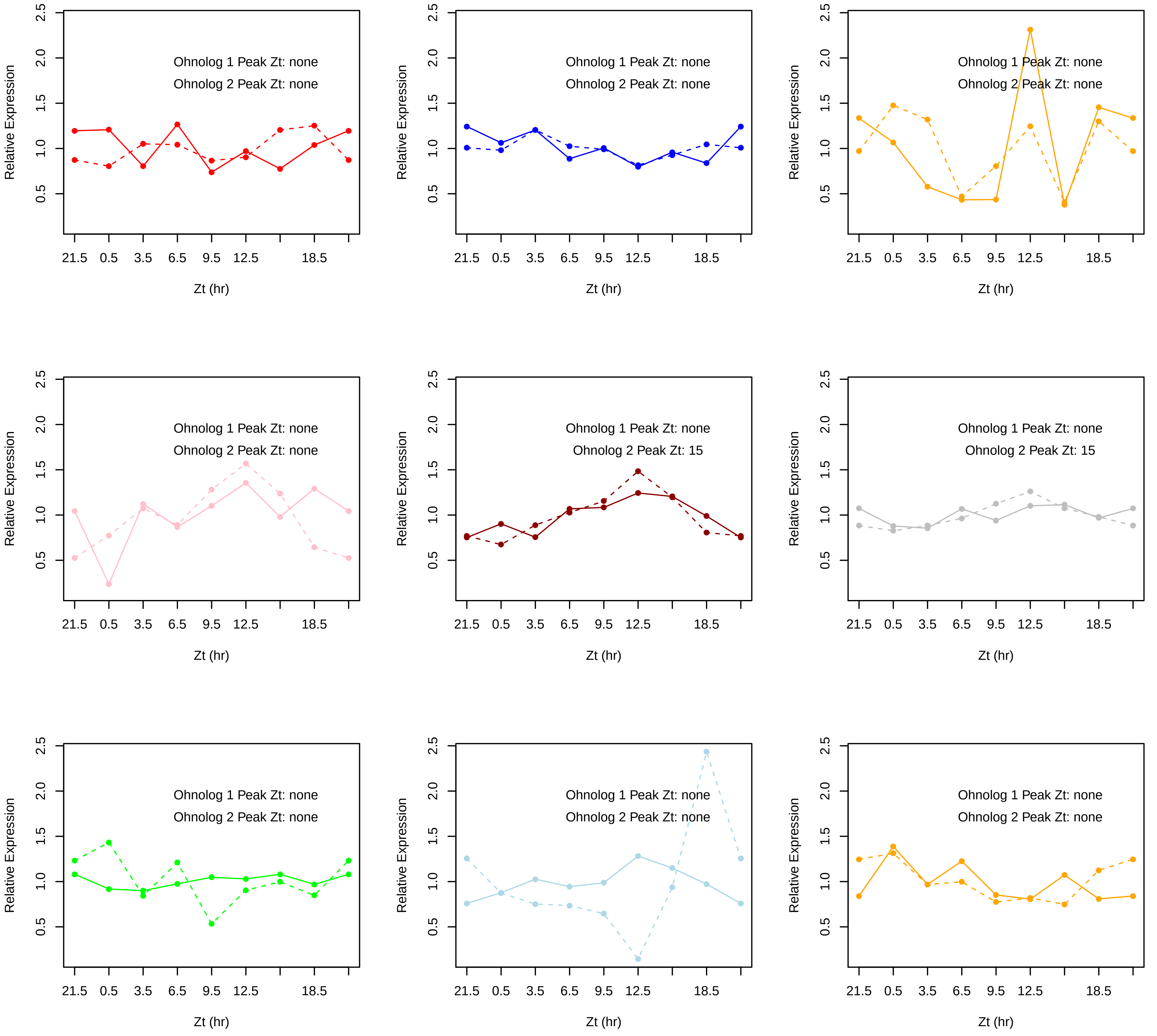

Ohnolog 1: ENSXMAG00000003262 mmp19
Ohnolog 2: ENSXMAG00000011637

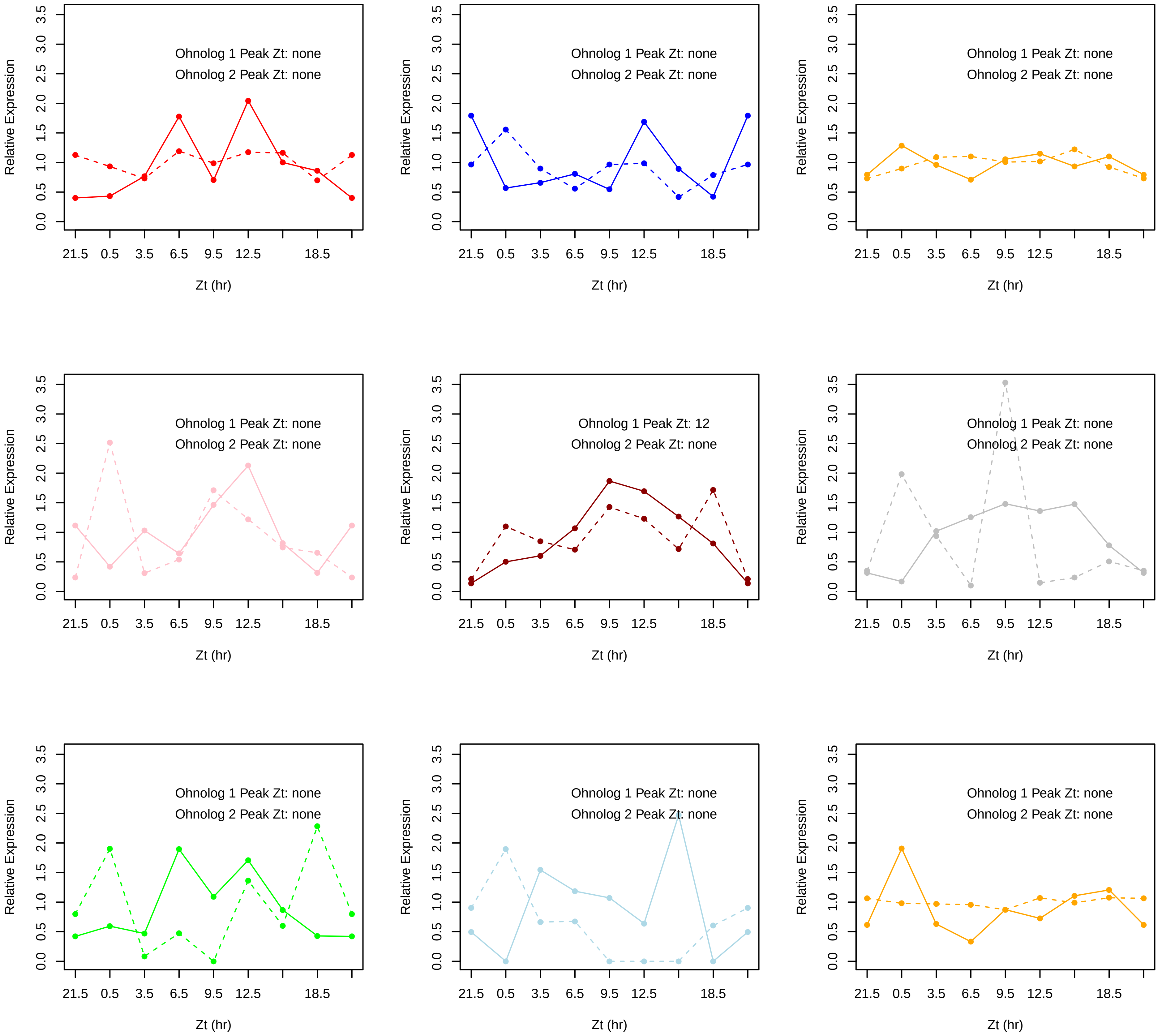

Ohnolog 1: ENSXMAG00000008353 slc25a36a (1 of many)
Ohnolog 2: ENSXMAG00000018866 slc25a36a (1 of many)

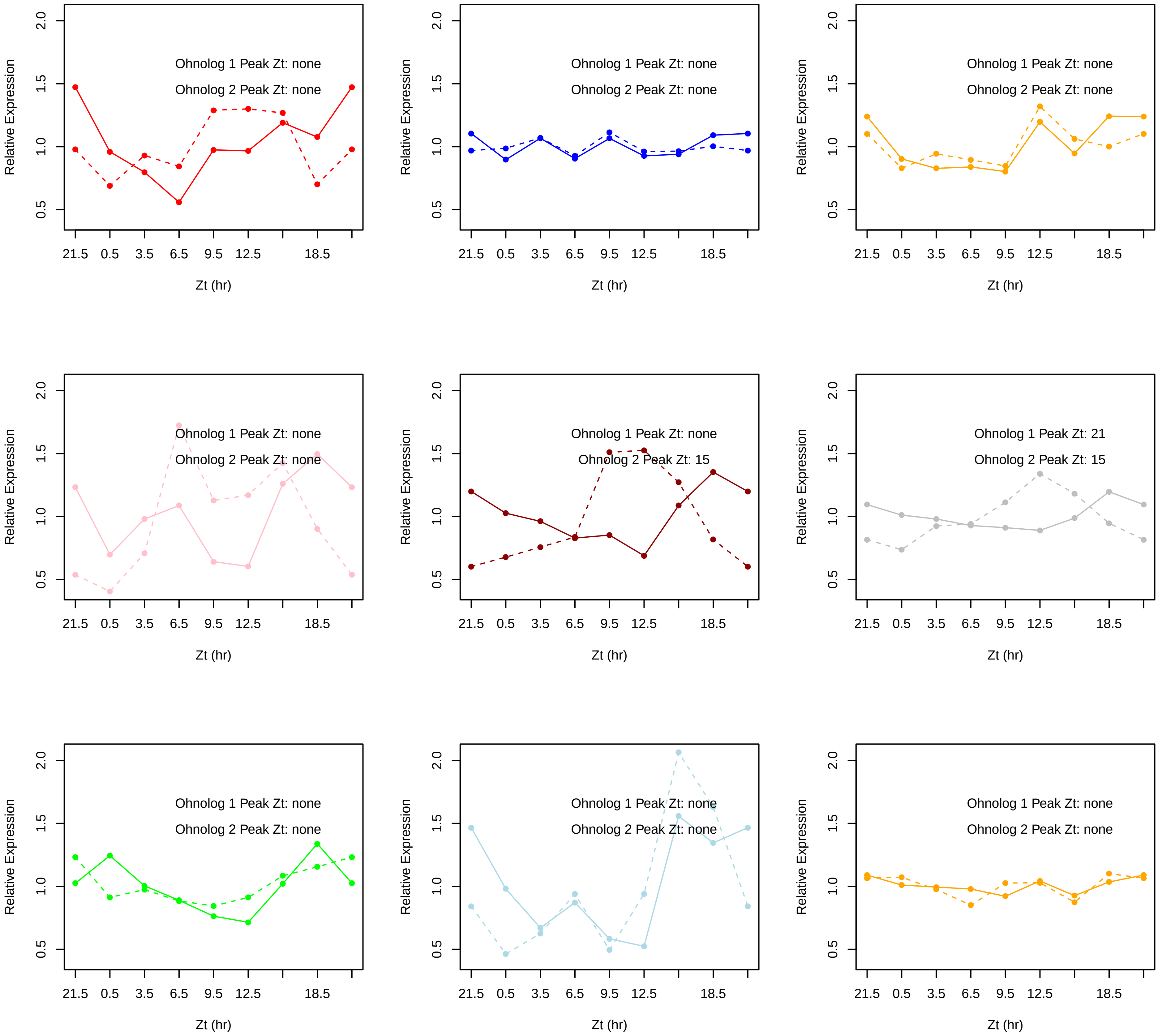

Ohnolog 1: ENSXMAG00000000544 arhgef18a
Ohnolog 2: ENSXMAG00000018641 arhgef18b

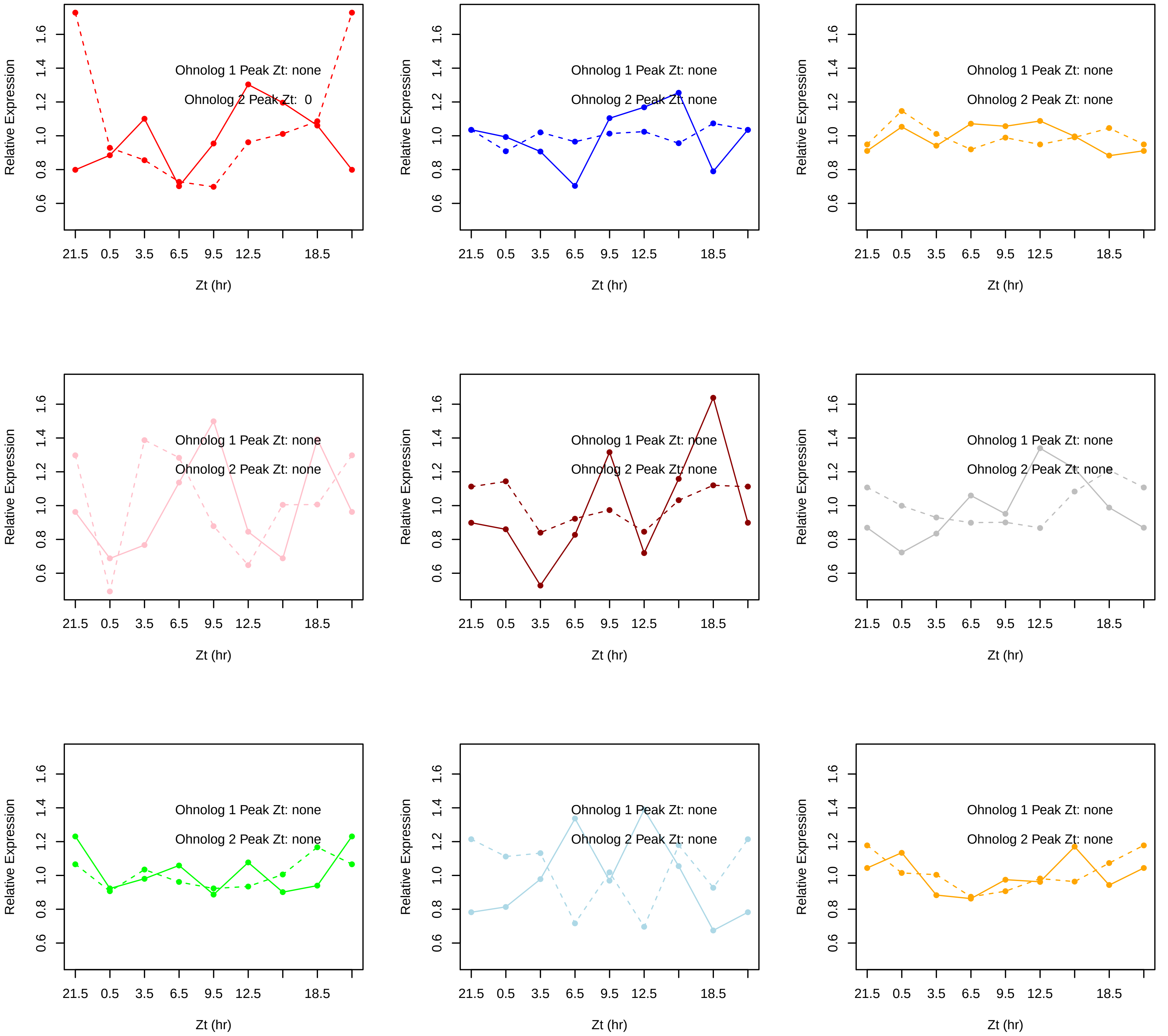

Ohnolog 1: ENSXMAG00000015051
Ohnolog 2: ENSXMAG00000024881 rps19

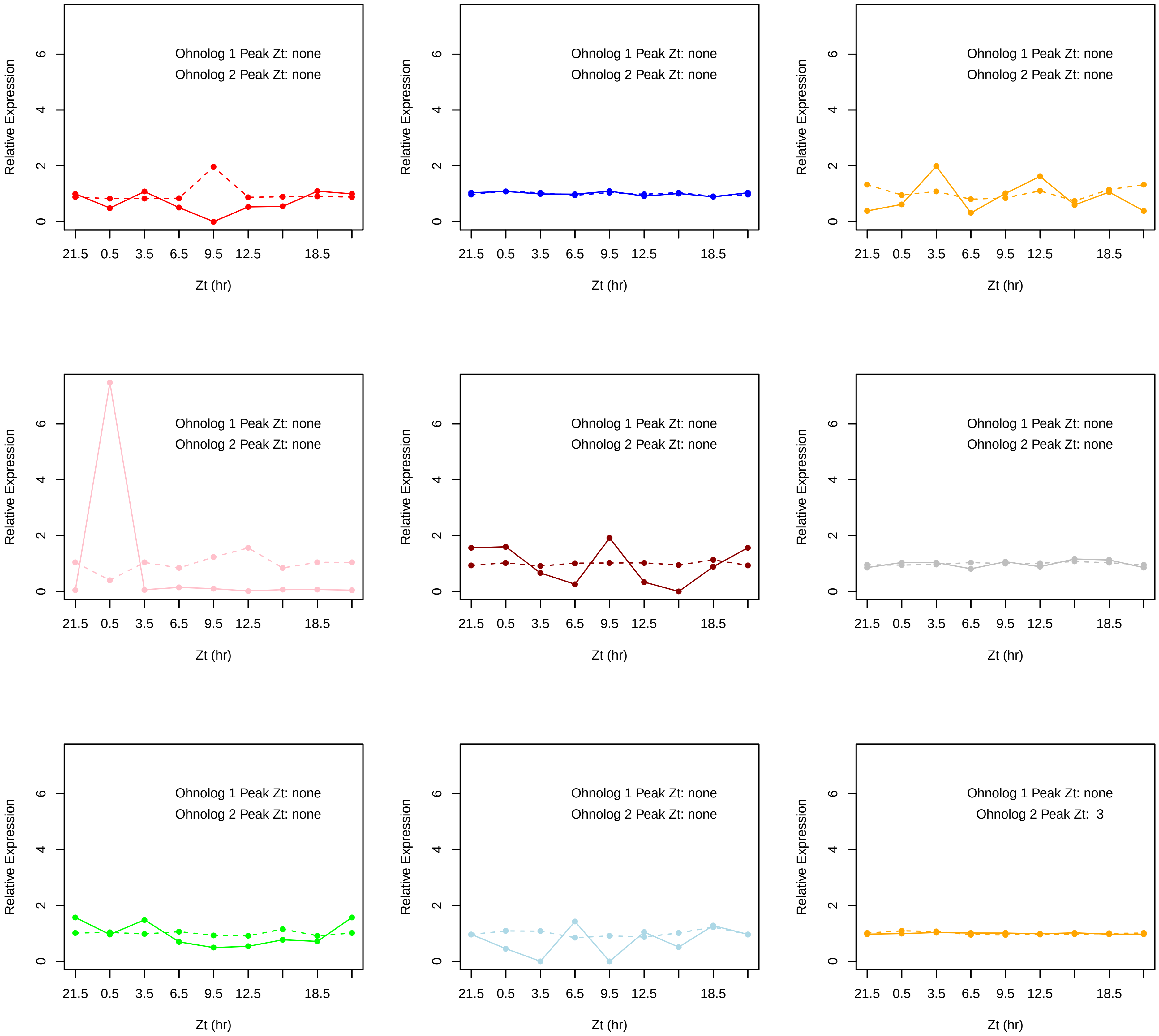

Ohnolog 1: ENSXMAG00000007022 syt9a
Ohnolog 2: ENSXMAG00000015409 syt9b

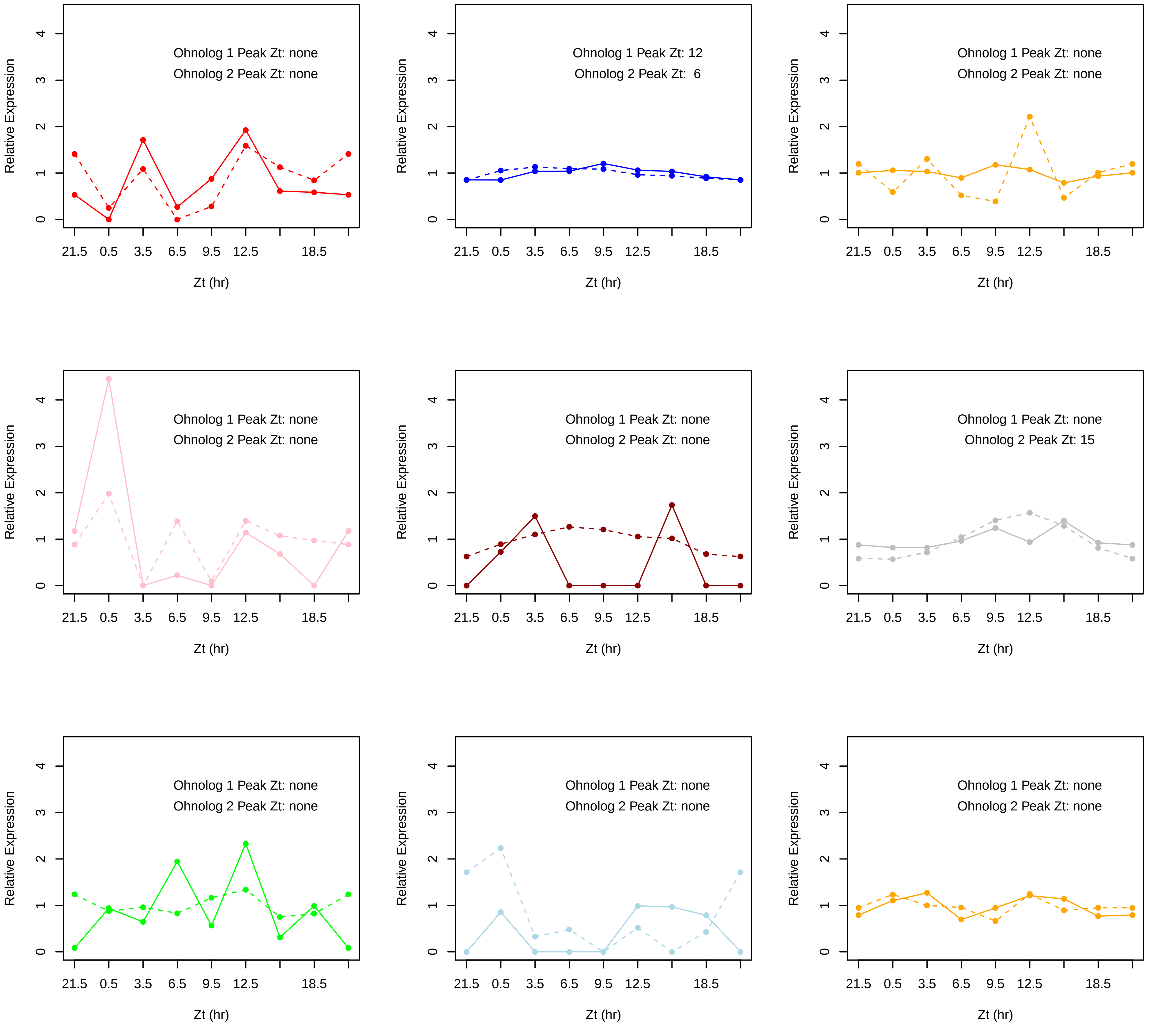

Ohnolog 1: ENSXMAG00000008630
Ohnolog 2: ENSXMAG00000011277 sept4a

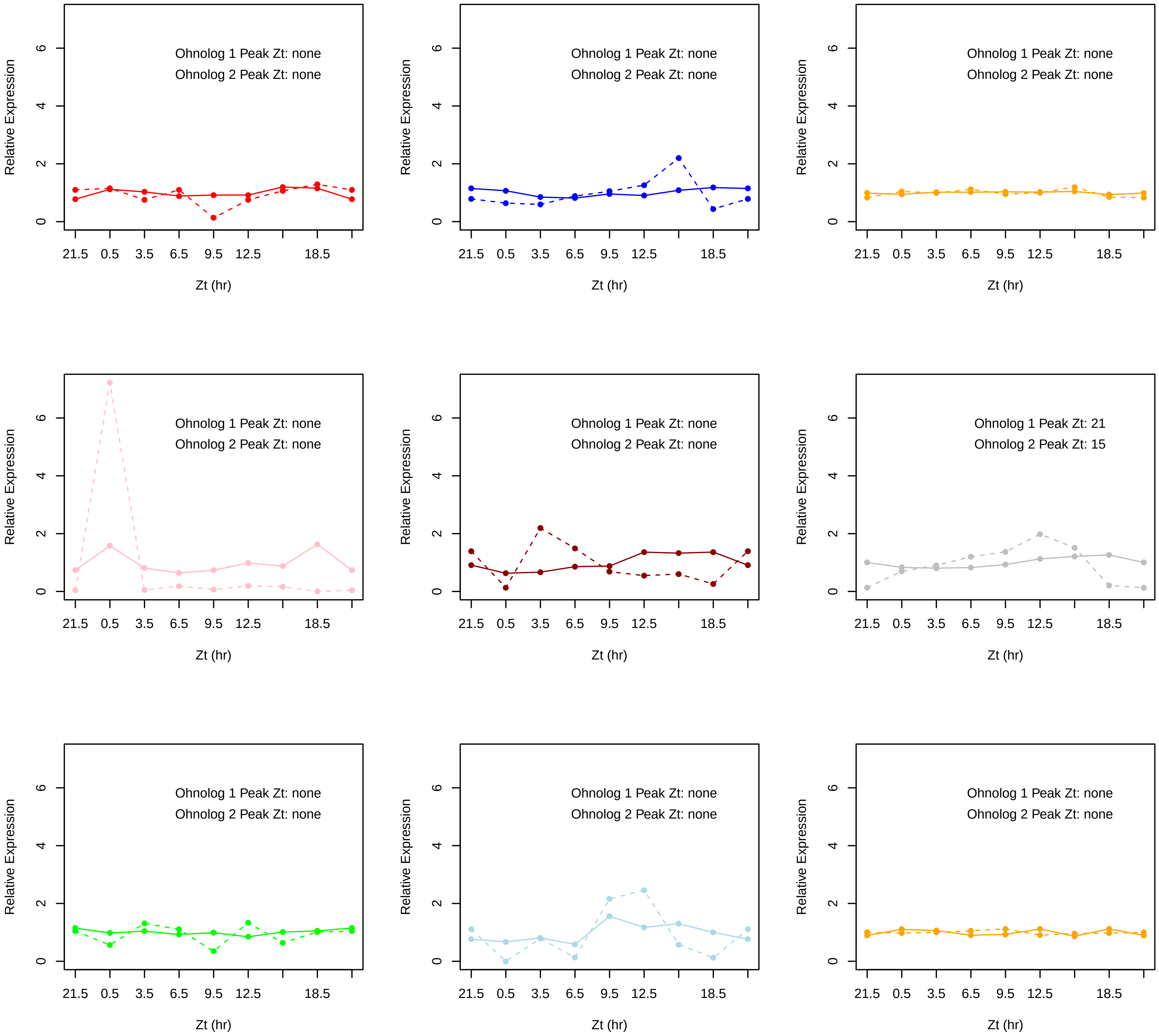

Ohnolog 1: ENSXMAG00000001259 myo1hb
Ohnolog 2: ENSXMAG00000007888 myo1ha

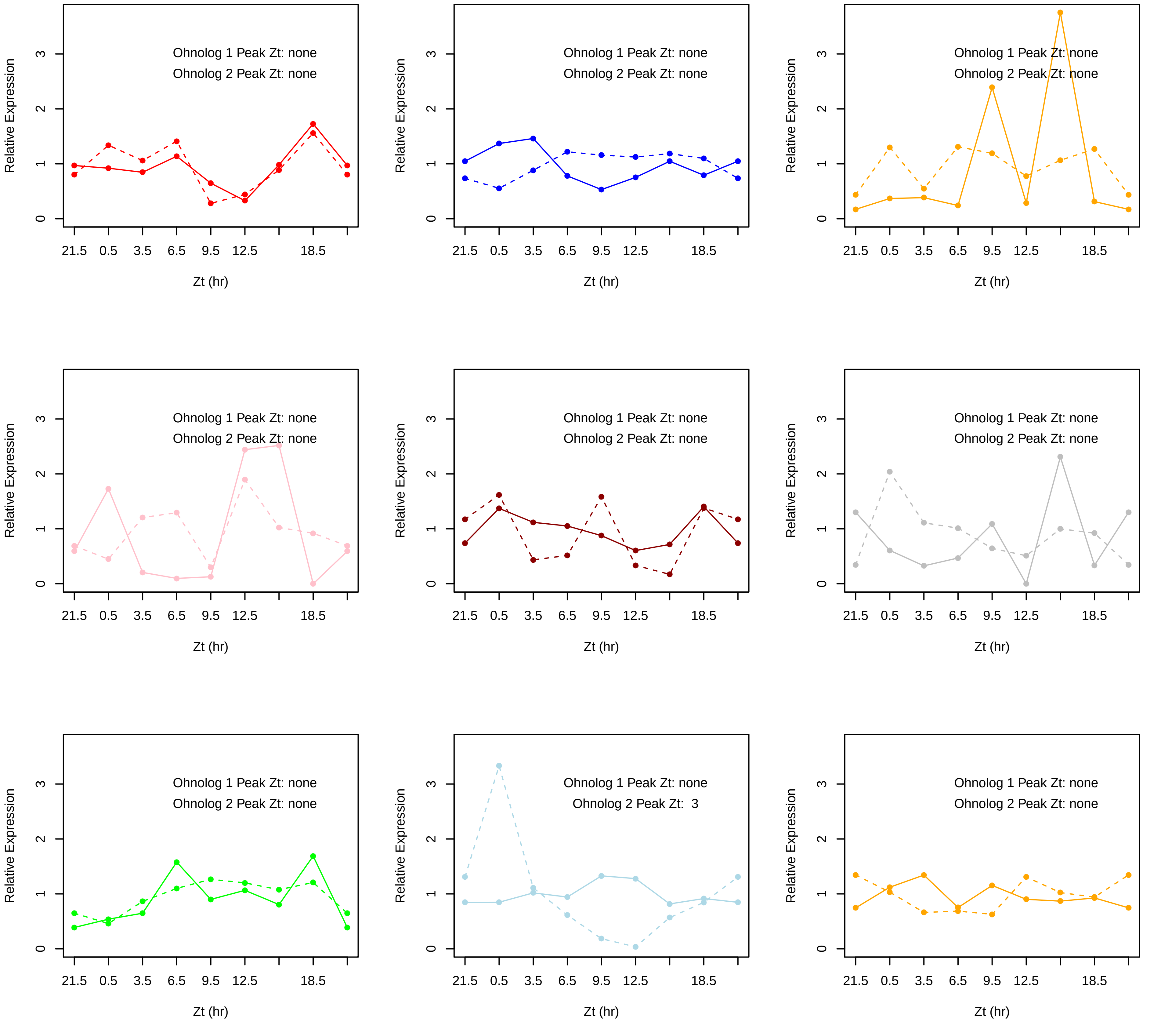

Ohnolog 1: ENSXMAG00000006651
Ohnolog 2: ENSXMAG00000016174 per2

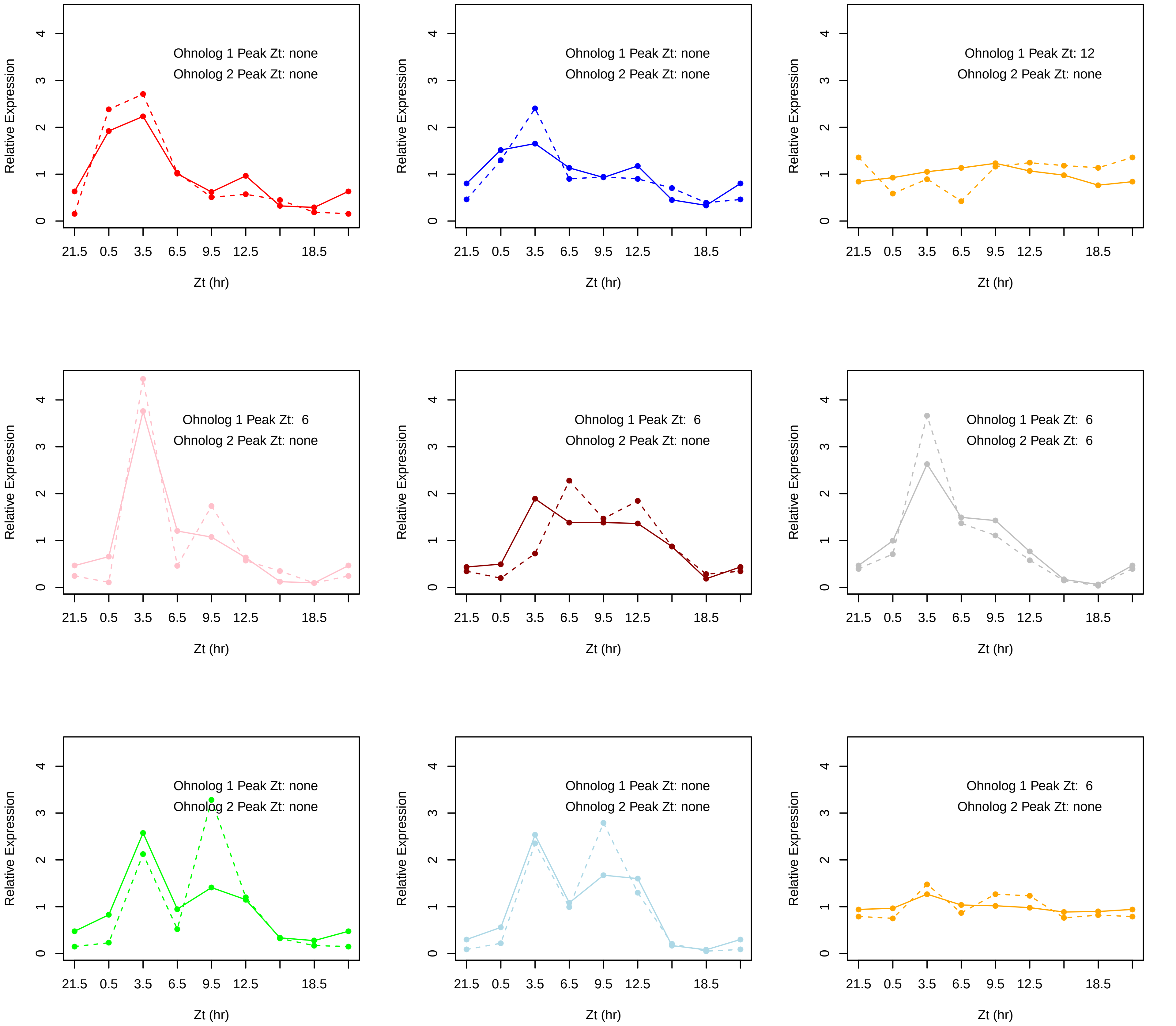

Ohnolog 1: ENSXMAG00000018511 timm44
Ohnolog 2: ENSXMAG00000018819

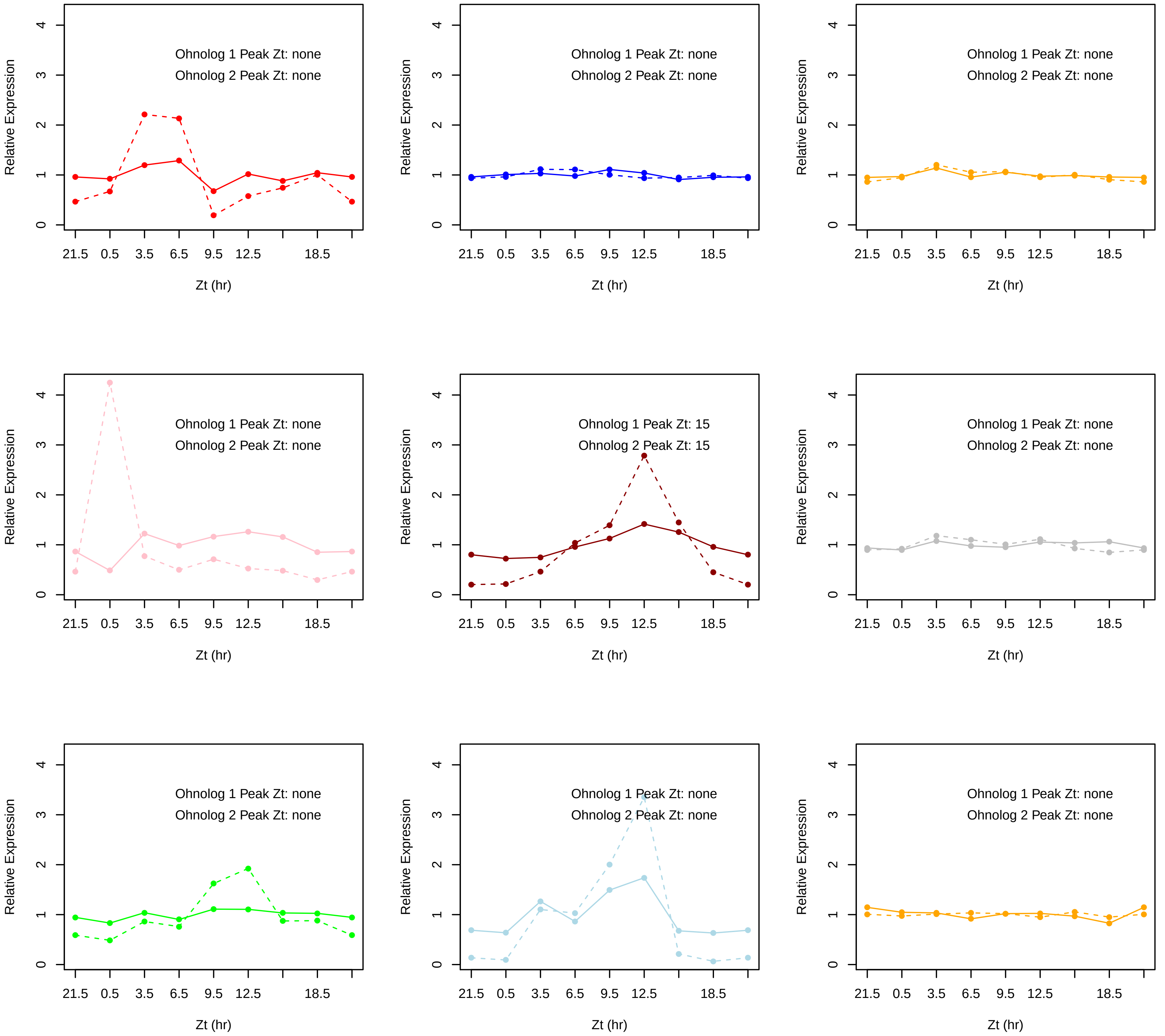

Ohnolog 1: ENSXMAG00000008604
Ohnolog 2: ENSXMAG00000011253 pafah1b1b

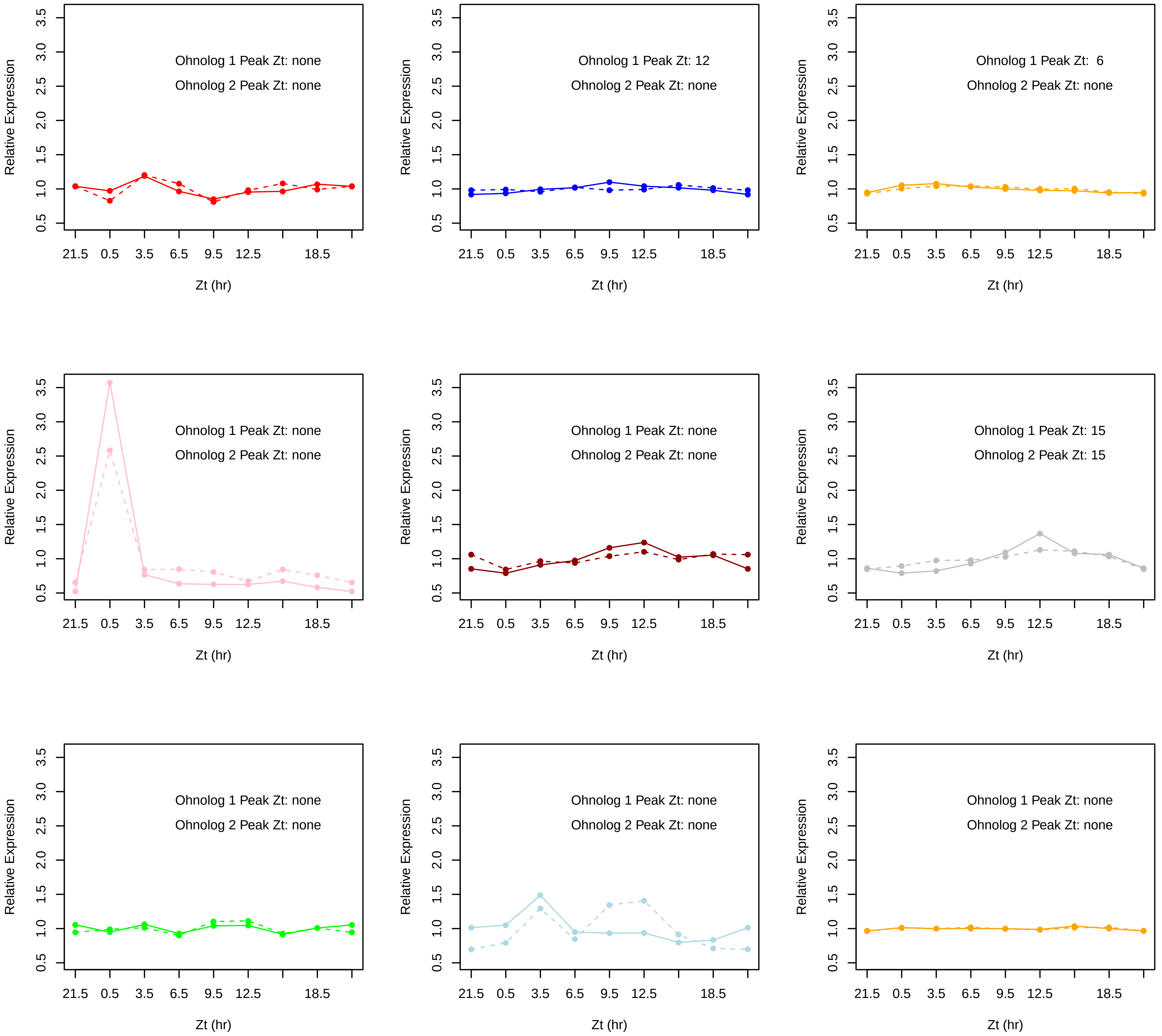

Ohnolog 1: ENSXMAG00000003490 MMD2 (1 of many)
Ohnolog 2: ENSXMAG00000028136 MMD2 (1 of many)

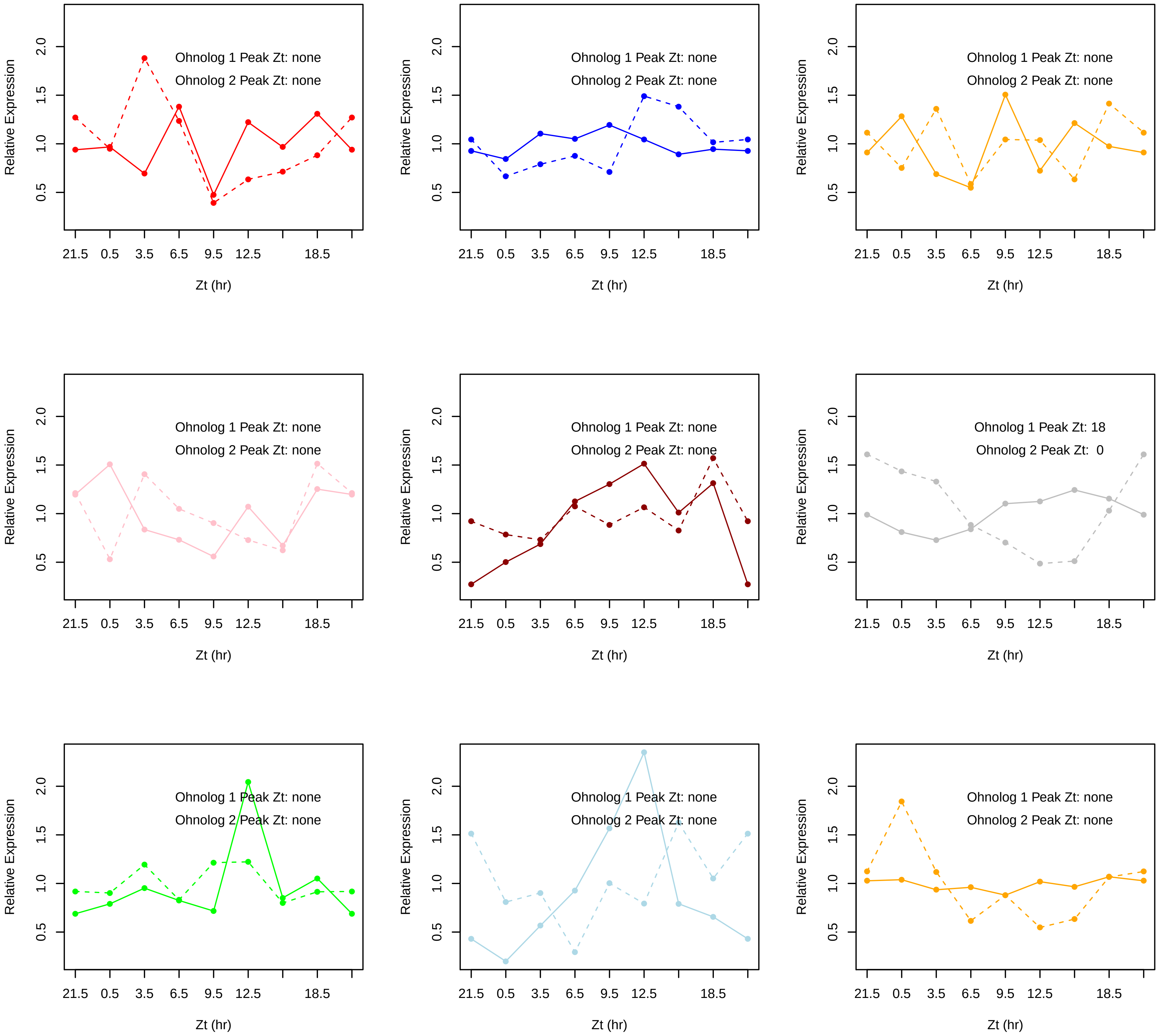

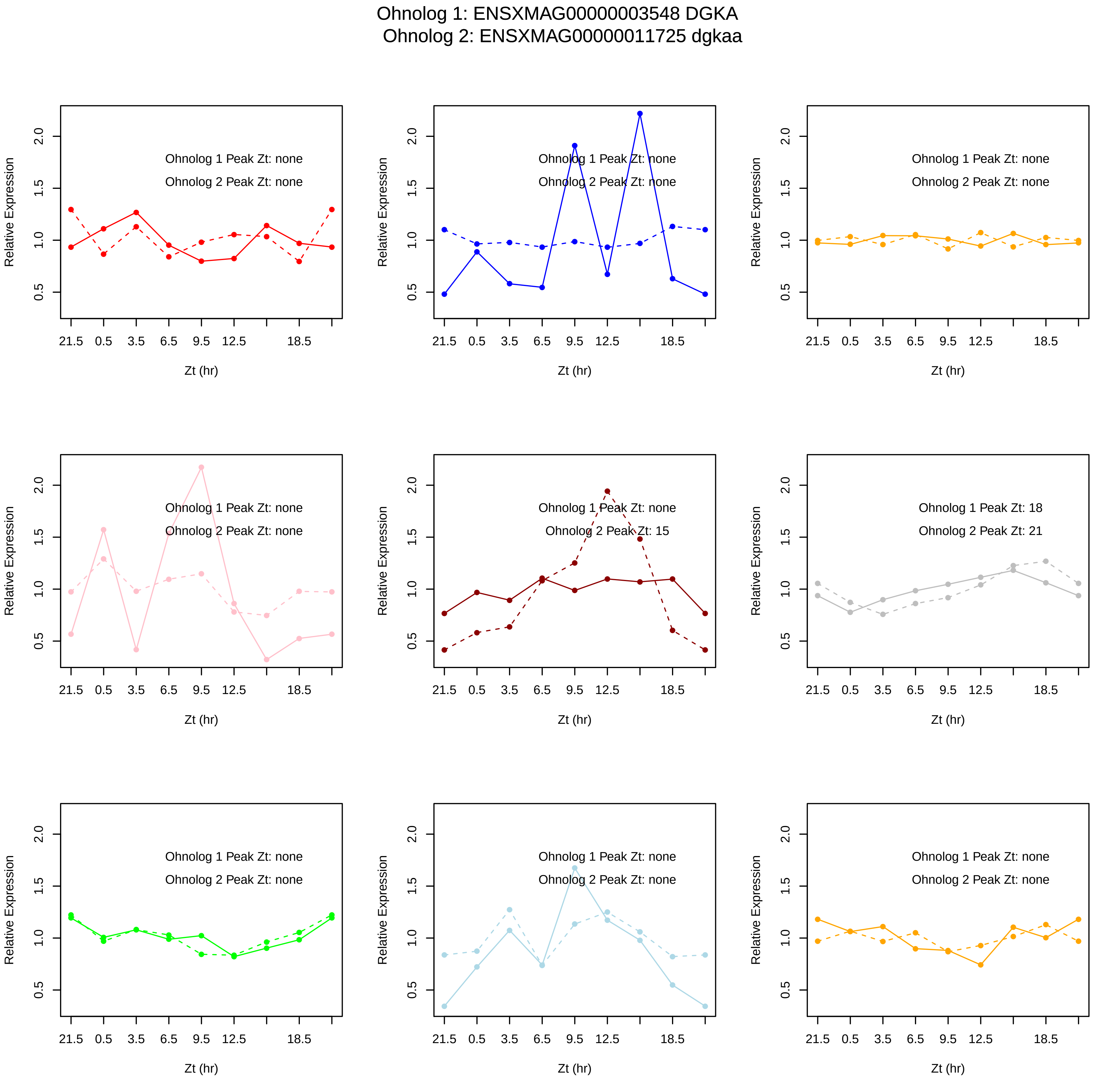
Ohnolog 1: ENSXMAG00000003548 DGKA
Ohnolog 2: ENSXMAG00000011725 dgkaa
Ohnolog 1 Peak Zt: none
Ohnolog 2 Peak Zt: none
Ohnolog 1 Peak Zt: none
Ohnolog 2 Peak Zt: none
Ohnolog 1 Peak Zt: none
Ohnolog 2 Peak Zt: none
Ohnolog 1 Peak Zt: none
Ohnolog 2 Peak Zt: none
Ohnolog 1 Peak Zt: none
Ohnolog 2 Peak Zt: 15
Ohnolog 1 Peak Zt: 18
Ohnolog 2 Peak Zt: 21
Ohnolog 1 Peak Zt: none
Ohnolog 2 Peak Zt: none
Ohnolog 1 Peak Zt: none
Ohnolog 2 Peak Zt: none
Ohnolog 1 Peak Zt: none
Ohnolog 2 Peak Zt: none
Relative Expression
Zt (hr)
21.5
0.5
3.5
6.5
9.5
12.5
18.5

Ohnolog 1: ENSXMAG00000009598 top1l
Ohnolog 2: ENSXMAG00000015345 top1

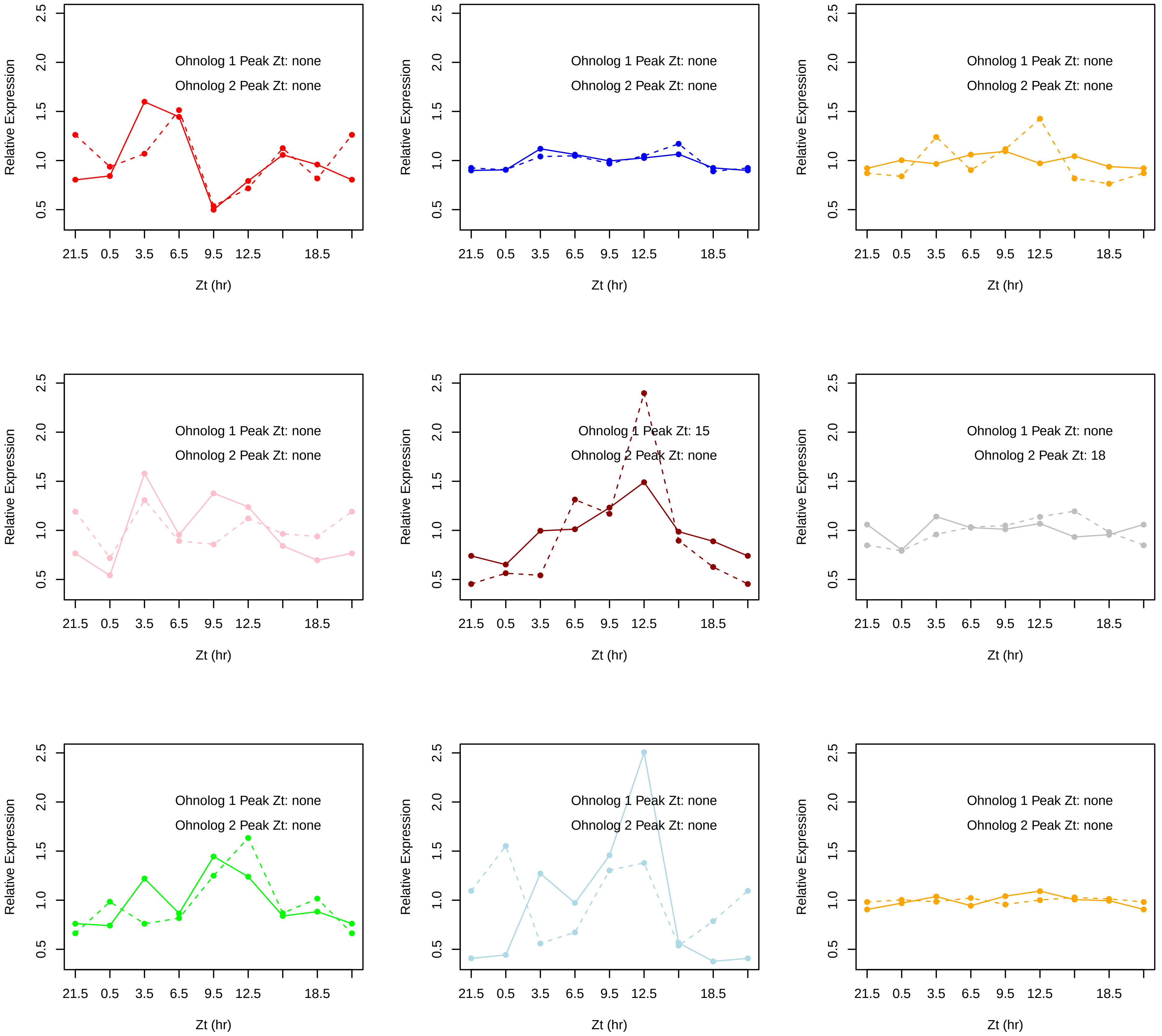

Ohnolog 1: ENSXMAG00000010823 pou2f2a
Ohnolog 2: ENSXMAG00000025547

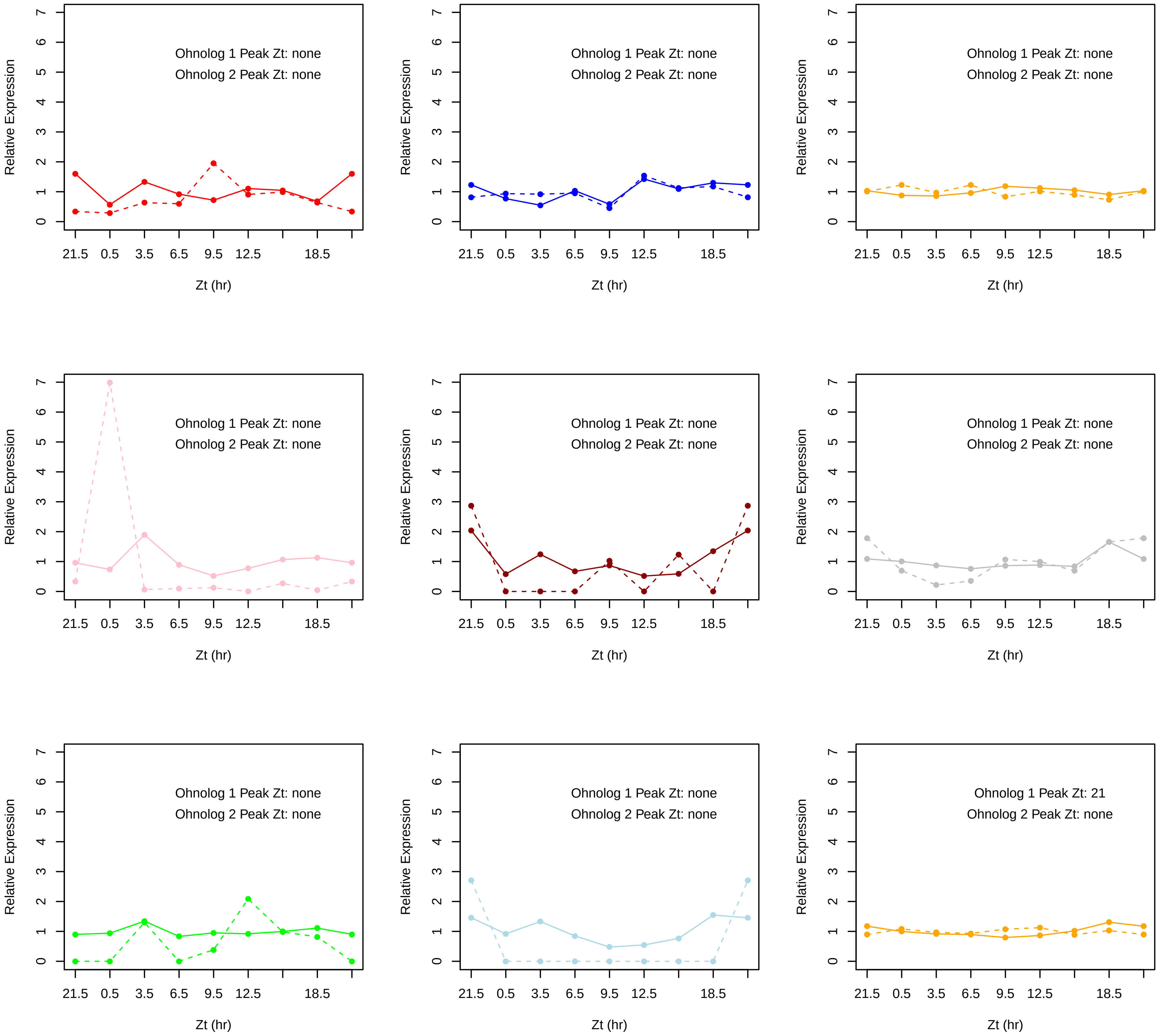

Ohnolog 1: ENSXMAG00000010816 erfl3
Ohnolog 2: ENSXMAG00000029798 erf

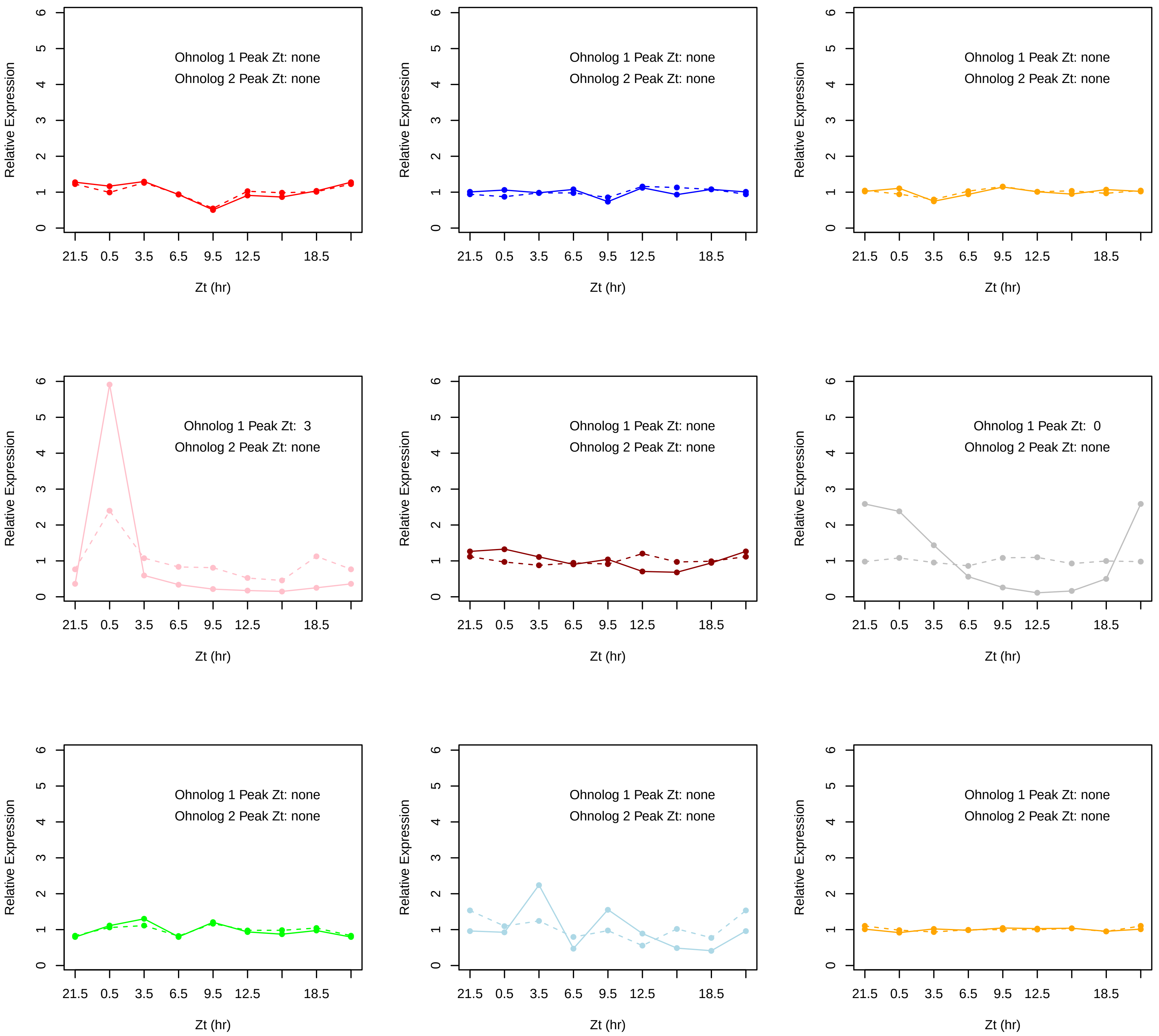

Ohnolog 1: ENSXMAG00000008861 ncam1a
Ohnolog 2: ENSXMAG00000029112 ncam1b

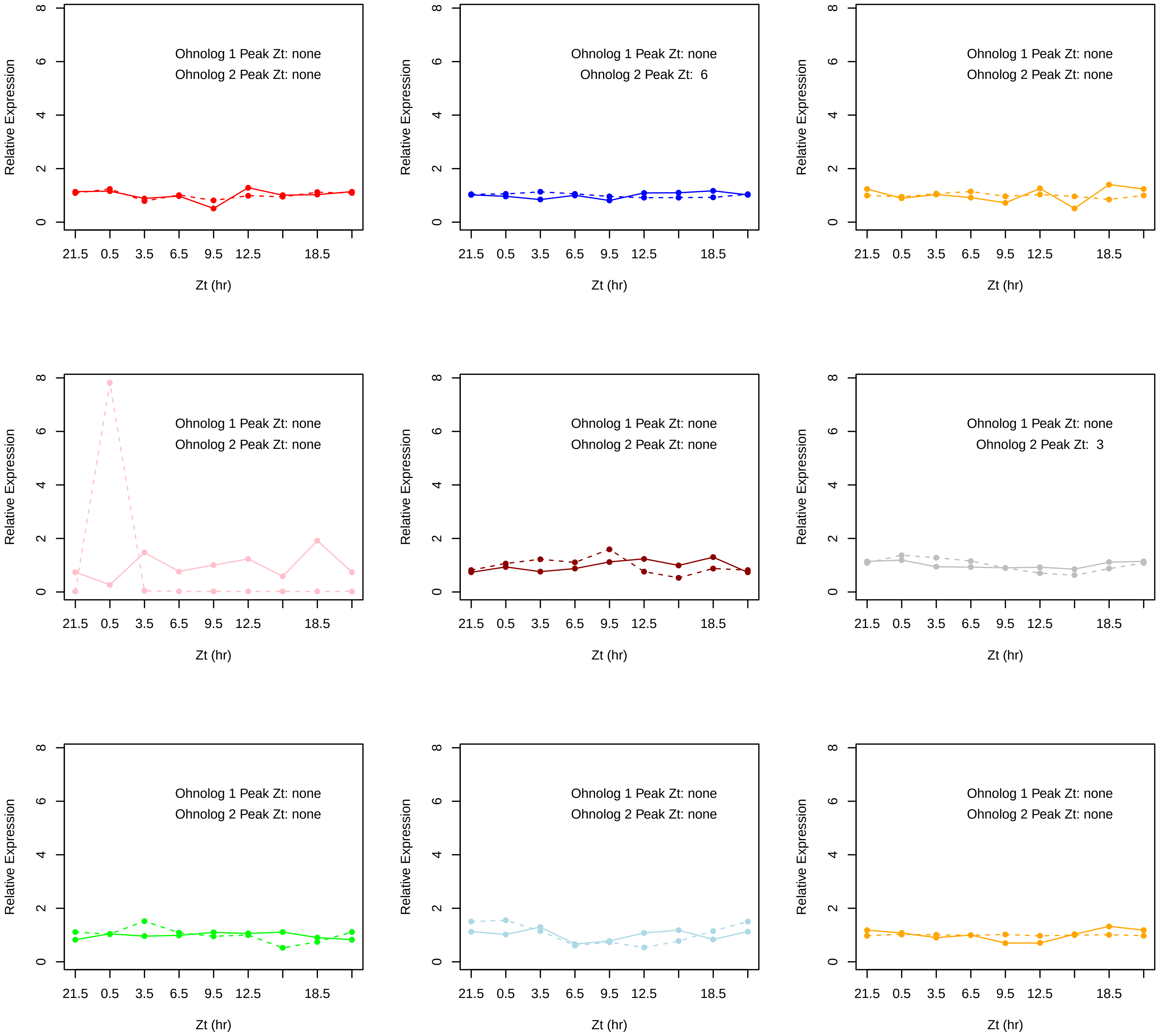

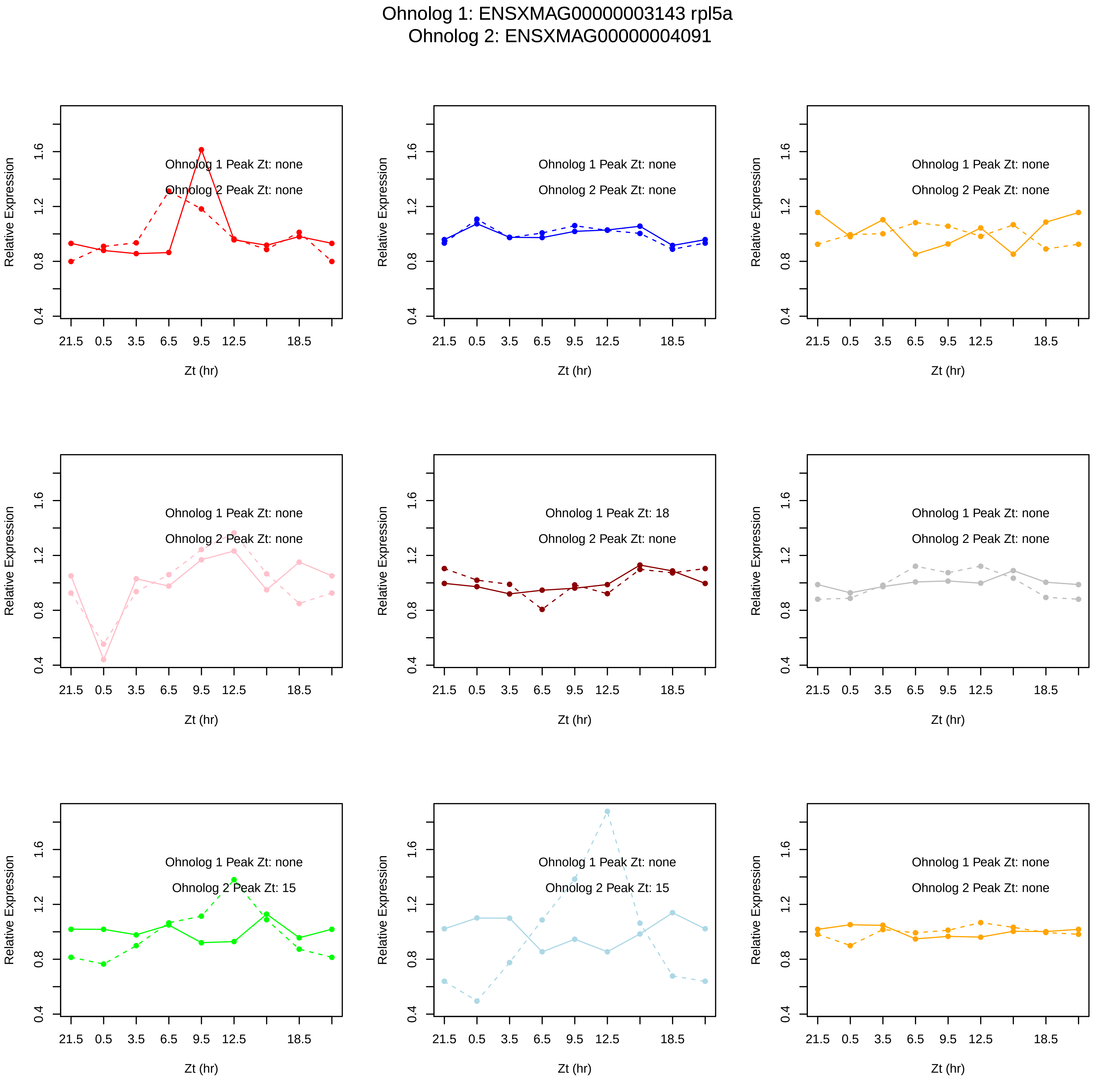
Ohnolog 1: ENSXMAG00000003143 rpl5a
Ohnolog 2: ENSXMAG00000004091
Ohnolog 1 Peak Zt: none
Ohnolog 2 Peak Zt: none
Ohnolog 1 Peak Zt: none
Ohnolog 2 Peak Zt: none
Ohnolog 1 Peak Zt: none
Ohnolog 2 Peak Zt: none
Ohnolog 1 Peak Zt: none
Ohnolog 2 Peak Zt: none
Ohnolog 1 Peak Zt: 18
Ohnolog 2 Peak Zt: none
Ohnolog 1 Peak Zt: none
Ohnolog 2 Peak Zt: none
Ohnolog 1 Peak Zt: none
Ohnolog 2 Peak Zt: 15
Ohnolog 1 Peak Zt: none
Ohnolog 2 Peak Zt: 15
Ohnolog 1 Peak Zt: none
Ohnolog 2 Peak Zt: none
Relative Expression
0.4
0.8
1.2
1.6
Zt (hr)
21.5
0.5
3.5
6.5
9.5
12.5
18.5

Ohnolog 1: ENSXMAG00000004123 glmna
Ohnolog 2: ENSXMAG00000010662 glmnb

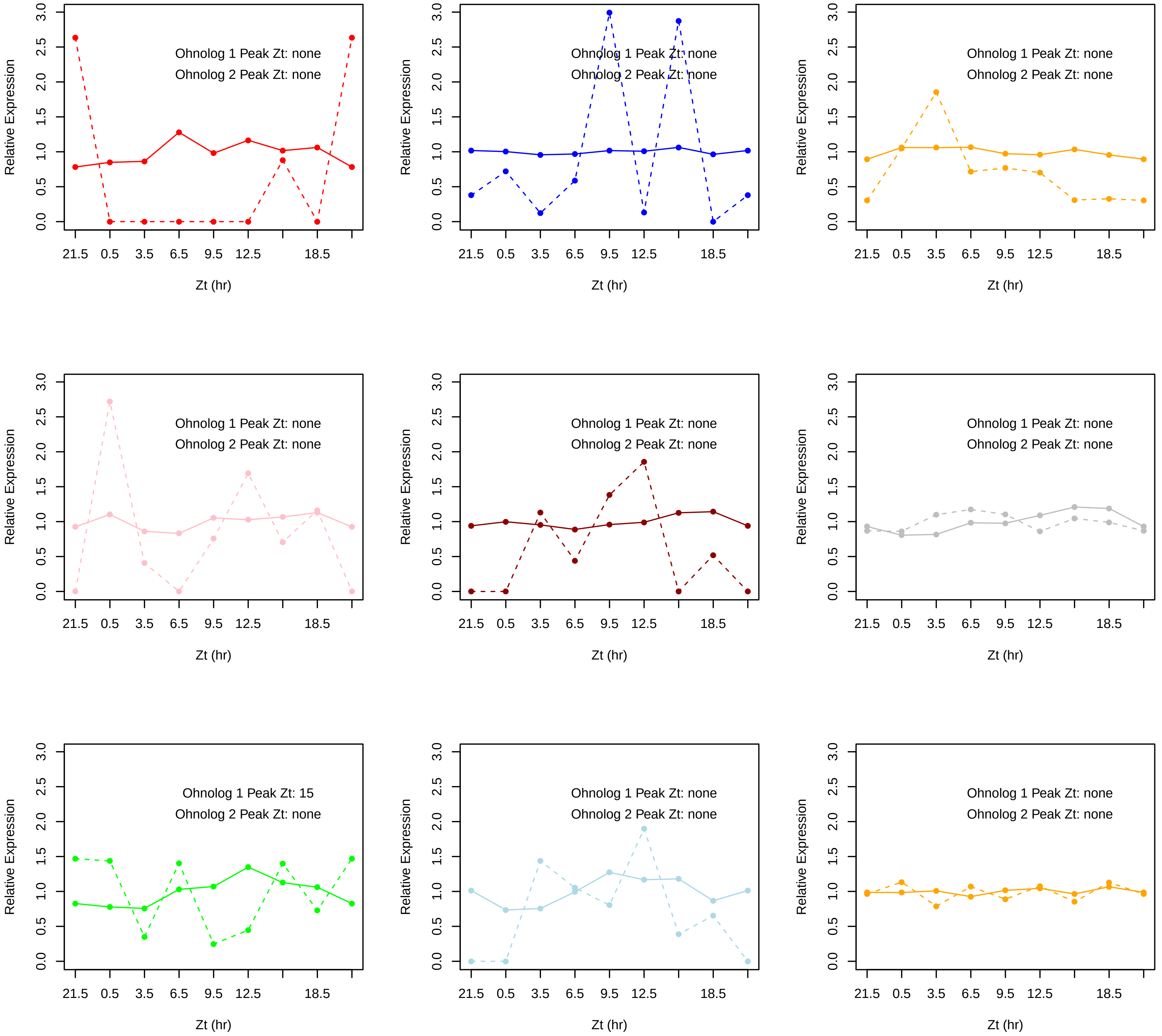

Ohnolog 1: ENSXMAG00000003226 kcnma1a
Ohnolog 2: ENSXMAG00000015365 KCNMA1

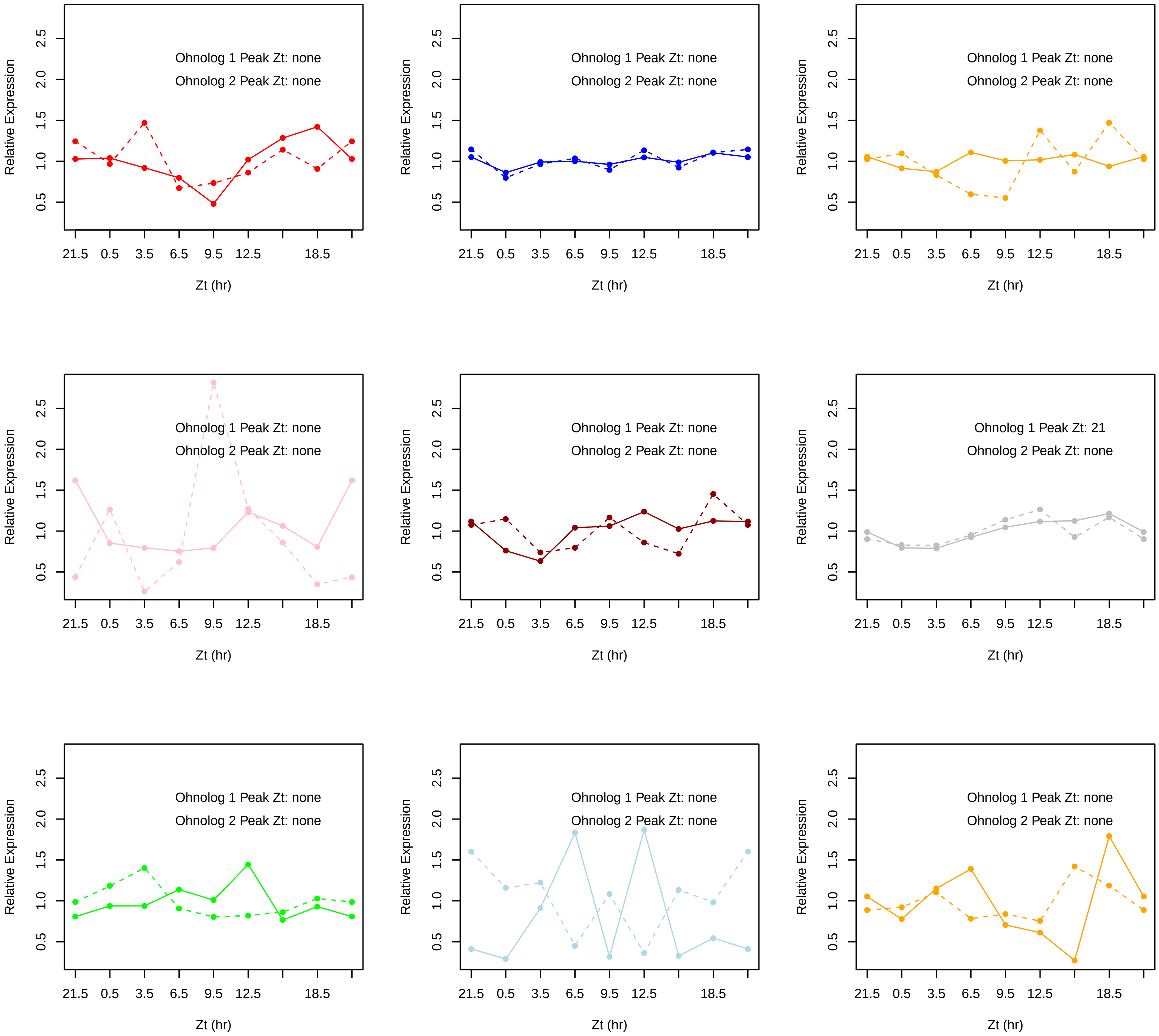

Ohnolog 1: ENSXMAG00000012702
Ohnolog 2: ENSXMAG00000018190 myorg

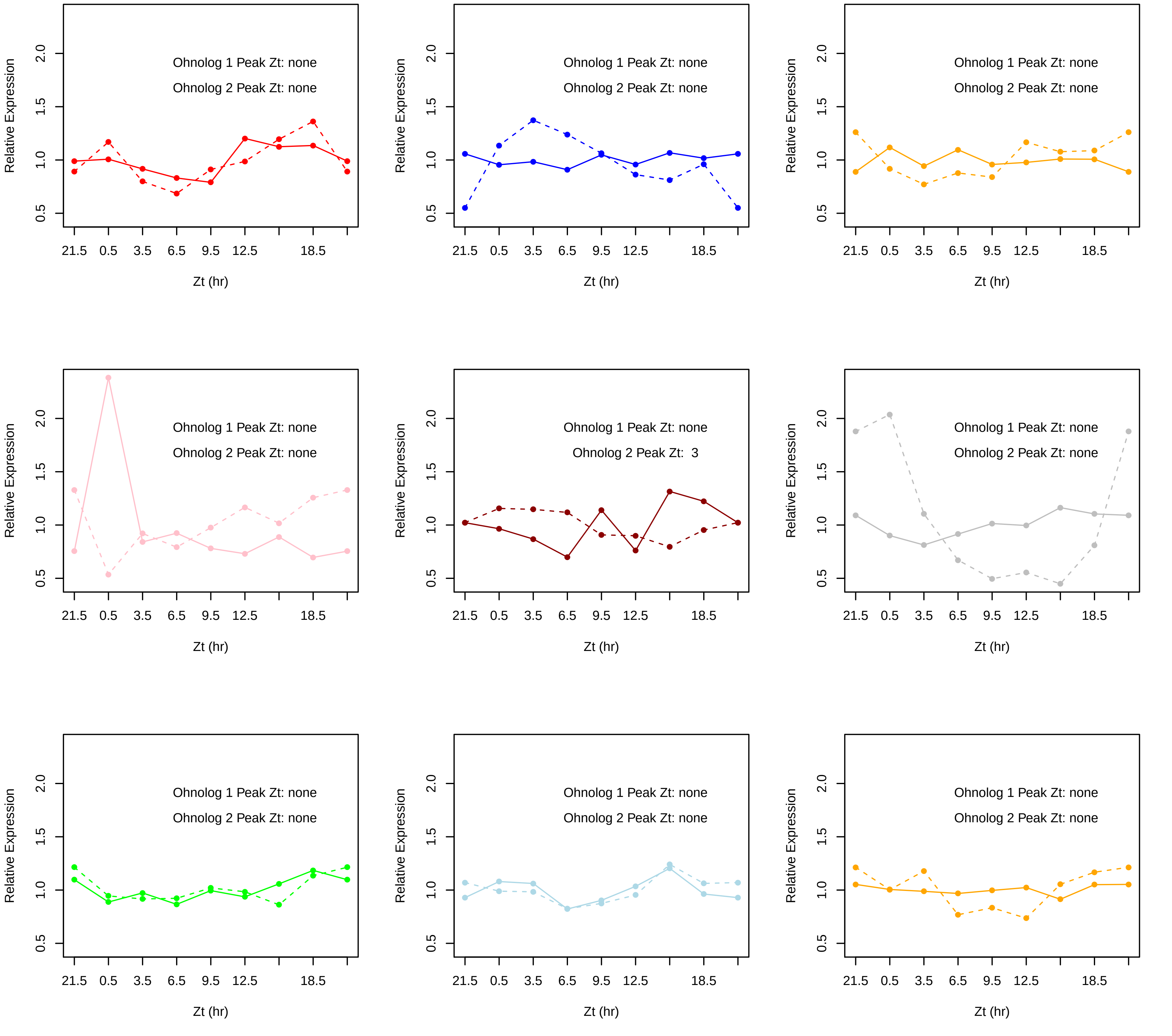

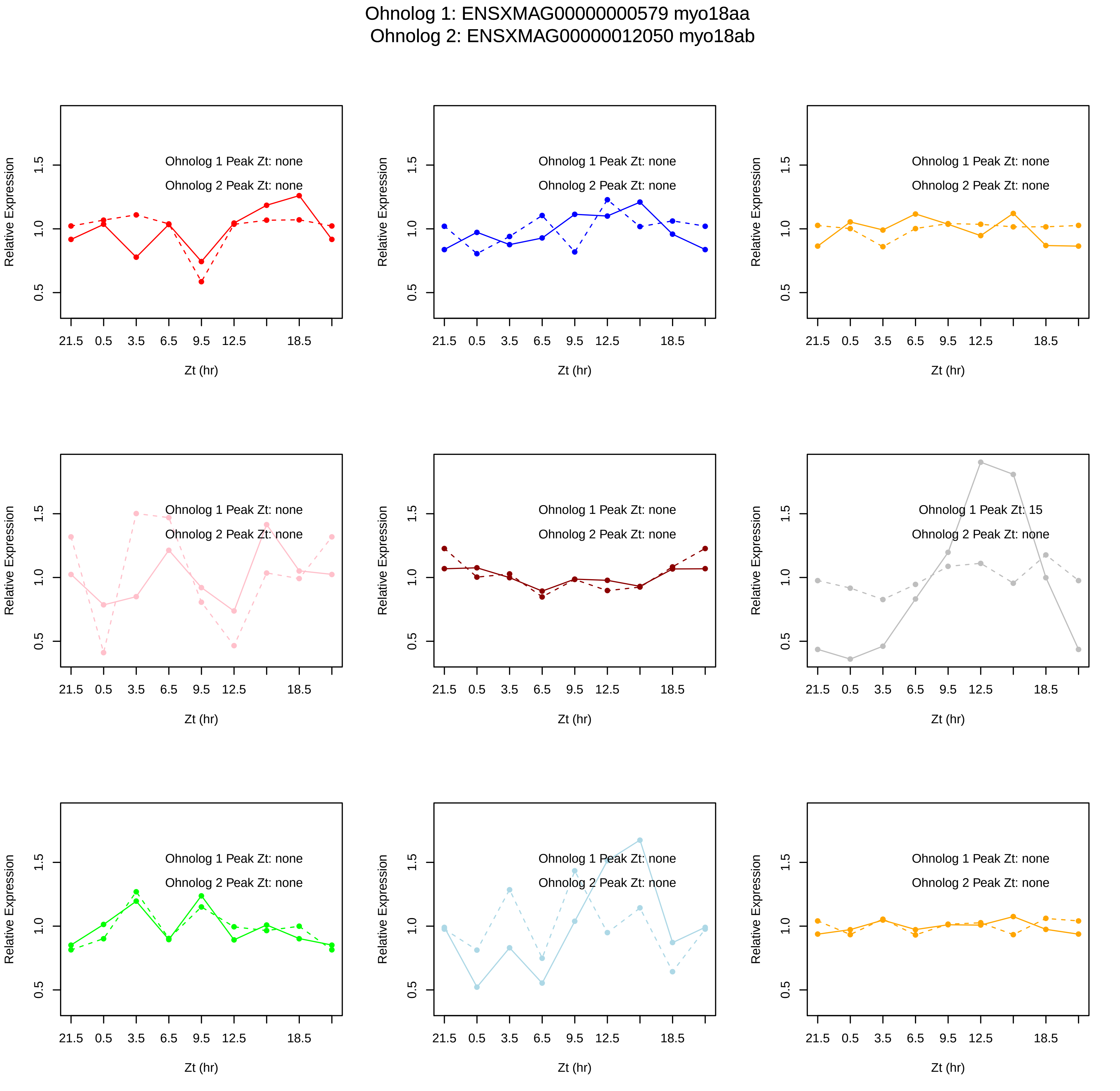
Ohnolog 1: ENSXMAG00000000579 myo18aa
Ohnolog 2: ENSXMAG00000012050 myo18ab
Ohnolog 1 Peak Zt: none
Ohnolog 2 Peak Zt: none
Ohnolog 1 Peak Zt: 15
Relative Expression
Zt (hr)
0.5
1.0
1.5
21.5
0.5
3.5
6.5
9.5
12.5
18.5

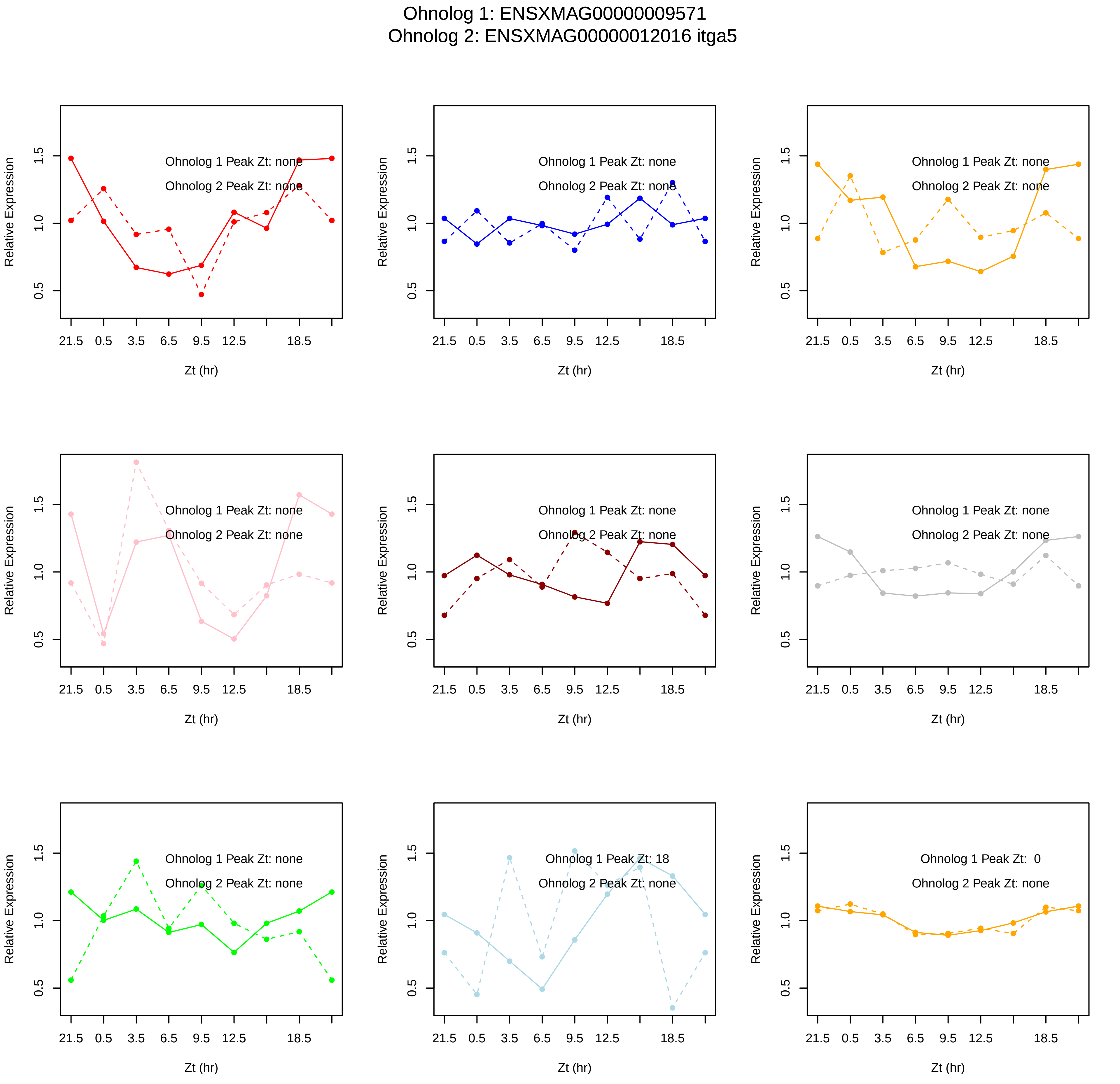
Ohnolog 1: ENSXMAG00000009571
Ohnolog 2: ENSXMAG00000012016 itga5
Ohnolog 1 Peak Zt: none
Ohnolog 2 Peak Zt: none
Ohnolog 1 Peak Zt: 18
Ohnolog 1 Peak Zt: 0
Relative Expression
Zt (hr)
21.5
0.5
3.5
6.5
9.5
12.5
18.5
0.5
1.0
1.5

Ohnolog 1: ENSXMAG00000003180 zcchc24
Ohnolog 2: ENSXMAG00000015304 ZCCHC24 (1 of many)

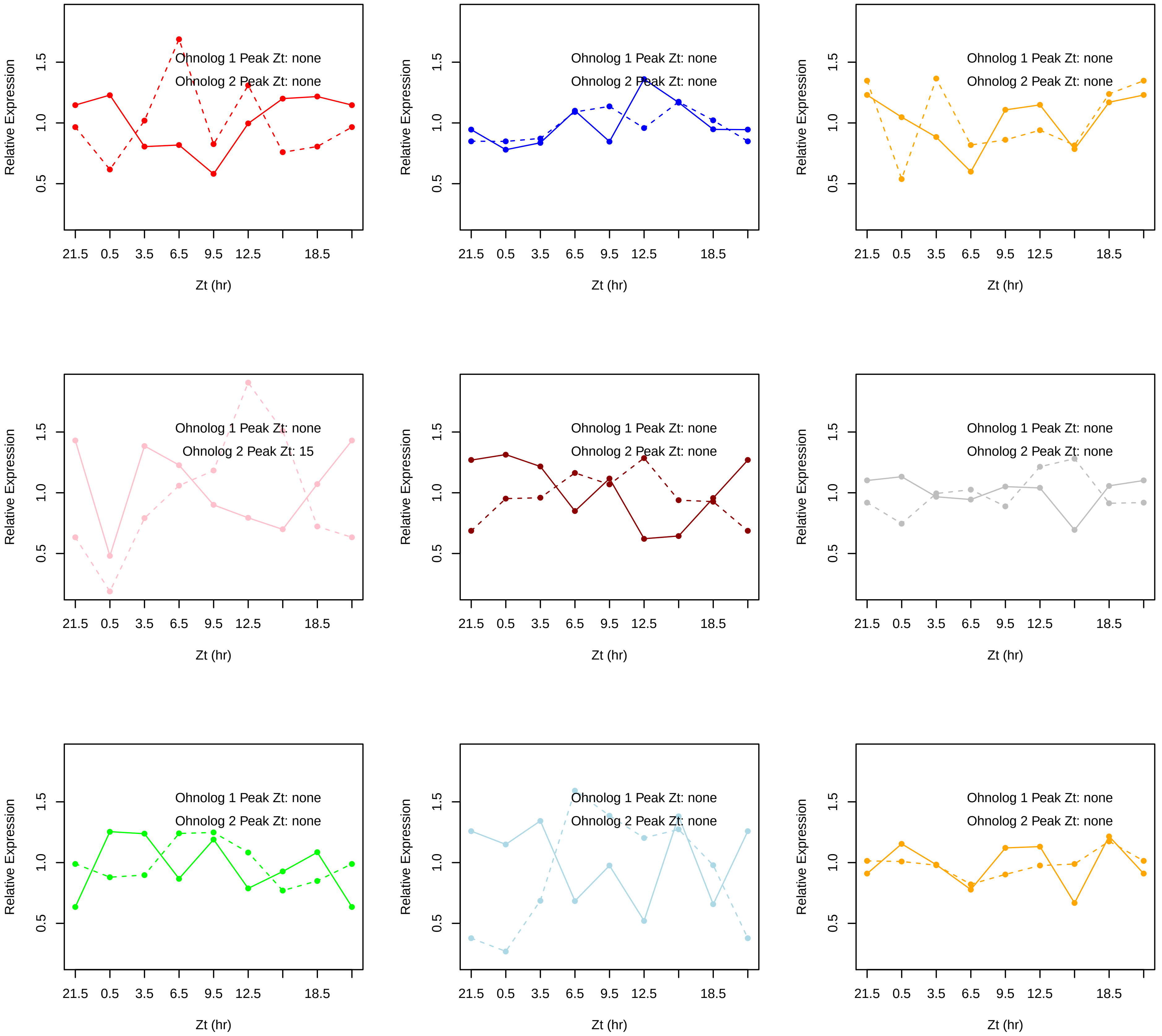

Ohnolog 1: ENSXMAG00000000530
Ohnolog 2: ENSXMAG00000024206 armc1

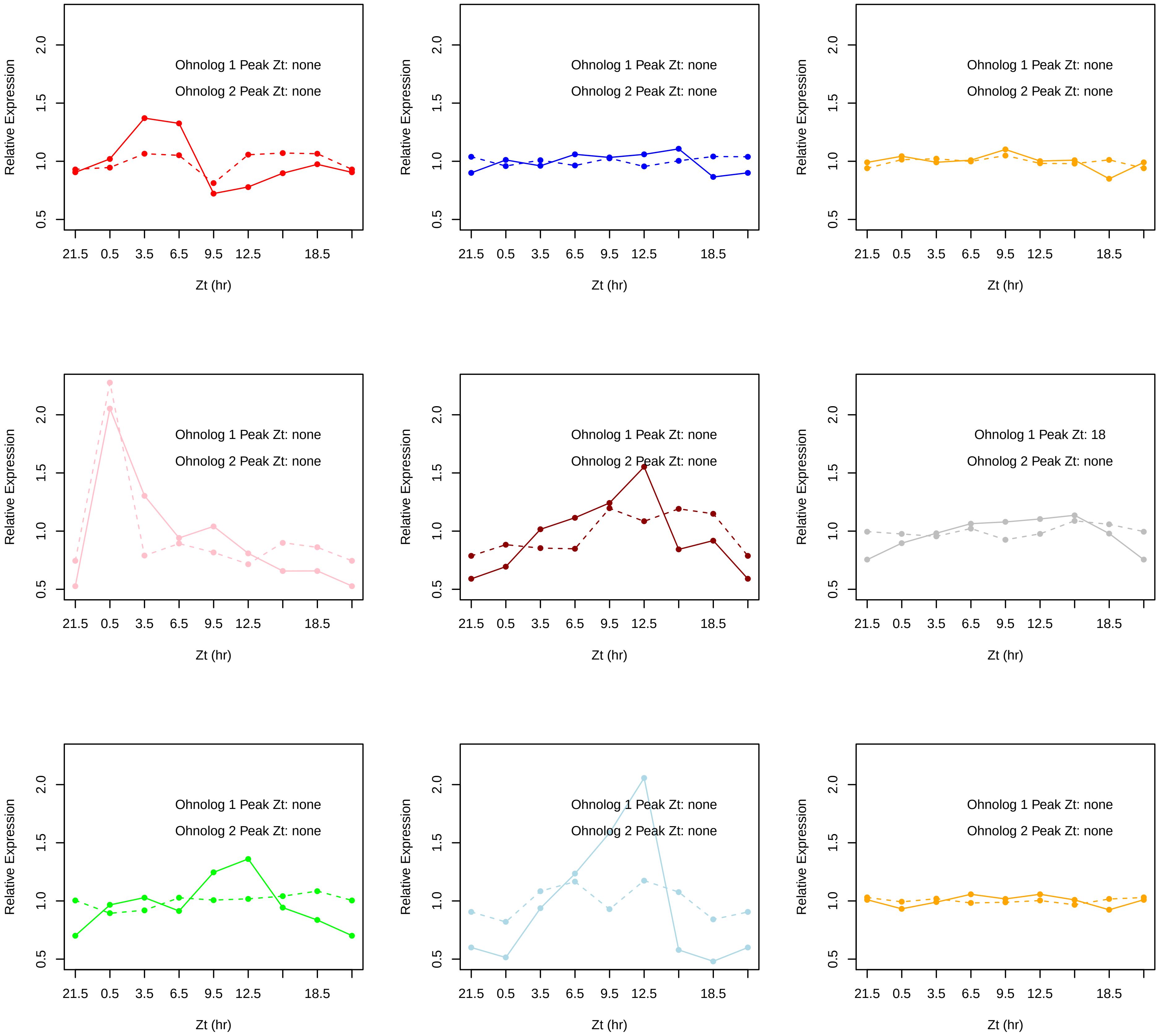

Ohnolog 1: ENSXMAG00000006042 JAM3 (1 of many)
Ohnolog 2: ENSXMAG00000028684 jam3a

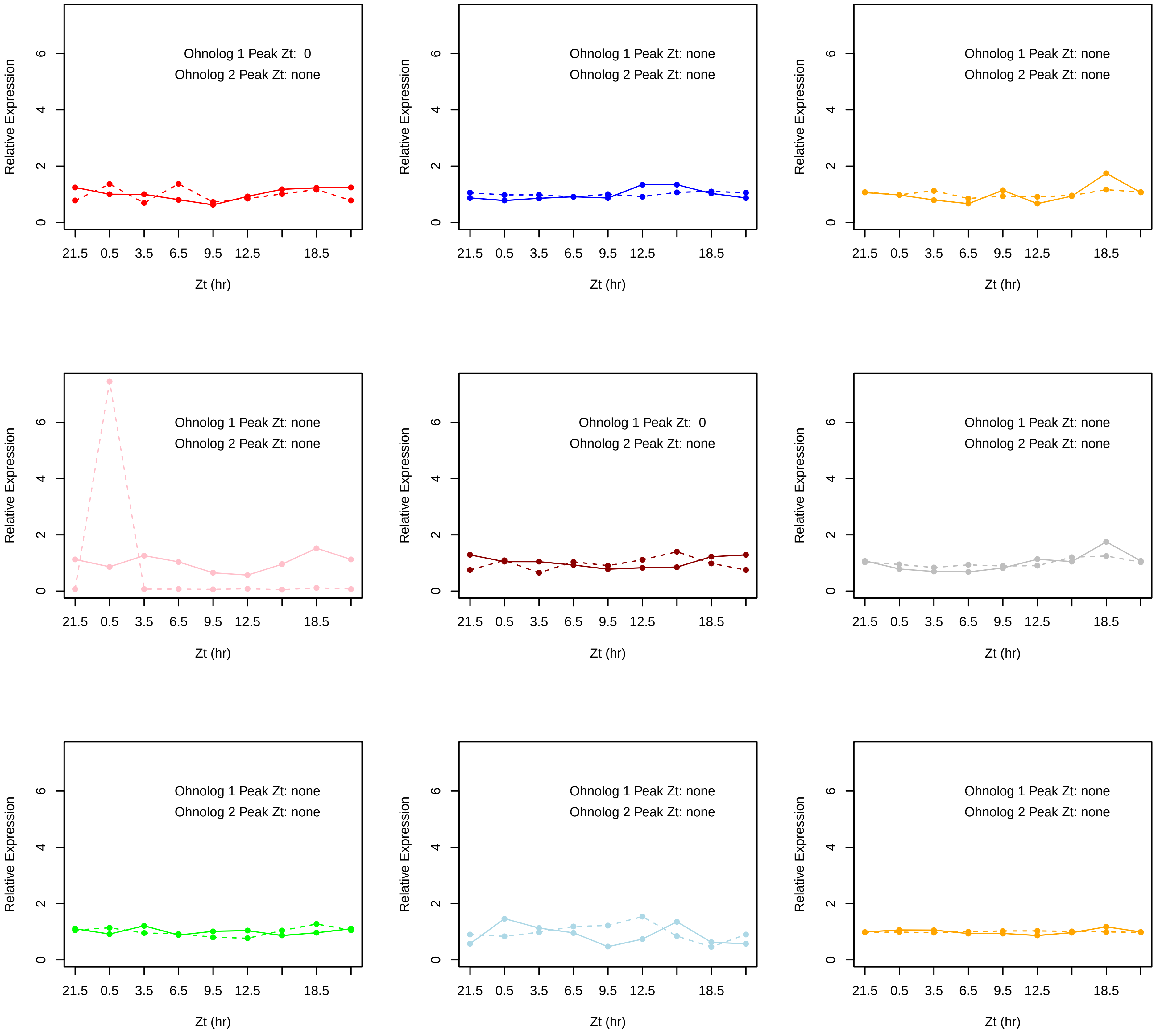

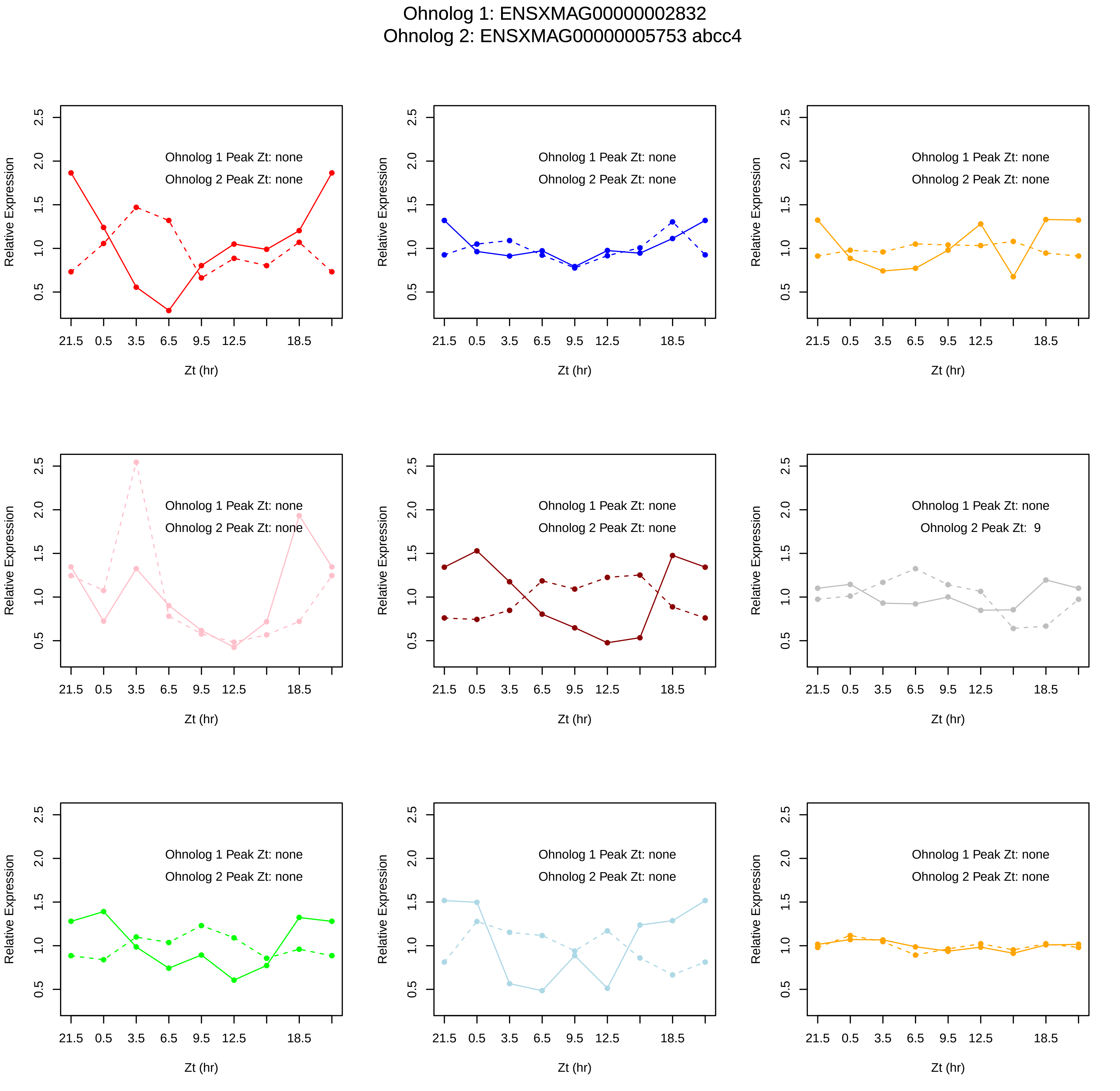

Ohnolog 1: ENSXMAG00000002832
Ohnolog 2: ENSXMAG00000005753 abcc4
Ohnolog 1 Peak Zt: none
Ohnolog 2 Peak Zt: none
Ohnolog 1 Peak Zt: none
Ohnolog 2 Peak Zt: none
Ohnolog 1 Peak Zt: none
Ohnolog 2 Peak Zt: none
Ohnolog 1 Peak Zt: none
Ohnolog 2 Peak Zt: none
Ohnolog 1 Peak Zt: none
Ohnolog 2 Peak Zt: none
Ohnolog 1 Peak Zt: none
Ohnolog 2 Peak Zt: 9
Ohnolog 1 Peak Zt: none
Ohnolog 2 Peak Zt: none
Ohnolog 1 Peak Zt: none
Ohnolog 2 Peak Zt: none
Ohnolog 1 Peak Zt: none
Ohnolog 2 Peak Zt: none
Relative Expression
Zt (hr)

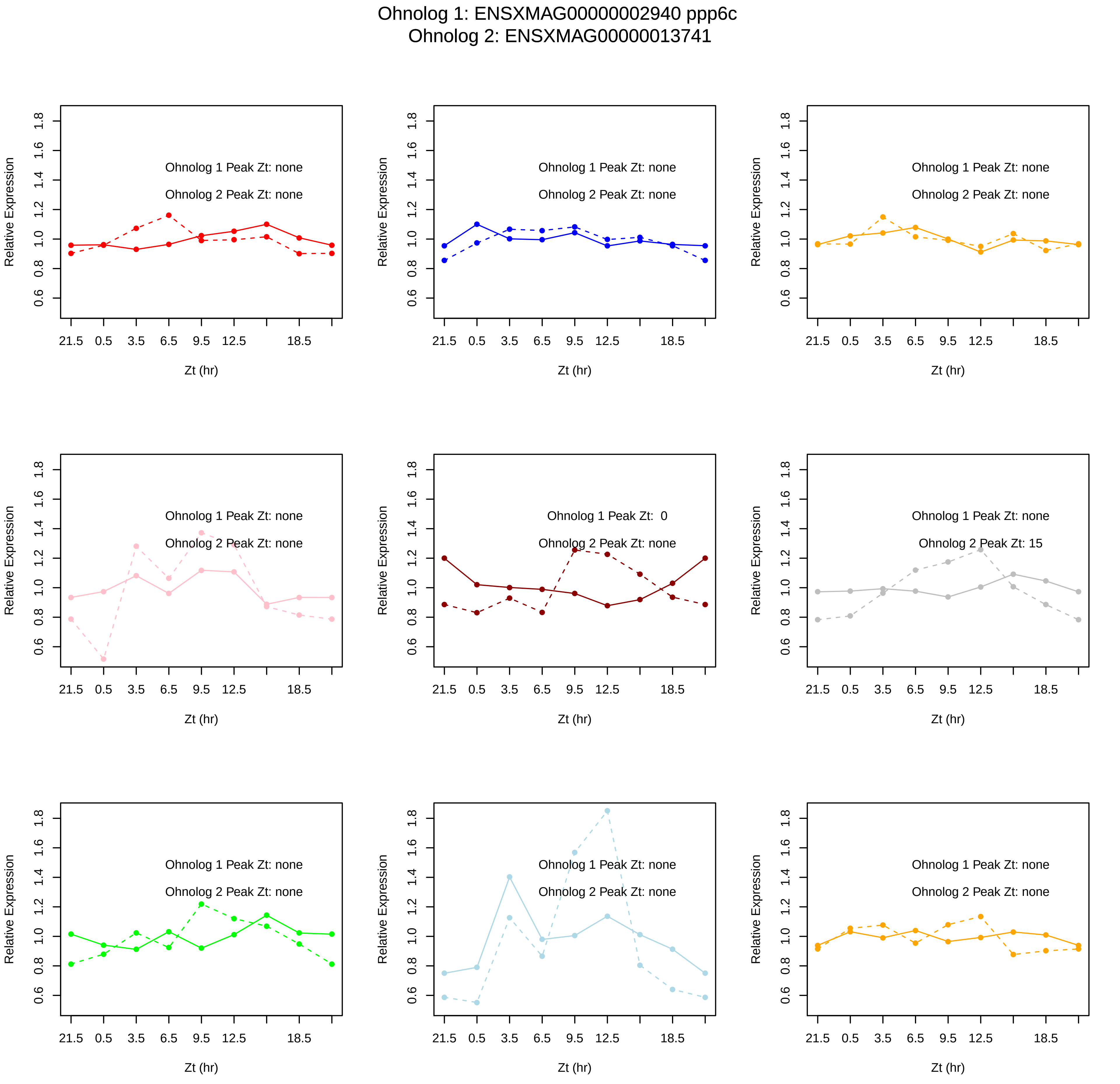
Ohnolog 1: ENSXMAG00000002940 ppp6c
Ohnolog 2: ENSXMAG00000013741
Ohnolog 1 Peak Zt: none
Ohnolog 2 Peak Zt: none
Ohnolog 1 Peak Zt: none
Ohnolog 2 Peak Zt: none
Ohnolog 1 Peak Zt: none
Ohnolog 2 Peak Zt: none
Ohnolog 1 Peak Zt: none
Ohnolog 2 Peak Zt: none
Ohnolog 1 Peak Zt: 0
Ohnolog 2 Peak Zt: none
Ohnolog 1 Peak Zt: none
Ohnolog 2 Peak Zt: 15
Ohnolog 1 Peak Zt: none
Ohnolog 2 Peak Zt: none
Ohnolog 1 Peak Zt: none
Ohnolog 2 Peak Zt: none
Ohnolog 1 Peak Zt: none
Ohnolog 2 Peak Zt: none
Relative Expression
Zt (hr)

Ohnolog 1: ENSXMAG00000021001 lrrc75bb
Ohnolog 2: ENSXMAG00000025138 lrrc75ba

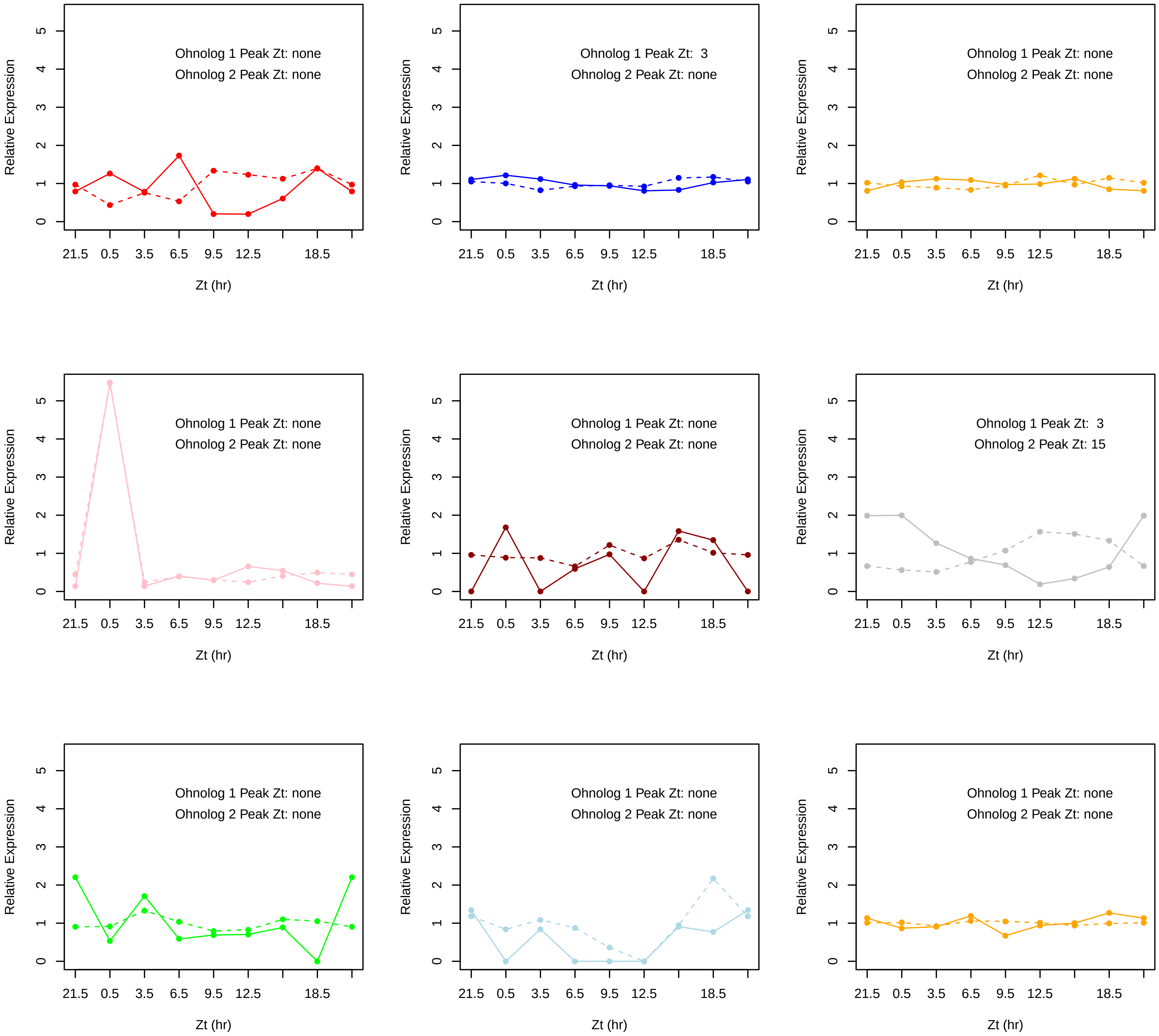

Ohnolog 1: ENSXMAG00000004449
Ohnolog 2: ENSXMAG00000018355

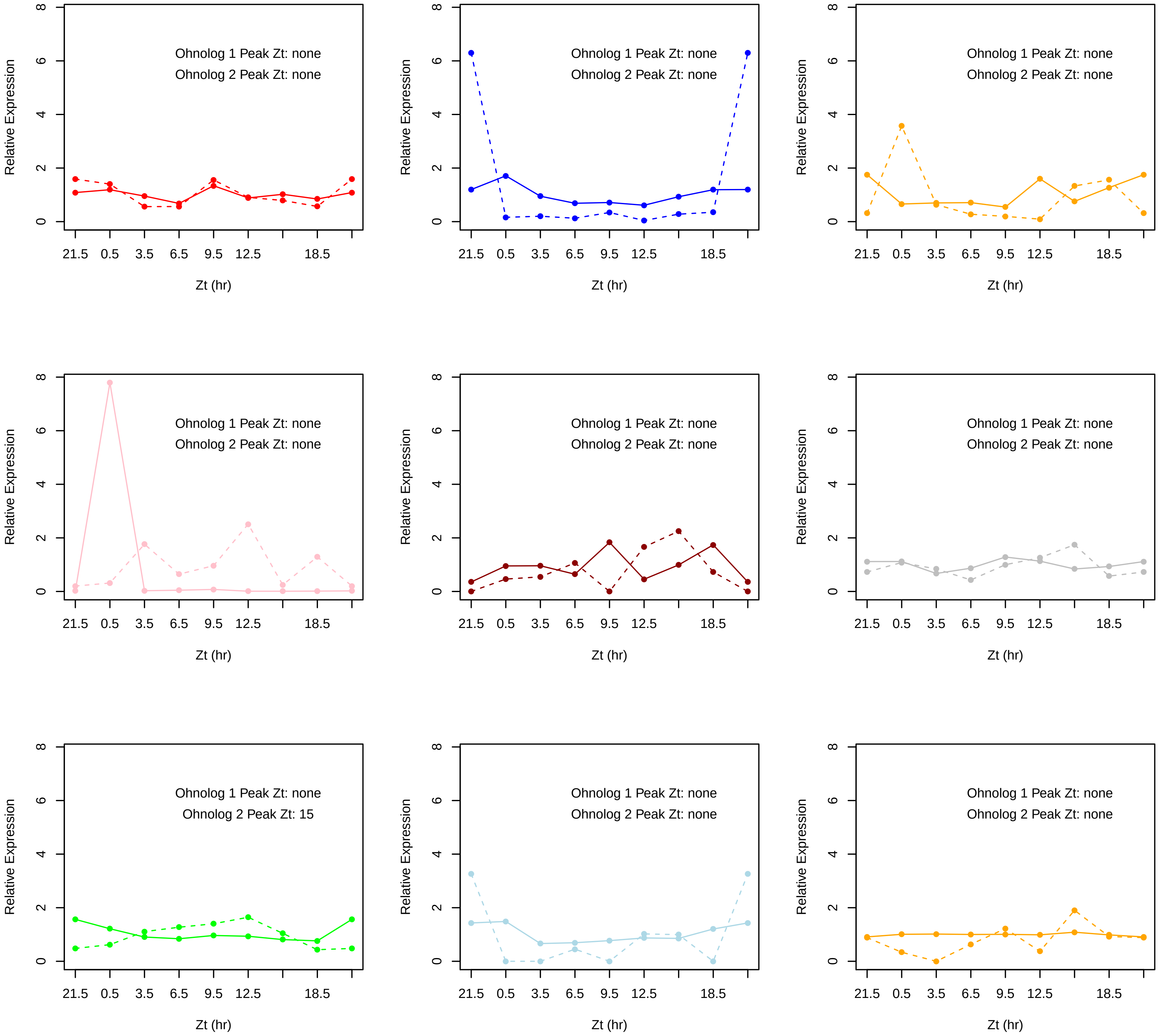

Ohnolog 1: ENSXMAG00000016824 col2a1a
Ohnolog 2: ENSXMAG00000017719

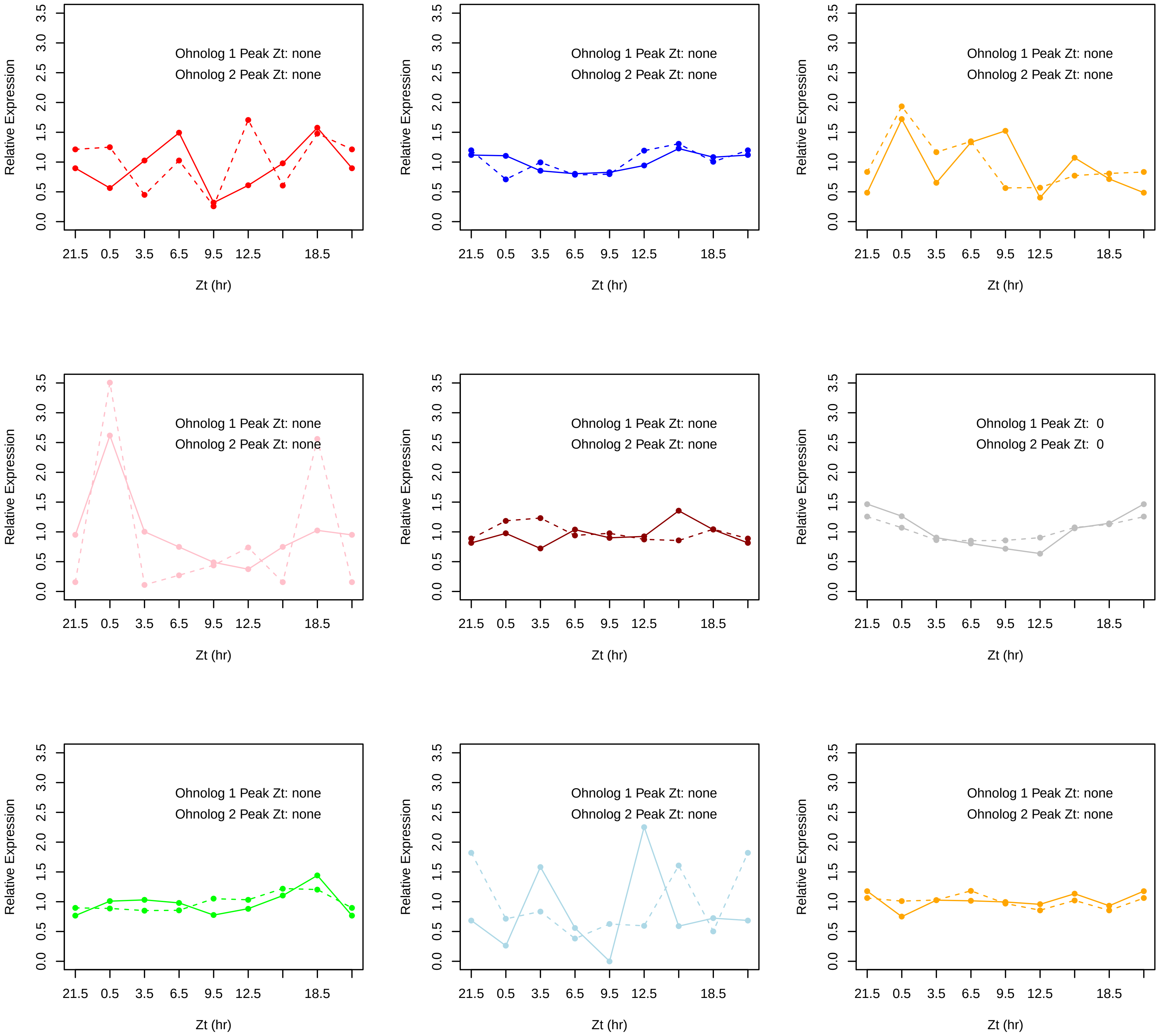

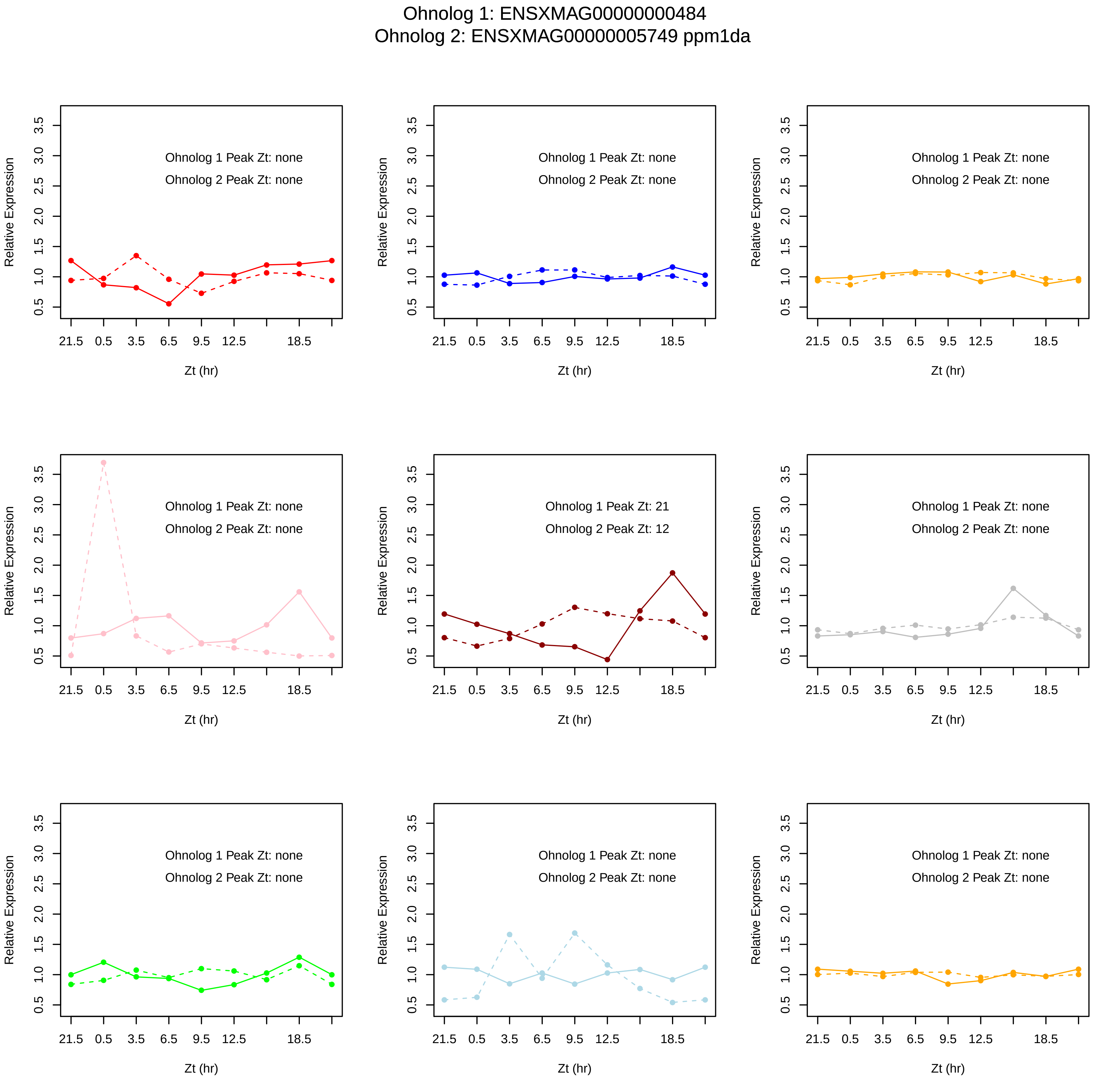
Ohnolog 1: ENSXMAG00000000484
Ohnolog 2: ENSXMAG00000005749 ppm1da
Ohnolog 1 Peak Zt: none
Ohnolog 2 Peak Zt: none
Ohnolog 1 Peak Zt: none
Ohnolog 2 Peak Zt: none
Ohnolog 1 Peak Zt: none
Ohnolog 2 Peak Zt: none
Ohnolog 1 Peak Zt: none
Ohnolog 2 Peak Zt: none
Ohnolog 1 Peak Zt: 21
Ohnolog 2 Peak Zt: 12
Ohnolog 1 Peak Zt: none
Ohnolog 2 Peak Zt: none
Ohnolog 1 Peak Zt: none
Ohnolog 2 Peak Zt: none
Ohnolog 1 Peak Zt: none
Ohnolog 2 Peak Zt: none
Ohnolog 1 Peak Zt: none
Ohnolog 2 Peak Zt: none
Relative Expression
Zt (hr)
0.5 1.0 1.5 2.0 2.5 3.0 3.5
21.5 0.5 3.5 6.5 9.5 12.5 18.5

Ohnolog 1: ENSXMAG00000000754 ano1
Ohnolog 2: ENSXMAG00000016443

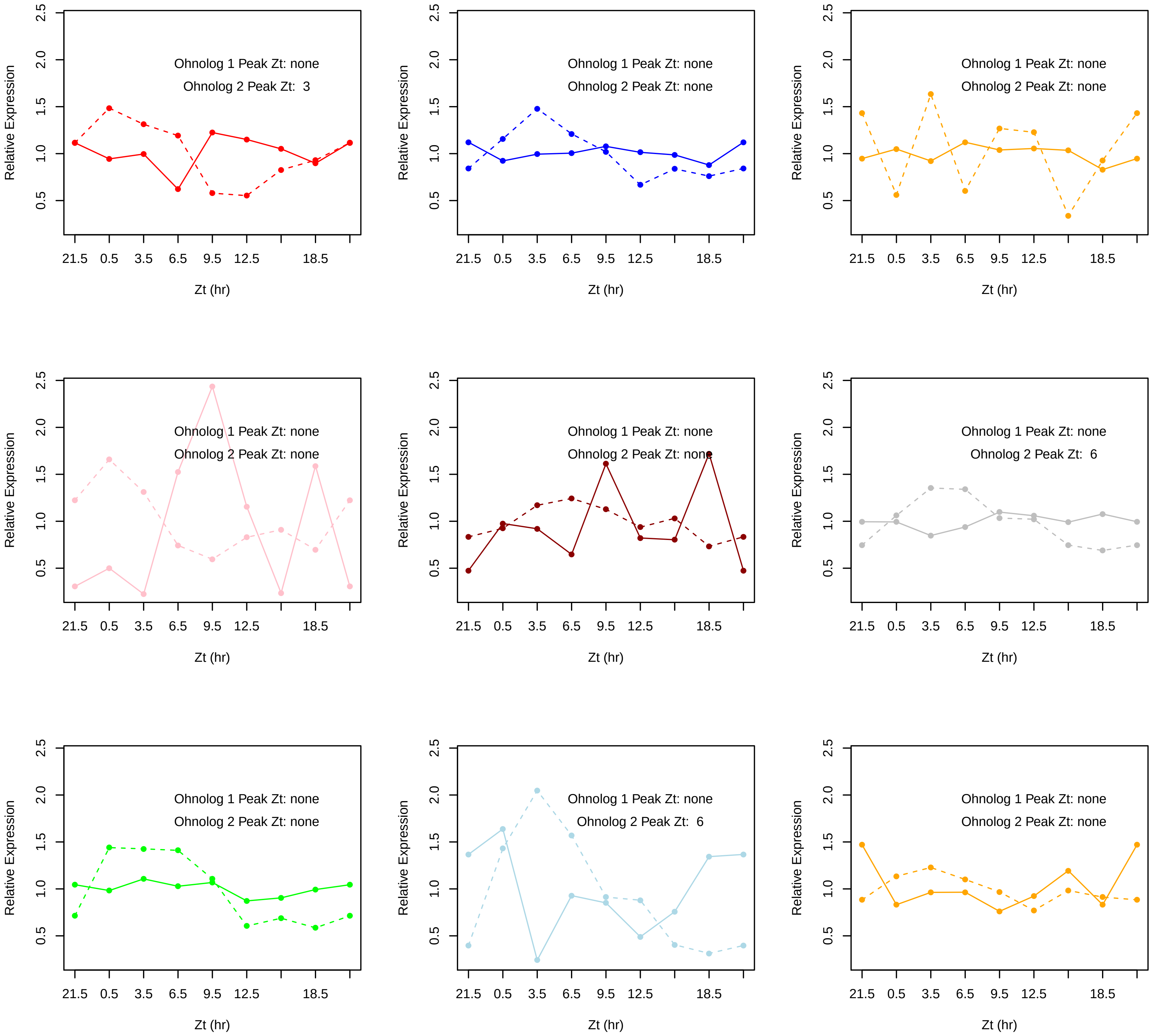

Ohnolog 1: ENSXMAG00000001732
Ohnolog 2: ENSXMAG00000006106 robo3

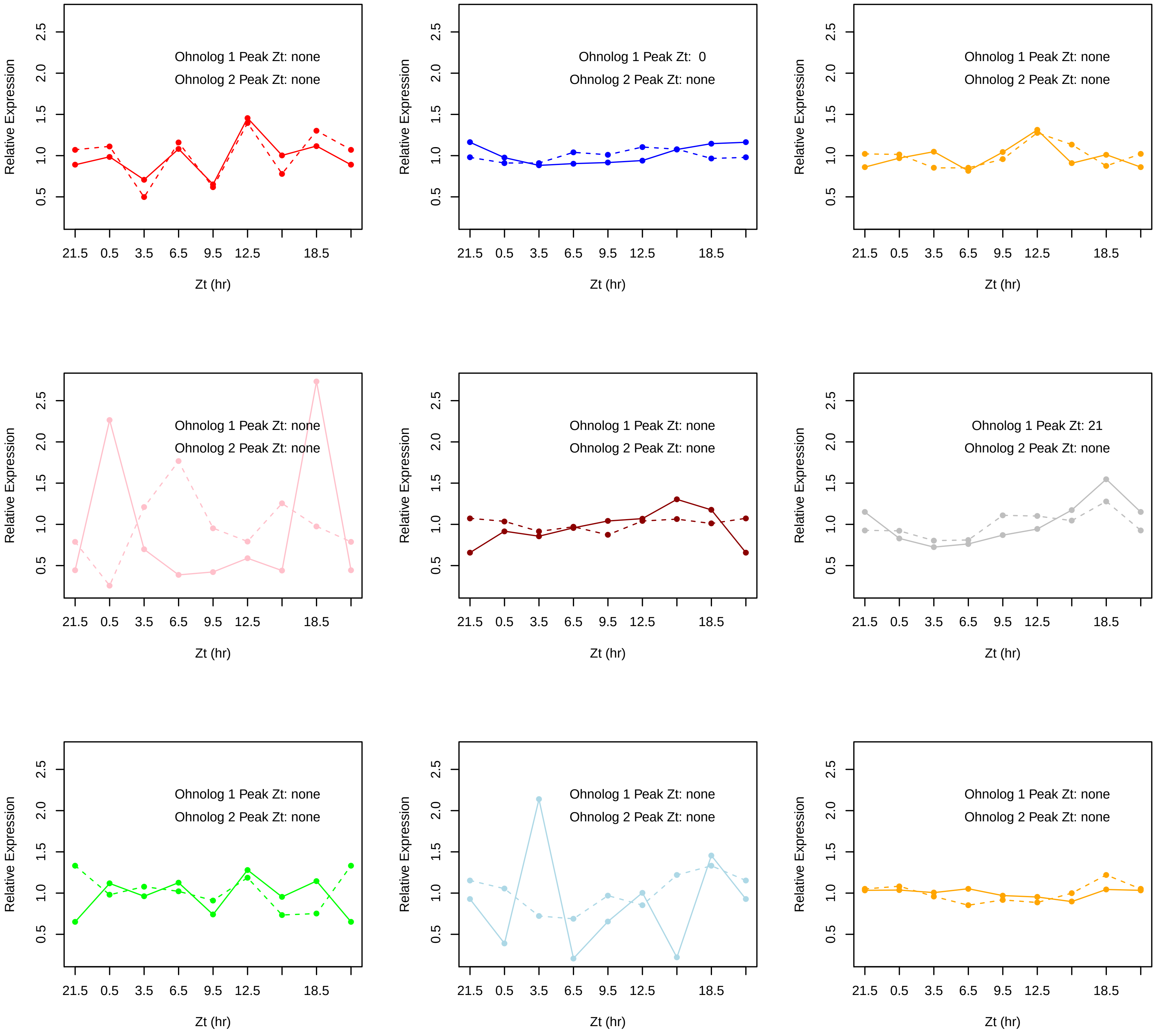

Ohnolog 1: ENSXMAG00000025011 slc48a1a
Ohnolog 2: ENSXMAG00000025467 slc48a1b

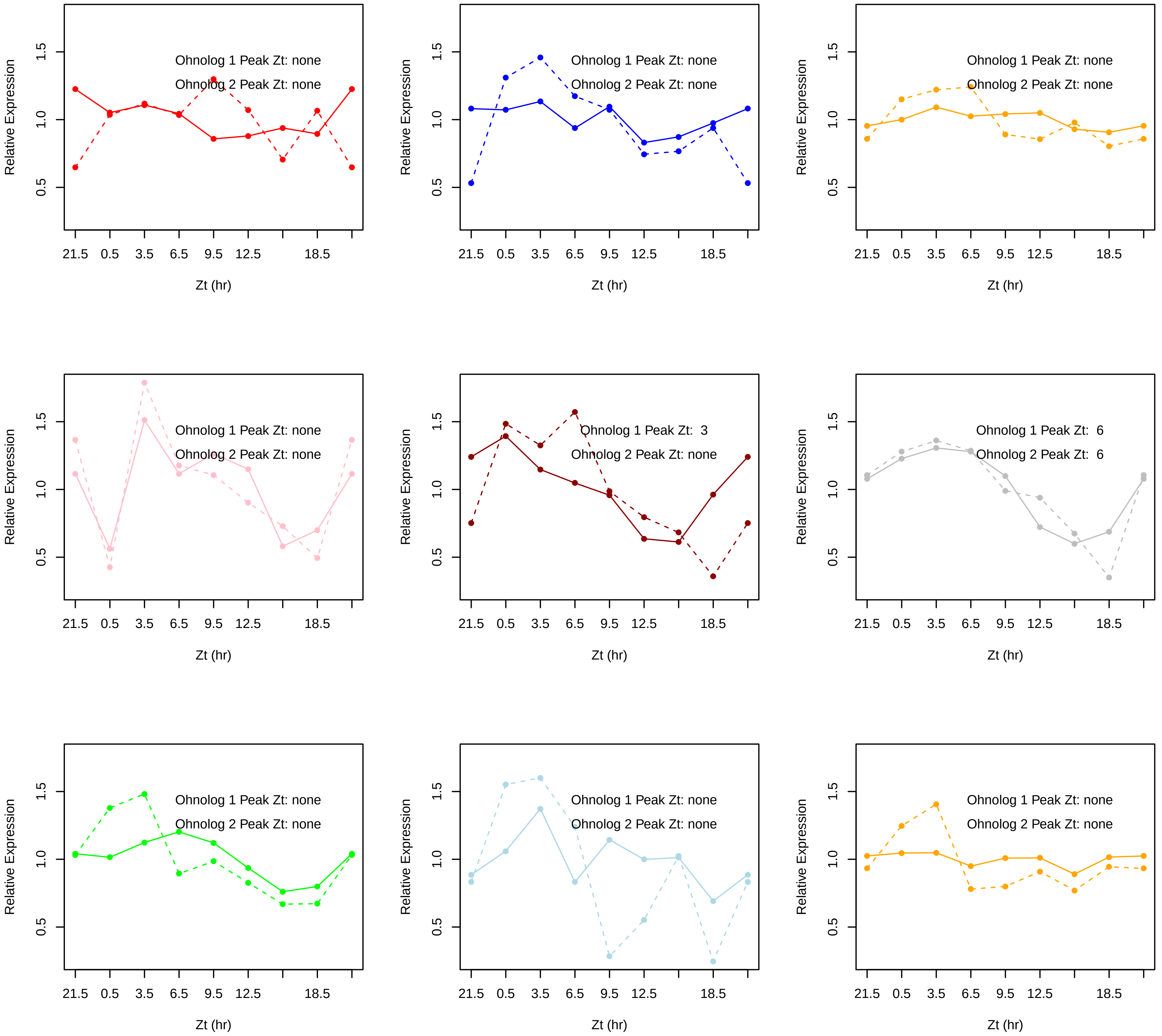

Ohnolog 1: ENSXMAG00000004547
Ohnolog 2: ENSXMAG00000028214 pcdh17

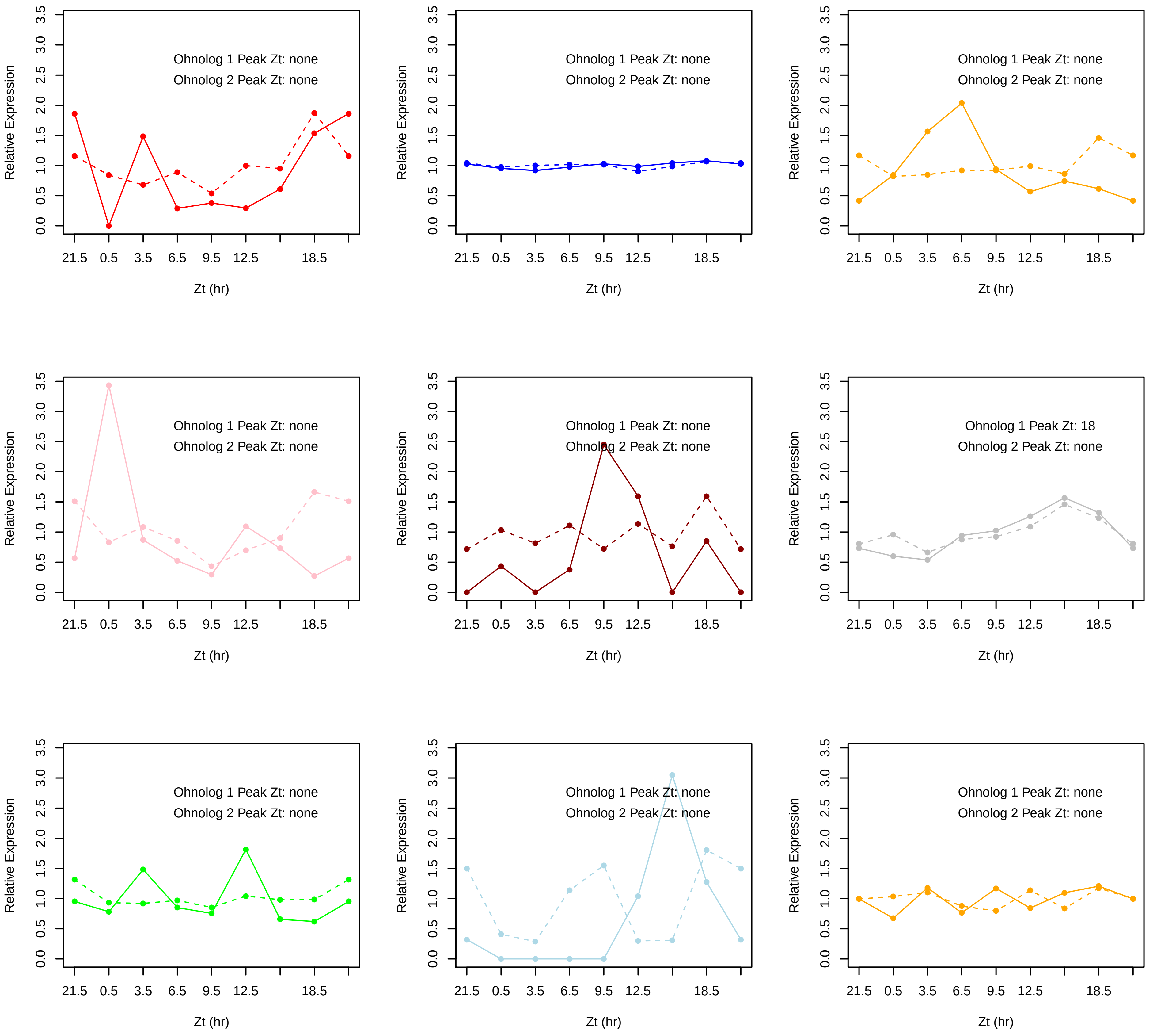

Ohnolog 1: ENSXMAG00000015893 amotl2b
Ohnolog 2: ENSXMAG00000016605 amotl2a

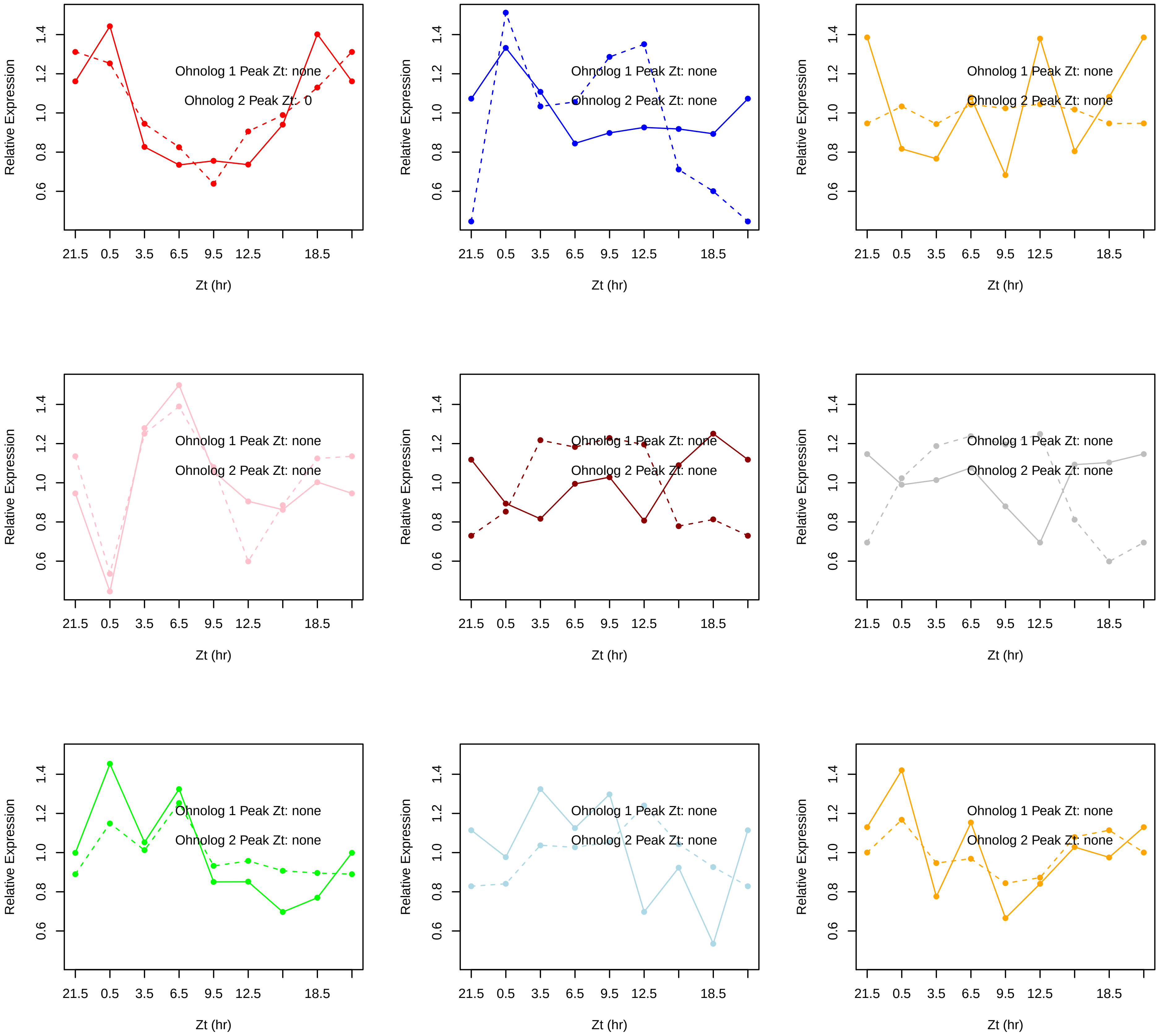

Ohnolog 1: ENSXMAG00000001285 rasgrf2a
Ohnolog 2: ENSXMAG00000016680 rasgrf2b

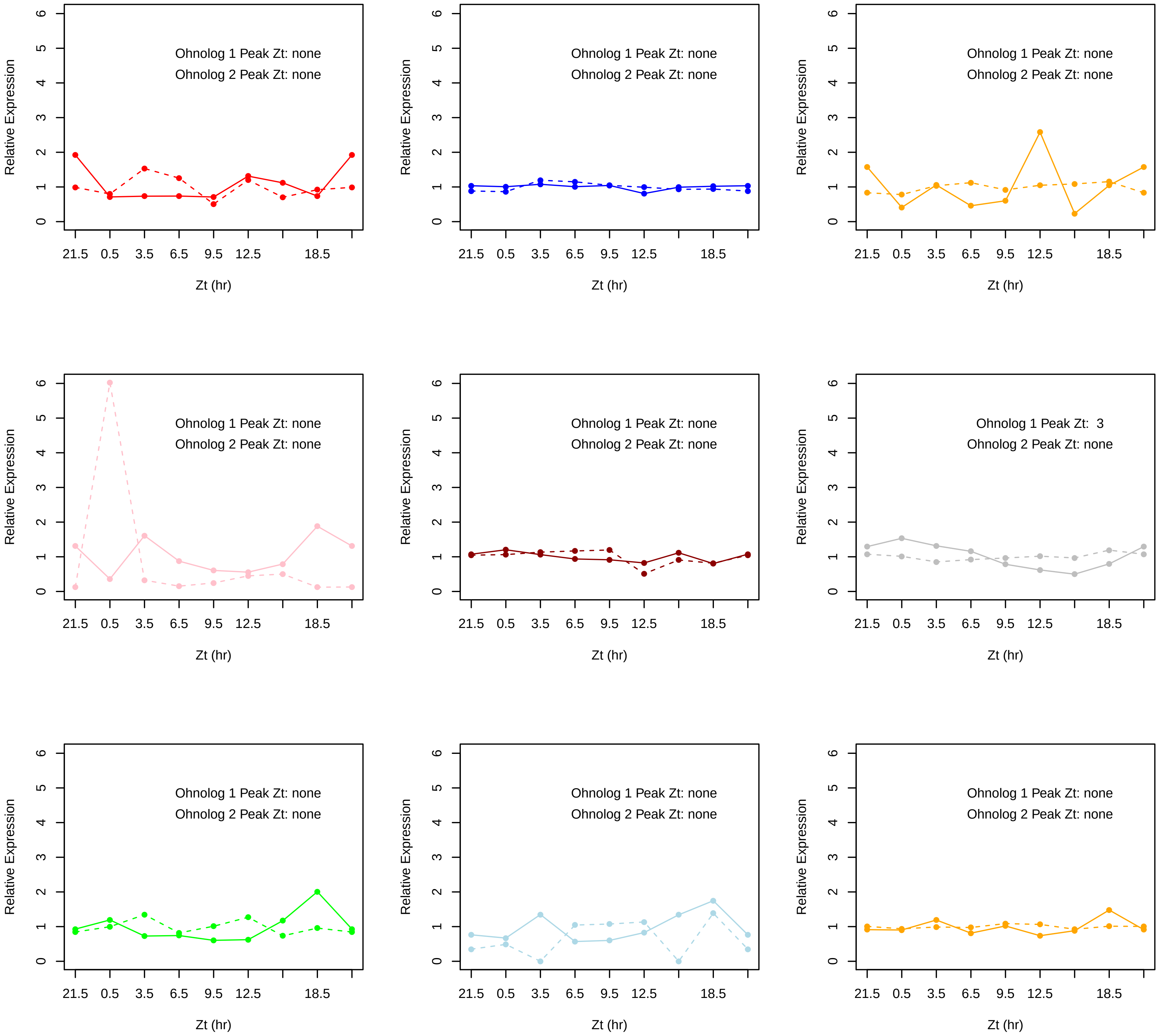

Ohnolog 1: ENSXMAG00000011605
Ohnolog 2: ENSXMAG00000016485 si:ch73−194h10.2

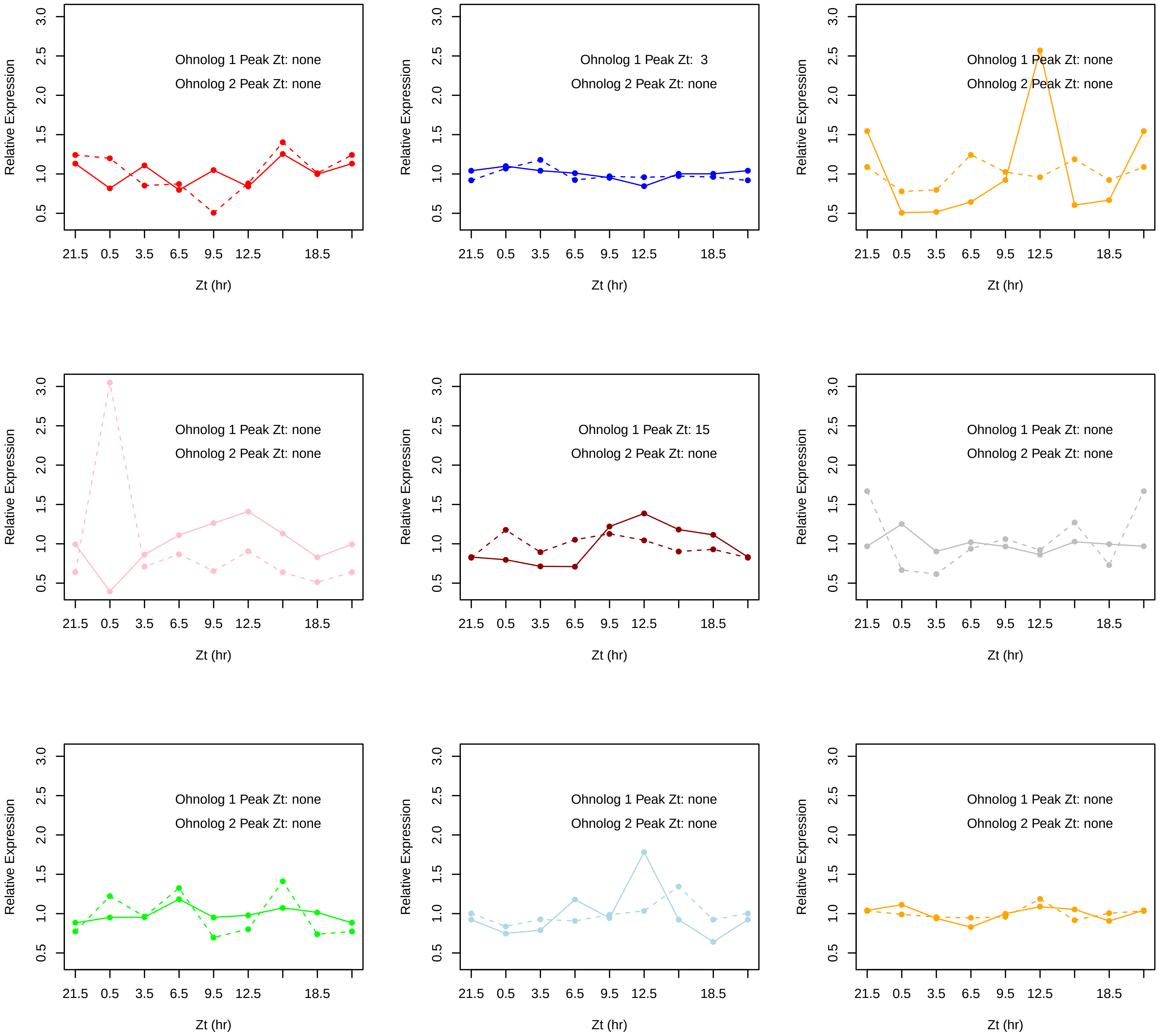

Ohnolog 1: ENSXMAG00000003095 dnajb12a
Ohnolog 2: ENSXMAG00000014960 dnajb12b

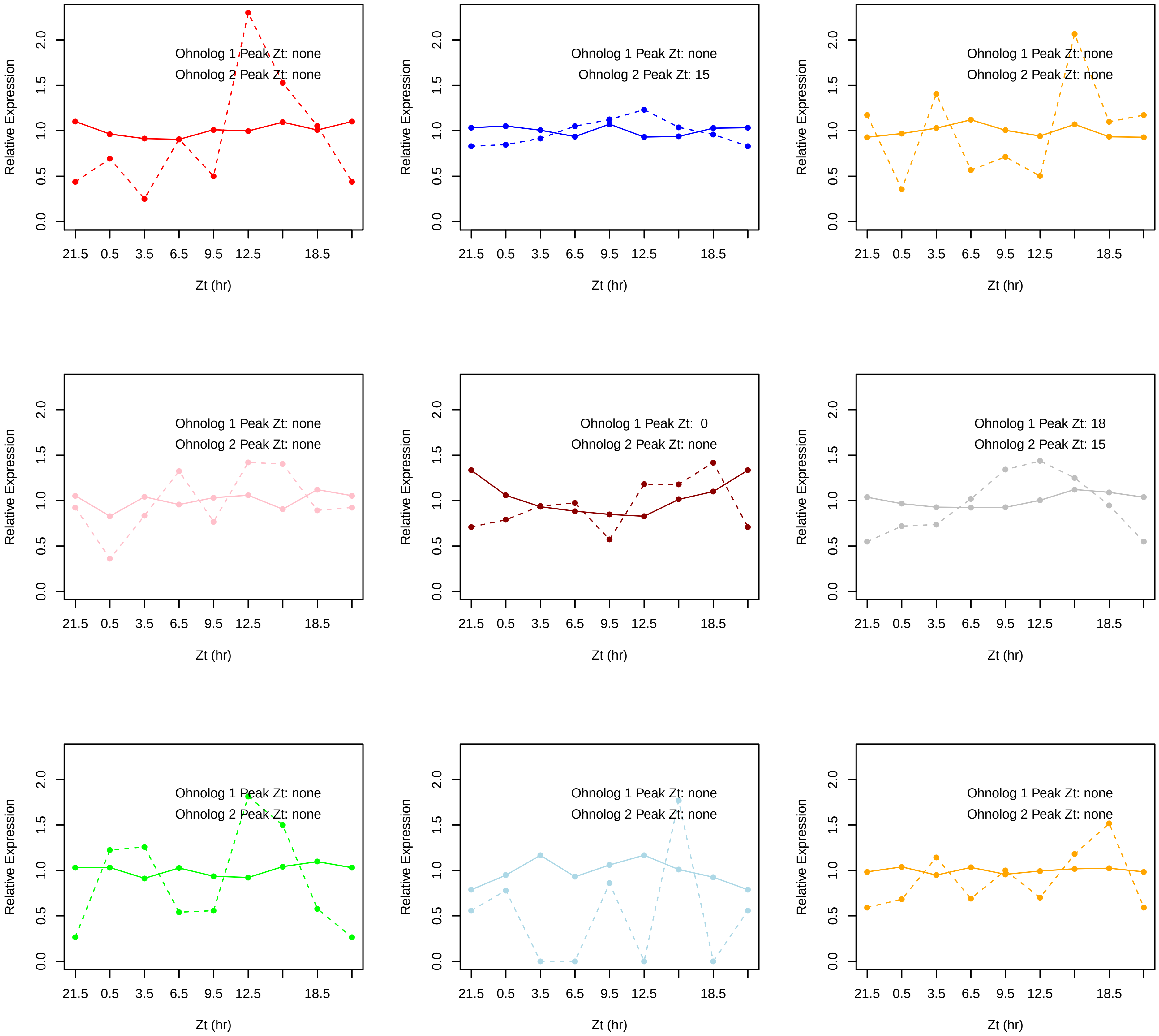

Ohnolog 1: ENSXMAG00000007098 usf2
Ohnolog 2: ENSXMAG00000025613

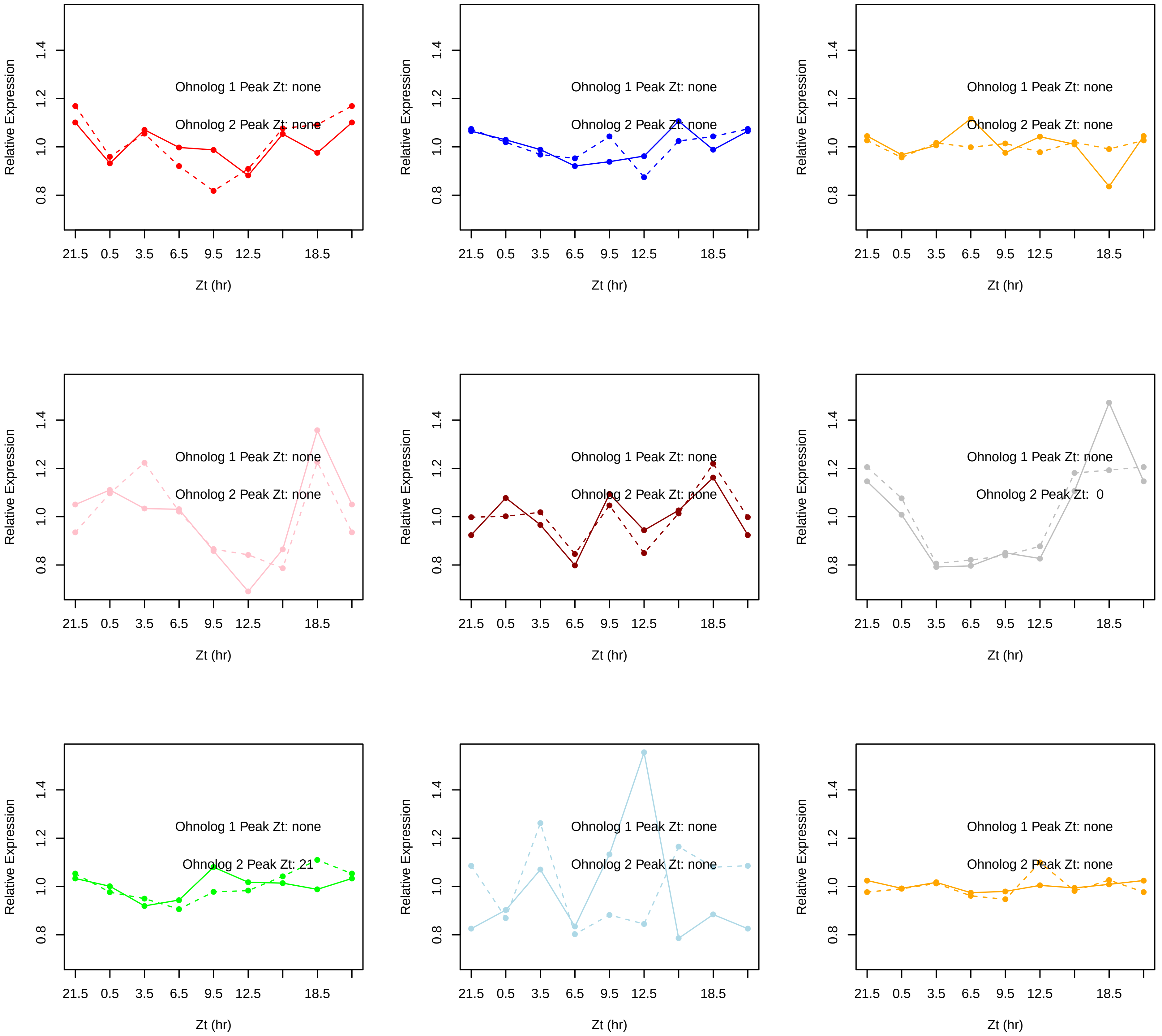

Ohnolog 1: ENSXMAG00000000134
Ohnolog 2: ENSXMAG00000001445 arhgap17b

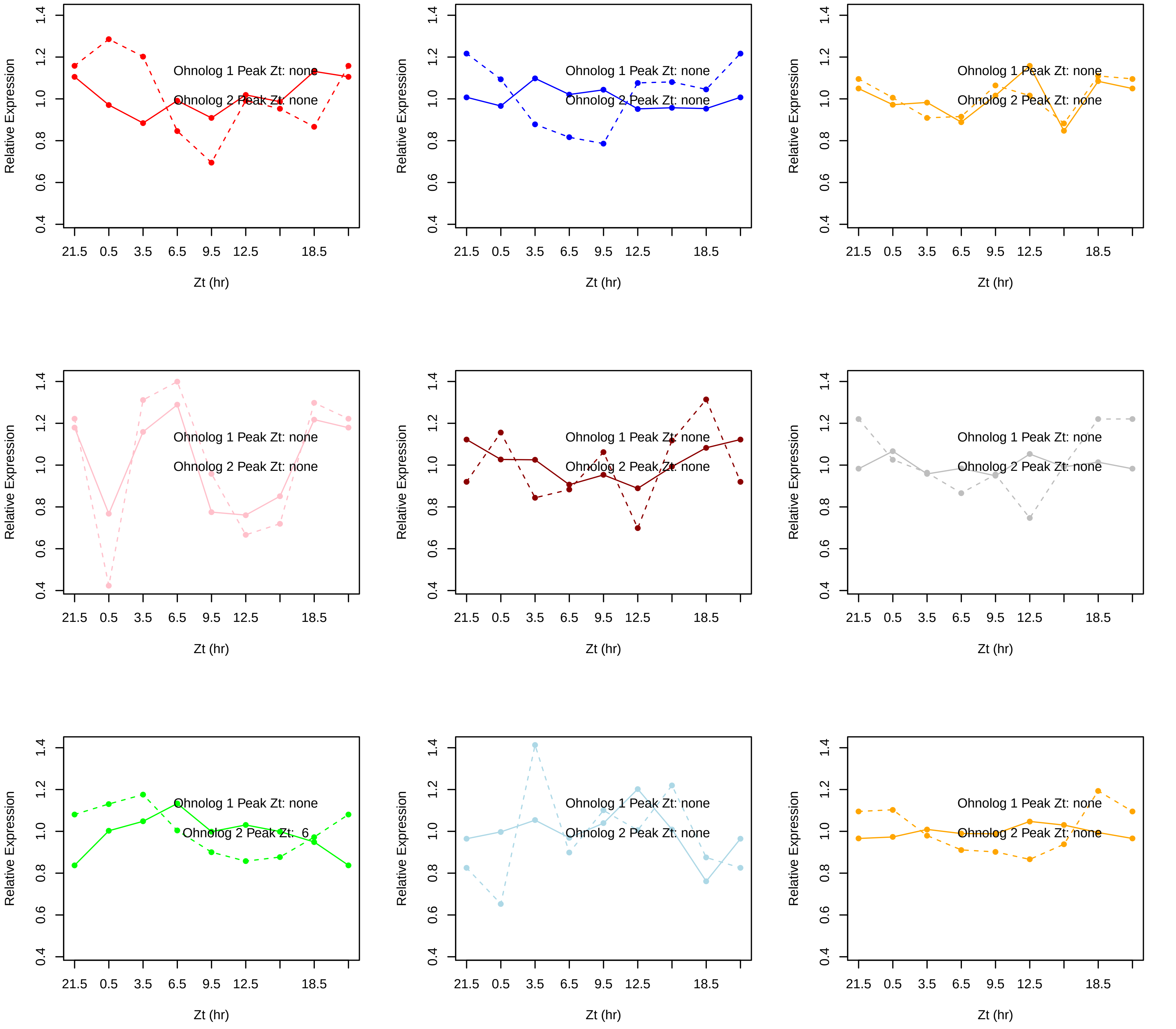

Ohnolog 1: ENSXMAG00000007034 COL4A3BP (1 of many)
Ohnolog 2: ENSXMAG00000018963 COL4A3BP (1 of many)

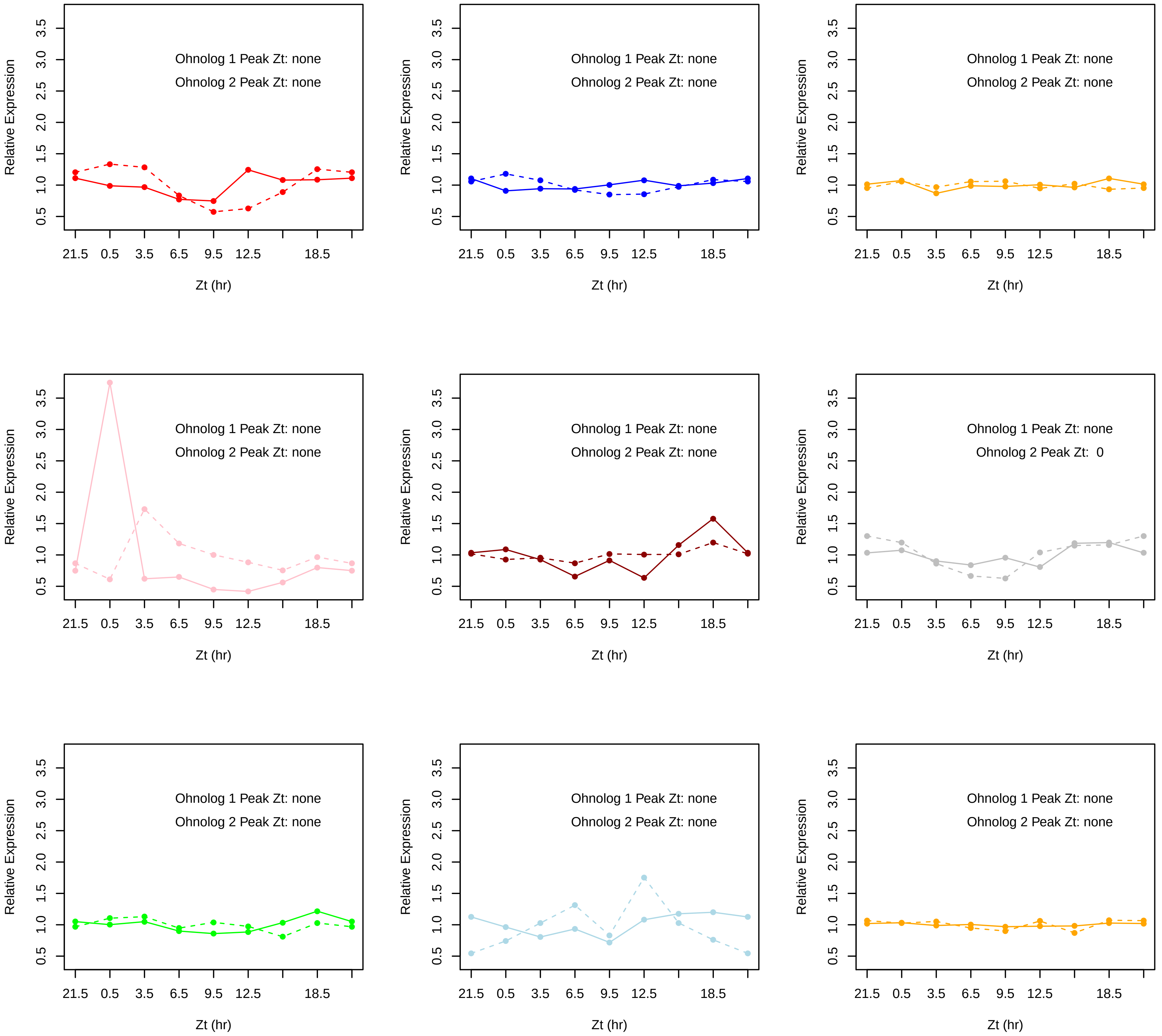

Ohnolog 1: ENSXMAG00000003022 cdhr1a
Ohnolog 2: ENSXMAG00000014849 CDHR1 (1 of many)

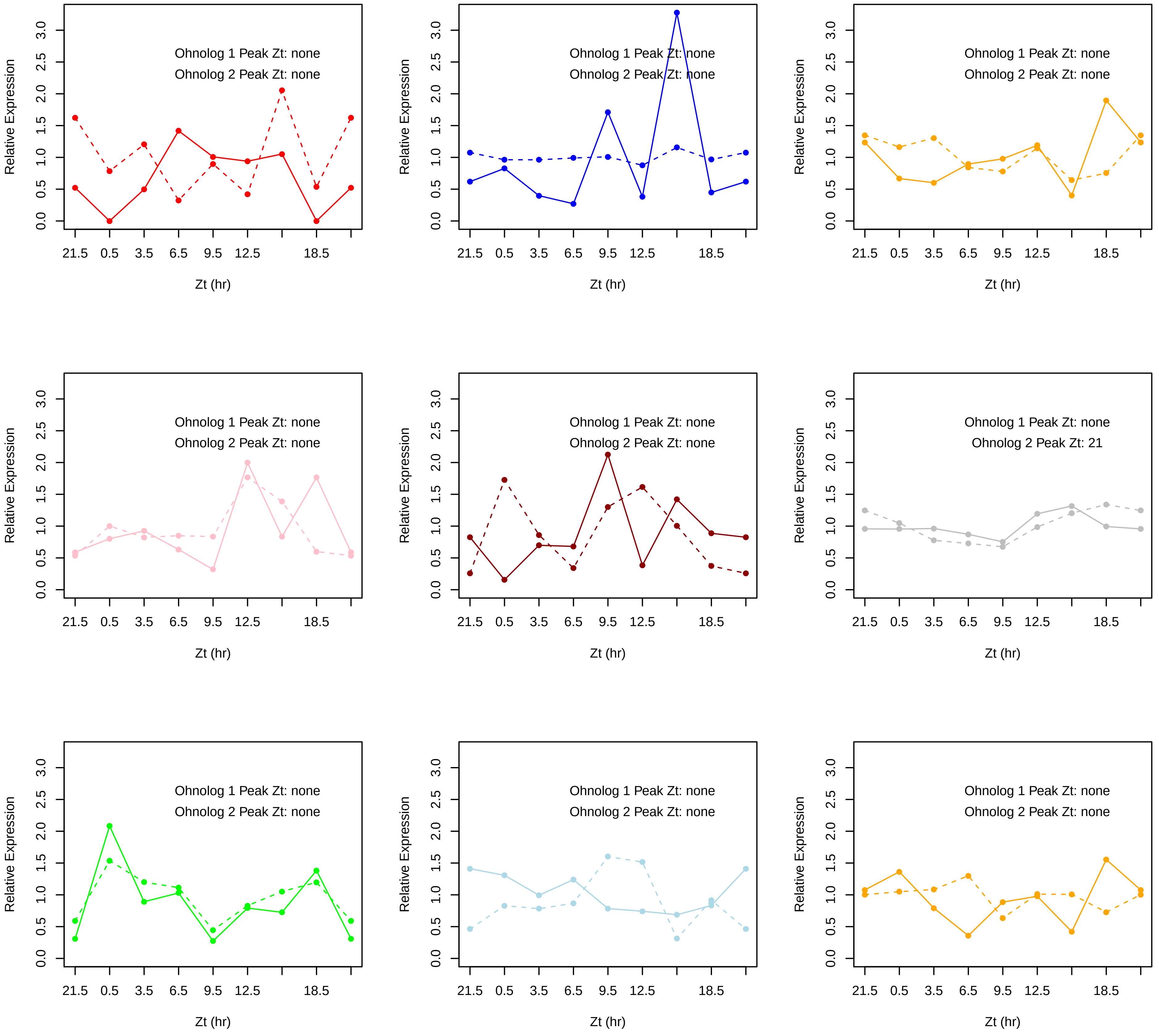

Ohnolog 1: ENSXMAG00000016777 map3k12
Ohnolog 2: ENSXMAG00000017771 MAP3K12 (1 of many)

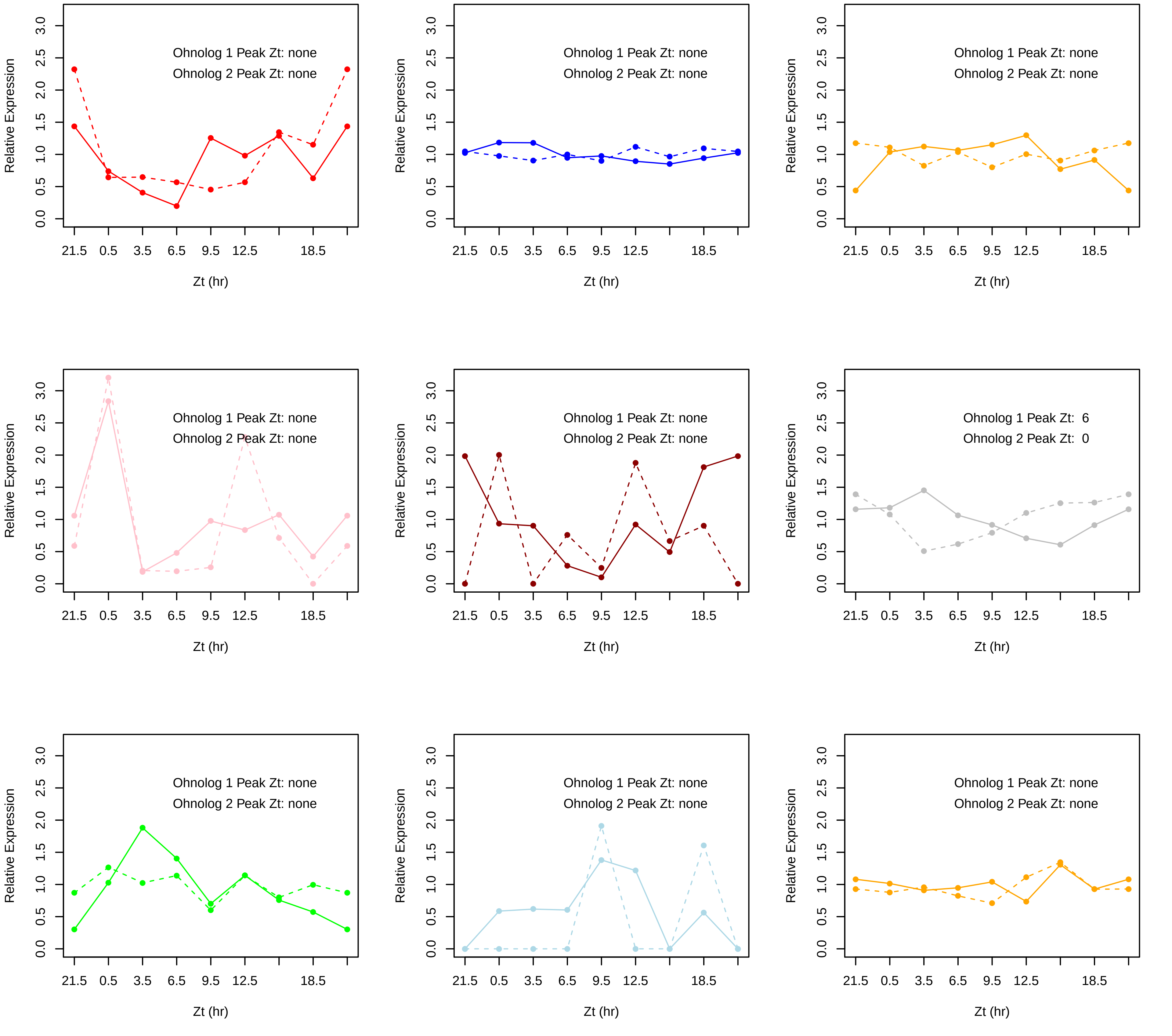

Ohnolog 1: ENSXMAG00000006118
Ohnolog 2: ENSXMAG00000015332 FMNL2

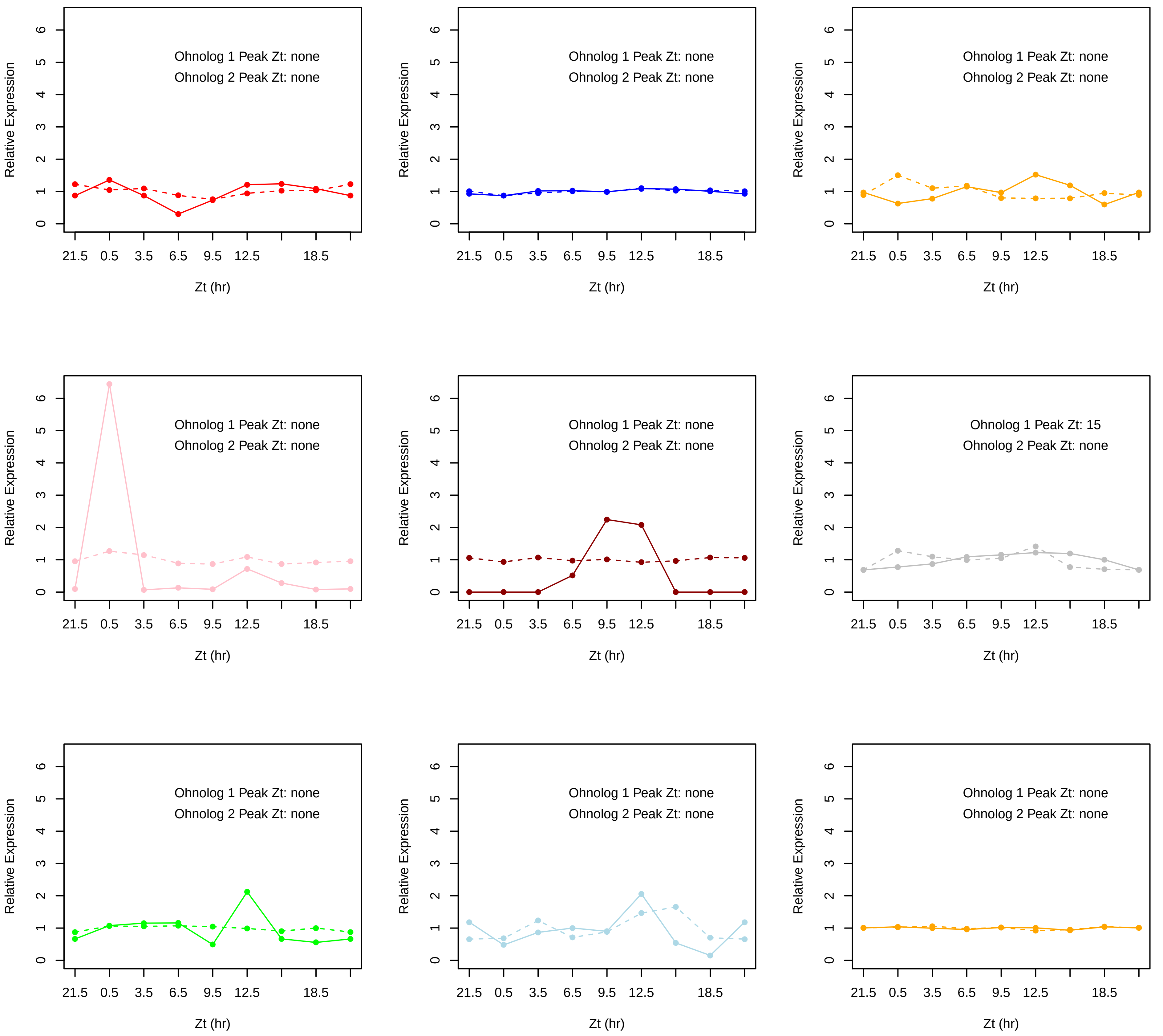

Ohnolog 1: ENSXMAG00000021752 pcbp2
Ohnolog 2: ENSXMAG00000023803 PCBP2

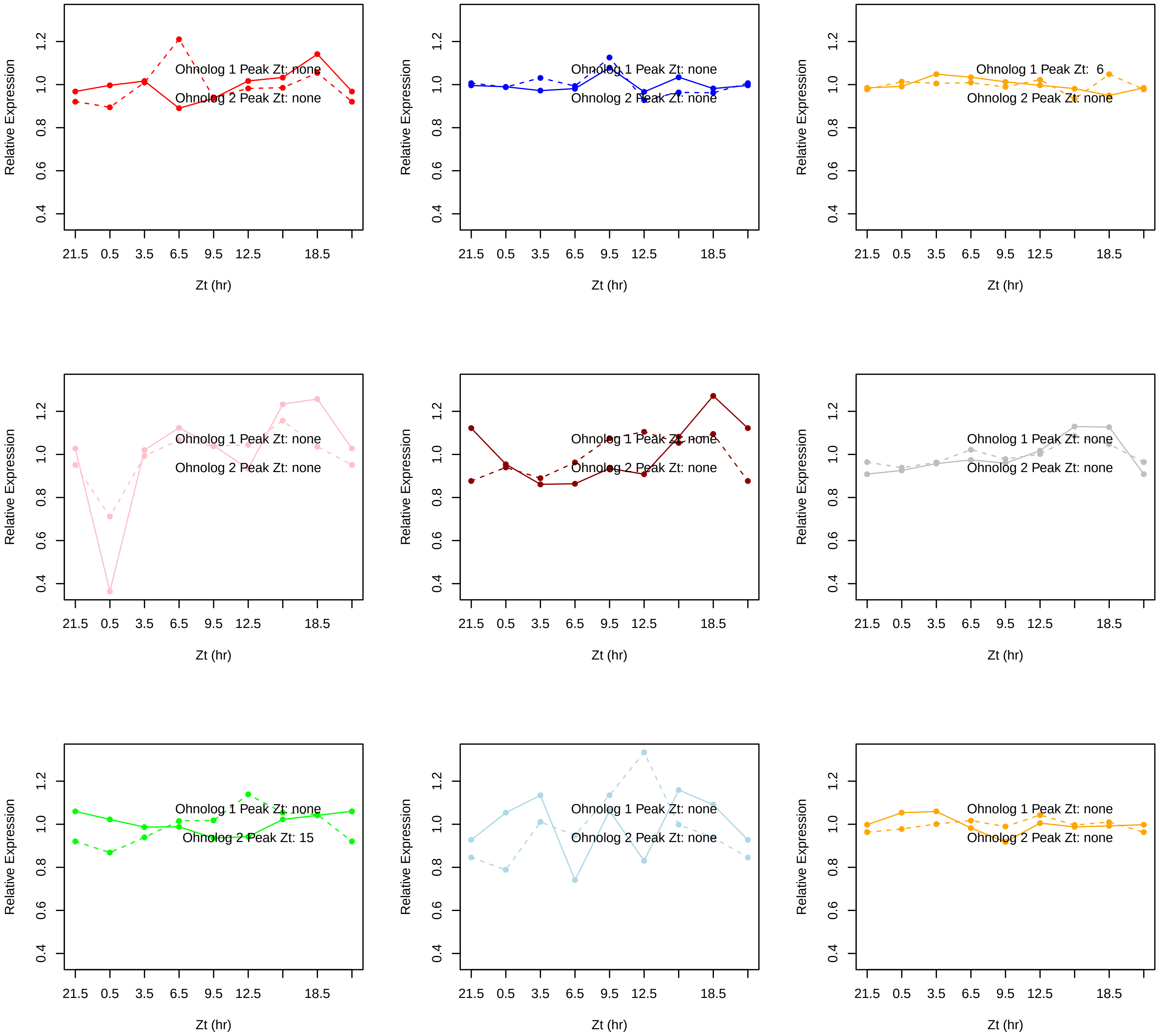

Ohnolog 1: ENSXMAG00000003019 lrit1a
Ohnolog 2: ENSXMAG00000014845 lrit1b

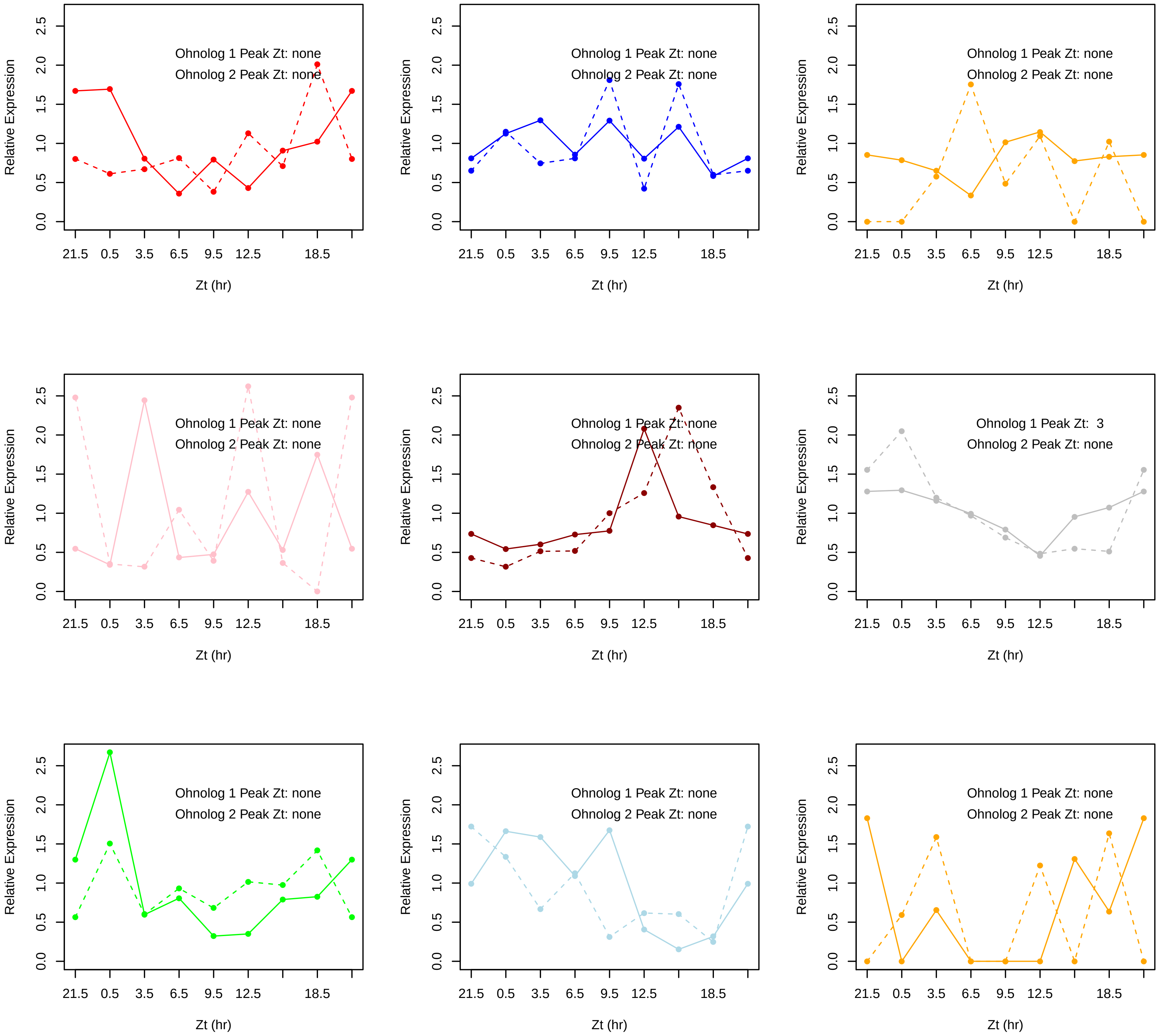

Ohnolog 1: ENSXMAG00000016619
Ohnolog 2: ENSXMAG00000018907 crhbp

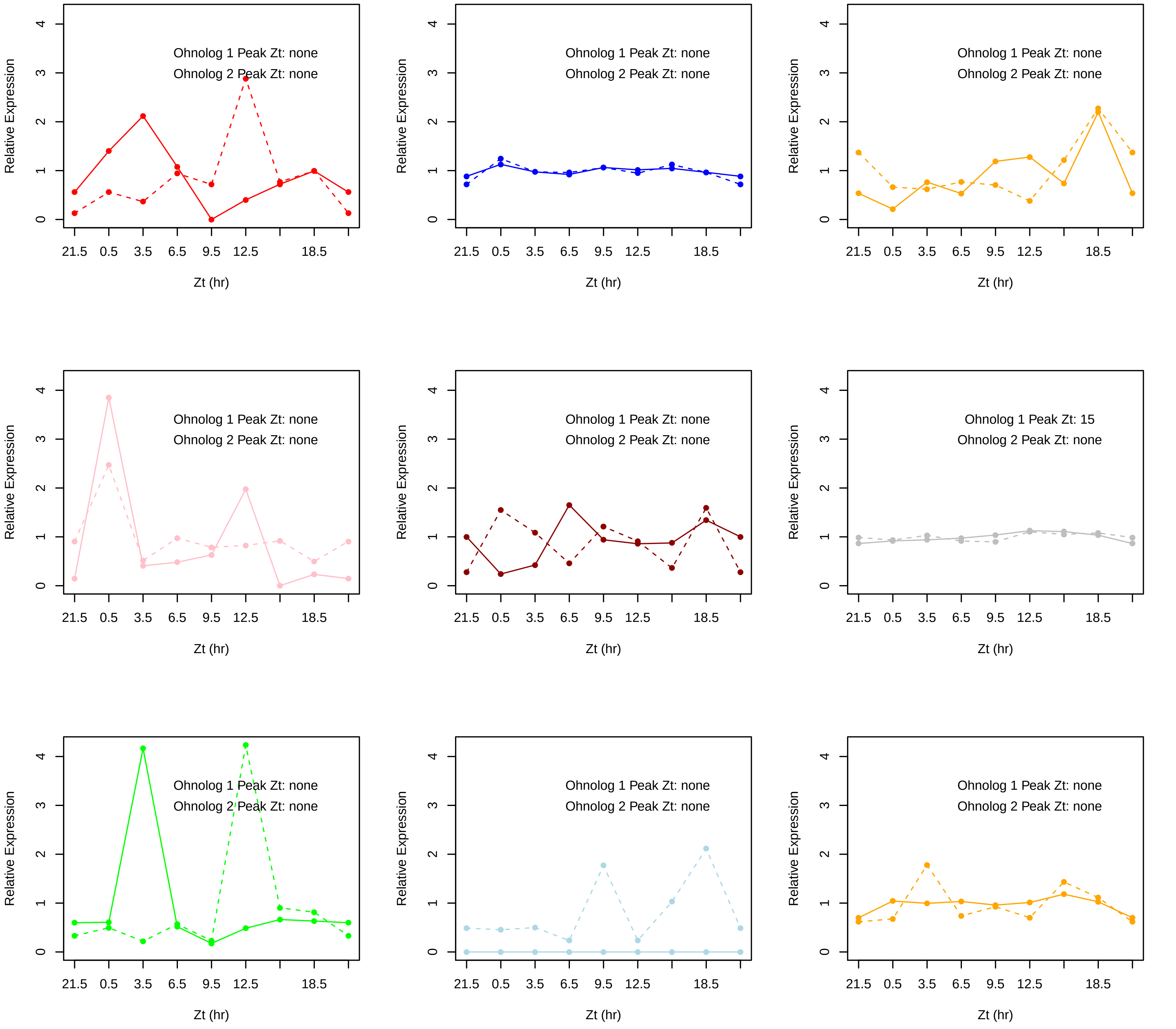

Ohnolog 1: ENSXMAG00000001997 si:ch211−251b21.1
Ohnolog 2: ENSXMAG00000021403 si:dkey−183j2.10

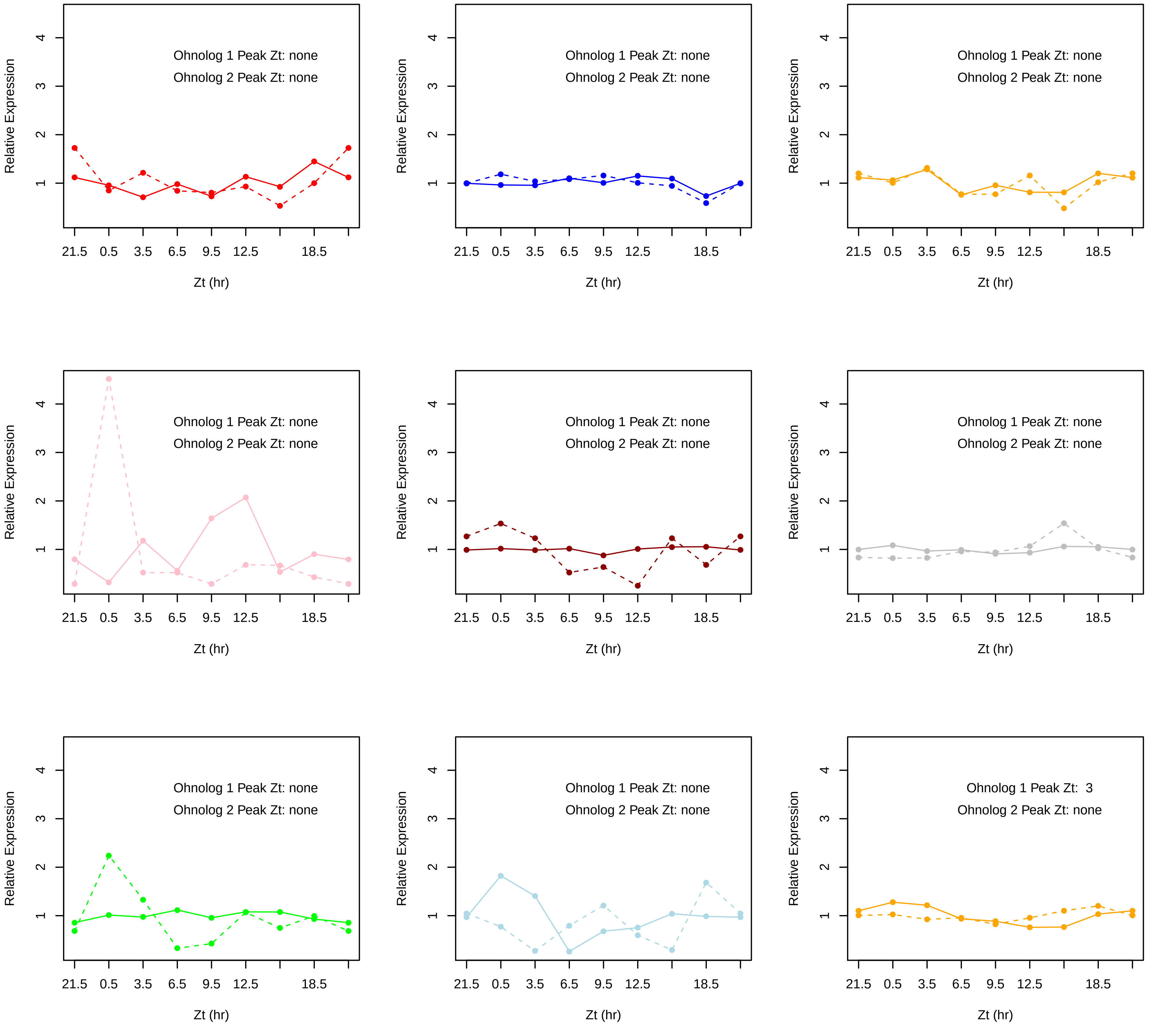

Ohnolog 1: ENSXMAG00000002955 myofl
Ohnolog 2: ENSXMAG00000016283 MYOF

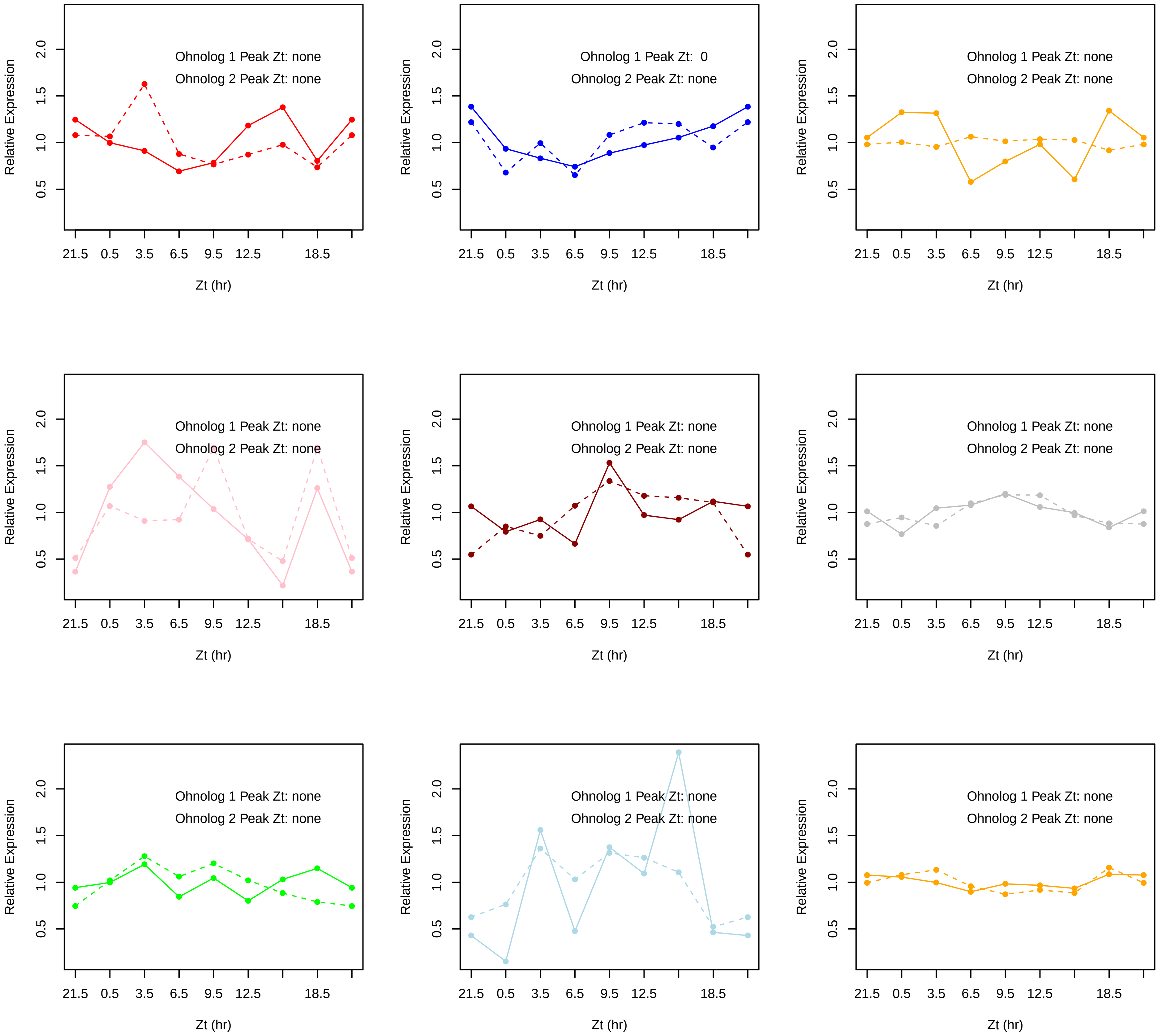

Ohnolog 1: ENSXMAG00000008707 caskin1
Ohnolog 2: ENSXMAG00000027891

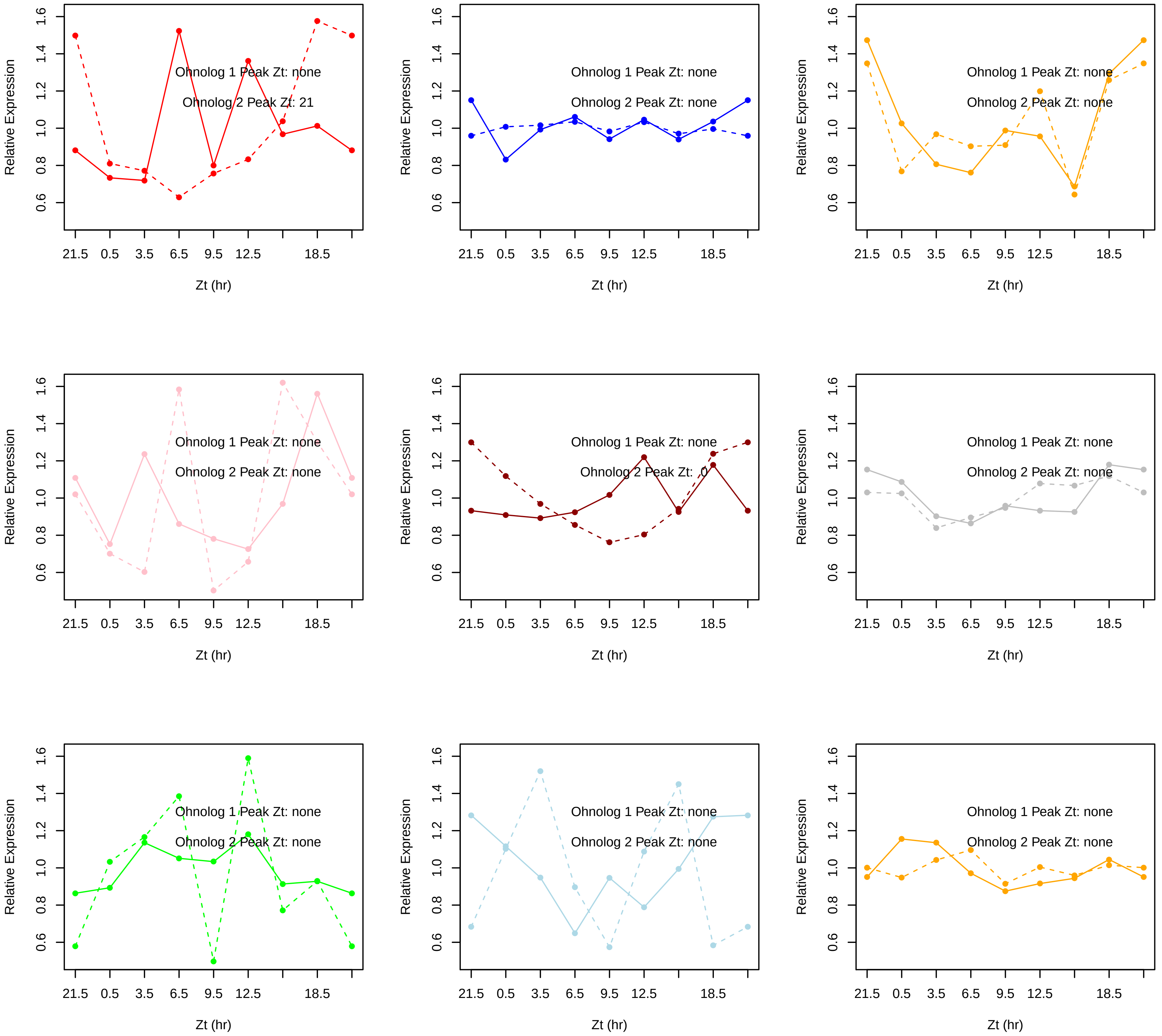

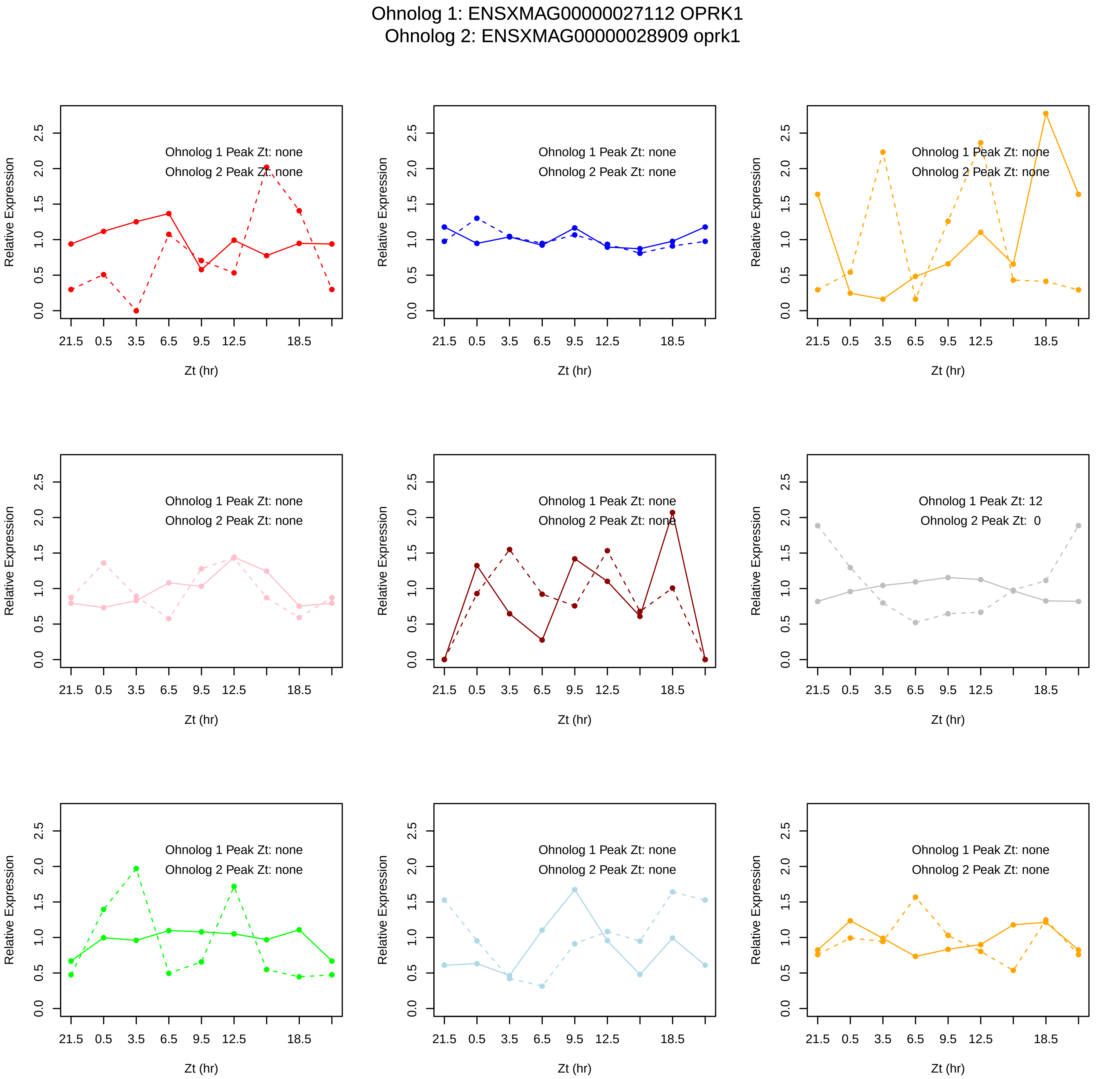
Ohnolog 1: ENSXMAG00000027112 OPRK1
Ohnolog 2: ENSXMAG00000028909 oprk1
Relative Expression
Zt (hr)
21.5
0.5
3.5
6.5
9.5
12.5
18.5
Ohnolog 1 Peak Zt: none
Ohnolog 2 Peak Zt: none
Ohnolog 1 Peak Zt: 12
Ohnolog 2 Peak Zt: 0

Ohnolog 1: ENSXMAG00000017874 KIF5A
Ohnolog 2: ENSXMAG00000018983 kif5aa

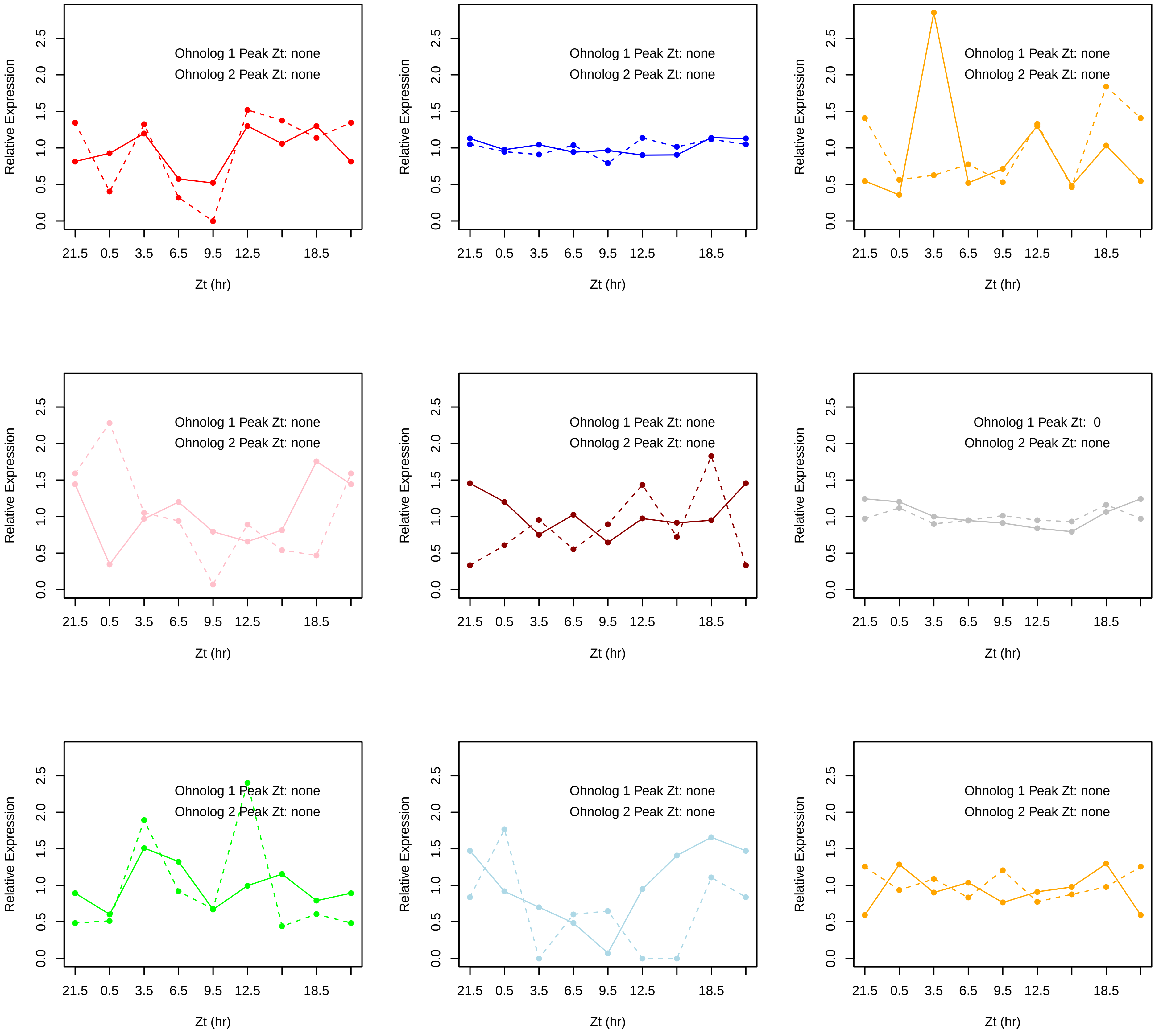

Ohnolog 1: ENSXMAG00000024922 SCAMP1
Ohnolog 2: ENSXMAG00000028164 scamp1

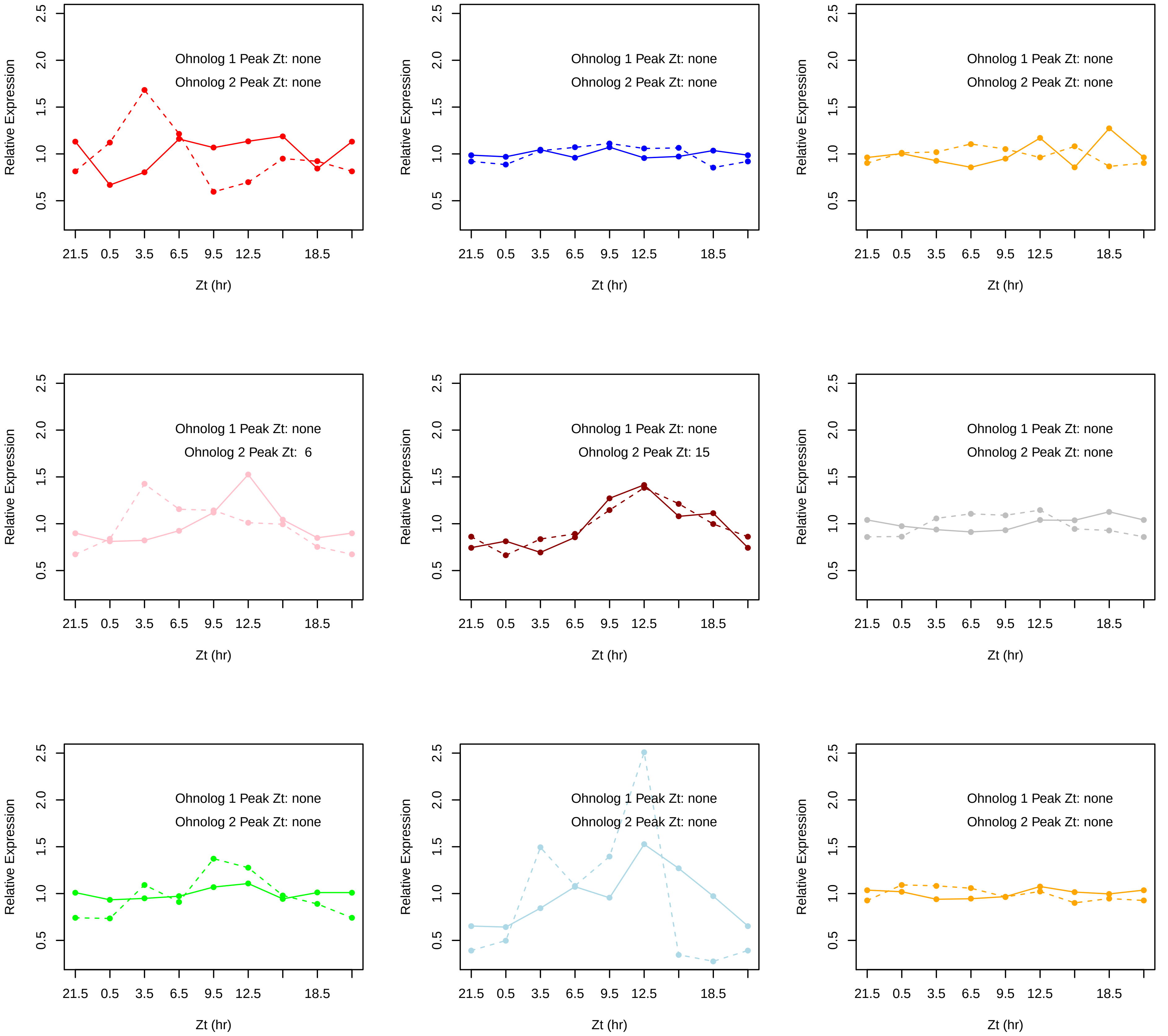

Ohnolog 1: ENSXMAG00000002830
Ohnolog 2: ENSXMAG00000008364 odf2a

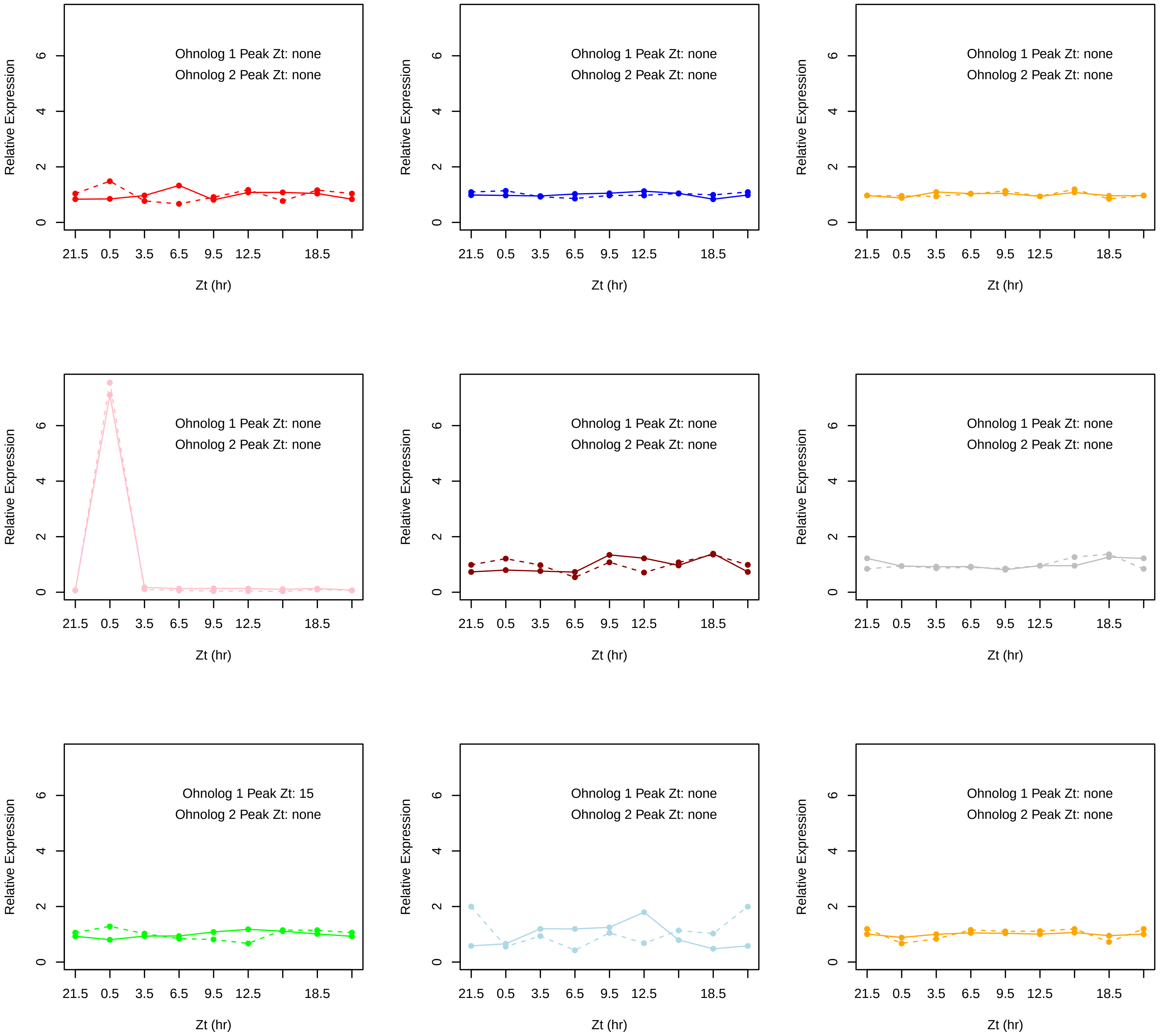

Ohnolog 1: ENSXMAG00000005071 thbs4b
Ohnolog 2: ENSXMAG00000007257 thbs4a

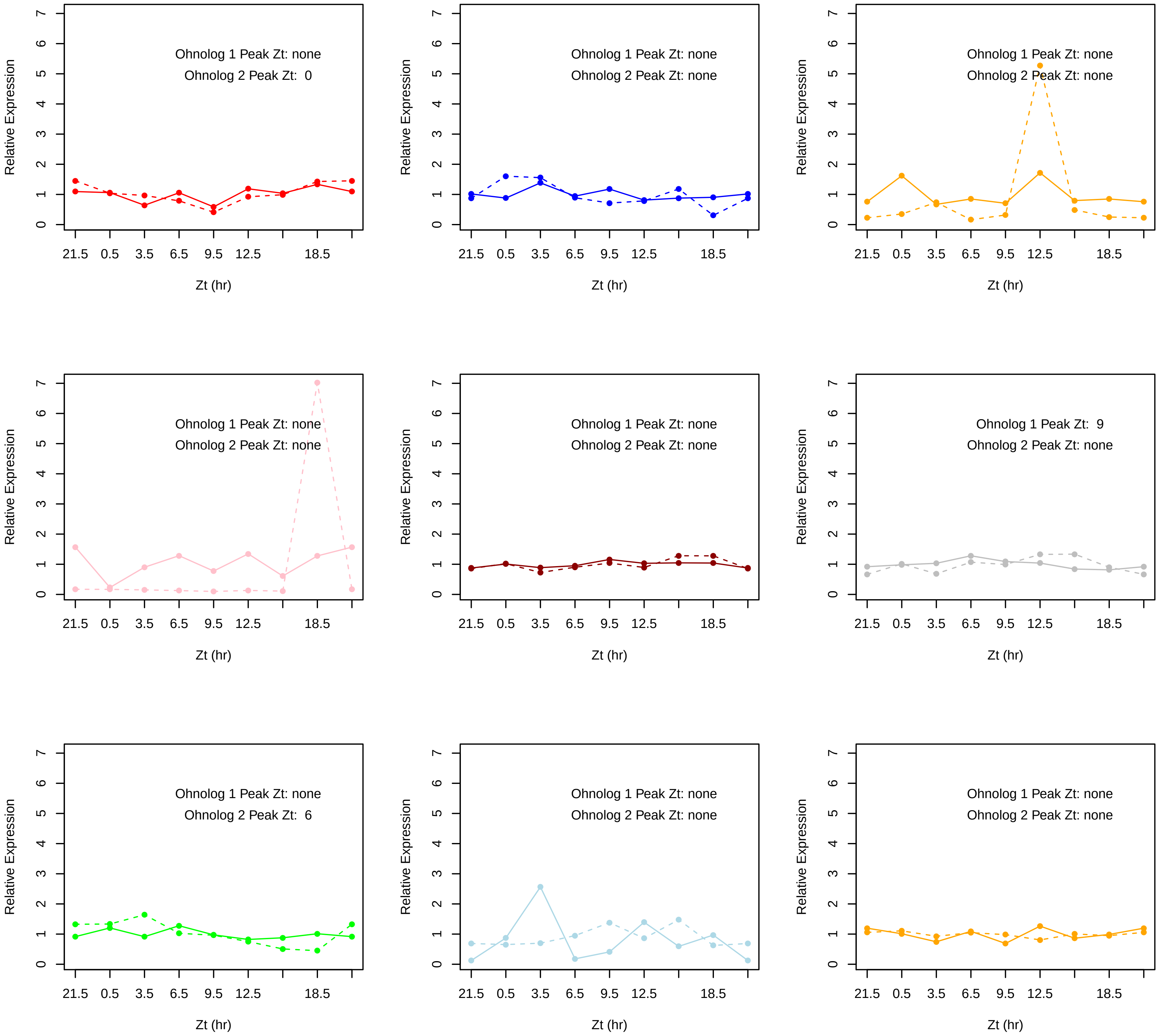

Ohnolog 1: ENSXMAG00000002879 ZDHHC12 (1 of many)
Ohnolog 2: ENSXMAG00000016743 zdhhc12b

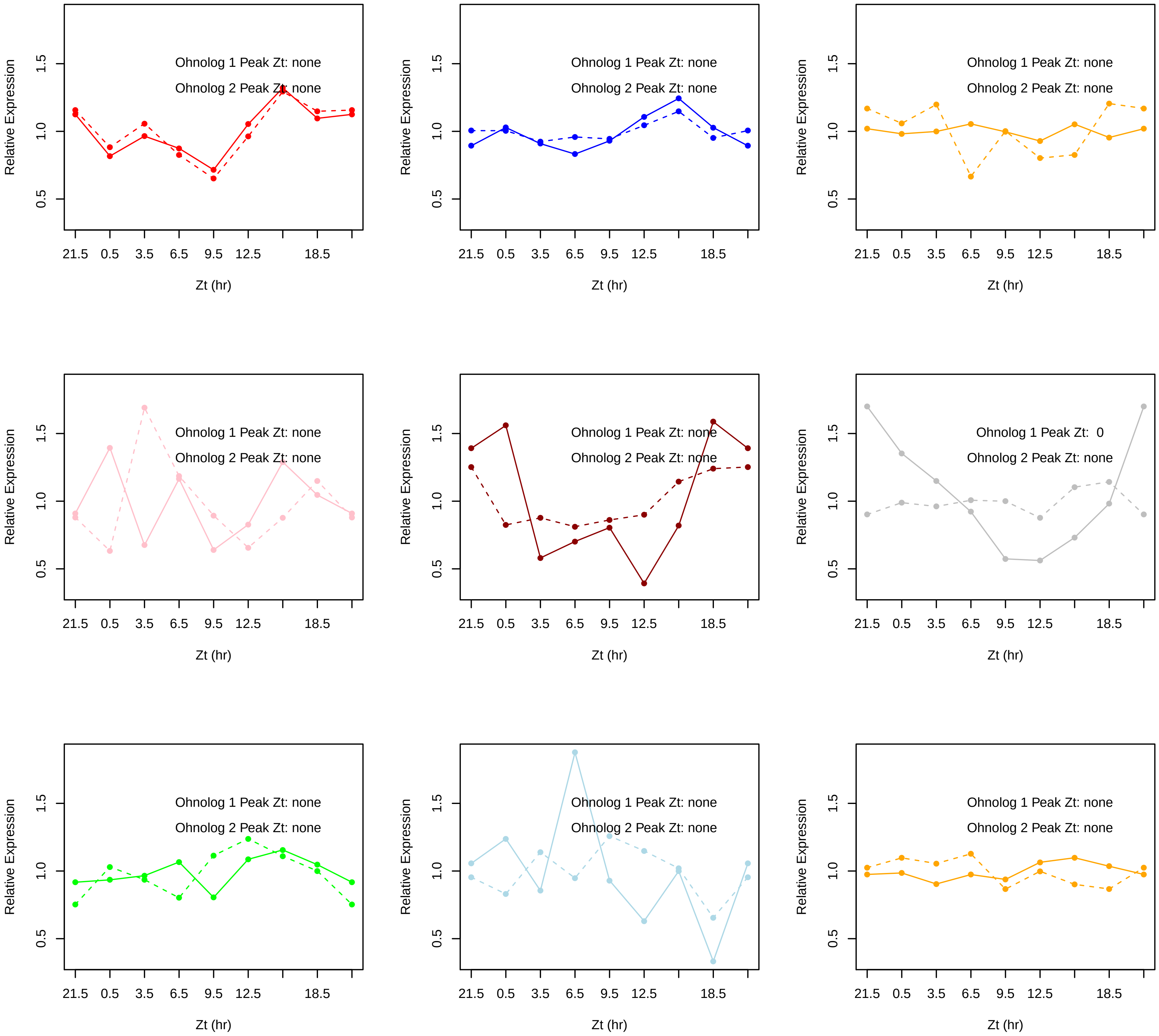

Ohnolog 1: ENSXMAG00000007125 calcrla
Ohnolog 2: ENSXMAG00000007989

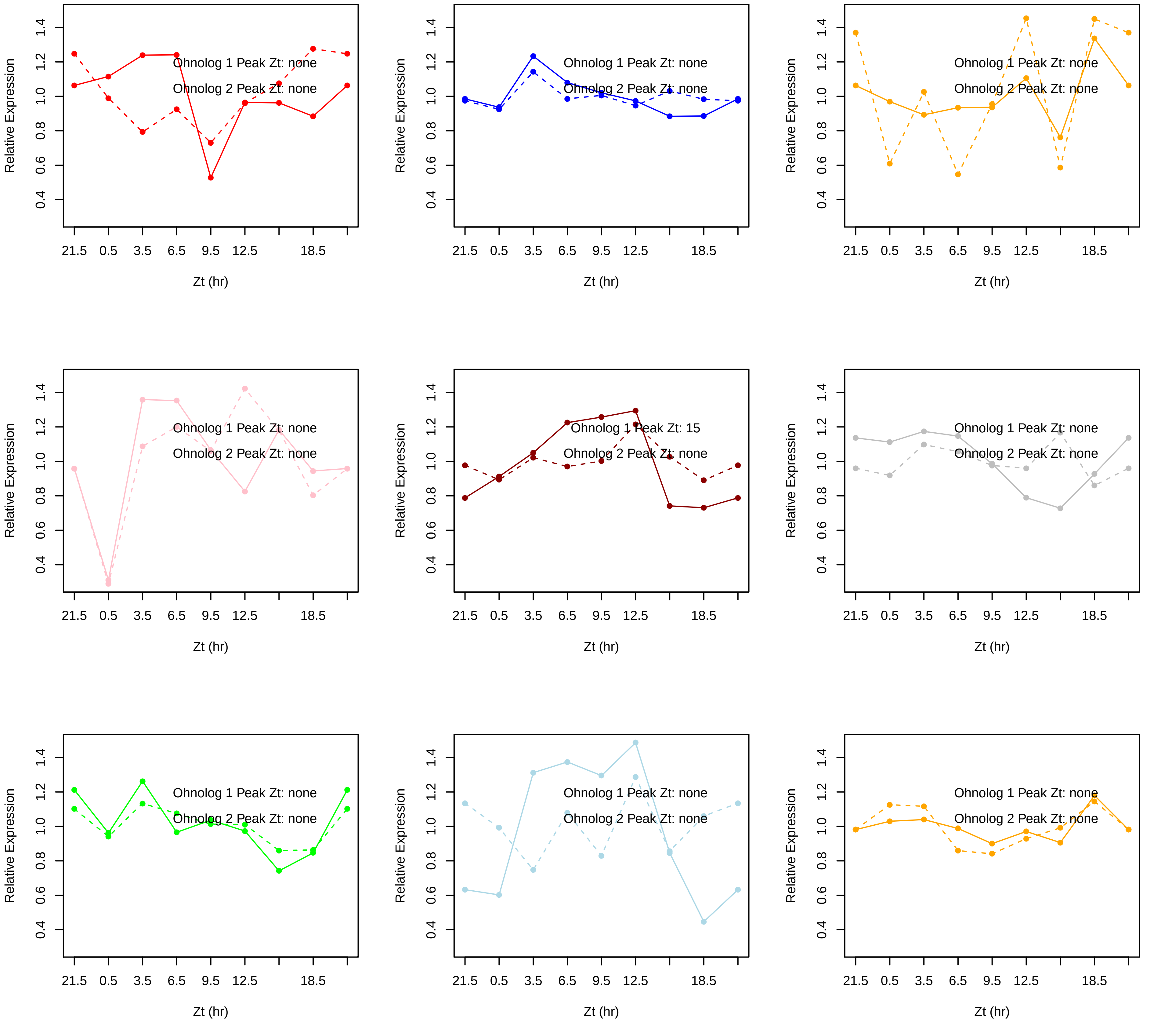

Ohnolog 1: ENSXMAG00000013868 colq
Ohnolog 2: ENSXMAG00000026168

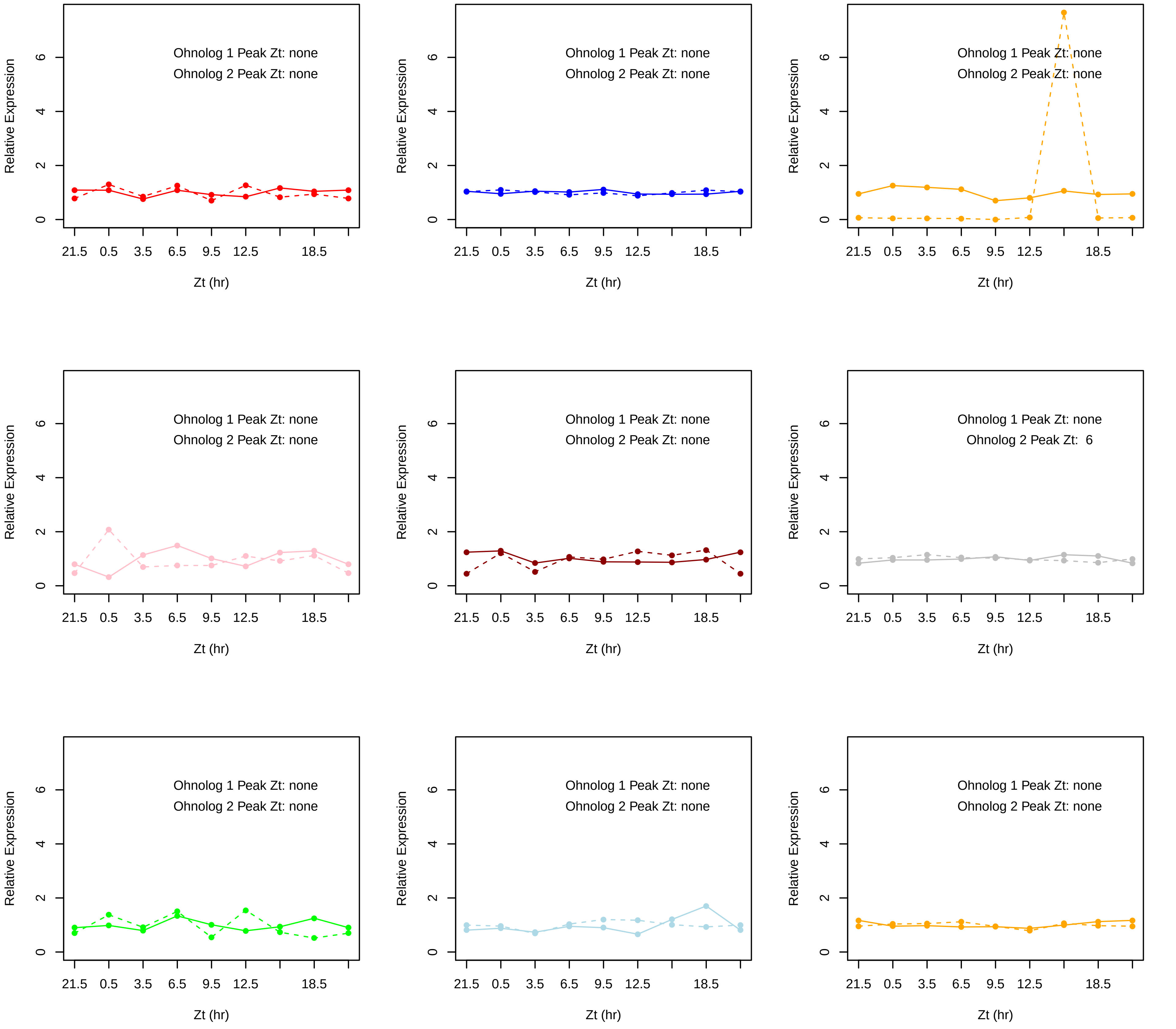

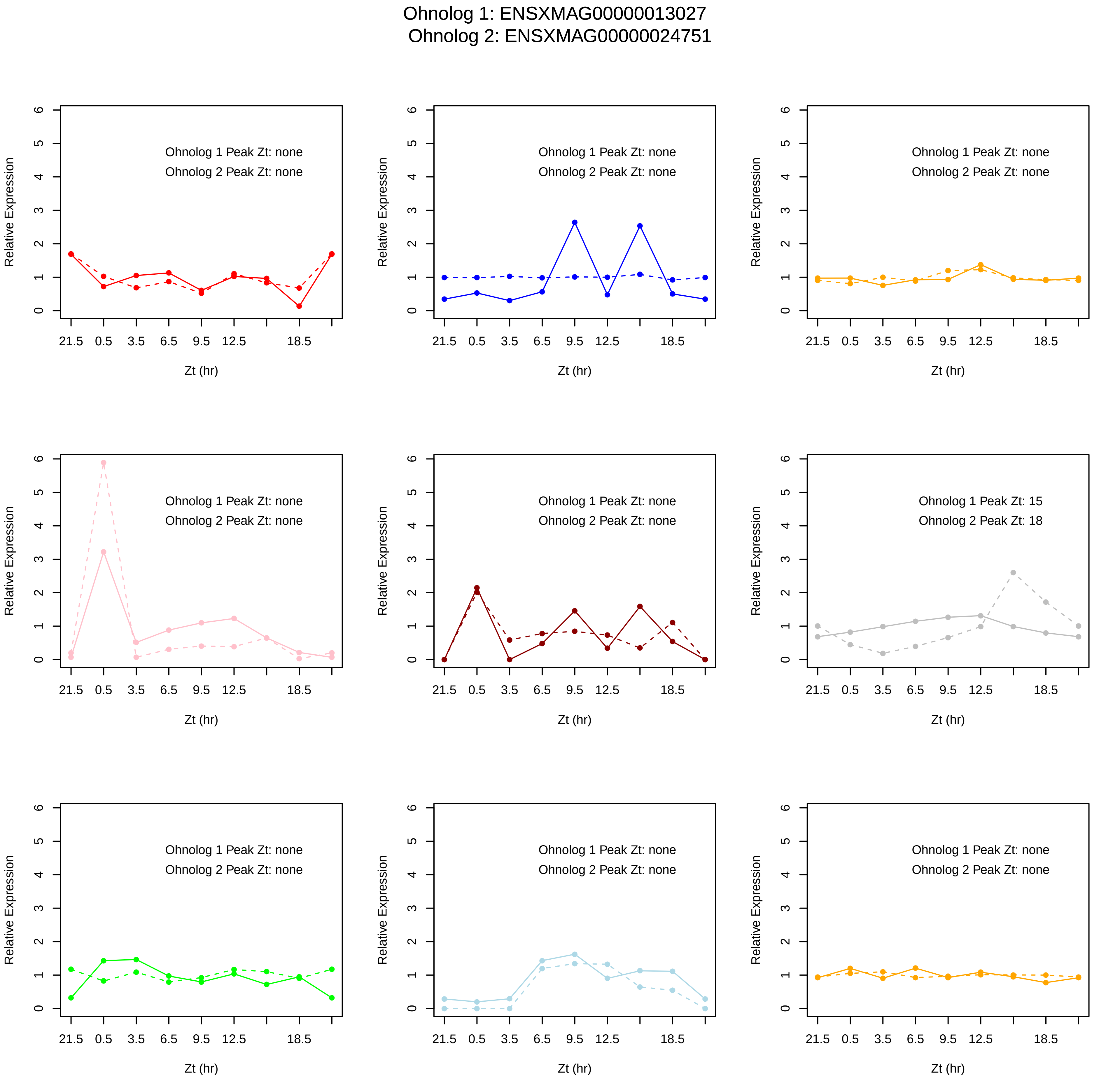

Ohnolog 1: ENSXMAG00000013027
Ohnolog 2: ENSXMAG00000024751
Ohnolog 1 Peak Zt: none
Ohnolog 2 Peak Zt: none
Ohnolog 1 Peak Zt: none
Ohnolog 2 Peak Zt: none
Ohnolog 1 Peak Zt: none
Ohnolog 2 Peak Zt: none
Ohnolog 1 Peak Zt: none
Ohnolog 2 Peak Zt: none
Ohnolog 1 Peak Zt: none
Ohnolog 2 Peak Zt: none
Ohnolog 1 Peak Zt: 15
Ohnolog 2 Peak Zt: 18
Ohnolog 1 Peak Zt: none
Ohnolog 2 Peak Zt: none
Ohnolog 1 Peak Zt: none
Ohnolog 2 Peak Zt: none
Ohnolog 1 Peak Zt: none
Ohnolog 2 Peak Zt: none
Relative Expression
Zt (hr)
21.5 0.5 3.5 6.5 9.5 12.5 18.5
0 1 2 3 4 5 6

Ohnolog 1: ENSXMAG00000011950 ucp3
Ohnolog 2: ENSXMAG00000011986

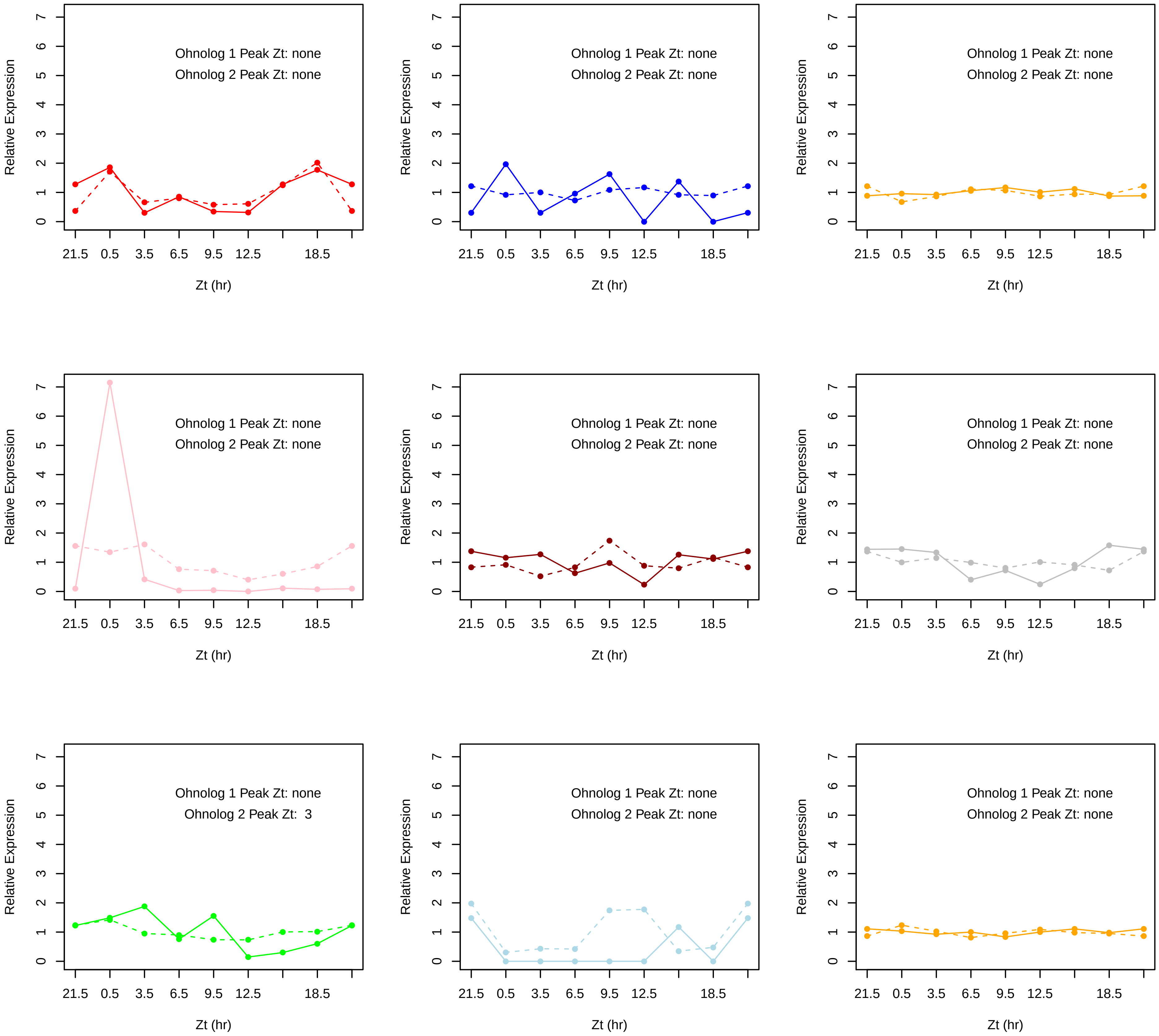

Ohnolog 1: ENSXMAG00000019295
Ohnolog 2: ENSXMAG00000027507

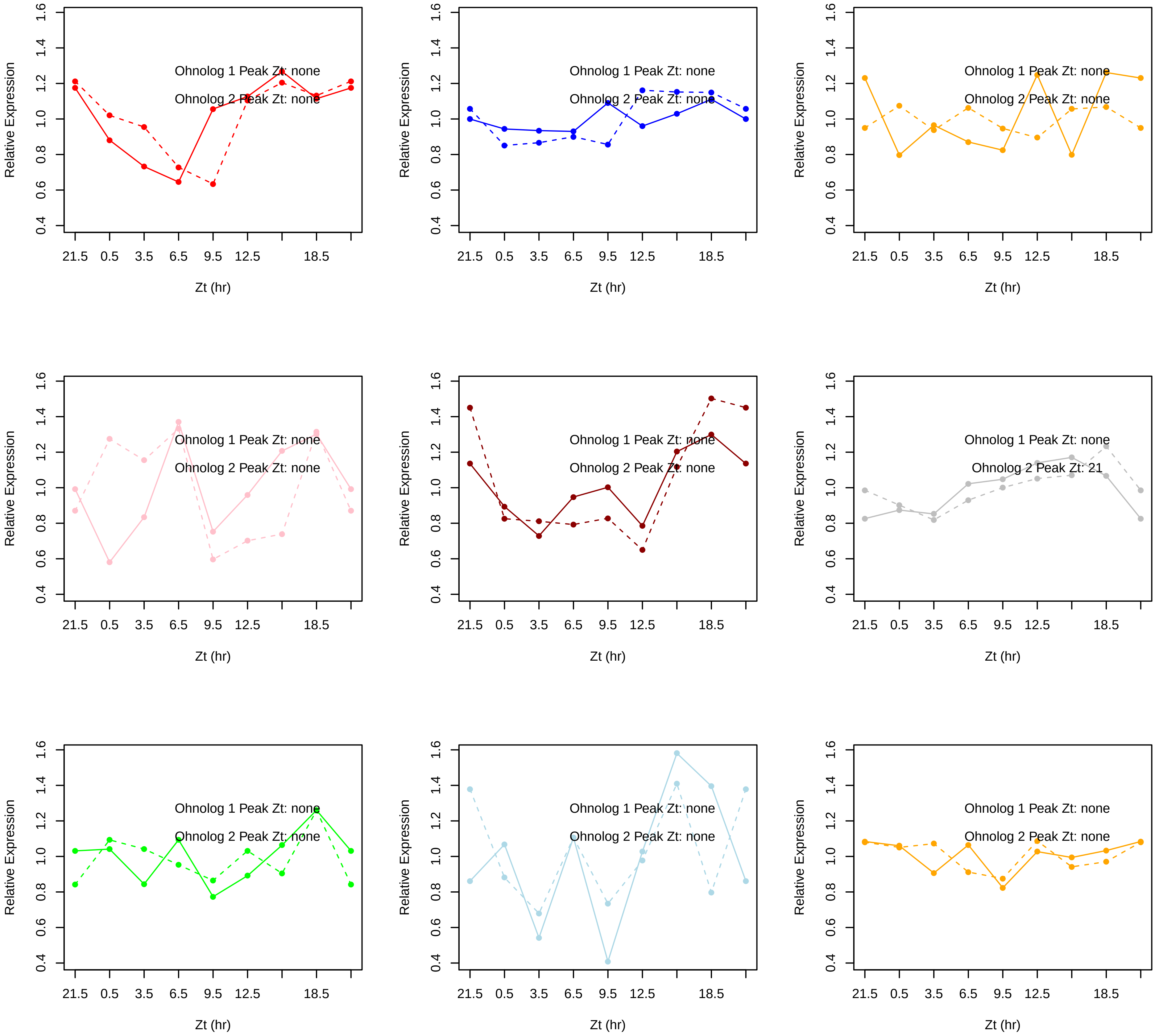

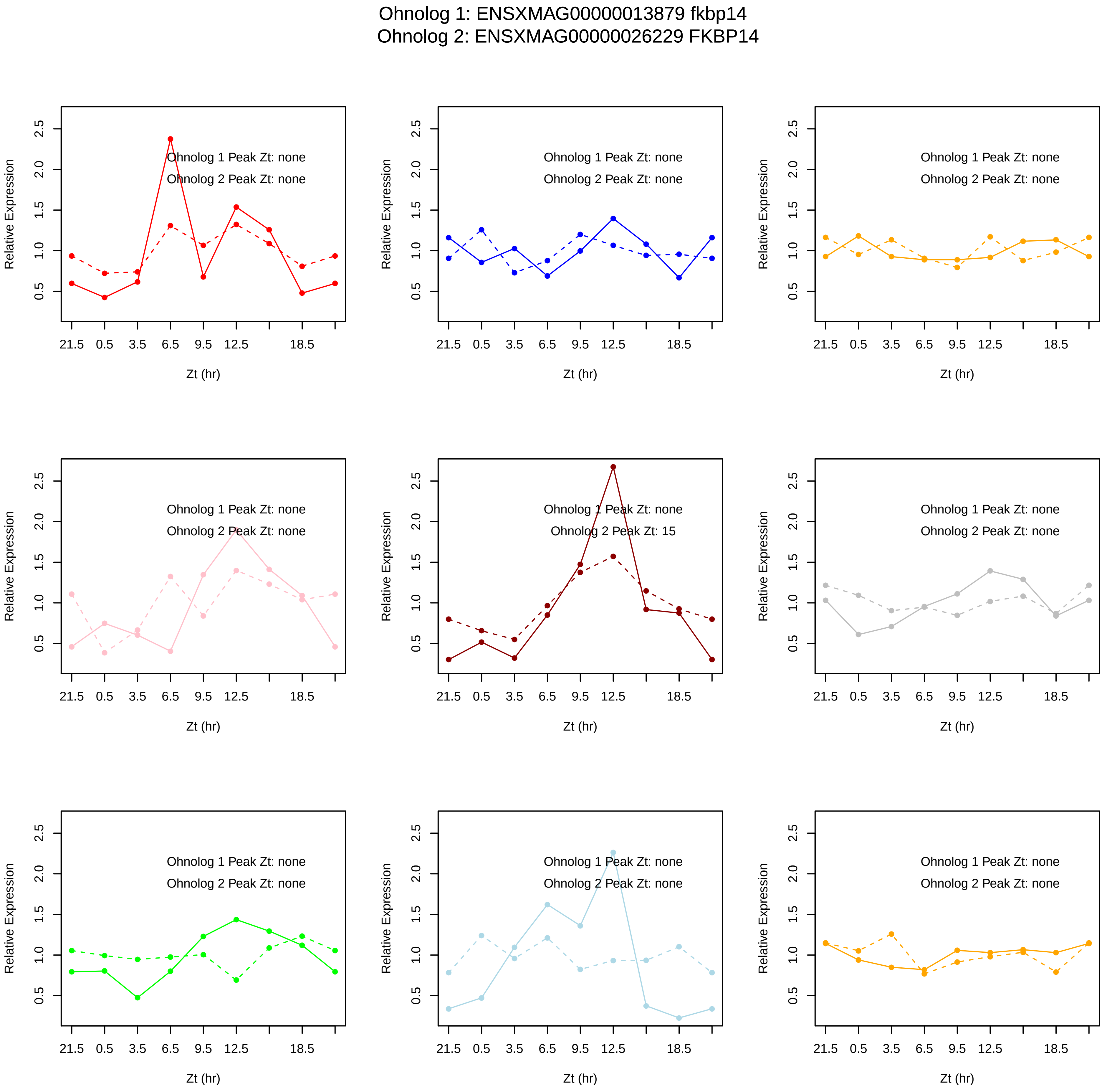
Ohnolog 1: ENSXMAG00000013879 fkbp14
Ohnolog 2: ENSXMAG00000026229 FKBP14
Ohnolog 1 Peak Zt: none
Ohnolog 2 Peak Zt: none
Ohnolog 1 Peak Zt: none
Ohnolog 2 Peak Zt: none
Ohnolog 1 Peak Zt: none
Ohnolog 2 Peak Zt: none
Ohnolog 1 Peak Zt: none
Ohnolog 2 Peak Zt: none
Ohnolog 1 Peak Zt: none
Ohnolog 2 Peak Zt: 15
Ohnolog 1 Peak Zt: none
Ohnolog 2 Peak Zt: none
Ohnolog 1 Peak Zt: none
Ohnolog 2 Peak Zt: none
Ohnolog 1 Peak Zt: none
Ohnolog 2 Peak Zt: none
Ohnolog 1 Peak Zt: none
Ohnolog 2 Peak Zt: none
Relative Expression
Zt (hr)
21.5 0.5 3.5 6.5 9.5 12.5 18.5
0.5 1.0 1.5 2.0 2.5

Ohnolog 1: ENSXMAG00000002891 fam102aa
Ohnolog 2: ENSXMAG00000028388 fam102ab

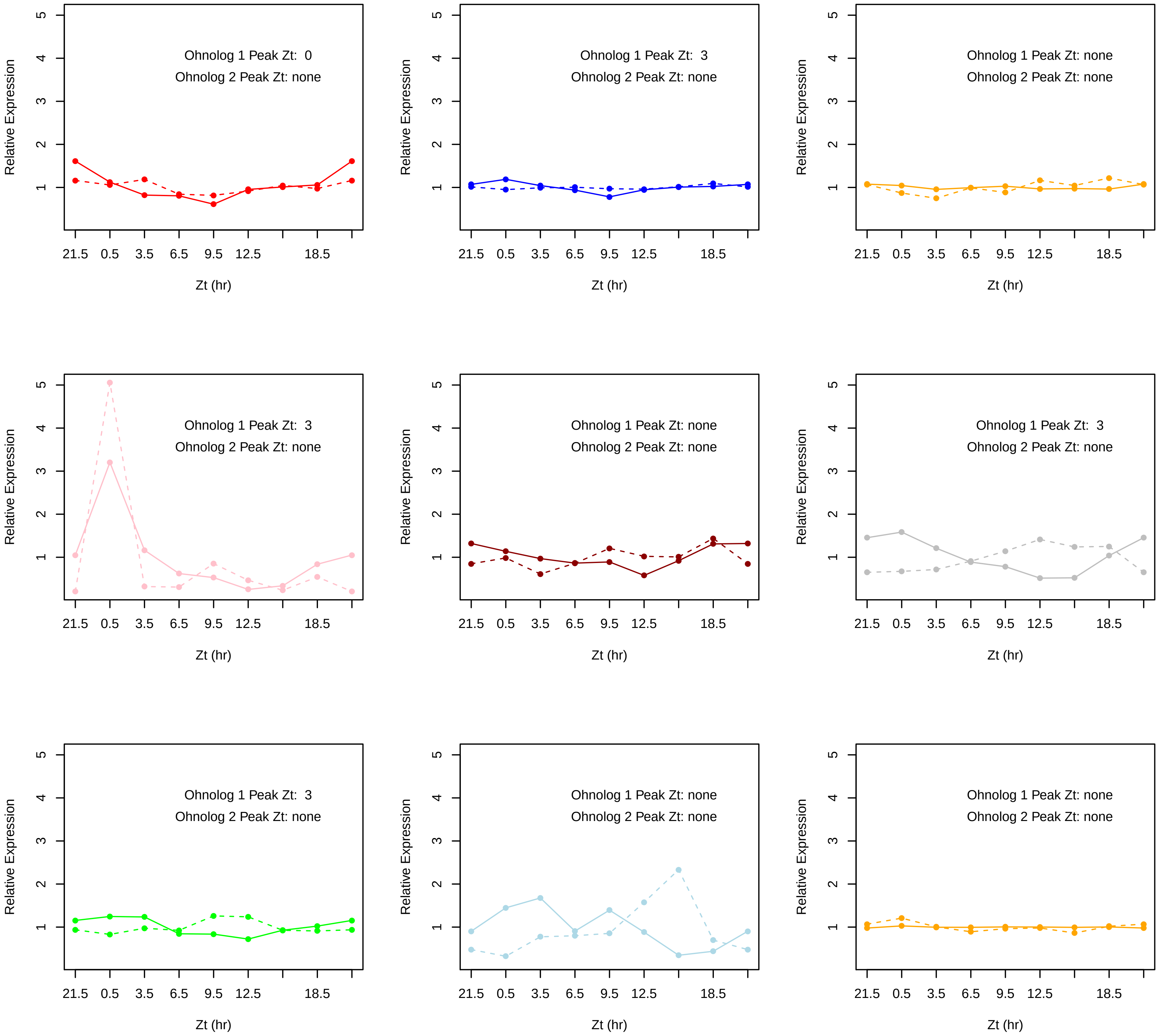

Ohnolog 1: ENSXMAG00000012793 TNPO2 (1 of many)
Ohnolog 2: ENSXMAG00000014217 TNPO2 (1 of many)

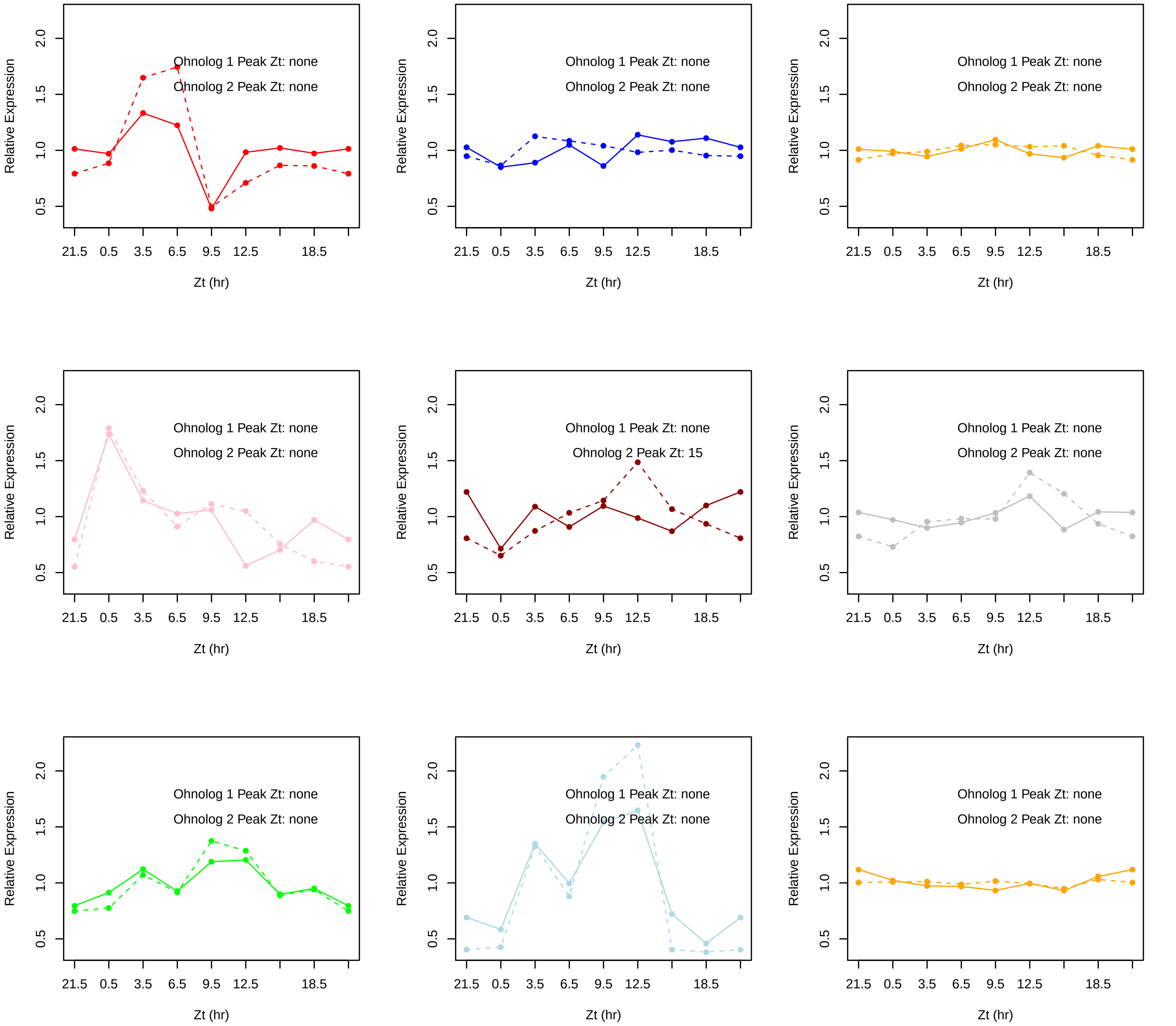

Ohnolog 1: ENSXMAG00000003122 slc1a7b
Ohnolog 2: ENSXMAG00000010563 slc1a7a

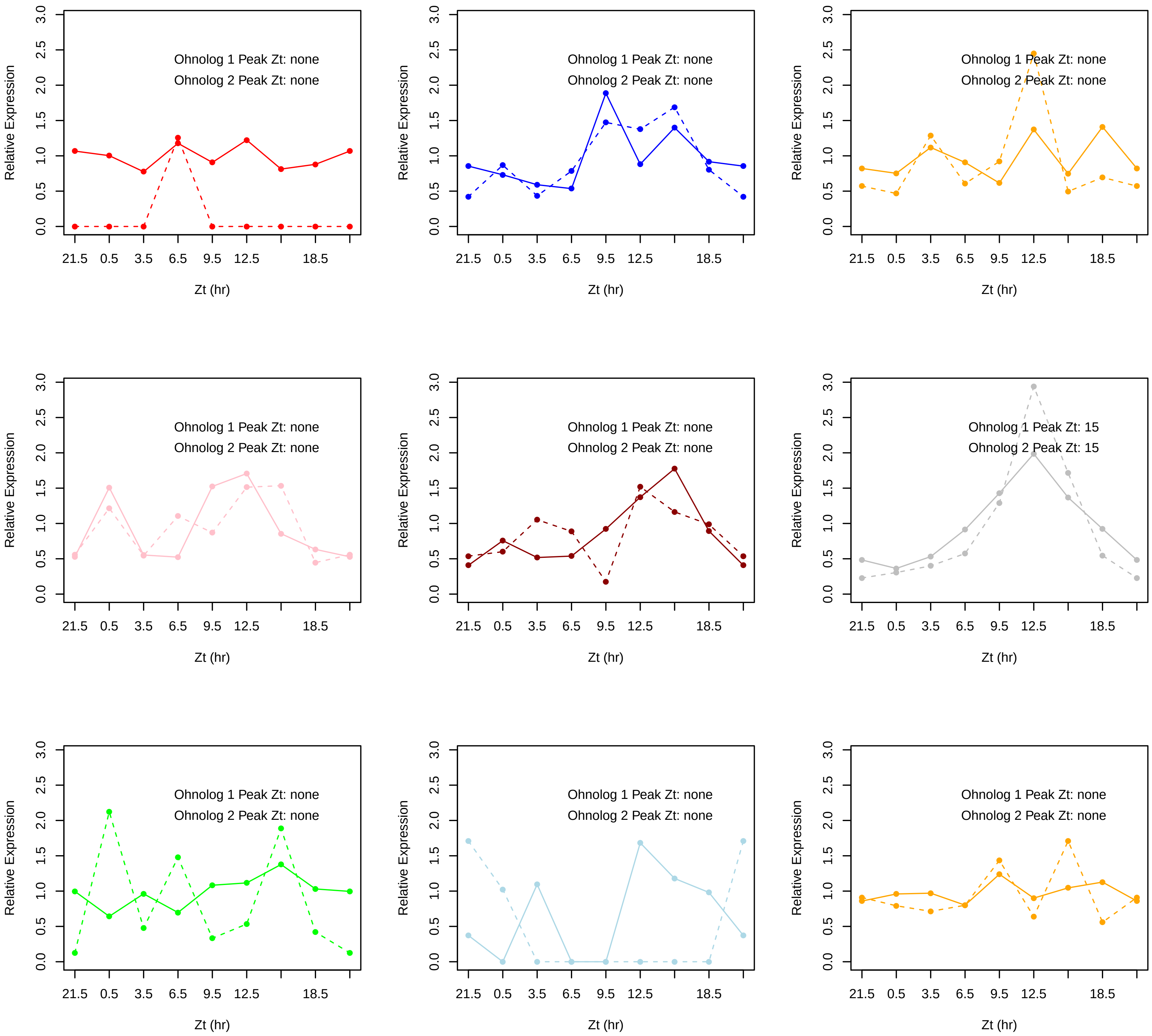

Ohnolog 1: ENSXMAG00000000222 grin2aa
Ohnolog 2: ENSXMAG00000000907 grin2ab

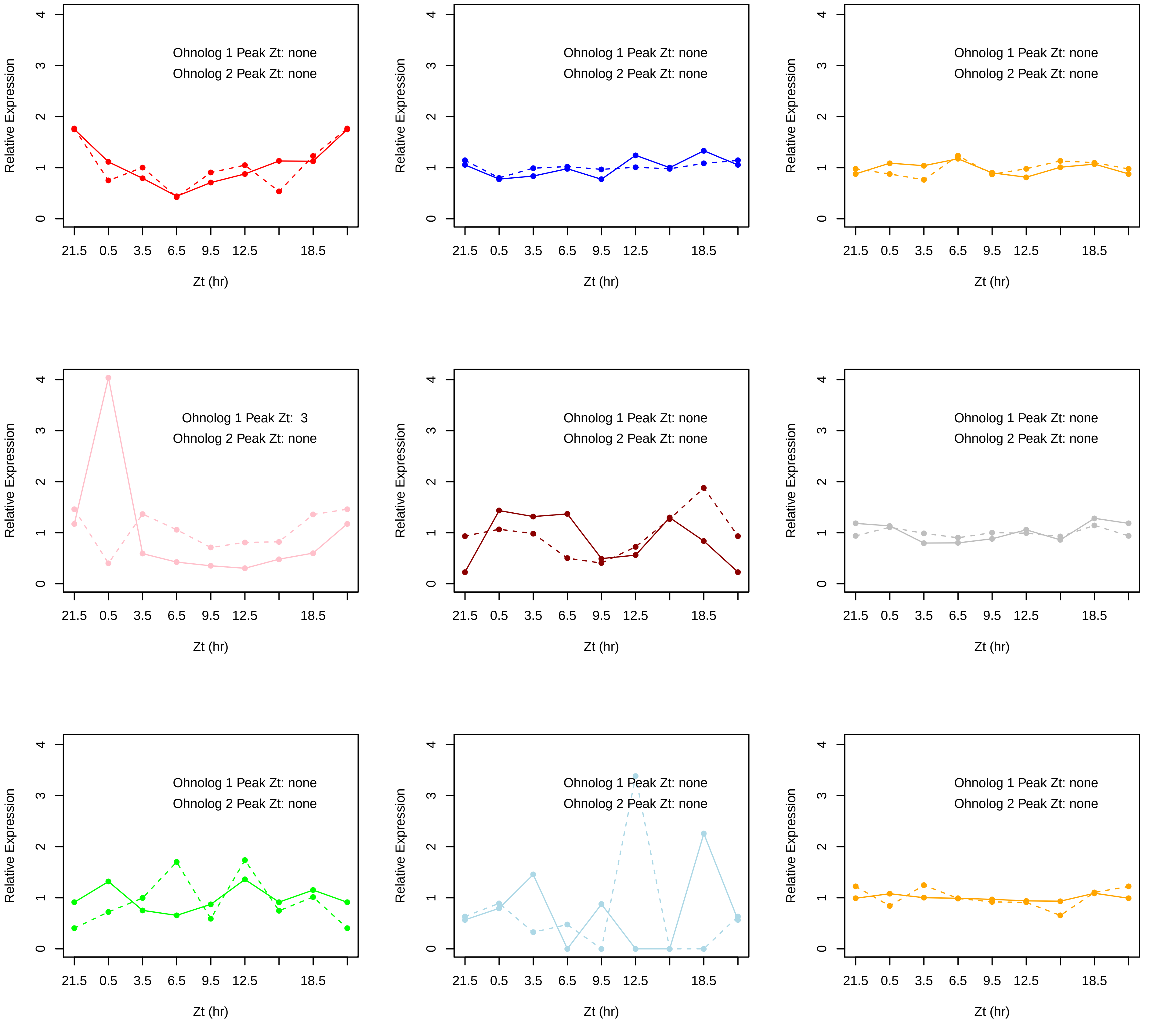

Ohnolog 1: ENSXMAG00000010169 shda
Ohnolog 2: ENSXMAG00000013357 shdb

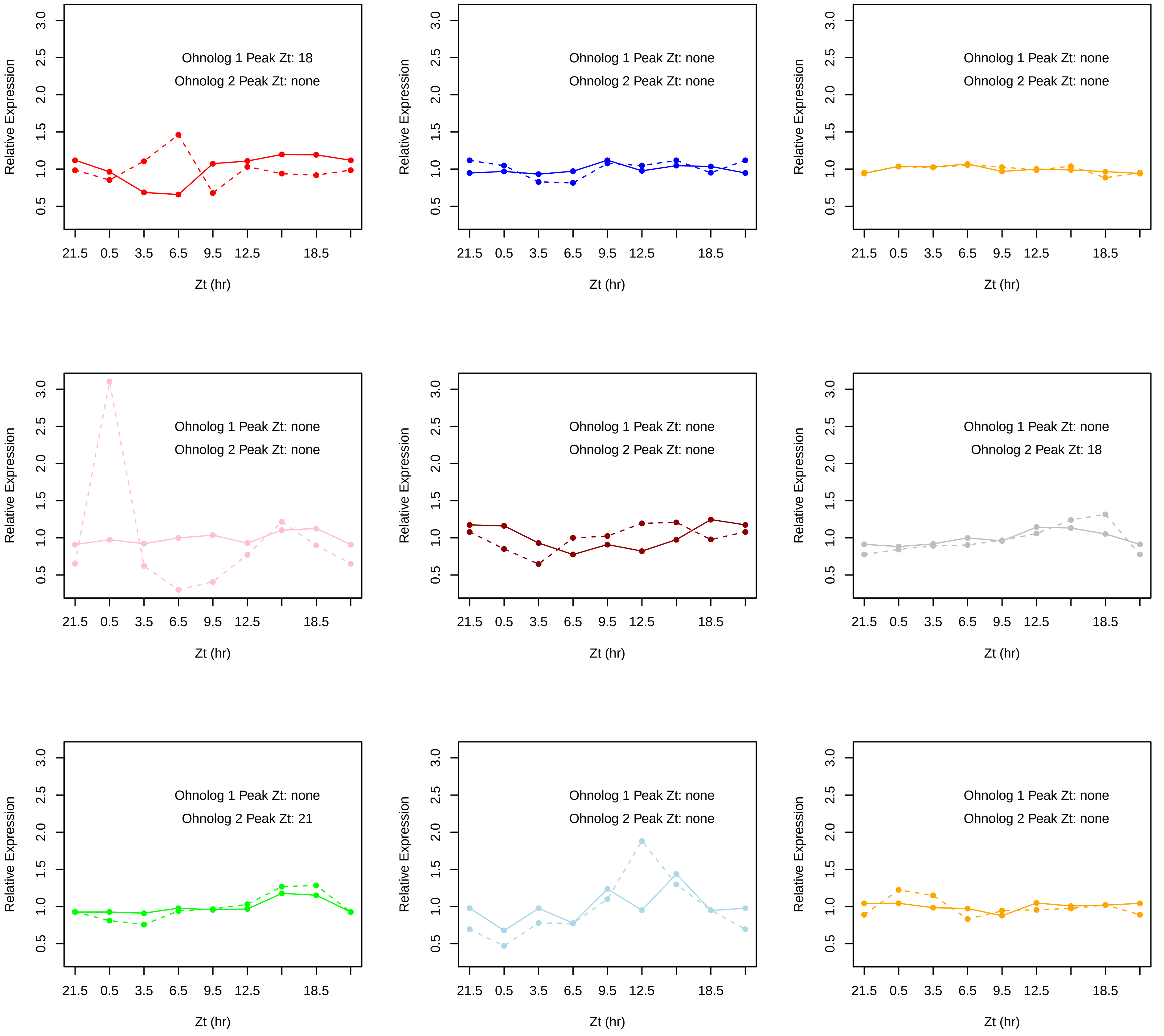

Ohnolog 1: ENSXMAG00000007837
Ohnolog 2: ENSXMAG00000010653 pcdh15a

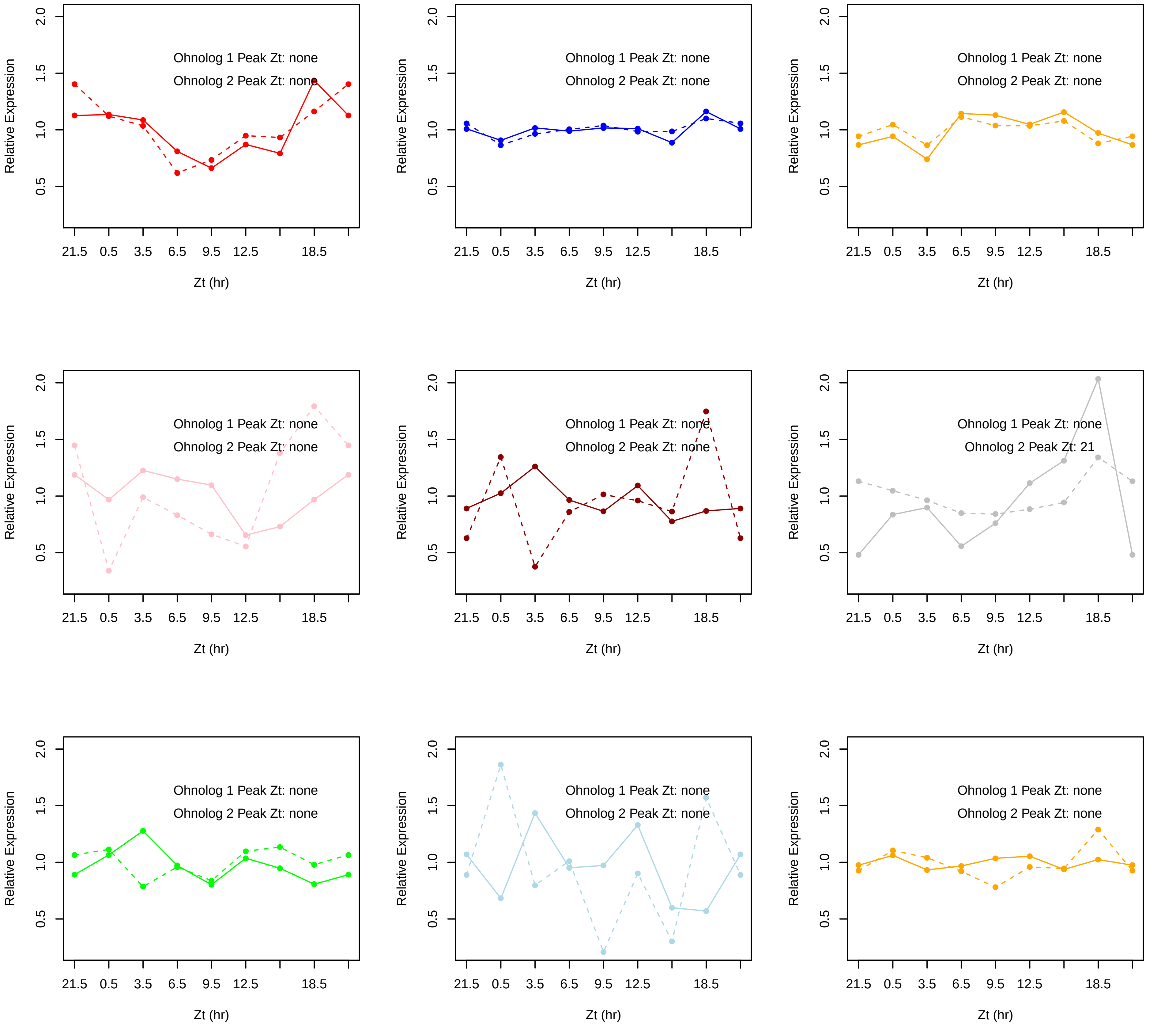

Ohnolog 1: ENSXMAG00000004102 rpa1
Ohnolog 2: ENSXMAG00000010333 RPA1 (1 of many)

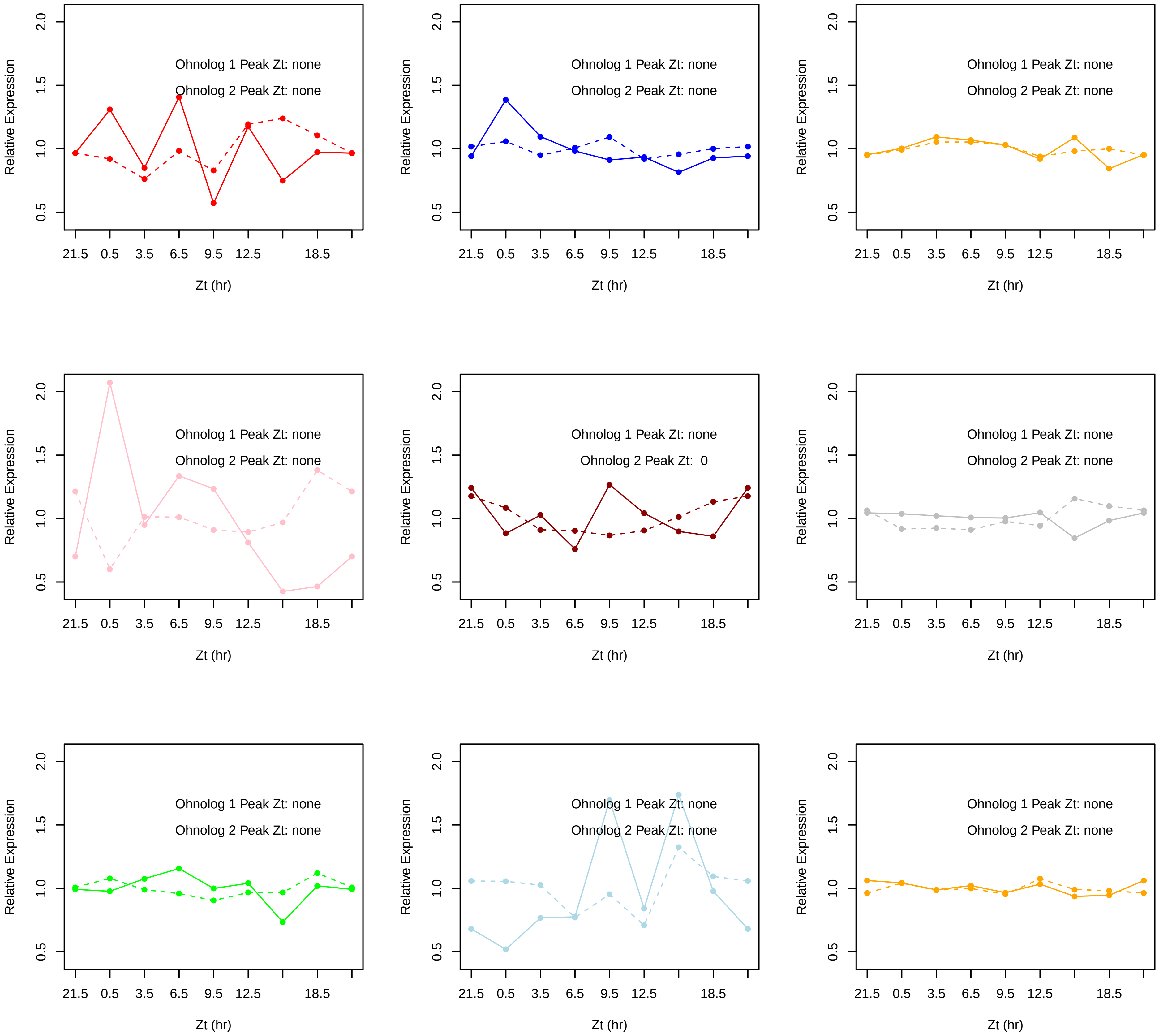

Ohnolog 1: ENSXMAG00000014642 crabp2b
Ohnolog 2: ENSXMAG00000016304 crabp2a

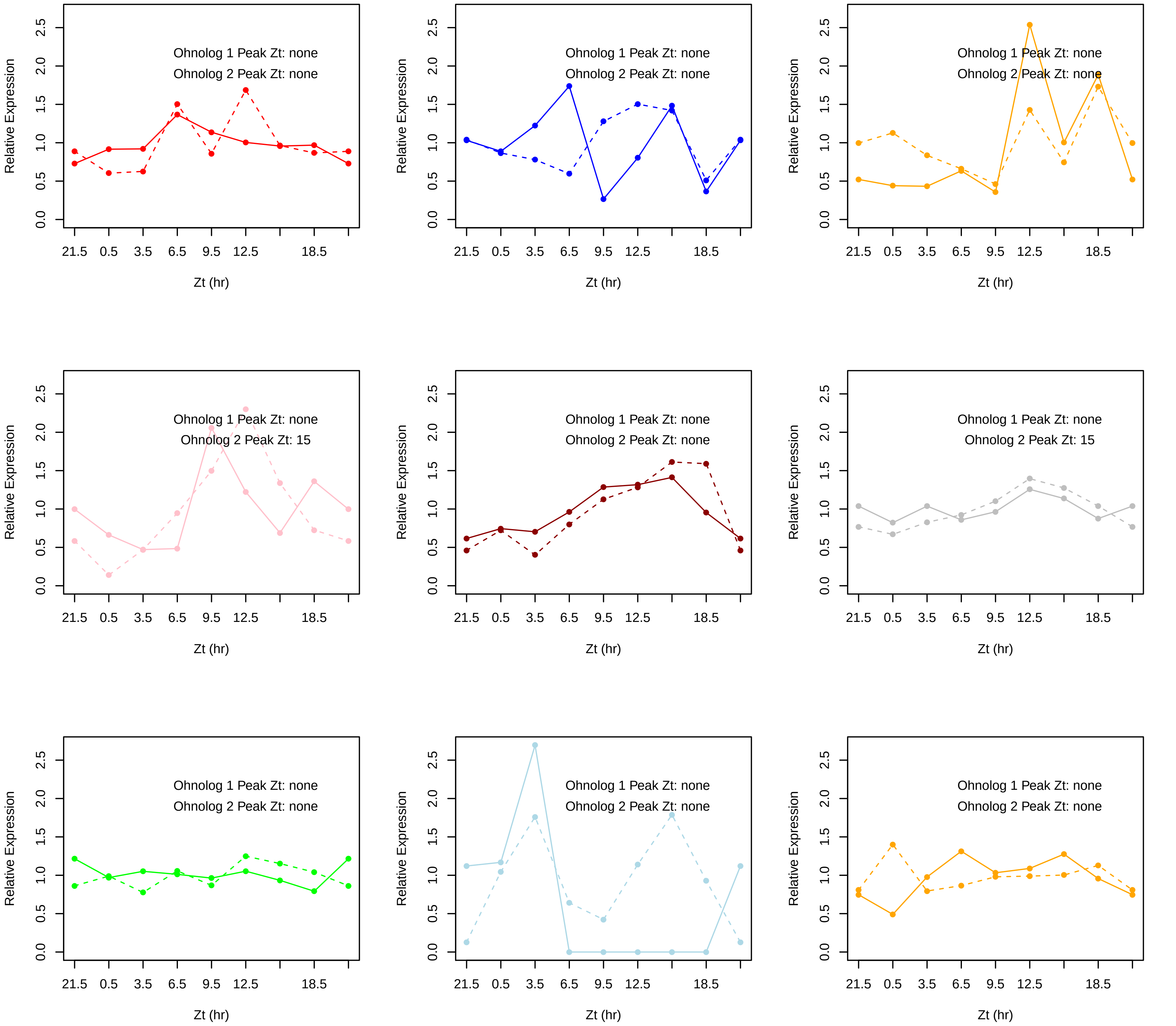

Ohnolog 1: ENSXMAG00000019305 cdc34b
Ohnolog 2: ENSXMAG00000023383 cdc34a

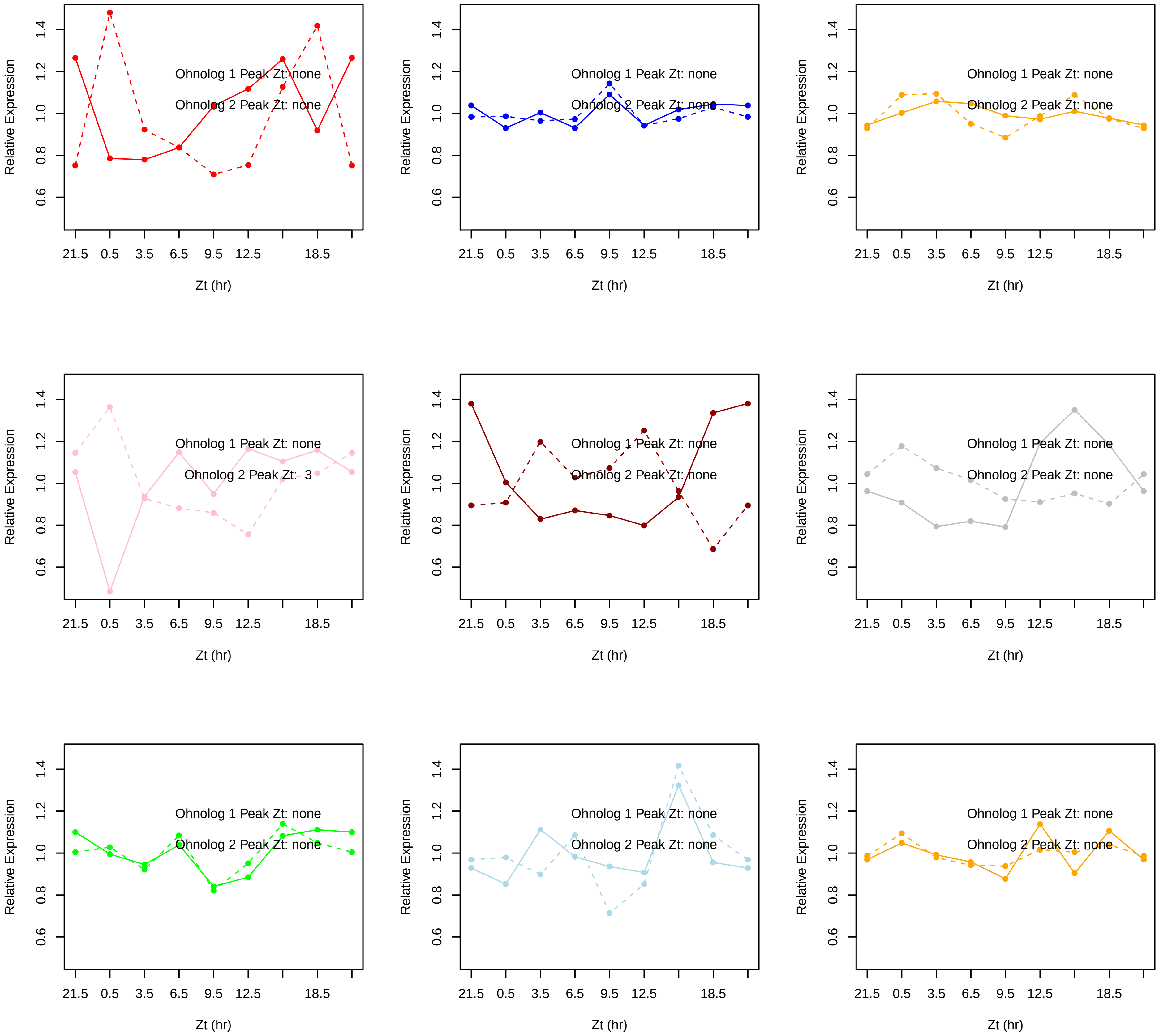

Ohnolog 1: ENSXMAG00000011017 osbpl1a
Ohnolog 2: ENSXMAG00000012174

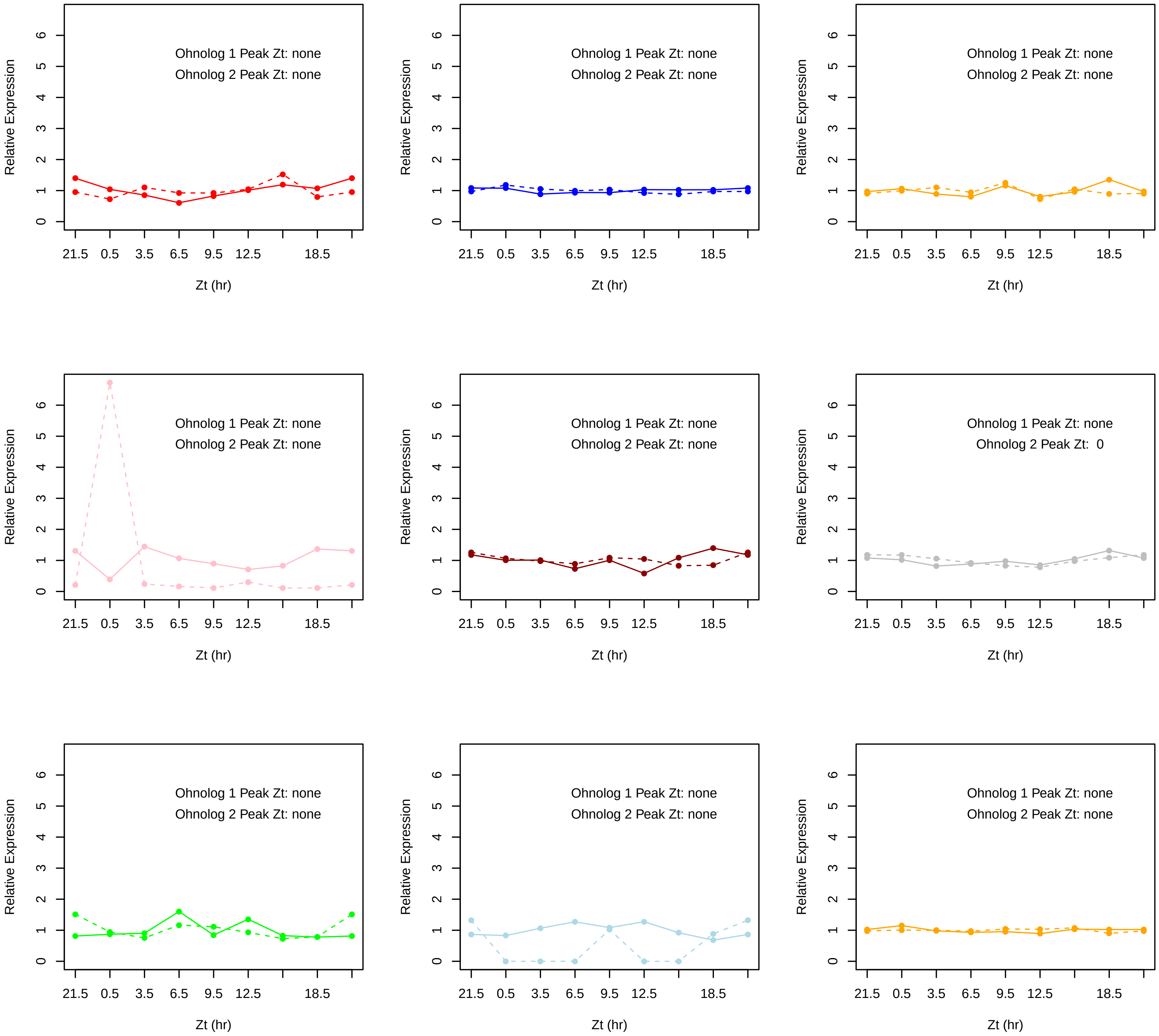

Ohnolog 1: ENSXMAG00000014619 celf3a
Ohnolog 2: ENSXMAG00000016475 celf3b

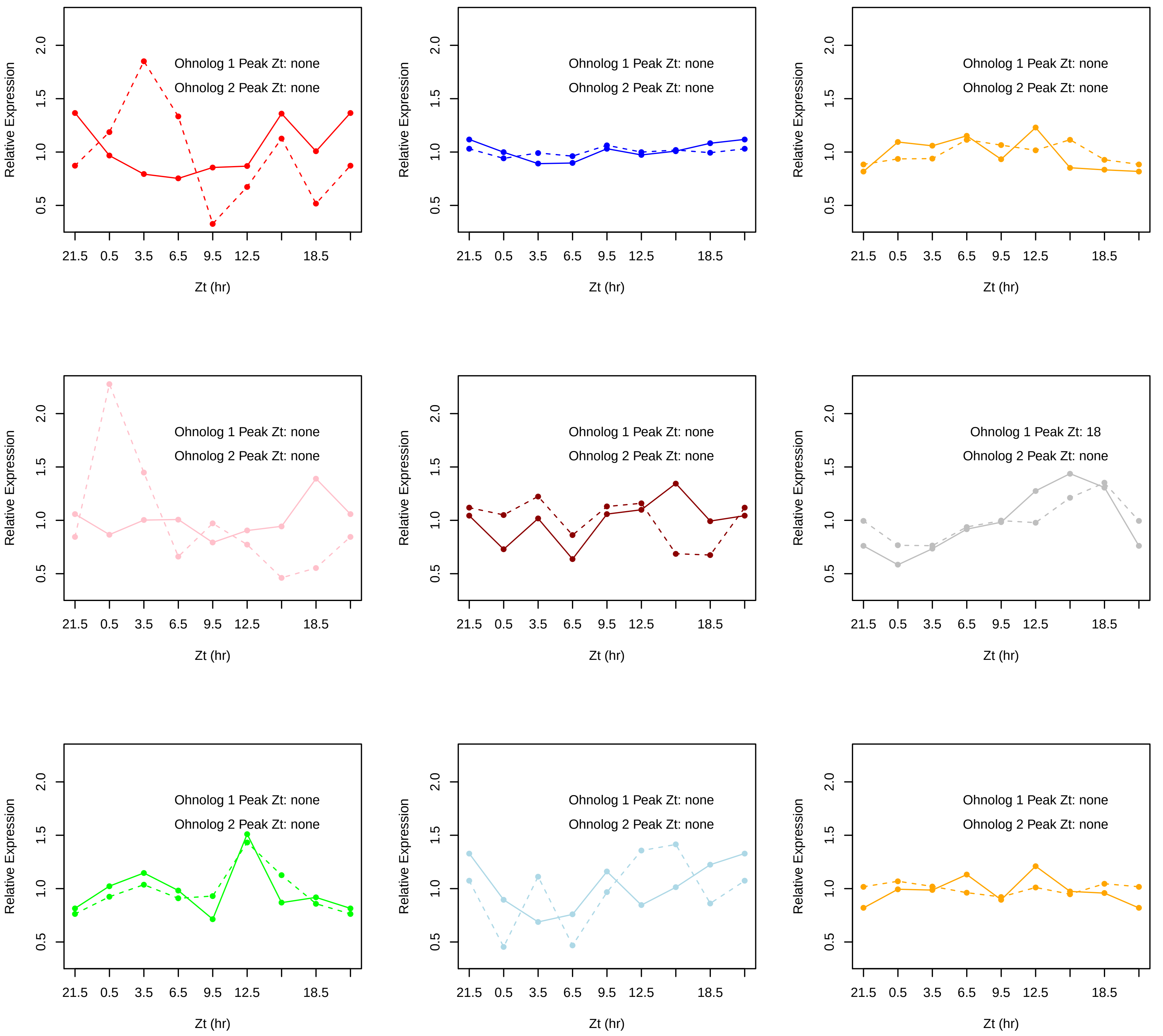

Ohnolog 1: ENSXMAG00000006561
Ohnolog 2: ENSXMAG00000008657 si:ch211−106h11.3

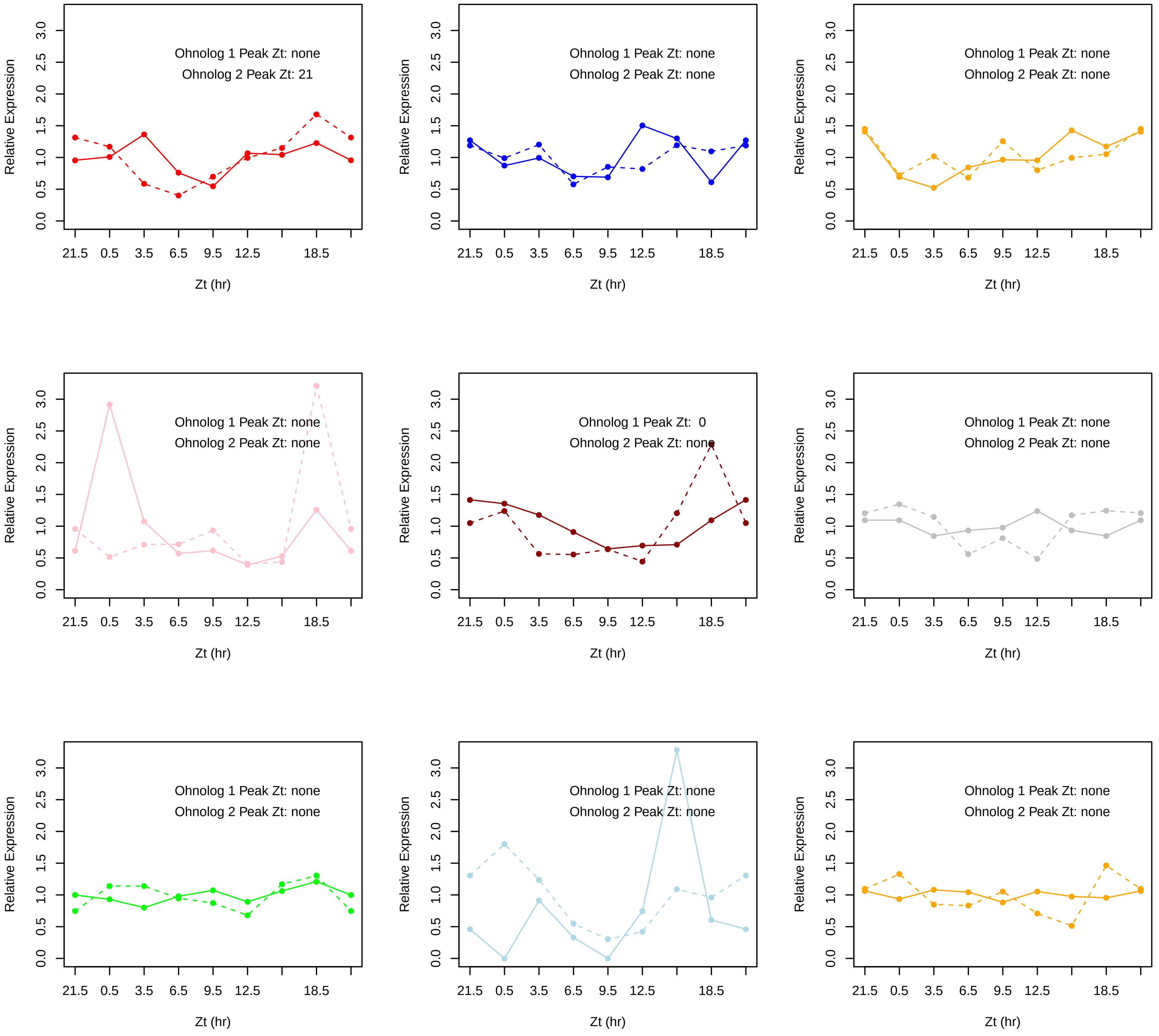

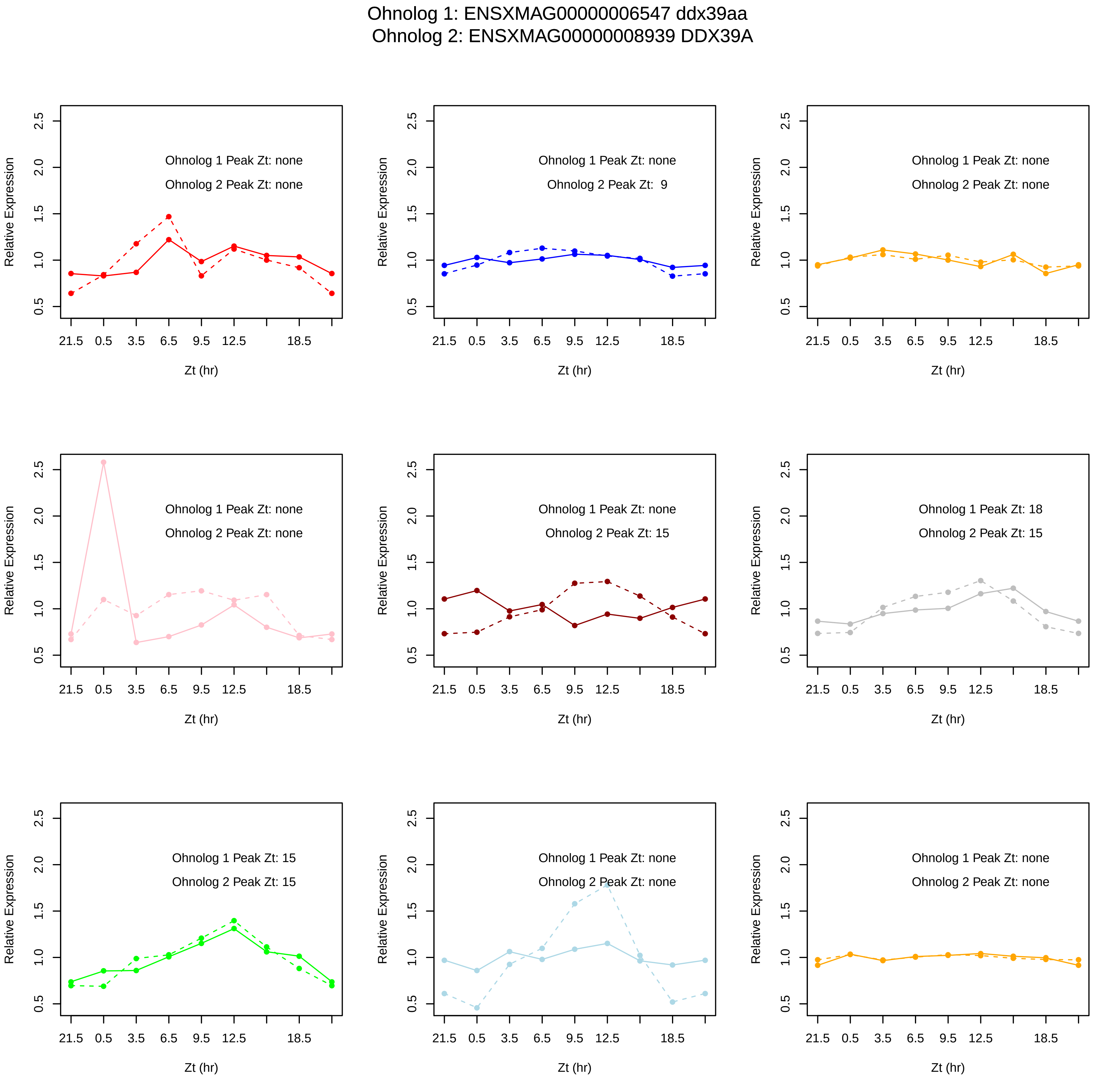
Ohnolog 1: ENSXMAG00000006547 ddx39aa
Ohnolog 2: ENSXMAG00000008939 DDX39A
Ohnolog 1 Peak Zt: none
Ohnolog 2 Peak Zt: none
Ohnolog 1 Peak Zt: none
Ohnolog 2 Peak Zt: 9
Ohnolog 1 Peak Zt: none
Ohnolog 2 Peak Zt: none
Ohnolog 1 Peak Zt: none
Ohnolog 2 Peak Zt: none
Ohnolog 1 Peak Zt: none
Ohnolog 2 Peak Zt: 15
Ohnolog 1 Peak Zt: 18
Ohnolog 2 Peak Zt: 15
Ohnolog 1 Peak Zt: 15
Ohnolog 2 Peak Zt: 15
Ohnolog 1 Peak Zt: none
Ohnolog 2 Peak Zt: none
Ohnolog 1 Peak Zt: none
Ohnolog 2 Peak Zt: none
Relative Expression
Zt (hr)
21.5
0.5
3.5
6.5
9.5
12.5
18.5
0.5
1.0
1.5
2.0
2.5

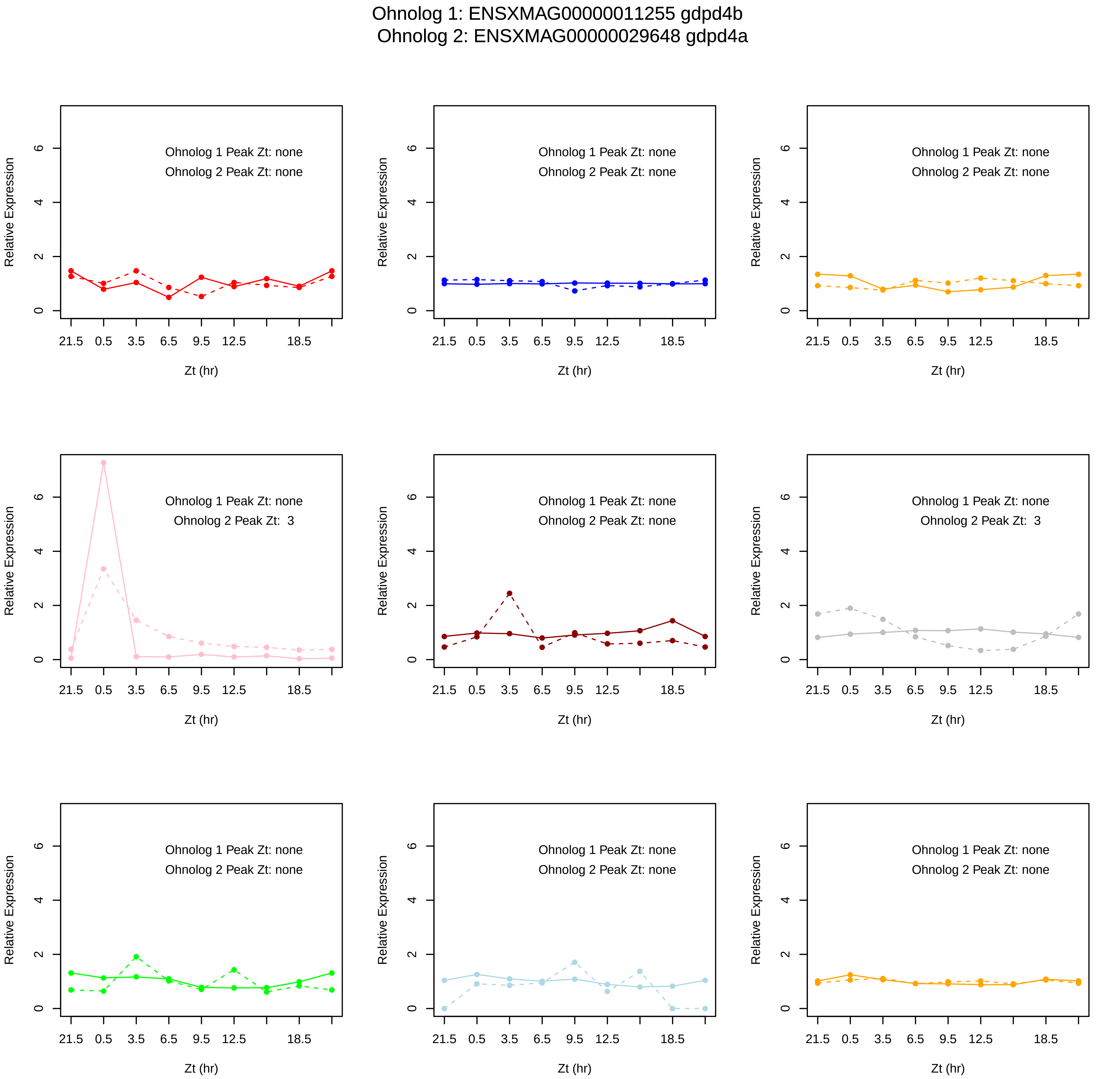

Ohnolog 1: ENSXMAG00000011255 gdpd4b
Ohnolog 2: ENSXMAG00000029648 gdpd4a
Ohnolog 1 Peak Zt: none
Ohnolog 2 Peak Zt: none
Ohnolog 1 Peak Zt: none
Ohnolog 2 Peak Zt: none
Ohnolog 1 Peak Zt: none
Ohnolog 2 Peak Zt: none
Ohnolog 1 Peak Zt: none
Ohnolog 2 Peak Zt: 3
Ohnolog 1 Peak Zt: none
Ohnolog 2 Peak Zt: none
Ohnolog 1 Peak Zt: none
Ohnolog 2 Peak Zt: 3
Ohnolog 1 Peak Zt: none
Ohnolog 2 Peak Zt: none
Ohnolog 1 Peak Zt: none
Ohnolog 2 Peak Zt: none
Ohnolog 1 Peak Zt: none
Ohnolog 2 Peak Zt: none
Relative Expression
Zt (hr)
0
2
4
6
21.5
0.5
3.5
6.5
9.5
12.5
18.5

Ohnolog 1: ENSXMAG00000011345 tardbpl
Ohnolog 2: ENSXMAG00000016713 tardbp

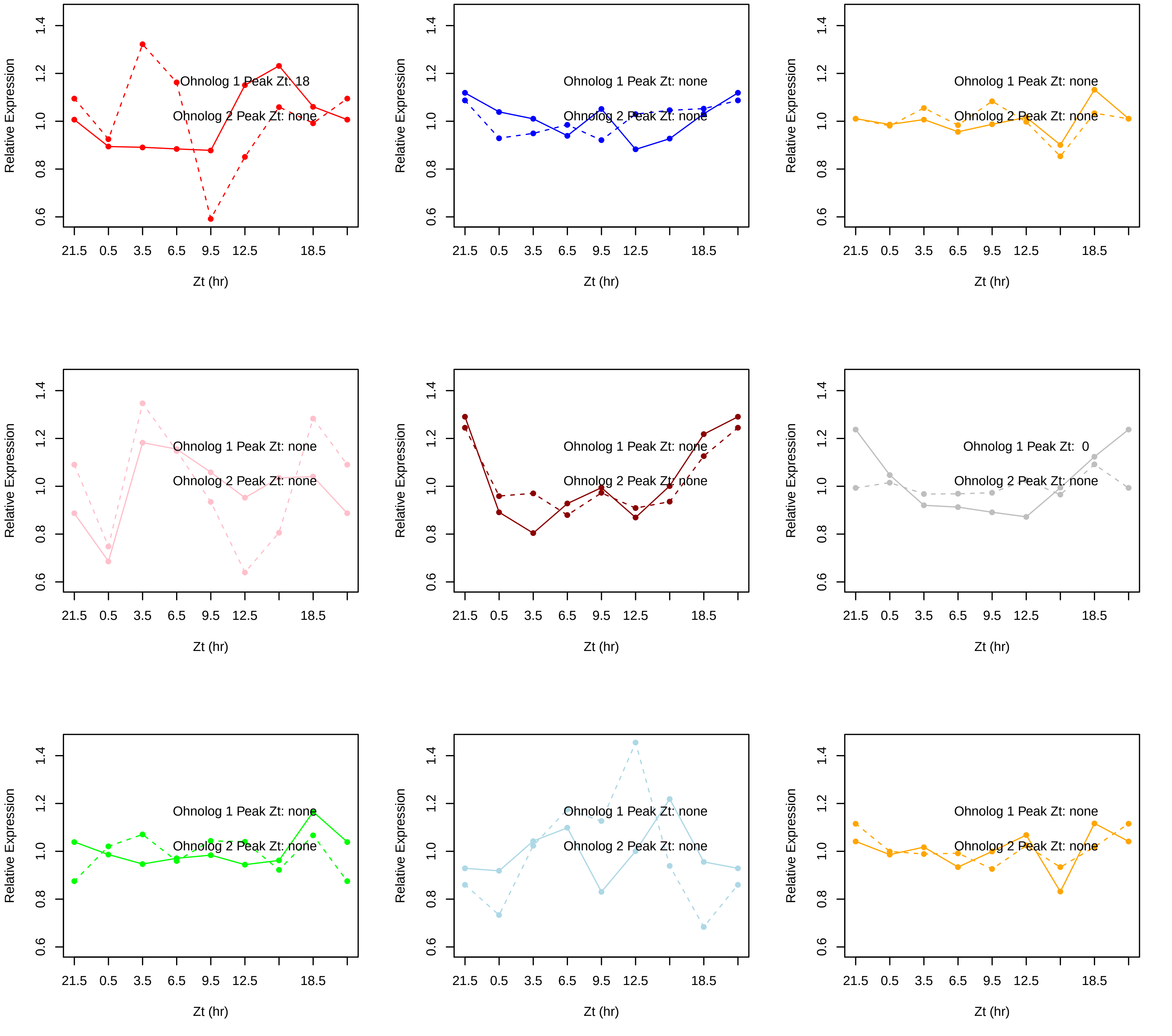

Ohnolog 1: ENSXMAG00000006429 pkn1a
Ohnolog 2: ENSXMAG00000008895 pkn1b

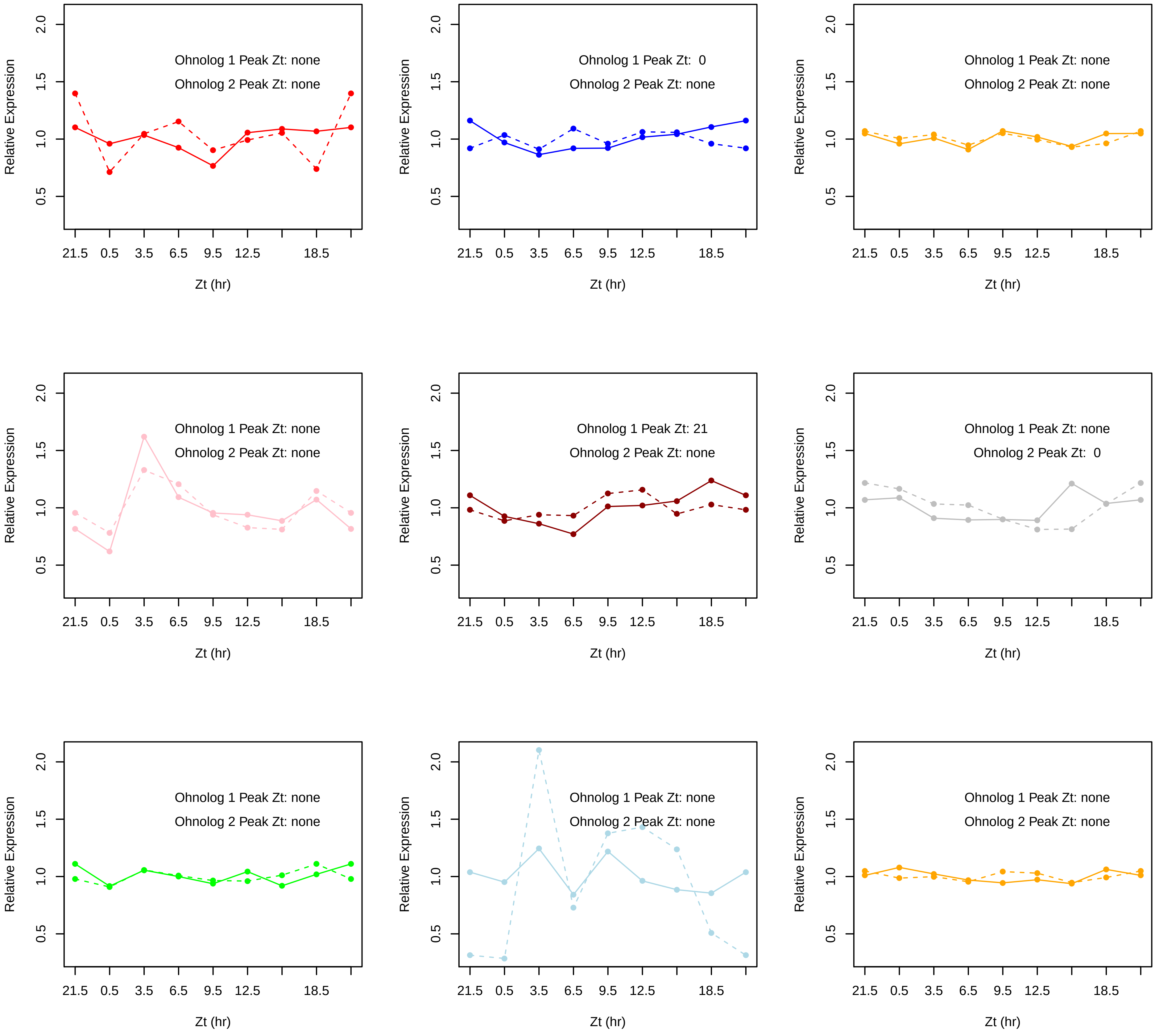

Ohnolog 1: ENSXMAG00000012106 ssh2a
Ohnolog 2: ENSXMAG00000023256 ssh2b

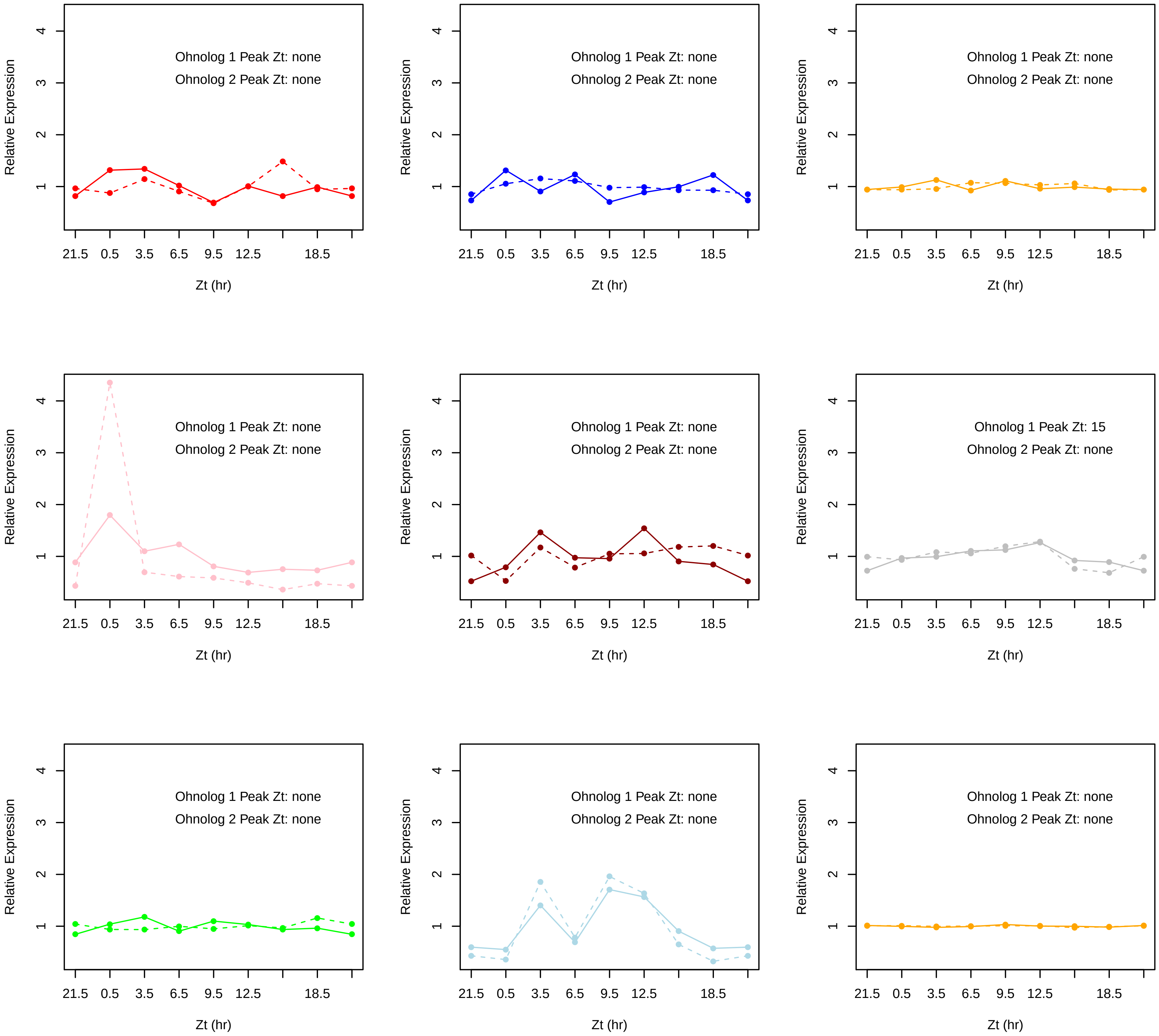

Ohnolog 1: ENSXMAG00000004628 myo7aa
Ohnolog 2: ENSXMAG00000011193 myo7ab

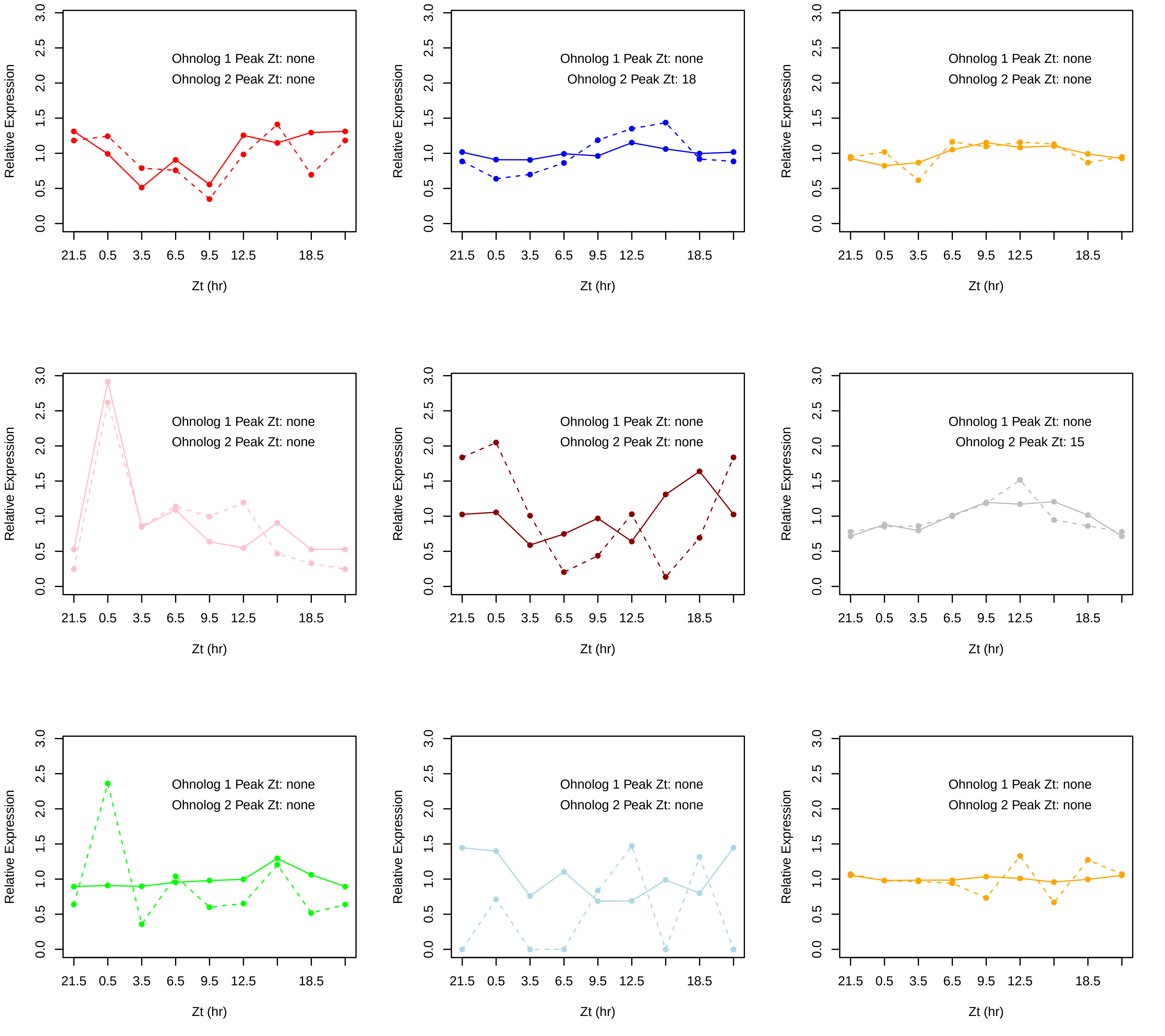

Ohnolog 1: ENSXMAG00000001137 ano9b
Ohnolog 2: ENSXMAG00000007047 ano9a

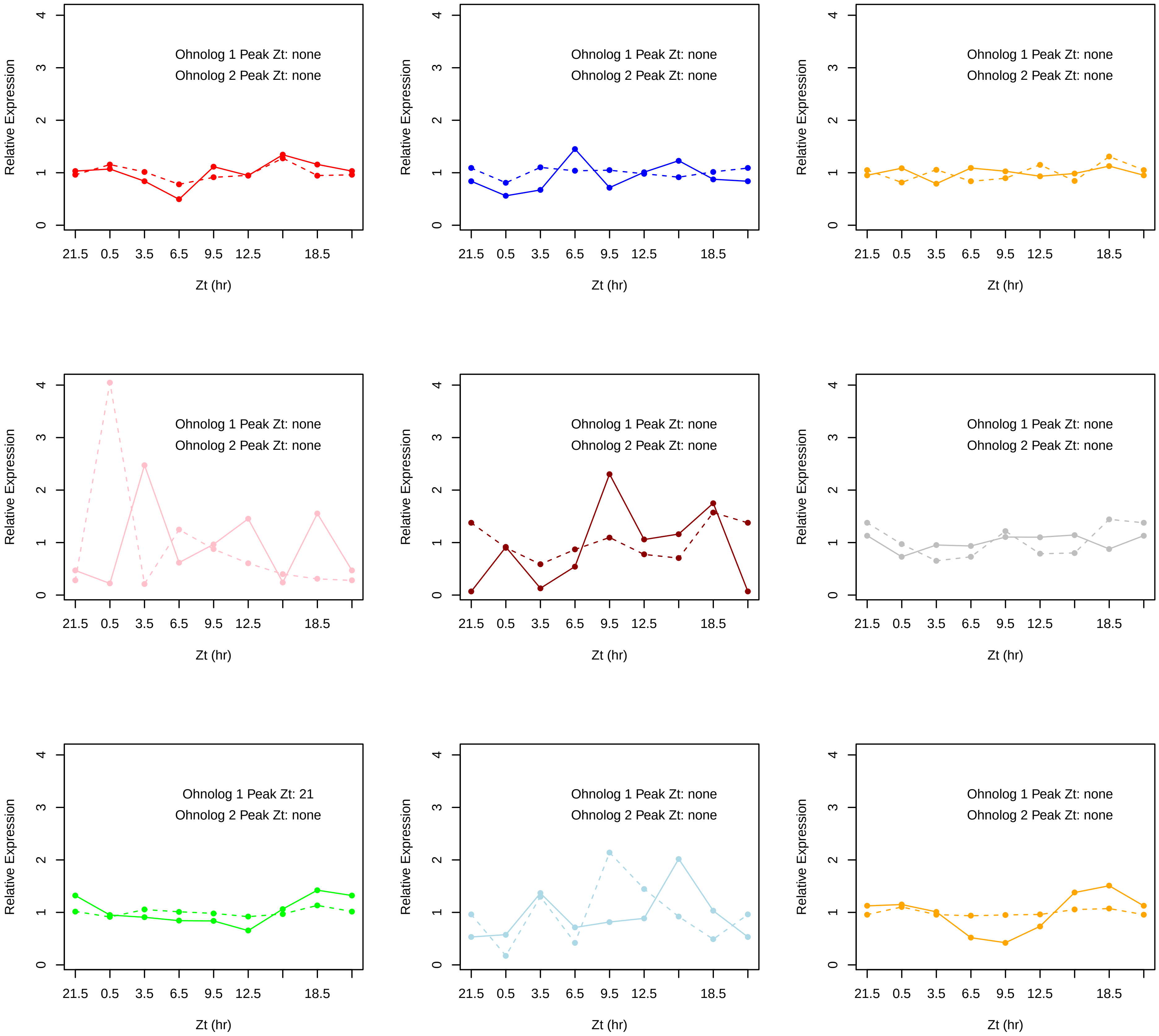

Ohnolog 1: ENSXMAG00000002738 SMARCB1
Ohnolog 2: ENSXMAG00000016803 smarcb1b

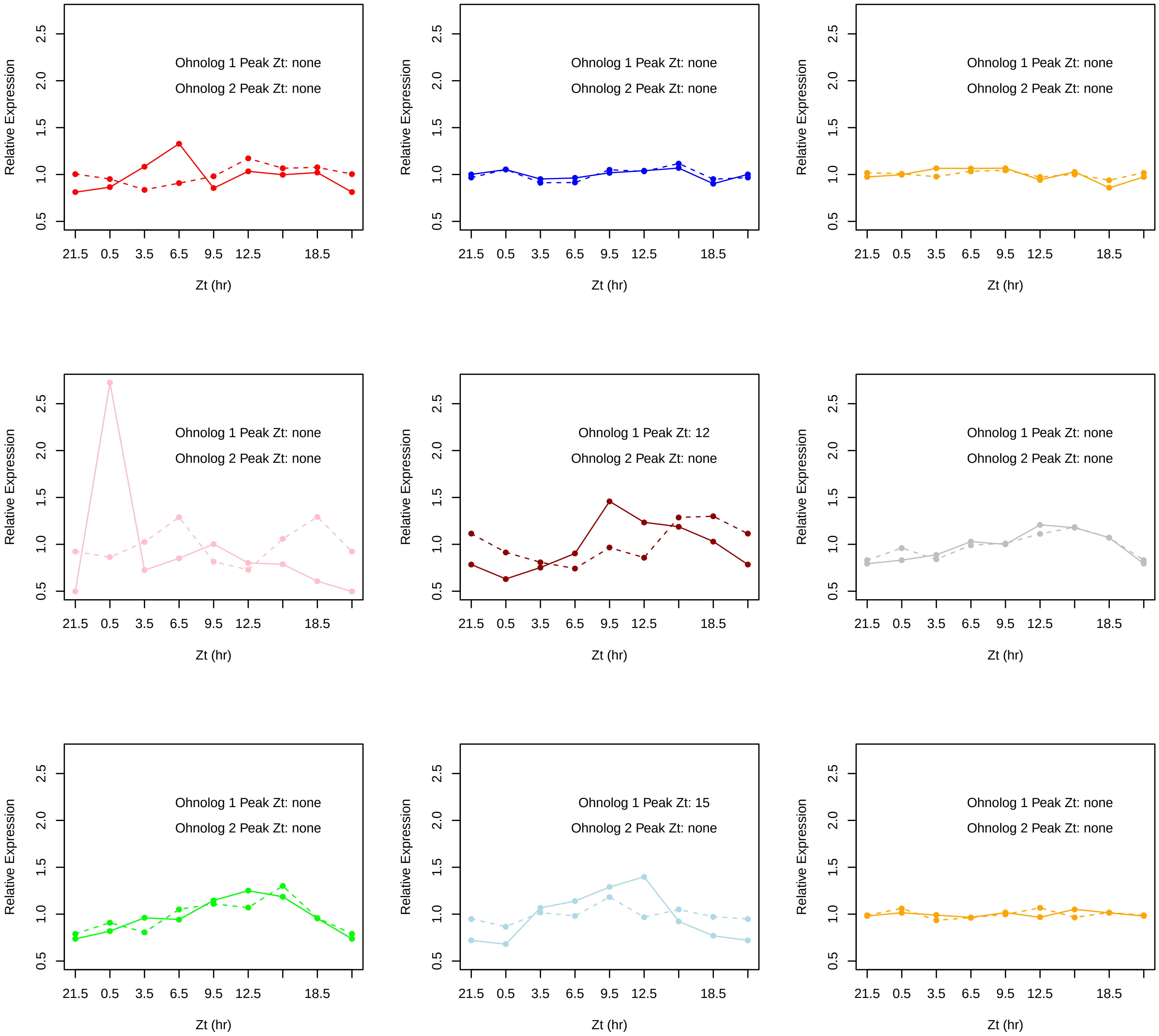

Ohnolog 1: ENSXMAG00000007080 cd81b
Ohnolog 2: ENSXMAG00000023163 cd81a

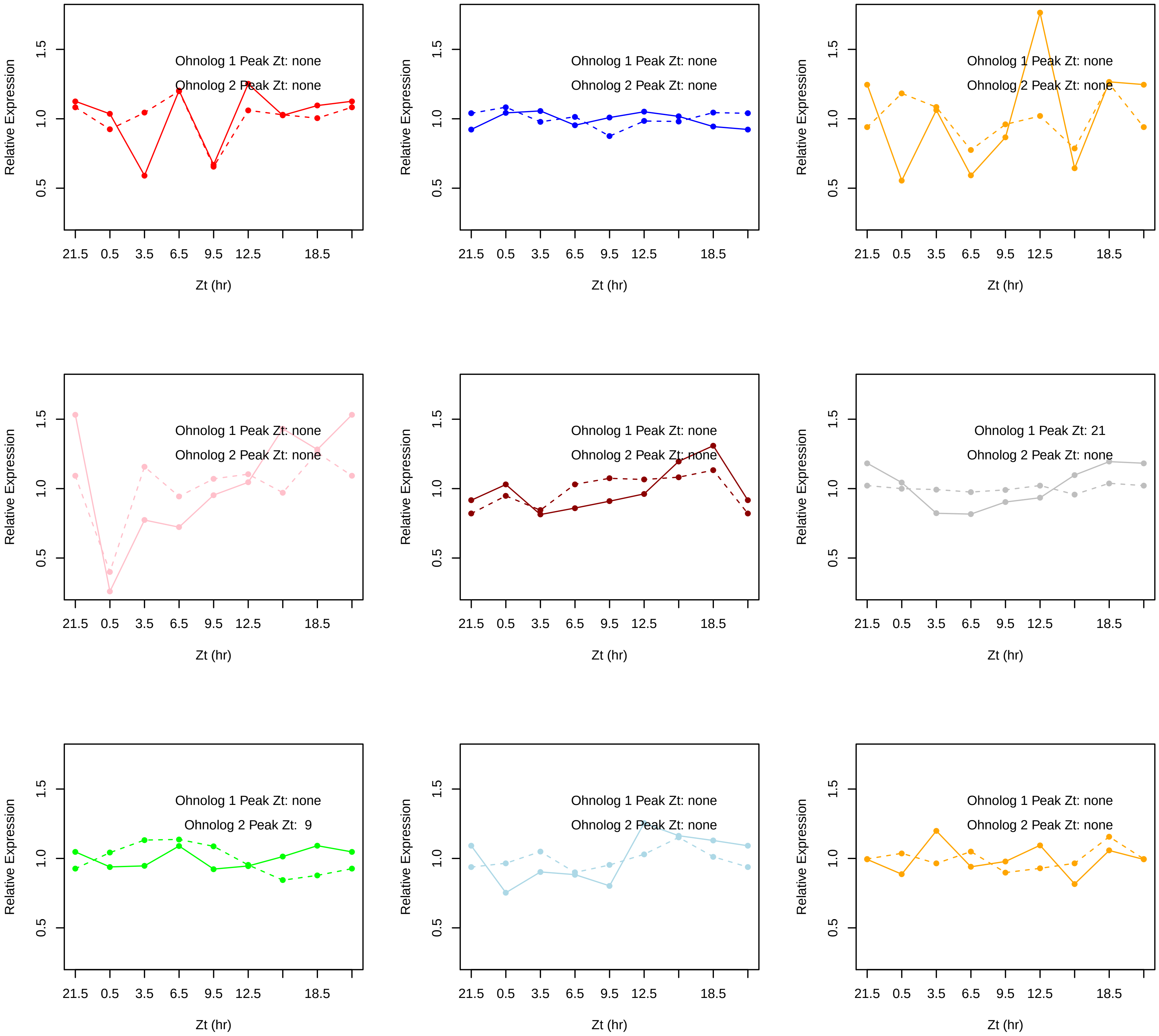

Ohnolog 1: ENSXMAG00000004486
Ohnolog 2: ENSXMAG00000012228 CUL5

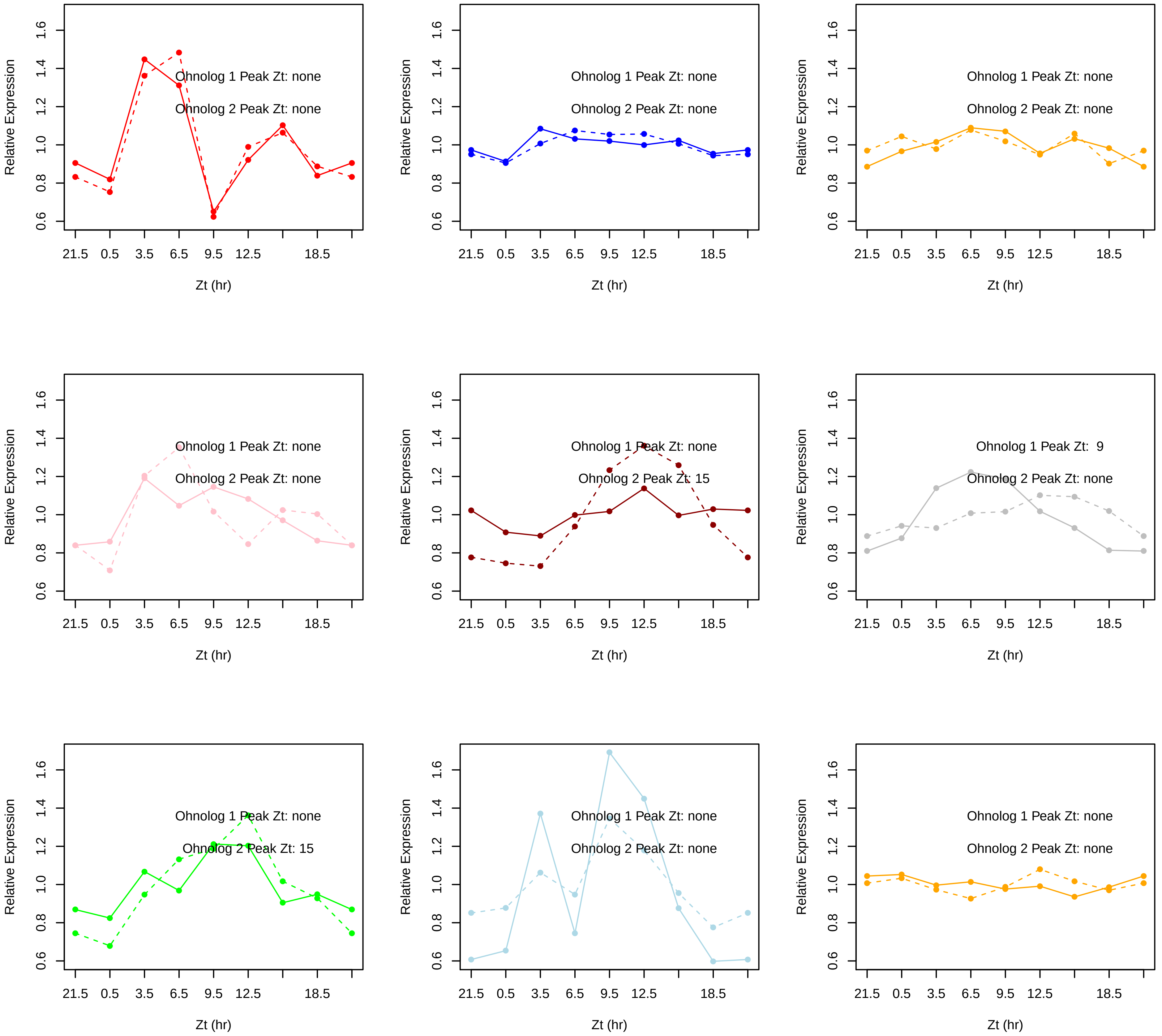

Ohnolog 1: ENSXMAG00000005262 si:dkey−217d24.6
Ohnolog 2: ENSXMAG00000008866 si:ch211−194b1.1

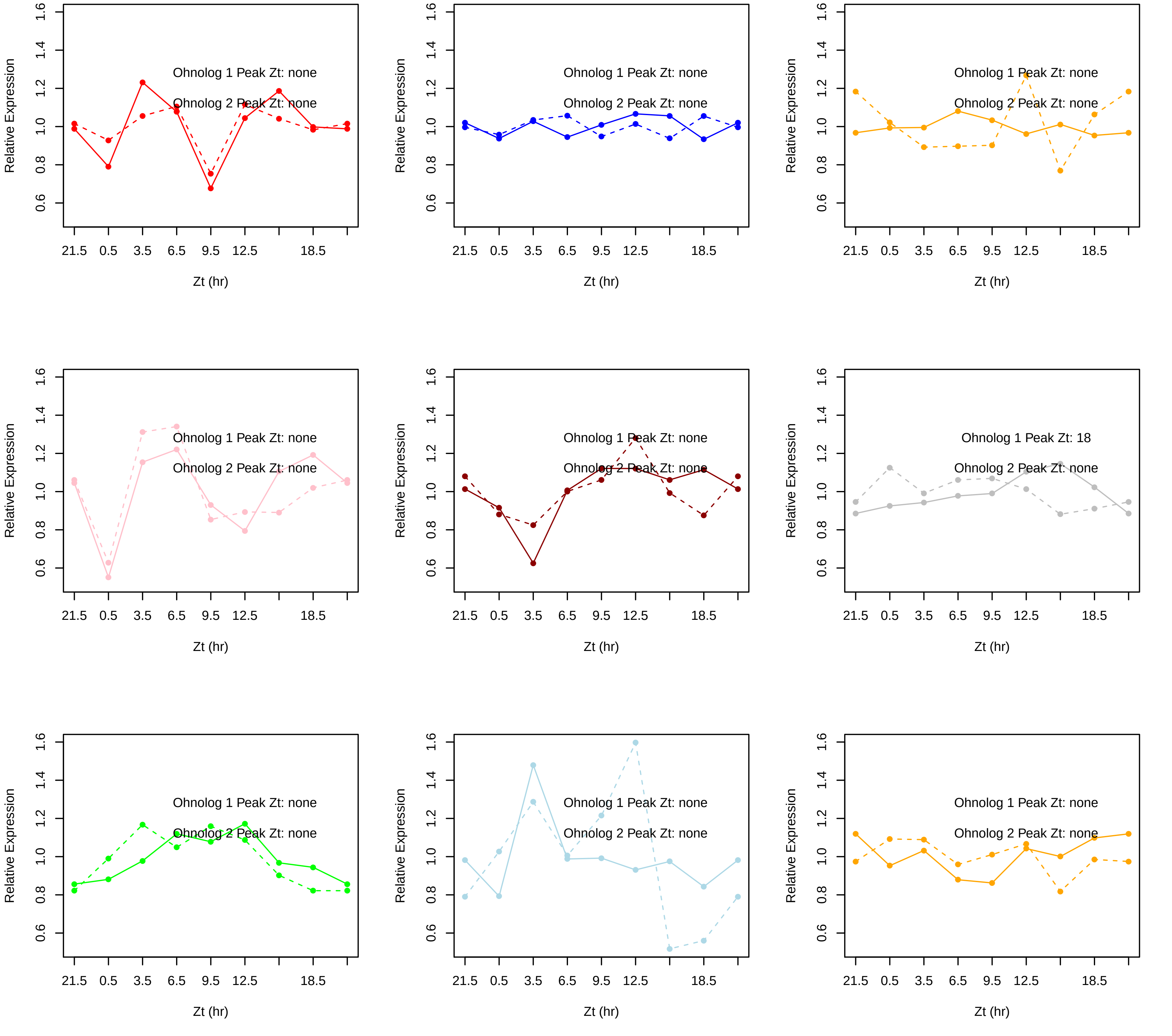

Ohnolog 1: ENSXMAG00000004522 slc35f2l
Ohnolog 2: ENSXMAG00000015958 slc35f2

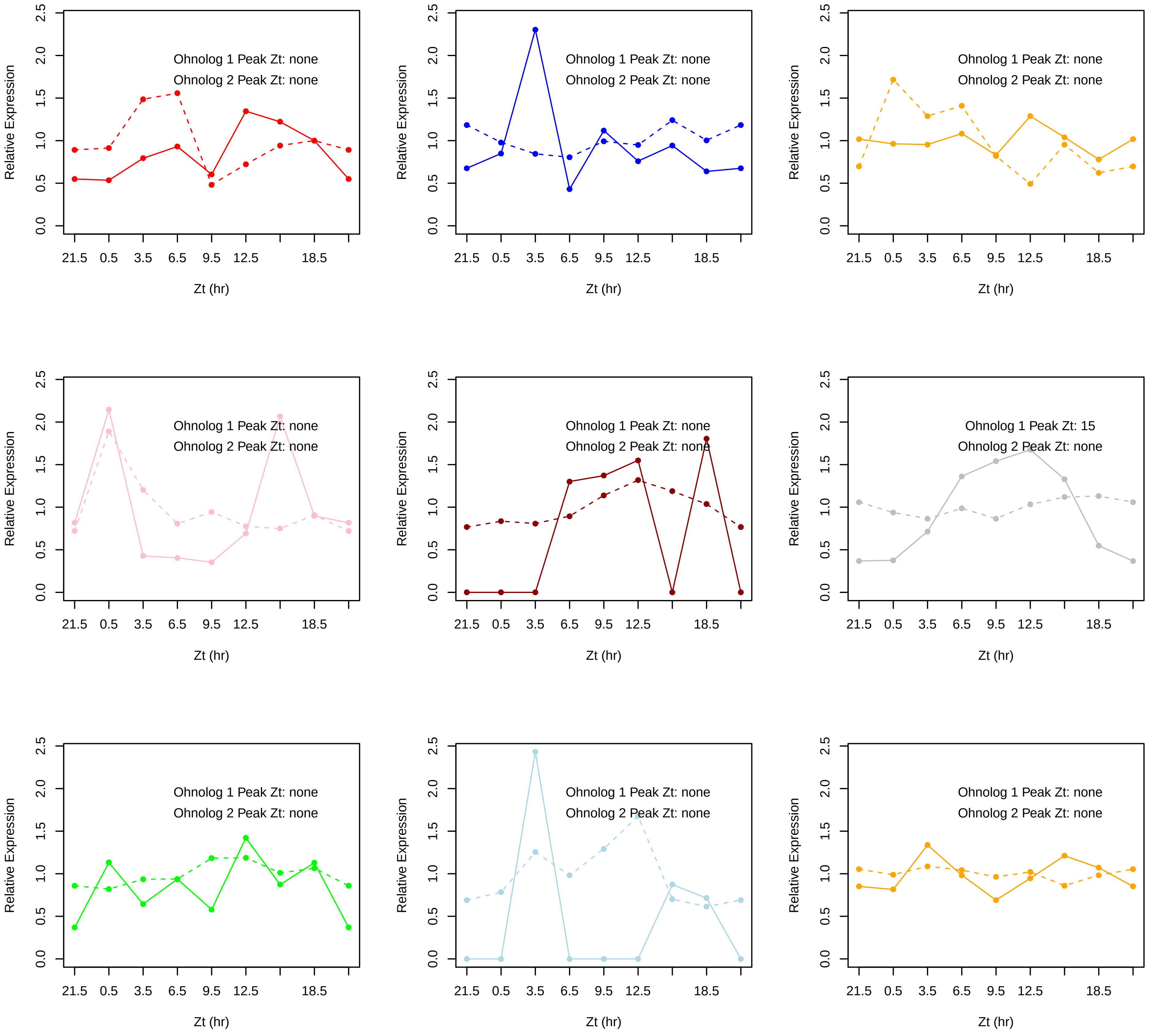

Ohnolog 1: ENSXMAG00000002228
Ohnolog 2: ENSXMAG00000007895 SCN8A

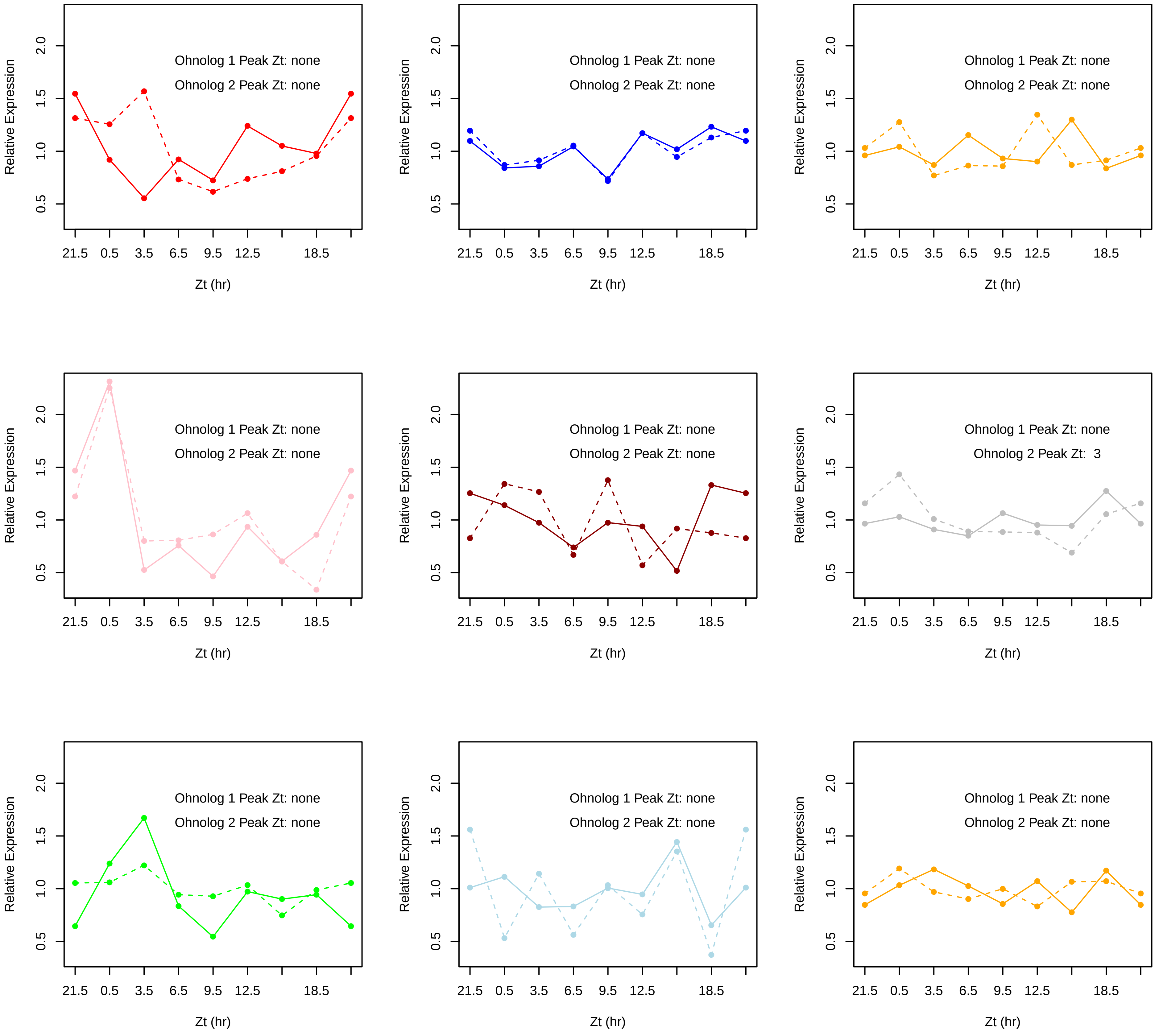

Ohnolog 1: ENSXMAG00000006835 rap1gap2b
Ohnolog 2: ENSXMAG00000012276

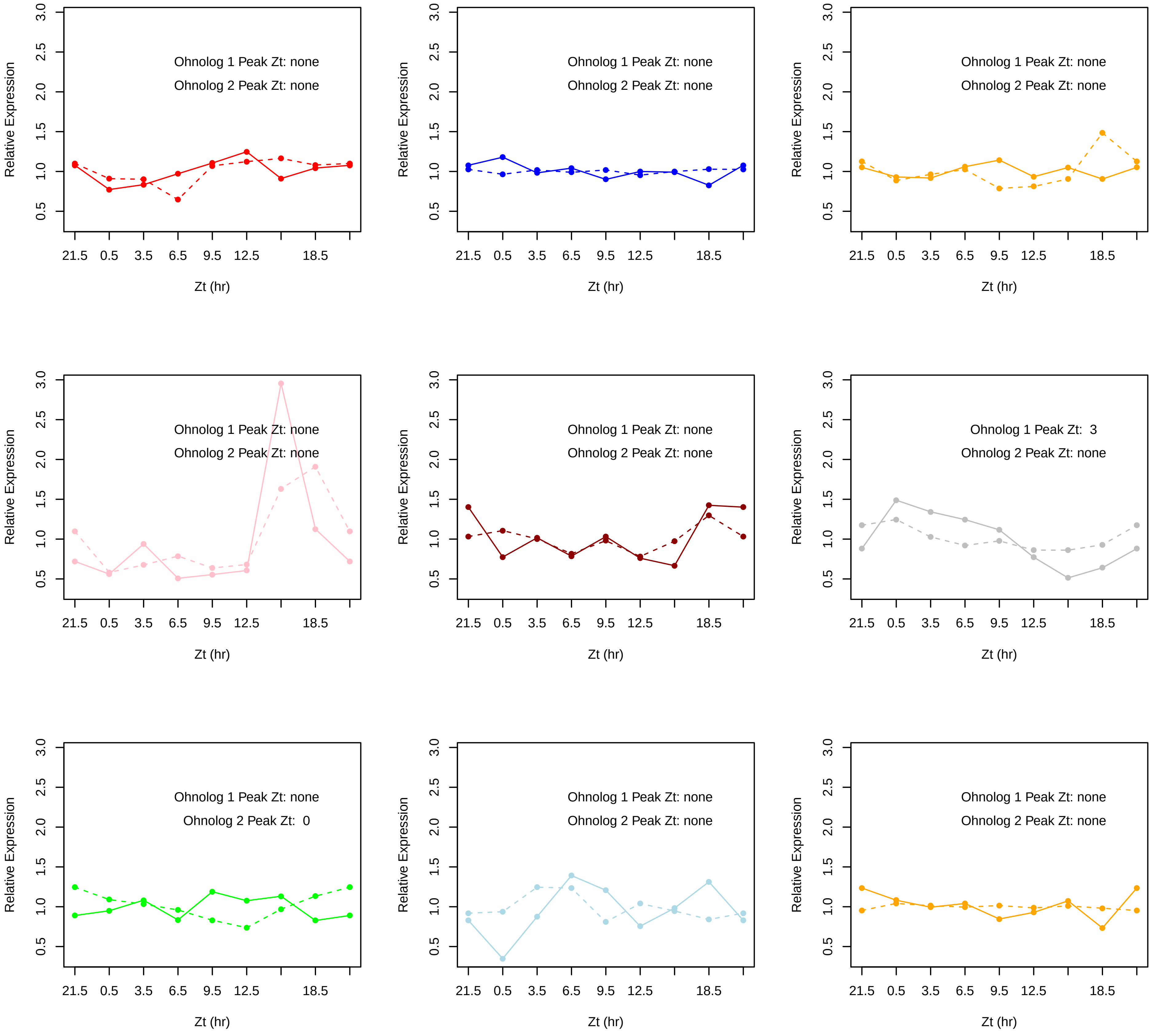

Ohnolog 1: ENSXMAG00000017106 nr1d2b
Ohnolog 2: ENSXMAG00000018709 nr1d2a

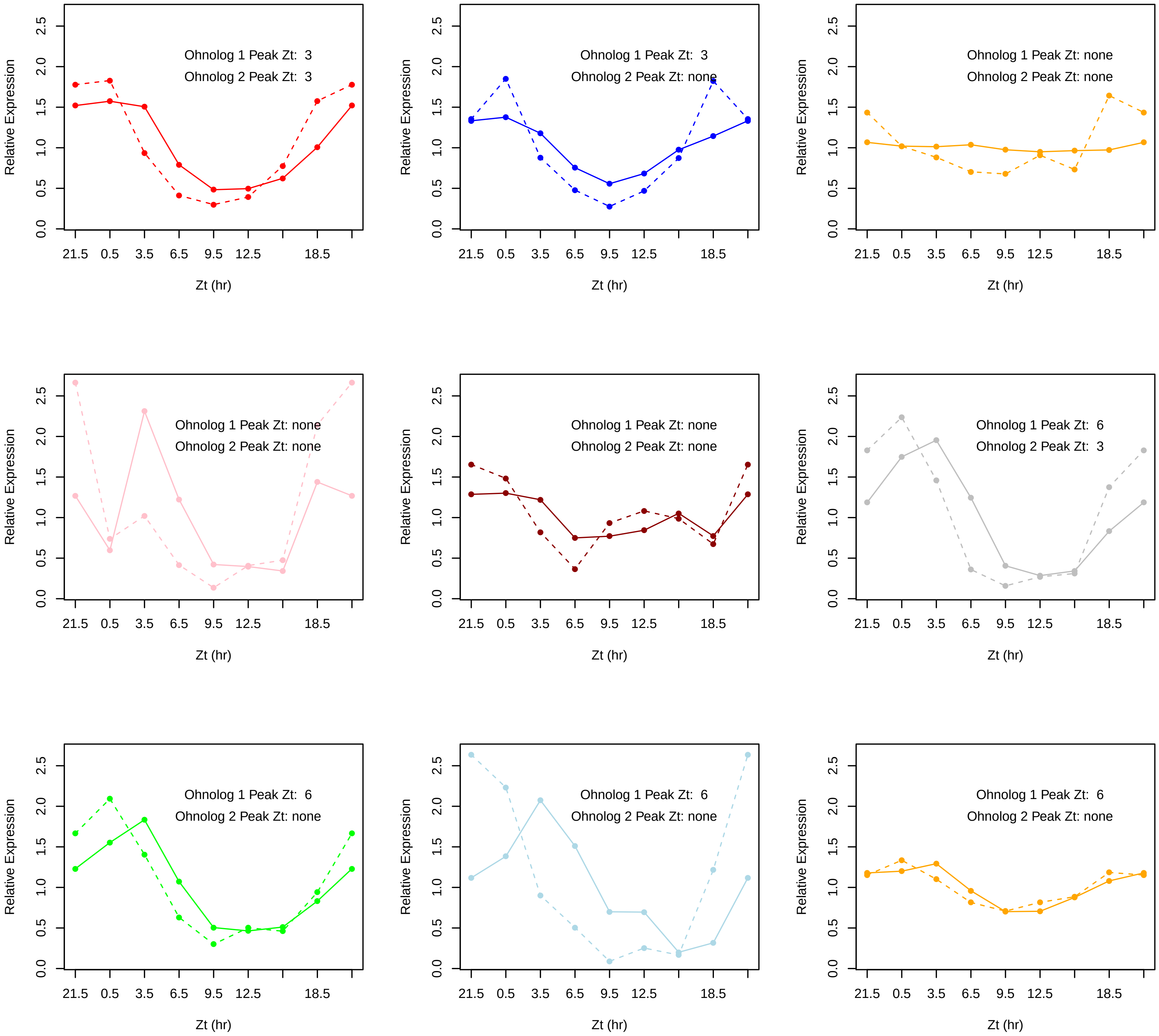

Ohnolog 1: ENSXMAG00000004660 RAB40C
Ohnolog 2: ENSXMAG00000013138 rab40c

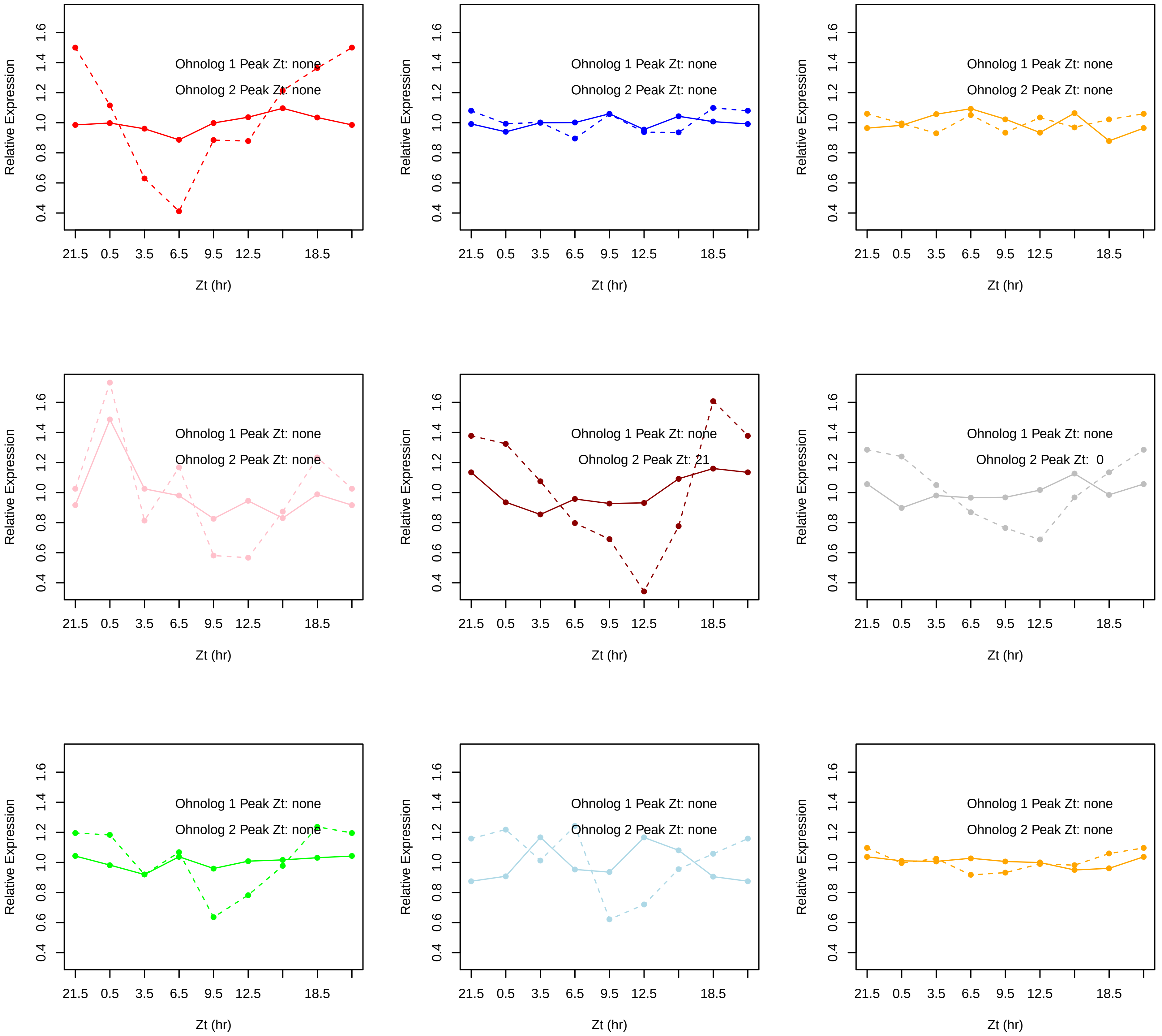

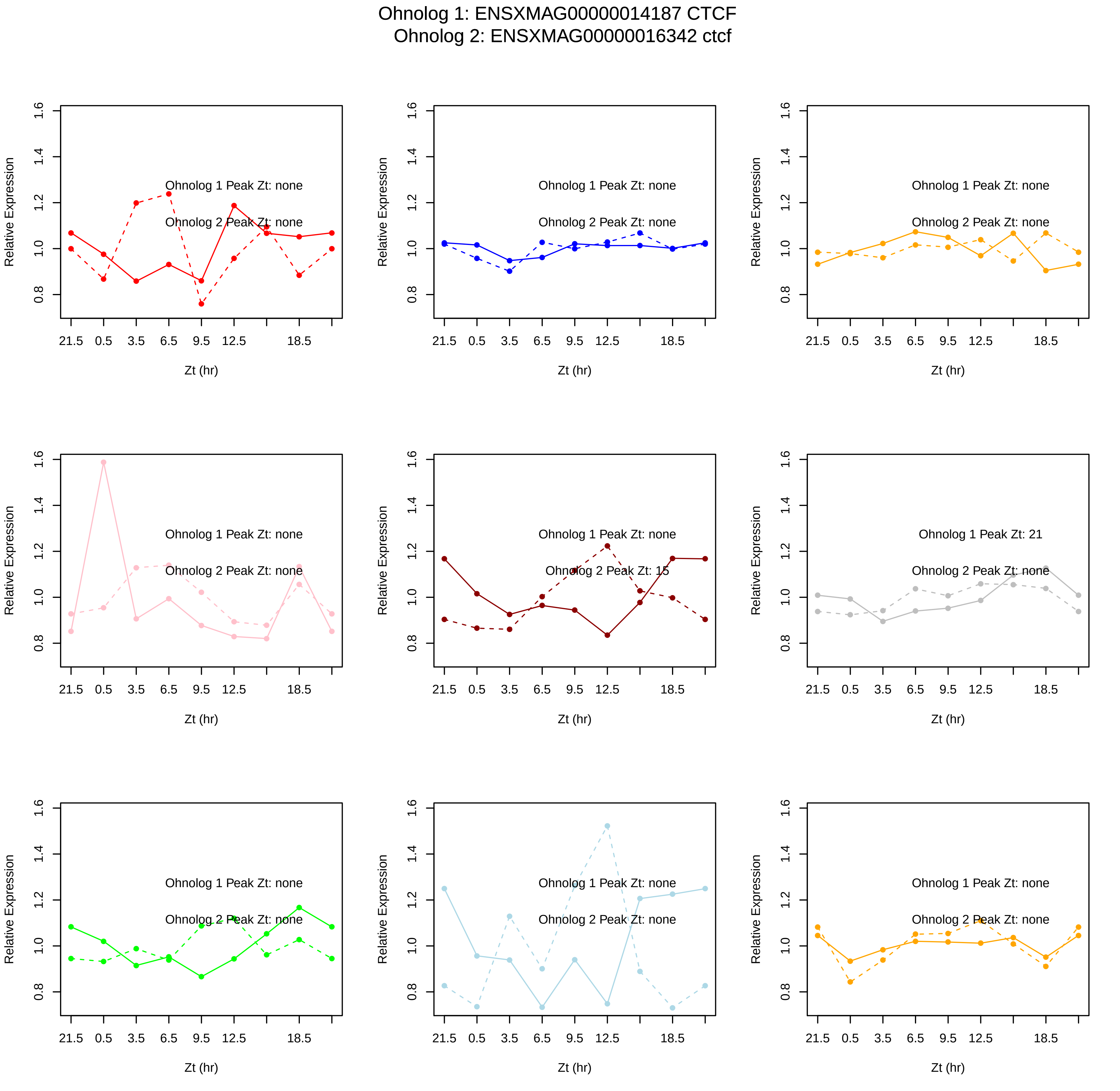
Ohnolog 1: ENSXMAG00000014187 CTCF
Ohnolog 2: ENSXMAG00000016342 ctcf
Ohnolog 1 Peak Zt: none
Ohnolog 2 Peak Zt: none
Ohnolog 1 Peak Zt: 21
Ohnolog 2 Peak Zt: 15
Relative Expression
Zt (hr)
21.5
0.5
3.5
6.5
9.5
12.5
18.5
0.8
1.0
1.2
1.4
1.6

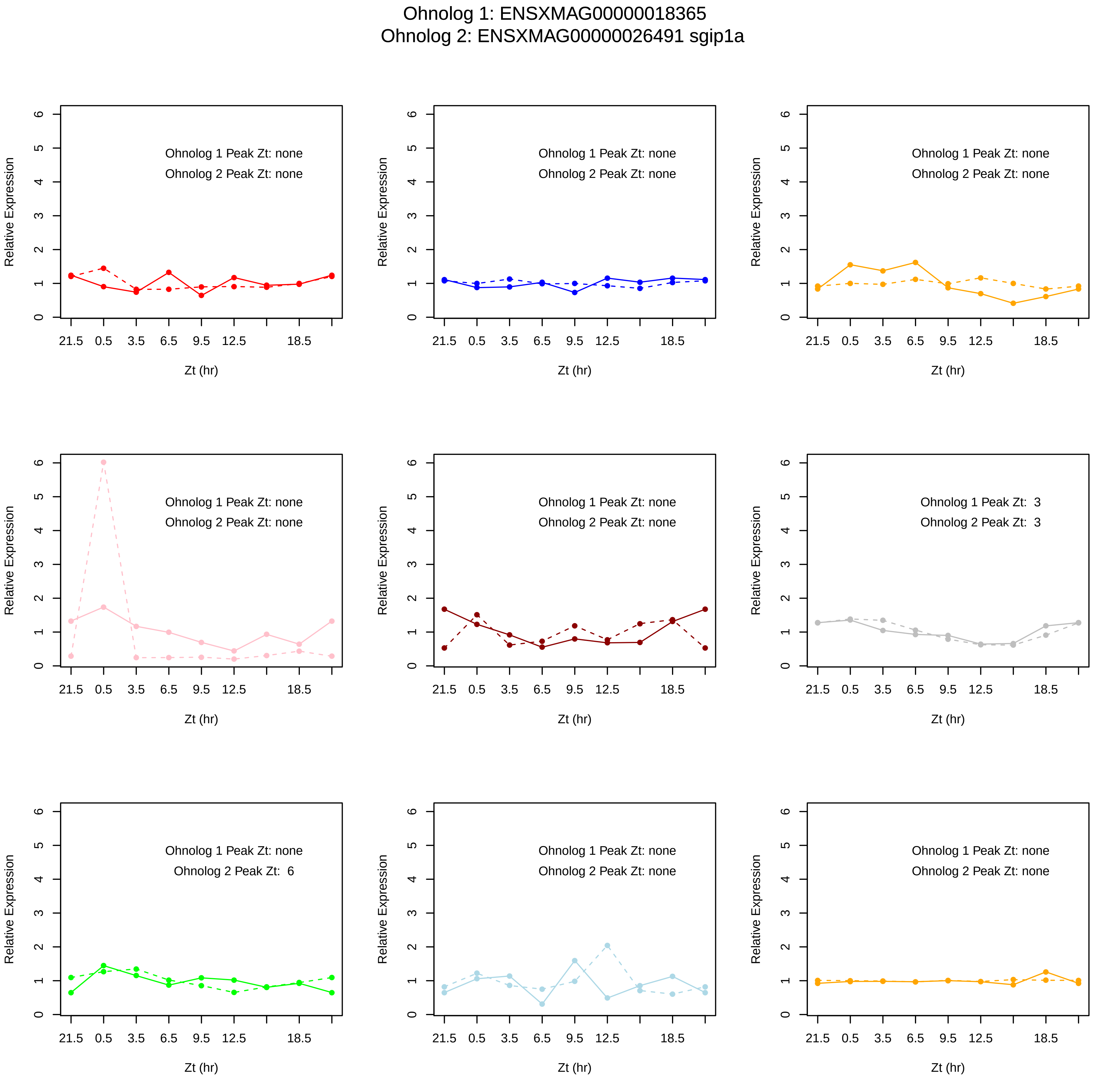
Ohnolog 1: ENSXMAG00000018365
Ohnolog 2: ENSXMAG00000026491 sgip1a
Ohnolog 1 Peak Zt: none
Ohnolog 2 Peak Zt: none
Ohnolog 1 Peak Zt: none
Ohnolog 2 Peak Zt: none
Ohnolog 1 Peak Zt: none
Ohnolog 2 Peak Zt: none
Ohnolog 1 Peak Zt: none
Ohnolog 2 Peak Zt: none
Ohnolog 1 Peak Zt: none
Ohnolog 2 Peak Zt: none
Ohnolog 1 Peak Zt: 3
Ohnolog 2 Peak Zt: 3
Ohnolog 1 Peak Zt: none
Ohnolog 2 Peak Zt: 6
Ohnolog 1 Peak Zt: none
Ohnolog 2 Peak Zt: none
Ohnolog 1 Peak Zt: none
Ohnolog 2 Peak Zt: none
Relative Expression
Zt (hr)
21.5 0.5 3.5 6.5 9.5 12.5 18.5
0 1 2 3 4 5 6

Ohnolog 1: ENSXMAG00000008529 gria4b
Ohnolog 2: ENSXMAG00000023735 gria4a

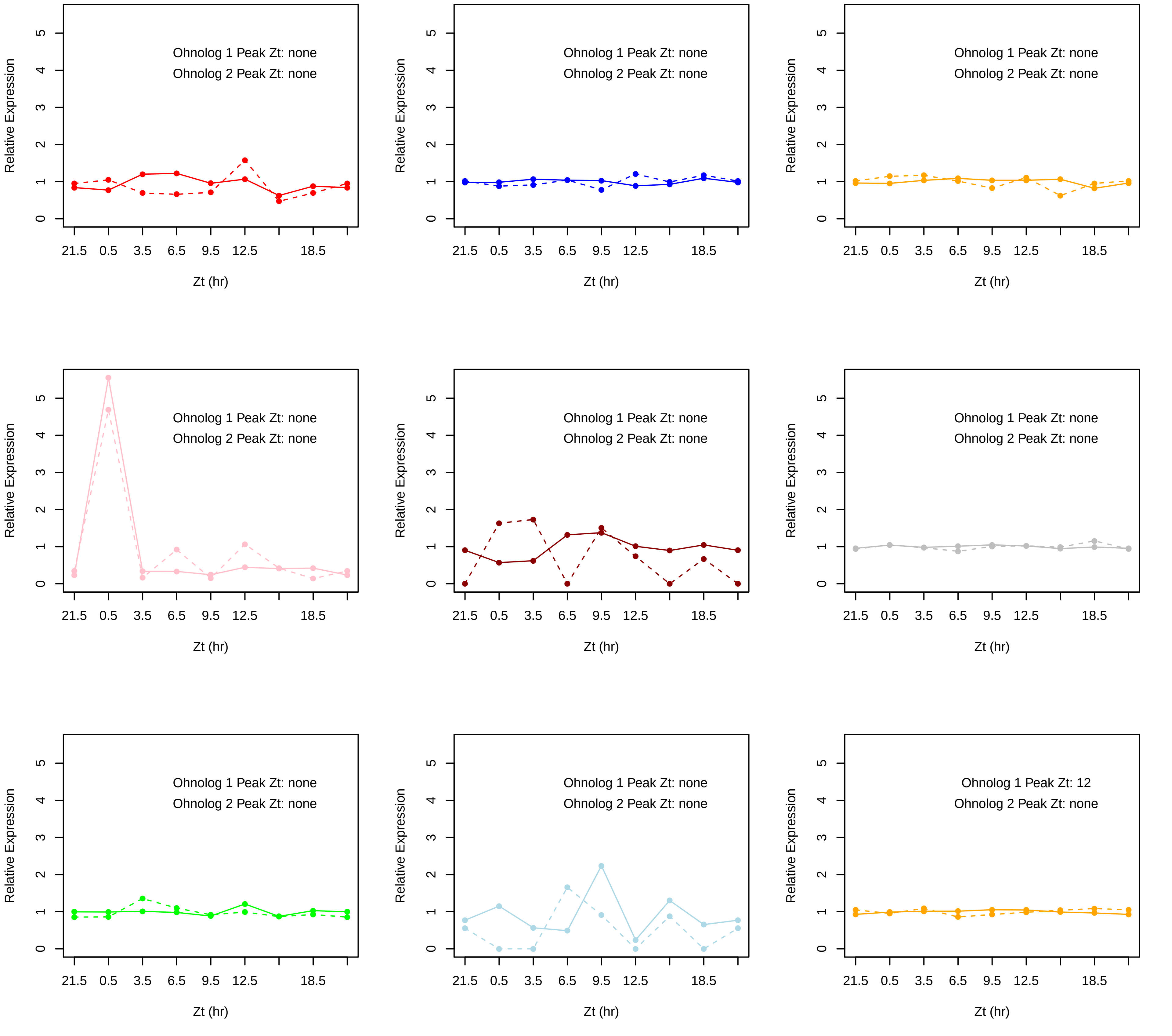

Ohnolog 1: ENSXMAG00000004881 xylt1 (1 of many)
Ohnolog 2: ENSXMAG00000028234 xylt1 (1 of many)

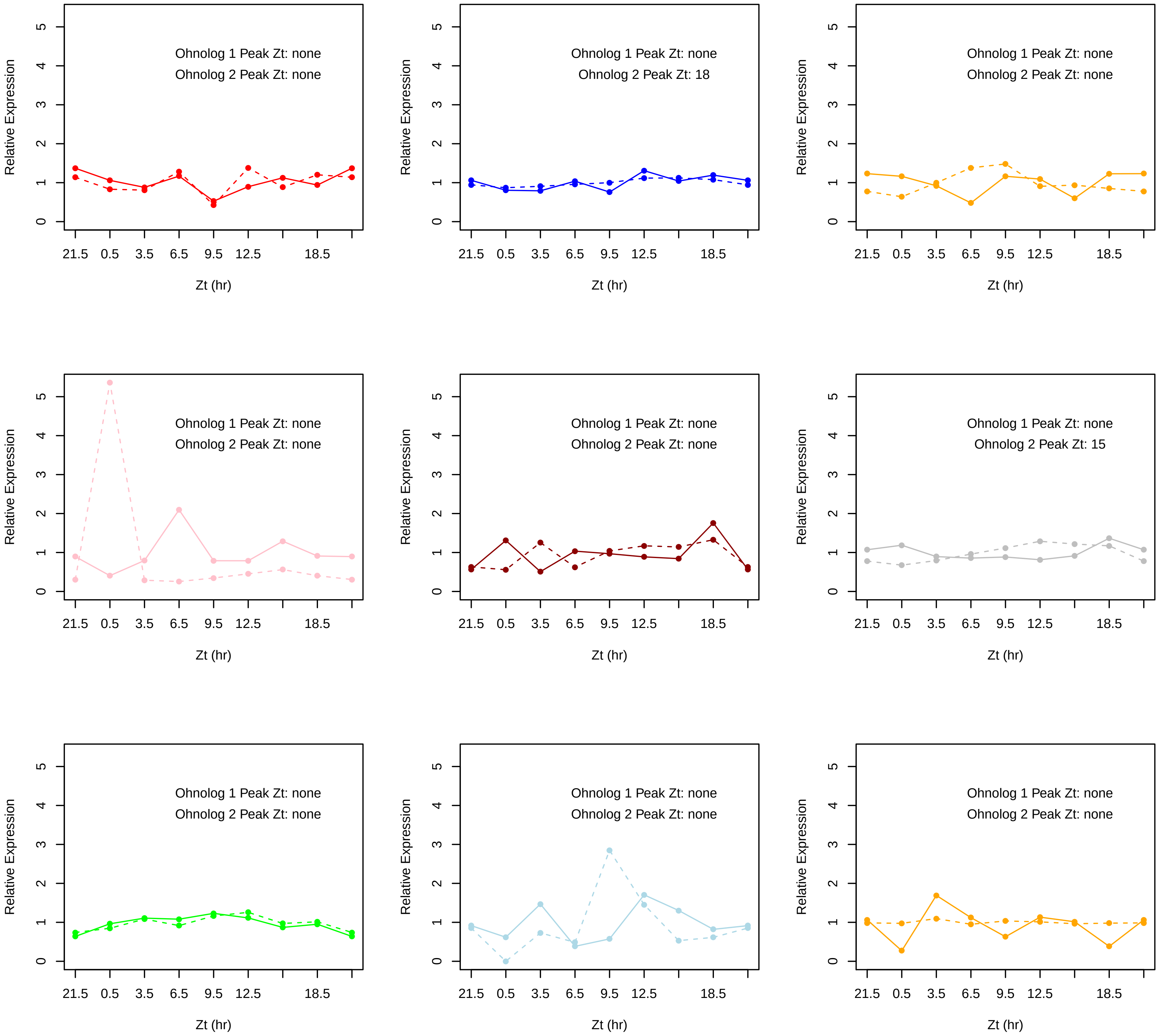

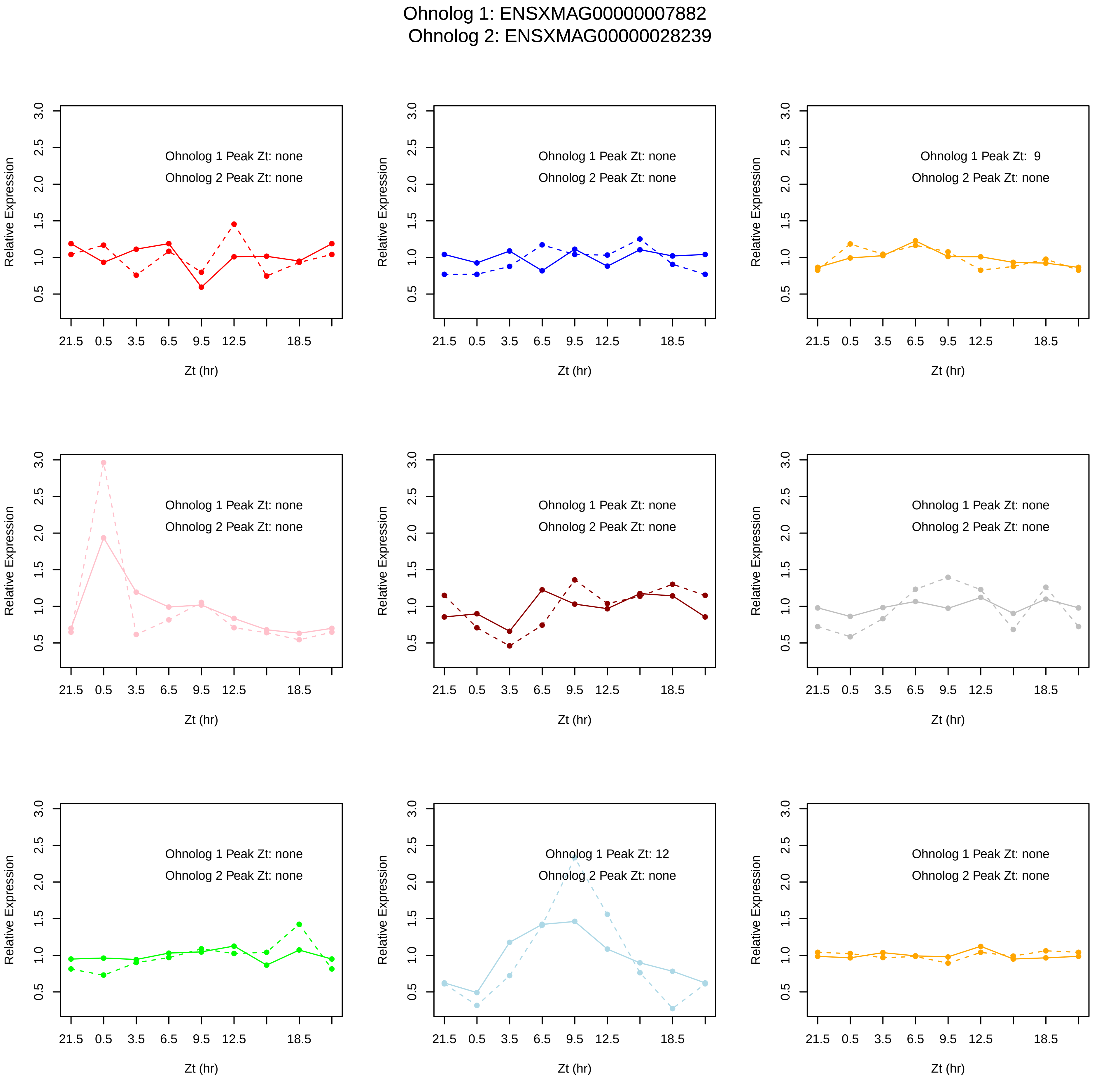

Ohnolog 1: ENSXMAG00000007882
Ohnolog 2: ENSXMAG00000028239
Ohnolog 1 Peak Zt: none
Ohnolog 2 Peak Zt: none
Ohnolog 1 Peak Zt: none
Ohnolog 2 Peak Zt: none
Ohnolog 1 Peak Zt: 9
Ohnolog 2 Peak Zt: none
Ohnolog 1 Peak Zt: none
Ohnolog 2 Peak Zt: none
Ohnolog 1 Peak Zt: none
Ohnolog 2 Peak Zt: none
Ohnolog 1 Peak Zt: none
Ohnolog 2 Peak Zt: none
Ohnolog 1 Peak Zt: none
Ohnolog 2 Peak Zt: none
Ohnolog 1 Peak Zt: 12
Ohnolog 2 Peak Zt: none
Ohnolog 1 Peak Zt: none
Ohnolog 2 Peak Zt: none
Relative Expression
Zt (hr)
21.5
0.5
3.5
6.5
9.5
12.5
18.5
0.5
1.0
1.5
2.0
2.5
3.0

Ohnolog 1: ENSXMAG00000007231
Ohnolog 2: ENSXMAG00000014814 pbxip1a

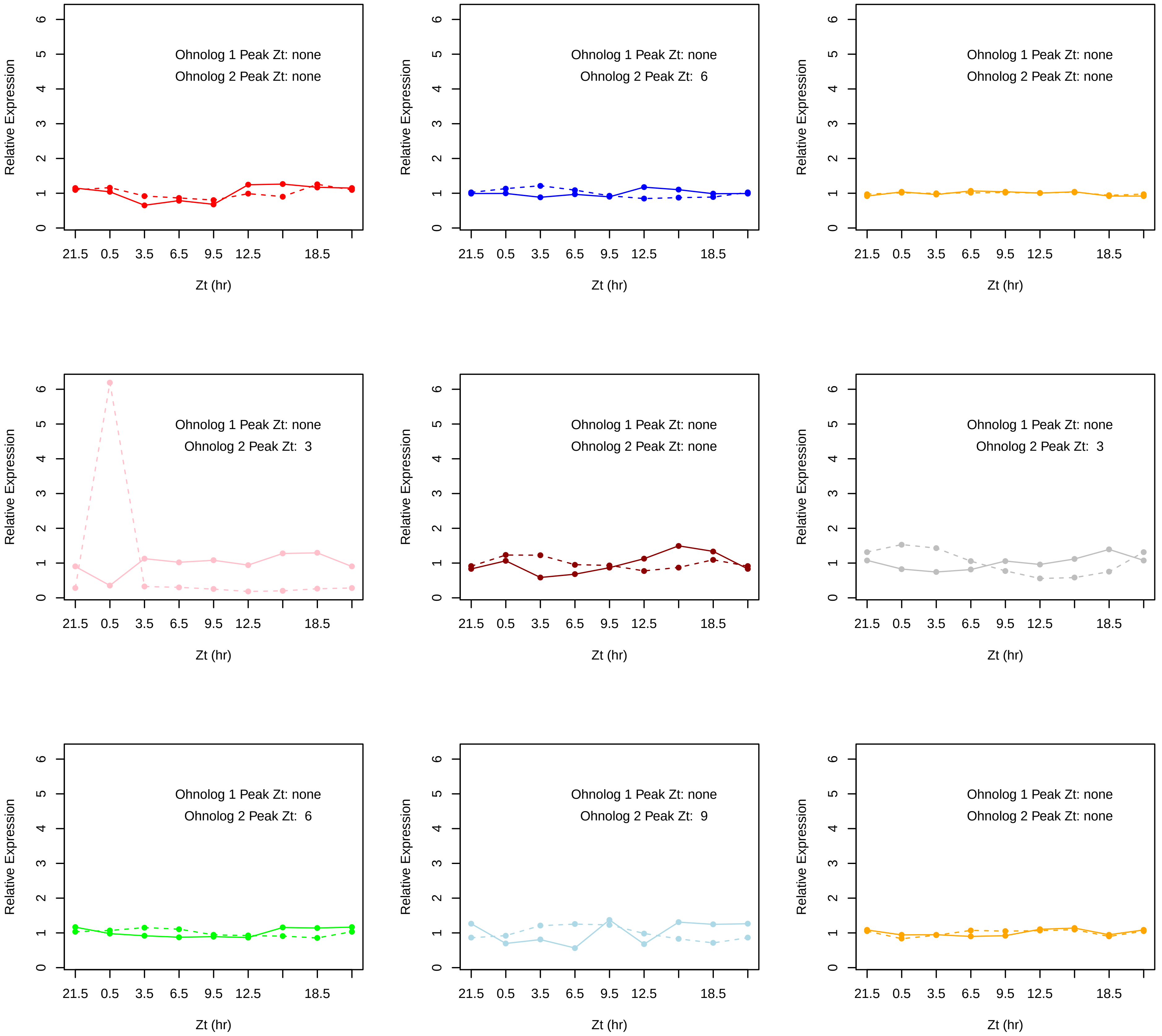

Ohnolog 1: ENSXMAG00000011686 hsd17b7
Ohnolog 2: ENSXMAG00000012536

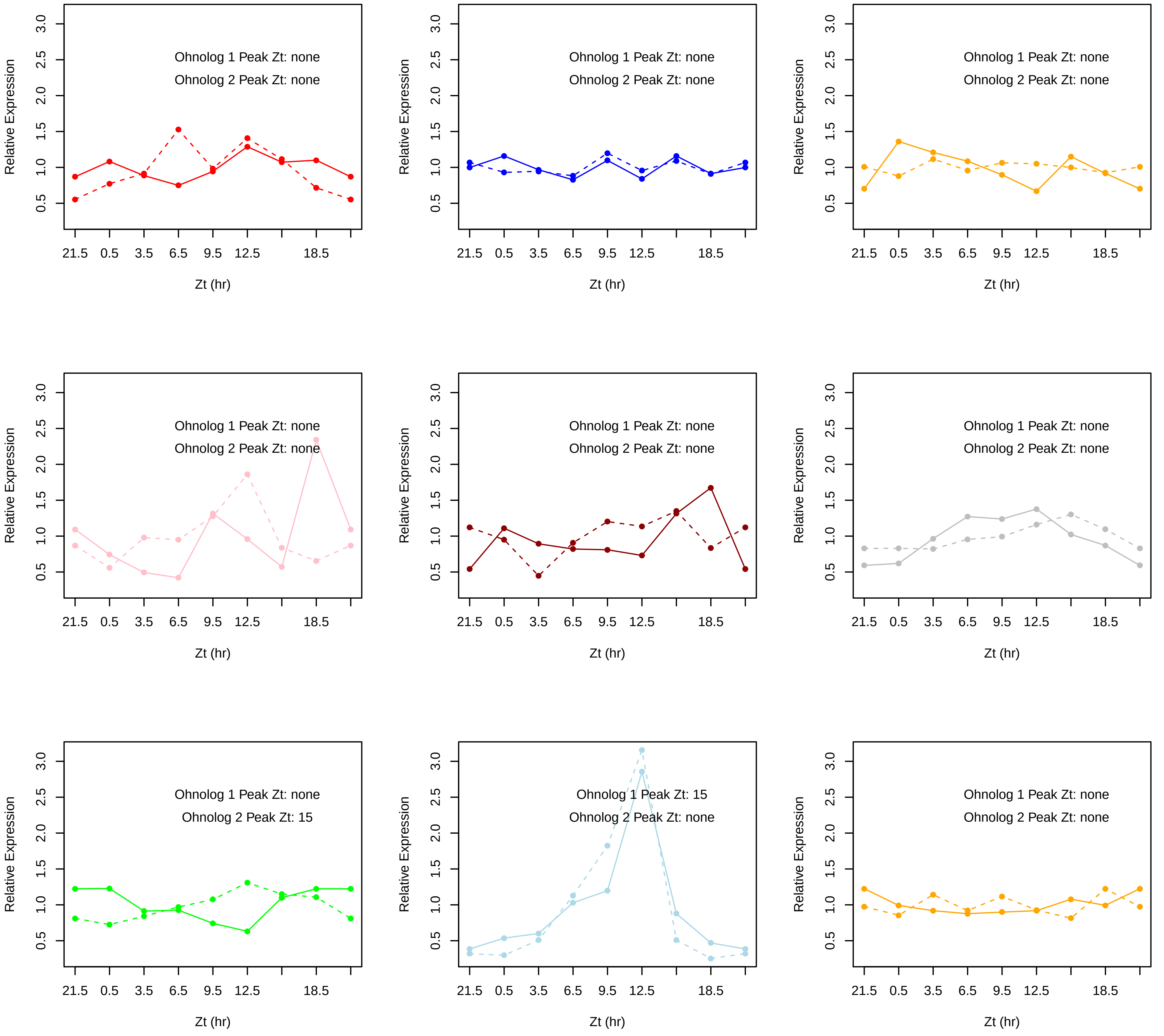

Ohnolog 1: ENSXMAG00000002271 rasgef1bb
Ohnolog 2: ENSXMAG00000010014 rasgef1ba

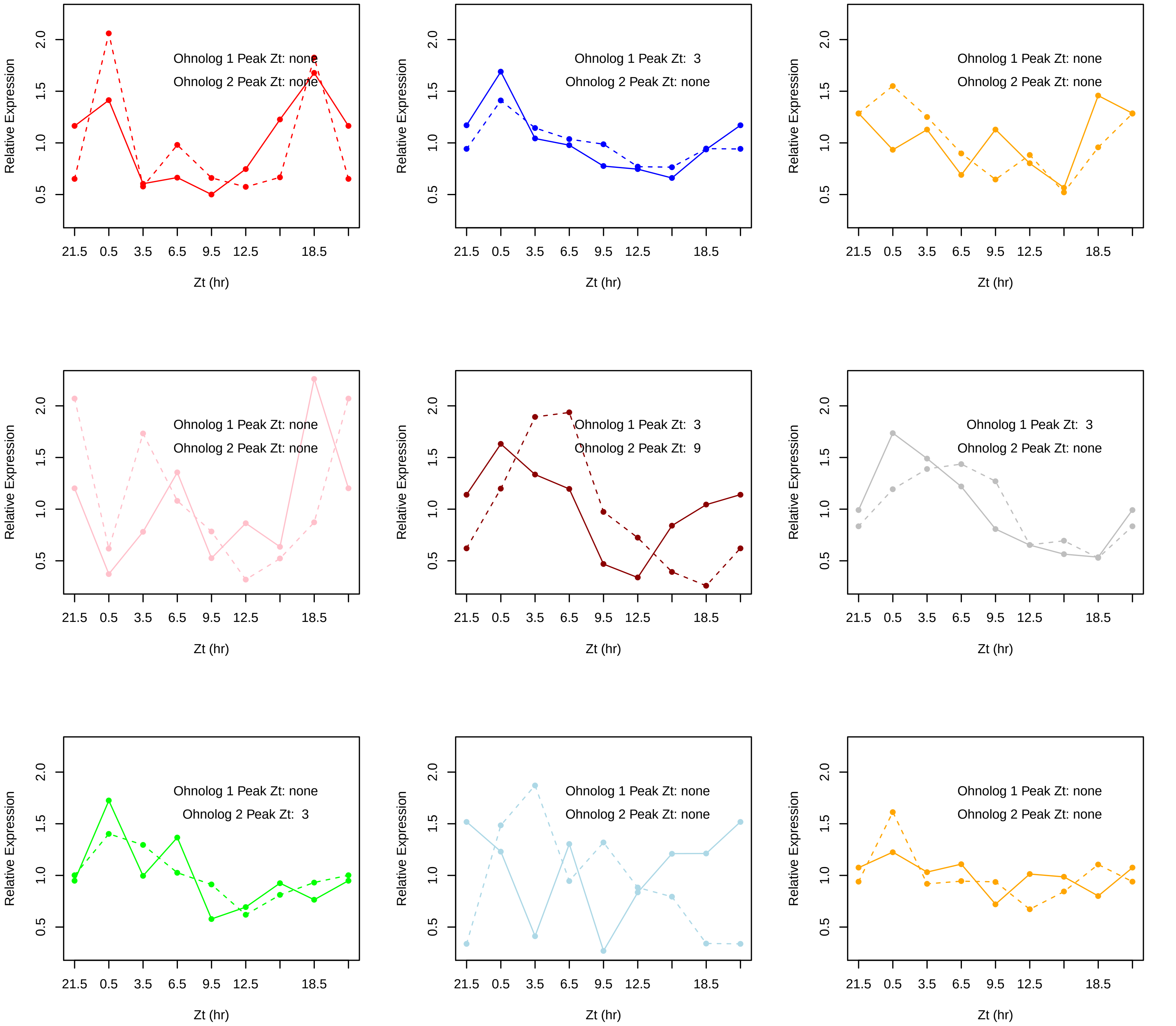

Ohnolog 1: ENSXMAG00000007142 rgs9a
Ohnolog 2: ENSXMAG00000017103 rgs9b

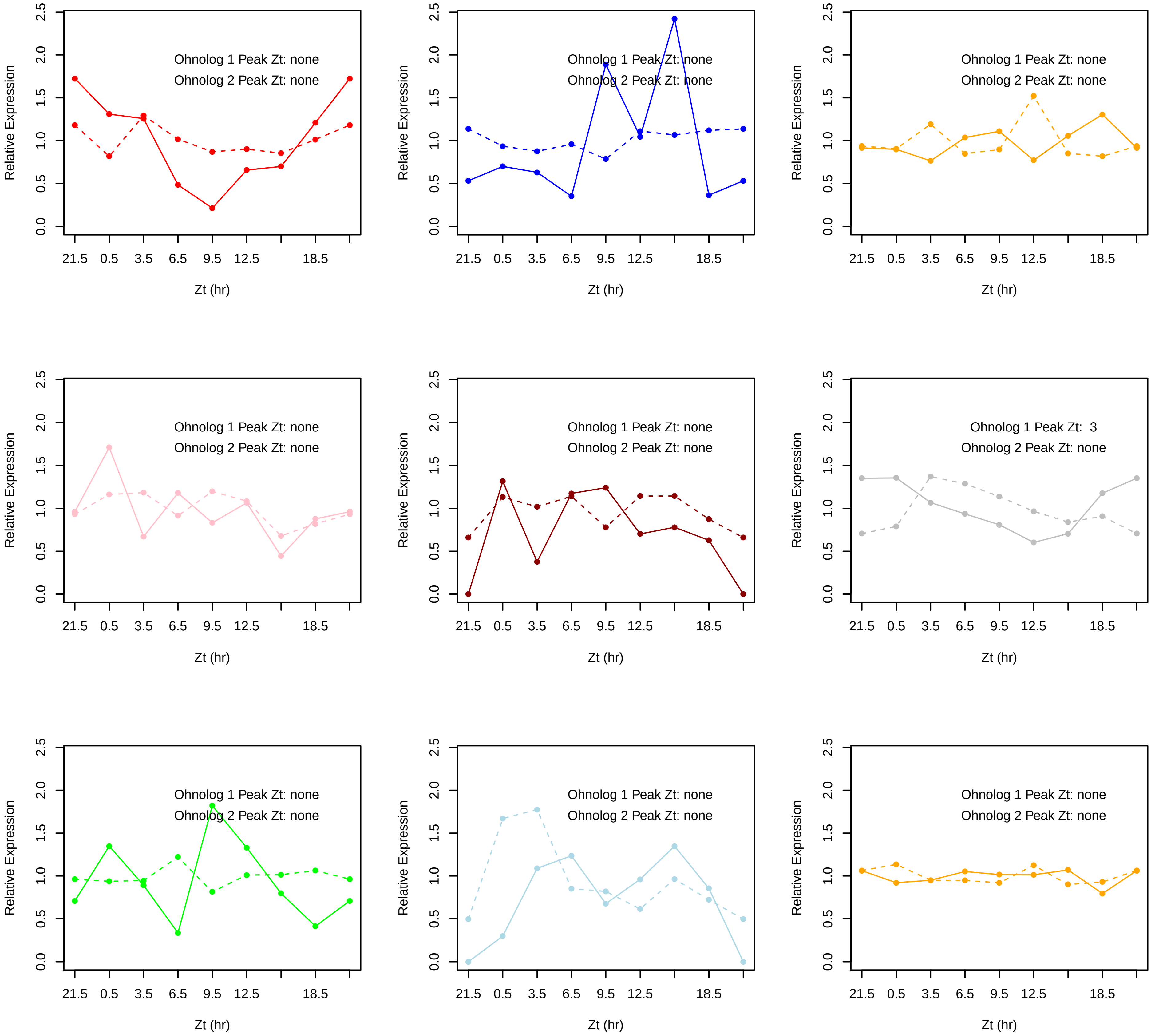

Ohnolog 1: ENSXMAG00000000413 lnpa
Ohnolog 2: ENSXMAG00000026819

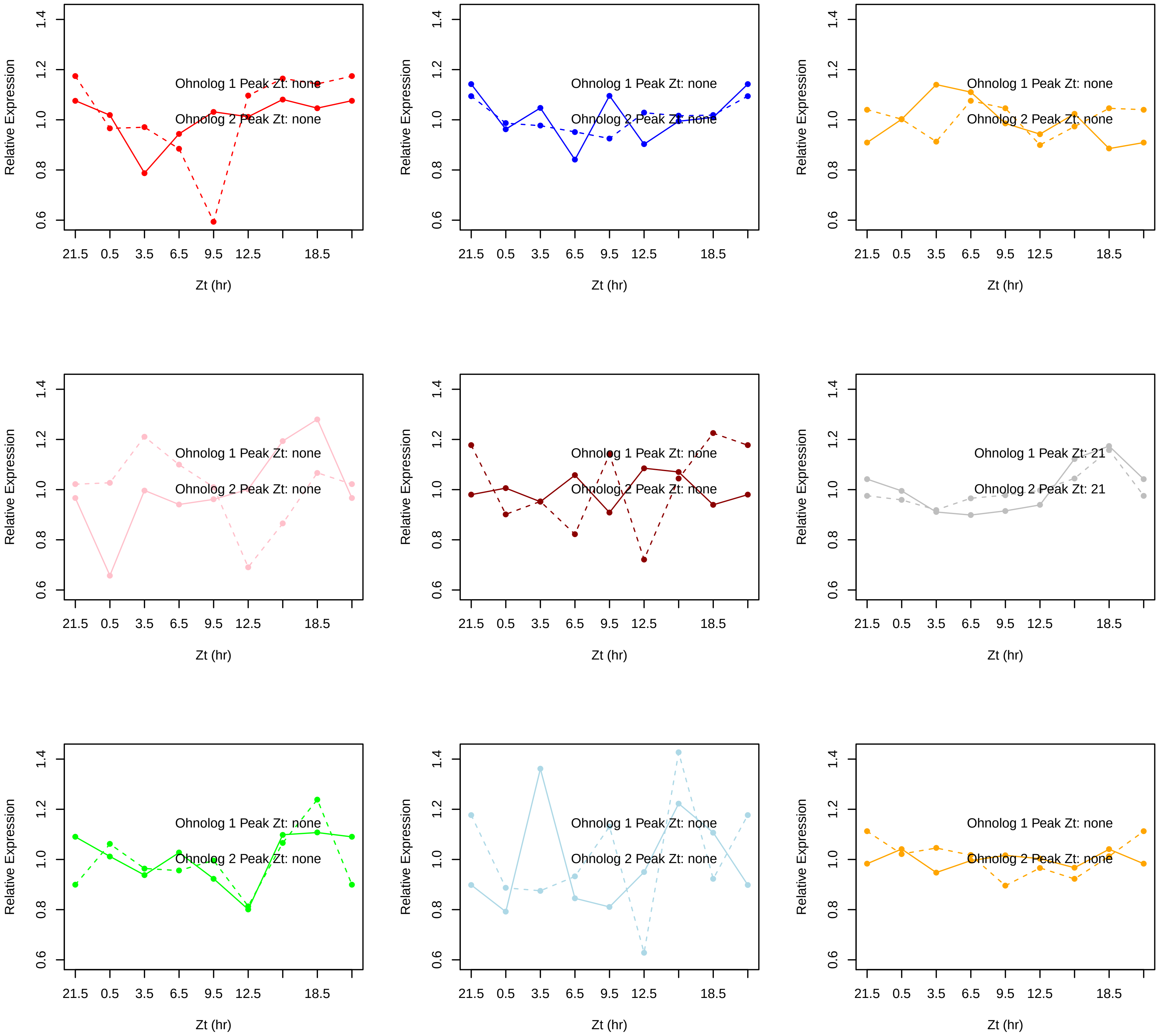

Ohnolog 1: ENSXMAG00000000410 atp5mc3b
Ohnolog 2: ENSXMAG00000019062

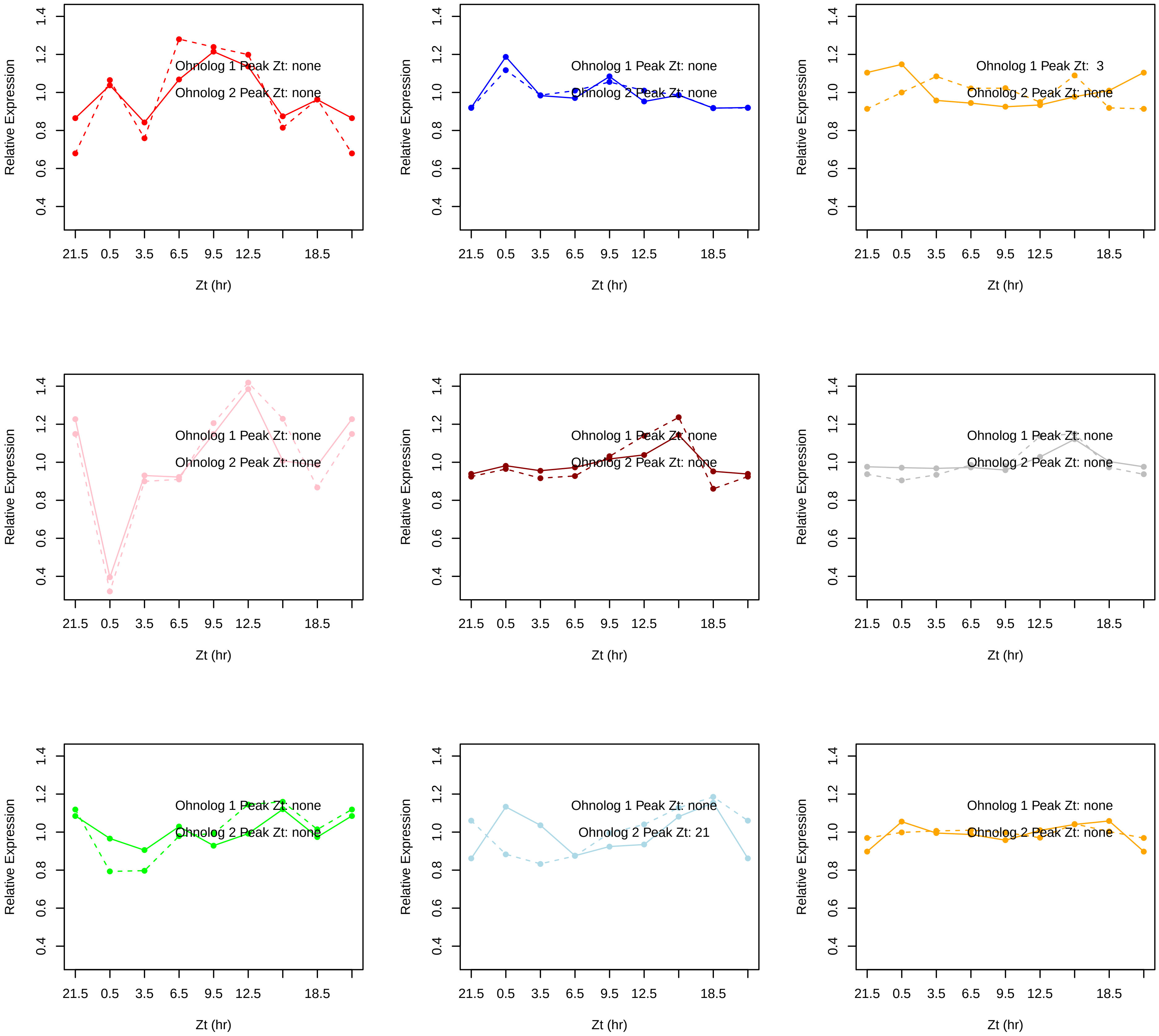

Ohnolog 1: ENSXMAG00000005615 rgs16
Ohnolog 2: ENSXMAG00000019234 rgs5b

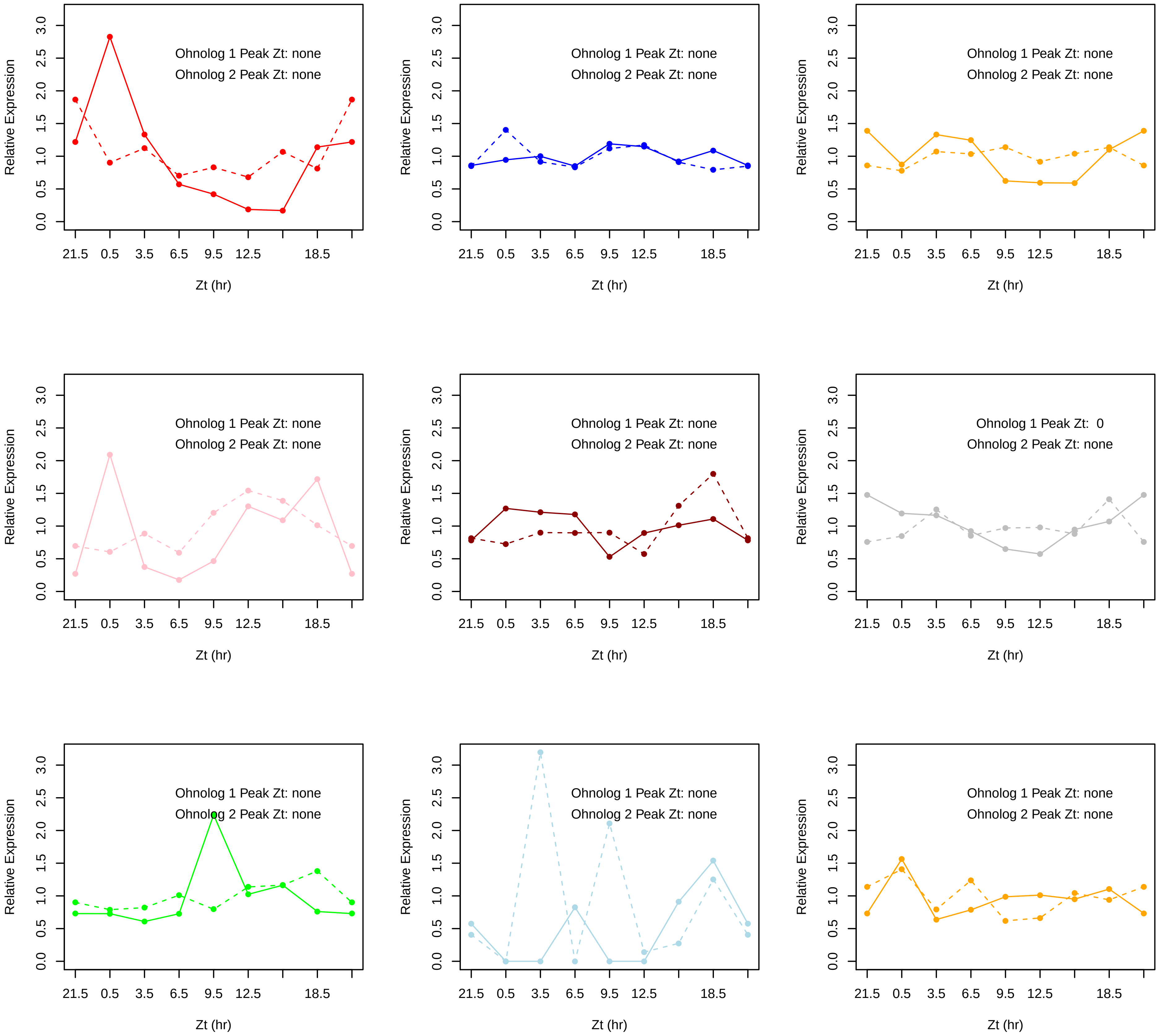

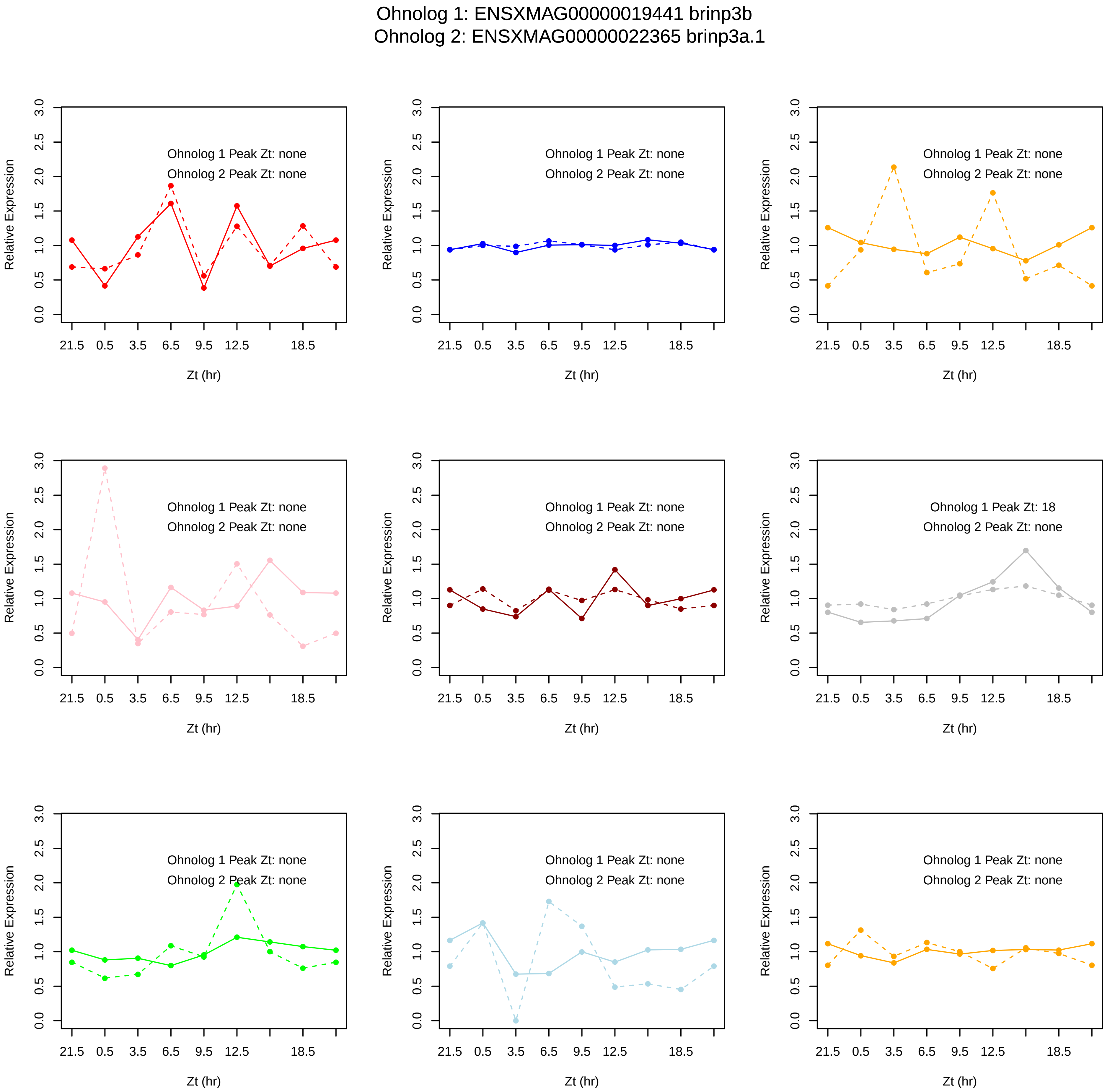

Ohnolog 1: ENSXMAG00000019441 brinp3b
Ohnolog 2: ENSXMAG00000022365 brinp3a.1
Ohnolog 1 Peak Zt: none
Ohnolog 2 Peak Zt: none
Ohnolog 1 Peak Zt: none
Ohnolog 2 Peak Zt: none
Ohnolog 1 Peak Zt: none
Ohnolog 2 Peak Zt: none
Ohnolog 1 Peak Zt: none
Ohnolog 2 Peak Zt: none
Ohnolog 1 Peak Zt: none
Ohnolog 2 Peak Zt: none
Ohnolog 1 Peak Zt: 18
Ohnolog 2 Peak Zt: none
Ohnolog 1 Peak Zt: none
Ohnolog 2 Peak Zt: none
Ohnolog 1 Peak Zt: none
Ohnolog 2 Peak Zt: none
Ohnolog 1 Peak Zt: none
Ohnolog 2 Peak Zt: none
Relative Expression
Zt (hr)
0.0
0.5
1.0
1.5
2.0
2.5
3.0
21.5
0.5
3.5
6.5
9.5
12.5
18.5

Ohnolog 1: ENSXMAG00000005488
Ohnolog 2: ENSXMAG00000005951

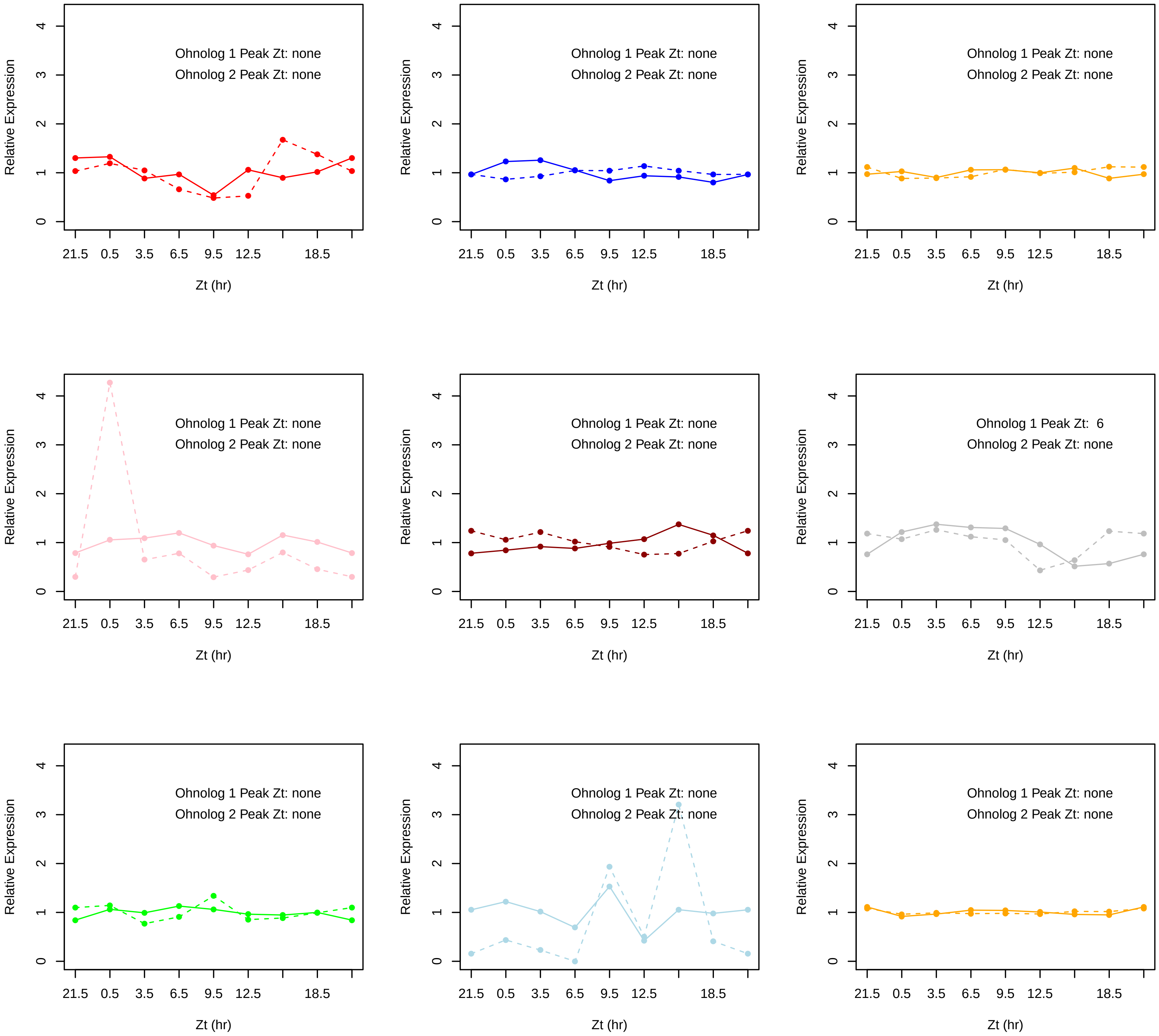

Ohnolog 1: ENSXMAG00000010616 ptges3a
Ohnolog 2: ENSXMAG00000017416 ptges3b

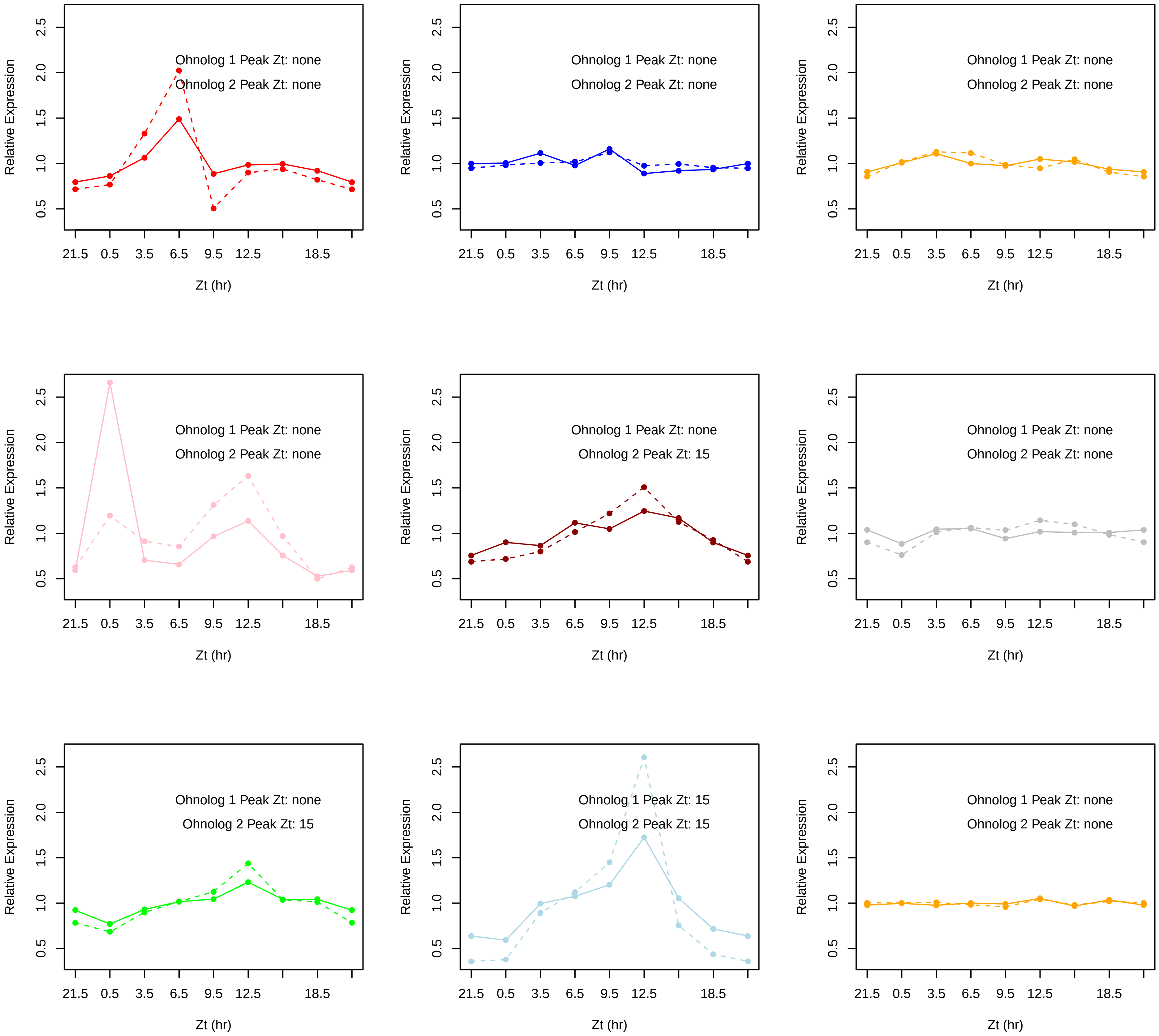

Ohnolog 1: ENSXMAG00000007574
Ohnolog 2: ENSXMAG00000022965 adgra1a

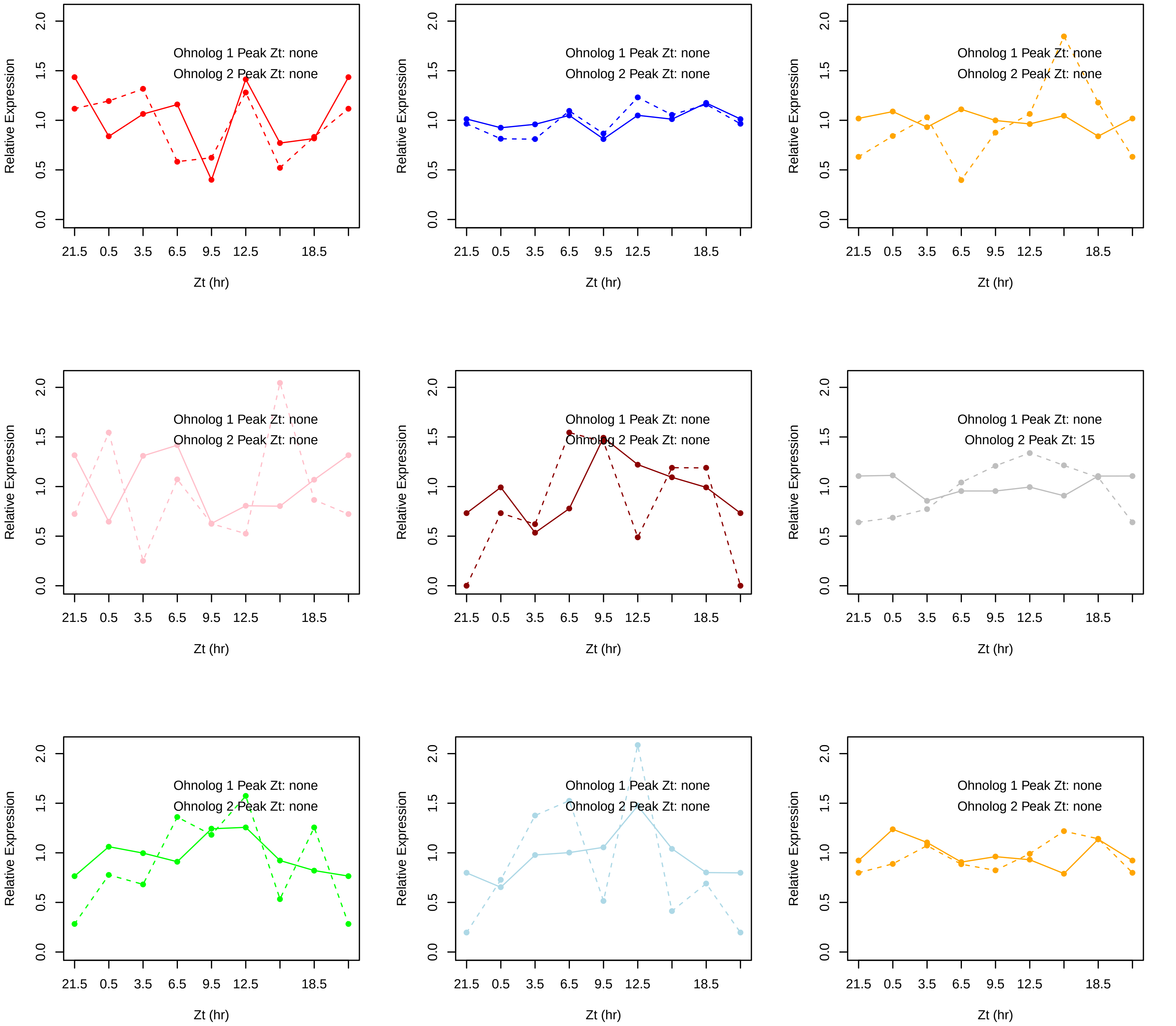

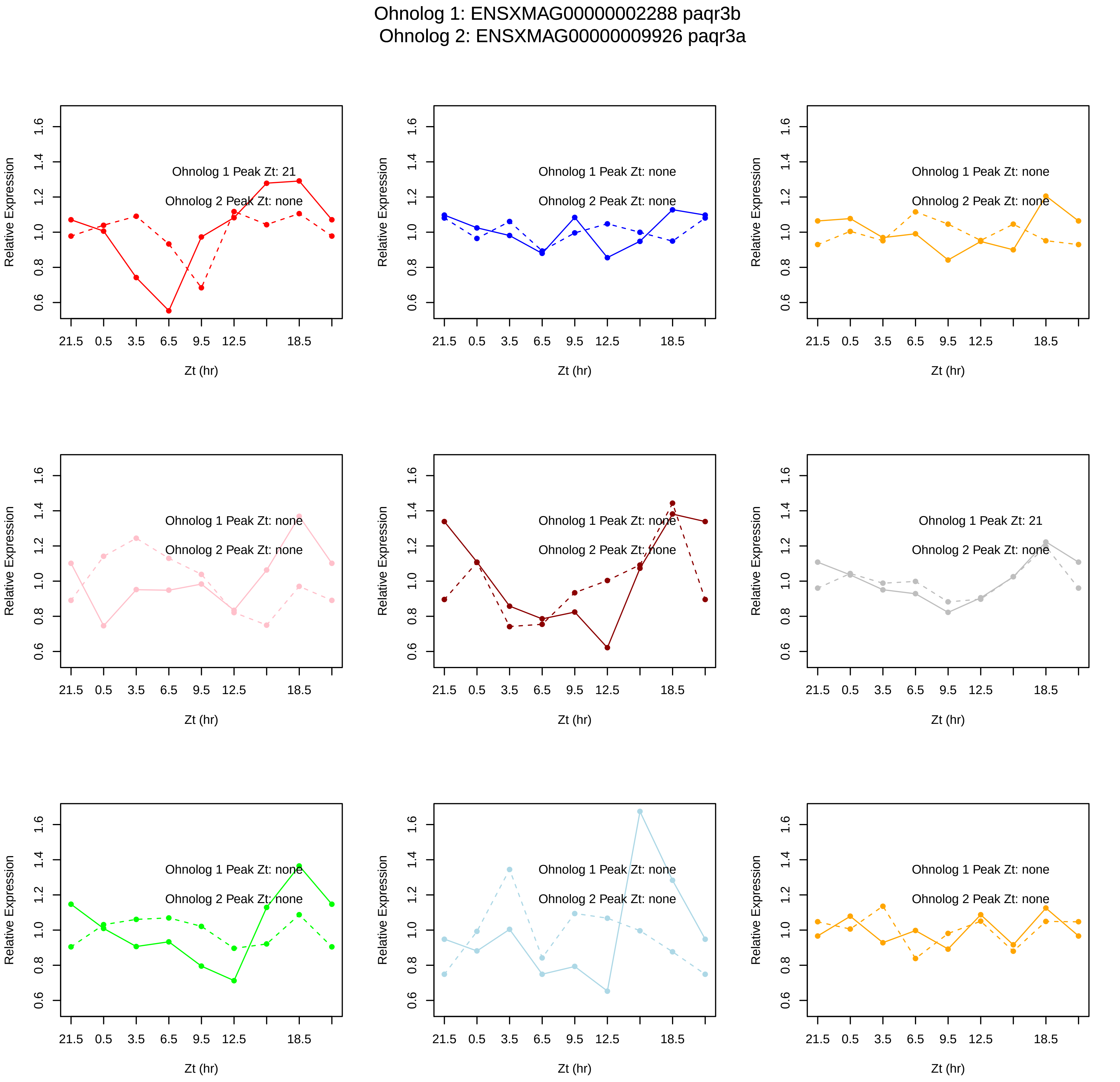
Ohnolog 1: ENSXMAG00000002288 paqr3b
Ohnolog 2: ENSXMAG00000009926 paqr3a
Ohnolog 1 Peak Zt: 21
Ohnolog 2 Peak Zt: none
Ohnolog 1 Peak Zt: none
Ohnolog 2 Peak Zt: none
Ohnolog 1 Peak Zt: none
Ohnolog 2 Peak Zt: none
Ohnolog 1 Peak Zt: none
Ohnolog 2 Peak Zt: none
Ohnolog 1 Peak Zt: none
Ohnolog 2 Peak Zt: none
Ohnolog 1 Peak Zt: 21
Ohnolog 2 Peak Zt: none
Ohnolog 1 Peak Zt: none
Ohnolog 2 Peak Zt: none
Ohnolog 1 Peak Zt: none
Ohnolog 2 Peak Zt: none
Ohnolog 1 Peak Zt: none
Ohnolog 2 Peak Zt: none
Relative Expression
0.6 0.8 1.0 1.2 1.4 1.6
21.5 0.5 3.5 6.5 9.5 12.5 18.5
Zt (hr)

Ohnolog 1: ENSXMAG00000004031 pptc7b
Ohnolog 2: ENSXMAG00000029877 pptc7a

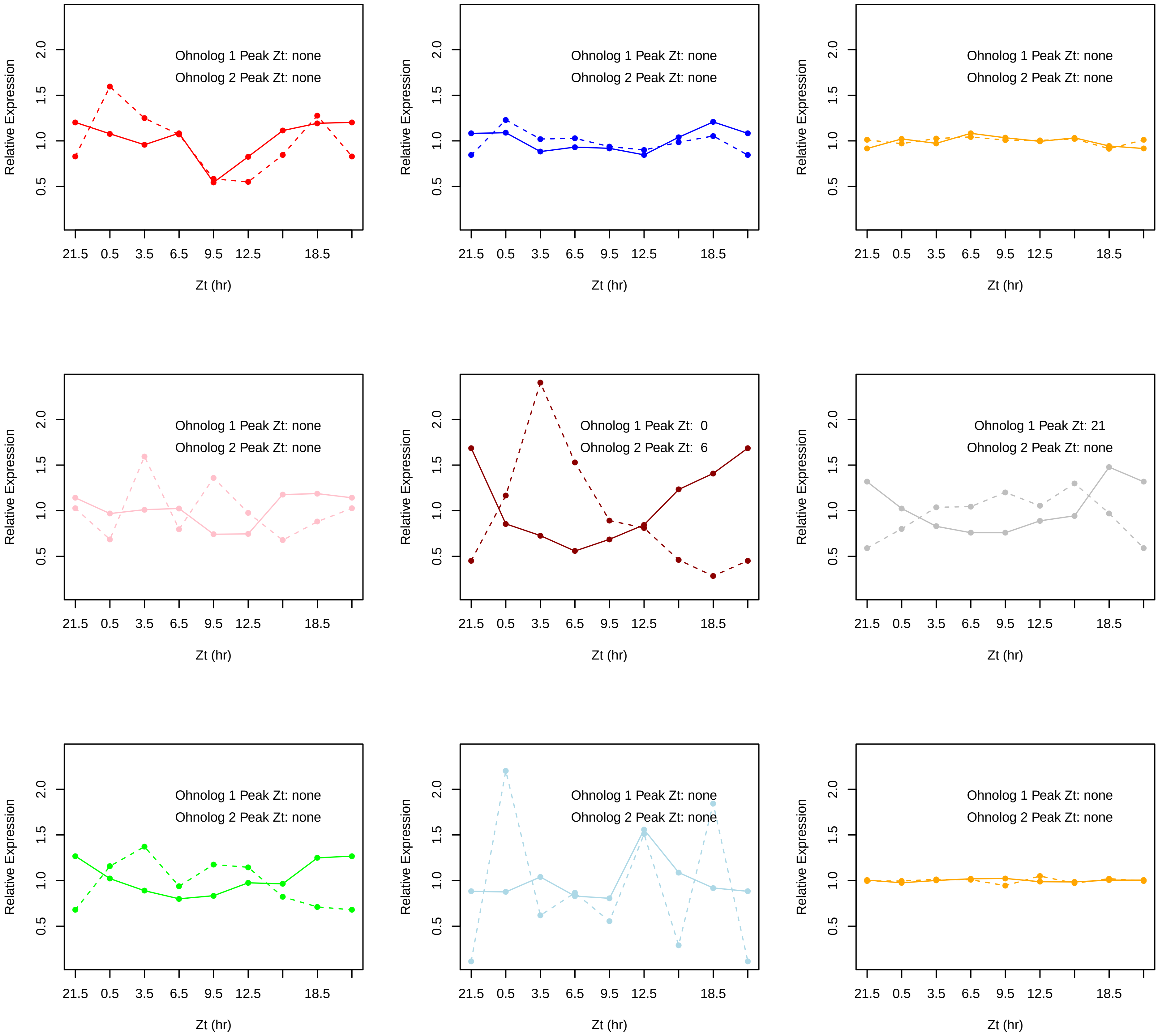

Ohnolog 1: ENSXMAG00000005021 gpr155b
Ohnolog 2: ENSXMAG00000005409 gpr155a

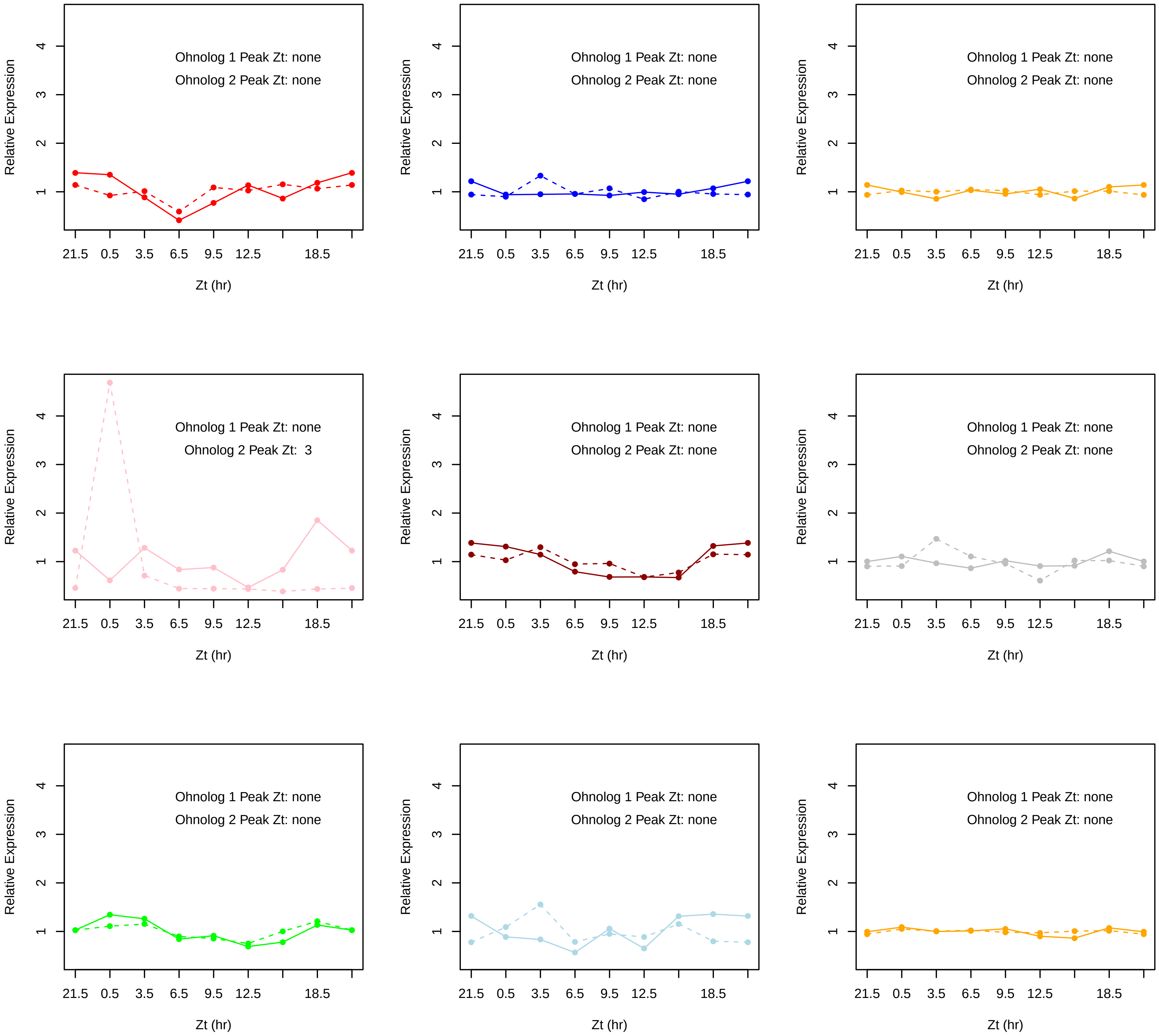

Ohnolog 1: ENSXMAG00000002712 RGS13 (1 of many)
Ohnolog 2: ENSXMAG00000024646 rgs13

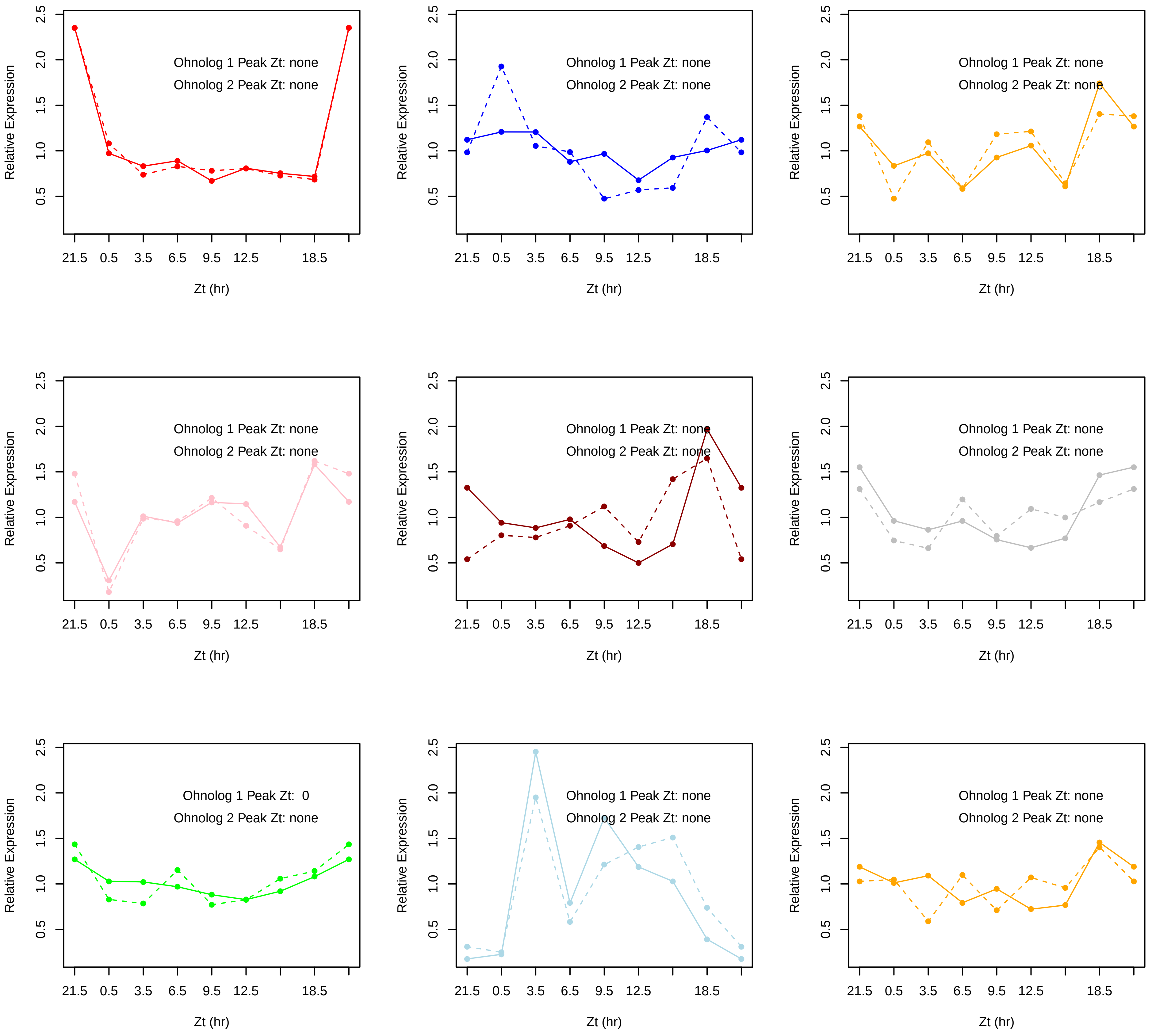

Ohnolog 1: ENSXMAG00000012988 si:dkeyp−72e1.7
Ohnolog 2: ENSXMAG00000029897

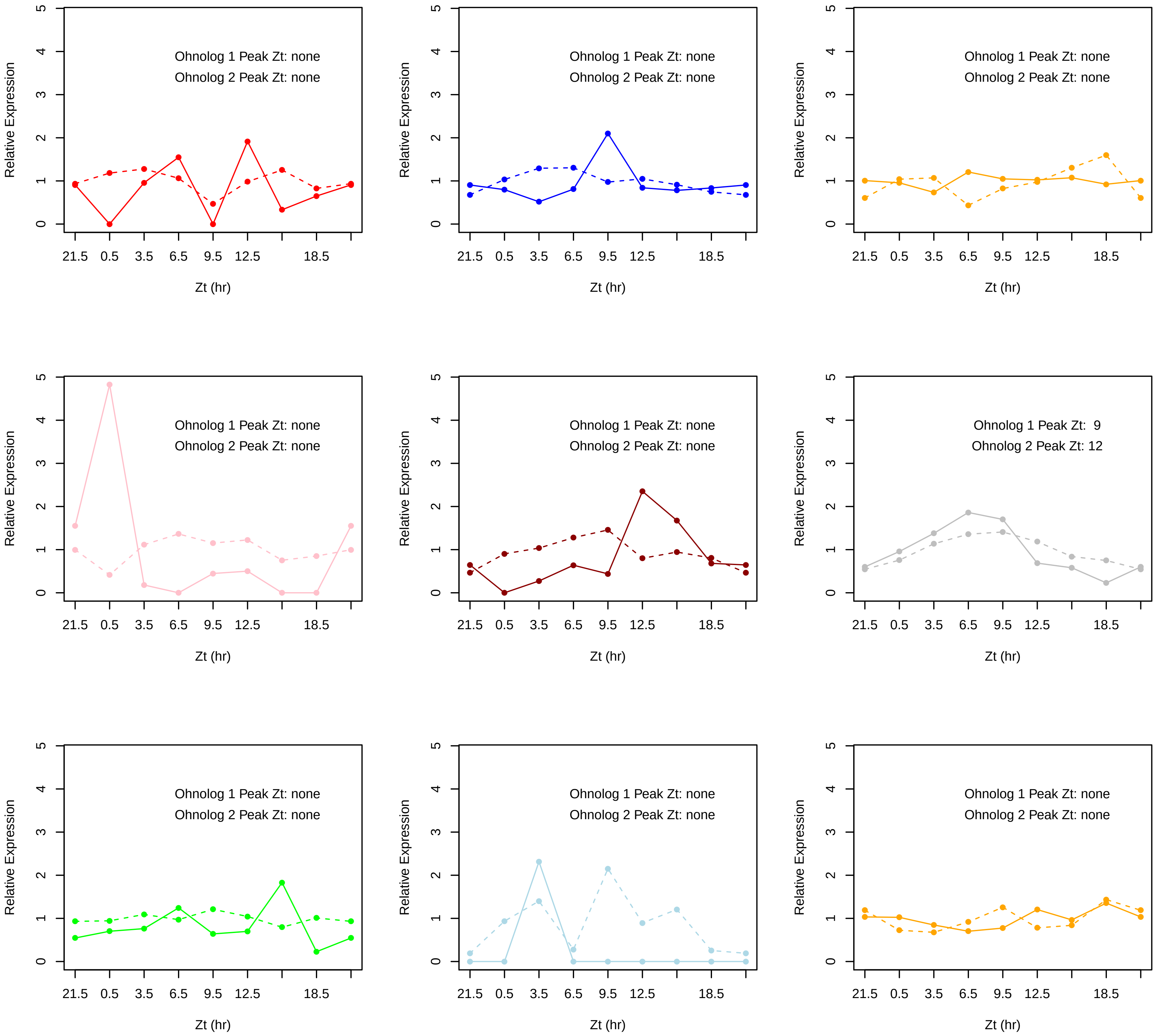

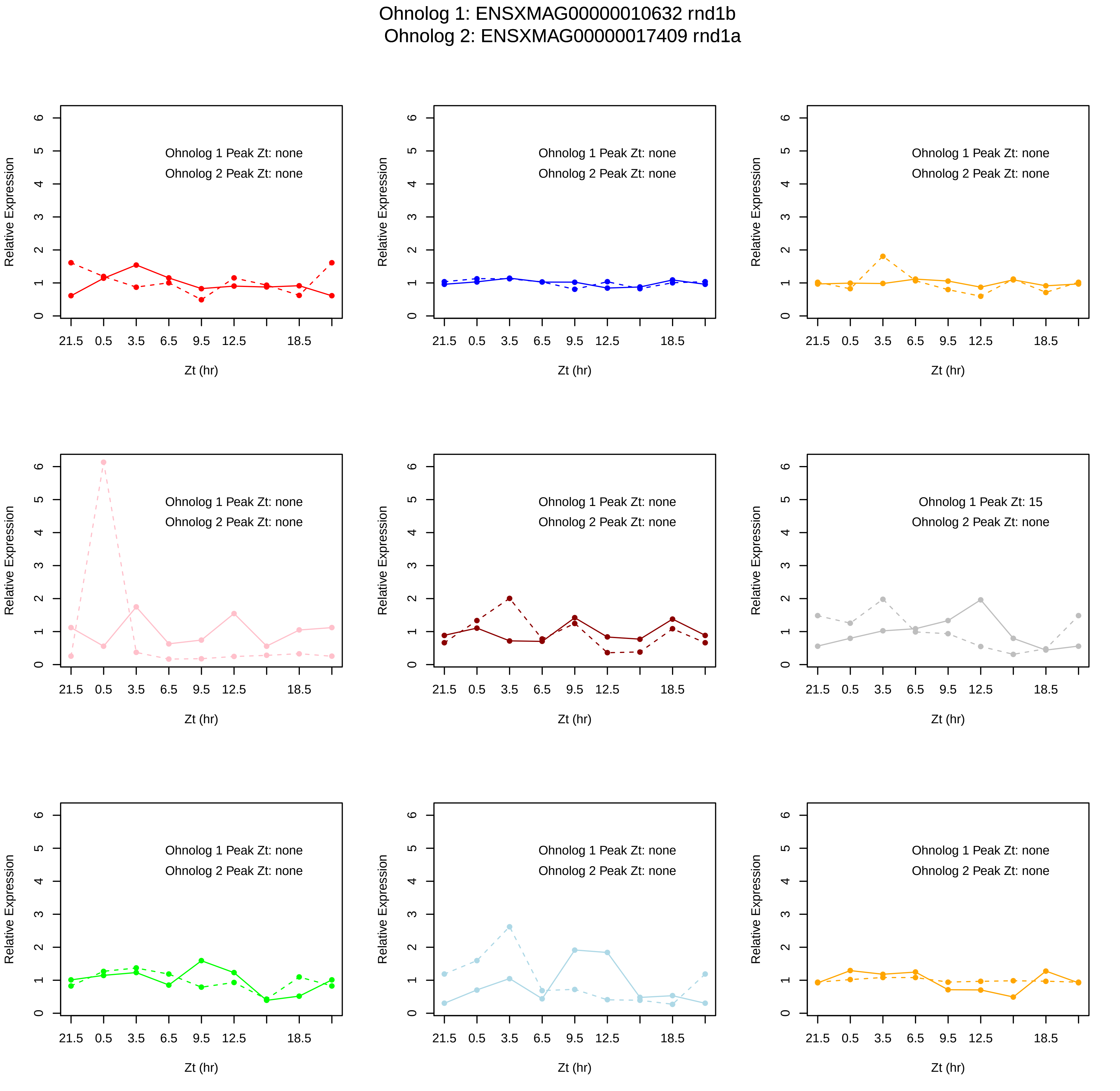

Ohnolog 1: ENSXMAG00000010632 rnd1b
Ohnolog 2: ENSXMAG00000017409 rnd1a
Ohnolog 1 Peak Zt: none
Ohnolog 2 Peak Zt: none
Ohnolog 1 Peak Zt: none
Ohnolog 2 Peak Zt: none
Ohnolog 1 Peak Zt: none
Ohnolog 2 Peak Zt: none
Ohnolog 1 Peak Zt: none
Ohnolog 2 Peak Zt: none
Ohnolog 1 Peak Zt: none
Ohnolog 2 Peak Zt: none
Ohnolog 1 Peak Zt: 15
Ohnolog 2 Peak Zt: none
Ohnolog 1 Peak Zt: none
Ohnolog 2 Peak Zt: none
Ohnolog 1 Peak Zt: none
Ohnolog 2 Peak Zt: none
Ohnolog 1 Peak Zt: none
Ohnolog 2 Peak Zt: none
Relative Expression
Zt (hr)
21.5 0.5 3.5 6.5 9.5 12.5 18.5

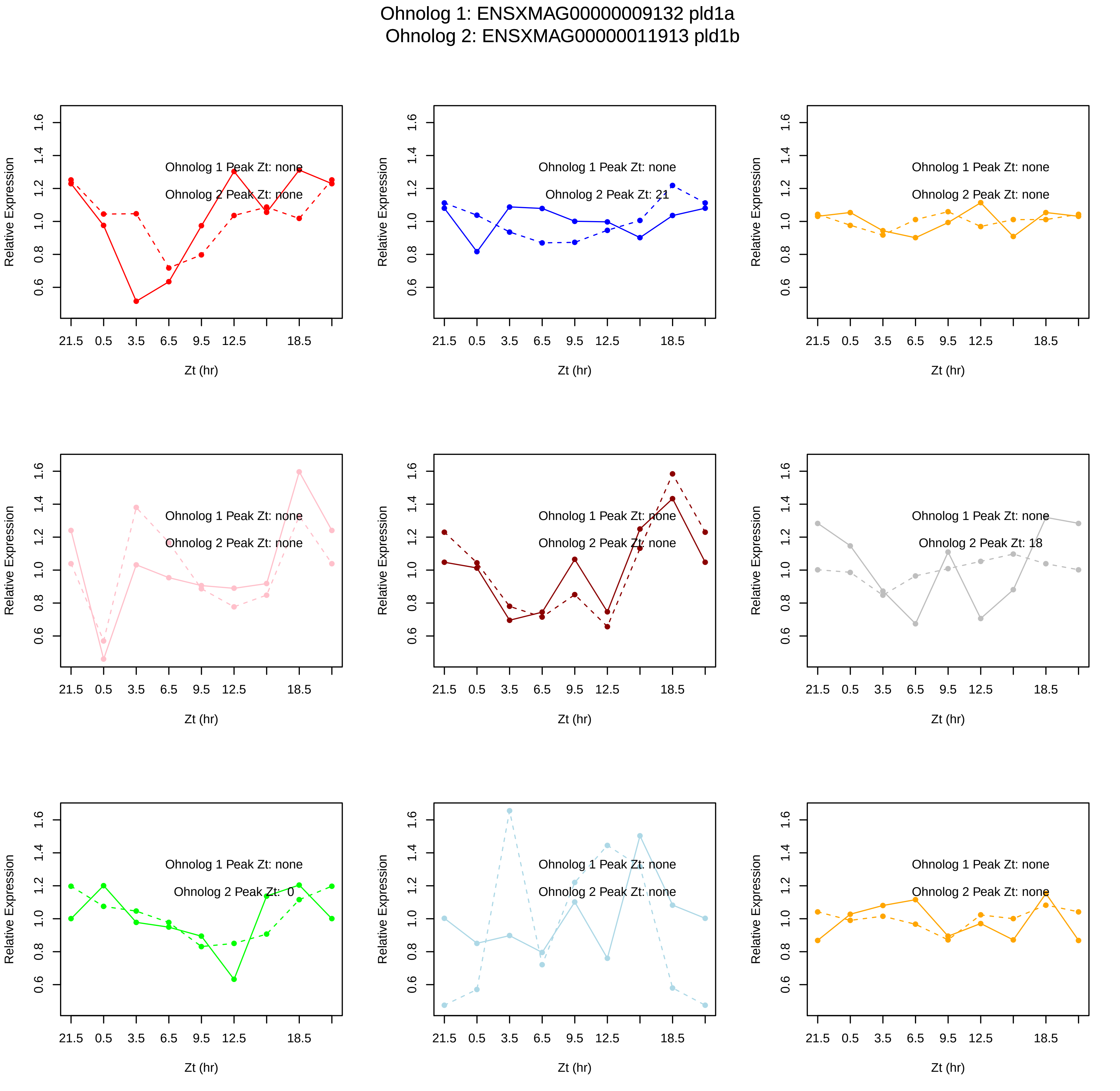
Ohnolog 1: ENSXMAG00000009132 pld1a
Ohnolog 2: ENSXMAG00000011913 pld1b
Ohnolog 1 Peak Zt: none
Ohnolog 2 Peak Zt: none
Ohnolog 1 Peak Zt: none
Ohnolog 2 Peak Zt: 21
Ohnolog 1 Peak Zt: none
Ohnolog 2 Peak Zt: none
Ohnolog 1 Peak Zt: none
Ohnolog 2 Peak Zt: none
Ohnolog 1 Peak Zt: none
Ohnolog 2 Peak Zt: none
Ohnolog 1 Peak Zt: none
Ohnolog 2 Peak Zt: 18
Ohnolog 1 Peak Zt: none
Ohnolog 2 Peak Zt: 0
Ohnolog 1 Peak Zt: none
Ohnolog 2 Peak Zt: none
Ohnolog 1 Peak Zt: none
Ohnolog 2 Peak Zt: none
Relative Expression
Zt (hr)
21.5
0.5
3.5
6.5
9.5
12.5
18.5
0.6
0.8
1.0
1.2
1.4
1.6

Ohnolog 1: ENSXMAG00000010656 adcy6b
Ohnolog 2: ENSXMAG00000017286 adcy6a

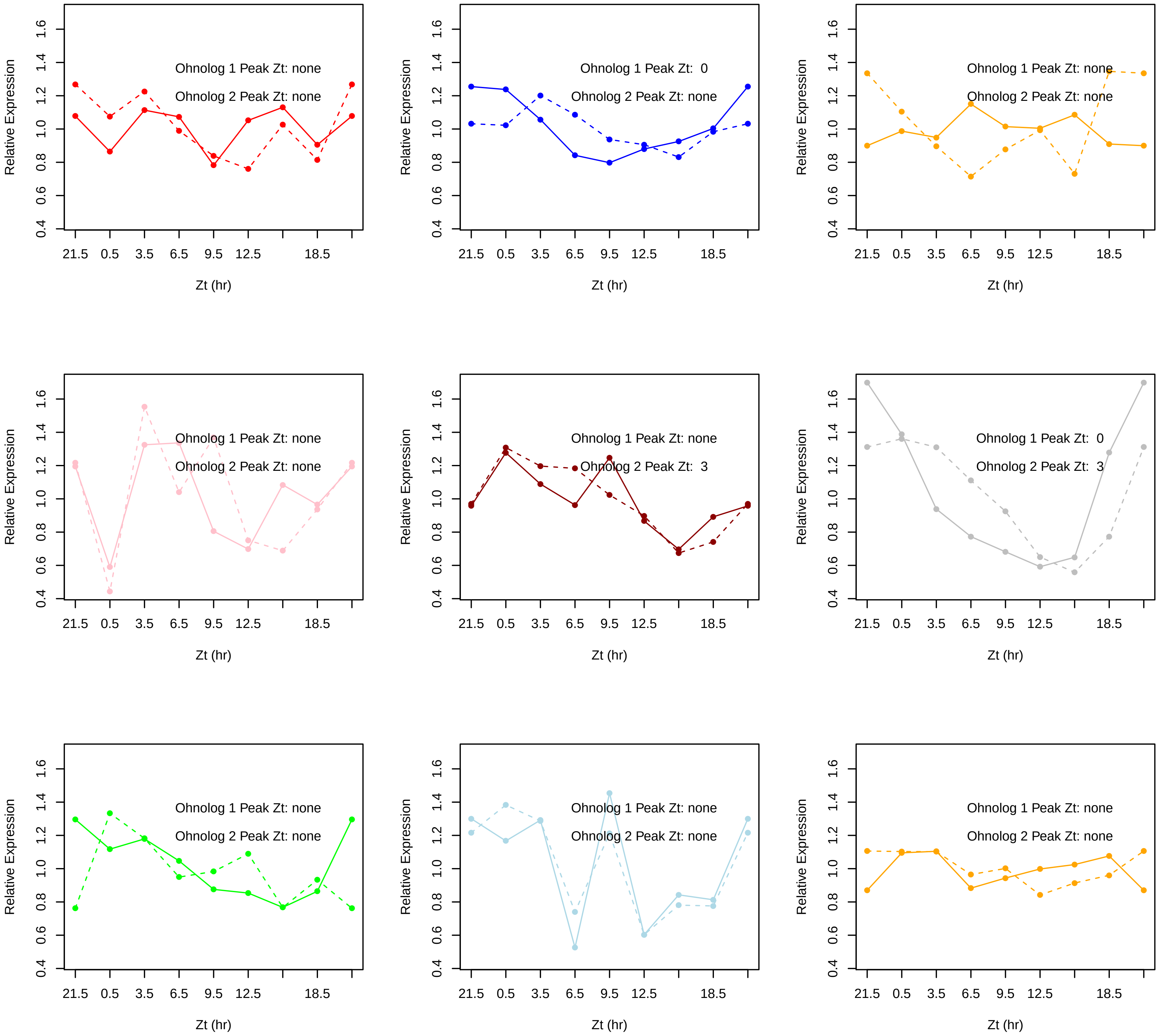

Ohnolog 1: ENSXMAG00000007284 gspt1l
Ohnolog 2: ENSXMAG00000022050 GSPT1

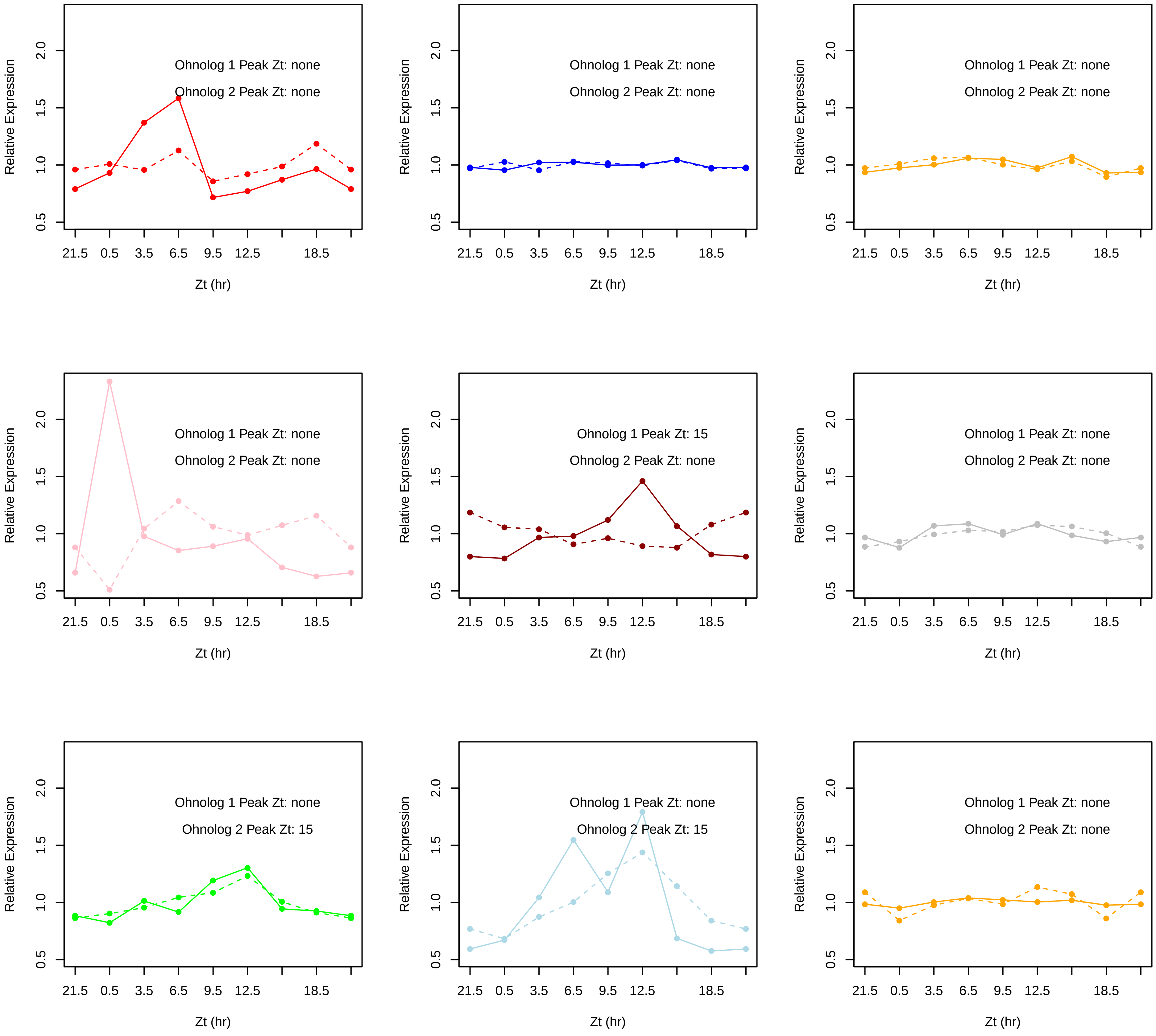

Ohnolog 1: ENSXMAG00000002447 crb1
Ohnolog 2: ENSXMAG00000004785

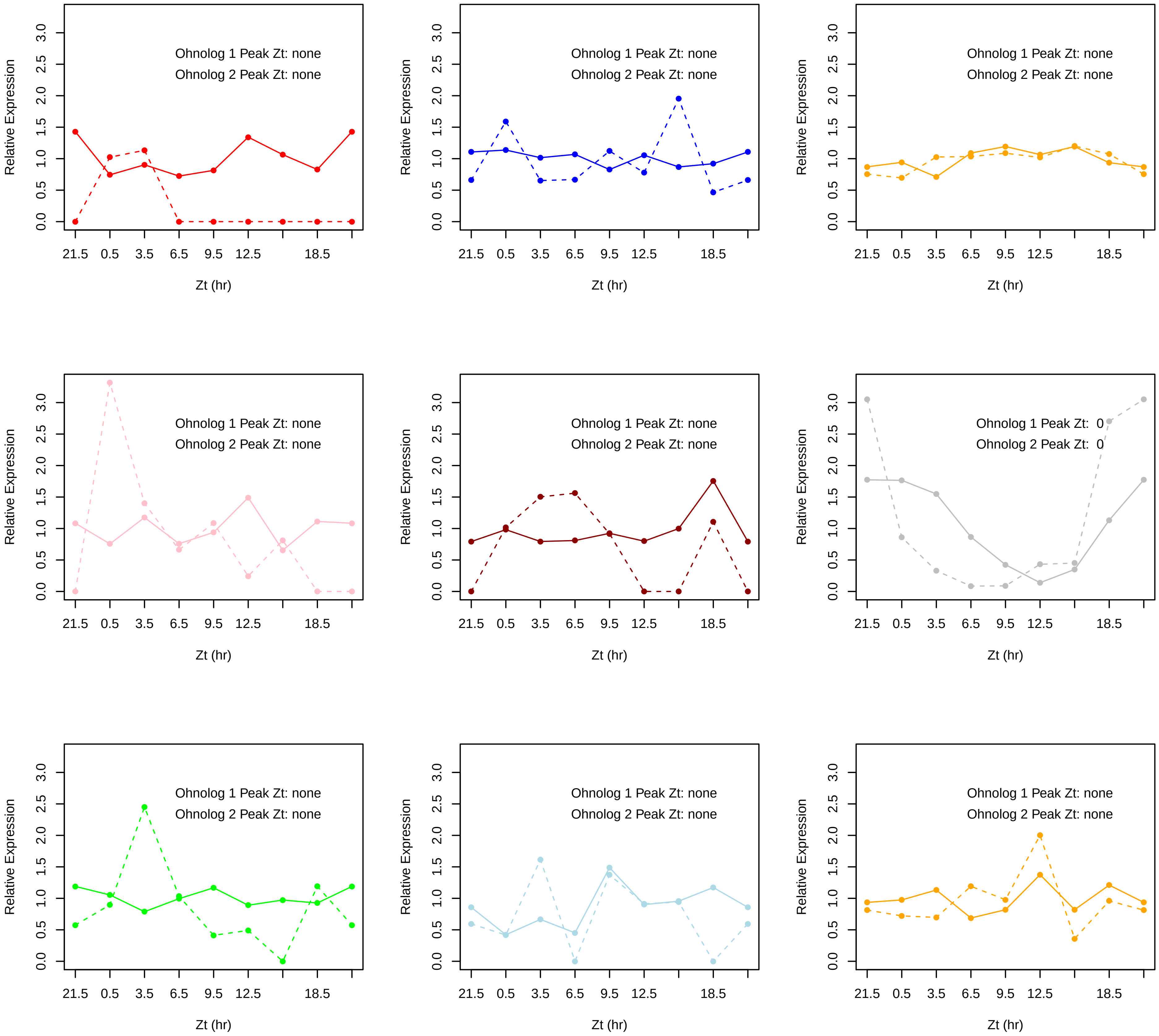

Ohnolog 1: ENSXMAG00000000208 pde4a
Ohnolog 2: ENSXMAG00000007185 PDE4A (1 of many)

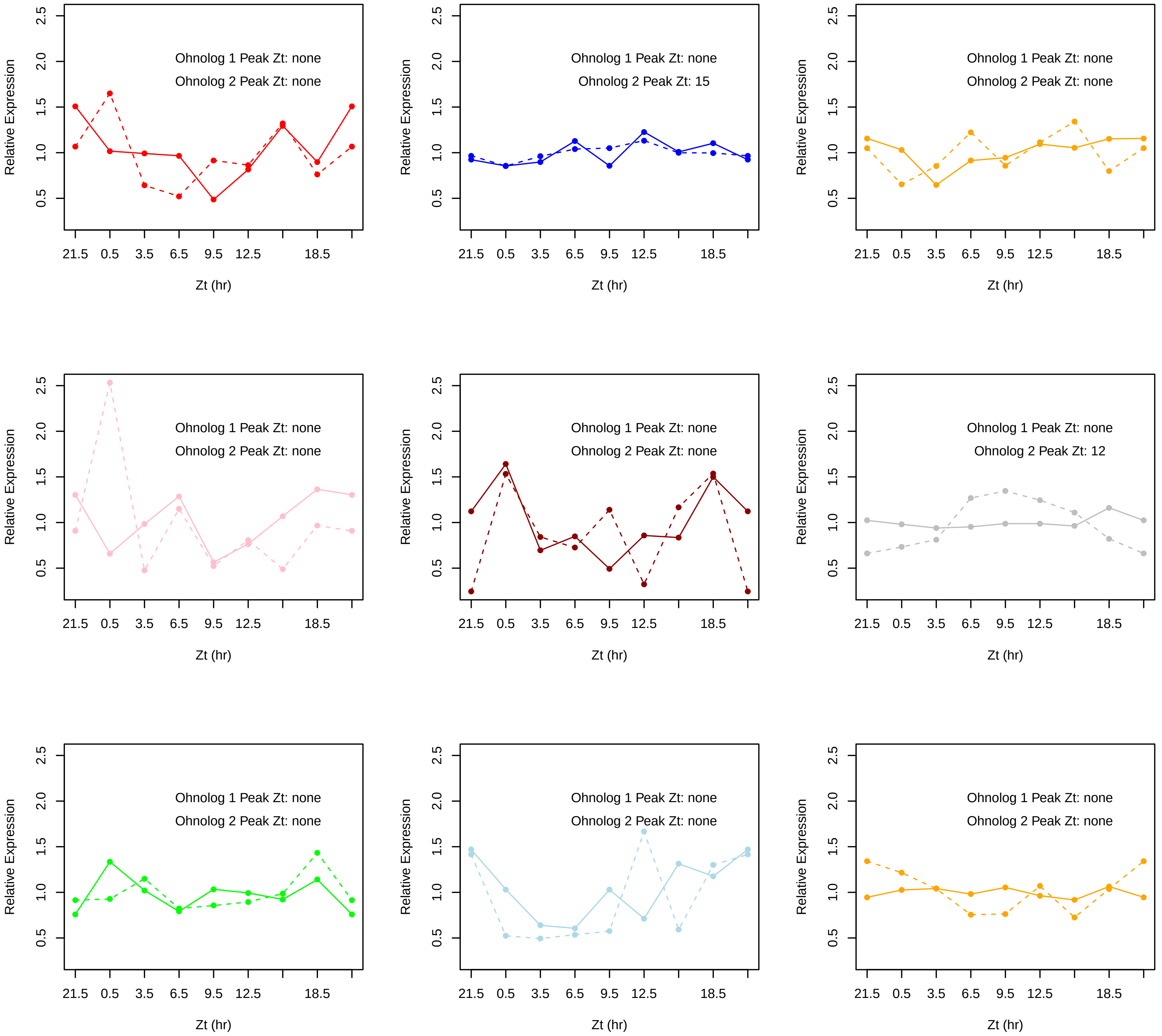

Ohnolog 1: ENSXMAG00000008340 acbd5a
Ohnolog 2: ENSXMAG00000022010 acbd5b

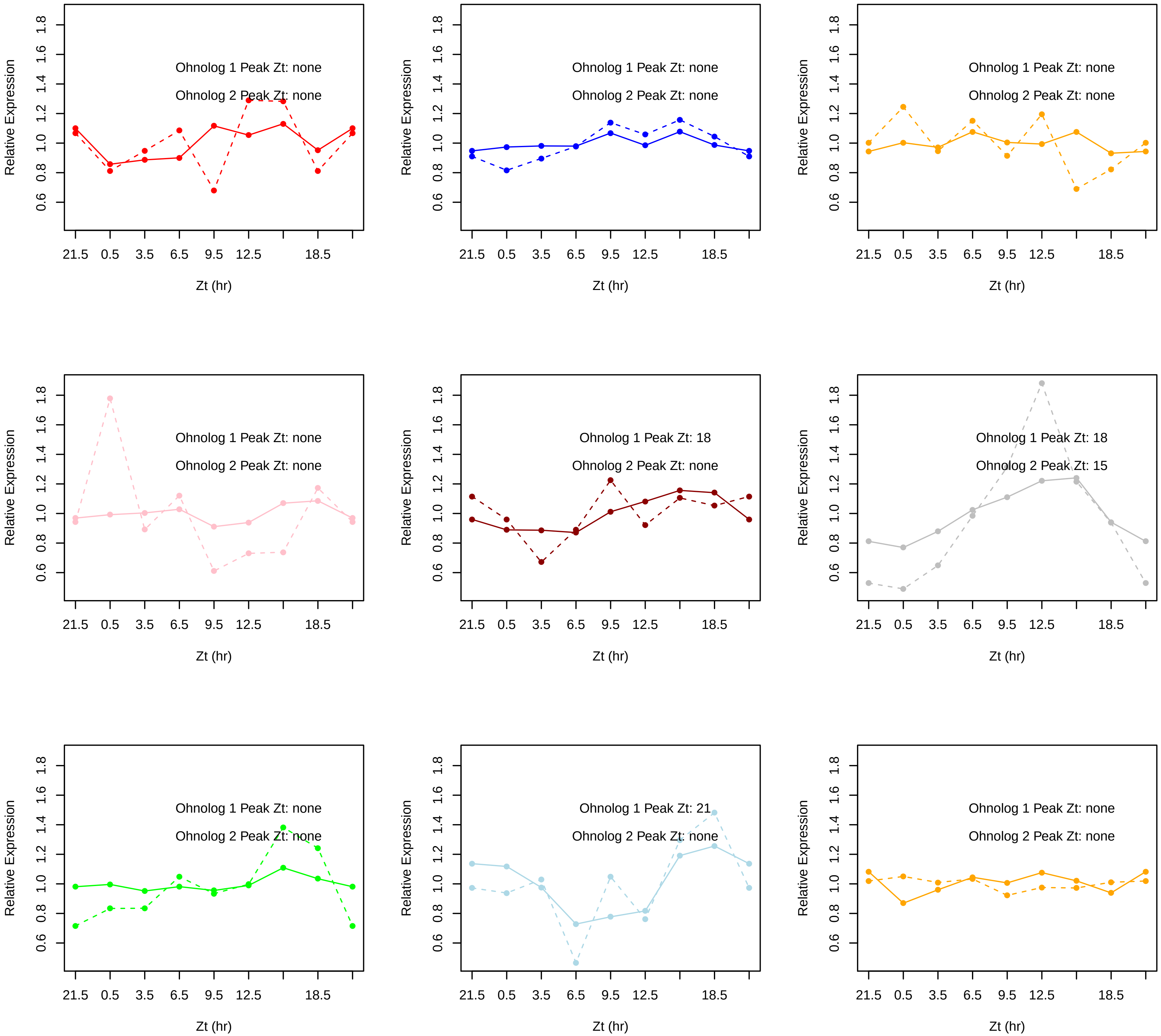

Ohnolog 1: ENSXMAG00000004822 vwa1
Ohnolog 2: ENSXMAG00000026357

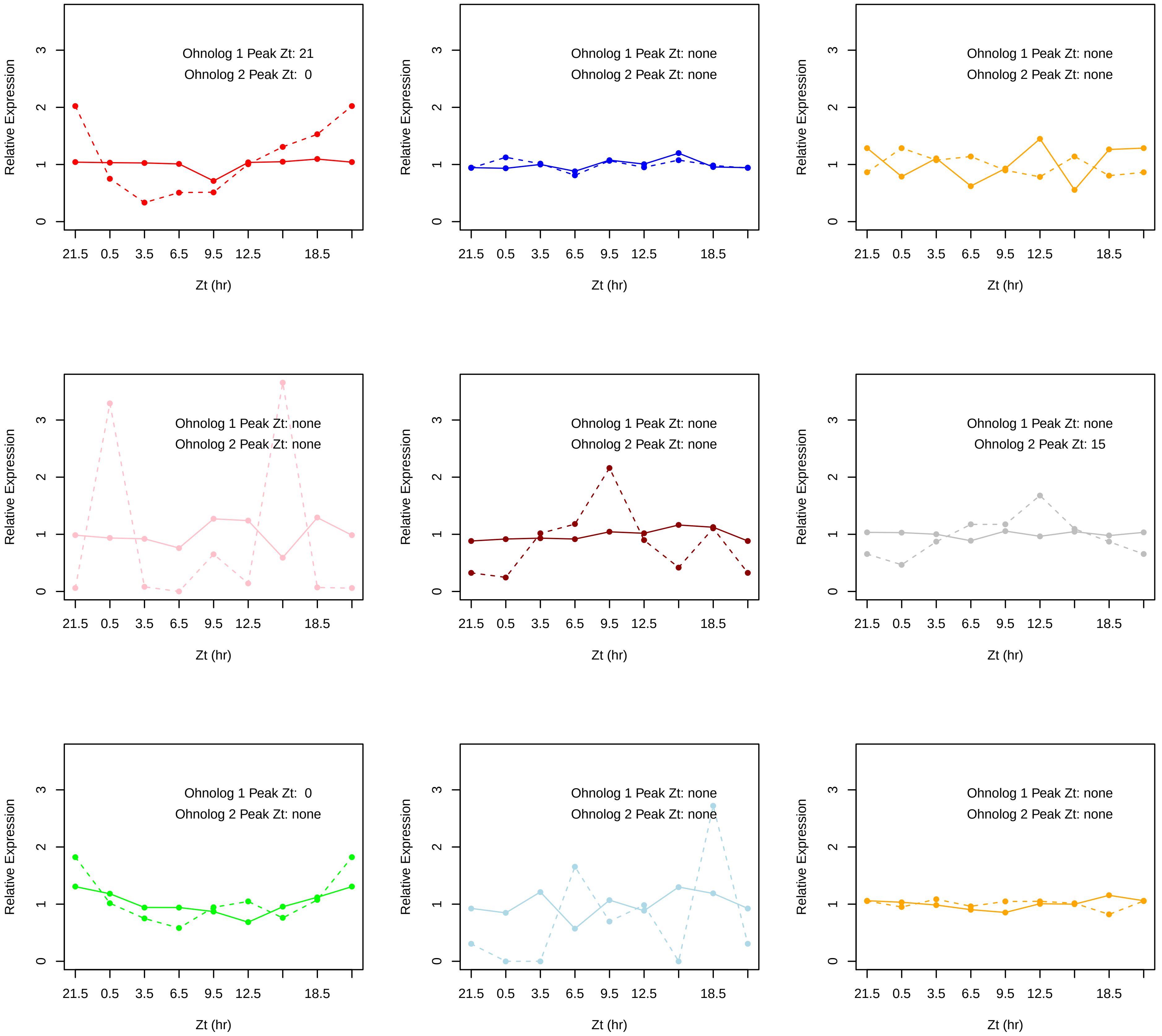

Ohnolog 1: ENSXMAG00000001967
Ohnolog 2: ENSXMAG00000004476 pla2g3

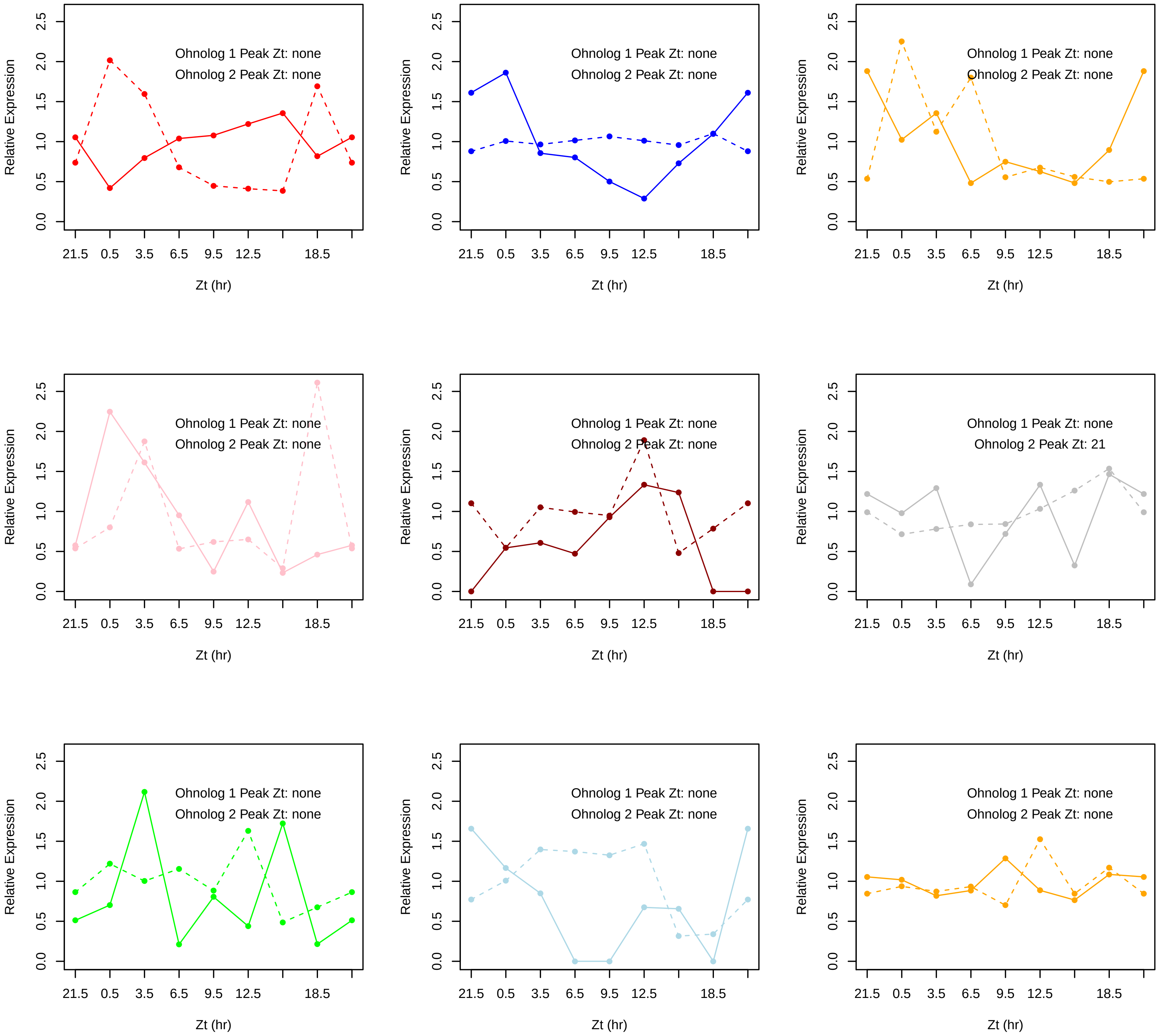

Ohnolog 1: ENSXMAG00000012673 ptpreb
Ohnolog 2: ENSXMAG00000016284 ptprea

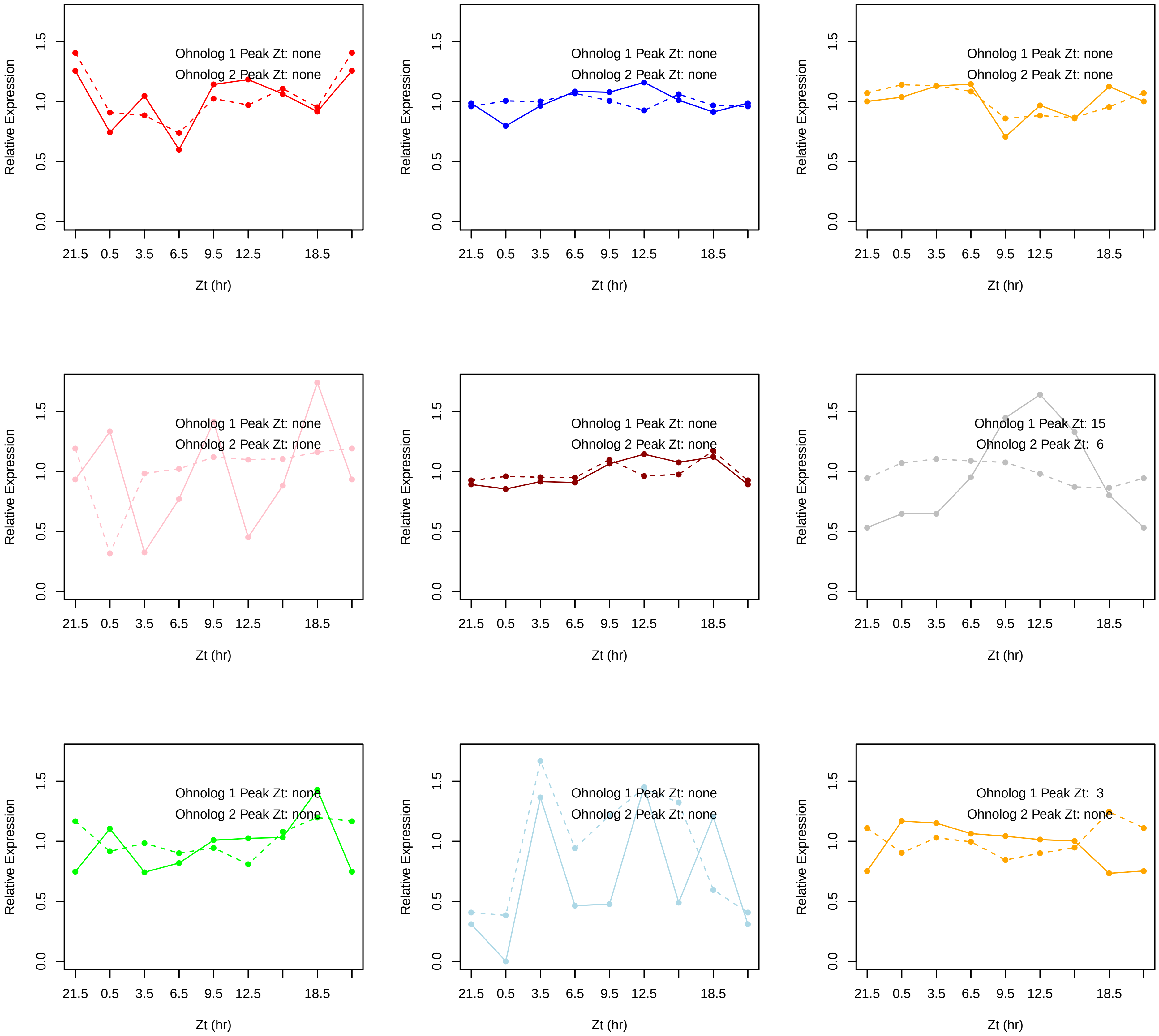

Ohnolog 1: ENSXMAG00000014446
Ohnolog 2: ENSXMAG00000015472 DENND3

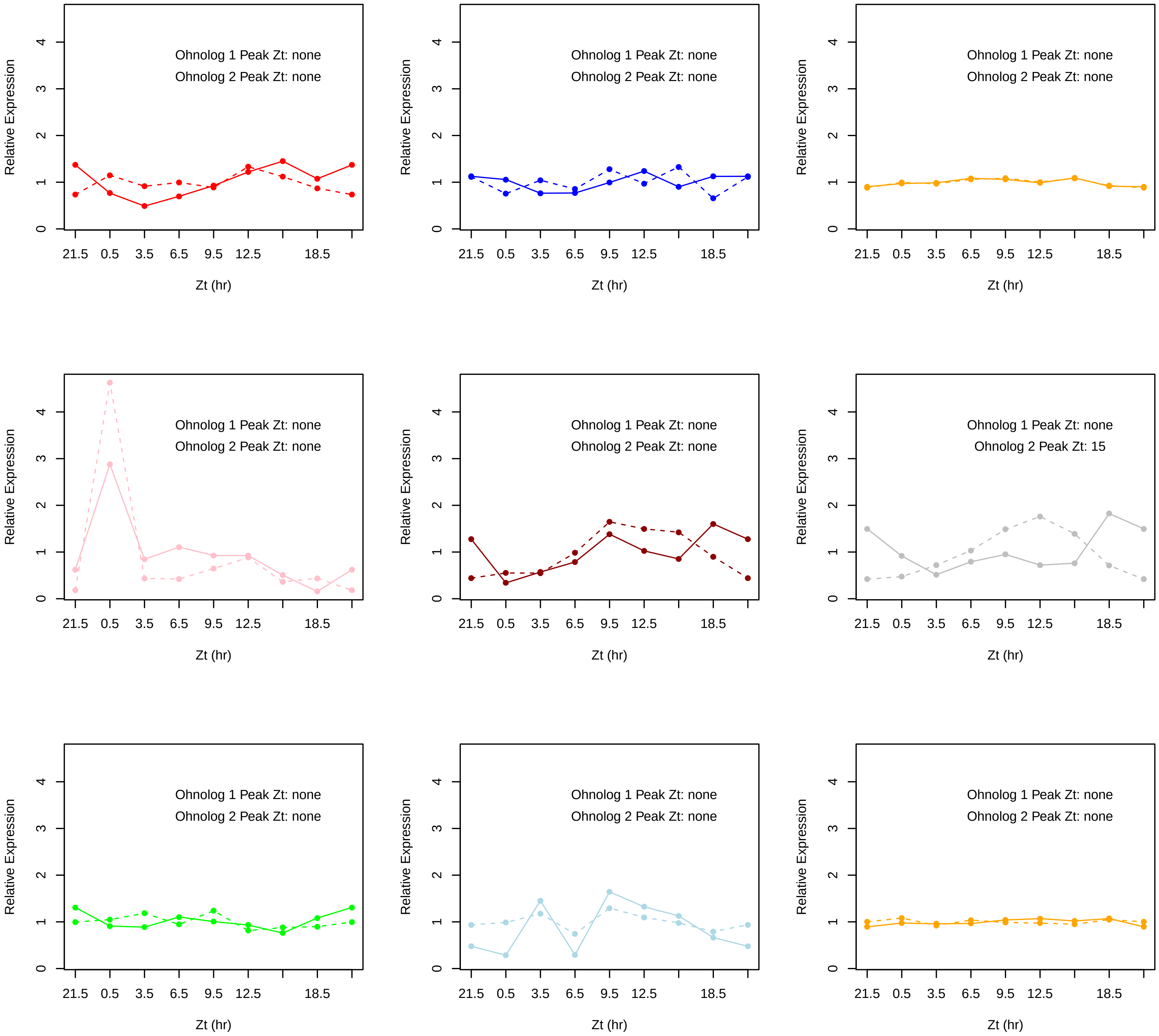

Ohnolog 1: ENSXMAG00000019058 SLC25A12 (1 of many)
Ohnolog 2: ENSXMAG00000028633 SLC25A12 (1 of many)

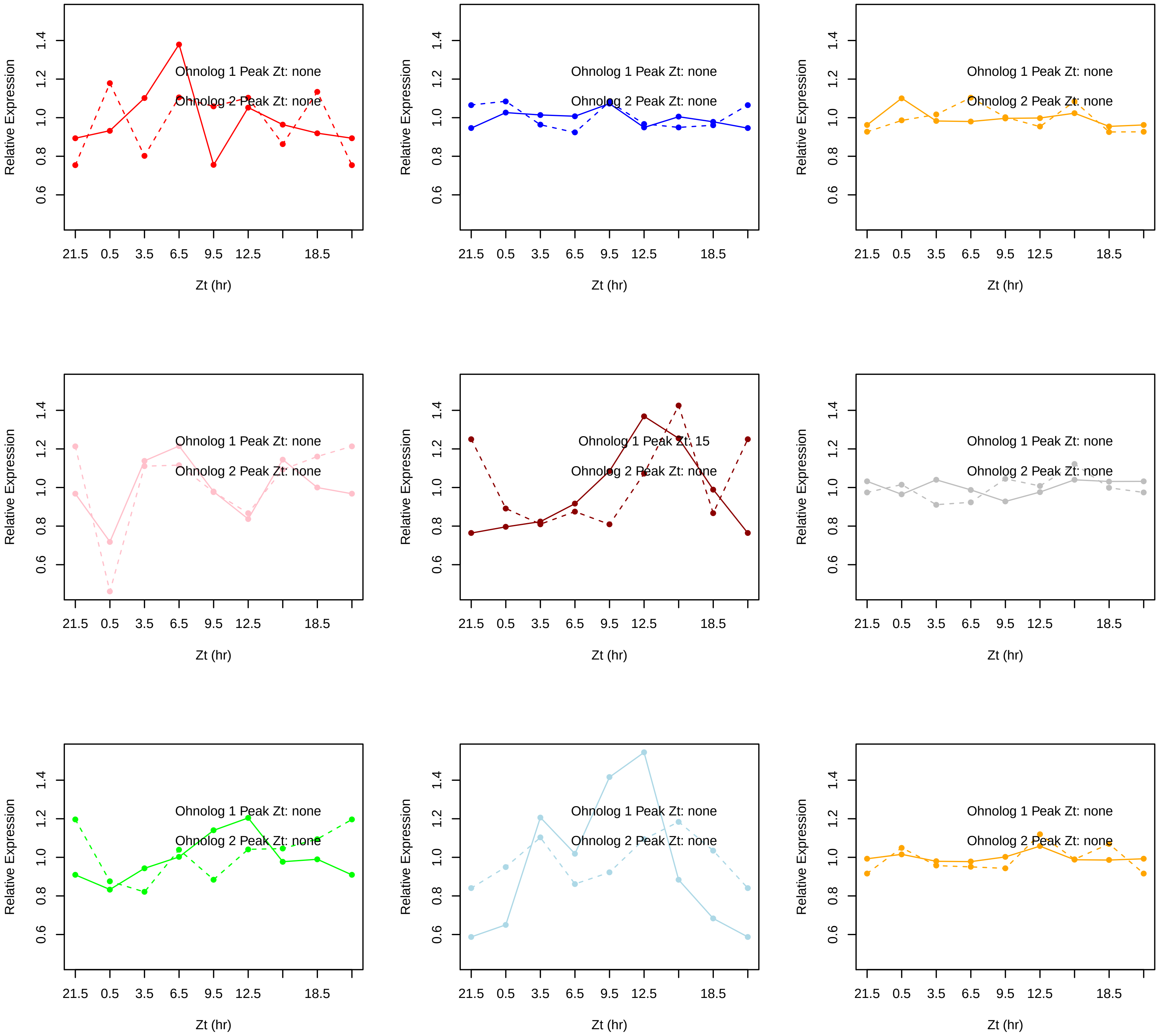

Ohnolog 1: ENSXMAG00000010911
Ohnolog 2: ENSXMAG00000015476 ptk2aa

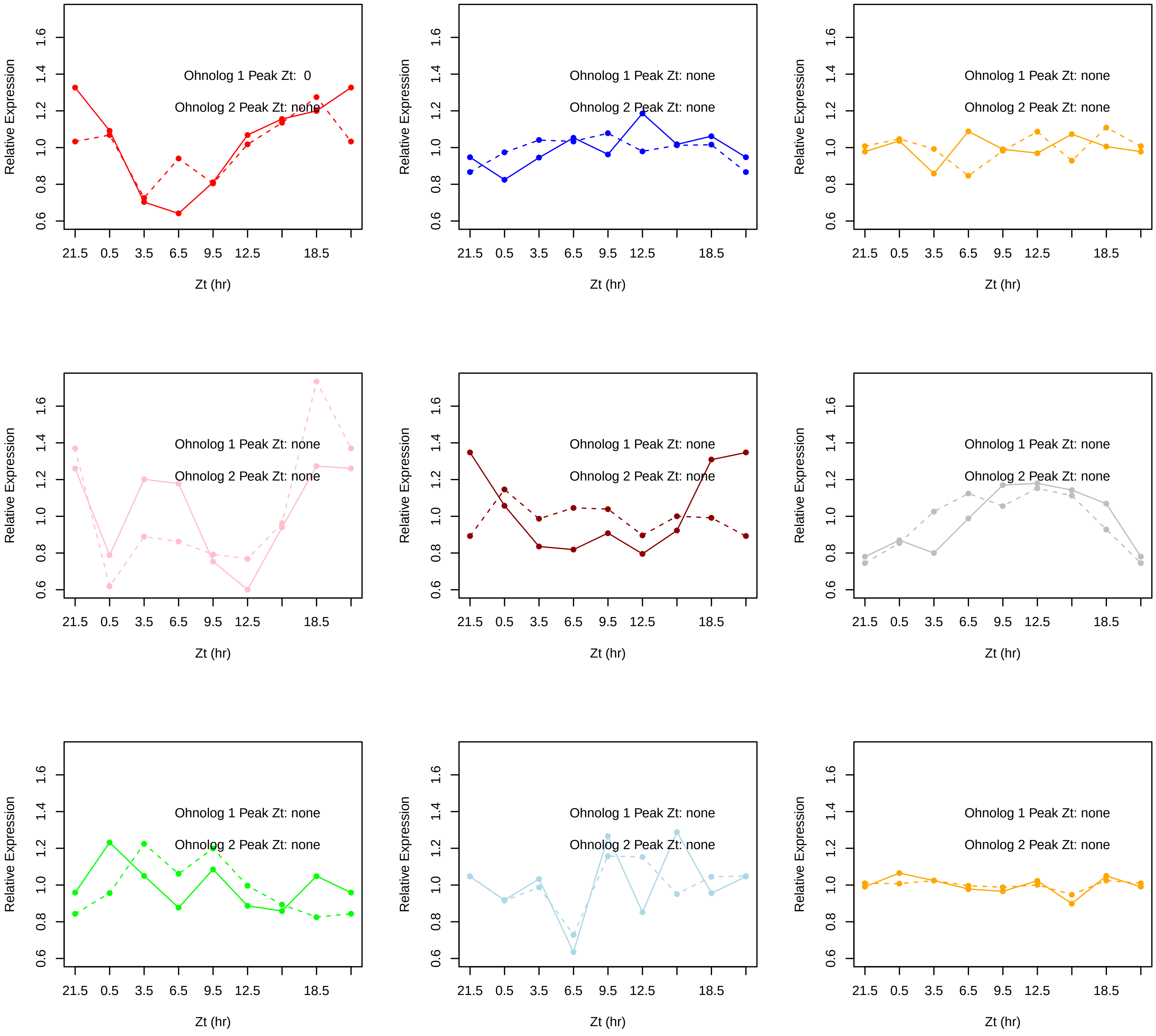

Ohnolog 1: ENSXMAG00000012600
Ohnolog 2: ENSXMAG00000016660 SDHB

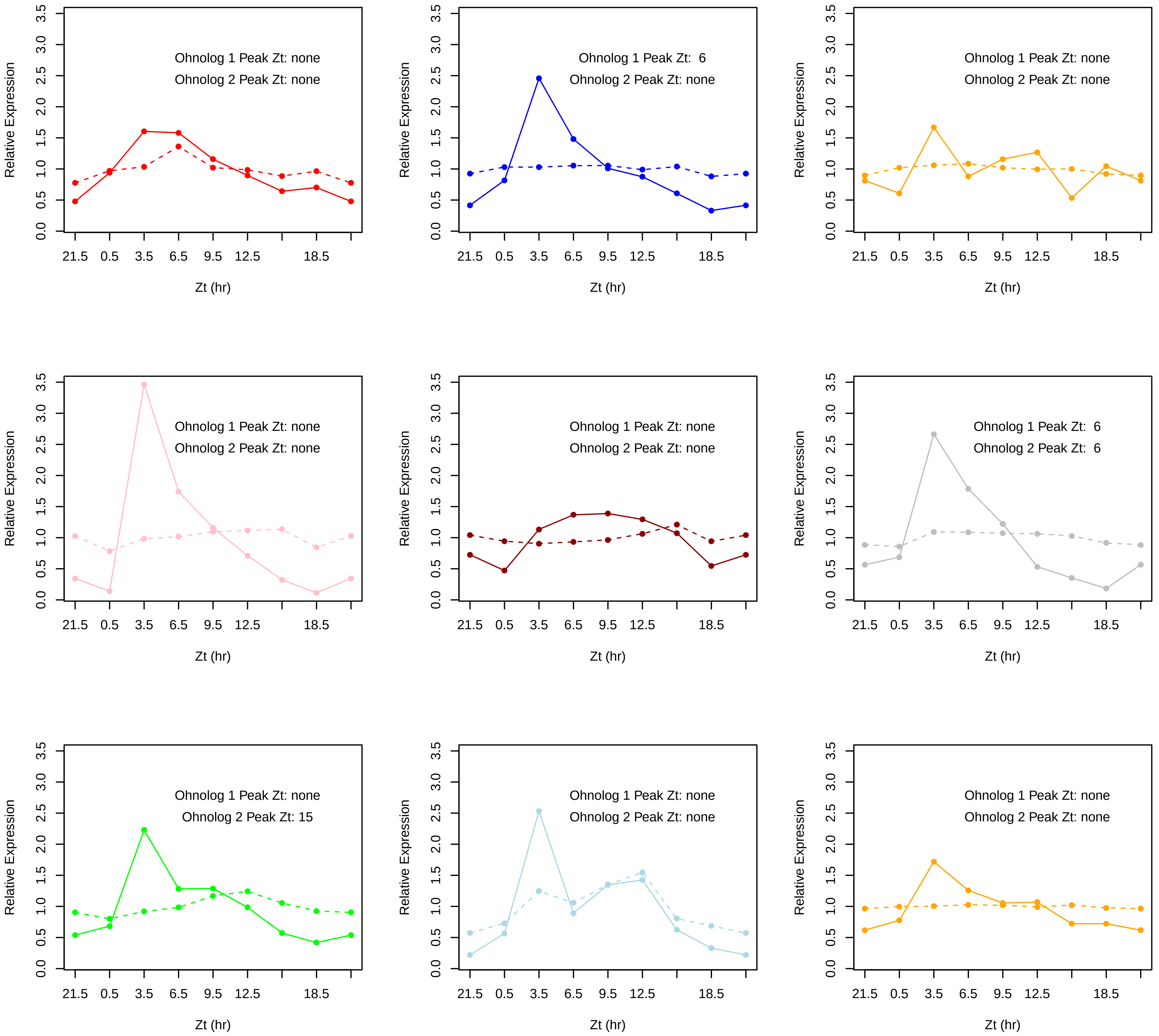

Ohnolog 1: ENSXMAG00000008421 gpr158a
Ohnolog 2: ENSXMAG00000011728 gpr158b

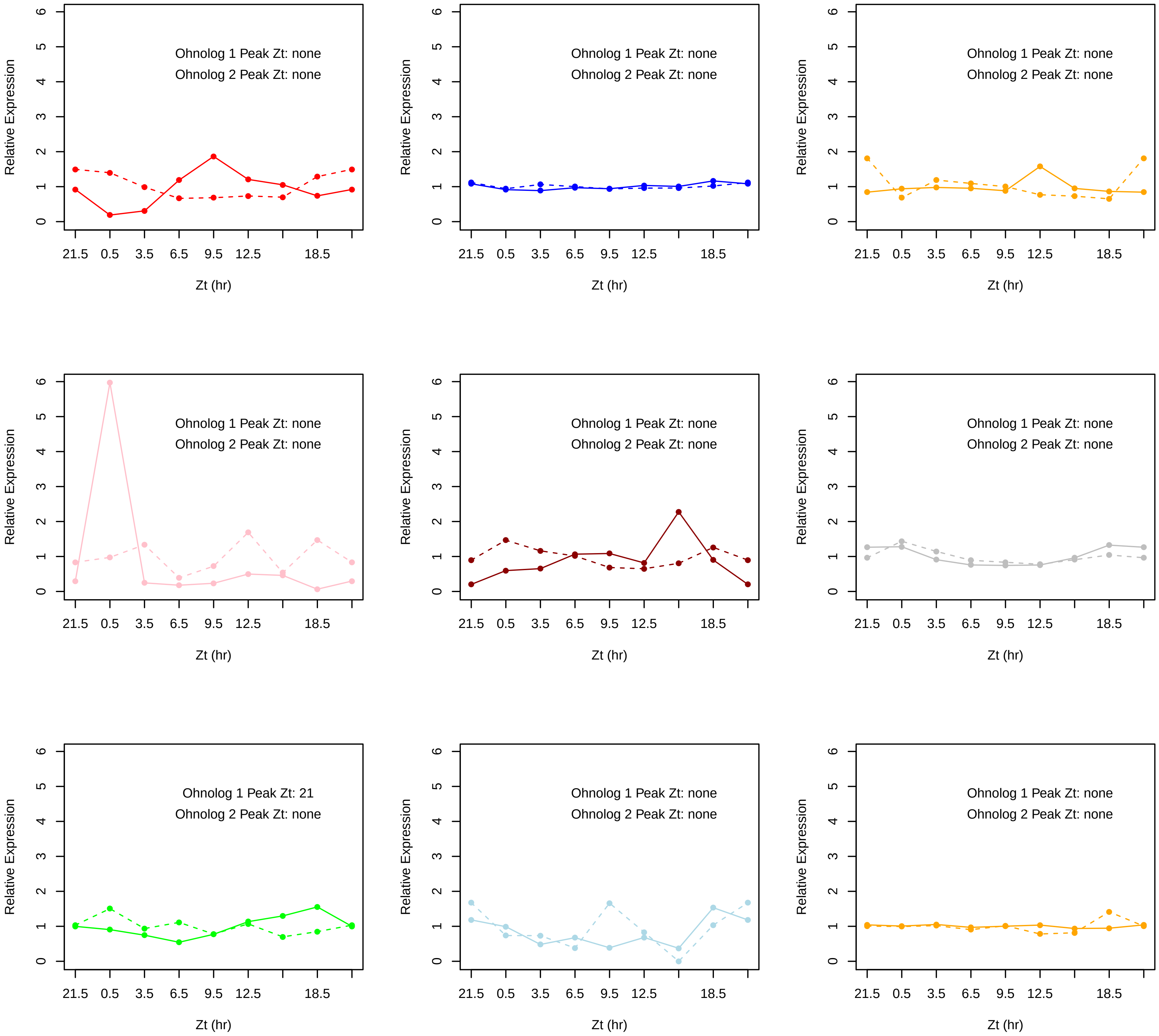

Ohnolog 1: ENSXMAG00000015605 ZSWIM6
Ohnolog 2: ENSXMAG00000019169 zswim6

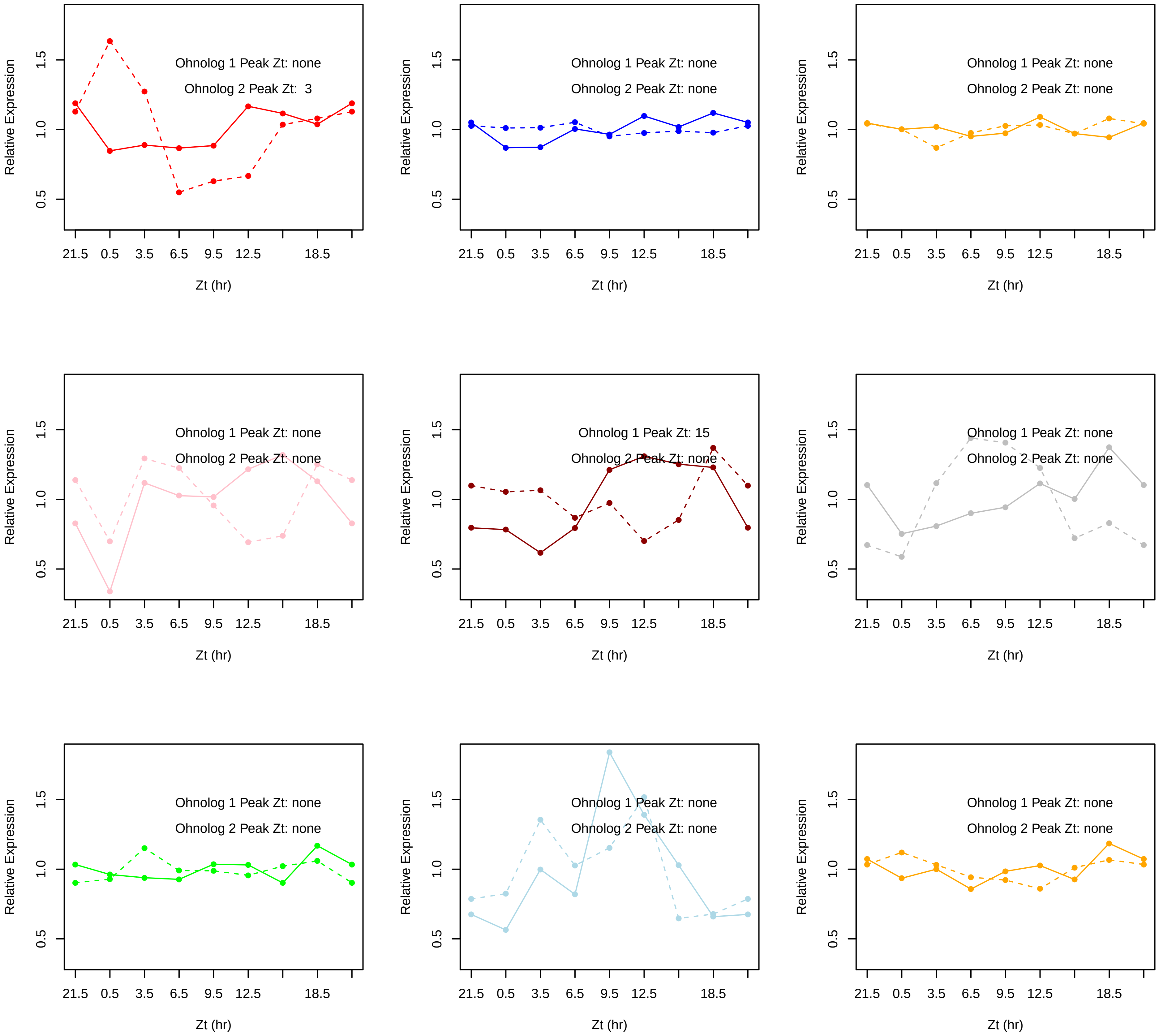

Ohnolog 1: ENSXMAG00000001916 PITPNB
Ohnolog 2: ENSXMAG00000030067 pitpnb

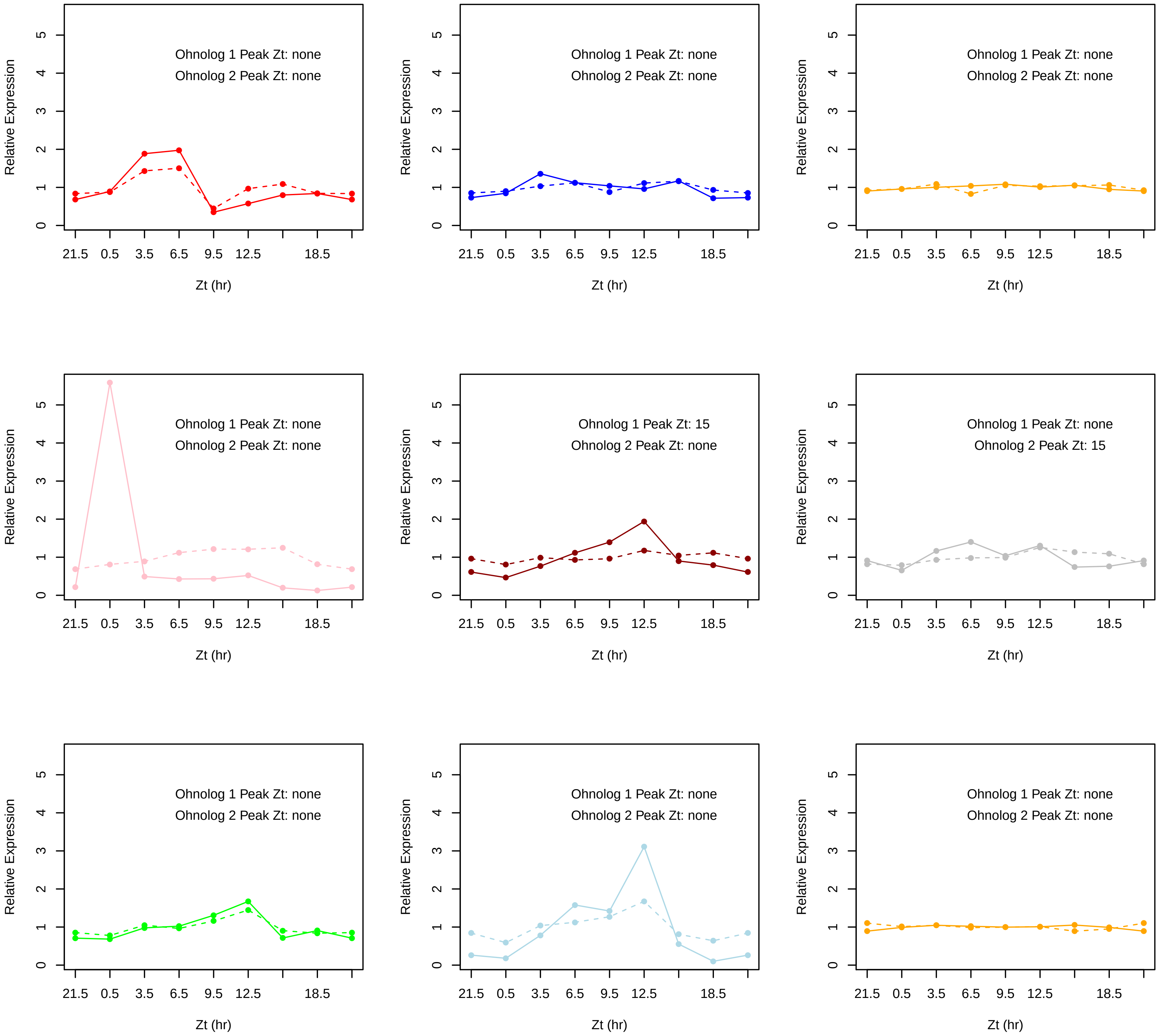

Ohnolog 1: ENSXMAG00000006321 smarca4a
Ohnolog 2: ENSXMAG00000007030

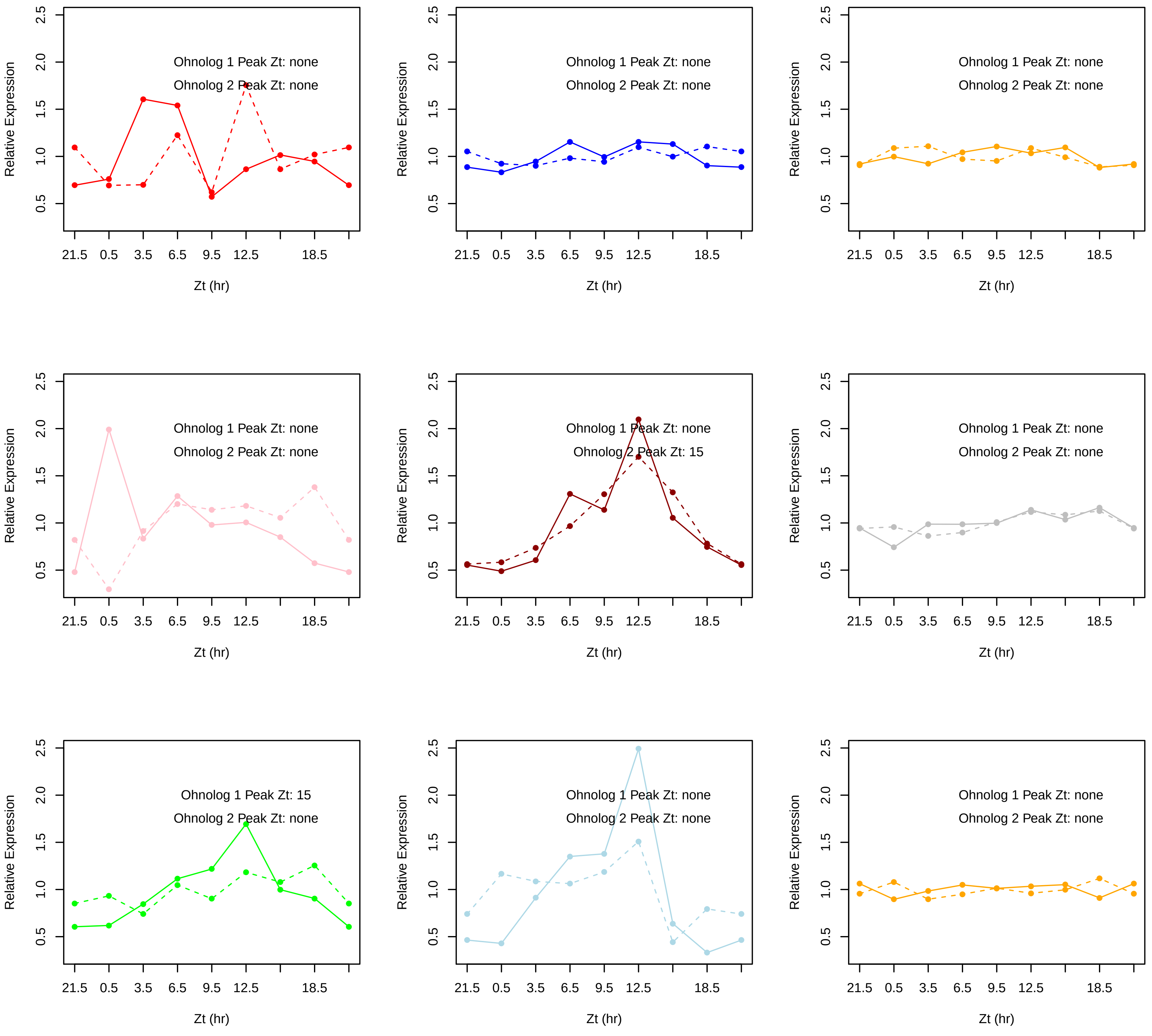

Ohnolog 1: ENSXMAG00000000740
Ohnolog 2: ENSXMAG00000007104 KIF2A

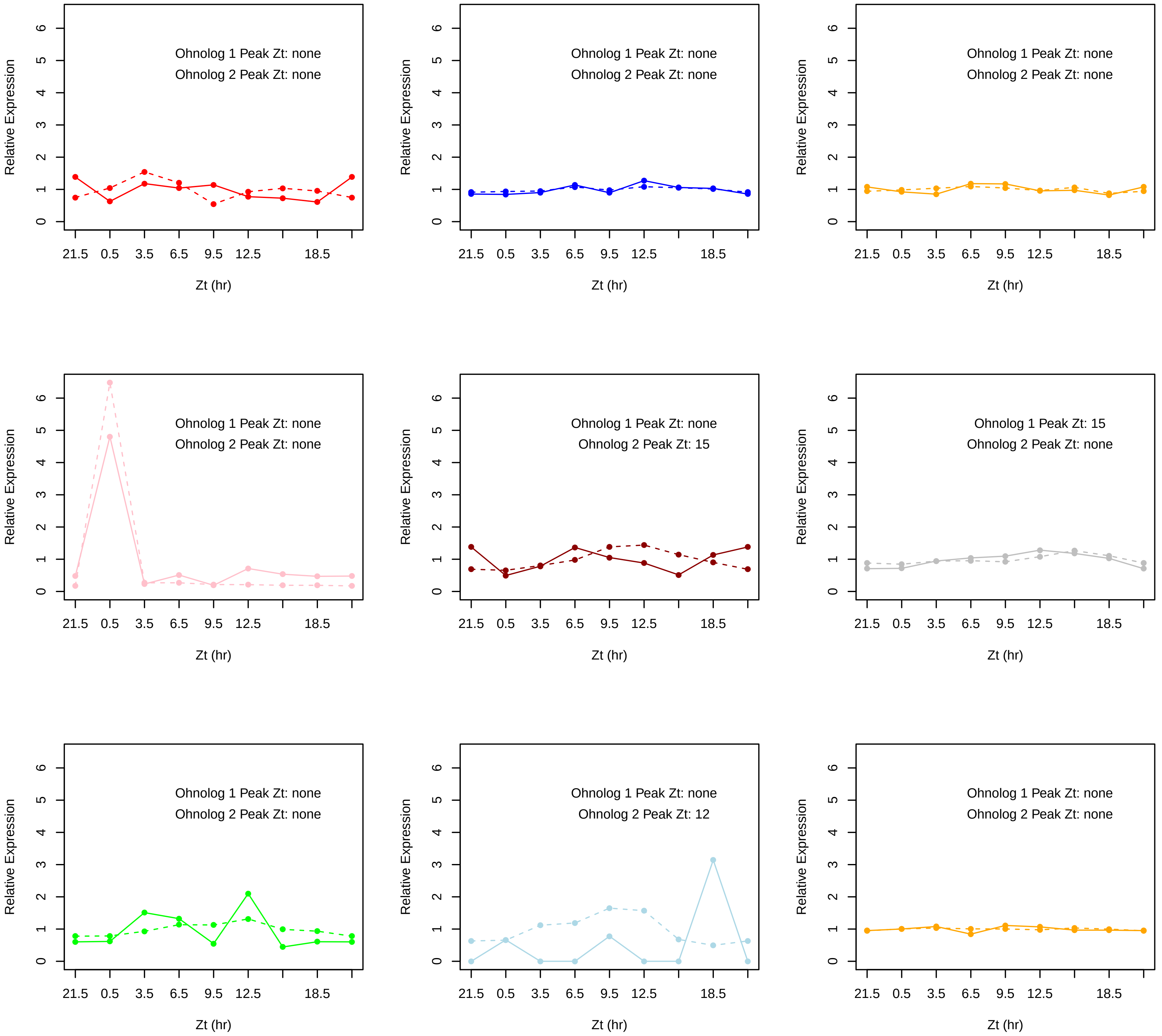

Ohnolog 1: ENSXMAG00000016721 SZRD1
Ohnolog 2: ENSXMAG00000025502 szrd1

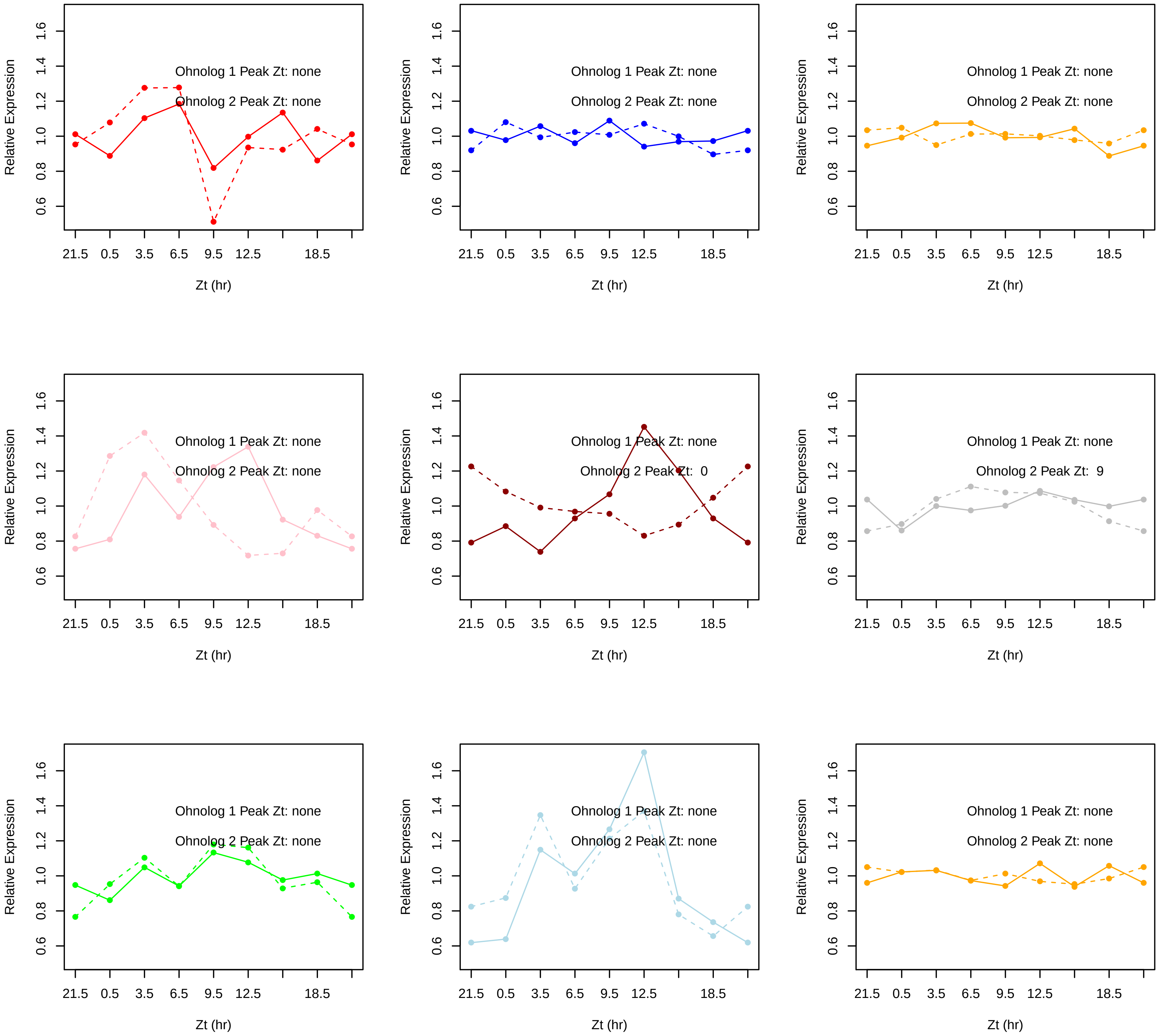

Ohnolog 1: ENSXMAG00000006970 mast1a
Ohnolog 2: ENSXMAG00000009142 mast1b

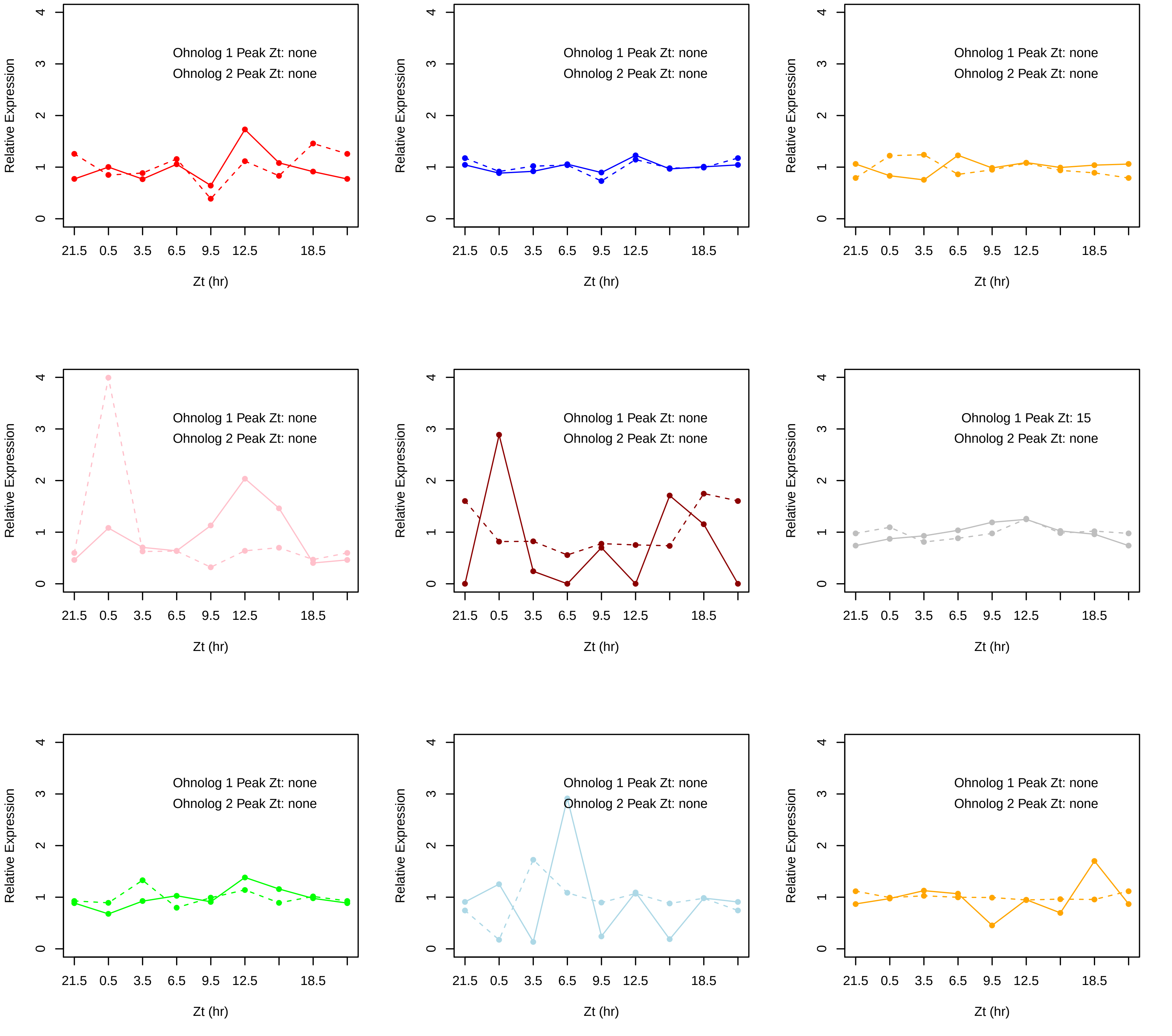

Ohnolog 1: ENSXMAG00000017772 inpp5jb
Ohnolog 2: ENSXMAG00000027563 inpp5ja

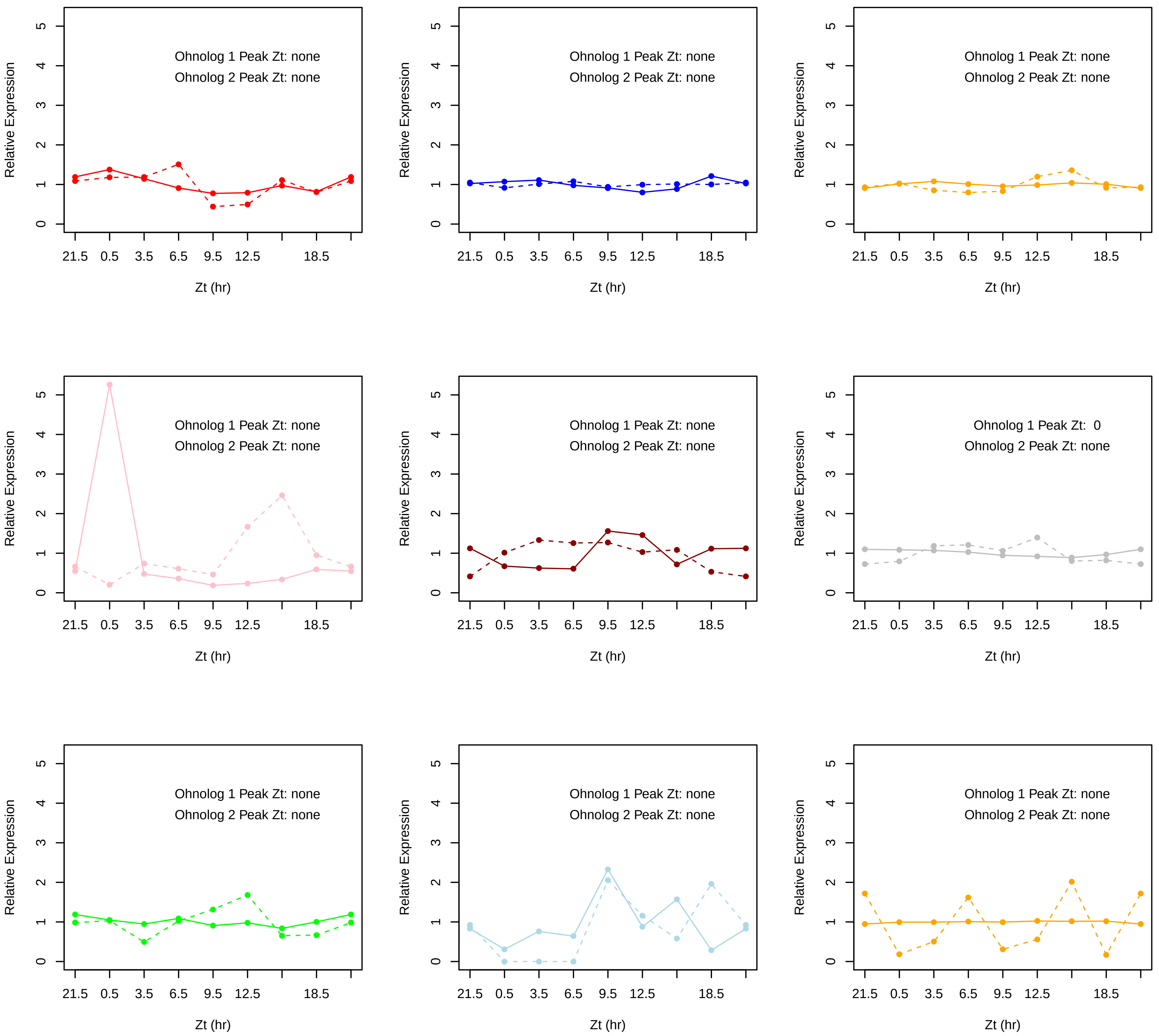

Ohnolog 1: ENSXMAG00000001163 tpi1a
Ohnolog 2: ENSXMAG00000011196 tpi1b

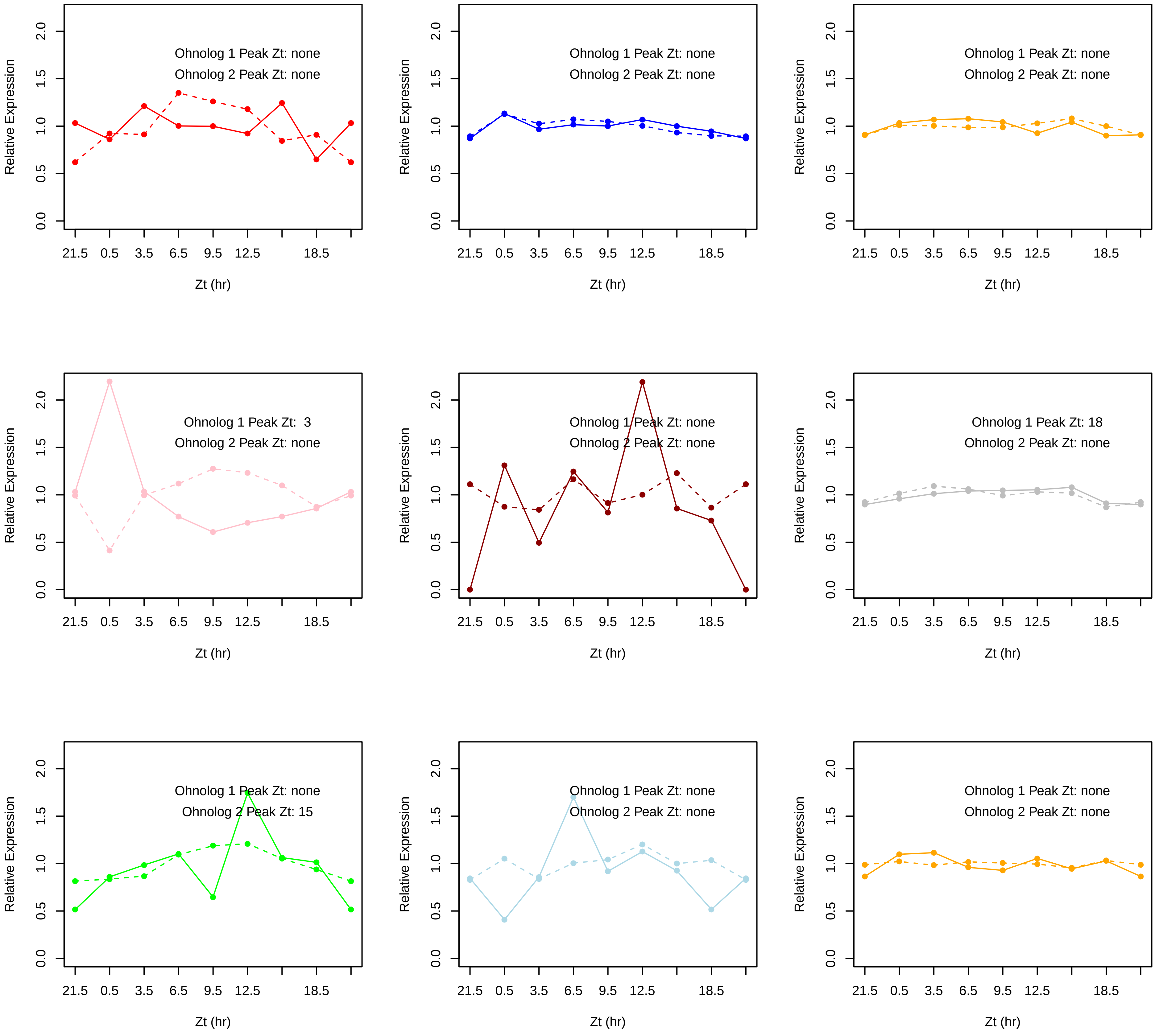

Ohnolog 1: ENSXMAG00000006158
Ohnolog 2: ENSXMAG00000030090 CYGB

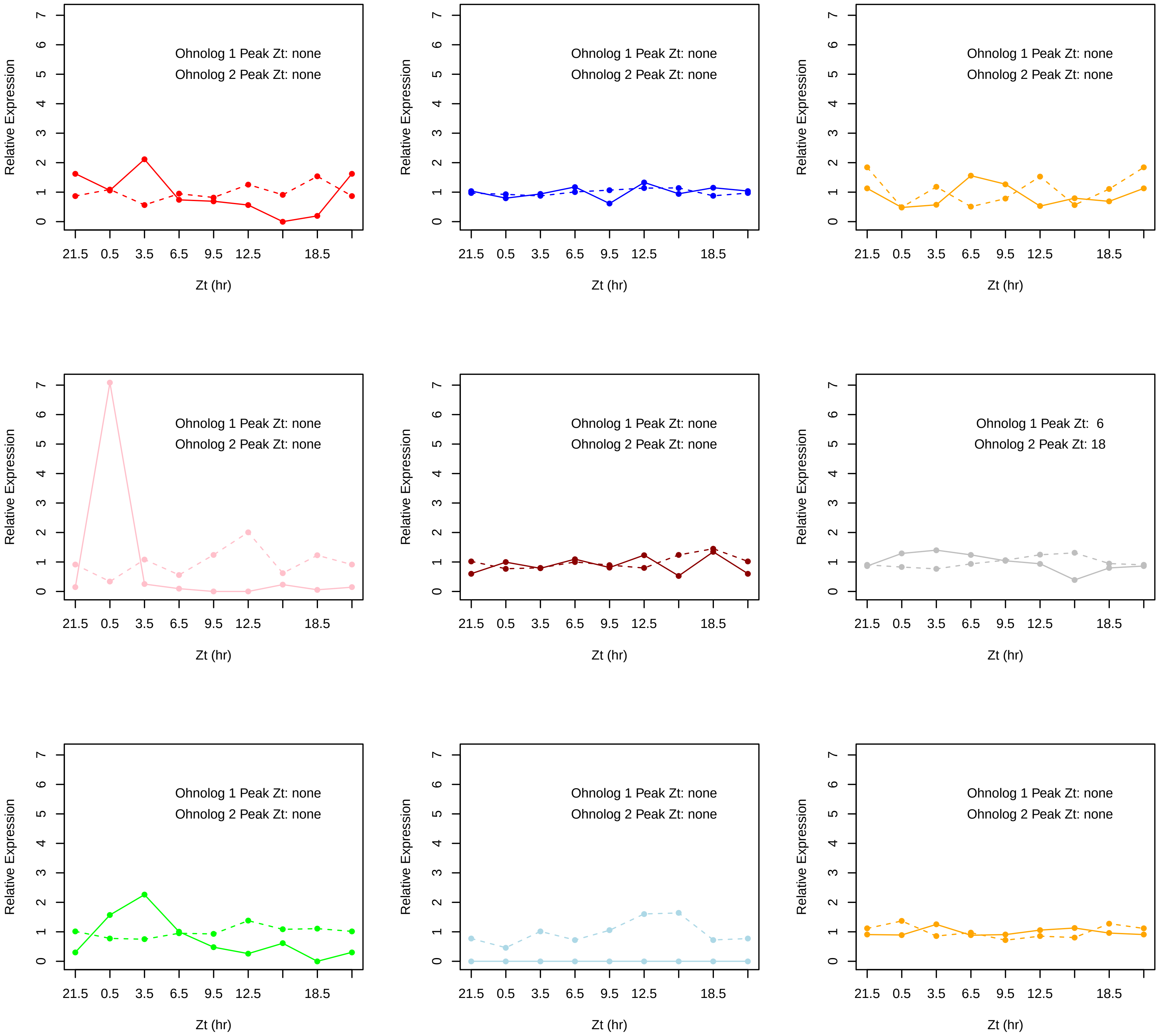

Ohnolog 1: ENSXMAG00000021384 spen
Ohnolog 2: ENSXMAG00000029887 si:ch1073−335m2.2

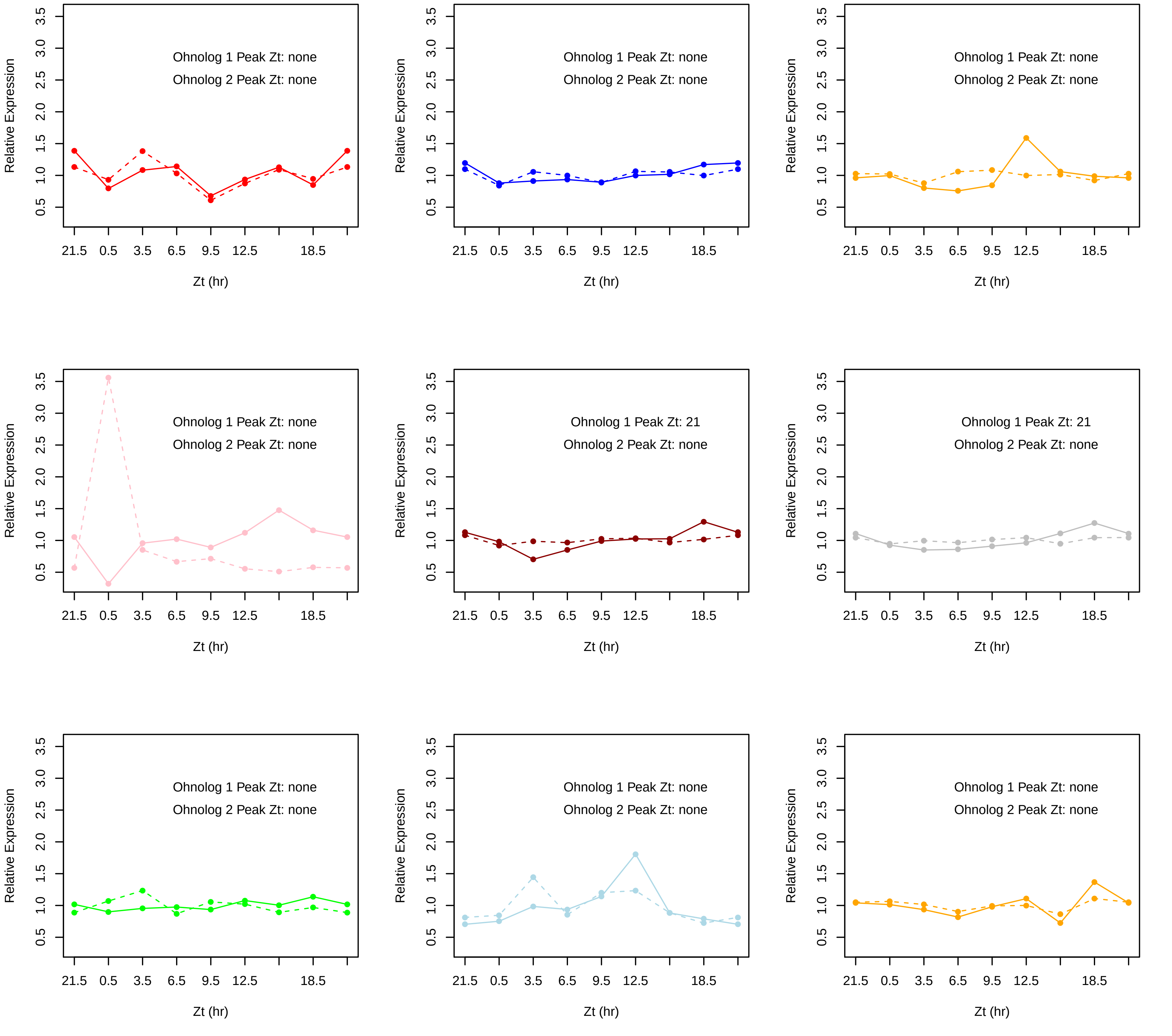

Ohnolog 1: ENSXMAG00000000320 selenoj
Ohnolog 2: ENSXMAG00000006441

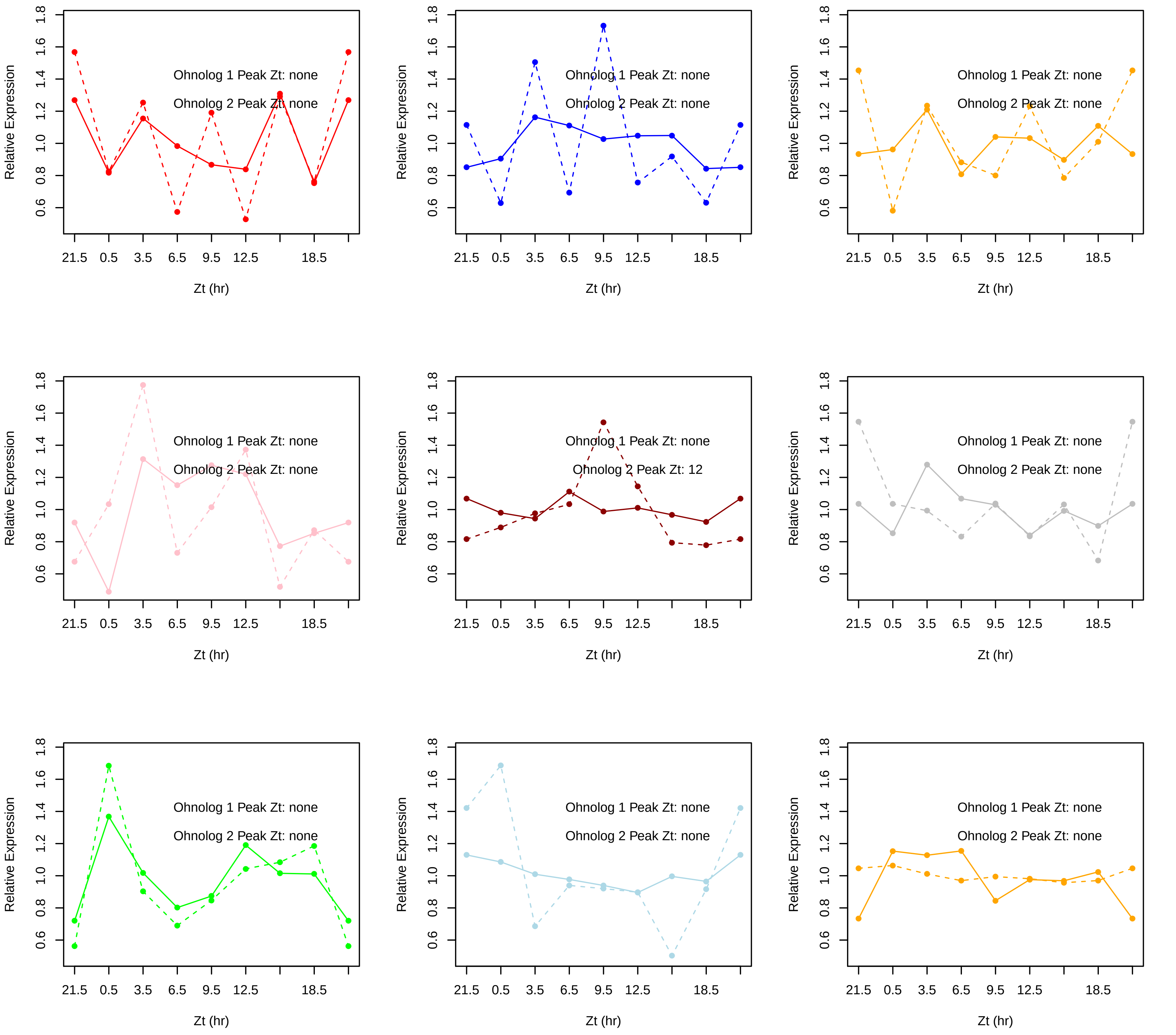

Ohnolog 1: ENSXMAG00000000476 vav3b
Ohnolog 2: ENSXMAG00000006442 VAV3 (1 of many)

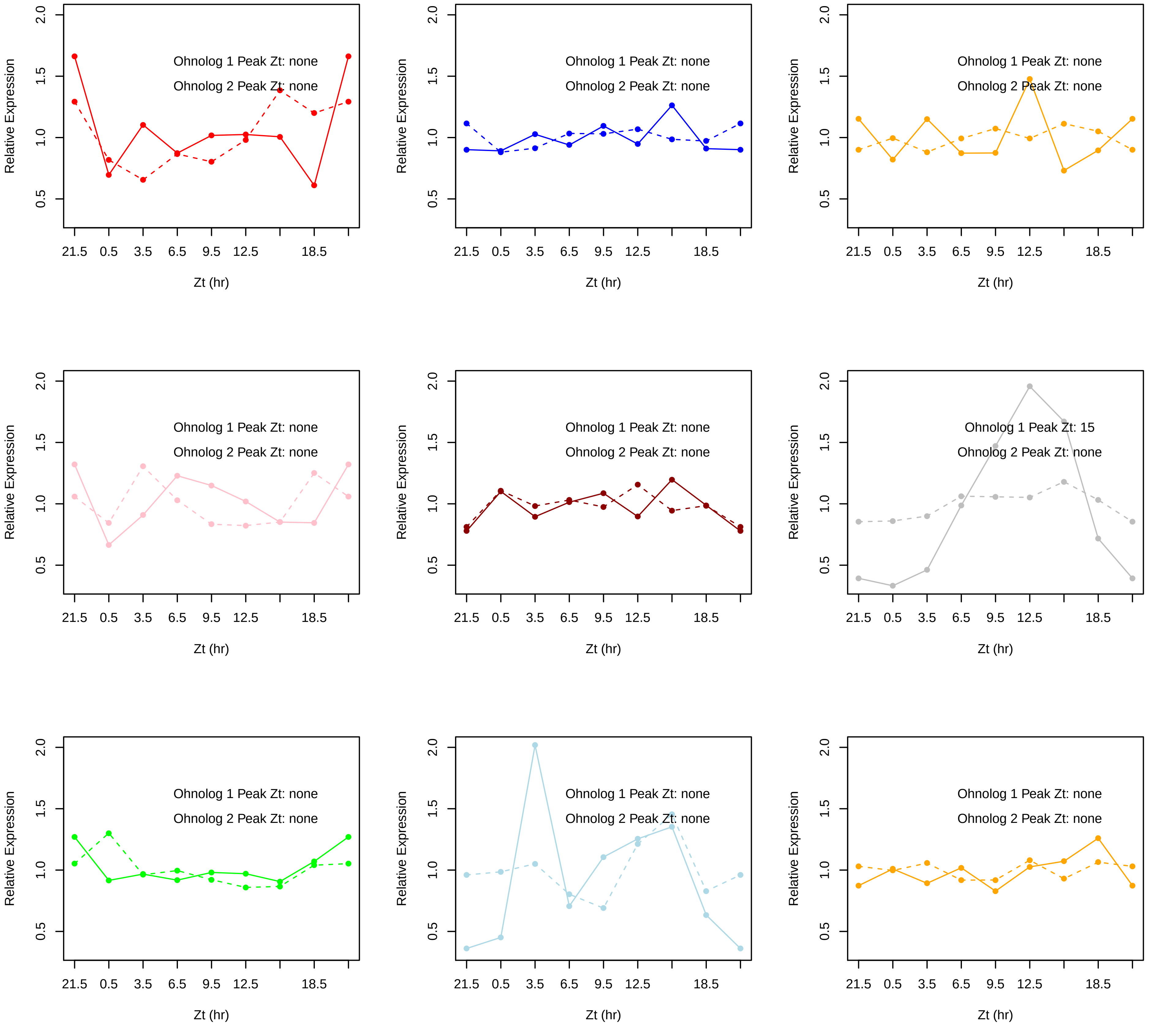

Ohnolog 1: ENSXMAG00000004335
Ohnolog 2: ENSXMAG00000010385 rrm1

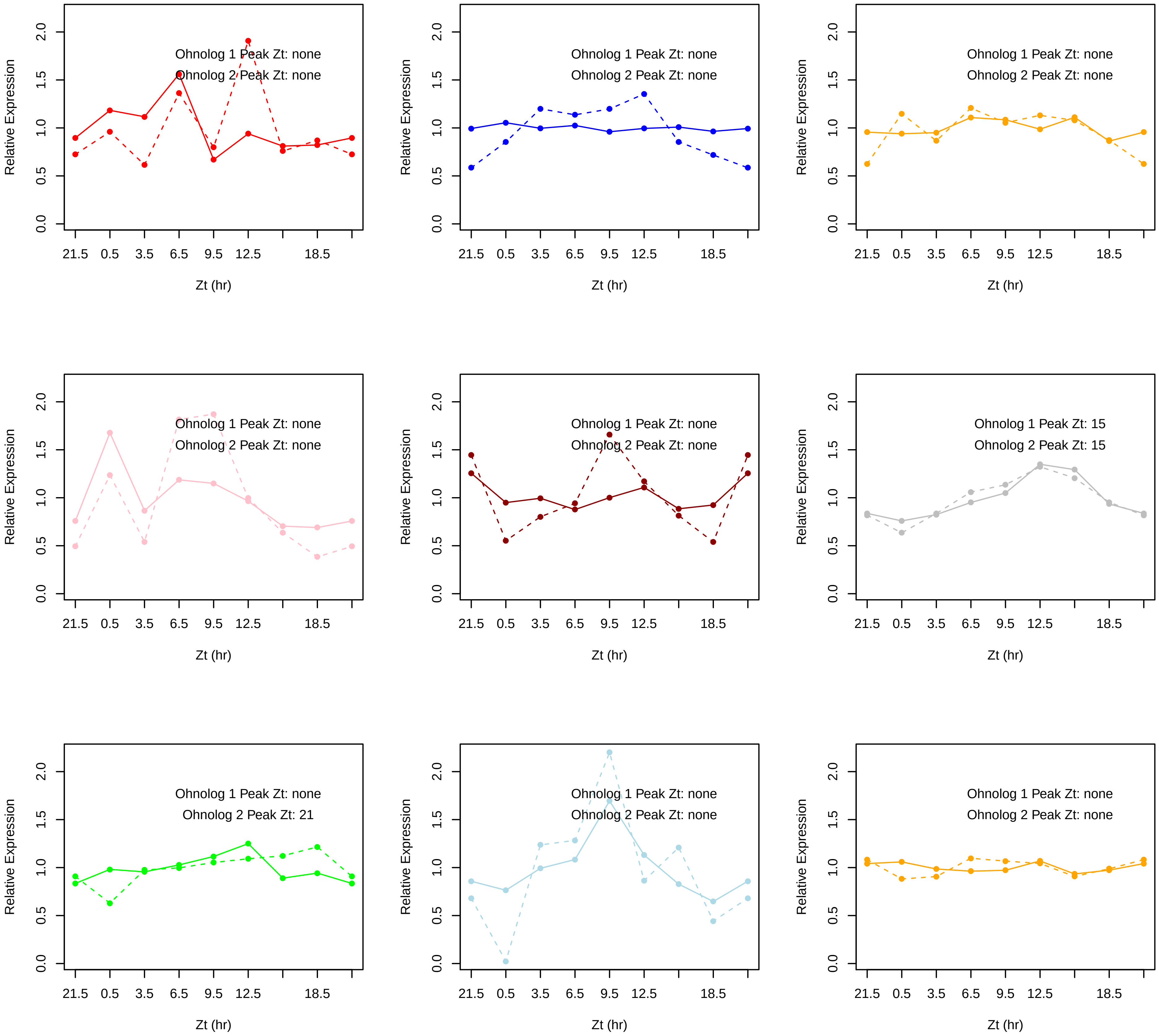

Ohnolog 1: ENSXMAG00000015852 c7a
Ohnolog 2: ENSXMAG00000016470 c7b

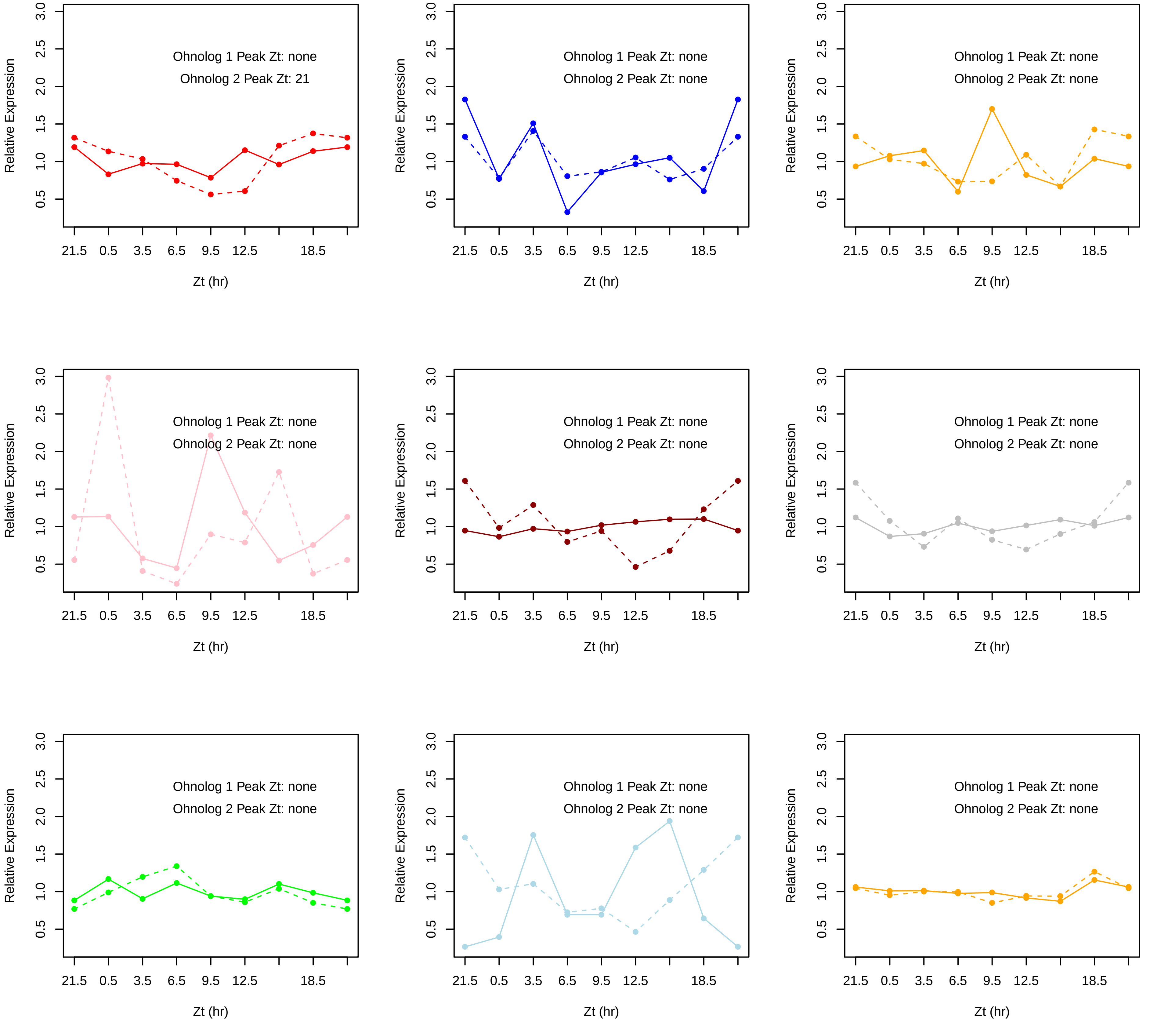

Ohnolog 1: ENSXMAG00000003851
Ohnolog 2: ENSXMAG00000009717 cabp2b

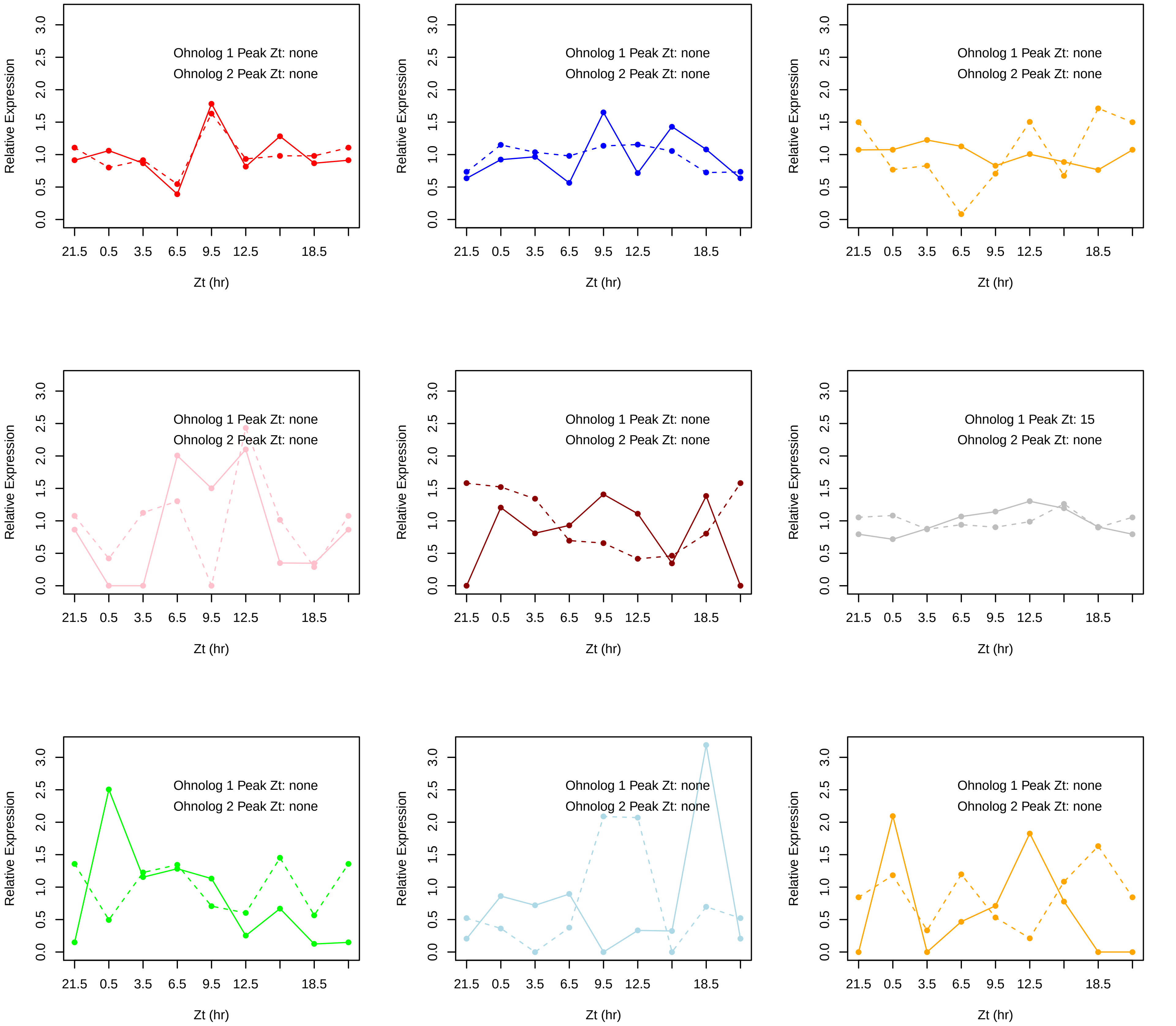

Ohnolog 1: ENSXMAG00000011034 si:ch211−154o6.2
Ohnolog 2: ENSXMAG00000016787 znf384l

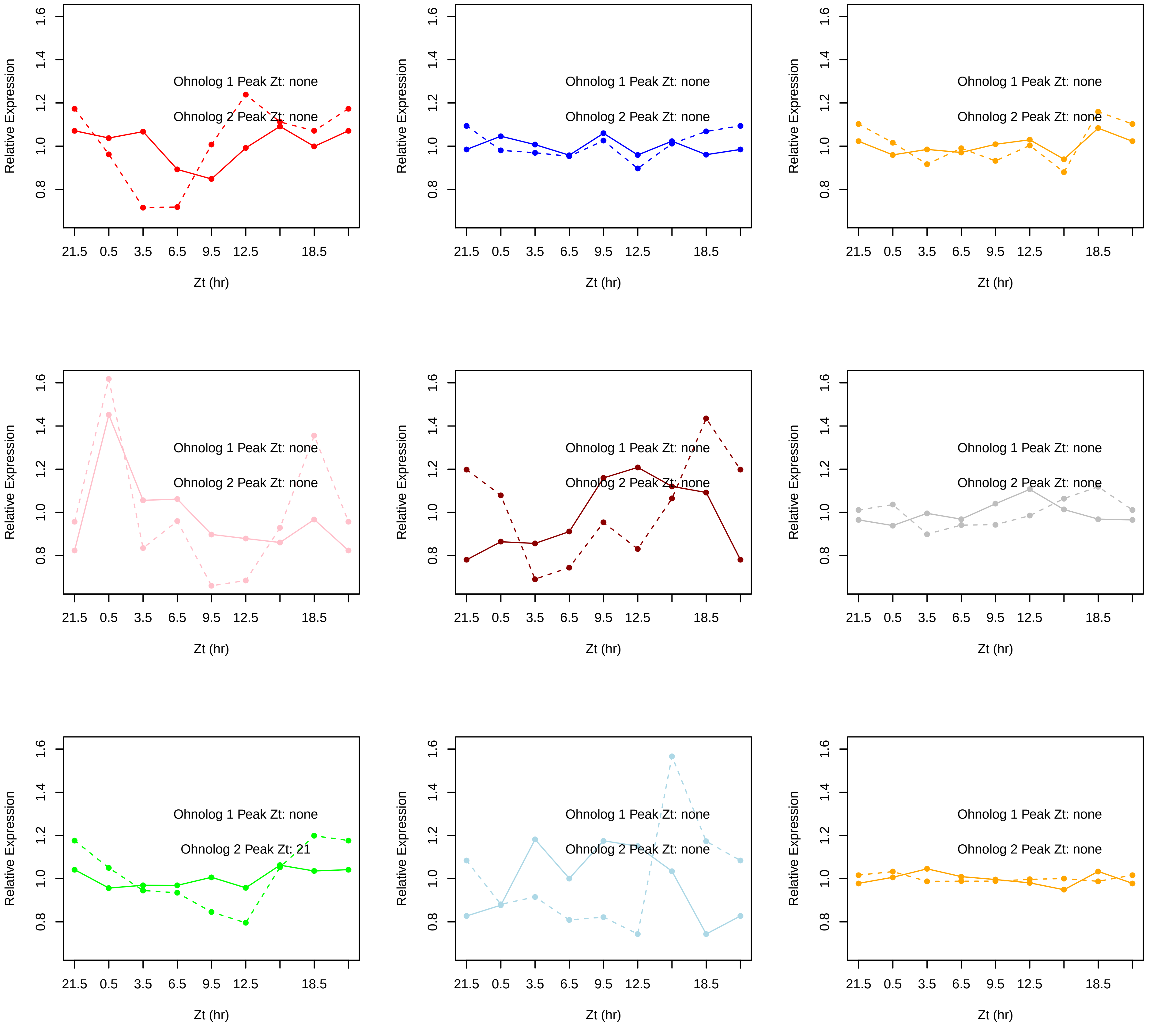

Ohnolog 1: ENSXMAG00000000677 olfm3b
Ohnolog 2: ENSXMAG00000025233 olfm3a

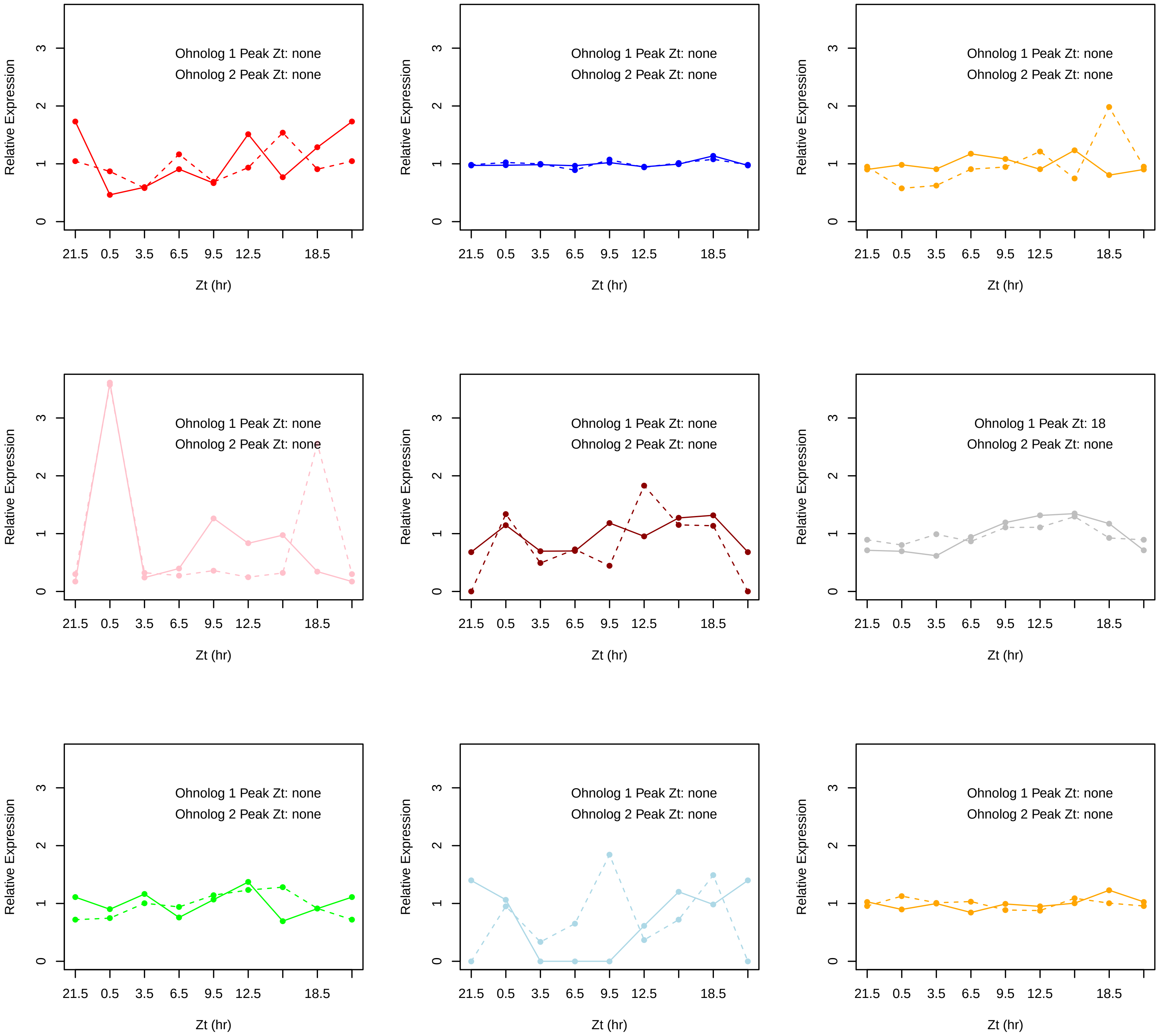

Ohnolog 1: ENSXMAG00000010903 grk7b
Ohnolog 2: ENSXMAG00000025417 grk7a

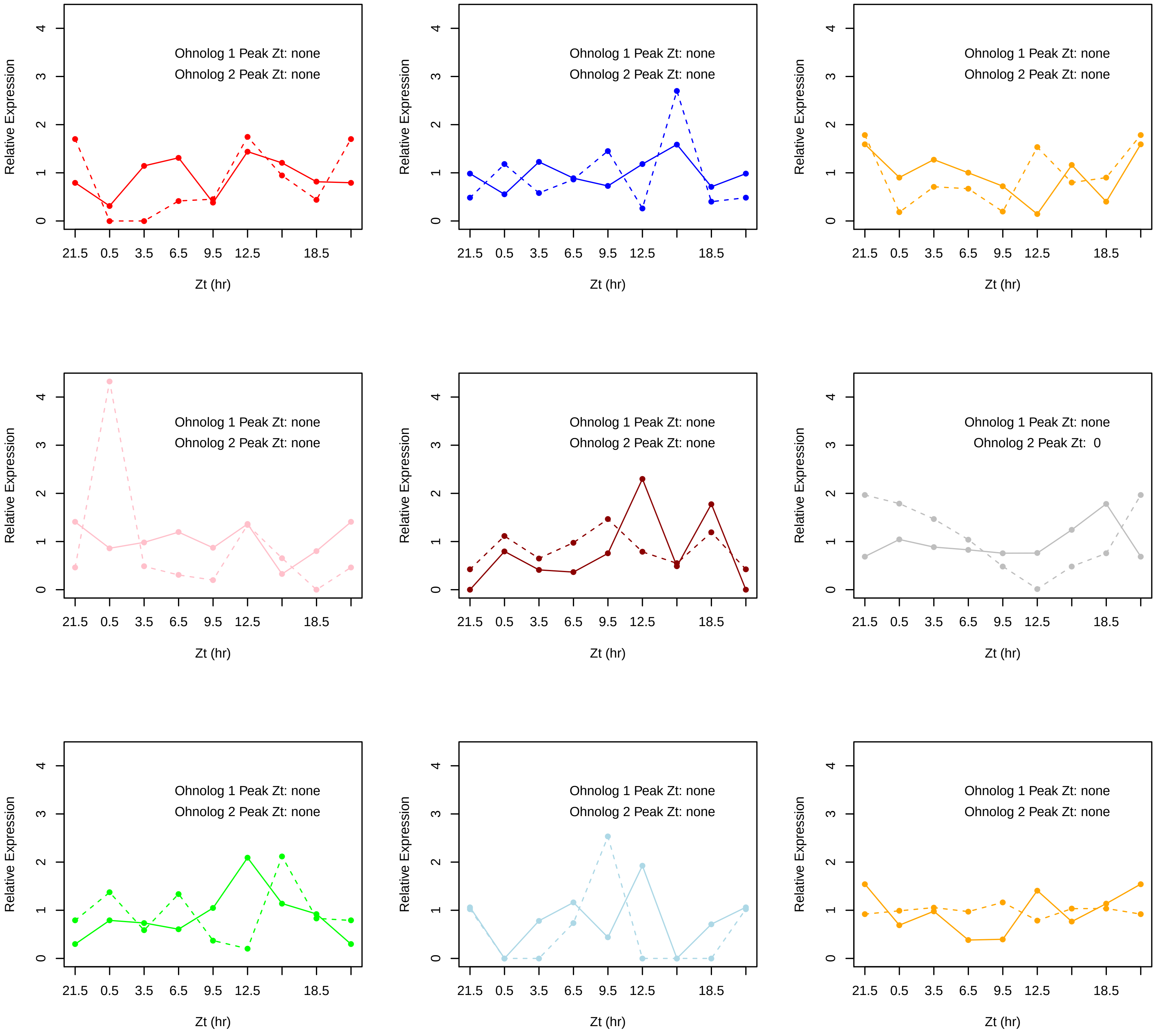

Ohnolog 1: ENSXMAG00000013970 klhl41b
Ohnolog 2: ENSXMAG00000014325 klhl41a

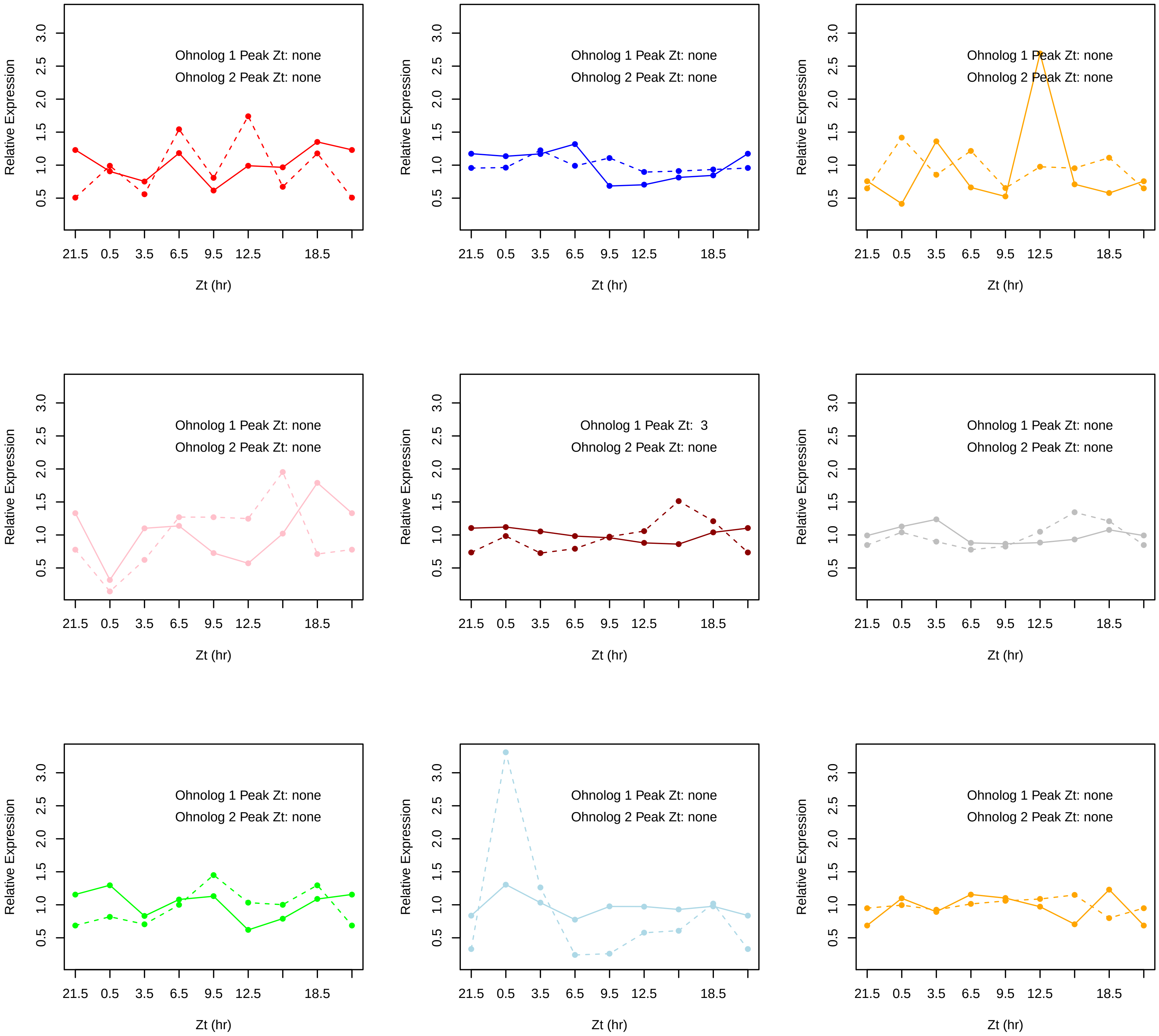

Ohnolog 1: ENSXMAG00000009803 tmx2a
Ohnolog 2: ENSXMAG00000013723

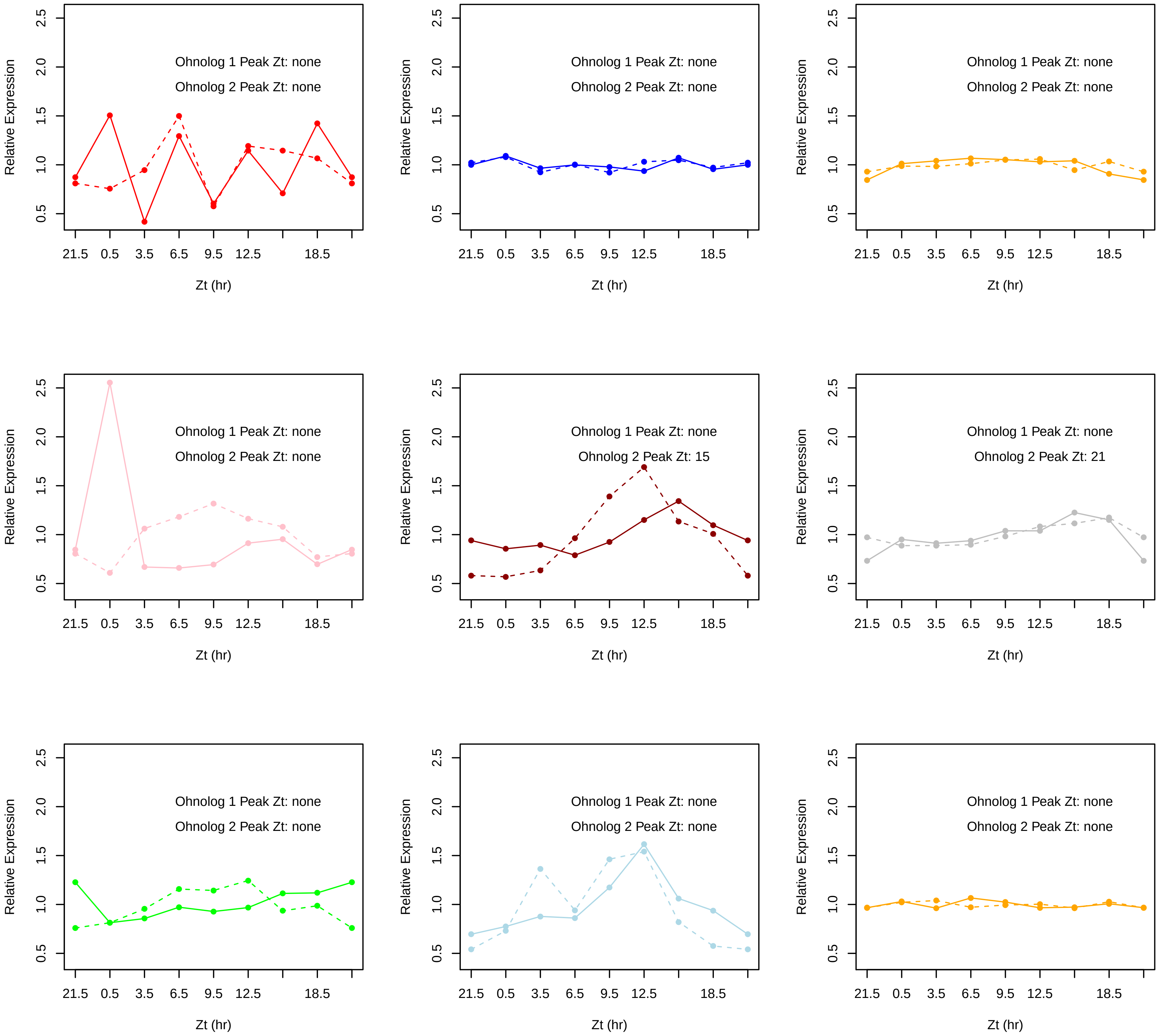

Ohnolog 1: ENSXMAG00000009791 zdhhc5b
Ohnolog 2: ENSXMAG00000013732 zdhhc5a

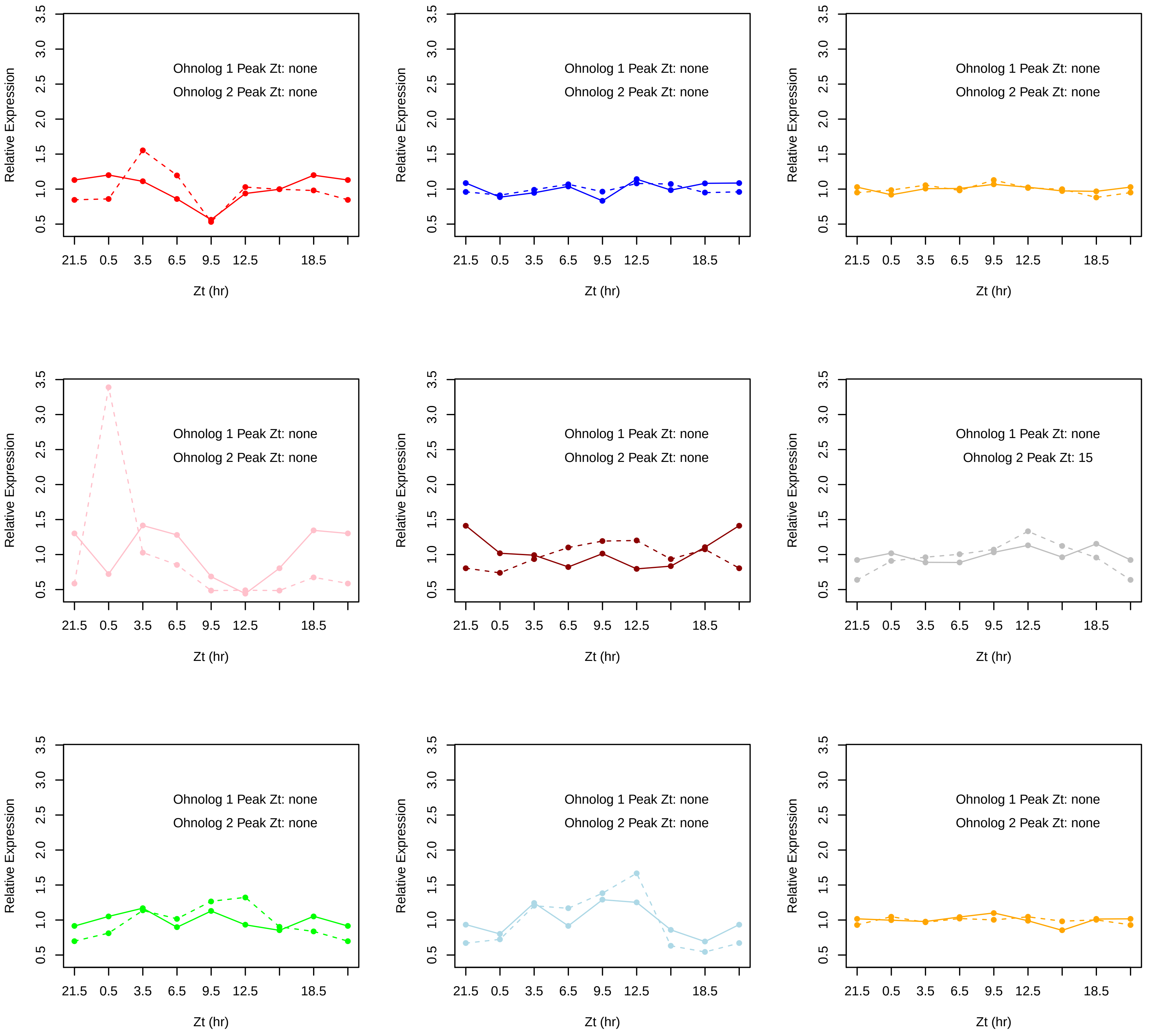

Ohnolog 1: ENSXMAG00000011911 epn1
Ohnolog 2: ENSXMAG00000014223 zgc:194578

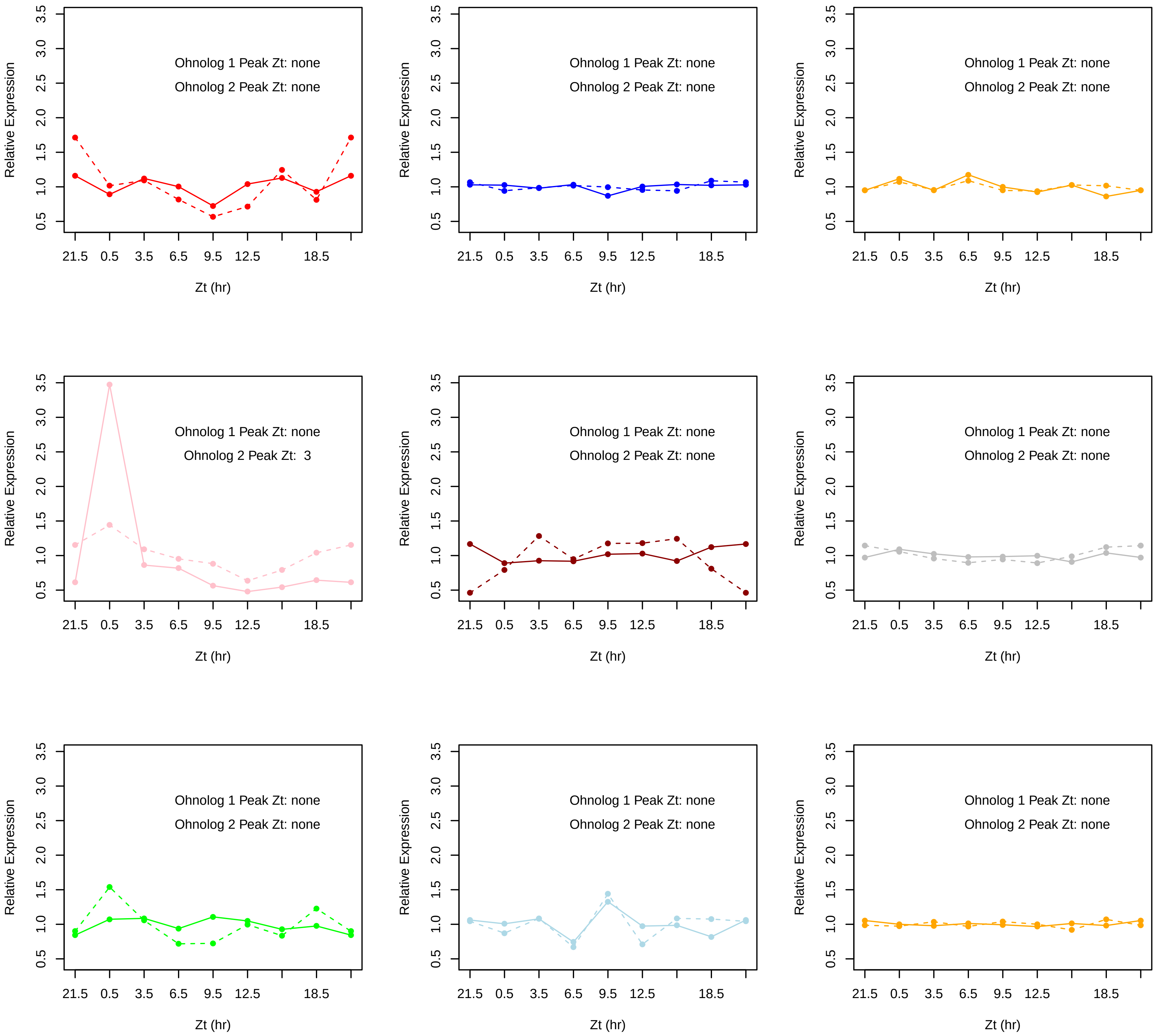

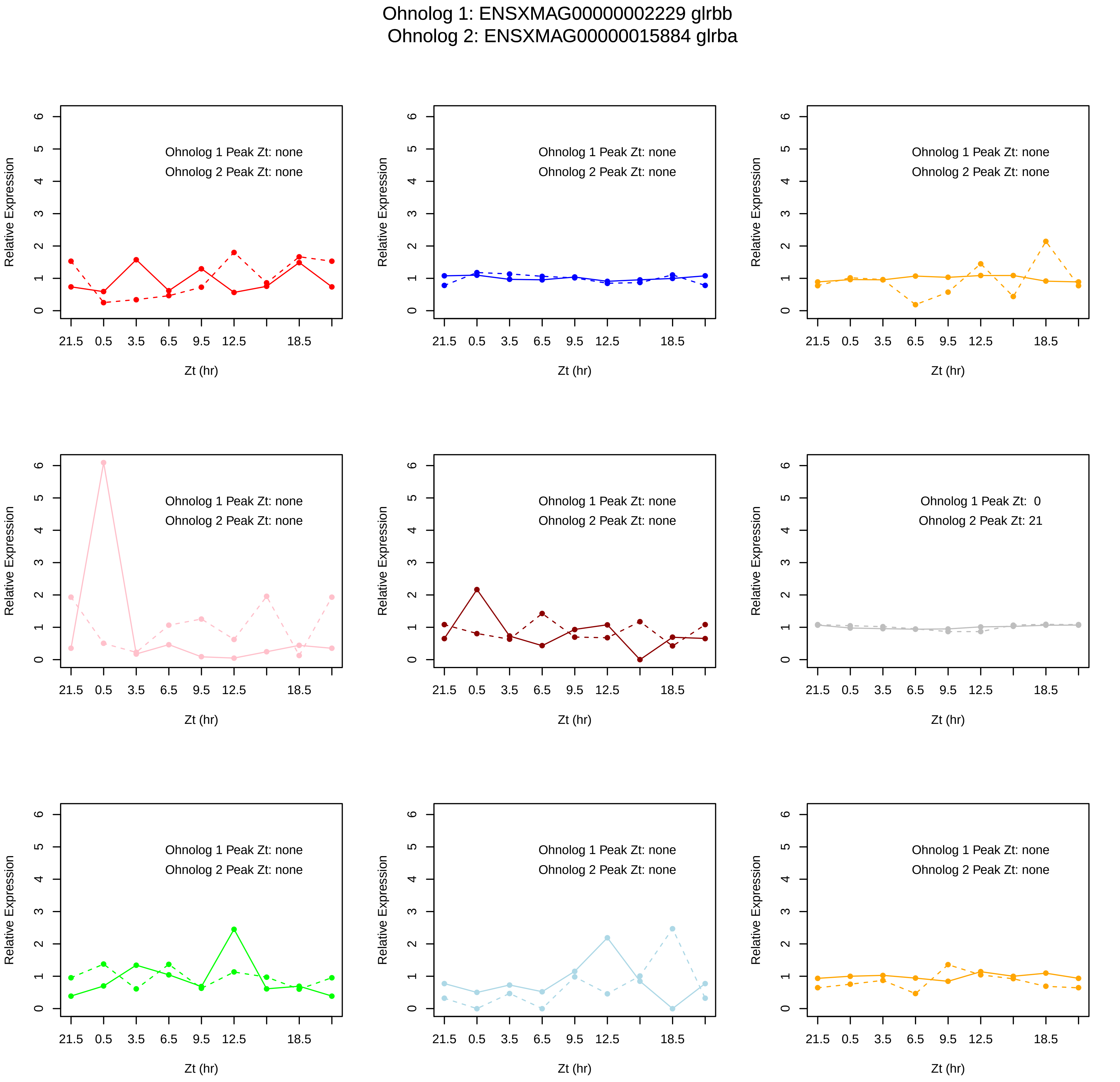

Ohnolog 1: ENSXMAG00000002229 glrbb
Ohnolog 2: ENSXMAG00000015884 glrba
Relative Expression
Zt (hr)
21.5
0.5
3.5
6.5
9.5
12.5
18.5
Ohnolog 1 Peak Zt: none
Ohnolog 2 Peak Zt: none
Ohnolog 1 Peak Zt: 0
Ohnolog 2 Peak Zt: 21

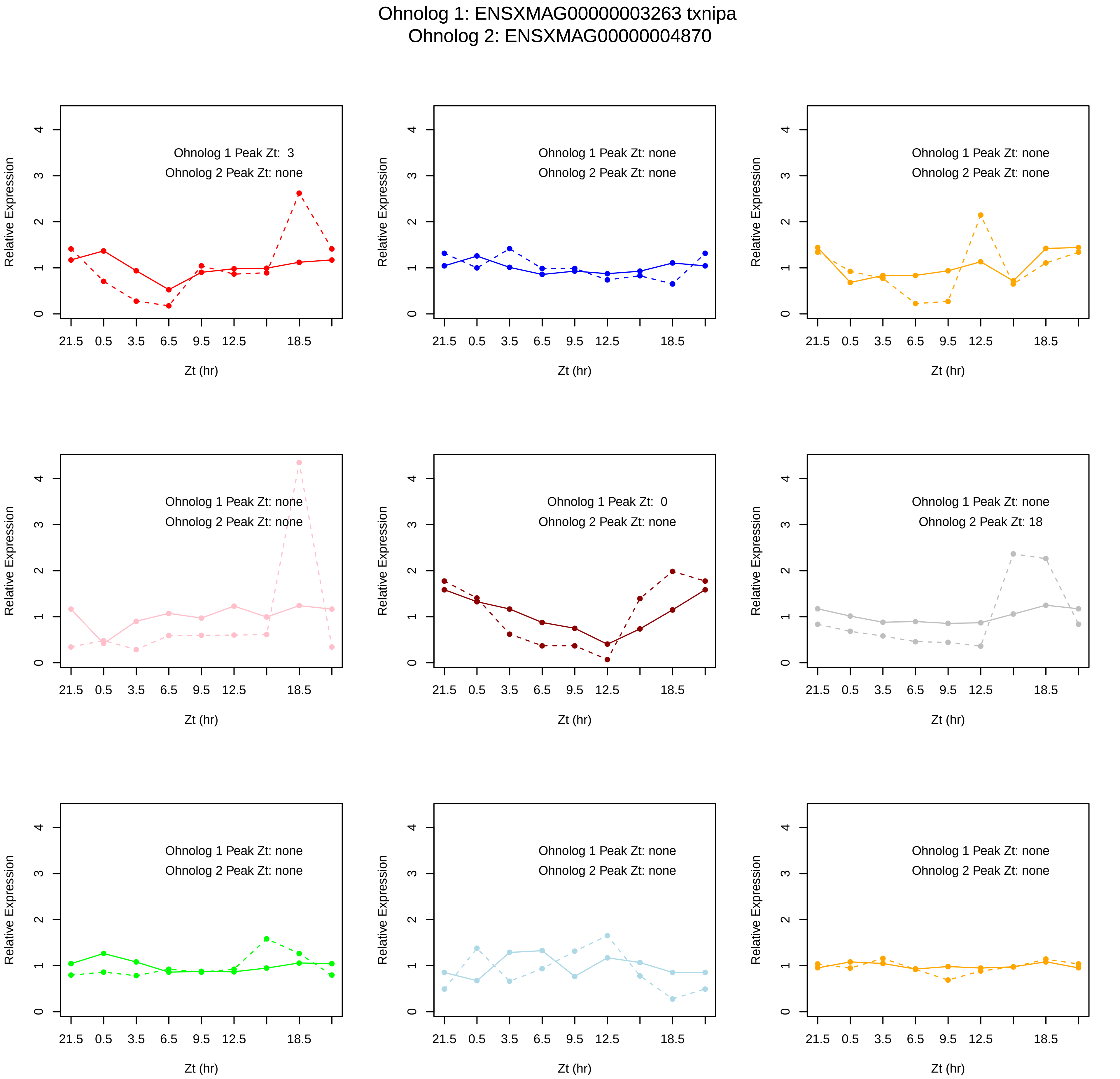

Ohnolog 1: ENSXMAG00000003263 txnipa
Ohnolog 2: ENSXMAG00000004870
Ohnolog 1 Peak Zt: 3
Ohnolog 2 Peak Zt: none
Ohnolog 1 Peak Zt: none
Ohnolog 2 Peak Zt: none
Ohnolog 1 Peak Zt: none
Ohnolog 2 Peak Zt: none
Ohnolog 1 Peak Zt: none
Ohnolog 2 Peak Zt: none
Ohnolog 1 Peak Zt: 0
Ohnolog 2 Peak Zt: none
Ohnolog 1 Peak Zt: none
Ohnolog 2 Peak Zt: 18
Ohnolog 1 Peak Zt: none
Ohnolog 2 Peak Zt: none
Ohnolog 1 Peak Zt: none
Ohnolog 2 Peak Zt: none
Ohnolog 1 Peak Zt: none
Ohnolog 2 Peak Zt: none
Relative Expression
Zt (hr)
0
1
2
3
4
21.5
0.5
3.5
6.5
9.5
12.5
18.5

Ohnolog 1: ENSXMAG00000010370 PLPPR4 (1 of many)
Ohnolog 2: ENSXMAG00000026909 plppr4b

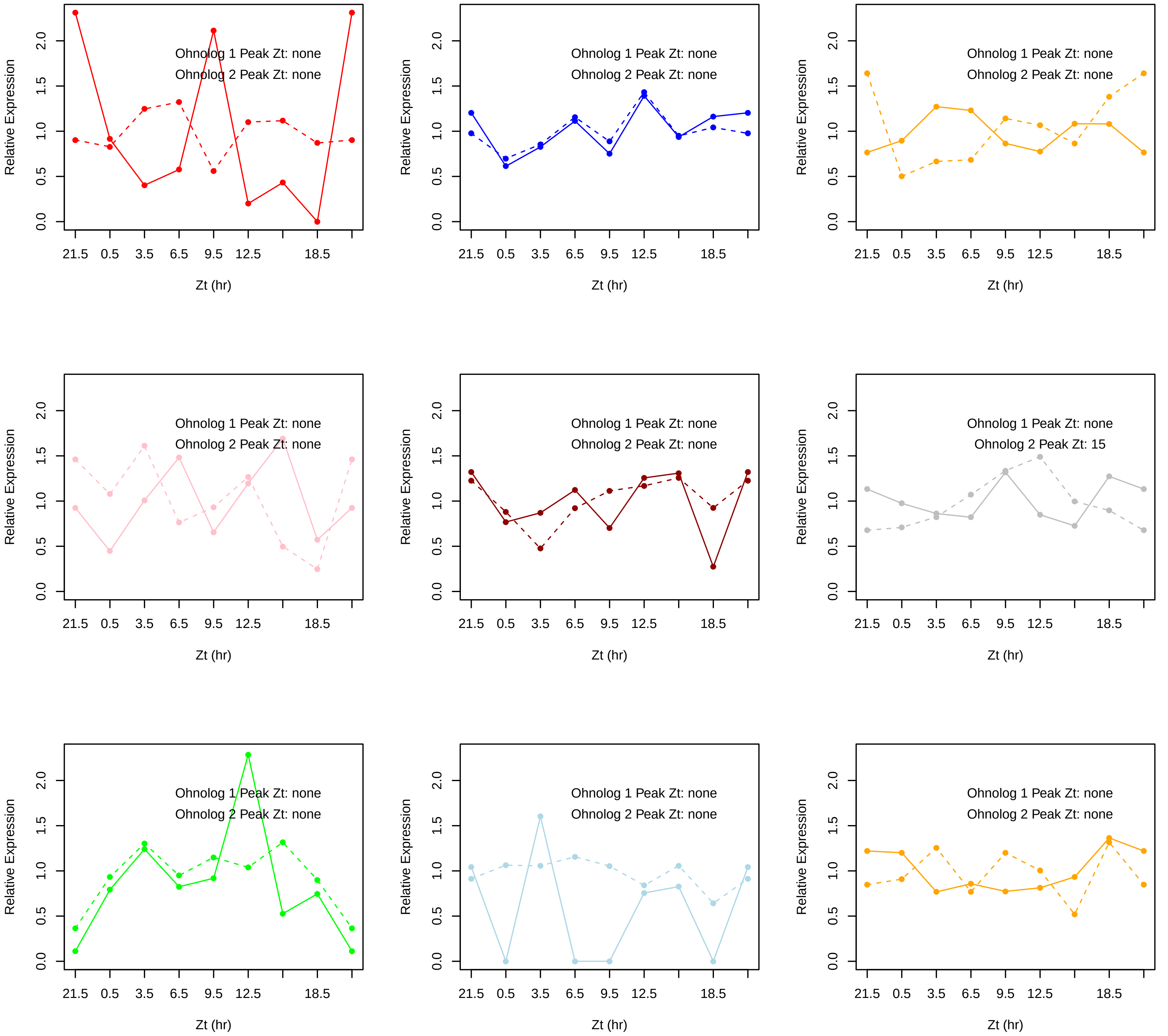

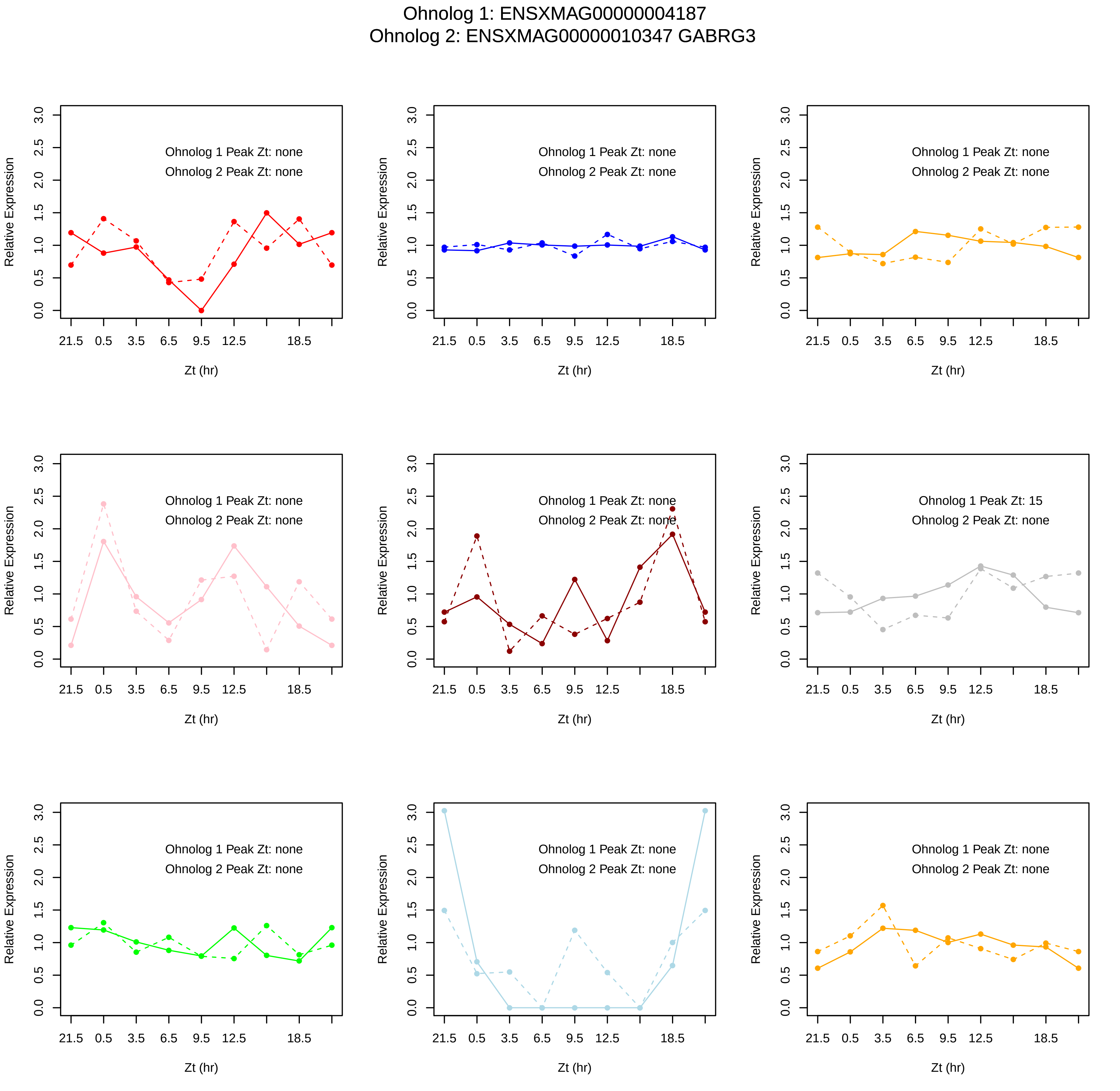
Ohnolog 1: ENSXMAG00000004187
Ohnolog 2: ENSXMAG00000010347 GABRG3
Ohnolog 1 Peak Zt: none
Ohnolog 2 Peak Zt: none
Ohnolog 1 Peak Zt: none
Ohnolog 2 Peak Zt: none
Ohnolog 1 Peak Zt: none
Ohnolog 2 Peak Zt: none
Ohnolog 1 Peak Zt: none
Ohnolog 2 Peak Zt: none
Ohnolog 1 Peak Zt: none
Ohnolog 2 Peak Zt: none
Ohnolog 1 Peak Zt: 15
Ohnolog 2 Peak Zt: none
Ohnolog 1 Peak Zt: none
Ohnolog 2 Peak Zt: none
Ohnolog 1 Peak Zt: none
Ohnolog 2 Peak Zt: none
Ohnolog 1 Peak Zt: none
Ohnolog 2 Peak Zt: none
Relative Expression
0.0
0.5
1.0
1.5
2.0
2.5
3.0
21.5
0.5
3.5
6.5
9.5
12.5
18.5
Zt (hr)

Ohnolog 1: ENSXMAG00000003635 dtnbp1b
Ohnolog 2: ENSXMAG00000015449

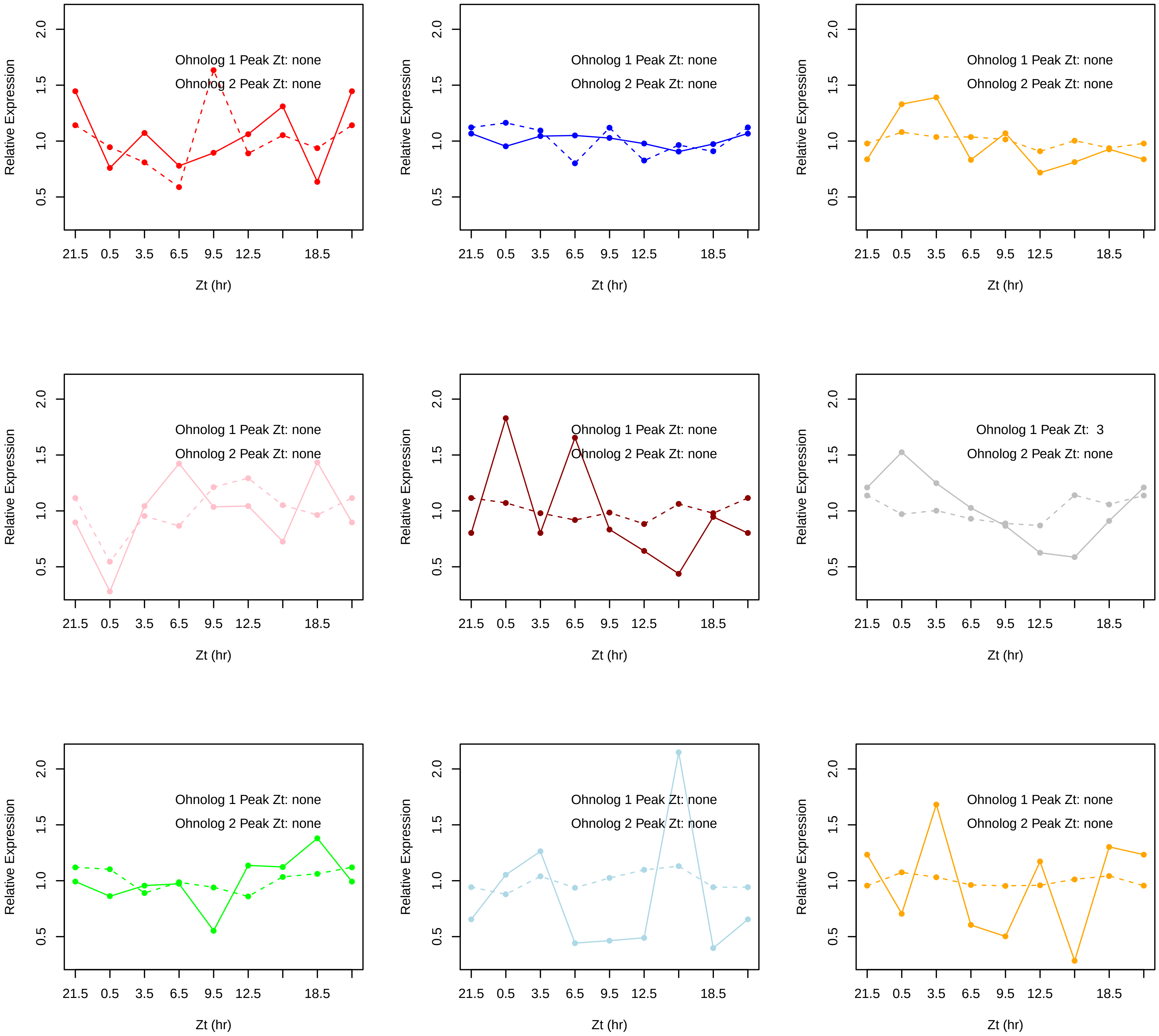

Ohnolog 1: ENSXMAG00000005153 slc44a2
Ohnolog 2: ENSXMAG00000011550

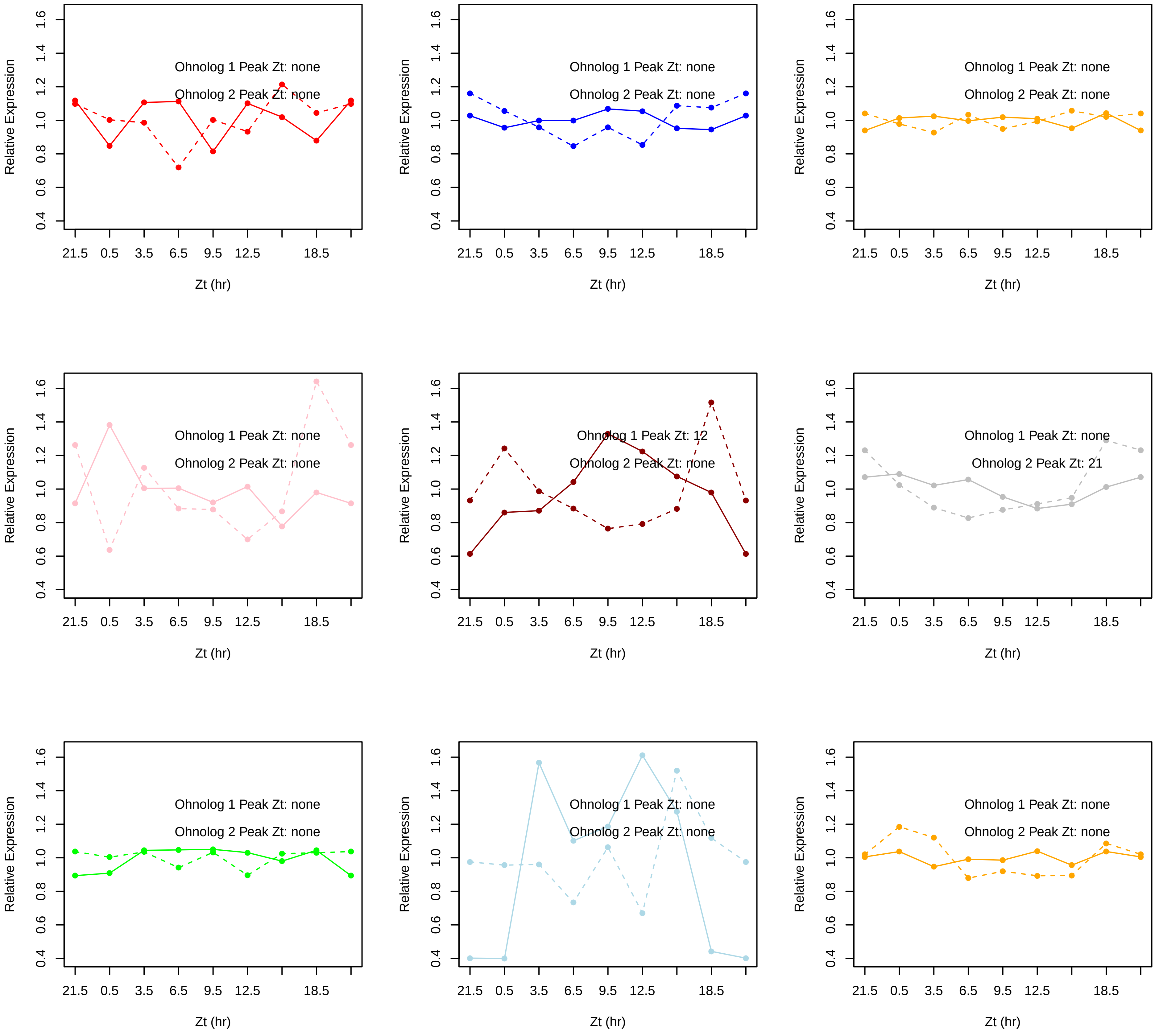

Ohnolog 1: ENSXMAG00000007084 dclk1b
Ohnolog 2: ENSXMAG00000008579 dclk1a

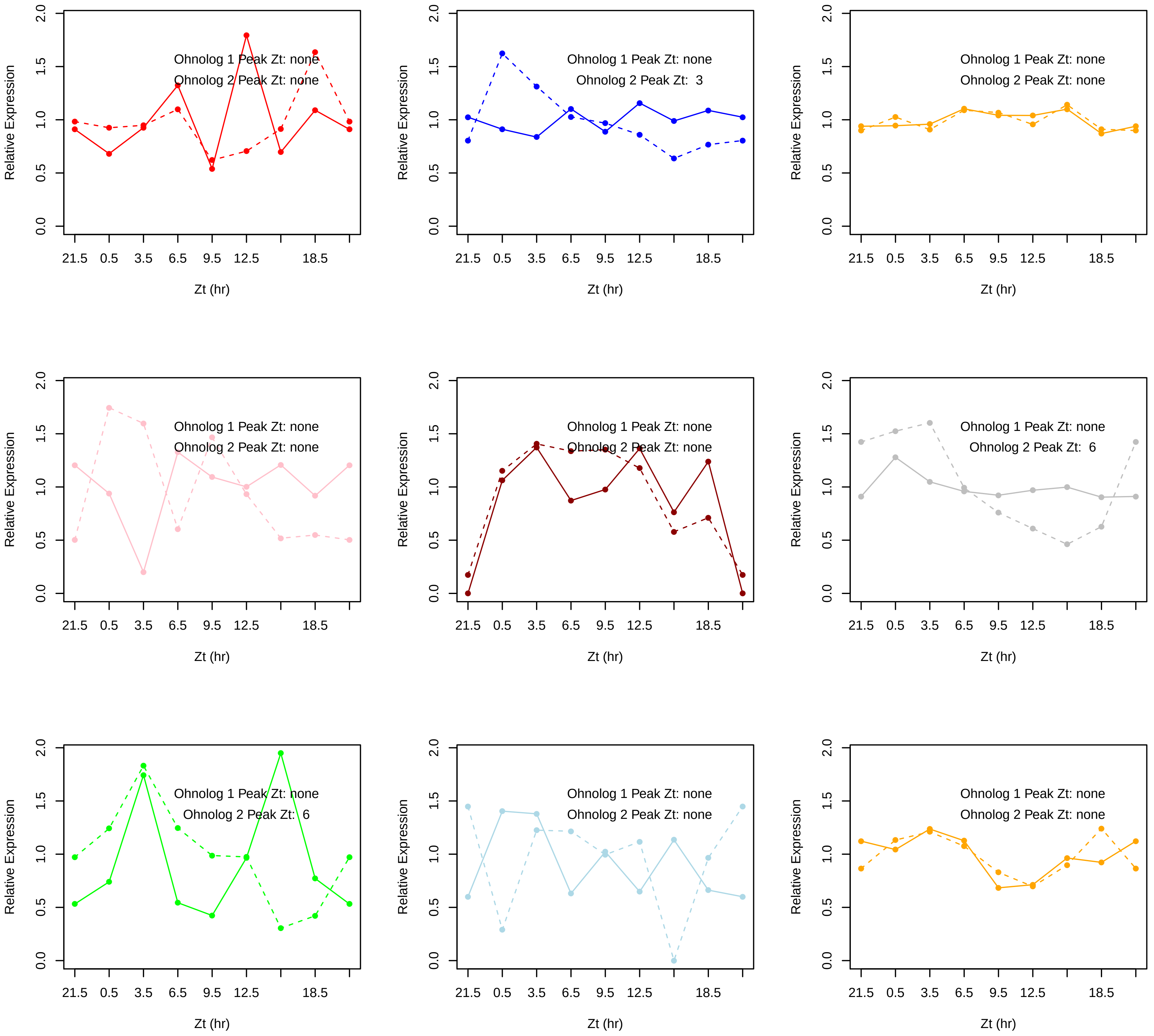

Ohnolog 1: ENSXMAG00000001119
Ohnolog 2: ENSXMAG00000014597

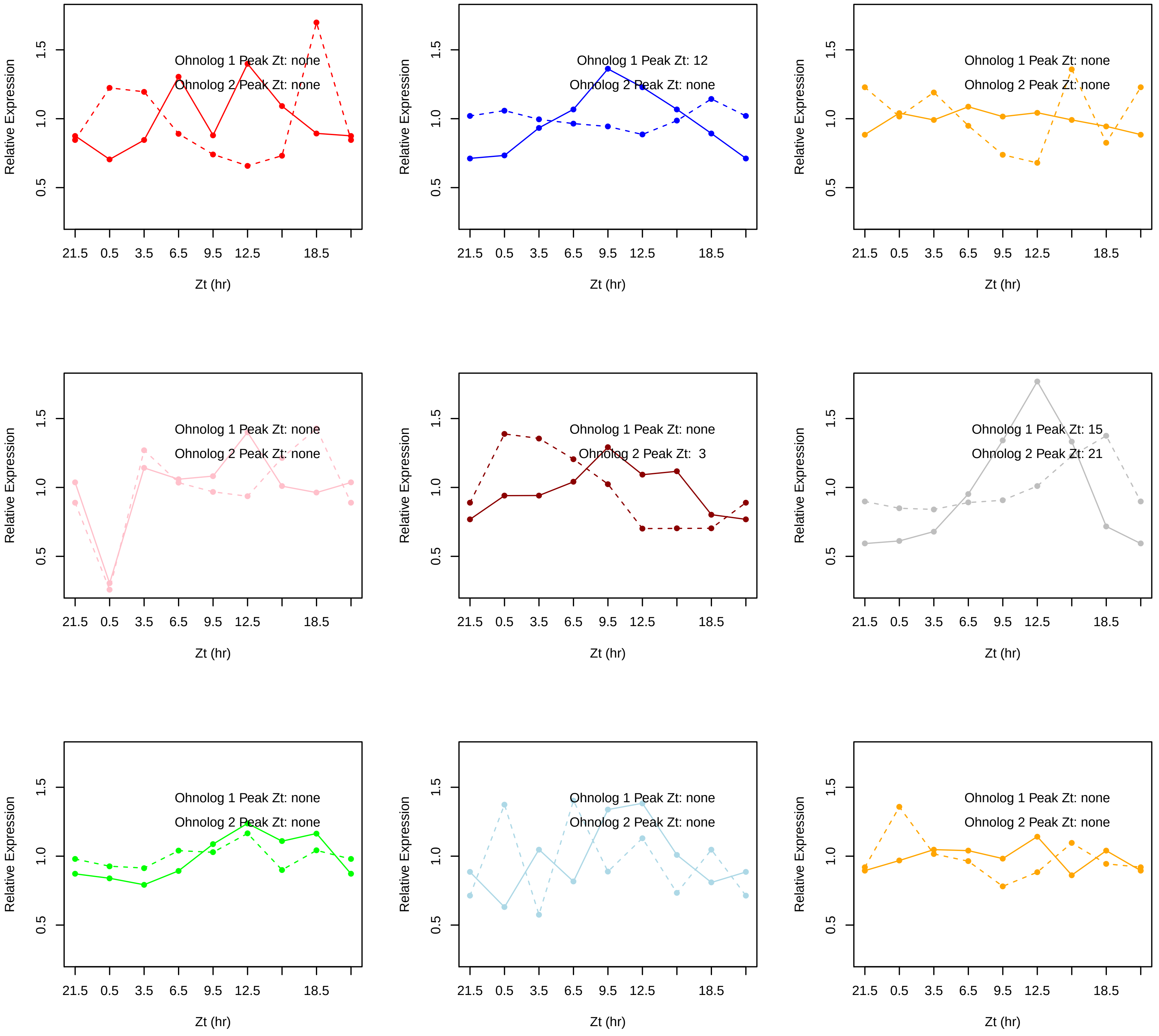

Ohnolog 1: ENSXMAG00000007108 spartb
Ohnolog 2: ENSXMAG00000008570 sparta

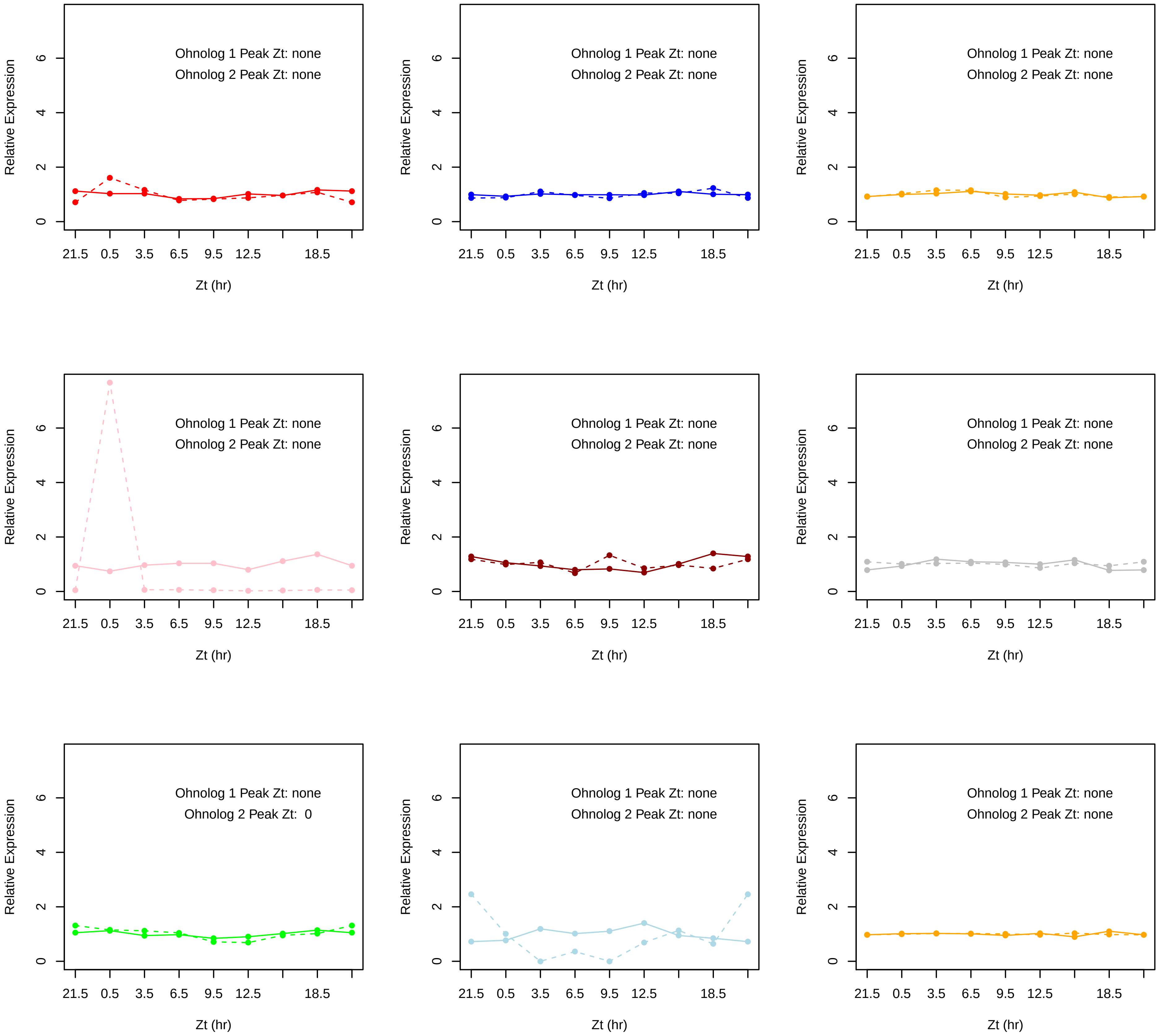

Ohnolog 1: ENSXMAG00000010707 zgc:100997
Ohnolog 2: ENSXMAG00000012164 clmn

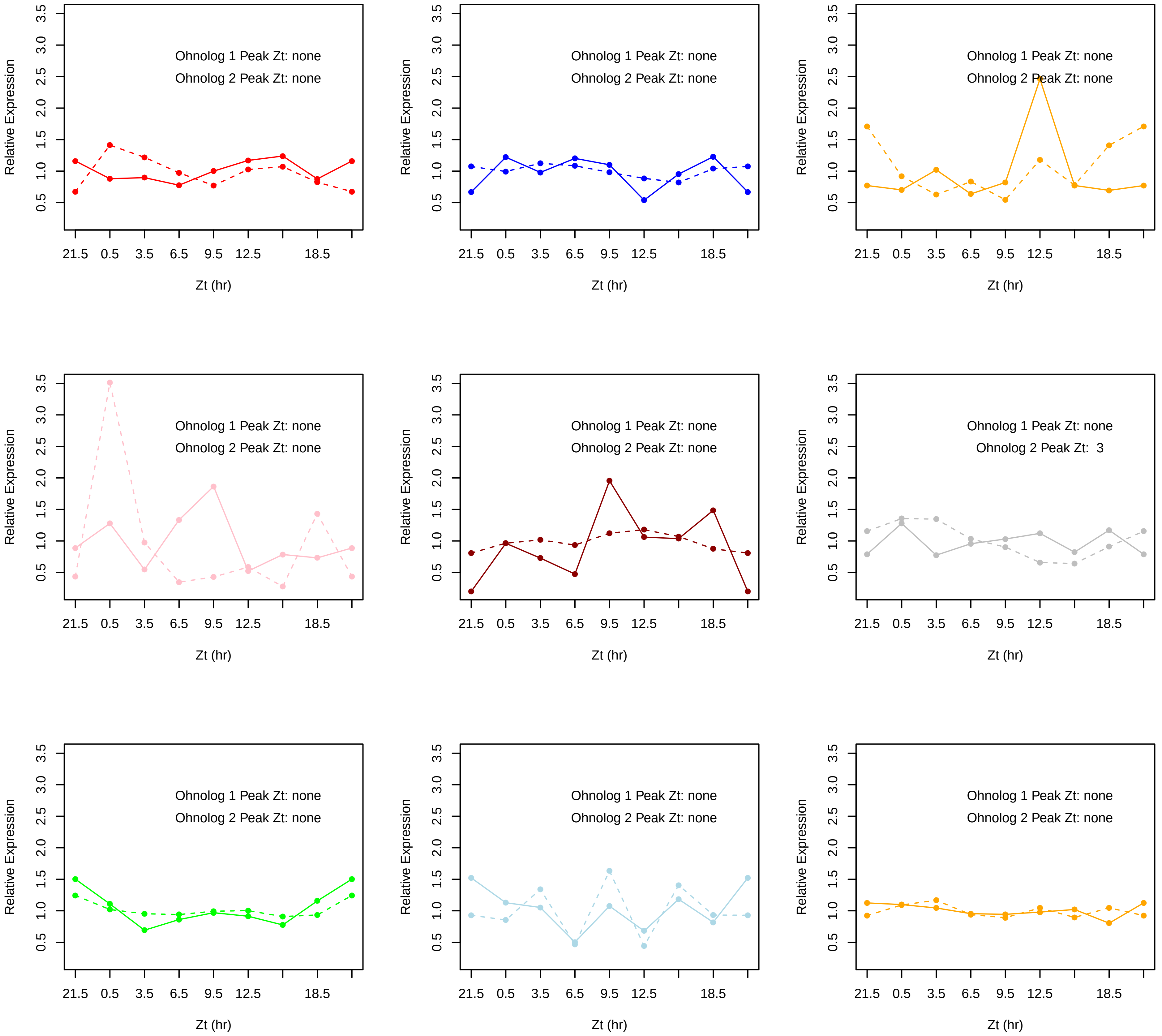

Ohnolog 1: ENSXMAG00000011435 slc4a10b
Ohnolog 2: ENSXMAG00000013798

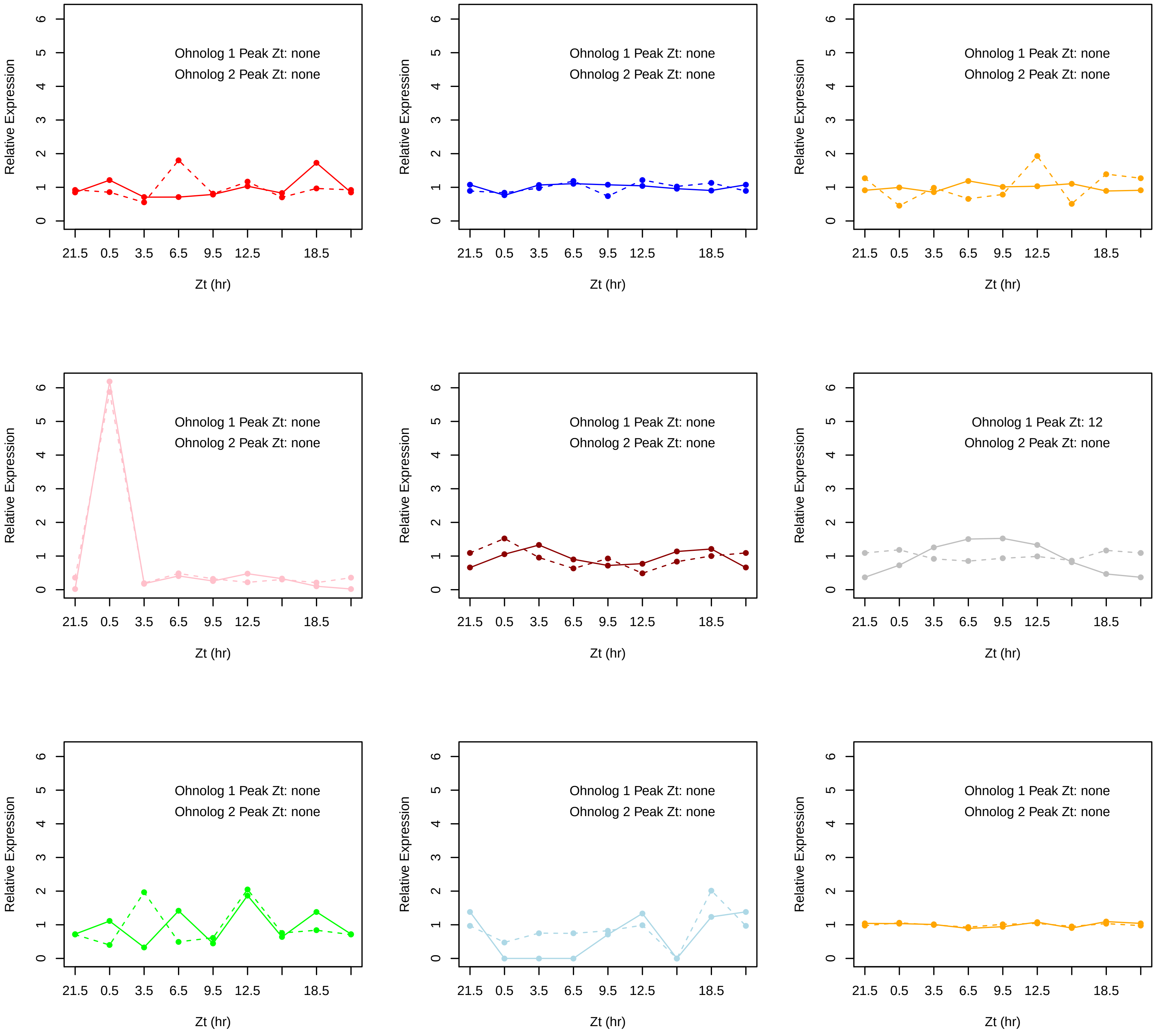

Ohnolog 1: ENSXMAG00000014657 pvalb8
Ohnolog 2: ENSXMAG00000014851 pvalb9

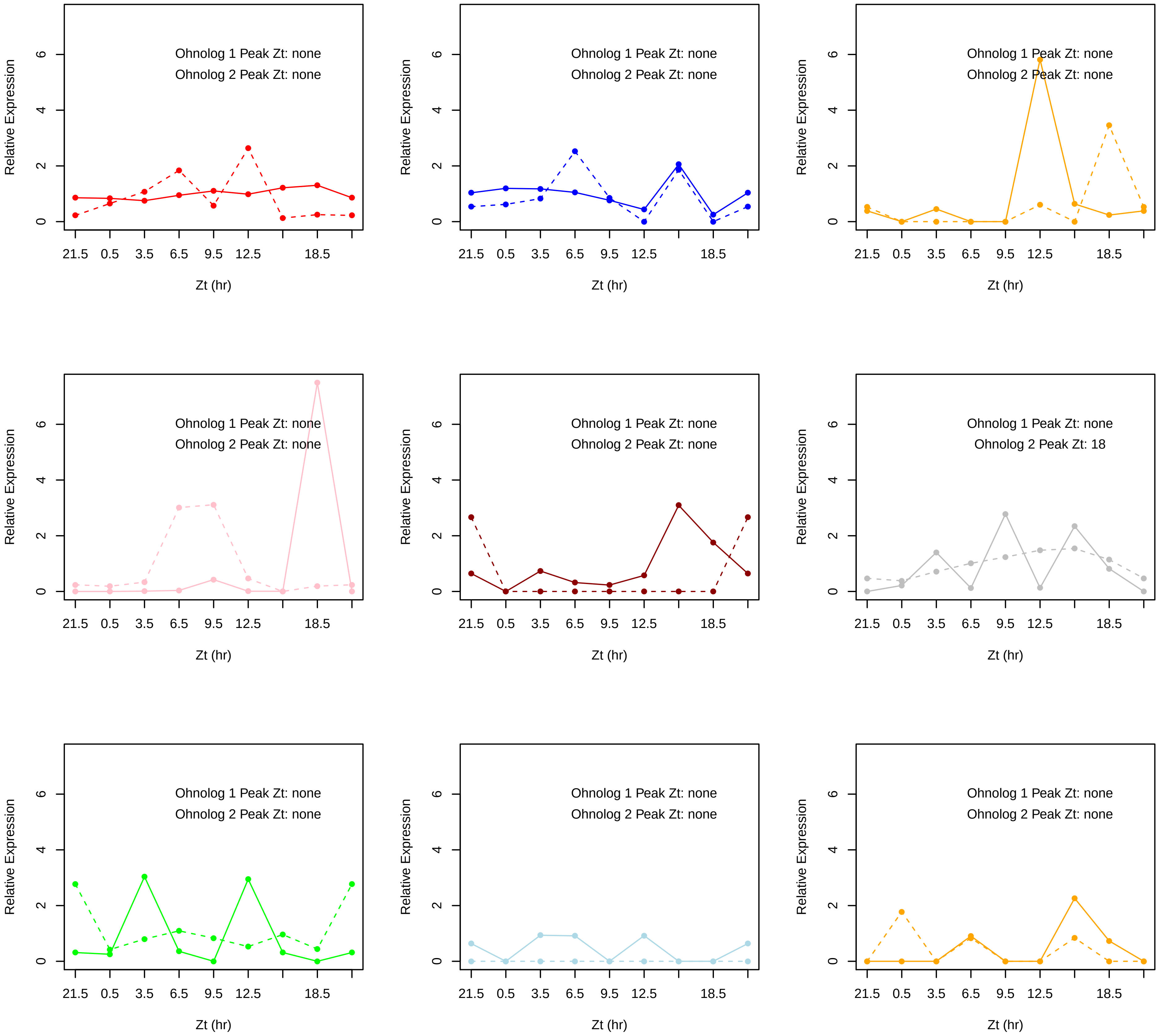

Ohnolog 1: ENSXMAG00000012028 slc27a1b
Ohnolog 2: ENSXMAG00000013238 slc27a1a

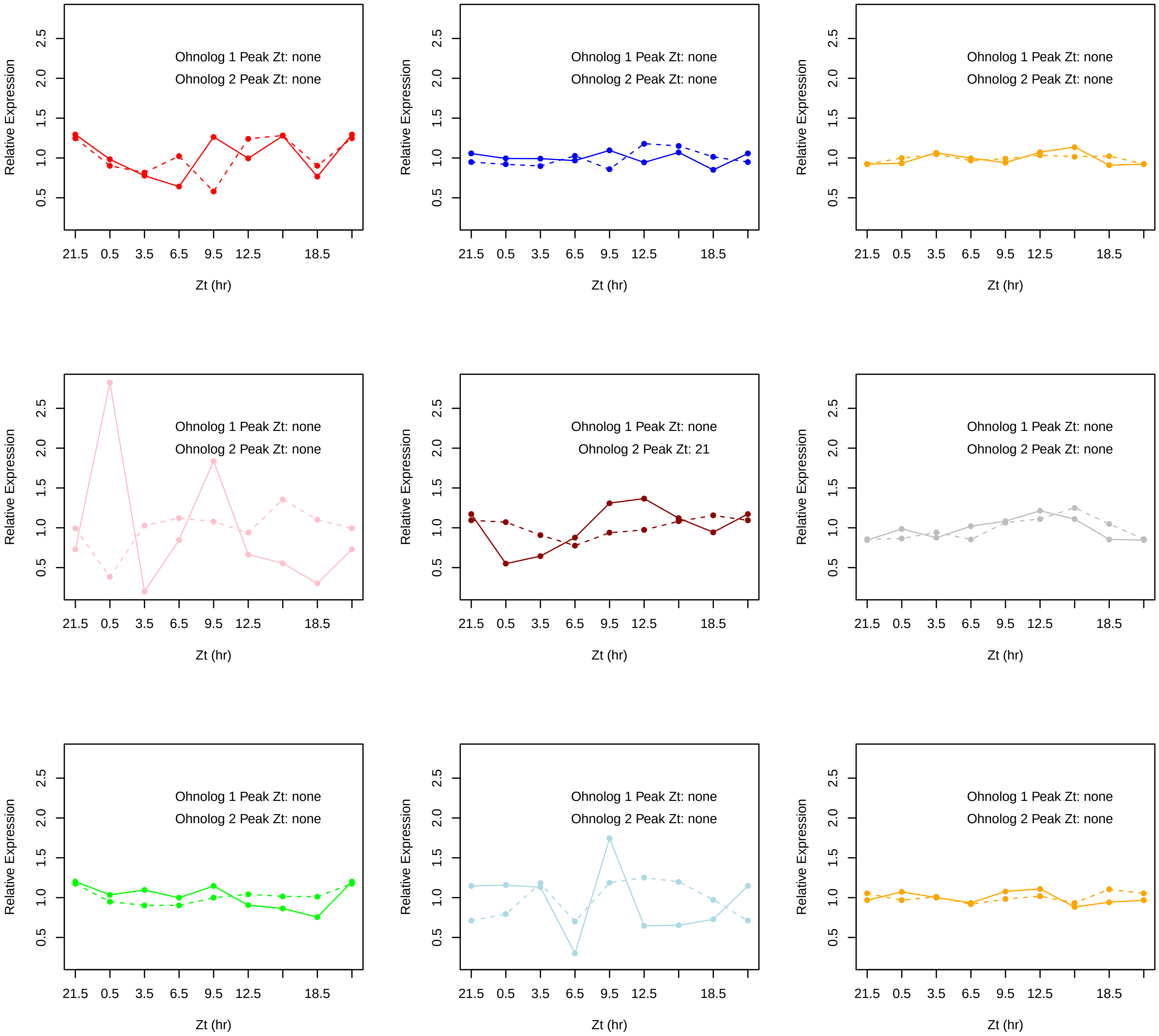

Ohnolog 1: ENSXMAG00000010736 GRAMD2B
Ohnolog 2: ENSXMAG00000029258

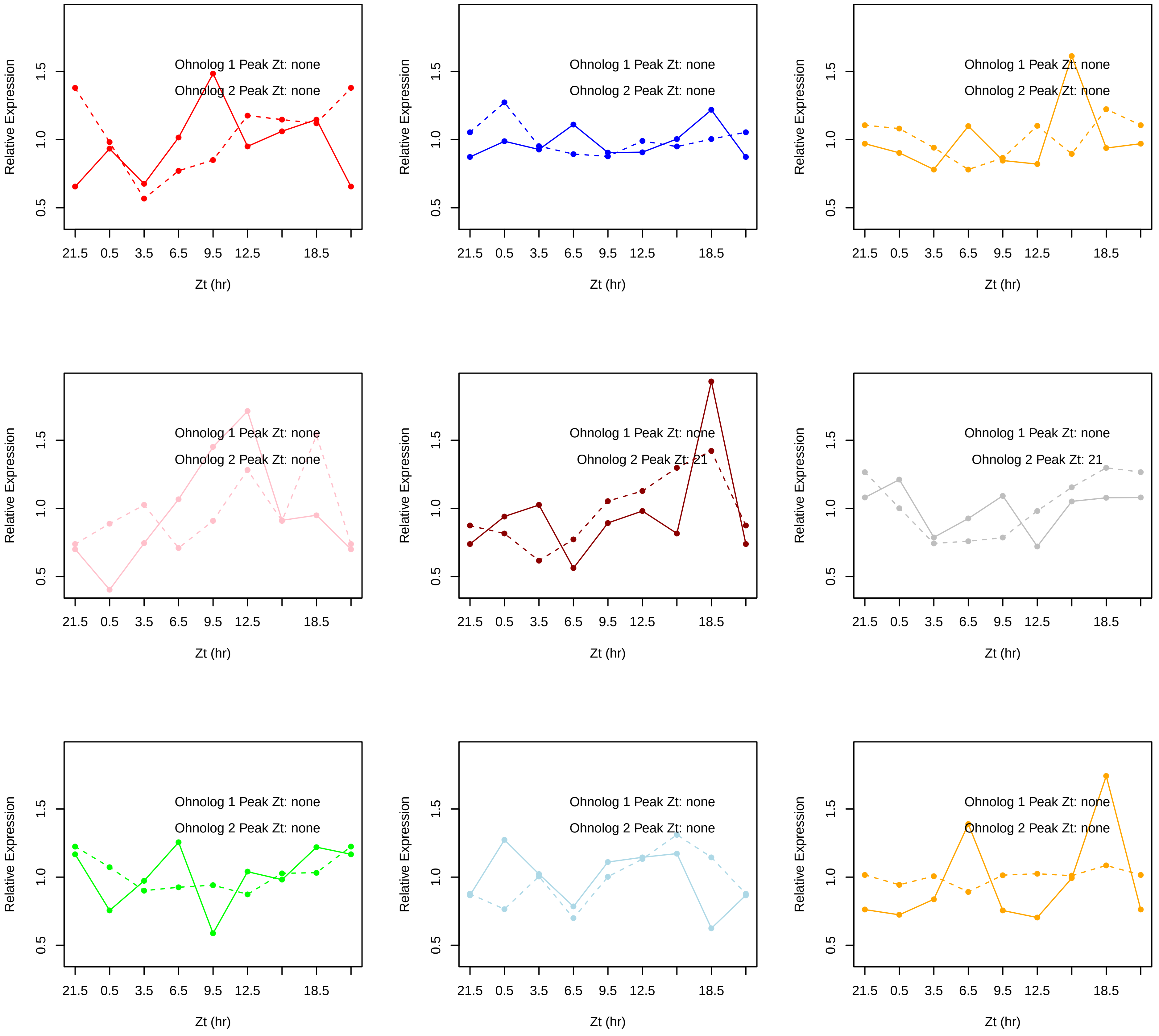

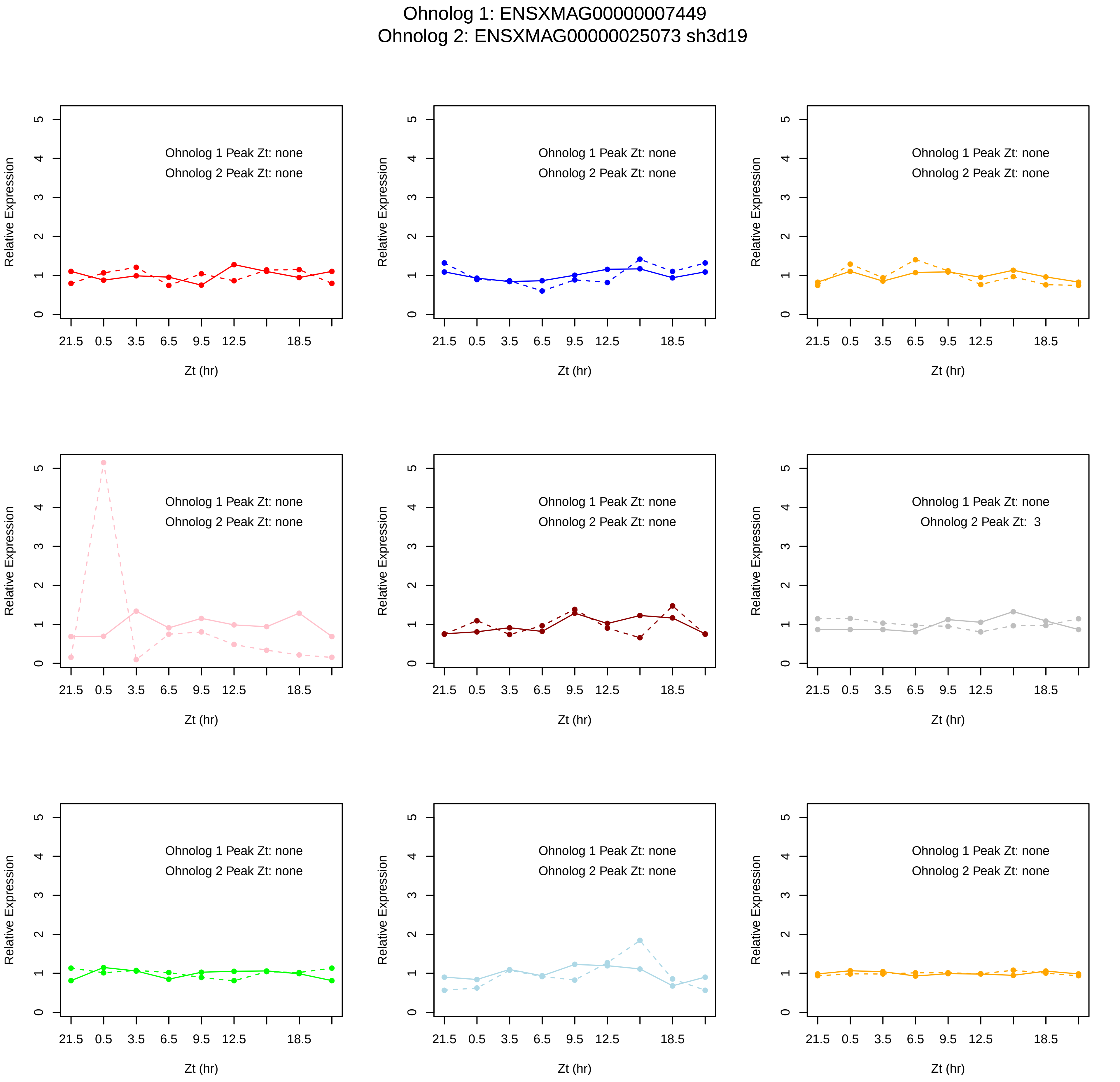
Ohnolog 1: ENSXMAG00000007449
Ohnolog 2: ENSXMAG00000025073 sh3d19
Ohnolog 1 Peak Zt: none
Ohnolog 2 Peak Zt: none
Ohnolog 1 Peak Zt: none
Ohnolog 2 Peak Zt: none
Ohnolog 1 Peak Zt: none
Ohnolog 2 Peak Zt: none
Ohnolog 1 Peak Zt: none
Ohnolog 2 Peak Zt: none
Ohnolog 1 Peak Zt: none
Ohnolog 2 Peak Zt: none
Ohnolog 1 Peak Zt: none
Ohnolog 2 Peak Zt: 3
Ohnolog 1 Peak Zt: none
Ohnolog 2 Peak Zt: none
Ohnolog 1 Peak Zt: none
Ohnolog 2 Peak Zt: none
Ohnolog 1 Peak Zt: none
Ohnolog 2 Peak Zt: none
Relative Expression
Zt (hr)
21.5 0.5 3.5 6.5 9.5 12.5 18.5
0 1 2 3 4 5

Ohnolog 1: ENSXMAG00000001802 rbms1a
Ohnolog 2: ENSXMAG00000011405 RBMS1 (1 of many)

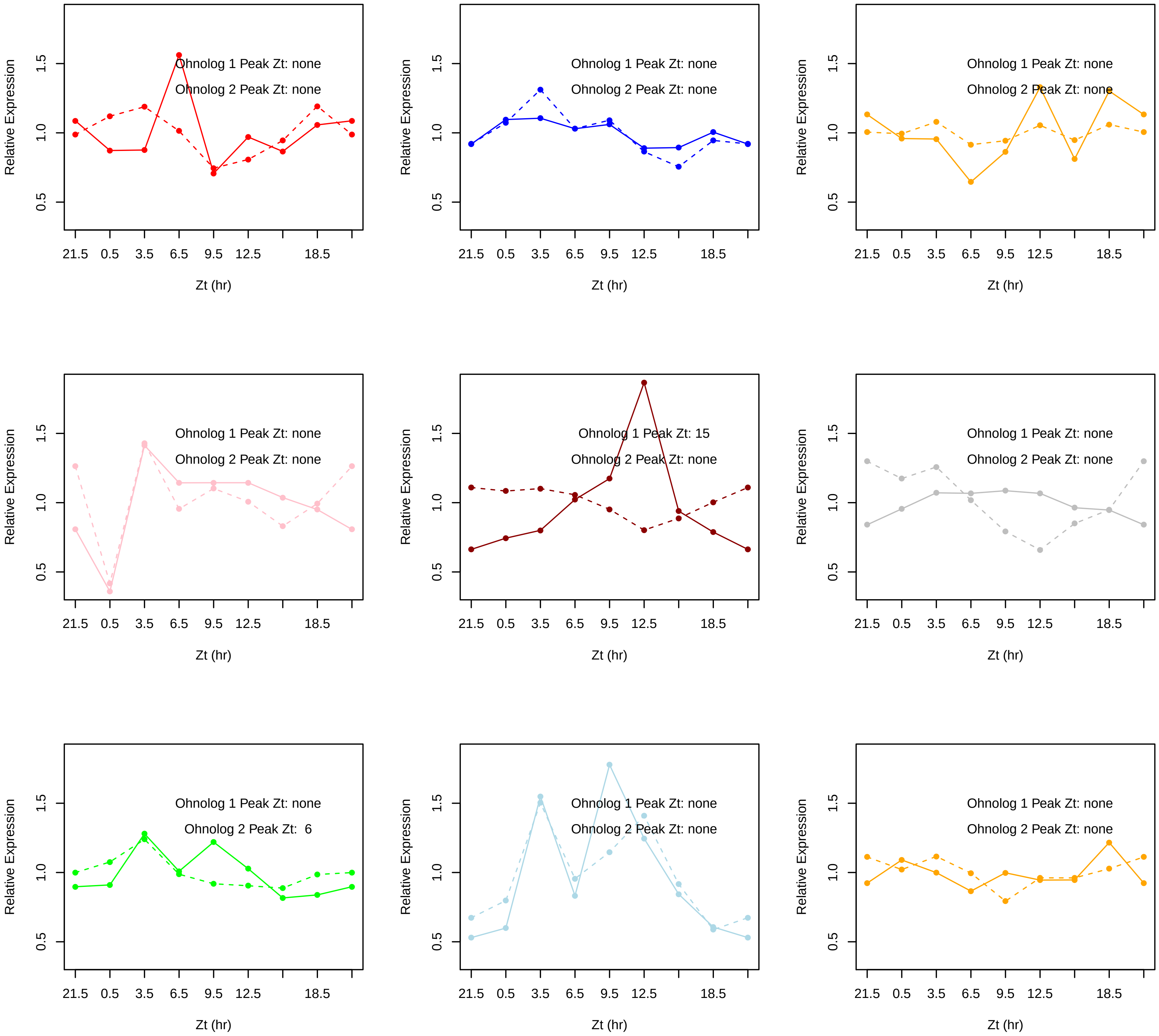

Ohnolog 1: ENSXMAG00000007202 trim3a
Ohnolog 2: ENSXMAG00000017483 trim3b

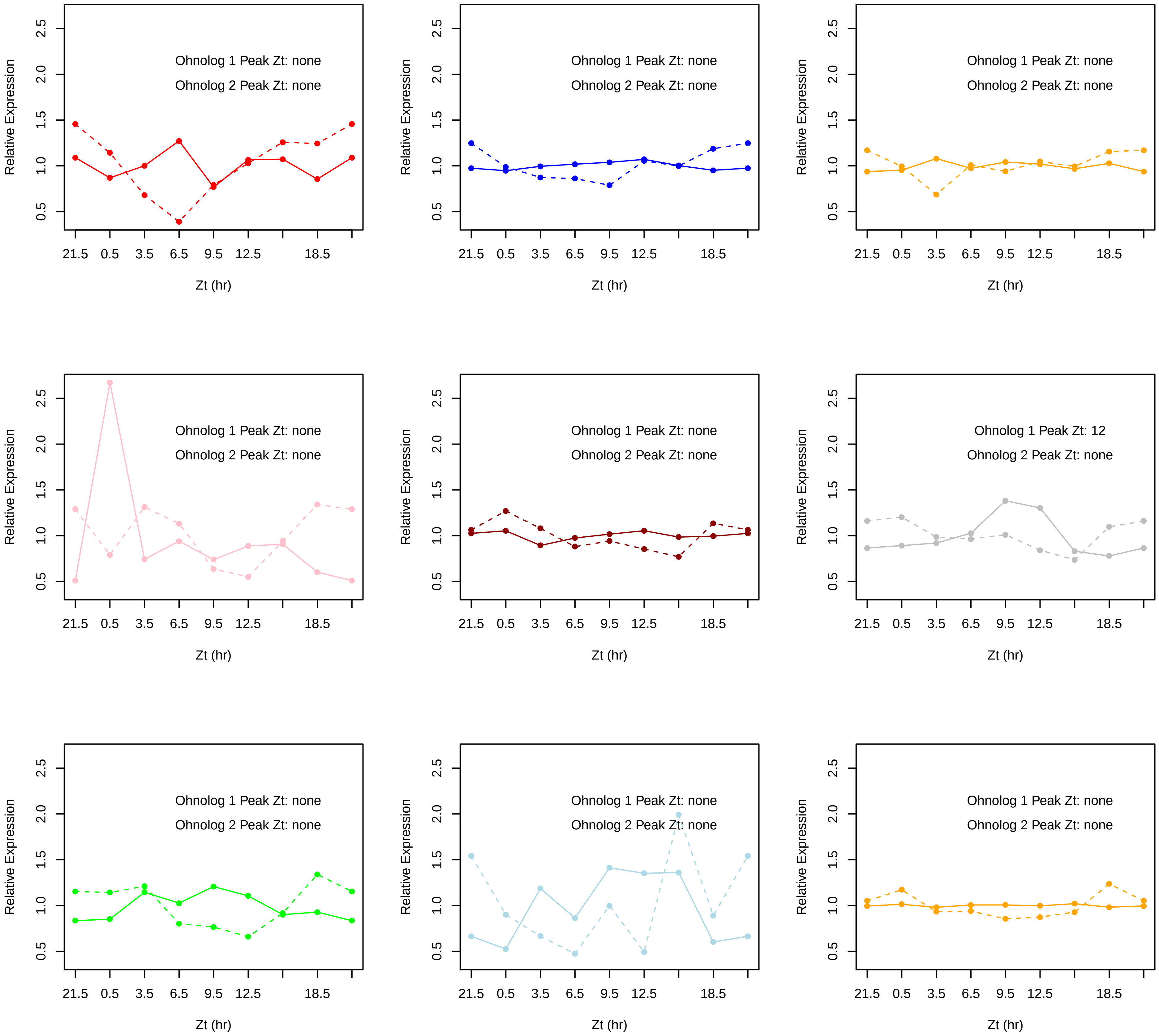

Ohnolog 1: ENSXMAG00000000364
Ohnolog 2: ENSXMAG00000010908 ism2b

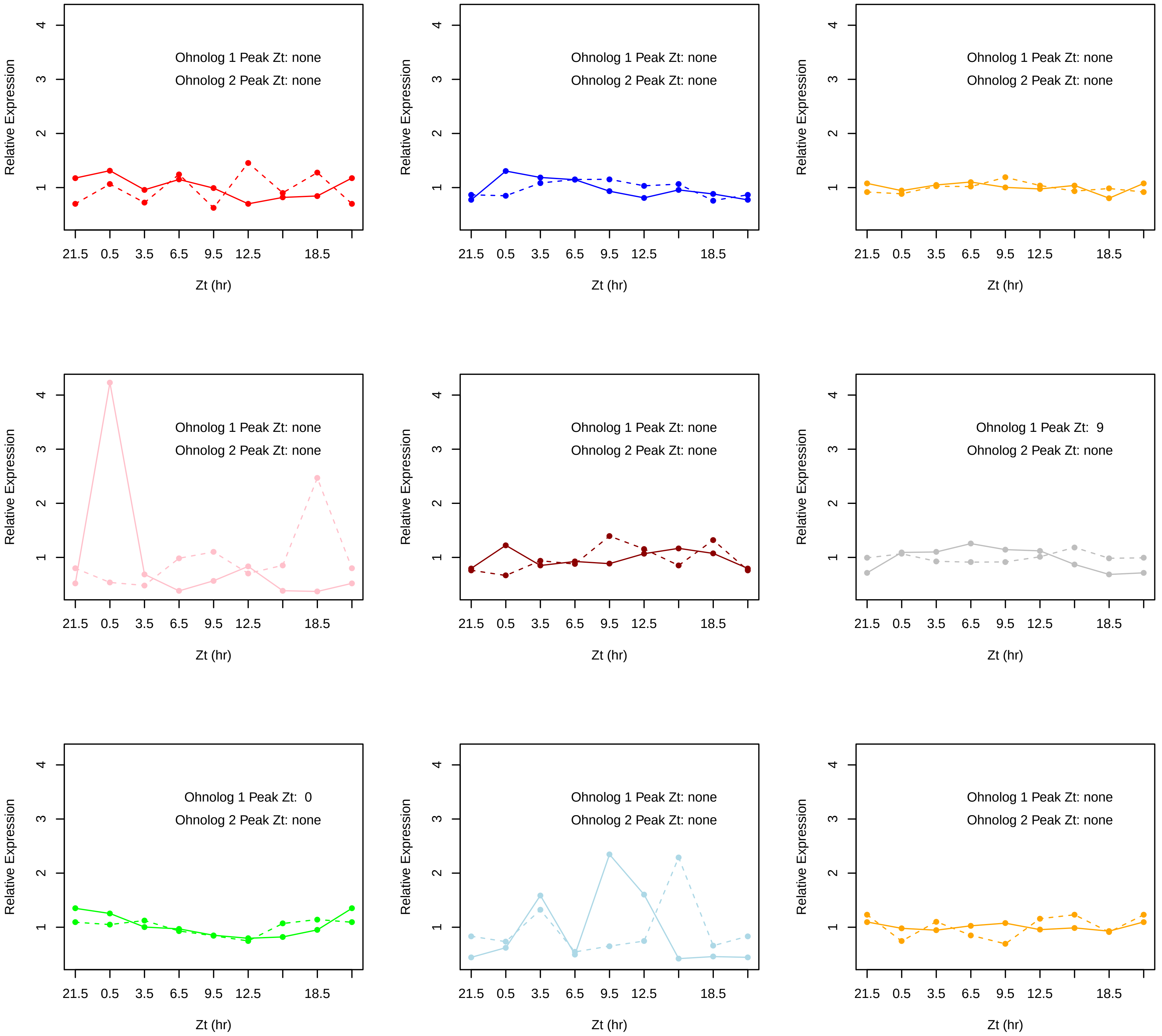

Ohnolog 1: ENSXMAG00000001820 TMEM184C (1 of many)
Ohnolog 2: ENSXMAG00000009576 tmem184c

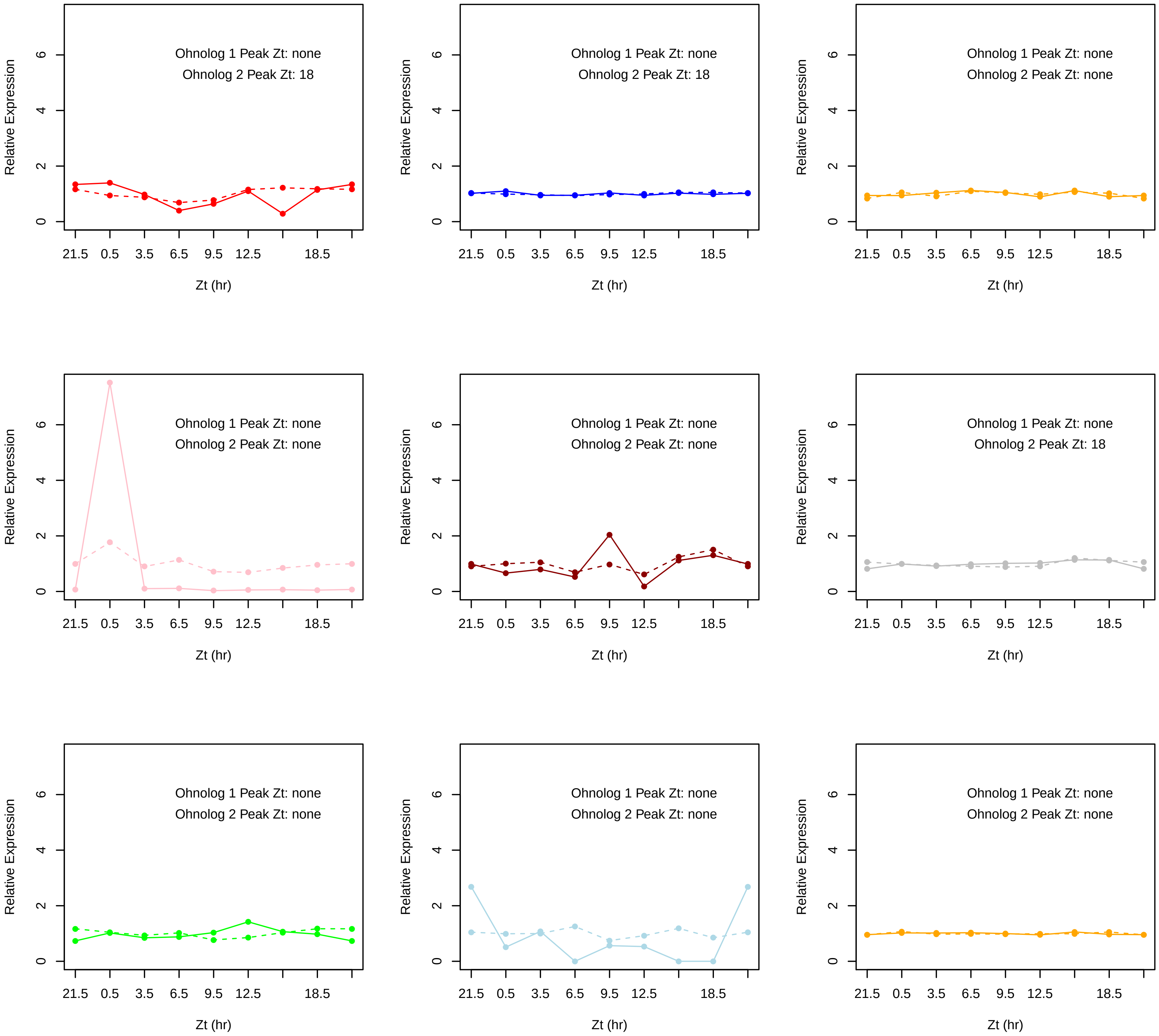

Ohnolog 1: ENSXMAG00000007371 slc25a15a
Ohnolog 2: ENSXMAG00000023673 slc25a15b

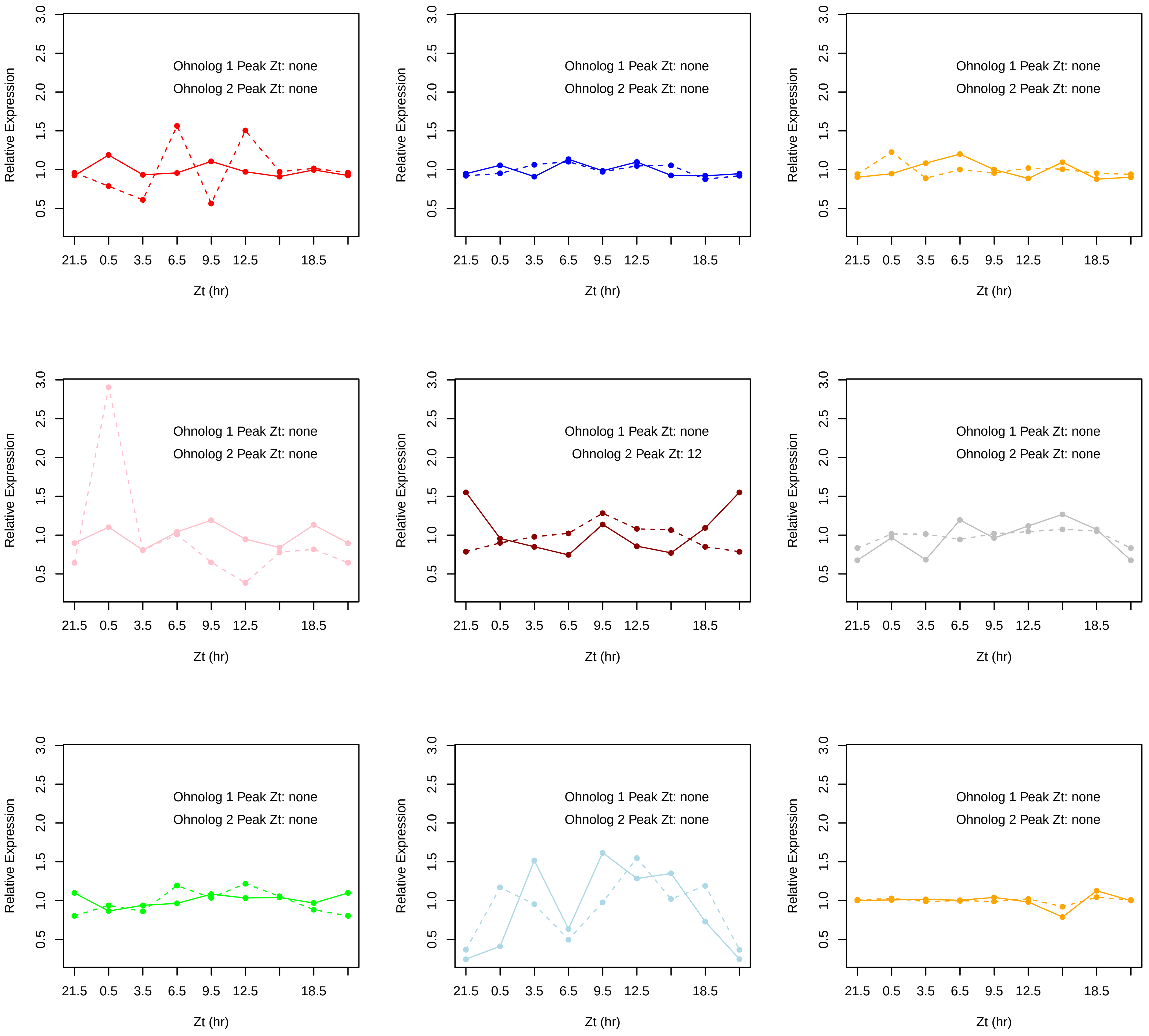

Ohnolog 1: ENSXMAG00000011165 plpp3
Ohnolog 2: ENSXMAG00000018151

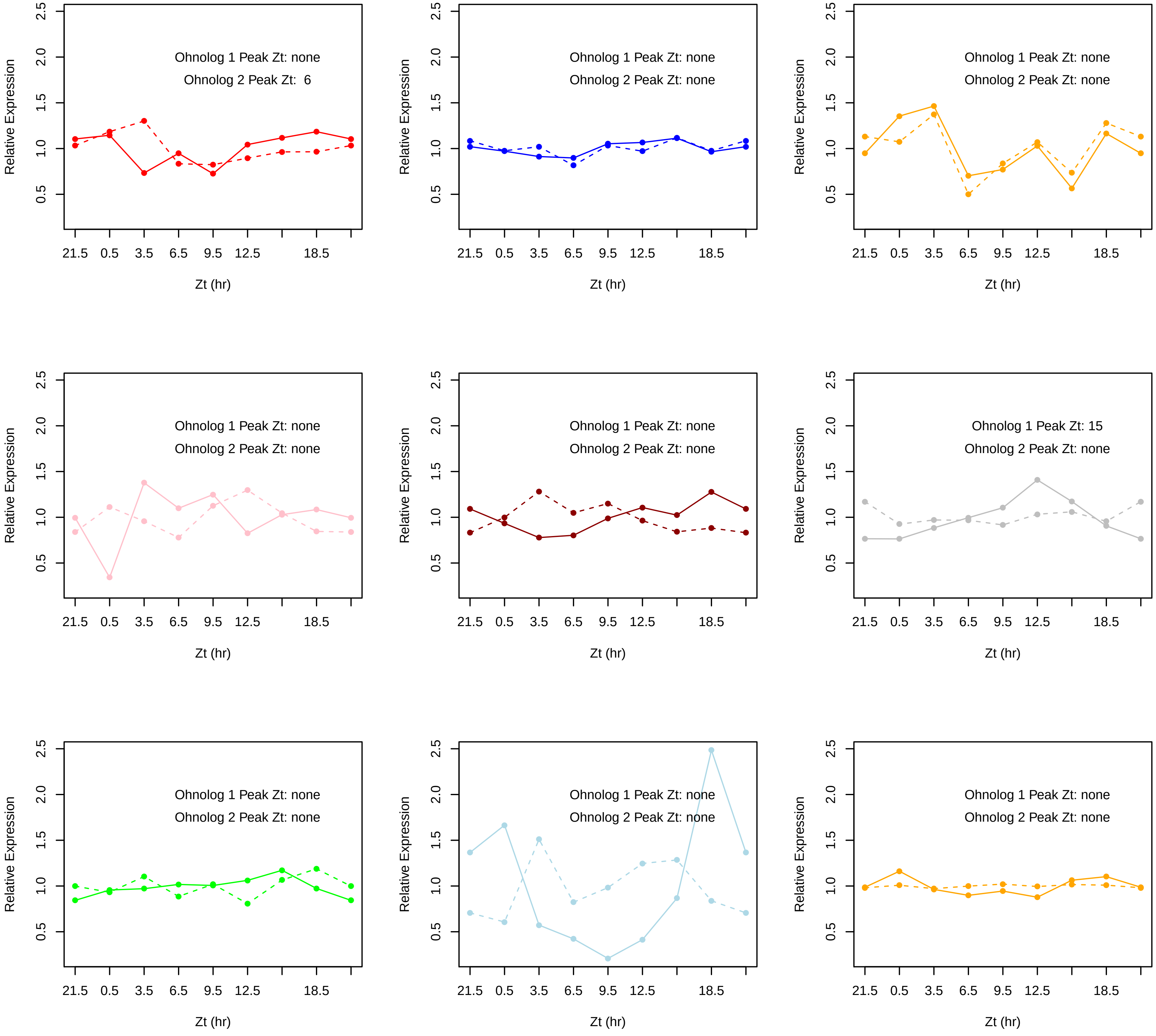

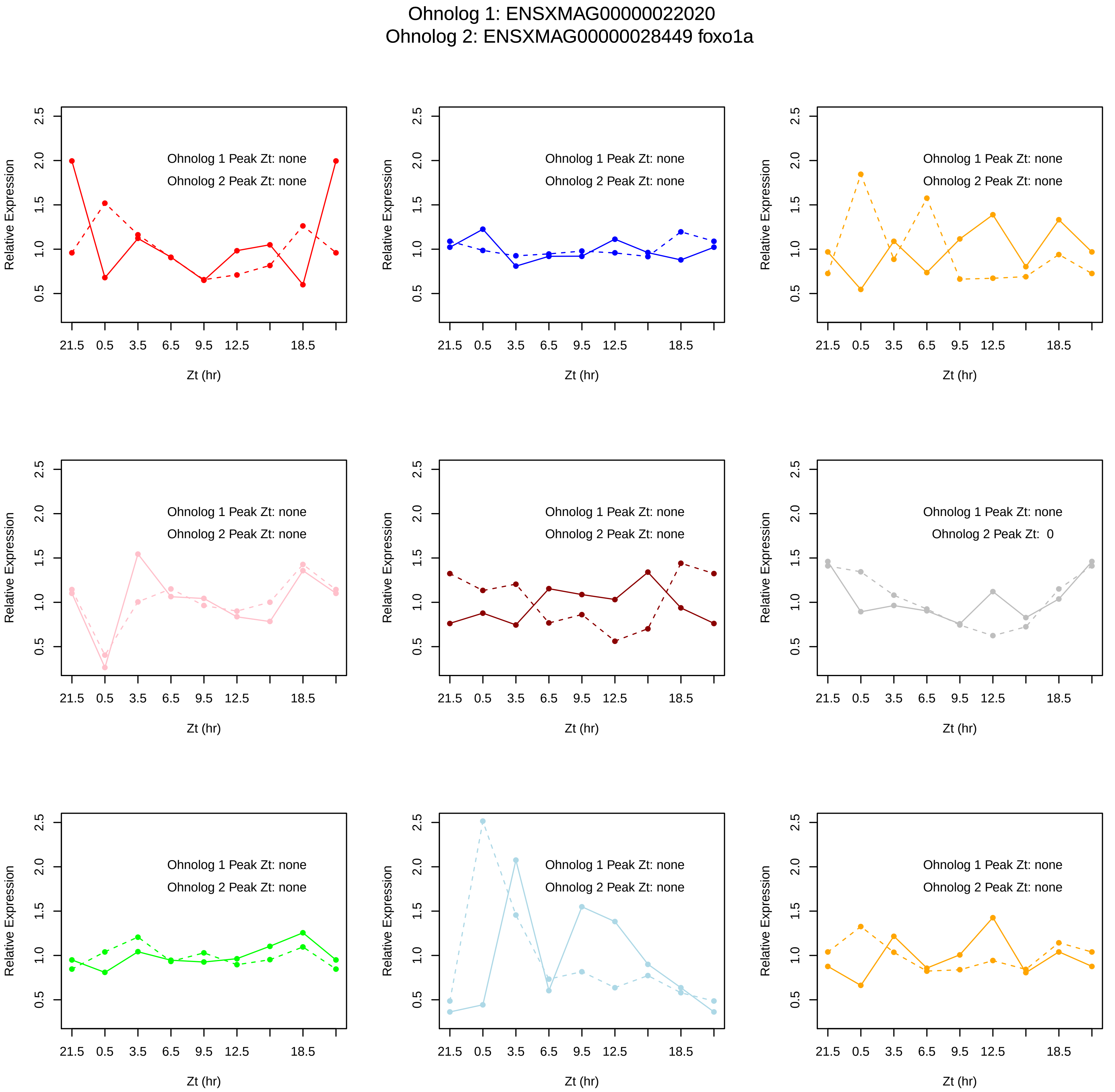
Ohnolog 1: ENSXMAG00000022020
Ohnolog 2: ENSXMAG00000028449 foxo1a
Ohnolog 1 Peak Zt: none
Ohnolog 2 Peak Zt: none
Ohnolog 1 Peak Zt: none
Ohnolog 2 Peak Zt: none
Ohnolog 1 Peak Zt: none
Ohnolog 2 Peak Zt: none
Ohnolog 1 Peak Zt: none
Ohnolog 2 Peak Zt: none
Ohnolog 1 Peak Zt: none
Ohnolog 2 Peak Zt: none
Ohnolog 1 Peak Zt: none
Ohnolog 2 Peak Zt: 0
Ohnolog 1 Peak Zt: none
Ohnolog 2 Peak Zt: none
Ohnolog 1 Peak Zt: none
Ohnolog 2 Peak Zt: none
Ohnolog 1 Peak Zt: none
Ohnolog 2 Peak Zt: none
Relative Expression
0.5
1.0
1.5
2.0
2.5
21.5
0.5
3.5
6.5
9.5
12.5
18.5
Zt (hr)

Ohnolog 1: ENSXMAG00000011184 dab1a
Ohnolog 2: ENSXMAG00000027199 dab1b

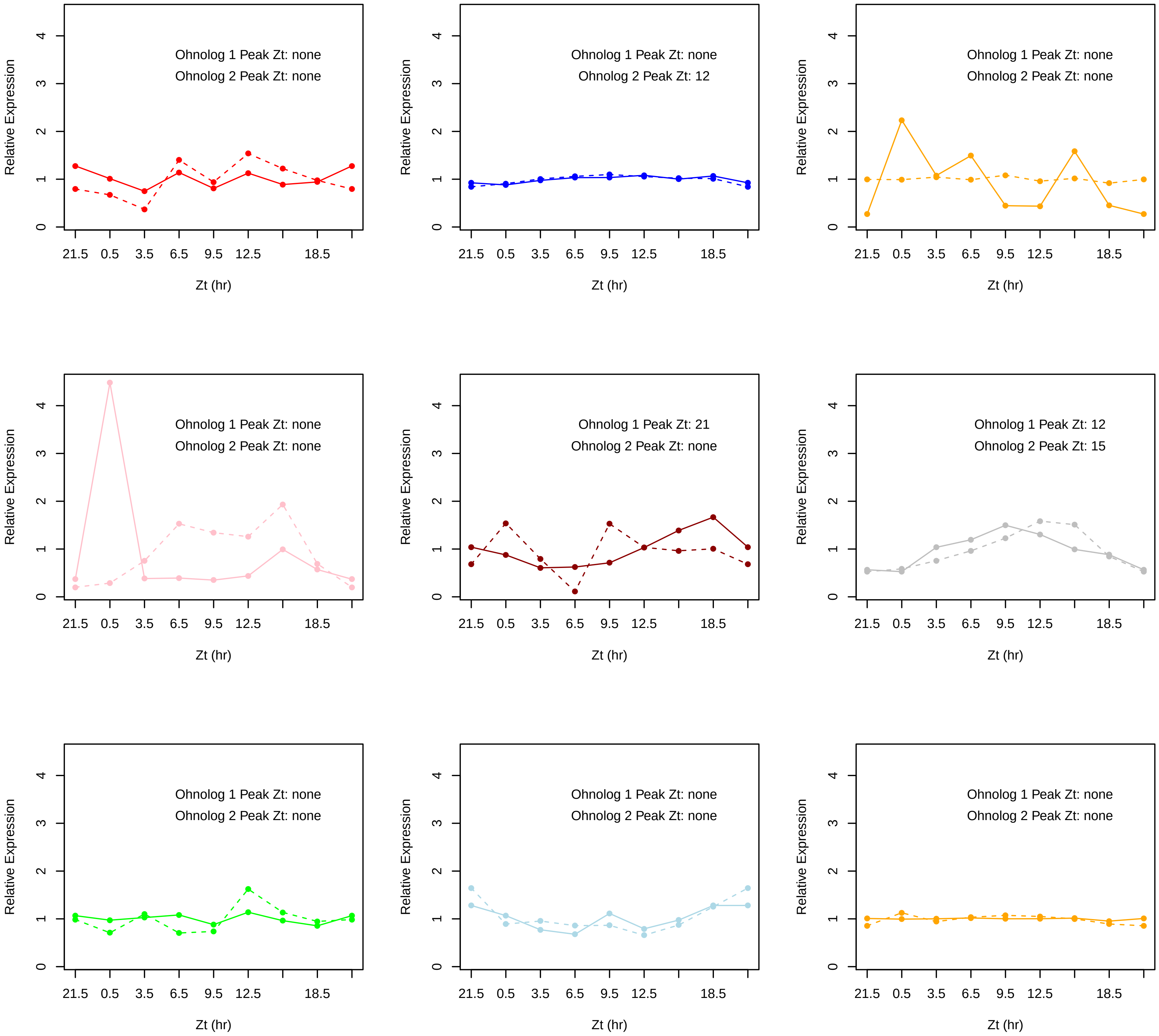

Ohnolog 1: ENSXMAG00000018972 shrprbck1r
Ohnolog 2: ENSXMAG00000022076 sharpin

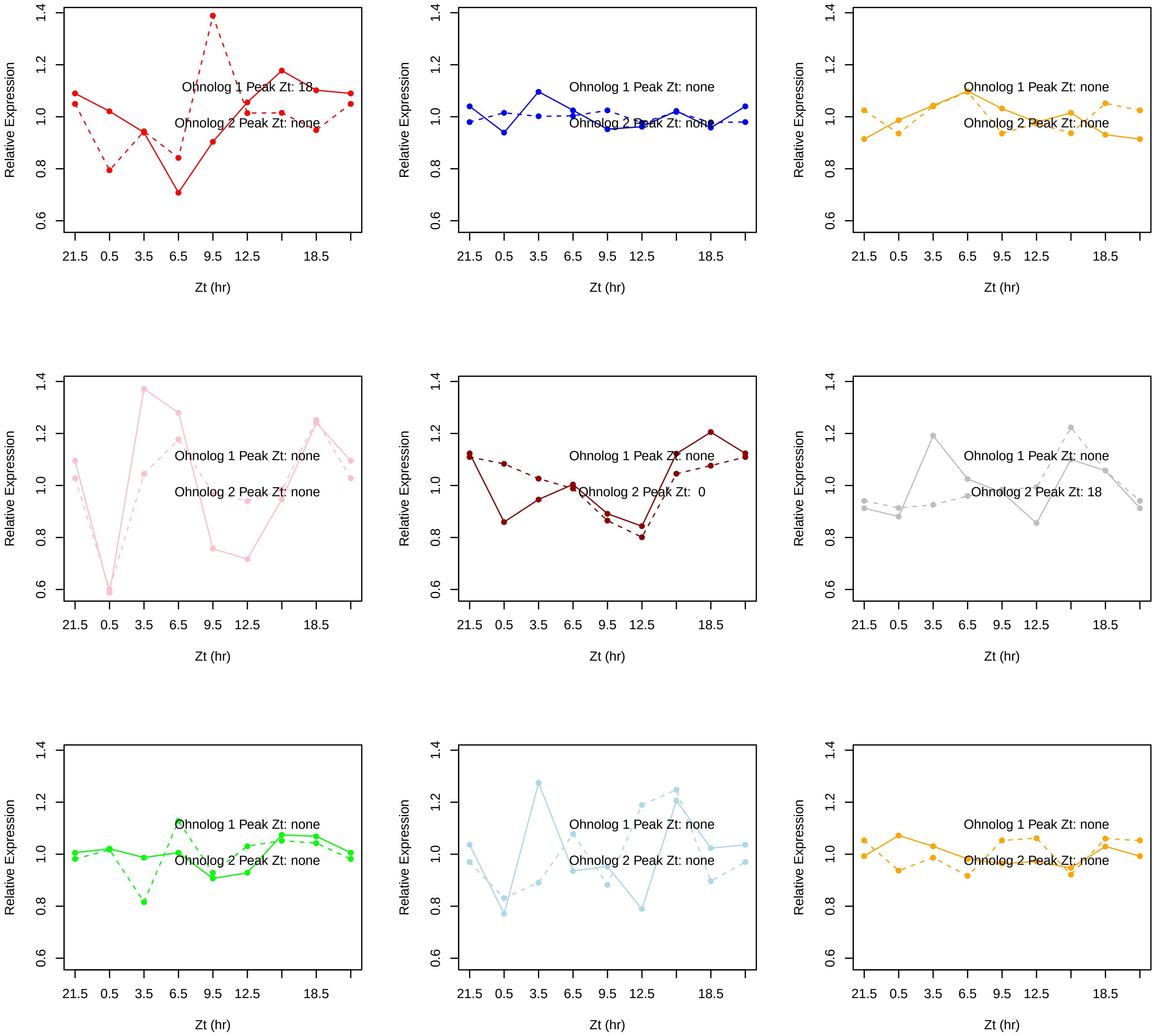

Ohnolog 1: ENSXMAG00000012169 dnm2b
Ohnolog 2: ENSXMAG00000013164 dnm2a

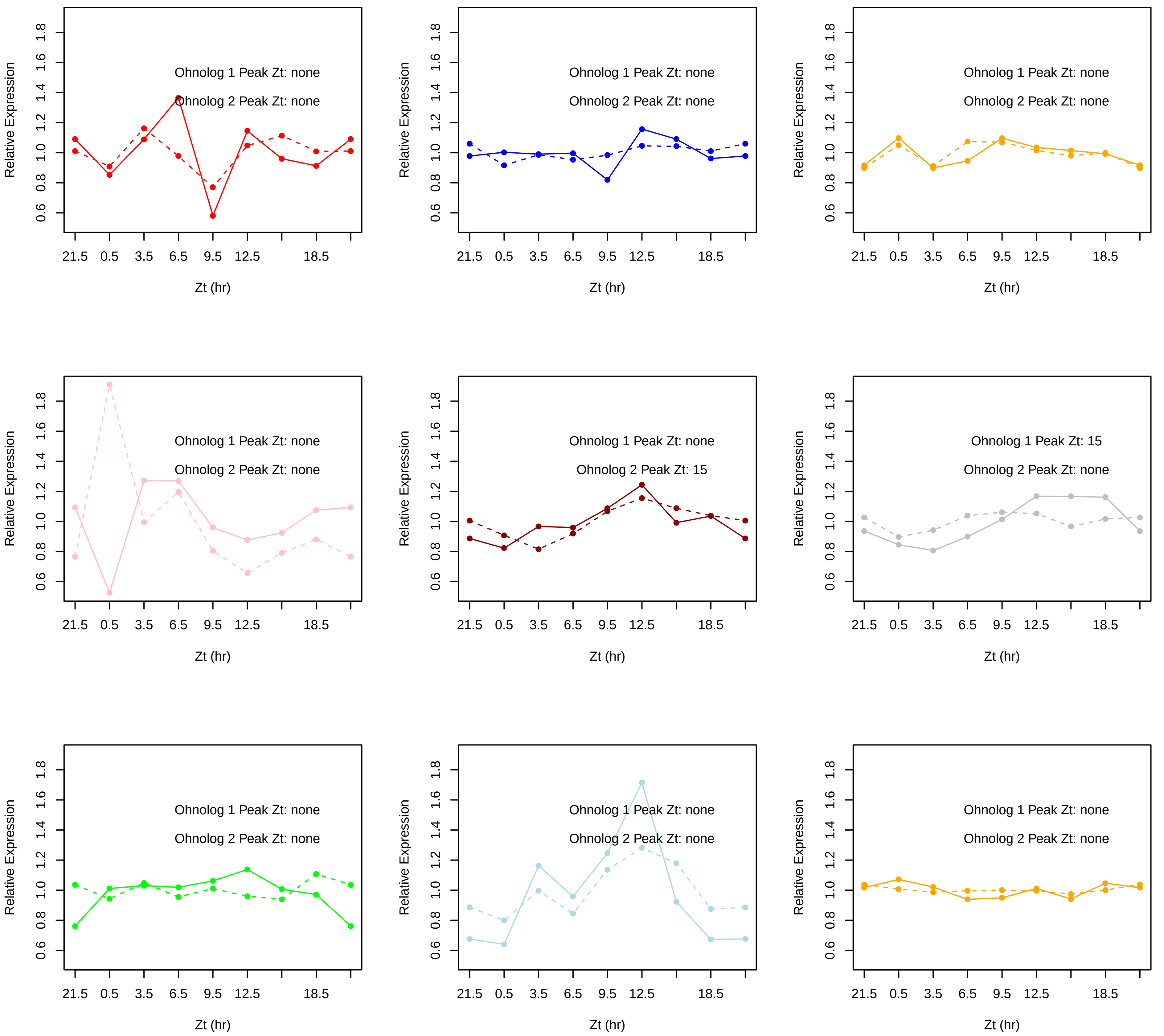

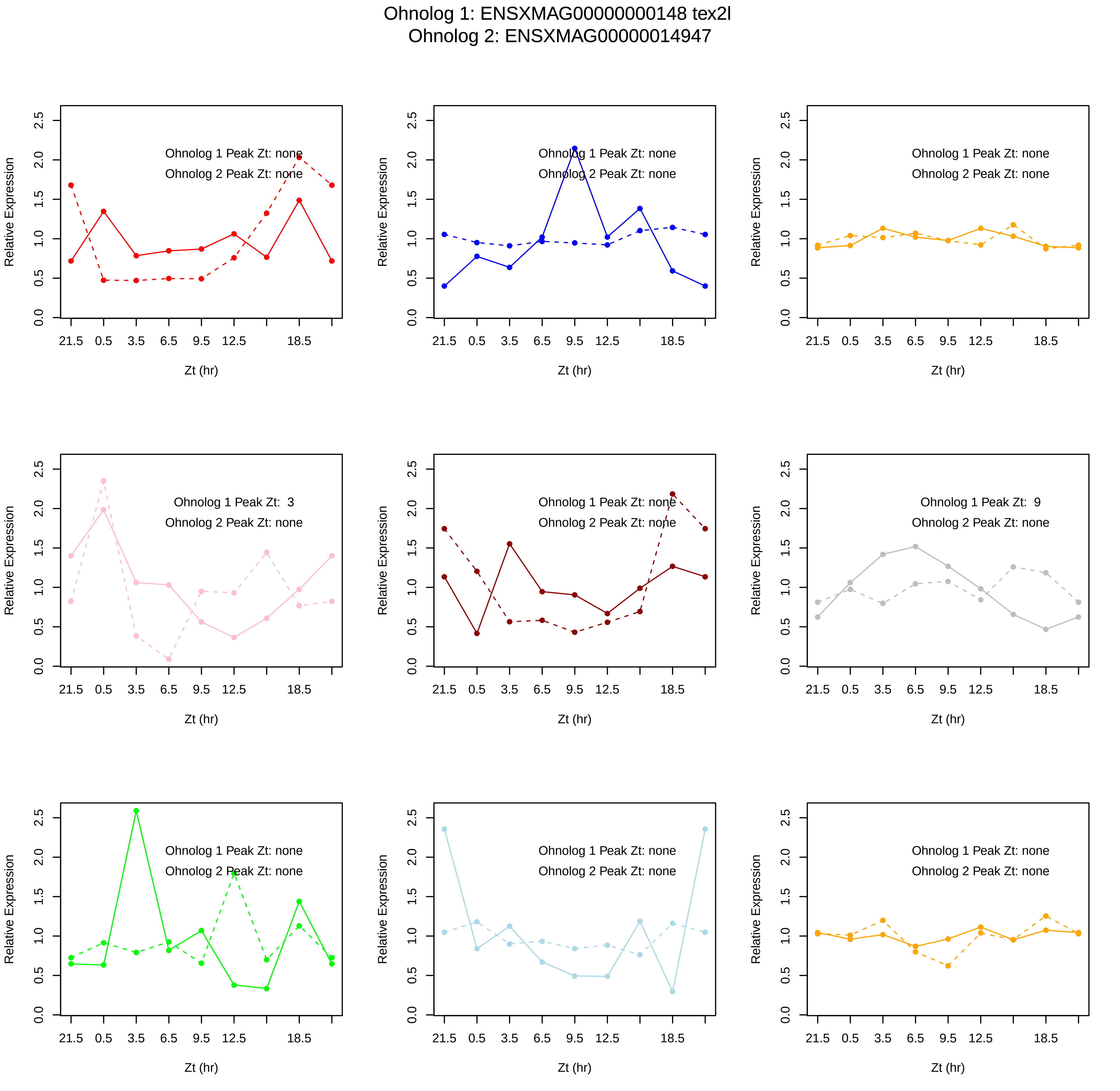

Ohnolog 1: ENSXMAG00000000148 tex2l
Ohnolog 2: ENSXMAG00000014947
Ohnolog 1 Peak Zt: none
Ohnolog 2 Peak Zt: none
Ohnolog 1 Peak Zt: none
Ohnolog 2 Peak Zt: none
Ohnolog 1 Peak Zt: none
Ohnolog 2 Peak Zt: none
Ohnolog 1 Peak Zt: 3
Ohnolog 2 Peak Zt: none
Ohnolog 1 Peak Zt: none
Ohnolog 2 Peak Zt: none
Ohnolog 1 Peak Zt: 9
Ohnolog 2 Peak Zt: none
Ohnolog 1 Peak Zt: none
Ohnolog 2 Peak Zt: none
Ohnolog 1 Peak Zt: none
Ohnolog 2 Peak Zt: none
Ohnolog 1 Peak Zt: none
Ohnolog 2 Peak Zt: none
Relative Expression
Zt (hr)
0.0 0.5 1.0 1.5 2.0 2.5
21.5 0.5 3.5 6.5 9.5 12.5 18.5

Ohnolog 1: ENSXMAG00000005256 nrsn1
Ohnolog 2: ENSXMAG00000029349

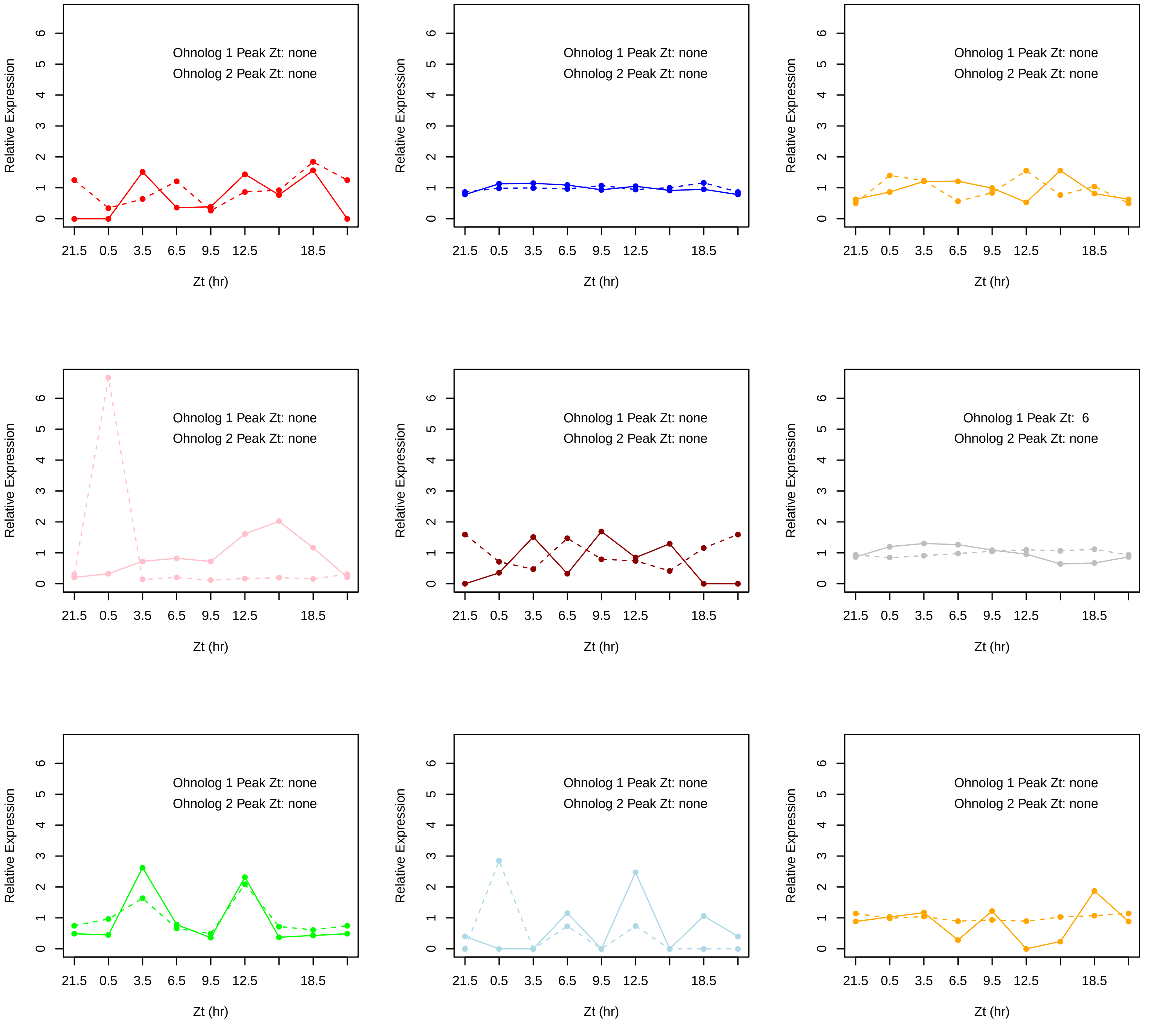

Ohnolog 1: ENSXMAG00000014989 MSRB1 (1 of many)
Ohnolog 2: ENSXMAG00000027955 msrb1b

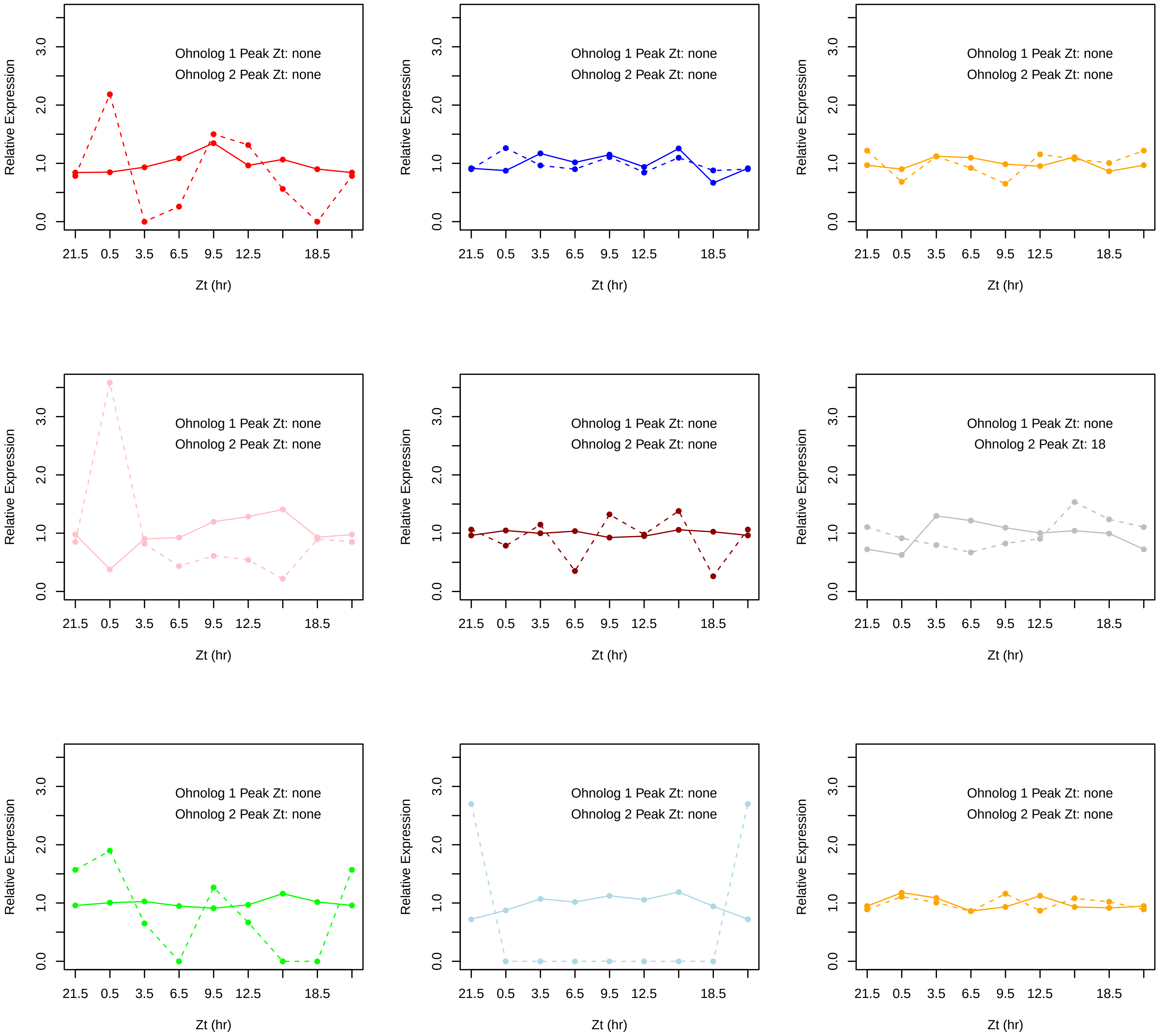

Ohnolog 1: ENSXMAG00000007339 SLC7A1
Ohnolog 2: ENSXMAG00000016068

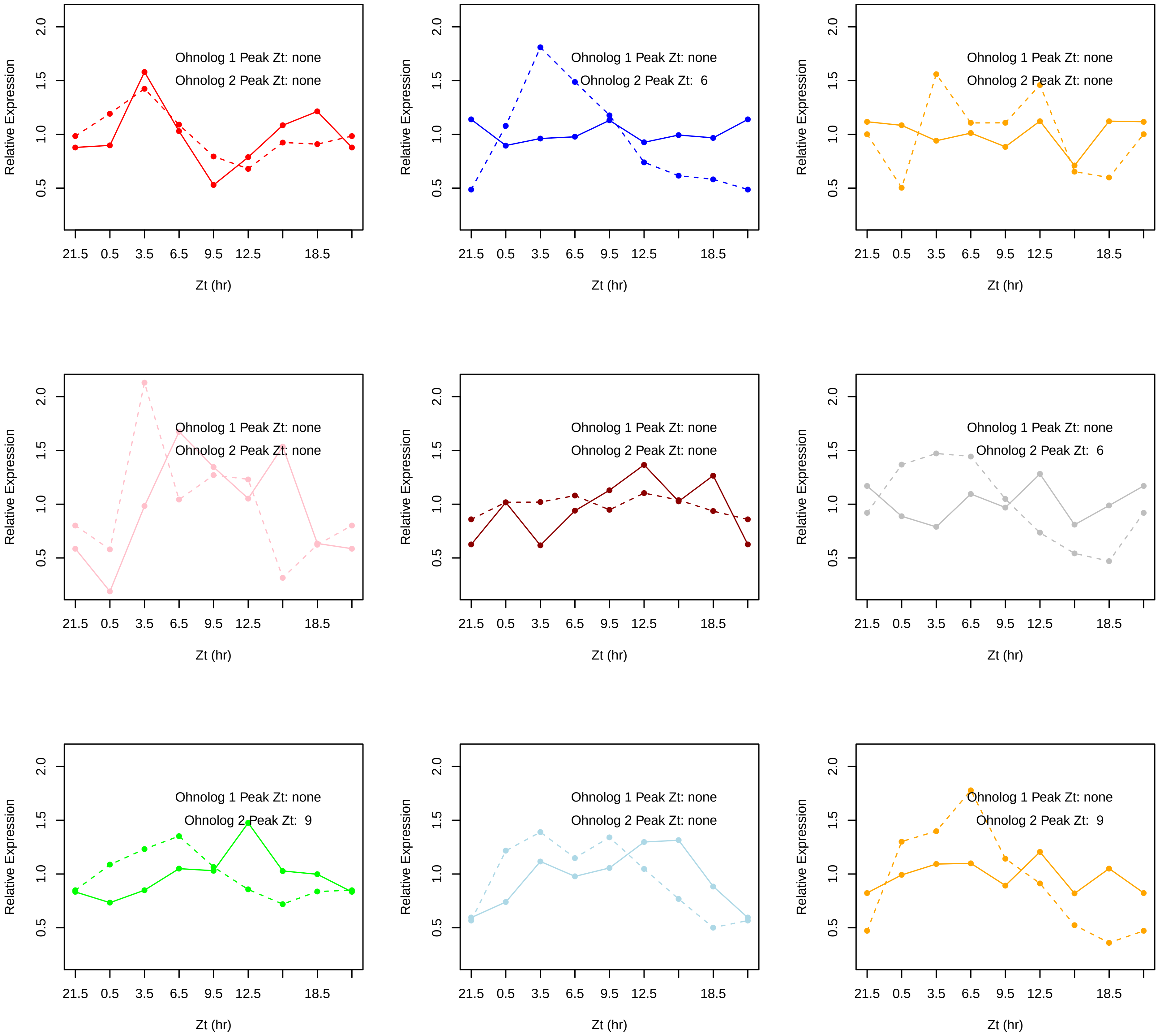

Ohnolog 1: ENSXMAG00000009861 ATP6V0C (1 of many)
Ohnolog 2: ENSXMAG00000023815 ATP6V0C

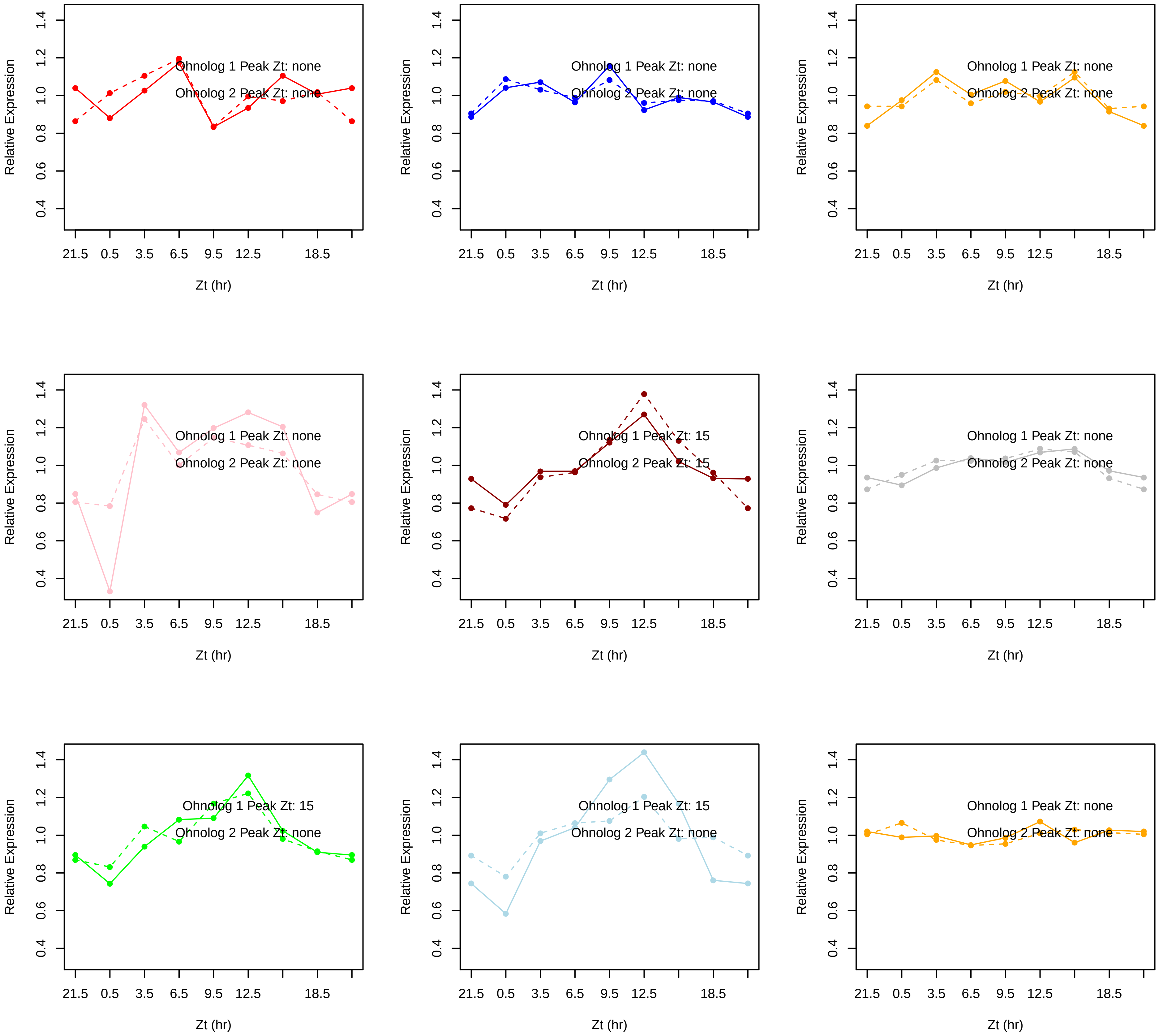

Ohnolog 1: ENSXMAG00000007326 cadm2b
Ohnolog 2: ENSXMAG00000010551

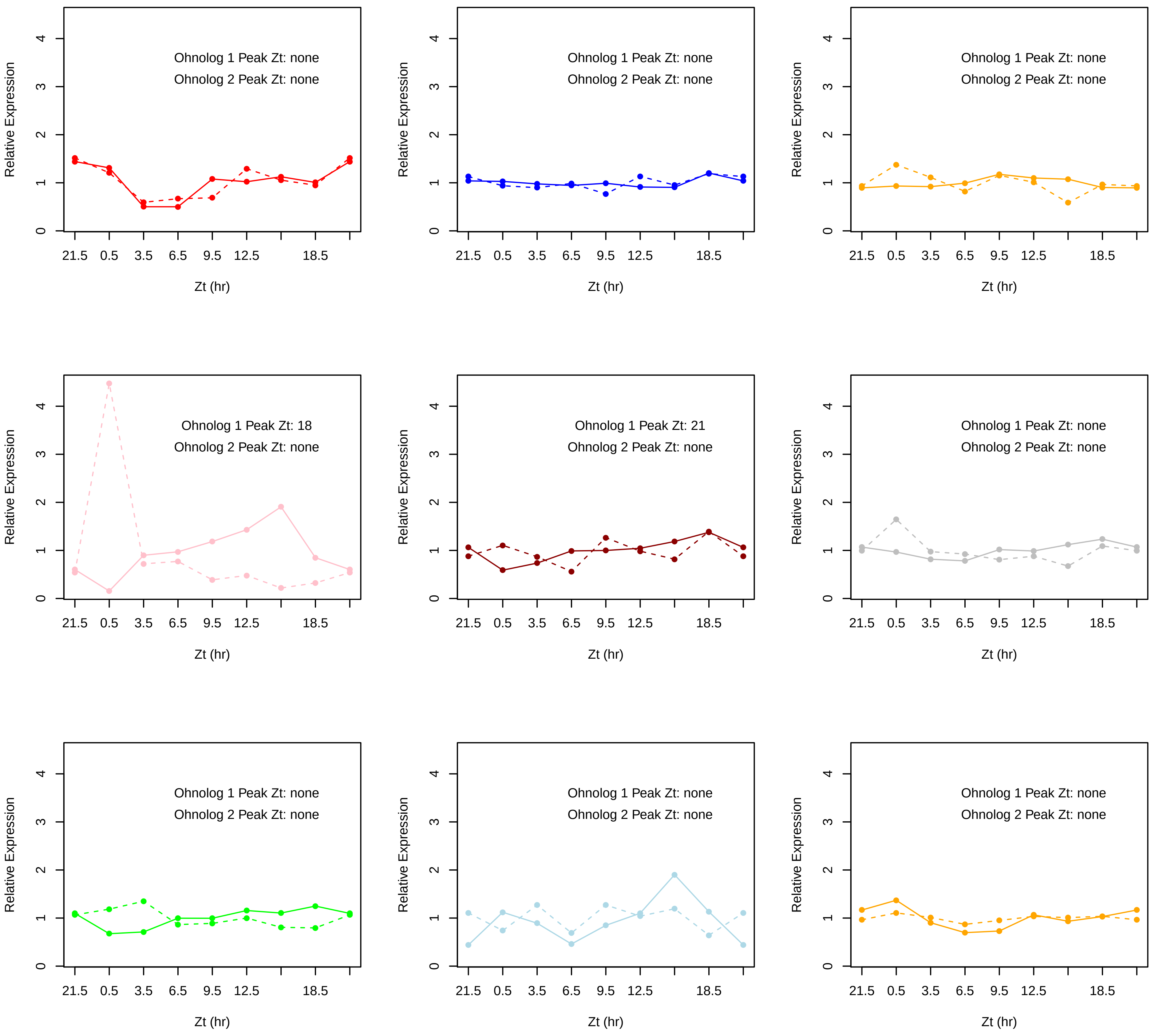

Ohnolog 1: ENSXMAG00000002580 slc25a28
Ohnolog 2: ENSXMAG00000005681 SLC25A28 (1 of many)

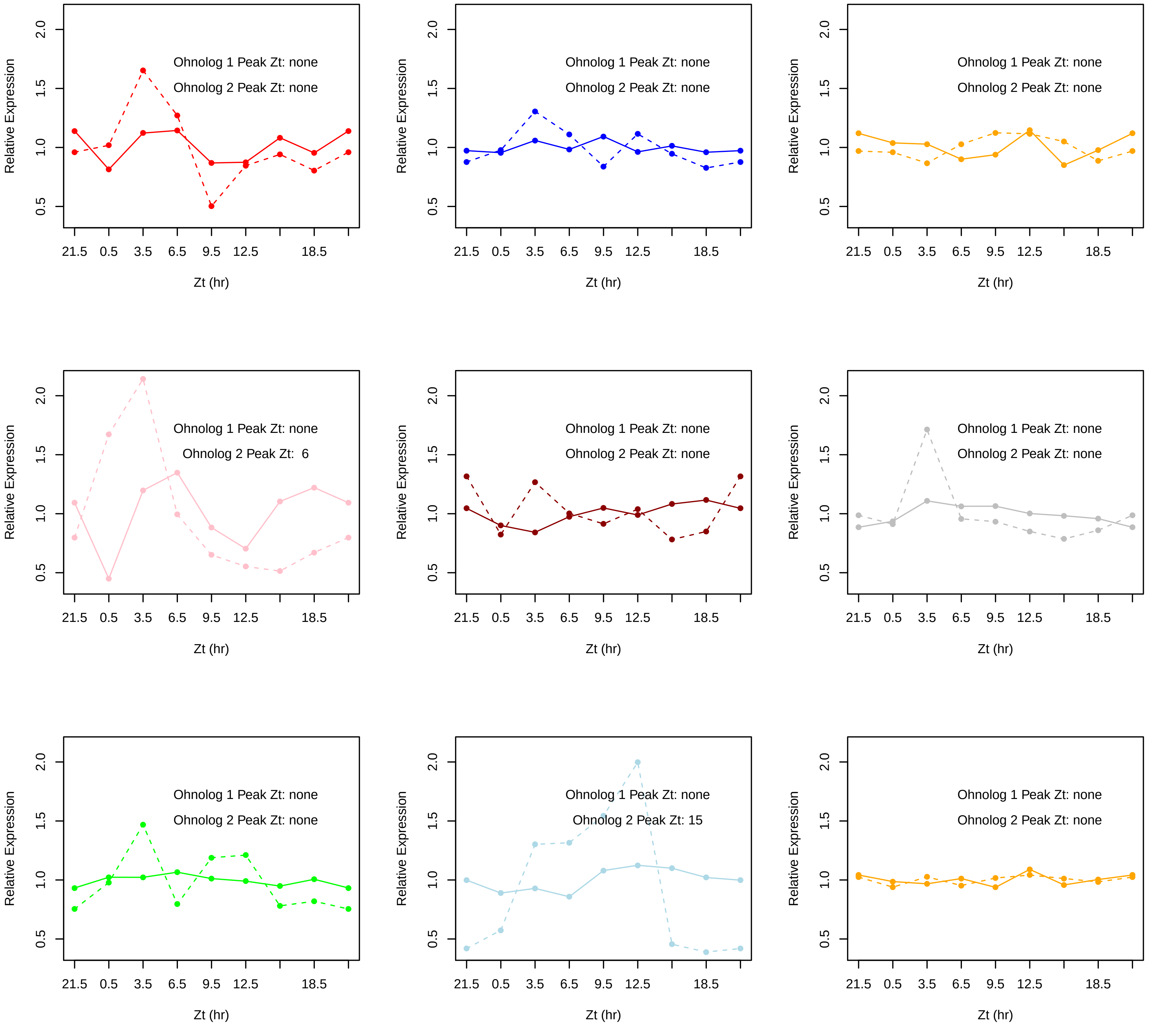

Ohnolog 1: ENSXMAG00000009808
Ohnolog 2: ENSXMAG00000026556 inpp4b

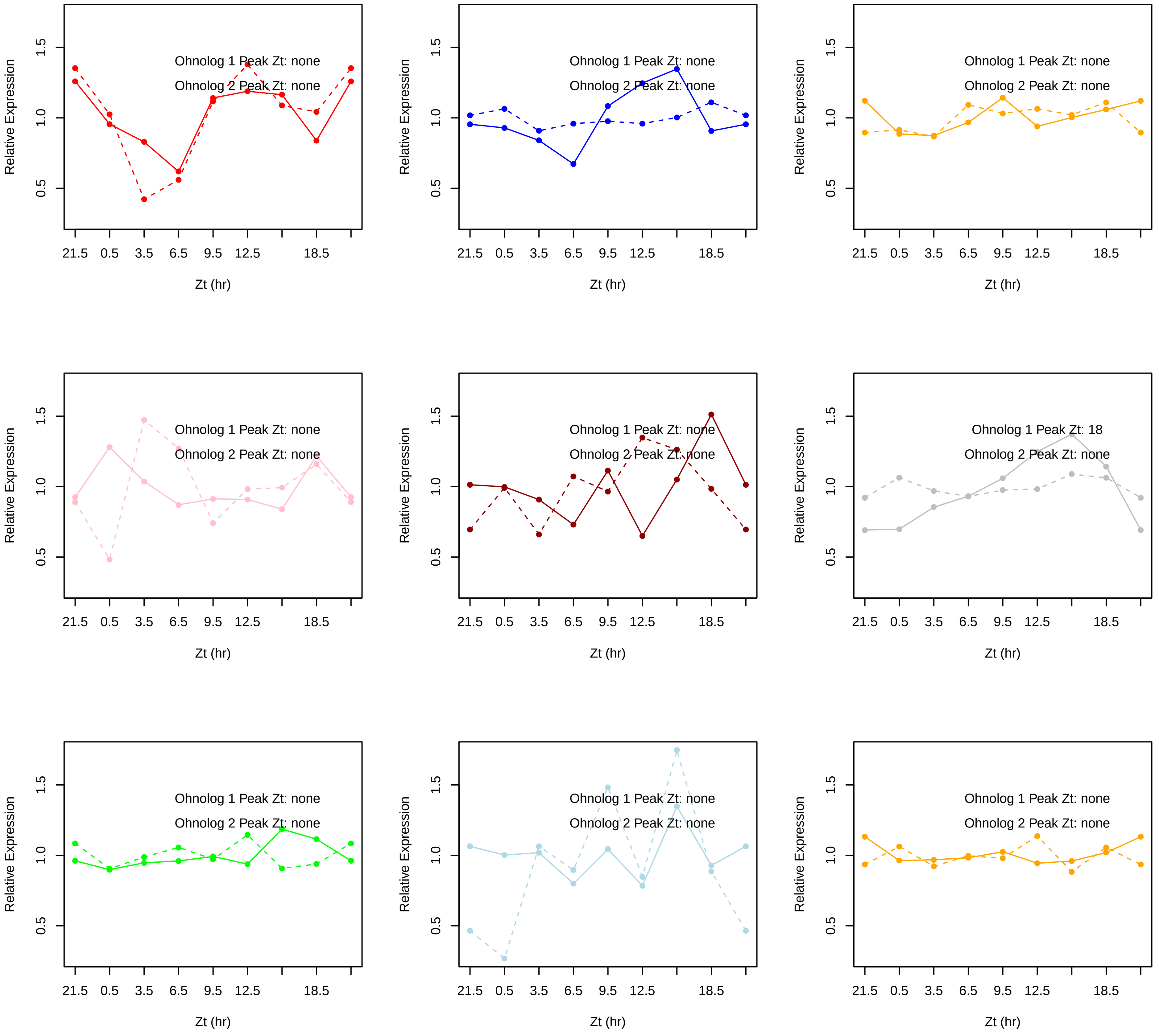

Ohnolog 1: ENSXMAG00000002566 CNNM1 (1 of many)
Ohnolog 2: ENSXMAG00000005657 CNNM1 (1 of many)

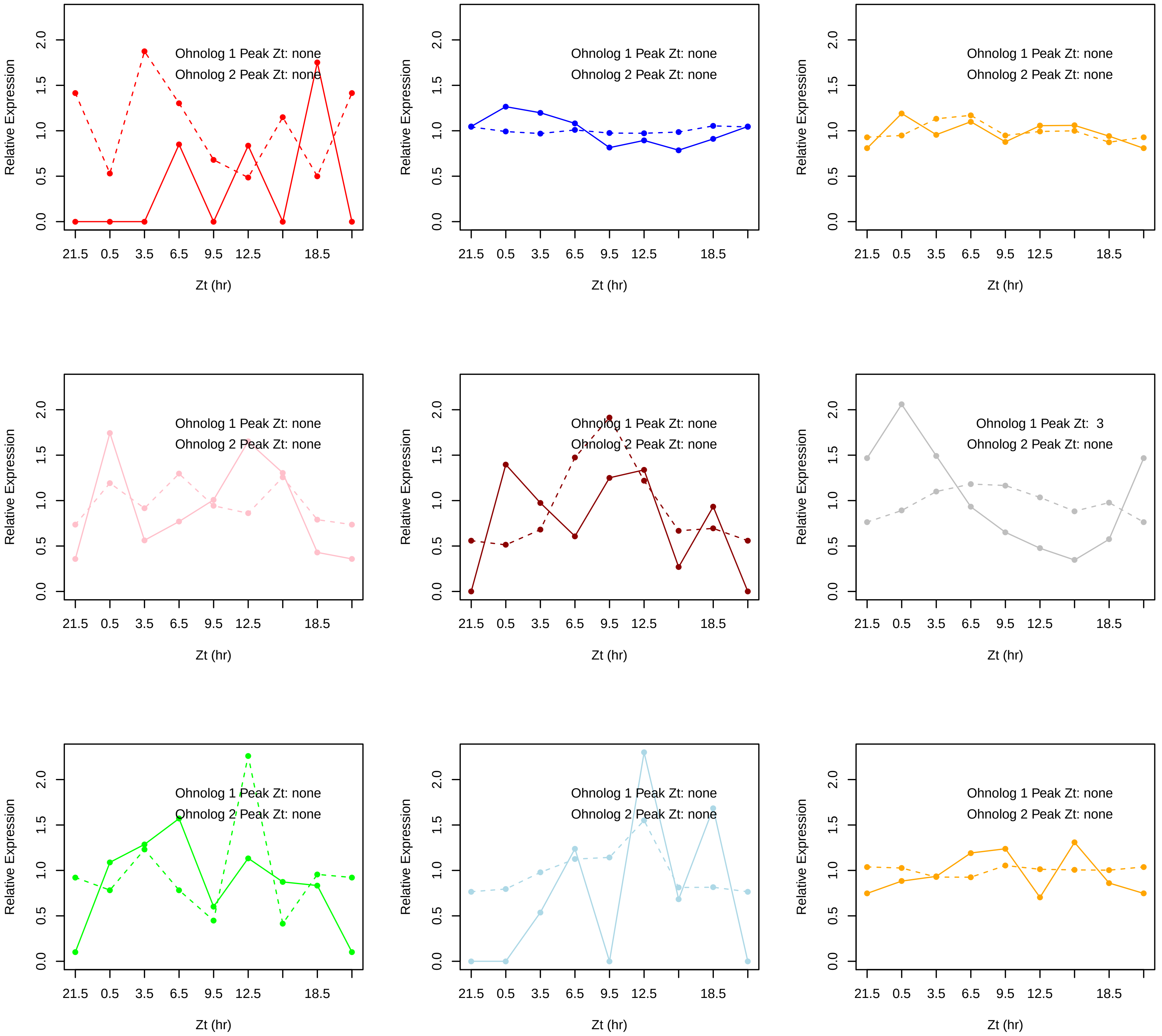

Ohnolog 1: ENSXMAG00000009793 rnf150a
Ohnolog 2: ENSXMAG00000026837 rnf150b

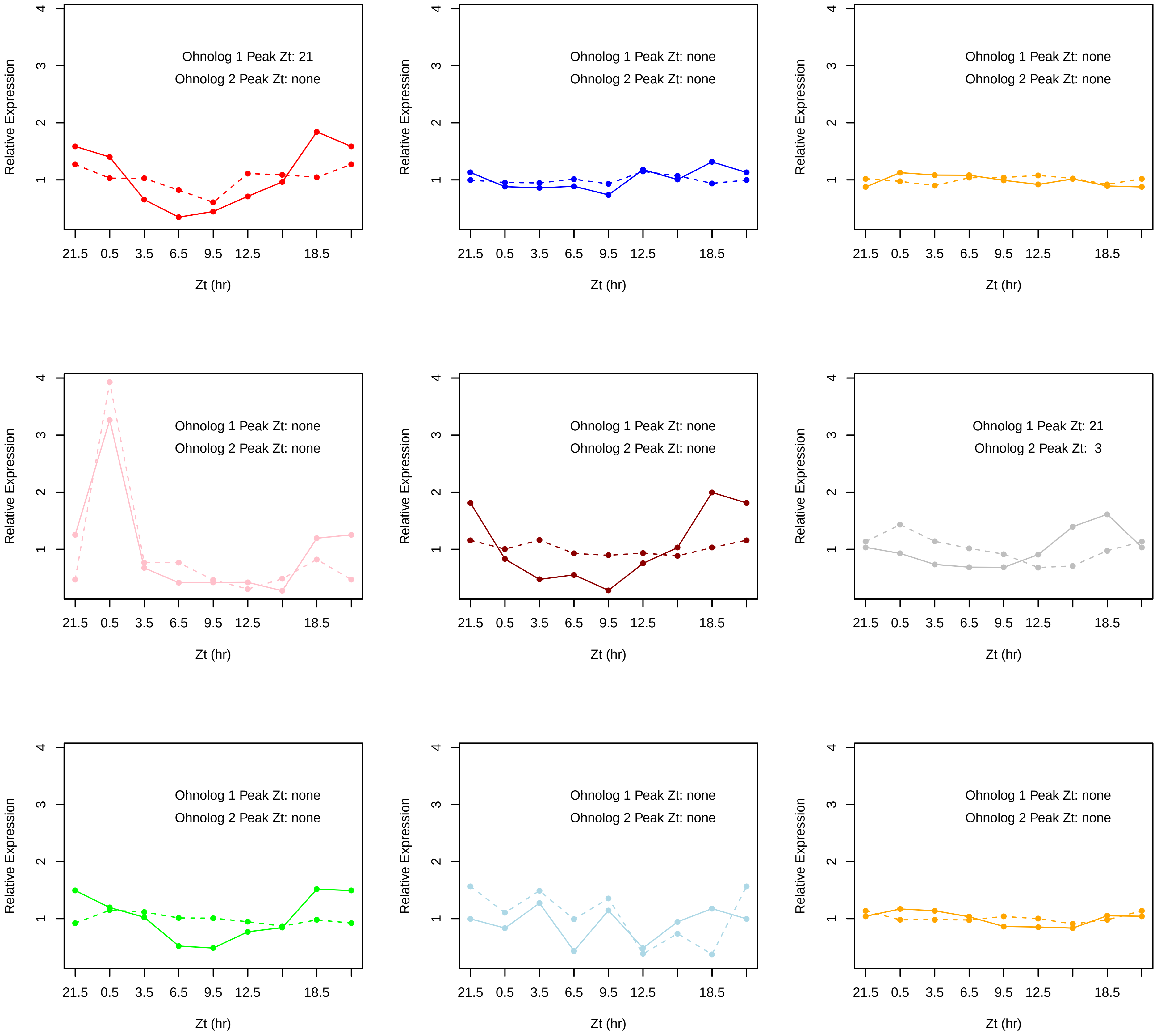

Ohnolog 1: ENSXMAG00000007309 bach1a
Ohnolog 2: ENSXMAG00000026281

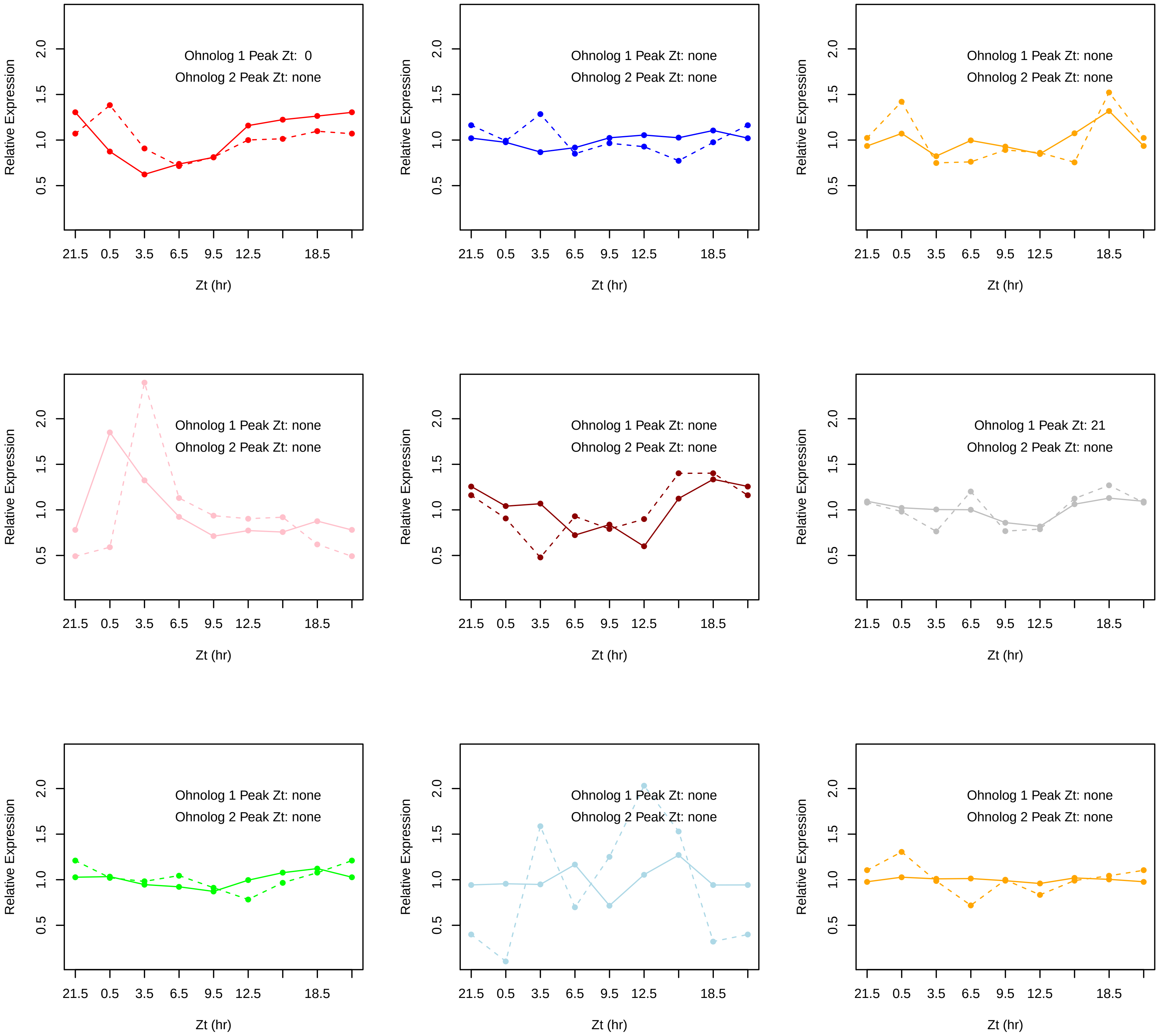

Ohnolog 1: ENSXMAG00000011392 atp11a
Ohnolog 2: ENSXMAG00000012027

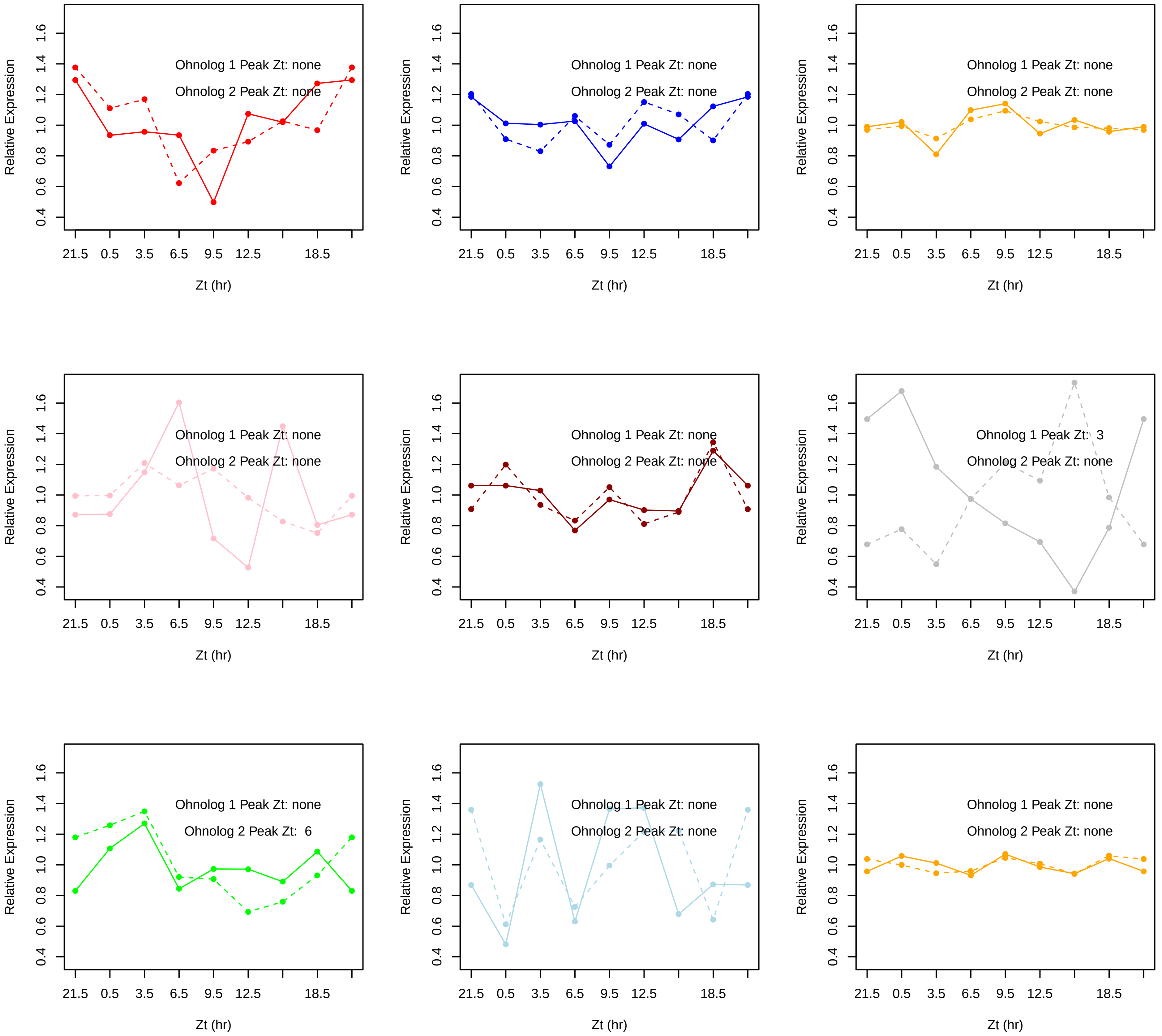

Ohnolog 1: ENSXMAG00000000294 CPEB4 (1 of many)
Ohnolog 2: ENSXMAG00000010247 cpeb4a

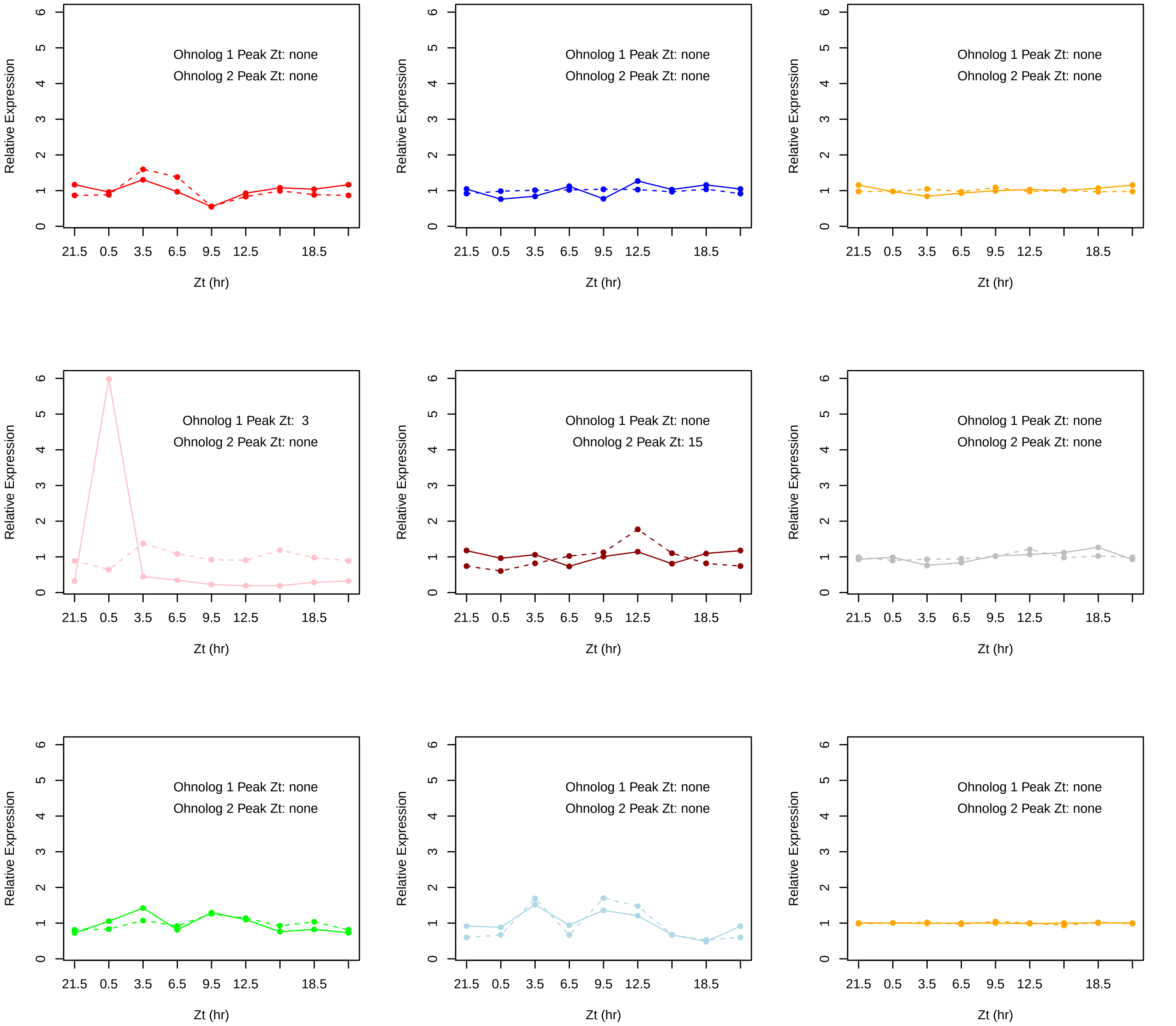

Ohnolog 1: ENSXMAG00000007277 tiam1b
Ohnolog 2: ENSXMAG00000017609 tiam1a

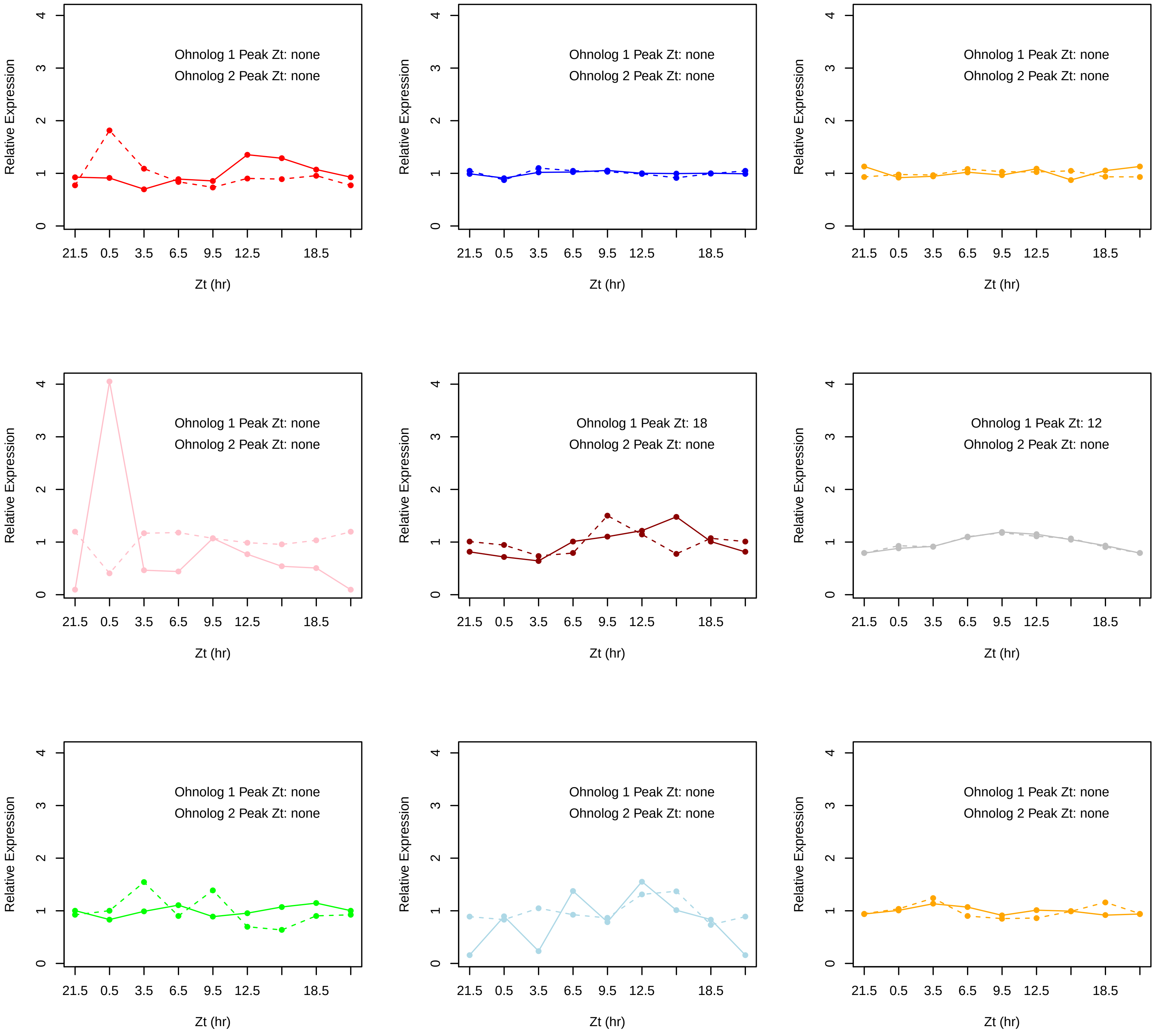

Ohnolog 1: ENSXMAG00000009398 nfkbiab
Ohnolog 2: ENSXMAG00000011763 nfkbiaa

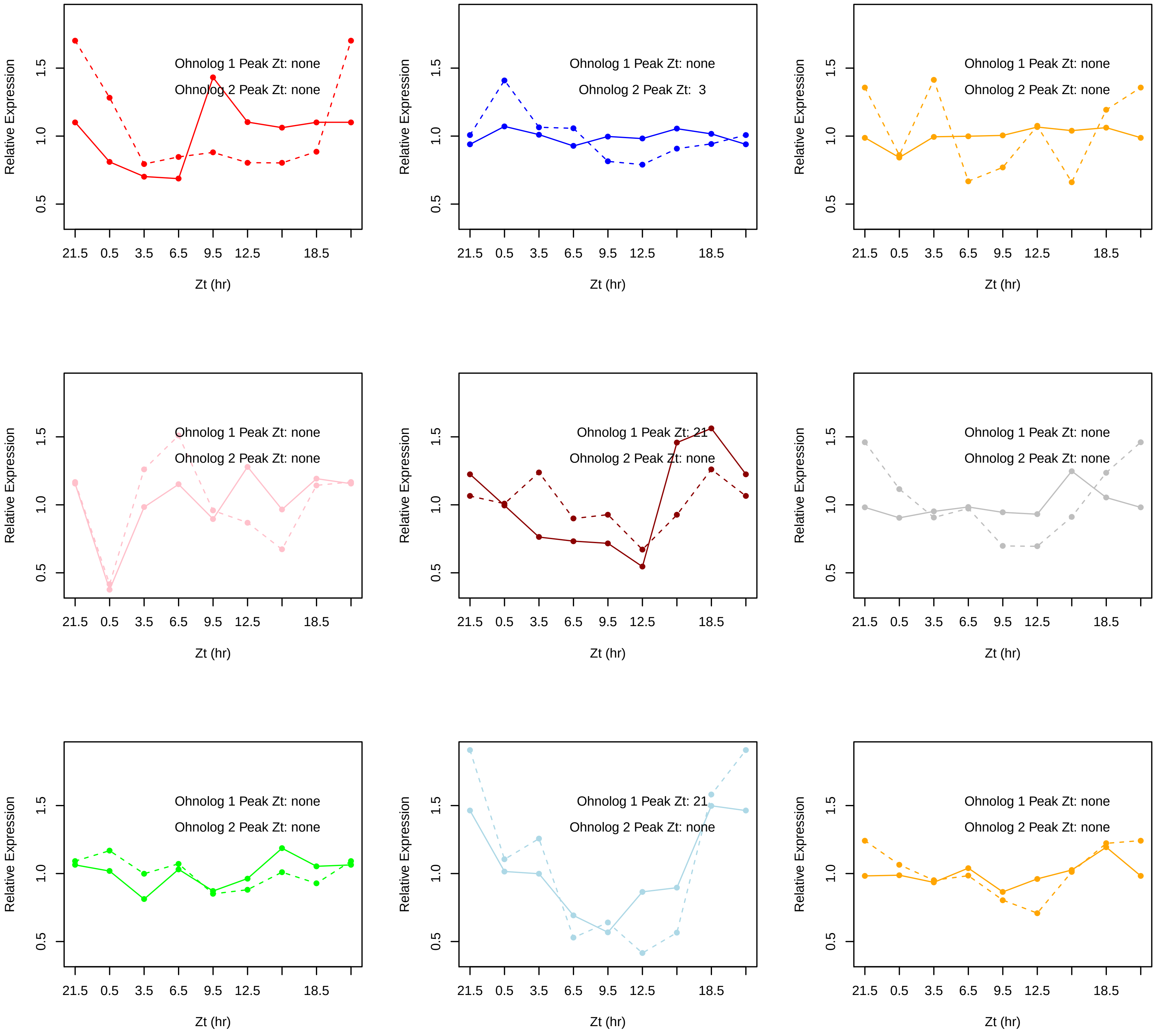

Ohnolog 1: ENSXMAG00000000497 bnip1b
Ohnolog 2: ENSXMAG00000023412 bnip1a

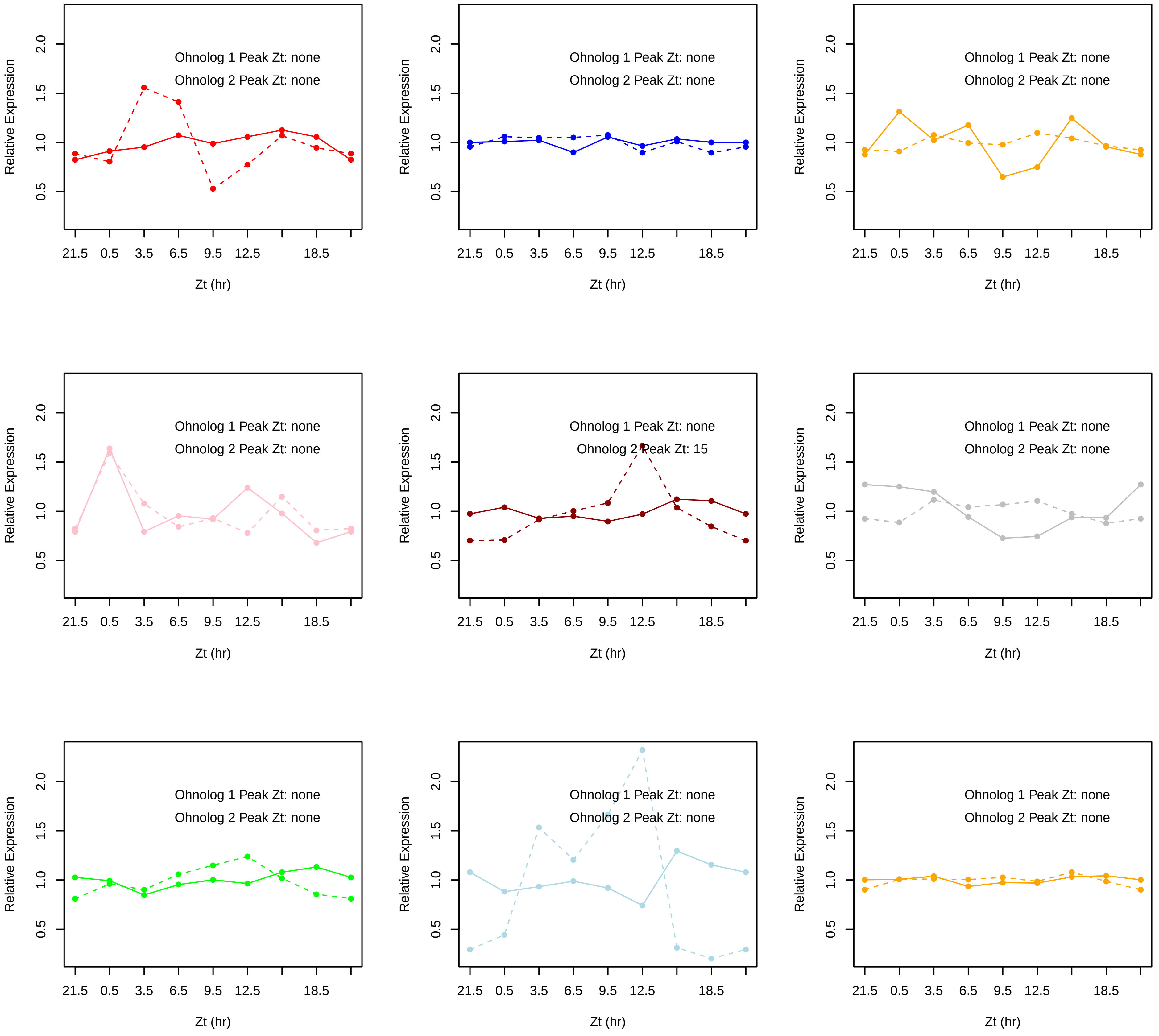

Ohnolog 1: ENSXMAG00000018473 guk1b
Ohnolog 2: ENSXMAG00000027618 guk1a

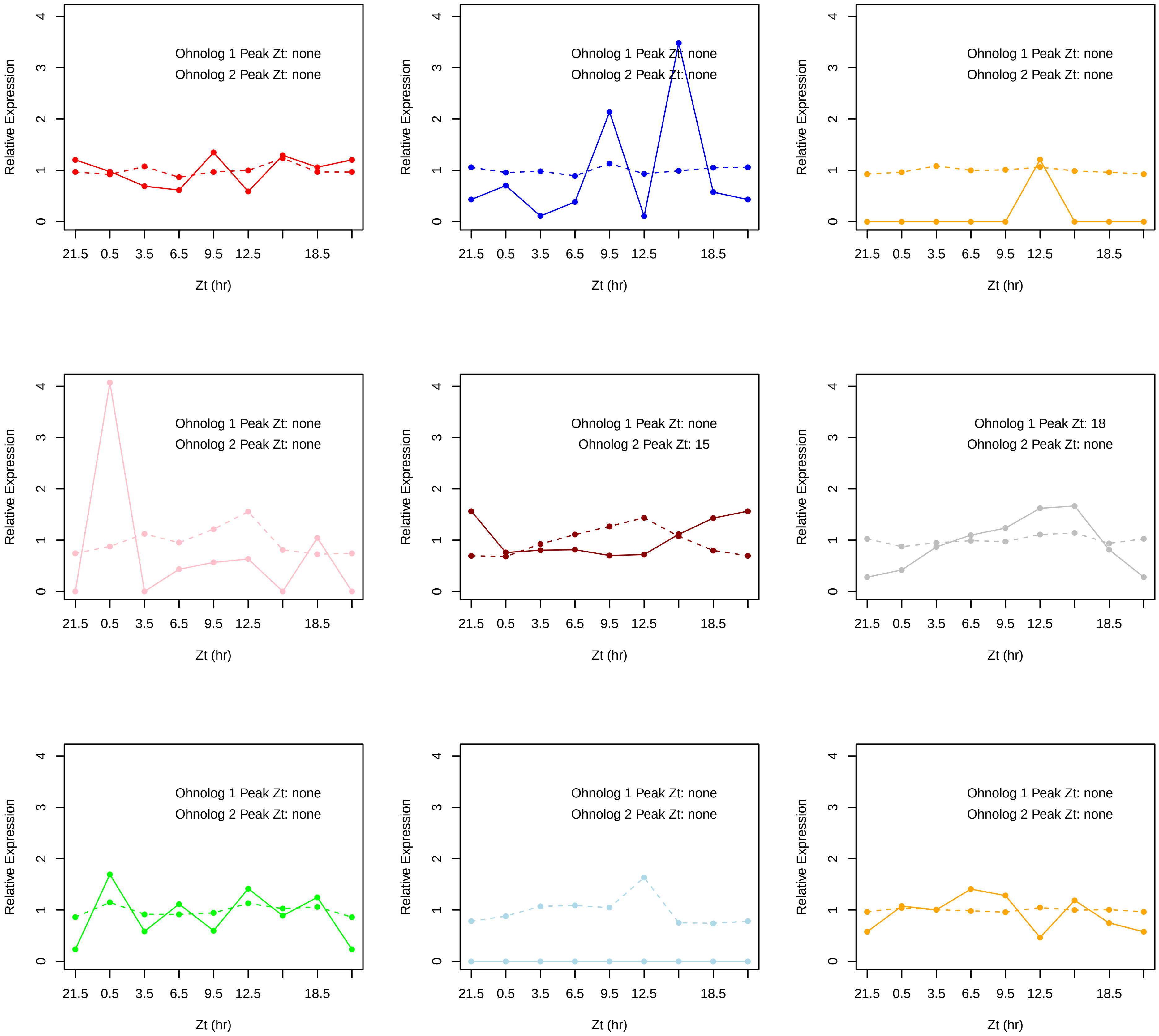

Ohnolog 1: ENSXMAG00000024734 igfbp2a
Ohnolog 2: ENSXMAG00000026555 igfbp2b

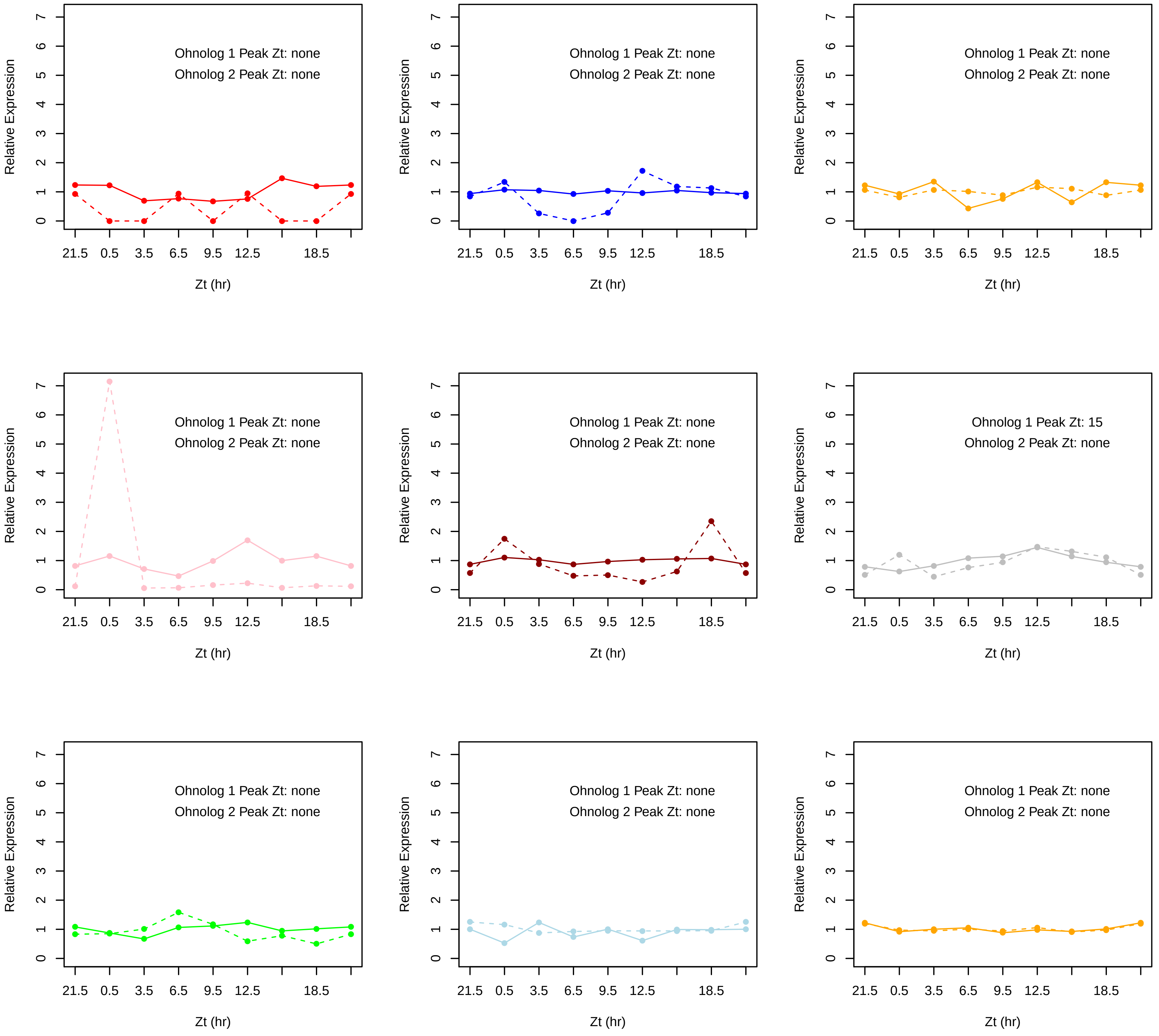

Ohnolog 1: ENSXMAG00000006943 guca1d
Ohnolog 2: ENSXMAG00000007435 guca1c

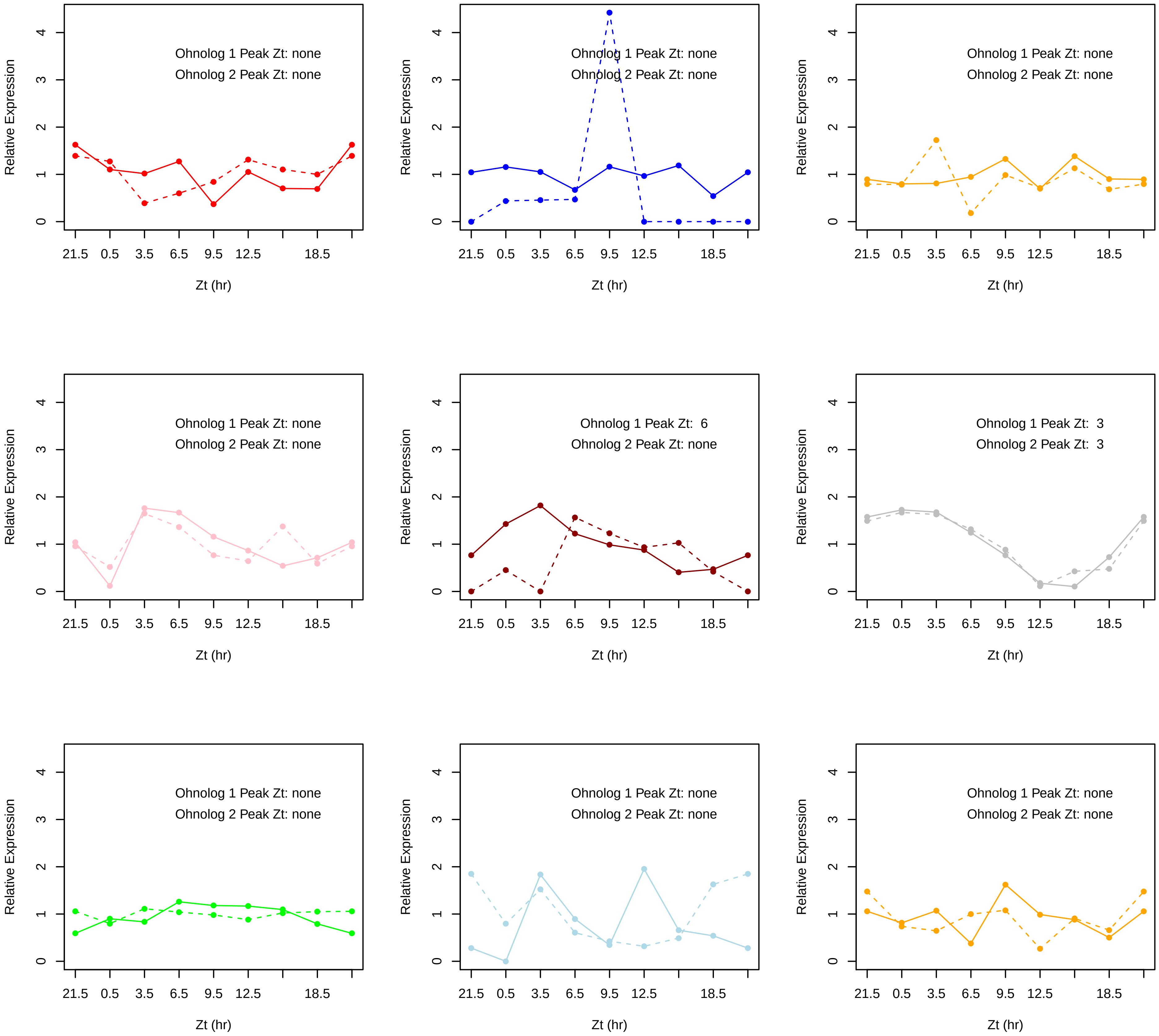

Ohnolog 1: ENSXMAG00000000532 CLINT1 (1 of many)
Ohnolog 2: ENSXMAG00000008223 clint1a

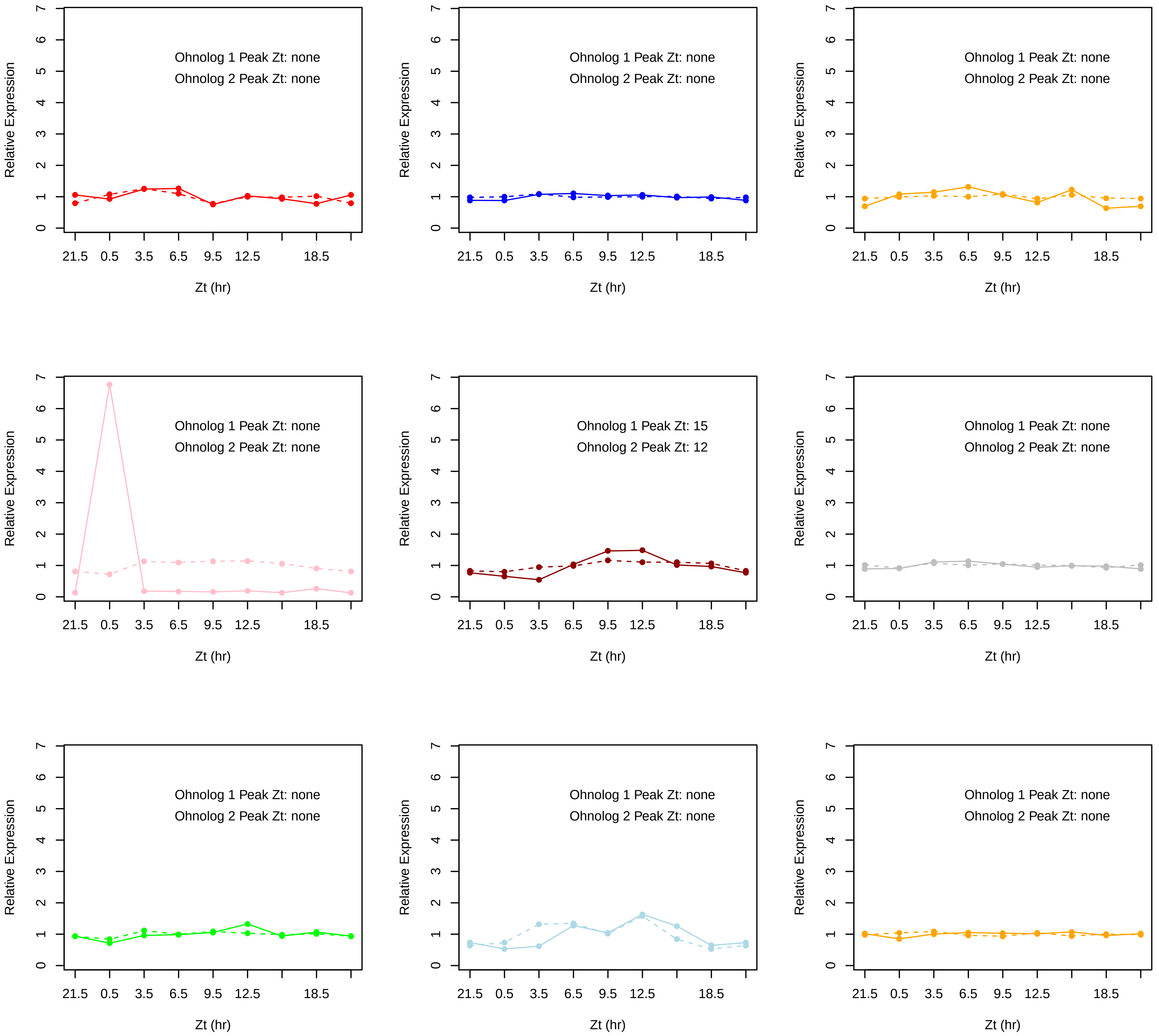

Ohnolog 1: ENSXMAG00000021113
Ohnolog 2: ENSXMAG00000024920 rab11fip5a

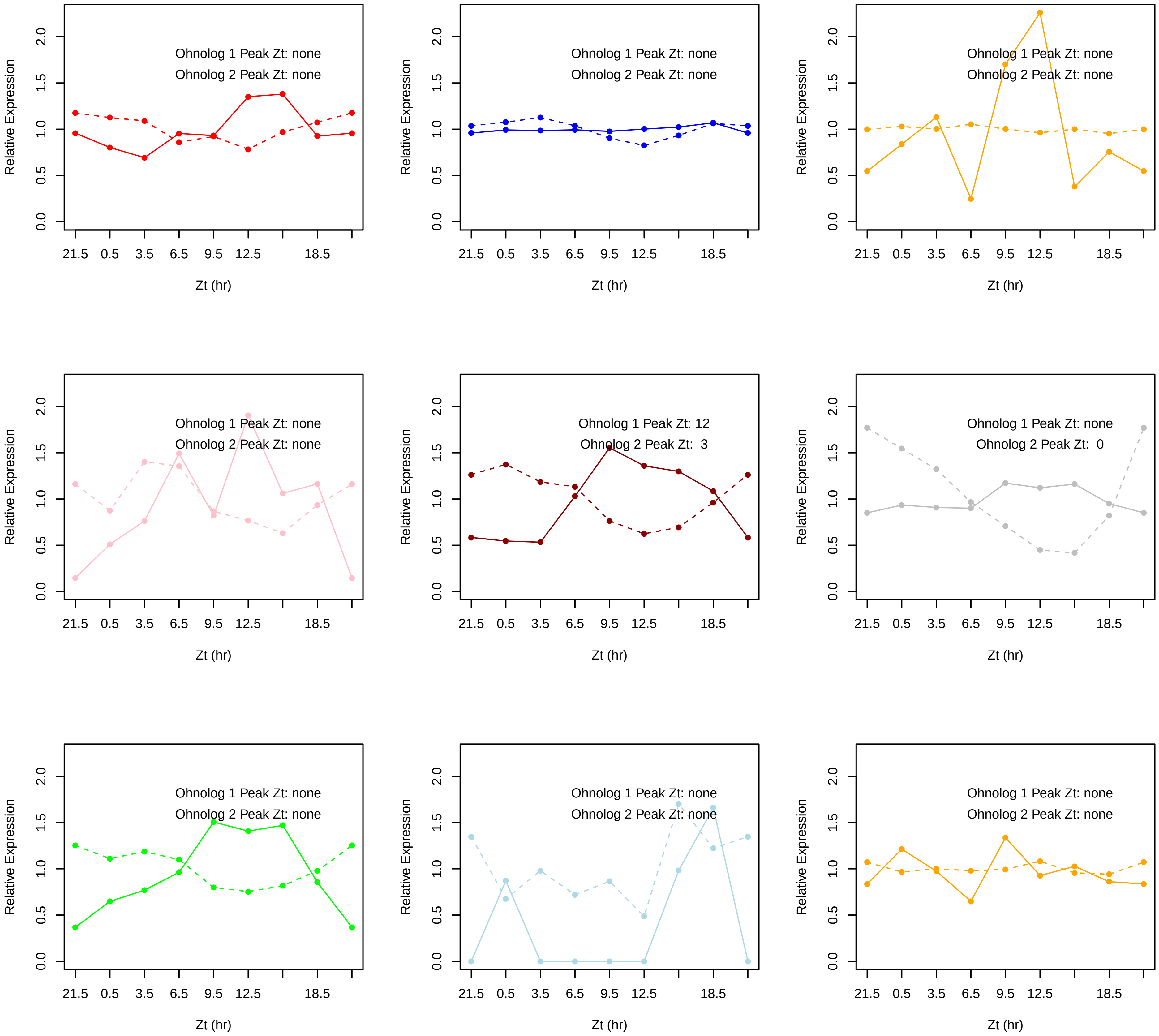

Ohnolog 1: ENSXMAG00000005008
Ohnolog 2: ENSXMAG00000022318 nectin3b

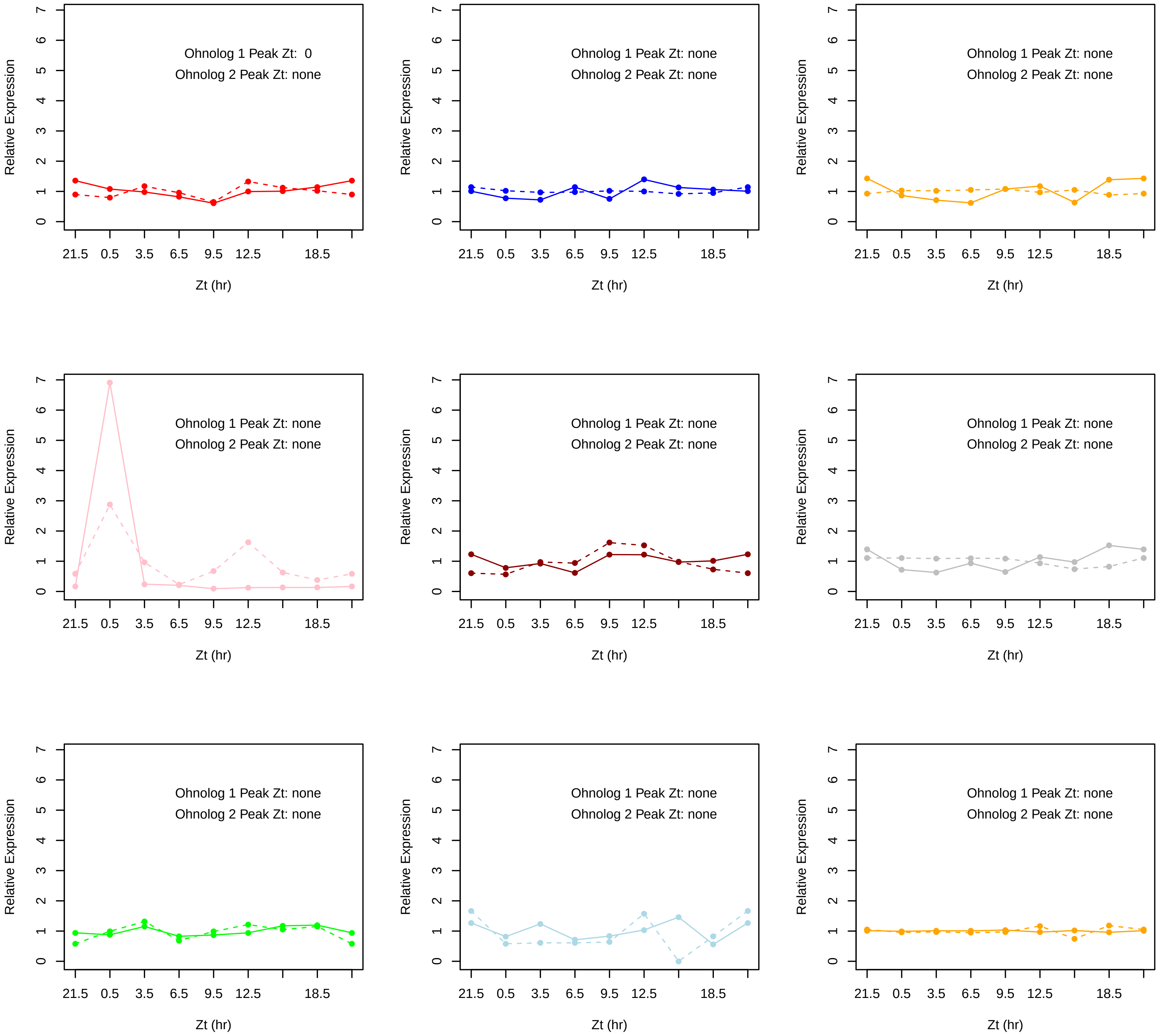

Ohnolog 1: ENSXMAG00000005018 pip5k1ba
Ohnolog 2: ENSXMAG00000007055

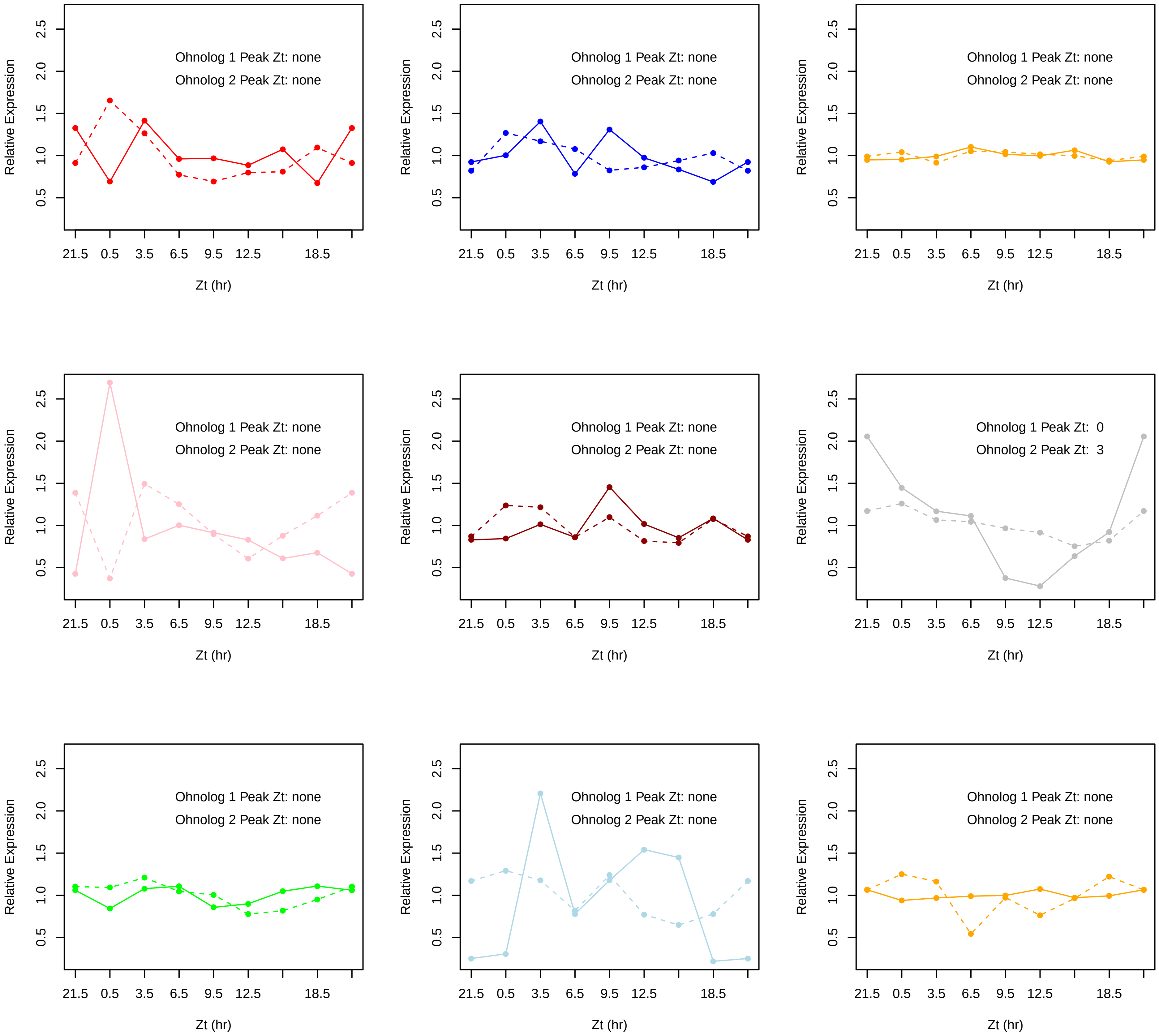

Ohnolog 1: ENSXMAG00000009757 myo10l1
Ohnolog 2: ENSXMAG00000015372

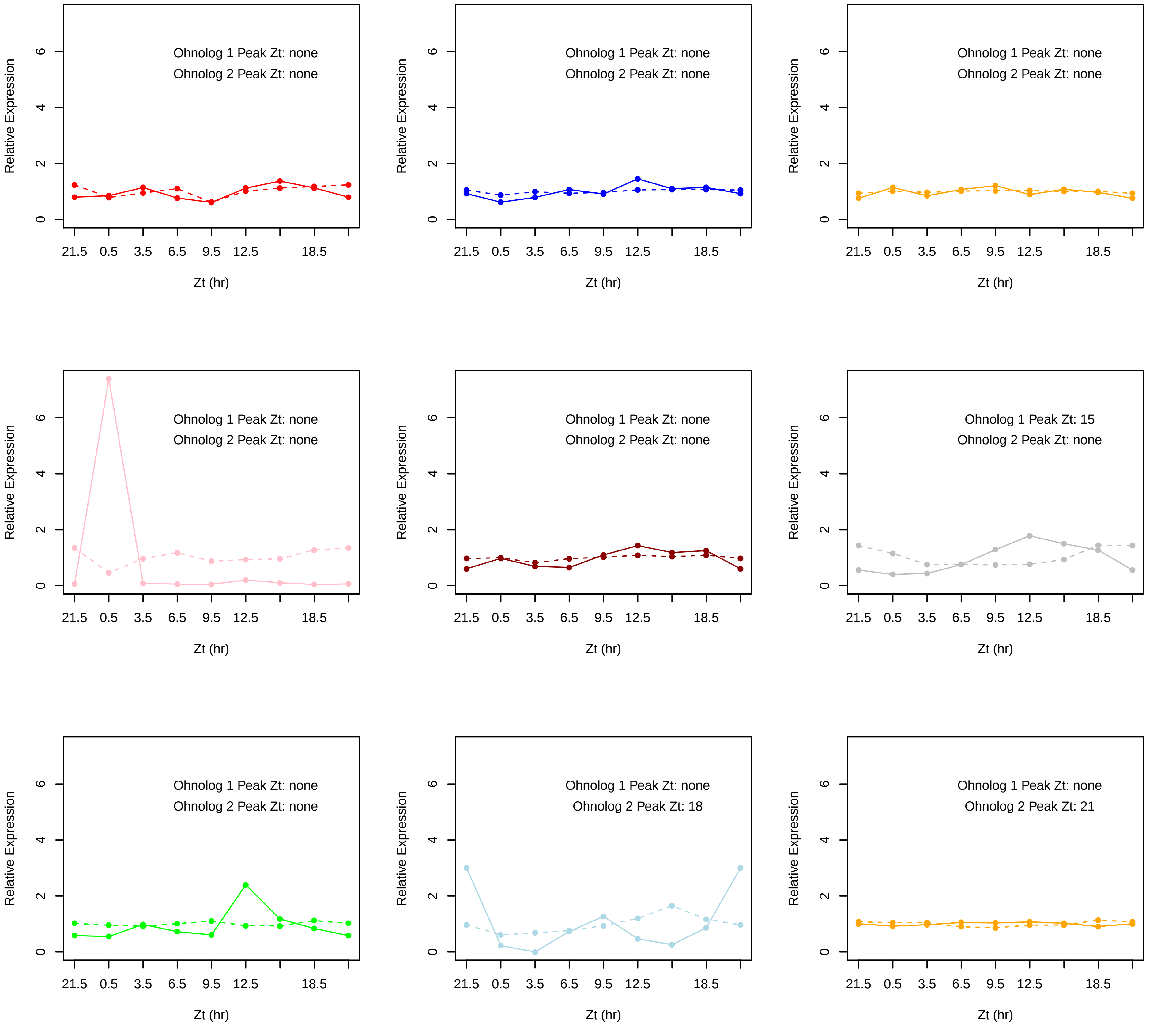

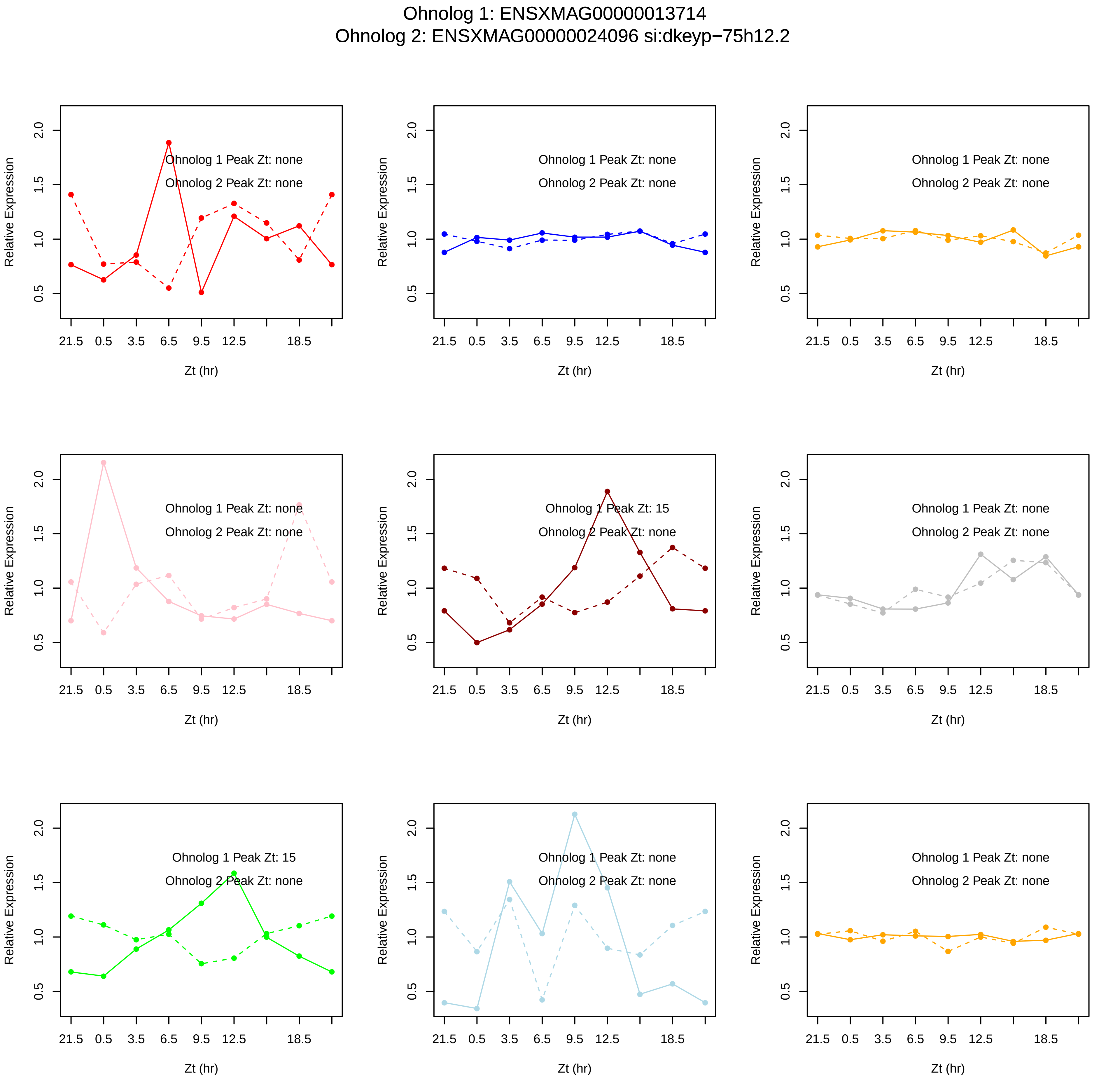
Ohnolog 1: ENSXMAG00000013714
Ohnolog 2: ENSXMAG00000024096 si:dkeyp−75h12.2
Ohnolog 1 Peak Zt: none
Ohnolog 2 Peak Zt: none
Ohnolog 1 Peak Zt: 15
Ohnolog 1 Peak Zt: 15
Relative Expression
Zt (hr)
21.5
0.5
3.5
6.5
9.5
12.5
18.5
0.5
1.0
1.5
2.0

Ohnolog 1: ENSXMAG00000012915
Ohnolog 2: ENSXMAG00000026622 gfra3

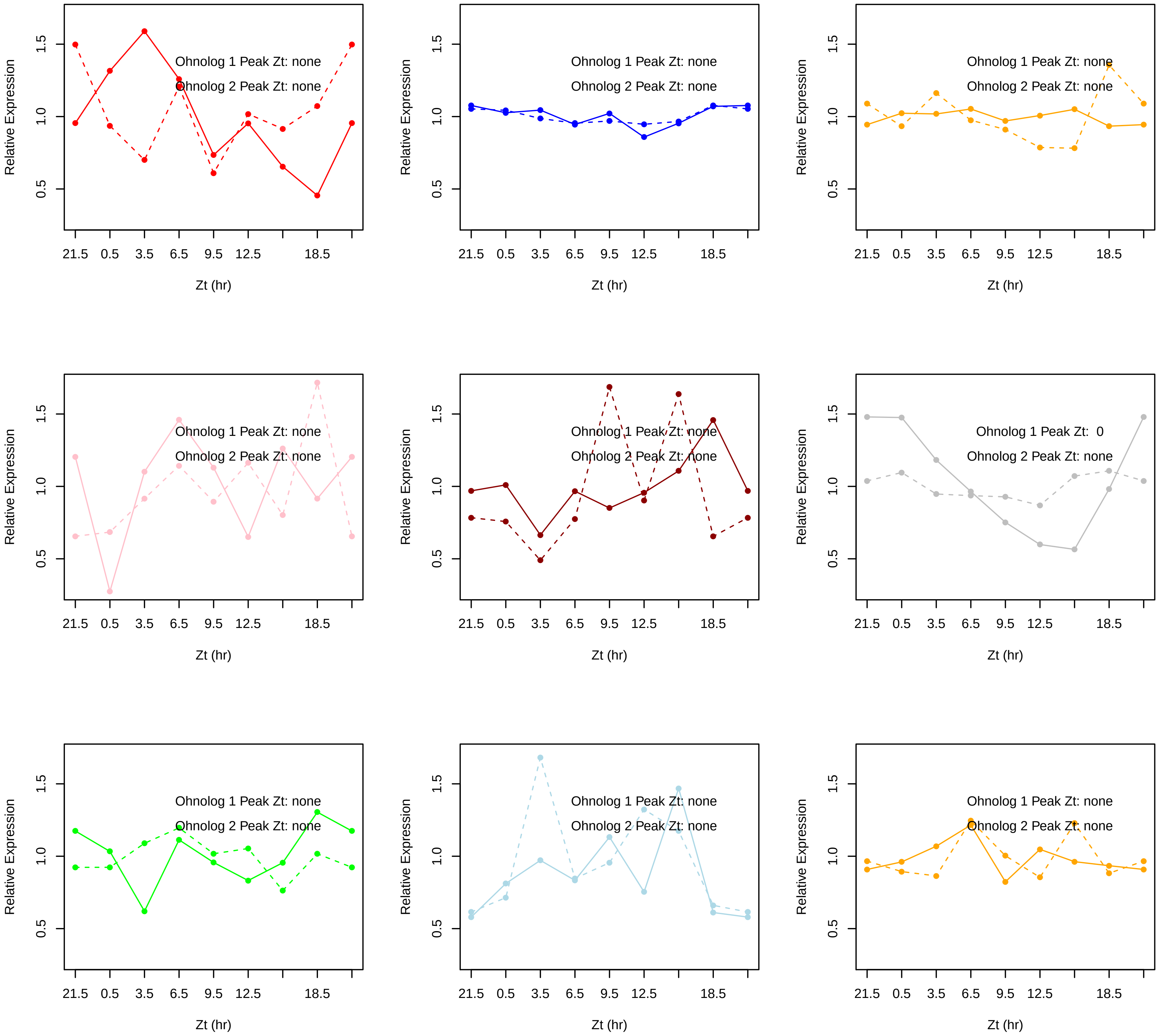

Ohnolog 1: ENSXMAG00000002596 ptpn2a
Ohnolog 2: ENSXMAG00000021556 ptpn2b

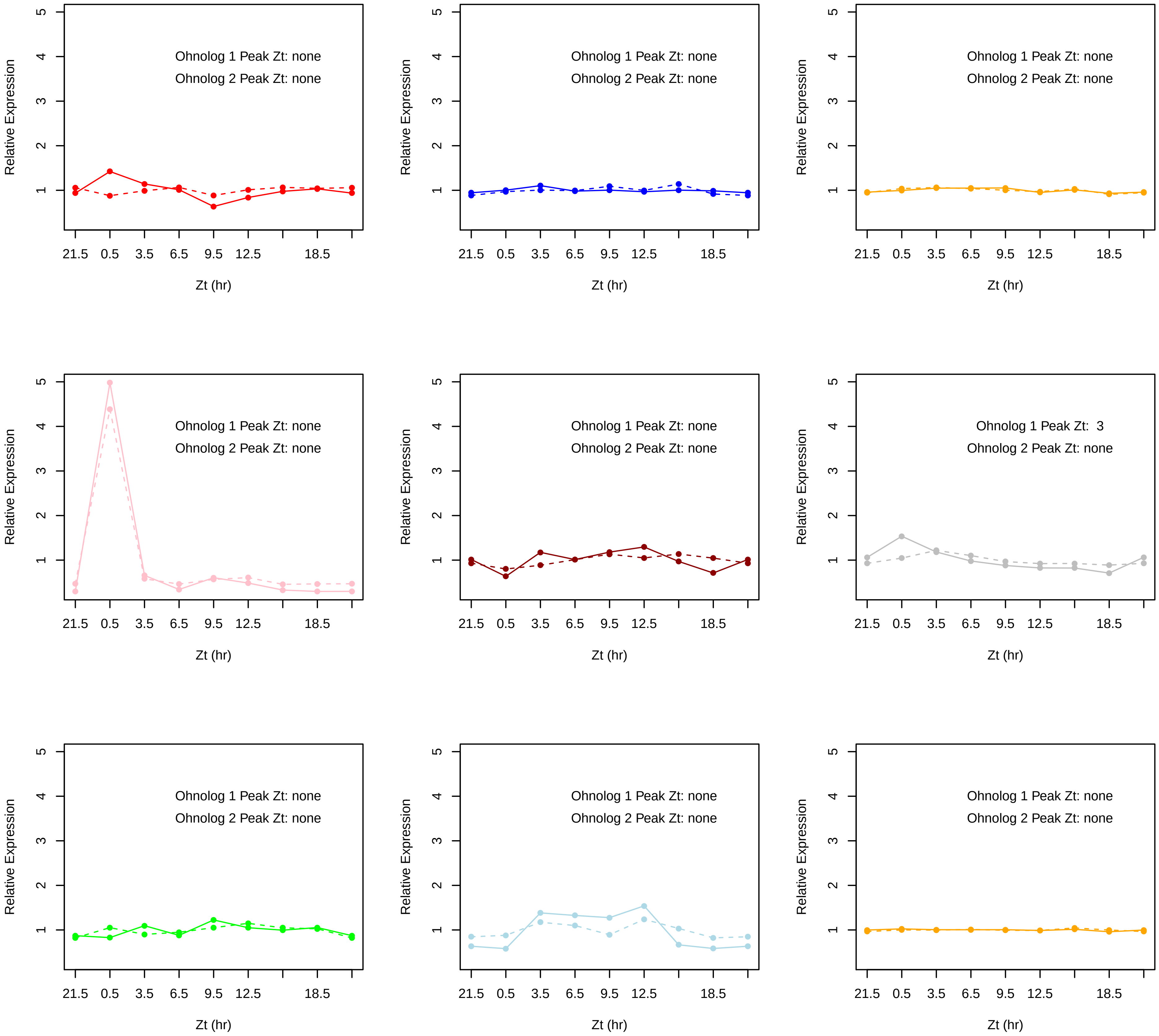

Ohnolog 1: ENSXMAG00000002574 grhl2a
Ohnolog 2: ENSXMAG00000003725 grhl2b

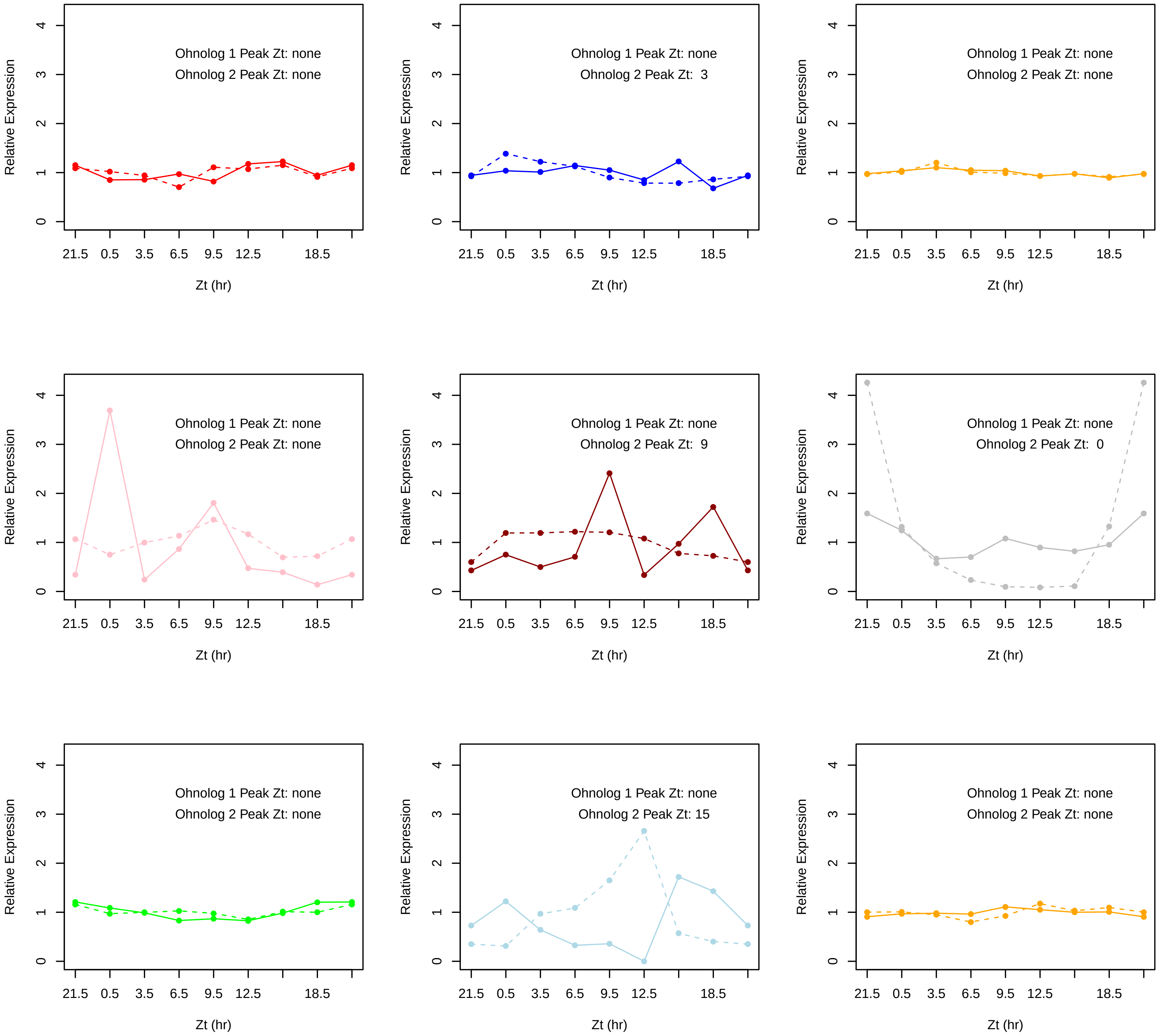

Ohnolog 1: ENSXMAG00000000293 klhl40a
Ohnolog 2: ENSXMAG00000009443 klhl40b

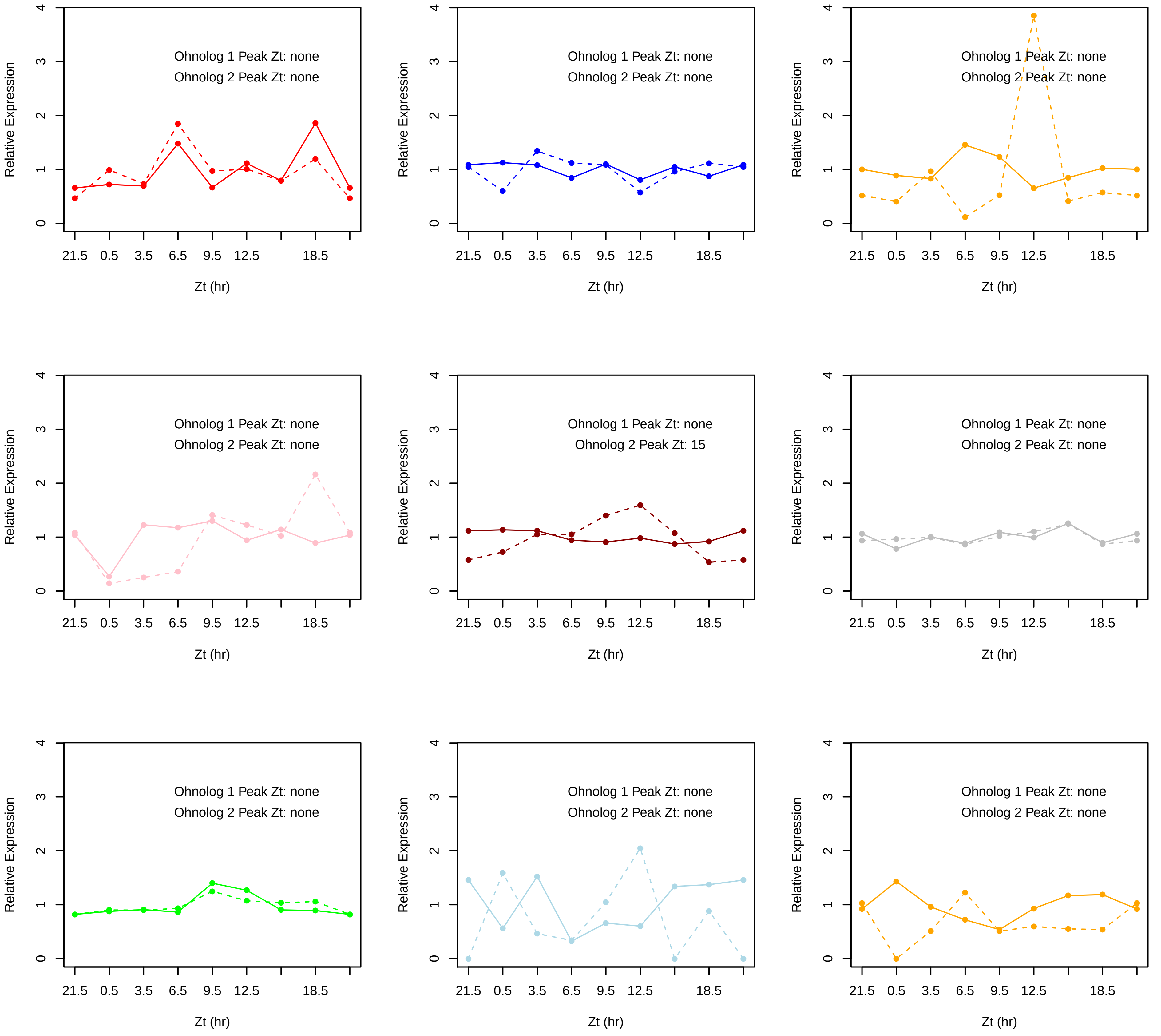

Ohnolog 1: ENSXMAG00000000405 kcnip1b
Ohnolog 2: ENSXMAG00000021925 KCNIP1

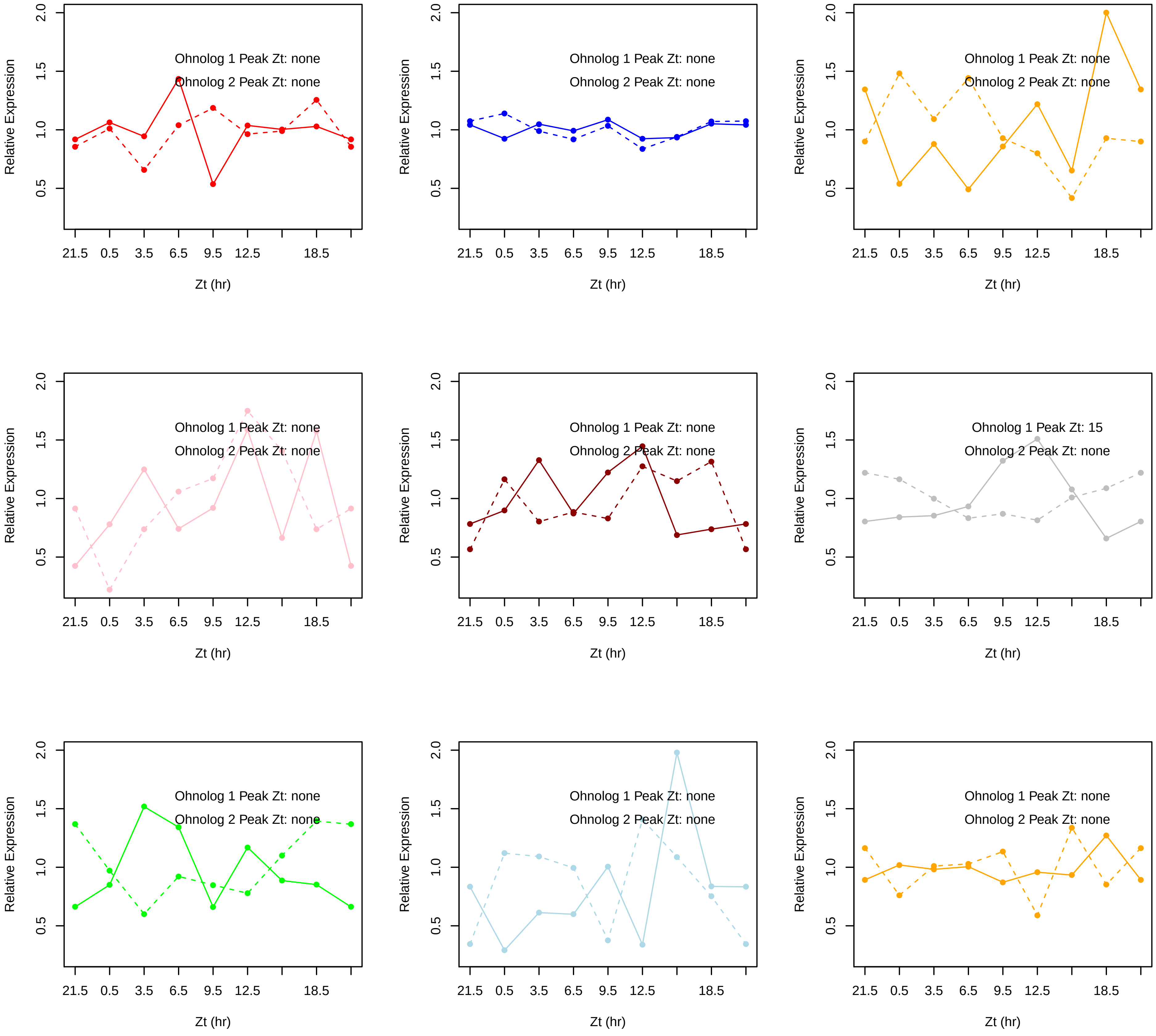

Ohnolog 1: ENSXMAG00000000230 plcd1b
Ohnolog 2: ENSXMAG00000009326 plcd1a

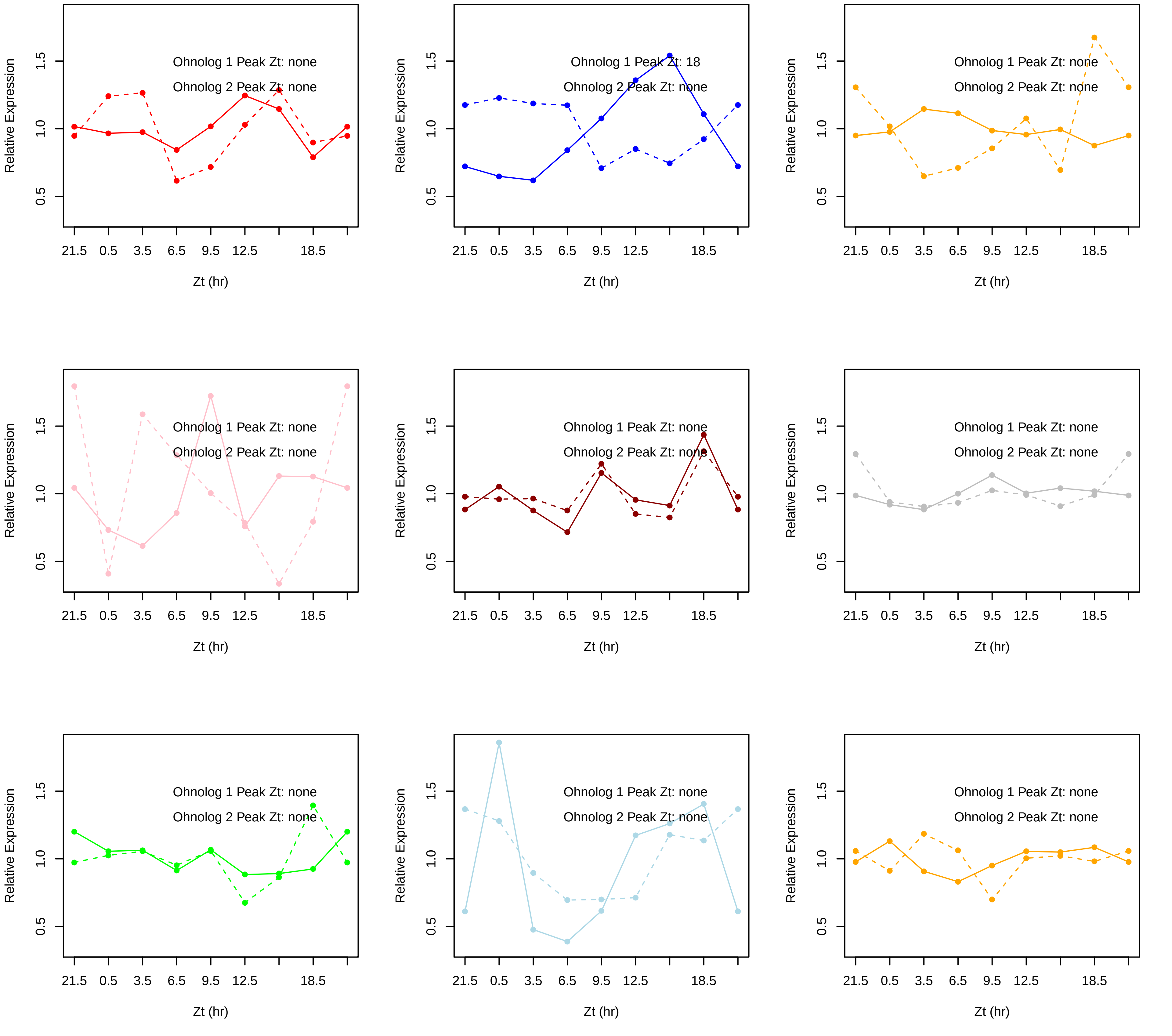

Ohnolog 1: ENSXMAG00000008152 NAV1 (1 of many)
Ohnolog 2: ENSXMAG00000018024 nav1b

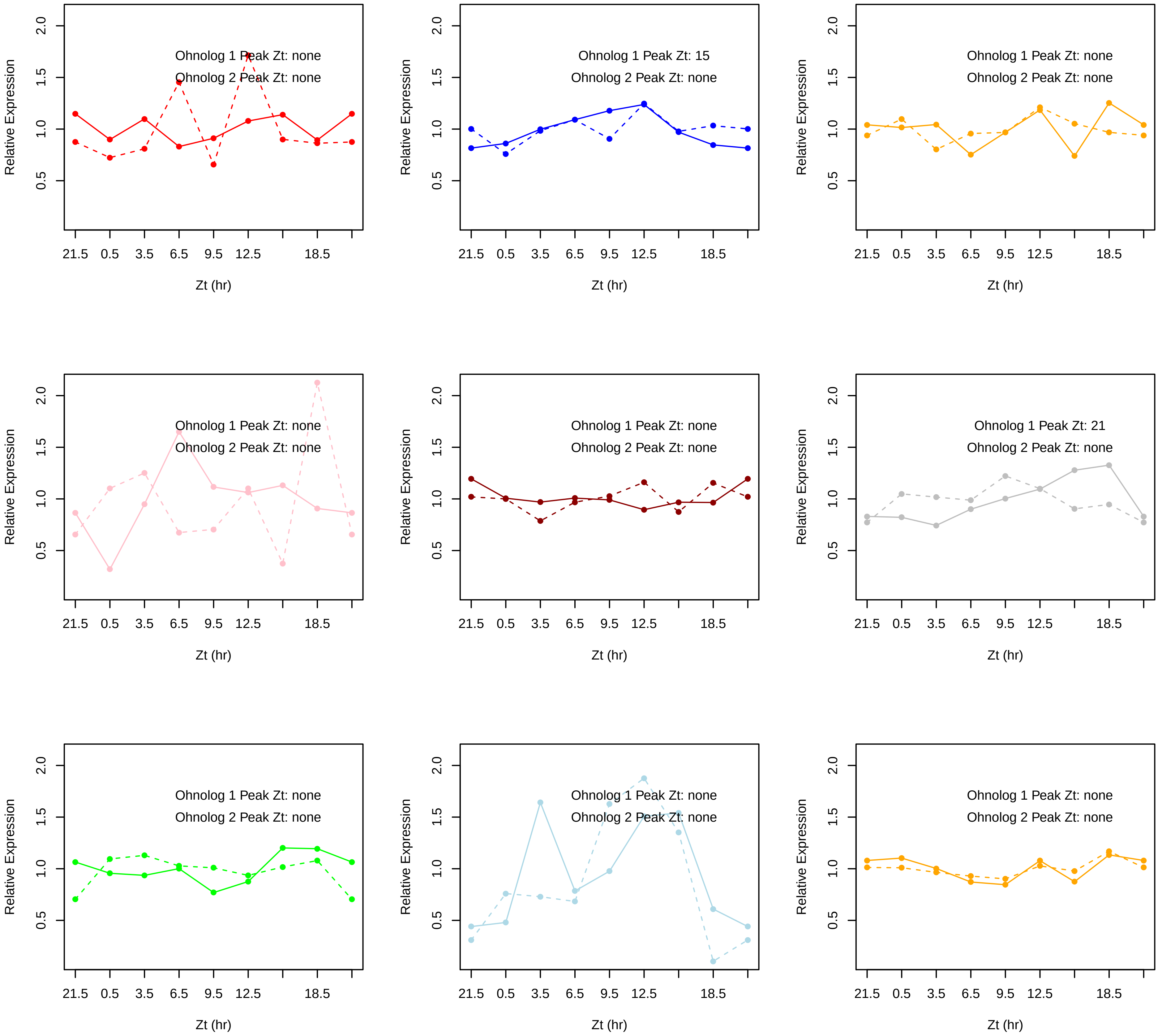

Ohnolog 1: ENSXMAG00000008184 PKP1 (1 of many)
Ohnolog 2: ENSXMAG00000018083 pkp1b

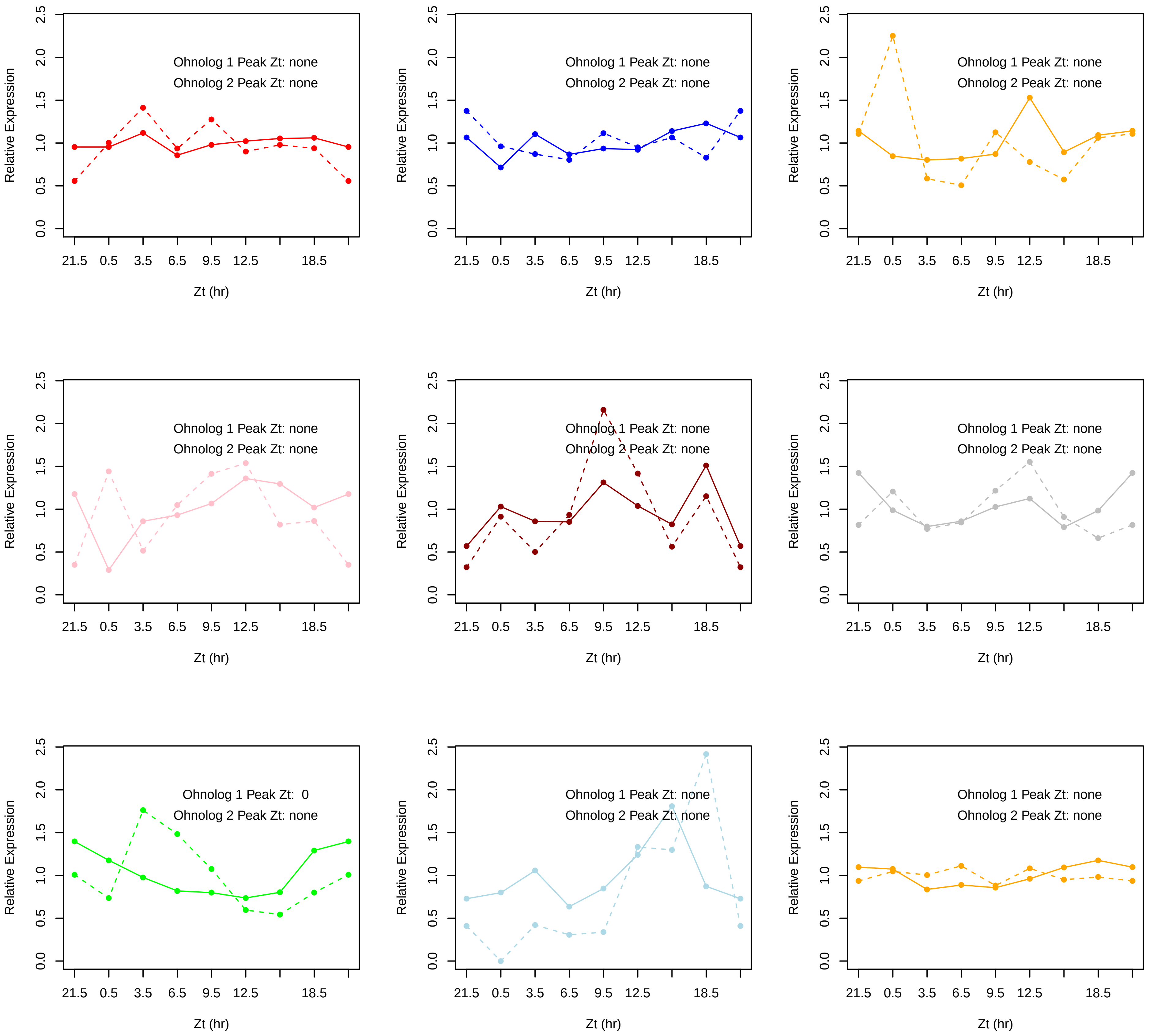

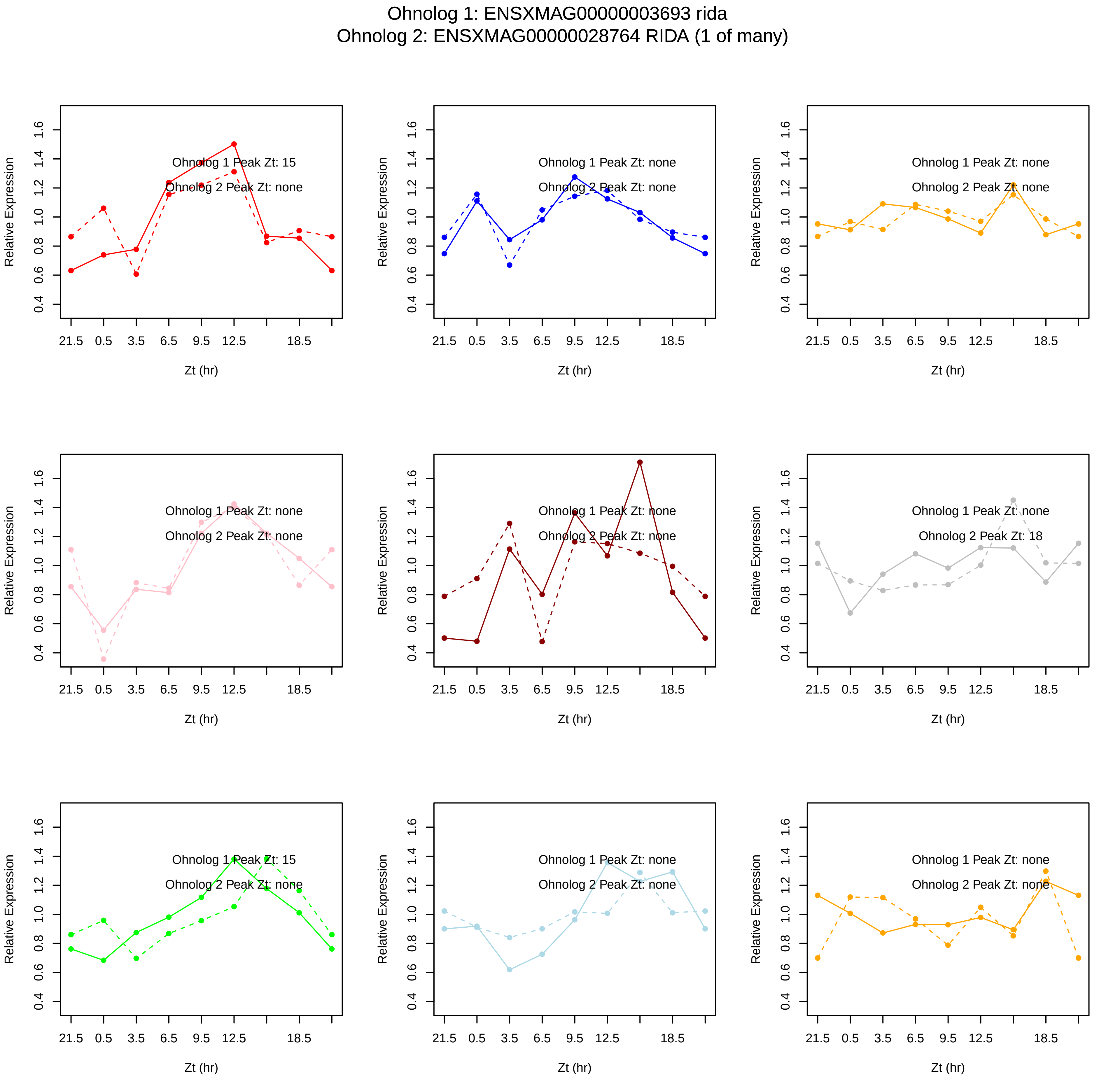

Ohnolog 1: ENSXMAG00000003693 rida
Ohnolog 2: ENSXMAG00000028764 RIDA (1 of many)
Ohnolog 1 Peak Zt: 15
Ohnolog 2 Peak Zt: none
Ohnolog 1 Peak Zt: none
Ohnolog 2 Peak Zt: none
Ohnolog 1 Peak Zt: none
Ohnolog 2 Peak Zt: none
Ohnolog 1 Peak Zt: none
Ohnolog 2 Peak Zt: none
Ohnolog 1 Peak Zt: none
Ohnolog 2 Peak Zt: none
Ohnolog 1 Peak Zt: none
Ohnolog 2 Peak Zt: 18
Ohnolog 1 Peak Zt: 15
Ohnolog 2 Peak Zt: none
Ohnolog 1 Peak Zt: none
Ohnolog 2 Peak Zt: none
Ohnolog 1 Peak Zt: none
Ohnolog 2 Peak Zt: none
Relative Expression
Zt (hr)
21.5
0.5
3.5
6.5
9.5
12.5
18.5
0.4
0.6
0.8
1.0
1.2
1.4
1.6

Ohnolog 1: ENSXMAG00000011144
Ohnolog 2: ENSXMAG00000017082 chl1b

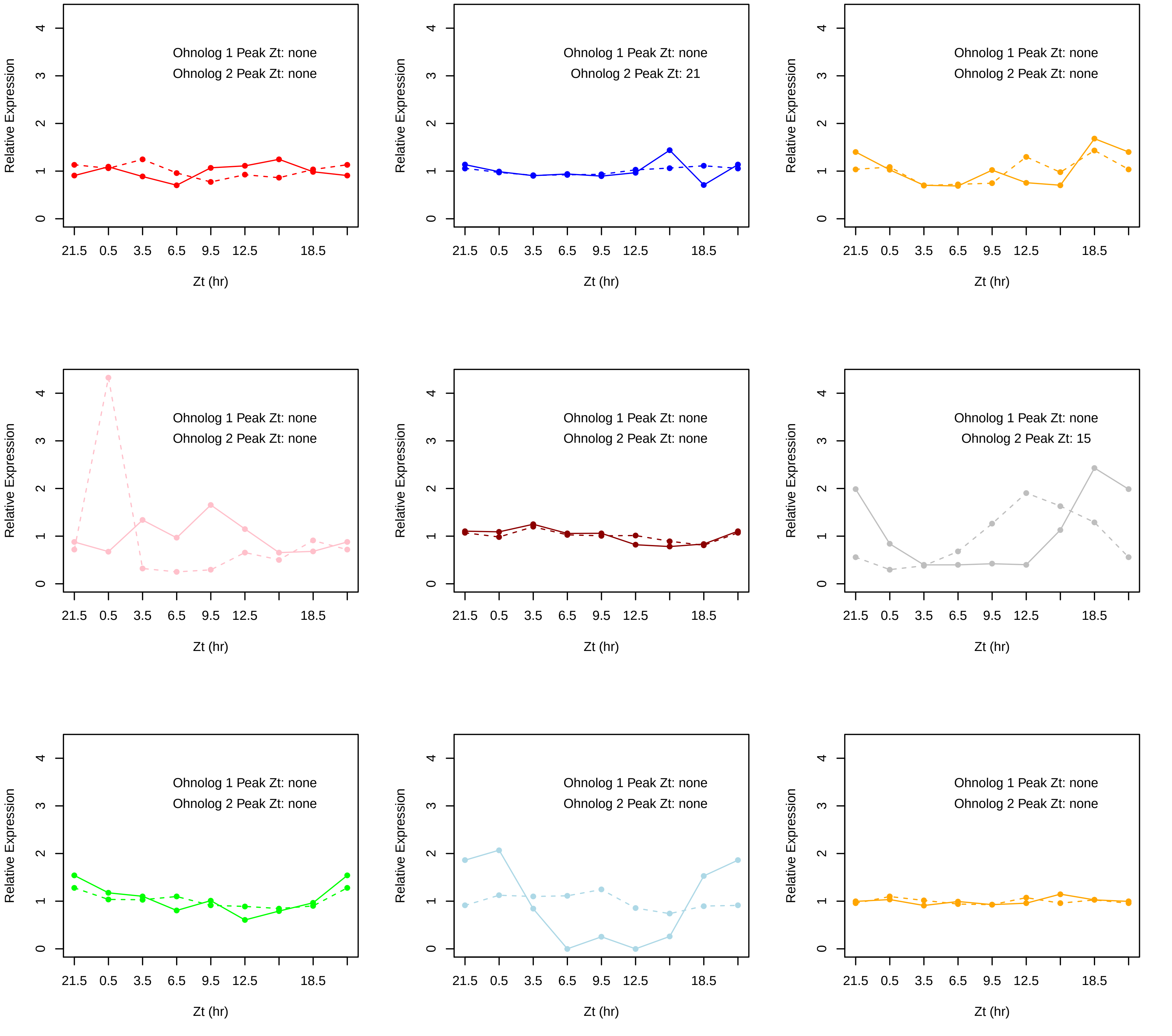

Ohnolog 1: ENSXMAG00000011659
Ohnolog 2: ENSXMAG00000019265 grk1a

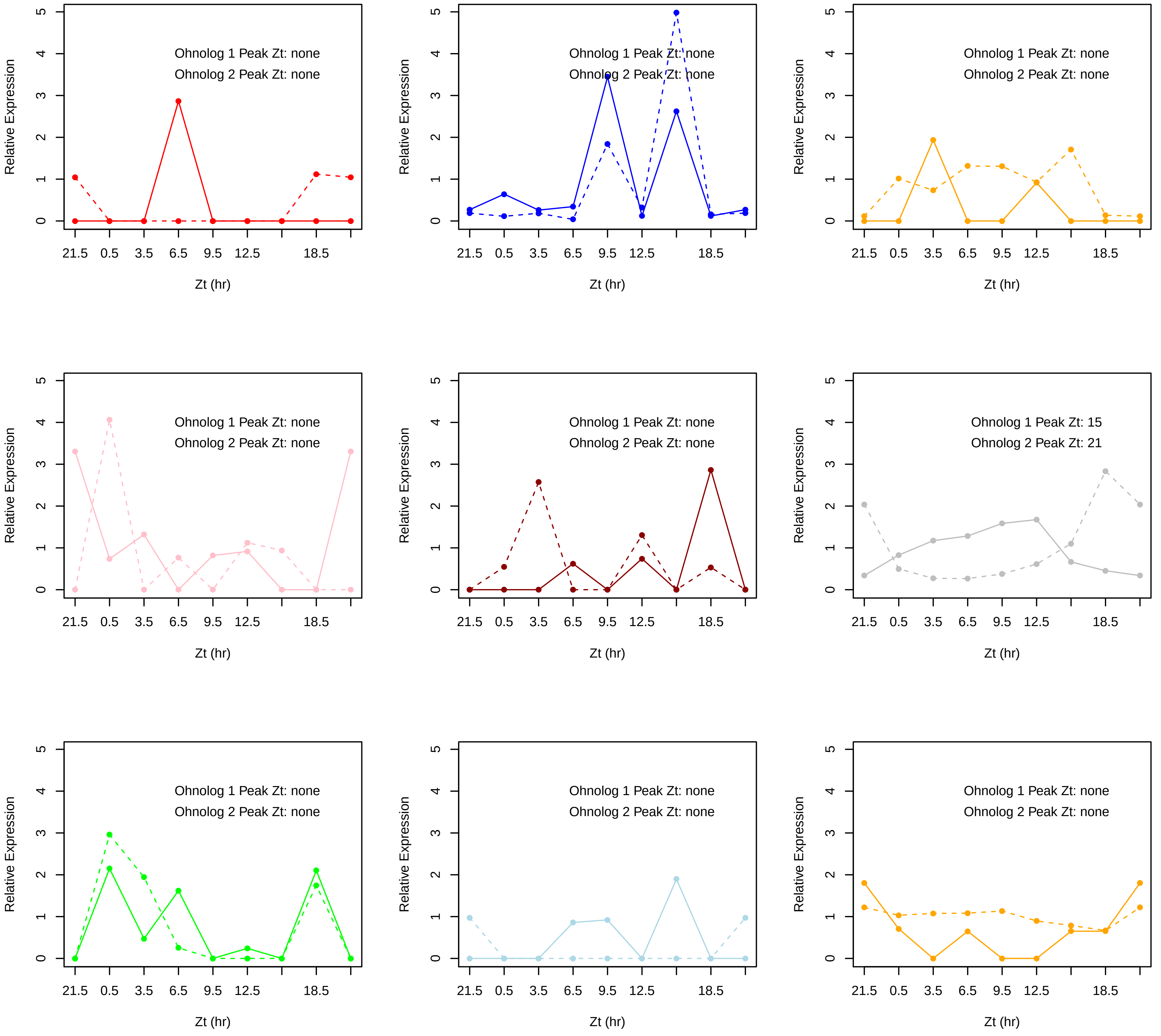

Ohnolog 1: ENSXMAG00000014092 acvr2ba
Ohnolog 2: ENSXMAG00000026459

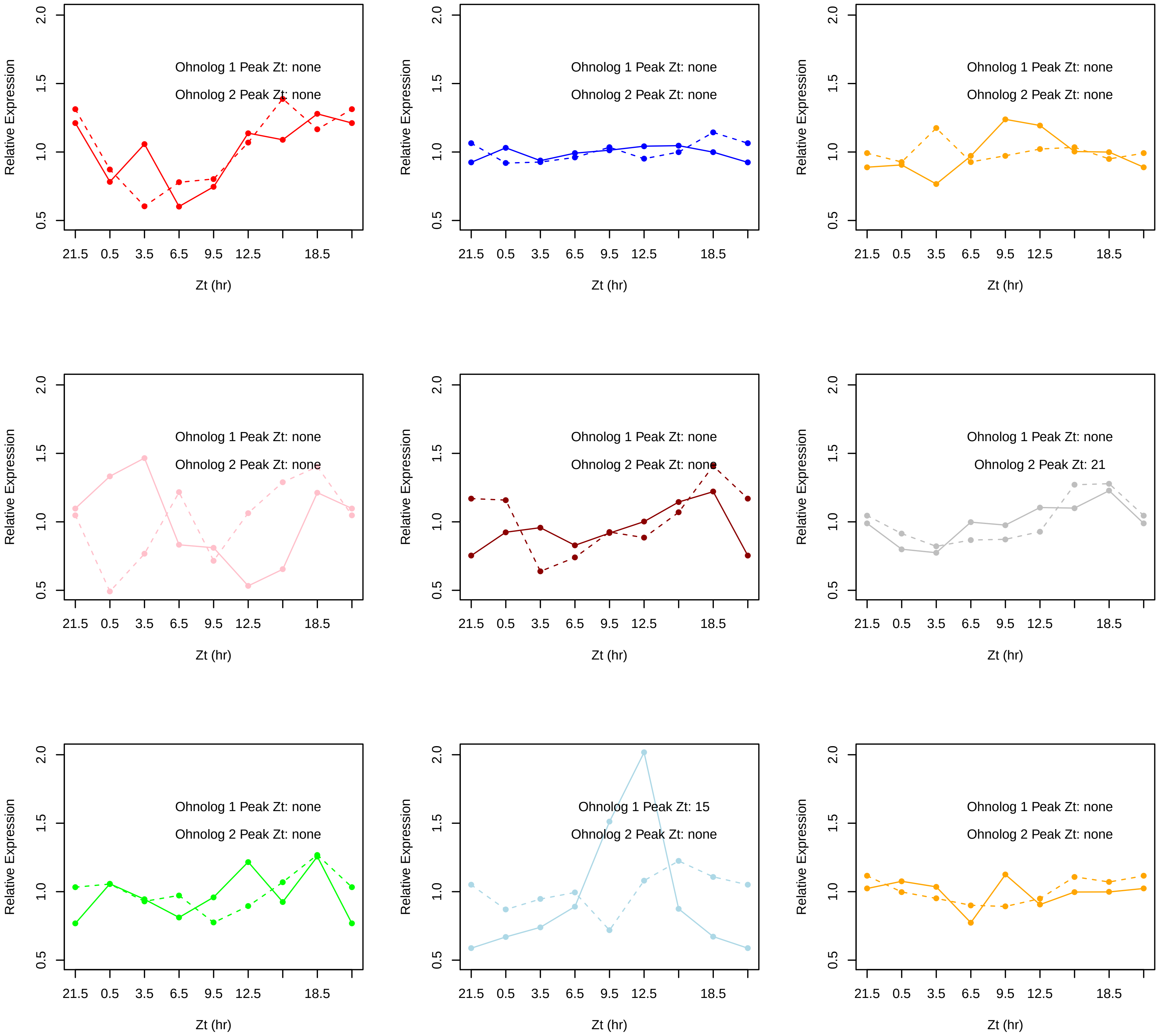

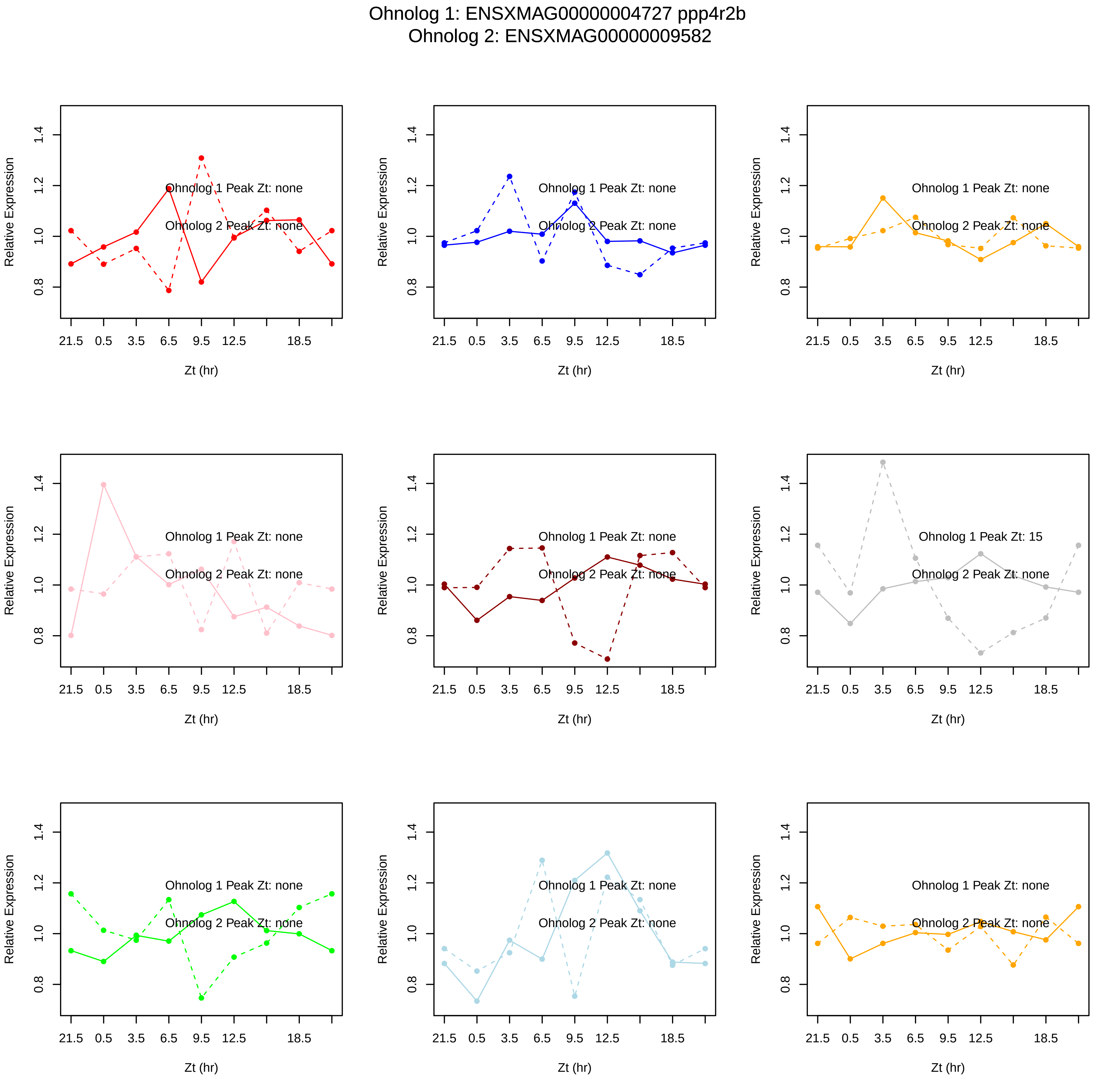
Ohnolog 1: ENSXMAG00000004727 ppp4r2b
Ohnolog 2: ENSXMAG00000009582
Ohnolog 1 Peak Zt: none
Ohnolog 2 Peak Zt: none
Ohnolog 1 Peak Zt: none
Ohnolog 2 Peak Zt: none
Ohnolog 1 Peak Zt: none
Ohnolog 2 Peak Zt: none
Ohnolog 1 Peak Zt: none
Ohnolog 2 Peak Zt: none
Ohnolog 1 Peak Zt: none
Ohnolog 2 Peak Zt: none
Ohnolog 1 Peak Zt: 15
Ohnolog 2 Peak Zt: none
Ohnolog 1 Peak Zt: none
Ohnolog 2 Peak Zt: none
Ohnolog 1 Peak Zt: none
Ohnolog 2 Peak Zt: none
Ohnolog 1 Peak Zt: none
Ohnolog 2 Peak Zt: none
Relative Expression
0.8
1.0
1.2
1.4
Zt (hr)
21.5
0.5
3.5
6.5
9.5
12.5
18.5

Ohnolog 1: ENSXMAG00000017940 zgc:101577
Ohnolog 2: ENSXMAG00000022264 C6orf106

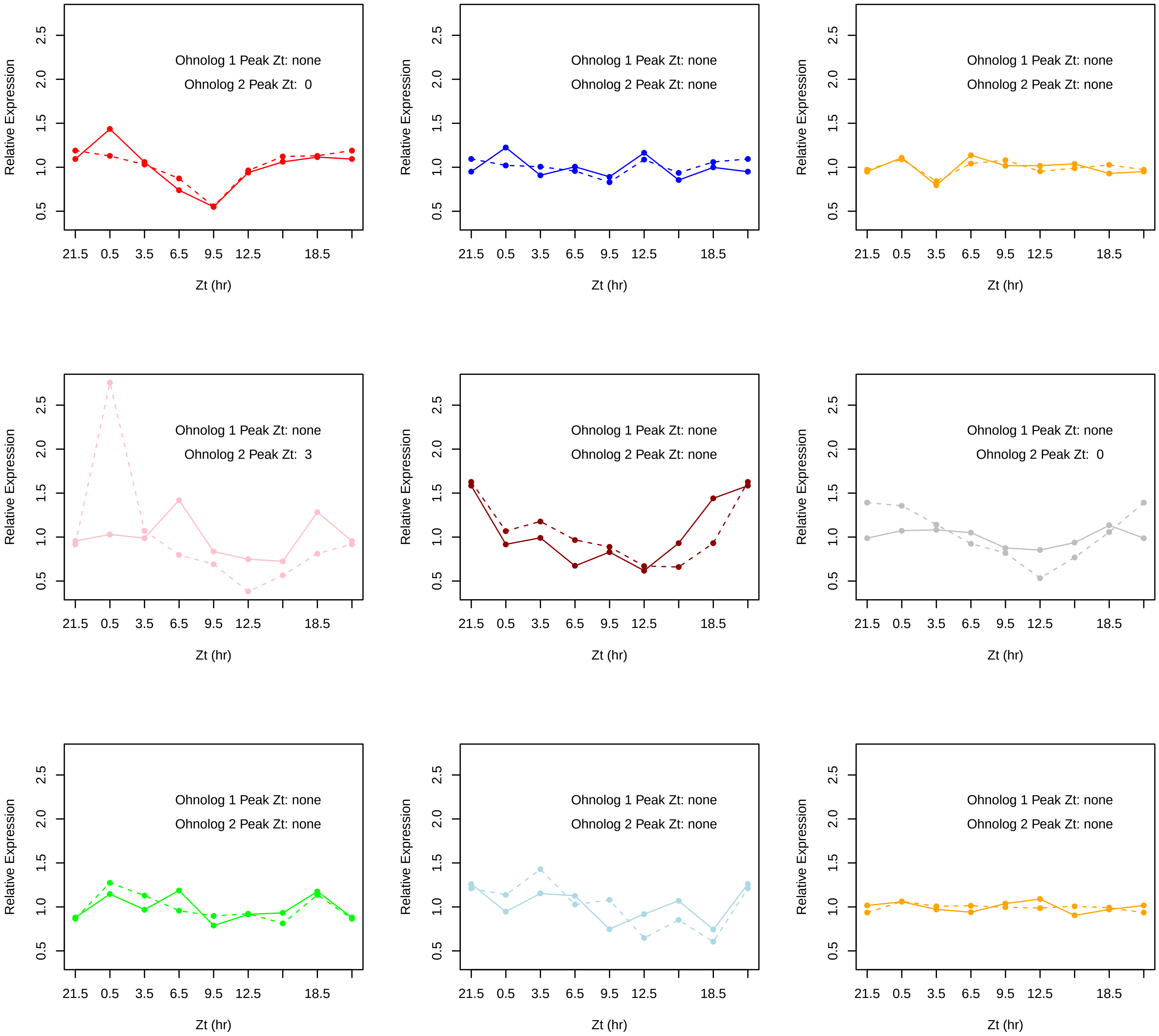

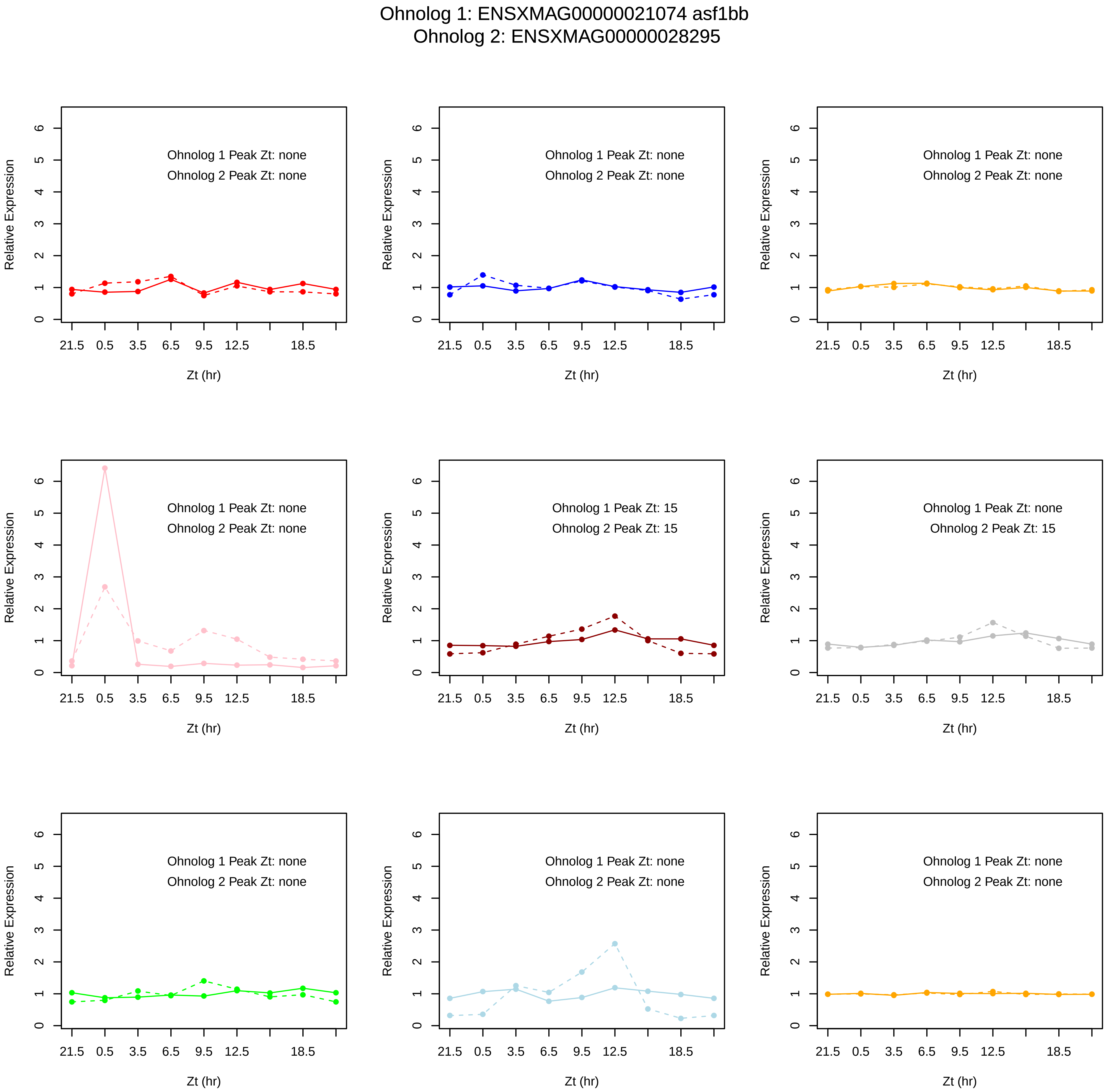
Ohnolog 1: ENSXMAG00000021074 asf1bb
Ohnolog 2: ENSXMAG00000028295
Ohnolog 1 Peak Zt: none
Ohnolog 2 Peak Zt: none
Ohnolog 1 Peak Zt: 15
Ohnolog 2 Peak Zt: 15
Ohnolog 2 Peak Zt: 15
Relative Expression
Zt (hr)
21.5
0.5
3.5
6.5
9.5
12.5
18.5
0
1
2
3
4
5
6

Ohnolog 1: ENSXMAG00000010468 uba3
Ohnolog 2: ENSXMAG00000016399 UBA3 (1 of many)

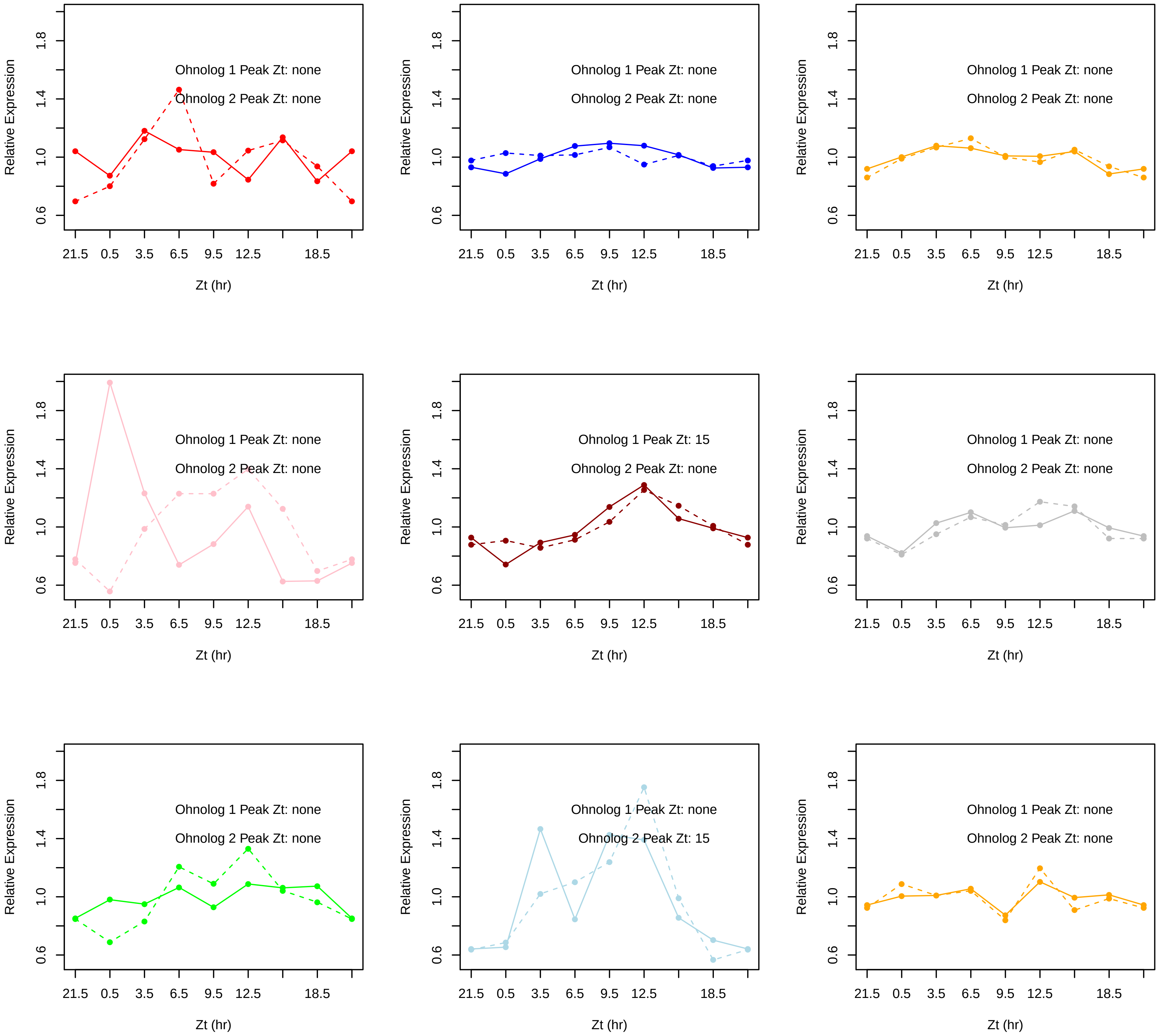

Ohnolog 1: ENSXMAG00000004439 WFS1 (1 of many)
Ohnolog 2: ENSXMAG00000012351 wfs1a

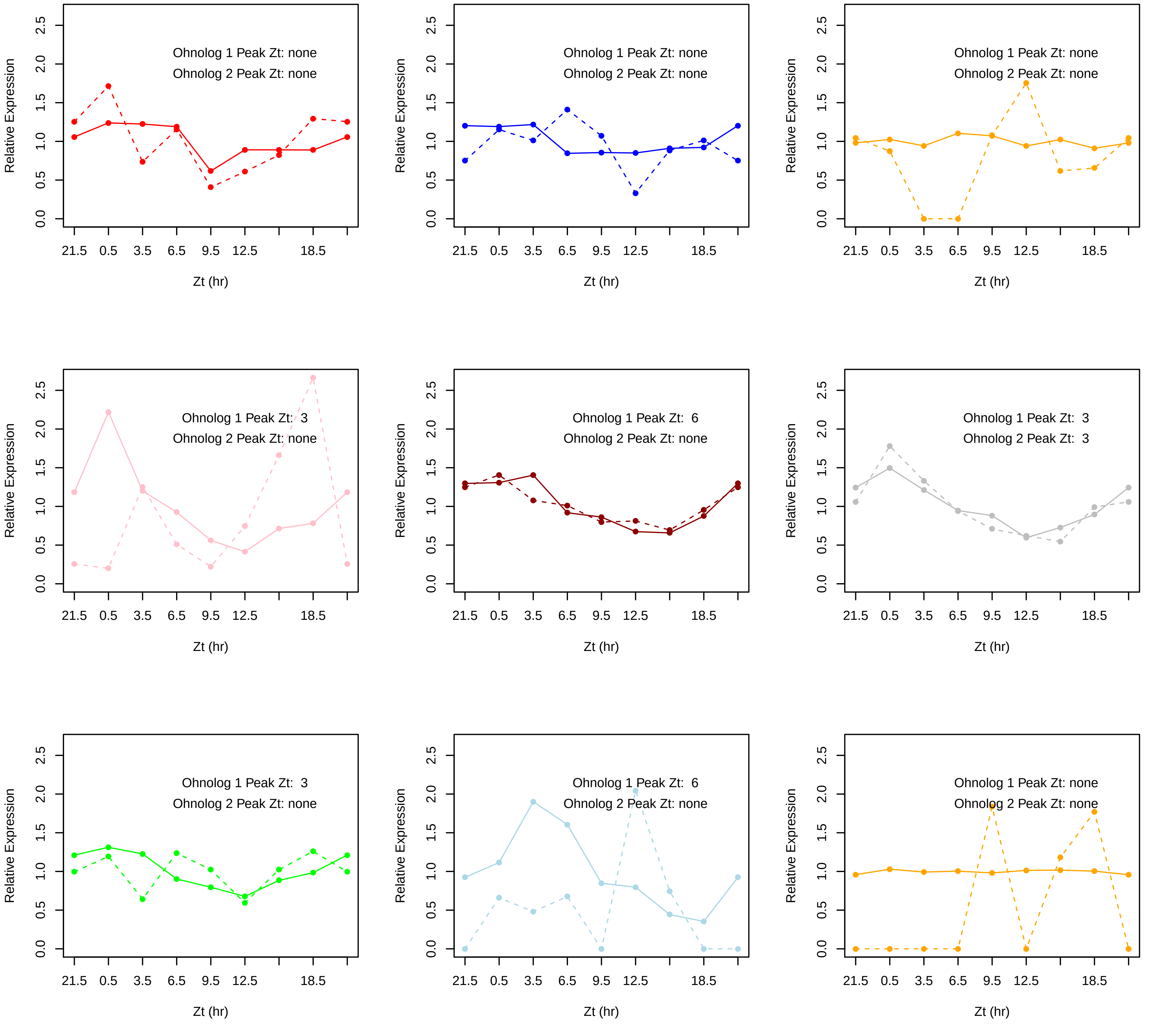

Ohnolog 1: ENSXMAG00000006854
Ohnolog 2: ENSXMAG00000016385 slc16a7

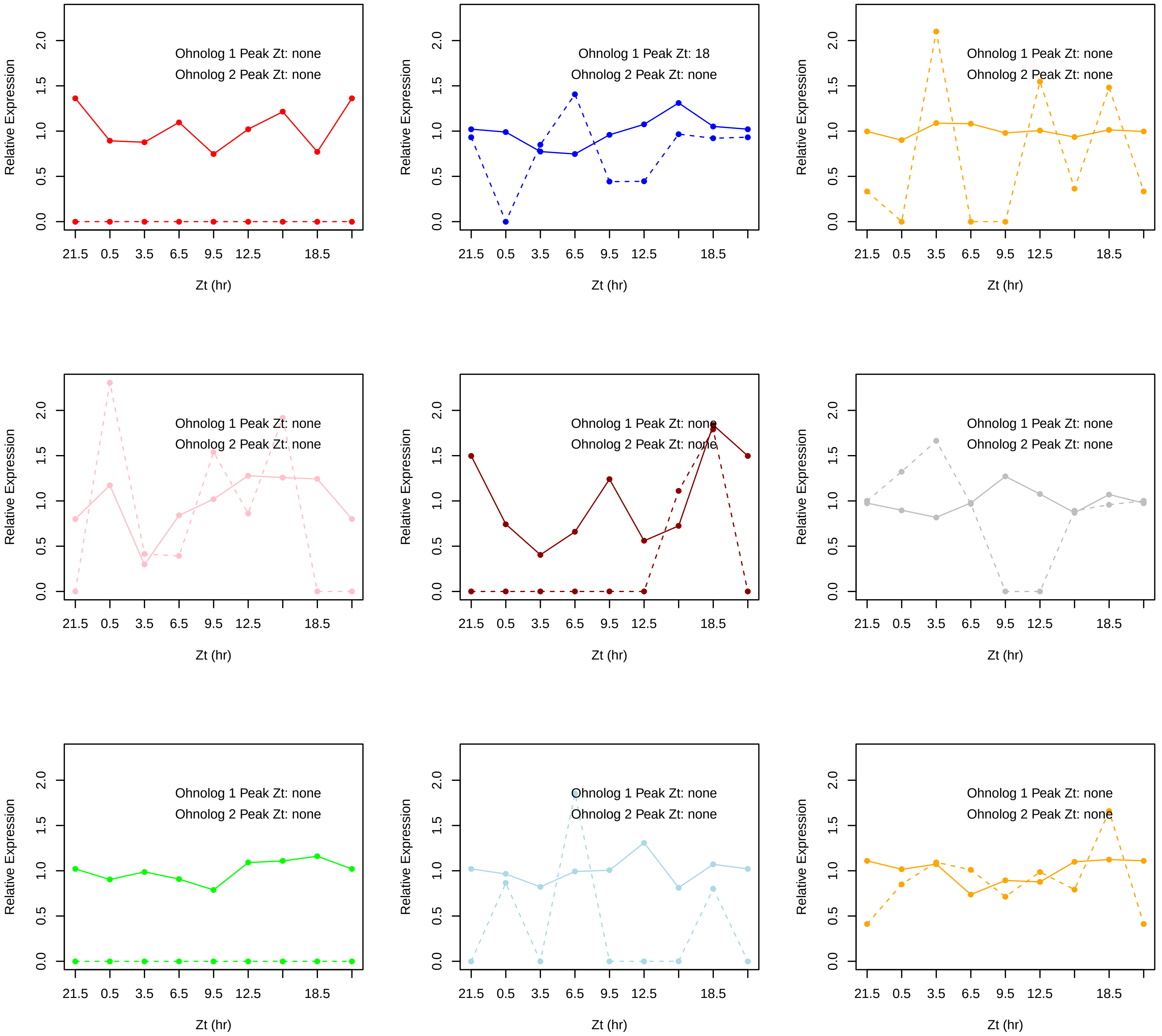

Ohnolog 1: ENSXMAG00000003470
Ohnolog 2: ENSXMAG00000004780 plpp1a

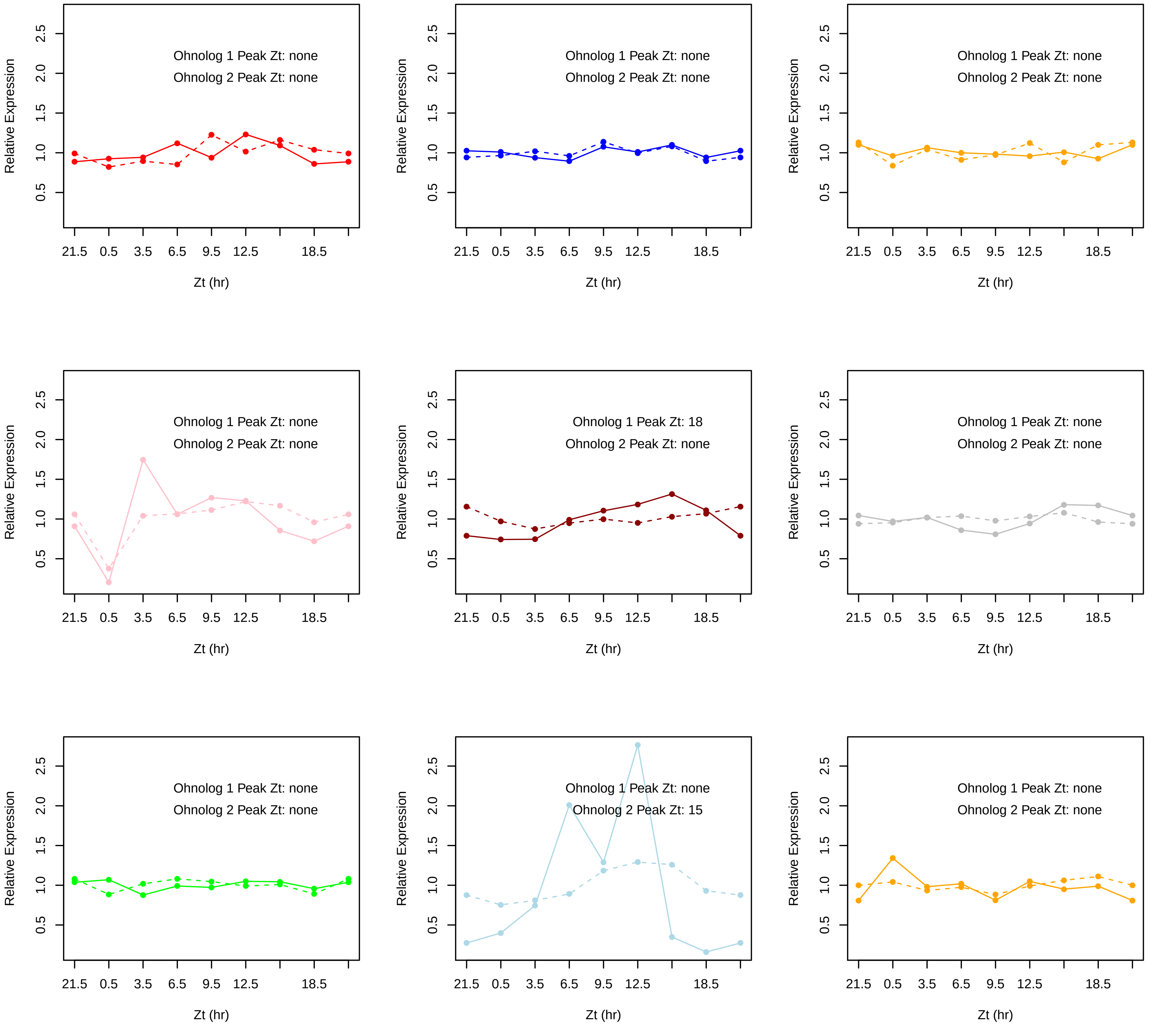

Ohnolog 1: ENSXMAG00000004941 sgpp1
Ohnolog 2: ENSXMAG00000022329 si:ch211−212g7.6

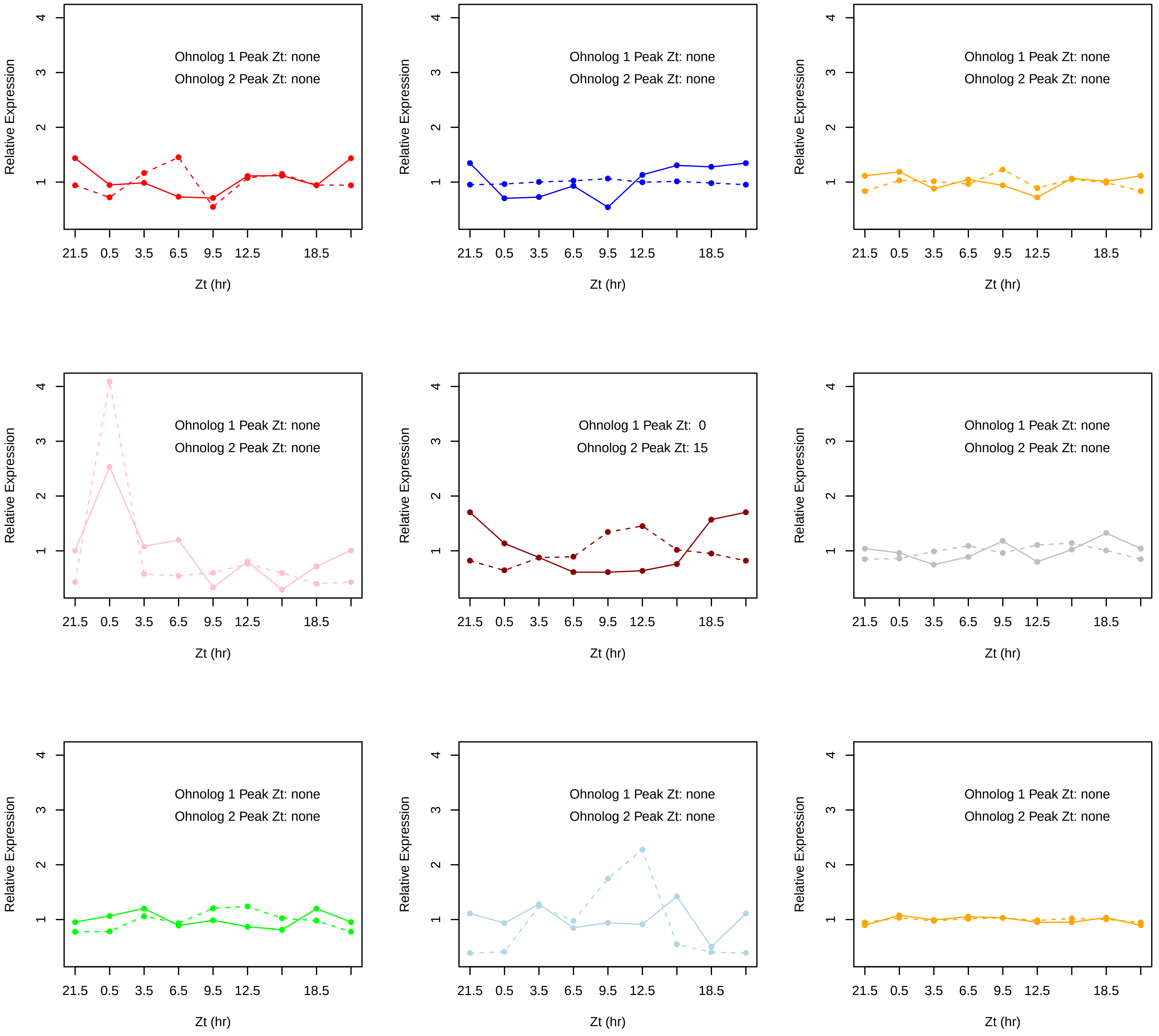

Ohnolog 1: ENSXMAG00000004514 sec22ba
Ohnolog 2: ENSXMAG00000025257 sec22bb

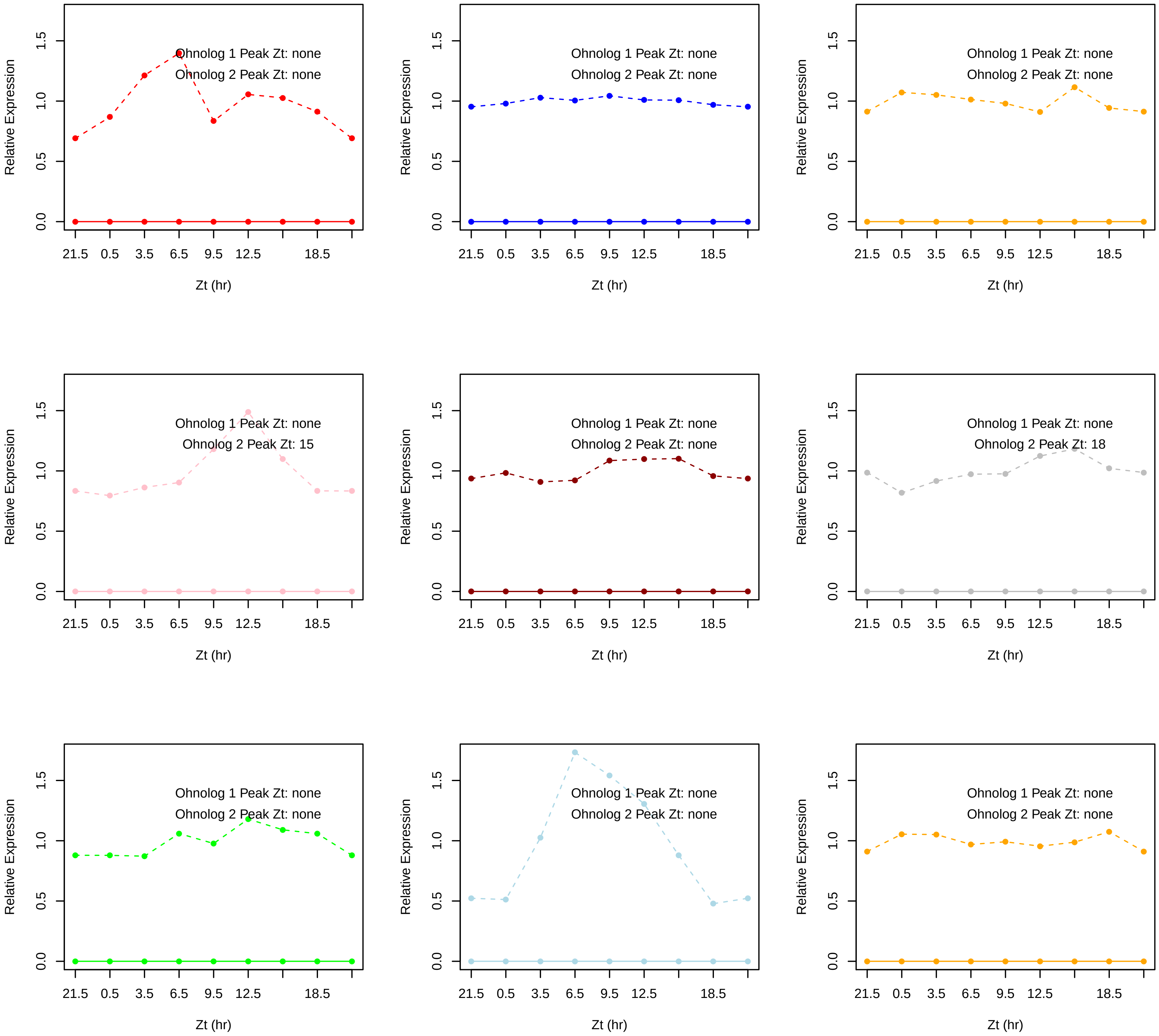

Ohnolog 1: ENSXMAG00000006136 esr2a
Ohnolog 2: ENSXMAG00000023811 esr2b

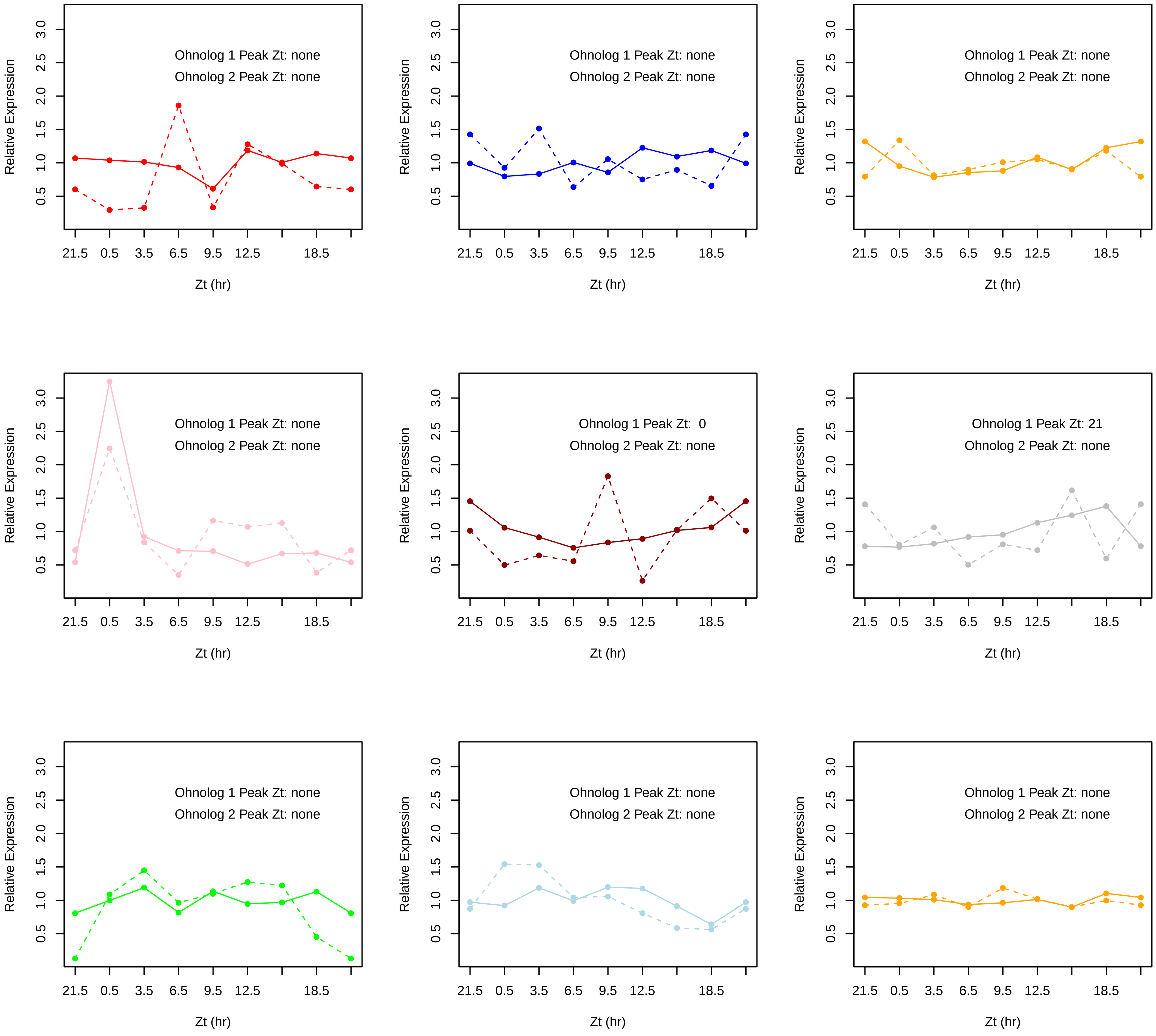

Ohnolog 1: ENSXMAG00000000875 mov10a
Ohnolog 2: ENSXMAG00000024782 mov10b.1

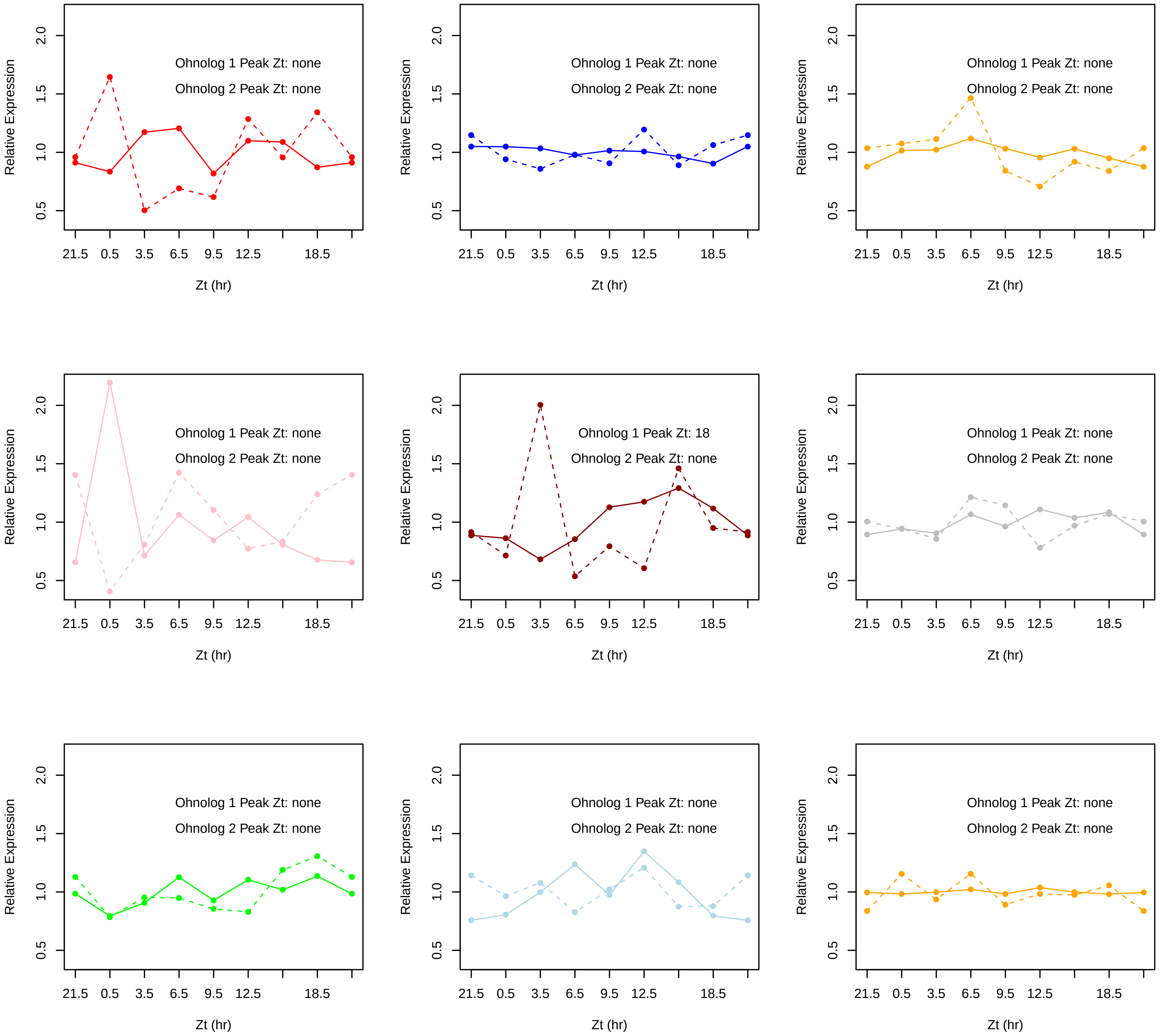

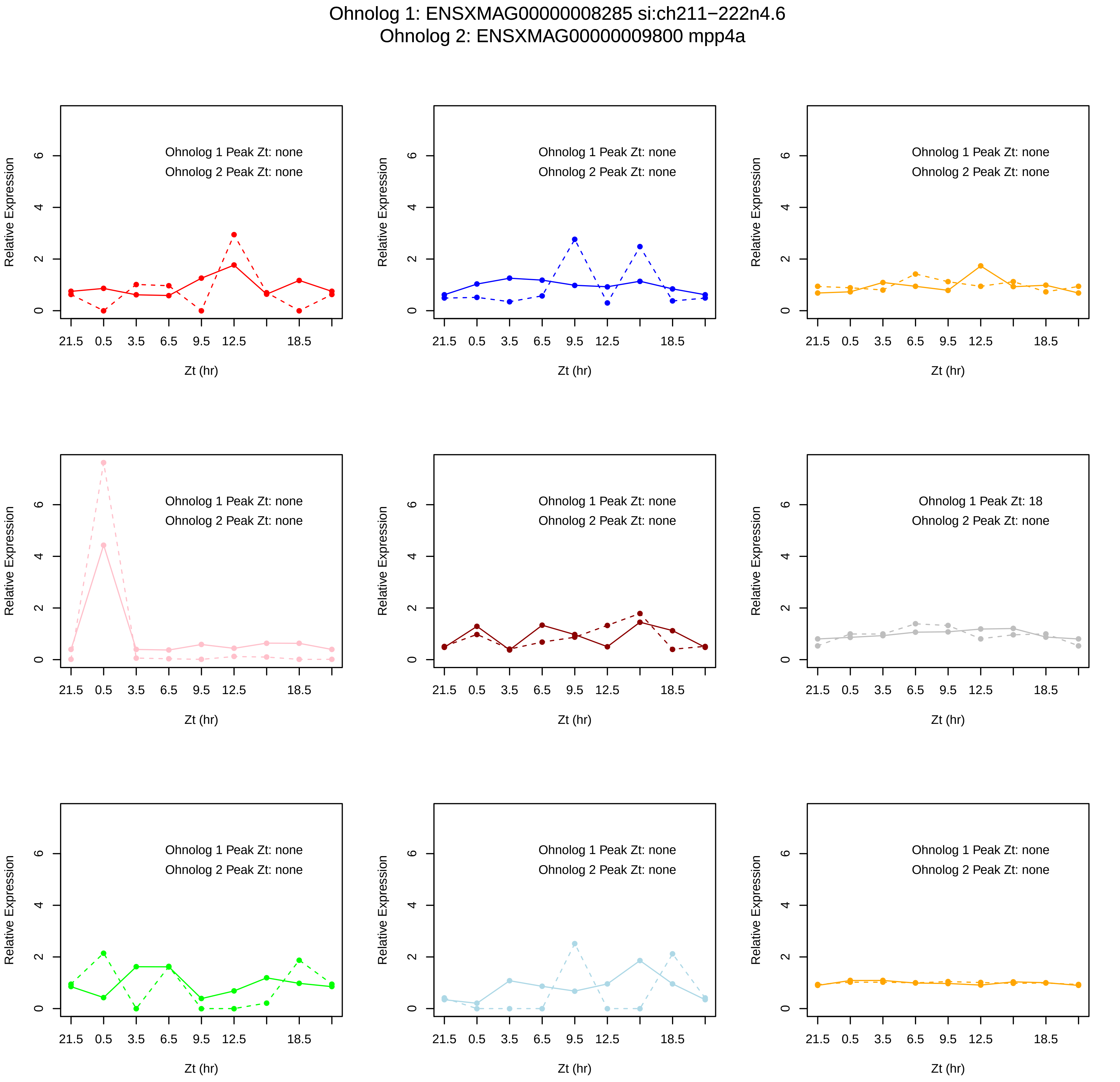

Ohnolog 1: ENSXMAG00000008285 si:ch211−222n4.6
Ohnolog 2: ENSXMAG00000009800 mpp4a
Ohnolog 1 Peak Zt: none
Ohnolog 2 Peak Zt: none
Ohnolog 1 Peak Zt: none
Ohnolog 2 Peak Zt: none
Ohnolog 1 Peak Zt: none
Ohnolog 2 Peak Zt: none
Ohnolog 1 Peak Zt: none
Ohnolog 2 Peak Zt: none
Ohnolog 1 Peak Zt: none
Ohnolog 2 Peak Zt: none
Ohnolog 1 Peak Zt: 18
Ohnolog 2 Peak Zt: none
Ohnolog 1 Peak Zt: none
Ohnolog 2 Peak Zt: none
Ohnolog 1 Peak Zt: none
Ohnolog 2 Peak Zt: none
Ohnolog 1 Peak Zt: none
Ohnolog 2 Peak Zt: none
Relative Expression
Zt (hr)
0
2
4
6
21.5
0.5
3.5
6.5
9.5
12.5
18.5

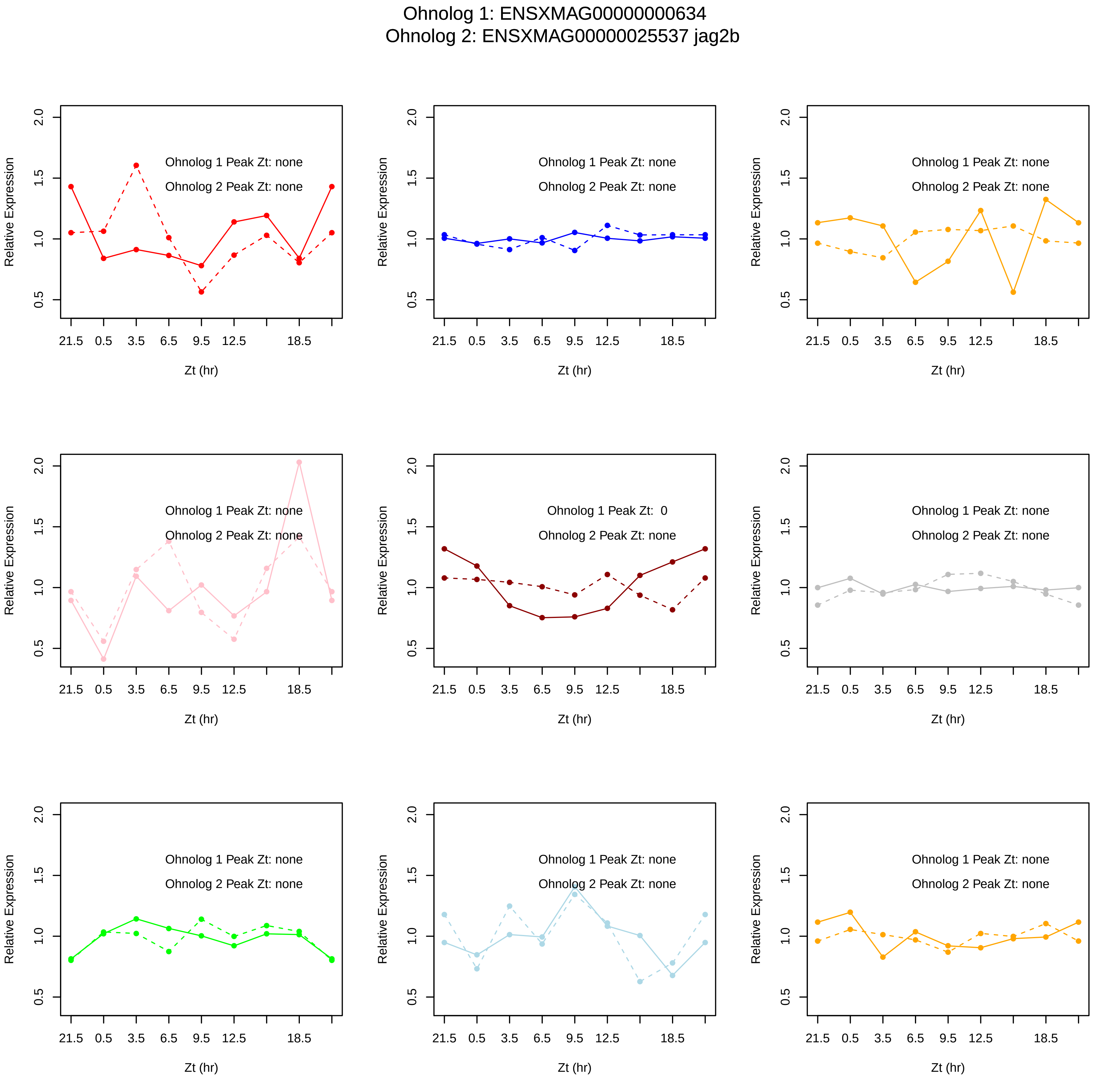

Ohnolog 1: ENSXMAG00000000634
Ohnolog 2: ENSXMAG00000025537 jag2b
Ohnolog 1 Peak Zt: none
Ohnolog 2 Peak Zt: none
Ohnolog 1 Peak Zt: 0
Relative Expression
Zt (hr)
21.5
0.5
3.5
6.5
9.5
12.5
18.5
0.5
1.0
1.5
2.0

Ohnolog 1: ENSXMAG00000012467 raph1b
Ohnolog 2: ENSXMAG00000030101 raph1a

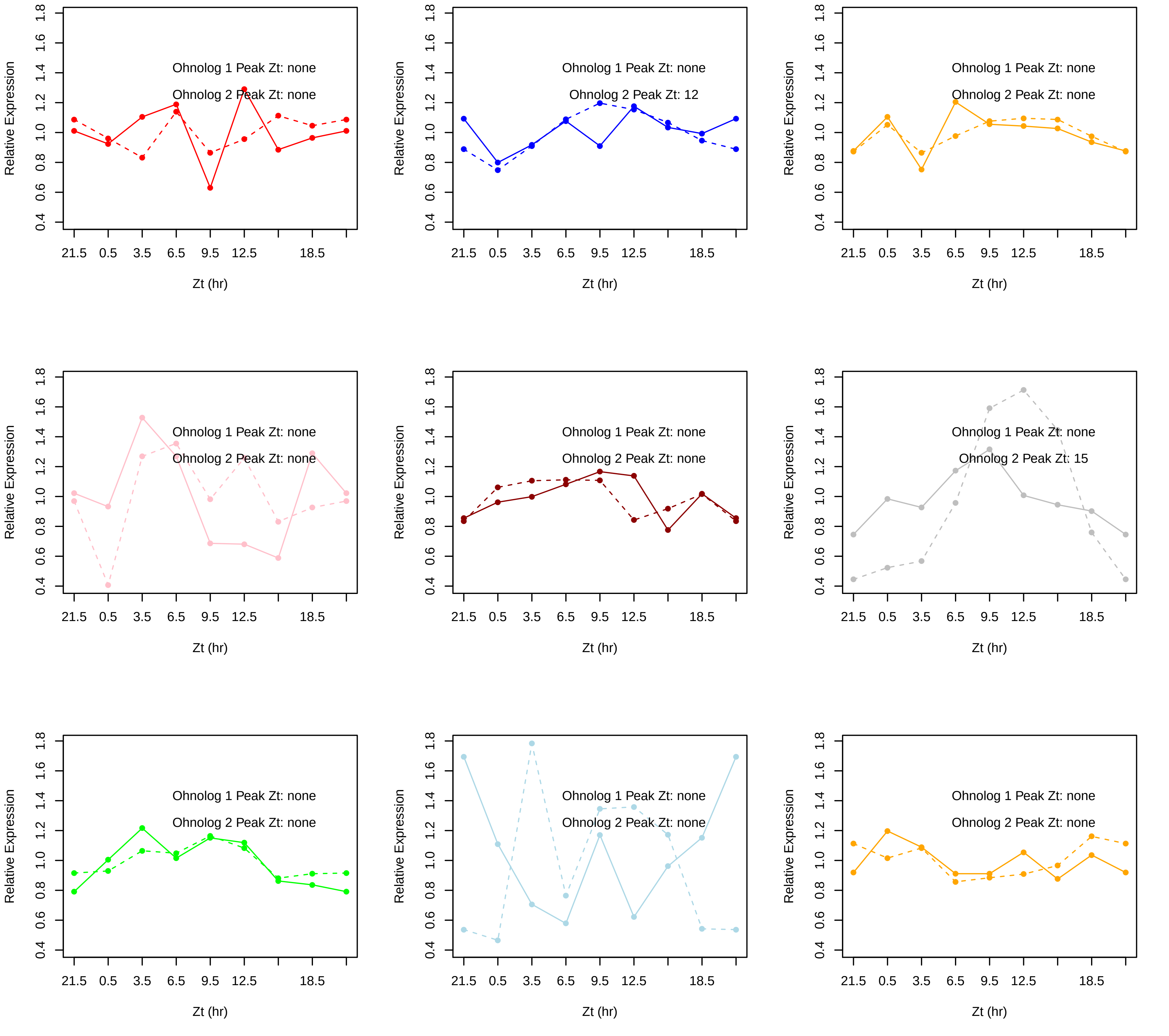

Ohnolog 1: ENSXMAG00000007579 kcnd3
Ohnolog 2: ENSXMAG00000019946 KCND3 (1 of many)

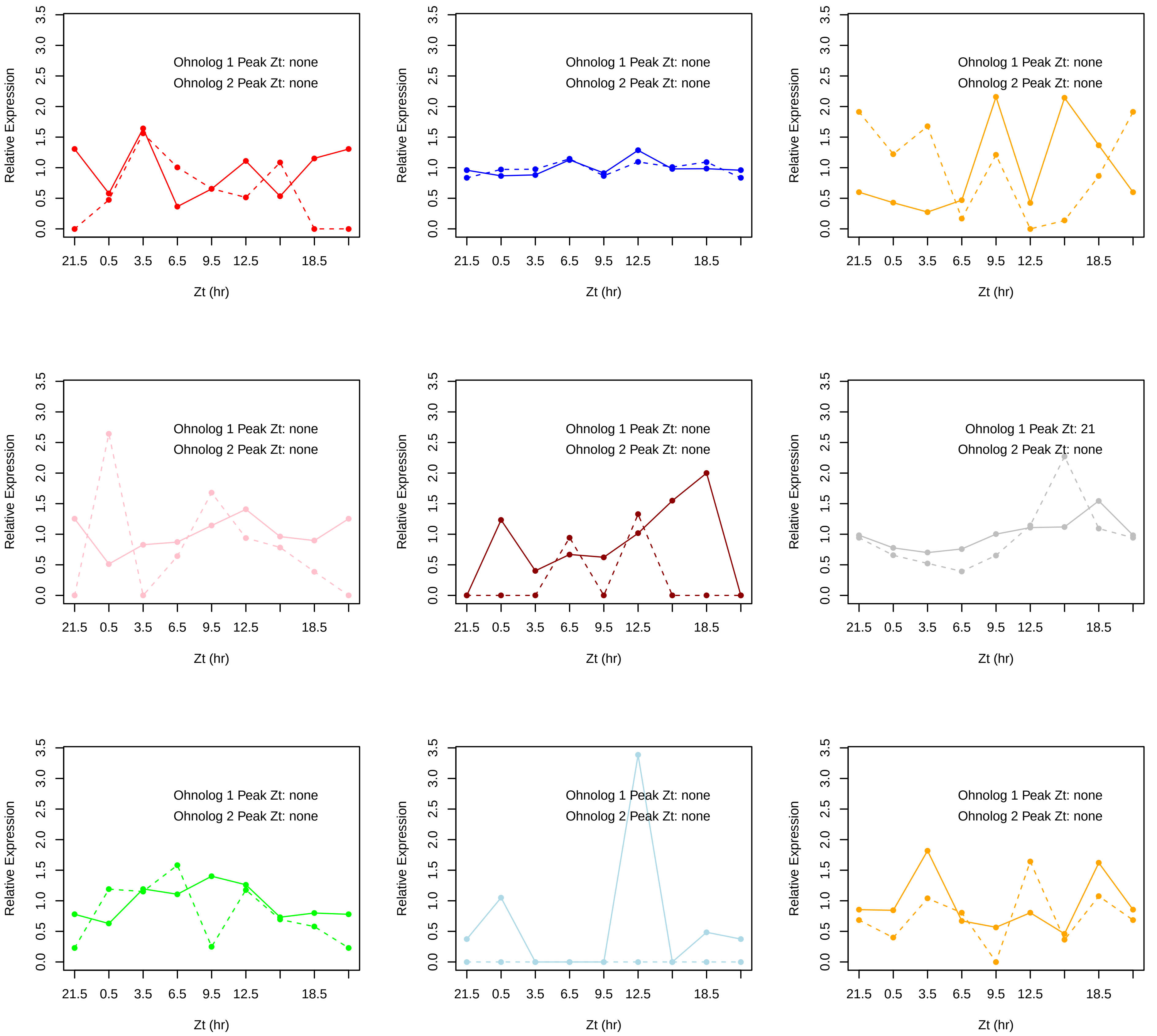

Ohnolog 1: ENSXMAG00000002387 brf1b
Ohnolog 2: ENSXMAG00000018476 BRF1 (1 of many)

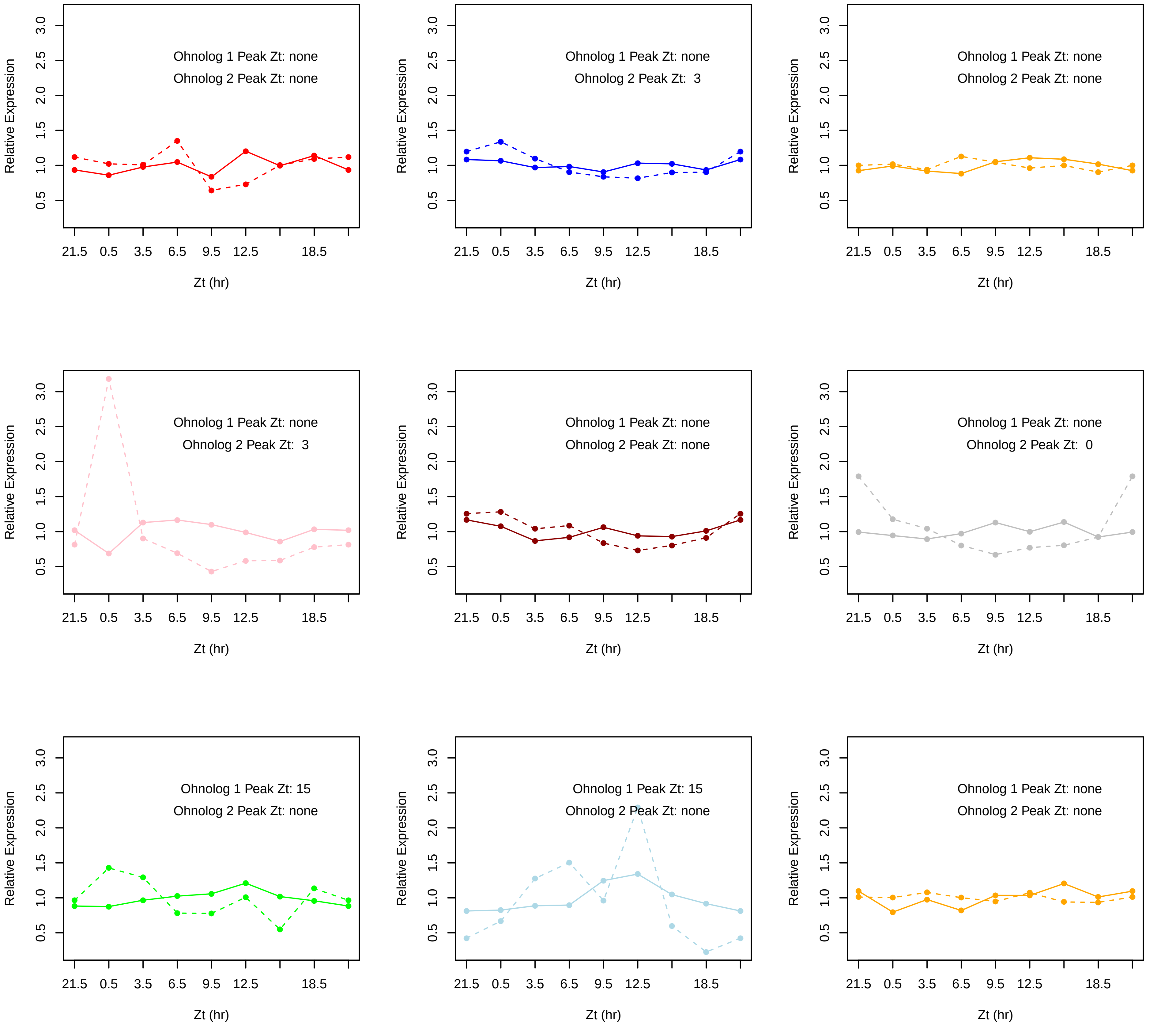

Ohnolog 1: ENSXMAG00000005986 rap1ab
Ohnolog 2: ENSXMAG00000025746

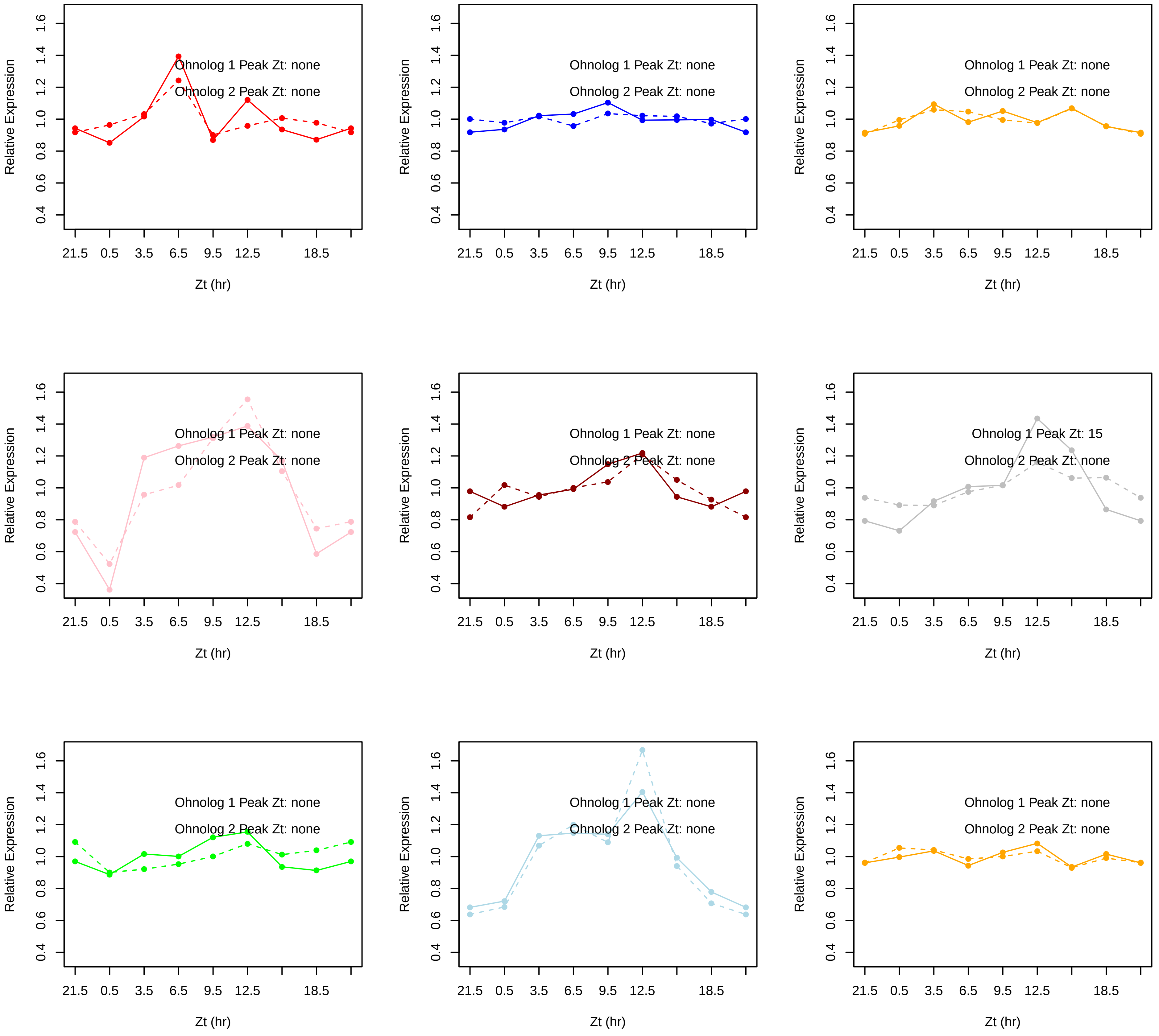

Ohnolog 1: ENSXMAG00000016541 mmp16b
Ohnolog 2: ENSXMAG00000027646 MMP16

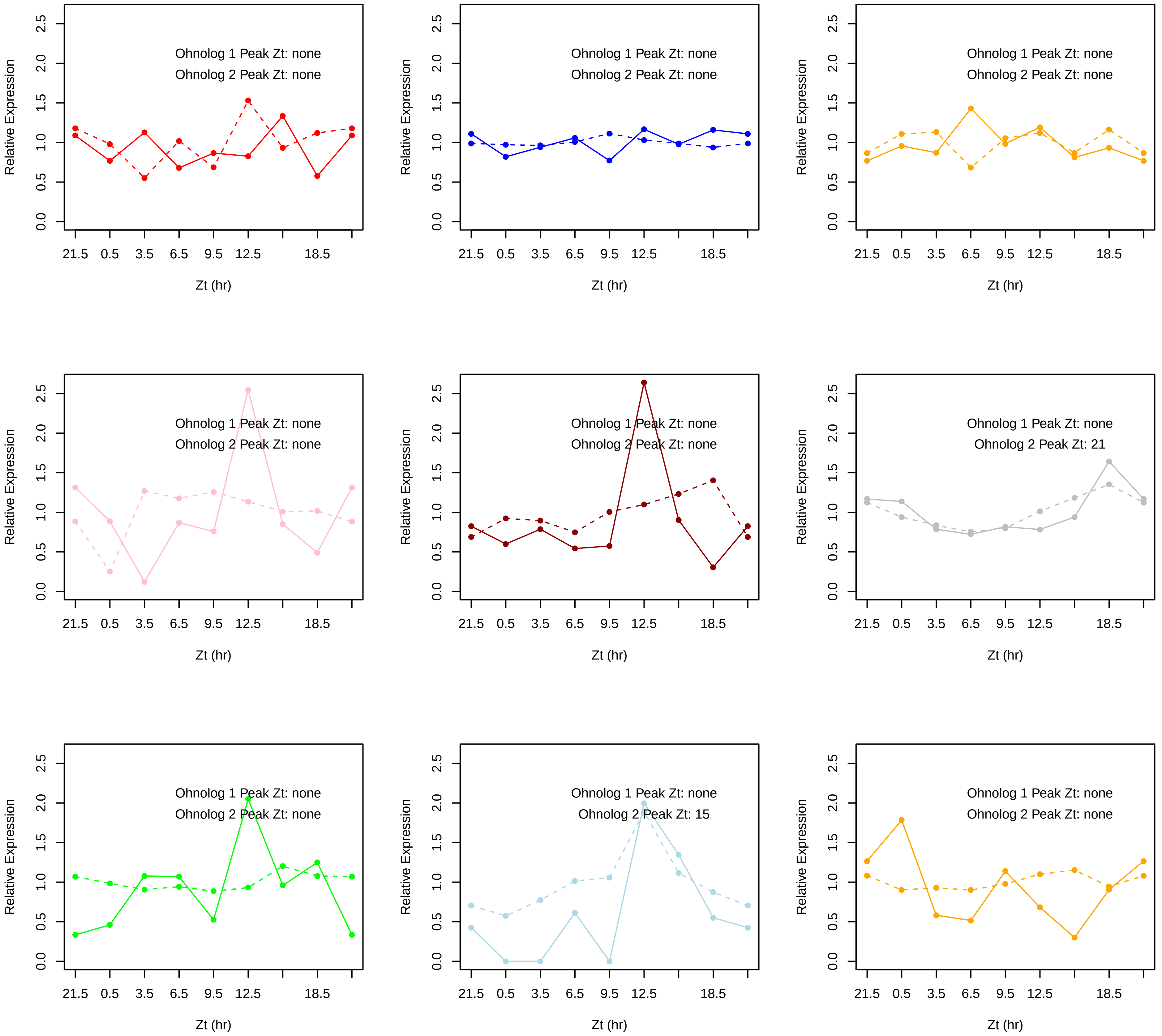

Ohnolog 1: ENSXMAG00000011330 myoc
Ohnolog 2: ENSXMAG00000028903

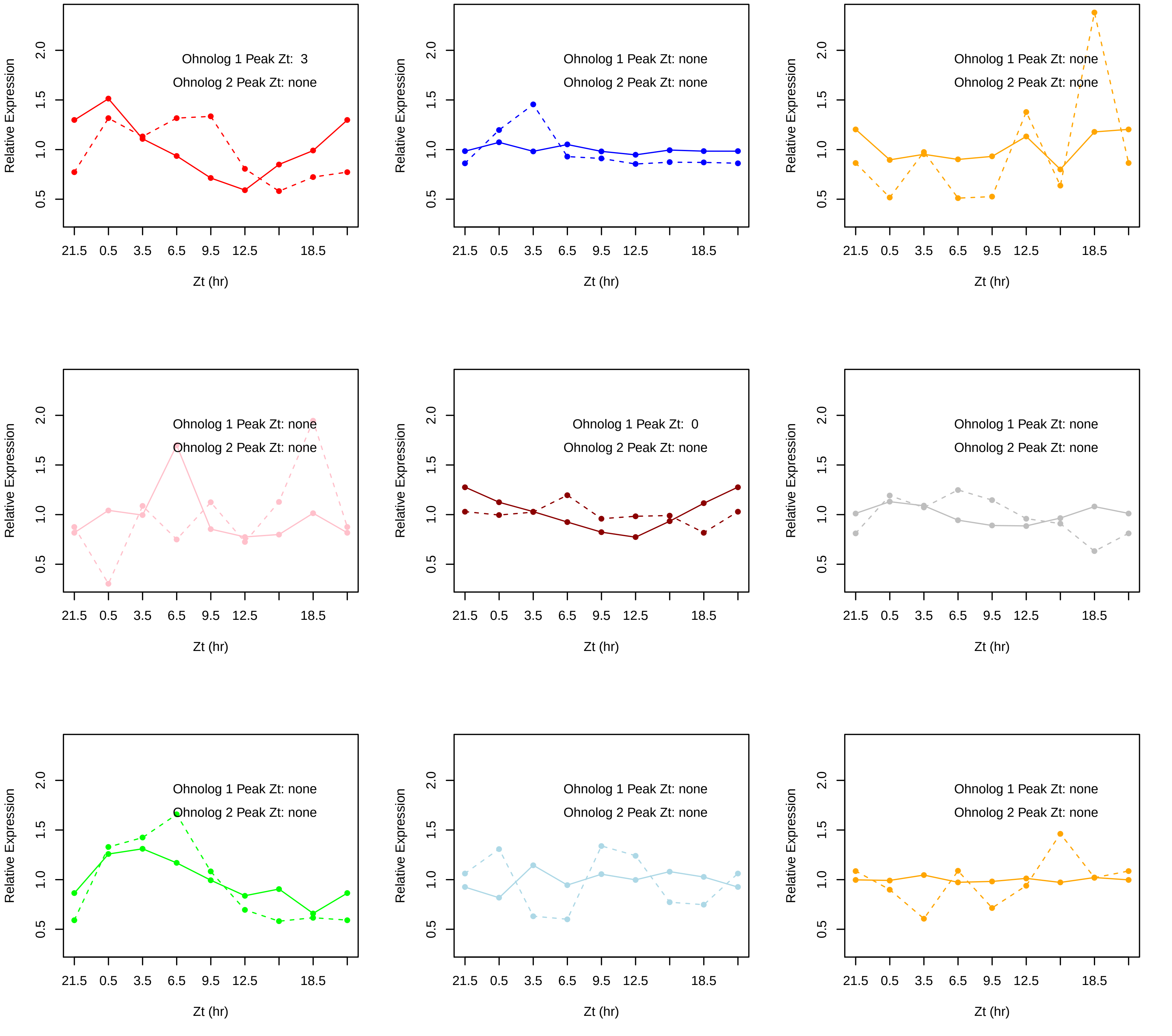

Ohnolog 1: ENSXMAG00000015437
Ohnolog 2: ENSXMAG00000023569 scoca

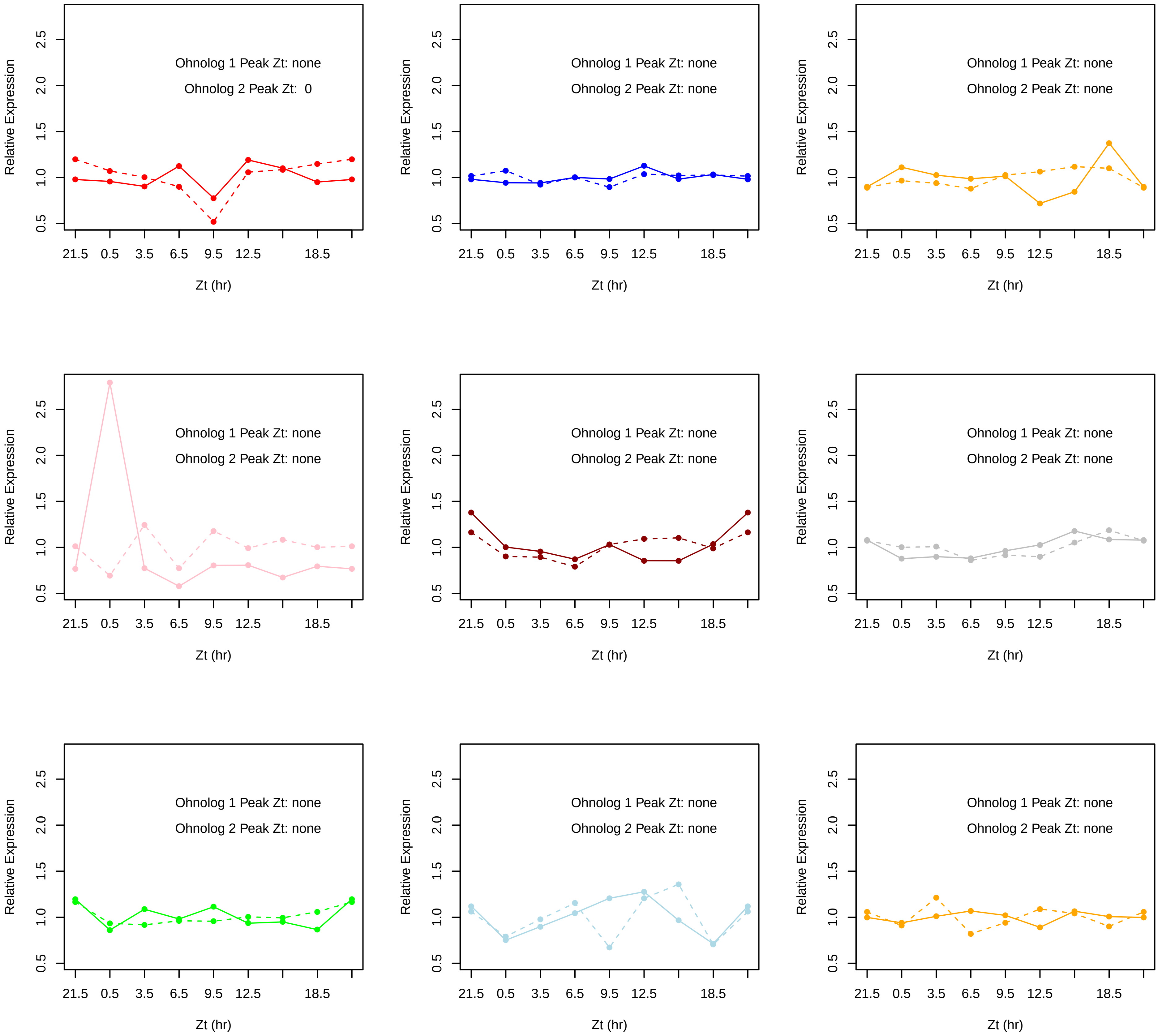

Ohnolog 1: ENSXMAG00000003333 olfml3b
Ohnolog 2: ENSXMAG00000012596 olfml3a

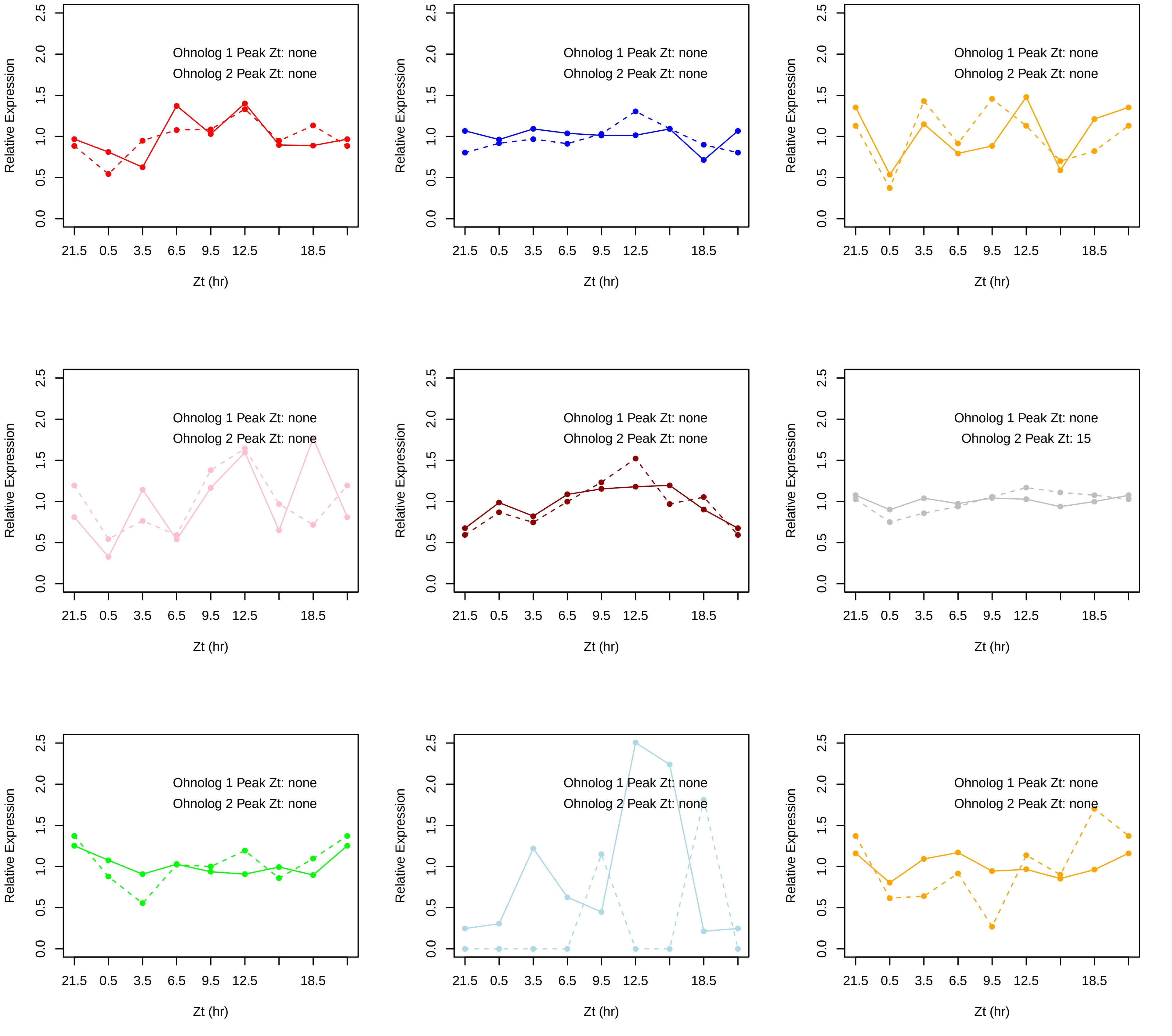

Ohnolog 1: ENSXMAG00000001522 NR3C1
Ohnolog 2: ENSXMAG00000009167 nr3c1

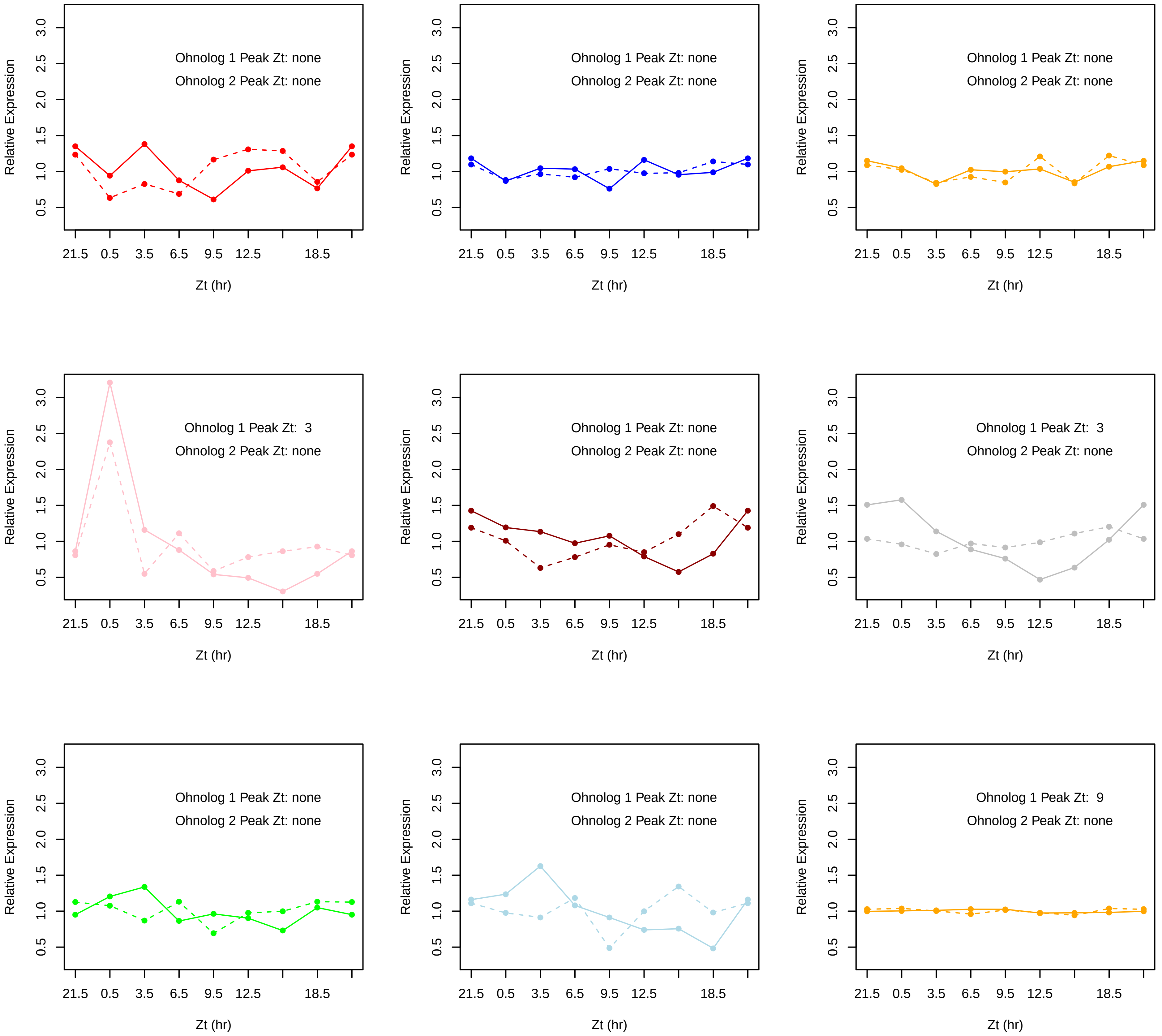

Ohnolog 1: ENSXMAG00000015104
Ohnolog 2: ENSXMAG00000018595 pik3r1

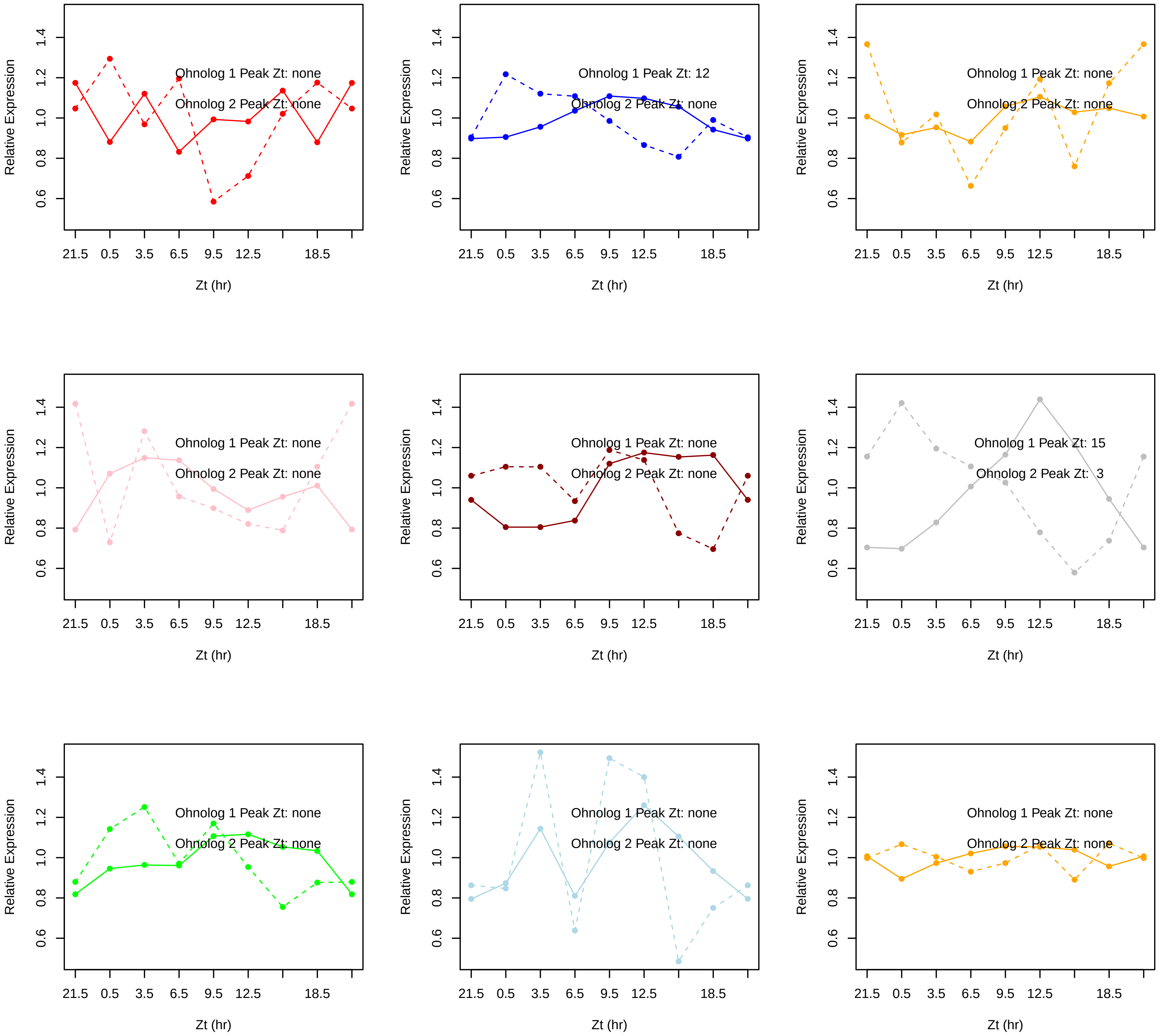

Ohnolog 1: ENSXMAG00000014805 jdp2a
Ohnolog 2: ENSXMAG00000025219 JDP2 (1 of many)

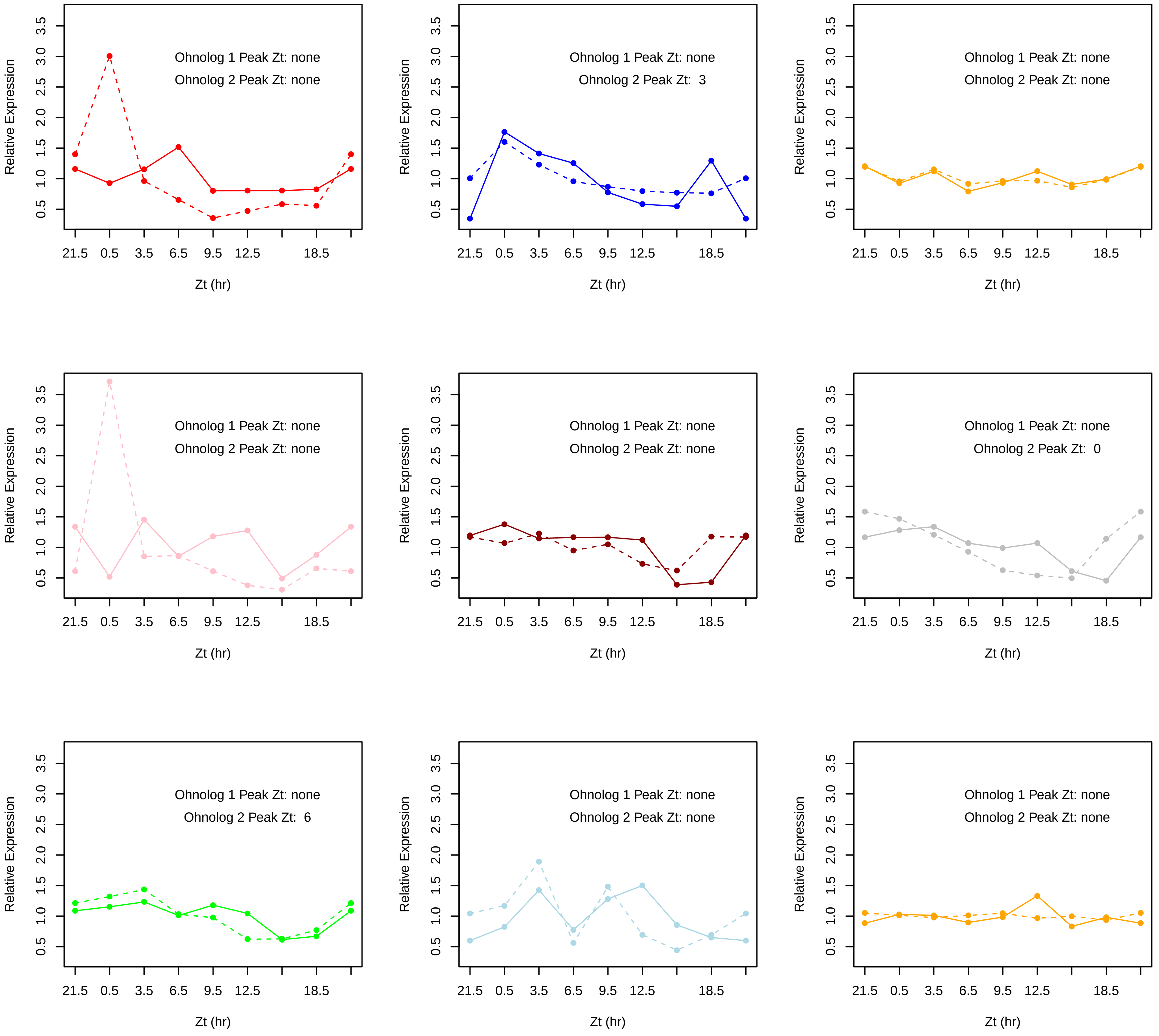

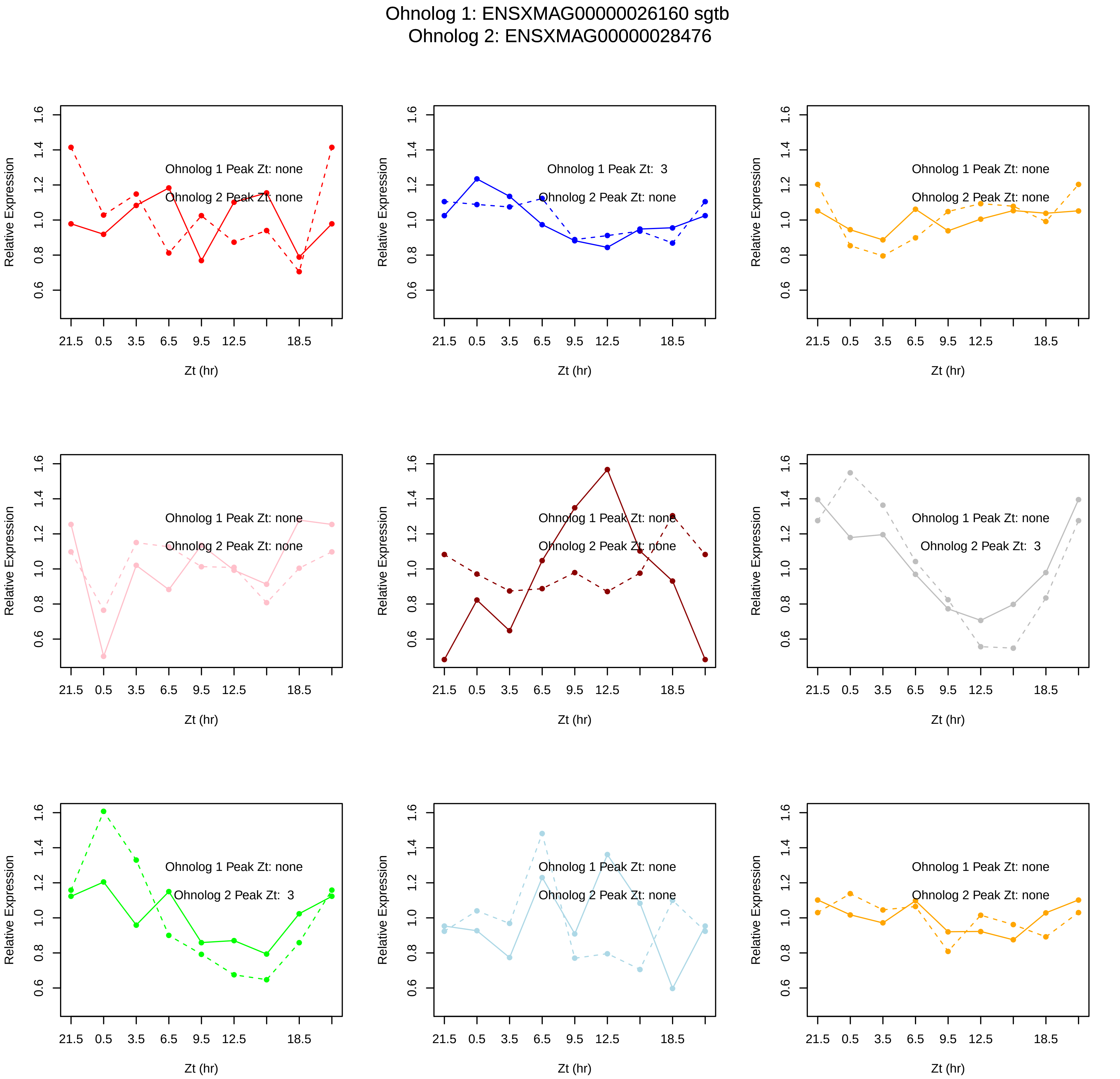
Ohnolog 1: ENSXMAG00000026160 sgtb
Ohnolog 2: ENSXMAG00000028476
Relative Expression
Zt (hr)
21.5 0.5 3.5 6.5 9.5 12.5 18.5
0.6 0.8 1.0 1.2 1.4 1.6
Ohnolog 1 Peak Zt: none
Ohnolog 2 Peak Zt: none
Ohnolog 1 Peak Zt: 3
Ohnolog 2 Peak Zt: none
Ohnolog 1 Peak Zt: none
Ohnolog 2 Peak Zt: none
Ohnolog 1 Peak Zt: none
Ohnolog 2 Peak Zt: none
Ohnolog 1 Peak Zt: none
Ohnolog 2 Peak Zt: none
Ohnolog 1 Peak Zt: none
Ohnolog 2 Peak Zt: 3
Ohnolog 1 Peak Zt: none
Ohnolog 2 Peak Zt: 3
Ohnolog 1 Peak Zt: none
Ohnolog 2 Peak Zt: none
Ohnolog 1 Peak Zt: none
Ohnolog 2 Peak Zt: none

Ohnolog 1: ENSXMAG00000005338 slc12a7b
Ohnolog 2: ENSXMAG00000010691

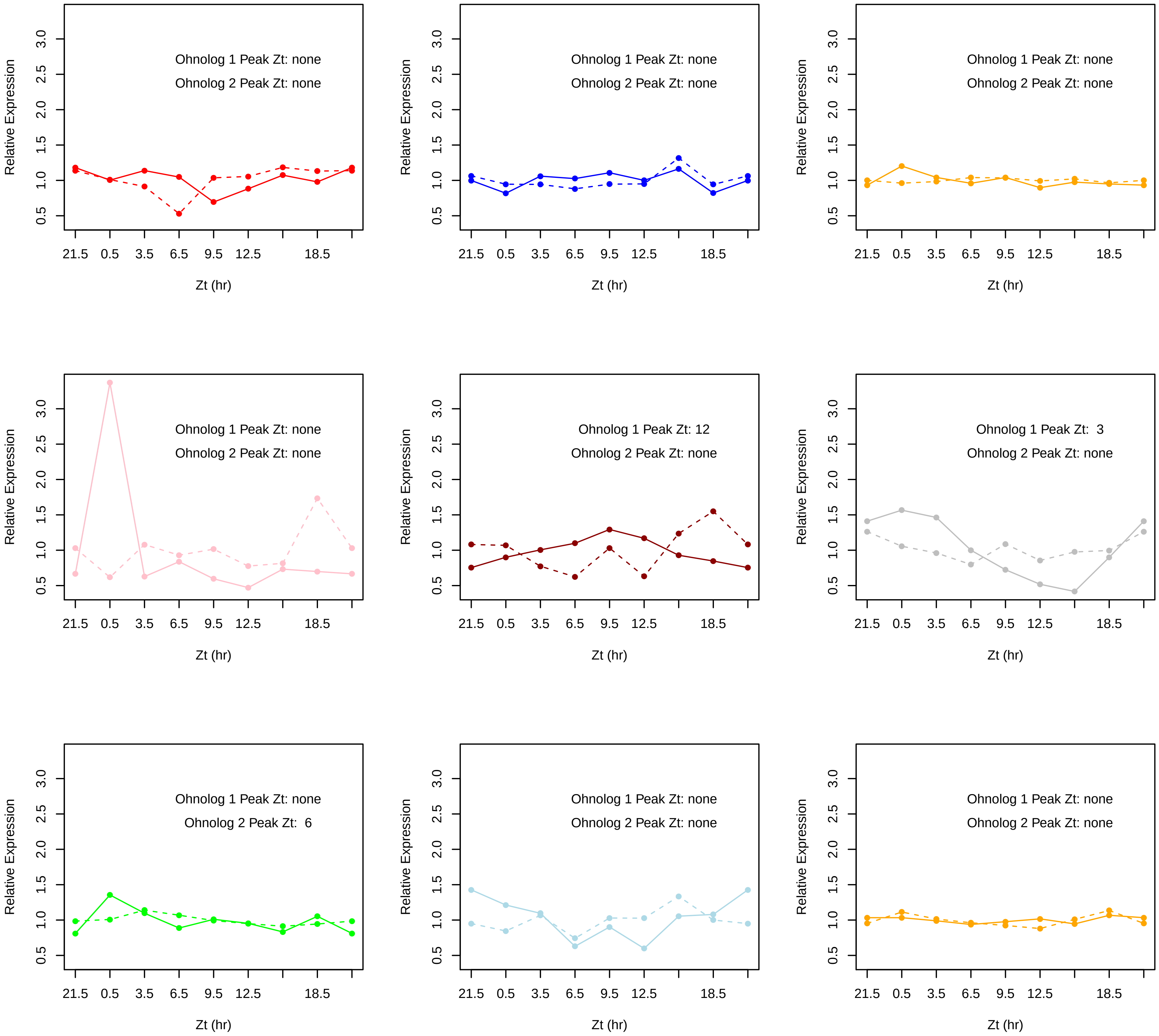

Ohnolog 1: ENSXMAG00000003970 TGFB3 (1 of many)
Ohnolog 2: ENSXMAG00000014713 tgfb3

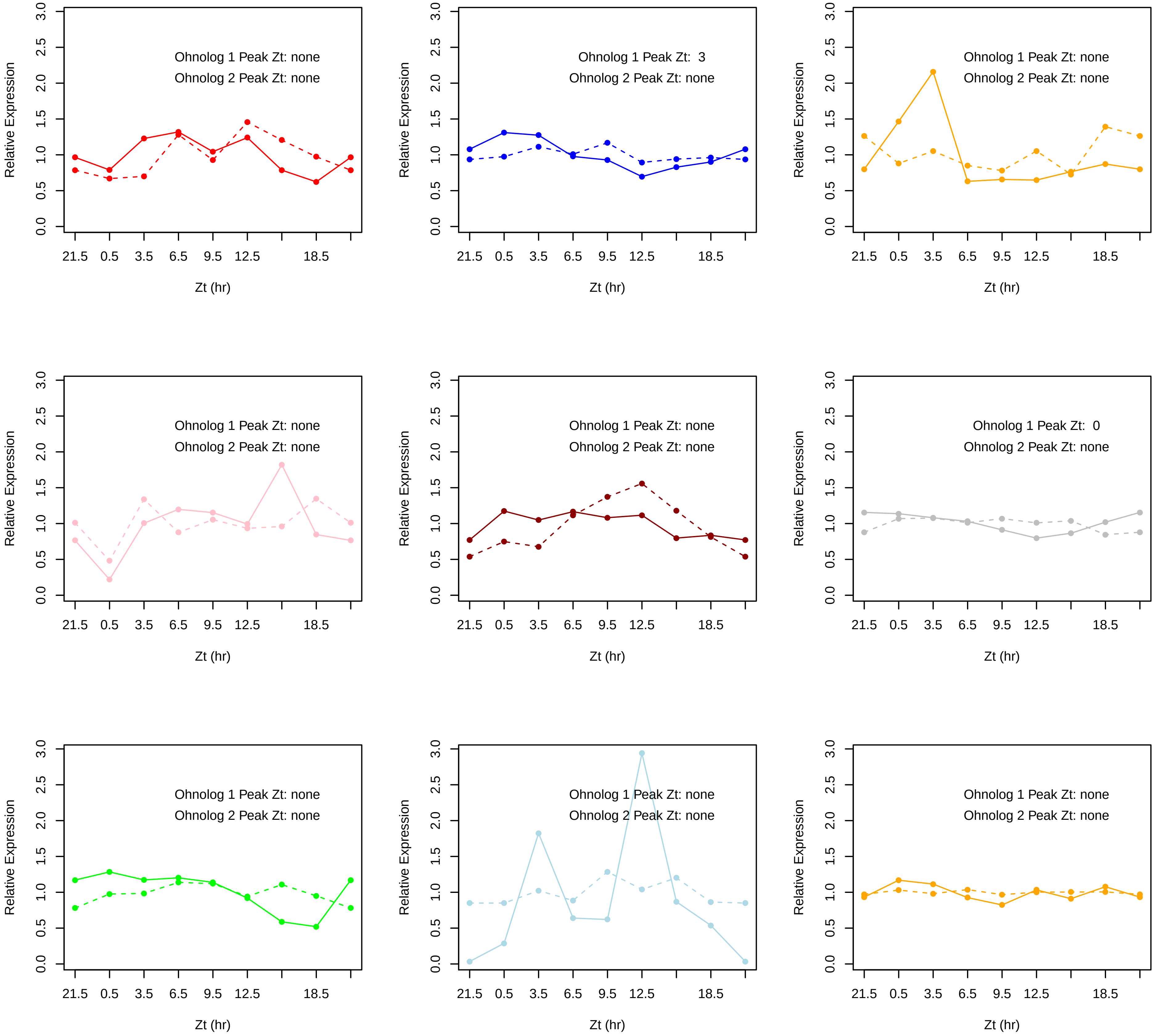

Ohnolog 1: ENSXMAG00000001608 hspa4a
Ohnolog 2: ENSXMAG00000009200 hspa4b

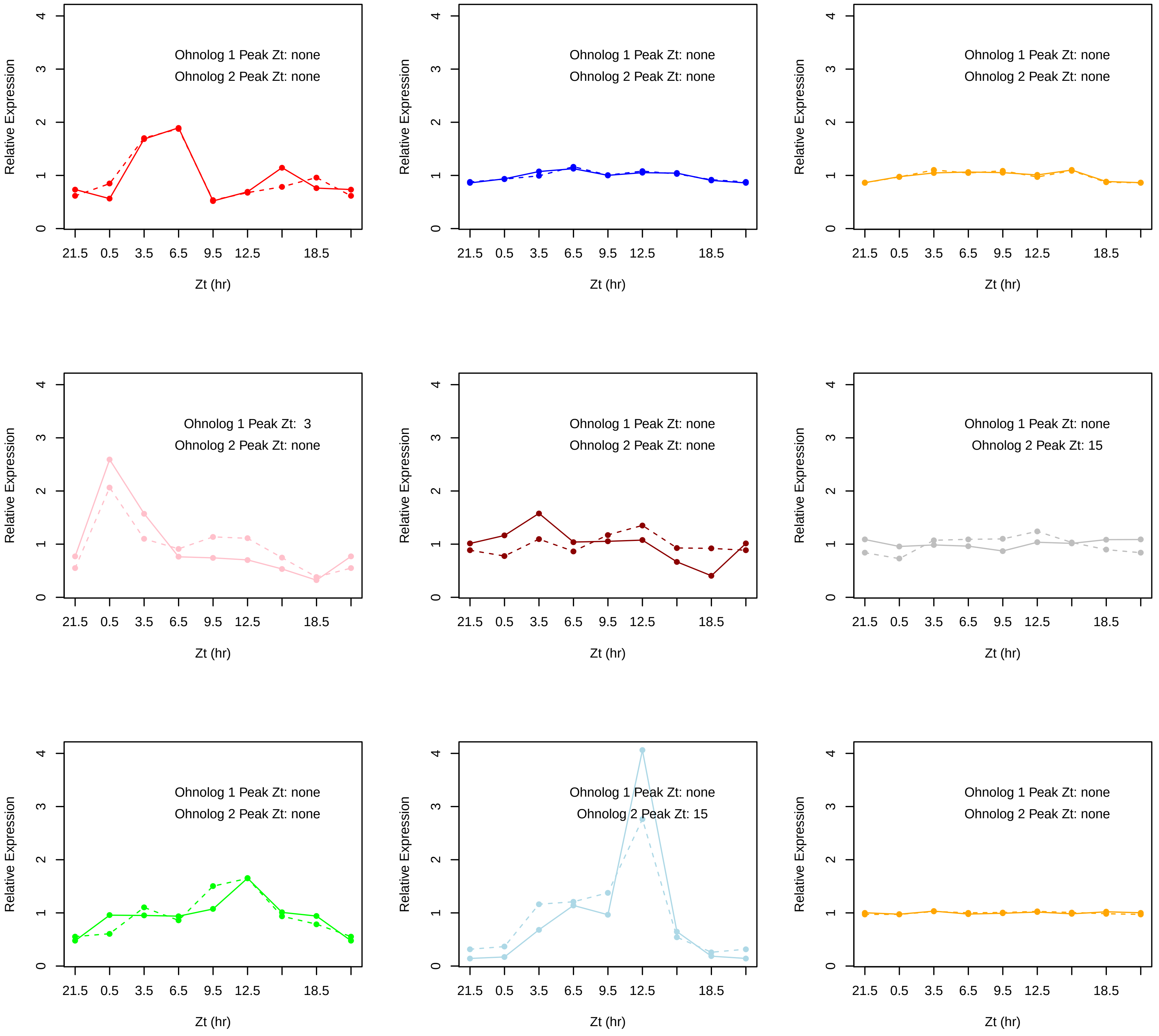

Ohnolog 1: ENSXMAG00000002816 tspan2a
Ohnolog 2: ENSXMAG00000026349

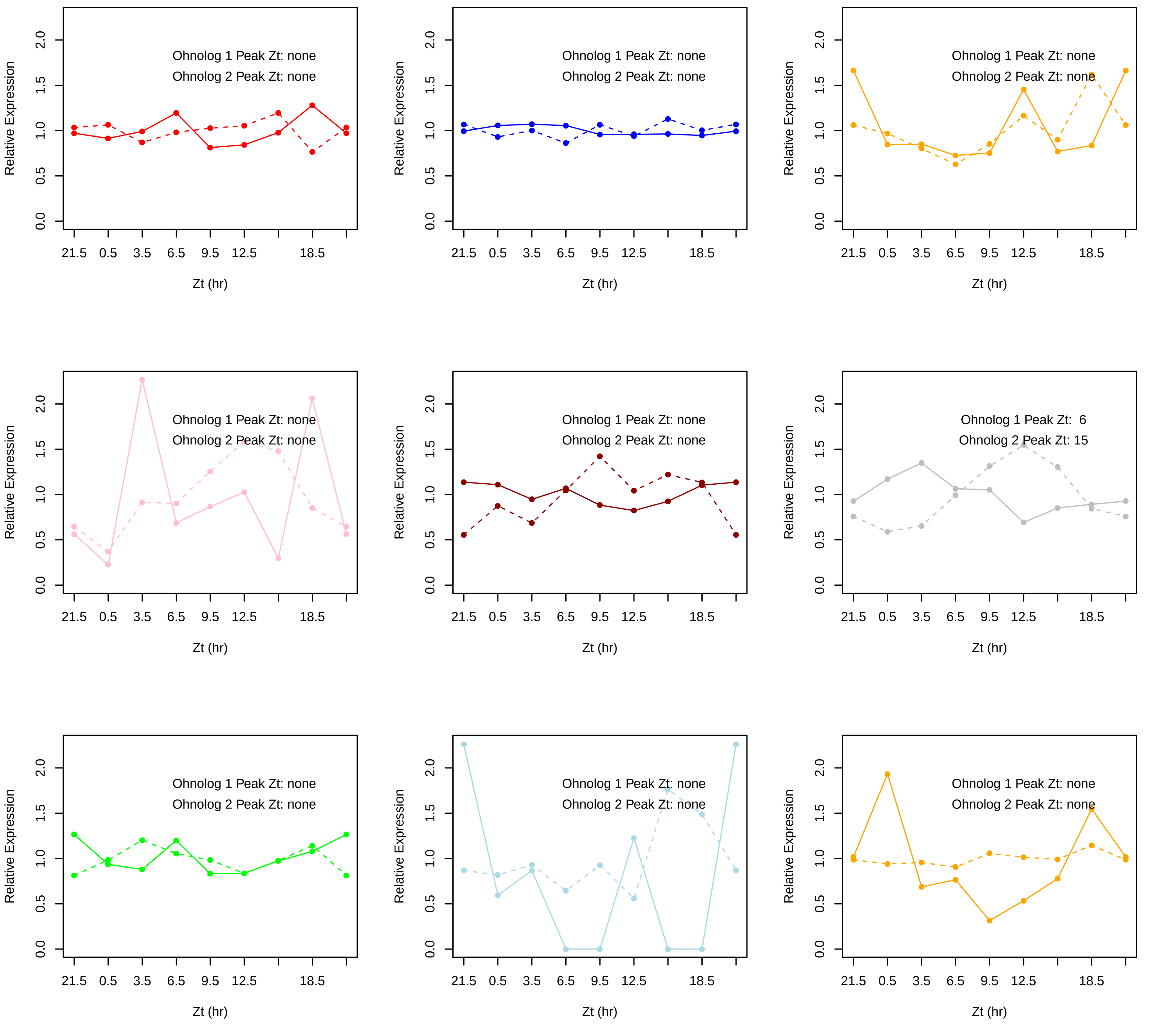

Ohnolog 1: ENSXMAG00000002805 slc25a55a
Ohnolog 2: ENSXMAG00000008253

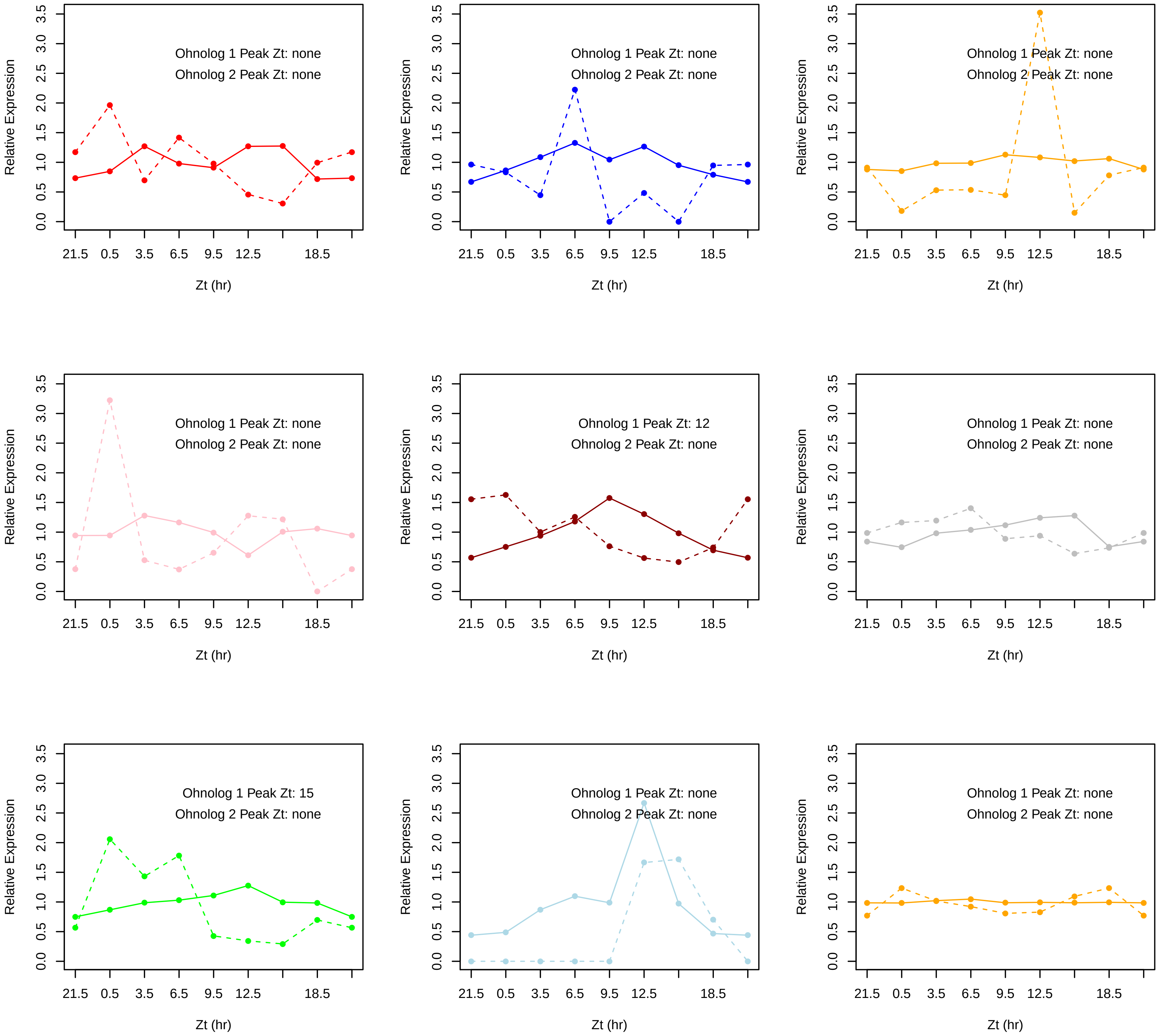

Ohnolog 1: ENSXMAG00000009263 gabra6a
Ohnolog 2: ENSXMAG00000012573 gabra6b

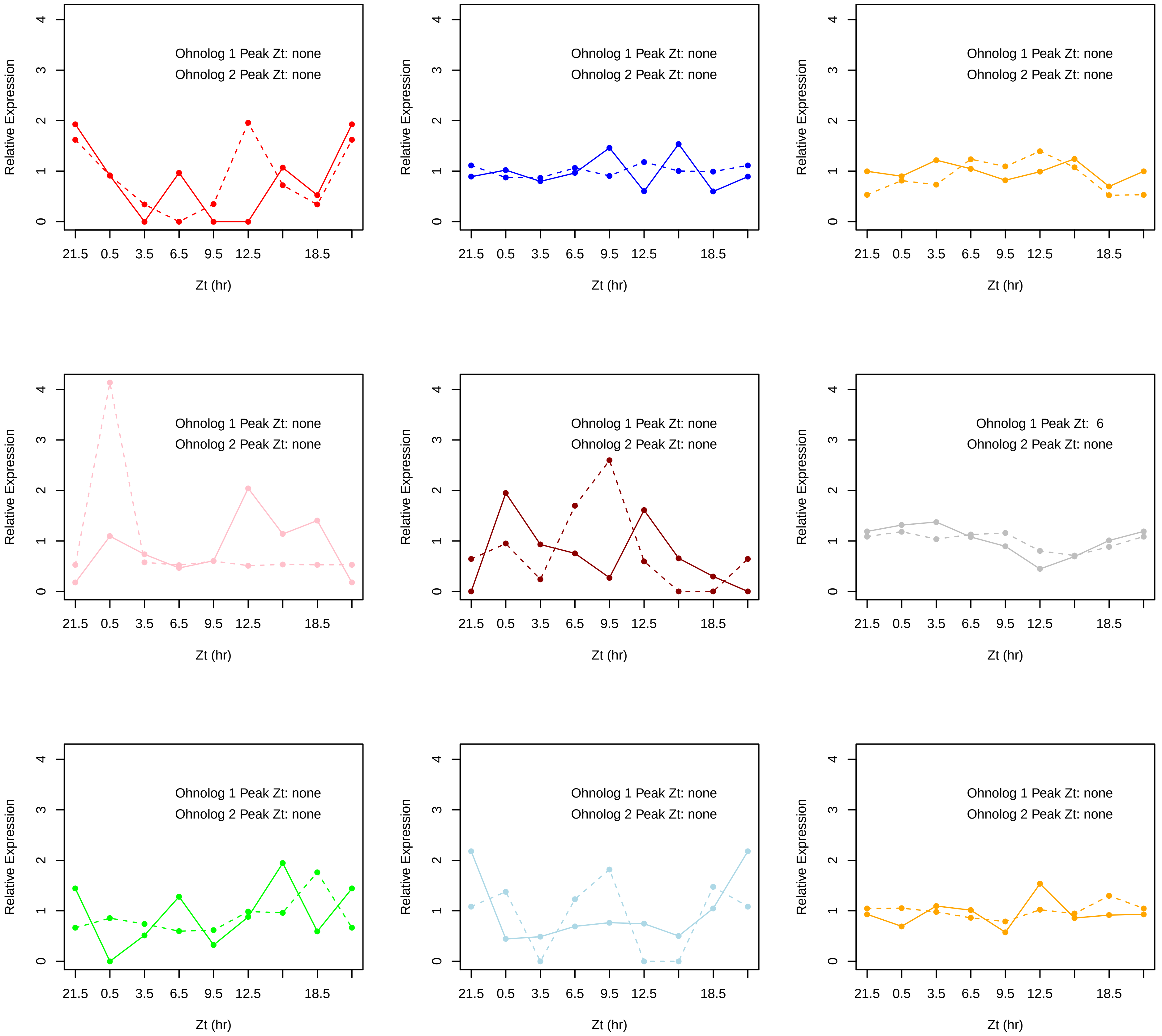

Ohnolog 1: ENSXMAG00000012670 atp5pf
Ohnolog 2: ENSXMAG00000021579

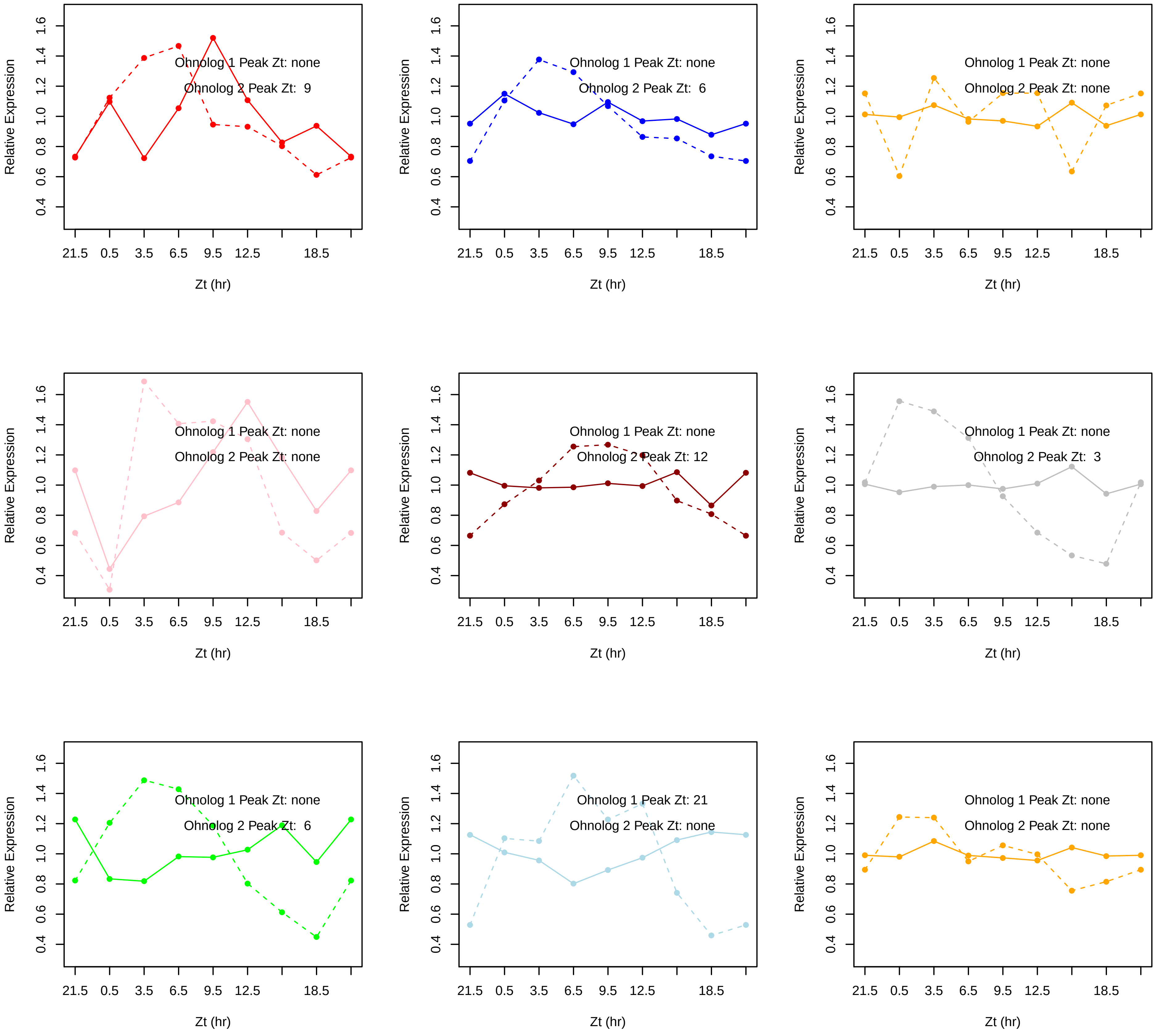

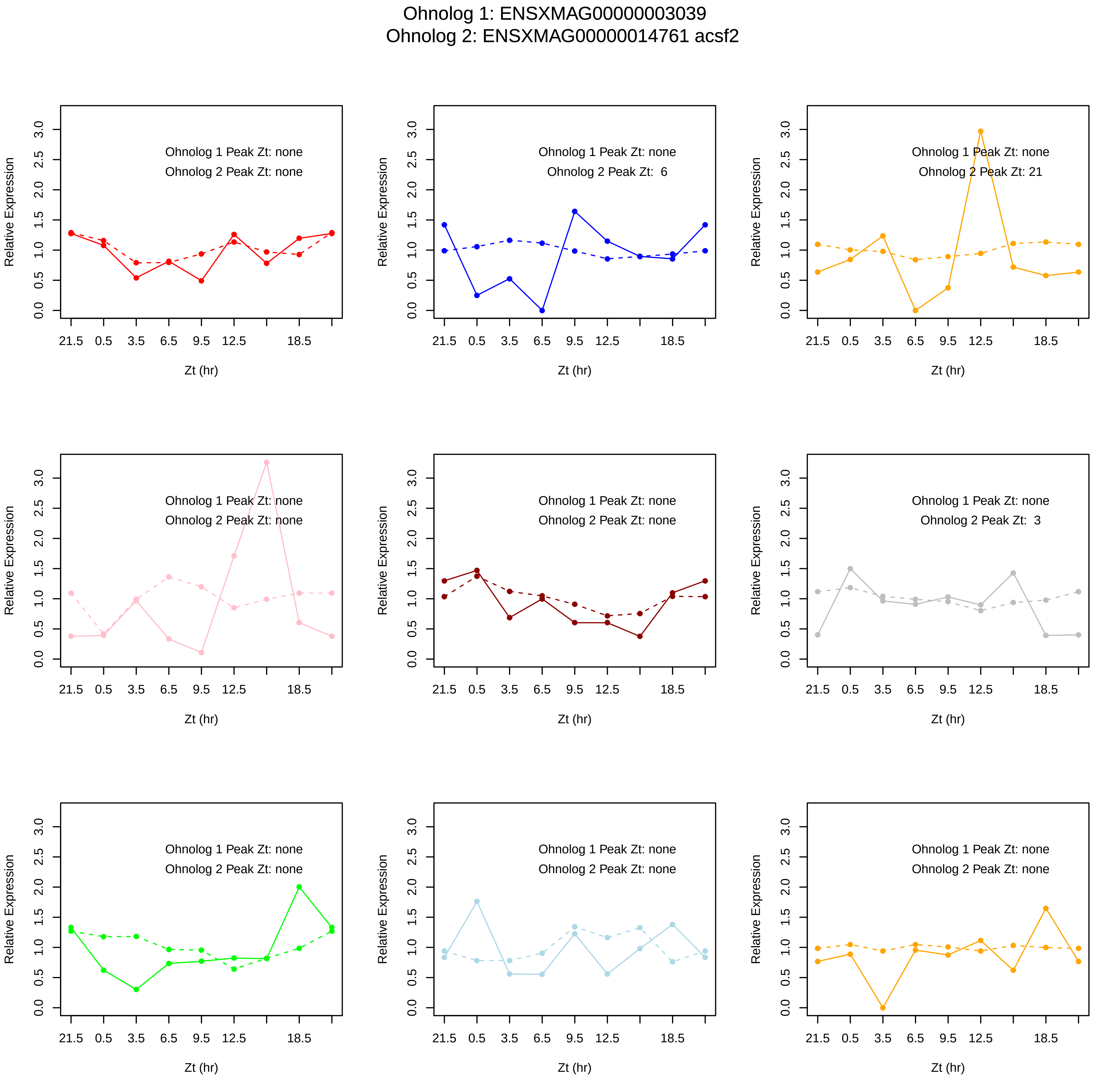
Ohnolog 1: ENSXMAG00000003039
Ohnolog 2: ENSXMAG00000014761 acsf2
Ohnolog 1 Peak Zt: none
Ohnolog 2 Peak Zt: none
Ohnolog 1 Peak Zt: none
Ohnolog 2 Peak Zt: 6
Ohnolog 1 Peak Zt: none
Ohnolog 2 Peak Zt: 21
Ohnolog 1 Peak Zt: none
Ohnolog 2 Peak Zt: none
Ohnolog 1 Peak Zt: none
Ohnolog 2 Peak Zt: none
Ohnolog 1 Peak Zt: none
Ohnolog 2 Peak Zt: 3
Ohnolog 1 Peak Zt: none
Ohnolog 2 Peak Zt: none
Ohnolog 1 Peak Zt: none
Ohnolog 2 Peak Zt: none
Ohnolog 1 Peak Zt: none
Ohnolog 2 Peak Zt: none
Relative Expression
Zt (hr)
21.5 0.5 3.5 6.5 9.5 12.5 18.5
0.0 0.5 1.0 1.5 2.0 2.5 3.0

Ohnolog 1: ENSXMAG00000000011 si:ch73−281f12.4
Ohnolog 2: ENSXMAG00000003234 si:ch211−176g6.2

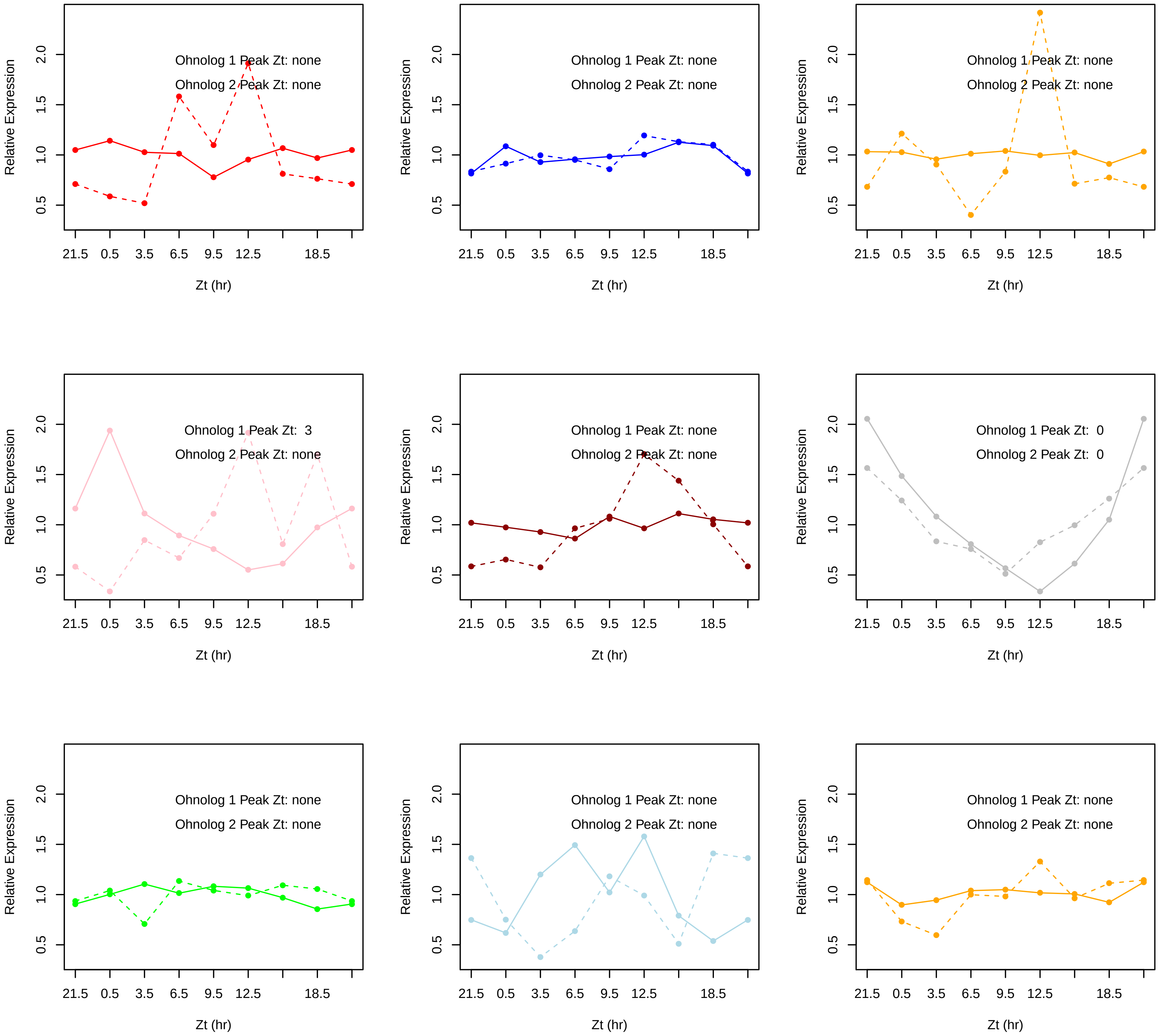

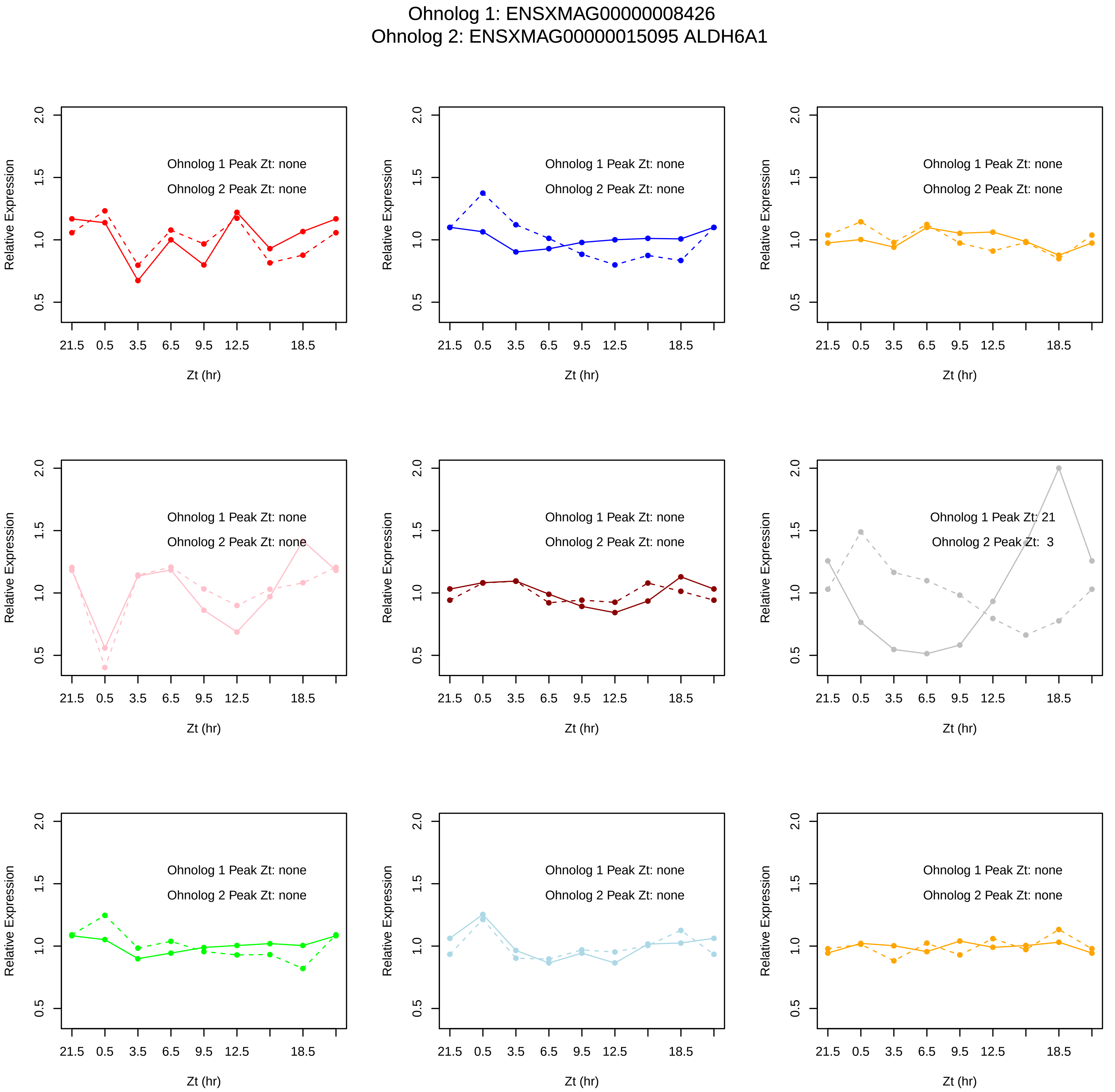
Ohnolog 1: ENSXMAG00000008426
Ohnolog 2: ENSXMAG00000015095 ALDH6A1
Ohnolog 1 Peak Zt: none
Ohnolog 2 Peak Zt: none
Ohnolog 1 Peak Zt: 21
Ohnolog 2 Peak Zt: 3
Relative Expression
Zt (hr)
21.5
0.5
3.5
6.5
9.5
12.5
18.5
0.5
1.0
1.5
2.0

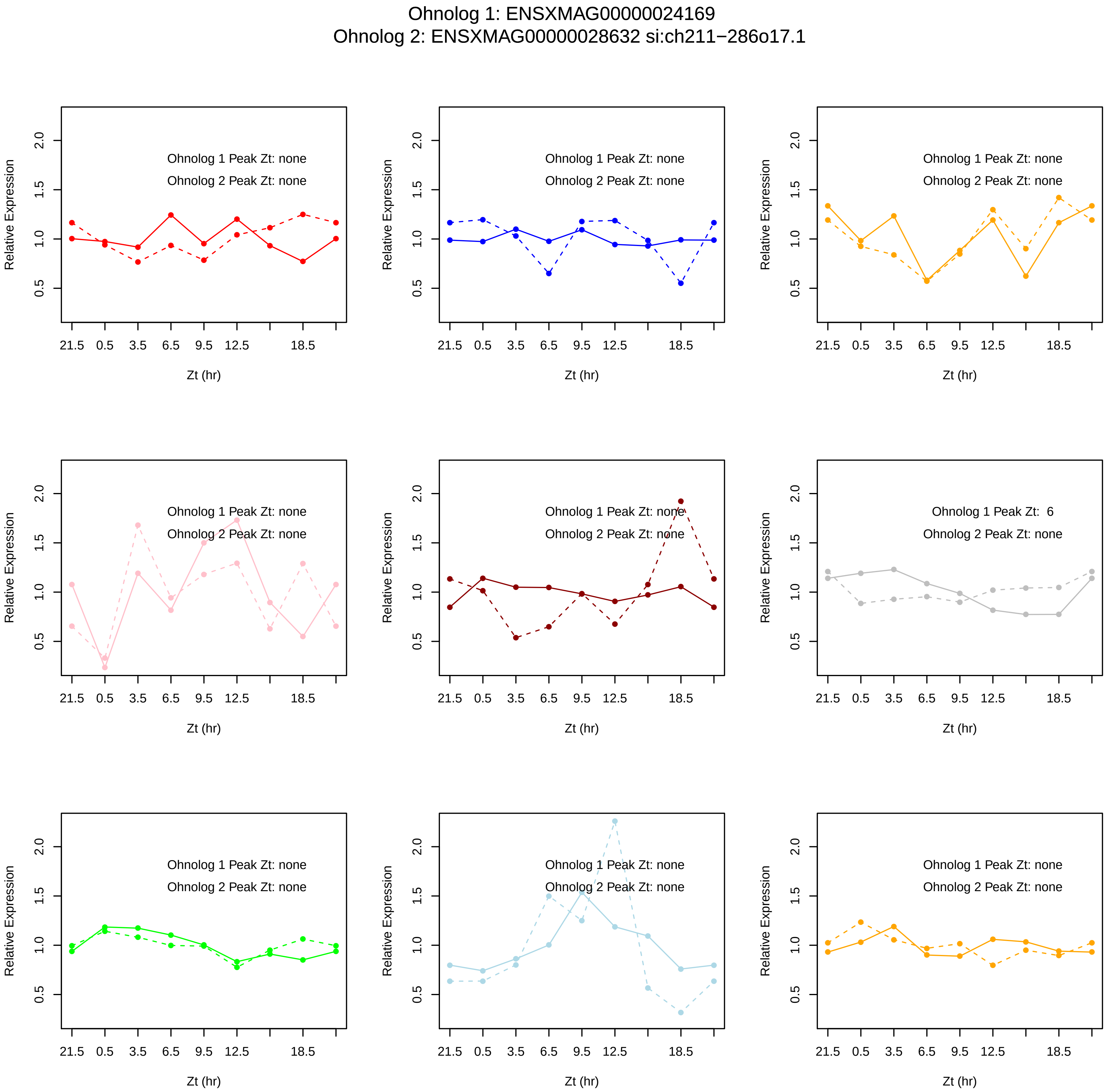
Ohnolog 1: ENSXMAG00000024169
Ohnolog 2: ENSXMAG00000028632 si:ch211−286o17.1
Ohnolog 1 Peak Zt: none
Ohnolog 2 Peak Zt: none
Ohnolog 1 Peak Zt: none
Ohnolog 2 Peak Zt: none
Ohnolog 1 Peak Zt: none
Ohnolog 2 Peak Zt: none
Ohnolog 1 Peak Zt: none
Ohnolog 2 Peak Zt: none
Ohnolog 1 Peak Zt: none
Ohnolog 2 Peak Zt: none
Ohnolog 1 Peak Zt: 6
Ohnolog 2 Peak Zt: none
Ohnolog 1 Peak Zt: none
Ohnolog 2 Peak Zt: none
Ohnolog 1 Peak Zt: none
Ohnolog 2 Peak Zt: none
Ohnolog 1 Peak Zt: none
Ohnolog 2 Peak Zt: none
Relative Expression
Zt (hr)
21.5 0.5 3.5 6.5 9.5 12.5 18.5
0.5 1.0 1.5 2.0

Ohnolog 1: ENSXMAG00000015236 cipca
Ohnolog 2: ENSXMAG00000025371 cipcb

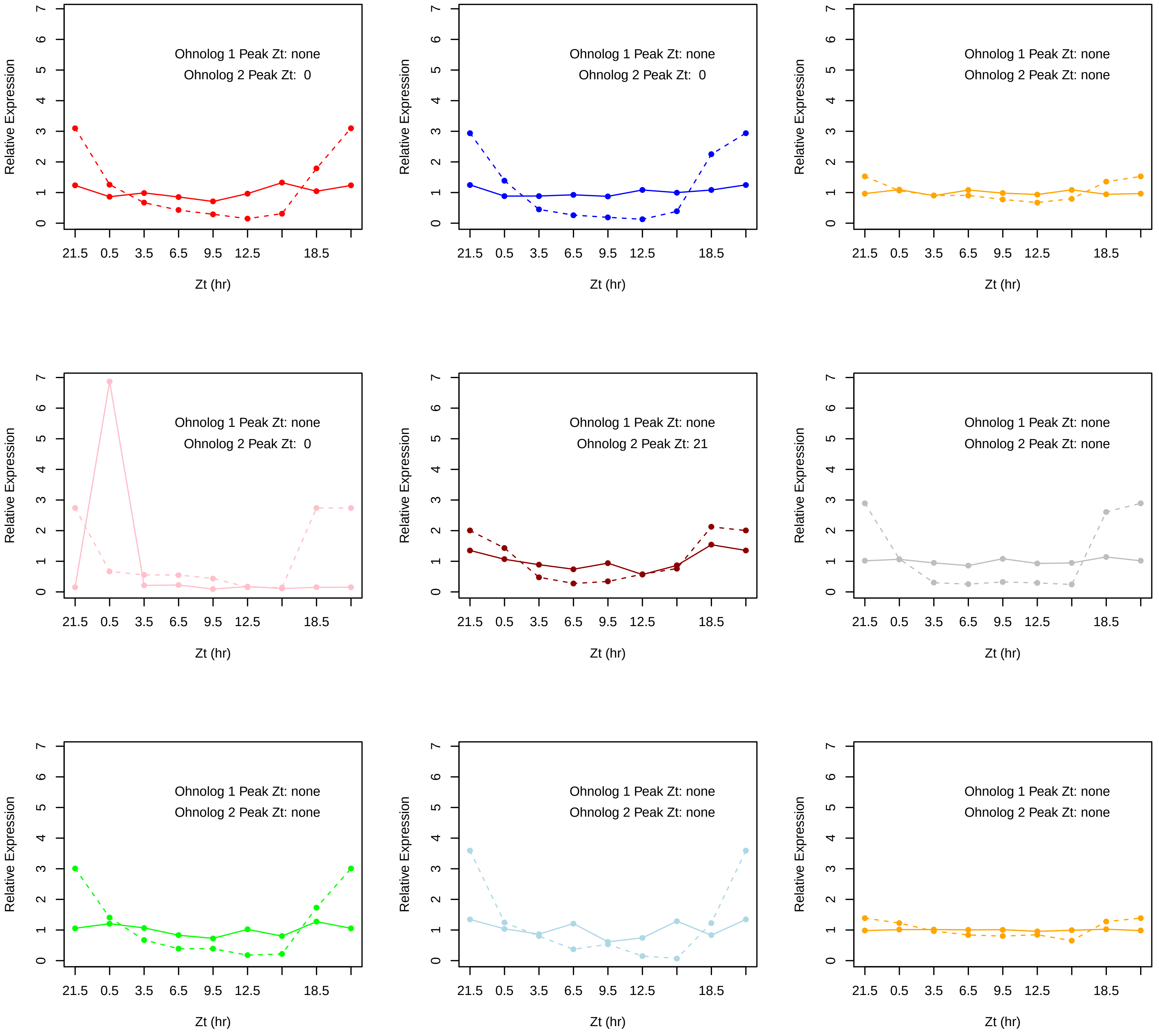

Ohnolog 1: ENSXMAG00000002157 LPIN2 (1 of many)
Ohnolog 2: ENSXMAG00000016718 lpin2

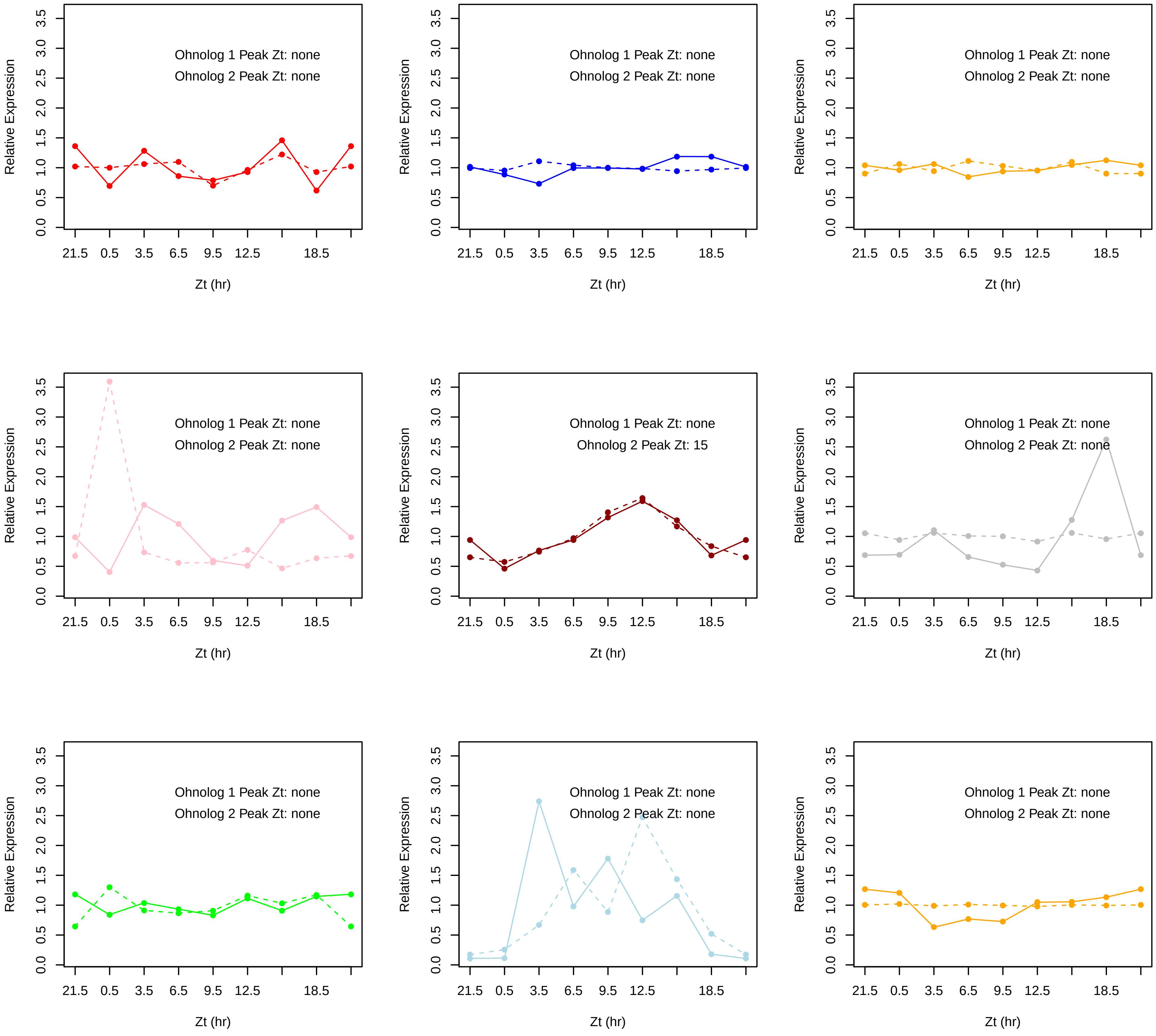

Ohnolog 1: ENSXMAG00000006394
Ohnolog 2: ENSXMAG00000008554 pdgfrb

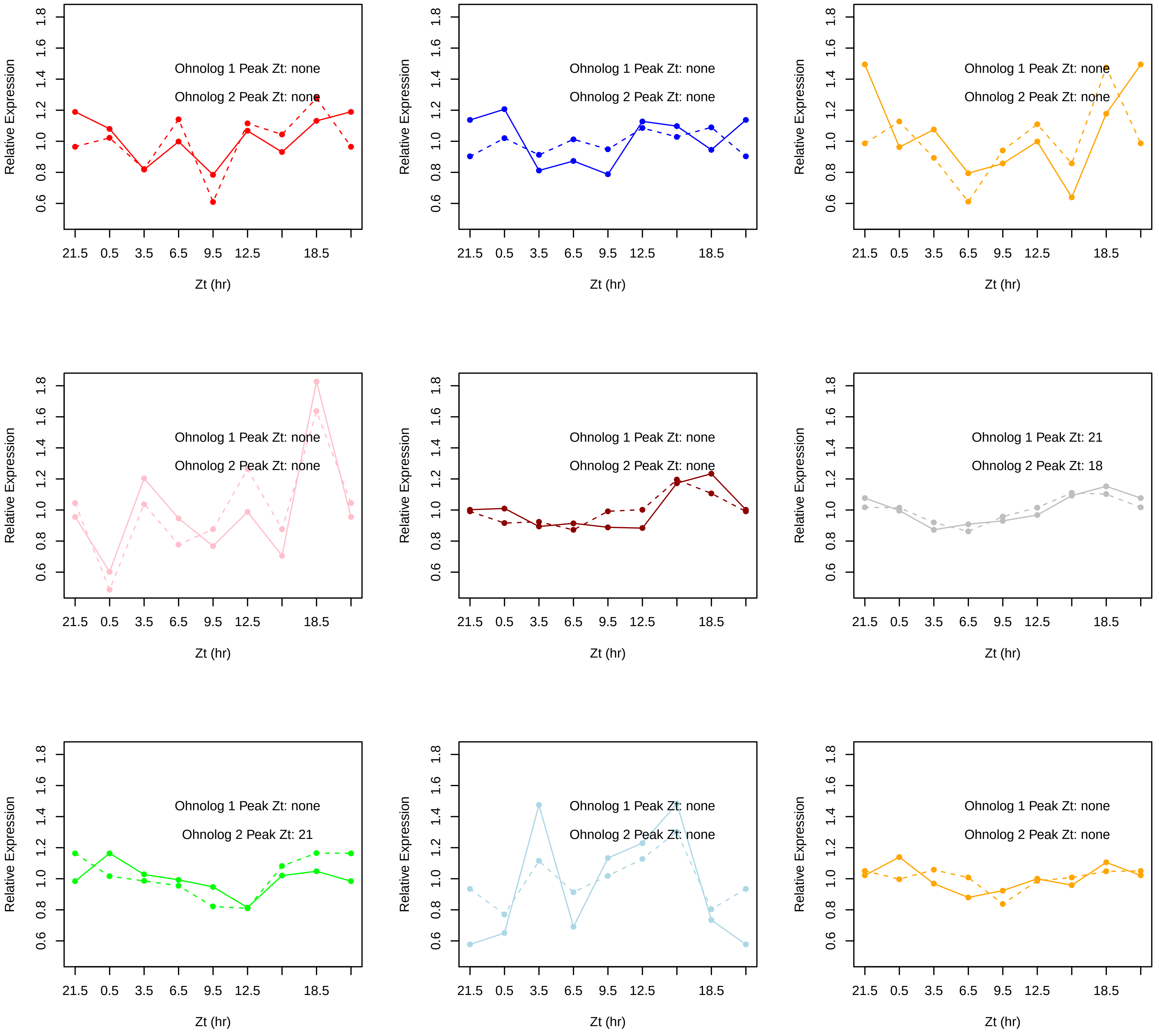

Ohnolog 1: ENSXMAG00000012966 grin2ca
Ohnolog 2: ENSXMAG00000017142

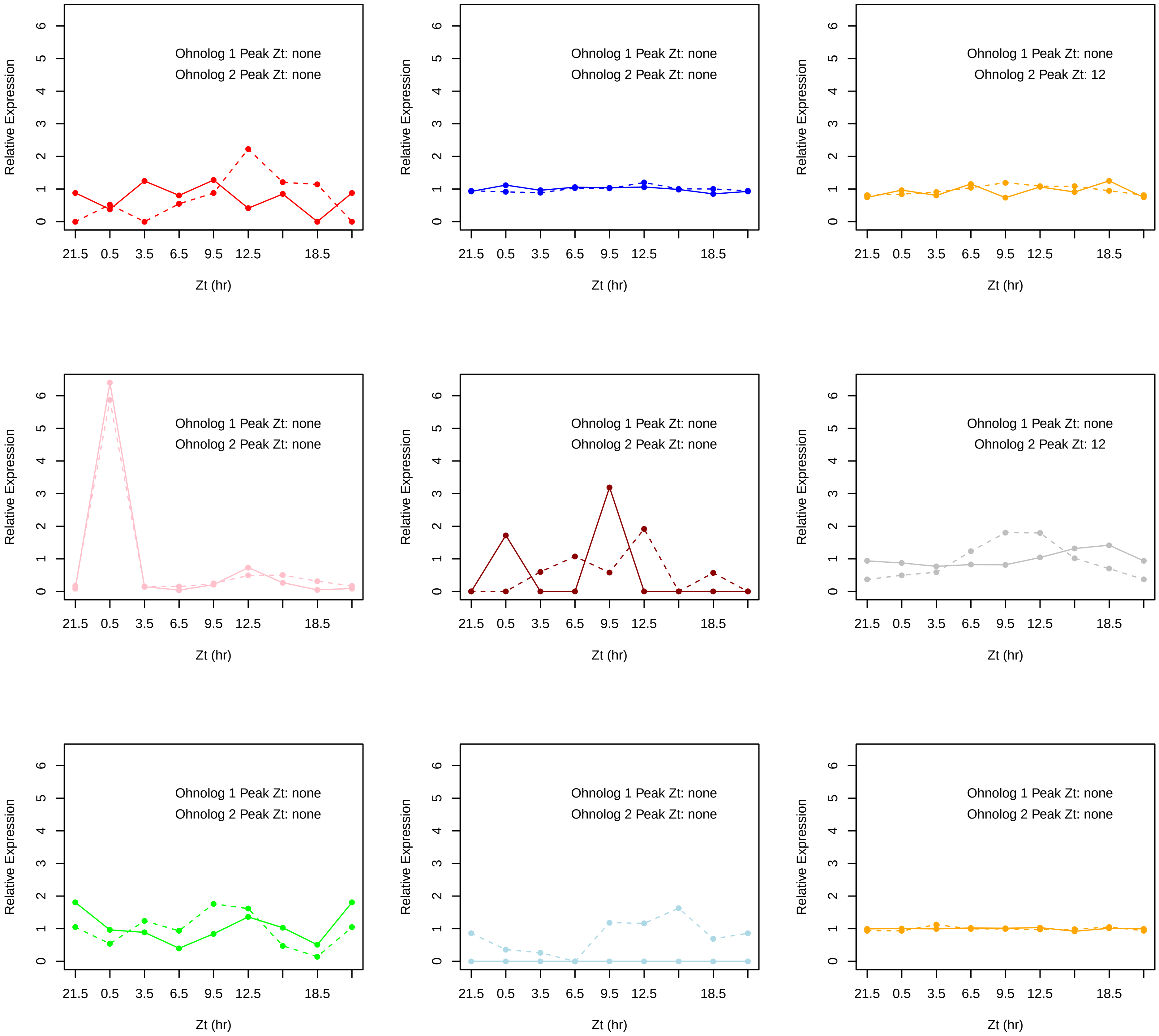

Ohnolog 1: ENSXMAG00000001881
Ohnolog 2: ENSXMAG00000022847 fgd5a

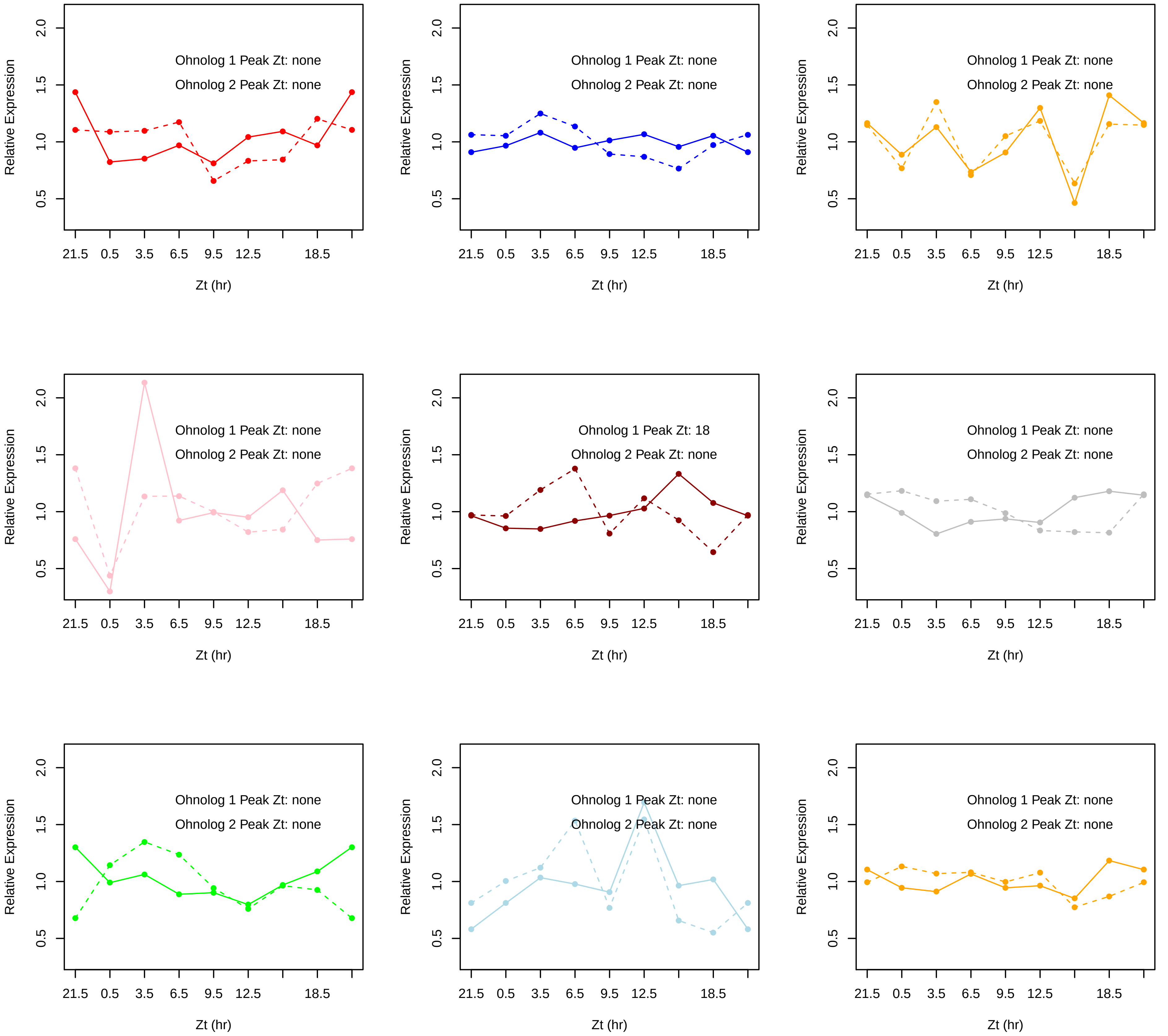

Ohnolog 1: ENSXMAG00000001160 atp5fa1 (1 of many)
Ohnolog 2: ENSXMAG00000003443 atp5fa1 (1 of many)

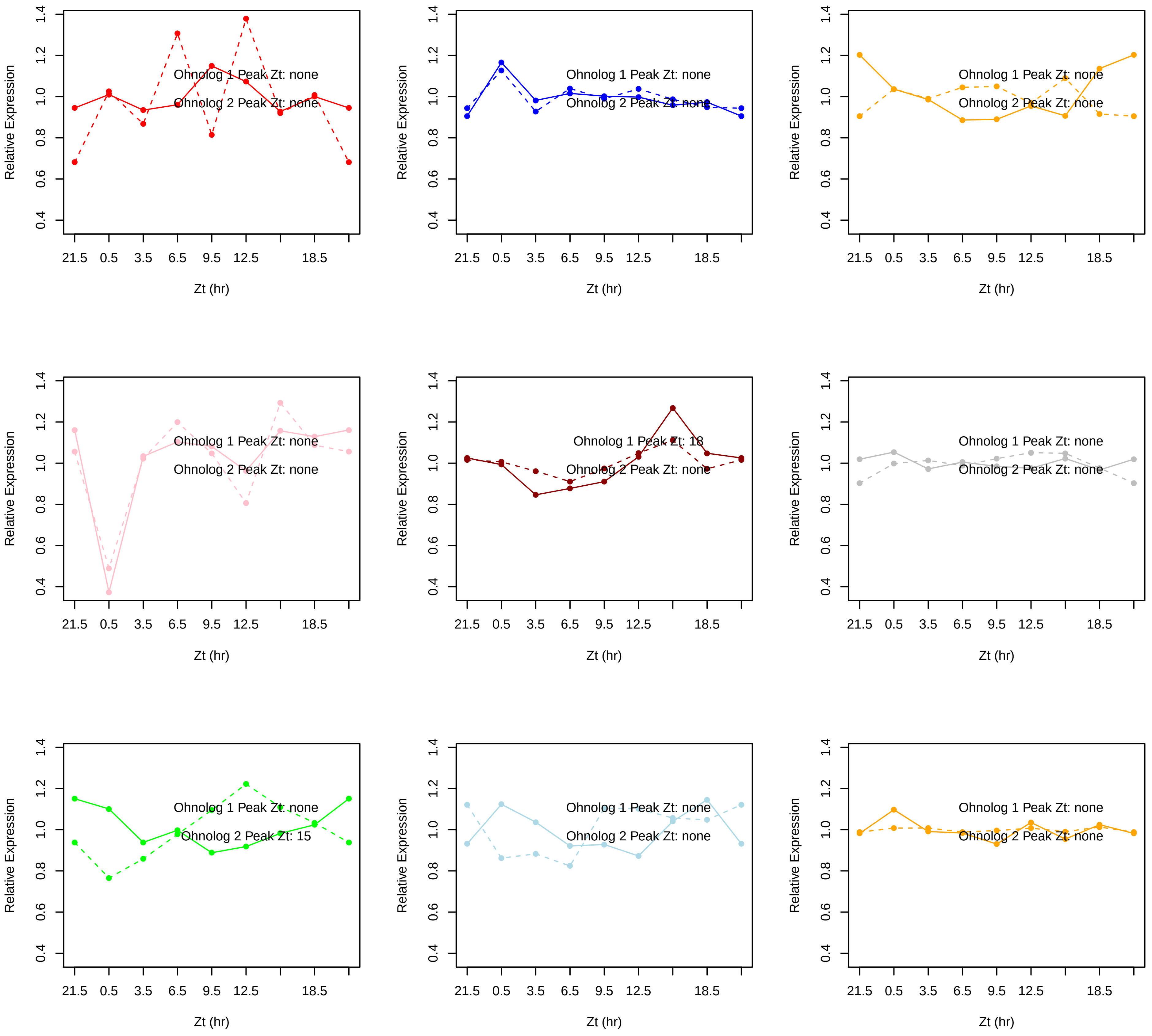

Ohnolog 1: ENSXMAG00000016769 thsd7aa
Ohnolog 2: ENSXMAG00000018876 thsd7ab

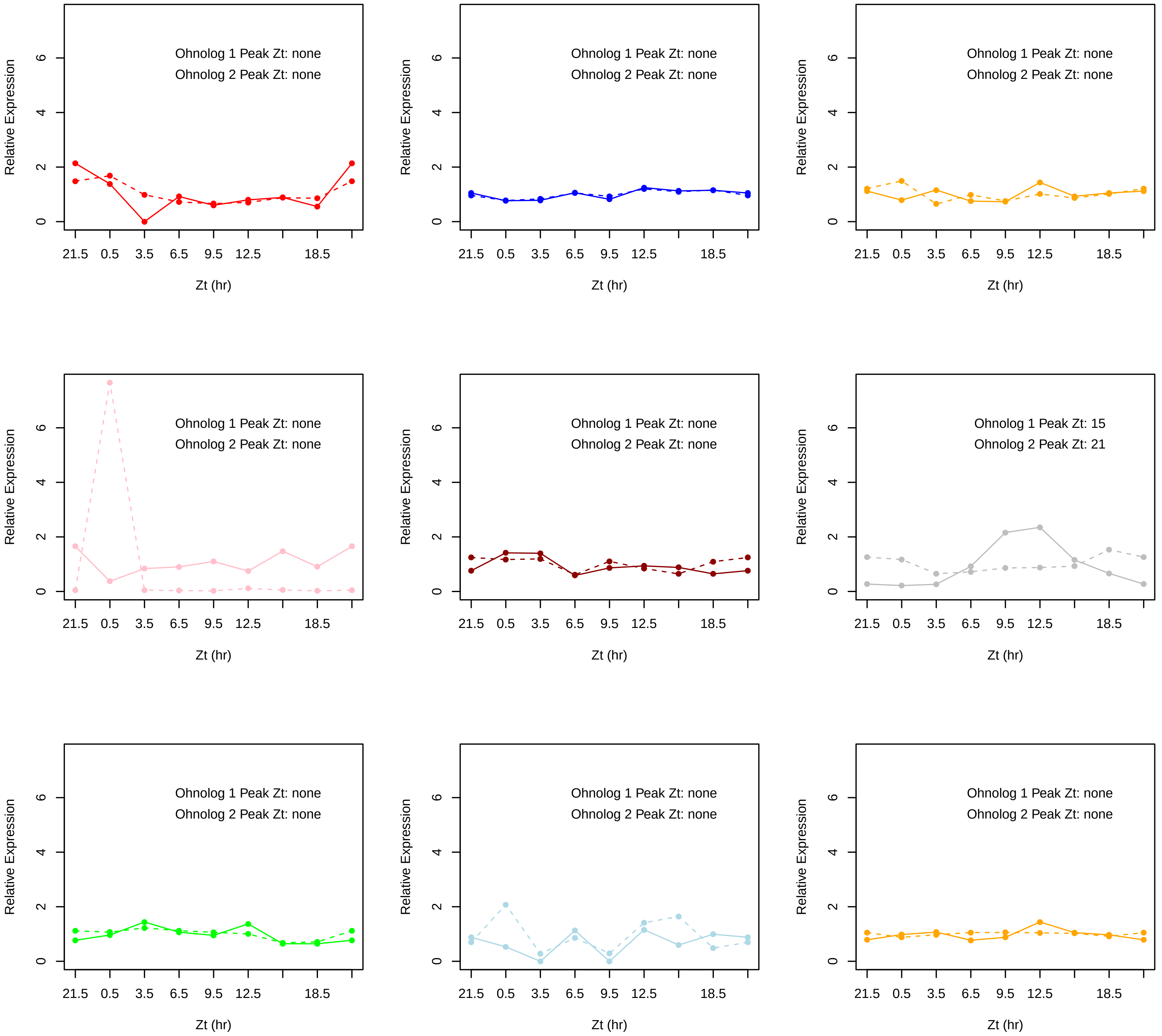

Ohnolog 1: ENSXMAG00000016760 tmem106bb
Ohnolog 2: ENSXMAG00000018910 tmem106ba

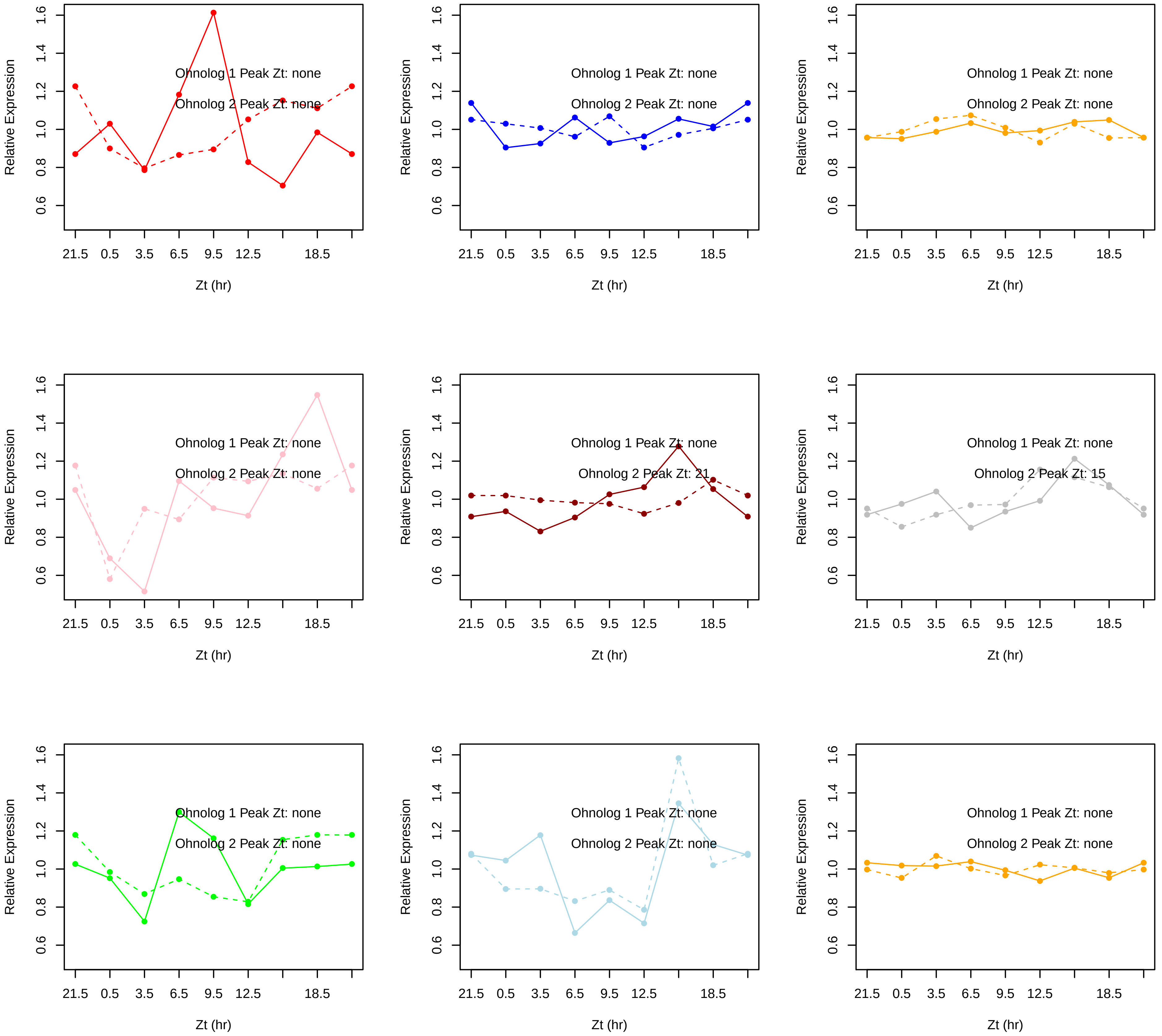

Ohnolog 1: ENSXMAG00000001406 camk1gb
Ohnolog 2: ENSXMAG00000018817 camk1ga

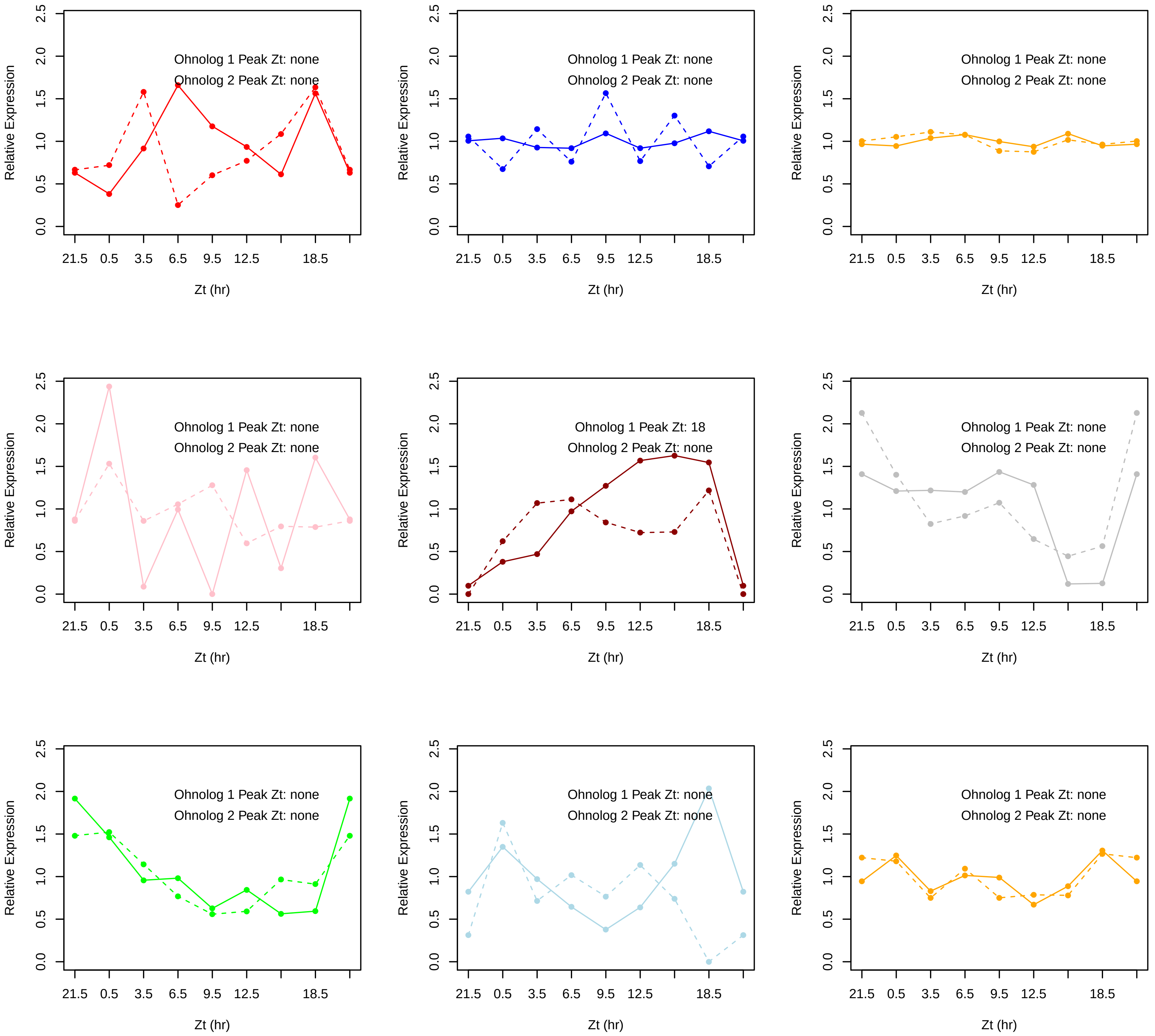

Ohnolog 1: ENSXMAG00000008551 vat1
Ohnolog 2: ENSXMAG00000010149 VAT1 (1 of many)

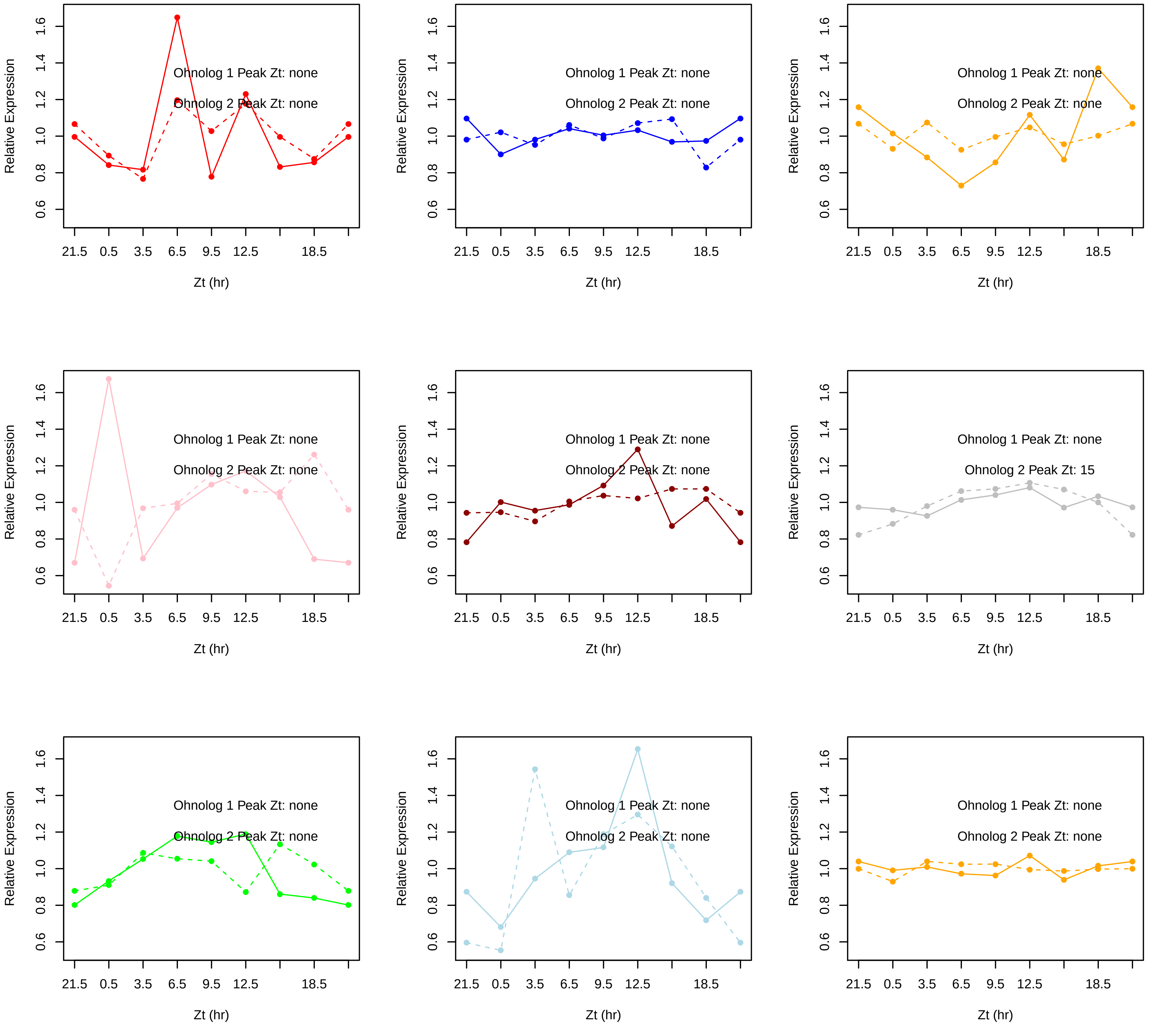

Ohnolog 1: ENSXMAG00000008197
Ohnolog 2: ENSXMAG00000023930

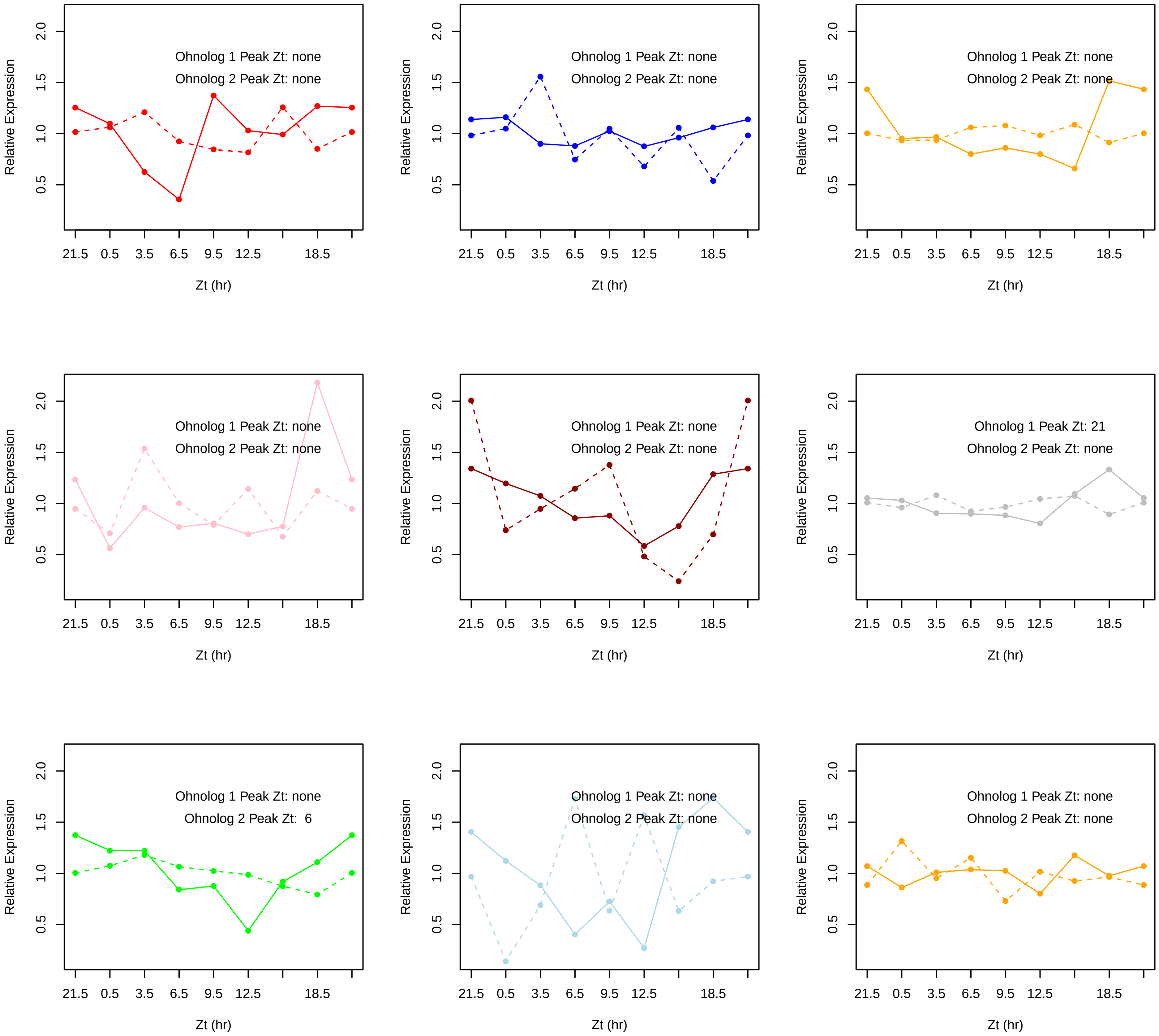

Ohnolog 1: ENSXMAG00000001200 pdgfbb
Ohnolog 2: ENSXMAG00000008034

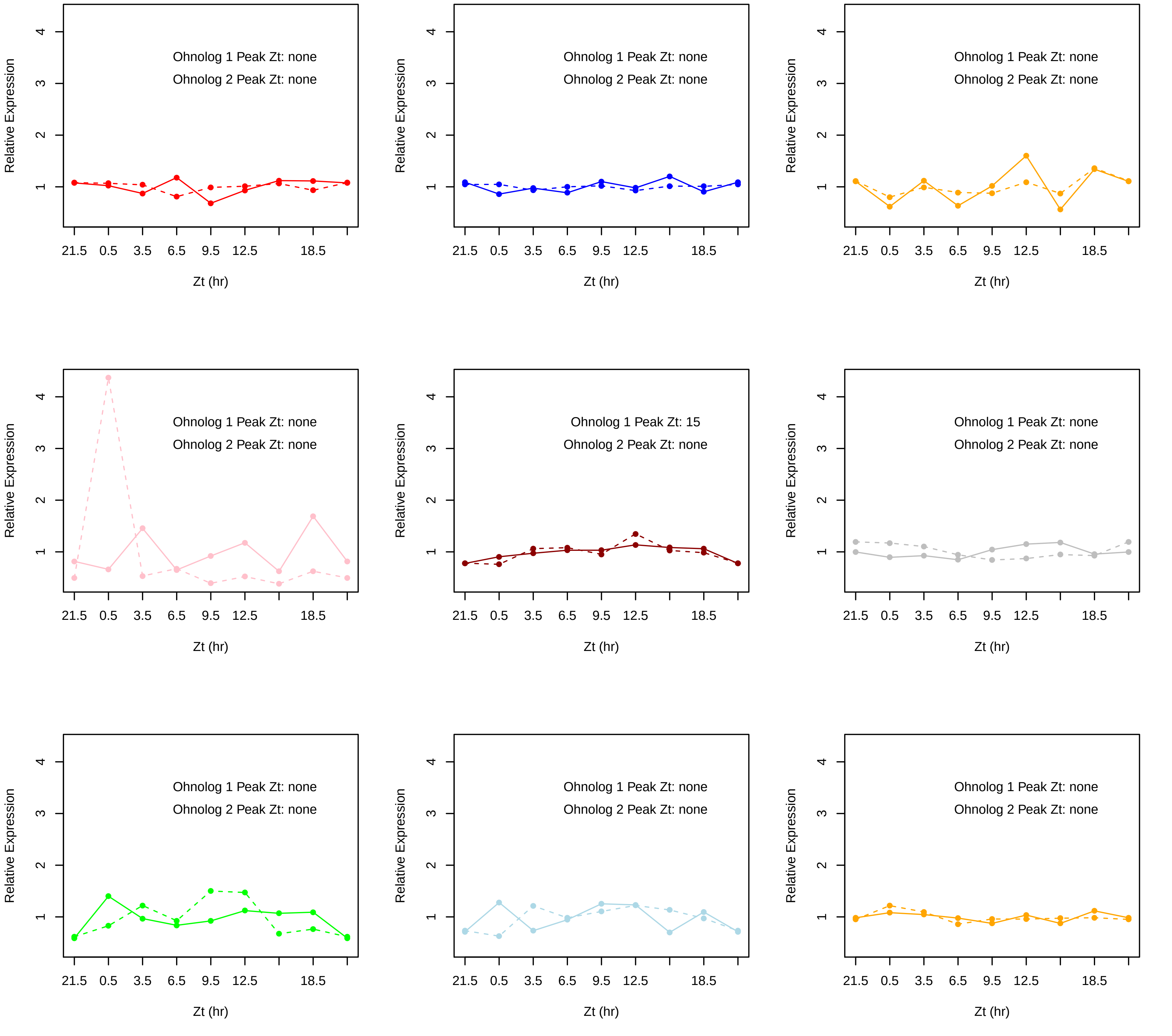

Ohnolog 1: ENSXMAG00000002178
Ohnolog 2: ENSXMAG00000025866 ADAM11

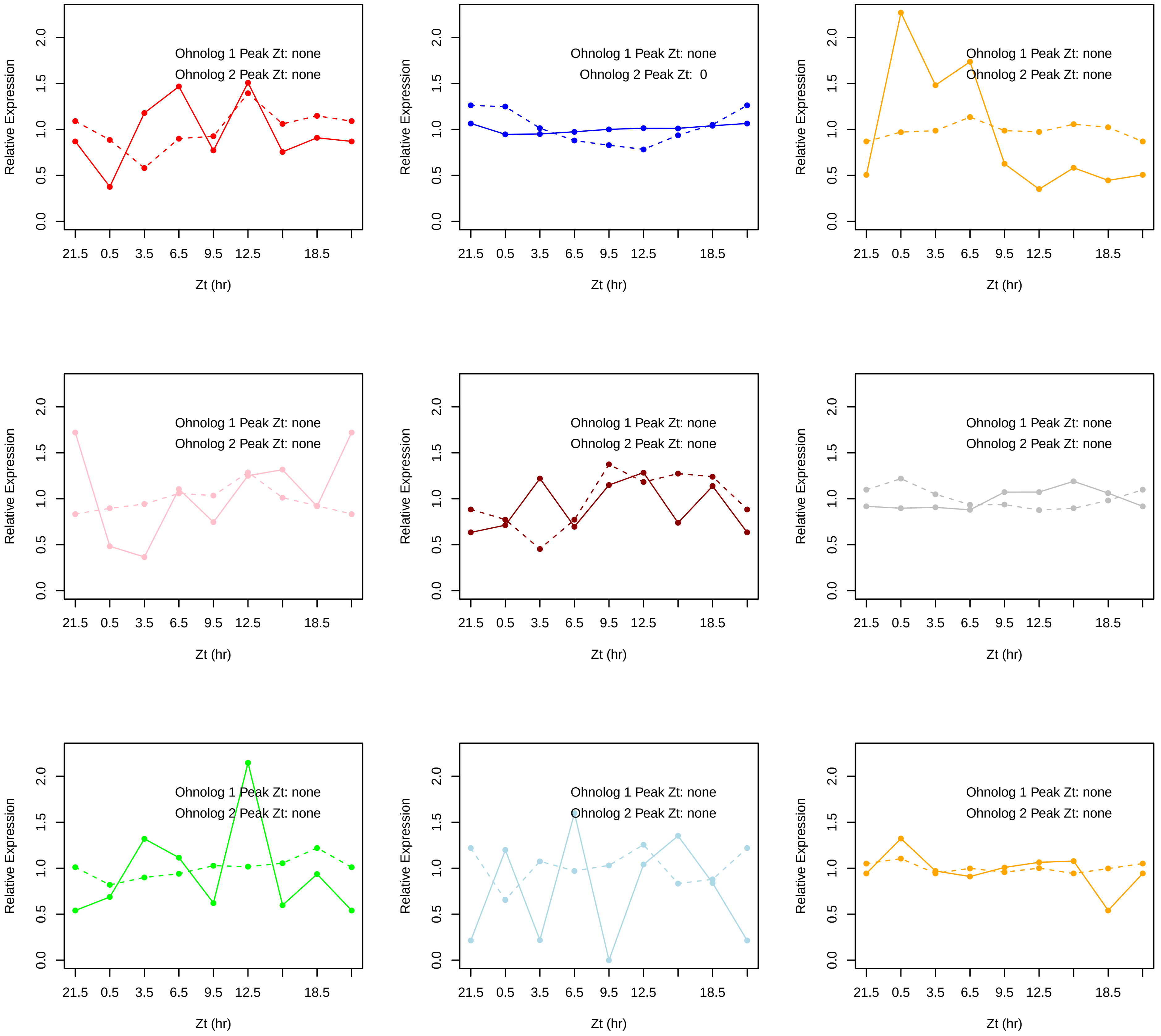

Ohnolog 1: ENSXMAG00000008387 PLCD3
Ohnolog 2: ENSXMAG00000014404 plcd3b

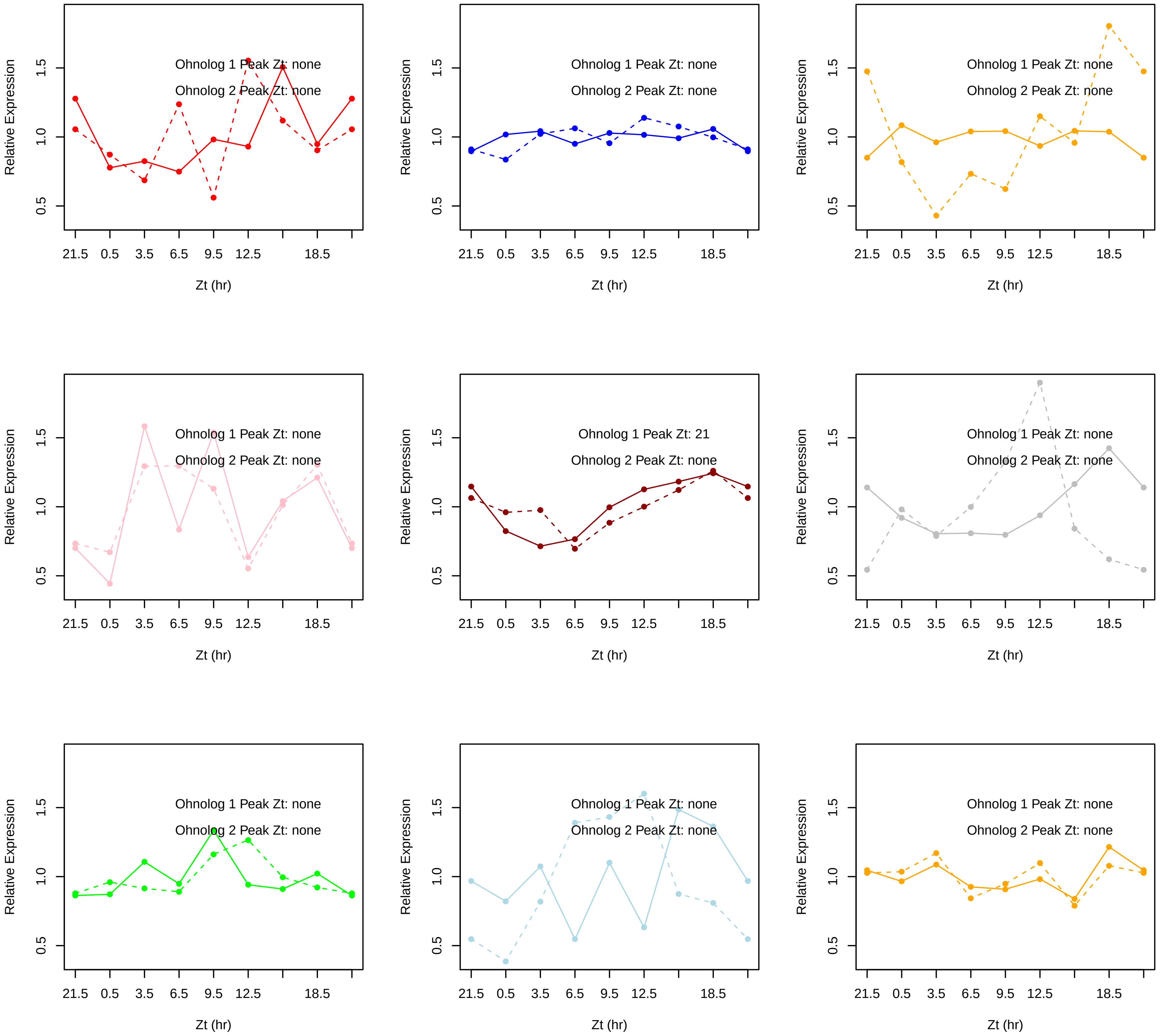

Ohnolog 1: ENSXMAG00000001279 desi1a
Ohnolog 2: ENSXMAG00000009685 desi1b

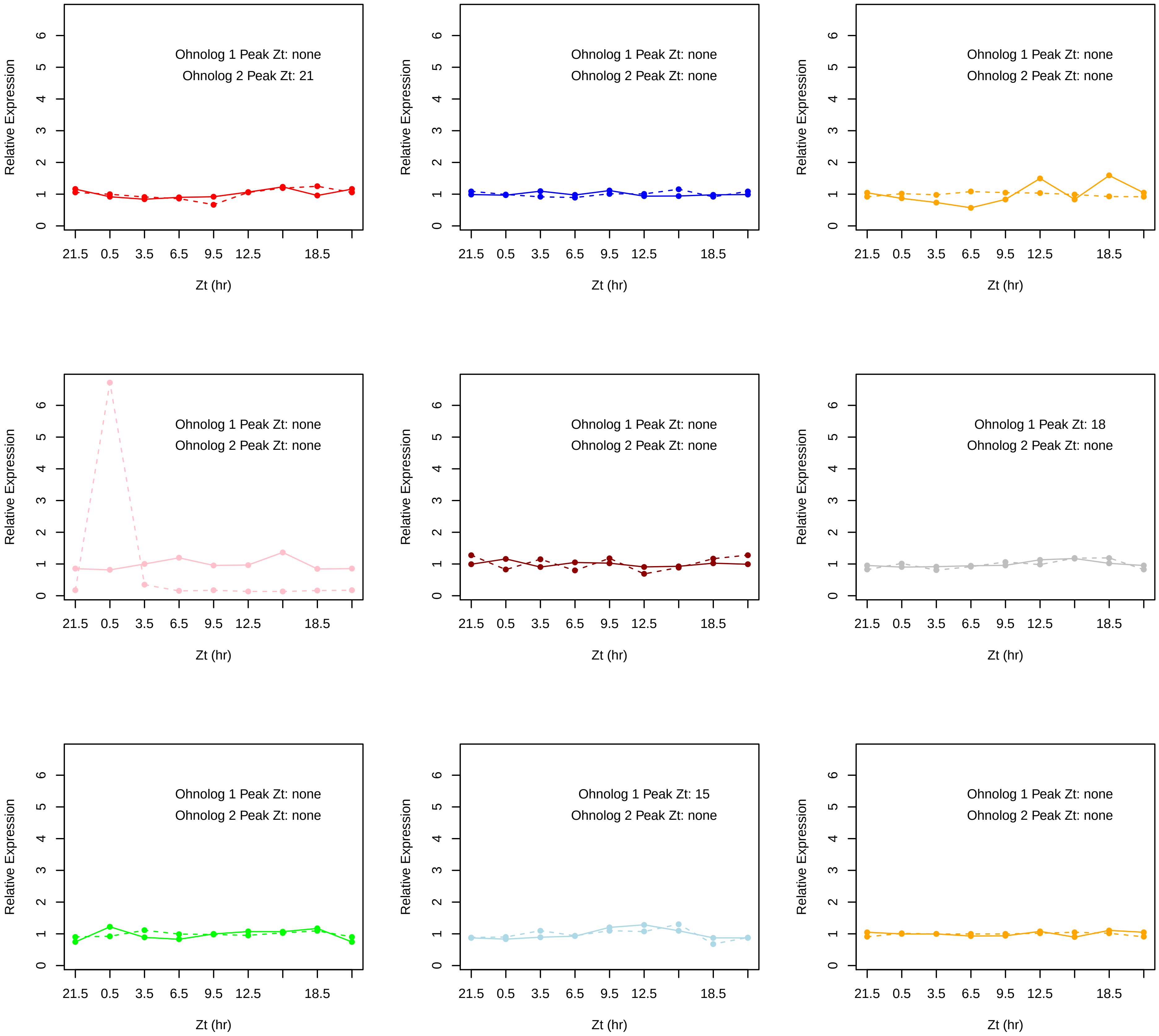

Ohnolog 1: ENSXMAG00000014677 slc44a1b
Ohnolog 2: ENSXMAG00000016107

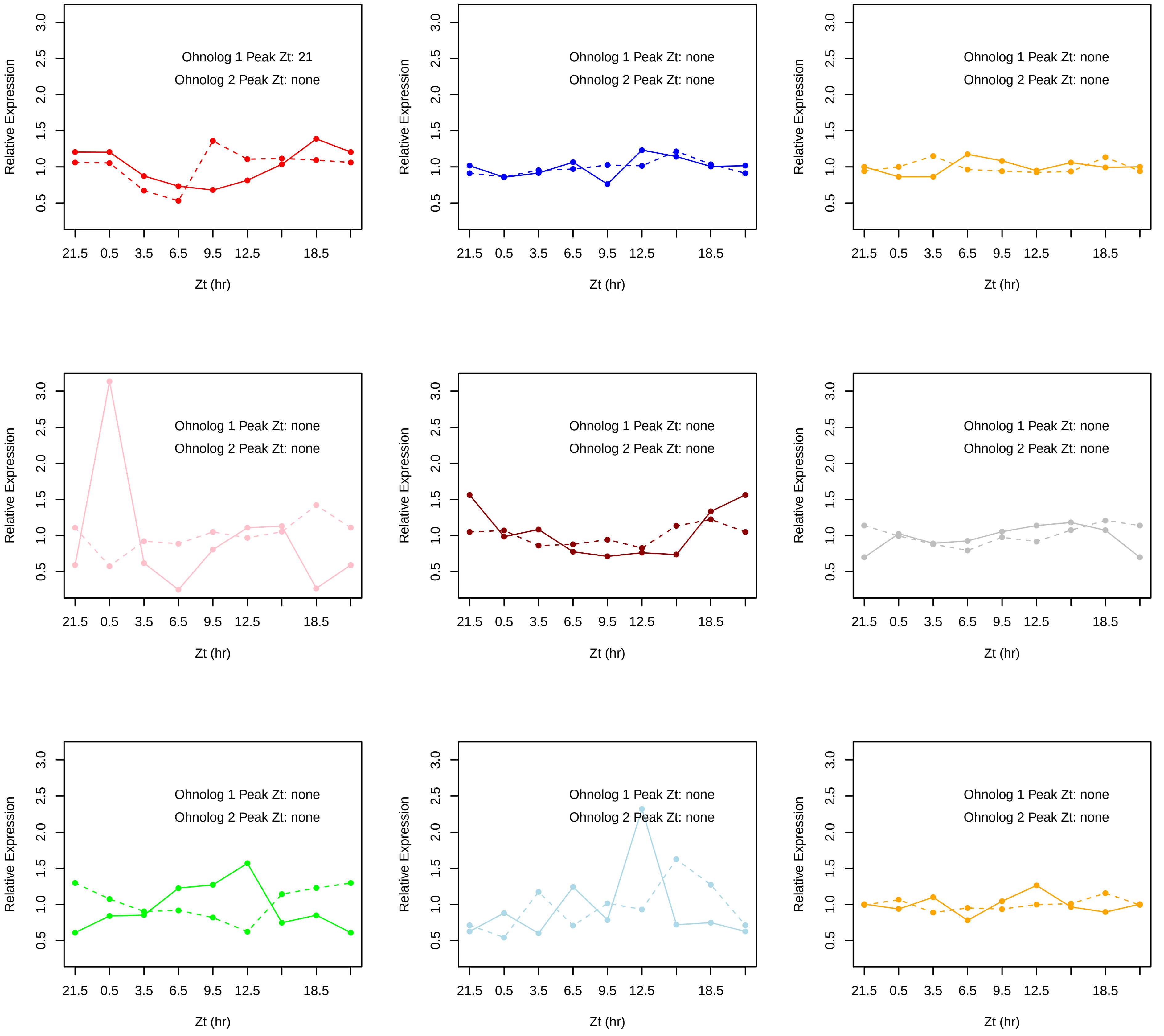

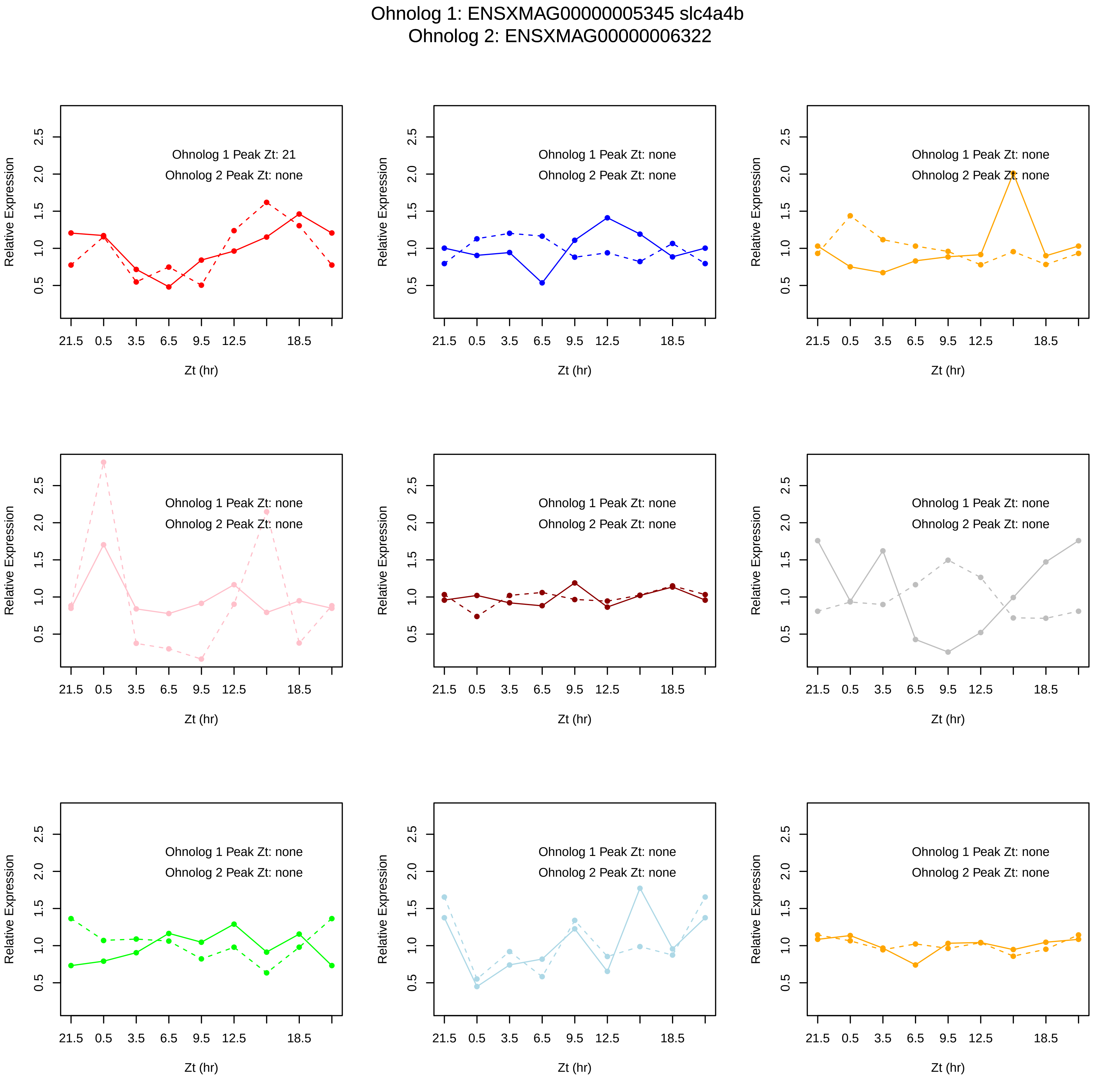
Ohnolog 1: ENSXMAG00000005345 slc4a4b
Ohnolog 2: ENSXMAG00000006322
Ohnolog 1 Peak Zt: 21
Ohnolog 2 Peak Zt: none
Ohnolog 1 Peak Zt: none
Ohnolog 2 Peak Zt: none
Relative Expression
Zt (hr)
21.5
0.5
3.5
6.5
9.5
12.5
18.5

Ohnolog 1: ENSXMAG00000000577 rapgef5a
Ohnolog 2: ENSXMAG00000015176 RAPGEF5

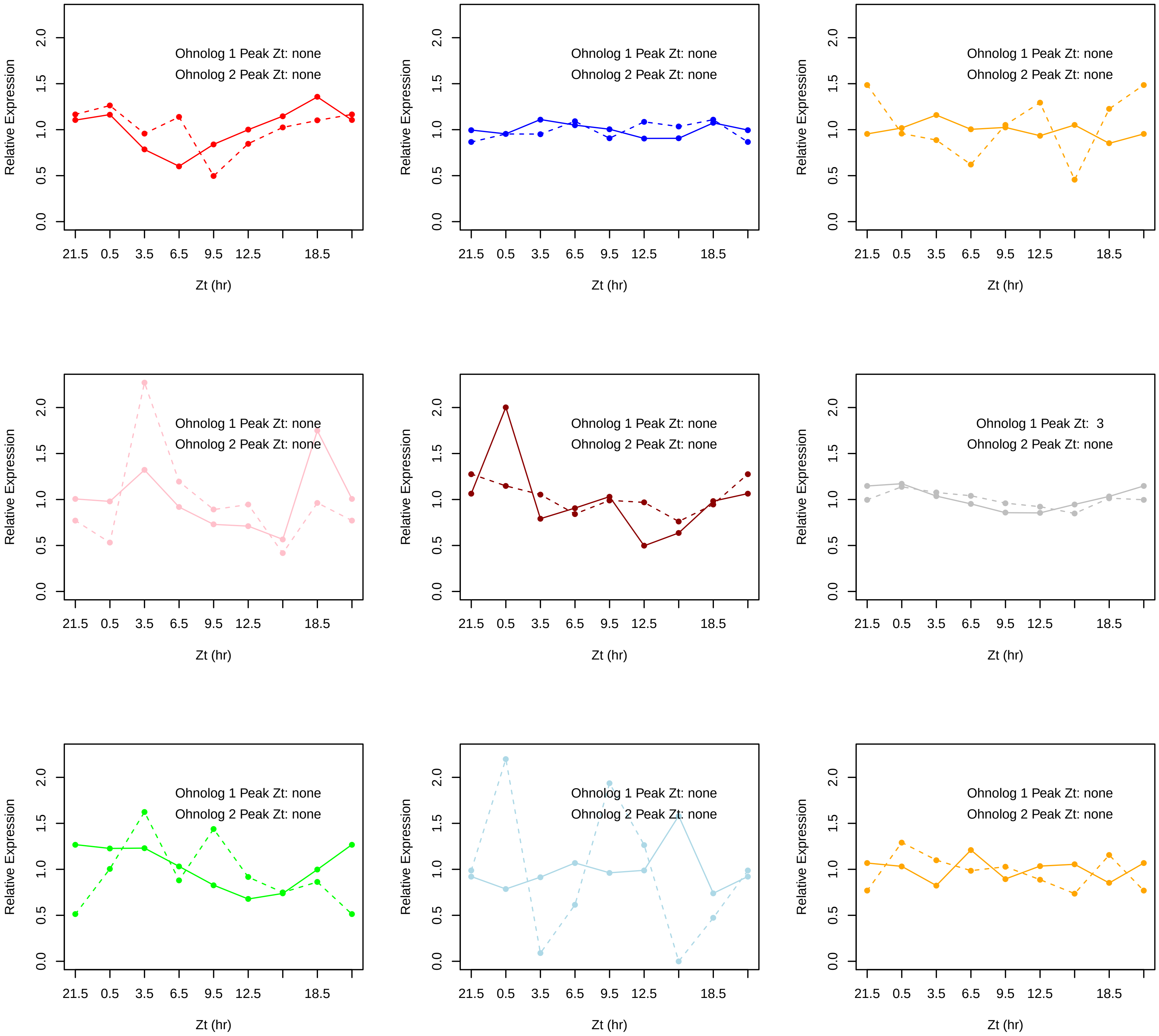

Ohnolog 1: ENSXMAG00000005844 smu1b
Ohnolog 2: ENSXMAG00000019096 smu1a

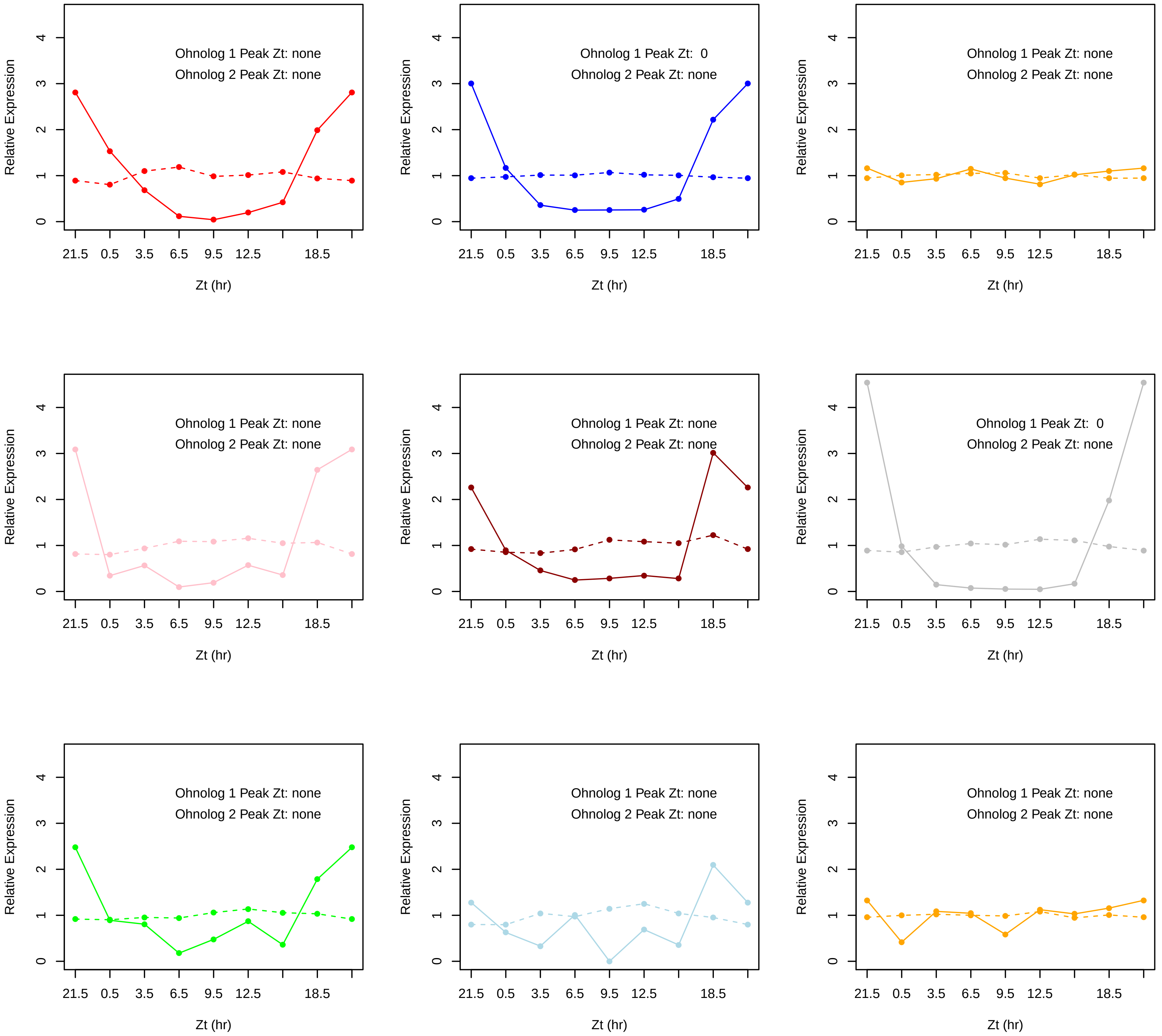

Ohnolog 1: ENSXMAG00000000663 rangap1b
Ohnolog 2: ENSXMAG00000003726 rangap1a

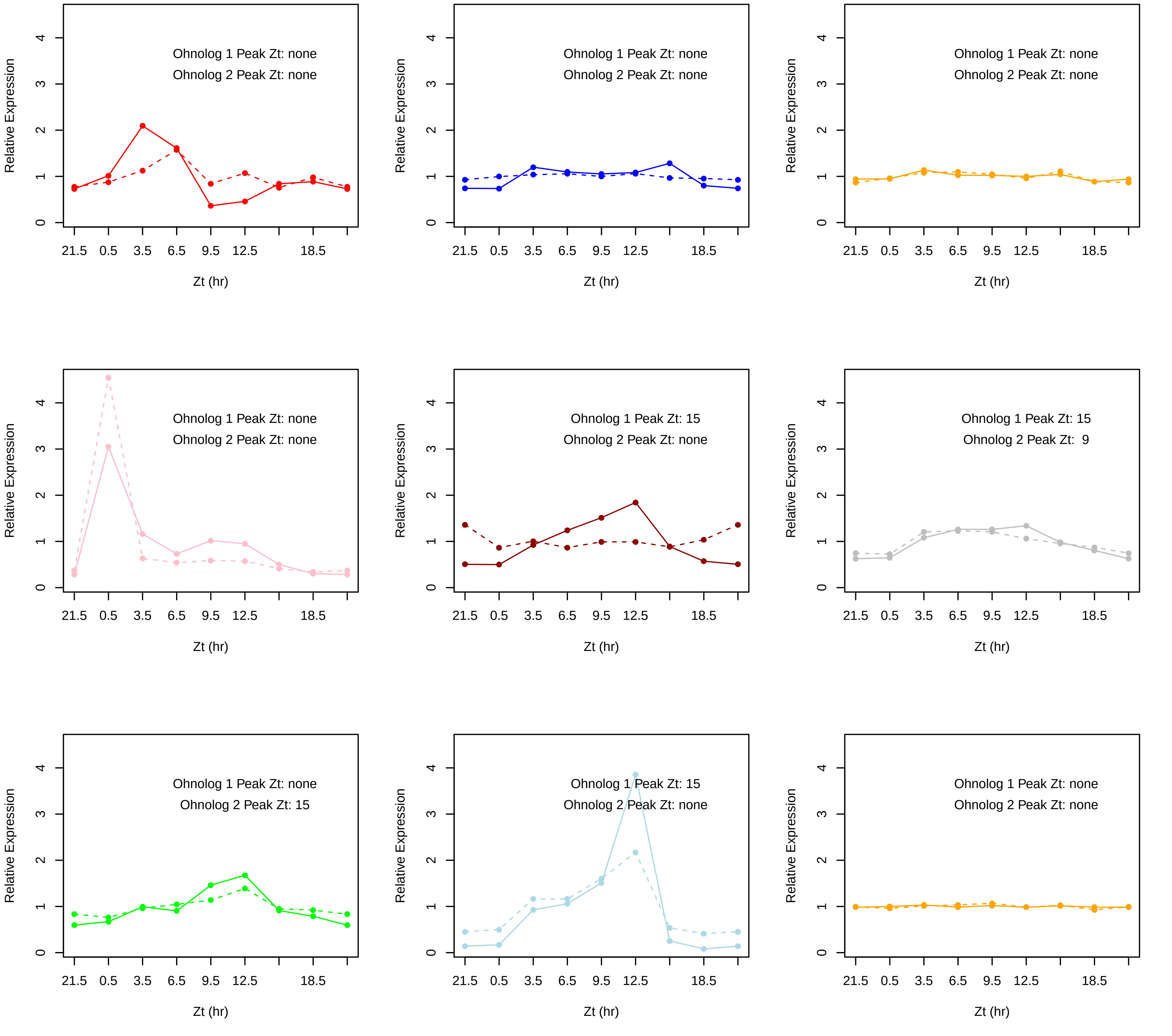

Ohnolog 1: ENSXMAG00000008317 znf281a
Ohnolog 2: ENSXMAG00000026693 znf281b

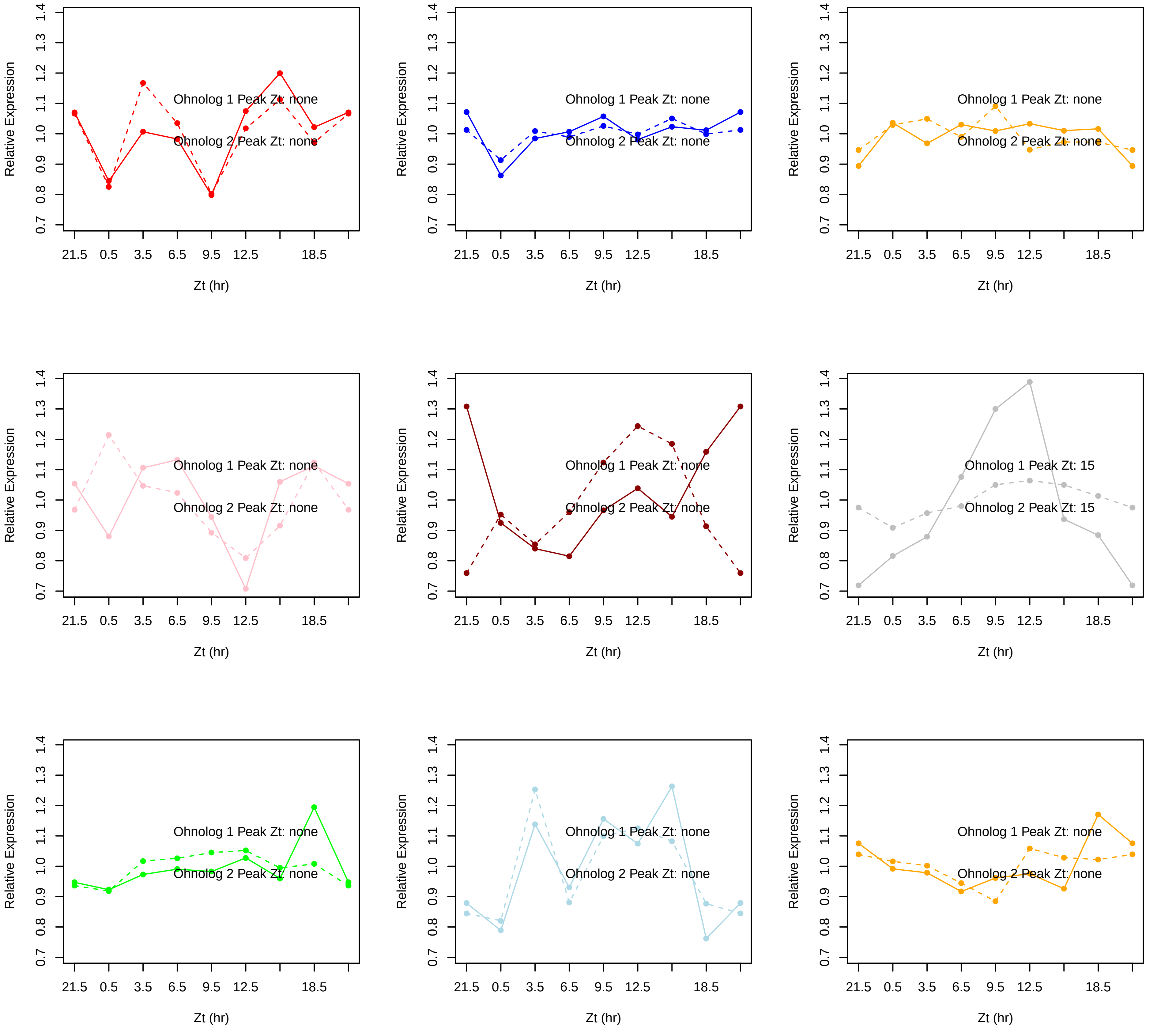

Ohnolog 1: ENSXMAG00000000678 tefa
Ohnolog 2: ENSXMAG00000026333 tefb

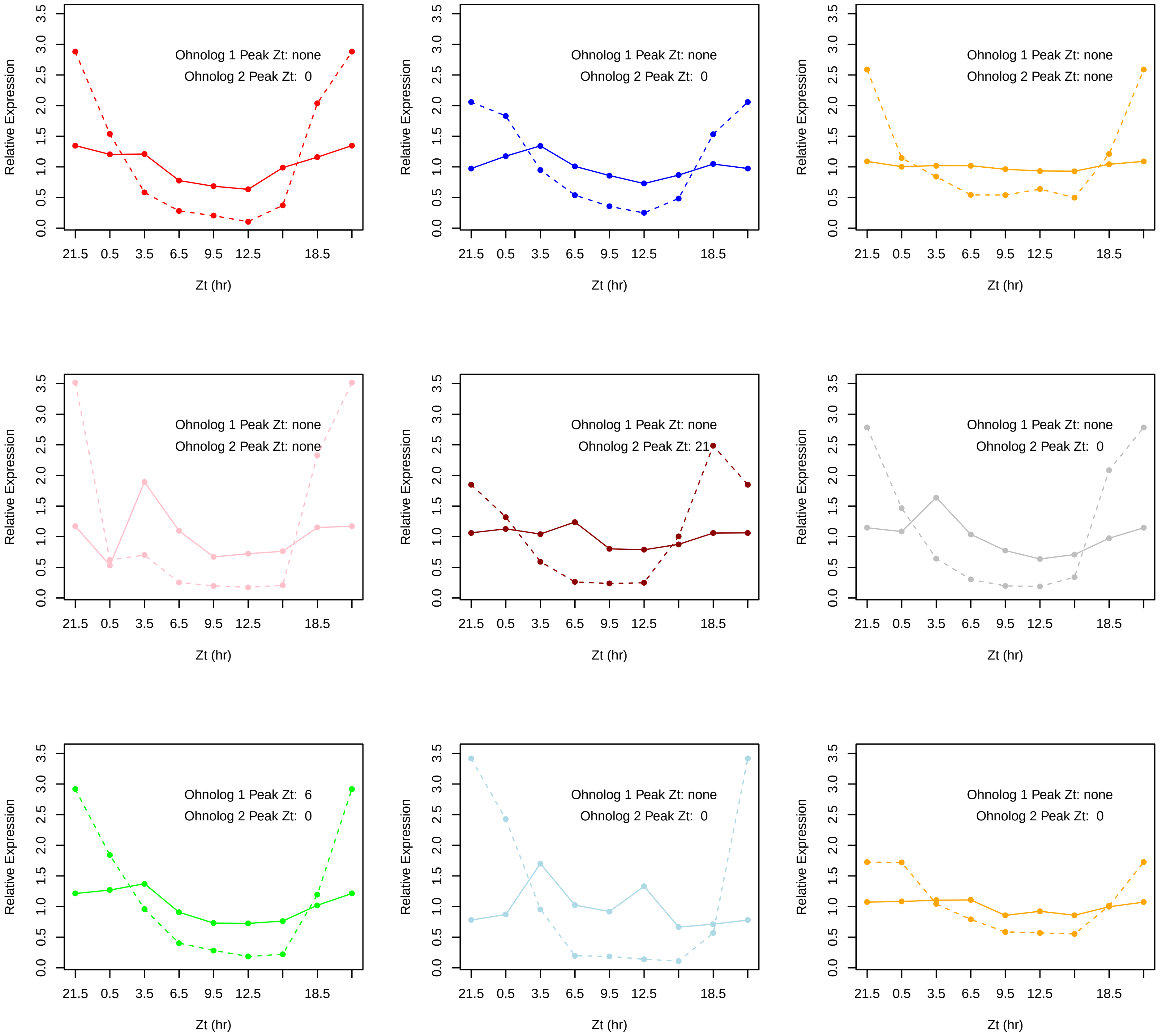

Ohnolog 1: ENSXMAG00000000683 aco2
Ohnolog 2: ENSXMAG00000006870 ACO2 (1 of many)

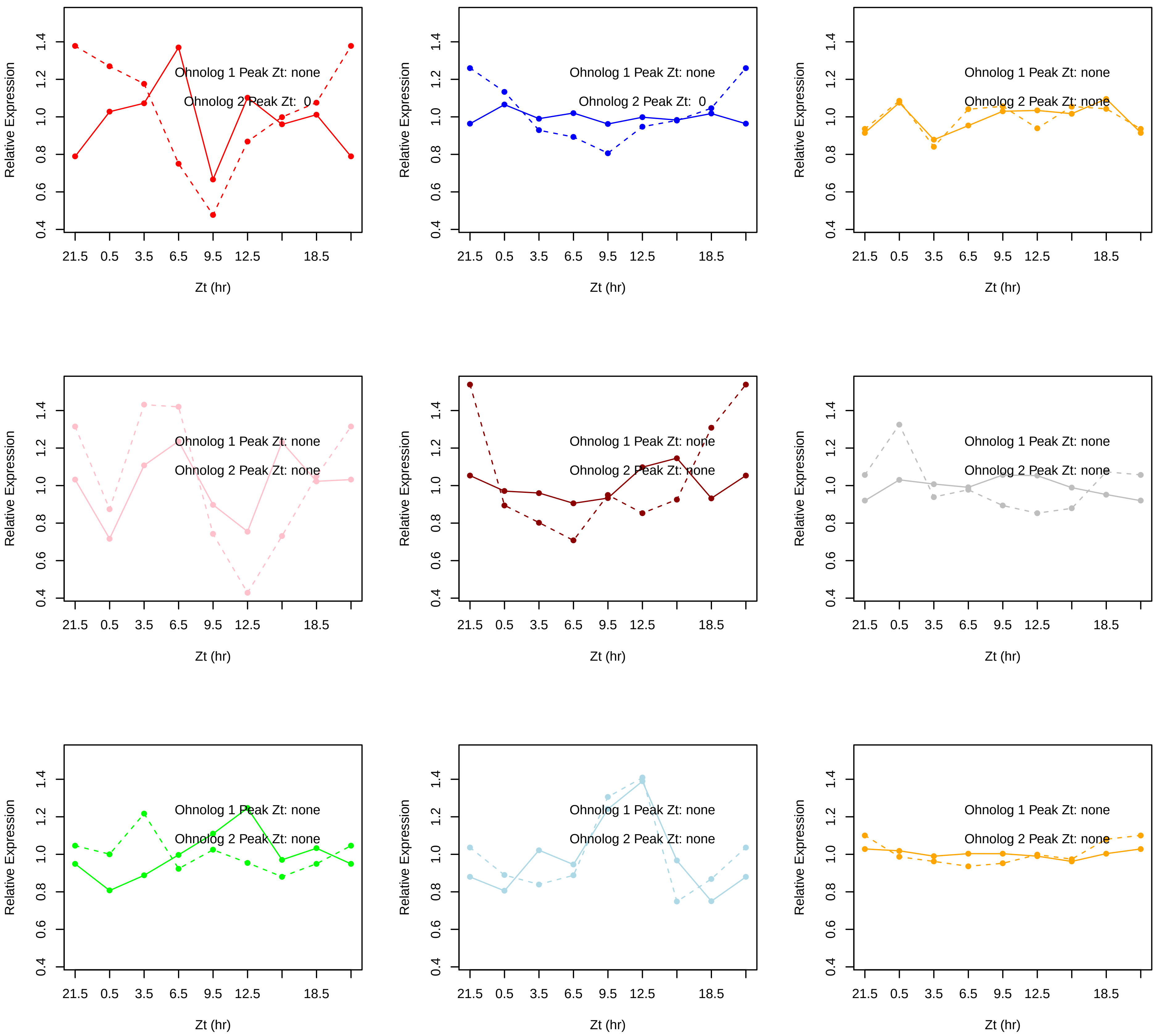

Ohnolog 1: ENSXMAG00000008446 evlb
Ohnolog 2: ENSXMAG00000024161 evla

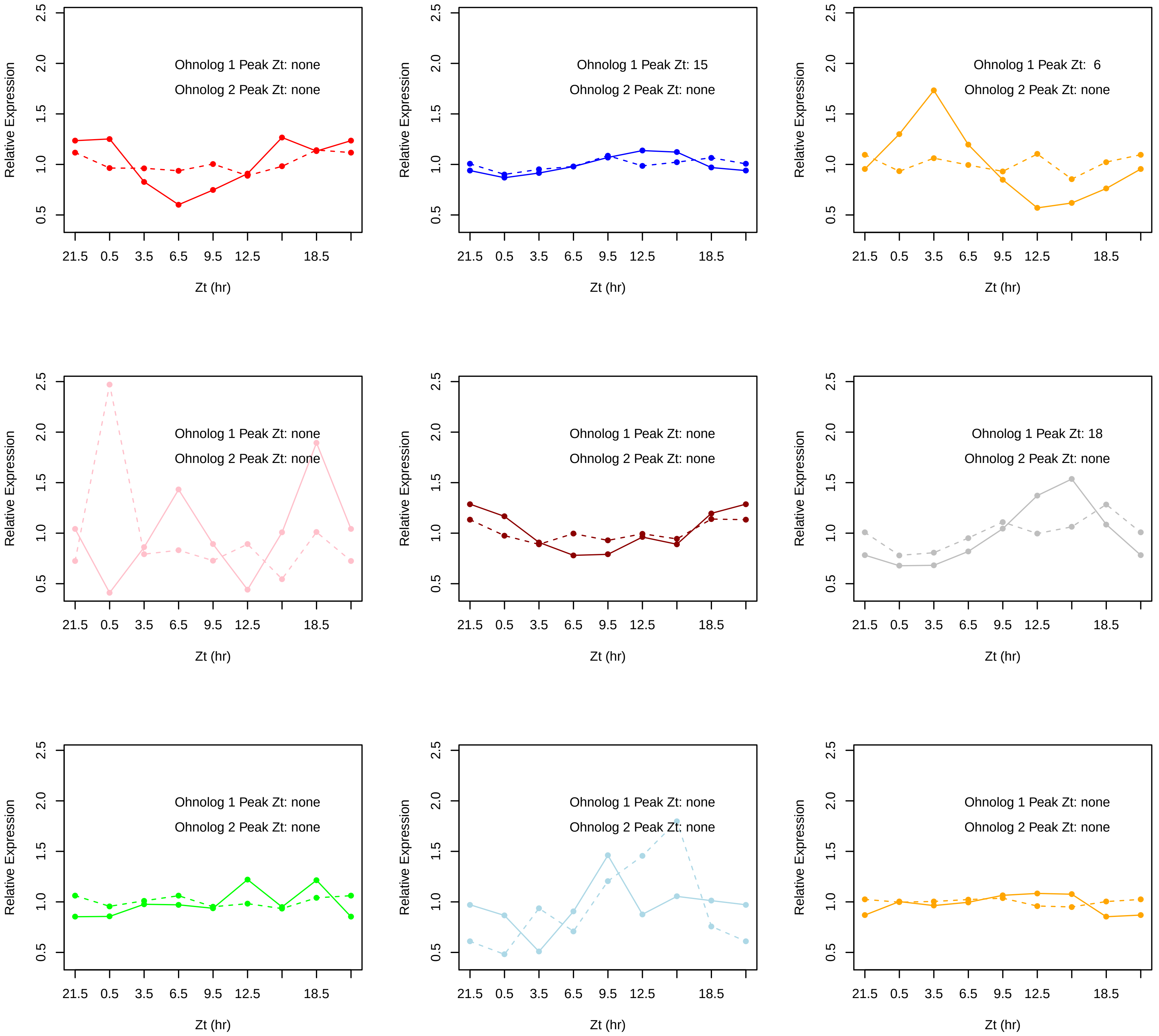

Ohnolog 1: ENSXMAG00000008287 mpp3a
Ohnolog 2: ENSXMAG00000016920 mpp3b

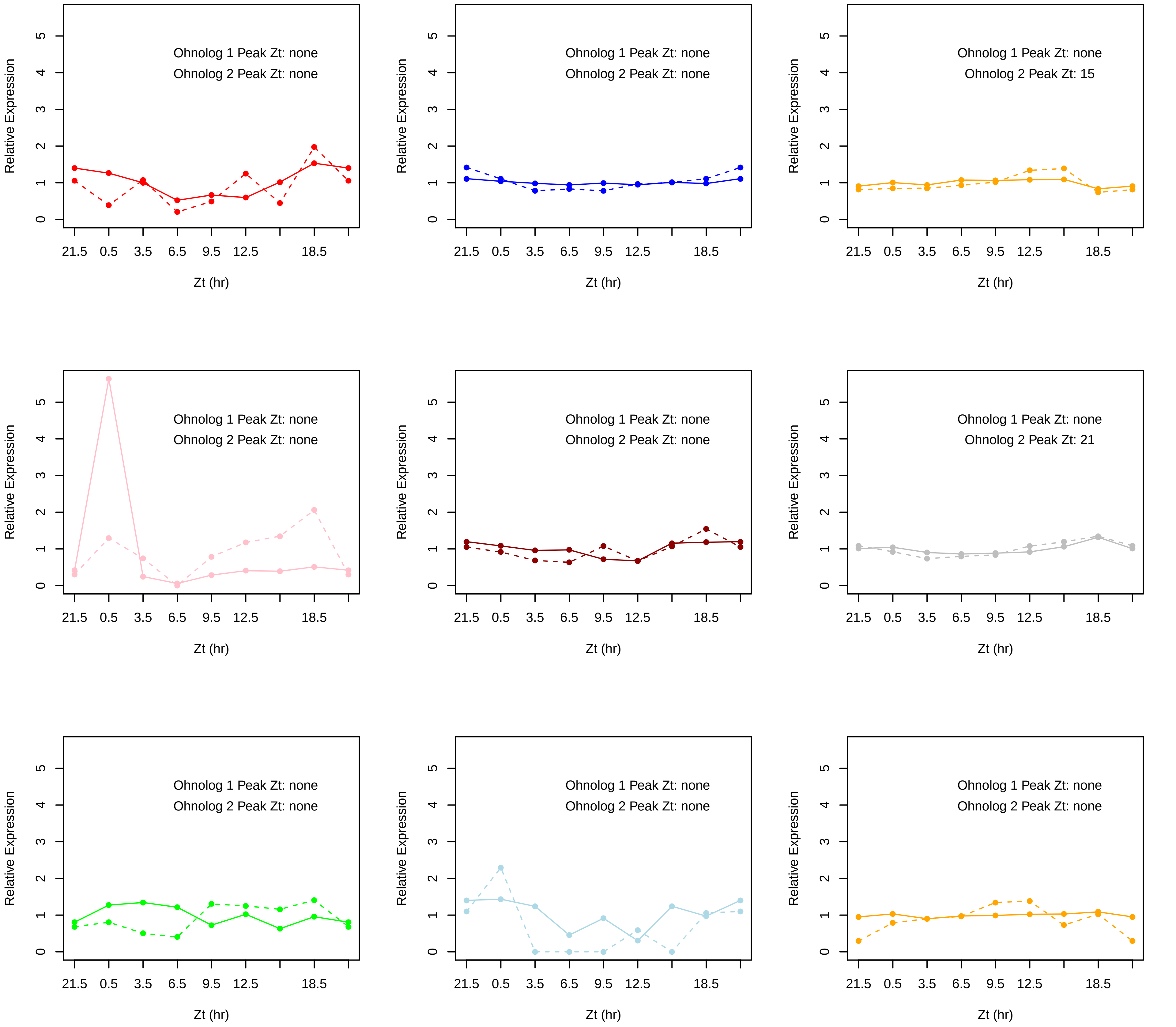

Ohnolog 1: ENSXMAG00000008204 ngfra
Ohnolog 2: ENSXMAG00000017049 ngfrb

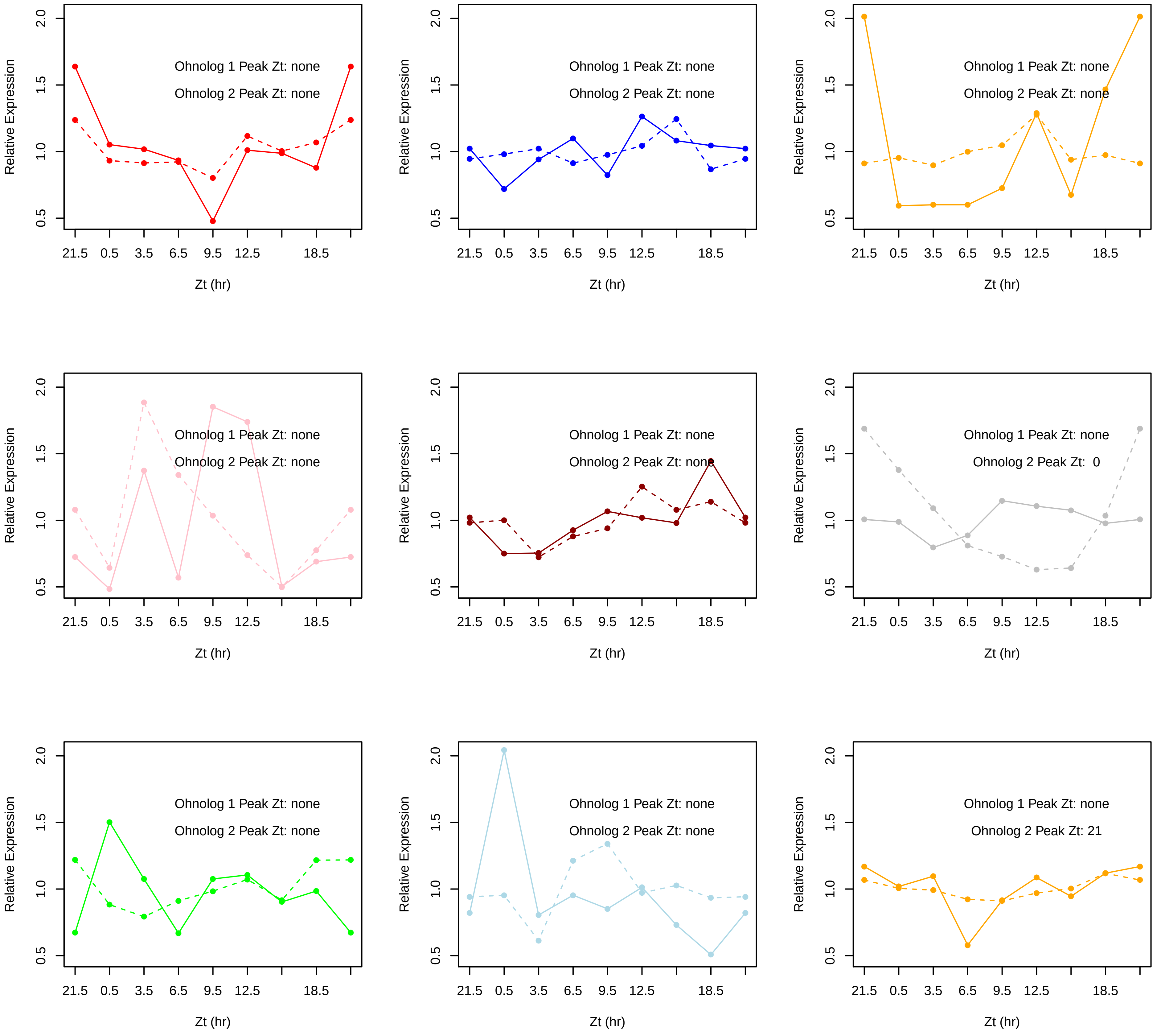

Ohnolog 1: ENSXMAG00000000115 slc2a15b
Ohnolog 2: ENSXMAG00000003577 slc2a15a

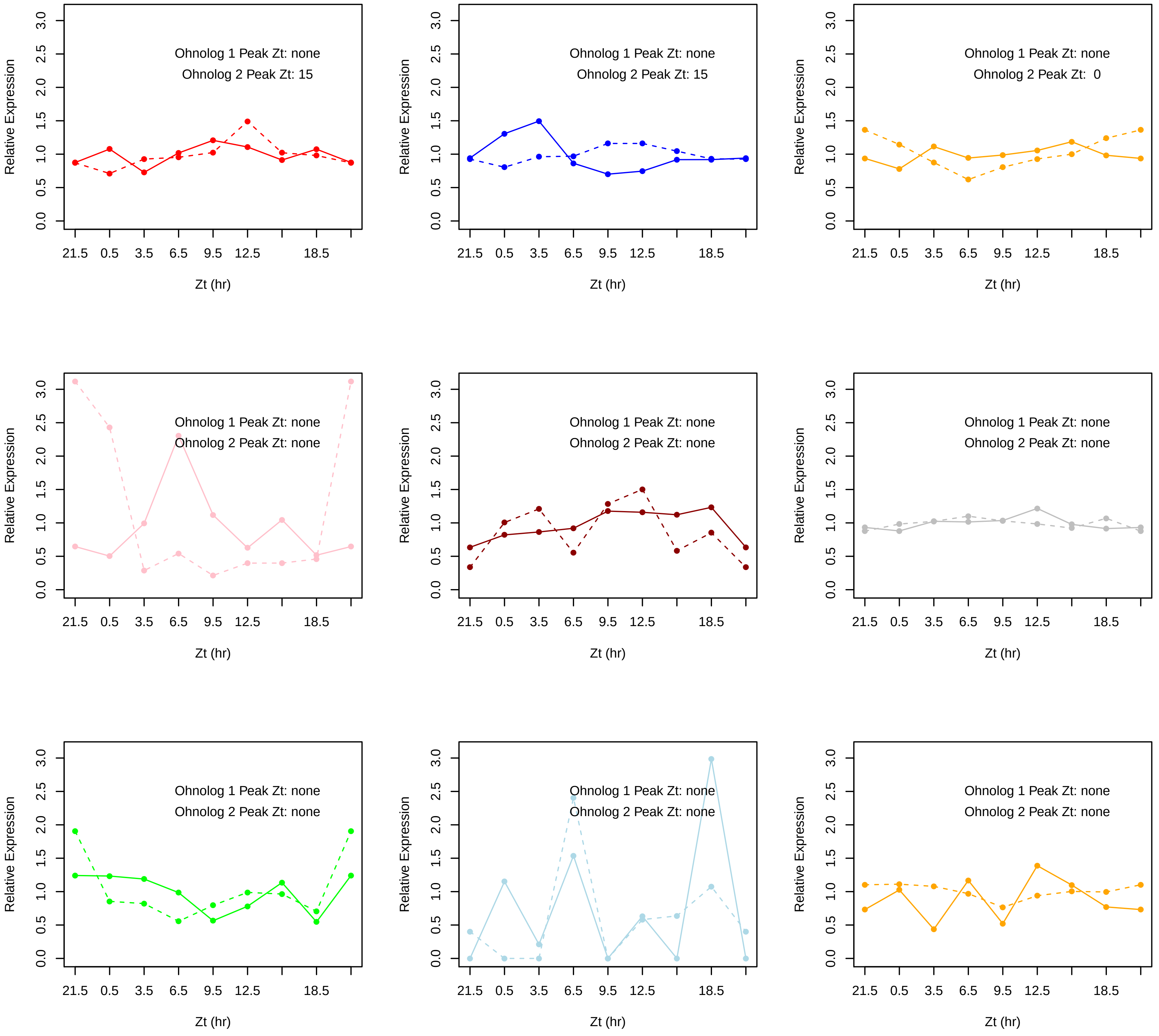

Ohnolog 1: ENSXMAG00000001083 osbpl3a
Ohnolog 2: ENSXMAG00000013928 osbpl3b

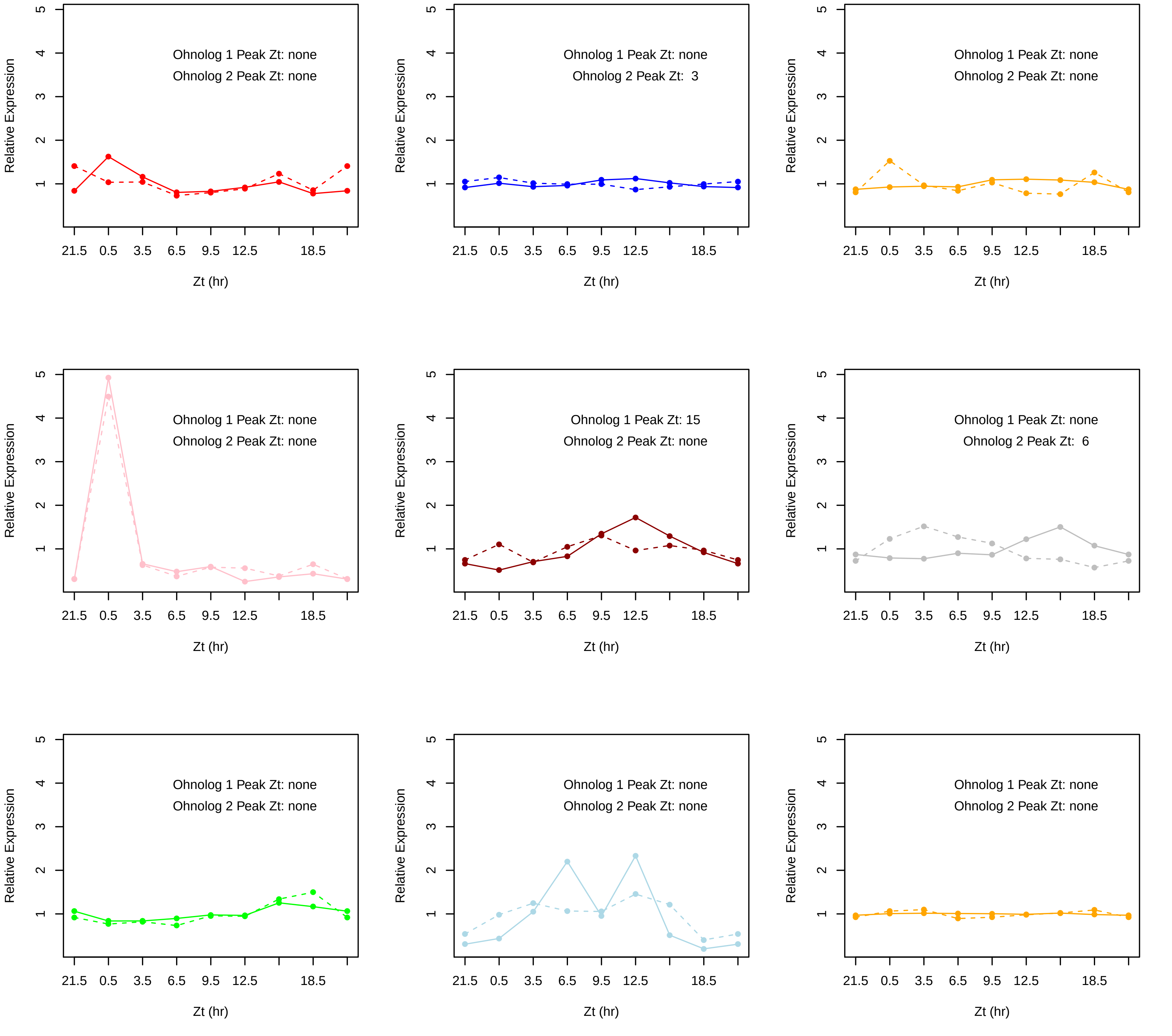

Ohnolog 1: ENSXMAG00000001077 si:dkey−46g23.1
Ohnolog 2: ENSXMAG00000012546 tnrc6b

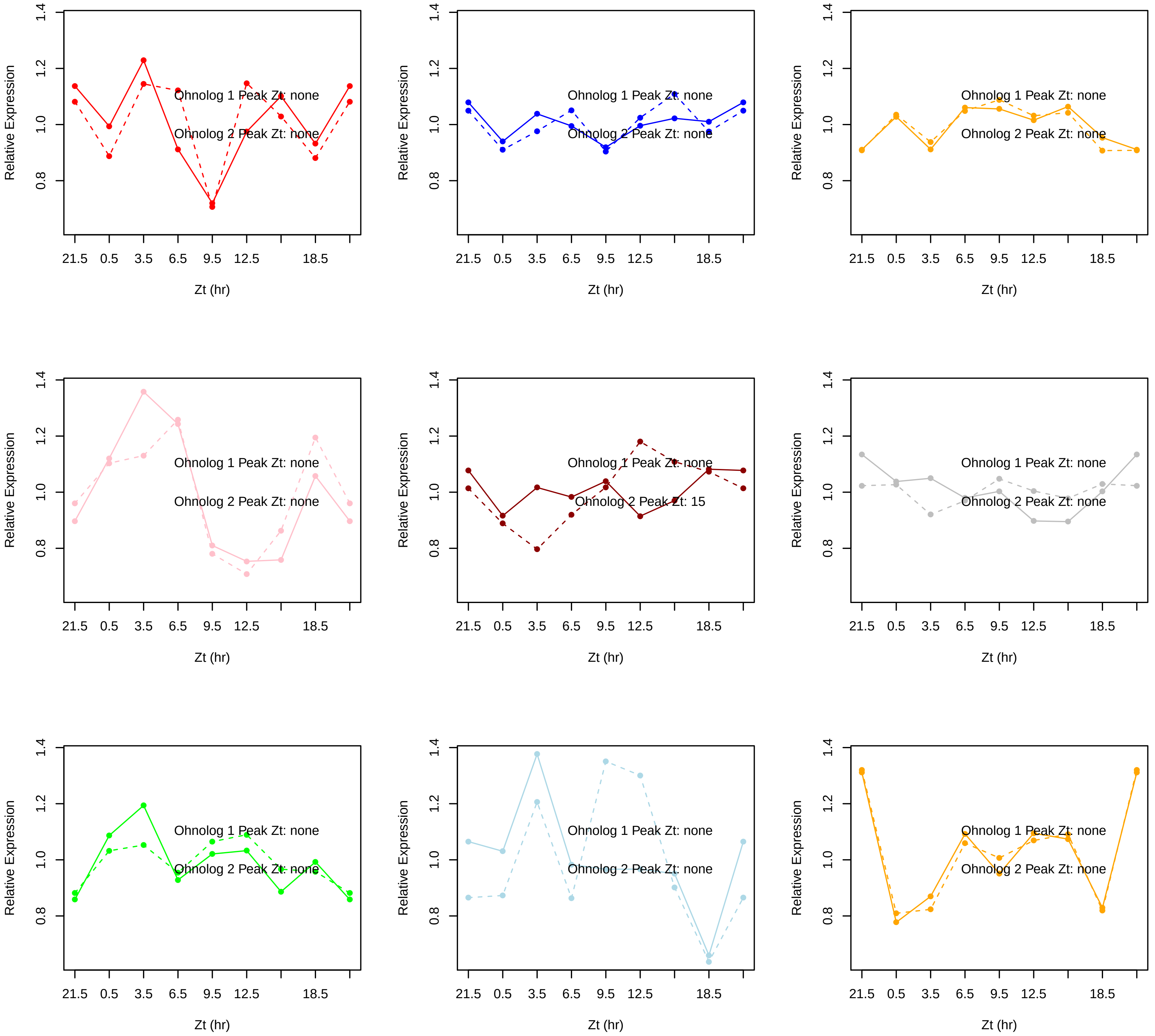

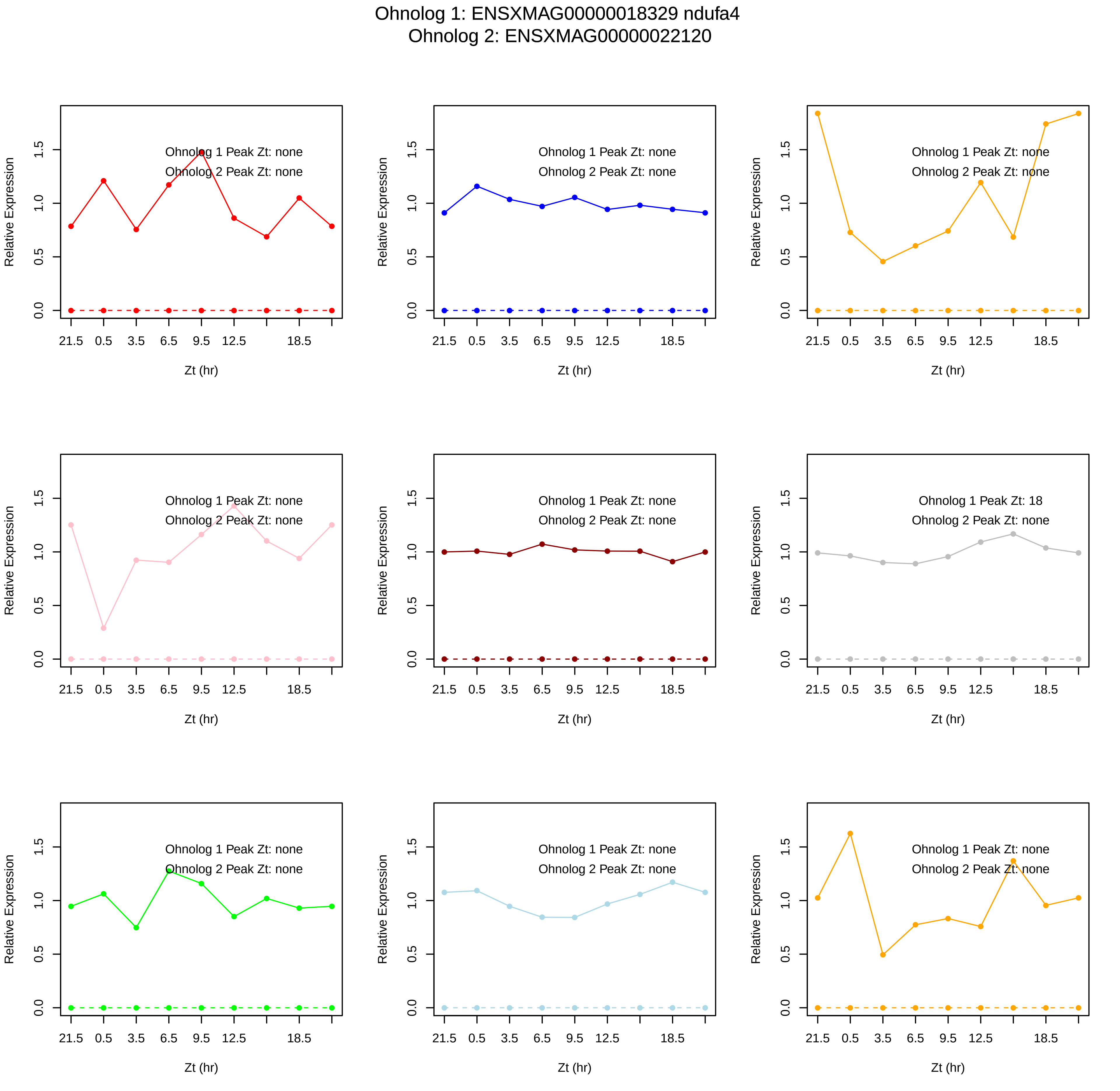
Ohnolog 1: ENSXMAG00000018329 ndufa4
Ohnolog 2: ENSXMAG00000022120
Relative Expression
Zt (hr)
21.5
0.5
3.5
6.5
9.5
12.5
18.5
0.0
0.5
1.0
1.5
Ohnolog 1 Peak Zt: none
Ohnolog 2 Peak Zt: none
Ohnolog 1 Peak Zt: 18

Ohnolog 1: ENSXMAG00000005784 nt5c2a
Ohnolog 2: ENSXMAG00000029312 nt5c2b

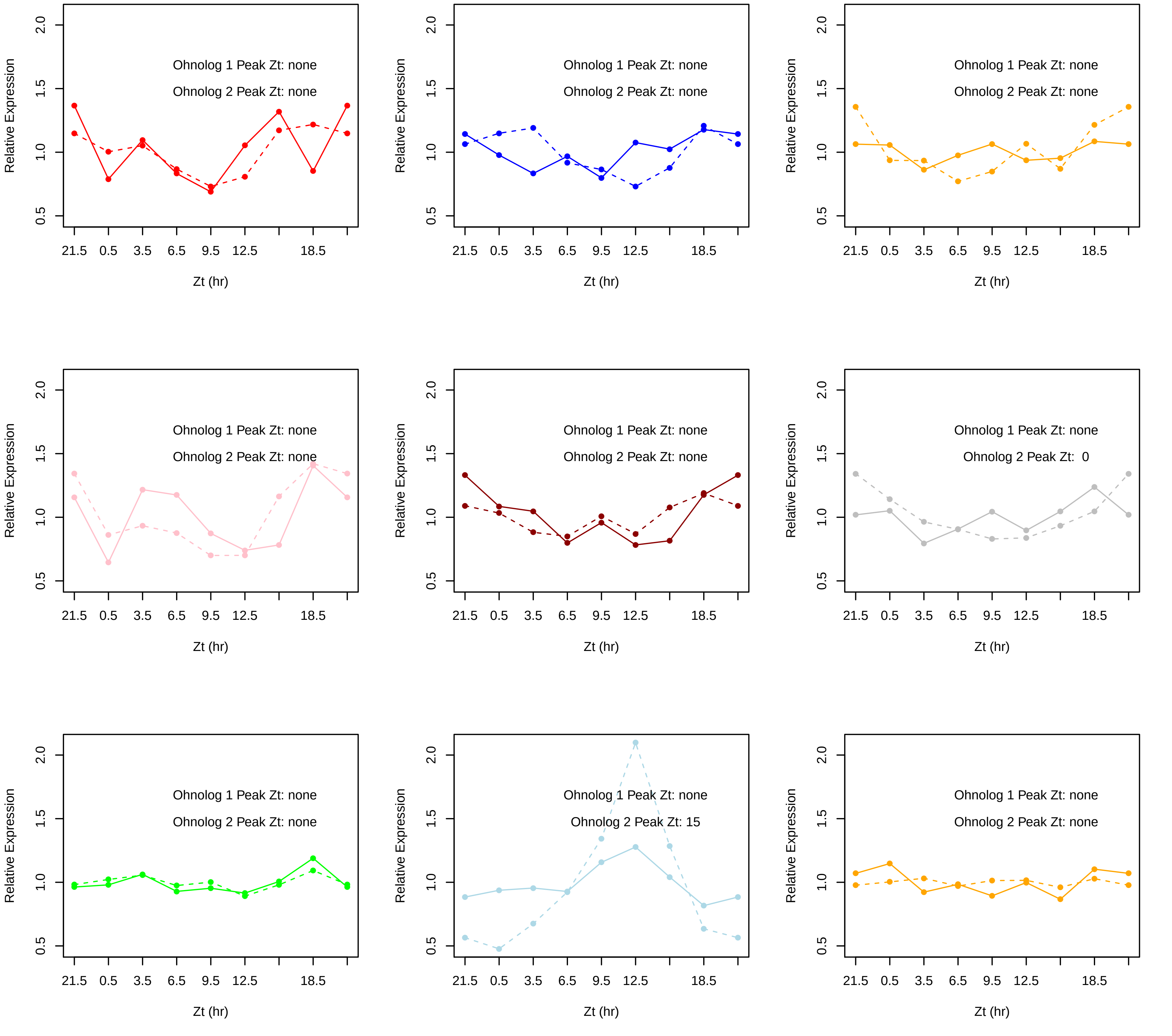

Ohnolog 1: ENSXMAG00000028422 hoxa10b
Ohnolog 2: ENSXMAG00000029054

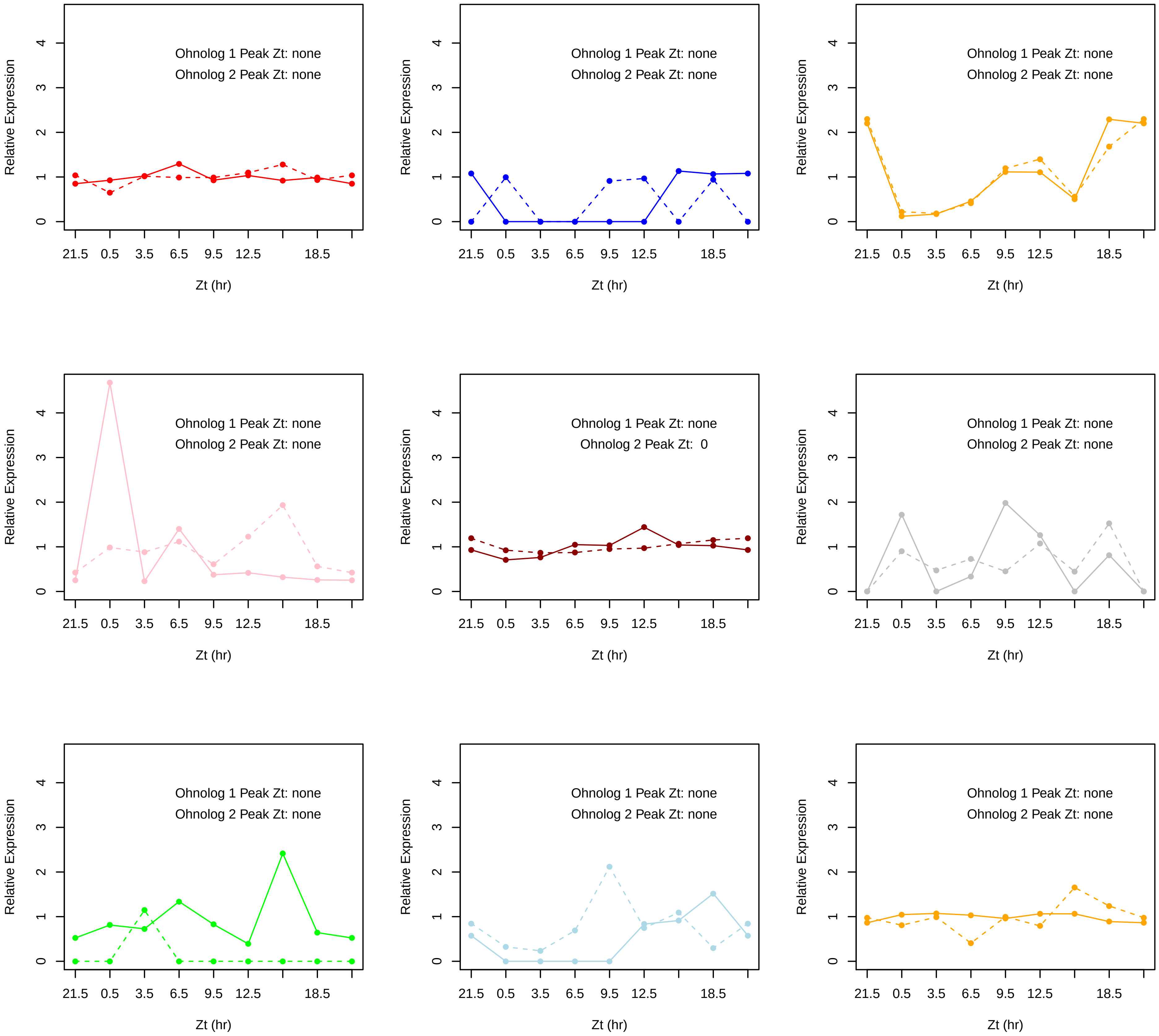

Ohnolog 1: ENSXMAG00000007352 si:dkey−51a16.9
Ohnolog 2: ENSXMAG00000026427 zgc:175214

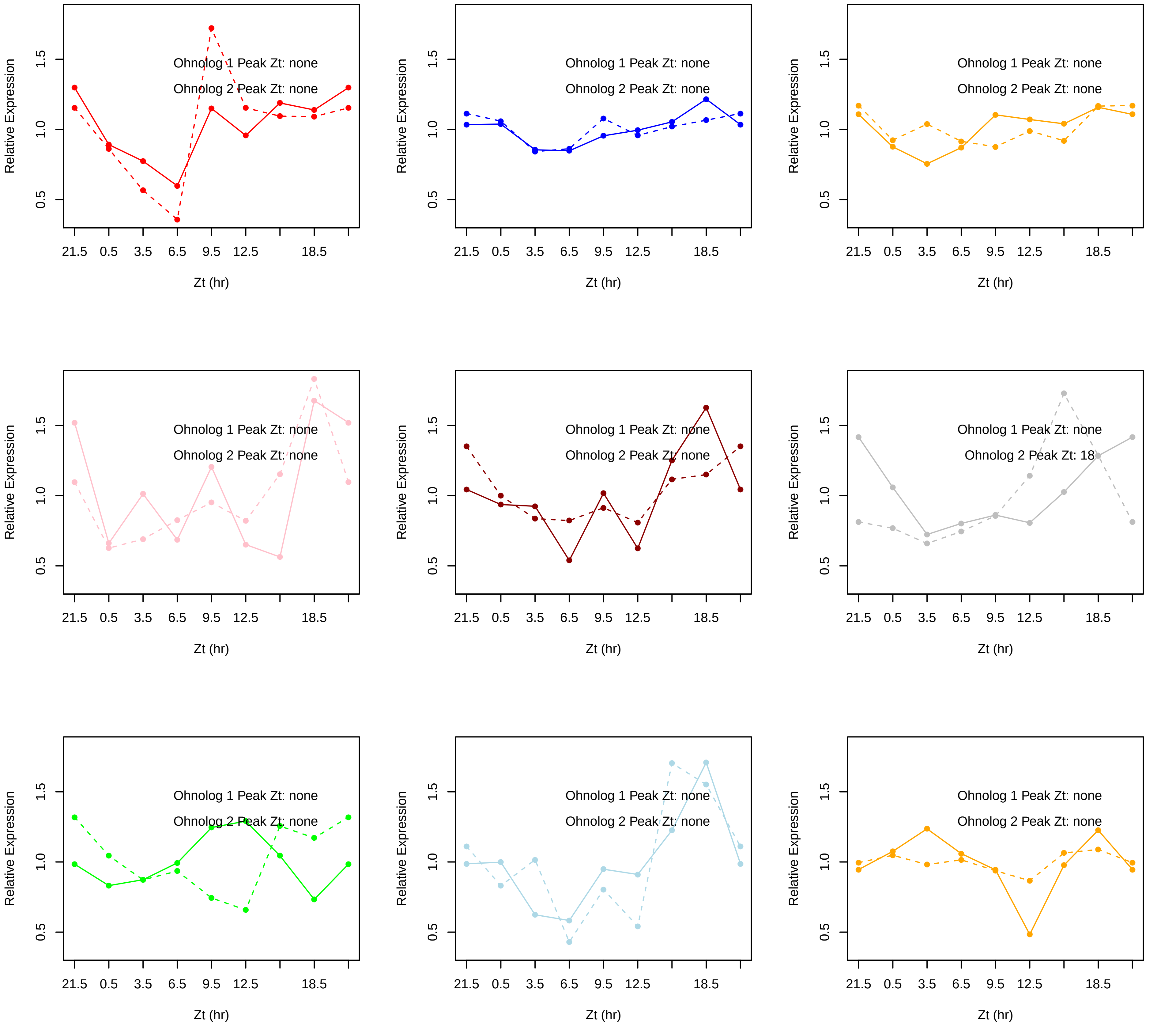

Ohnolog 1: ENSXMAG00000013824 fmnl1a
Ohnolog 2: ENSXMAG00000016886

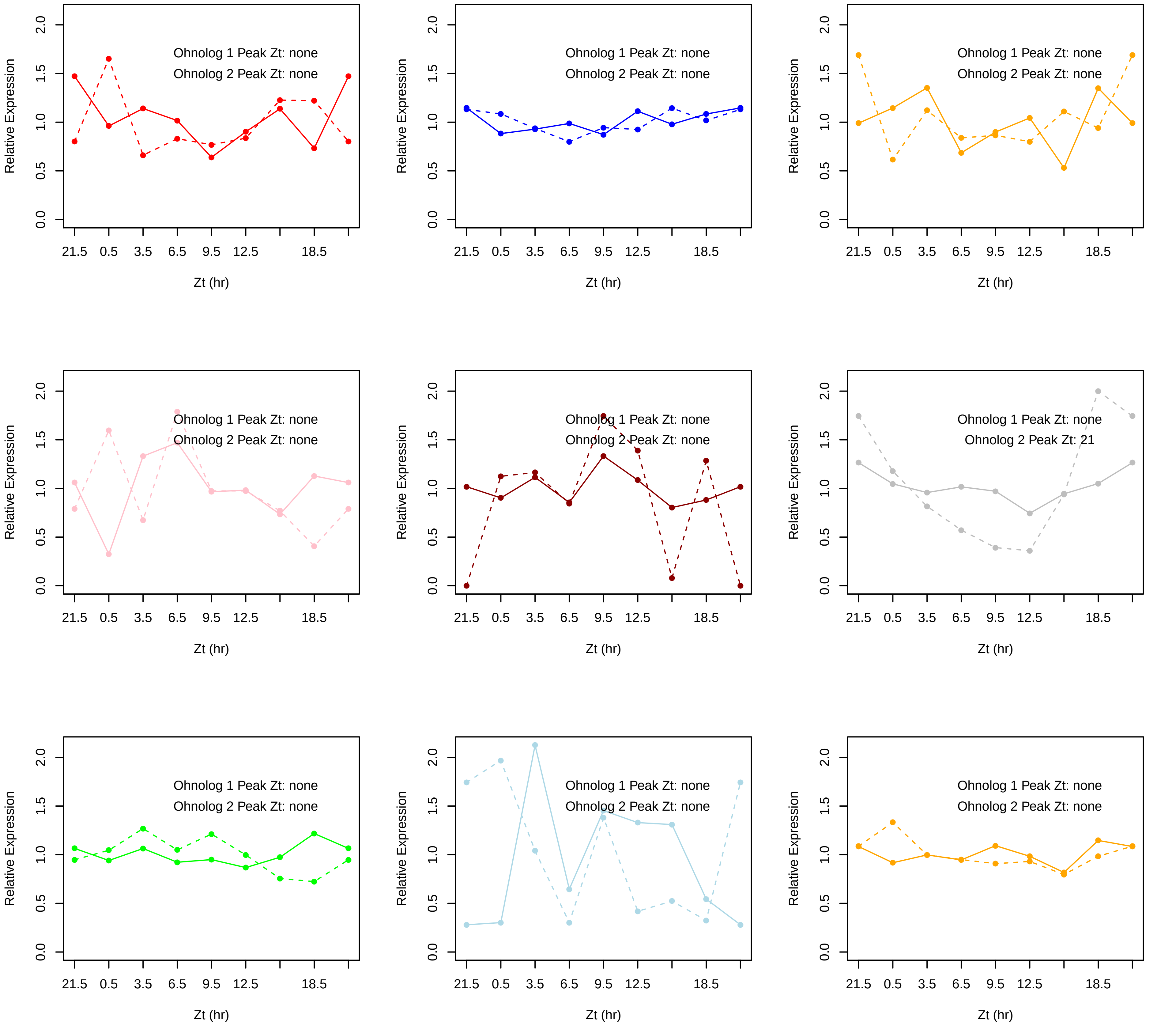

Ohnolog 1: ENSXMAG00000005912 cnga1a
Ohnolog 2: ENSXMAG00000010681 cnga1b

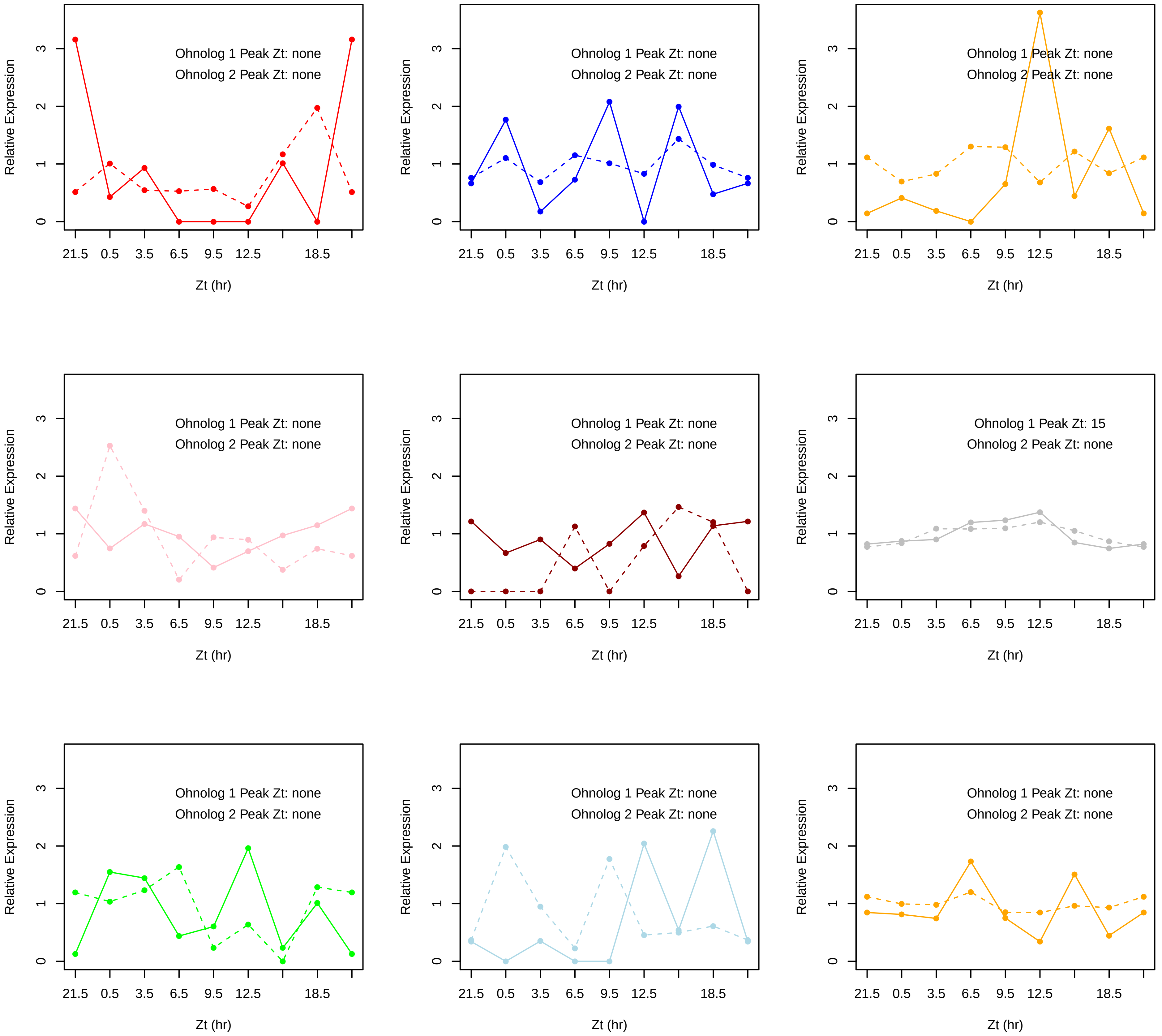

Ohnolog 1: ENSXMAG00000006407
Ohnolog 2: ENSXMAG00000008955 myh9b

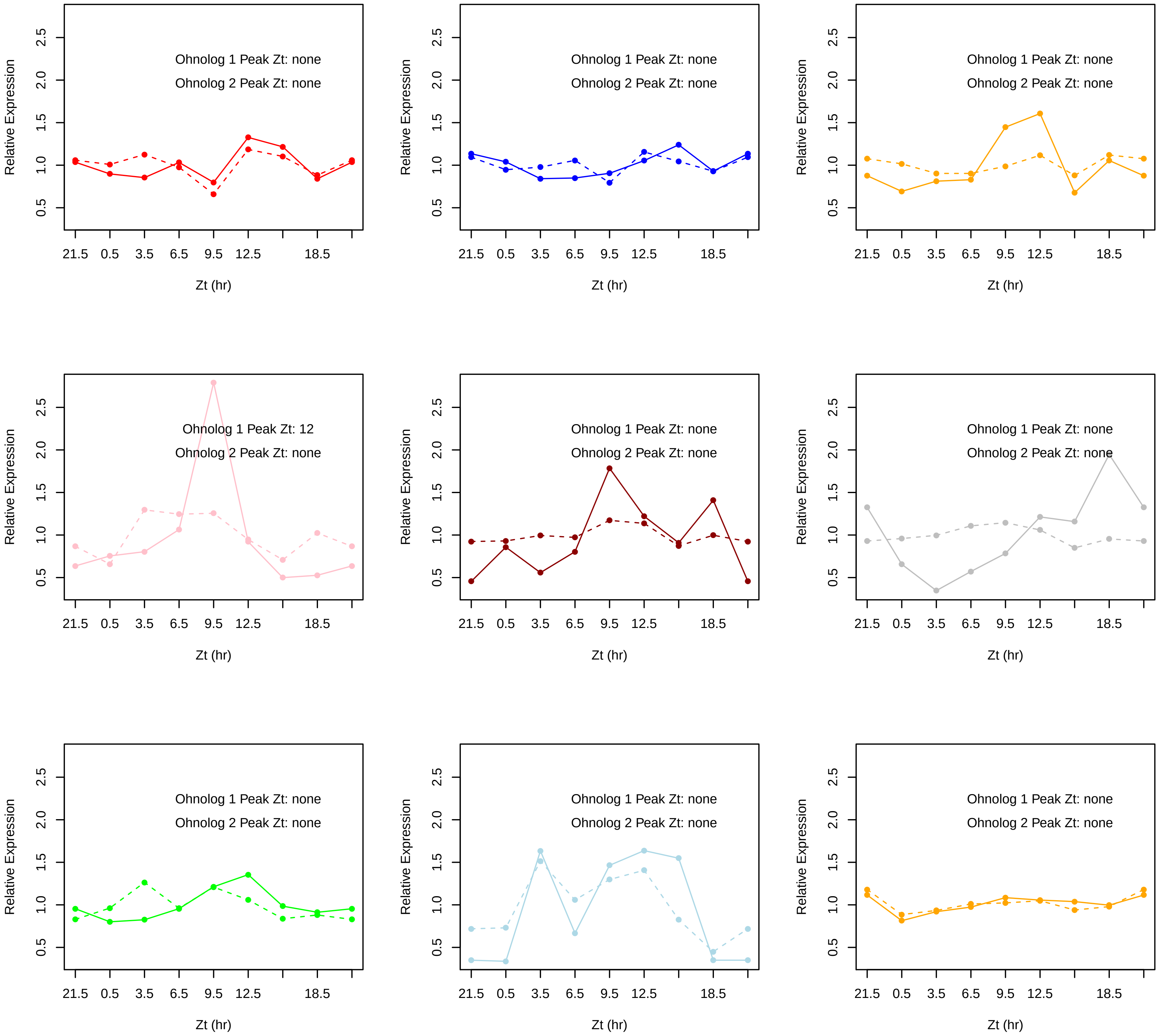

Ohnolog 1: ENSXMAG00000006405 slc25a38b
Ohnolog 2: ENSXMAG00000009093 SLC25A38

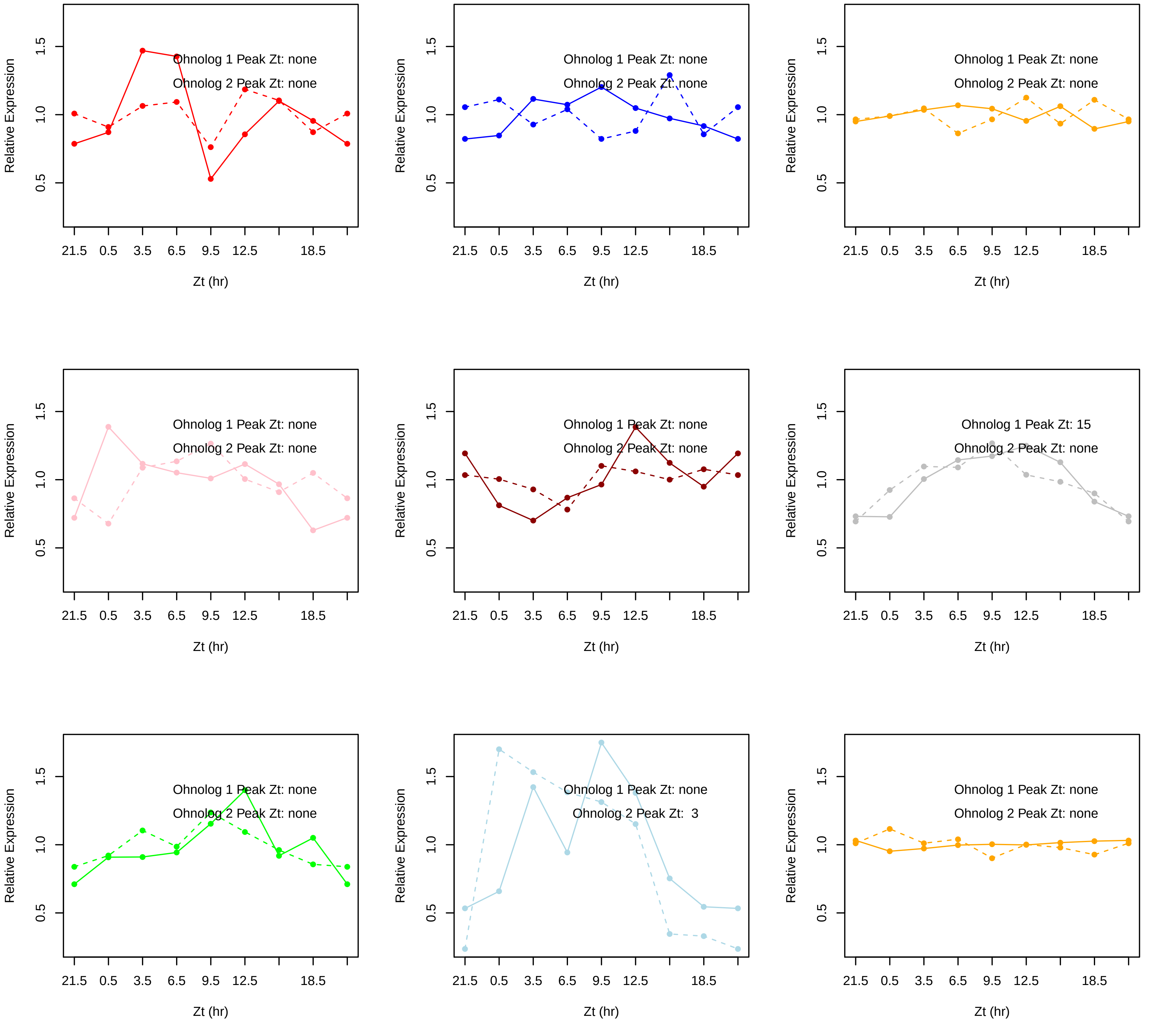

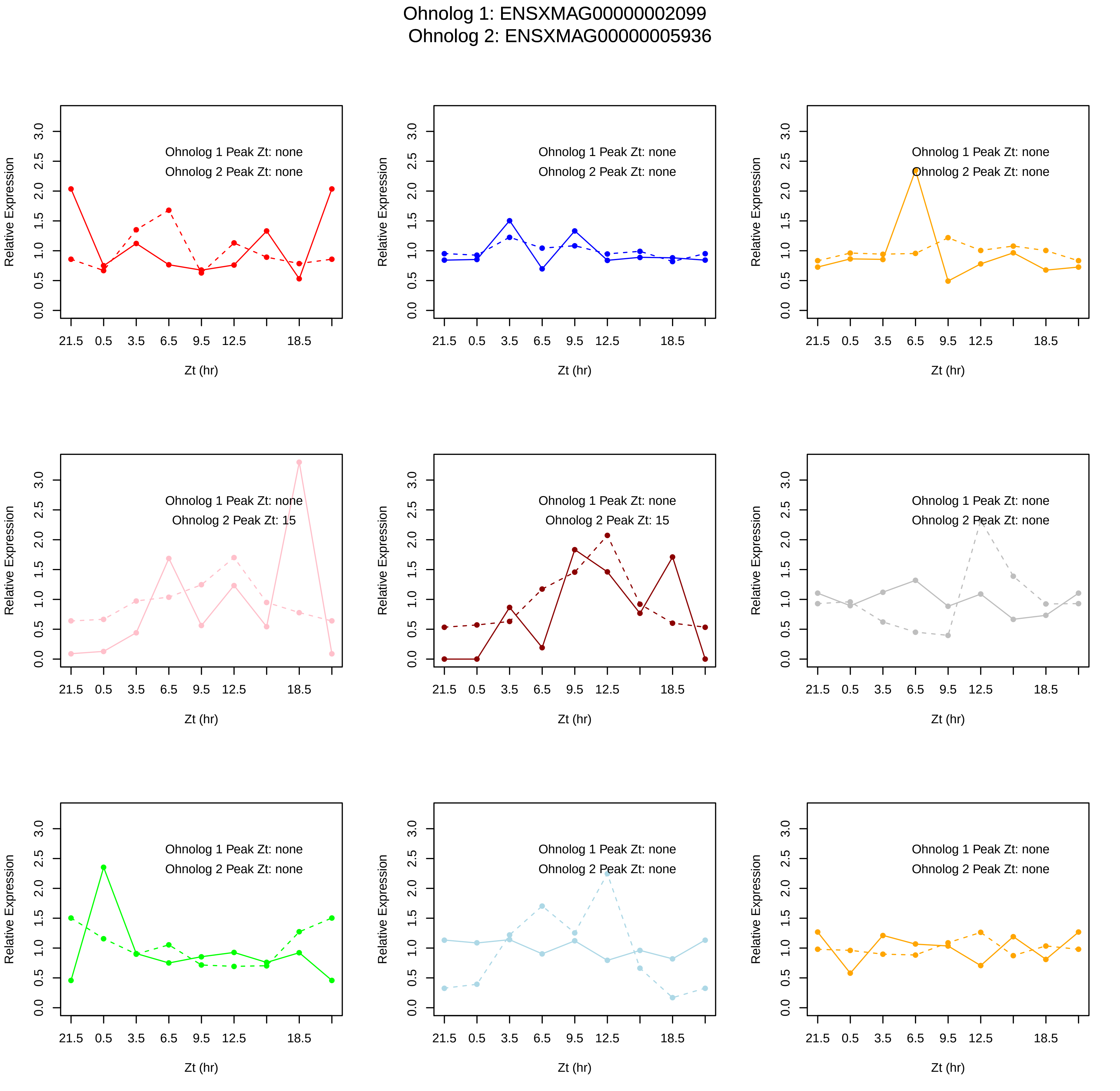
Ohnolog 1: ENSXMAG00000002099
Ohnolog 2: ENSXMAG00000005936
Ohnolog 1 Peak Zt: none
Ohnolog 2 Peak Zt: none
Ohnolog 1 Peak Zt: none
Ohnolog 2 Peak Zt: none
Ohnolog 1 Peak Zt: none
Ohnolog 2 Peak Zt: none
Ohnolog 1 Peak Zt: none
Ohnolog 2 Peak Zt: 15
Ohnolog 1 Peak Zt: none
Ohnolog 2 Peak Zt: 15
Ohnolog 1 Peak Zt: none
Ohnolog 2 Peak Zt: none
Ohnolog 1 Peak Zt: none
Ohnolog 2 Peak Zt: none
Ohnolog 1 Peak Zt: none
Ohnolog 2 Peak Zt: none
Ohnolog 1 Peak Zt: none
Ohnolog 2 Peak Zt: none
Relative Expression
Zt (hr)

Ohnolog 1: ENSXMAG00000009106 ubn2b
Ohnolog 2: ENSXMAG00000020944 ubn2a

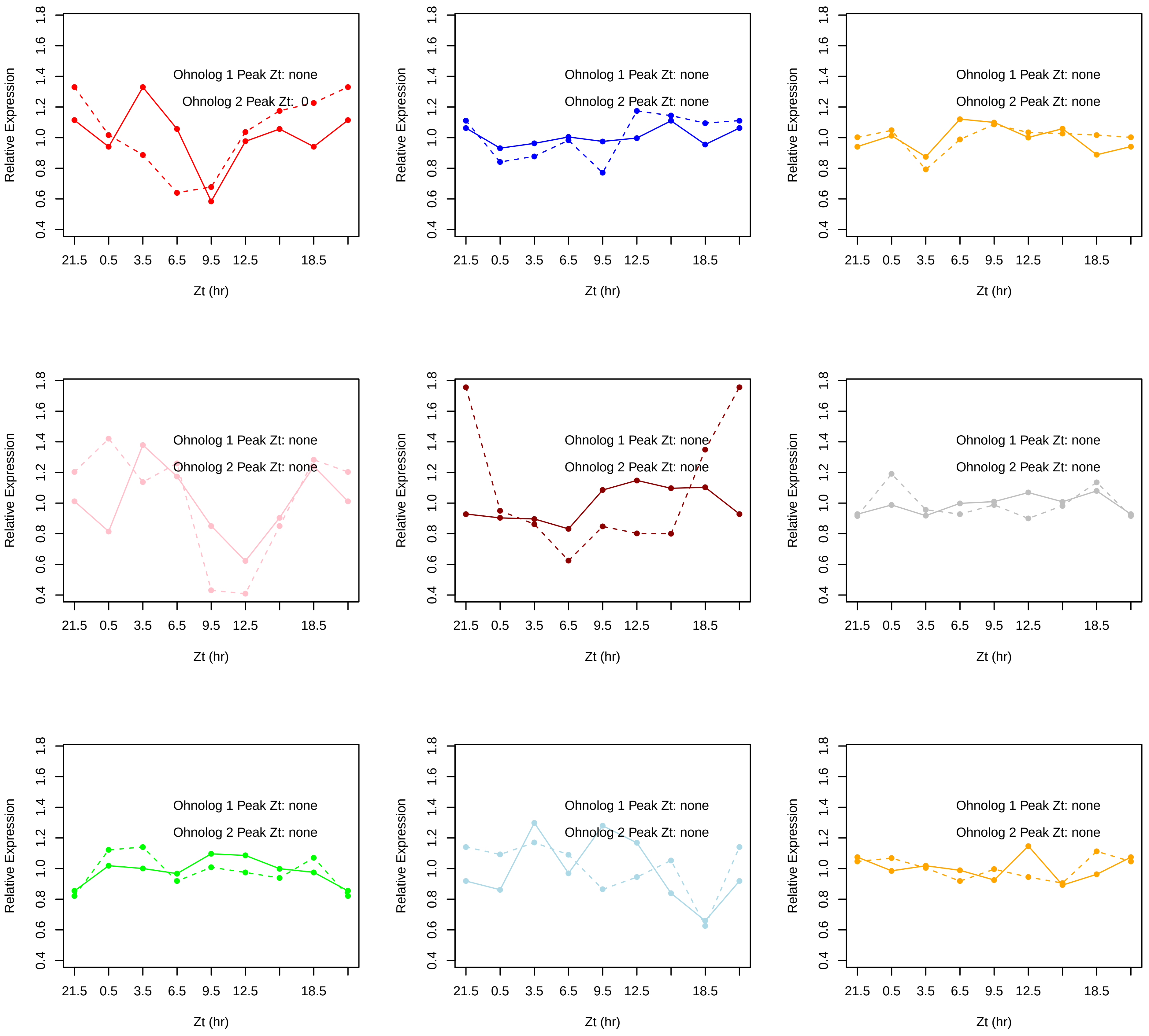

Ohnolog 1: ENSXMAG00000004607
Ohnolog 2: ENSXMAG00000005288

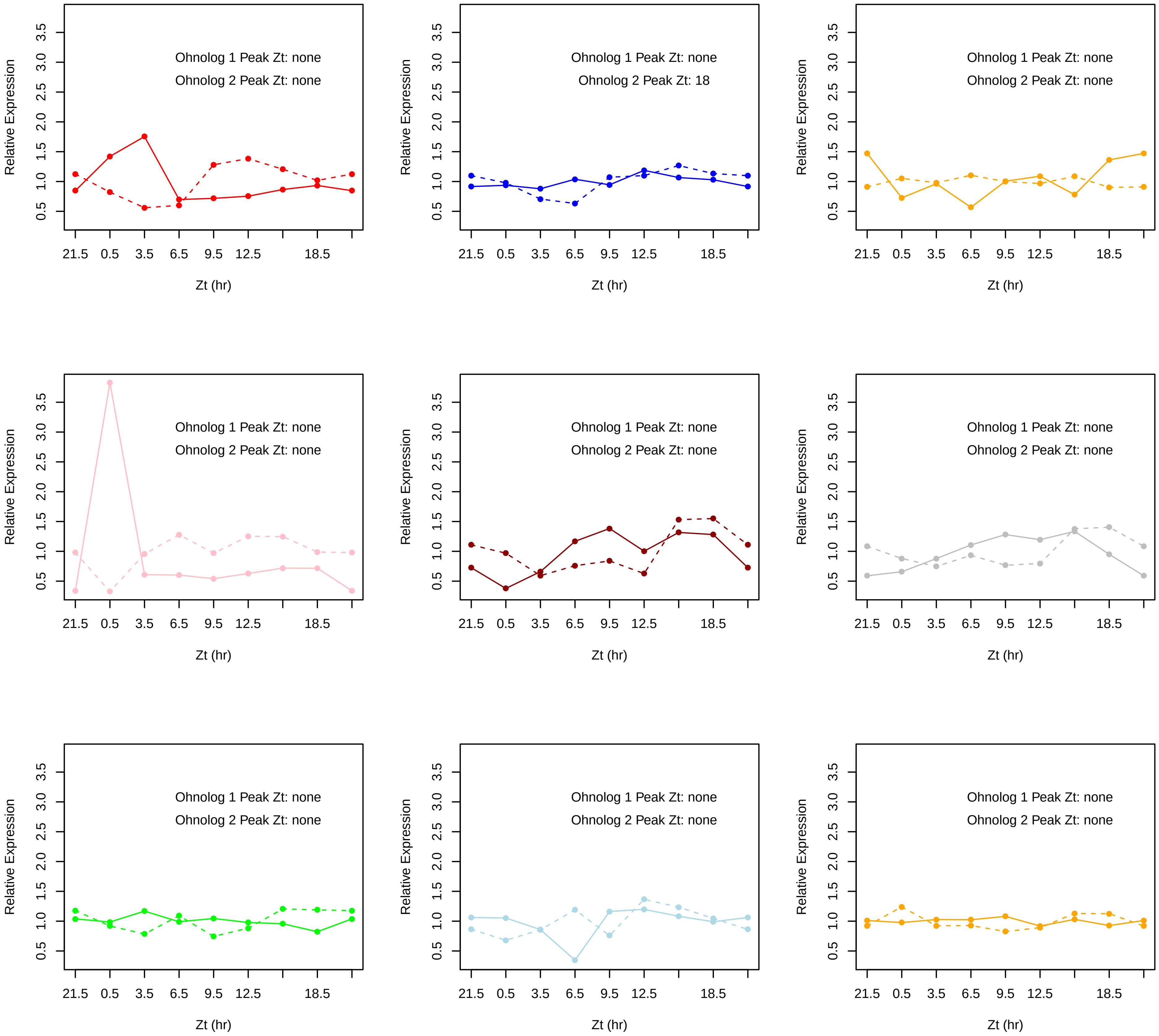

Ohnolog 1: ENSXMAG00000013166 SHISA6
Ohnolog 2: ENSXMAG00000027975

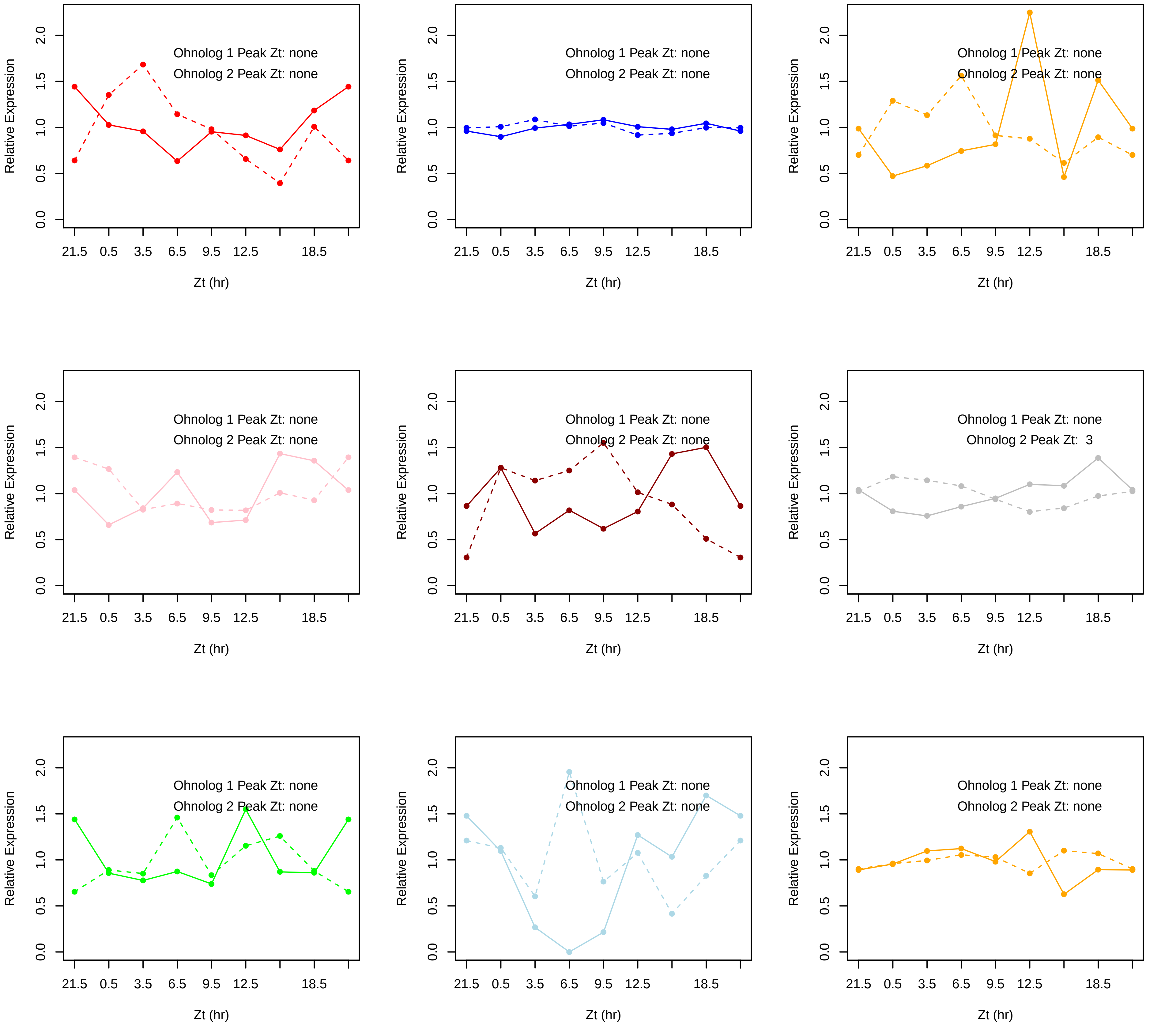

Ohnolog 1: ENSXMAG00000002337
Ohnolog 2: ENSXMAG00000015293 eif4eb

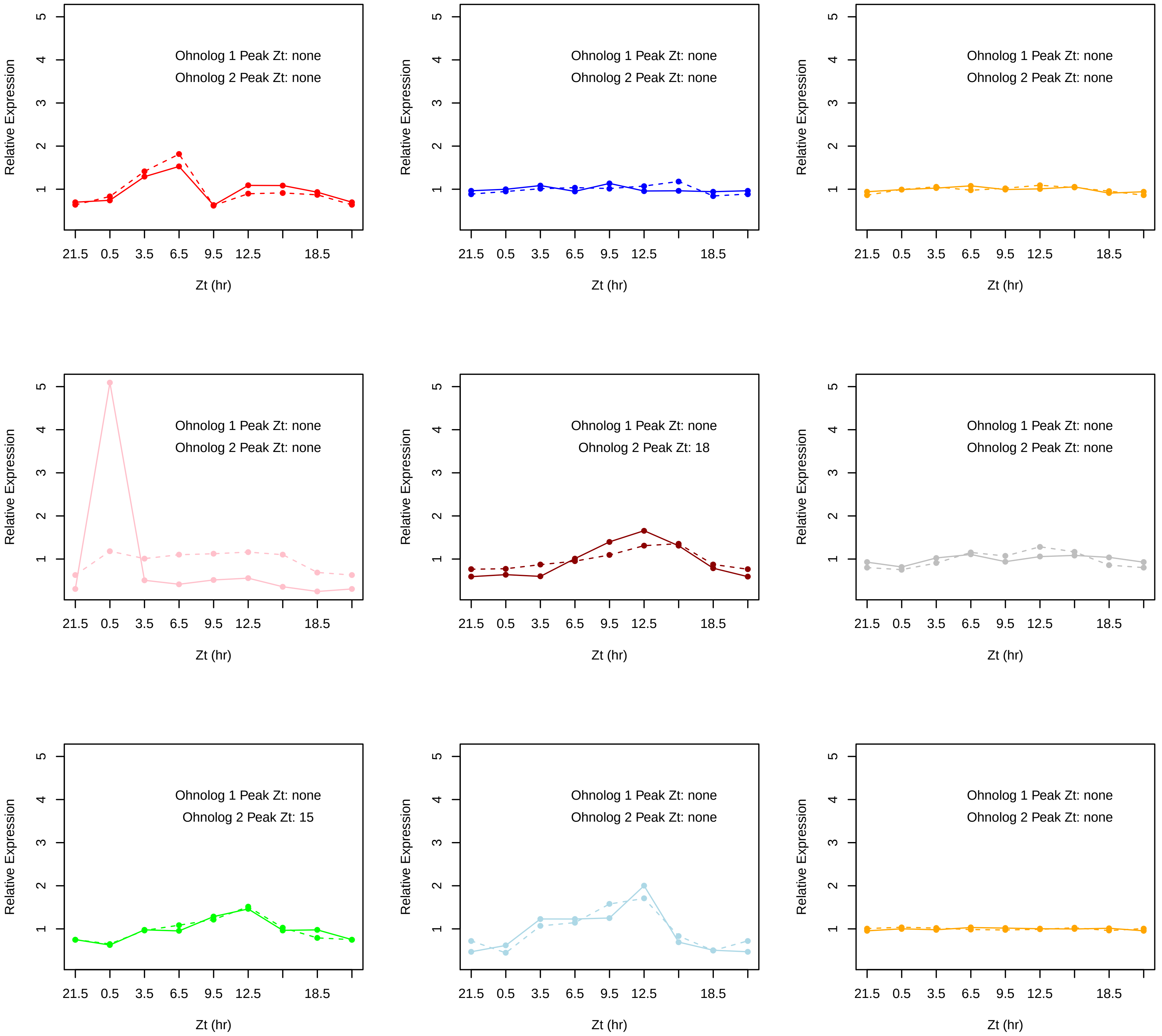

Ohnolog 1: ENSXMAG00000011644 foxk2
Ohnolog 2: ENSXMAG00000013158 si:ch211−262e15.1

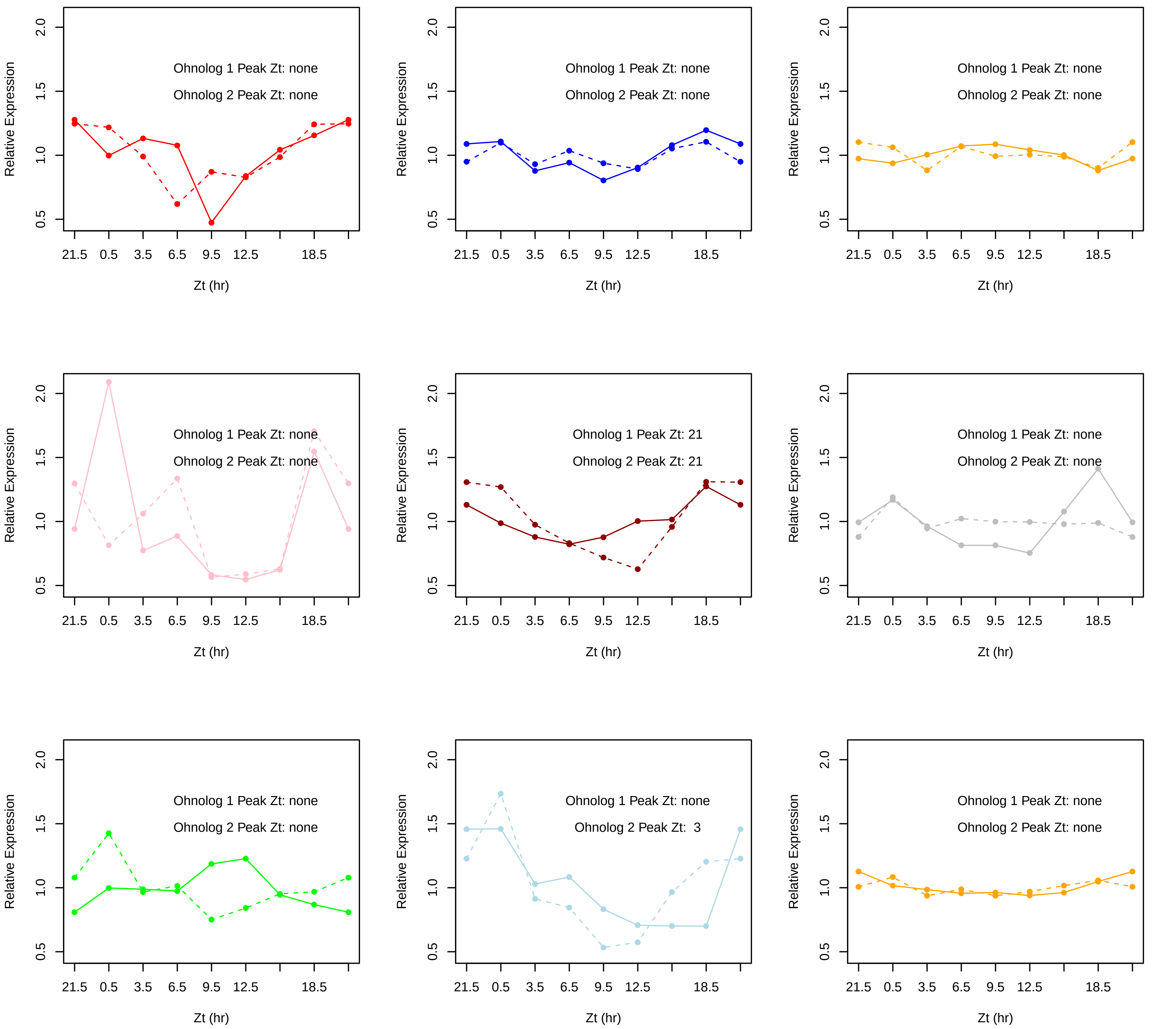

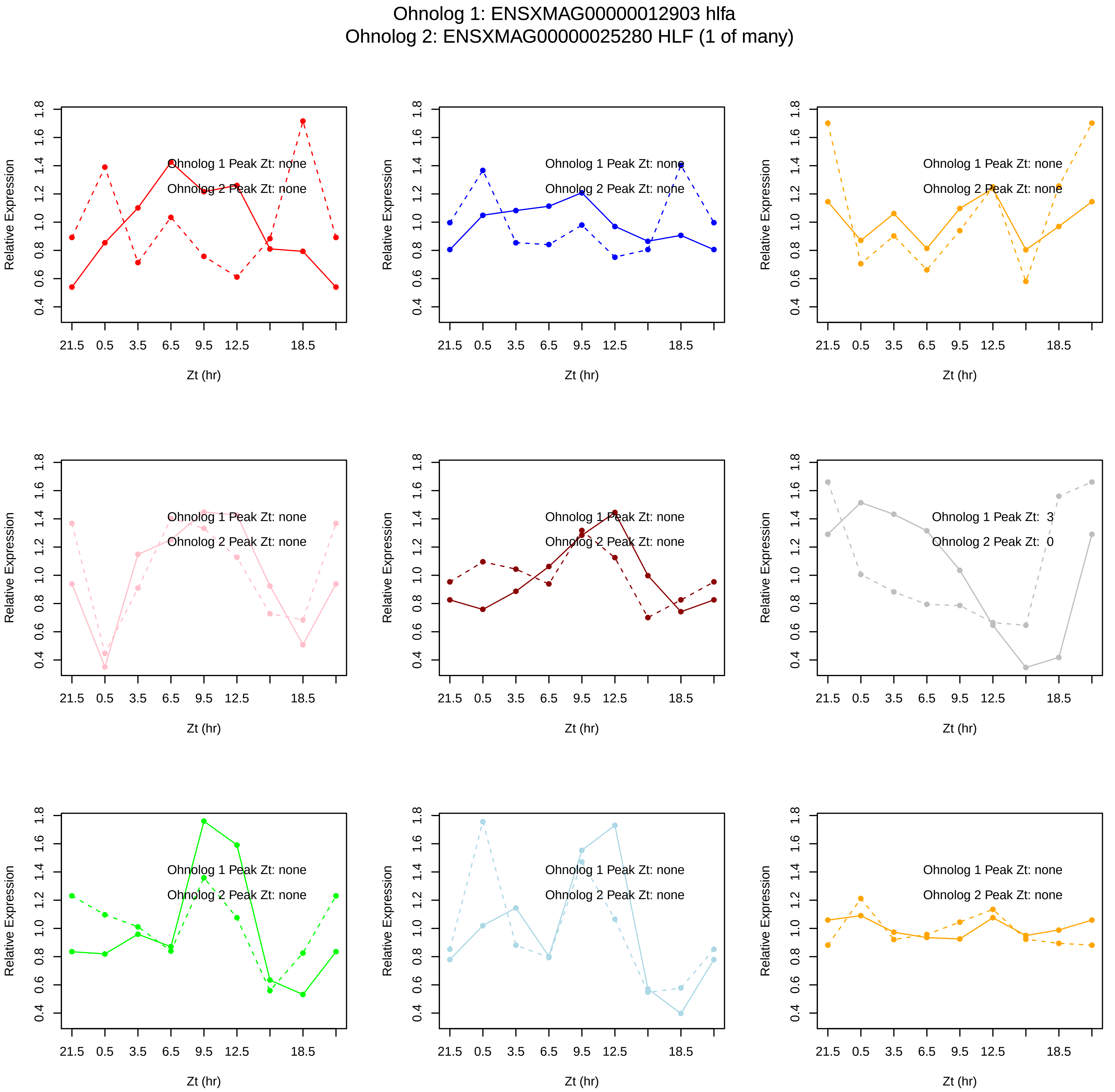
Ohnolog 1: ENSXMAG00000012903 hlfa
Ohnolog 2: ENSXMAG00000025280 HLF (1 of many)
Relative Expression
Zt (hr)
21.5
0.5
3.5
6.5
9.5
12.5
18.5
0.4
0.6
0.8
1.0
1.2
1.4
1.6
1.8
Ohnolog 1 Peak Zt: none
Ohnolog 2 Peak Zt: none
Ohnolog 1 Peak Zt: 3
Ohnolog 2 Peak Zt: 0

Ohnolog 1: ENSXMAG00000004645 adarb2
Ohnolog 2: ENSXMAG00000006770

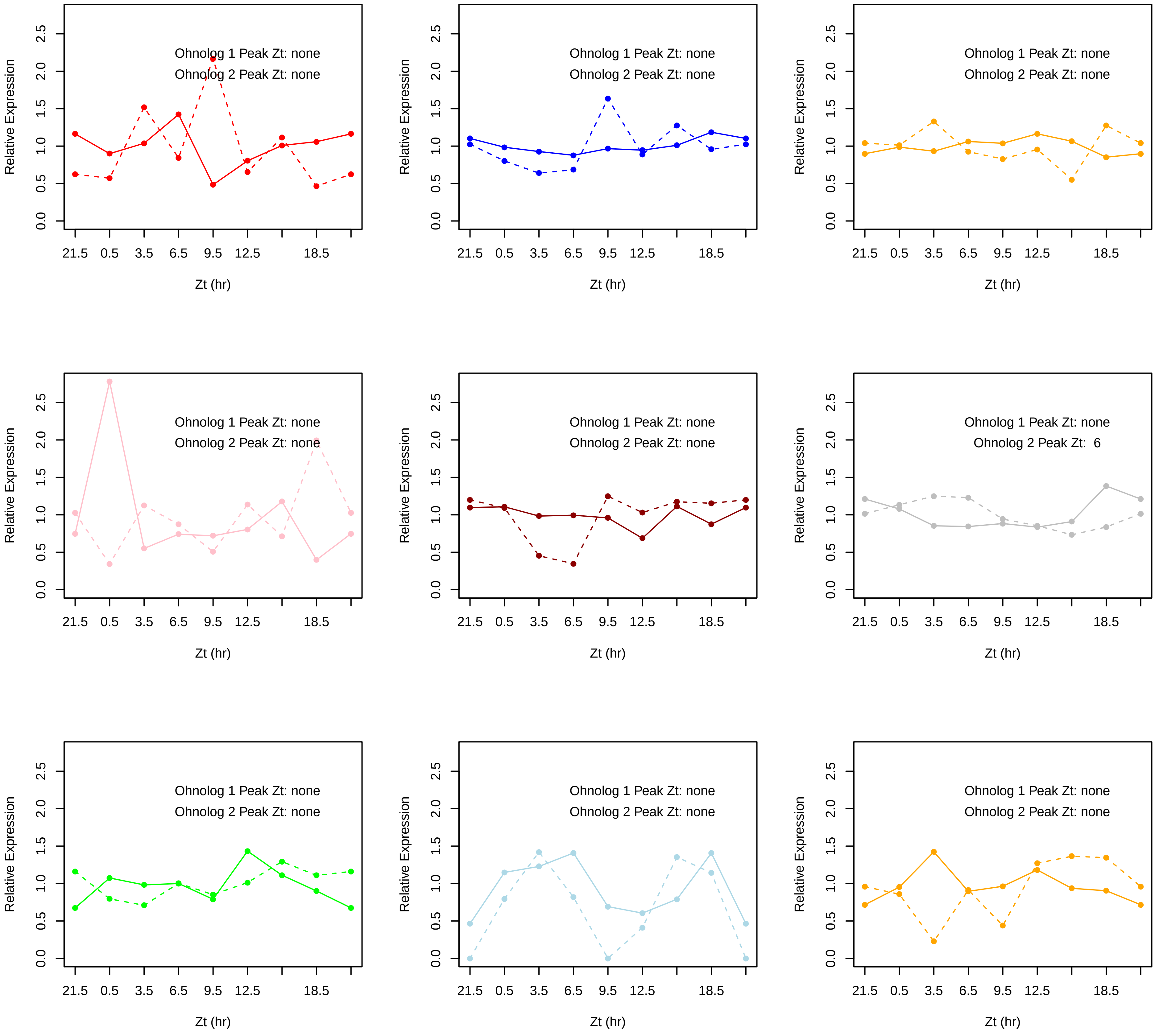

Ohnolog 1: ENSXMAG00000006812 zfyve1
Ohnolog 2: ENSXMAG00000014436 ZFYVE1 (1 of many)

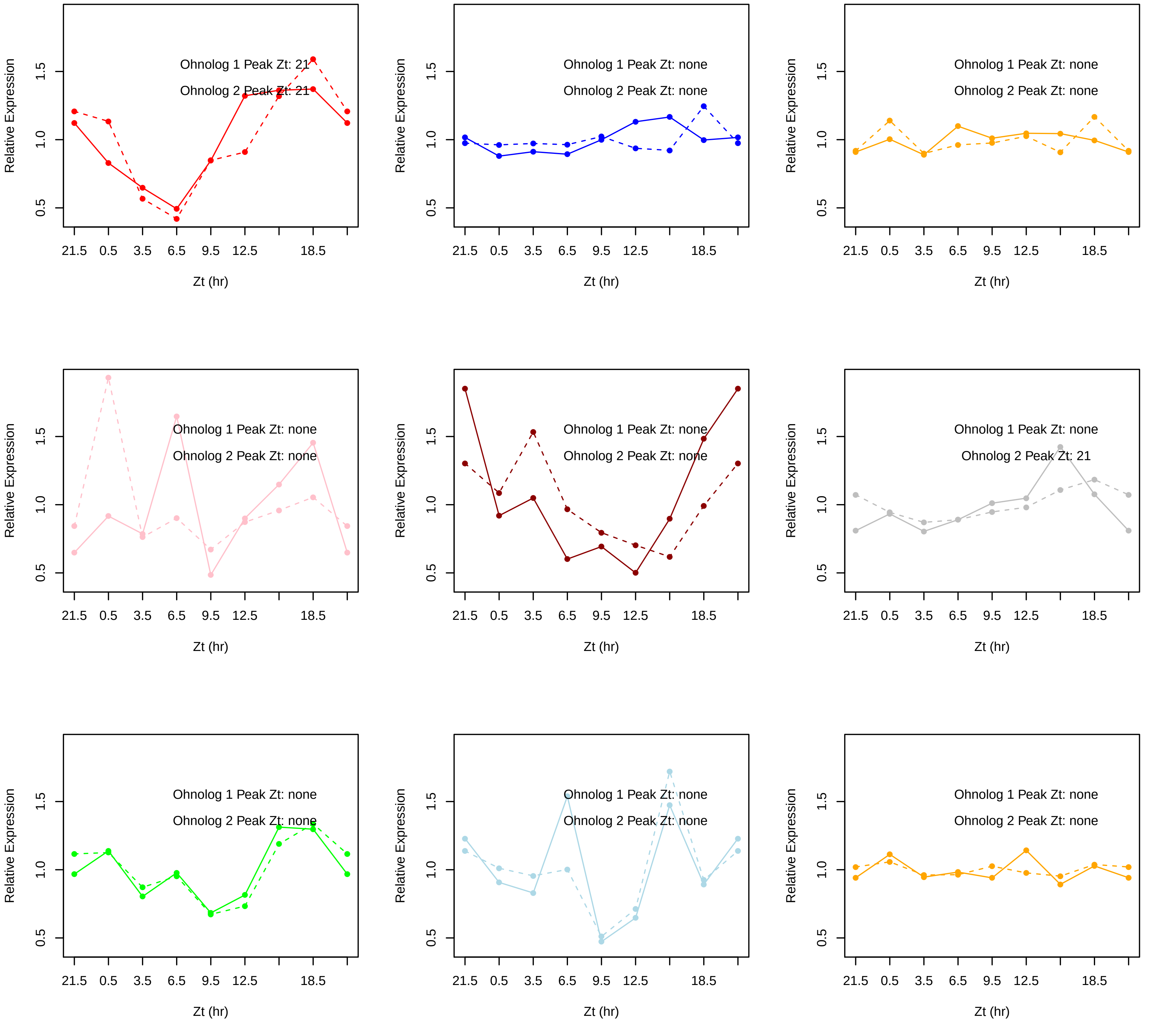

Ohnolog 1: ENSXMAG00000017382 tanc2a
Ohnolog 2: ENSXMAG00000017606 TANC2

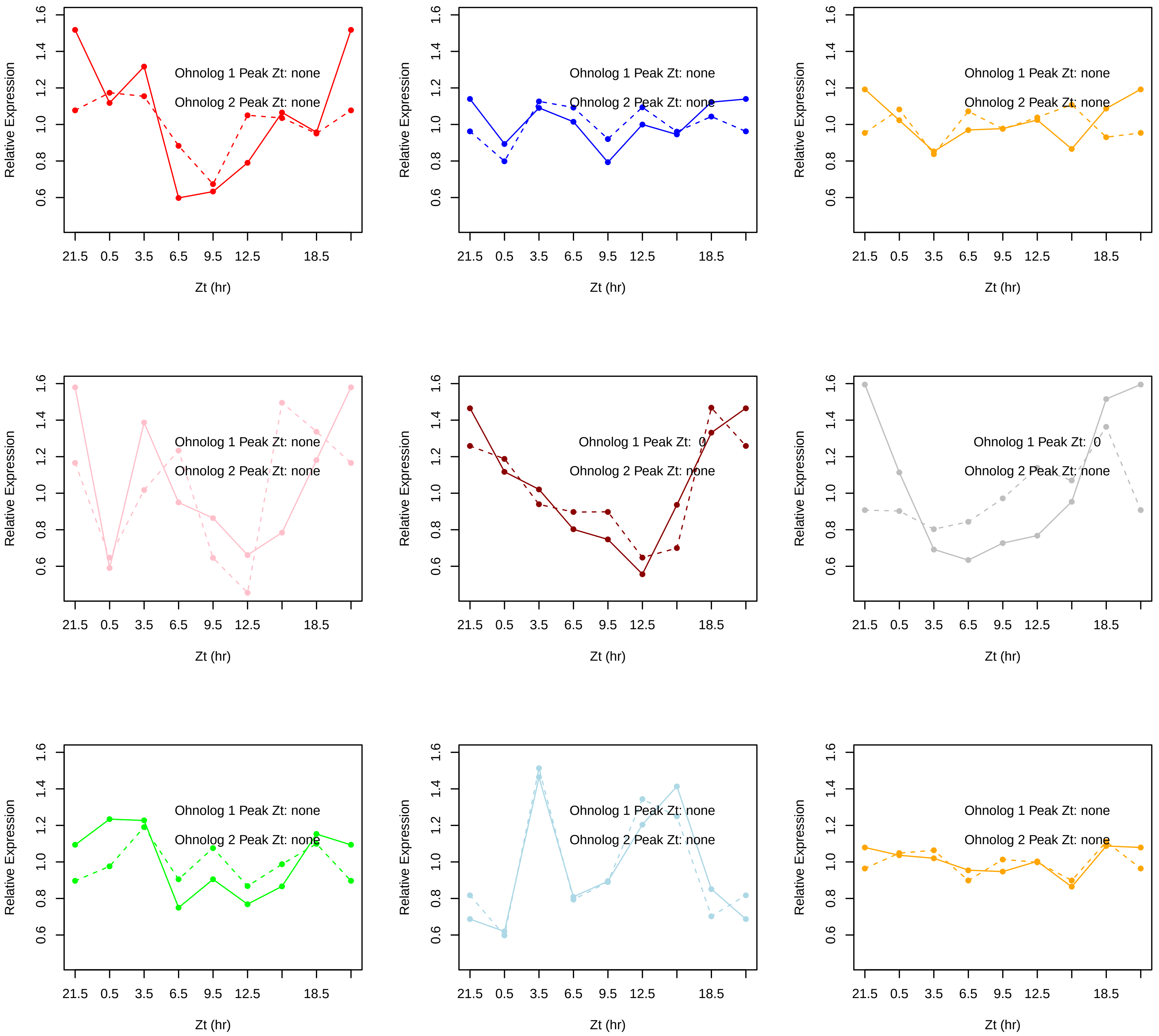

Ohnolog 1: ENSXMAG00000007056 mxi1
Ohnolog 2: ENSXMAG00000012758 MXI1 (1 of many)

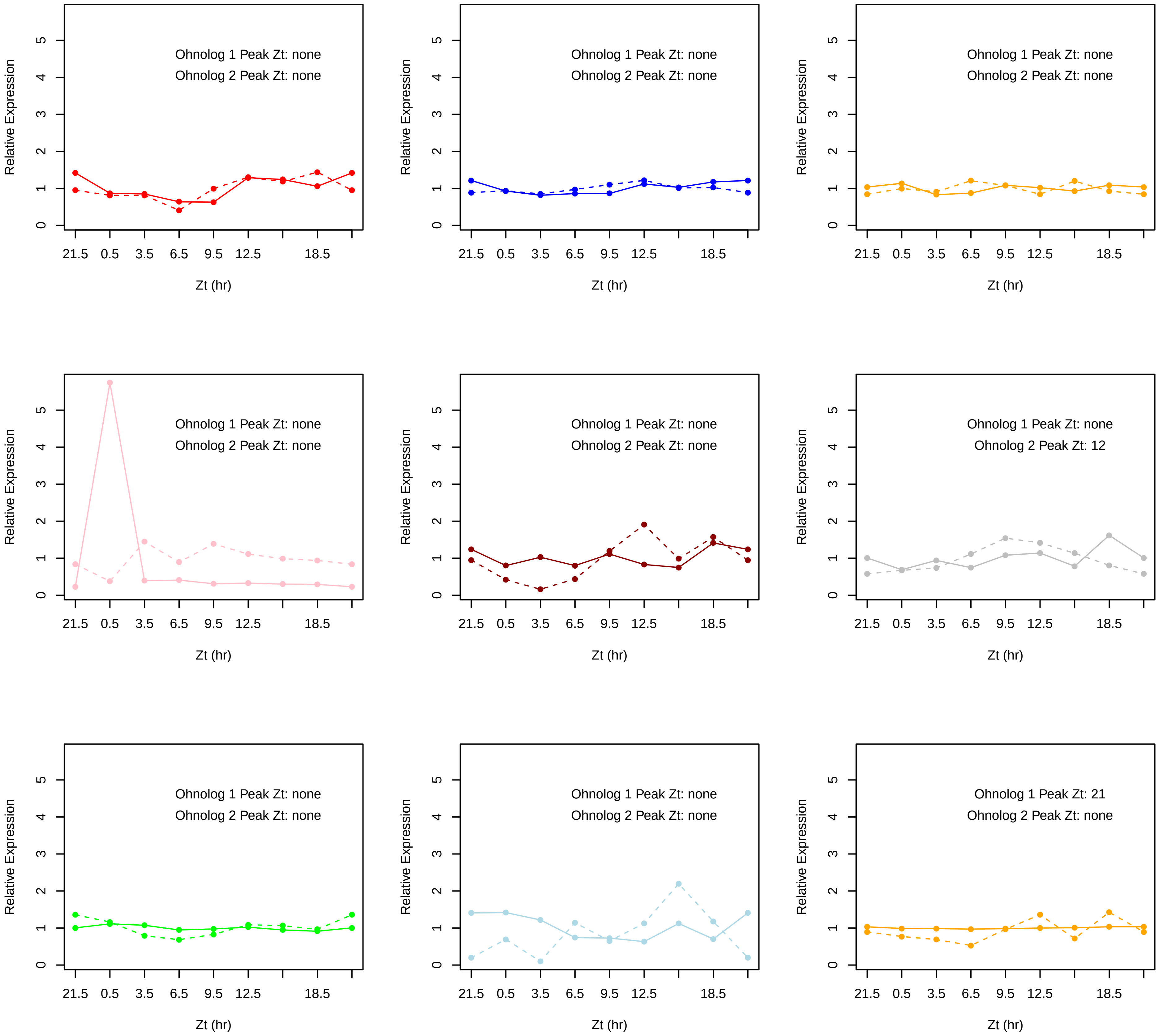

Ohnolog 1: ENSXMAG00000014401 rgs6
Ohnolog 2: ENSXMAG00000027736 RGS6

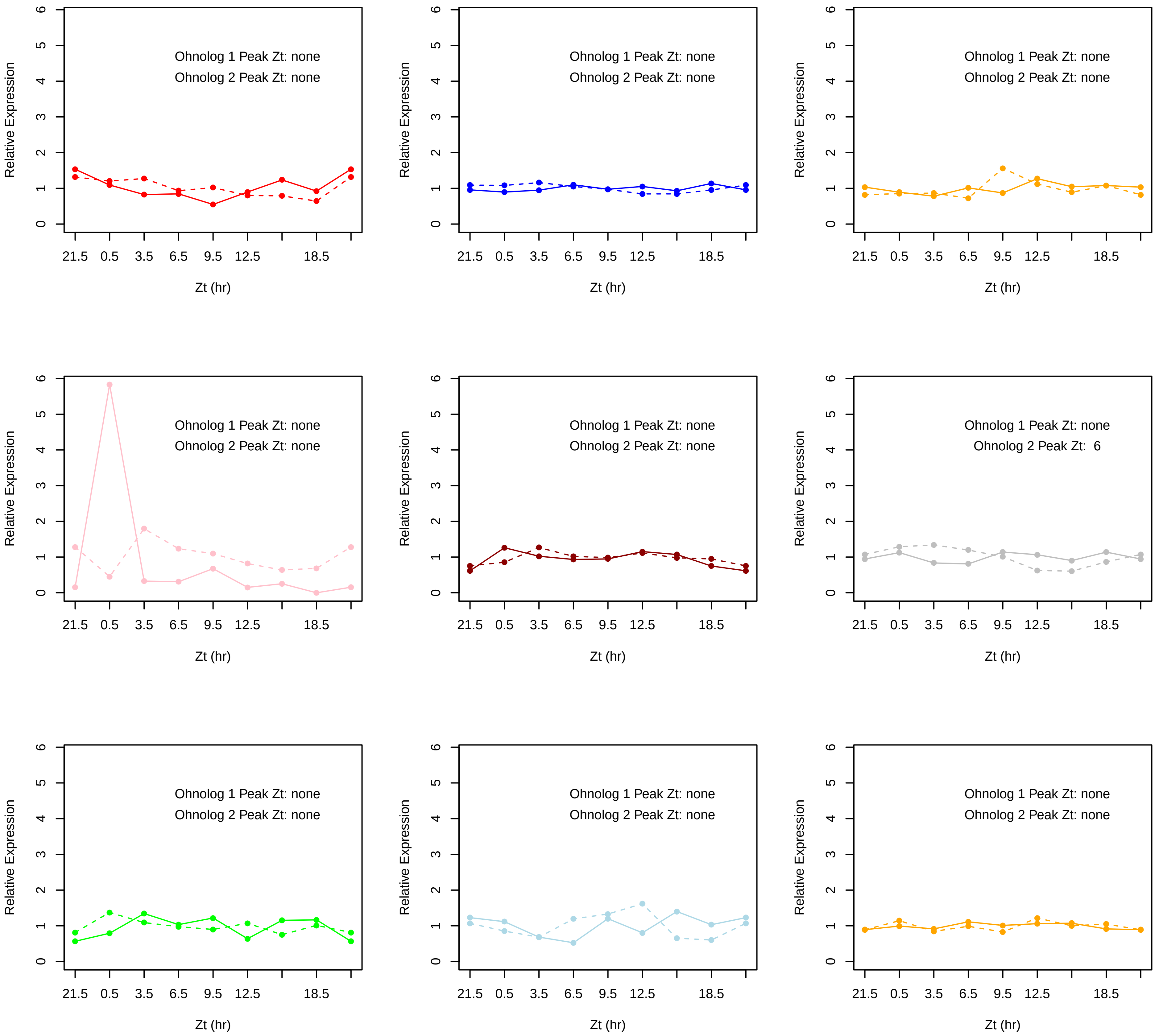

Ohnolog 1: ENSXMAG00000024355
Ohnolog 2: ENSXMAG00000029848 WNK4

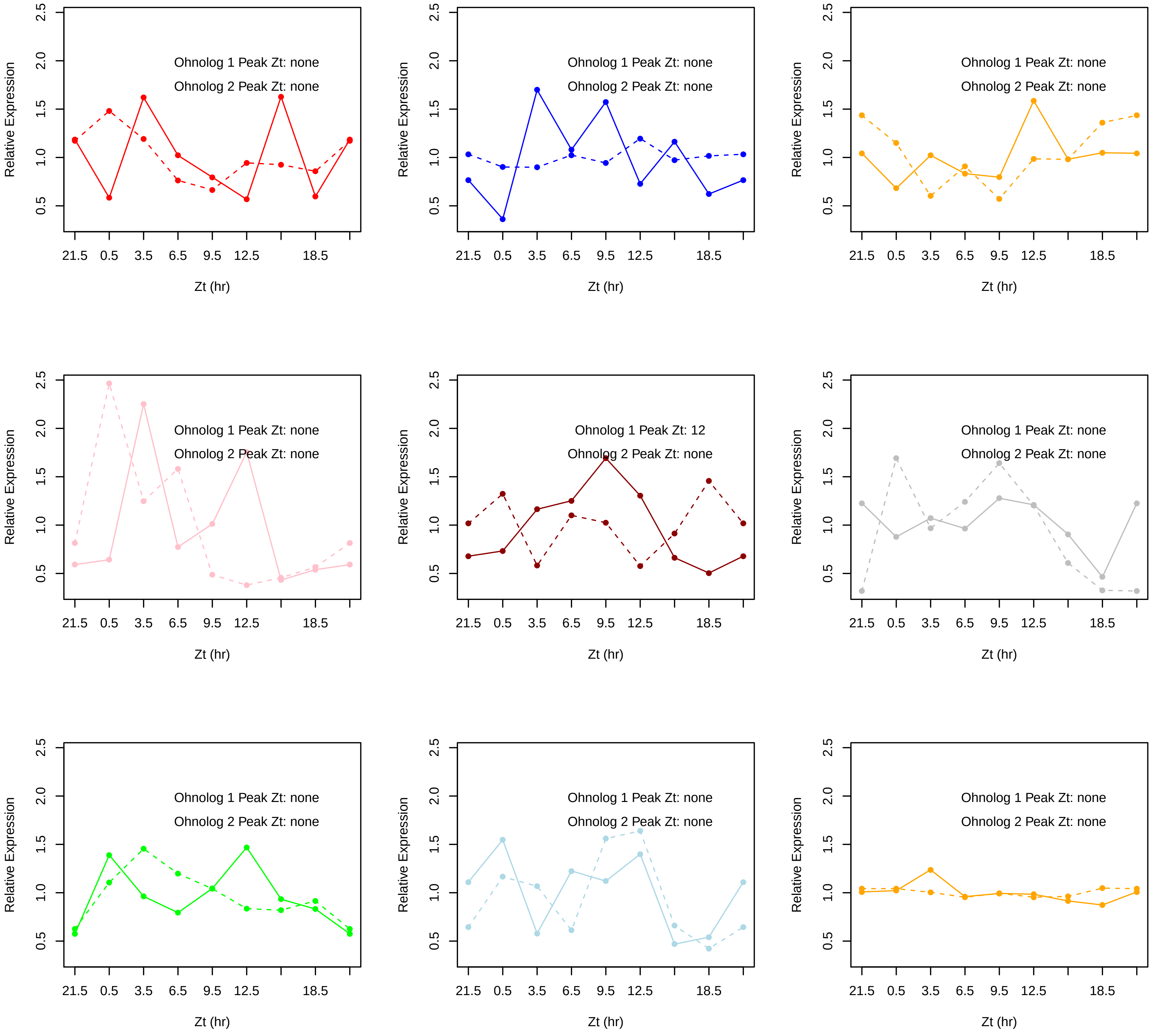

Ohnolog 1: ENSXMAG00000002363 esyt2b
Ohnolog 2: ENSXMAG00000003829 esyt2a

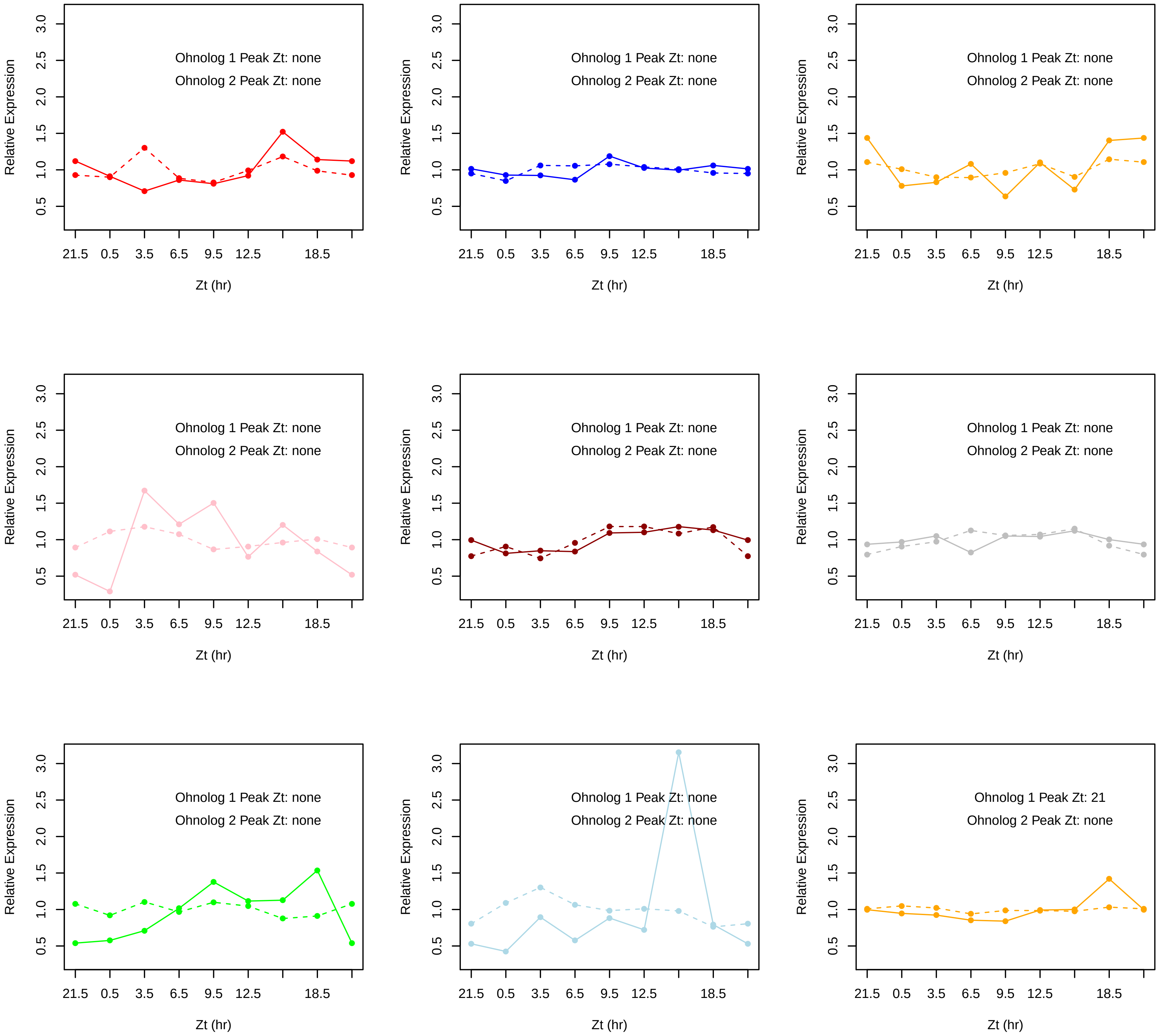

Ohnolog 1: ENSXMAG00000007059 add3a
Ohnolog 2: ENSXMAG00000012762 add3b

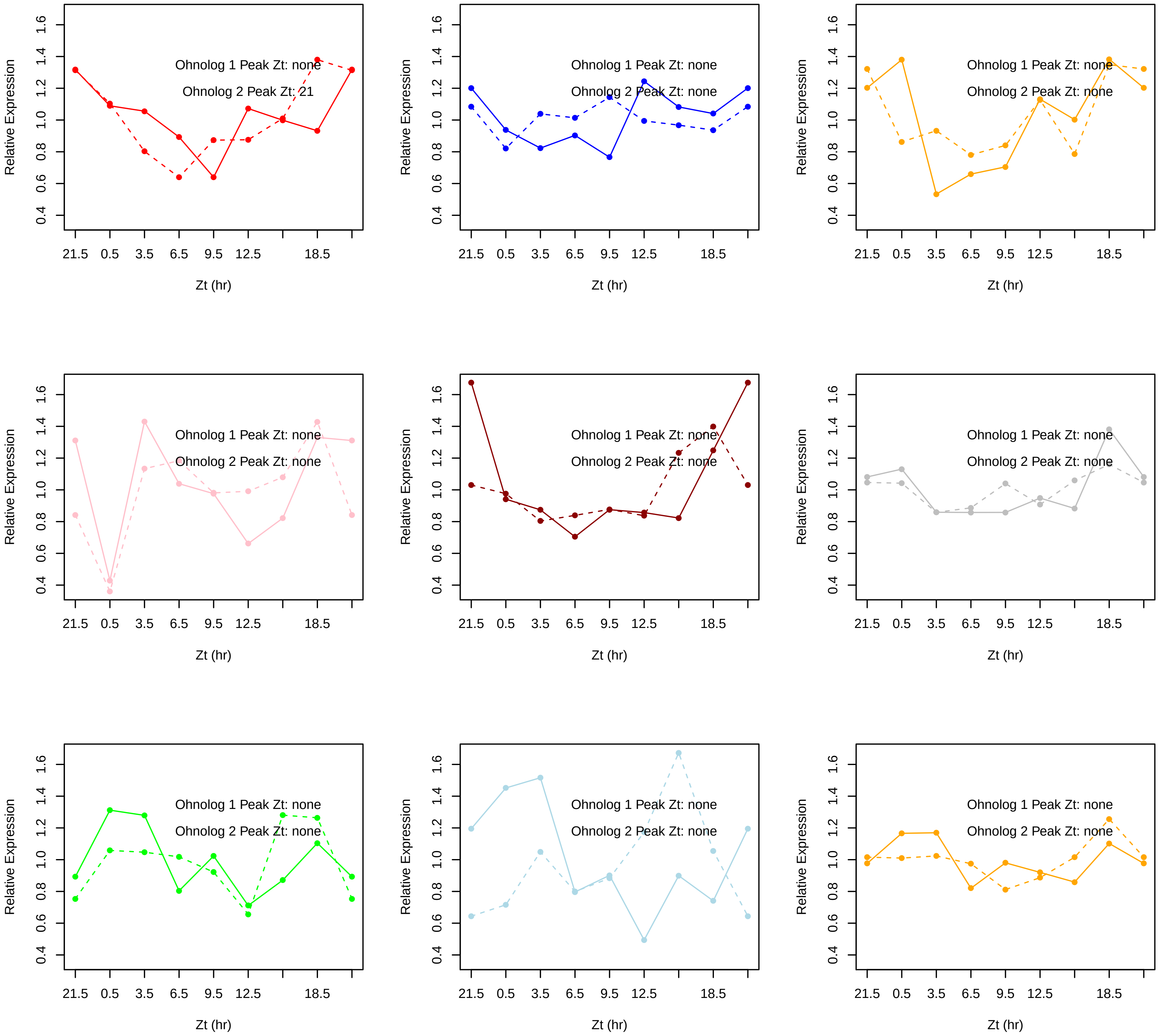

Ohnolog 1: ENSXMAG00000015502 GABRA2
Ohnolog 2: ENSXMAG00000025635

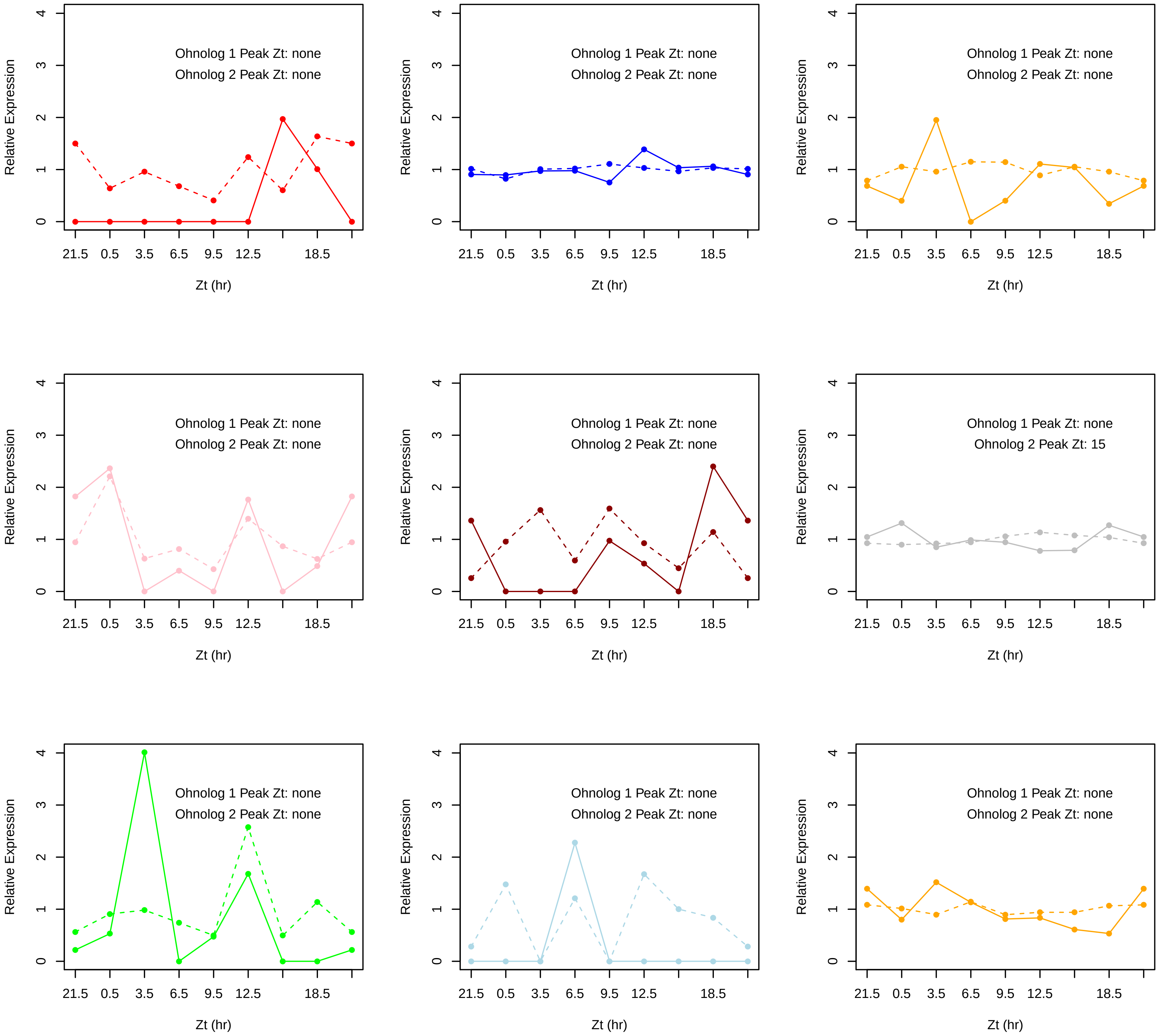

Ohnolog 1: ENSXMAG00000007856 sipa1l1
Ohnolog 2: ENSXMAG00000014370 SIPA1L1 (1 of many)

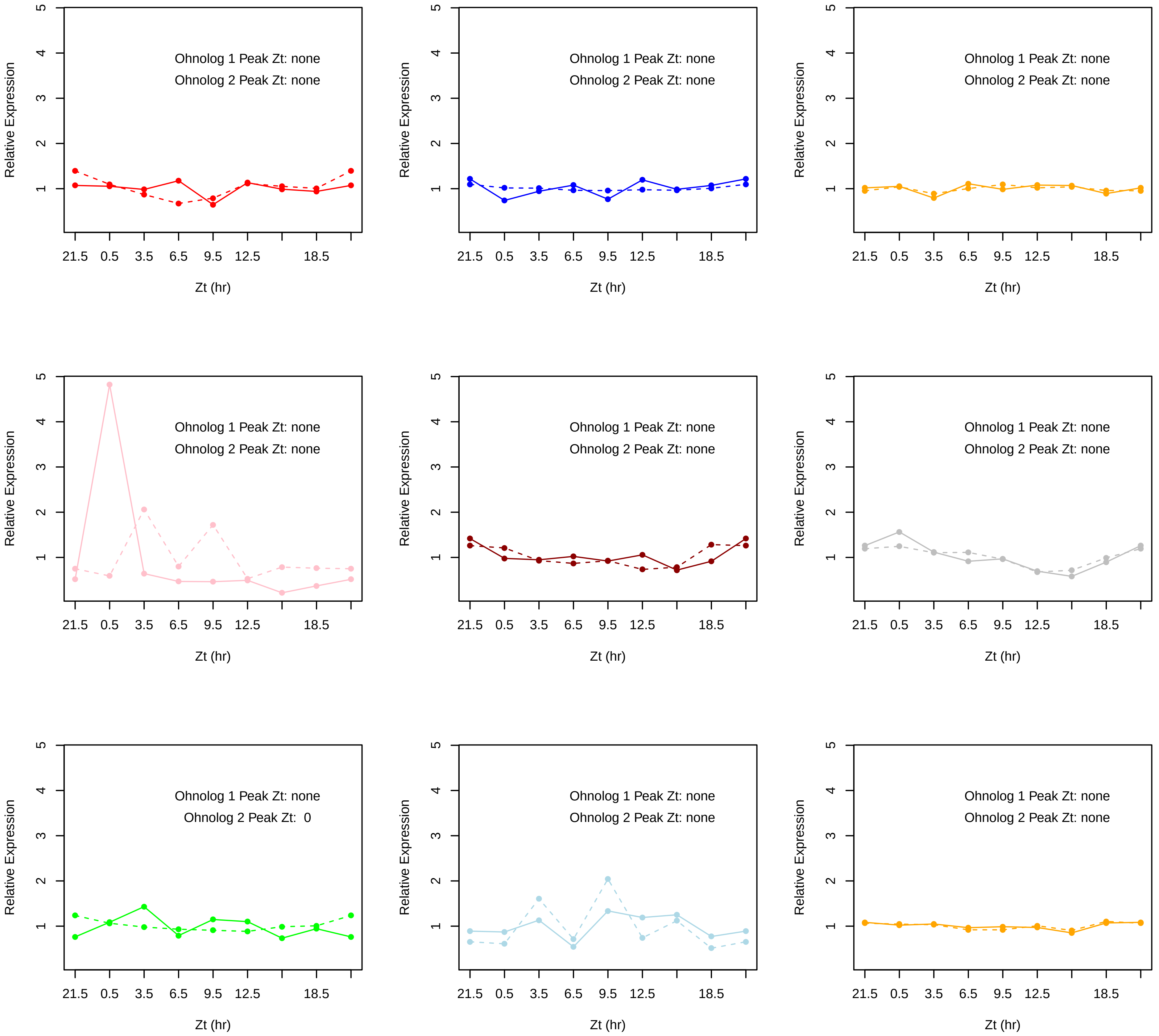

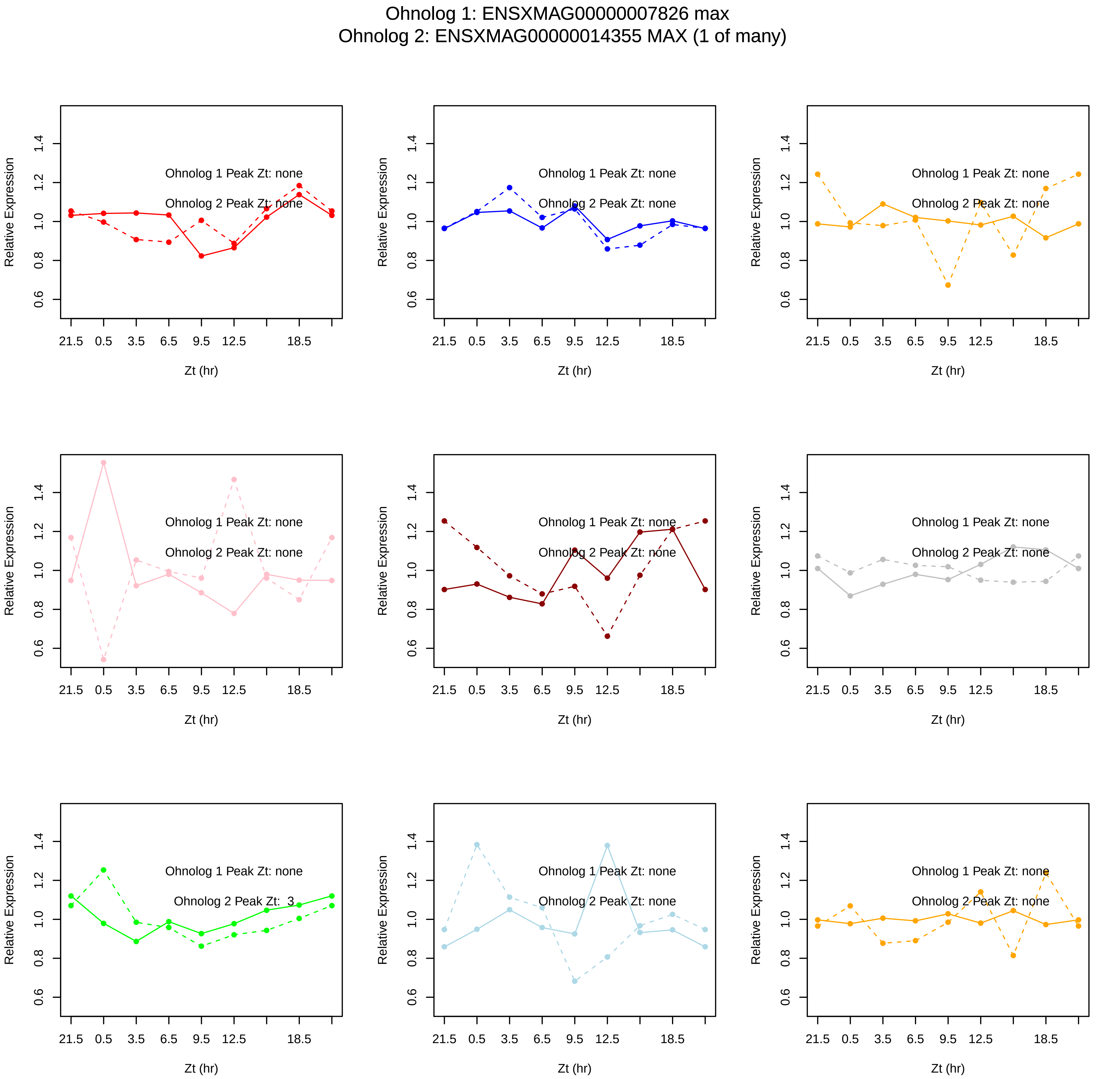

Ohnolog 1: ENSXMAG00000007826 max
Ohnolog 2: ENSXMAG00000014355 MAX (1 of many)
Ohnolog 1 Peak Zt: none
Ohnolog 2 Peak Zt: none
Ohnolog 1 Peak Zt: none
Ohnolog 2 Peak Zt: none
Ohnolog 1 Peak Zt: none
Ohnolog 2 Peak Zt: none
Ohnolog 1 Peak Zt: none
Ohnolog 2 Peak Zt: none
Ohnolog 1 Peak Zt: none
Ohnolog 2 Peak Zt: none
Ohnolog 1 Peak Zt: none
Ohnolog 2 Peak Zt: none
Ohnolog 1 Peak Zt: none
Ohnolog 2 Peak Zt: 3
Ohnolog 1 Peak Zt: none
Ohnolog 2 Peak Zt: none
Ohnolog 1 Peak Zt: none
Ohnolog 2 Peak Zt: none
Relative Expression
Zt (hr)
21.5
0.5
3.5
6.5
9.5
12.5
18.5
0.6
0.8
1.0
1.2
1.4

Ohnolog 1: ENSXMAG00000007798 susd6
Ohnolog 2: ENSXMAG00000014341

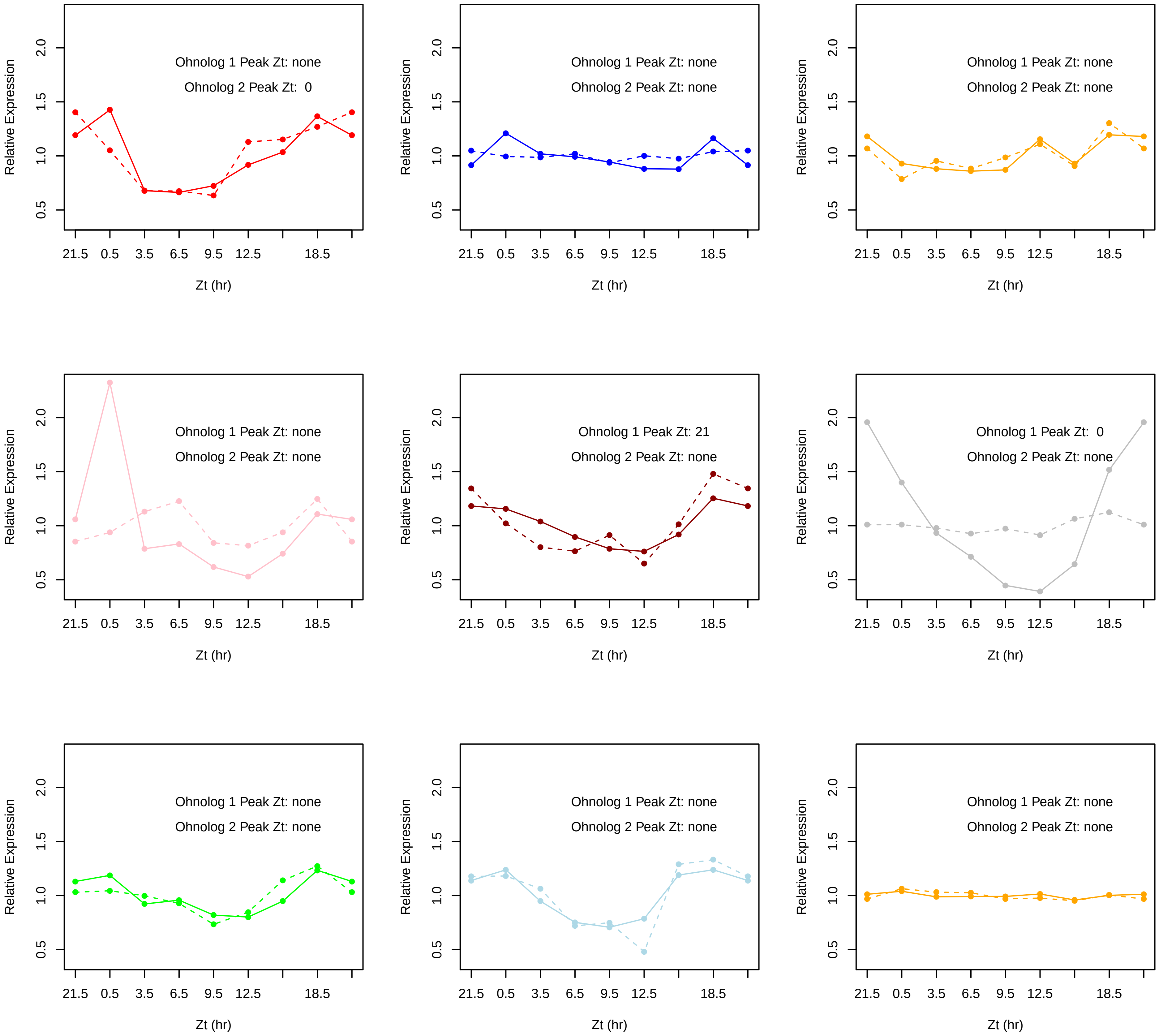

Ohnolog 1: ENSXMAG00000007770 katnbl1
Ohnolog 2: ENSXMAG00000025396

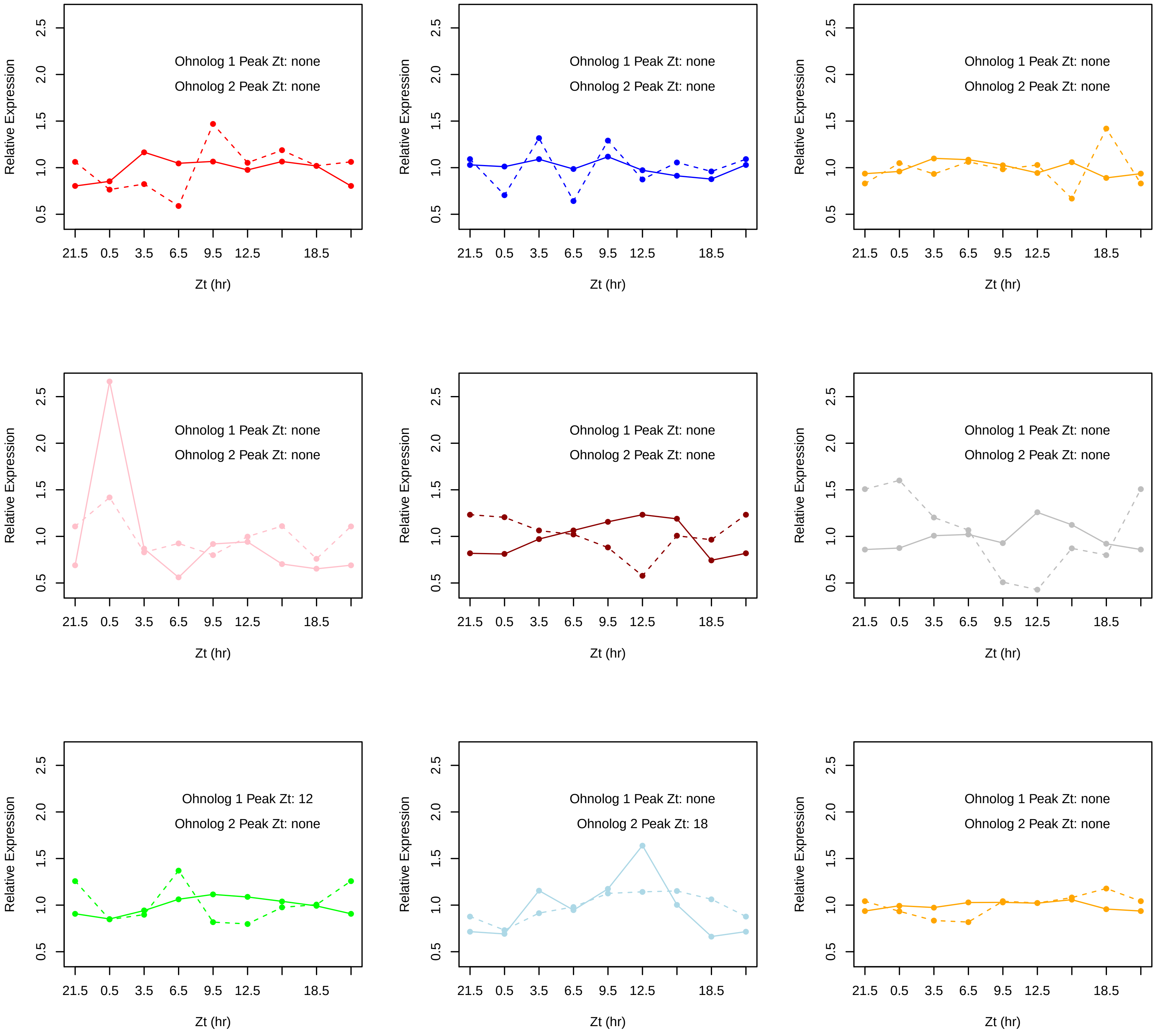

Ohnolog 1: ENSXMAG00000002918 kcnv2a
Ohnolog 2: ENSXMAG00000015430 kcnv2b

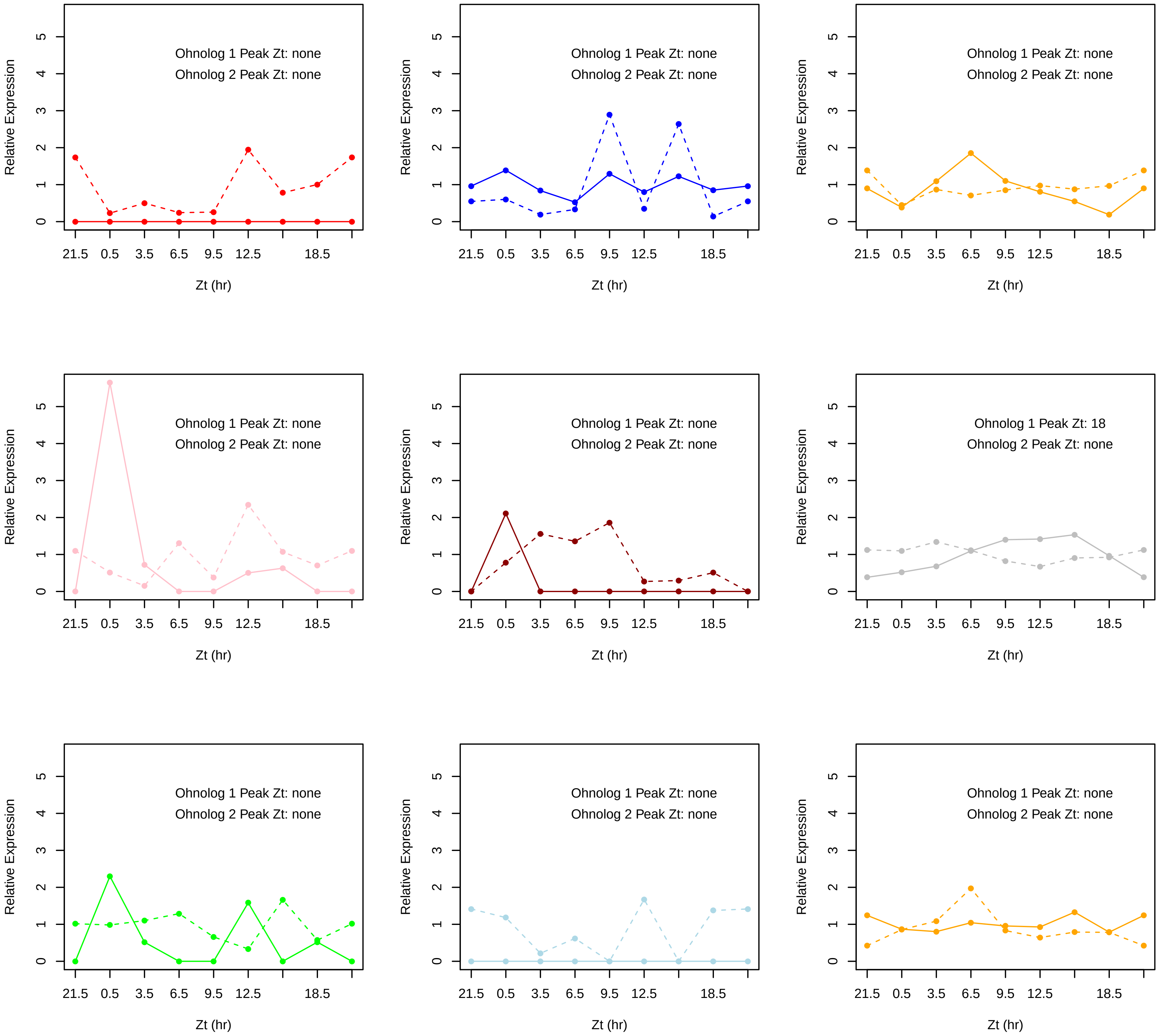

Ohnolog 1: ENSXMAG00000021695 zgc:64051
Ohnolog 2: ENSXMAG00000026852

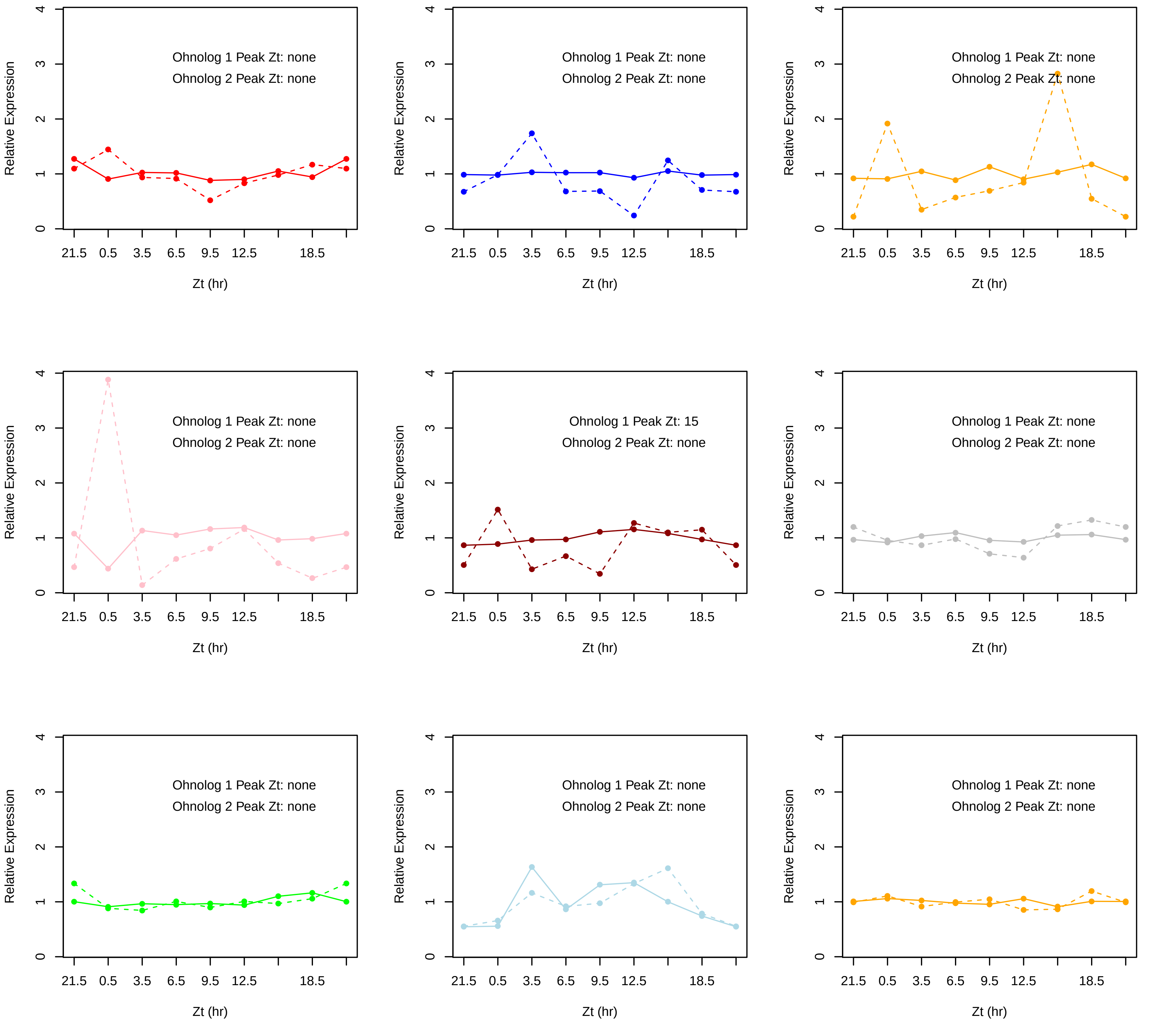

Ohnolog 1: ENSXMAG00000005692 usp53b
Ohnolog 2: ENSXMAG00000021530 USP53

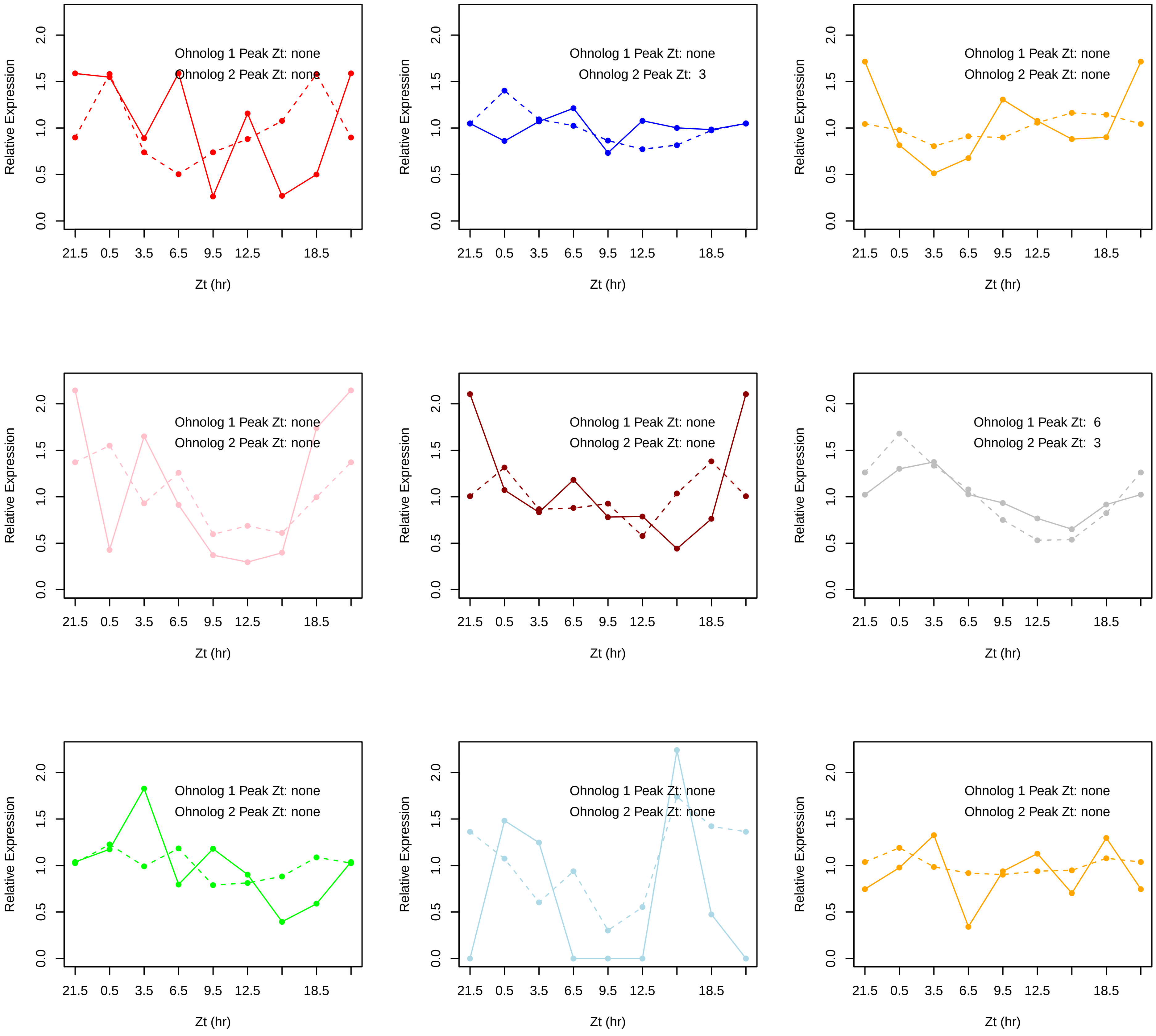

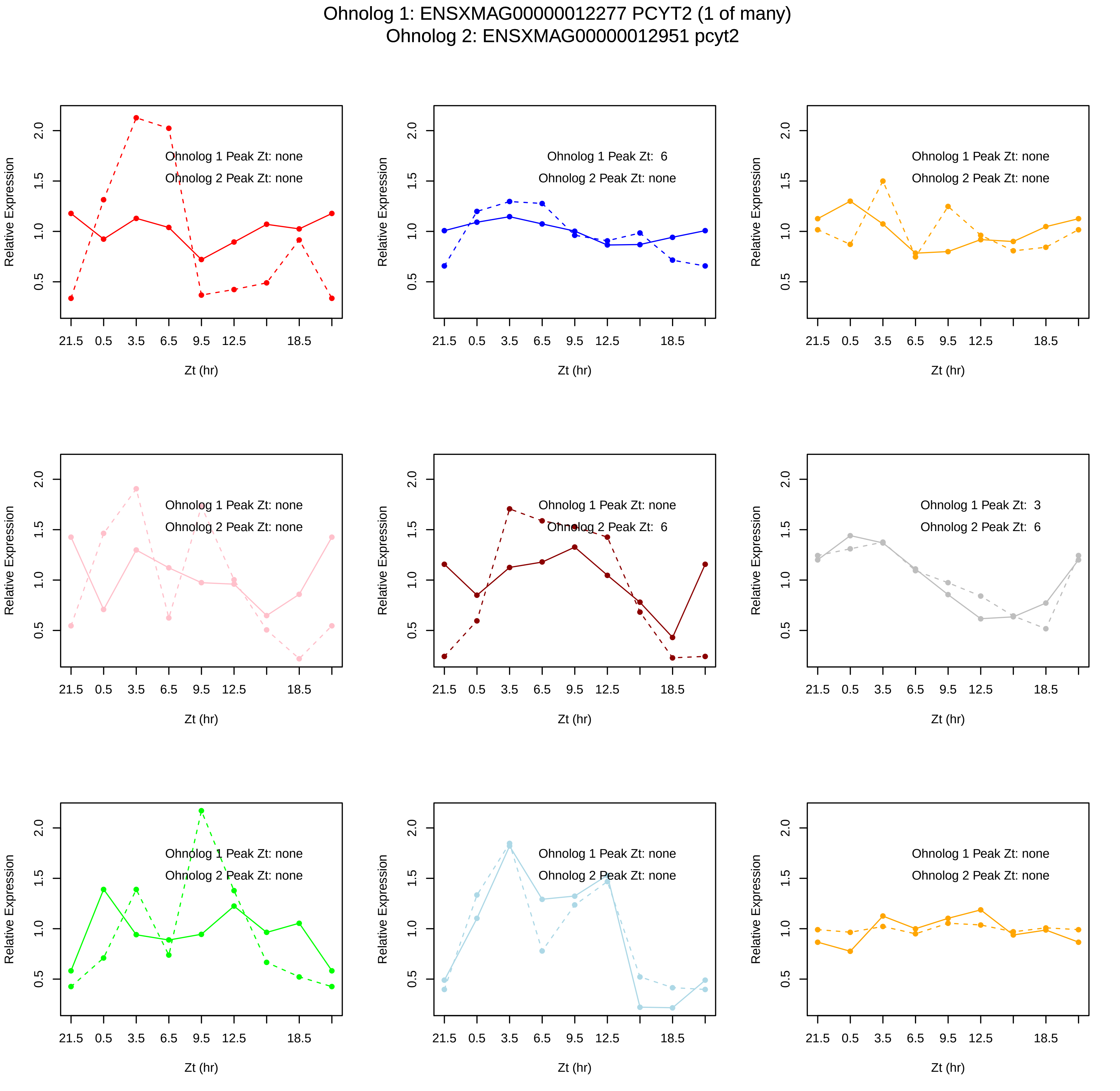

Ohnolog 1: ENSXMAG00000012277 PCYT2 (1 of many)
Ohnolog 2: ENSXMAG00000012951 pcyt2
Ohnolog 1 Peak Zt: none
Ohnolog 2 Peak Zt: none
Ohnolog 1 Peak Zt: 6
Ohnolog 2 Peak Zt: none
Ohnolog 1 Peak Zt: none
Ohnolog 2 Peak Zt: none
Ohnolog 1 Peak Zt: none
Ohnolog 2 Peak Zt: none
Ohnolog 1 Peak Zt: none
Ohnolog 2 Peak Zt: 6
Ohnolog 1 Peak Zt: 3
Ohnolog 2 Peak Zt: 6
Ohnolog 1 Peak Zt: none
Ohnolog 2 Peak Zt: none
Ohnolog 1 Peak Zt: none
Ohnolog 2 Peak Zt: none
Ohnolog 1 Peak Zt: none
Ohnolog 2 Peak Zt: none
Relative Expression
Zt (hr)
0.5 1.0 1.5 2.0
21.5 0.5 3.5 6.5 9.5 12.5 18.5

Ohnolog 1: ENSXMAG00000008886 znf385c
Ohnolog 2: ENSXMAG00000010258

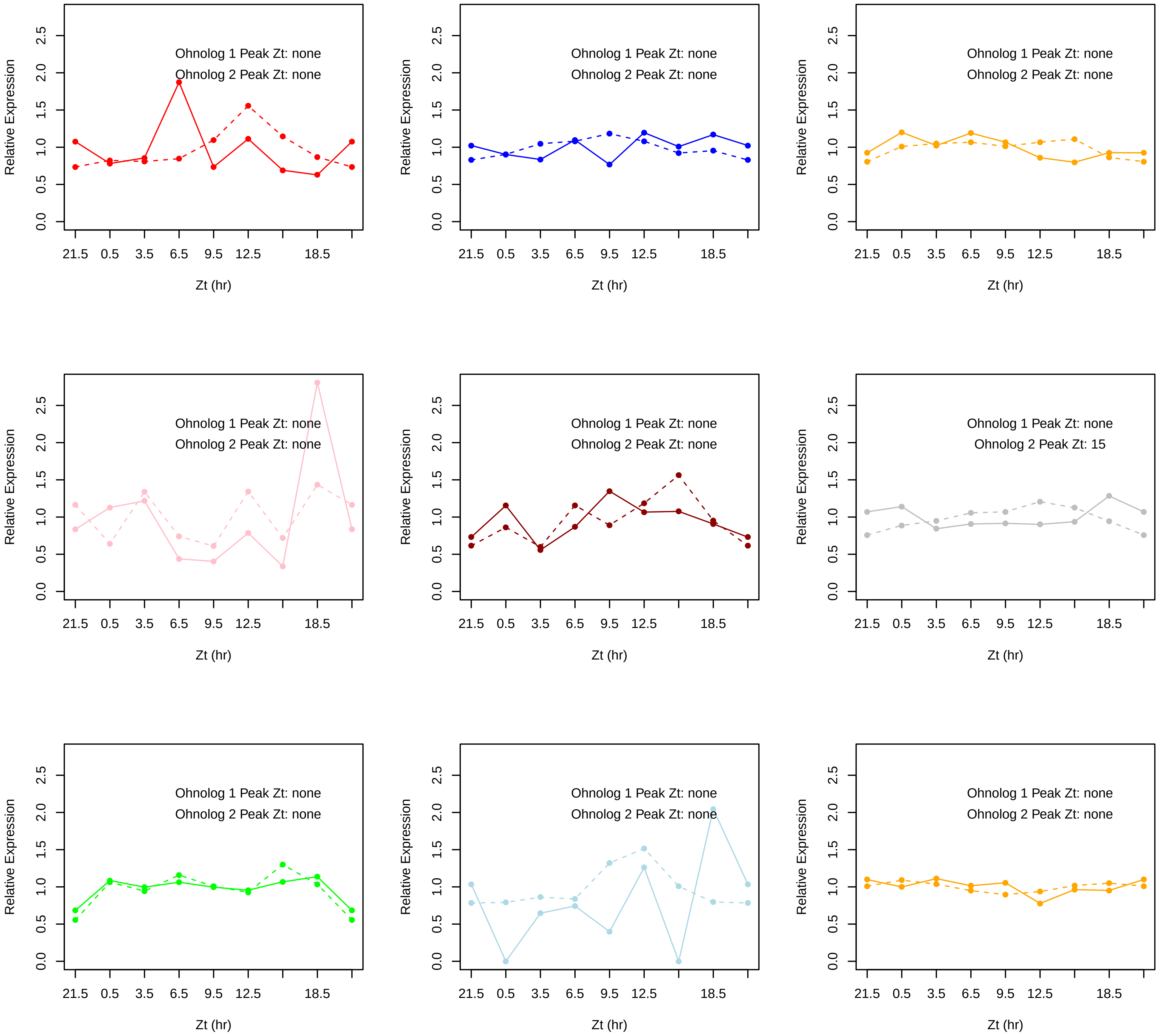

Ohnolog 1: ENSXMAG00000022730 zgc:100829
Ohnolog 2: ENSXMAG00000027090

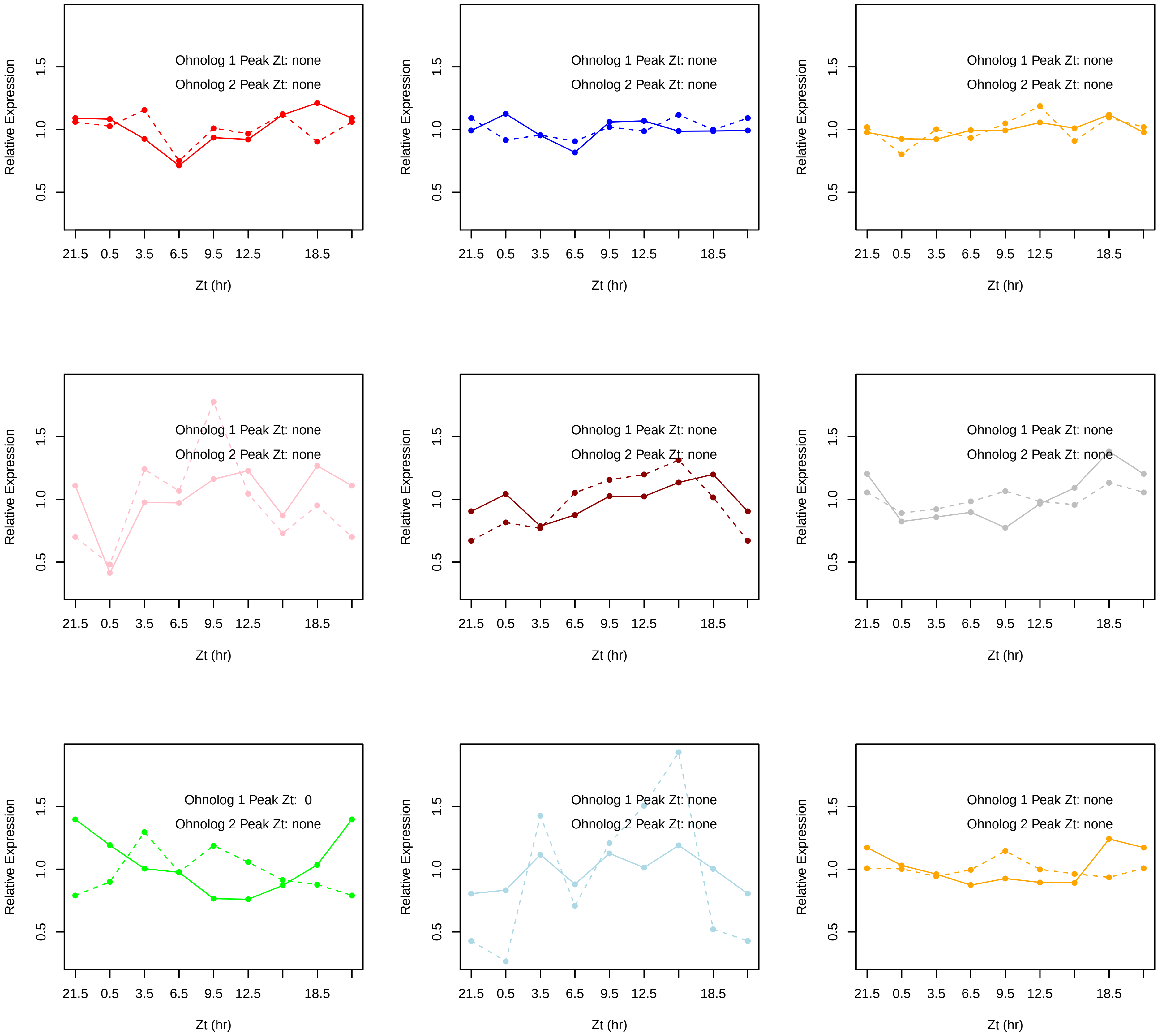

Ohnolog 1: ENSXMAG00000008856 dnajc7
Ohnolog 2: ENSXMAG00000010242

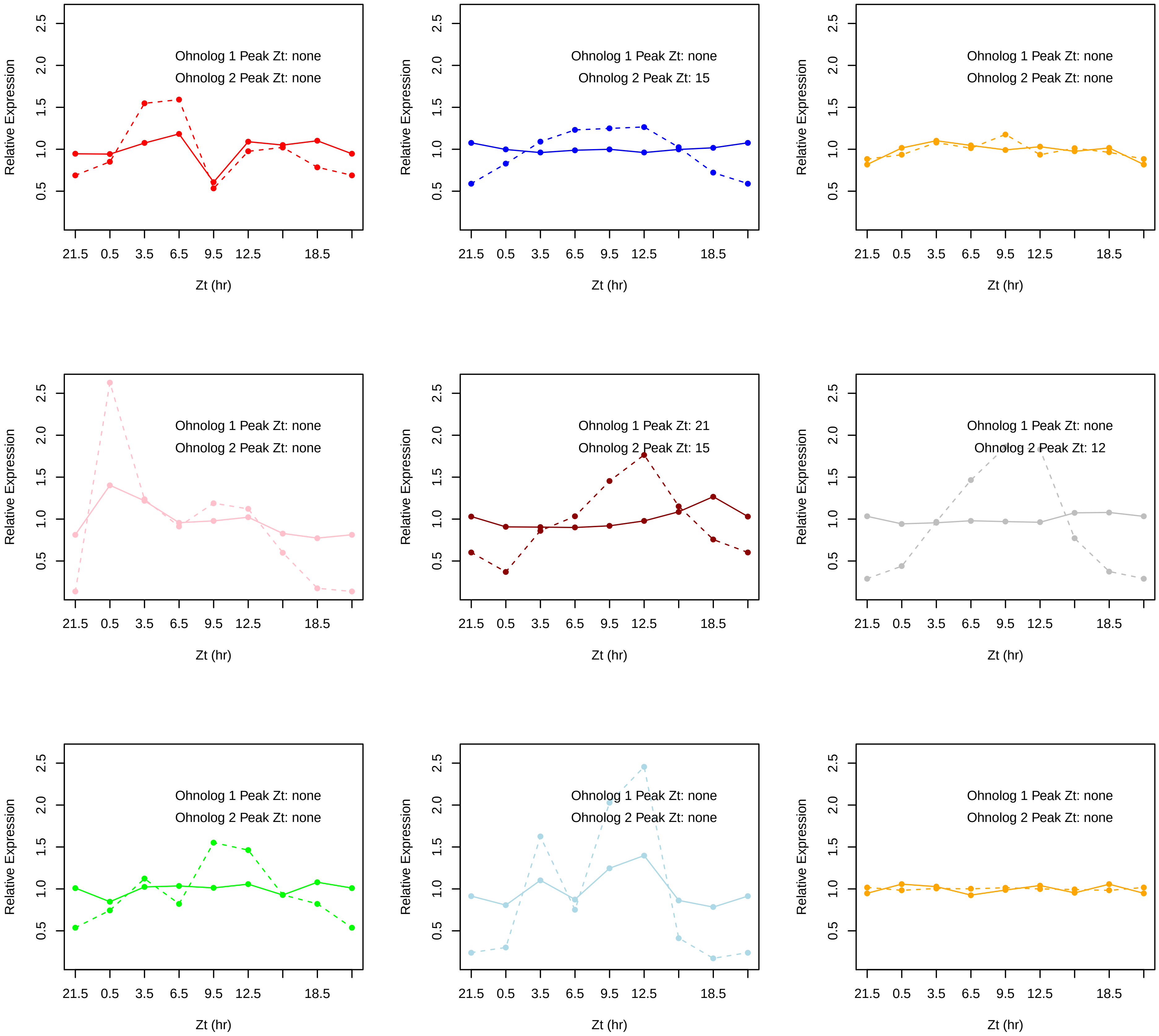

Ohnolog 1: ENSXMAG00000003899
Ohnolog 2: ENSXMAG00000016494

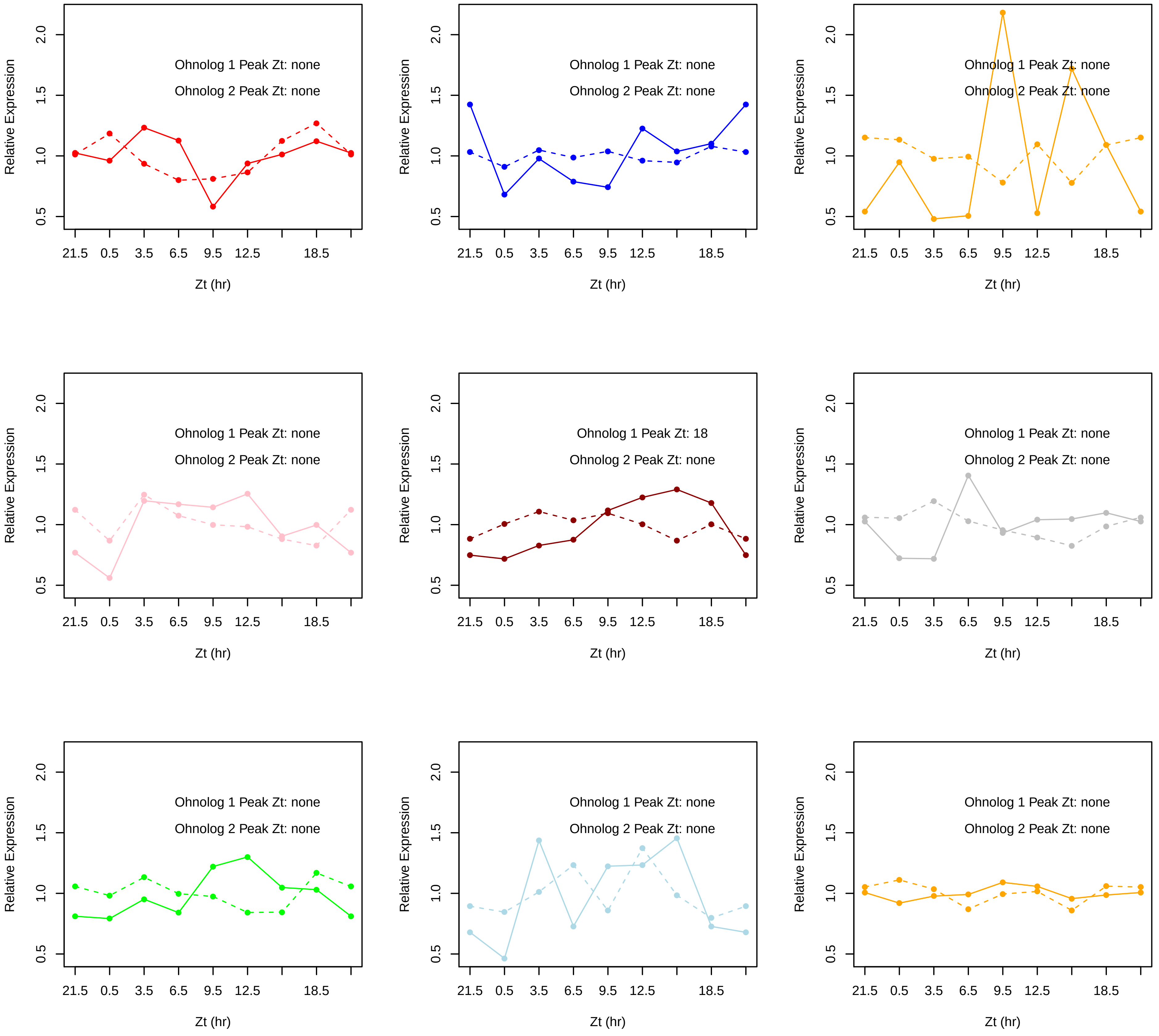

Ohnolog 1: ENSXMAG00000002862
Ohnolog 2: ENSXMAG00000017646 sema4gb

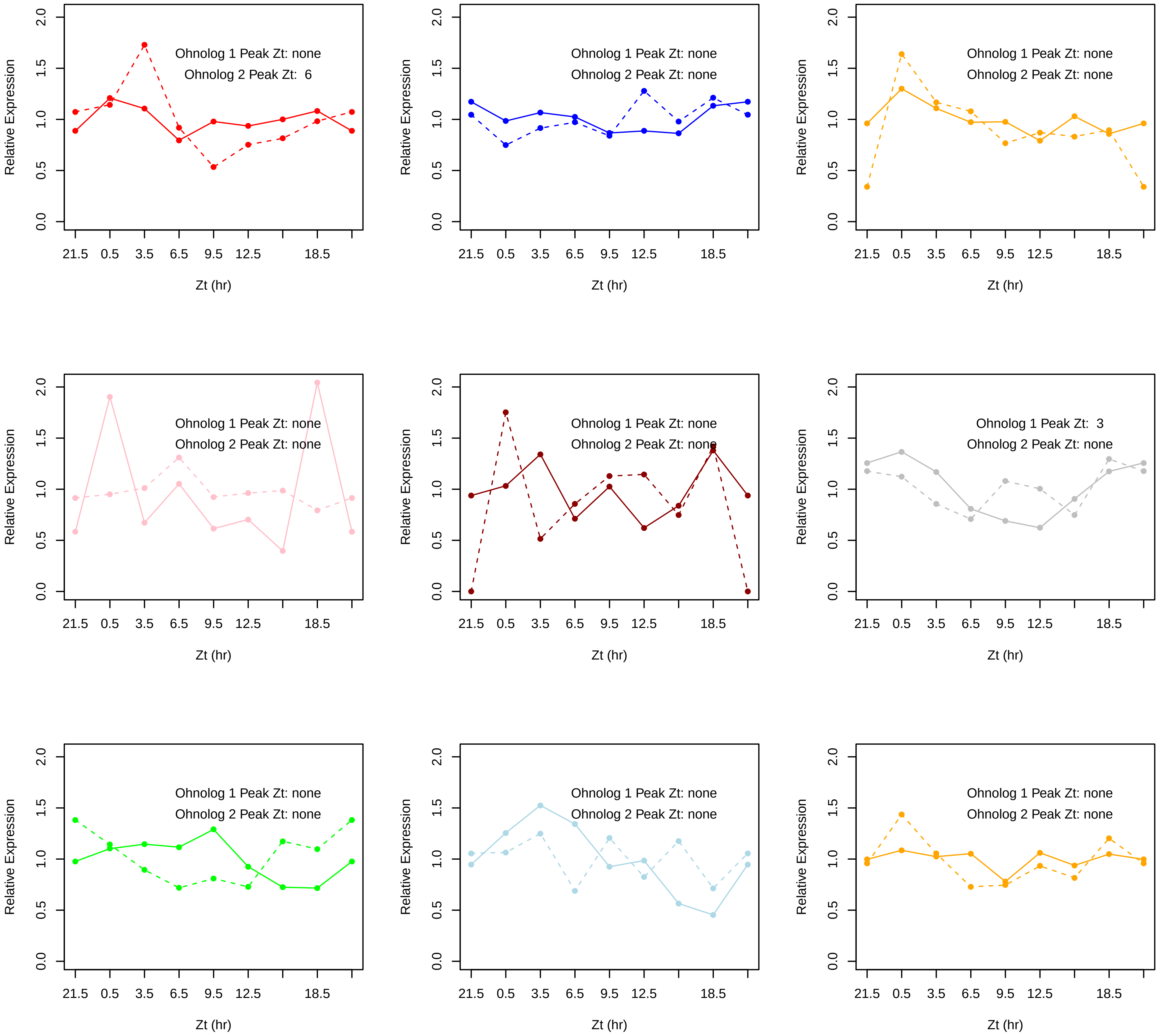

Ohnolog 1: ENSXMAG00000008800 aclya
Ohnolog 2: ENSXMAG00000010204 aclyb

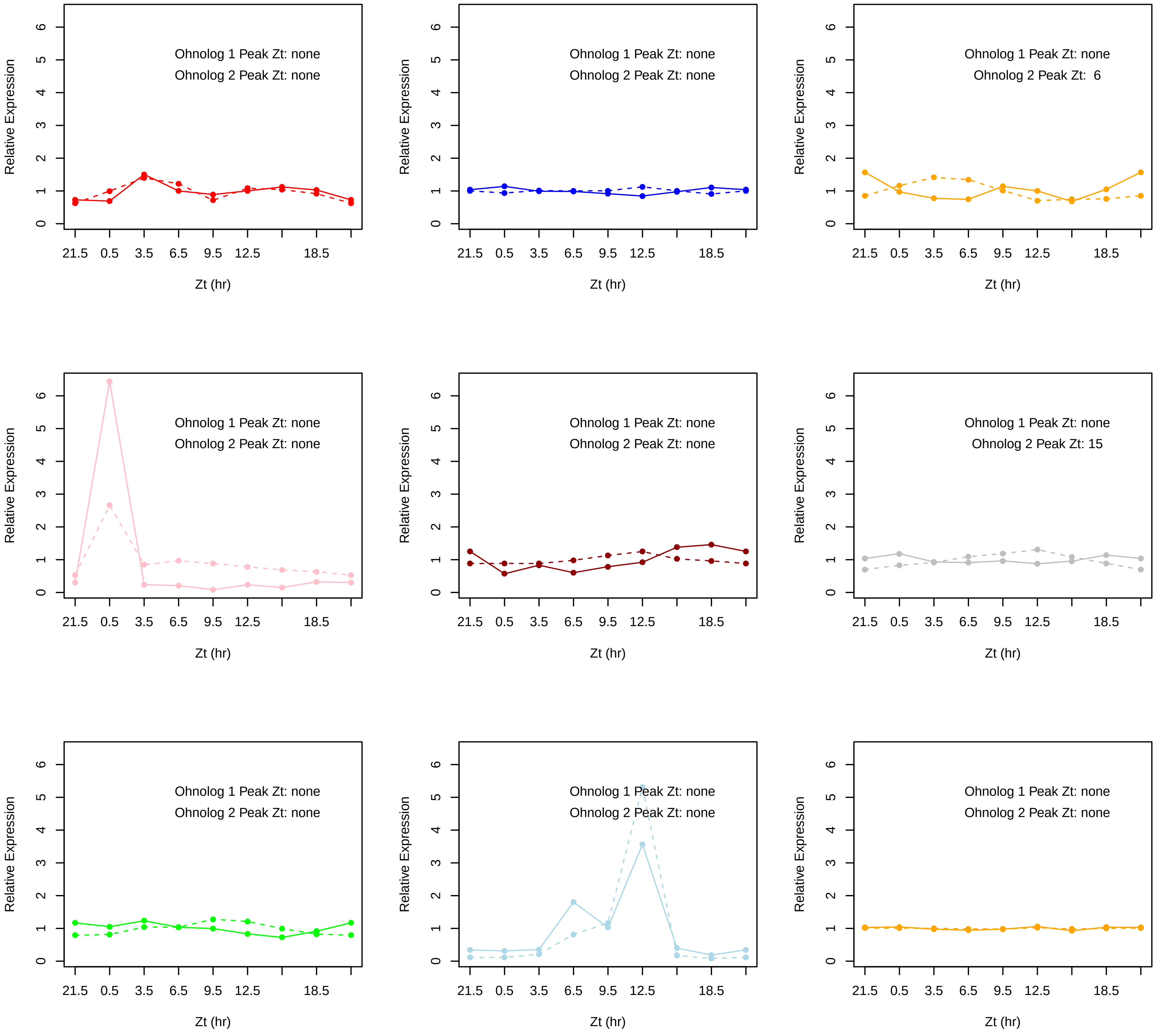

Ohnolog 1: ENSXMAG00000008750 fkbp10a
Ohnolog 2: ENSXMAG00000025831 fkbp10b

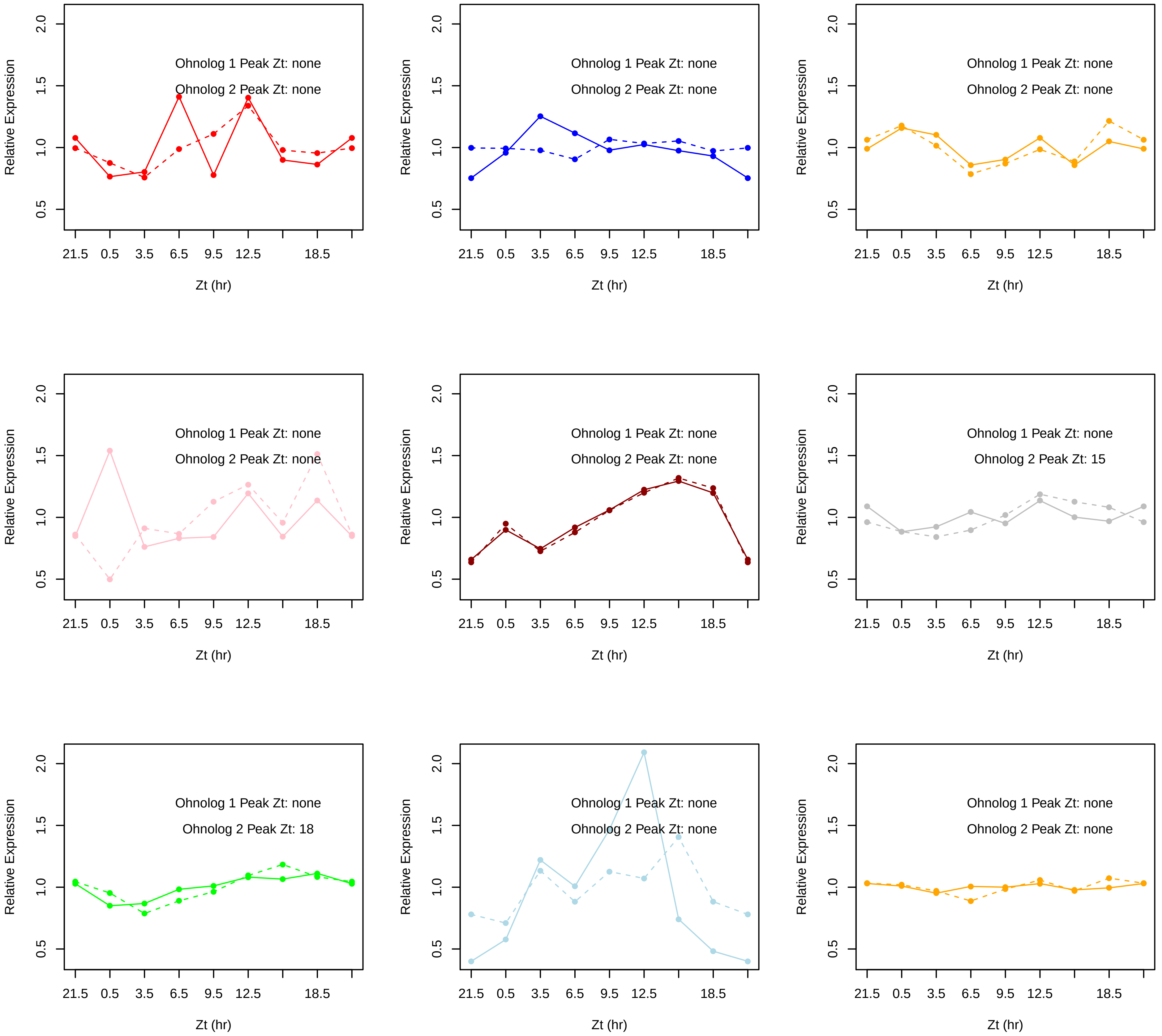

Ohnolog 1: ENSXMAG00000010255 aimp1
Ohnolog 2: ENSXMAG00000021224 zgc:101853

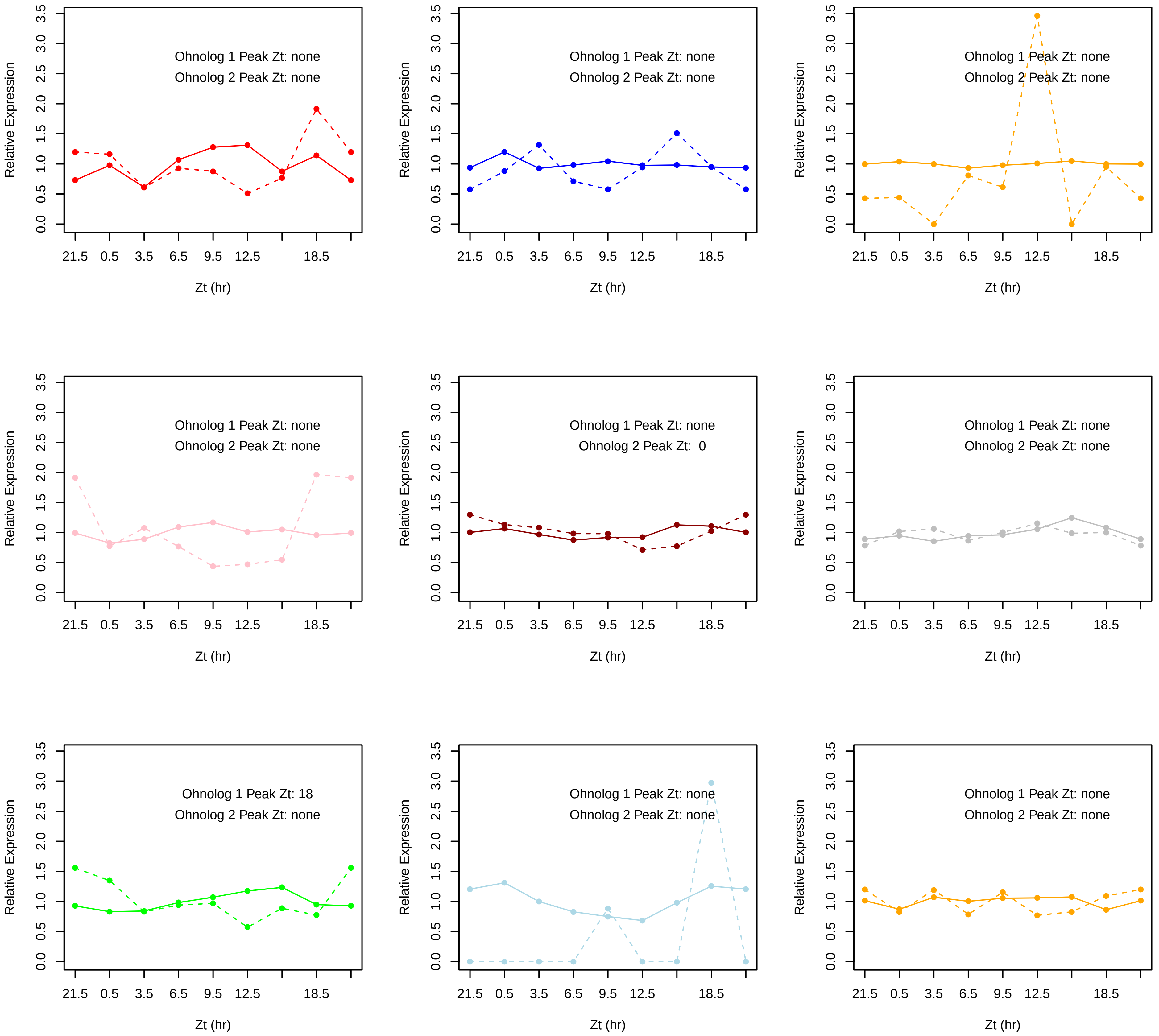

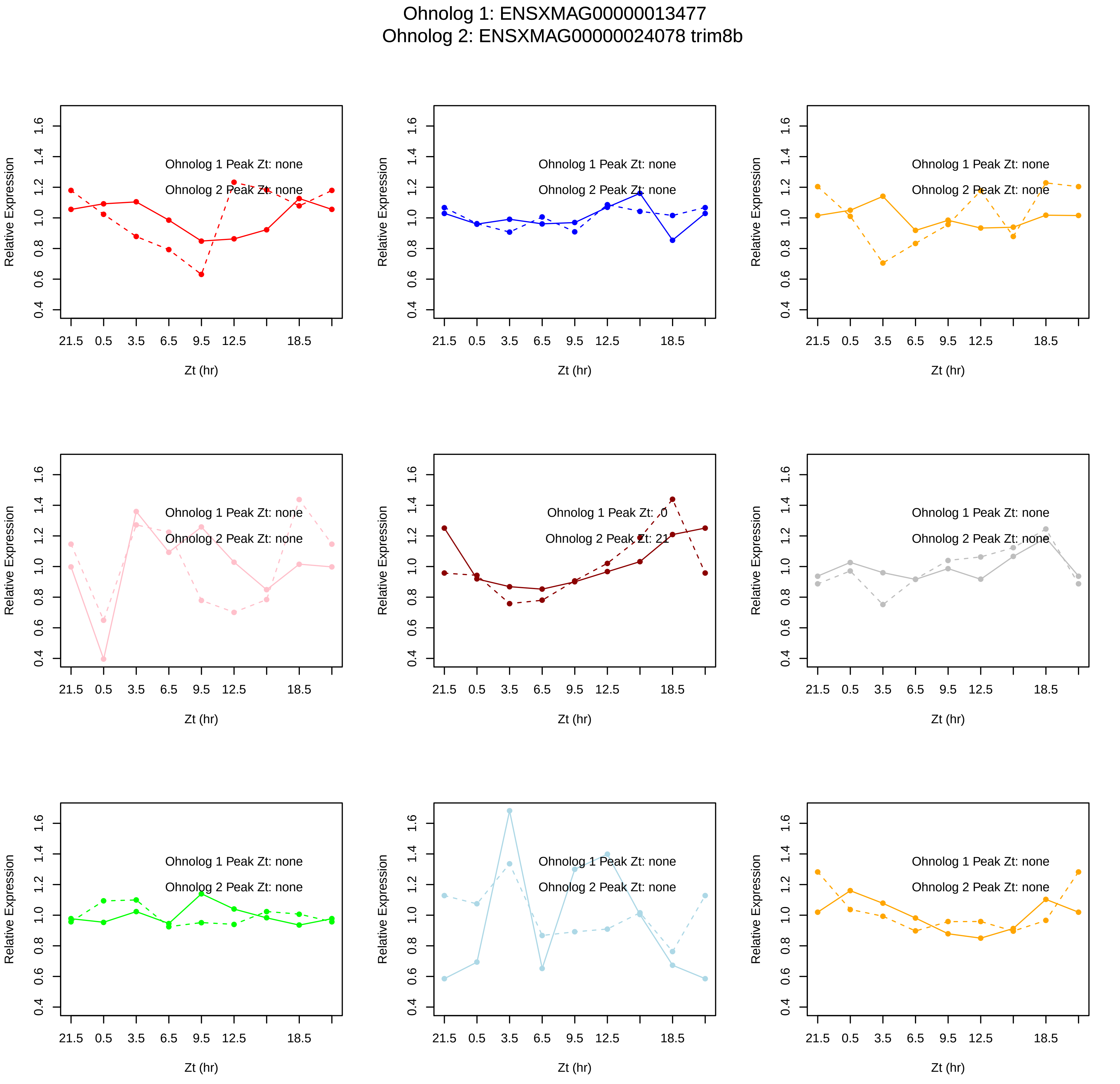
Ohnolog 1: ENSXMAG00000013477
Ohnolog 2: ENSXMAG00000024078 trim8b
Ohnolog 1 Peak Zt: none
Ohnolog 2 Peak Zt: none
Ohnolog 1 Peak Zt: 0
Ohnolog 2 Peak Zt: 21
Relative Expression
Zt (hr)
21.5
0.5
3.5
6.5
9.5
12.5
18.5
0.4
0.6
0.8
1.0
1.2
1.4
1.6

Ohnolog 1: ENSXMAG00000000246 cant1b
Ohnolog 2: ENSXMAG00000012376 cant1a

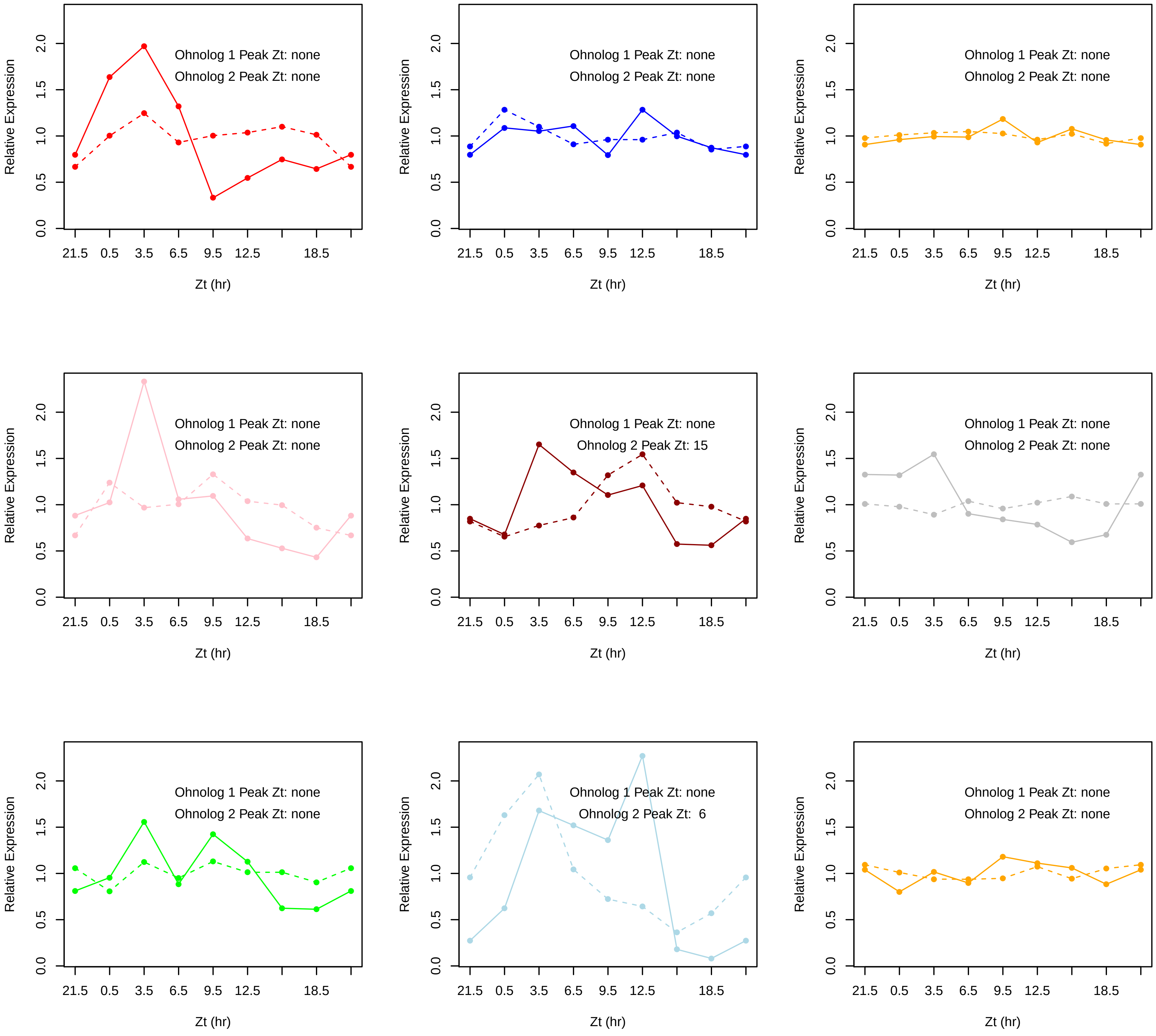

Ohnolog 1: ENSXMAG00000011299
Ohnolog 2: ENSXMAG00000018369

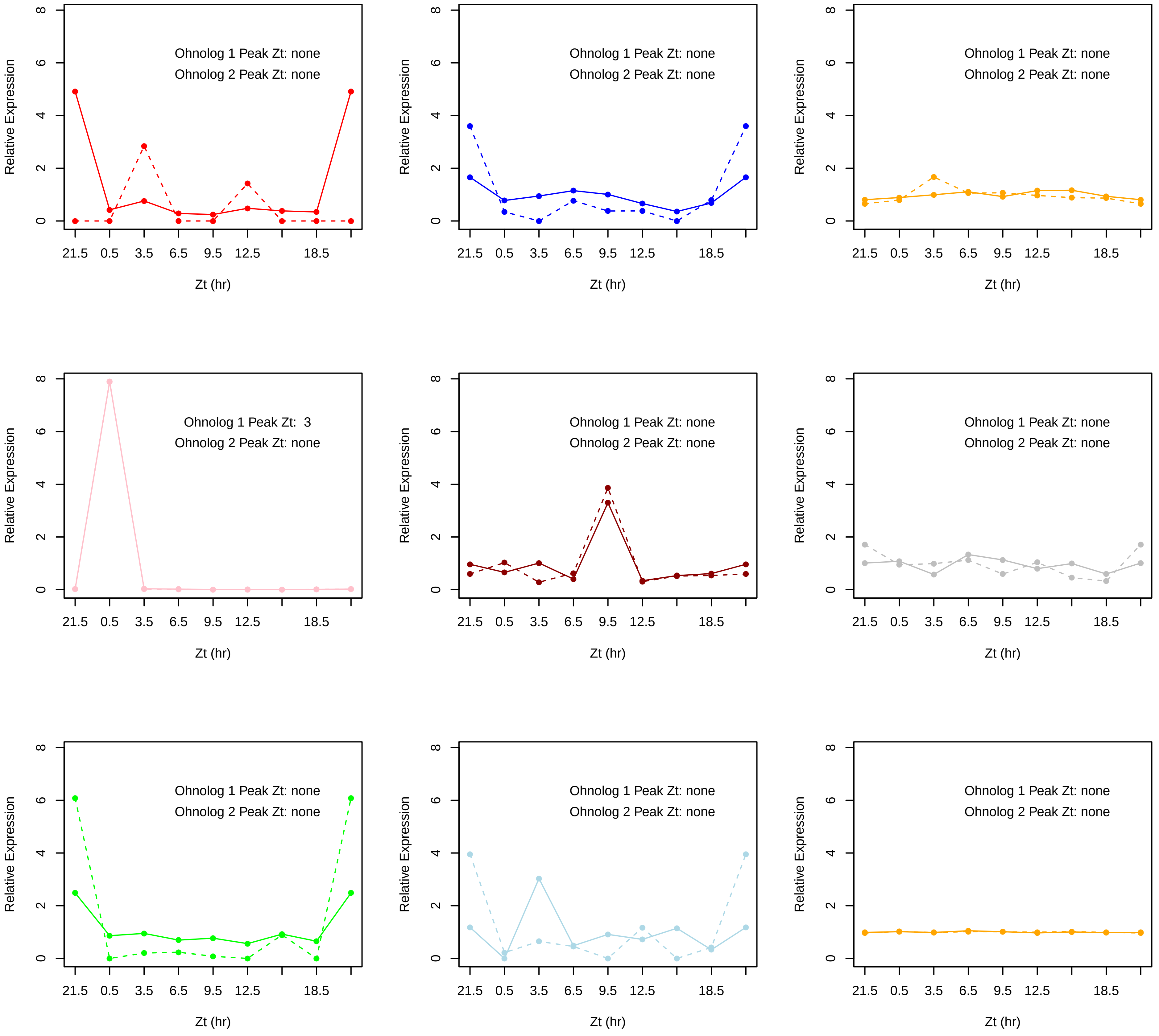

Ohnolog 1: ENSXMAG00000021192 cyth1b
Ohnolog 2: ENSXMAG00000027086 cyth1a

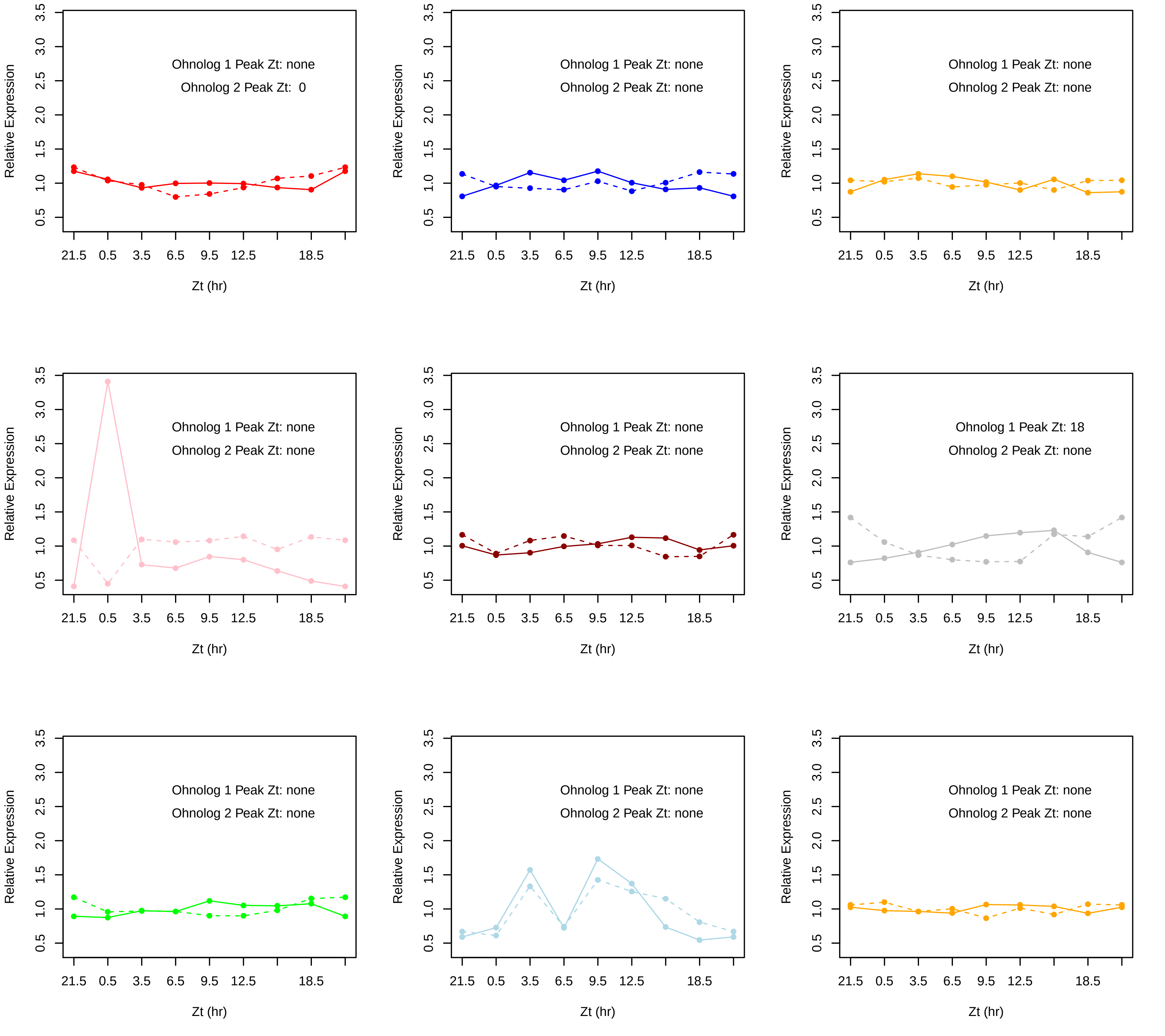

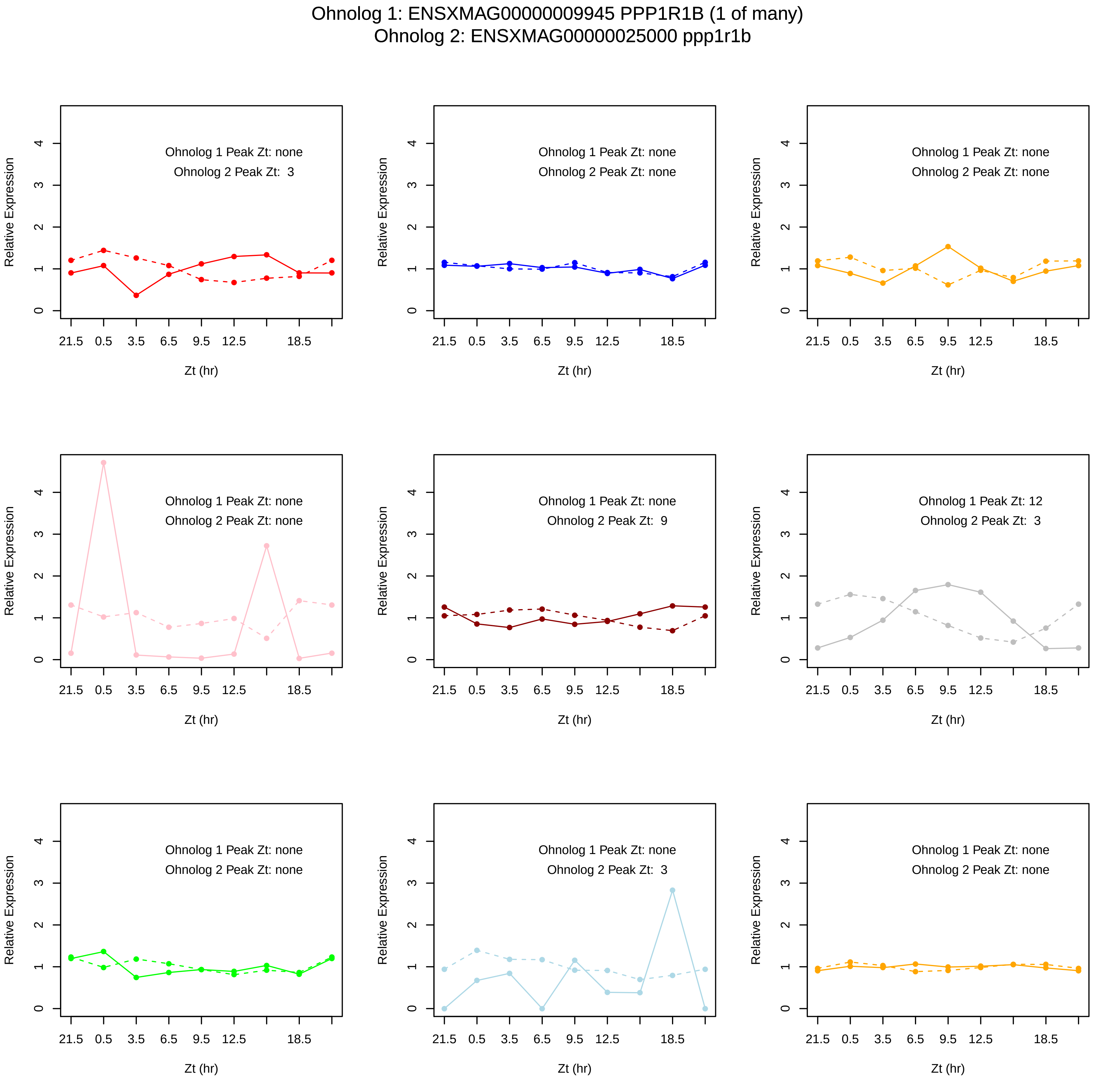

Ohnolog 1: ENSXMAG00000009945 PPP1R1B (1 of many)
Ohnolog 2: ENSXMAG00000025000 ppp1r1b
Ohnolog 1 Peak Zt: none
Ohnolog 2 Peak Zt: 3
Ohnolog 1 Peak Zt: none
Ohnolog 2 Peak Zt: none
Ohnolog 1 Peak Zt: none
Ohnolog 2 Peak Zt: none
Ohnolog 1 Peak Zt: none
Ohnolog 2 Peak Zt: none
Ohnolog 1 Peak Zt: none
Ohnolog 2 Peak Zt: 9
Ohnolog 1 Peak Zt: 12
Ohnolog 2 Peak Zt: 3
Ohnolog 1 Peak Zt: none
Ohnolog 2 Peak Zt: none
Ohnolog 1 Peak Zt: none
Ohnolog 2 Peak Zt: 3
Ohnolog 1 Peak Zt: none
Ohnolog 2 Peak Zt: none
Relative Expression
Zt (hr)
21.5
0.5
3.5
6.5
9.5
12.5
18.5
0
1
2
3
4

Ohnolog 1: ENSXMAG00000001349
Ohnolog 2: ENSXMAG00000016875 lmbrd2a

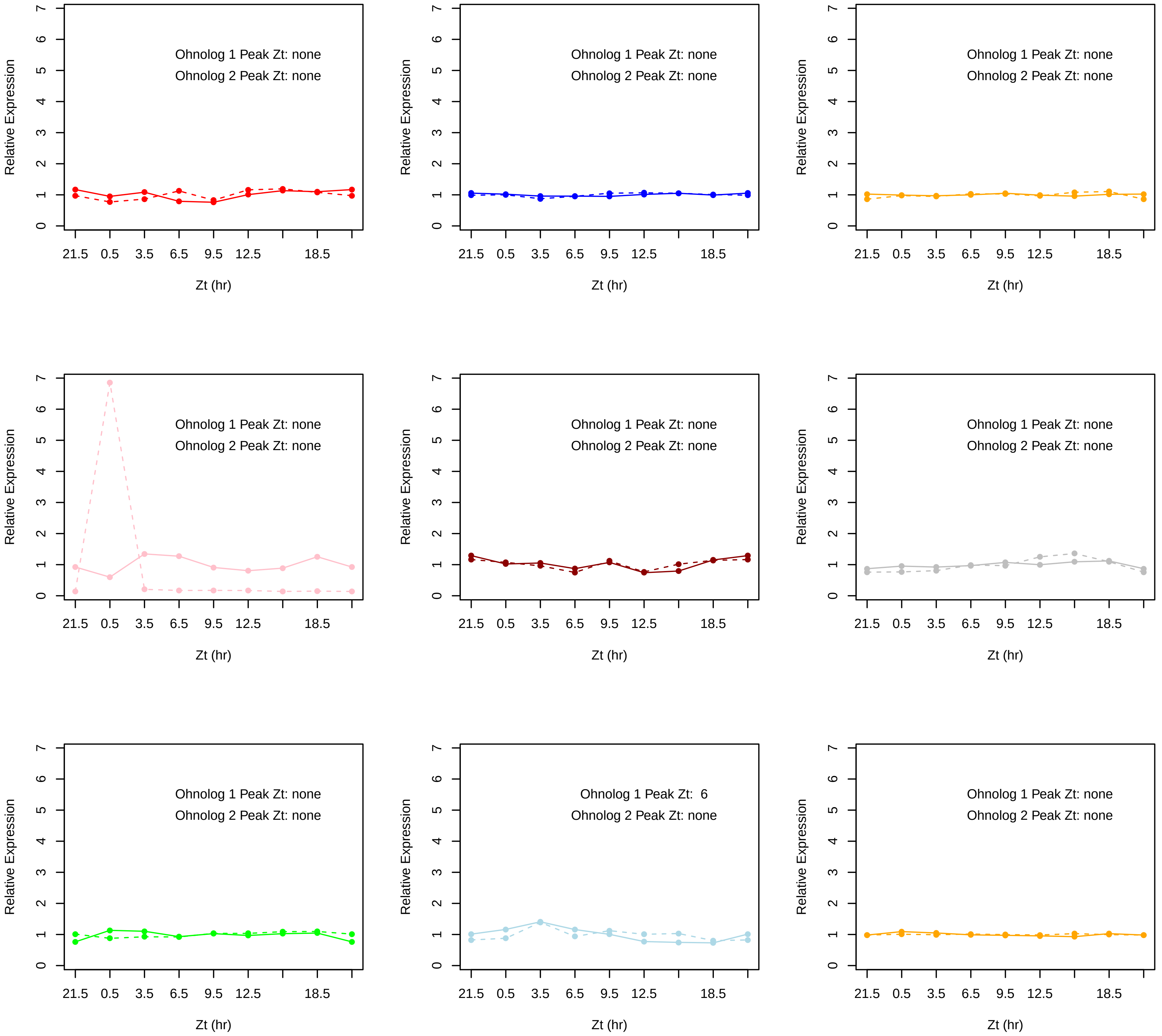

Ohnolog 1: ENSXMAG00000007614
Ohnolog 2: ENSXMAG00000008396

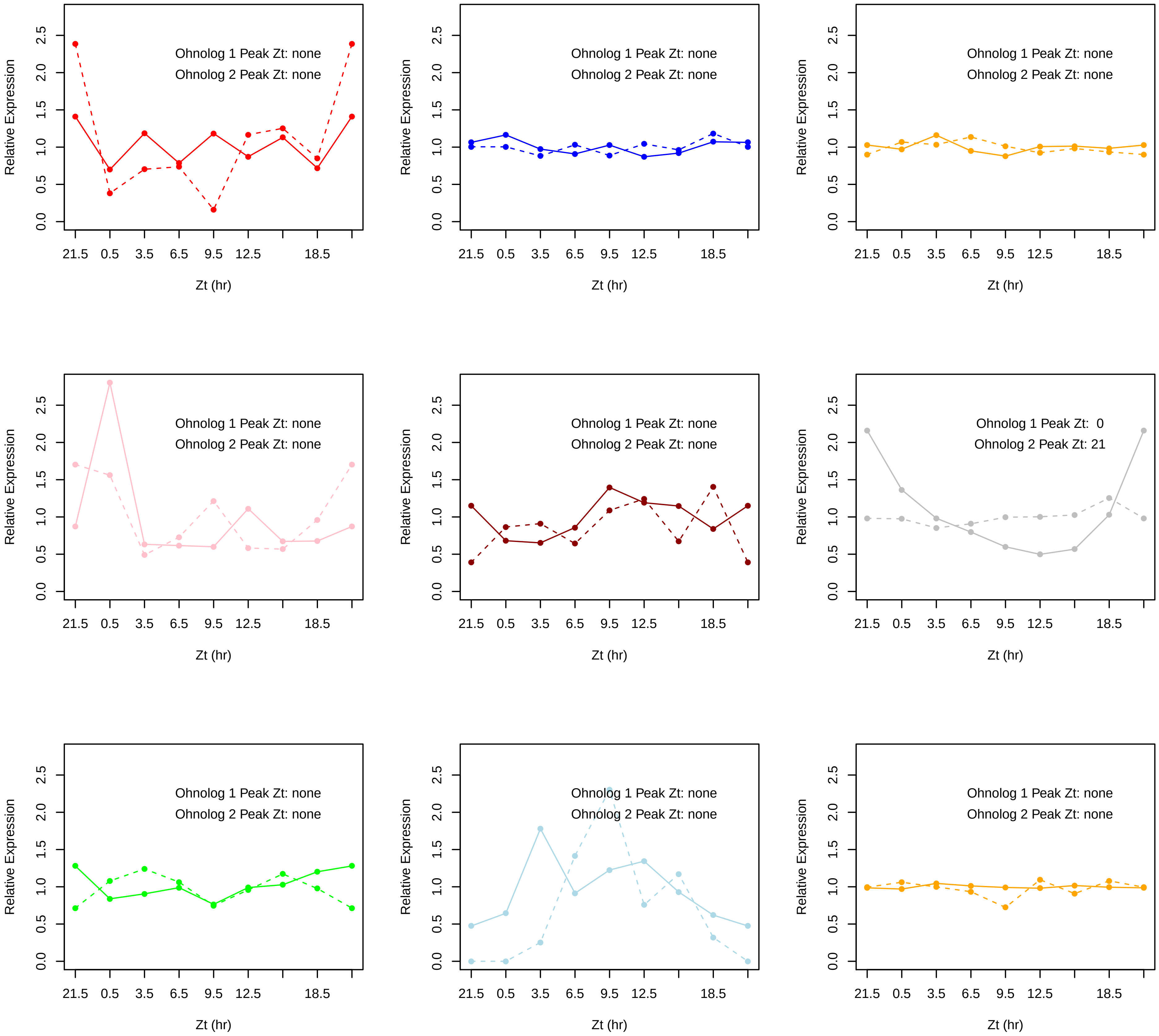

Ohnolog 1: ENSXMAG00000013379
Ohnolog 2: ENSXMAG00000014472 arhgap19

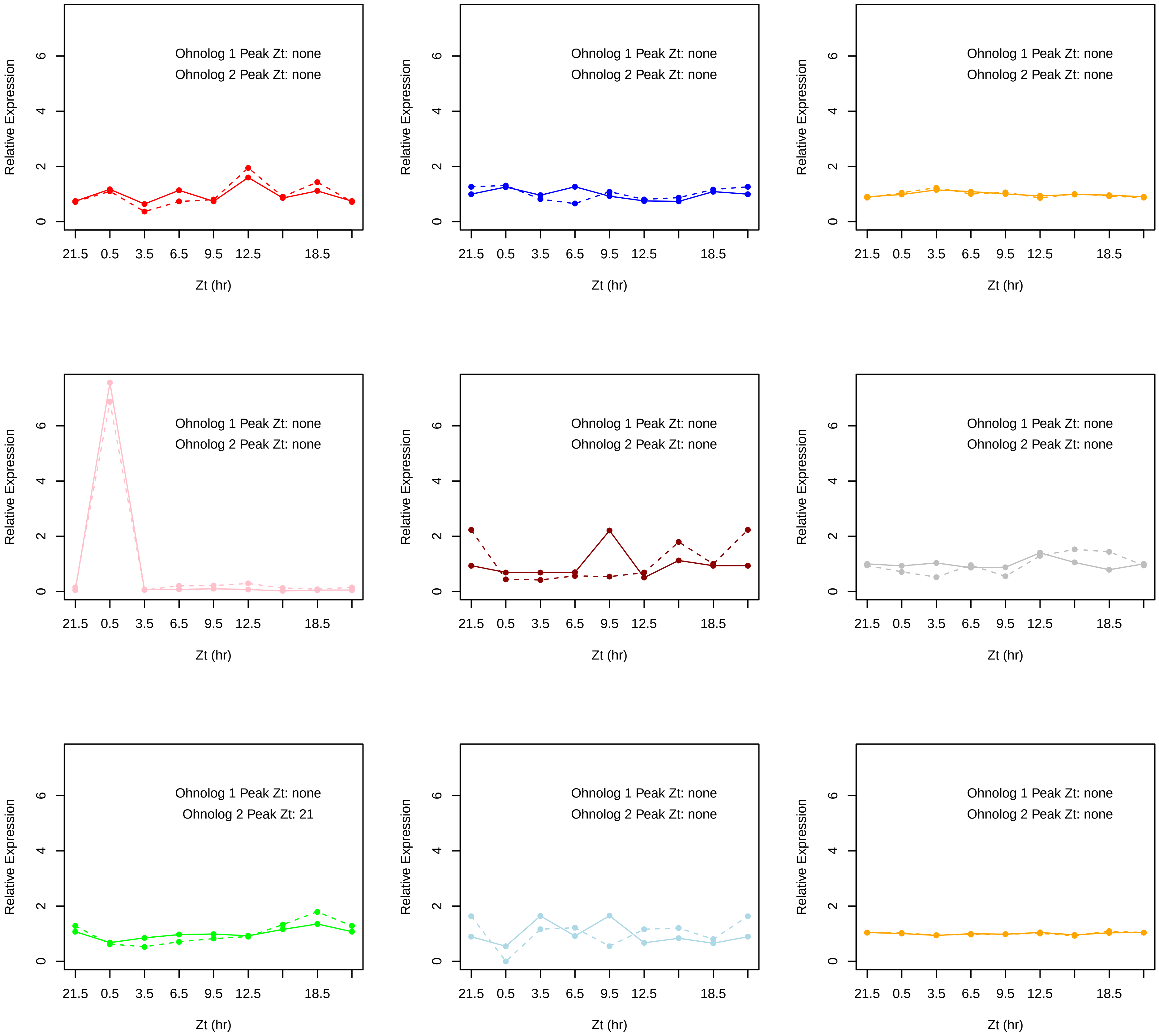

Ohnolog 1: ENSXMAG00000008471 rpl19
Ohnolog 2: ENSXMAG00000025571 RPL19 (1 of many)

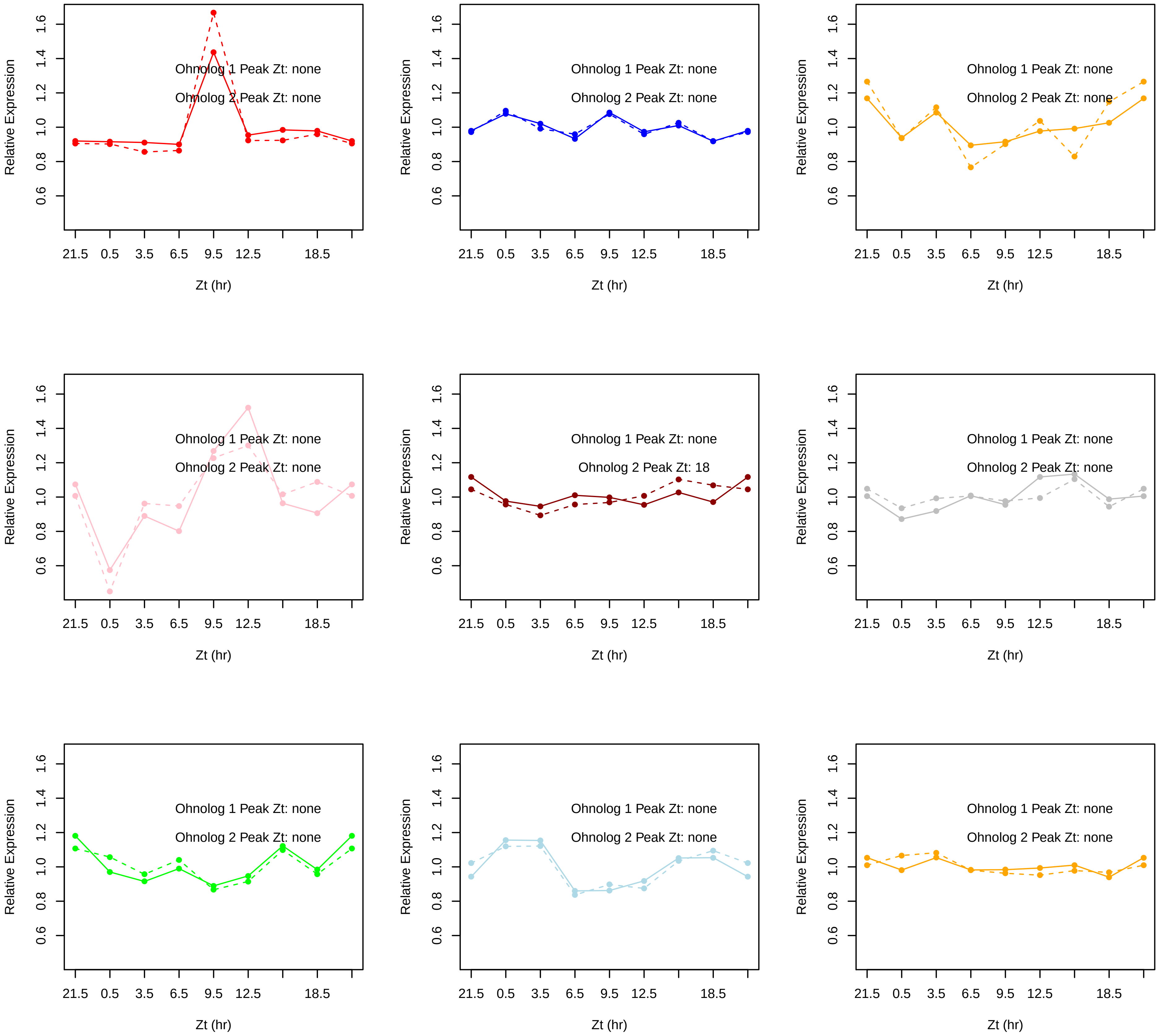

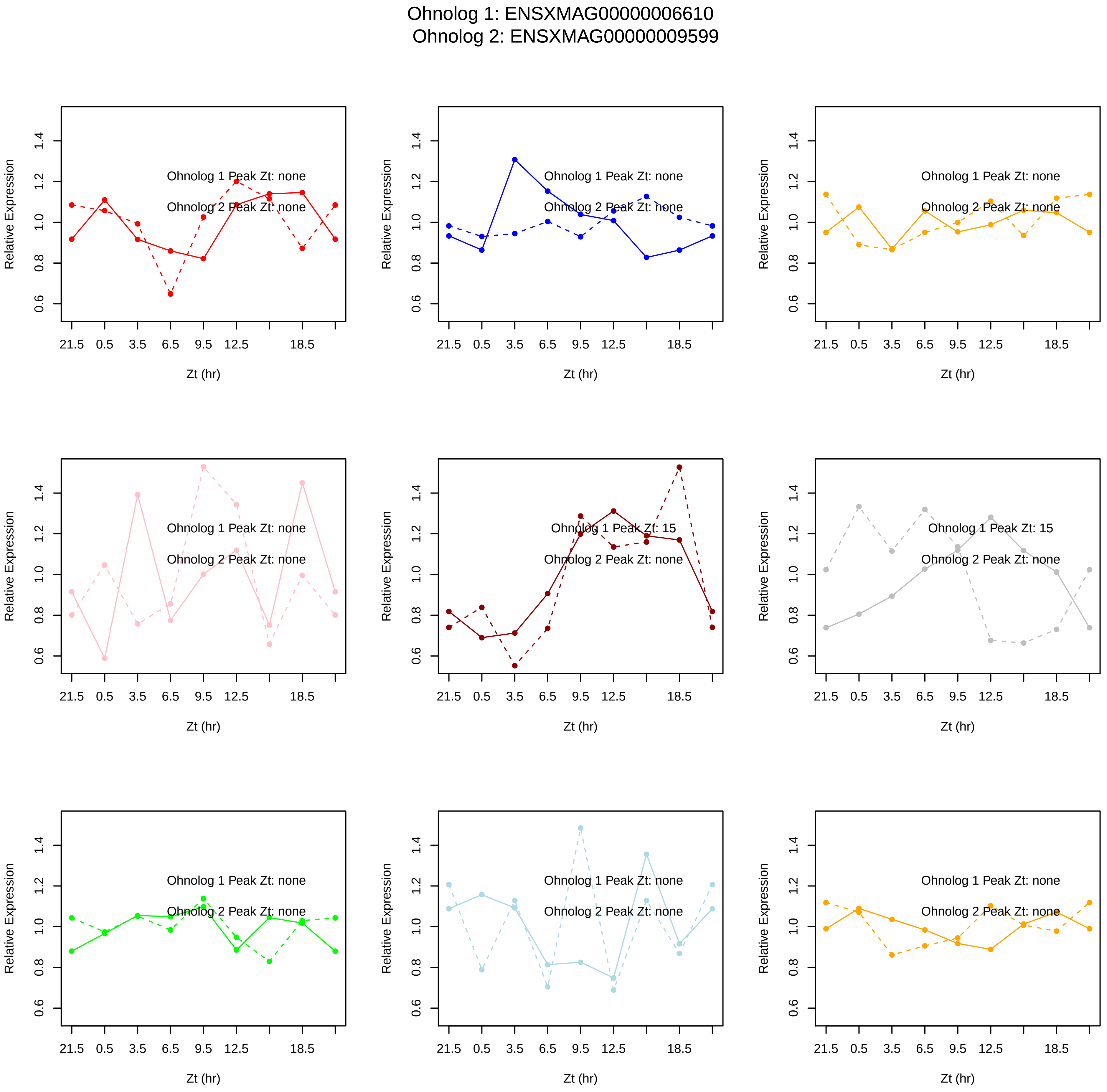
Ohnolog 1: ENSXMAG00000006610
Ohnolog 2: ENSXMAG00000009599
Relative Expression
Zt (hr)
0.6
0.8
1.0
1.2
1.4
21.5
0.5
3.5
6.5
9.5
12.5
18.5
Ohnolog 1 Peak Zt: none
Ohnolog 2 Peak Zt: none
Ohnolog 1 Peak Zt: none
Ohnolog 2 Peak Zt: none
Ohnolog 1 Peak Zt: none
Ohnolog 2 Peak Zt: none
Ohnolog 1 Peak Zt: none
Ohnolog 2 Peak Zt: none
Ohnolog 1 Peak Zt: 15
Ohnolog 2 Peak Zt: none
Ohnolog 1 Peak Zt: 15
Ohnolog 2 Peak Zt: none
Ohnolog 1 Peak Zt: none
Ohnolog 2 Peak Zt: none
Ohnolog 1 Peak Zt: none
Ohnolog 2 Peak Zt: none
Ohnolog 1 Peak Zt: none
Ohnolog 2 Peak Zt: none

Ohnolog 1: ENSXMAG00000006597 spint1a
Ohnolog 2: ENSXMAG00000009603

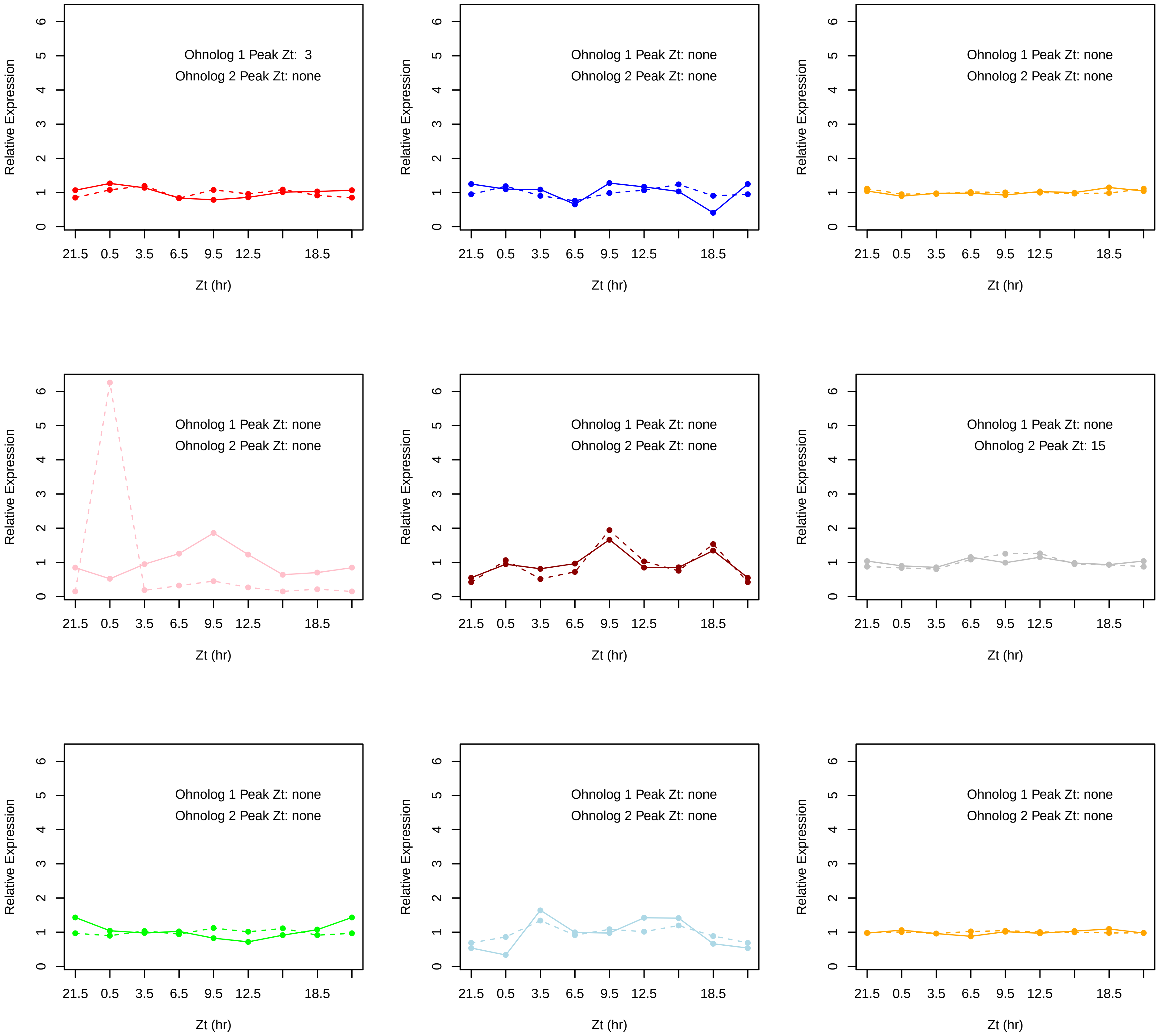

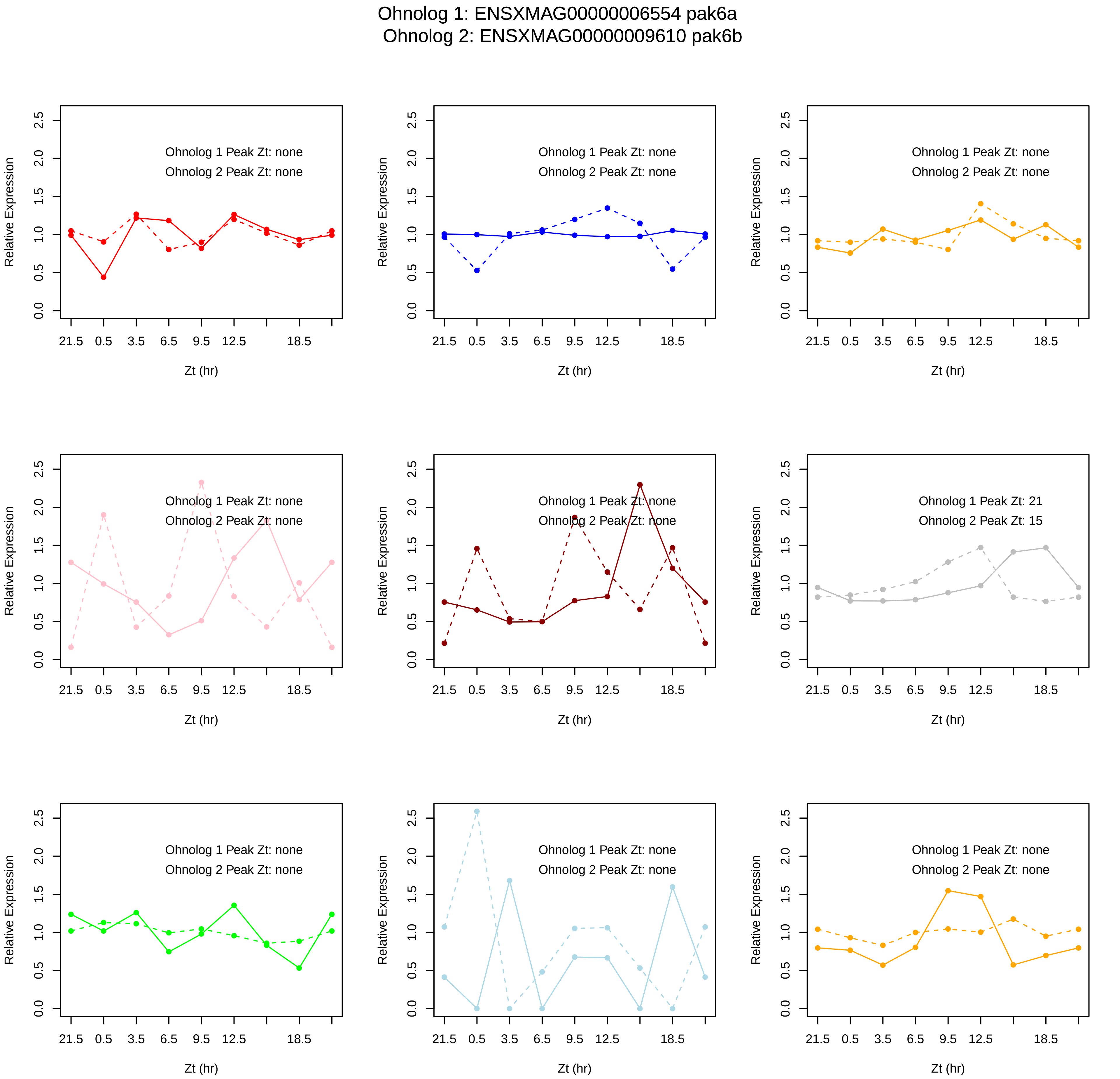
Ohnolog 1: ENSXMAG00000006554 pak6a
Ohnolog 2: ENSXMAG00000009610 pak6b
Ohnolog 1 Peak Zt: none
Ohnolog 2 Peak Zt: none
Ohnolog 1 Peak Zt: none
Ohnolog 2 Peak Zt: none
Ohnolog 1 Peak Zt: none
Ohnolog 2 Peak Zt: none
Ohnolog 1 Peak Zt: none
Ohnolog 2 Peak Zt: none
Ohnolog 1 Peak Zt: none
Ohnolog 2 Peak Zt: none
Ohnolog 1 Peak Zt: 21
Ohnolog 2 Peak Zt: 15
Ohnolog 1 Peak Zt: none
Ohnolog 2 Peak Zt: none
Ohnolog 1 Peak Zt: none
Ohnolog 2 Peak Zt: none
Ohnolog 1 Peak Zt: none
Ohnolog 2 Peak Zt: none
Relative Expression
Zt (hr)
21.5
0.5
3.5
6.5
9.5
12.5
18.5
0.0
0.5
1.0
1.5
2.0
2.5

Ohnolog 1: ENSXMAG00000006477 mthfd1b
Ohnolog 2: ENSXMAG00000009631

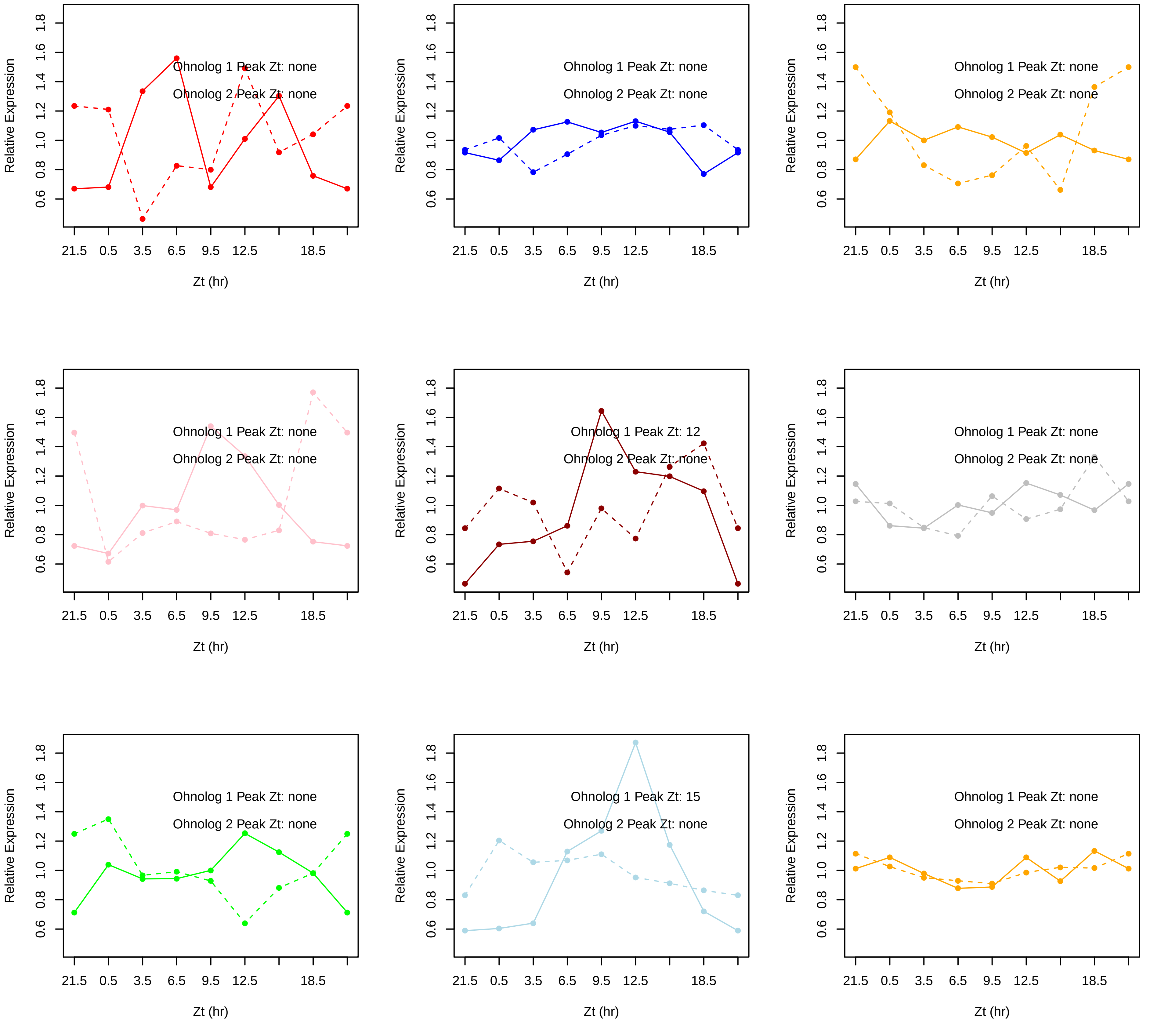

Ohnolog 1: ENSXMAG00000012739 chmp6a
Ohnolog 2: ENSXMAG00000014473 CHMP6 (1 of many)

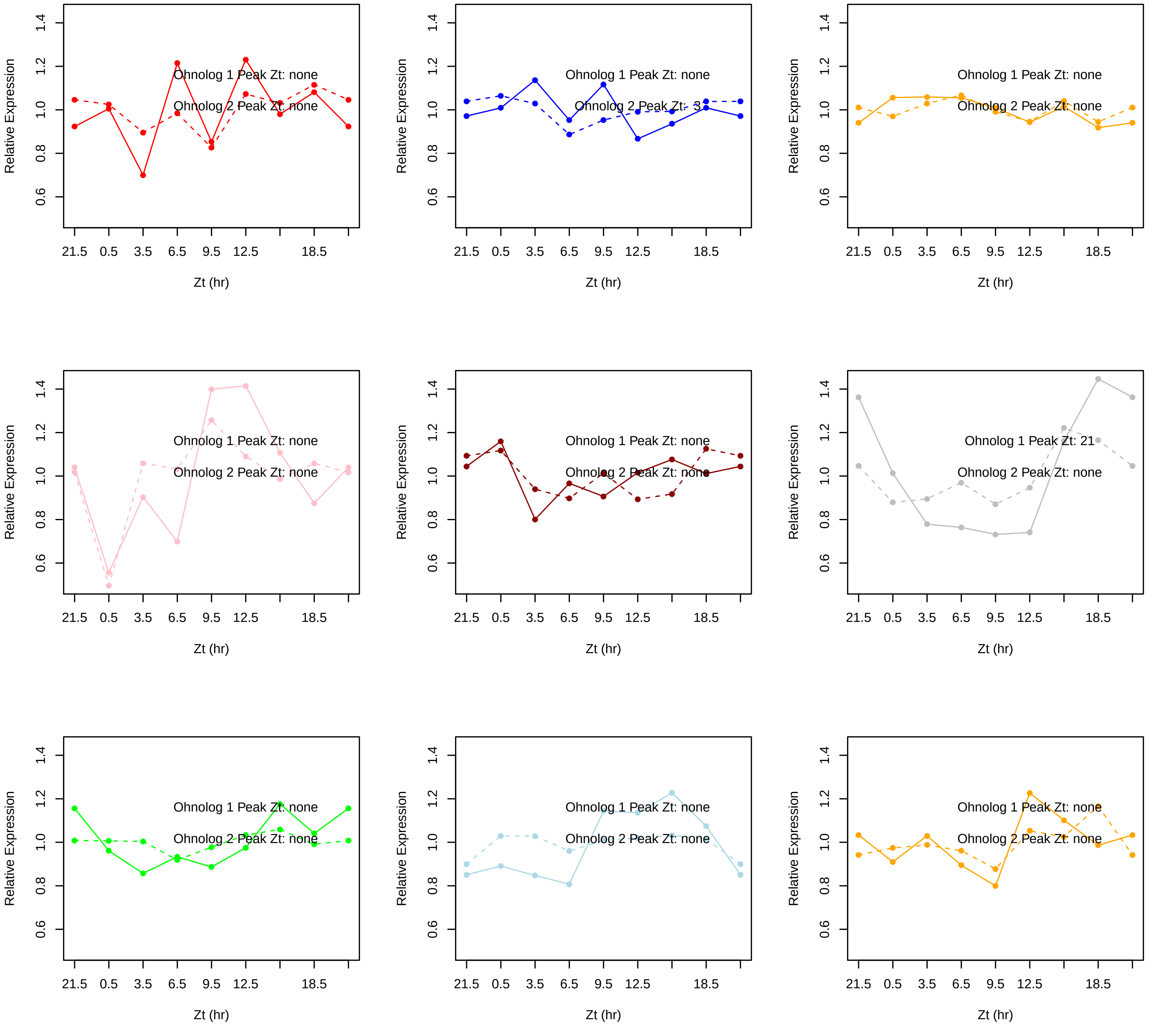

Ohnolog 1: ENSXMAG00000001628 pdlim5b
Ohnolog 2: ENSXMAG00000021636 pdlim5a

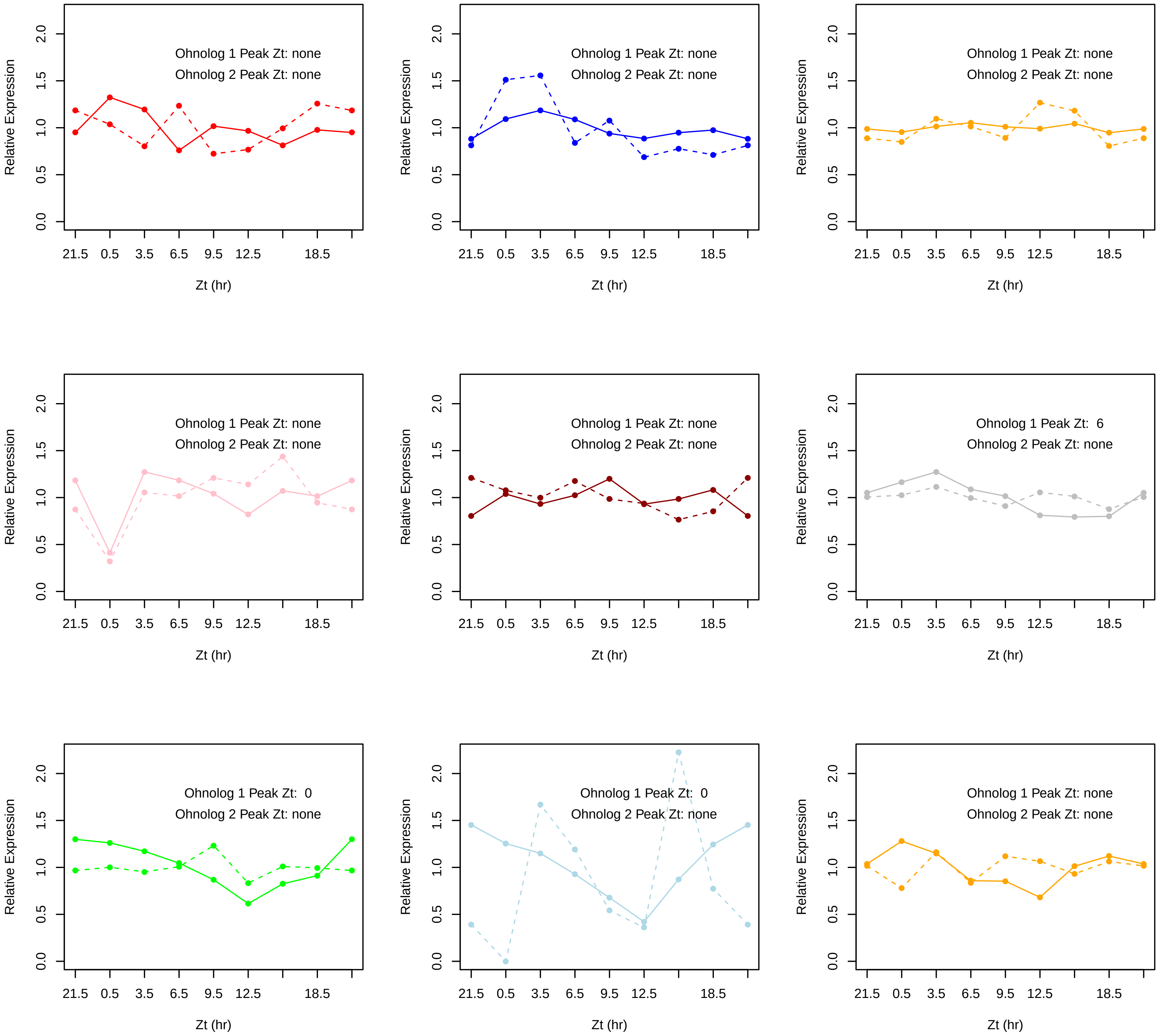

Ohnolog 1: ENSXMAG00000001635 bmpr1bb
Ohnolog 2: ENSXMAG00000017124 bmpr1ba

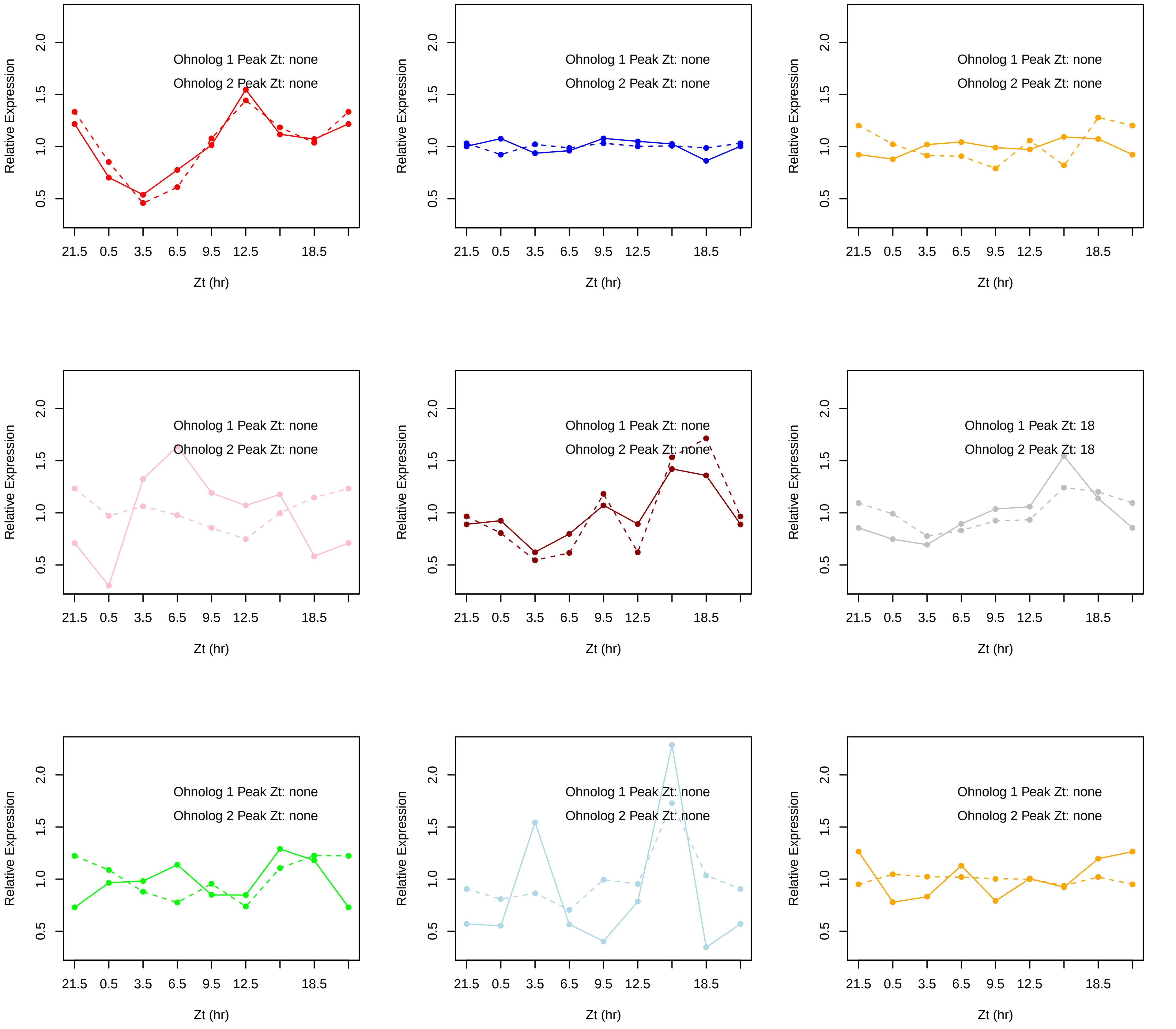

Ohnolog 1: ENSXMAG00000013225 fyco1a
Ohnolog 2: ENSXMAG00000016929 fyco1b

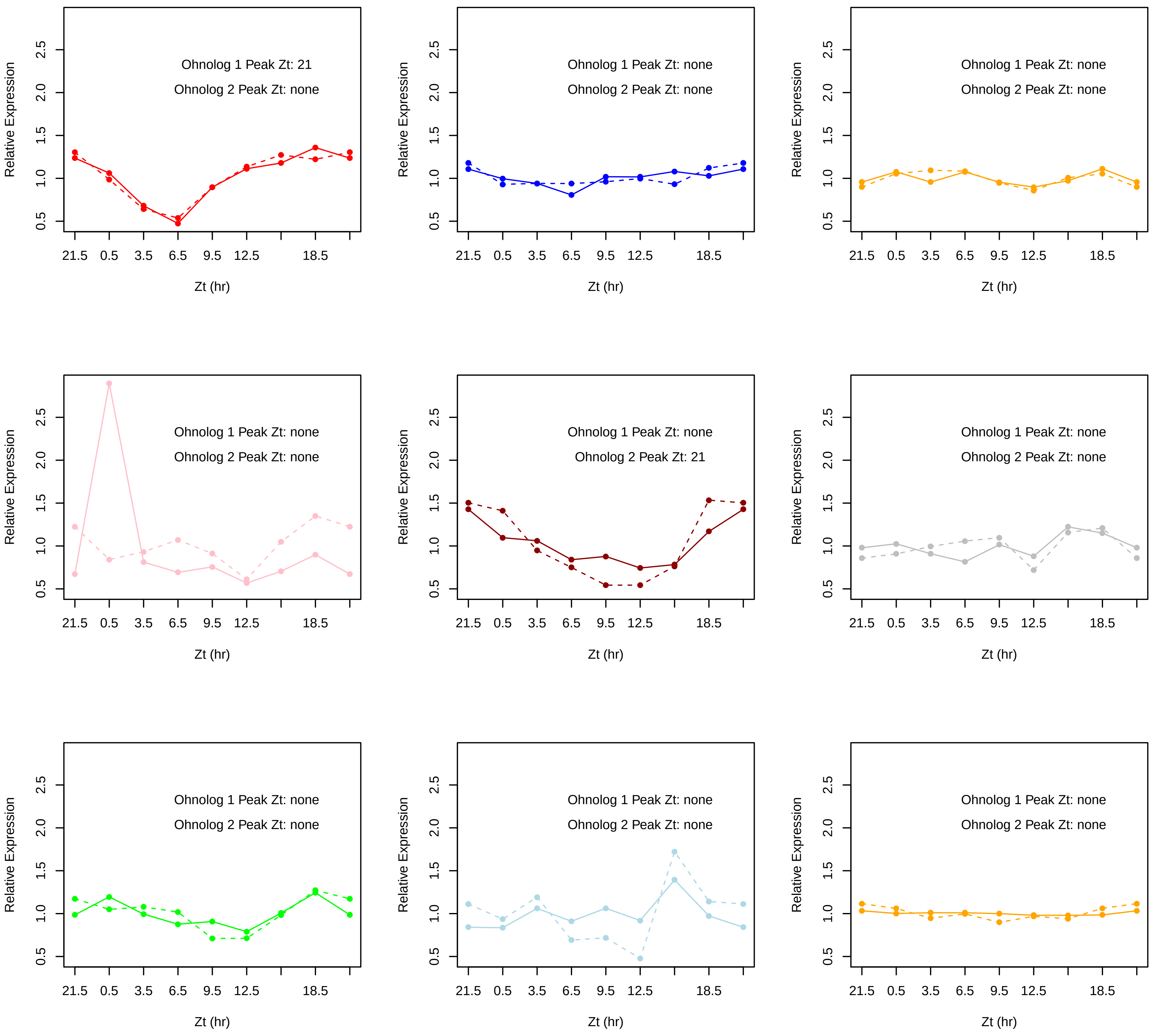

Ohnolog 1: ENSXMAG00000023445 gfra1b
Ohnolog 2: ENSXMAG00000027096 GFRA1

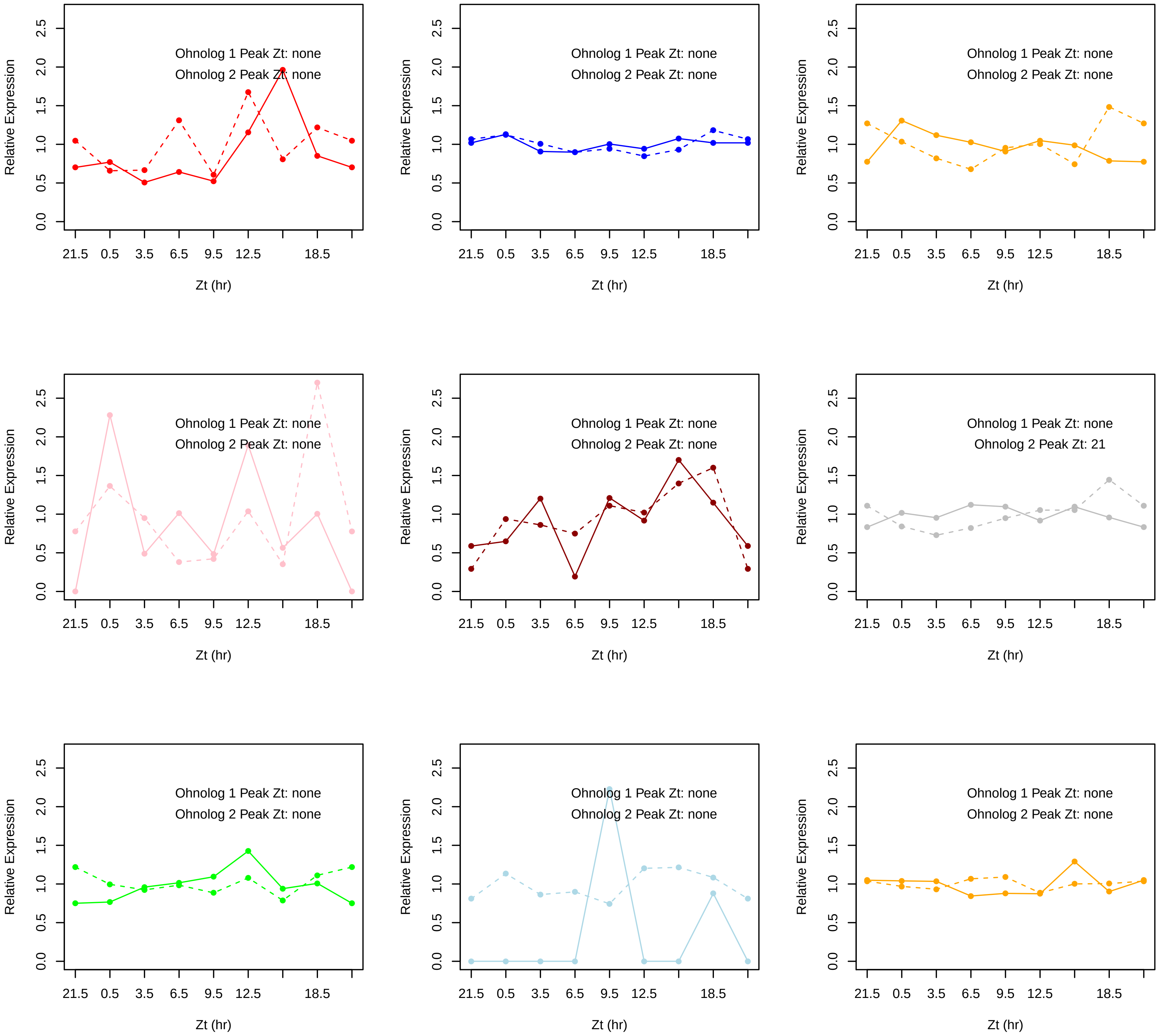

Ohnolog 1: ENSXMAG00000010812 nsfa
Ohnolog 2: ENSXMAG00000017938 nsfb

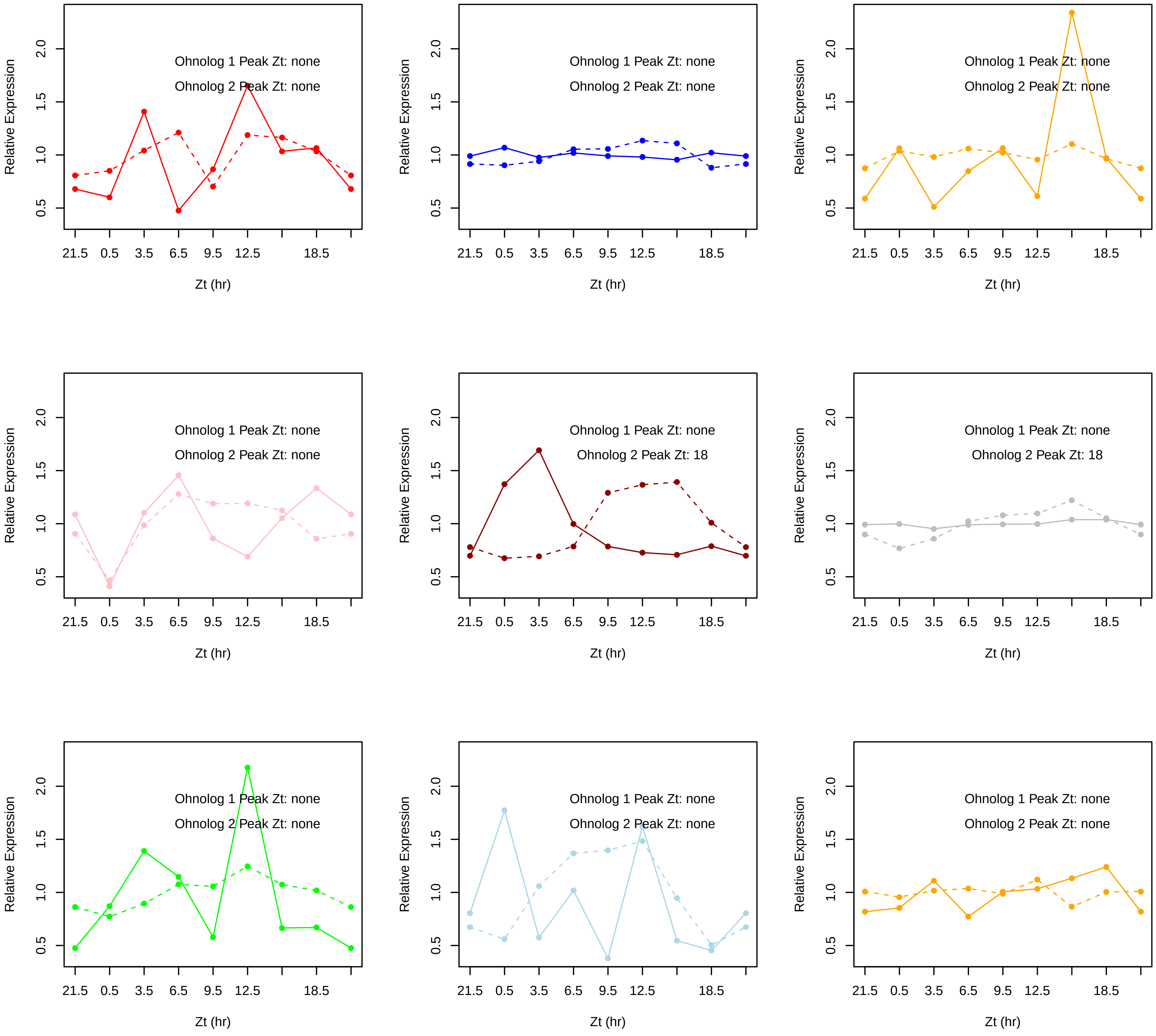

Ohnolog 1: ENSXMAG00000008384
Ohnolog 2: ENSXMAG00000009679 chrna9

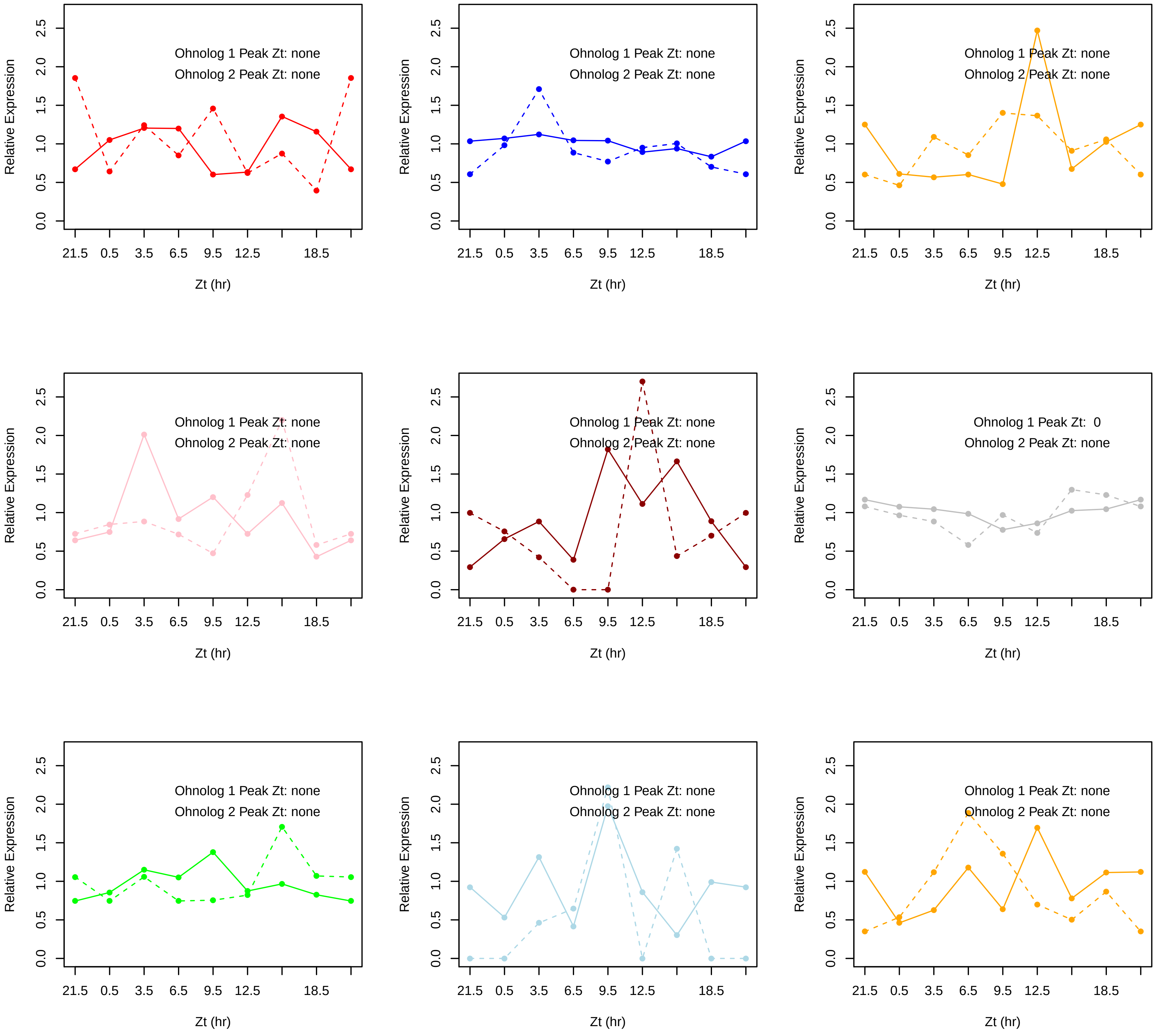

Ohnolog 1: ENSXMAG00000006166 mylk4b
Ohnolog 2: ENSXMAG00000016372 mylk4a

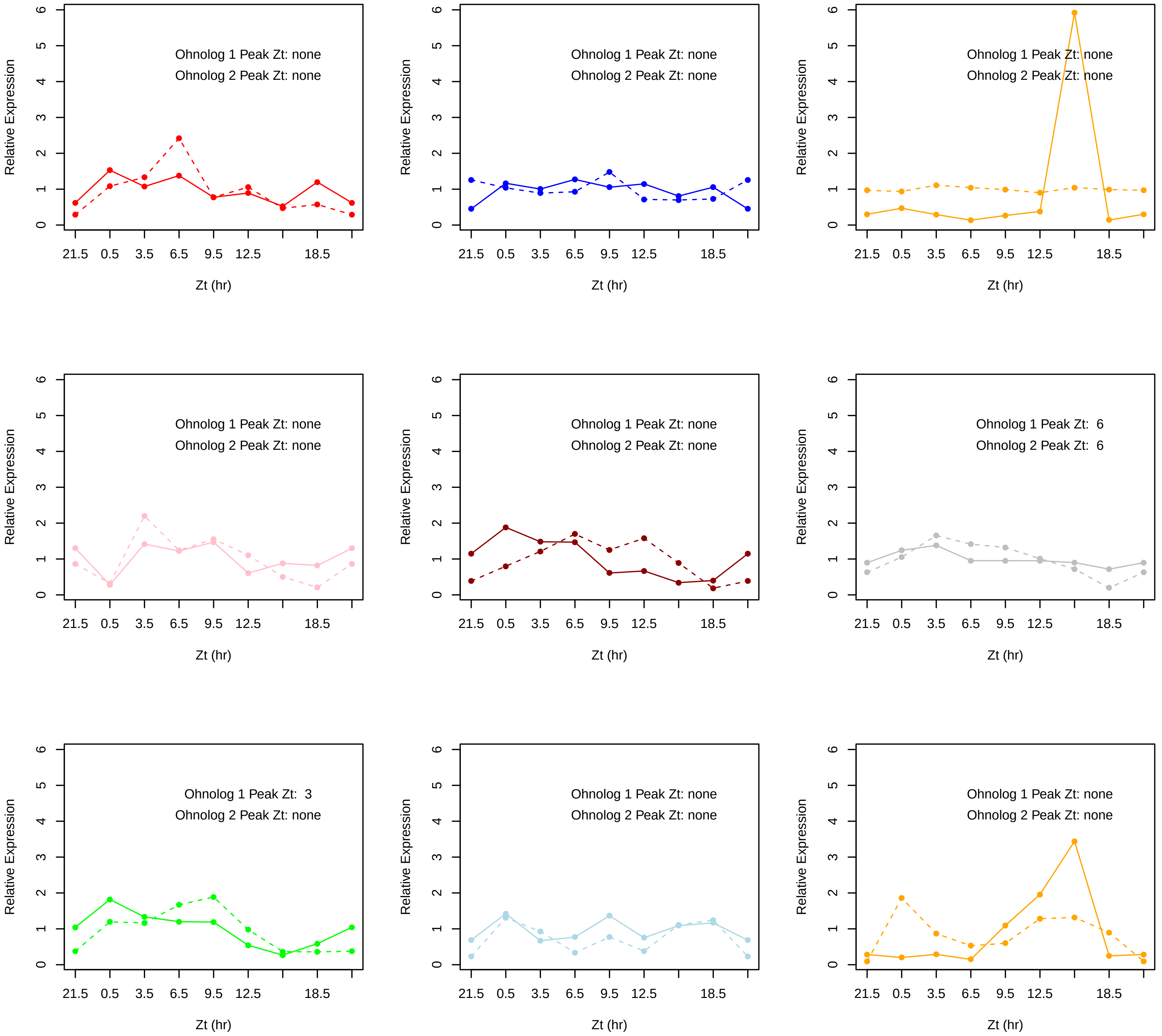

Ohnolog 1: ENSXMAG00000010829
Ohnolog 2: ENSXMAG00000017922

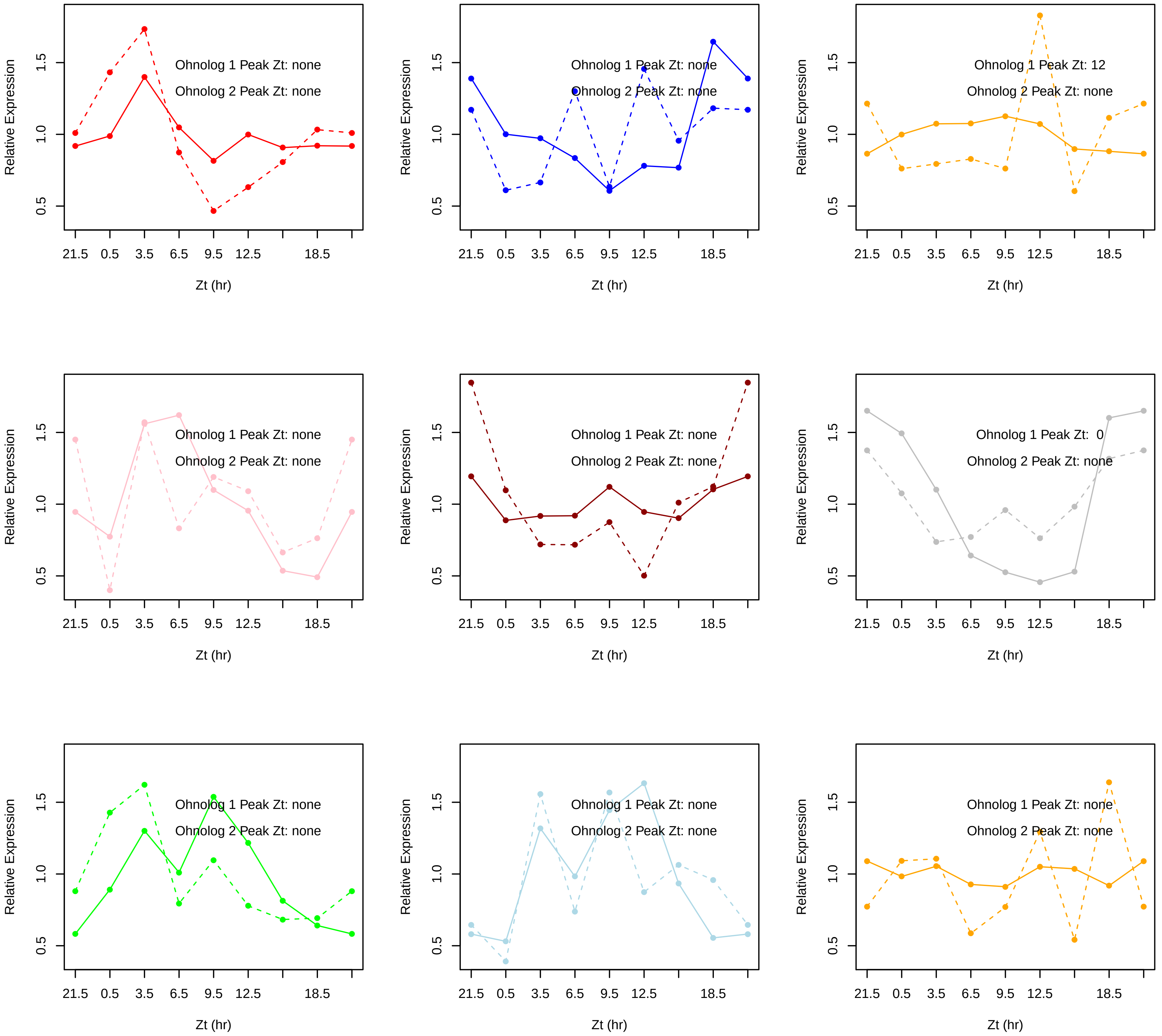

Ohnolog 1: ENSXMAG00000006258
Ohnolog 2: ENSXMAG00000023204 dusp22b

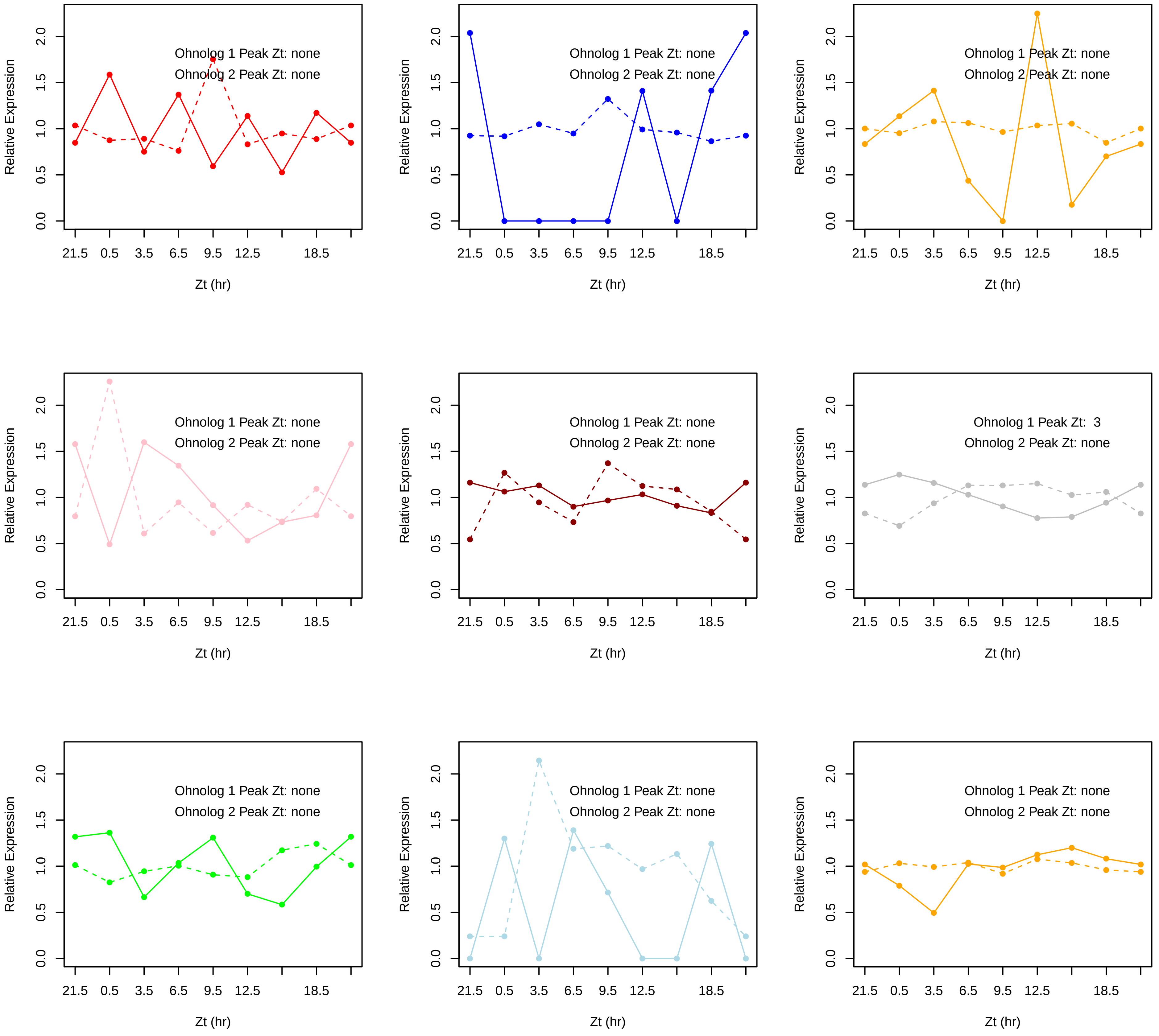

Ohnolog 1: ENSXMAG00000016931 tmcc1b
Ohnolog 2: ENSXMAG00000029828 TMCC1 (1 of many)

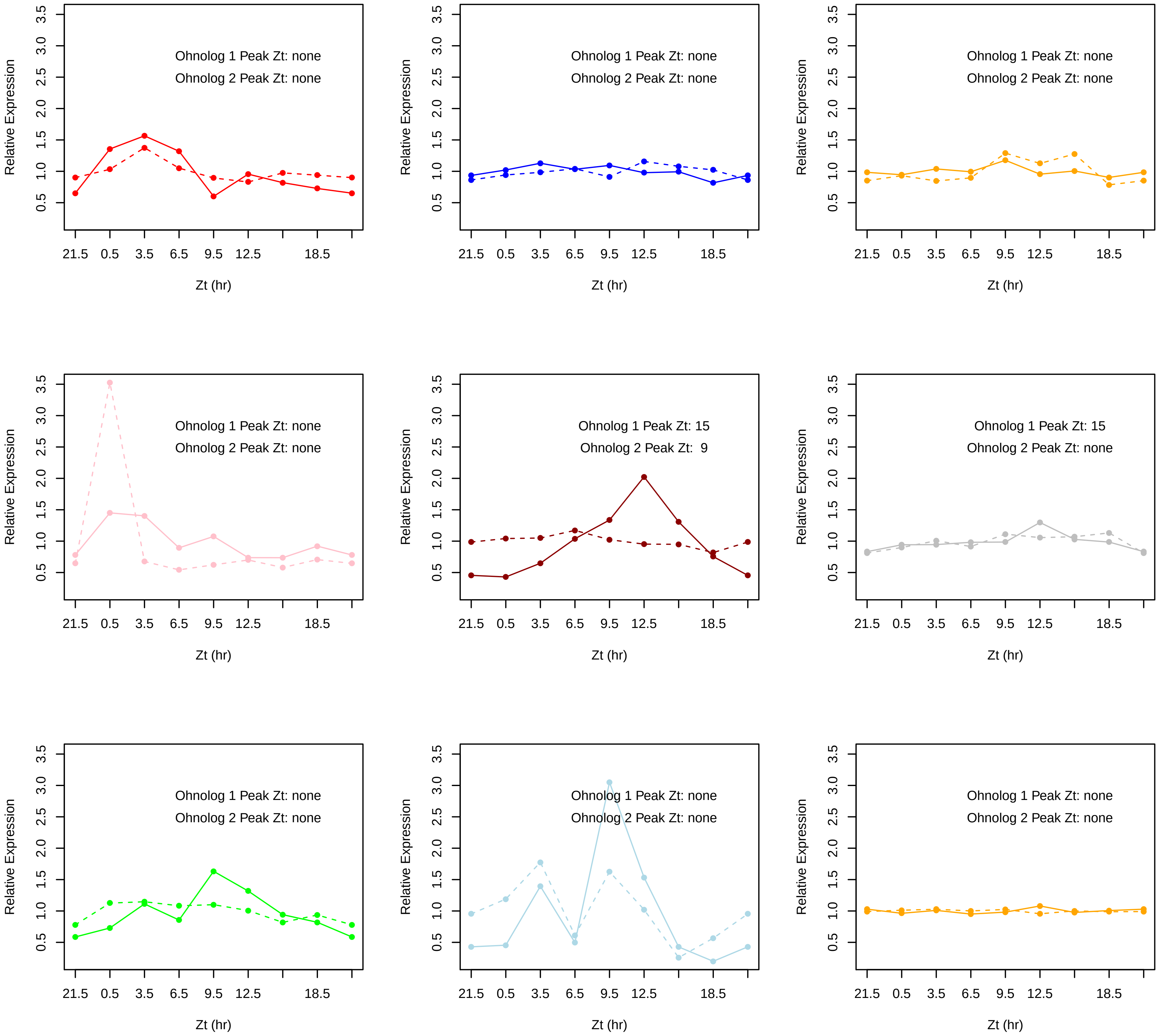

Ohnolog 1: ENSXMAG00000006335 TNS3 (1 of many)
Ohnolog 2: ENSXMAG00000016847 TNS3 (1 of many)

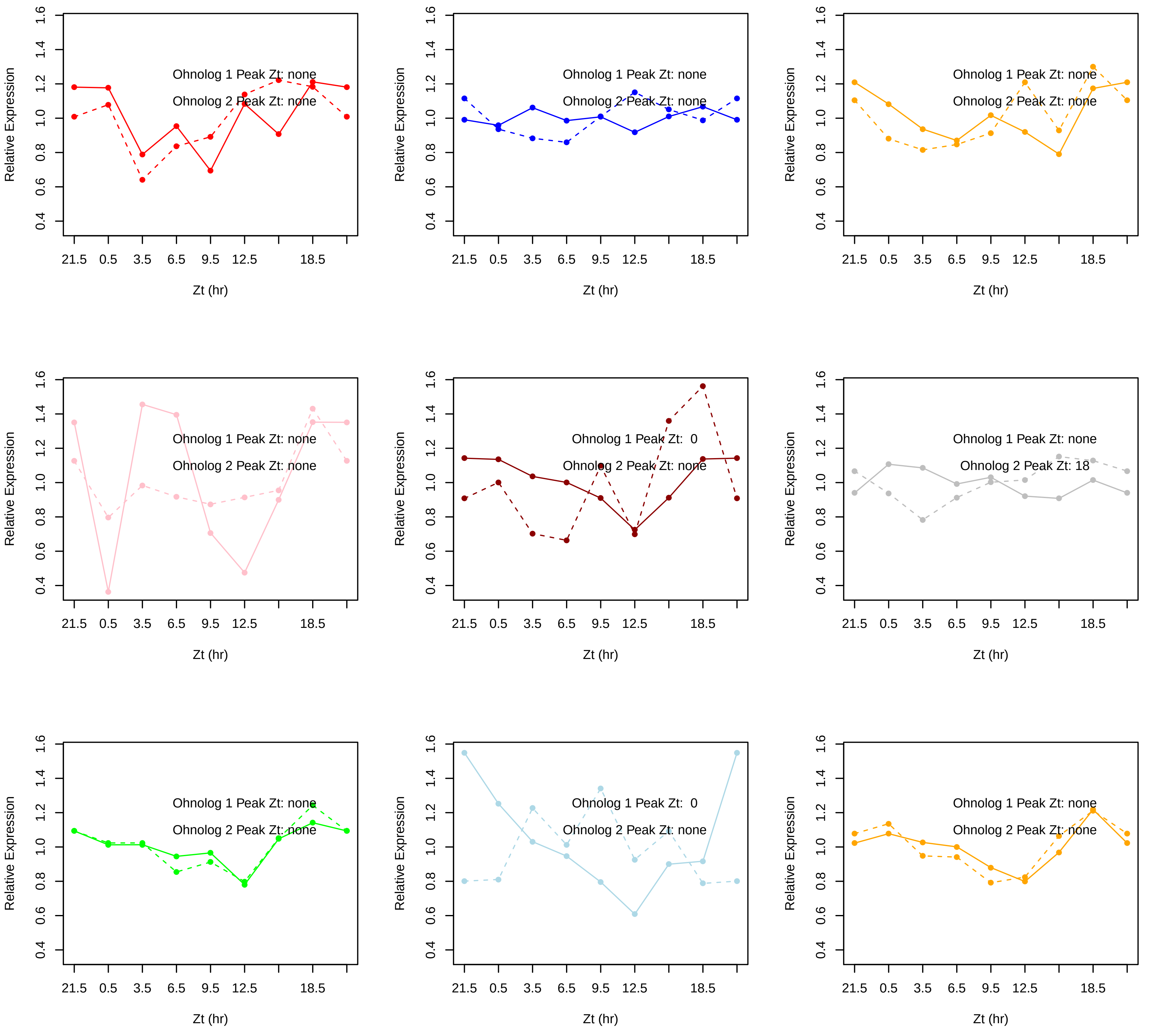

Ohnolog 1: ENSXMAG00000007470
Ohnolog 2: ENSXMAG00000017432 kbtbd12

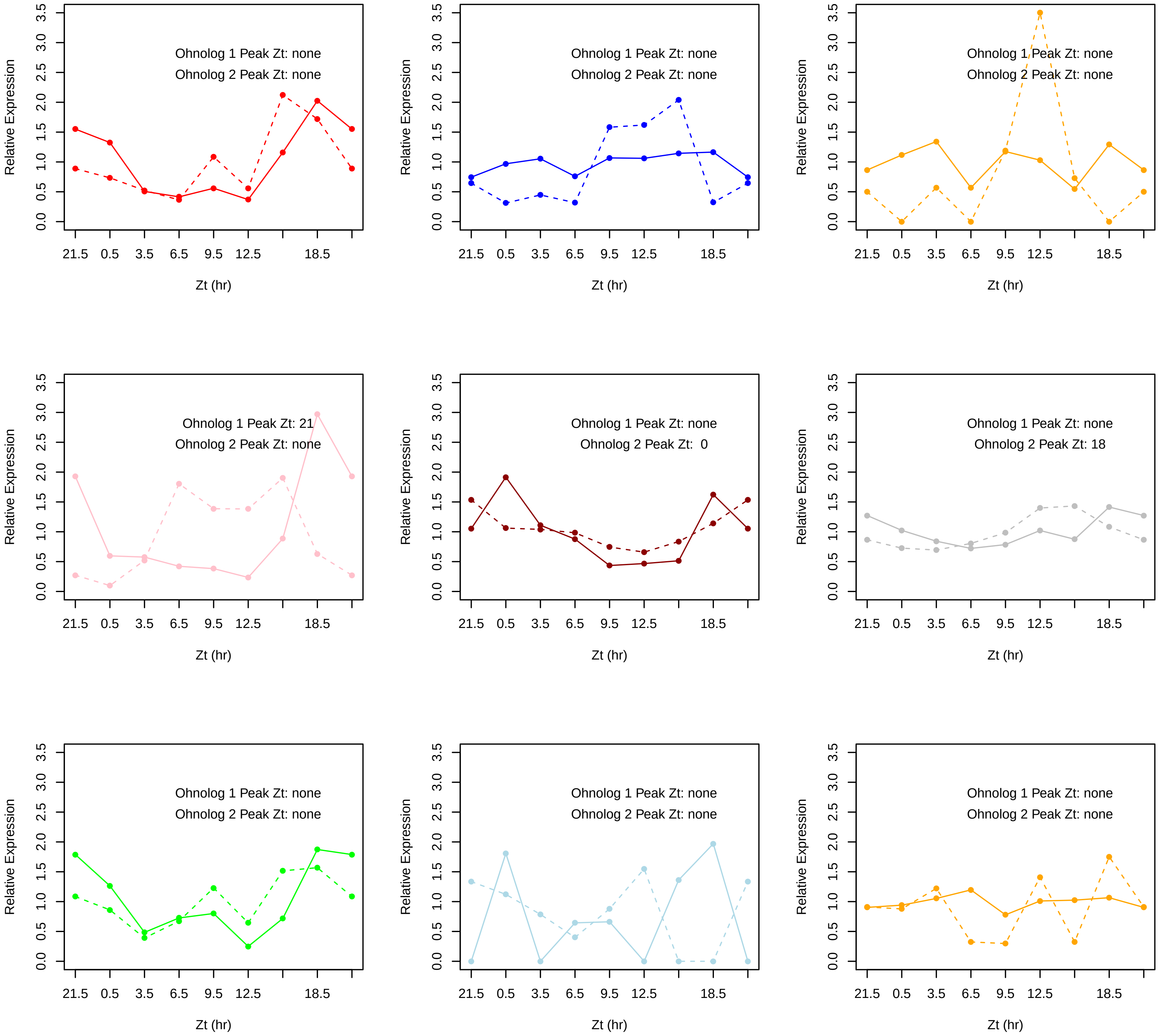

Ohnolog 1: ENSXMAG00000006331 igfbp3
Ohnolog 2: ENSXMAG00000028732 IGFBP3

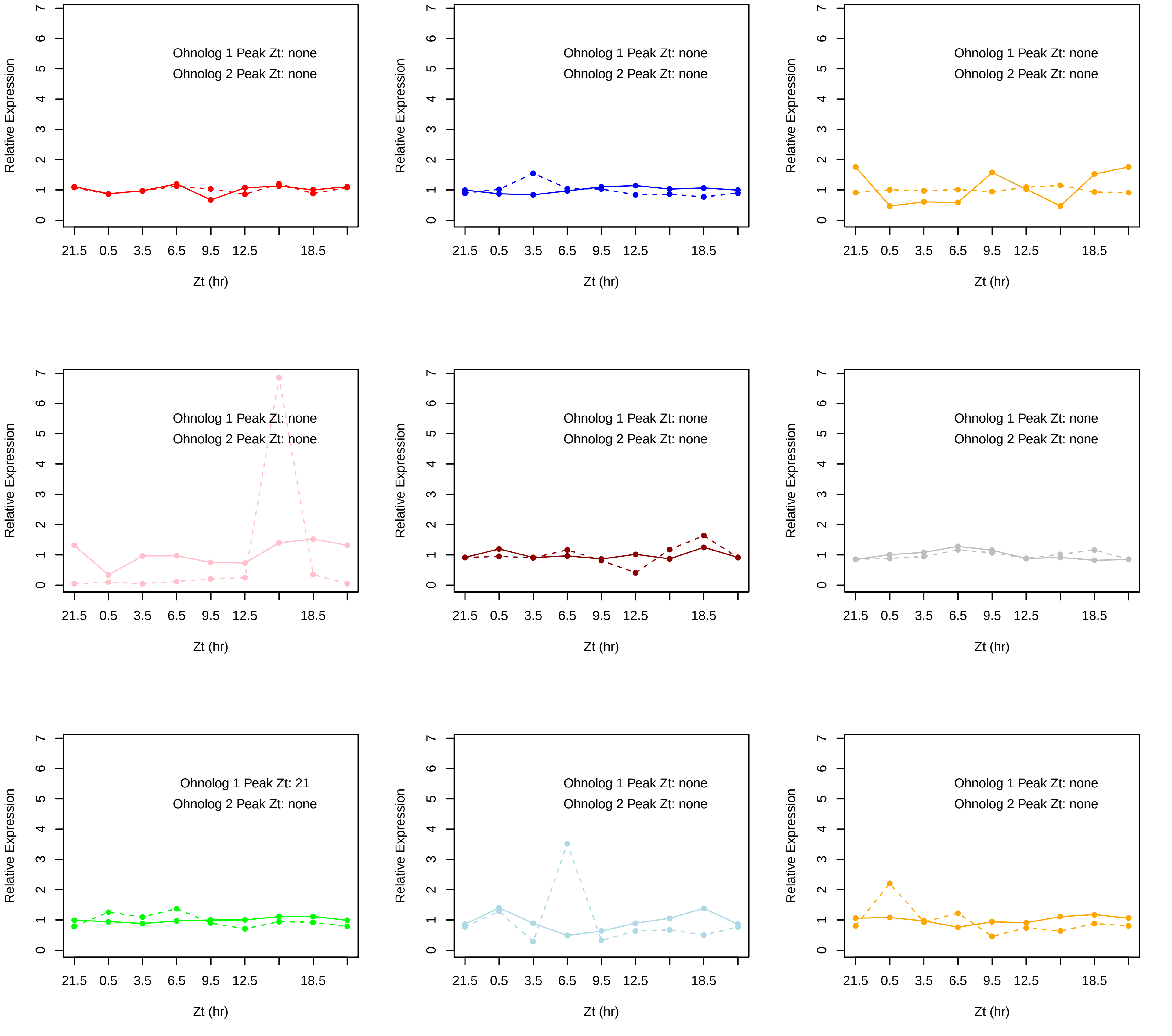

Ohnolog 1: ENSXMAG00000012564 HID1 (1 of many)
Ohnolog 2: ENSXMAG00000016830 HID1 (1 of many)

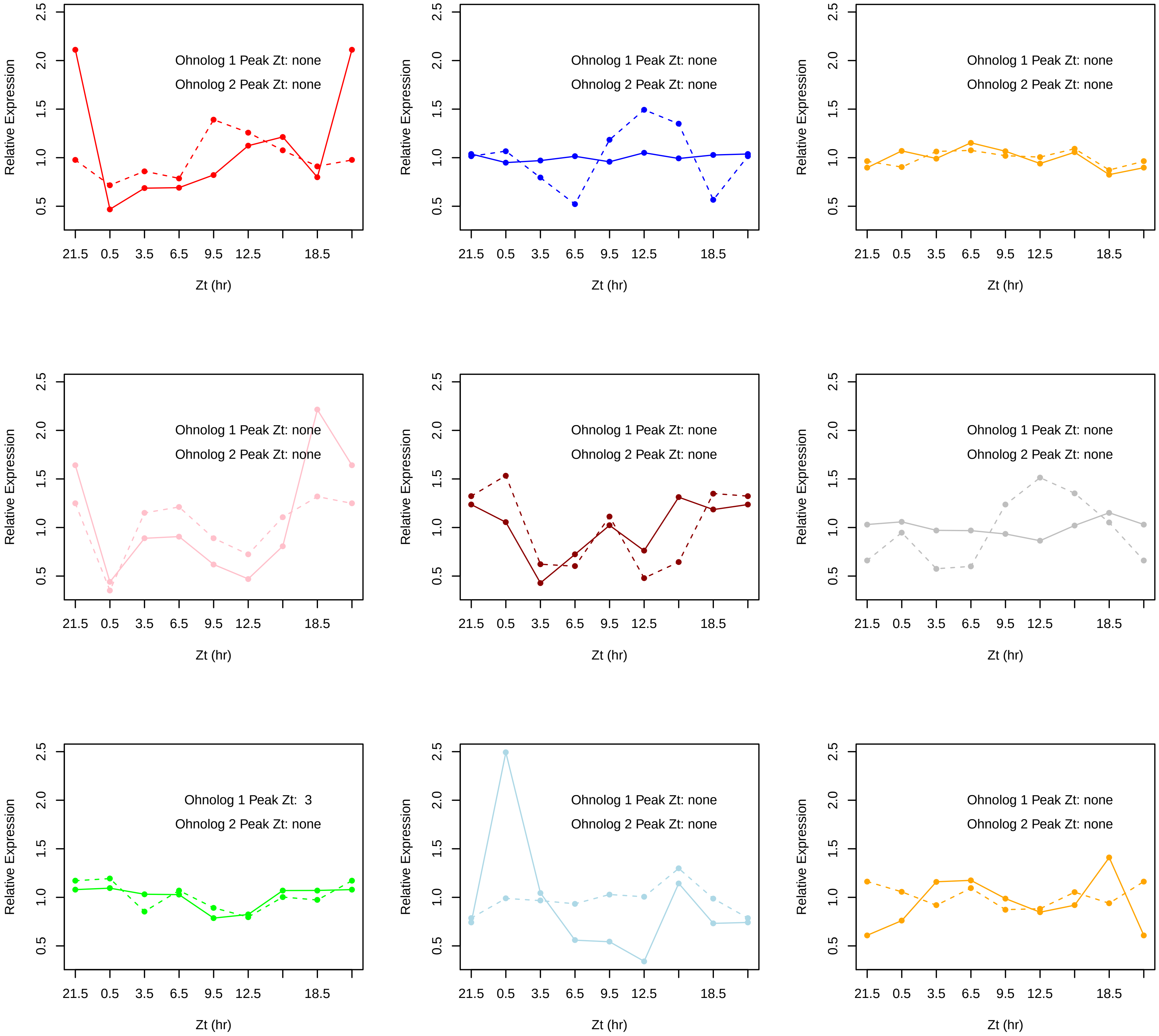

Ohnolog 1: ENSXMAG00000006293 phactr1
Ohnolog 2: ENSXMAG00000016948

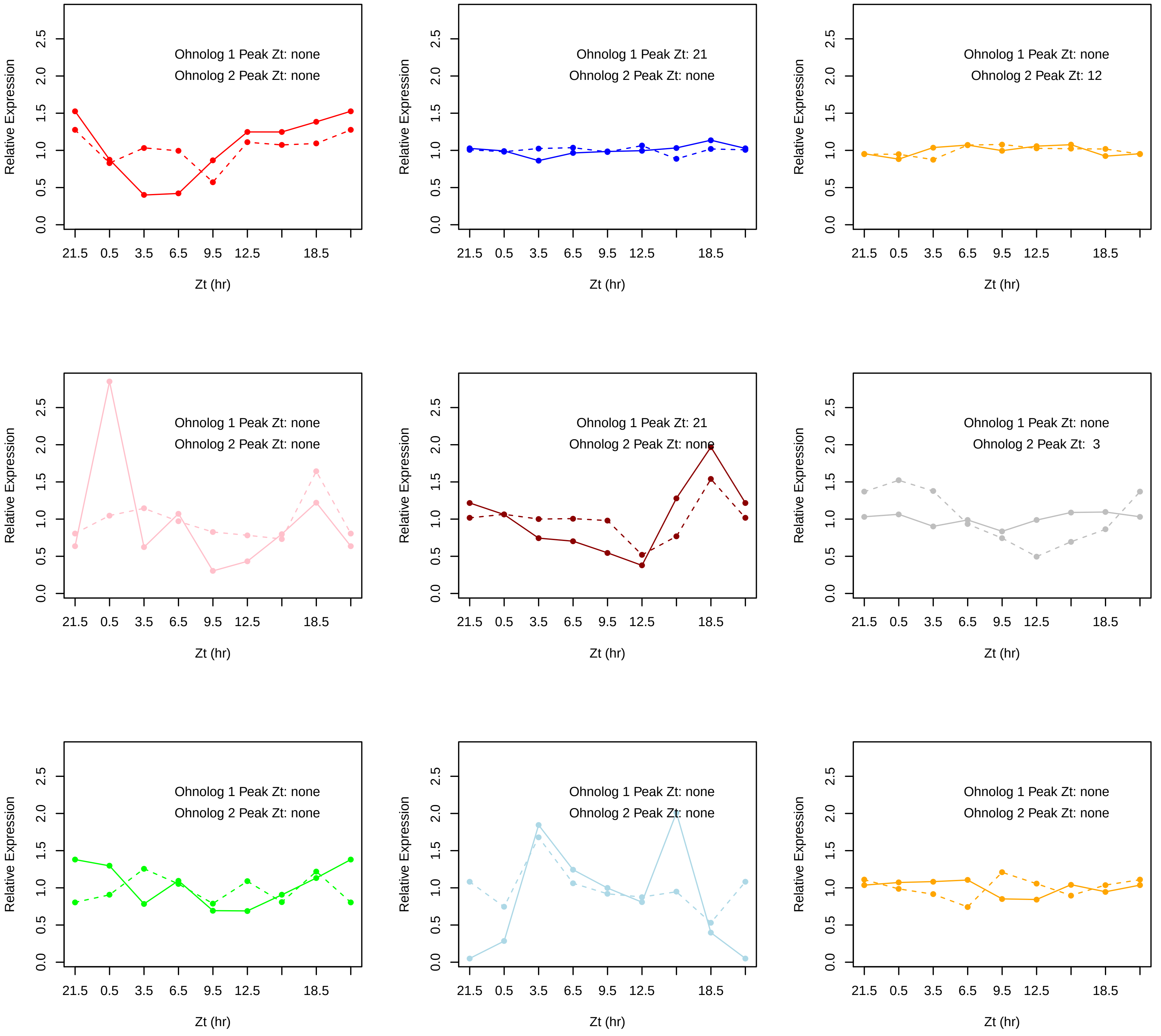

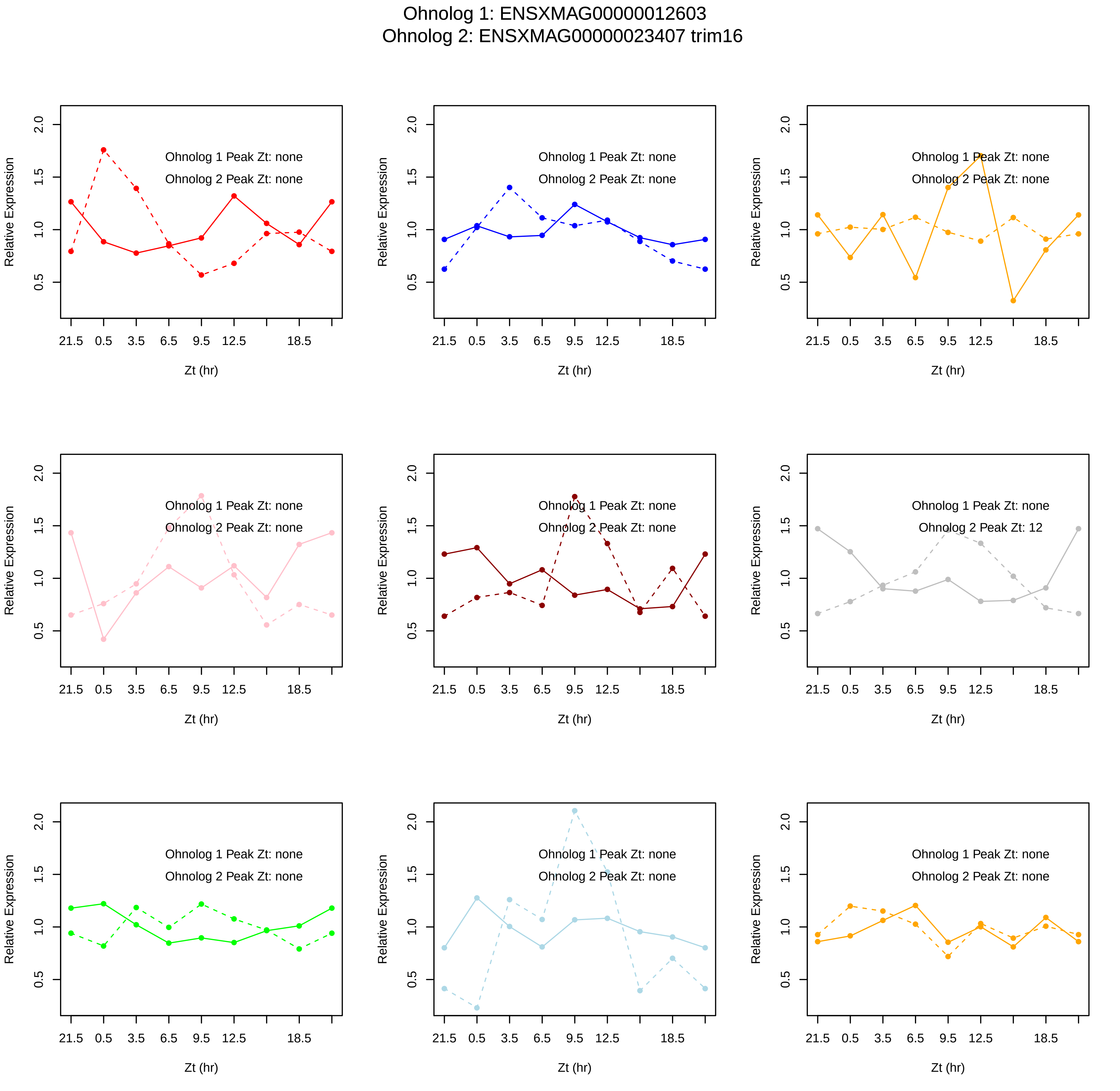
Ohnolog 1: ENSXMAG00000012603
Ohnolog 2: ENSXMAG00000023407 trim16
Ohnolog 1 Peak Zt: none
Ohnolog 2 Peak Zt: none
Ohnolog 1 Peak Zt: none
Ohnolog 2 Peak Zt: 12
Relative Expression
Zt (hr)
21.5
0.5
3.5
6.5
9.5
12.5
18.5
0.5
1.0
1.5
2.0

Ohnolog 1: ENSXMAG00000012781 si:ch211−114m9.1
Ohnolog 2: ENSXMAG00000014836

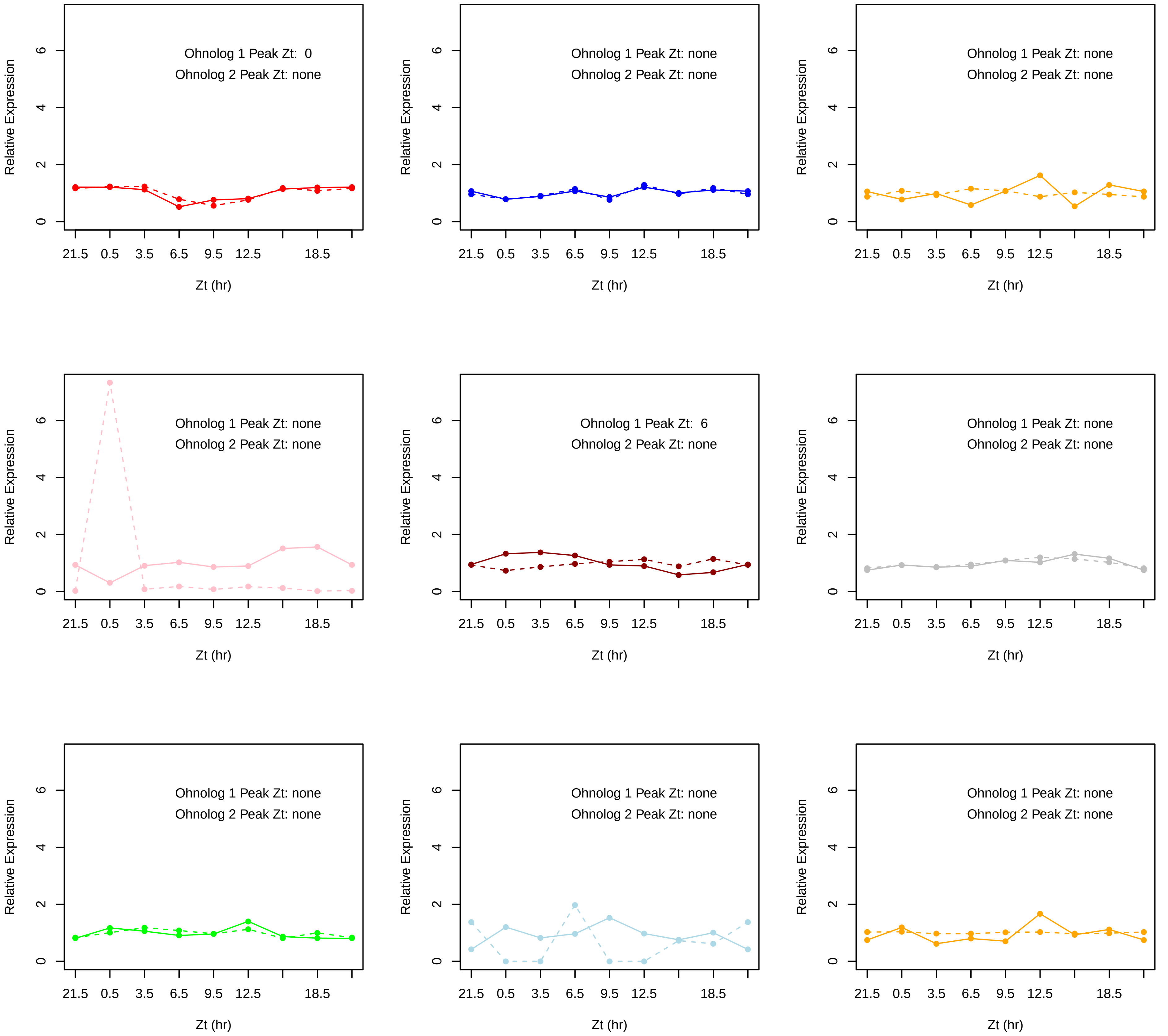

Ohnolog 1: ENSXMAG00000025314 kctd6a
Ohnolog 2: ENSXMAG00000025640 kctd6b

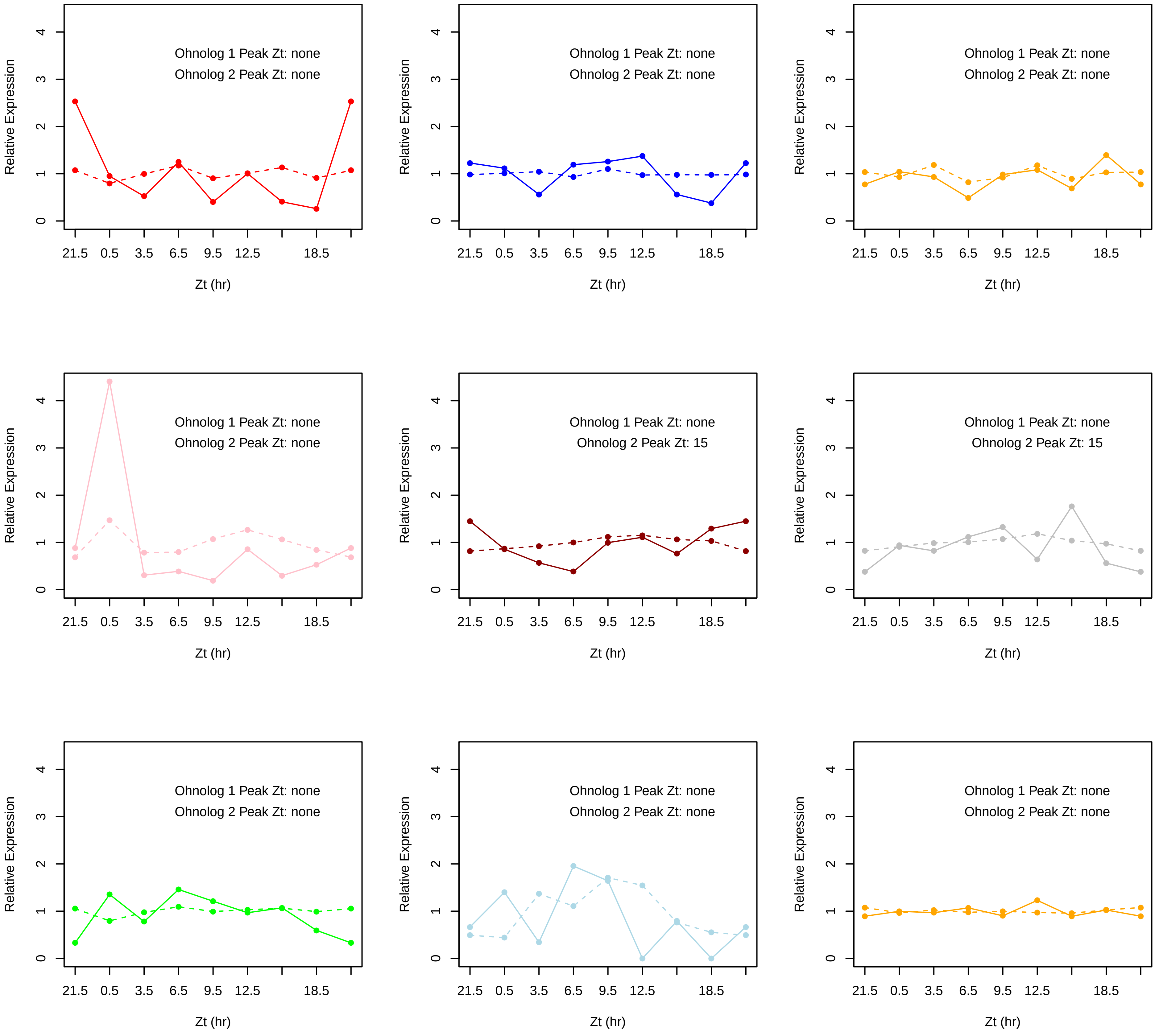

Ohnolog 1: ENSXMAG00000012493
Ohnolog 2: ENSXMAG00000023015 DCTN6

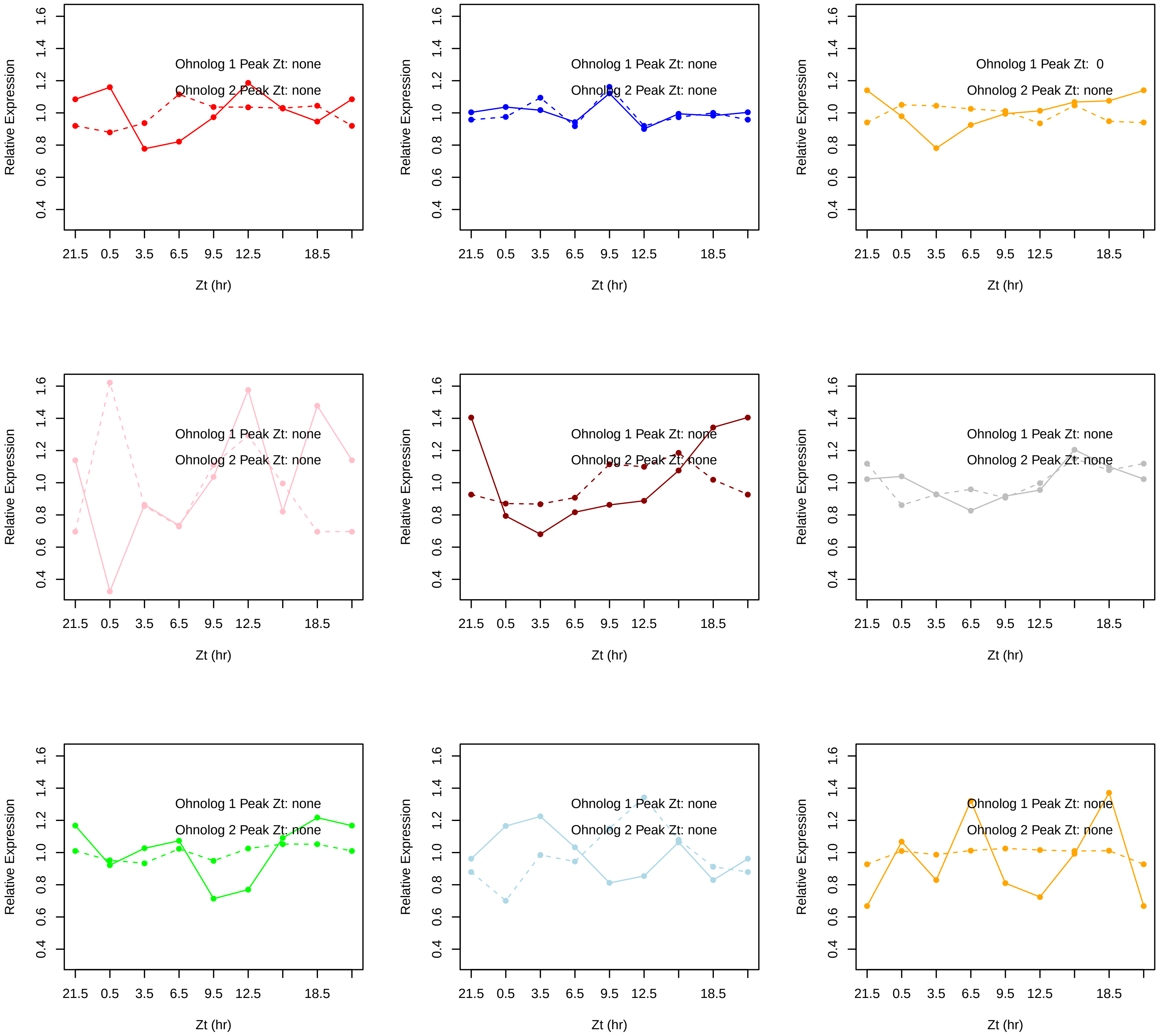

Ohnolog 1: ENSXMAG00000004882
Ohnolog 2: ENSXMAG00000012102 adgrl3.1

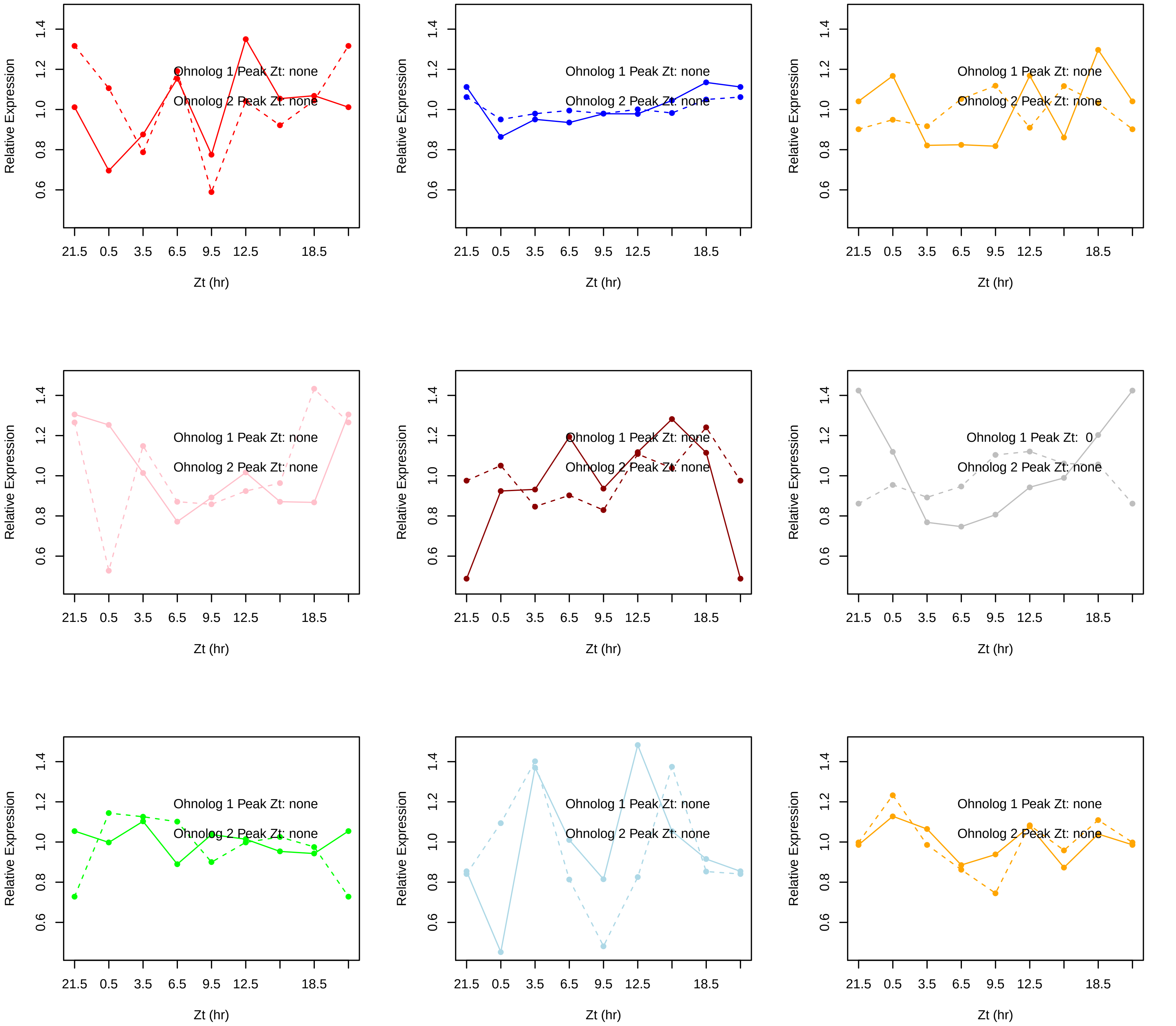

Ohnolog 1: ENSXMAG00000013776 atp6ap1lb
Ohnolog 2: ENSXMAG00000025029

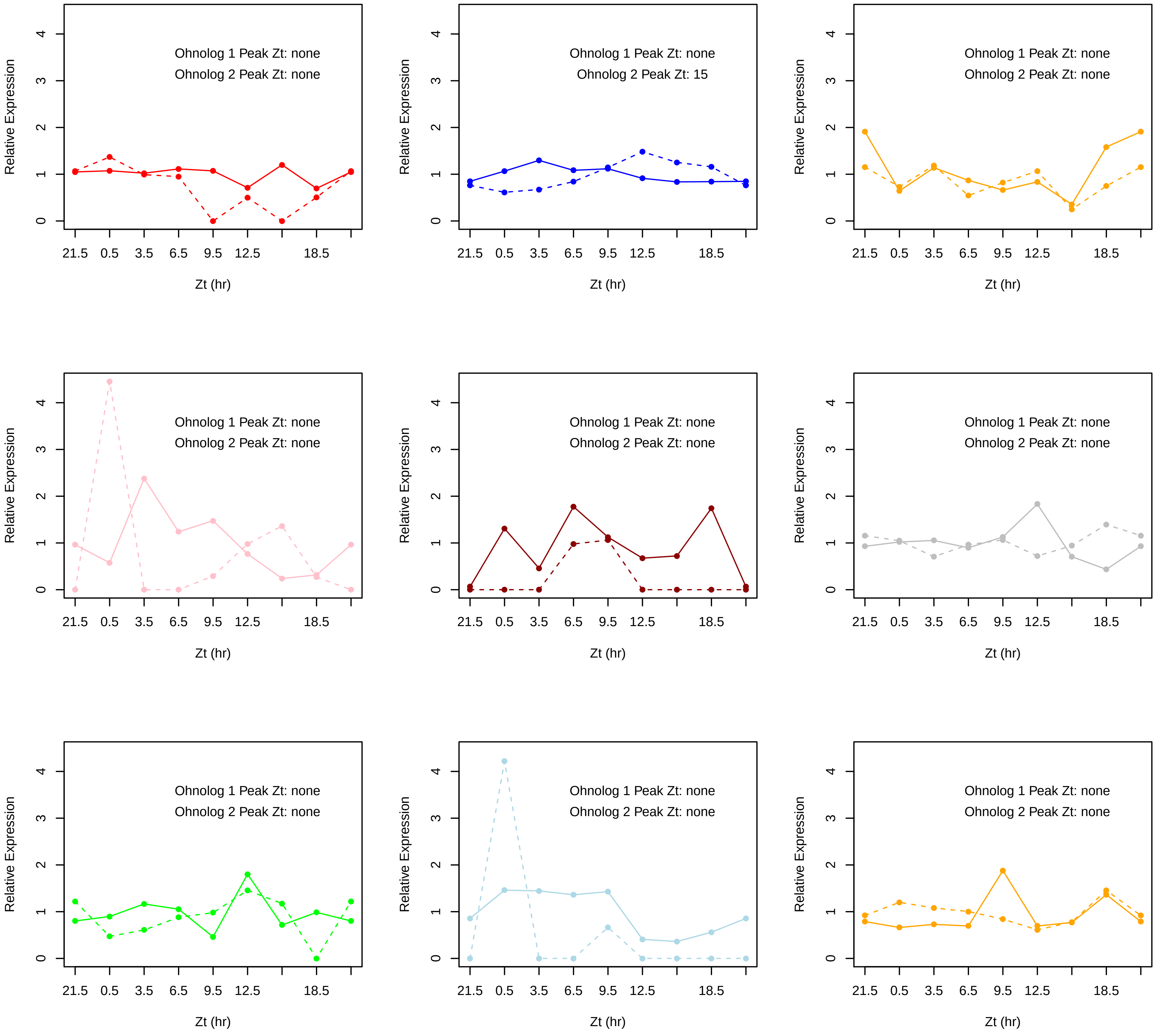

Ohnolog 1: ENSXMAG00000007410 cfap52 (1 of many)
Ohnolog 2: ENSXMAG00000026073 cfap52 (1 of many)

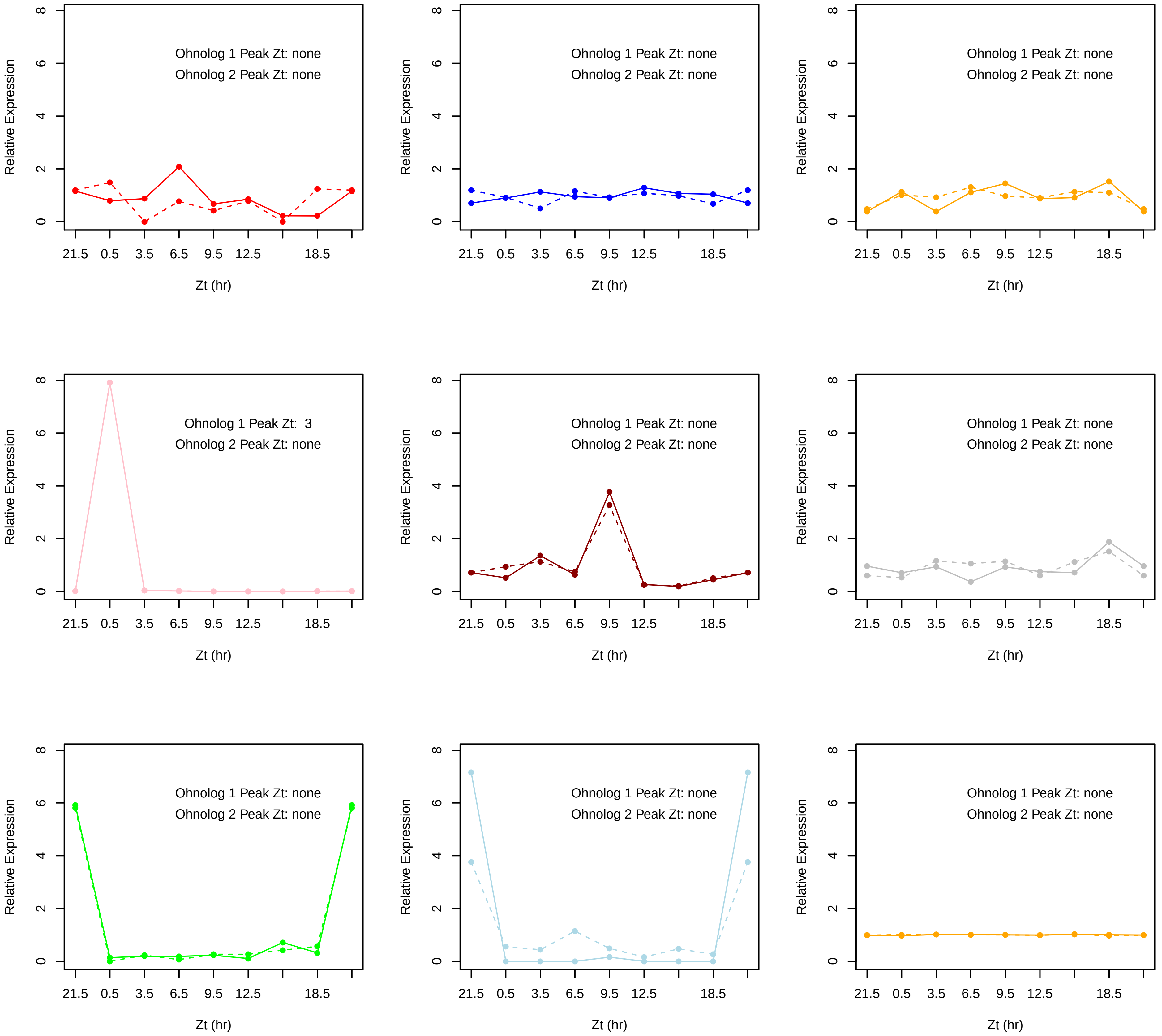

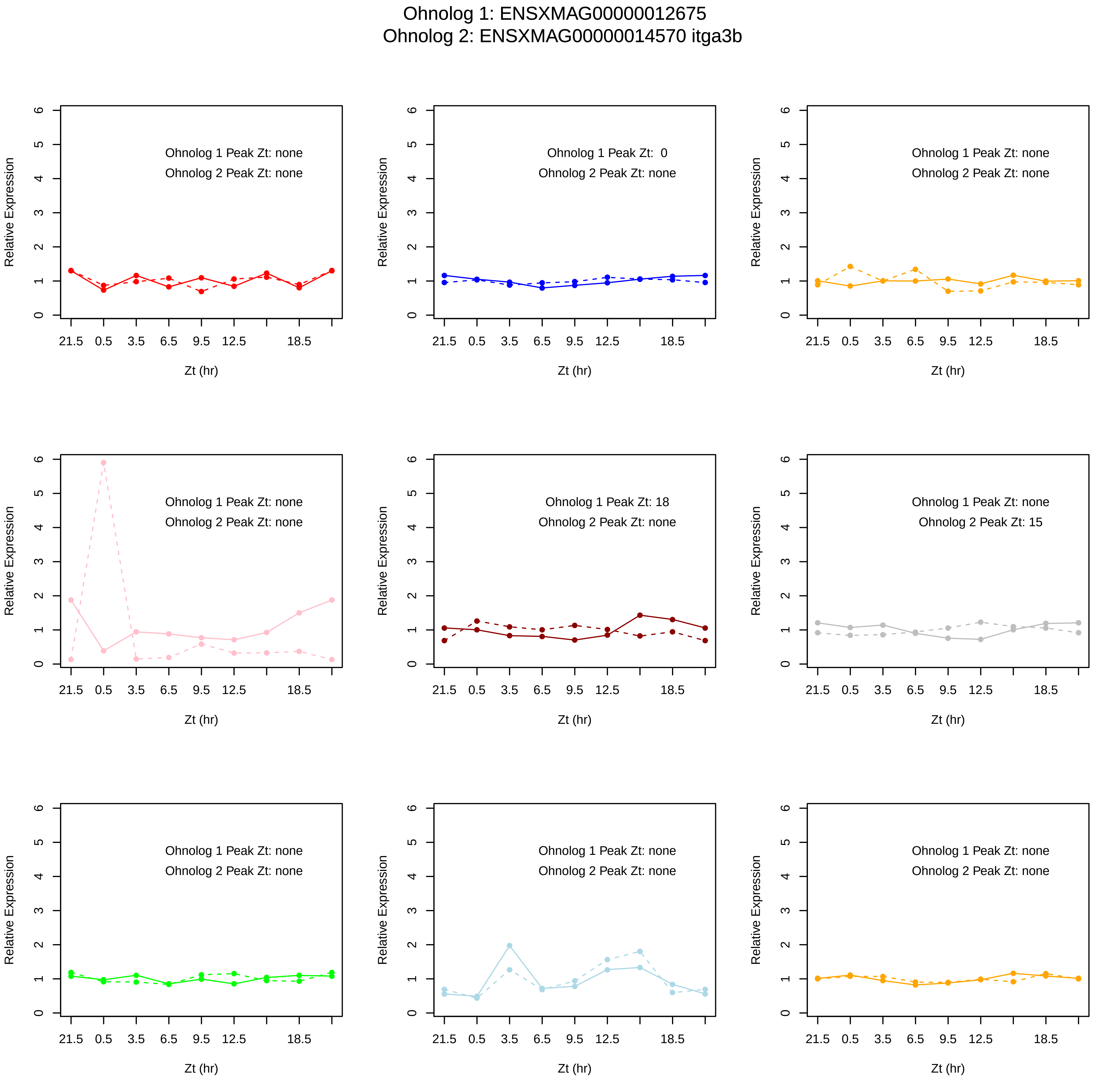

Ohnolog 1: ENSXMAG00000012675
Ohnolog 2: ENSXMAG00000014570 itga3b
Ohnolog 1 Peak Zt: none
Ohnolog 2 Peak Zt: none
Ohnolog 1 Peak Zt: 0
Ohnolog 2 Peak Zt: none
Ohnolog 1 Peak Zt: none
Ohnolog 2 Peak Zt: none
Ohnolog 1 Peak Zt: none
Ohnolog 2 Peak Zt: none
Ohnolog 1 Peak Zt: 18
Ohnolog 2 Peak Zt: none
Ohnolog 1 Peak Zt: none
Ohnolog 2 Peak Zt: 15
Ohnolog 1 Peak Zt: none
Ohnolog 2 Peak Zt: none
Ohnolog 1 Peak Zt: none
Ohnolog 2 Peak Zt: none
Ohnolog 1 Peak Zt: none
Ohnolog 2 Peak Zt: none
Relative Expression
Zt (hr)
21.5
0.5
3.5
6.5
9.5
12.5
18.5

Ohnolog 1: ENSXMAG00000003371 paplnb
Ohnolog 2: ENSXMAG00000009186 paplna

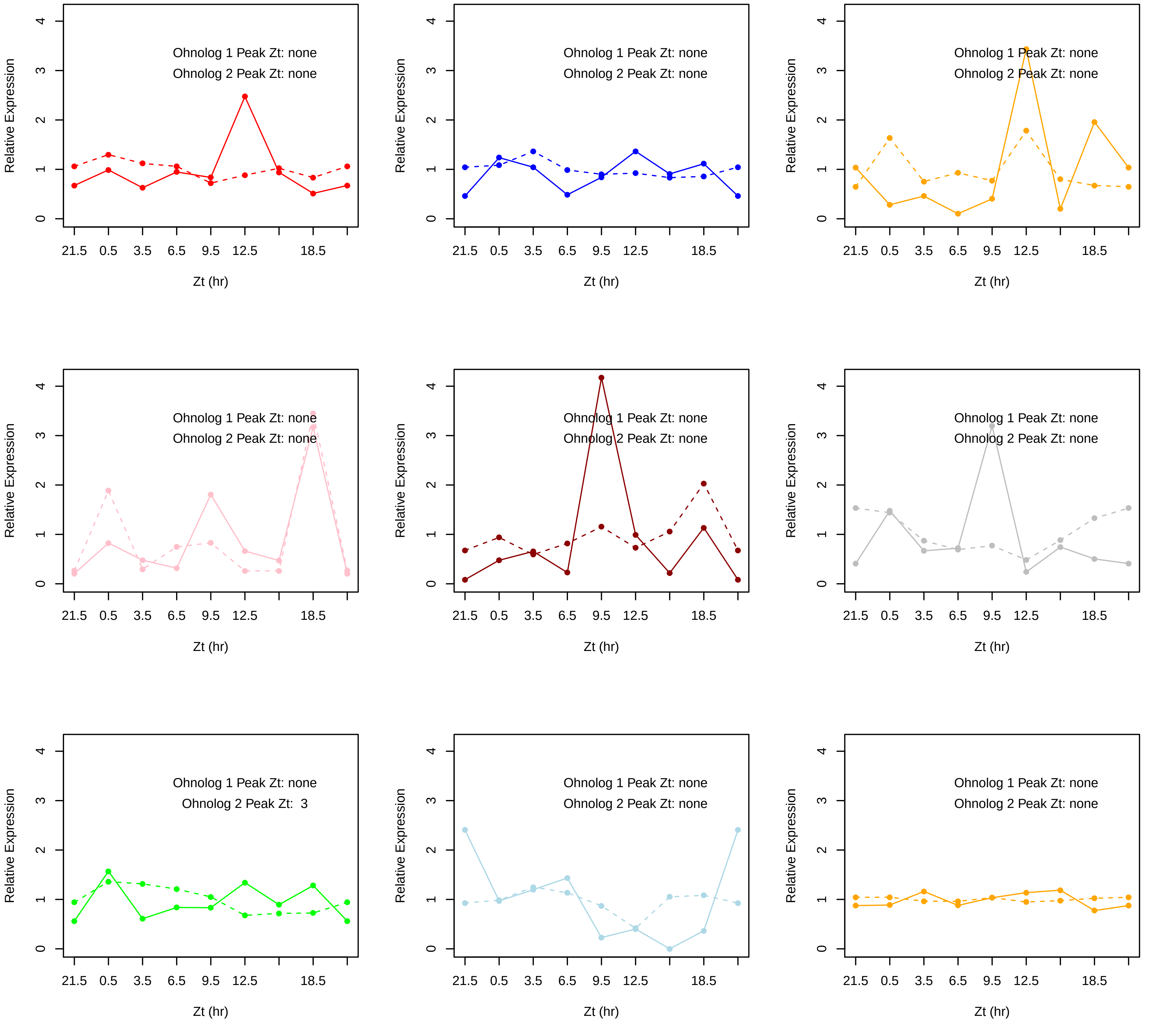

Ohnolog 1: ENSXMAG00000006711 jpt1a
Ohnolog 2: ENSXMAG00000014551 jpt1b

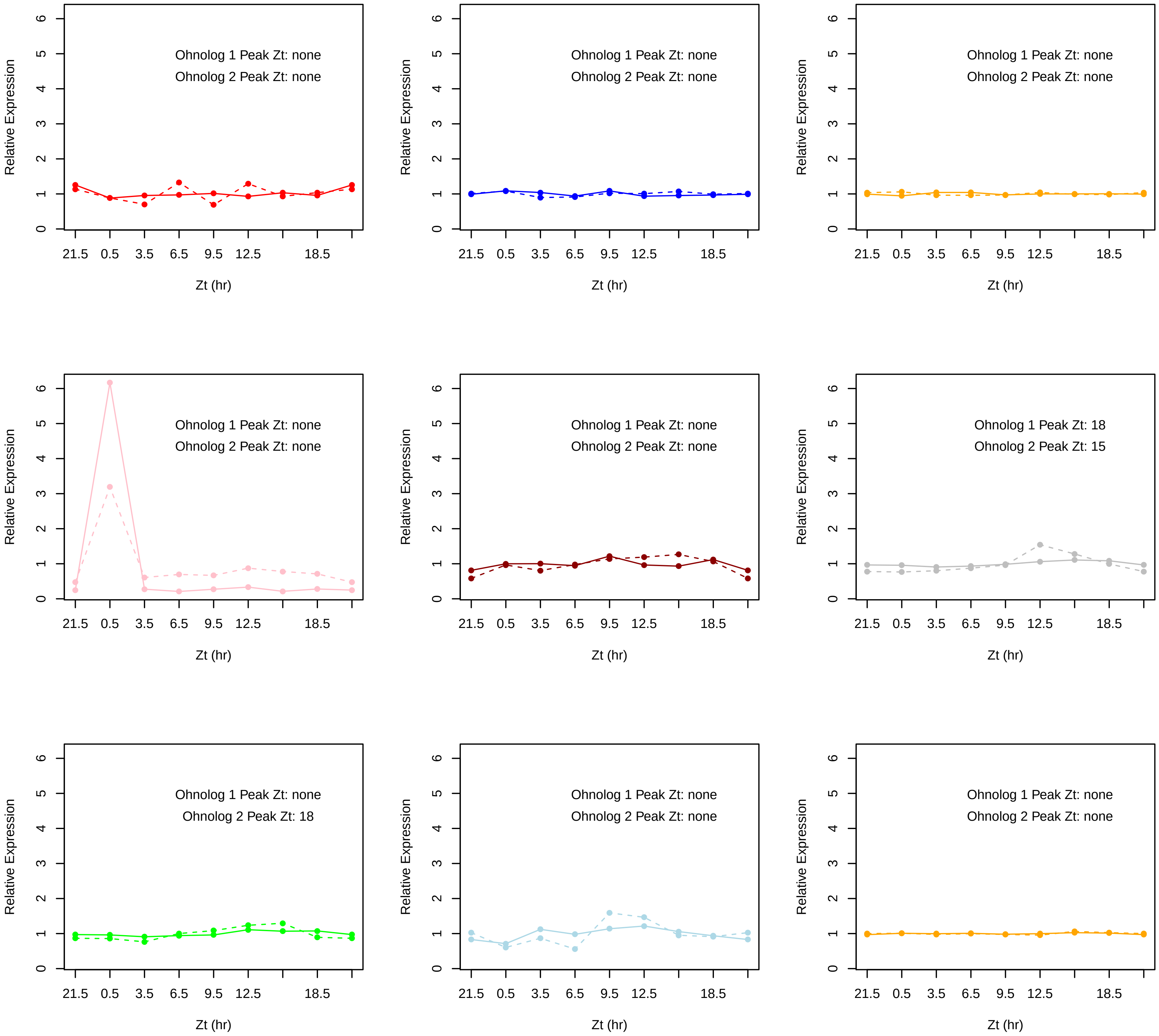

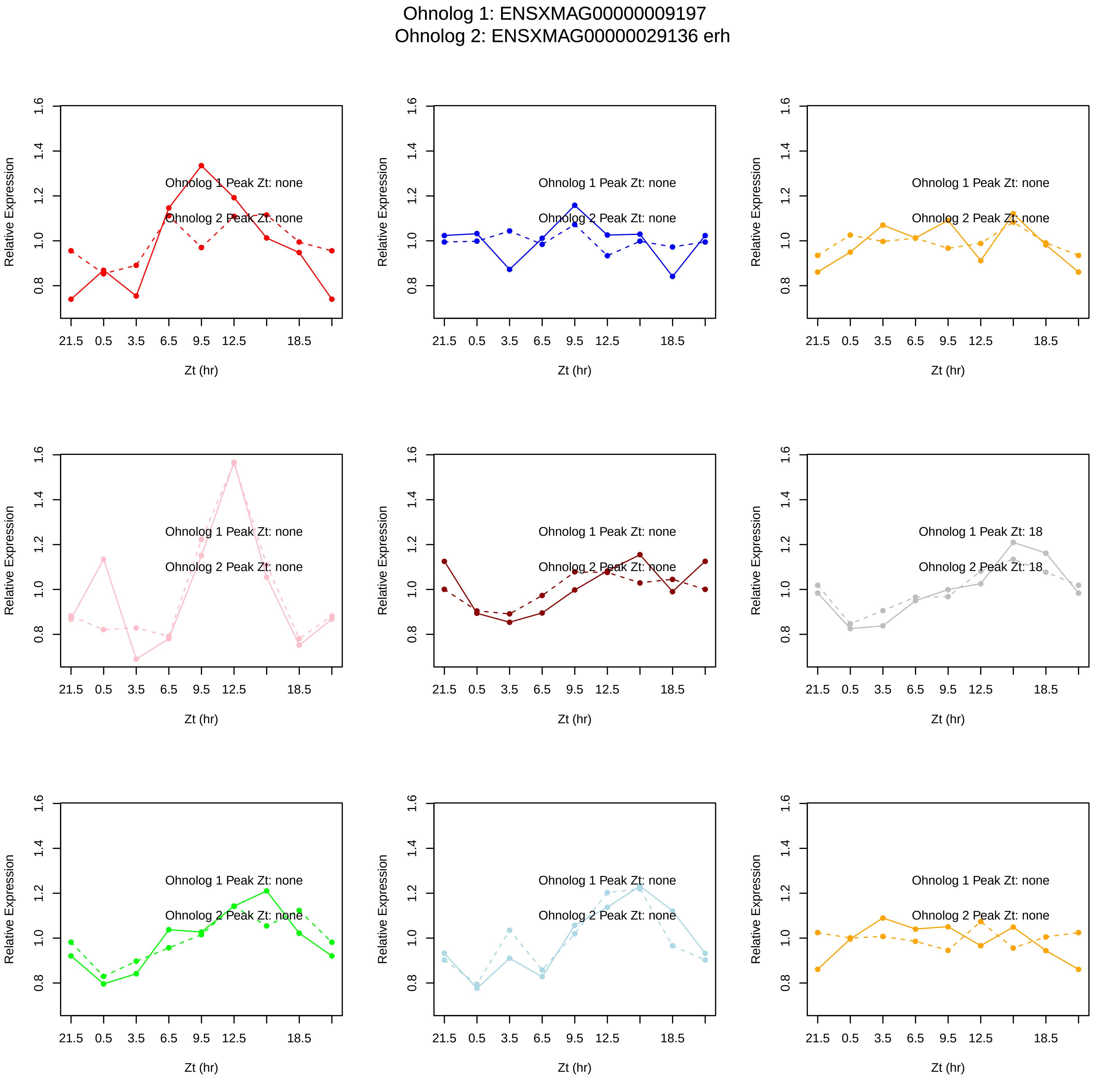
Ohnolog 1: ENSXMAG00000009197
Ohnolog 2: ENSXMAG00000029136 erh
Ohnolog 1 Peak Zt: none
Ohnolog 2 Peak Zt: none
Ohnolog 1 Peak Zt: none
Ohnolog 2 Peak Zt: none
Ohnolog 1 Peak Zt: none
Ohnolog 2 Peak Zt: none
Ohnolog 1 Peak Zt: none
Ohnolog 2 Peak Zt: none
Ohnolog 1 Peak Zt: none
Ohnolog 2 Peak Zt: none
Ohnolog 1 Peak Zt: 18
Ohnolog 2 Peak Zt: 18
Ohnolog 1 Peak Zt: none
Ohnolog 2 Peak Zt: none
Ohnolog 1 Peak Zt: none
Ohnolog 2 Peak Zt: none
Ohnolog 1 Peak Zt: none
Ohnolog 2 Peak Zt: none
Relative Expression
Zt (hr)
21.5 0.5 3.5 6.5 9.5 12.5 18.5
0.8 1.0 1.2 1.4 1.6

Ohnolog 1: ENSXMAG00000014393 SEPT9
Ohnolog 2: ENSXMAG00000028828 sept9b

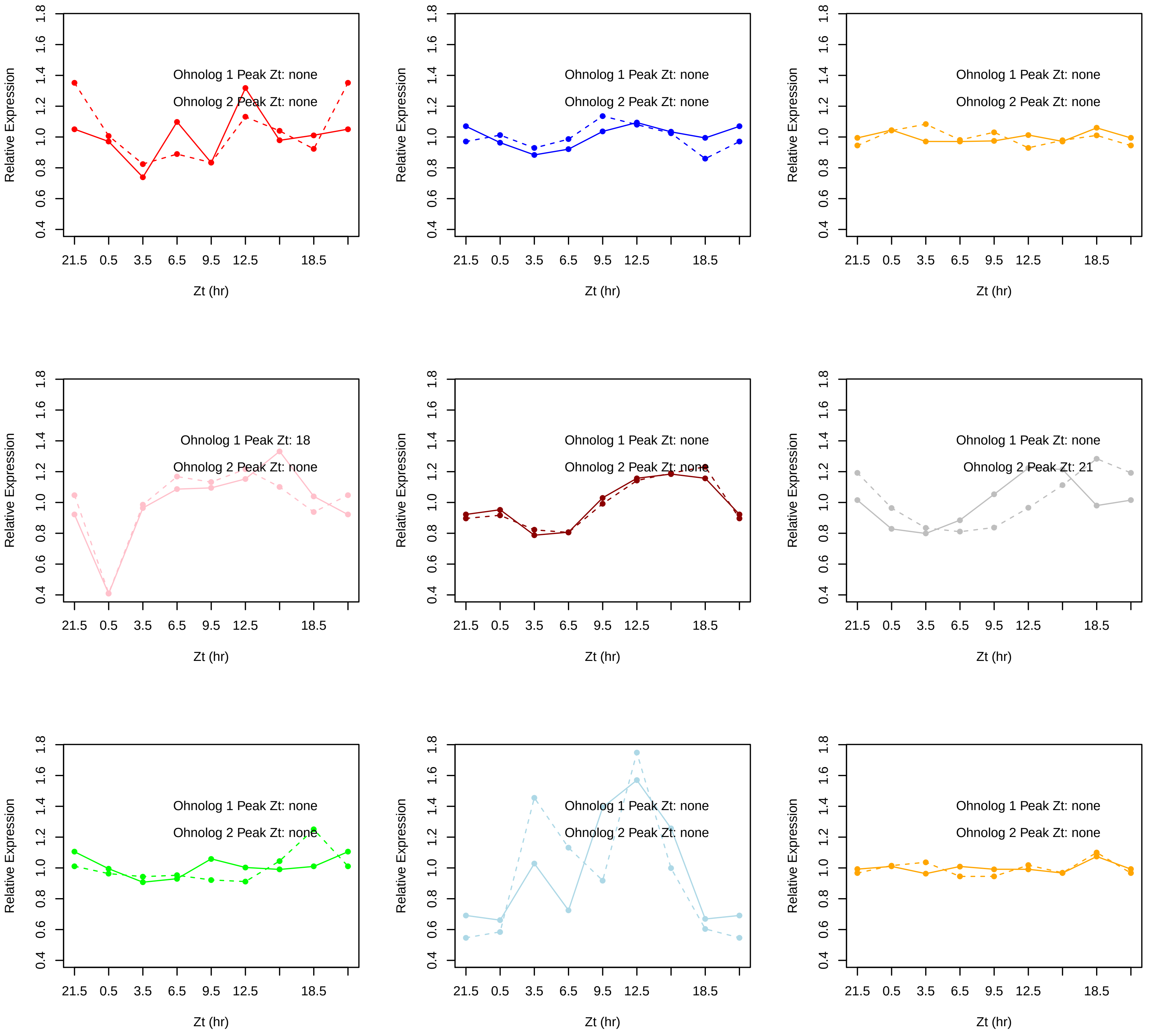

Ohnolog 1: ENSXMAG00000012250 dag1
Ohnolog 2: ENSXMAG00000015708

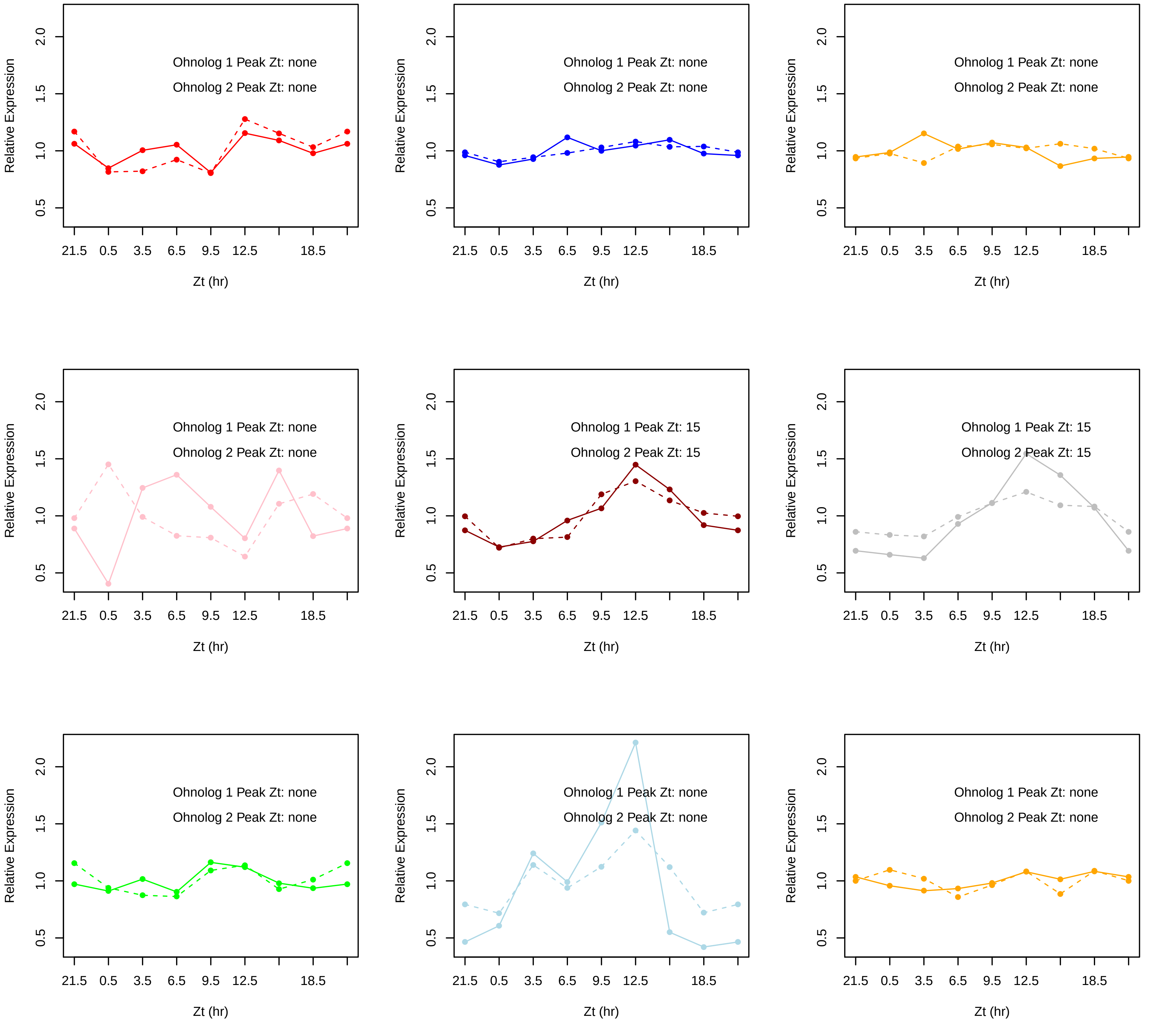

Ohnolog 1: ENSXMAG00000000363 kdm6bb
Ohnolog 2: ENSXMAG00000008979 kdm6ba

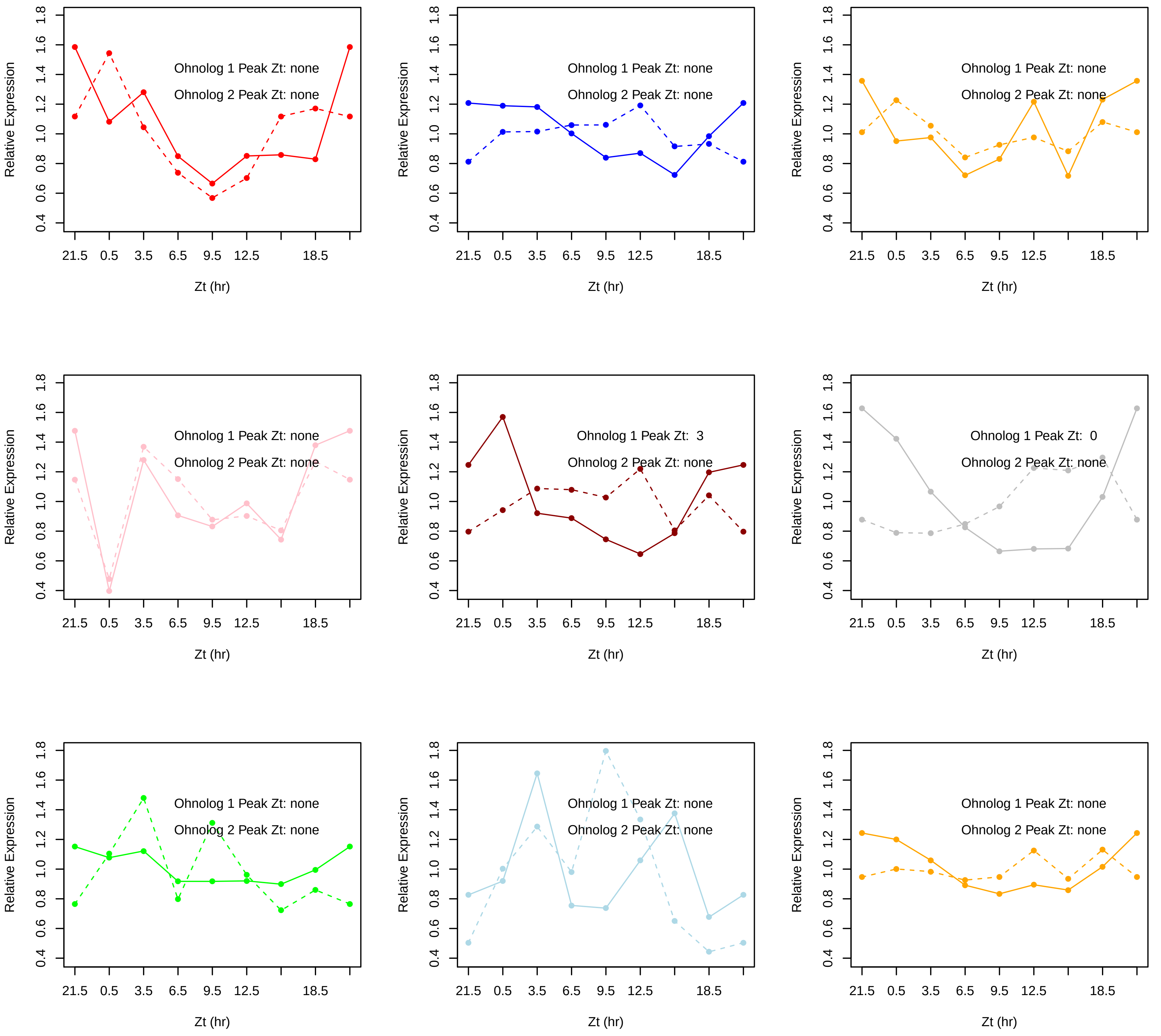

Ohnolog 1: ENSXMAG00000009792
Ohnolog 2: ENSXMAG00000009932 ppm1k

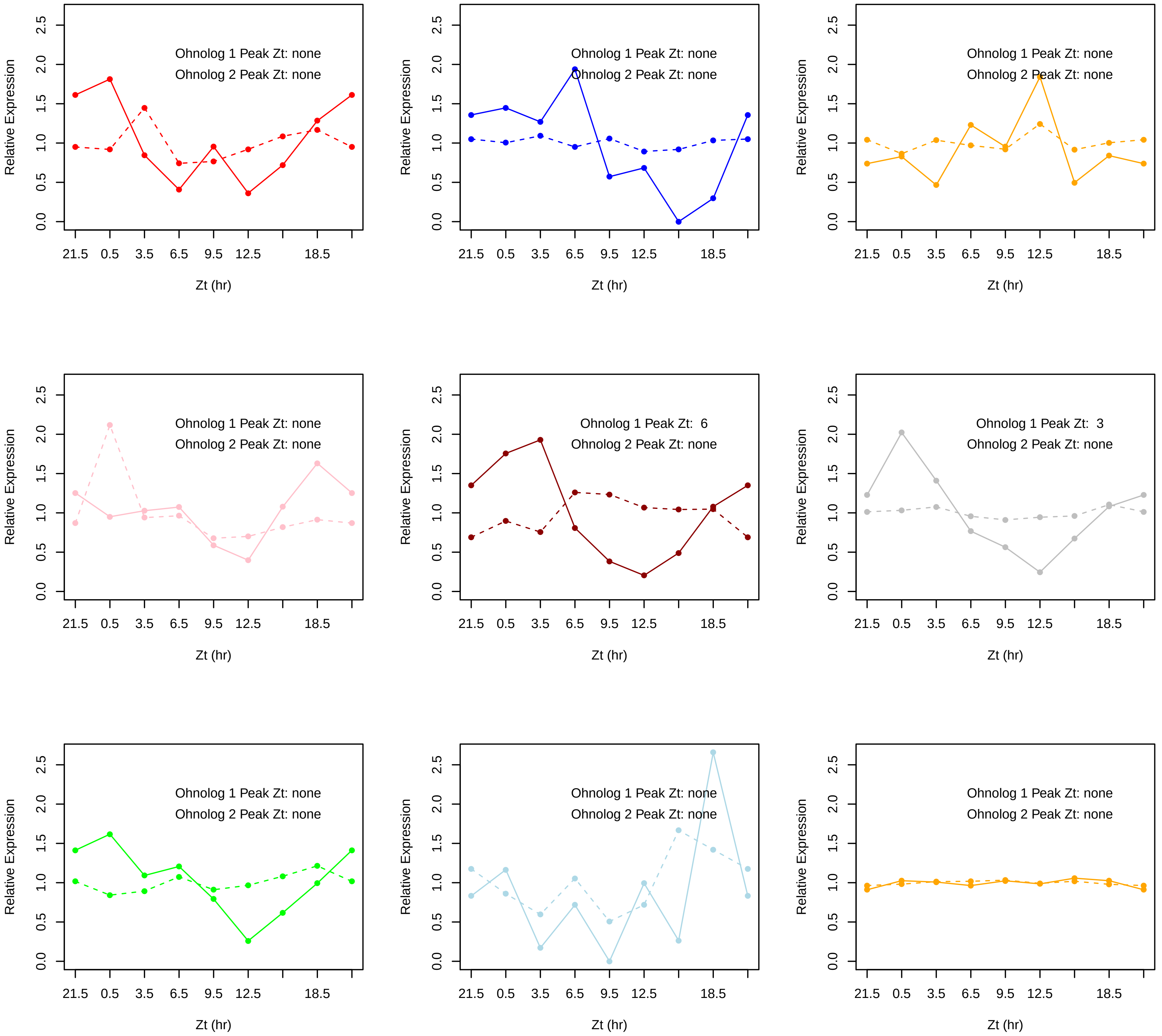

Ohnolog 1: ENSXMAG00000005972
Ohnolog 2: ENSXMAG00000015781 DCAF1

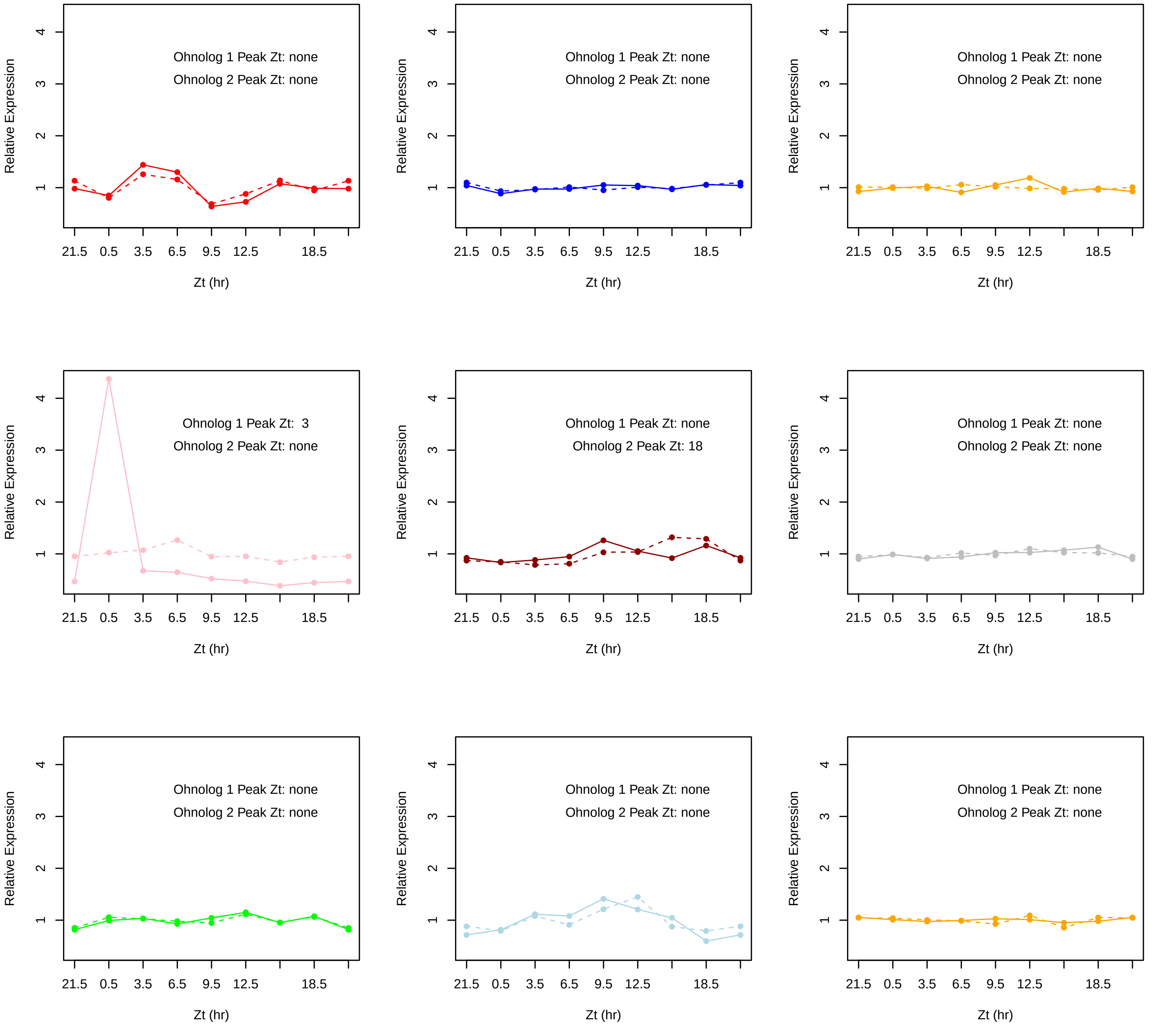

Ohnolog 1: ENSXMAG00000012369 SPHK1 (1 of many)
Ohnolog 2: ENSXMAG00000025799 SPHK1 (1 of many)

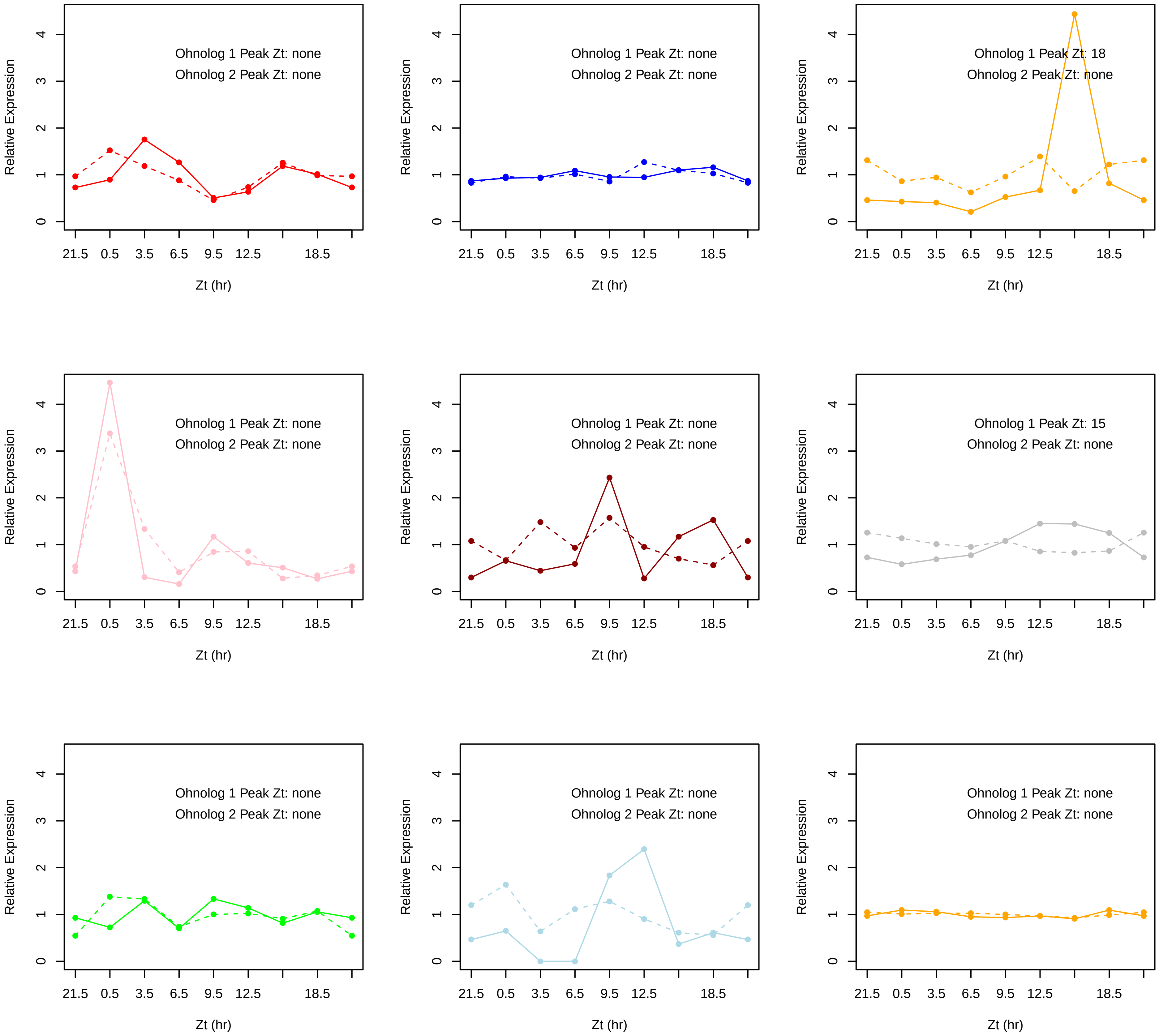

Ohnolog 1: ENSXMAG00000006679 prtgb
Ohnolog 2: ENSXMAG00000014280 PRTG

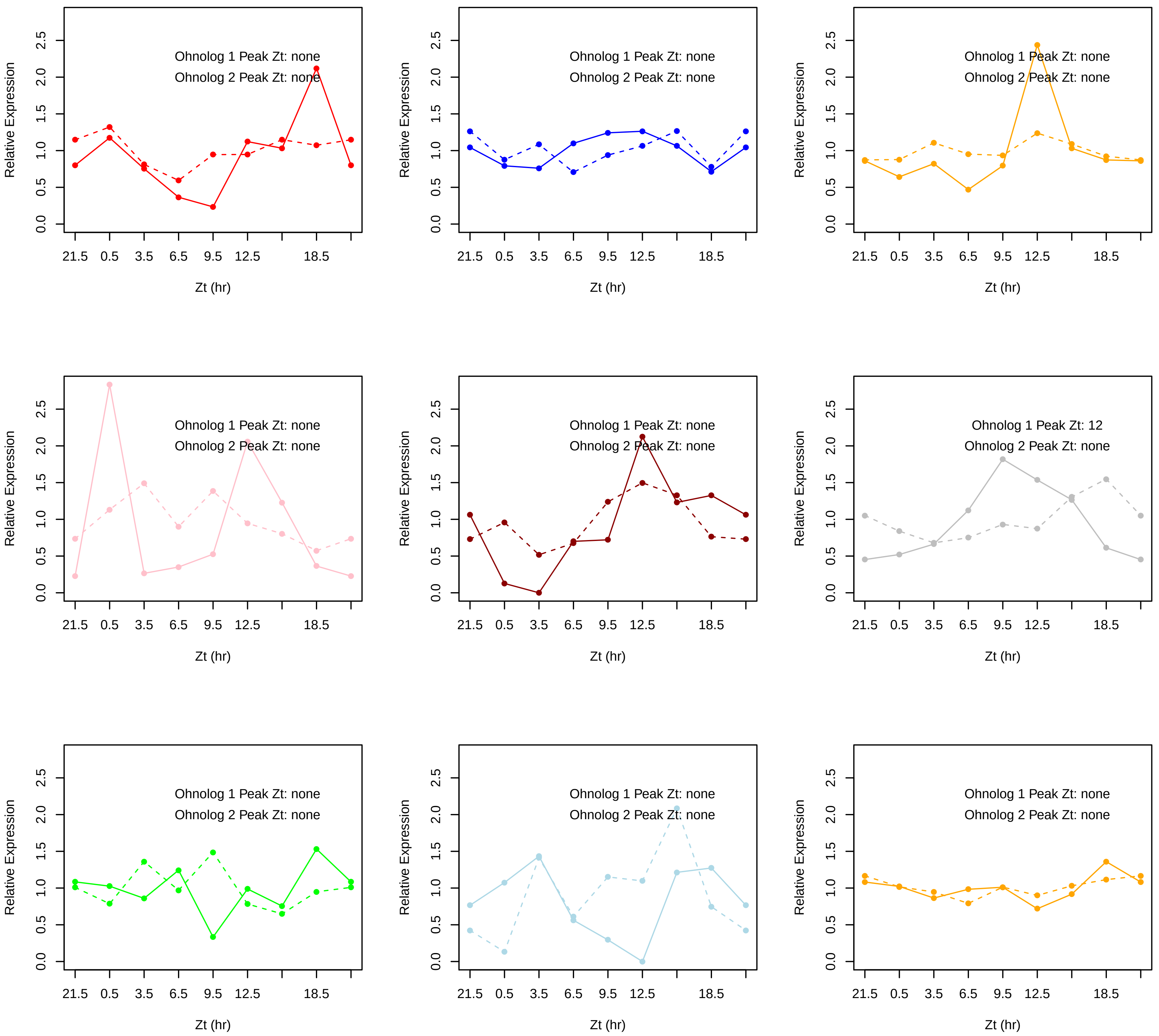

Ohnolog 1: ENSXMAG00000015790 ssuh2rs1
Ohnolog 2: ENSXMAG00000024535

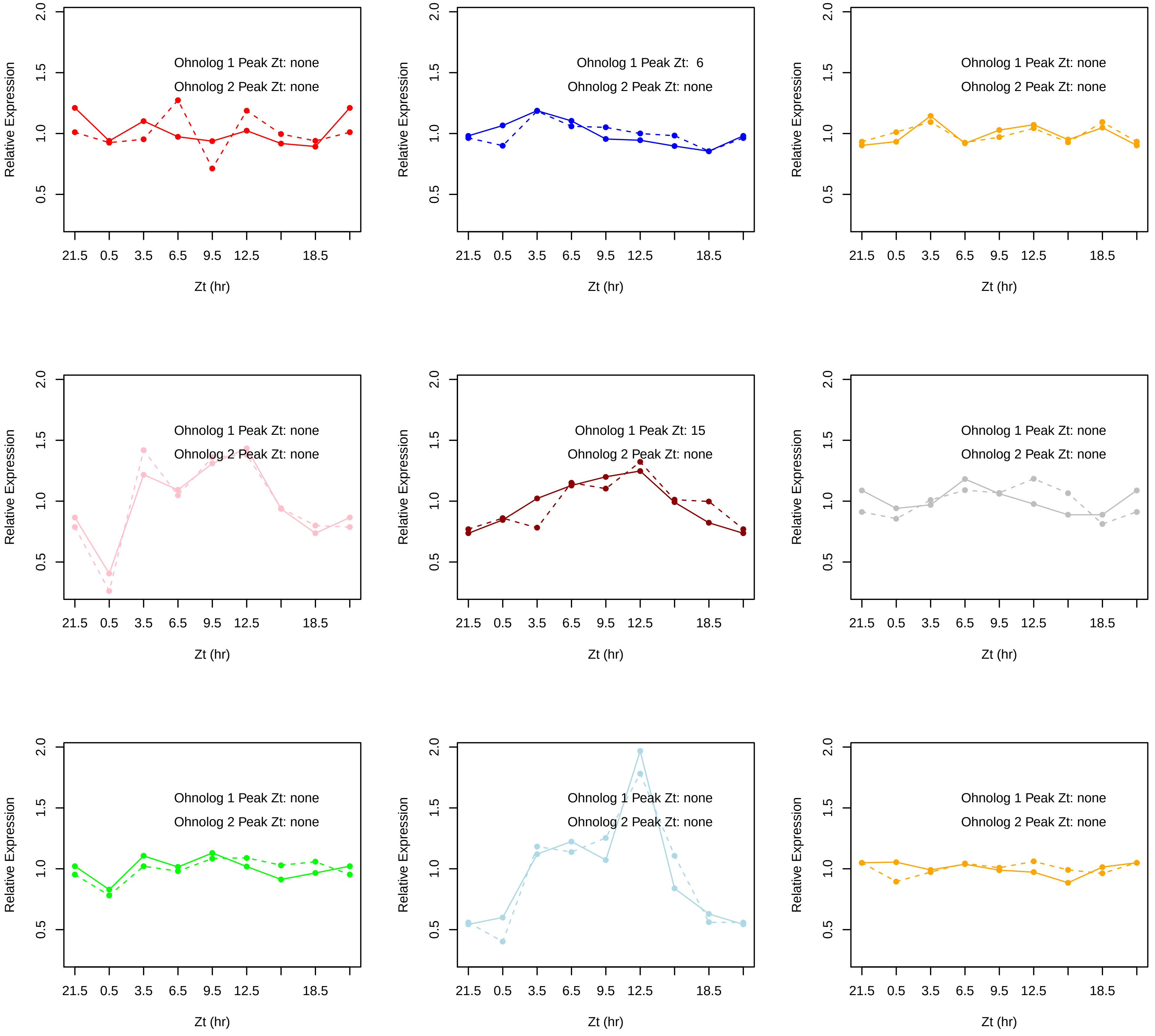

Ohnolog 1: ENSXMAG00000012511 gucy2d
Ohnolog 2: ENSXMAG00000013383

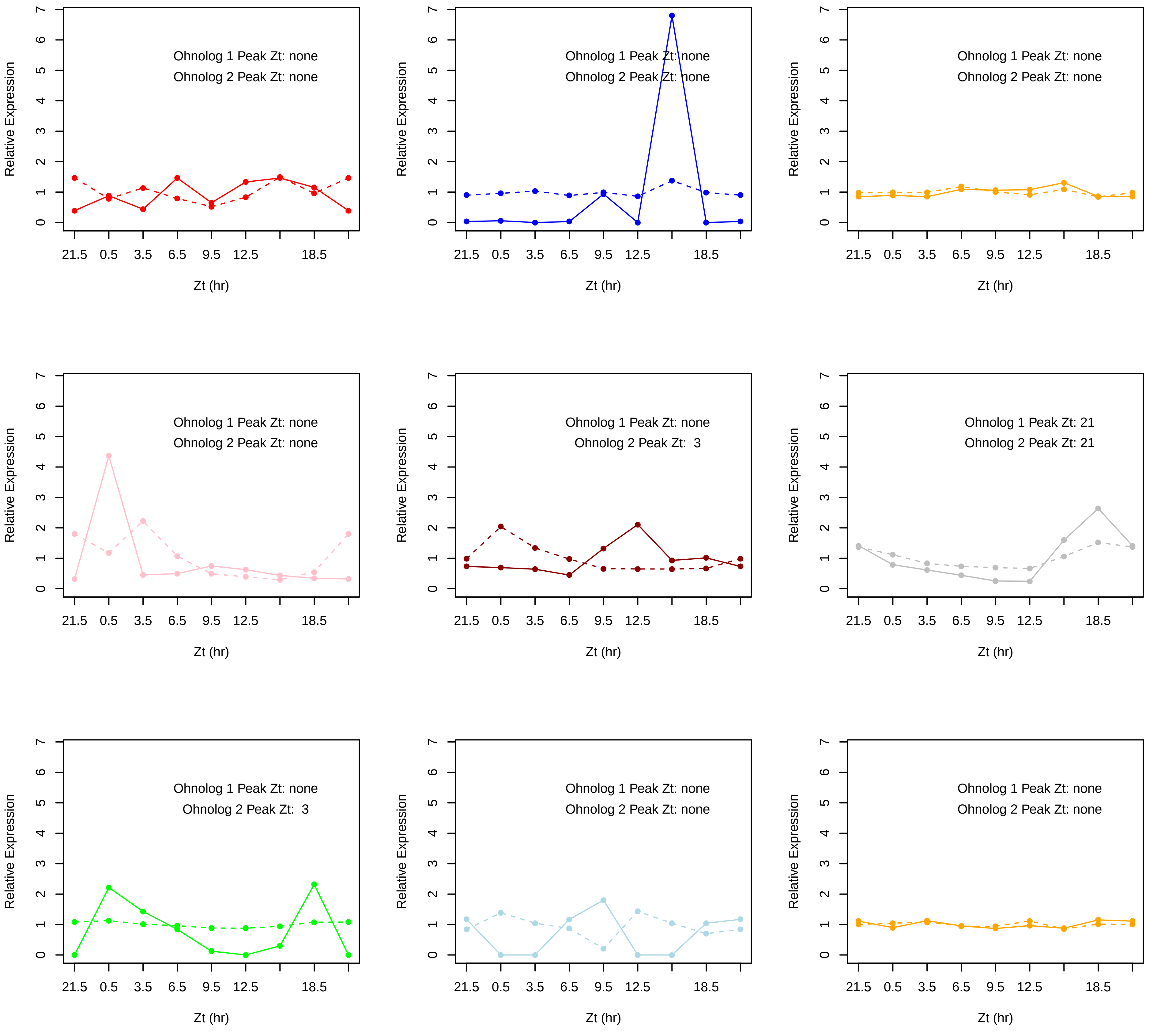

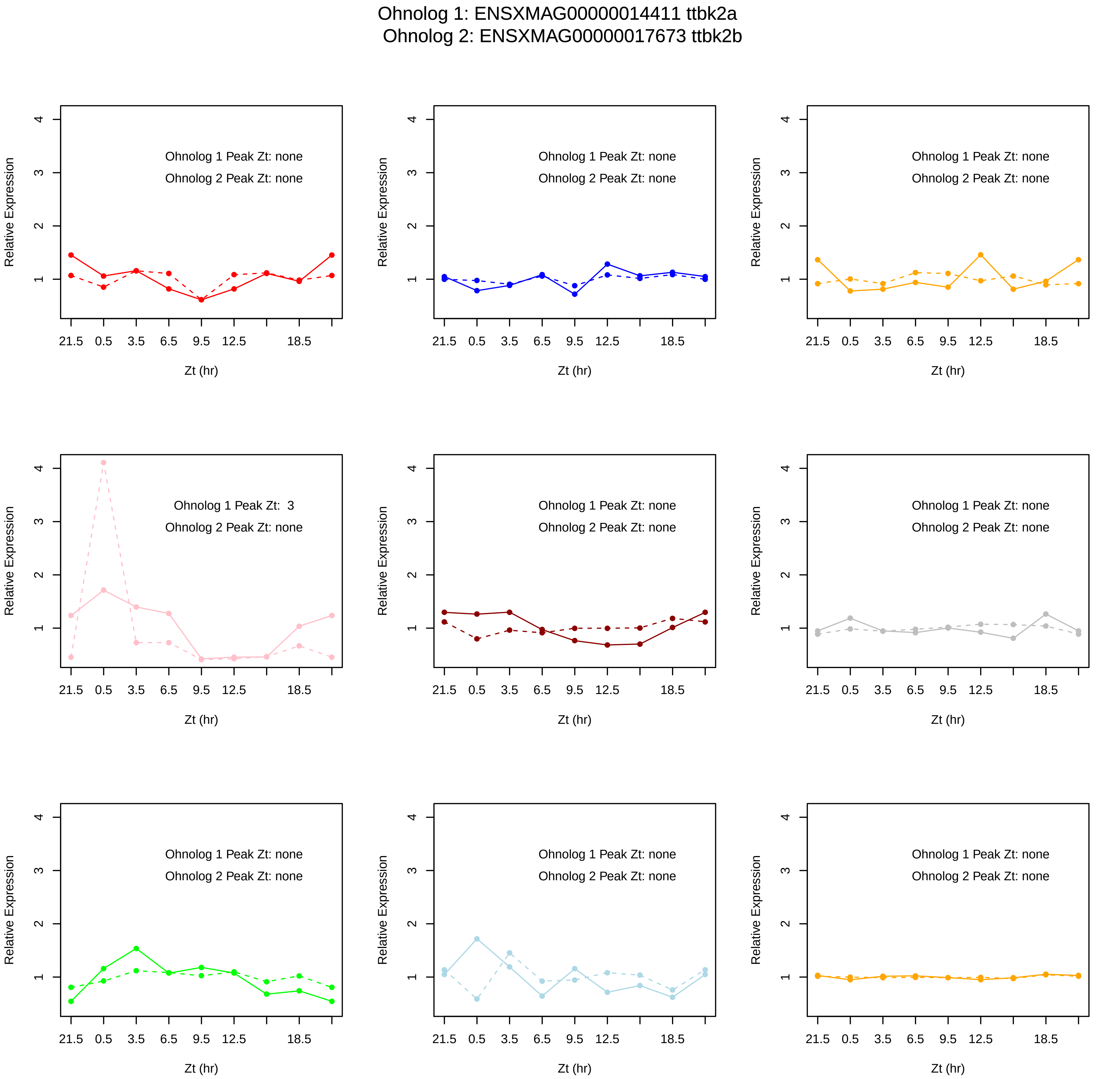

Ohnolog 1: ENSXMAG00000014411 ttbk2a
Ohnolog 2: ENSXMAG00000017673 ttbk2b
Ohnolog 1 Peak Zt: none
Ohnolog 2 Peak Zt: none
Ohnolog 1 Peak Zt: 3
Relative Expression
Zt (hr)
21.5
0.5
3.5
6.5
9.5
12.5
18.5

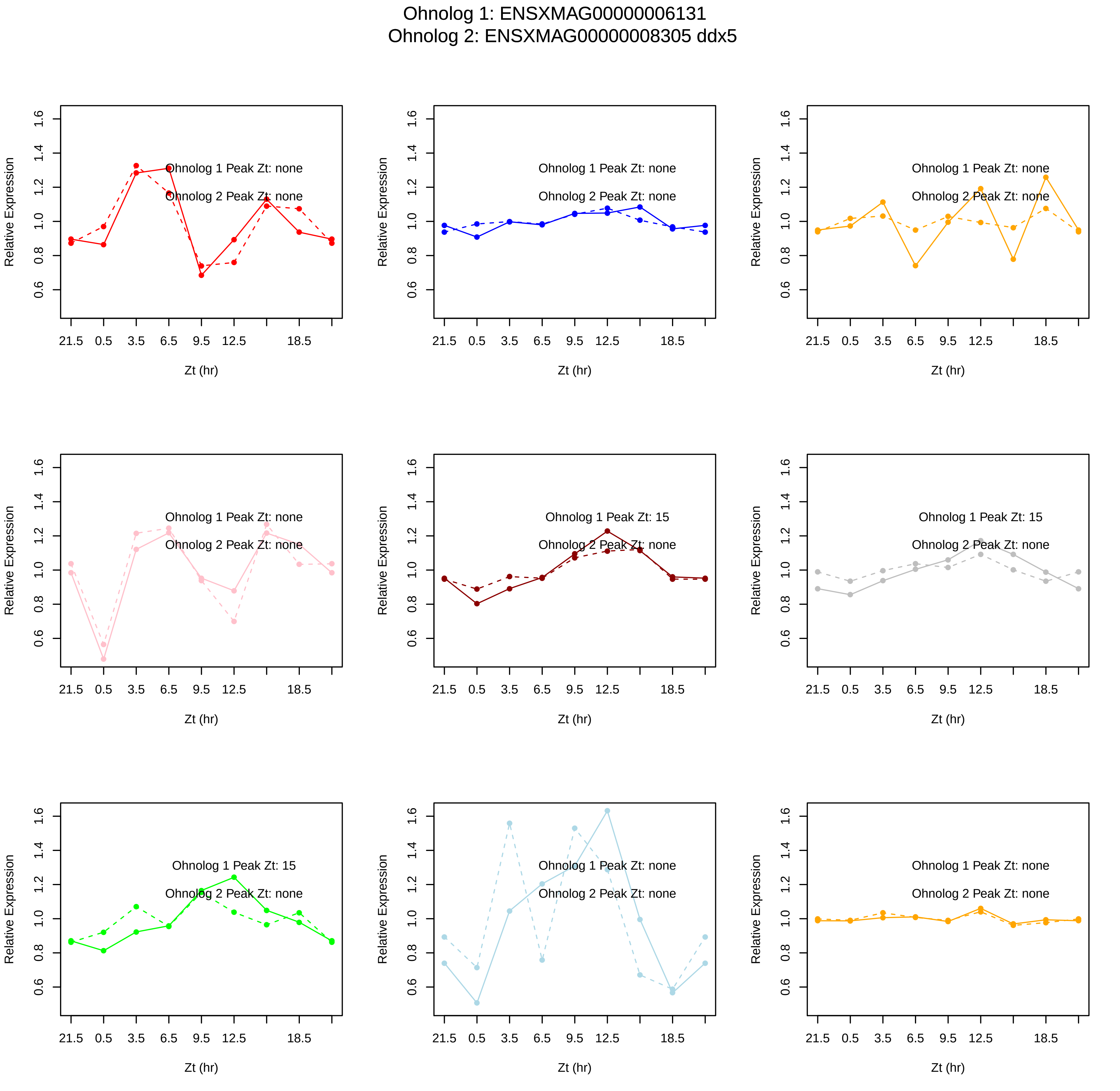
Ohnolog 1: ENSXMAG00000006131
Ohnolog 2: ENSXMAG00000008305 ddx5
Ohnolog 1 Peak Zt: none
Ohnolog 2 Peak Zt: none
Ohnolog 1 Peak Zt: none
Ohnolog 2 Peak Zt: none
Ohnolog 1 Peak Zt: none
Ohnolog 2 Peak Zt: none
Ohnolog 1 Peak Zt: none
Ohnolog 2 Peak Zt: none
Ohnolog 1 Peak Zt: 15
Ohnolog 2 Peak Zt: none
Ohnolog 1 Peak Zt: 15
Ohnolog 2 Peak Zt: none
Ohnolog 1 Peak Zt: 15
Ohnolog 2 Peak Zt: none
Ohnolog 1 Peak Zt: none
Ohnolog 2 Peak Zt: none
Ohnolog 1 Peak Zt: none
Ohnolog 2 Peak Zt: none
Relative Expression
Zt (hr)
0.6
0.8
1.0
1.2
1.4
1.6
21.5
0.5
3.5
6.5
9.5
12.5
18.5

Ohnolog 1: ENSXMAG00000009165 grip2b
Ohnolog 2: ENSXMAG00000015803 grip2a

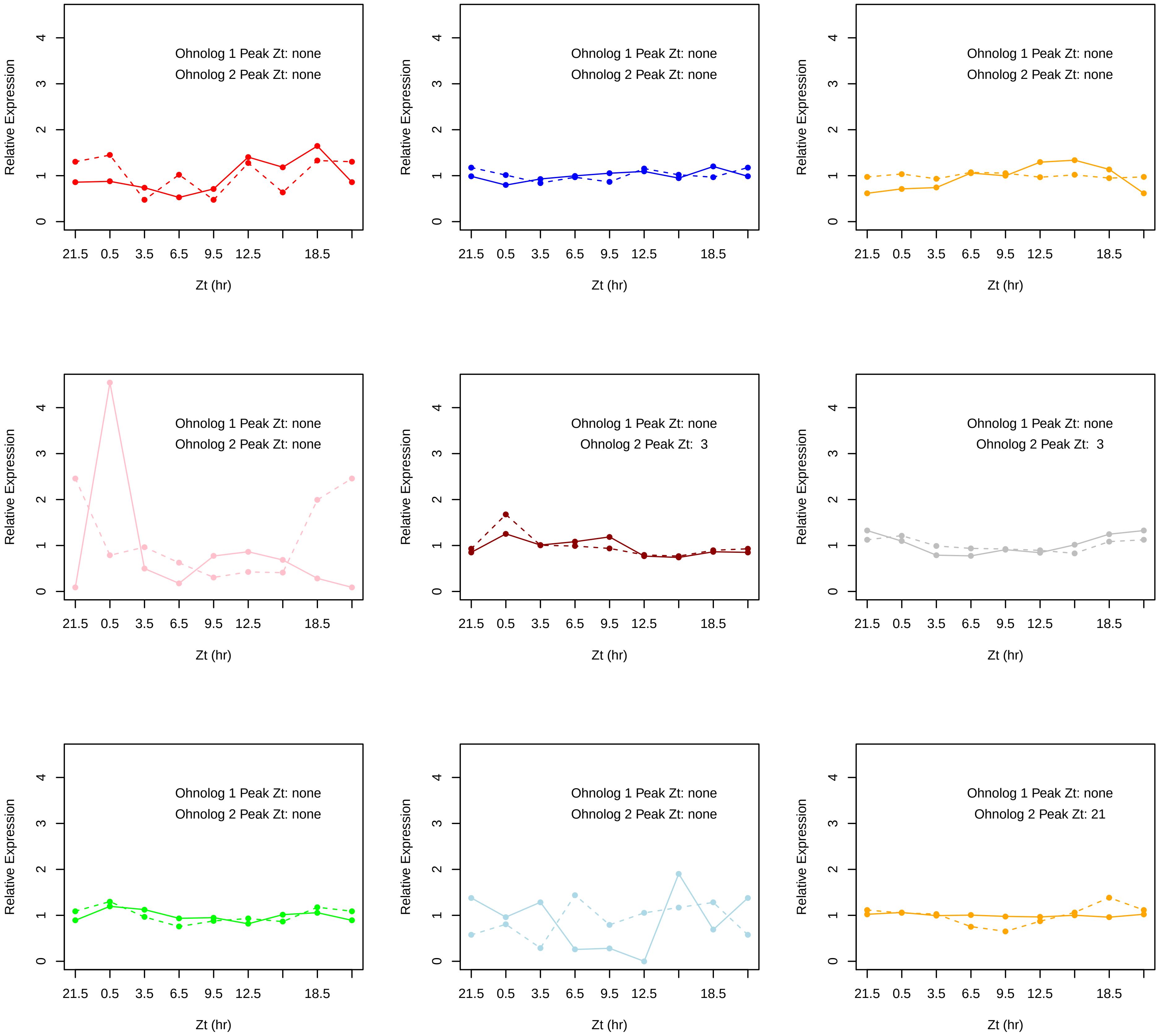

Ohnolog 1: ENSXMAG00000015849 elmsan1b
Ohnolog 2: ENSXMAG00000017677 elmsan1a

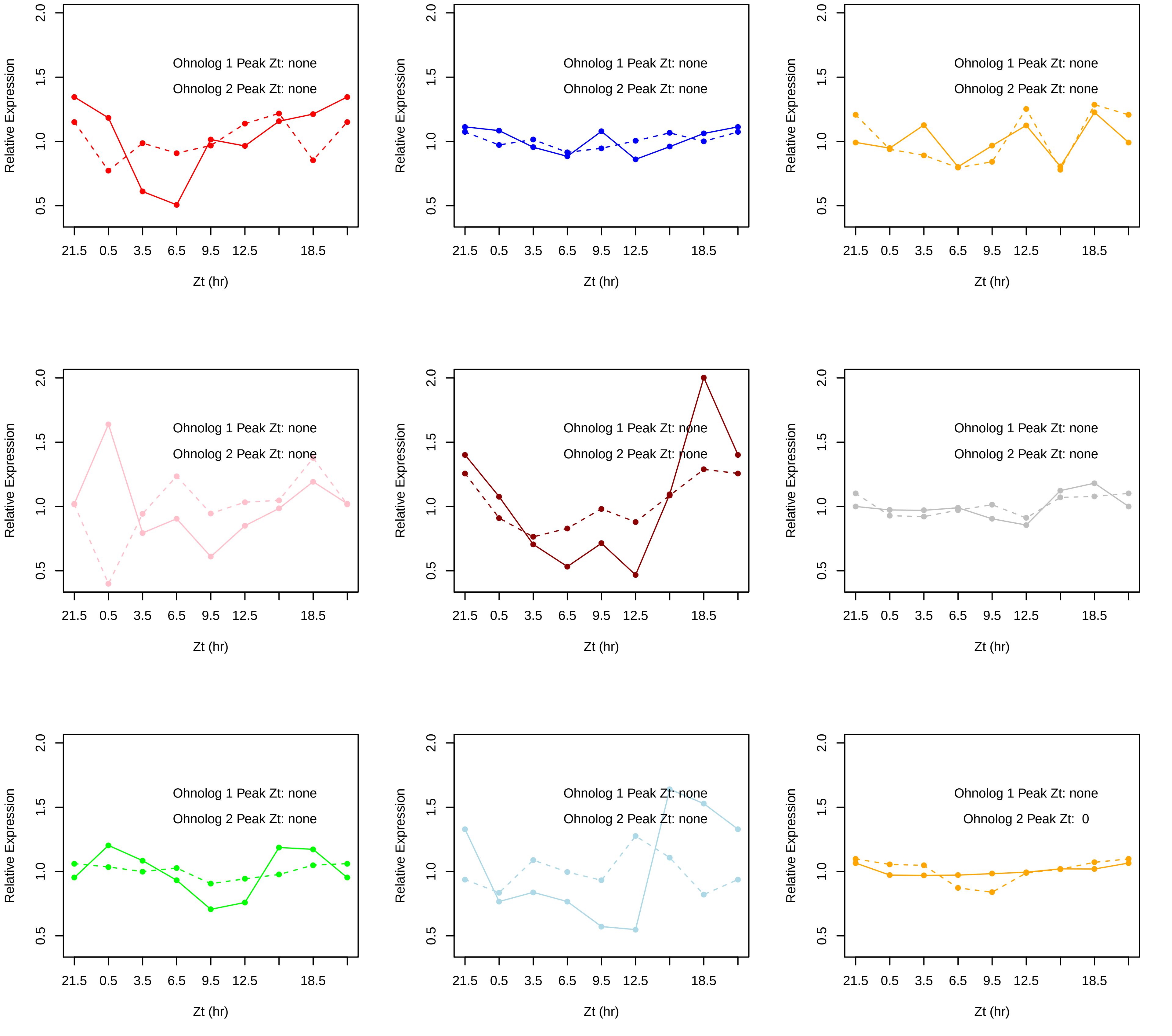

Ohnolog 1: ENSXMAG00000002642 smarce1
Ohnolog 2: ENSXMAG00000018179 SMARCE1

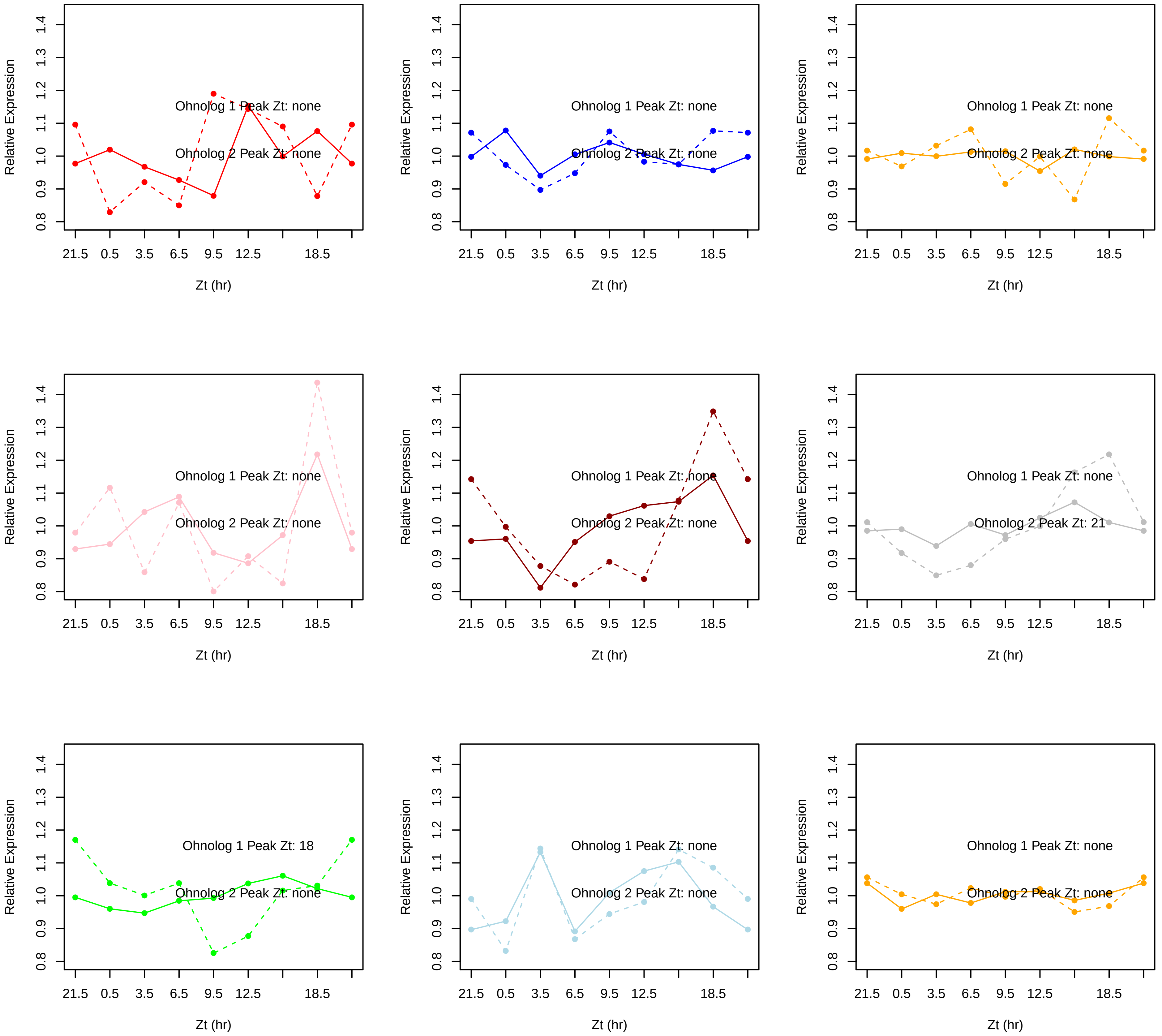

Ohnolog 1: ENSXMAG00000006616 aqp9b
Ohnolog 2: ENSXMAG00000029098

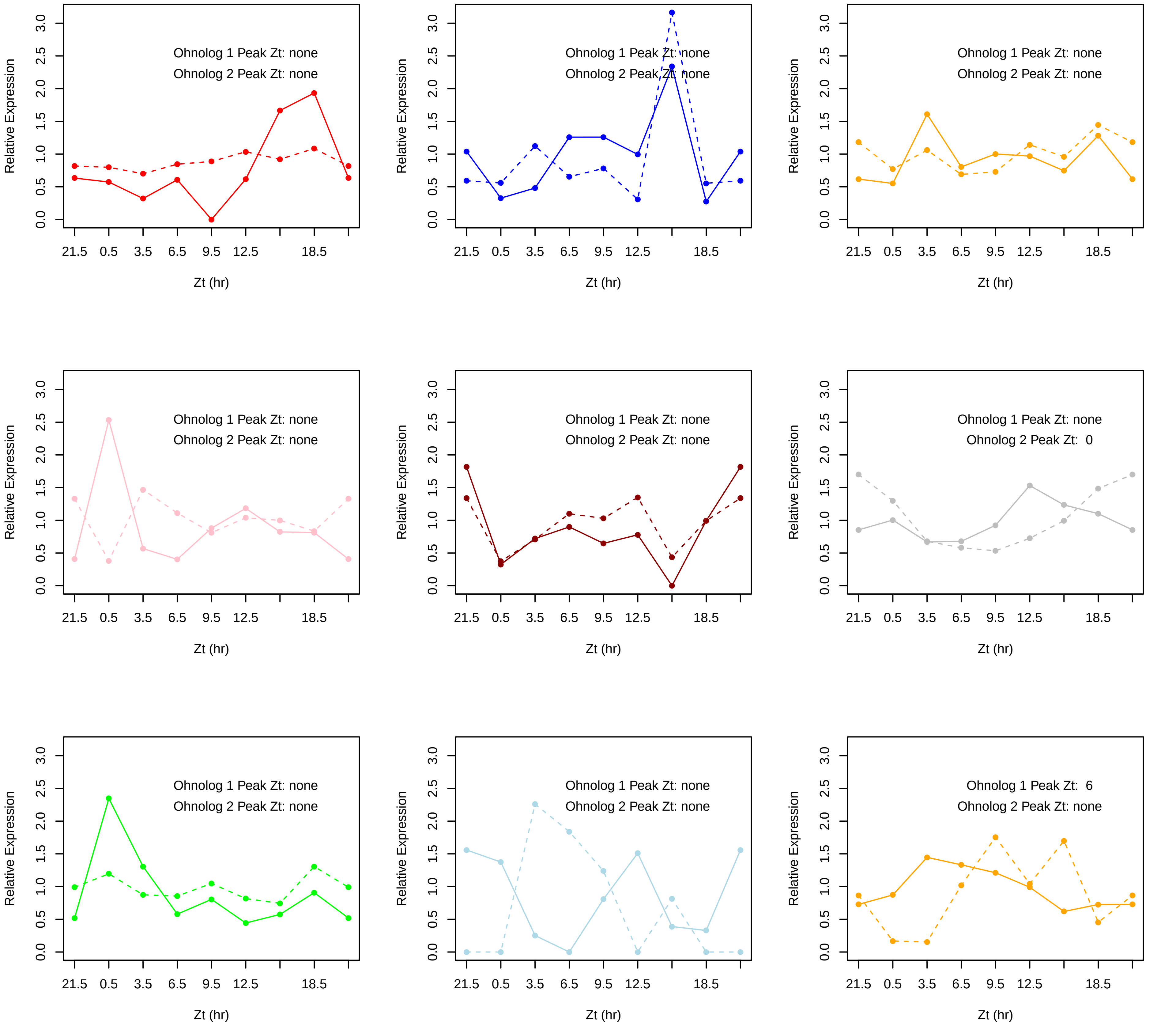

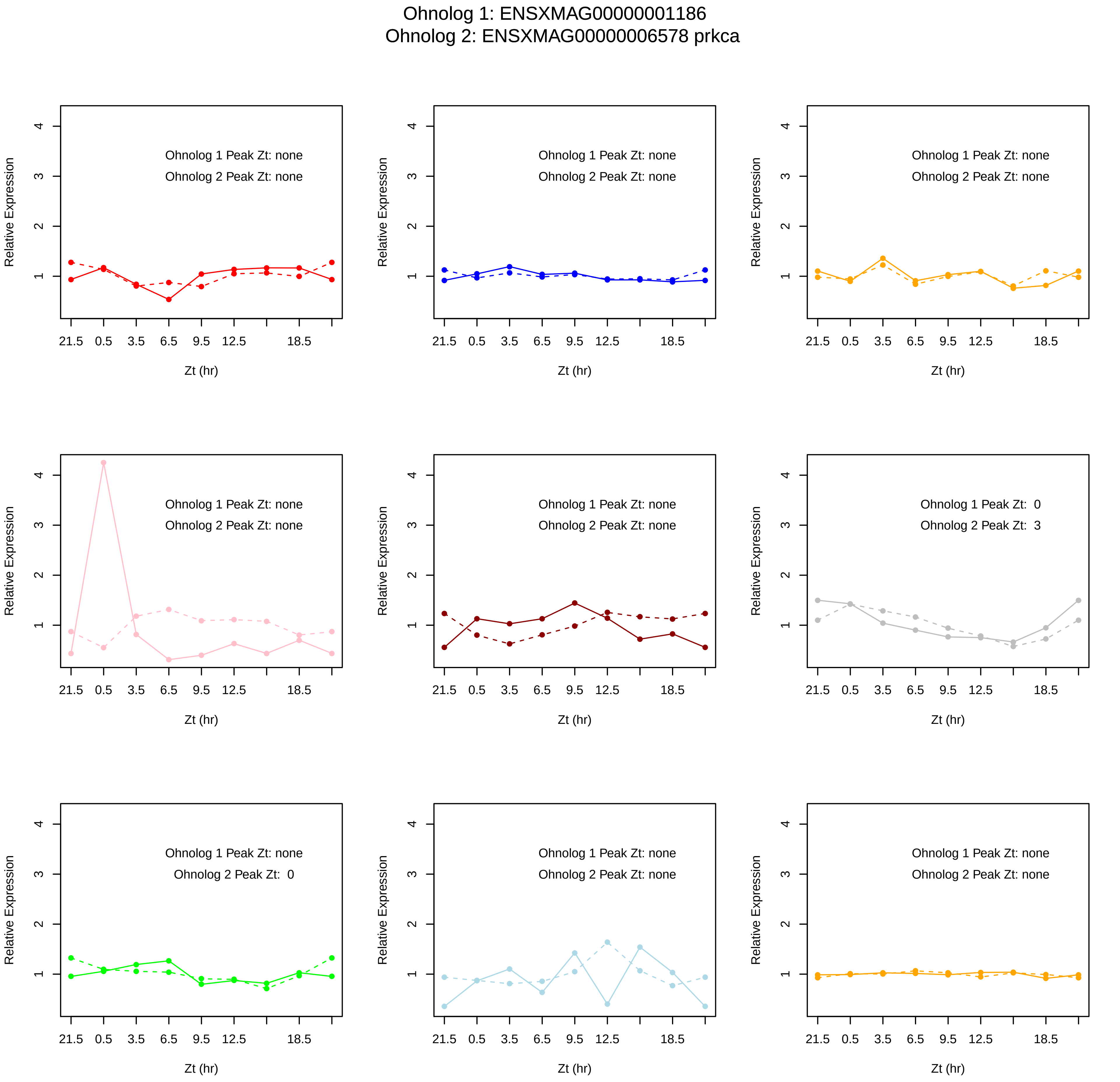
Ohnolog 1: ENSXMAG00000001186
Ohnolog 2: ENSXMAG00000006578 prkca
Ohnolog 1 Peak Zt: none
Ohnolog 2 Peak Zt: none
Ohnolog 1 Peak Zt: none
Ohnolog 2 Peak Zt: none
Ohnolog 1 Peak Zt: none
Ohnolog 2 Peak Zt: none
Ohnolog 1 Peak Zt: none
Ohnolog 2 Peak Zt: none
Ohnolog 1 Peak Zt: none
Ohnolog 2 Peak Zt: none
Ohnolog 1 Peak Zt: 0
Ohnolog 2 Peak Zt: 3
Ohnolog 1 Peak Zt: none
Ohnolog 2 Peak Zt: 0
Ohnolog 1 Peak Zt: none
Ohnolog 2 Peak Zt: none
Ohnolog 1 Peak Zt: none
Ohnolog 2 Peak Zt: none
Relative Expression
Zt (hr)
21.5
0.5
3.5
6.5
9.5
12.5
18.5
1
2
3
4

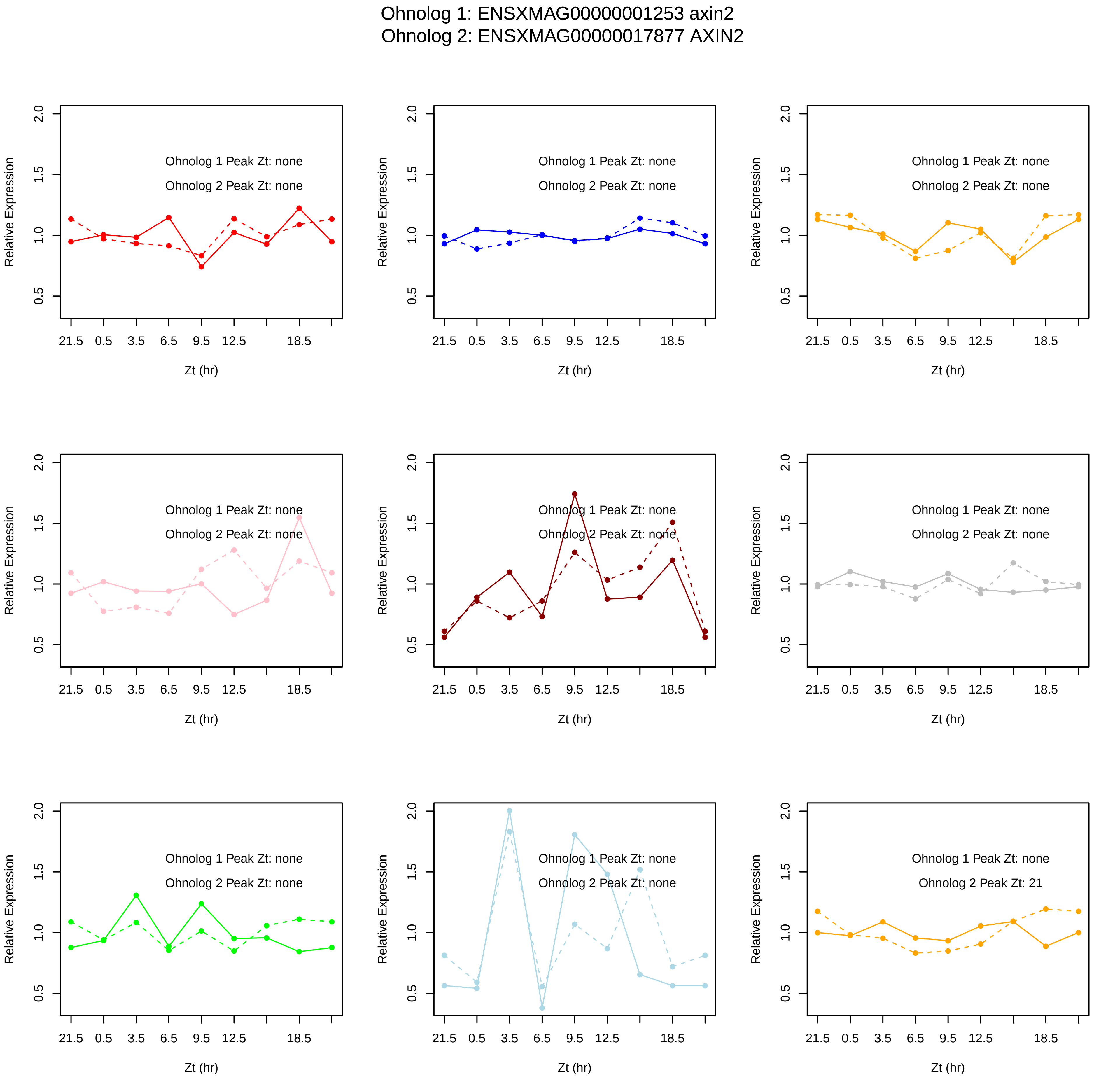
Ohnolog 1: ENSXMAG00000001253 axin2
Ohnolog 2: ENSXMAG00000017877 AXIN2
Ohnolog 1 Peak Zt: none
Ohnolog 2 Peak Zt: none
Ohnolog 1 Peak Zt: none
Ohnolog 2 Peak Zt: none
Ohnolog 1 Peak Zt: none
Ohnolog 2 Peak Zt: none
Ohnolog 1 Peak Zt: none
Ohnolog 2 Peak Zt: none
Ohnolog 1 Peak Zt: none
Ohnolog 2 Peak Zt: none
Ohnolog 1 Peak Zt: none
Ohnolog 2 Peak Zt: none
Ohnolog 1 Peak Zt: none
Ohnolog 2 Peak Zt: none
Ohnolog 1 Peak Zt: none
Ohnolog 2 Peak Zt: none
Ohnolog 1 Peak Zt: none
Ohnolog 2 Peak Zt: 21
Relative Expression
Zt (hr)
21.5 0.5 3.5 6.5 9.5 12.5 18.5
0.5 1.0 1.5 2.0

Ohnolog 1: ENSXMAG00000001283 tex2
Ohnolog 2: ENSXMAG00000017886

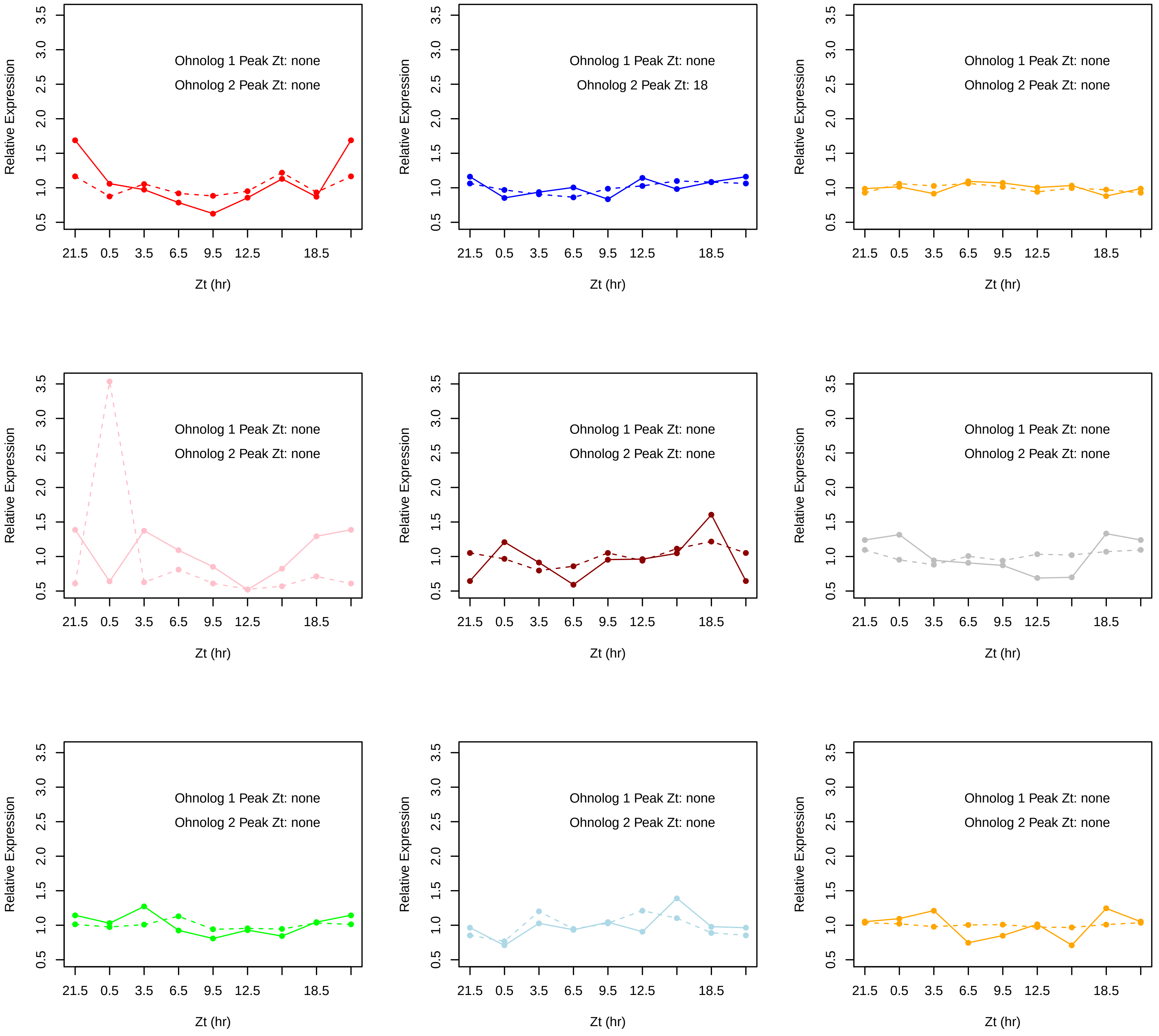

Ohnolog 1: ENSXMAG00000006814 rhot1b
Ohnolog 2: ENSXMAG00000014316 rhot1a

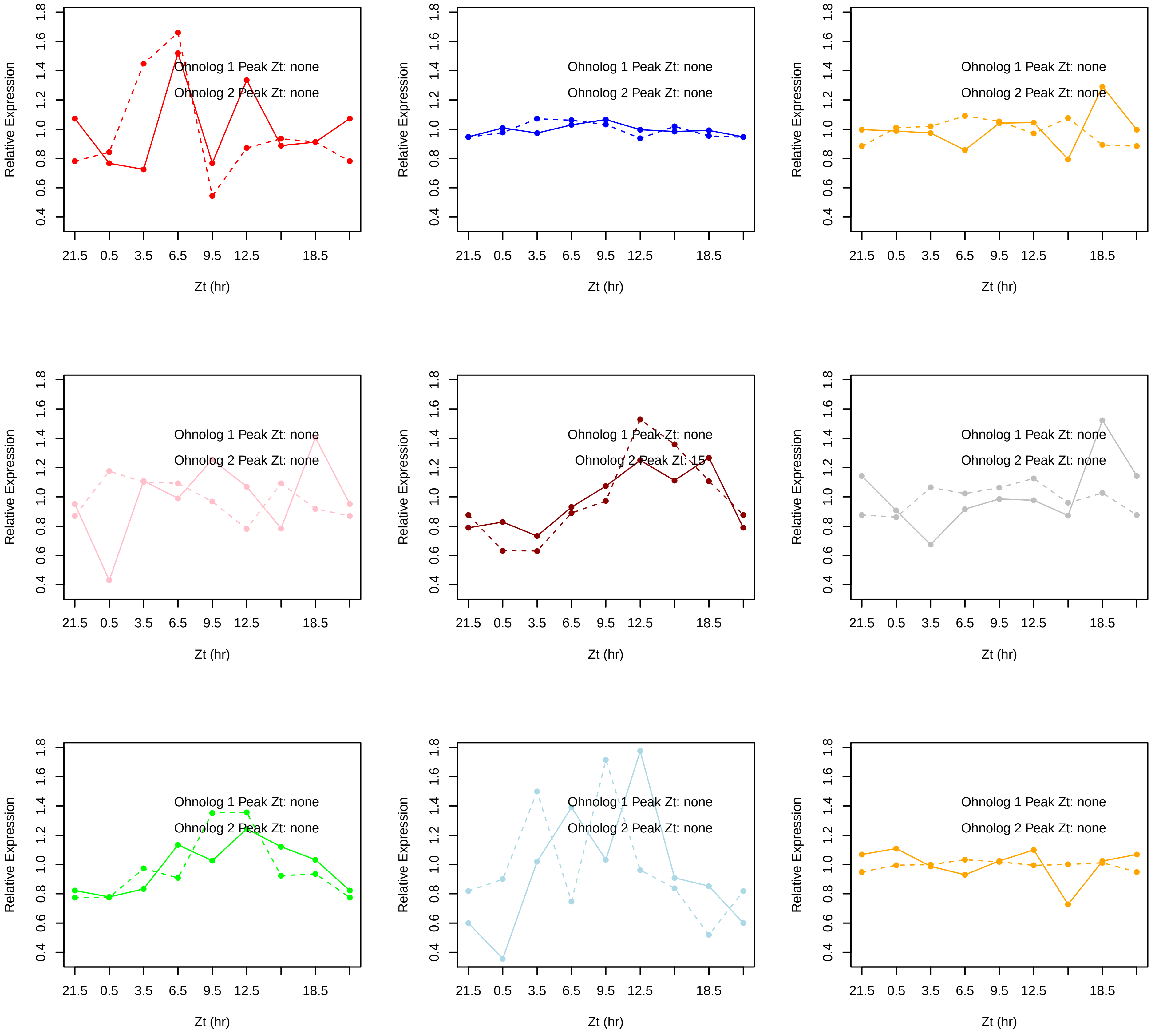

Ohnolog 1: ENSXMAG00000000385 fgf11b
Ohnolog 2: ENSXMAG00000013668 fgf11a

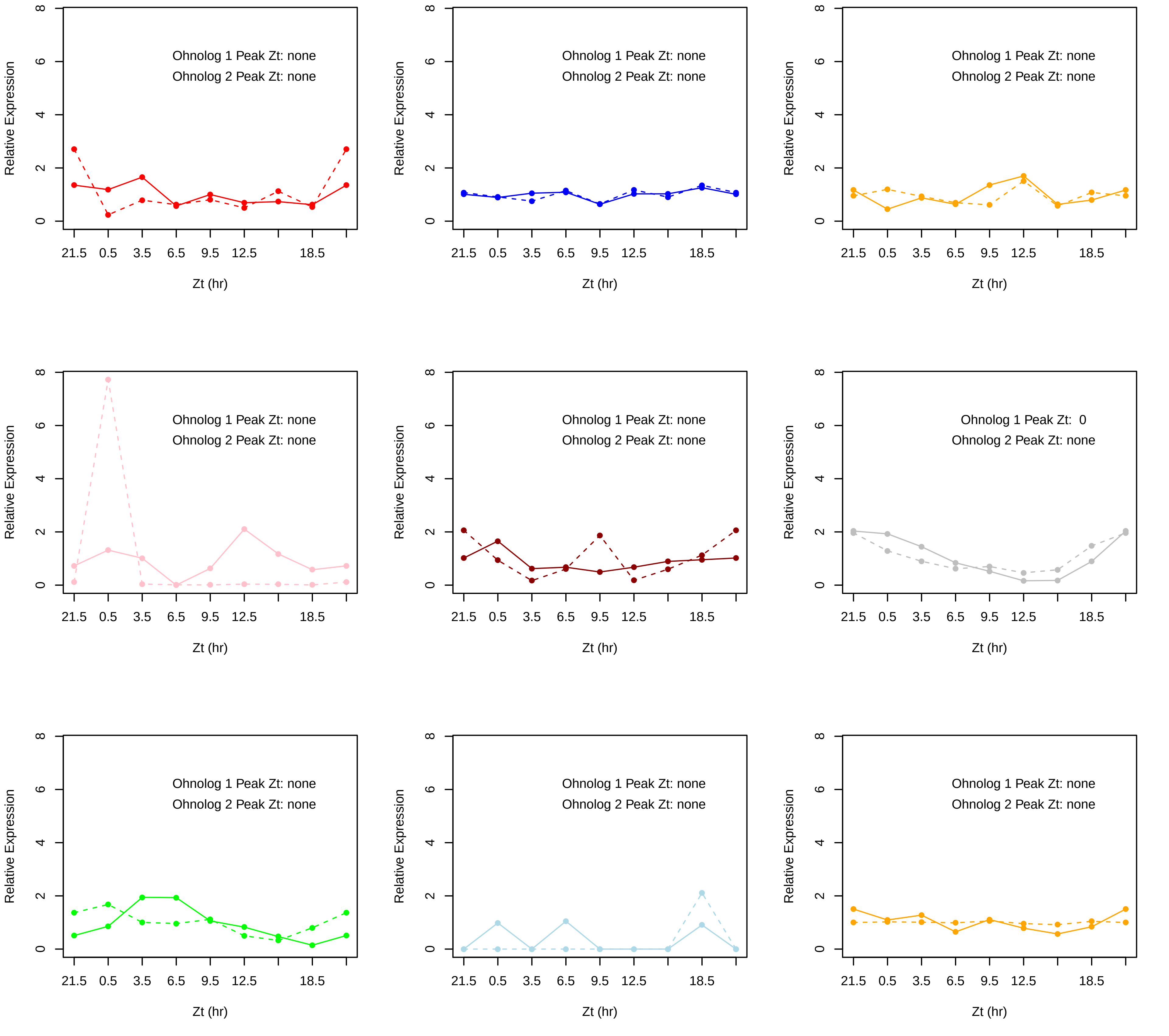

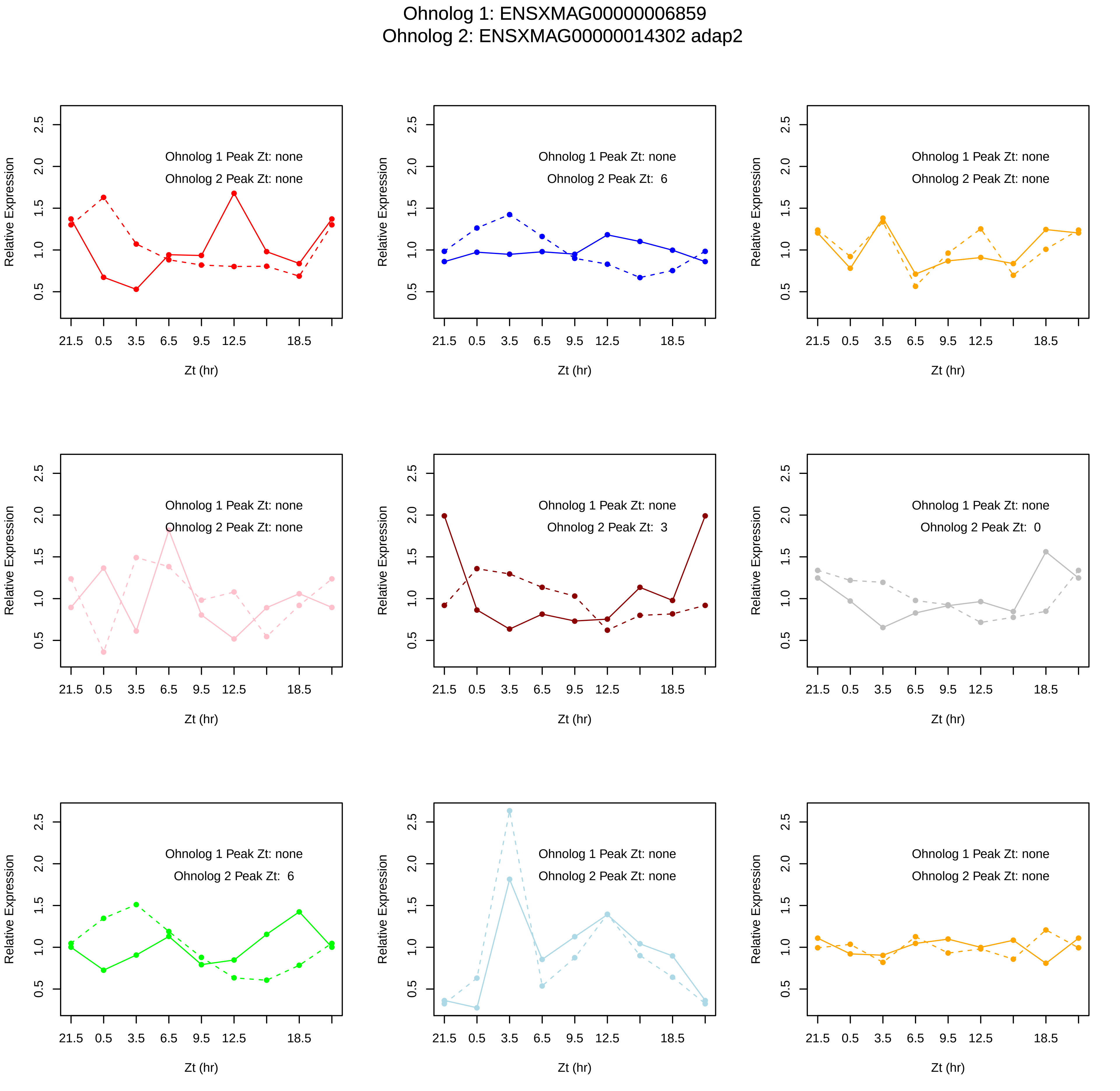
Ohnolog 1: ENSXMAG00000006859
Ohnolog 2: ENSXMAG00000014302 adap2
Ohnolog 1 Peak Zt: none
Ohnolog 2 Peak Zt: none
Ohnolog 1 Peak Zt: none
Ohnolog 2 Peak Zt: 6
Ohnolog 1 Peak Zt: none
Ohnolog 2 Peak Zt: none
Ohnolog 1 Peak Zt: none
Ohnolog 2 Peak Zt: none
Ohnolog 1 Peak Zt: none
Ohnolog 2 Peak Zt: 3
Ohnolog 1 Peak Zt: none
Ohnolog 2 Peak Zt: 0
Ohnolog 1 Peak Zt: none
Ohnolog 2 Peak Zt: 6
Ohnolog 1 Peak Zt: none
Ohnolog 2 Peak Zt: none
Ohnolog 1 Peak Zt: none
Ohnolog 2 Peak Zt: none
Relative Expression
Zt (hr)
21.5
0.5
3.5
6.5
9.5
12.5
18.5
0.5
1.0
1.5
2.0
2.5

Ohnolog 1: ENSXMAG00000012979 suz12a
Ohnolog 2: ENSXMAG00000017579 SUZ12

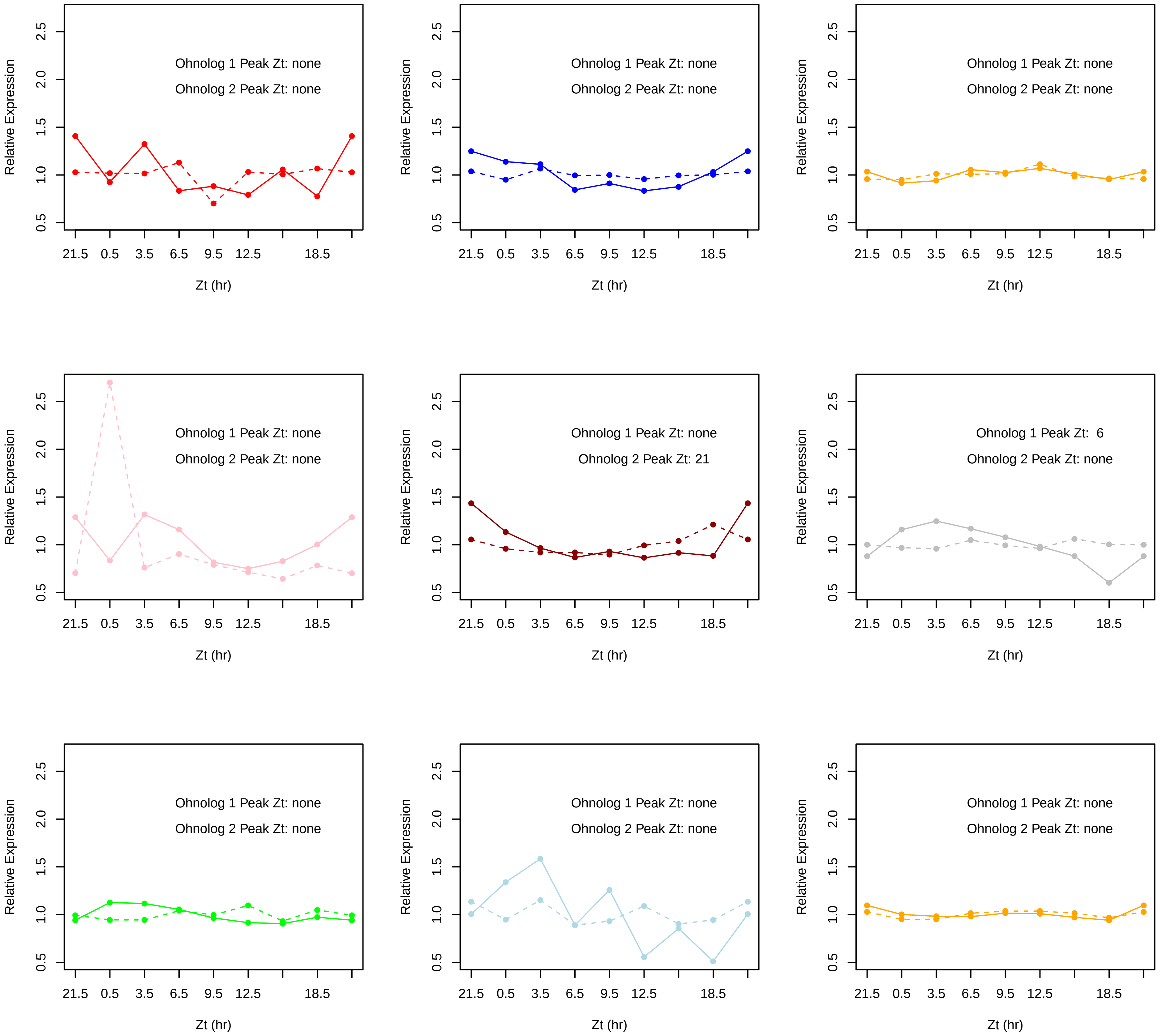

Ohnolog 1: ENSXMAG00000017789
Ohnolog 2: ENSXMAG00000023607 dusp7

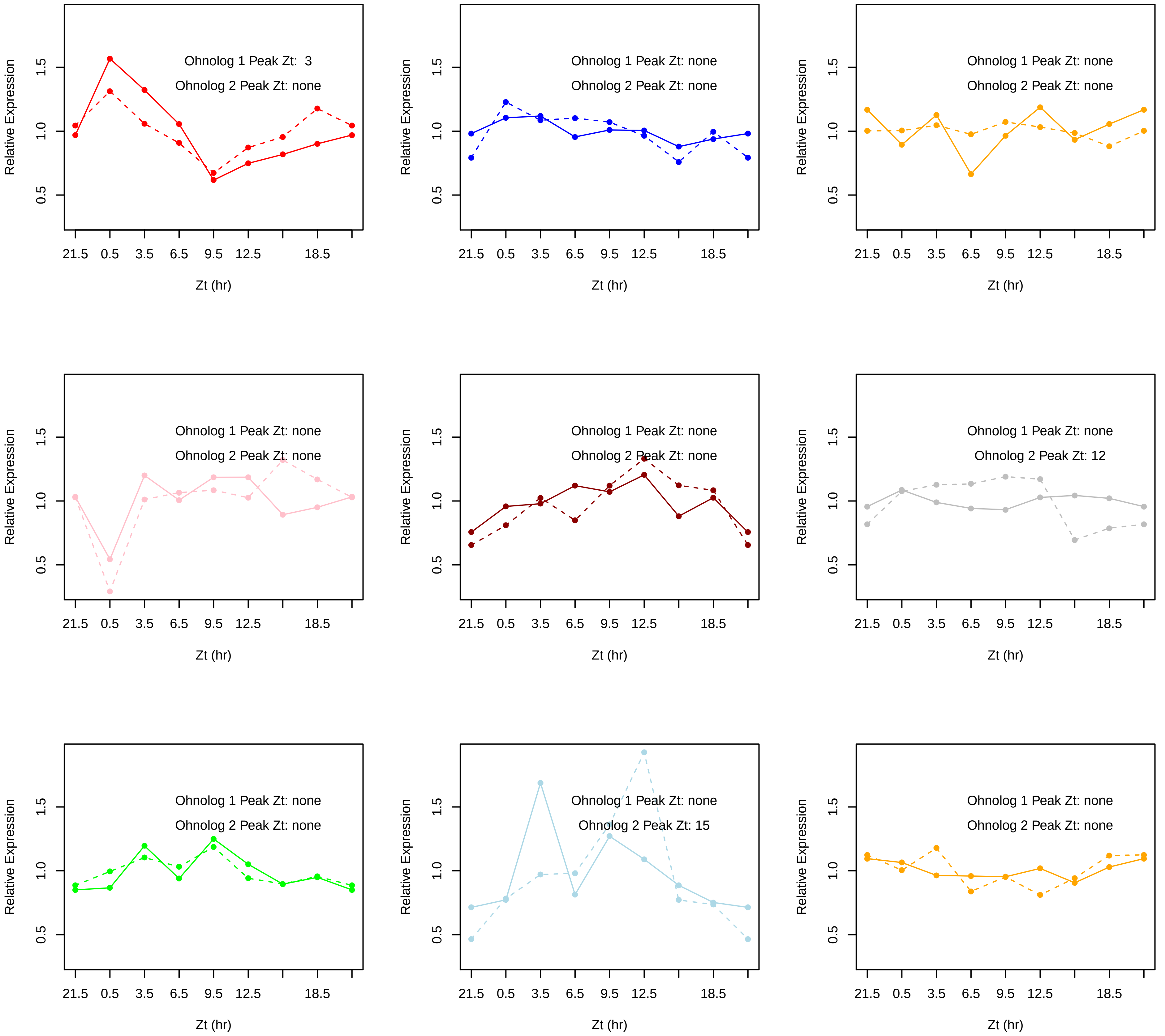

Ohnolog 1: ENSXMAG00000001429 cbx8a
Ohnolog 2: ENSXMAG00000021089 cbx8b

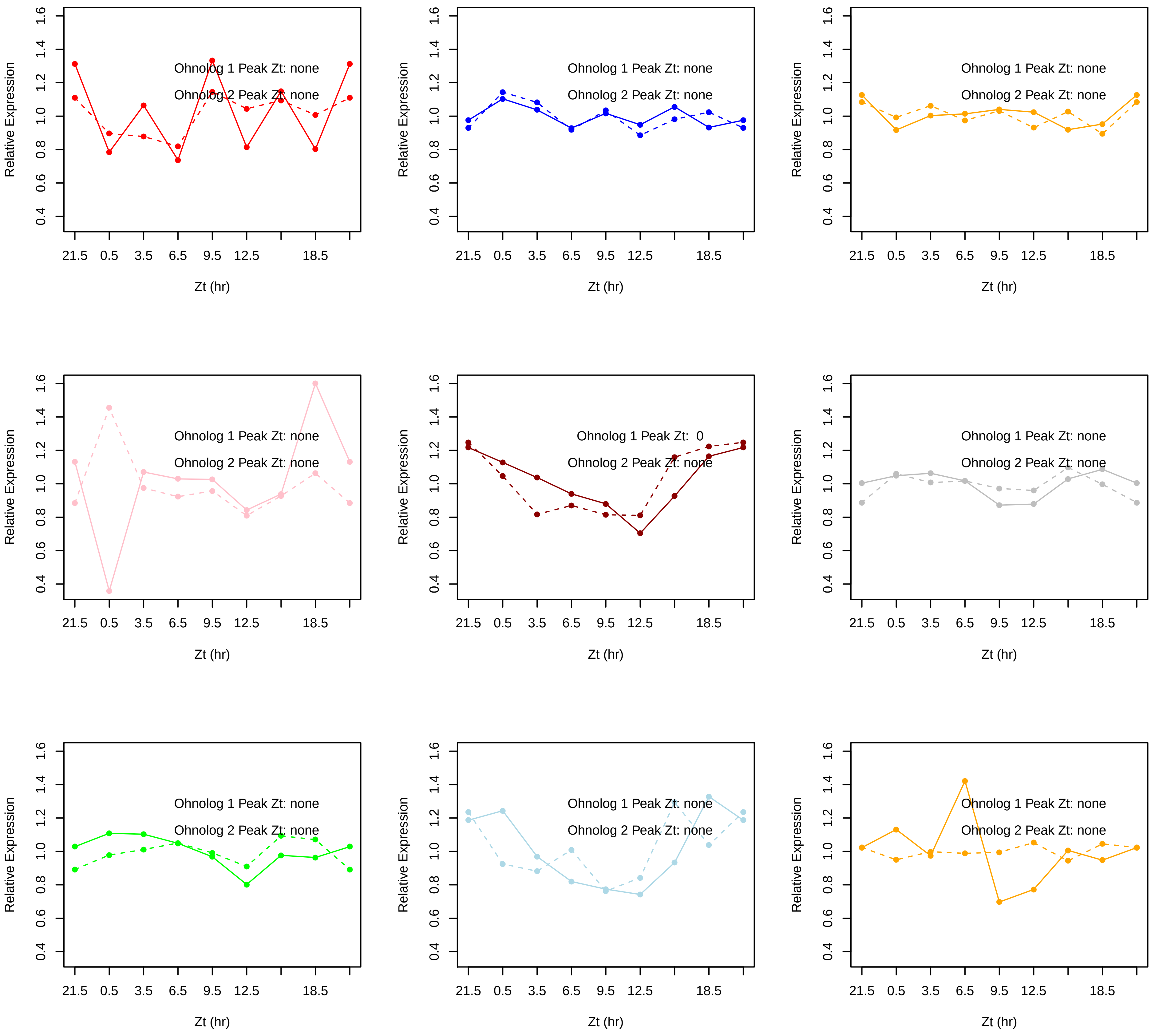

Ohnolog 1: ENSXMAG00000010483 ogna
Ohnolog 2: ENSXMAG00000024264 ognb

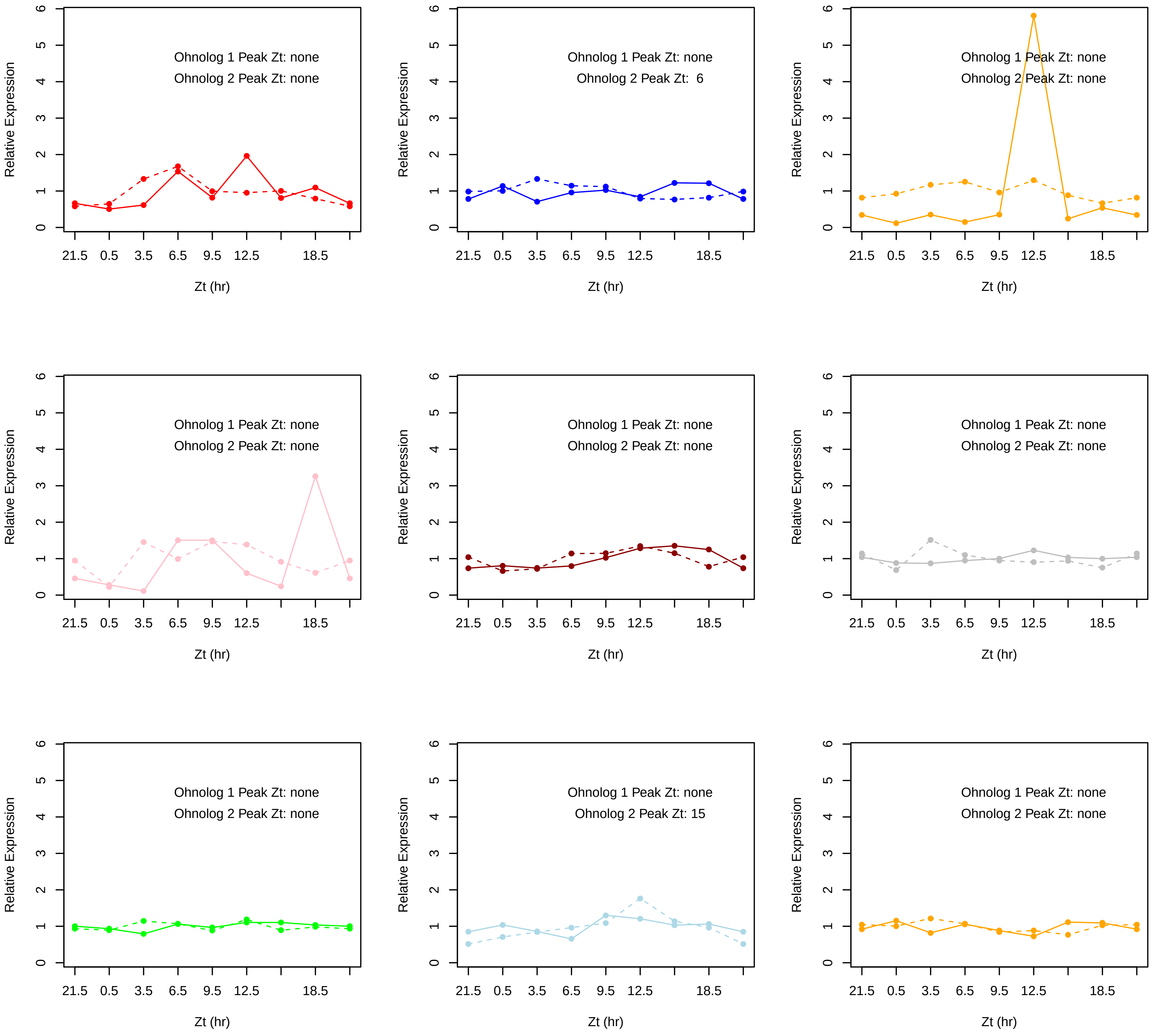

Ohnolog 1: ENSXMAG00000012443
Ohnolog 2: ENSXMAG00000017656

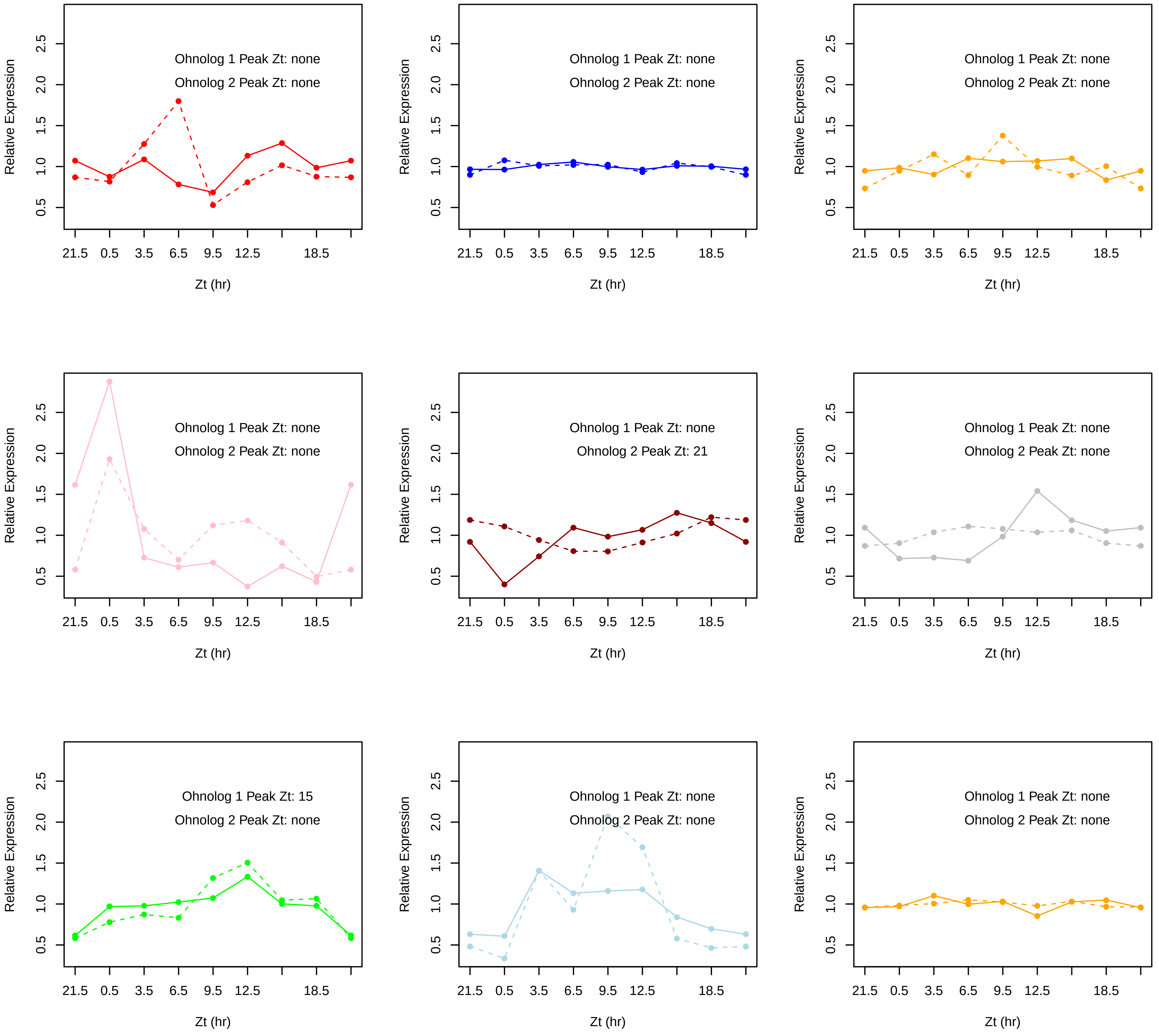

Ohnolog 1: ENSXMAG00000007522 ptpdc1a
Ohnolog 2: ENSXMAG00000017581 ptpdc1b

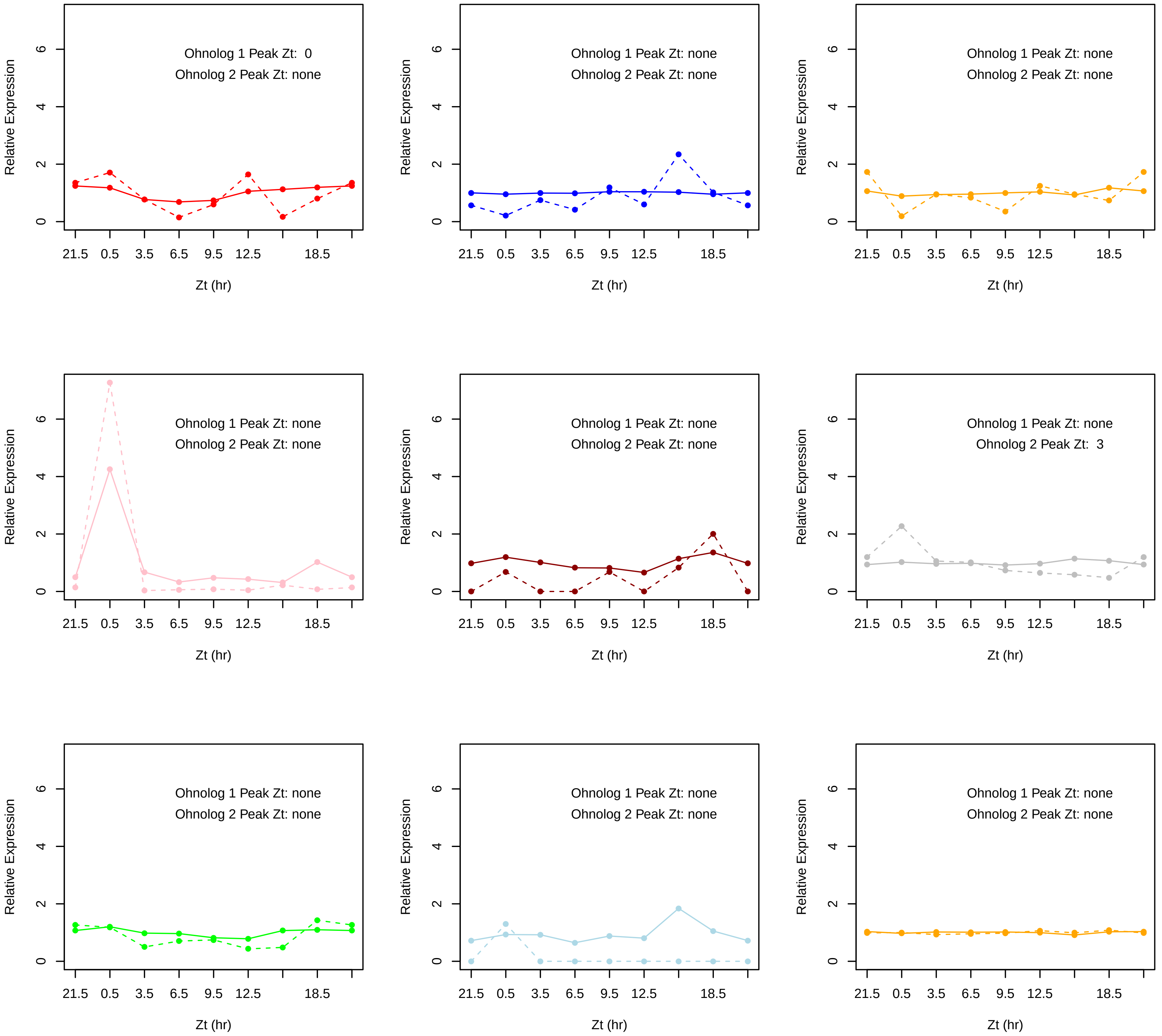

Ohnolog 1: ENSXMAG00000025916
Ohnolog 2: ENSXMAG00000026041 si:dkey−175g6.2

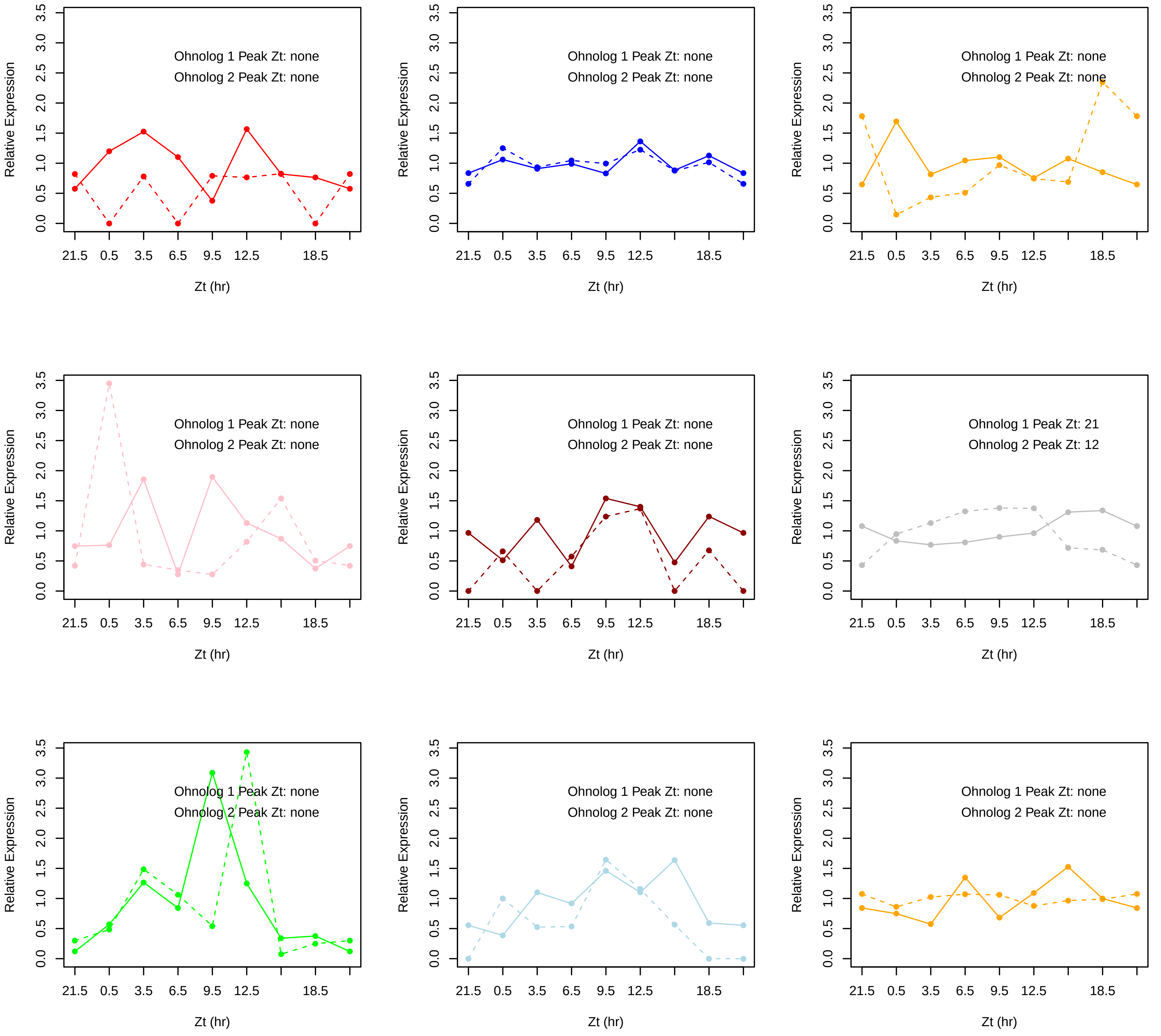

Ohnolog 1: ENSXMAG00000017090 NINJ1 (1 of many)
Ohnolog 2: ENSXMAG00000022037 NINJ1 (1 of many)

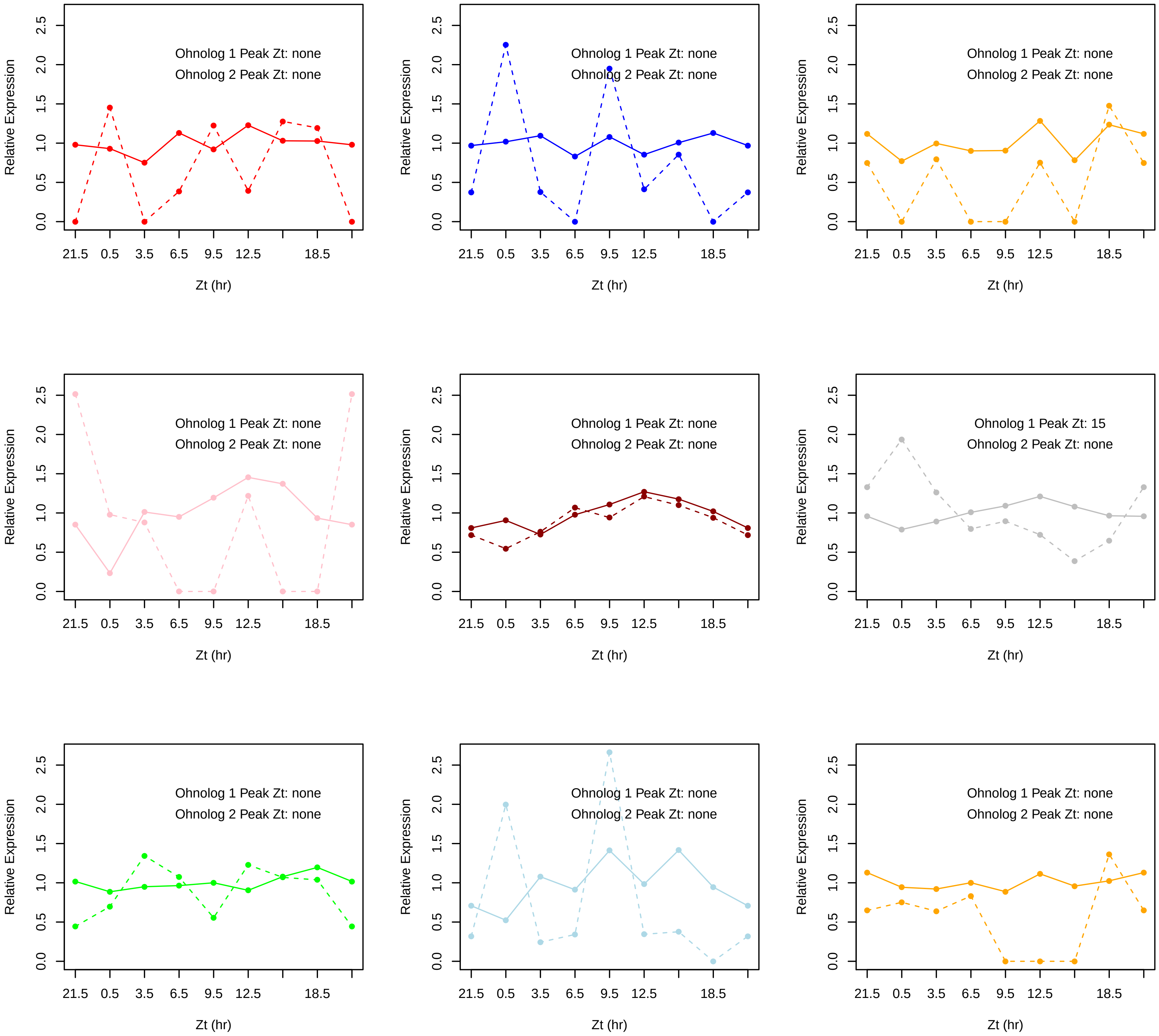

Ohnolog 1: ENSXMAG00000006869
Ohnolog 2: ENSXMAG00000018049 prc1b

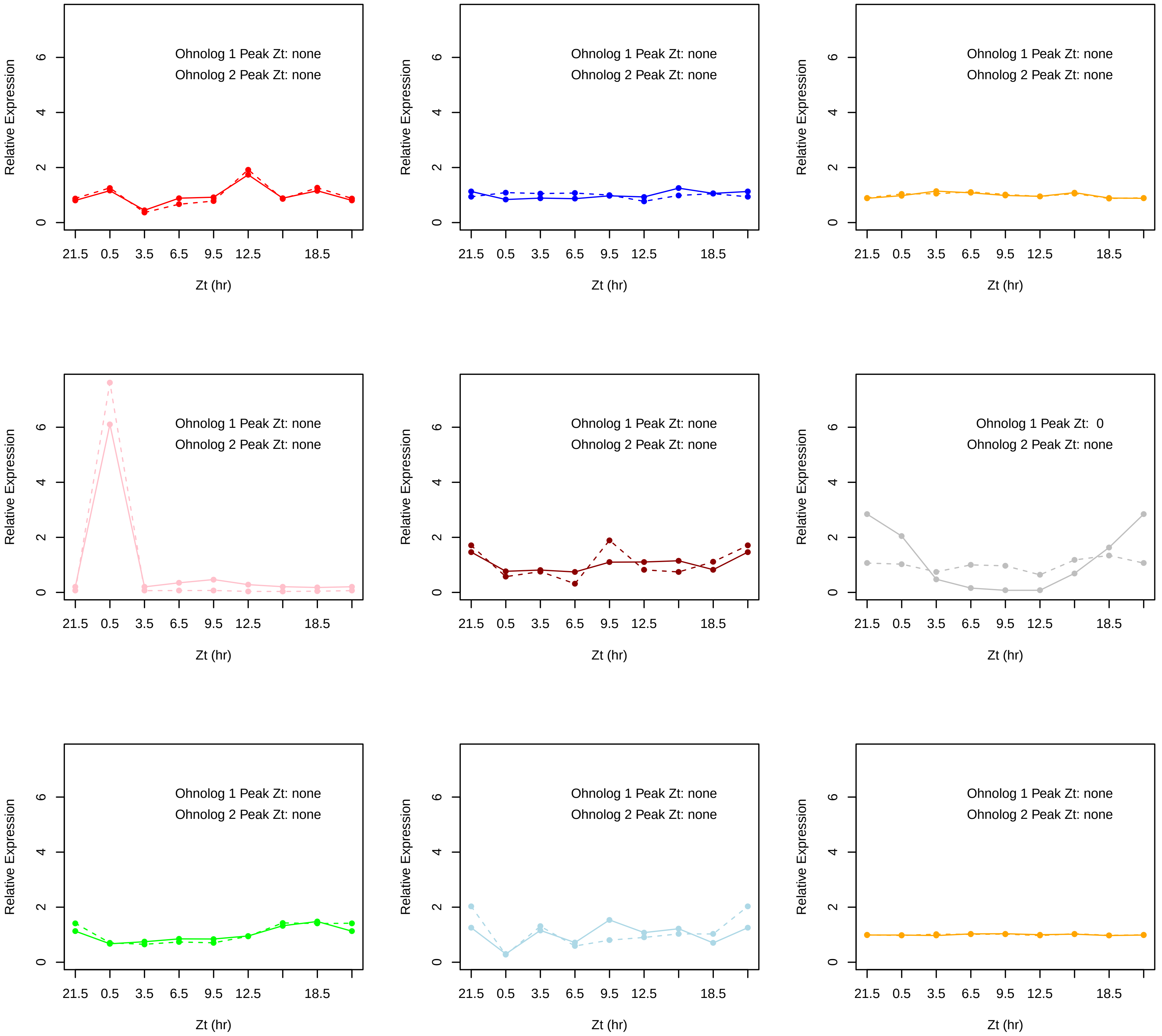

Ohnolog 1: ENSXMAG00000000160
Ohnolog 2: ENSXMAG00000017247 mpp5b

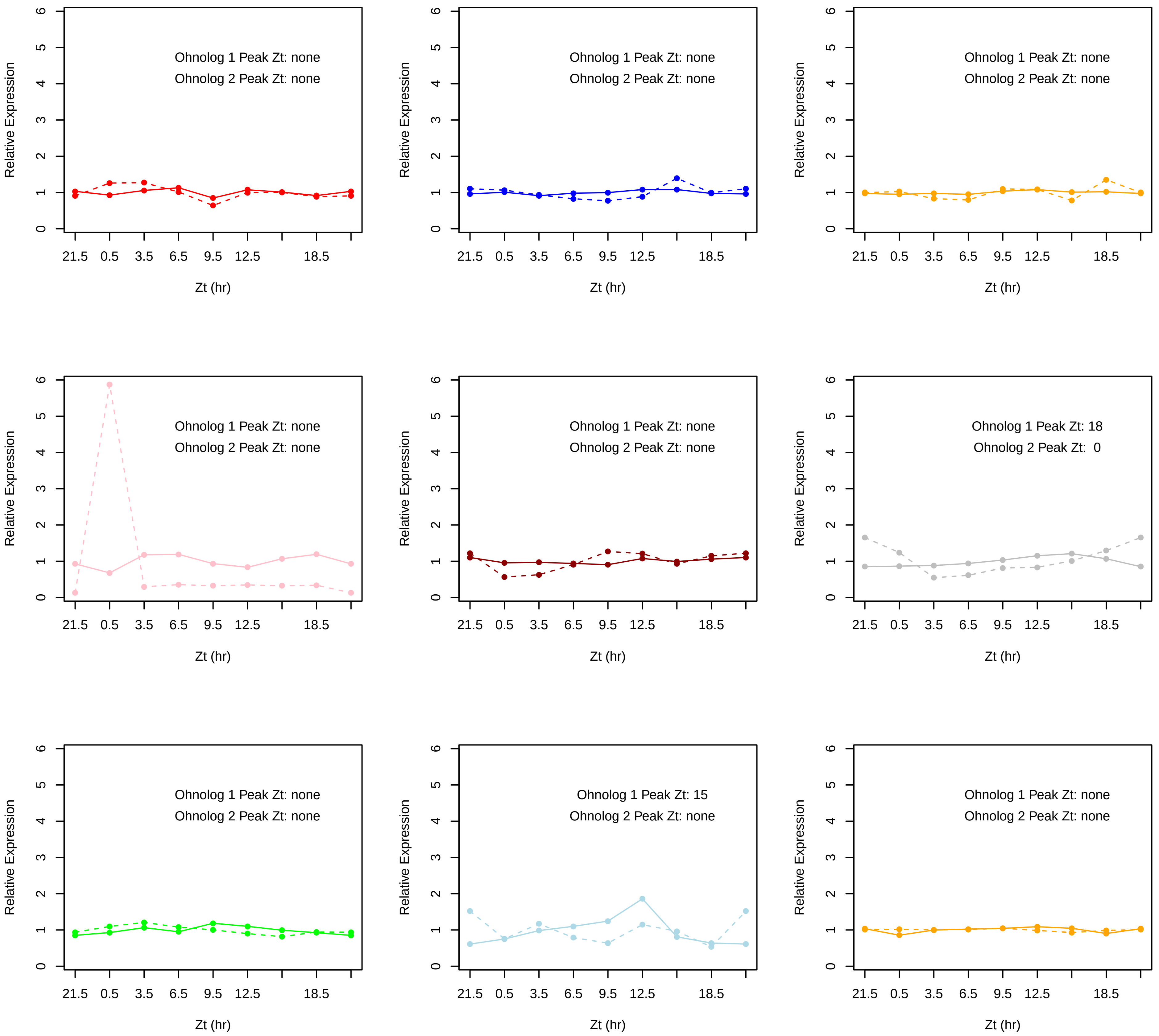

Ohnolog 1: ENSXMAG00000013503 kita
Ohnolog 2: ENSXMAG00000017634 kitb

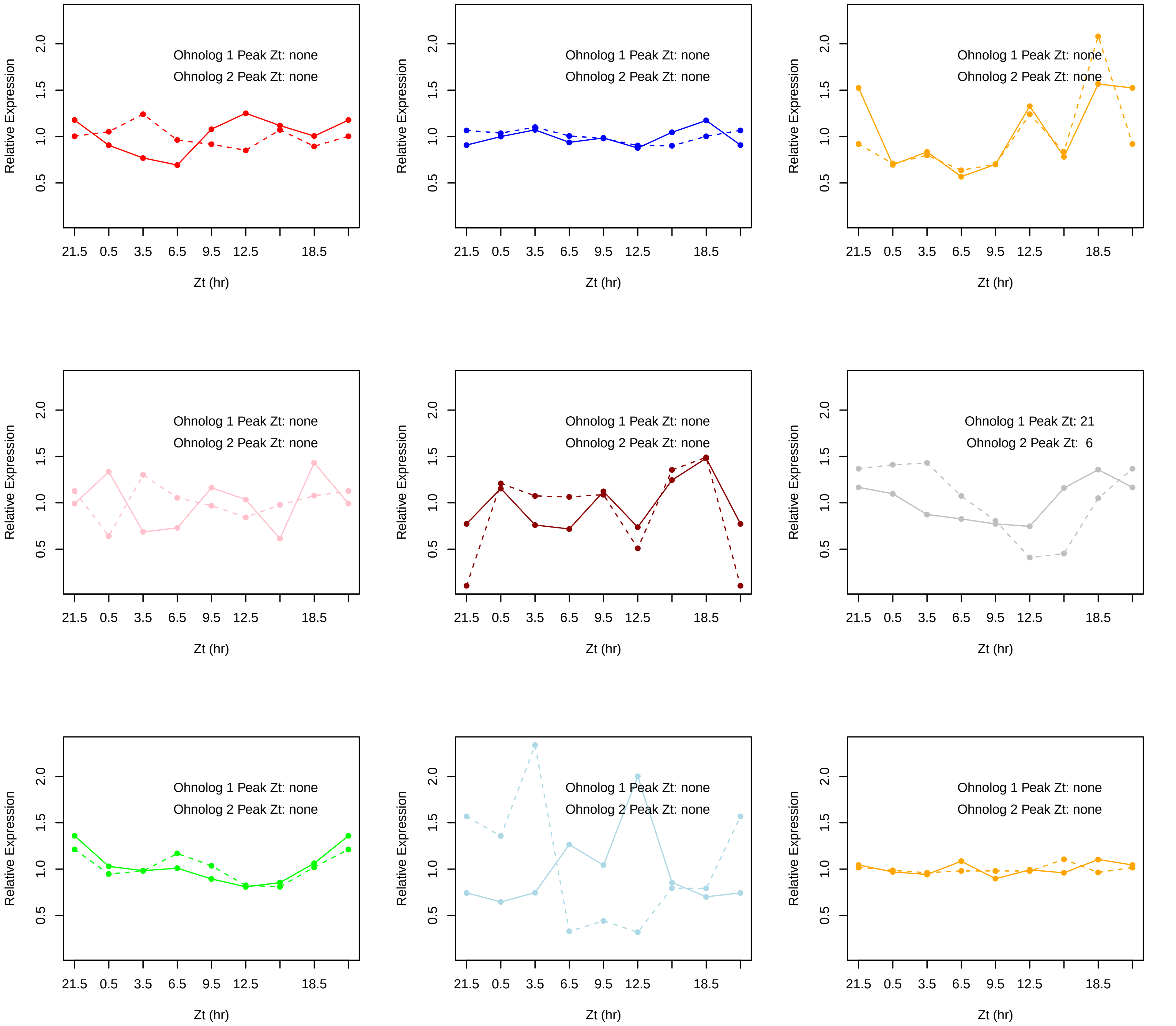

Ohnolog 1: ENSXMAG00000010771 ehd2a
Ohnolog 2: ENSXMAG00000029134 ehd2b

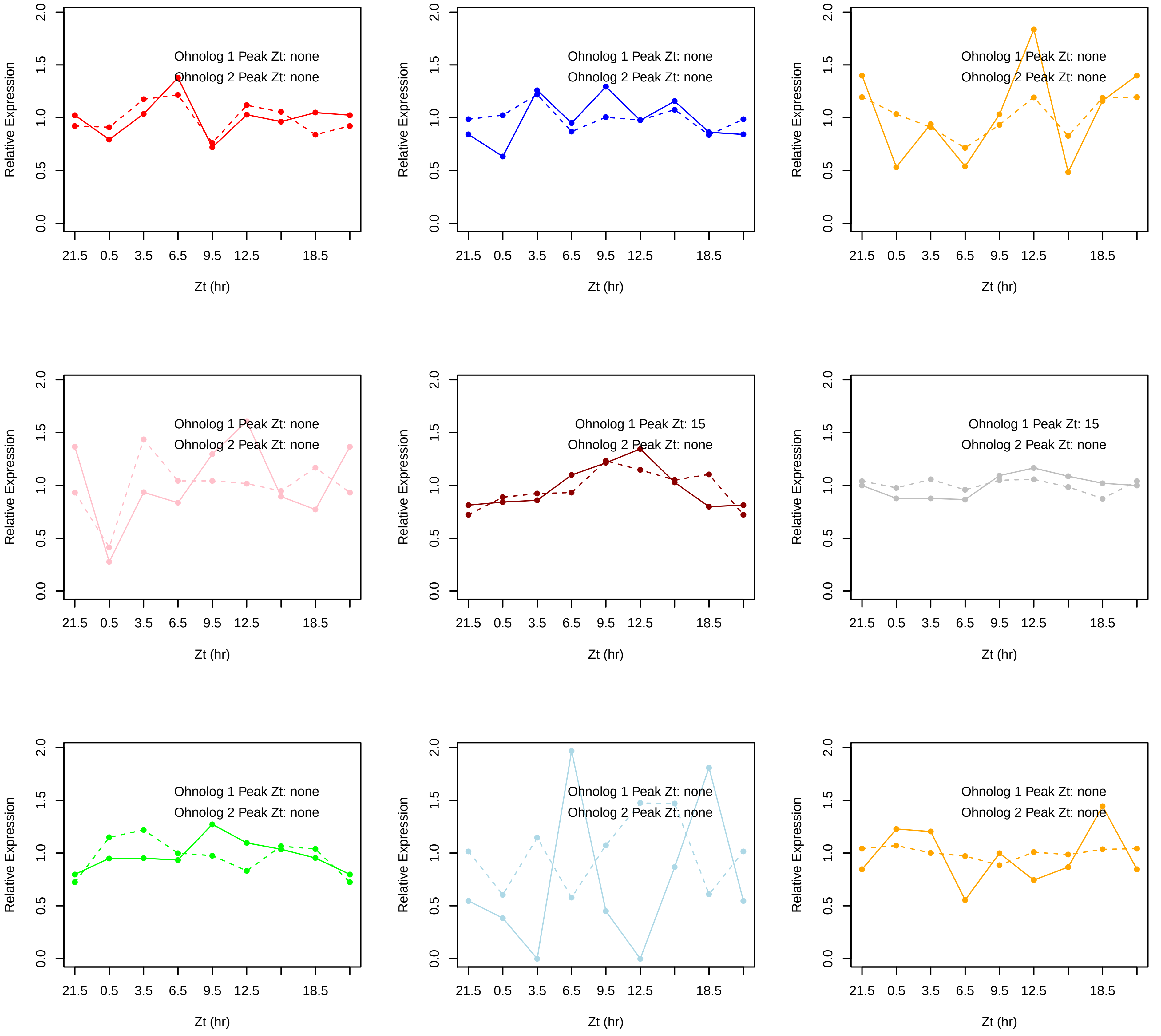

Ohnolog 1: ENSXMAG00000012976 ppip5k1b
Ohnolog 2: ENSXMAG00000018275 ppip5k1a

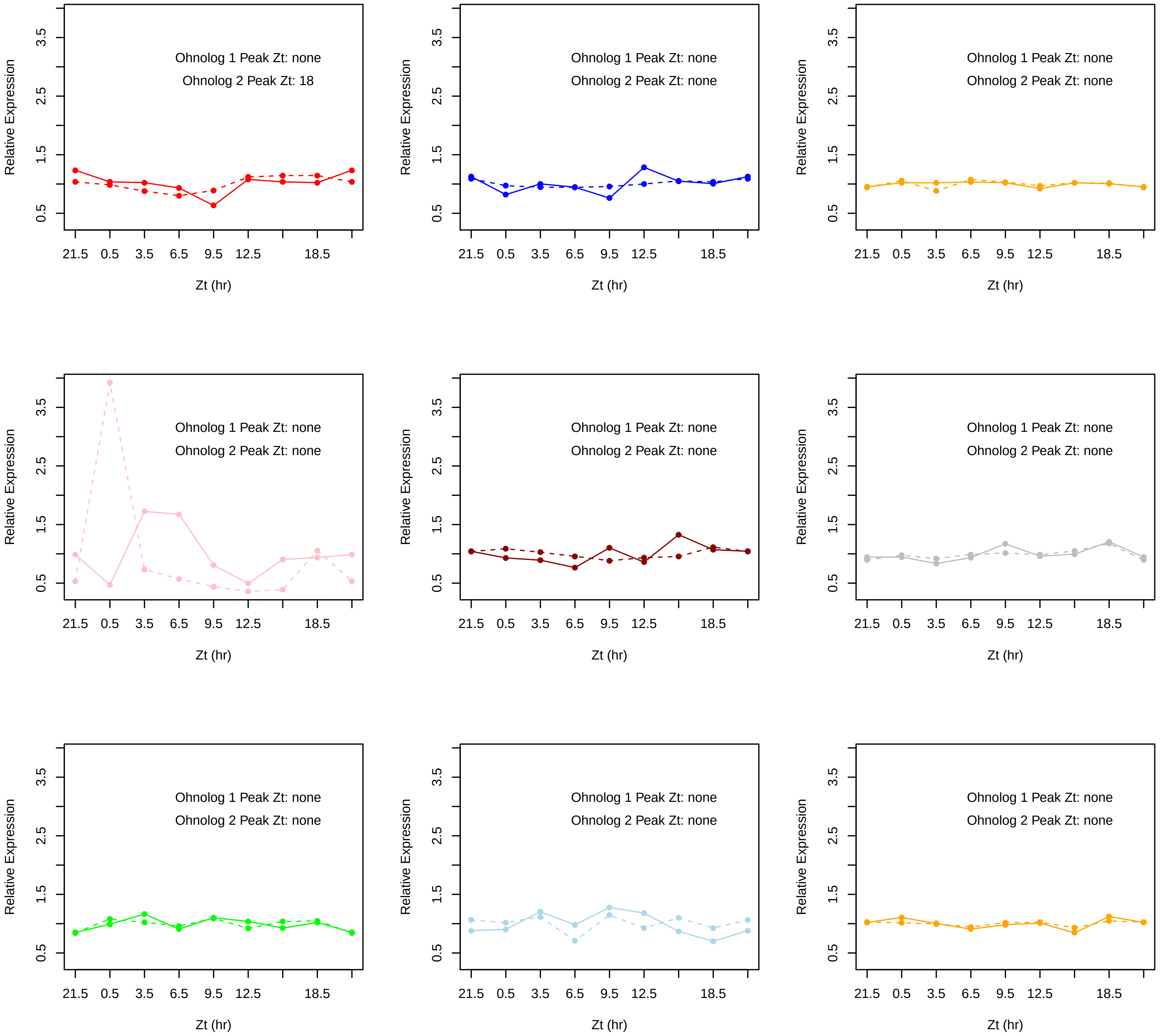

Ohnolog 1: ENSXMAG00000013036 mfge8b
Ohnolog 2: ENSXMAG00000024531

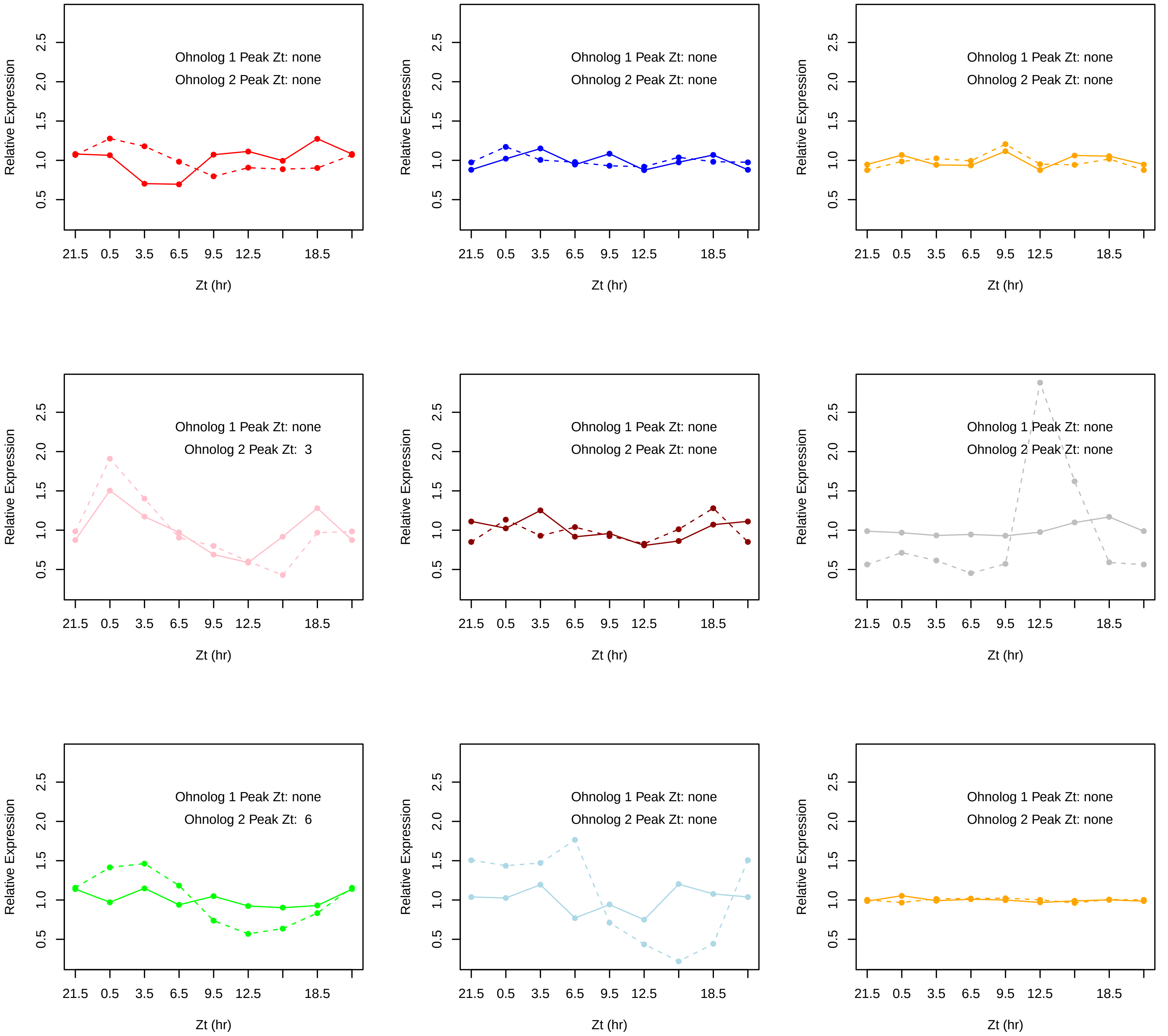

Ohnolog 1: ENSXMAG00000002892 RASGRF1
Ohnolog 2: ENSXMAG00000017009

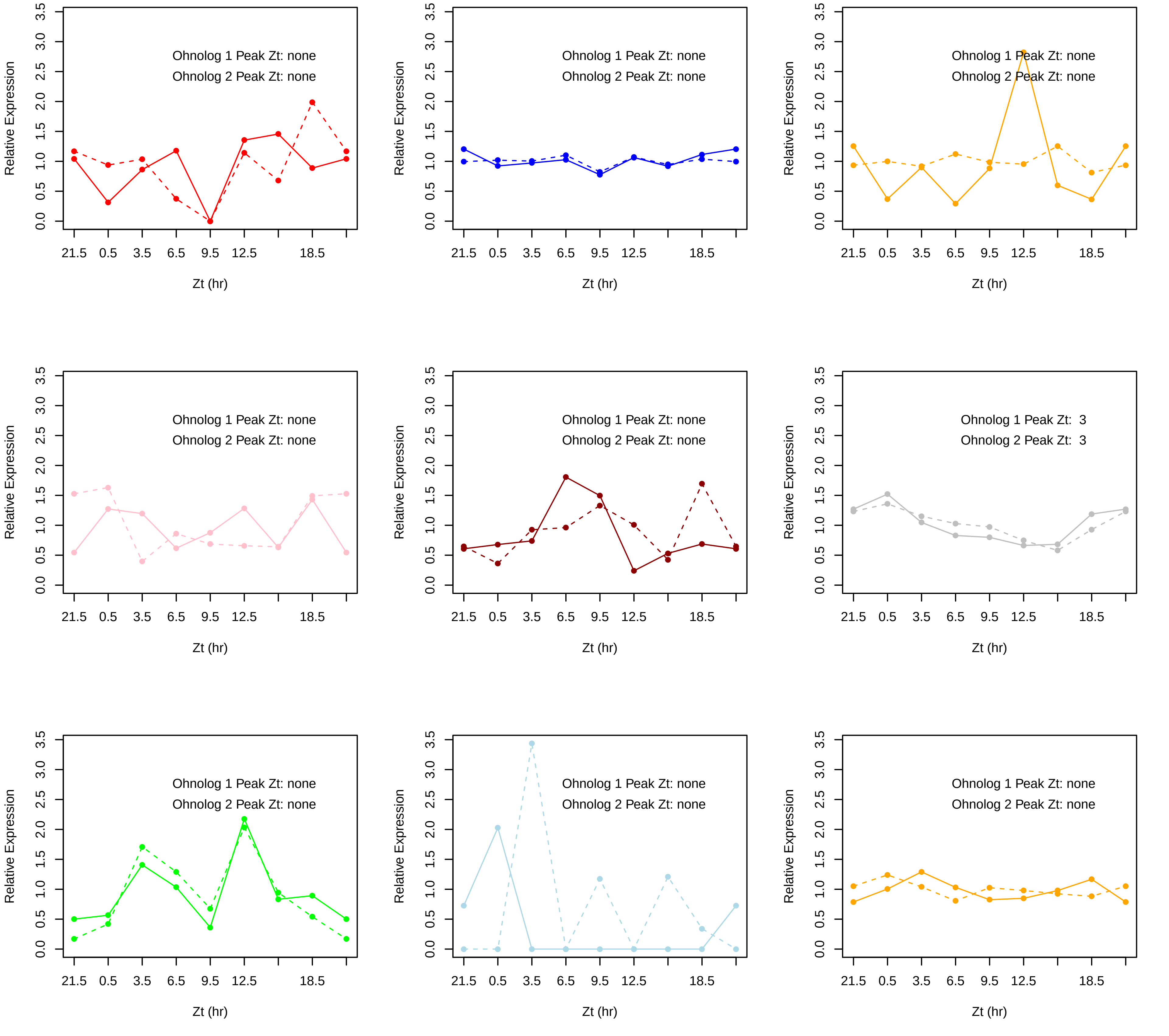

Ohnolog 1: ENSXMAG00000002886
Ohnolog 2: ENSXMAG00000016987 si:ch211−107o10.3

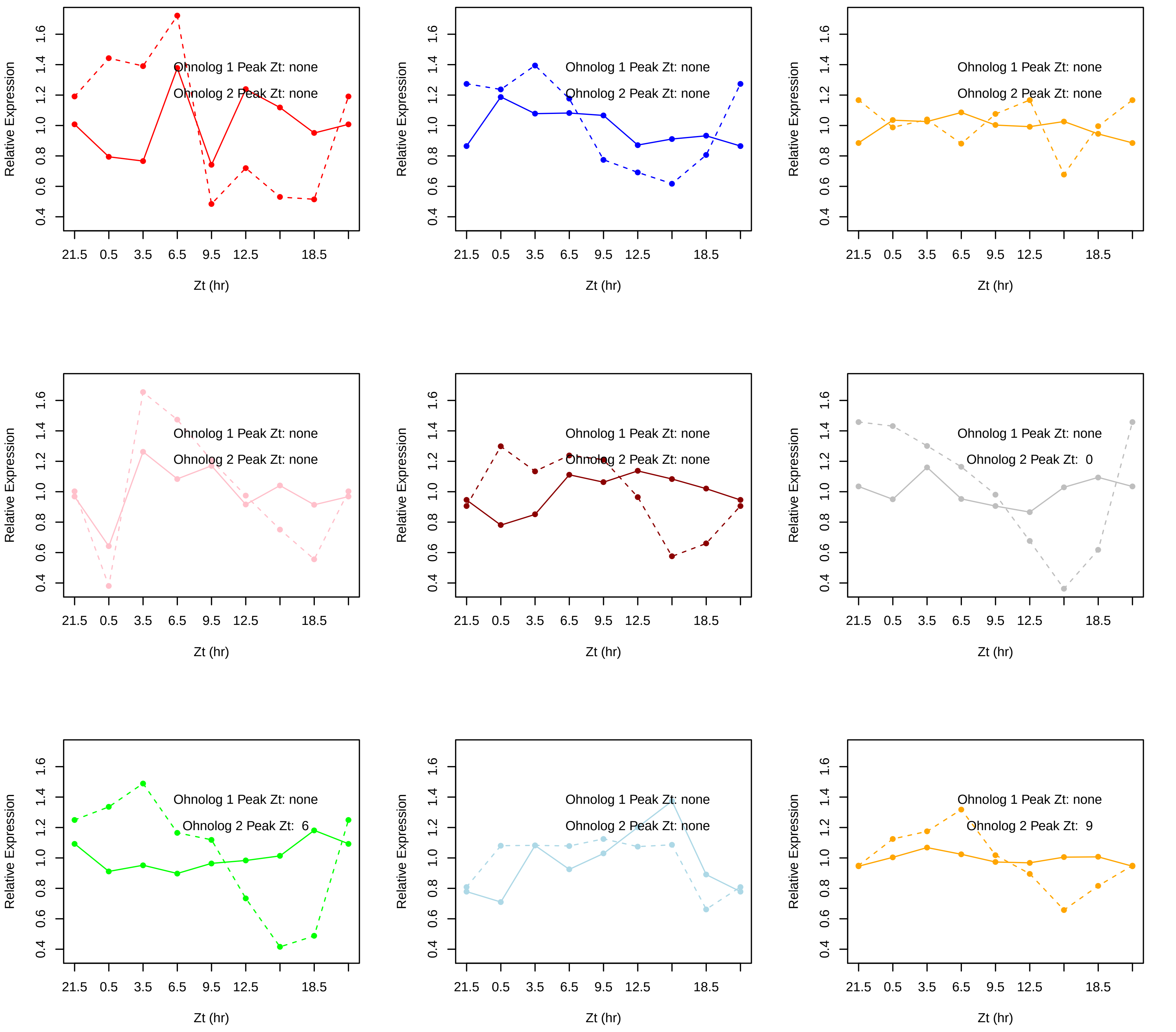

Ohnolog 1: ENSXMAG00000007547
Ohnolog 2: ENSXMAG00000017369 p4htm

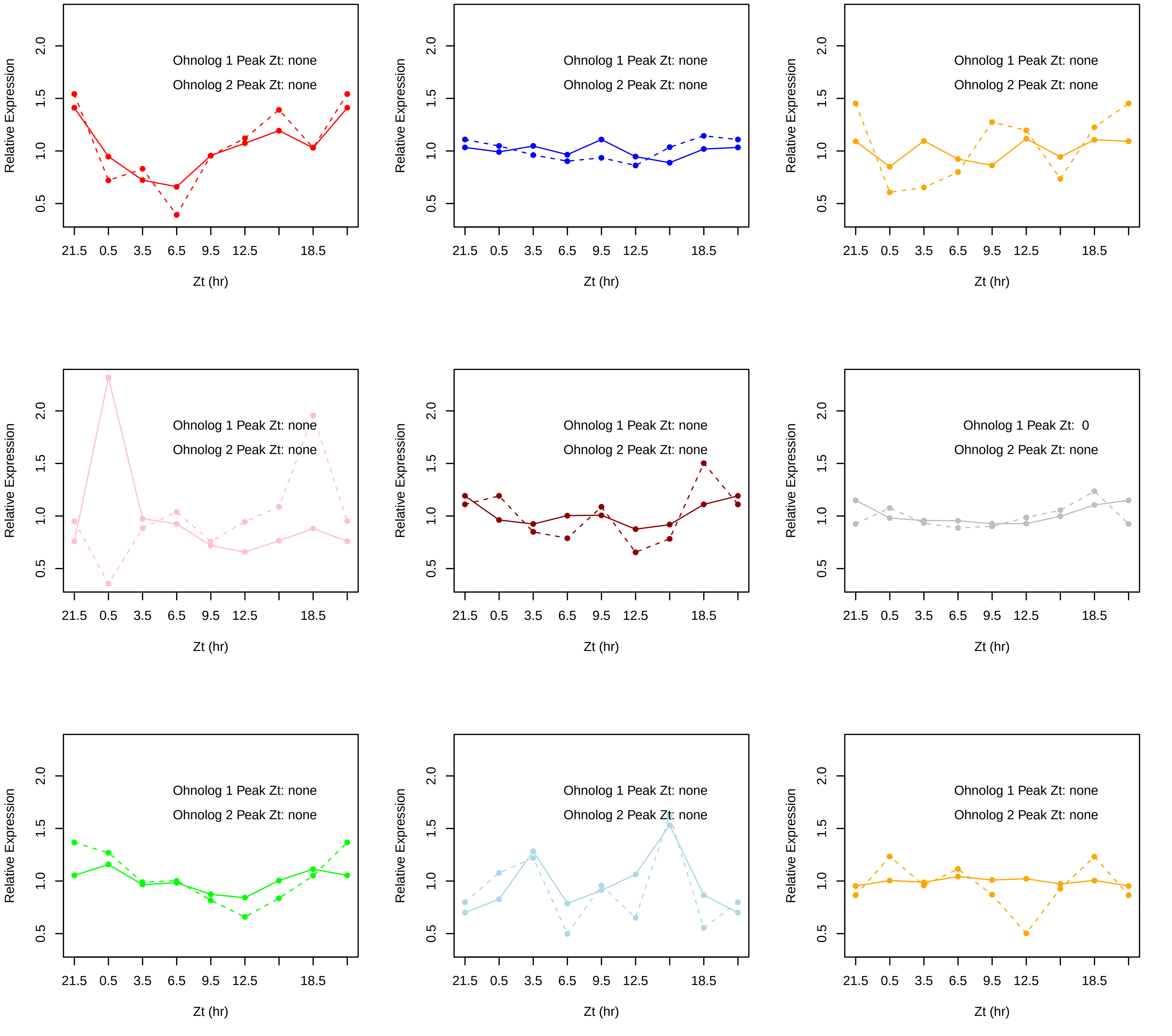

Ohnolog 1: ENSXMAG00000001747 ip6k2b
Ohnolog 2: ENSXMAG00000023264 ip6k2a

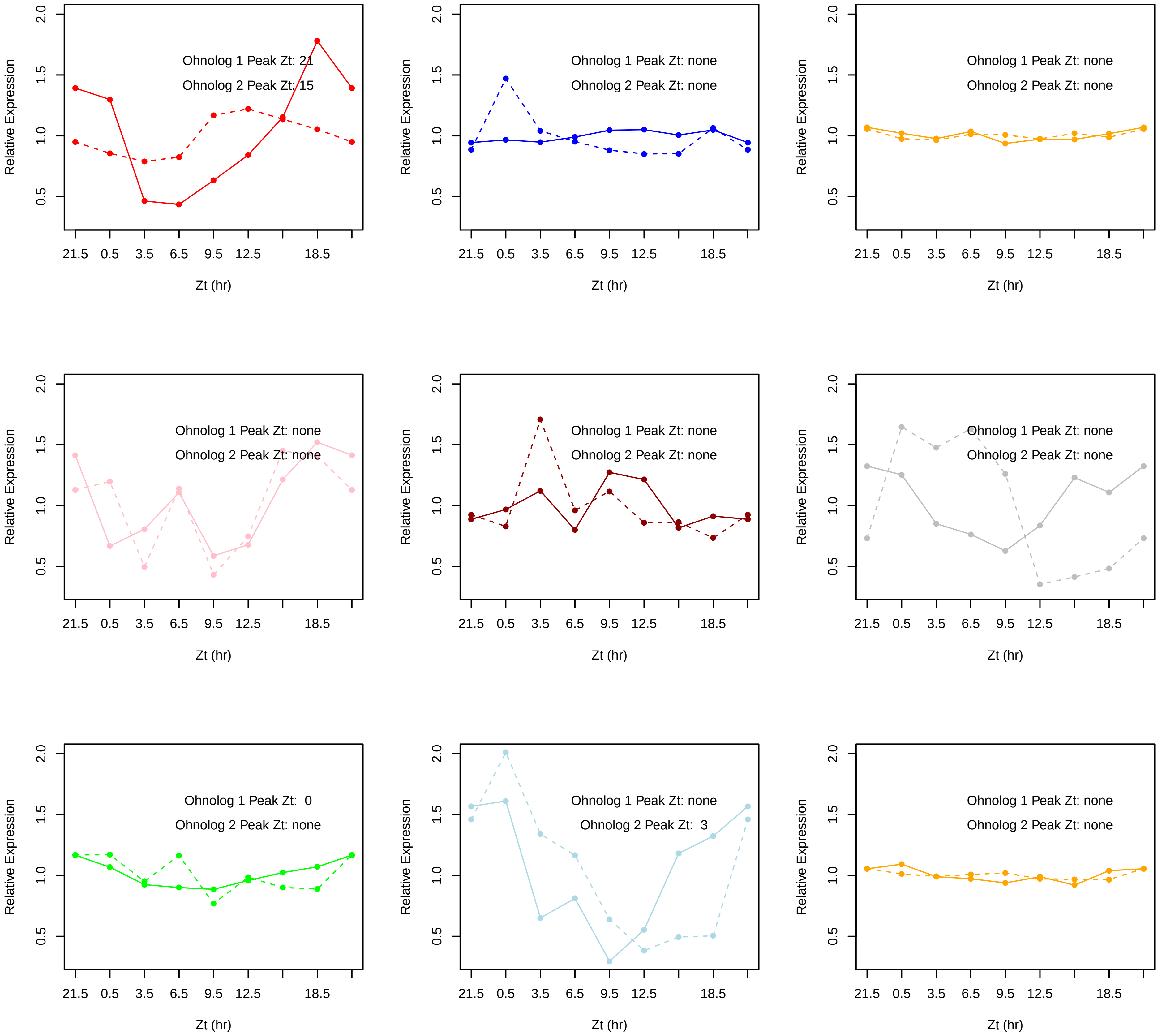

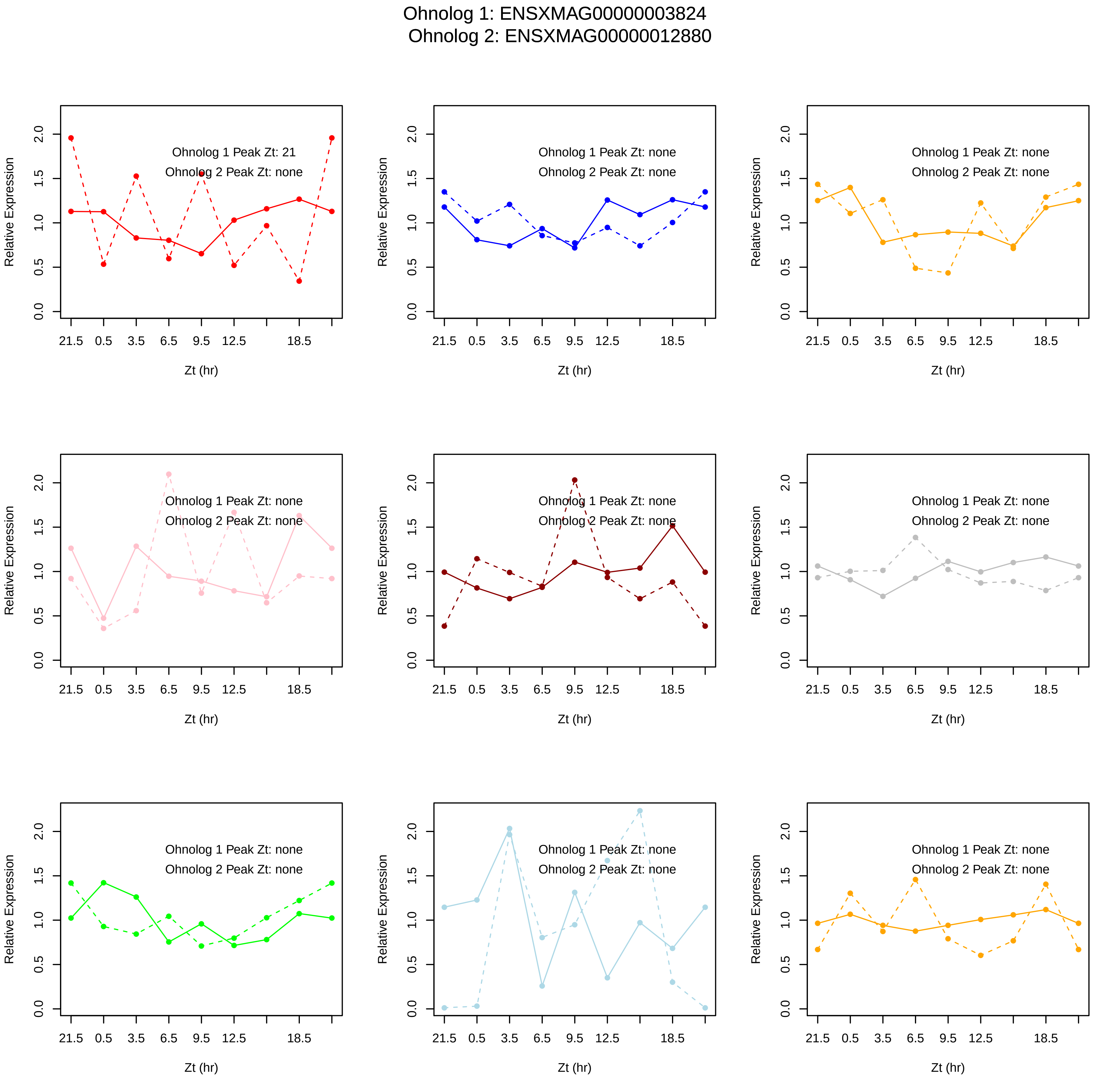
Ohnolog 1: ENSXMAG00000003824
Ohnolog 2: ENSXMAG00000012880
Ohnolog 1 Peak Zt: 21
Ohnolog 2 Peak Zt: none
Ohnolog 1 Peak Zt: none
Ohnolog 2 Peak Zt: none
Relative Expression
Zt (hr)
21.5
0.5
3.5
6.5
9.5
12.5
18.5
0.0
0.5
1.0
1.5
2.0

Ohnolog 1: ENSXMAG00000001861 prkcda
Ohnolog 2: ENSXMAG00000010429 prkcdb

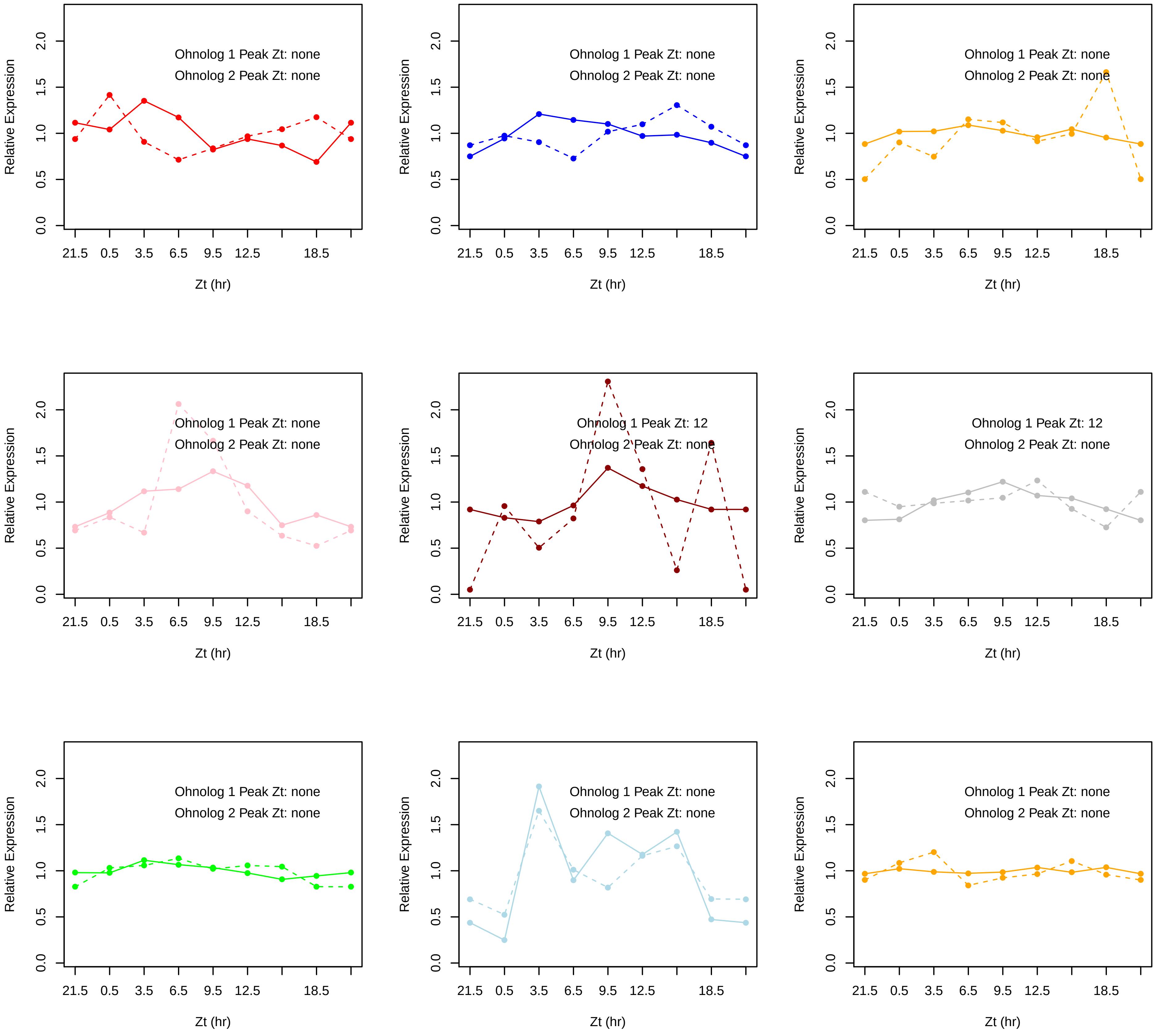

Ohnolog 1: ENSXMAG00000000035 nptnb
Ohnolog 2: ENSXMAG00000007414 nptna

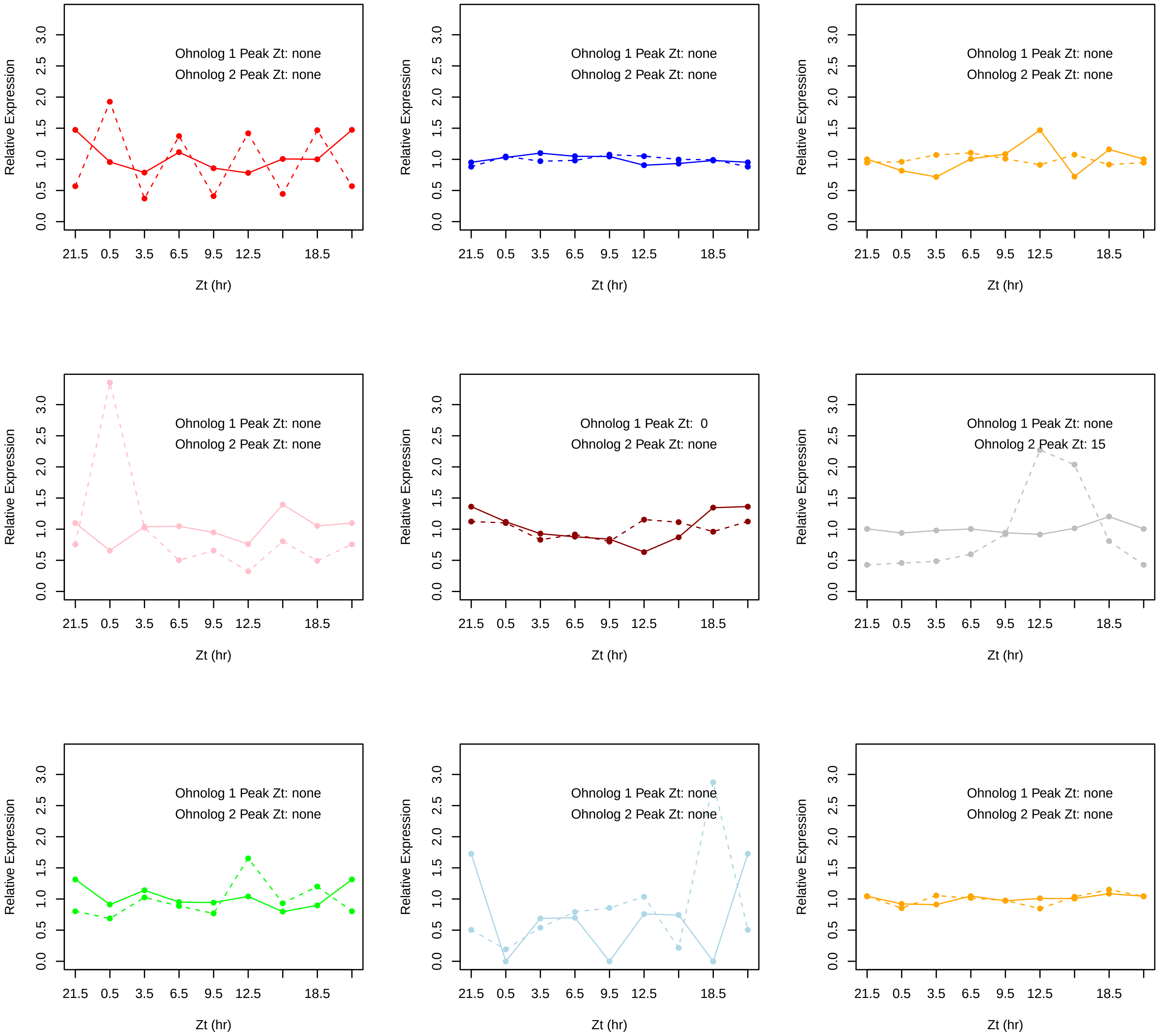

Ohnolog 1: ENSXMAG00000023285 rab7
Ohnolog 2: ENSXMAG00000024665 RAB7A

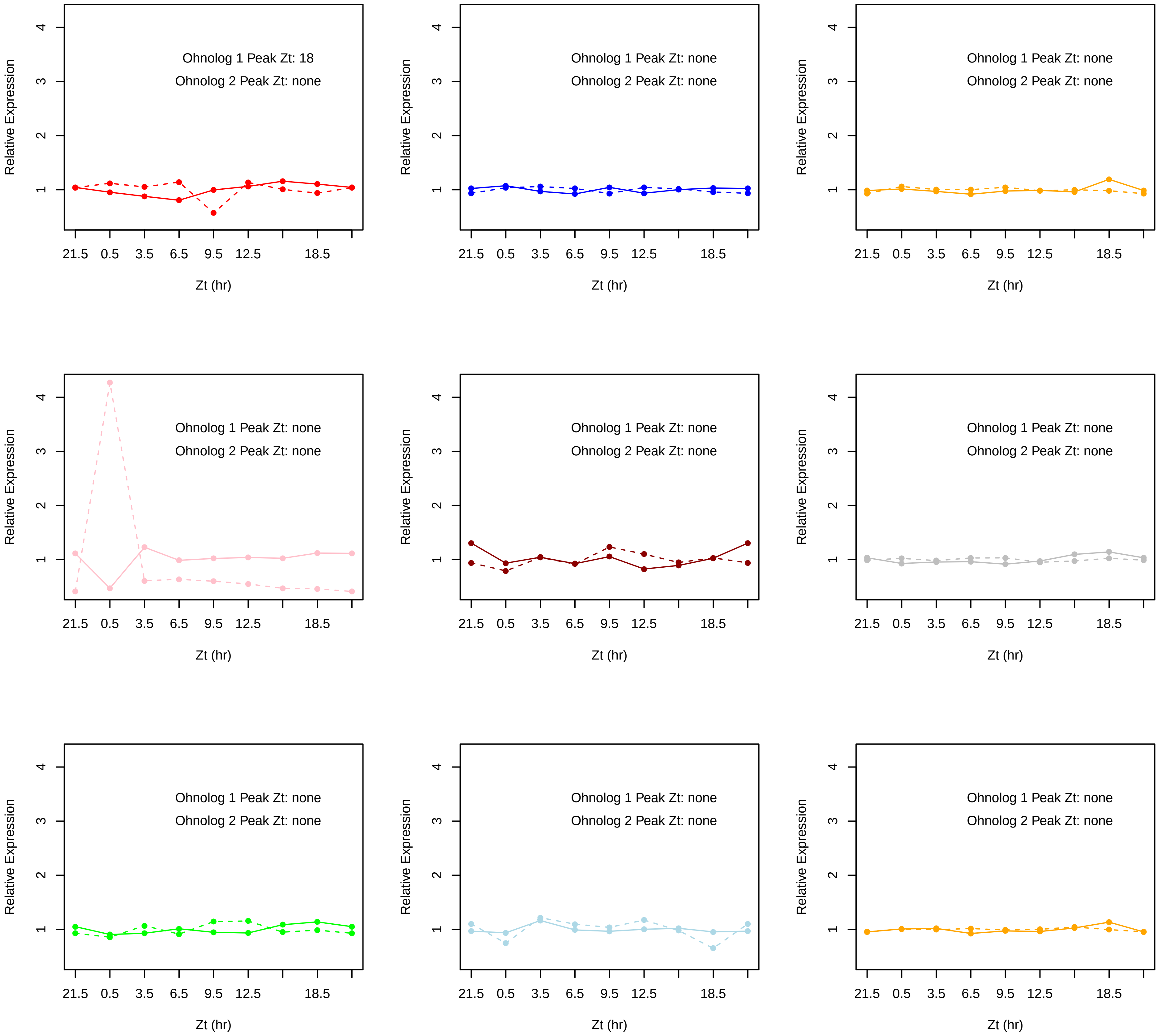

Ohnolog 1: ENSXMAG00000002966
Ohnolog 2: ENSXMAG00000017063 frmd5

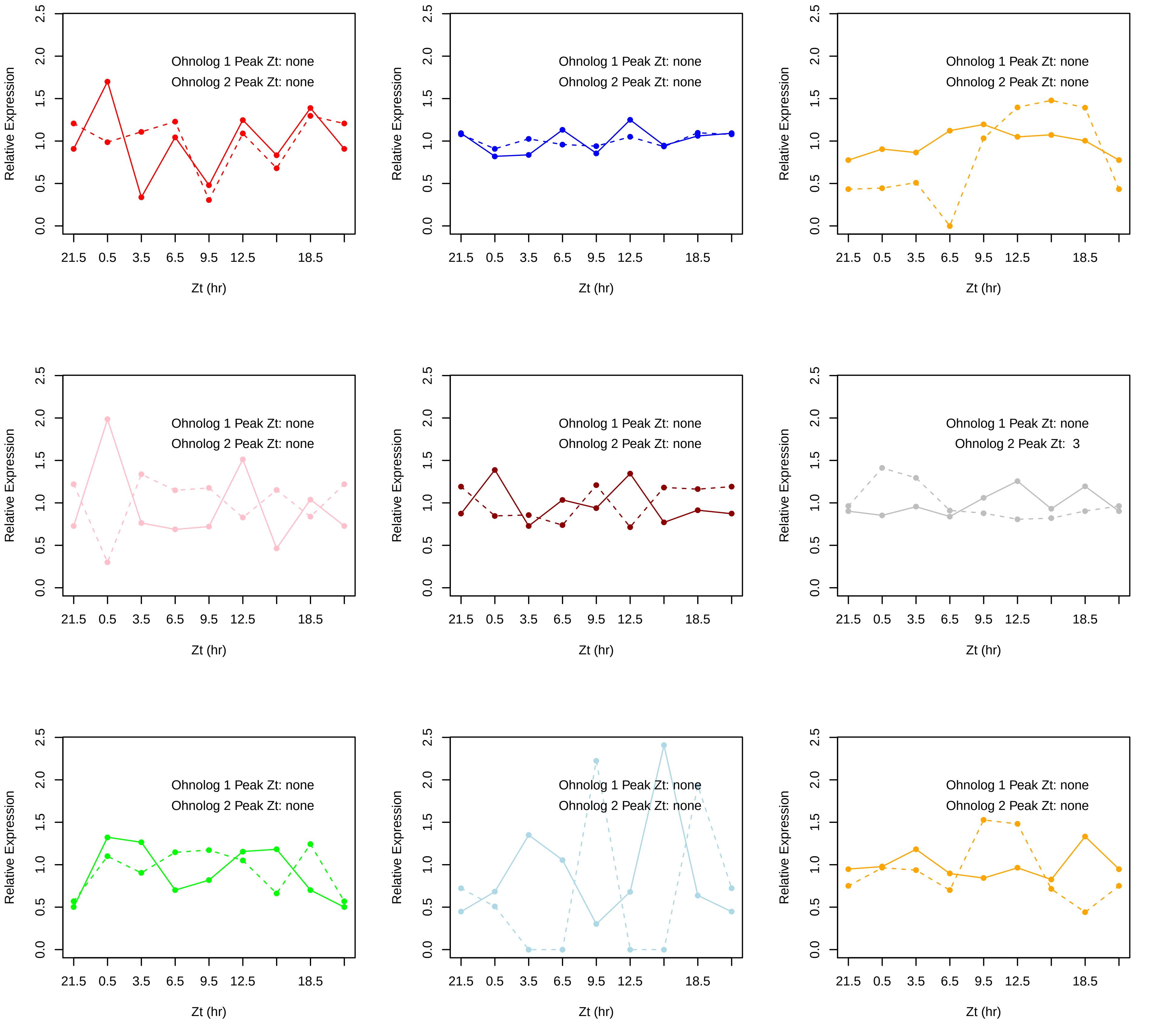

Ohnolog 1: ENSXMAG00000008911
Ohnolog 2: ENSXMAG00000027672 iqsec2b

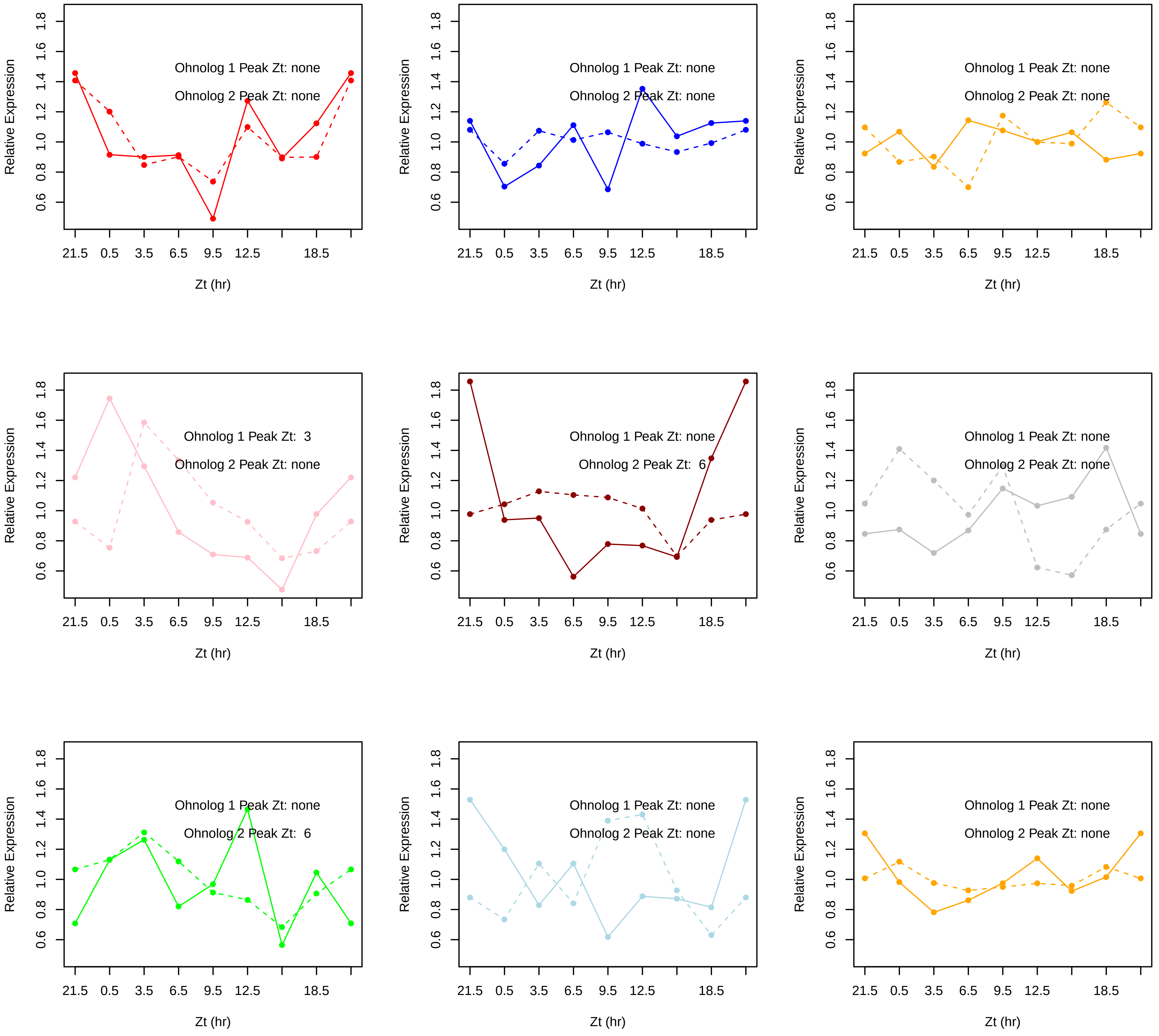

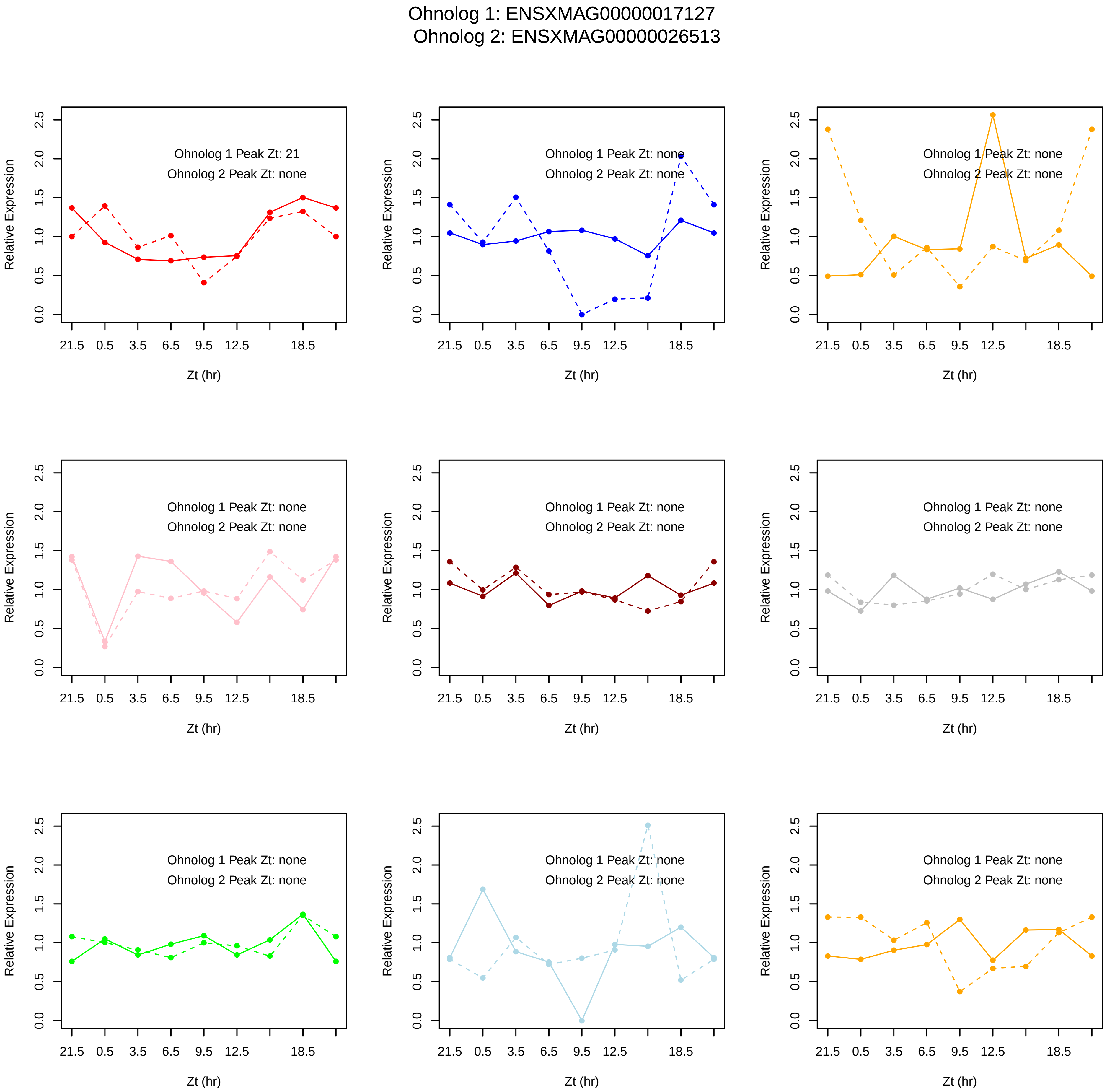
Ohnolog 1: ENSXMAG00000017127
Ohnolog 2: ENSXMAG00000026513
Ohnolog 1 Peak Zt: 21
Ohnolog 2 Peak Zt: none
Ohnolog 1 Peak Zt: none
Ohnolog 2 Peak Zt: none
Ohnolog 1 Peak Zt: none
Ohnolog 2 Peak Zt: none
Ohnolog 1 Peak Zt: none
Ohnolog 2 Peak Zt: none
Ohnolog 1 Peak Zt: none
Ohnolog 2 Peak Zt: none
Ohnolog 1 Peak Zt: none
Ohnolog 2 Peak Zt: none
Ohnolog 1 Peak Zt: none
Ohnolog 2 Peak Zt: none
Ohnolog 1 Peak Zt: none
Ohnolog 2 Peak Zt: none
Ohnolog 1 Peak Zt: none
Ohnolog 2 Peak Zt: none
Relative Expression
Zt (hr)

Ohnolog 1: ENSXMAG00000006418 mapk8ip1a
Ohnolog 2: ENSXMAG00000027053

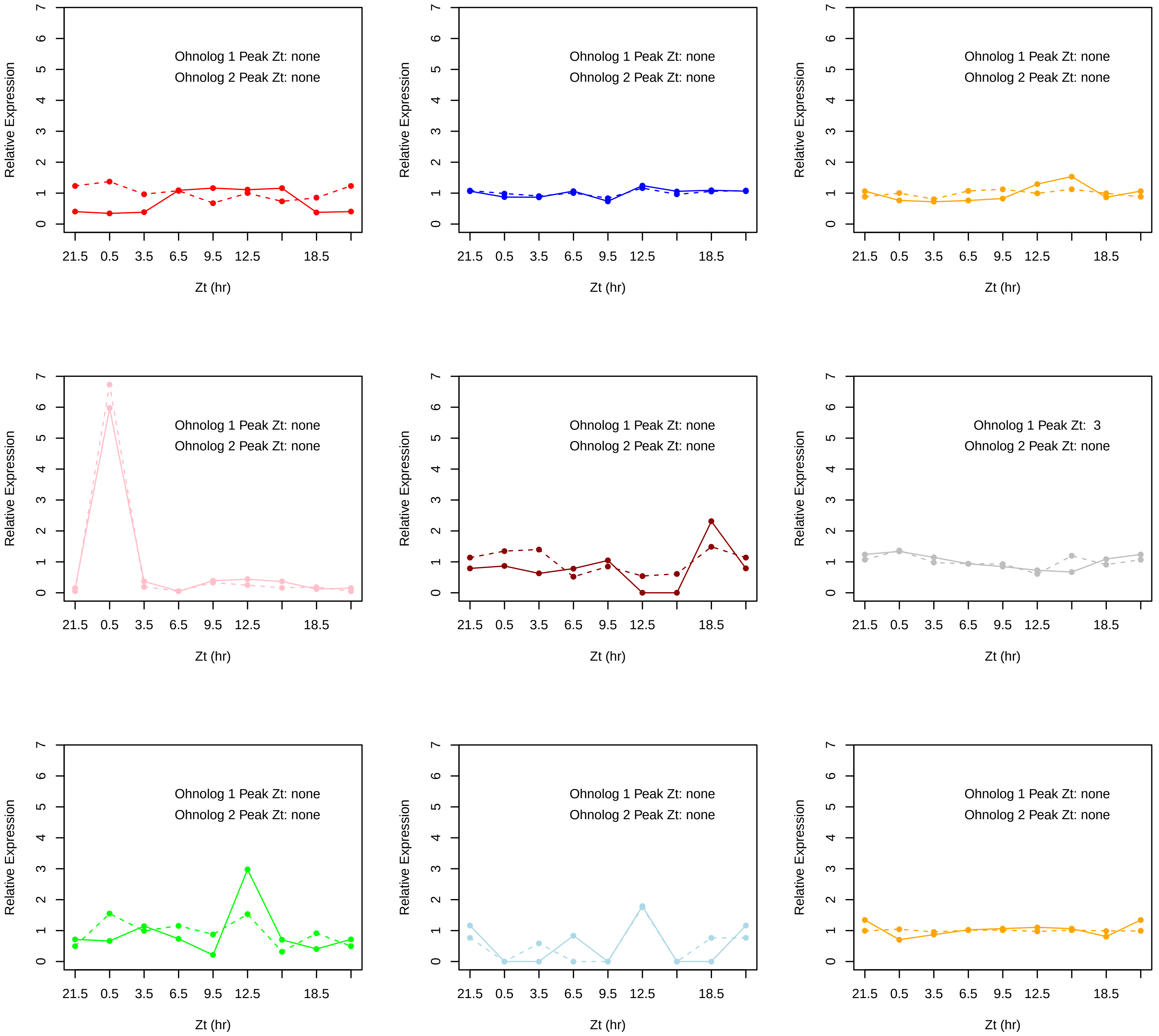

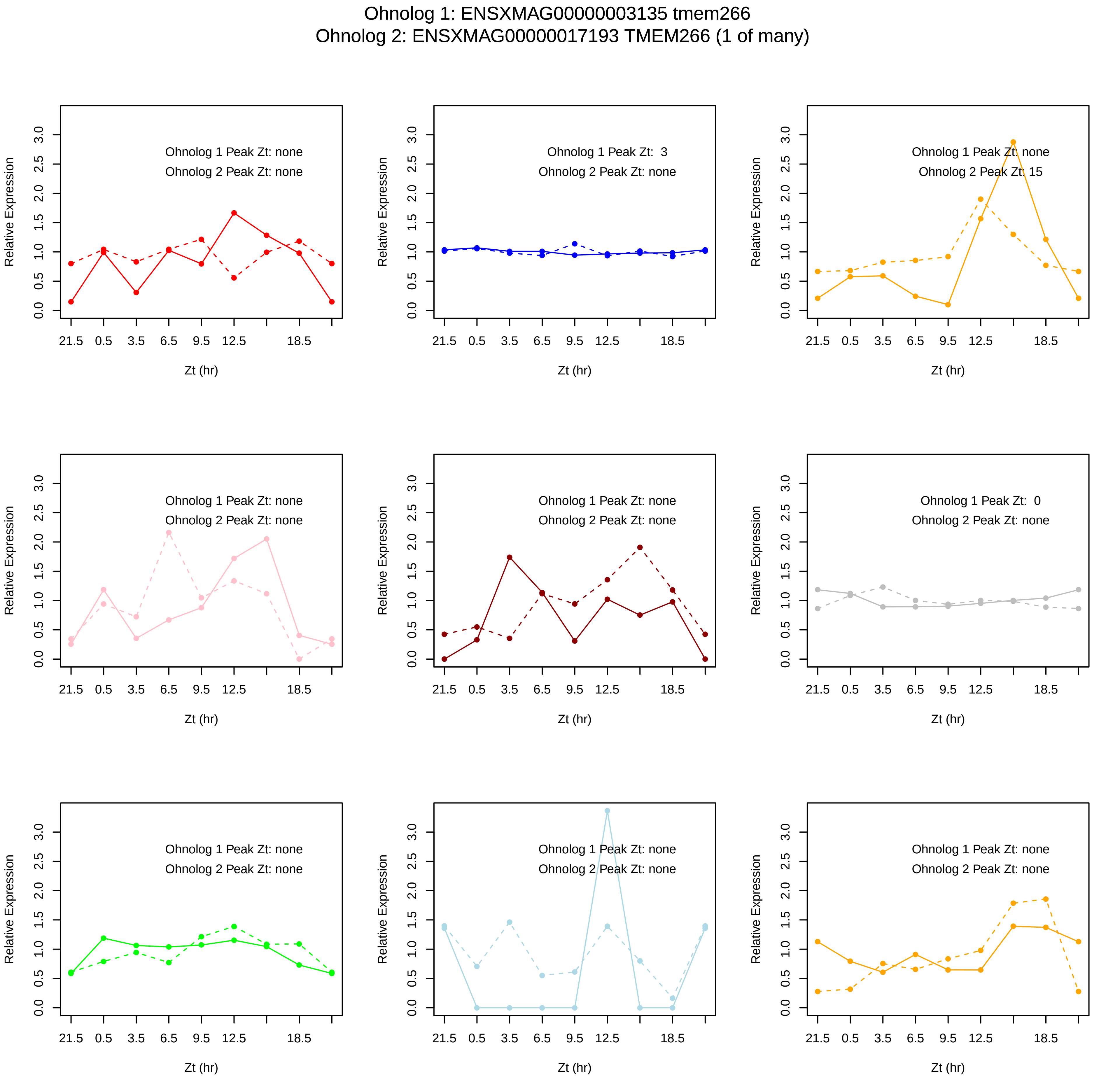

Ohnolog 1: ENSXMAG00000003135 tmem266
Ohnolog 2: ENSXMAG00000017193 TMEM266 (1 of many)
Ohnolog 1 Peak Zt: none
Ohnolog 2 Peak Zt: none
Ohnolog 1 Peak Zt: 3
Ohnolog 2 Peak Zt: none
Ohnolog 1 Peak Zt: none
Ohnolog 2 Peak Zt: 15
Ohnolog 1 Peak Zt: none
Ohnolog 2 Peak Zt: none
Ohnolog 1 Peak Zt: none
Ohnolog 2 Peak Zt: none
Ohnolog 1 Peak Zt: 0
Ohnolog 2 Peak Zt: none
Ohnolog 1 Peak Zt: none
Ohnolog 2 Peak Zt: none
Ohnolog 1 Peak Zt: none
Ohnolog 2 Peak Zt: none
Ohnolog 1 Peak Zt: none
Ohnolog 2 Peak Zt: none
Relative Expression
Zt (hr)
21.5 0.5 3.5 6.5 9.5 12.5 18.5
0.0 0.5 1.0 1.5 2.0 2.5 3.0

Ohnolog 1: ENSXMAG00000003228 roraa
Ohnolog 2: ENSXMAG00000018442

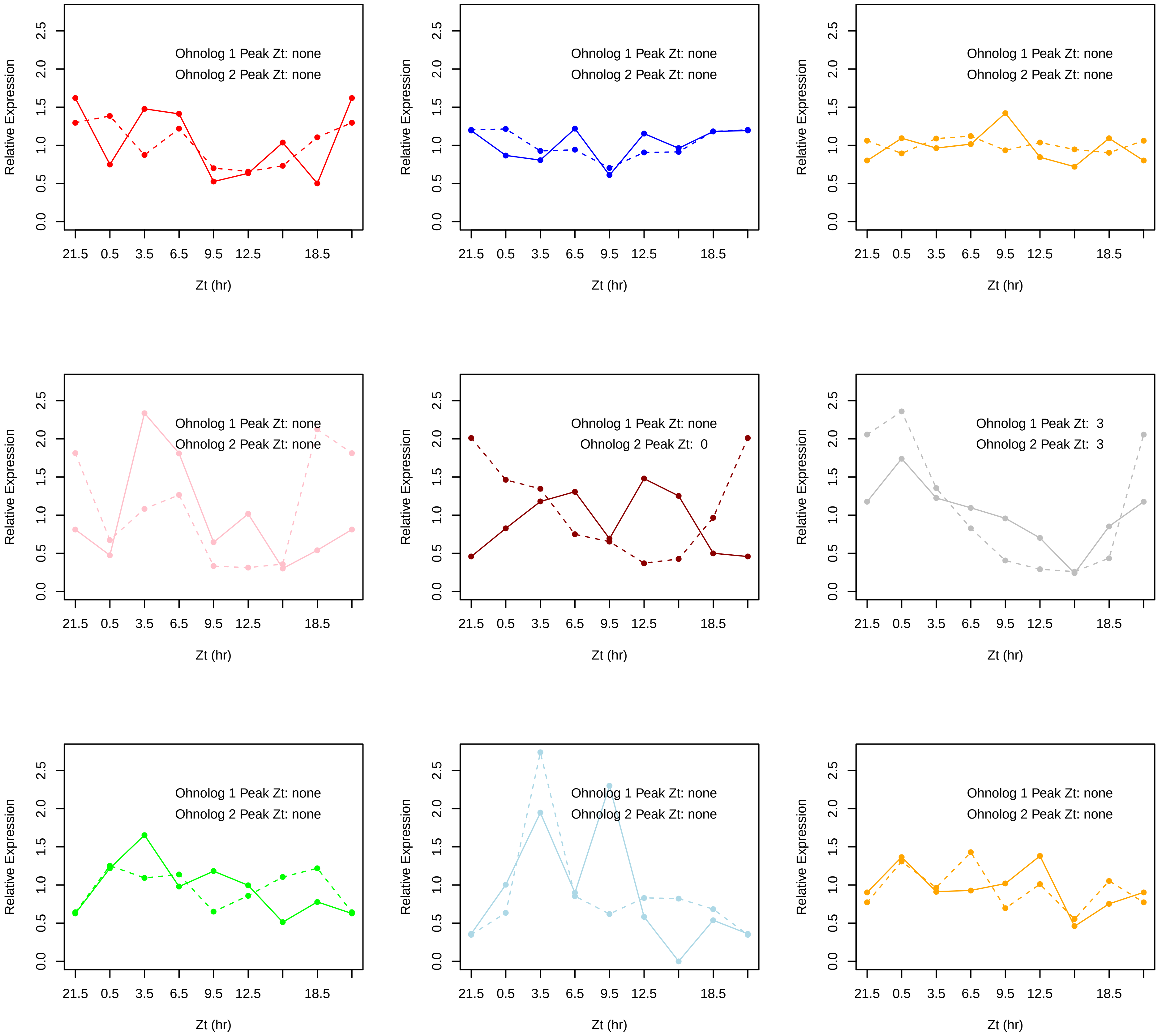

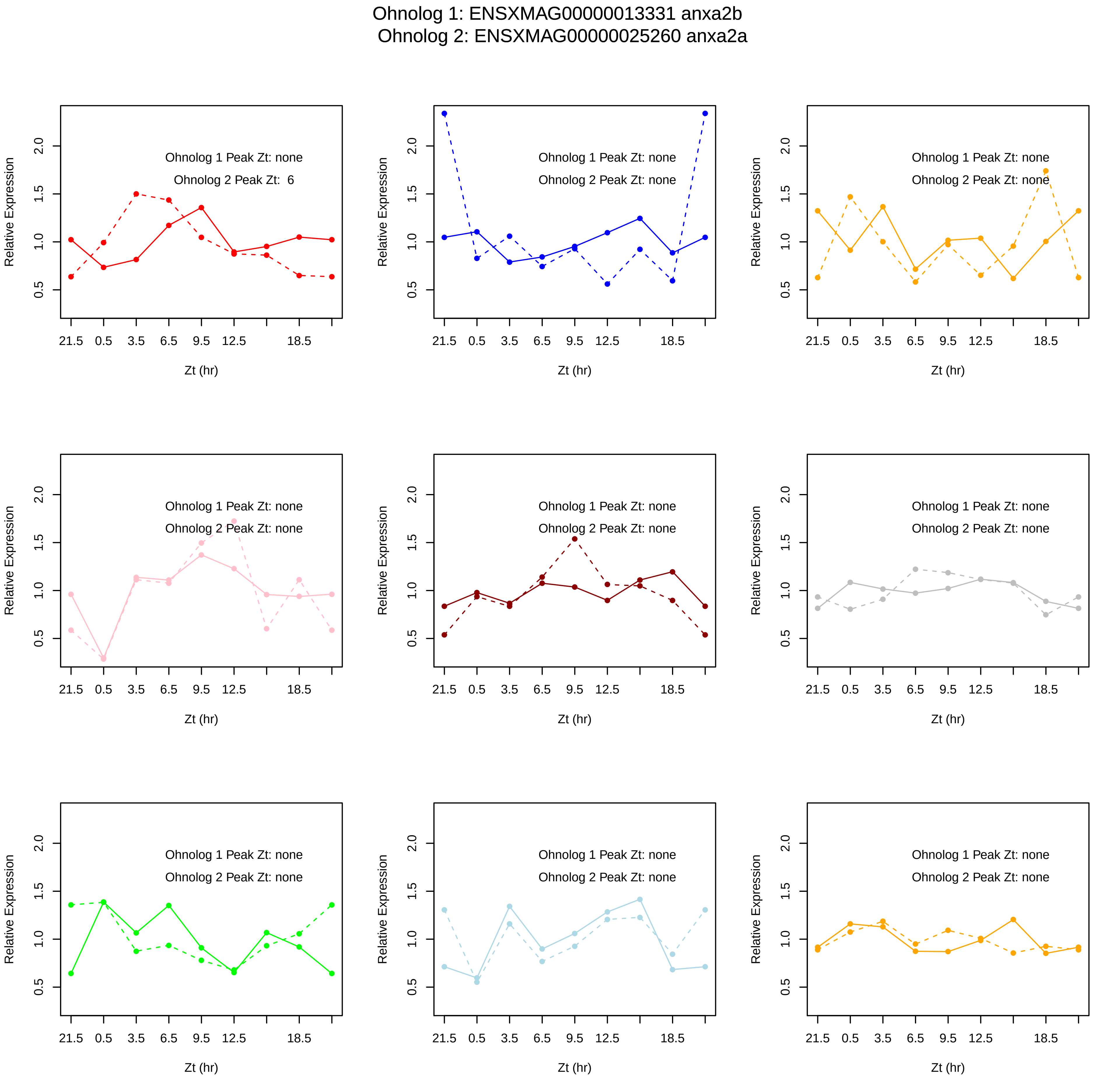
Ohnolog 1: ENSXMAG00000013331 anxa2b
Ohnolog 2: ENSXMAG00000025260 anxa2a
Ohnolog 1 Peak Zt: none
Ohnolog 2 Peak Zt: 6
Ohnolog 1 Peak Zt: none
Ohnolog 2 Peak Zt: none
Ohnolog 1 Peak Zt: none
Ohnolog 2 Peak Zt: none
Ohnolog 1 Peak Zt: none
Ohnolog 2 Peak Zt: none
Ohnolog 1 Peak Zt: none
Ohnolog 2 Peak Zt: none
Ohnolog 1 Peak Zt: none
Ohnolog 2 Peak Zt: none
Ohnolog 1 Peak Zt: none
Ohnolog 2 Peak Zt: none
Ohnolog 1 Peak Zt: none
Ohnolog 2 Peak Zt: none
Ohnolog 1 Peak Zt: none
Ohnolog 2 Peak Zt: none
Relative Expression
Zt (hr)

Ohnolog 1: ENSXMAG00000000226
Ohnolog 2: ENSXMAG00000010902 l1cama

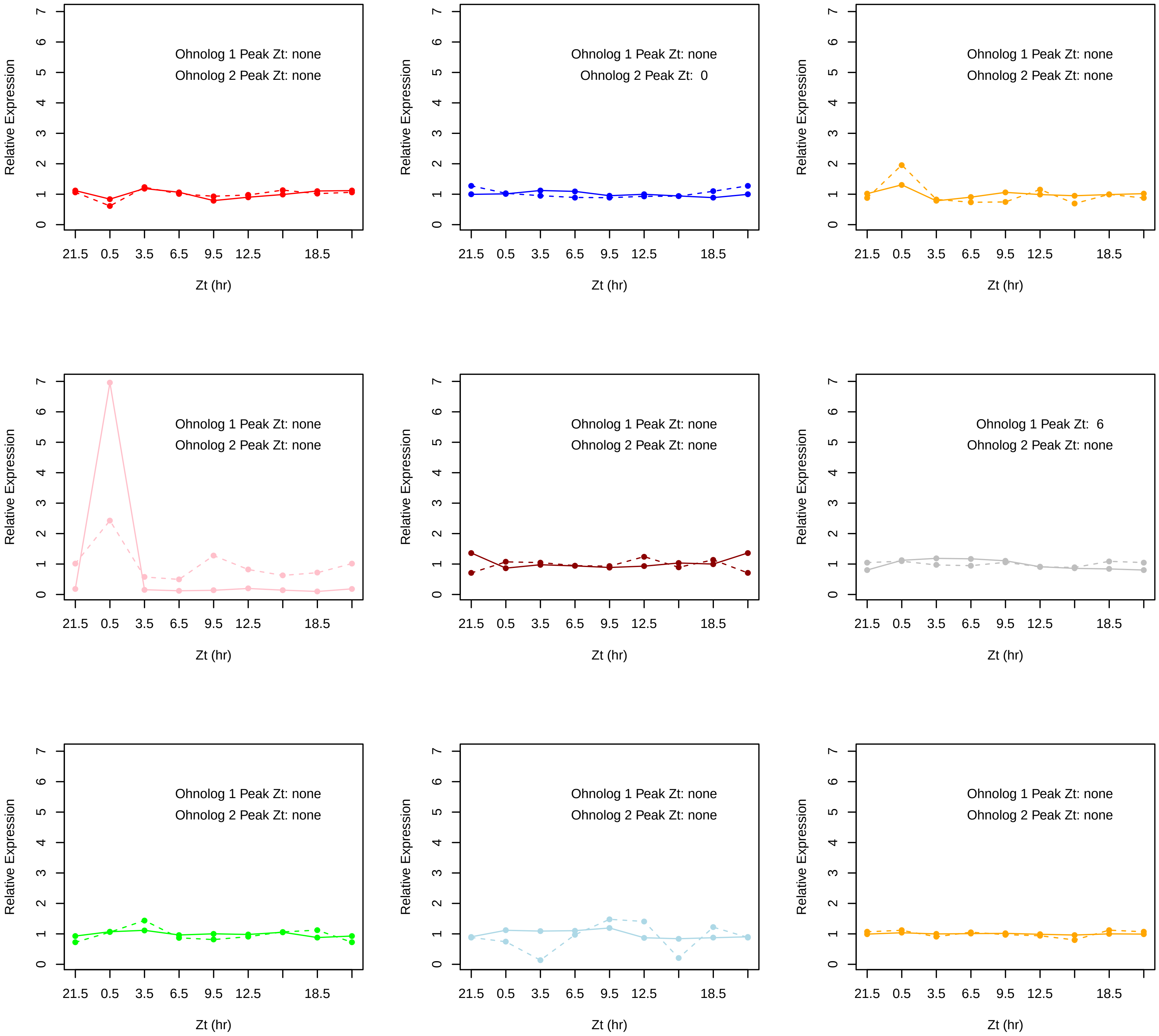

Ohnolog 1: ENSXMAG00000001773 ppm1na
Ohnolog 2: ENSXMAG00000012008 ppm1nb

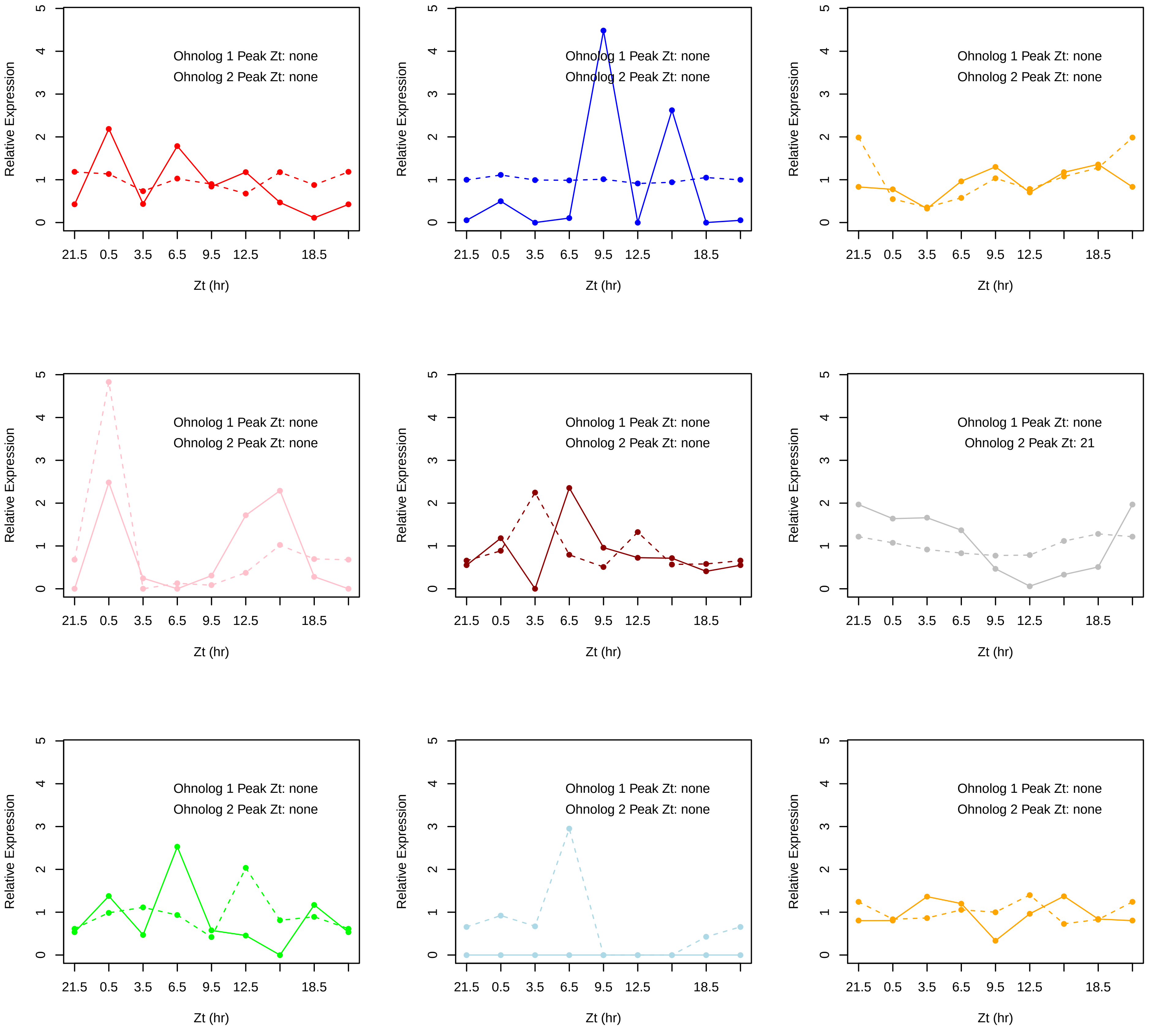

Ohnolog 1: ENSXMAG00000000900 bmp1a
Ohnolog 2: ENSXMAG00000003972 bmp1b

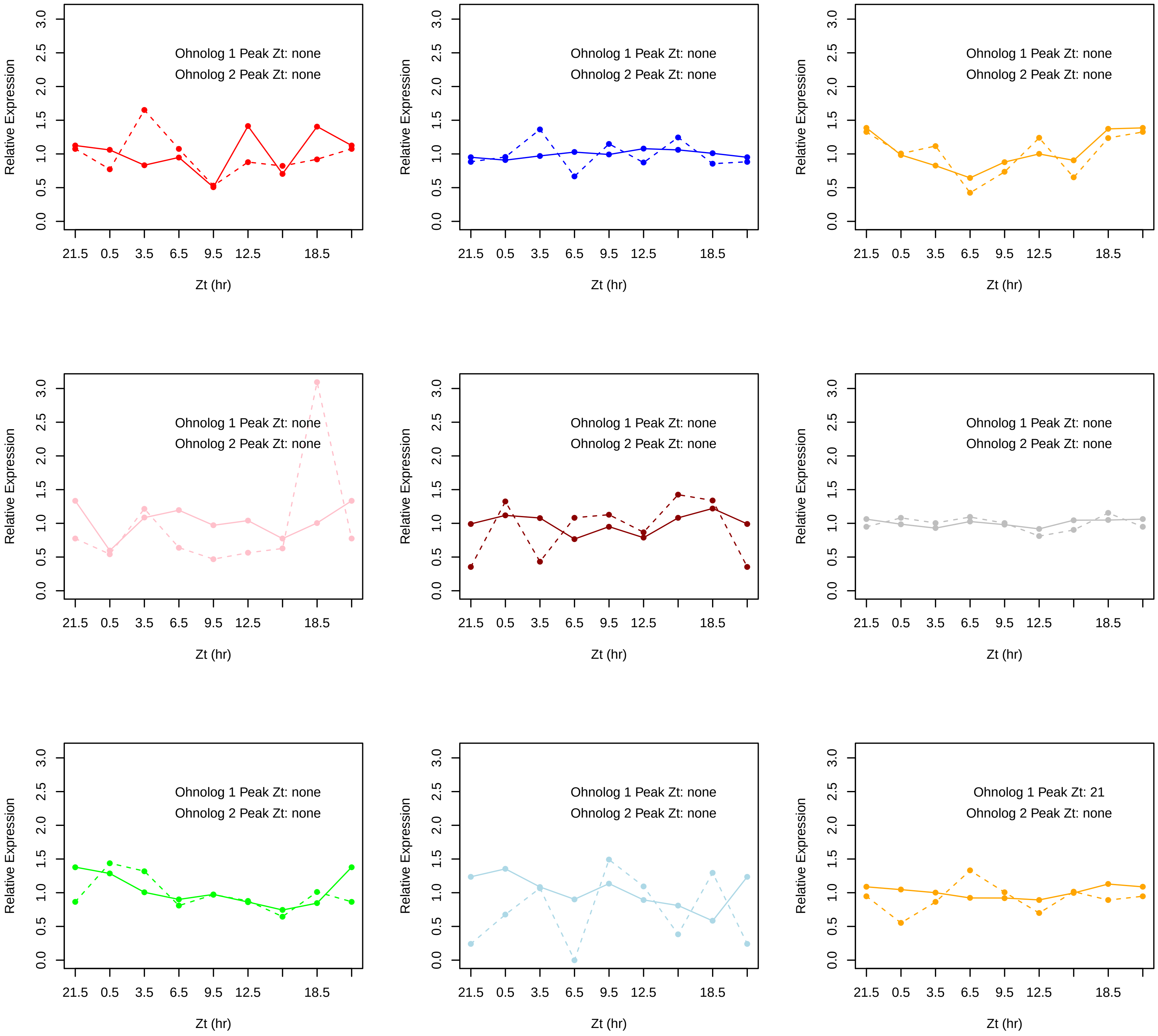

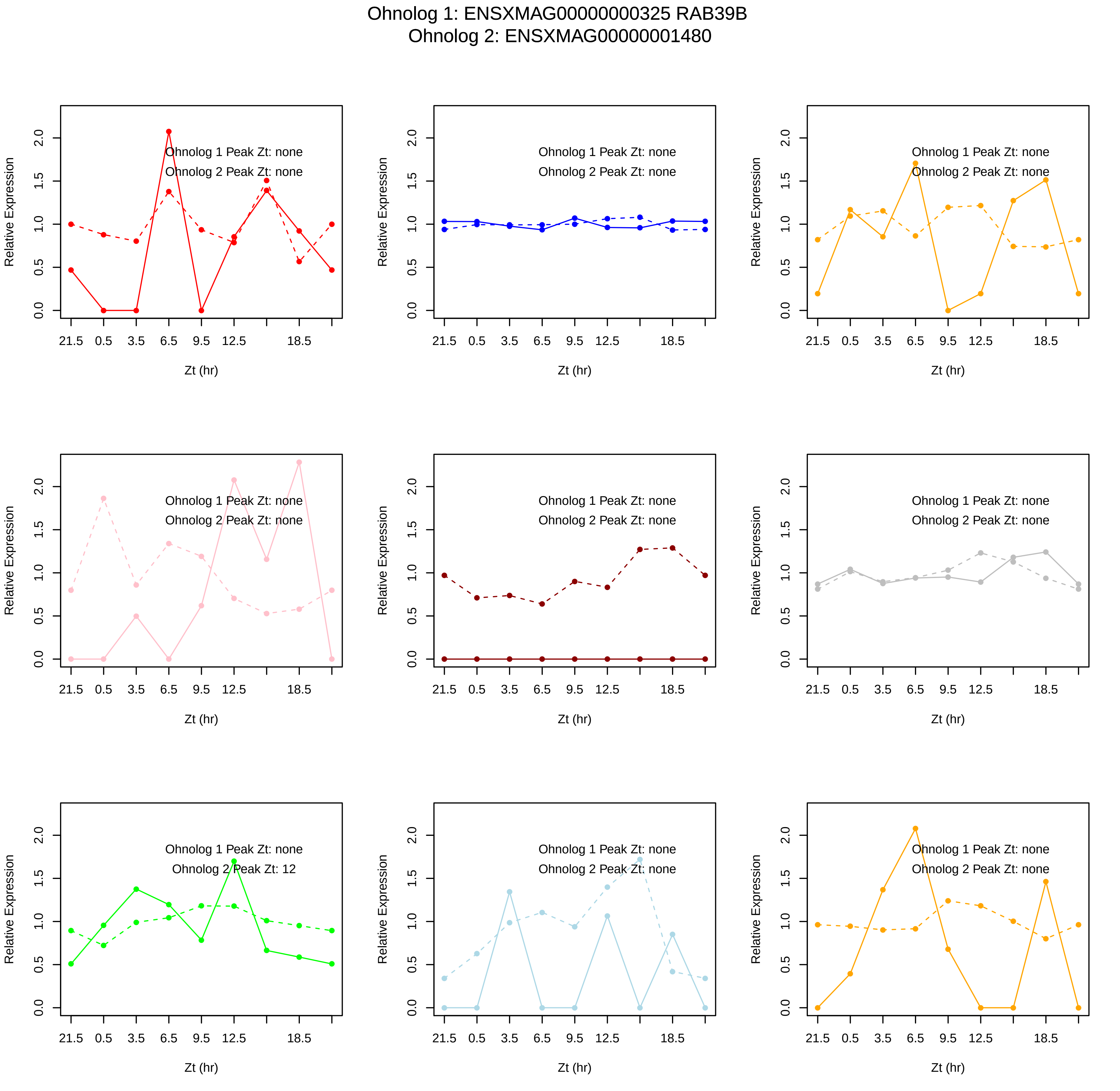

Ohnolog 1: ENSXMAG00000000325 RAB39B
Ohnolog 2: ENSXMAG00000001480
Ohnolog 1 Peak Zt: none
Ohnolog 2 Peak Zt: none
Ohnolog 2 Peak Zt: 12
Relative Expression
Zt (hr)
0.0
0.5
1.0
1.5
2.0
21.5
0.5
3.5
6.5
9.5
12.5
18.5

Ohnolog 1: ENSXMAG00000012952 scamp2
Ohnolog 2: ENSXMAG00000025884 SCAMP2

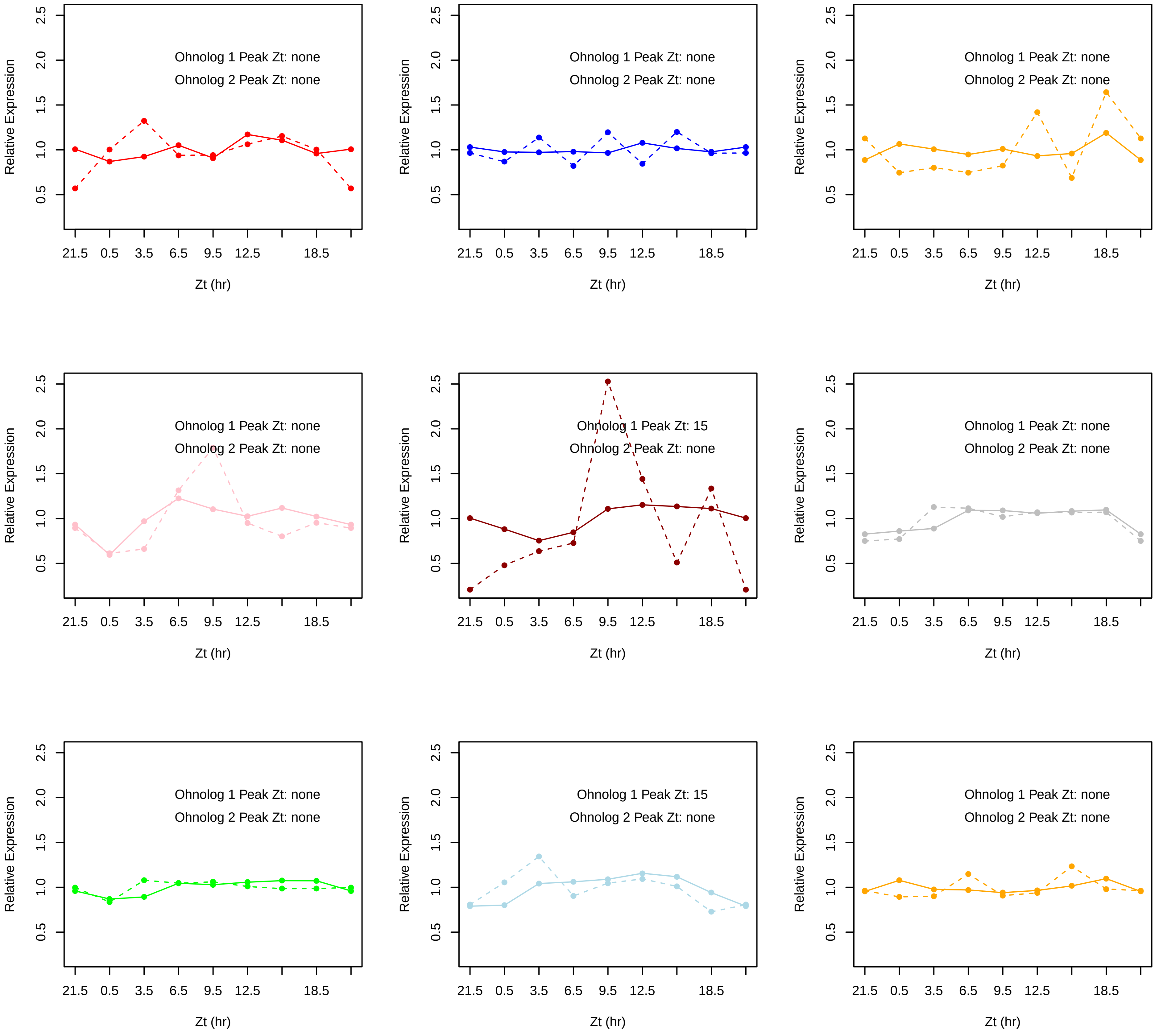

Ohnolog 1: ENSXMAG00000001846 AAK1 (1 of many)
Ohnolog 2: ENSXMAG00000006860 aak1b

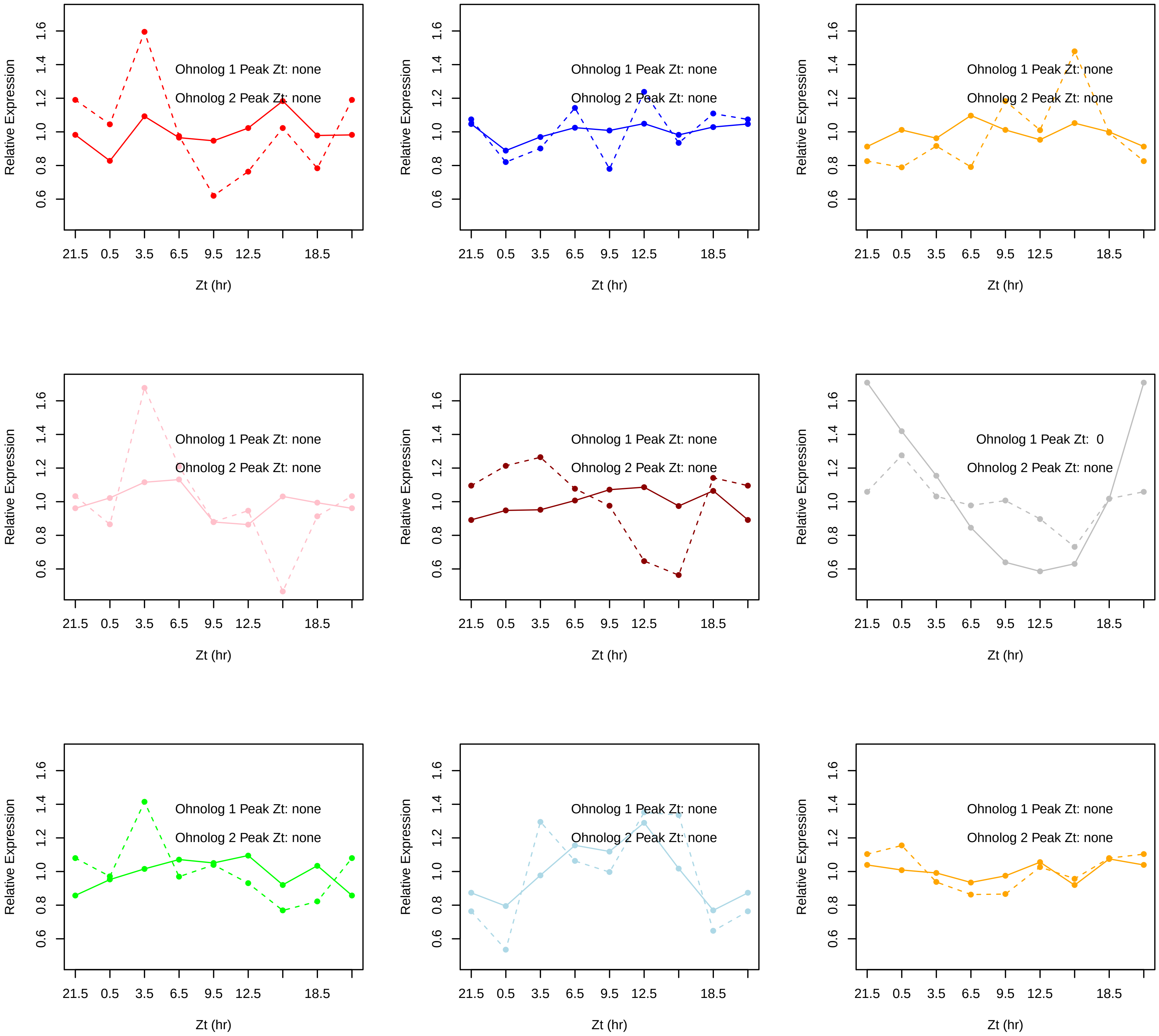

Ohnolog 1: ENSXMAG00000010311
Ohnolog 2: ENSXMAG00000015842 nr2e3

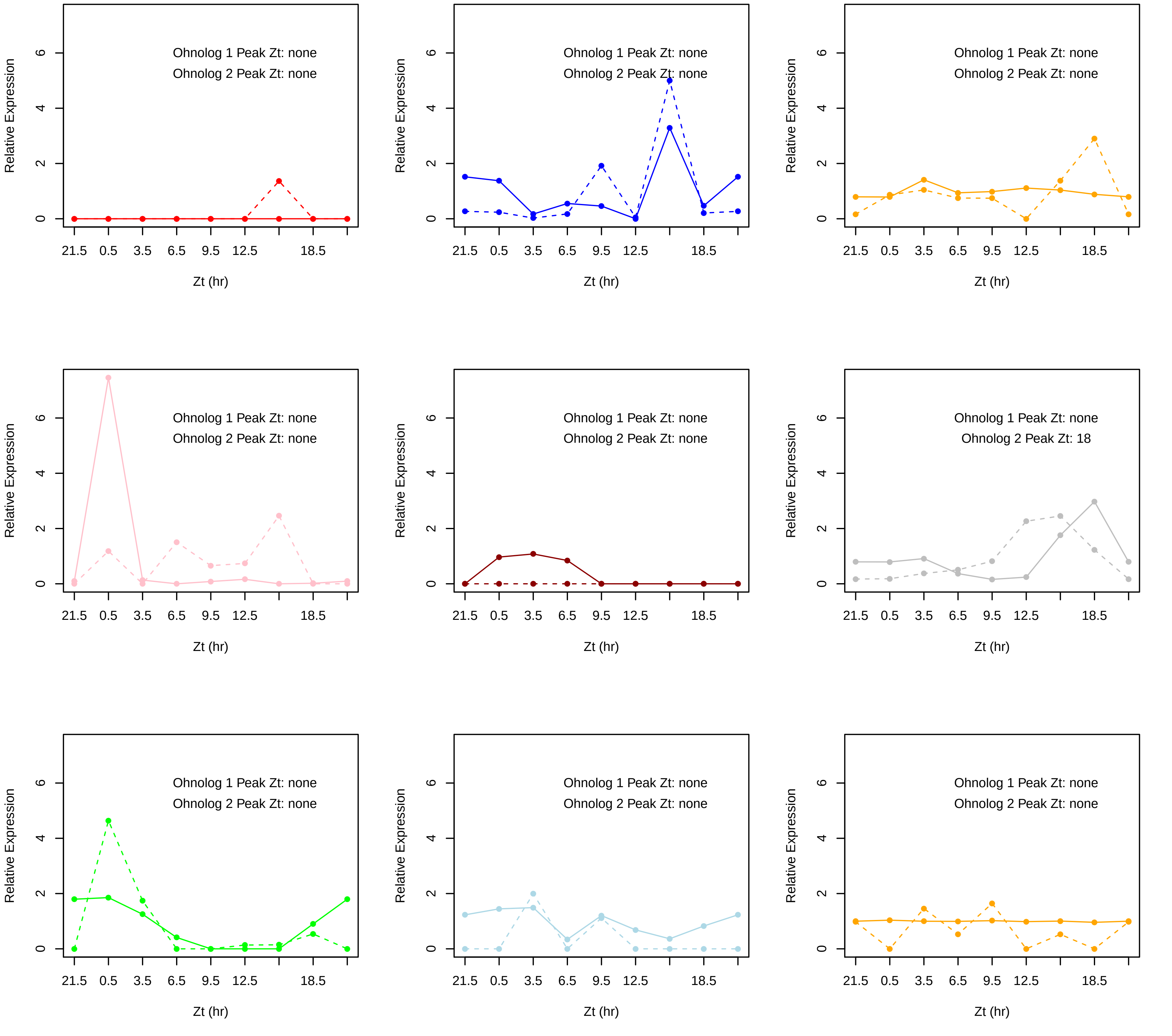

Ohnolog 1: ENSXMAG00000000027 slc25a44b
Ohnolog 2: ENSXMAG00000002204 slc25a44a

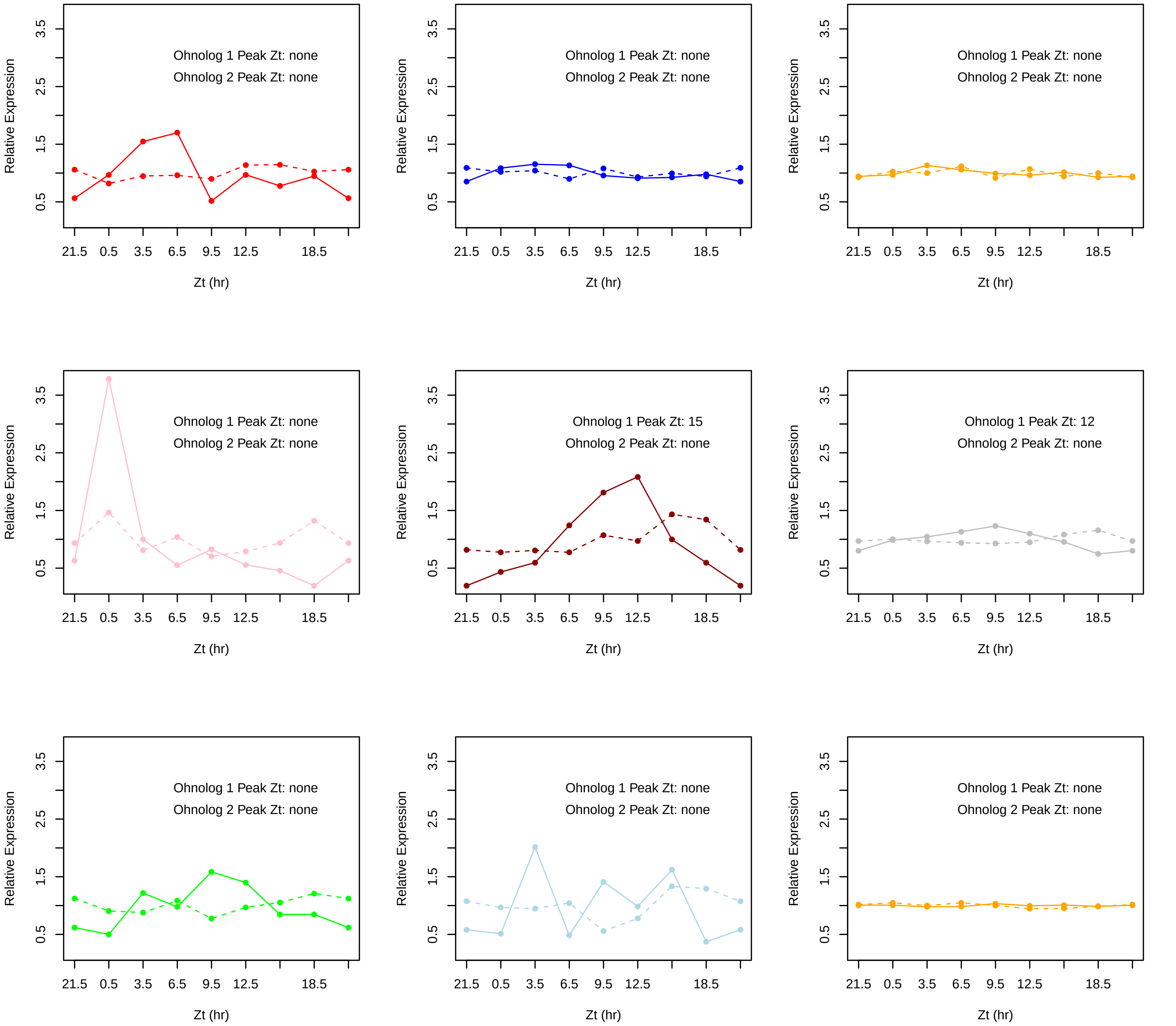

Ohnolog 1: ENSXMAG00000002217 crabp1b
Ohnolog 2: ENSXMAG00000027139

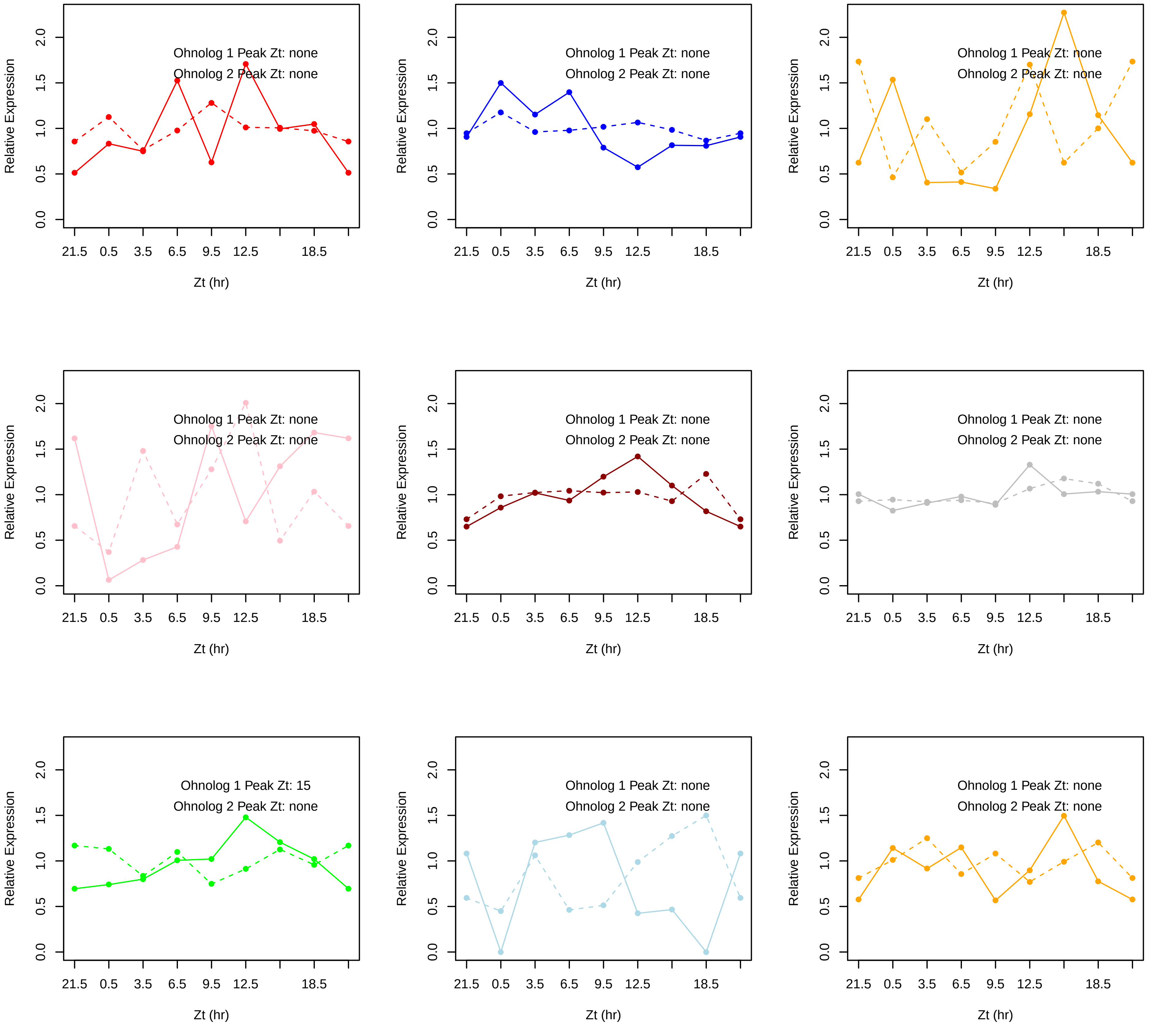

Ohnolog 1: ENSXMAG00000017435
Ohnolog 2: ENSXMAG00000023145

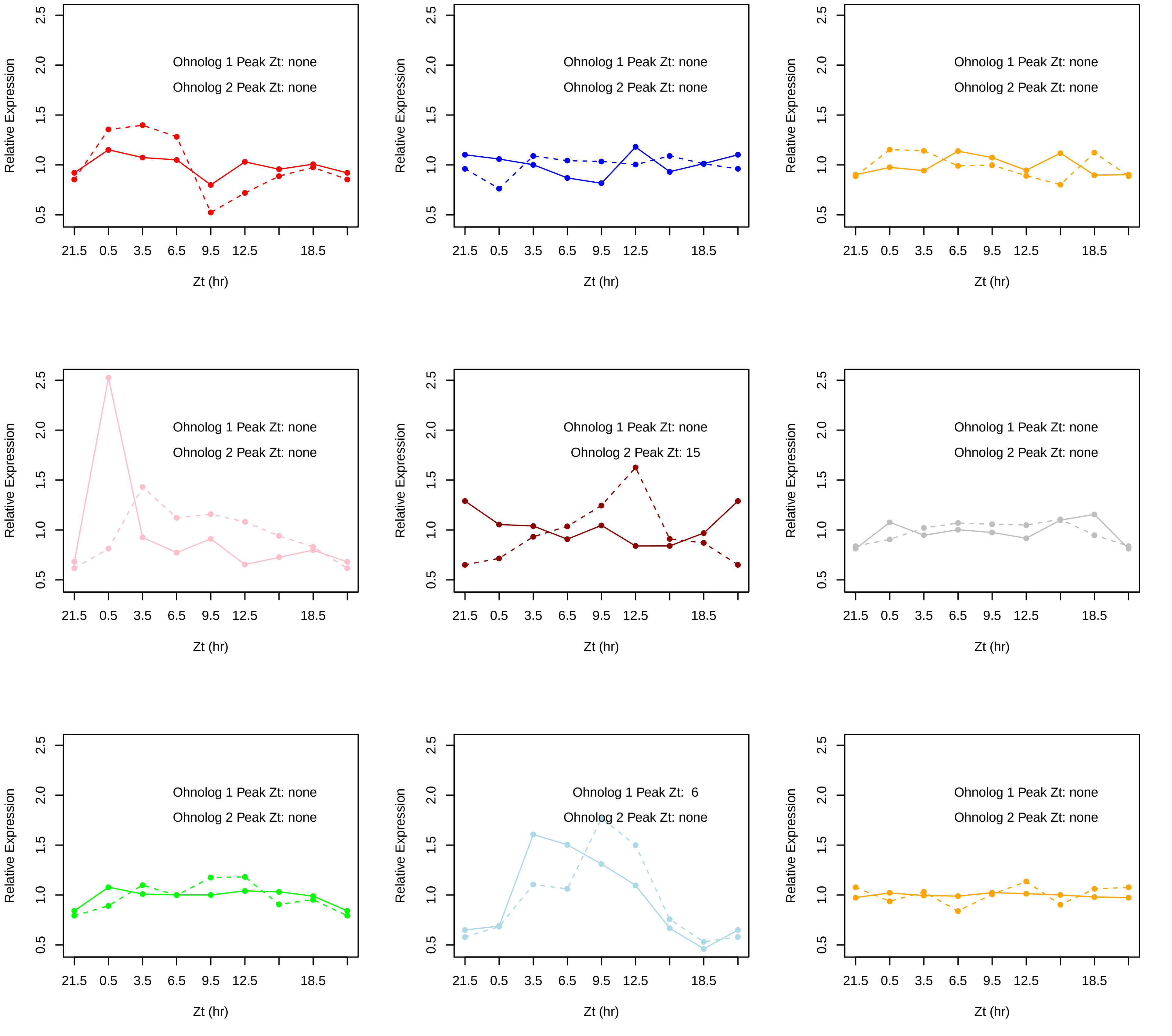

Ohnolog 1: ENSXMAG00000023425 cox5aa
Ohnolog 2: ENSXMAG00000029675

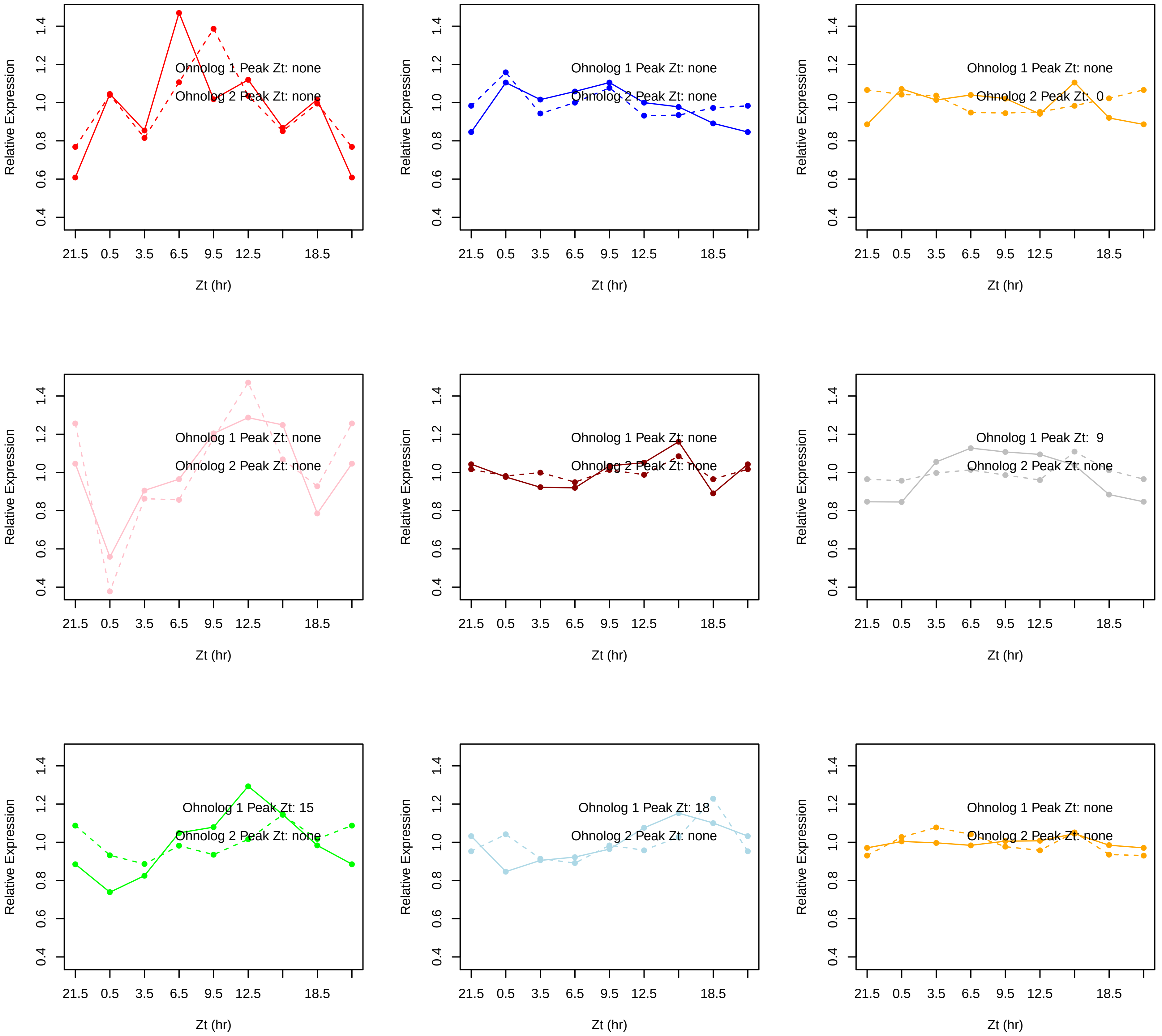

Ohnolog 1: ENSXMAG00000000404 mcf2b
Ohnolog 2: ENSXMAG00000013325 mcf2a

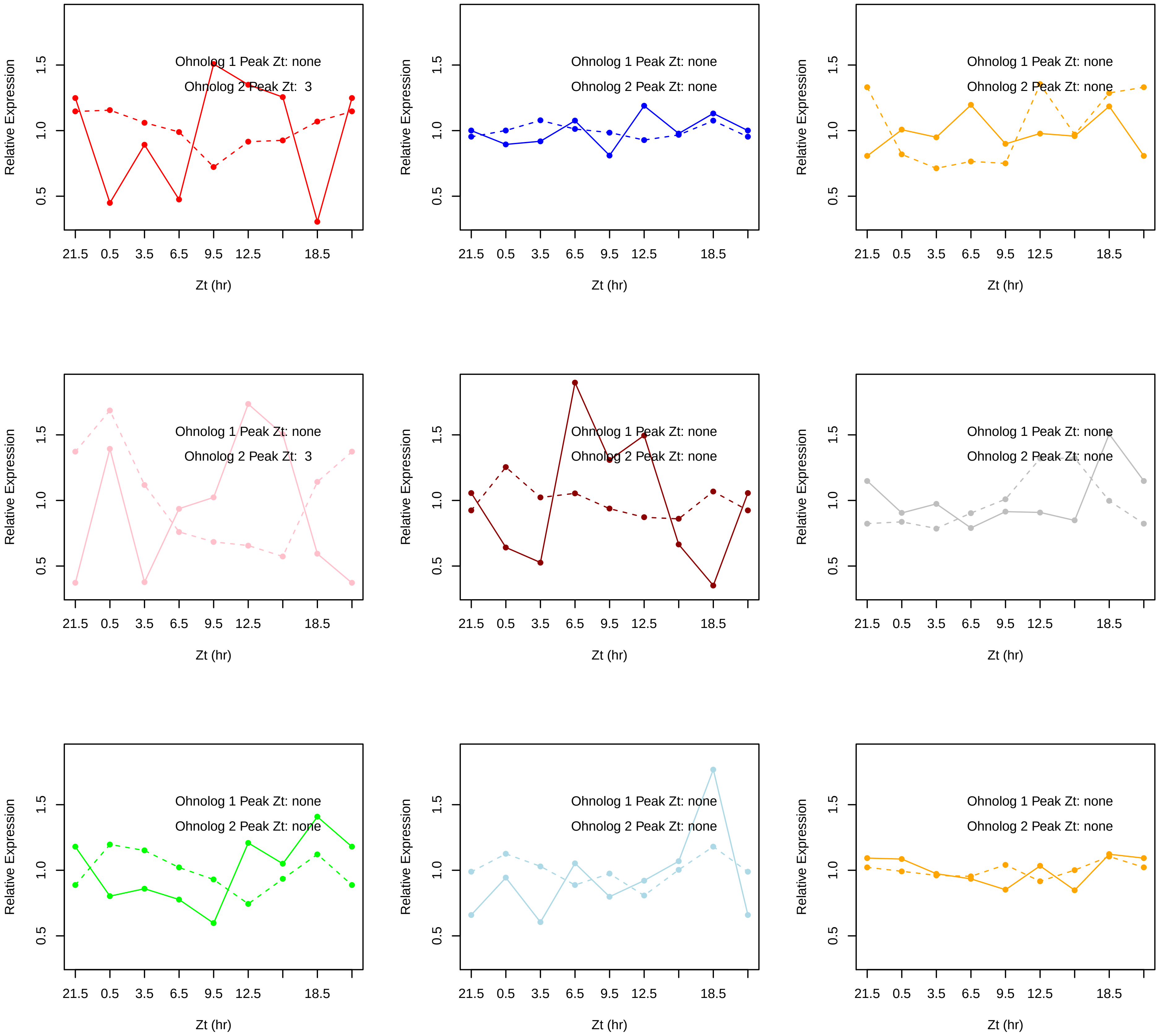

Ohnolog 1: ENSXMAG00000001482 arntl2
Ohnolog 2: ENSXMAG00000008892 ARNTL2 (1 of many)

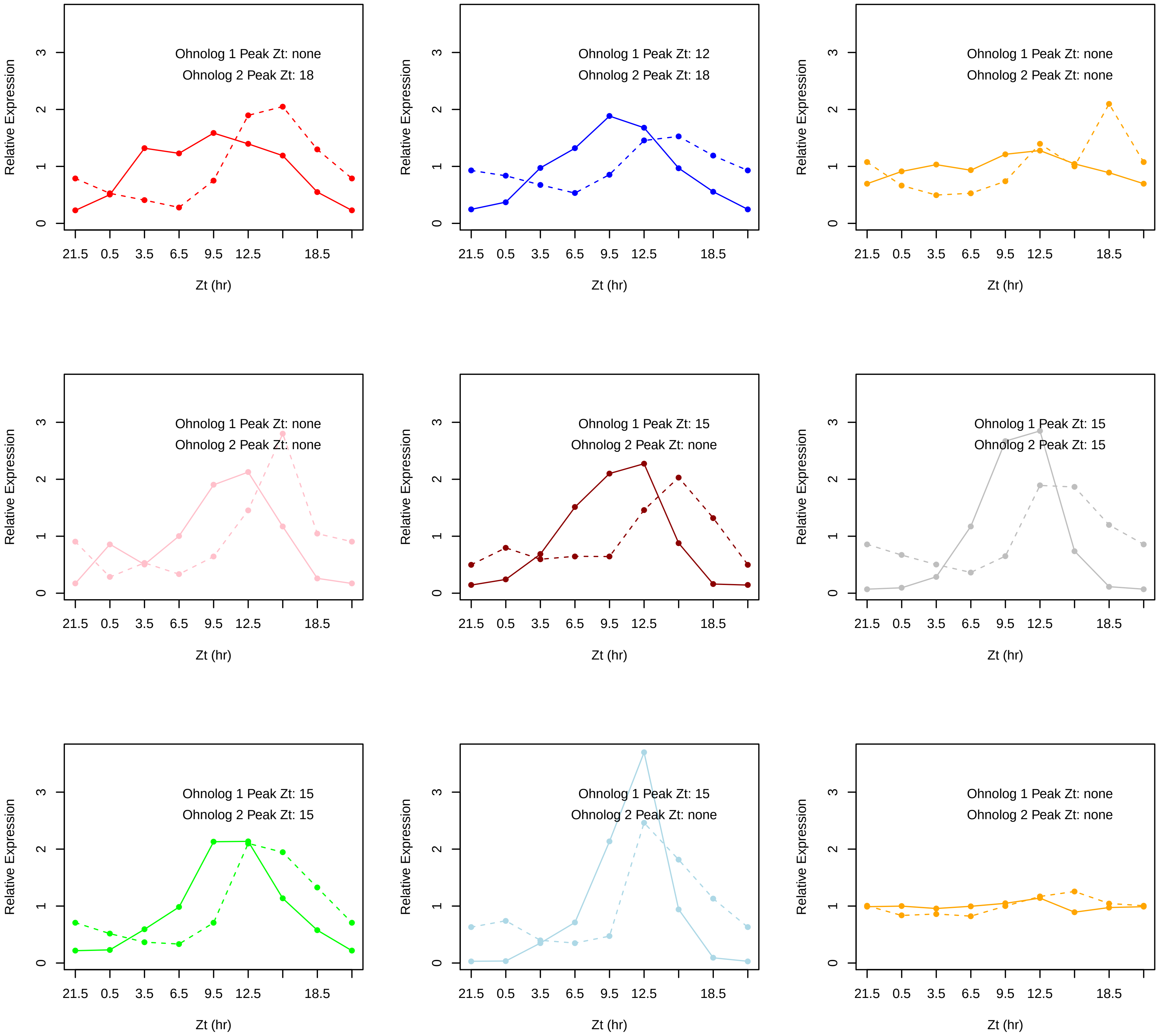

Ohnolog 1: ENSXMAG00000002364 ank1b
Ohnolog 2: ENSXMAG00000017872

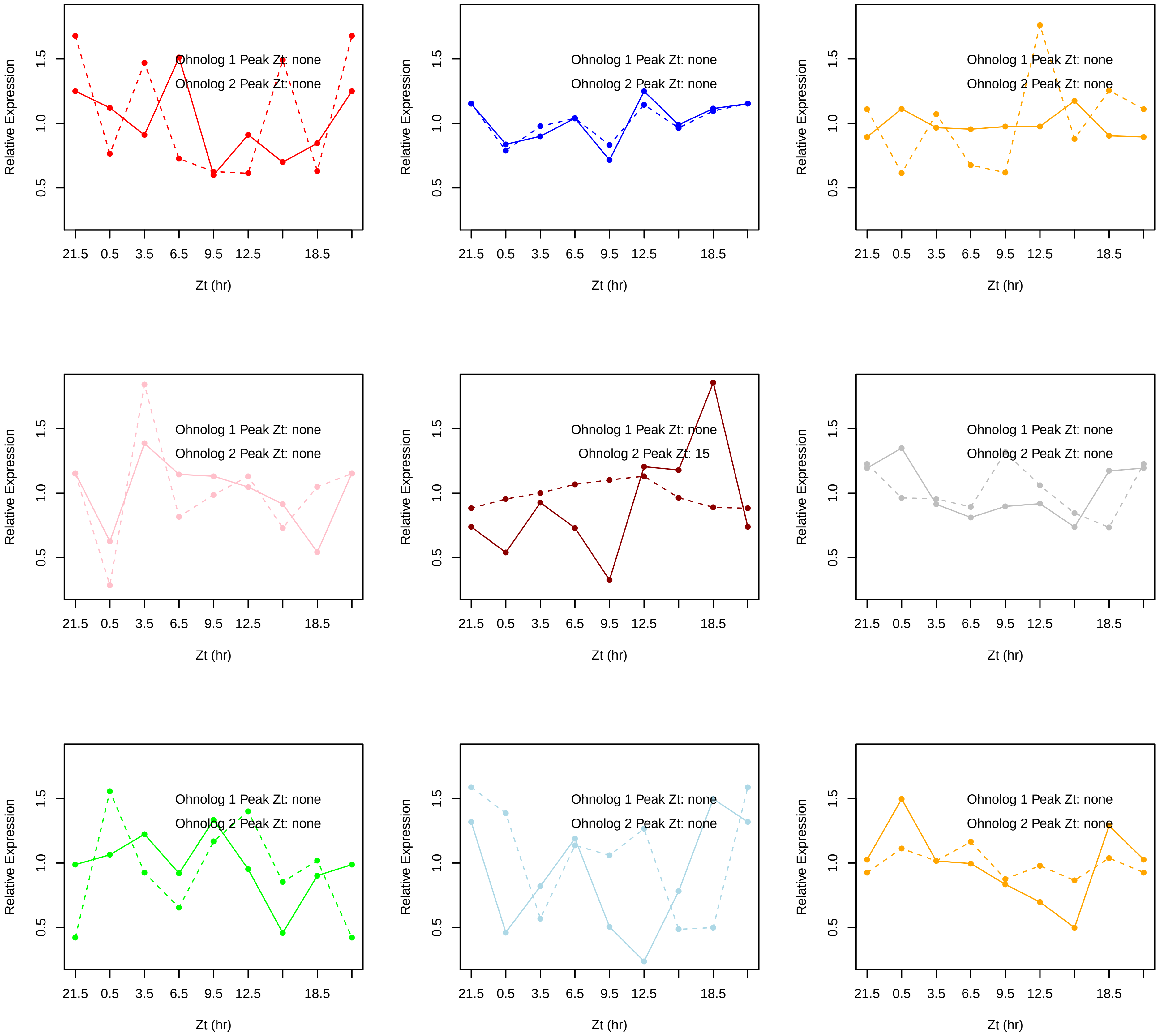

Ohnolog 1: ENSXMAG00000005432 lman2la
Ohnolog 2: ENSXMAG00000024814 lman2lb

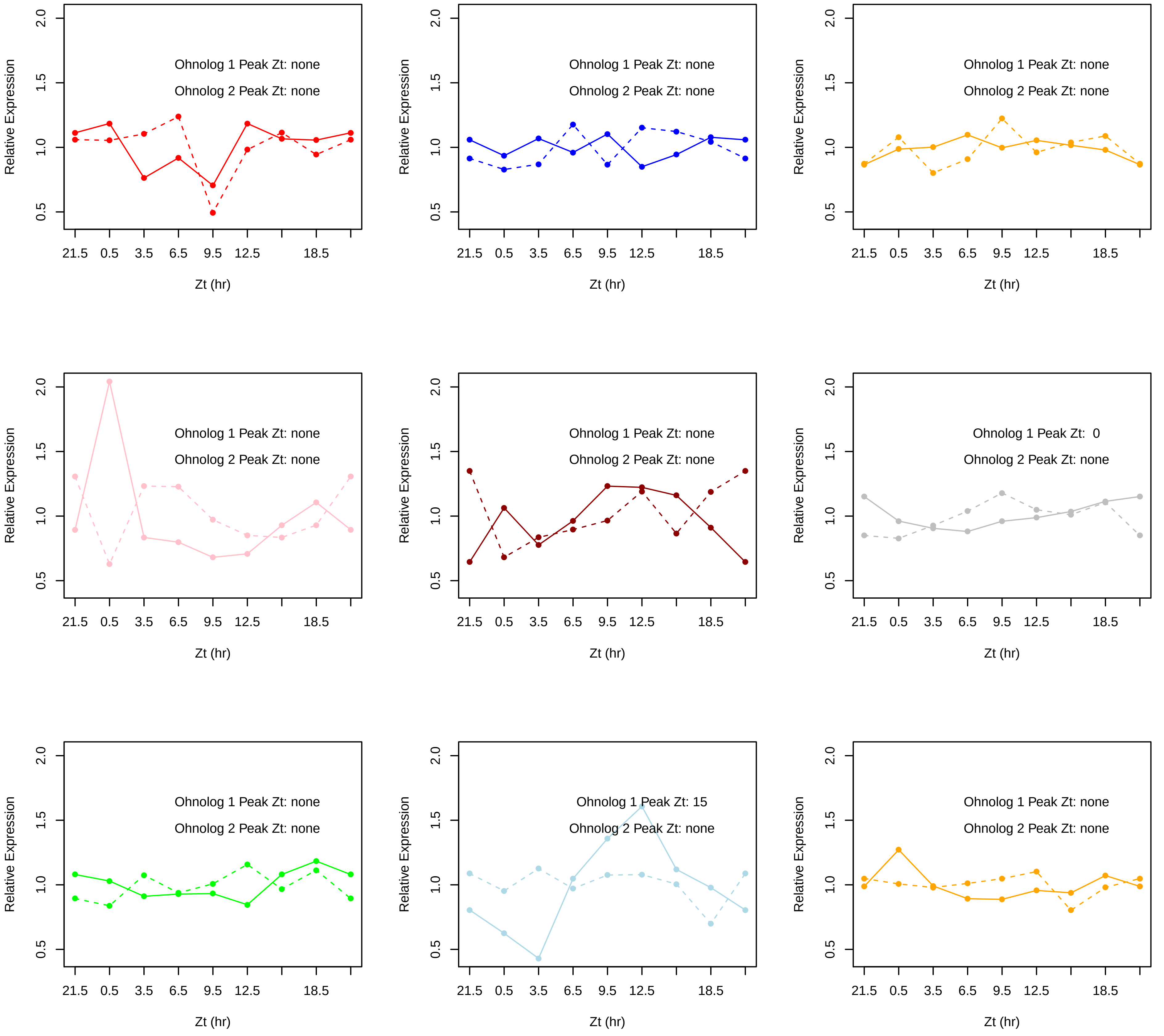

Ohnolog 1: ENSXMAG00000001744 cry1ab
Ohnolog 2: ENSXMAG00000017466

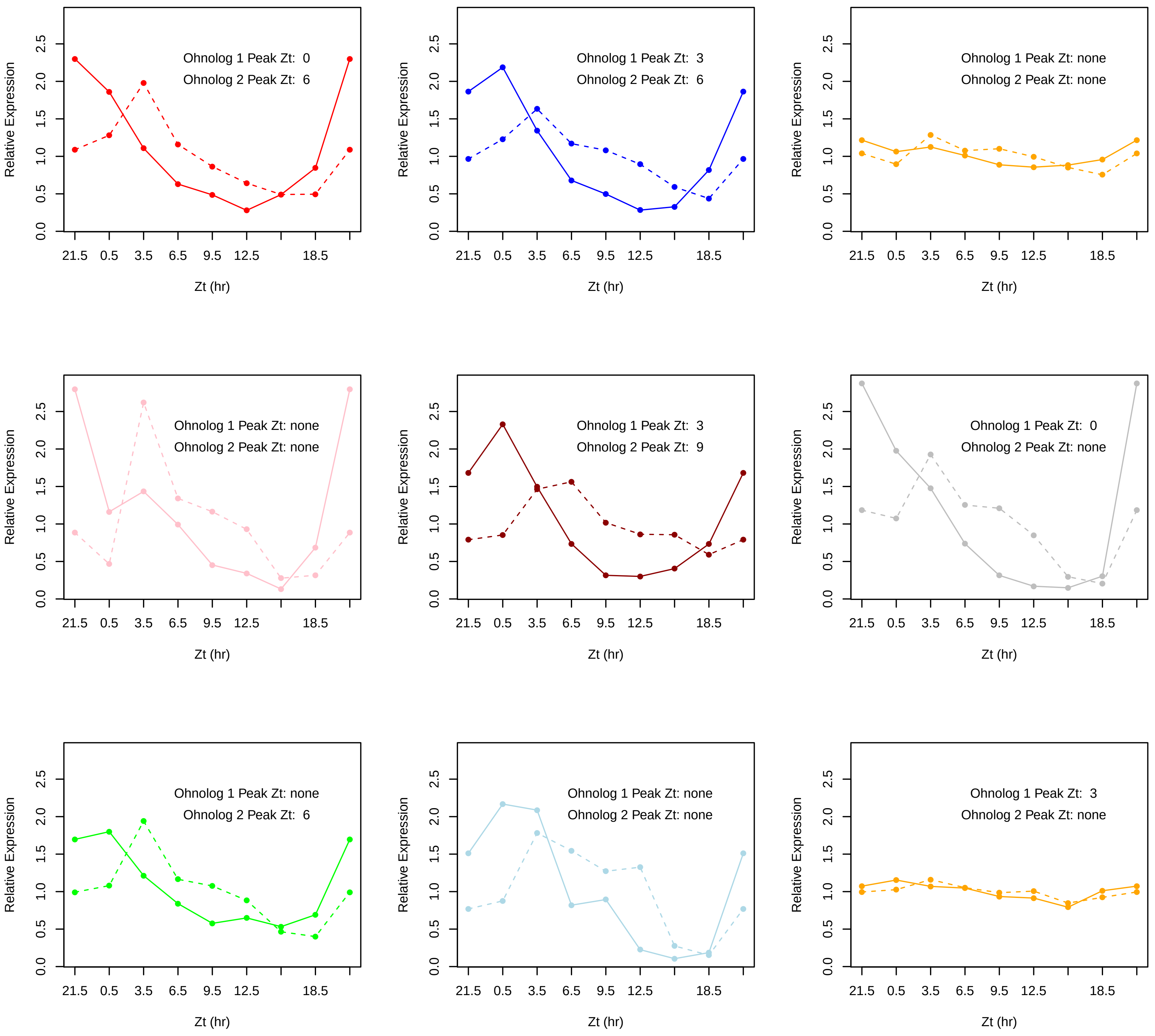

Ohnolog 1: ENSXMAG00000002689
Ohnolog 2: ENSXMAG00000025444

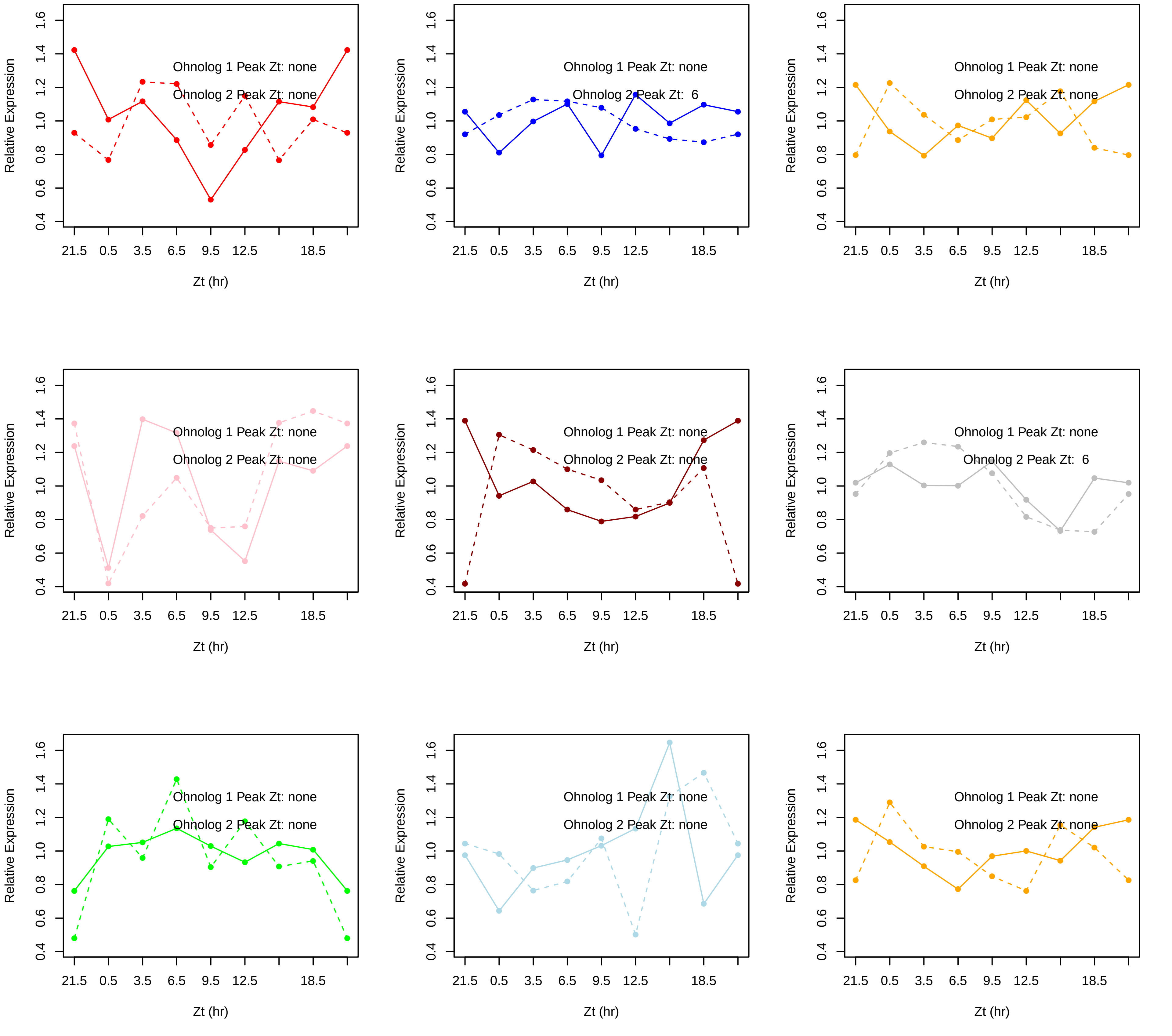

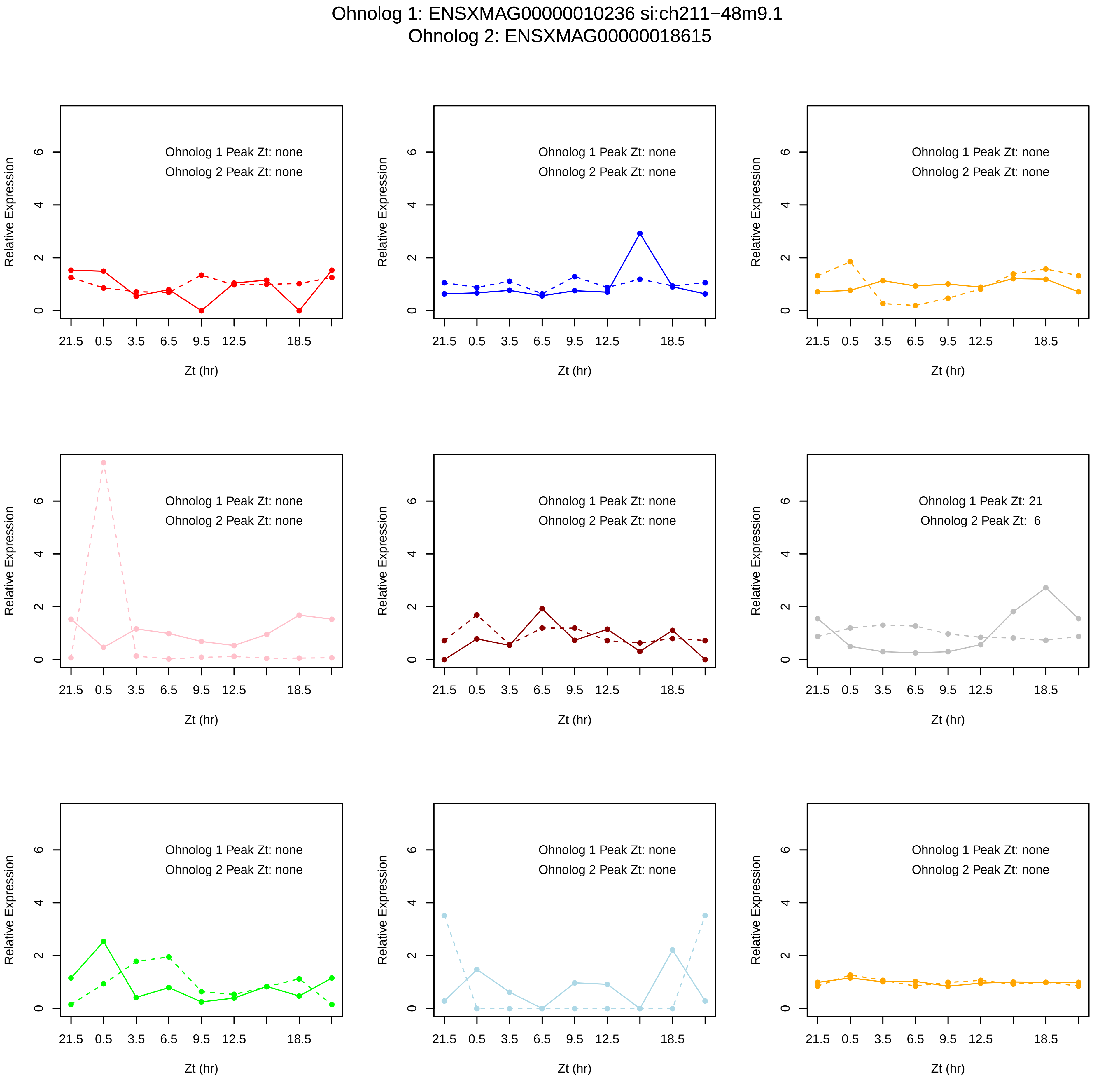
Ohnolog 1: ENSXMAG00000010236 si:ch211−48m9.1
Ohnolog 2: ENSXMAG00000018615
Ohnolog 1 Peak Zt: none
Ohnolog 2 Peak Zt: none
Ohnolog 1 Peak Zt: 21
Ohnolog 2 Peak Zt: 6
Relative Expression
Zt (hr)
21.5
0.5
3.5
6.5
9.5
12.5
18.5

Ohnolog 1: ENSXMAG00000001294
Ohnolog 2: ENSXMAG00000016966 ldhbb

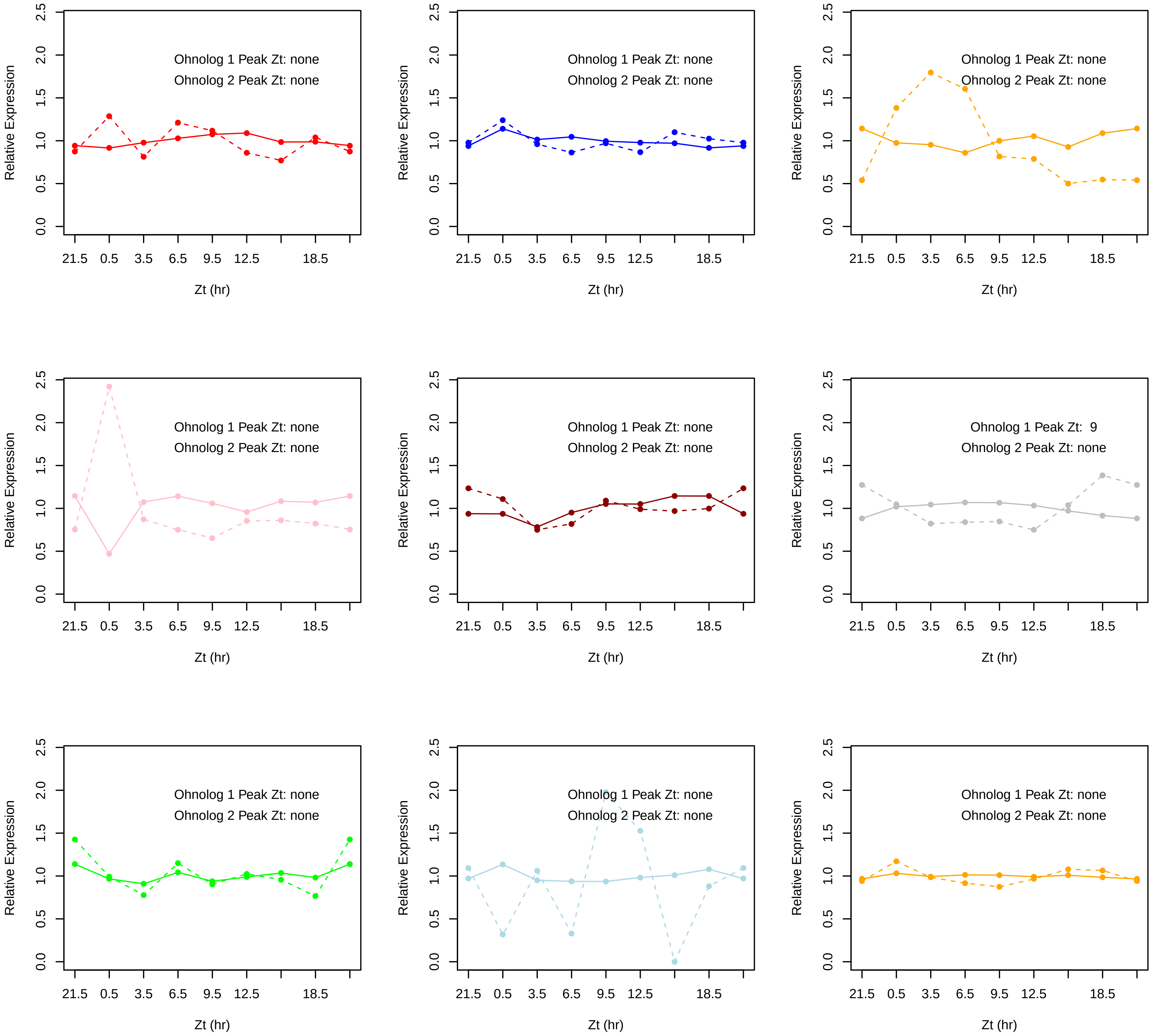

Ohnolog 1: ENSXMAG00000001328 aebp2
Ohnolog 2: ENSXMAG00000026296 AEBP2

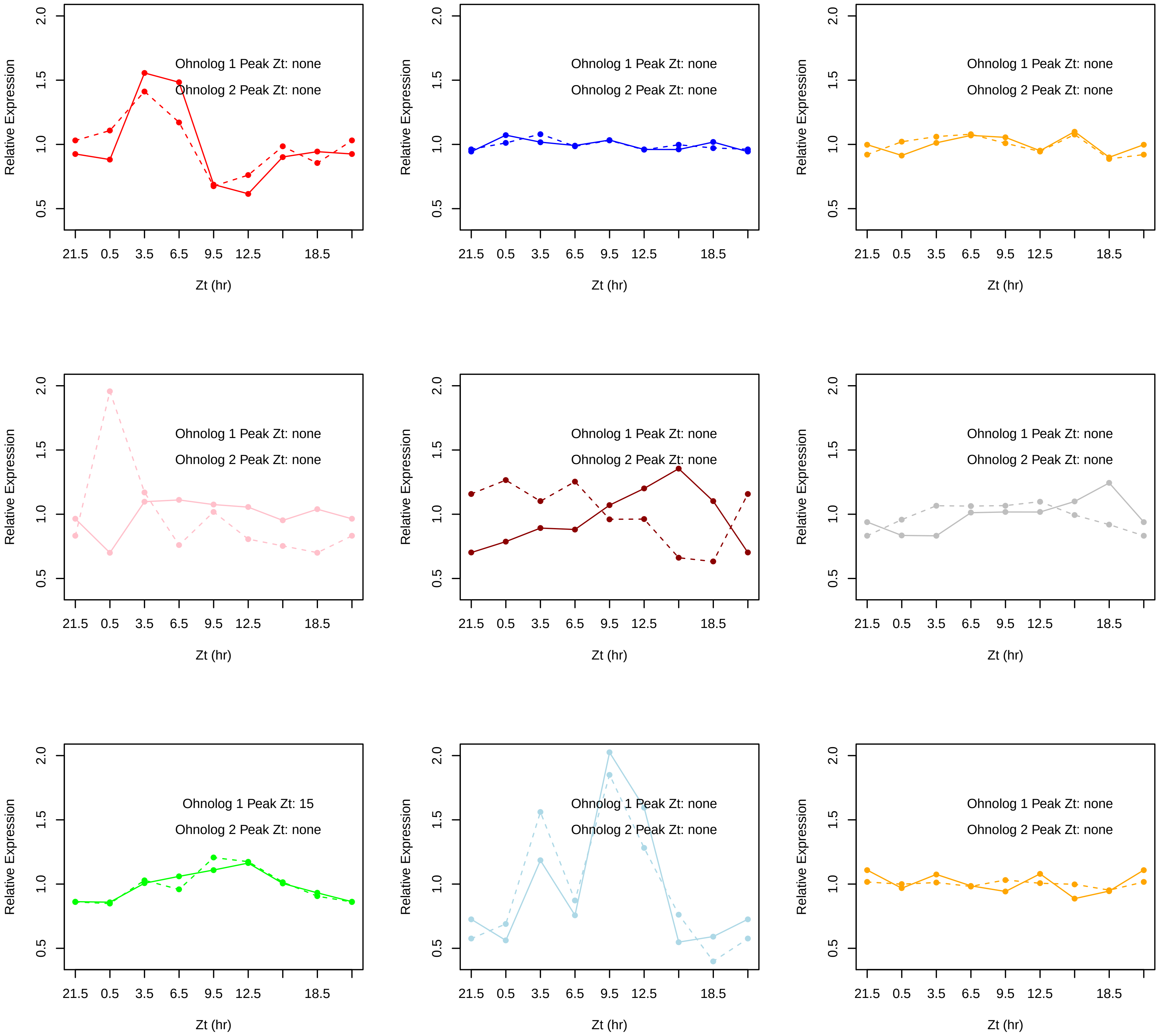

Ohnolog 1: ENSXMAG00000001309
Ohnolog 2: ENSXMAG00000007144 PLEKHA5

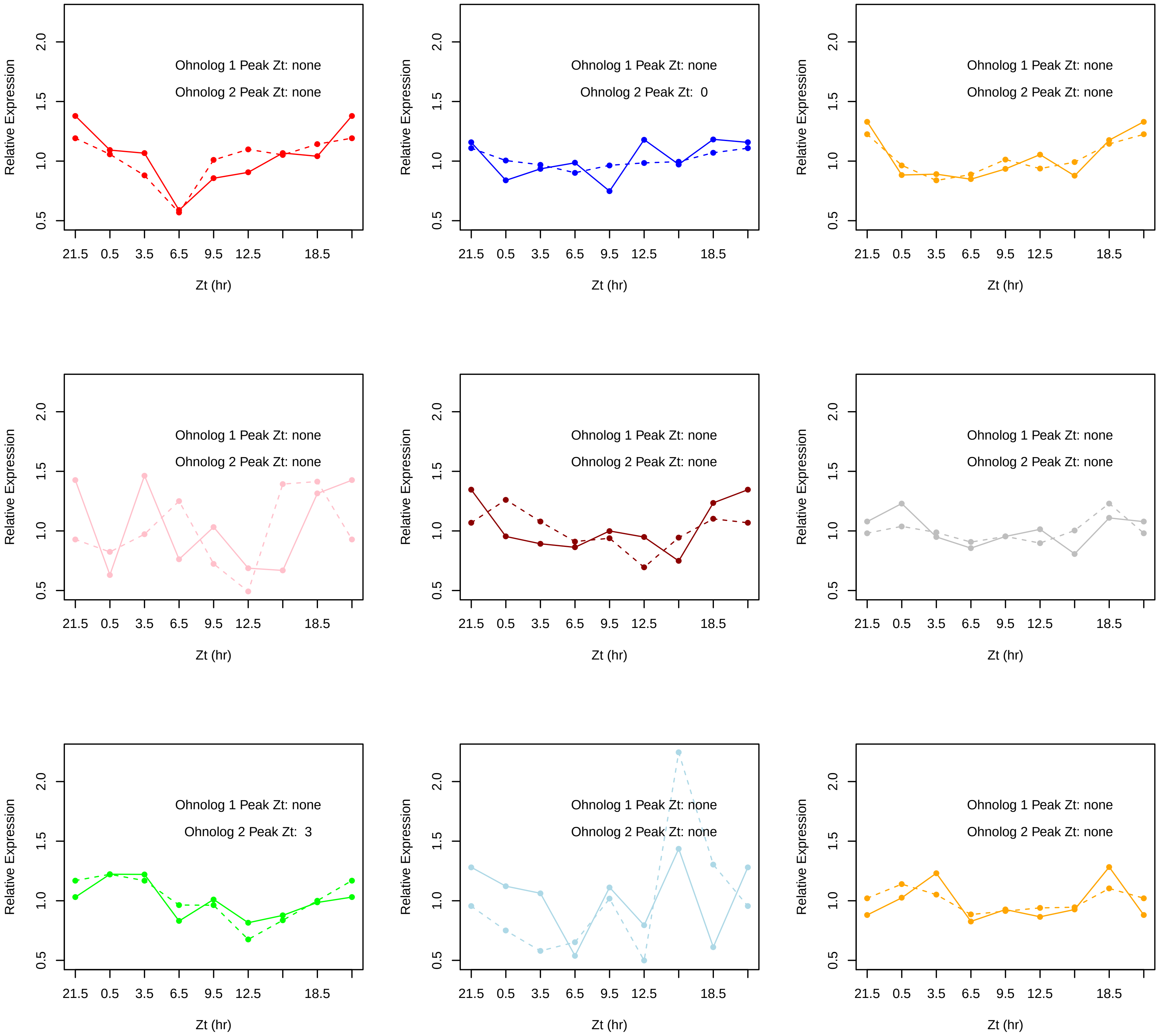

Ohnolog 1: ENSXMAG00000010470 loxl2b
Ohnolog 2: ENSXMAG00000018377 loxl2a

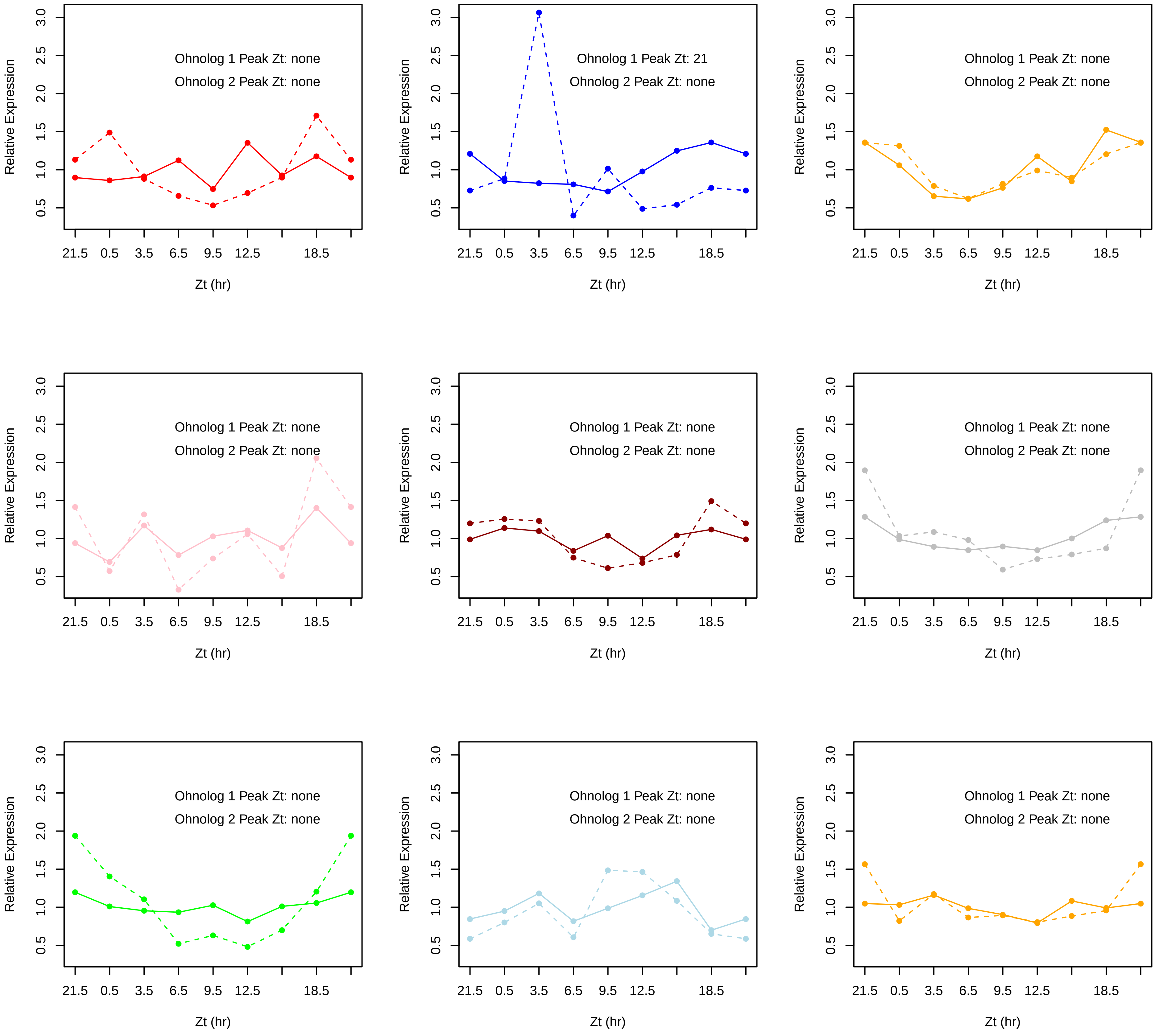

Ohnolog 1: ENSXMAG00000010490 hdr
Ohnolog 2: ENSXMAG00000022145

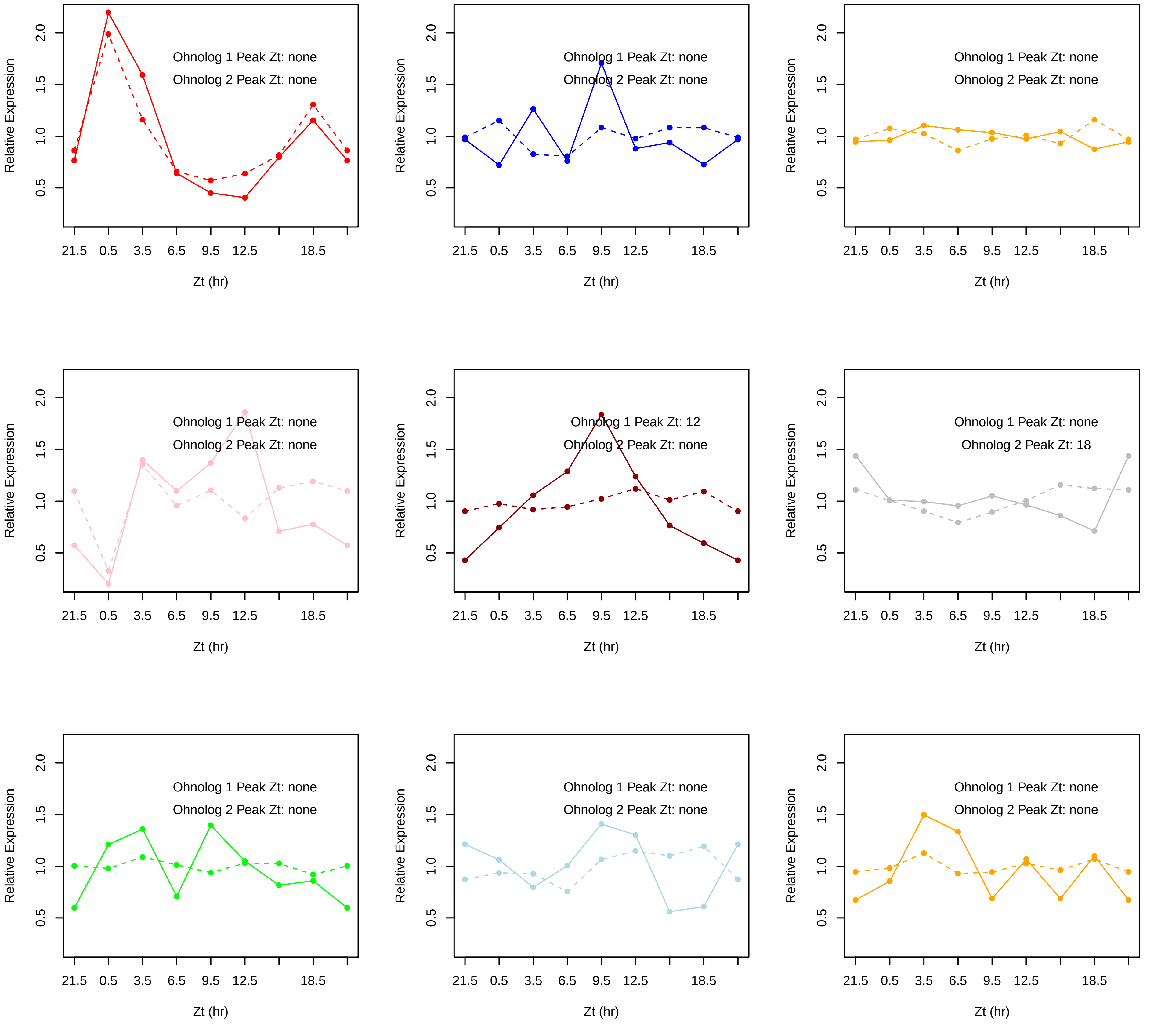

Ohnolog 1: ENSXMAG00000014461 dla
Ohnolog 2: ENSXMAG00000014878

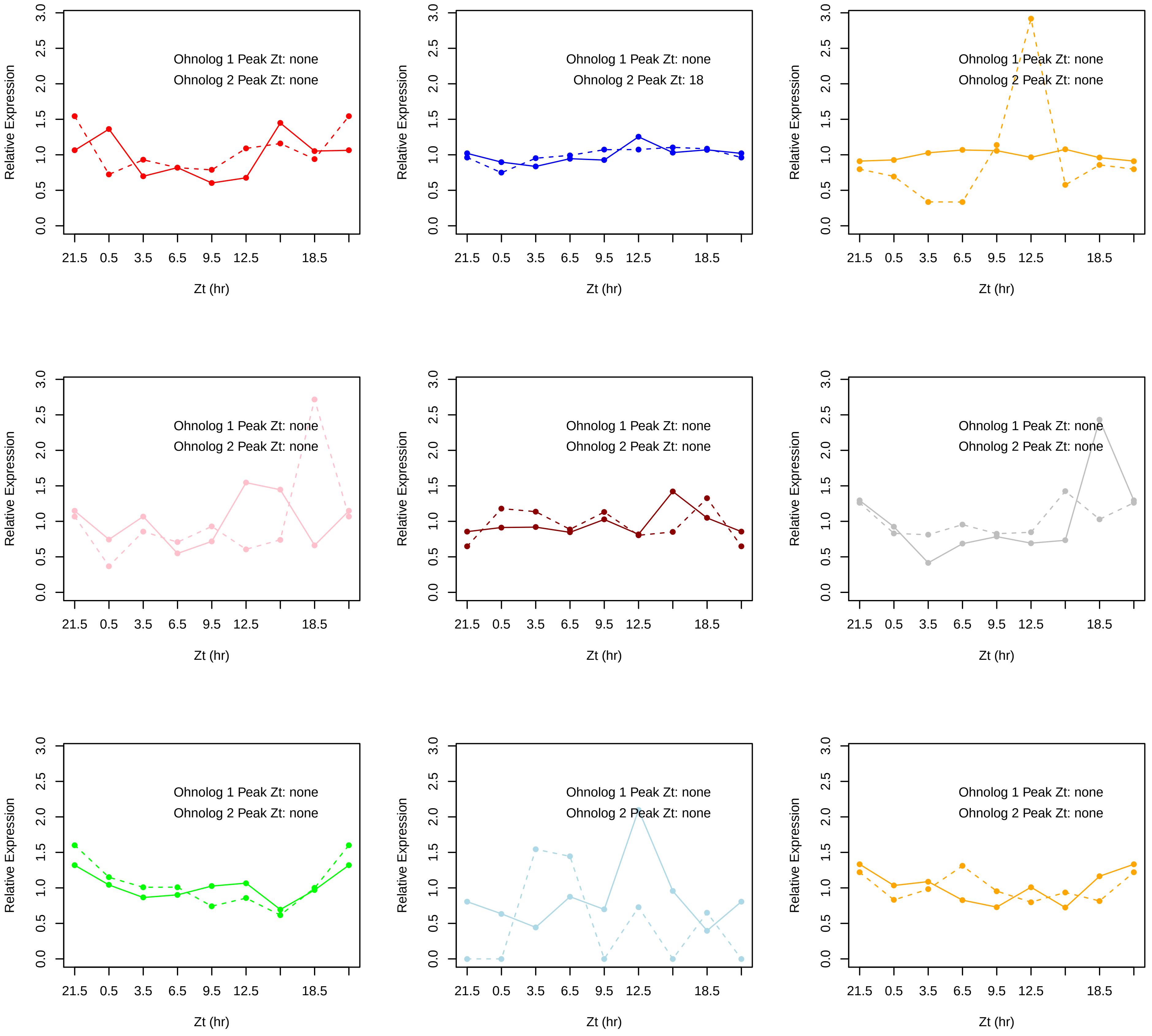

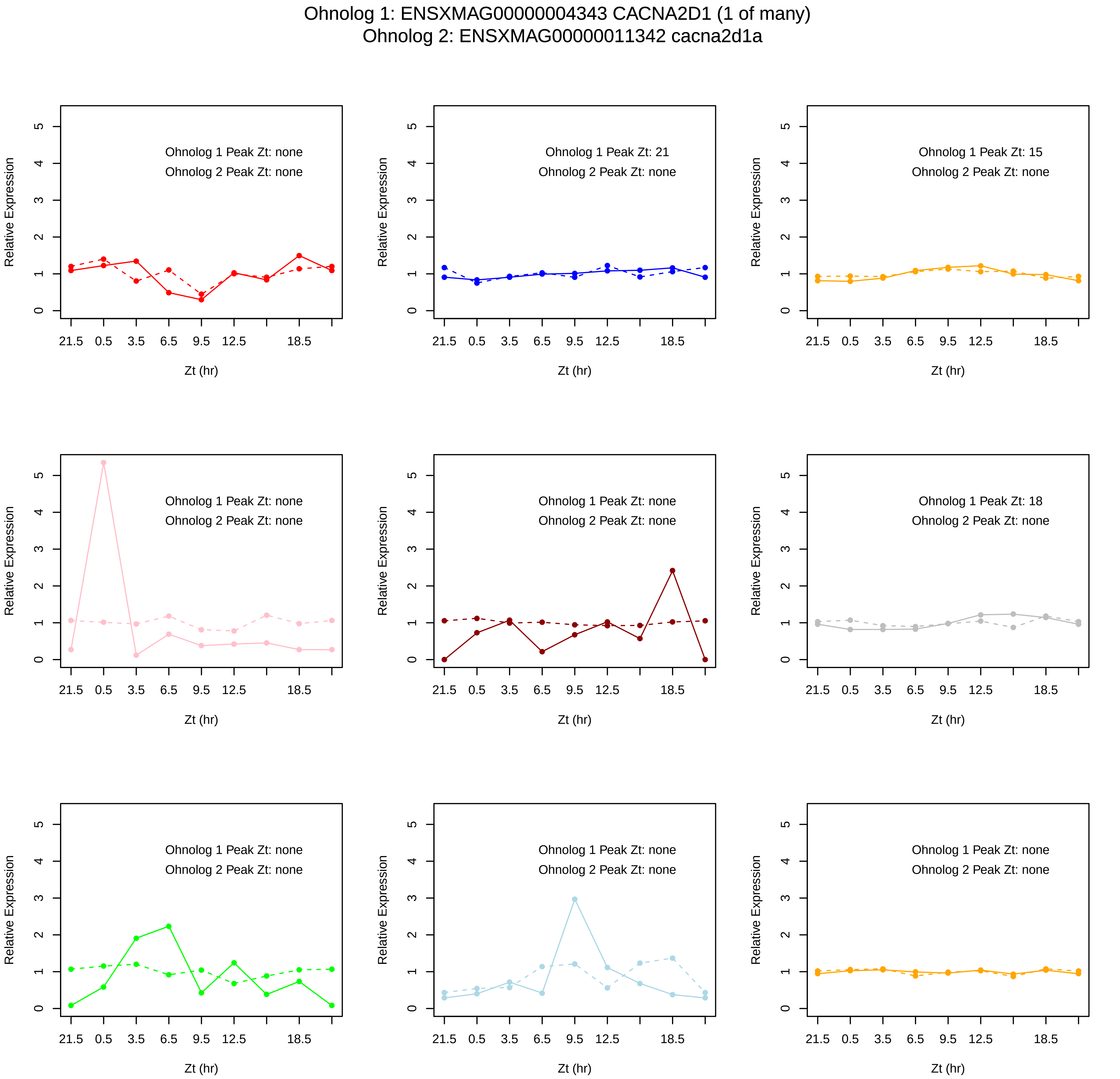

Ohnolog 1: ENSXMAG00000004343 CACNA2D1 (1 of many)
Ohnolog 2: ENSXMAG00000011342 cacna2d1a
Ohnolog 1 Peak Zt: none
Ohnolog 2 Peak Zt: none
Ohnolog 1 Peak Zt: 21
Ohnolog 2 Peak Zt: none
Ohnolog 1 Peak Zt: 15
Ohnolog 2 Peak Zt: none
Ohnolog 1 Peak Zt: none
Ohnolog 2 Peak Zt: none
Ohnolog 1 Peak Zt: none
Ohnolog 2 Peak Zt: none
Ohnolog 1 Peak Zt: 18
Ohnolog 2 Peak Zt: none
Ohnolog 1 Peak Zt: none
Ohnolog 2 Peak Zt: none
Ohnolog 1 Peak Zt: none
Ohnolog 2 Peak Zt: none
Ohnolog 1 Peak Zt: none
Ohnolog 2 Peak Zt: none
Relative Expression
Zt (hr)
21.5 0.5 3.5 6.5 9.5 12.5 18.5
0 1 2 3 4 5

Ohnolog 1: ENSXMAG00000004015
Ohnolog 2: ENSXMAG00000030049 KCNIP3

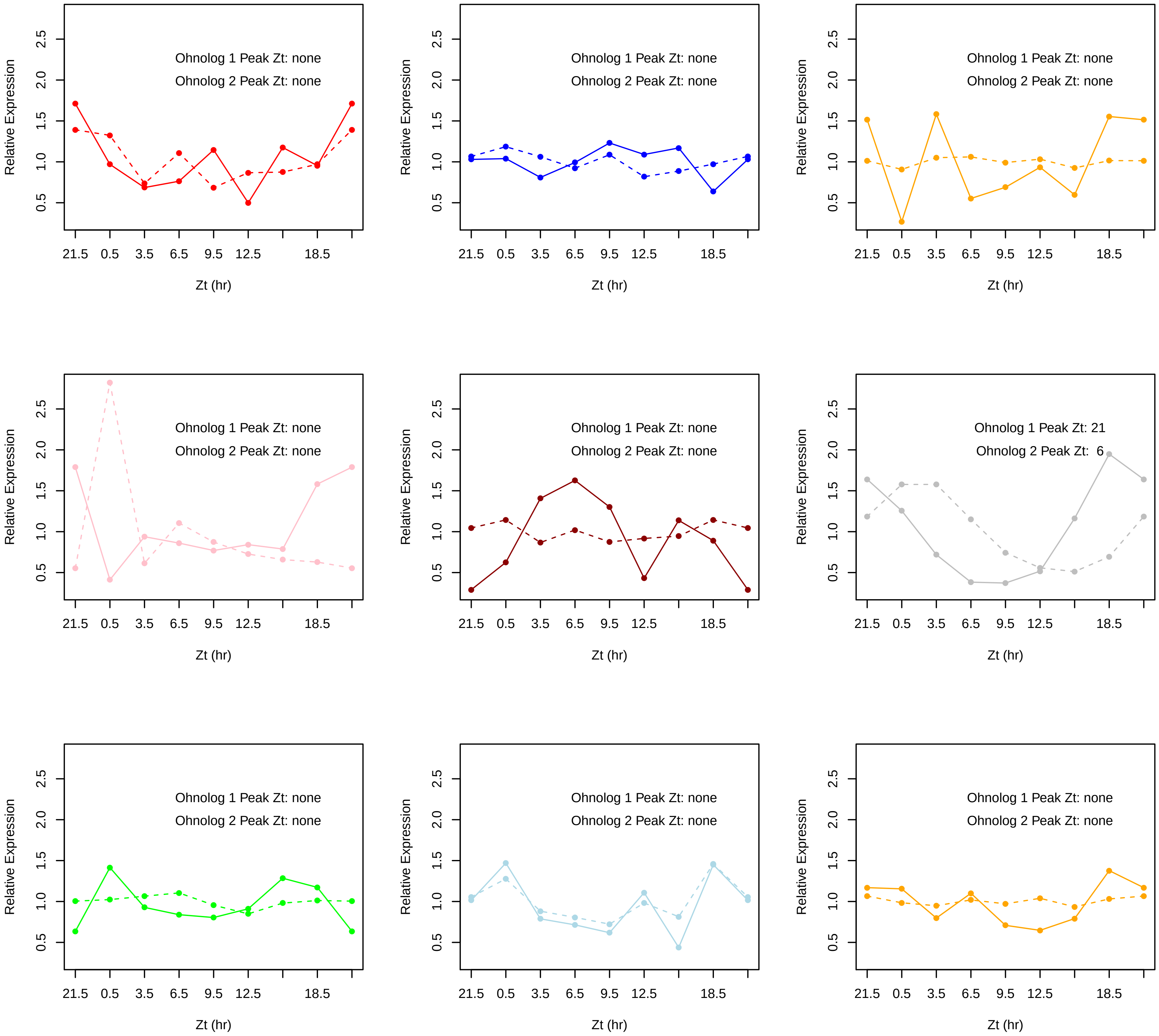

Ohnolog 1: ENSXMAG00000016859
Ohnolog 2: ENSXMAG00000024223 celf2

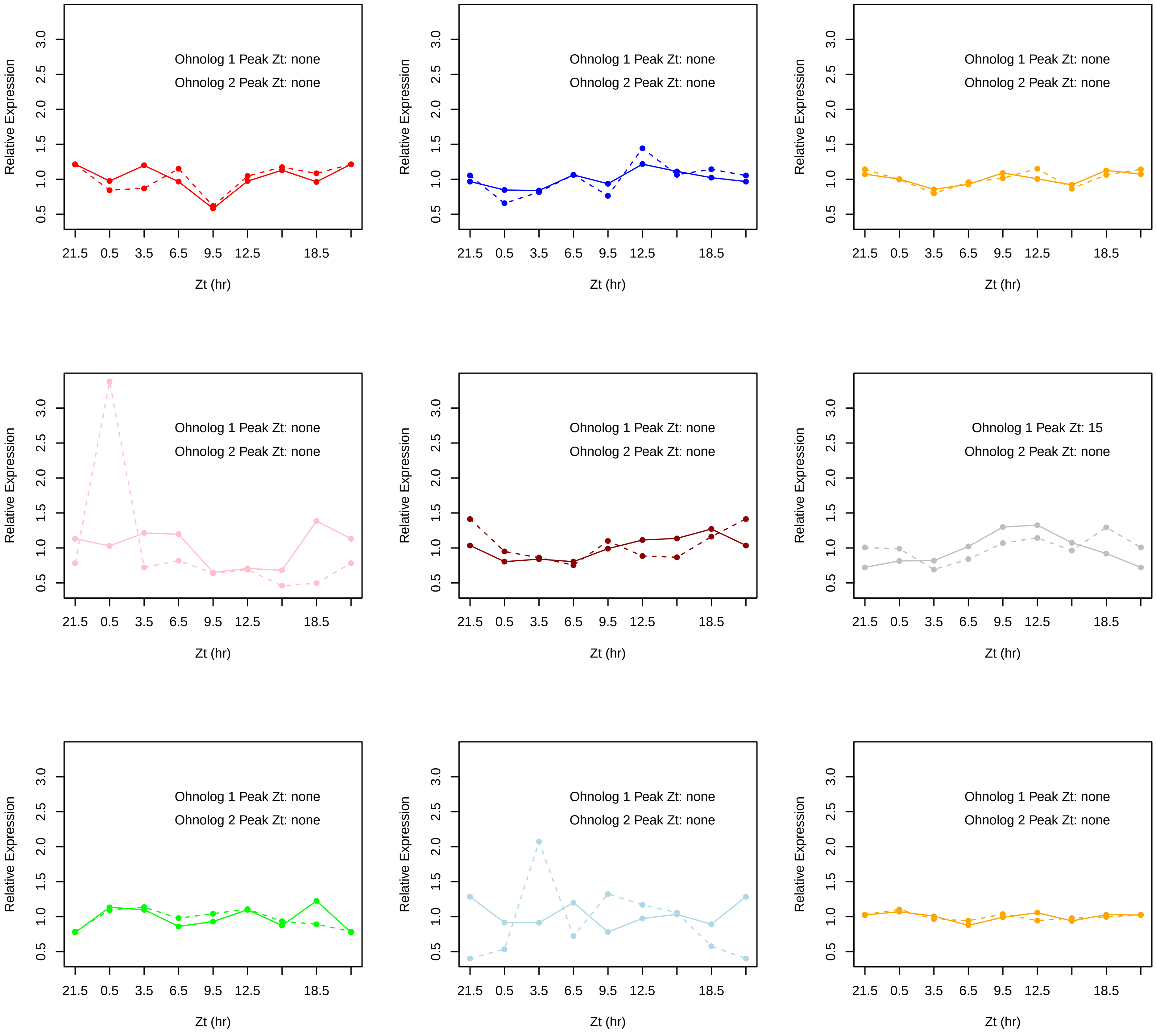

Ohnolog 1: ENSXMAG00000015733 slc8a1a
Ohnolog 2: ENSXMAG00000017251 slc8a1b

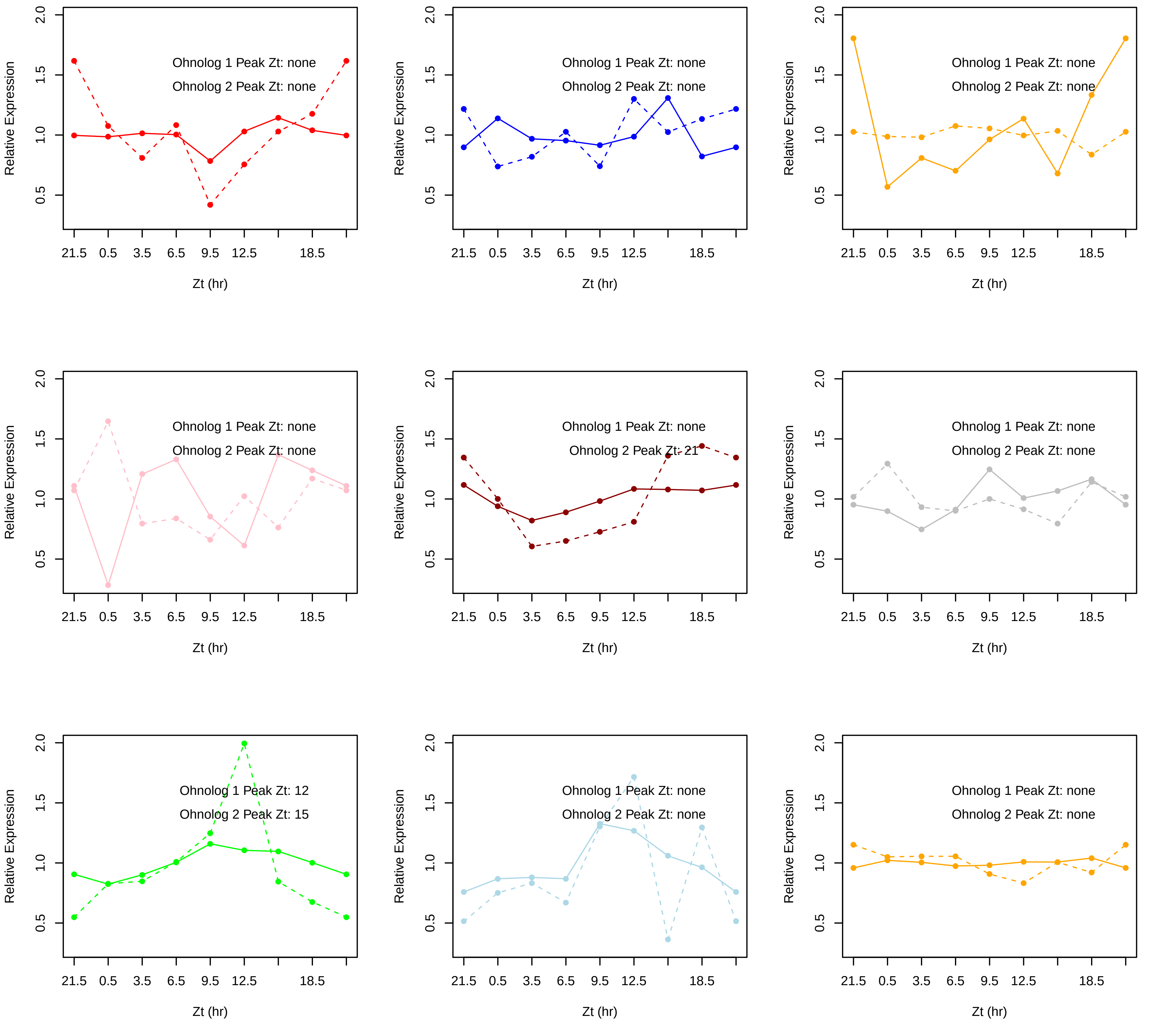

Ohnolog 1: ENSXMAG00000007586
Ohnolog 2: ENSXMAG00000016455 hbp1

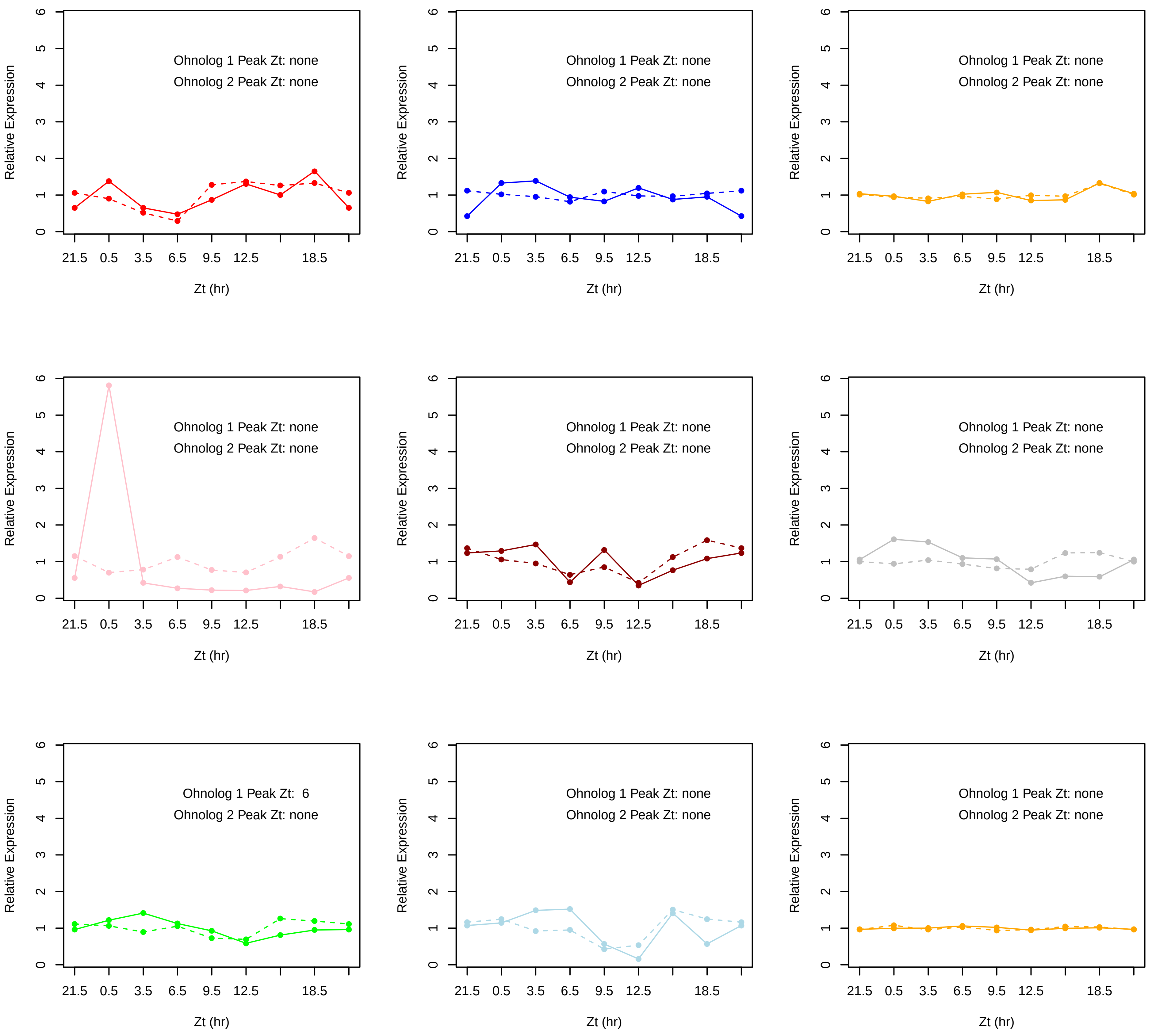

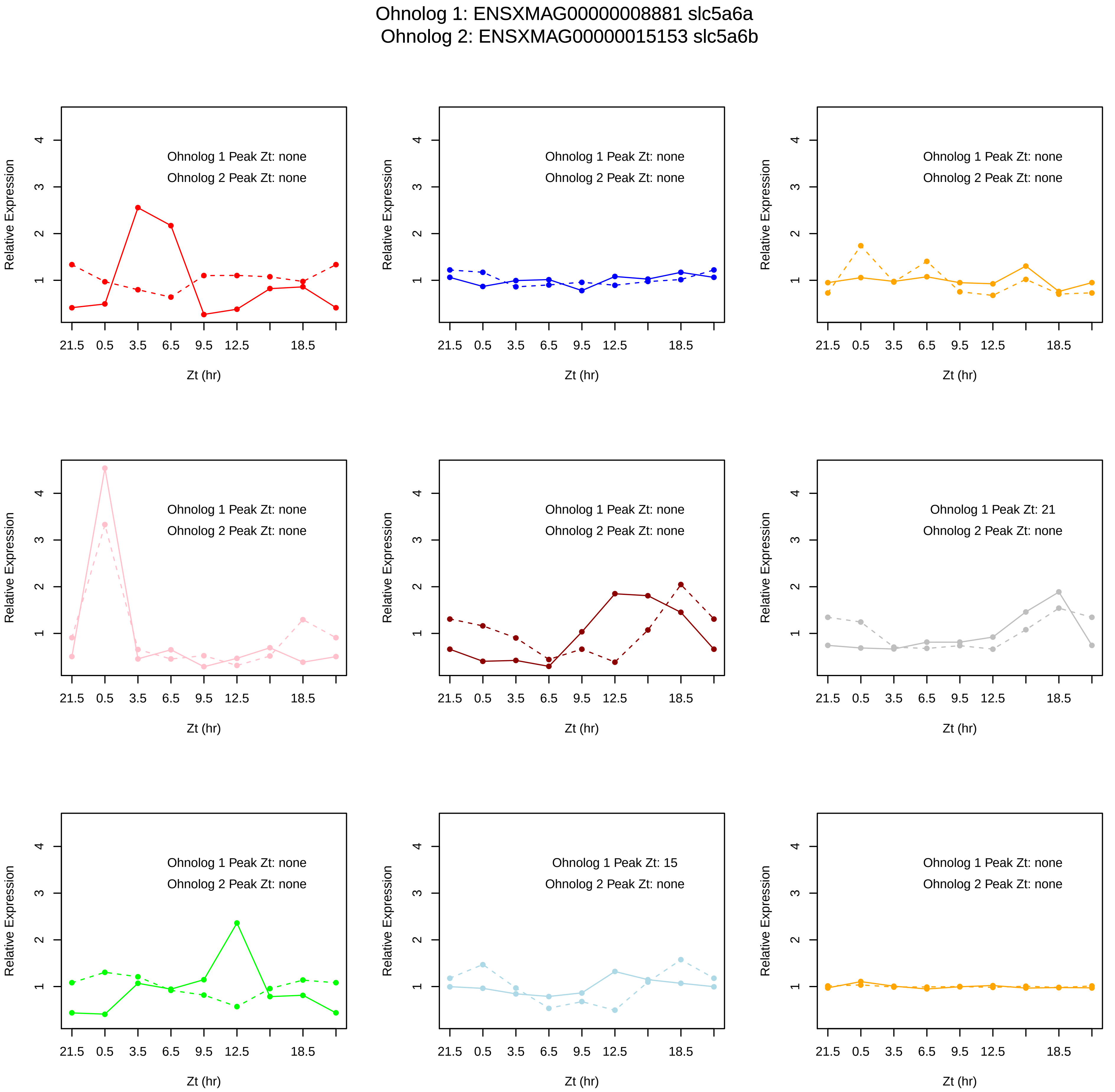
Ohnolog 1: ENSXMAG00000008881 slc5a6a
Ohnolog 2: ENSXMAG00000015153 slc5a6b
Ohnolog 1 Peak Zt: none
Ohnolog 2 Peak Zt: none
Ohnolog 1 Peak Zt: none
Ohnolog 2 Peak Zt: none
Ohnolog 1 Peak Zt: none
Ohnolog 2 Peak Zt: none
Ohnolog 1 Peak Zt: none
Ohnolog 2 Peak Zt: none
Ohnolog 1 Peak Zt: none
Ohnolog 2 Peak Zt: none
Ohnolog 1 Peak Zt: 21
Ohnolog 2 Peak Zt: none
Ohnolog 1 Peak Zt: none
Ohnolog 2 Peak Zt: none
Ohnolog 1 Peak Zt: 15
Ohnolog 2 Peak Zt: none
Ohnolog 1 Peak Zt: none
Ohnolog 2 Peak Zt: none
Relative Expression
Zt (hr)
21.5 0.5 3.5 6.5 9.5 12.5 18.5
1 2 3 4

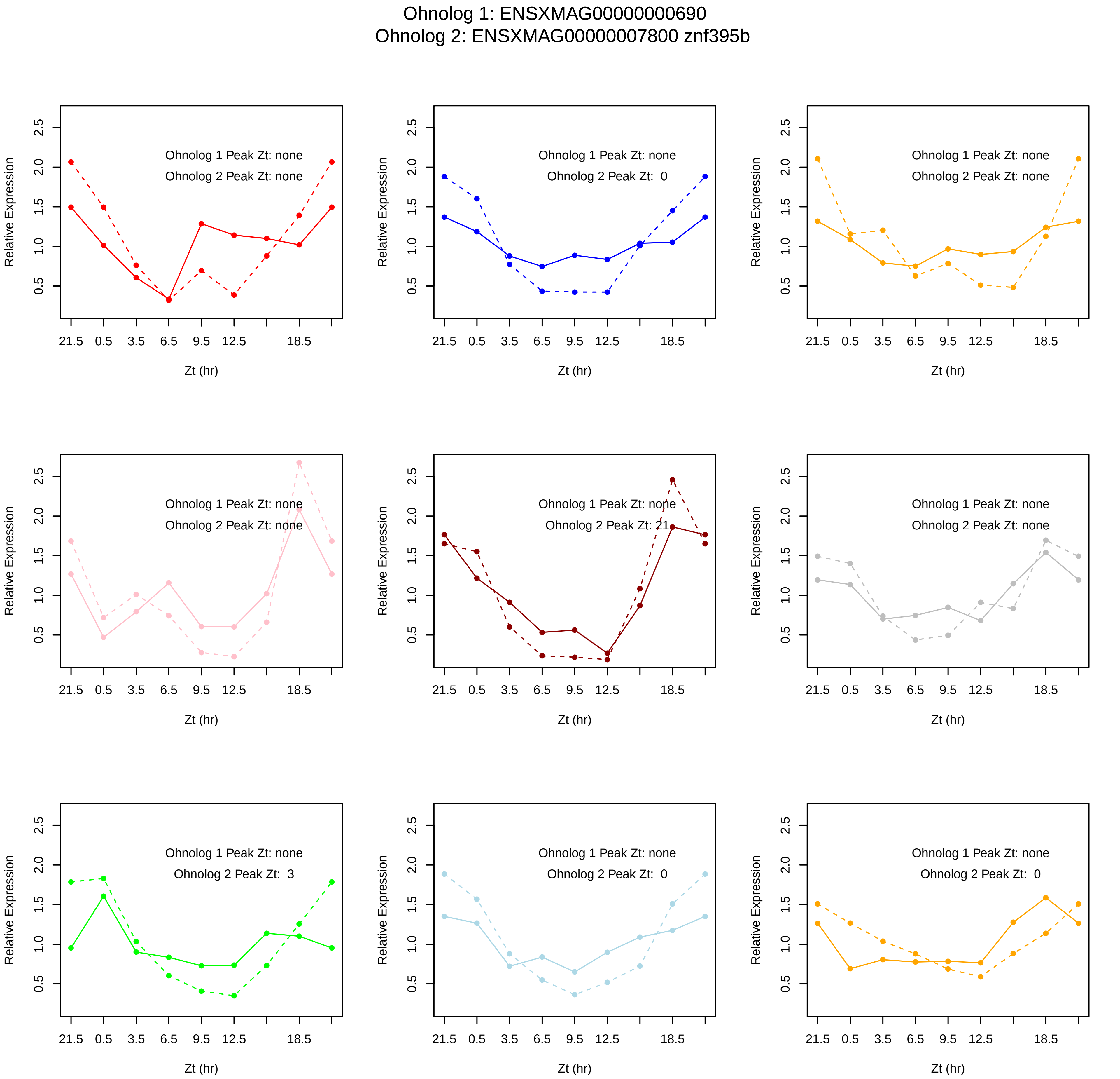

Ohnolog 1: ENSXMAG00000000690
Ohnolog 2: ENSXMAG00000007800 znf395b
Ohnolog 1 Peak Zt: none
Ohnolog 2 Peak Zt: none
Ohnolog 1 Peak Zt: none
Ohnolog 2 Peak Zt: 0
Ohnolog 1 Peak Zt: none
Ohnolog 2 Peak Zt: none
Ohnolog 1 Peak Zt: none
Ohnolog 2 Peak Zt: none
Ohnolog 1 Peak Zt: none
Ohnolog 2 Peak Zt: 21
Ohnolog 1 Peak Zt: none
Ohnolog 2 Peak Zt: none
Ohnolog 1 Peak Zt: none
Ohnolog 2 Peak Zt: 3
Ohnolog 1 Peak Zt: none
Ohnolog 2 Peak Zt: 0
Ohnolog 1 Peak Zt: none
Ohnolog 2 Peak Zt: 0
Relative Expression
Zt (hr)
21.5 0.5 3.5 6.5 9.5 12.5 18.5
0.5 1.0 1.5 2.0 2.5

Ohnolog 1: ENSXMAG00000027974 fgl2a
Ohnolog 2: ENSXMAG00000028171

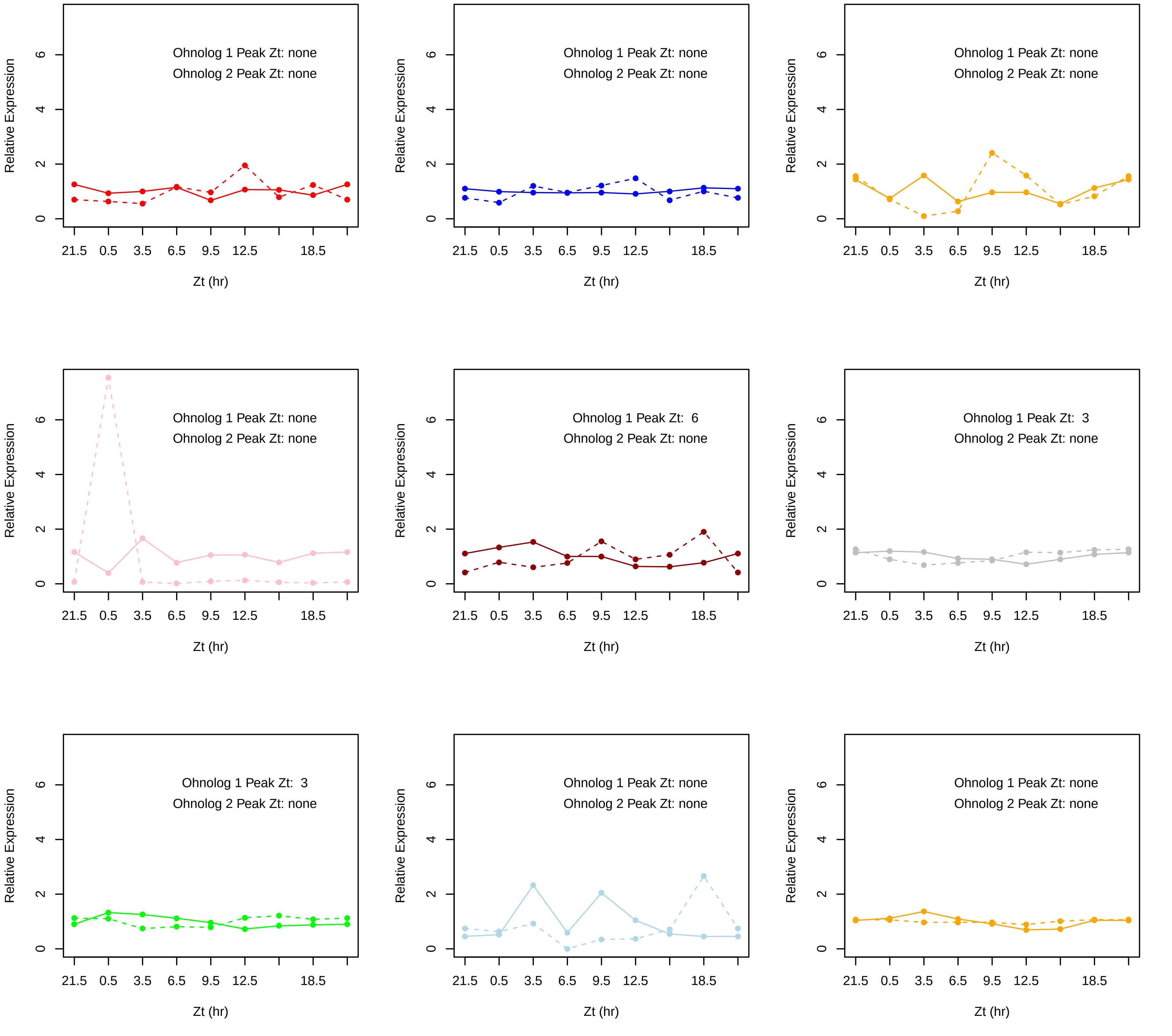

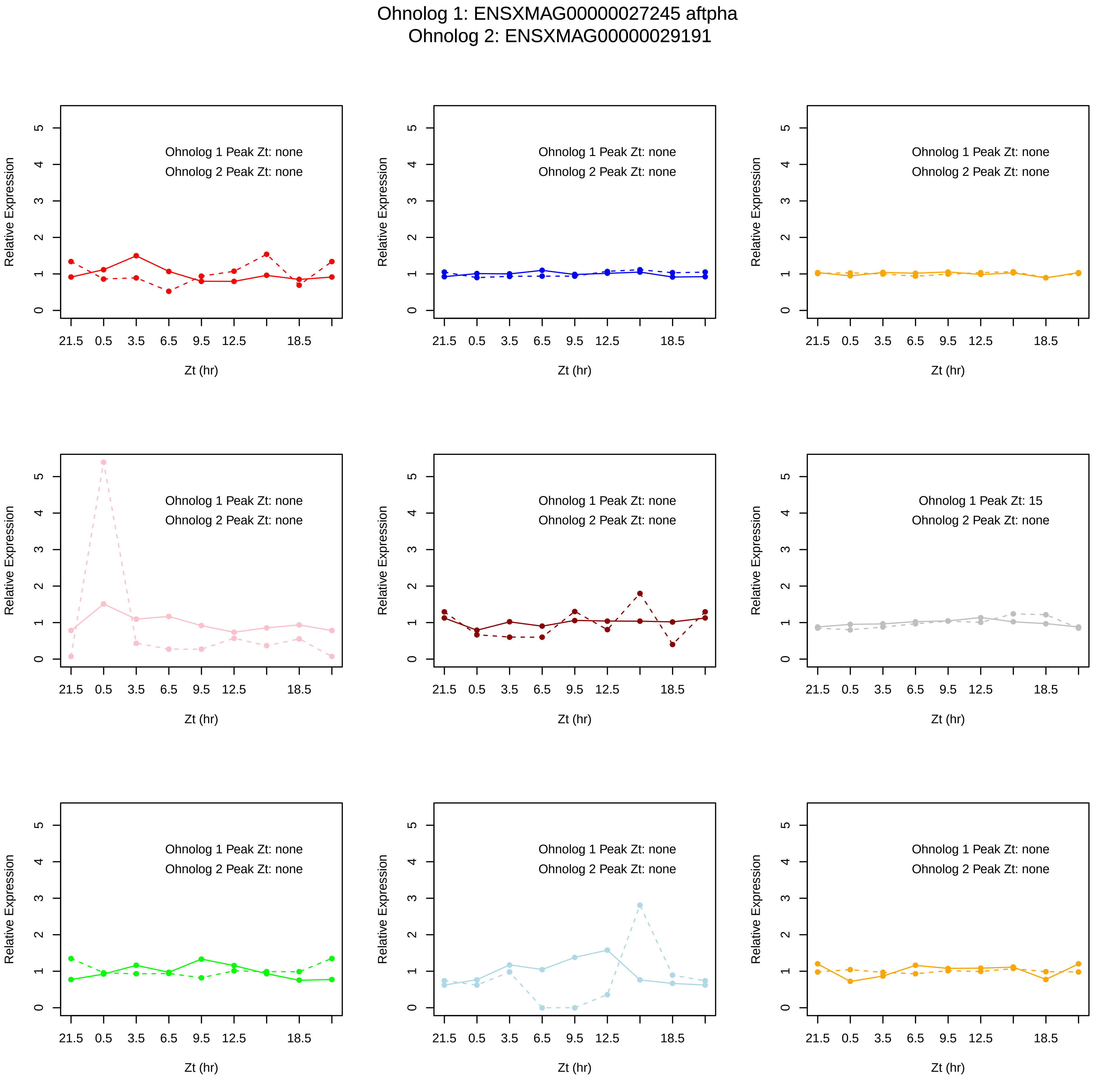
Ohnolog 1: ENSXMAG00000027245 aftpha
Ohnolog 2: ENSXMAG00000029191
Ohnolog 1 Peak Zt: none
Ohnolog 2 Peak Zt: none
Ohnolog 1 Peak Zt: 15
Relative Expression
Zt (hr)
21.5
0.5
3.5
6.5
9.5
12.5
18.5

Ohnolog 1: ENSXMAG00000005970 lgalsla
Ohnolog 2: ENSXMAG00000011845 lgalslb

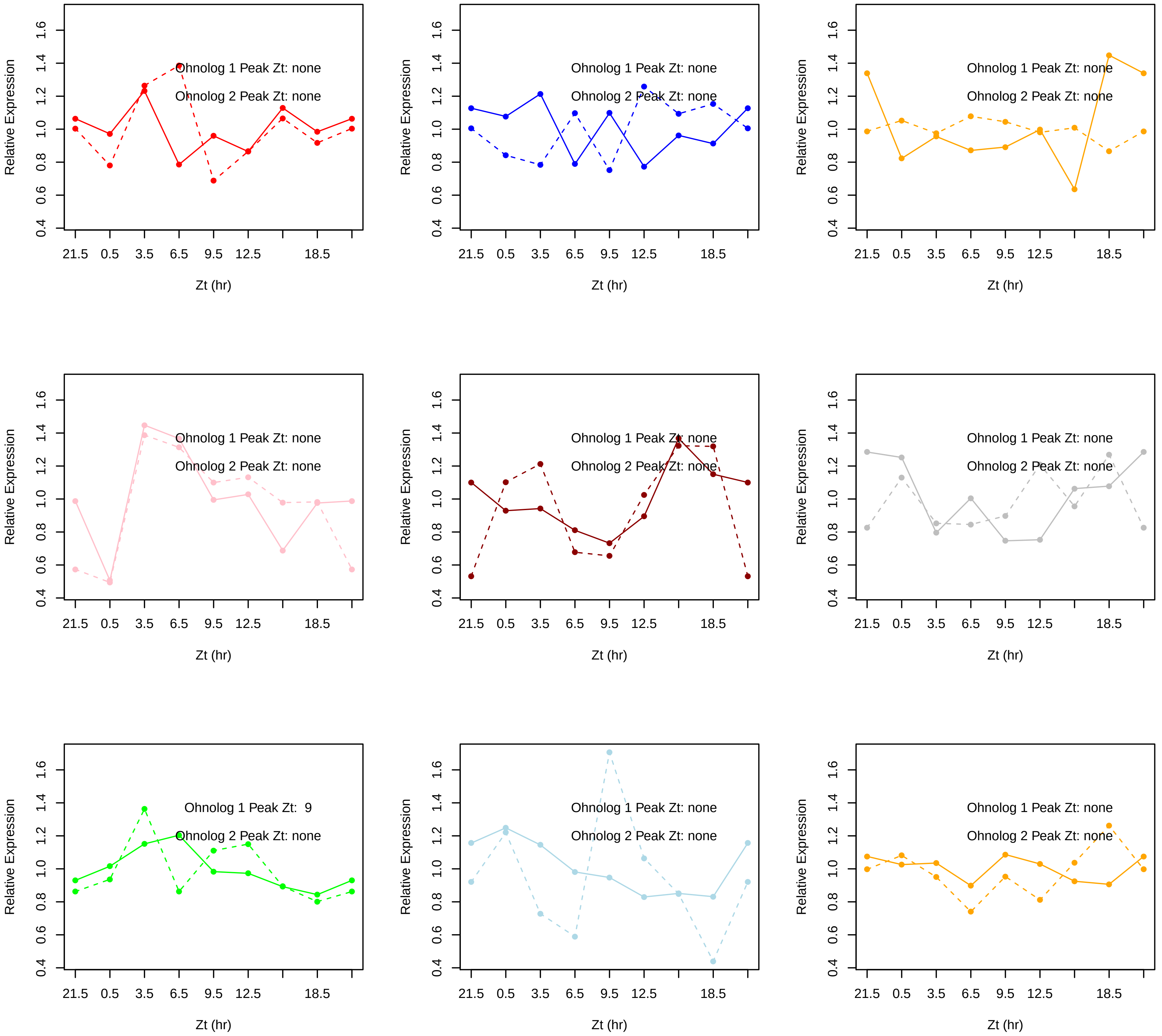

Ohnolog 1: ENSXMAG00000020875 cerk
Ohnolog 2: ENSXMAG00000029787

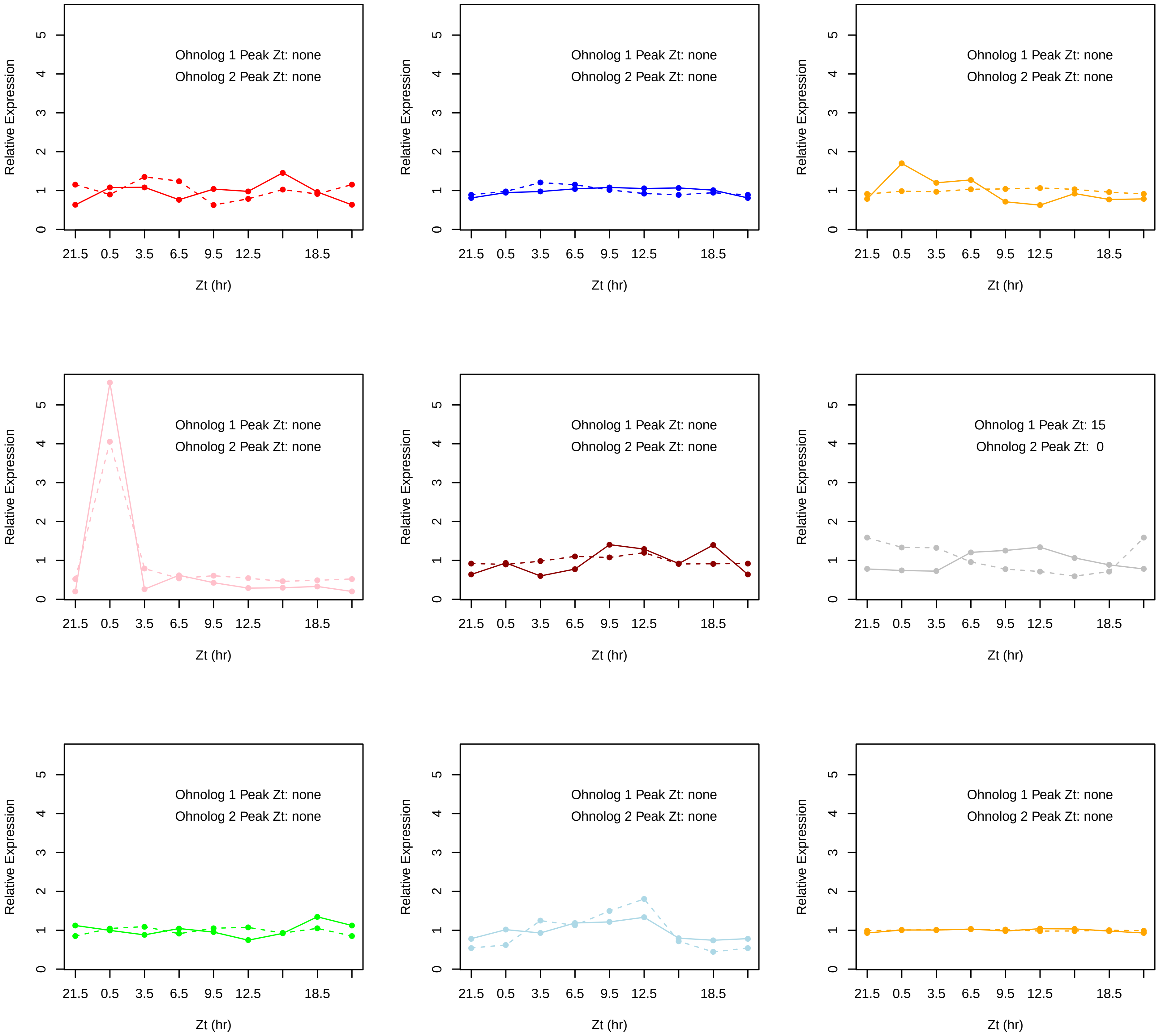

Ohnolog 1: ENSXMAG00000017888
Ohnolog 2: ENSXMAG00000025049 eif4ebp1

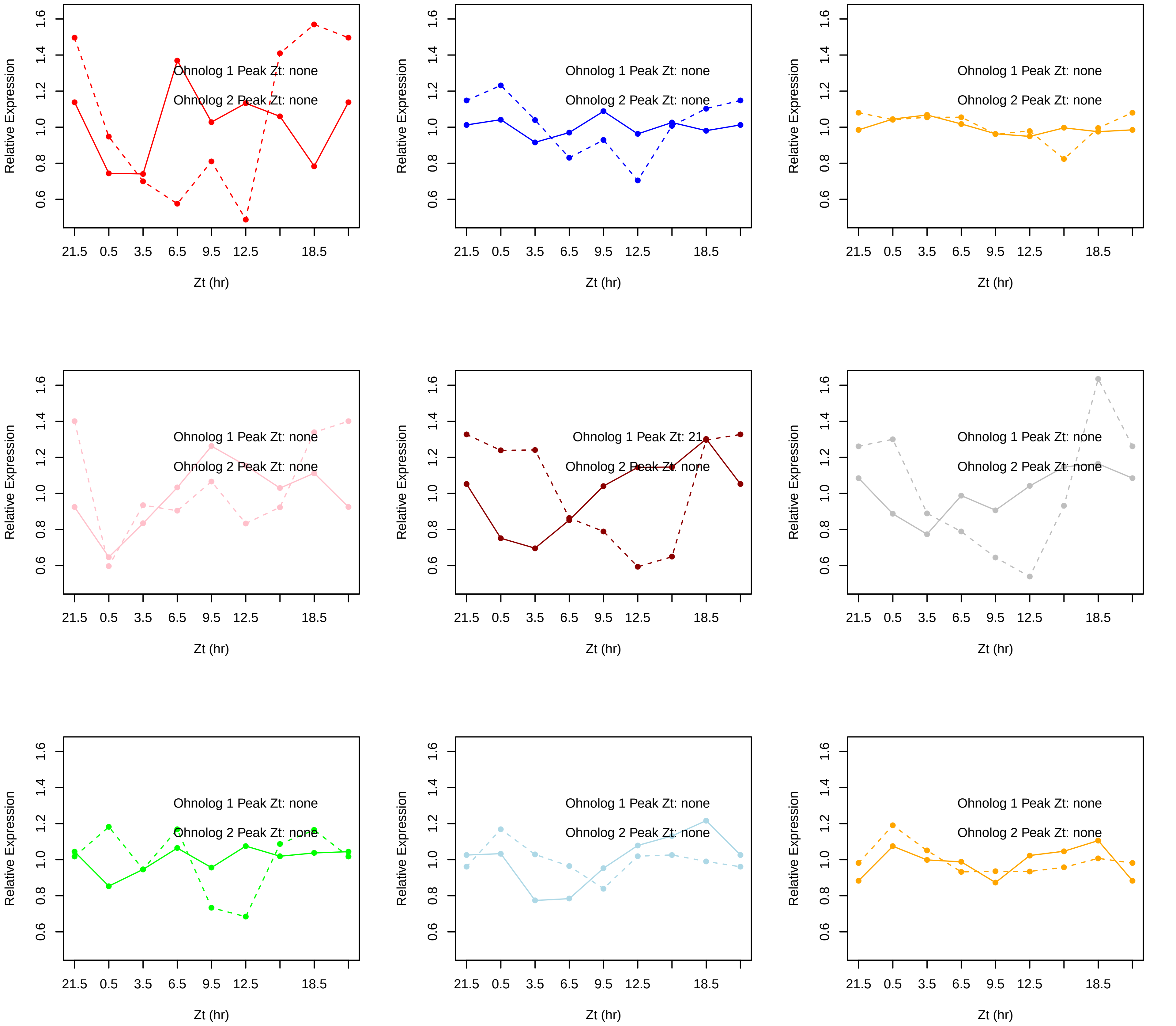

Ohnolog 1: ENSXMAG00000002043 igsf9b
Ohnolog 2: ENSXMAG00000028682 igsf9a

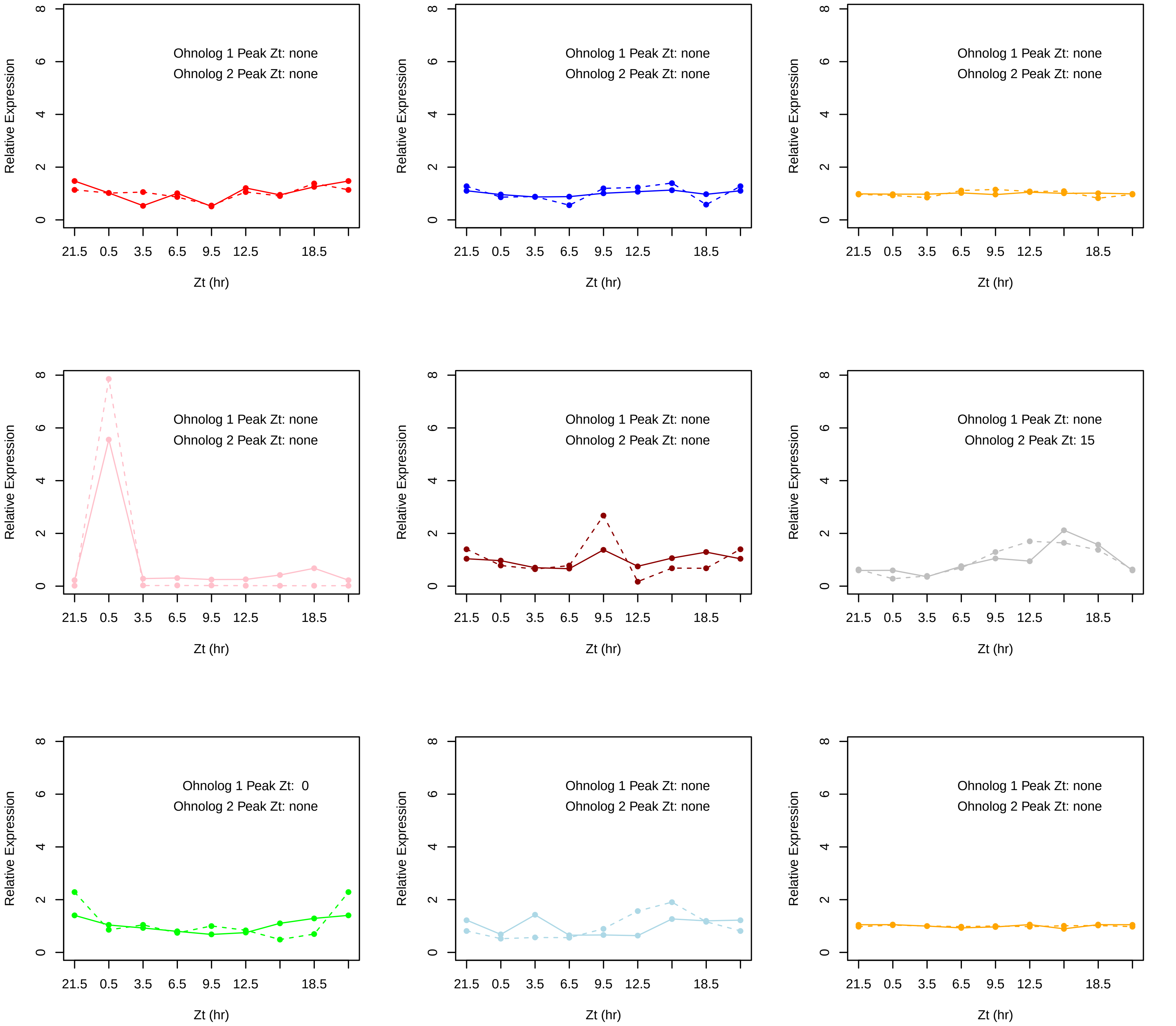

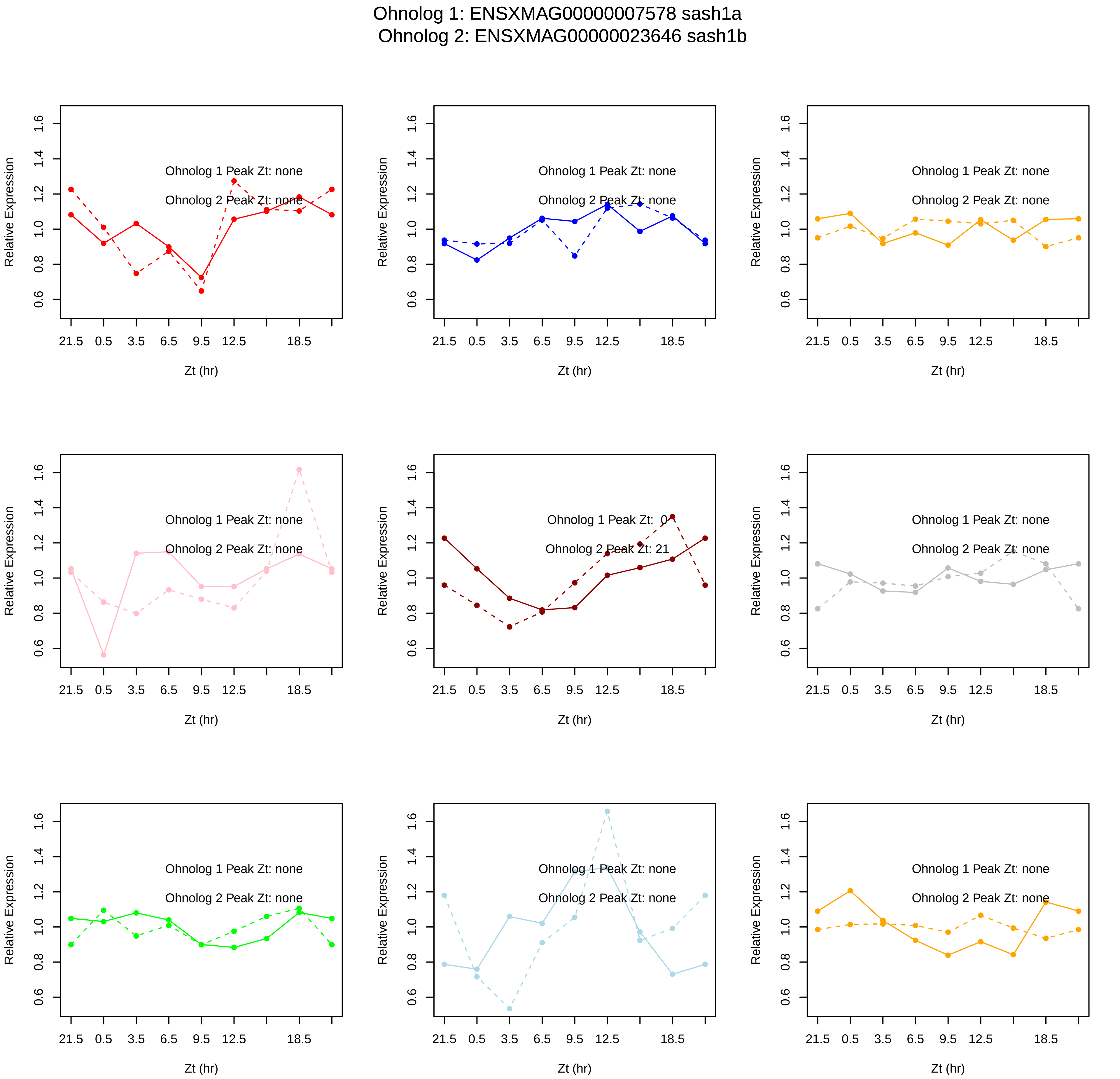
Ohnolog 1: ENSXMAG00000007578 sash1a
Ohnolog 2: ENSXMAG00000023646 sash1b
Ohnolog 1 Peak Zt: none
Ohnolog 2 Peak Zt: none
Ohnolog 1 Peak Zt: none
Ohnolog 2 Peak Zt: none
Ohnolog 1 Peak Zt: none
Ohnolog 2 Peak Zt: none
Ohnolog 1 Peak Zt: none
Ohnolog 2 Peak Zt: none
Ohnolog 1 Peak Zt: 0
Ohnolog 2 Peak Zt: 21
Ohnolog 1 Peak Zt: none
Ohnolog 2 Peak Zt: none
Ohnolog 1 Peak Zt: none
Ohnolog 2 Peak Zt: none
Ohnolog 1 Peak Zt: none
Ohnolog 2 Peak Zt: none
Ohnolog 1 Peak Zt: none
Ohnolog 2 Peak Zt: none
Relative Expression
Zt (hr)
21.5 0.5 3.5 6.5 9.5 12.5 18.5
0.6 0.8 1.0 1.2 1.4 1.6

Ohnolog 1: ENSXMAG00000005658 ccdc85a
Ohnolog 2: ENSXMAG00000023108 ccdc85al

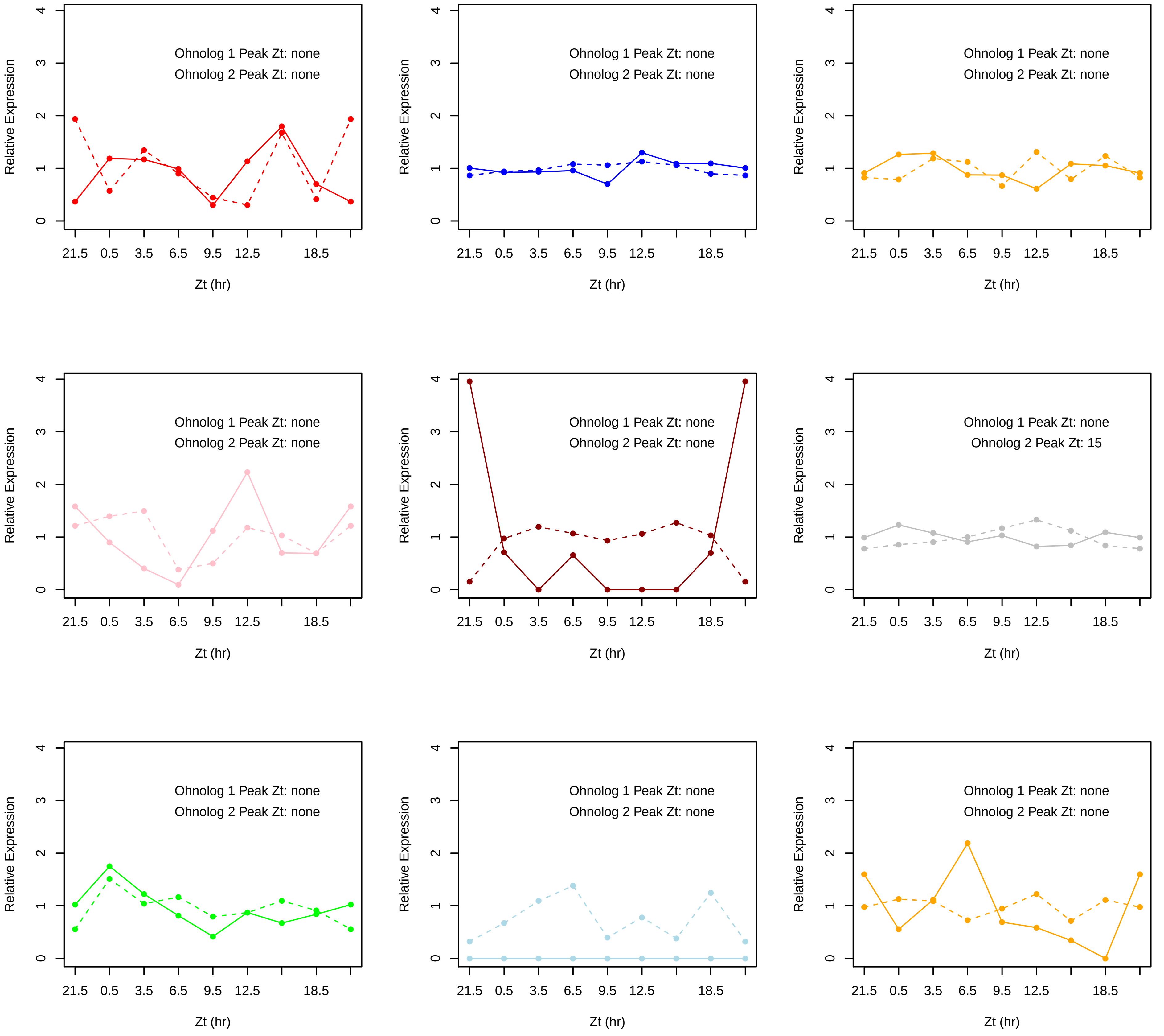

Ohnolog 1: ENSXMAG00000012035
Ohnolog 2: ENSXMAG00000024491 kcnq5a

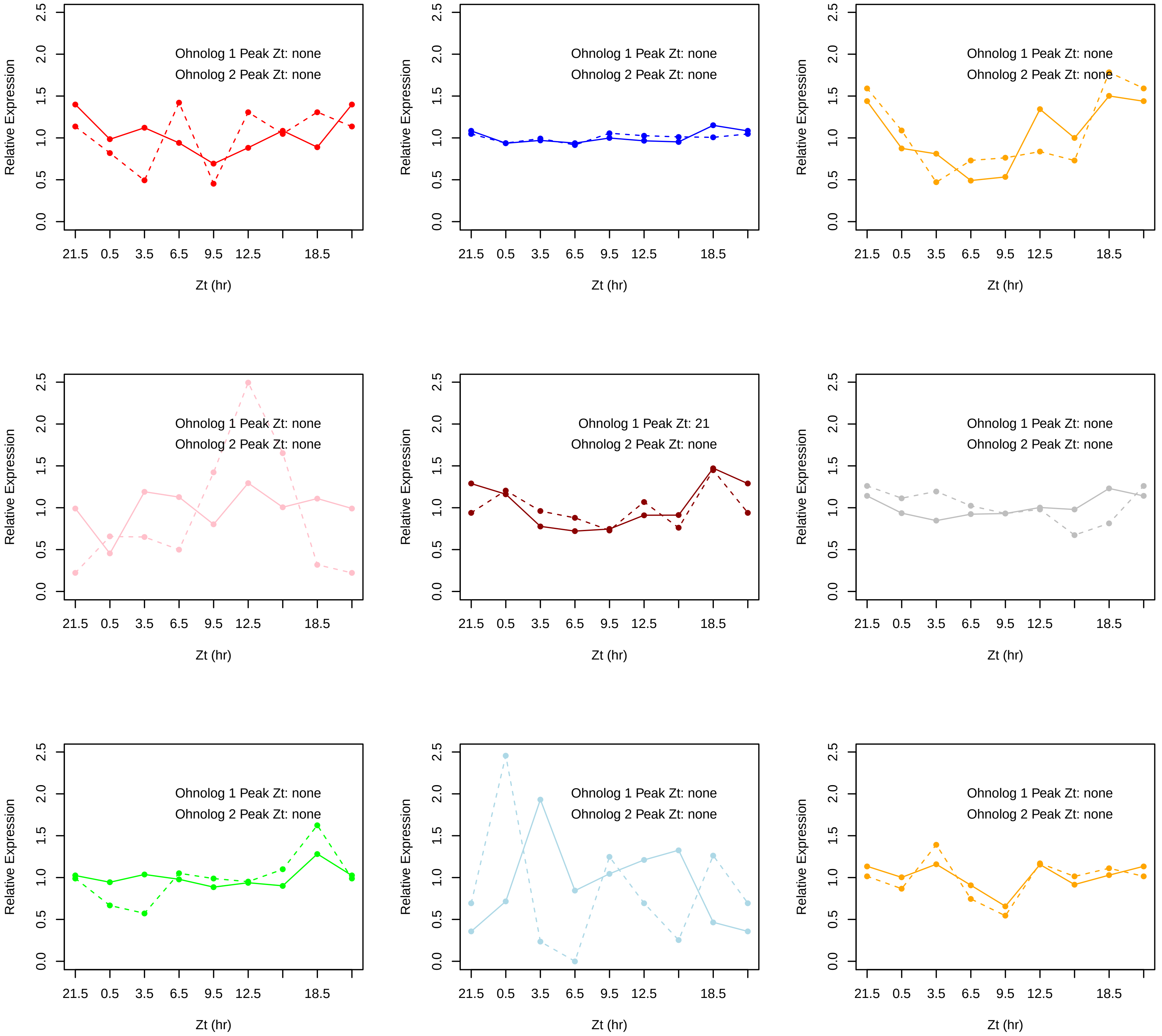

Ohnolog 1: ENSXMAG00000007246 rims1a
Ohnolog 2: ENSXMAG00000018211 RIMS1 (1 of many)

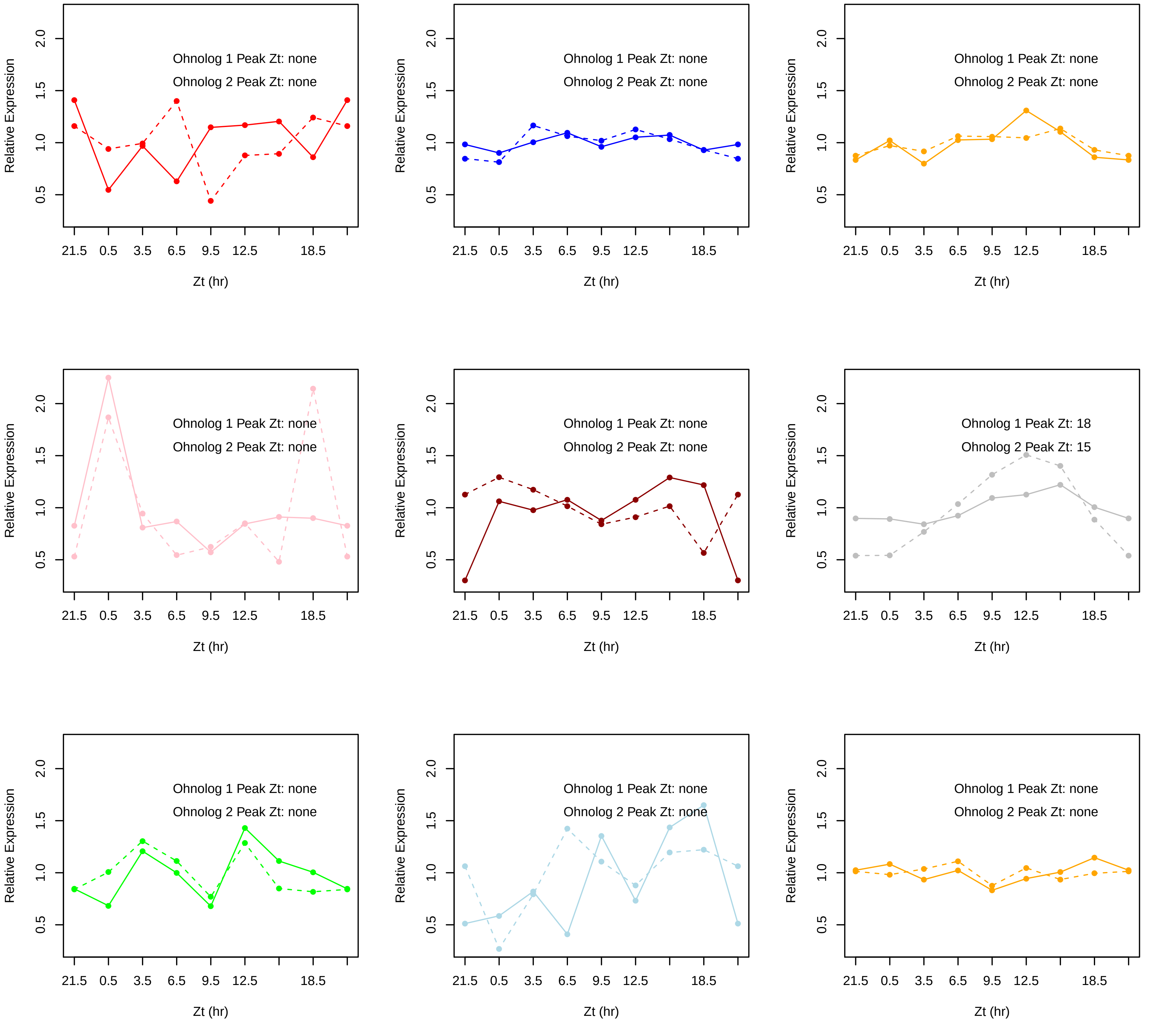

Ohnolog 1: ENSXMAG00000016365
Ohnolog 2: ENSXMAG00000028202

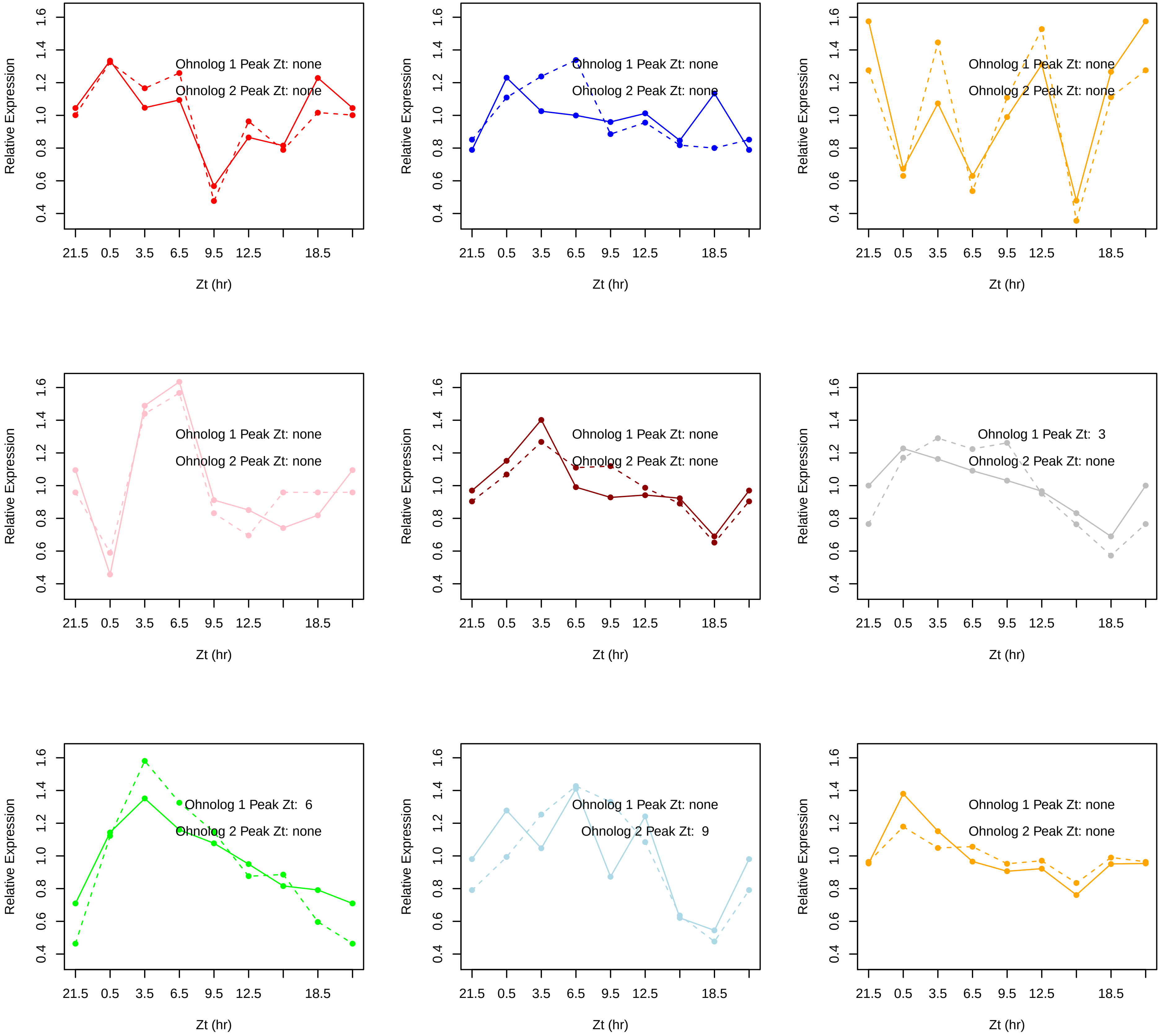

Ohnolog 1: ENSXMAG00000003229 tulp4a
Ohnolog 2: ENSXMAG00000015466 tulp4b

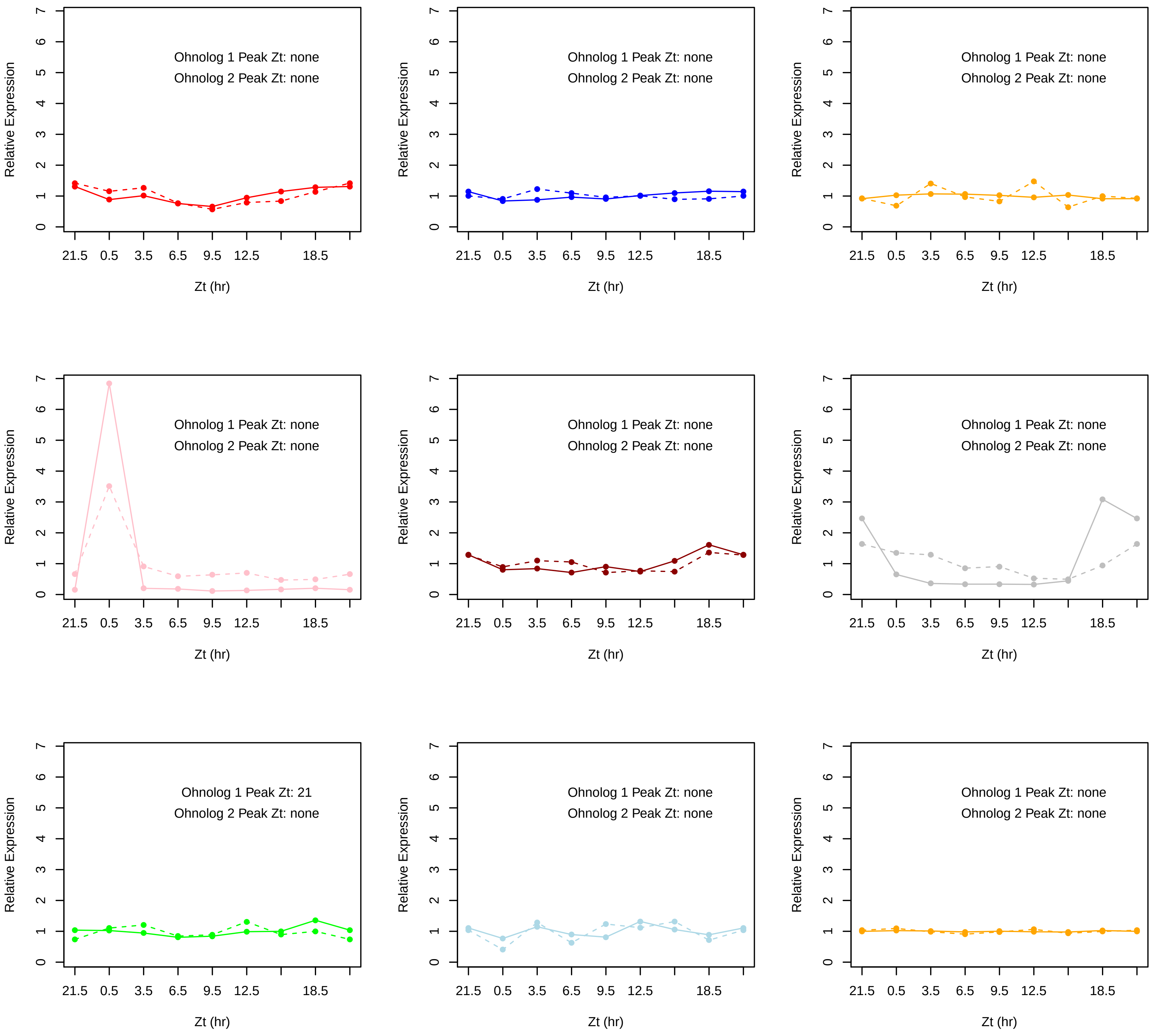

Ohnolog 1: ENSXMAG00000003189 myct1a
Ohnolog 2: ENSXMAG00000024565 myct1b

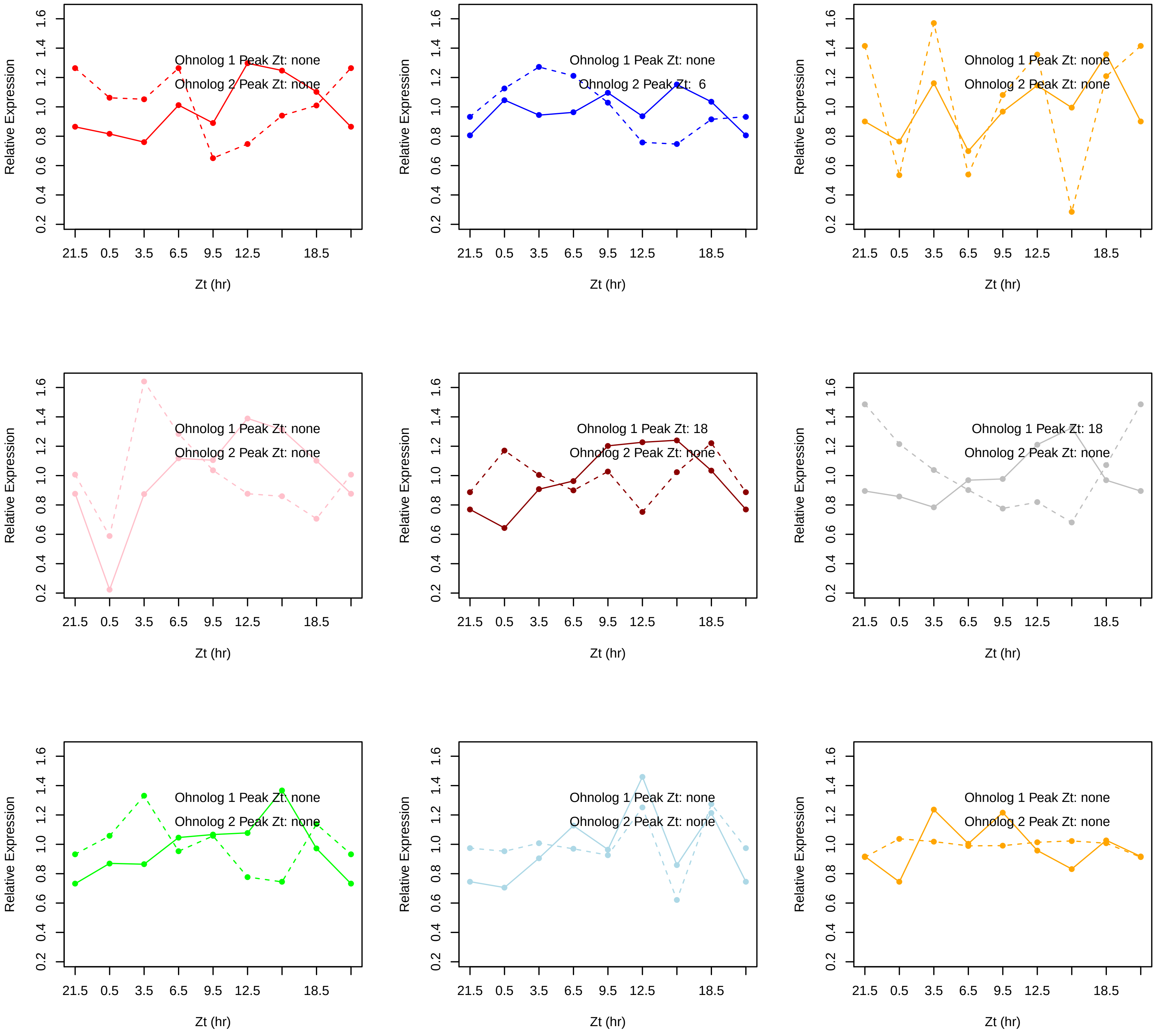

Ohnolog 1: ENSXMAG00000010885
Ohnolog 2: ENSXMAG00000016119 NRXN1

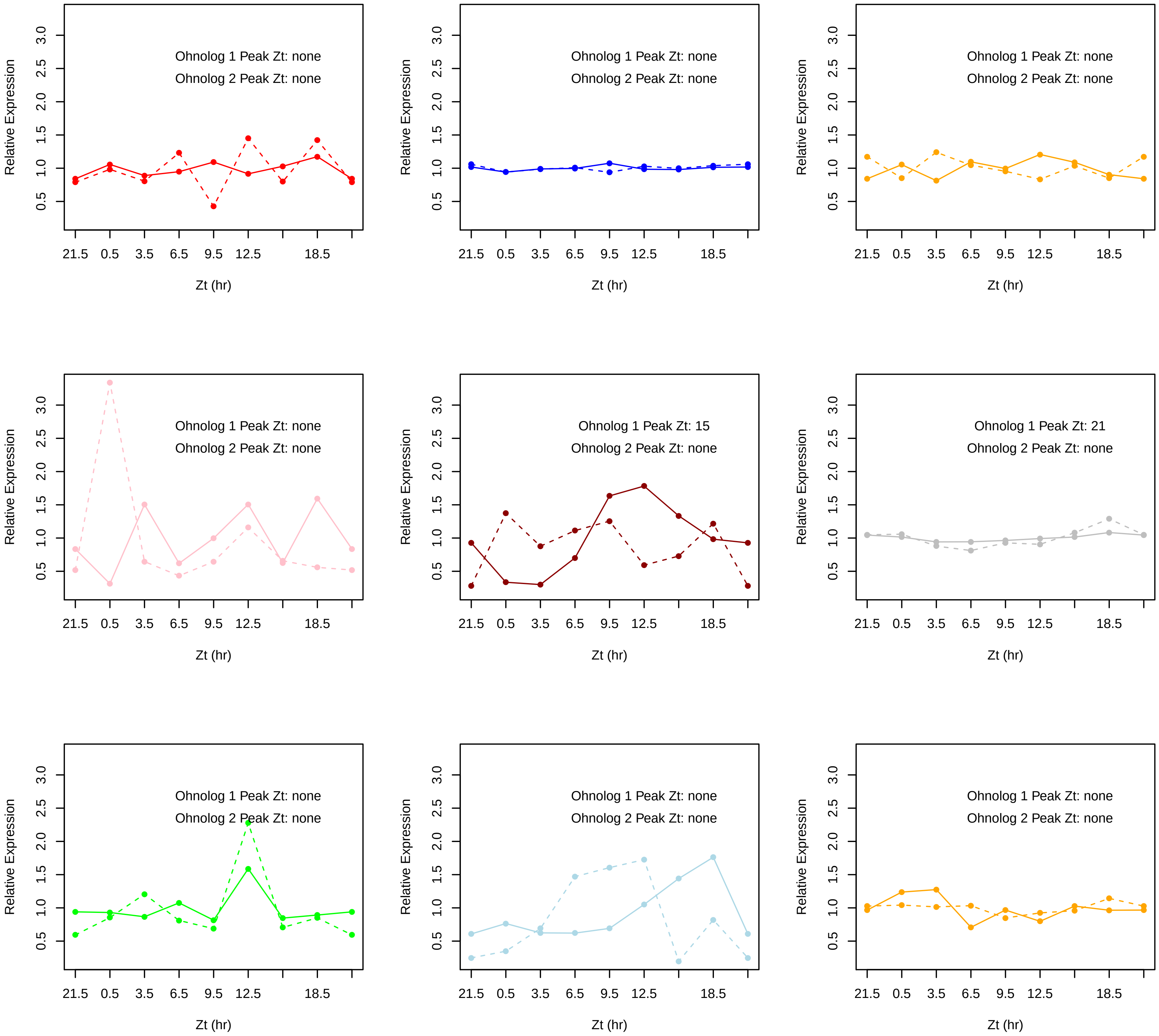

Ohnolog 1: ENSXMAG00000015620
Ohnolog 2: ENSXMAG00000021324 dnmt3ab

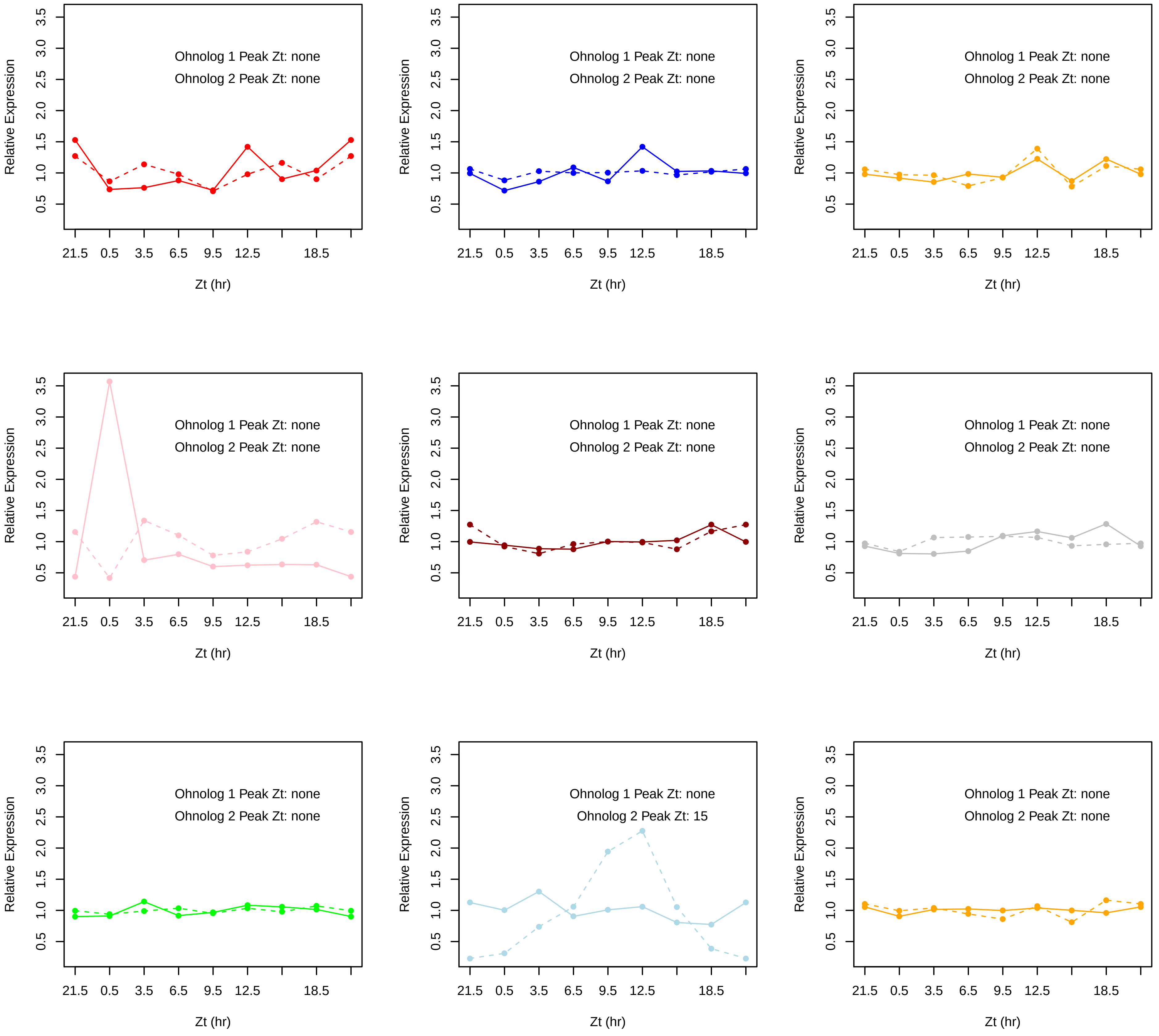

Ohnolog 1: ENSXMAG00000003995
Ohnolog 2: ENSXMAG00000018765 kitlga

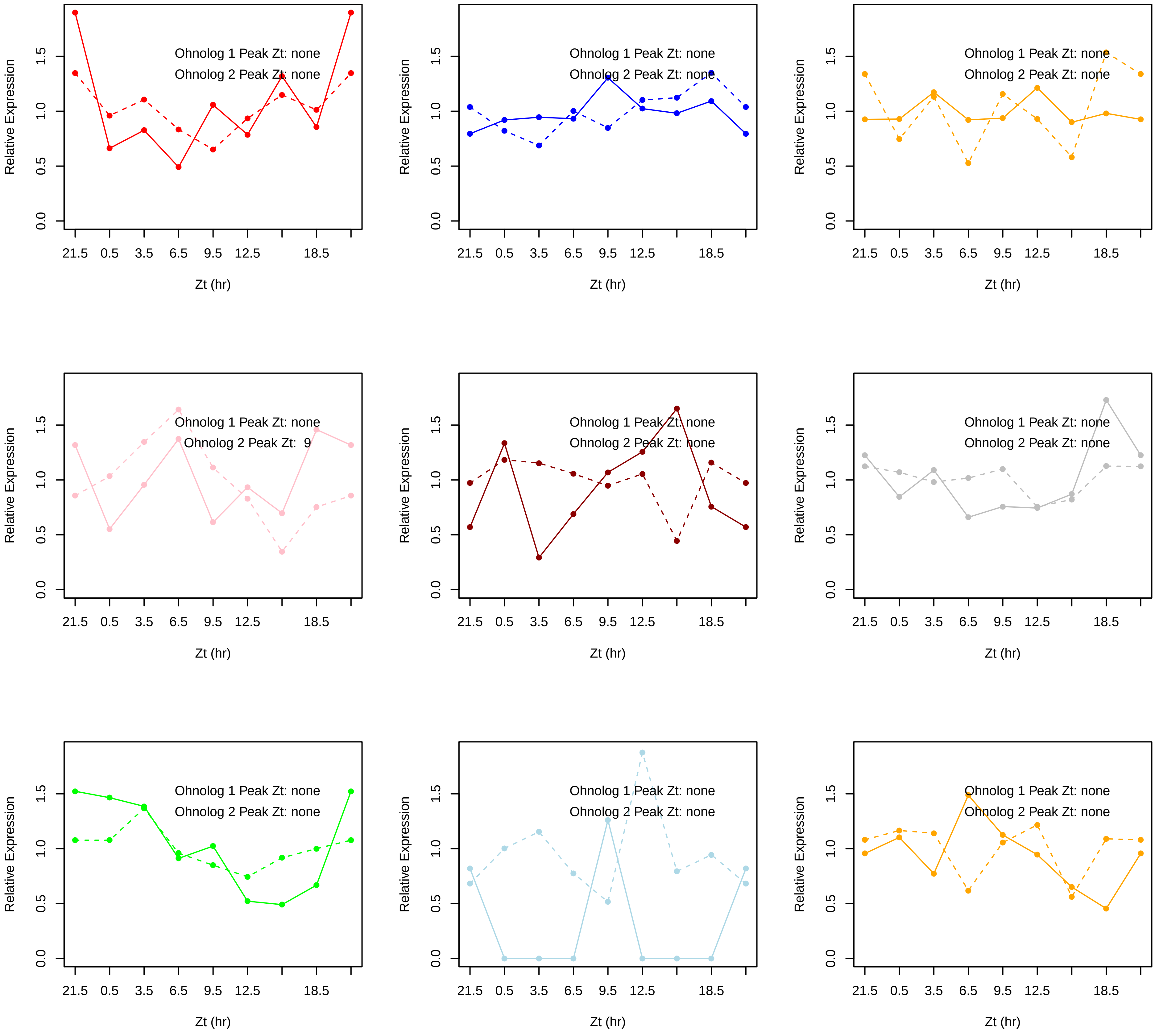

Ohnolog 1: ENSXMAG00000009952
Ohnolog 2: ENSXMAG00000017405 kcnk3a

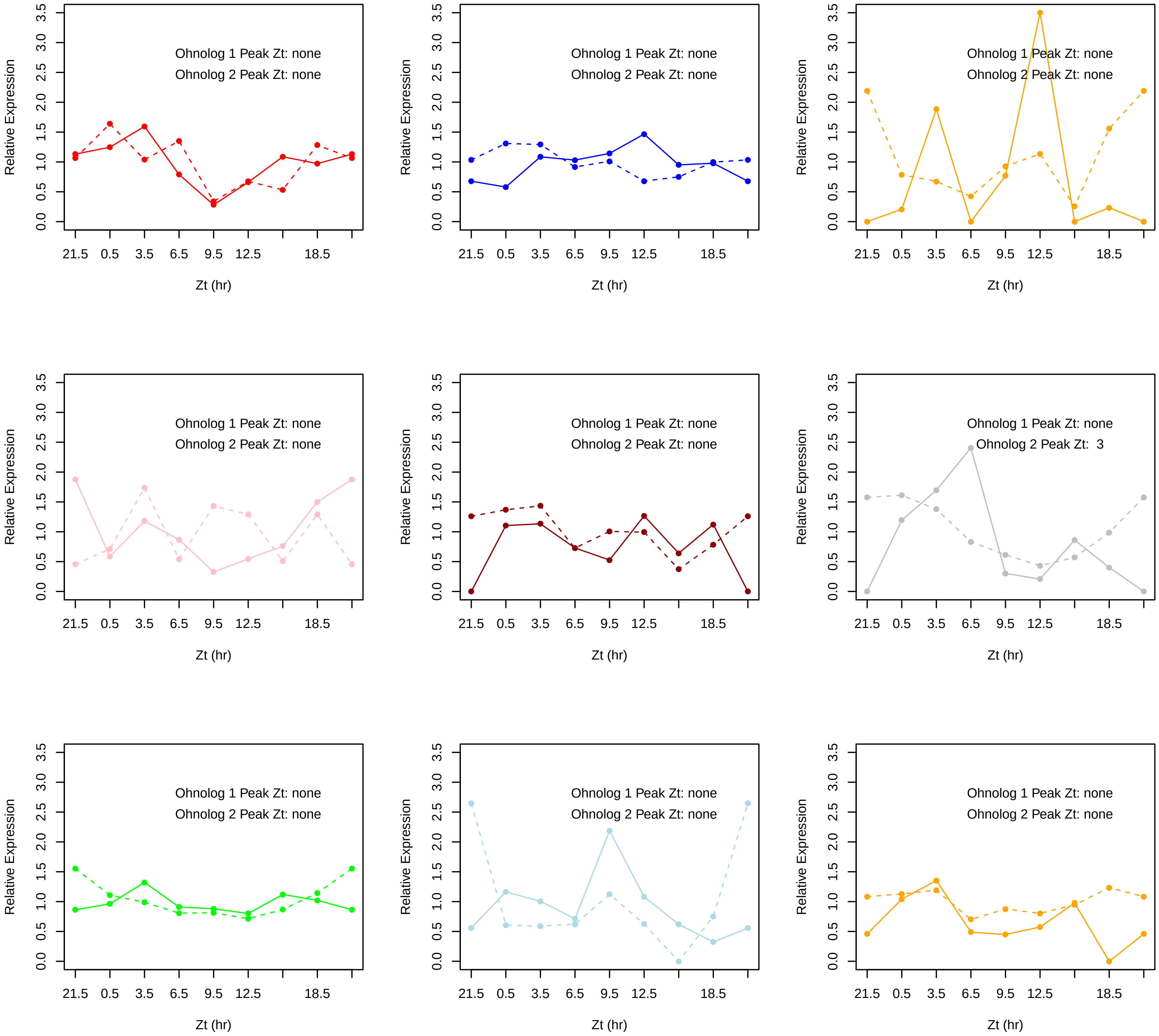

Ohnolog 1: ENSXMAG00000010752 vsnl1a
Ohnolog 2: ENSXMAG00000015237

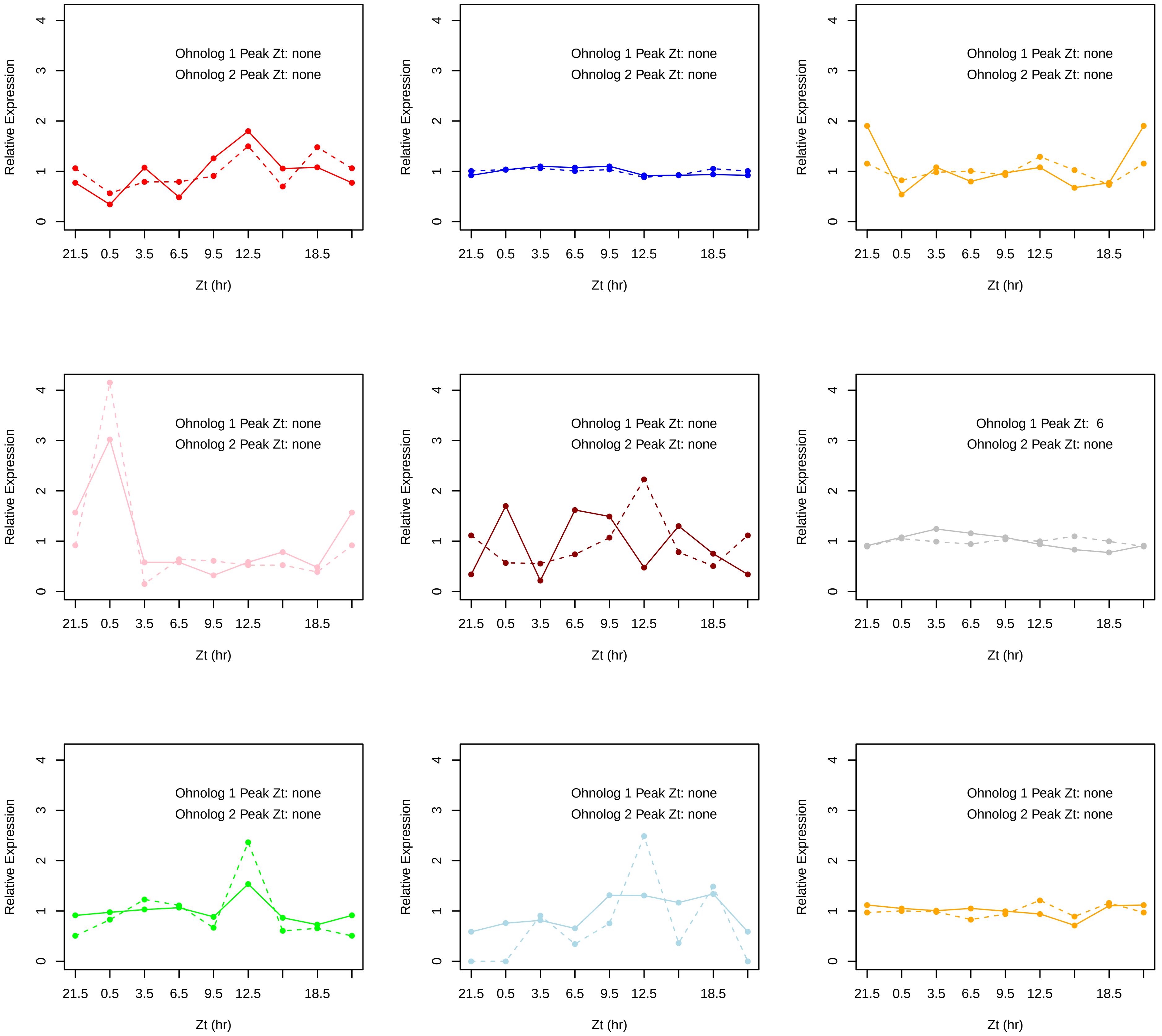

Ohnolog 1: ENSXMAG00000021354 rspo3
Ohnolog 2: ENSXMAG00000025978

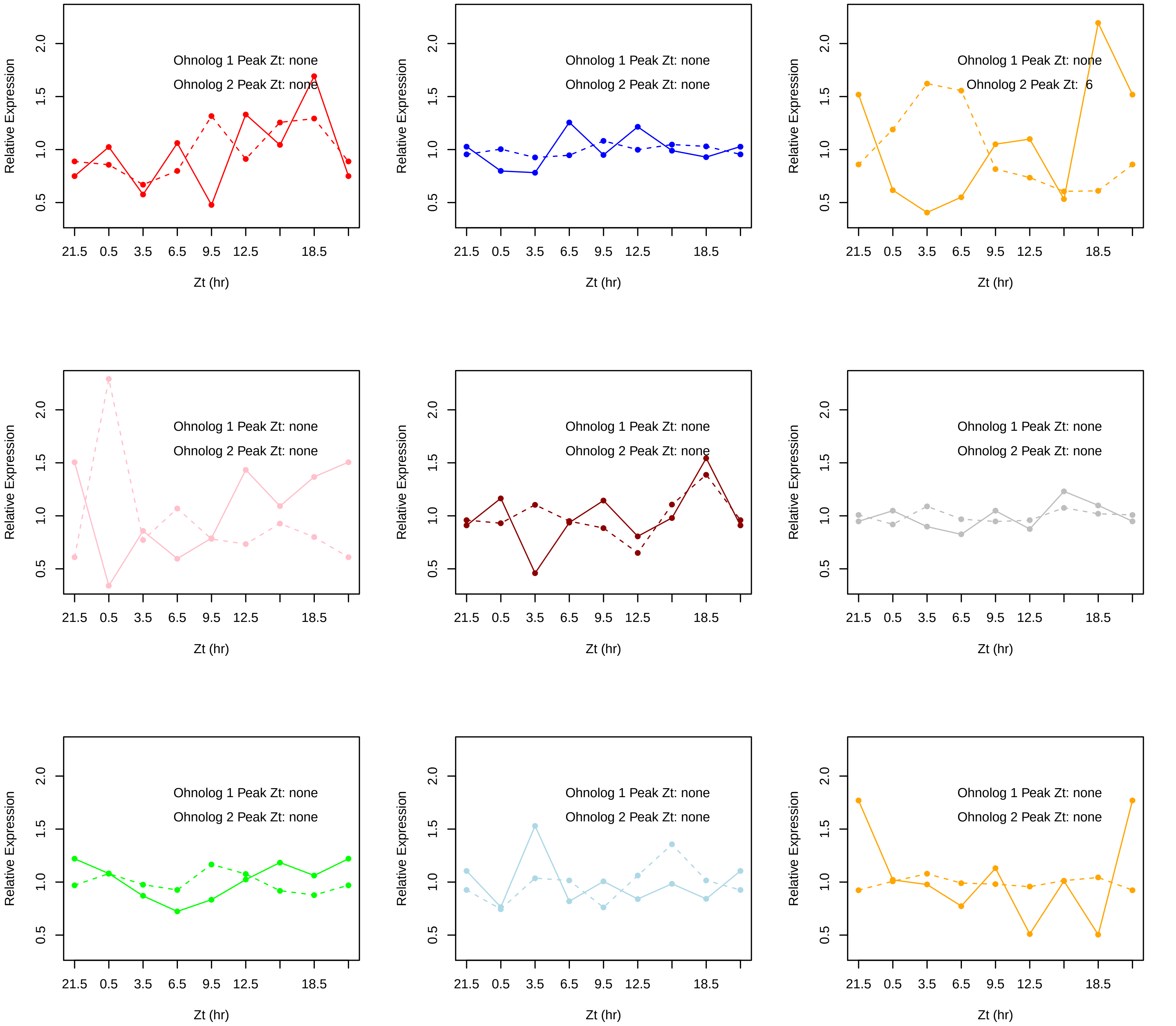

Ohnolog 1: ENSXMAG00000016785 sim1a
Ohnolog 2: ENSXMAG00000019075

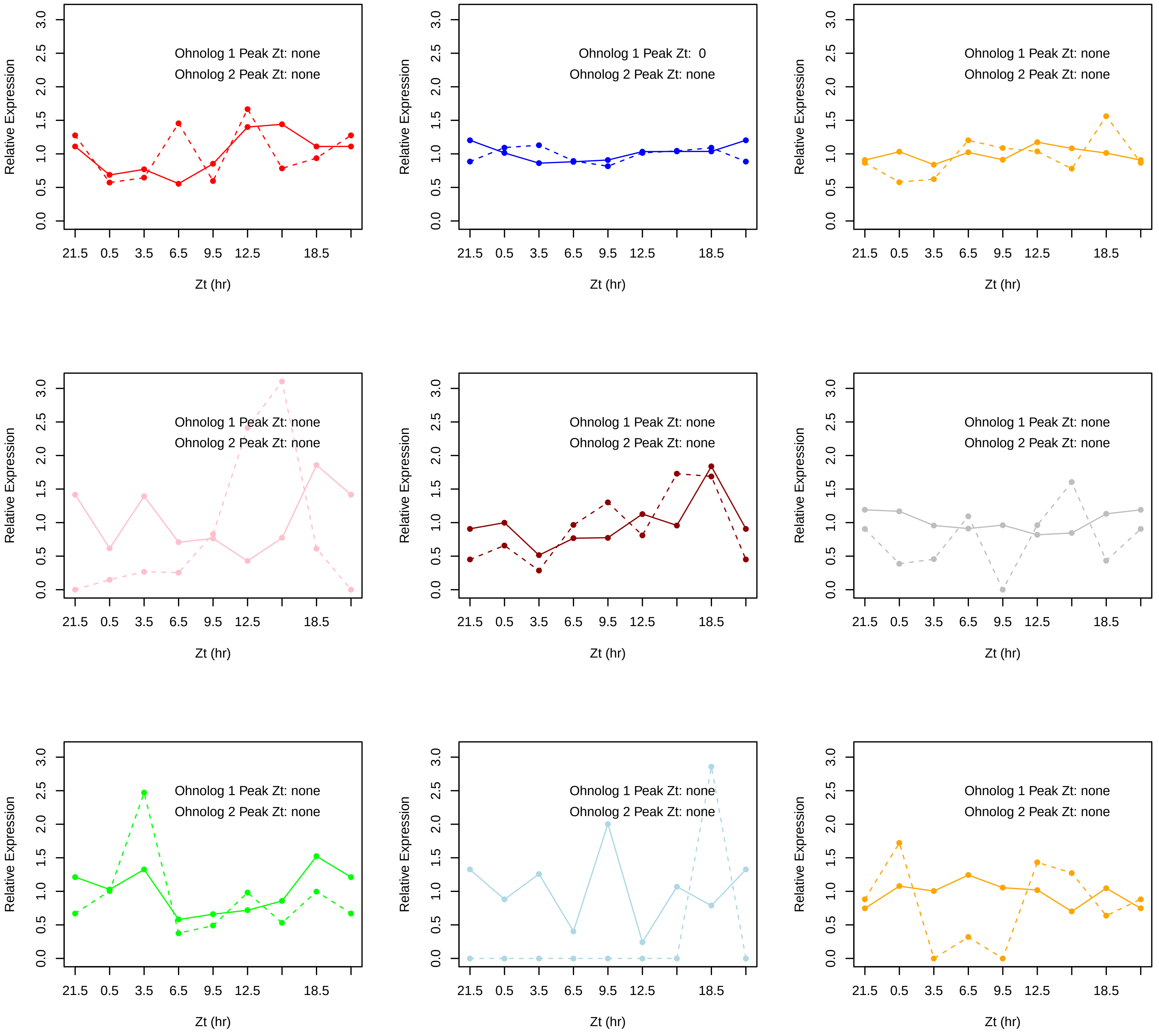

Ohnolog 1: ENSXMAG00000011188 lrtm2a
Ohnolog 2: ENSXMAG00000025768

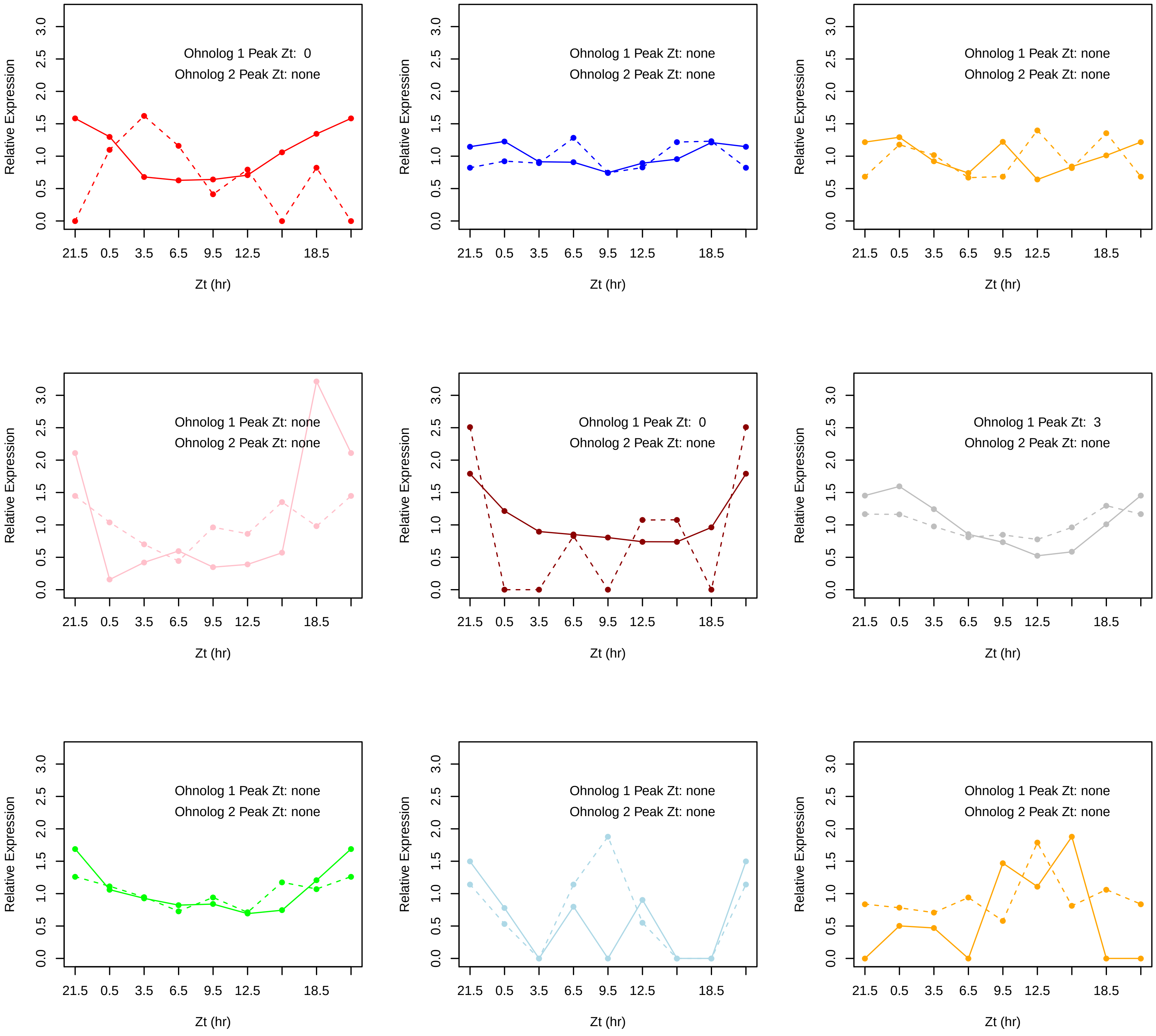

Ohnolog 1: ENSXMAG00000023214
Ohnolog 2: ENSXMAG00000028462 cnstb

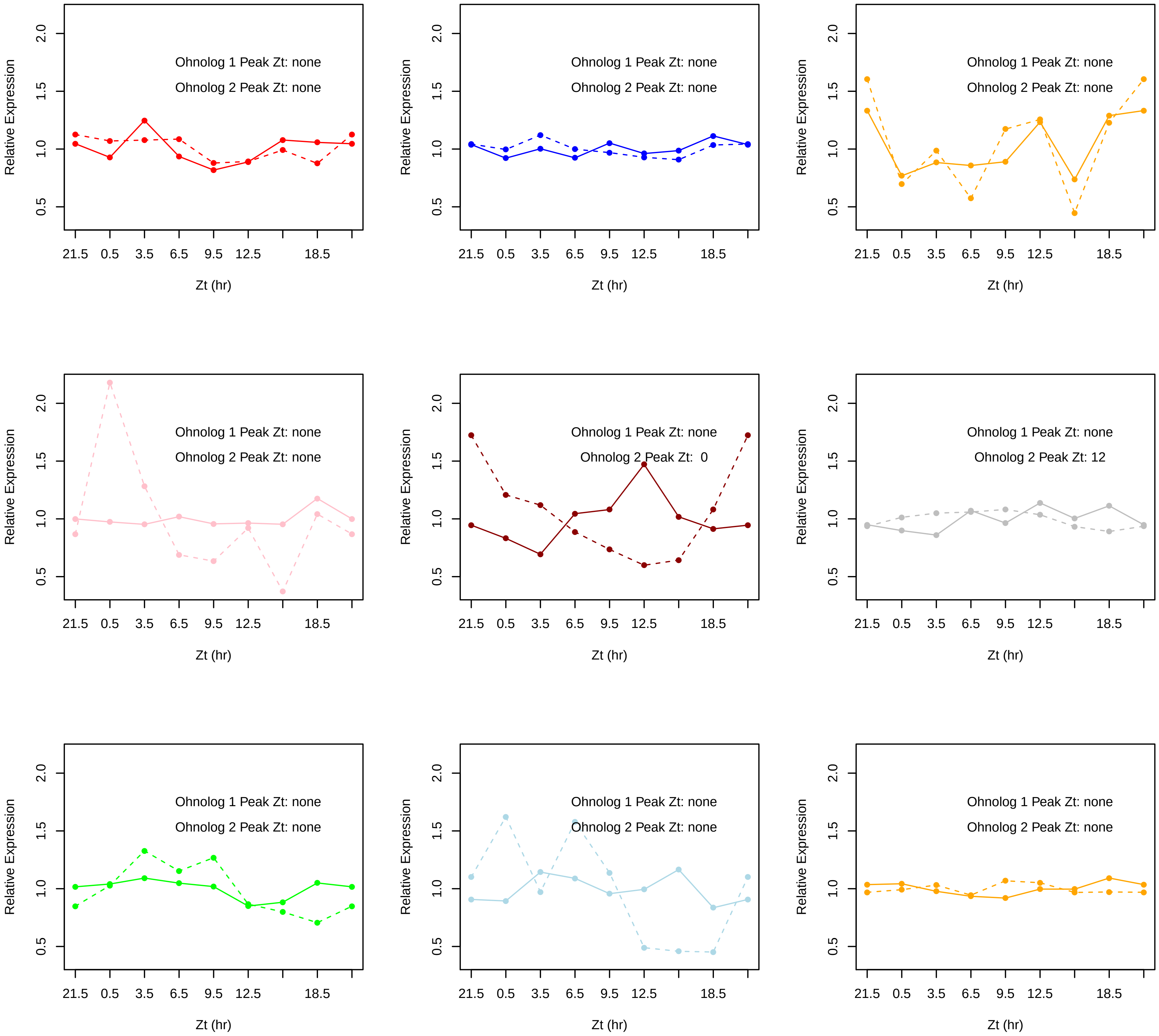

Ohnolog 1: ENSXMAG00000007048 coq8a
Ohnolog 2: ENSXMAG00000015084

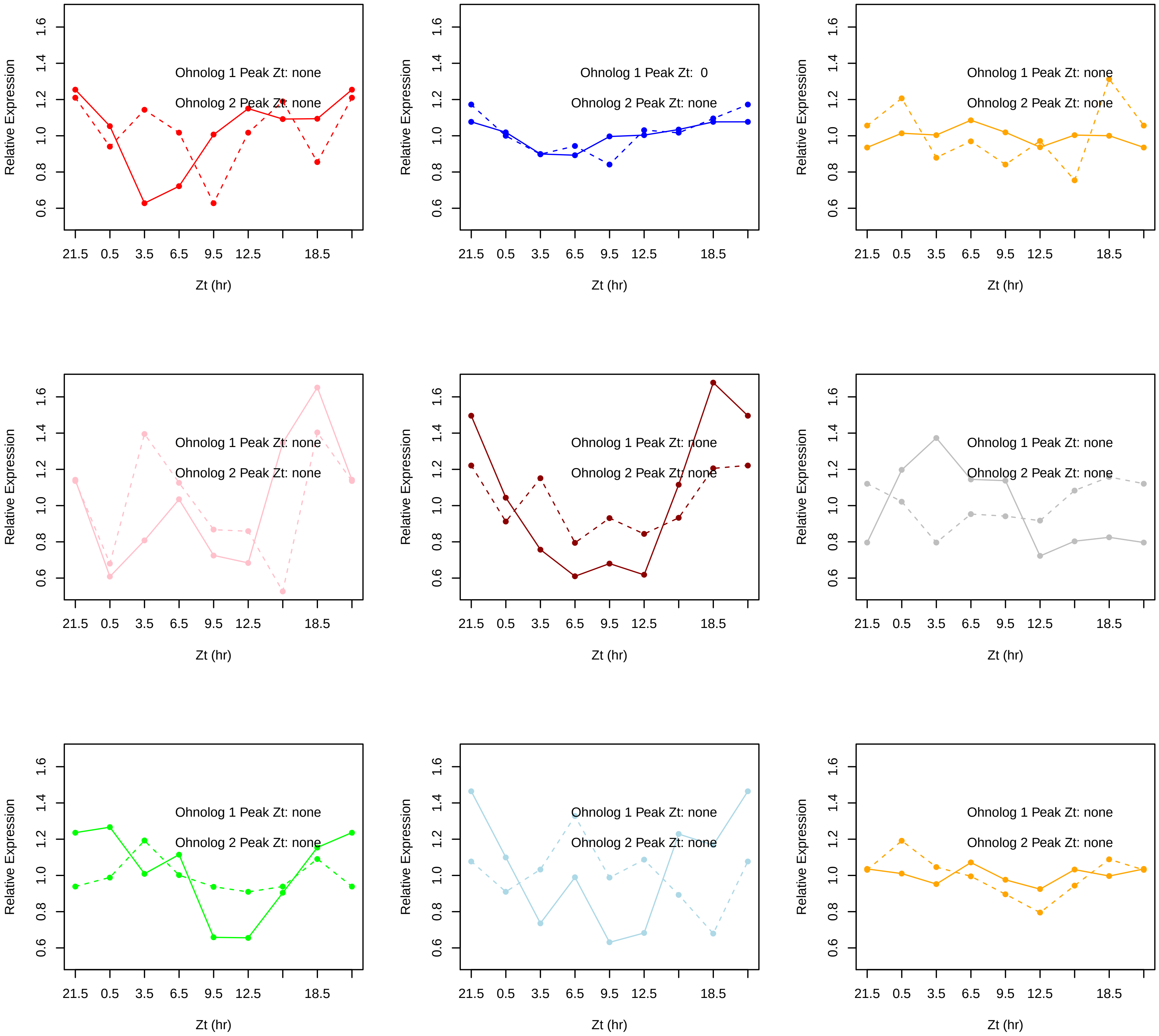

Ohnolog 1: ENSXMAG00000002960
Ohnolog 2: ENSXMAG00000009810 adam17b

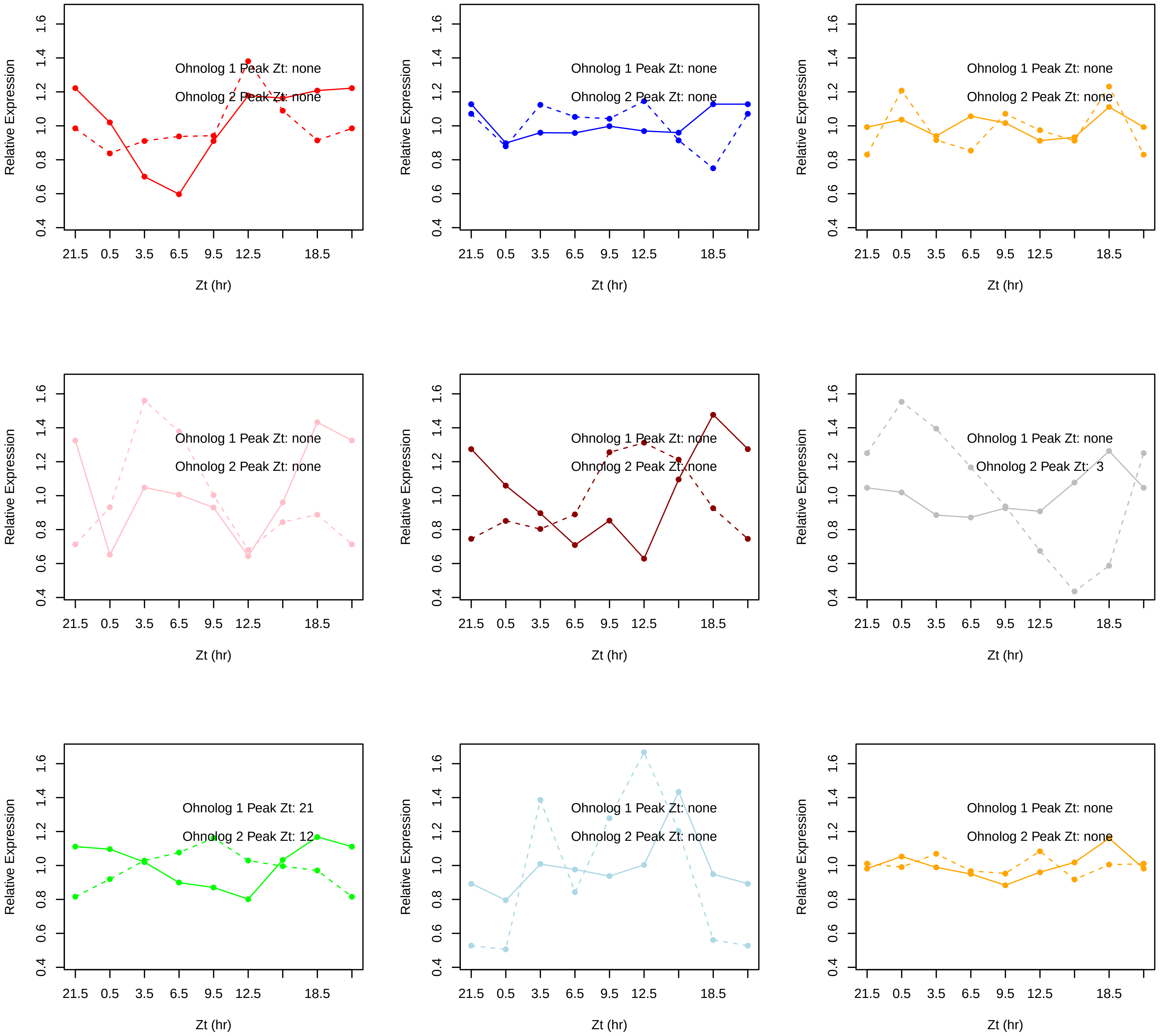

Ohnolog 1: ENSXMAG00000007446 ZDHHC14 (1 of many)
Ohnolog 2: ENSXMAG00000009927 zdhhc14

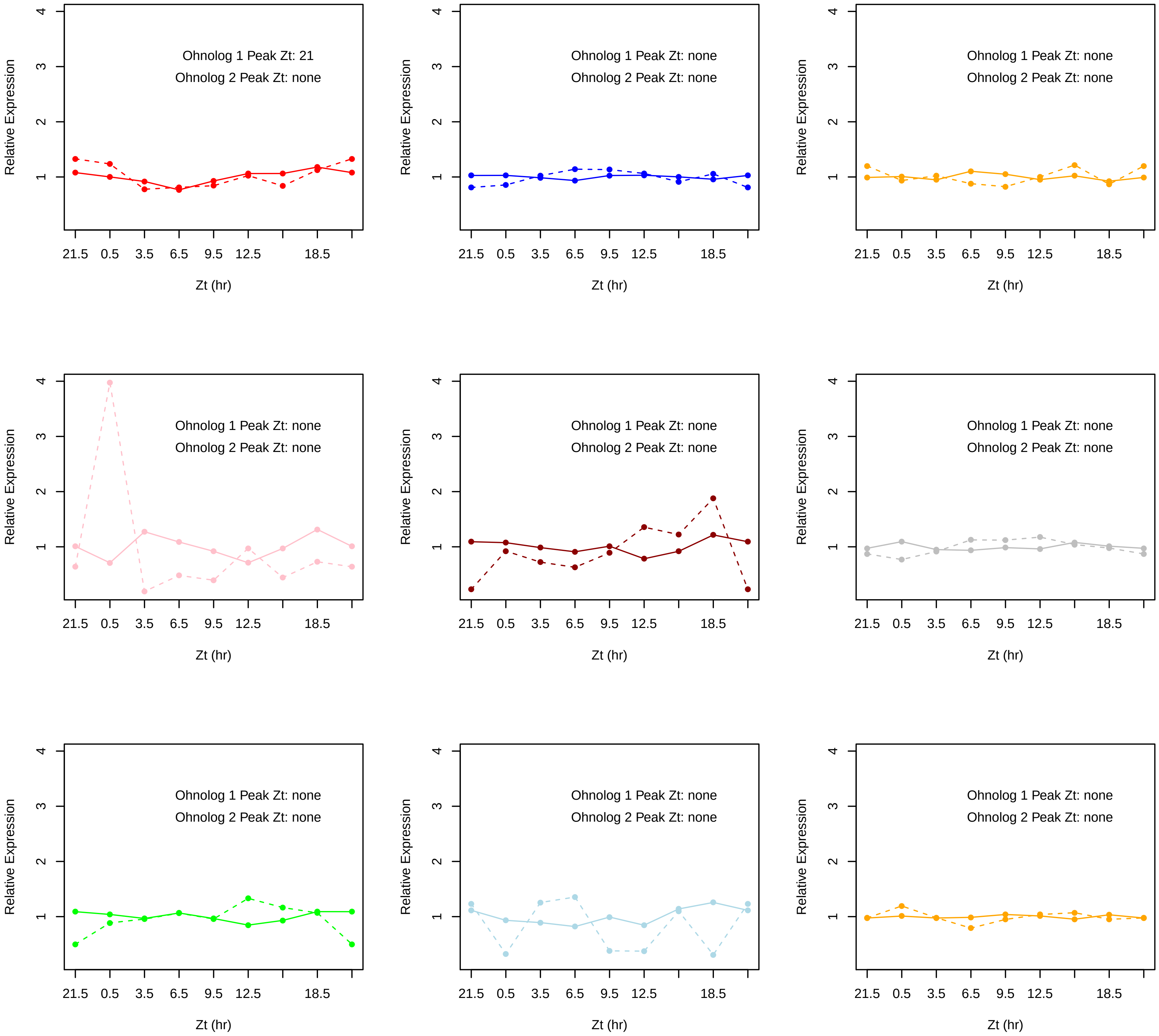

Ohnolog 1: ENSXMAG00000006338
Ohnolog 2: ENSXMAG00000009940 arid1b

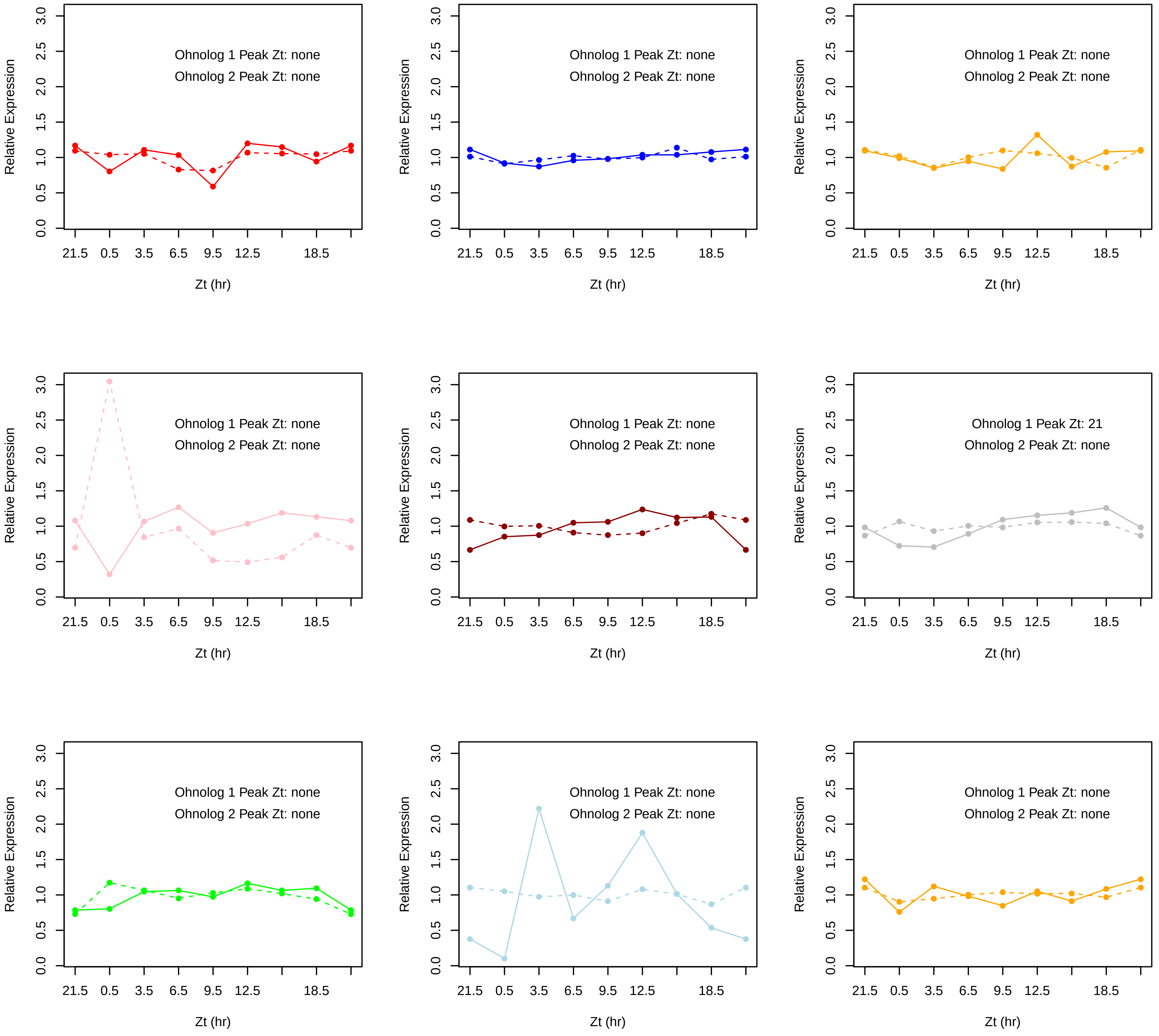

Ohnolog 1: ENSXMAG00000009976 TIAM2 (1 of many)
Ohnolog 2: ENSXMAG00000019210 tiam2a

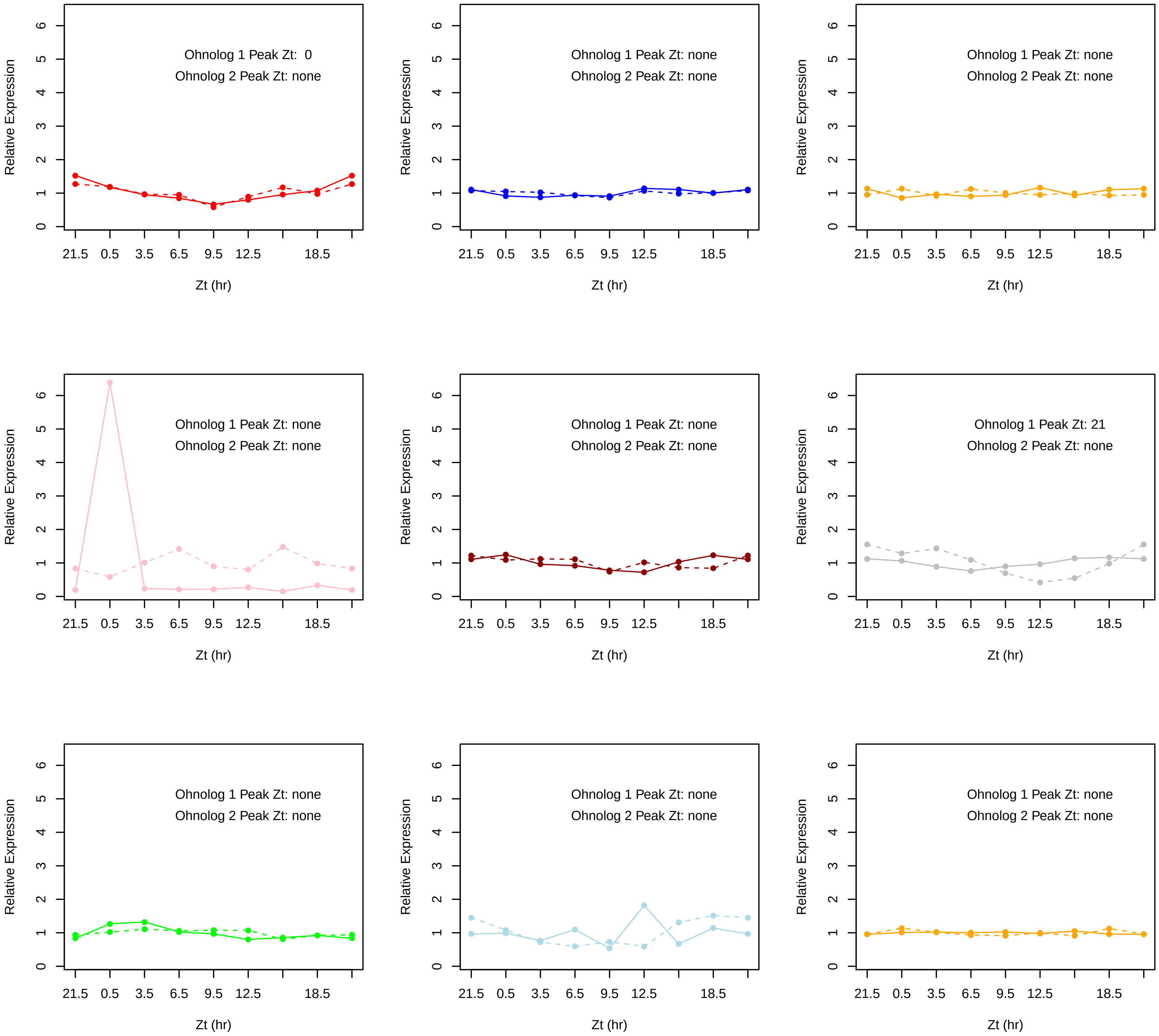

Ohnolog 1: ENSXMAG00000010212 fam83b
Ohnolog 2: ENSXMAG00000014414

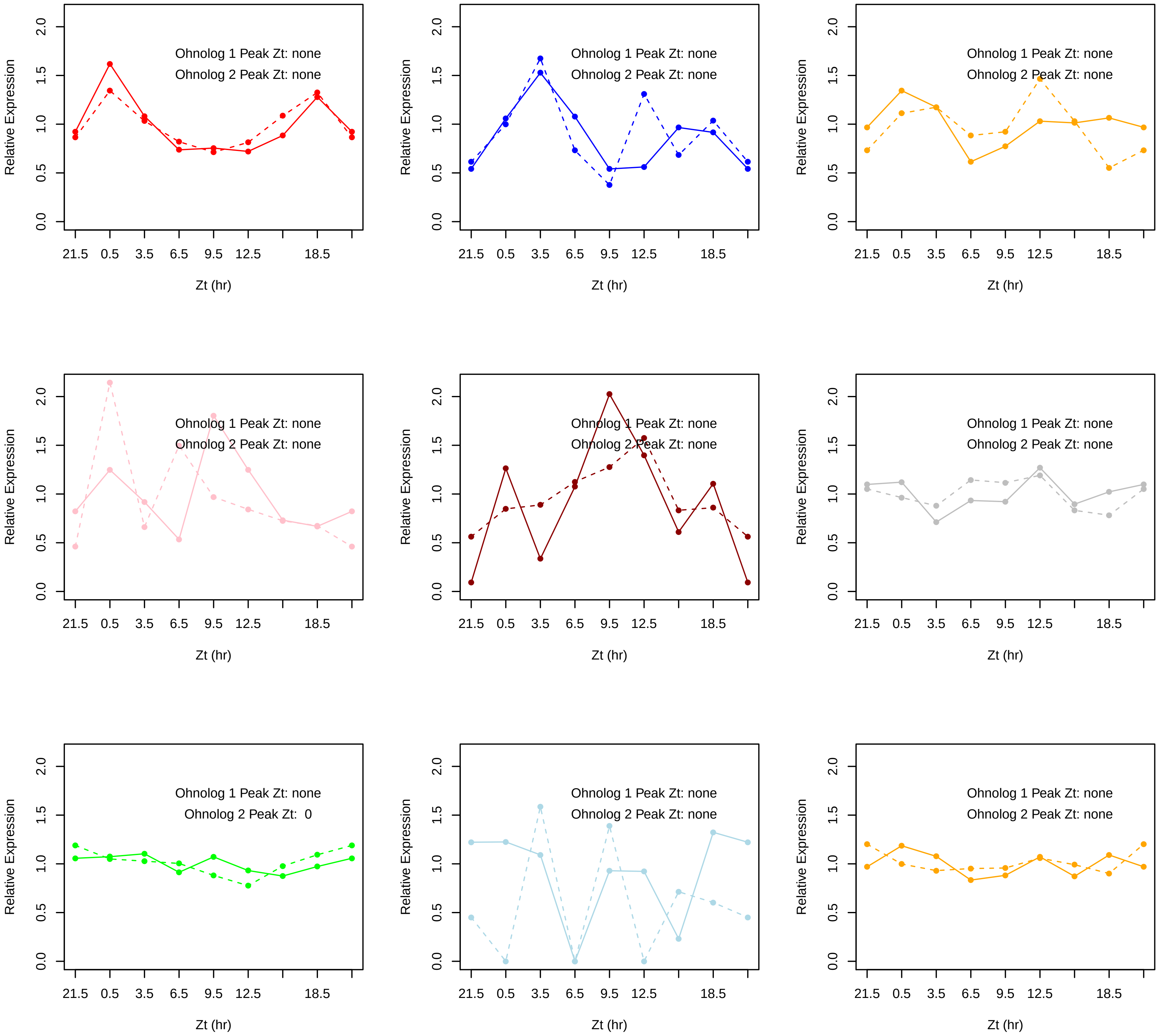

Ohnolog 1: ENSXMAG00000009234 gabrr2a
Ohnolog 2: ENSXMAG00000027658

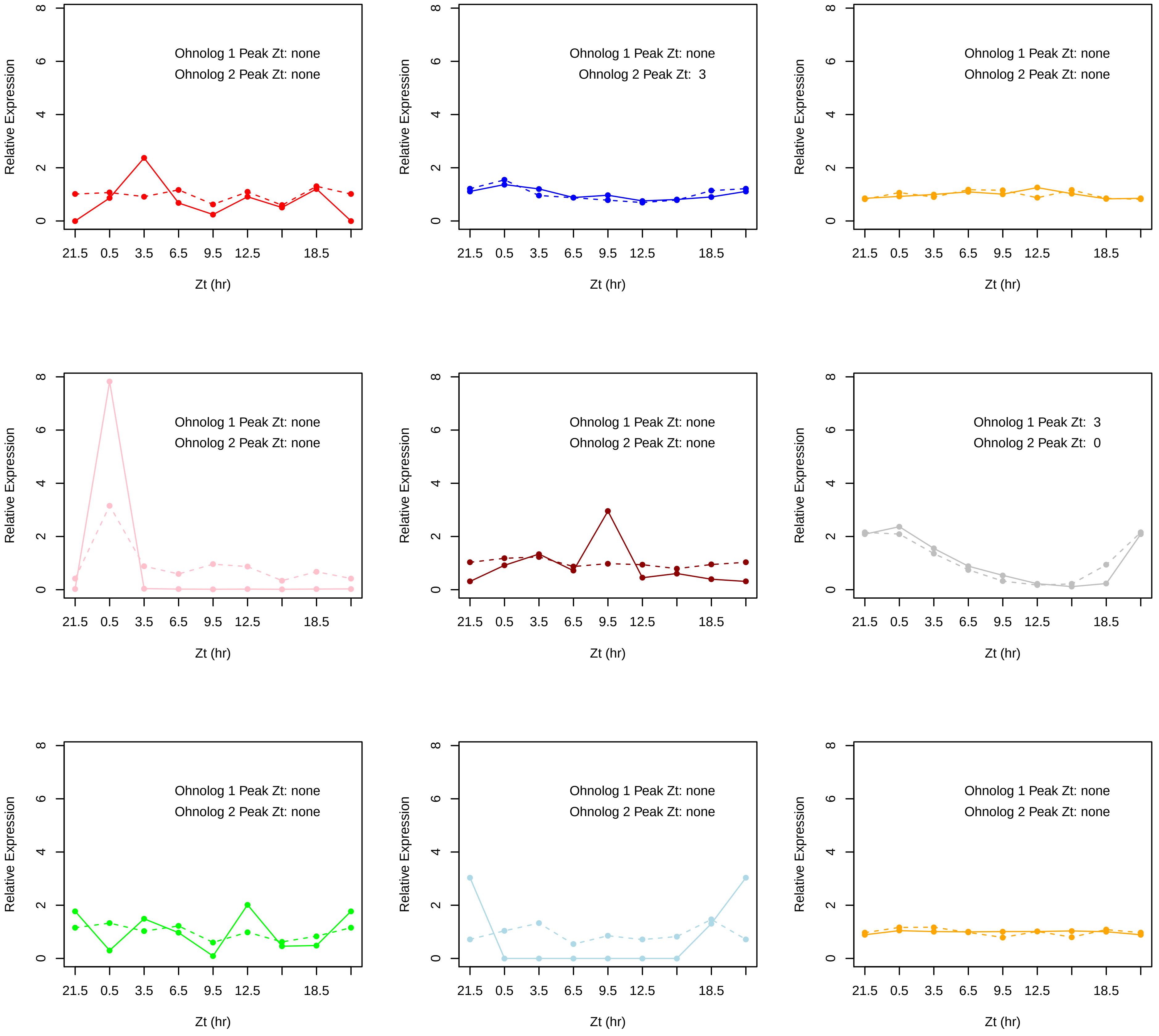

Ohnolog 1: ENSXMAG00000003764 ANO2
Ohnolog 2: ENSXMAG00000008729

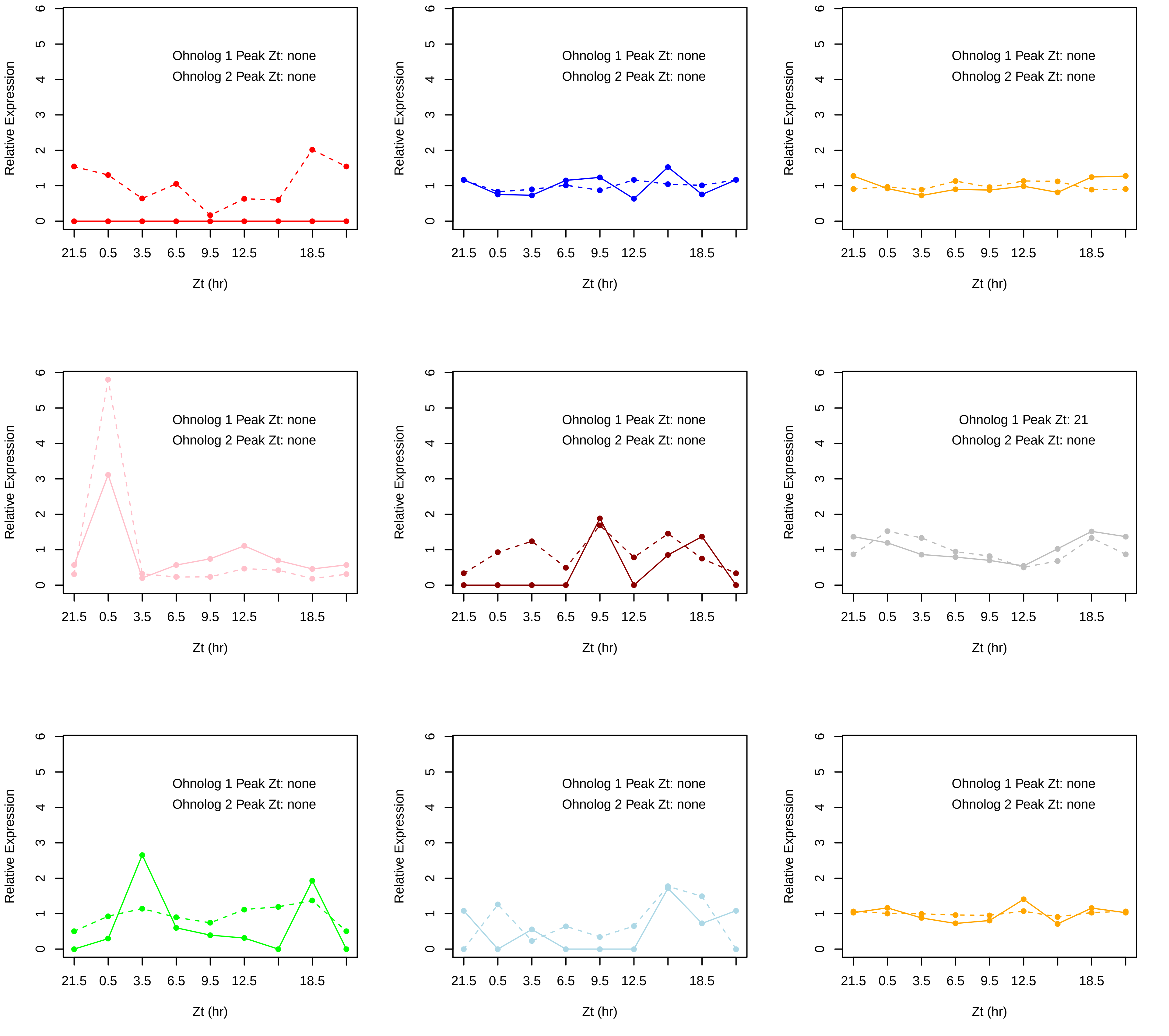

Ohnolog 1: ENSXMAG00000010369 kcnk5b
Ohnolog 2: ENSXMAG00000018611 kcnk5a

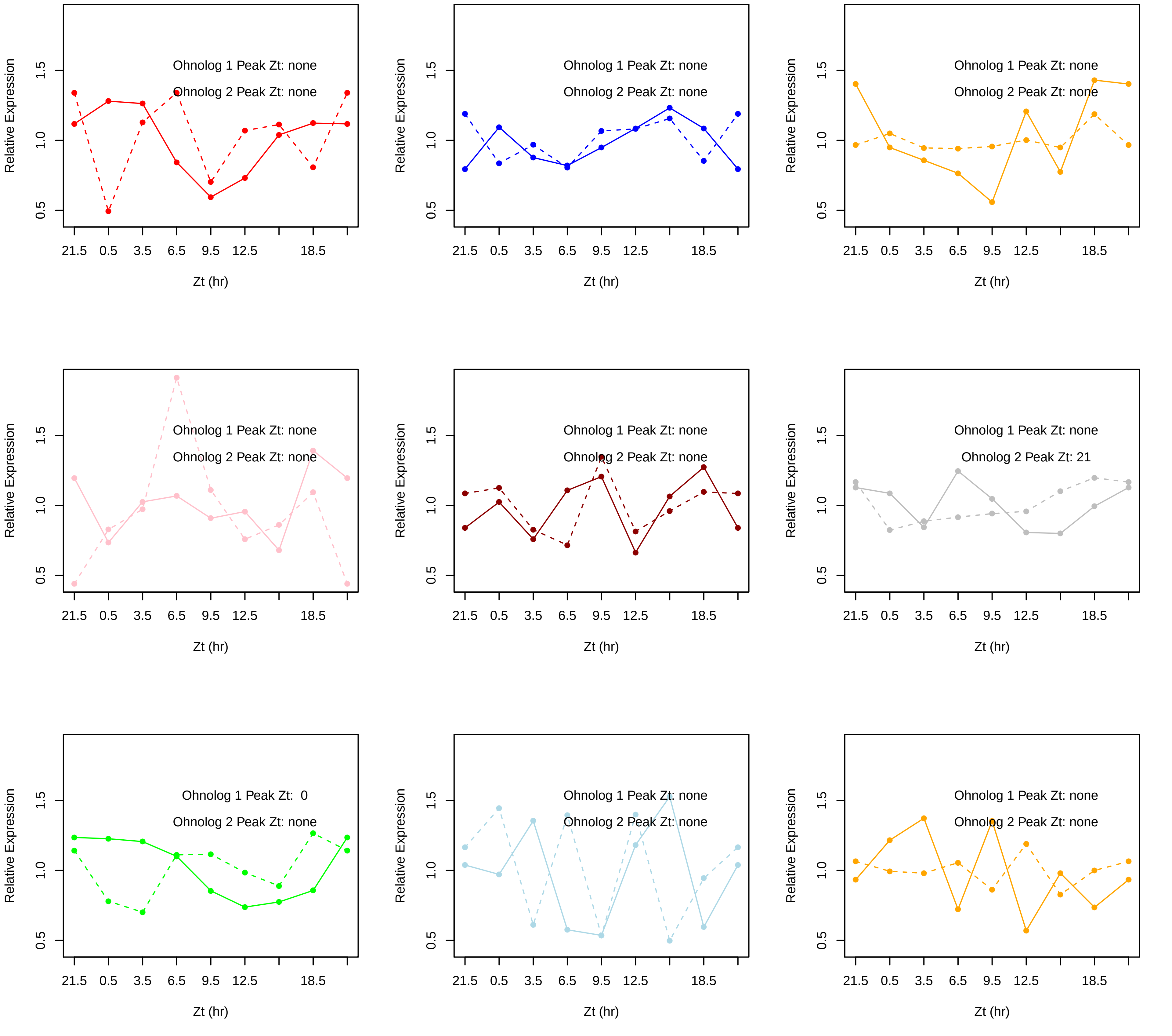

Ohnolog 1: ENSXMAG00000021472 RDH14 (1 of many)
Ohnolog 2: ENSXMAG00000022080 rdh14b

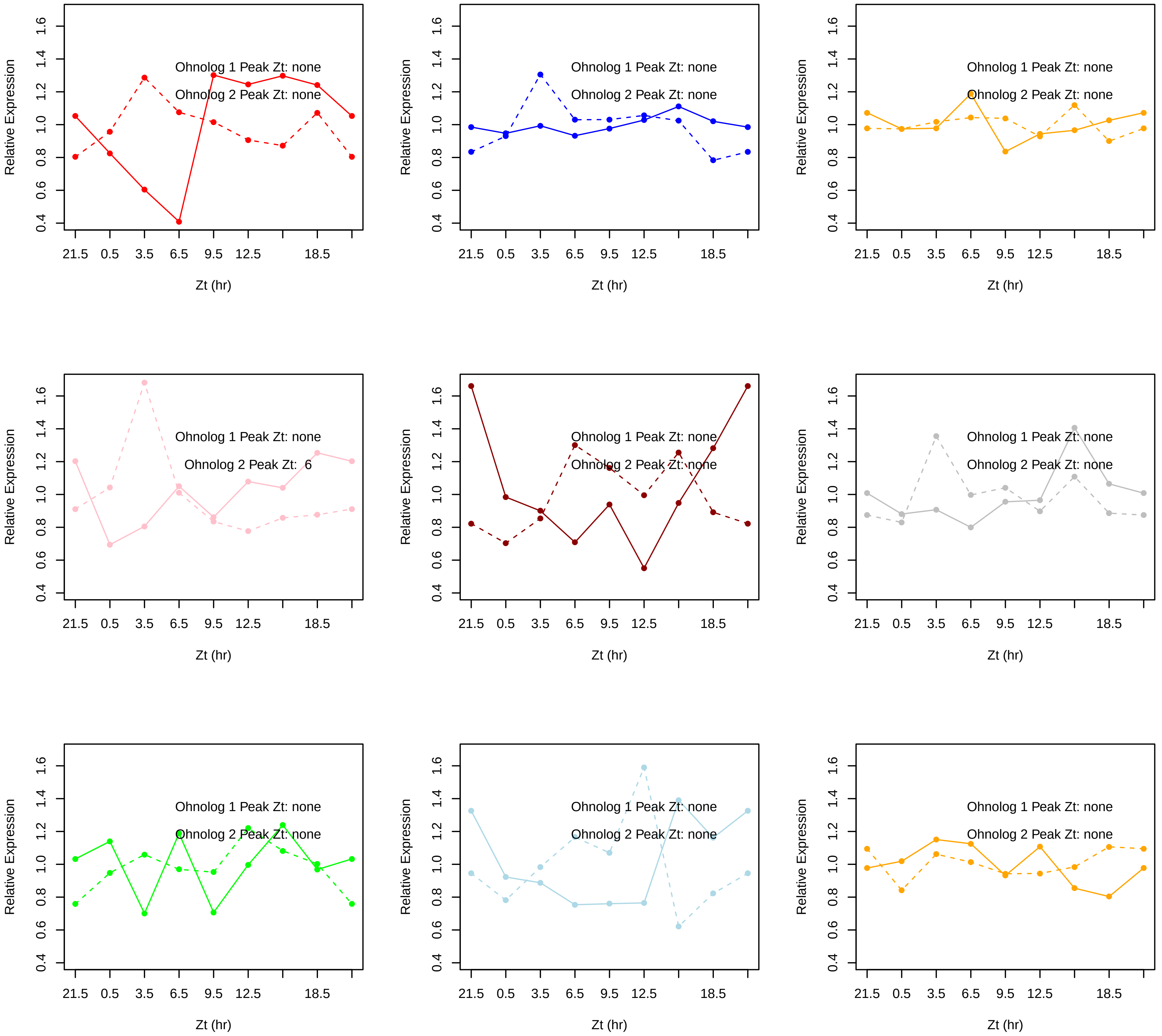

Ohnolog 1: ENSXMAG00000002367 myt1la
Ohnolog 2: ENSXMAG00000028196 MYT1L (1 of many)

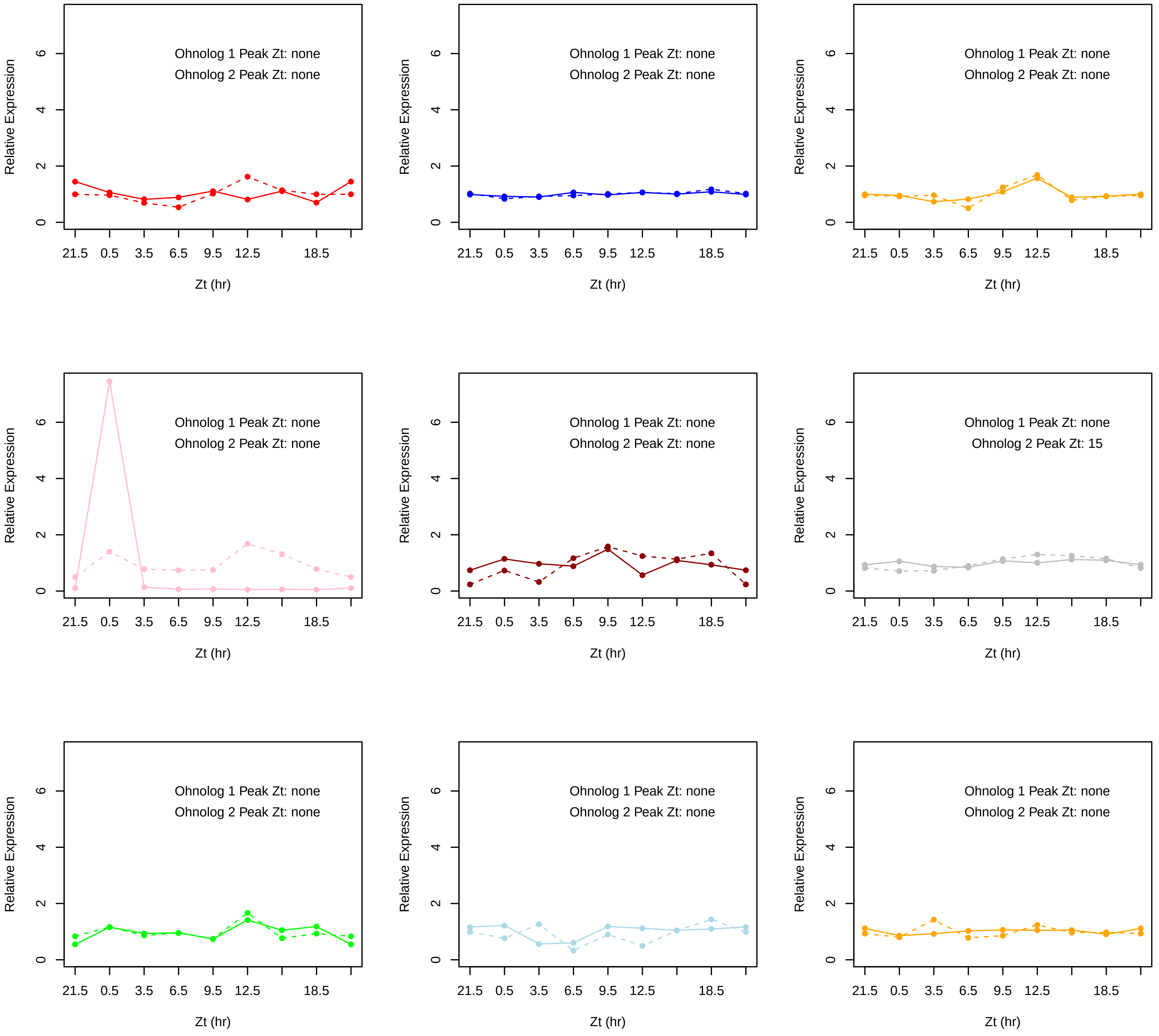

Ohnolog 1: ENSXMAG00000021883
Ohnolog 2: ENSXMAG00000024962 mylpfa

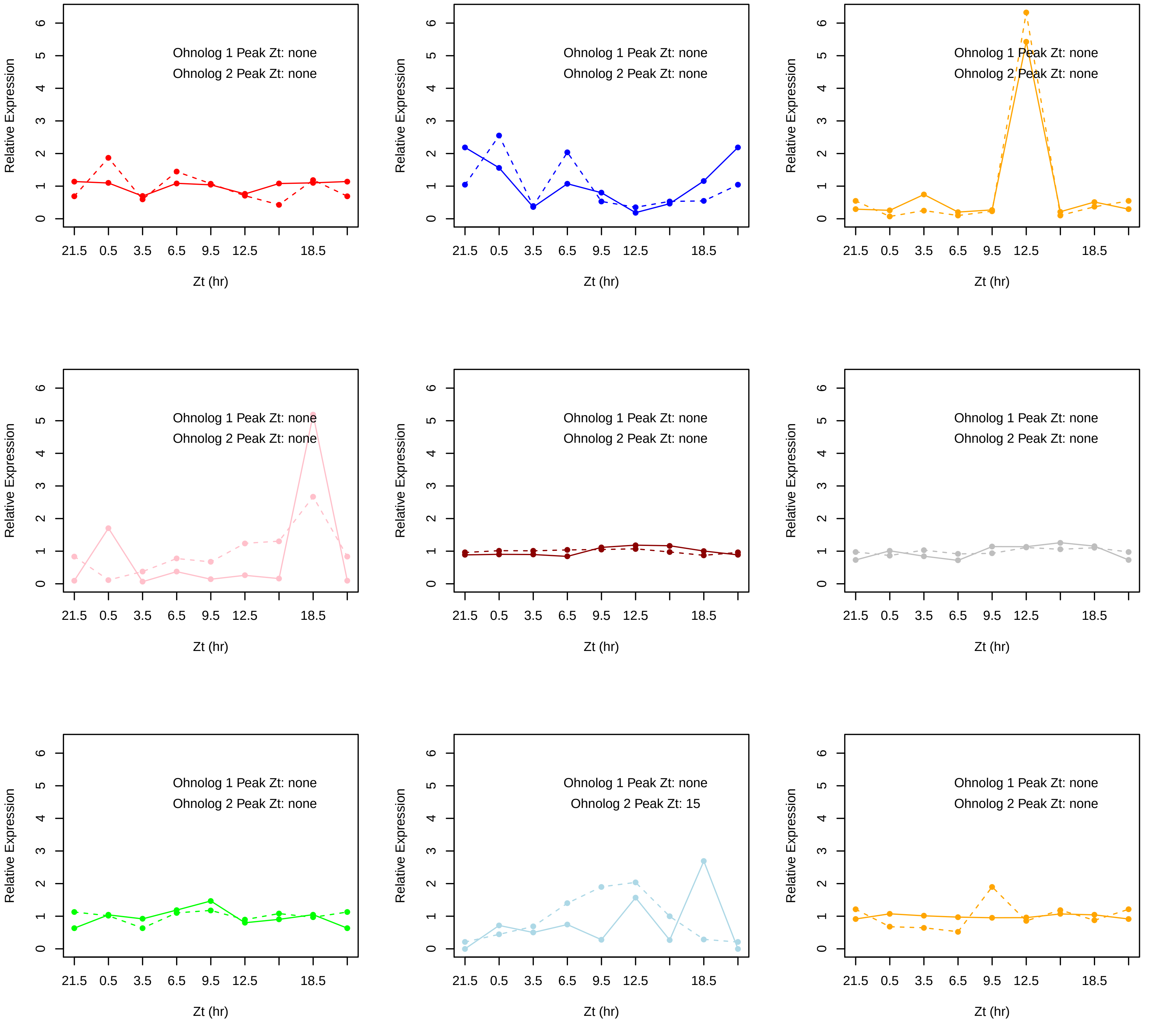

Ohnolog 1: ENSXMAG00000021789
Ohnolog 2: ENSXMAG00000028778

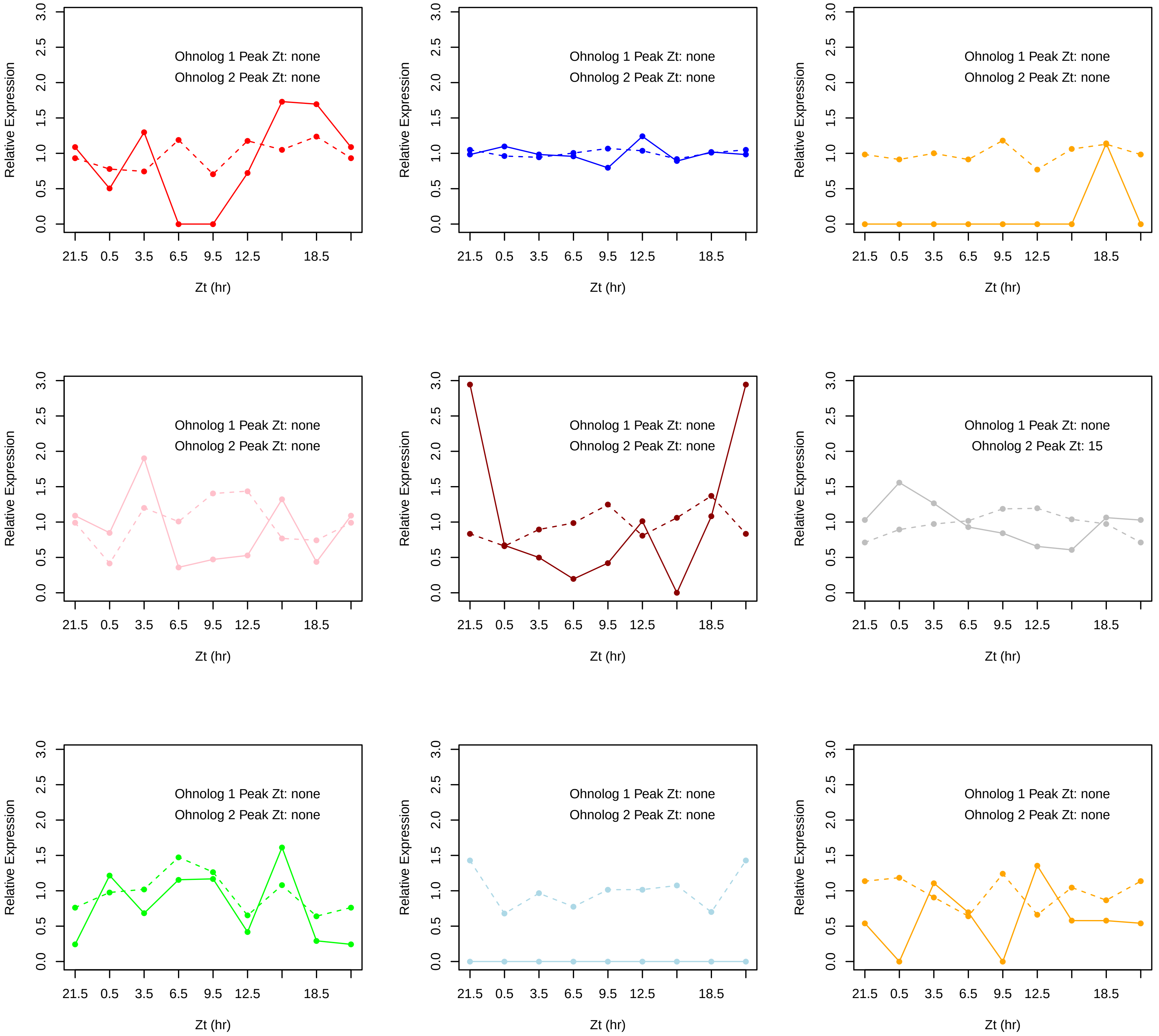

Ohnolog 1: ENSXMAG00000019016
Ohnolog 2: ENSXMAG00000029906

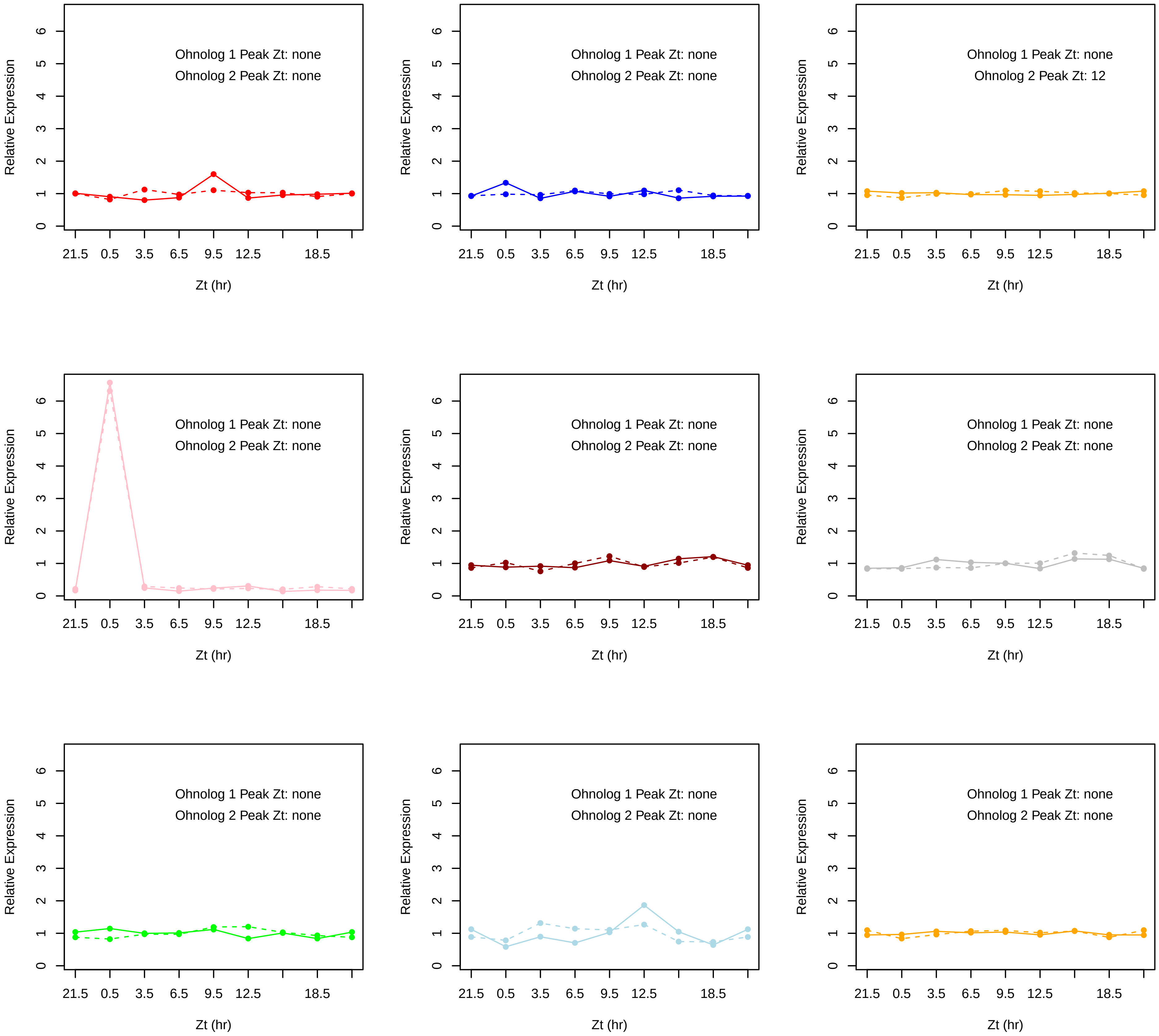

Ohnolog 1: ENSXMAG00000020084 JUND
Ohnolog 2: ENSXMAG00000025875

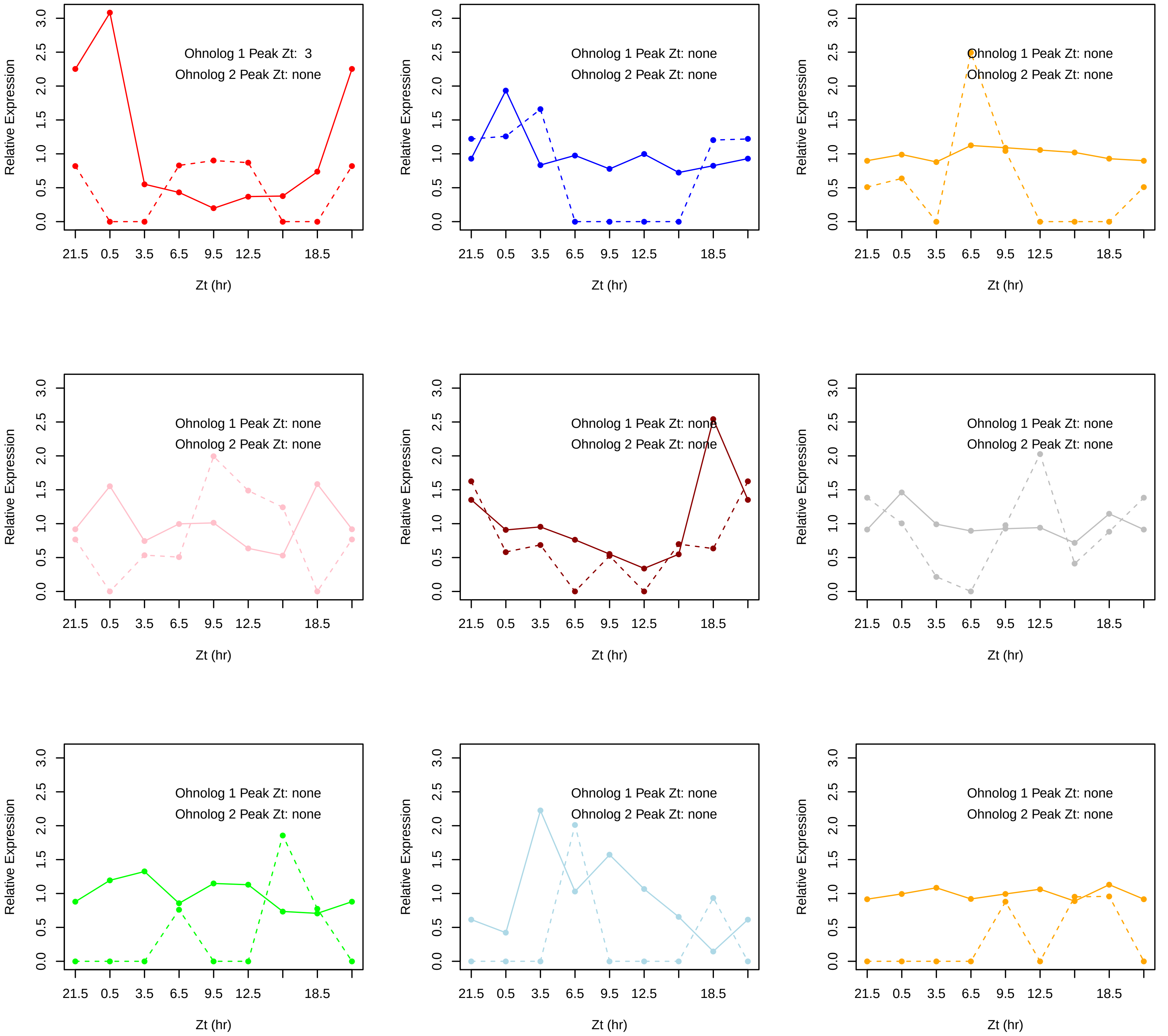

Ohnolog 1: ENSXMAG00000019679 flrt2
Ohnolog 2: ENSXMAG00000020345 flrt2

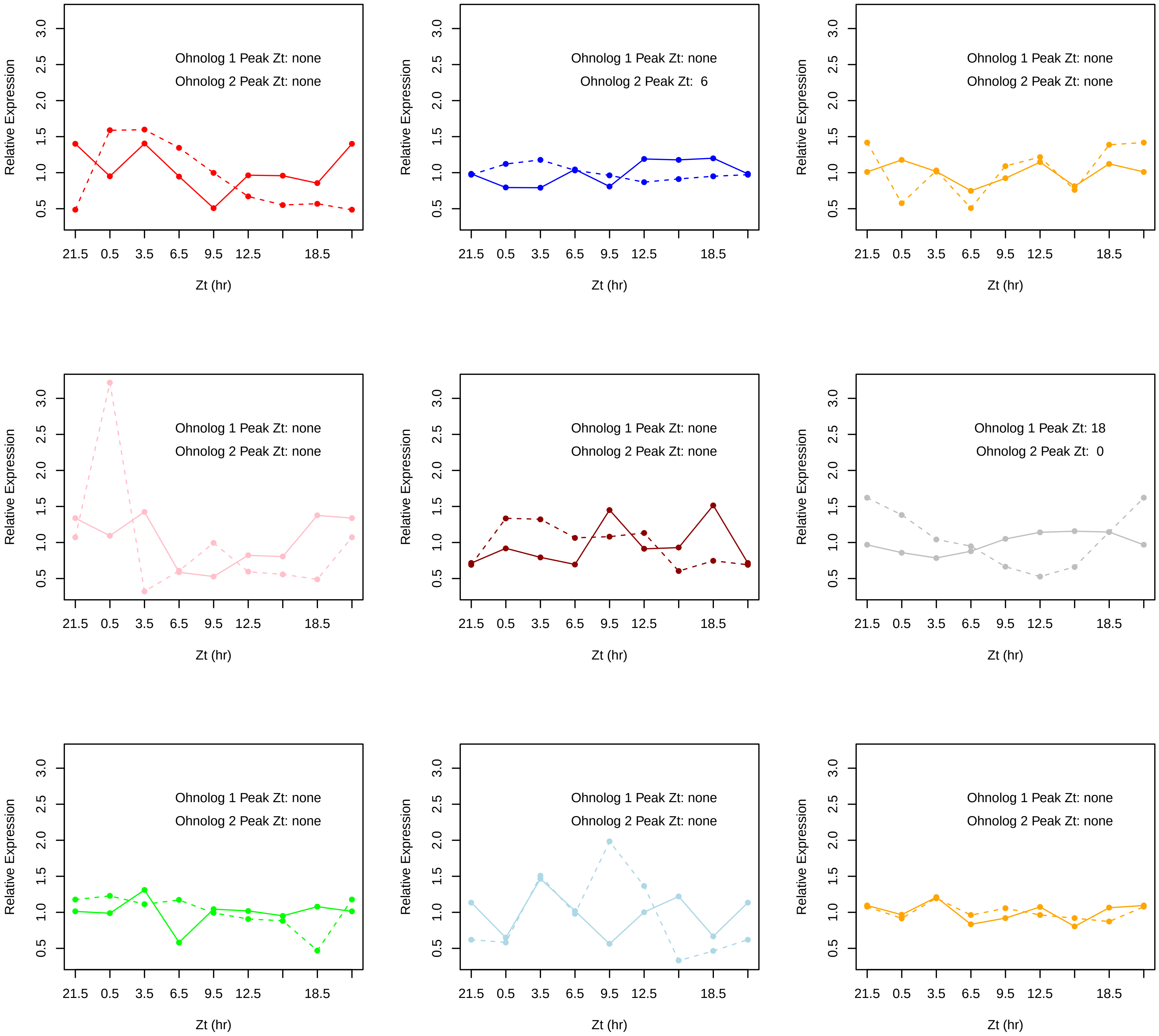

Ohnolog 1: ENSXMAG00000011339 TMEM125
Ohnolog 2: ENSXMAG00000019647 tmem125b

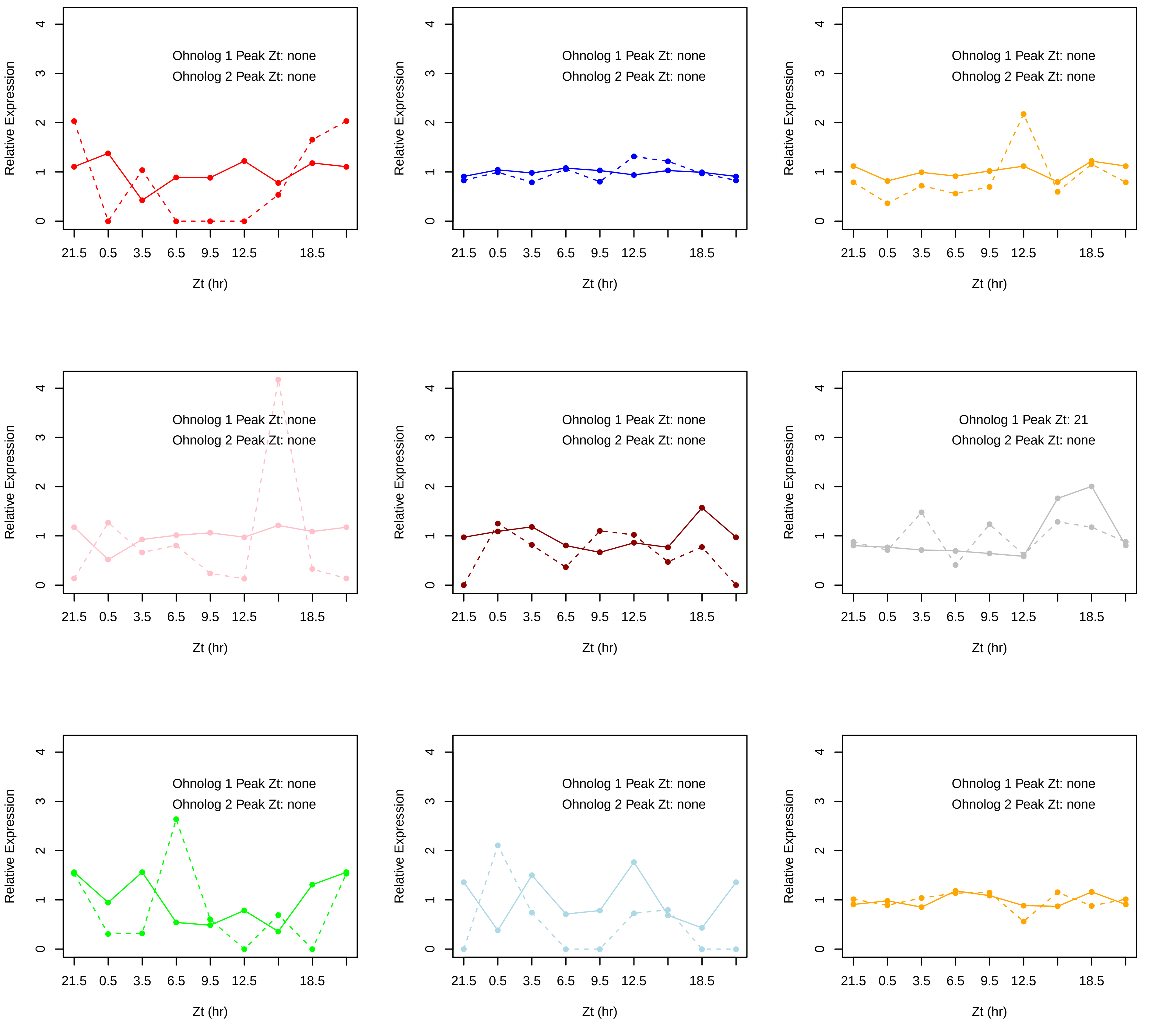

Ohnolog 1: ENSXMAG00000019972
Ohnolog 2: ENSXMAG00000028941 jun

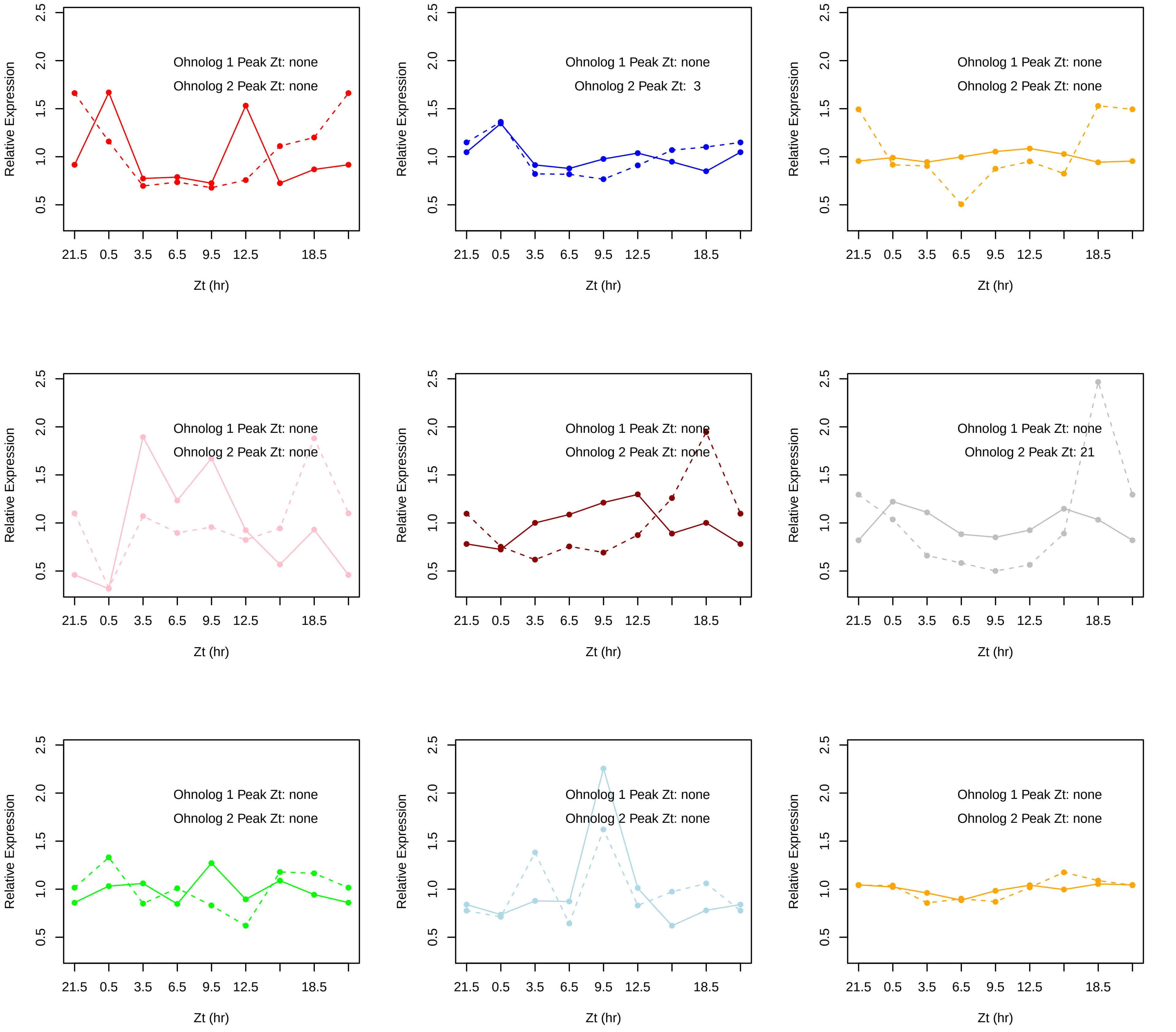

Ohnolog 1: ENSXMAG00000019454 cdc42ep4a
Ohnolog 2: ENSXMAG00000019871 cdc42ep4b

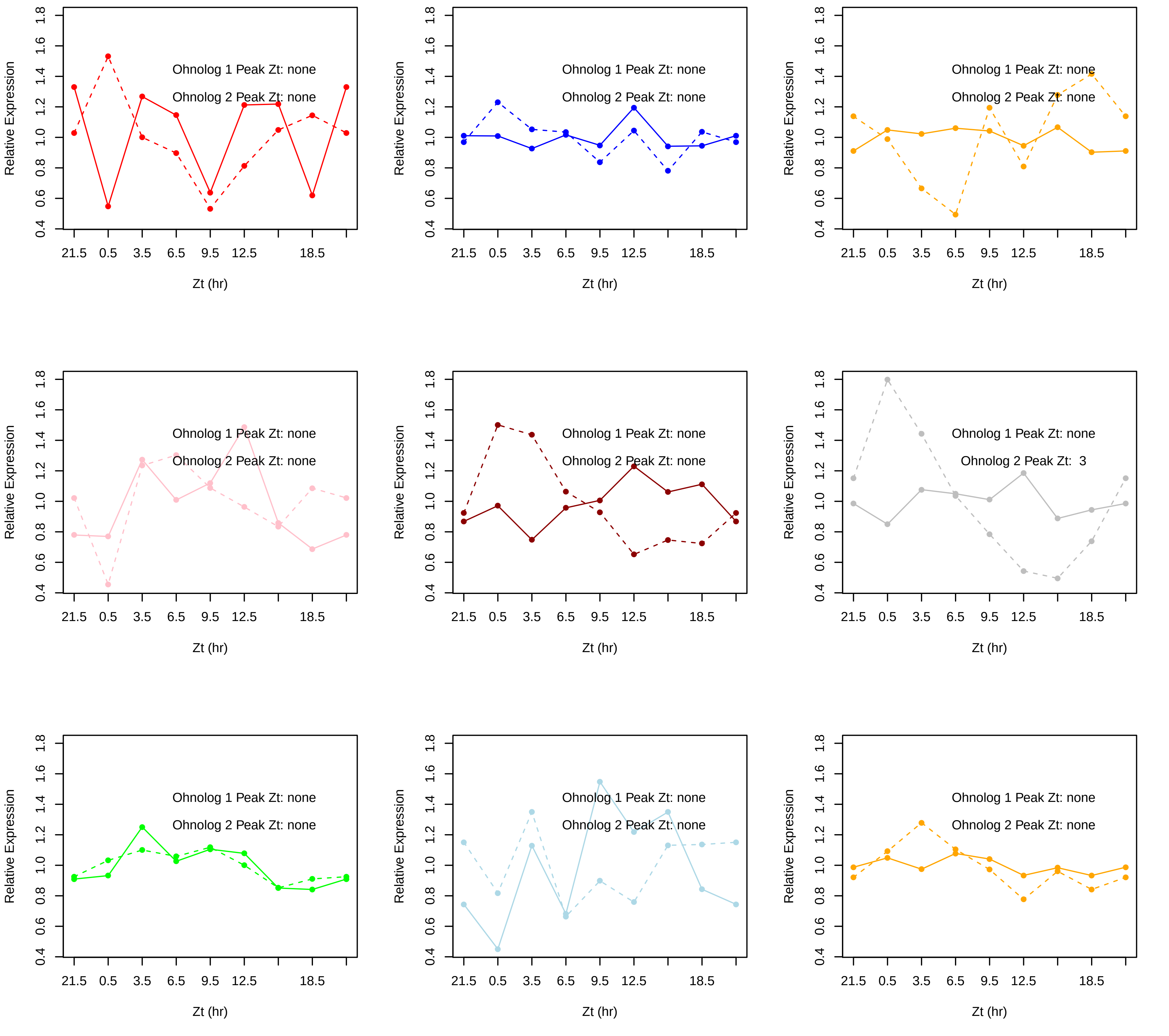

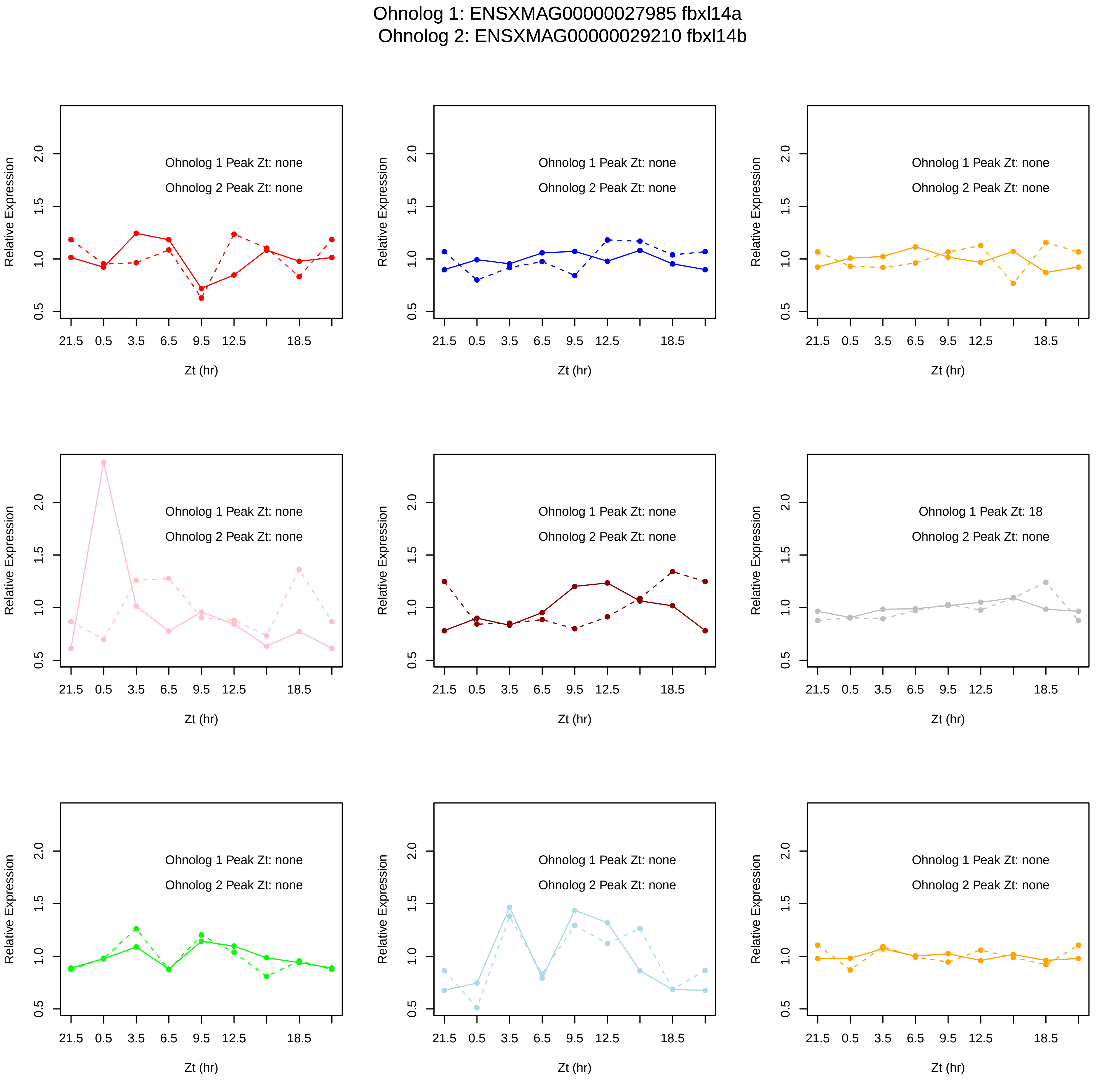
Ohnolog 1: ENSXMAG00000027985 fbxl14a
Ohnolog 2: ENSXMAG00000029210 fbxl14b
Ohnolog 1 Peak Zt: none
Ohnolog 2 Peak Zt: none
Ohnolog 1 Peak Zt: none
Ohnolog 2 Peak Zt: none
Ohnolog 1 Peak Zt: none
Ohnolog 2 Peak Zt: none
Ohnolog 1 Peak Zt: none
Ohnolog 2 Peak Zt: none
Ohnolog 1 Peak Zt: none
Ohnolog 2 Peak Zt: none
Ohnolog 1 Peak Zt: 18
Ohnolog 2 Peak Zt: none
Ohnolog 1 Peak Zt: none
Ohnolog 2 Peak Zt: none
Ohnolog 1 Peak Zt: none
Ohnolog 2 Peak Zt: none
Ohnolog 1 Peak Zt: none
Ohnolog 2 Peak Zt: none
Relative Expression
Zt (hr)
0.5
1.0
1.5
2.0
21.5
0.5
3.5
6.5
9.5
12.5
18.5

Ohnolog 1: ENSXMAG00000009062
Ohnolog 2: ENSXMAG00000020192 rbm15

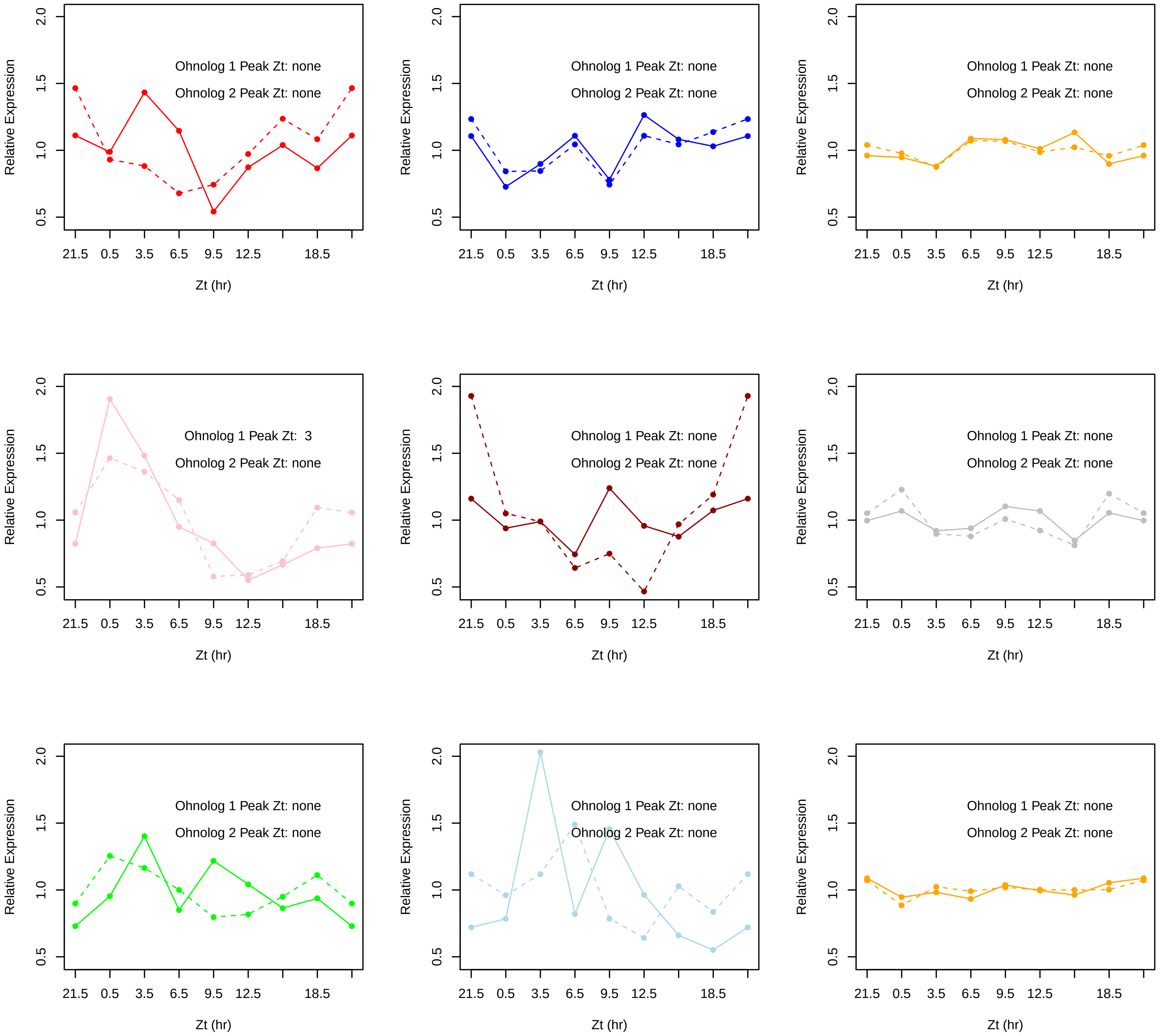

Ohnolog 1: ENSXMAG00000019909 ADRA2C
Ohnolog 2: ENSXMAG00000028511 adra2c

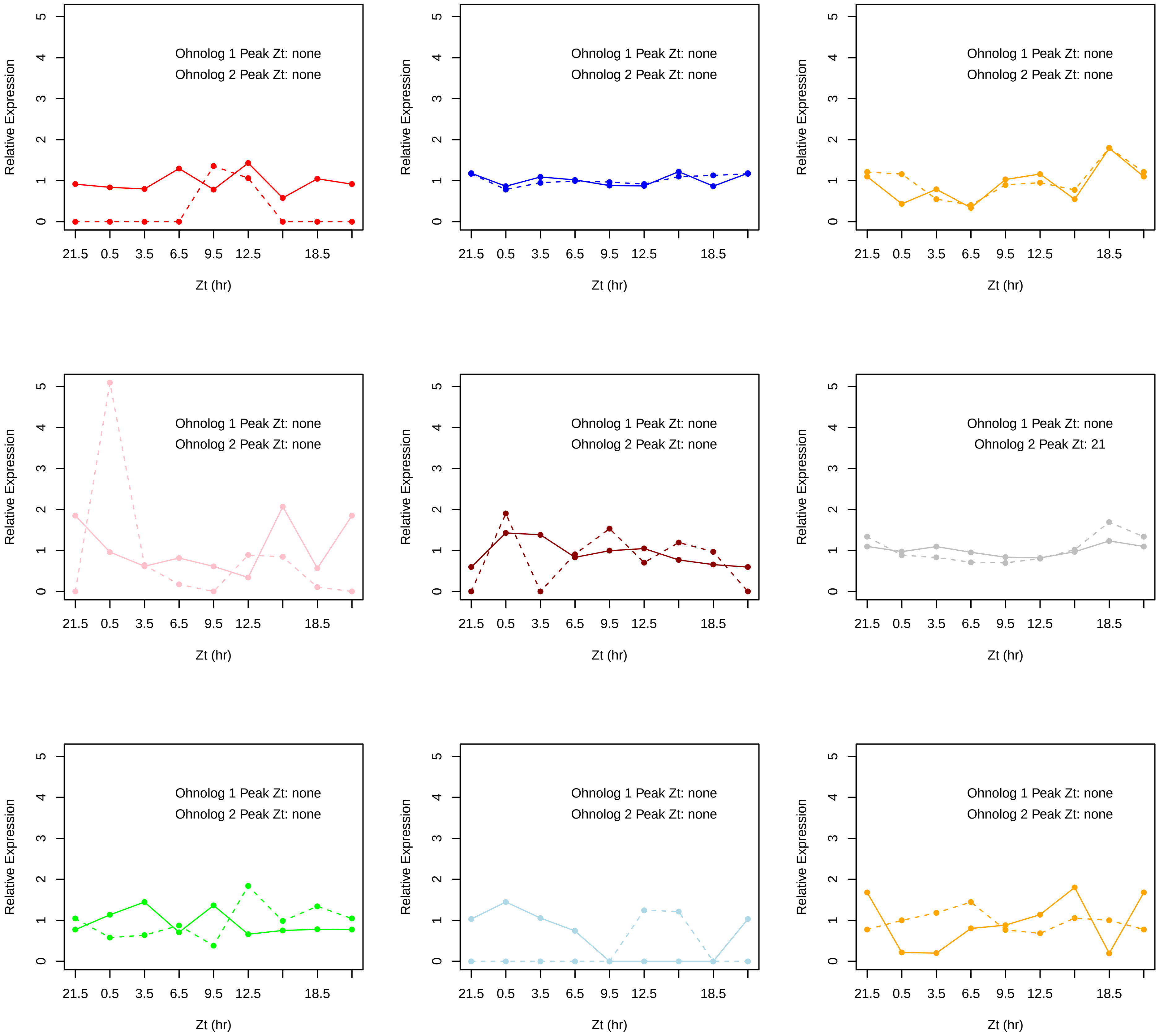

Ohnolog 1: ENSXMAG00000010941
Ohnolog 2: ENSXMAG00000018562

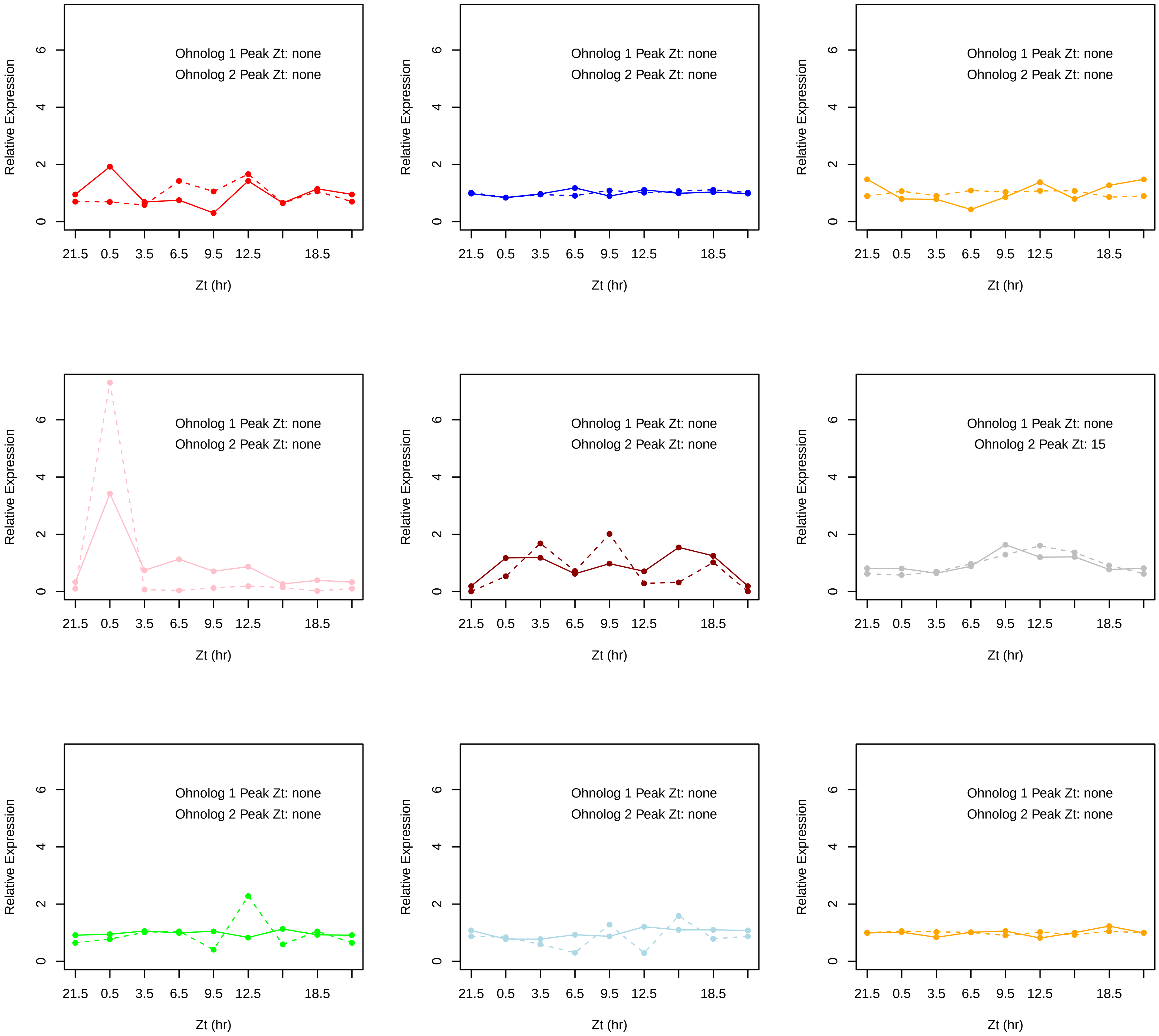

Ohnolog 1: ENSXMAG00000016113 cldn5b
Ohnolog 2: ENSXMAG00000023193 cldn5a

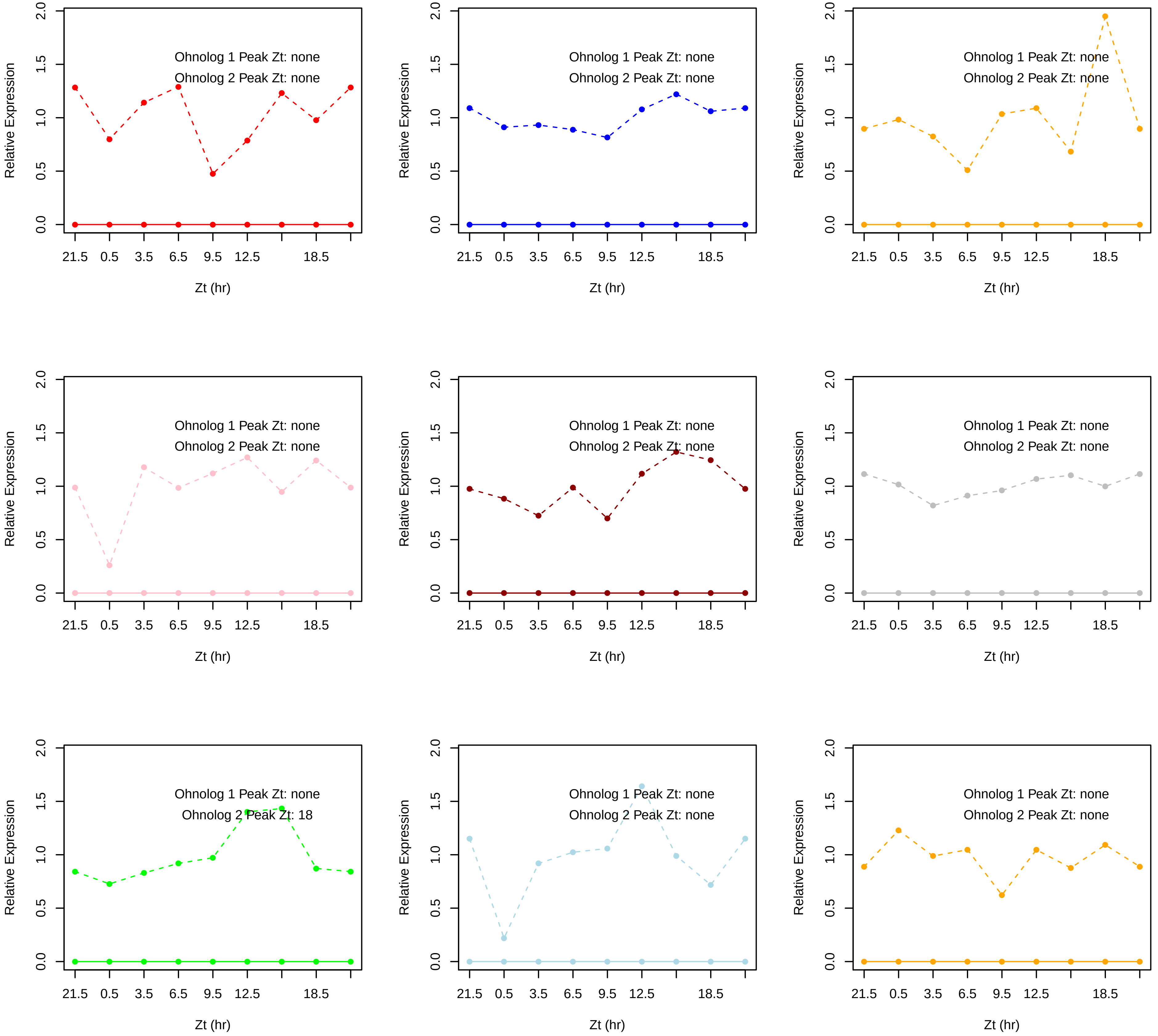

Ohnolog 1: ENSXMAG00000020272
Ohnolog 2: ENSXMAG00000027447 neurod4

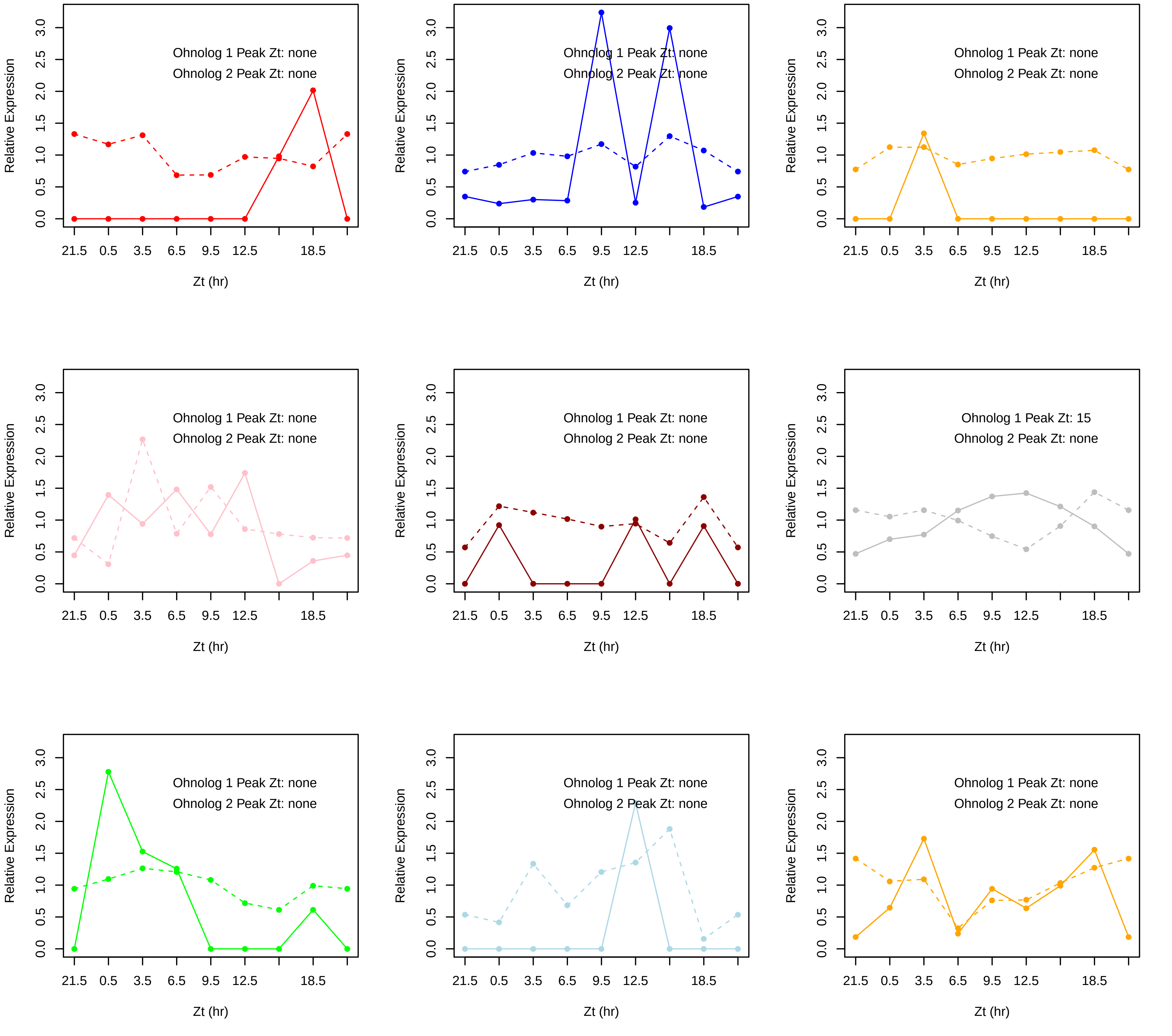

Ohnolog 1: ENSXMAG00000019856 SLC18A3
Ohnolog 2: ENSXMAG00000020102 slc18a3a

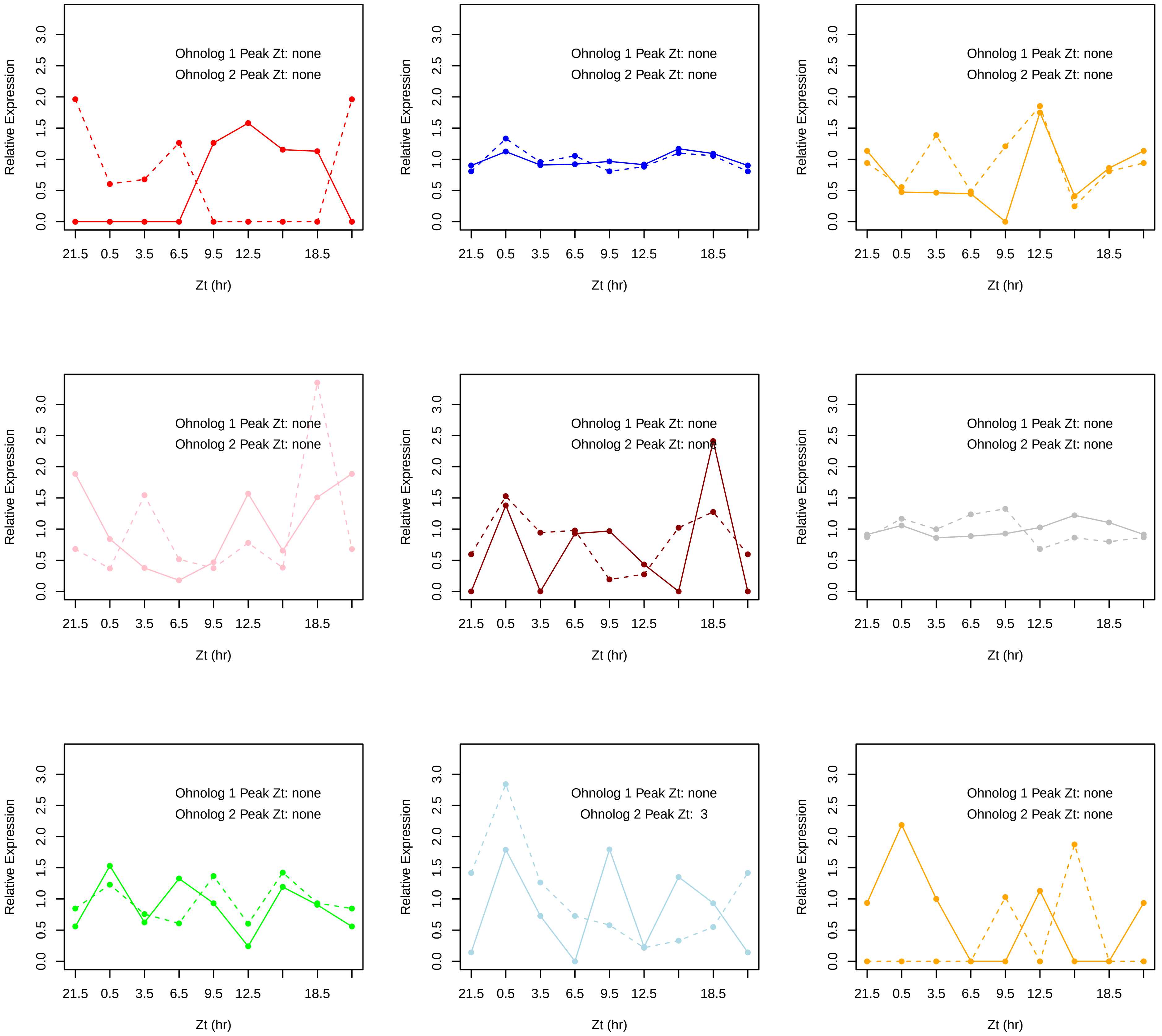

Ohnolog 1: ENSXMAG00000014234 adoa
Ohnolog 2: ENSXMAG00000020001 adob

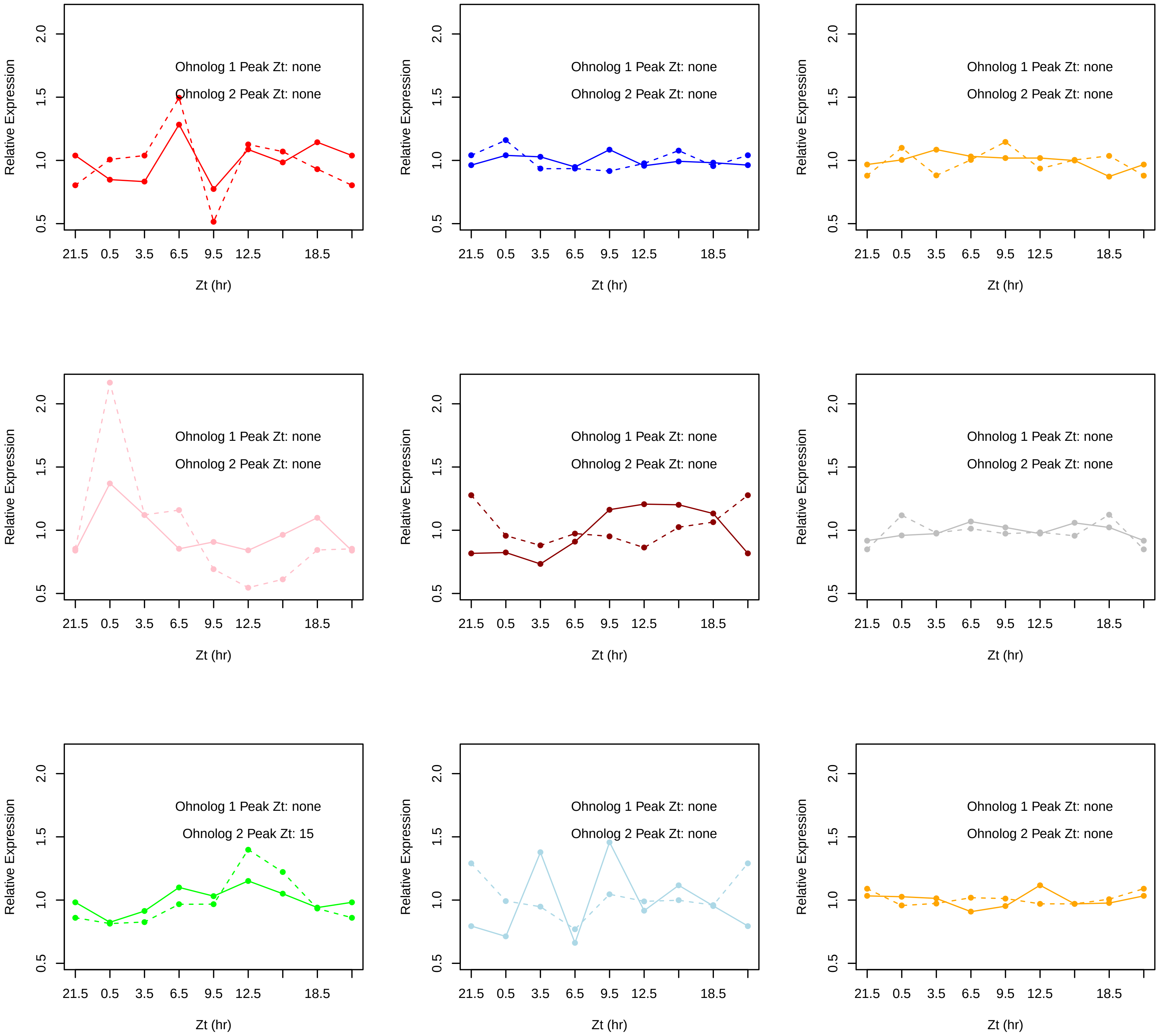

Ohnolog 1: ENSXMAG00000005127 nfil3−5
Ohnolog 2: ENSXMAG00000019530

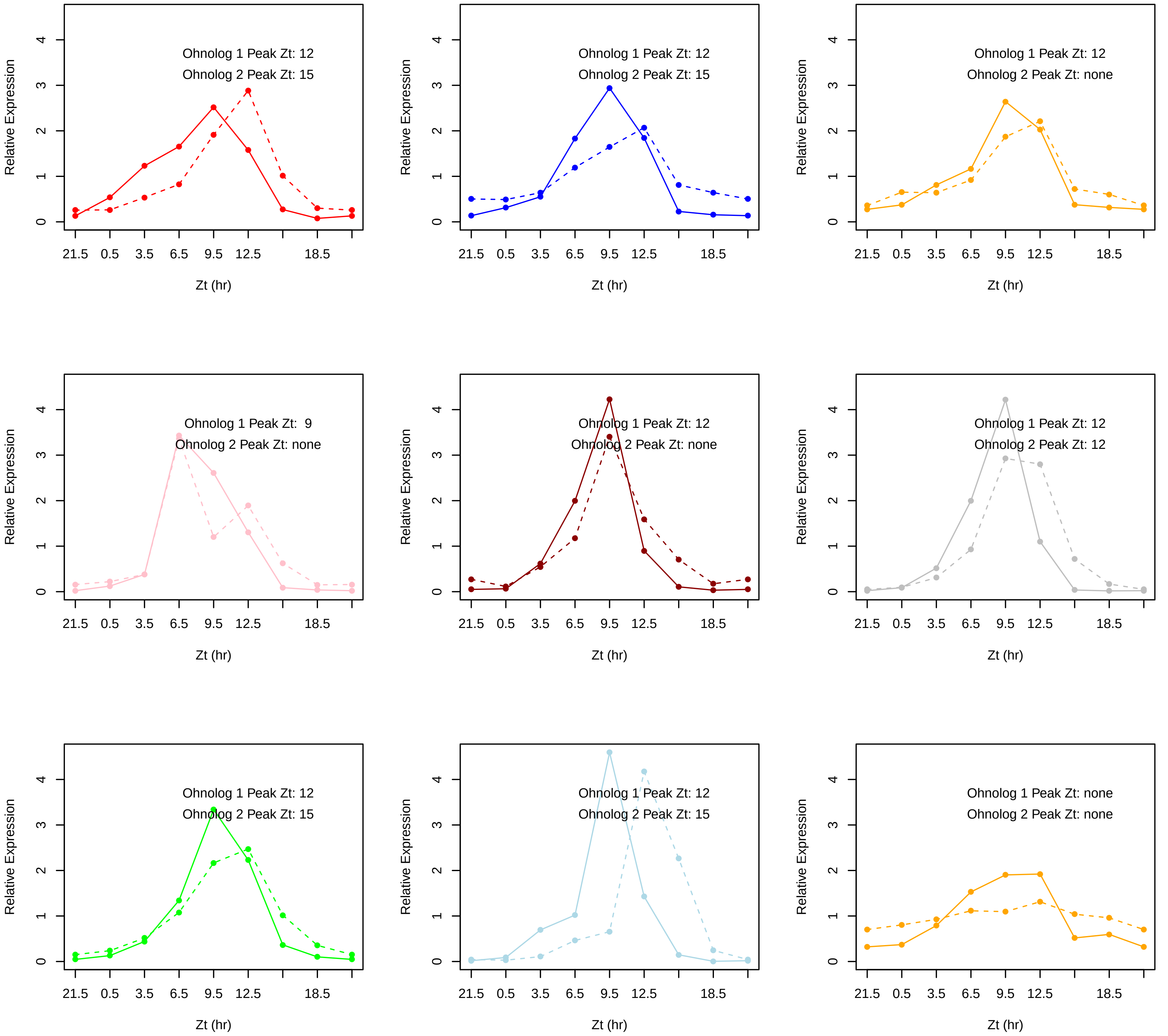

Ohnolog 1: ENSXMAG00000025105 si:ch1073−44g3.1
Ohnolog 2: ENSXMAG00000025956 zgc:77058

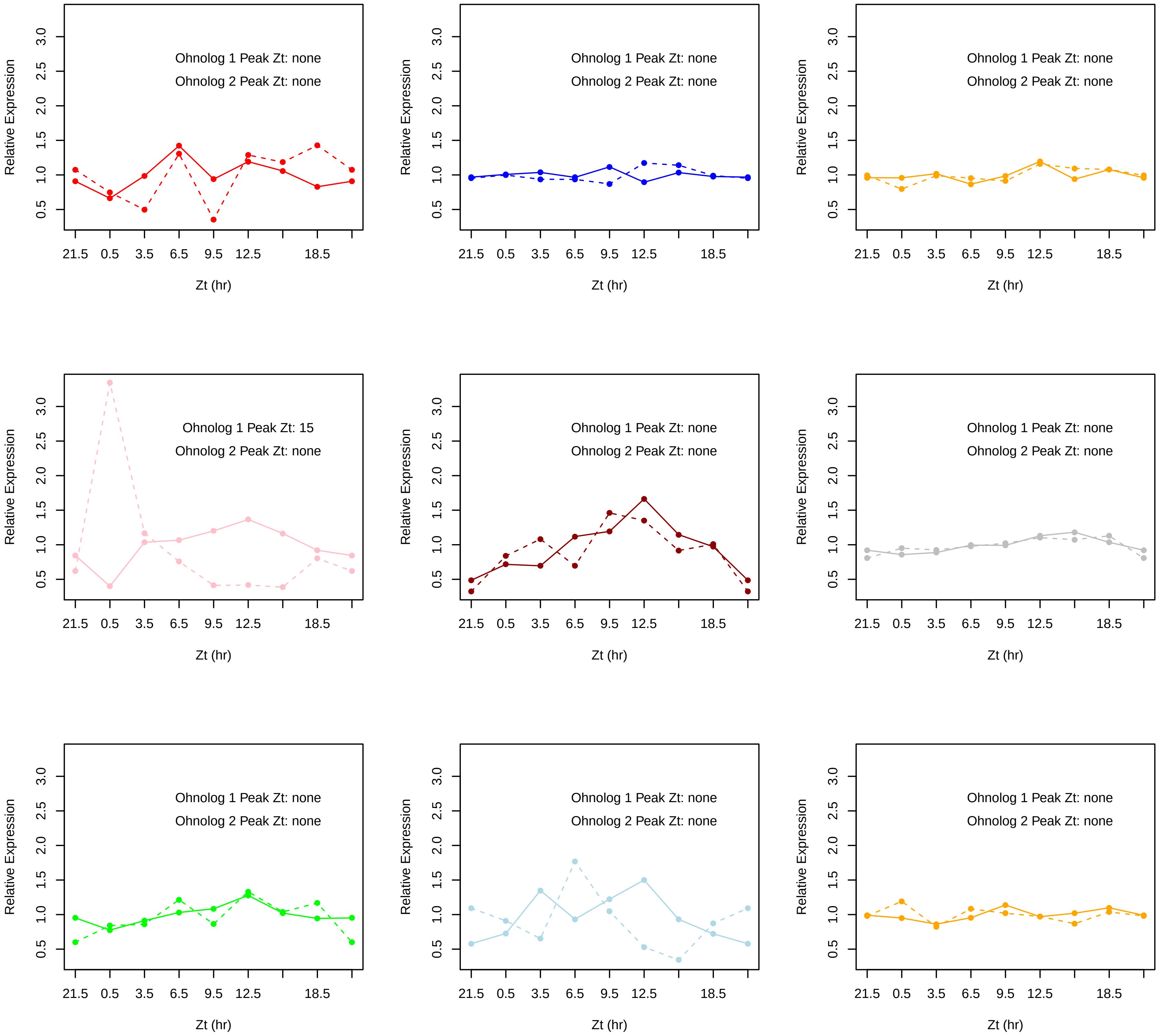

Ohnolog 1: ENSXMAG00000008654 zgc:110182
Ohnolog 2: ENSXMAG00000020332 fam84b

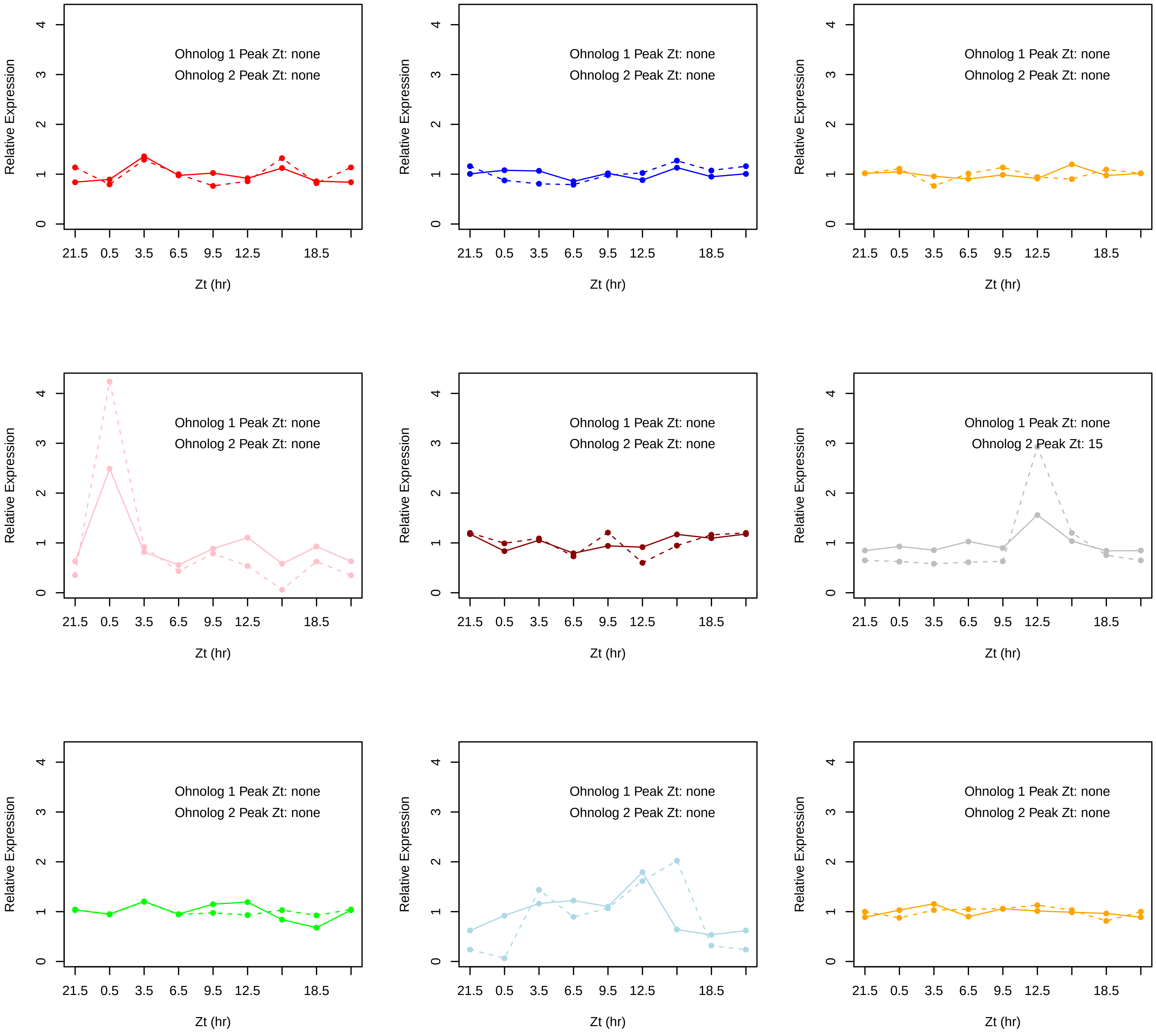

Ohnolog 1: ENSXMAG00000002730
Ohnolog 2: ENSXMAG00000019568 slc5a3b

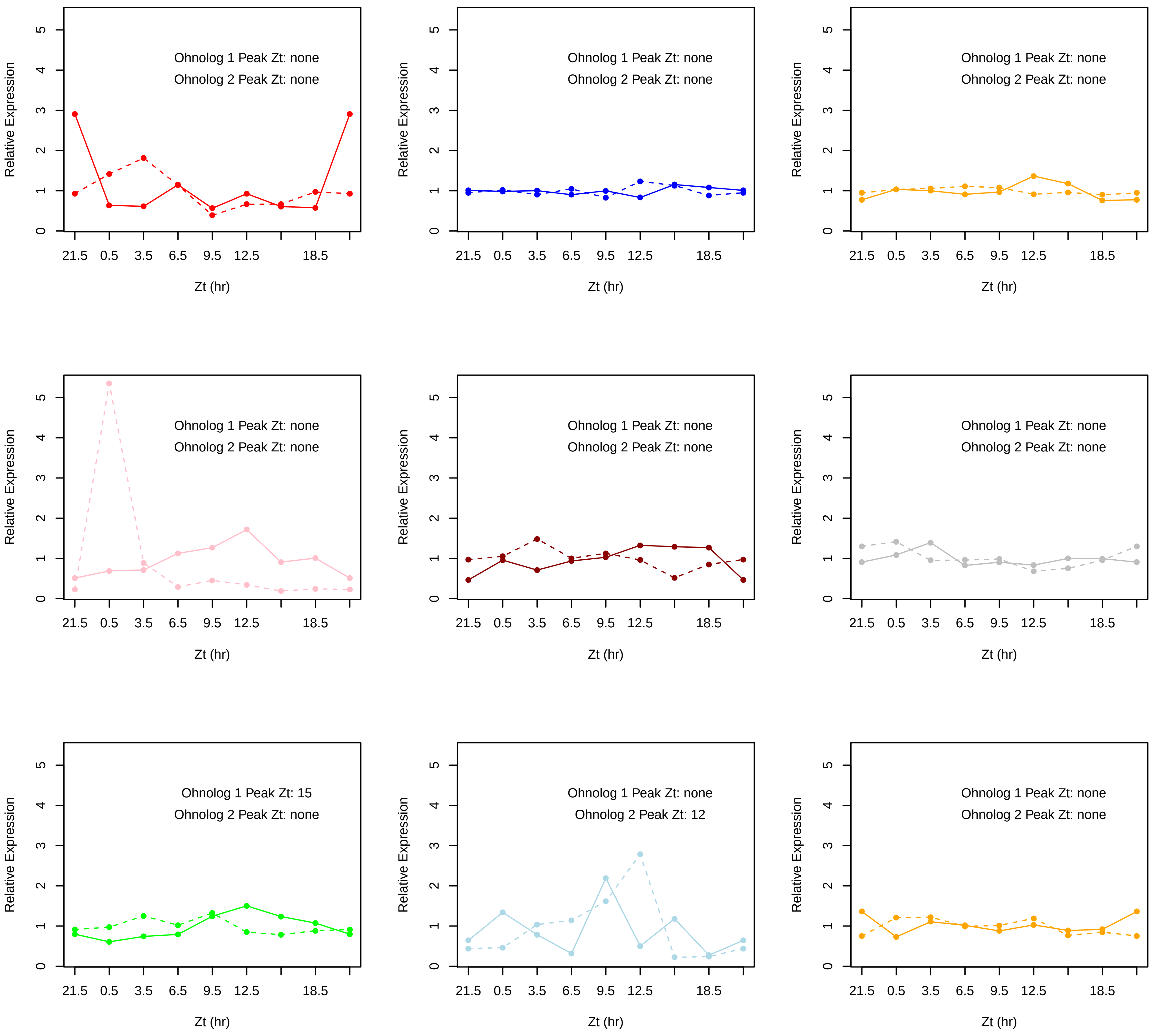

Supplement: Supplementary file 1 — Additional file 1: Supplement Figure 1. Sample collection and experimental setup: 9 organs (skin, brain, liver, gill, heart, muscle, testis, ovary and eye) were collected at 5 am, 8 am, 11 am, 2 pm, 5 pm, 8 pm, 11 pm and 2 am. Duplicate samples of total RNA from each organ of individual fish was isolated for gene expression profiling. Supplement Figure 2. Flowchart depicting the configuration of the gene expression browser. Establishment of the gene expression browser includes 2 main steps: 1. Sample collection and gene expression profiling, and Gene ID and Gene name conversion: Gene expression data were normalized and scaled and stored in the XGSC website; All Xiphophorus Ensembl gene ID and corresponding gene name, as well as other bioinformatics information were downloaded into a custom table and stored in the XGSC website; 2. Configuration of the user interface and server: user interface was designed to convert common gene name to Ensembl gene ID to precisely identify the gene of interest, and to show basal gene expression pattern. These tools are stored in both shiny.io and Github for user to launch the browser remotely or locally. Supplement Figure 3. Illustration of the gene ID converter. The user interface (UI) contains a gene ID conversion tool. Users can input a common gene name as a key word to search through the whole genome to identify genes that may be of study interest. An example of “per” for period gene family is used in the Fig. 9 lines of data were returned. Among those two are gper1, a GPCR gene, and three are perp, a TP53 apoptosis effector gene that are all not relevant to the per gene family, and 4 per gene family members: per1b, per2, per3 and a per2 paralog (ENSMAG00000006651). Supplement Figure 4. Illustration of the gene expression browser user interface. Once in the web tool at the Xiphophorus Genetic Stock Center web page, an outside user can enter a proper Ensembl gene ID as input in order to generate a bar graph representing the organ- [file SupplementFig7 Revision.pdf › SupplementFig7 Revision.pdf]
