# Supplementary material for: Global assessment of organ specific basal gene expression over a diurnal cycle with analyses of gene copies exhibiting cyclic expression patterns
Source: BMC Genomics. 2020 Nov 11;21:787. doi: 10.1186/s12864-020-07202-9 (PMC7659085; doi:10.1186/s12864-020-07202-9)
Supplement: Supplementary file 1 — Additional file 1: Supplement Figure 1. Sample collection and experimental setup: 9 organs (skin, brain, liver, gill, heart, muscle, testis, ovary and eye) were collected at 5 am, 8 am, 11 am, 2 pm, 5 pm, 8 pm, 11 pm and 2 am. Duplicate samples of total RNA from each organ of individual fish was isolated for gene expression profiling. Supplement Figure 2. Flowchart depicting the configuration of the gene expression browser. Establishment of the gene expression browser includes 2 main steps: 1. Sample collection and gene expression profiling, and Gene ID and Gene name conversion: Gene expression data were normalized and scaled and stored in the XGSC website; All Xiphophorus Ensembl gene ID and corresponding gene name, as well as other bioinformatics information were downloaded into a custom table and stored in the XGSC website; 2. Configuration of the user interface and server: user interface was designed to convert common gene name to Ensembl gene ID to precisely identify the gene of interest, and to show basal gene expression pattern. These tools are stored in both shiny.io and Github for user to launch the browser remotely or locally. Supplement Figure 3. Illustration of the gene ID converter. The user interface (UI) contains a gene ID conversion tool. Users can input a common gene name as a key word to search through the whole genome to identify genes that may be of study interest. An example of “per” for period gene family is used in the Fig. 9 lines of data were returned. Among those two are gper1, a GPCR gene, and three are perp, a TP53 apoptosis effector gene that are all not relevant to the per gene family, and 4 per gene family members: per1b, per2, per3 and a per2 paralog (ENSMAG00000006651). Supplement Figure 4. Illustration of the gene expression browser user interface. Once in the web tool at the Xiphophorus Genetic Stock Center web page, an outside user can enter a proper Ensembl gene ID as input in order to generate a bar graph representing the organ- [file SupplementFig8 Revision.pdf › SupplementFig8 Revision.pdf]

Singeleton: ENSXMAG00000028248 b4galt3

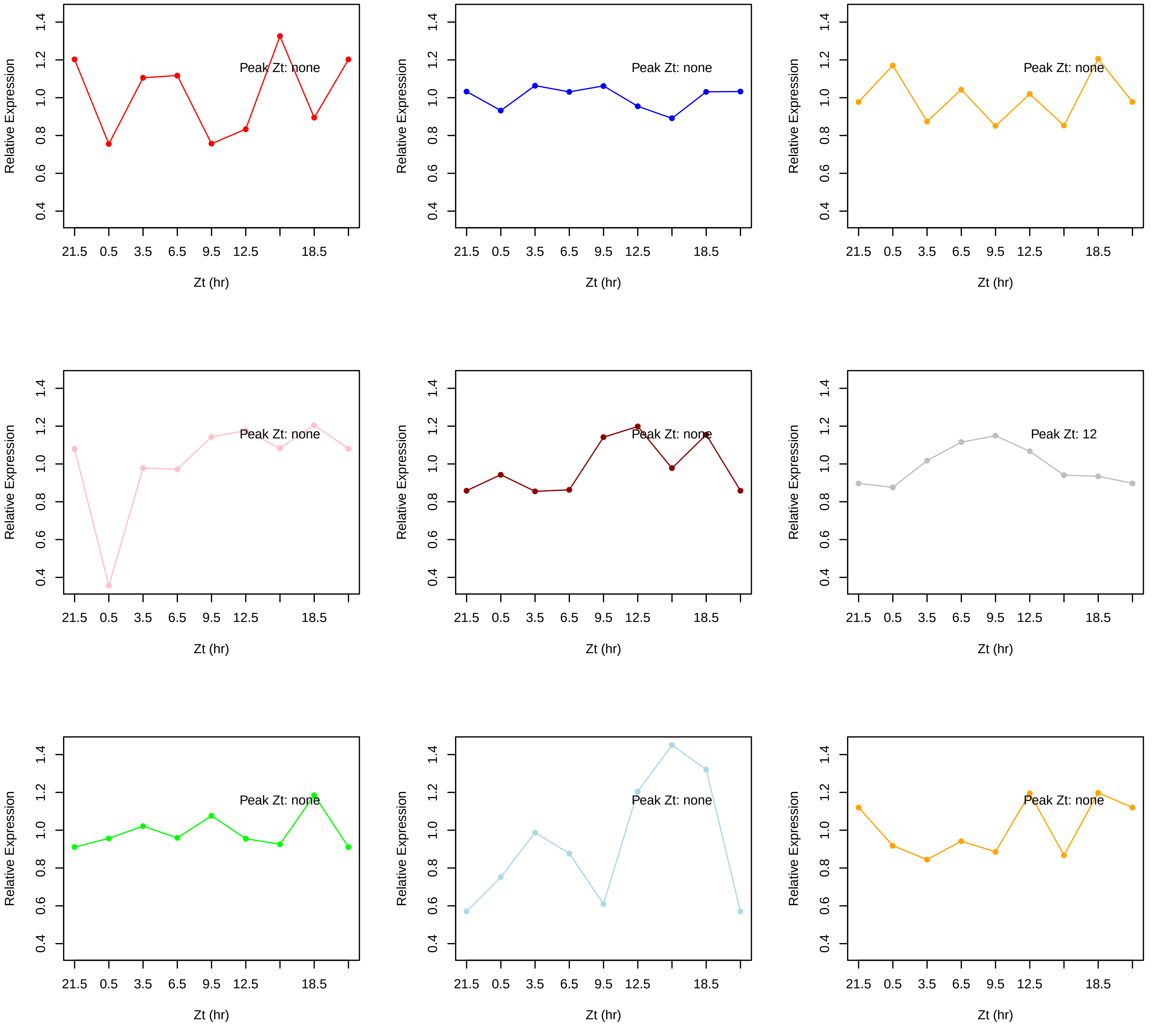

Singeleton: ENSXMAG00000028803 gatd1

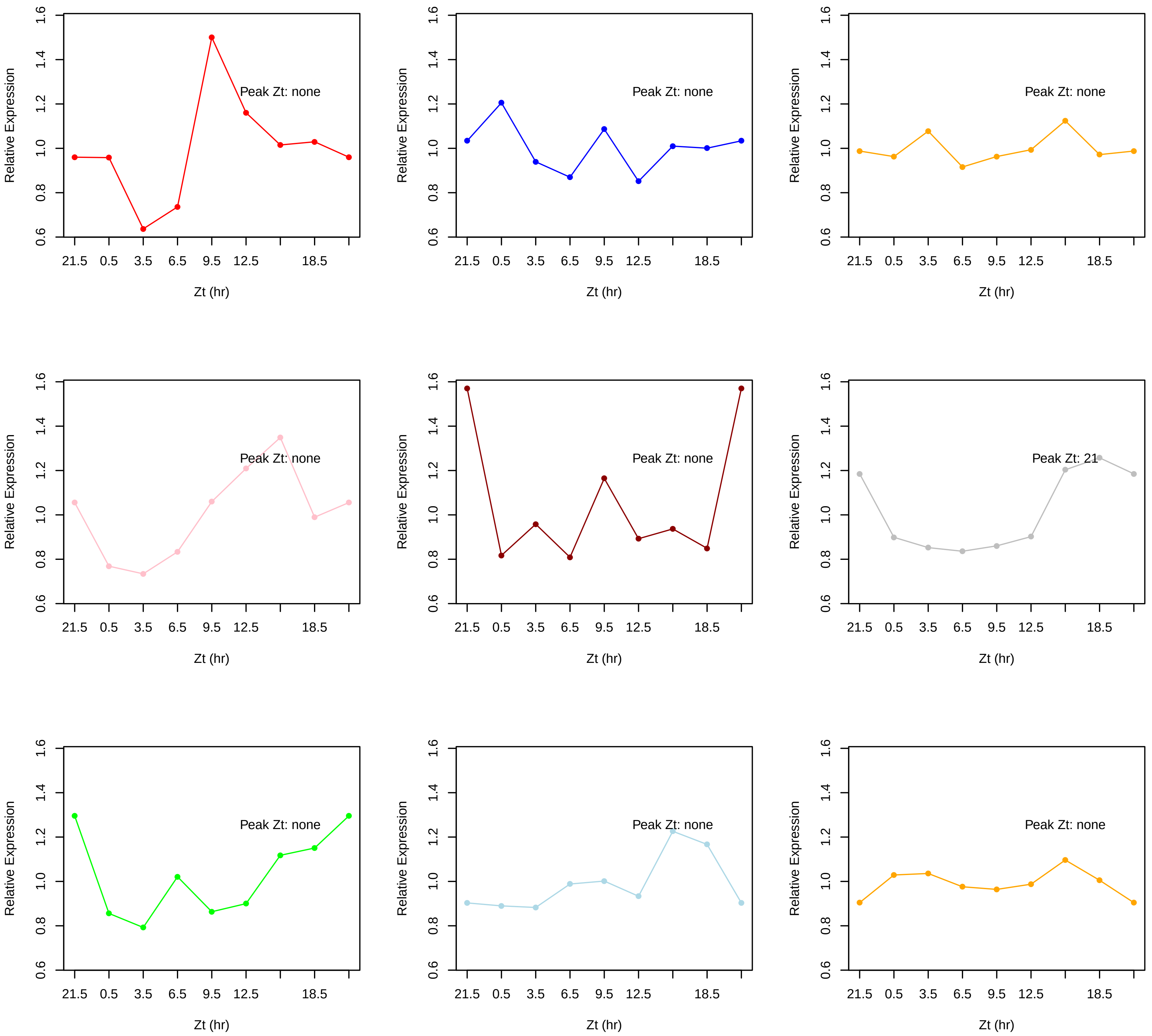

Singeleton: ENSXMAG00000012272 prpf31

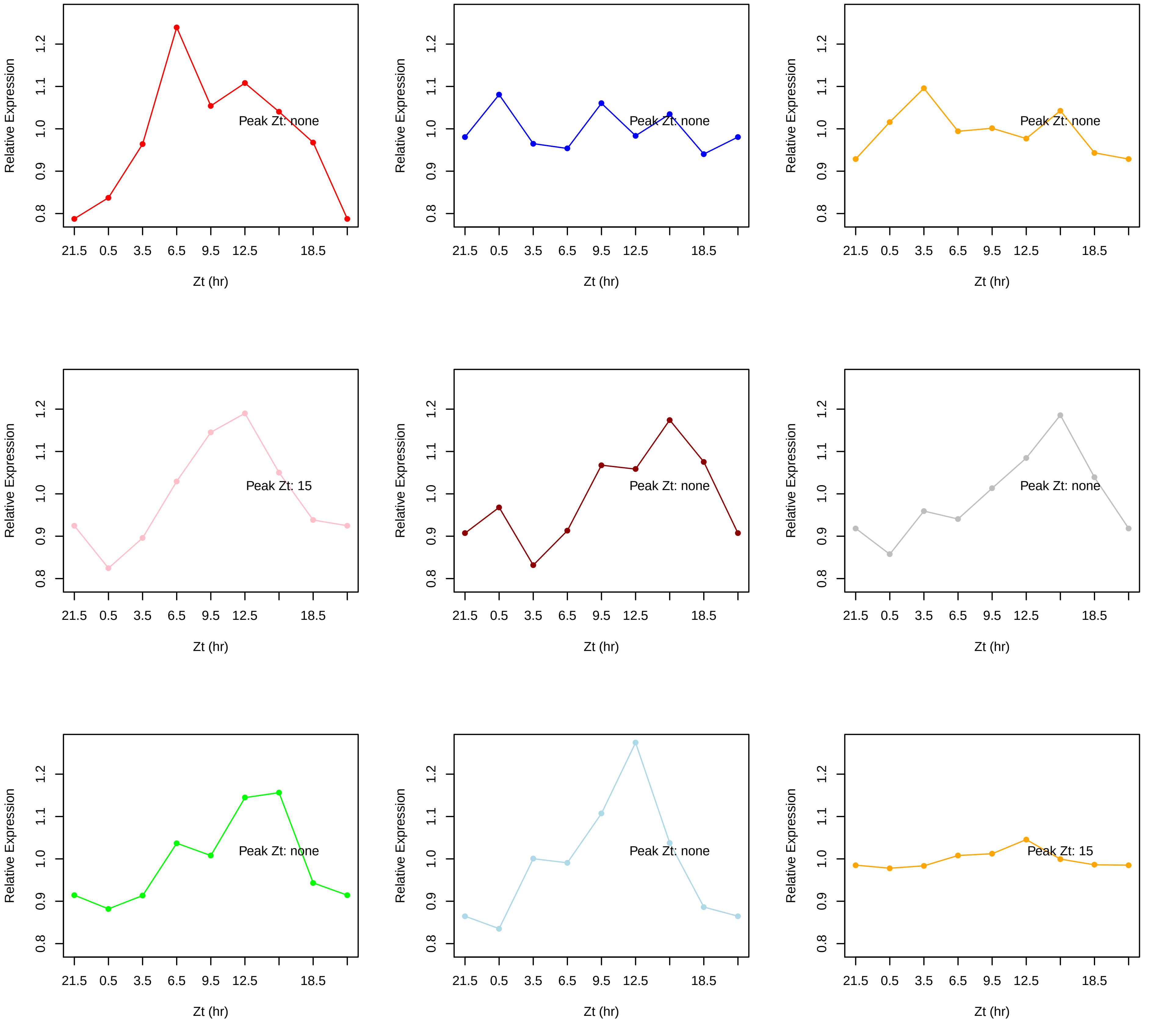

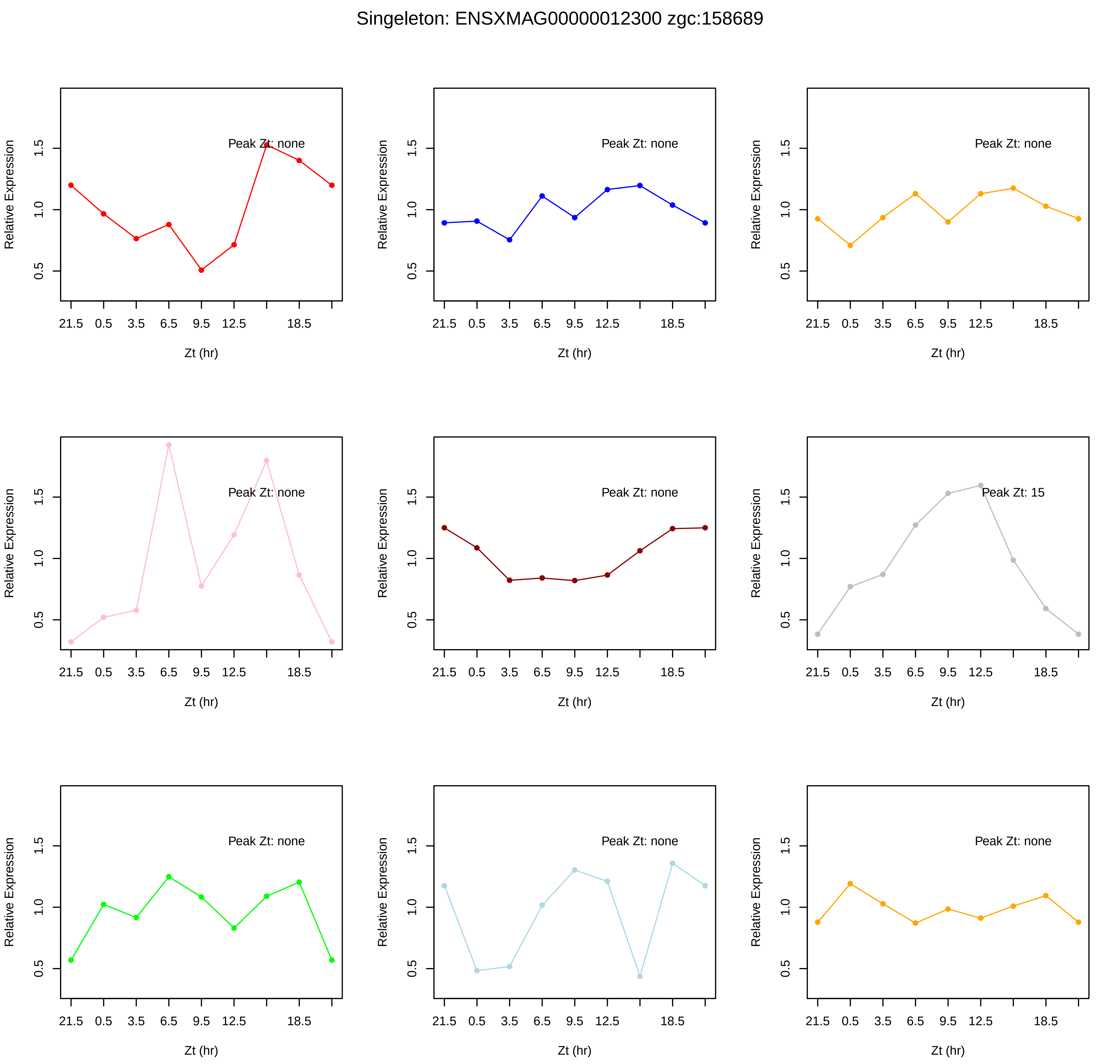

Singeleton: ENSXMAG00000012300 zgc:158689
Peak Zt: none
Peak Zt: none
Peak Zt: none
Peak Zt: none
Peak Zt: none
Peak Zt: 15
Peak Zt: none
Peak Zt: none
Peak Zt: none
Relative Expression
Zt (hr)
21.5
0.5
3.5
6.5
9.5
12.5
18.5

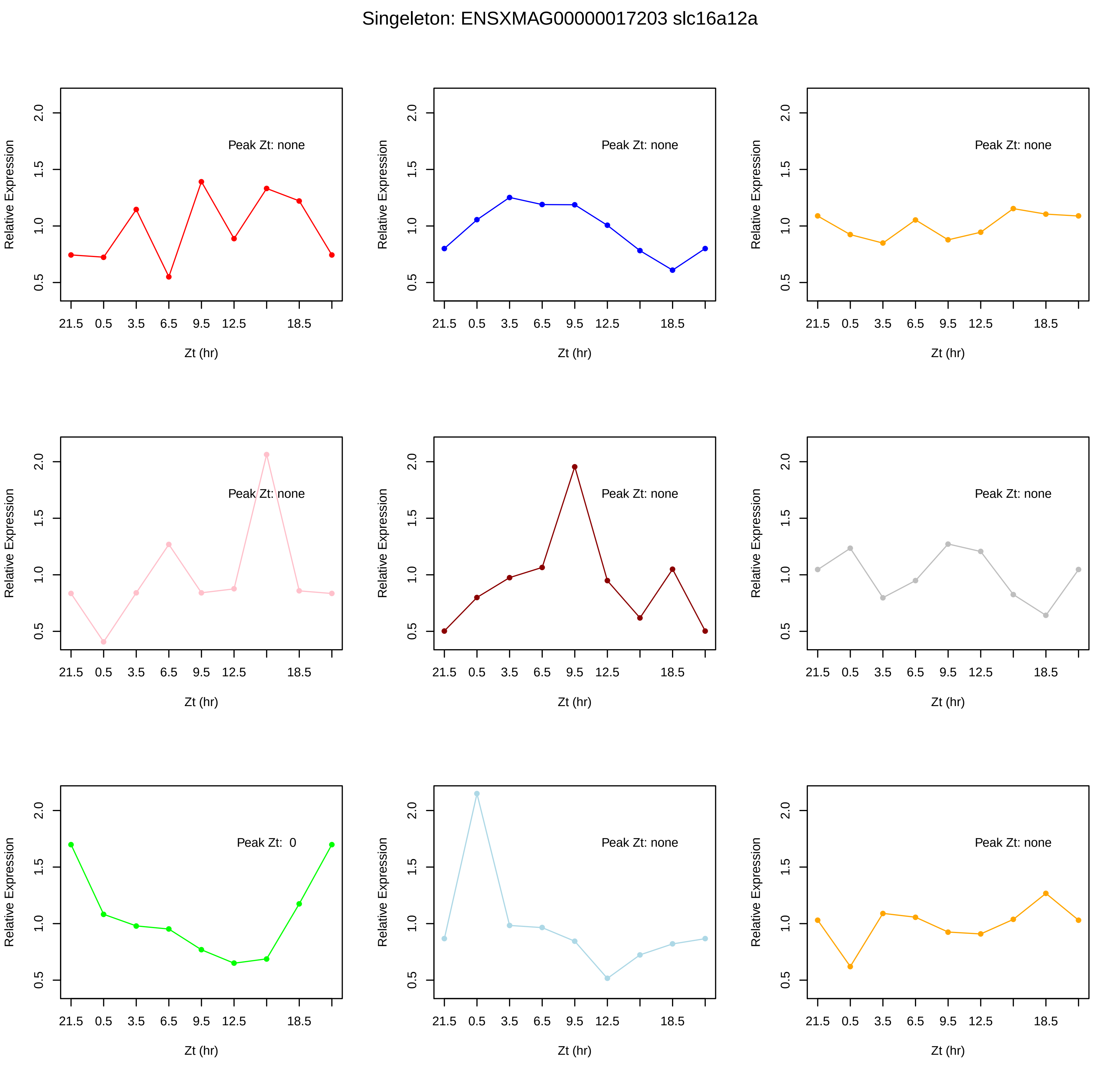
Singleton: ENSXMAG00000017203 slc16a12a
Relative Expression
Zt (hr)
21.5 0.5 3.5 6.5 9.5 12.5 18.5
0.5 1.0 1.5 2.0
Peak Zt: none
Peak Zt: none
Peak Zt: none
Peak Zt: none
Peak Zt: none
Peak Zt: none
Peak Zt: 0
Peak Zt: none
Peak Zt: none

Singeleton: ENSXMAG00000015470 zgc:65997

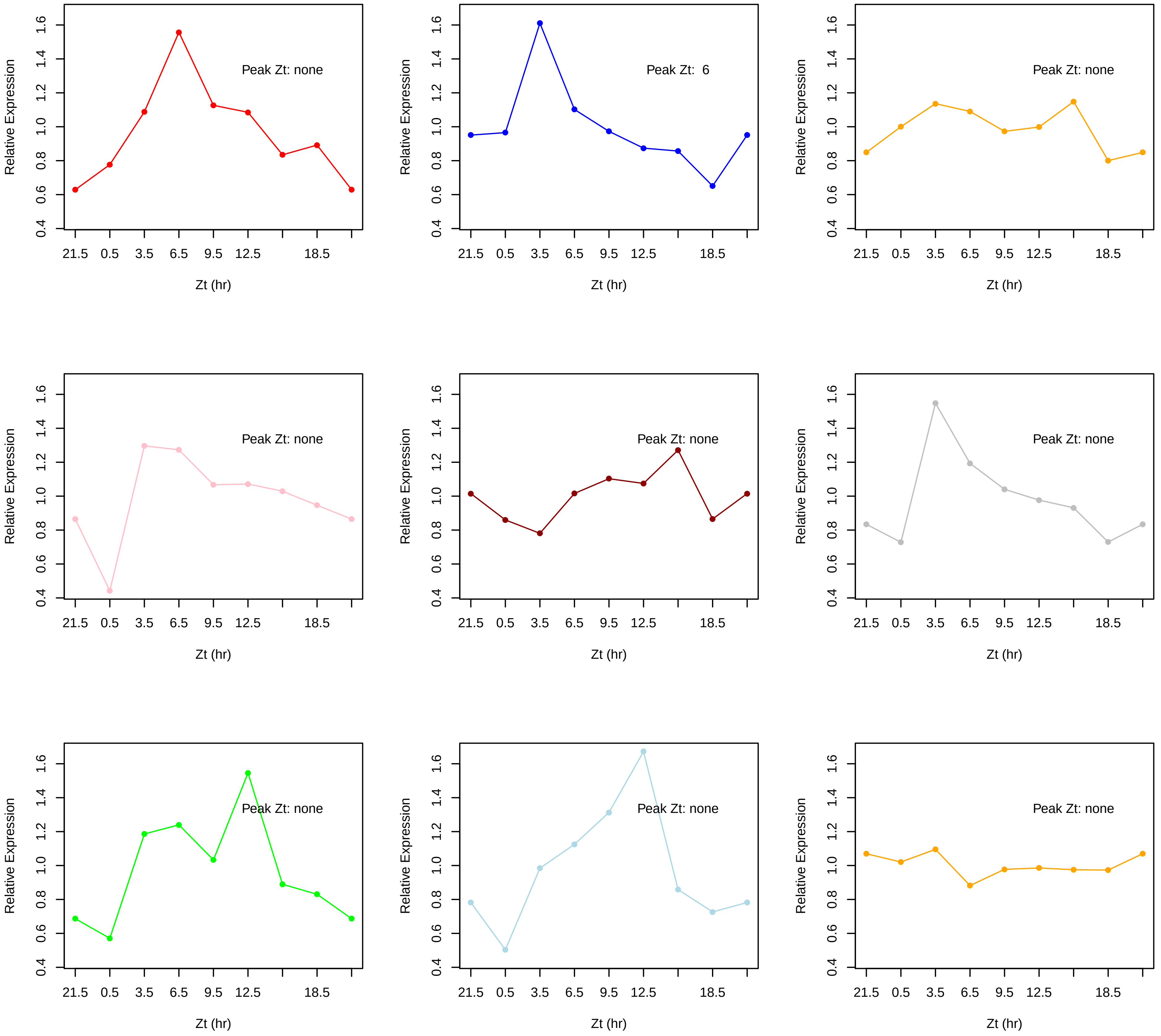

Singeleton: ENSXMAG00000004868 ankrd1a

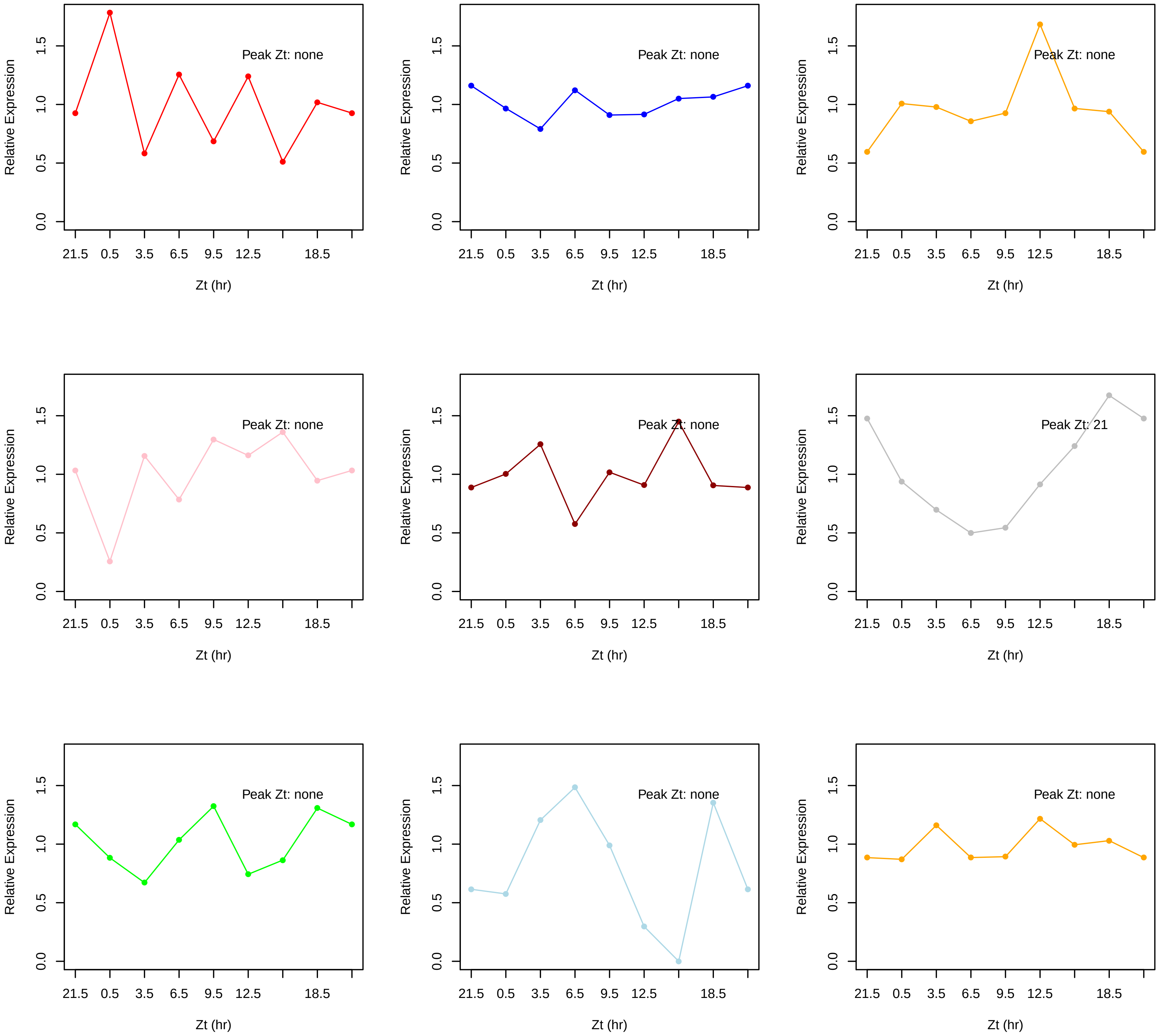

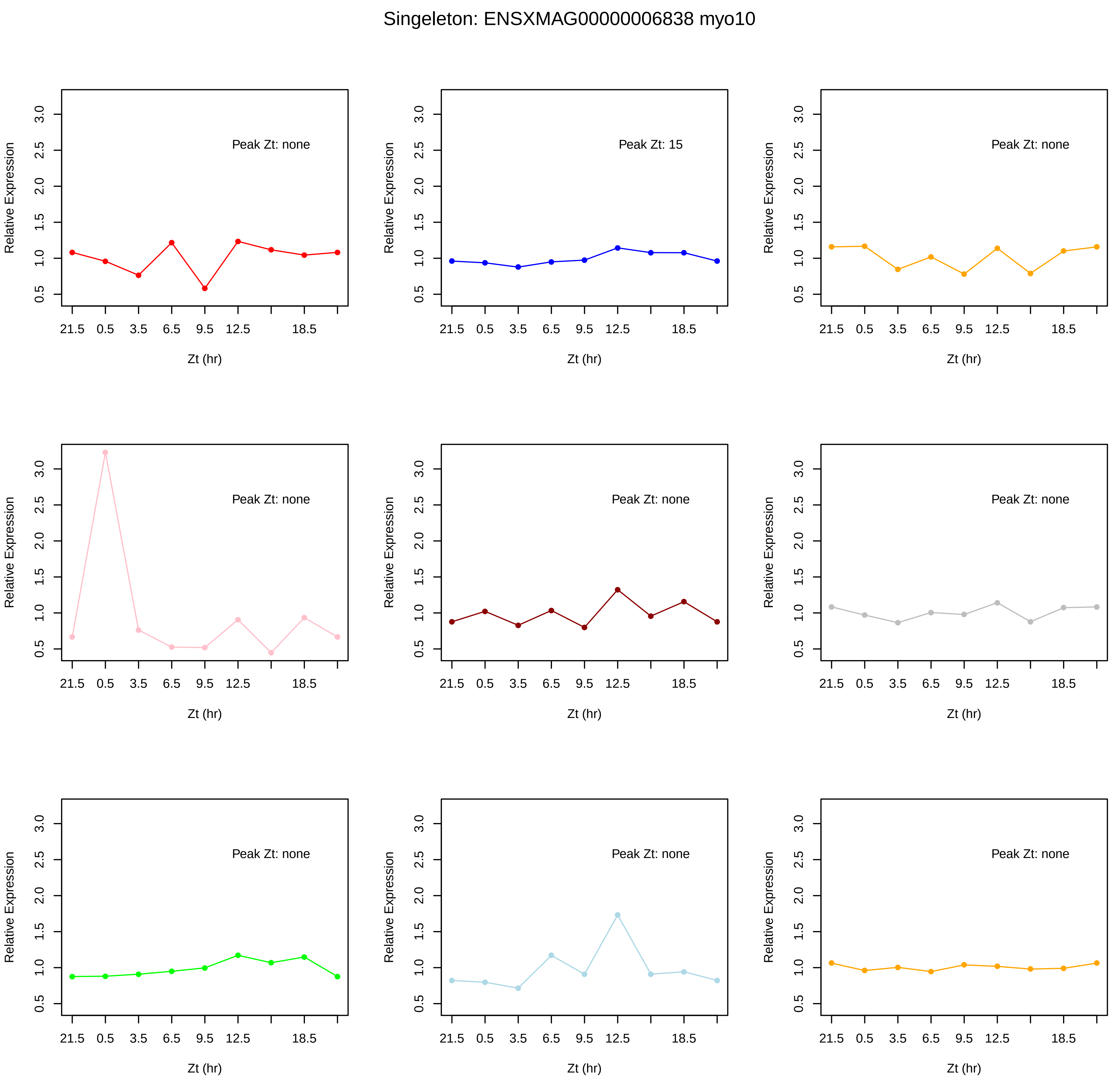
Singeleton: ENSXMAG00000006838 myo10
Peak Zt: none
Peak Zt: 15
Peak Zt: none
Peak Zt: none
Peak Zt: none
Peak Zt: none
Peak Zt: none
Peak Zt: none
Peak Zt: none
Relative Expression
Zt (hr)
21.5
0.5
3.5
6.5
9.5
12.5
18.5

Singeleton: ENSXMAG00000004878 pcgf5a

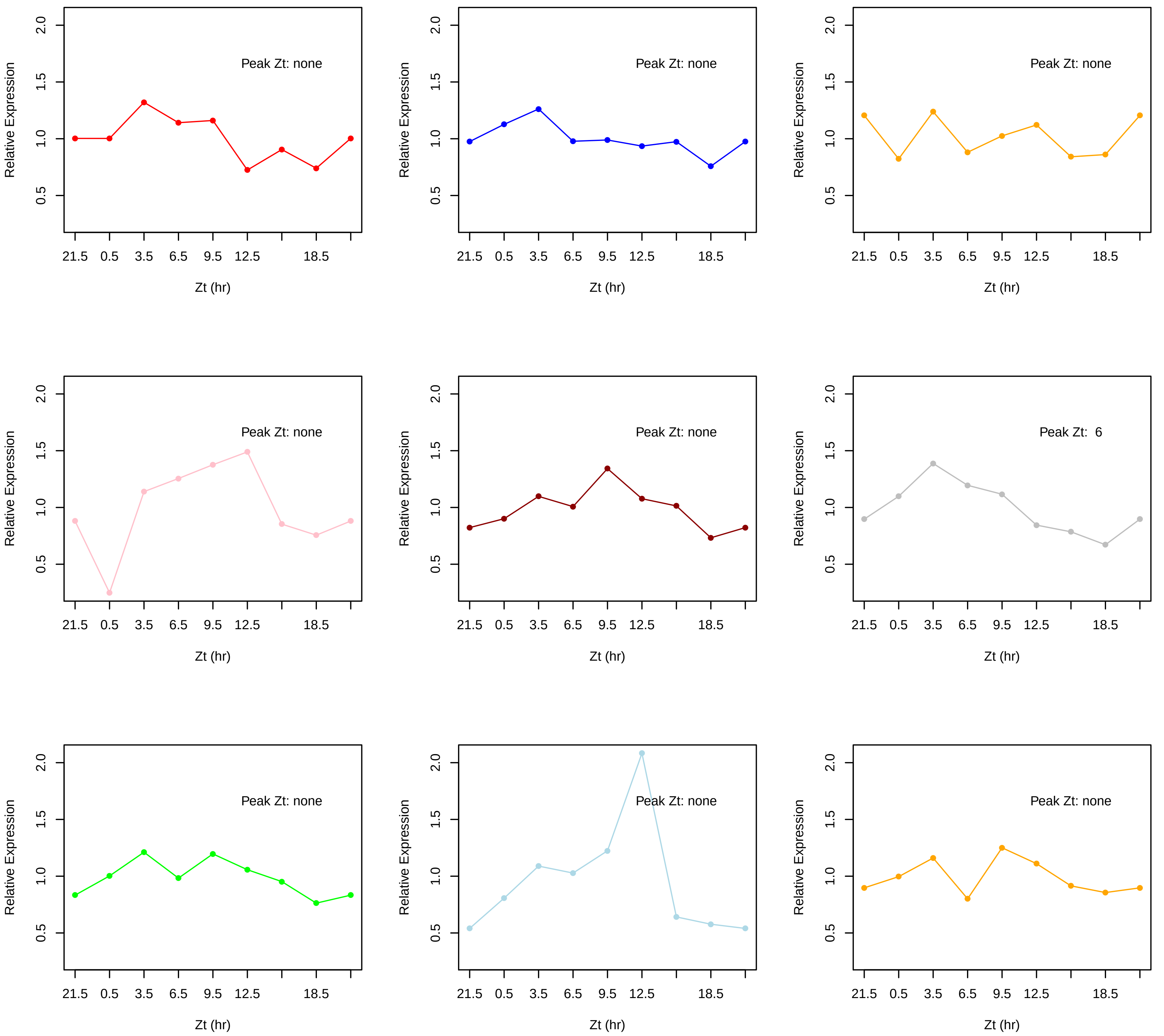

Singeleton: ENSXMAG00000012825 hectd2

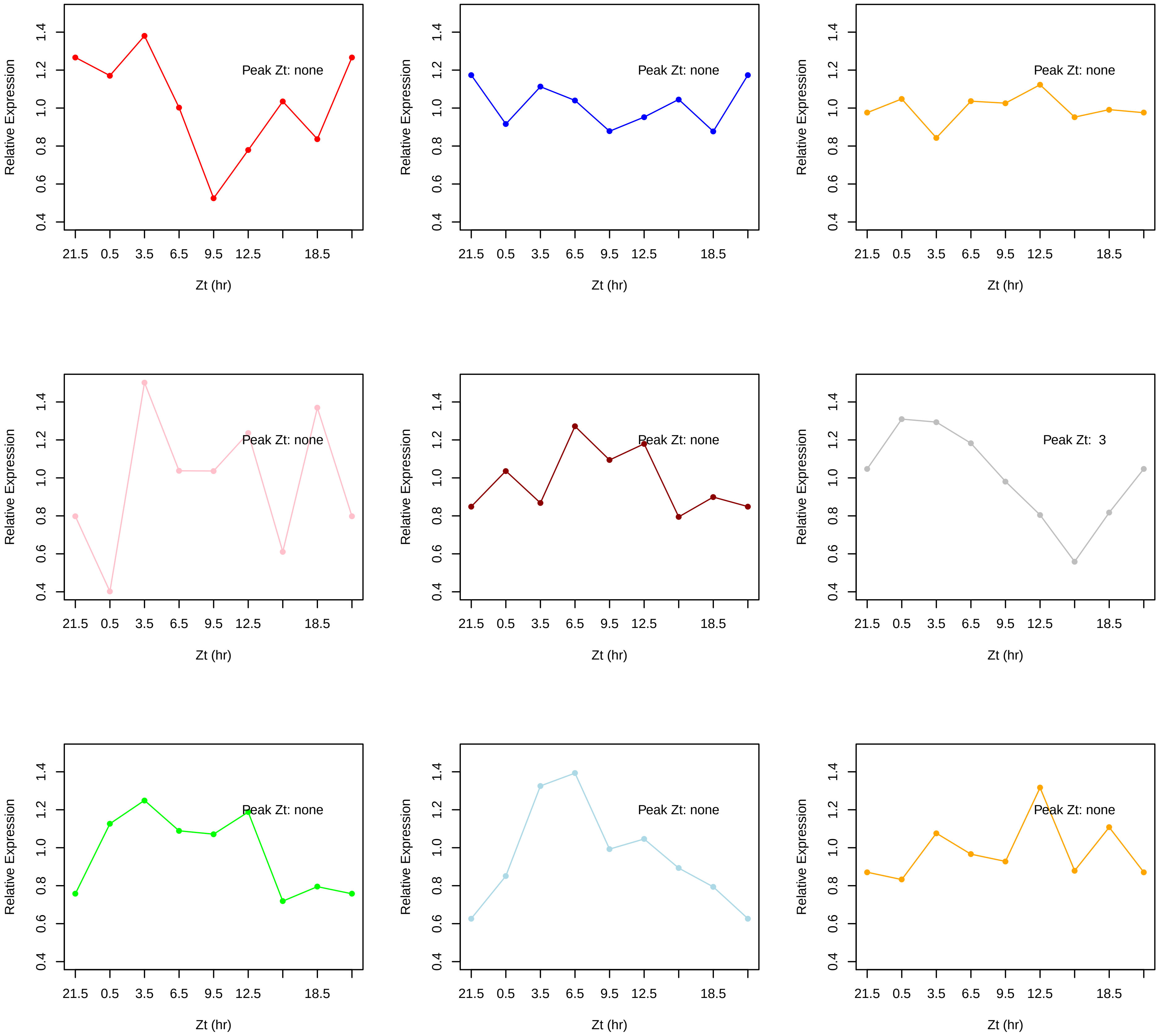

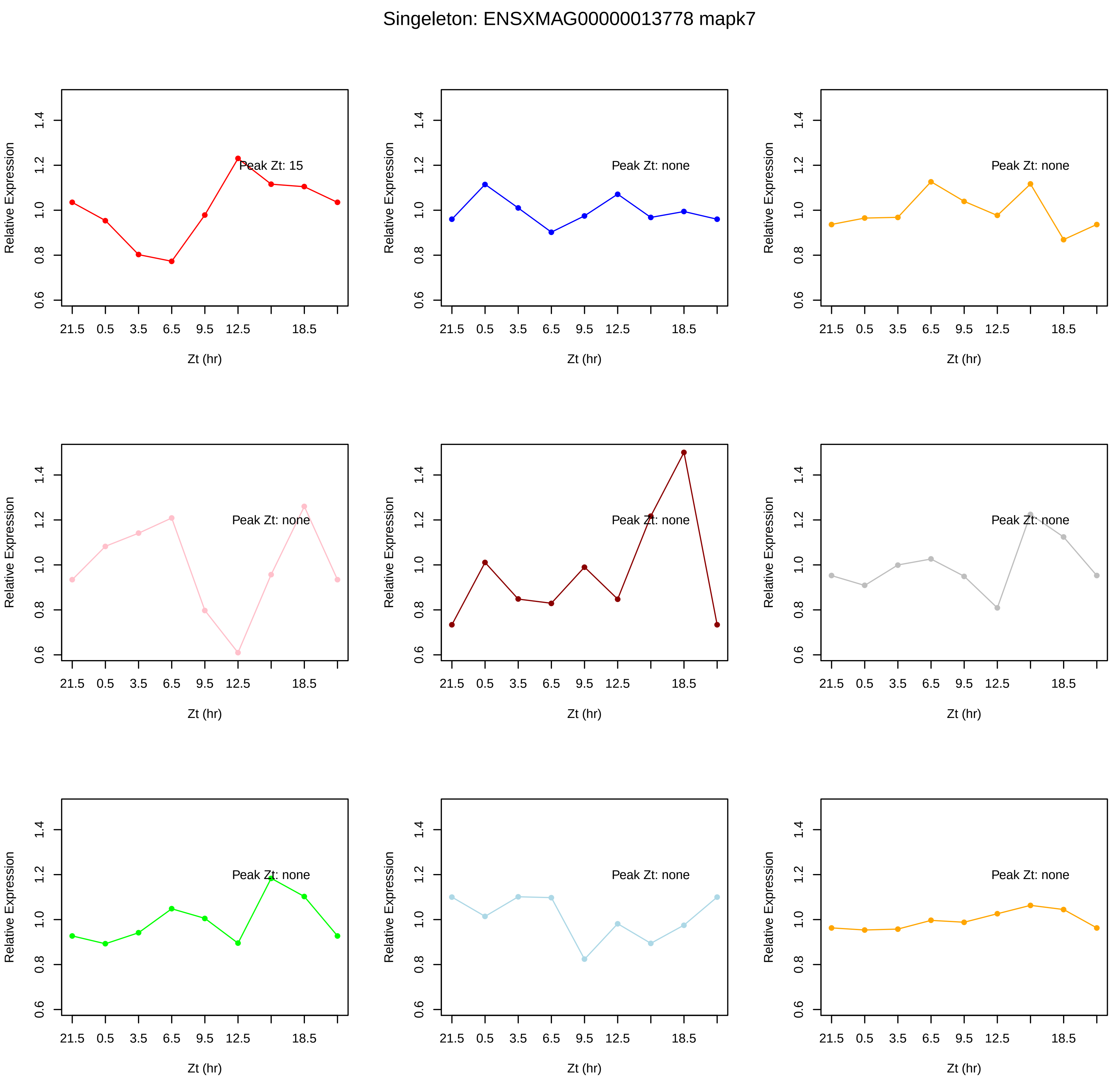

Singeleton: ENSXMAG00000013778 mapk7
Peak Zt: 15
Peak Zt: none
Relative Expression
Zt (hr)
0.6
0.8
1.0
1.2
1.4
21.5
0.5
3.5
6.5
9.5
12.5
18.5

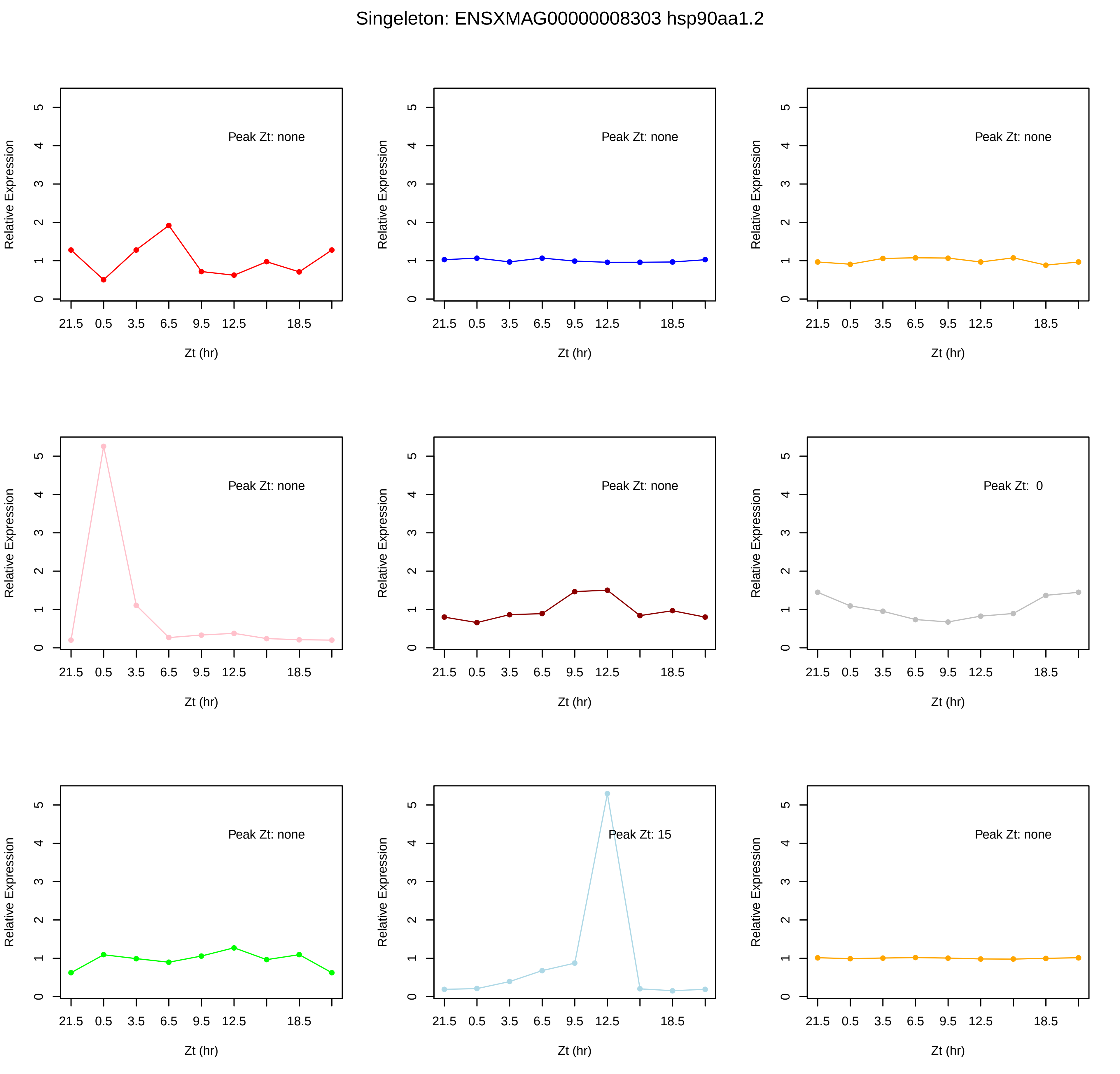
Singeleton: ENSXMAG00000008303 hsp90aa1.2
Peak Zt: none
Peak Zt: none
Peak Zt: none
Peak Zt: none
Peak Zt: none
Peak Zt: 0
Peak Zt: none
Peak Zt: 15
Peak Zt: none
Relative Expression
Zt (hr)
21.5
0.5
3.5
6.5
9.5
12.5
18.5
0
1
2
3
4
5

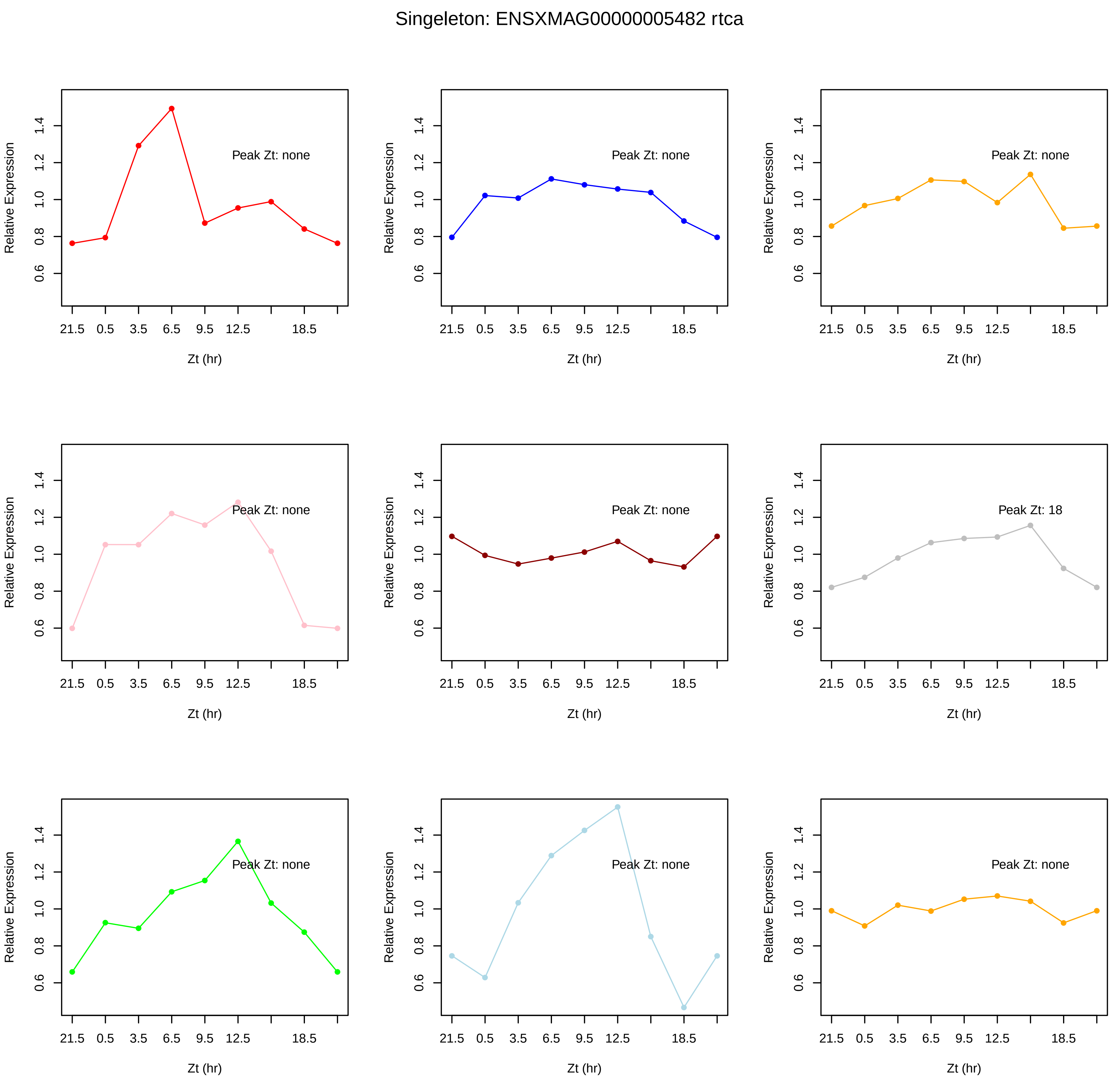
Singeleton: ENSXMAG00000005482 rtca
Peak Zt: none
Peak Zt: none
Peak Zt: none
Peak Zt: none
Peak Zt: none
Peak Zt: 18
Peak Zt: none
Peak Zt: none
Peak Zt: none
Relative Expression
Zt (hr)
21.5
0.5
3.5
6.5
9.5
12.5
18.5
0.6
0.8
1.0
1.2
1.4

Singeleton: ENSXMAG00000022282

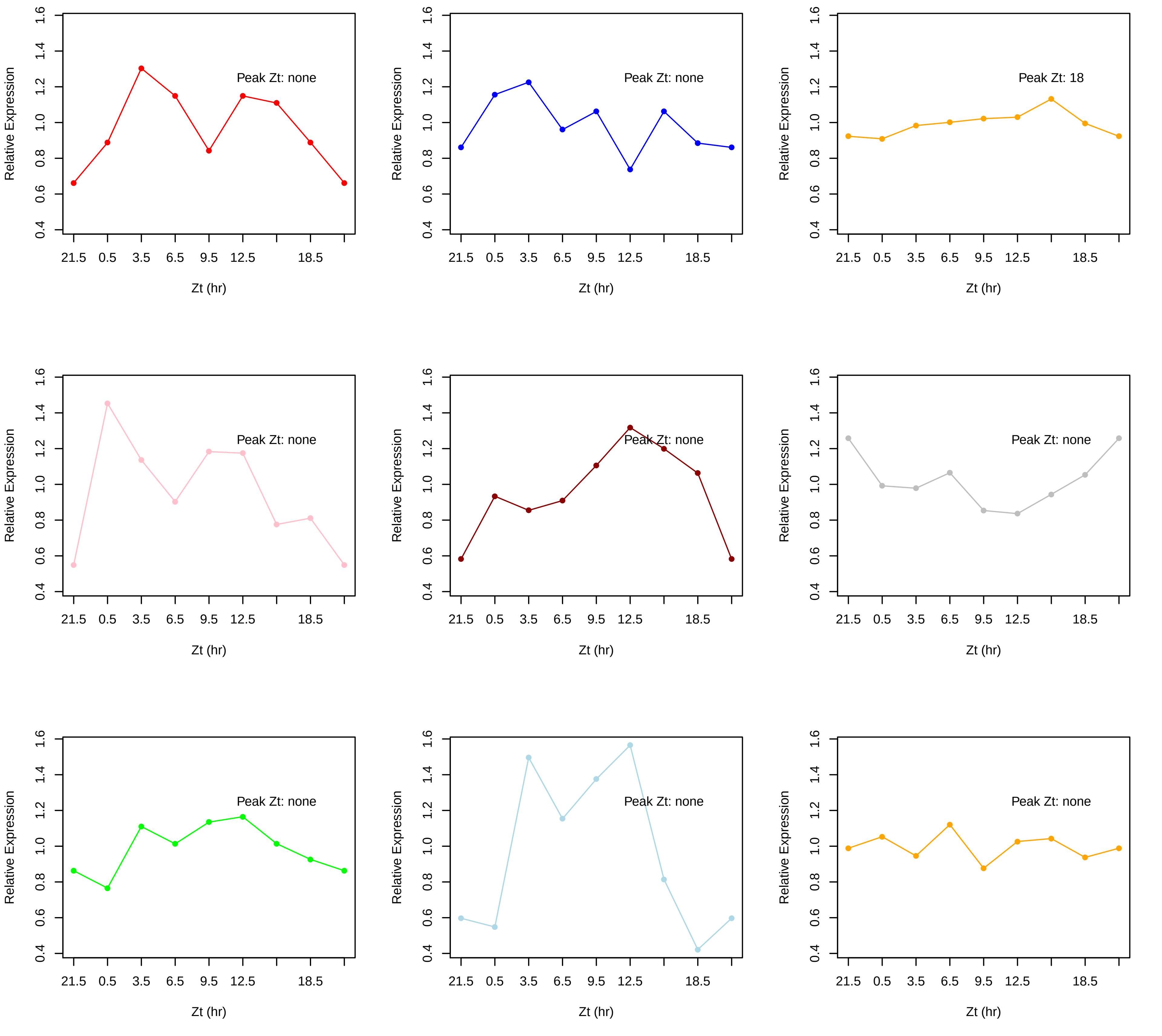

Singeleton: ENSXMAG00000006551 kdm4b

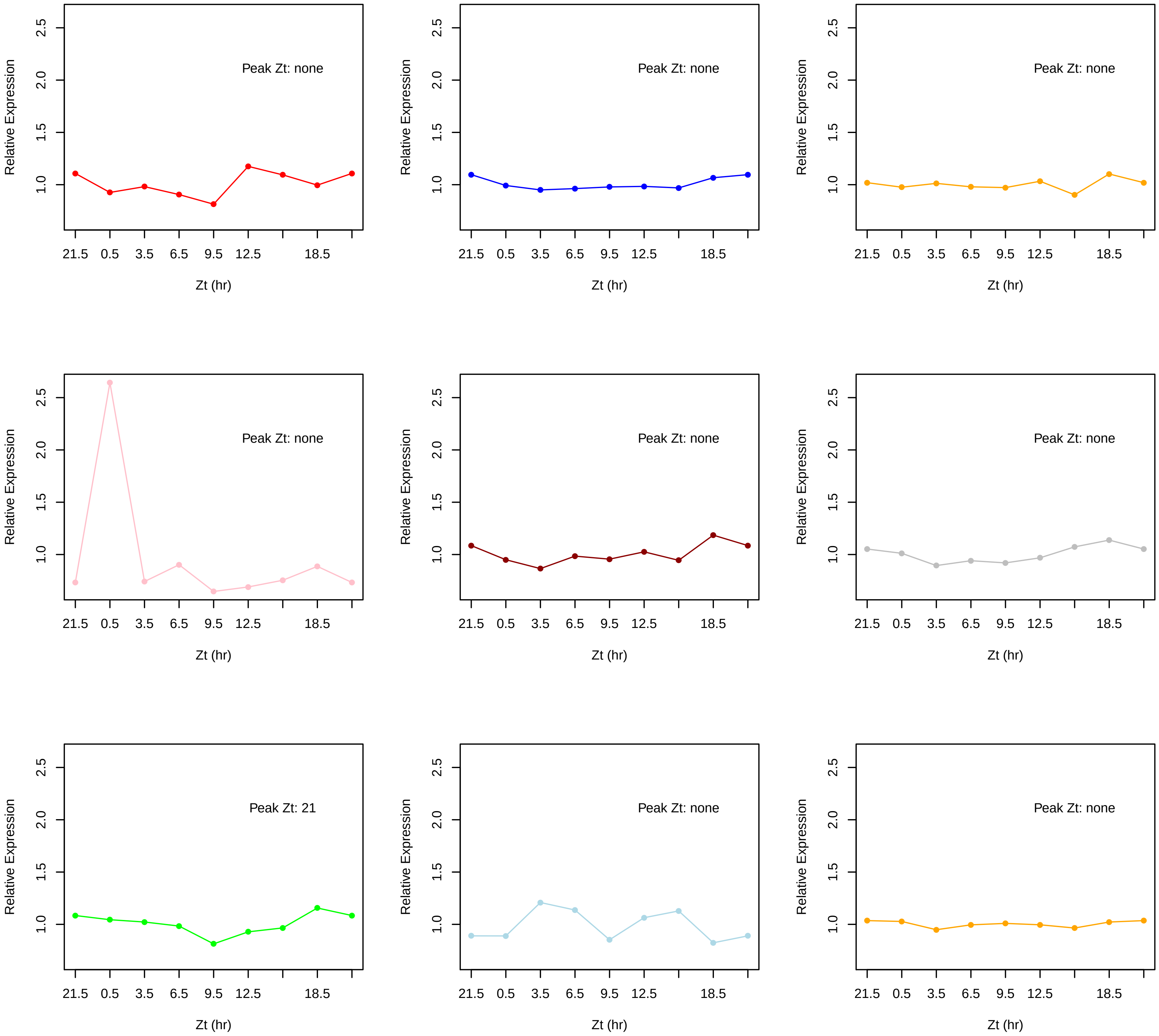

Singeleton: ENSXMAG00000008399 ubl3a

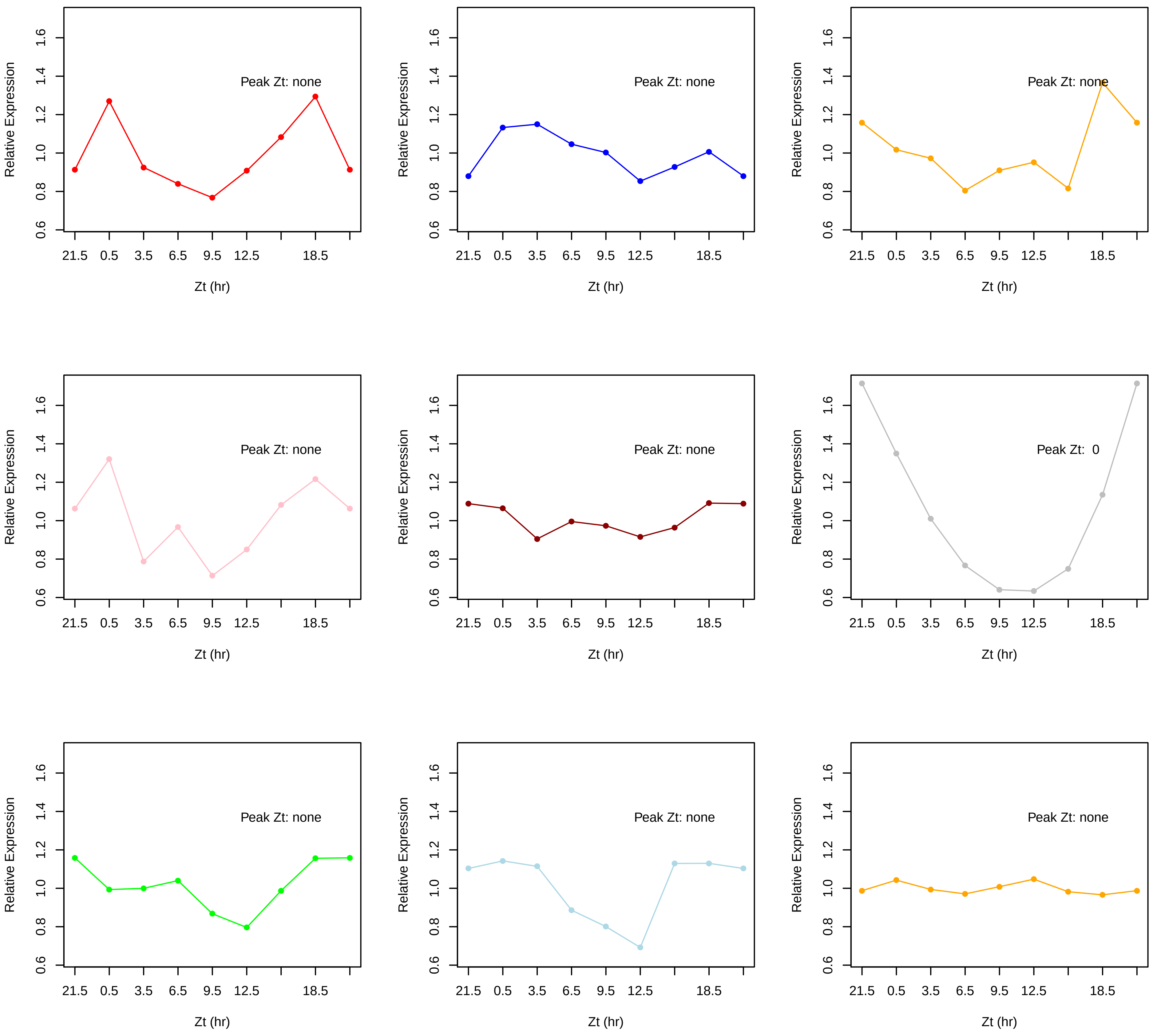

Singleton: ENSXMAG00000010583 rnf25

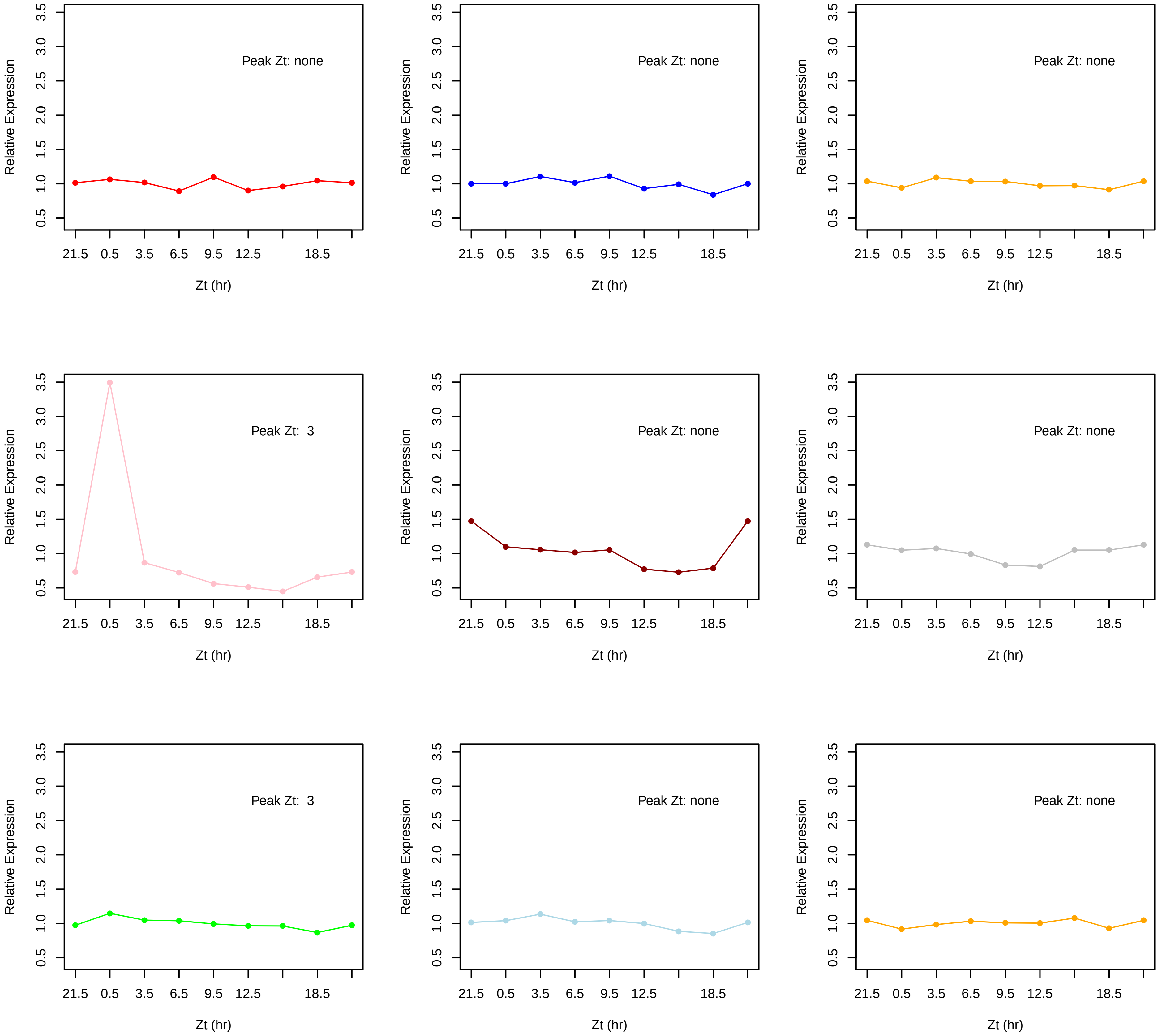

Singeleton: ENSXMAG00000005525 vcam1b

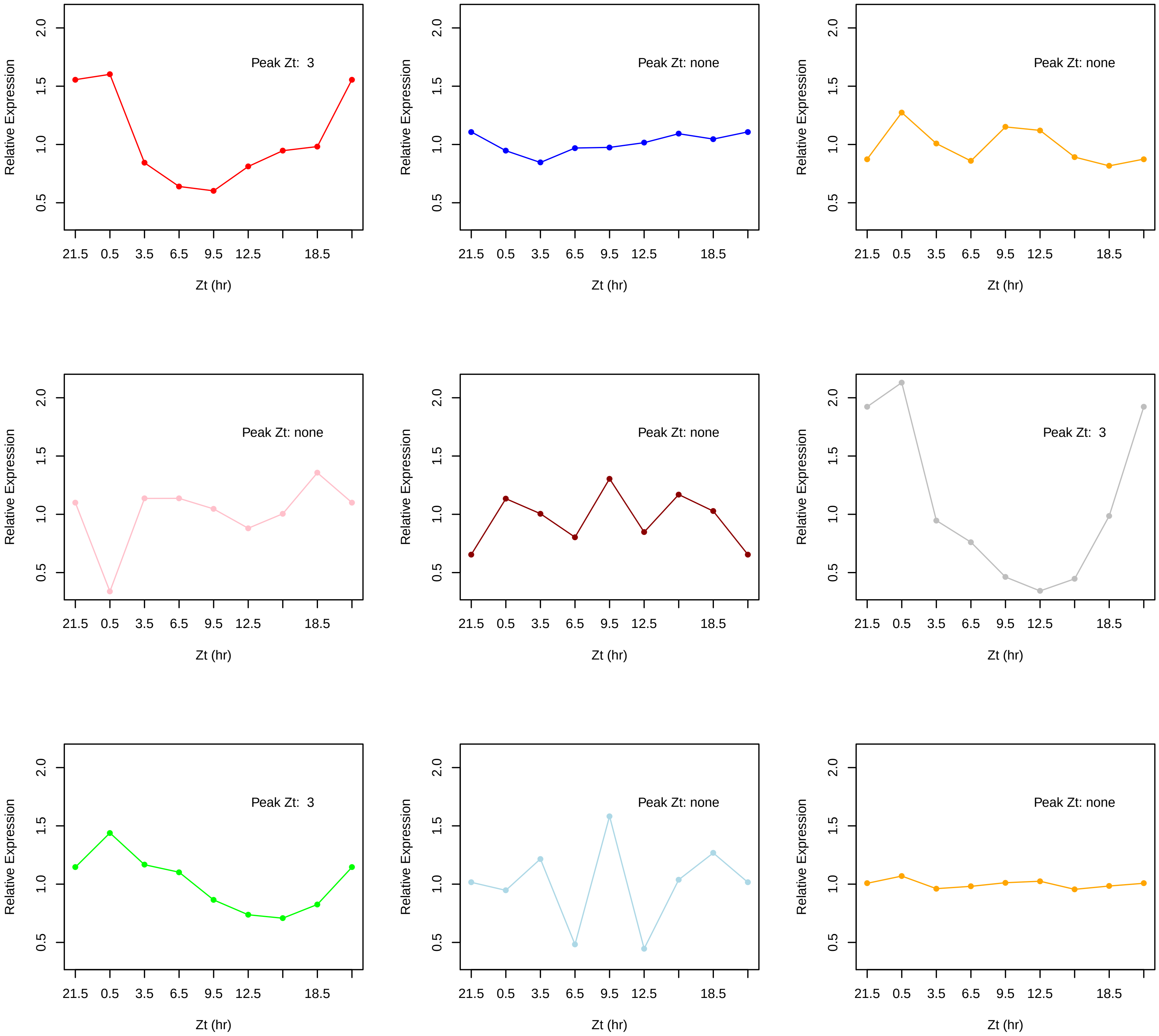

Singeleton: ENSXMAG00000014065 slc44a4

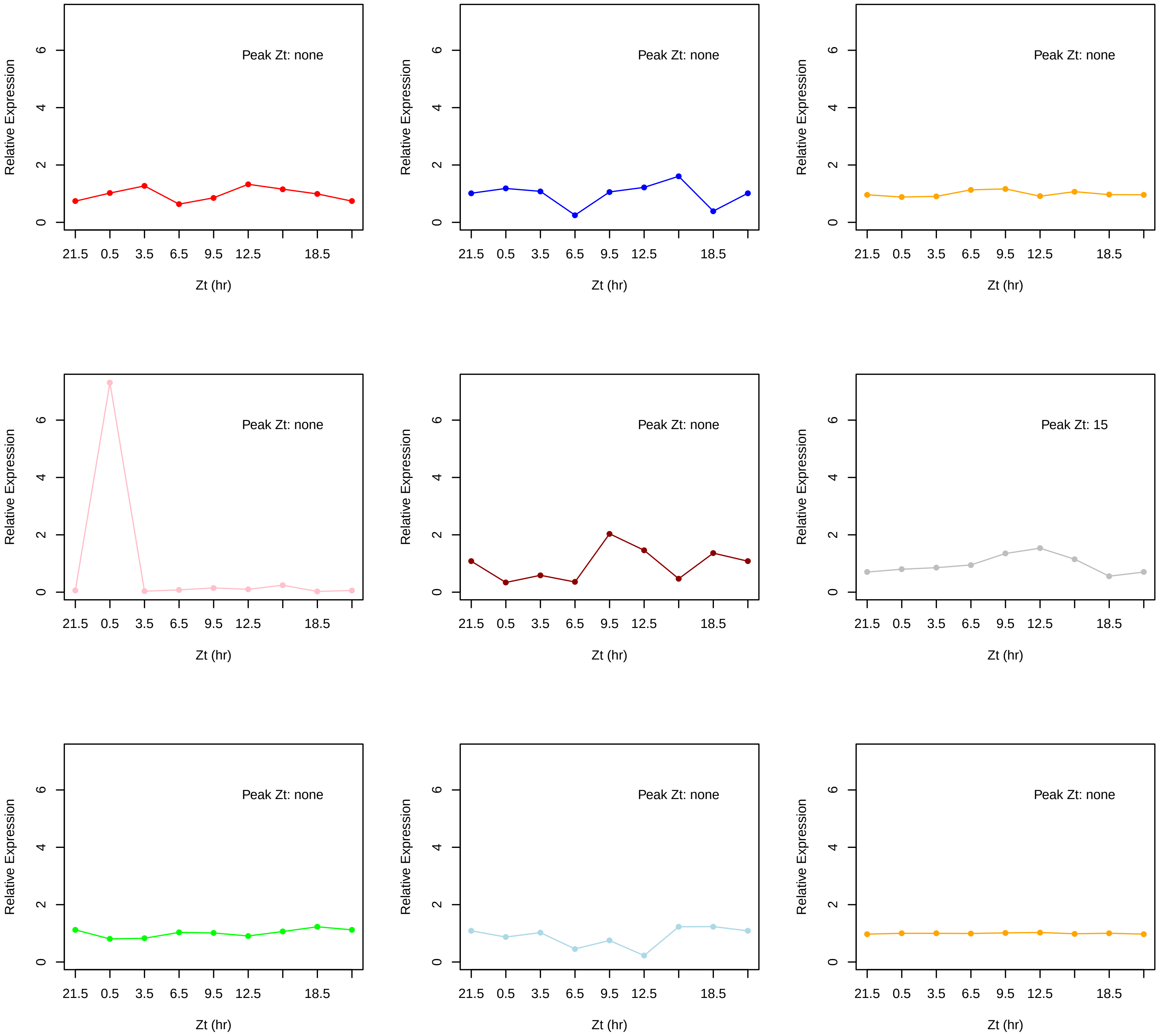

Singeleton: ENSXMAG00000011172 katnal1

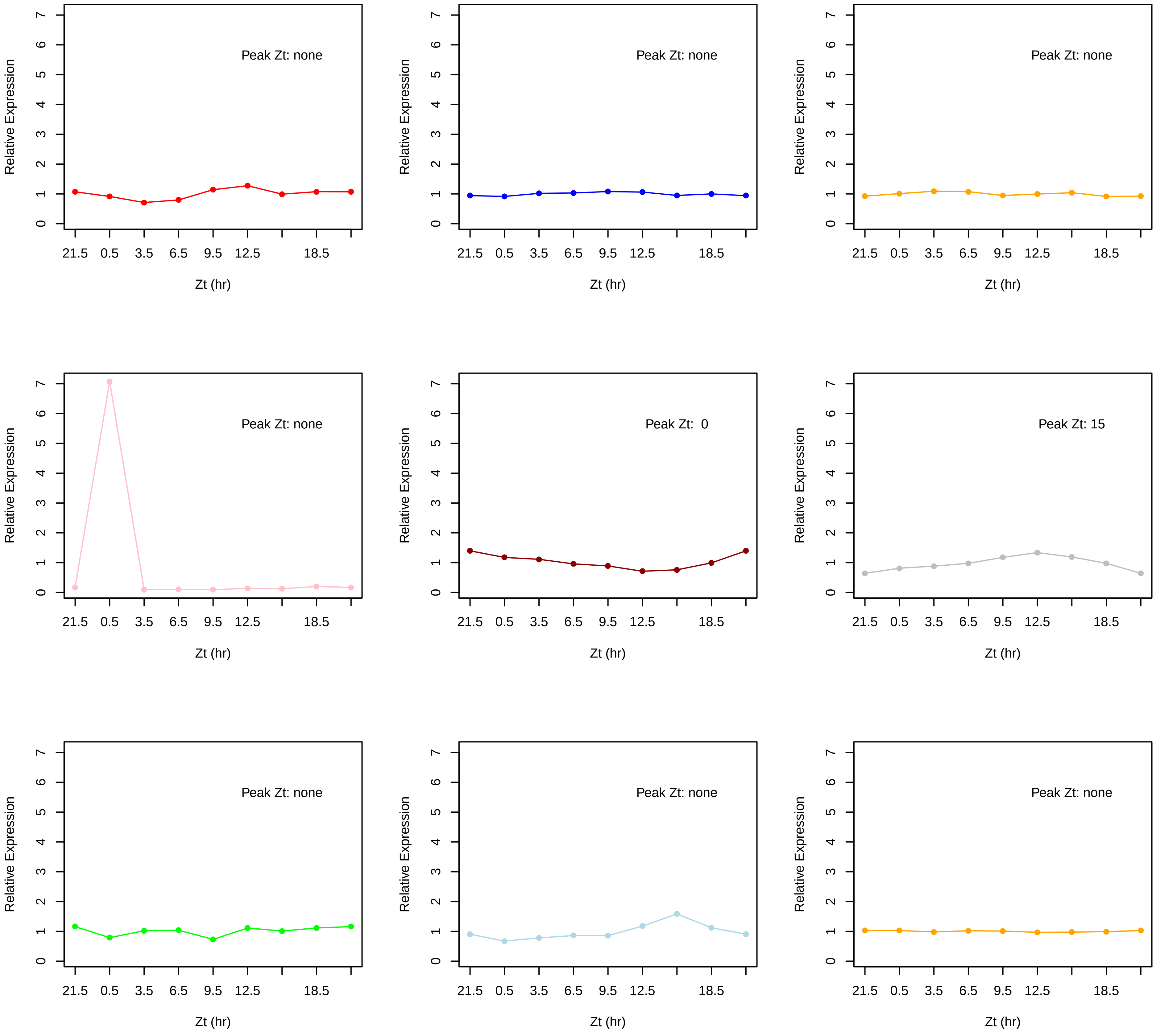

Singleton: ENSXMAG00000024583 ky

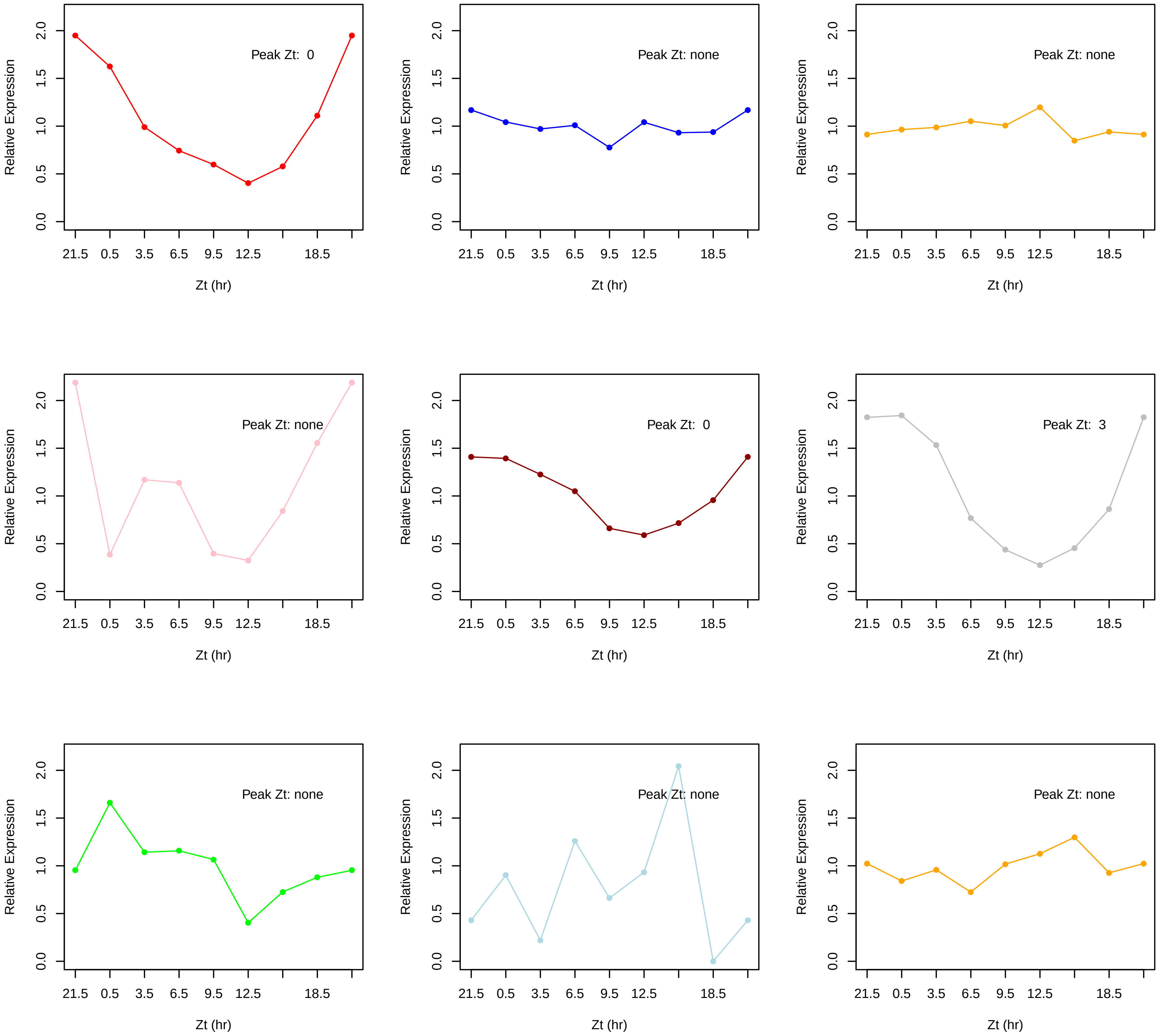

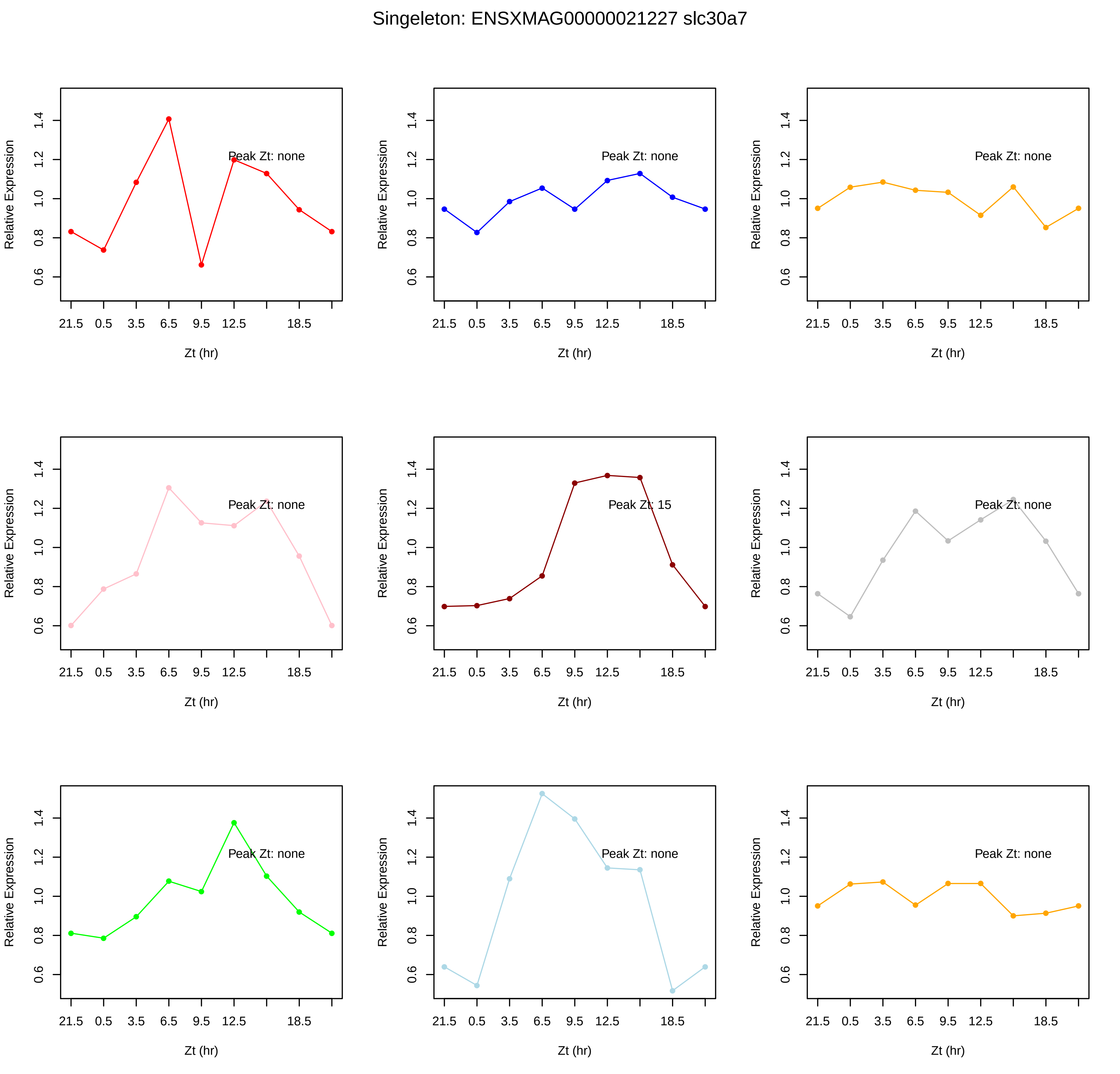

Singeleton: ENSXMAG00000021227 slc30a7
Relative Expression
Zt (hr)
21.5 0.5 3.5 6.5 9.5 12.5 18.5
0.6 0.8 1.0 1.2 1.4
Peak Zt: none
Peak Zt: 15

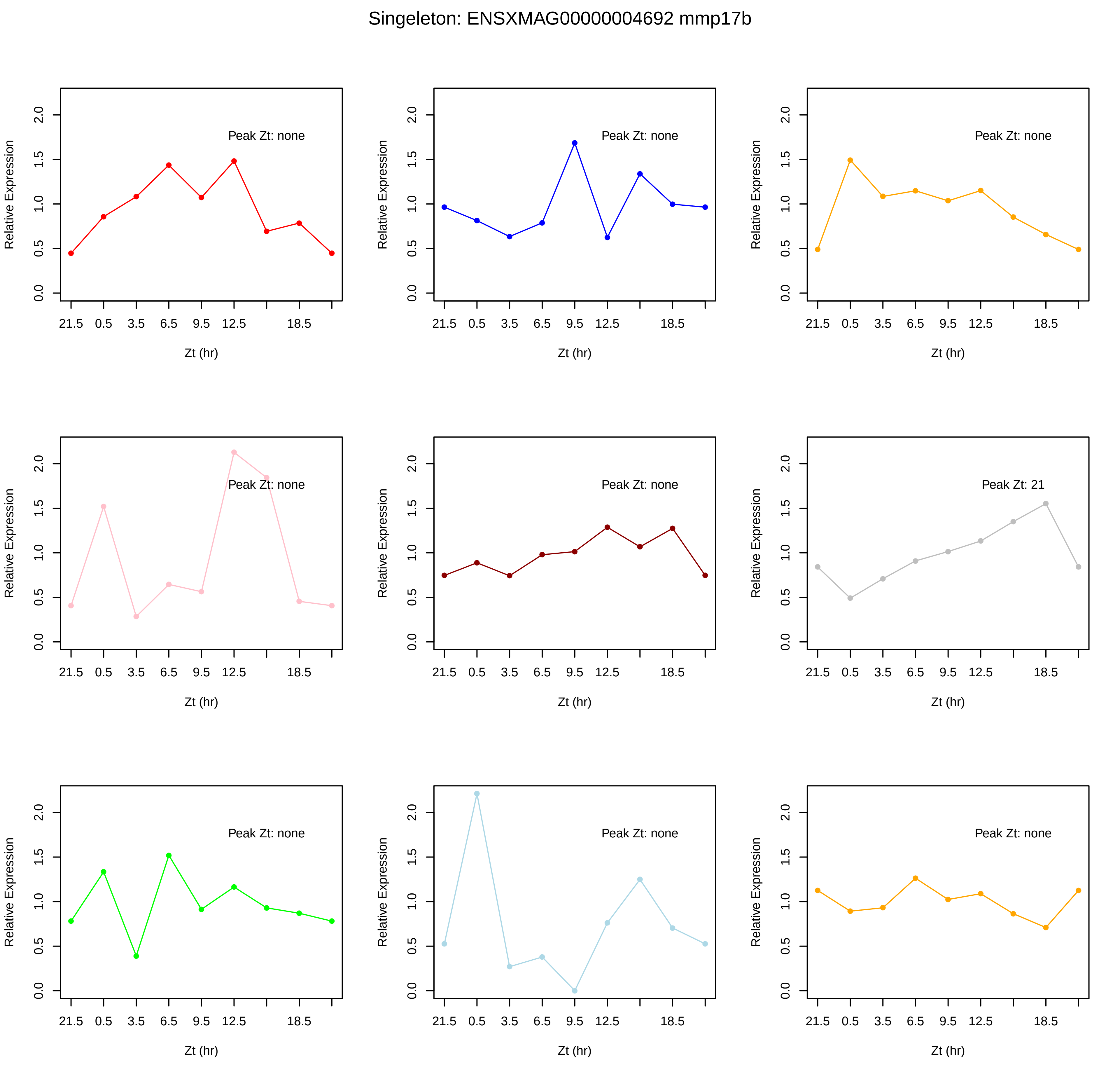
Singeleton: ENSXMAG00000004692 mmp17b
Peak Zt: none
Peak Zt: none
Peak Zt: none
Peak Zt: none
Peak Zt: none
Peak Zt: 21
Peak Zt: none
Peak Zt: none
Peak Zt: none
Relative Expression
Zt (hr)
21.5
0.5
3.5
6.5
9.5
12.5
18.5

Singeleton: ENSXMAG00000026608 dph5

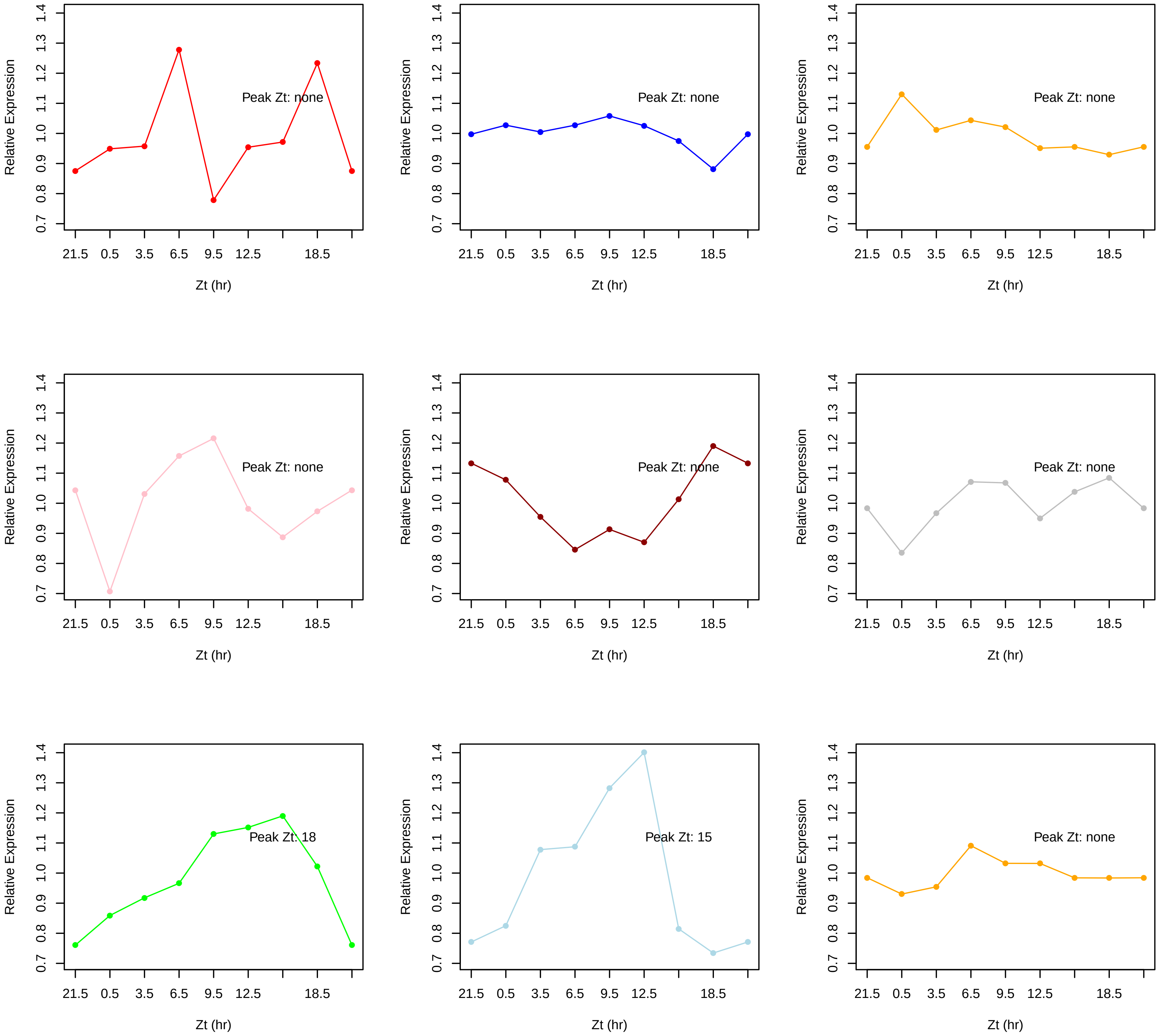

Singeleton: ENSXMAG00000015670 zgc:110366

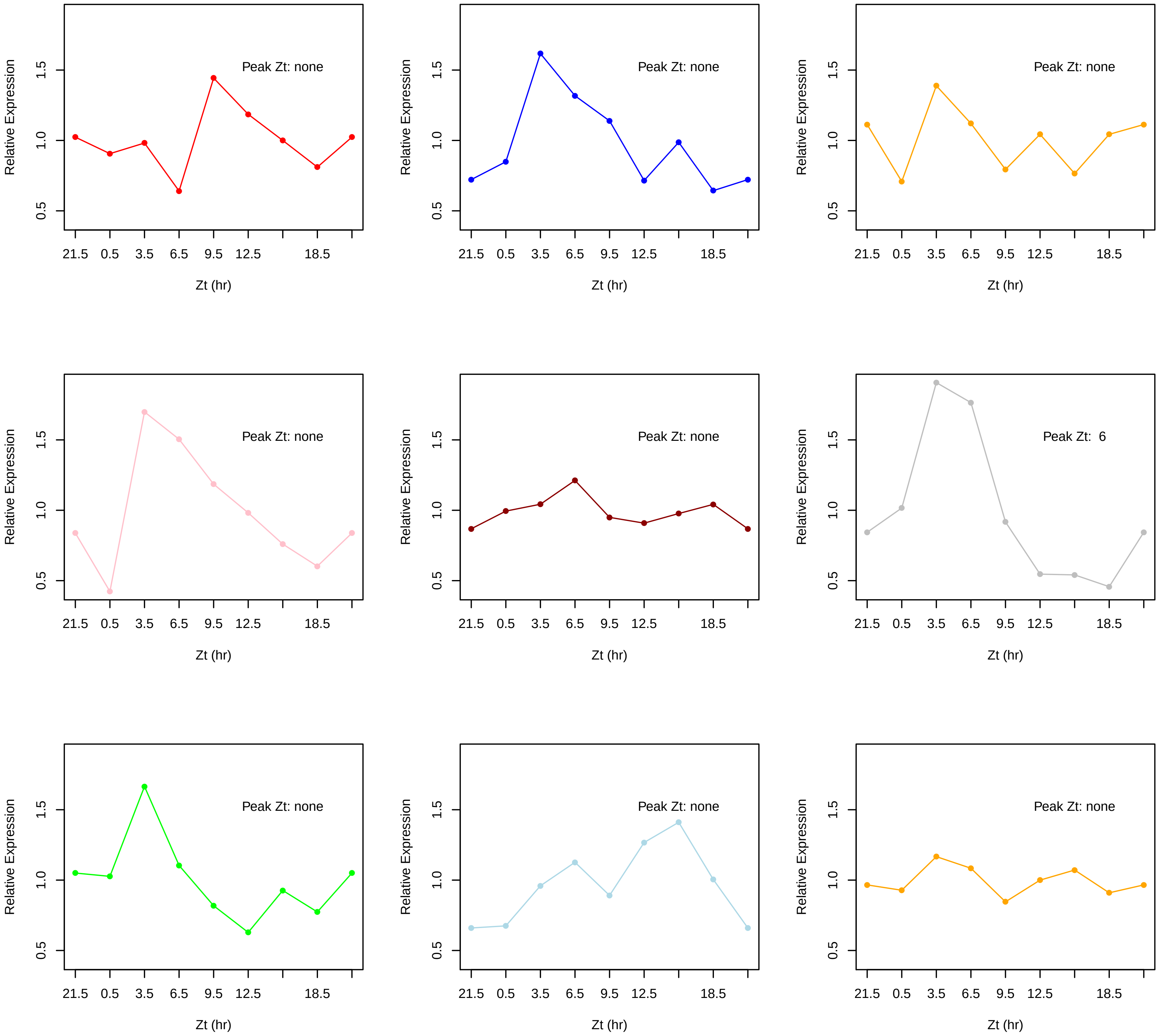

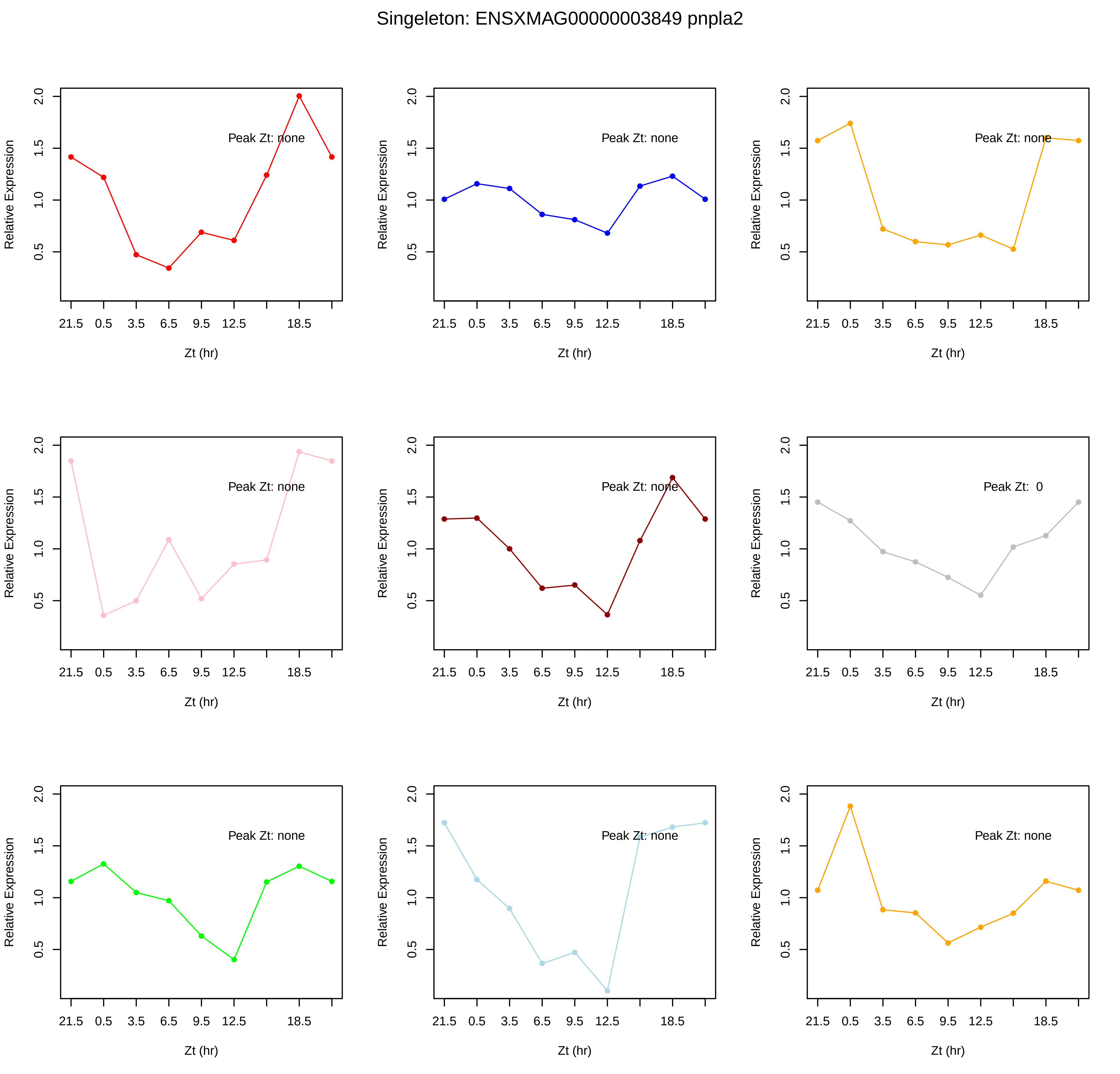
Singeleton: ENSXMAG00000003849 pnpla2
Peak Zt: none
Peak Zt: none
Peak Zt: none
Peak Zt: none
Peak Zt: none
Peak Zt: 0
Peak Zt: none
Peak Zt: none
Peak Zt: none
Relative Expression
Zt (hr)
21.5
0.5
3.5
6.5
9.5
12.5
18.5
0.5
1.0
1.5
2.0

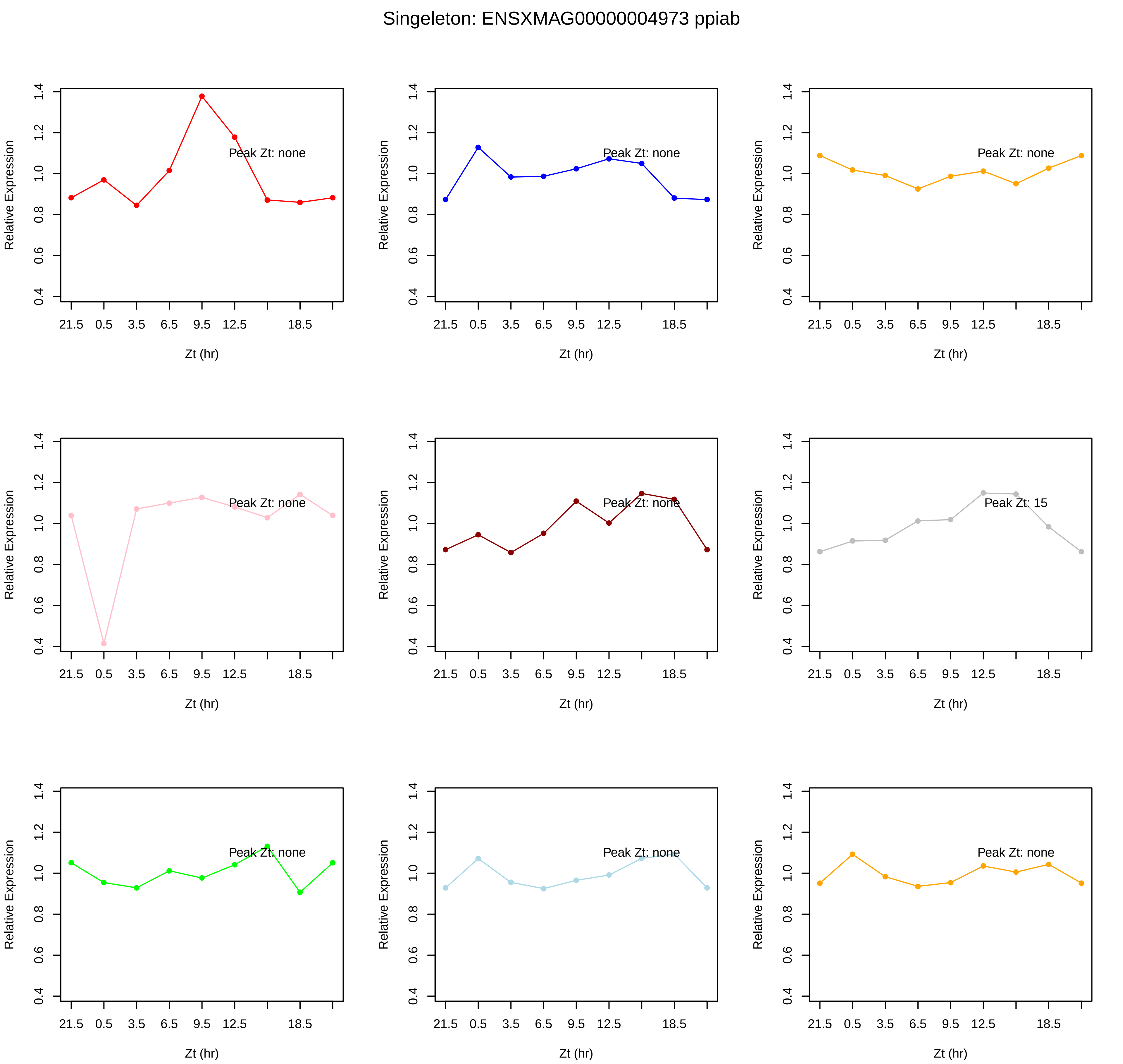
Singleton: ENSXMAG00000004973 ppiab
Relative Expression
Zt (hr)
21.5
0.5
3.5
6.5
9.5
12.5
18.5
0.4
0.6
0.8
1.0
1.2
1.4
Peak Zt: none
Peak Zt: 15

Singeleton: ENSXMAG00000015673 rc3h1b

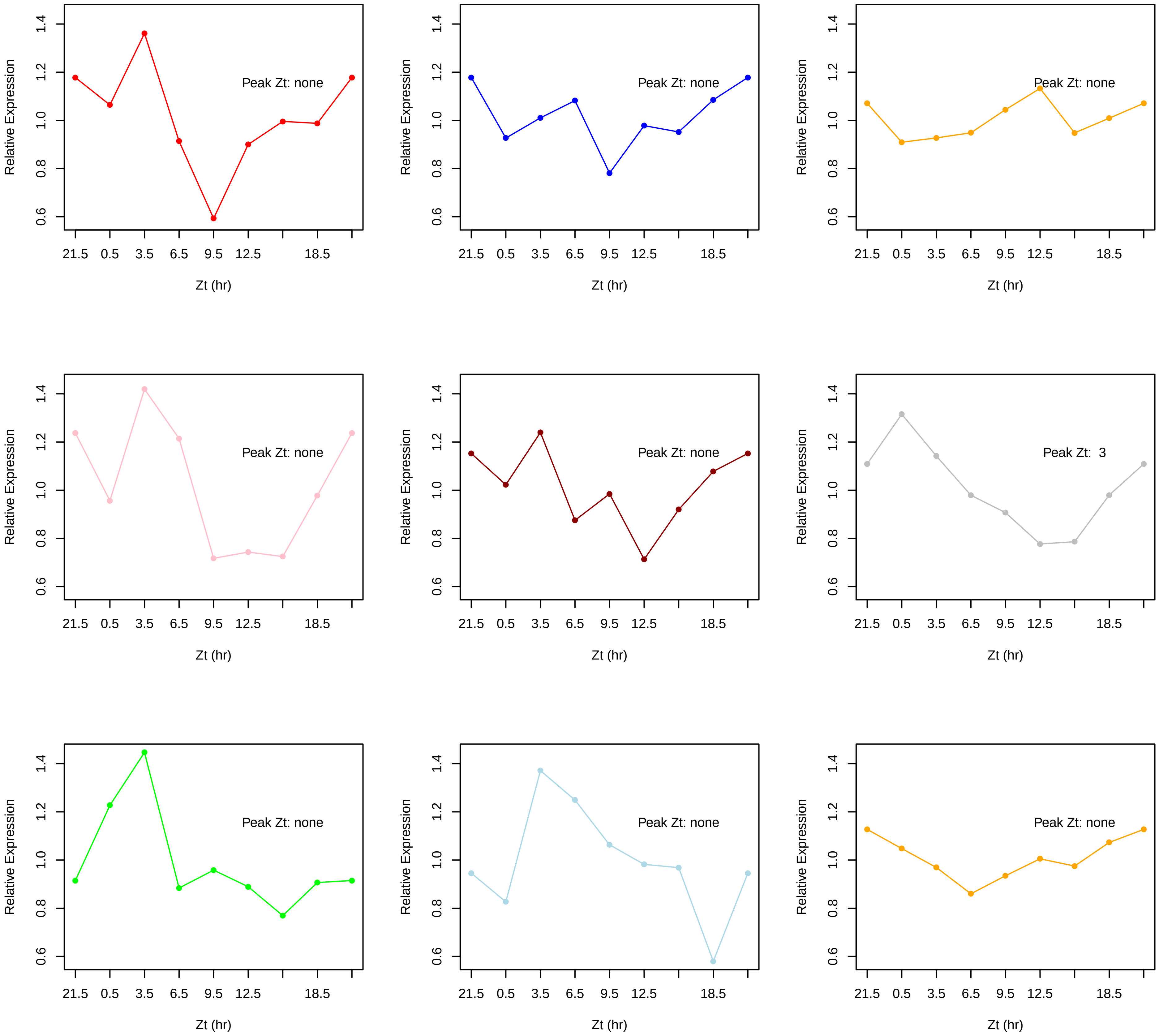

Singleton: ENSXMAG00000023054 wdr95

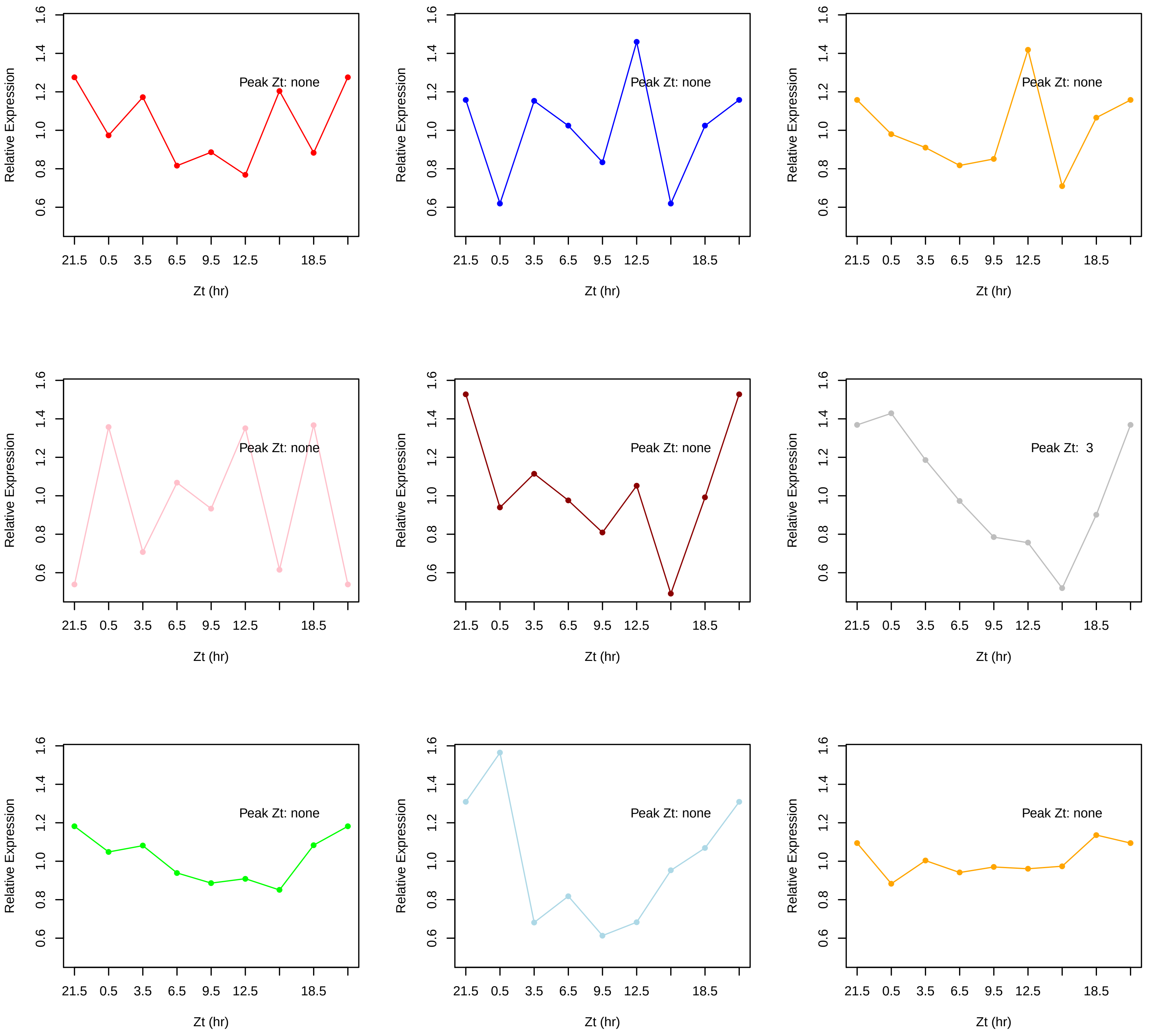

Singeleton: ENSXMAG00000011116 cep152

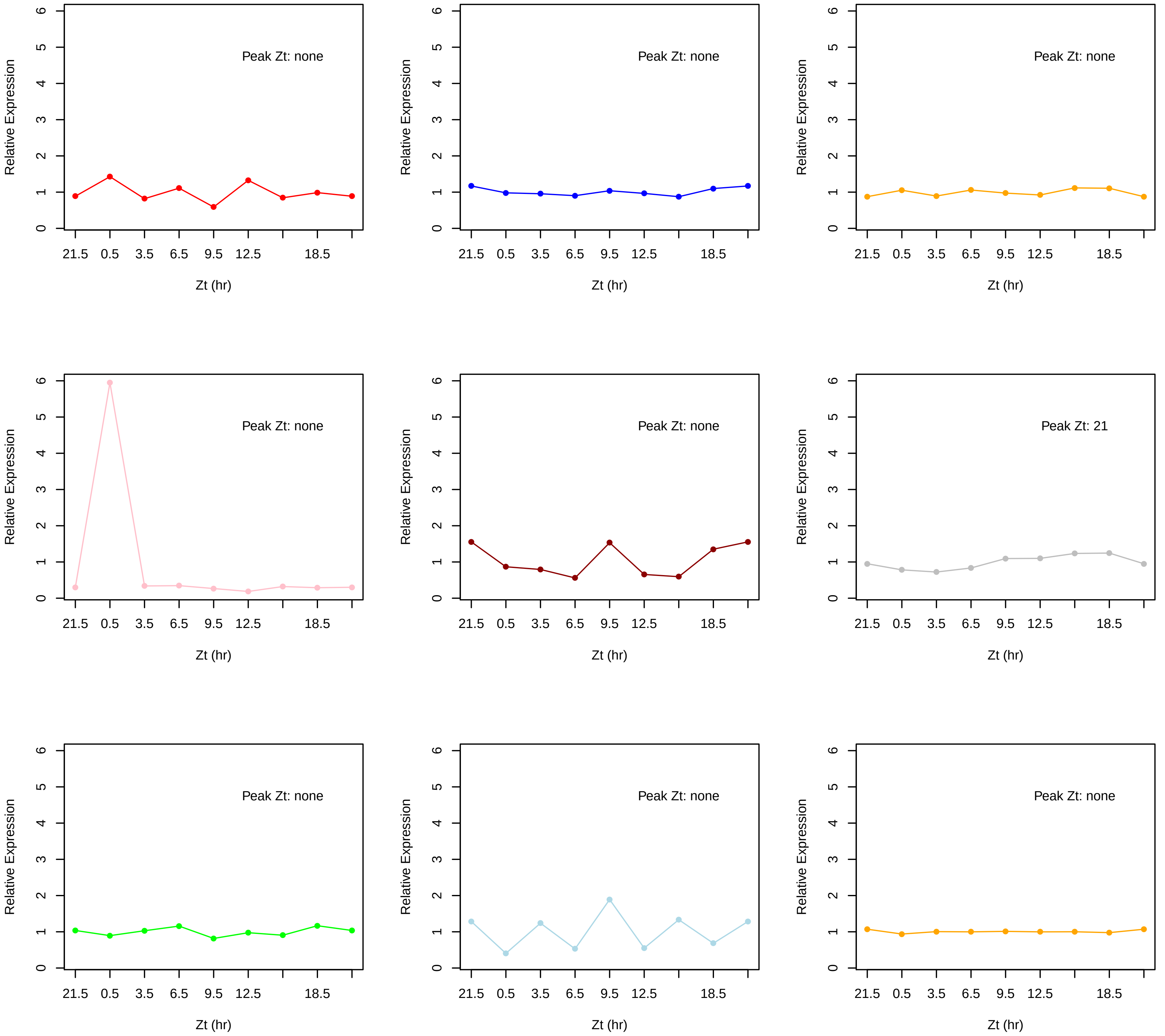

Singeleton: ENSXMAG00000010778 dbt

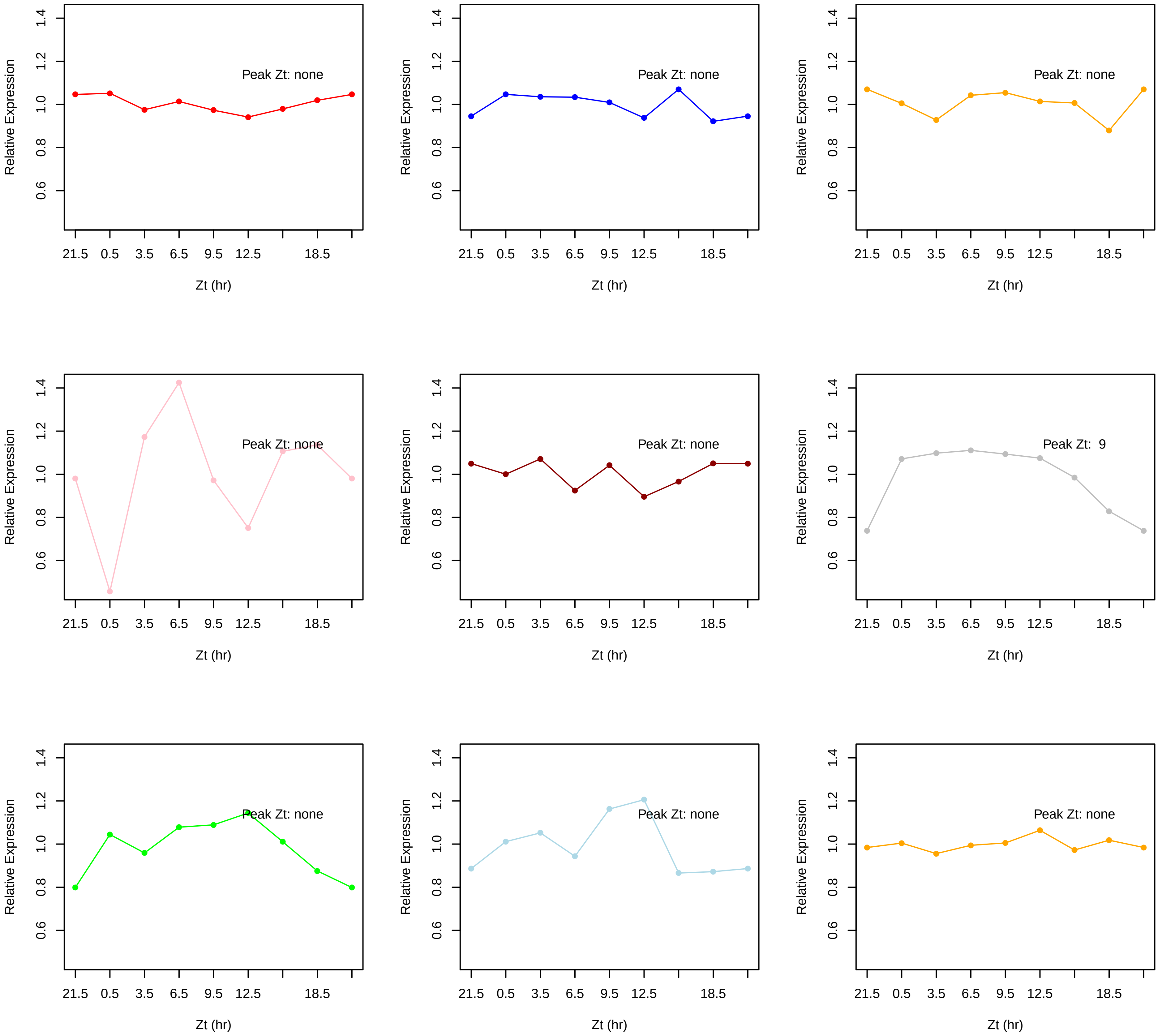

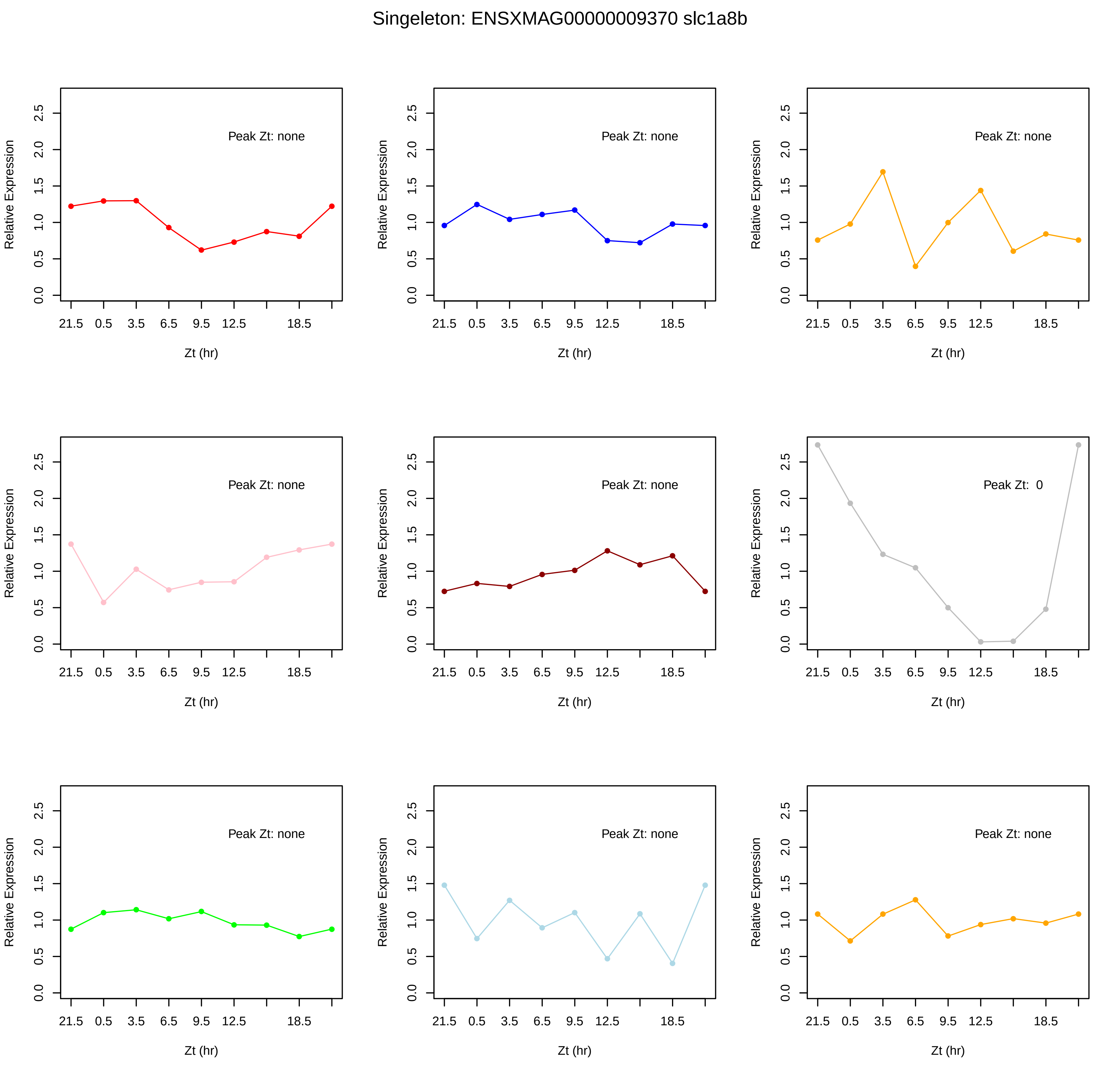

Singeleton: ENSXMAG00000009370 slc1a8b
Peak Zt: none
Peak Zt: none
Peak Zt: none
Peak Zt: none
Peak Zt: none
Peak Zt: 0
Peak Zt: none
Peak Zt: none
Peak Zt: none
Relative Expression
Zt (hr)
21.5
0.5
3.5
6.5
9.5
12.5
18.5
0.0
0.5
1.0
1.5
2.0
2.5

Singeleton: ENSXMAG00000018280 lin7c

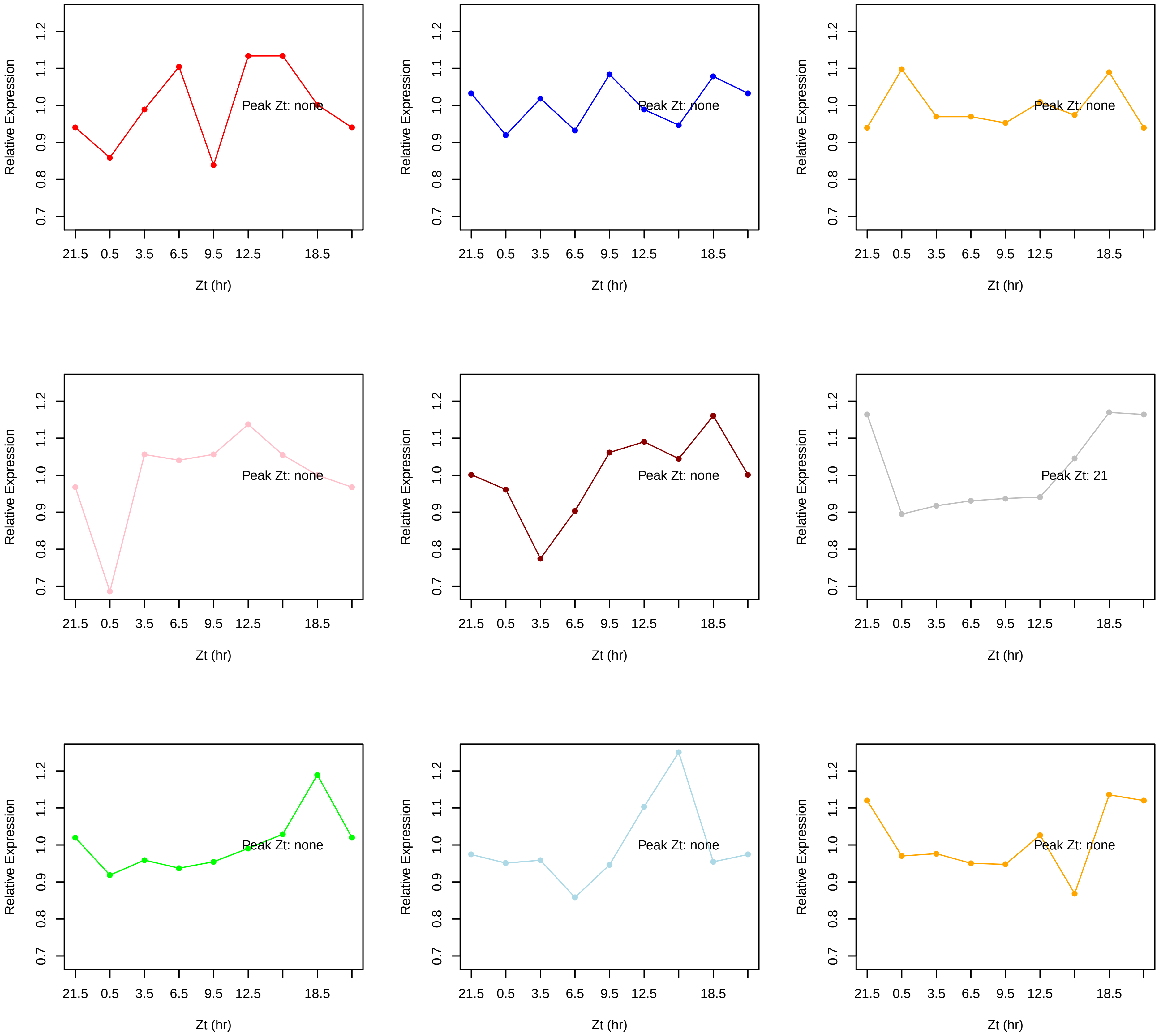

Singleton: ENSXMAG00000016897 bpnt1

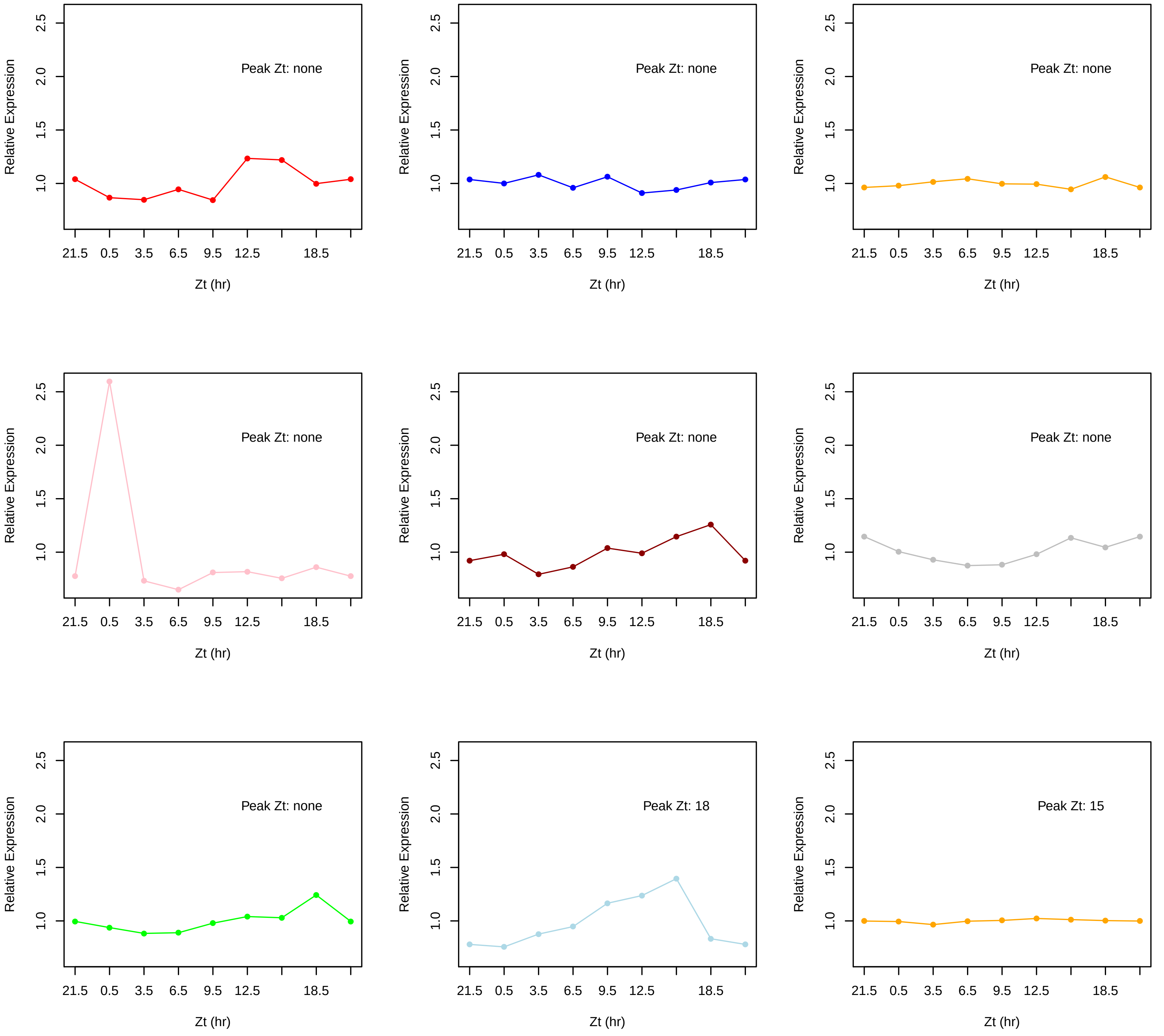

Singeleton: ENSXMAG00000012244 nf1a

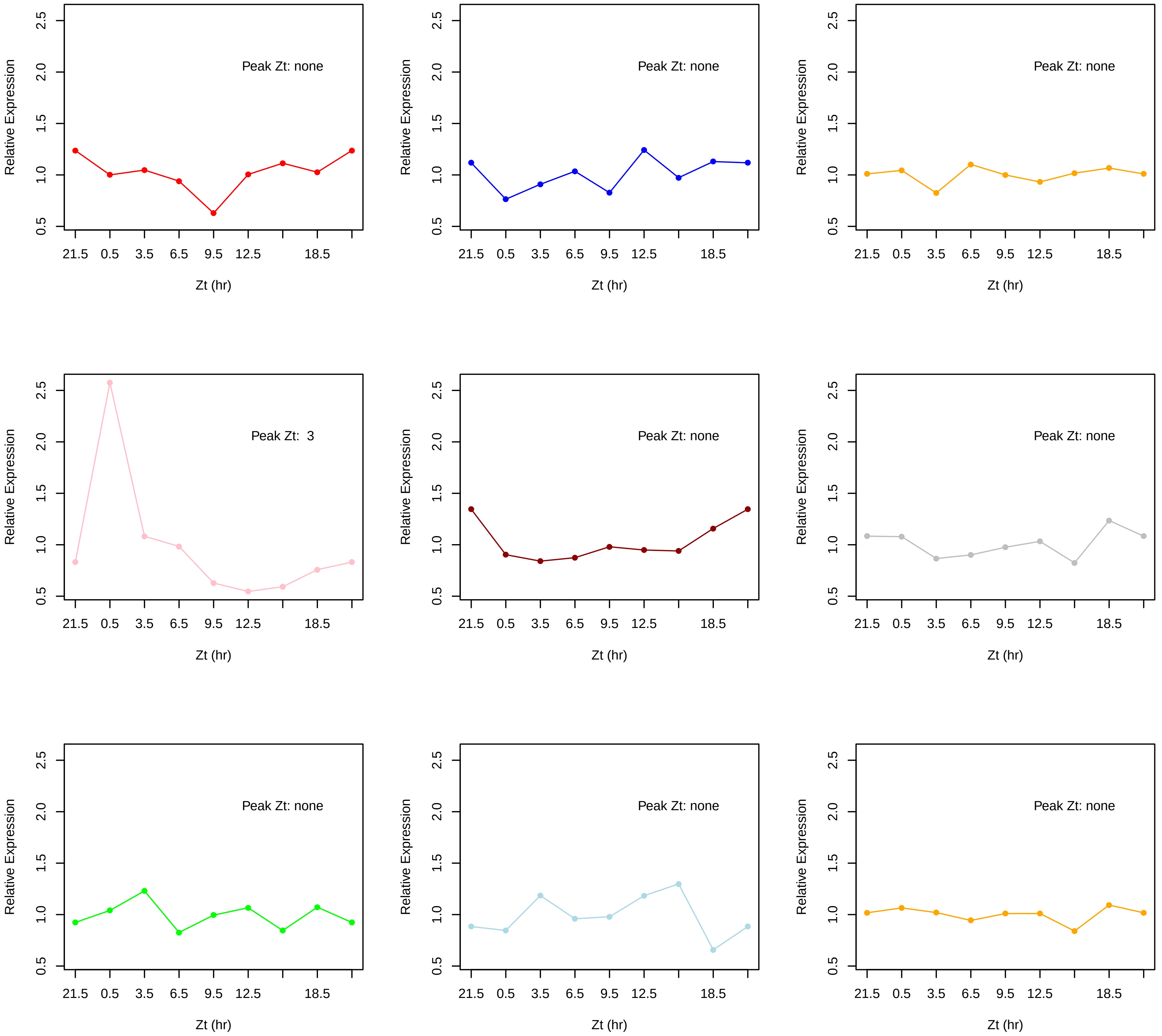

Singleton: ENSXMAG00000021235 si:ch211−11k18.4

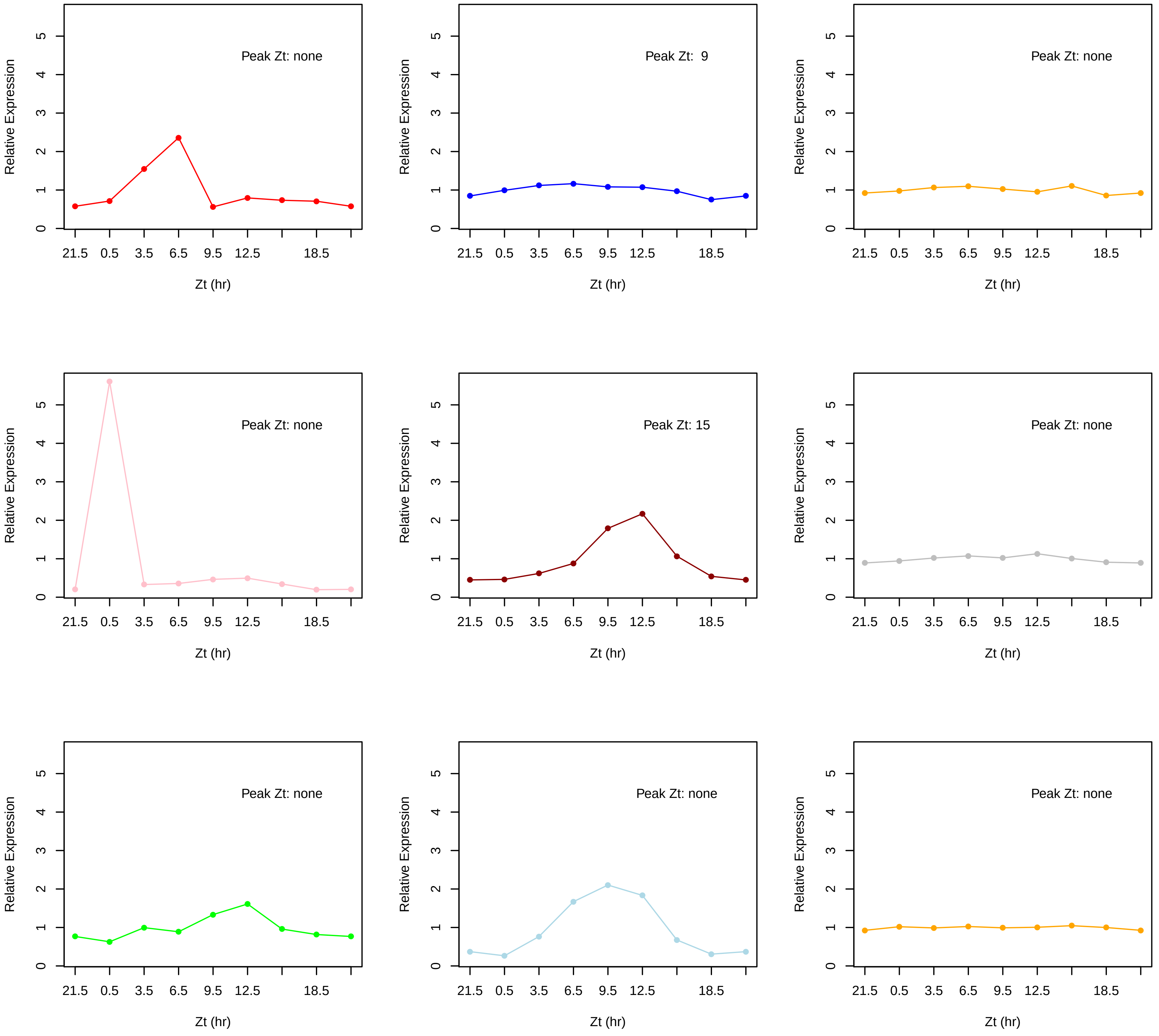

Singleton: ENSXMAG00000017658 zmp:0000000711

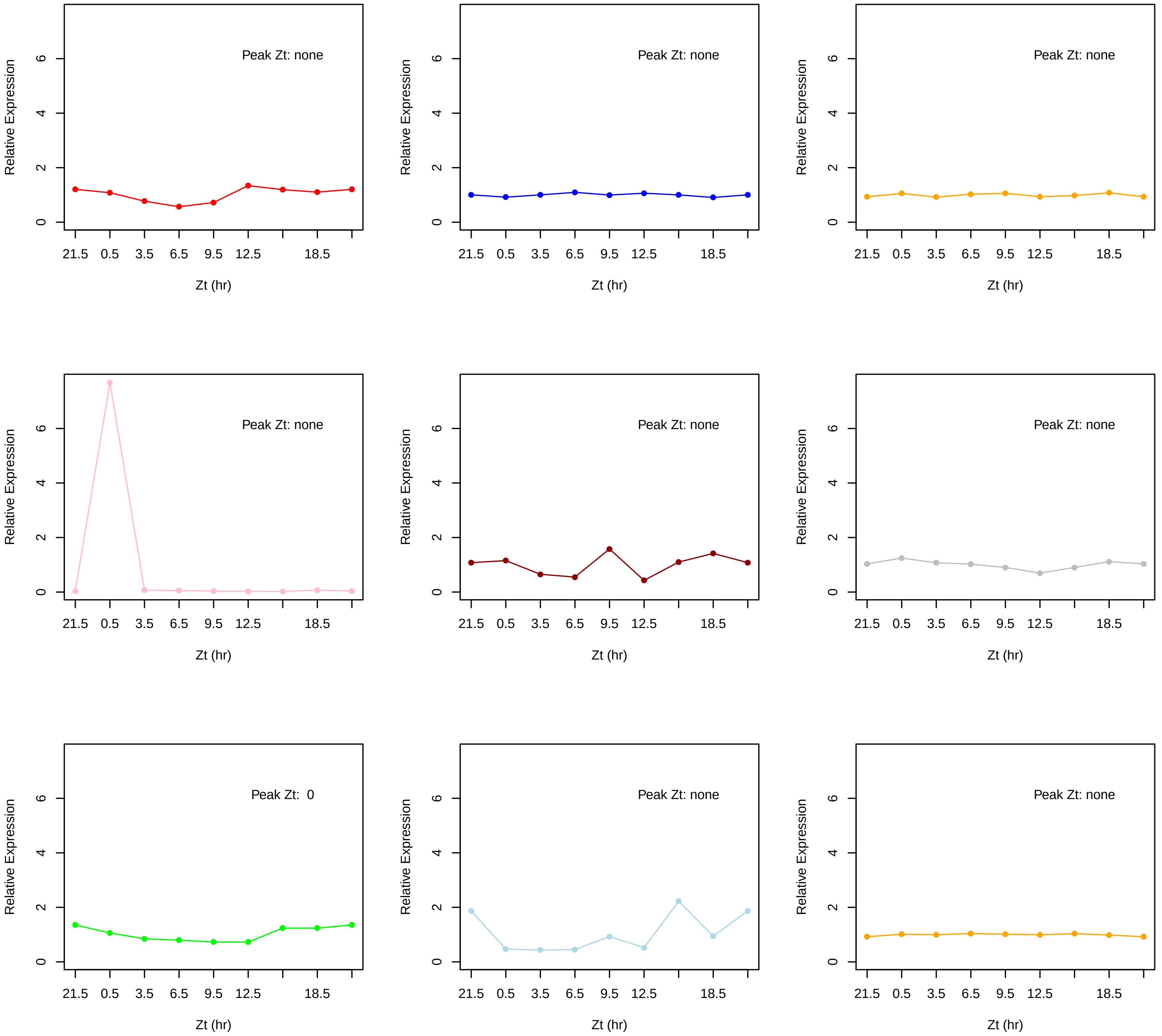

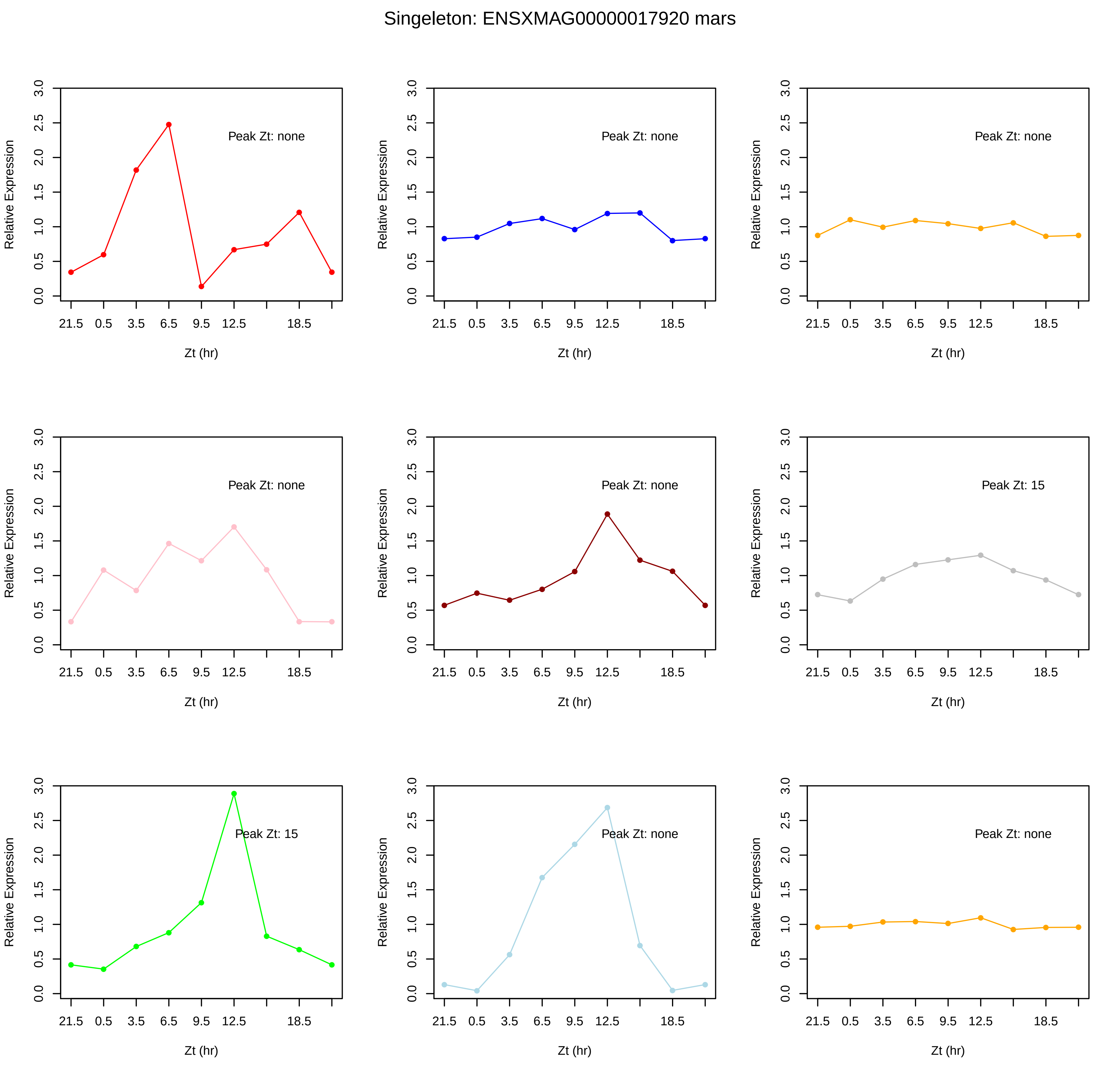
Singeleton: ENSXMAG00000017920 mars
Peak Zt: none
Peak Zt: none
Peak Zt: none
Peak Zt: none
Peak Zt: none
Peak Zt: 15
Peak Zt: 15
Peak Zt: none
Peak Zt: none
Relative Expression
Zt (hr)
21.5
0.5
3.5
6.5
9.5
12.5
18.5

Singleton: ENSXMAG00000010769 lrrc39

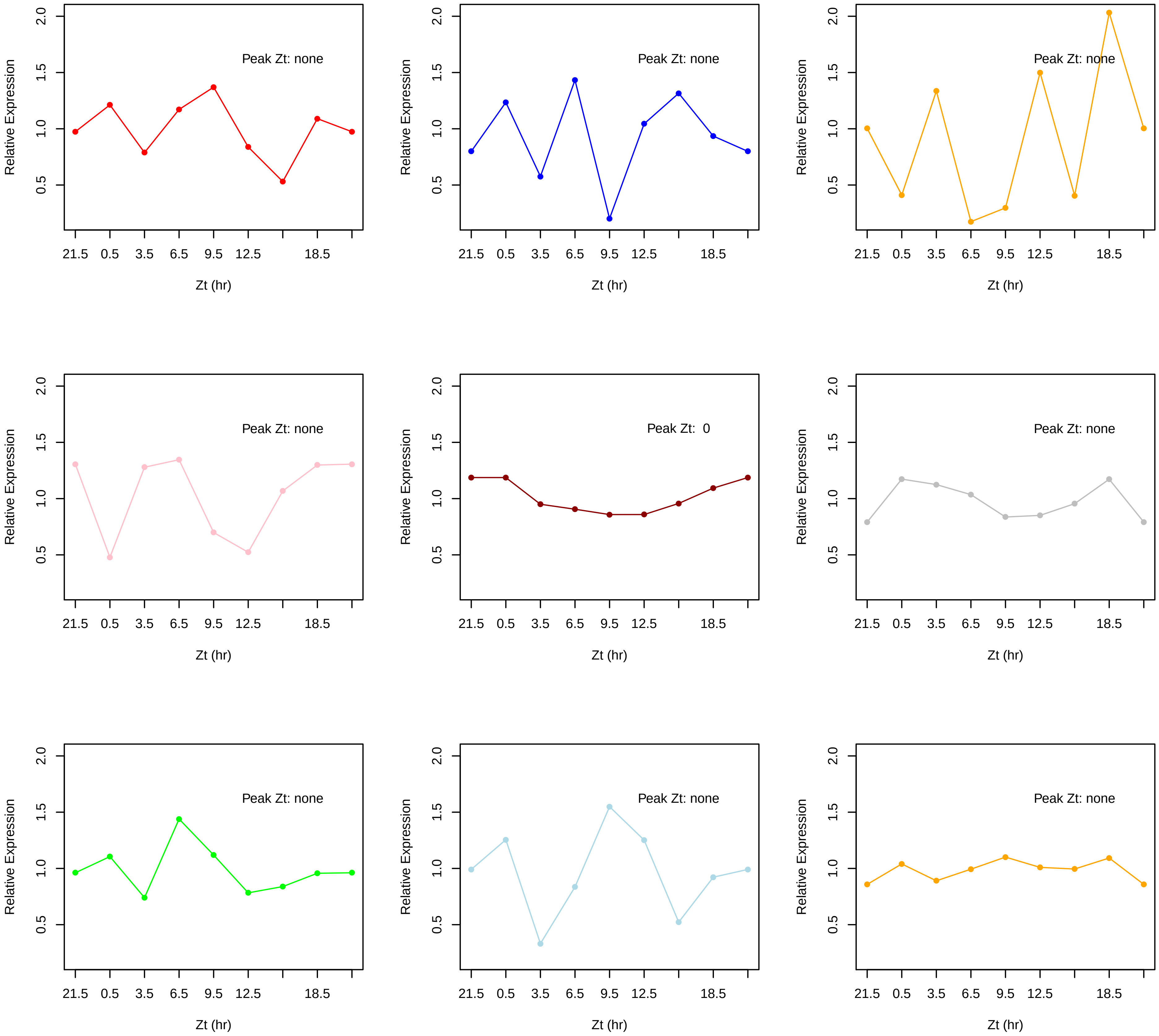

Singeleton: ENSXMAG00000010765 trmt13

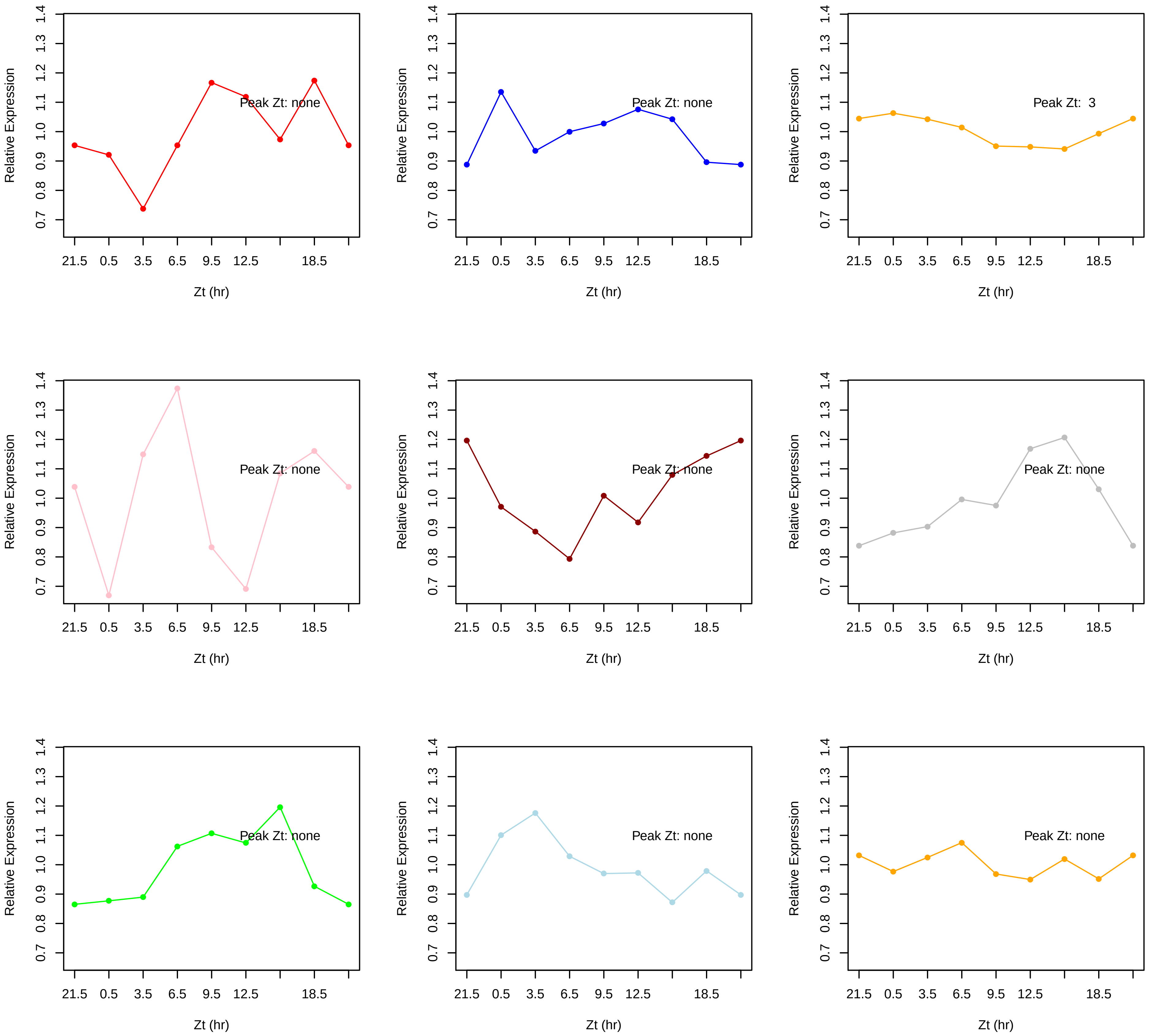

Singeleton: ENSXMAG00000023265 pla2g15

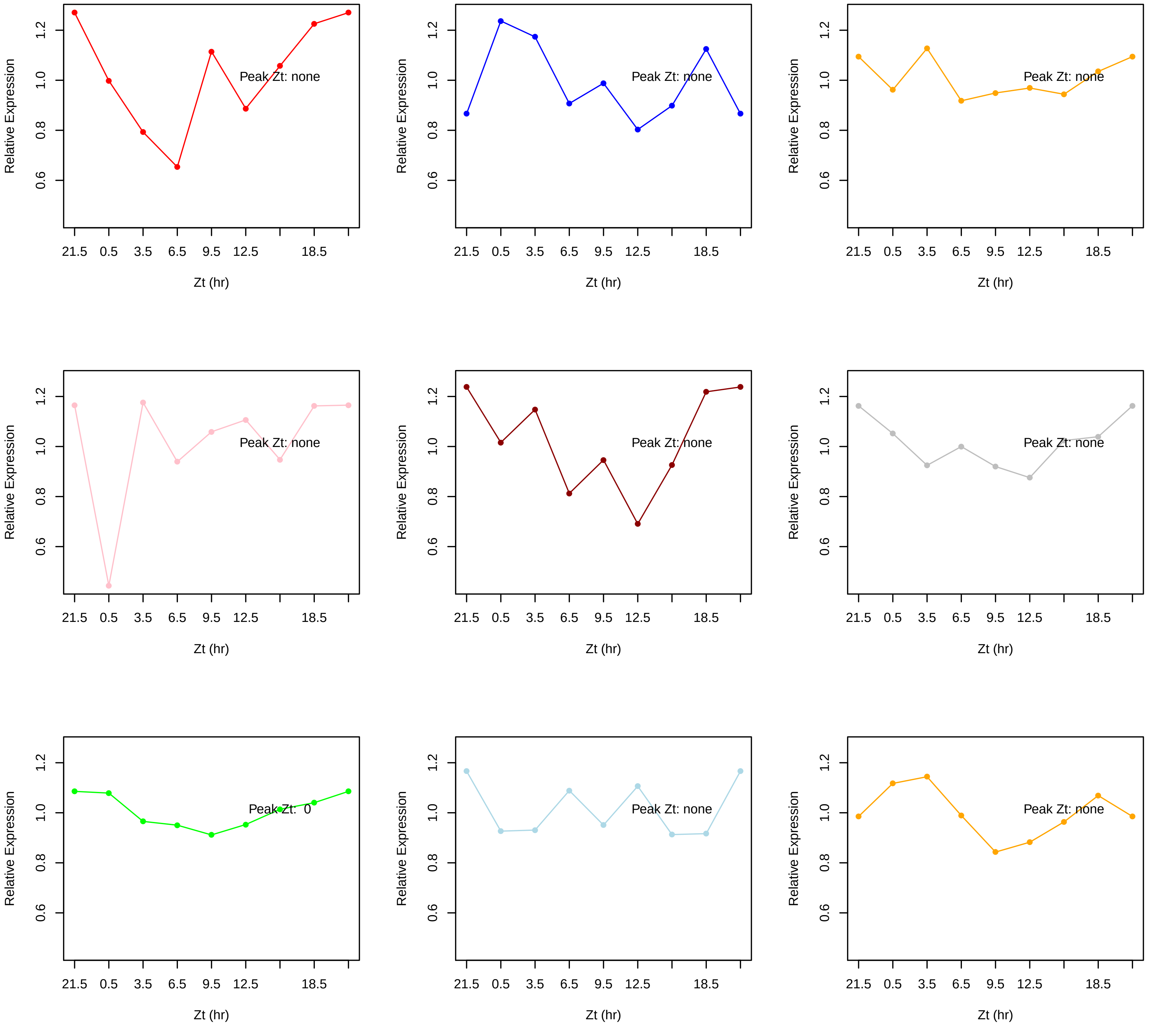

Singeleton: ENSXMAG00000013564 pbx2

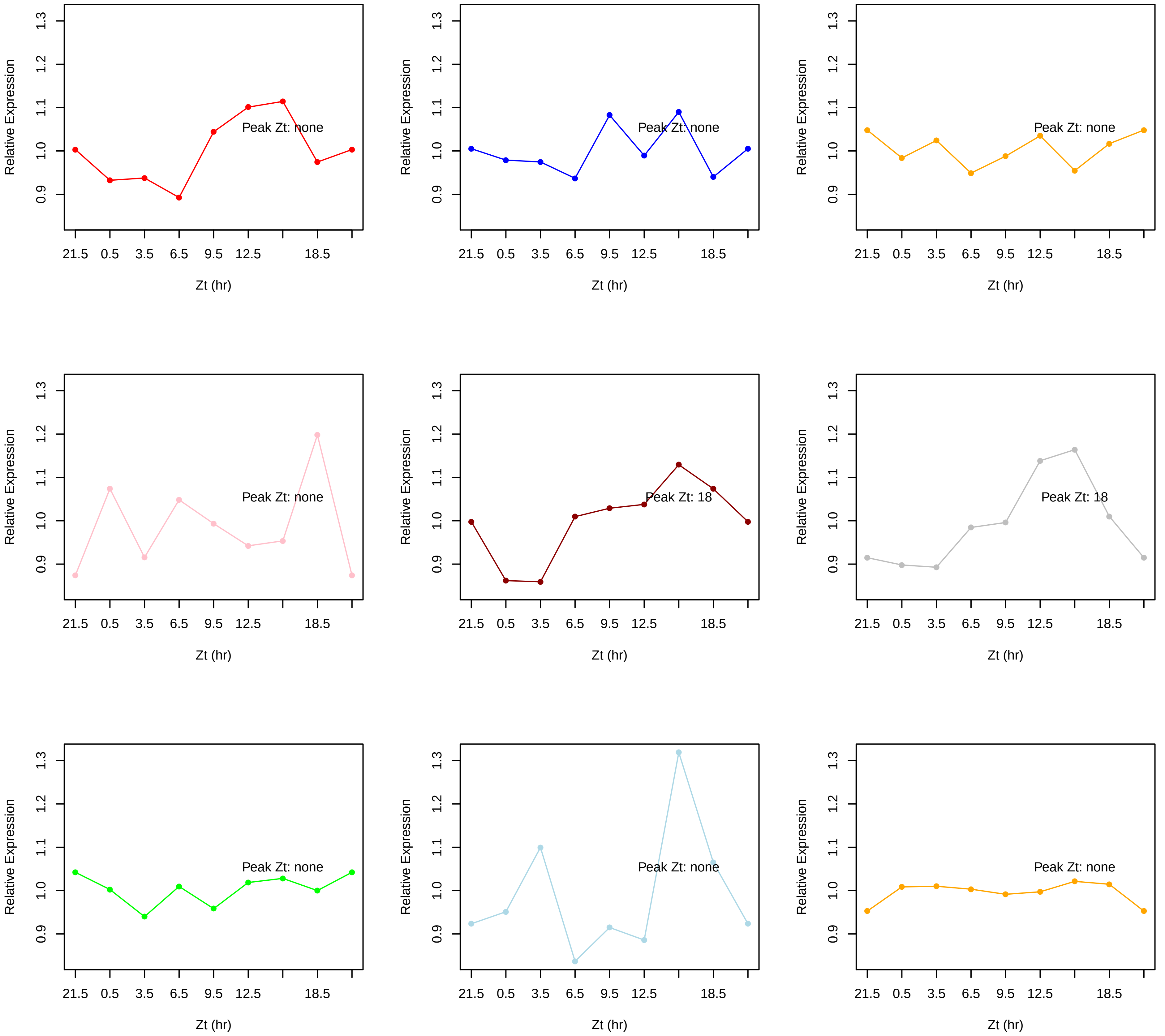

Singeleton: ENSXMAG00000016060 ttc14

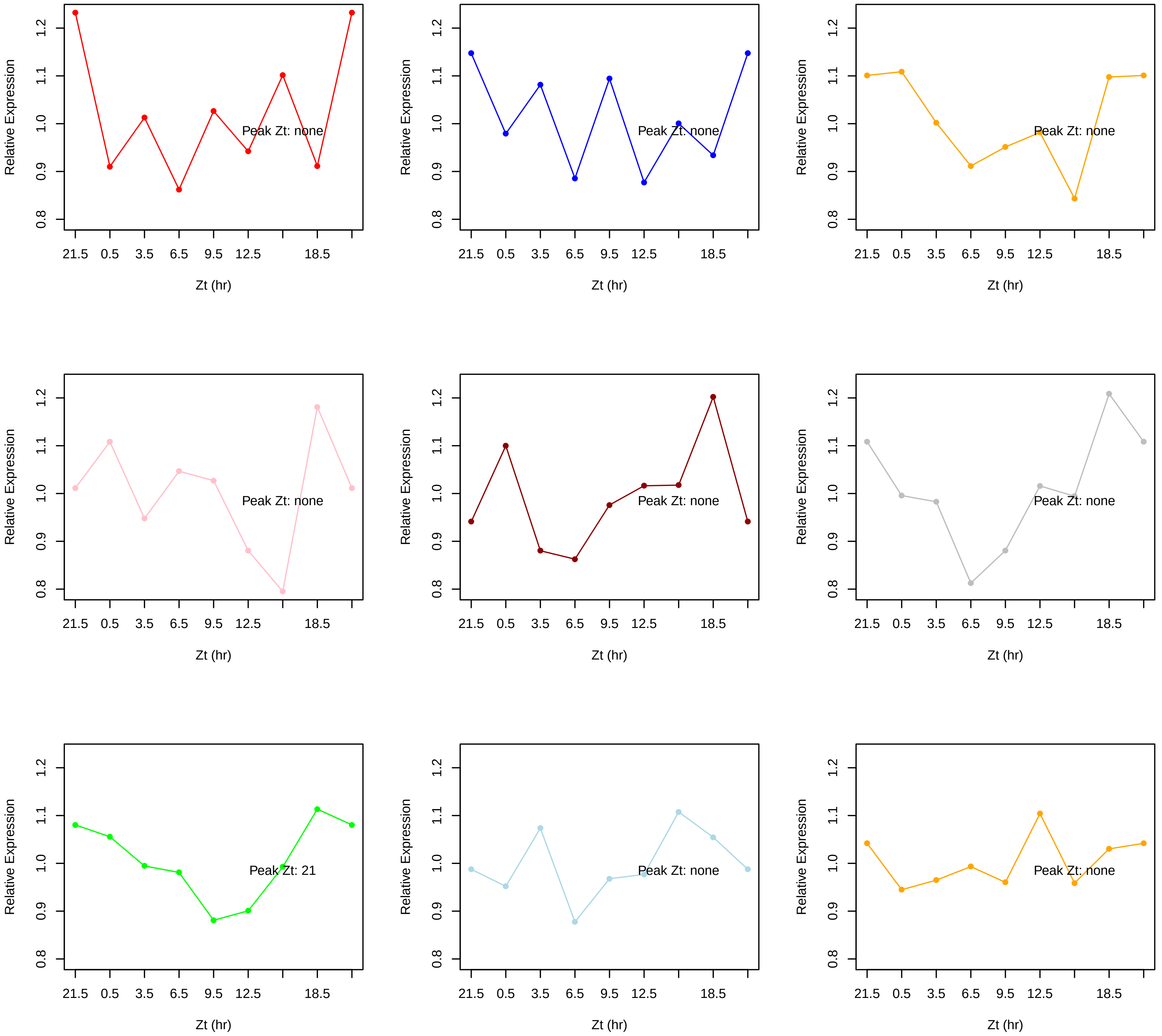

Singeleton: ENSXMAG00000024800 mto1

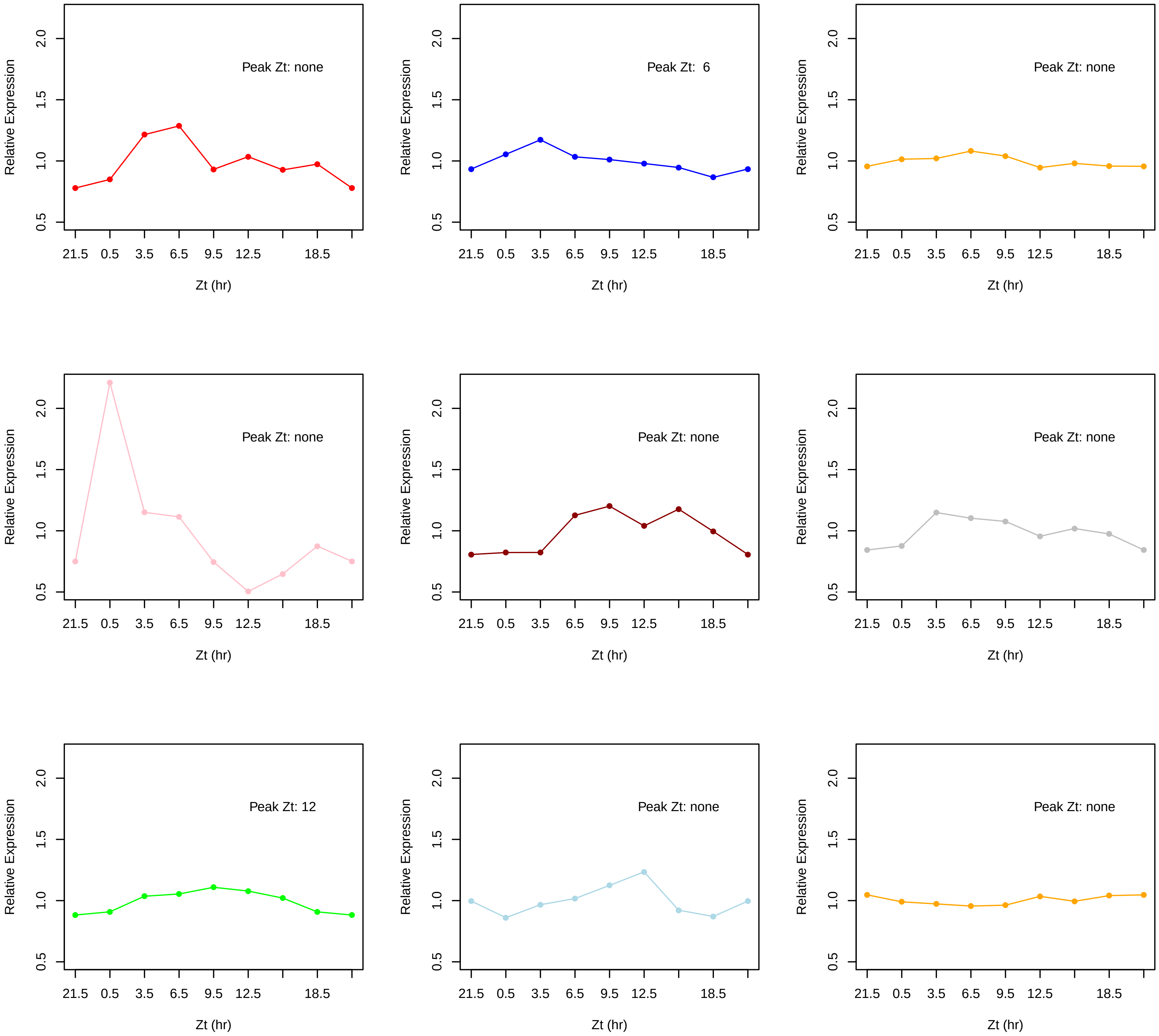

Singleton: ENSXMAG00000022074 pfdn6

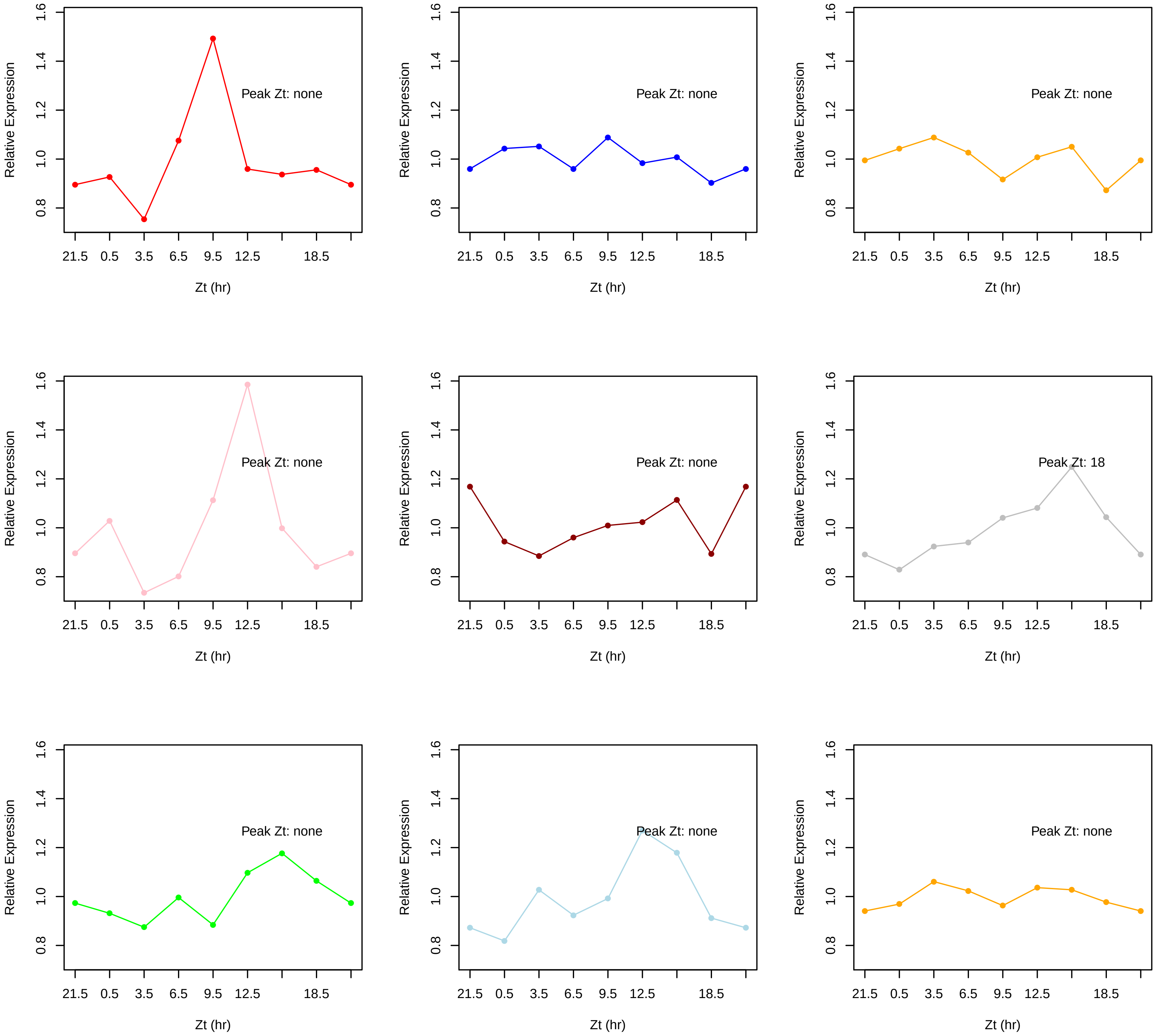

Singeleton: ENSXMAG00000024687 usb1

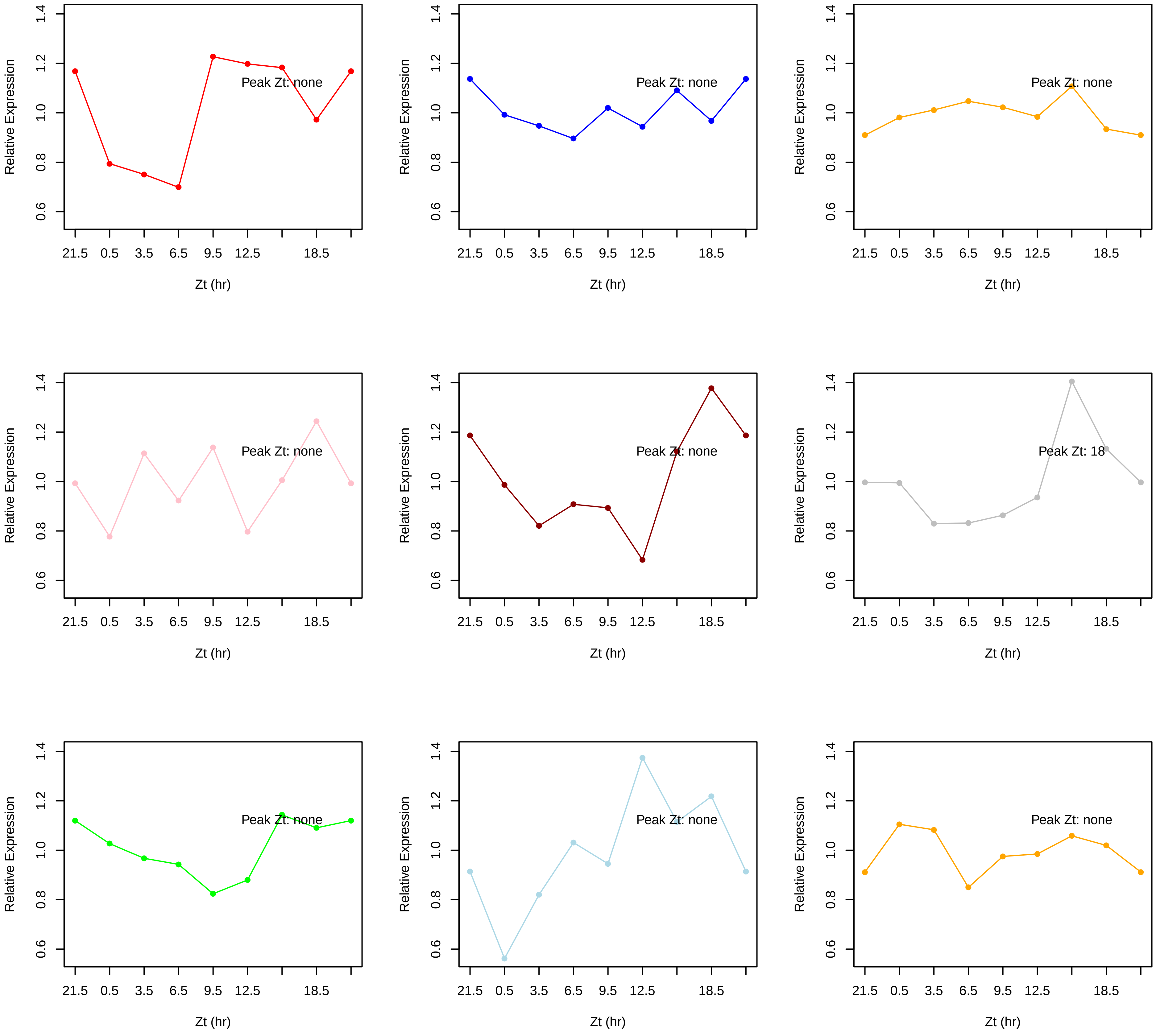

Singeleton: ENSXMAG00000014018 zgc:92275

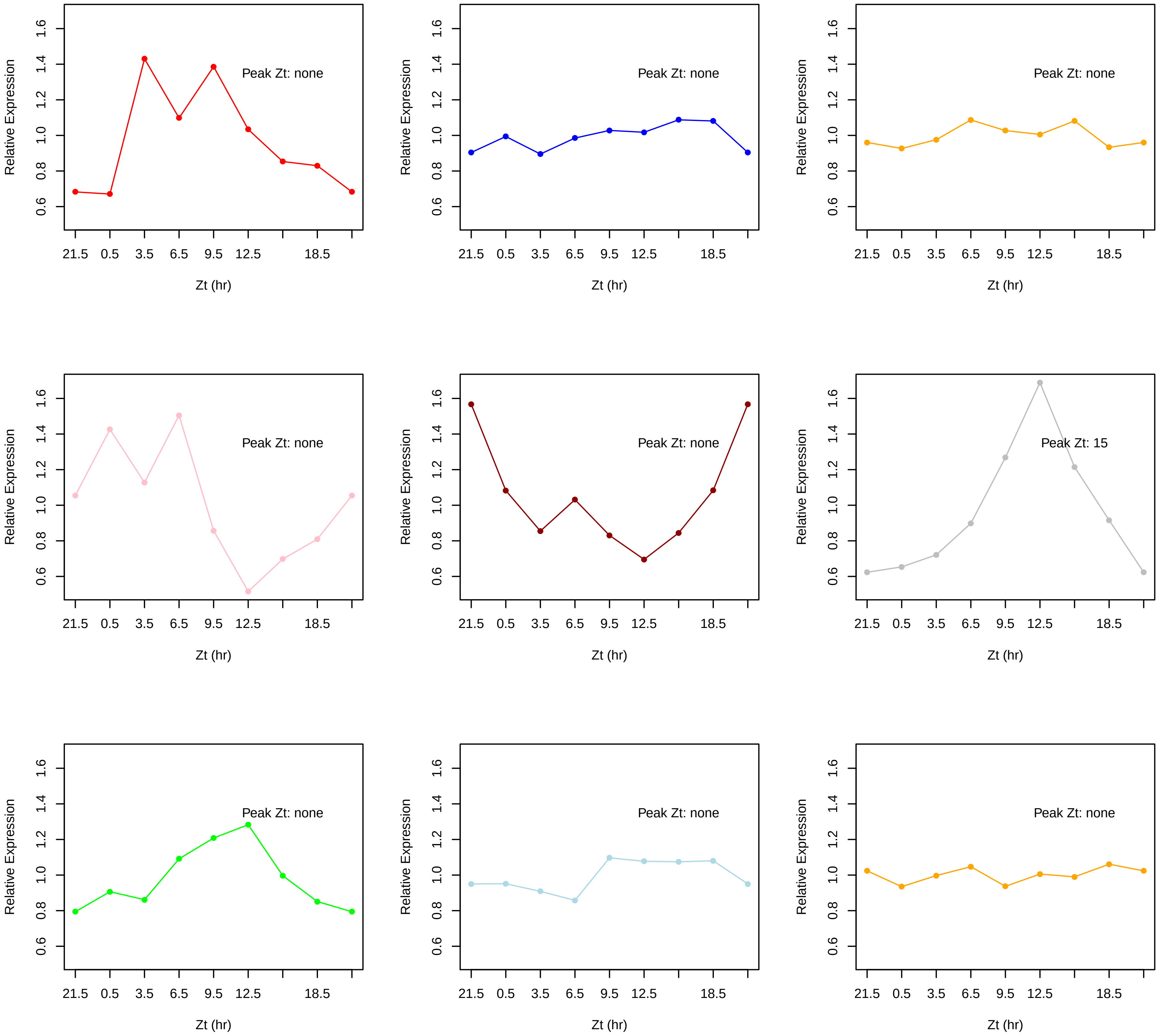

Singeleton: ENSXMAG00000027218 phrf1

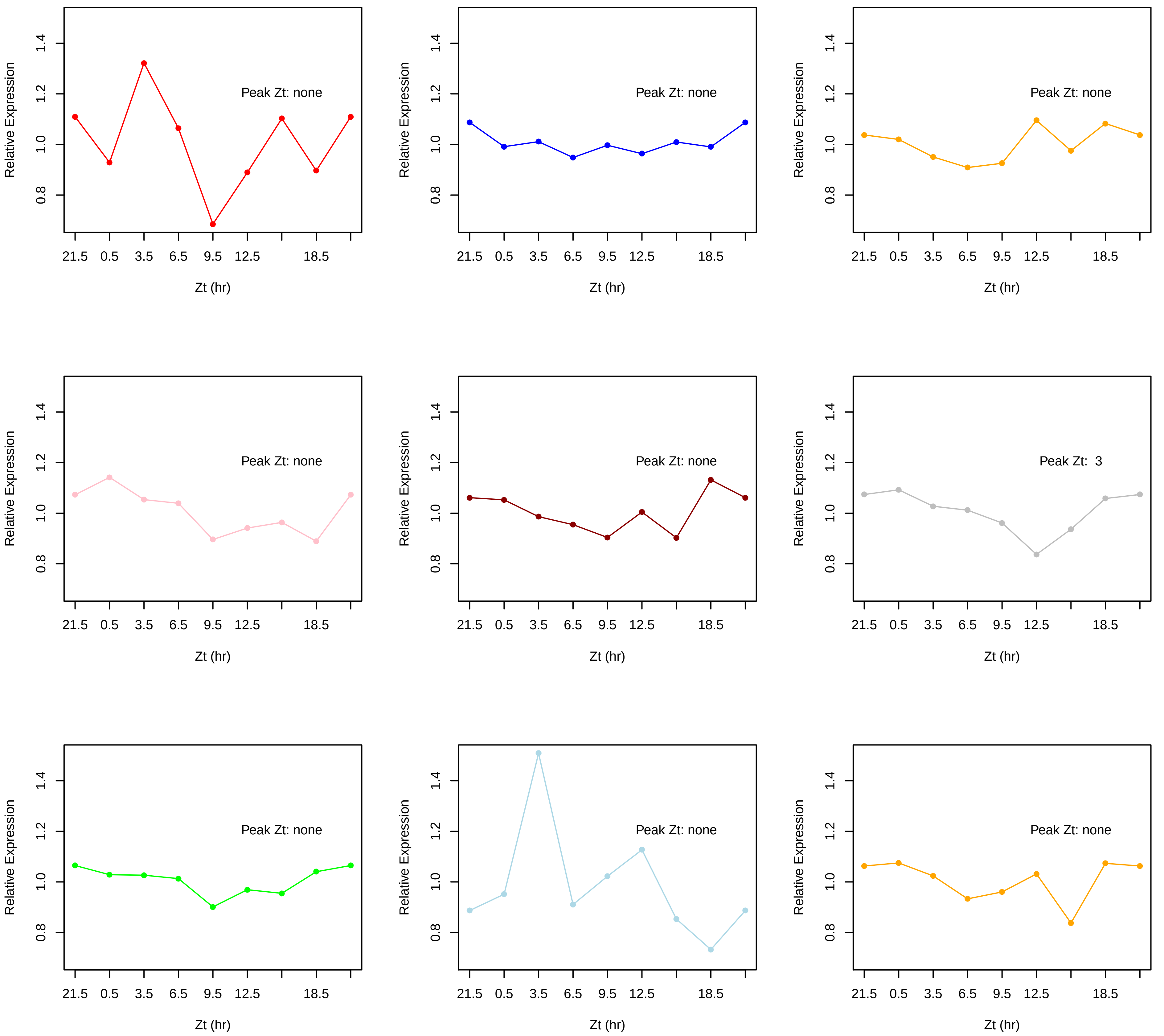

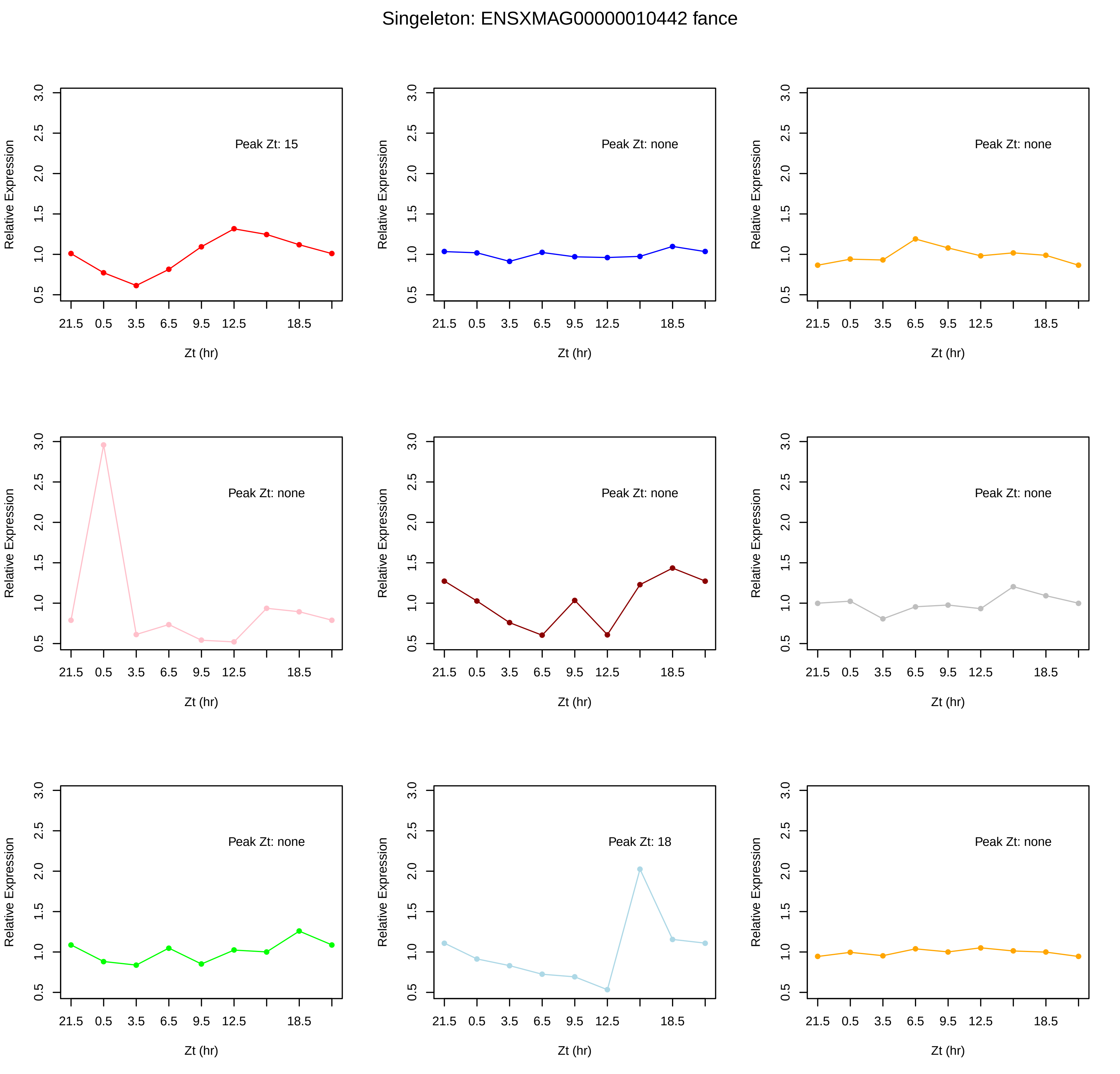
Singeleton: ENSXMAG00000010442 fance
Peak Zt: 15
Peak Zt: none
Peak Zt: none
Peak Zt: none
Peak Zt: none
Peak Zt: none
Peak Zt: none
Peak Zt: 18
Peak Zt: none
Relative Expression
Zt (hr)

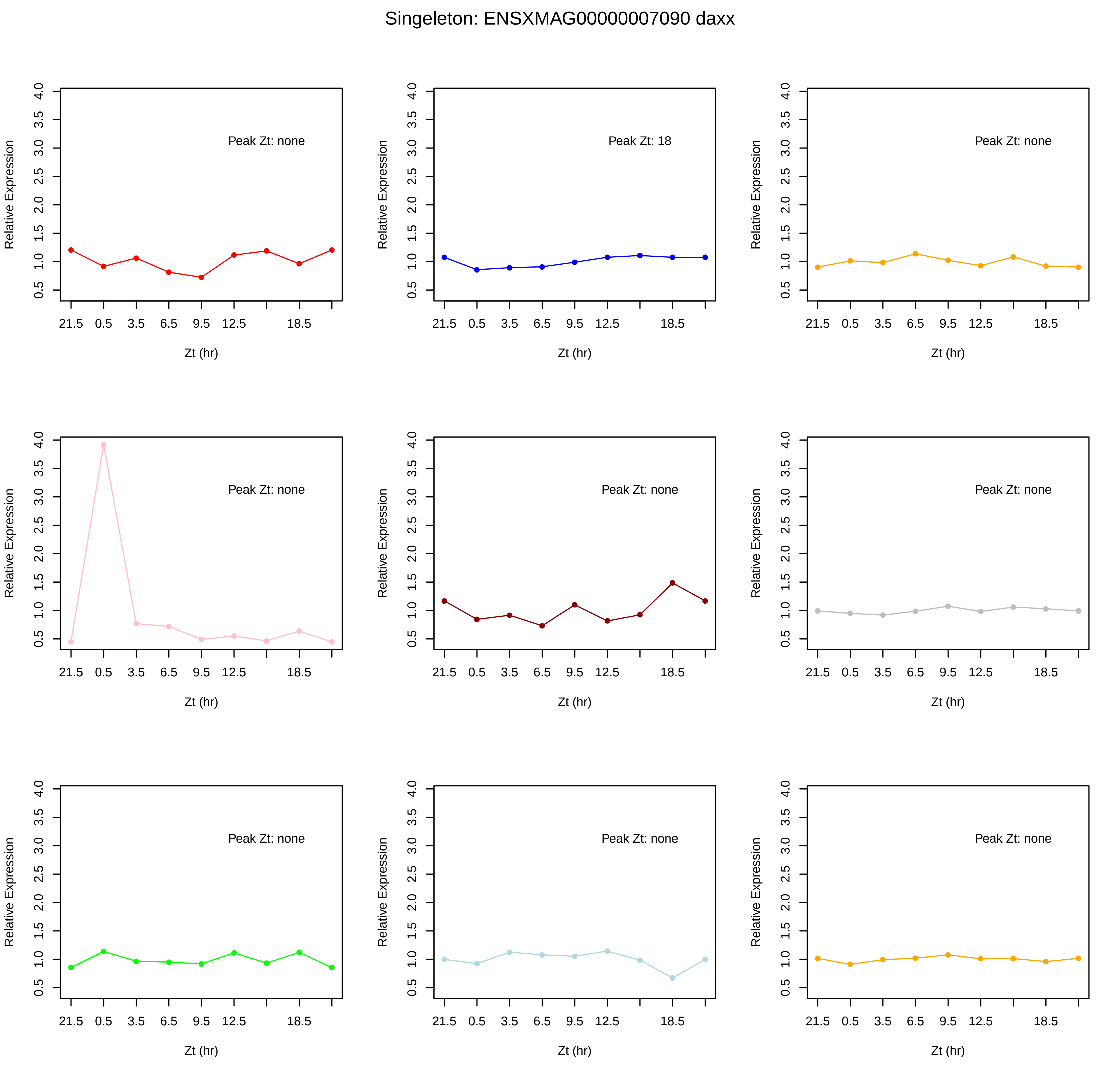

Singeleton: ENSXMAG00000007090 daxx
Peak Zt: none
Peak Zt: 18
Peak Zt: none
Peak Zt: none
Peak Zt: none
Peak Zt: none
Peak Zt: none
Peak Zt: none
Peak Zt: none
Relative Expression
Zt (hr)
21.5
0.5
3.5
6.5
9.5
12.5
18.5
0.5
1.0
1.5
2.0
2.5
3.0
3.5
4.0

Singleton: ENSXMAG00000012520 pdap1a

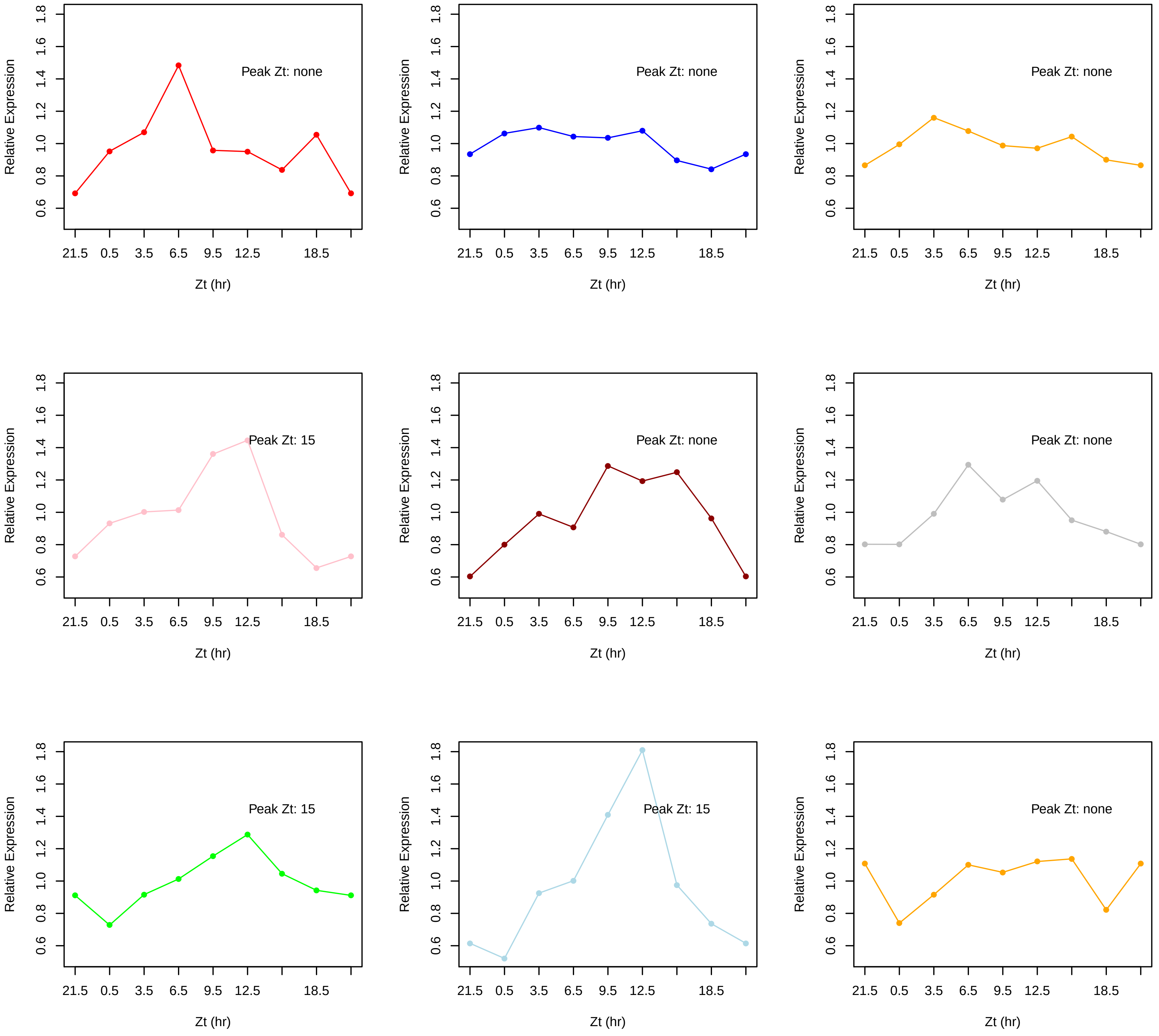

Singleton: ENSXMAG00000027119 si:dkey−17e16.10

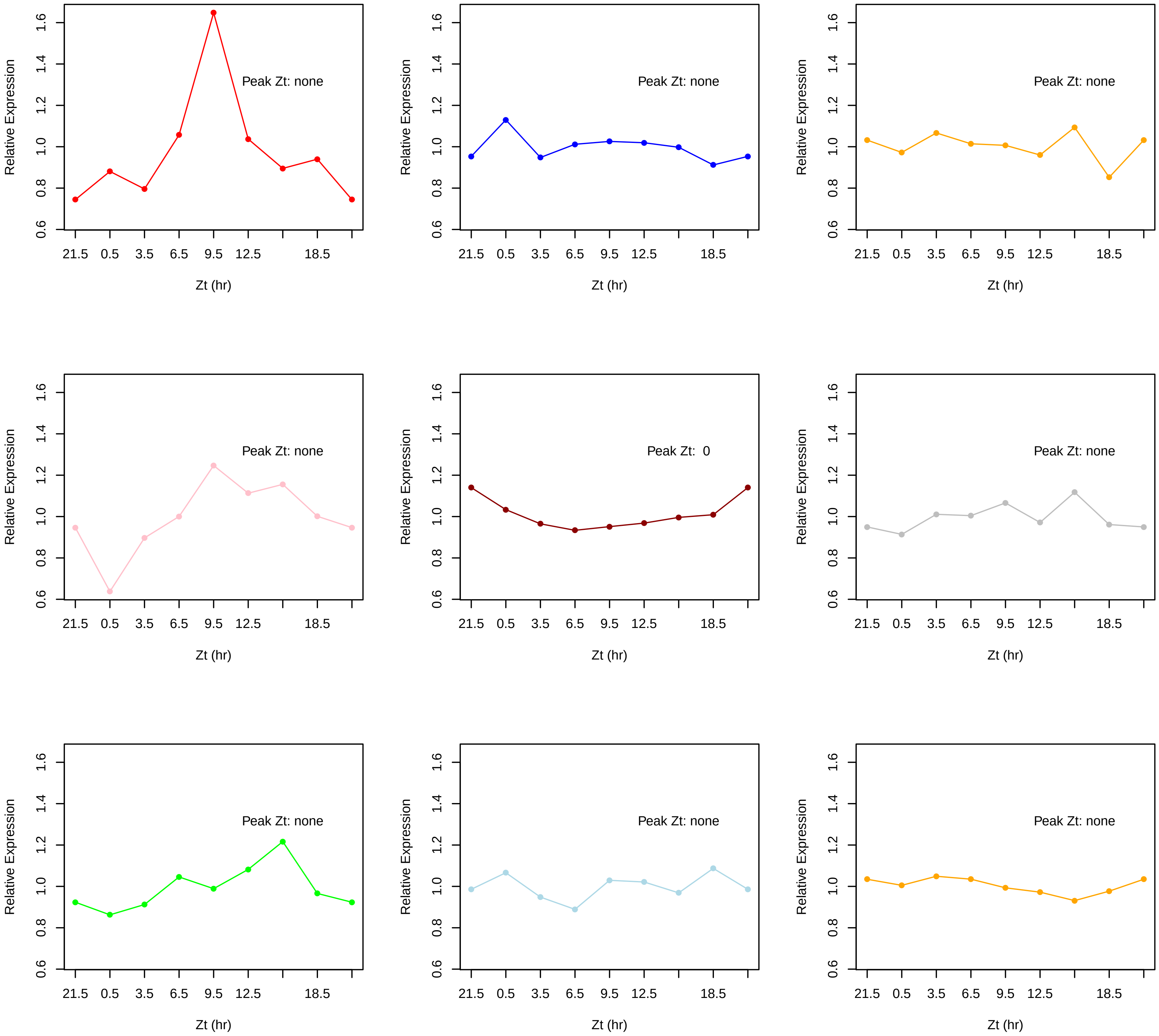

Singeleton: ENSXMAG00000004603 rtel1

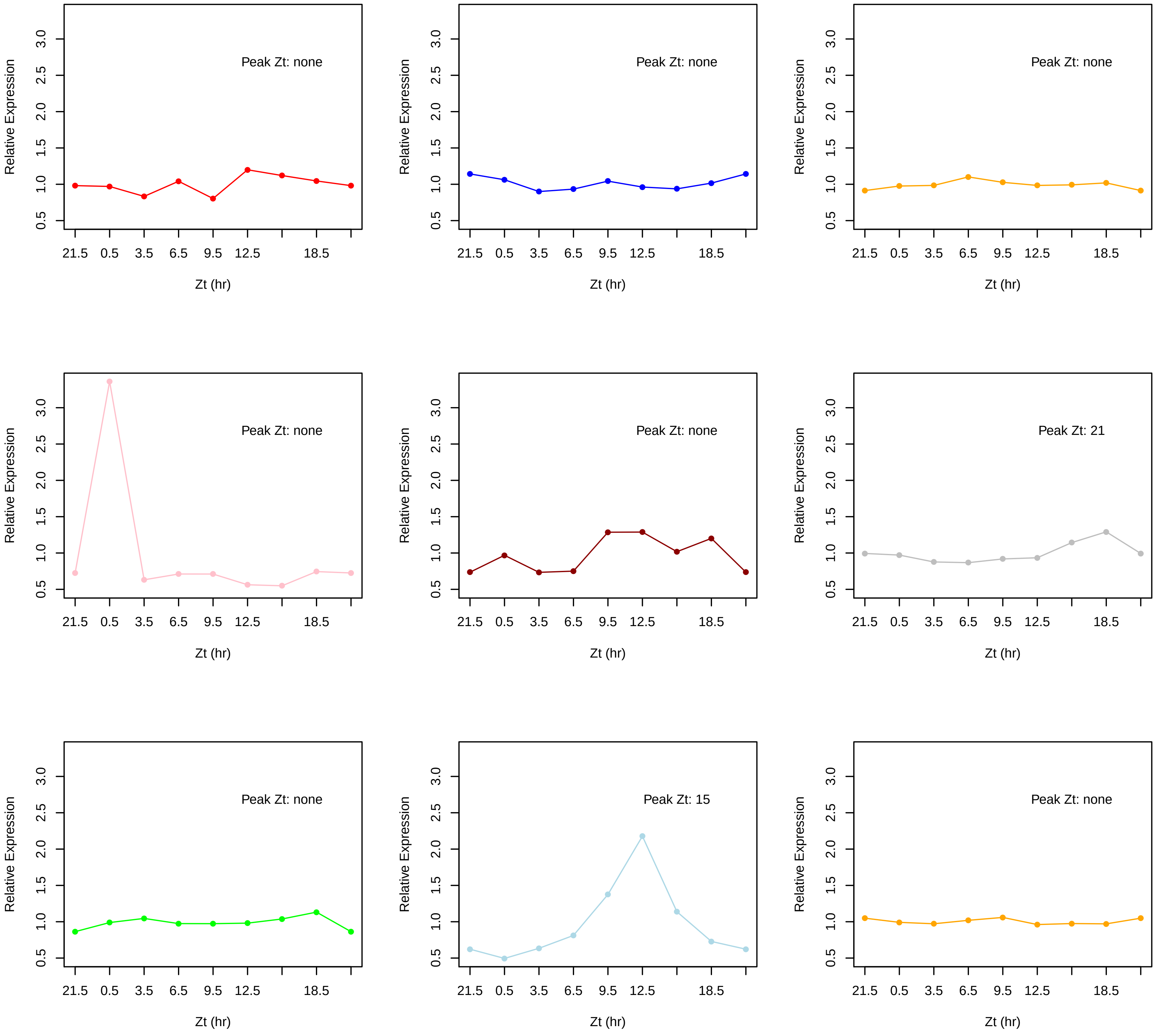

Singleton: ENSXMAG00000007664 cdc26

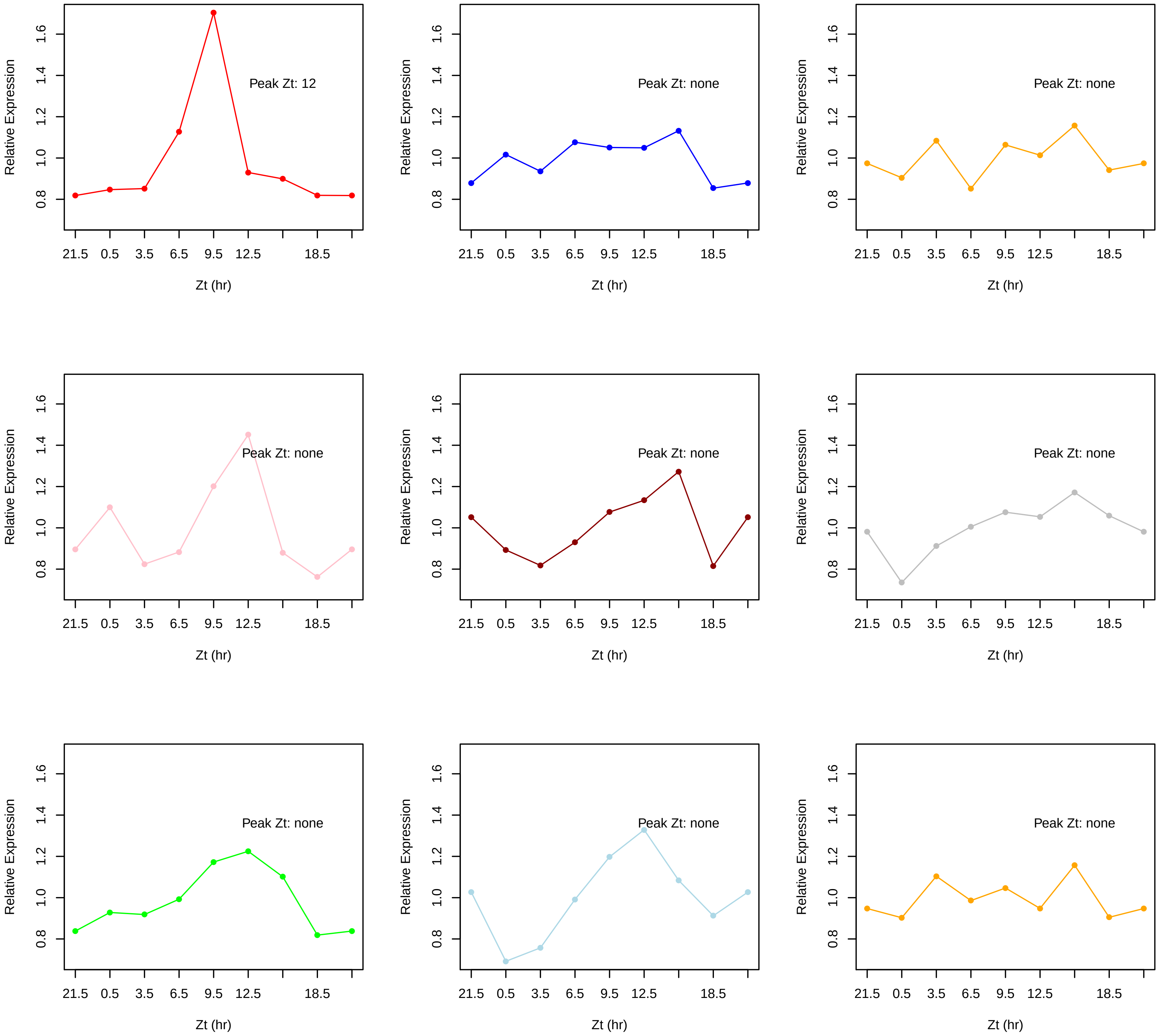

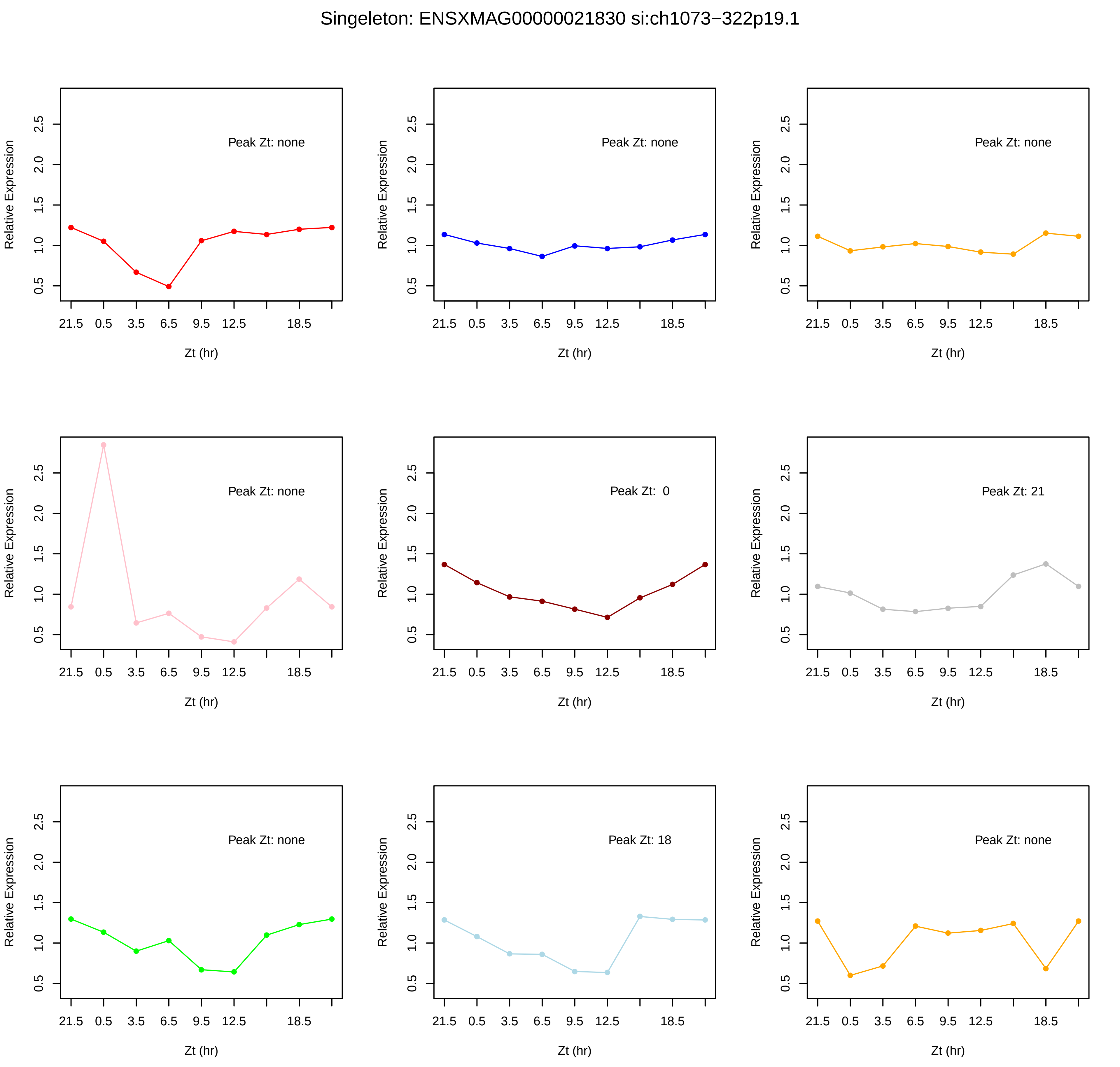
Singeleton: ENSXMAG00000021830 si:ch1073−322p19.1
Peak Zt: none
Peak Zt: none
Peak Zt: none
Peak Zt: none
Peak Zt: 0
Peak Zt: 21
Peak Zt: none
Peak Zt: 18
Peak Zt: none
Relative Expression
Zt (hr)
21.5
0.5
3.5
6.5
9.5
12.5
18.5

Singeleton: ENSXMAG00000004576

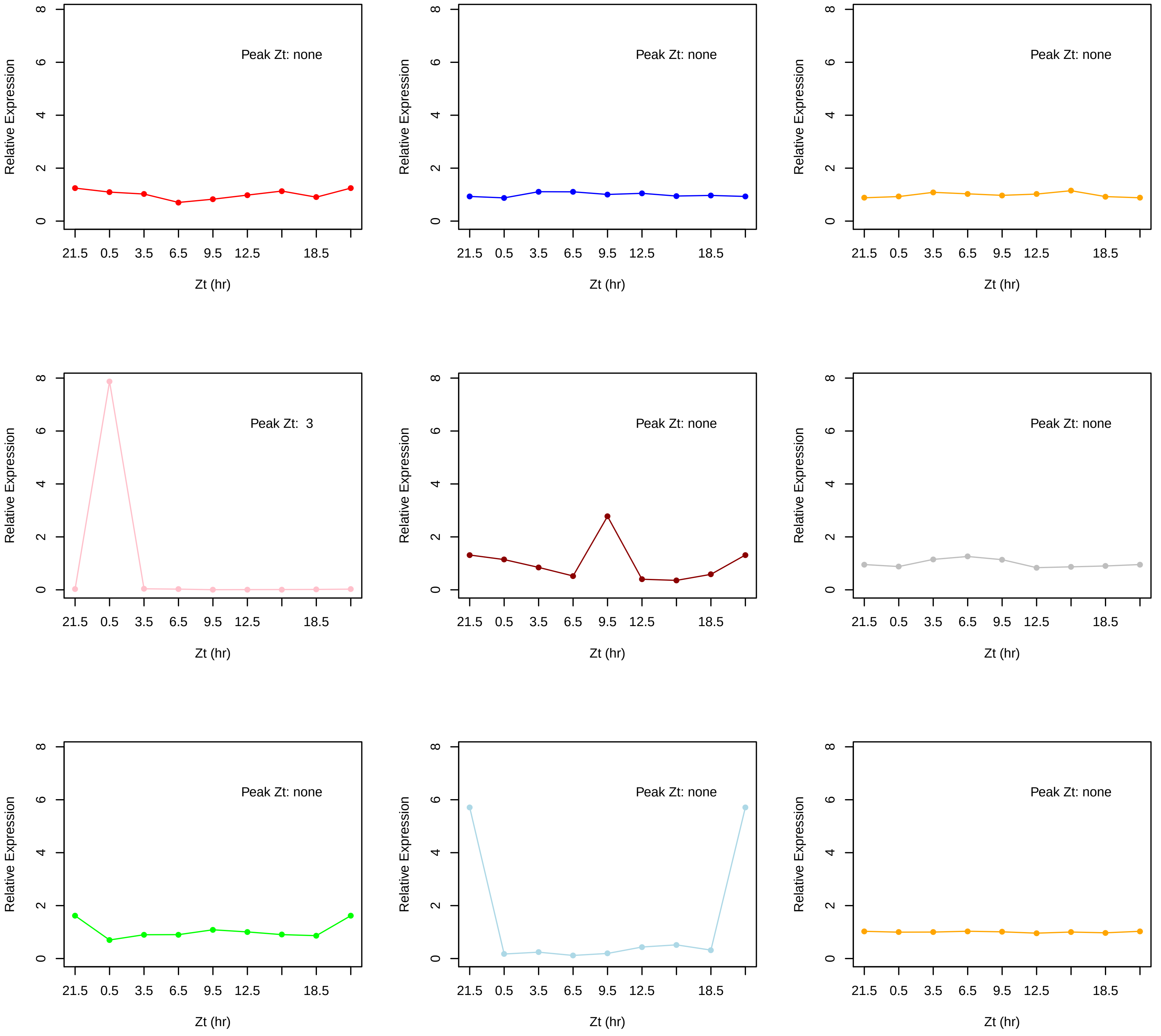

Singleton: ENSXMAG00000006361 crema

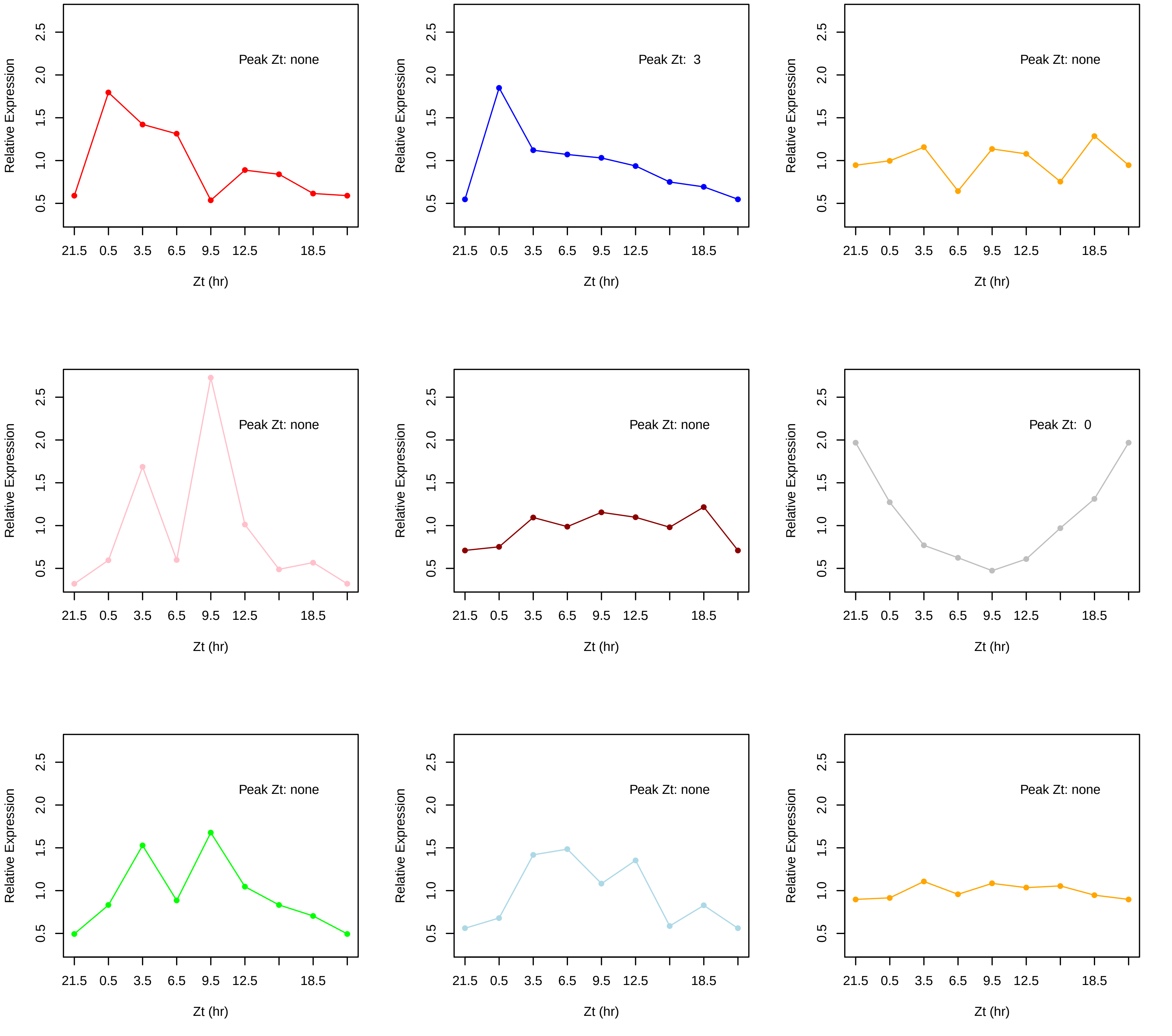

Singeleton: ENSXMAG00000002750 prpf4

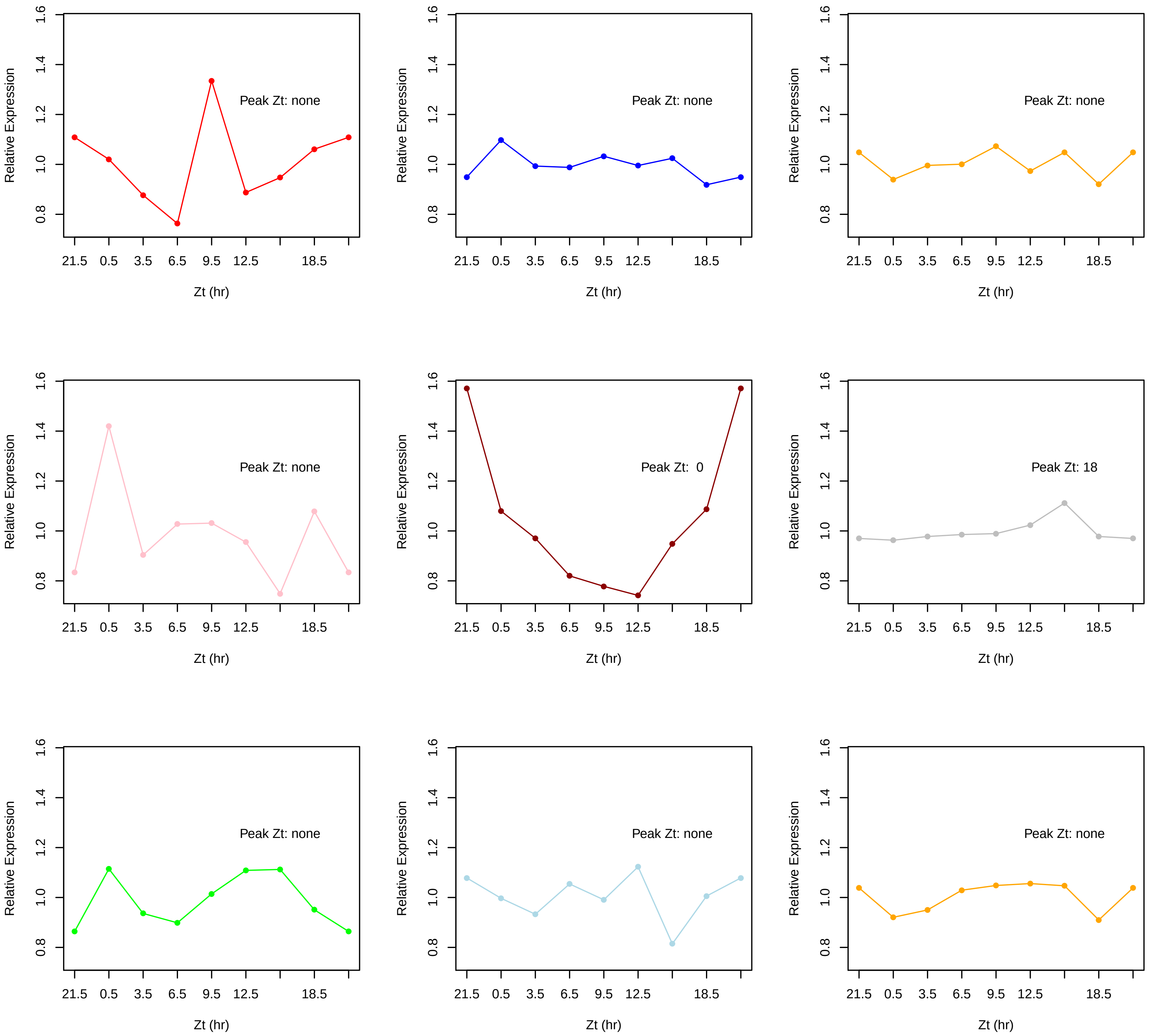

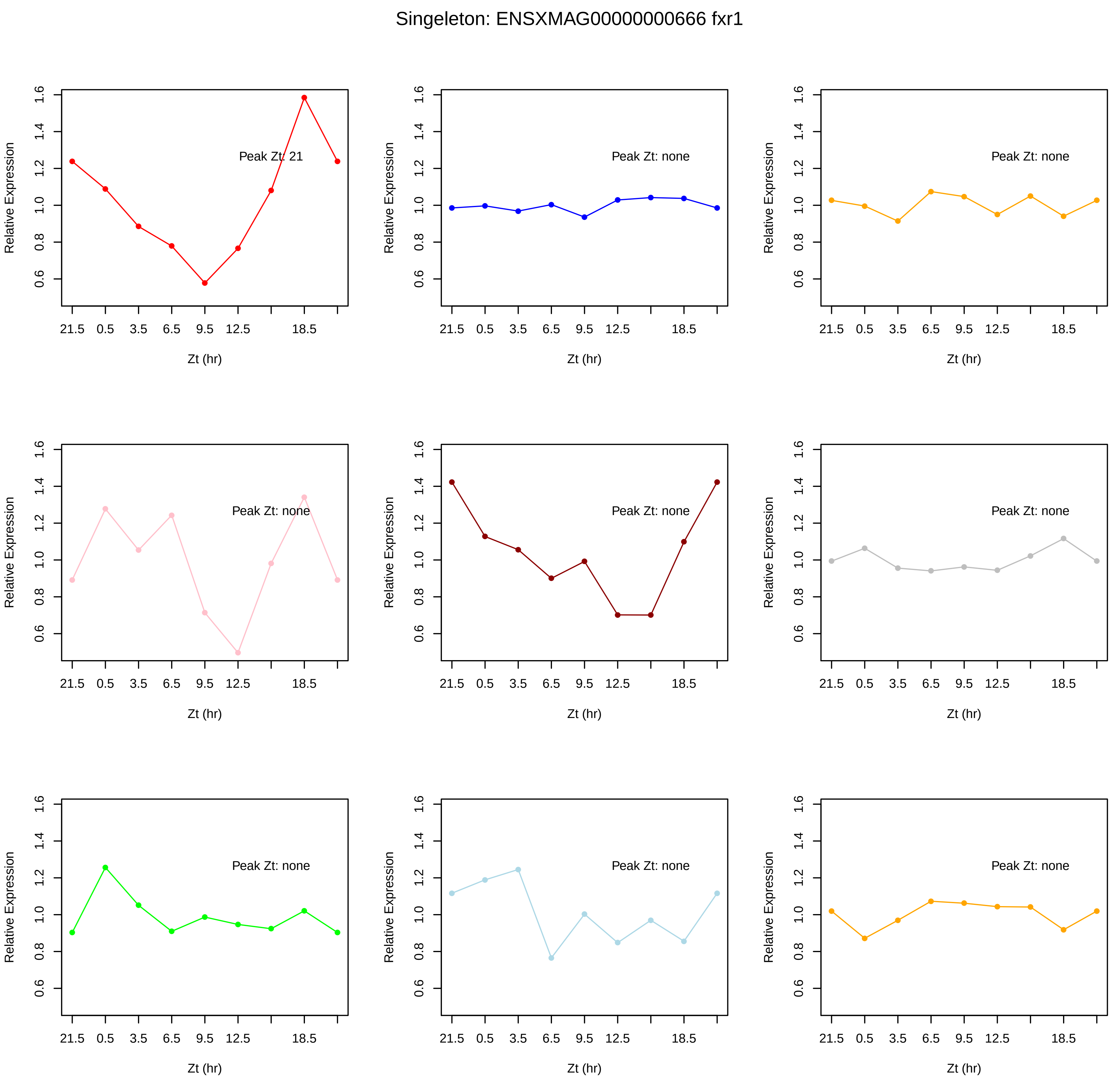
Singeleton: ENSXMAG00000000666 fxr1
Peak Zt: 21
Peak Zt: none
Peak Zt: none
Peak Zt: none
Peak Zt: none
Peak Zt: none
Peak Zt: none
Peak Zt: none
Peak Zt: none
Relative Expression
Zt (hr)
21.5
0.5
3.5
6.5
9.5
12.5
18.5

Singleton: ENSXMAG00000025038 reep6

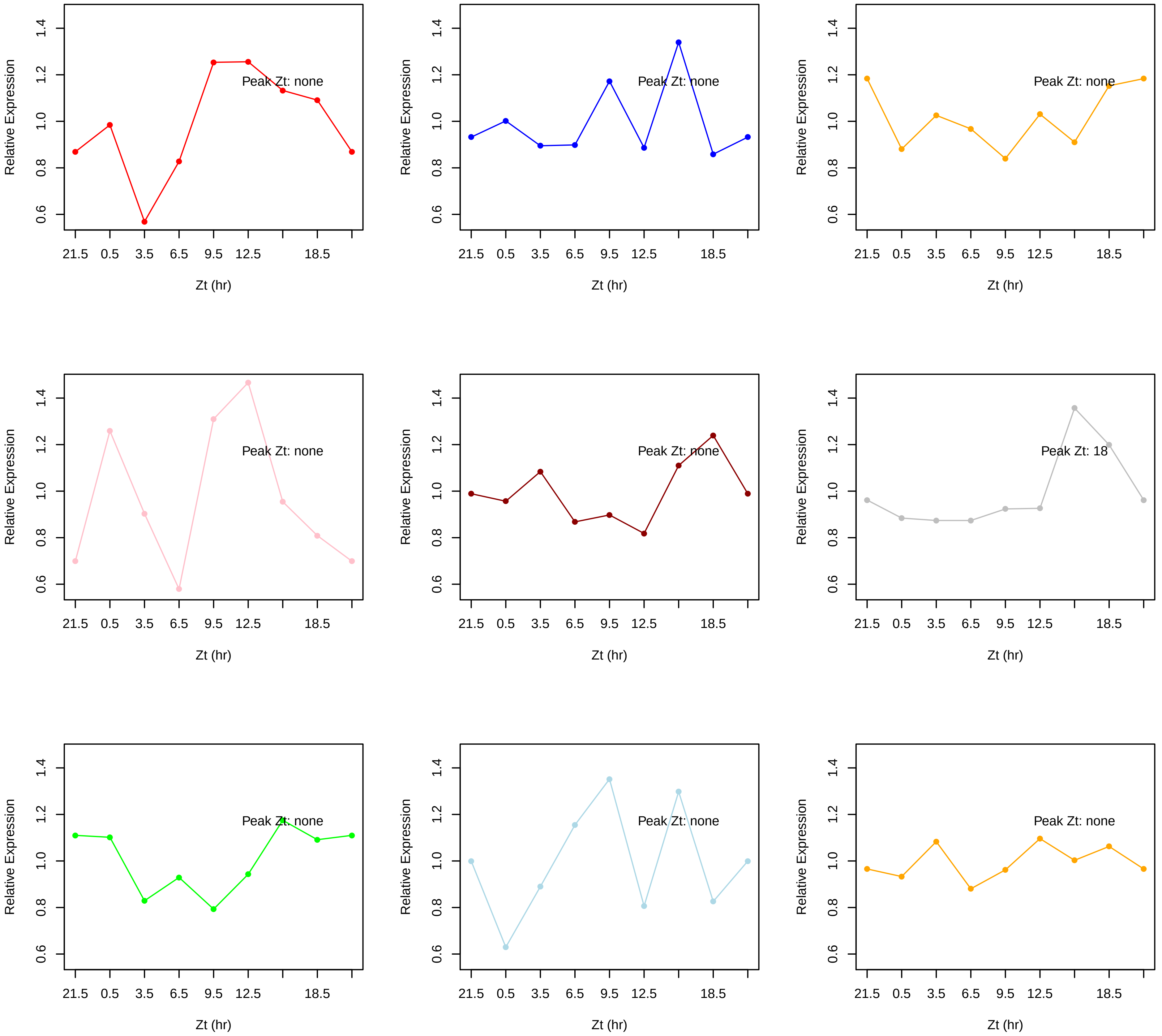

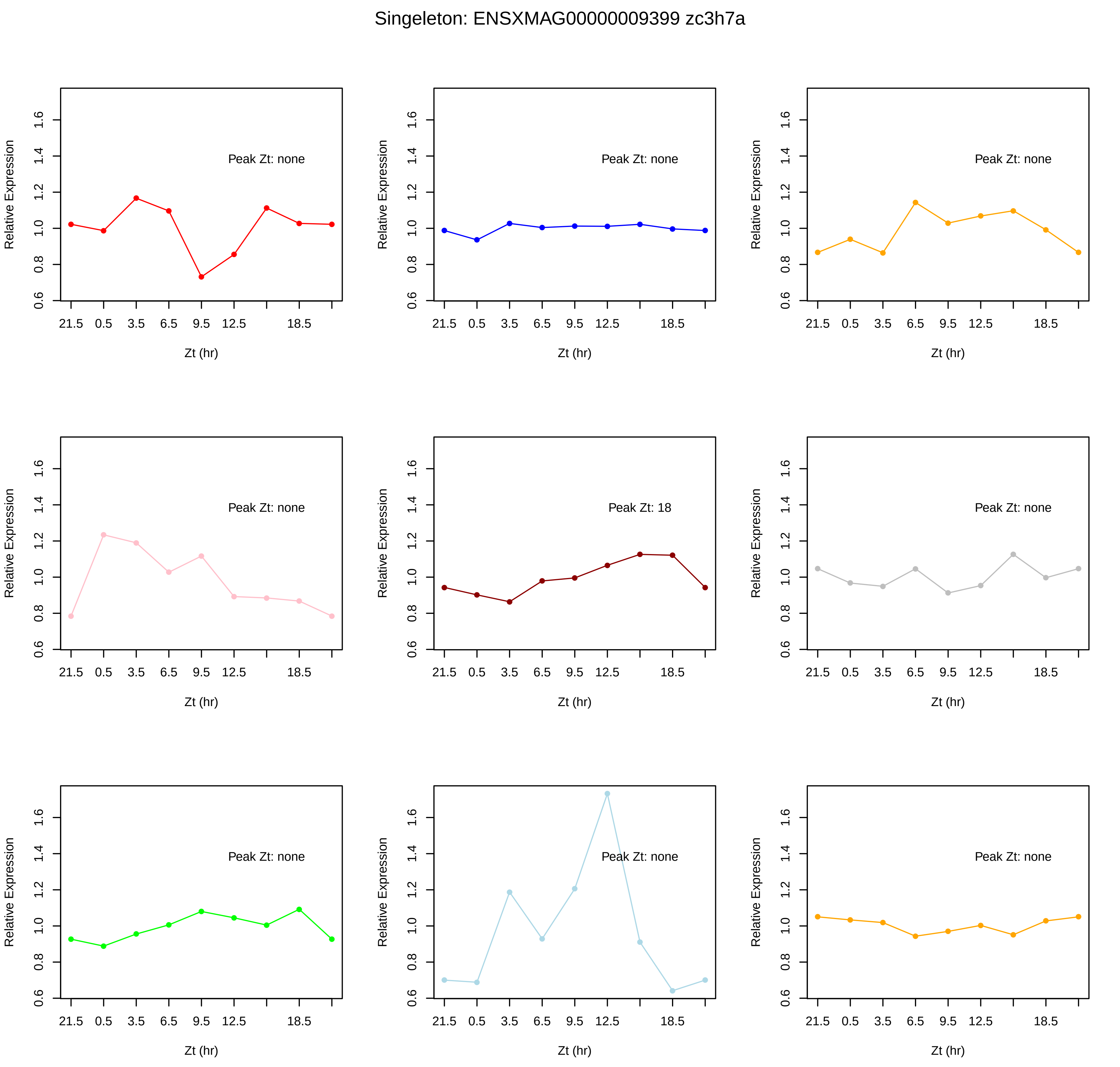
Singeleton: ENSXMAG00000009399 zc3h7a
Peak Zt: none
Peak Zt: none
Peak Zt: none
Peak Zt: none
Peak Zt: 18
Peak Zt: none
Peak Zt: none
Peak Zt: none
Peak Zt: none
Relative Expression
Zt (hr)
21.5 0.5 3.5 6.5 9.5 12.5 18.5
0.6 0.8 1.0 1.2 1.4 1.6

Singeleton: ENSXMAG00000023368 mrpl11

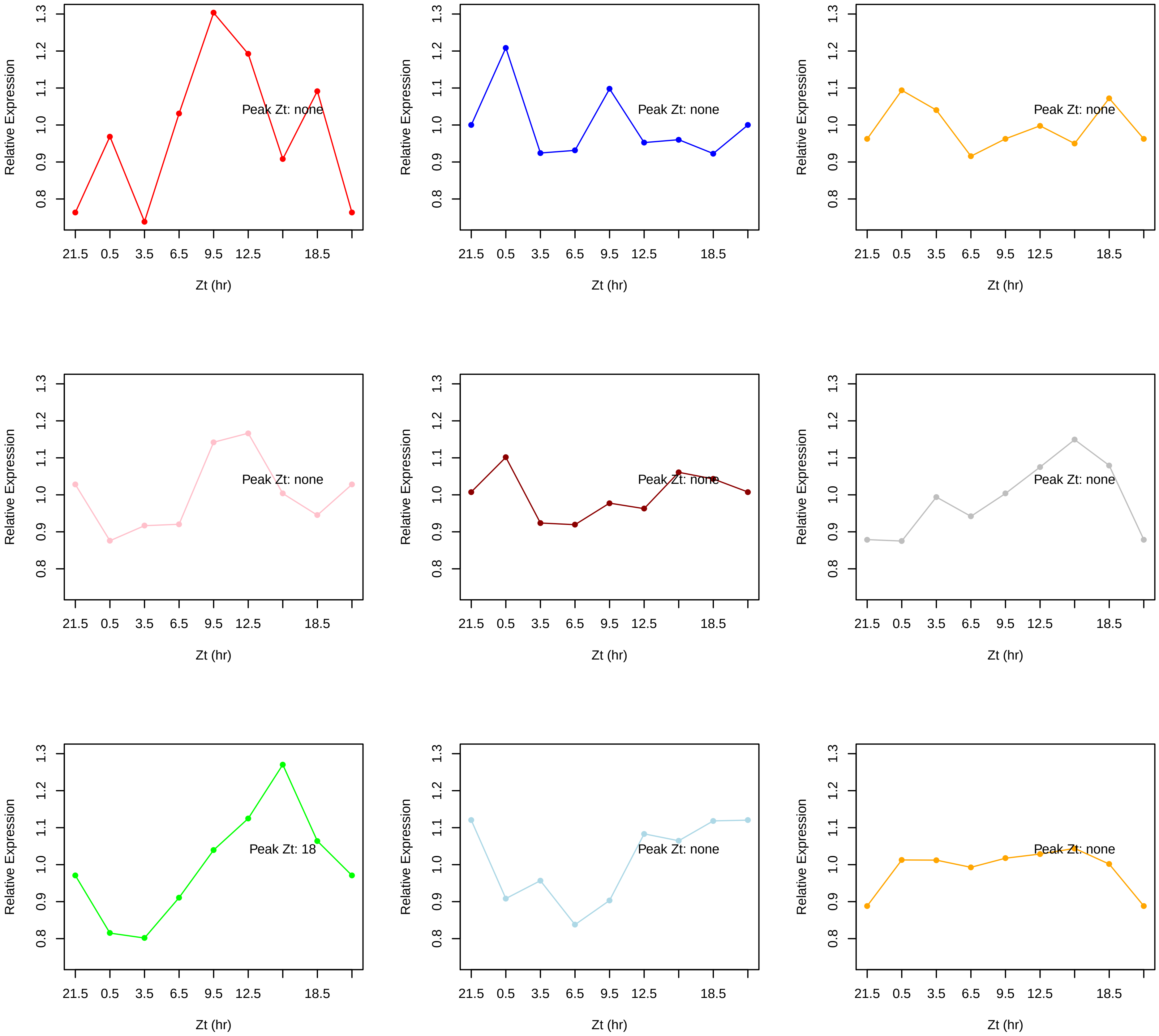

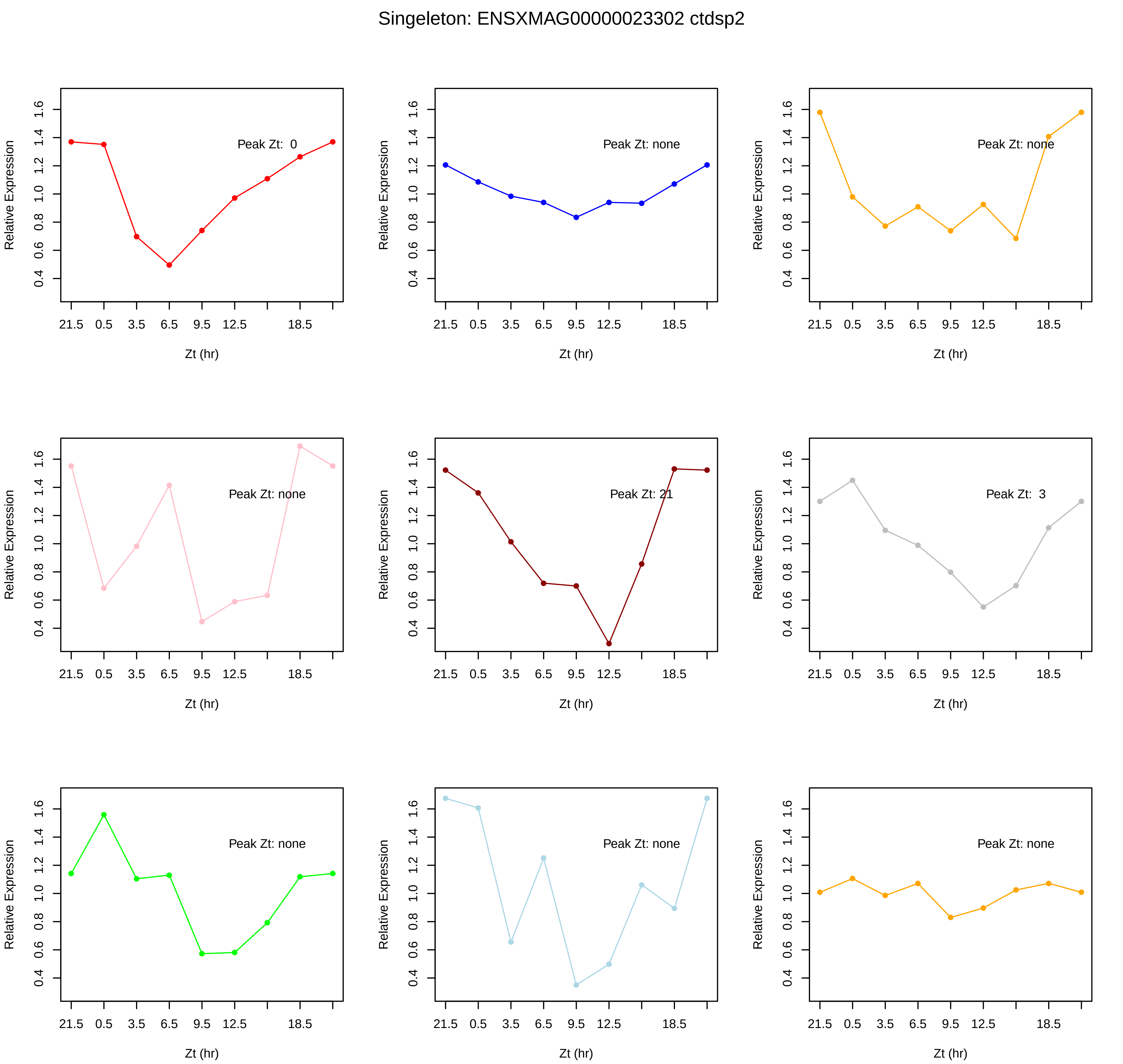
Singeleton: ENSXMAG00000023302 ctdsp2
Peak Zt: 0
Peak Zt: none
Peak Zt: none
Peak Zt: none
Peak Zt: 21
Peak Zt: 3
Peak Zt: none
Peak Zt: none
Peak Zt: none
Relative Expression
Zt (hr)
21.5
0.5
3.5
6.5
9.5
12.5
18.5

Singeleton: ENSXMAG00000015130 LRRC56

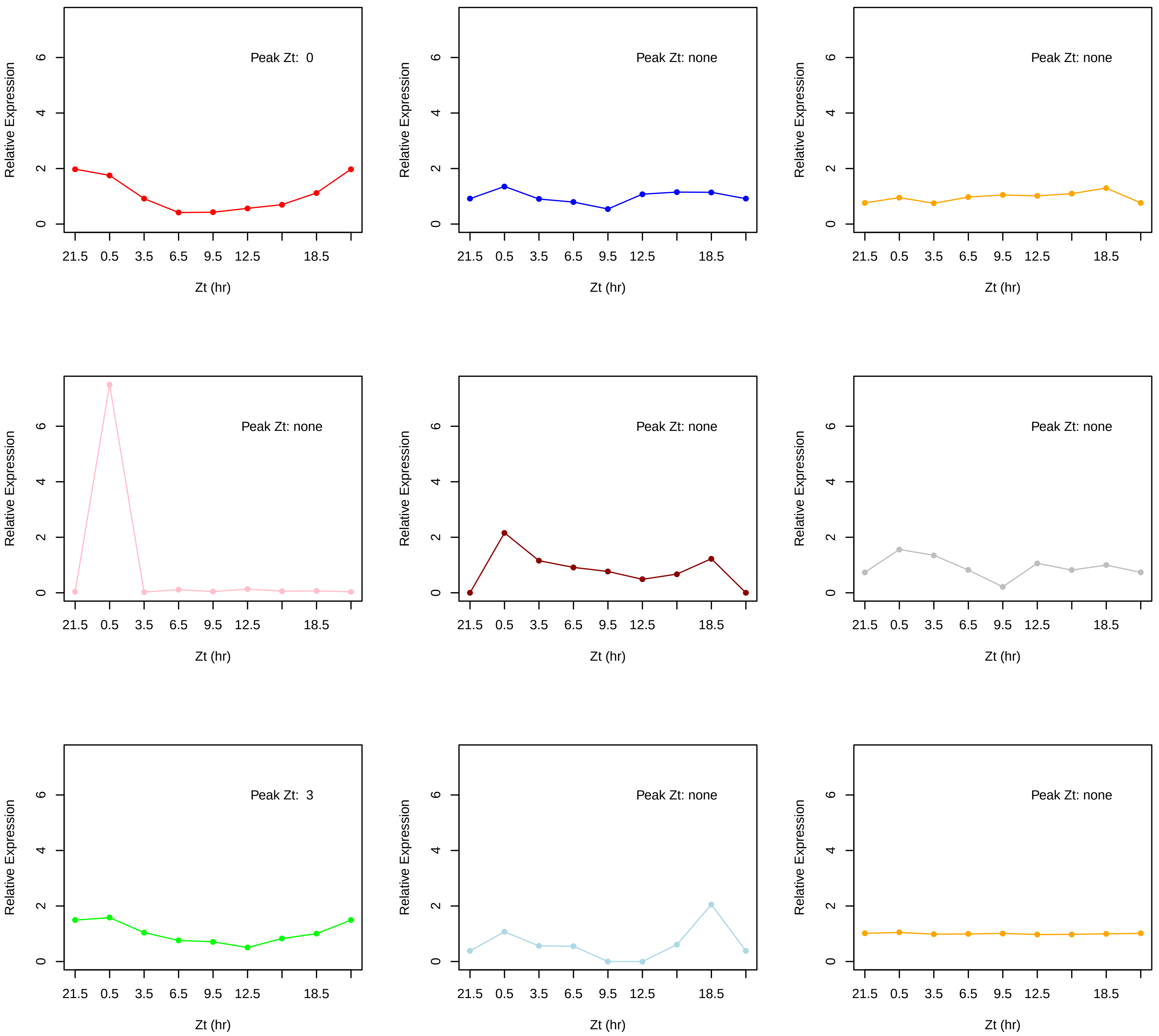

Singleton: ENSXMAG00000009391 zgc:92162

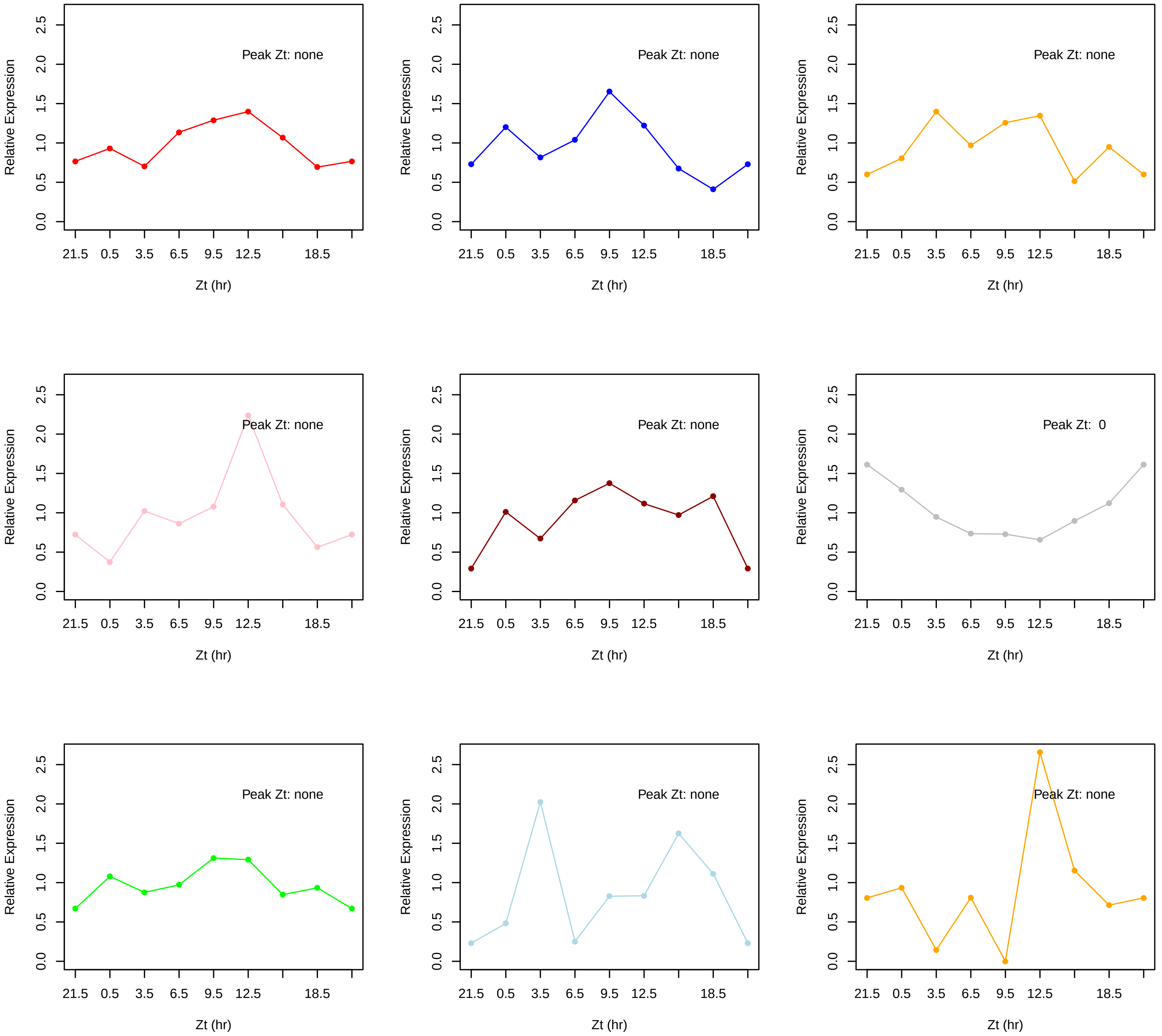

Singeleton: ENSXMAG00000008655 alad

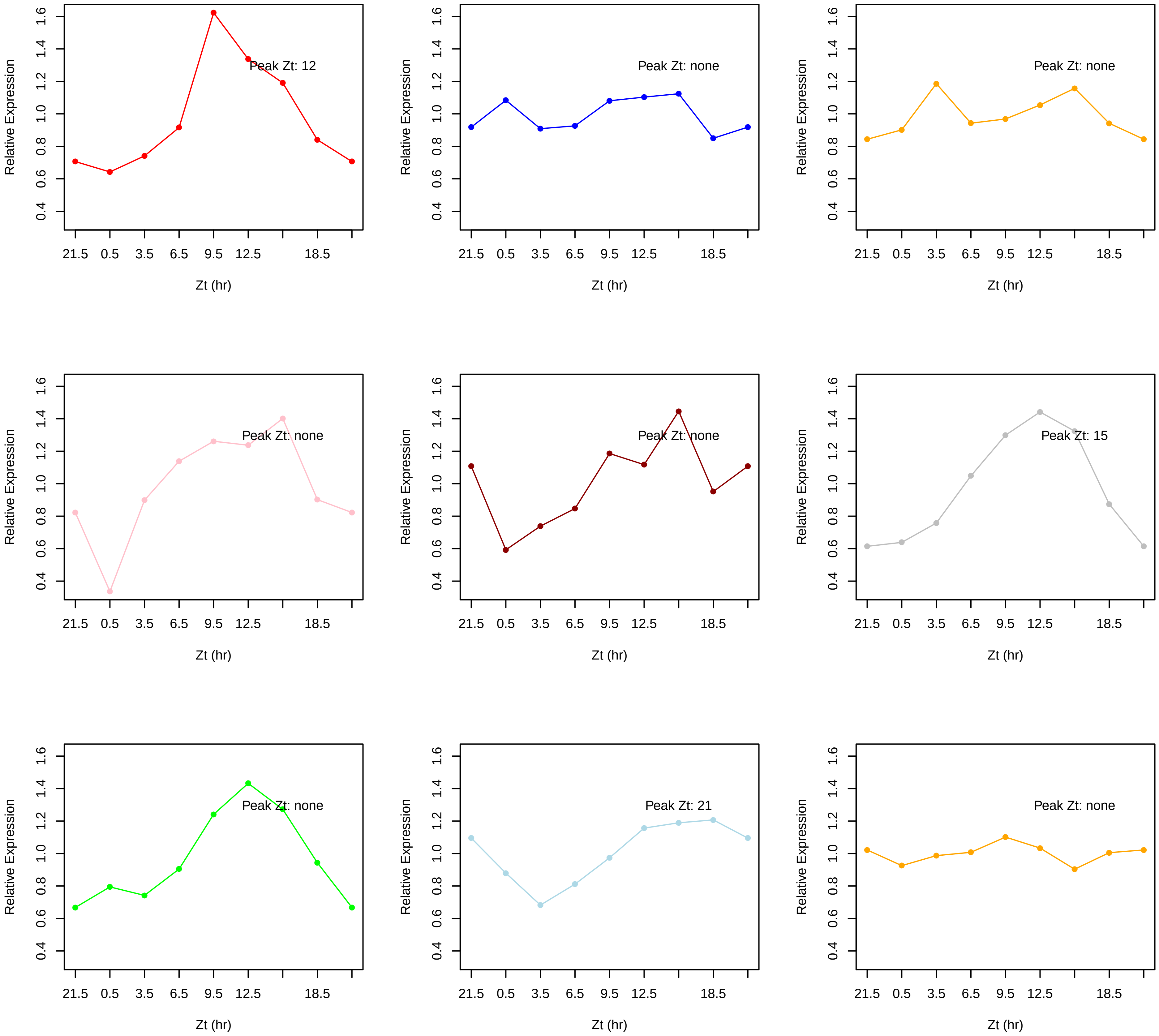

Singleton: ENSXMAG00000015700 sfxn2

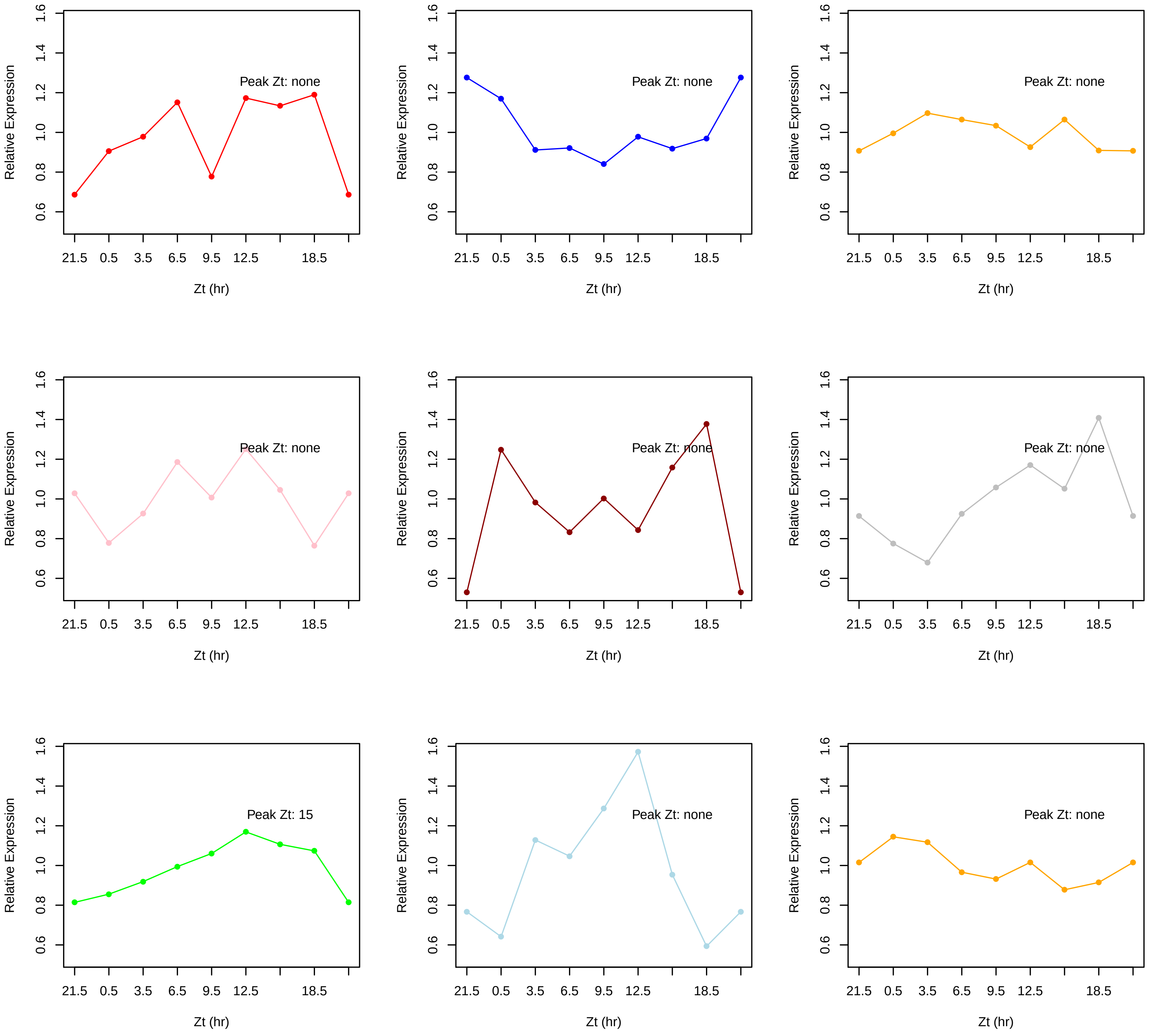

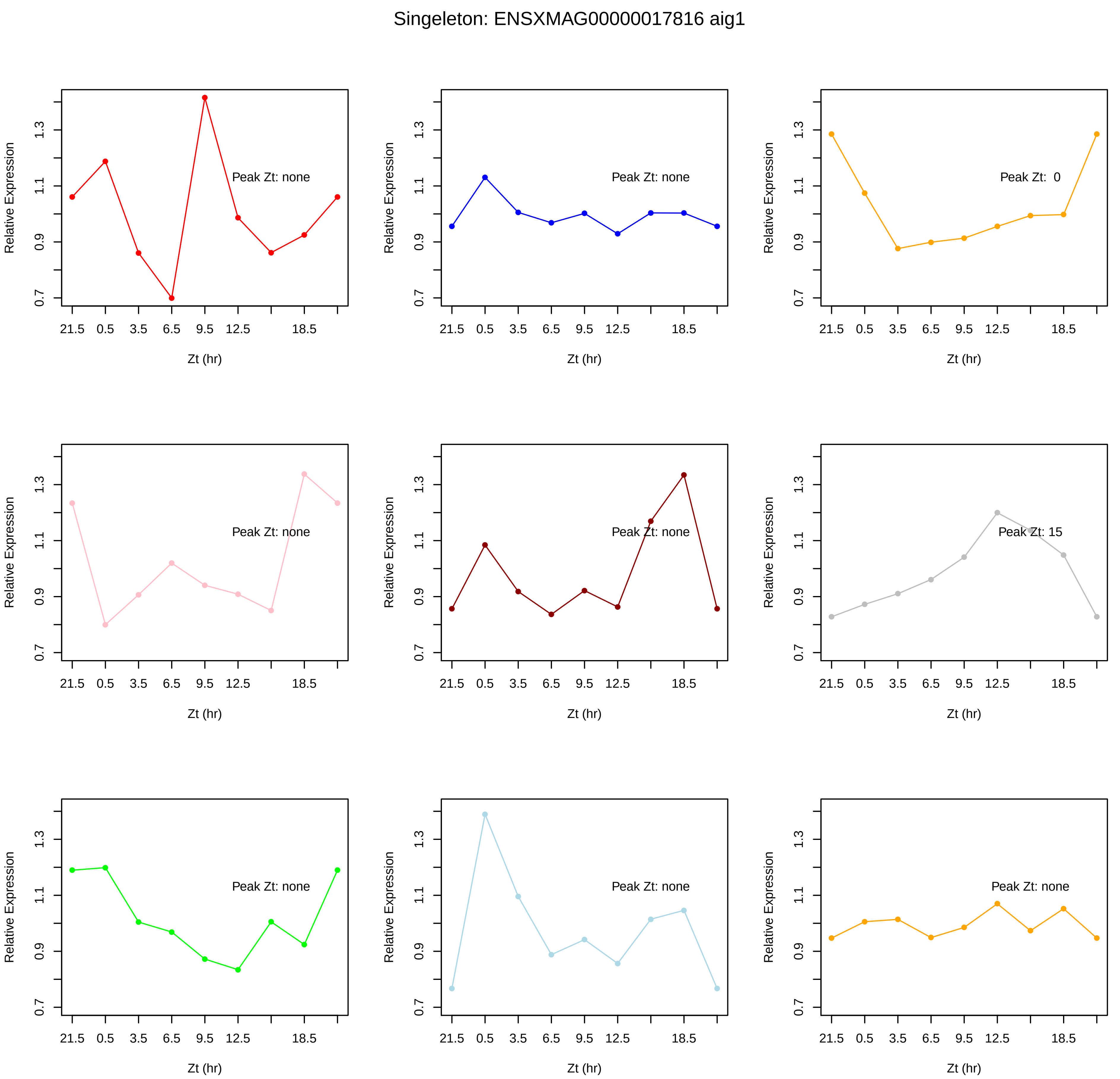
Singeleton: ENSXMAG00000017816 aig1
Relative Expression
Zt (hr)
Peak Zt: none
Peak Zt: none
Peak Zt: 0
Peak Zt: none
Peak Zt: none
Peak Zt: 15
Peak Zt: none
Peak Zt: none
Peak Zt: none

Singeleton: ENSXMAG00000013161 efemp2a

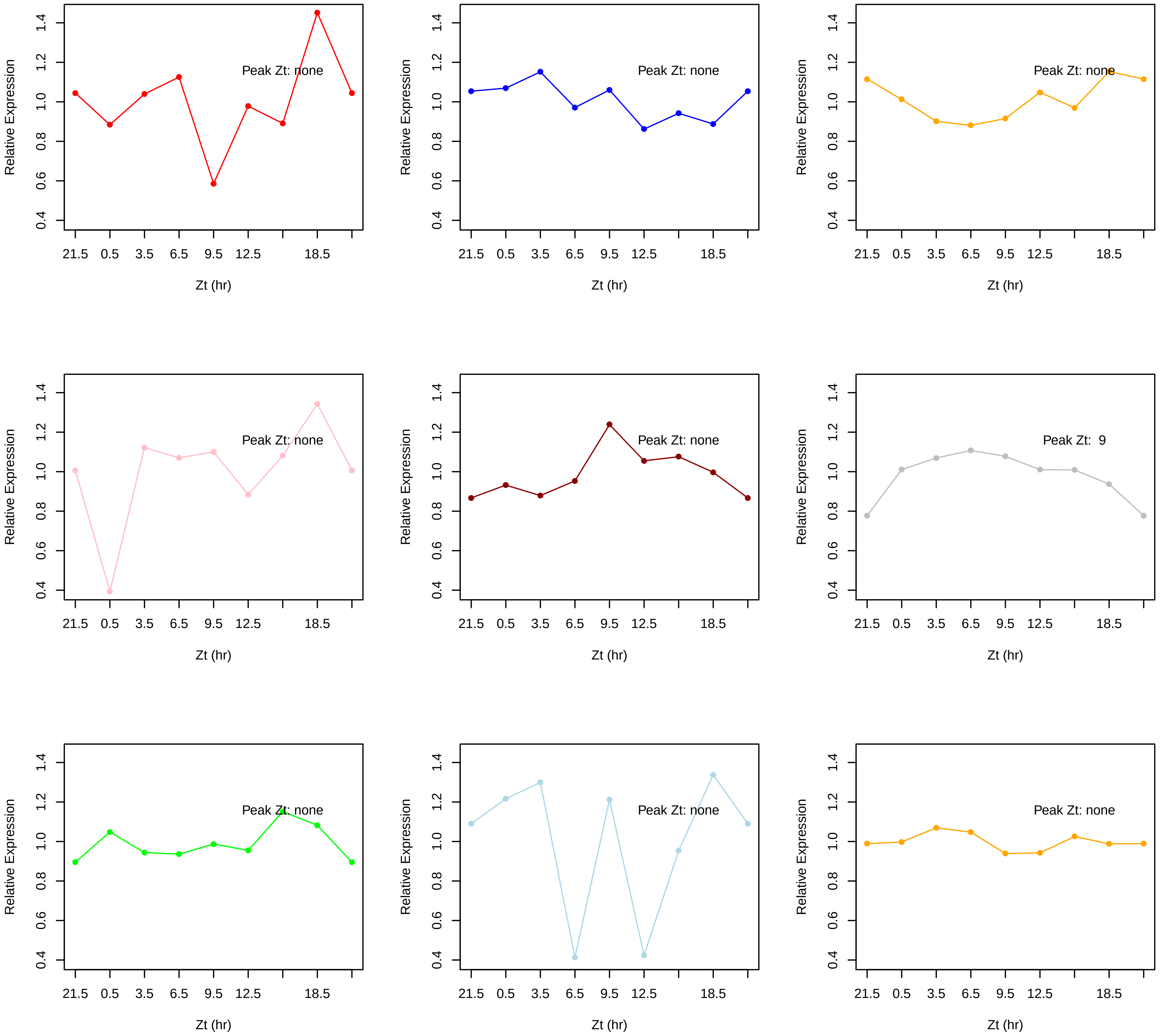

Singeleton: ENSXMAG00000011577

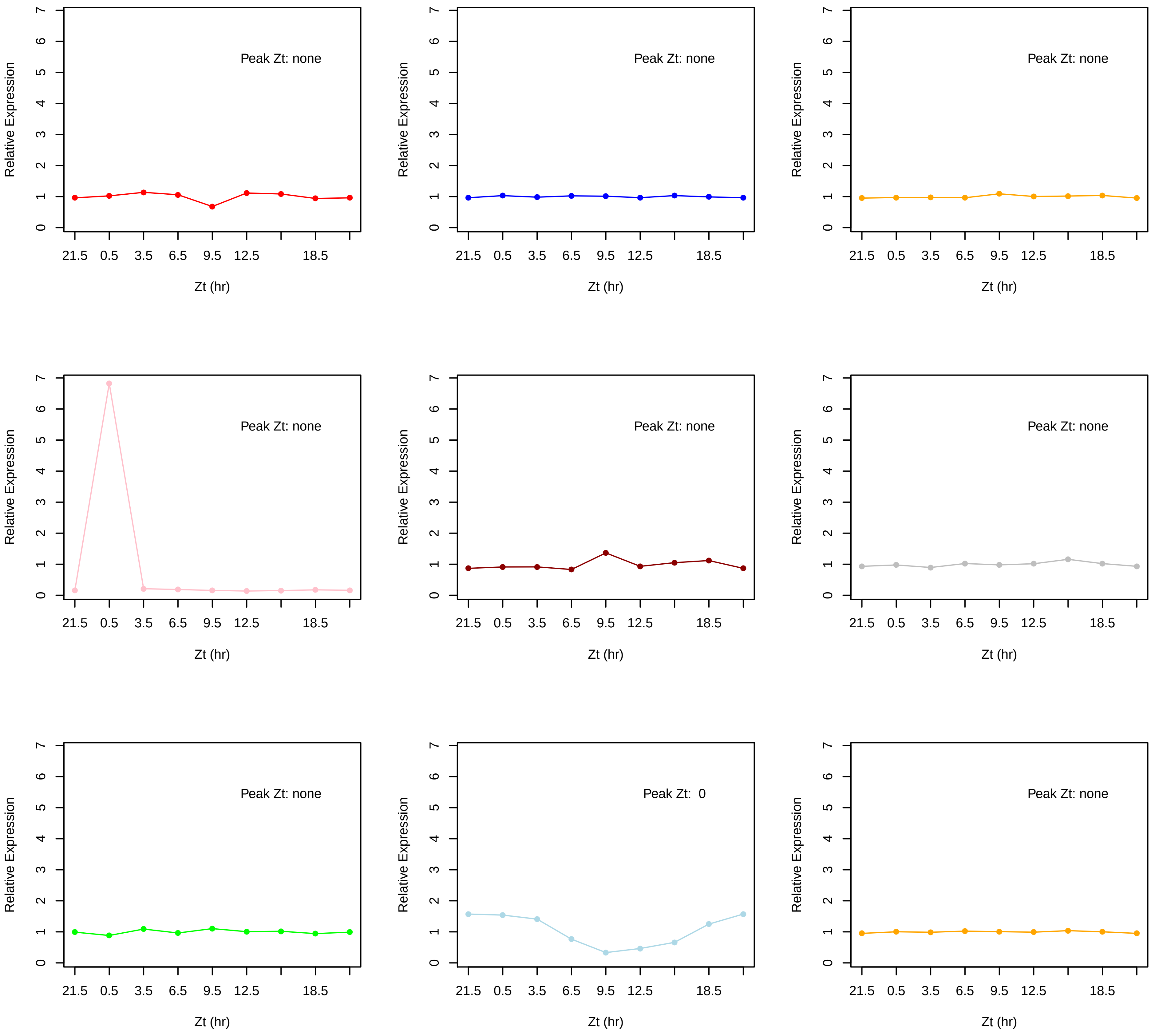

Singeleton: ENSXMAG00000007333 tprn

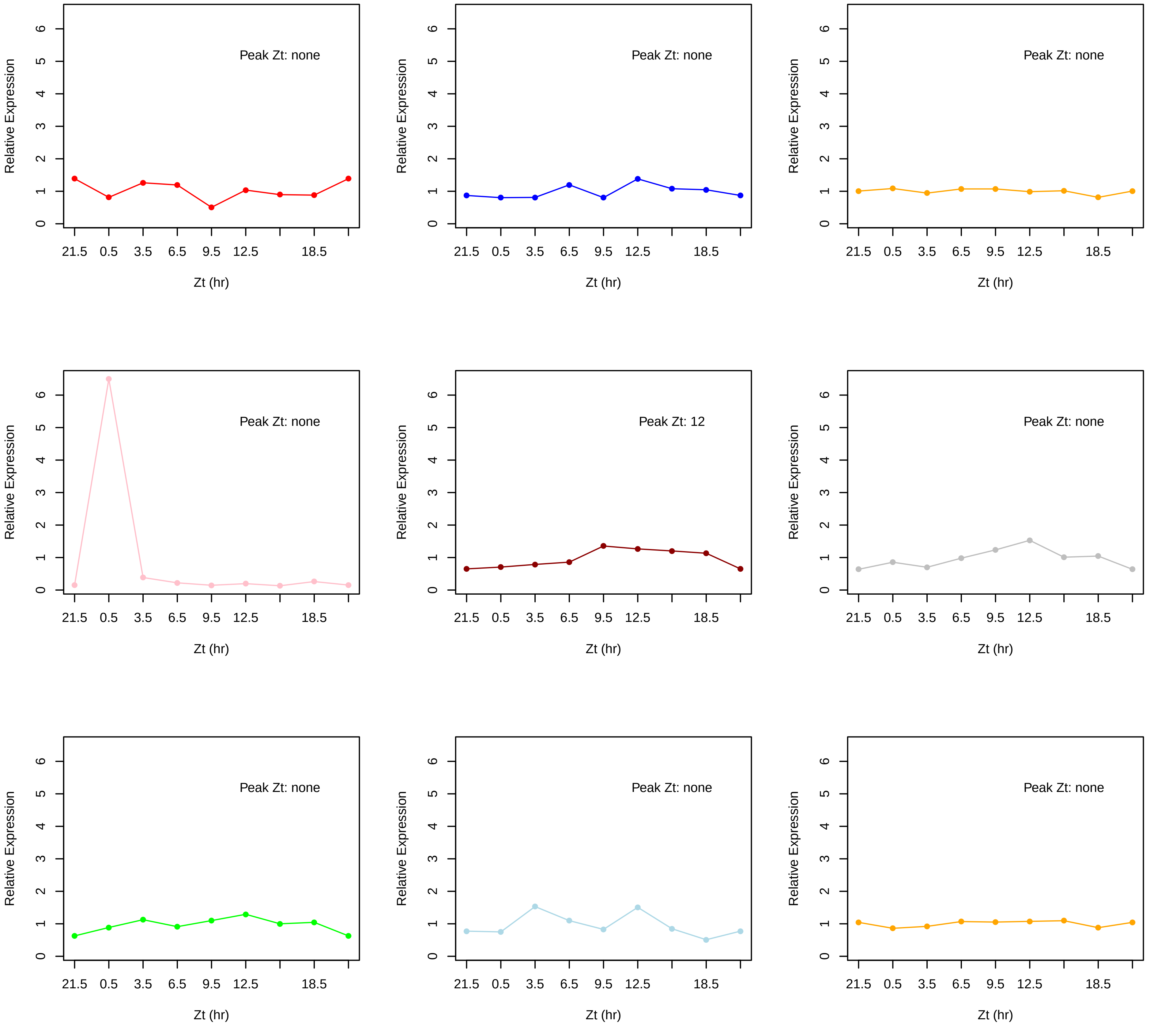

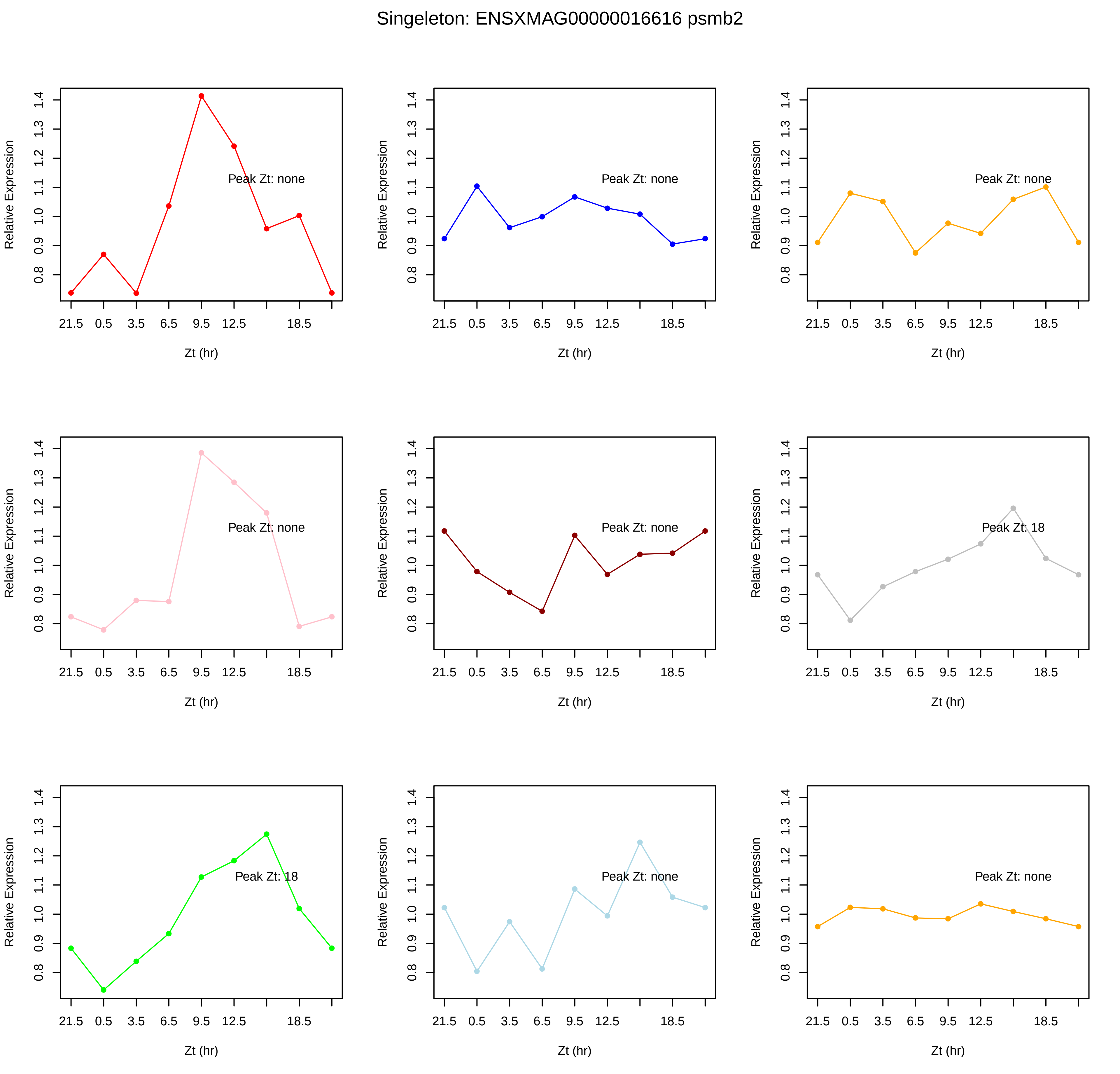

Singeleton: ENSXMAG00000016616 psmb2
Peak Zt: none
Peak Zt: none
Peak Zt: none
Peak Zt: none
Peak Zt: none
Peak Zt: 18
Peak Zt: 18
Peak Zt: none
Peak Zt: none
Relative Expression
Zt (hr)

Singleton: ENSXMAG00000003129 atmin

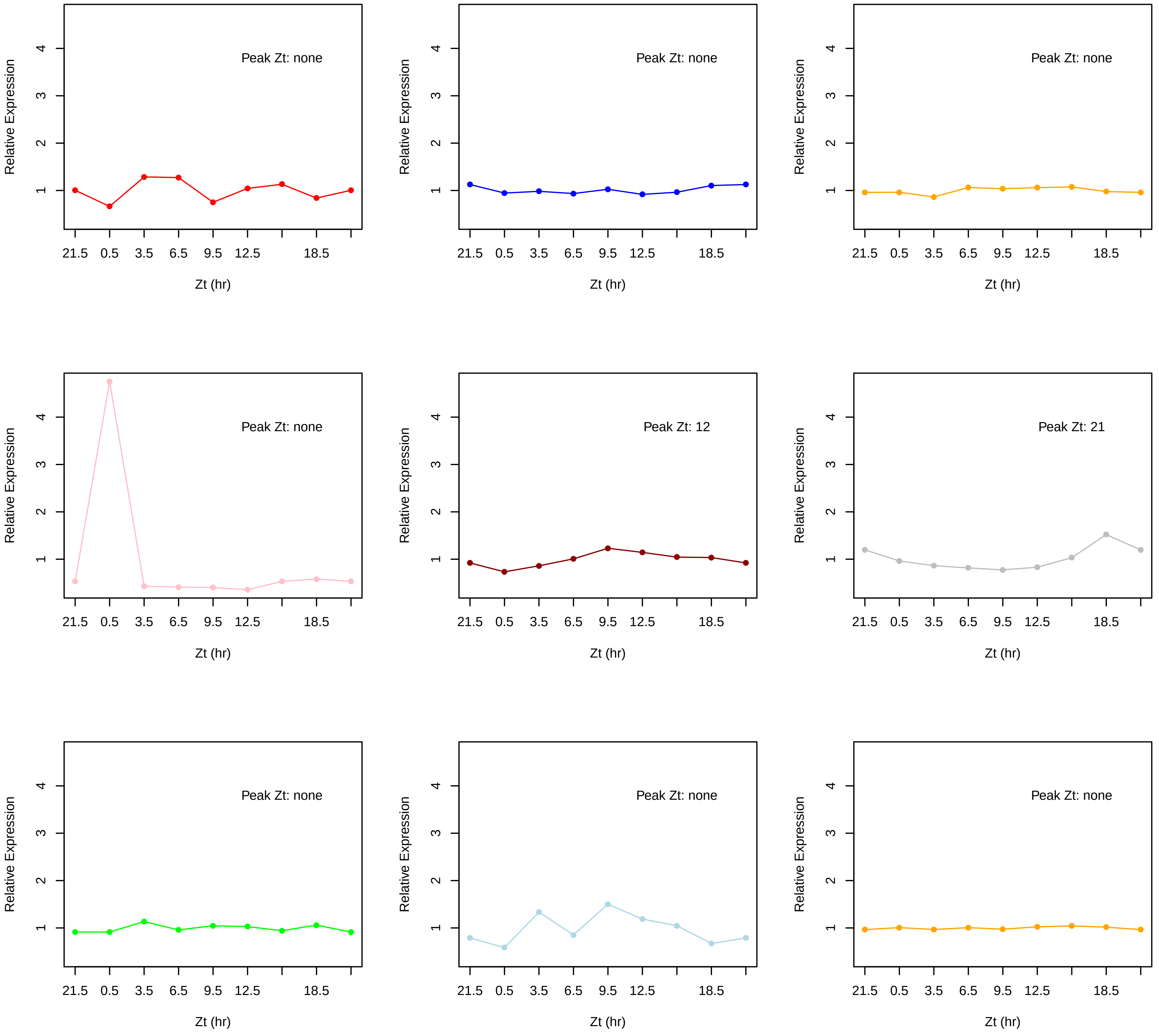

Singeleton: ENSXMAG00000015140 tsg101a

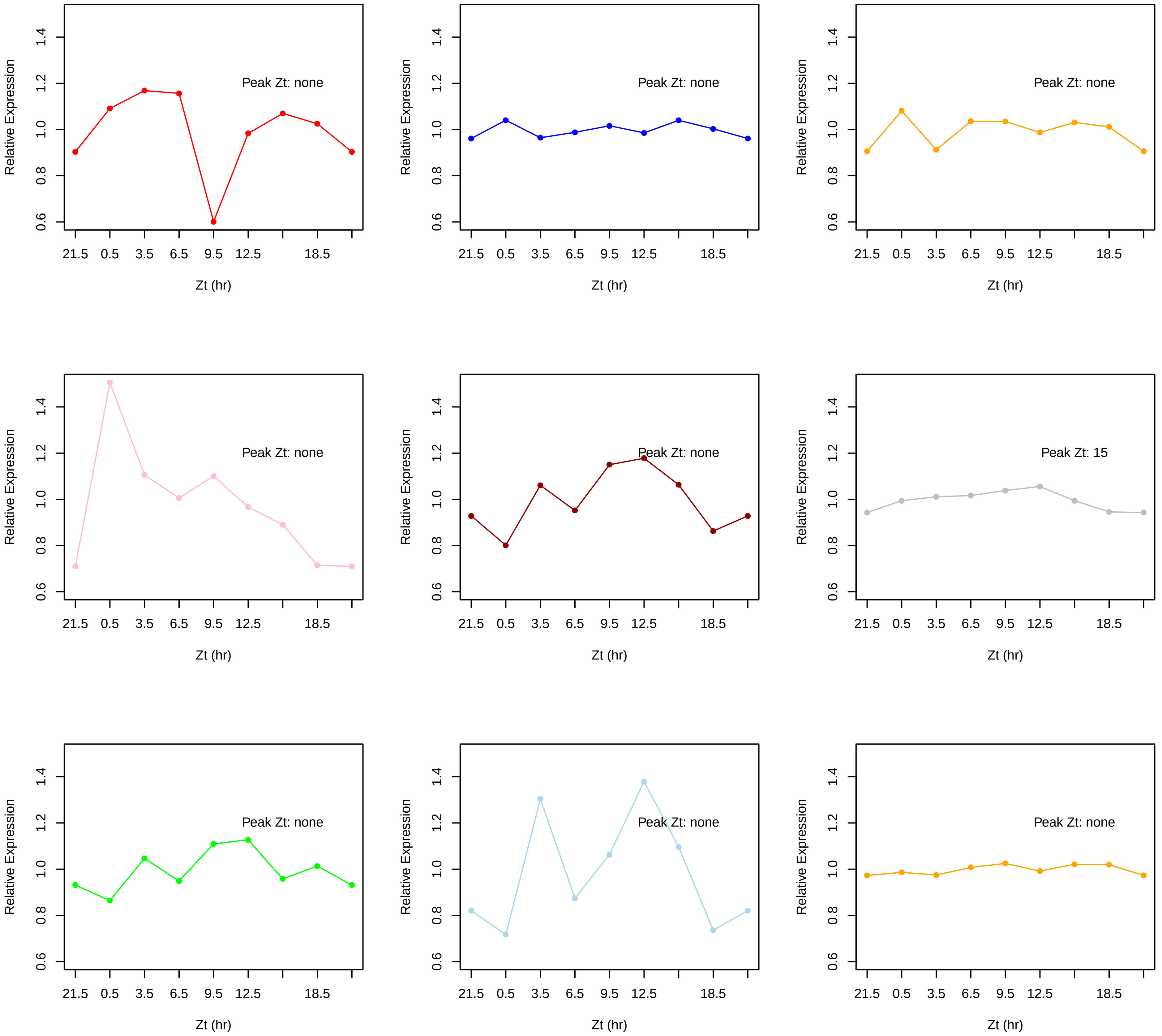

Singeleton: ENSXMAG00000014783 parn

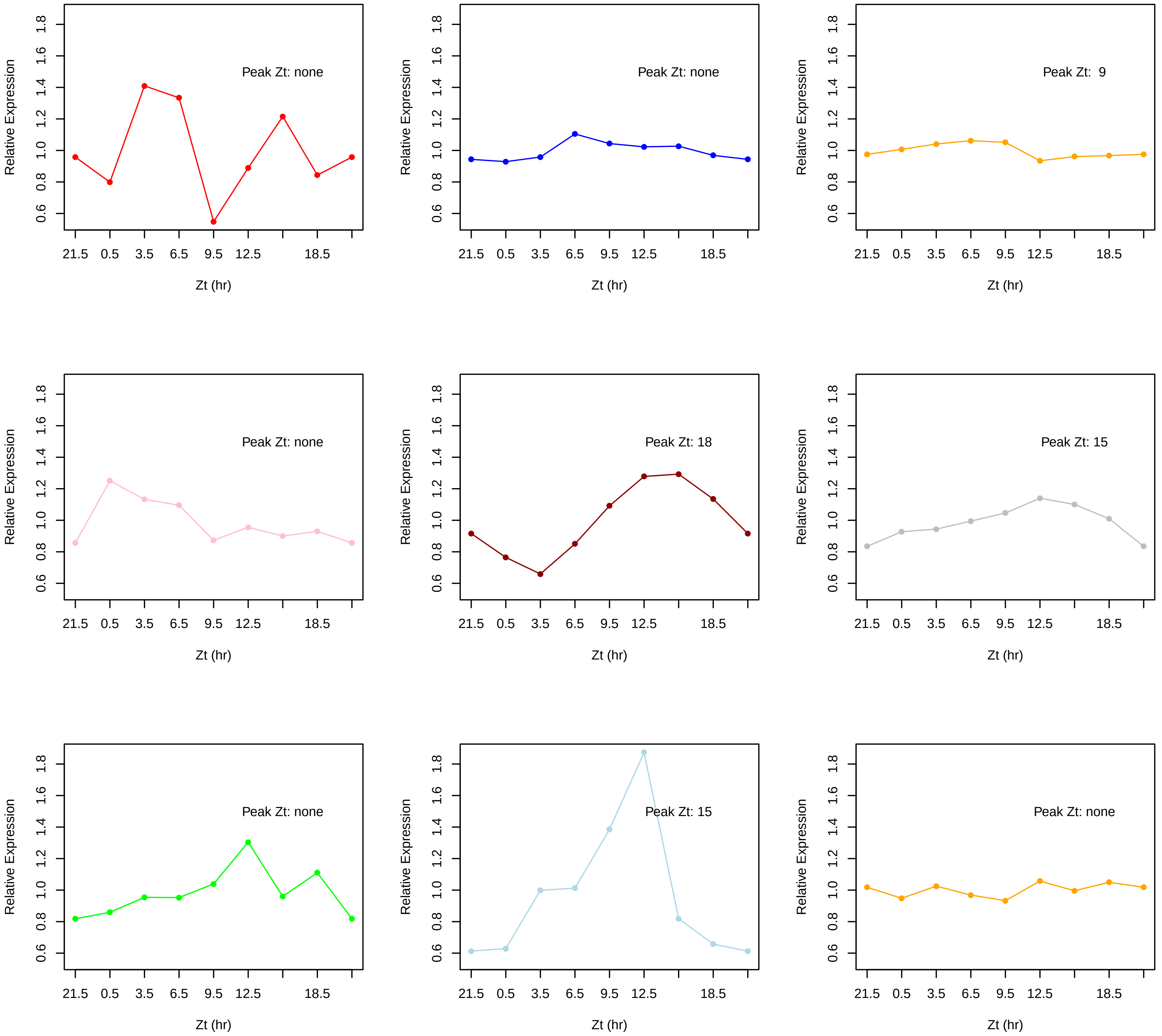

Singeleton: ENSXMAG00000026922 prrt1

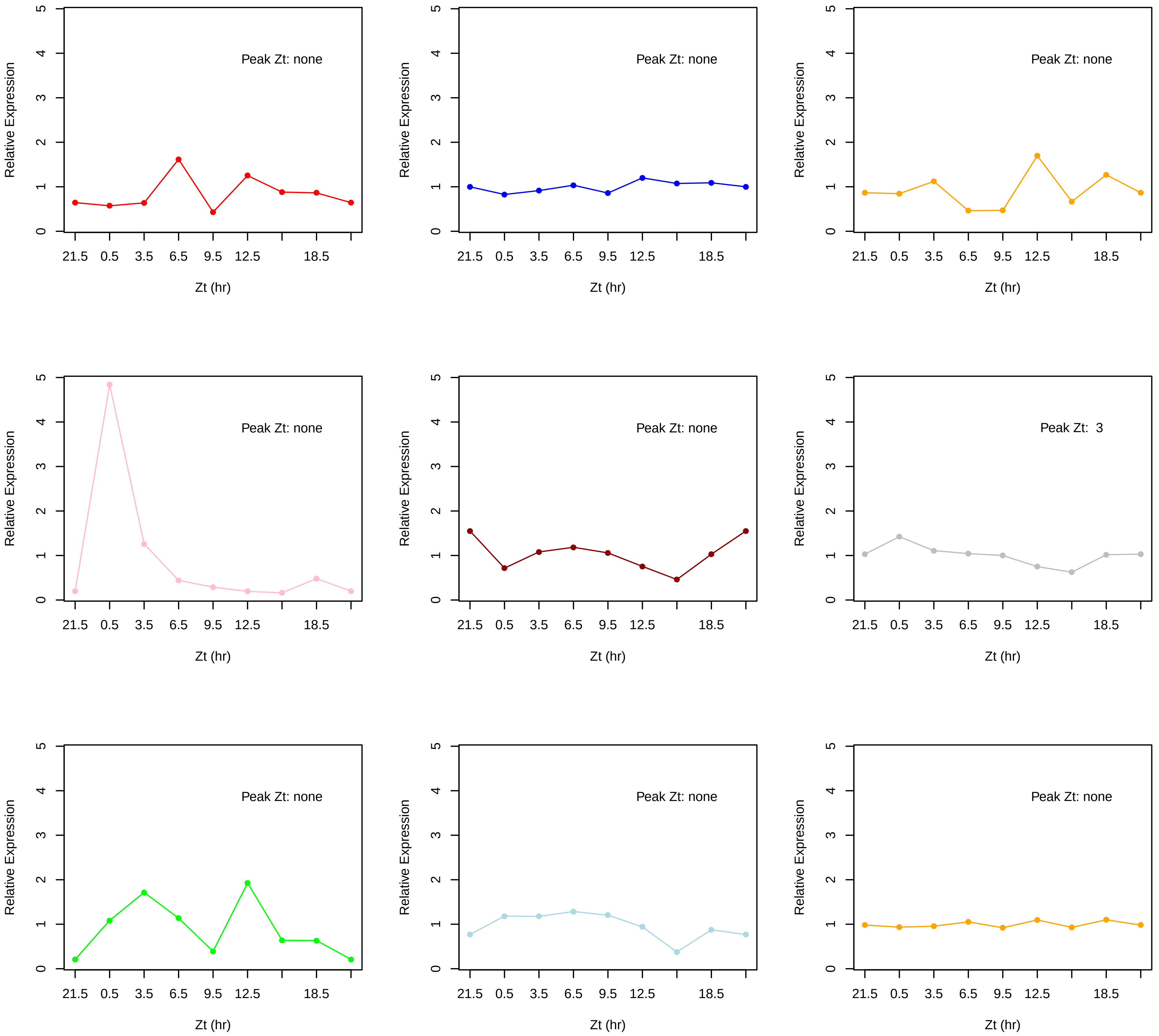

Singeleton: ENSXMAG00000013834

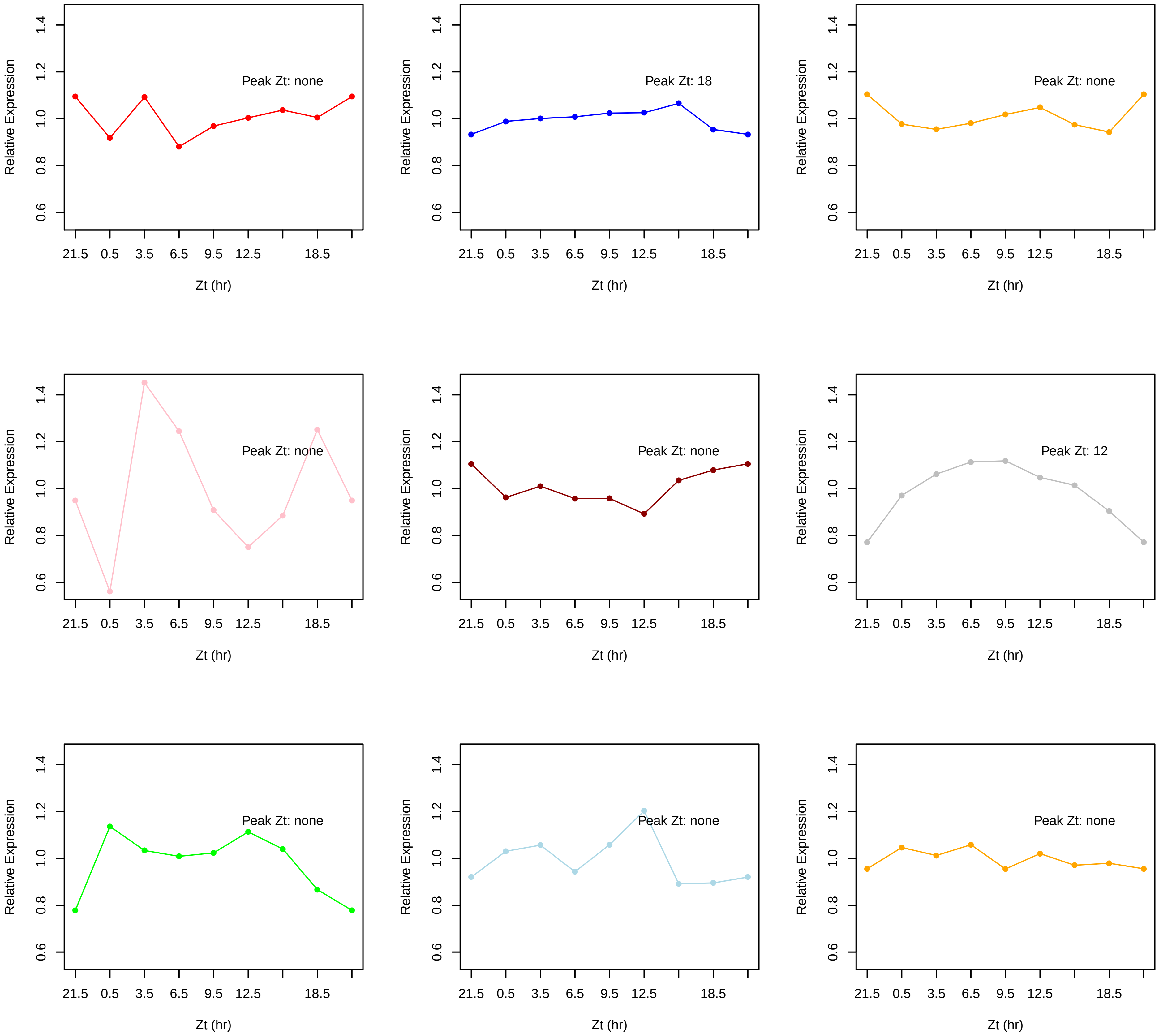

Singeleton: ENSXMAG00000000282

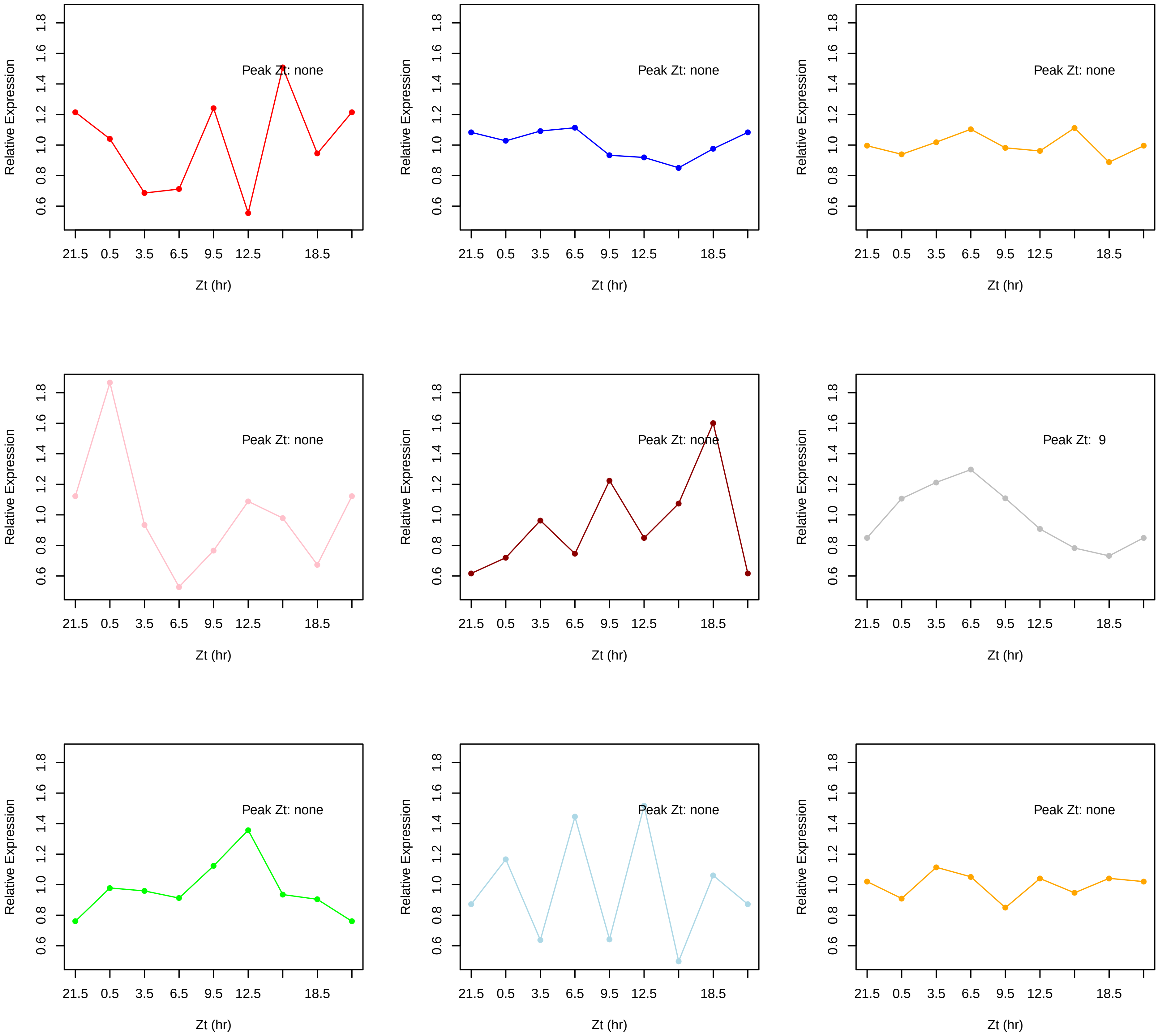

Singleton: ENSXMAG00000003446 tfap2e

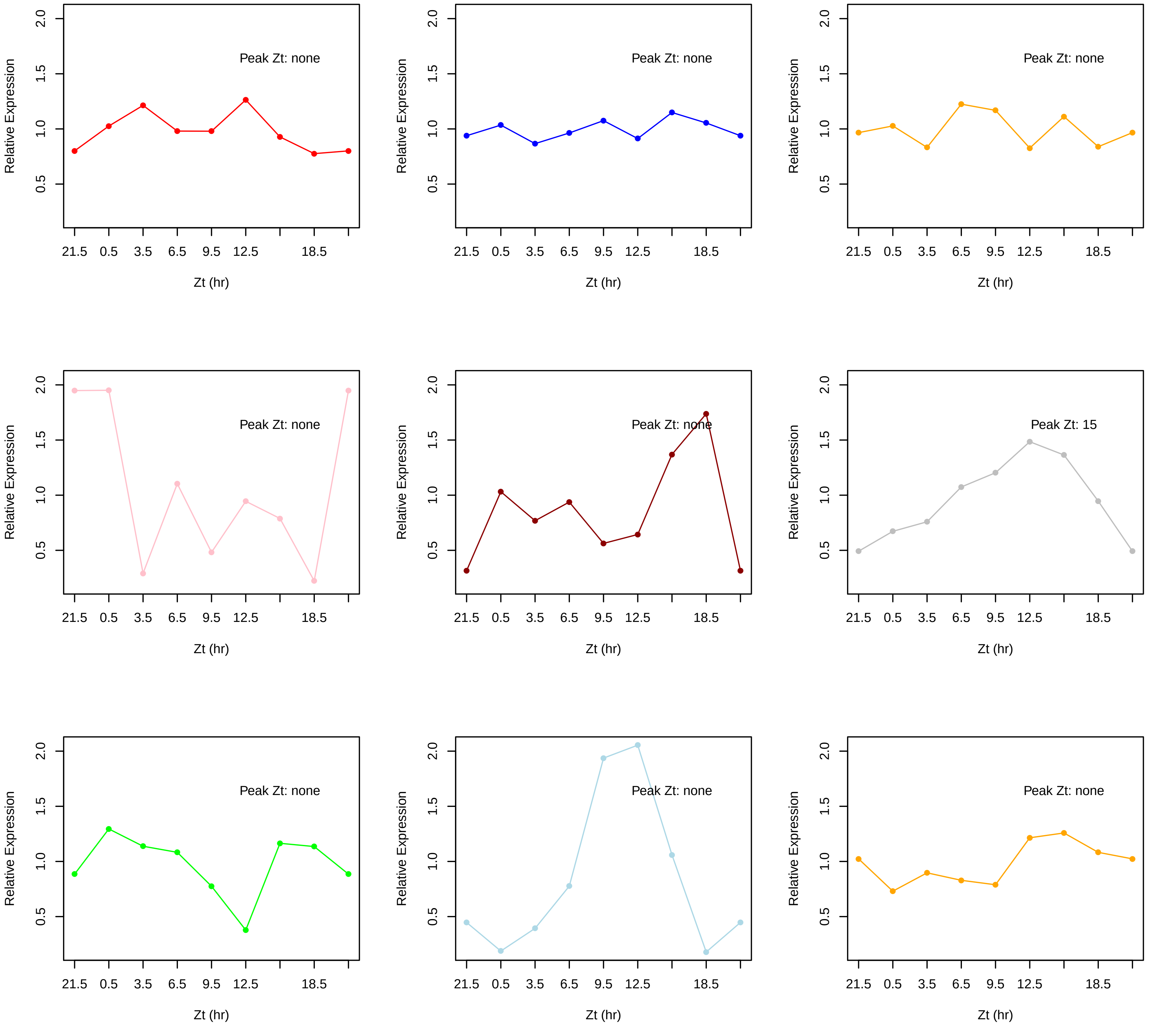

Singeleton: ENSXMAG00000021480 pex3

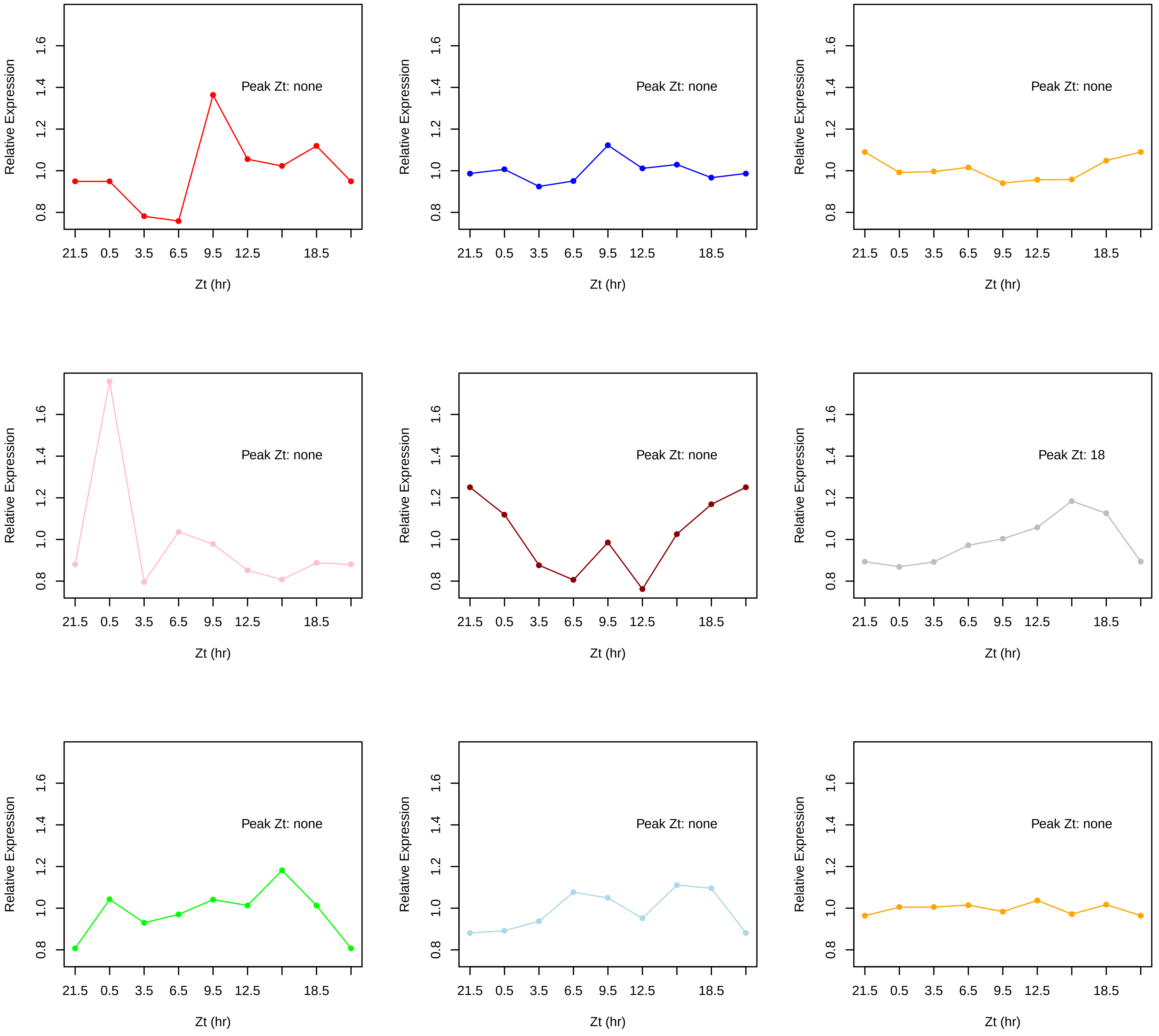

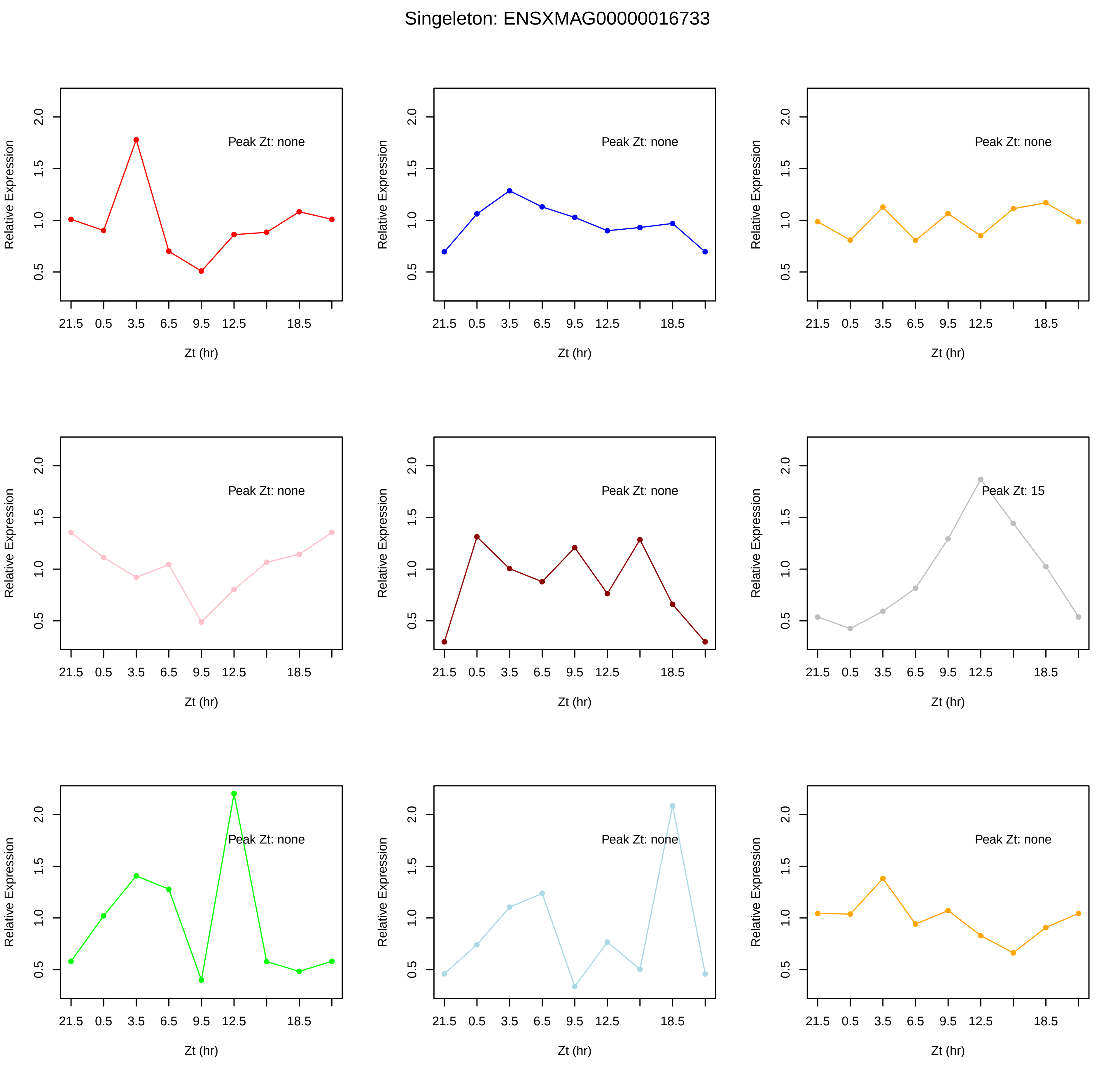
Singeleton: ENSXMAG00000016733
Peak Zt: none
Peak Zt: 15
Relative Expression
Zt (hr)
21.5
0.5
3.5
6.5
9.5
12.5
18.5

Singleton: ENSXMAG00000014183 ddah2

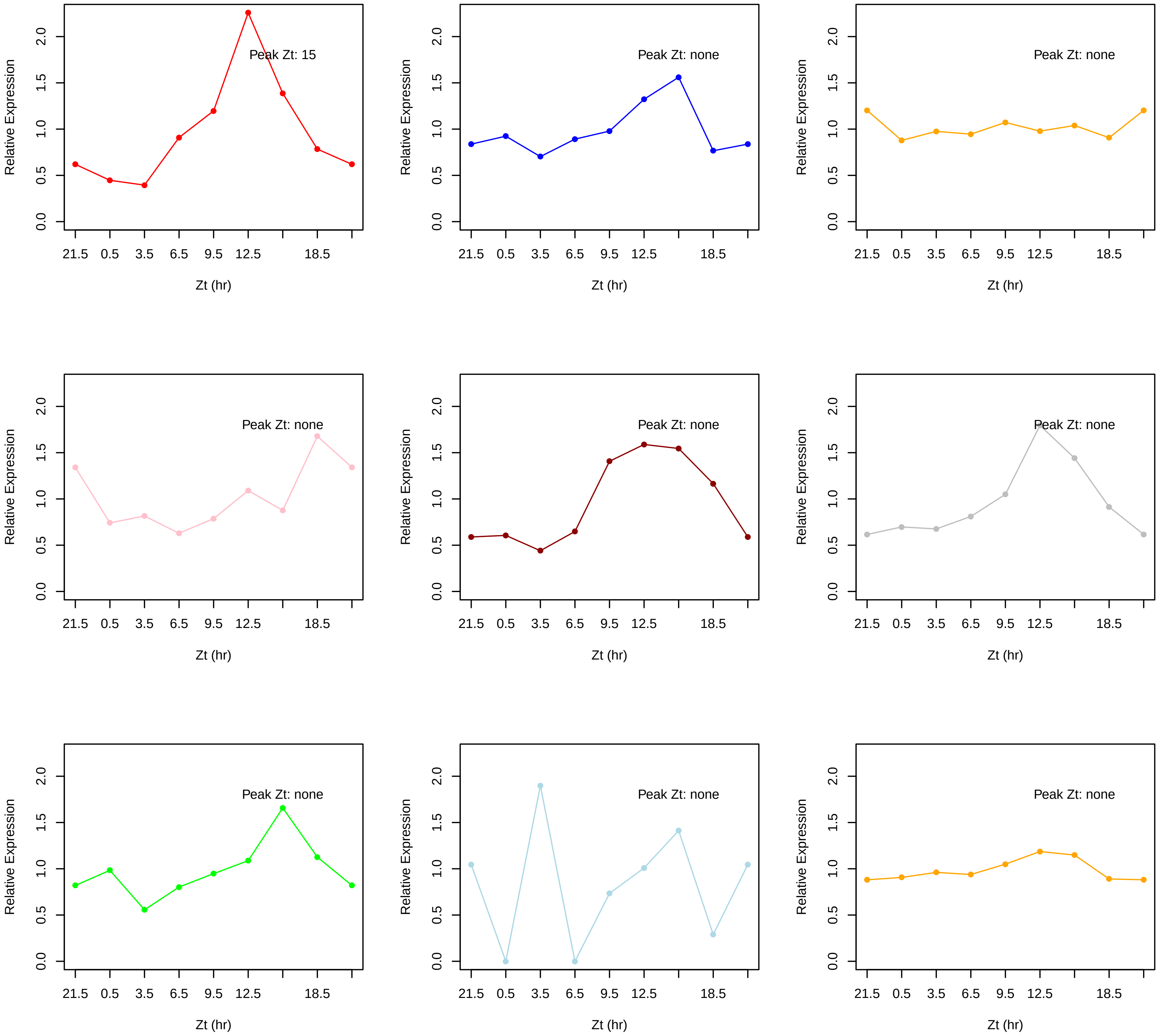

Singleton: ENSXMAG00000013033 gadd45bb

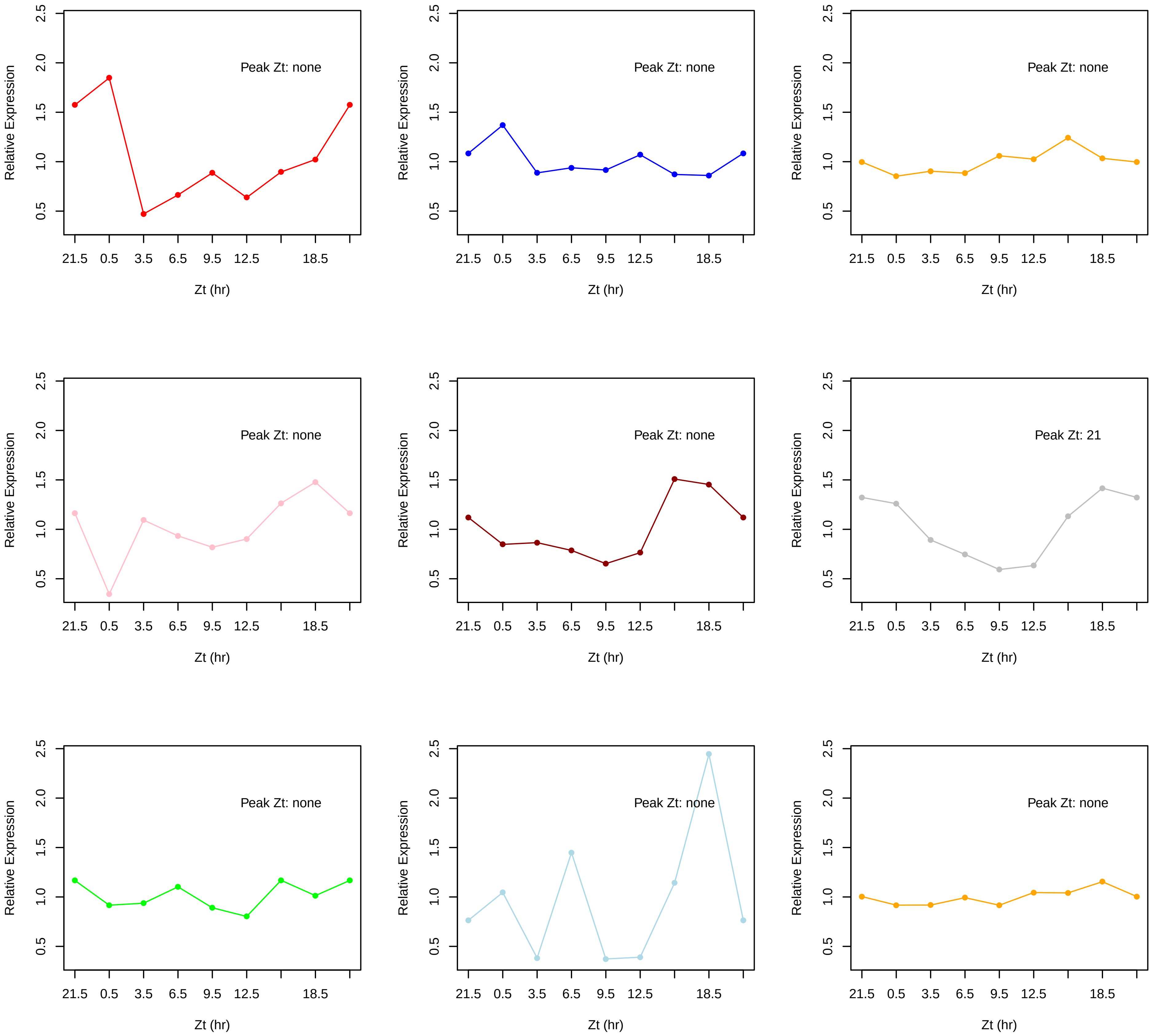

Singeleton: ENSXMAG00000027698 ncdn

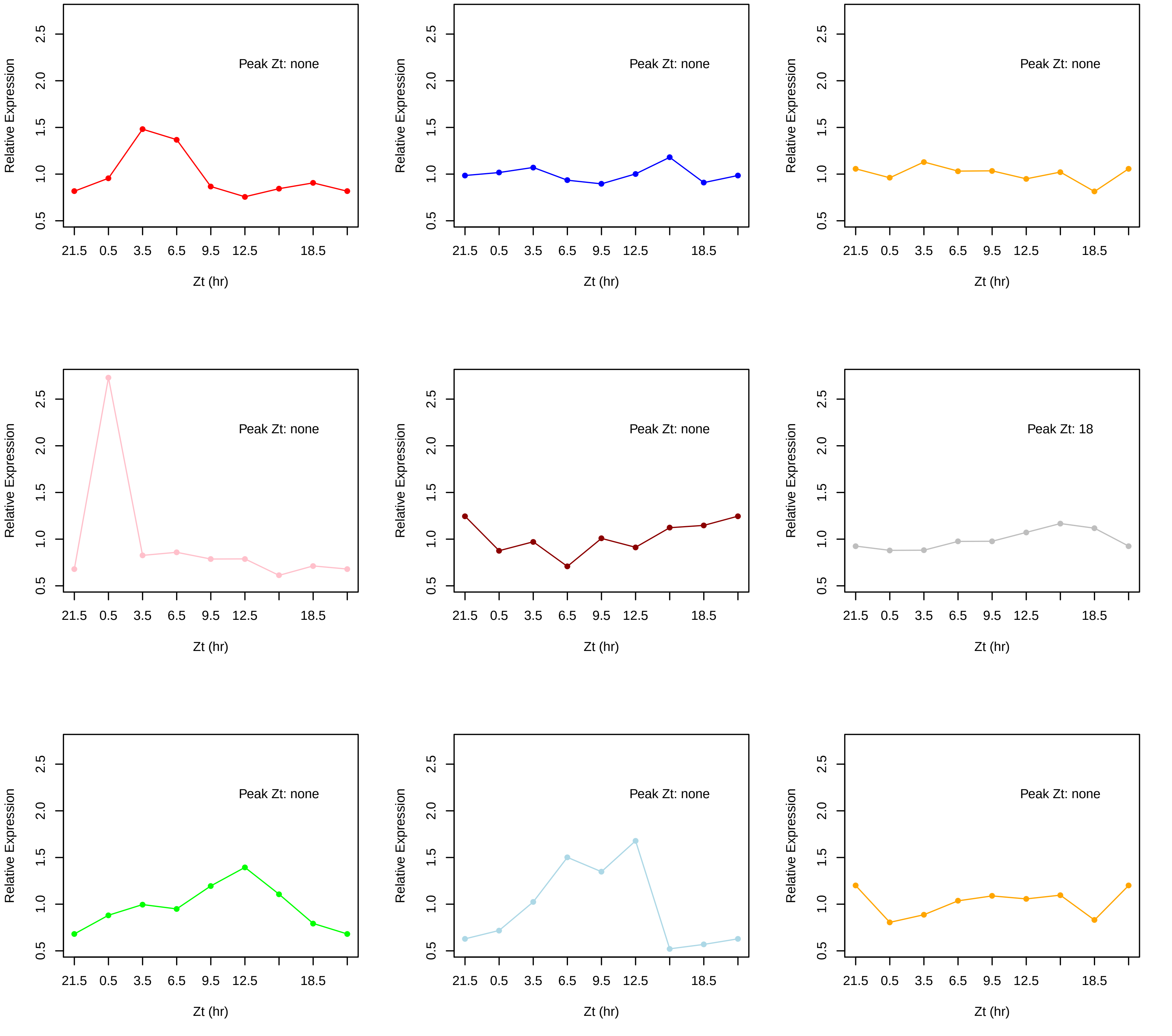

Singeleton: ENSXMAG00000003625 gmppaa

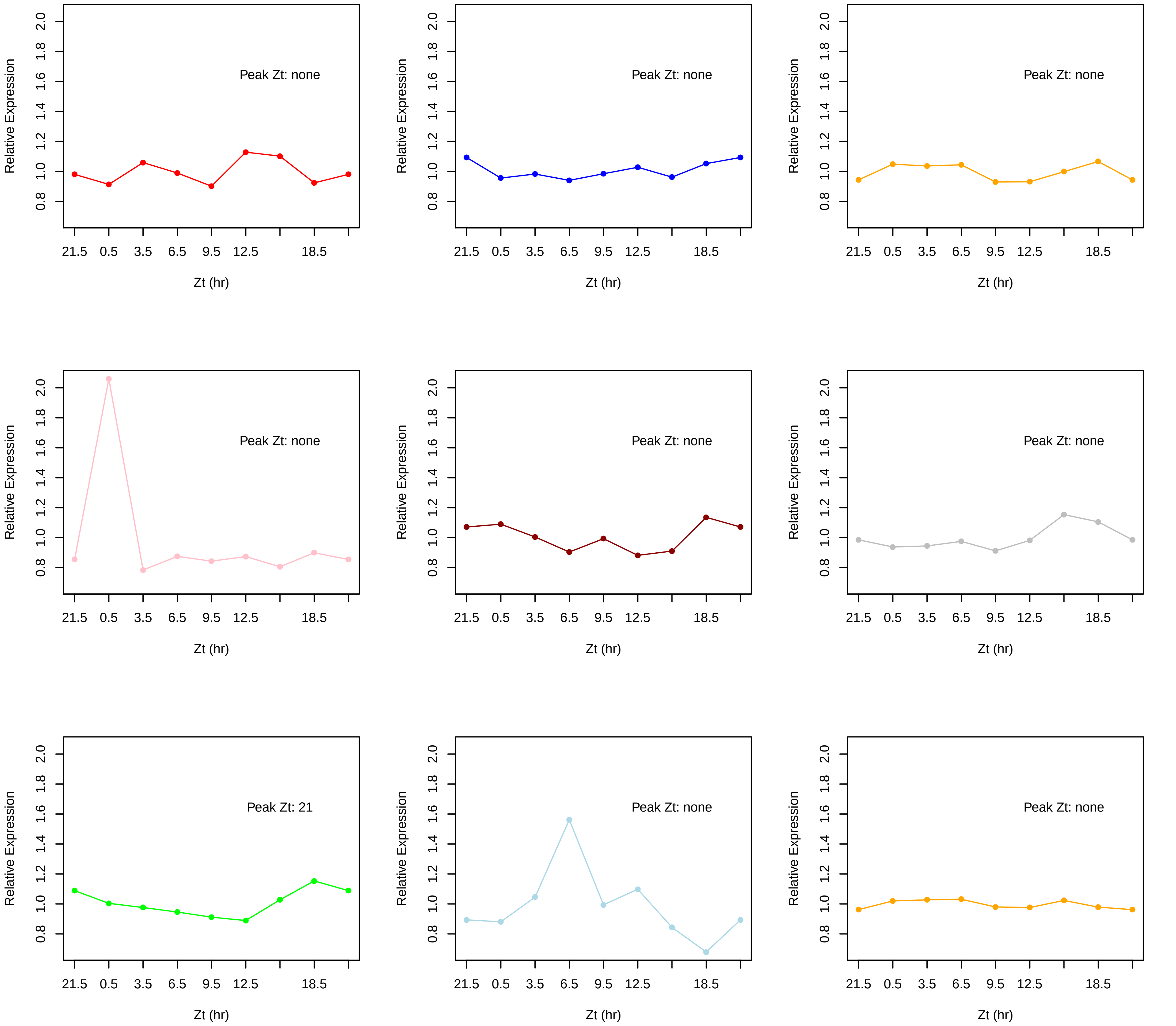

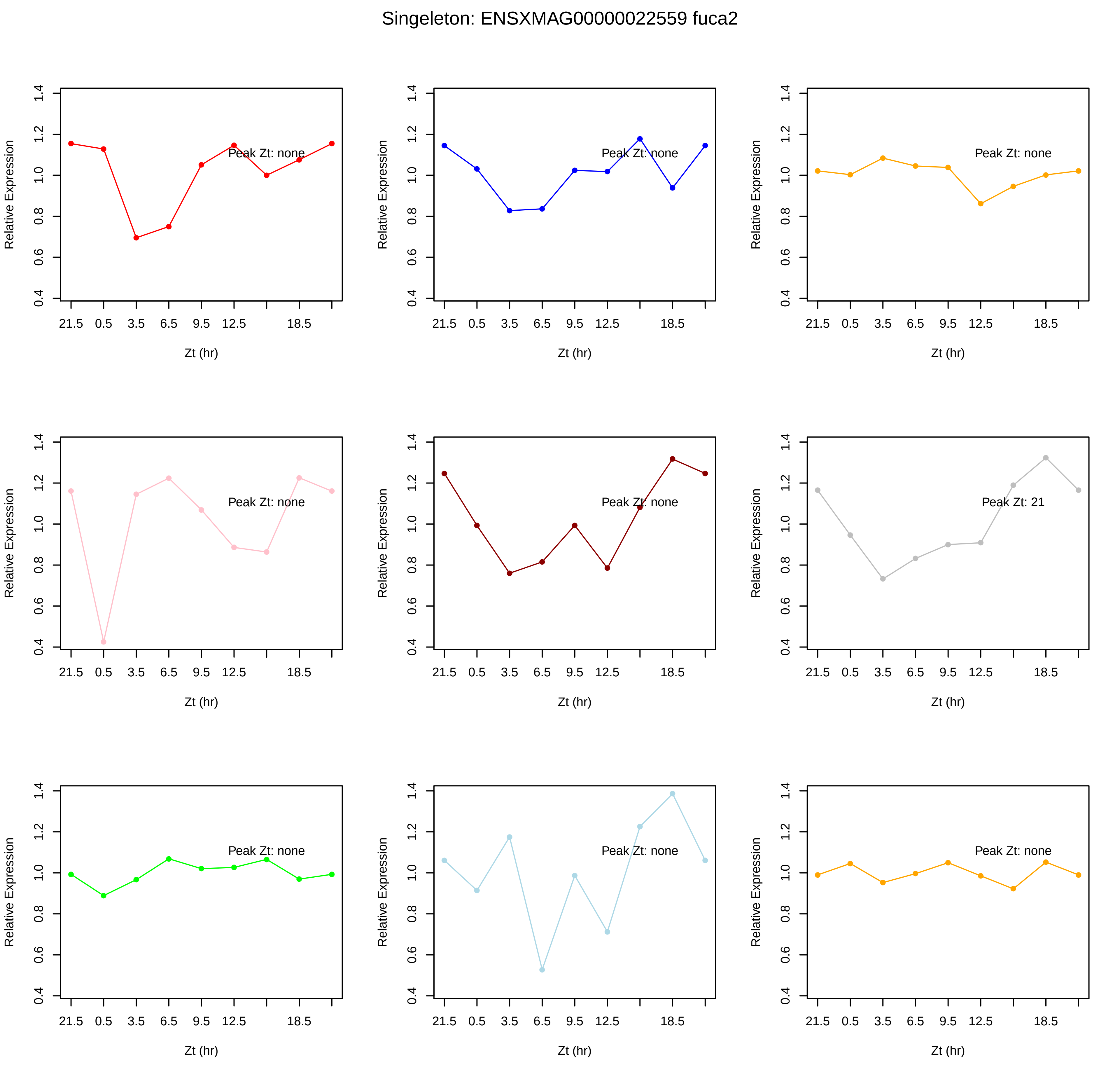
Singleton: ENSXMAG00000022559 fuca2
Relative Expression
Zt (hr)
21.5
0.5
3.5
6.5
9.5
12.5
18.5
0.4
0.6
0.8
1.0
1.2
1.4
Peak Zt: none
Peak Zt: 21

Singleton: ENSXMAG00000013157 fosl1a

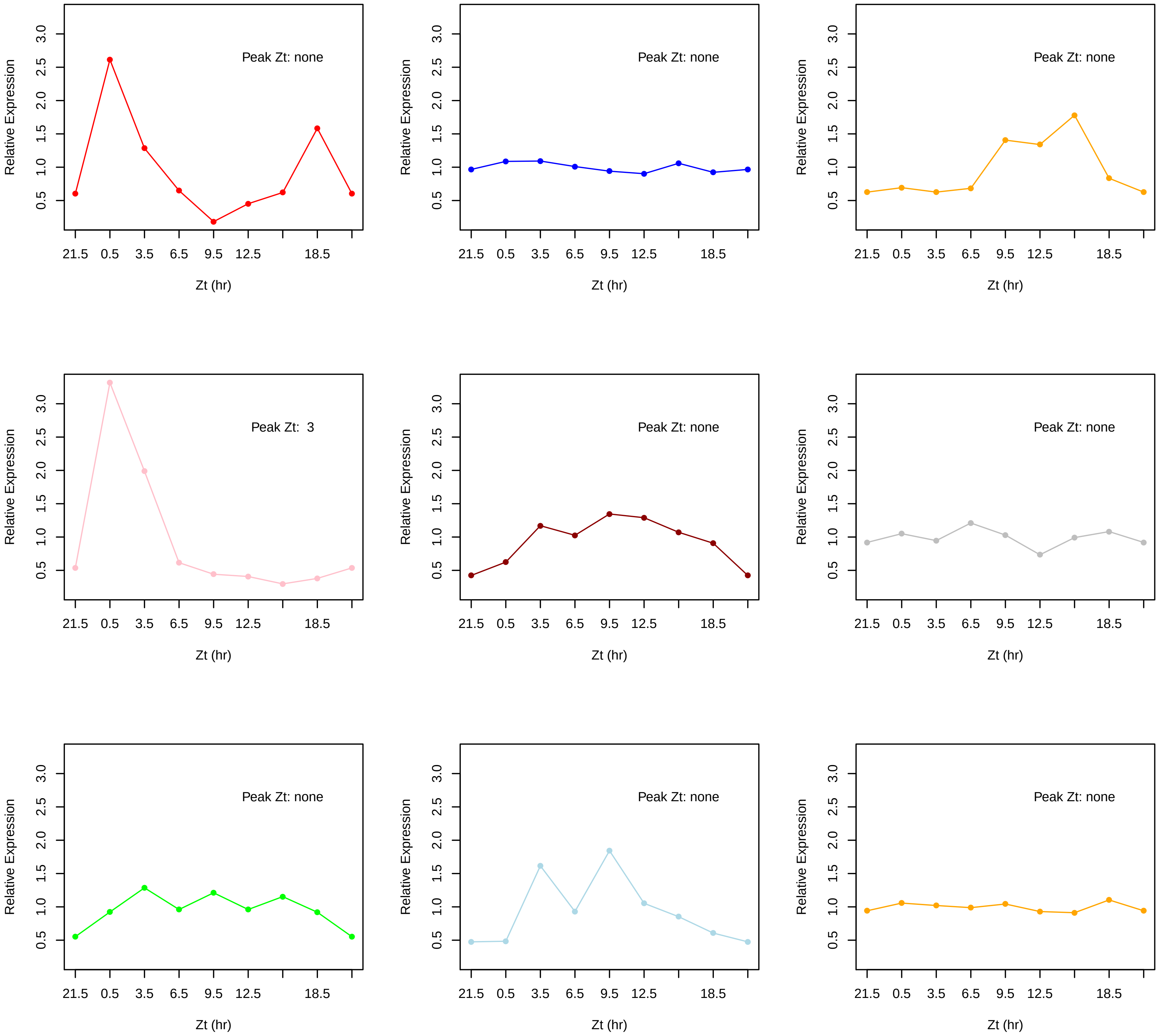

Singleton: ENSXMAG00000003454 si:ch211−193k19.1

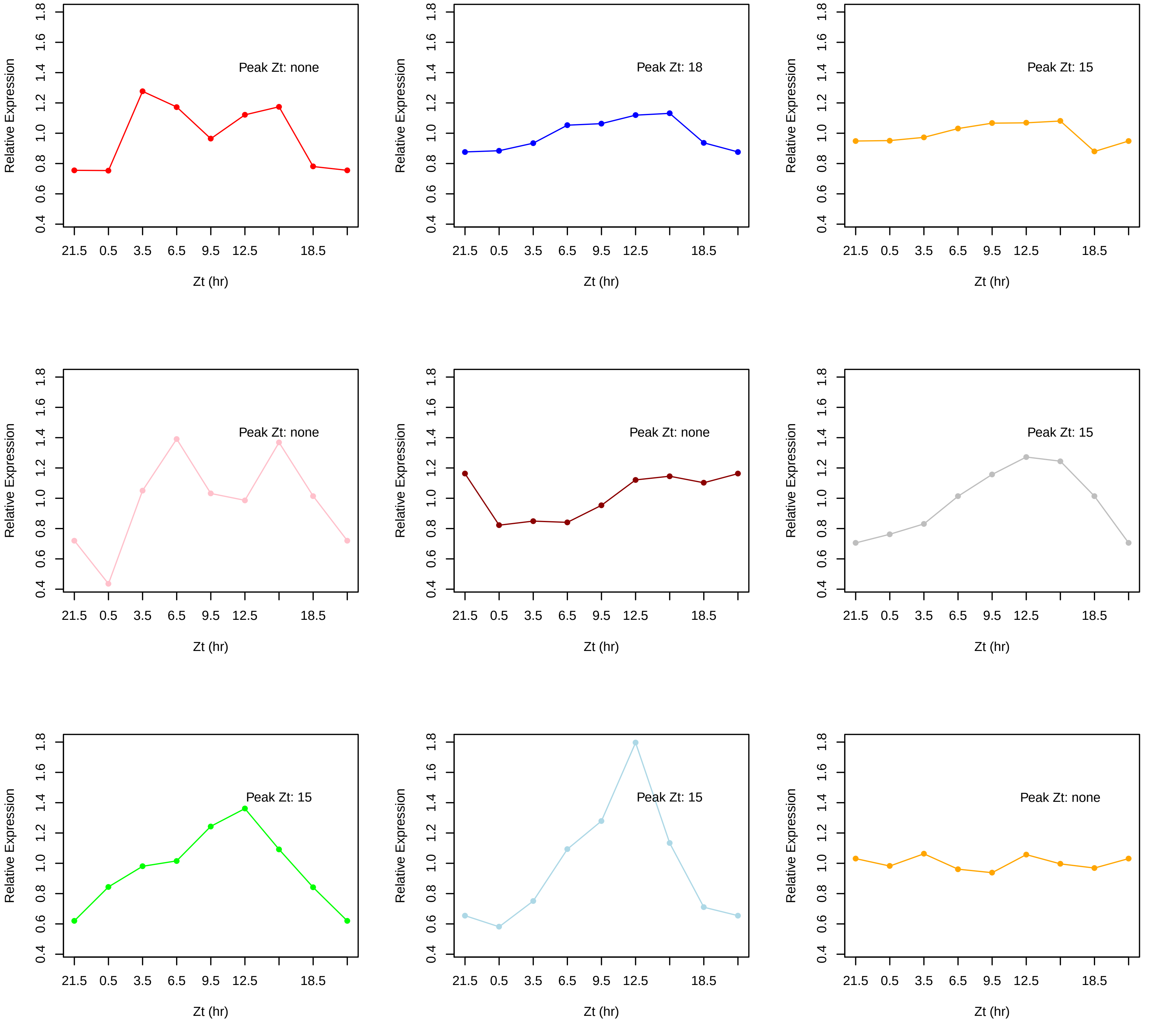

Singeleton: ENSXMAG00000002454 poldip2

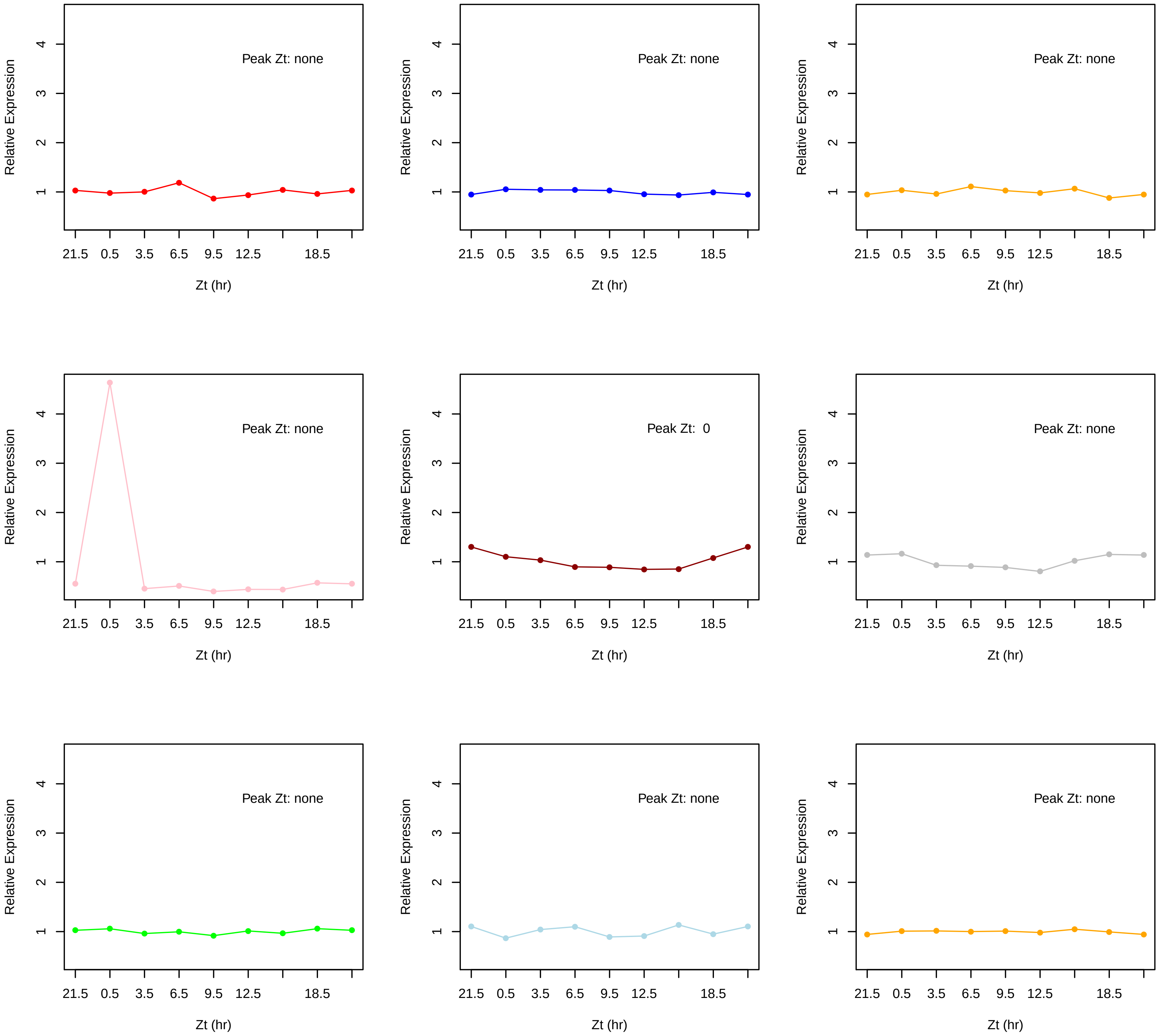

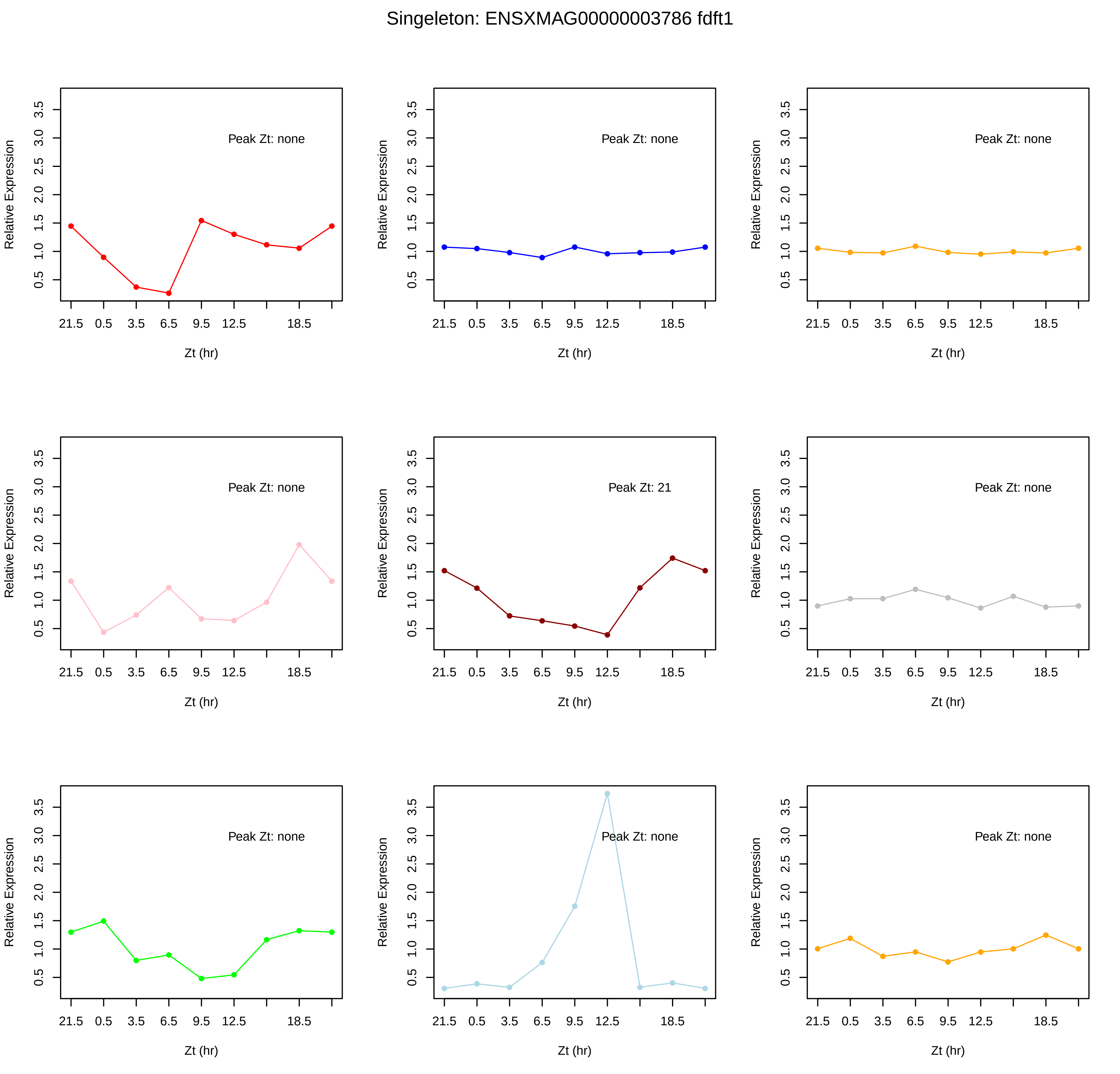

Singeleton: ENSXMAG00000003786 fdft1
Peak Zt: none
Peak Zt: none
Peak Zt: none
Peak Zt: none
Peak Zt: 21
Peak Zt: none
Peak Zt: none
Peak Zt: none
Peak Zt: none
Relative Expression
Zt (hr)
21.5
0.5
3.5
6.5
9.5
12.5
18.5

Singeleton: ENSXMAG00000006196 pabpn1

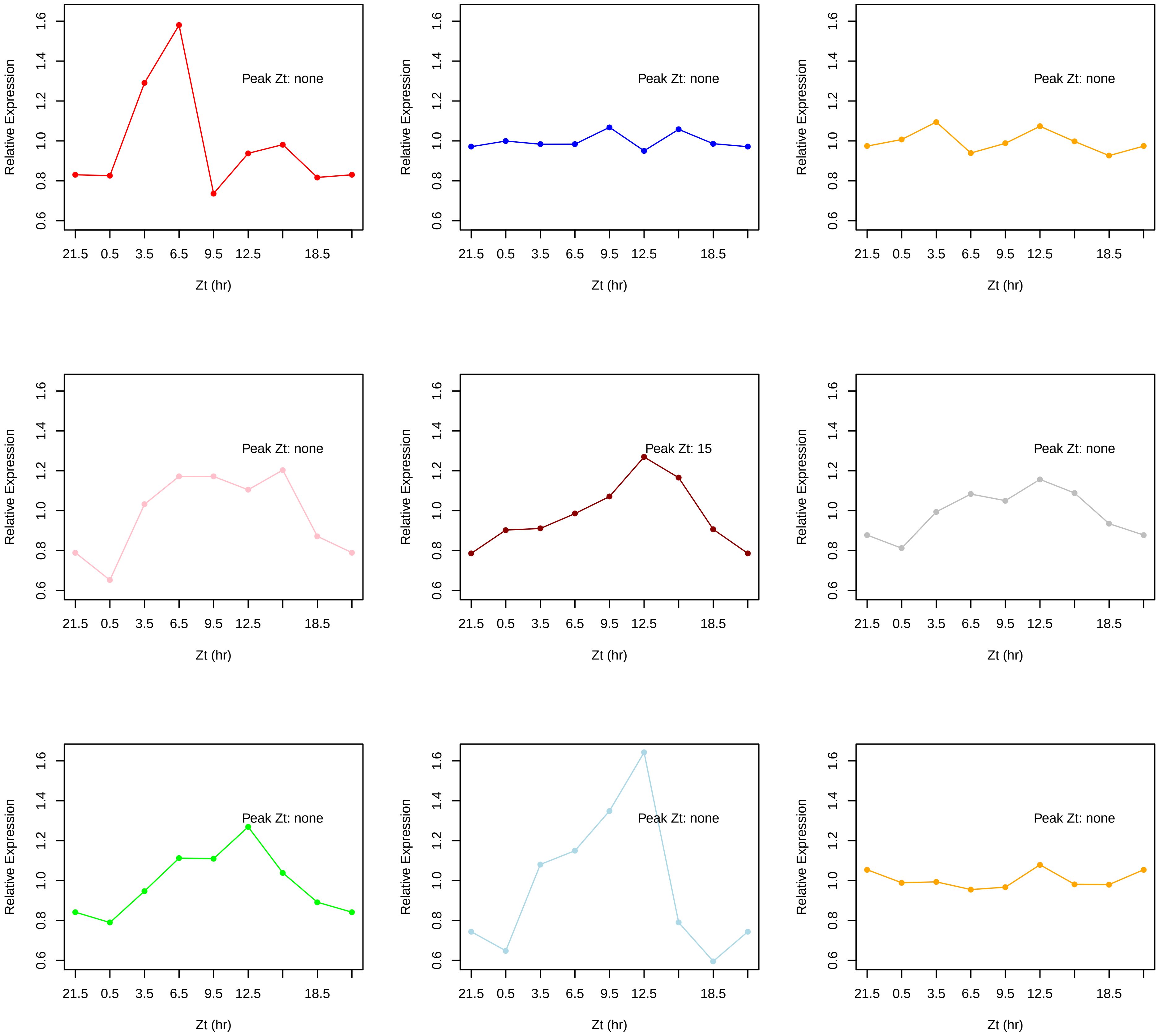

Singeleton: ENSXMAG00000024411 adprhl2

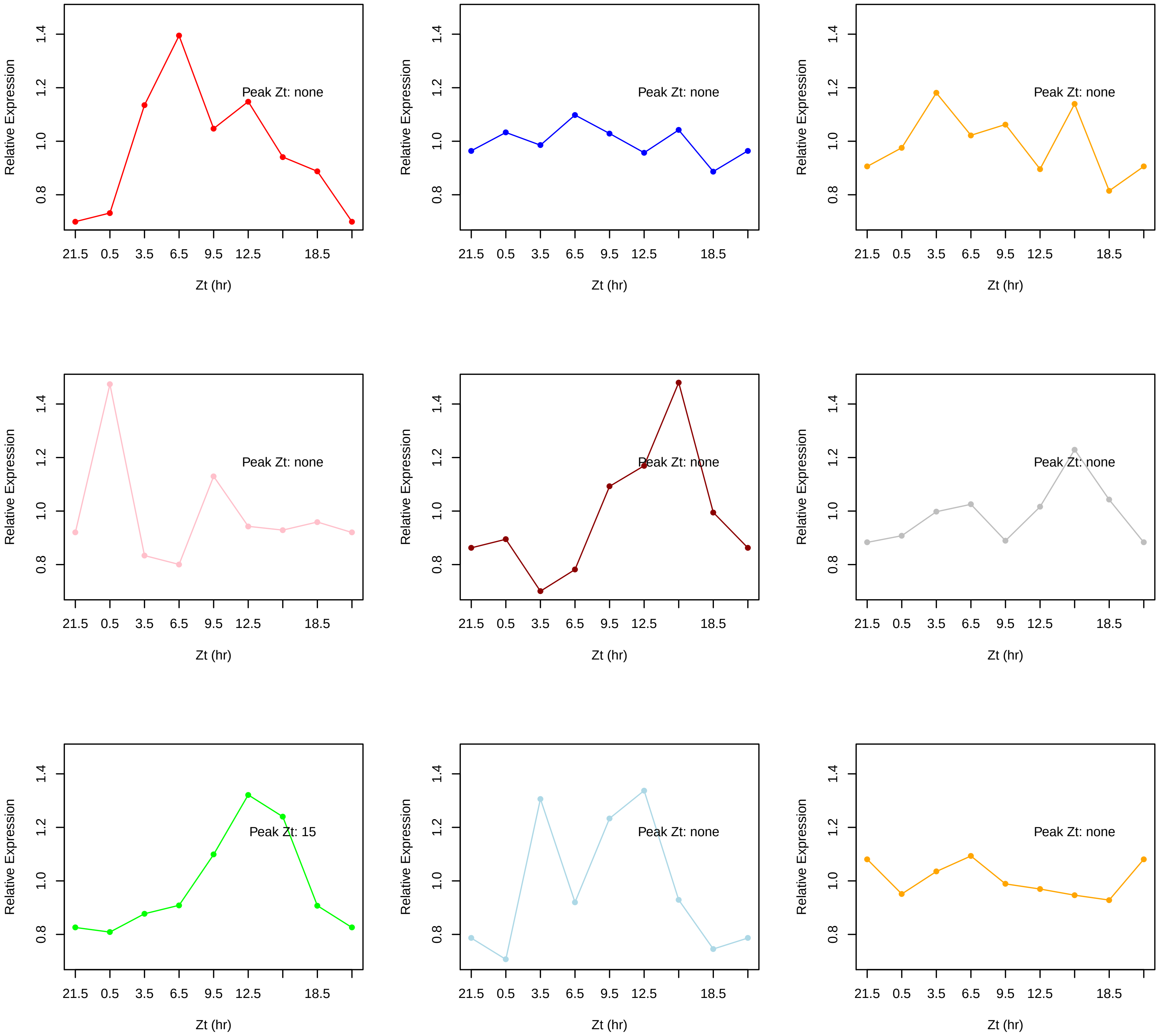

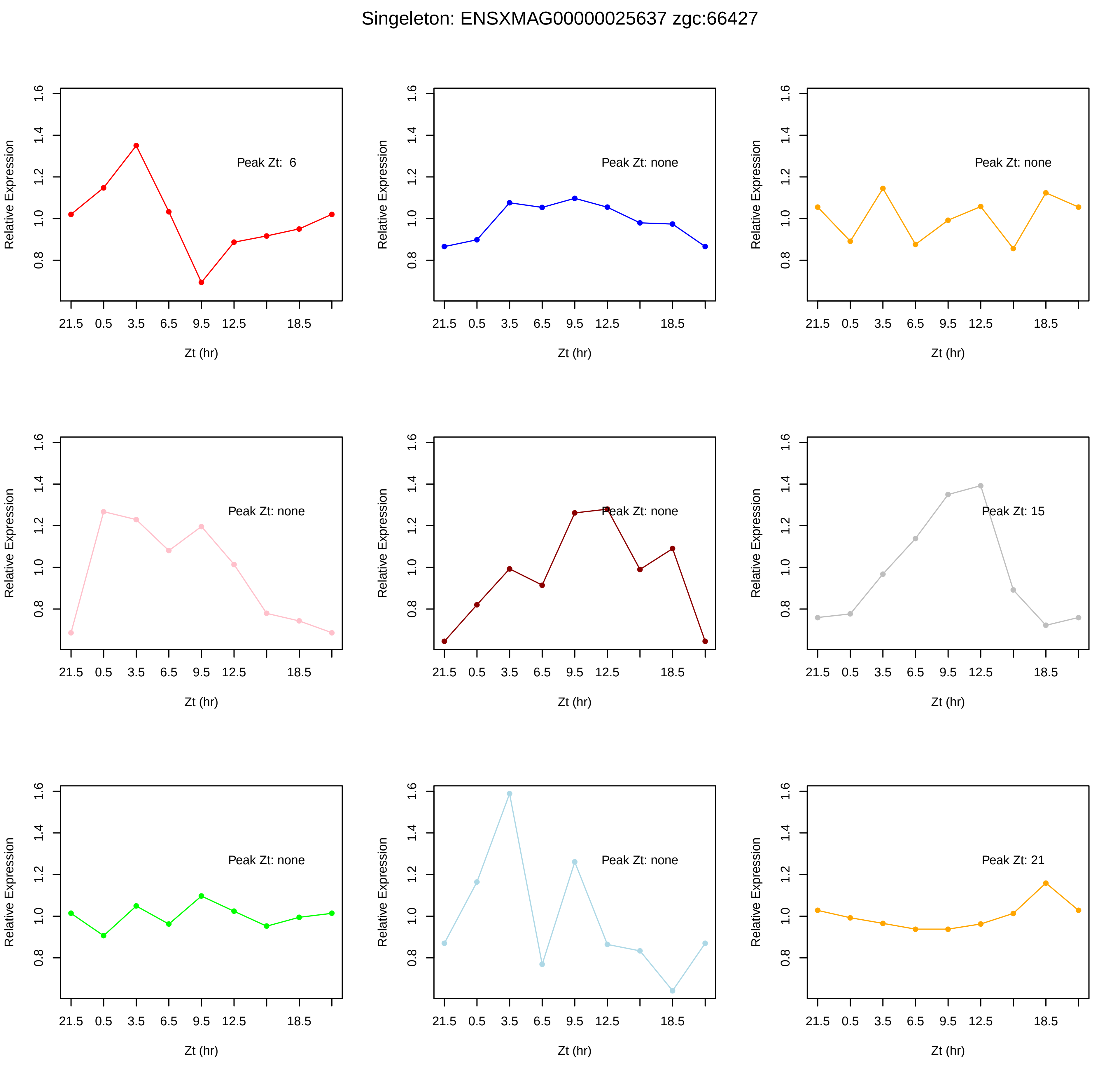
Singeleton: ENSXMAG00000025637 zgc:66427
Peak Zt: 6
Peak Zt: none
Peak Zt: none
Peak Zt: none
Peak Zt: none
Peak Zt: 15
Peak Zt: none
Peak Zt: none
Peak Zt: 21
Relative Expression
Zt (hr)
21.5
0.5
3.5
6.5
9.5
12.5
18.5
0.8
1.0
1.2
1.4
1.6

Singeleton: ENSXMAG00000026118 atf3

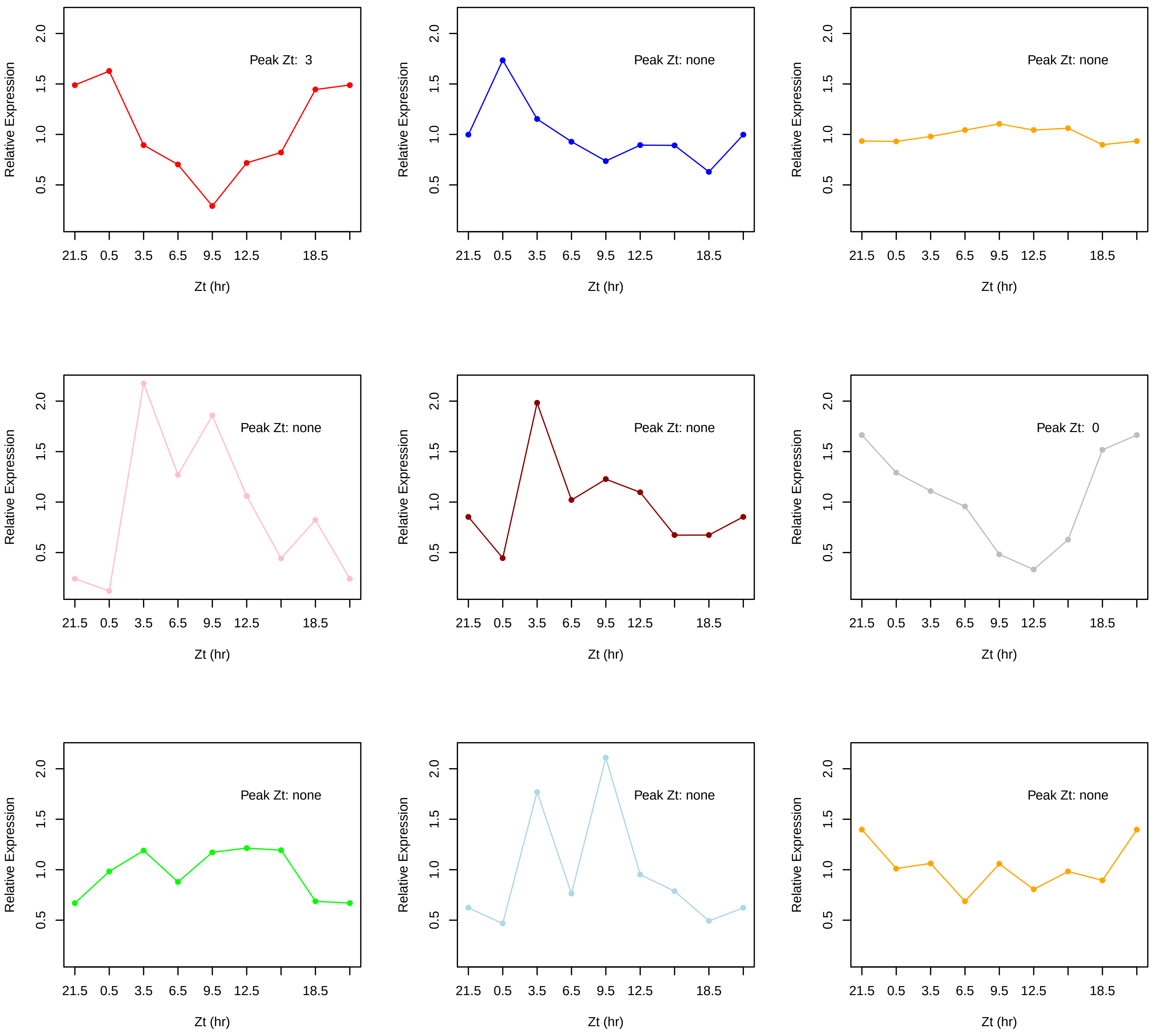

Singeleton: ENSXMAG00000007630 bcl9l

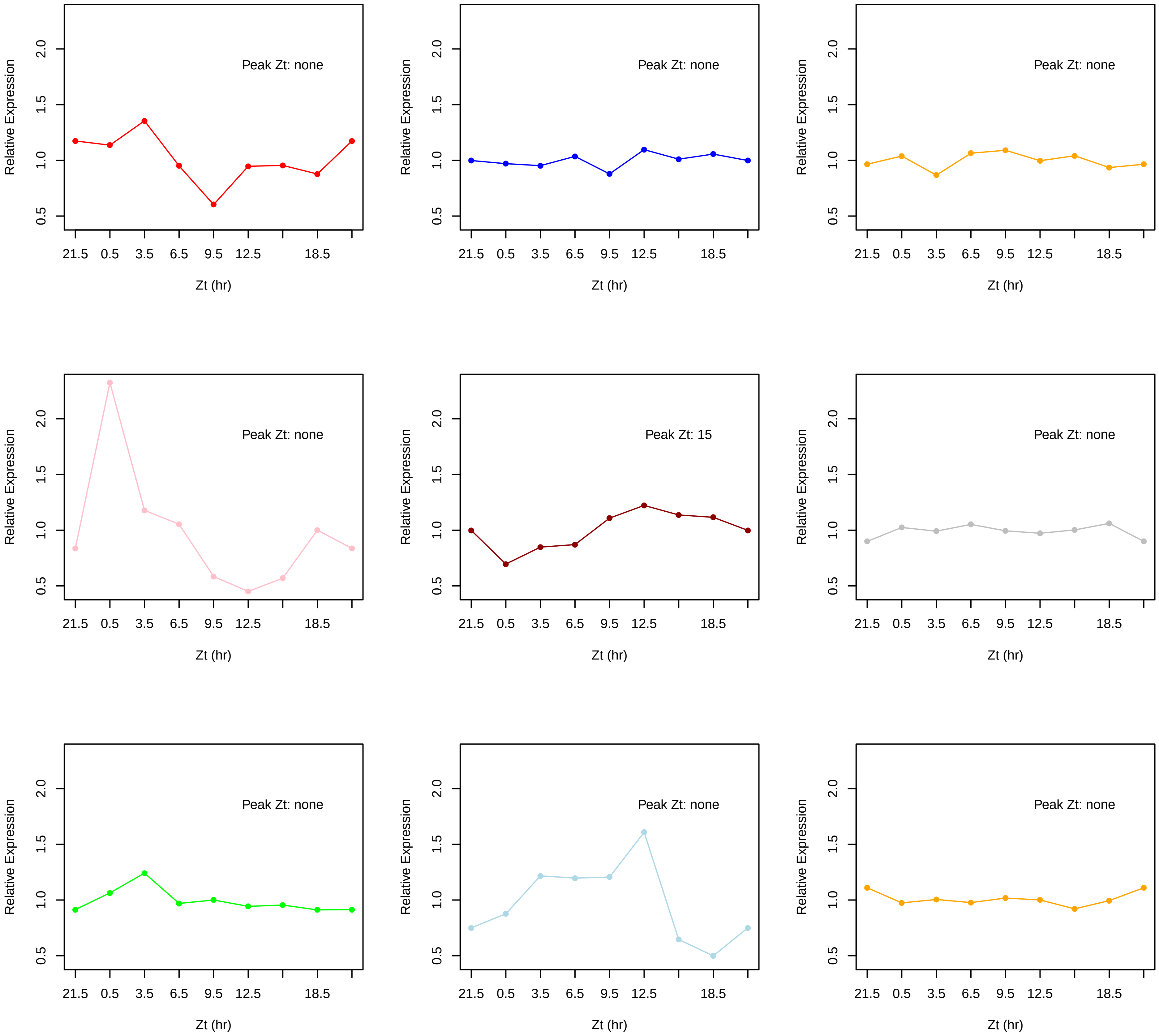

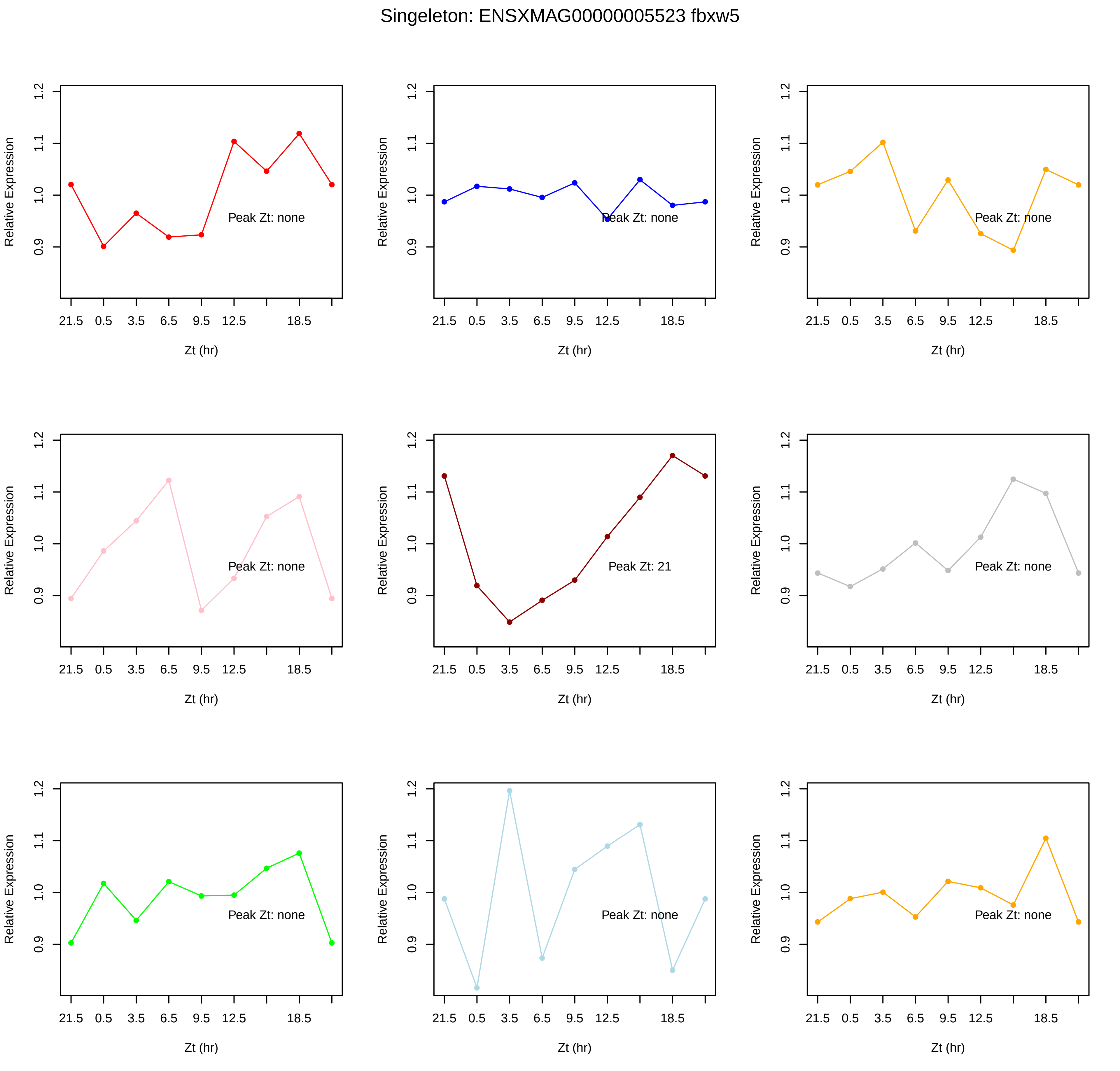
Singleton: ENSXMAG00000005523 fbxw5
Relative Expression
Zt (hr)
21.5
0.5
3.5
6.5
9.5
12.5
18.5
0.9
1.0
1.1
1.2
Peak Zt: none
Peak Zt: 21

Singeleton: ENSXMAG00000028529 cap1

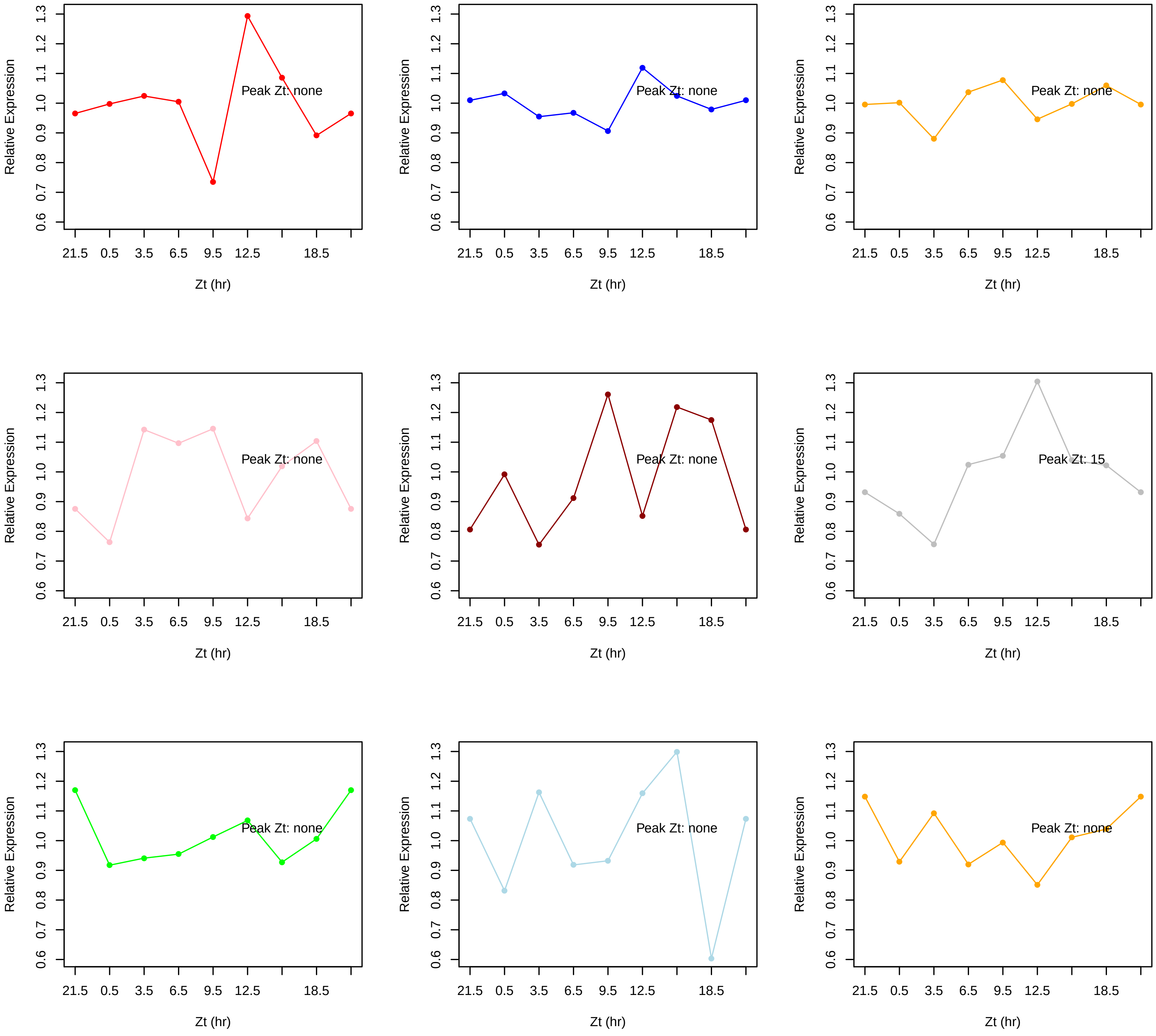

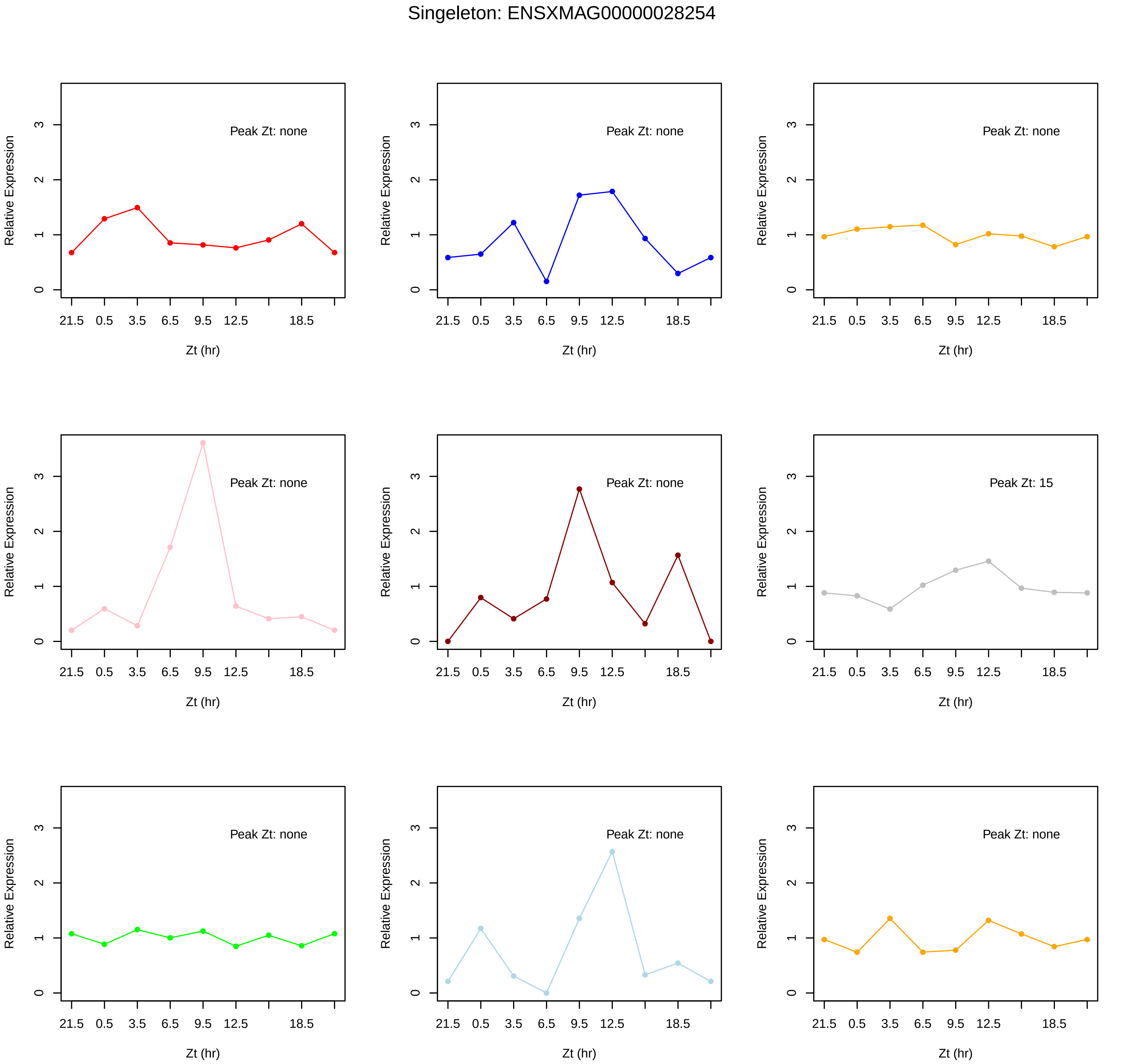
Singeleton: ENSXMAG00000028254
Peak Zt: none
Peak Zt: none
Peak Zt: none
Peak Zt: none
Peak Zt: none
Peak Zt: 15
Peak Zt: none
Peak Zt: none
Peak Zt: none
Relative Expression
Zt (hr)
21.5
0.5
3.5
6.5
9.5
12.5
18.5
0
1
2
3

Singeleton: ENSXMAG00000000609

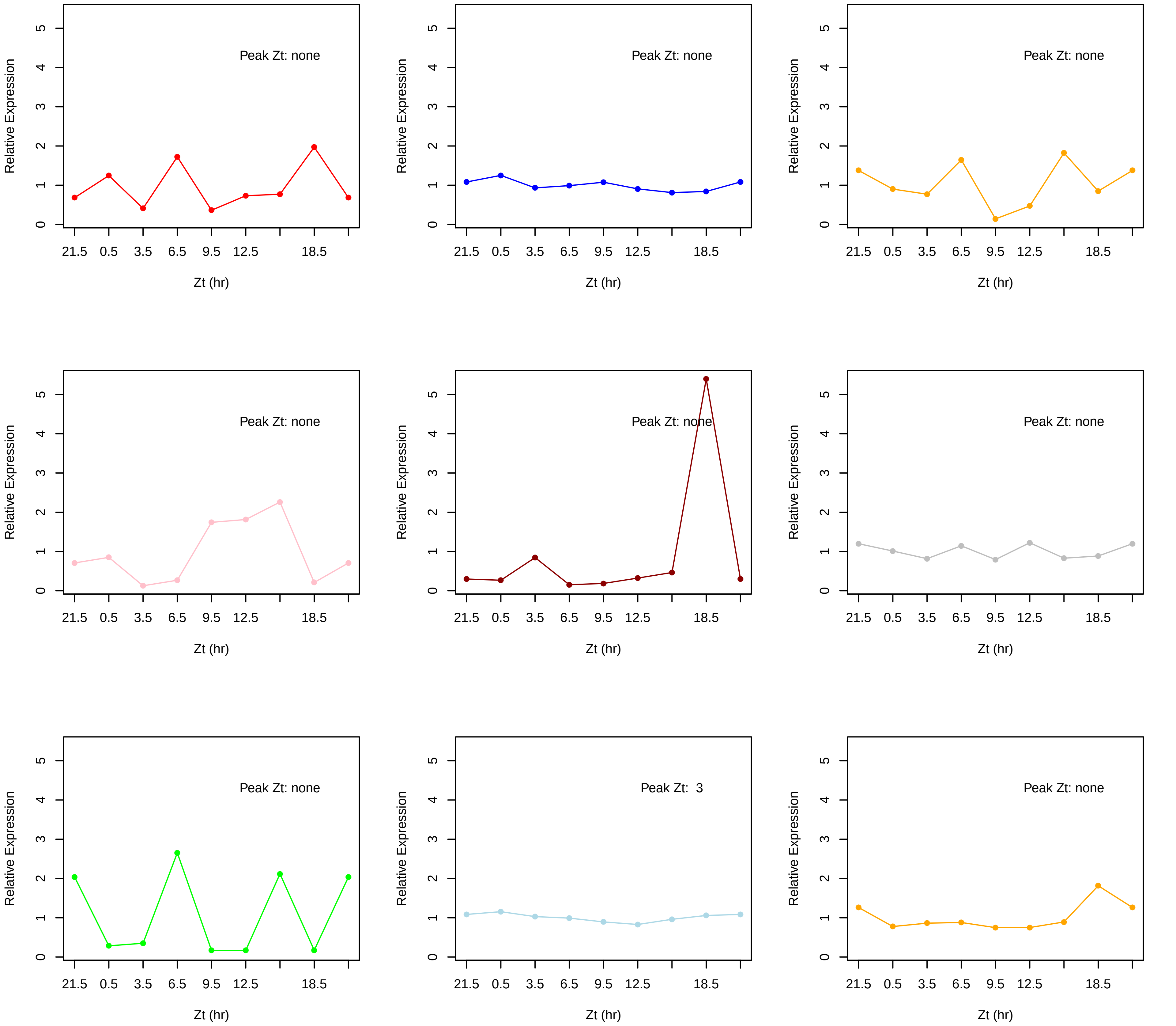

Singeleton: ENSXMAG00000000545 ptdss2

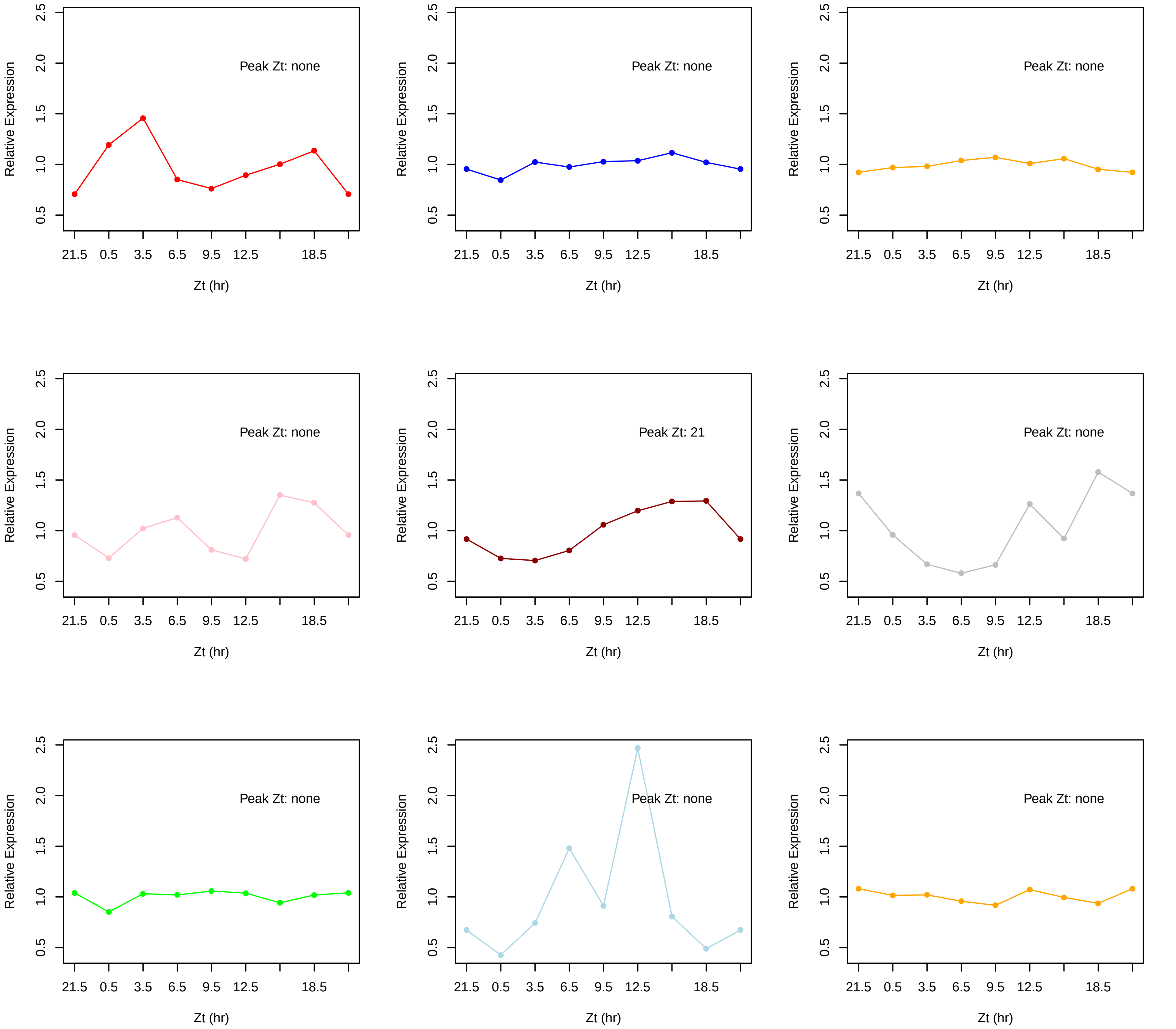

Singeleton: ENSXMAG00000010660 nle1

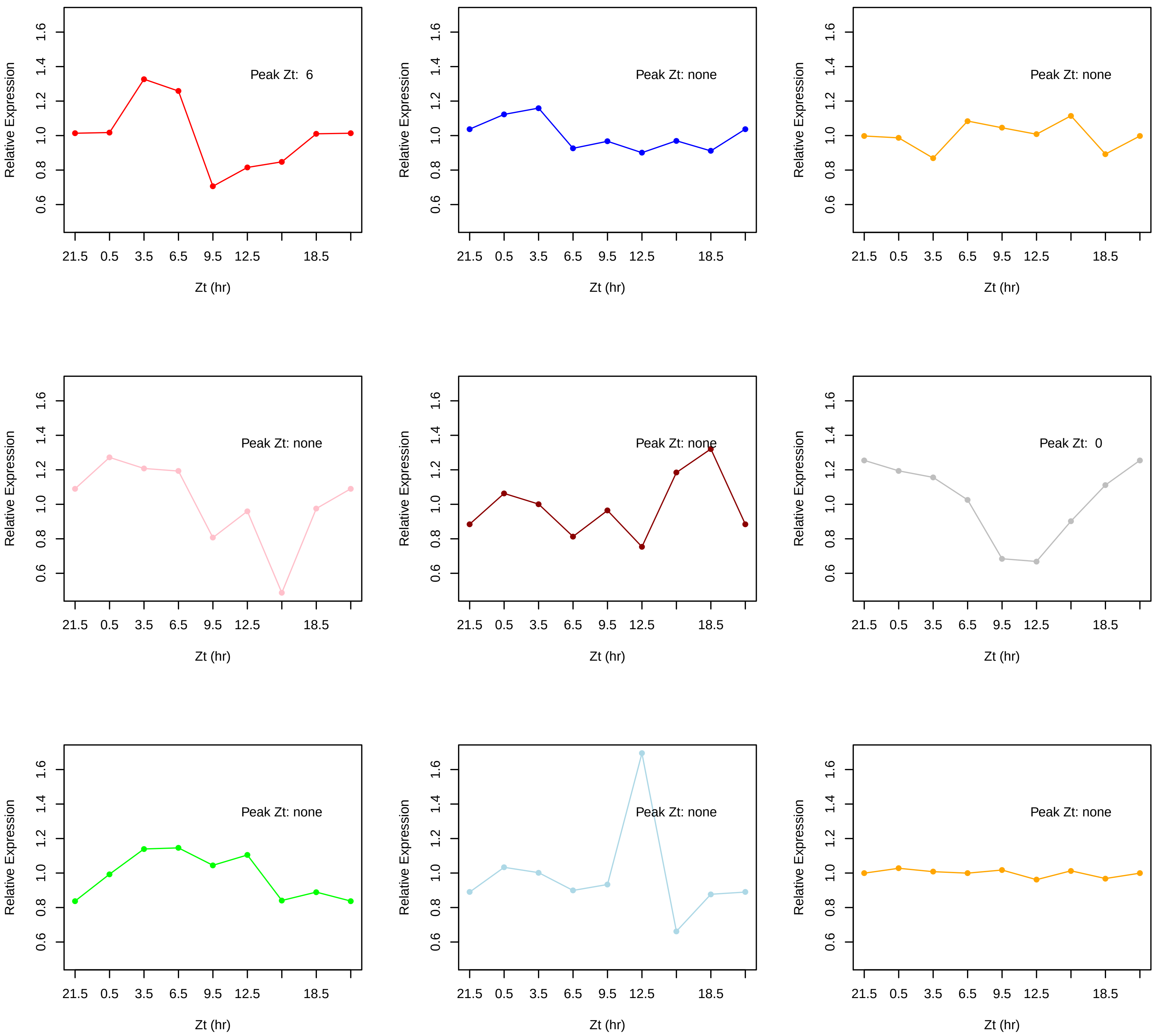

Singeleton: ENSXMAG00000029183 batf3

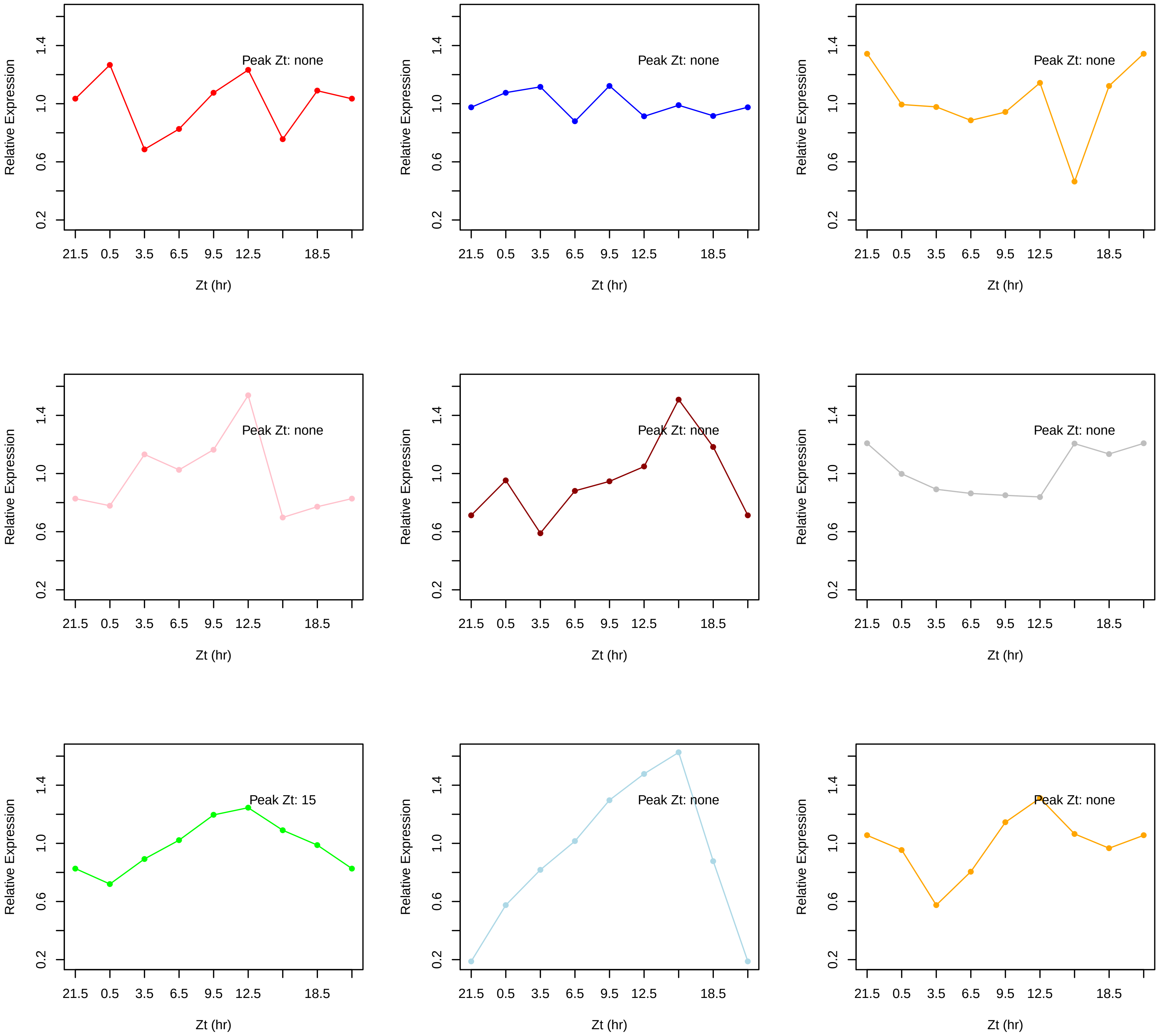

Singleton: ENSXMAG00000014035 usp25

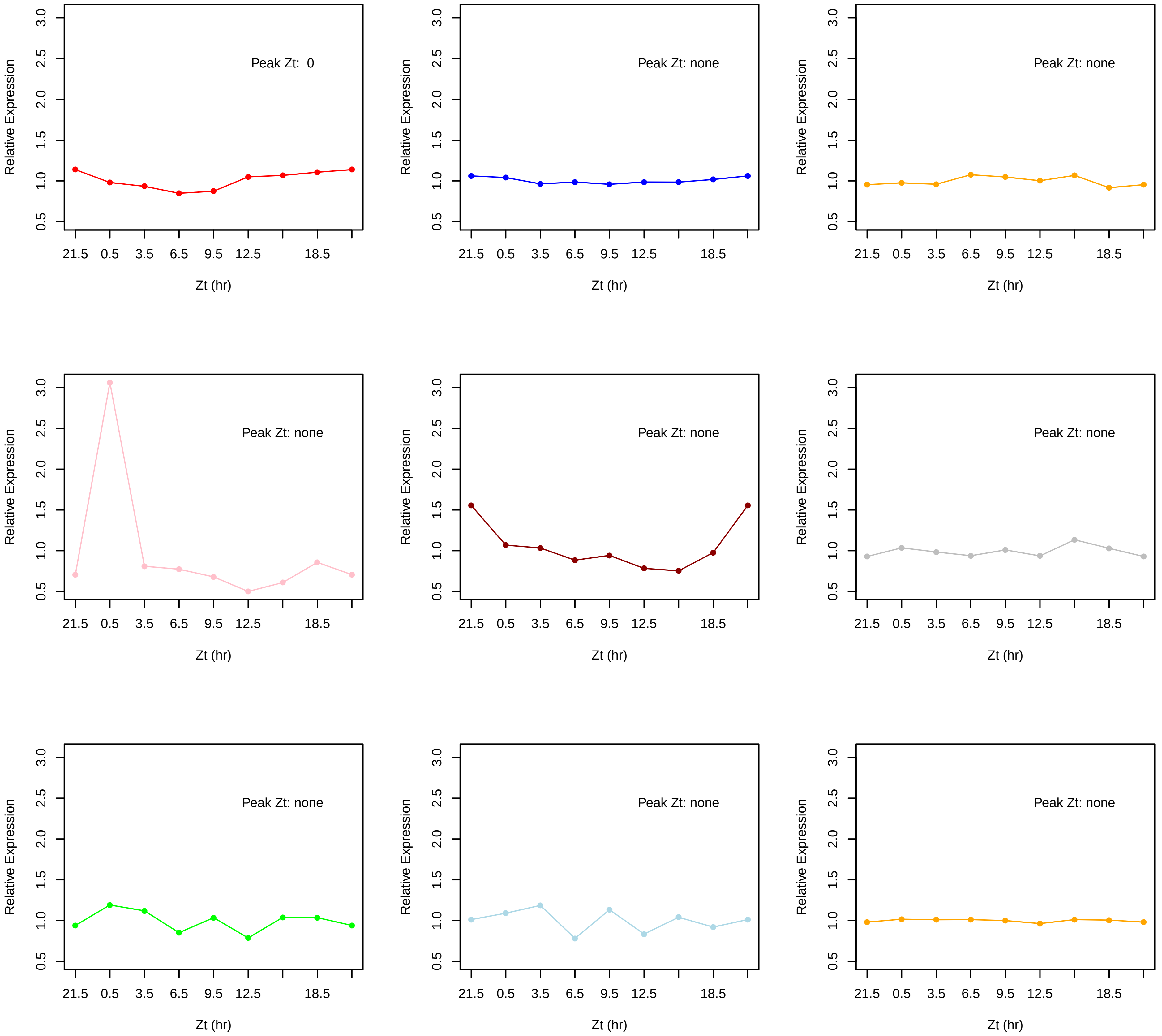

Singleton: ENSXMAG00000003413 xkr5a

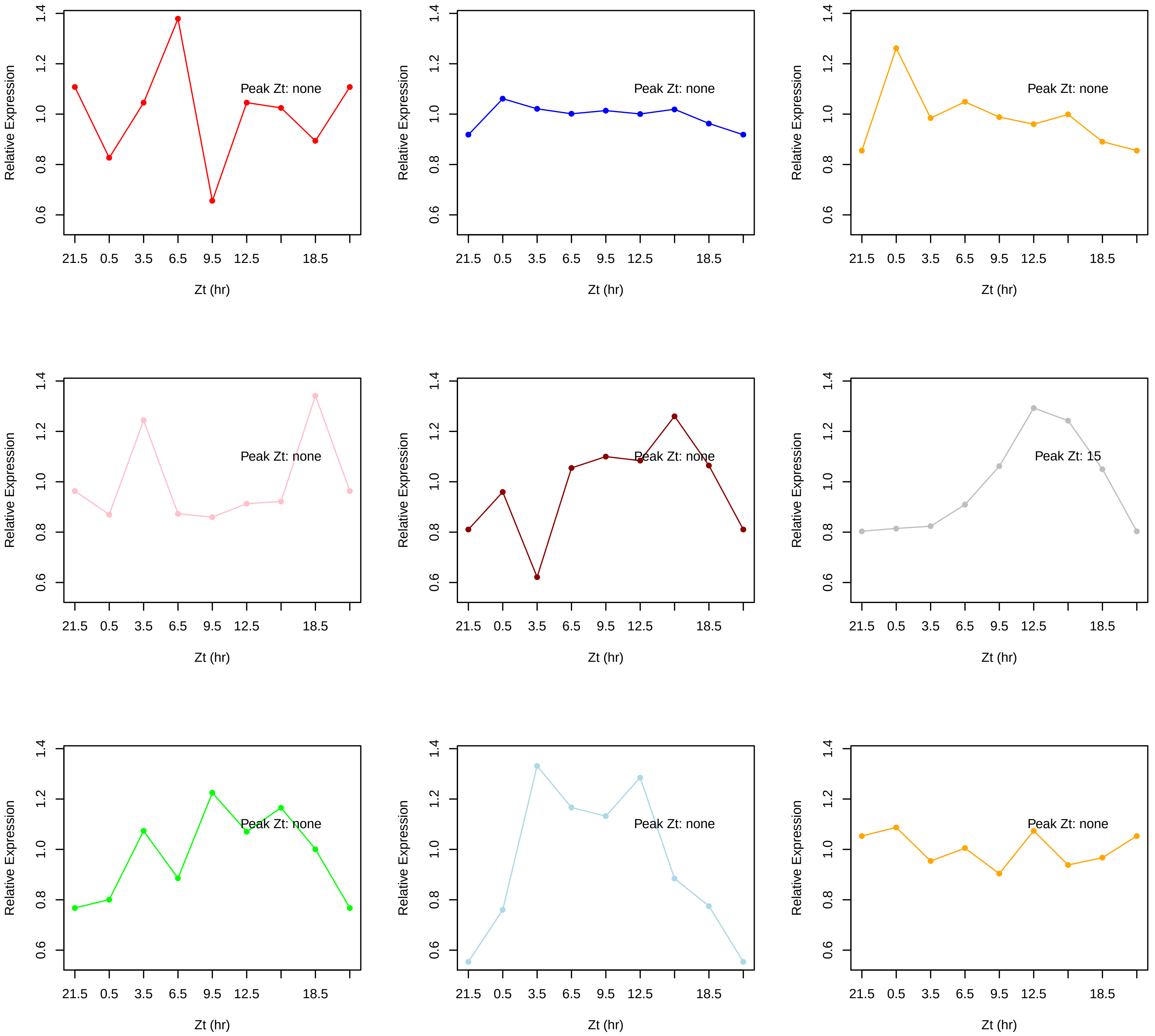

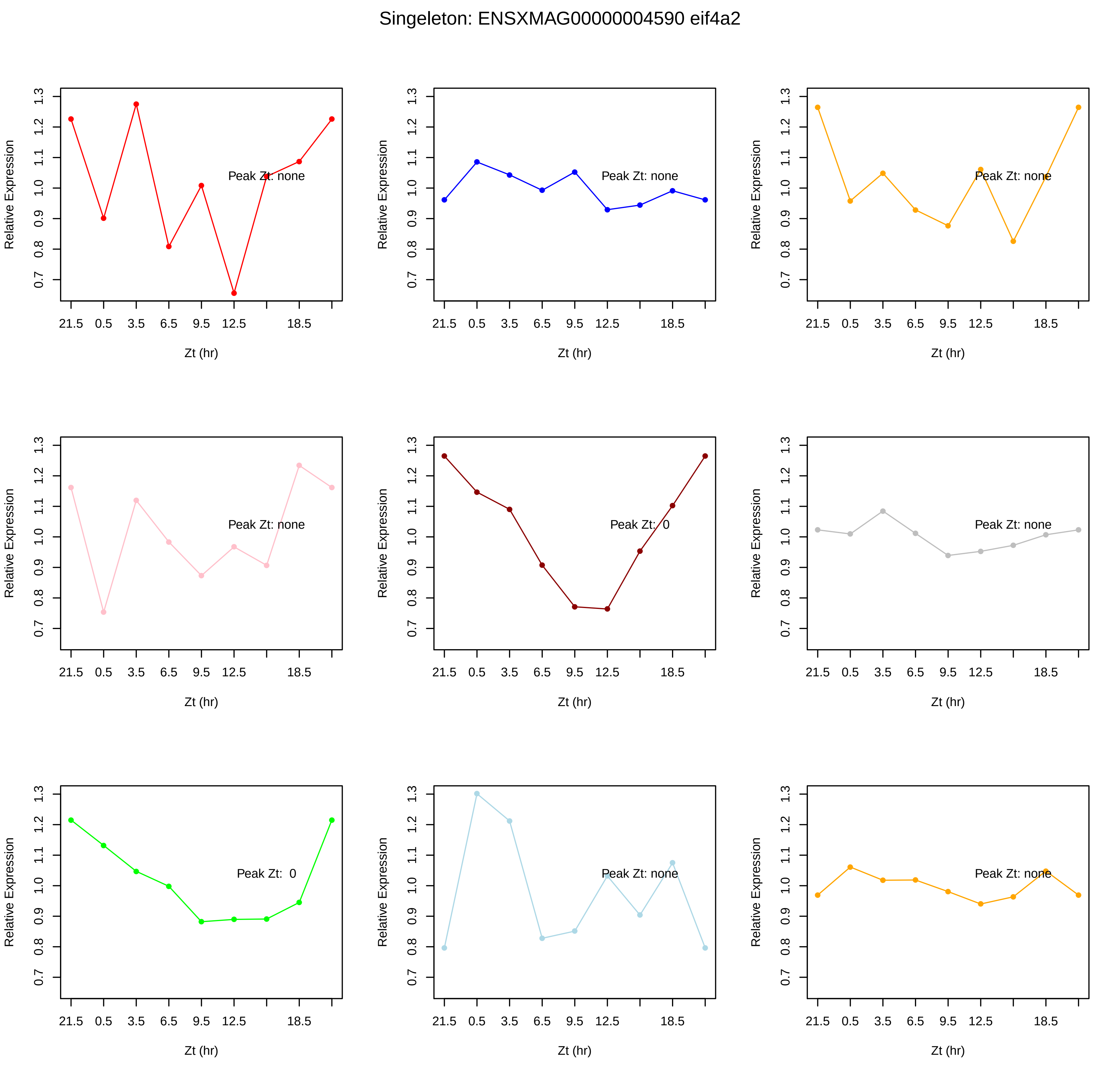
Singleton: ENSXMAG00000004590 eif4a2
Relative Expression
Zt (hr)
21.5
0.5
3.5
6.5
9.5
12.5
18.5
0.7
0.8
0.9
1.0
1.1
1.2
1.3
Peak Zt: none
Peak Zt: none
Peak Zt: none
Peak Zt: none
Peak Zt: 0
Peak Zt: none
Peak Zt: 0
Peak Zt: none
Peak Zt: none

Singleton: ENSXMAG00000022745

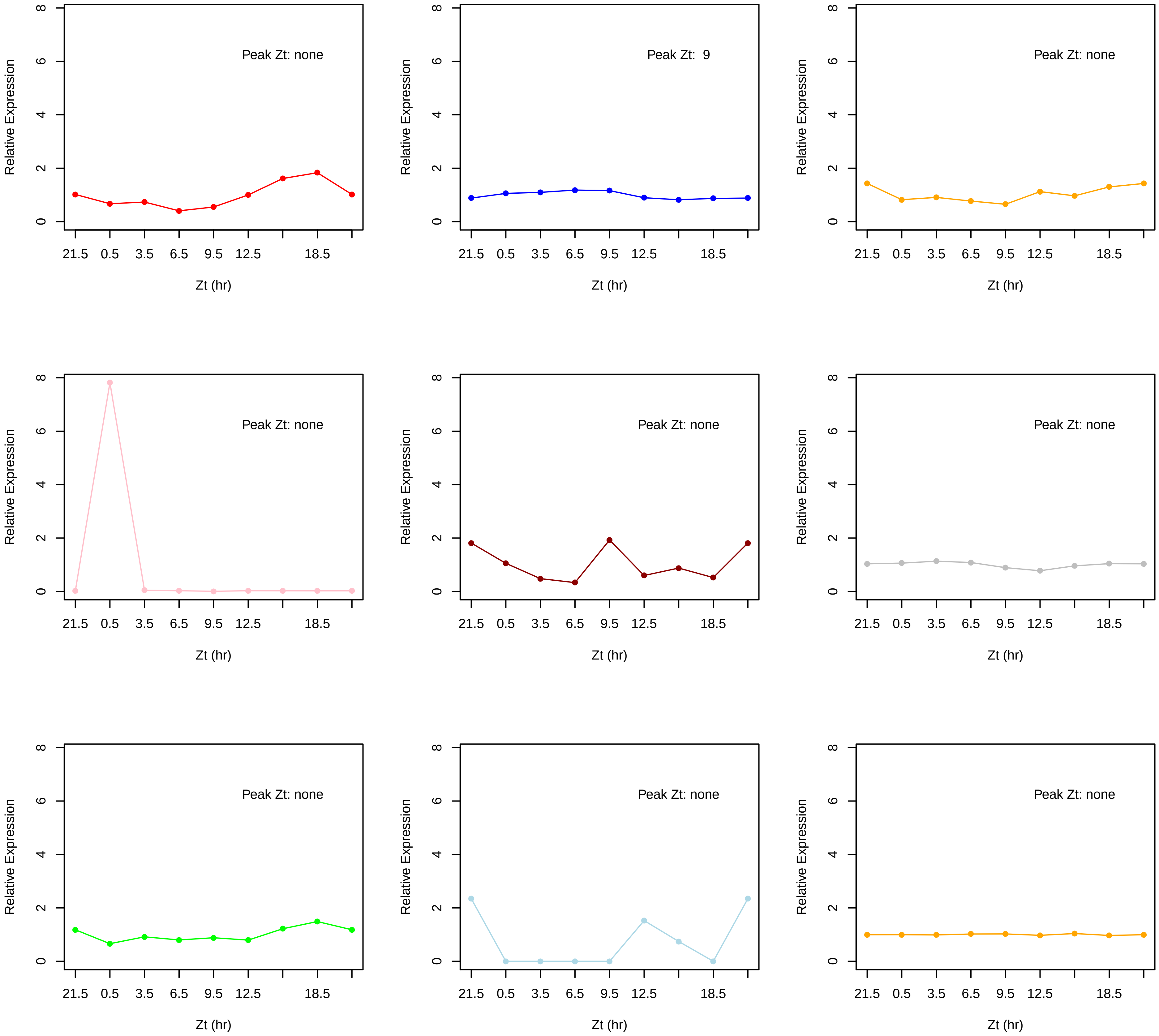

Singeleton: ENSXMAG00000026052 thumpd1

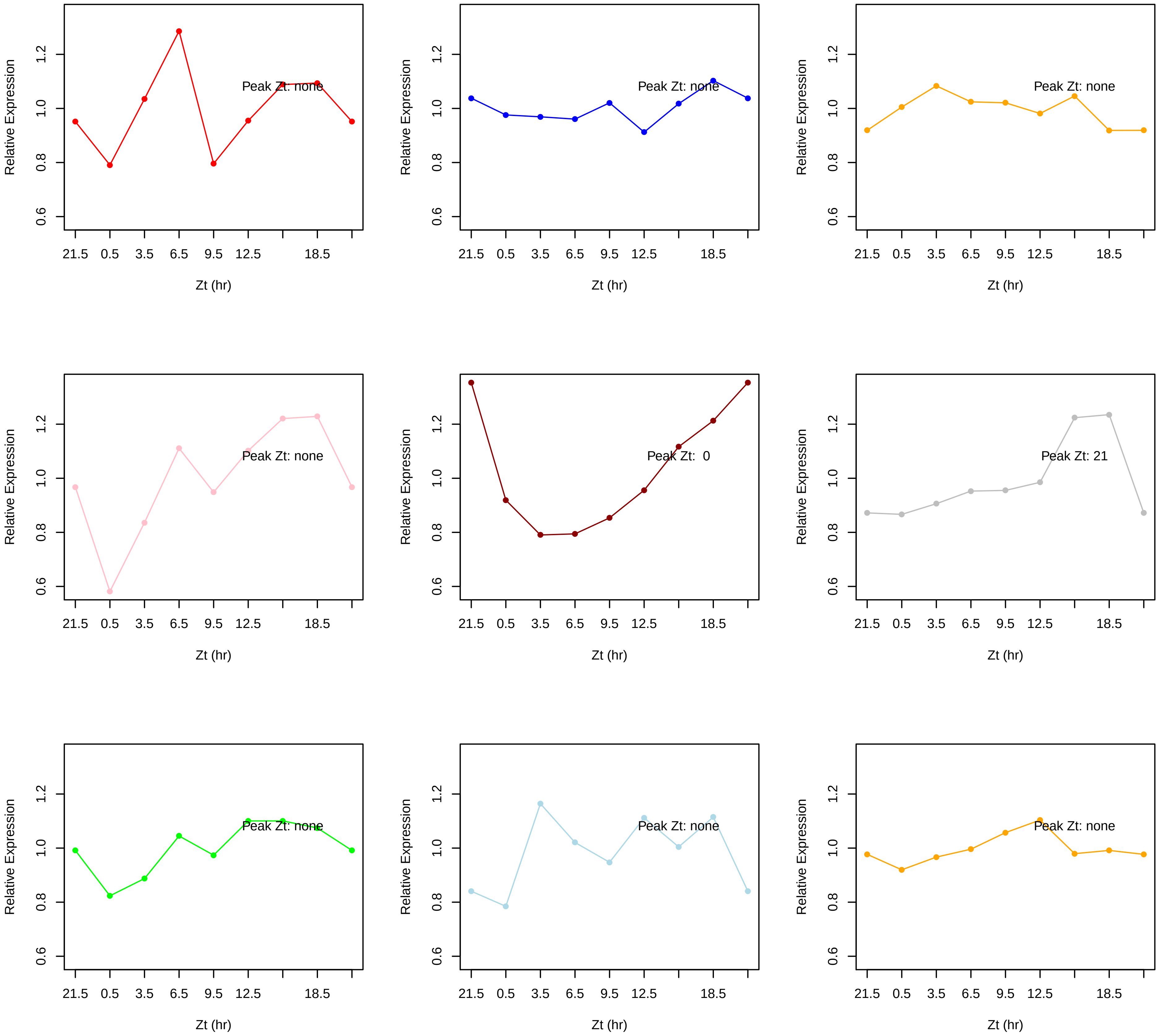

Singeleton: ENSXMAG00000023031 bpgm

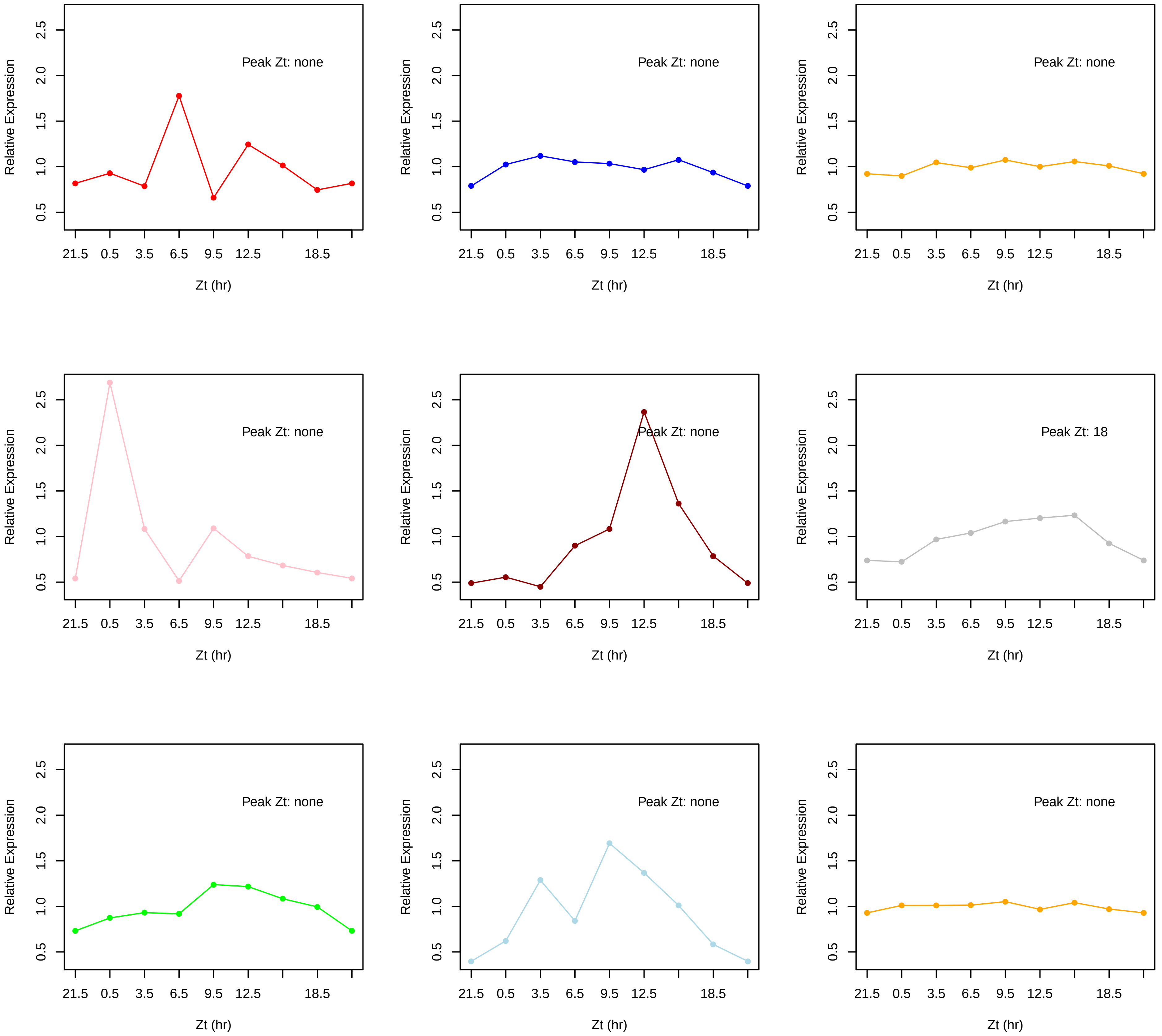

Singleton: ENSXMAG00000026738 COQ4

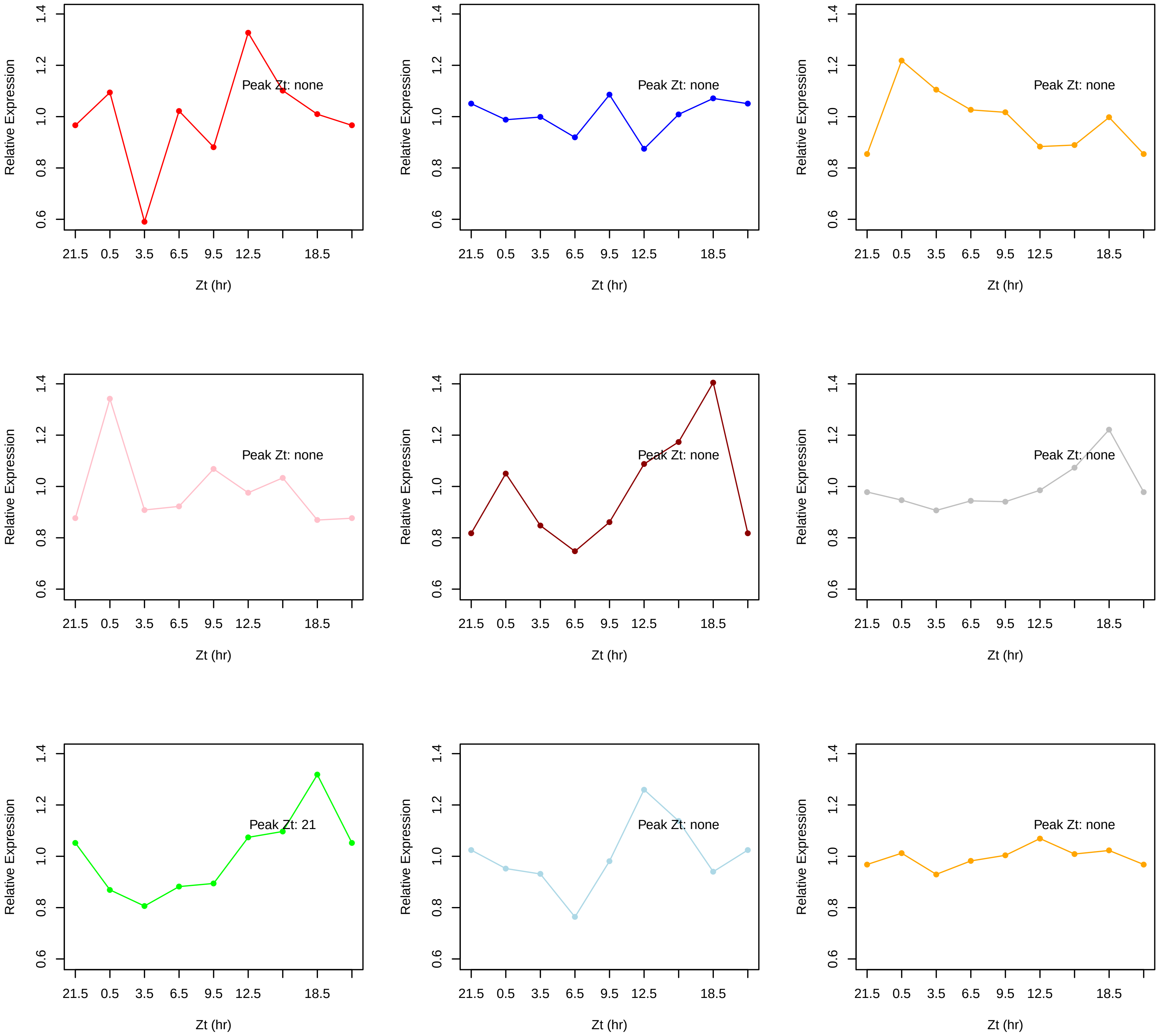

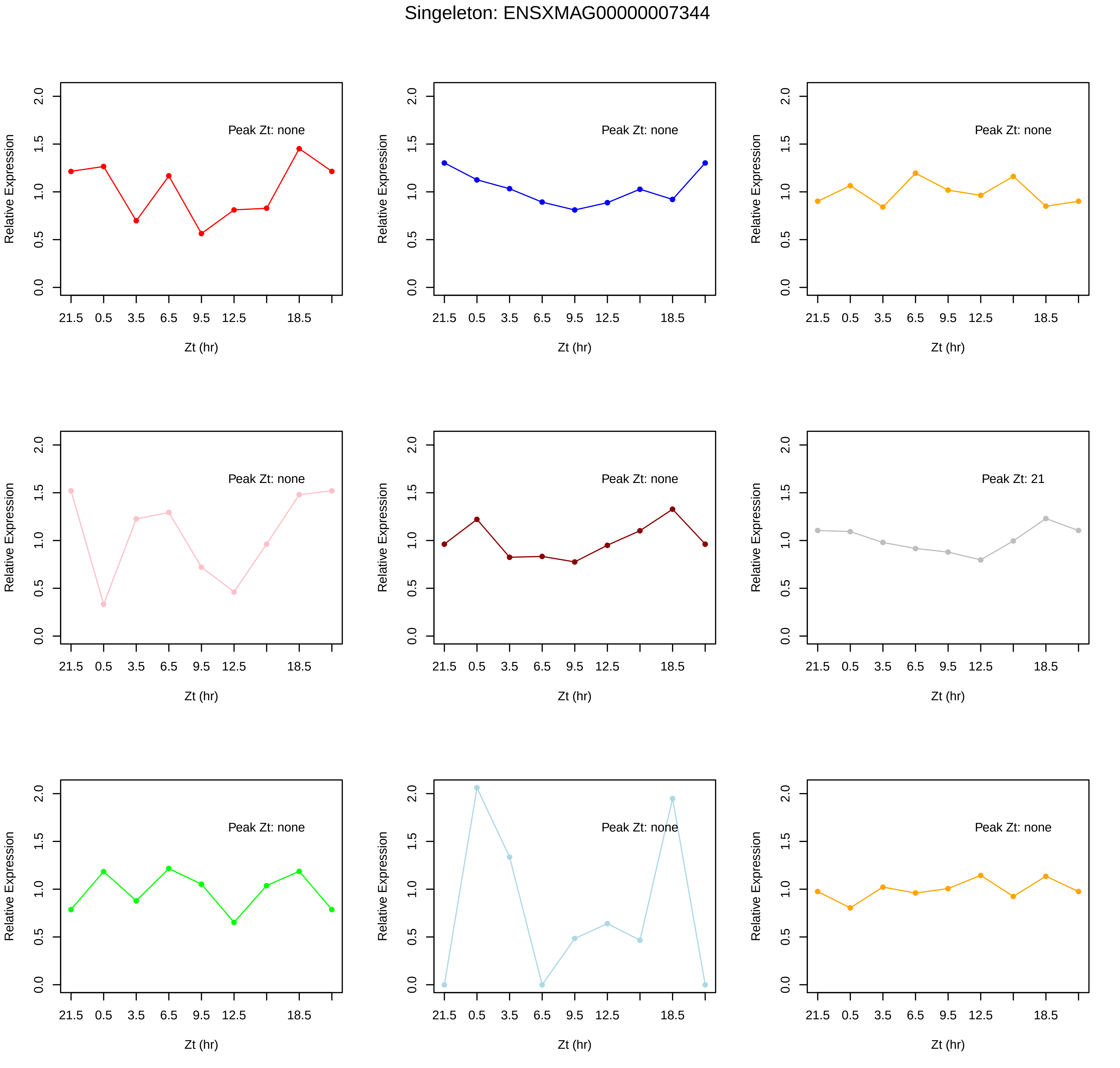
Singeleton: ENSXMAG00000007344
Peak Zt: none
Peak Zt: none
Peak Zt: none
Peak Zt: none
Peak Zt: none
Peak Zt: 21
Peak Zt: none
Peak Zt: none
Peak Zt: none
Relative Expression
Zt (hr)
21.5
0.5
3.5
6.5
9.5
12.5
18.5
0.0
0.5
1.0
1.5
2.0

Singleton: ENSXMAG00000003399 tram2

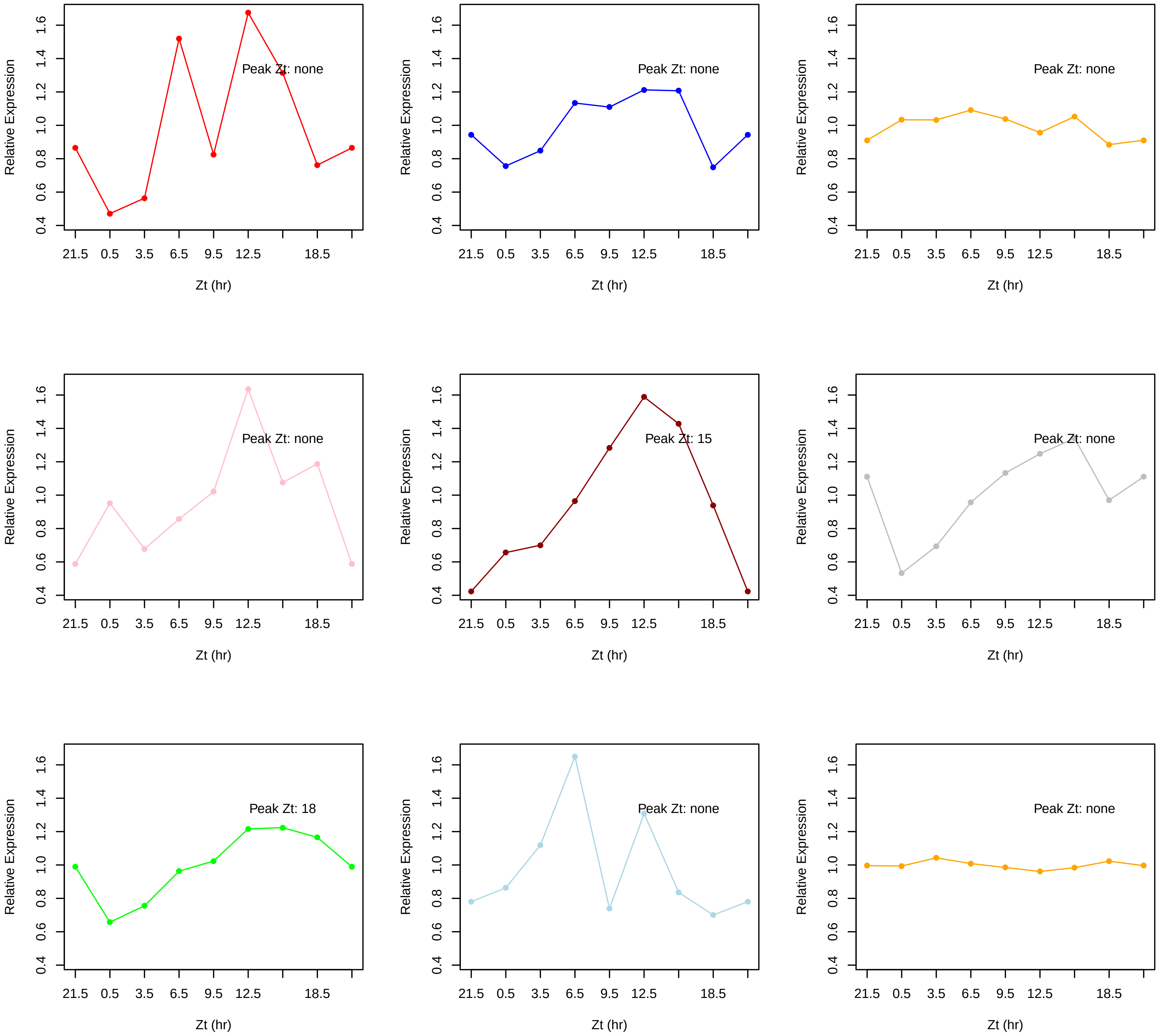

Singeleton: ENSXMAG00000014333 eif4h

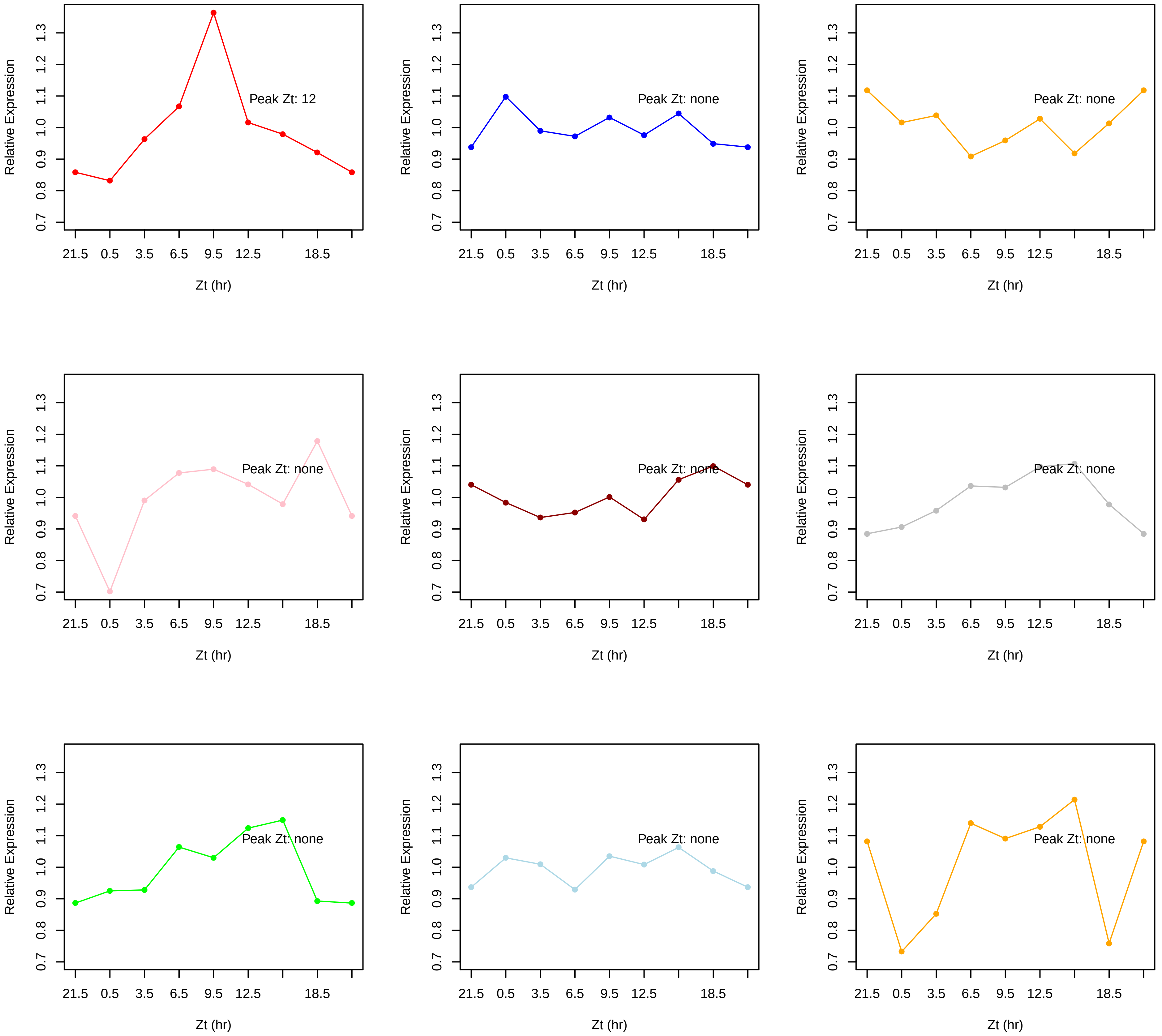

Singeleton: ENSXMAG00000008552 zgc:153372

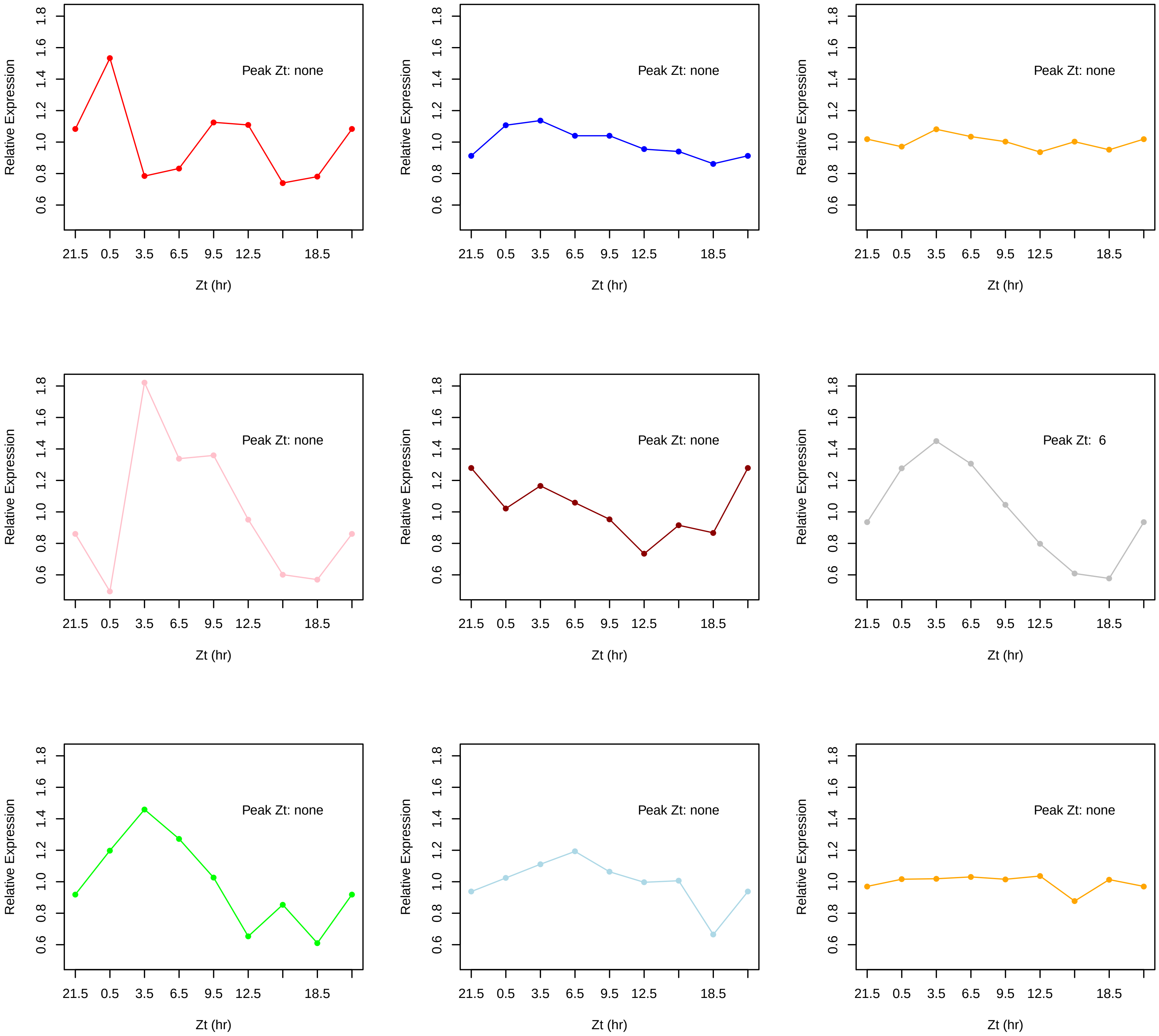

Singeleton: ENSXMAG00000016257 si:ch1073−513e17.1

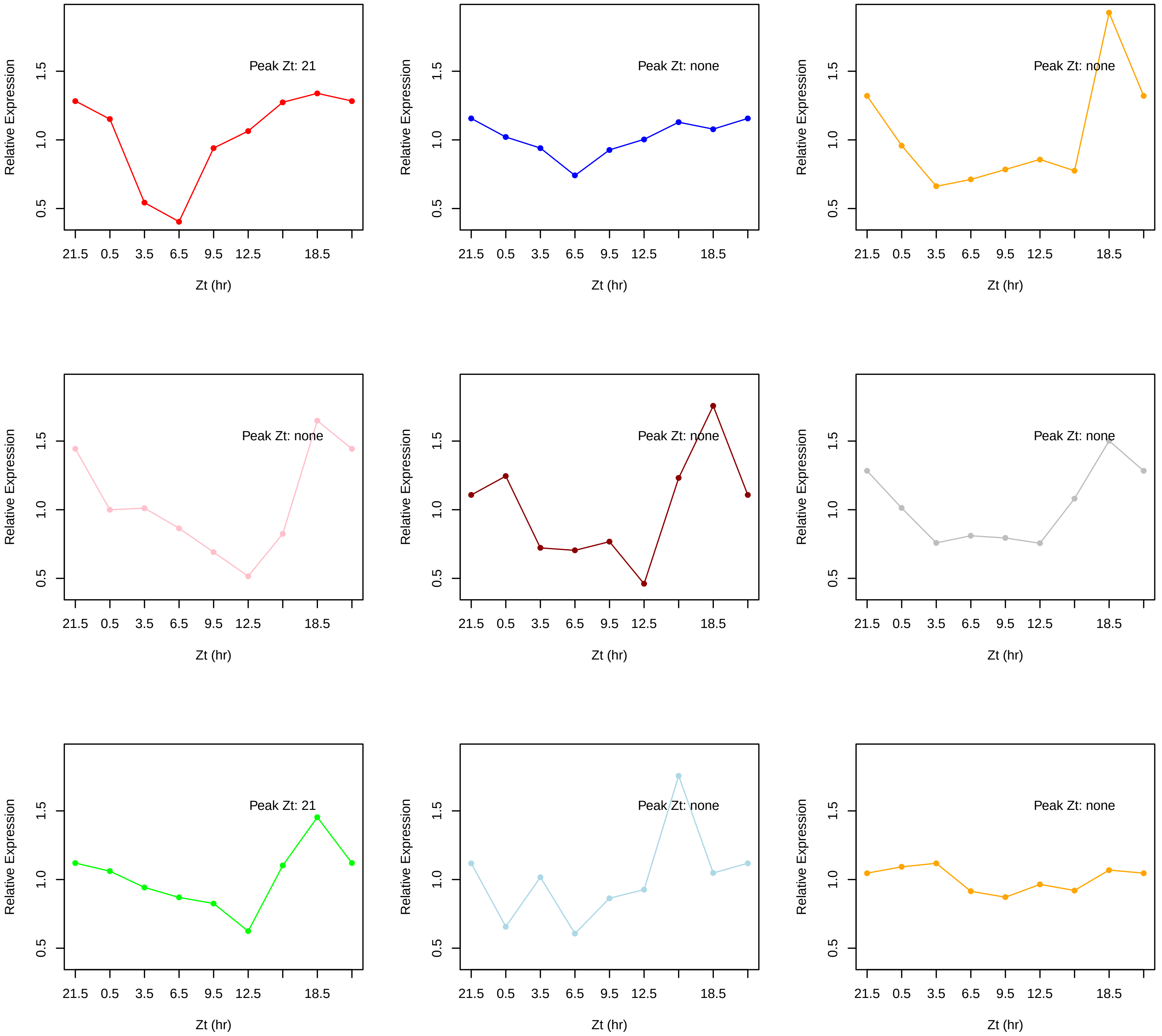

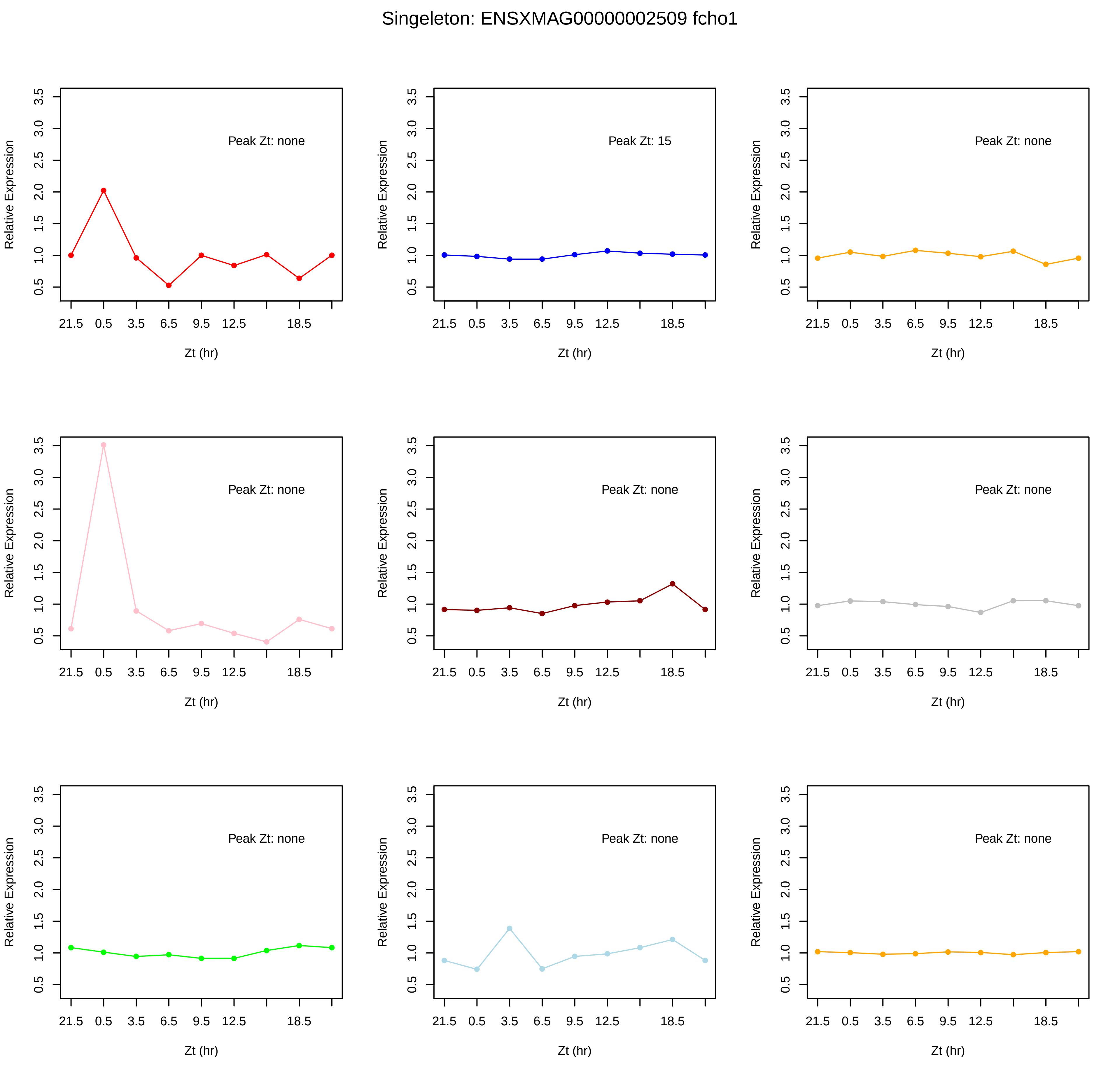
Singeleton: ENSXMAG00000002509 fcho1
Peak Zt: none
Peak Zt: 15
Peak Zt: none
Peak Zt: none
Peak Zt: none
Peak Zt: none
Peak Zt: none
Peak Zt: none
Peak Zt: none
Relative Expression
Zt (hr)
21.5
0.5
3.5
6.5
9.5
12.5
18.5

Singeleton: ENSXMAG00000024616 mcf2l2

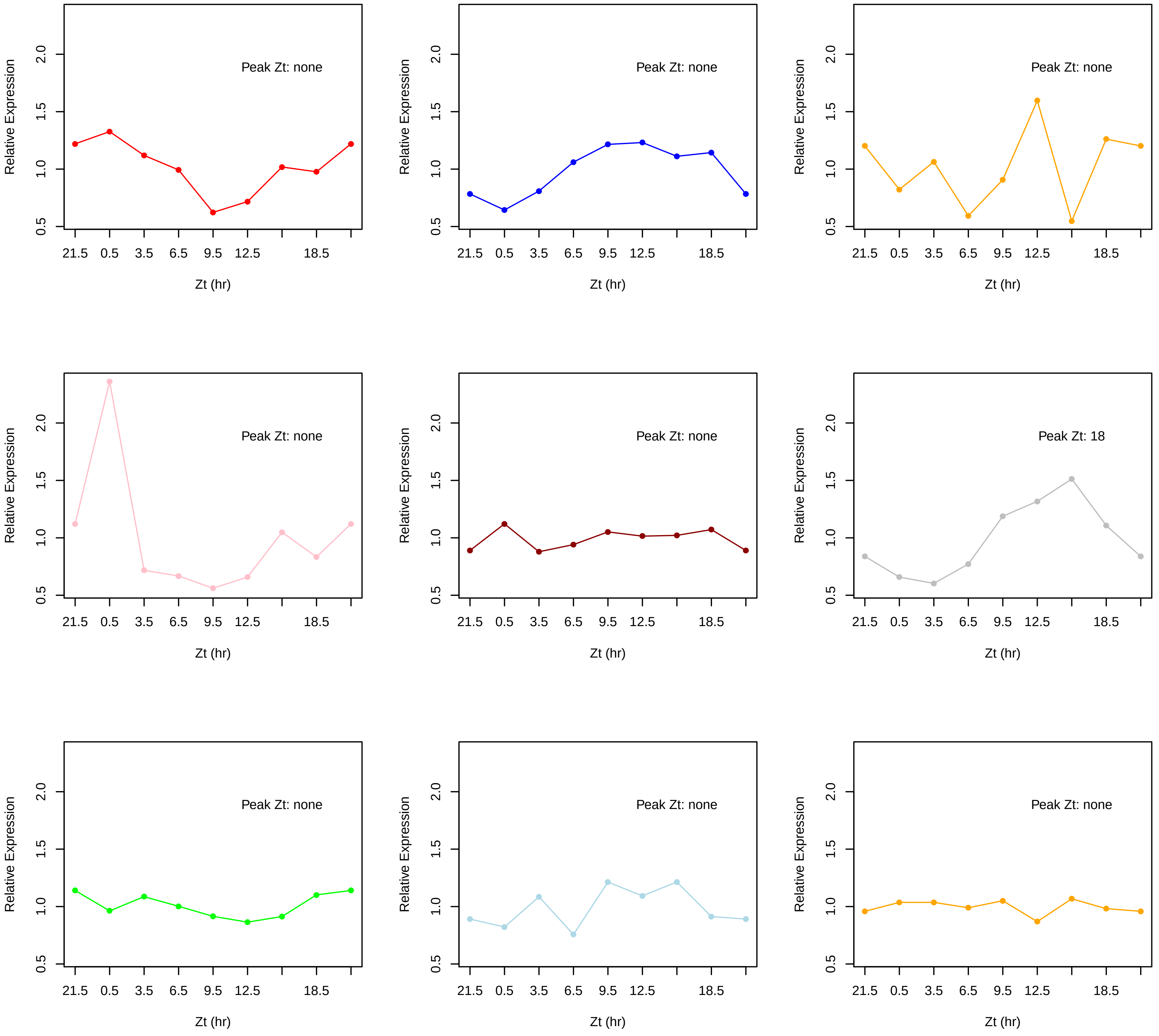

Singleton: ENSXMAG00000003396 efhc1

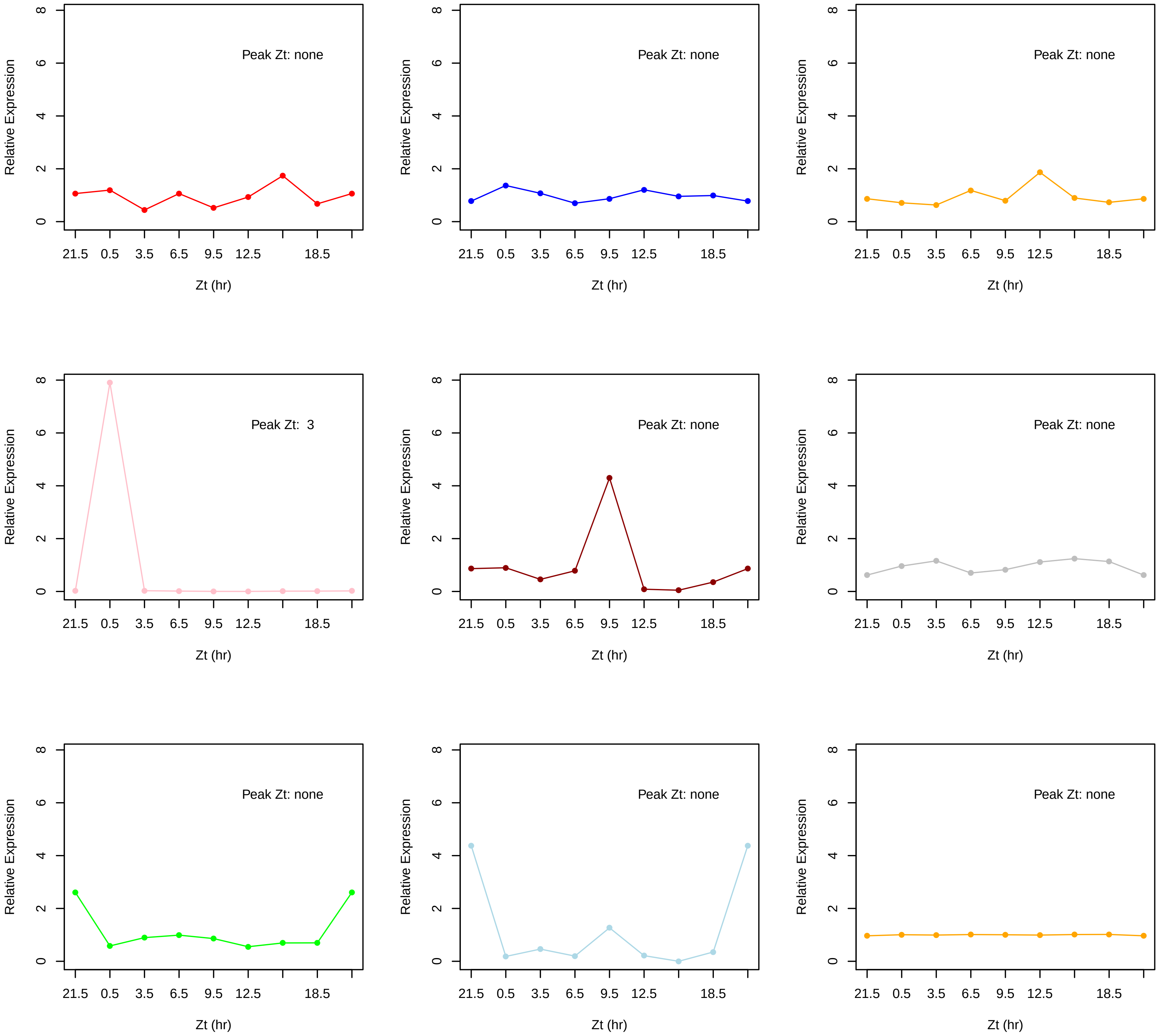

Singeleton: ENSXMAG00000013149 yif1a

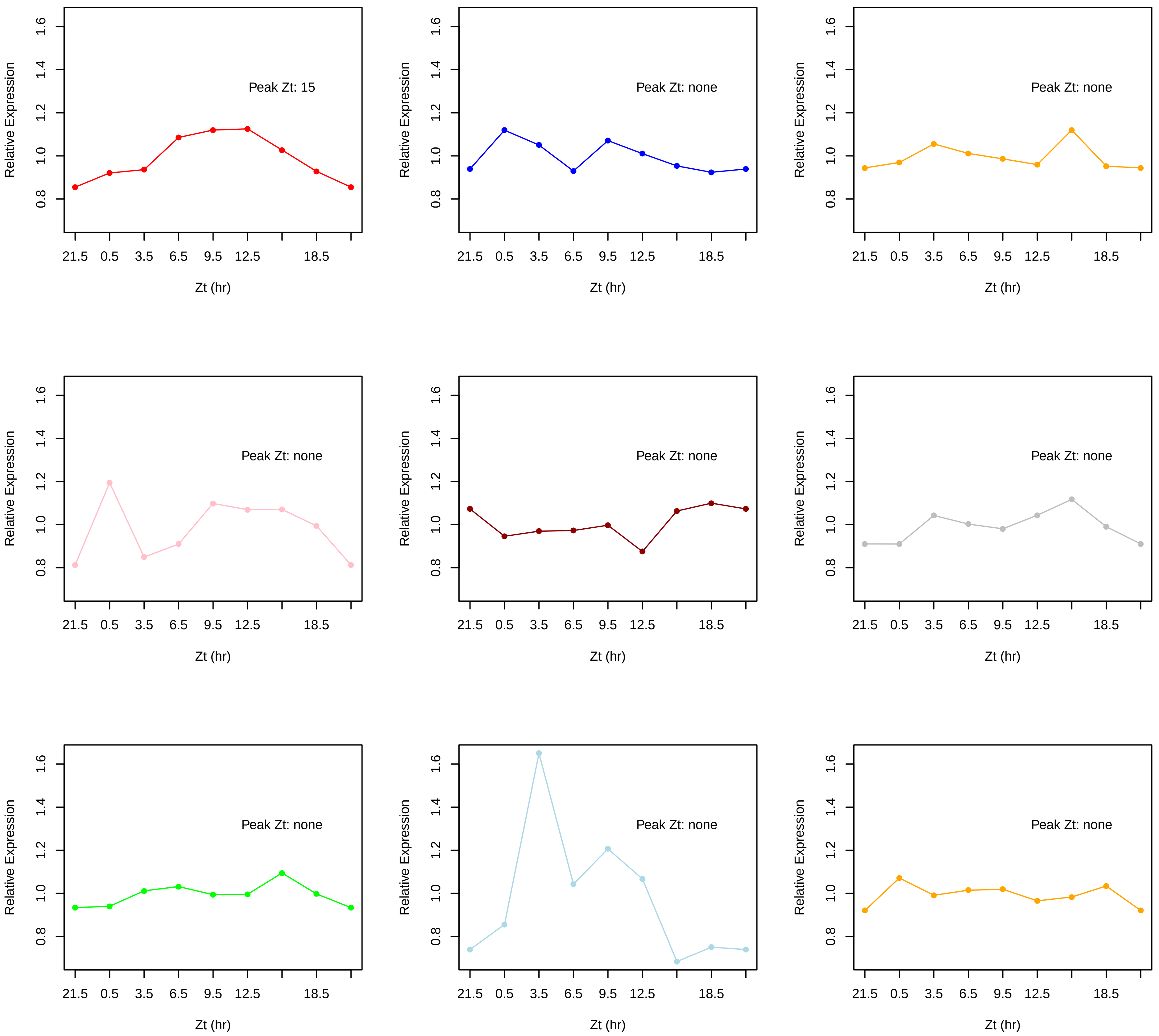

Singleton: ENSXMAG00000005200 vash2

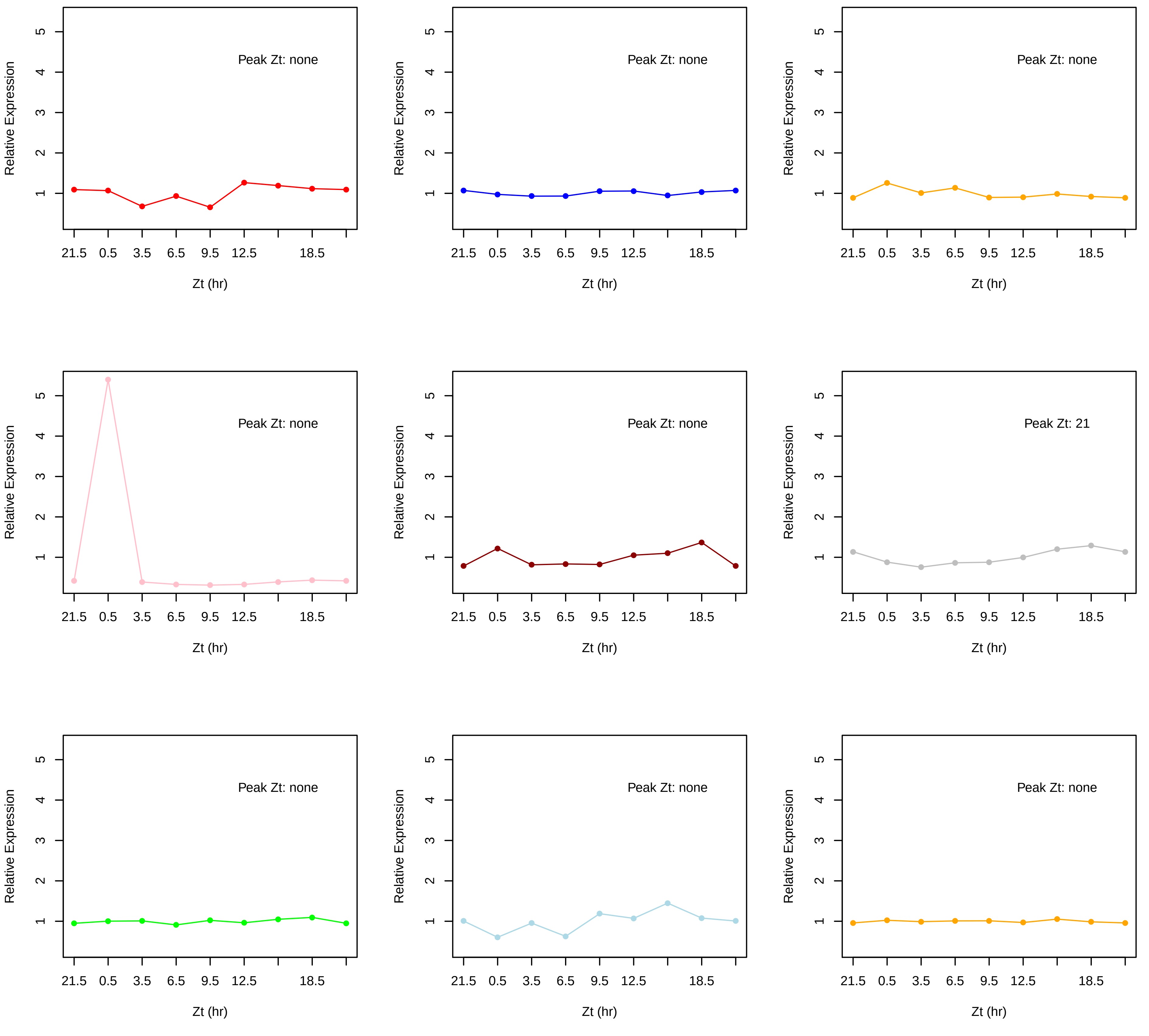

Singleton: ENSXMAG00000005301 ehd1a

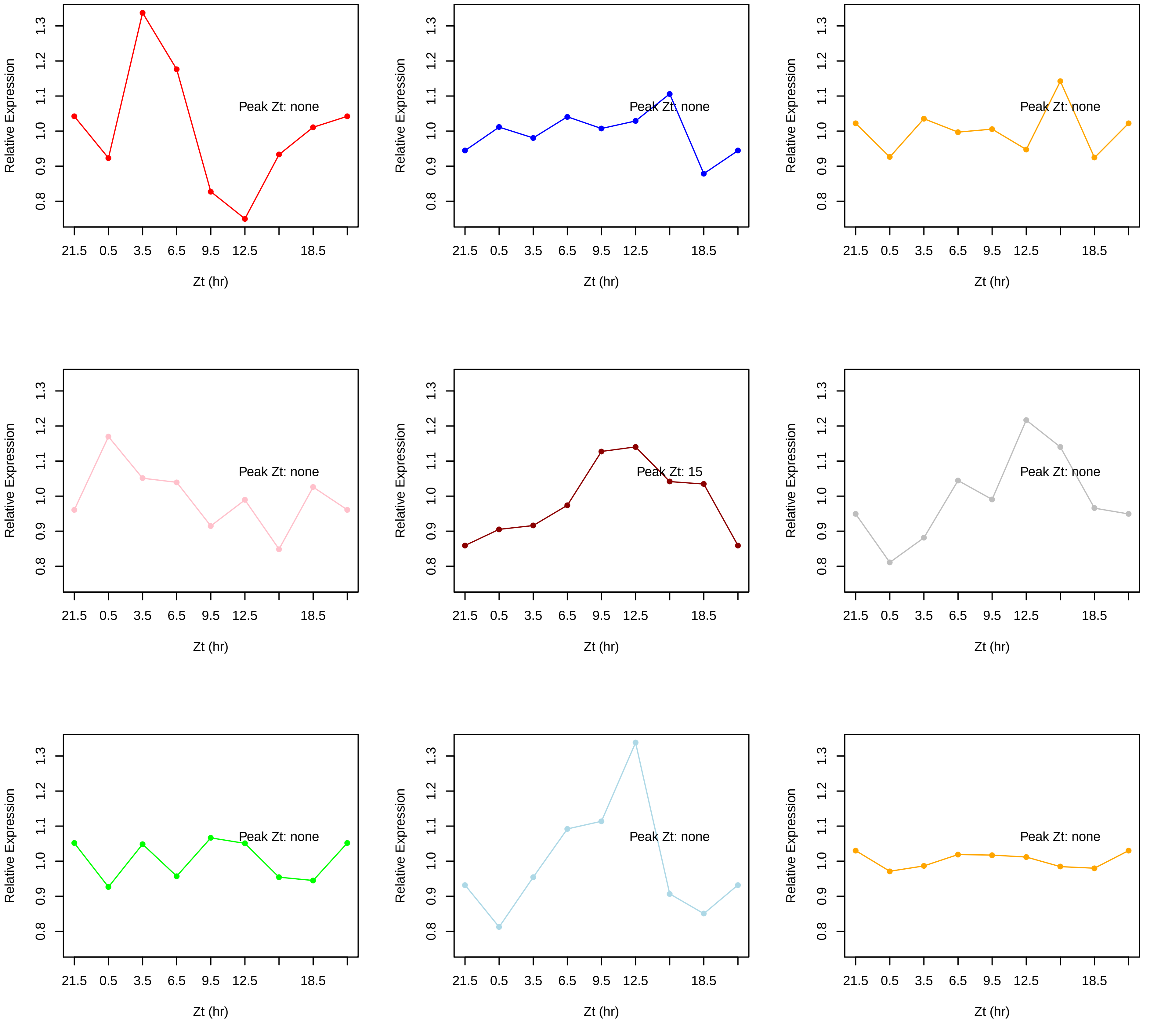

Singleton: ENSXMAG00000016322

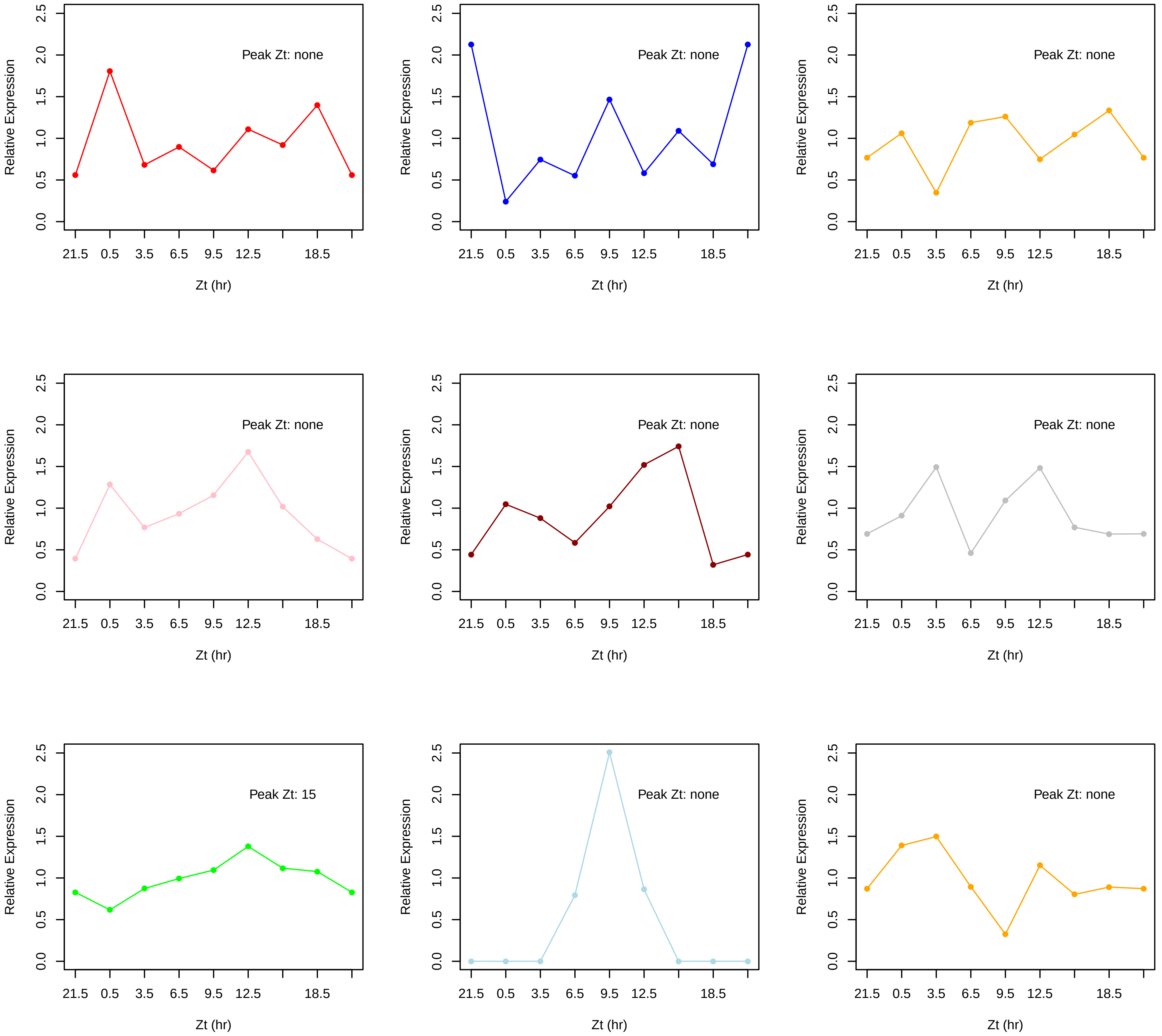

Singeleton: ENSXMAG00000002601 p3h1

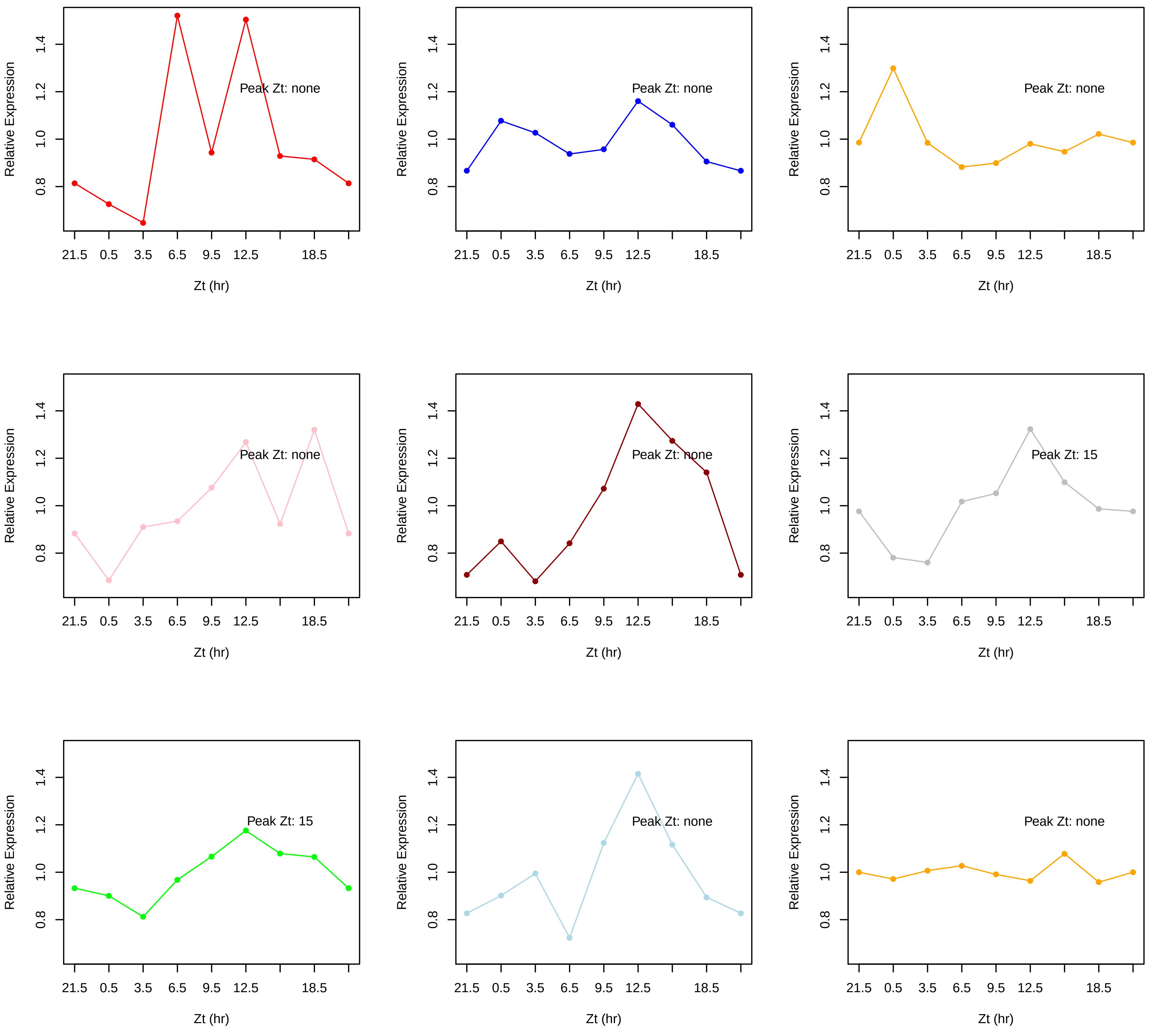

Singeleton: ENSXMAG00000025699 cobl

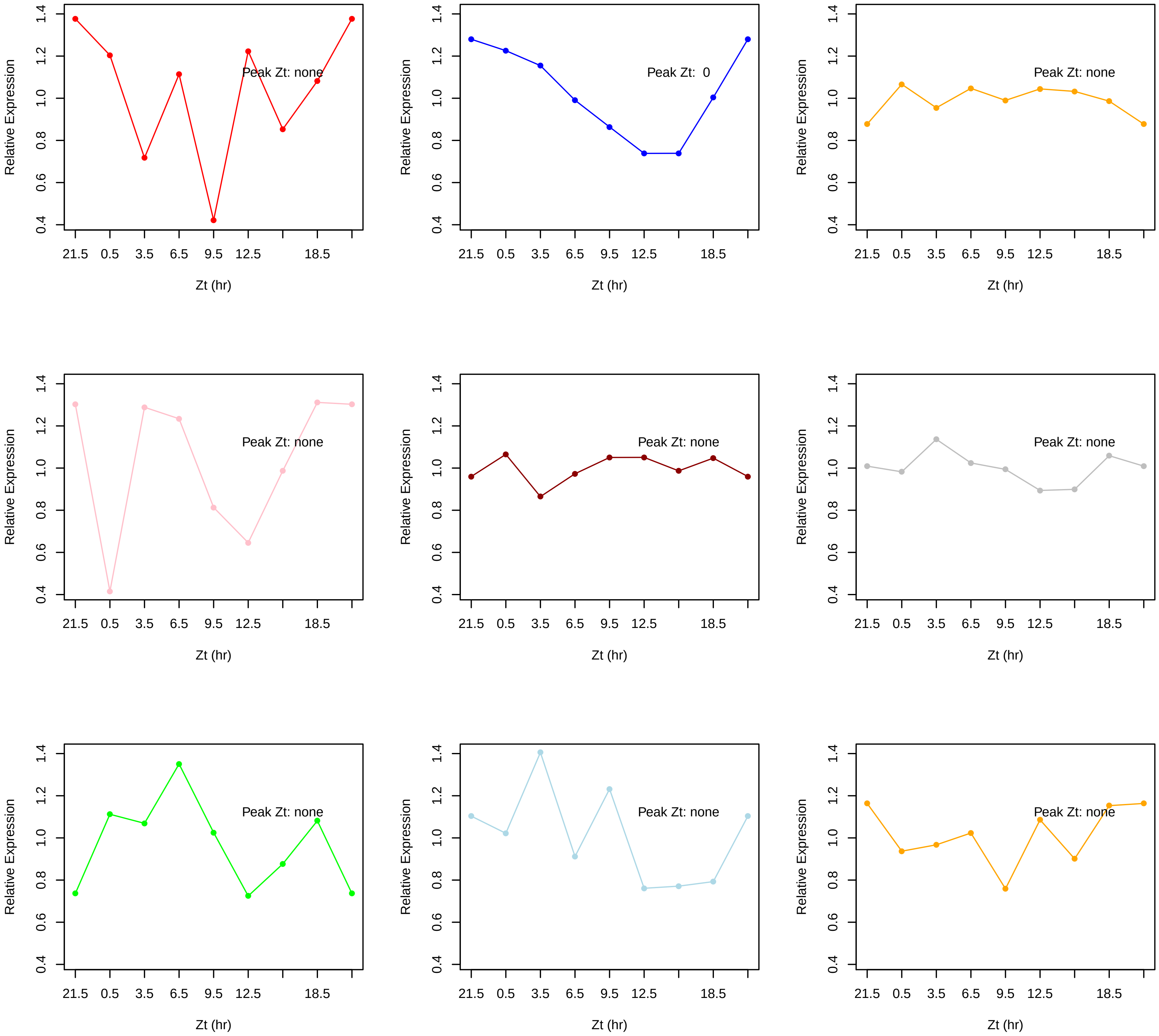

Singeleton: ENSXMAG00000003010 abcb6a

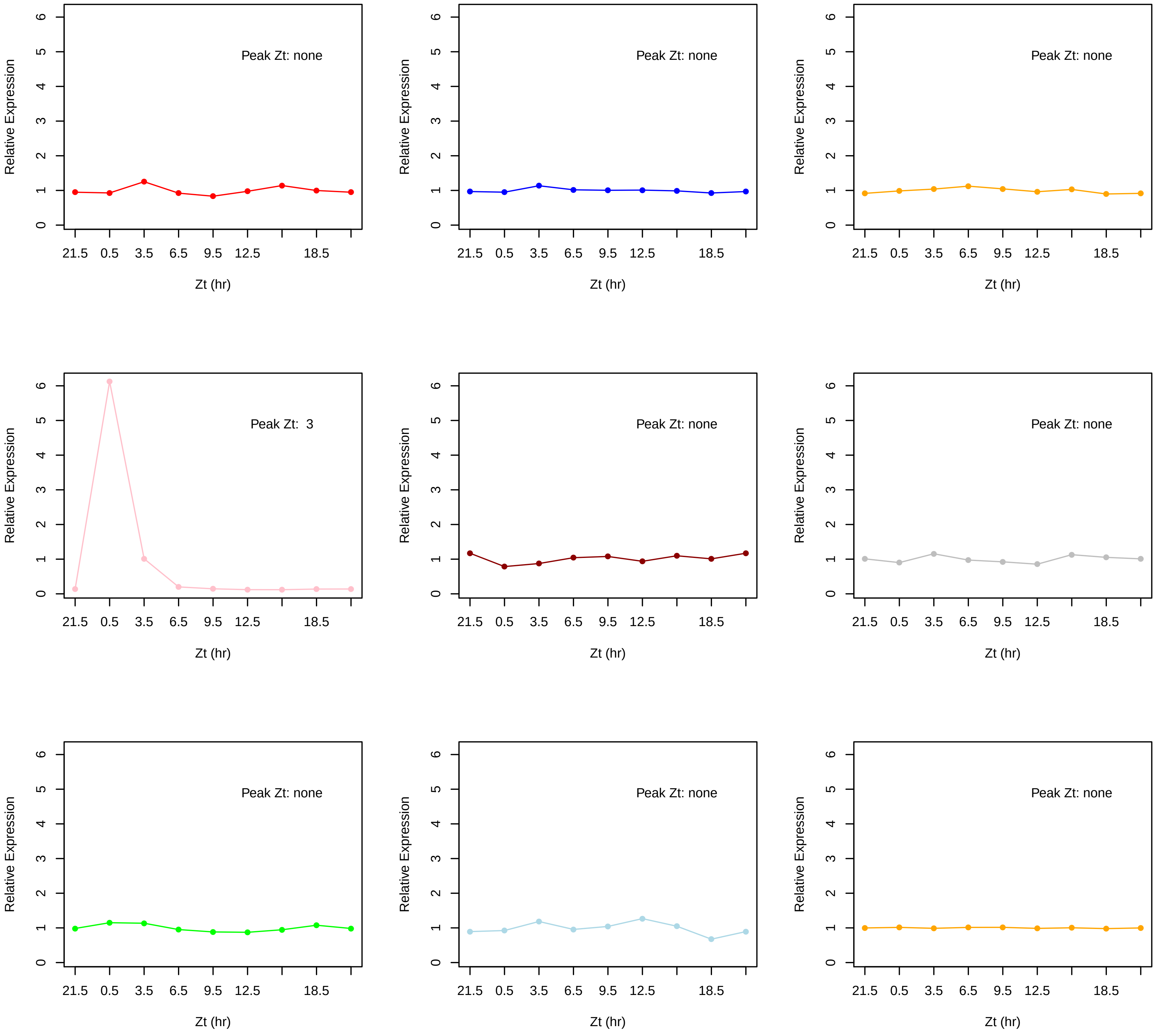

Singeleton: ENSXMAG00000011043 zgc:77880

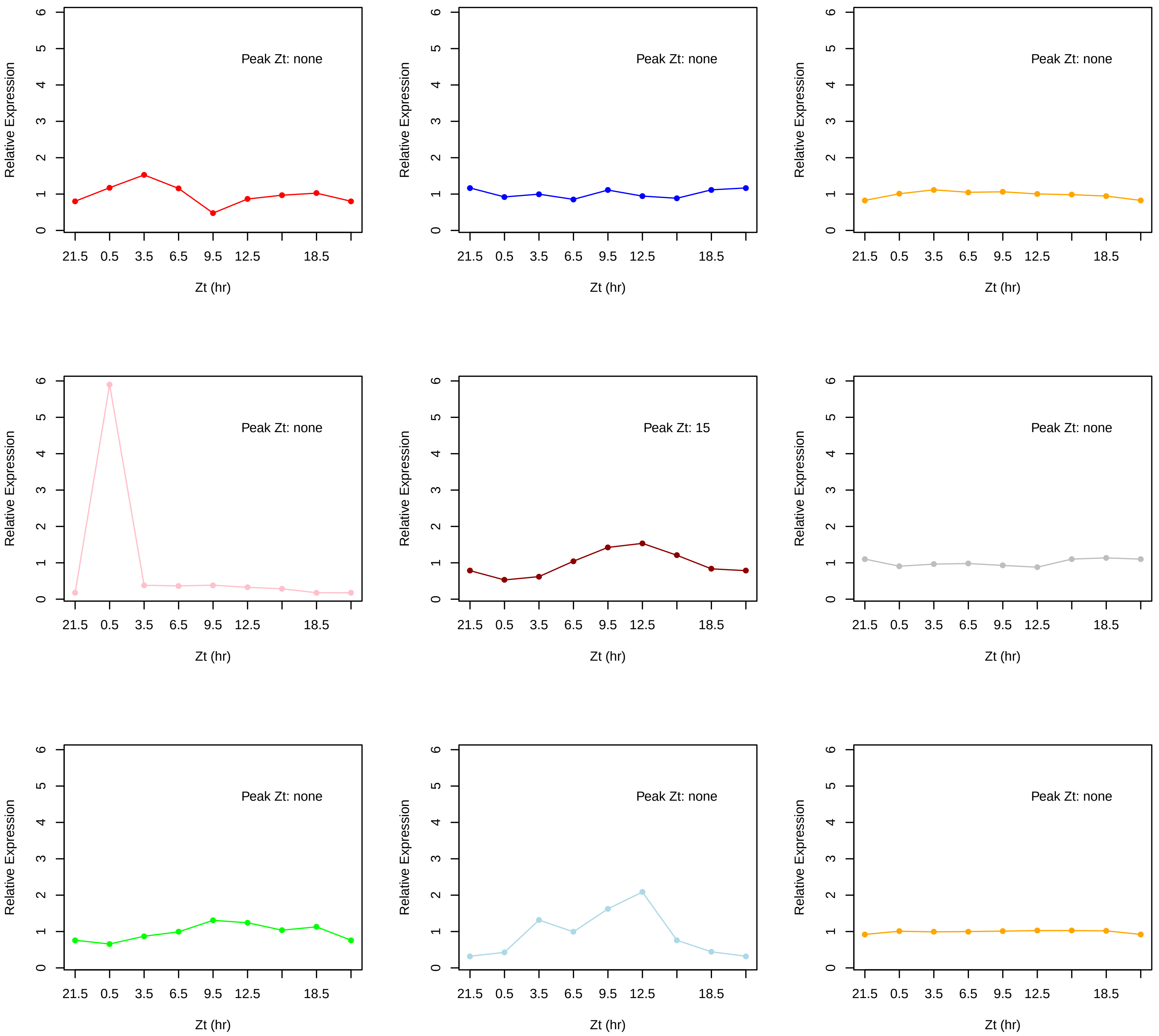

Singleton: ENSXMAG00000002550 acsbg2

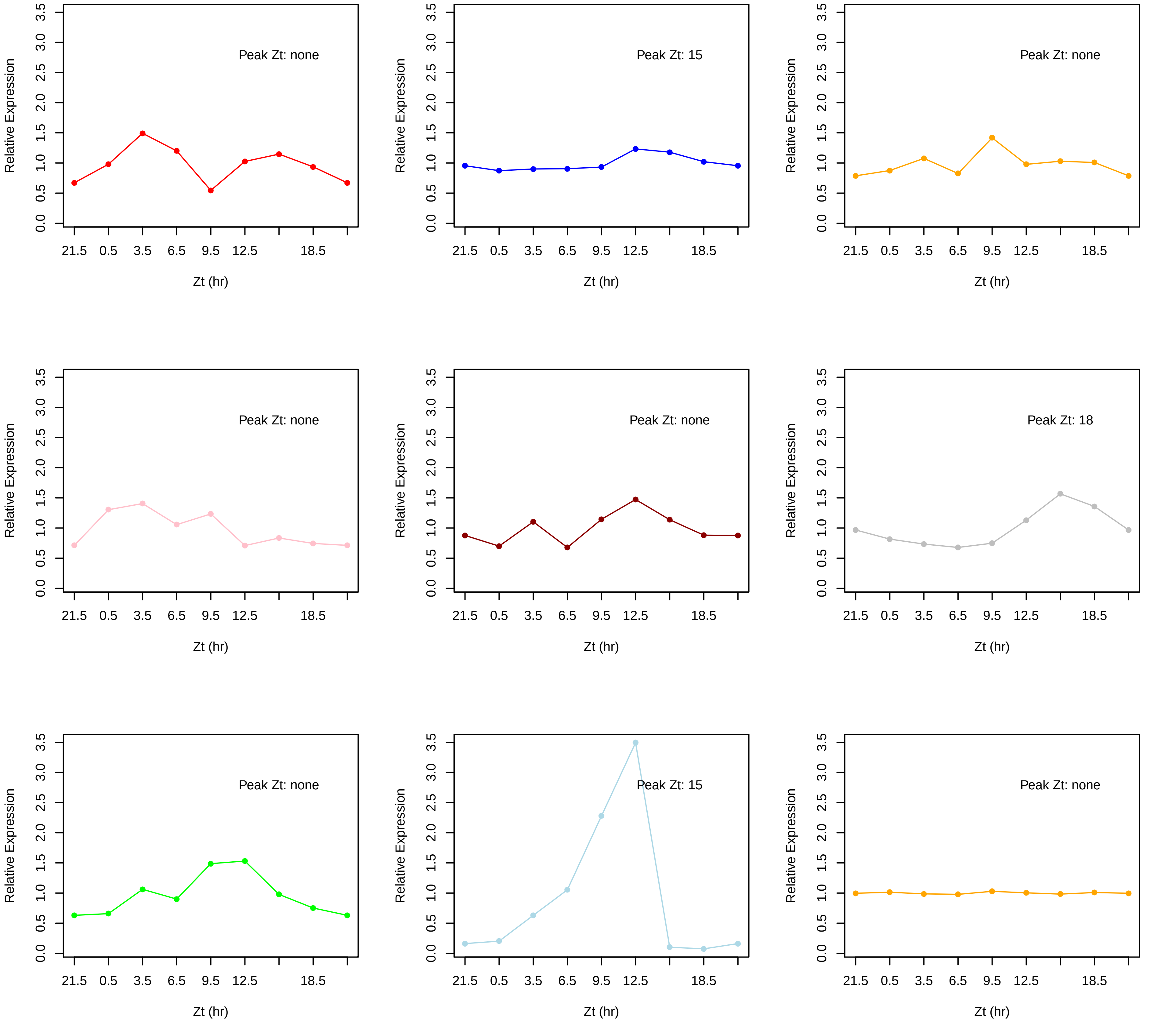

Singeleton: ENSXMAG00000003225 zgc:153018

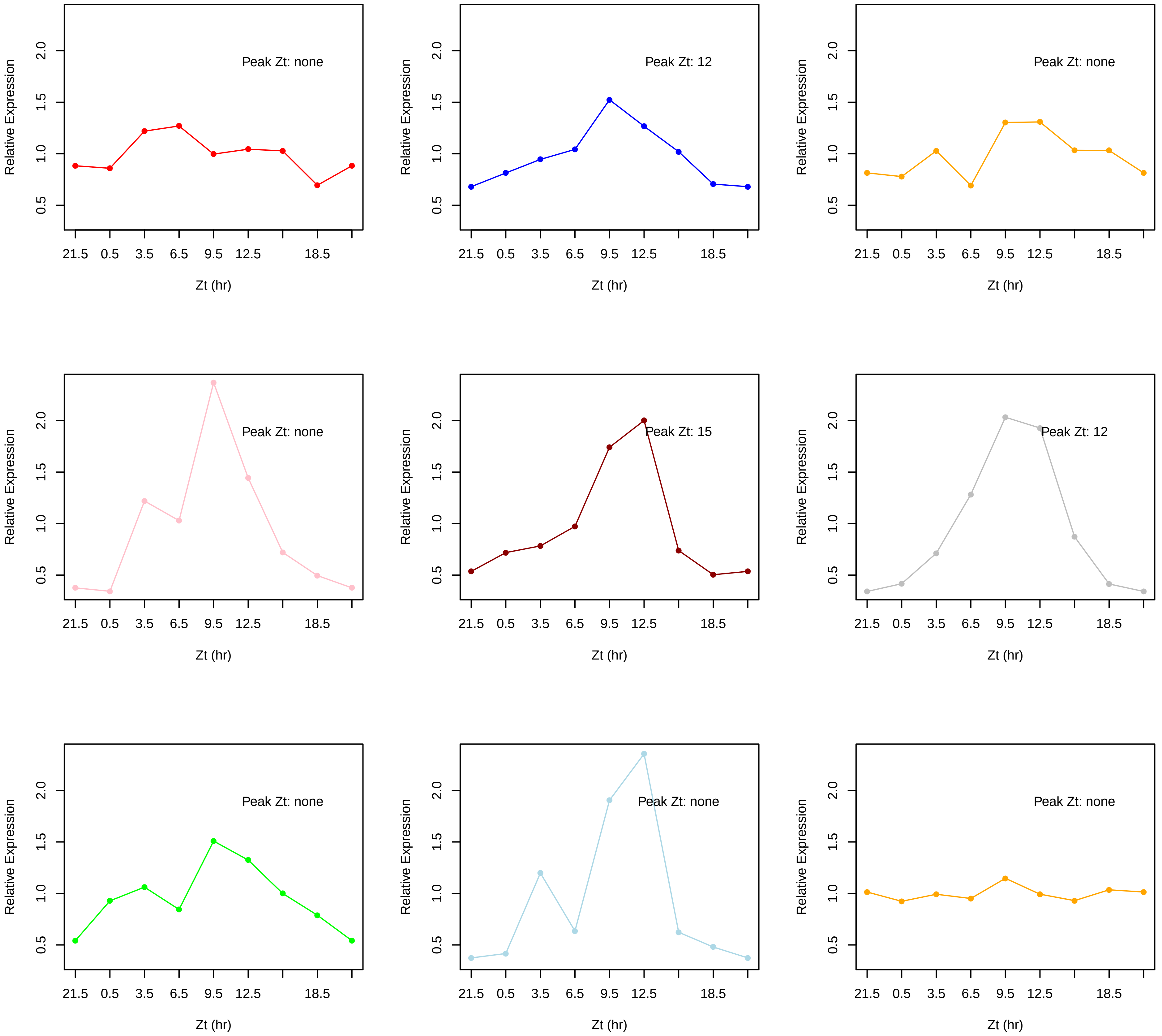

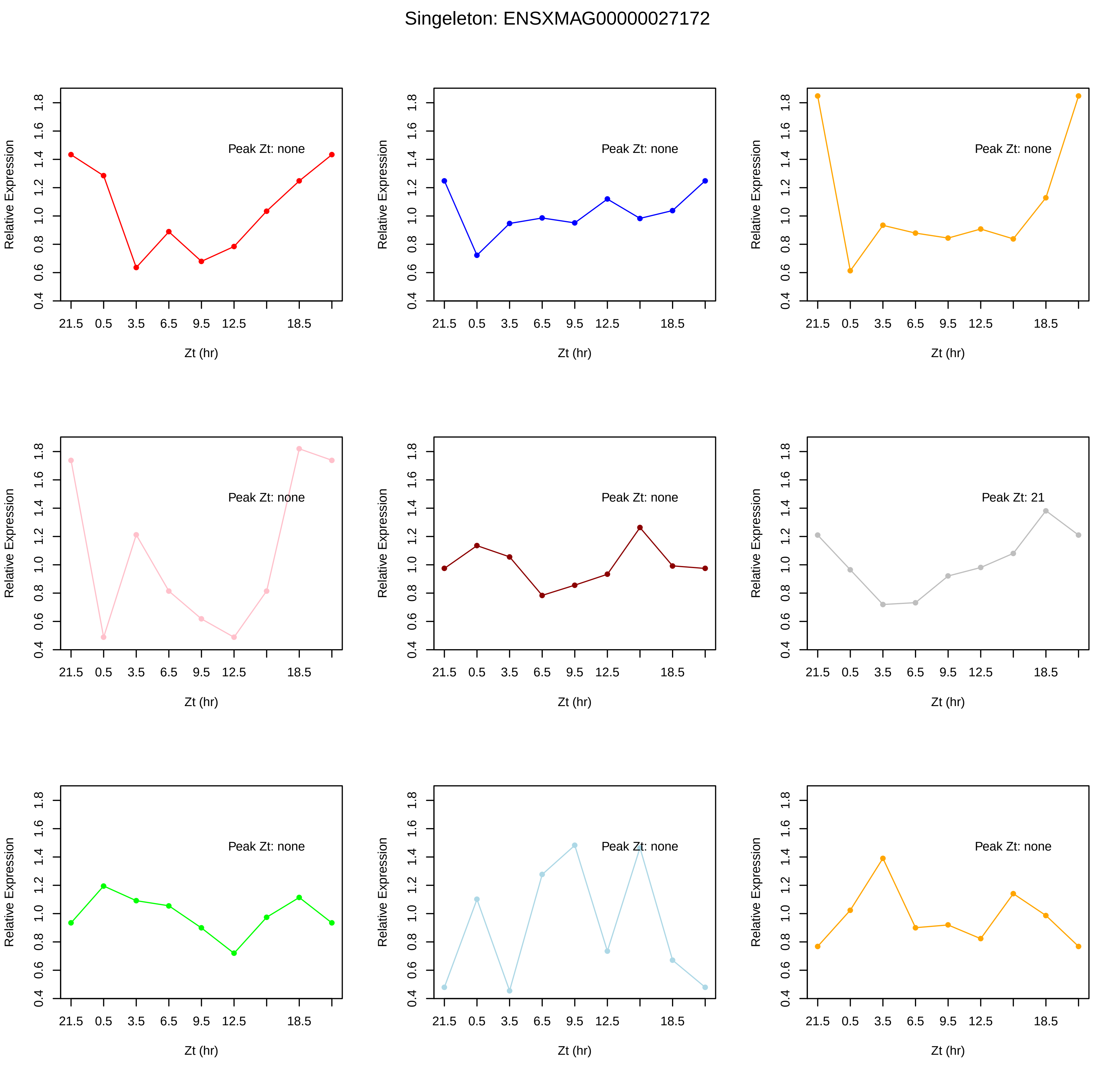

Singeleton: ENSXMAG00000027172
Peak Zt: none
Peak Zt: none
Peak Zt: none
Peak Zt: none
Peak Zt: none
Peak Zt: 21
Peak Zt: none
Peak Zt: none
Peak Zt: none
Relative Expression
Zt (hr)
21.5 0.5 3.5 6.5 9.5 12.5 18.5
0.4 0.6 0.8 1.0 1.2 1.4 1.6 1.8

Singeleton: ENSXMAG00000004731 nlgn1

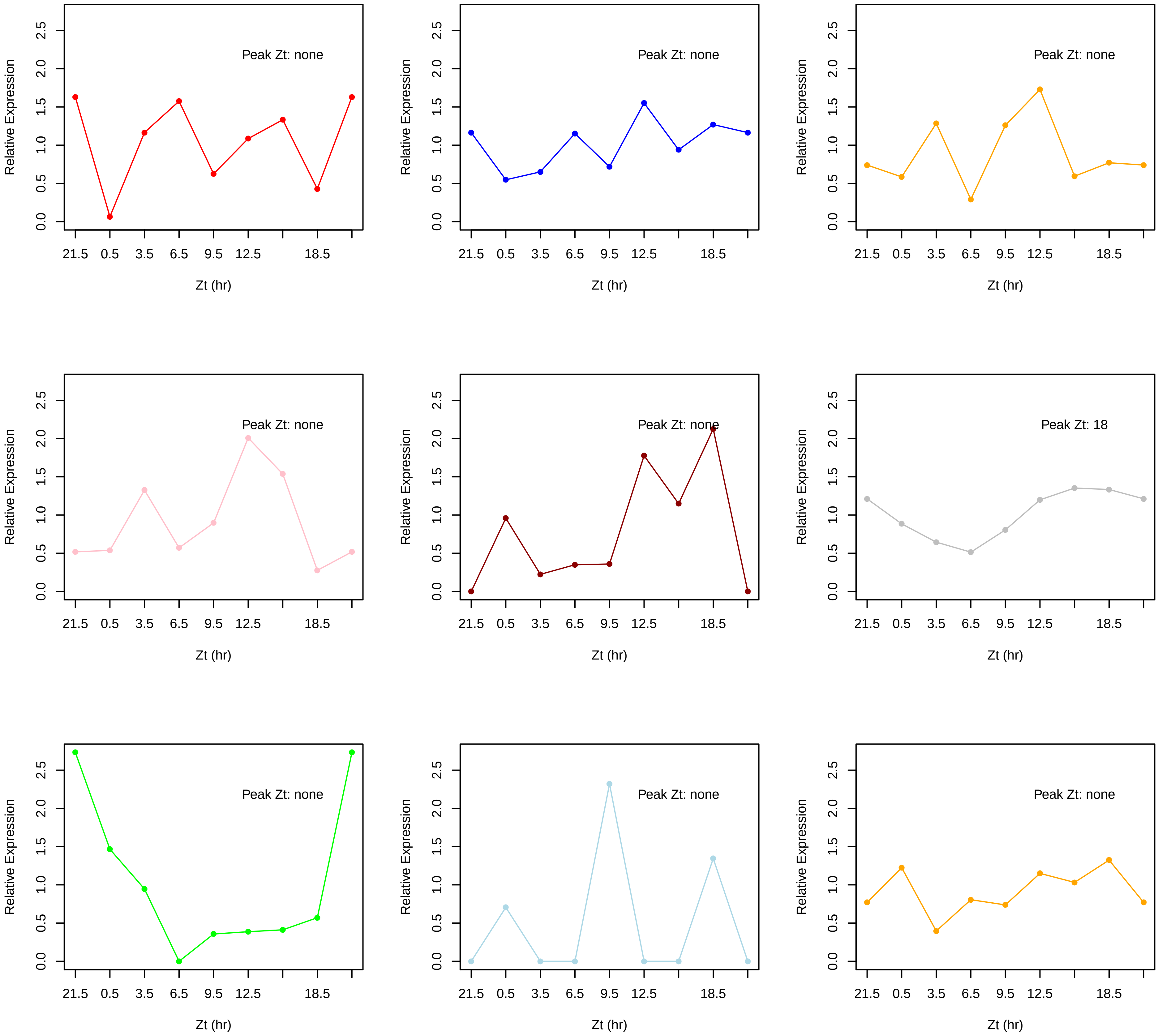

Singeleton: ENSXMAG00000005492 tmem141

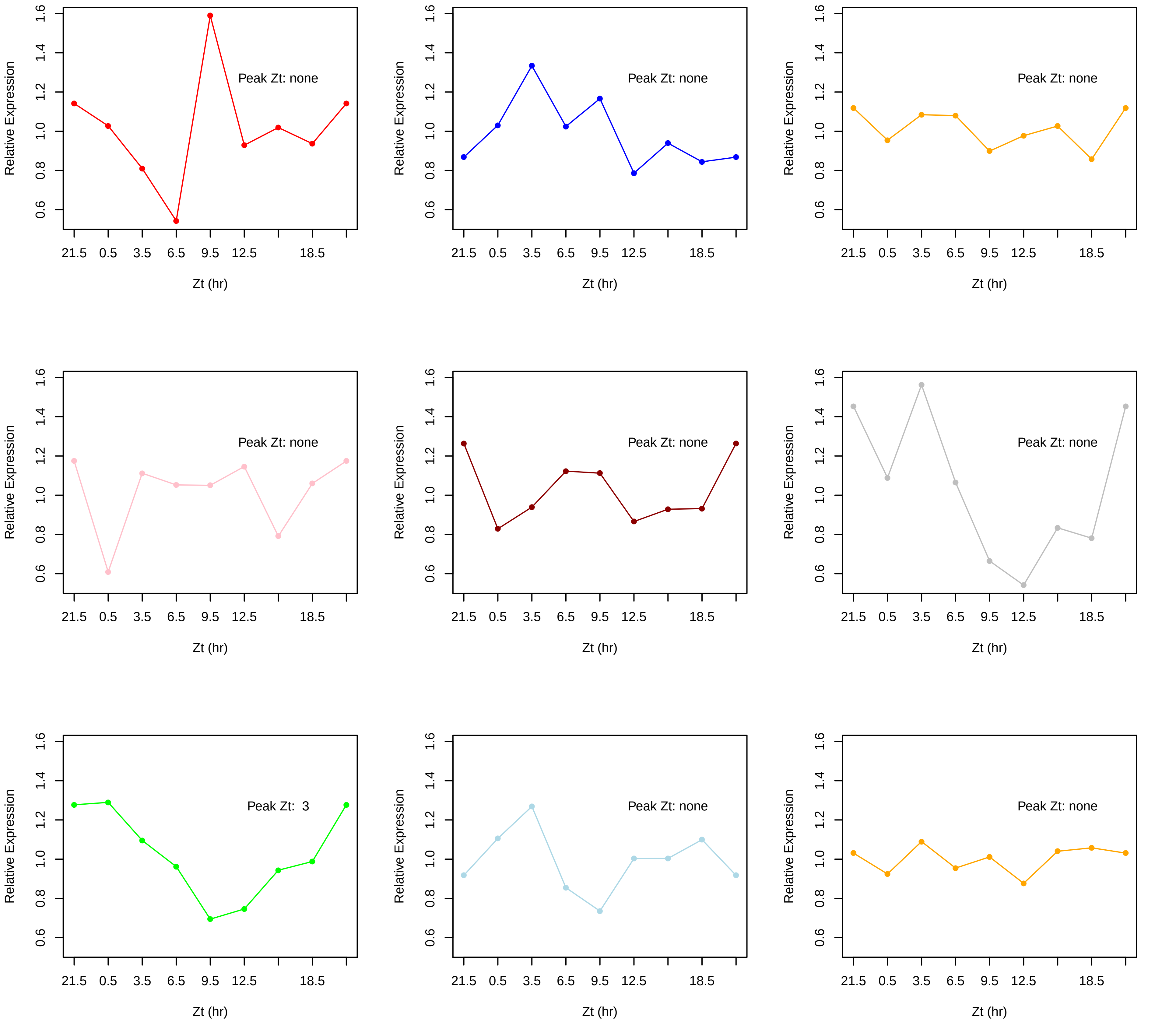

Singeleton: ENSXMAG00000007211 si:dkeyp−72e1.9

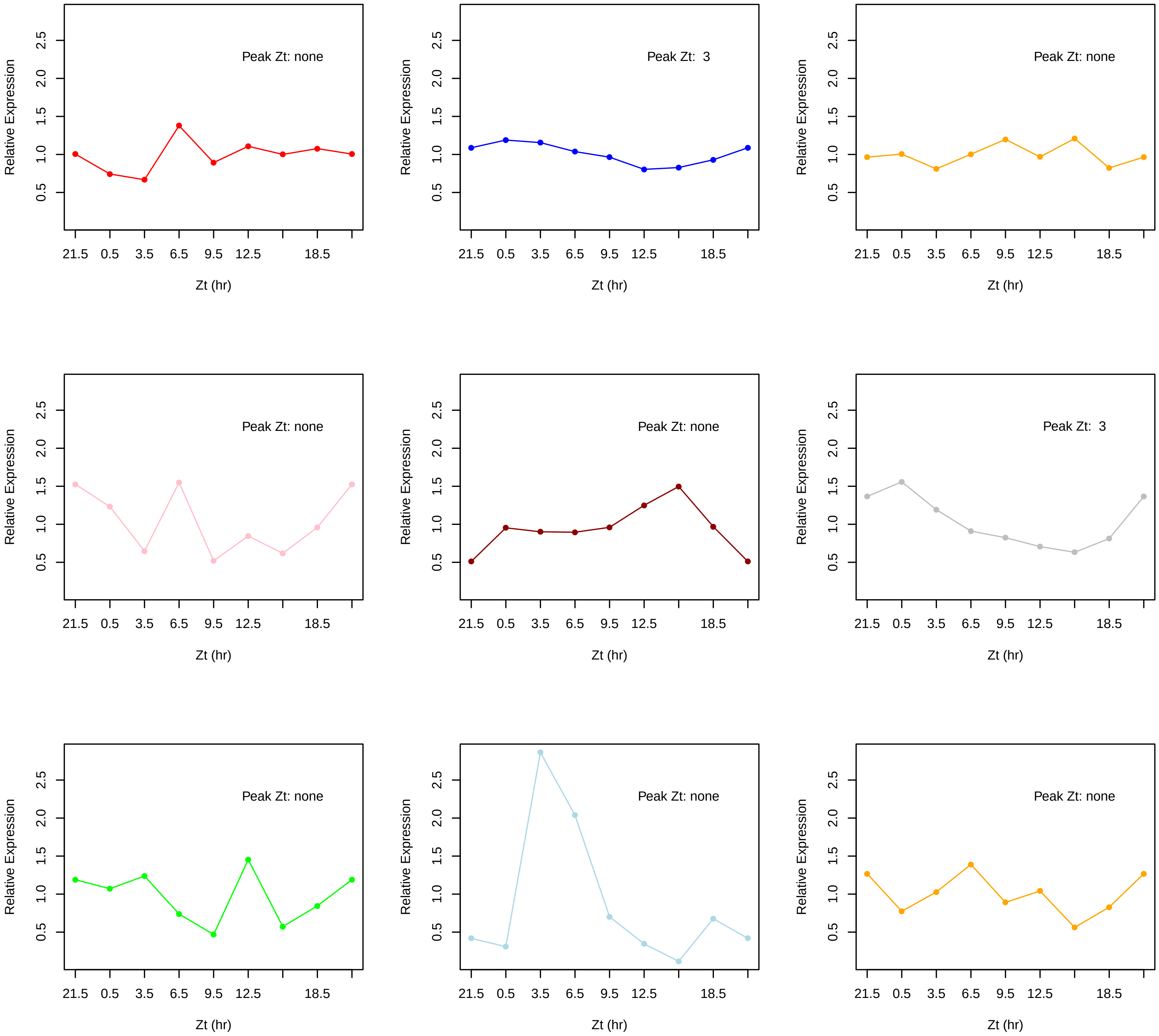

Singeleton: ENSXMAG00000022456 SLC22A17

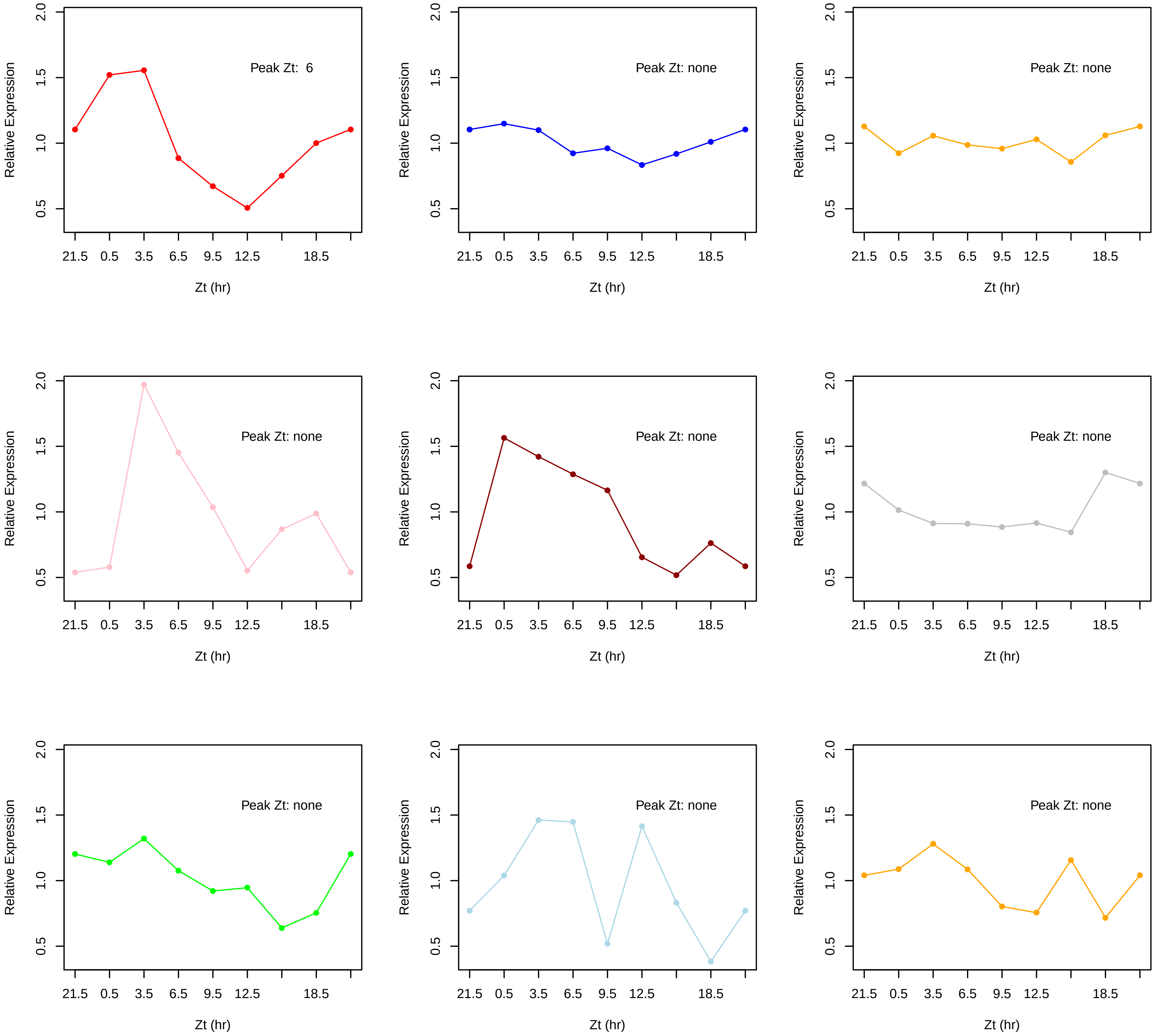

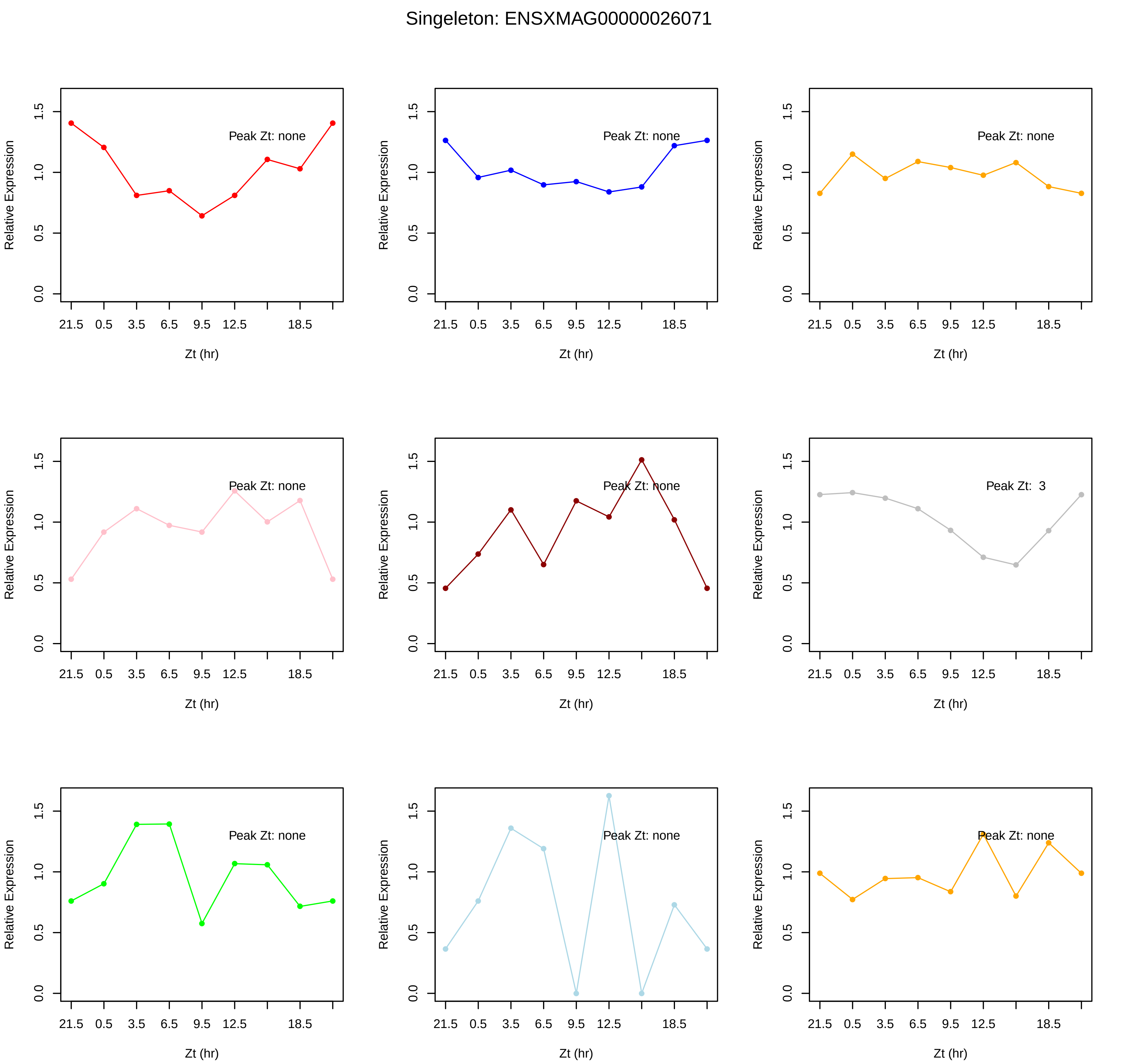
Singeleton: ENSXMAG00000026071
Peak Zt: none
Peak Zt: none
Peak Zt: none
Peak Zt: none
Peak Zt: none
Peak Zt: 3
Peak Zt: none
Peak Zt: none
Peak Zt: none
Relative Expression
Zt (hr)
21.5
0.5
3.5
6.5
9.5
12.5
18.5
0.0
0.5
1.0
1.5

Singleton: ENSXMAG00000001685 gucy2f

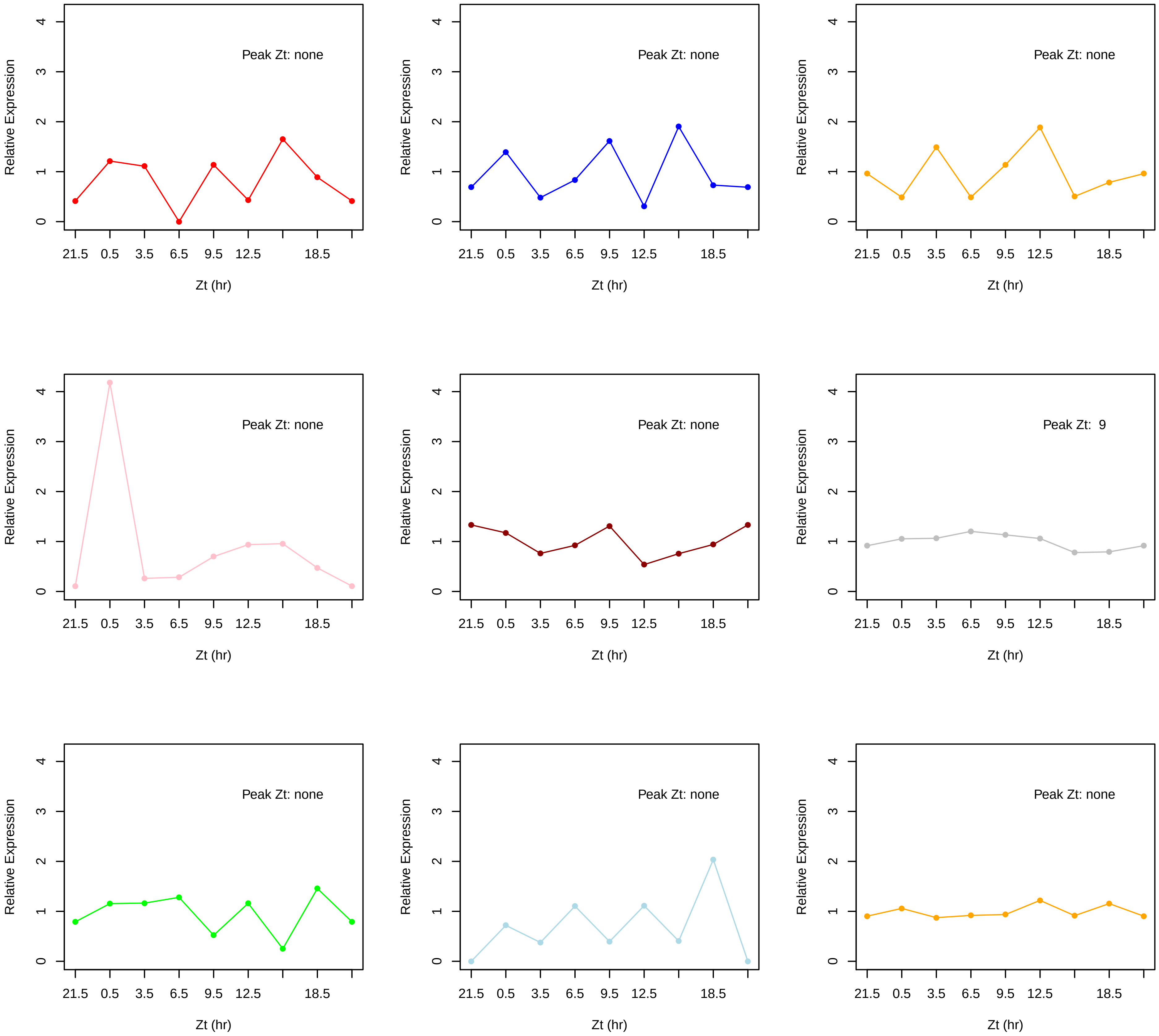

Singeleton: ENSXMAG00000011594 spopla

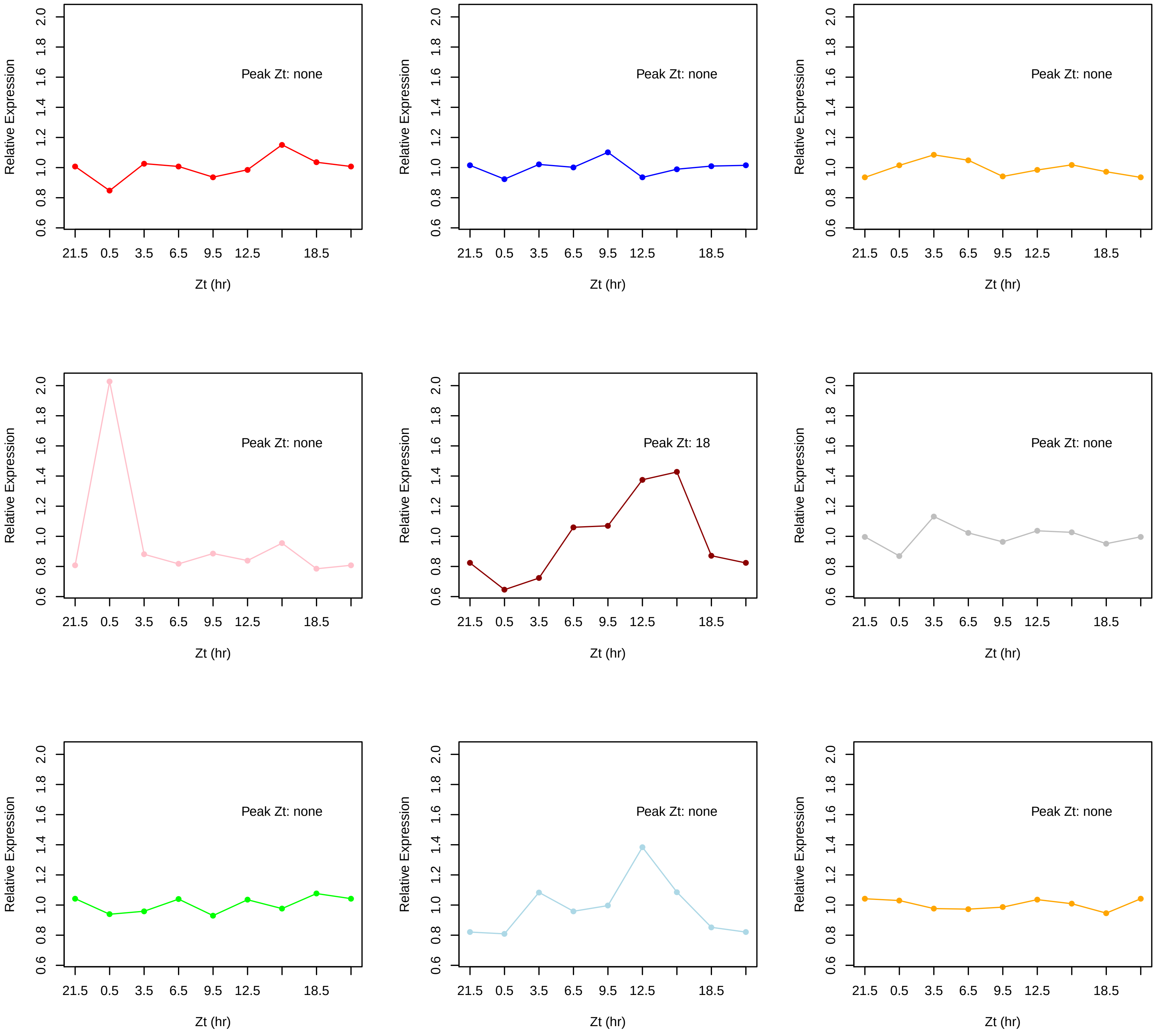

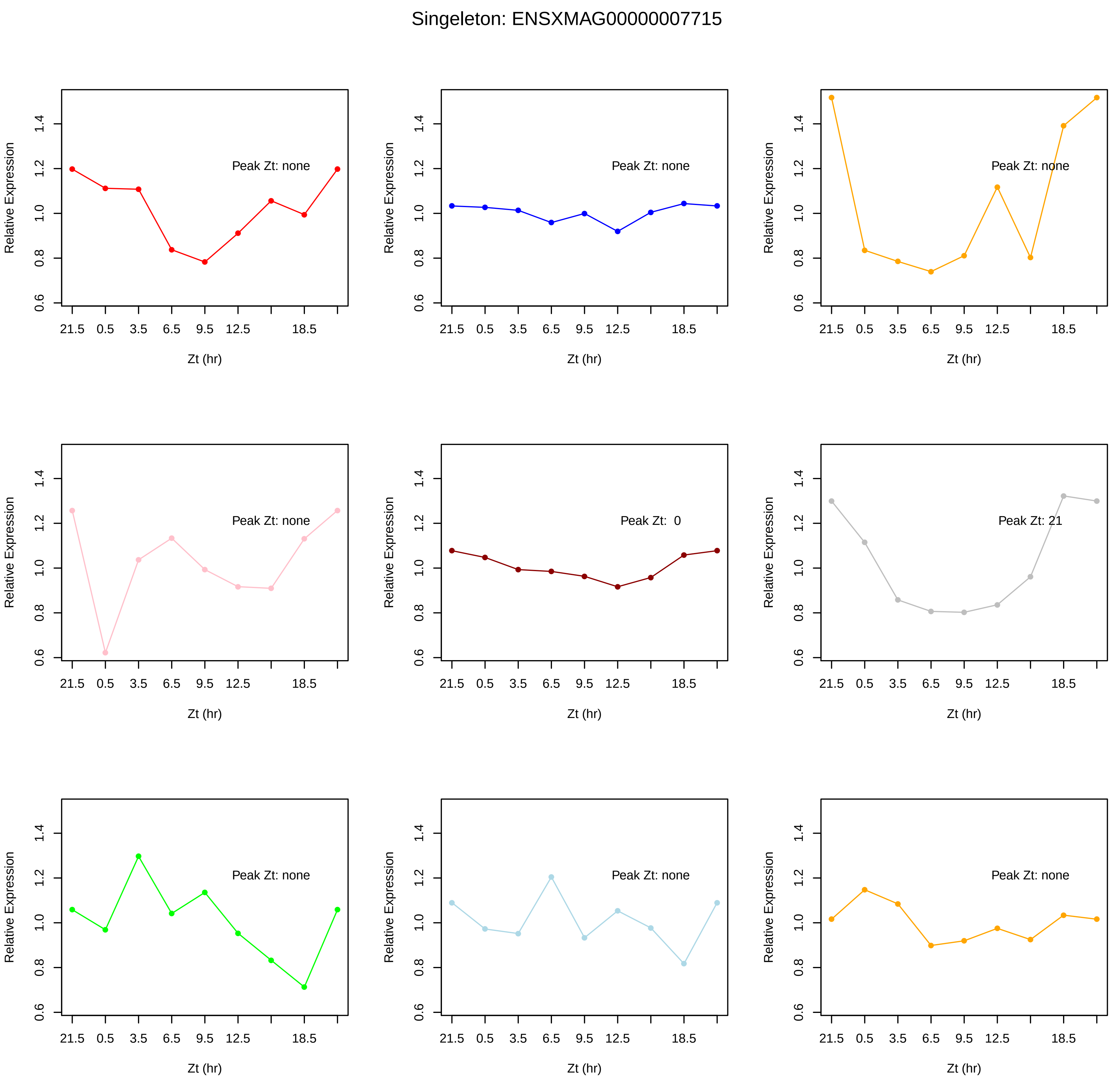
Singleton: ENSXMAG00000007715
Peak Zt: none
Peak Zt: none
Peak Zt: none
Peak Zt: none
Peak Zt: 0
Peak Zt: 21
Peak Zt: none
Peak Zt: none
Peak Zt: none
Relative Expression
Zt (hr)
21.5
0.5
3.5
6.5
9.5
12.5
18.5
0.6
0.8
1.0
1.2
1.4

Singeleton: ENSXMAG00000004767 NAALADL2

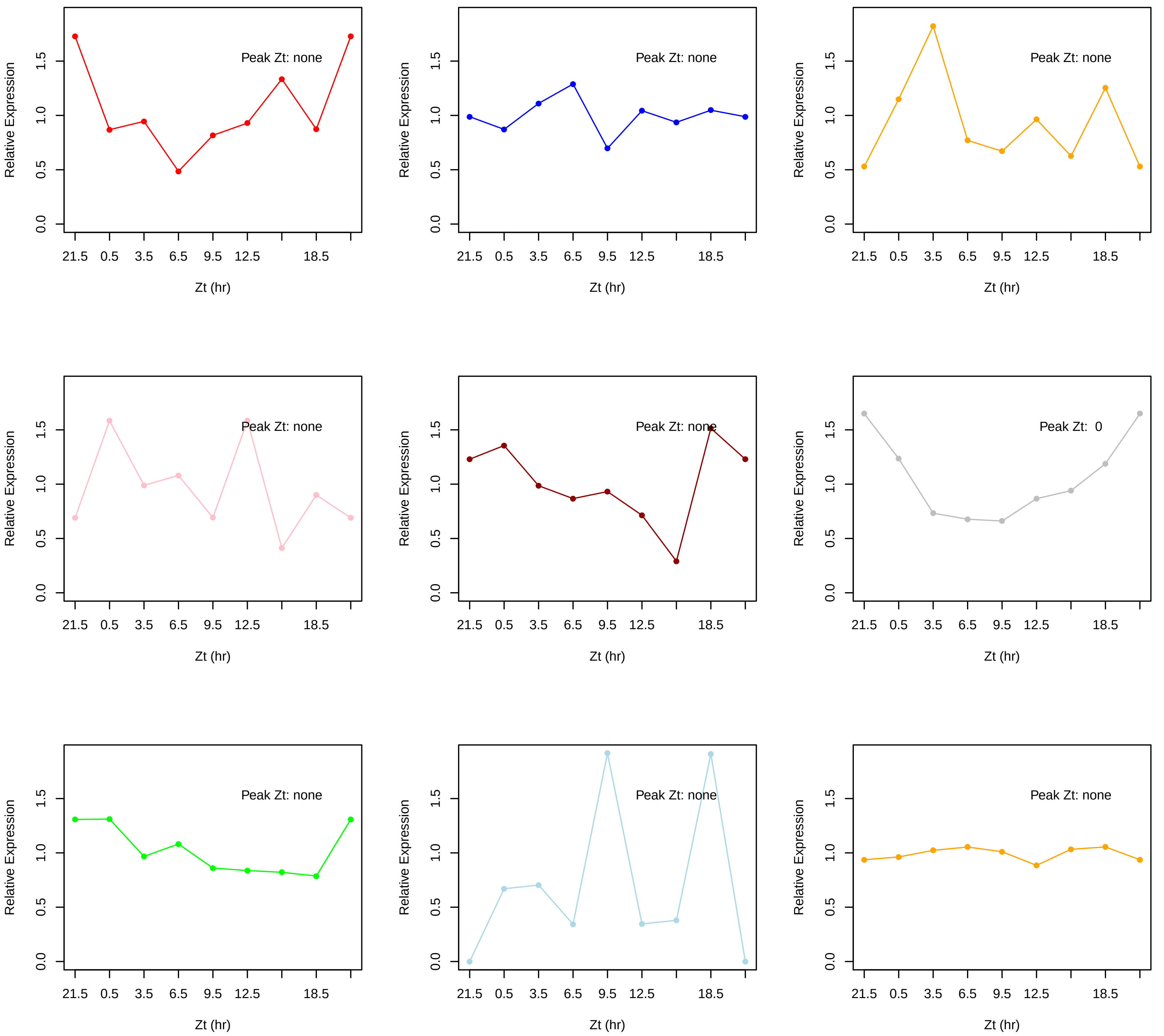

Singleton: ENSXMAG00000002576 acer1

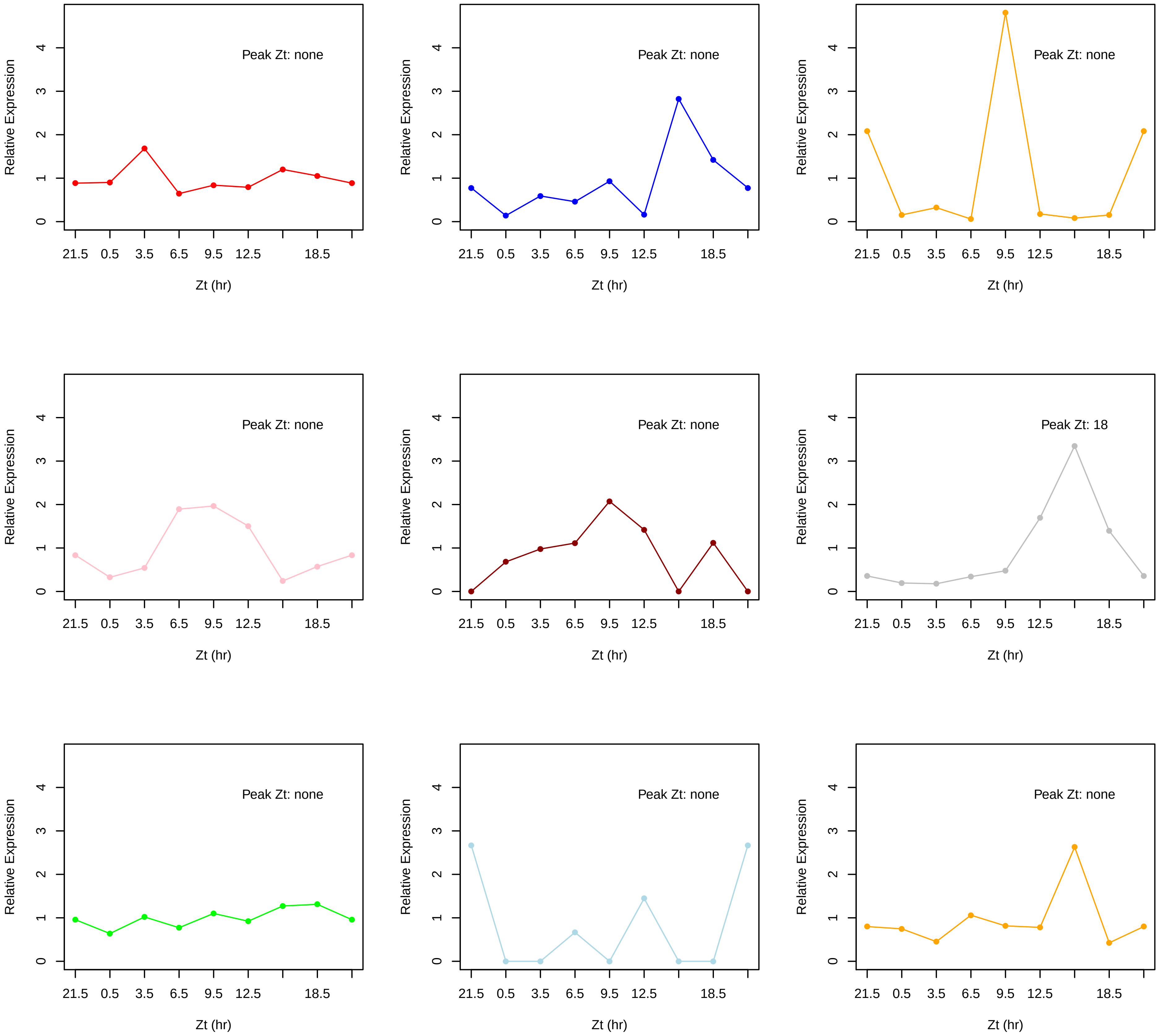

Singeleton: ENSXMAG00000014943 ppl

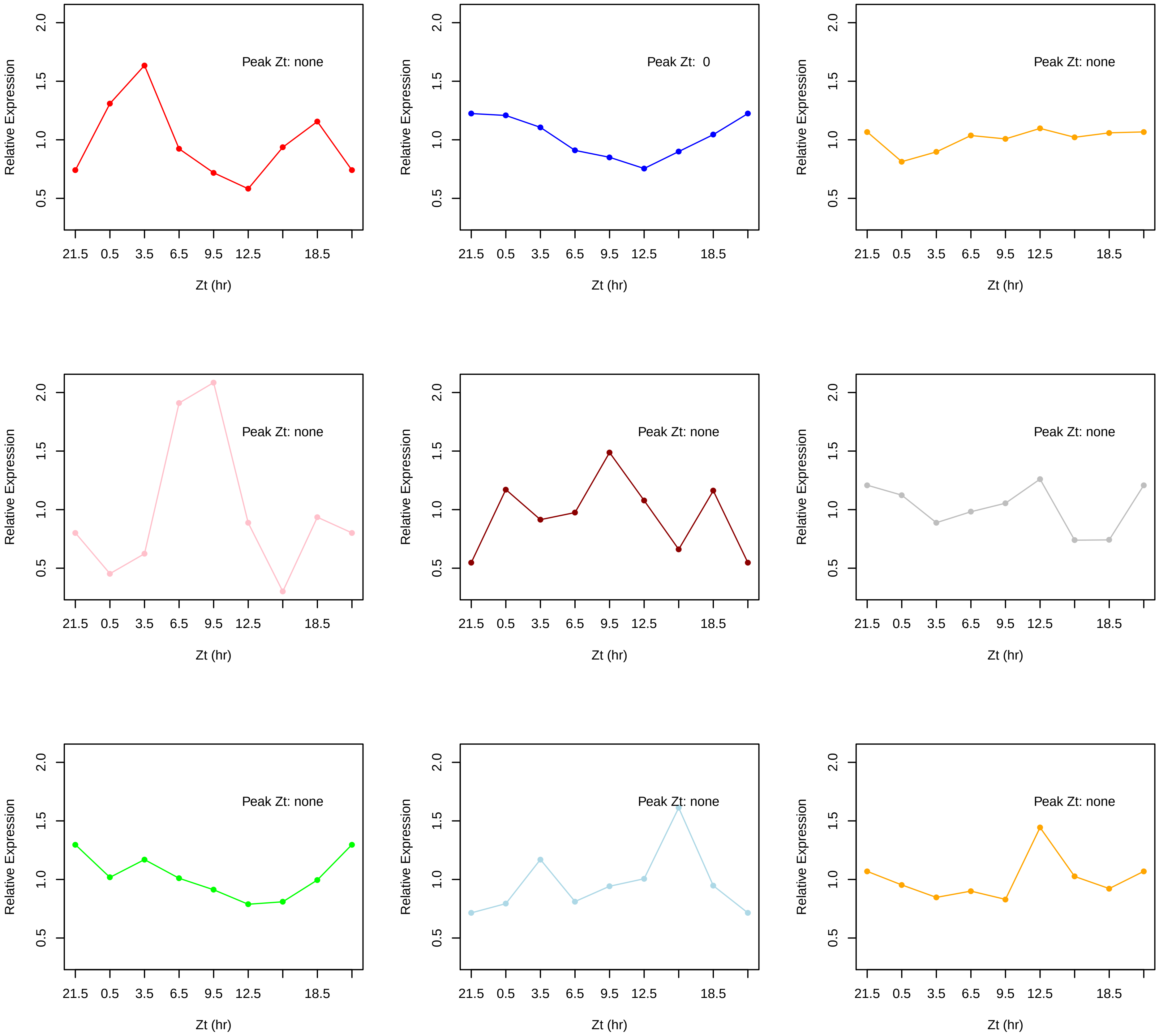

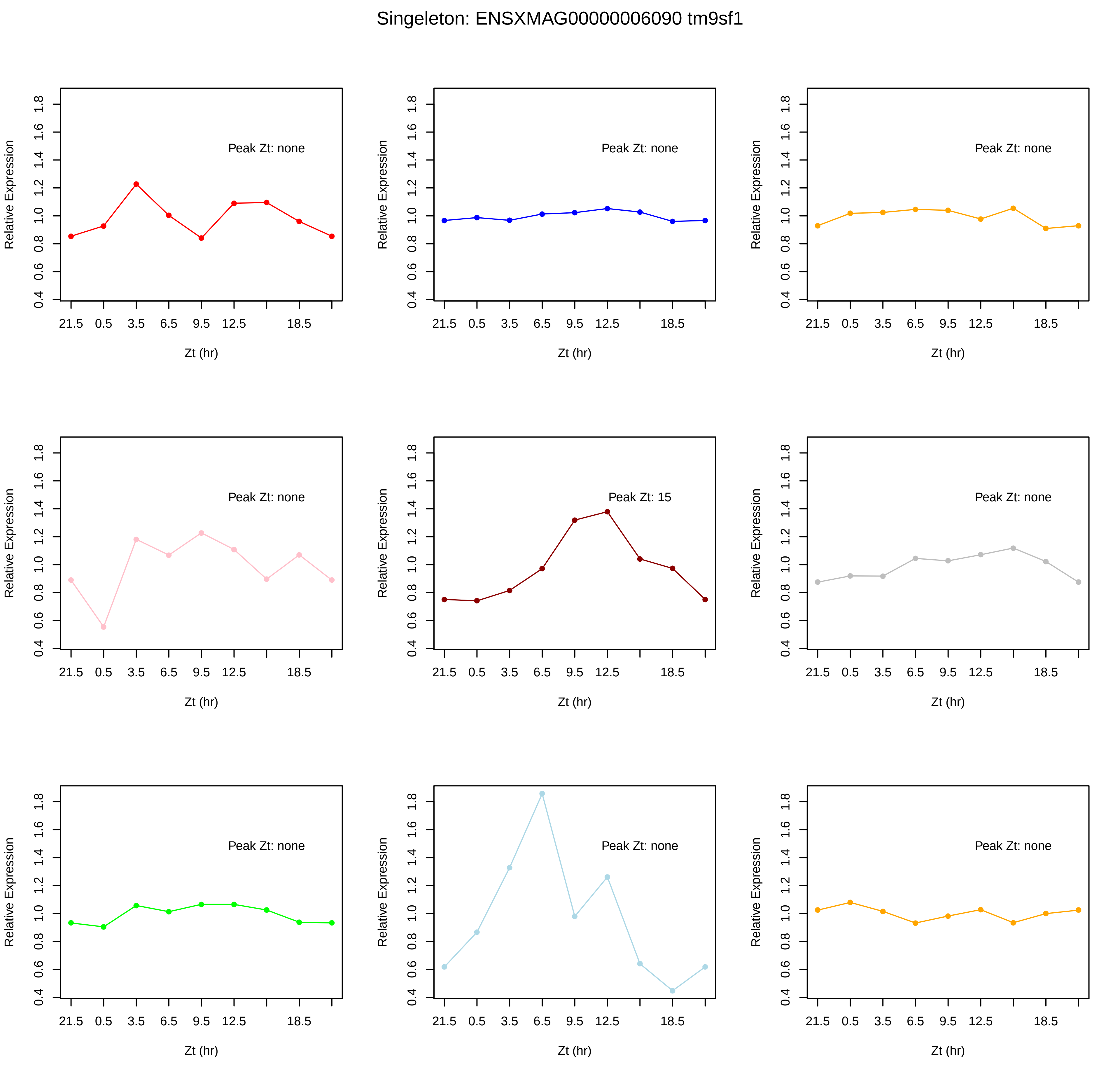
Singeleton: ENSXMAG00000006090 tm9sf1
Peak Zt: none
Peak Zt: none
Peak Zt: none
Peak Zt: none
Peak Zt: 15
Peak Zt: none
Peak Zt: none
Peak Zt: none
Peak Zt: none
Relative Expression
Zt (hr)

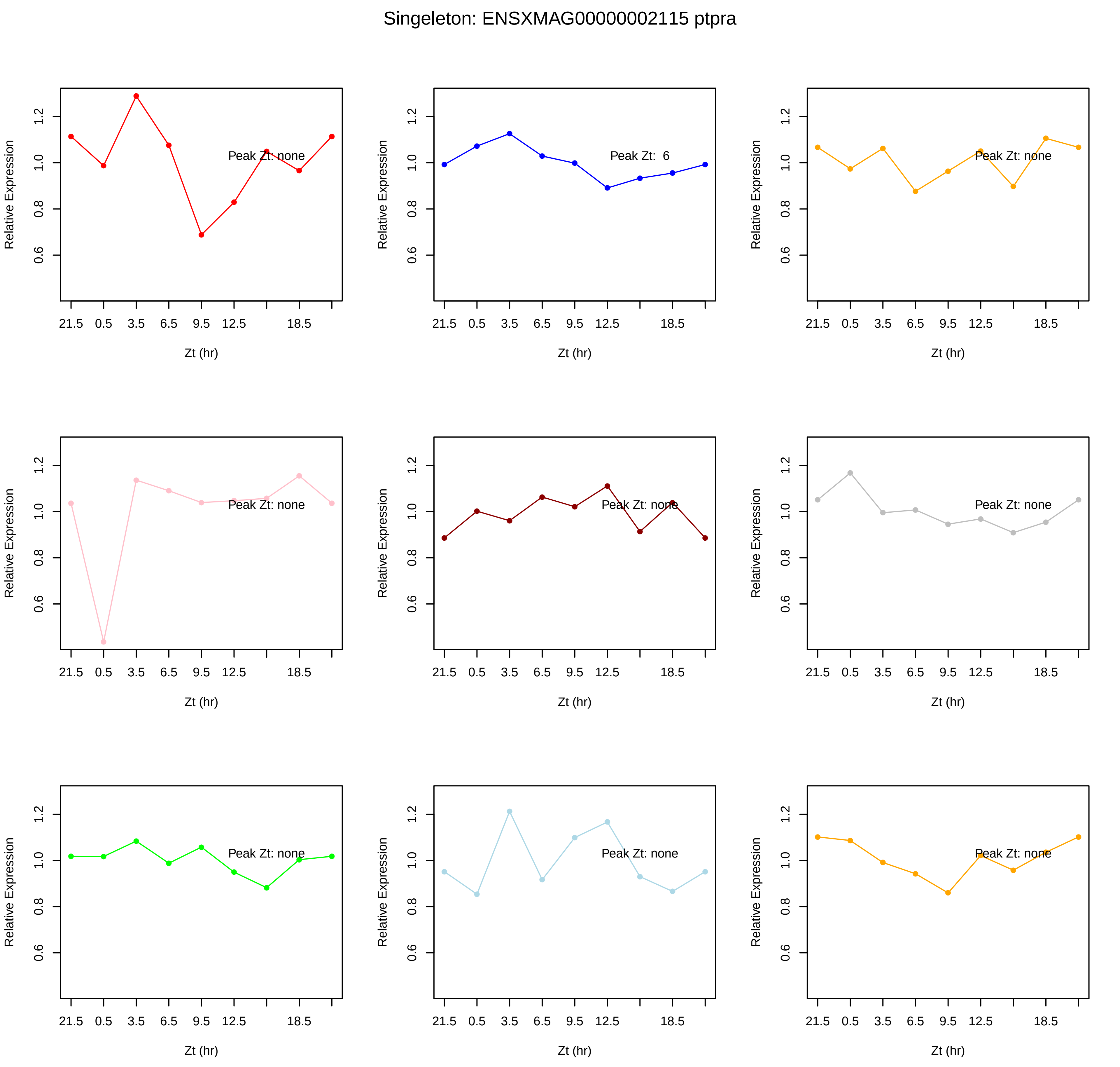

Singeleton: ENSXMAG00000002115 ptpra
Relative Expression
Zt (hr)
21.5 0.5 3.5 6.5 9.5 12.5 18.5
0.6 0.8 1.0 1.2
Peak Zt: none
Peak Zt: 6
Peak Zt: none
Peak Zt: none
Peak Zt: none
Peak Zt: none
Peak Zt: none
Peak Zt: none
Peak Zt: none

Singleton: ENSXMAG00000026139 ap5s1

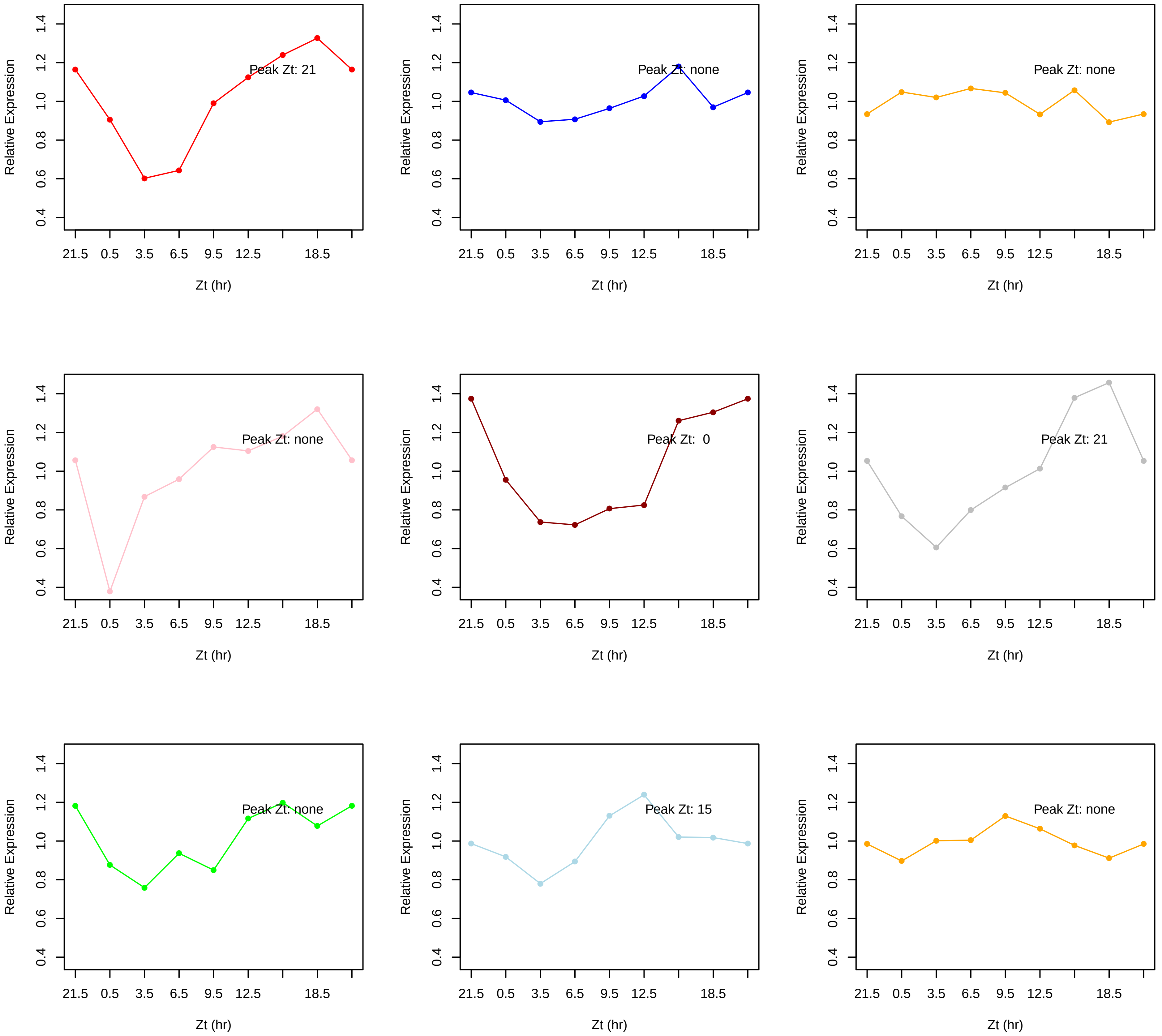

Singleton: ENSXMAG00000016371 lypla2

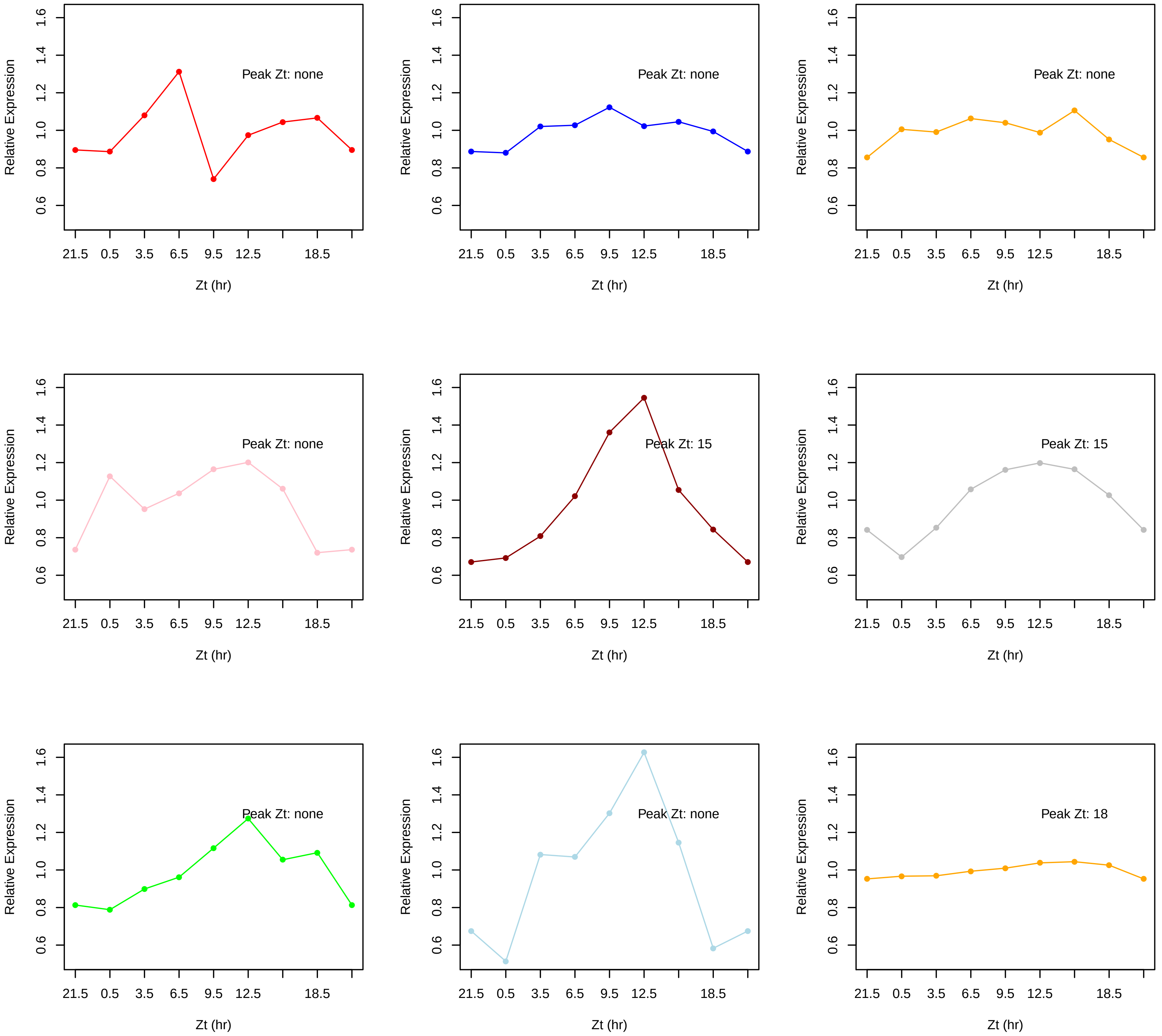

Singeleton: ENSXMAG00000001682 tsku

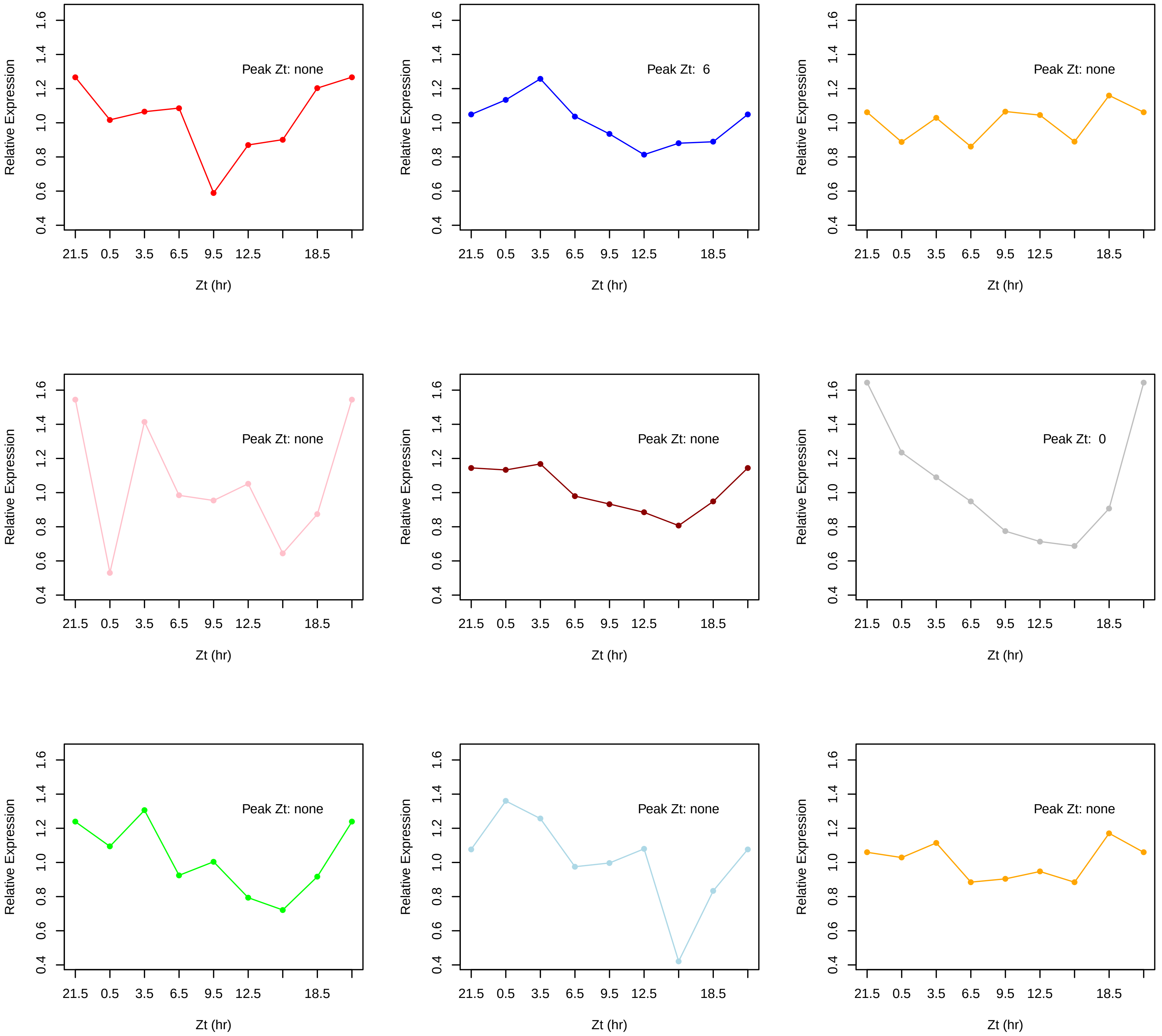

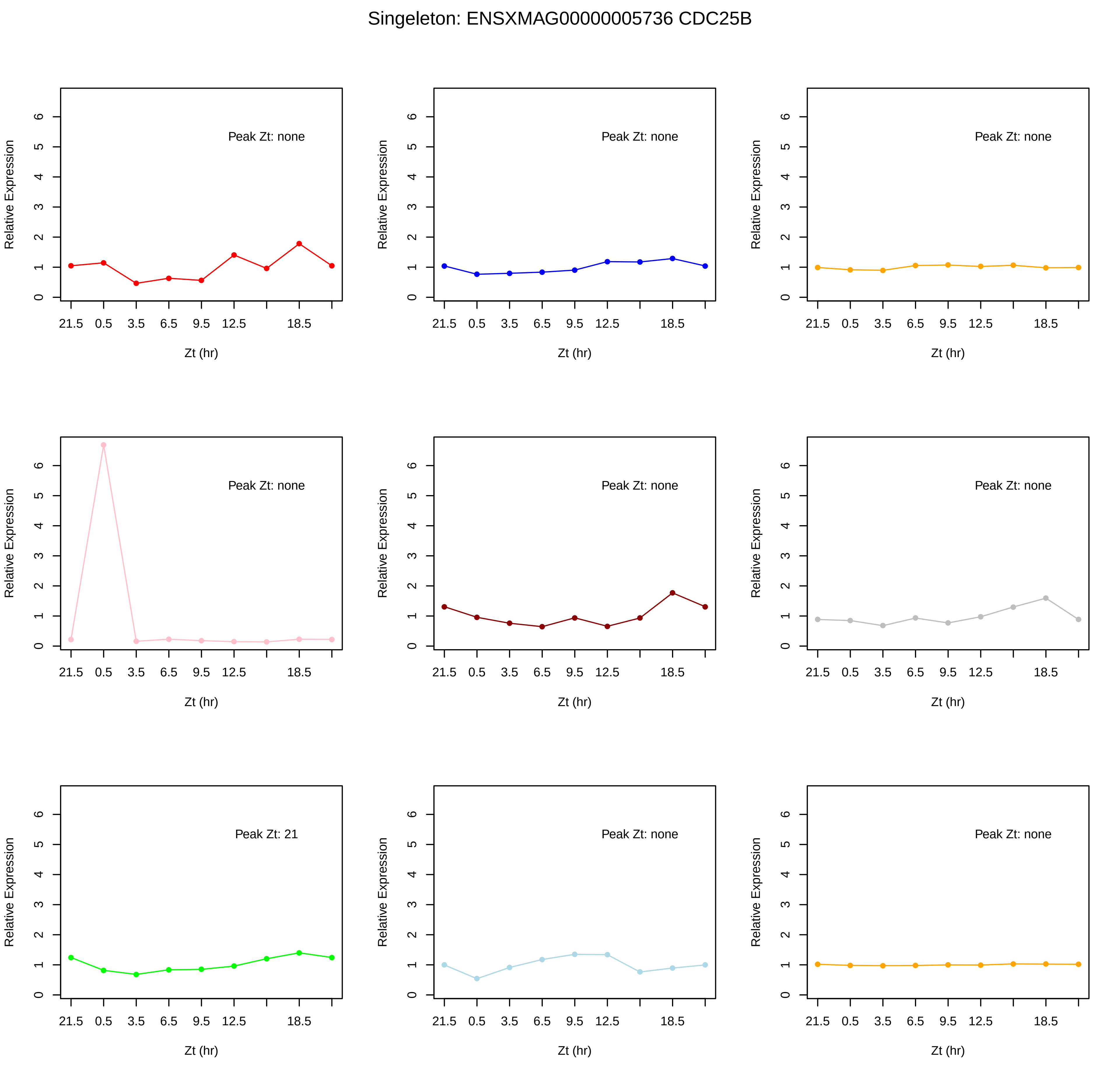
Singeleton: ENSXMAG00000005736 CDC25B
Peak Zt: none
Peak Zt: none
Peak Zt: none
Peak Zt: none
Peak Zt: none
Peak Zt: none
Peak Zt: 21
Peak Zt: none
Peak Zt: none
Relative Expression
Zt (hr)
21.5
0.5
3.5
6.5
9.5
12.5
18.5

Singeleton: ENSXMAG00000028910

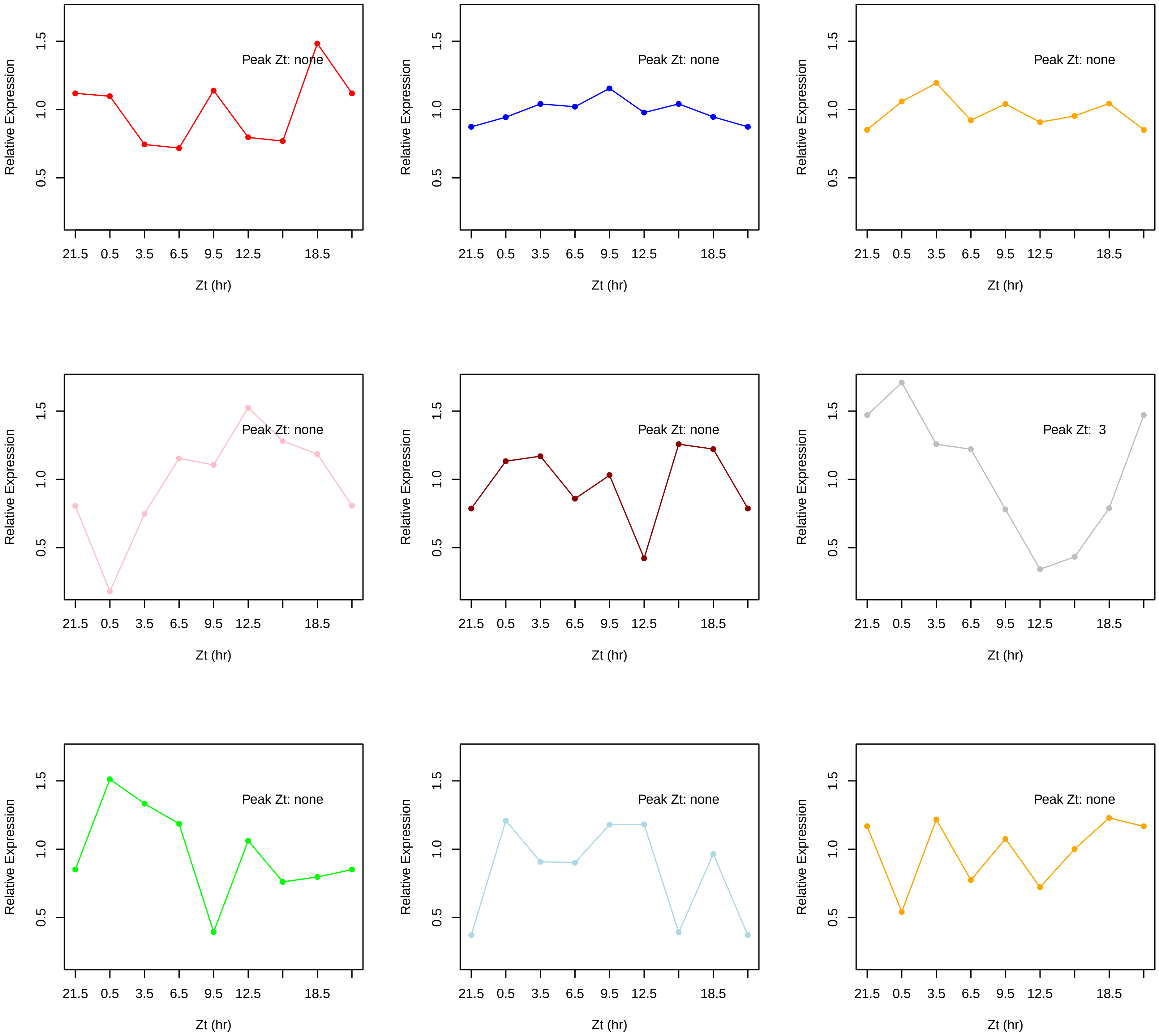

Singleton: ENSXMAG00000014002 acer3

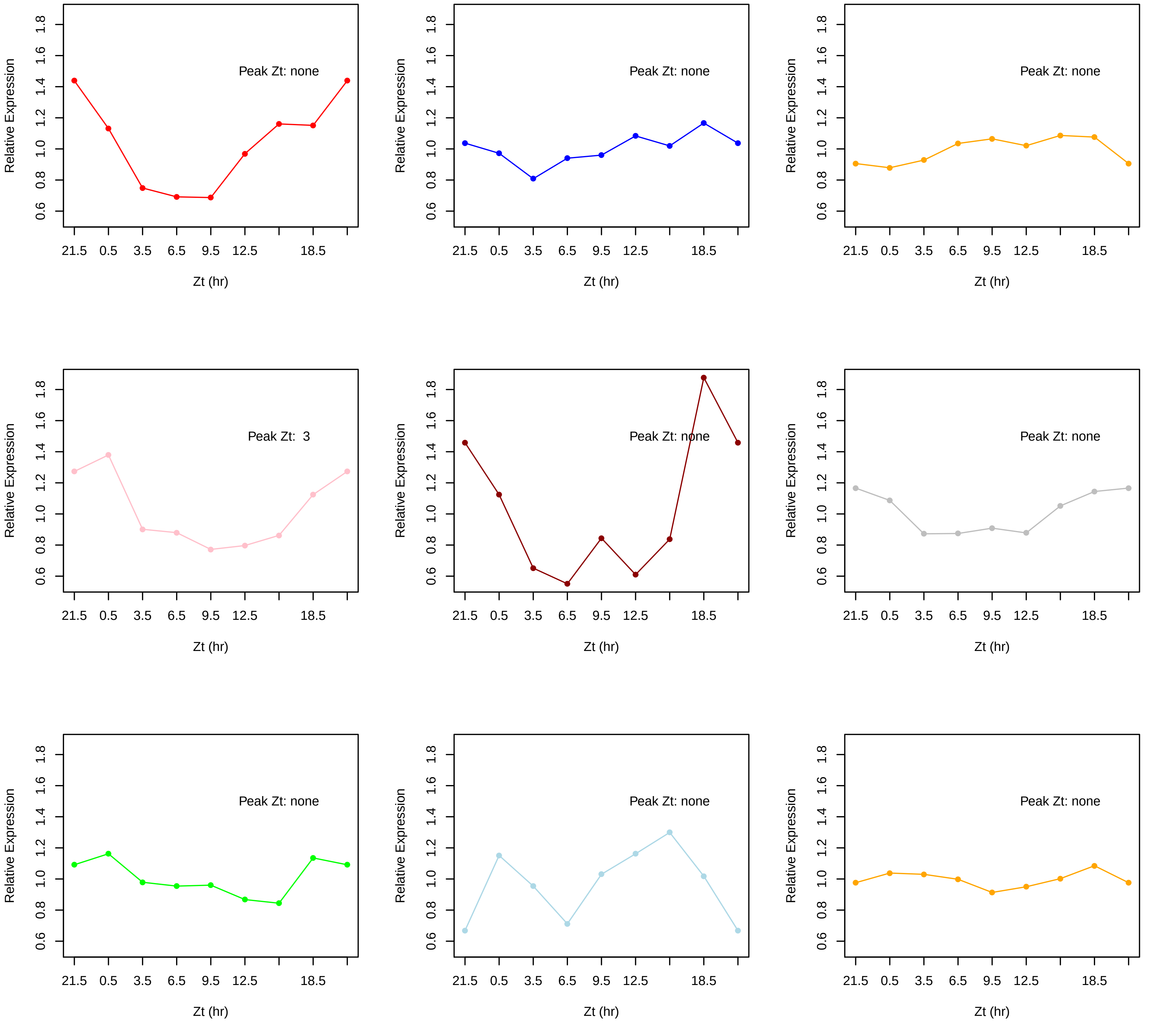

Singeleton: ENSXMAG00000003067 mthfsd

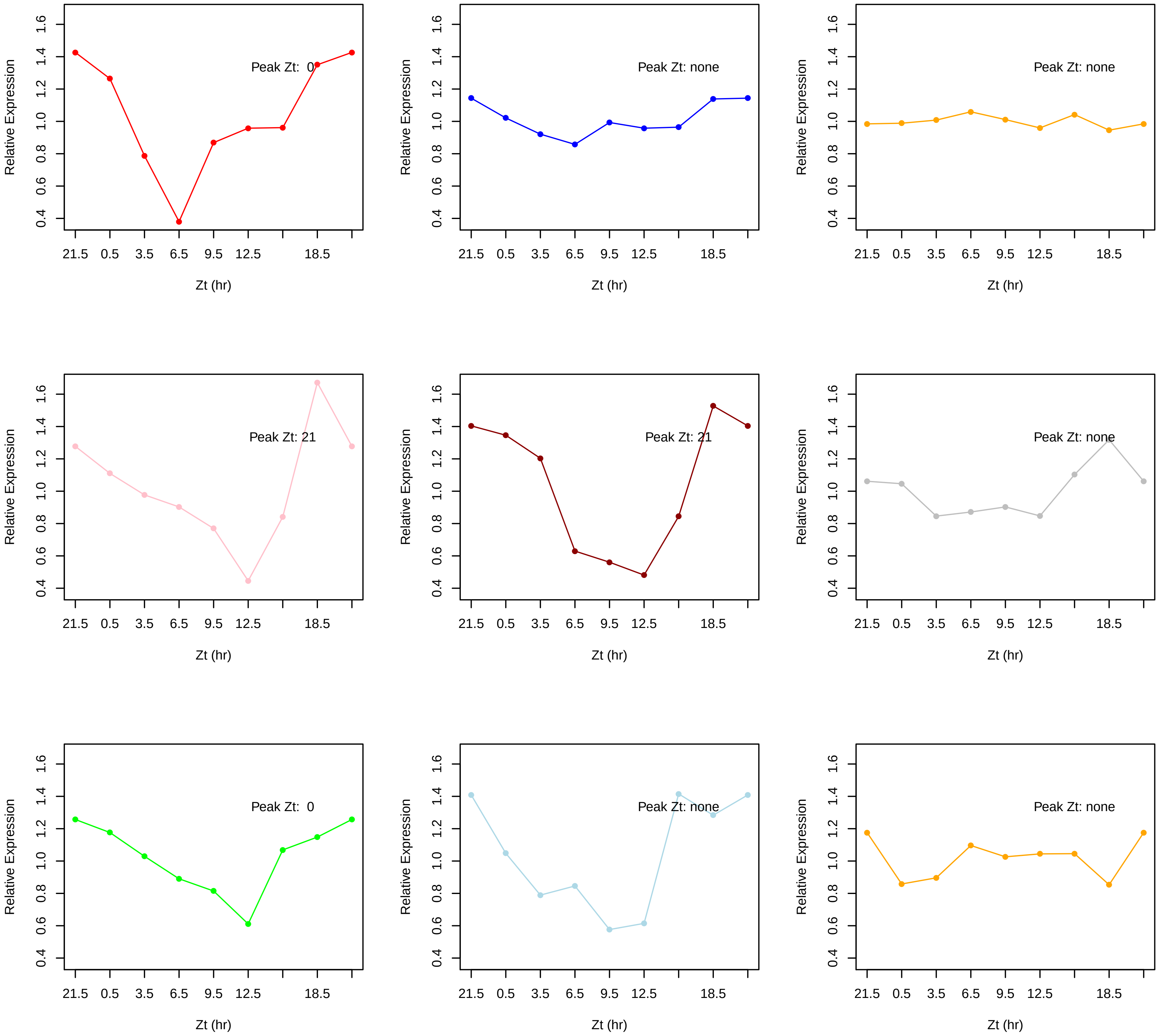

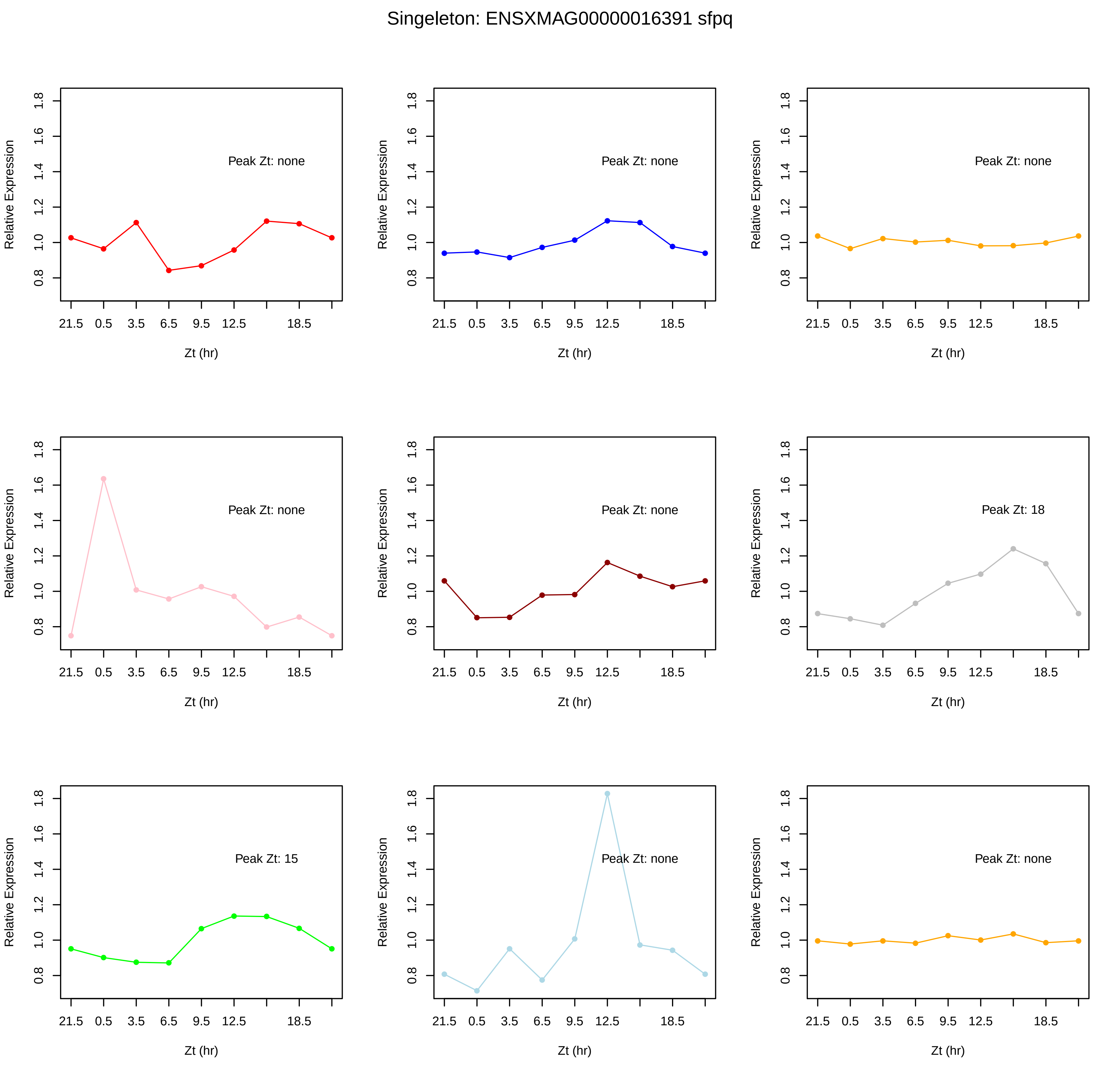
Singeleton: ENSXMAG00000016391 sfpq
Peak Zt: none
Peak Zt: none
Peak Zt: none
Peak Zt: none
Peak Zt: none
Peak Zt: 18
Peak Zt: 15
Peak Zt: none
Peak Zt: none
Relative Expression
Zt (hr)
21.5
0.5
3.5
6.5
9.5
12.5
18.5
0.8
1.0
1.2
1.4
1.6
1.8

Singeleton: ENSXMAG00000004678 ctnnbl1

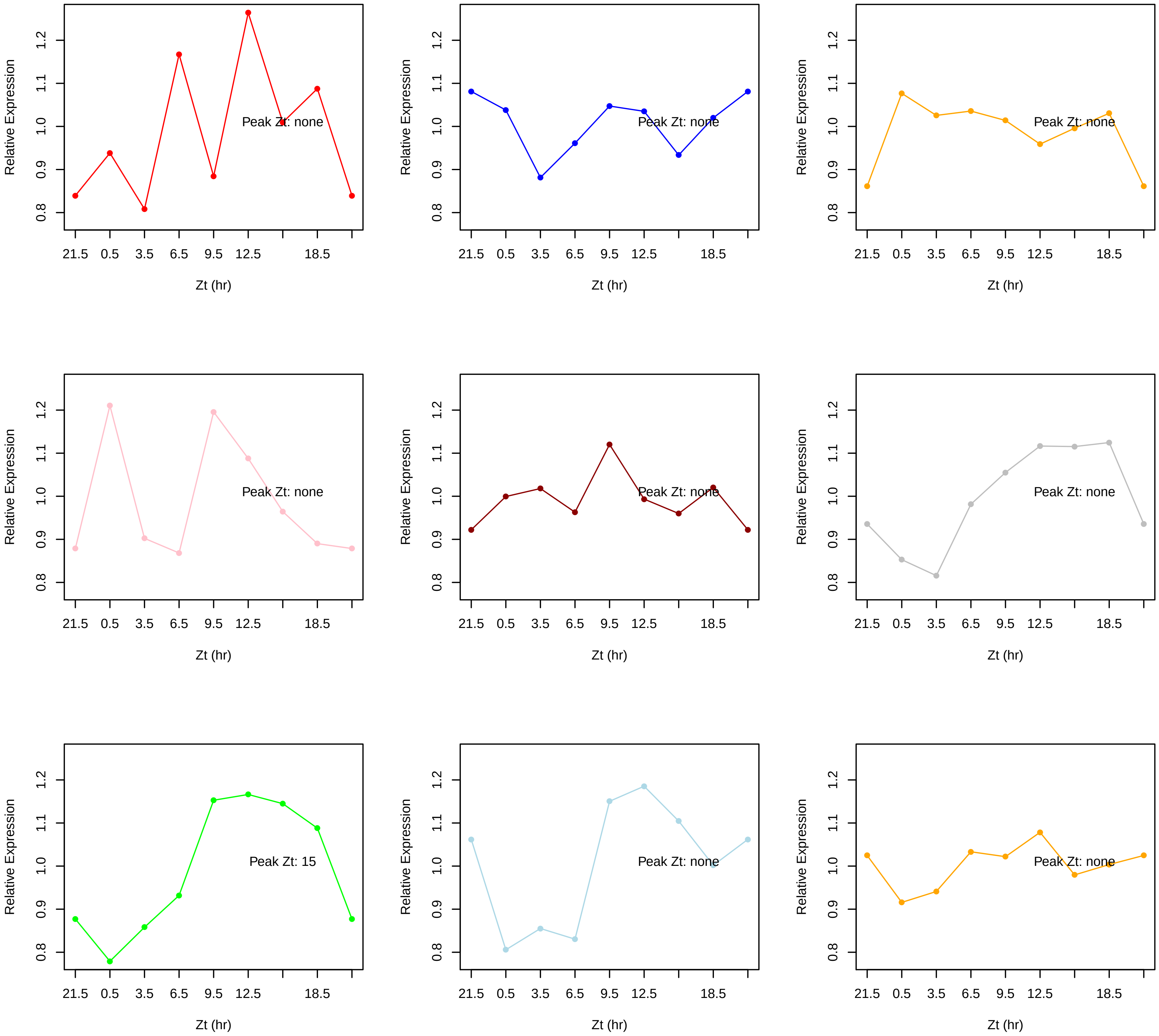

Singeleton: ENSXMAG00000005217 rps6ka4

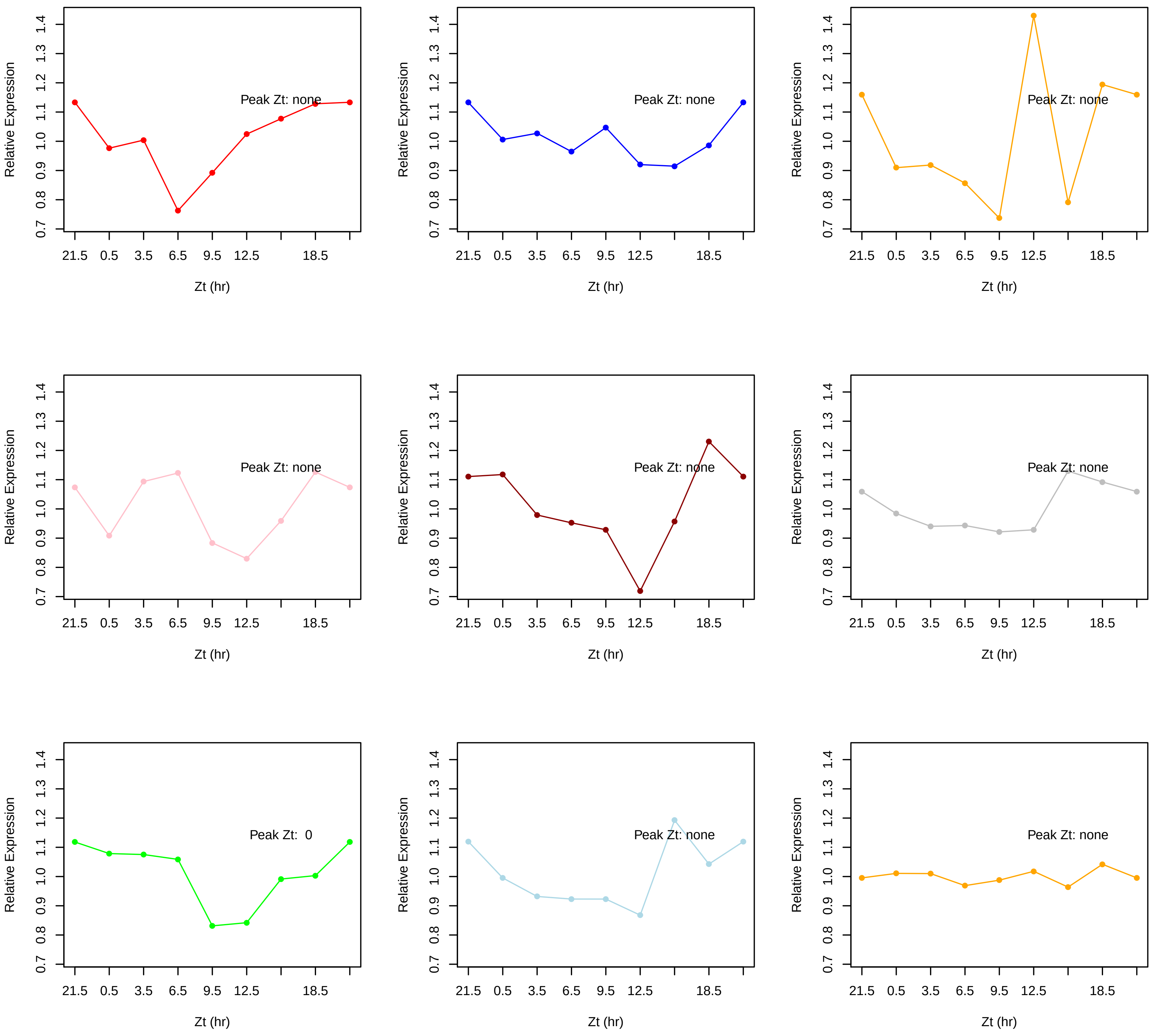

Singleton: ENSXMAG00000025804 cdc42

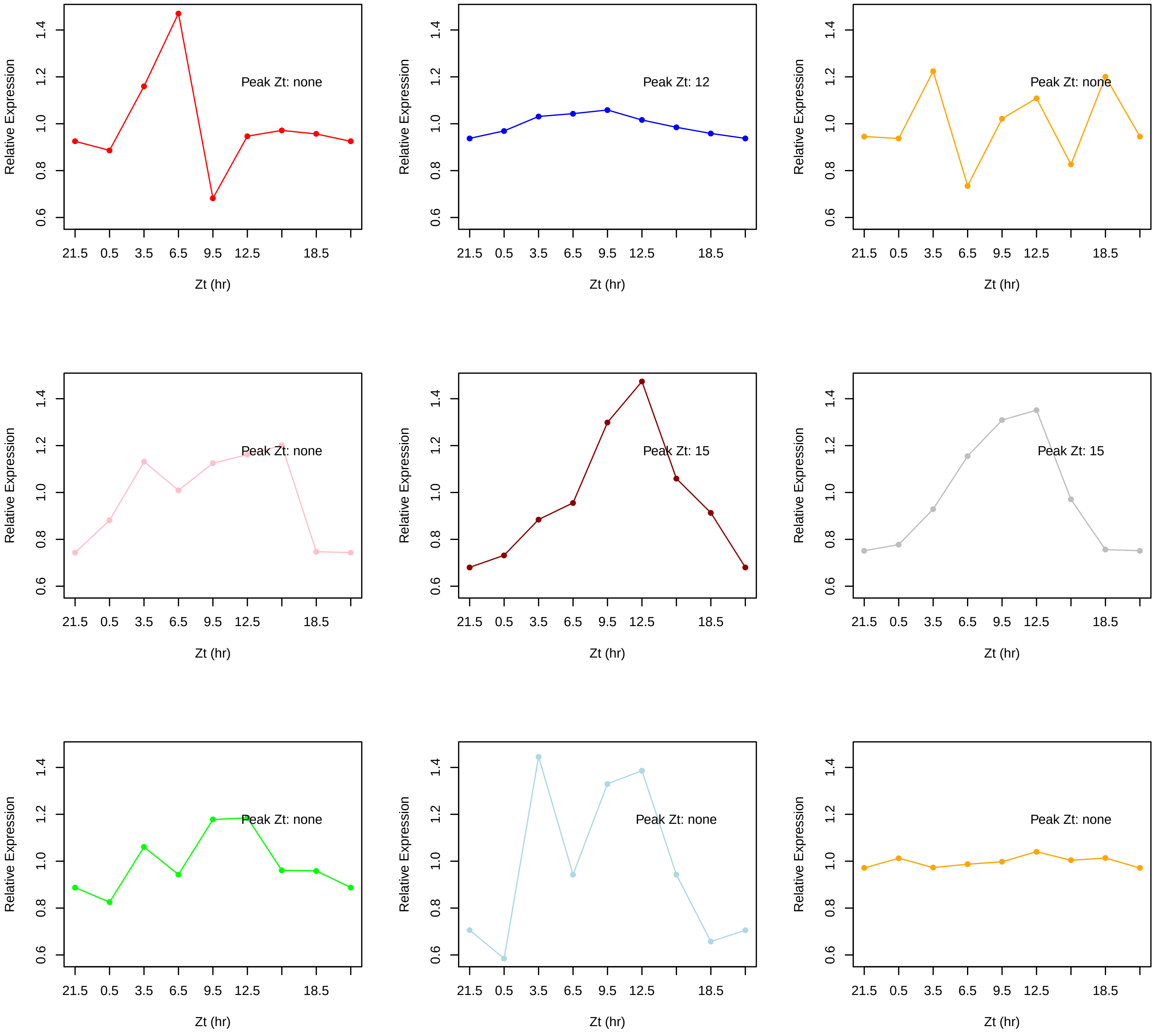

Singeleton: ENSXMAG00000006626 commd9

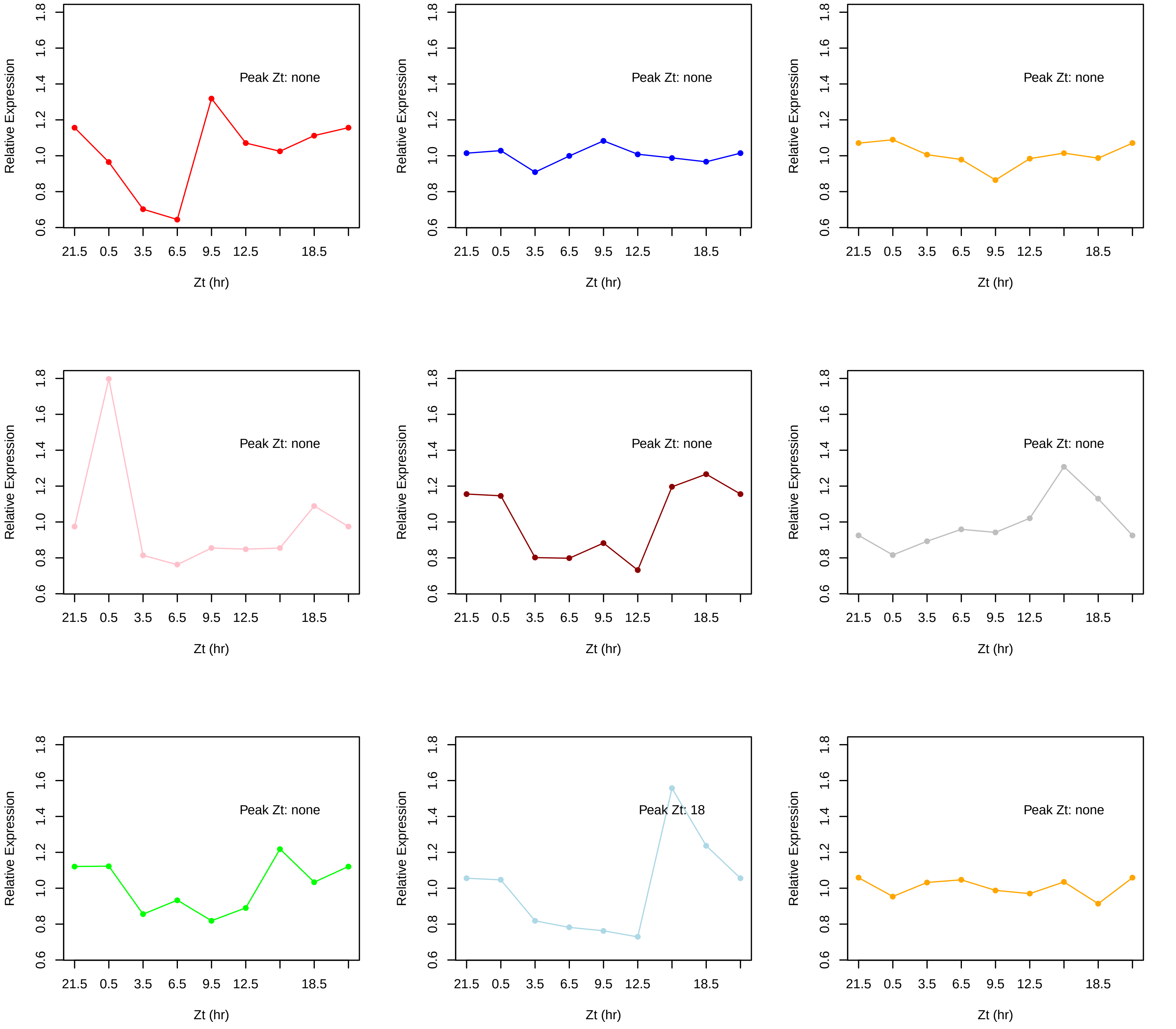

Singeleton: ENSXMAG00000013450

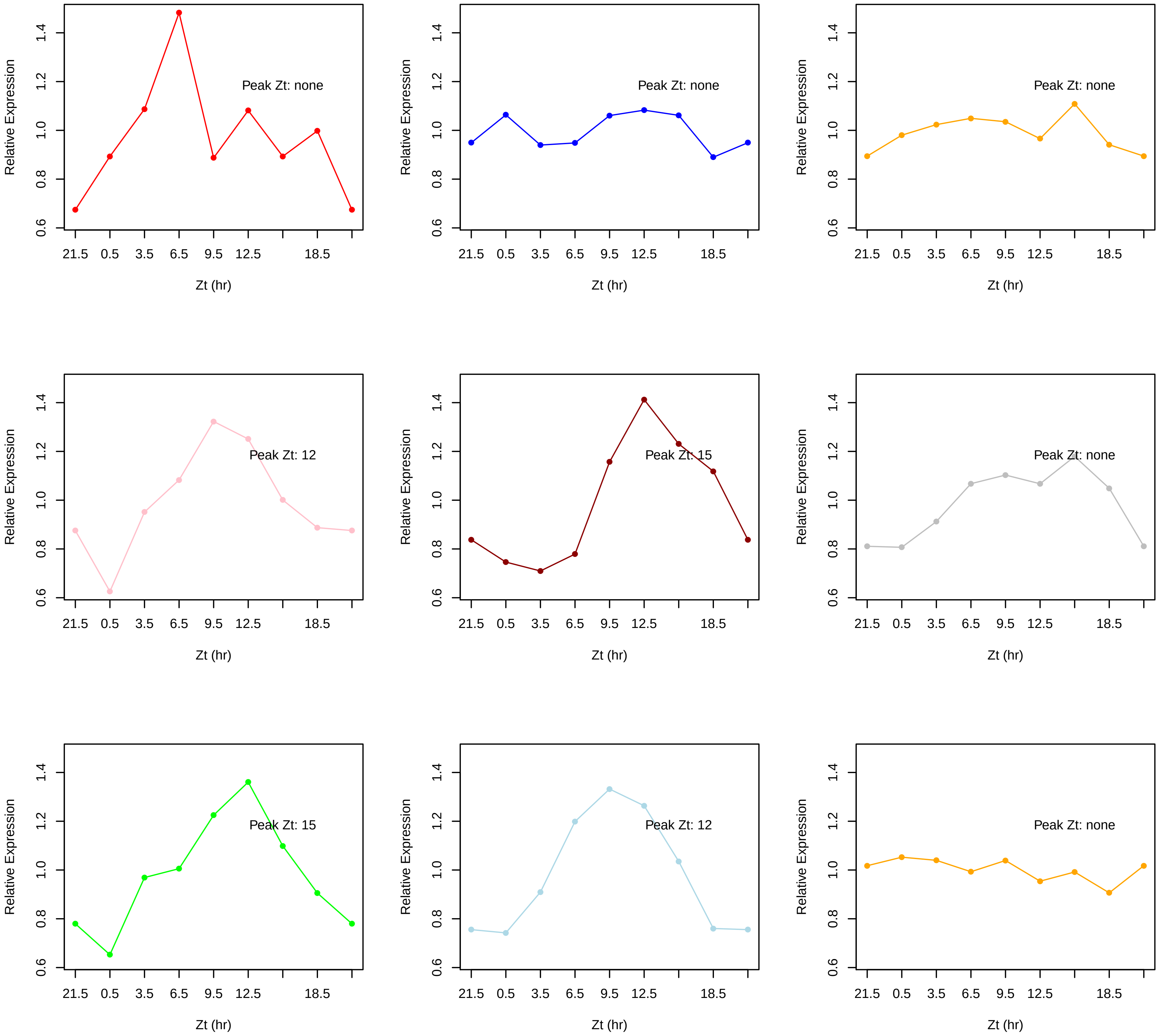

Singleton: ENSXMAG00000002101 idh3b

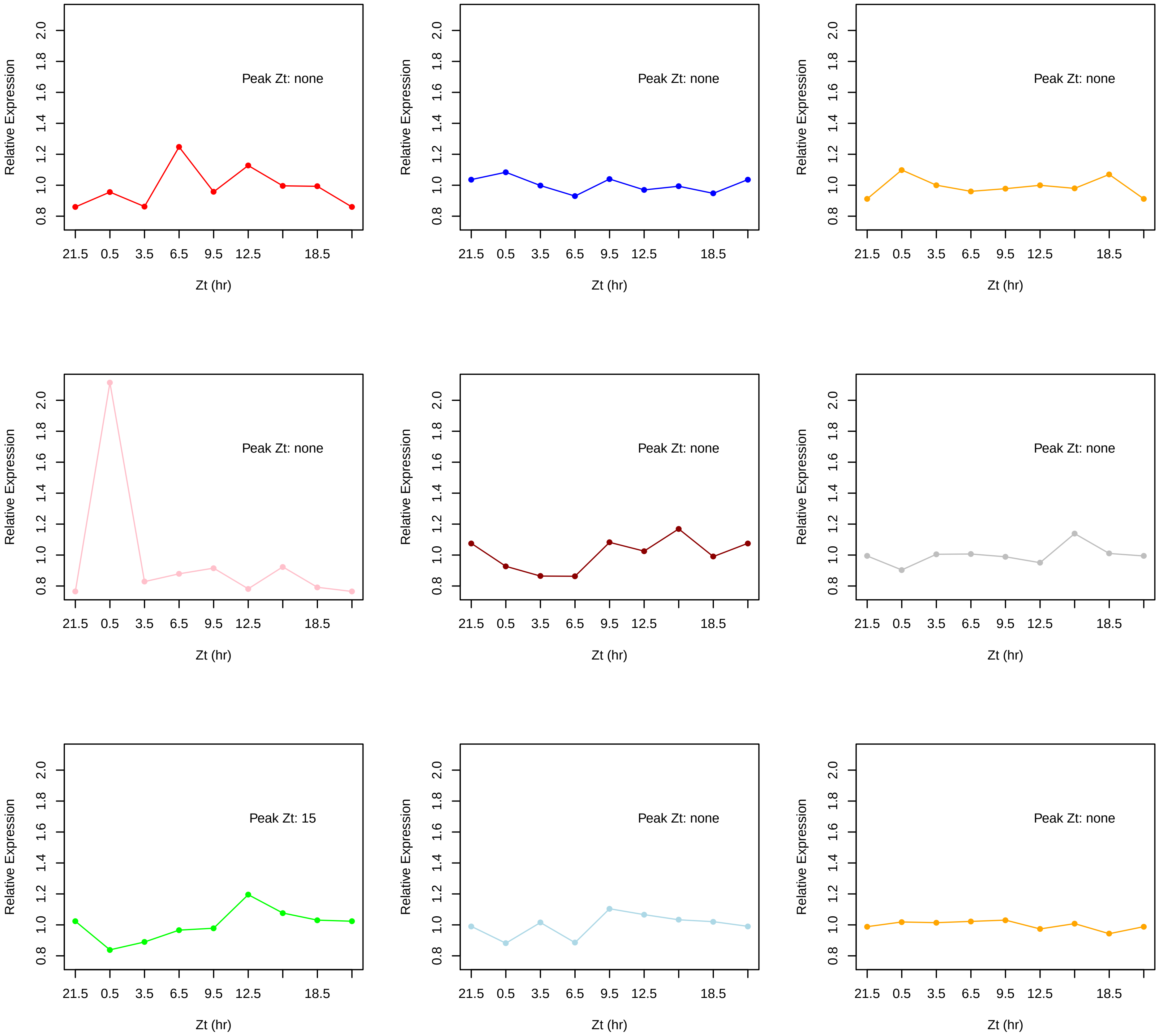

Singeleton: ENSXMAG00000023381 thtpa

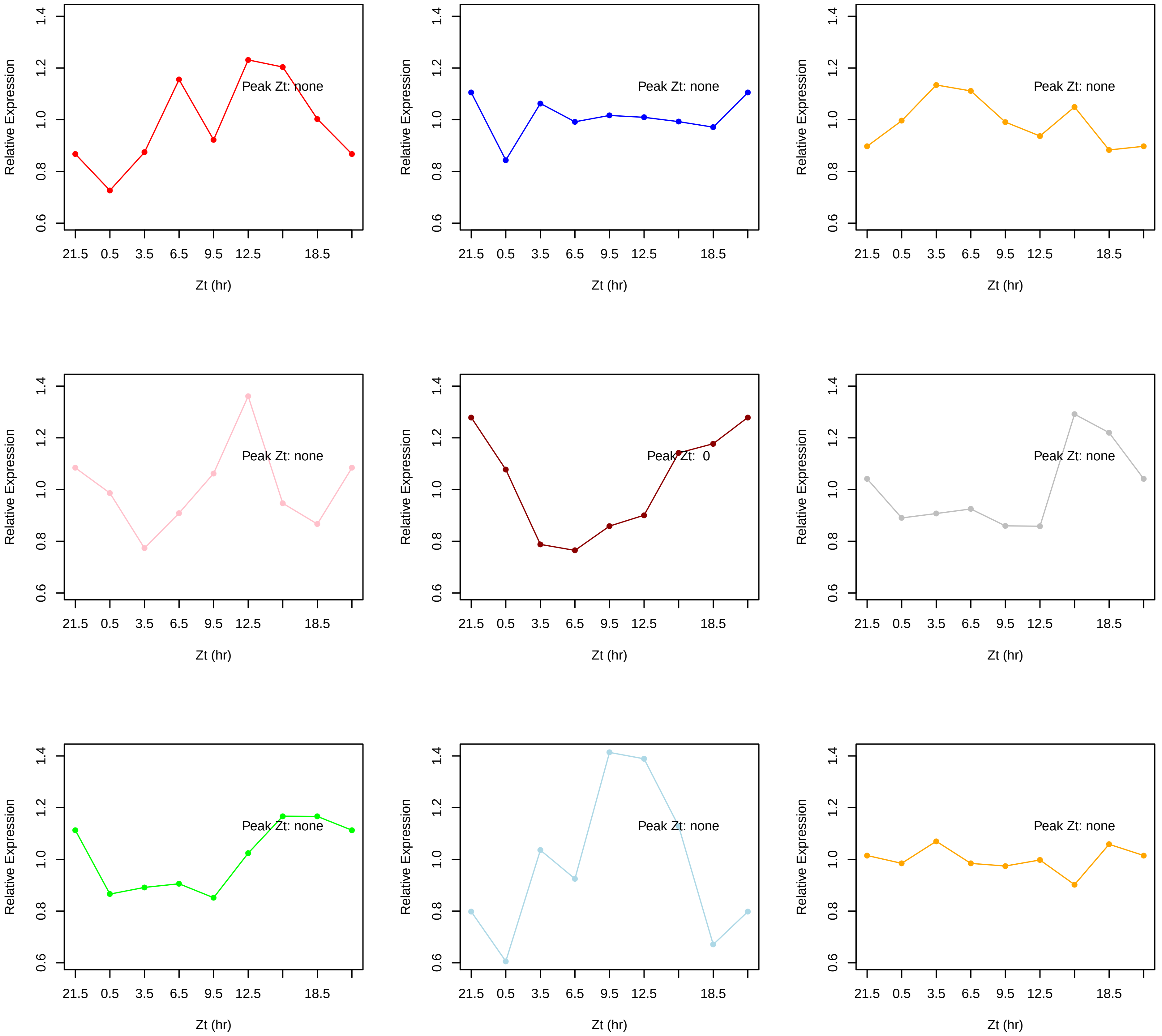

Singleton: ENSXMAG00000019220 nsun5

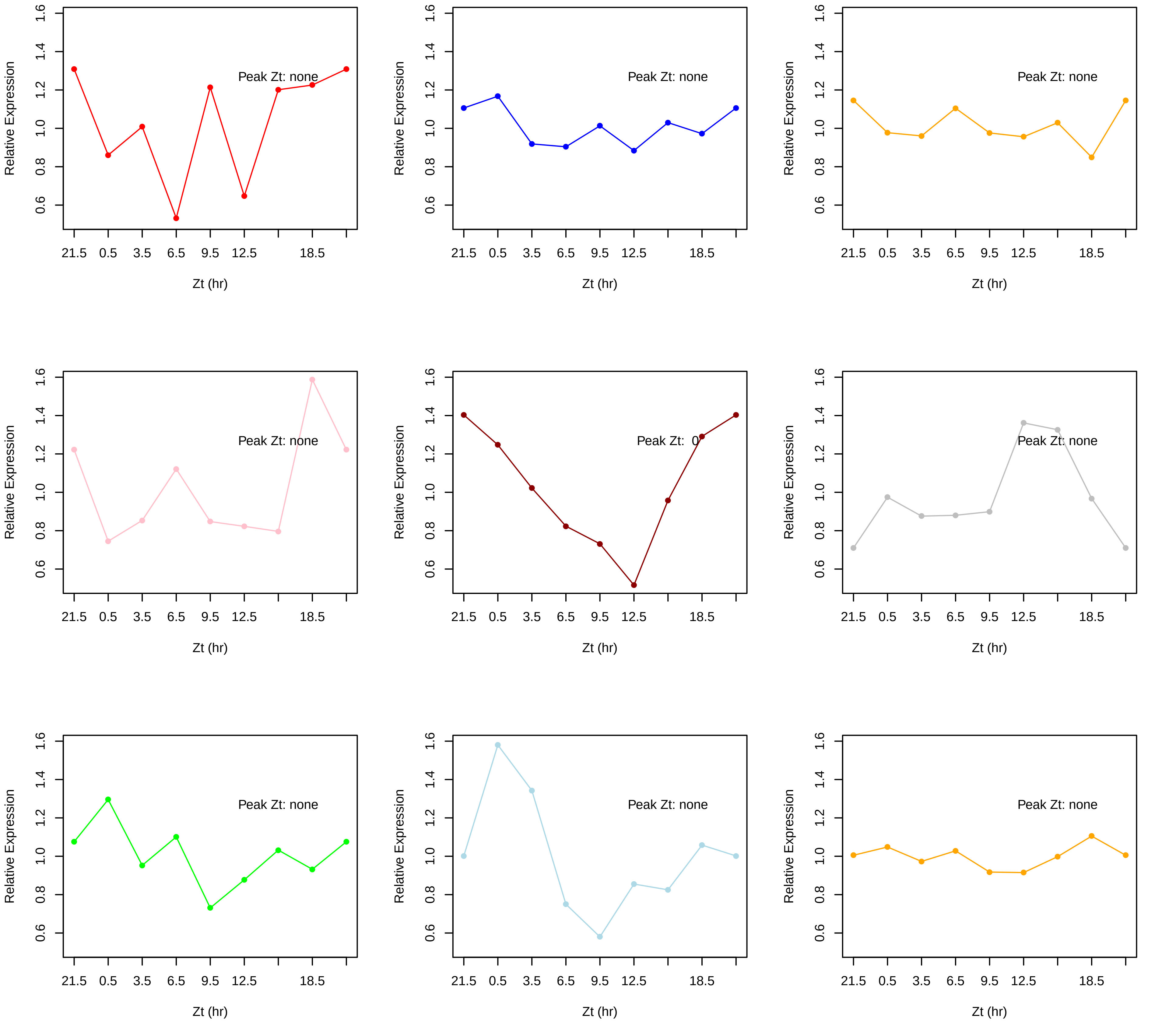

Singeleton: ENSXMAG00000015002 glyr1

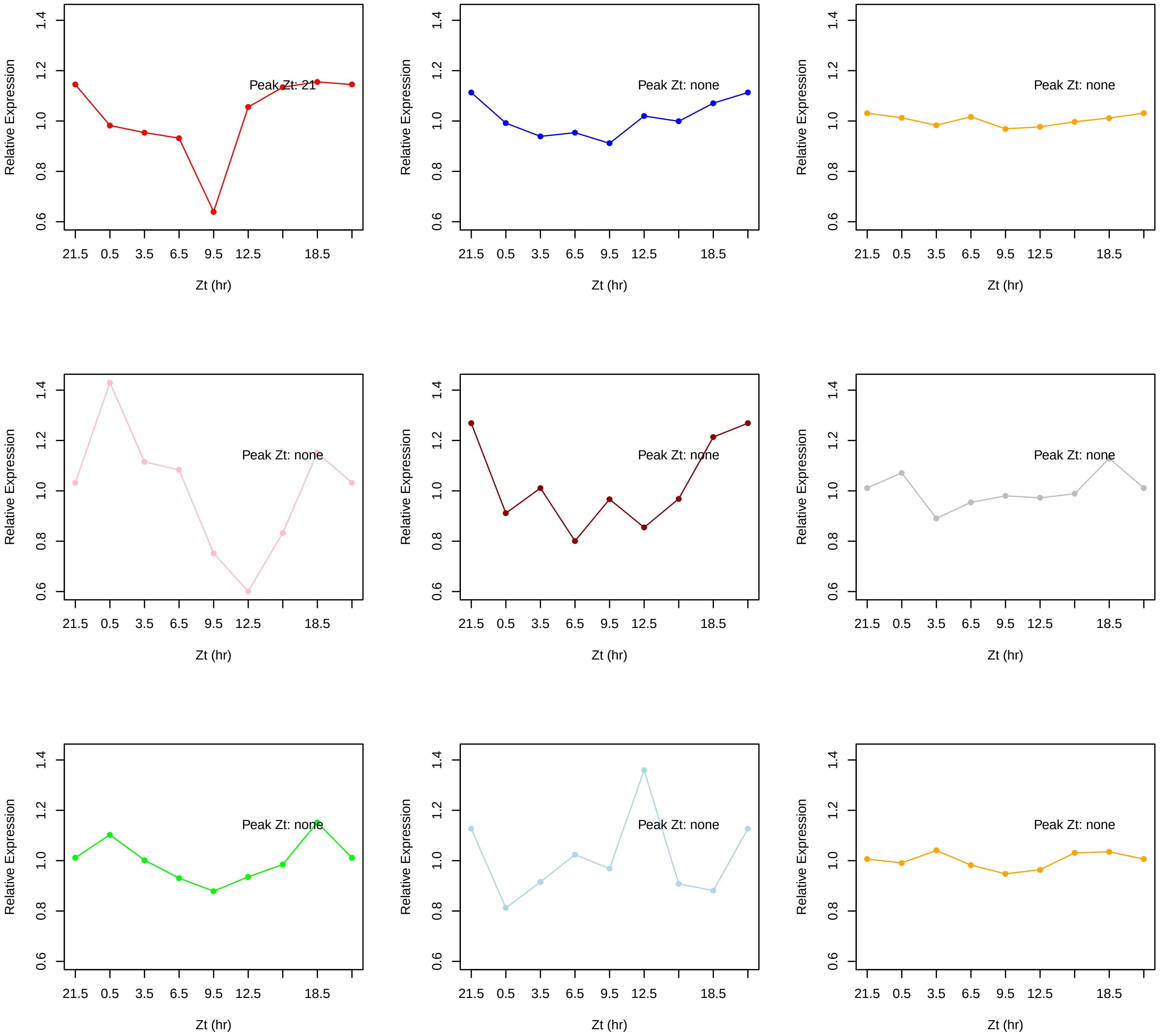

Singeleton: ENSXMAG00000003066 DBNDD1

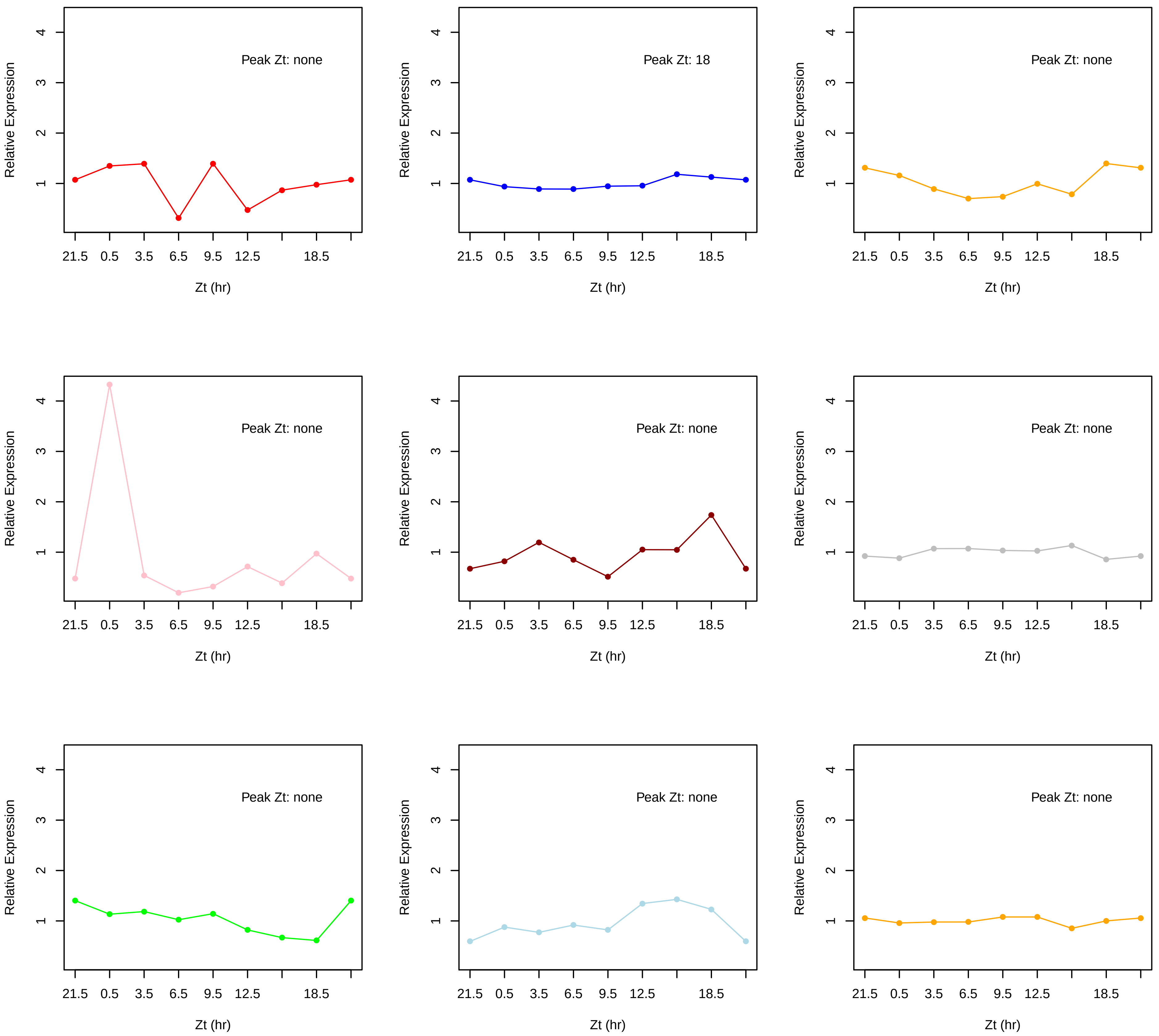

Singeleton: ENSXMAG00000002760 snrka

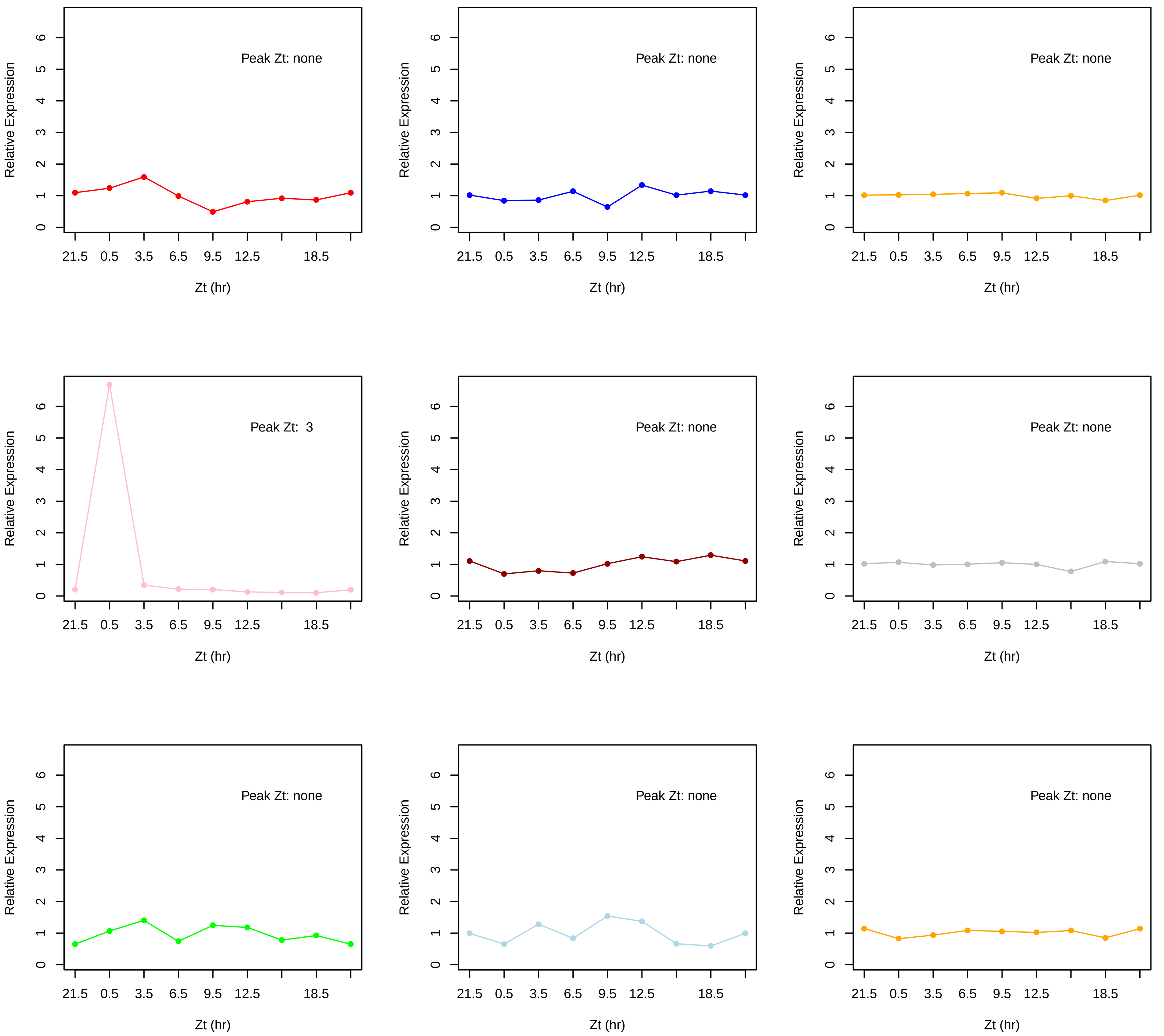

Singeleton: ENSXMAG00000004653 si:ch73−21k16.5

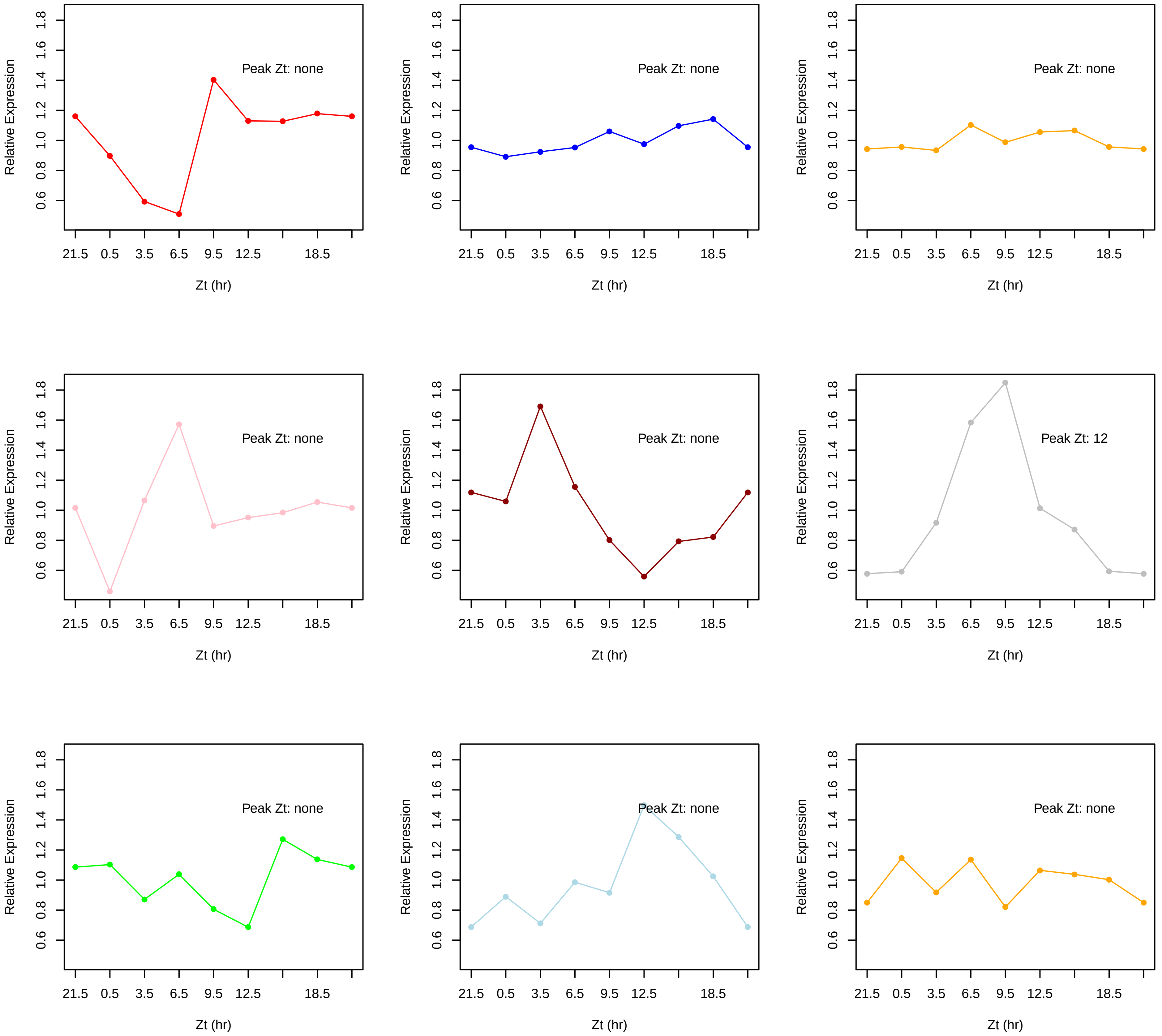

Singleton: ENSXMAG00000005240 ganab

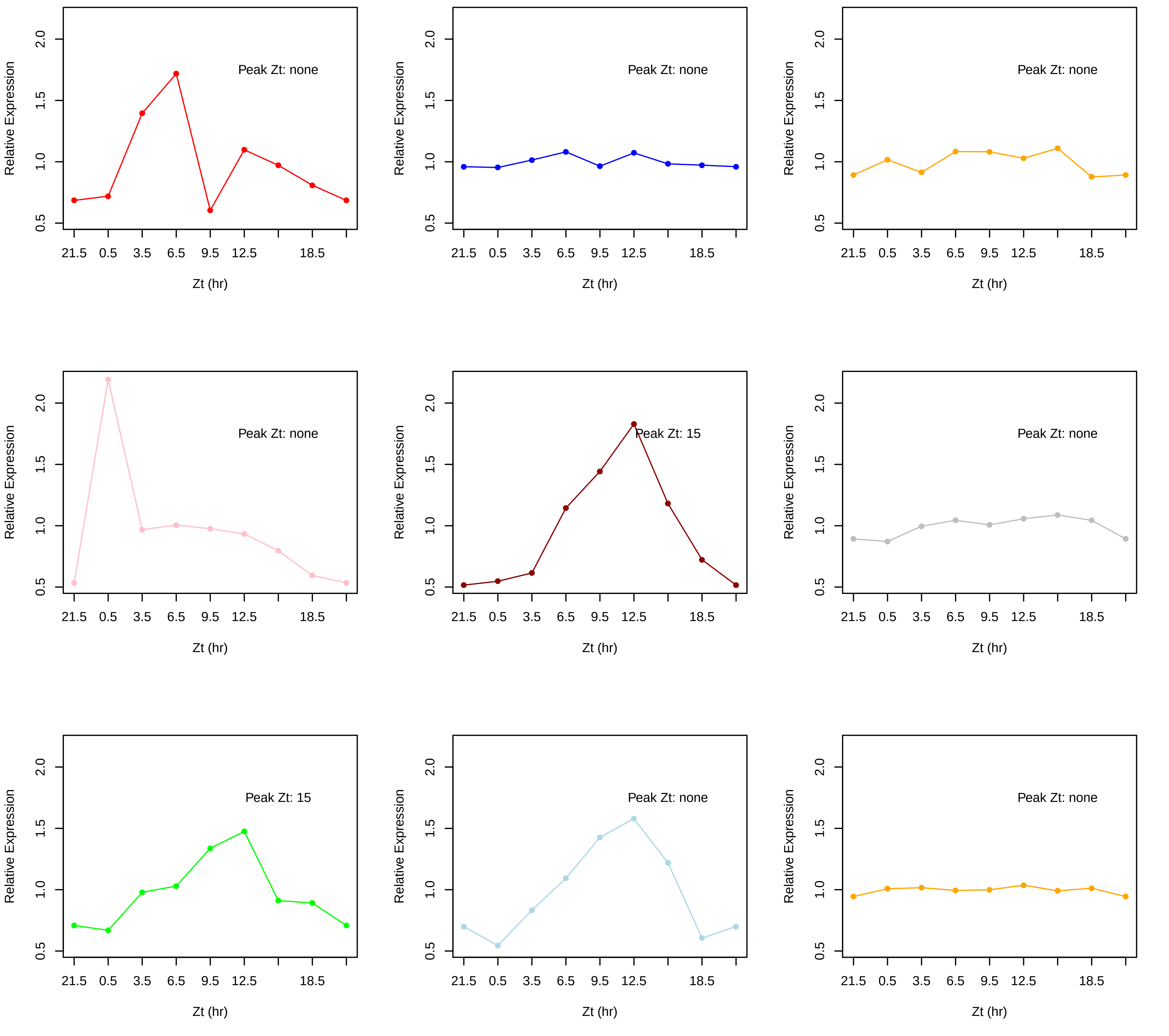

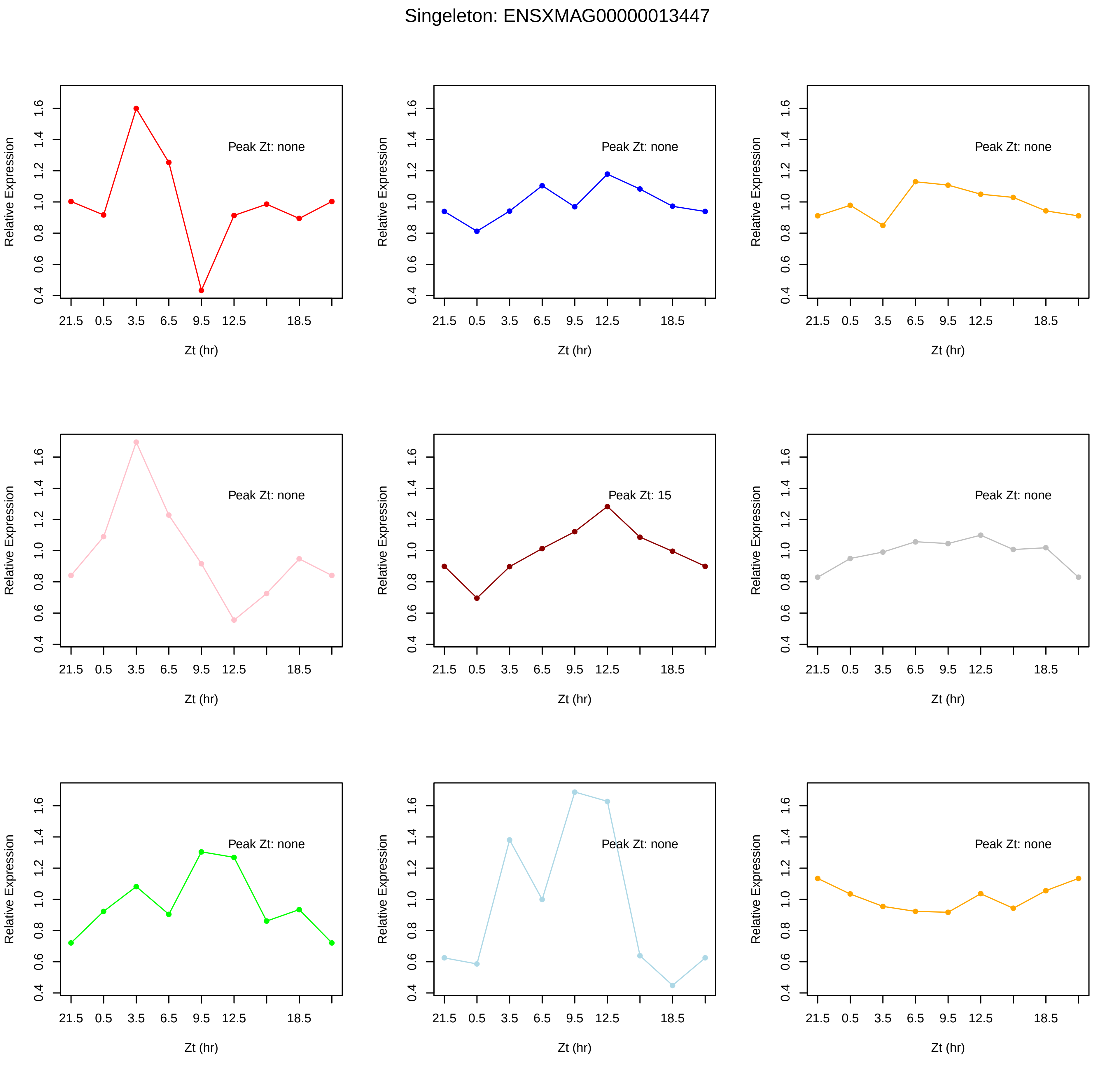

Singeleton: ENSXMAG00000013447
Peak Zt: none
Peak Zt: none
Peak Zt: none
Peak Zt: none
Peak Zt: 15
Peak Zt: none
Peak Zt: none
Peak Zt: none
Peak Zt: none
Relative Expression
Zt (hr)
0.4
0.6
0.8
1.0
1.2
1.4
1.6
21.5
0.5
3.5
6.5
9.5
12.5
18.5

Singeleton: ENSXMAG00000004725 tti1

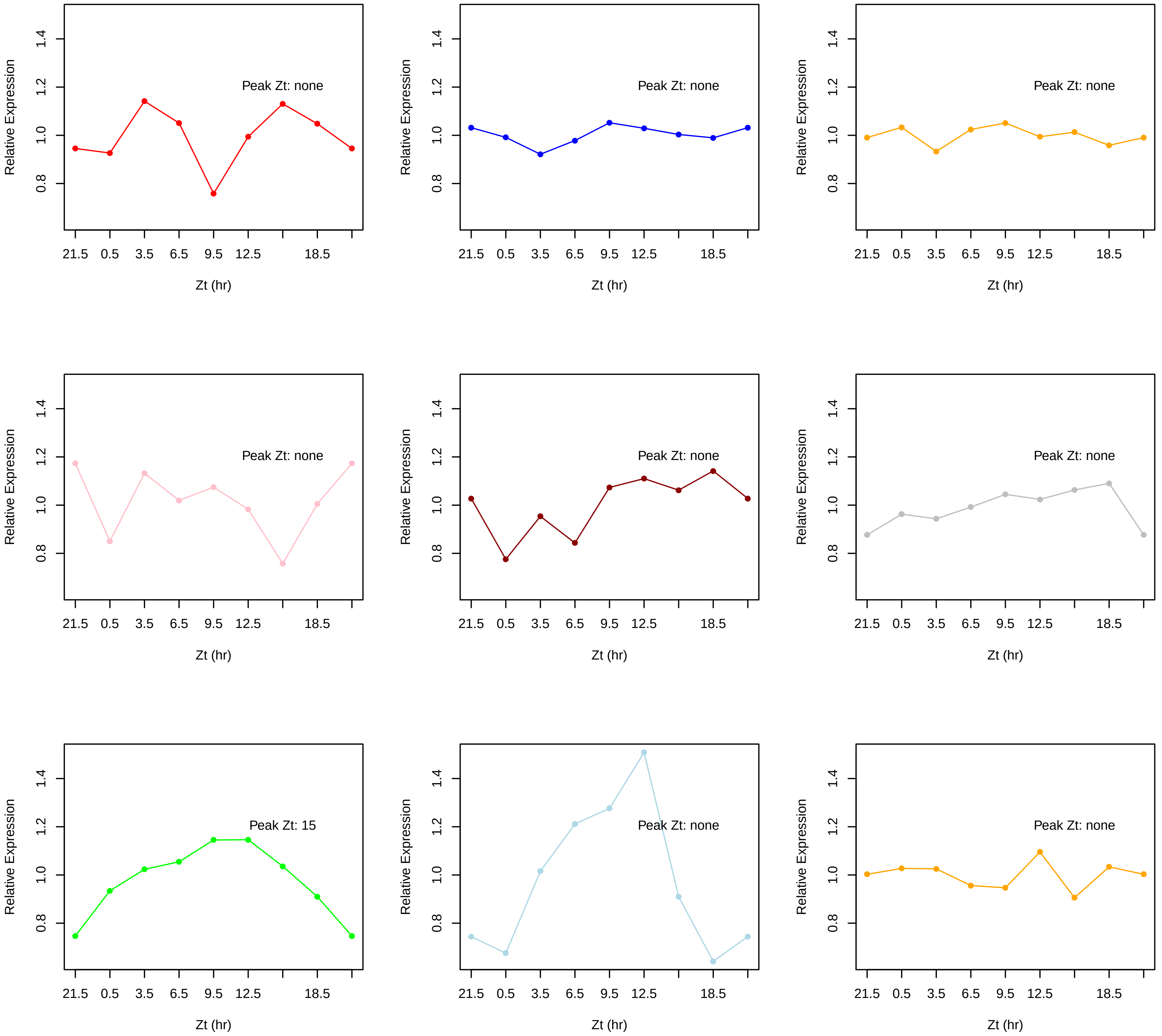

Singeleton: ENSXMAG00000003041

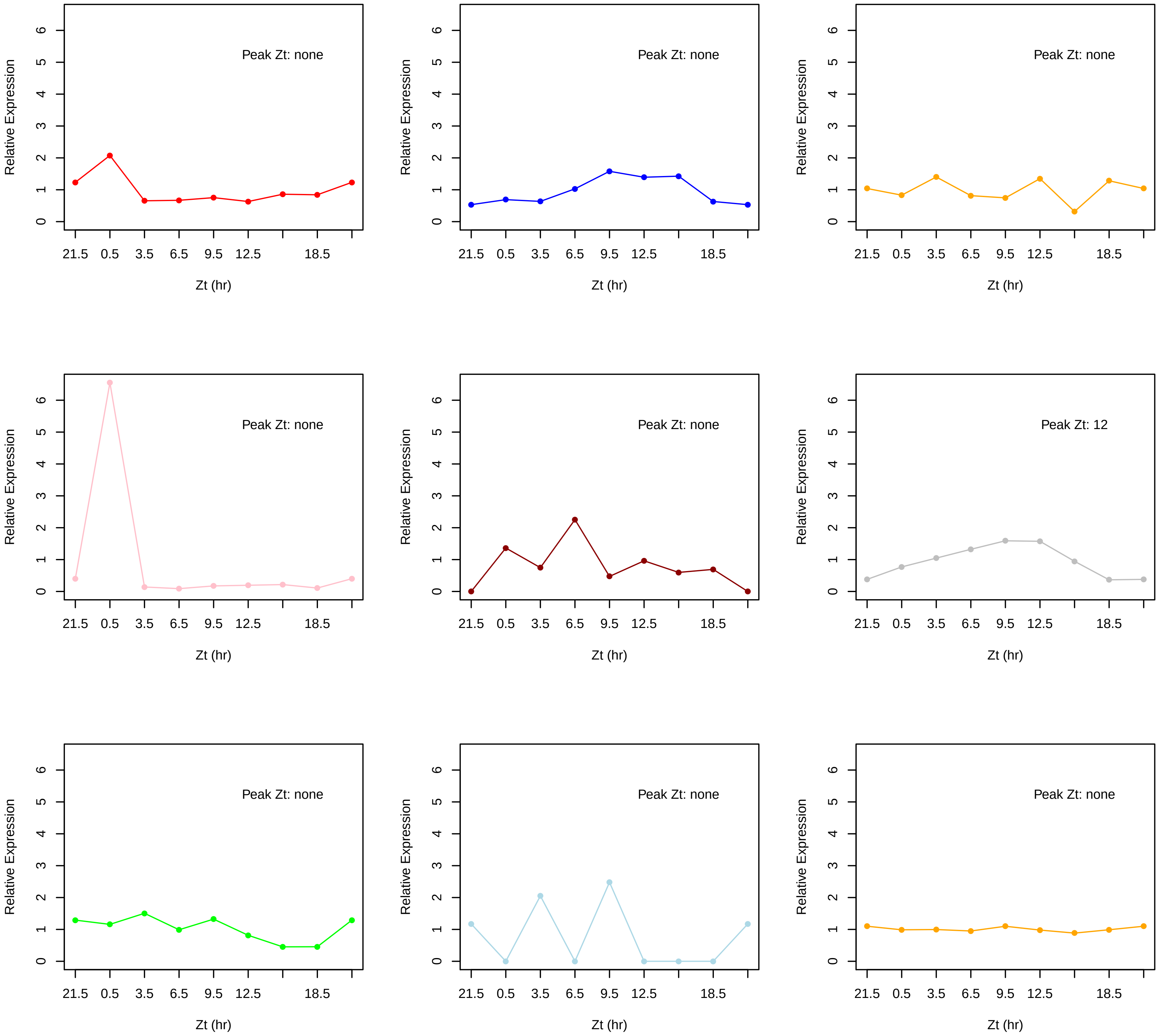

Singeleton: ENSXMAG00000012462 api5

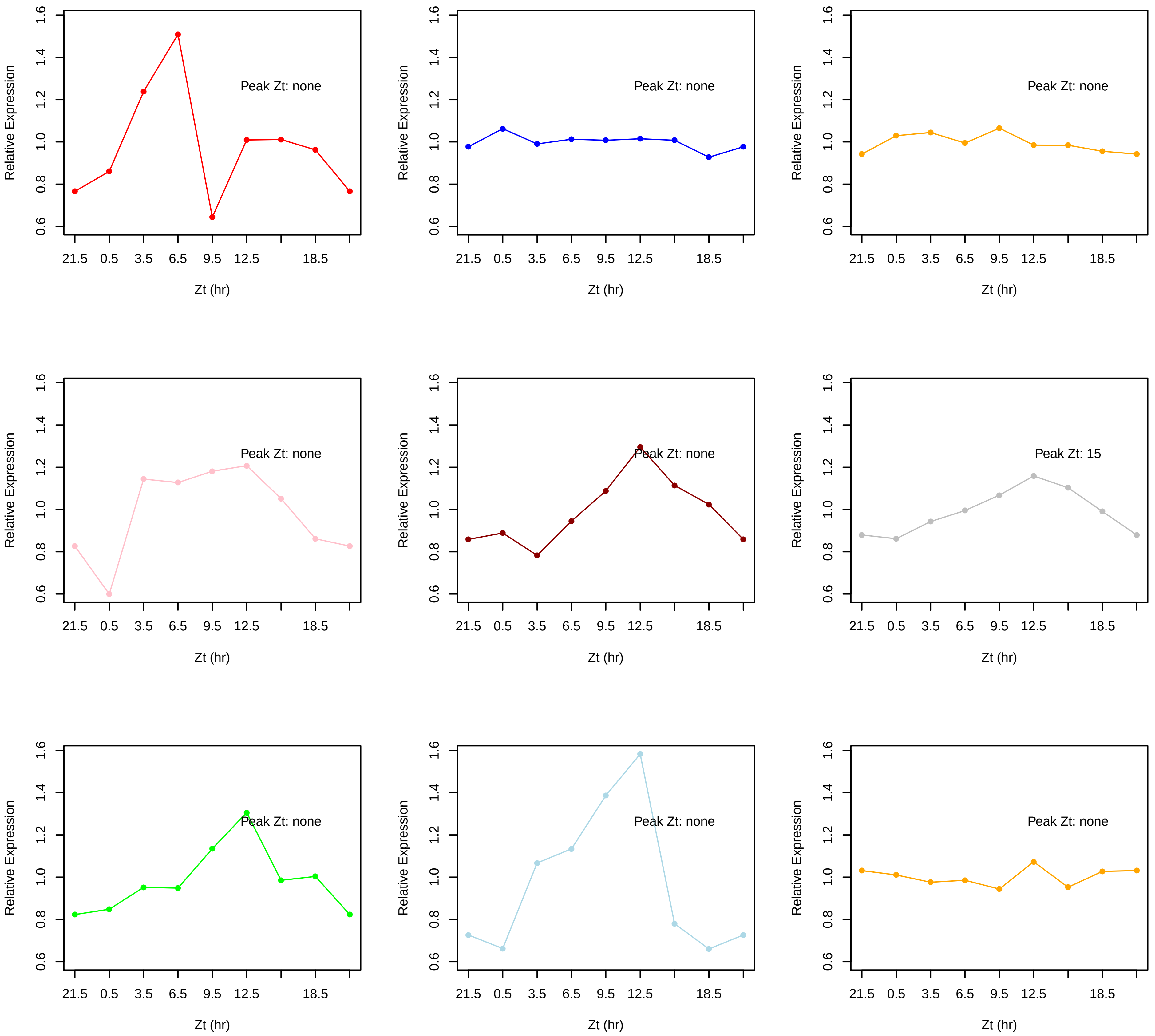

Singeleton: ENSXMAG00000003220 nudt22

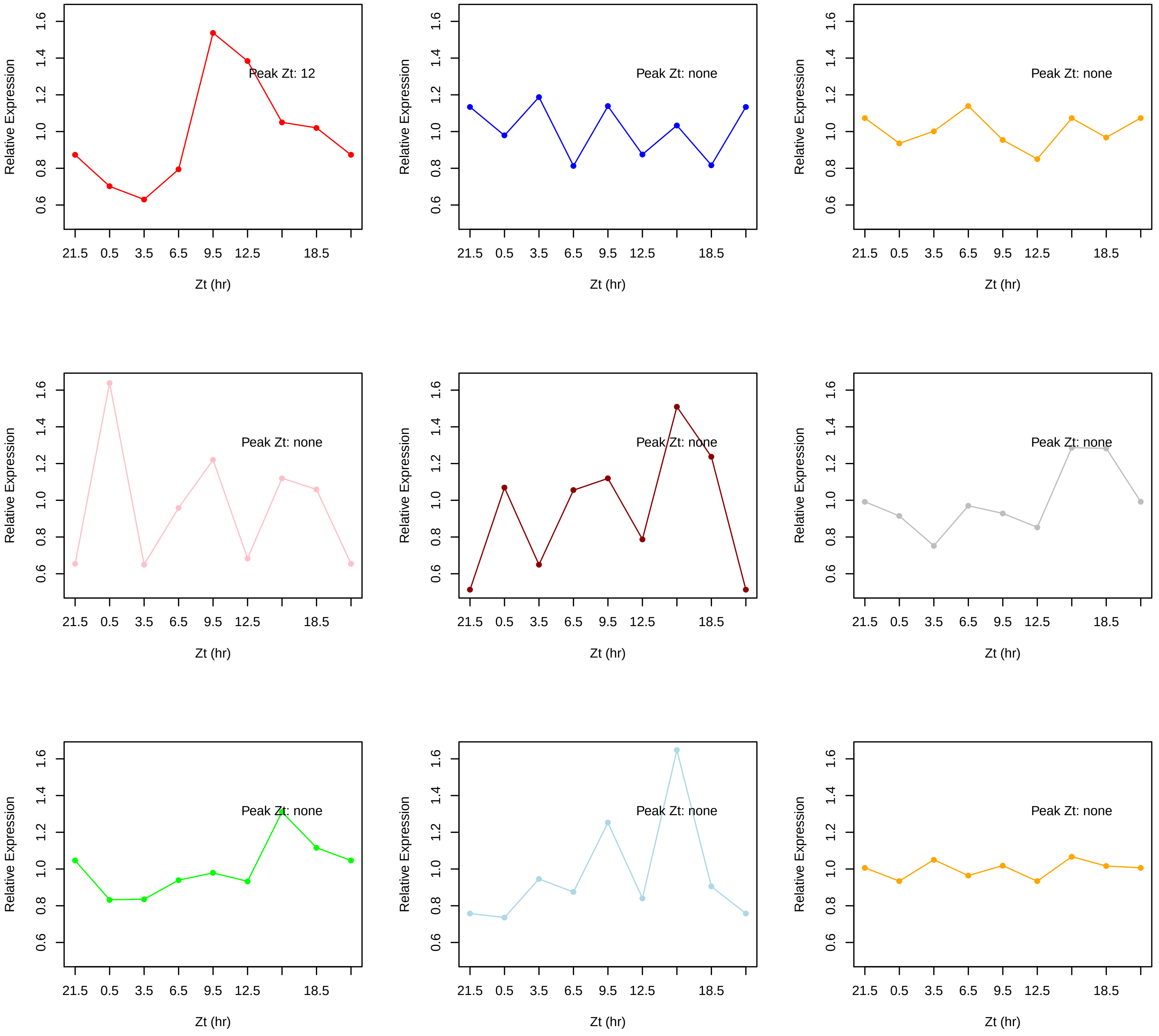

Singeleton: ENSXMAG00000018945 rprd1b

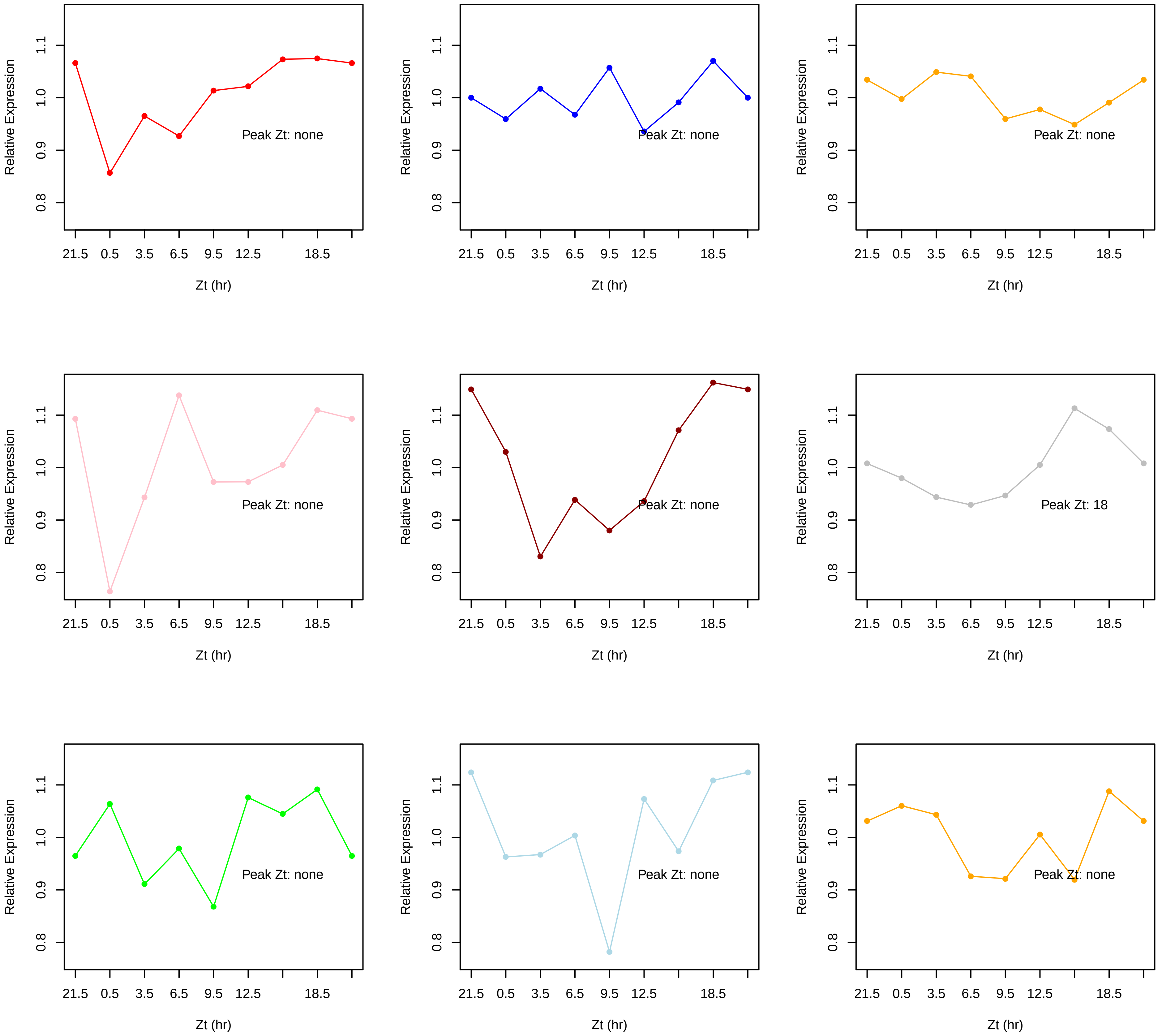

Singeleton: ENSXMAG00000001668 ano10a

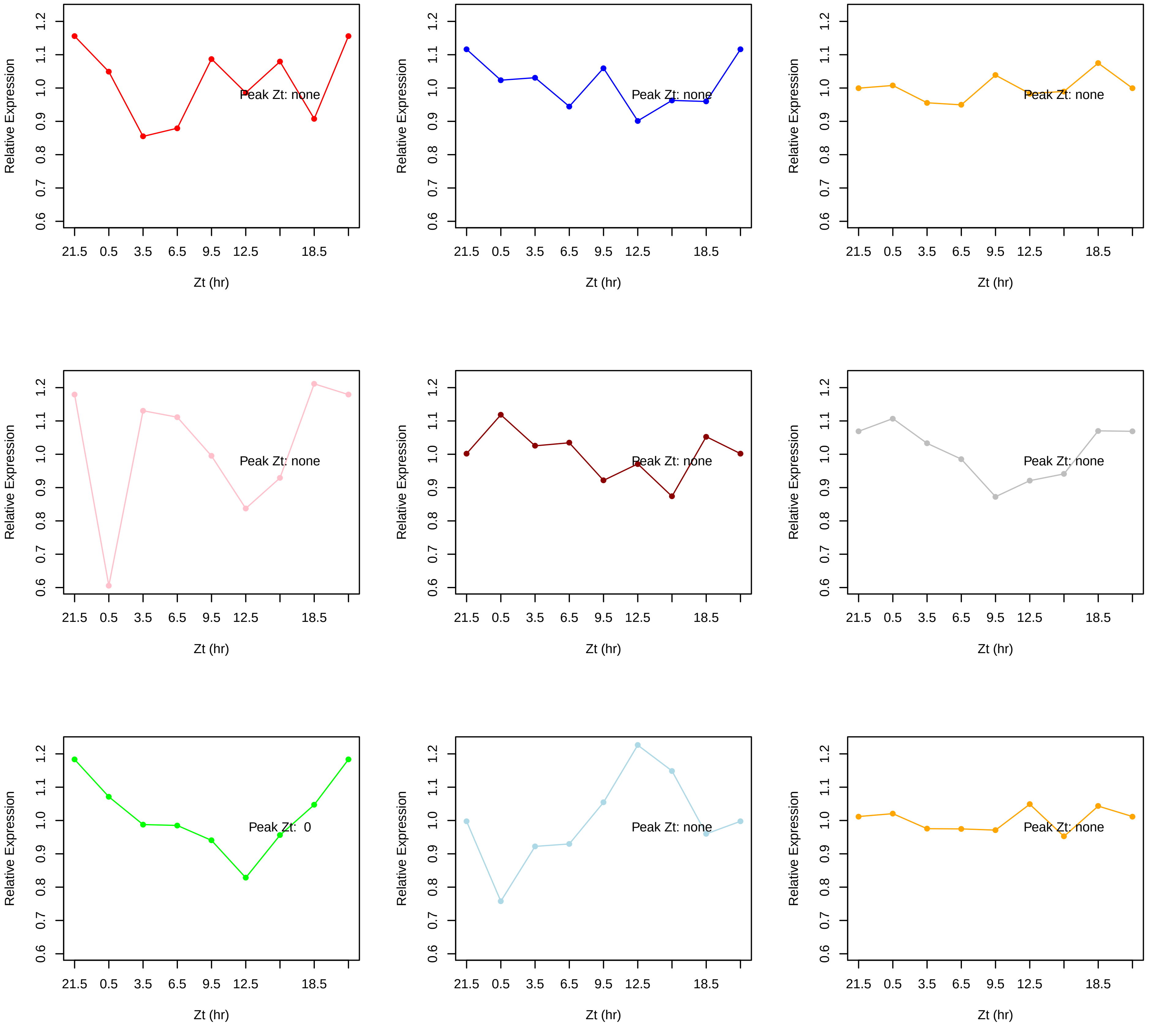

Singeleton: ENSXMAG00000000570 dysf

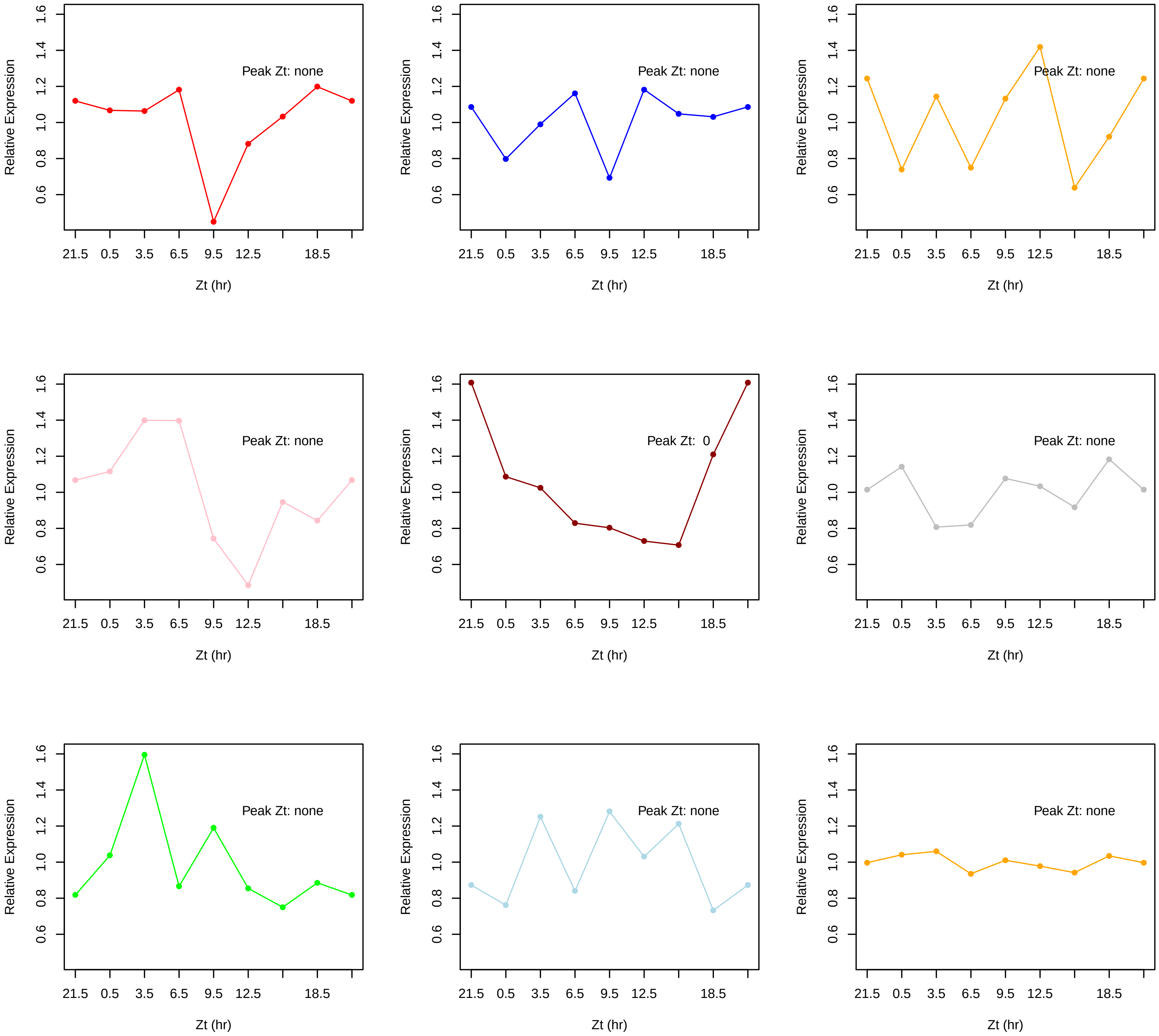

Singeleton: ENSXMAG00000003061 phpt1

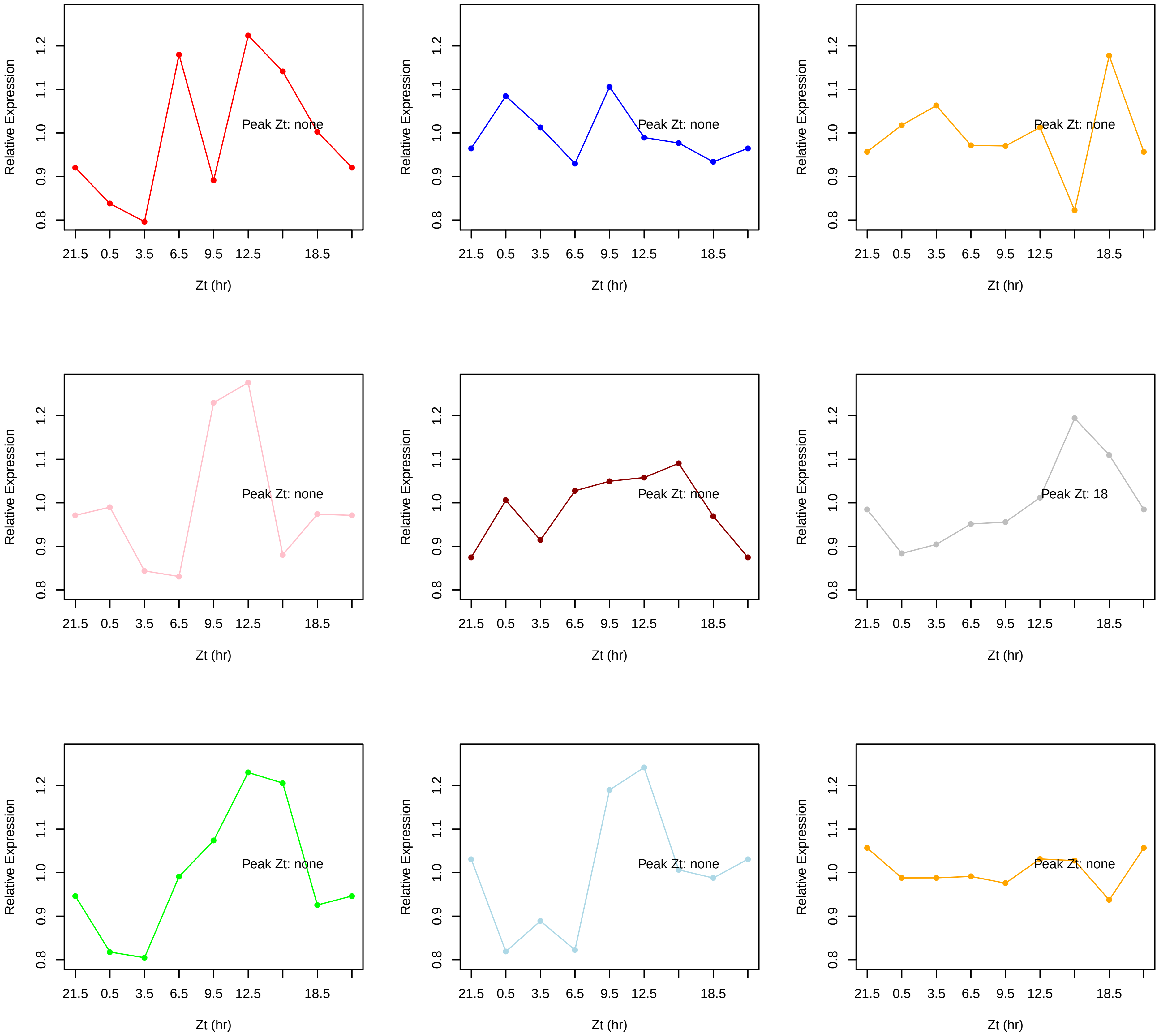

Singeleton: ENSXMAG00000002664 mob3a

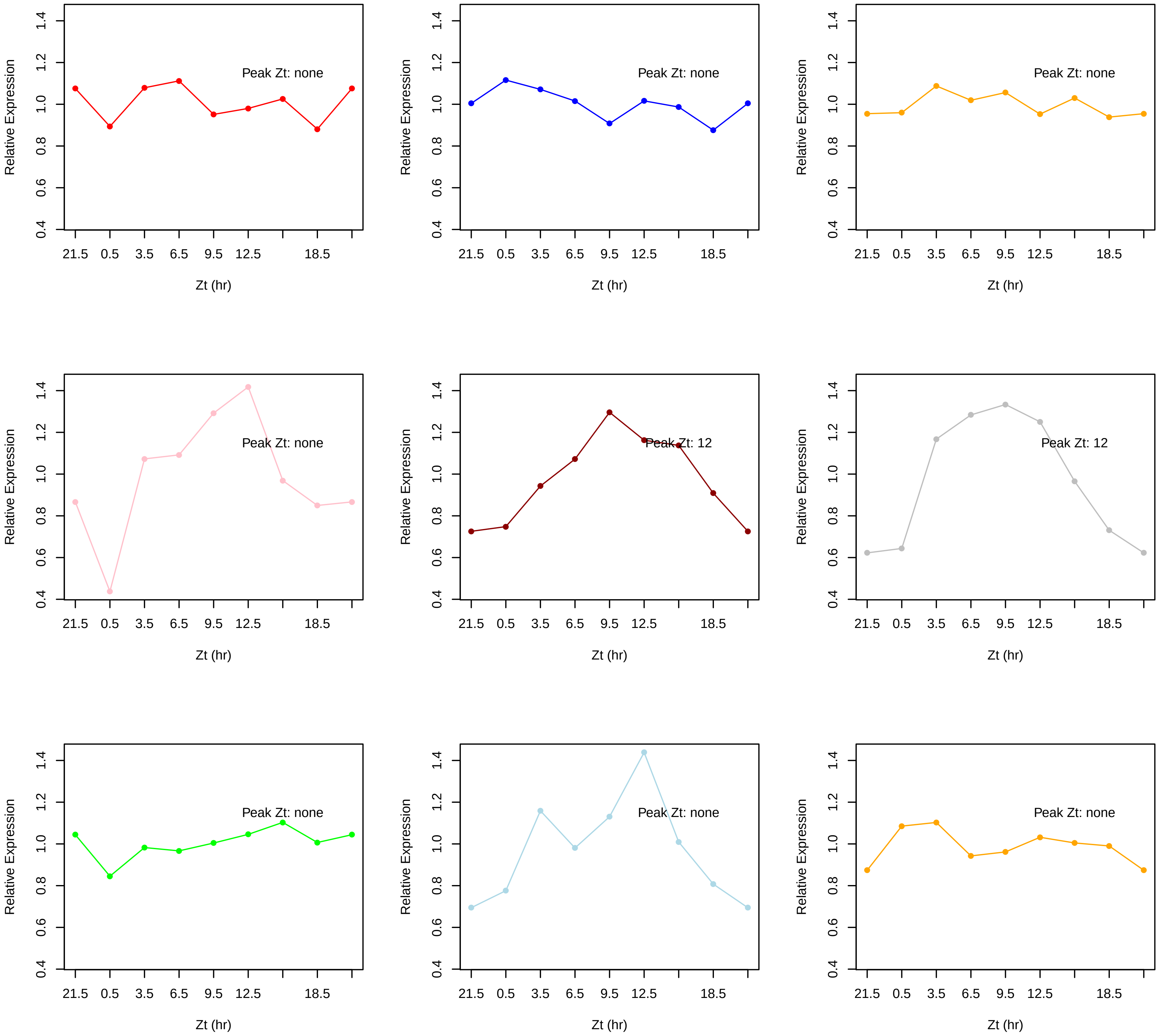

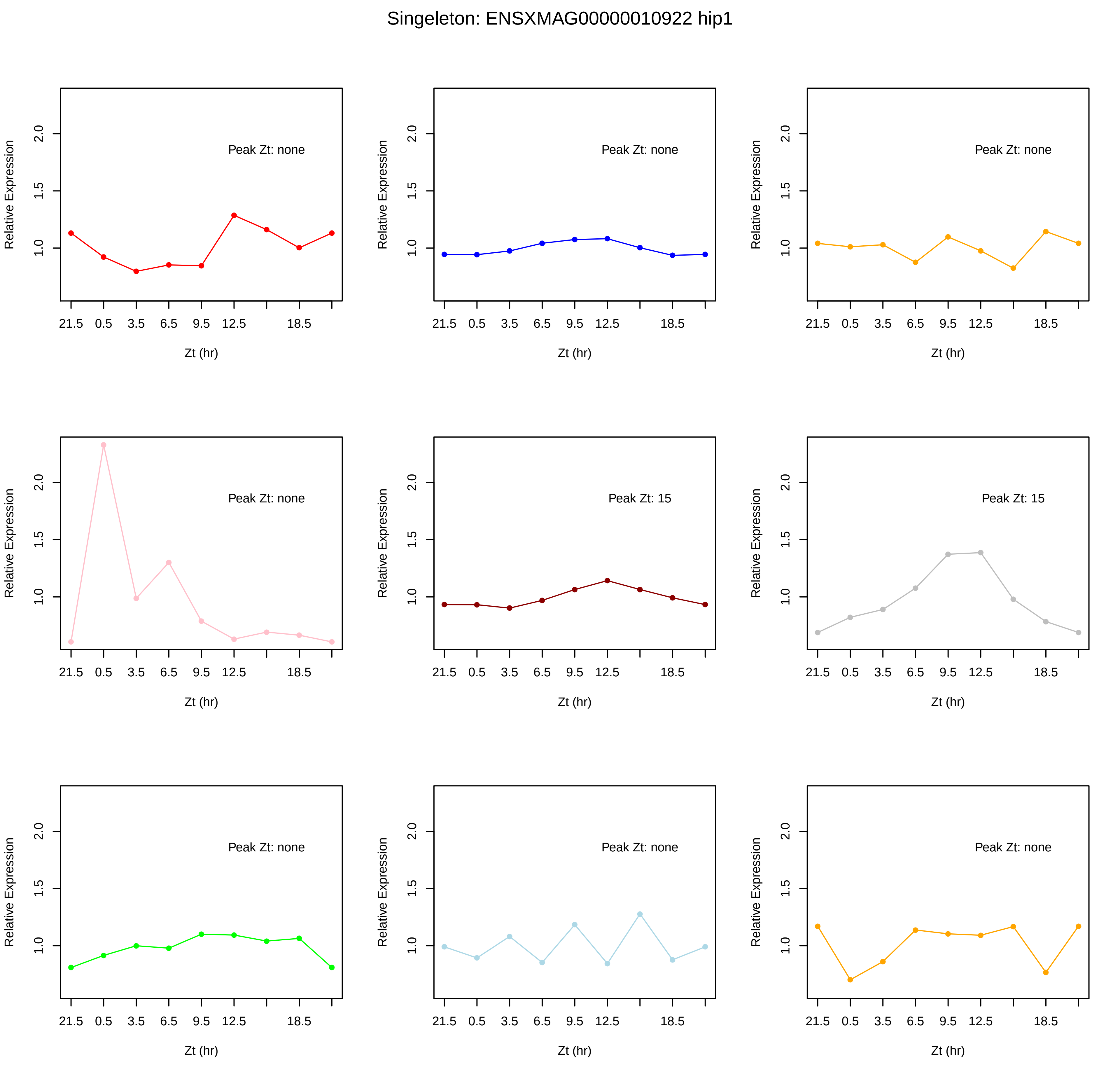

Singeleton: ENSXMAG00000010922 hip1
Relative Expression
Zt (hr)
21.5
0.5
3.5
6.5
9.5
12.5
18.5
1.0
1.5
2.0
Peak Zt: none
Peak Zt: none
Peak Zt: none
Peak Zt: none
Peak Zt: 15
Peak Zt: 15
Peak Zt: none
Peak Zt: none
Peak Zt: none

Singleton: ENSXMAG00000003200 stx5a

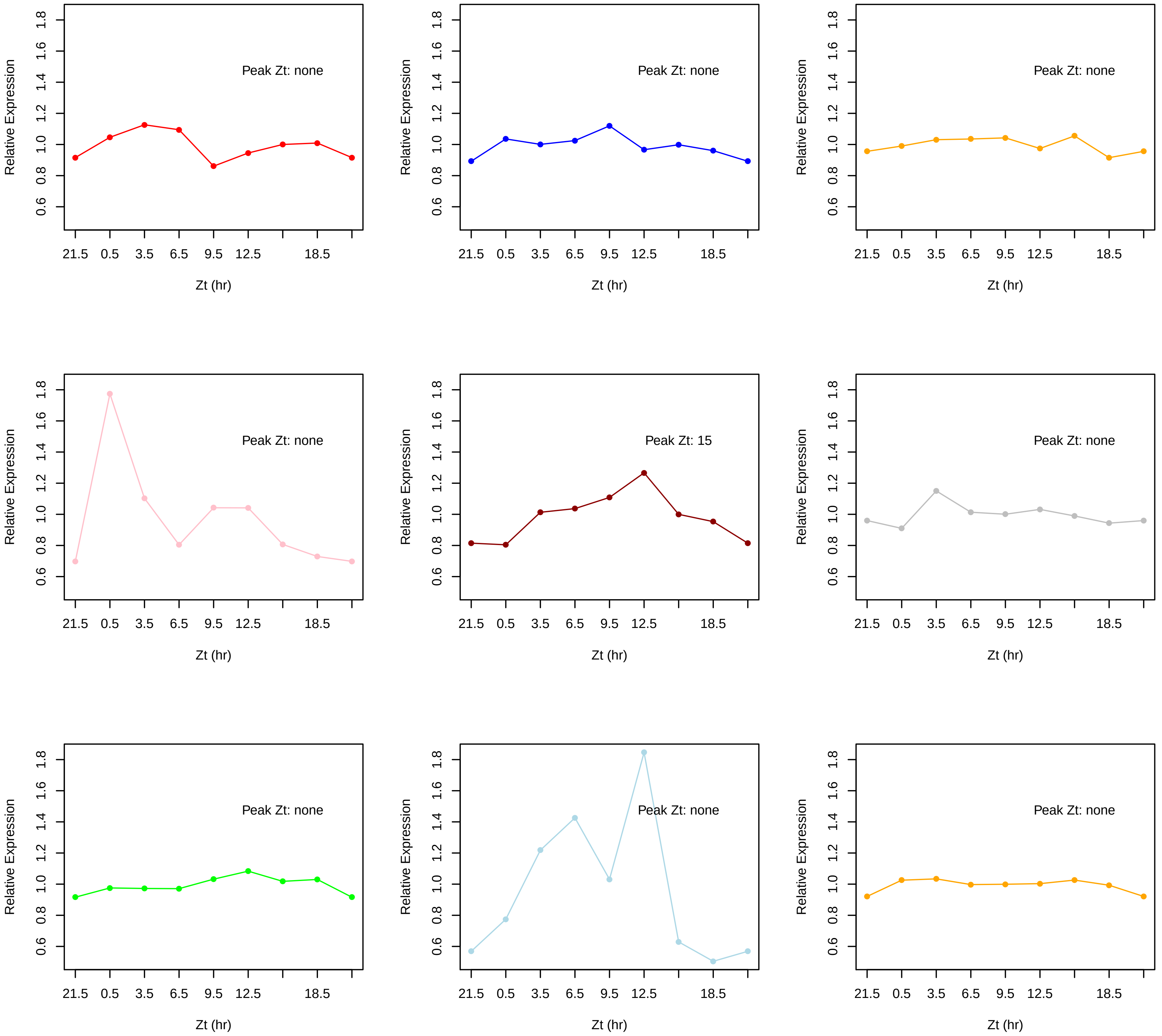

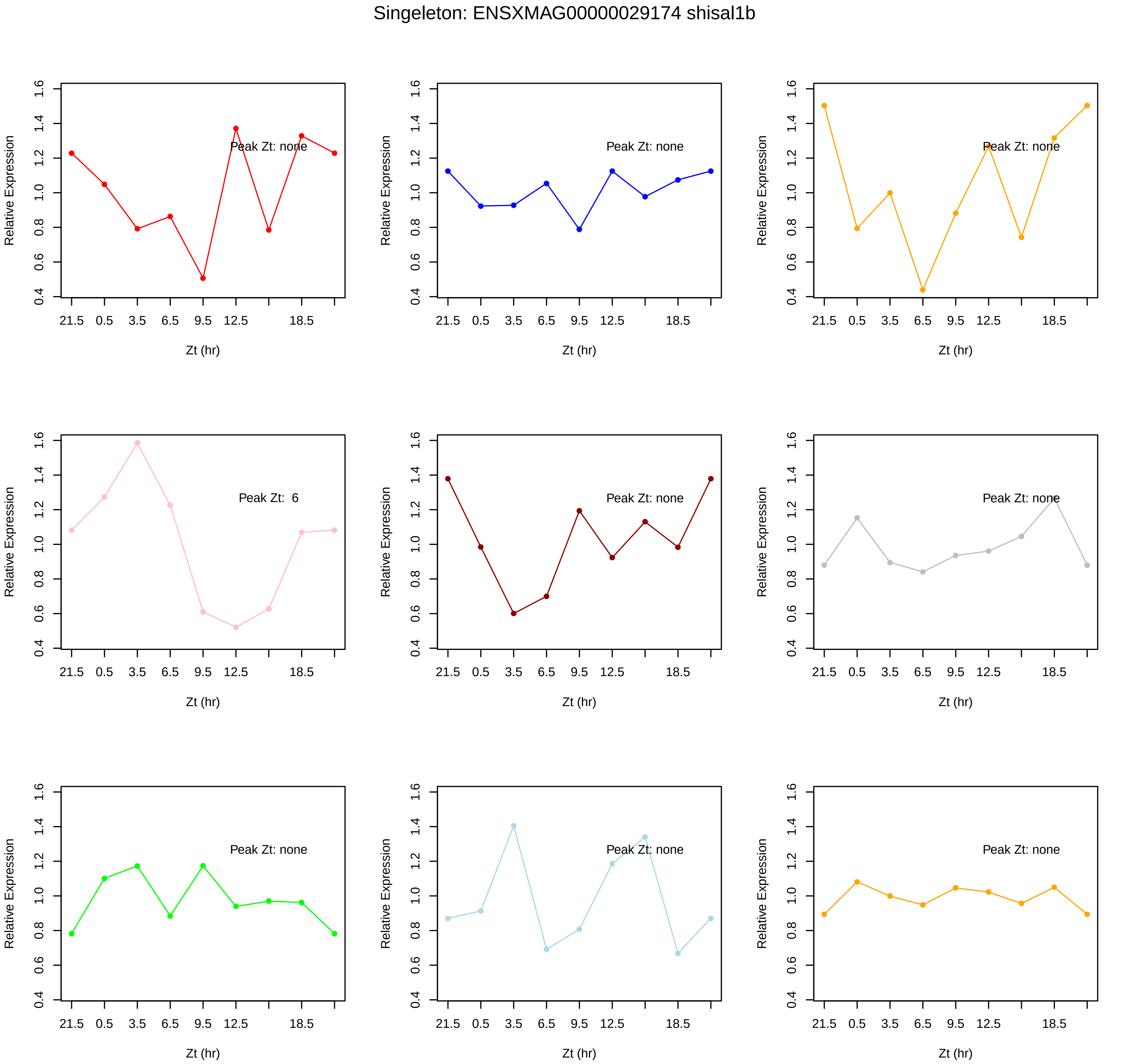

Singeleton: ENSXMAG00000029174 shisal1b
Peak Zt: none
Peak Zt: 6
Relative Expression
Zt (hr)
21.5 0.5 3.5 6.5 9.5 12.5 18.5
0.4 0.6 0.8 1.0 1.2 1.4 1.6

Singeleton: ENSXMAG00000024123 trim62

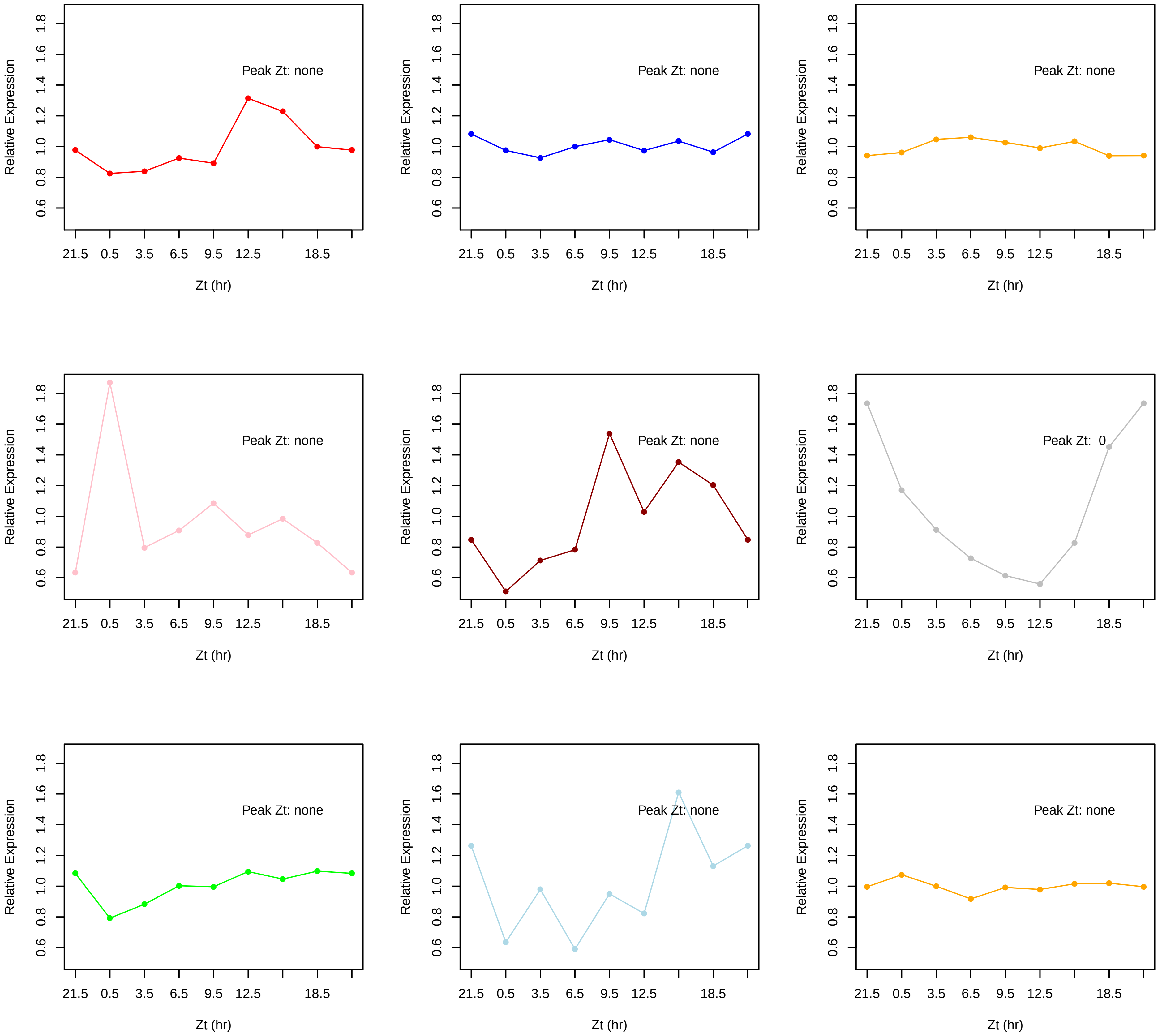

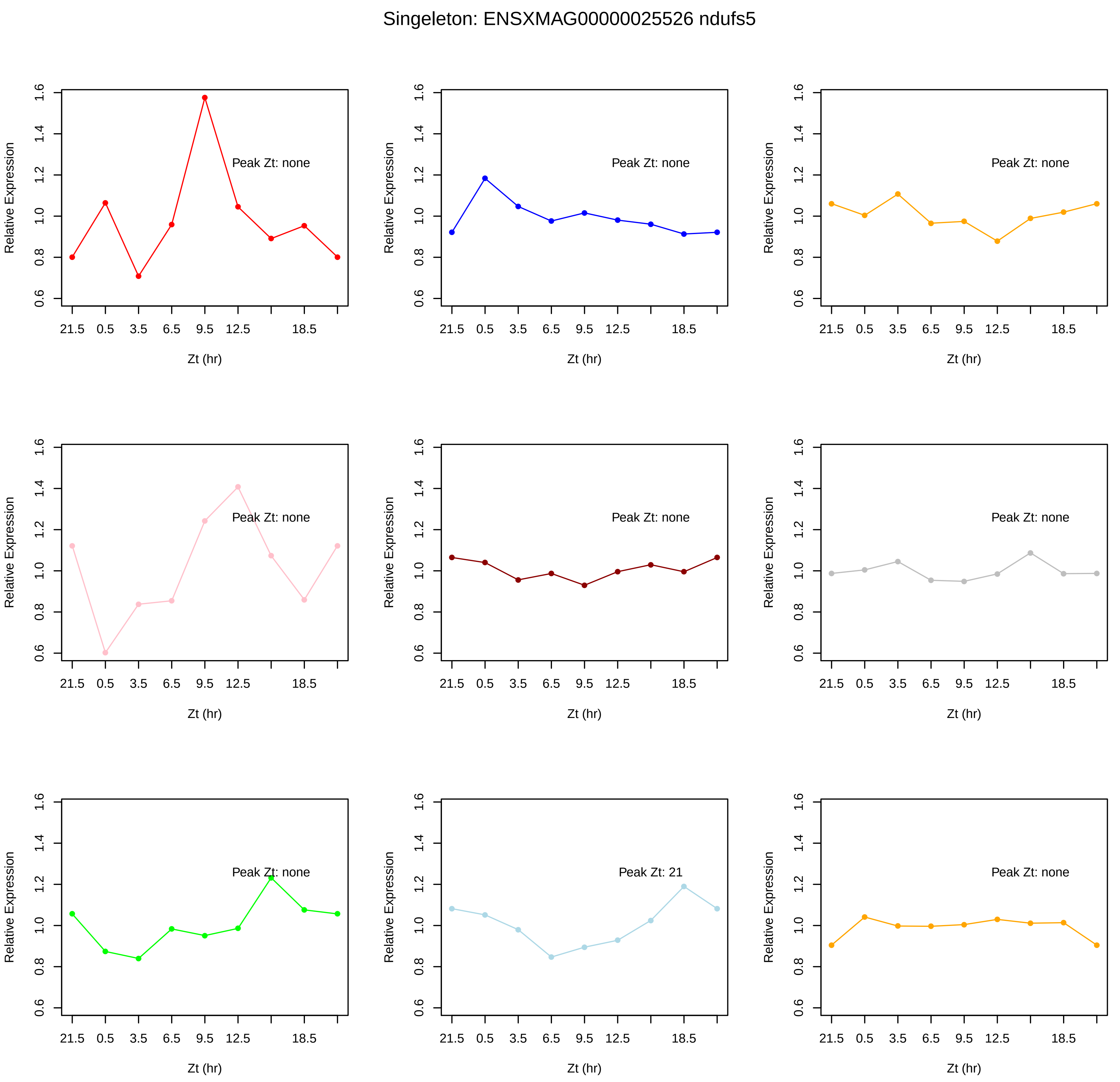
Singeleton: ENSXMAG00000025526 ndufs5
Relative Expression
Zt (hr)
21.5
0.5
3.5
6.5
9.5
12.5
18.5
0.6
0.8
1.0
1.2
1.4
1.6
Peak Zt: none
Peak Zt: none
Peak Zt: none
Peak Zt: none
Peak Zt: none
Peak Zt: none
Peak Zt: none
Peak Zt: 21
Peak Zt: none

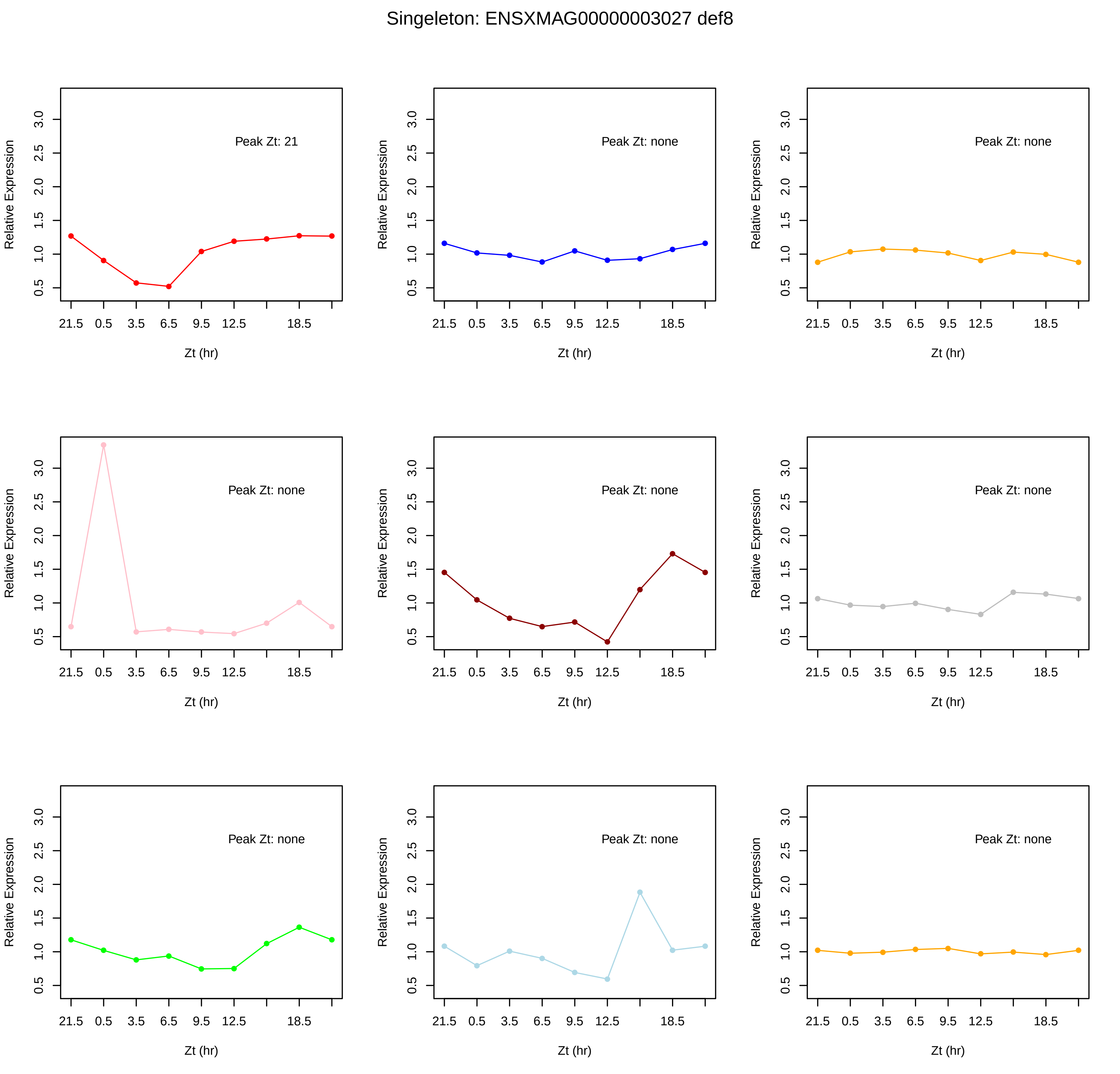
Singeleton: ENSXMAG00000003027 def8
Peak Zt: 21
Peak Zt: none
Peak Zt: none
Peak Zt: none
Peak Zt: none
Peak Zt: none
Peak Zt: none
Peak Zt: none
Peak Zt: none
Relative Expression
Zt (hr)
21.5 0.5 3.5 6.5 9.5 12.5 18.5
0.5 1.0 1.5 2.0 2.5 3.0

Singleton: ENSXMAG00000018589 tgm2b

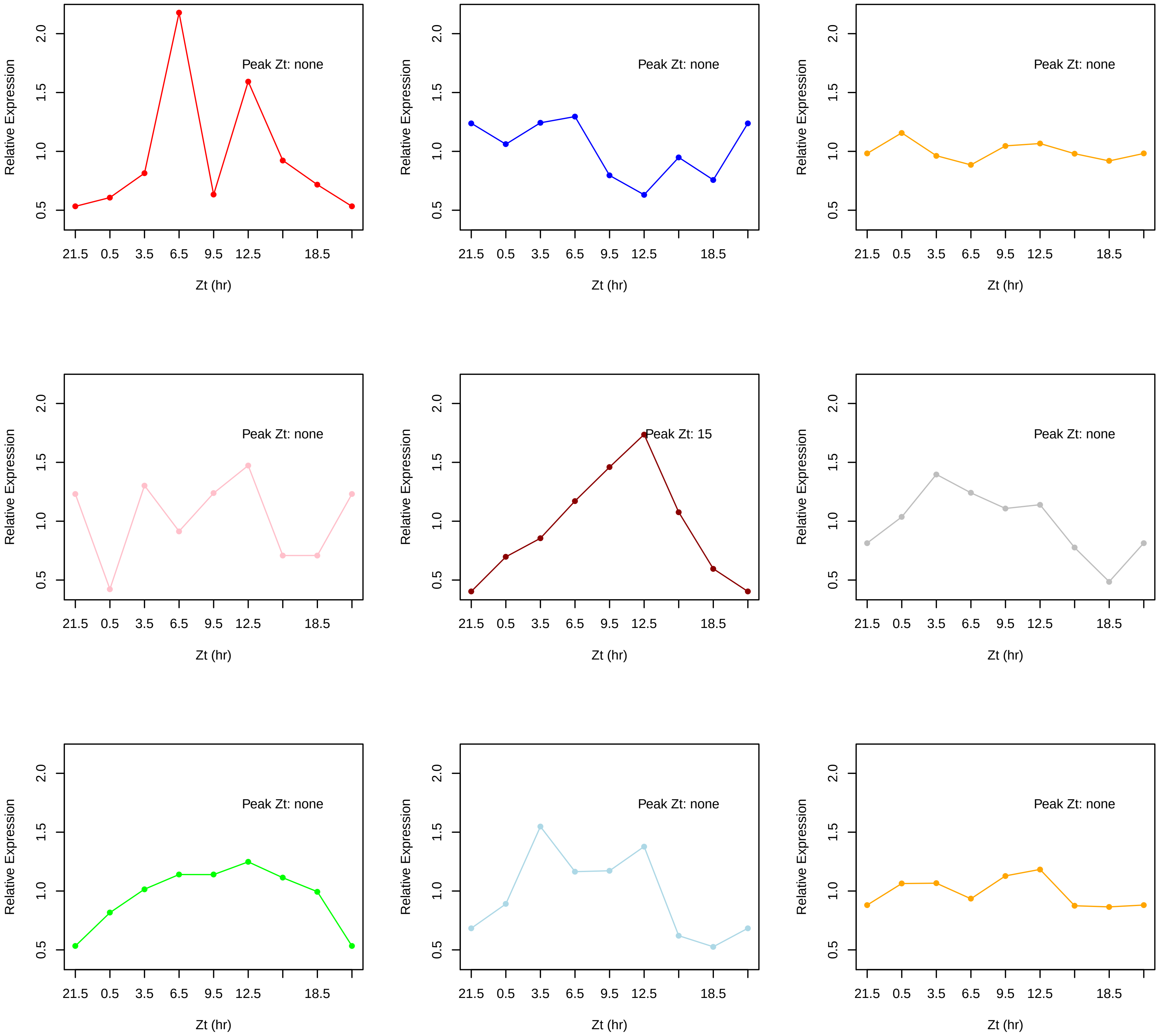

Singeleton: ENSXMAG00000026129 mknk2b

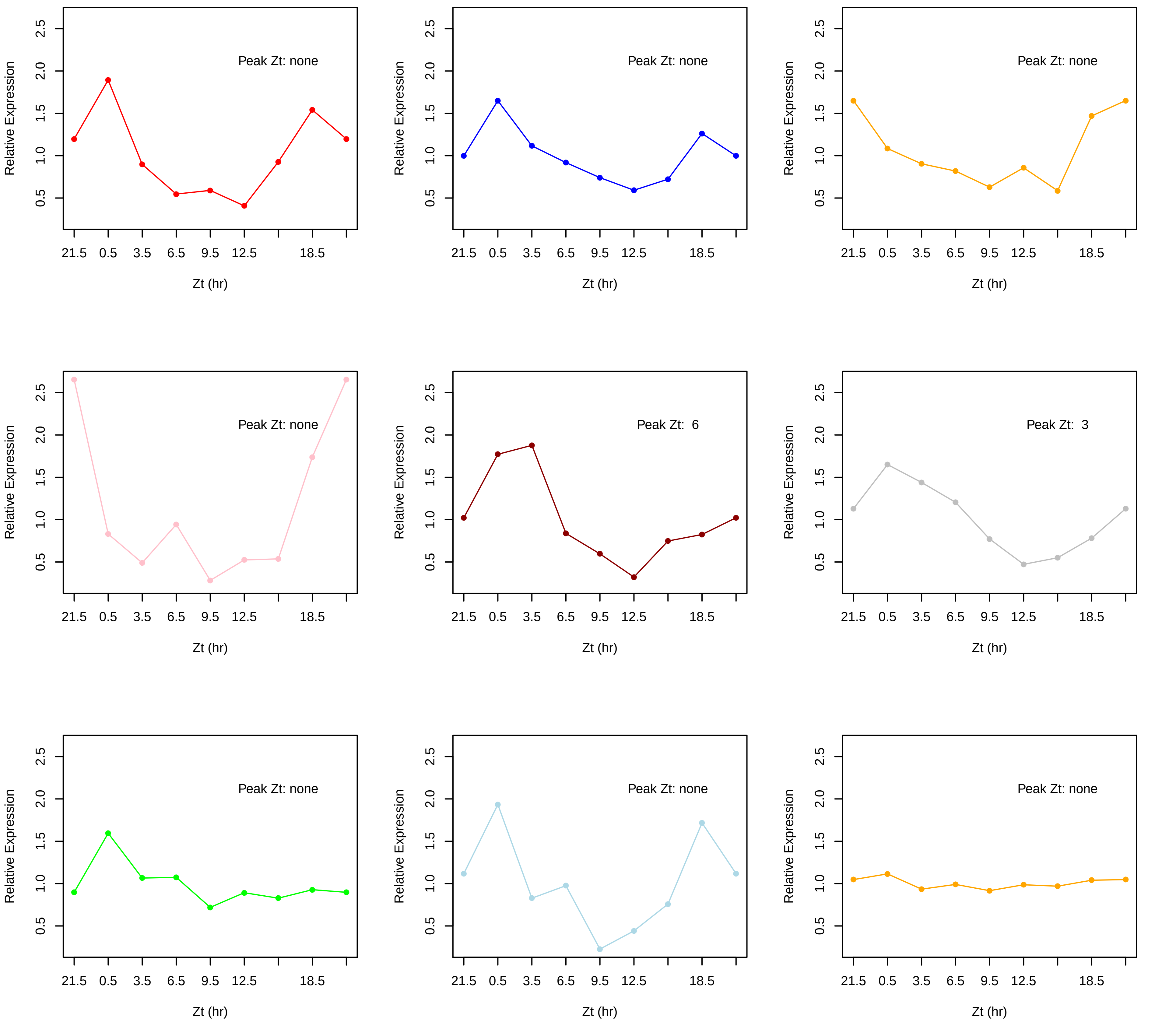

Singleton: ENSXMAG00000010684 thnsl2

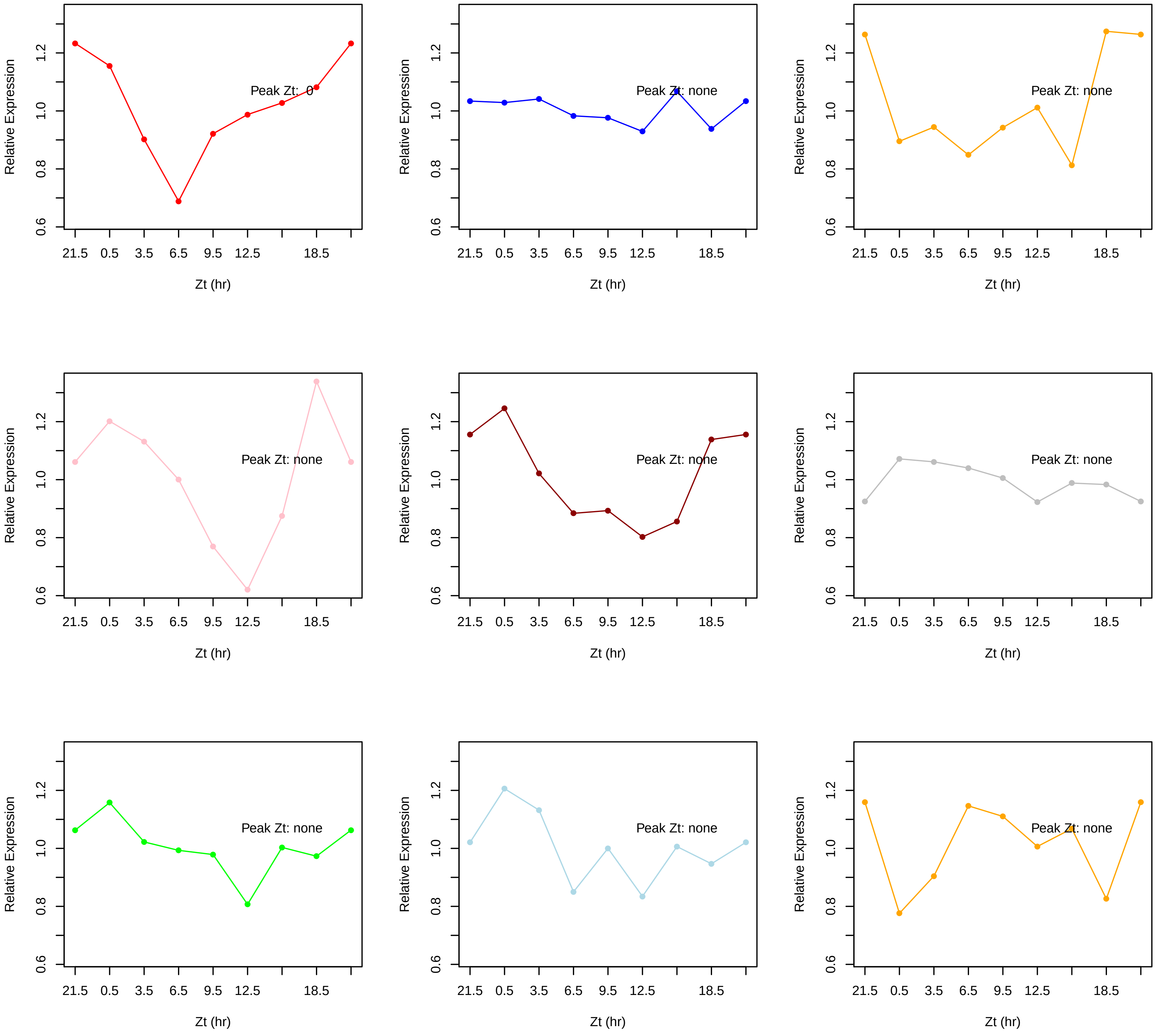

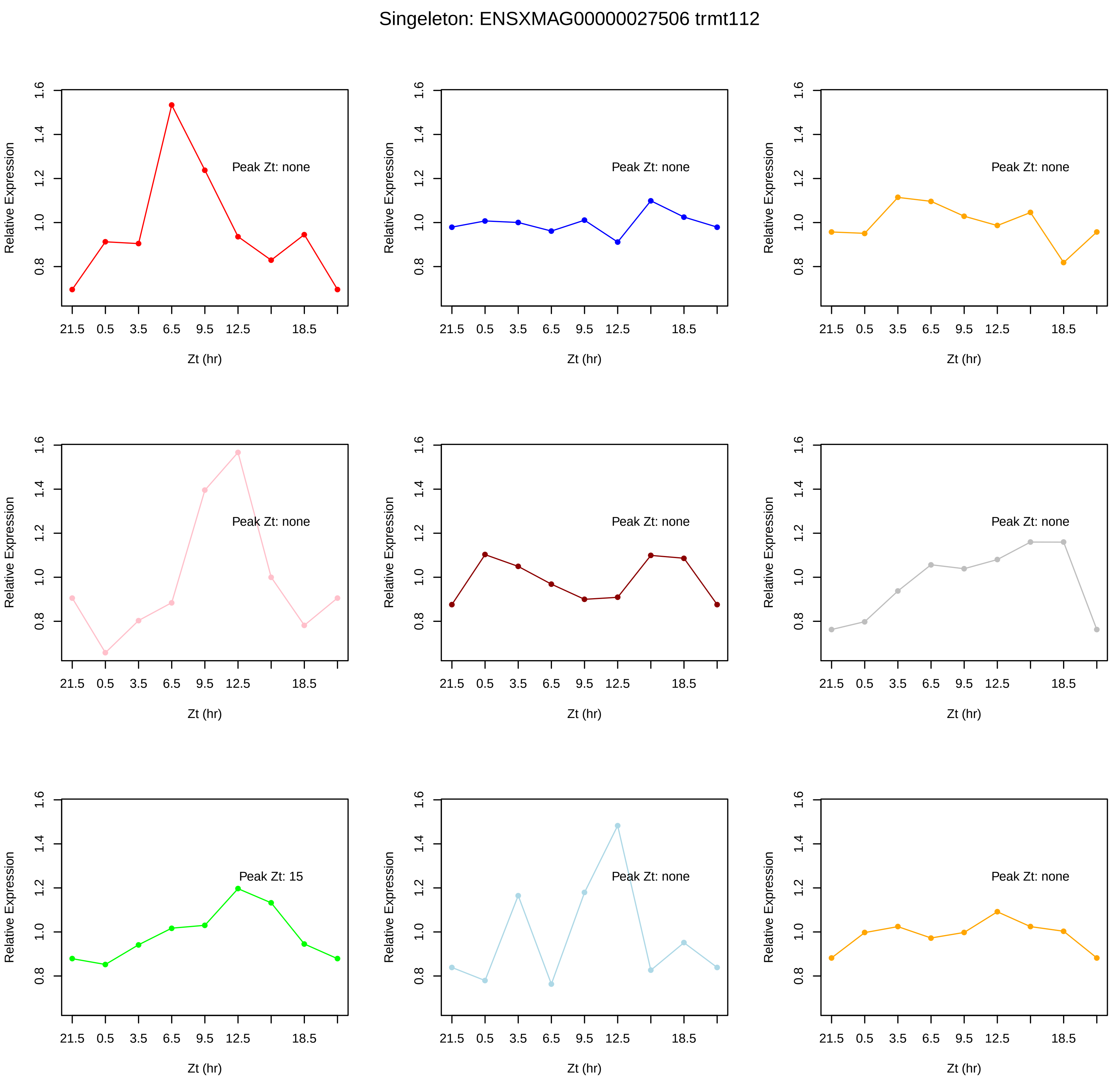

Singeleton: ENSXMAG00000027506 trmt112
Peak Zt: none
Peak Zt: none
Peak Zt: none
Peak Zt: none
Peak Zt: none
Peak Zt: none
Peak Zt: 15
Peak Zt: none
Peak Zt: none
Relative Expression
Zt (hr)
21.5
0.5
3.5
6.5
9.5
12.5
18.5
0.8
1.0
1.2
1.4
1.6

Singeleton: ENSXMAG00000018570 quo

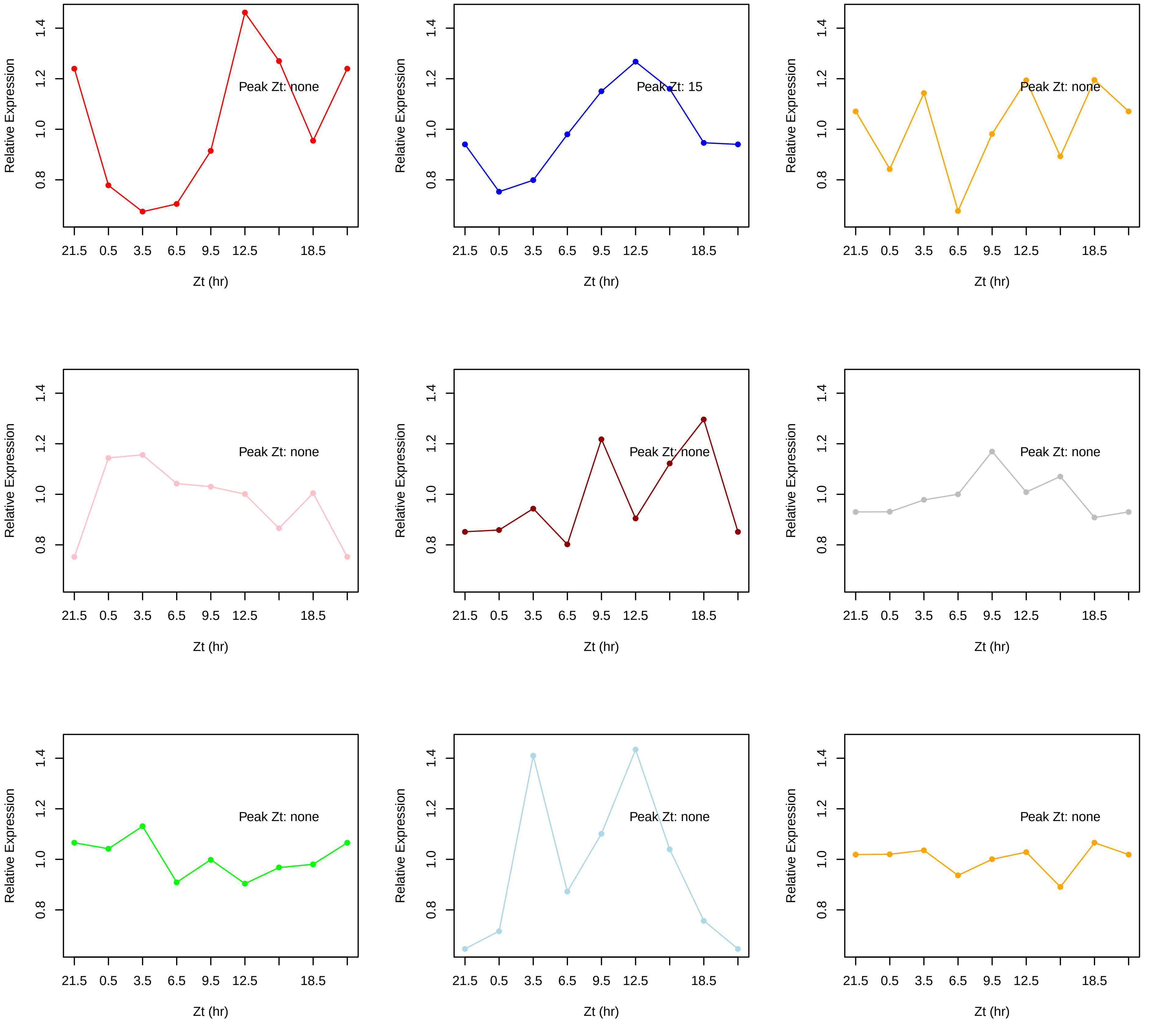

Singeleton: ENSXMAG00000010954 ypel2a

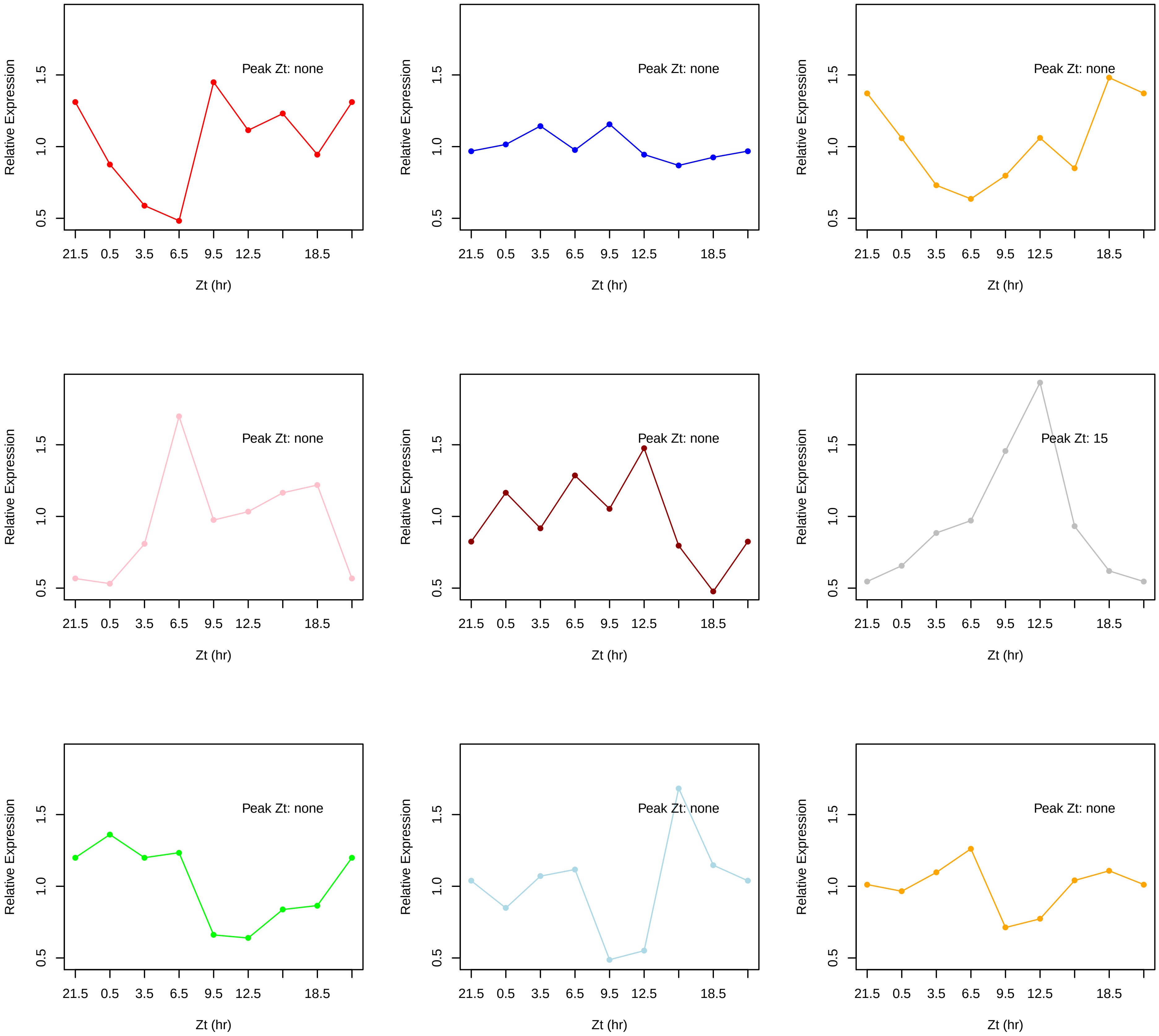

Singleton: ENSXMAG00000024981 emc9

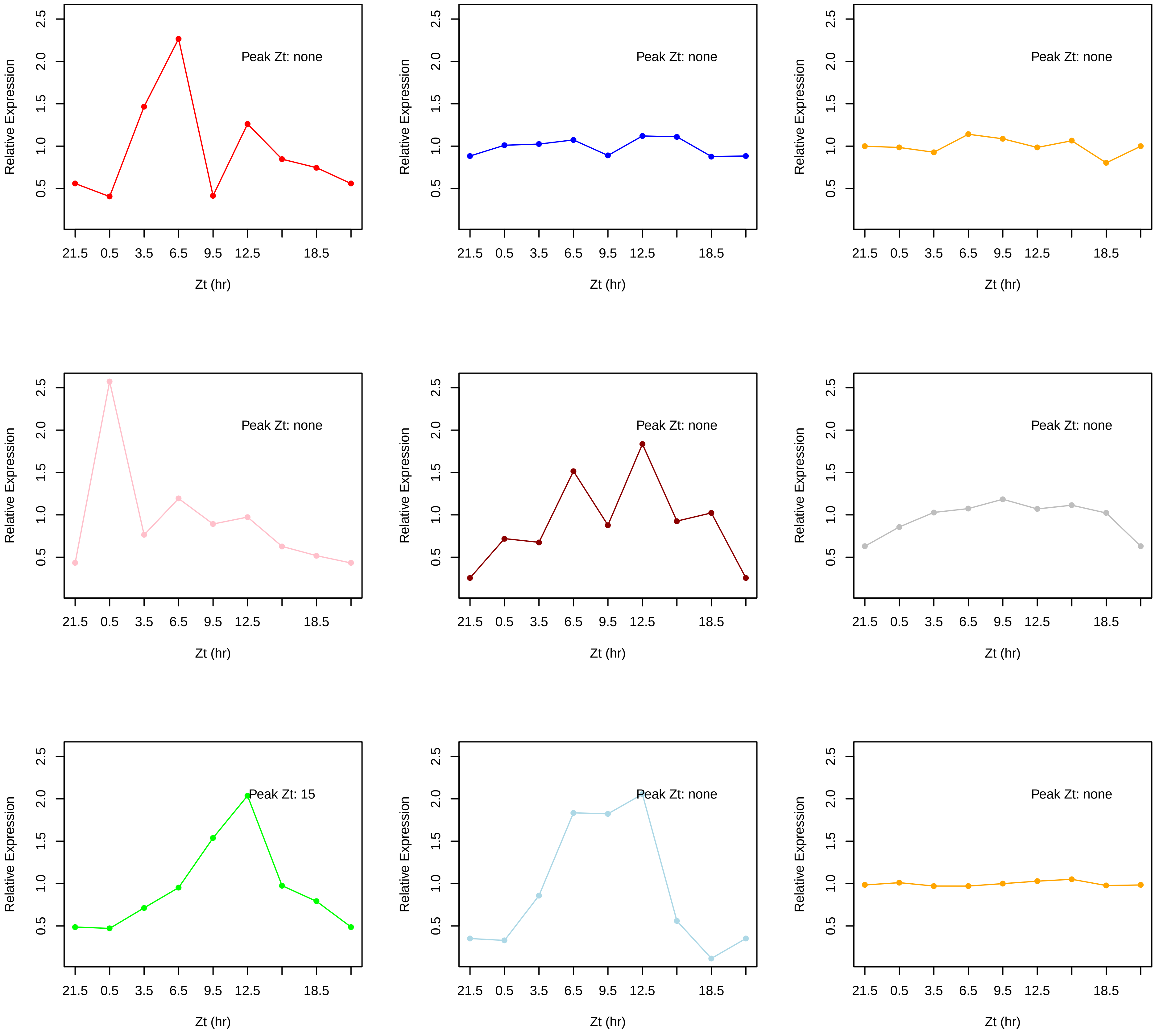

Singeleton: ENSXMAG00000002681 rexo1

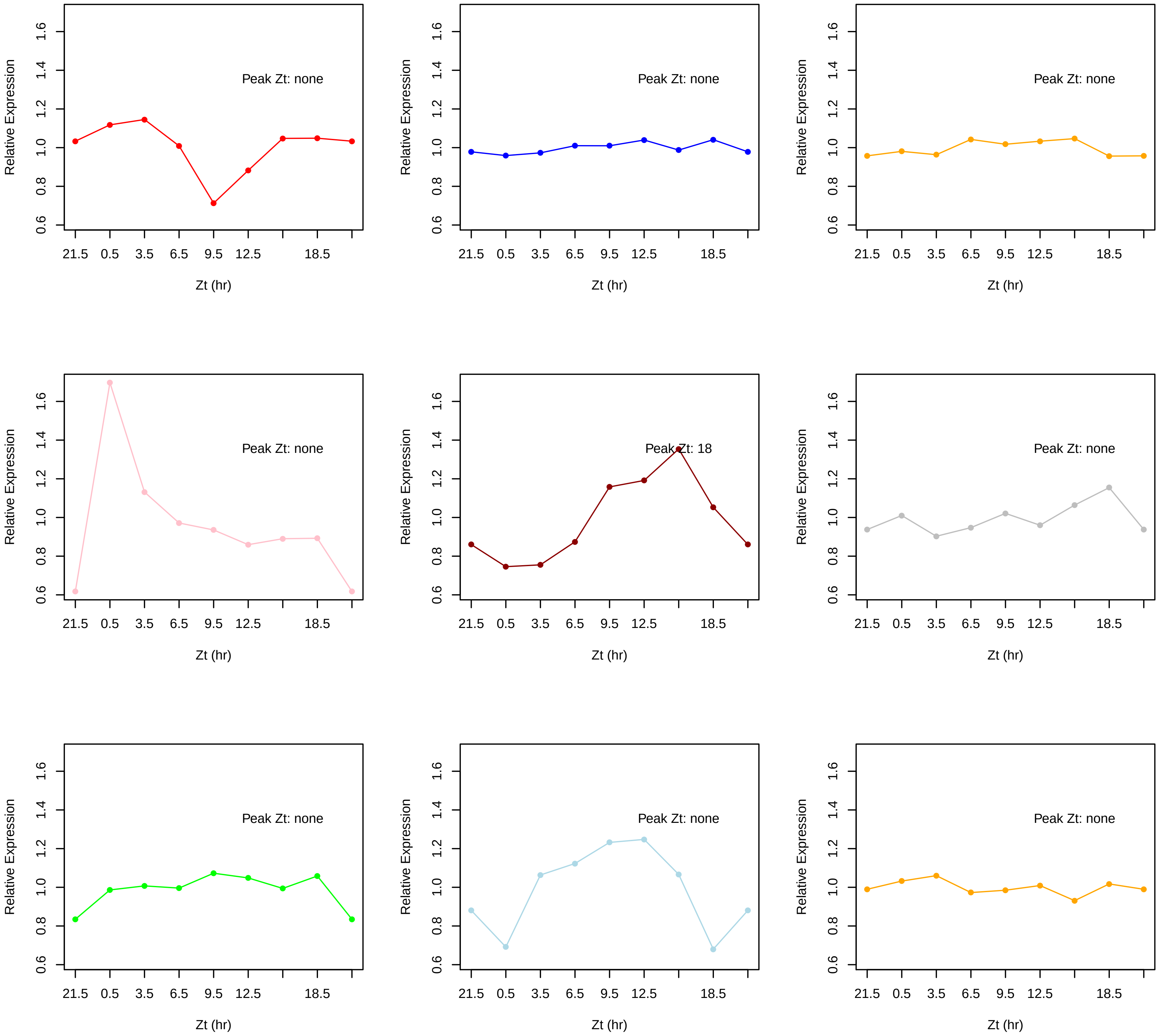

Singeleton: ENSXMAG00000028689 abracl

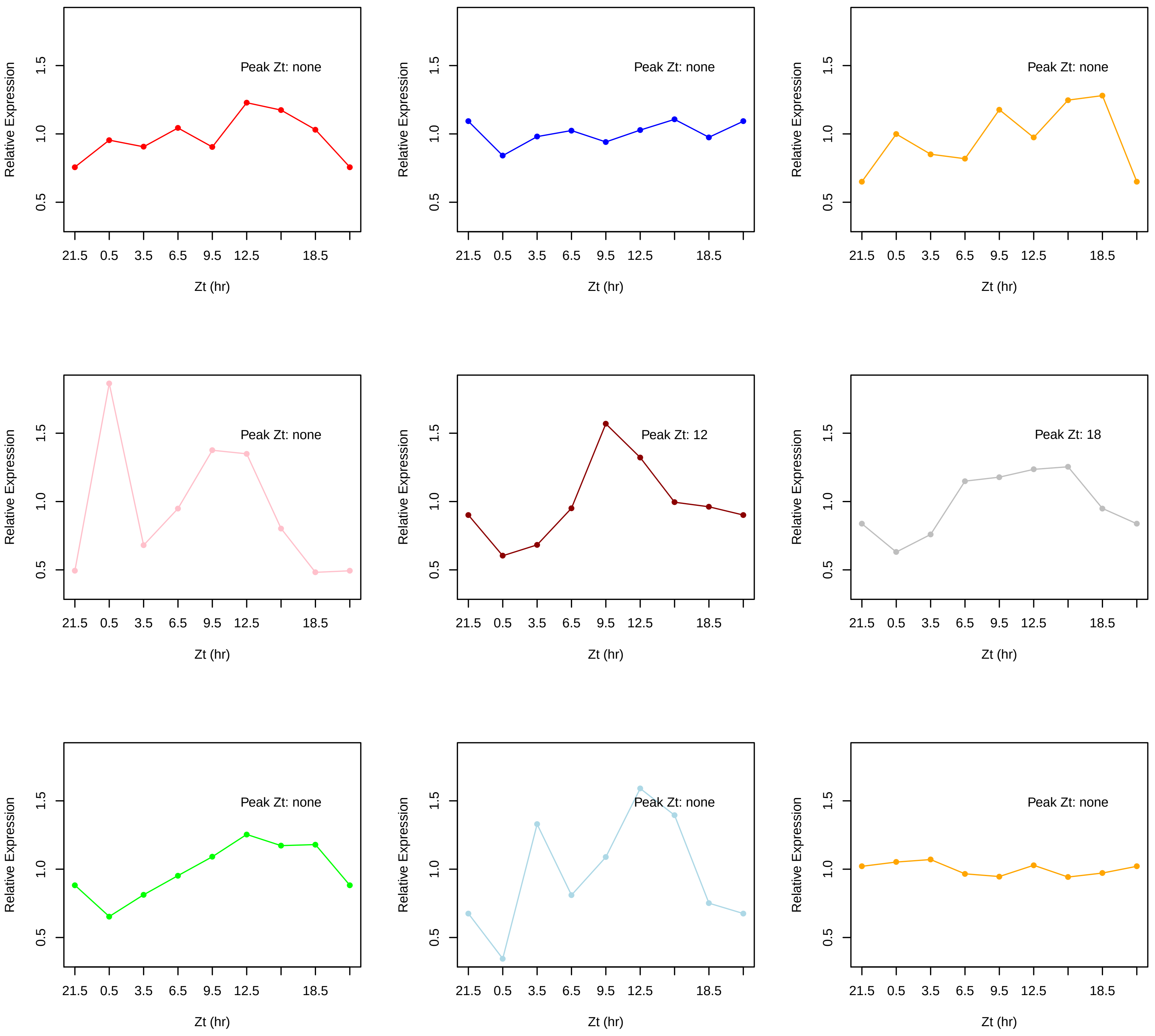

Singleton: ENSXMAG00000027488

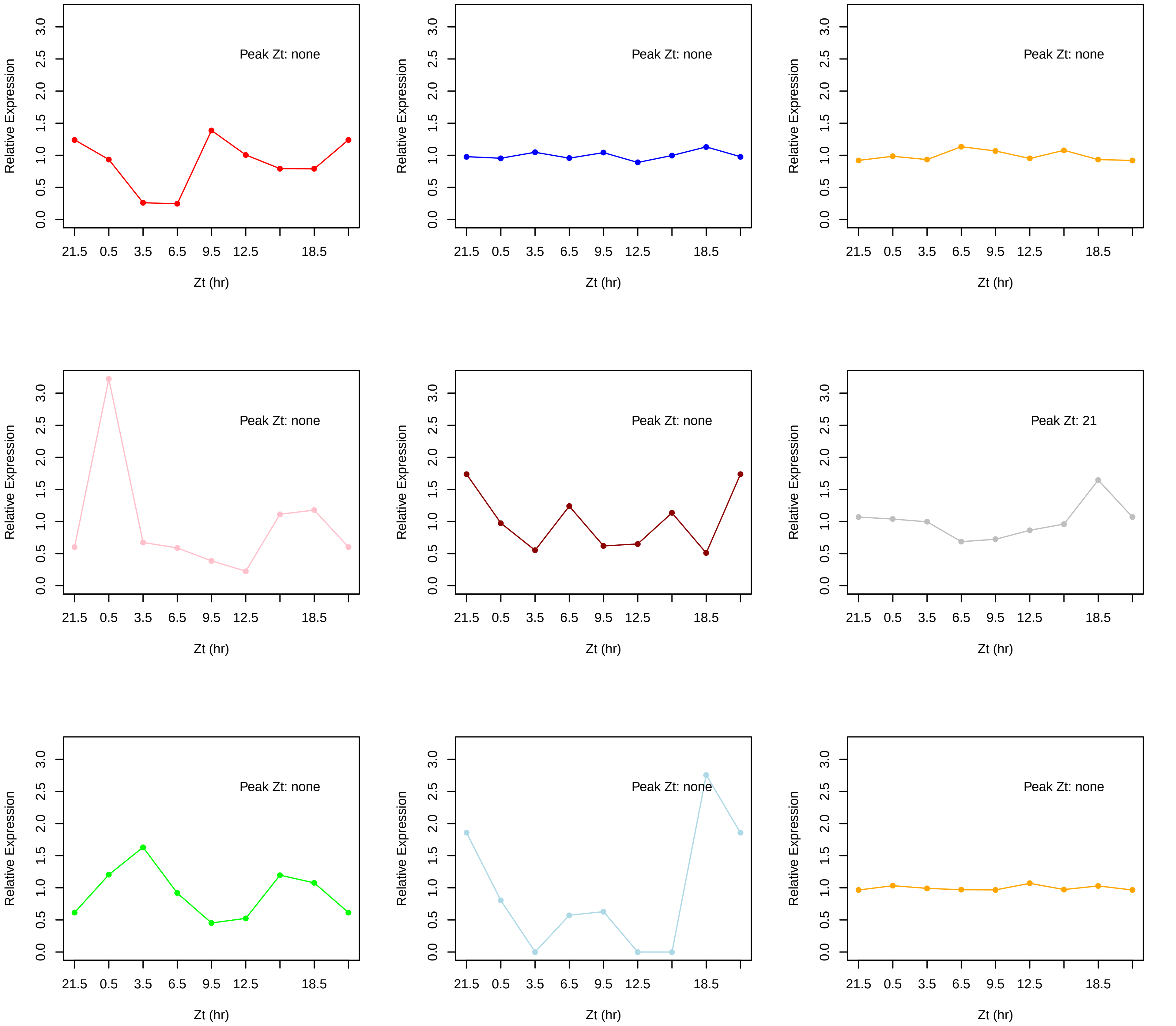

Singeleton: ENSXMAG00000007667 heca

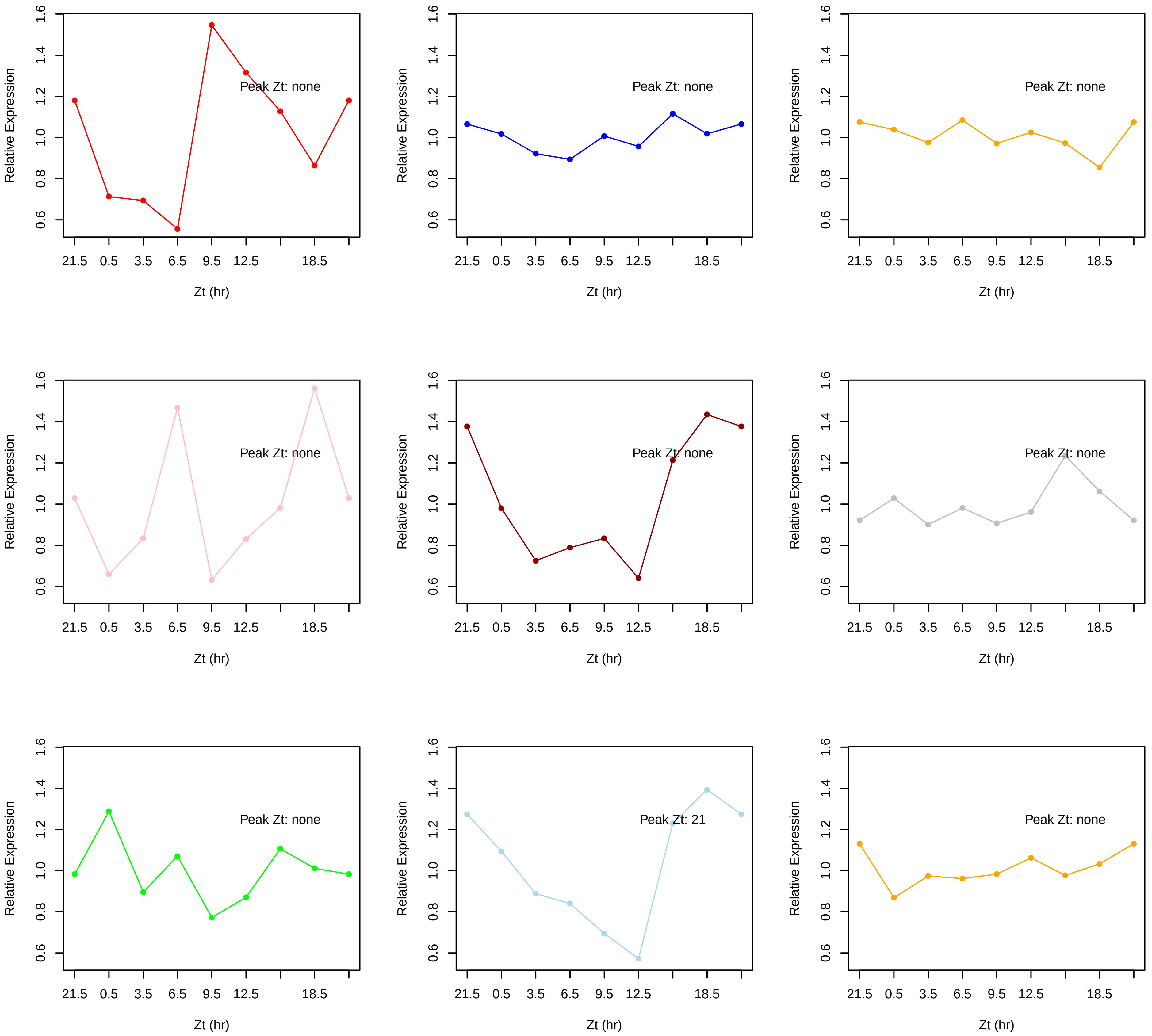

Singleton: ENSXMAG00000013072

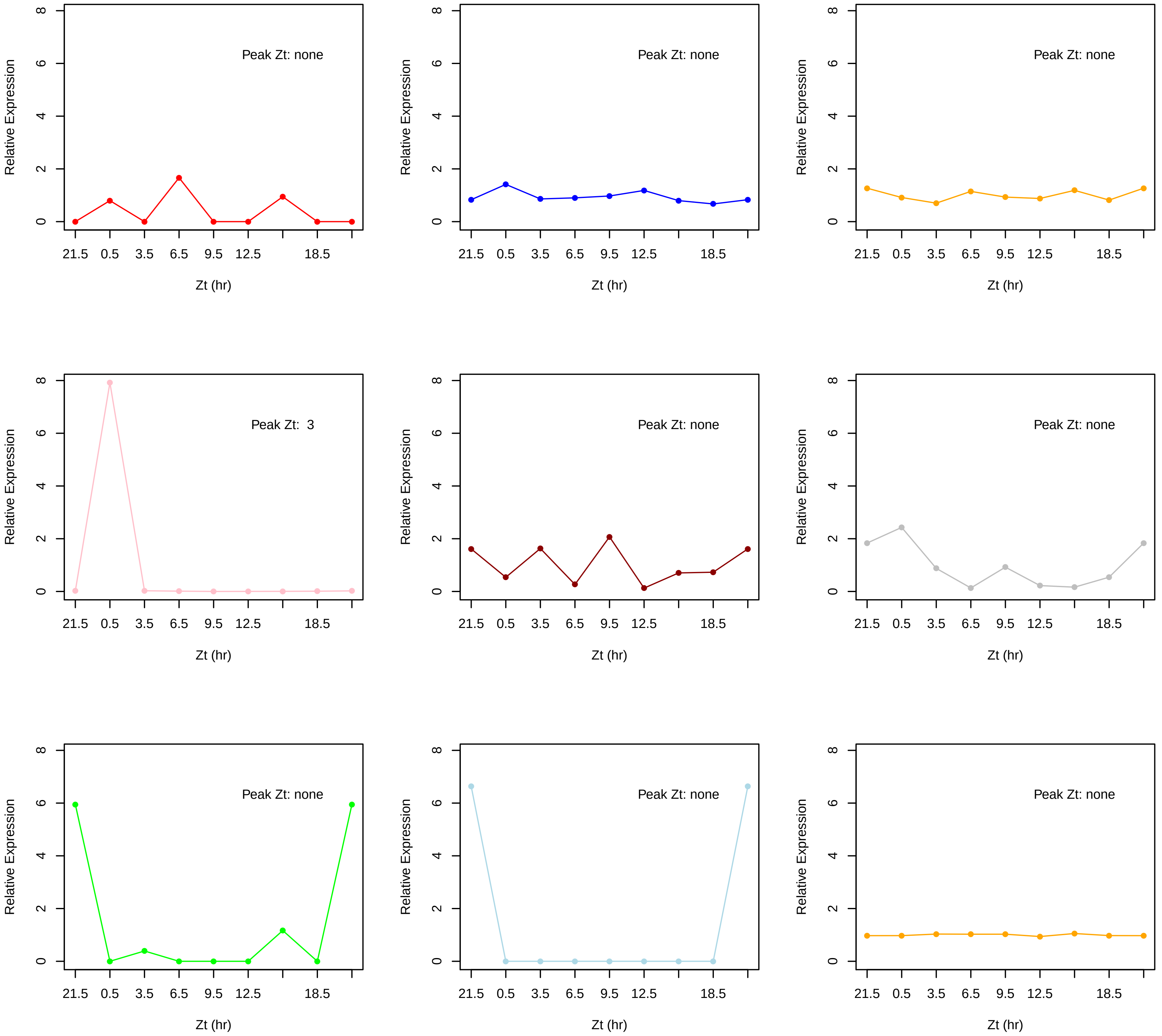

Singeleton: ENSXMAG00000009027 smg8

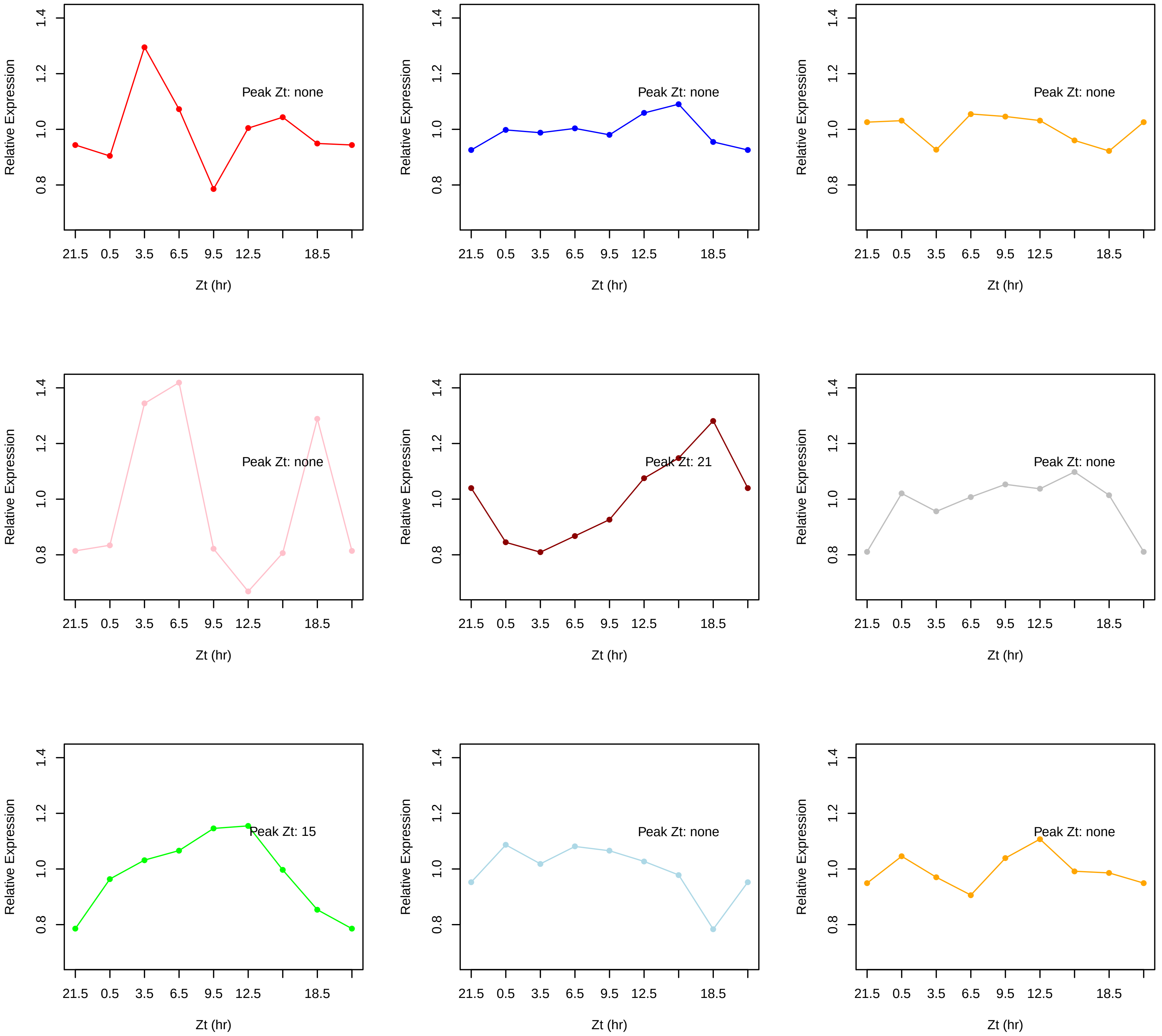

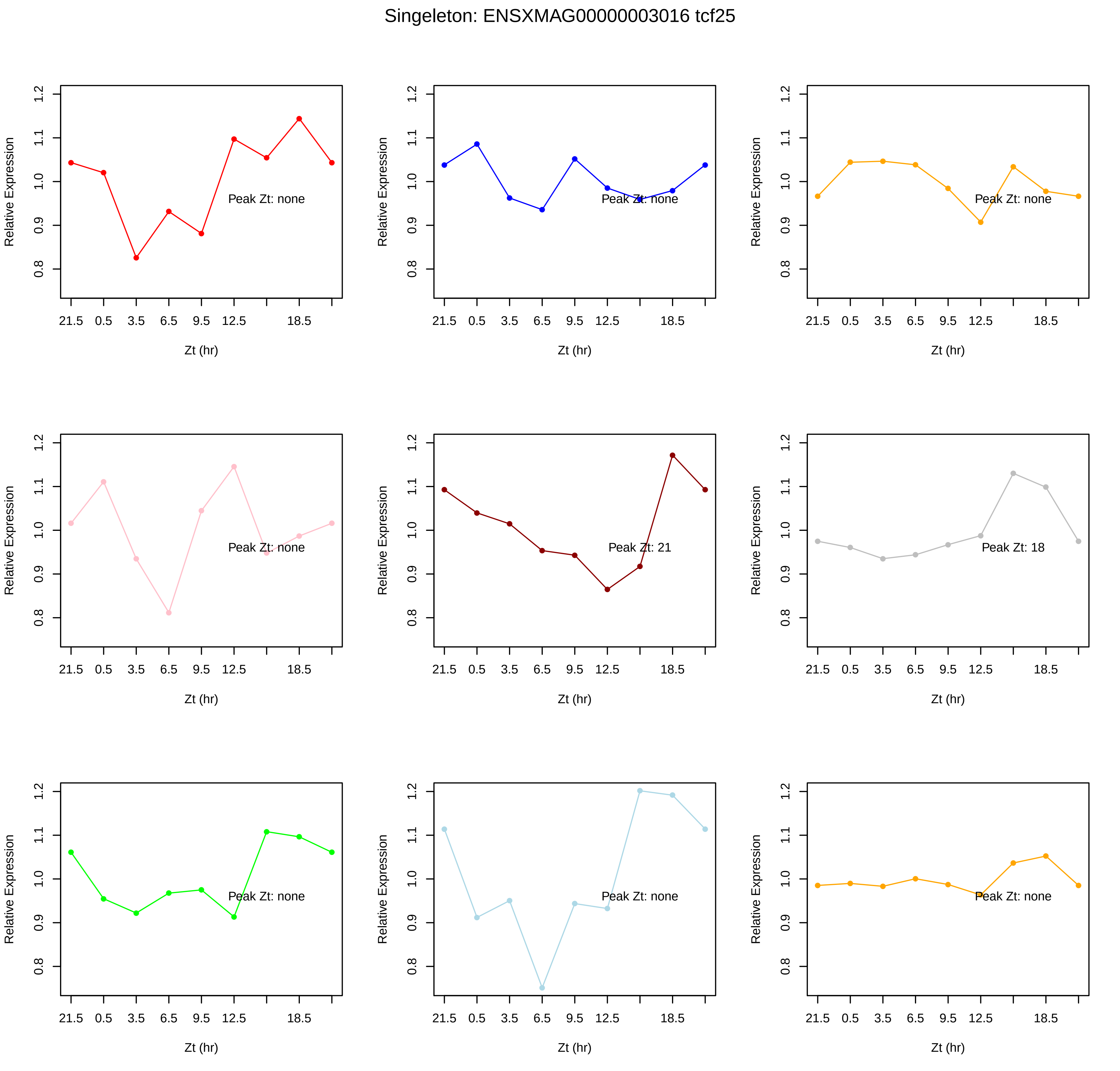
Singeleton: ENSXMAG00000003016 tcf25
Relative Expression
Zt (hr)
21.5
0.5
3.5
6.5
9.5
12.5
18.5
0.8
0.9
1.0
1.1
1.2
Peak Zt: none
Peak Zt: 21
Peak Zt: 18

Singleton: ENSXMAG00000015223 tcaim

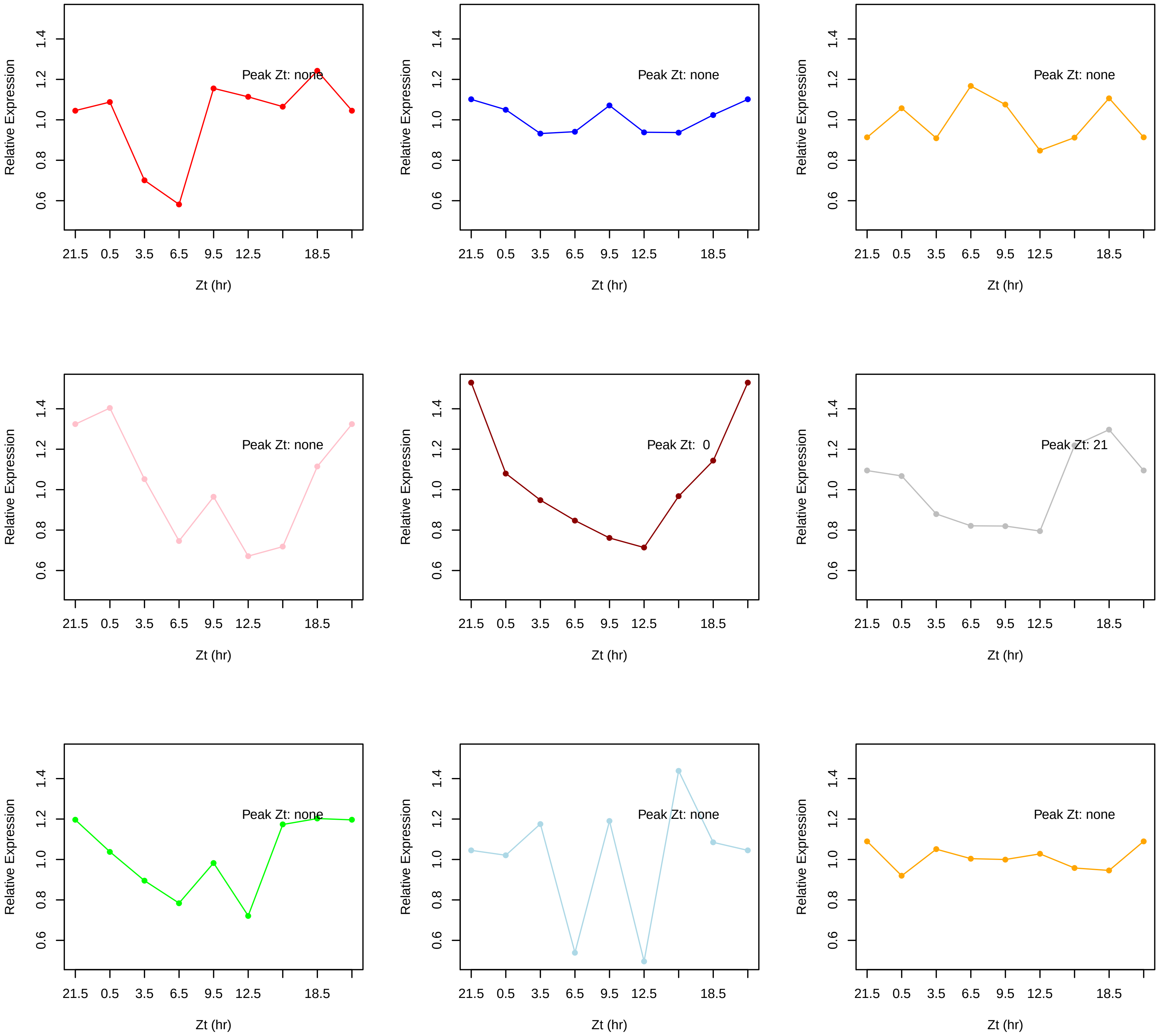

Singeleton: ENSXMAG00000000554 cyp26b1

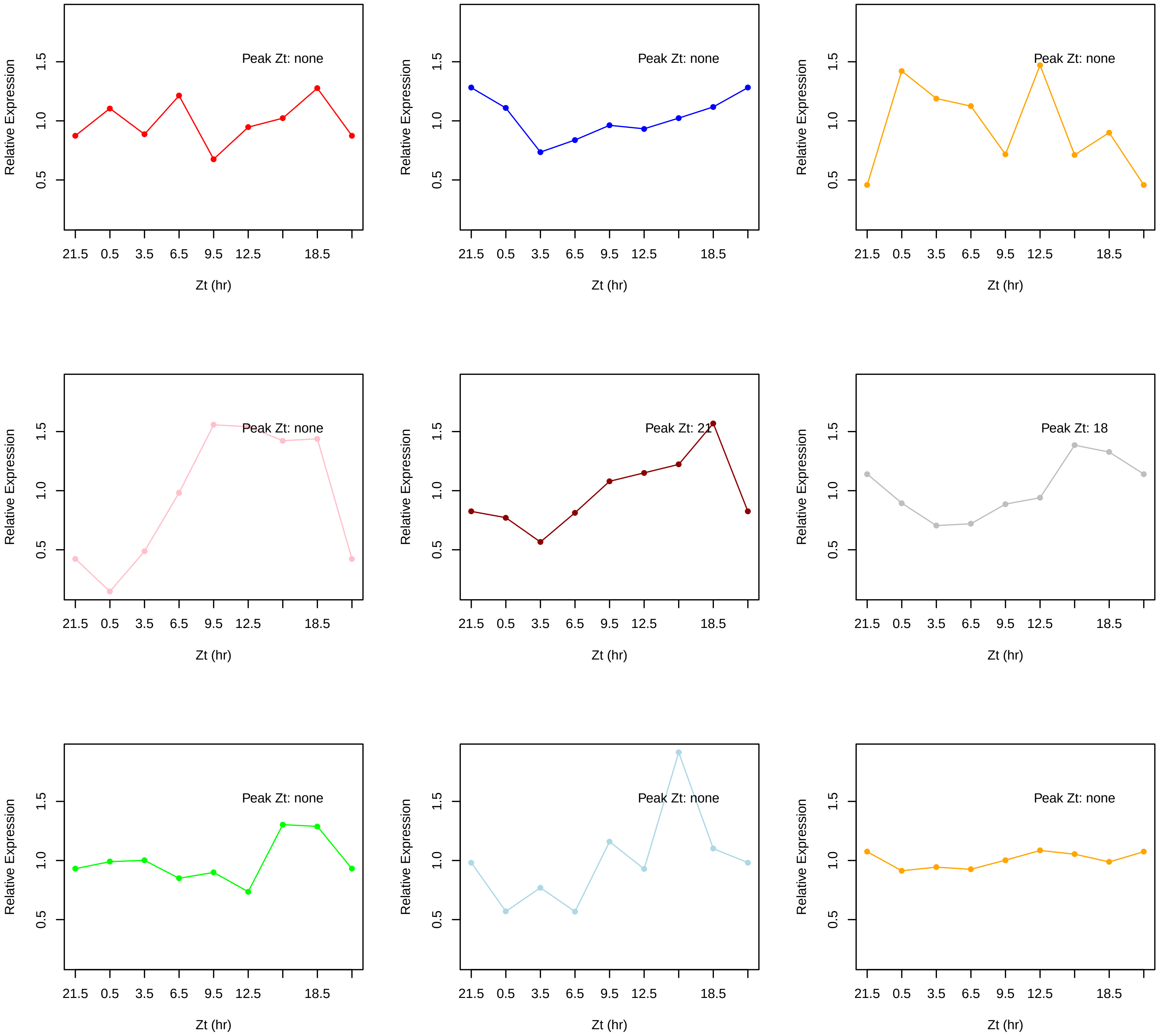

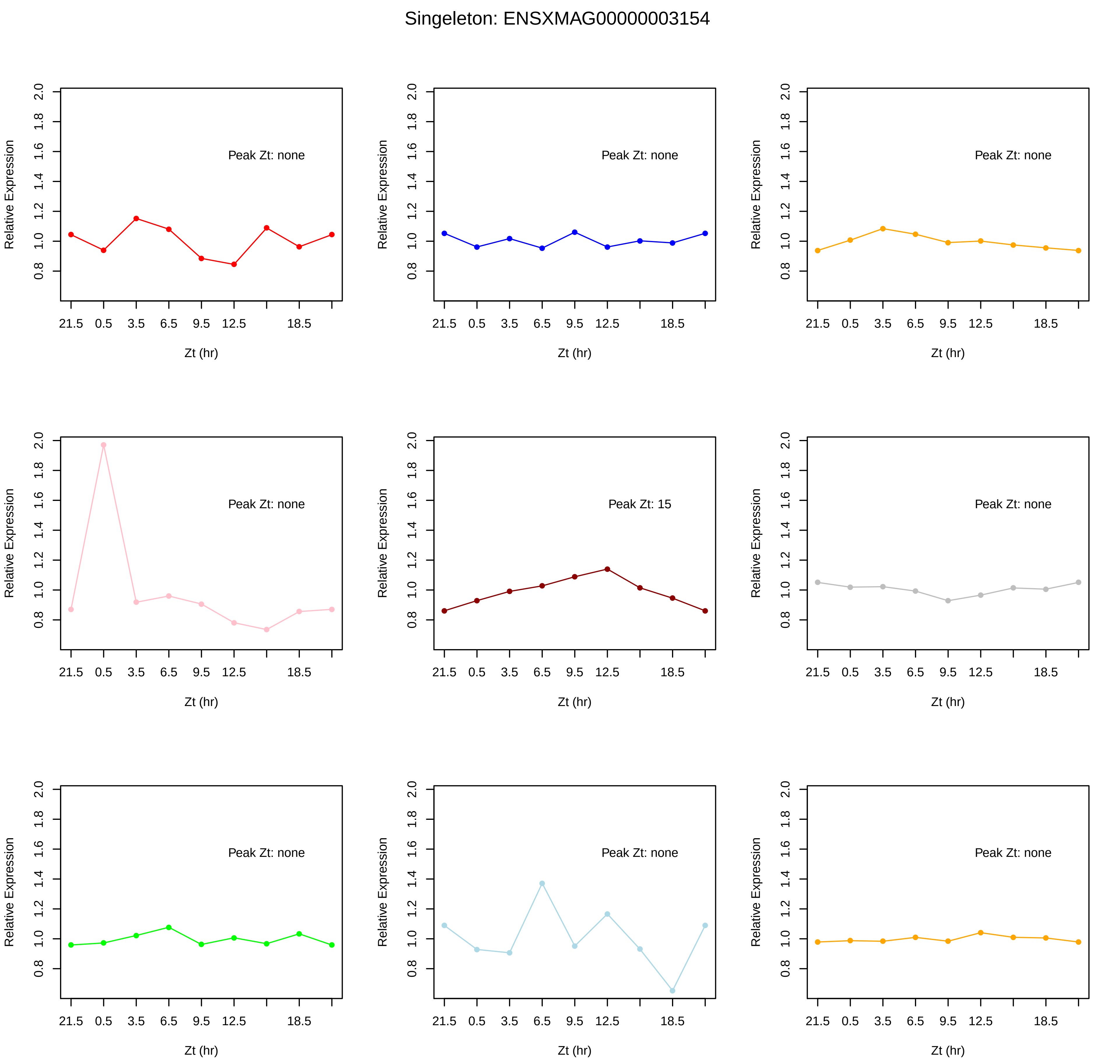
Singeleton: ENSXMAG00000003154
Peak Zt: none
Peak Zt: none
Peak Zt: none
Peak Zt: none
Peak Zt: 15
Peak Zt: none
Peak Zt: none
Peak Zt: none
Peak Zt: none
Relative Expression
Zt (hr)
21.5
0.5
3.5
6.5
9.5
12.5
18.5
0.8
1.0
1.2
1.4
1.6
1.8
2.0

Singeleton: ENSXMAG00000018223 actl6a

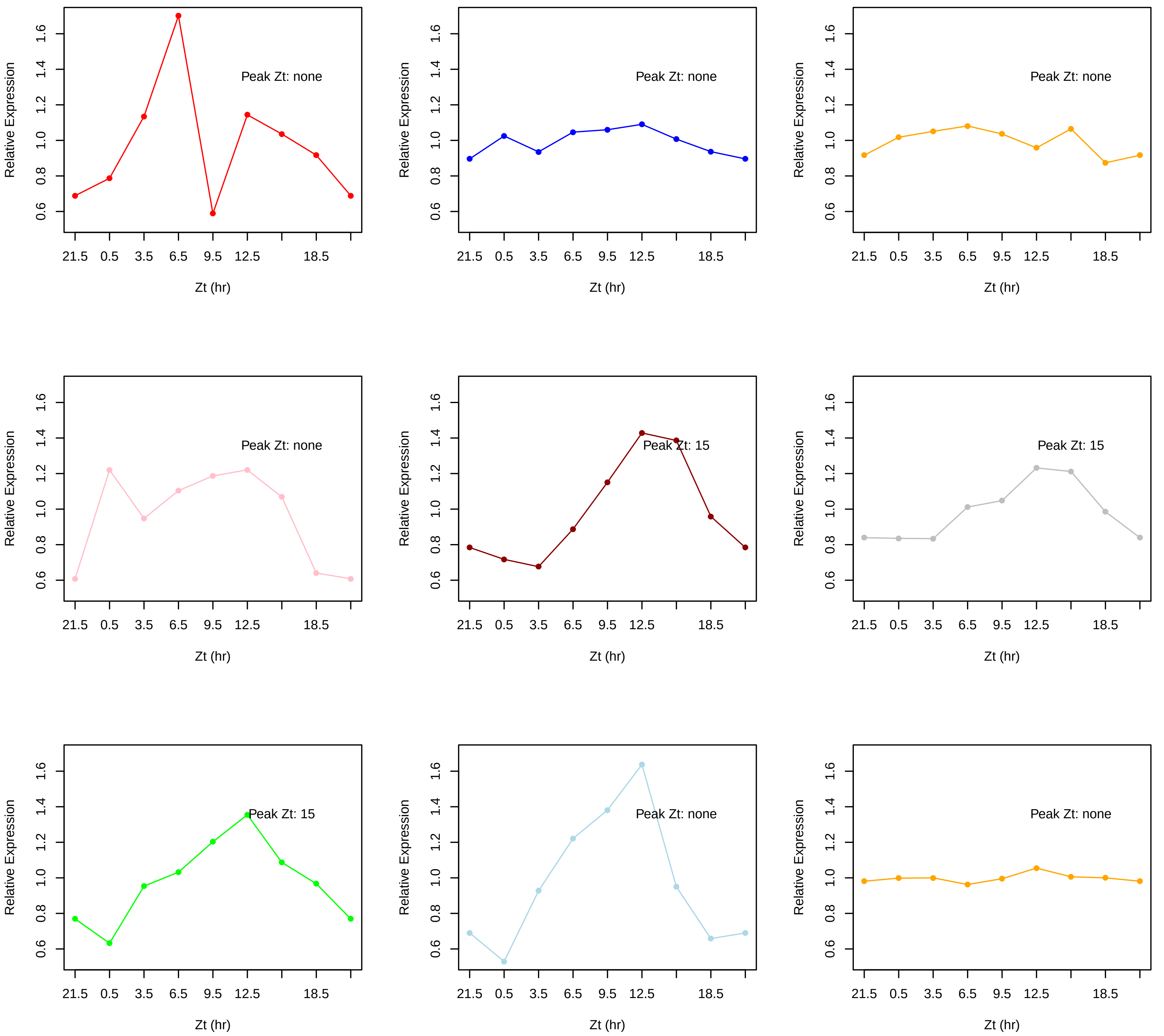

Singeleton: ENSXMAG00000003013 cyba

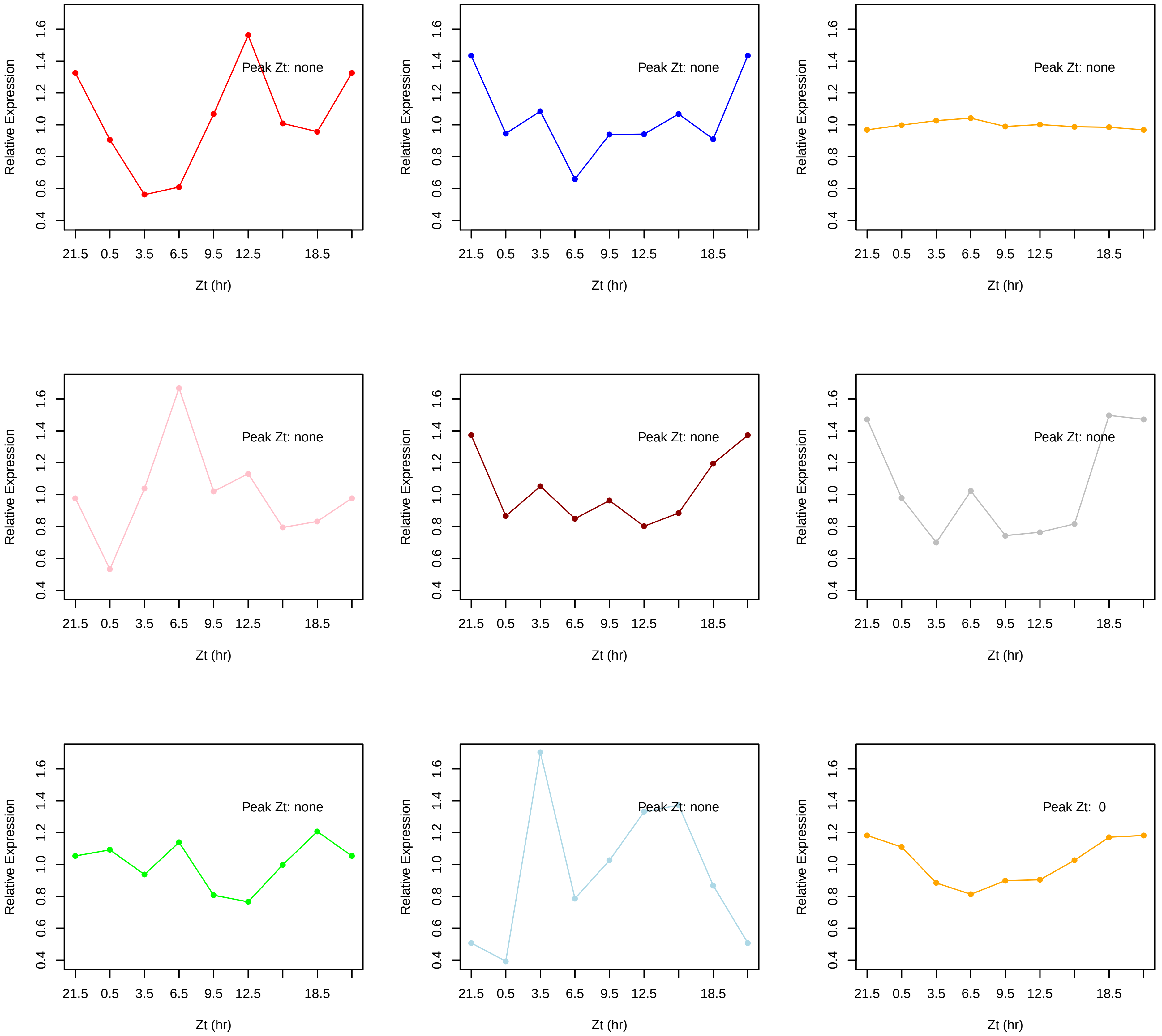

Singeleton: ENSXMAG00000018868 mfsd12a

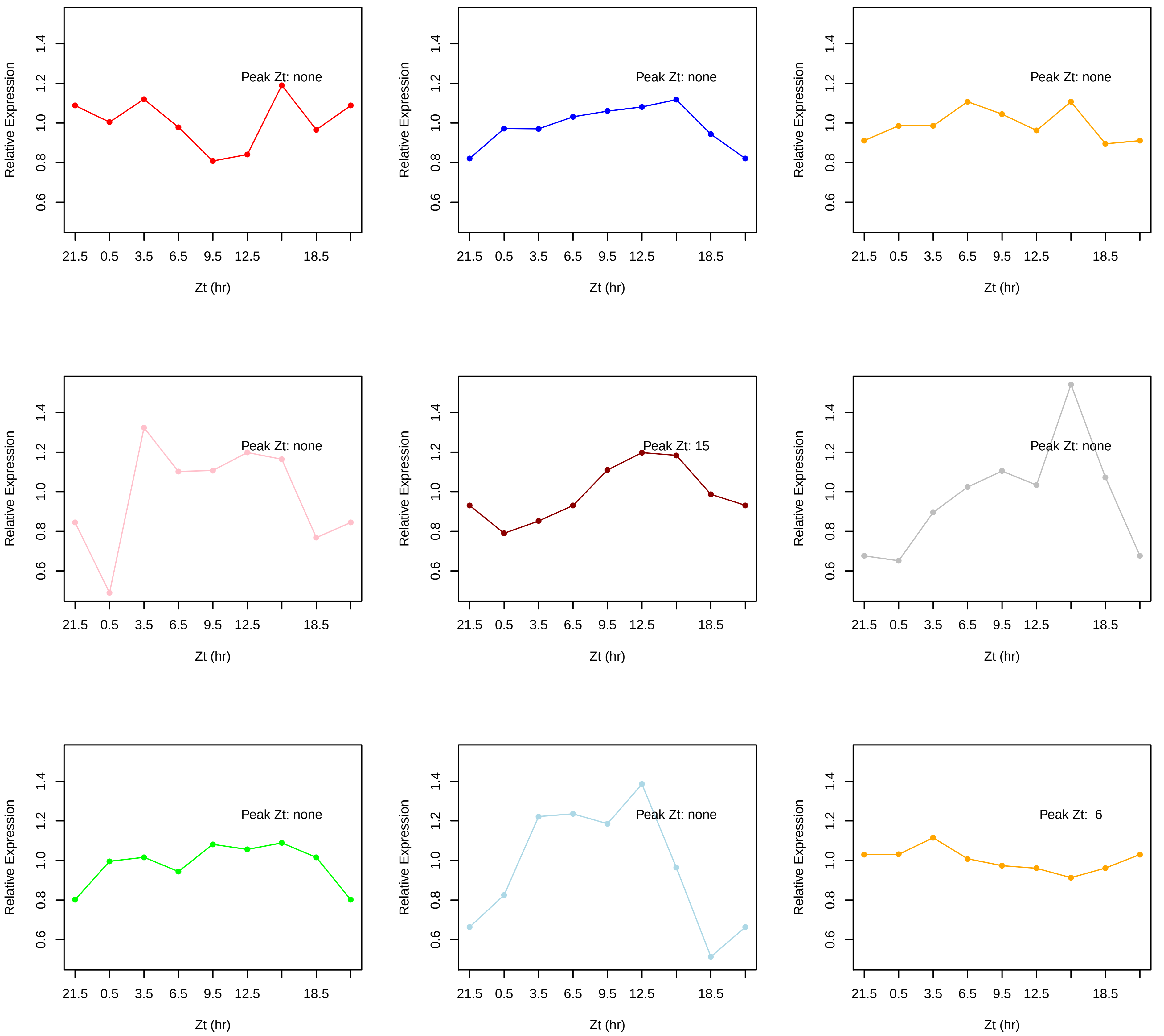

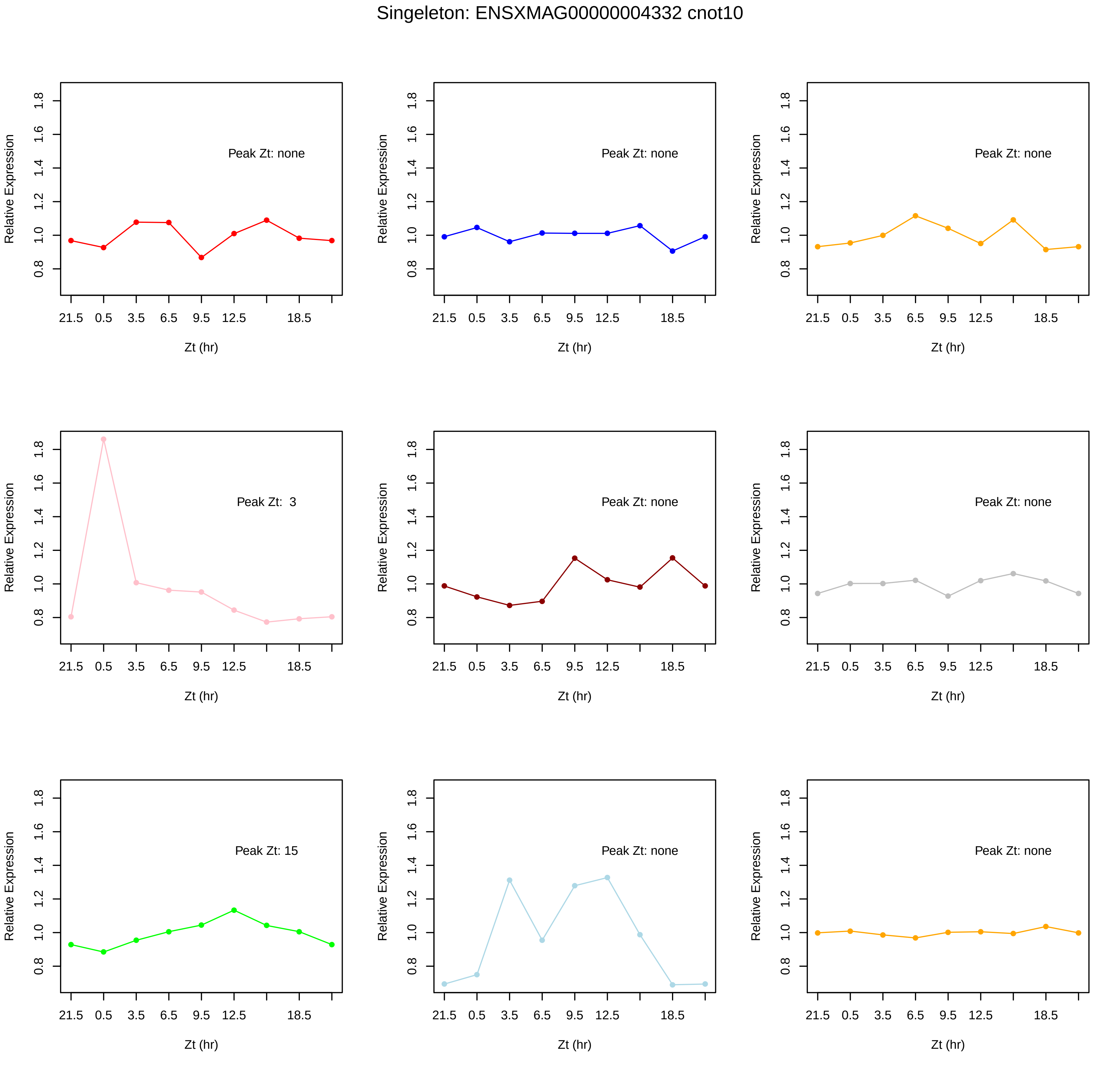

Singeleton: ENSXMAG00000004332 cnot10
Peak Zt: none
Peak Zt: none
Peak Zt: none
Peak Zt: 3
Peak Zt: none
Peak Zt: none
Peak Zt: 15
Peak Zt: none
Peak Zt: none
Relative Expression
Zt (hr)
21.5
0.5
3.5
6.5
9.5
12.5
18.5
0.8
1.0
1.2
1.4
1.6
1.8

Singeleton: ENSXMAG00000013231 atf6

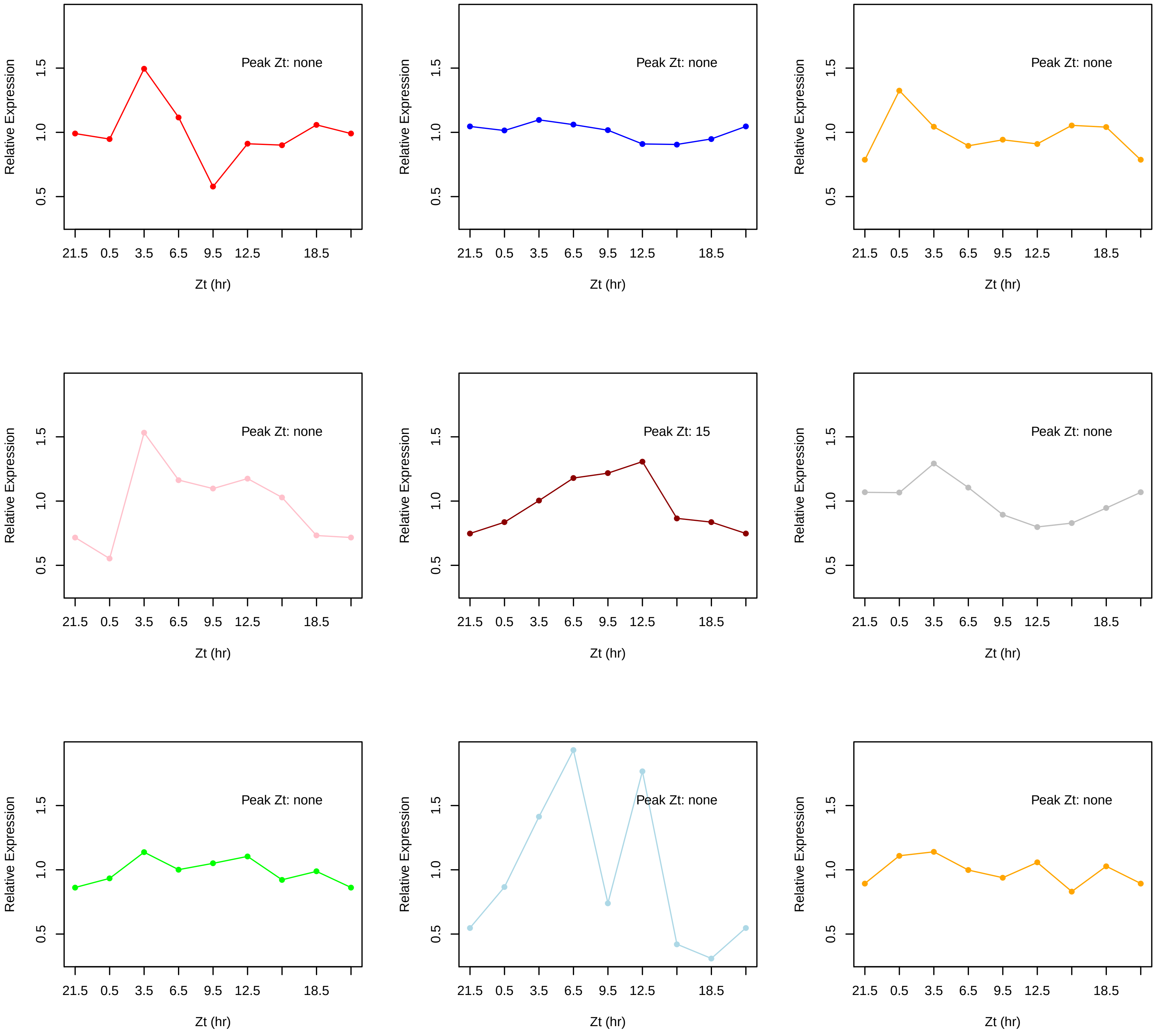

Singeleton: ENSXMAG00000012114 sik1

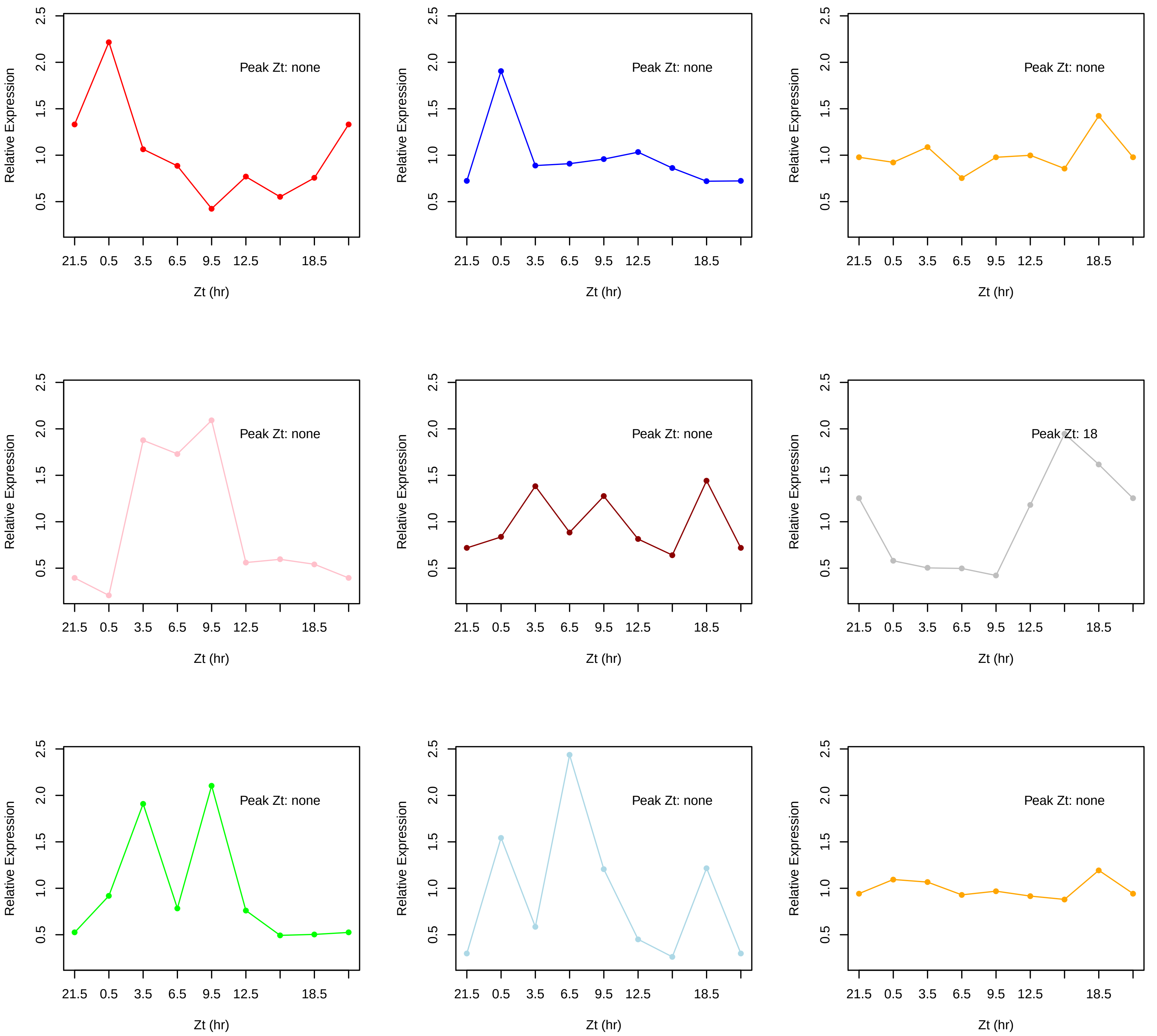

Singeleton: ENSXMAG00000003630 nfic

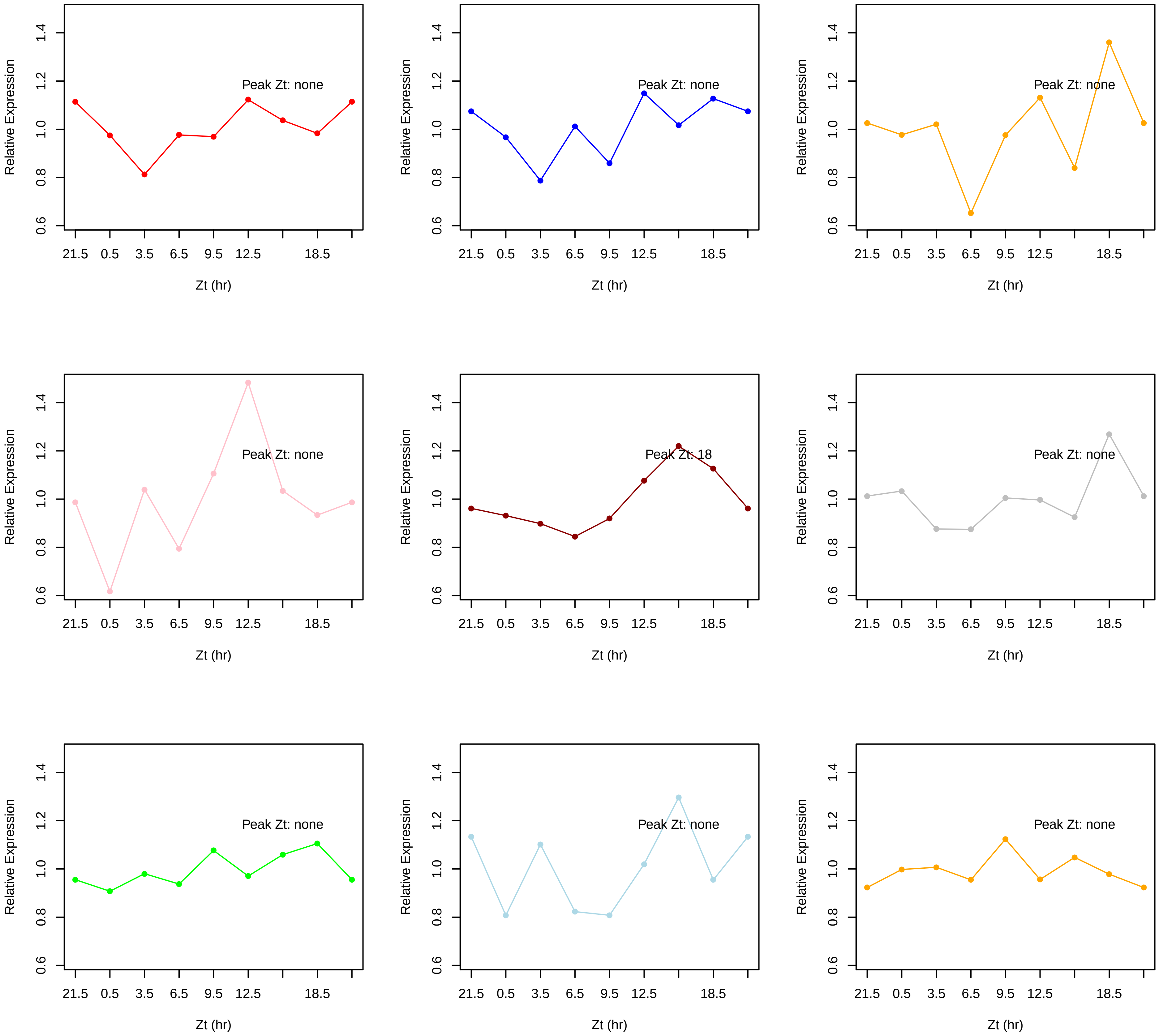

Singeleton: ENSXMAG00000019572 FASTKD5

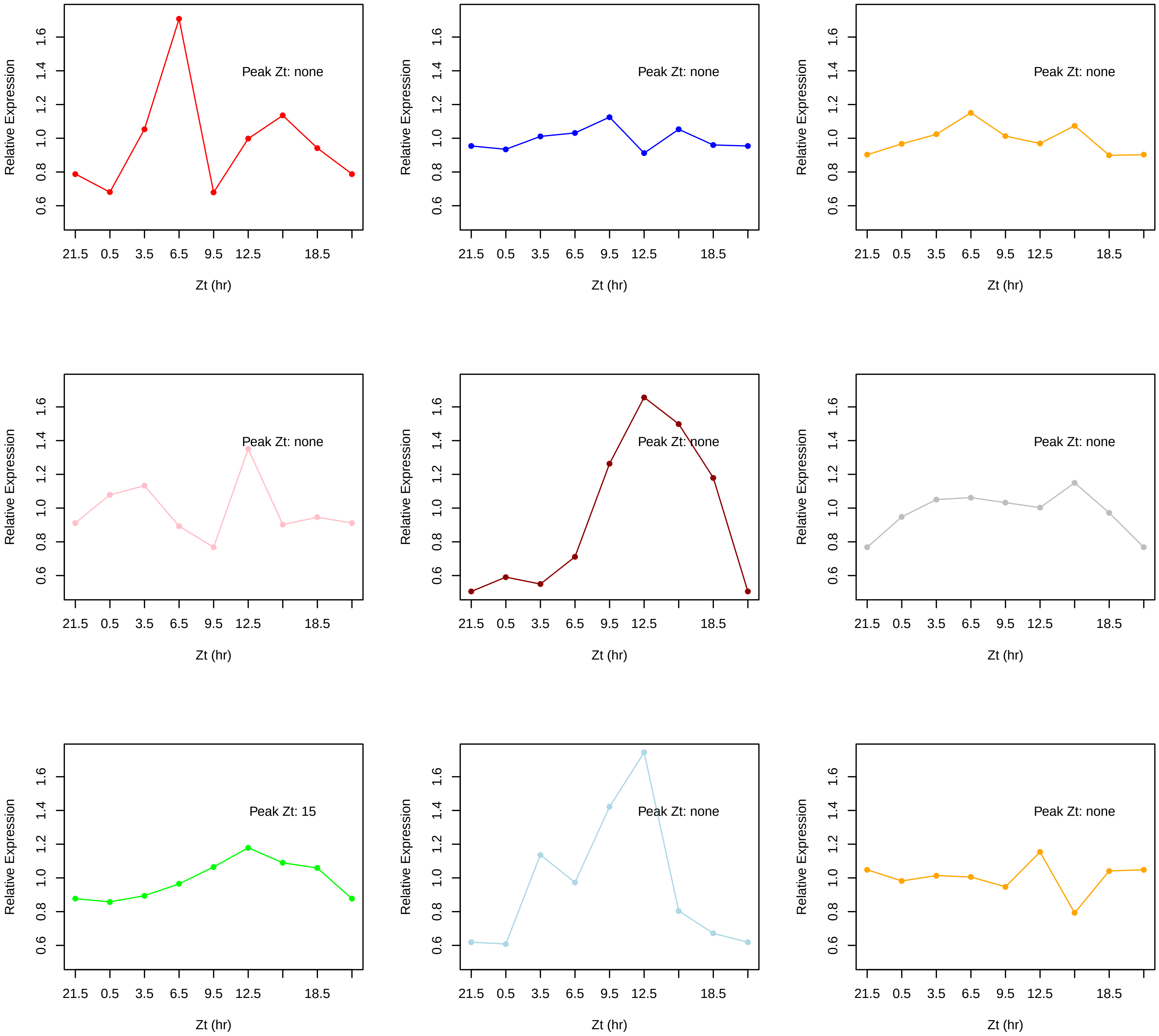

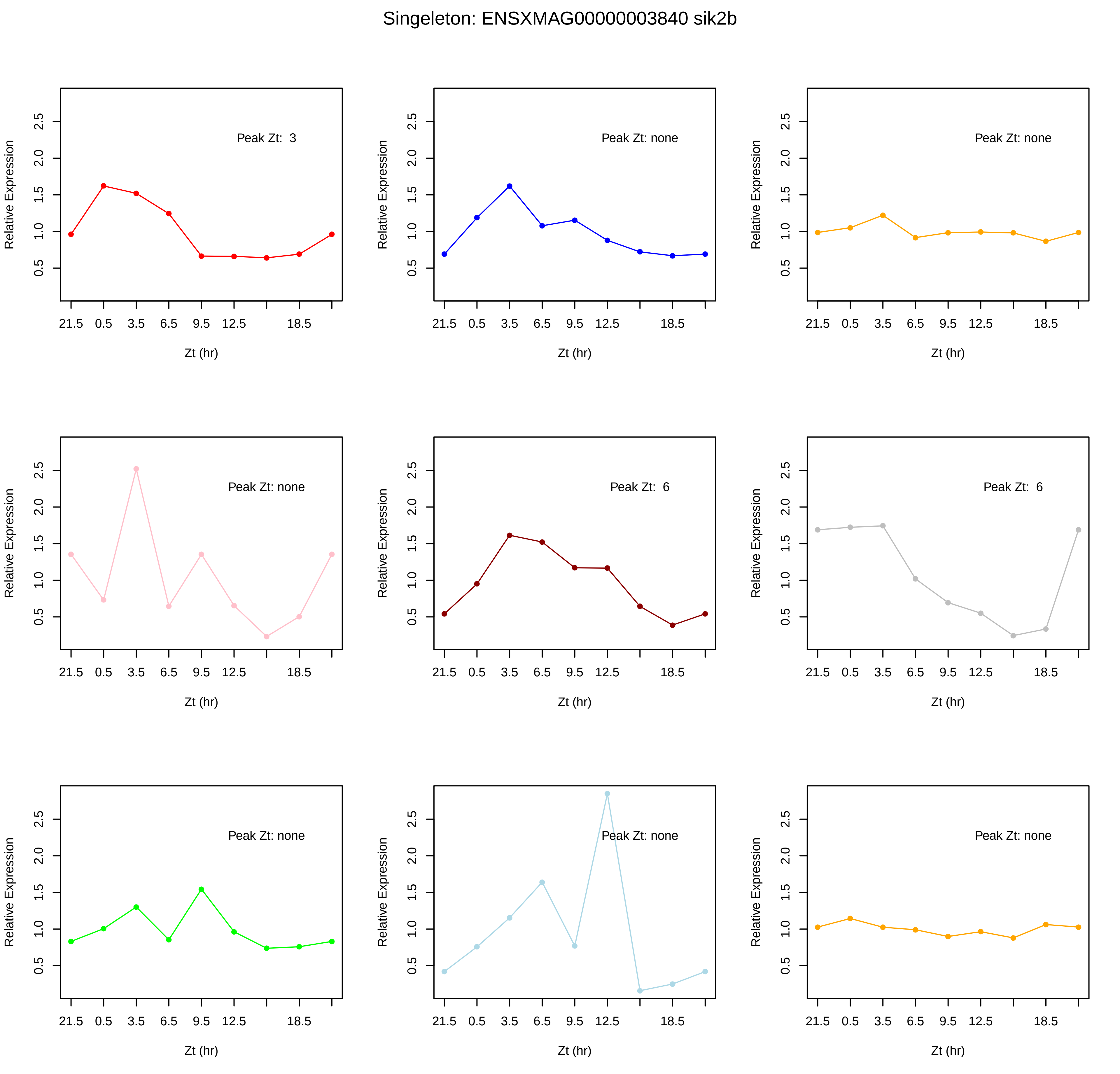
Singeleton: ENSXMAG00000003840 sik2b
Peak Zt: 3
Peak Zt: none
Peak Zt: none
Peak Zt: none
Peak Zt: 6
Peak Zt: 6
Peak Zt: none
Peak Zt: none
Peak Zt: none
Relative Expression
Zt (hr)

Singleton: ENSXMAG00000018249 mrpl47

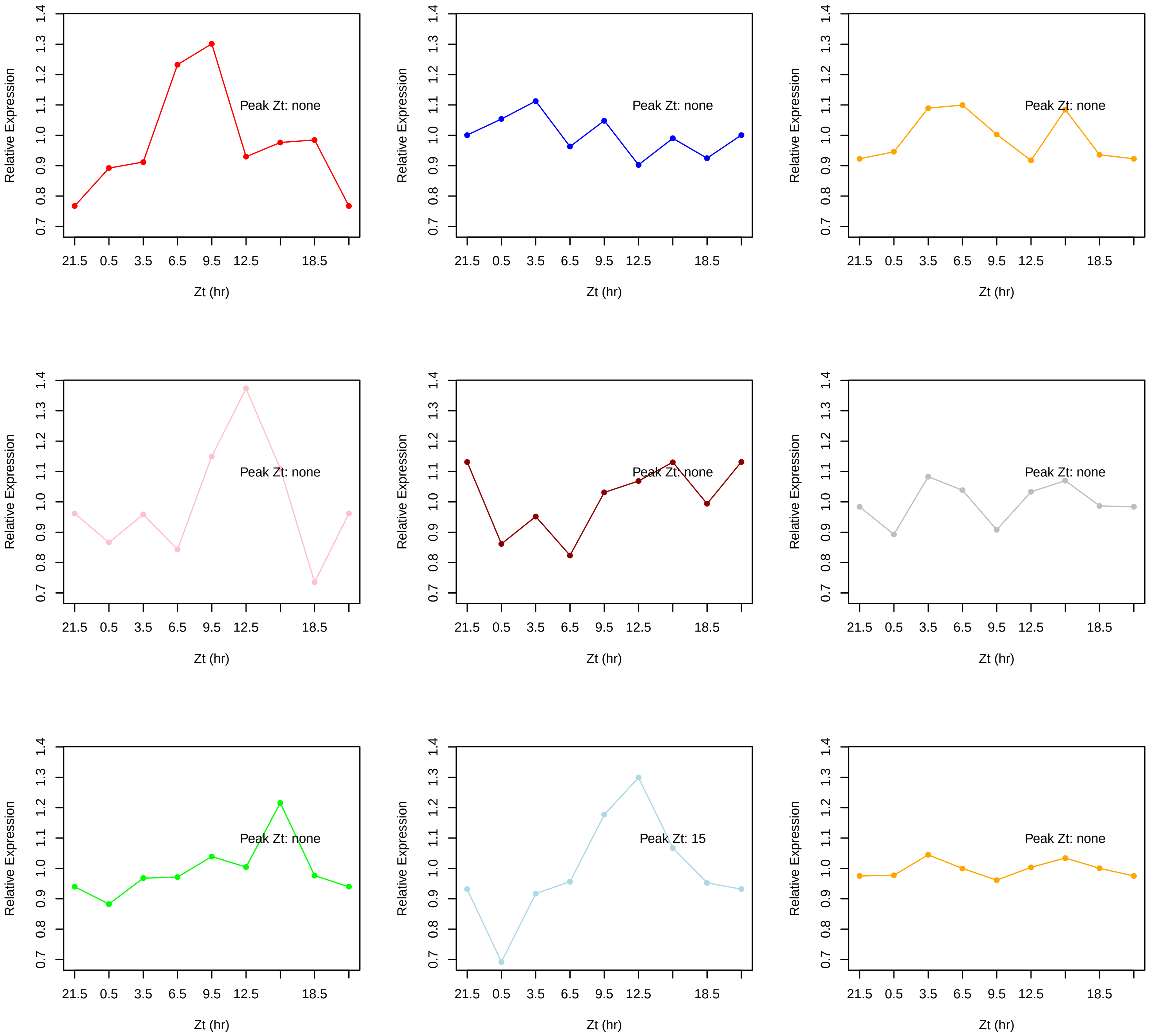

Singeleton: ENSXMAG00000024208 gng3

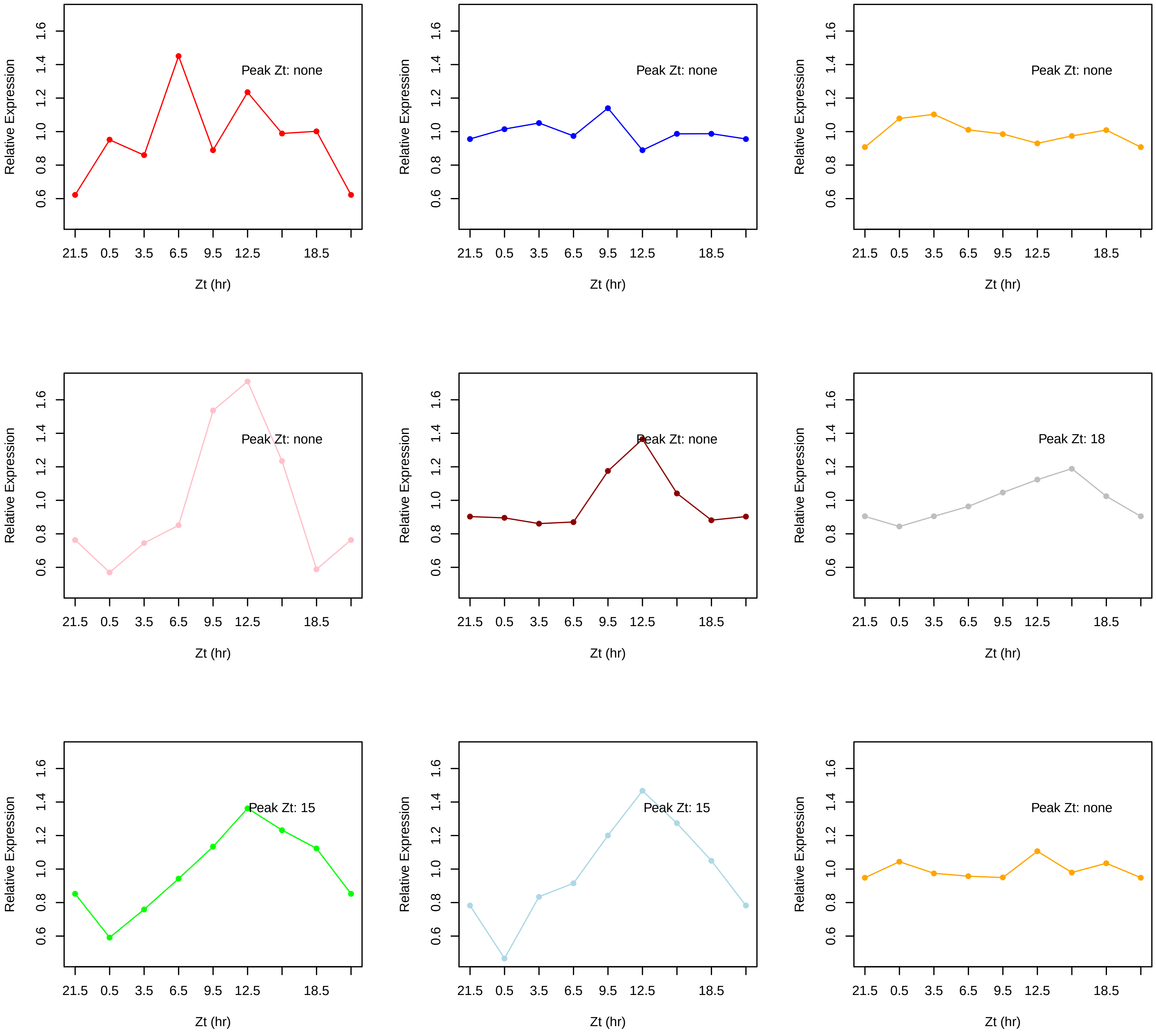

Singeleton: ENSXMAG00000018523 zgc:123305

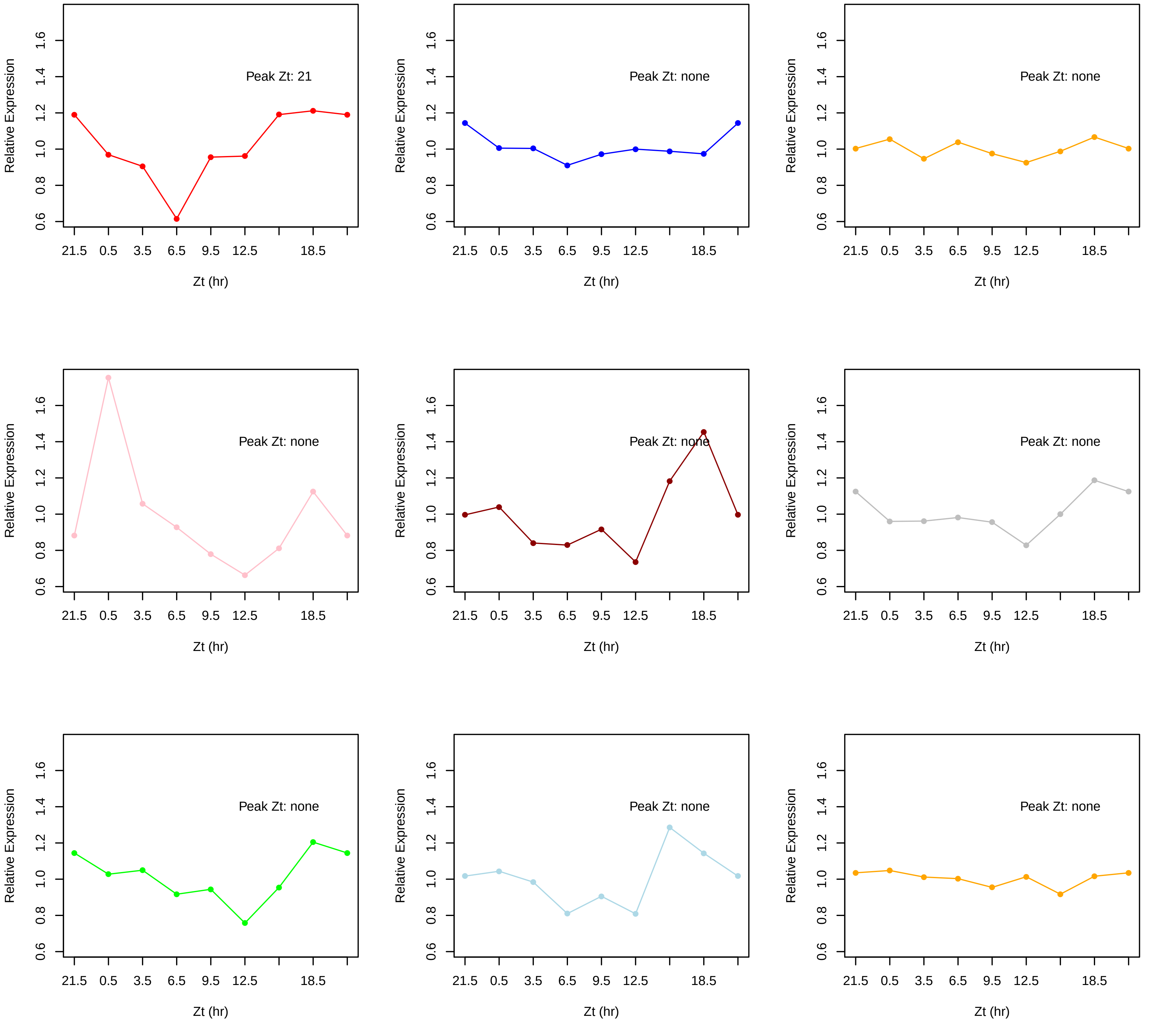

Singeleton: ENSXMAG00000014994

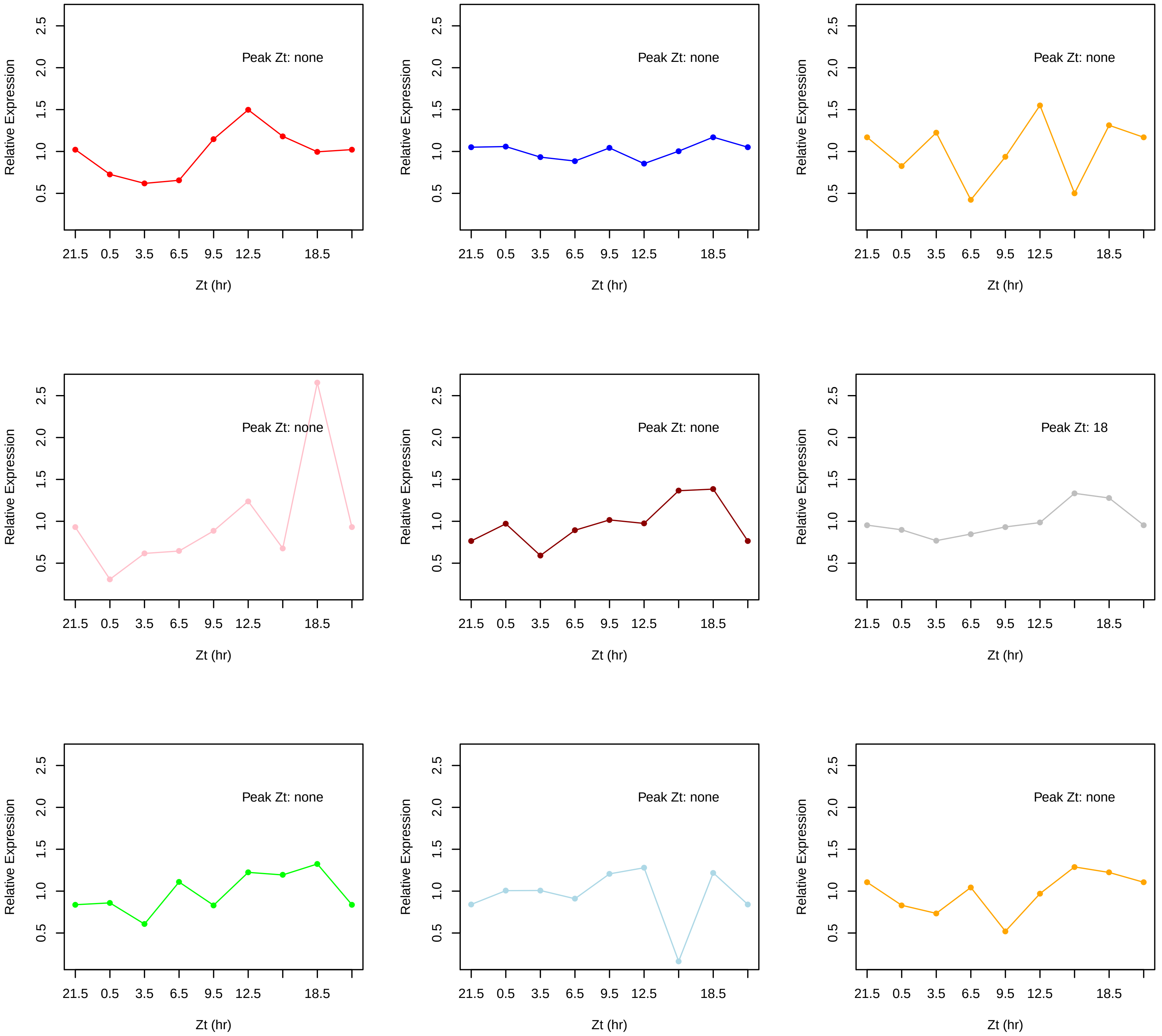

Singeleton: ENSXMAG00000018257 ndufb5

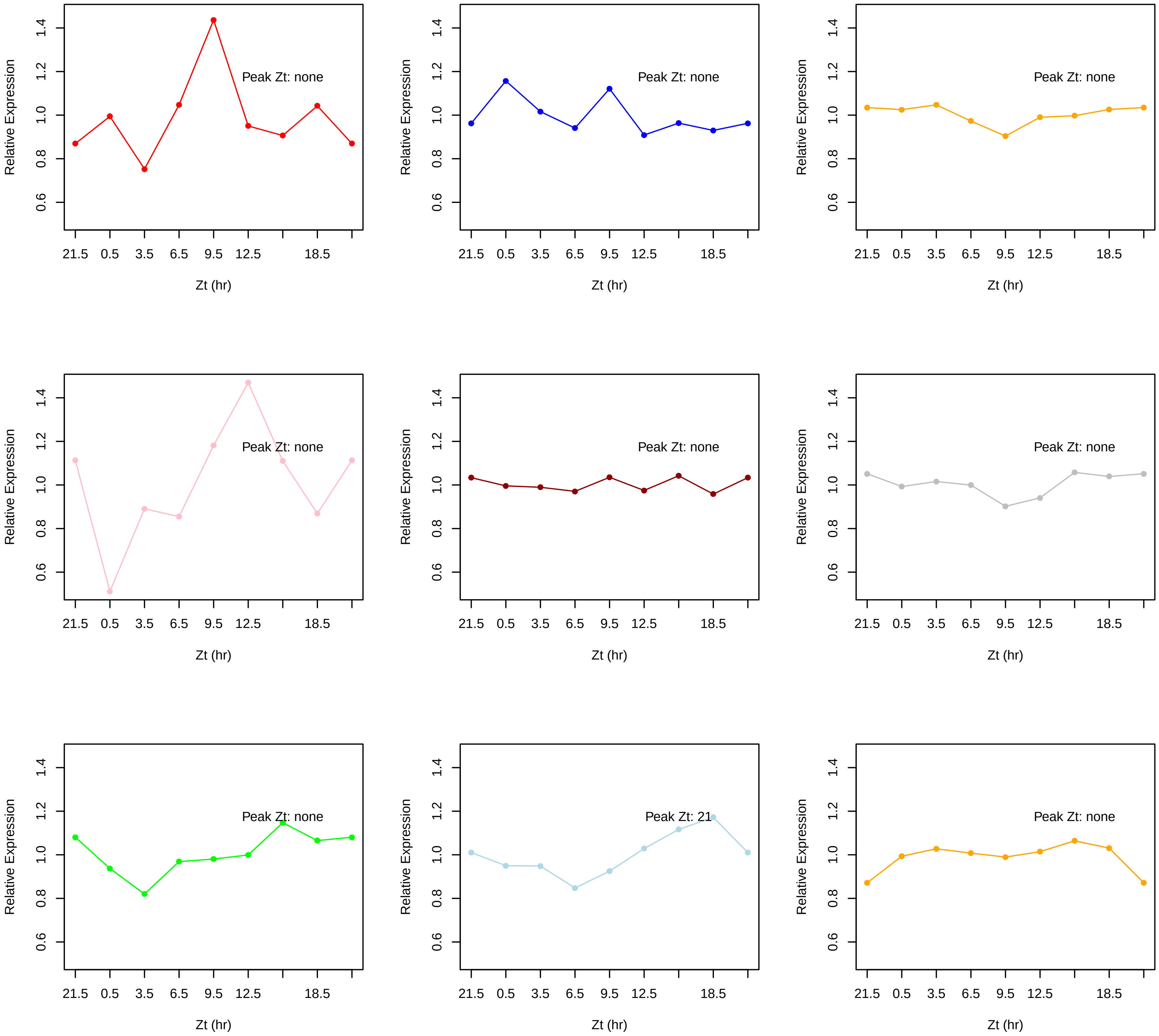

Singeleton: ENSXMAG00000005060 adssl

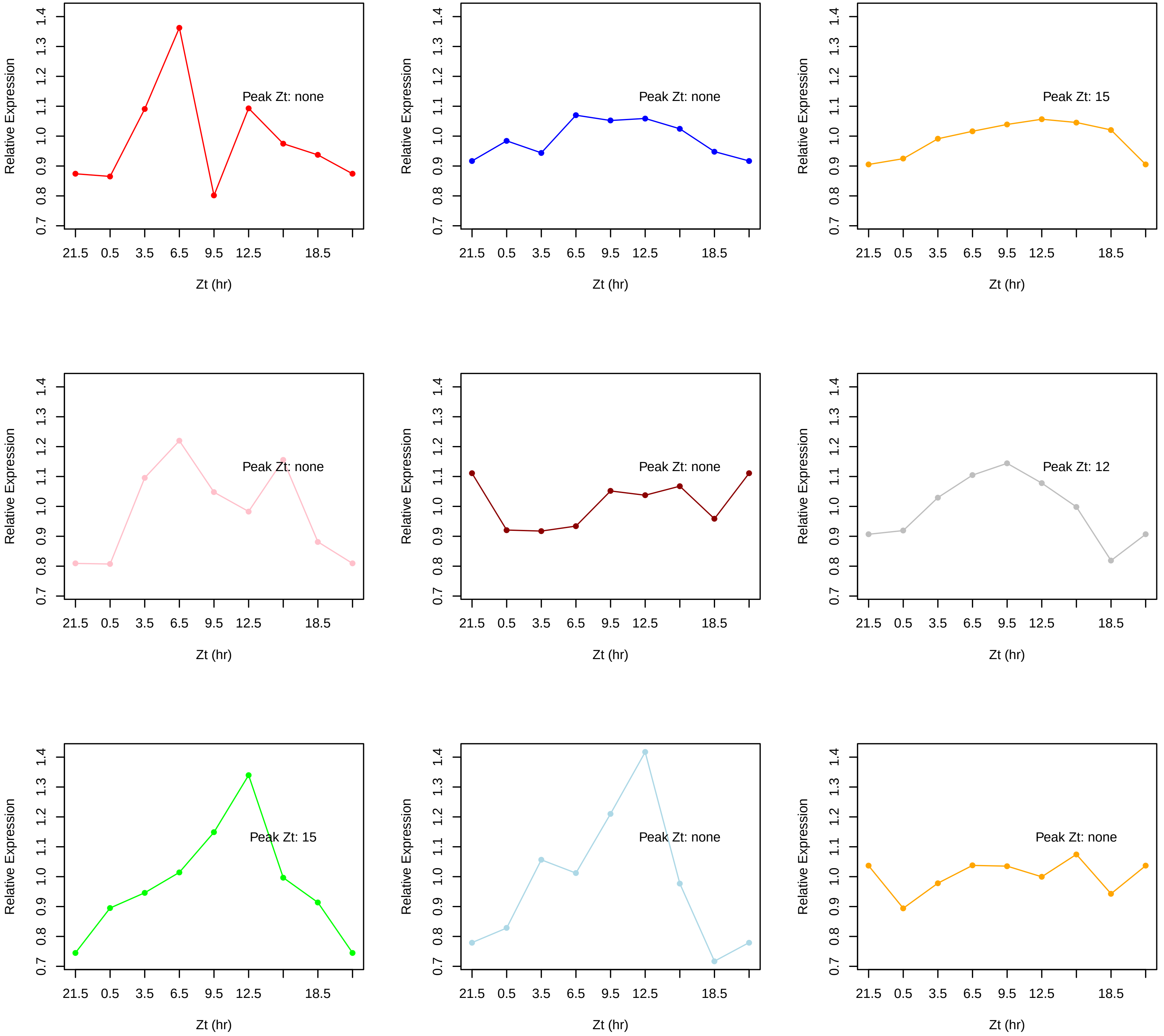

Singleton: ENSXMAG00000028674 ppm1e

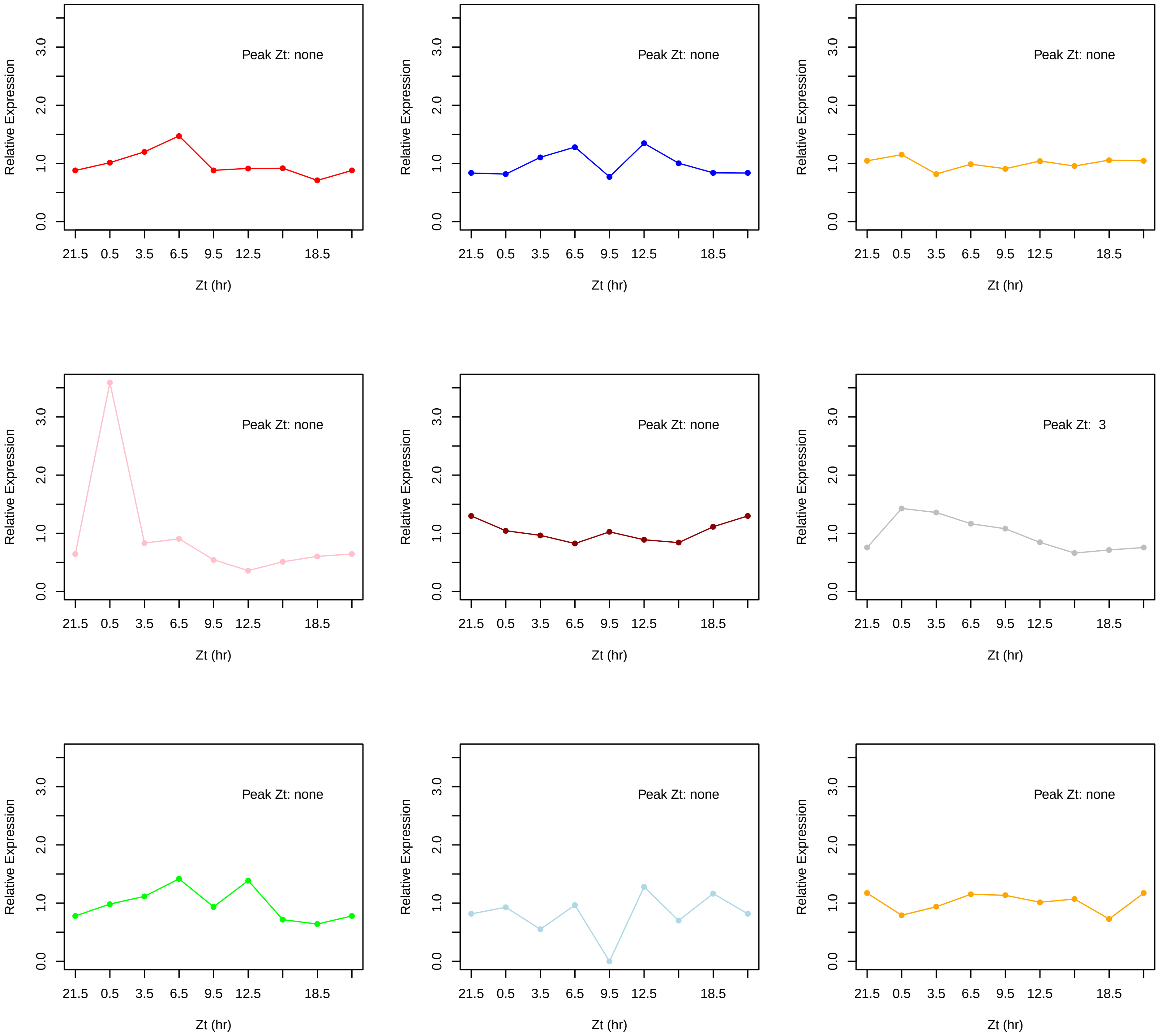

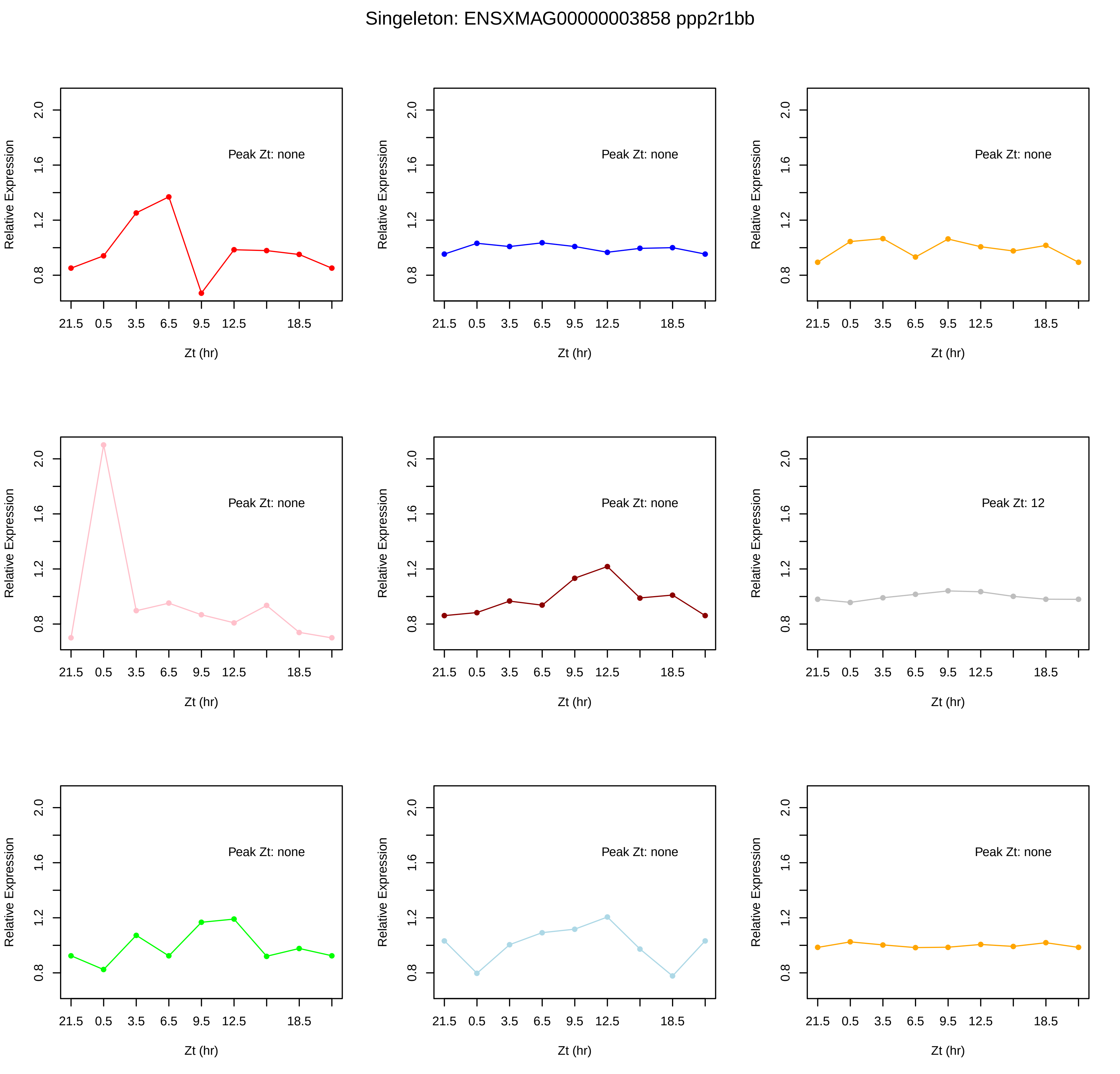
Singeleton: ENSXMAG00000003858 ppp2r1bb
Peak Zt: none
Peak Zt: none
Peak Zt: none
Peak Zt: none
Peak Zt: none
Peak Zt: 12
Peak Zt: none
Peak Zt: none
Peak Zt: none
Relative Expression
Zt (hr)
21.5
0.5
3.5
6.5
9.5
12.5
18.5
0.8
1.2
1.6
2.0

Singeleton: ENSXMAG00000003194 TINAGL1

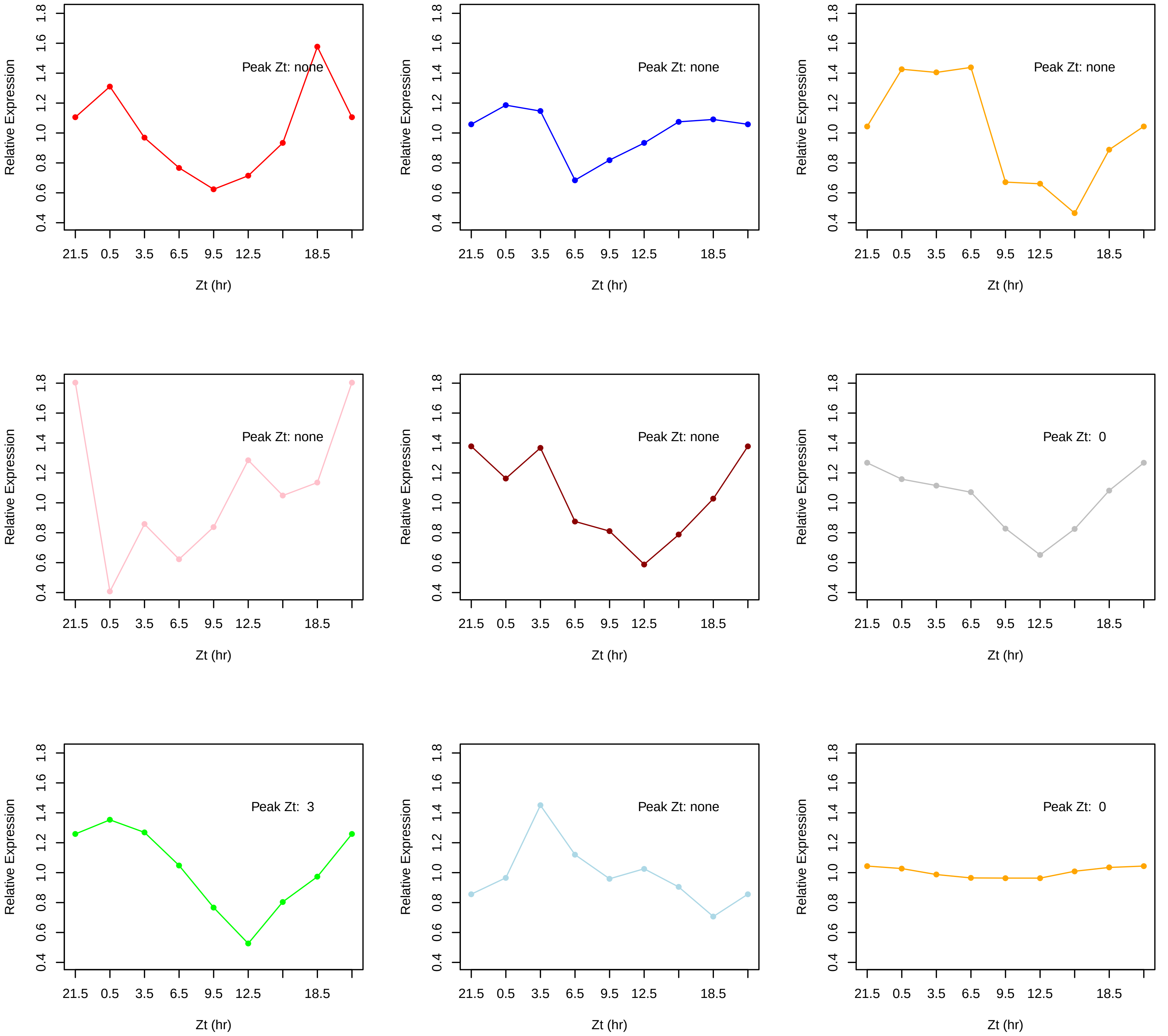

Singleton: ENSXMAG00000018261 usp13

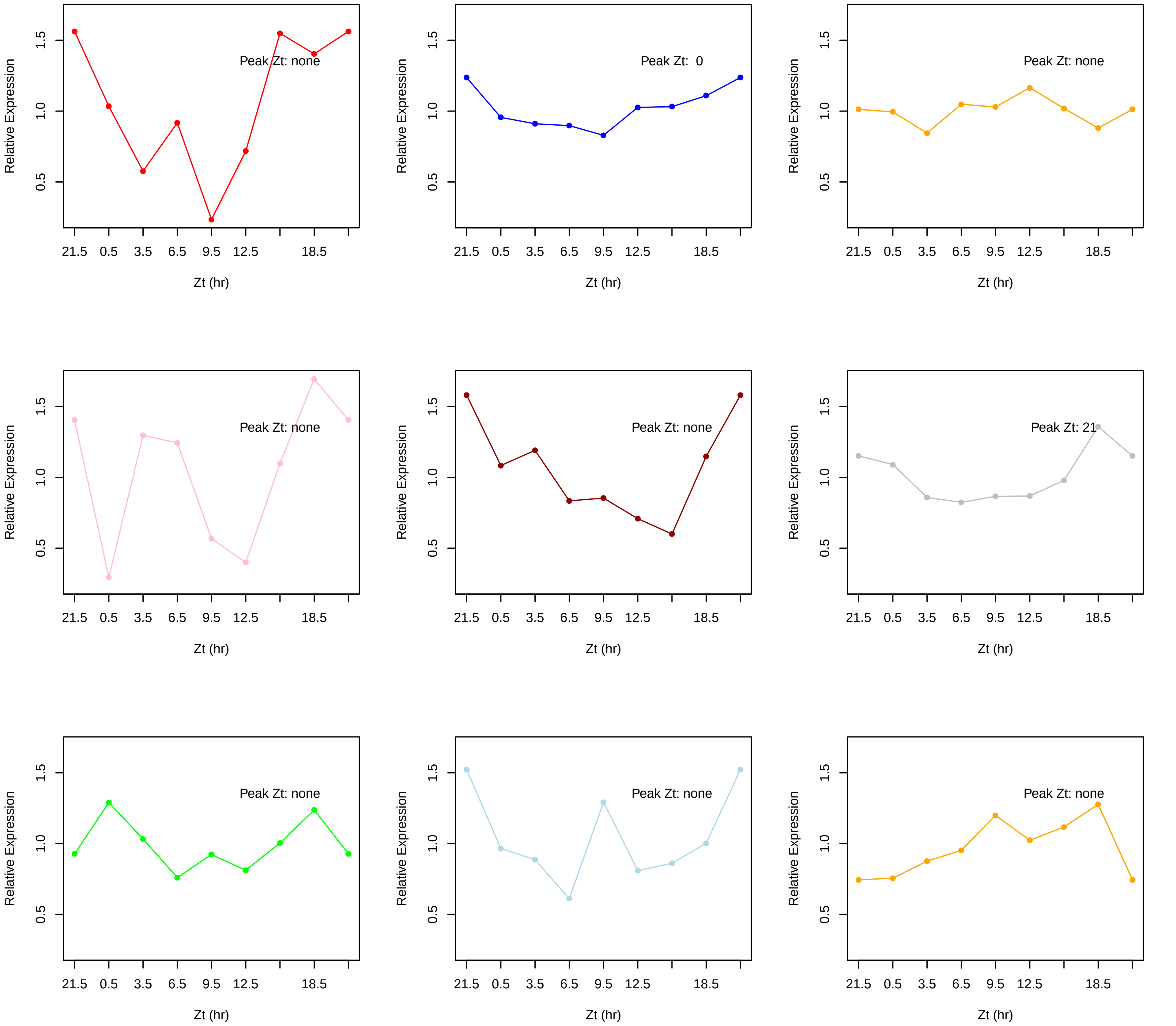

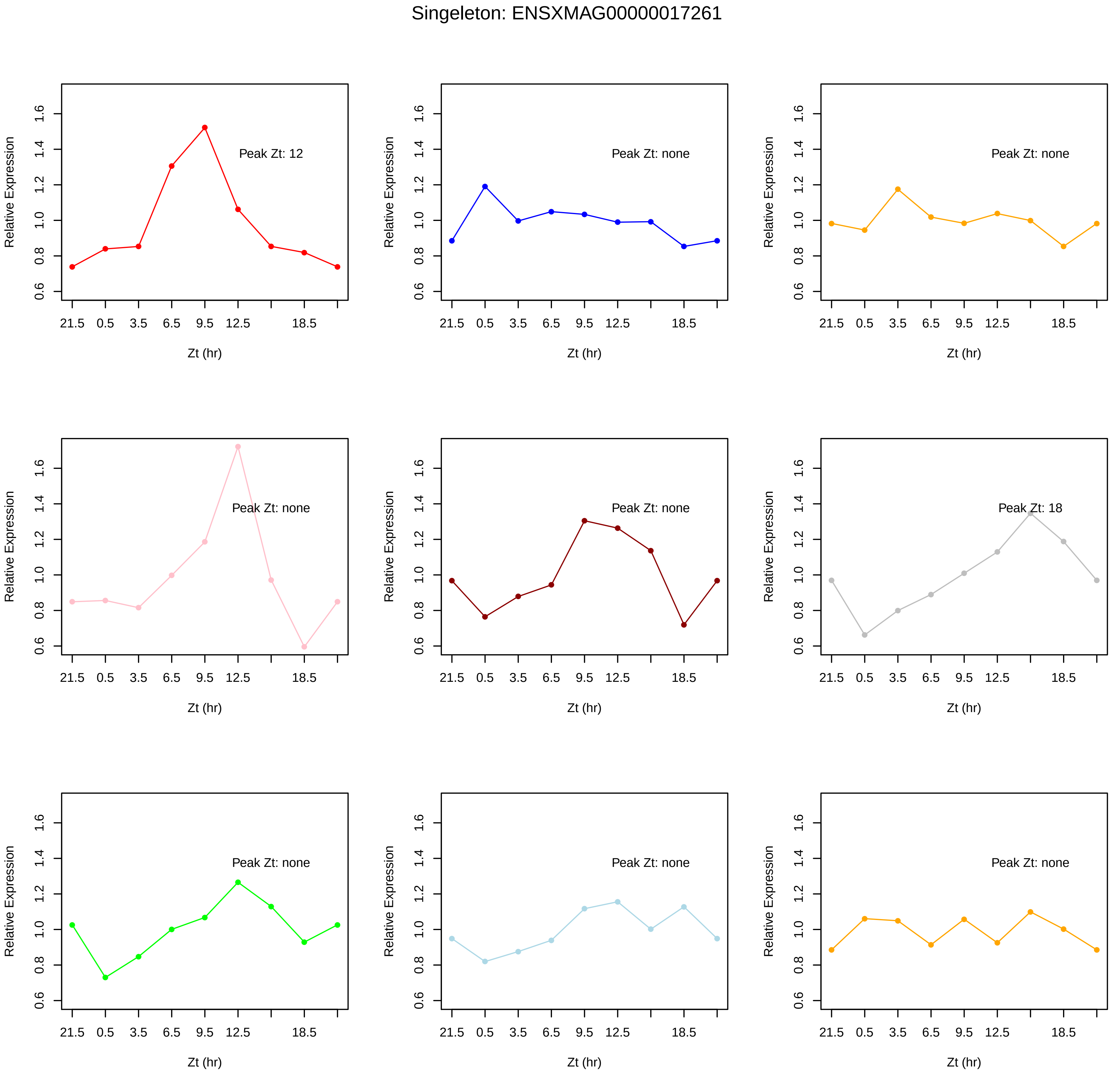
Singeleton: ENSXMAG00000017261
Peak Zt: 12
Peak Zt: none
Peak Zt: none
Peak Zt: none
Peak Zt: none
Peak Zt: 18
Peak Zt: none
Peak Zt: none
Peak Zt: none
Relative Expression
Zt (hr)
21.5
0.5
3.5
6.5
9.5
12.5
18.5
0.6
0.8
1.0
1.2
1.4
1.6

Singeleton: ENSXMAG00000022830 dohh

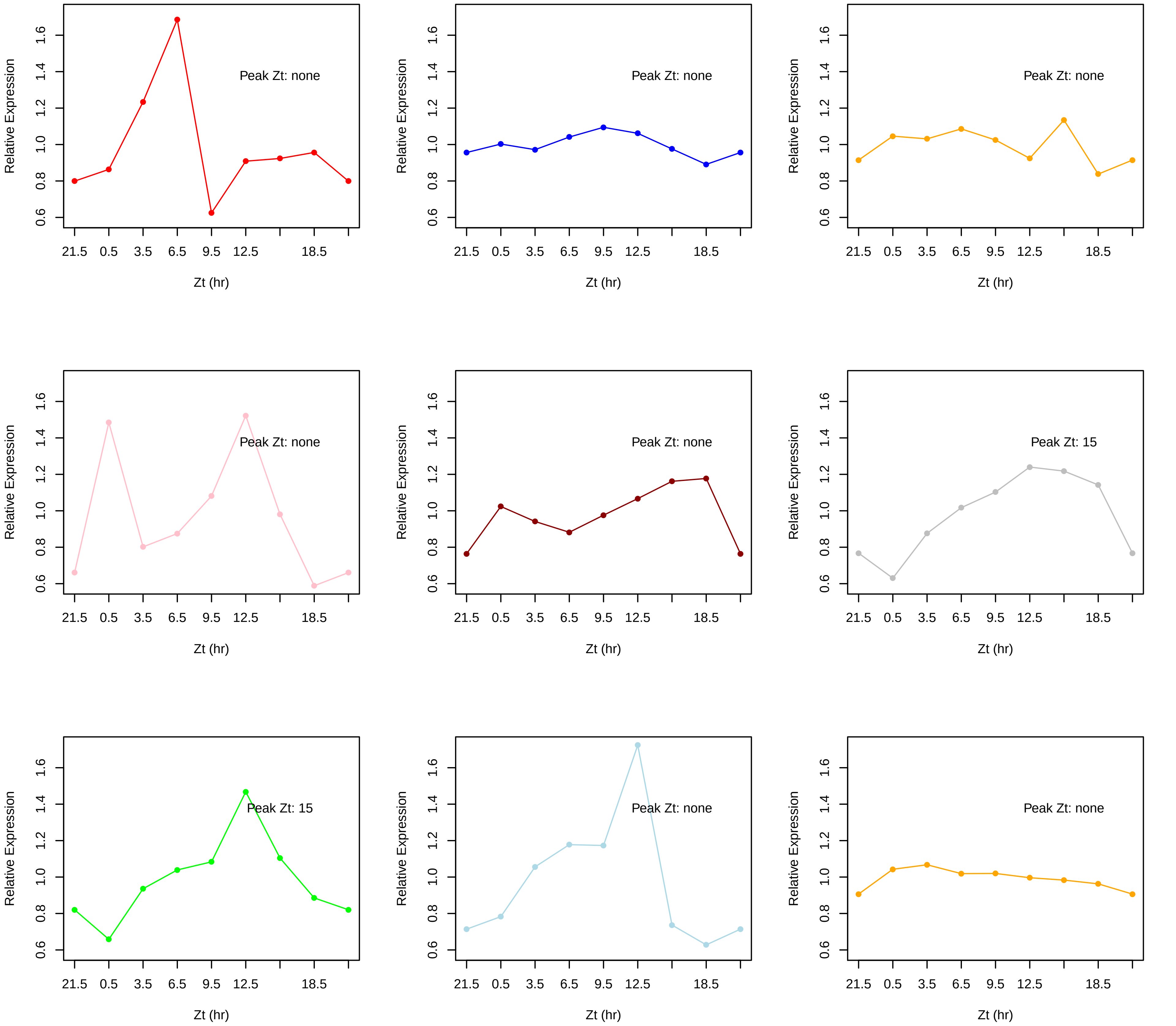

Singeleton: ENSXMAG00000001487 apip

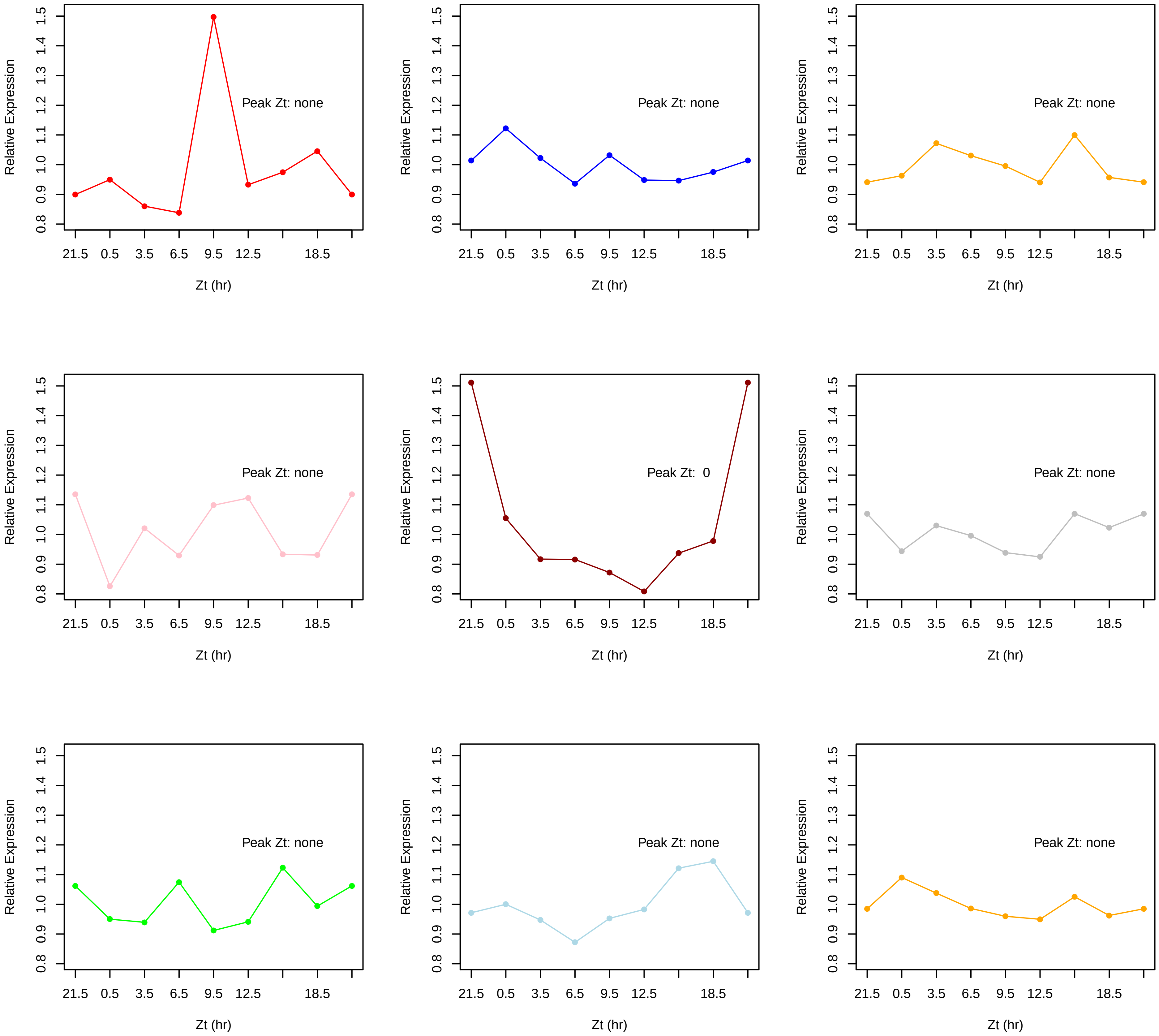

Singeleton: ENSXMAG00000001695 slc7a8a

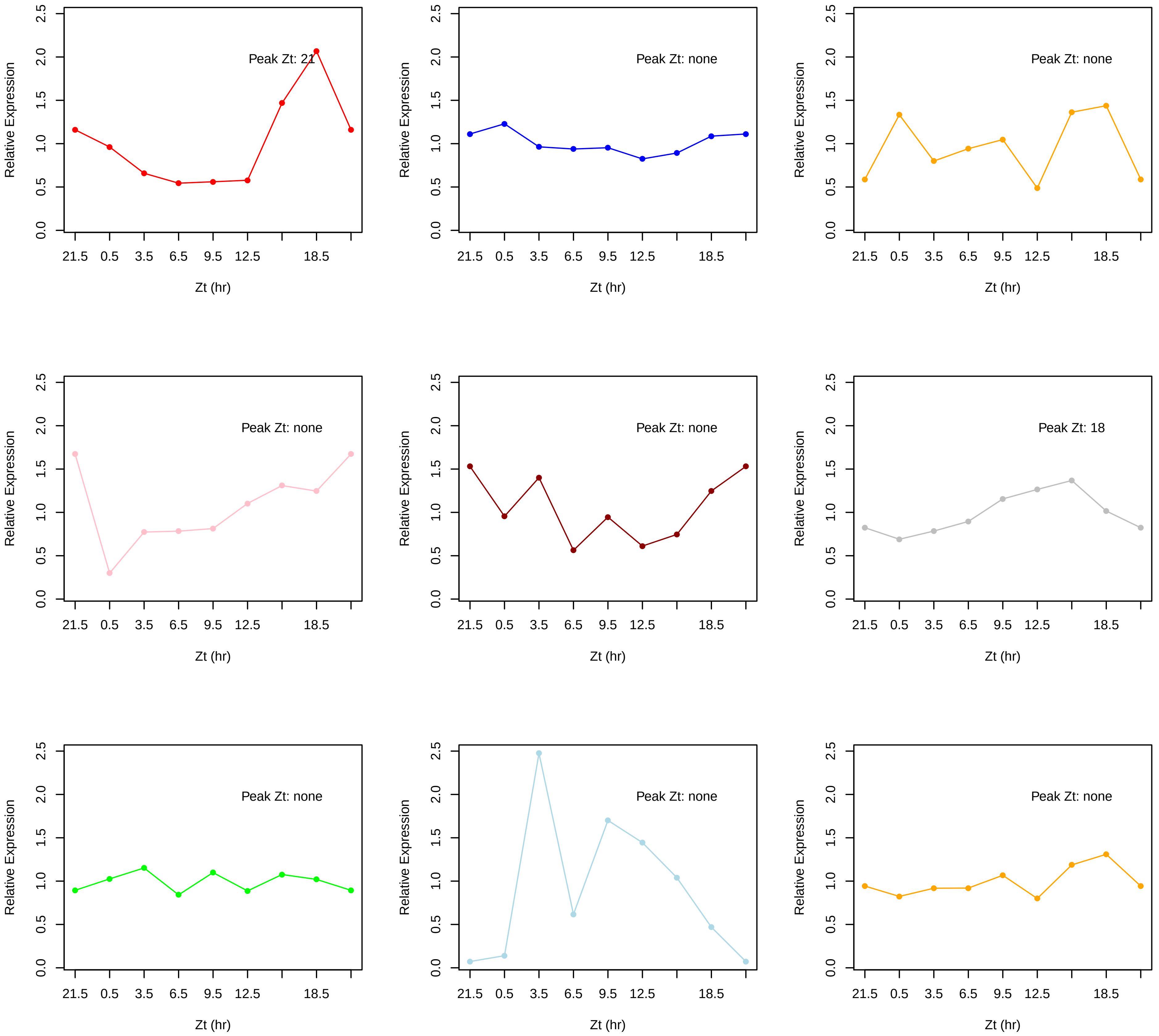

Singeleton: ENSXMAG00000008725 ulk4

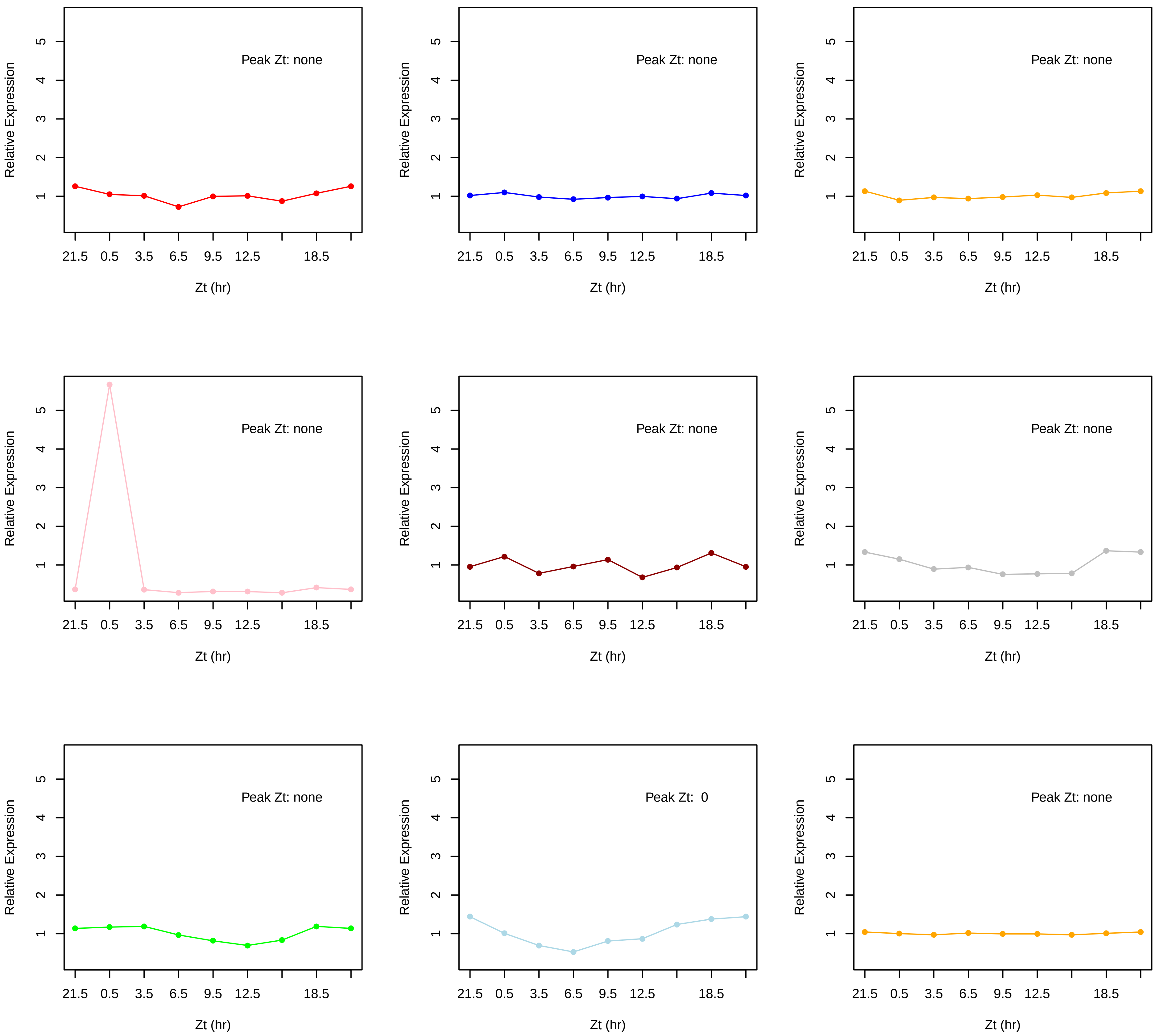

Singeleton: ENSXMAG00000022062 paip2b

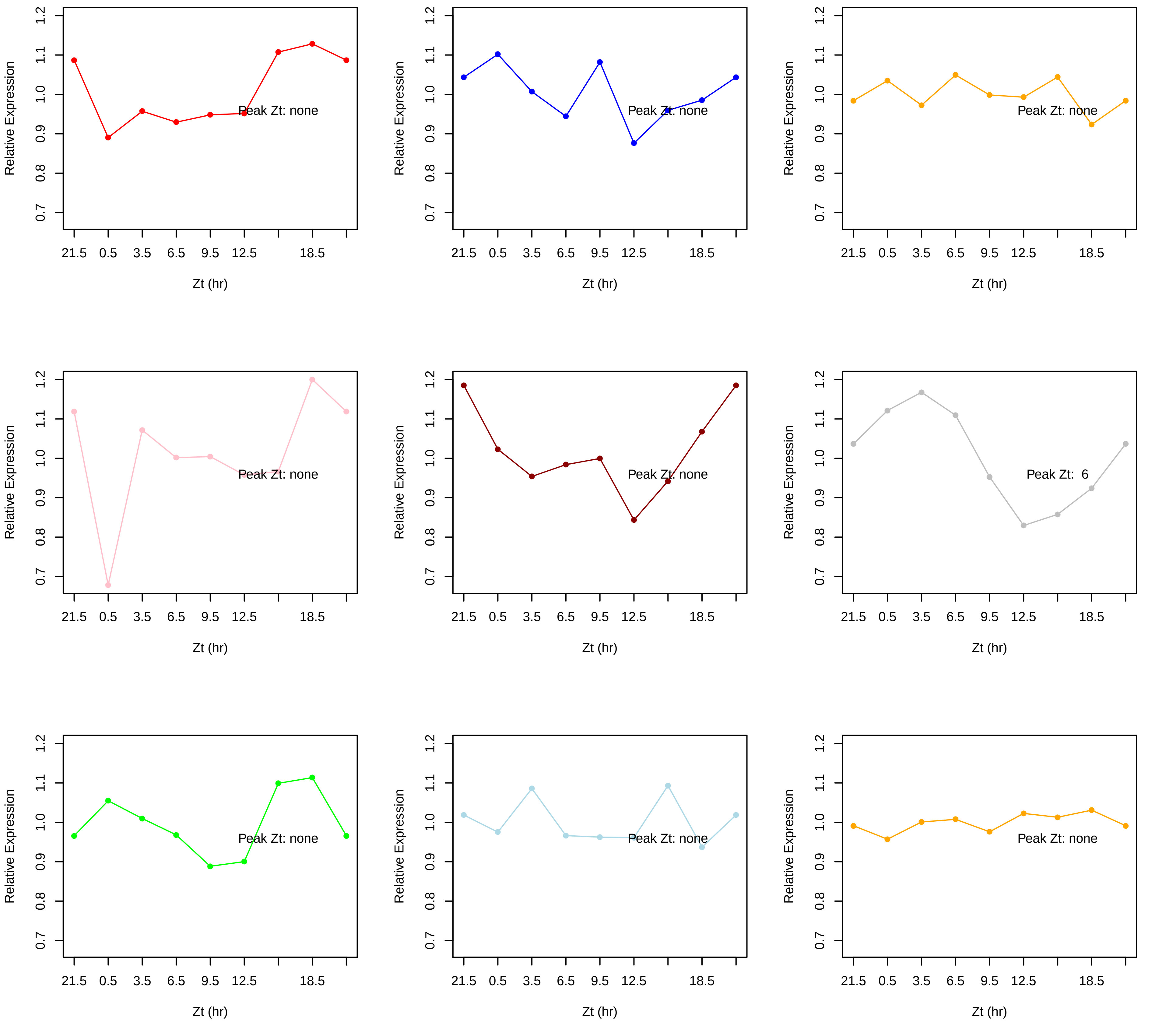

Singeleton: ENSXMAG00000003184 KCNQ4

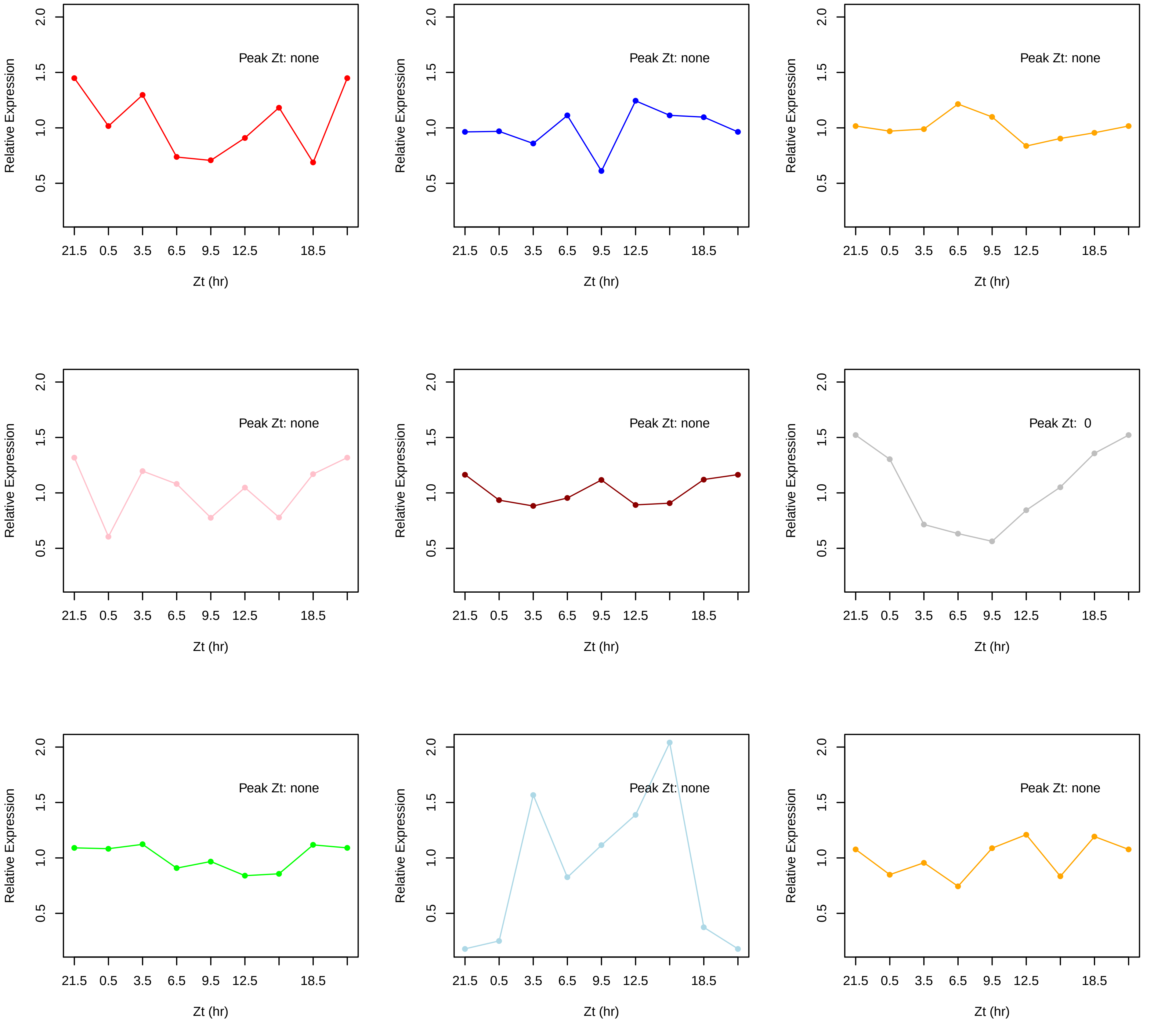

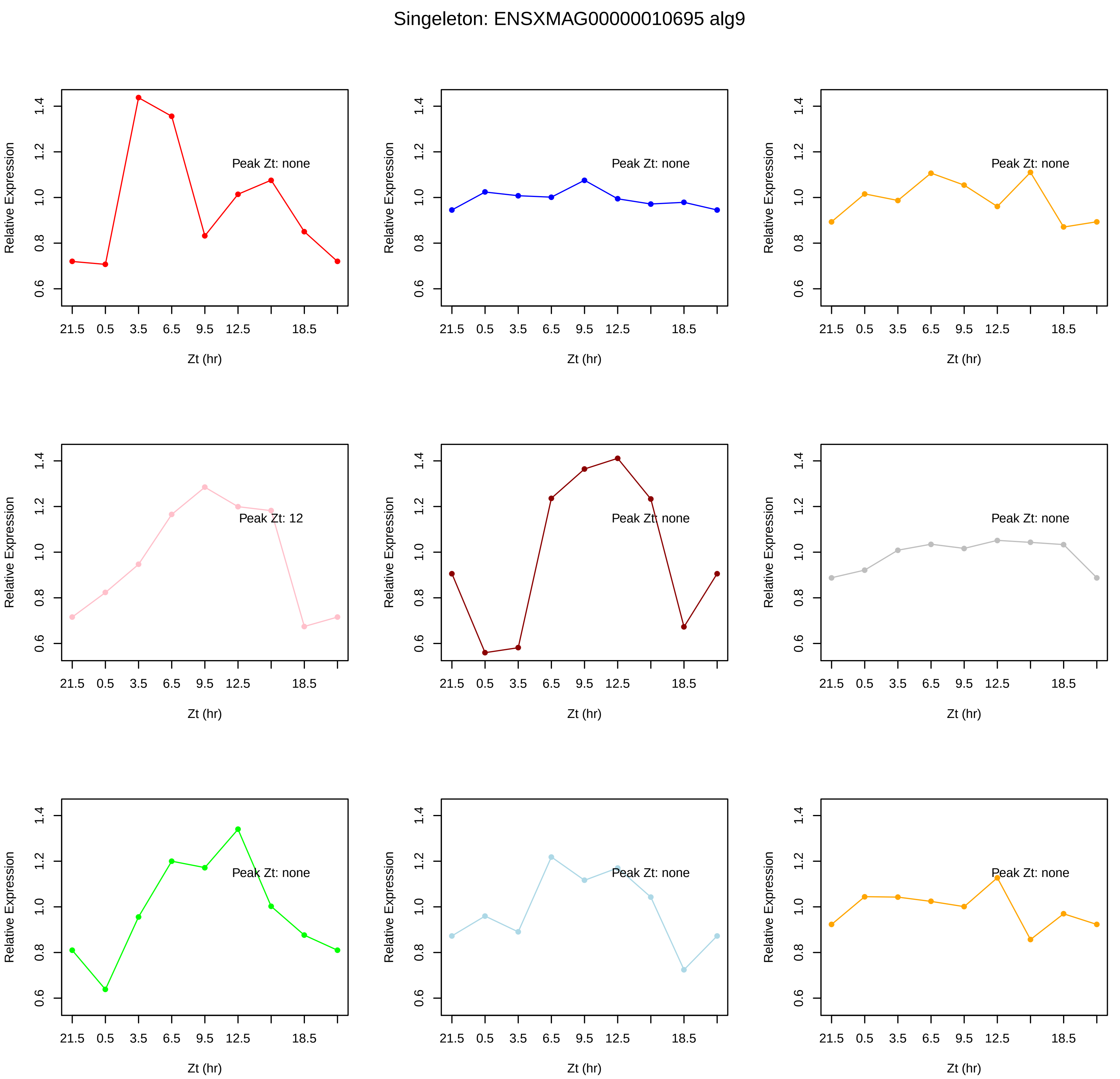

Singeleton: ENSXMAG00000010695 alg9
Relative Expression
Zt (hr)
21.5
0.5
3.5
6.5
9.5
12.5
18.5
0.6
0.8
1.0
1.2
1.4
Peak Zt: none
Peak Zt: 12

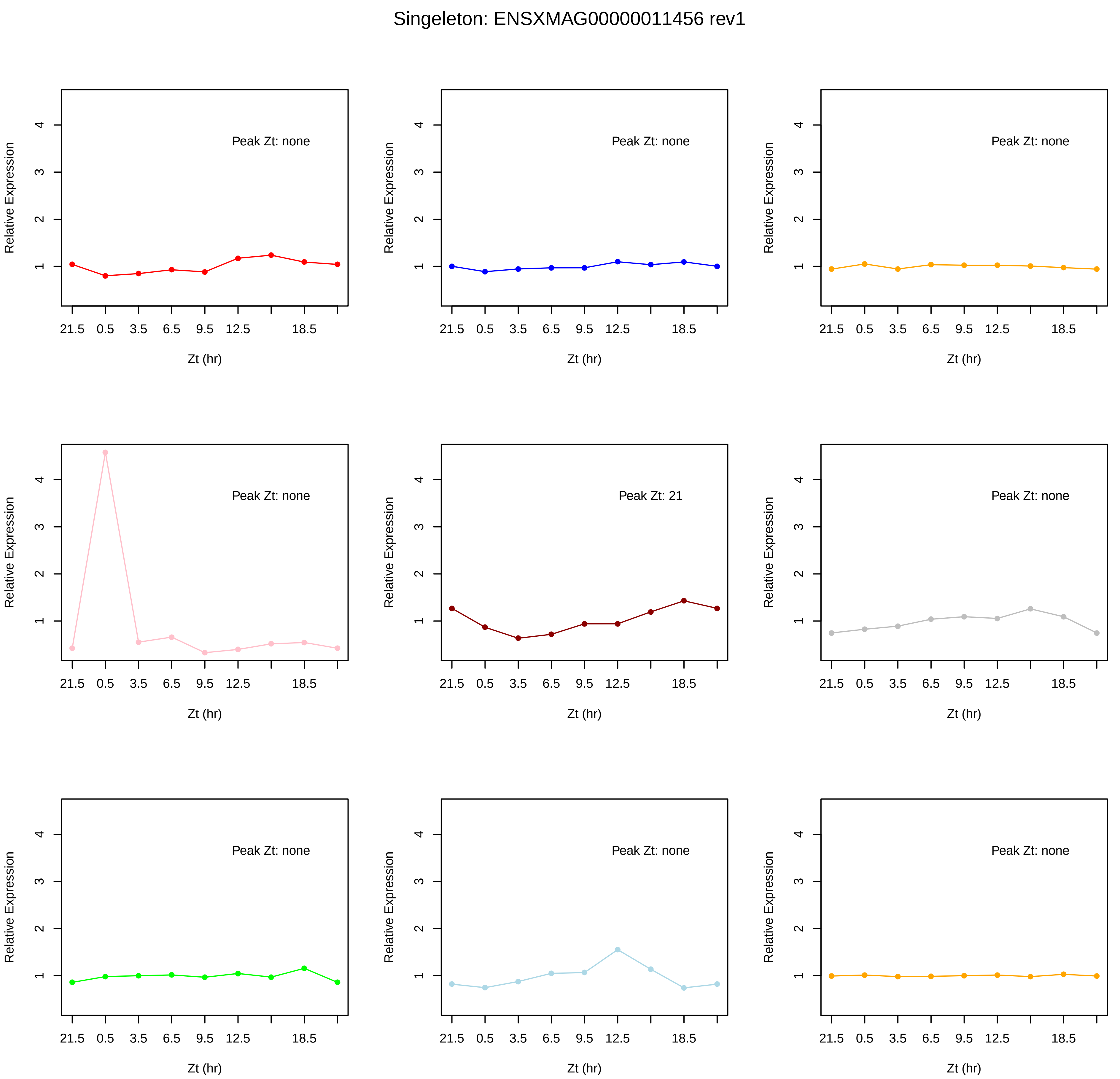
Singeleton: ENSXMAG00000011456 rev1
Peak Zt: none
Peak Zt: none
Peak Zt: none
Peak Zt: none
Peak Zt: 21
Peak Zt: none
Peak Zt: none
Peak Zt: none
Peak Zt: none
Relative Expression
Zt (hr)
21.5 0.5 3.5 6.5 9.5 12.5 18.5
1 2 3 4

Singeleton: ENSXMAG00000029144

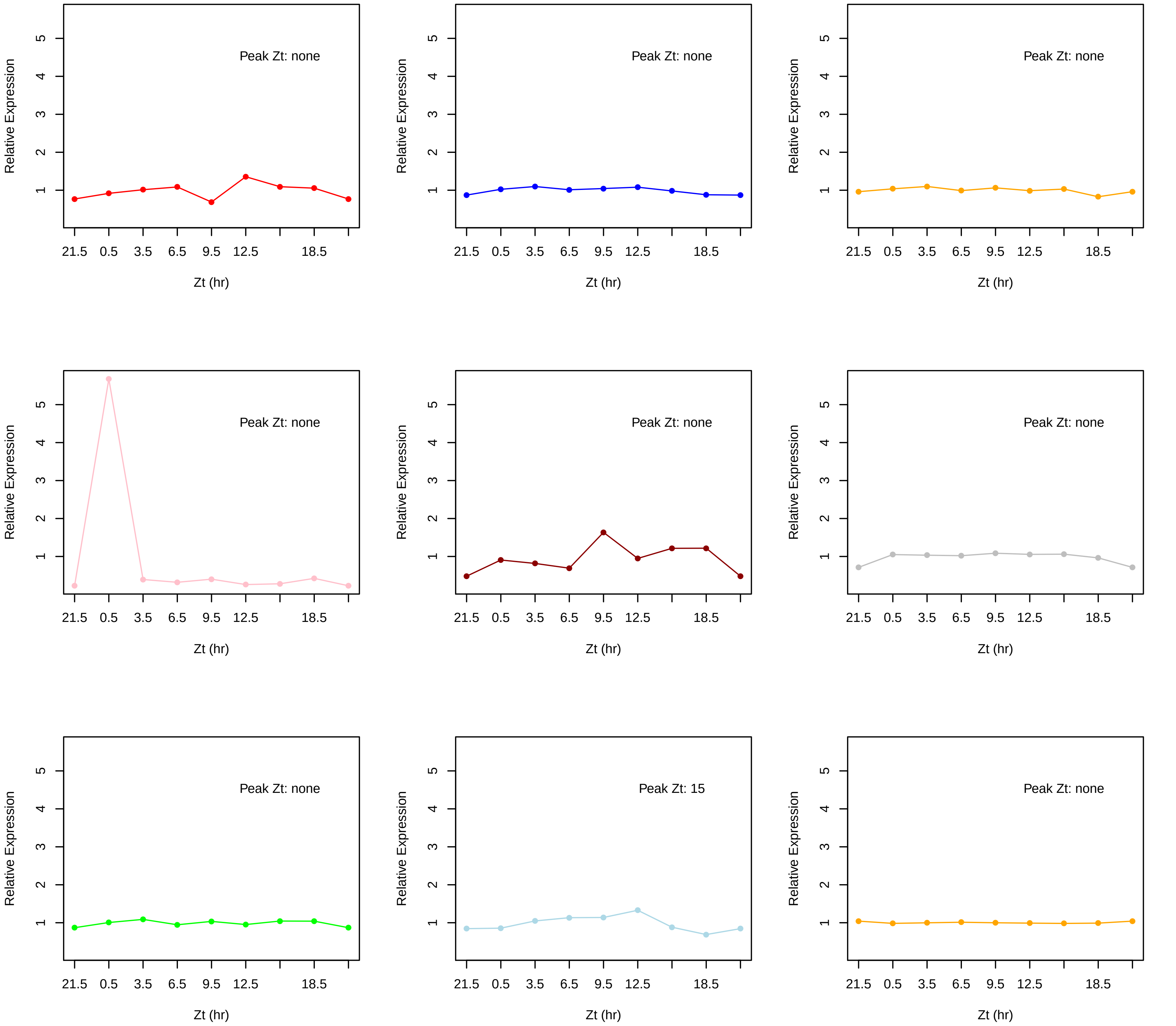

Singeleton: ENSXMAG00000017282 GXYLT1

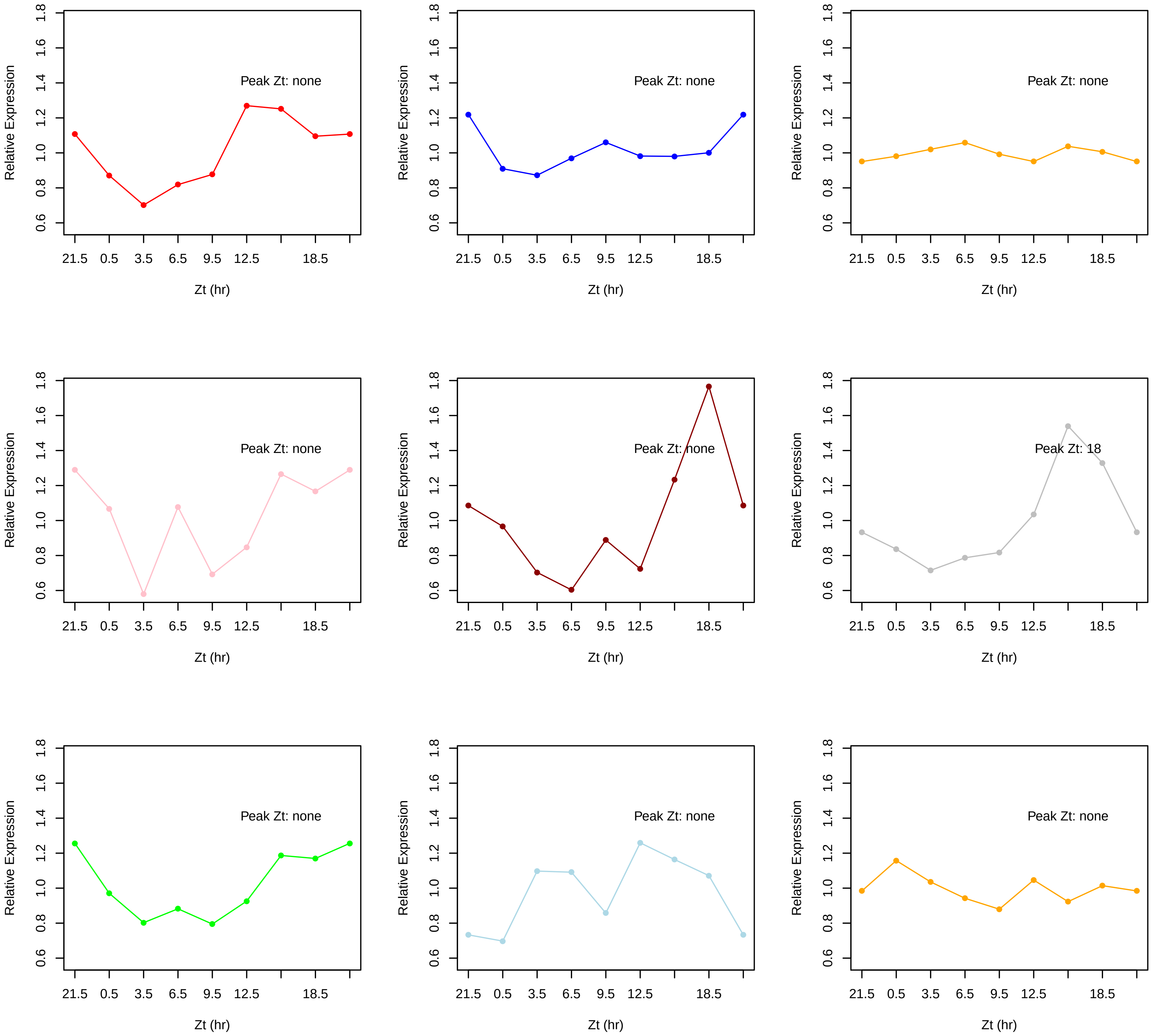

Singeleton: ENSXMAG00000008819 nagk

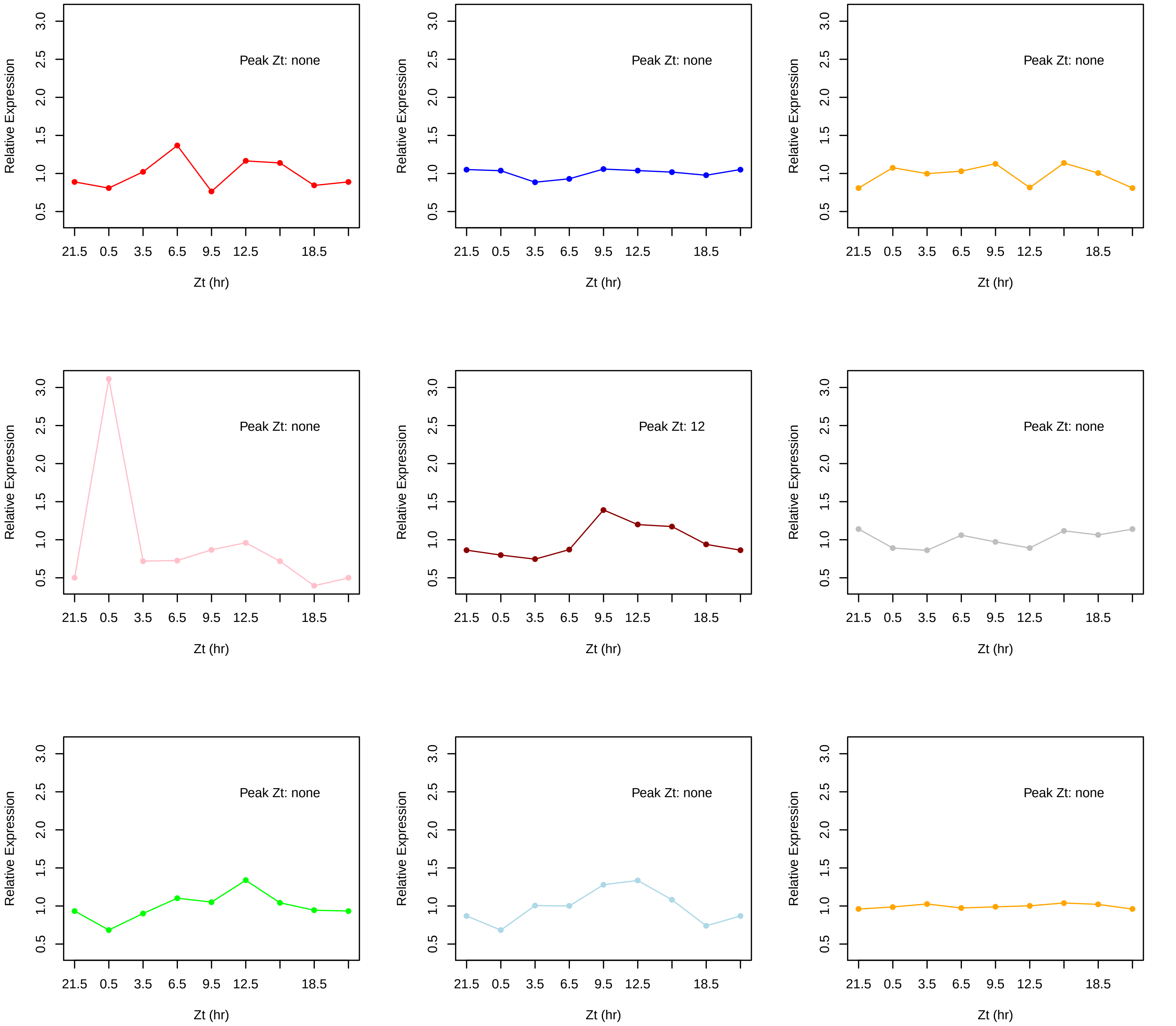

Singleton: ENSXMAG00000019942 si:dkey−237j11.3

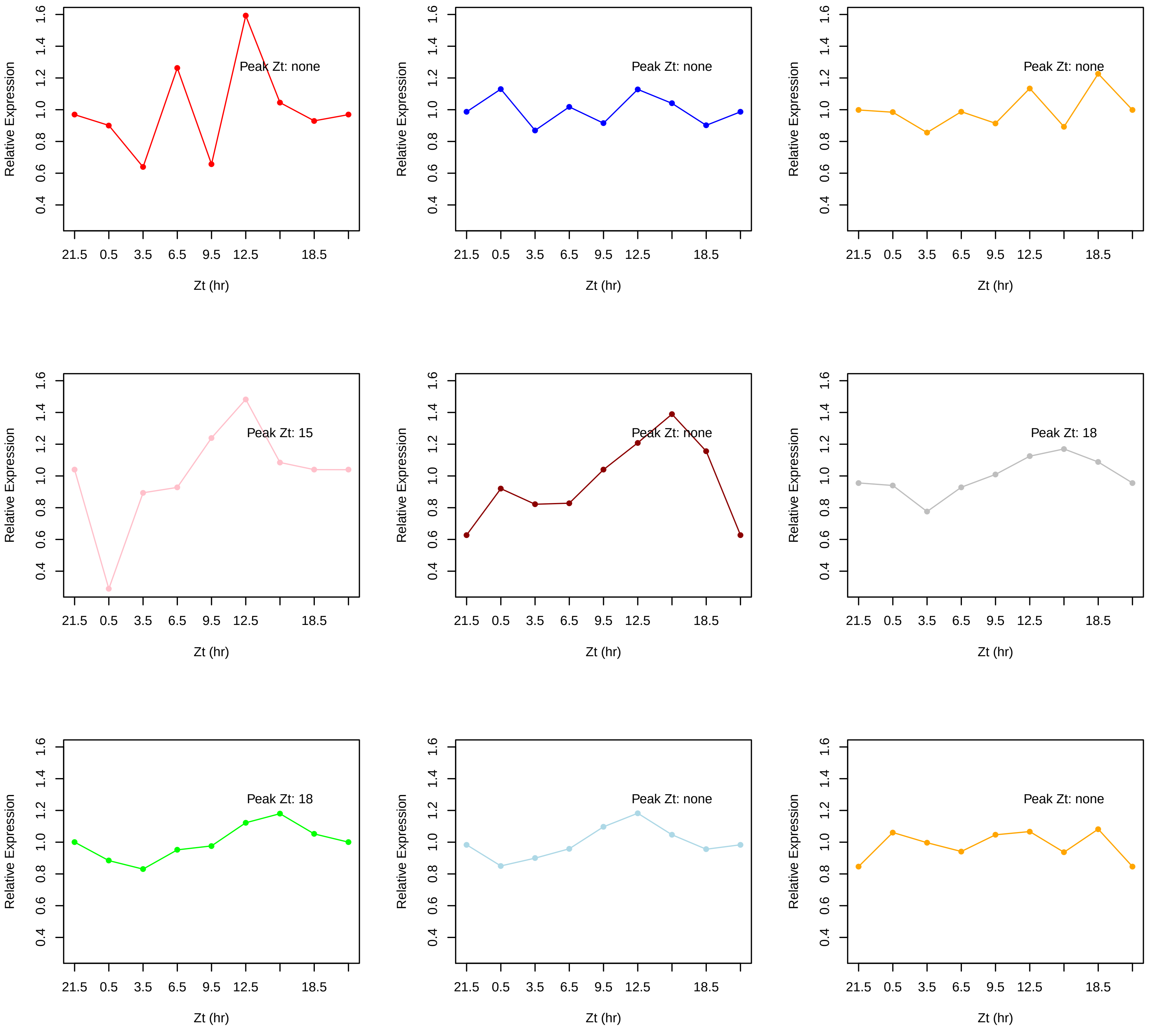

Singeleton: ENSXMAG00000010615 orc4

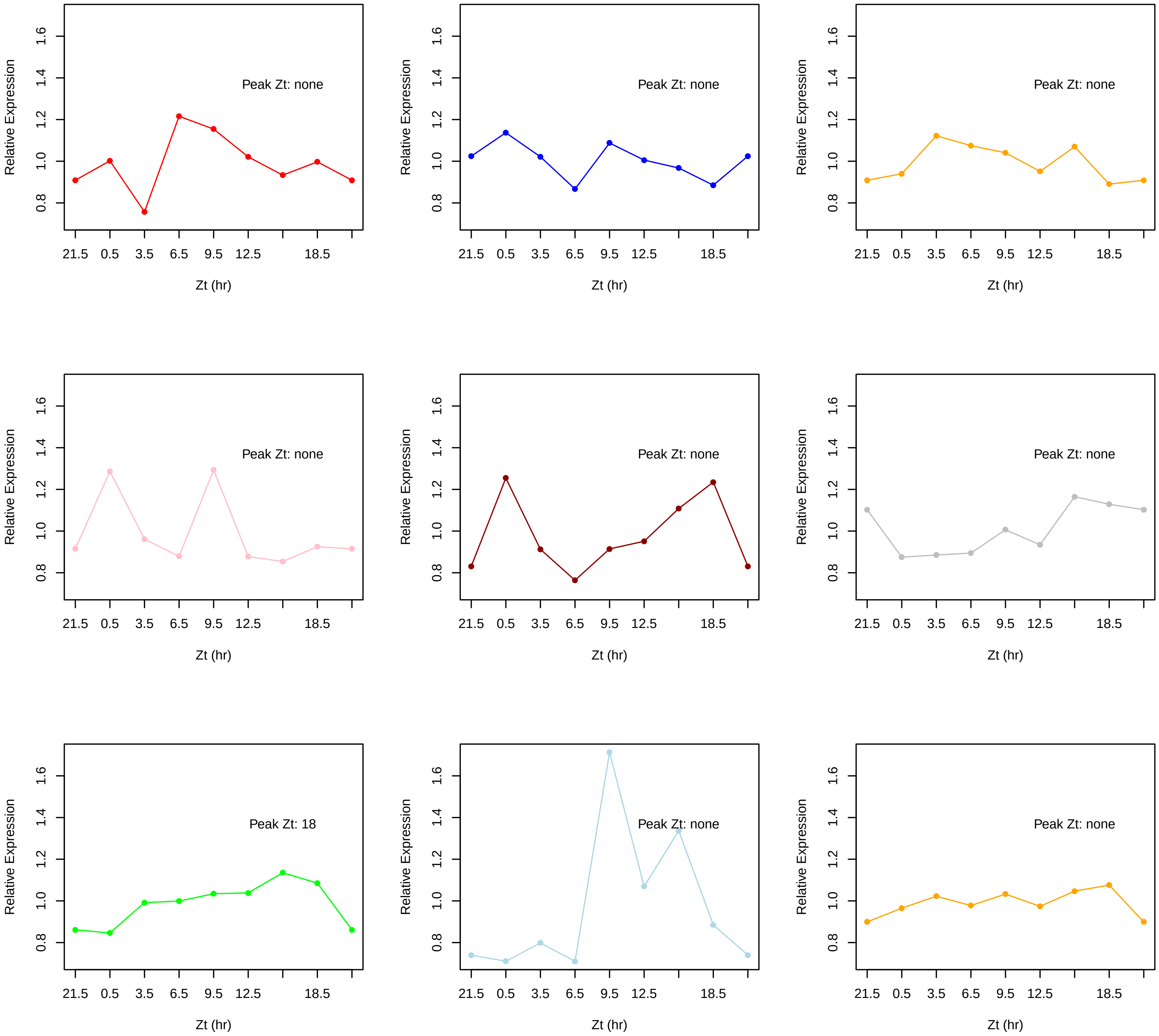

Singeleton: ENSXMAG00000025433 yaf2

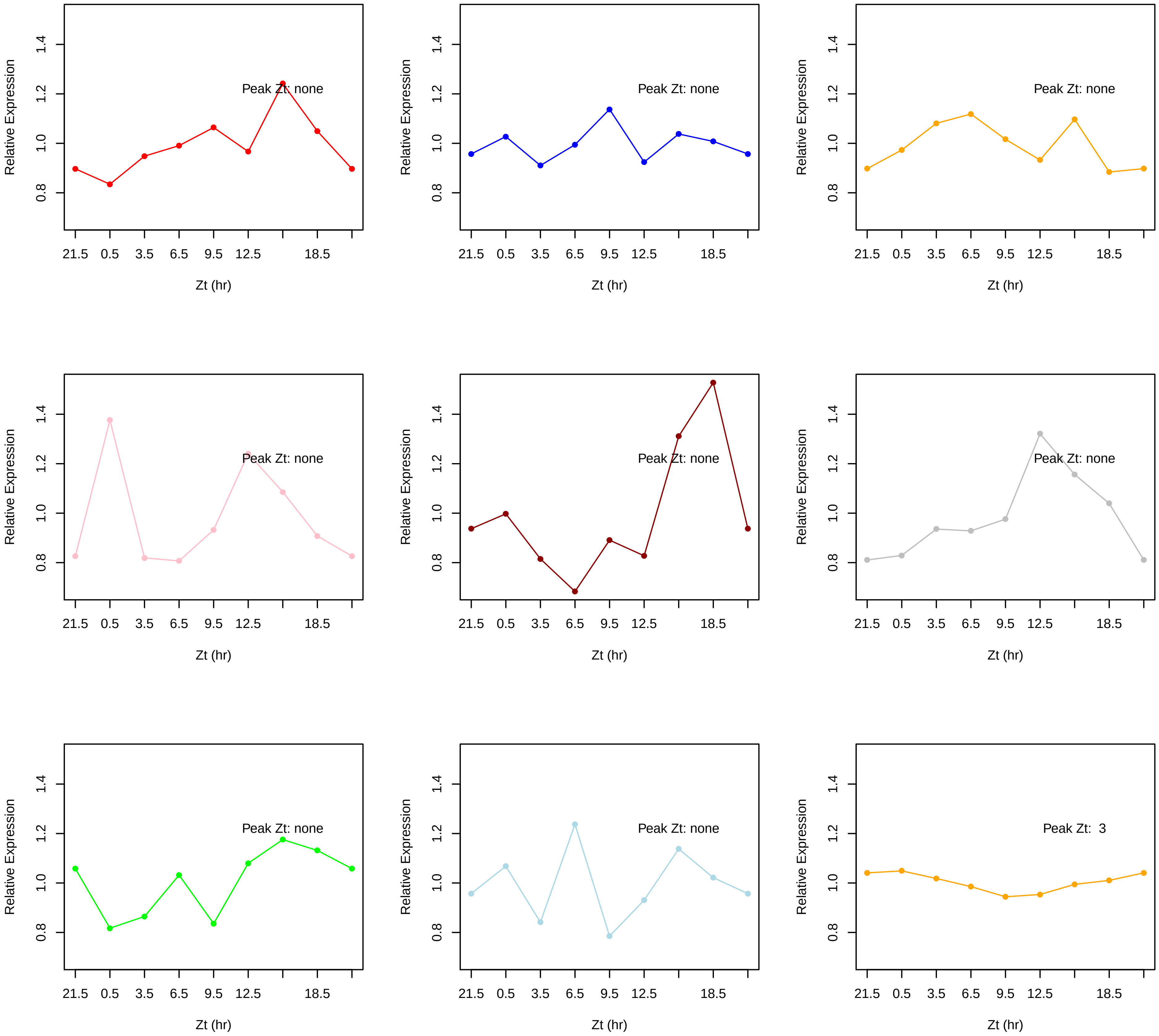

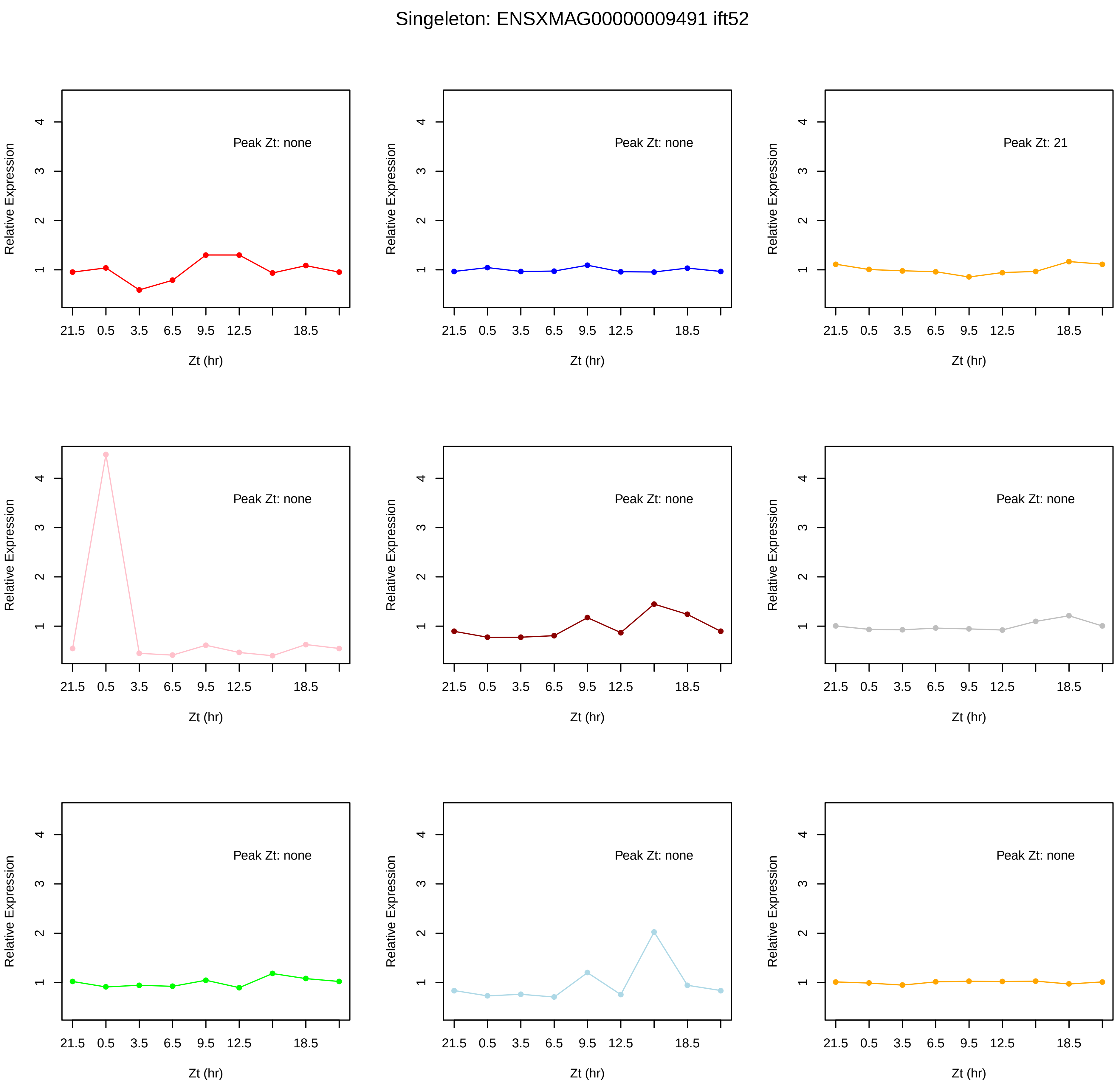

Singeleton: ENSXMAG00000009491 ift52
Peak Zt: none
Peak Zt: none
Peak Zt: 21
Peak Zt: none
Peak Zt: none
Peak Zt: none
Peak Zt: none
Peak Zt: none
Peak Zt: none
Relative Expression
Zt (hr)
21.5
0.5
3.5
6.5
9.5
12.5
18.5

Singeleton: ENSXMAG00000003179 nfyc

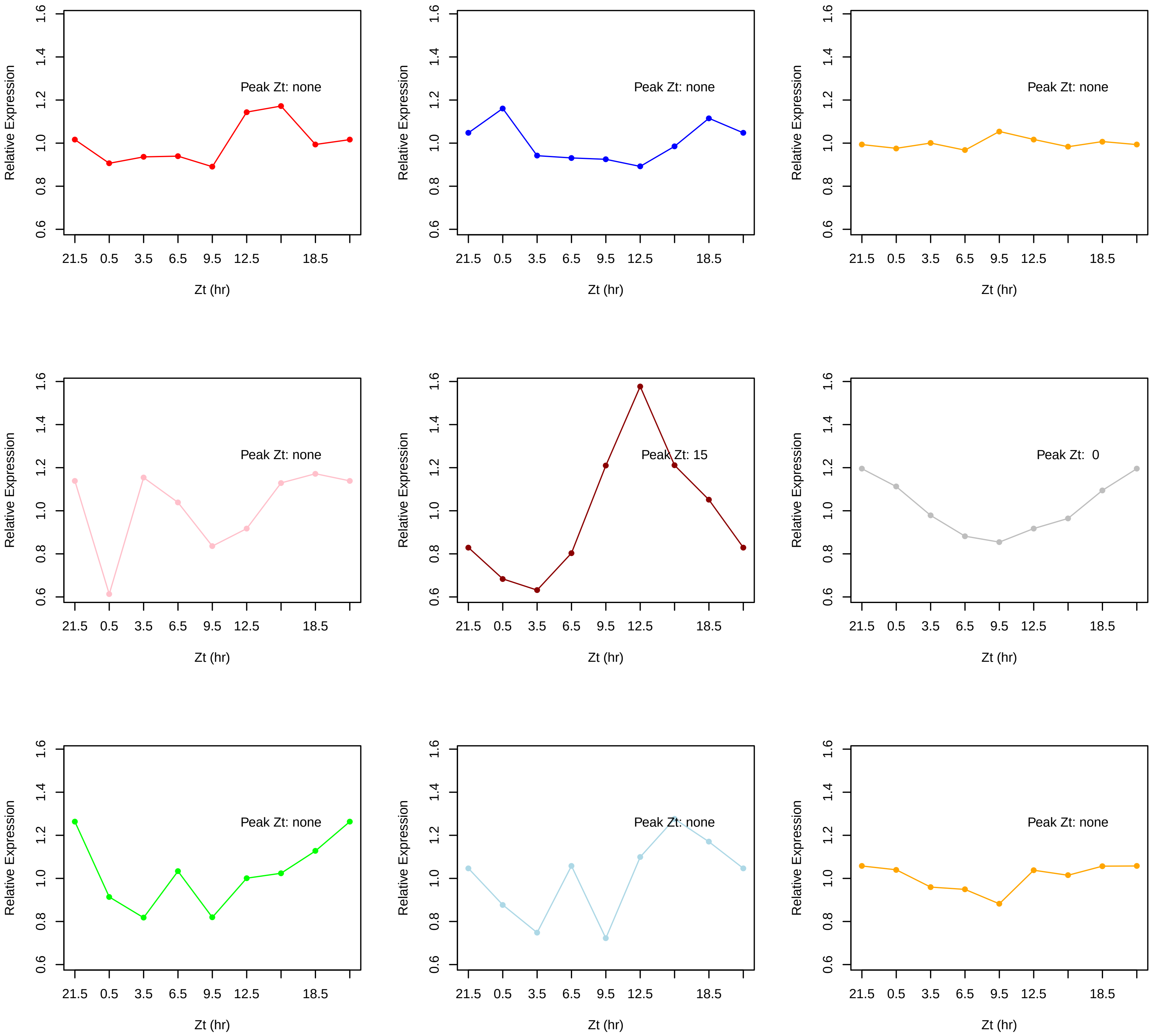

Singeleton: ENSXMAG00000008710 ctnnb1

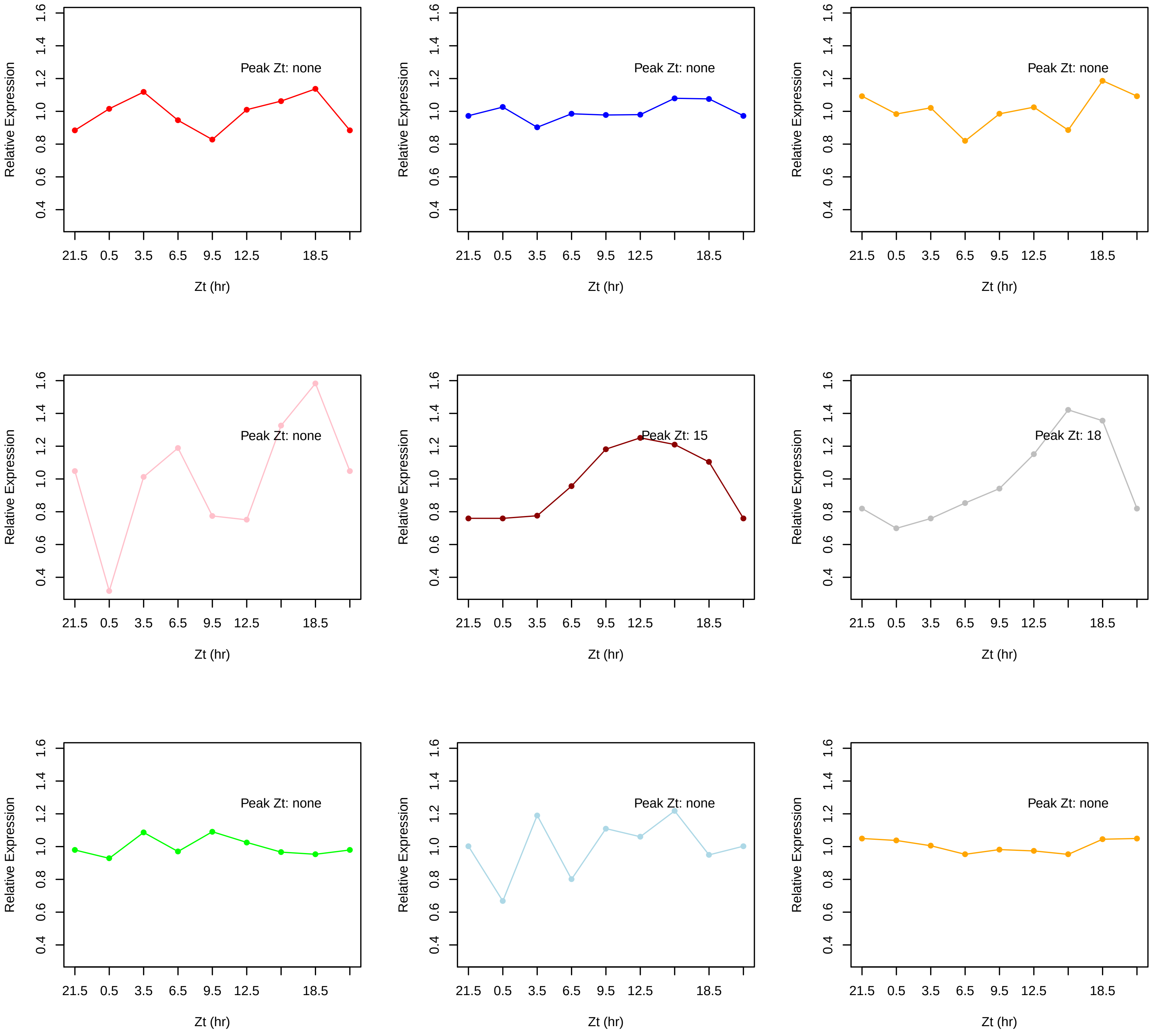

Singeleton: ENSXMAG00000000733 si:ch211−43f4.1

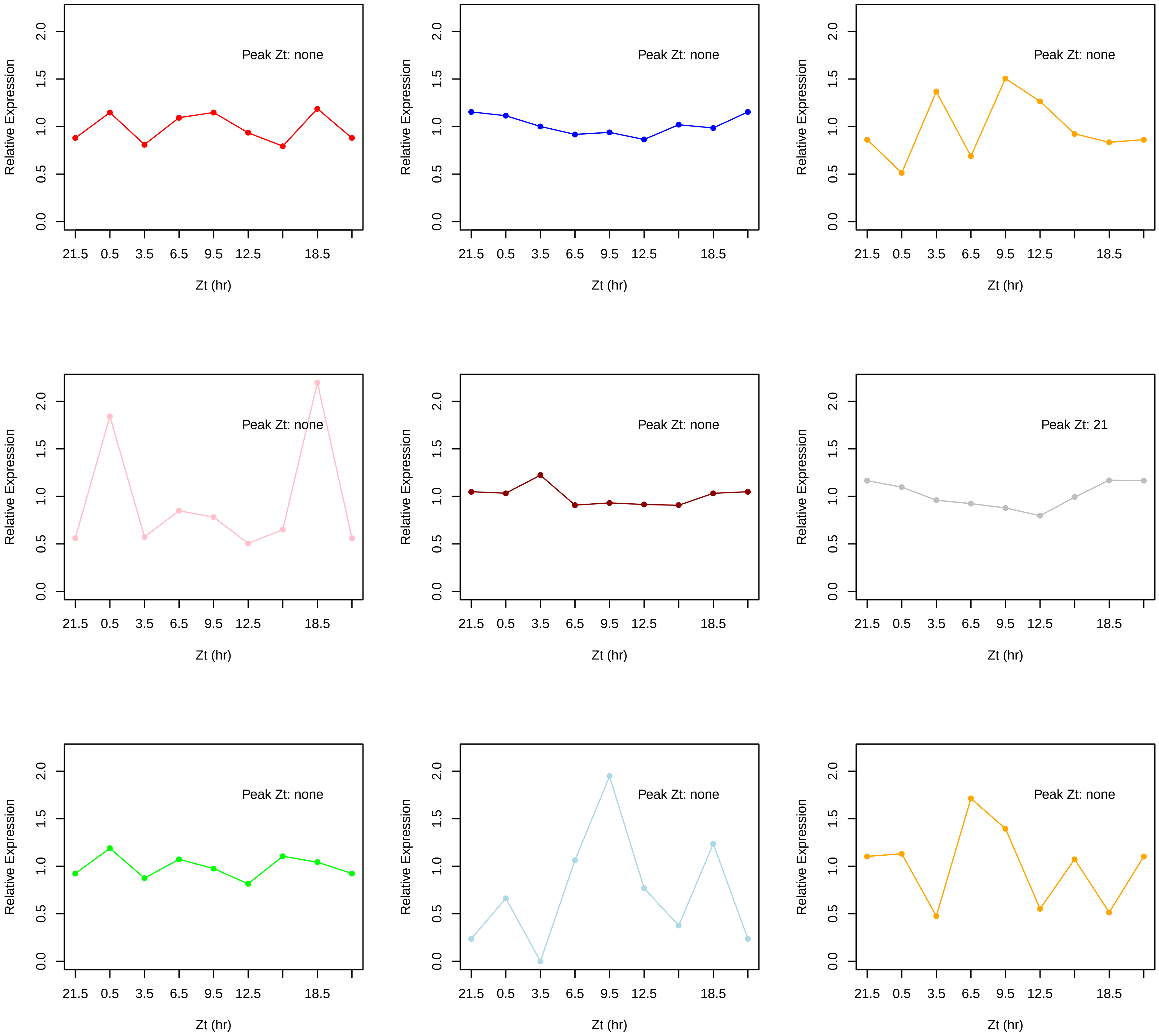

Singleton: ENSXMAG00000003875 phb2a

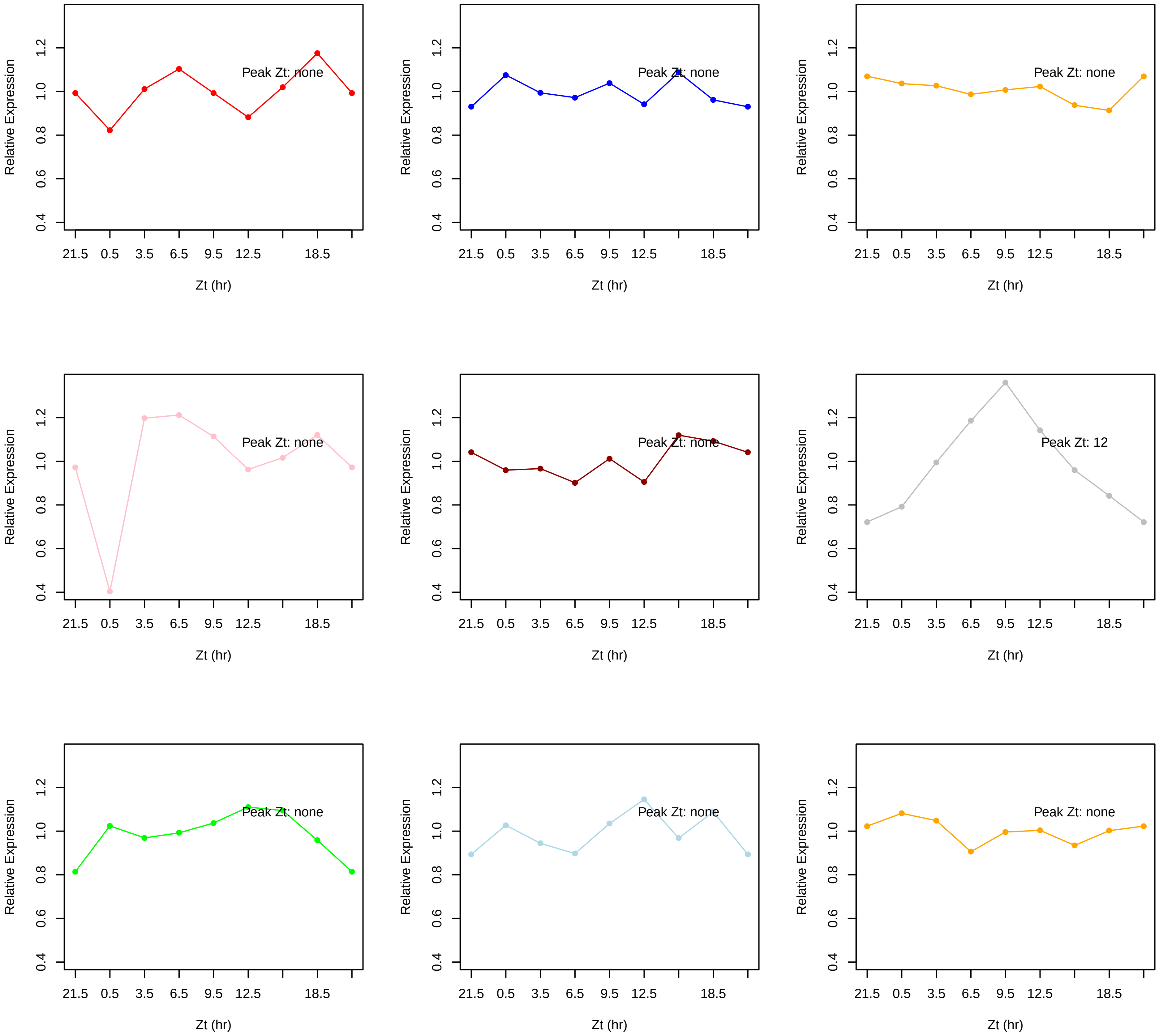

Singeleton: ENSXMAG00000014070 mad1l1

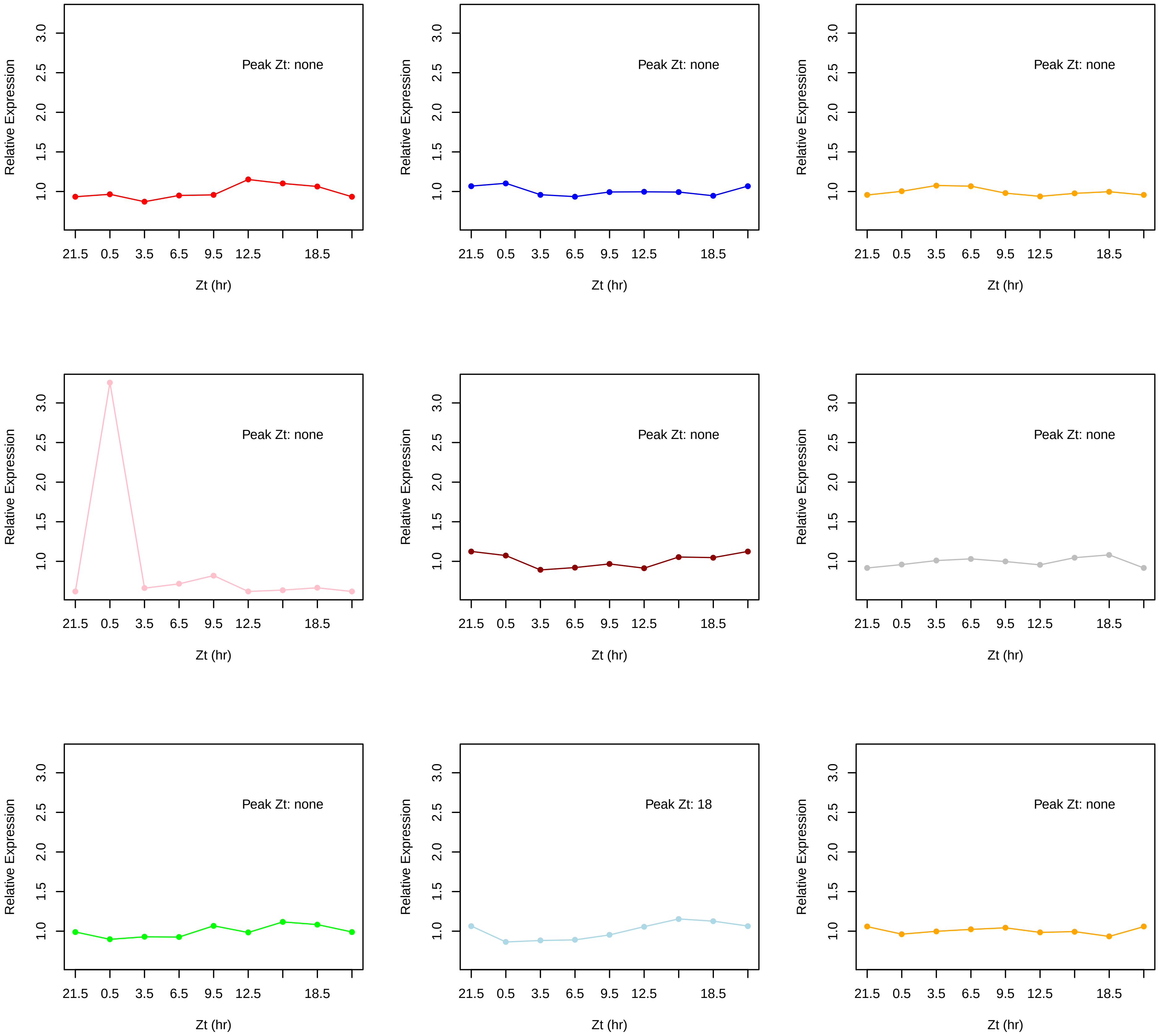

Singleton: ENSXMAG00000022203 AHNAK

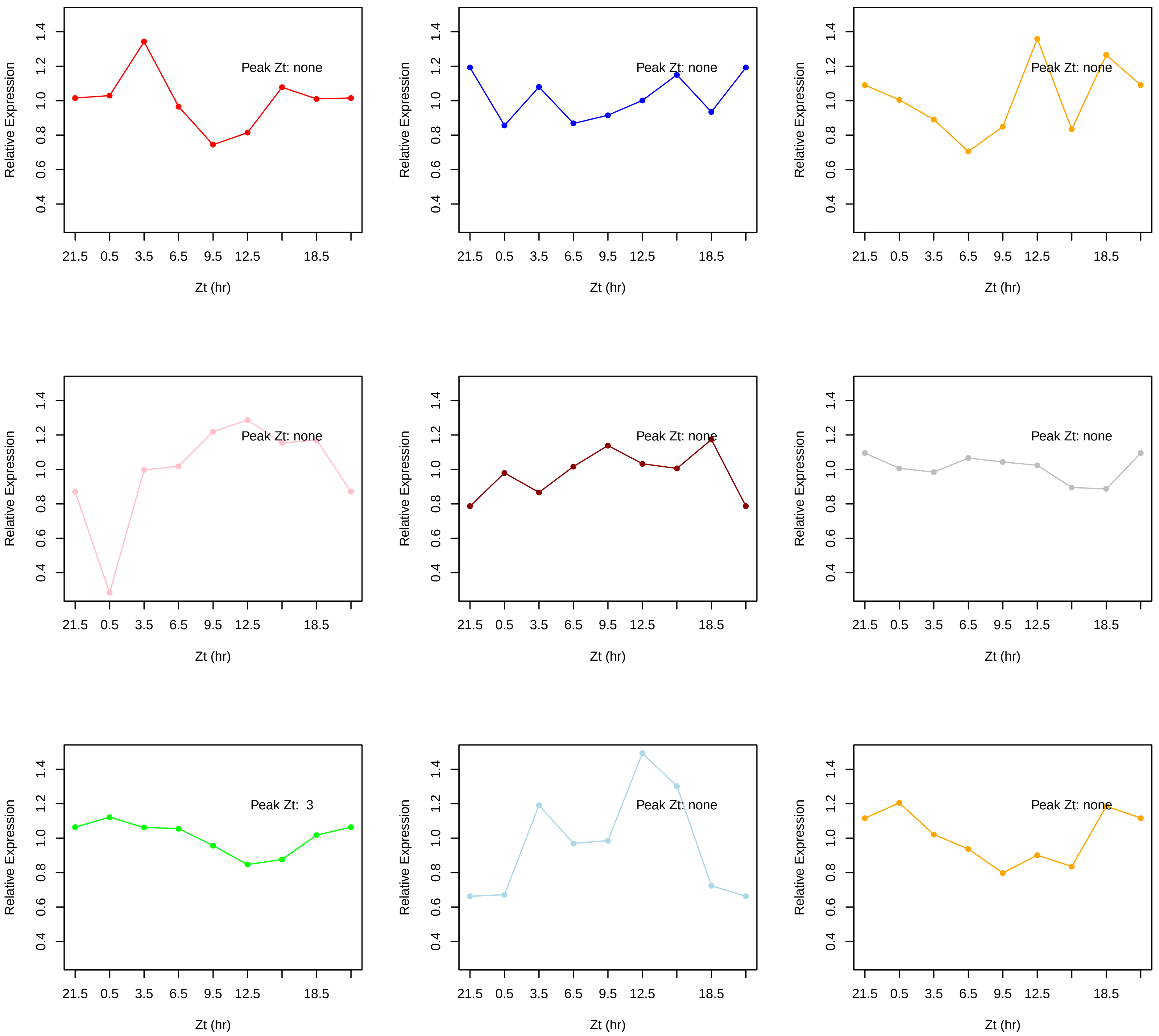

Singeleton: ENSXMAG00000026998 cog8

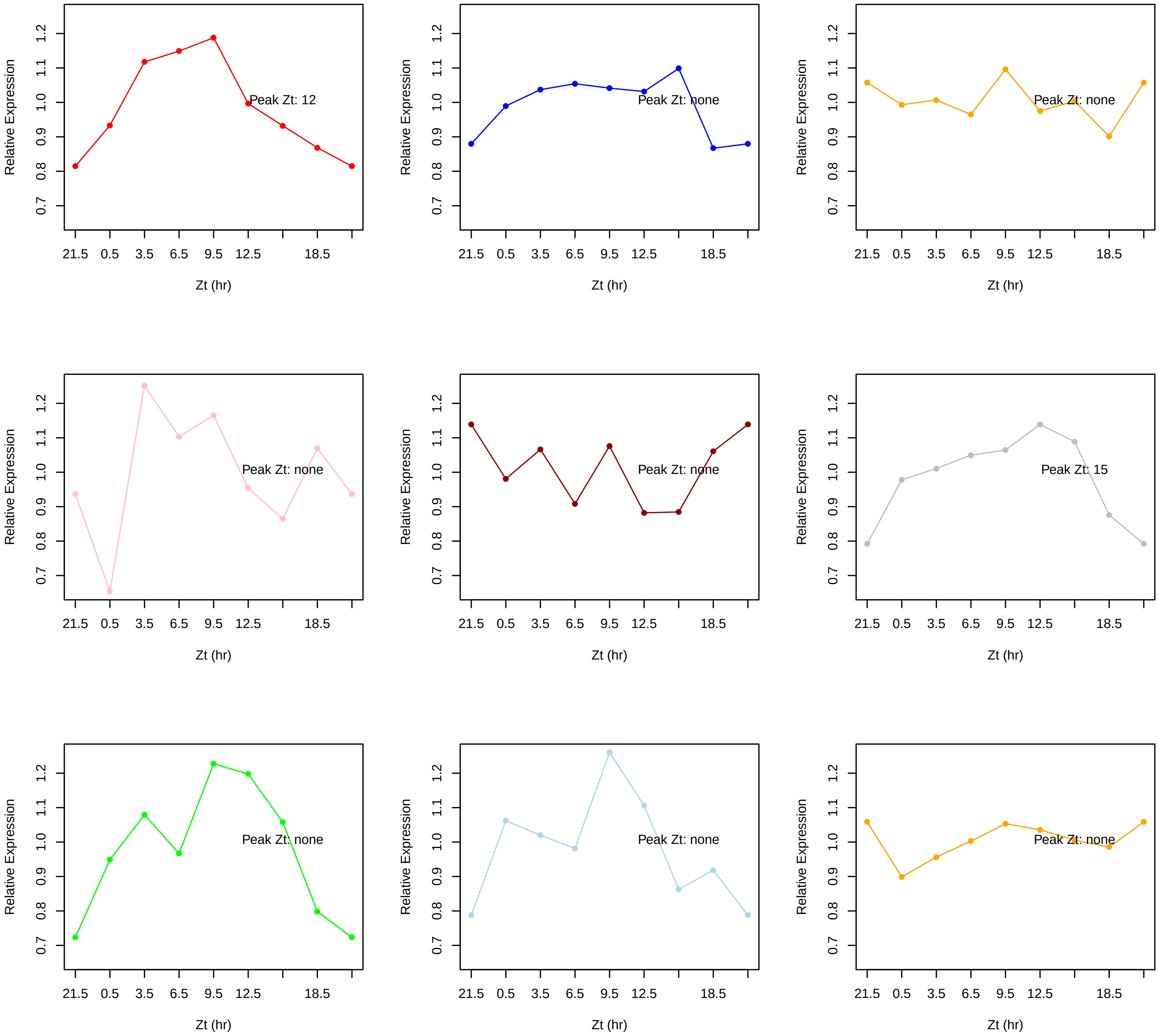

Singeleton: ENSXMAG00000000737 cmtm8b

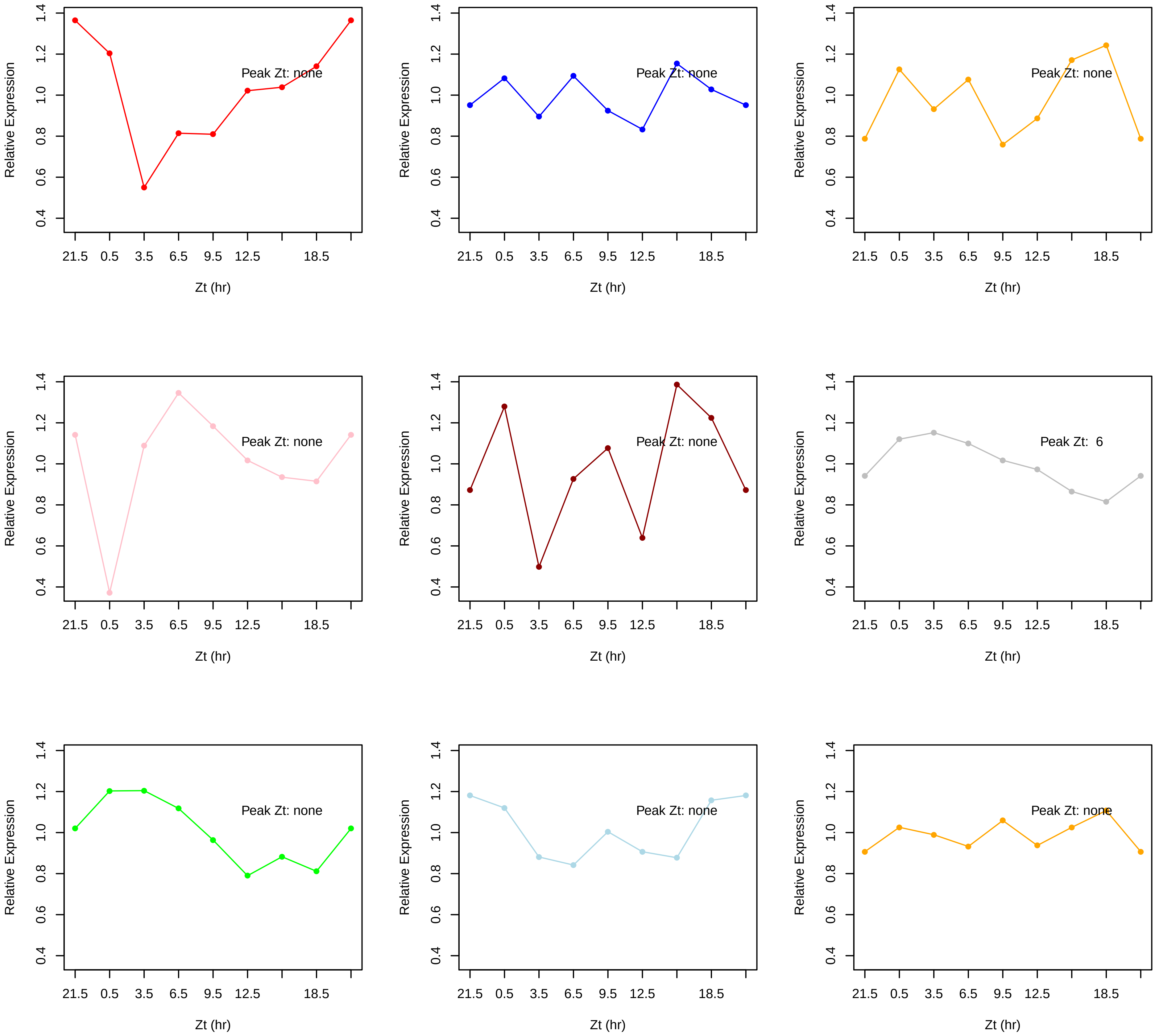

Singeleton: ENSXMAG00000010929 fdxacb1

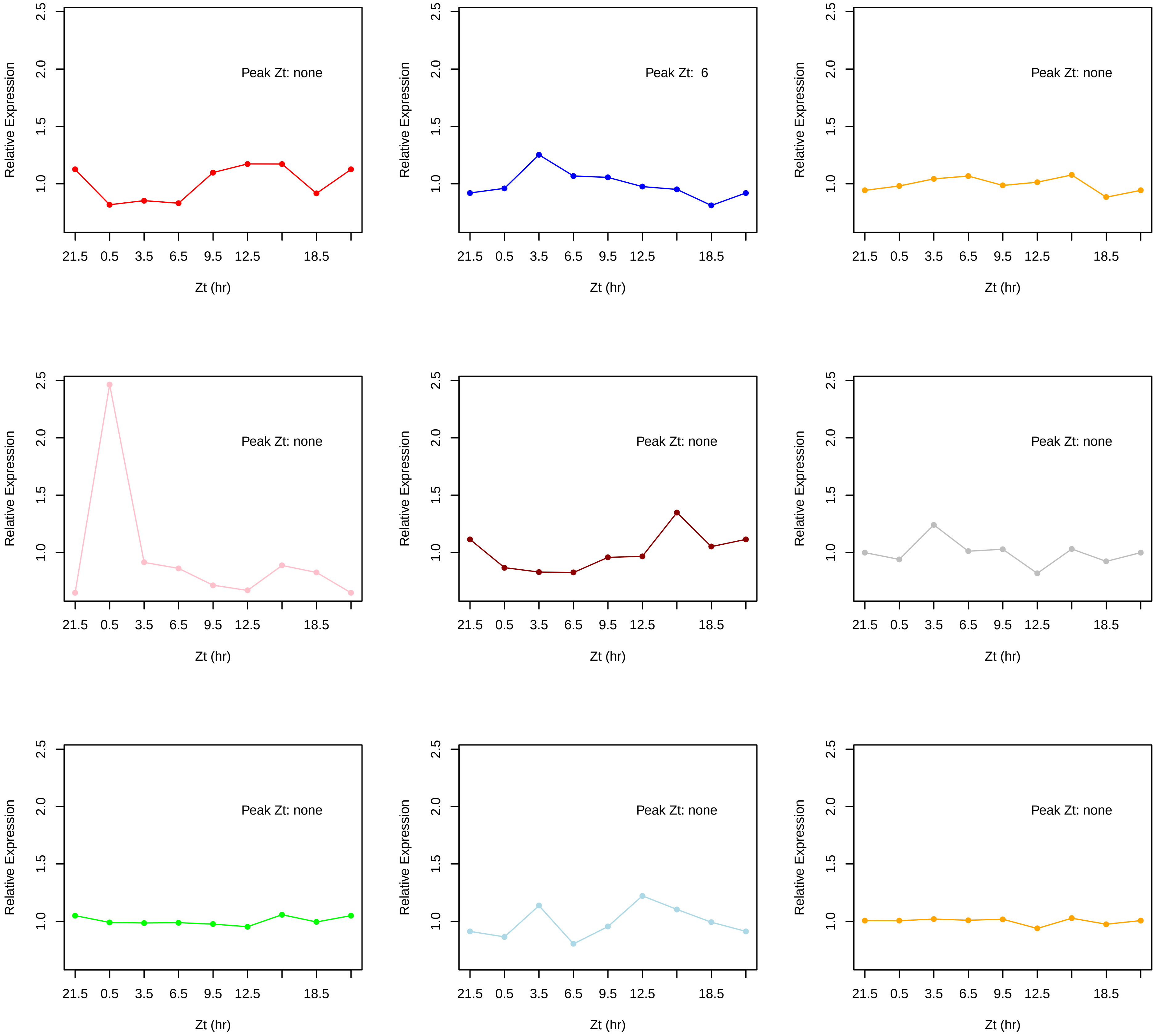

Singeleton: ENSXMAG00000009359 zcrb1

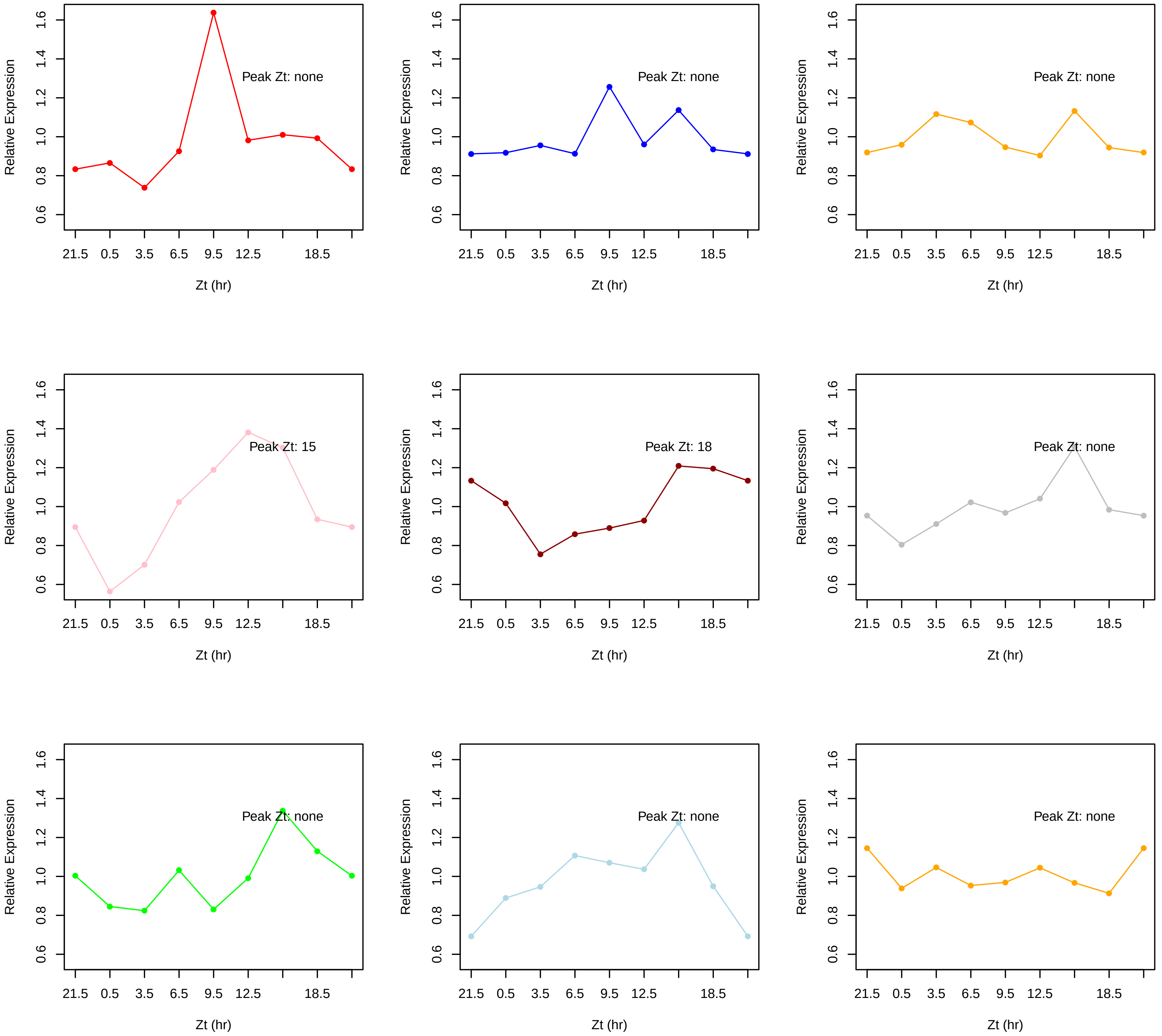

Singeleton: ENSXMAG00000017371 nob1

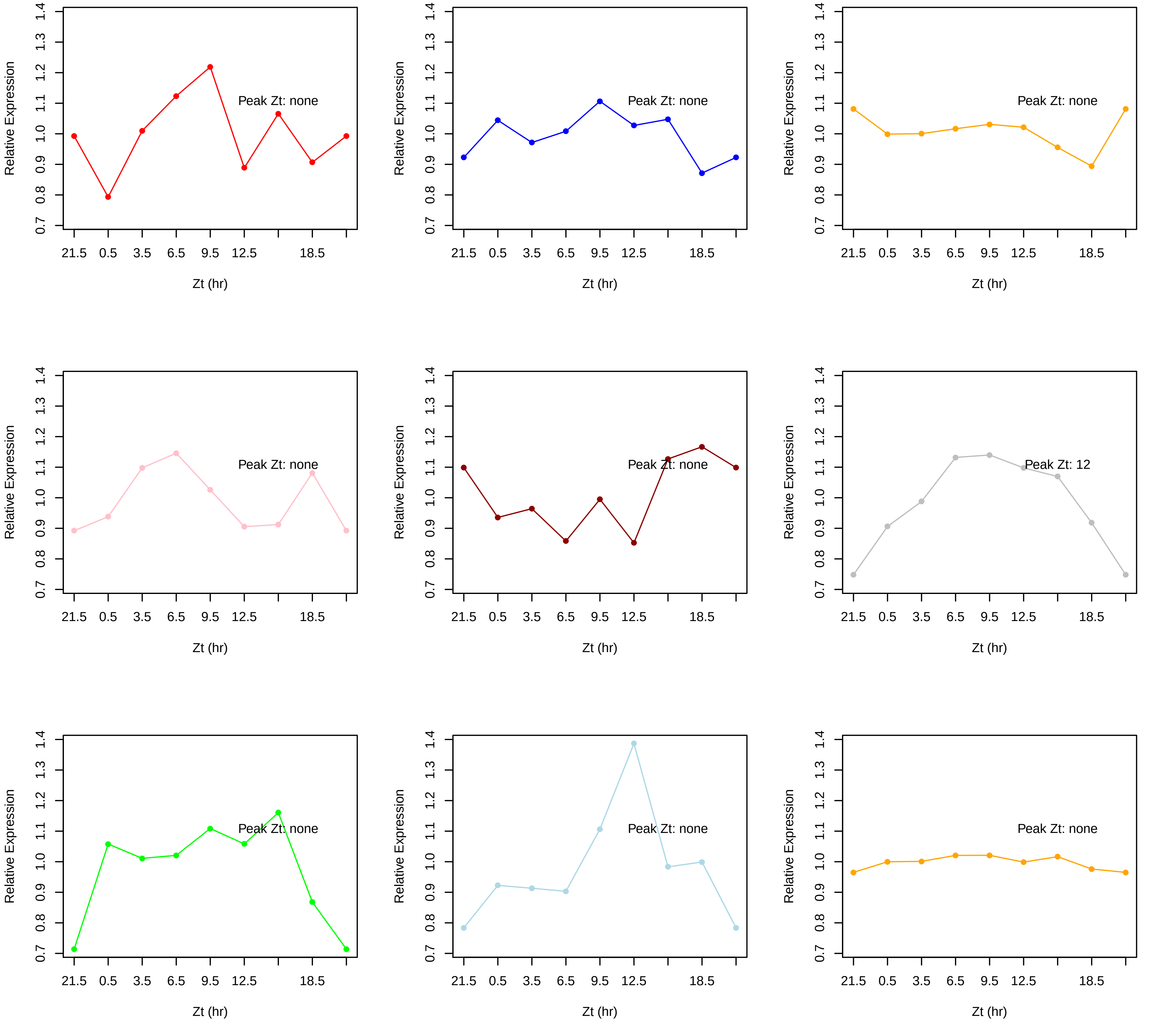

Singeleton: ENSXMAG00000021253

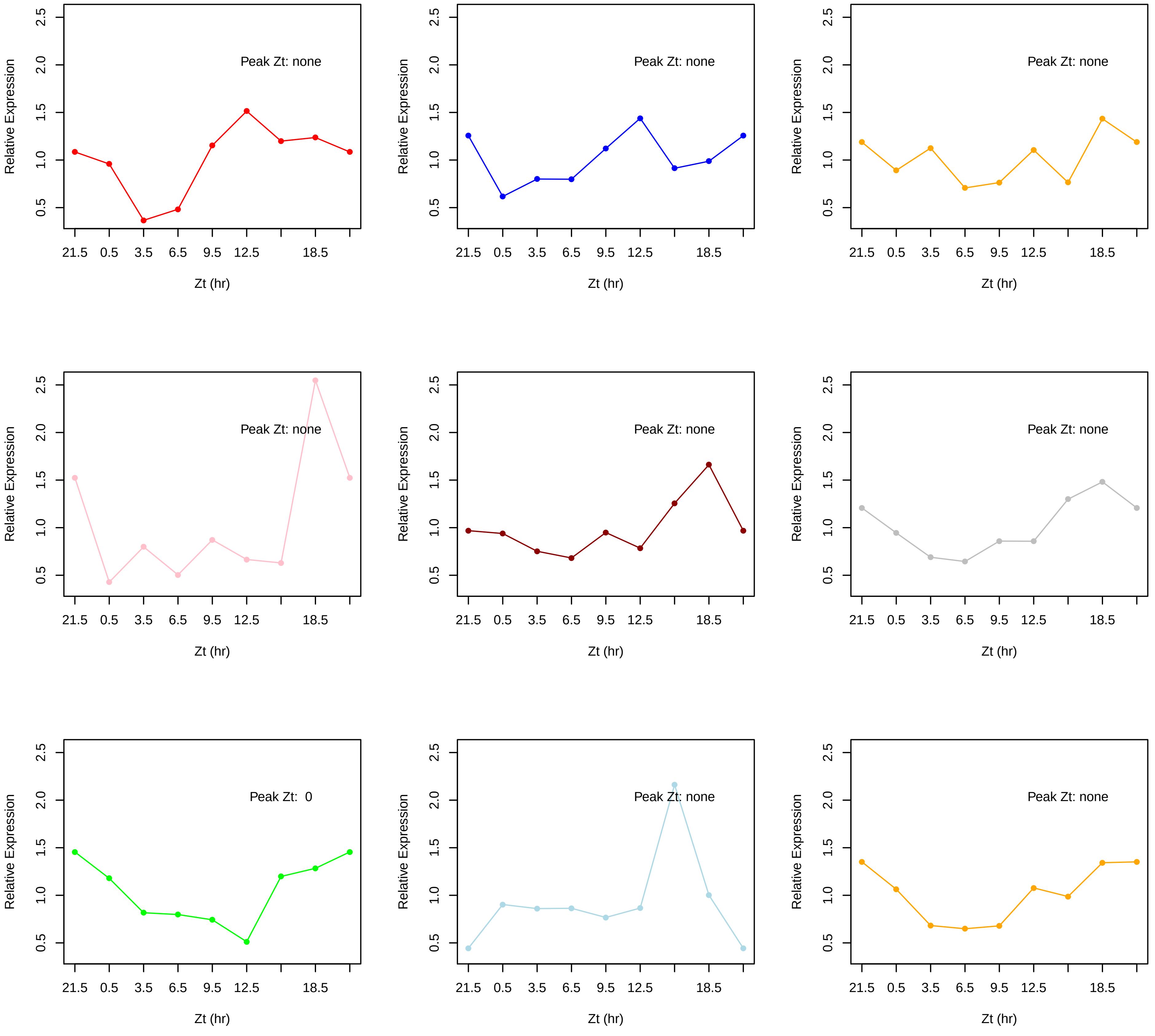

Singleton: ENSXMAG00000024238 kctd7

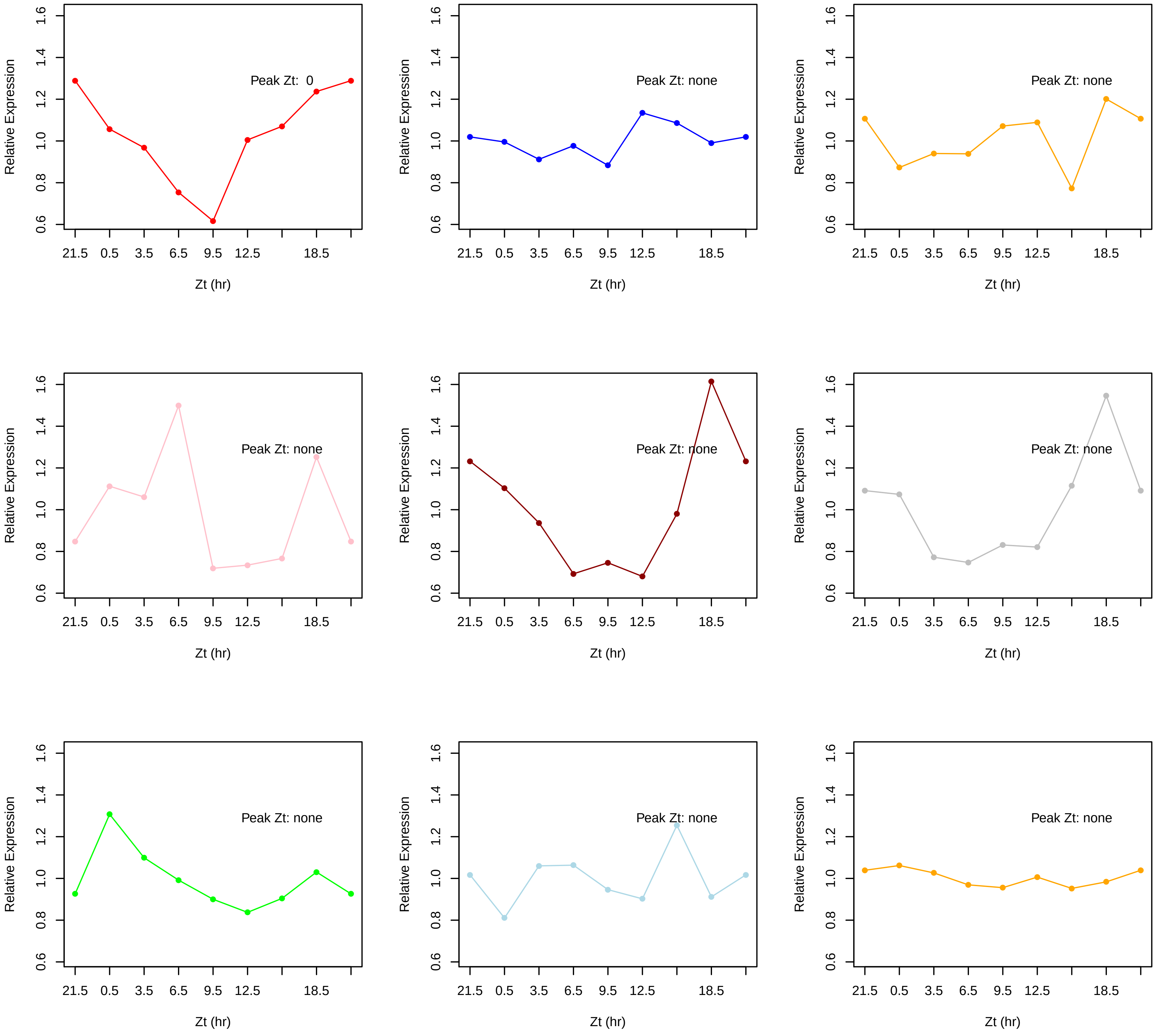

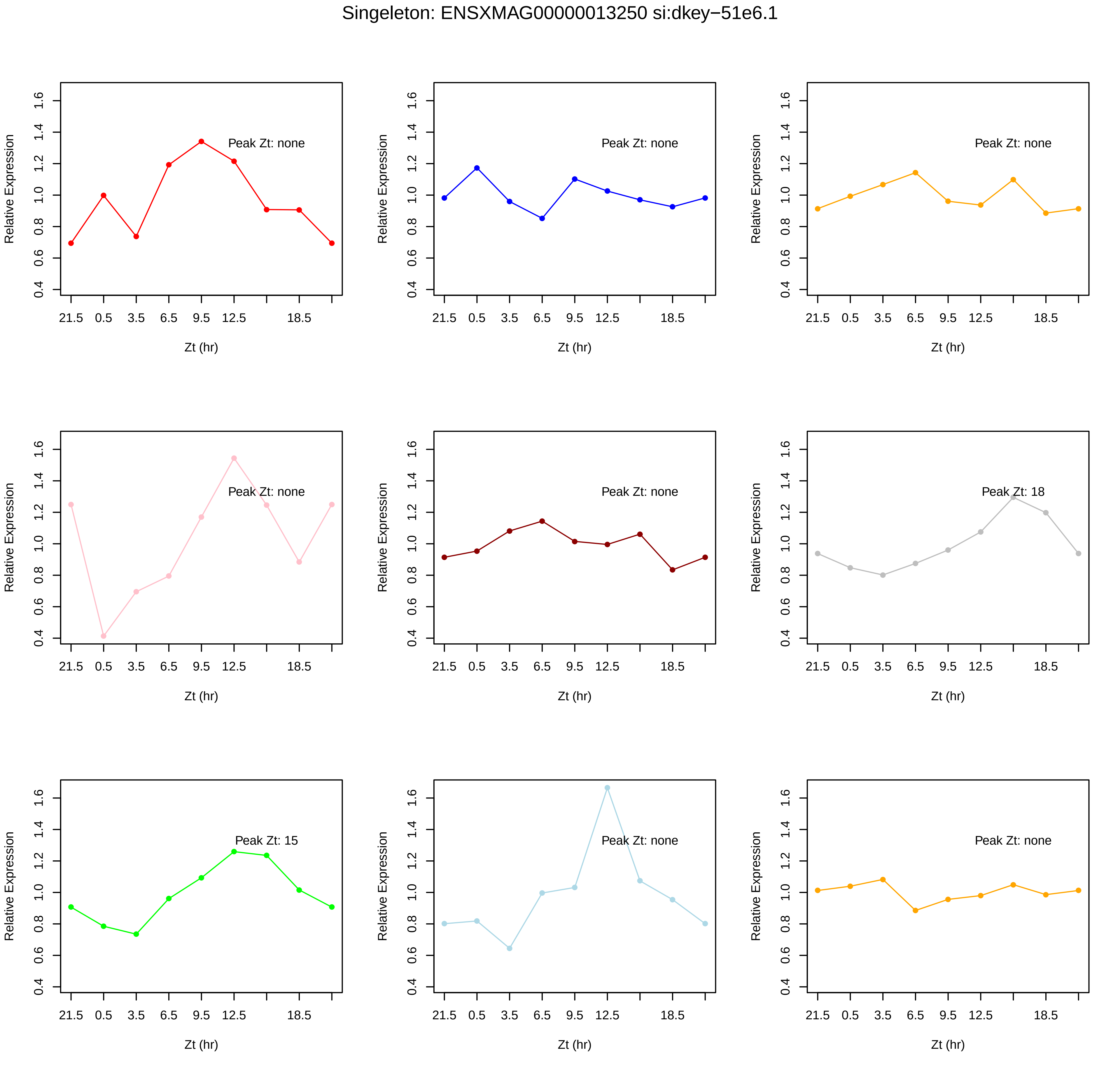
Singeleton: ENSXMAG00000013250 si:dkey−51e6.1
Peak Zt: none
Peak Zt: none
Peak Zt: none
Peak Zt: none
Peak Zt: none
Peak Zt: 18
Peak Zt: 15
Peak Zt: none
Peak Zt: none
Relative Expression
Zt (hr)

Singeleton: ENSXMAG00000012069 ttyh1

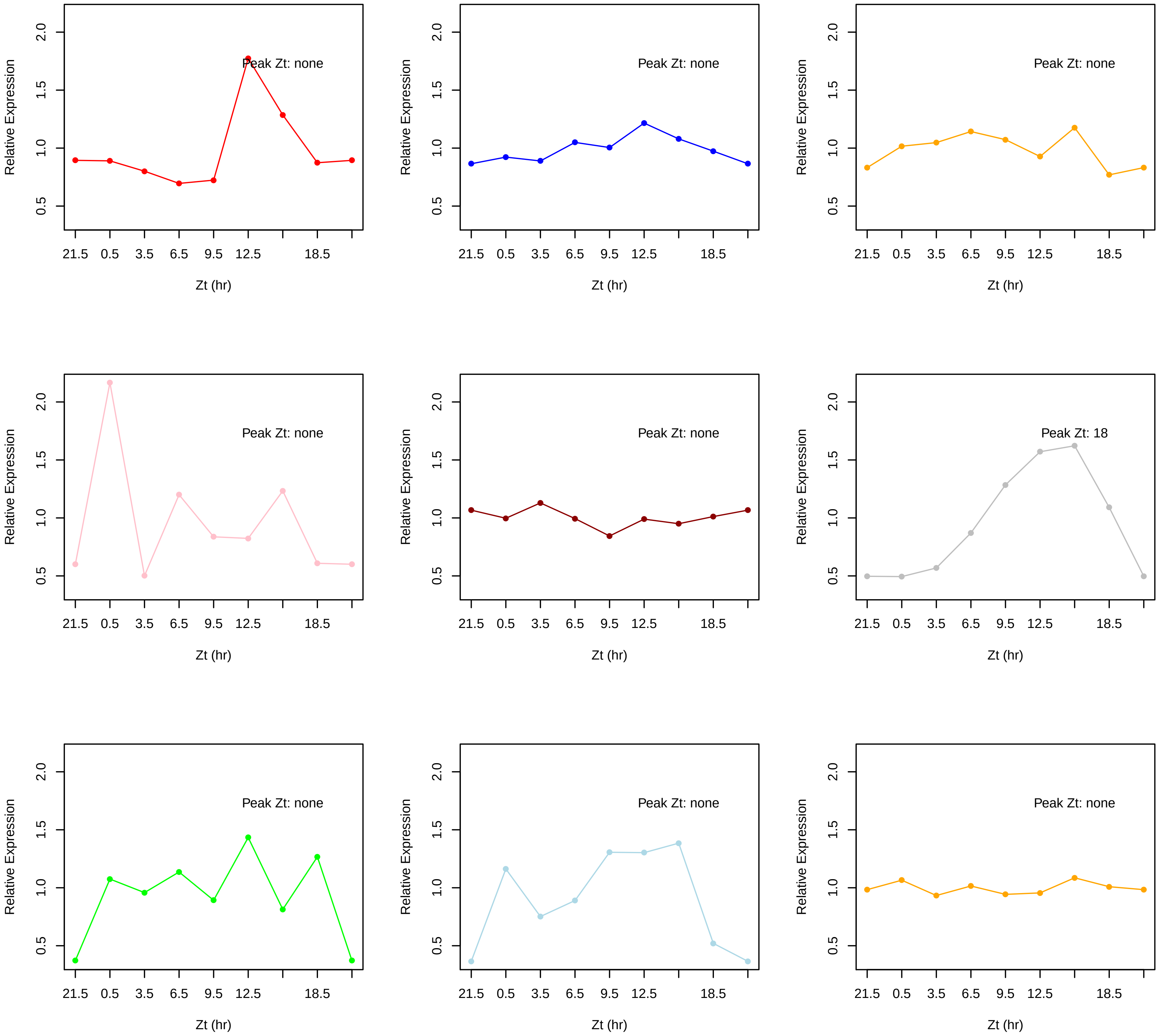

Singeleton: ENSXMAG00000000806

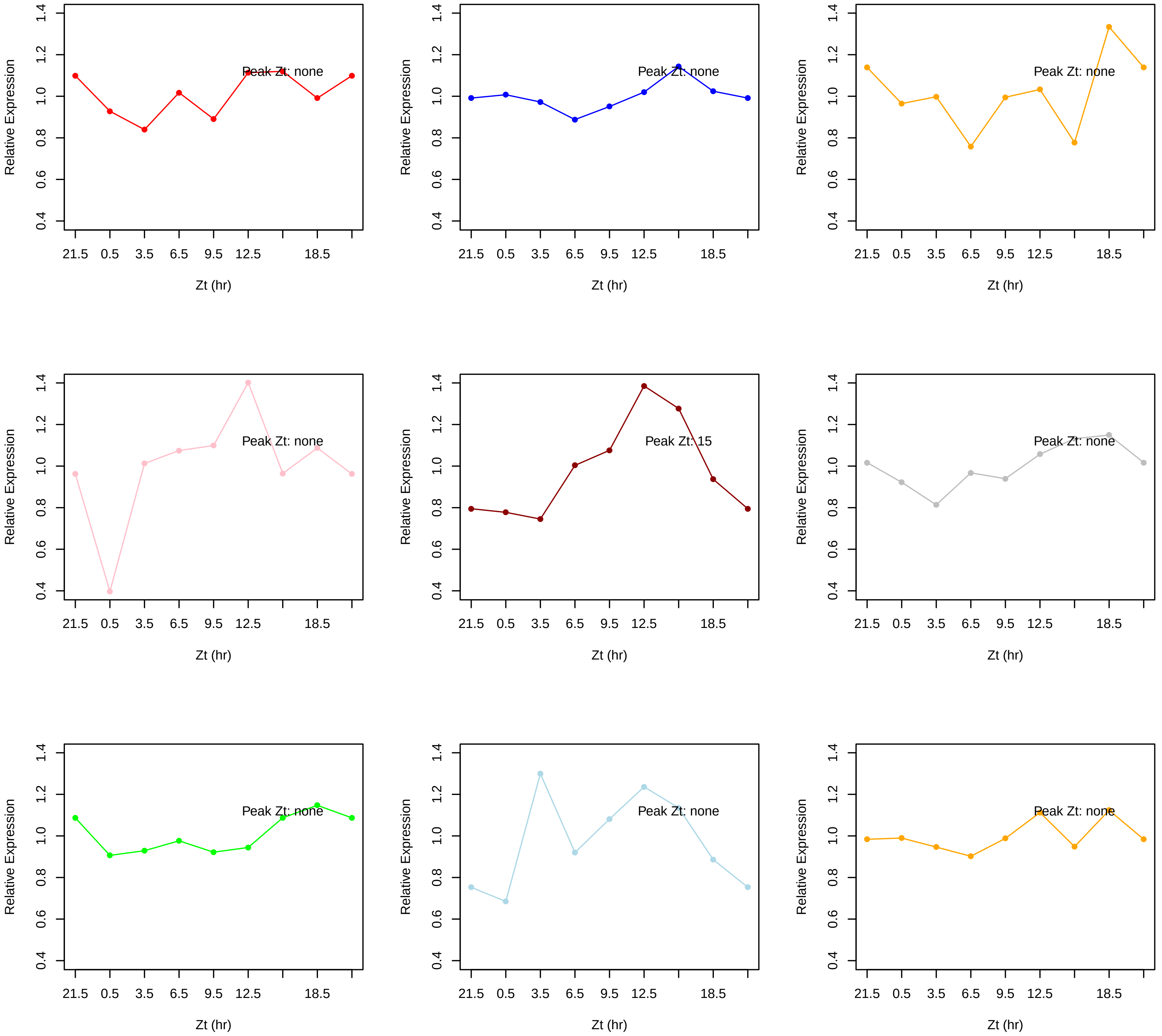

Singleton: ENSXMAG00000009314 mrm2

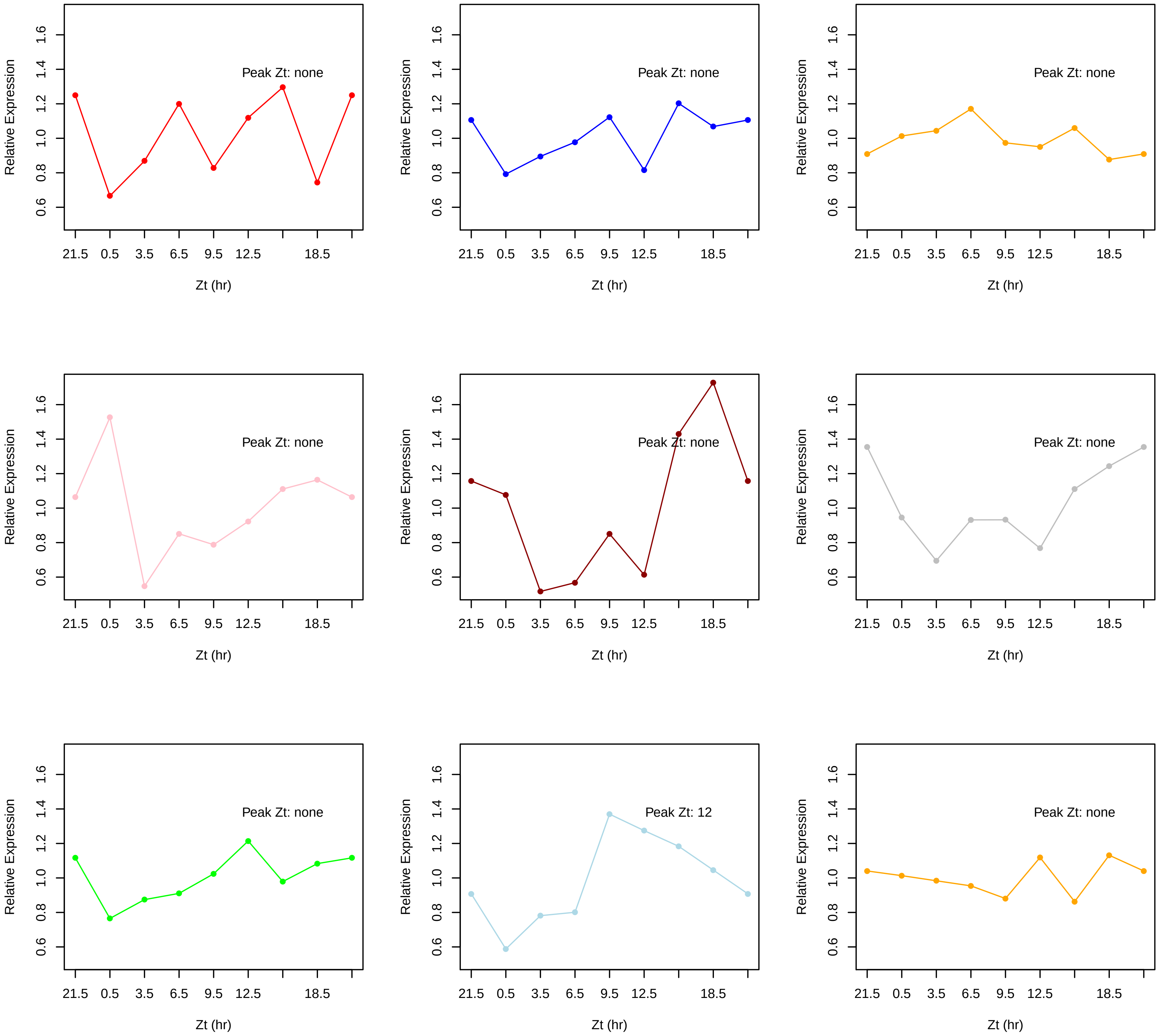

Singeleton: ENSXMAG00000024725

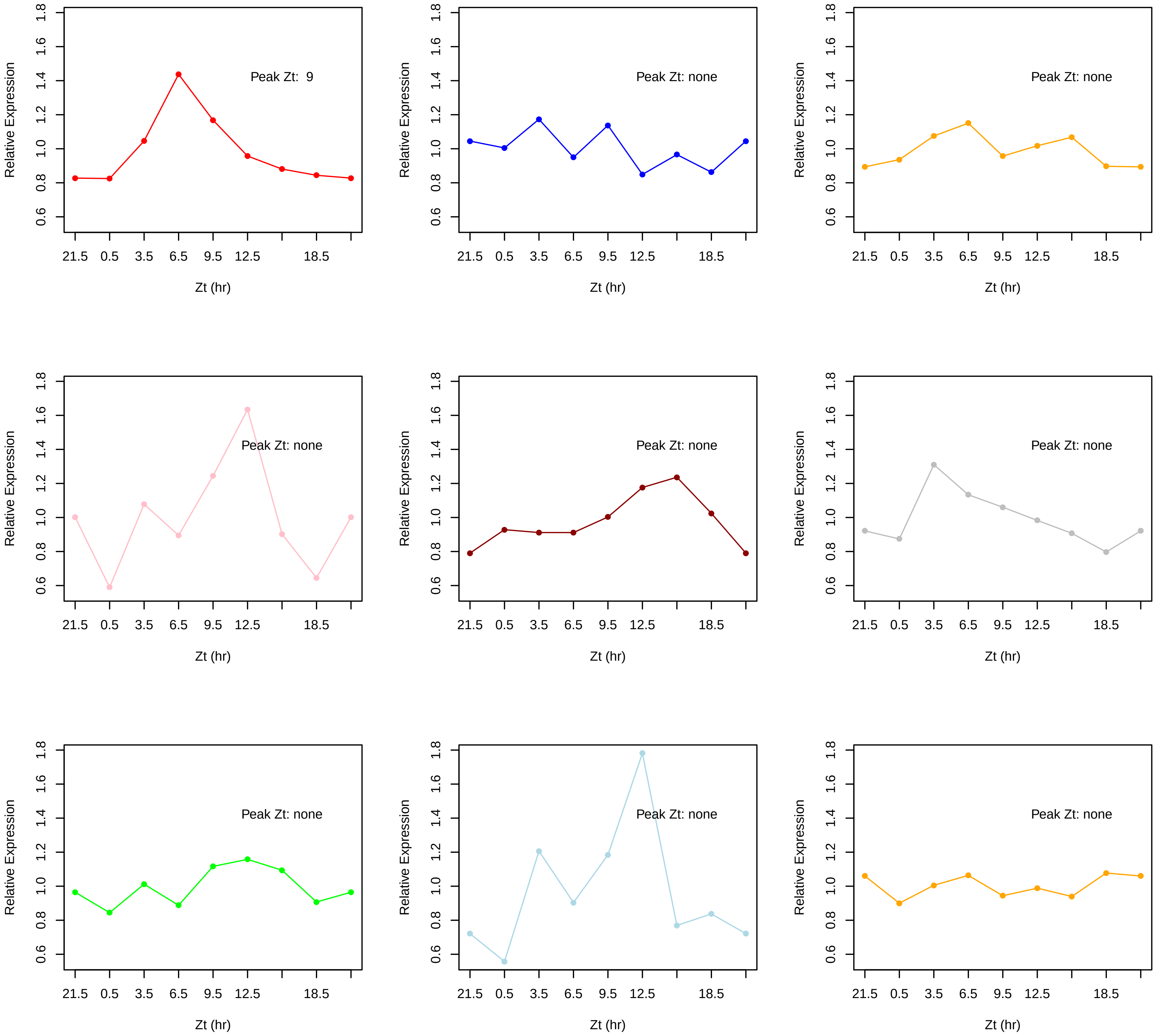

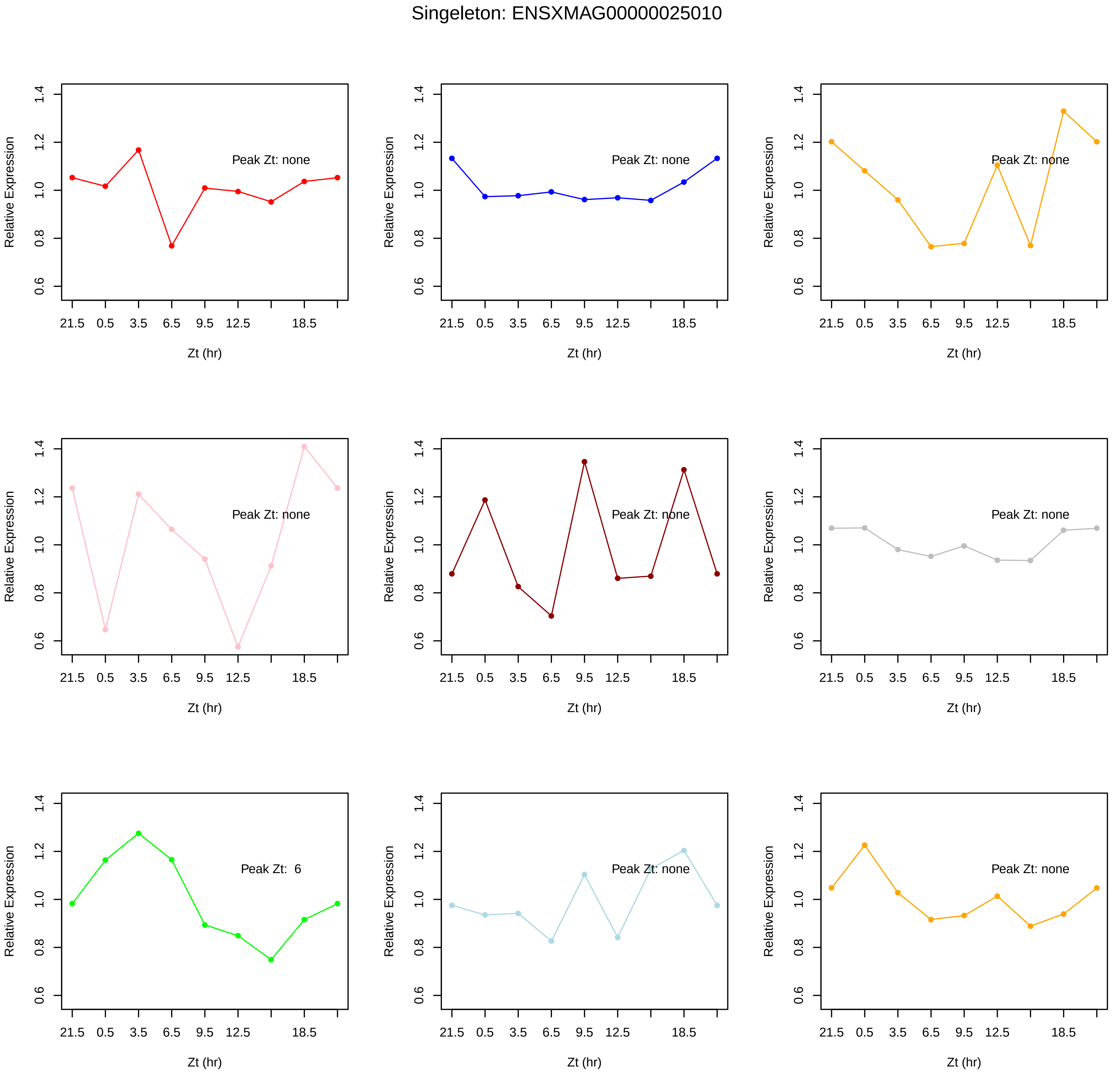
Singeleton: ENSXMAG00000025010
Peak Zt: none
Peak Zt: none
Peak Zt: none
Peak Zt: none
Peak Zt: none
Peak Zt: none
Peak Zt: 6
Peak Zt: none
Peak Zt: none
Relative Expression
Zt (hr)
21.5 0.5 3.5 6.5 9.5 12.5 18.5
0.6 0.8 1.0 1.2 1.4

Singleton: ENSXMAG00000000769 LDLRAD3

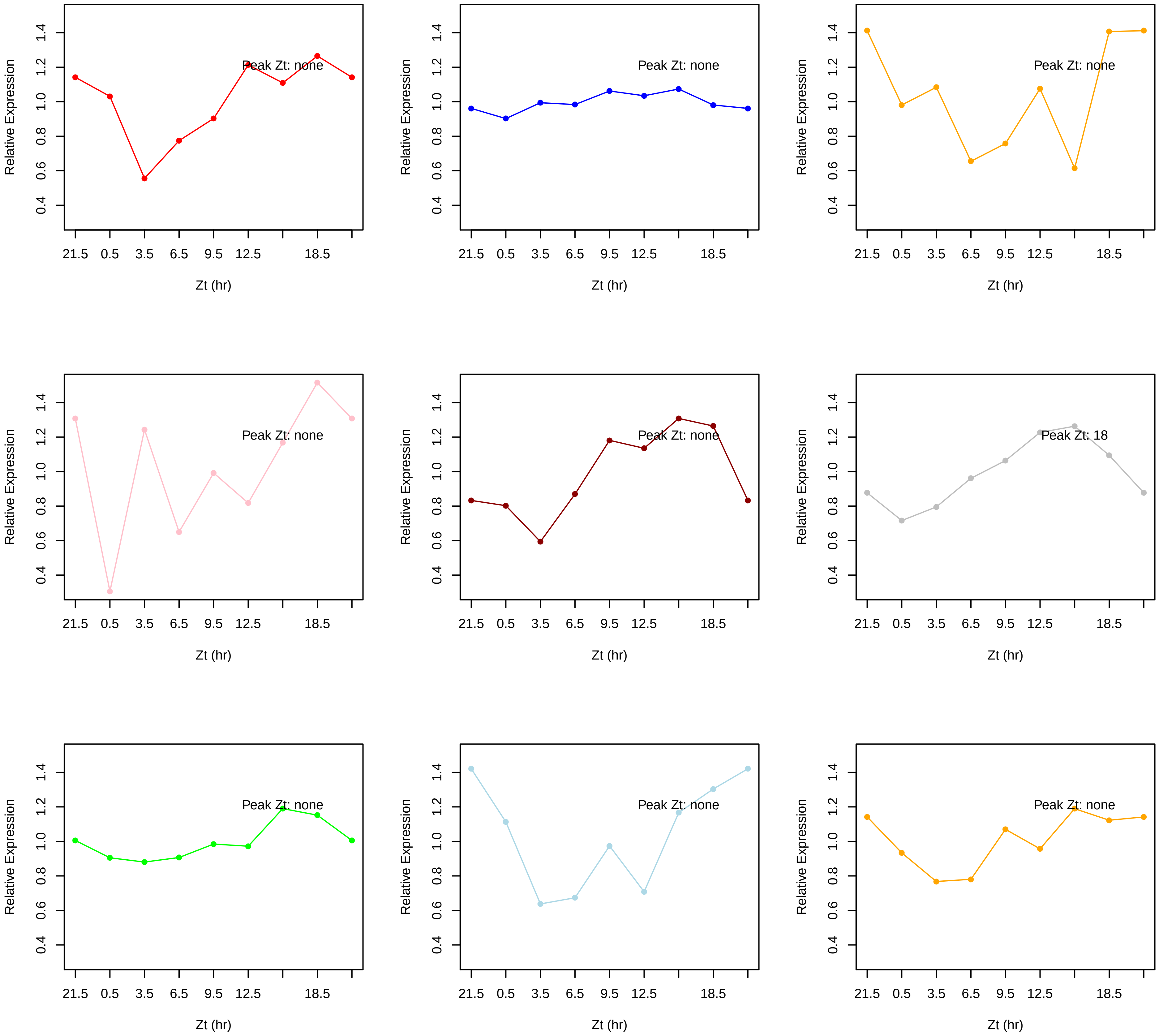

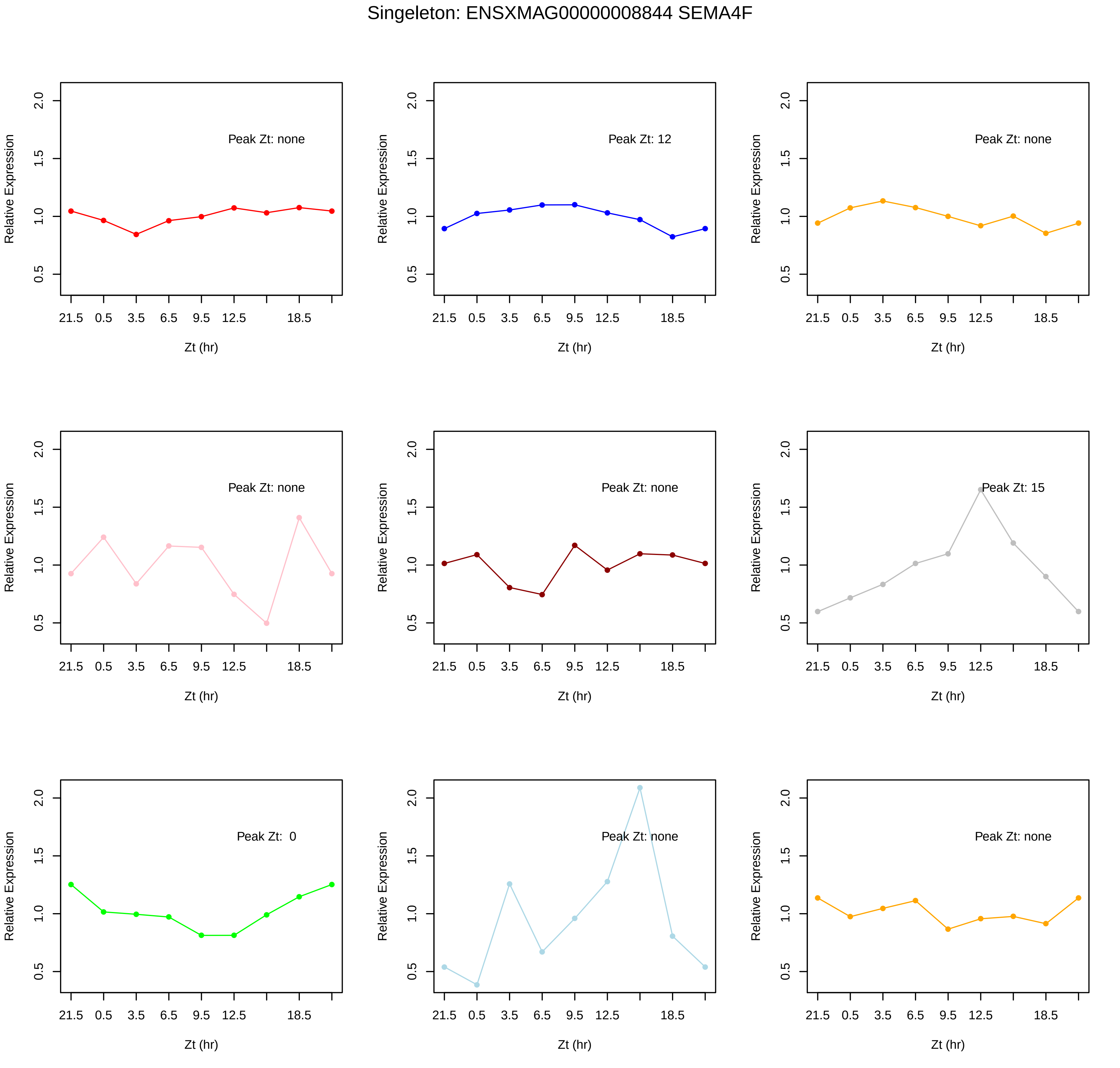

Singeleton: ENSXMAG00000008844 SEMA4F
Peak Zt: none
Peak Zt: 12
Peak Zt: none
Peak Zt: none
Peak Zt: none
Peak Zt: 15
Peak Zt: 0
Peak Zt: none
Peak Zt: none
Relative Expression
Zt (hr)
21.5
0.5
3.5
6.5
9.5
12.5
18.5

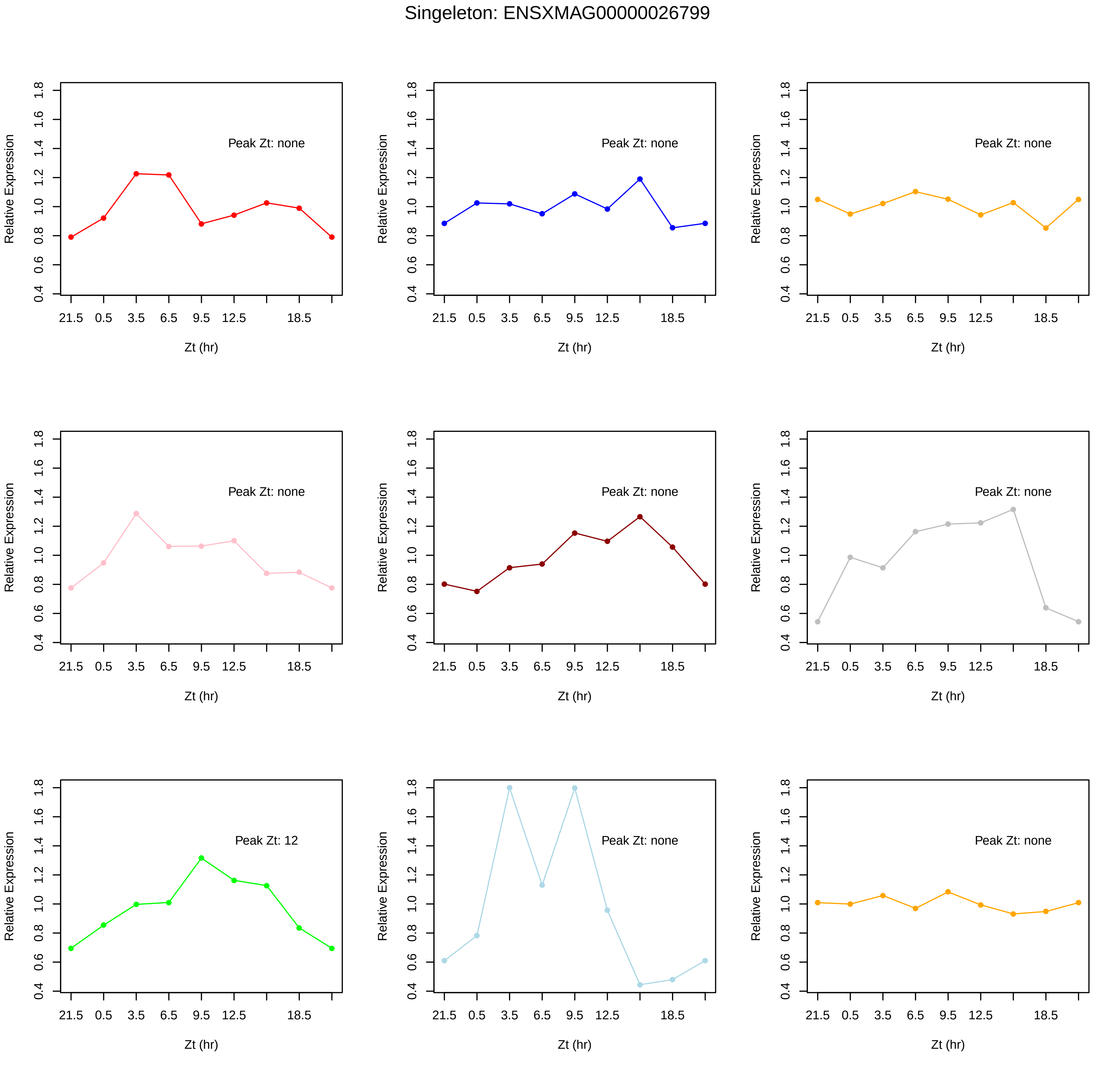
Singeleton: ENSXMAG00000026799
Peak Zt: none
Peak Zt: none
Peak Zt: none
Peak Zt: none
Peak Zt: none
Peak Zt: none
Peak Zt: 12
Peak Zt: none
Peak Zt: none
Relative Expression
Zt (hr)
21.5
0.5
3.5
6.5
9.5
12.5
18.5
0.4
0.6
0.8
1.0
1.2
1.4
1.6
1.8

Singeleton: ENSXMAG00000007919

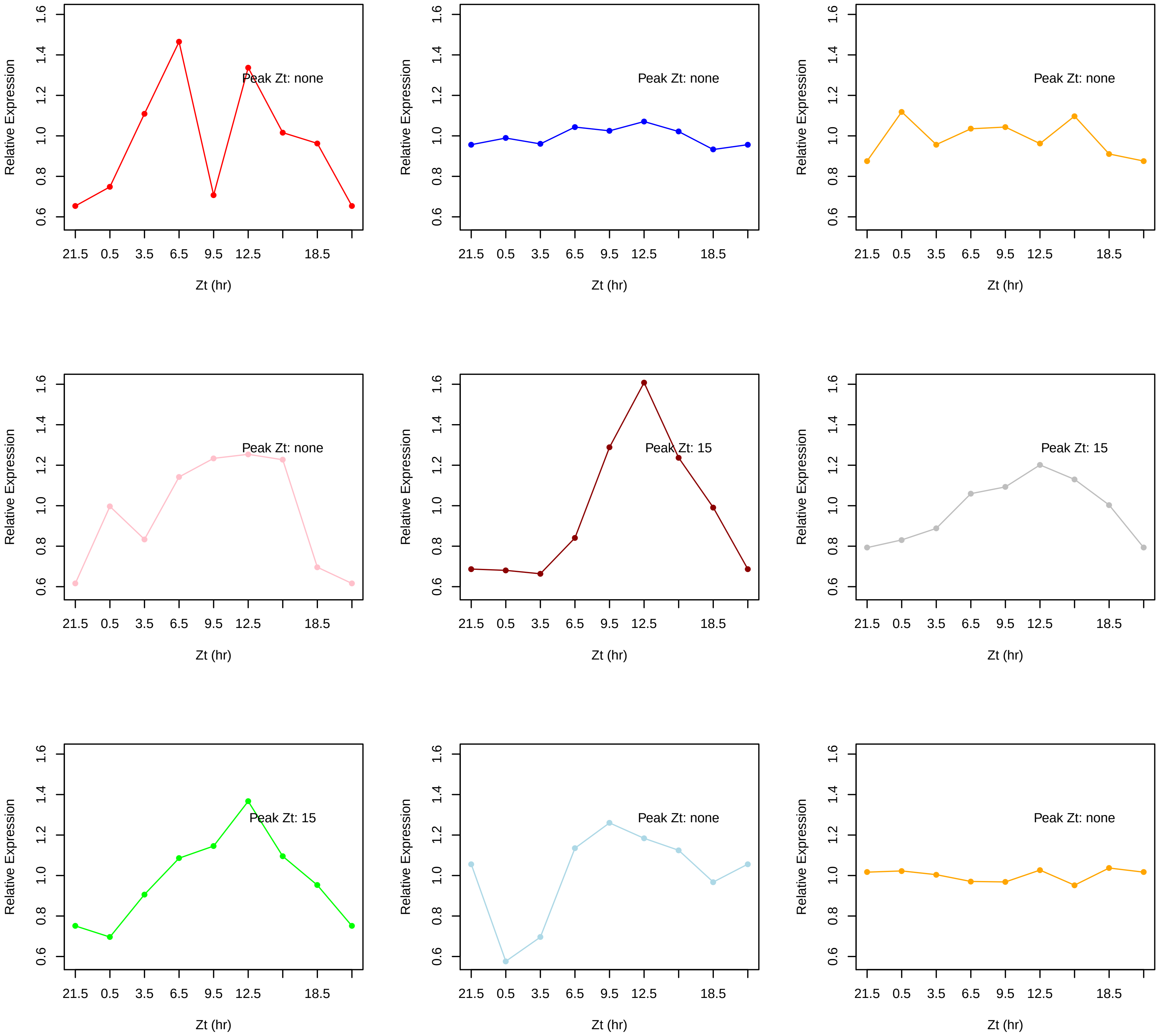

Singeleton: ENSXMAG00000011080 slc22a6l

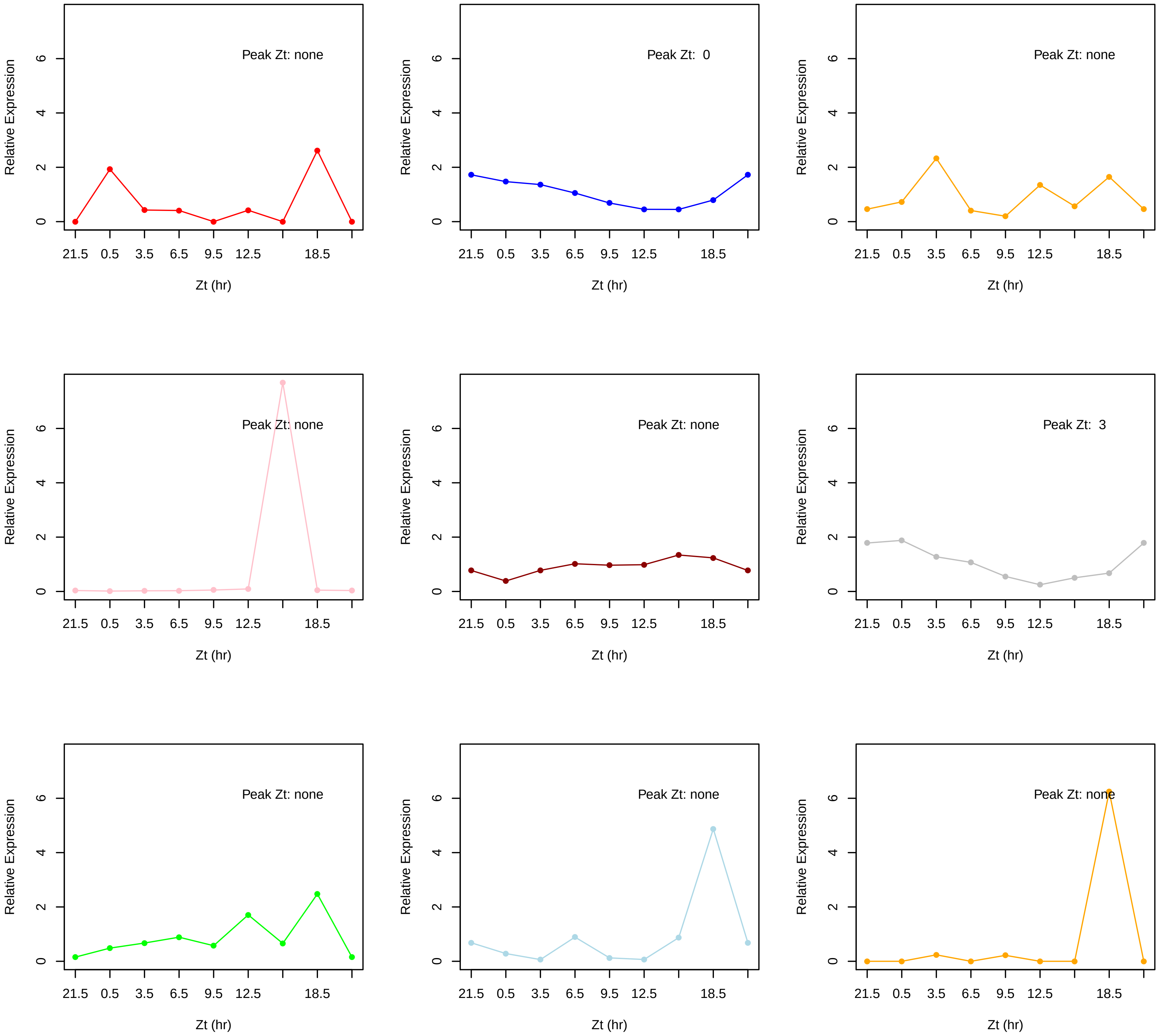

Singeleton: ENSXMAG00000013264 scyl3

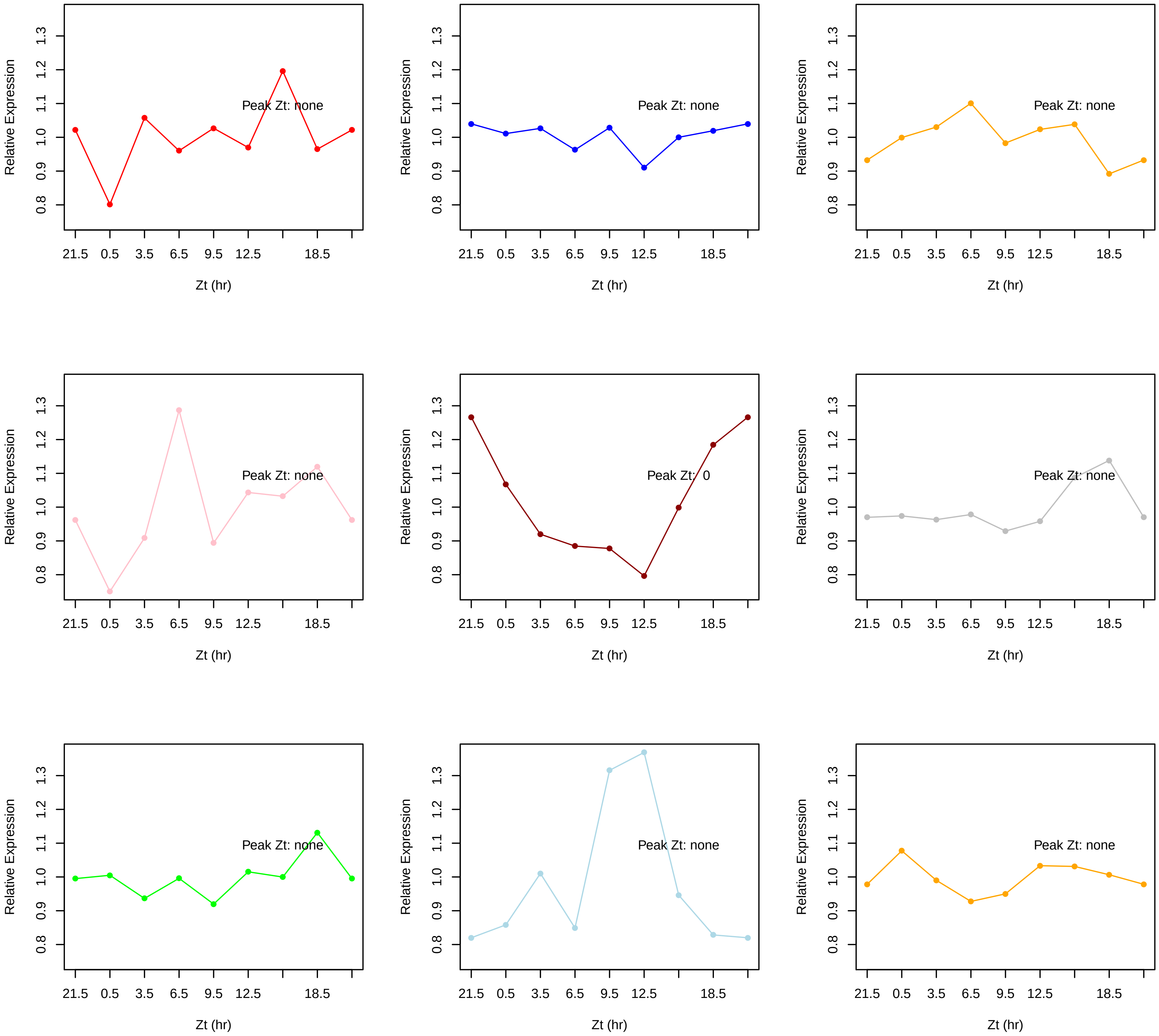

Singleton: ENSXMAG00000018500 bud23

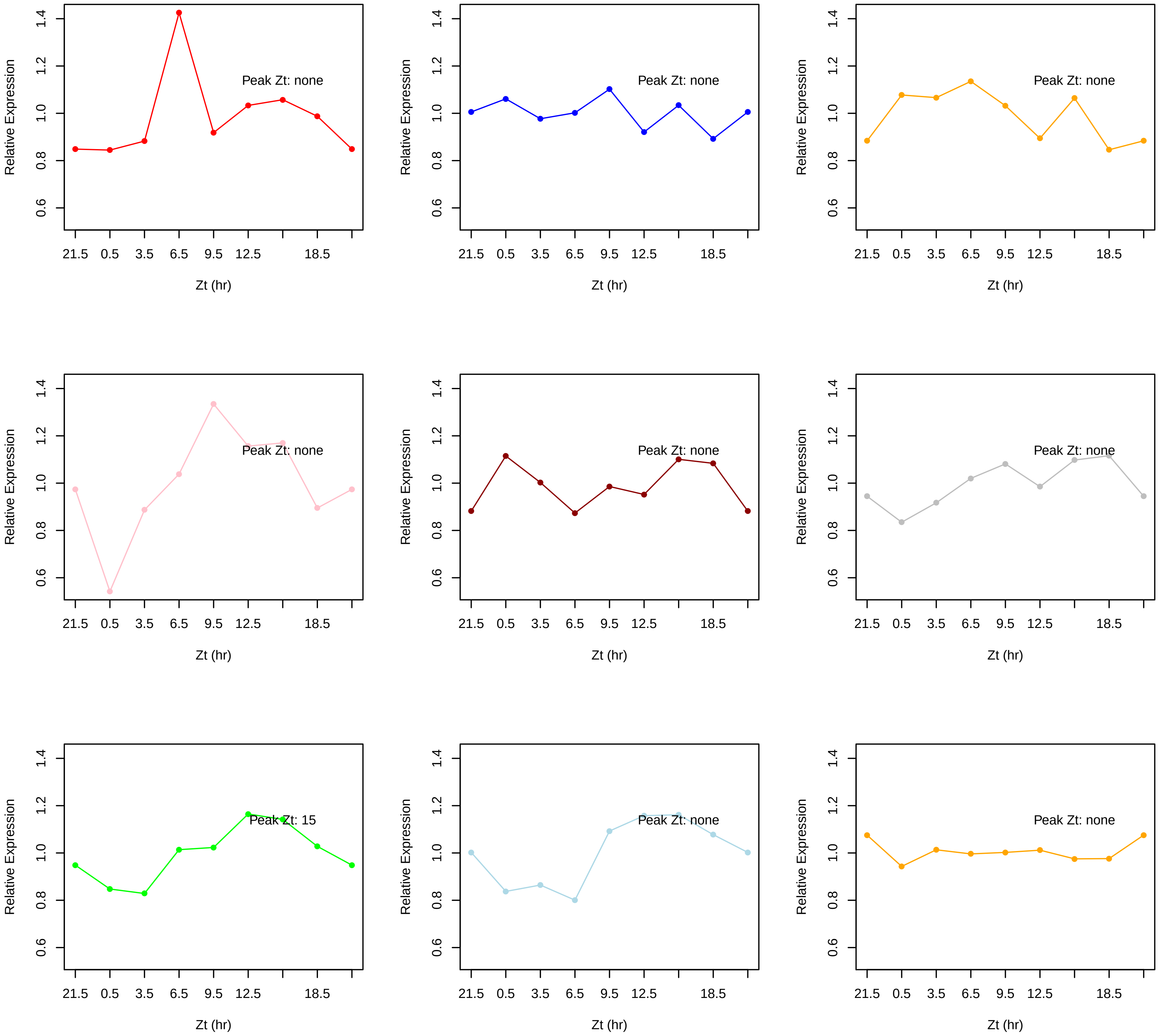

Singeleton: ENSXMAG00000004228 dpp7

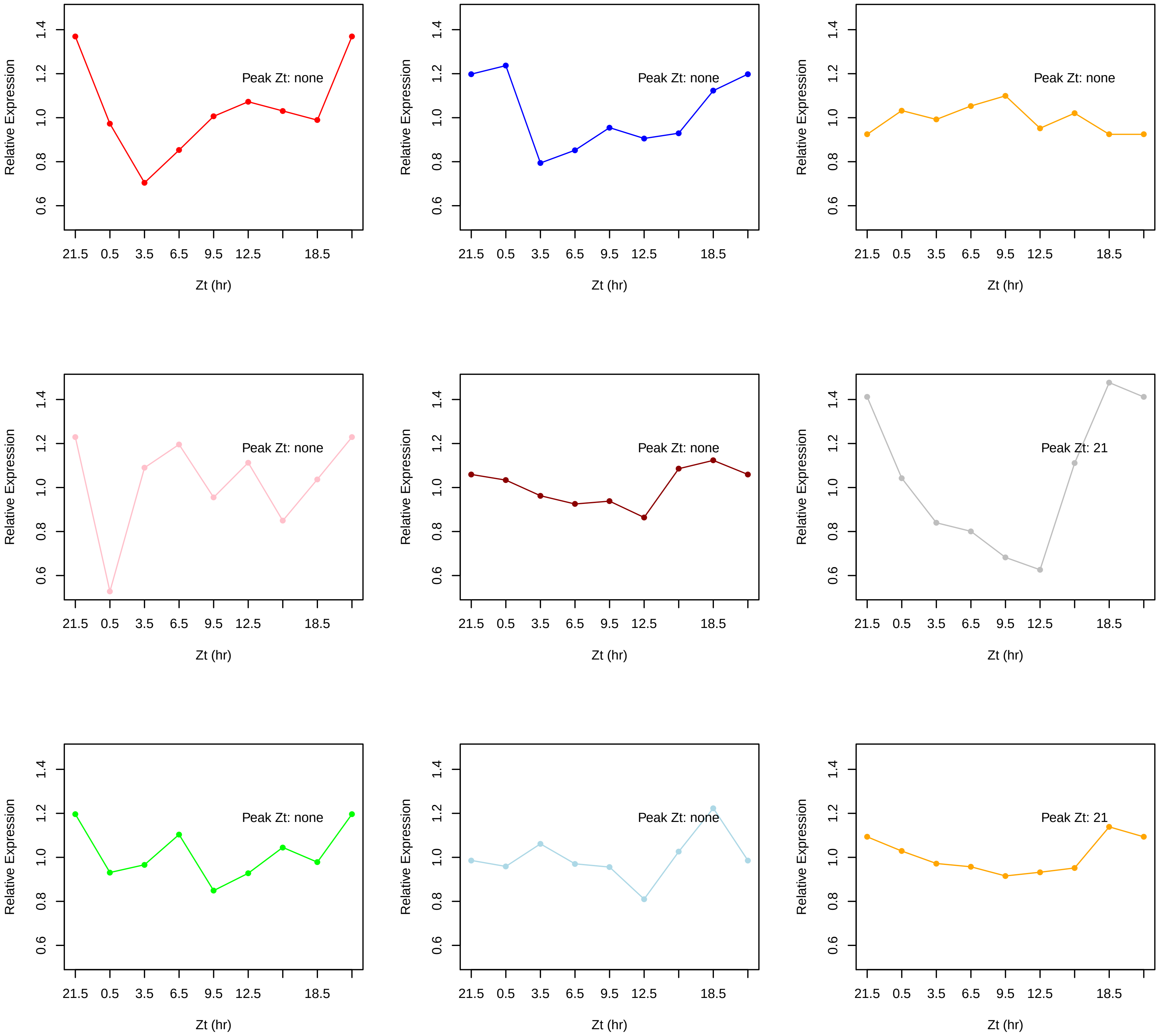

Singeleton: ENSXMAG00000011272 mast3a

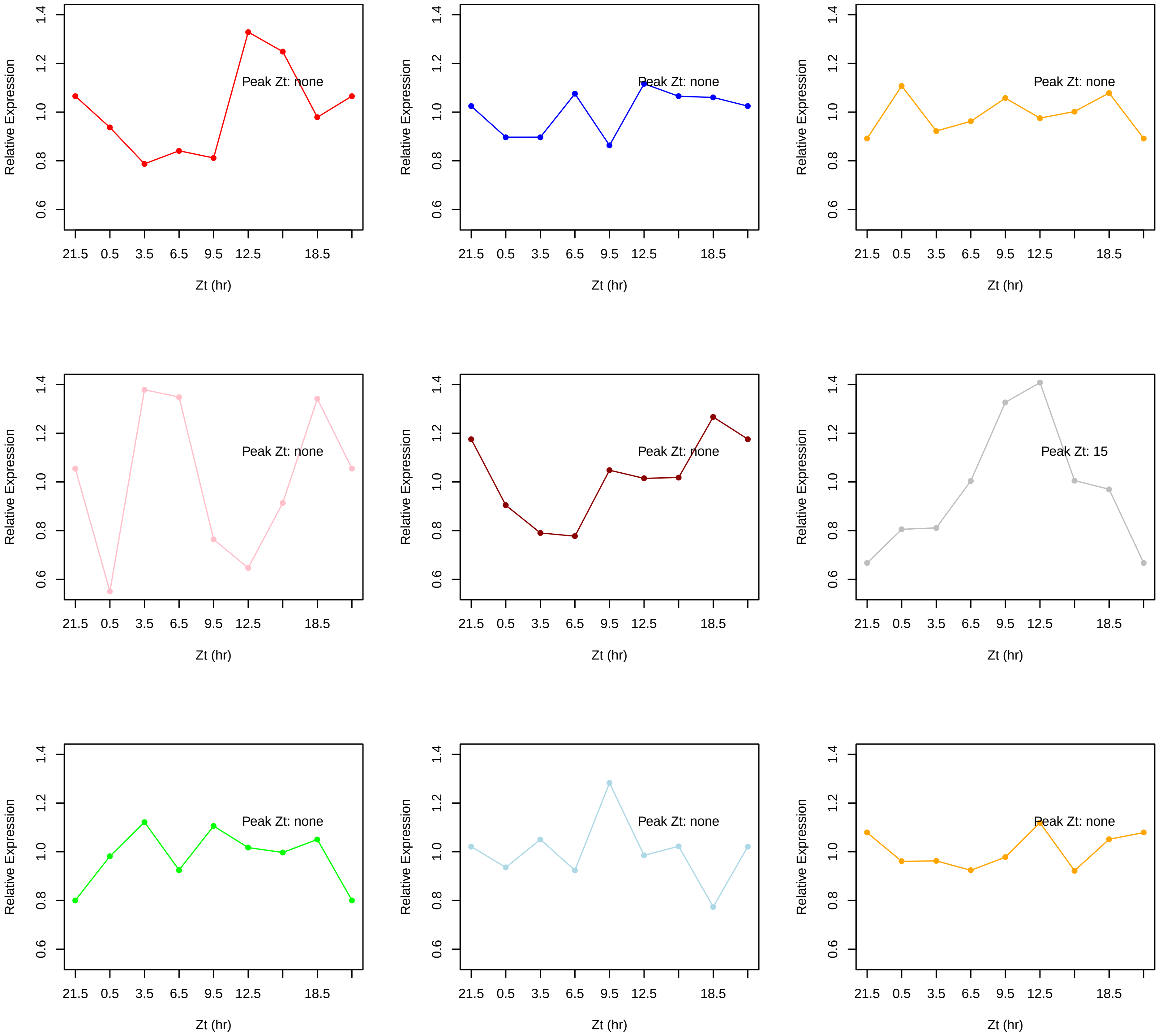

Singeleton: ENSXMAG00000020294 MRPL21

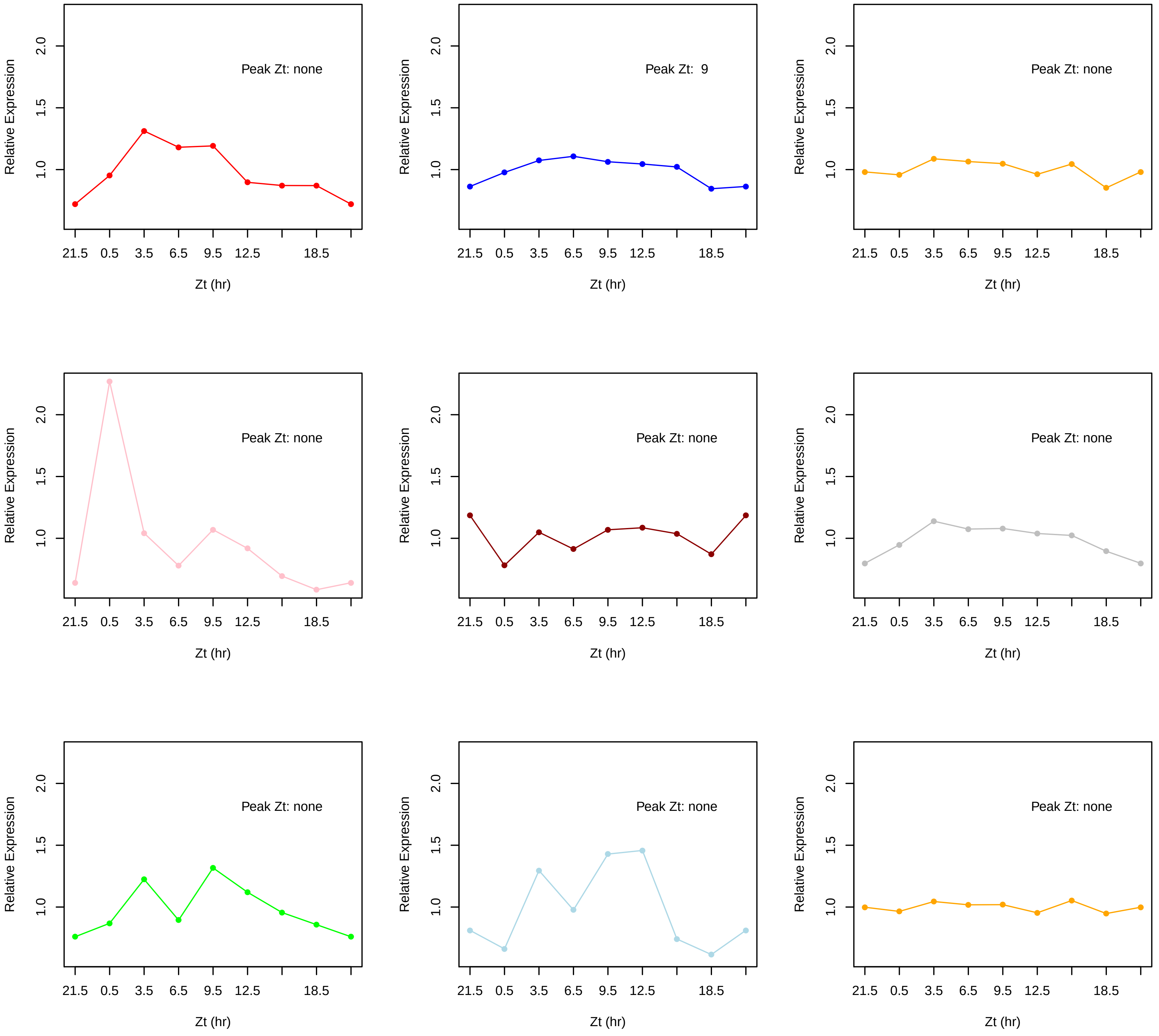

Singeleton: ENSXMAG00000002672

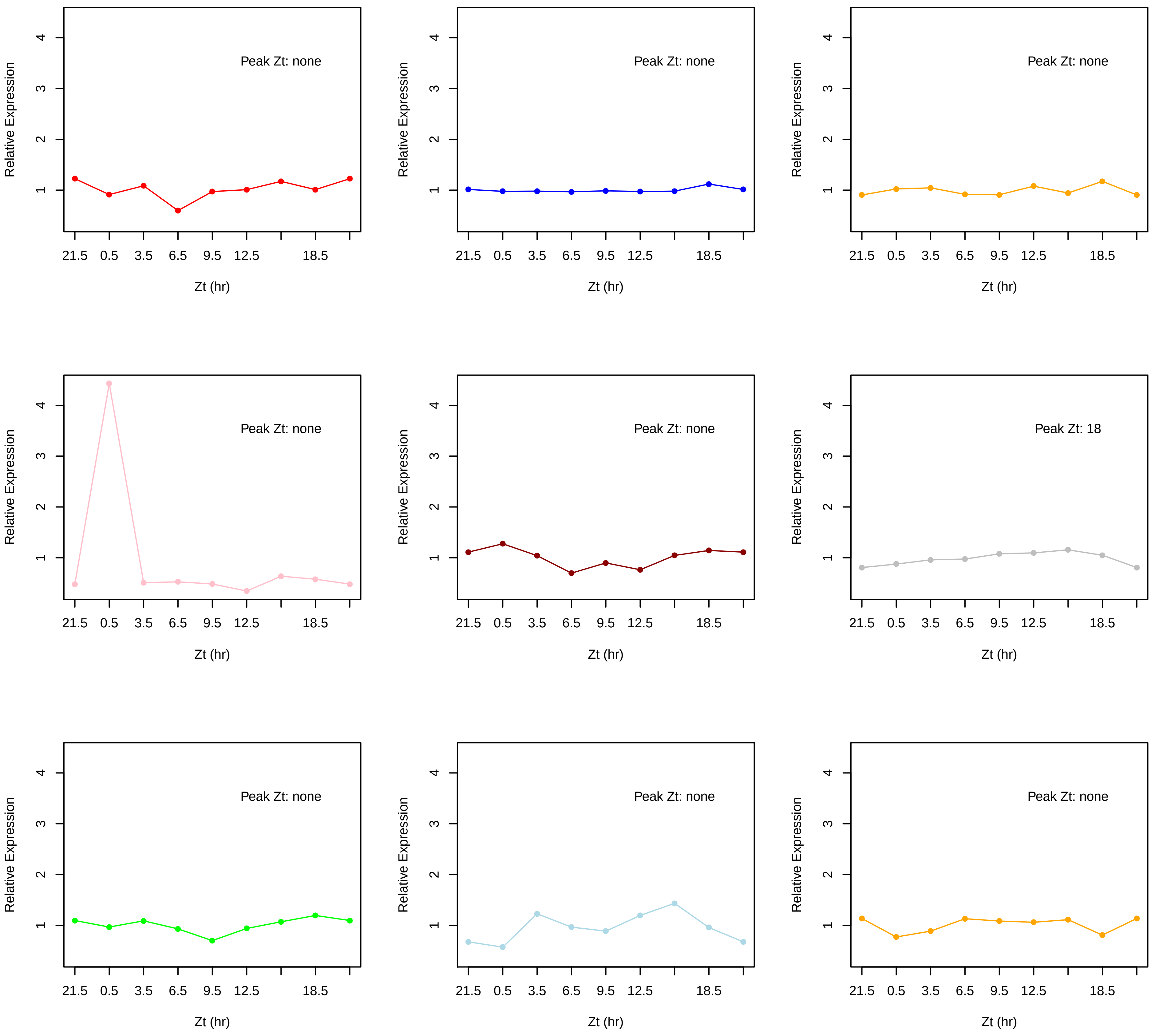

Singeleton: ENSXMAG00000019400 ttc37

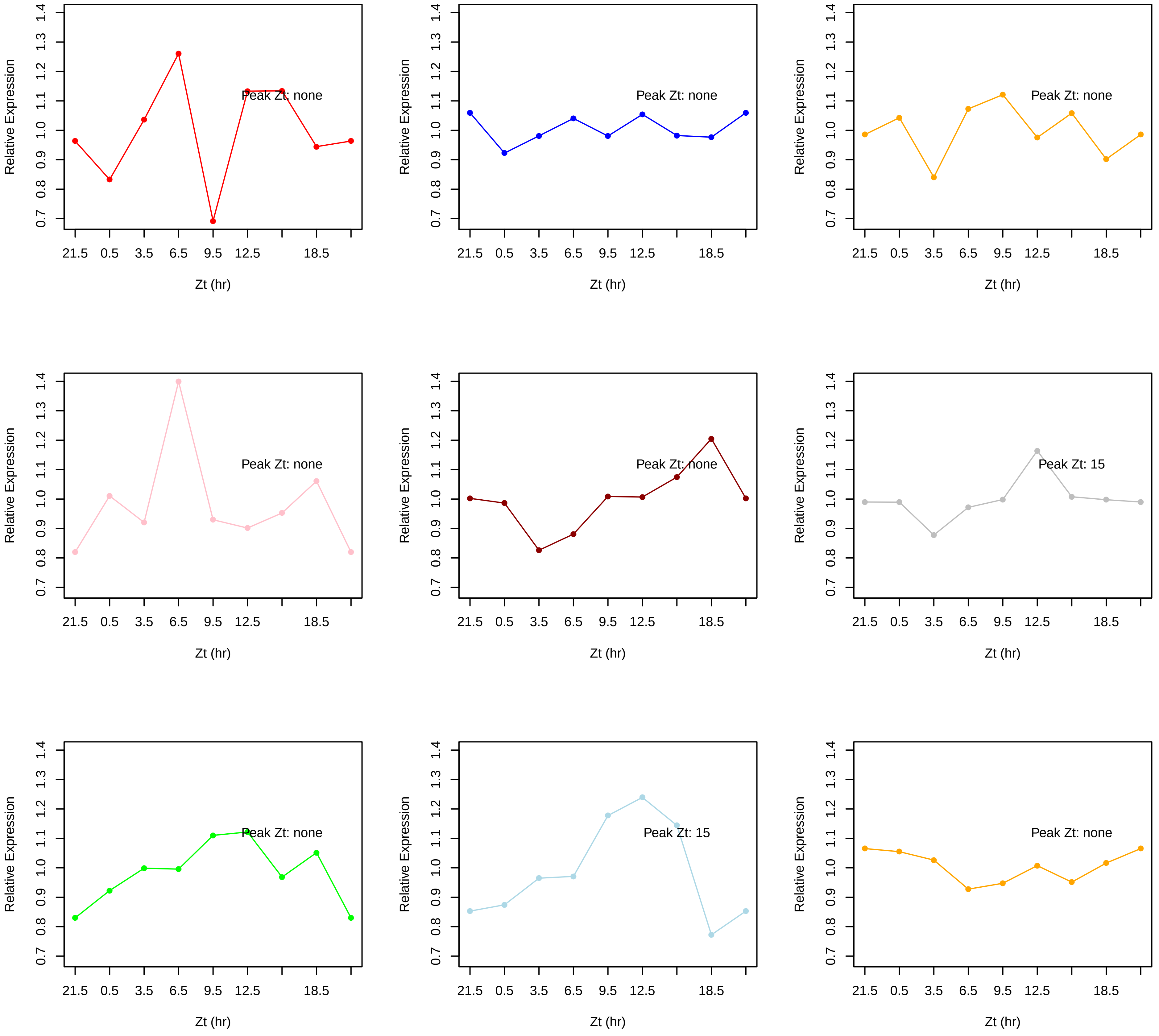

Singeleton: ENSXMAG00000017324 ADAMTS20

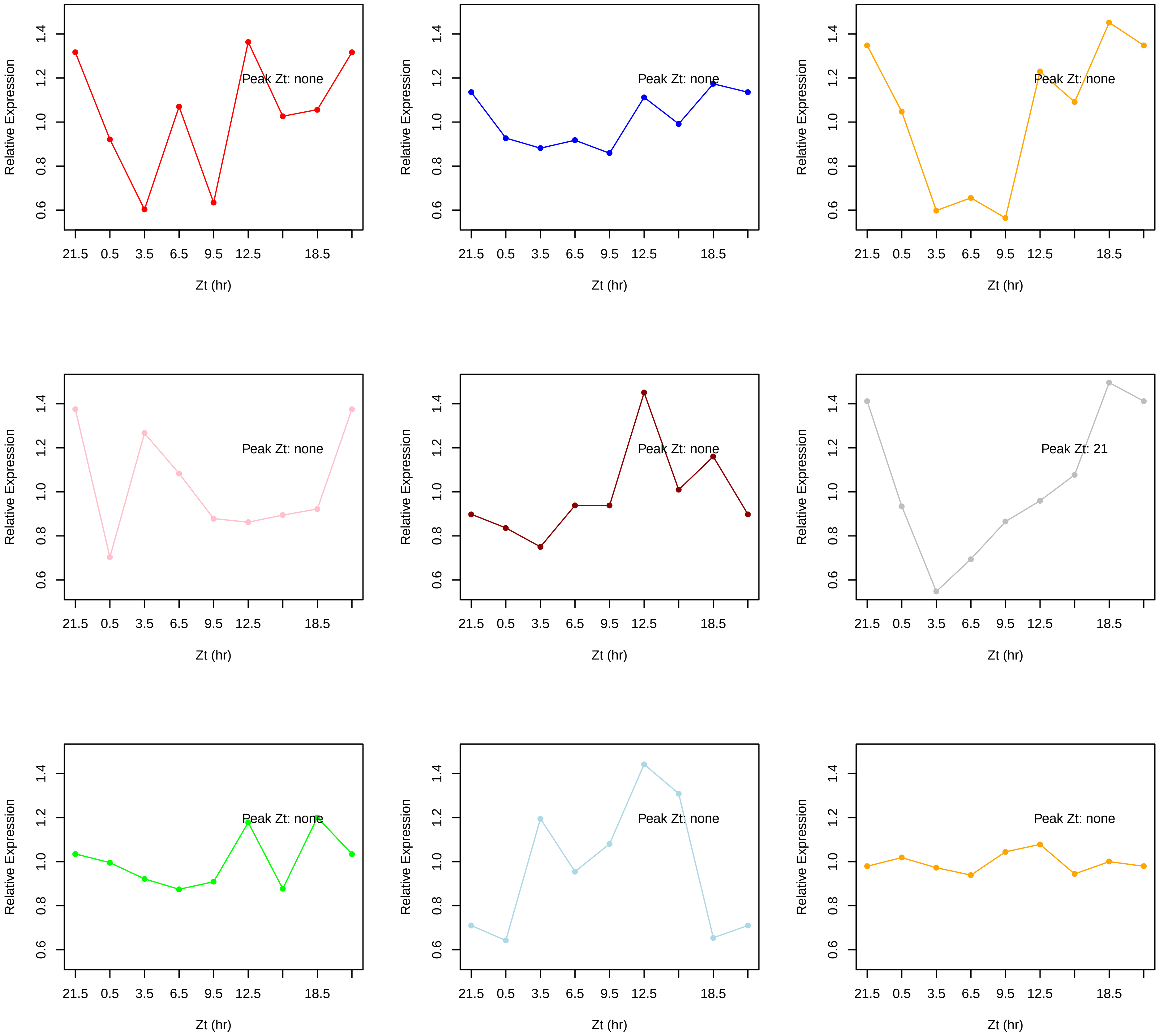

Singeleton: ENSXMAG00000003705 ighmbp2

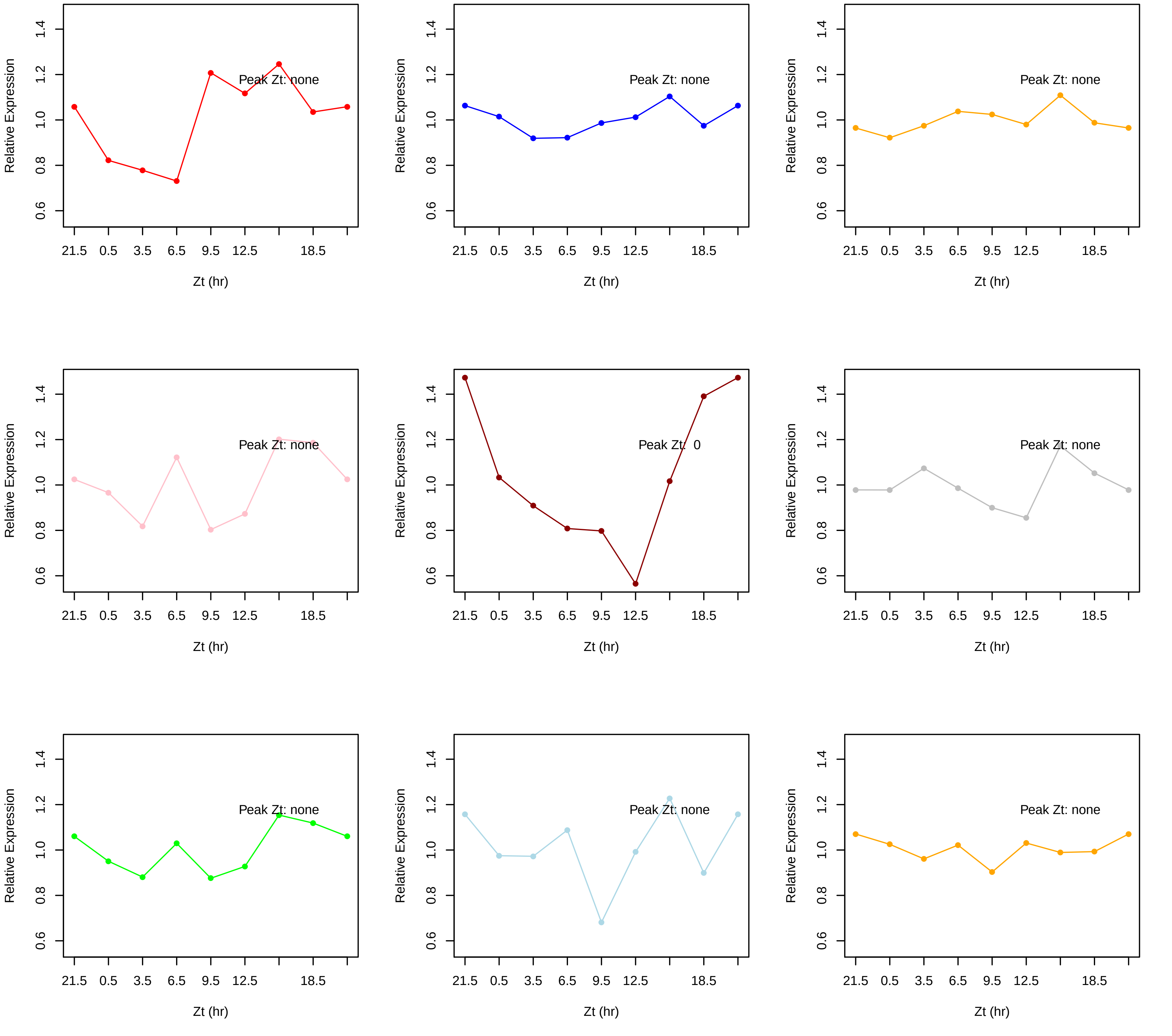

Singeleton: ENSXMAG00000015541 EIF4ENIF1

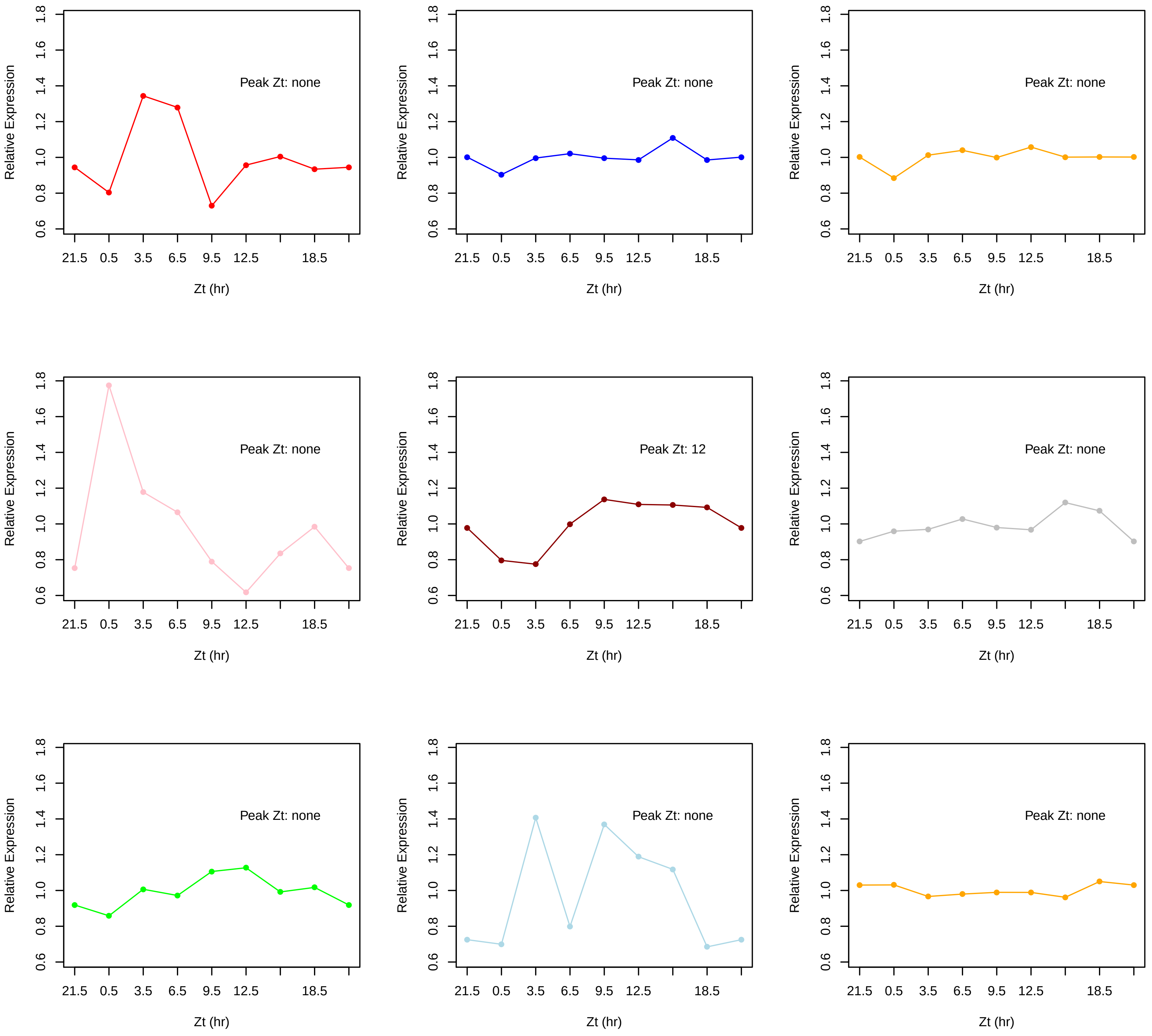

Singeleton: ENSXMAG00000008449 pacs1a

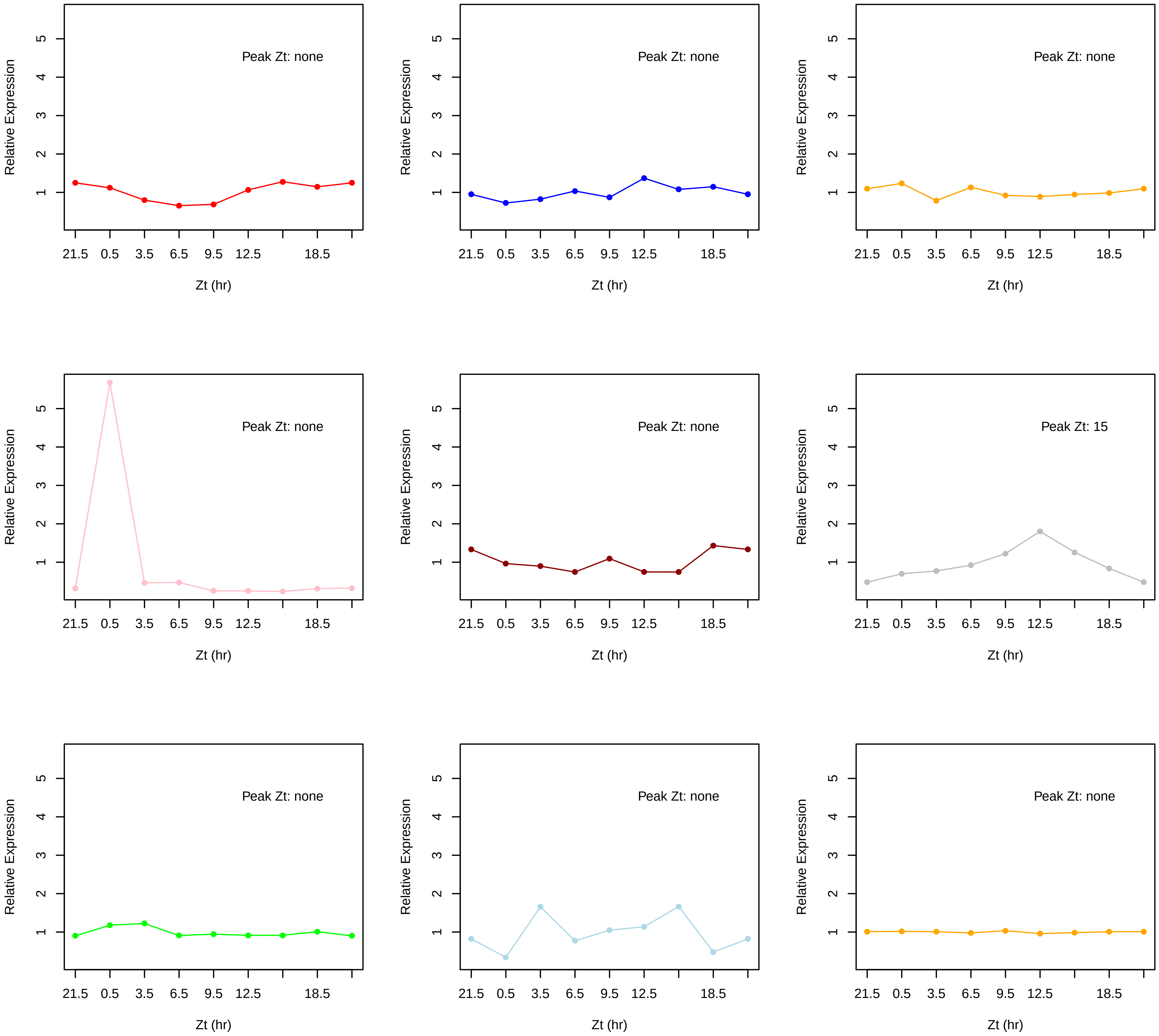

Singeleton: ENSXMAG00000019146 syt12

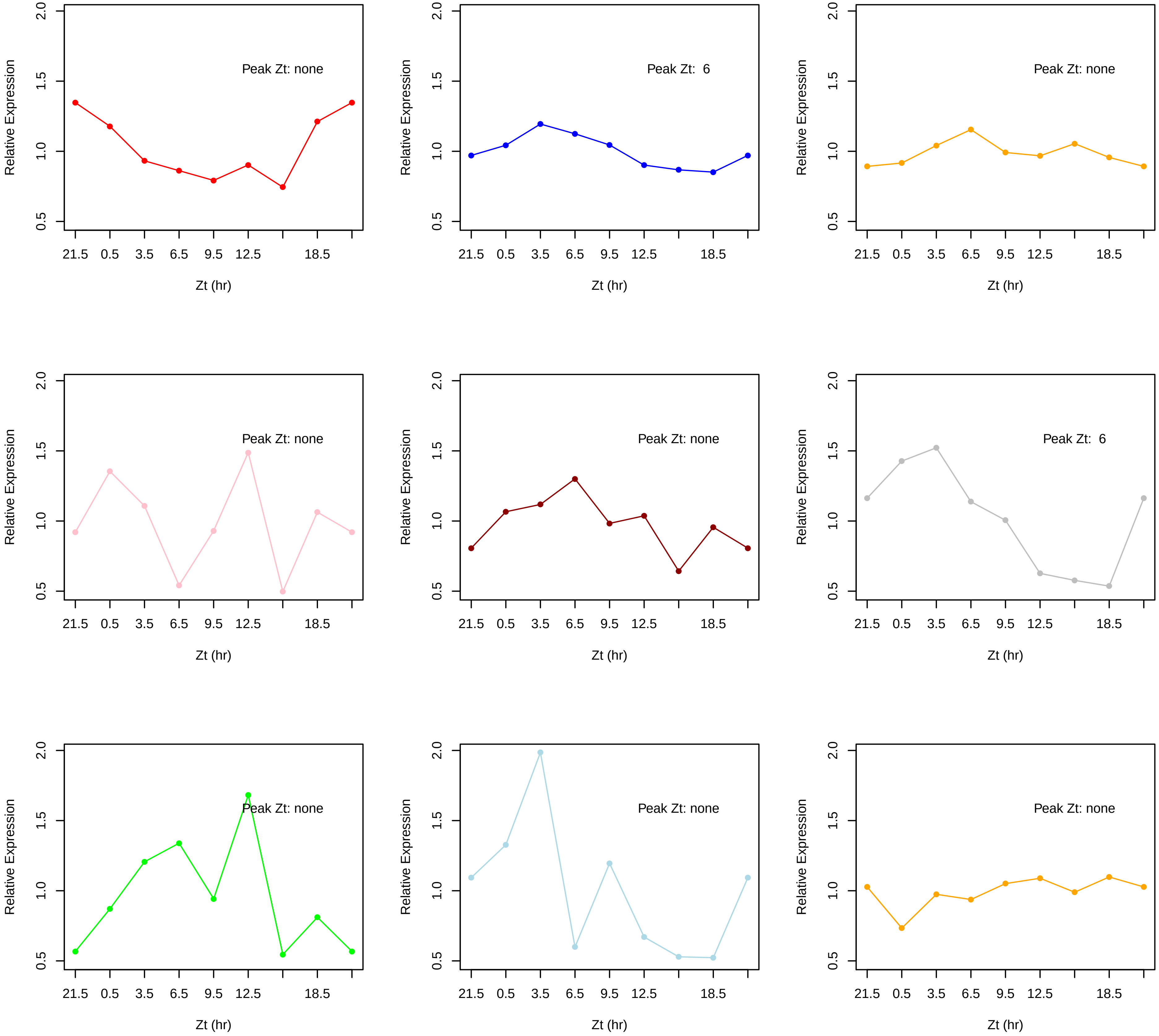

Singeleton: ENSXMAG00000011470 creg2

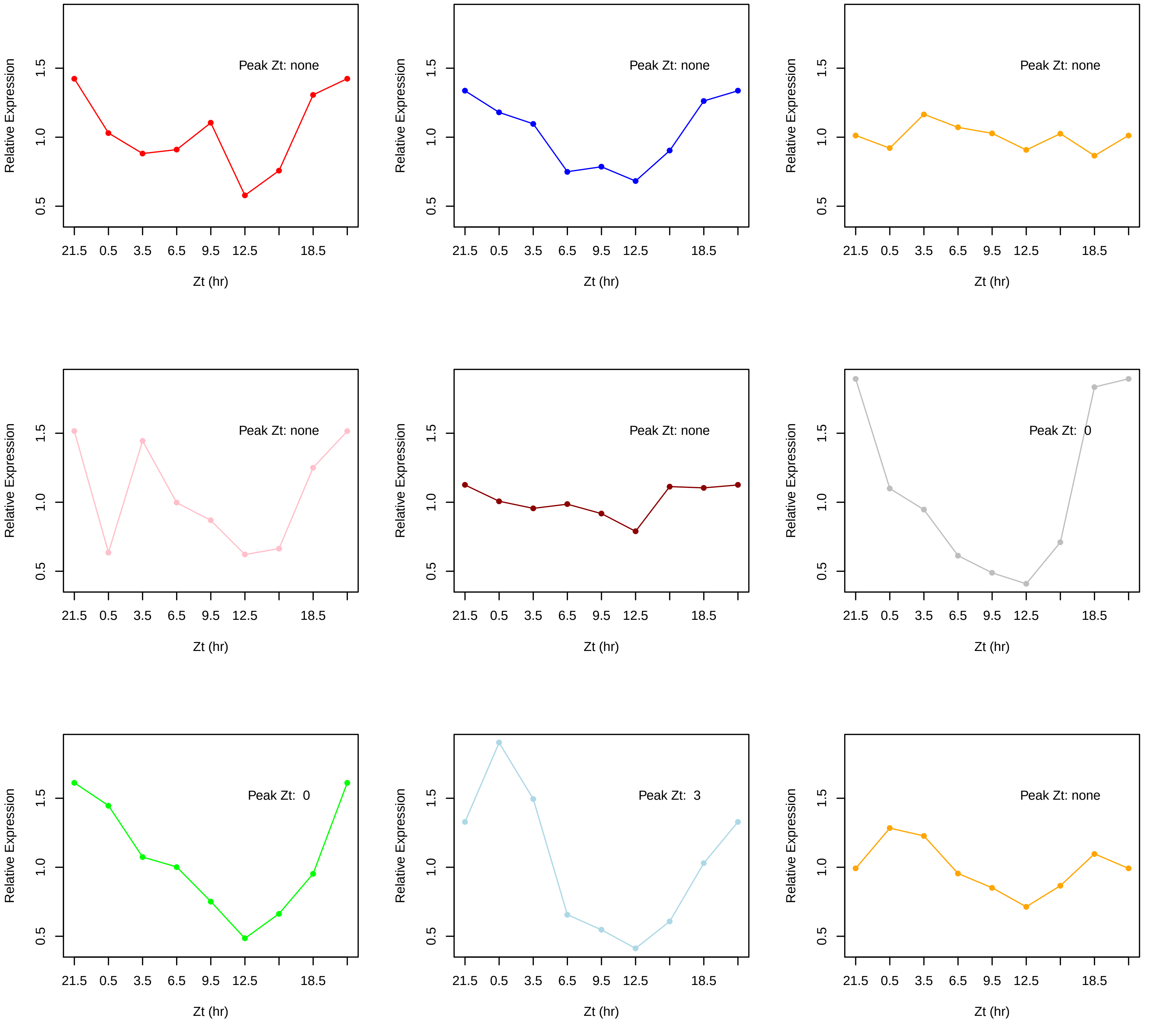

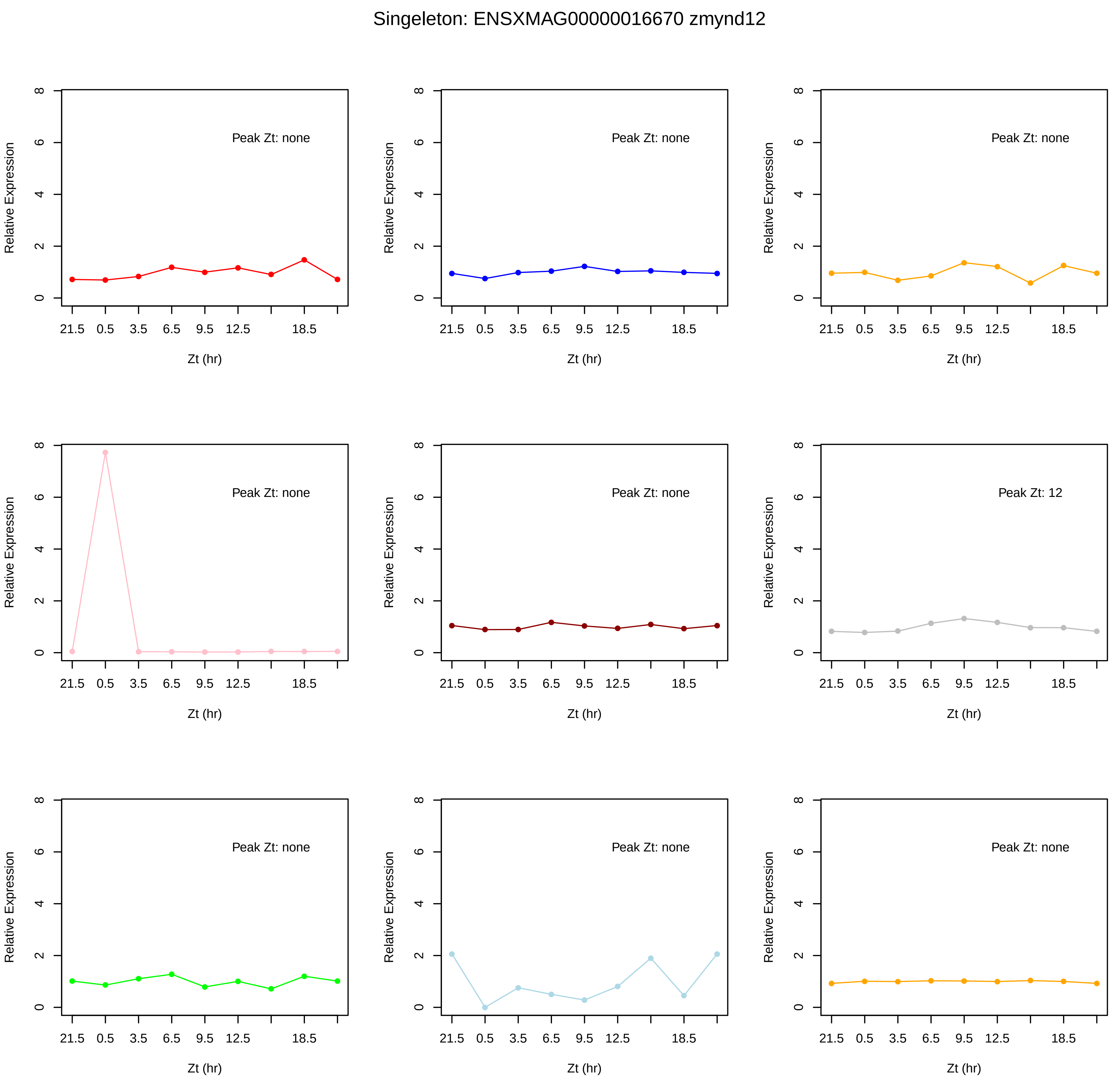
Singeleton: ENSXMAG00000016670 zmynd12
Peak Zt: none
Peak Zt: none
Peak Zt: none
Peak Zt: none
Peak Zt: none
Peak Zt: 12
Peak Zt: none
Peak Zt: none
Peak Zt: none
Relative Expression
Zt (hr)
21.5
0.5
3.5
6.5
9.5
12.5
18.5

Singeleton: ENSXMAG00000024777 zdhhc24

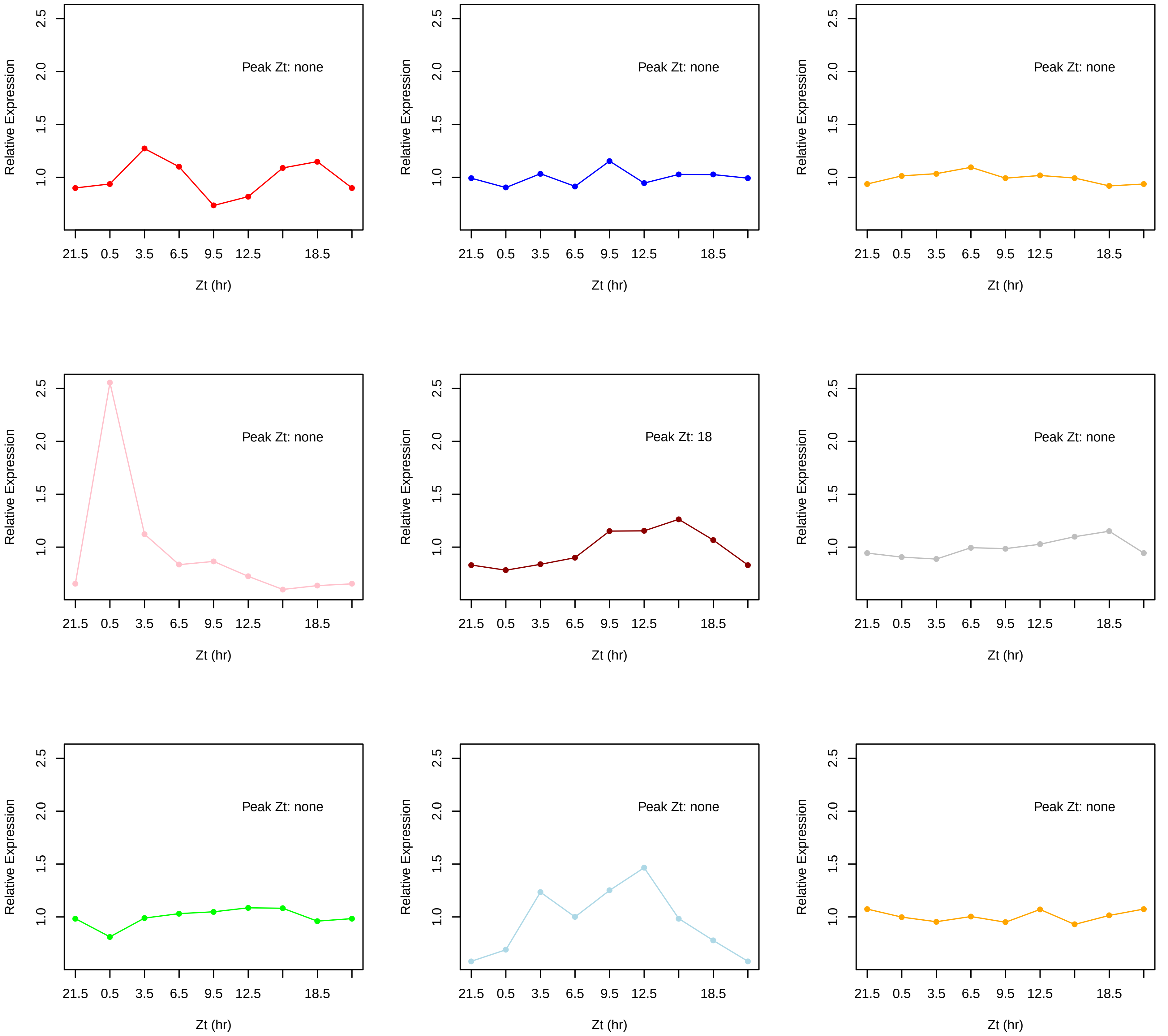

Singeleton: ENSXMAG00000011475 mgat4a

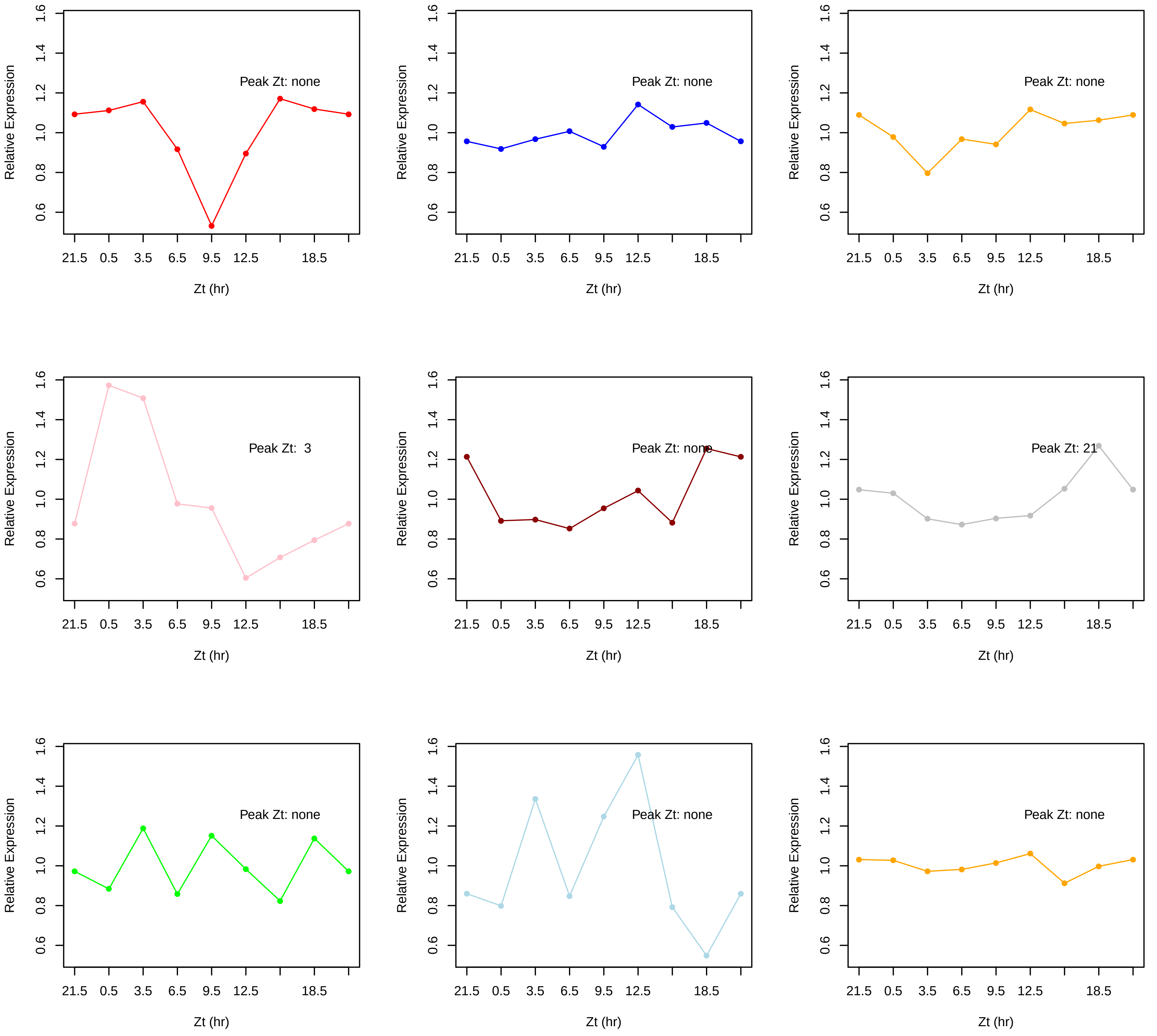

Singeleton: ENSXMAG00000028502 prex1

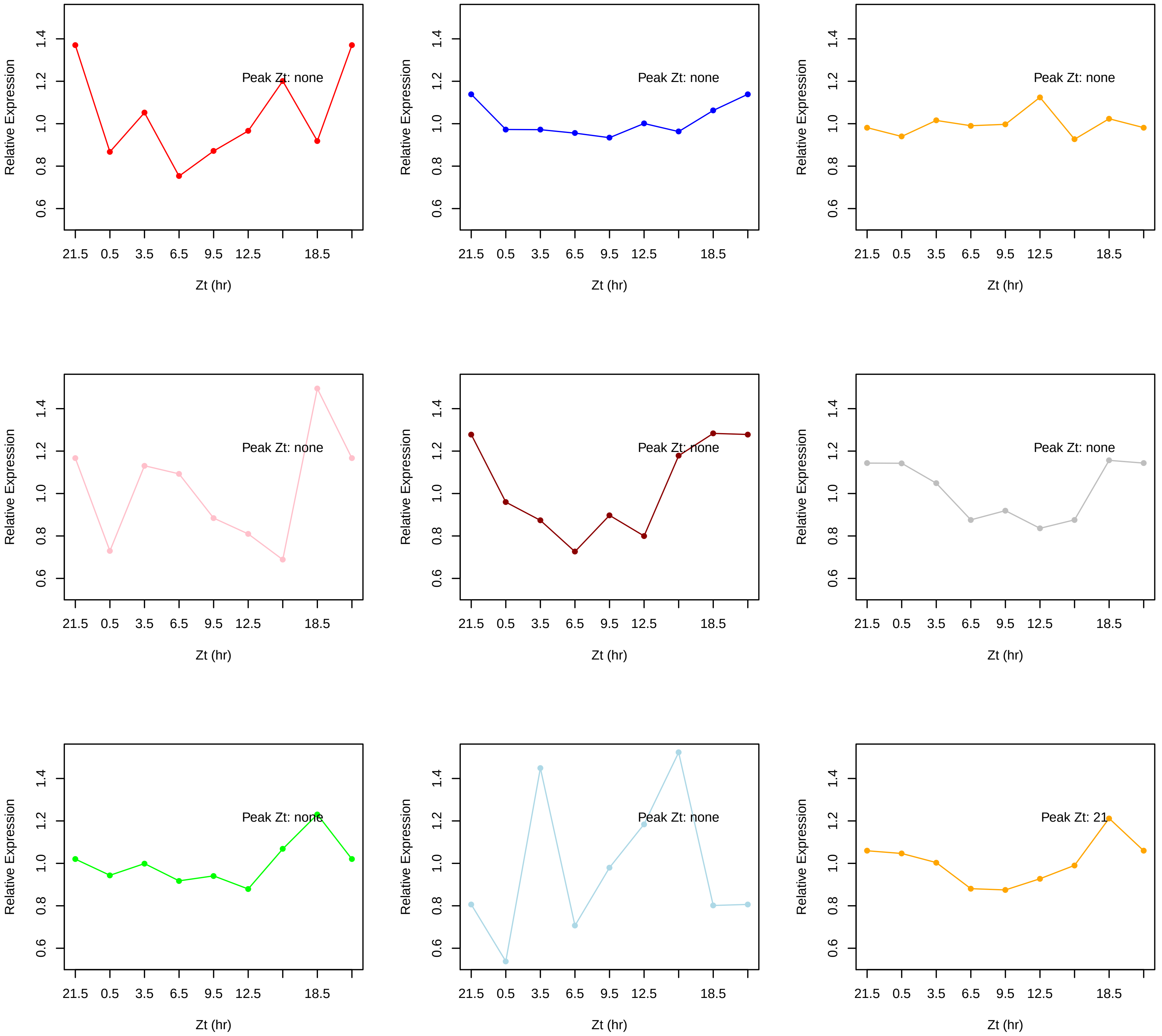

Singeleton: ENSXMAG00000018668 odr4

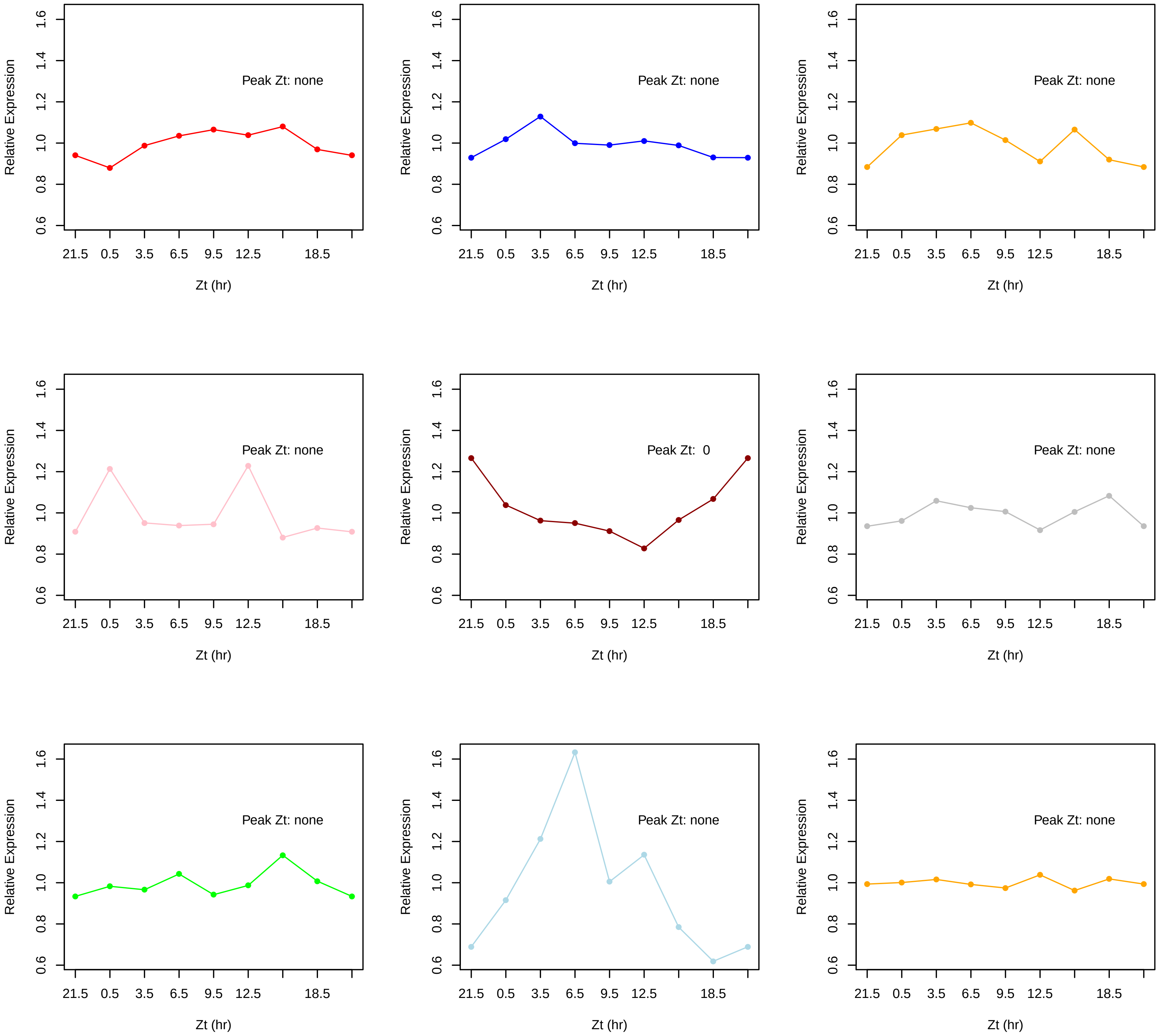

Singleton: ENSXMAG00000002309

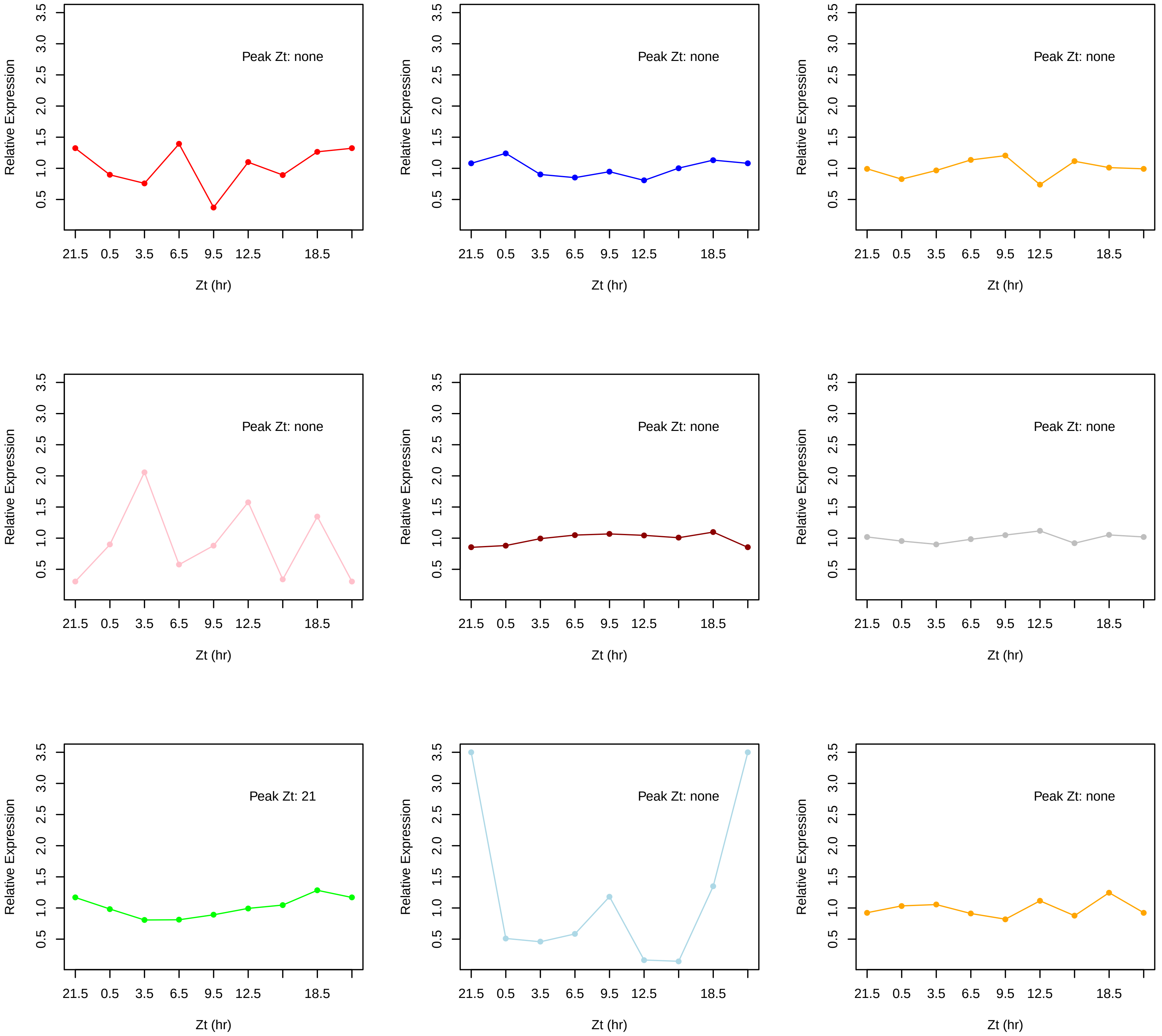

Singeleton: ENSXMAG00000005675 cadm3

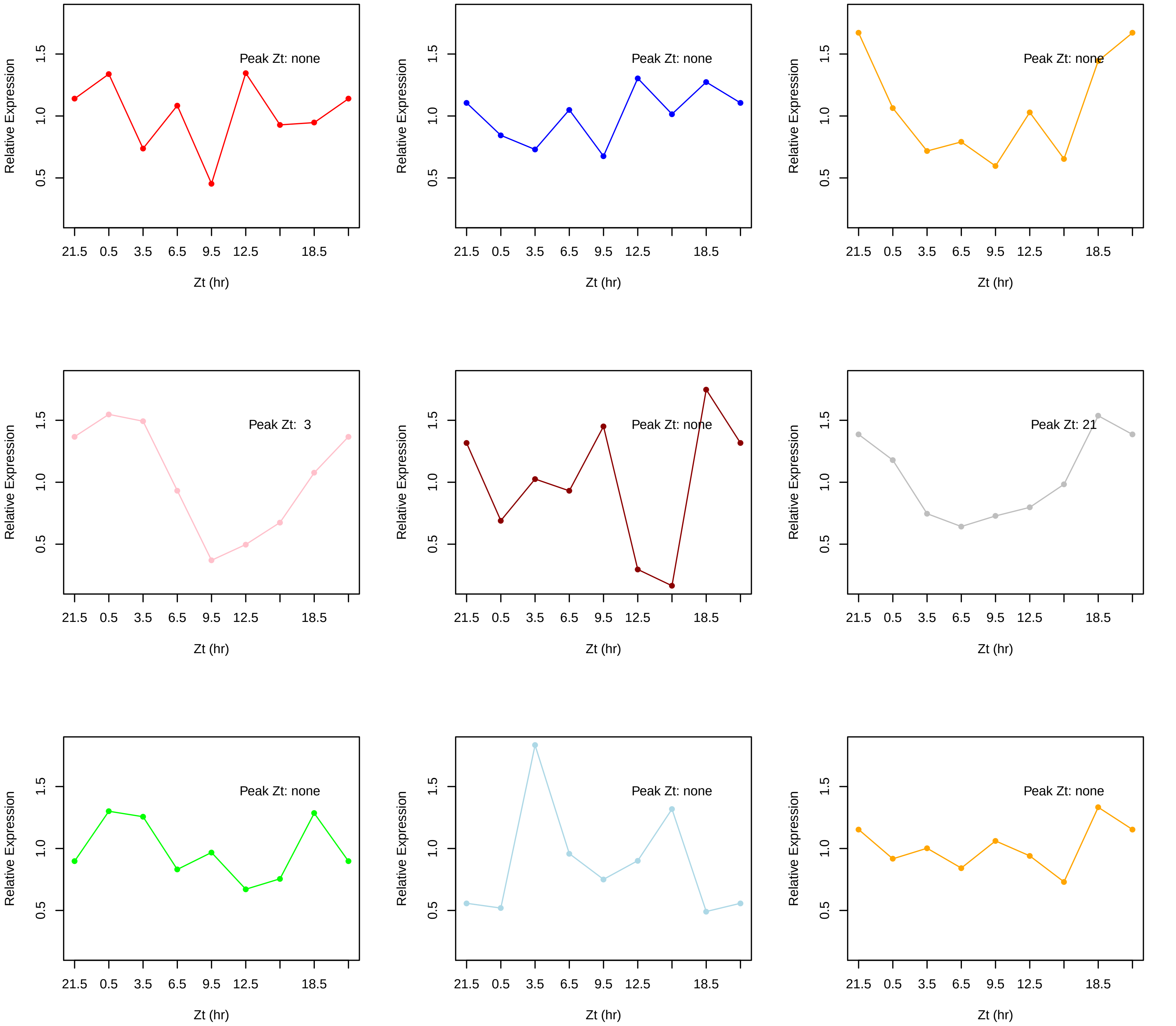

Singeleton: ENSXMAG00000008438 zgc:175248

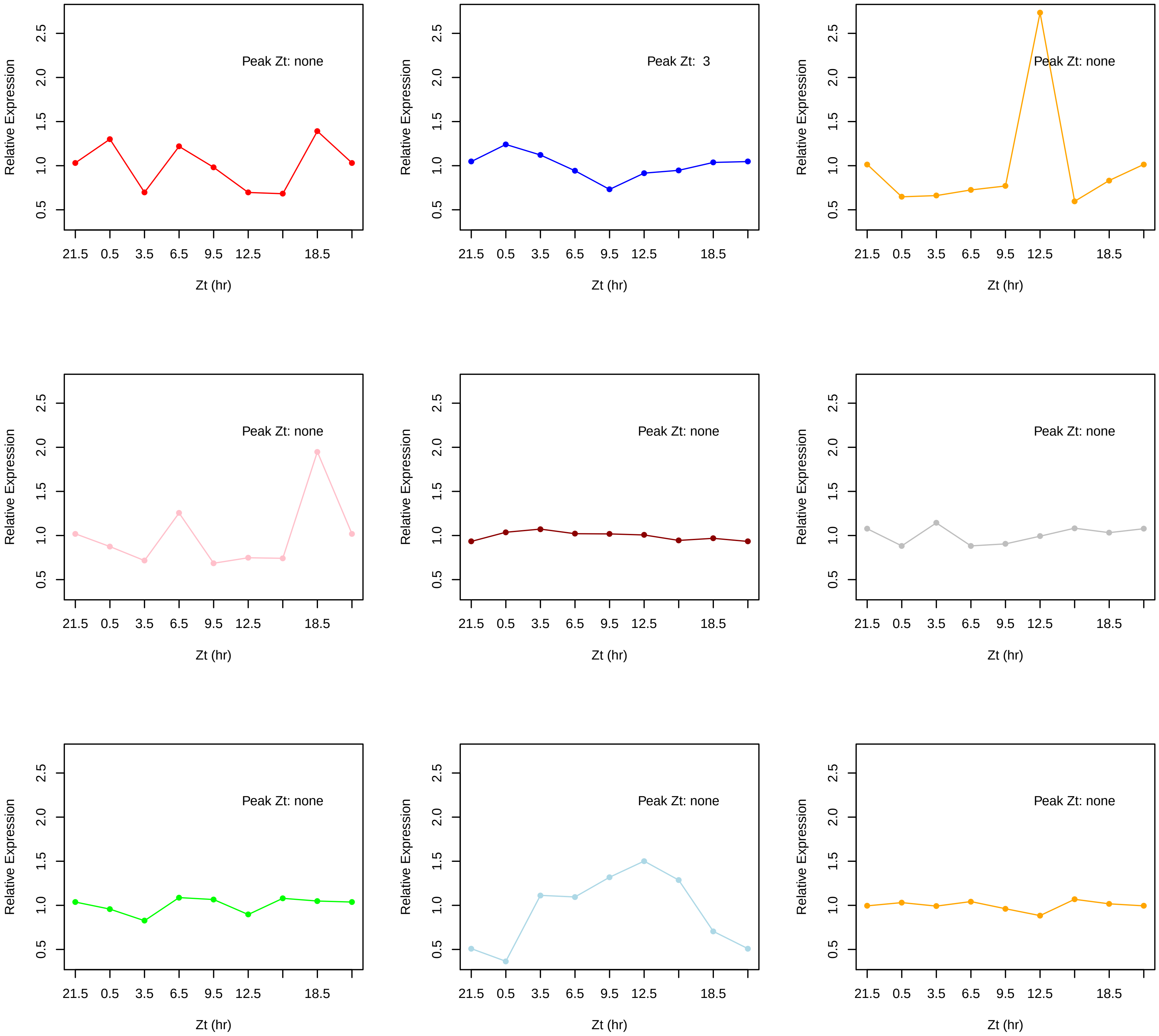

Singleton: ENSXMAG00000007915 rnd3a

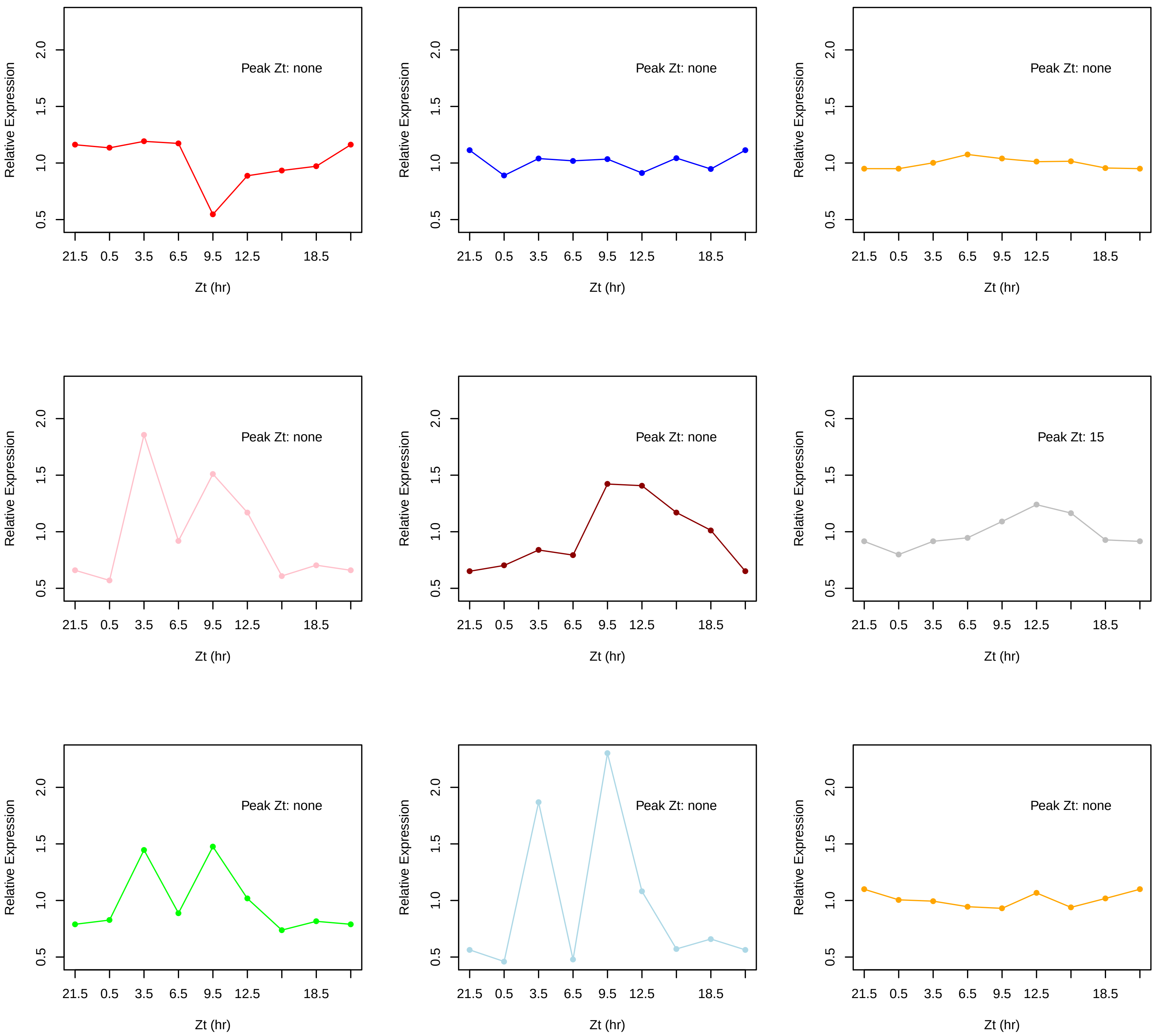

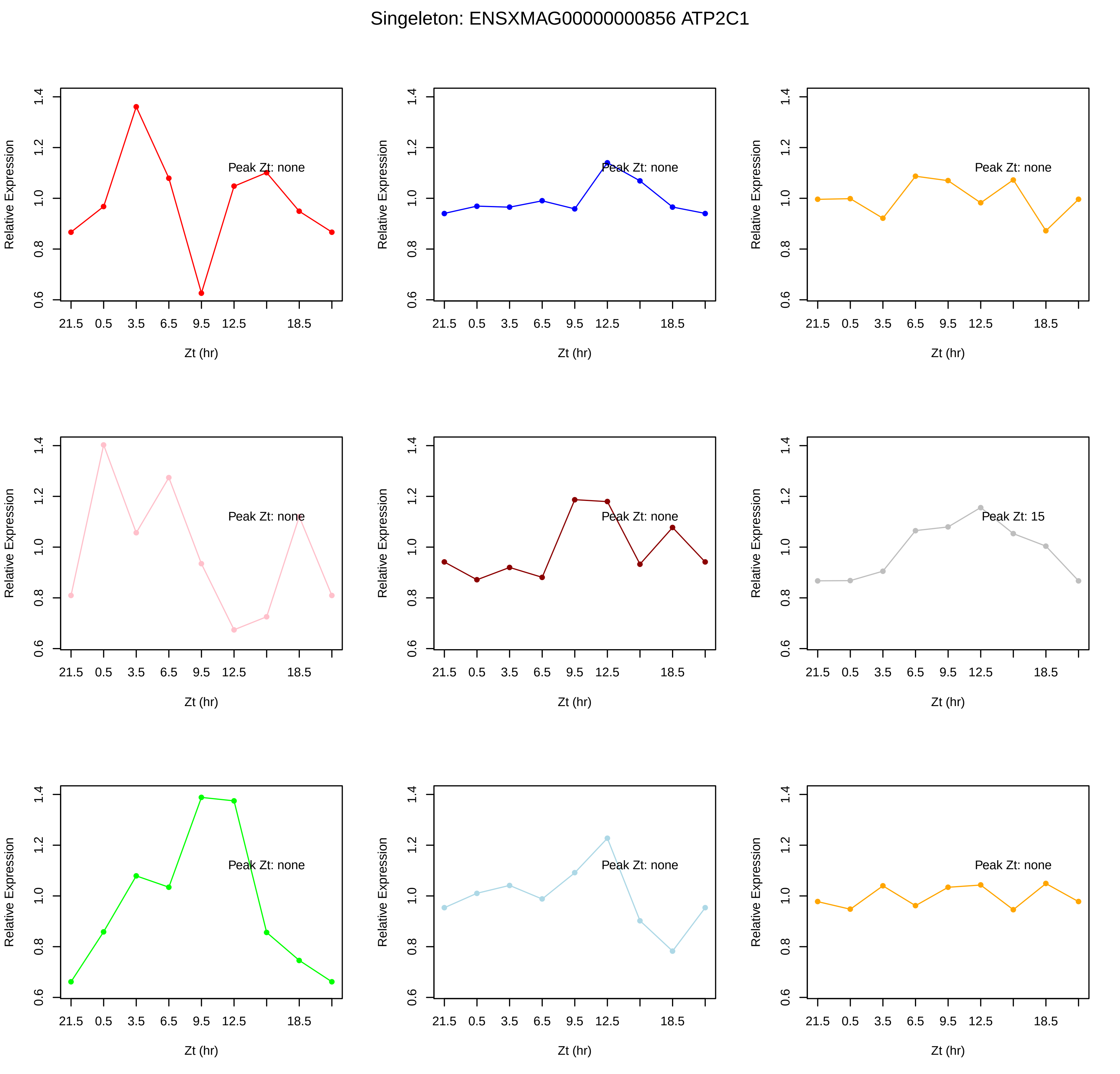

Singeleton: ENSXMAG00000000856 ATP2C1
Relative Expression
Zt (hr)
21.5
0.5
3.5
6.5
9.5
12.5
18.5
0.6
0.8
1.0
1.2
1.4
Peak Zt: none
Peak Zt: 15

Singeleton: ENSXMAG00000024820 slc35e1

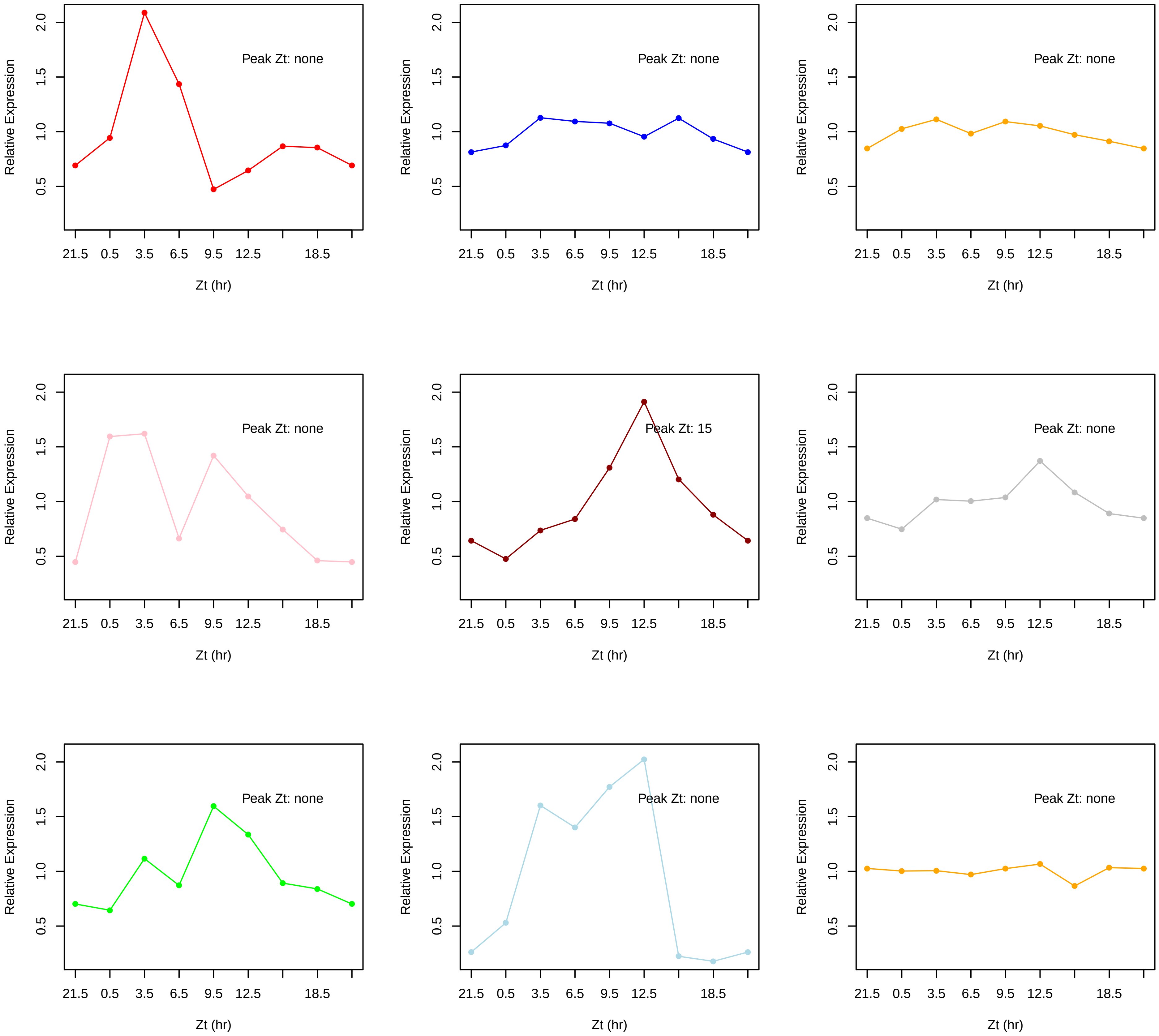

Singleton: ENSXMAG00000027813 ptpn5

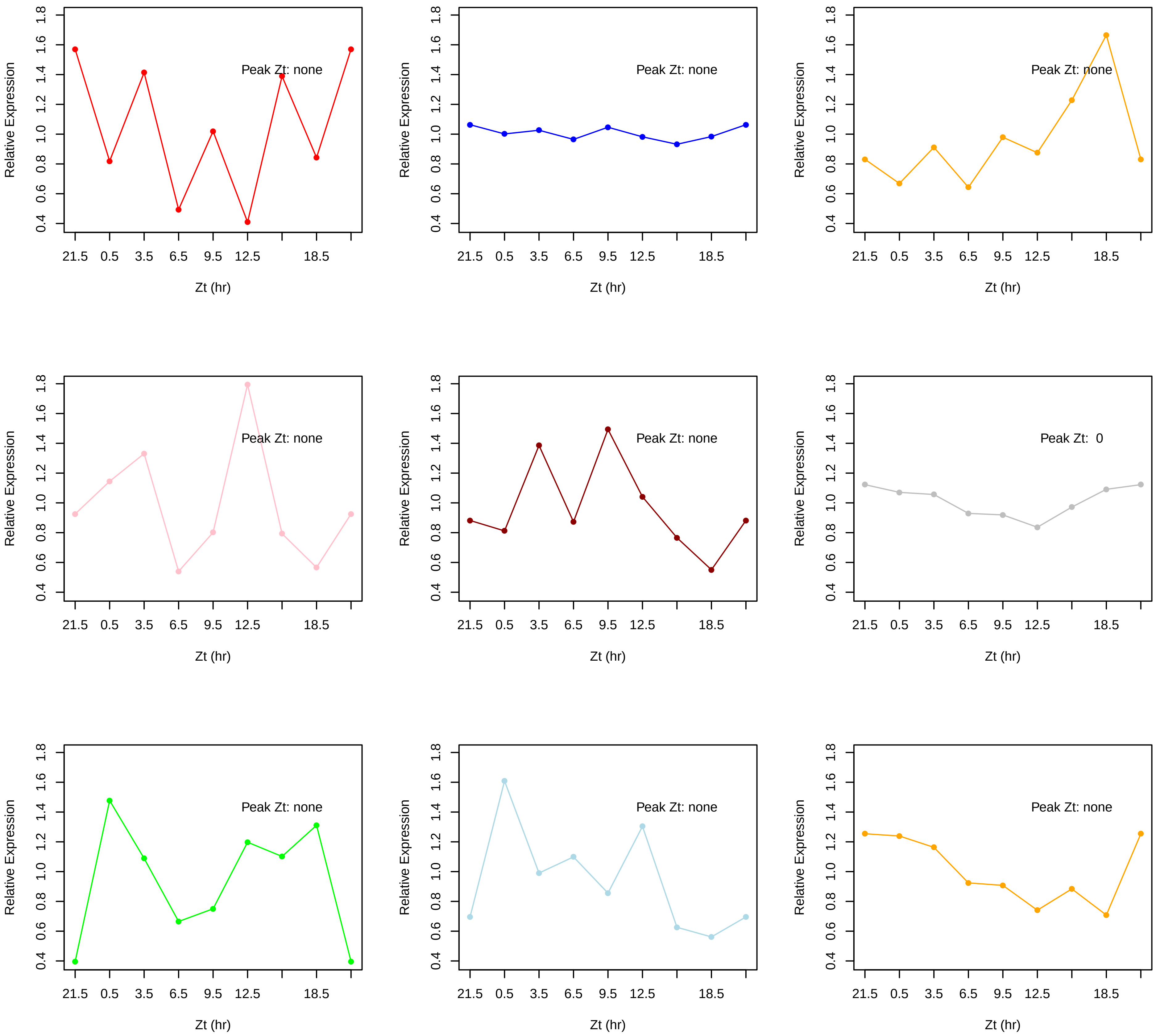

Singleton: ENSXMAG00000029274 COL26A1

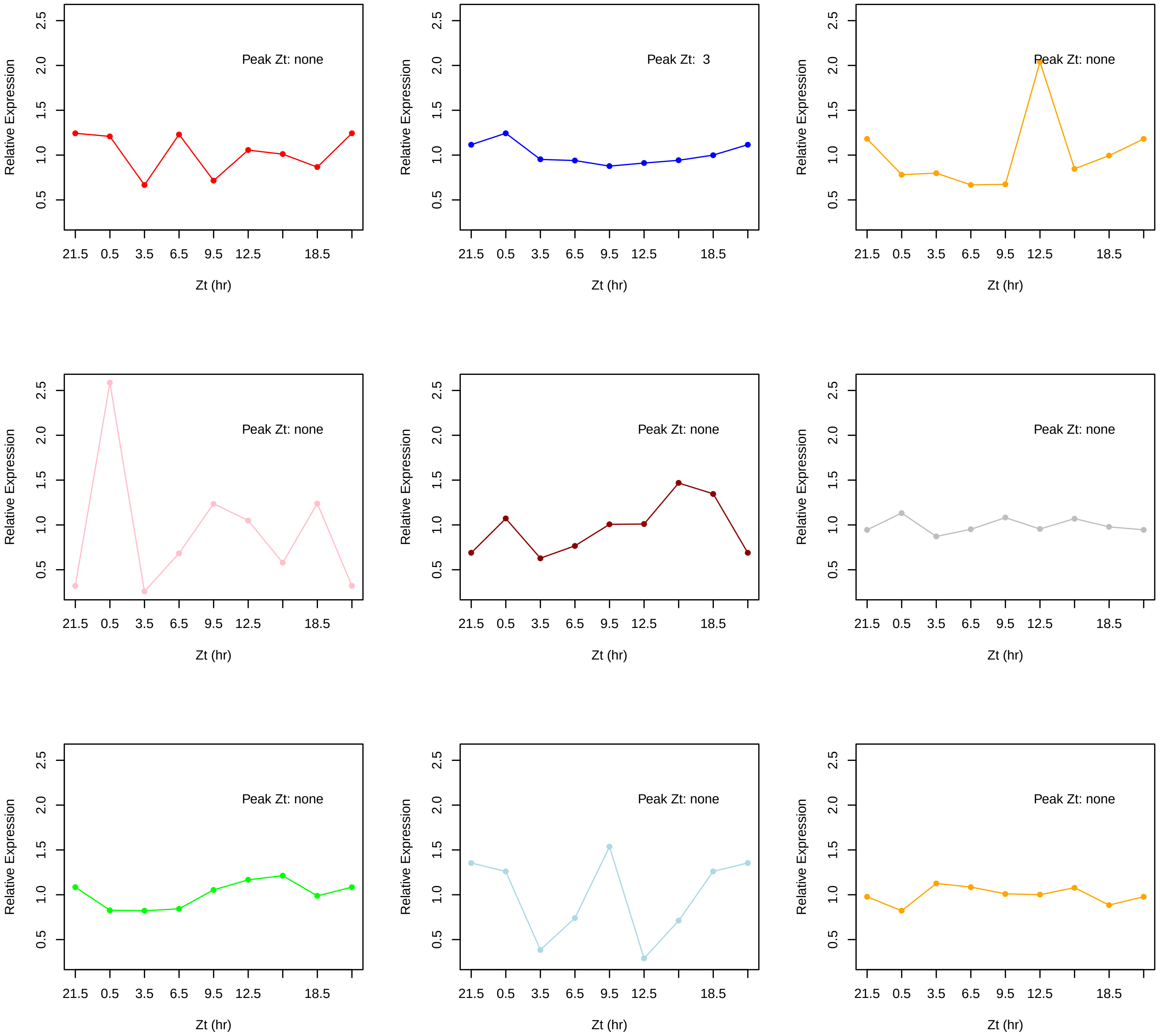

Singeleton: ENSXMAG00000024543

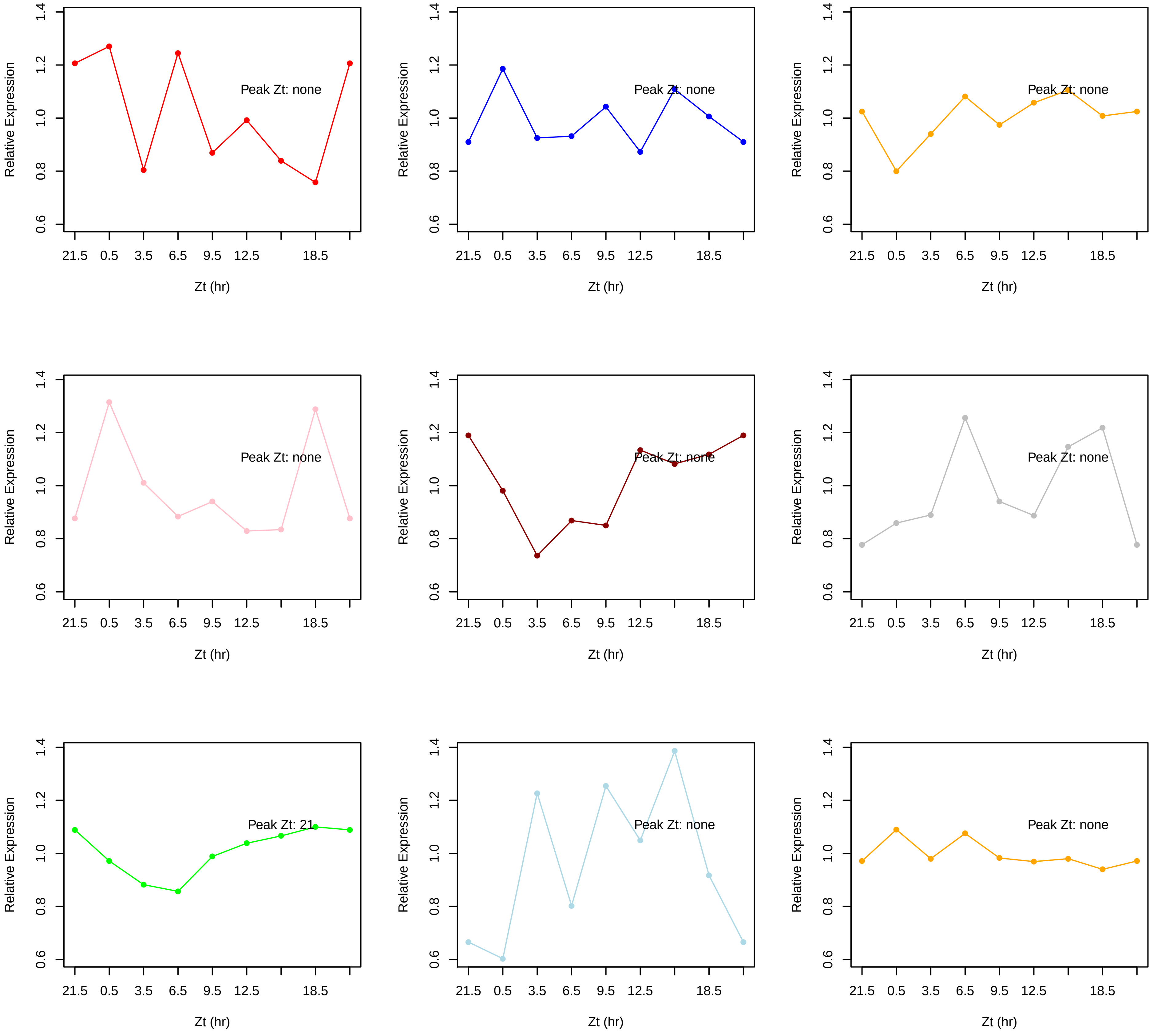

Singeleton: ENSXMAG00000009376 pus7l

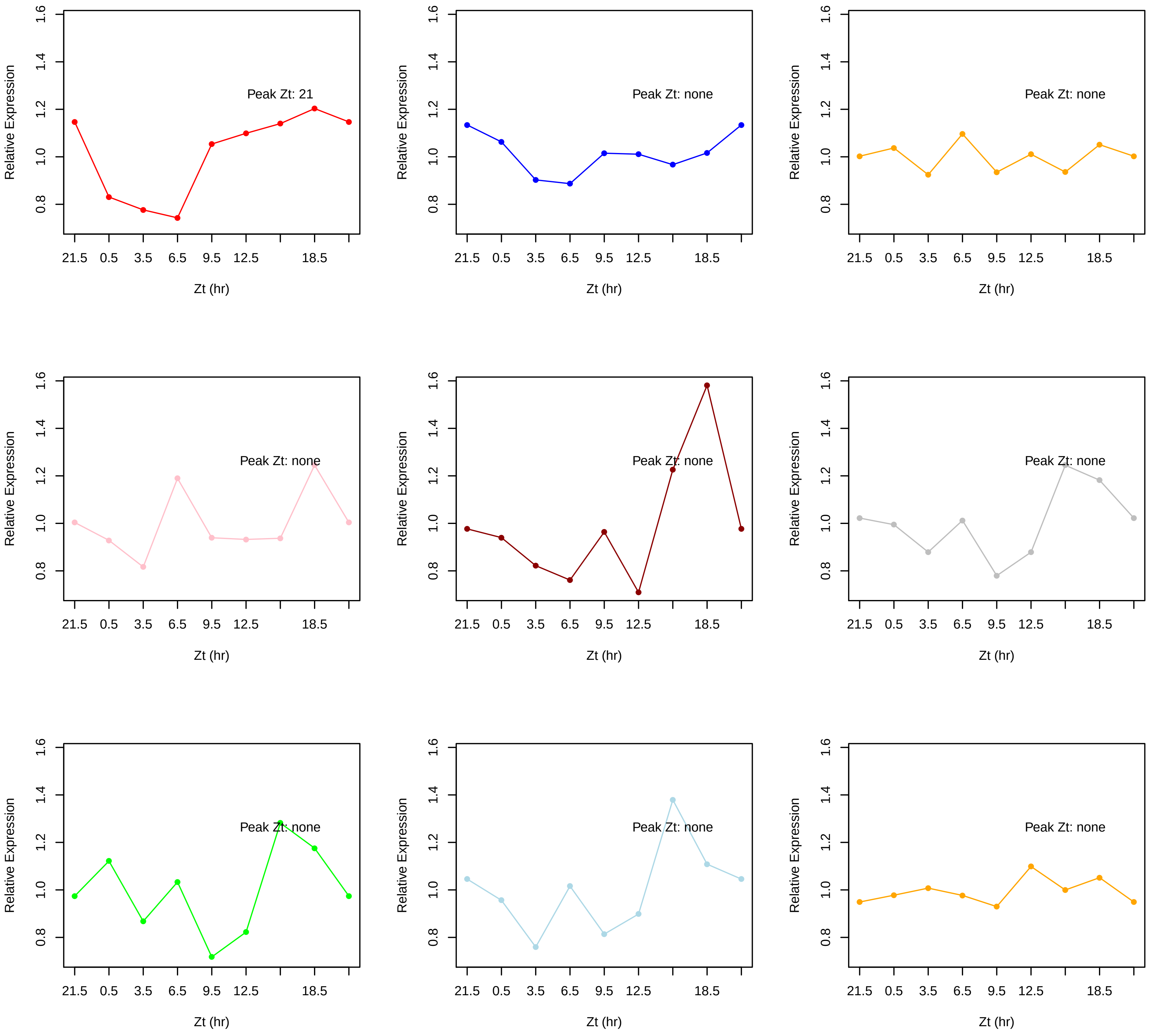

Singeleton: ENSXMAG00000010985 mmp28

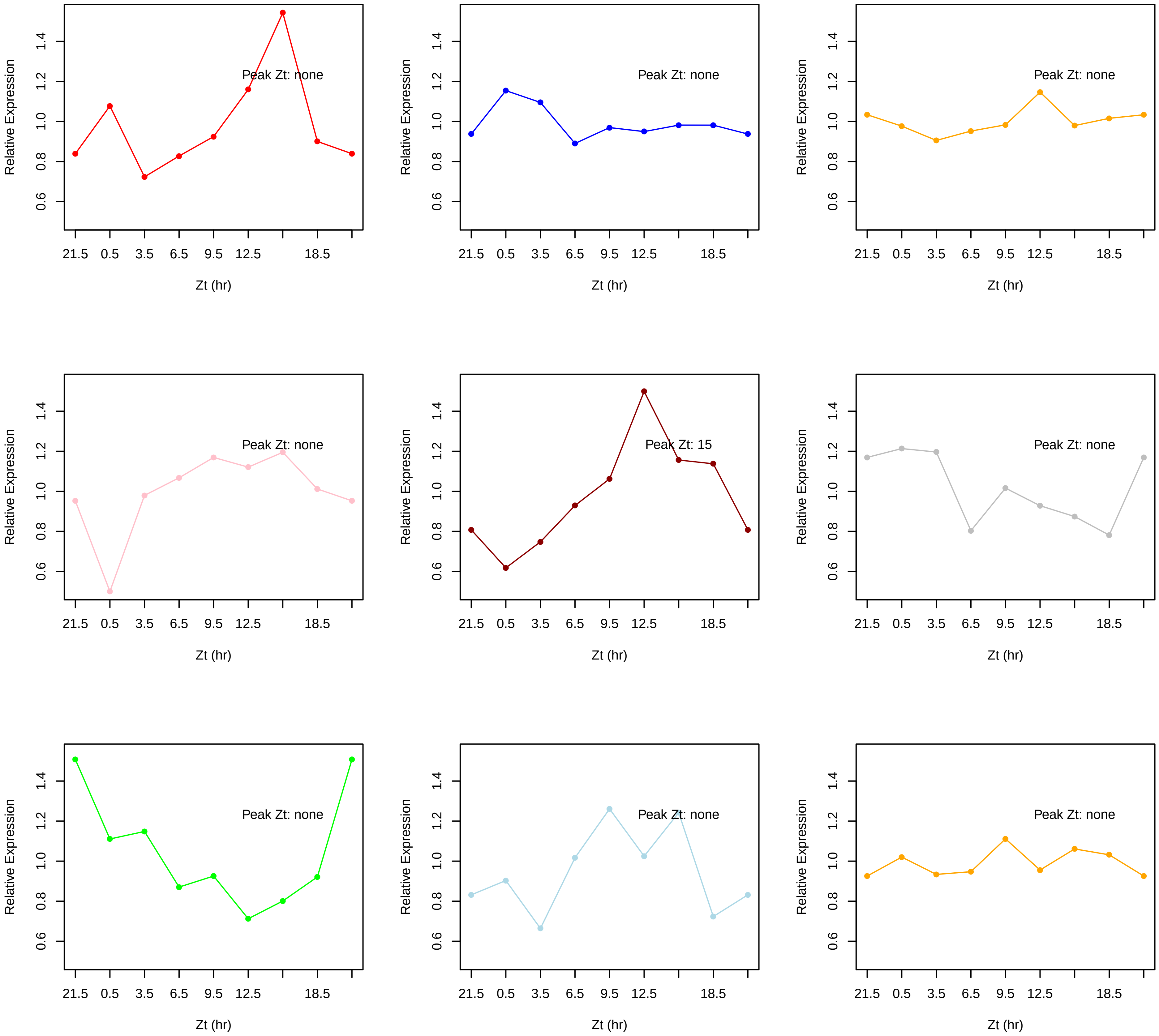

Singeleton: ENSXMAG00000002418 ptgs2b

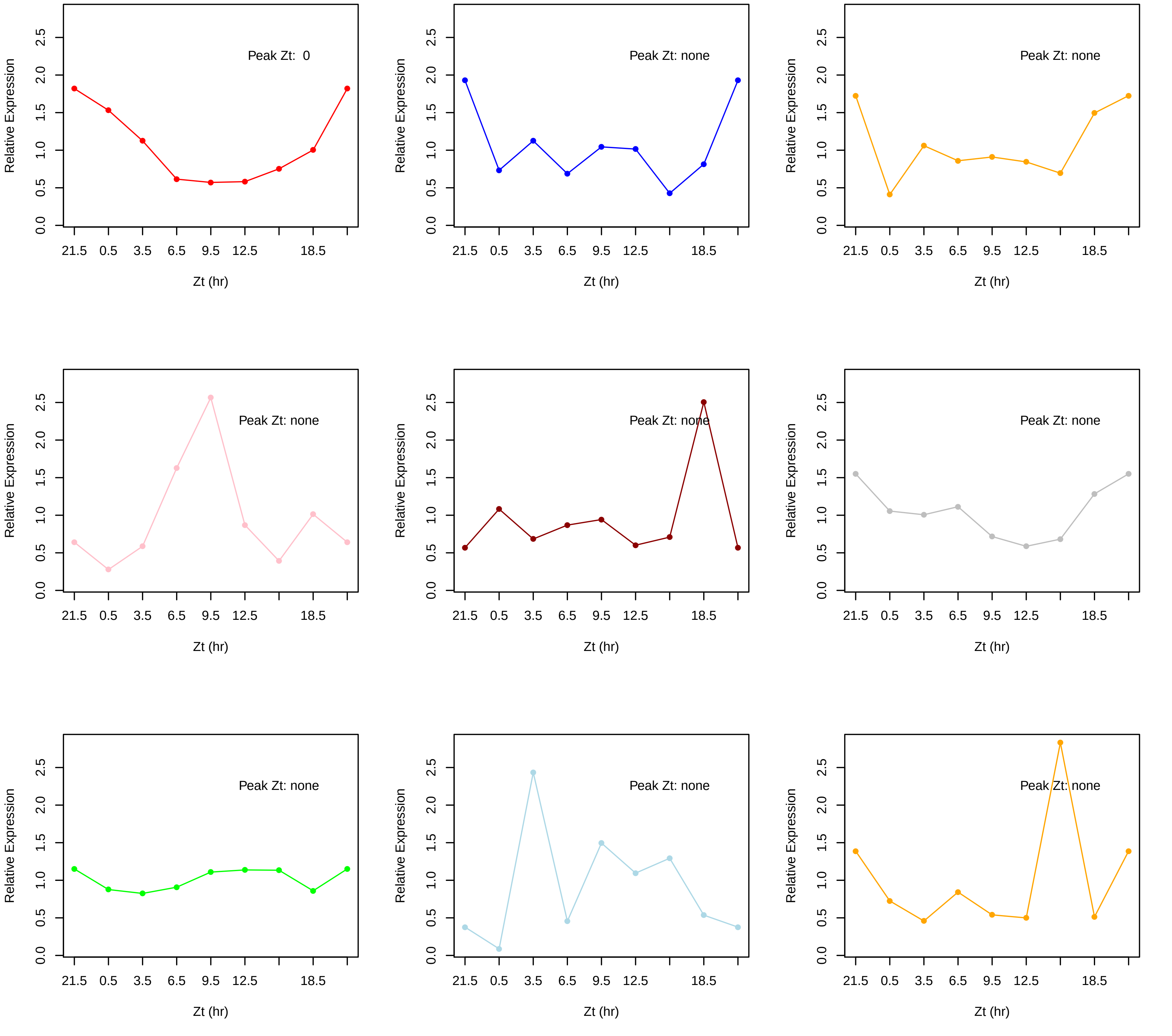

Singeleton: ENSXMAG00000006688

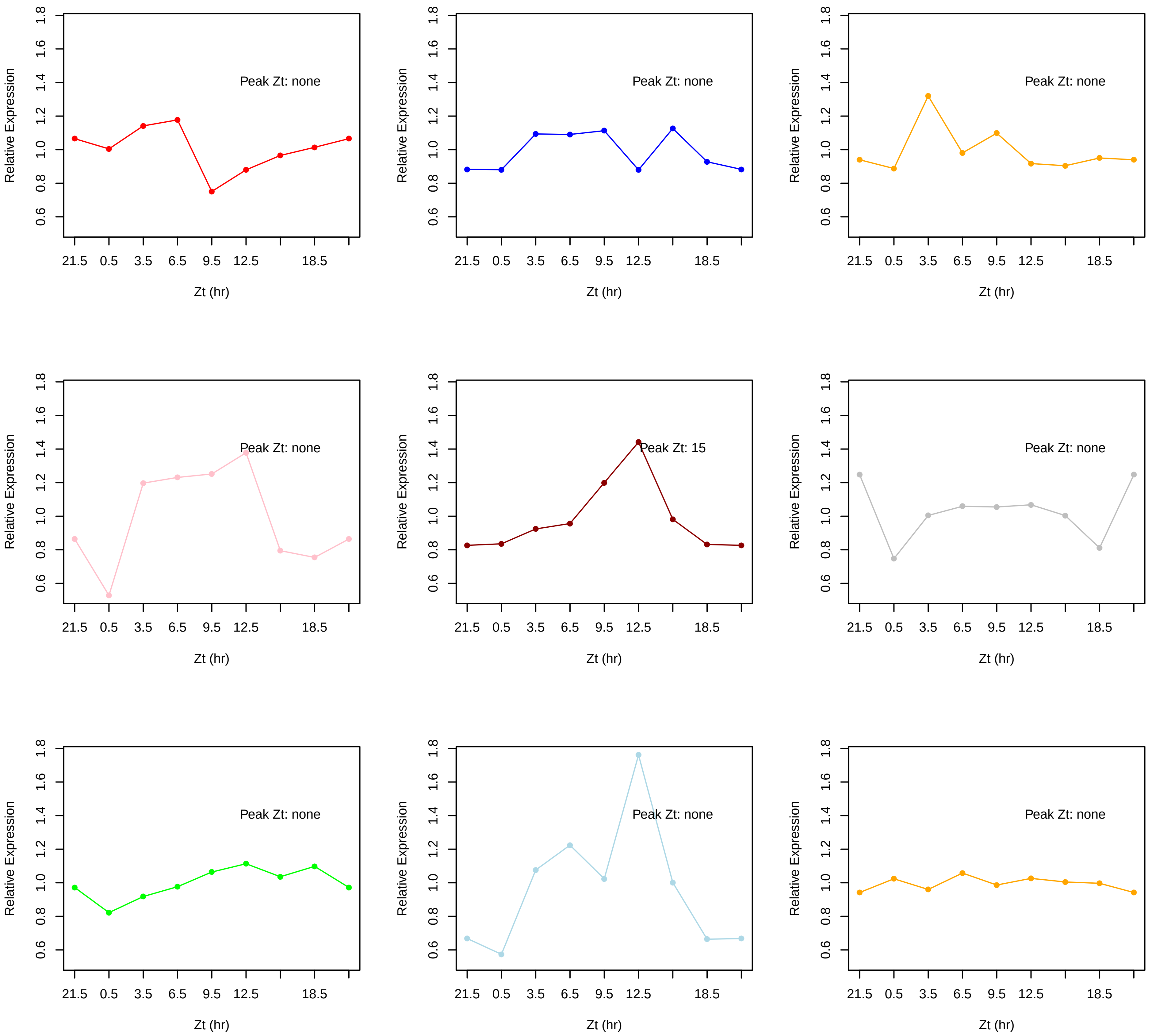

Singeleton: ENSXMAG00000005799 mpz

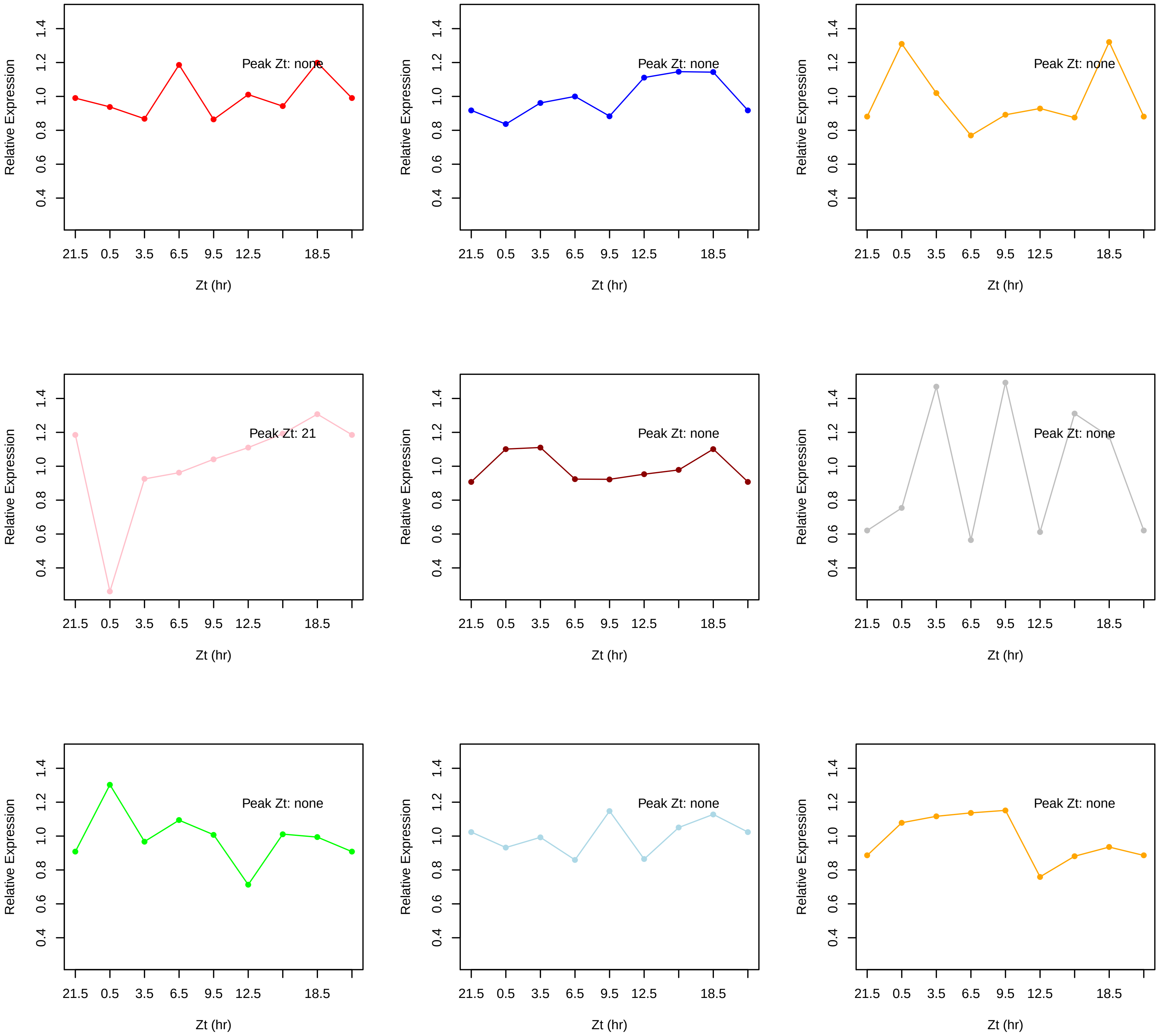

Singeleton: ENSXMAG00000000726

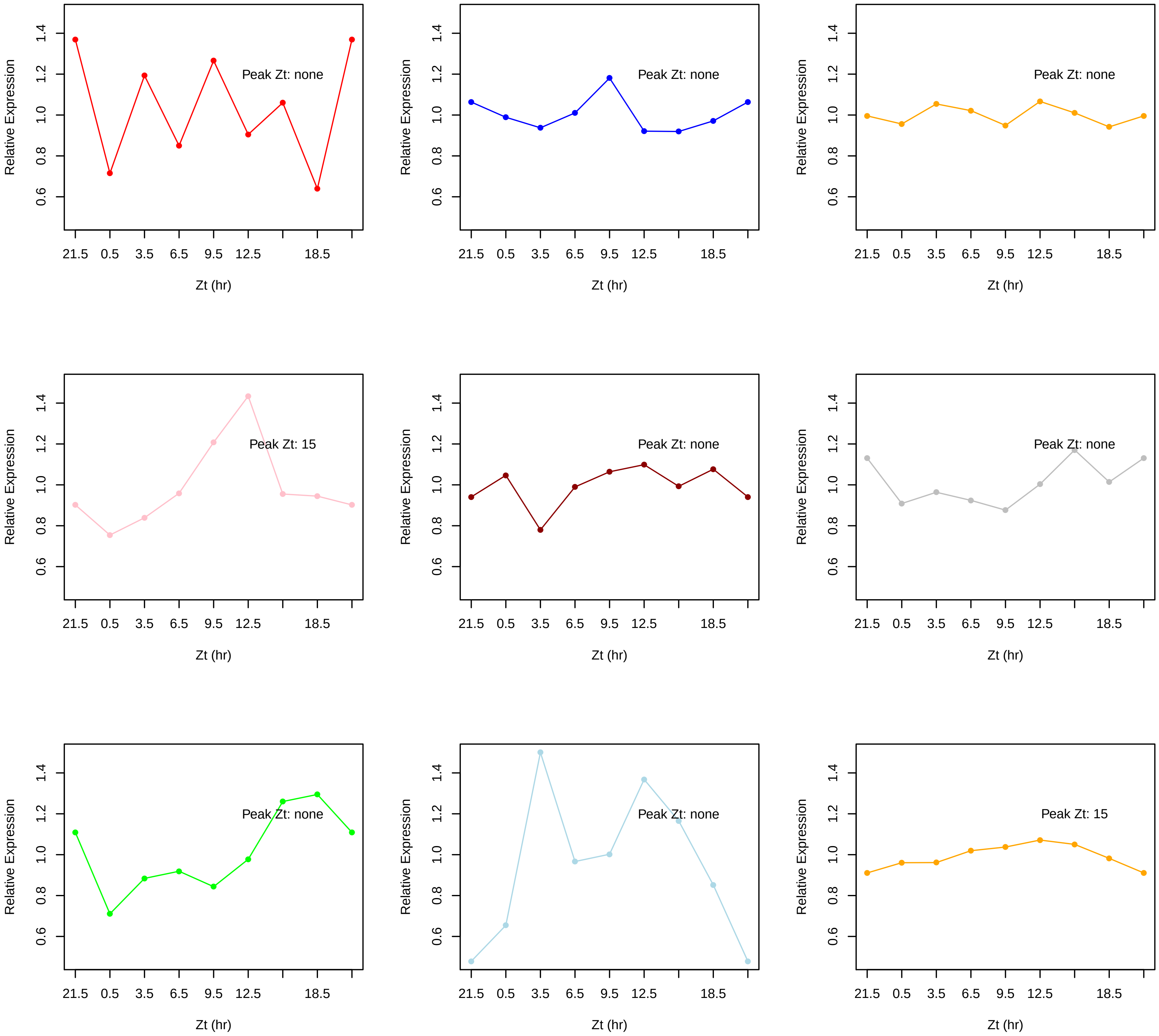

Singeleton: ENSXMAG00000004507 DNAAF5

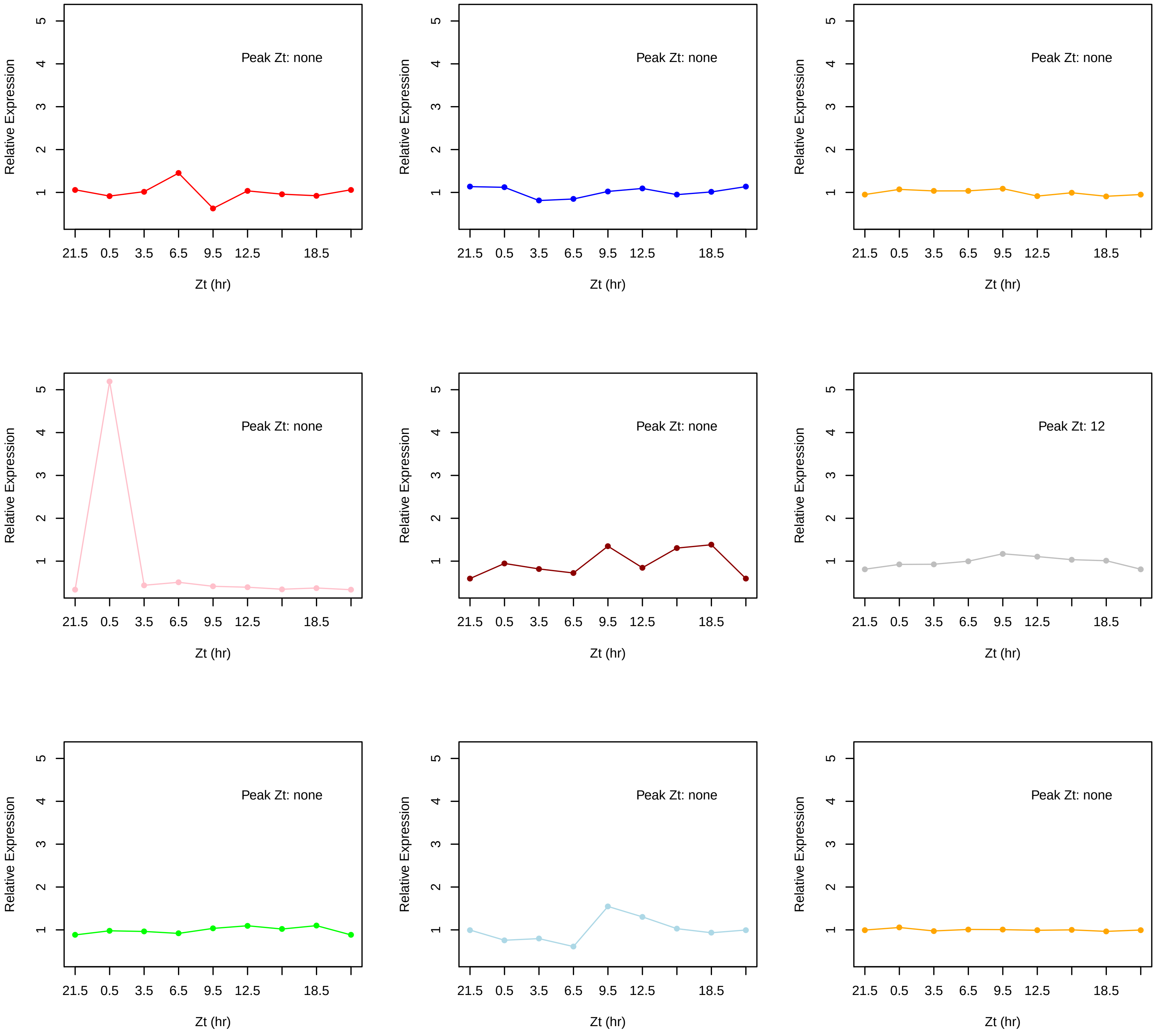

Singeleton: ENSXMAG00000012883 rmc1

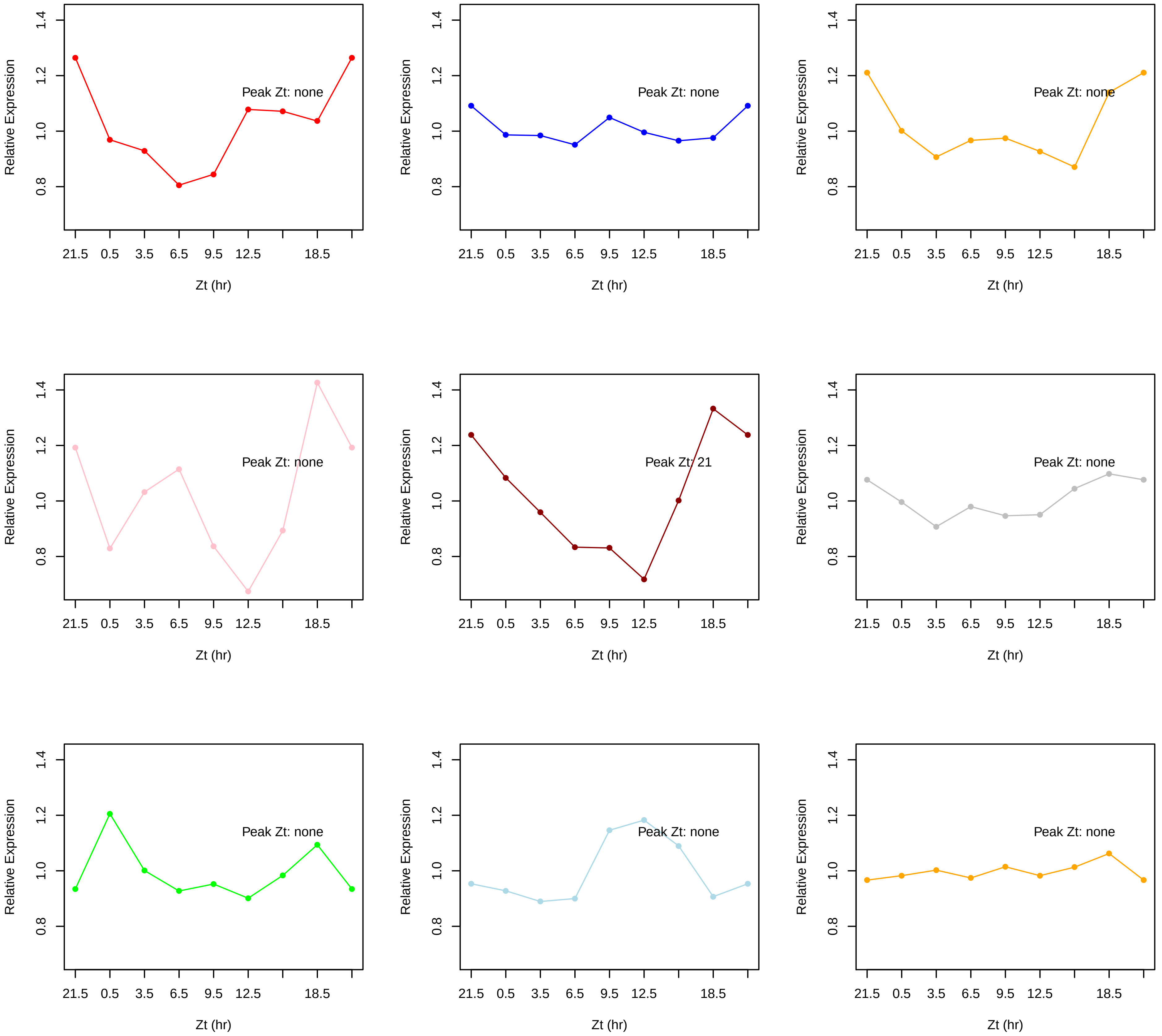

Singeleton: ENSXMAG00000010983 taf15

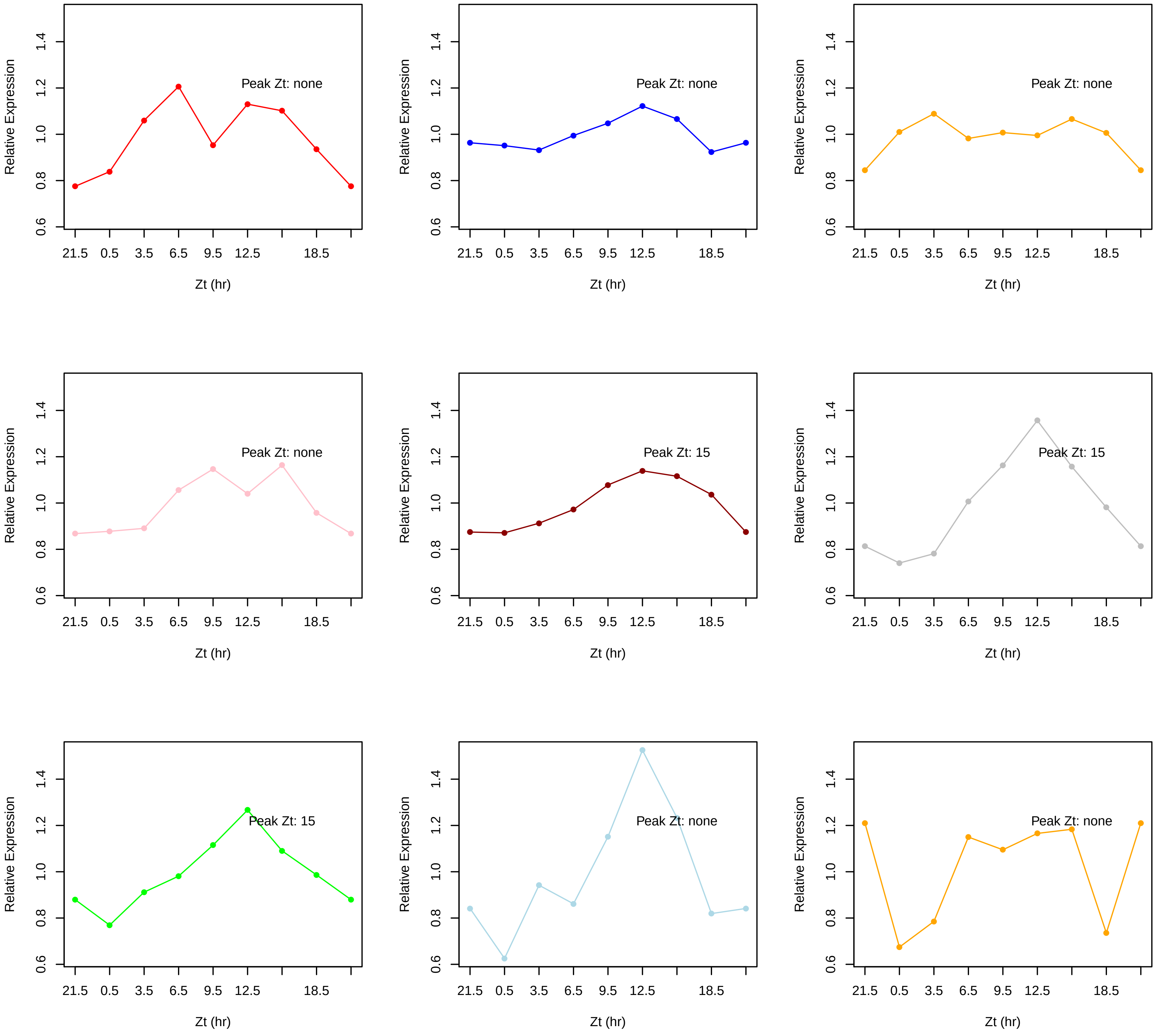

Singeleton: ENSXMAG00000019403

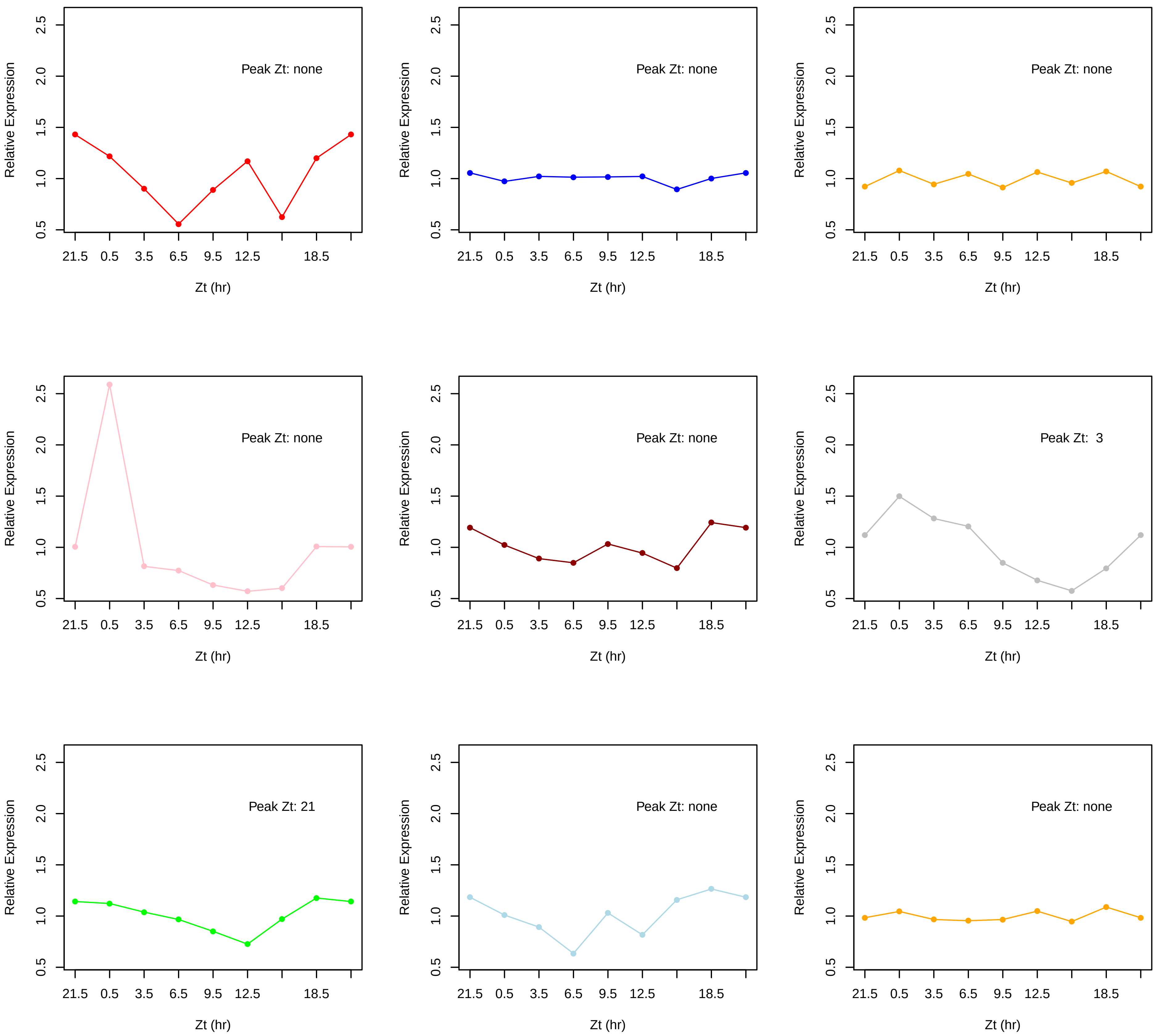

Singeleton: ENSXMAG00000004026 bicdl1

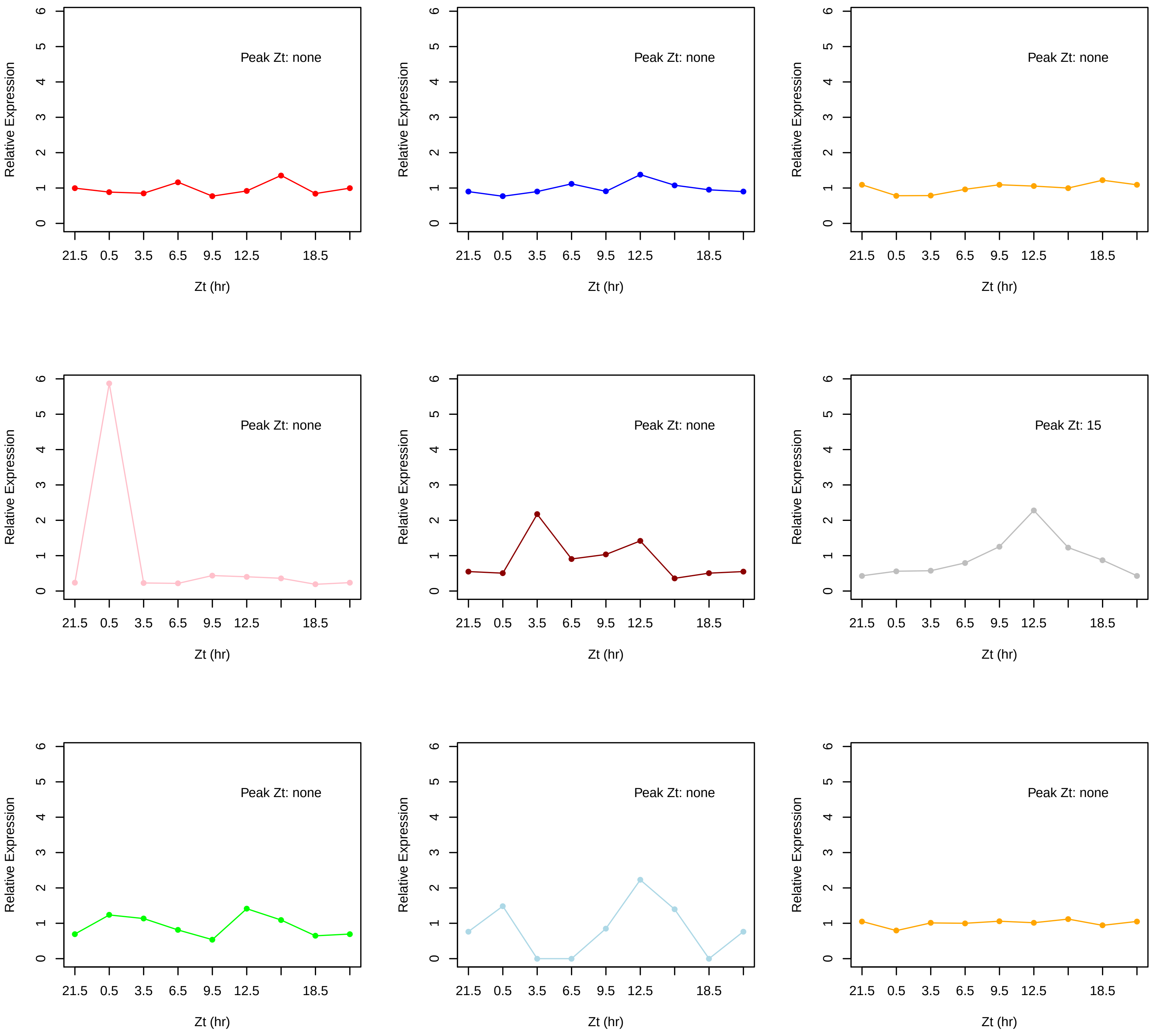

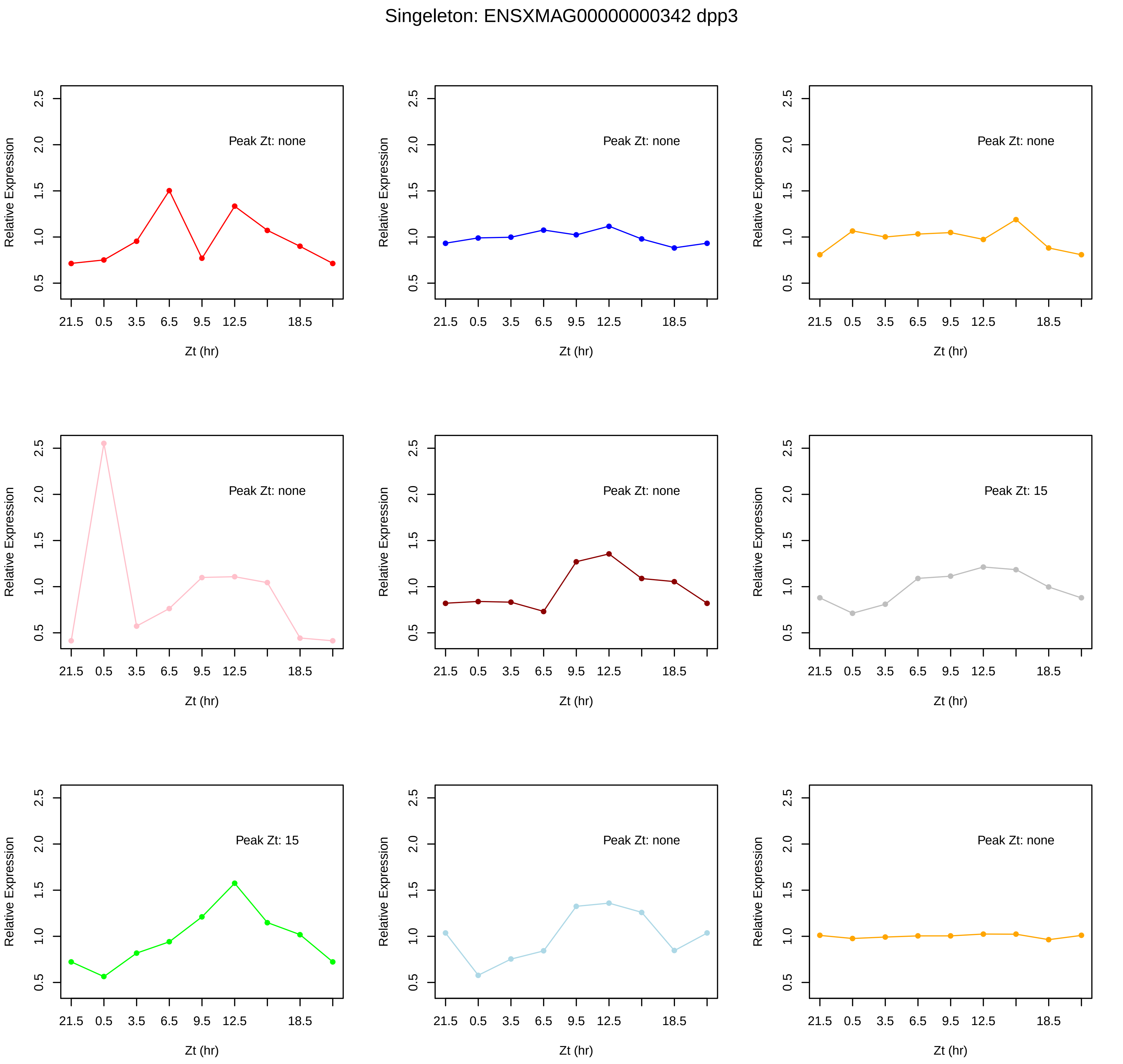

Singeleton: ENSXMAG00000000342 dpp3
Peak Zt: none
Peak Zt: none
Peak Zt: none
Peak Zt: none
Peak Zt: none
Peak Zt: 15
Peak Zt: 15
Peak Zt: none
Peak Zt: none
Relative Expression
Zt (hr)
21.5
0.5
3.5
6.5
9.5
12.5
18.5
0.5
1.0
1.5
2.0
2.5

Singeleton: ENSXMAG00000018609 osbpl5

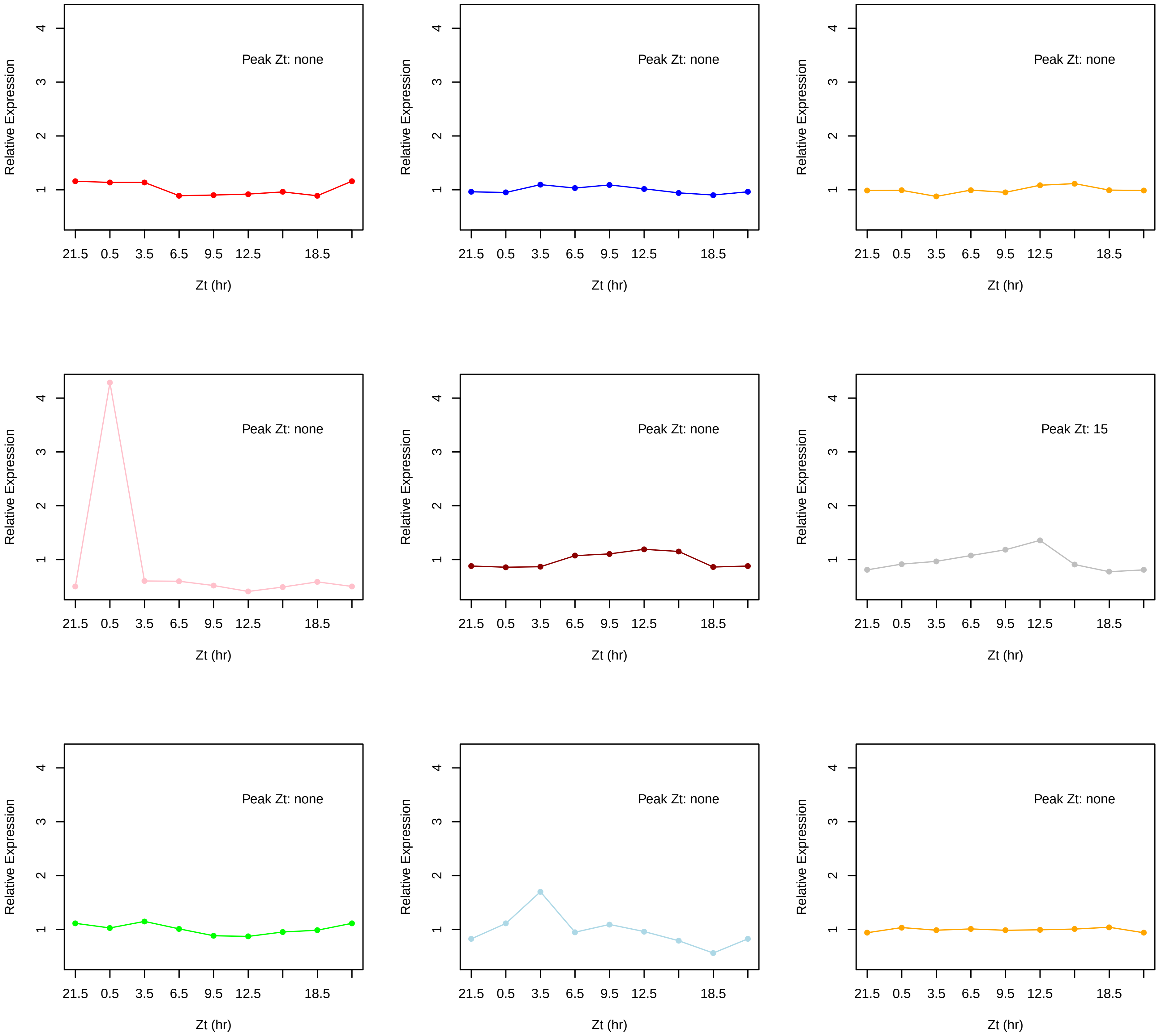

Singeleton: ENSXMAG00000019404 fam172a

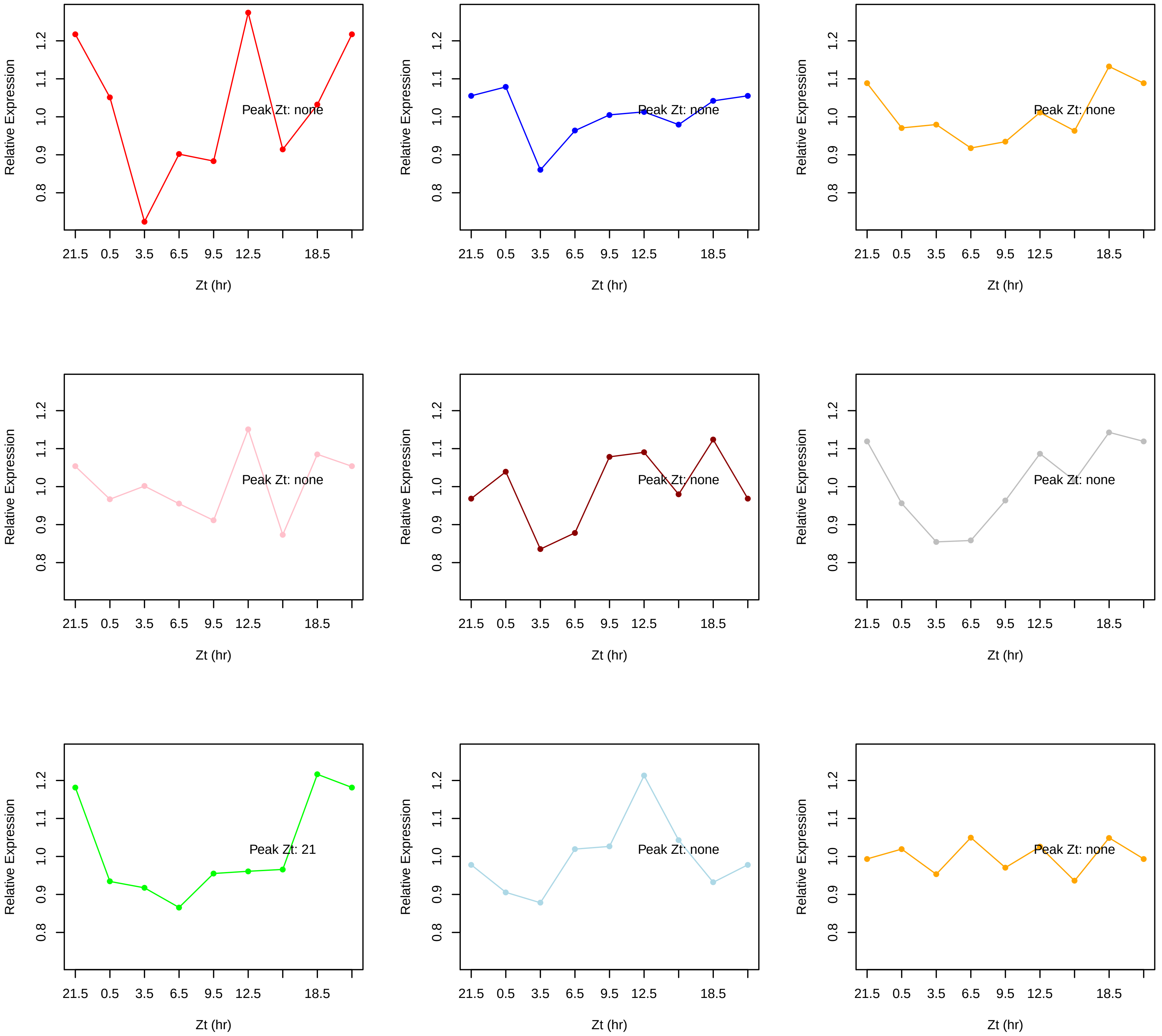

Singeleton: ENSXMAG00000001178 MIPEP

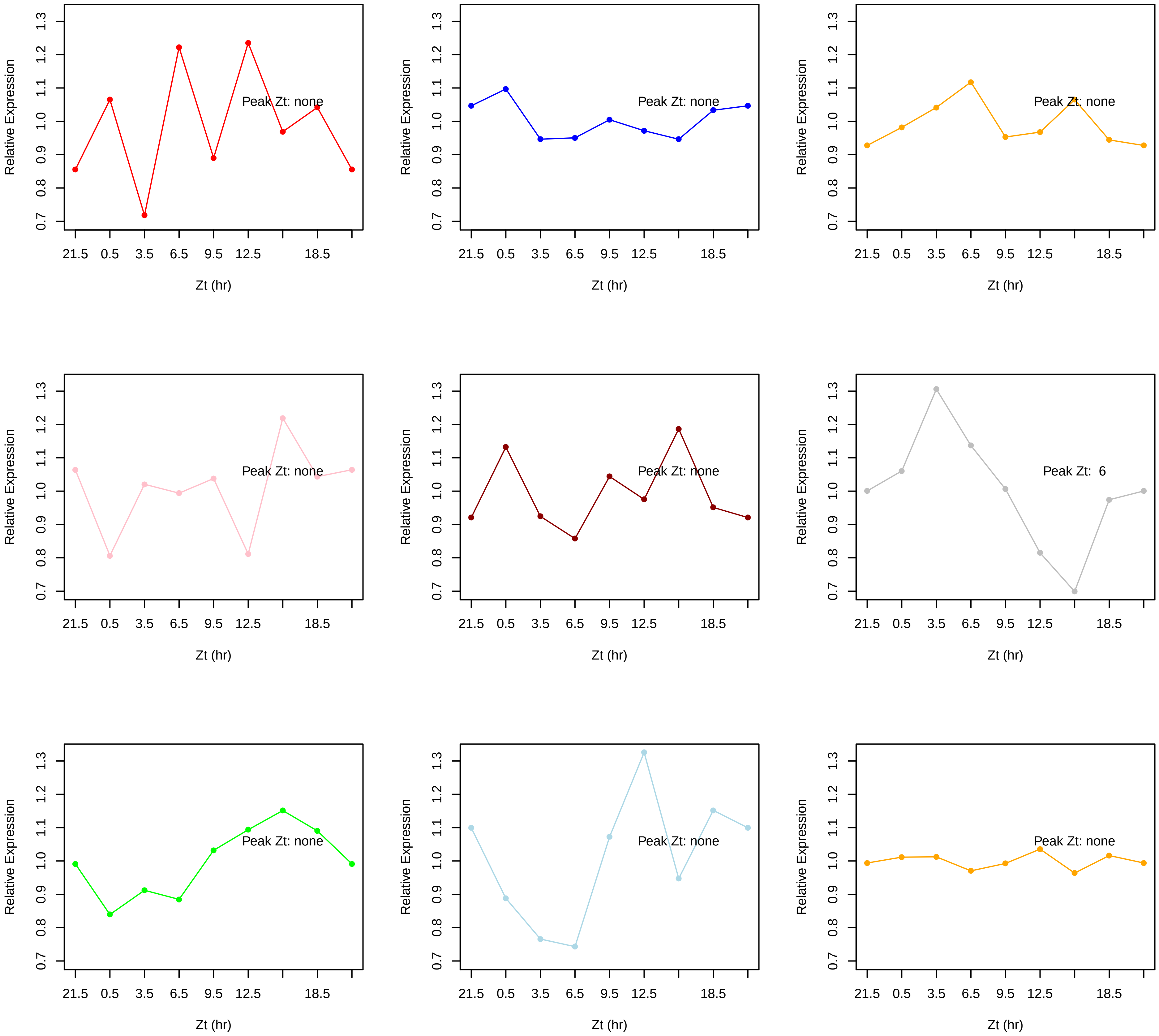

Singeleton: ENSXMAG00000004772 chrna4b

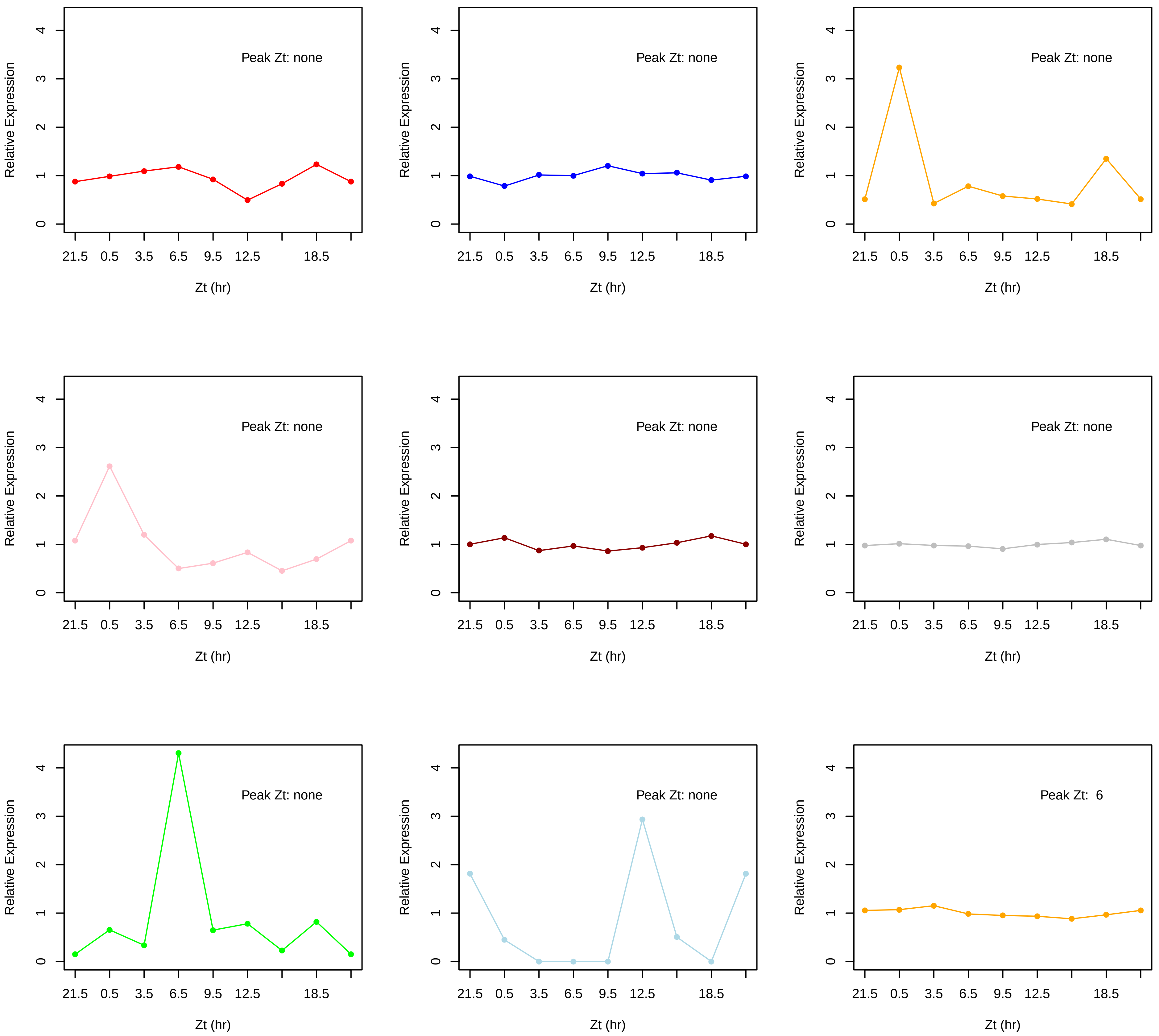

Singeleton: ENSXMAG00000010634 trappc3

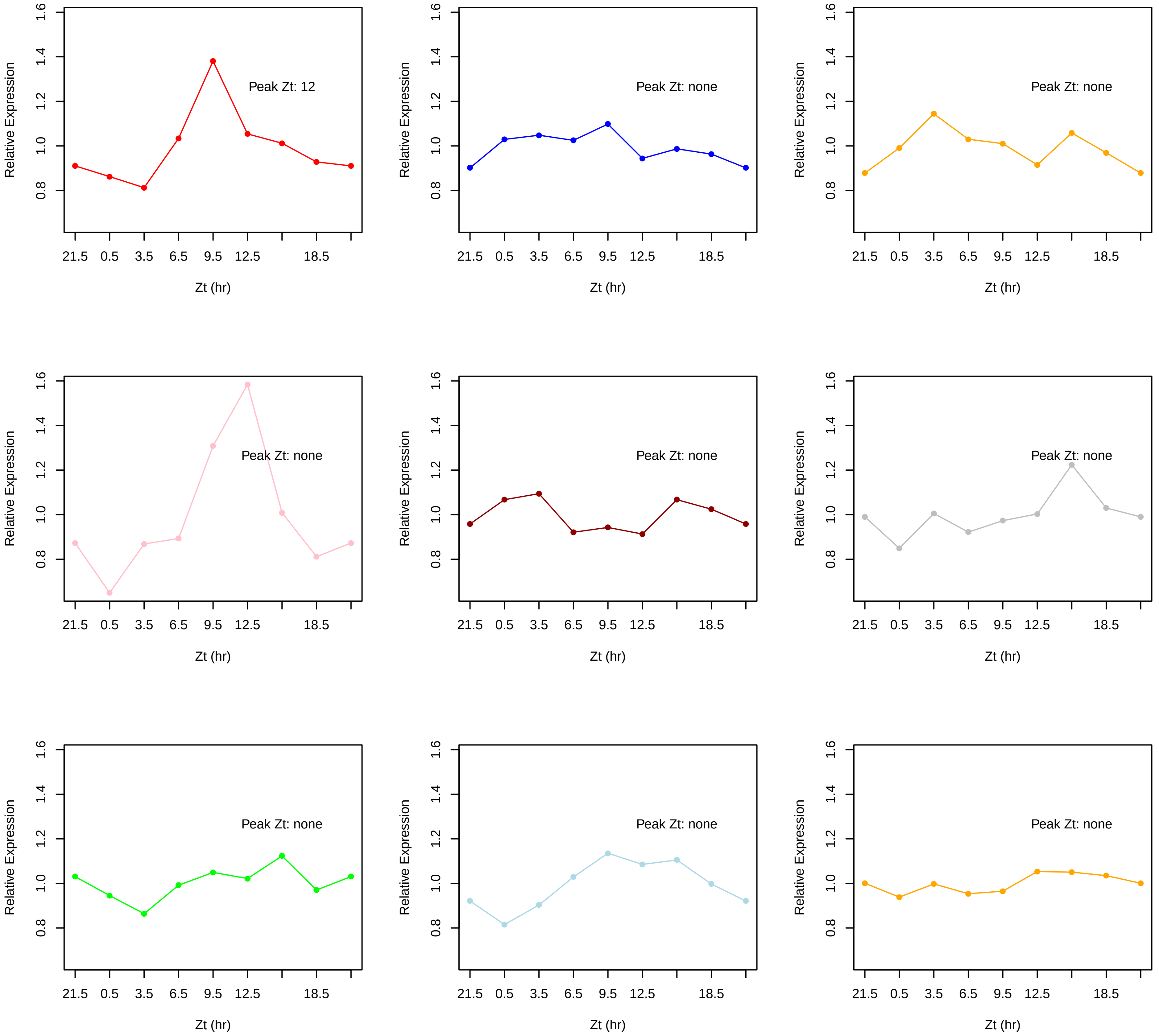

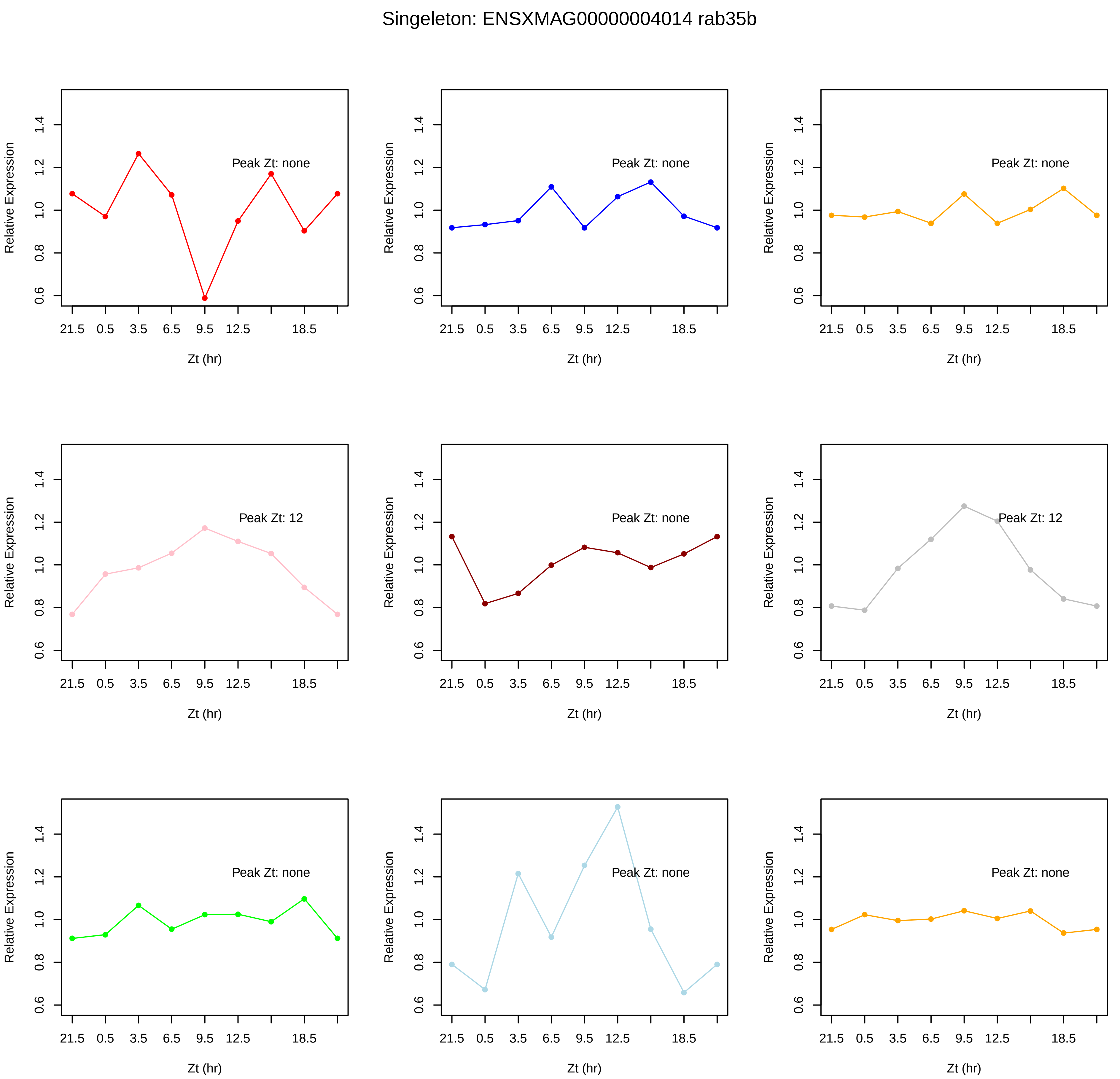
Singeleton: ENSXMAG00000004014 rab35b
Peak Zt: none
Peak Zt: none
Peak Zt: none
Peak Zt: 12
Peak Zt: none
Peak Zt: 12
Peak Zt: none
Peak Zt: none
Peak Zt: none
Relative Expression
Zt (hr)
21.5
0.5
3.5
6.5
9.5
12.5
18.5
0.6
0.8
1.0
1.2
1.4

Singeleton: ENSXMAG00000017399 twf1a

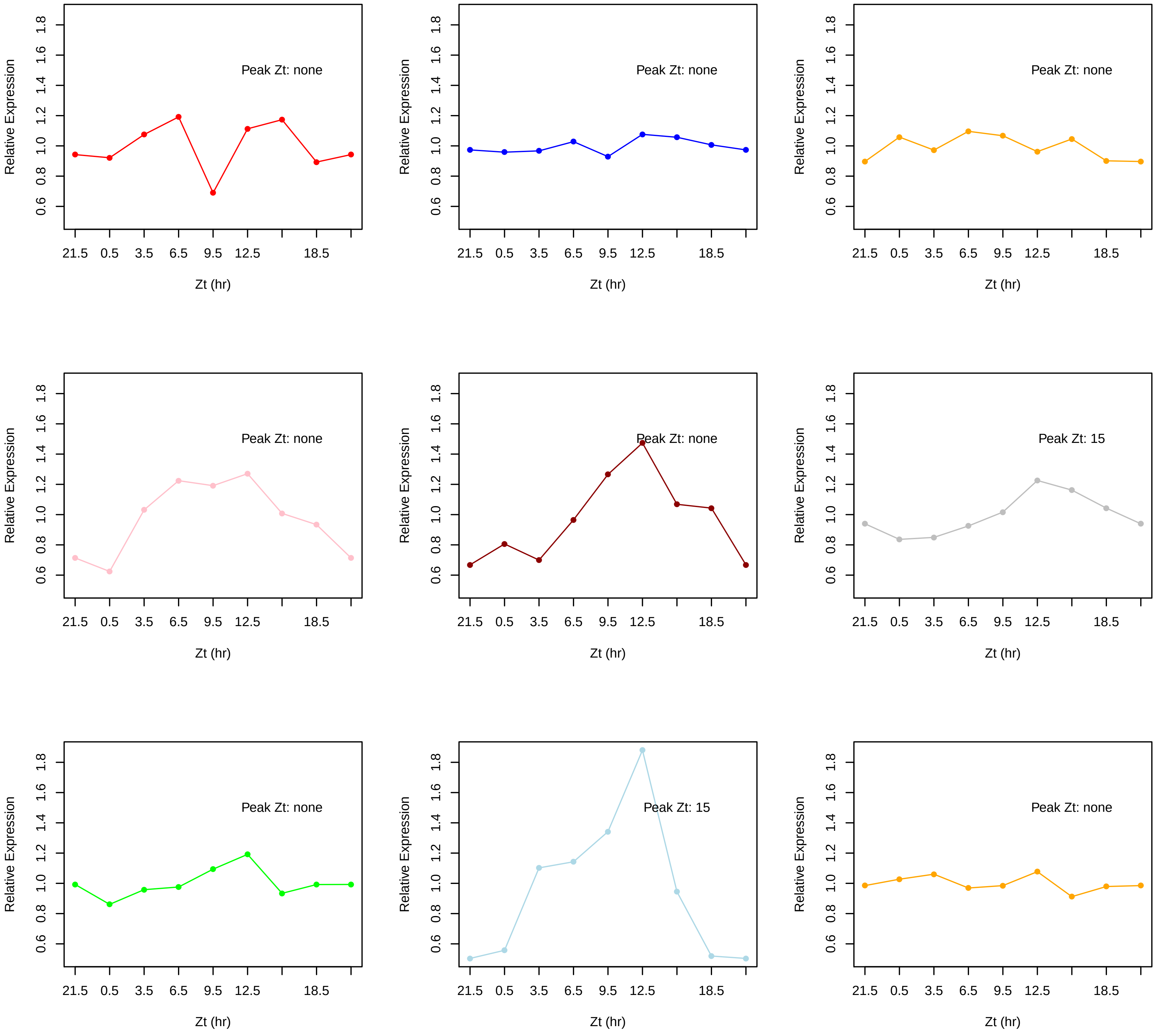

Singeleton: ENSXMAG00000010967 porb

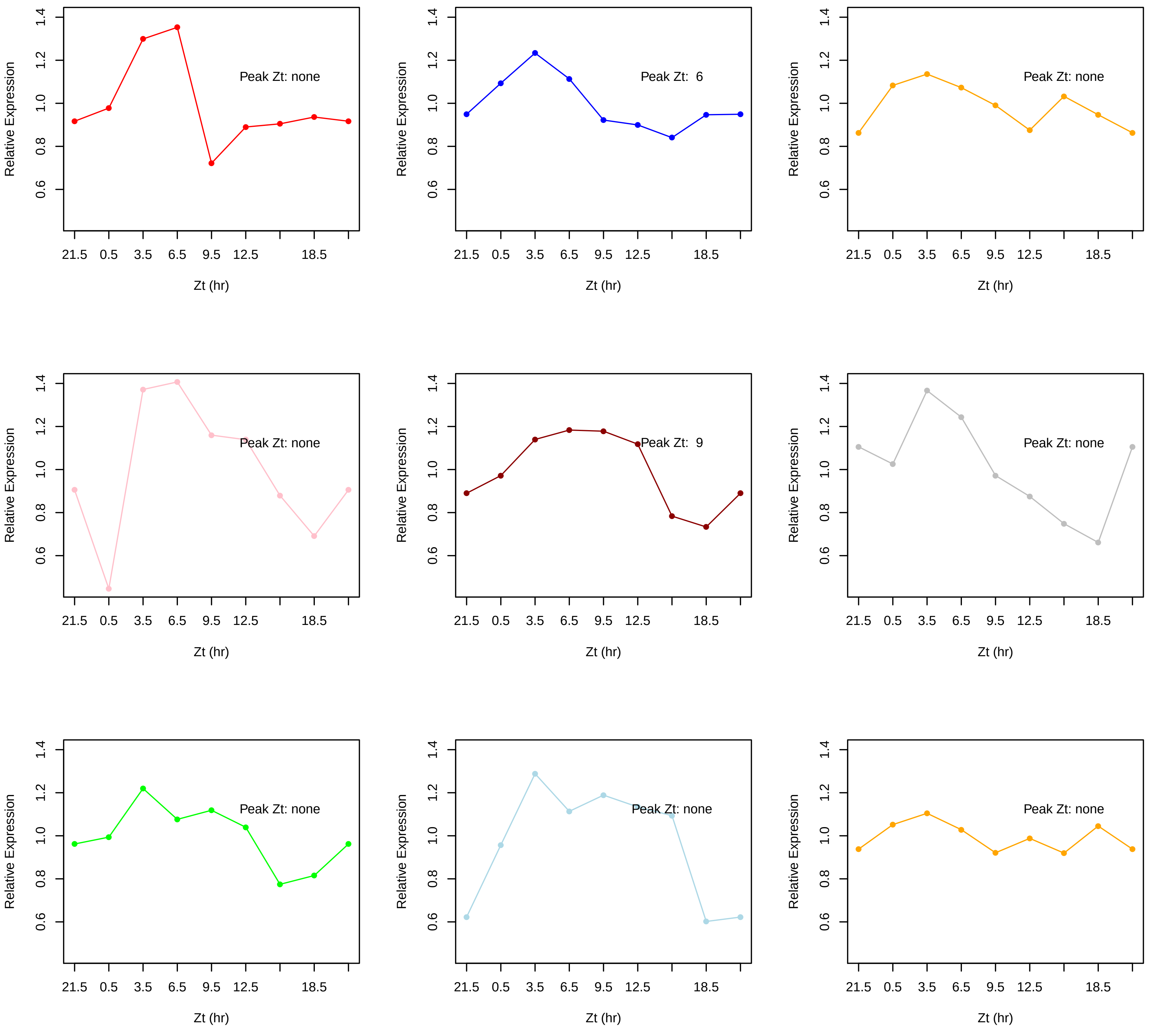

Singleton: ENSXMAG00000002998 pdcd5

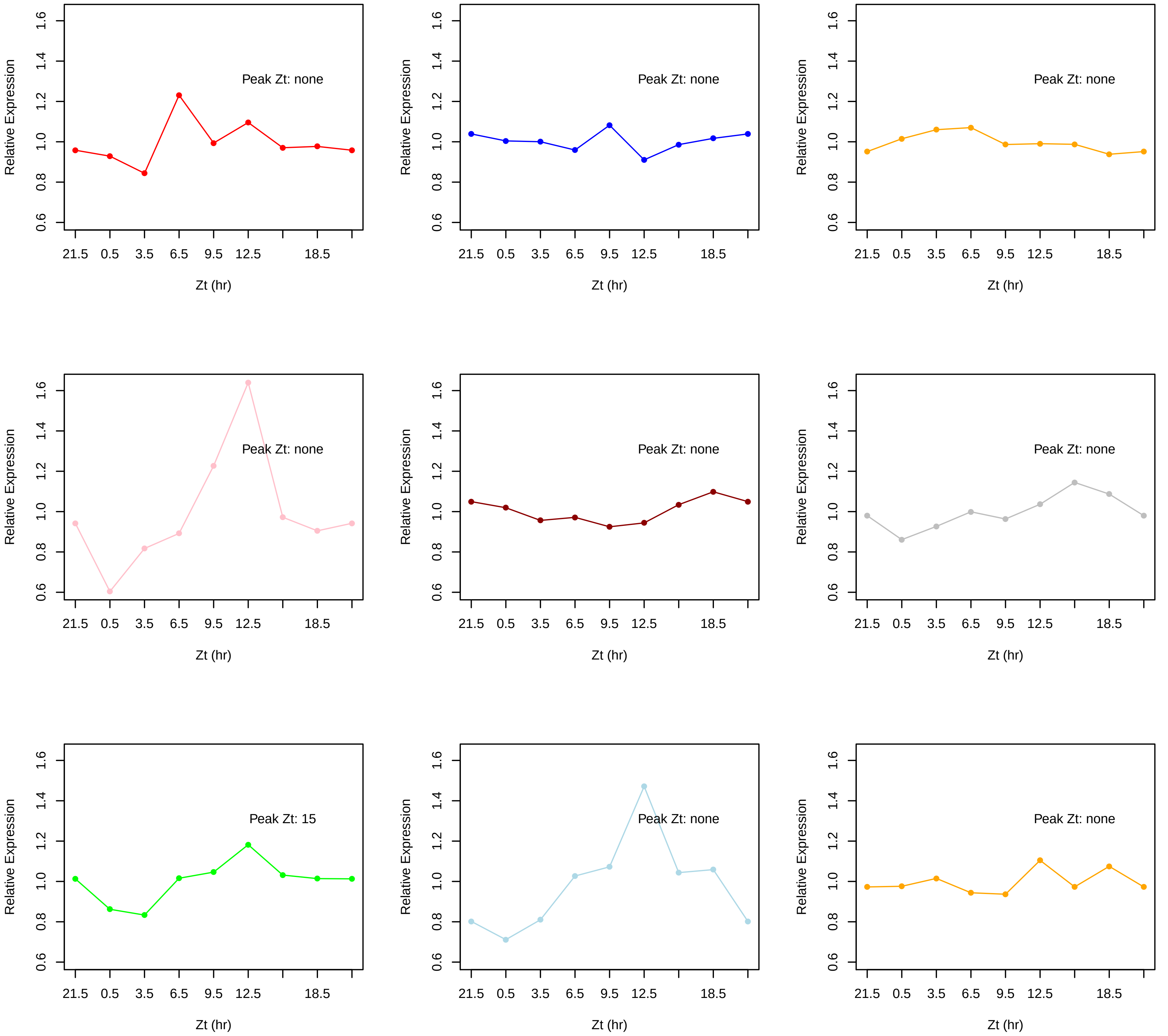

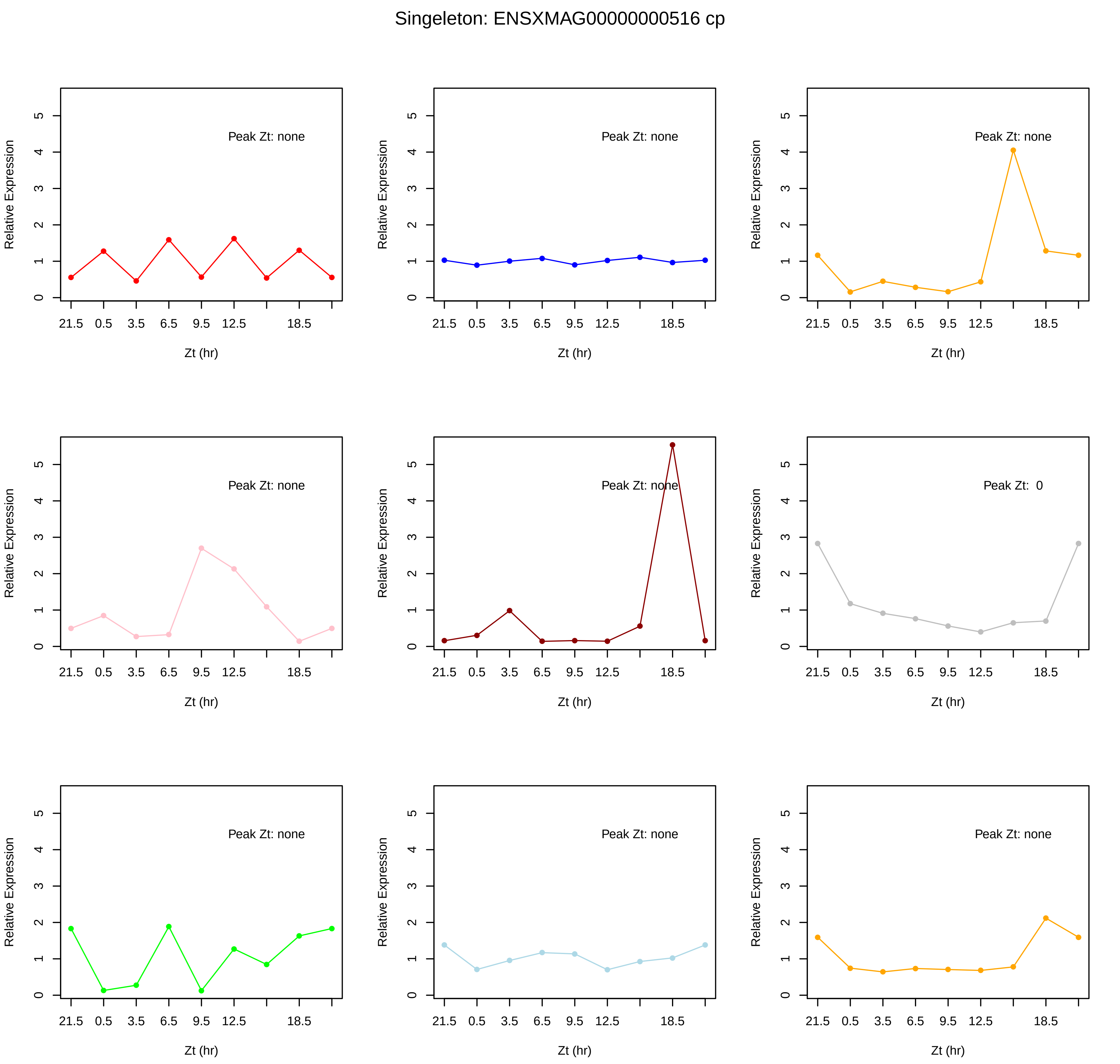
Singeleton: ENSXMAG00000000516 cp
Peak Zt: none
Peak Zt: none
Peak Zt: none
Peak Zt: none
Peak Zt: none
Peak Zt: 0
Peak Zt: none
Peak Zt: none
Peak Zt: none
Relative Expression
Zt (hr)
21.5
0.5
3.5
6.5
9.5
12.5
18.5

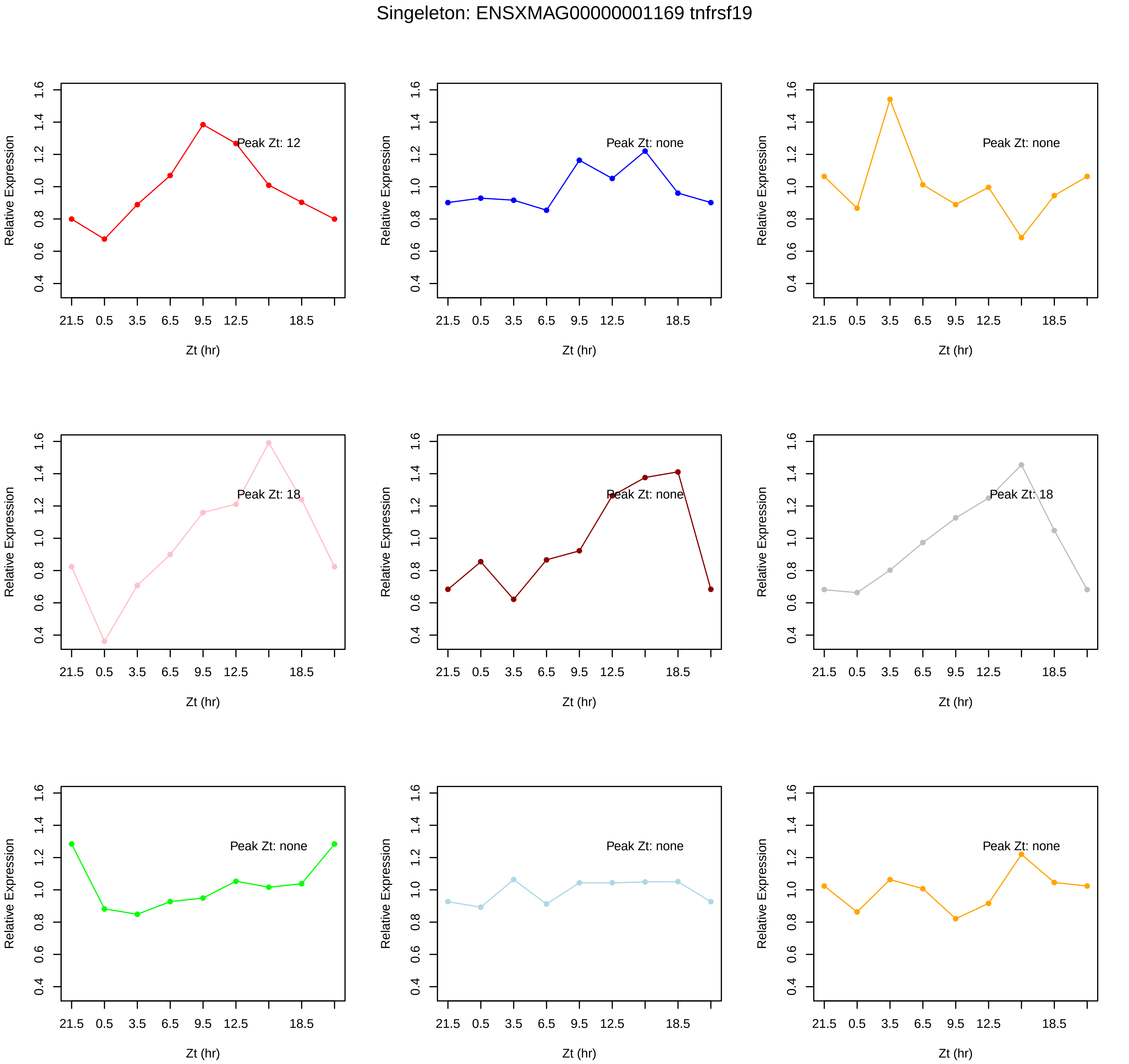
Singeleton: ENSXMAG00000001169 tnfrsf19
Peak Zt: 12
Peak Zt: none
Peak Zt: none
Peak Zt: 18
Peak Zt: none
Peak Zt: 18
Peak Zt: none
Peak Zt: none
Peak Zt: none
Relative Expression
Zt (hr)
21.5 0.5 3.5 6.5 9.5 12.5 18.5
0.4 0.6 0.8 1.0 1.2 1.4 1.6

Singleton: ENSXMAG00000024776 si:dkey−205h13.1

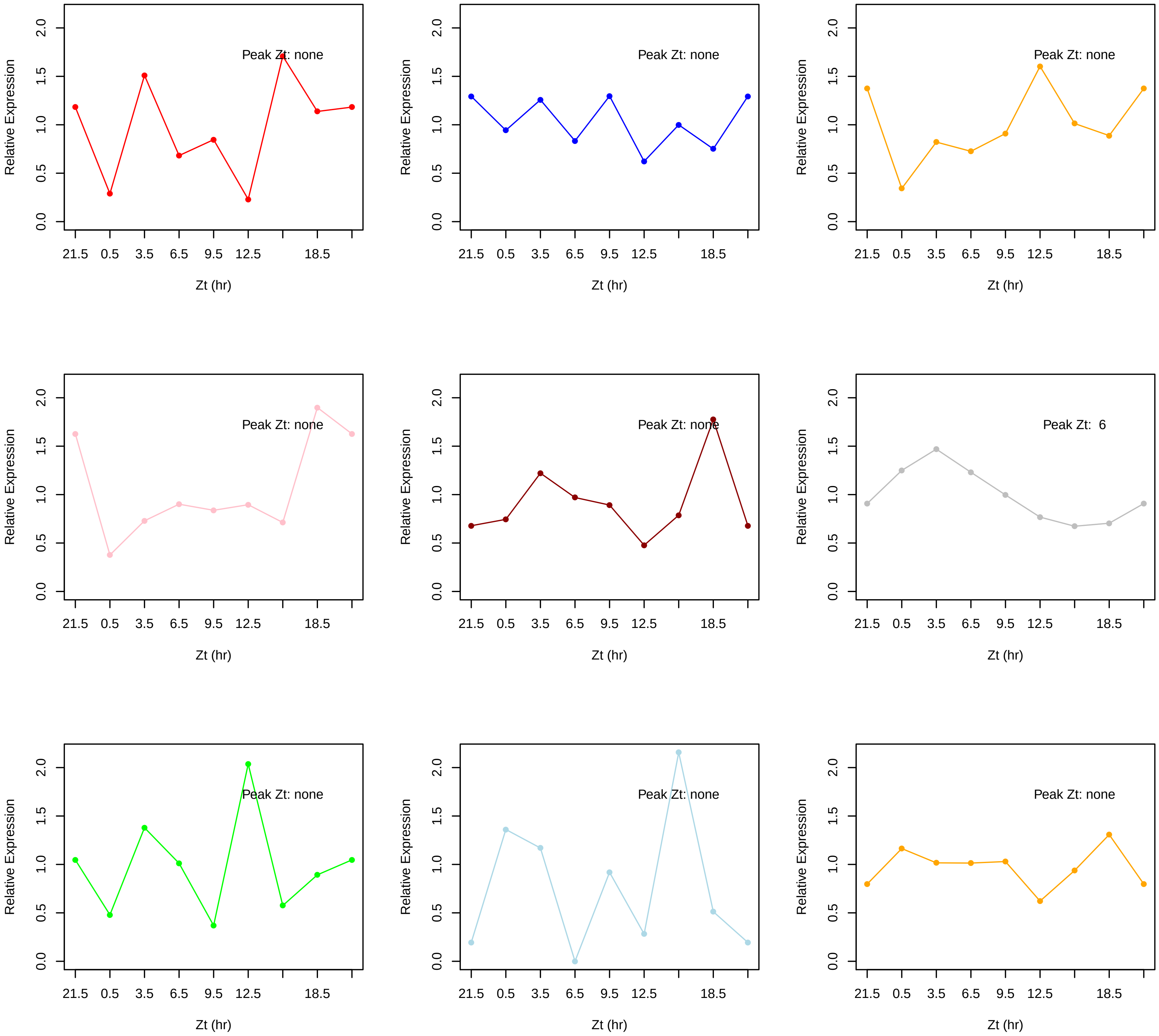

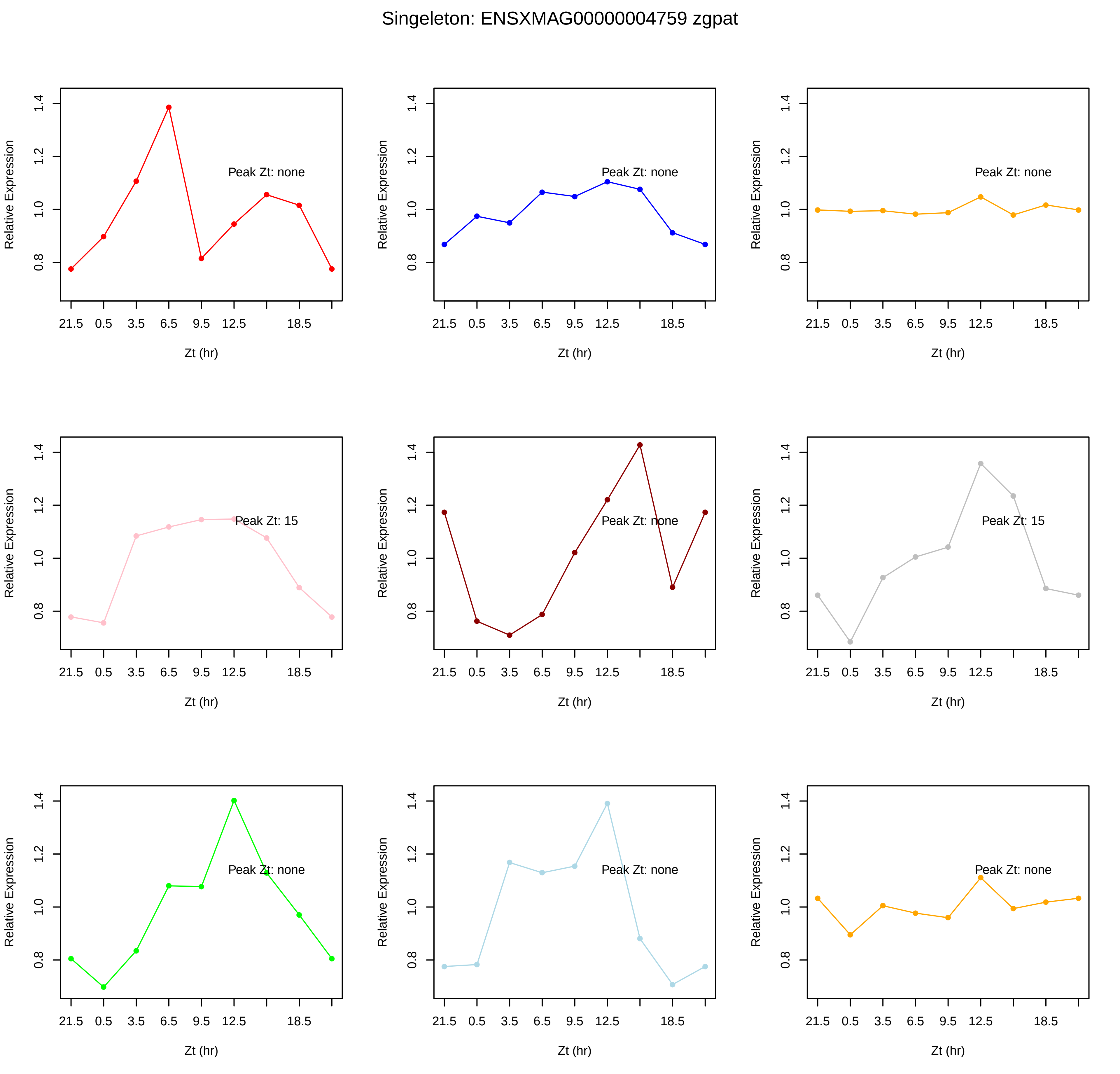
Singeleton: ENSXMAG00000004759 zgpat
Relative Expression
Zt (hr)
Peak Zt: none
Peak Zt: none
Peak Zt: none
Peak Zt: 15
Peak Zt: none
Peak Zt: 15
Peak Zt: none
Peak Zt: none
Peak Zt: none

Singeleton: ENSXMAG00000010324 slc22a18

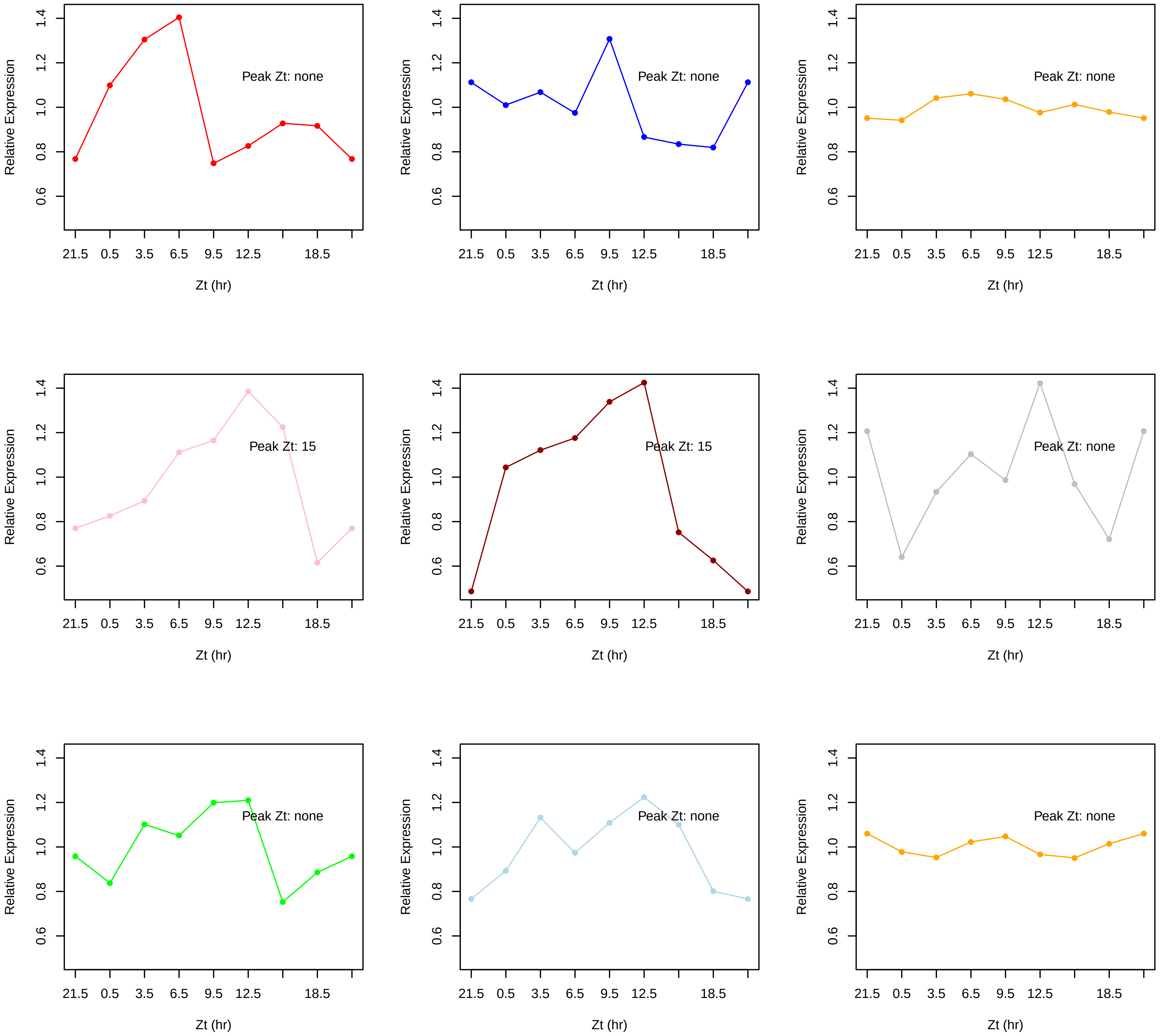

Singeleton: ENSXMAG00000009654 get4

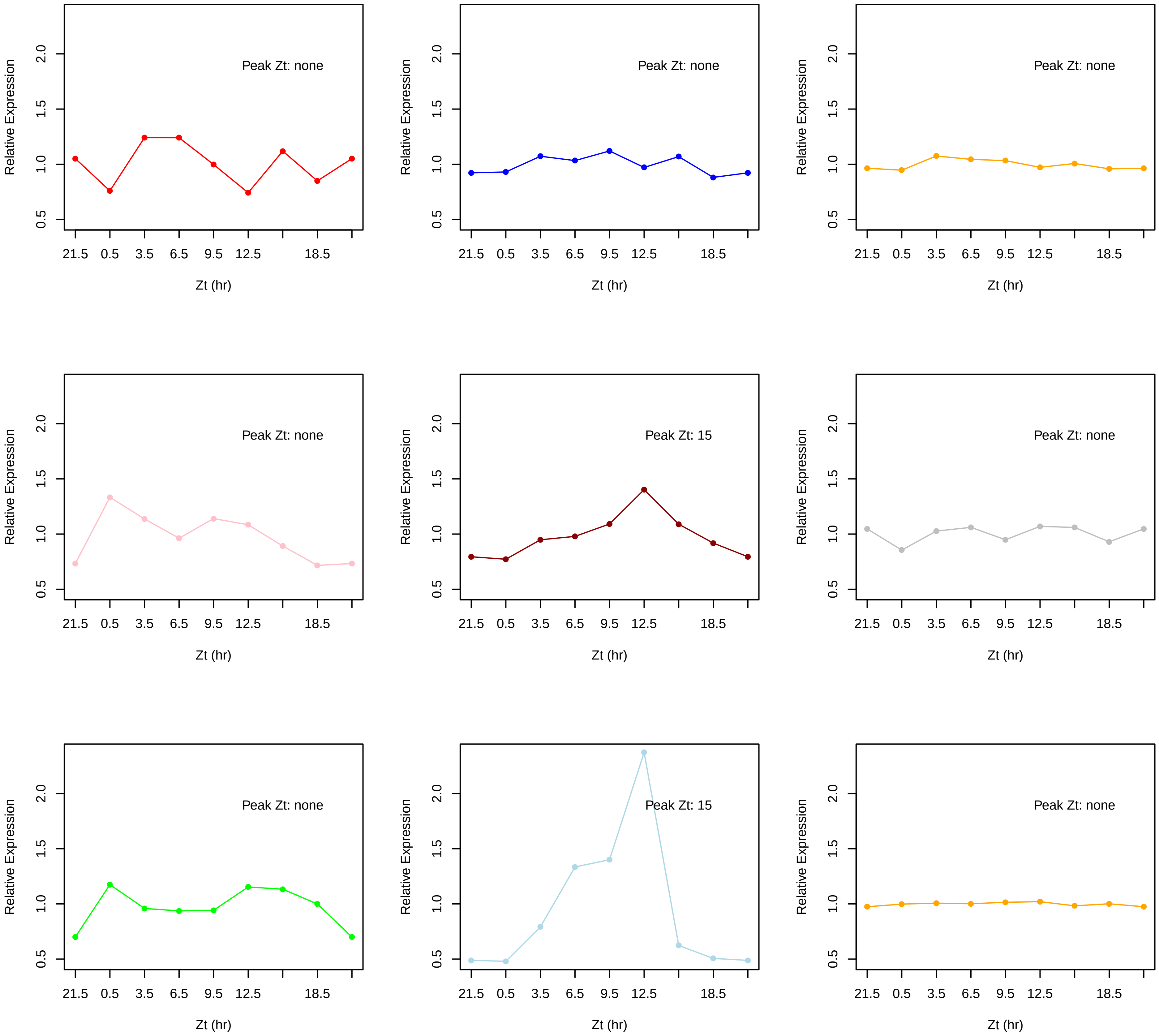

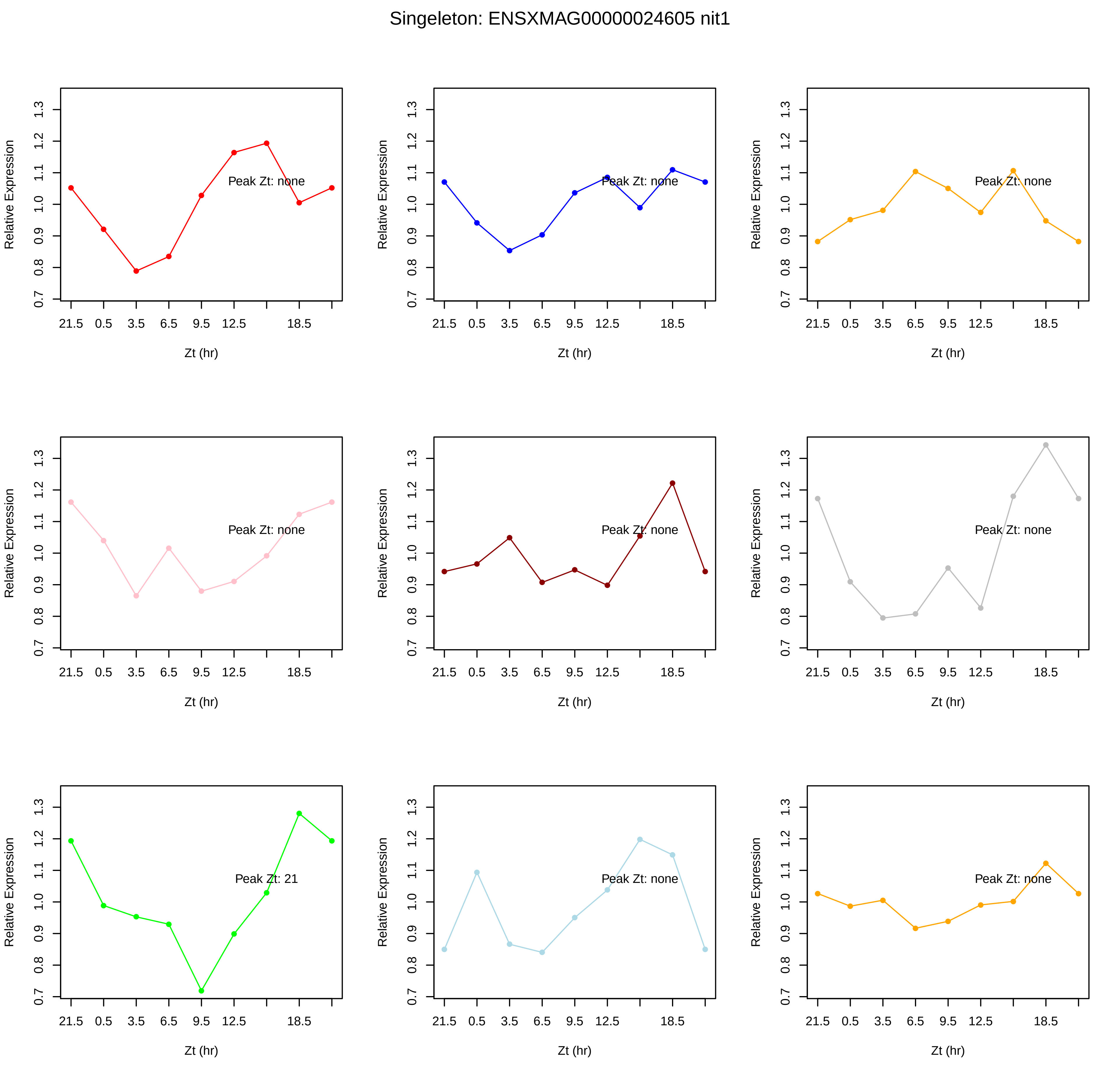

Singeleton: ENSXMAG00000024605 nit1
Relative Expression
Zt (hr)
21.5
0.5
3.5
6.5
9.5
12.5
18.5
0.7
0.8
0.9
1.0
1.1
1.2
1.3
Peak Zt: none
Peak Zt: 21

Singleton: ENSXMAG00000007139 f5

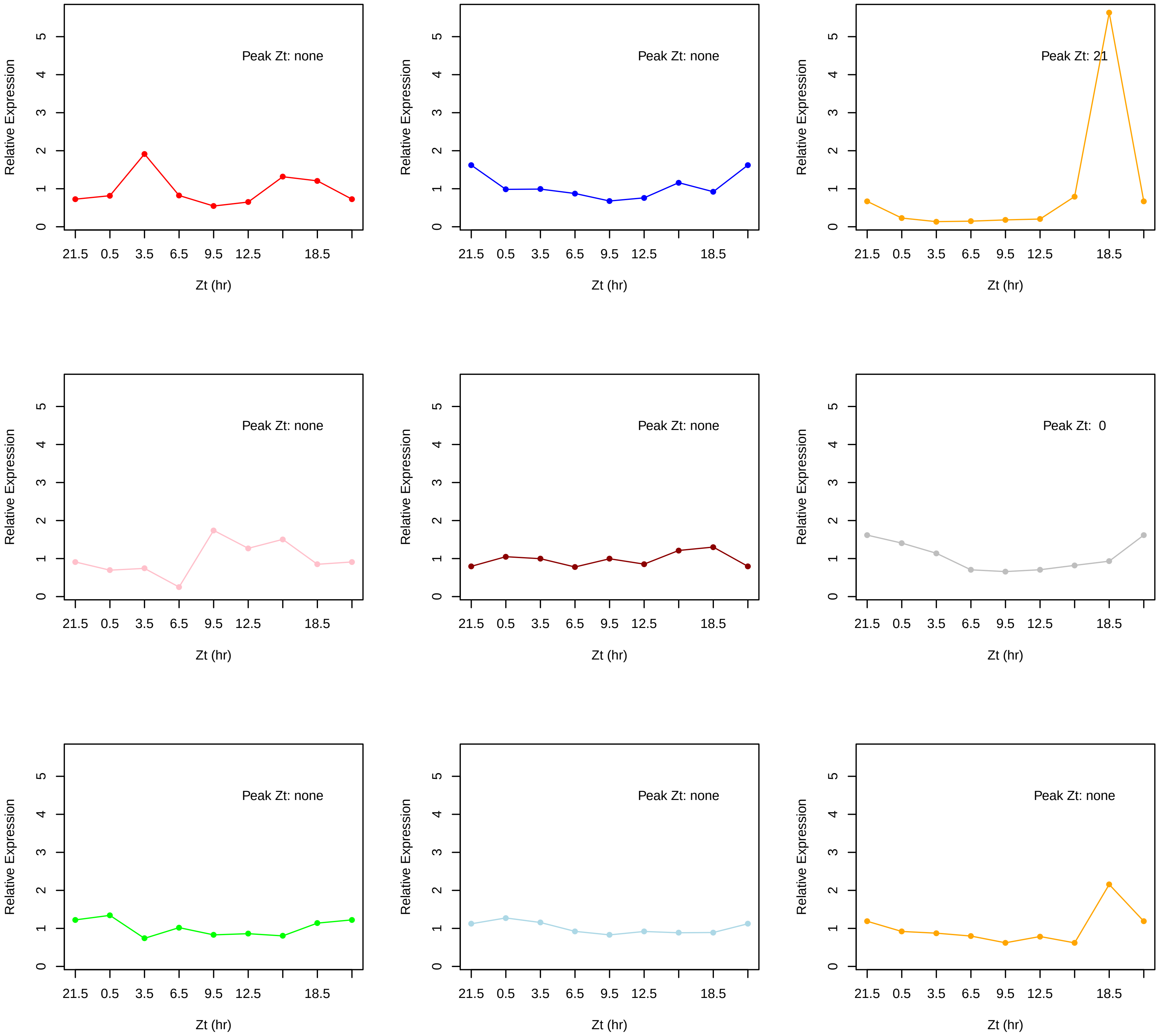

Singleton: ENSXMAG00000002627 arfrp1

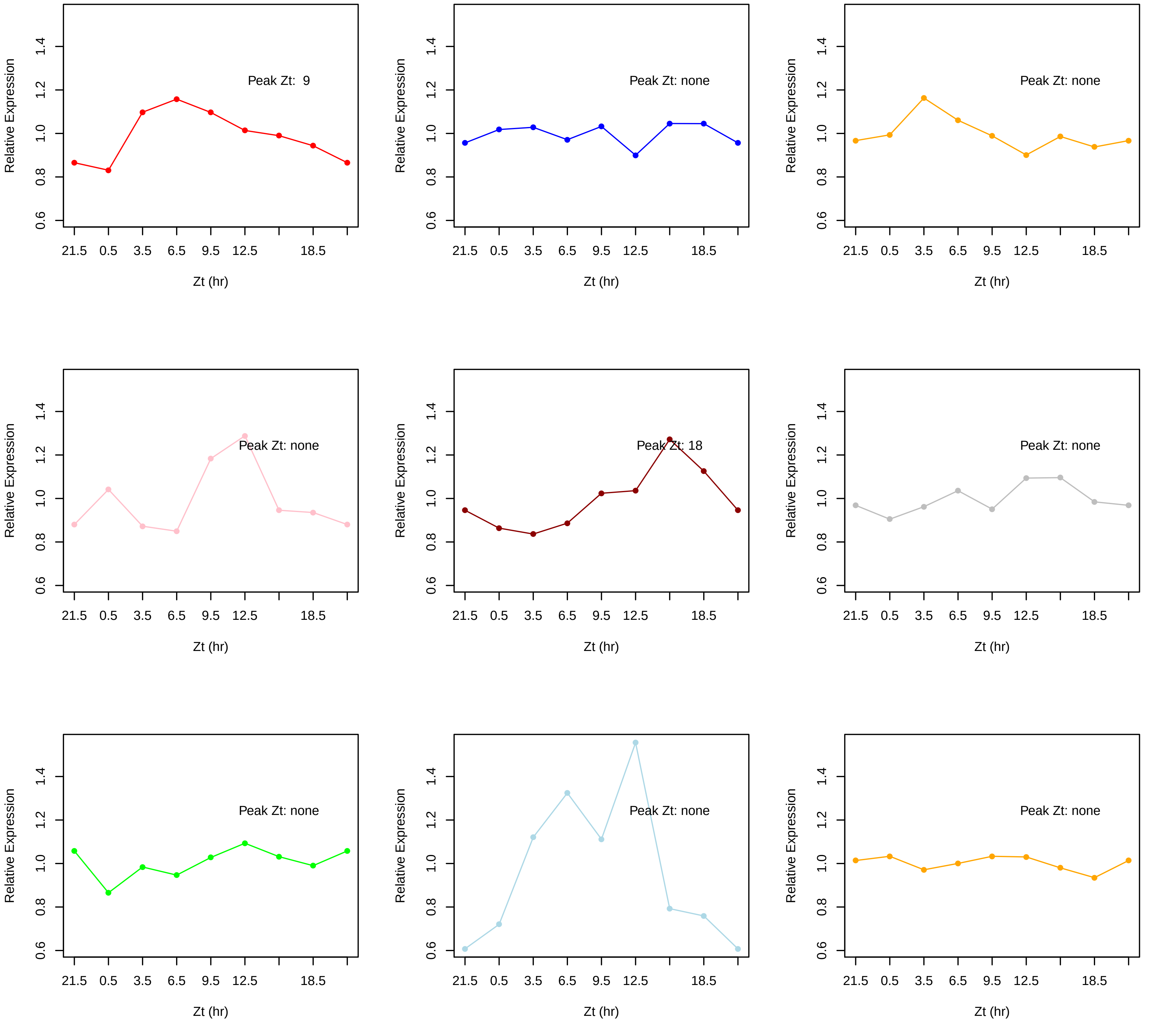

Singeleton: ENSXMAG00000029859 tm4sf18

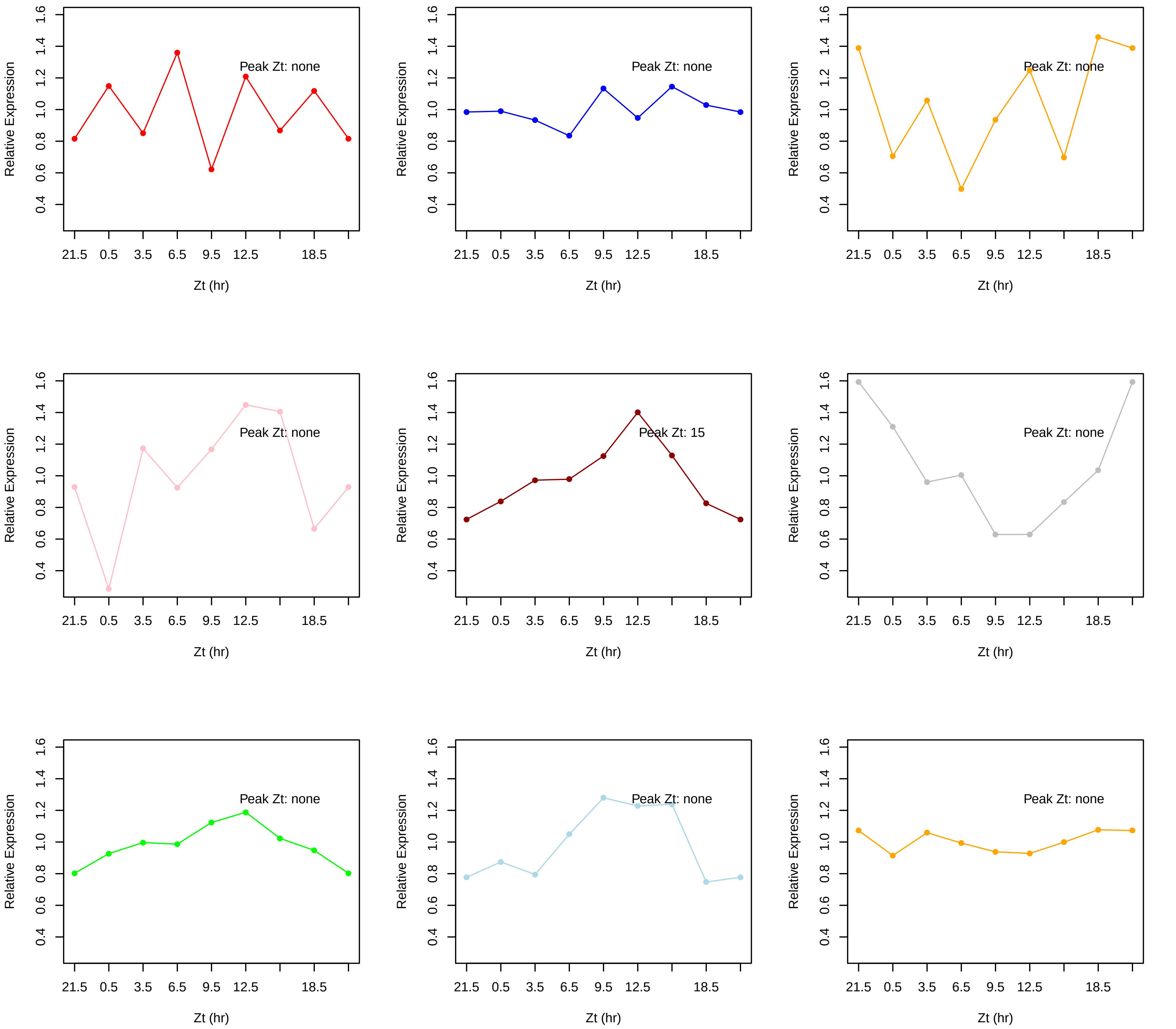

Singeleton: ENSXMAG00000026908

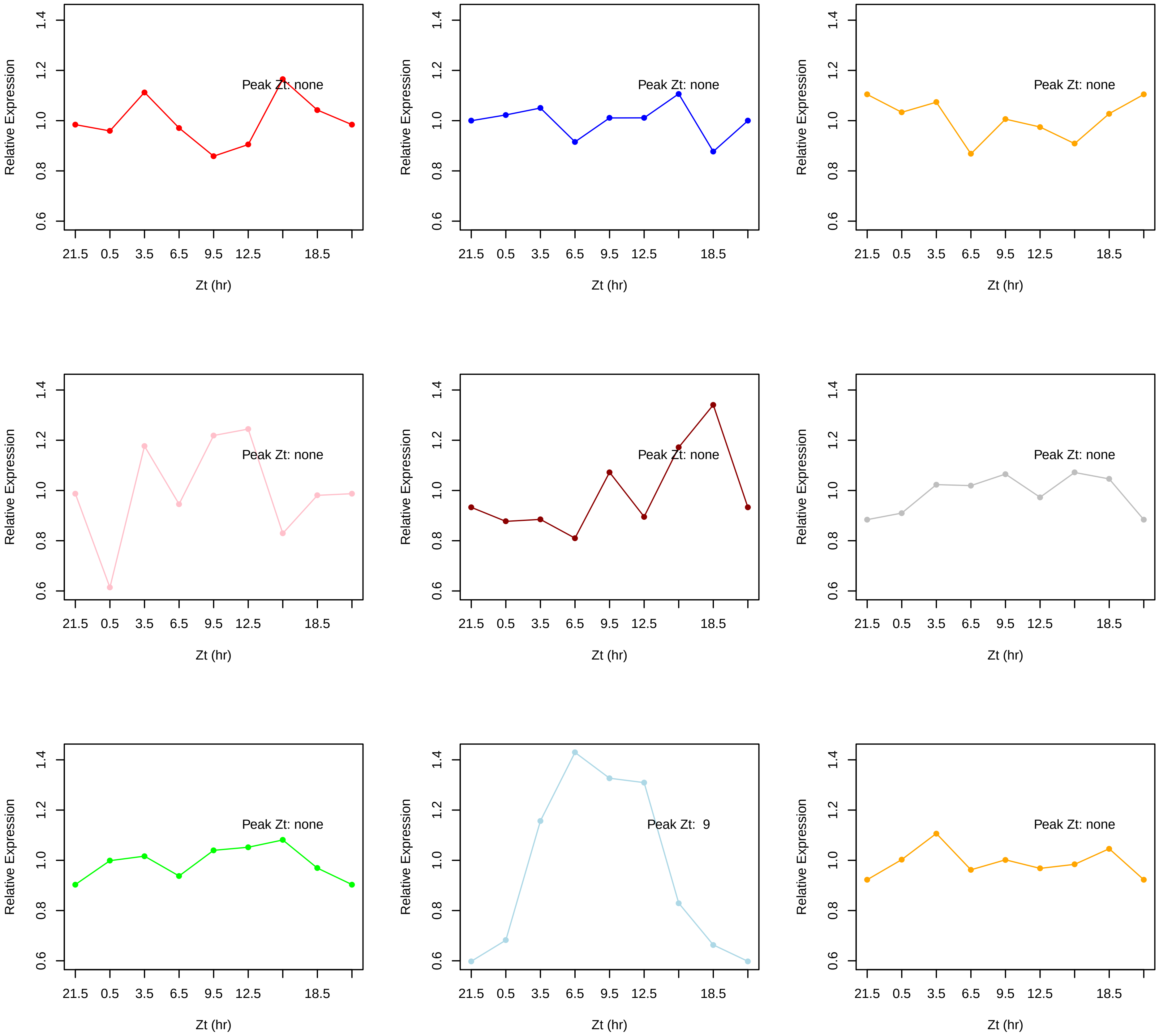

Singeleton: ENSXMAG00000023905 arrdc1b

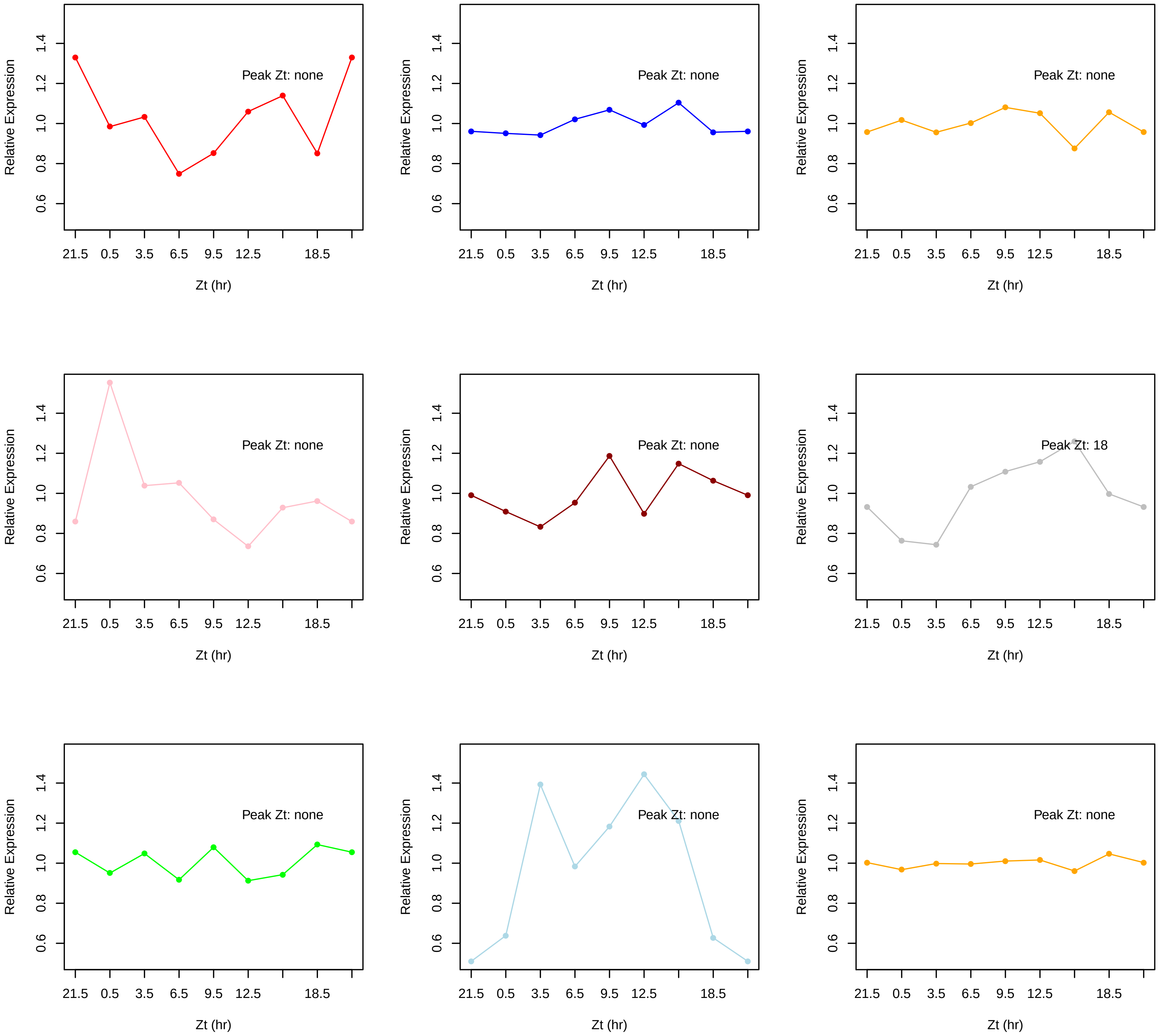

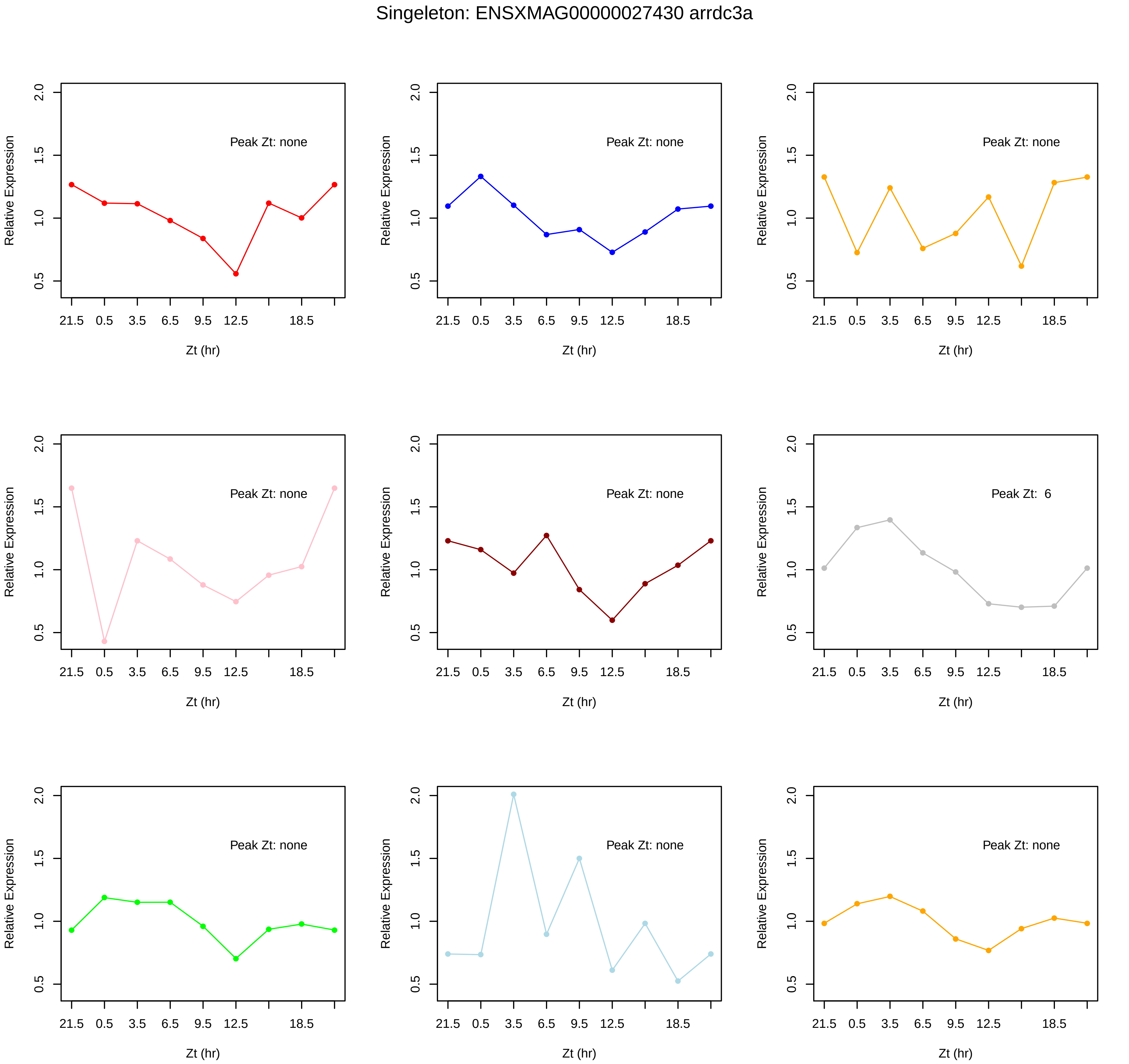
Singeleton: ENSXMAG00000027430 arrdc3a
Peak Zt: none
Peak Zt: none
Peak Zt: none
Peak Zt: none
Peak Zt: none
Peak Zt: 6
Peak Zt: none
Peak Zt: none
Peak Zt: none
Relative Expression
Zt (hr)
21.5
0.5
3.5
6.5
9.5
12.5
18.5
0.5
1.0
1.5
2.0

Singleton: ENSXMAG00000015925 ptbp1a

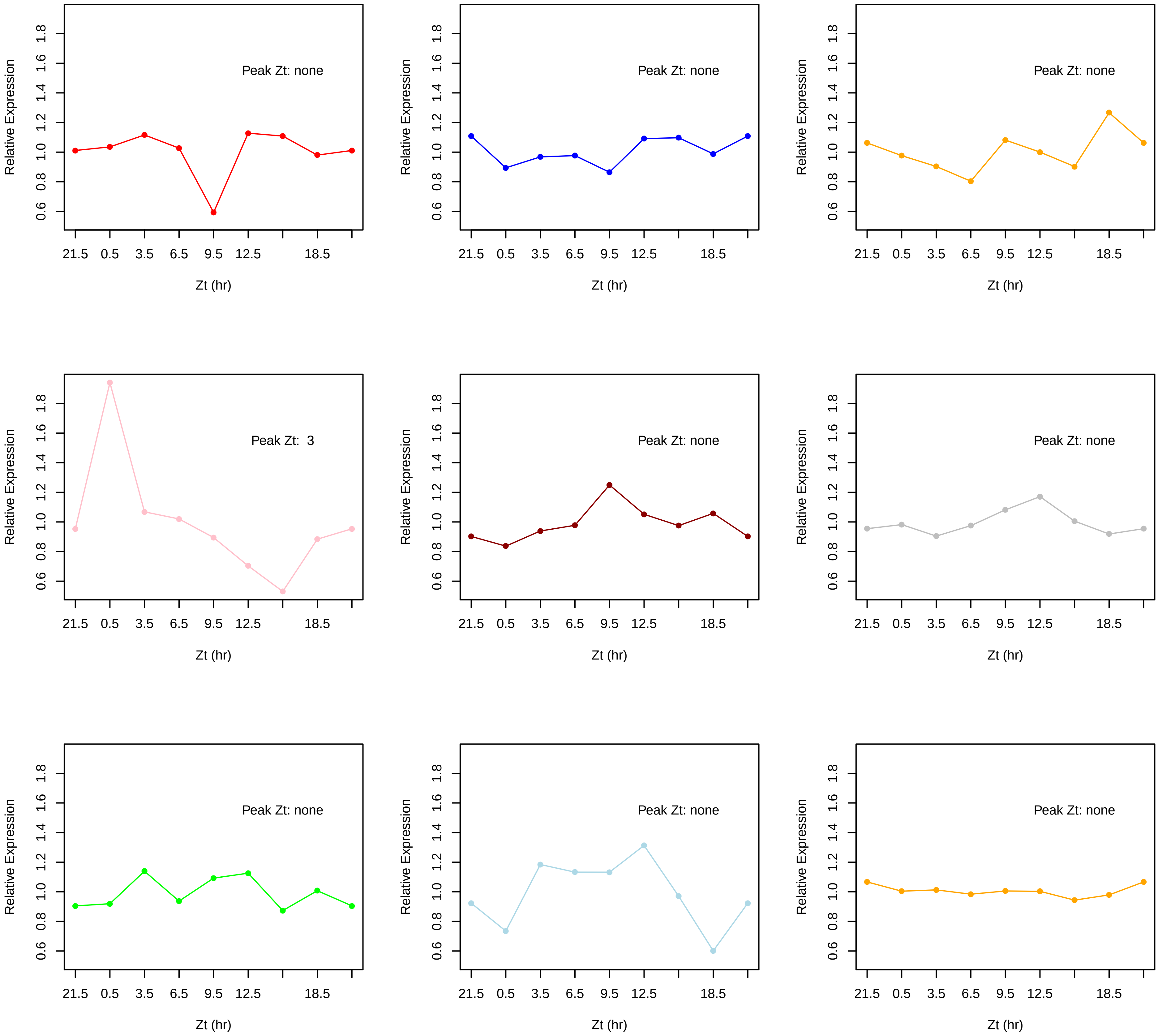

Singeleton: ENSXMAG00000001141 sgcg

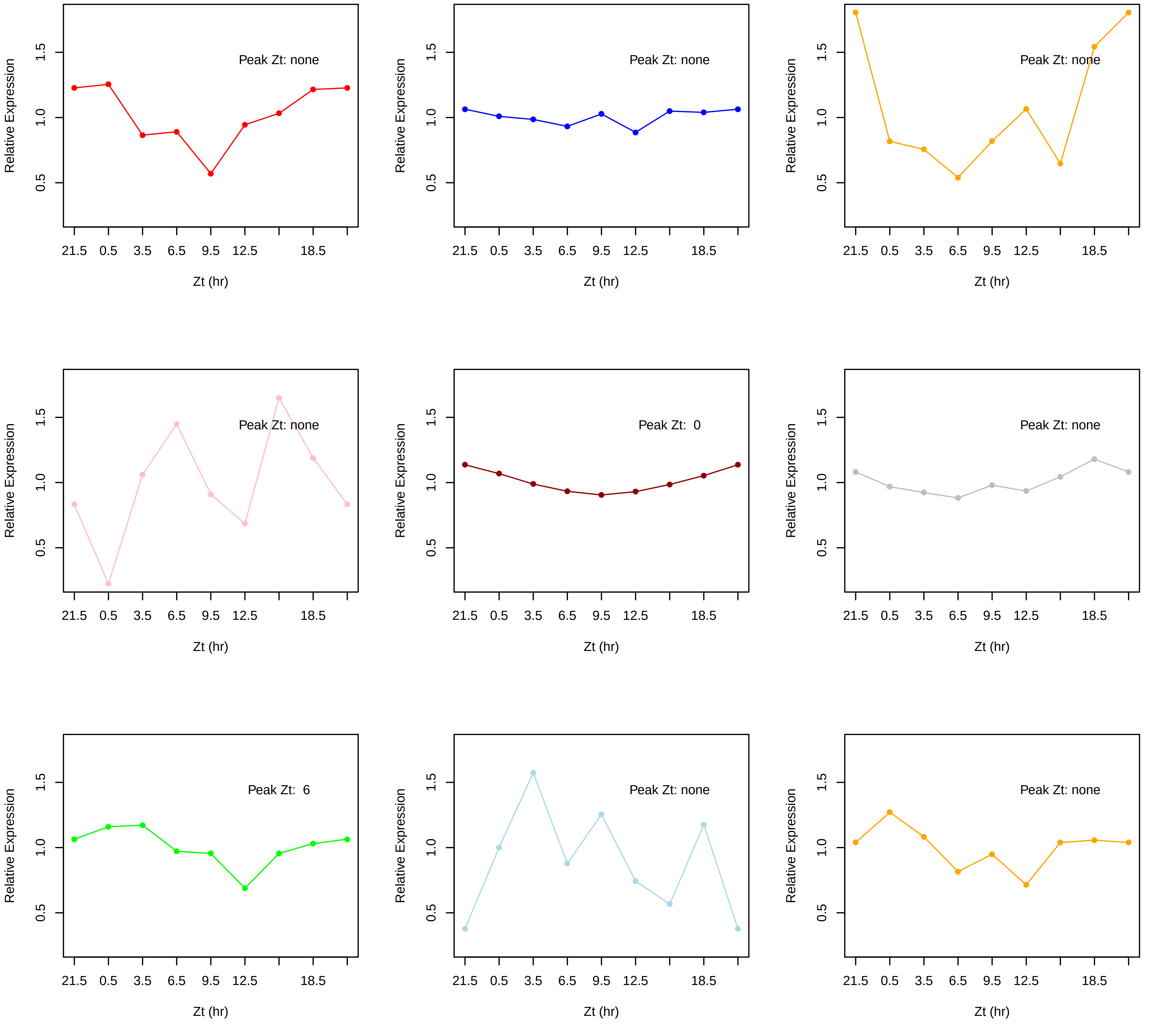

Singeleton: ENSXMAG00000021244 cables1

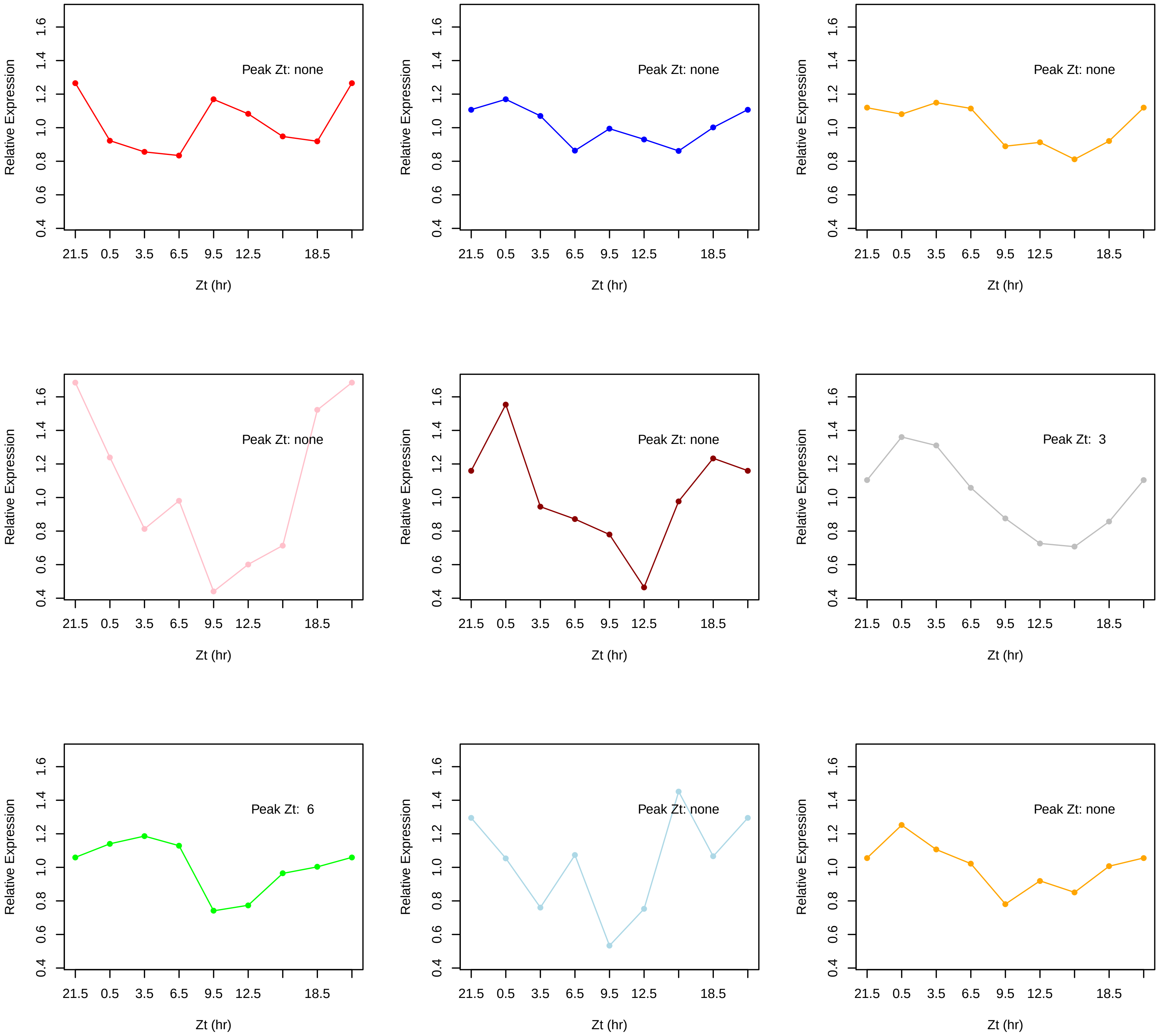

Singeleton: ENSXMAG00000000223 adgrv1

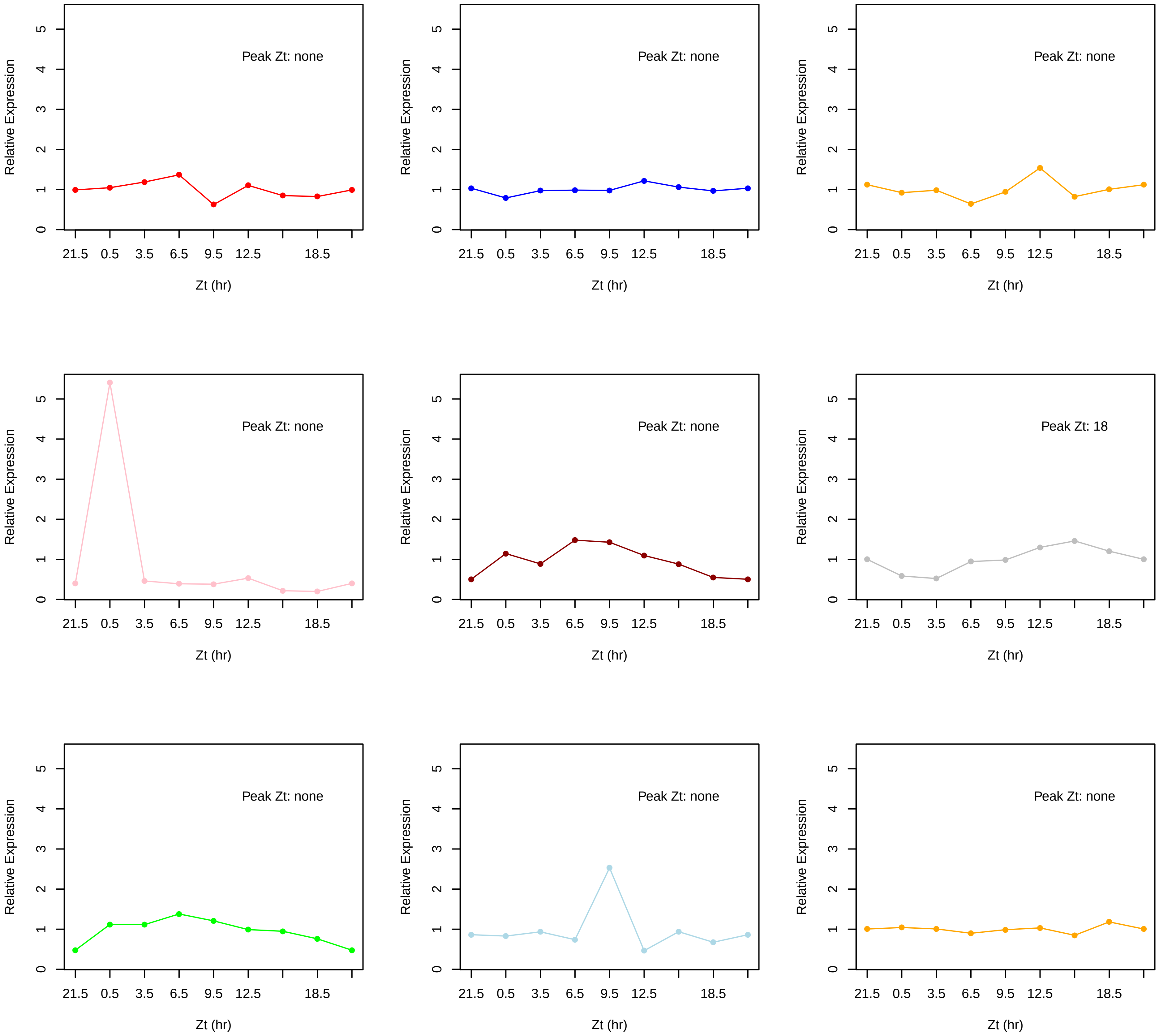

Singeleton: ENSXMAG00000002928 ctu1

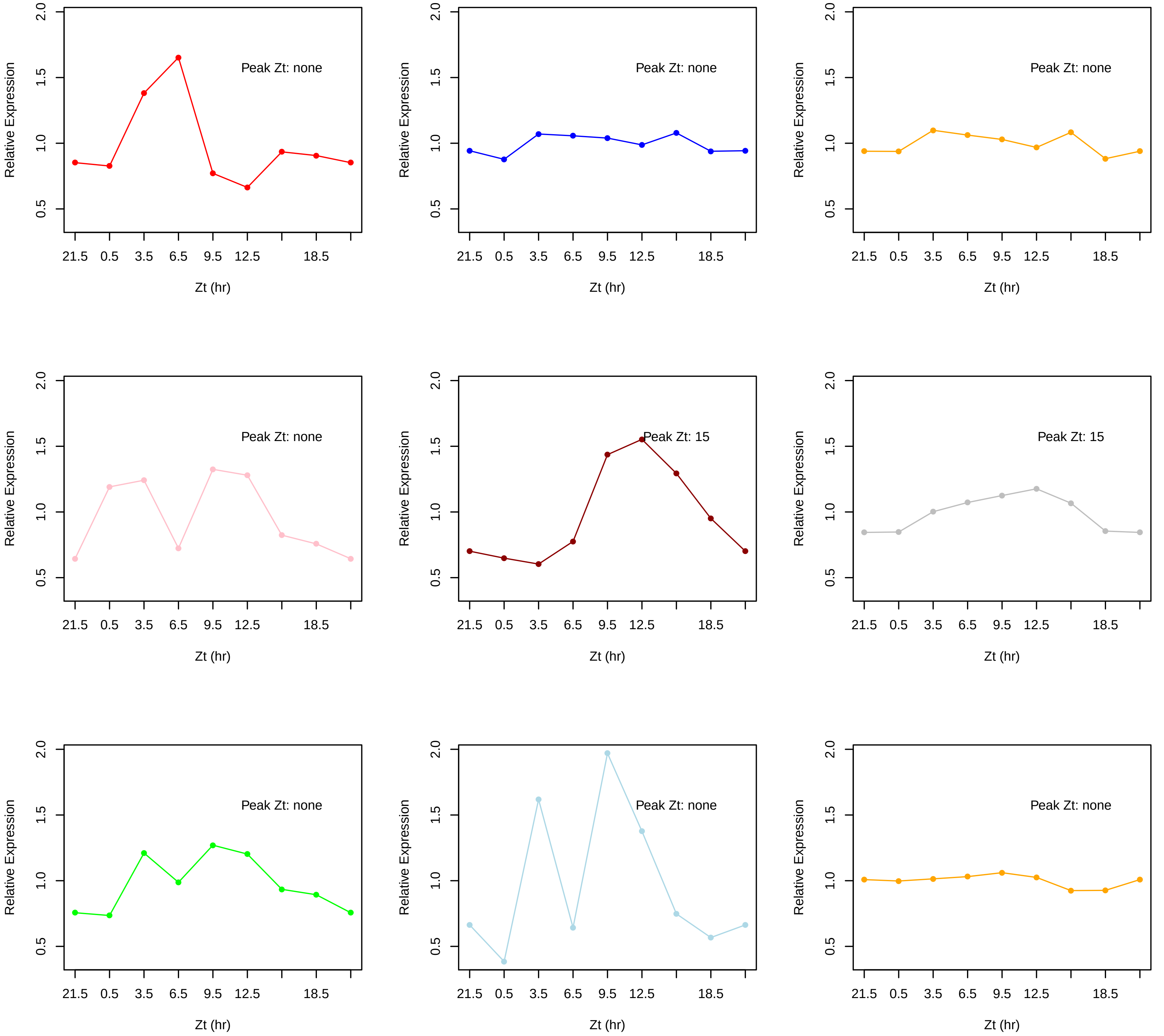

Singeleton: ENSXMAG00000008164 pef1

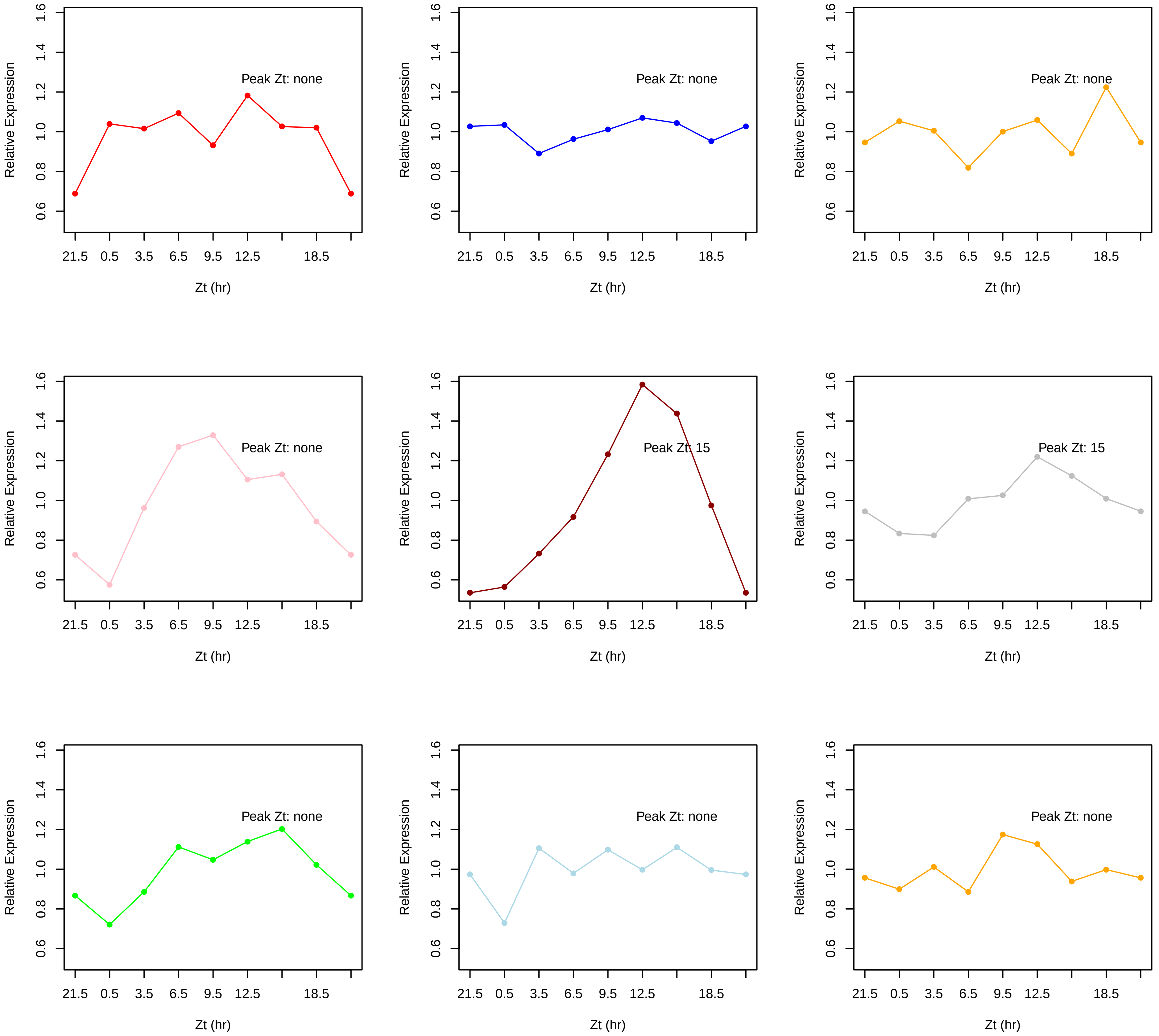

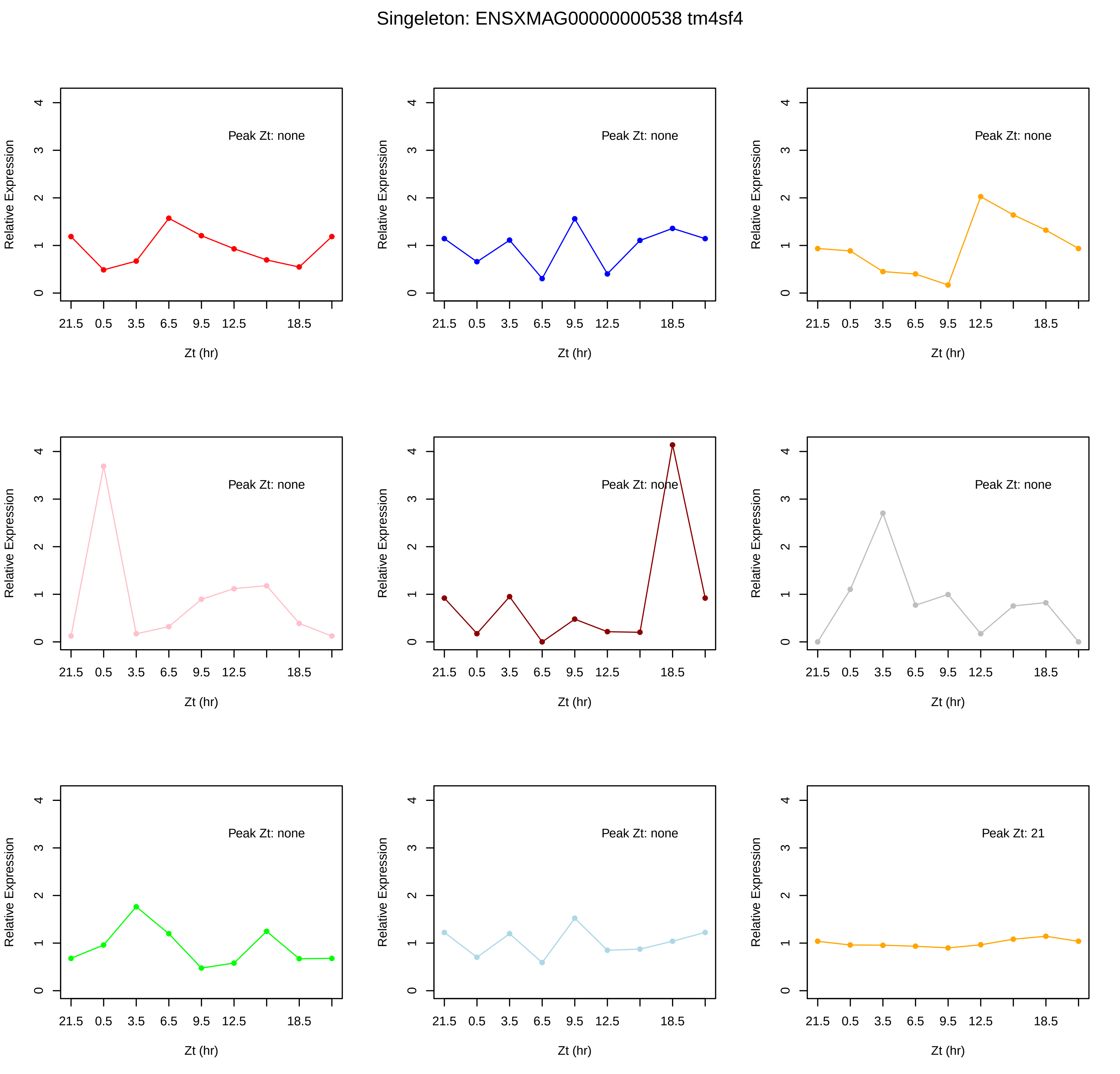

Singeleton: ENSXMAG00000000538 tm4sf4
Peak Zt: none
Peak Zt: none
Peak Zt: none
Peak Zt: none
Peak Zt: none
Peak Zt: none
Peak Zt: none
Peak Zt: none
Peak Zt: 21
Relative Expression
Zt (hr)
21.5
0.5
3.5
6.5
9.5
12.5
18.5
0
1
2
3
4

Singleton: ENSXMAG00000002313 ivns1abpa

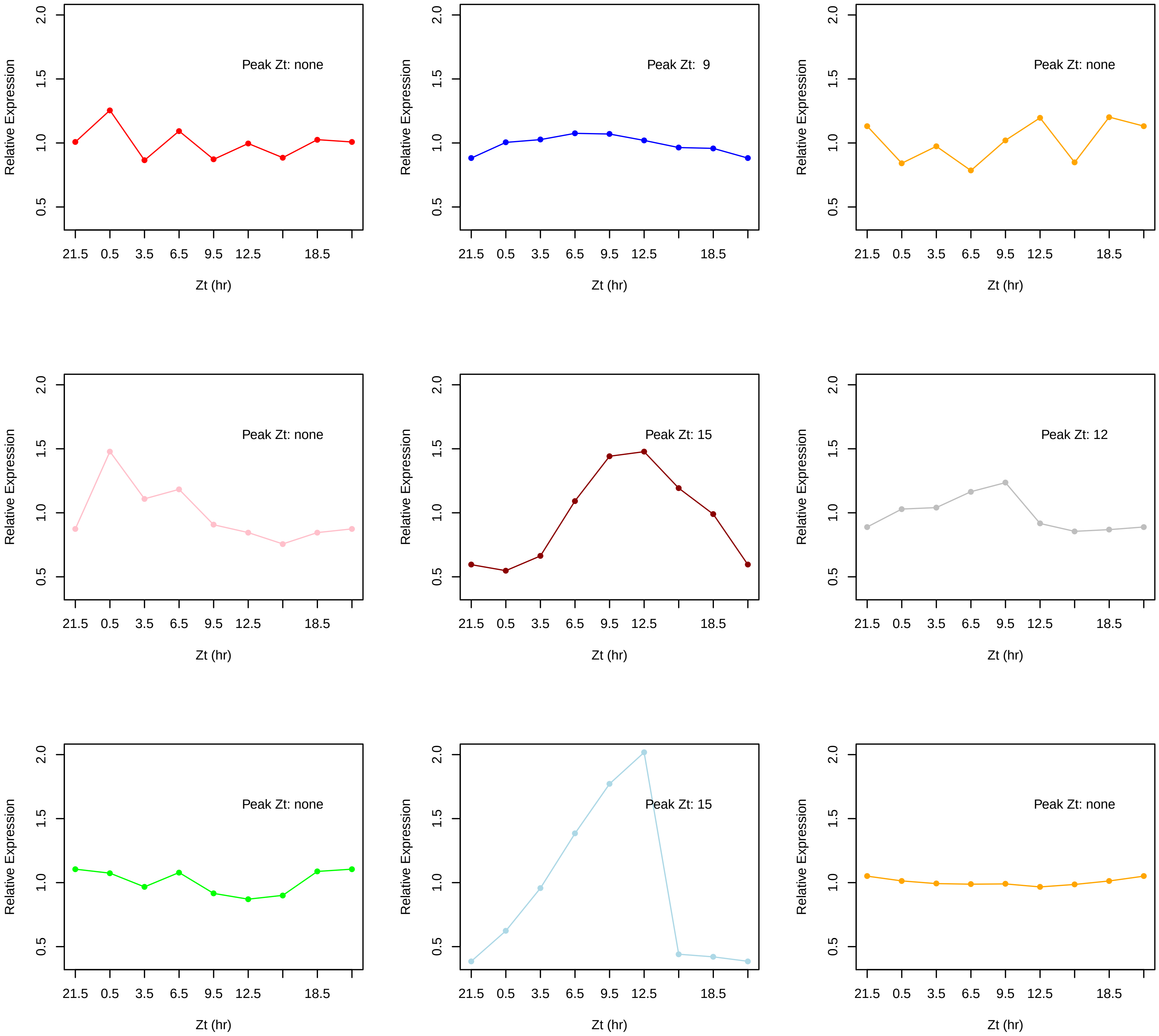

Singeleton: ENSXMAG00000018667 scarb1

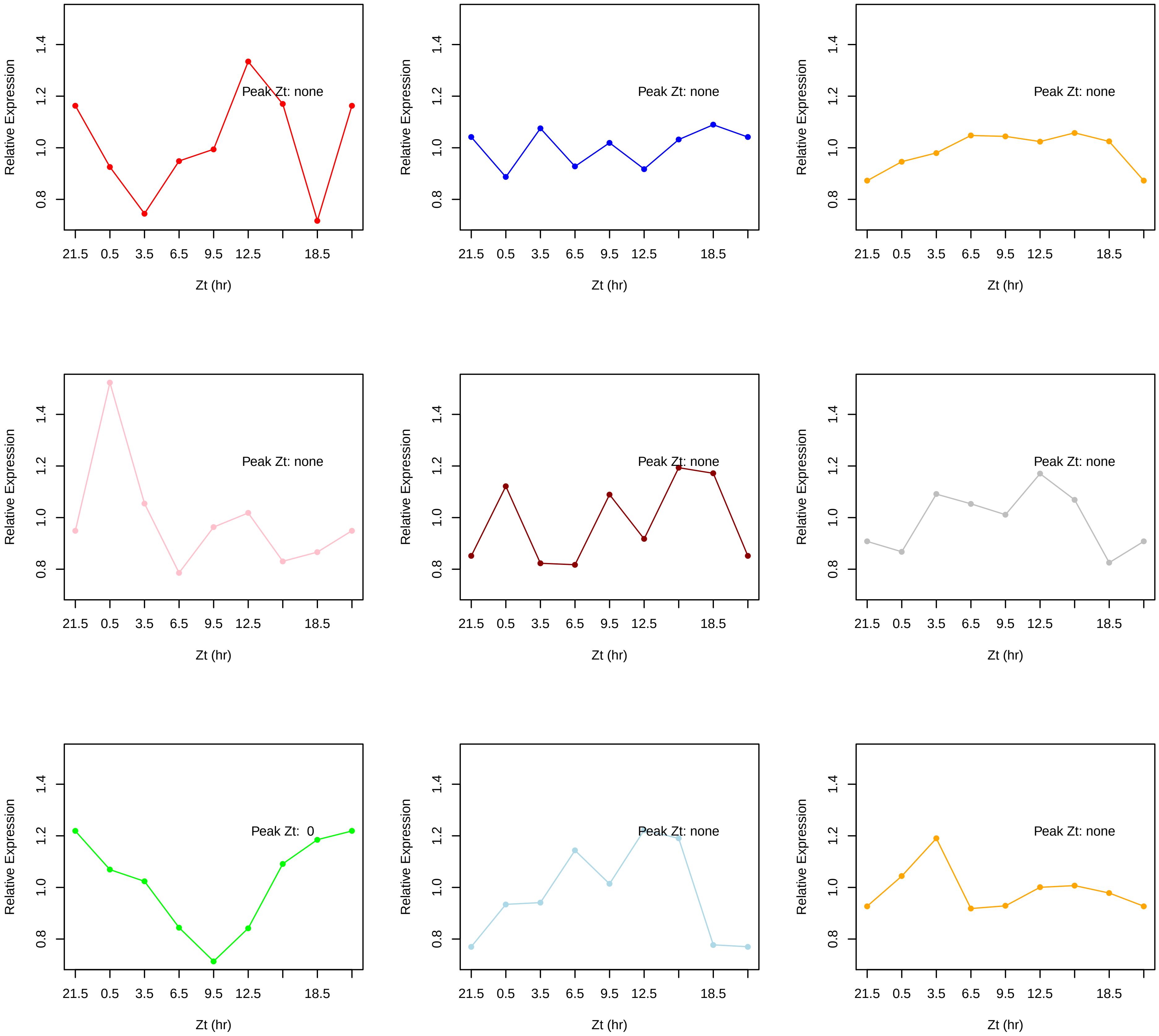

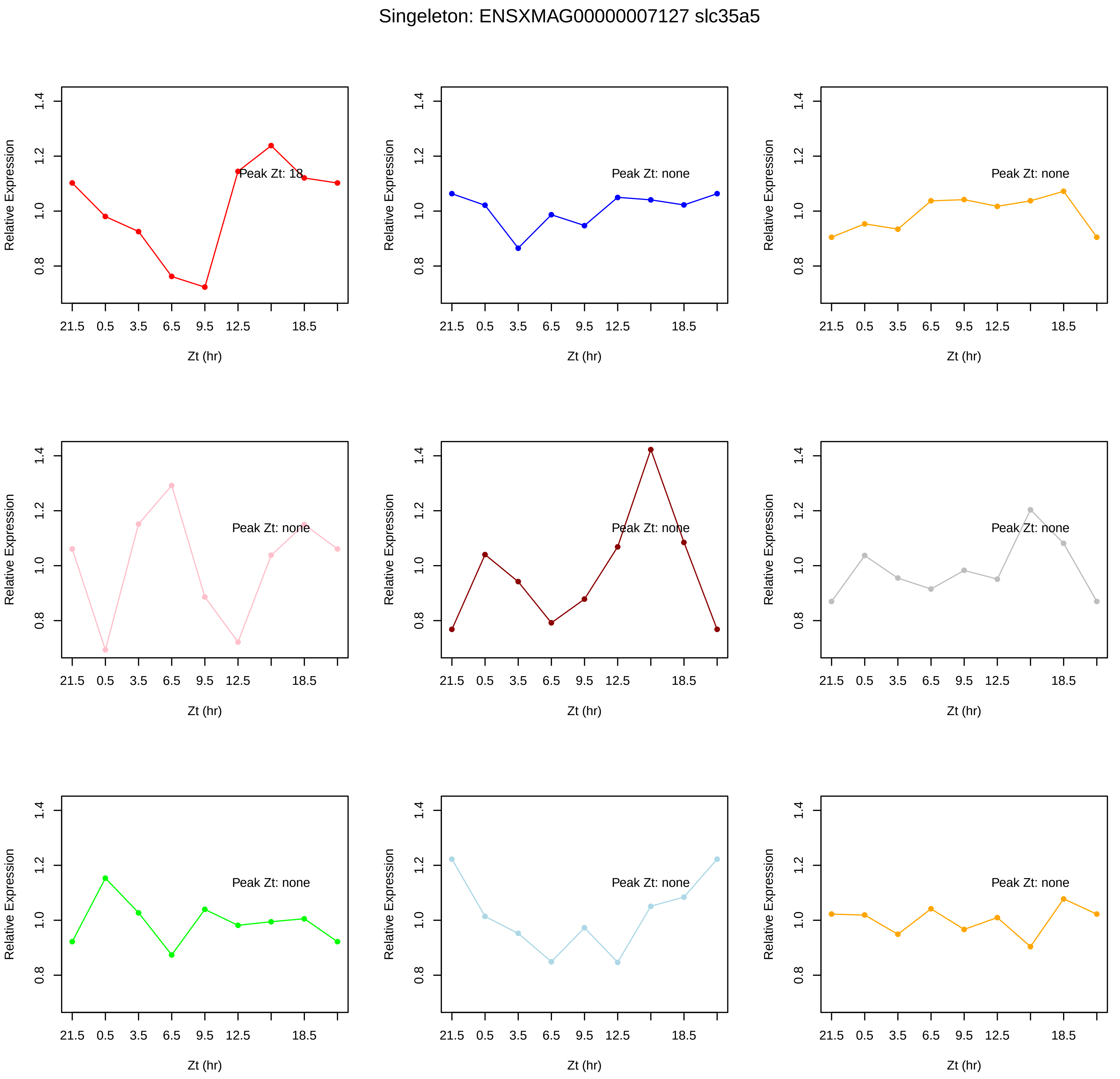
Singeleton: ENSXMAG00000007127 slc35a5
Peak Zt: 18
Peak Zt: none
Relative Expression
Zt (hr)
21.5
0.5
3.5
6.5
9.5
12.5
18.5
0.8
1.0
1.2
1.4

Singeleton: ENSXMAG00000015395 cpt1ab

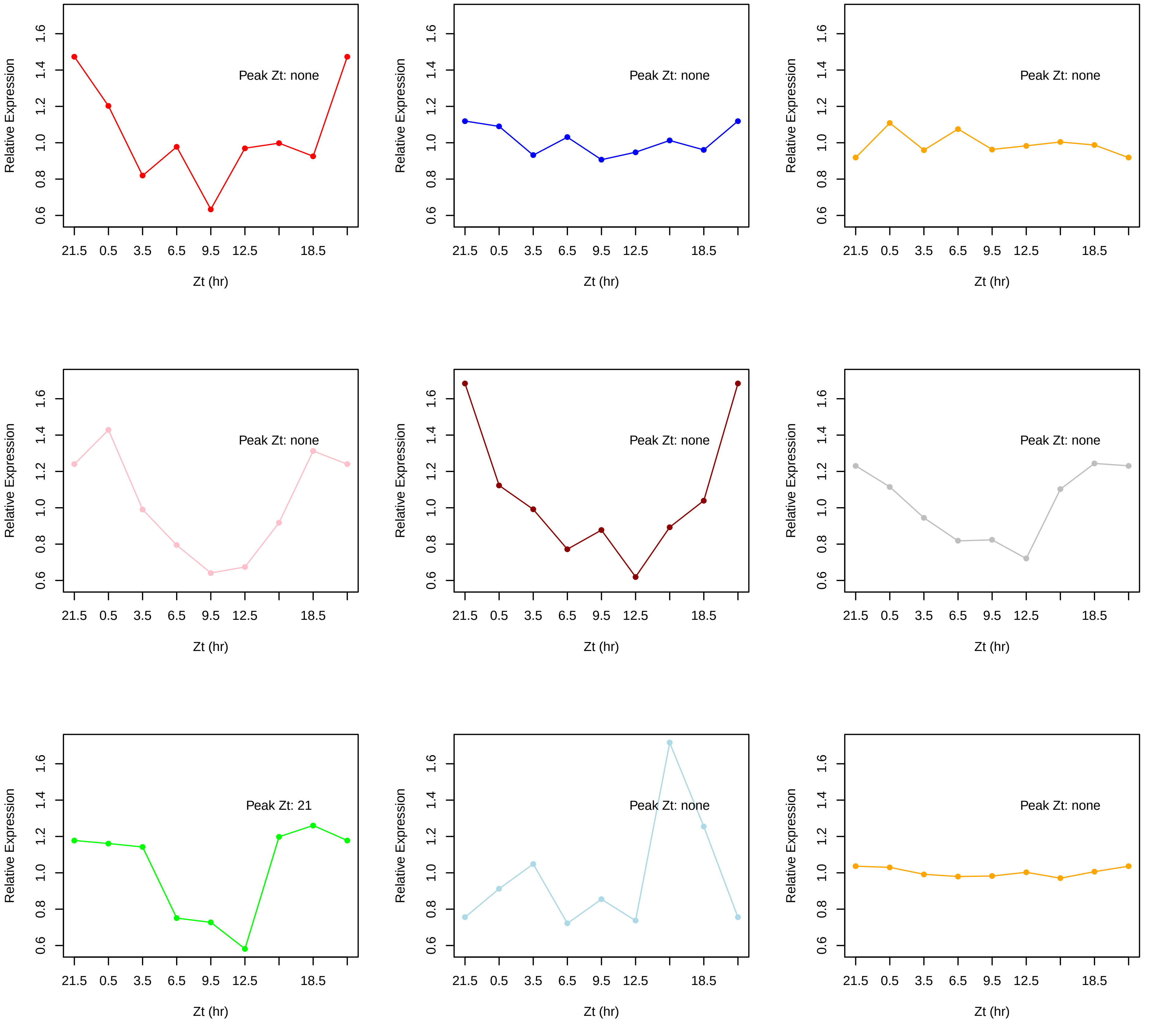

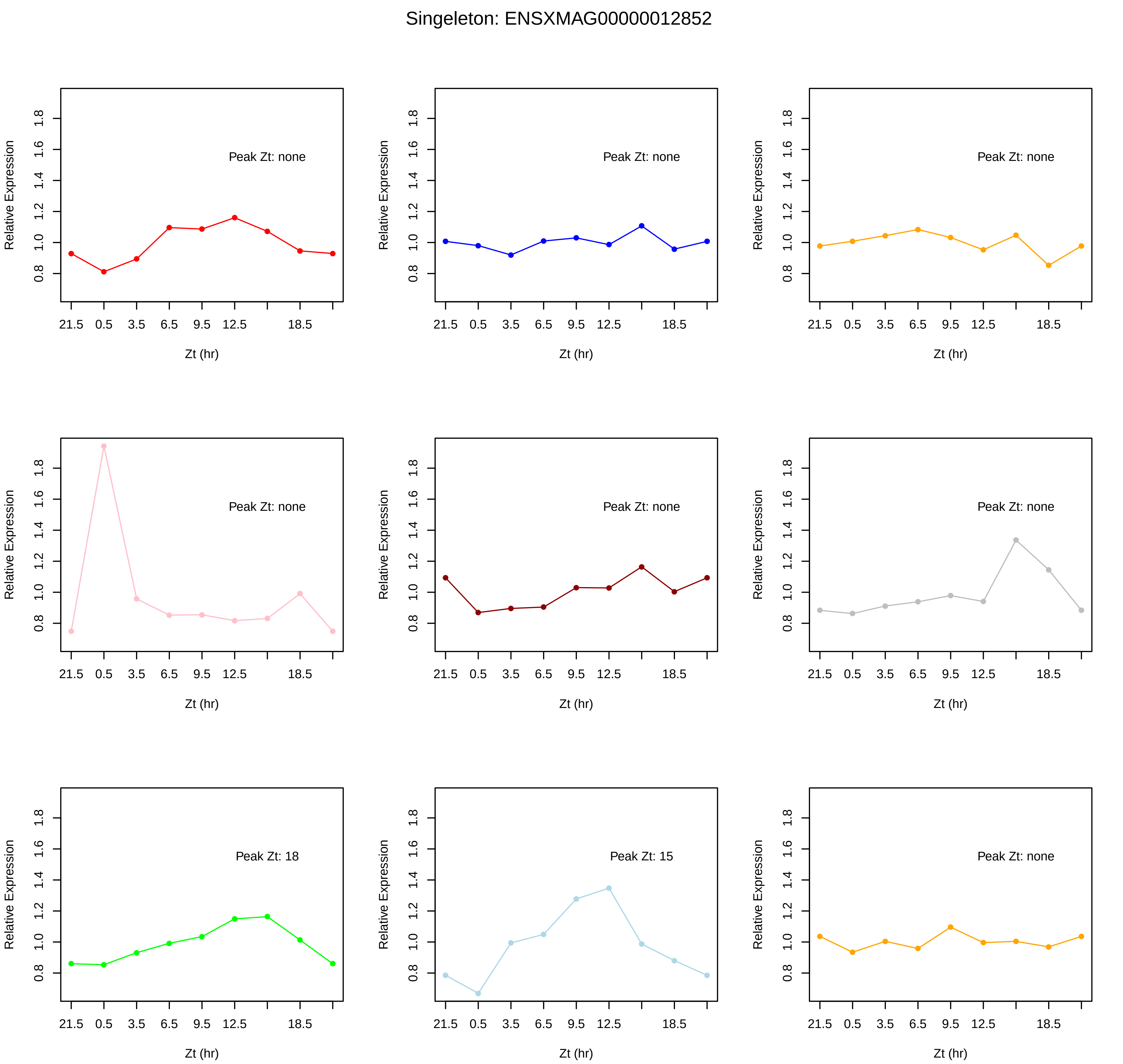
Singeleton: ENSXMAG00000012852
Peak Zt: none
Peak Zt: none
Peak Zt: none
Peak Zt: none
Peak Zt: none
Peak Zt: none
Peak Zt: 18
Peak Zt: 15
Peak Zt: none
Relative Expression
Zt (hr)
21.5 0.5 3.5 6.5 9.5 12.5 18.5
0.8 1.0 1.2 1.4 1.6 1.8

Singleton: ENSXMAG00000018701 micu2

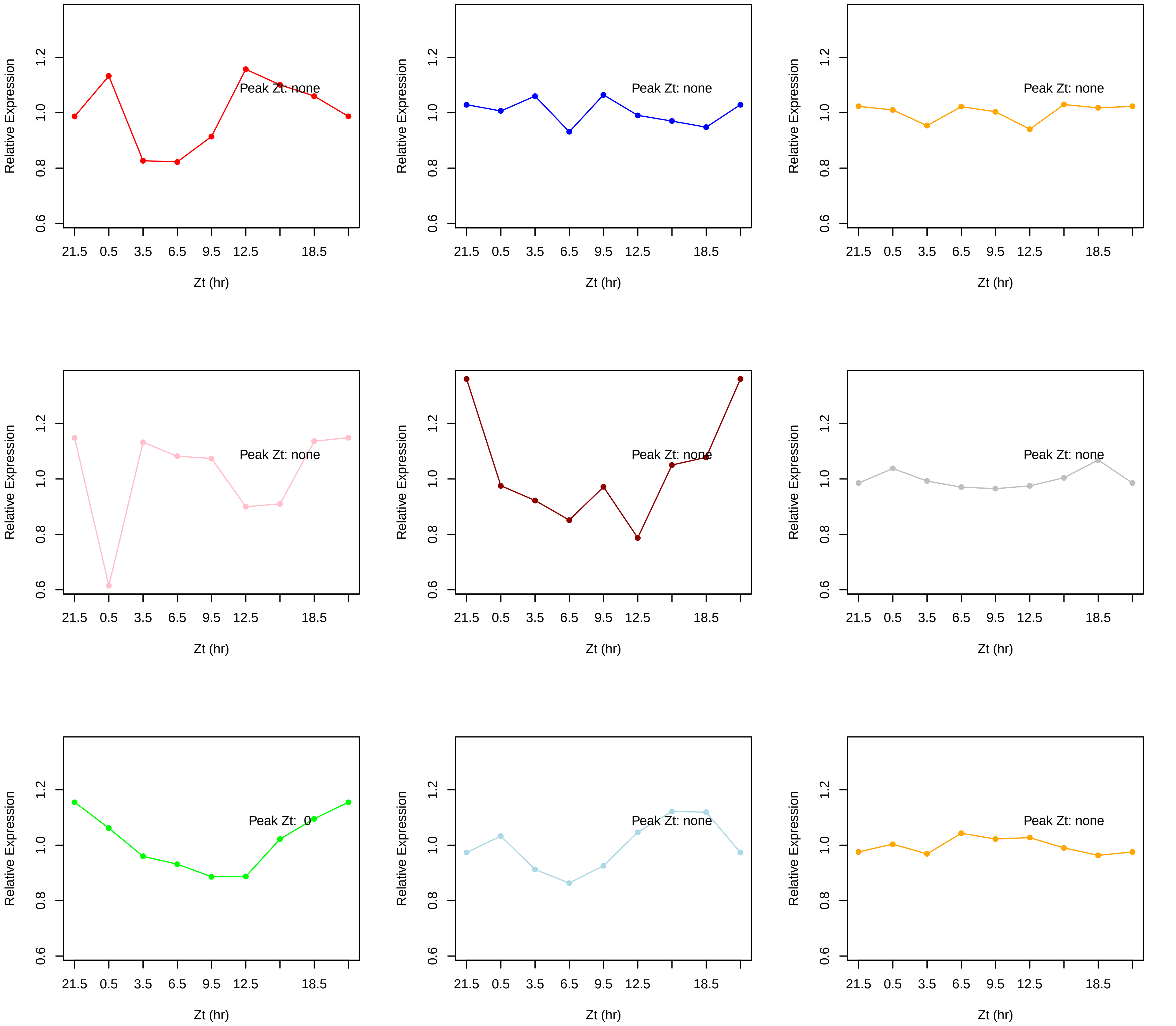

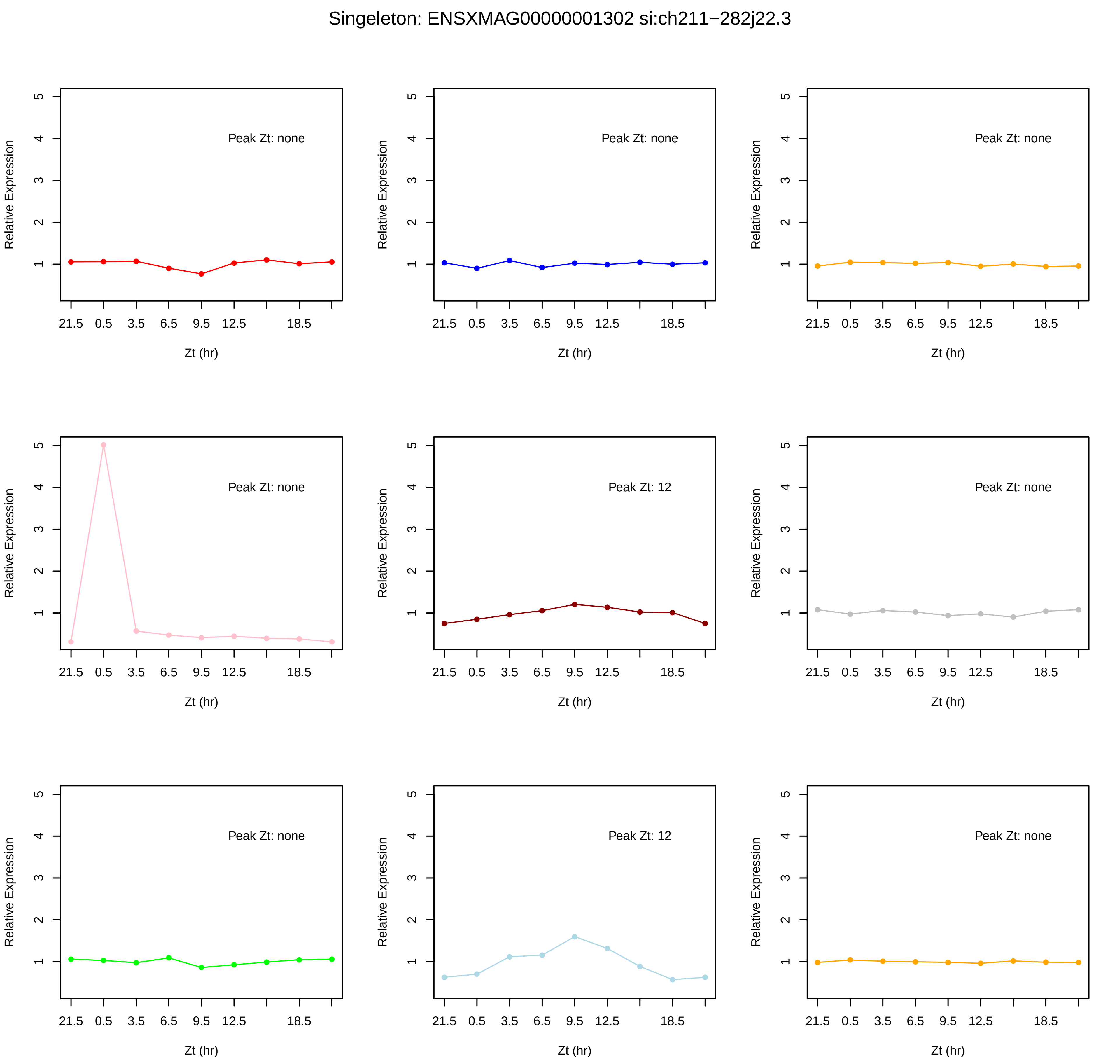

Singeleton: ENSXMAG00000001302 si:ch211−282j22.3
Peak Zt: none
Peak Zt: none
Peak Zt: none
Peak Zt: none
Peak Zt: 12
Peak Zt: none
Peak Zt: none
Peak Zt: 12
Peak Zt: none
Relative Expression
Zt (hr)
21.5
0.5
3.5
6.5
9.5
12.5
18.5

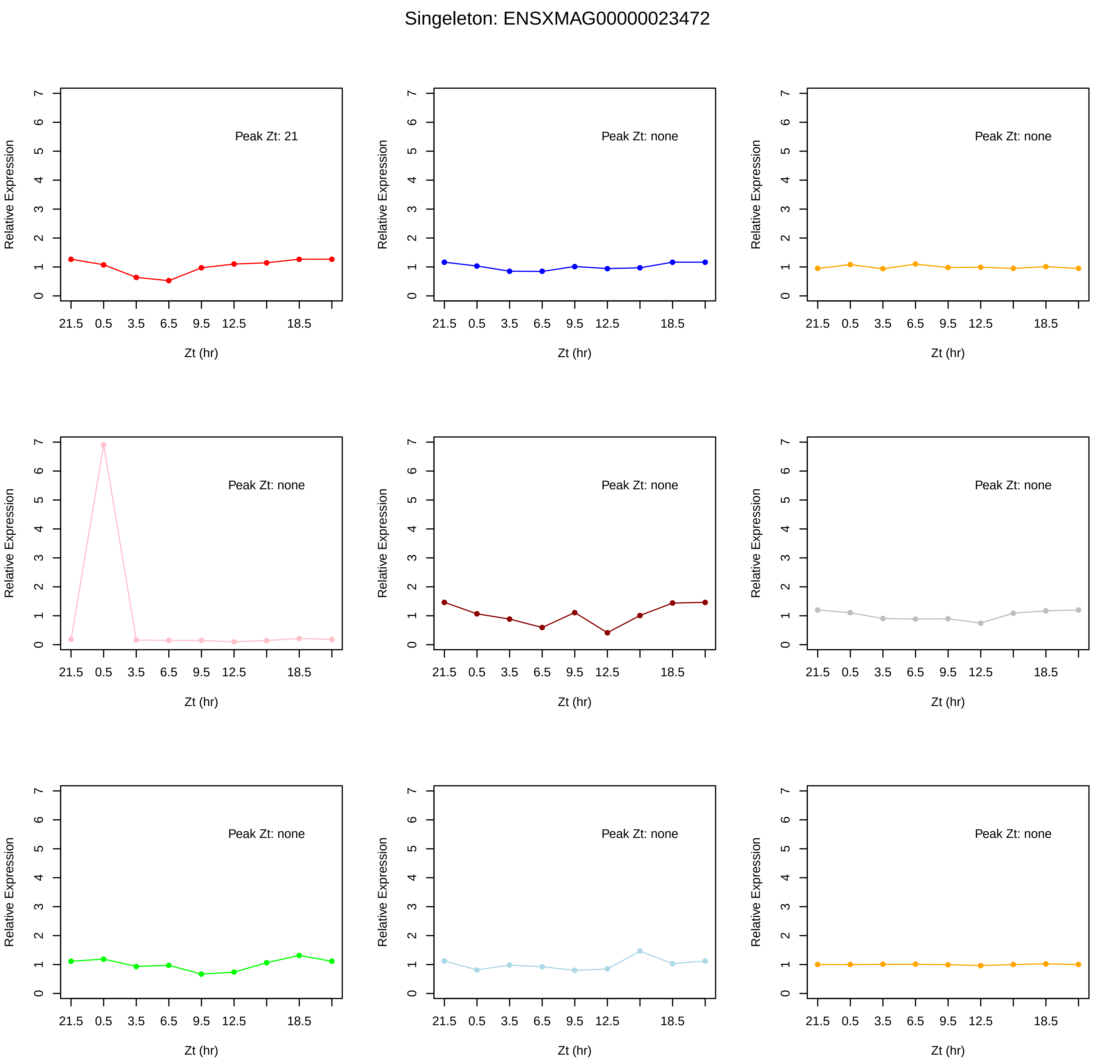
Singleton: ENSXMAG00000023472
Peak Zt: 21
Peak Zt: none
Peak Zt: none
Peak Zt: none
Peak Zt: none
Peak Zt: none
Peak Zt: none
Peak Zt: none
Peak Zt: none
Relative Expression
Zt (hr)
21.5
0.5
3.5
6.5
9.5
12.5
18.5

Singeleton: ENSXMAG00000004827 RNPS1

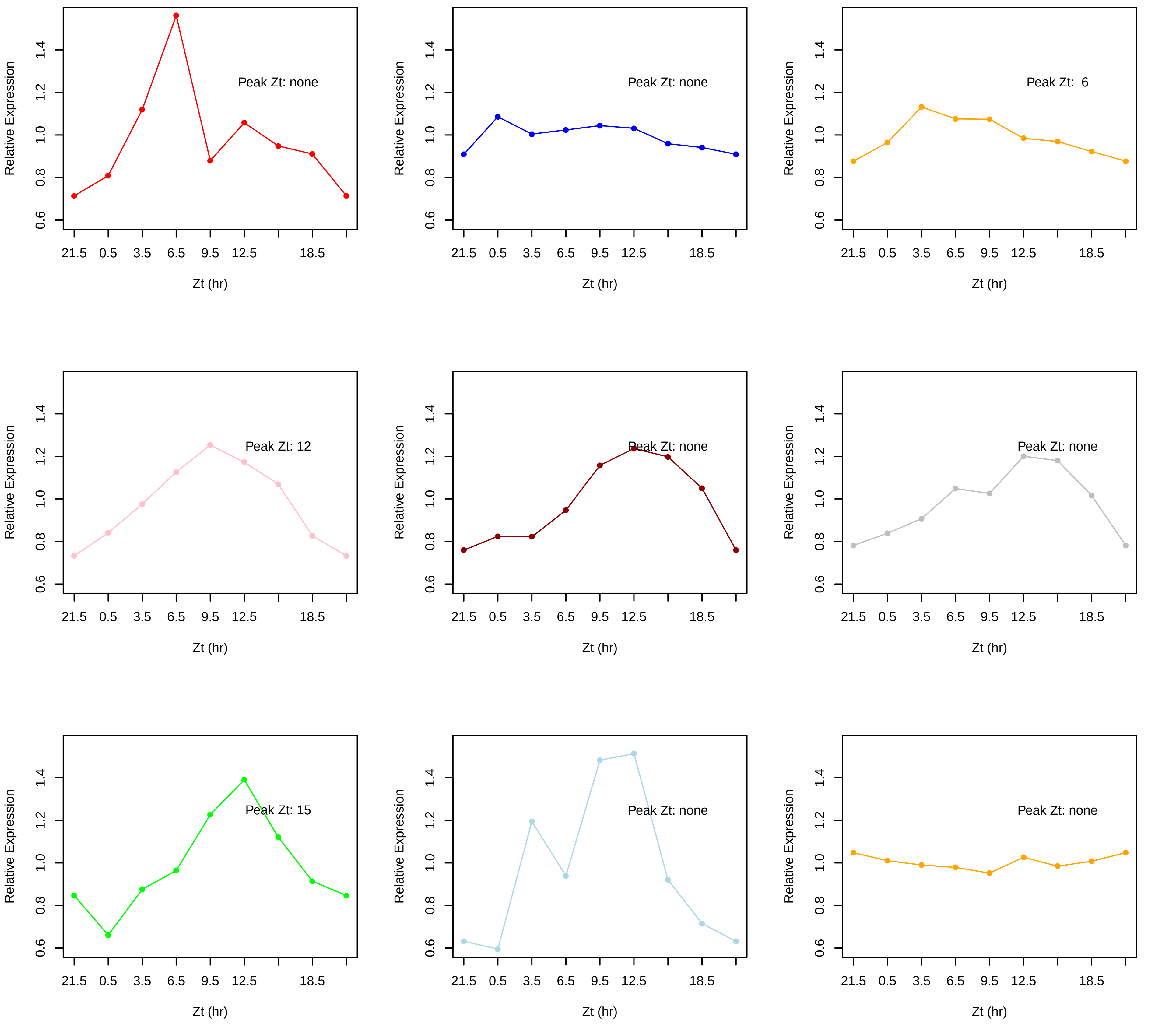

Singeleton: ENSXMAG00000005698 atp1a2a

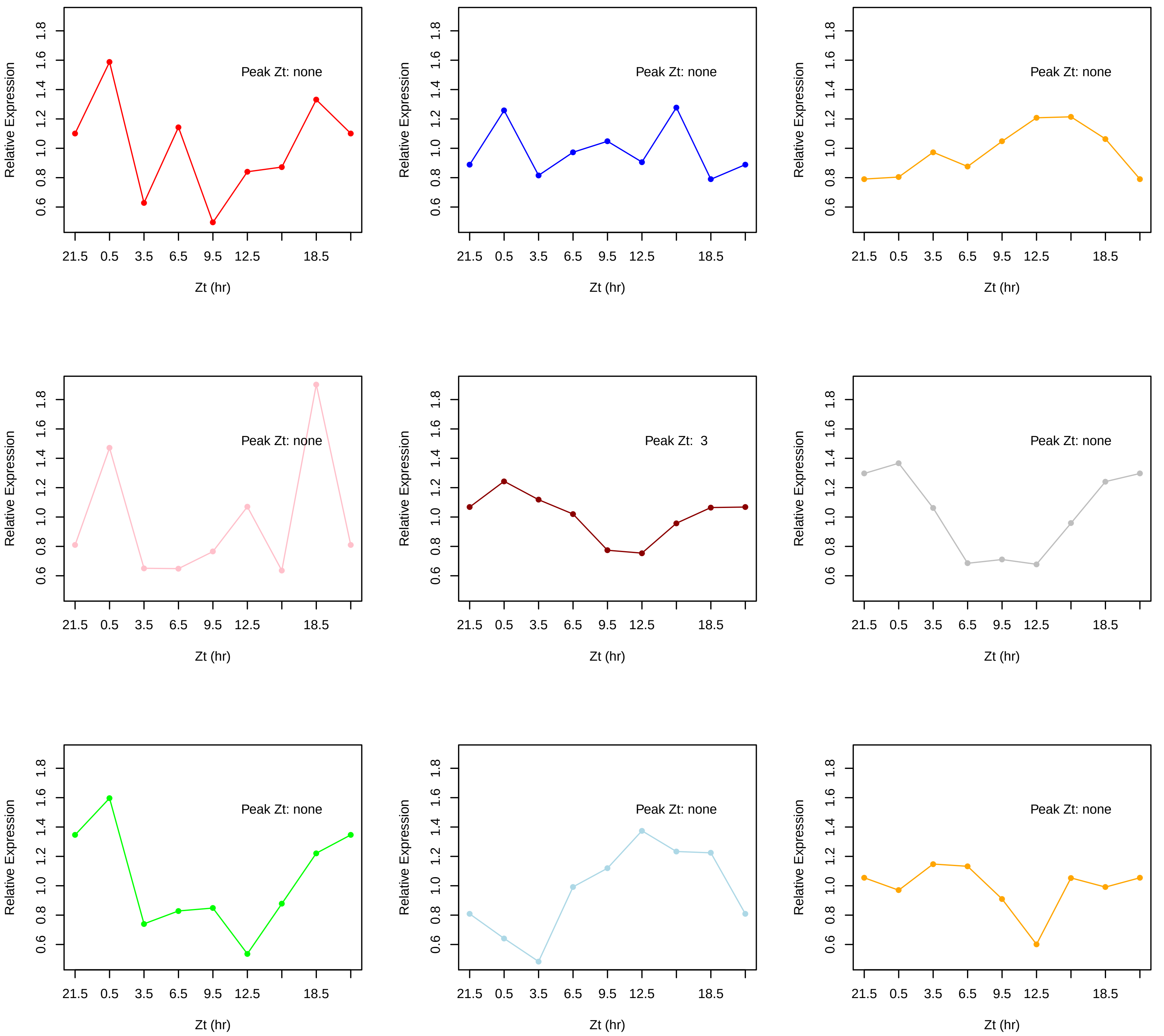

Singeleton: ENSXMAG00000008732 mark2b

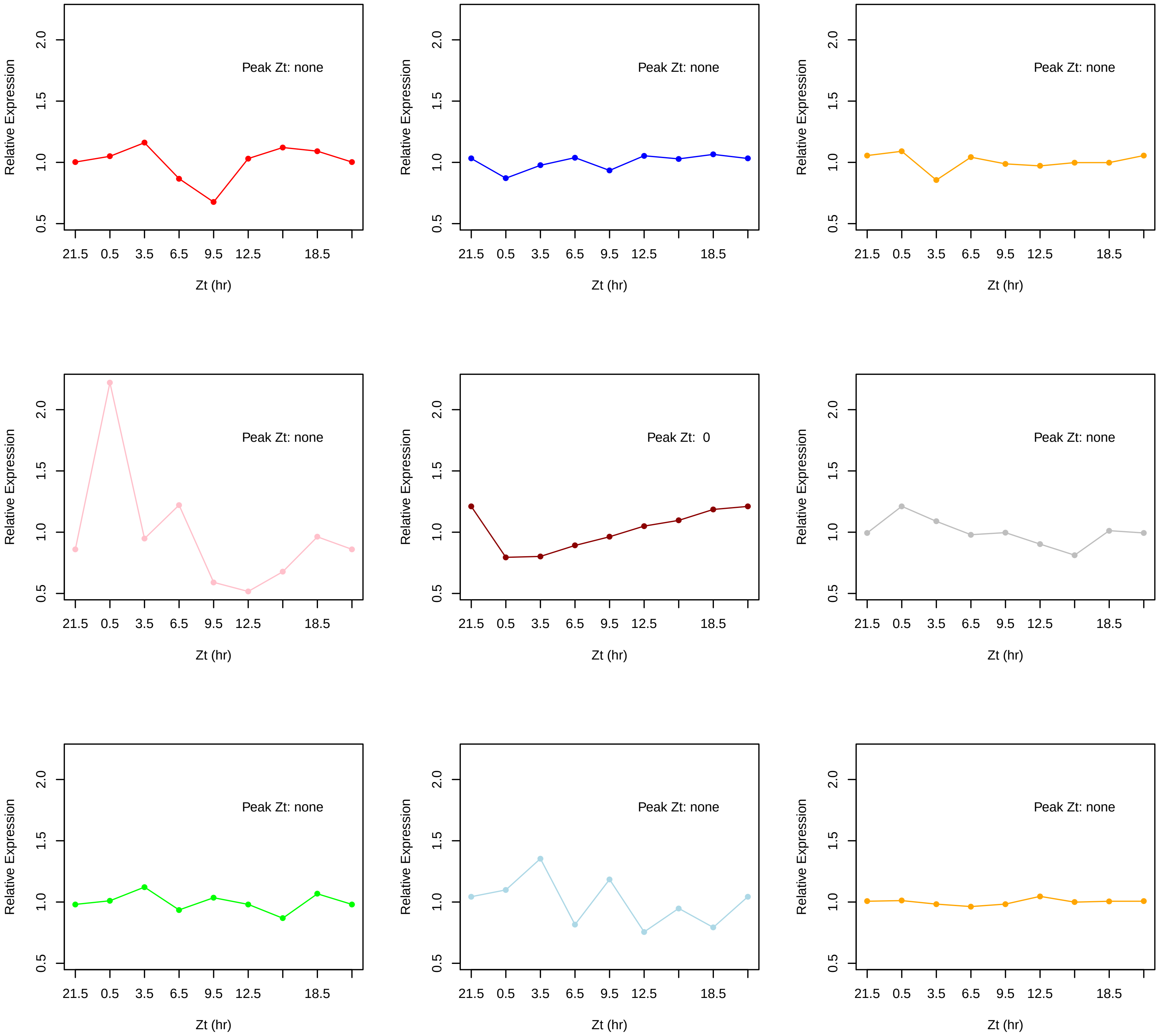

Singeleton: ENSXMAG00000007110 ccdc80

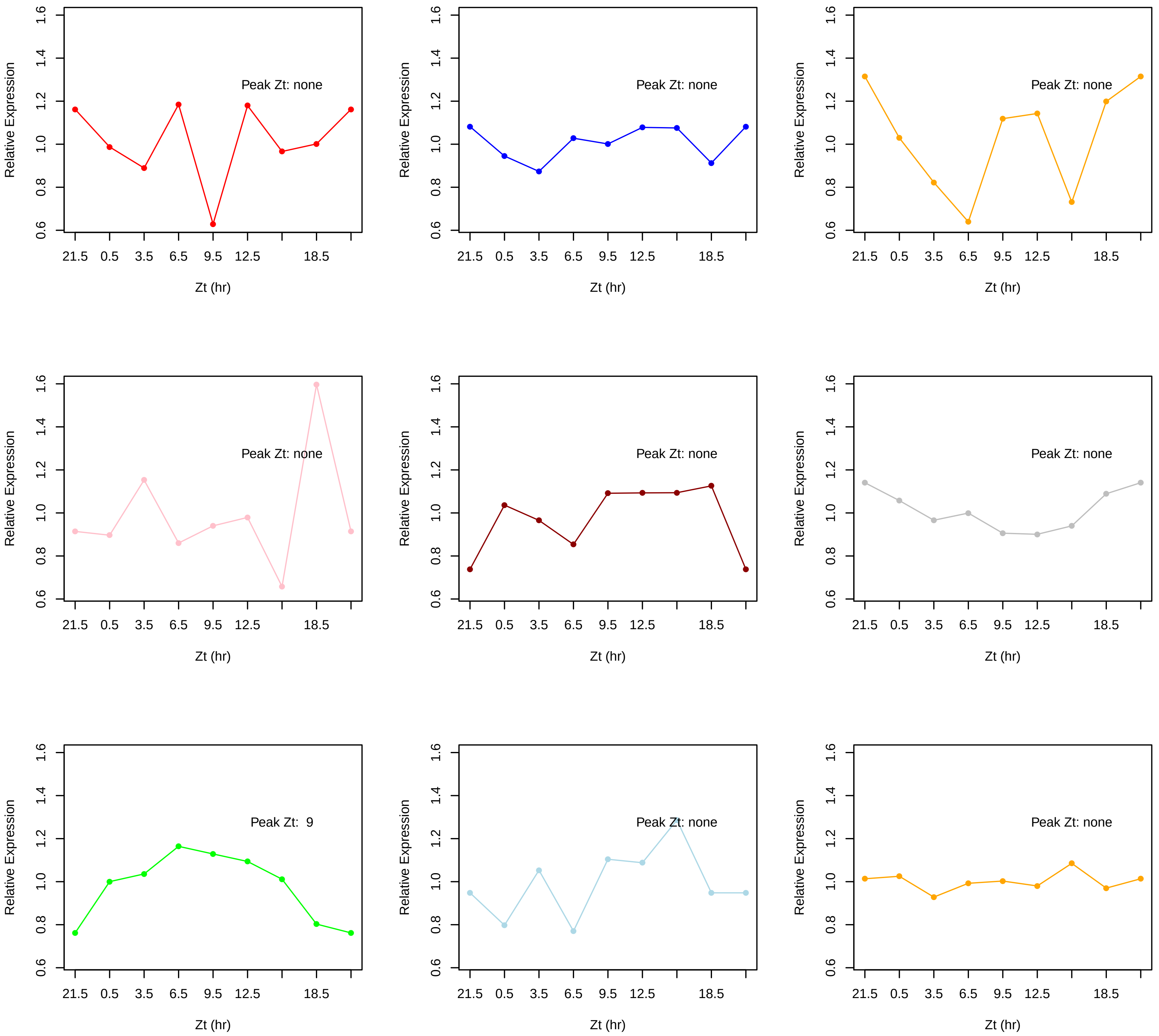

Singleton: ENSXMAG00000011946 rhpn2

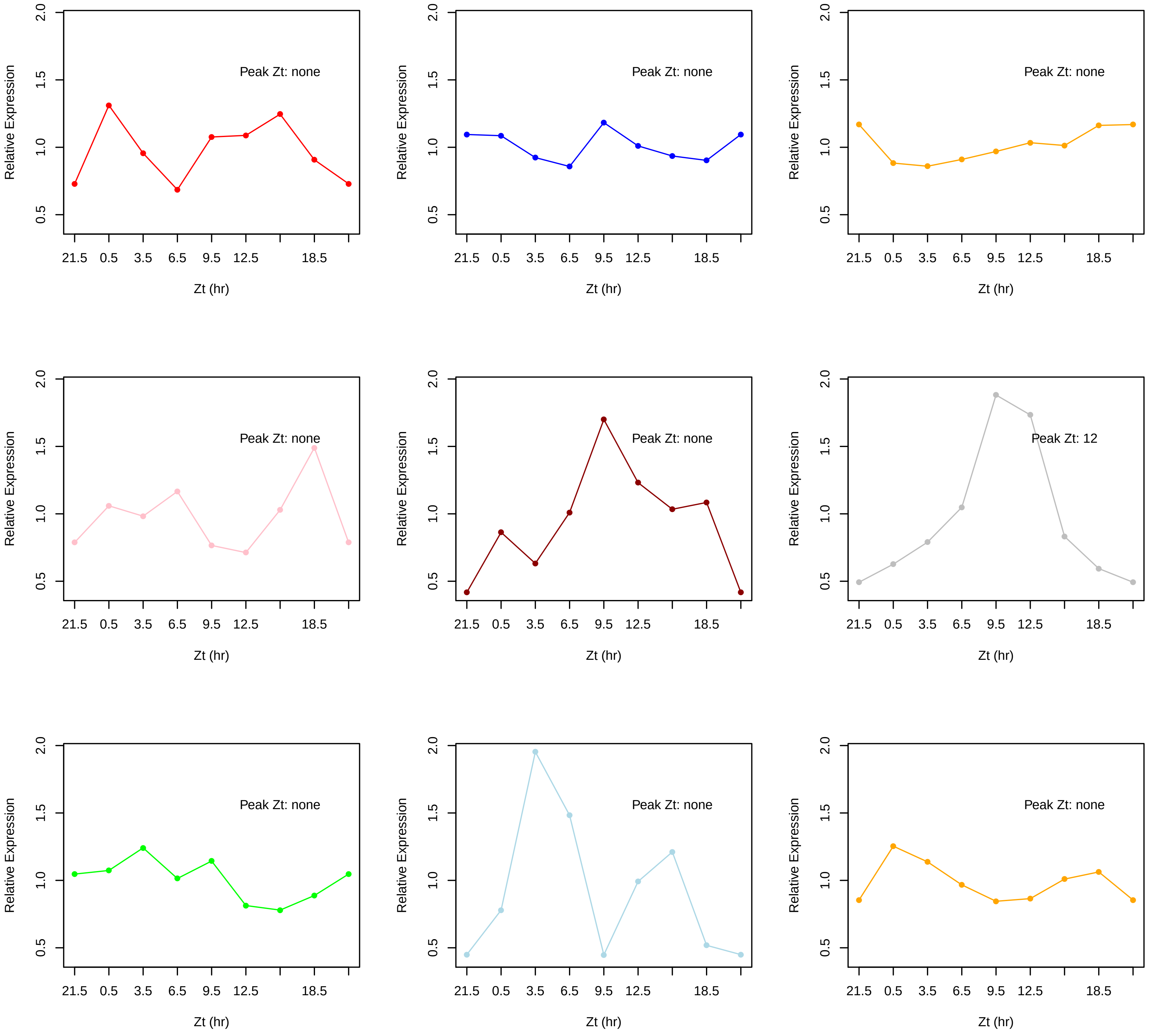

Singeleton: ENSXMAG00000019056 gale

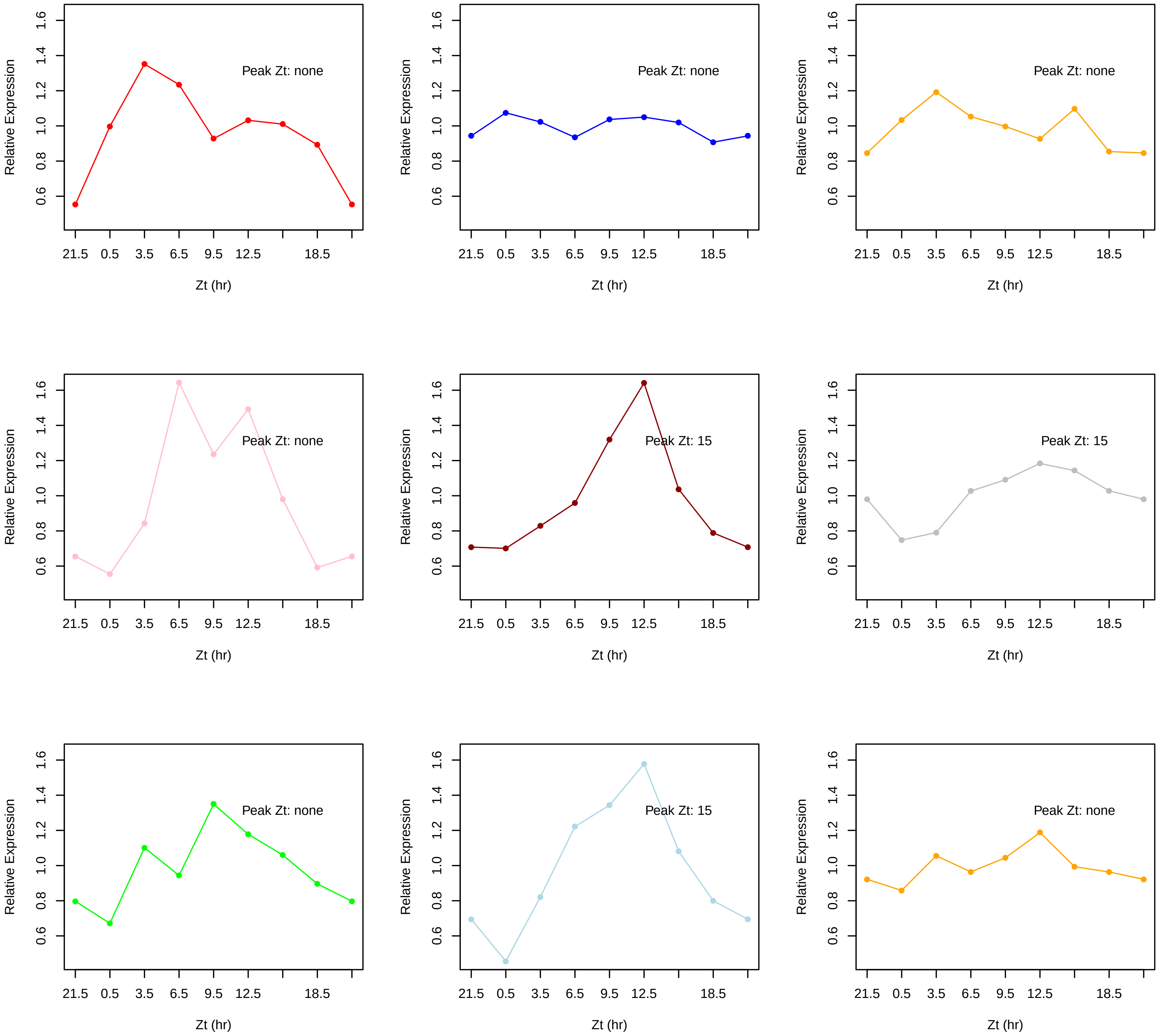

Singleton: ENSXMAG00000024652 pdk3a

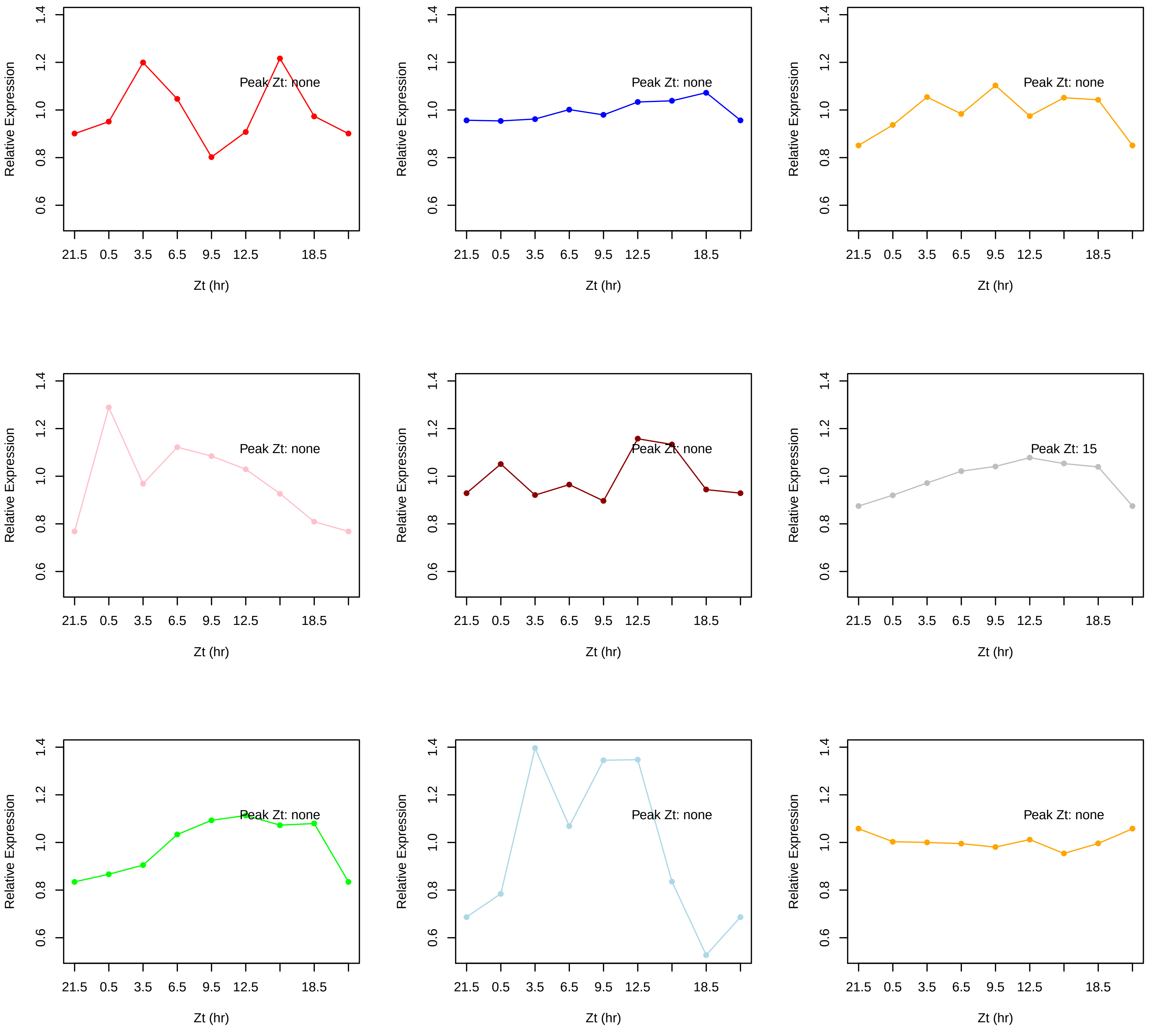

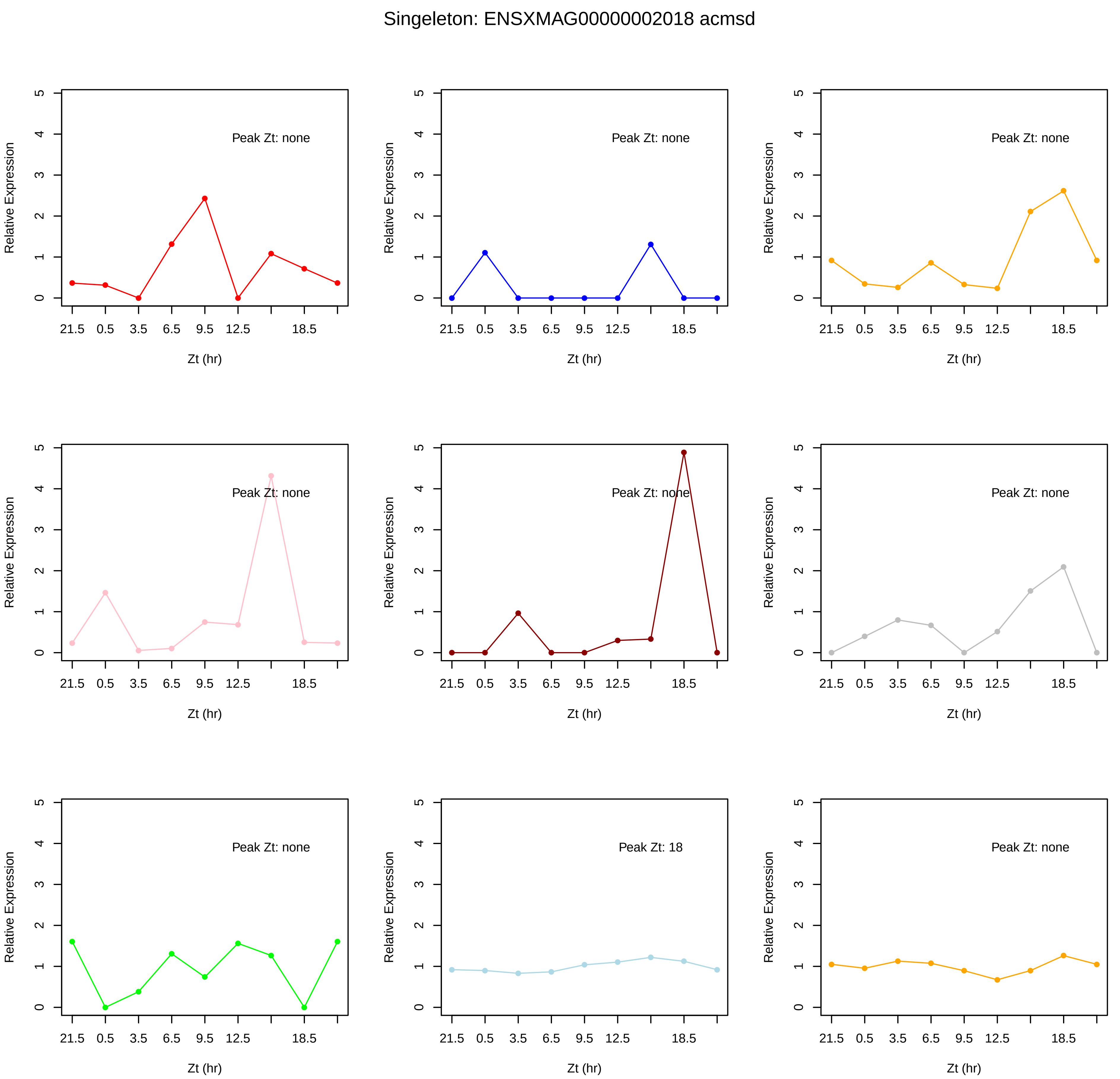
Singeleton: ENSXMAG00000002018 acmsd
Peak Zt: none
Peak Zt: none
Peak Zt: none
Peak Zt: none
Peak Zt: none
Peak Zt: none
Peak Zt: none
Peak Zt: 18
Peak Zt: none
Relative Expression
Zt (hr)
21.5 0.5 3.5 6.5 9.5 12.5 18.5

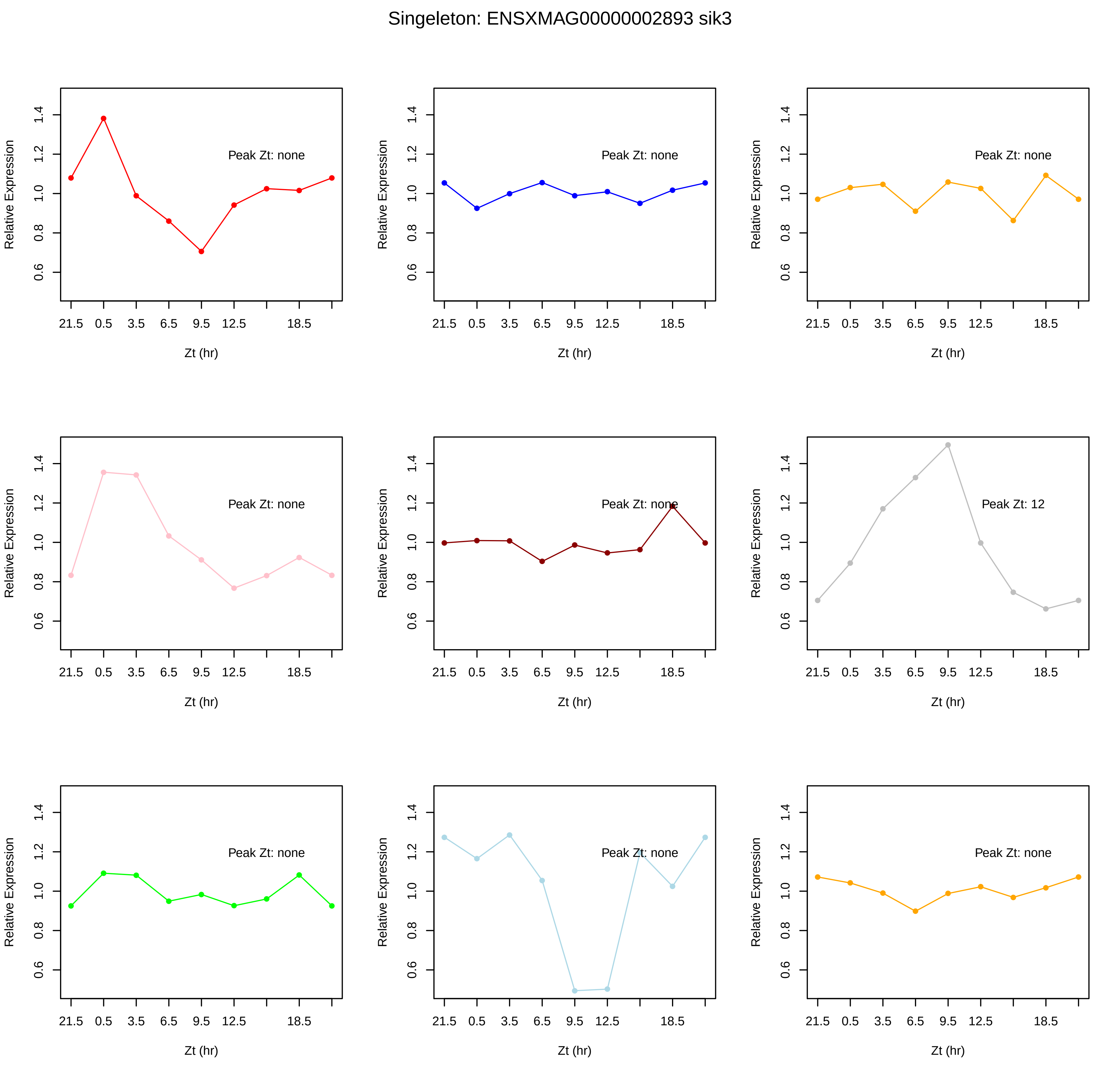

Singeleton: ENSXMAG00000002893 sik3
Relative Expression
Zt (hr)
21.5
0.5
3.5
6.5
9.5
12.5
18.5
0.6
0.8
1.0
1.2
1.4
Peak Zt: none
Peak Zt: 12

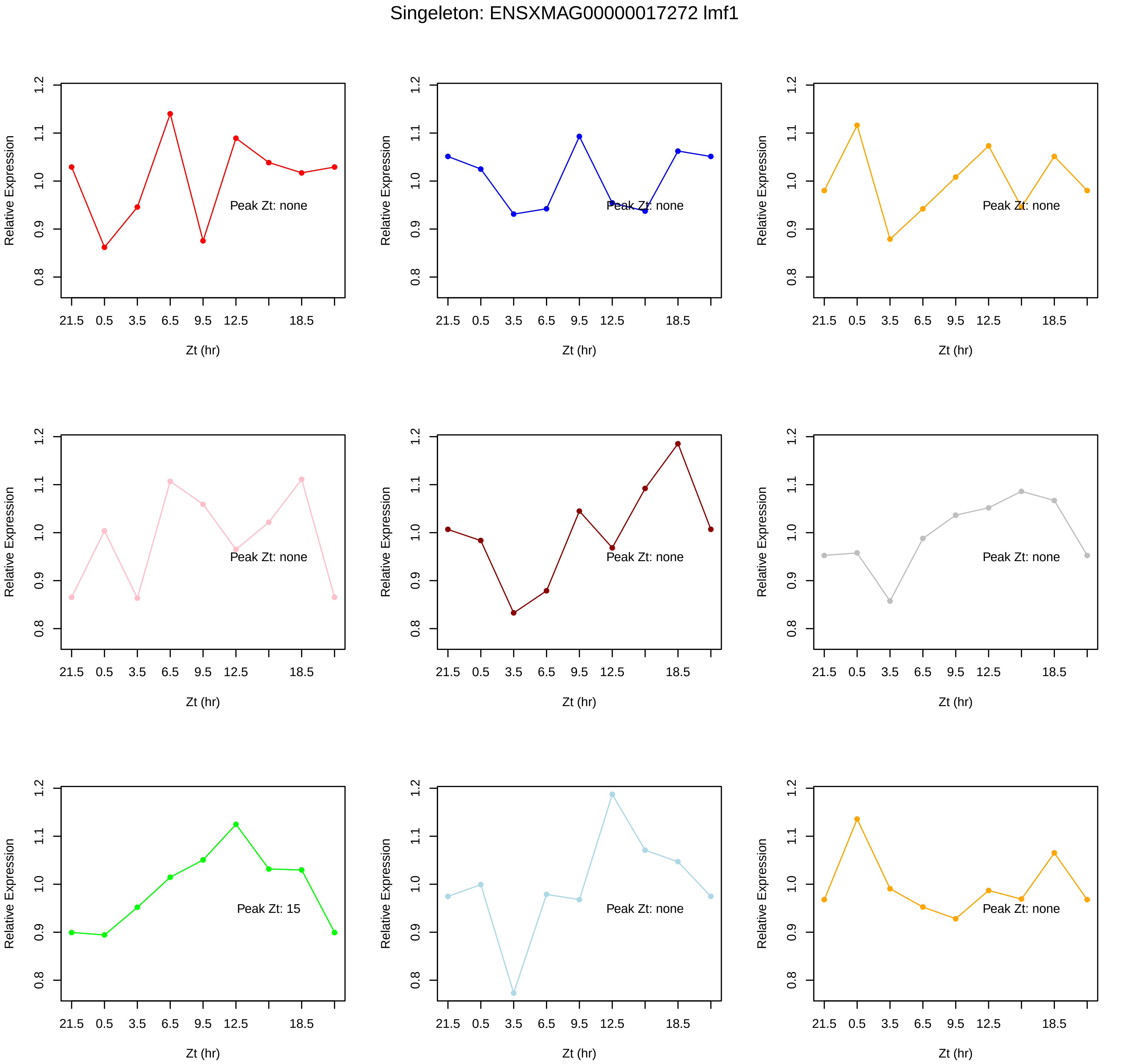
Singeleton: ENSXMAG00000017272 lmf1
Relative Expression
Zt (hr)
21.5
0.5
3.5
6.5
9.5
12.5
18.5
0.8
0.9
1.0
1.1
1.2
Peak Zt: none
Peak Zt: 15

Singeleton: ENSXMAG00000010740 med16

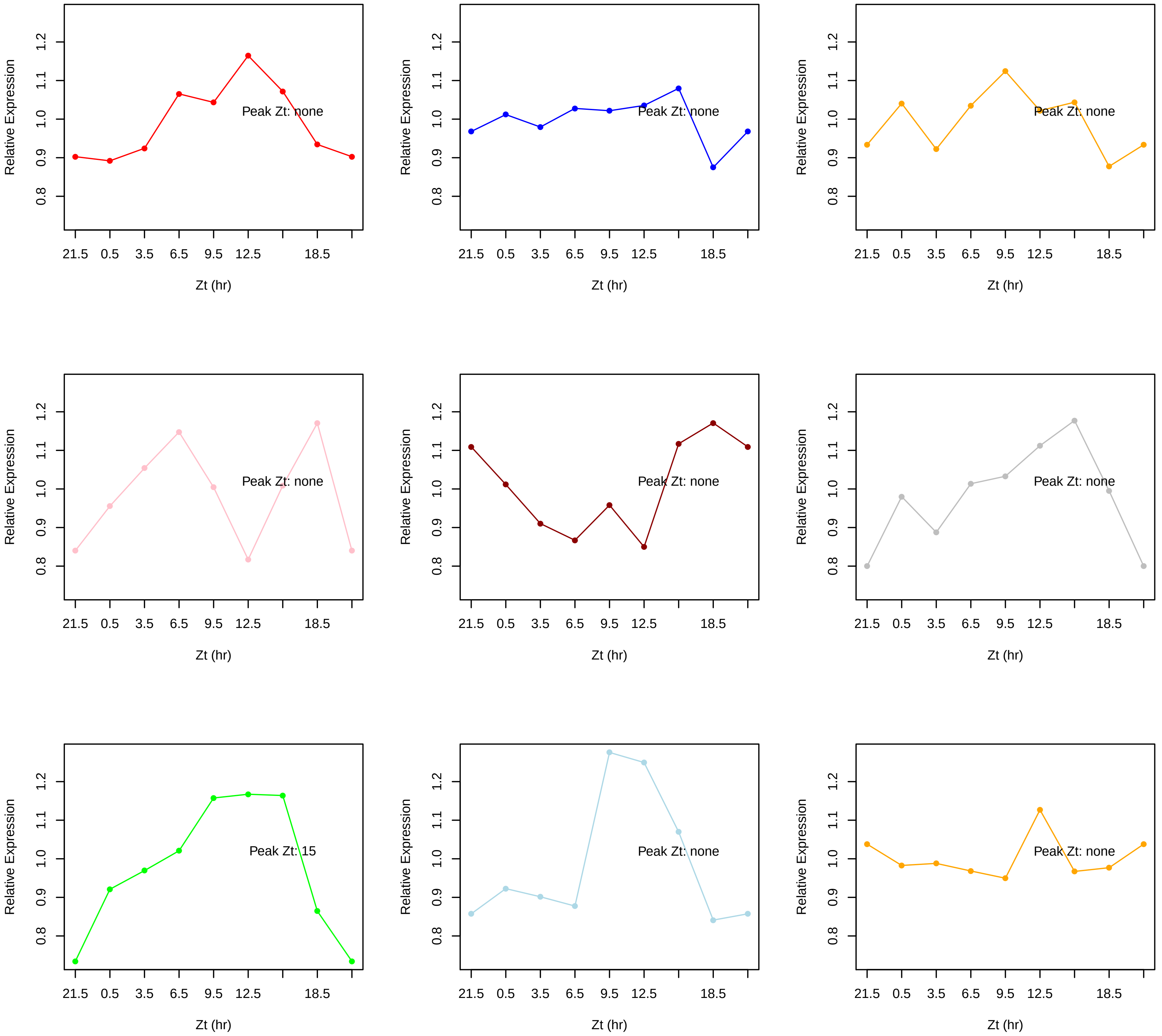

Singeleton: ENSXMAG00000007684 adam19a

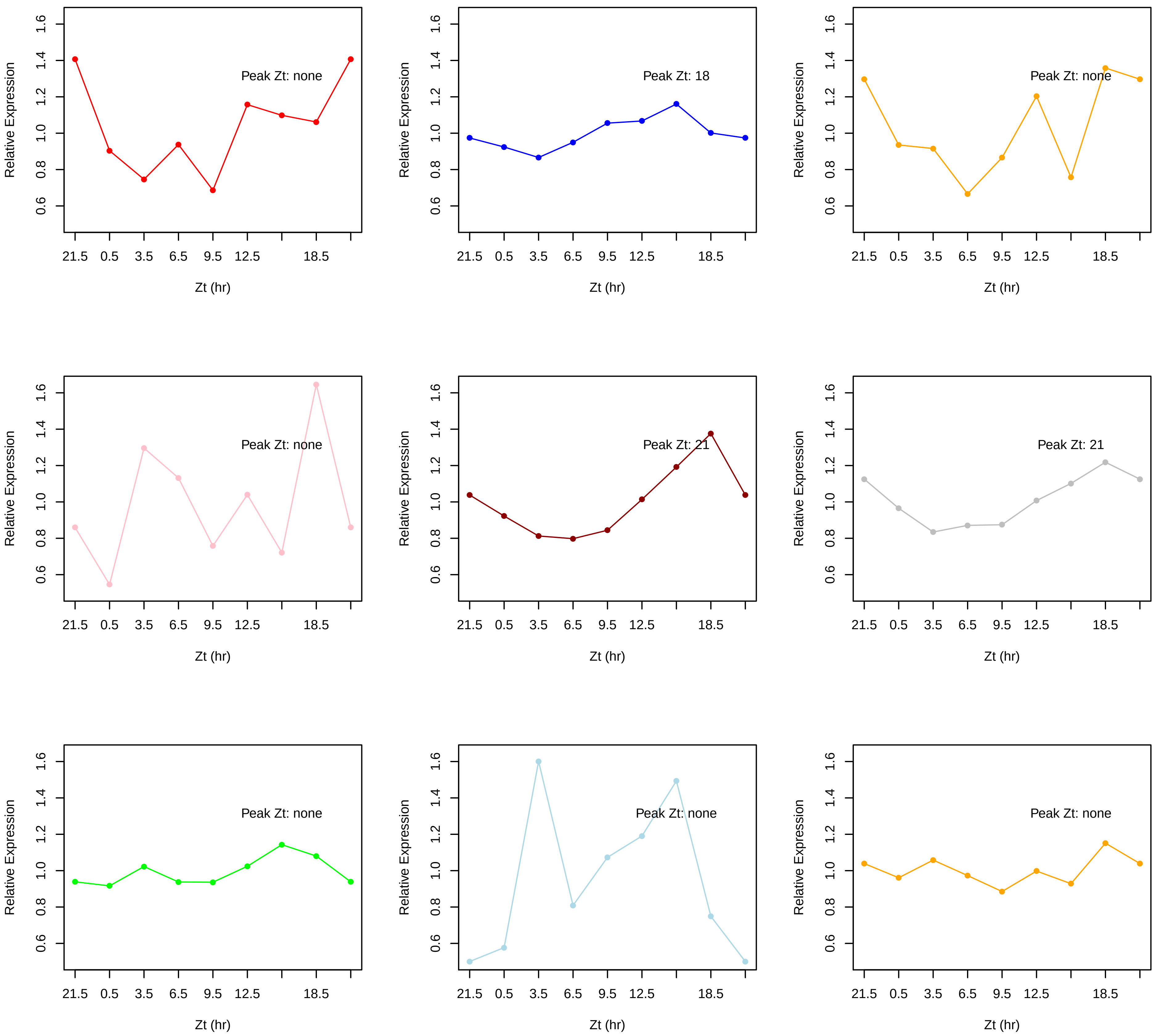

Singeleton: ENSXMAG00000008815 orai2

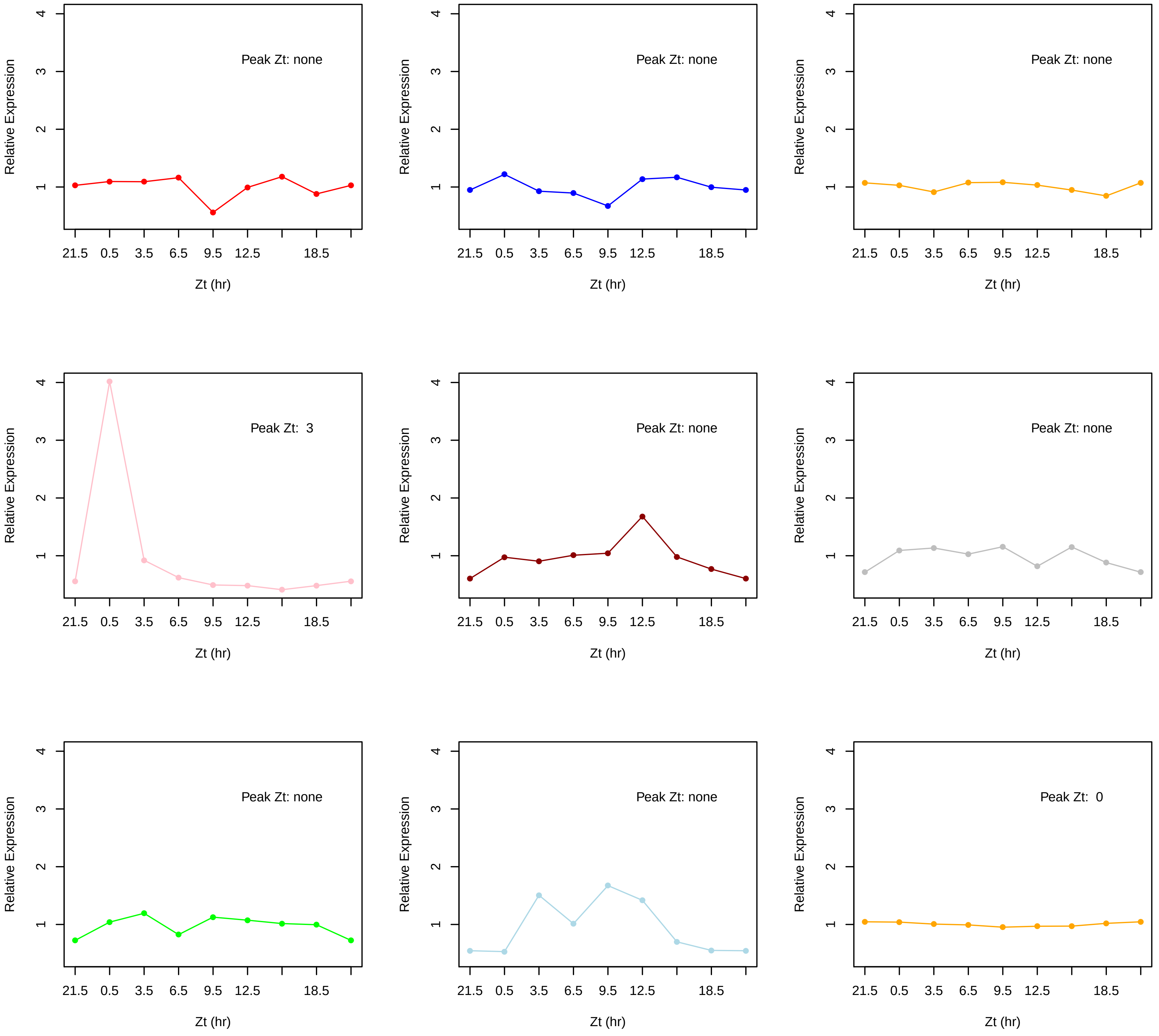

Singeleton: ENSXMAG00000012847 stk35

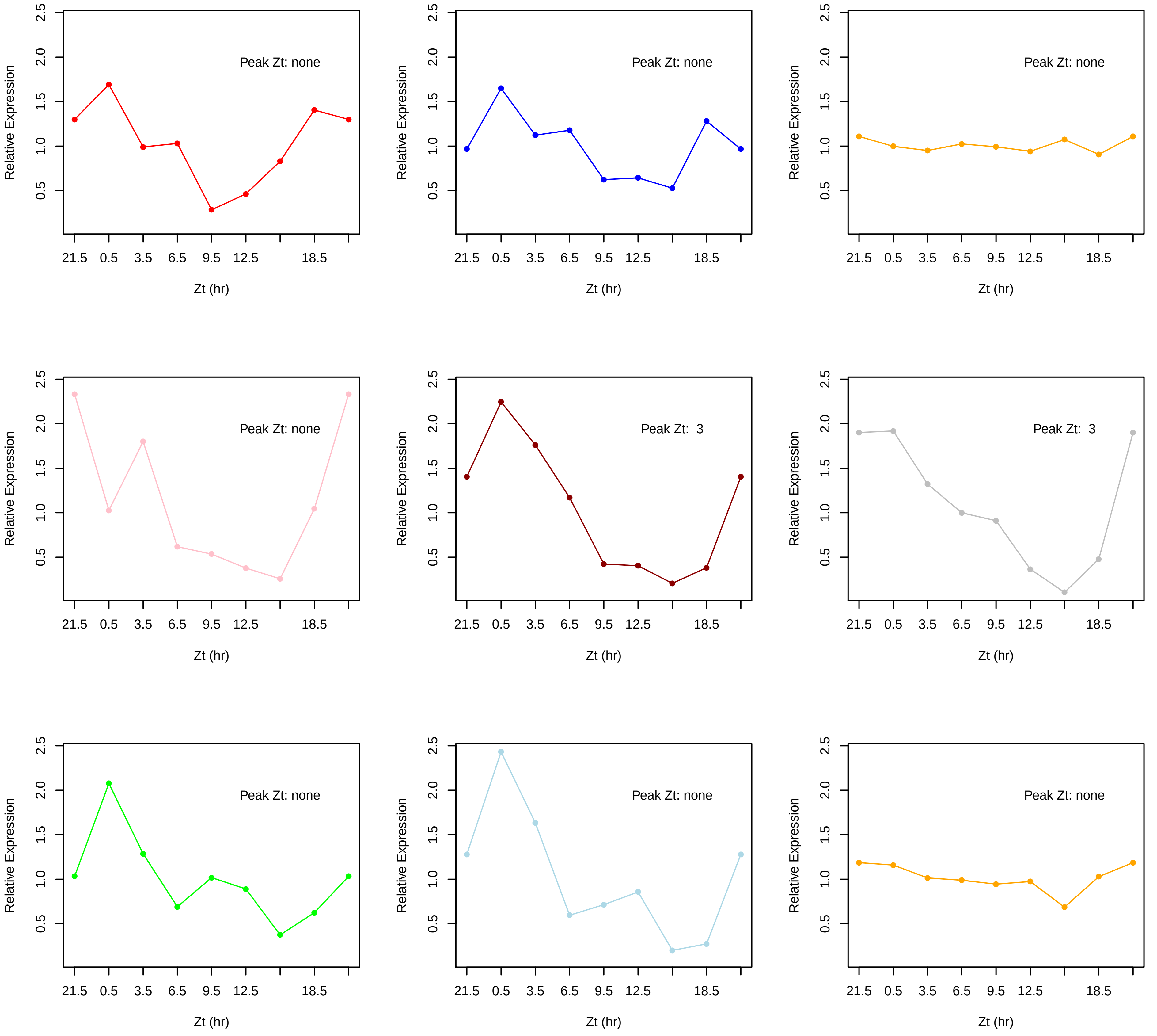

Singleton: ENSXMAG00000007846 mgat5

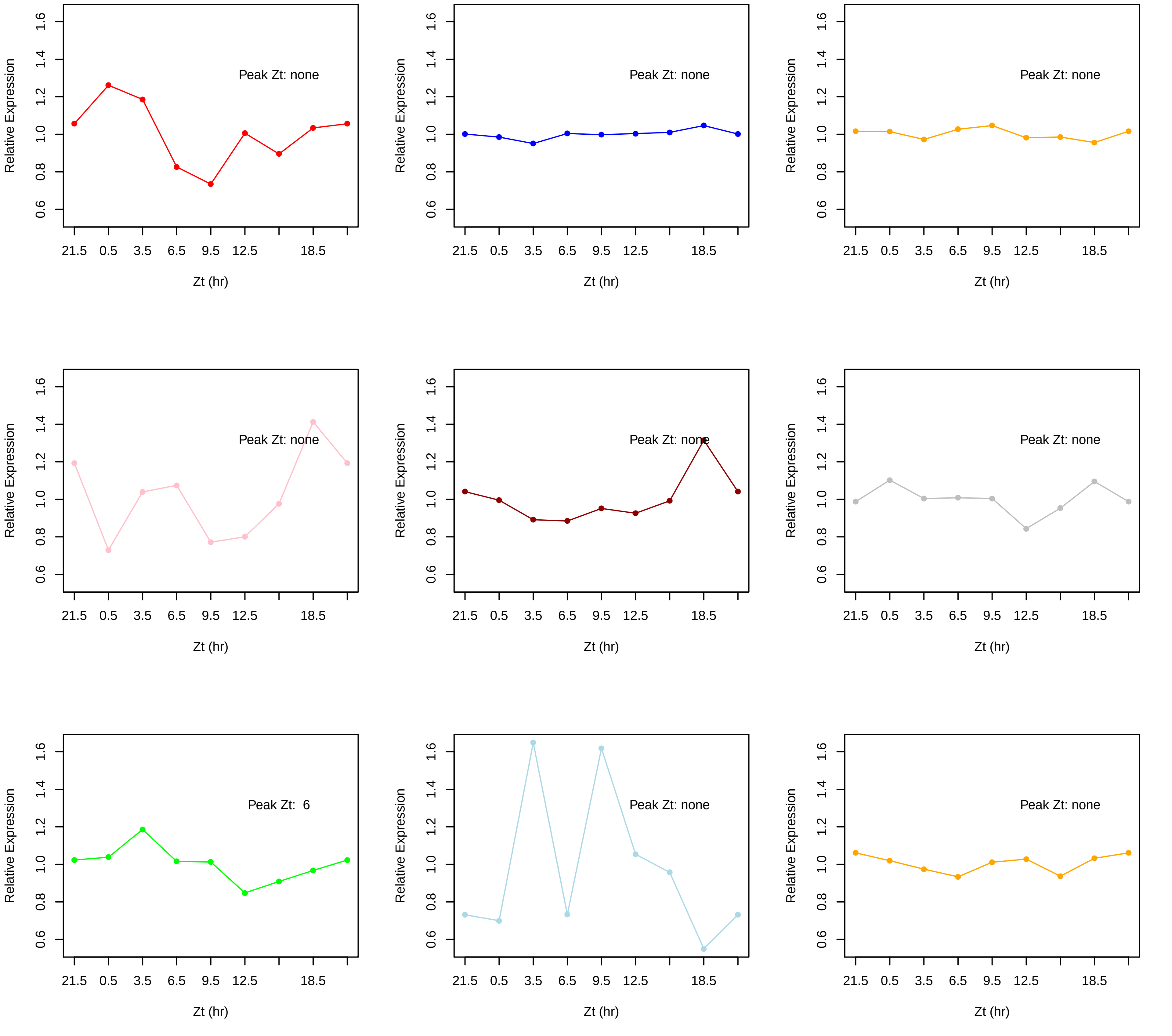

Singeleton: ENSXMAG00000026772 pdyn

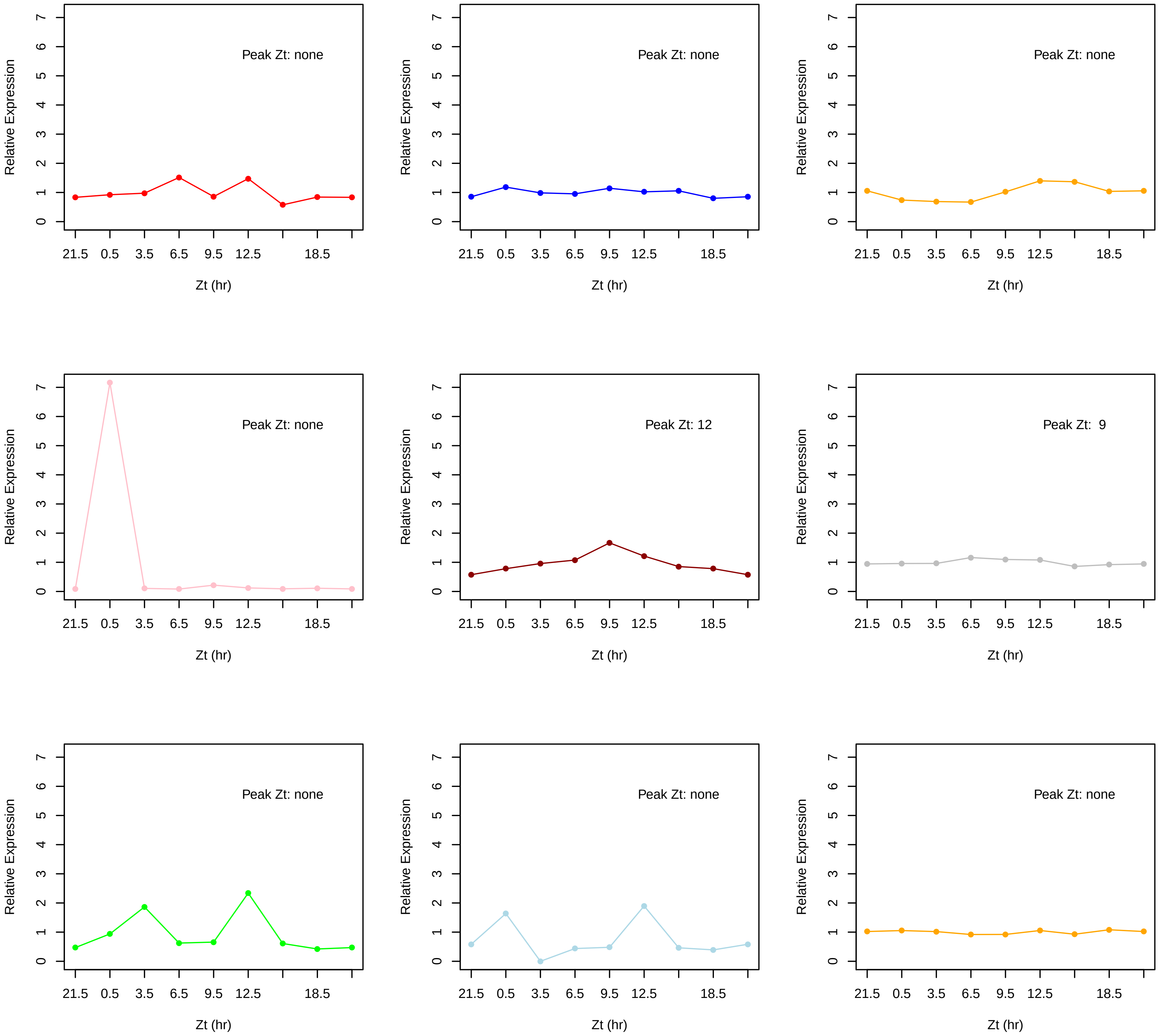

Singeleton: ENSXMAG00000011819 tmem259

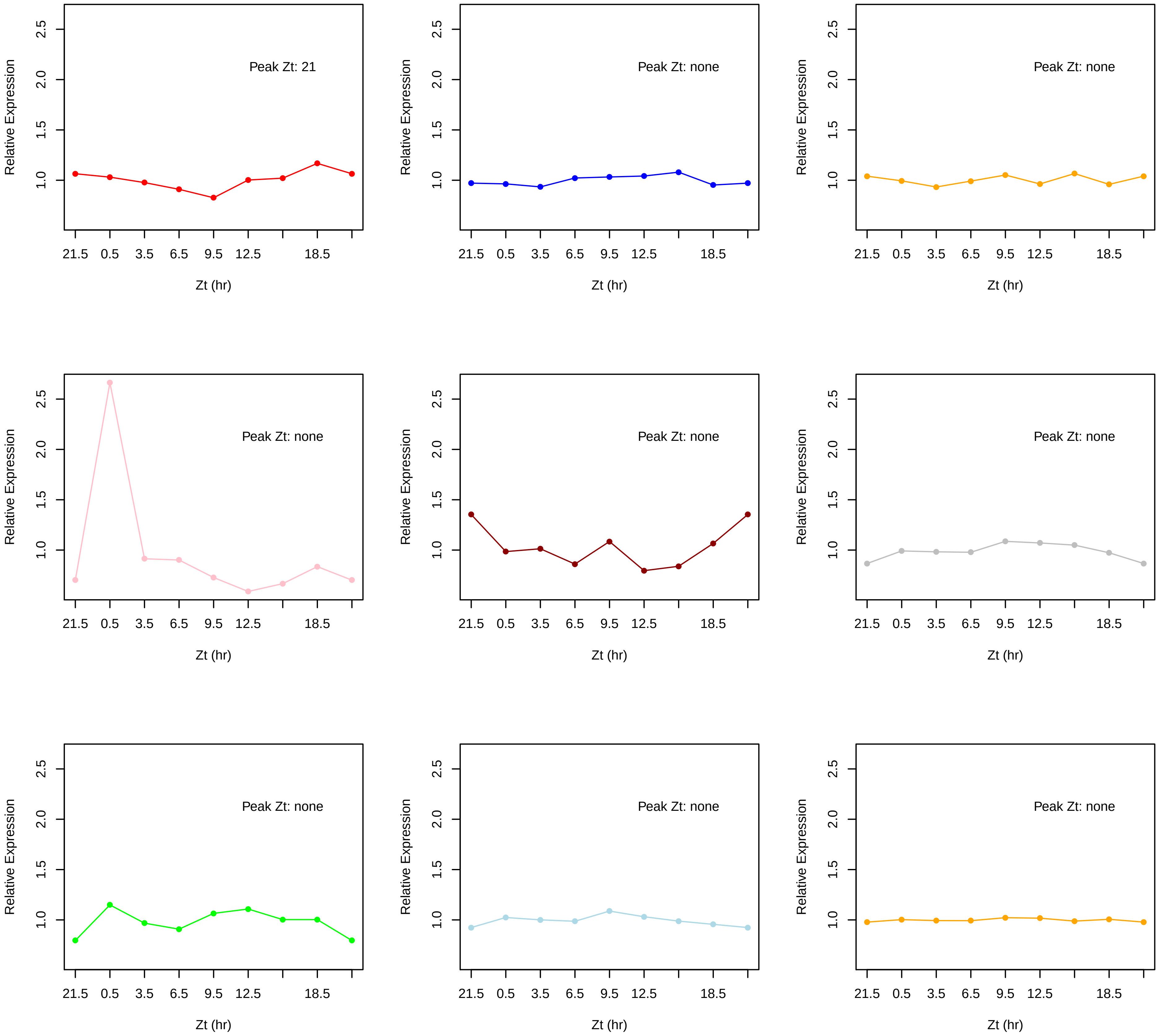

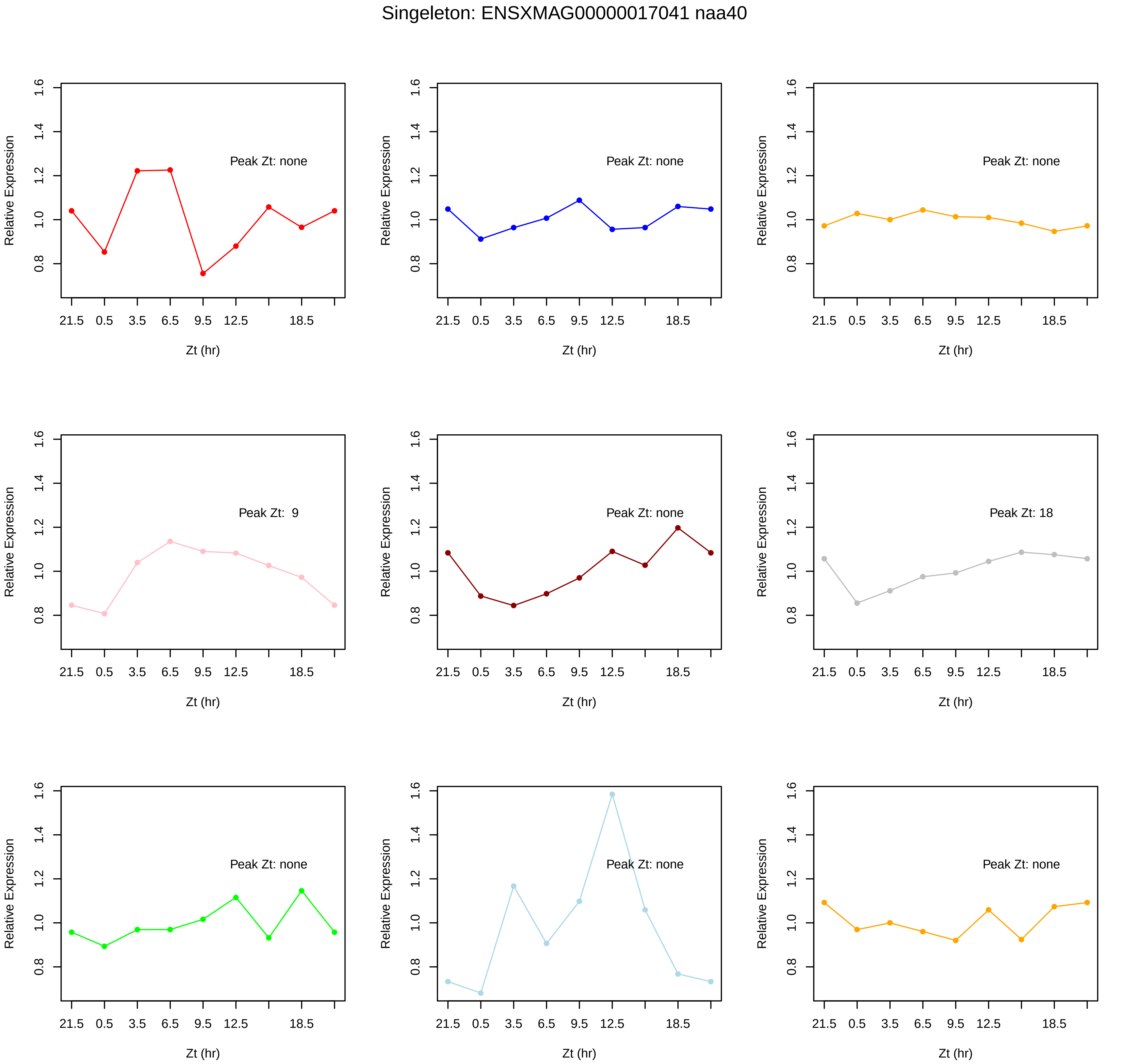
Singleton: ENSXMAG00000017041 naa40
Peak Zt: none
Peak Zt: none
Peak Zt: none
Peak Zt: 9
Peak Zt: none
Peak Zt: 18
Peak Zt: none
Peak Zt: none
Peak Zt: none
Relative Expression
Zt (hr)
21.5
0.5
3.5
6.5
9.5
12.5
18.5
0.8
1.0
1.2
1.4
1.6

Singeleton: ENSXMAG00000019414 mef2cb

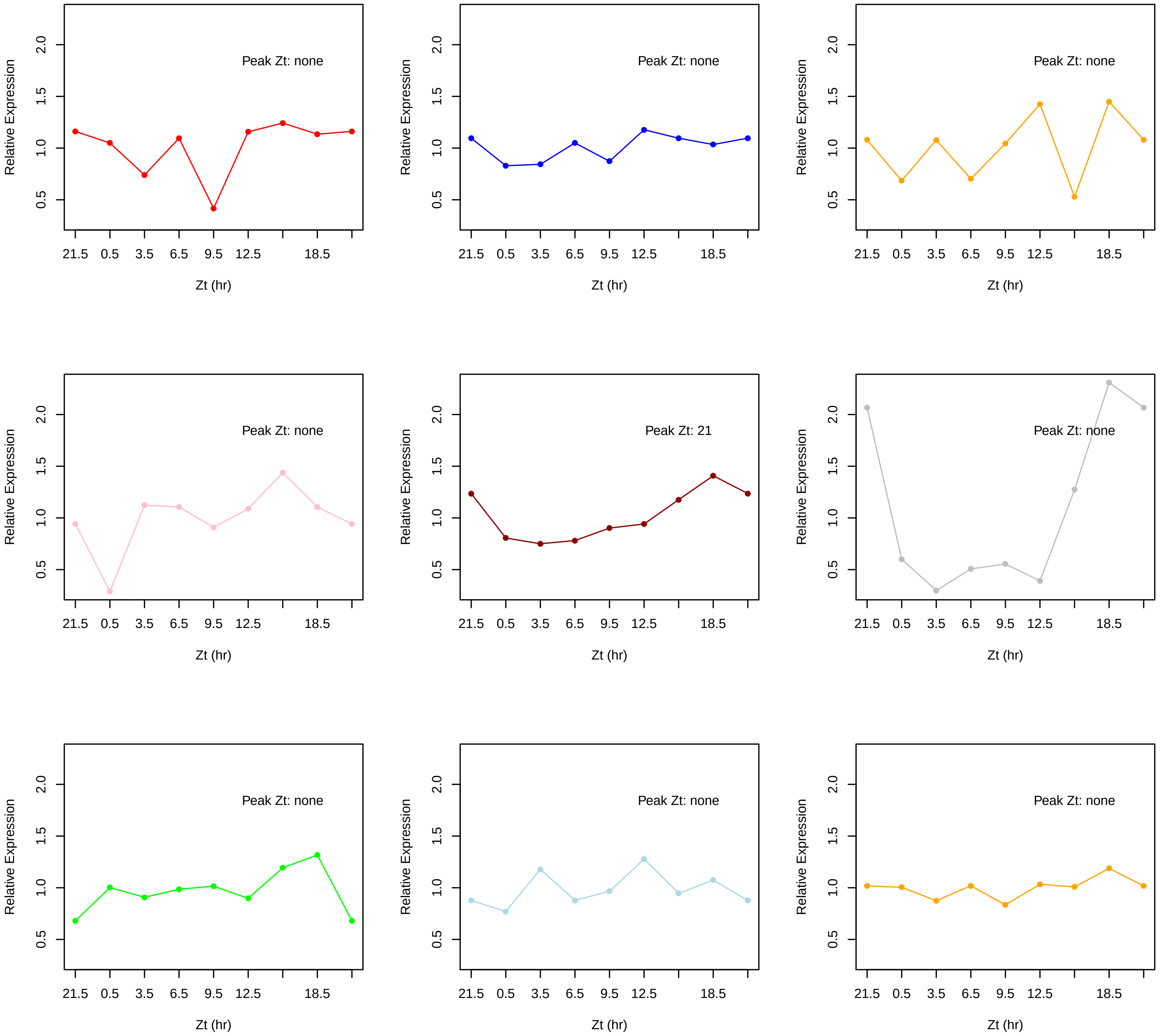

Singeleton: ENSXMAG00000011885 pafah1b2

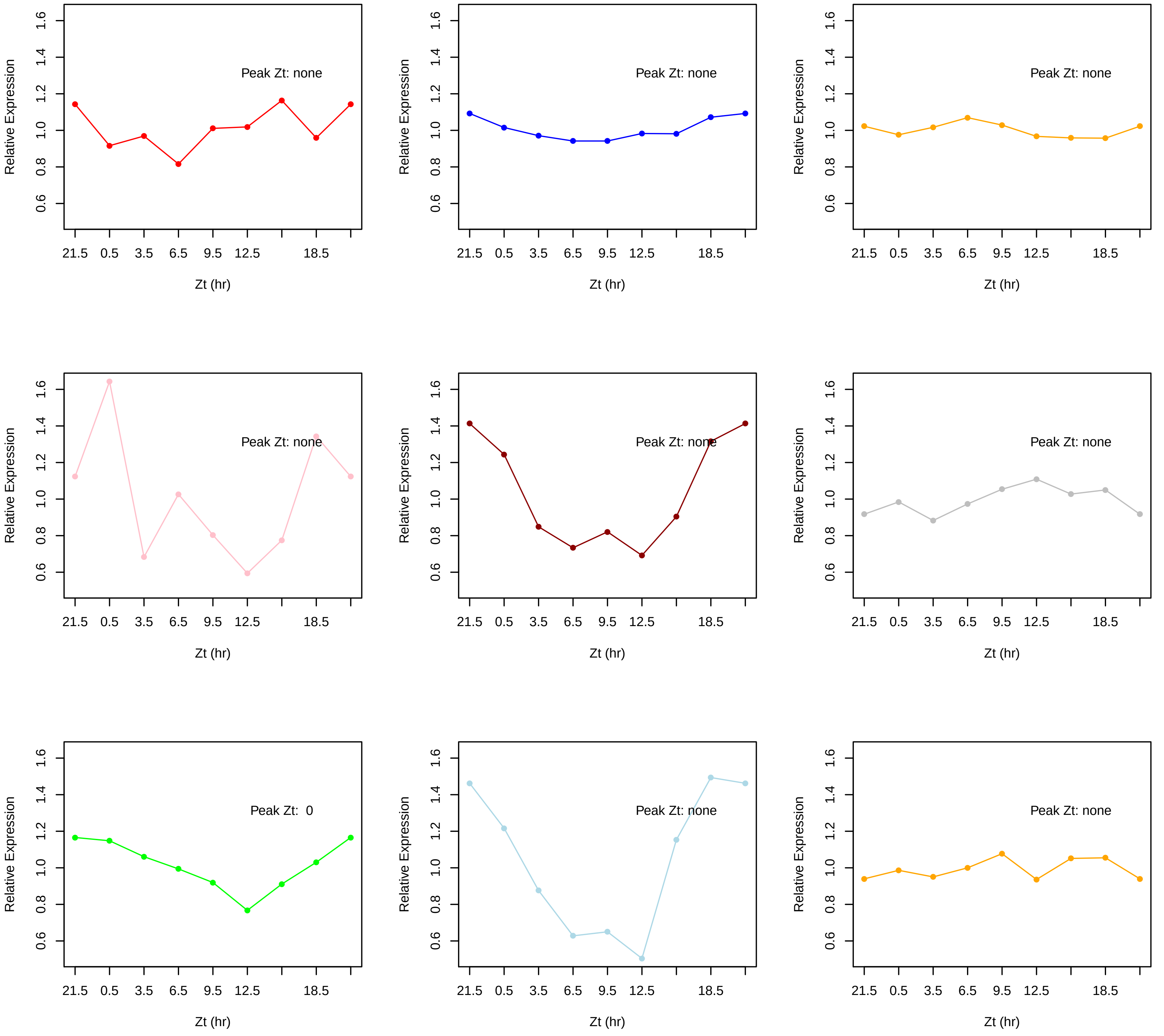

Singeleton: ENSXMAG00000010642

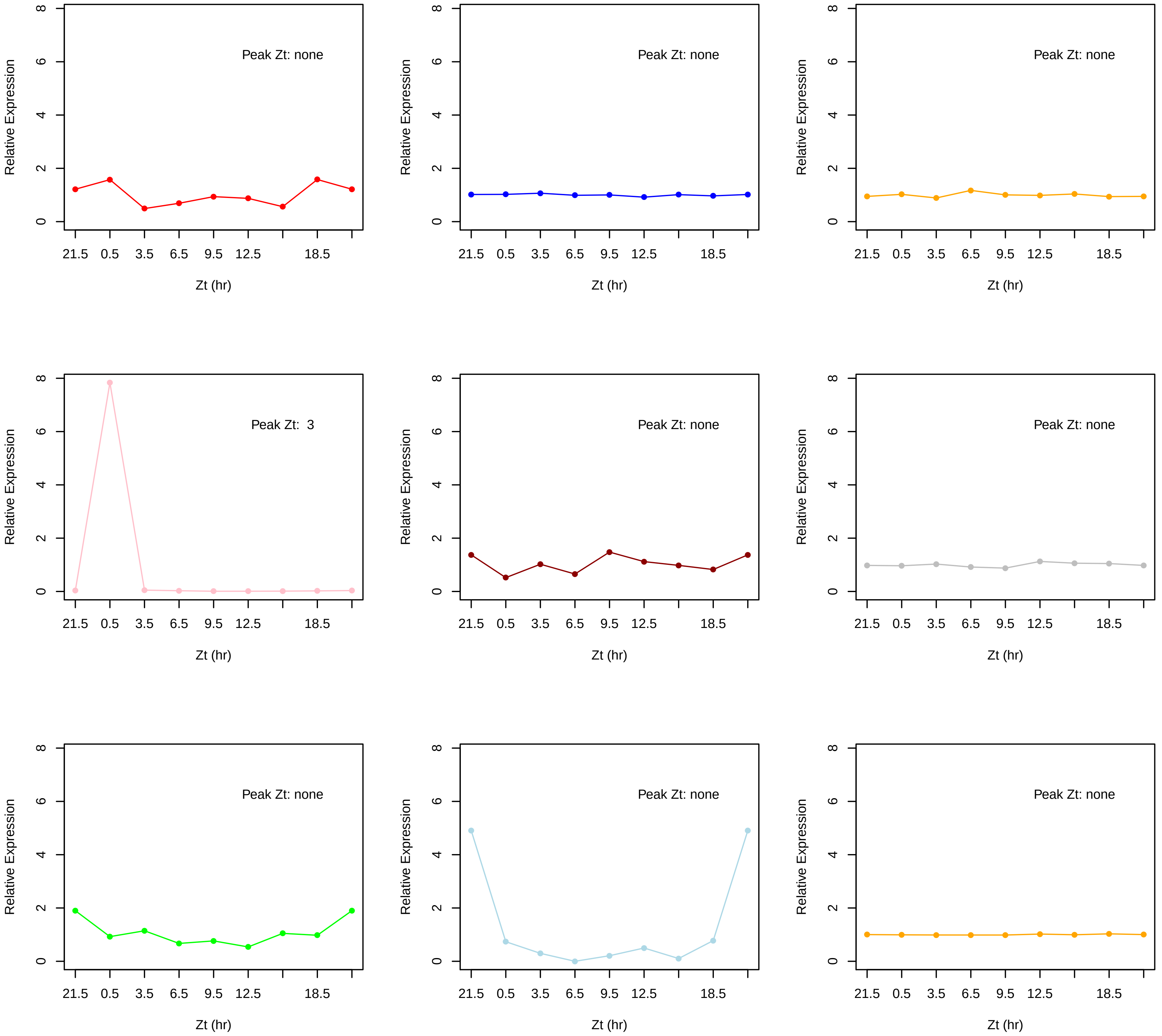

Singleton: ENSXMAG00000008786 lrwd1

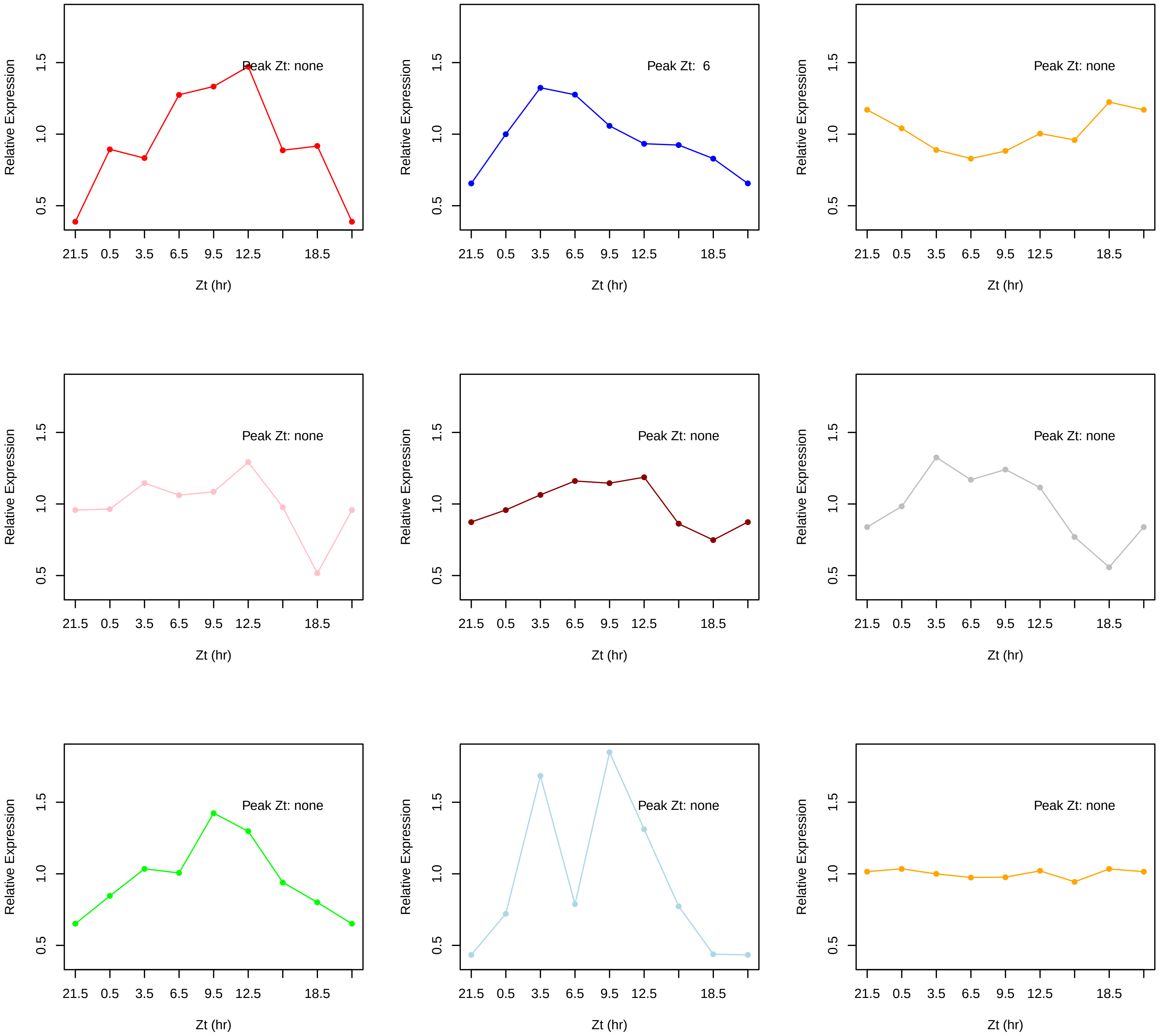

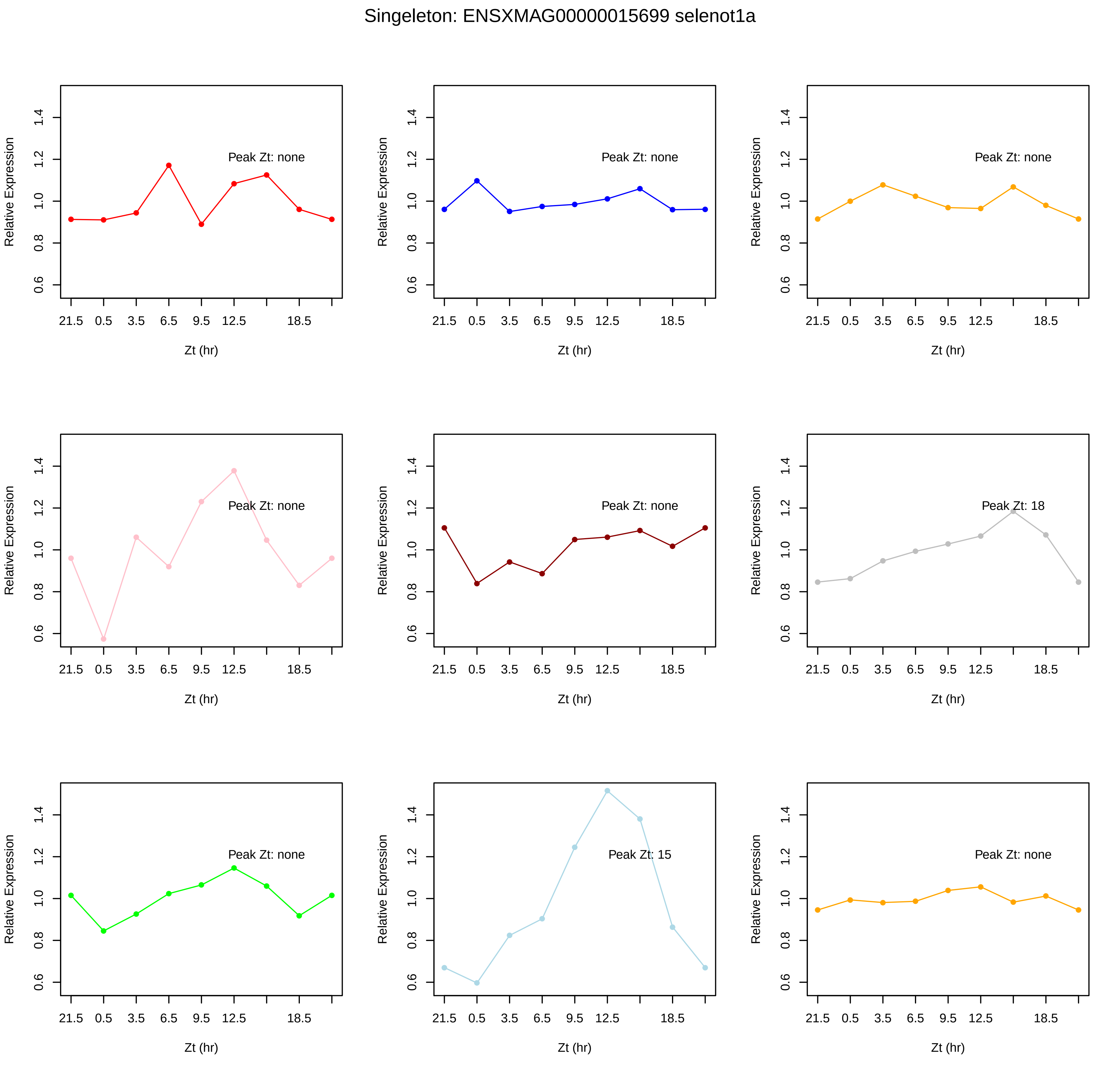

Singeleton: ENSXMAG00000015699 selenot1a
Peak Zt: none
Peak Zt: none
Peak Zt: none
Peak Zt: none
Peak Zt: none
Peak Zt: 18
Peak Zt: none
Peak Zt: 15
Peak Zt: none
Relative Expression
Zt (hr)
21.5
0.5
3.5
6.5
9.5
12.5
18.5
0.6
0.8
1.0
1.2
1.4

Singeleton: ENSXMAG00000002882

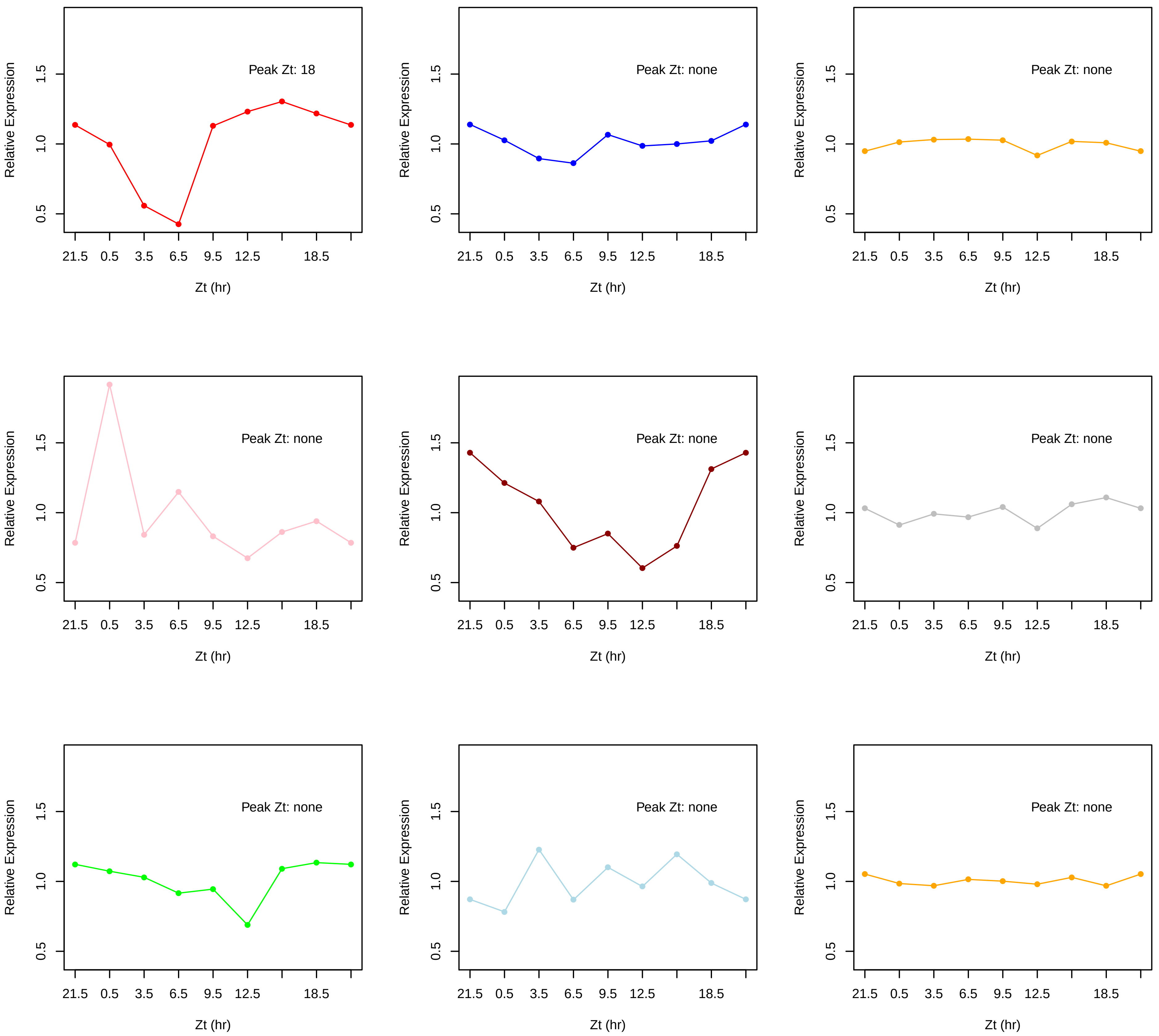

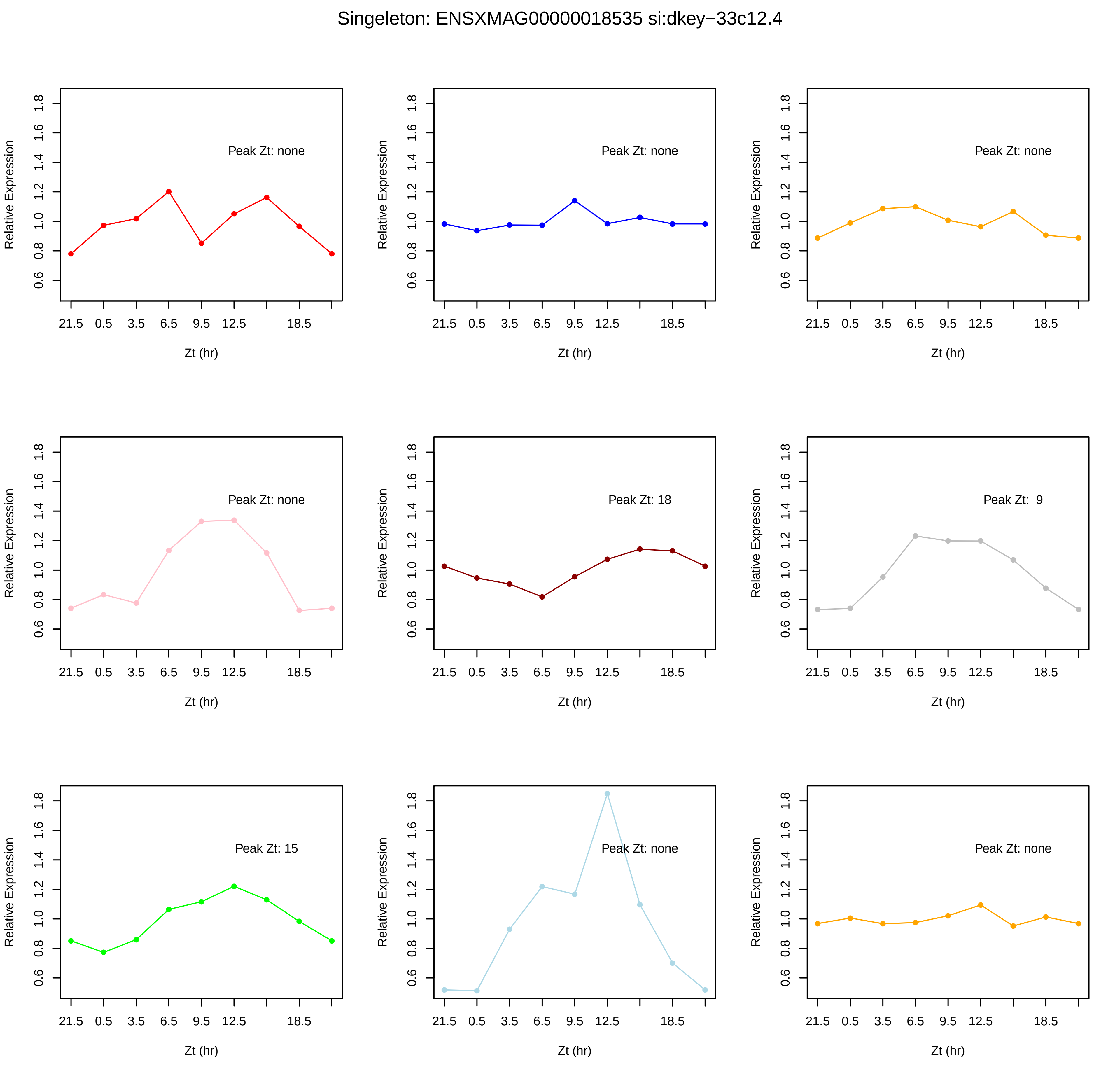

Singeleton: ENSXMAG00000018535 si:dkey−33c12.4
Peak Zt: none
Peak Zt: none
Peak Zt: none
Peak Zt: none
Peak Zt: 18
Peak Zt: 9
Peak Zt: 15
Peak Zt: none
Peak Zt: none
Relative Expression
Zt (hr)
21.5
0.5
3.5
6.5
9.5
12.5
18.5
0.6
0.8
1.0
1.2
1.4
1.6
1.8

Singeleton: ENSXMAG00000028054 mettl11b

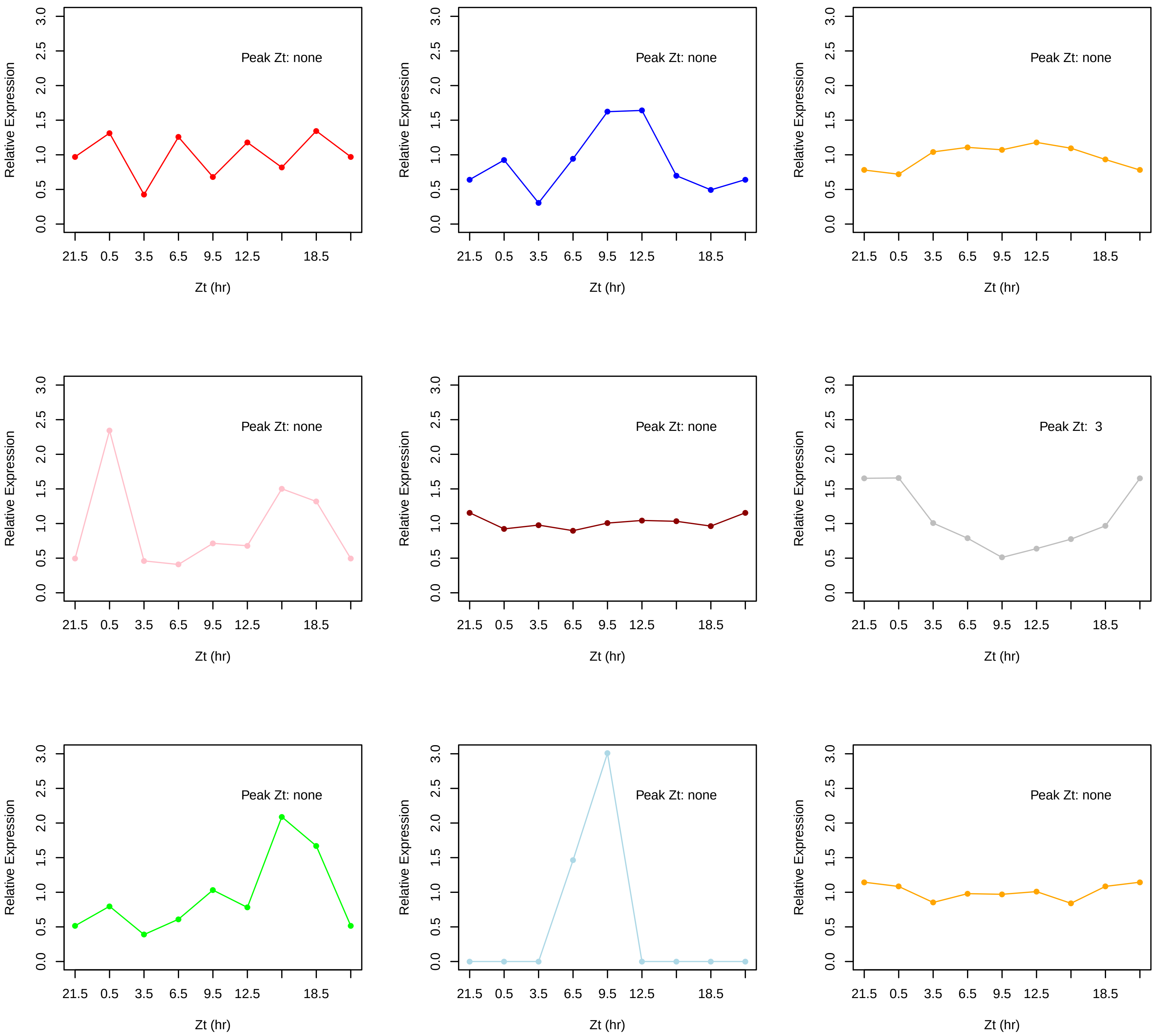

Singleton: ENSXMAG00000013306 EAF1

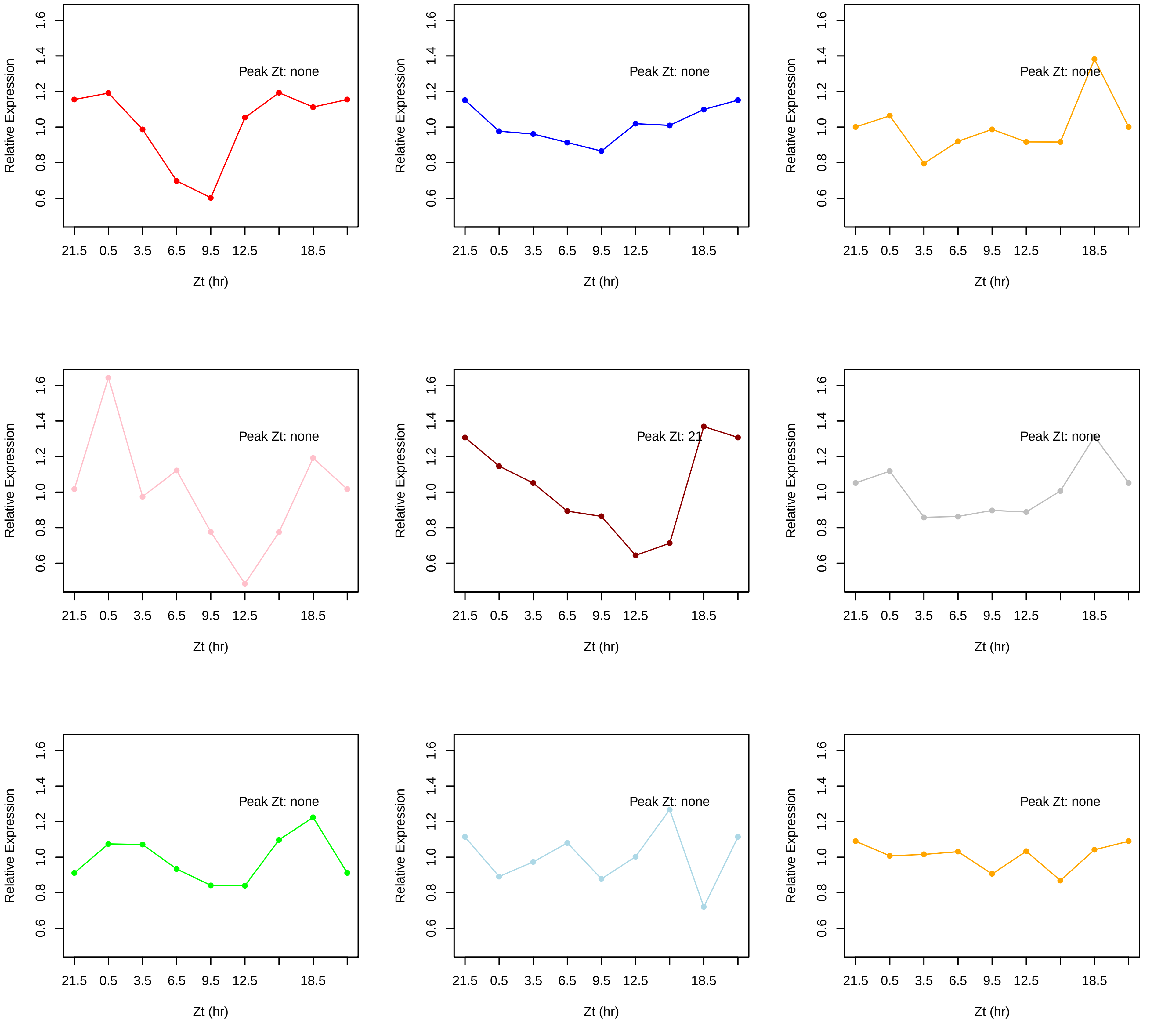

Singeleton: ENSXMAG00000019415 tmem161b

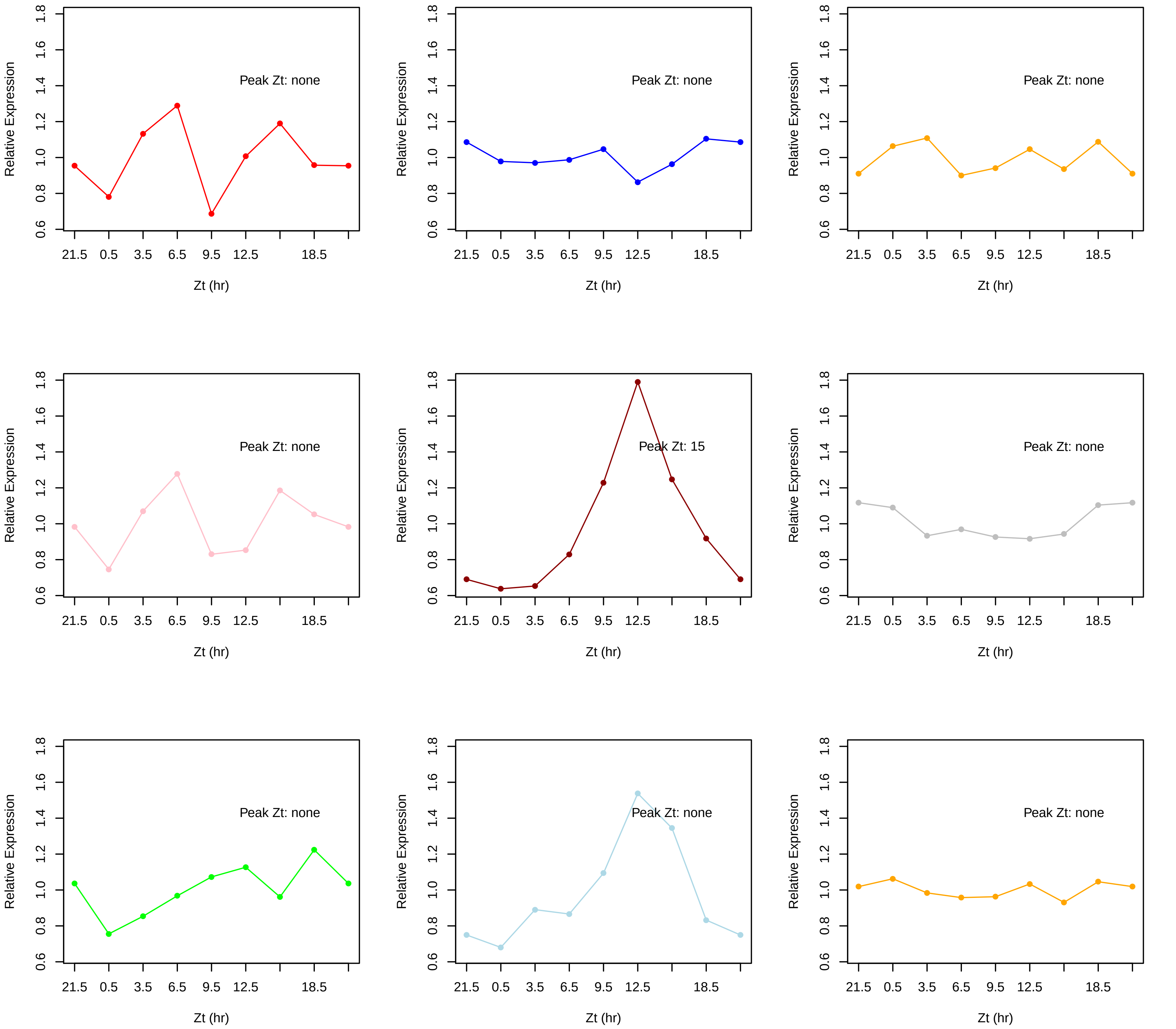

Singleton: ENSXMAG00000005917 abhd3

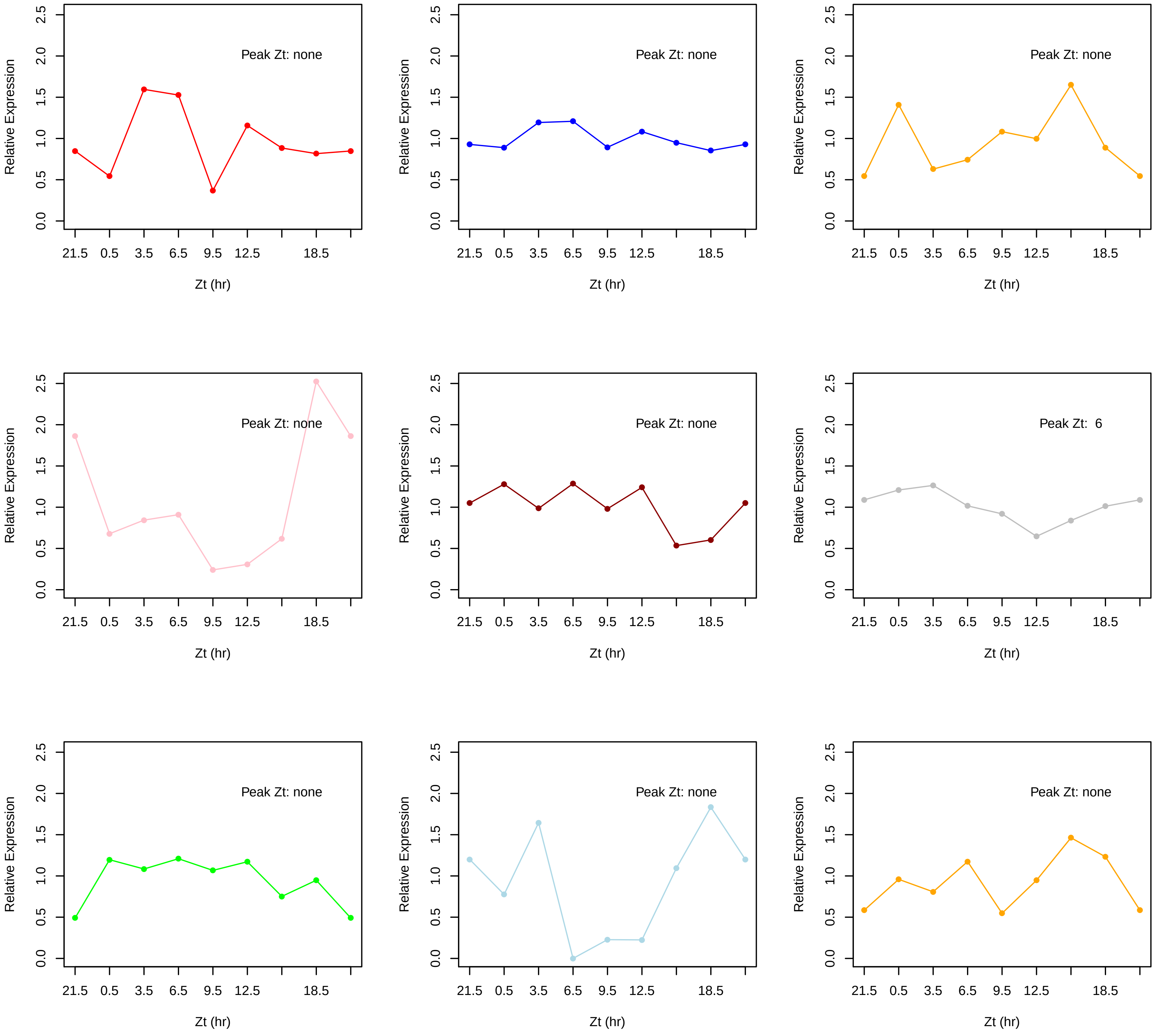

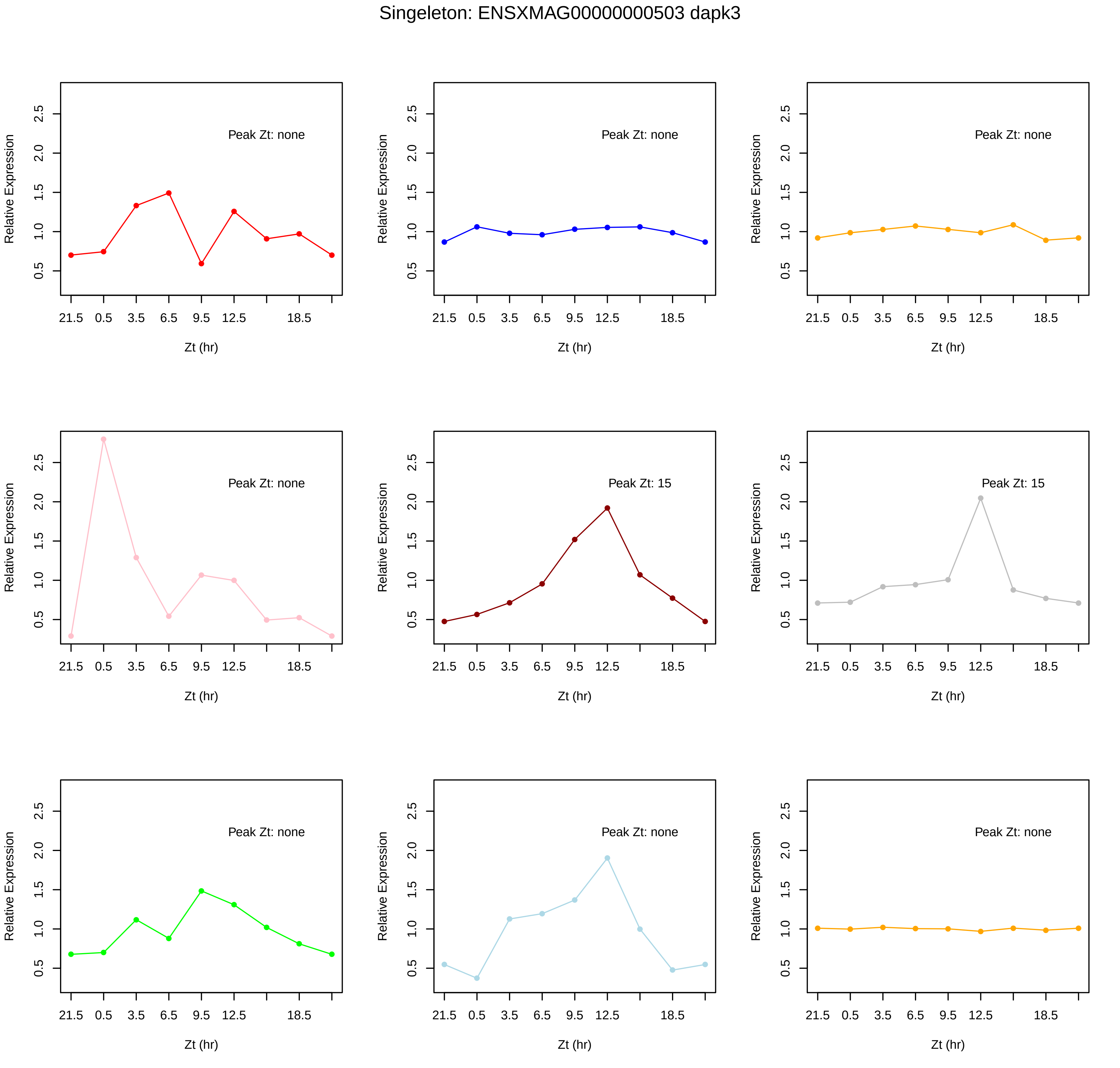
Singeleton: ENSXMAG00000000503 dapk3
Peak Zt: none
Peak Zt: none
Peak Zt: none
Peak Zt: none
Peak Zt: 15
Peak Zt: 15
Peak Zt: none
Peak Zt: none
Peak Zt: none
Relative Expression
Zt (hr)
21.5 0.5 3.5 6.5 9.5 12.5 18.5
0.5 1.0 1.5 2.0 2.5

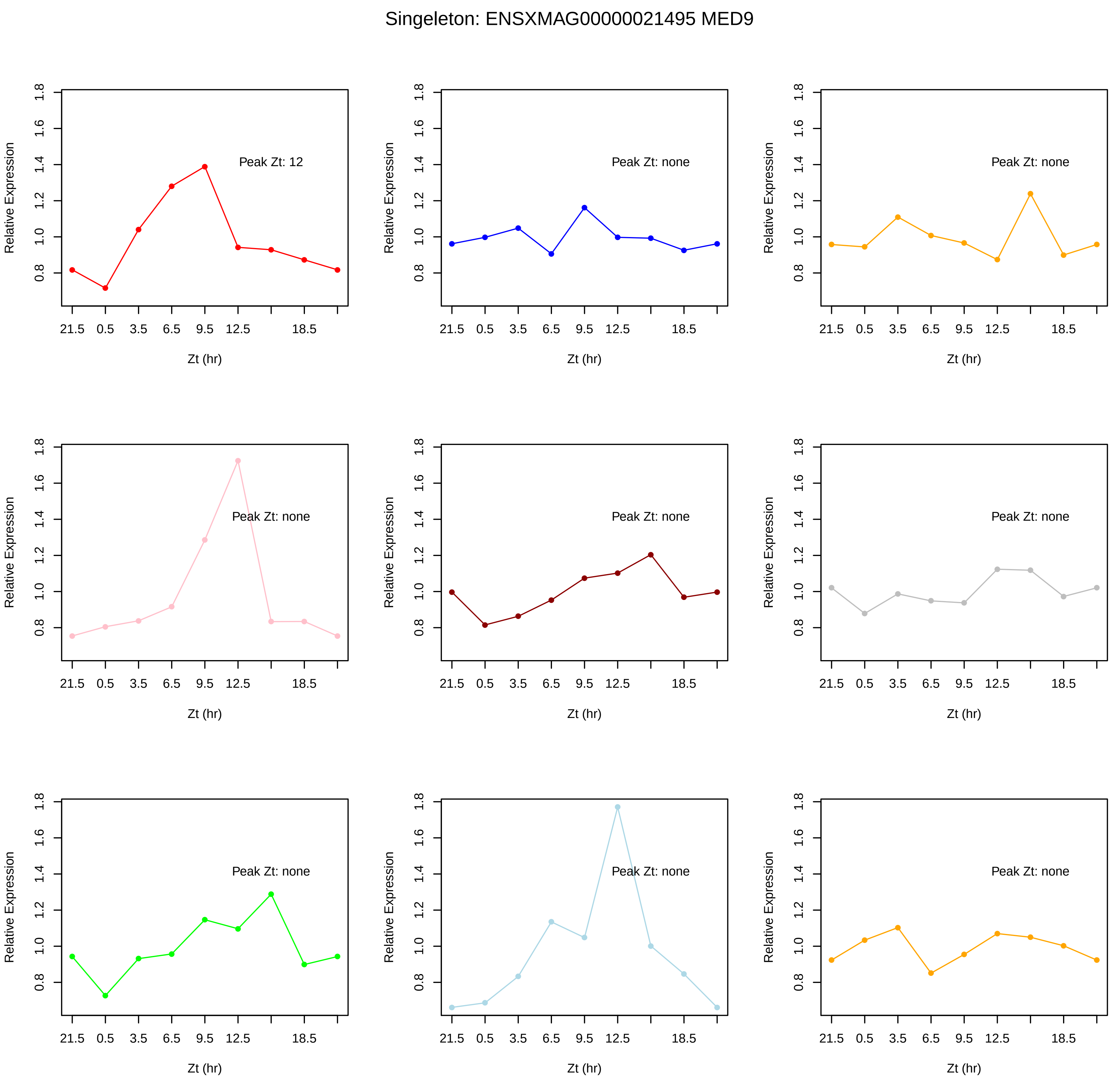
Singleton: ENSXMAG00000021495 MED9
Relative Expression
Zt (hr)
21.5 0.5 3.5 6.5 9.5 12.5 18.5
0.8 1.0 1.2 1.4 1.6 1.8
Peak Zt: 12
Peak Zt: none
Peak Zt: none
Peak Zt: none
Peak Zt: none
Peak Zt: none
Peak Zt: none
Peak Zt: none
Peak Zt: none

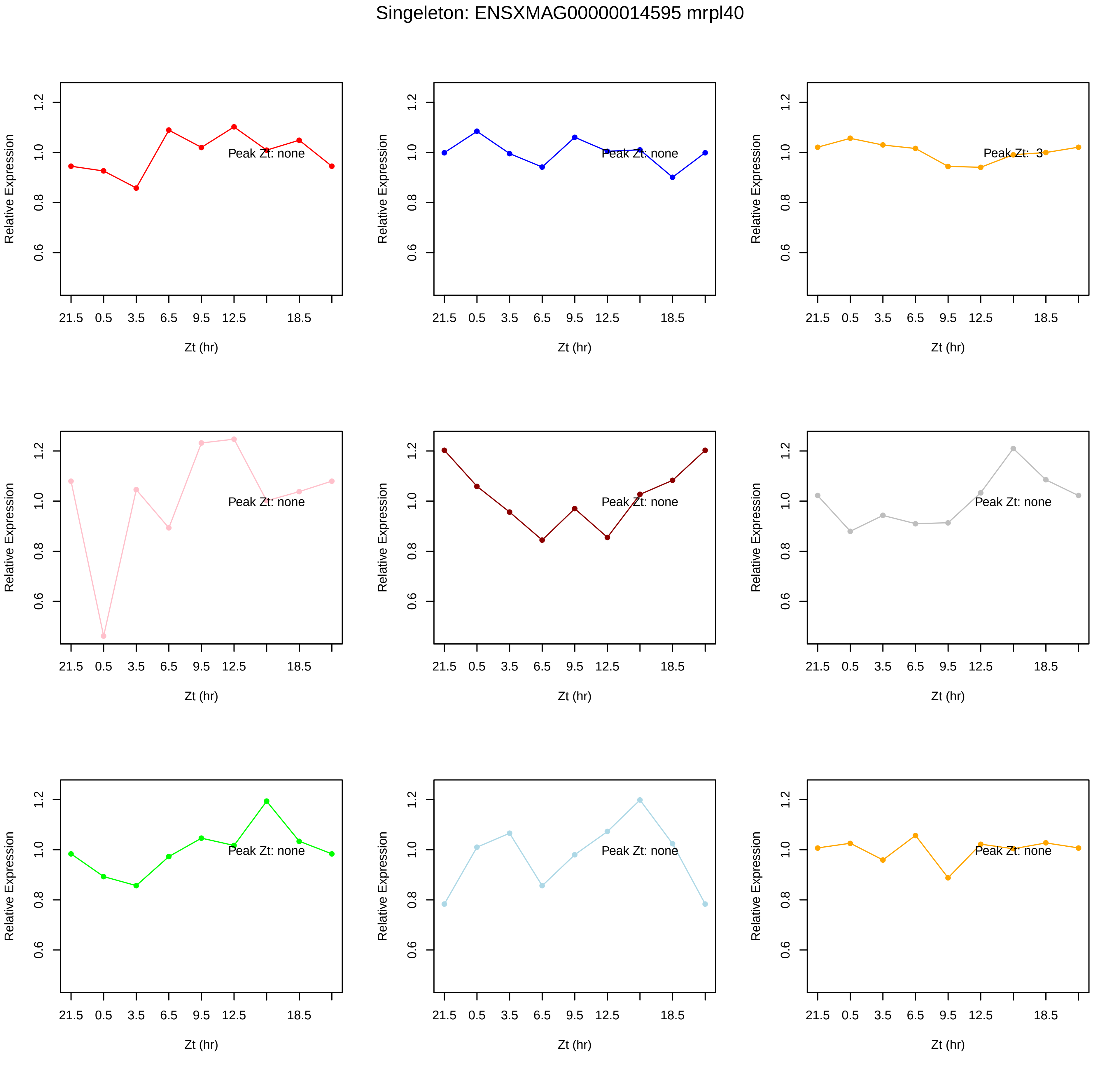

Singeleton: ENSXMAG00000014595 mrpl40
Relative Expression
Zt (hr)
21.5
0.5
3.5
6.5
9.5
12.5
18.5
0.6
0.8
1.0
1.2
Peak Zt: none
Peak Zt: 3

Singleton: ENSXMAG00000000452 mturn

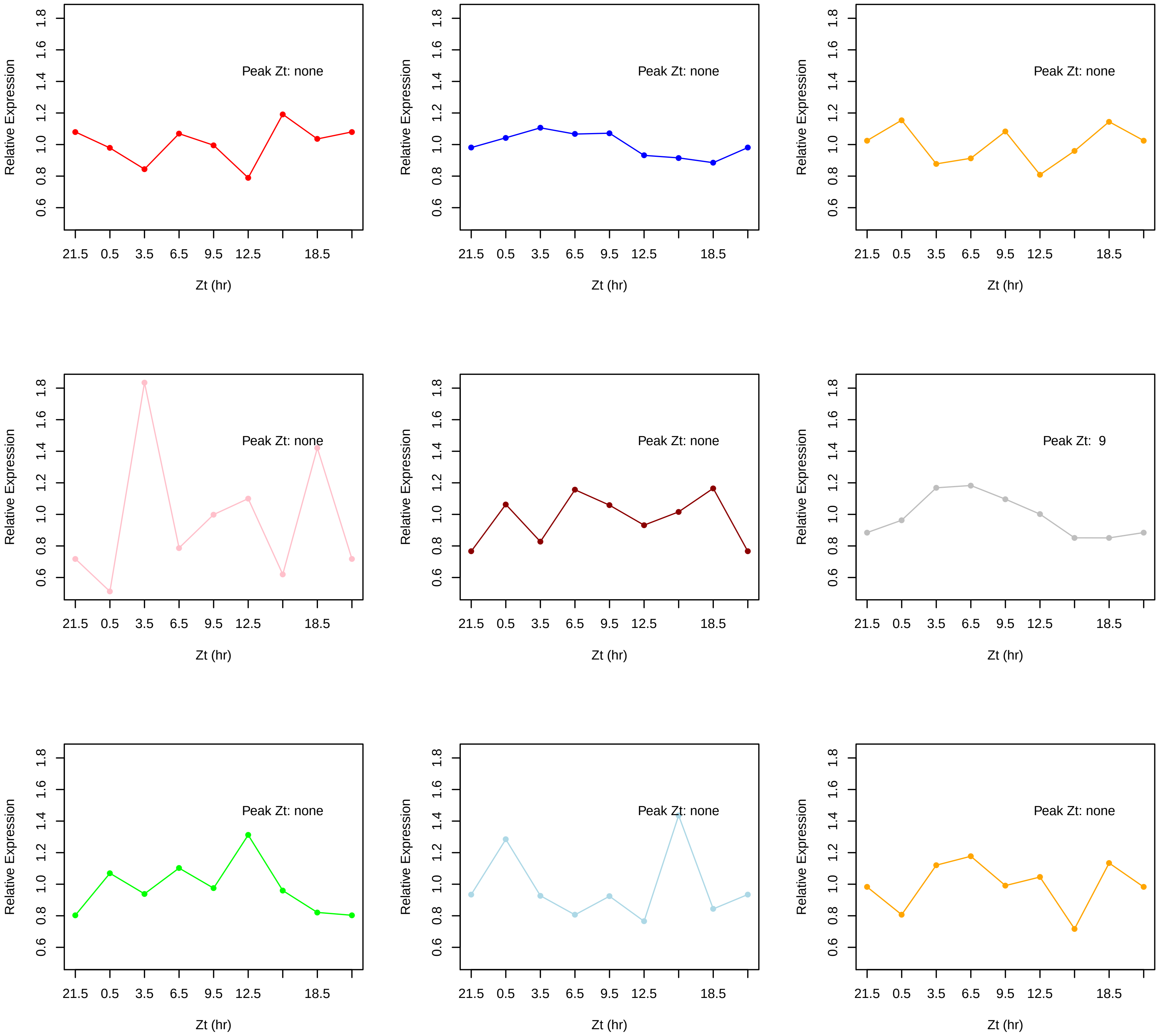

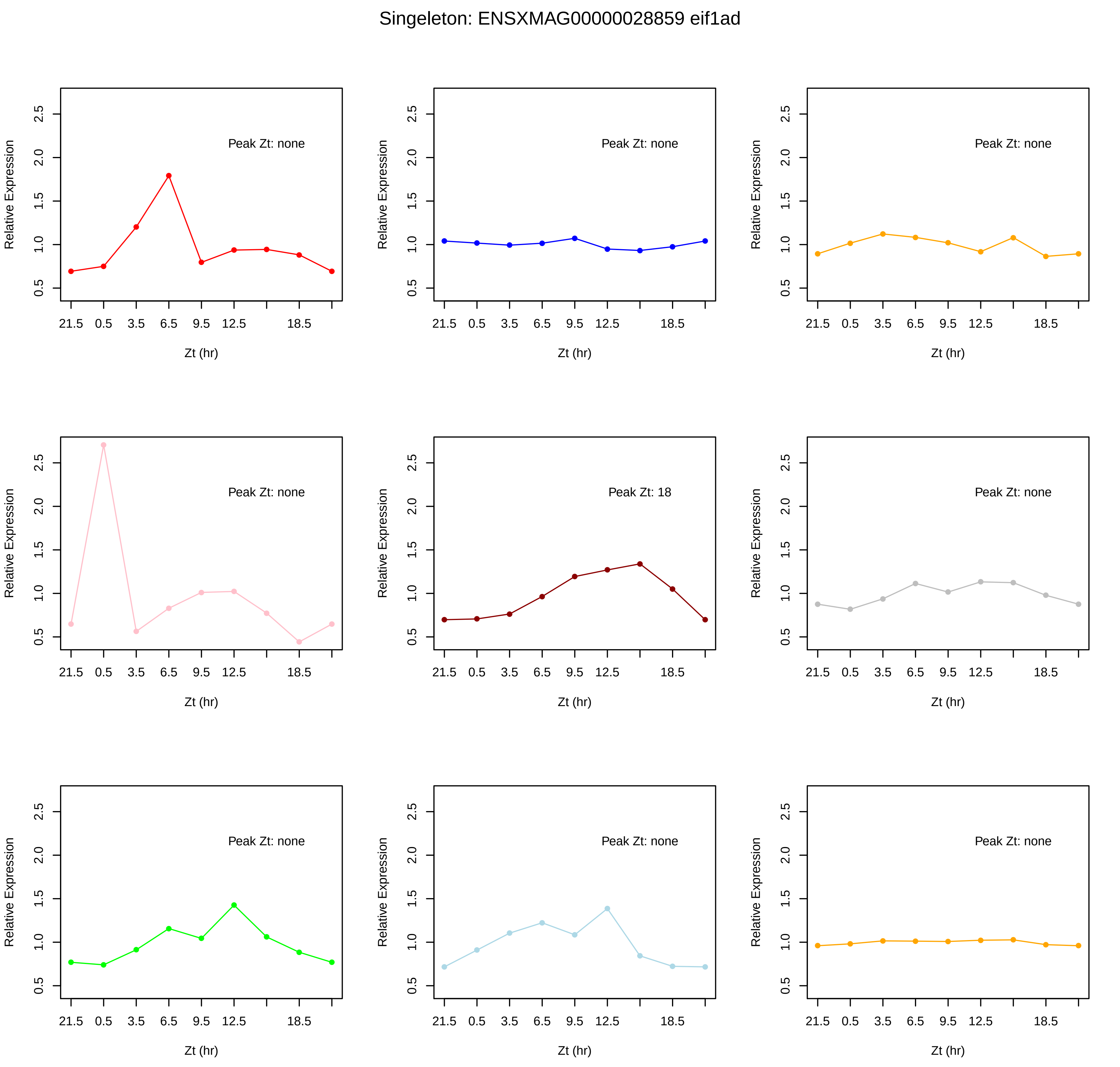

Singleton: ENSXMAG00000028859 eif1ad
Peak Zt: none
Peak Zt: none
Peak Zt: none
Peak Zt: none
Peak Zt: 18
Peak Zt: none
Peak Zt: none
Peak Zt: none
Peak Zt: none
Relative Expression
Zt (hr)
21.5
0.5
3.5
6.5
9.5
12.5
18.5
0.5
1.0
1.5
2.0
2.5

Singeleton: ENSXMAG00000012422 polr2j

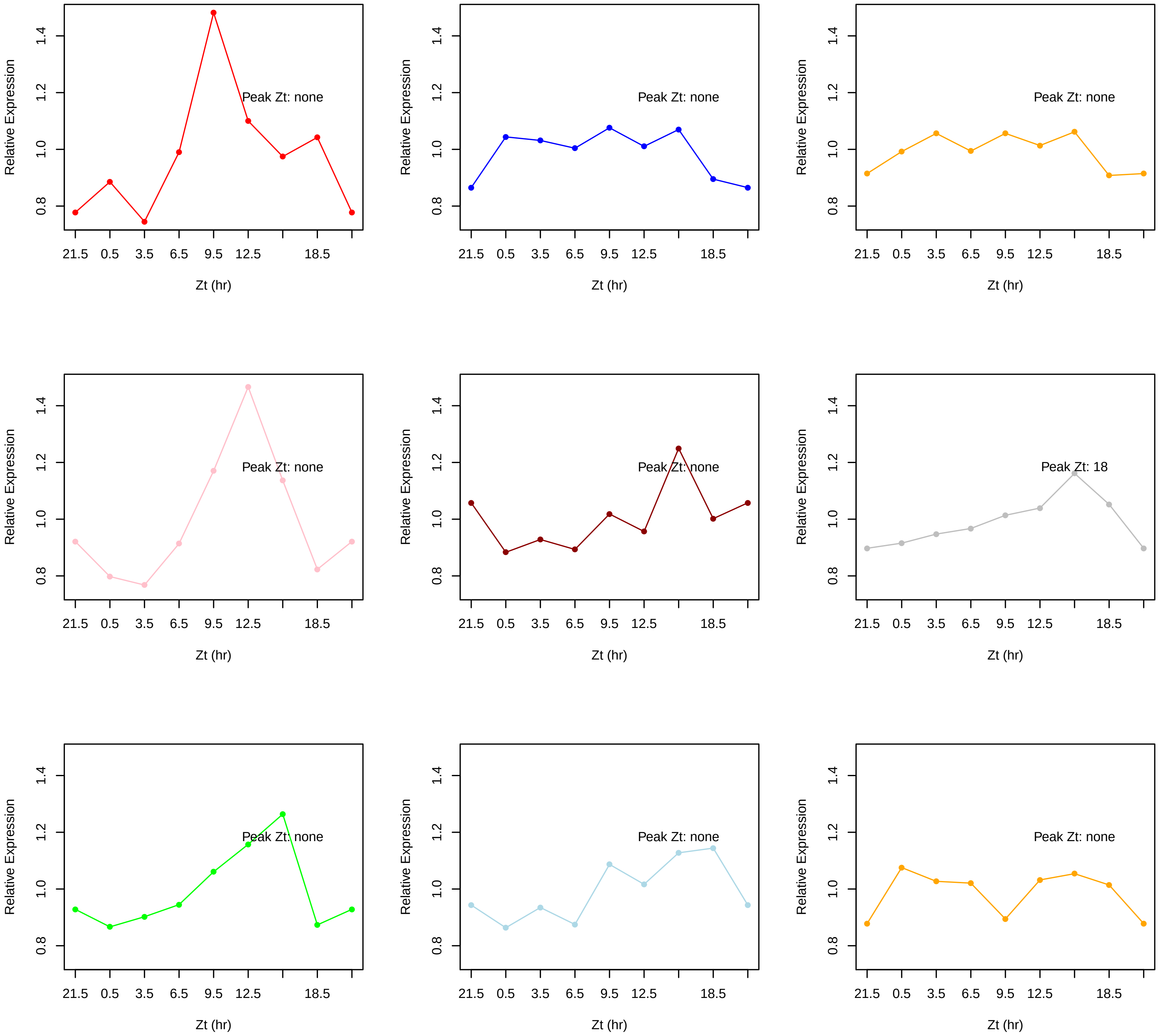

Singeleton: ENSXMAG00000024266 tsen34

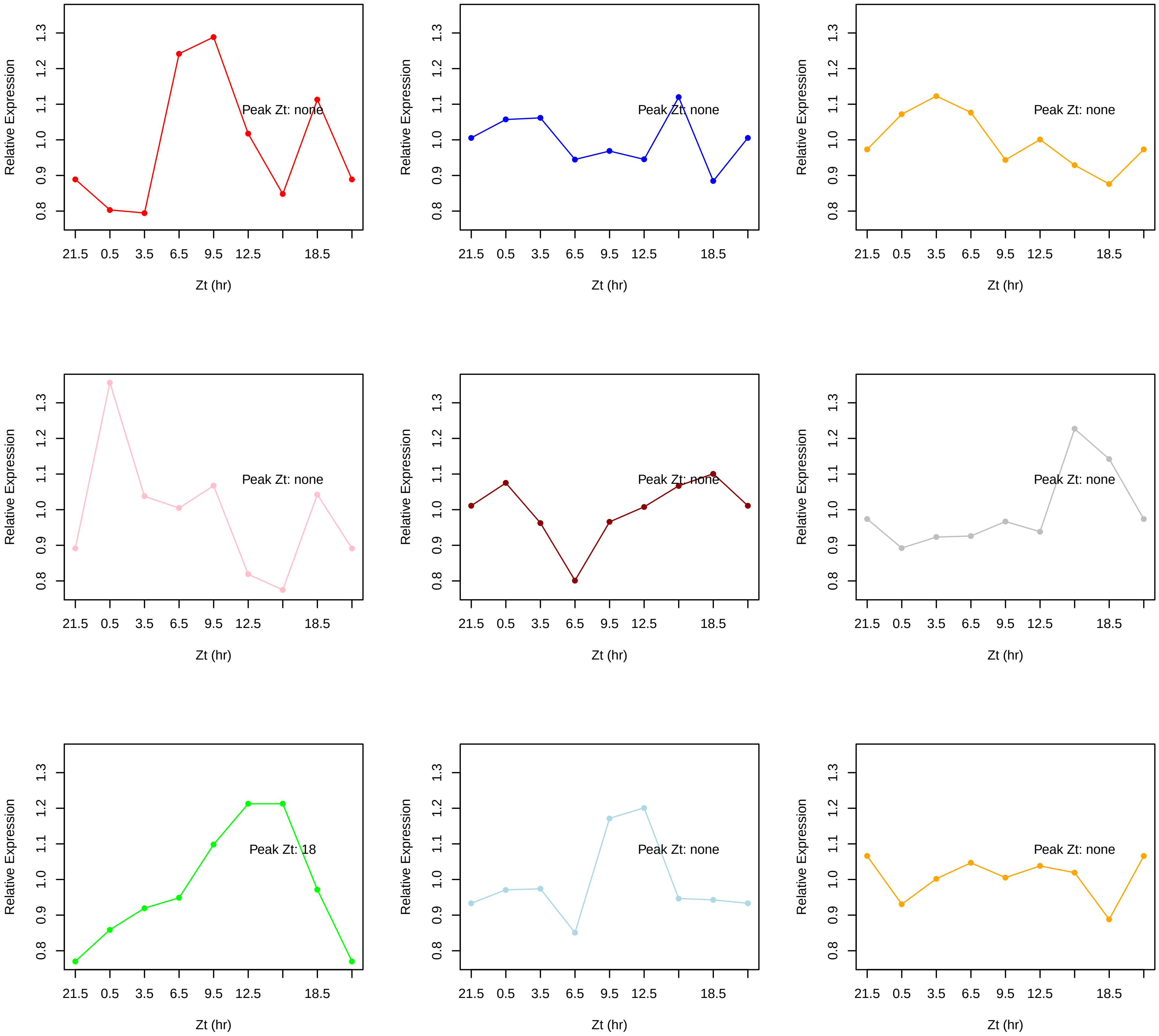

Singeleton: ENSXMAG00000005462 copa

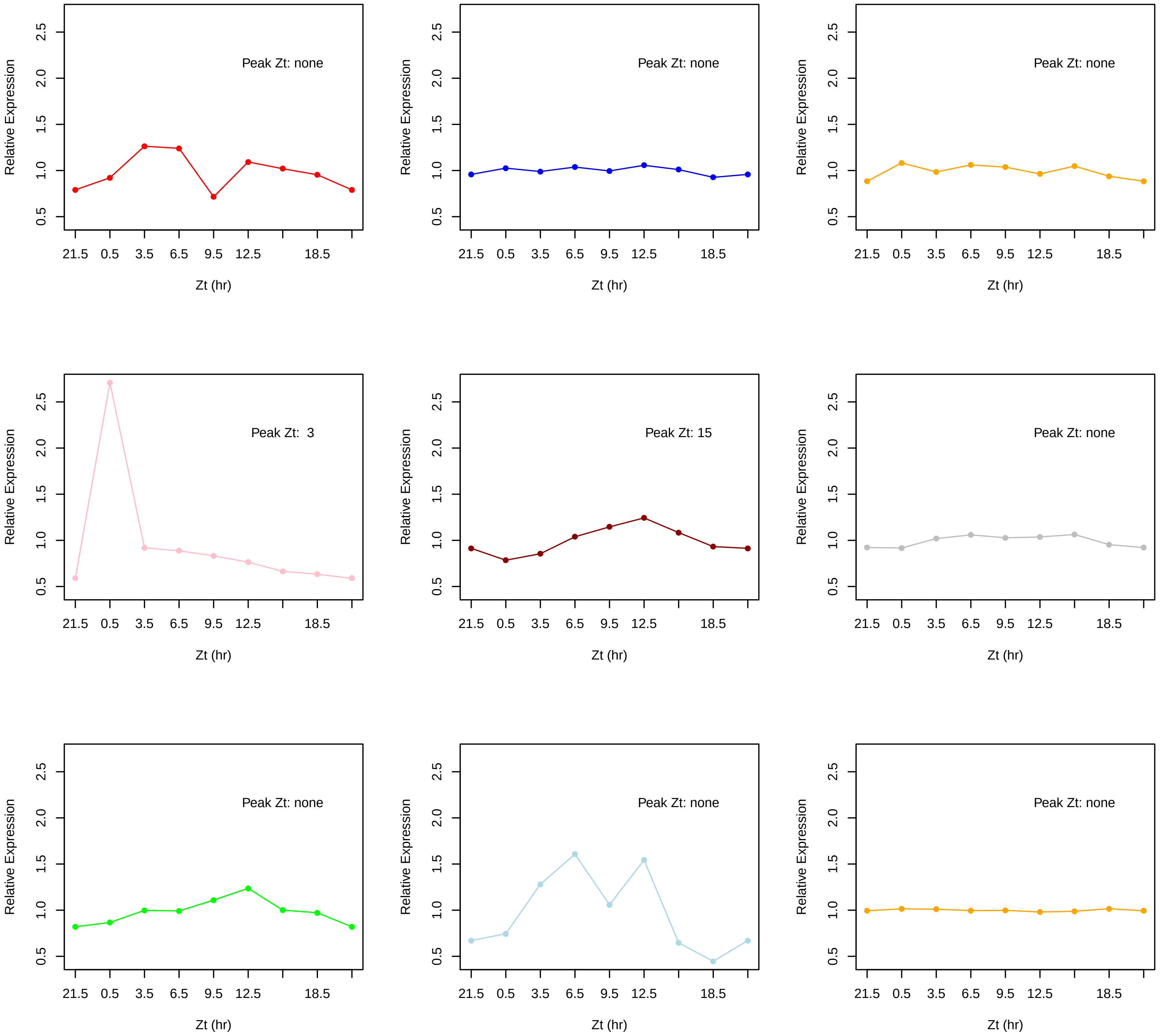

Singleton: ENSXMAG00000005444 prrx1b

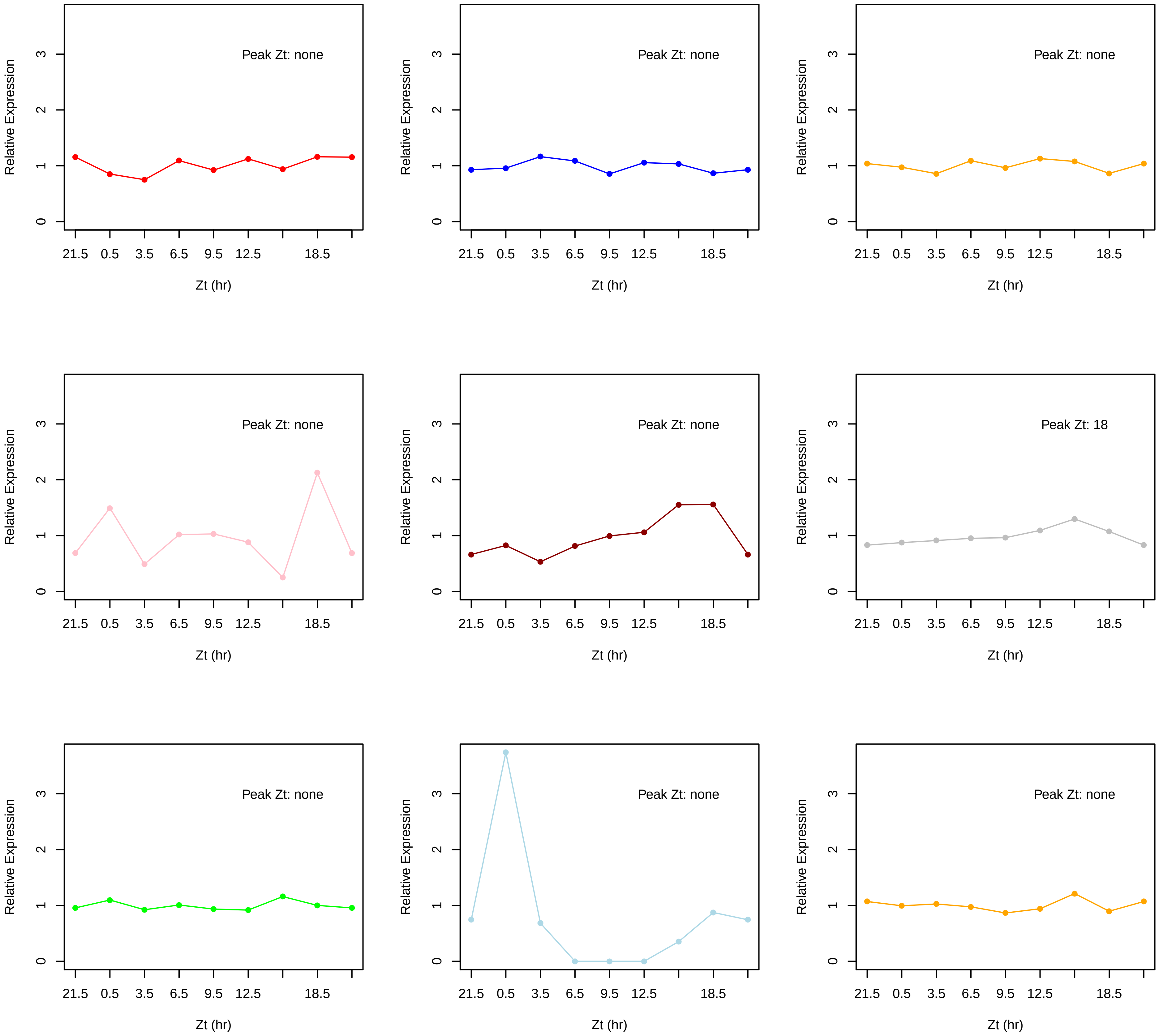

Singleton: ENSXMAG00000027492 znrf2b

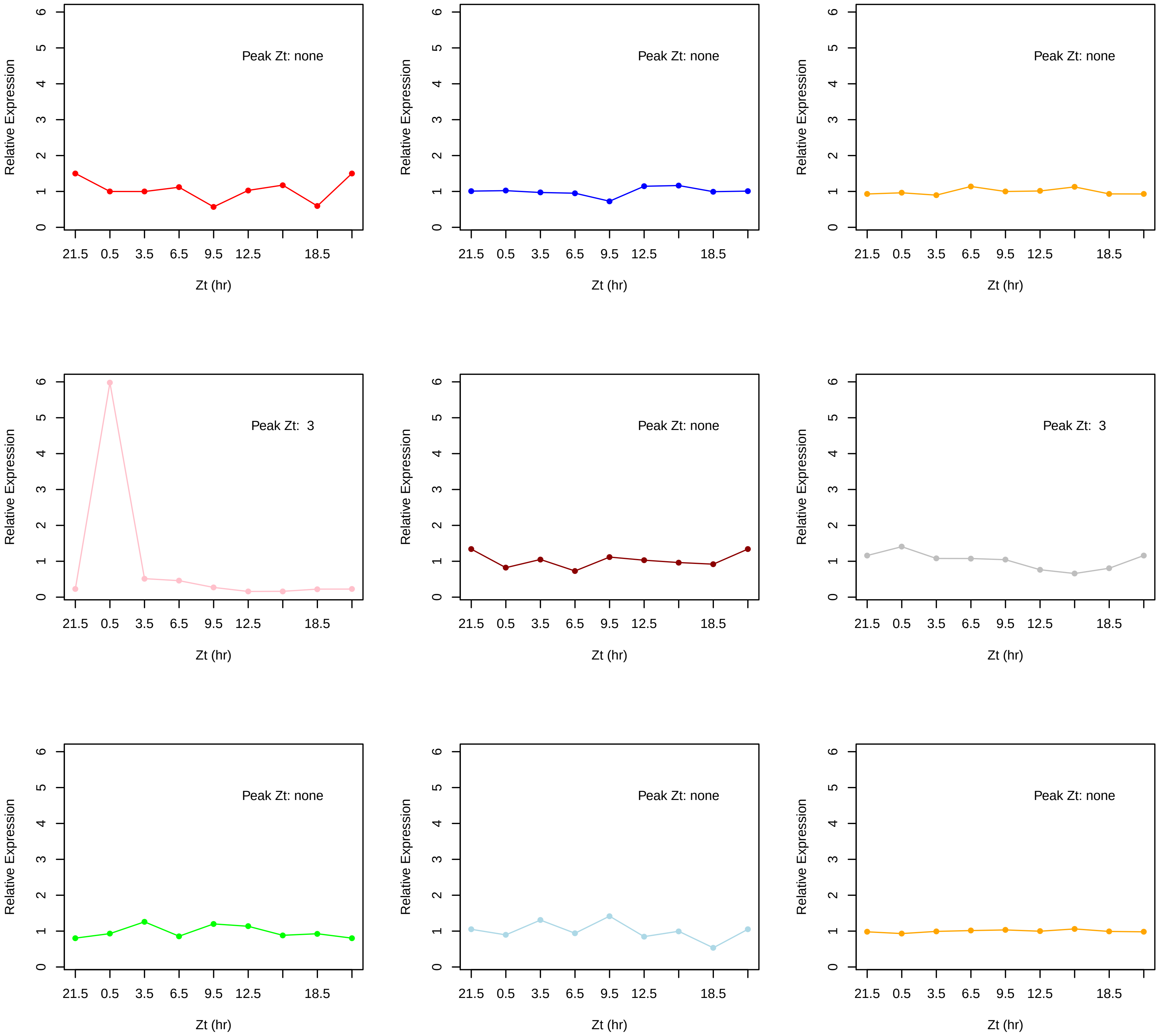

Singeleton: ENSXMAG00000000044 hira

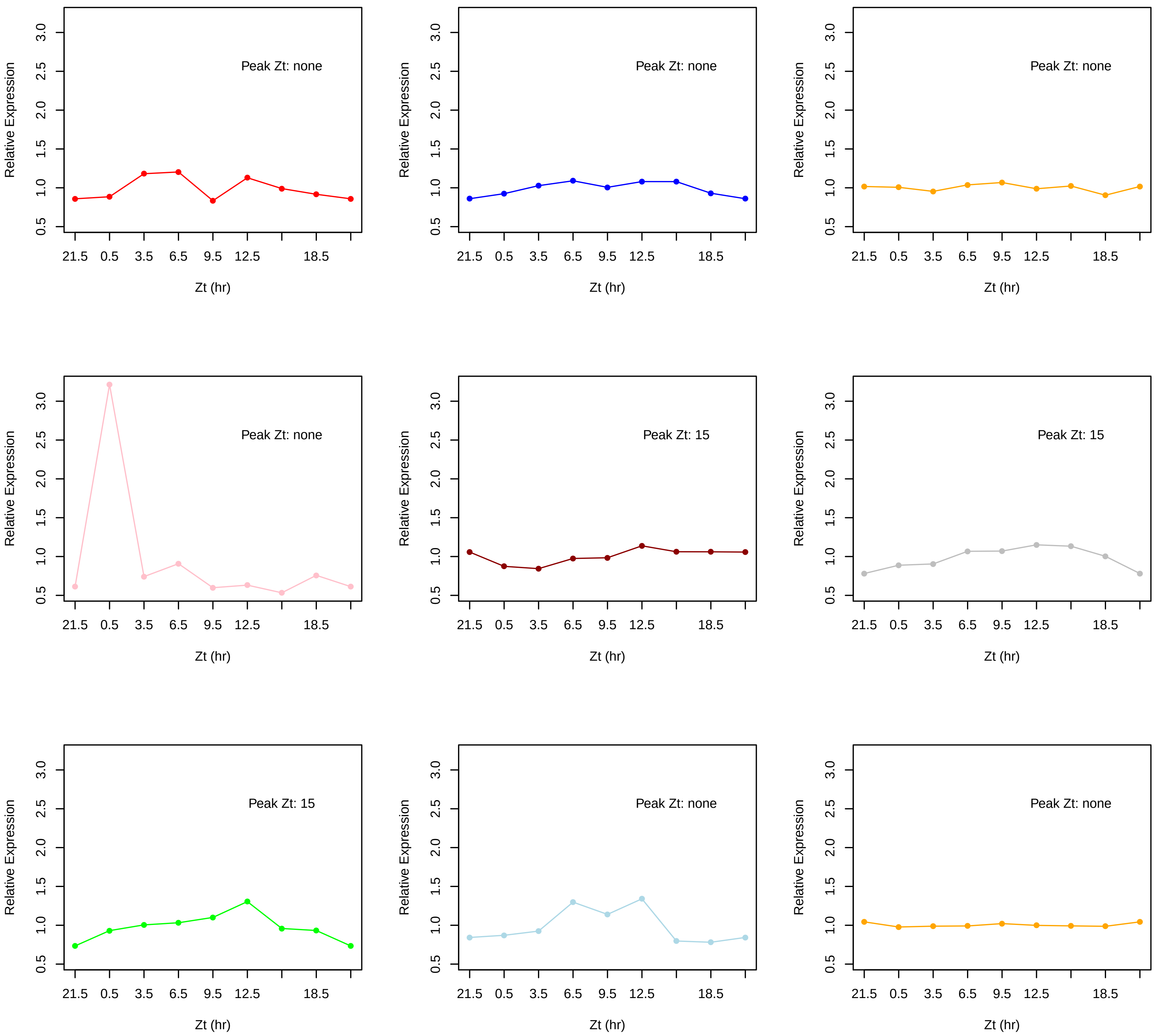

Singeleton: ENSXMAG00000001932 ptpn4a

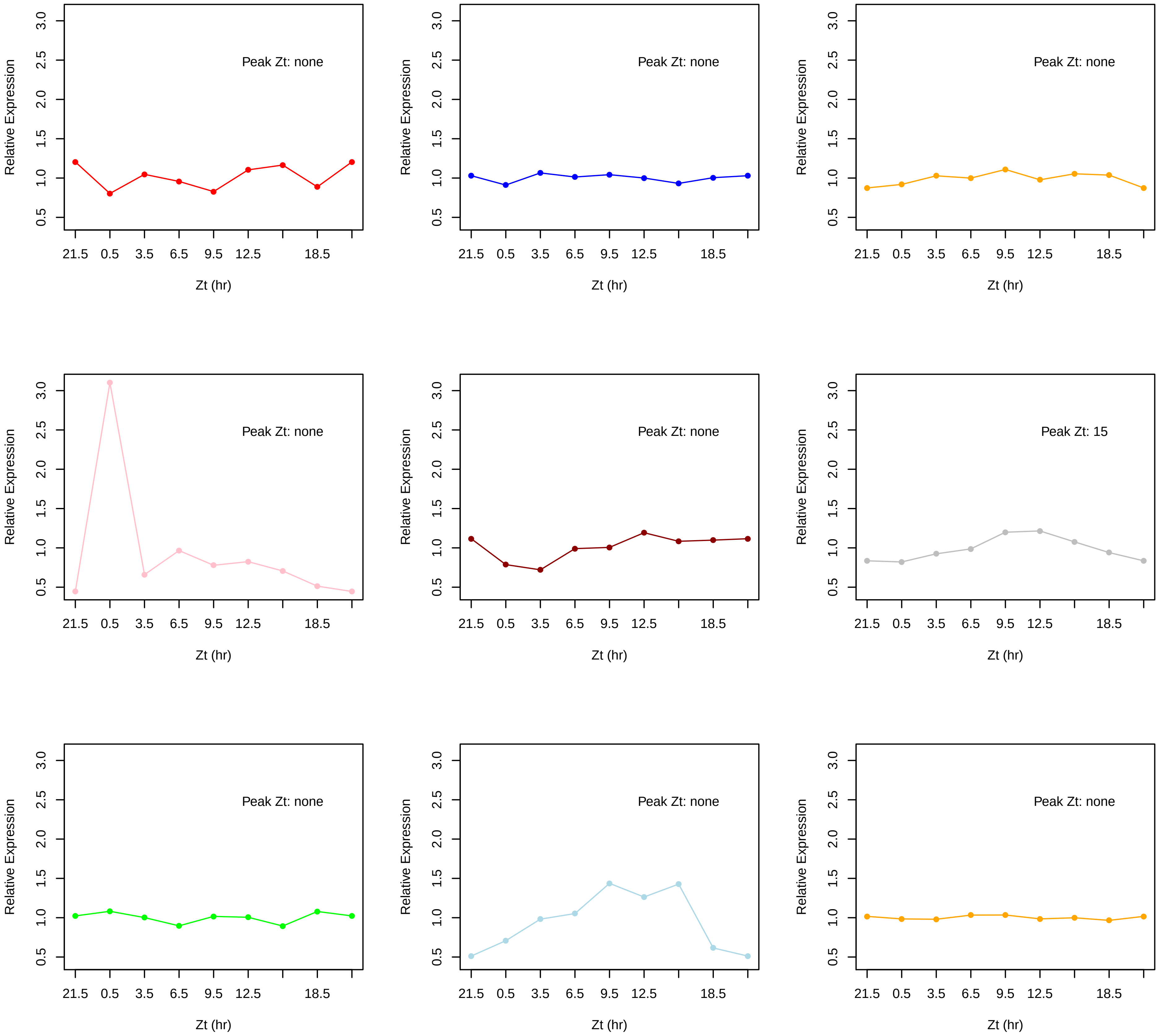

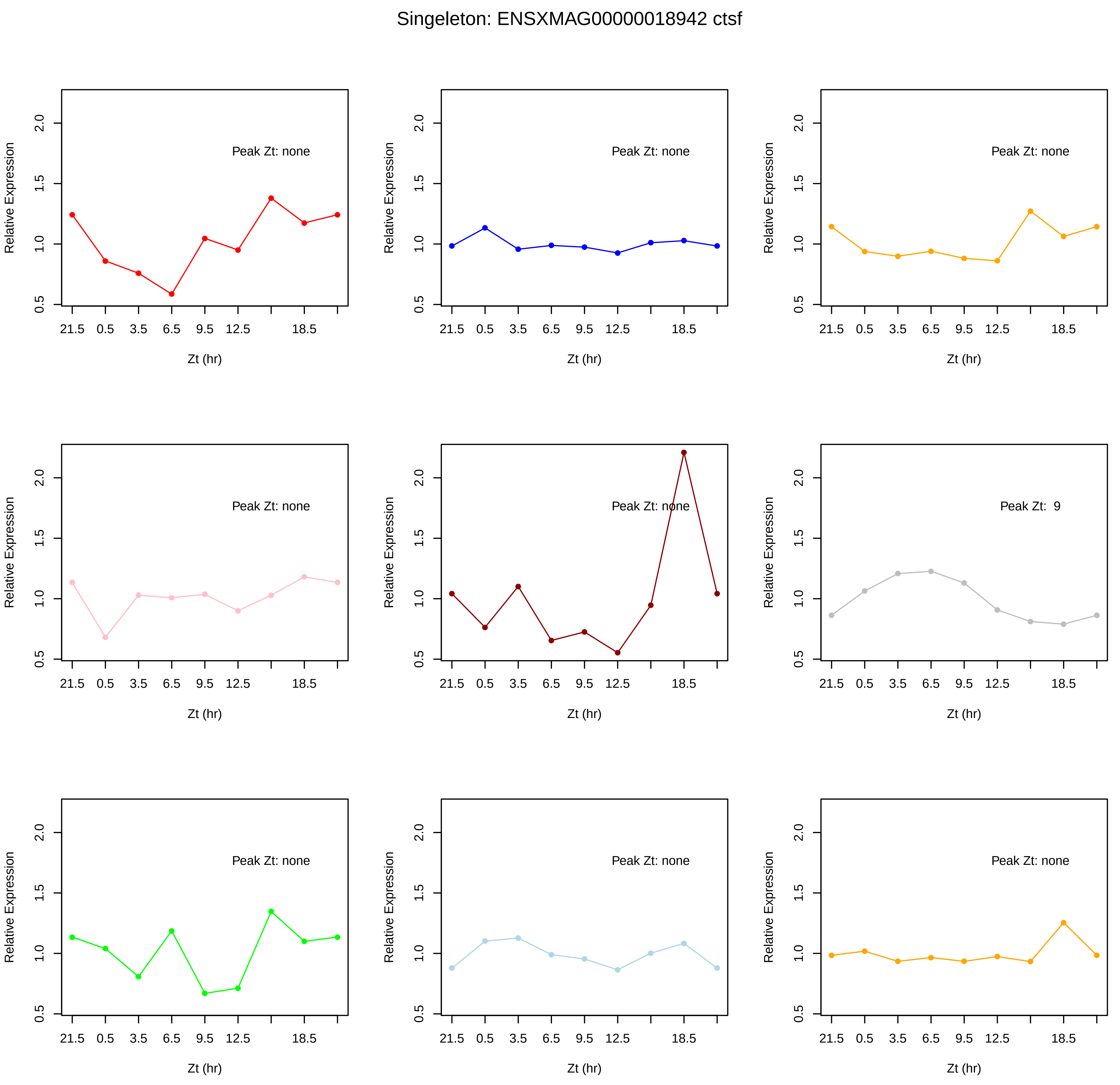

Singeleton: ENSXMAG00000018942 ctsf
Peak Zt: none
Peak Zt: none
Peak Zt: none
Peak Zt: none
Peak Zt: none
Peak Zt: 9
Peak Zt: none
Peak Zt: none
Peak Zt: none
Relative Expression
Zt (hr)
21.5
0.5
3.5
6.5
9.5
12.5
18.5
0.5
1.0
1.5
2.0

Singeleton: ENSXMAG00000001293 pou5f3

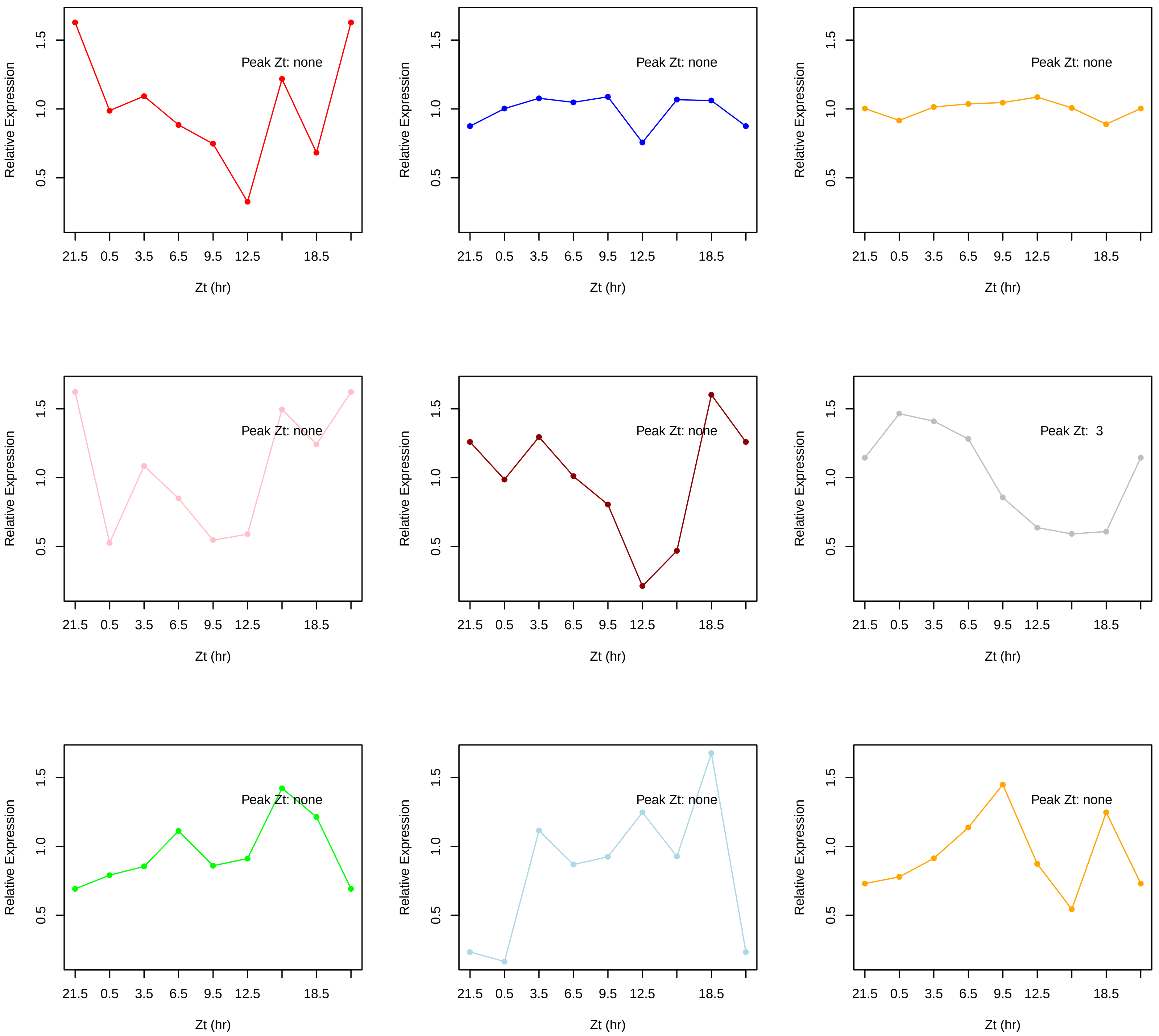

Singeleton: ENSXMAG00000024700 ccnh

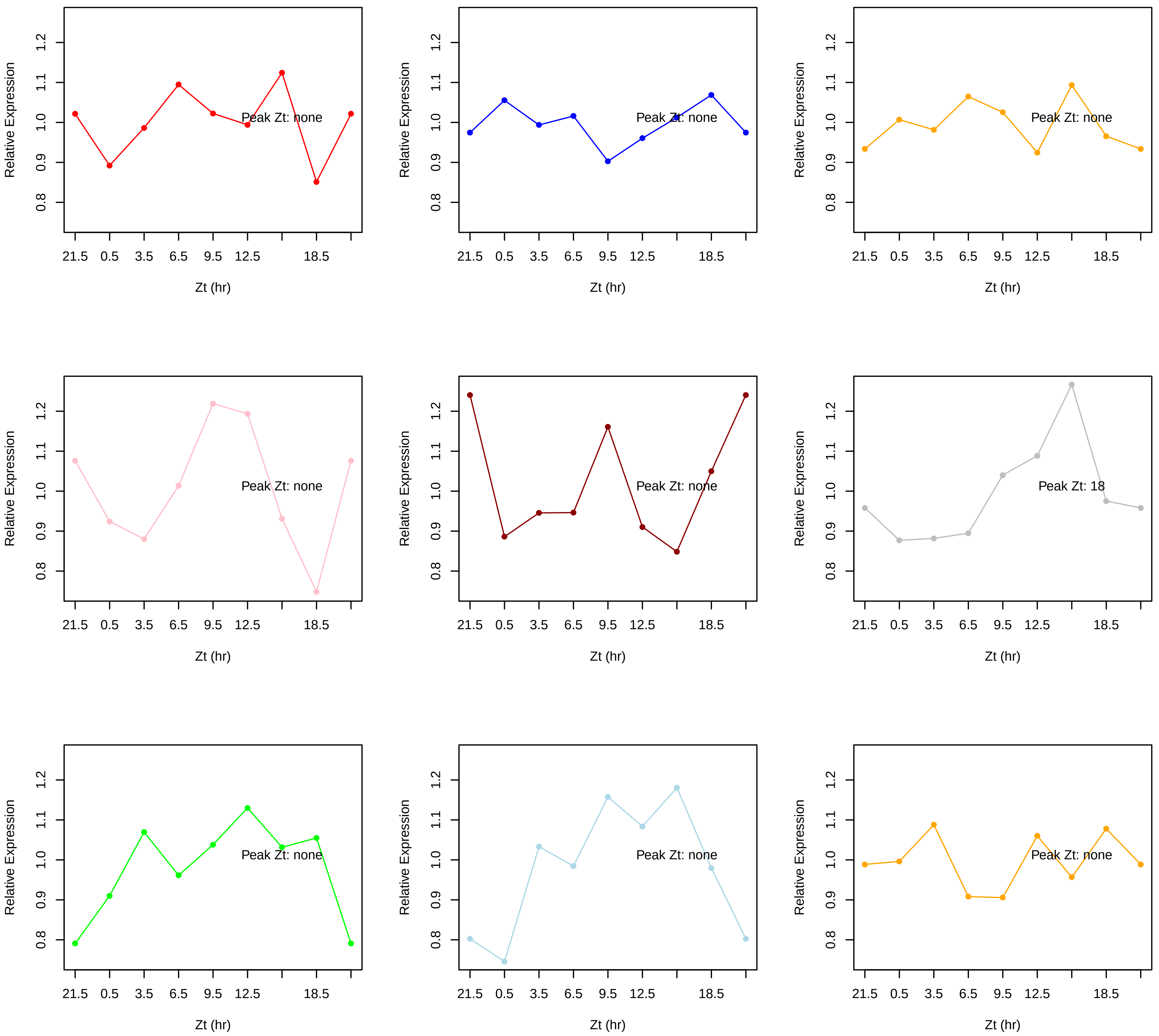

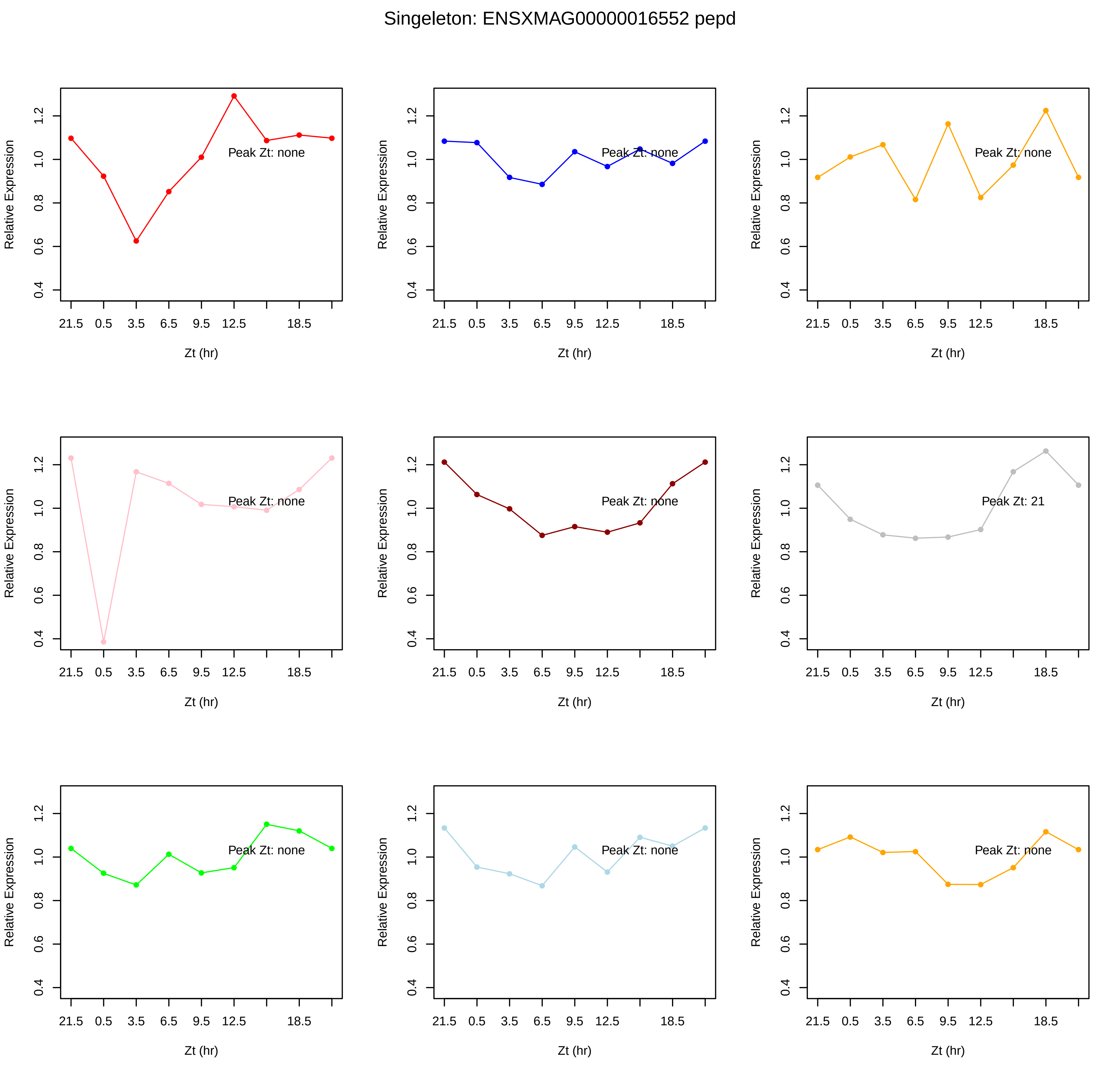
Singeleton: ENSXMAG00000016552 pepd
Relative Expression
Zt (hr)
Peak Zt: none
Peak Zt: 21
21.5
0.5
3.5
6.5
9.5
12.5
18.5
0.4
0.6
0.8
1.0
1.2

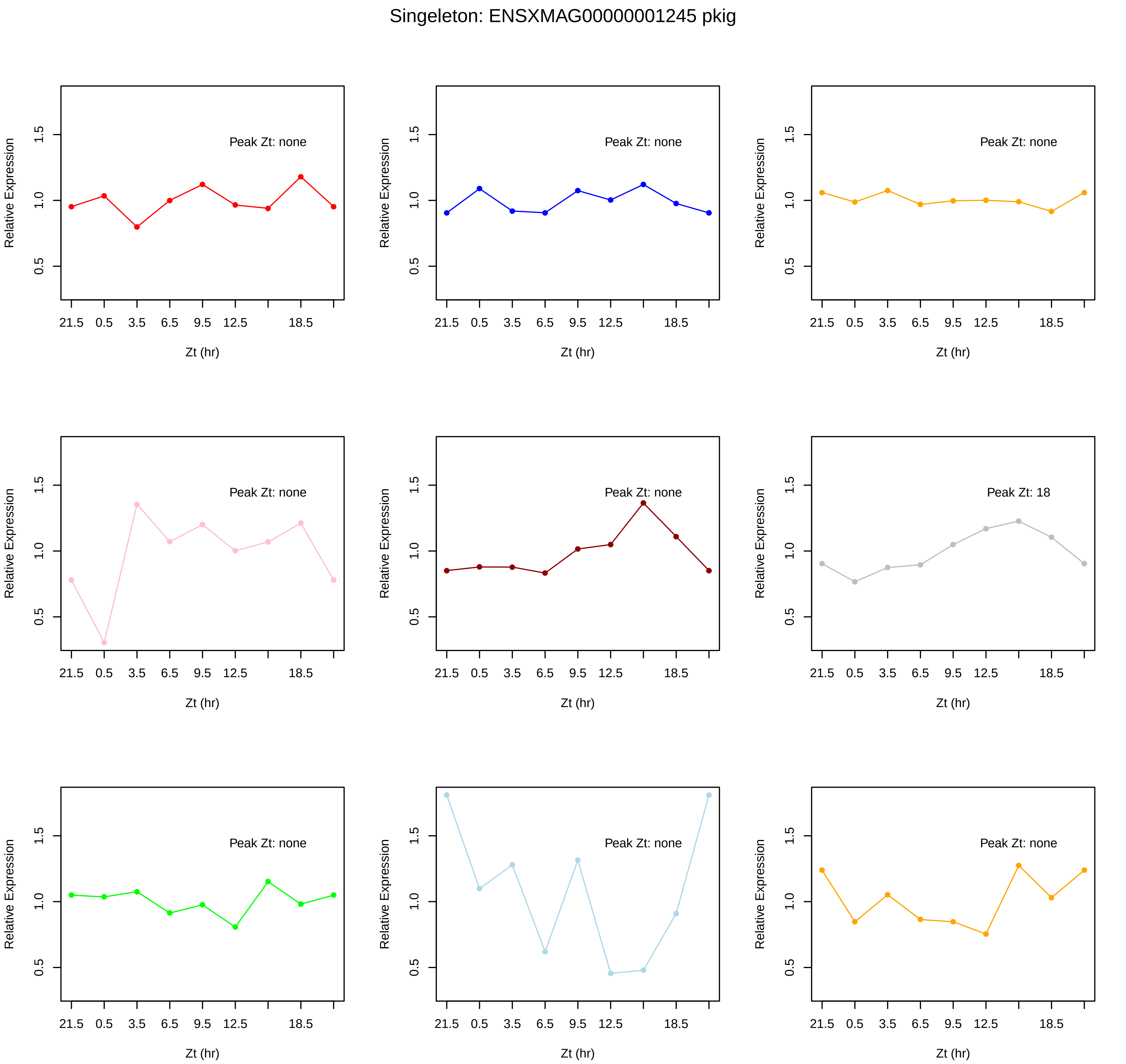
Singeleton: ENSXMAG00000001245 pkig
Peak Zt: none
Peak Zt: none
Peak Zt: none
Peak Zt: none
Peak Zt: none
Peak Zt: 18
Peak Zt: none
Peak Zt: none
Peak Zt: none
Relative Expression
Zt (hr)
21.5
0.5
3.5
6.5
9.5
12.5
18.5
0.5
1.0
1.5

Singeleton: ENSXMAG00000028853 arxa

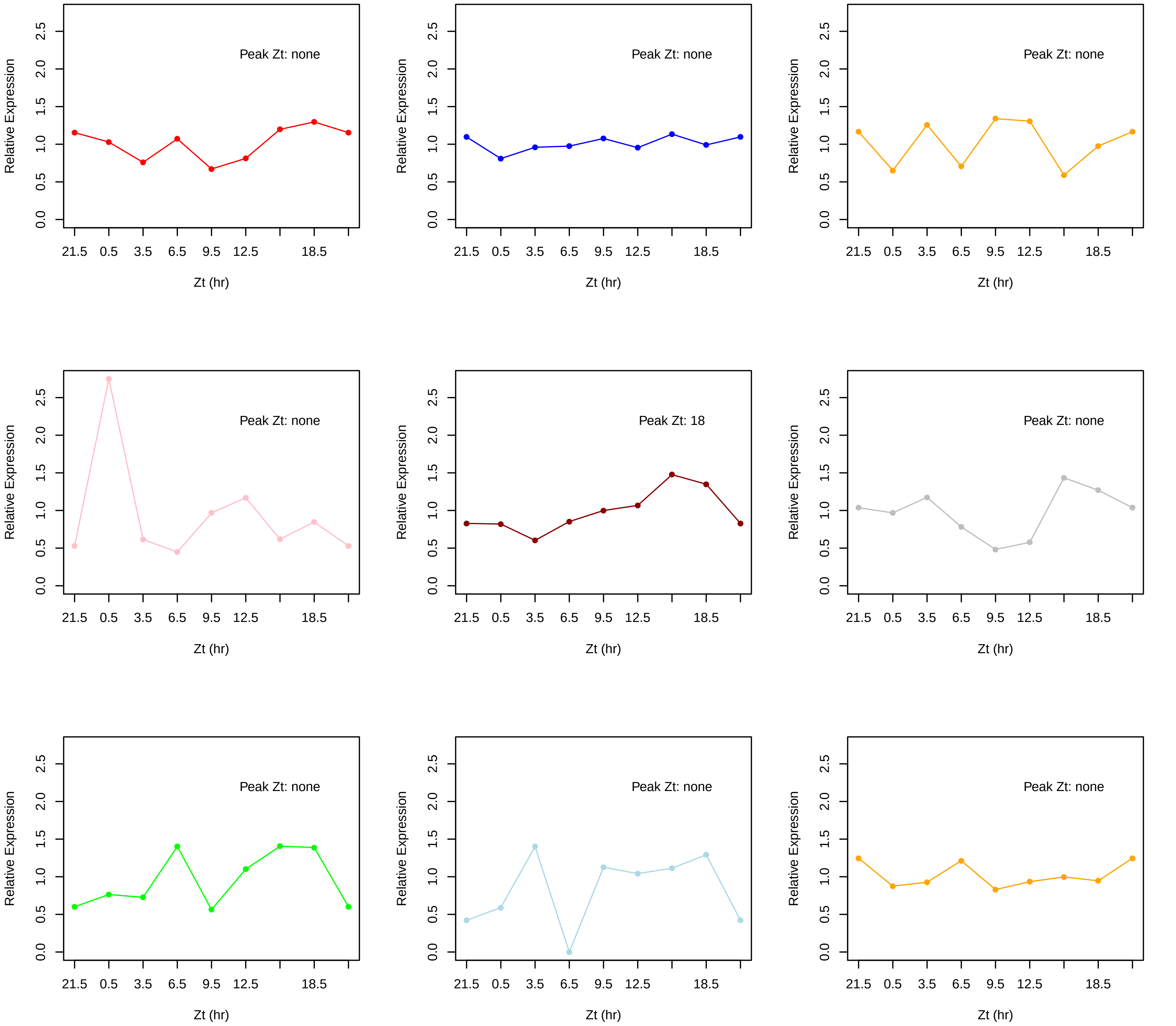

Singleton: ENSXMAG00000005874 greb1l

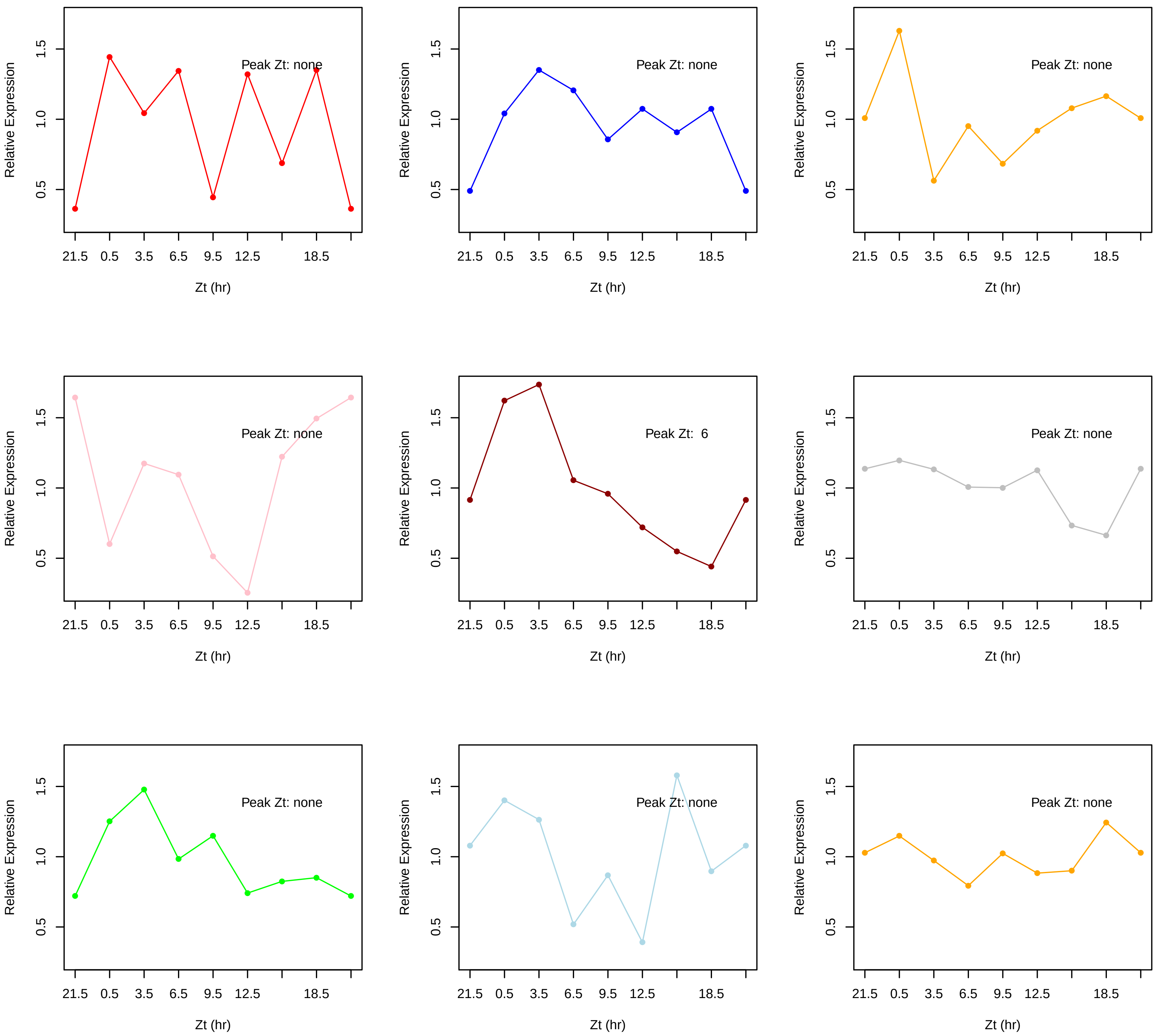

Singeleton: ENSXMAG00000029954

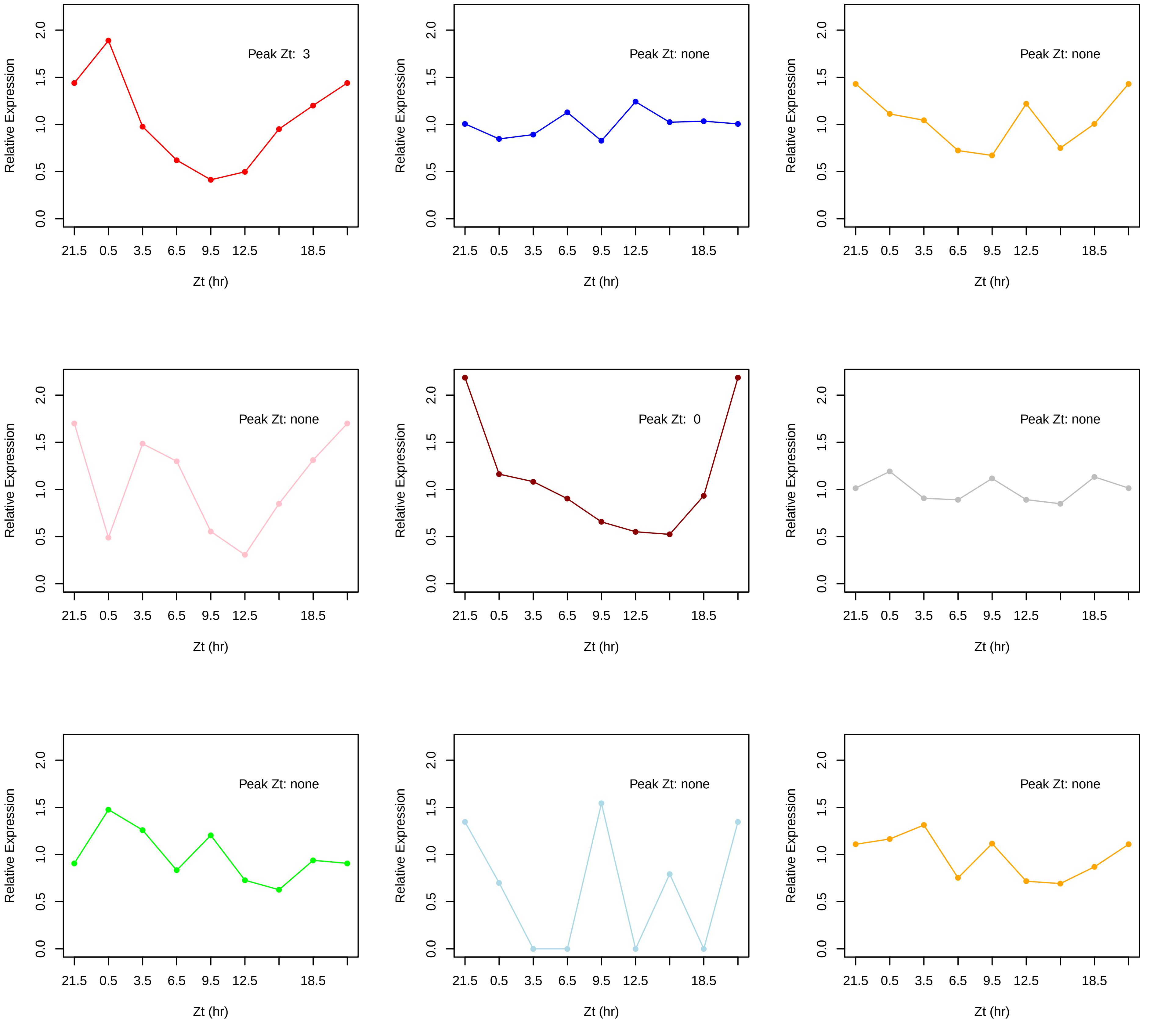

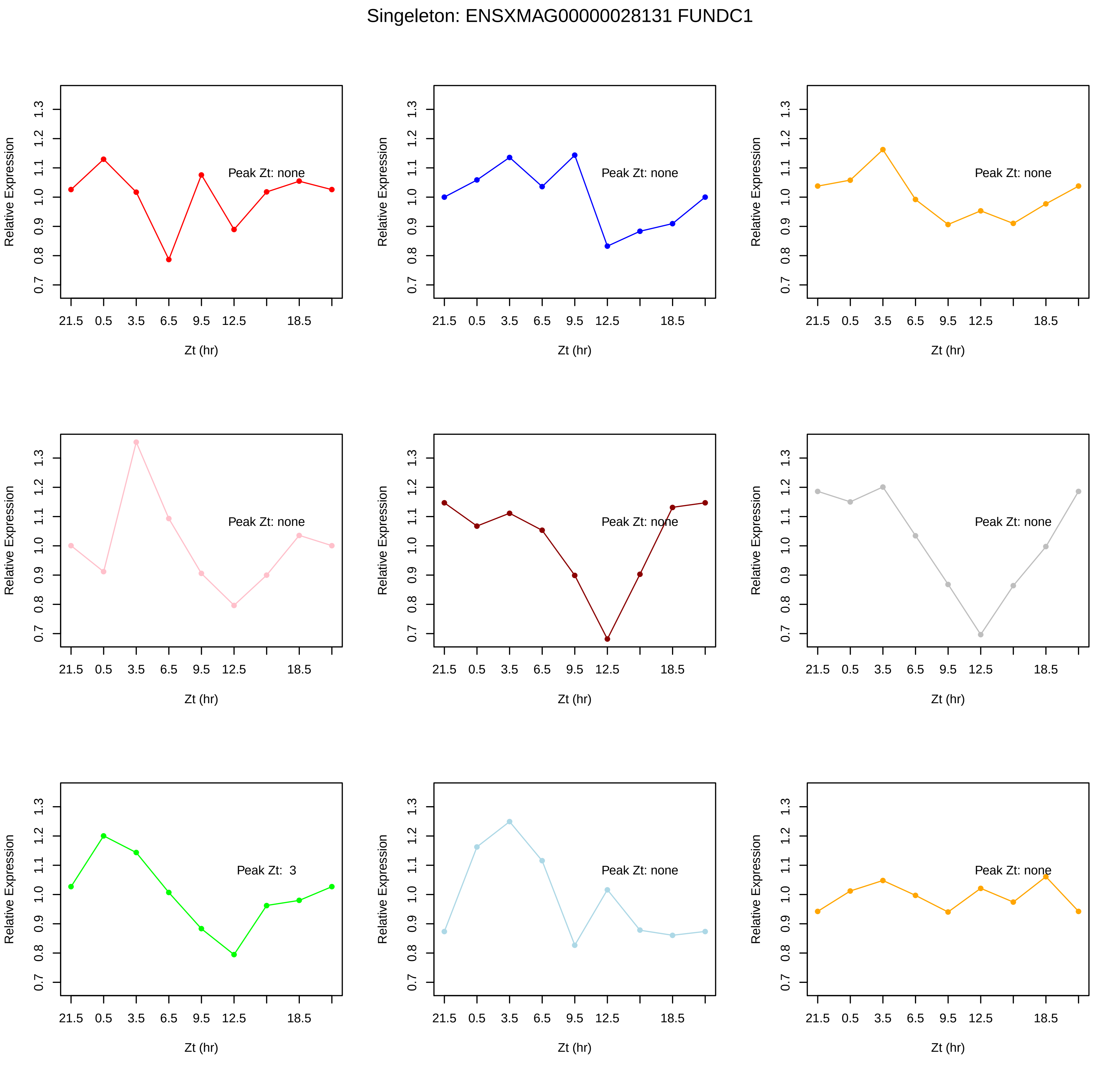
Singeleton: ENSXMAG00000028131 FUNDC1
Relative Expression
Zt (hr)
21.5
0.5
3.5
6.5
9.5
12.5
18.5
0.7
0.8
0.9
1.0
1.1
1.2
1.3
Peak Zt: none
Peak Zt: 3

Singleton: ENSXMAG00000012356 agmat

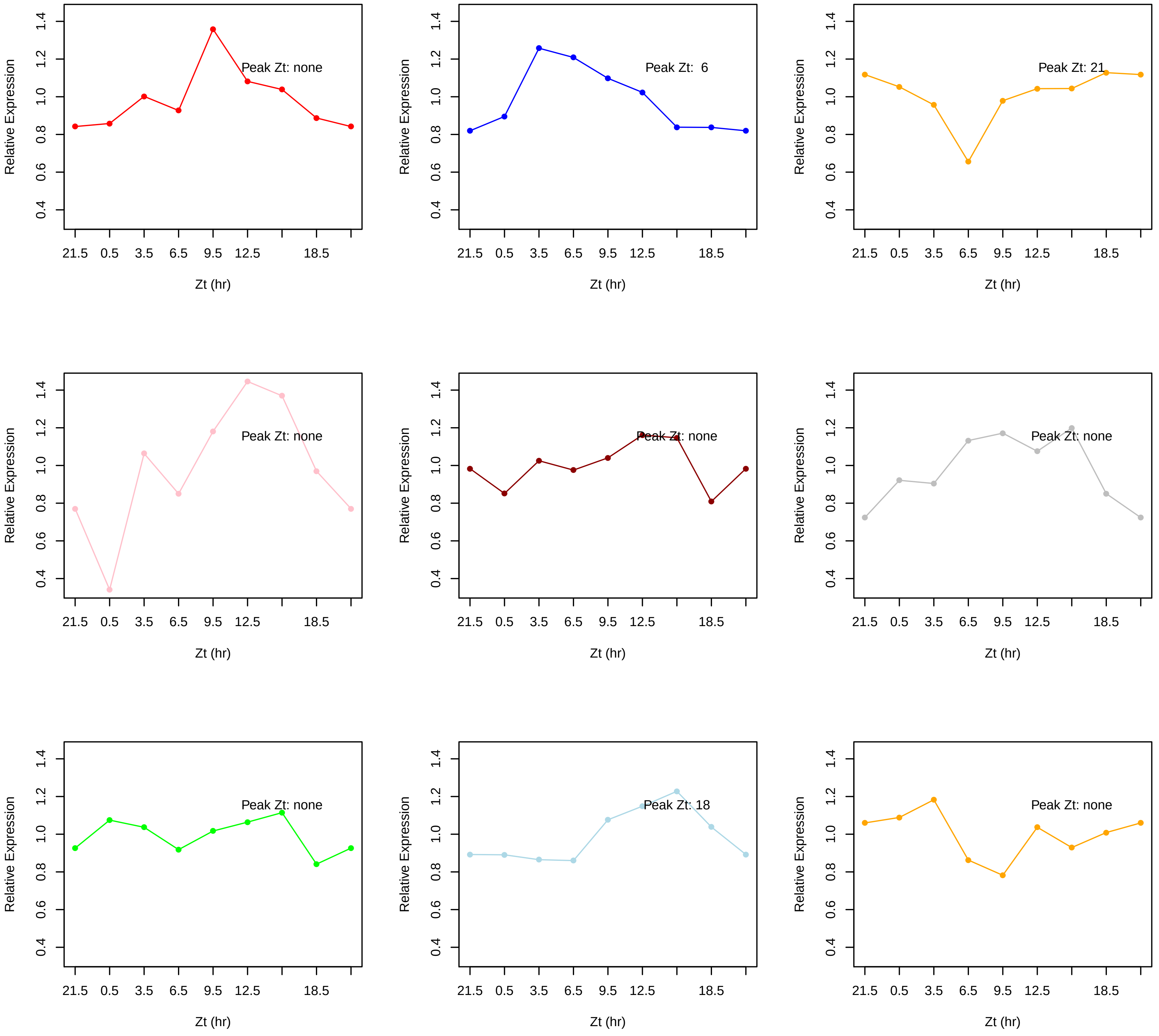

Singeleton: ENSXMAG00000015336 tmem86a

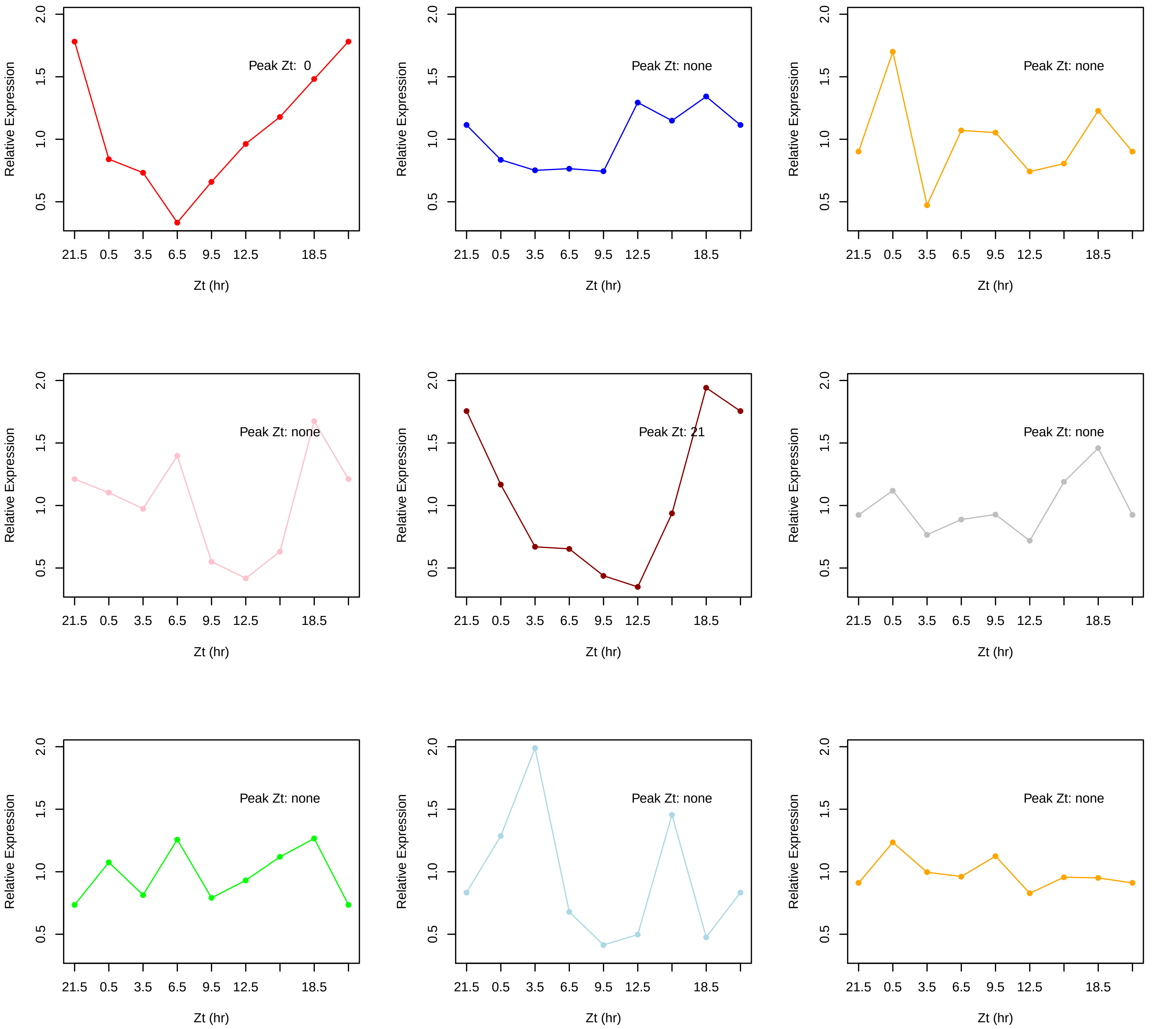

Singeleton: ENSXMAG00000008574 pcxb

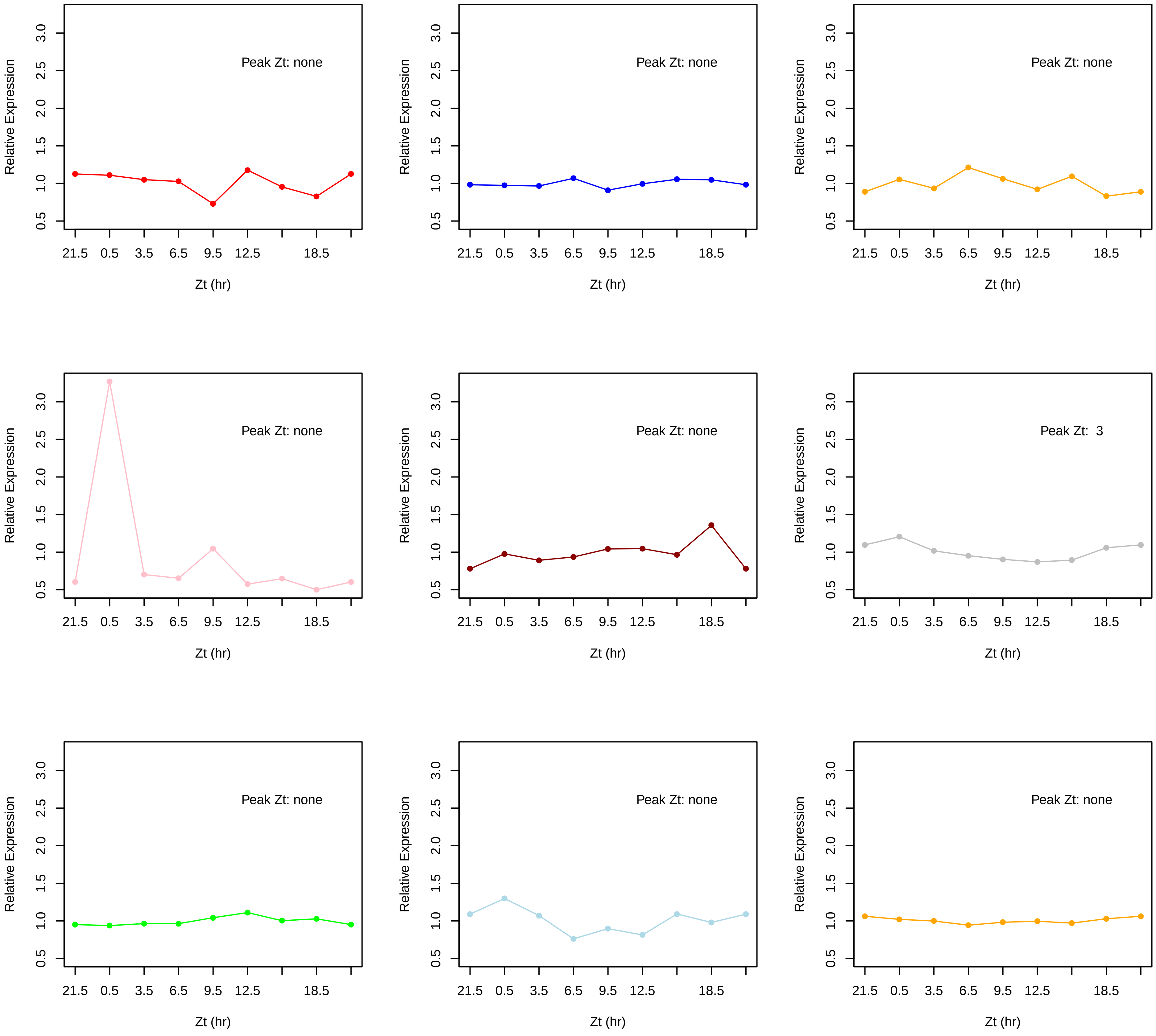

Singleton: ENSXMAG00000010930 TOMM40L

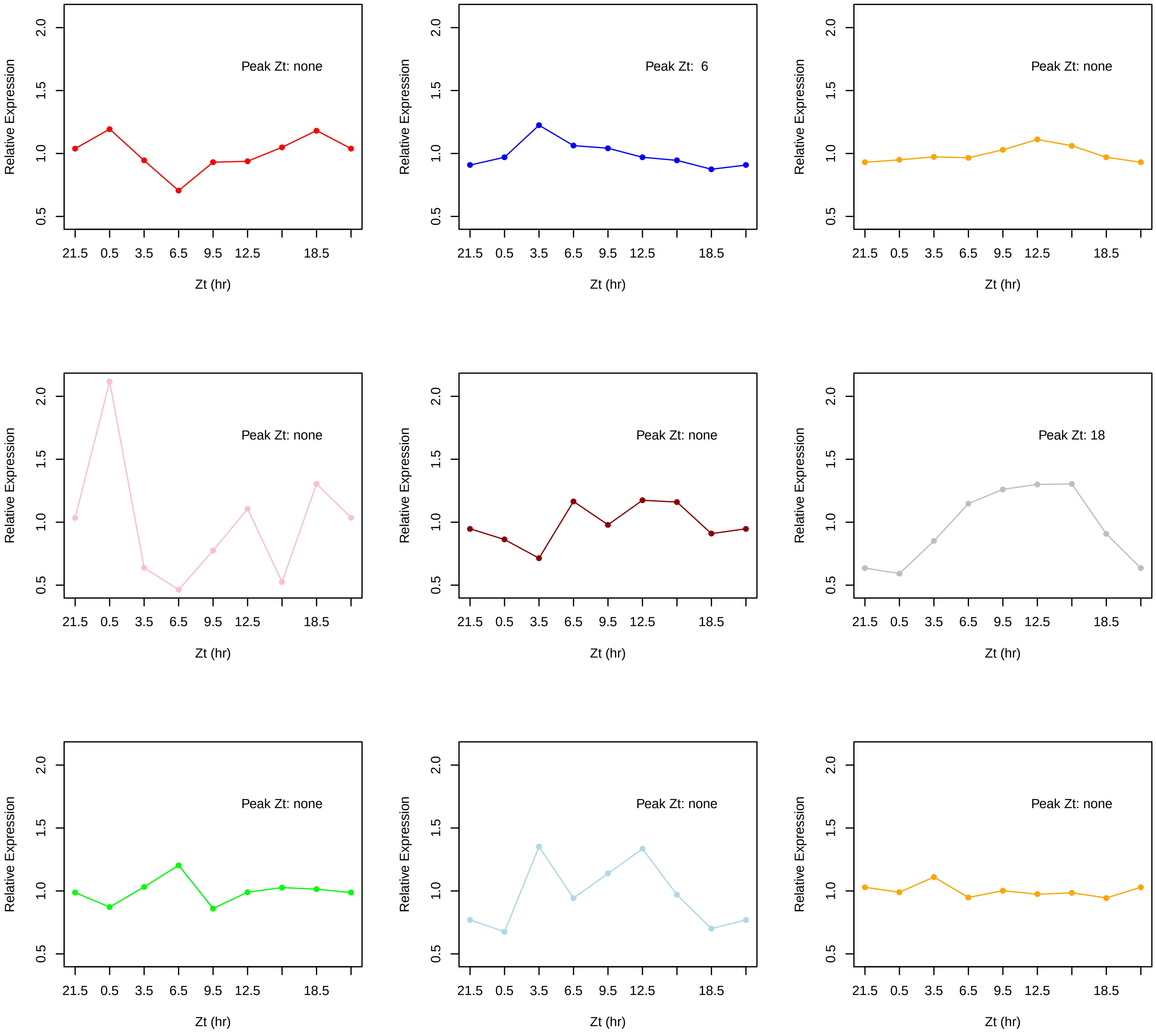

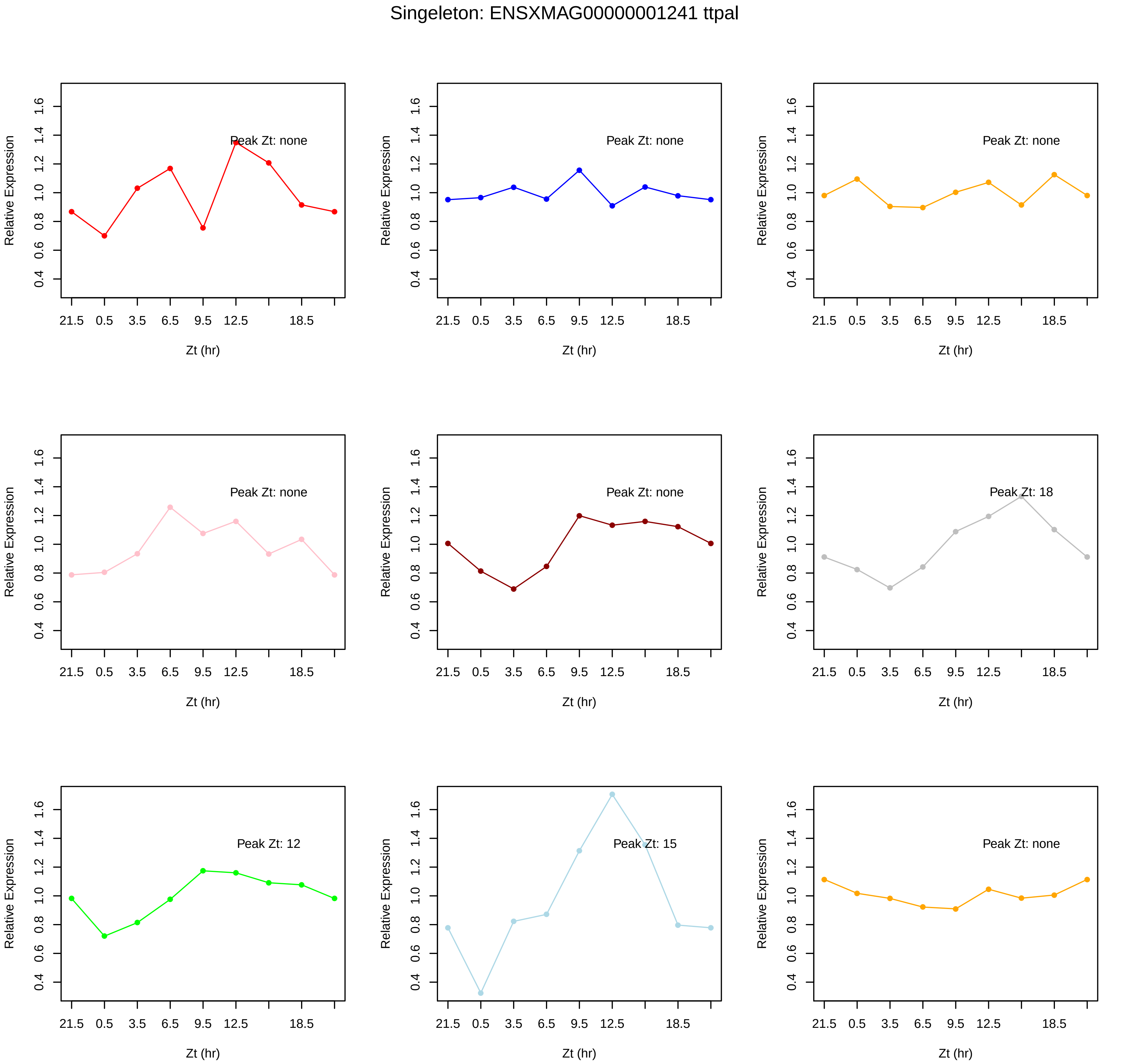
Singeleton: ENSXMAG00000001241 ttpal
Peak Zt: none
Peak Zt: none
Peak Zt: none
Peak Zt: none
Peak Zt: none
Peak Zt: 18
Peak Zt: 12
Peak Zt: 15
Peak Zt: none
Relative Expression
Zt (hr)
21.5 0.5 3.5 6.5 9.5 12.5 18.5
0.4 0.6 0.8 1.0 1.2 1.4 1.6

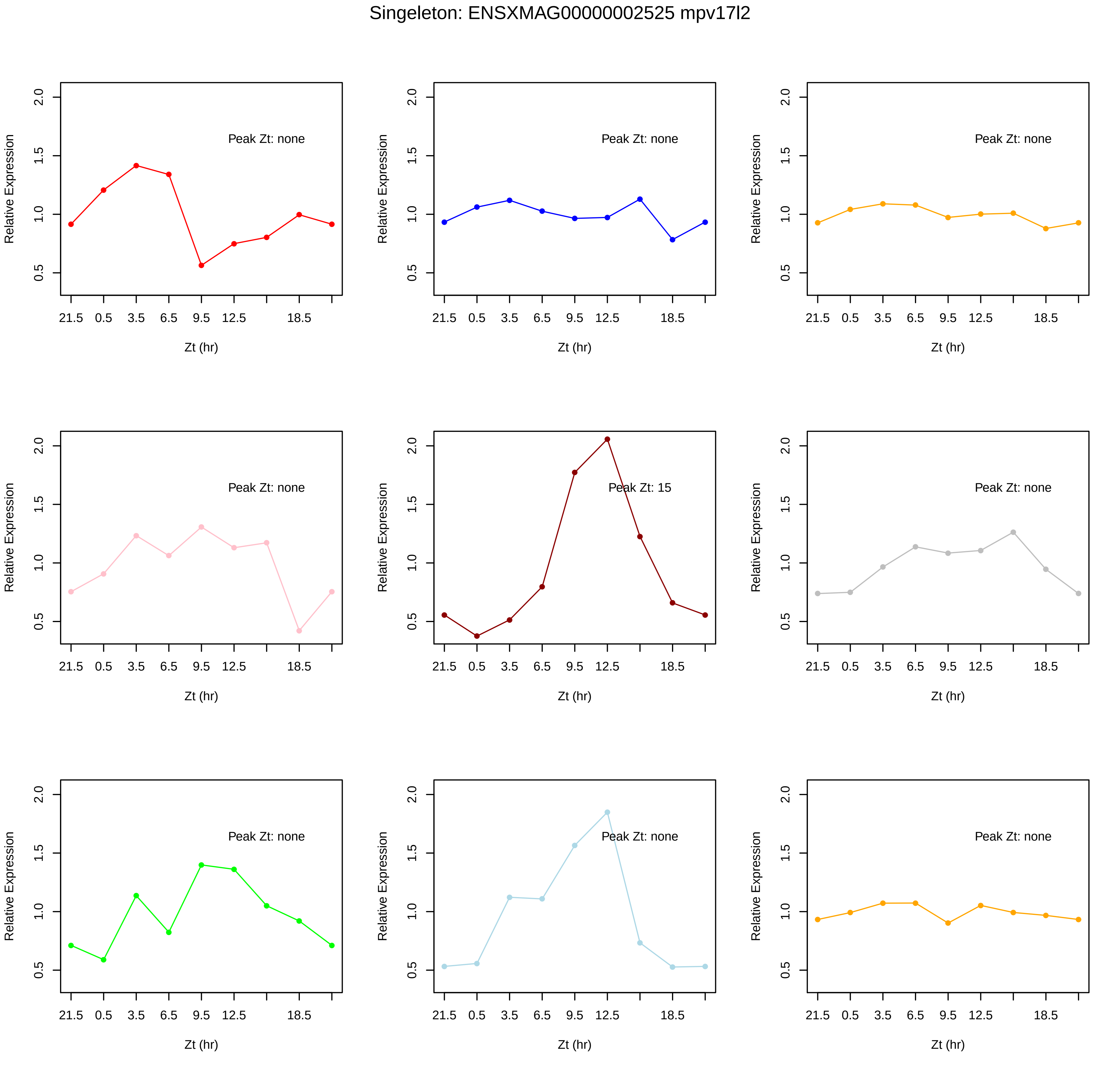
Singeleton: ENSXMAG00000002525 mpv17l2
Peak Zt: none
Peak Zt: none
Peak Zt: none
Peak Zt: none
Peak Zt: 15
Peak Zt: none
Peak Zt: none
Peak Zt: none
Peak Zt: none
Relative Expression
Zt (hr)
21.5 0.5 3.5 6.5 9.5 12.5 18.5
0.5 1.0 1.5 2.0

Singeleton: ENSXMAG00000001986 npm1b

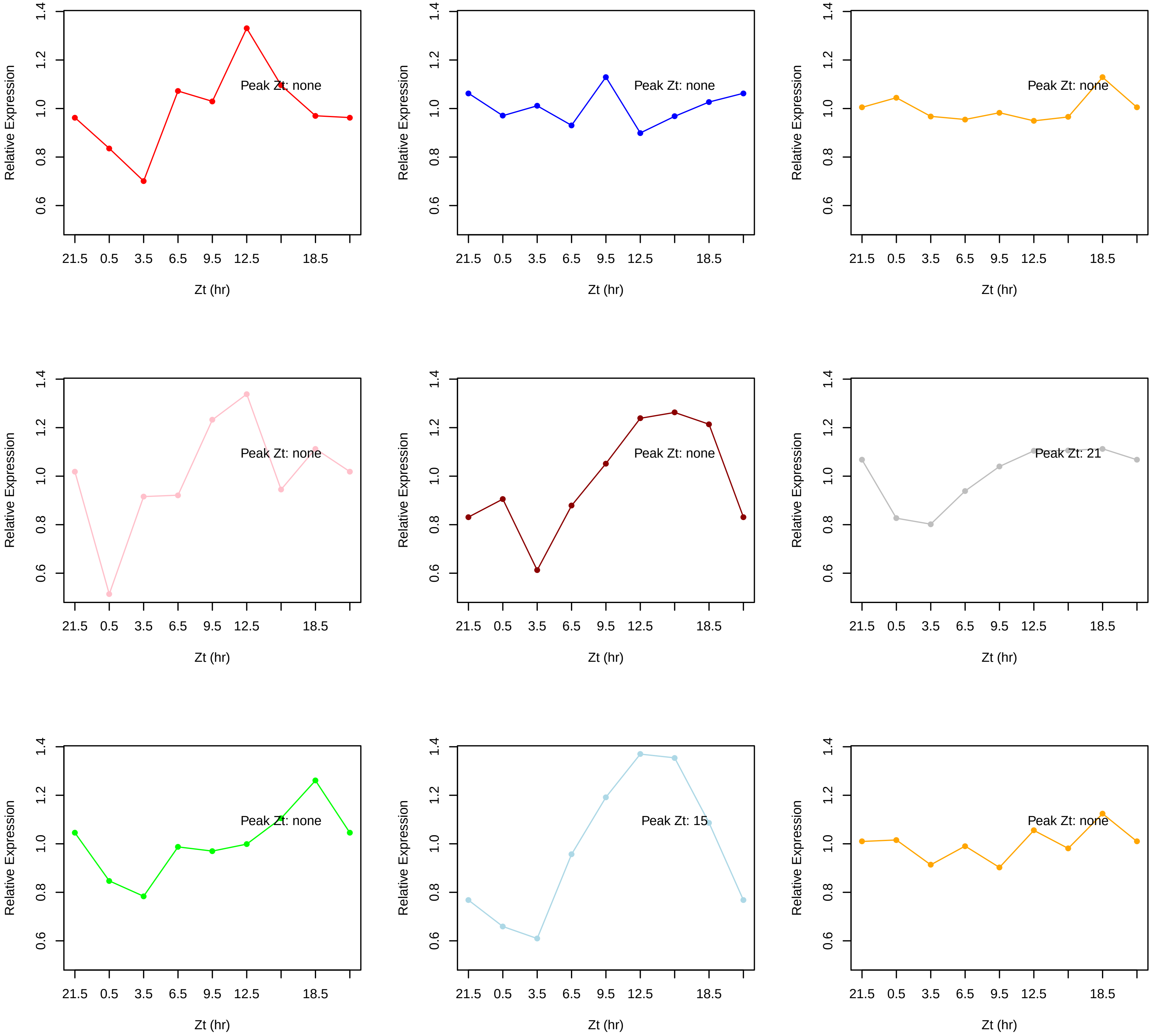

Singeleton: ENSXMAG00000028971 castor2

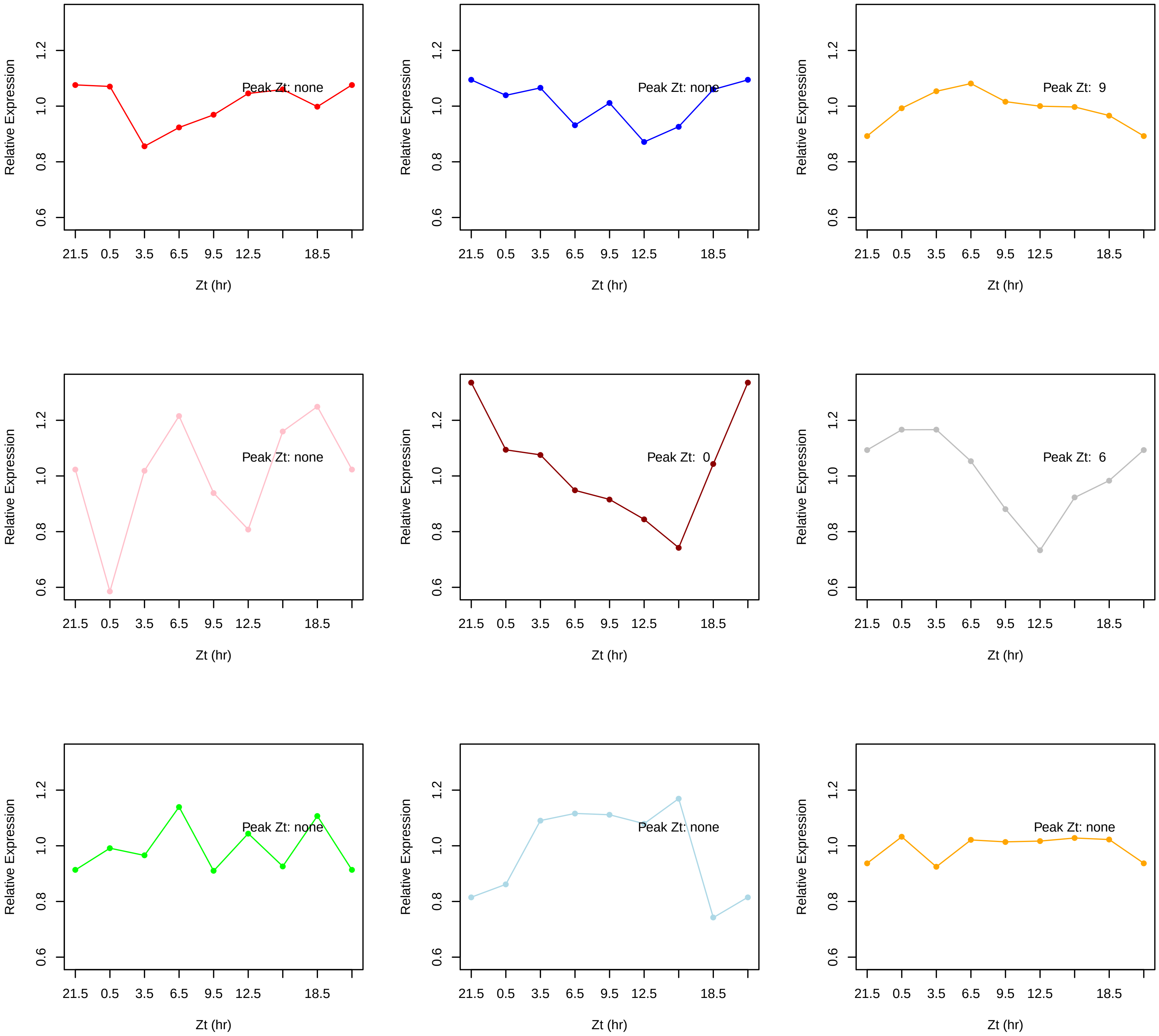

Singeleton: ENSXMAG00000029888 PRKAB2

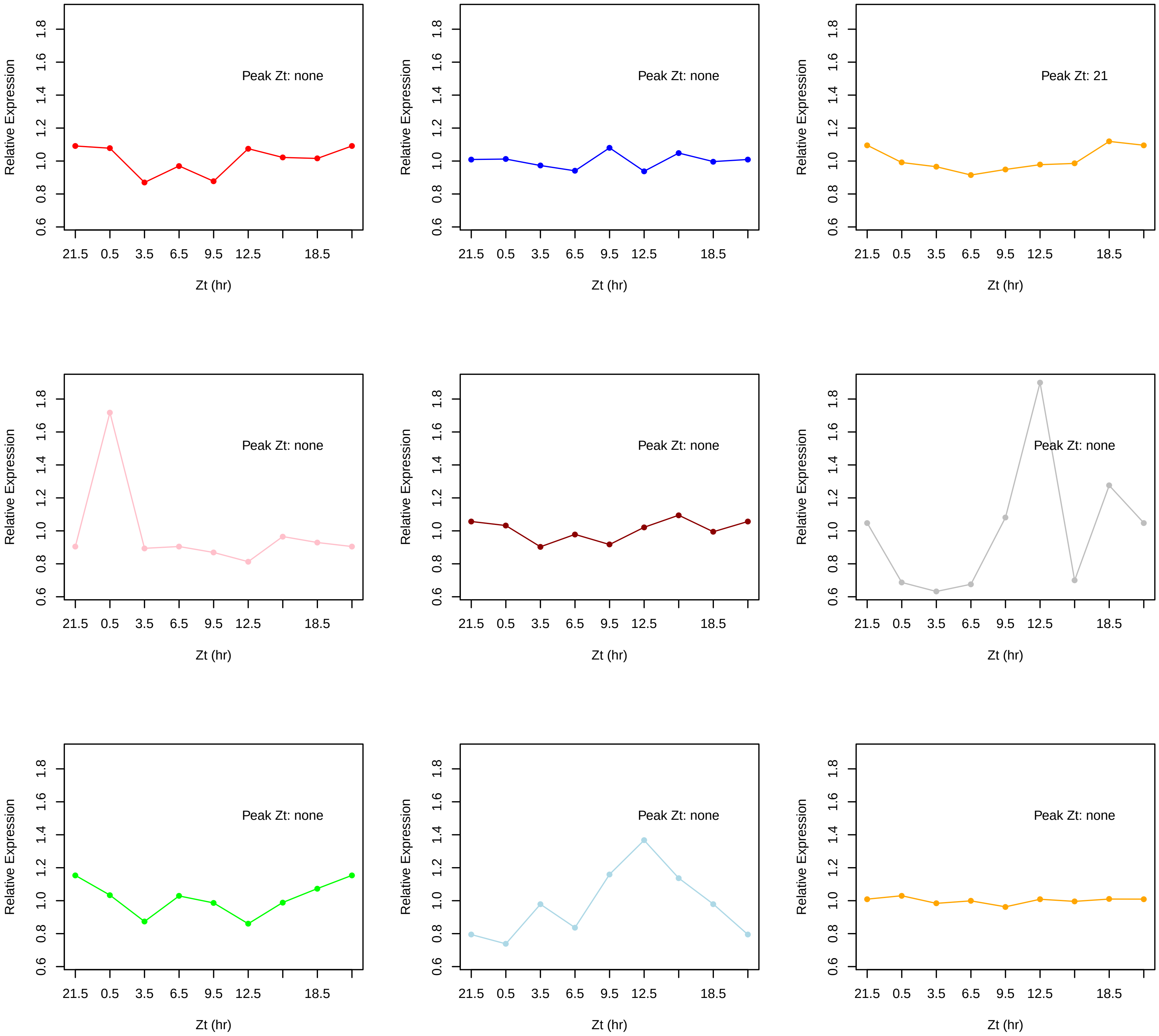

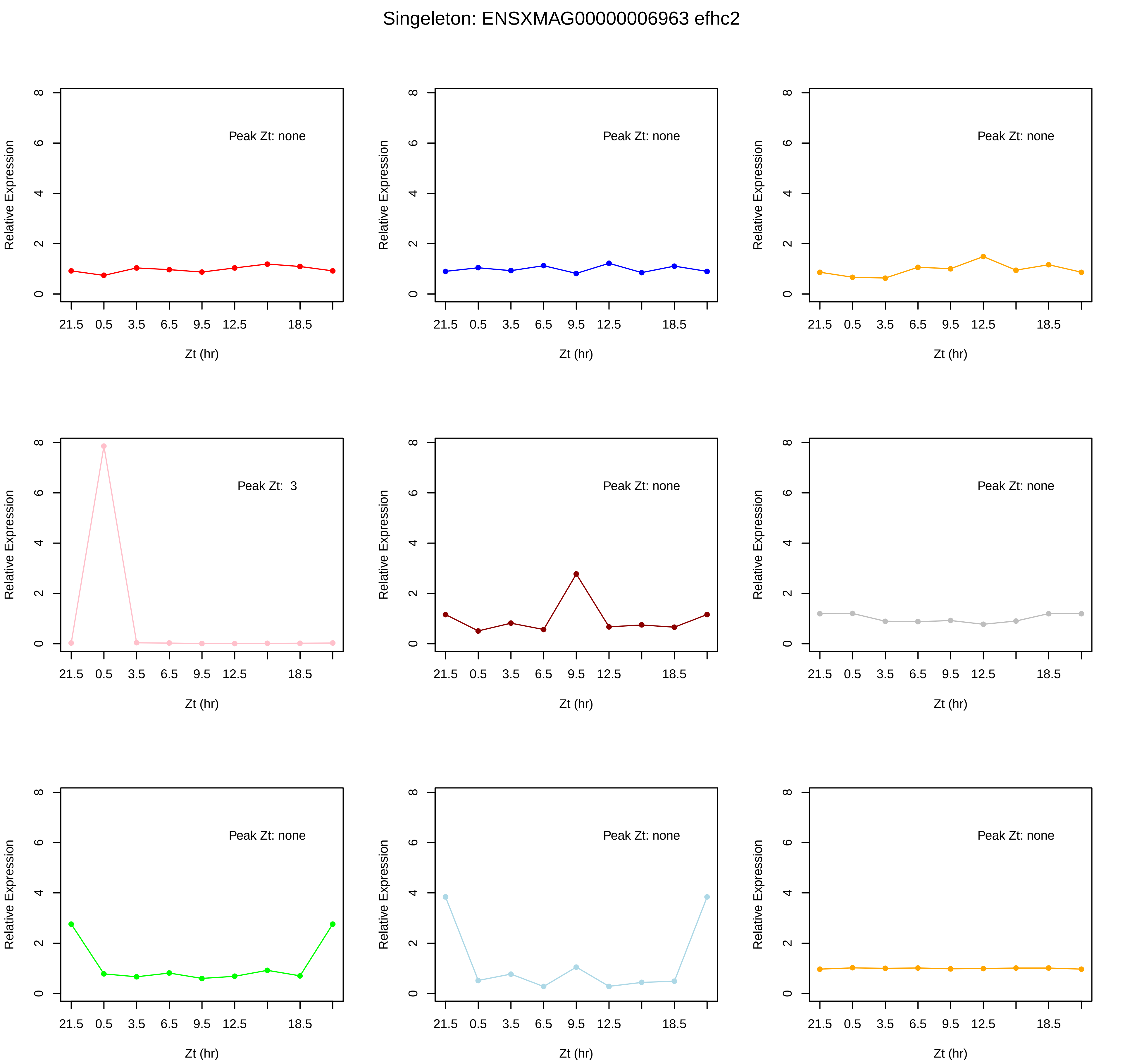
Singeleton: ENSXMAG00000006963 efhc2
Peak Zt: none
Peak Zt: none
Peak Zt: none
Peak Zt: 3
Peak Zt: none
Peak Zt: none
Peak Zt: none
Peak Zt: none
Peak Zt: none
Relative Expression
Zt (hr)
21.5 0.5 3.5 6.5 9.5 12.5 18.5
0 2 4 6 8

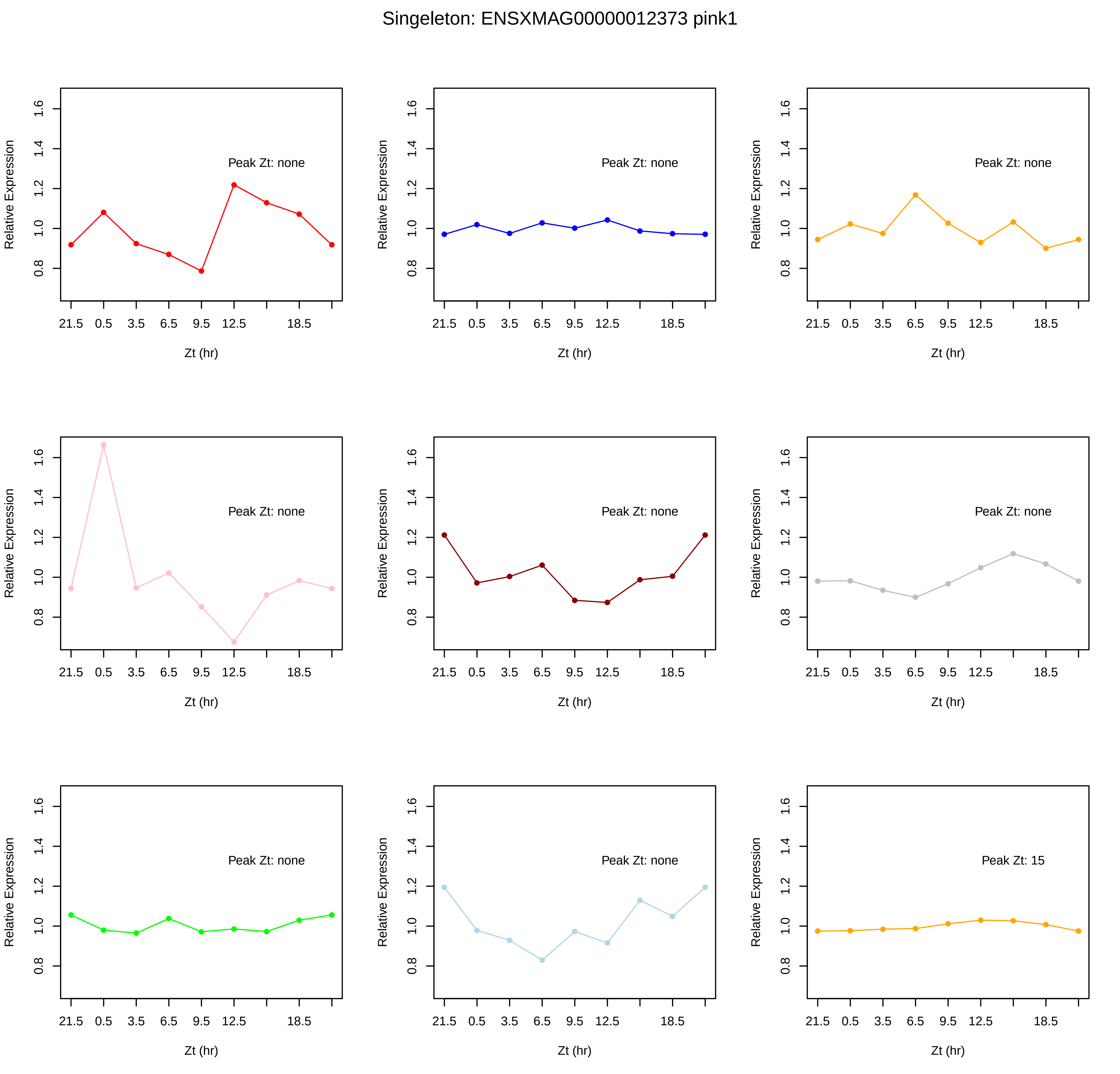

Singeleton: ENSXMAG00000012373 pink1
Relative Expression
Zt (hr)
21.5
0.5
3.5
6.5
9.5
12.5
18.5
0.8
1.0
1.2
1.4
1.6
Peak Zt: none
Peak Zt: 15

Singleton: ENSXMAG00000019418 cox7c

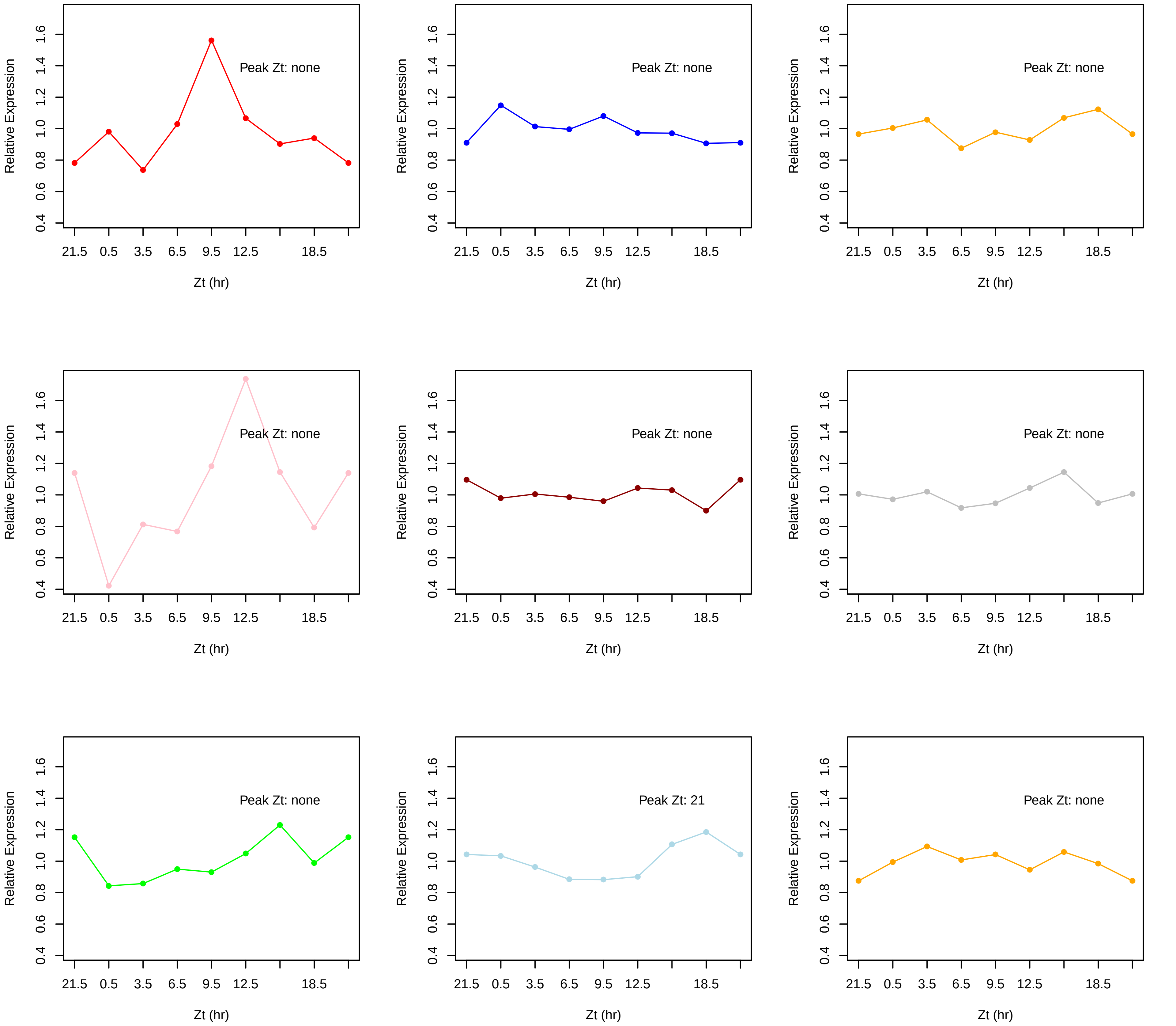

Singeleton: ENSXMAG00000028406 b4galnt4a

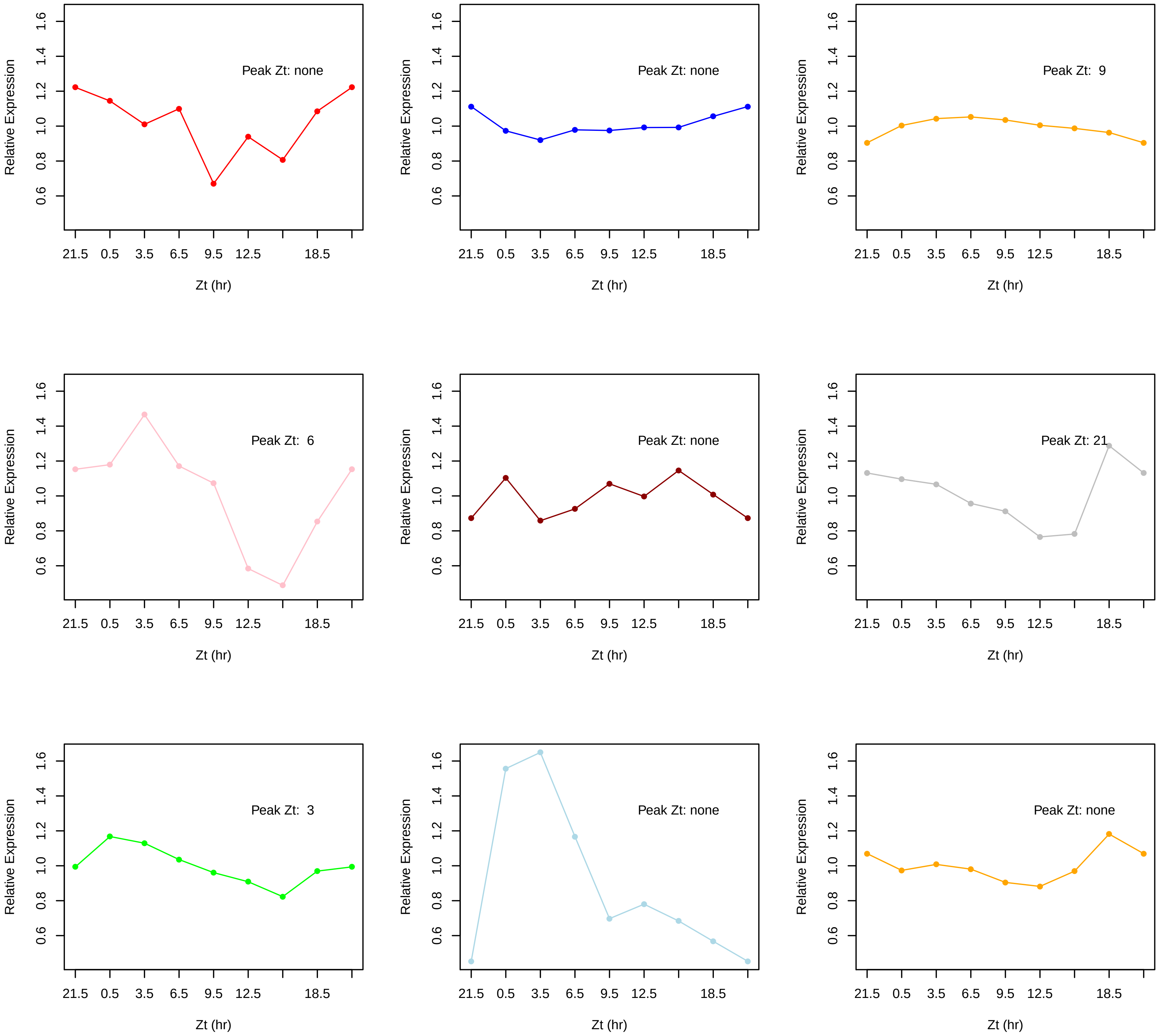

Singleton: ENSXMAG00000024139 lrfn4b

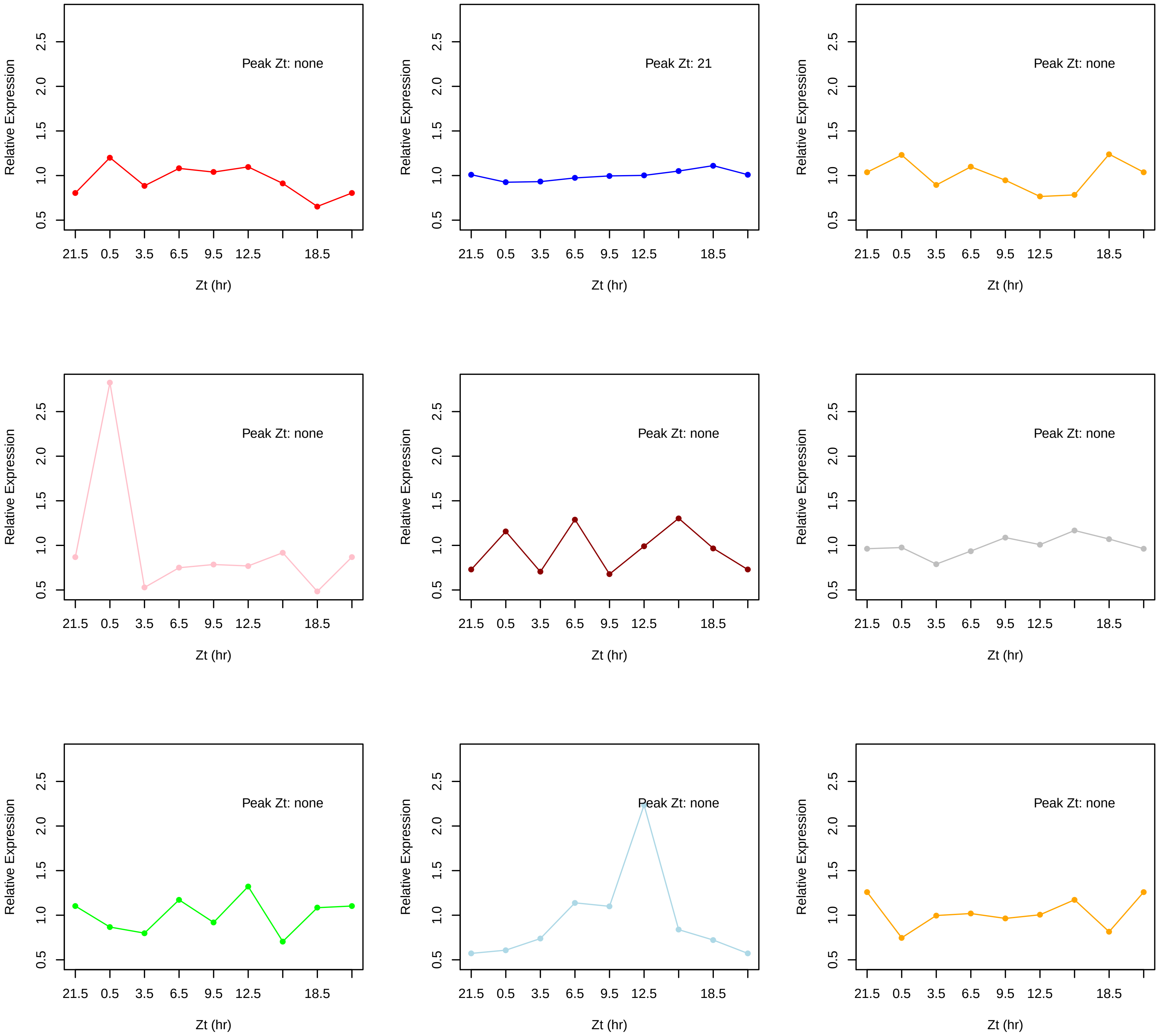

Singleton: ENSXMAG00000025625 edil3a

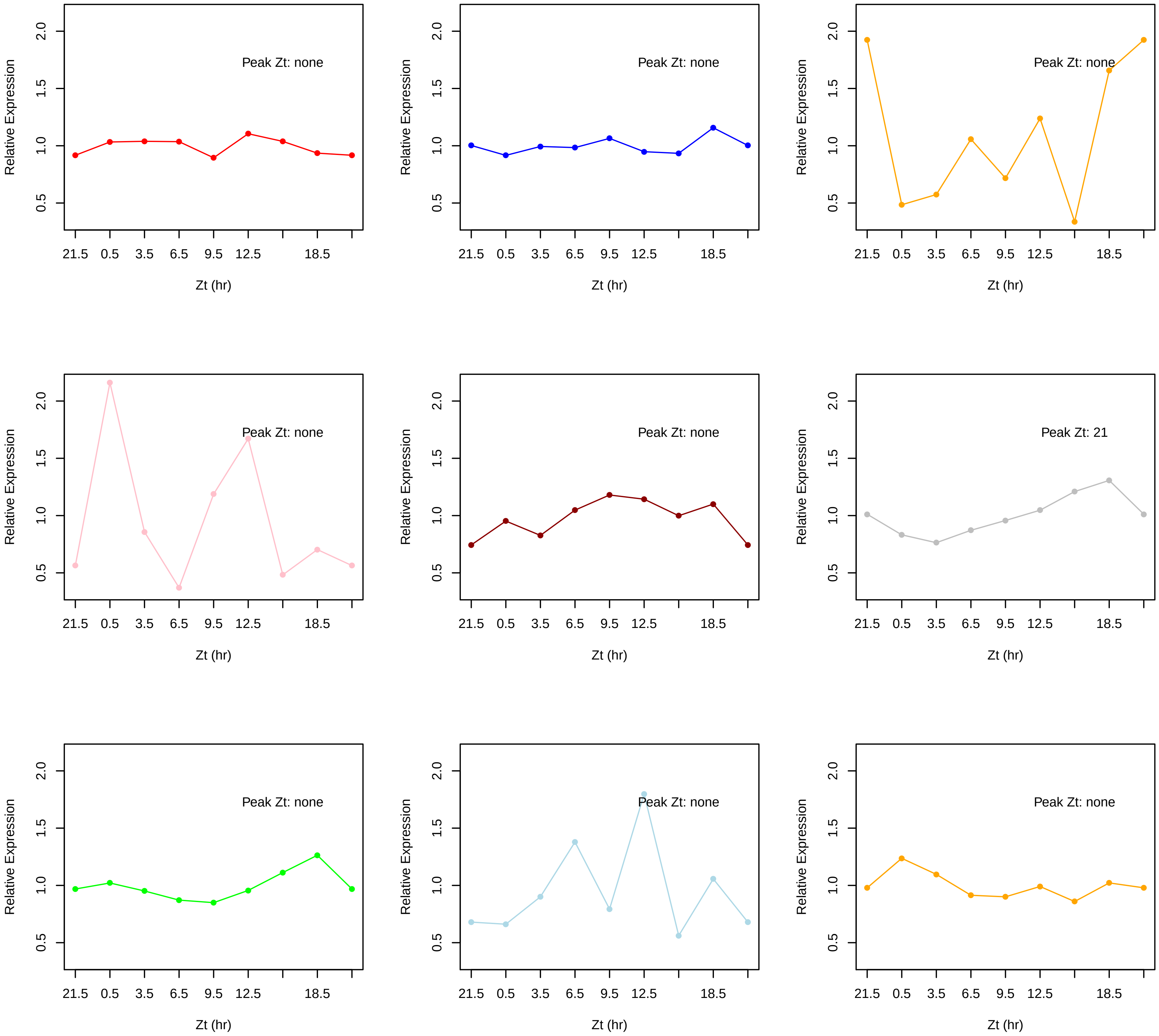

Singeleton: ENSXMAG00000028457 dph7

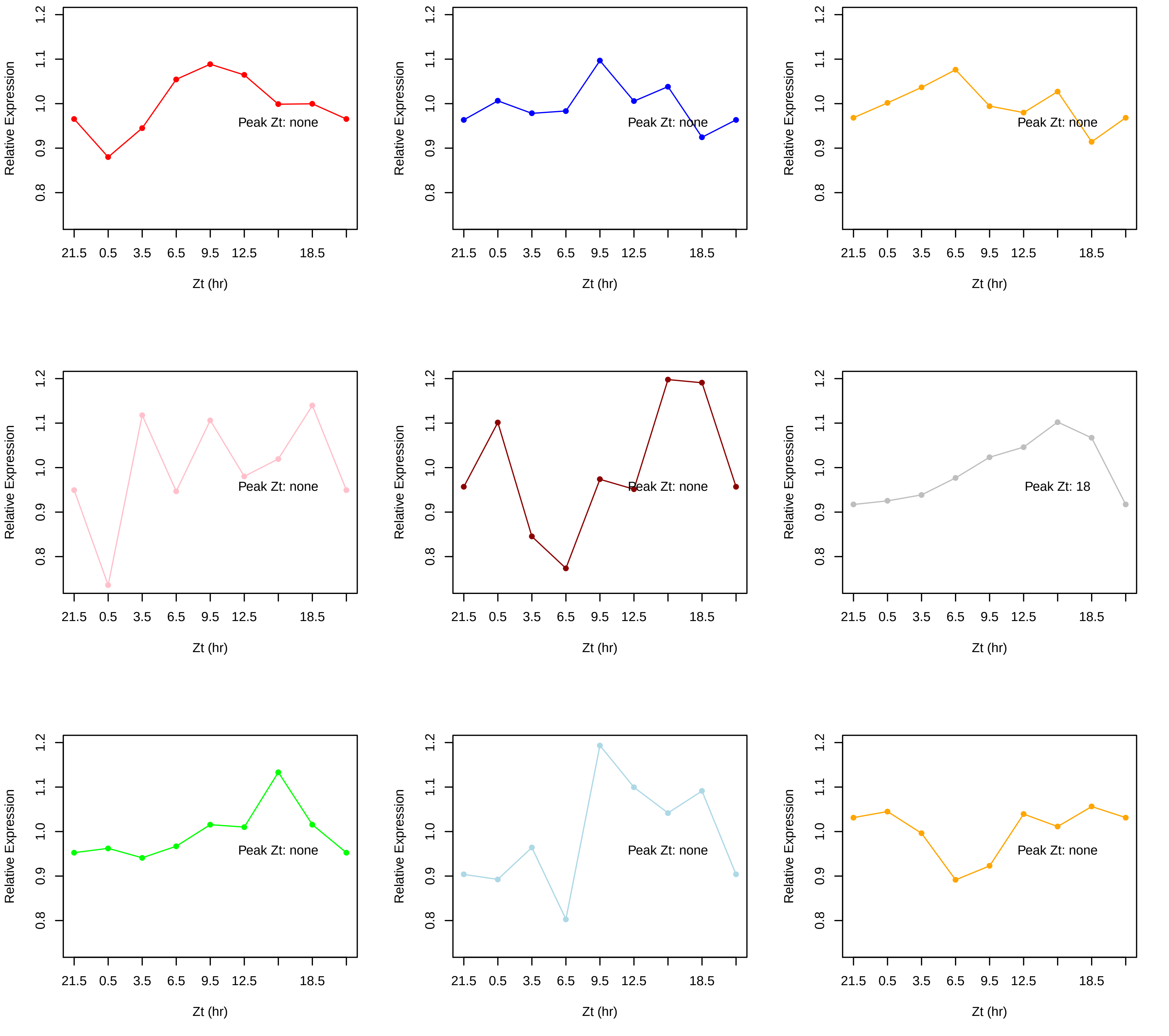

Singeleton: ENSXMAG00000025671

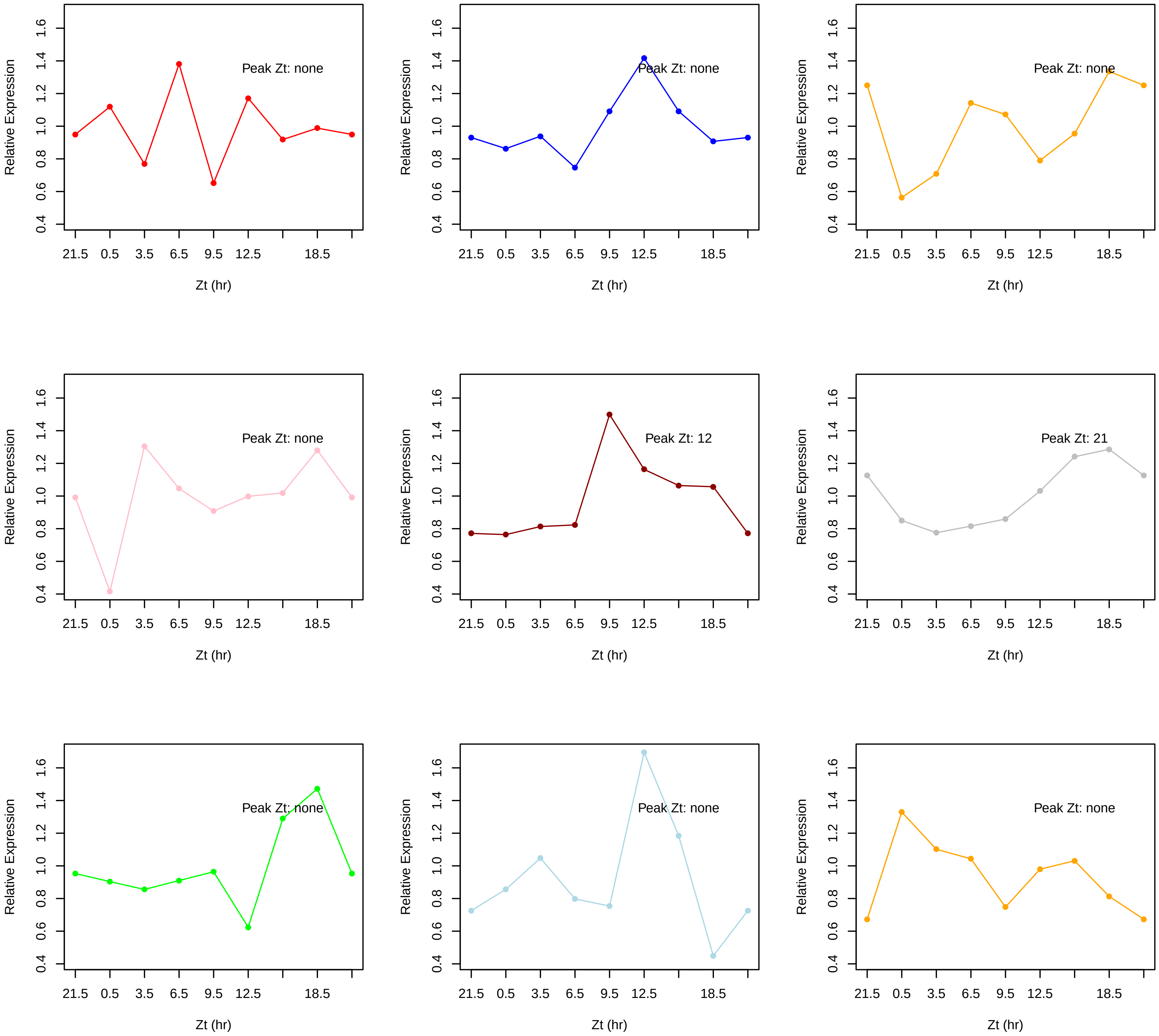

Singeleton: ENSXMAG00000029412 cdab

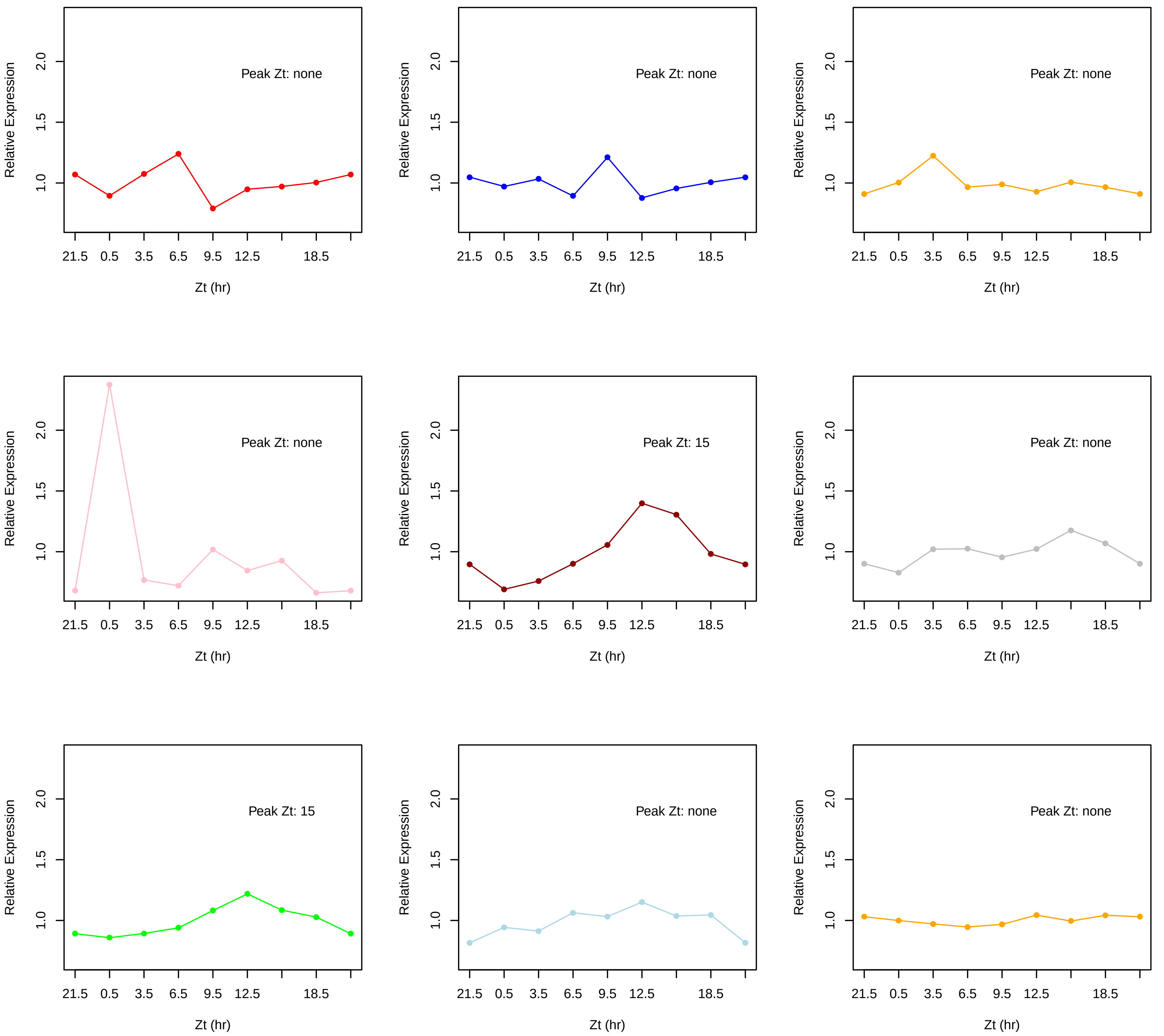

Singeleton: ENSXMAG00000008666 plcb3

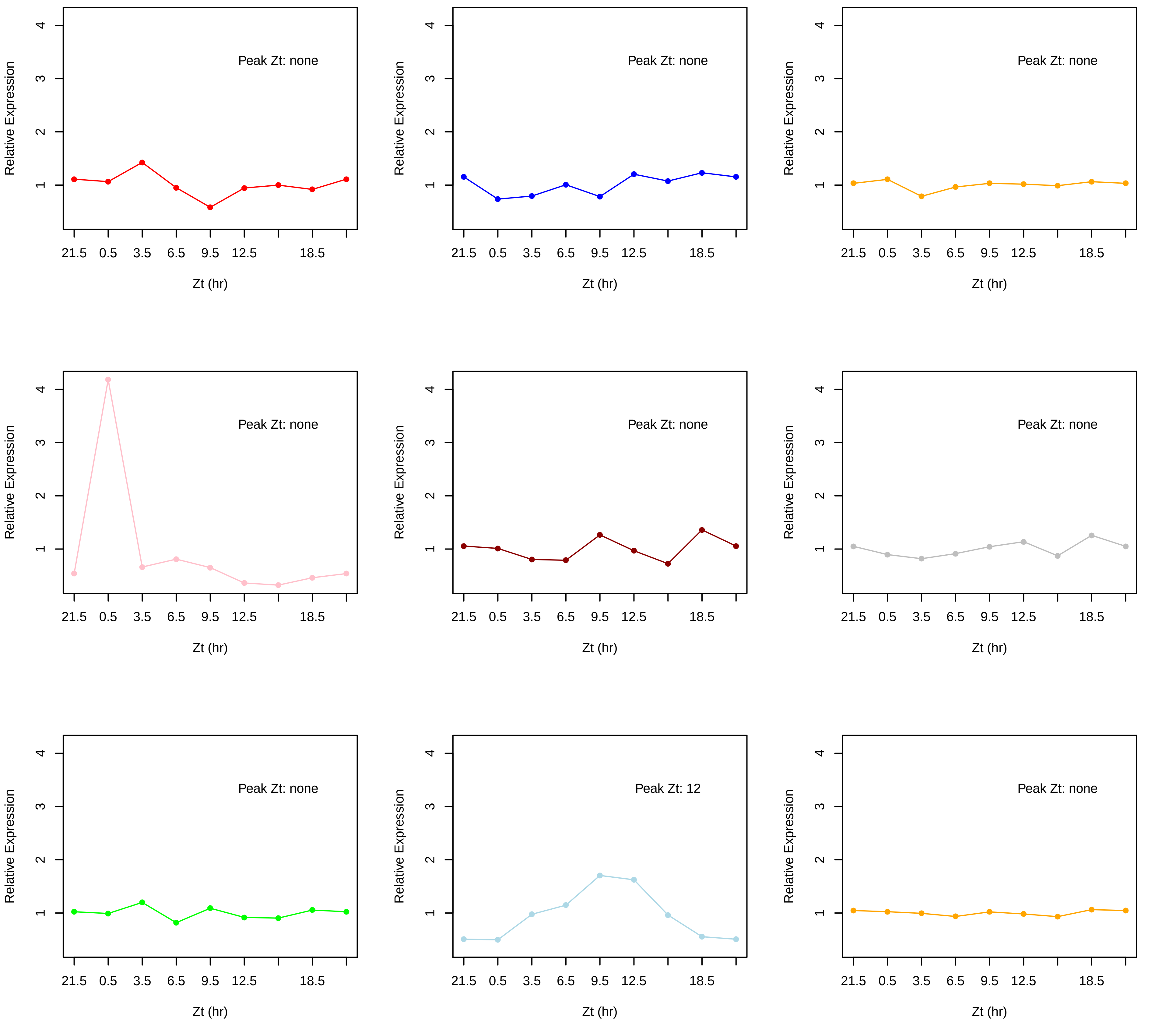

Singeleton: ENSXMAG00000024891 ndp

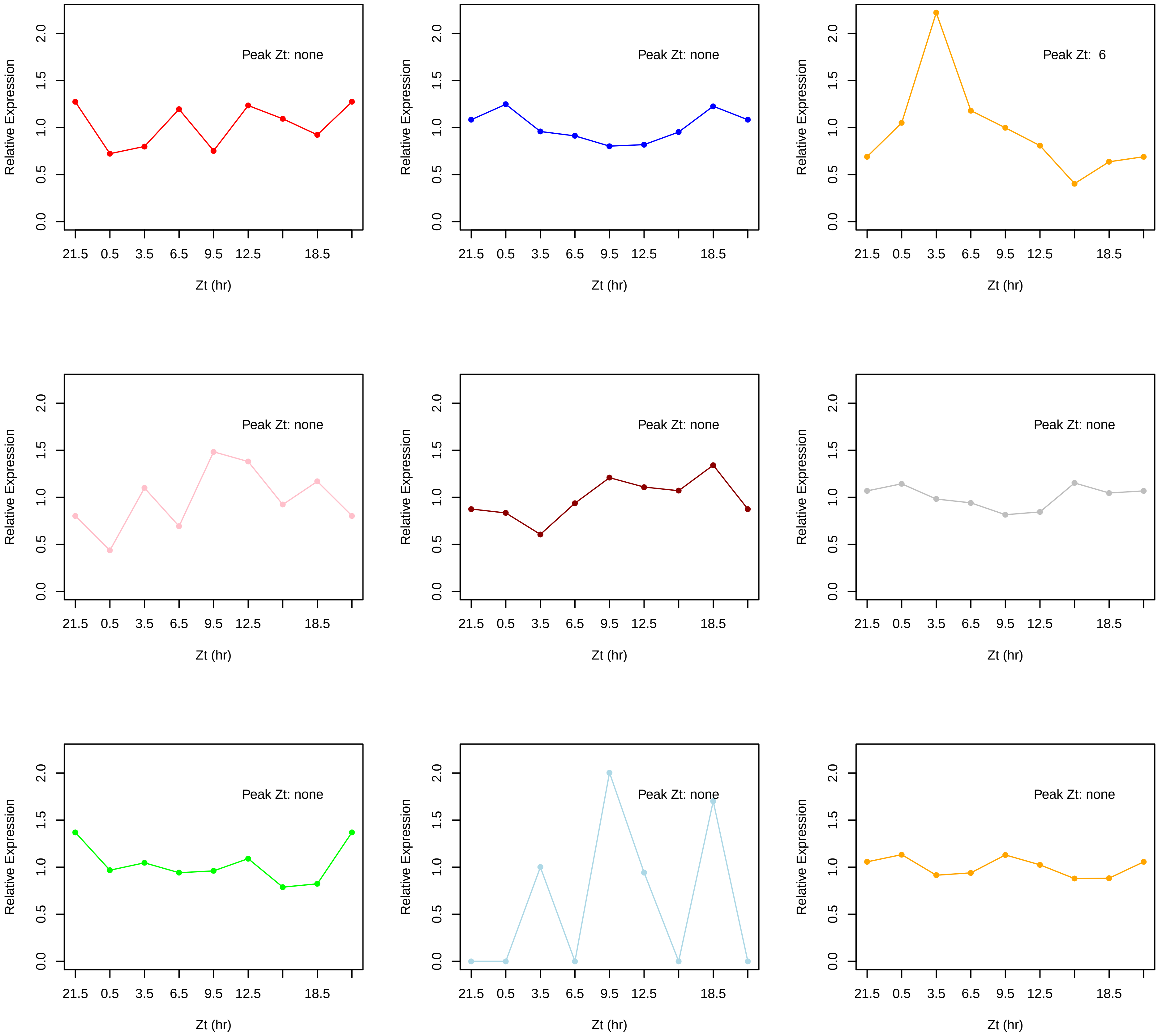

Singeleton: ENSXMAG00000021656

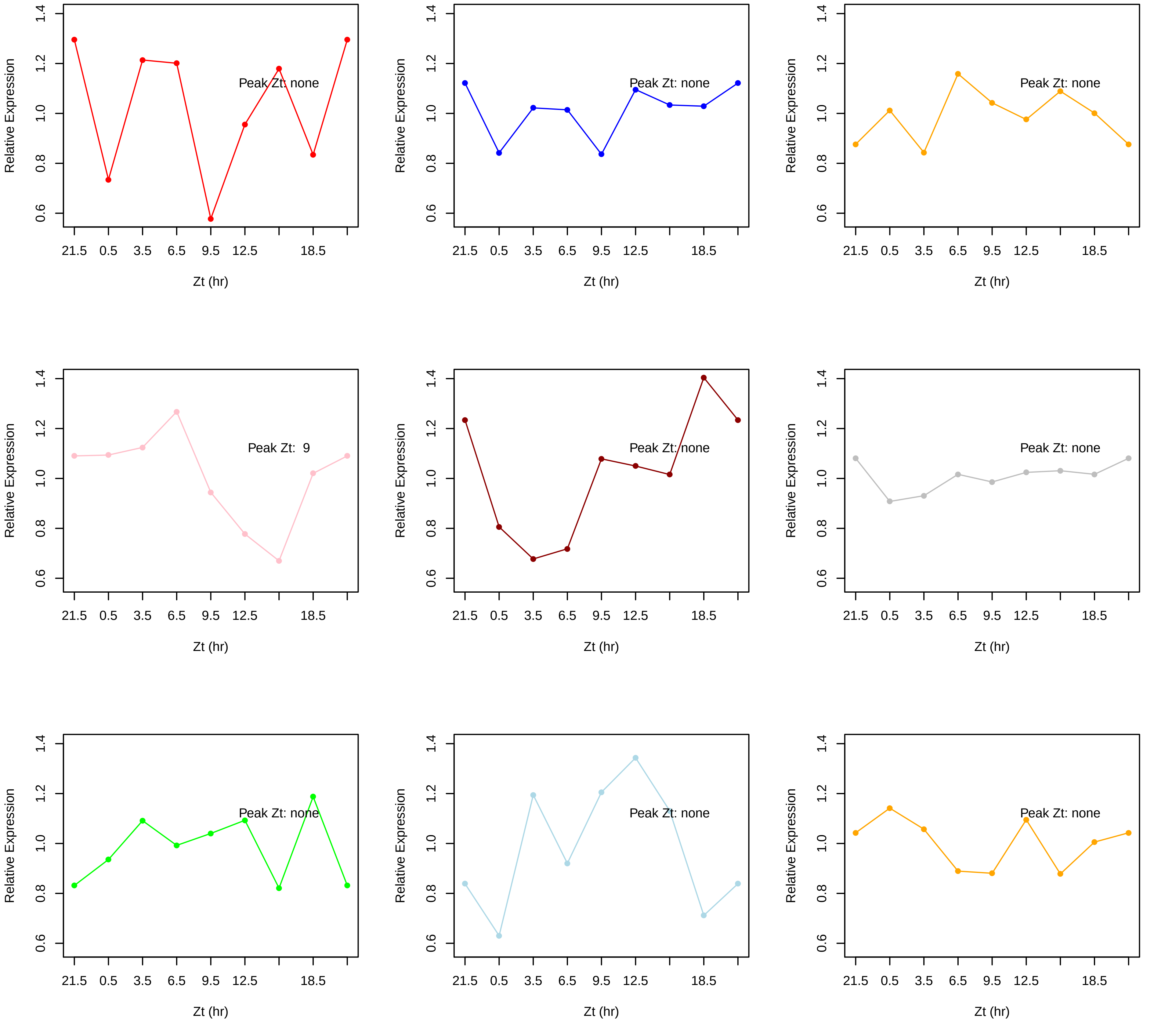

Singeleton: ENSXMAG00000022692 hpca

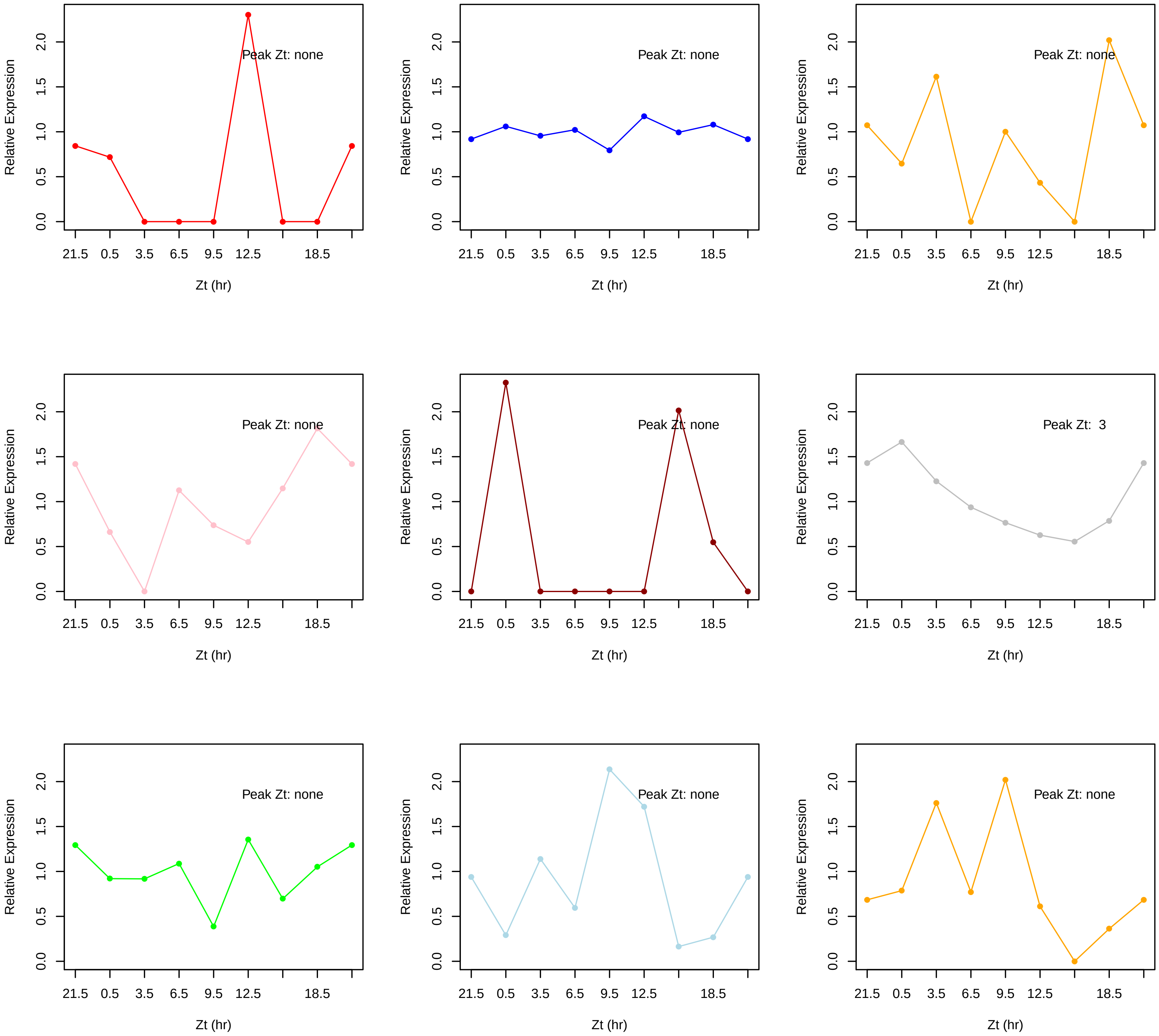

Singeleton: ENSXMAG00000006915 mao

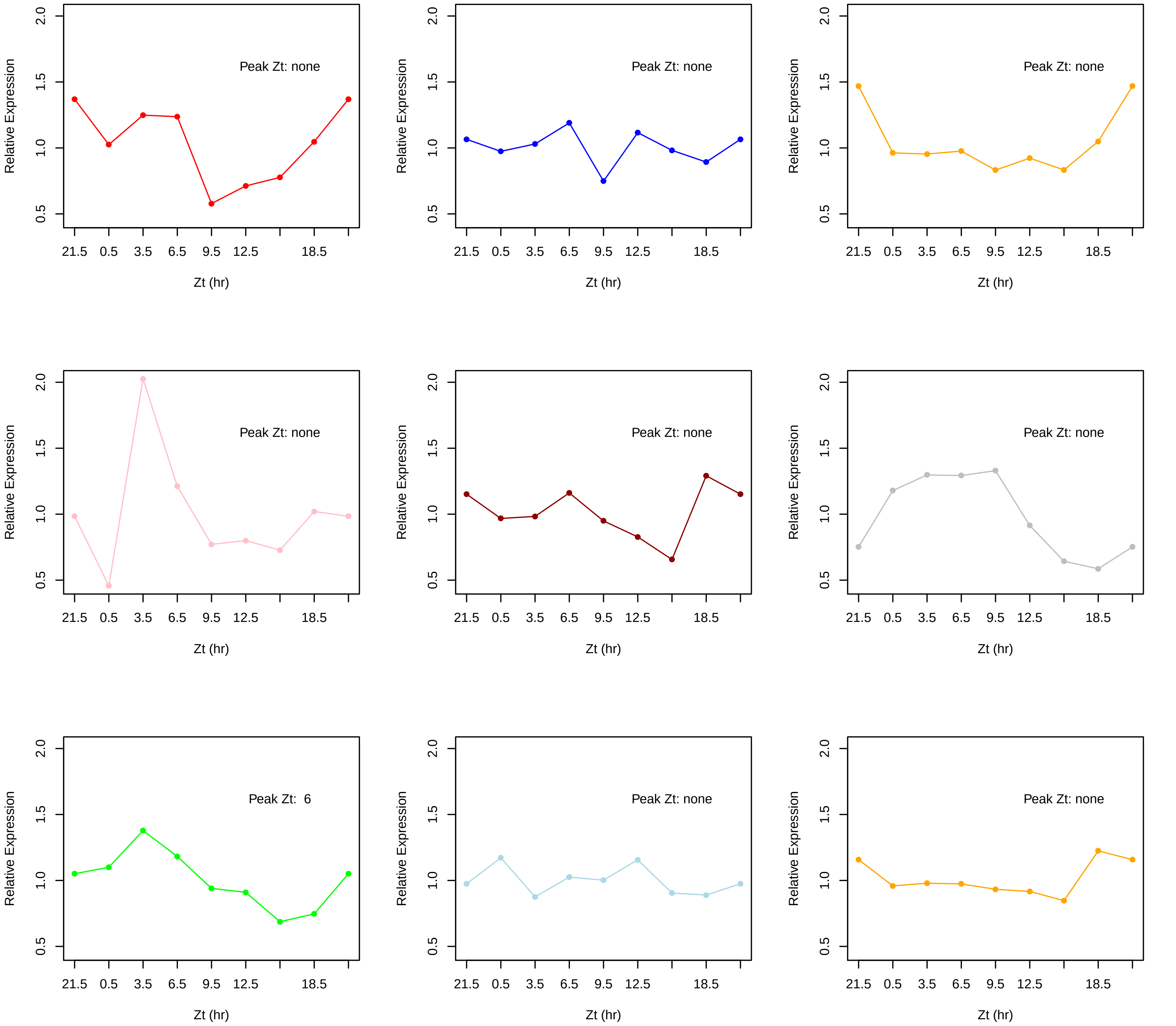

Singleton: ENSXMAG00000011720 igsf10

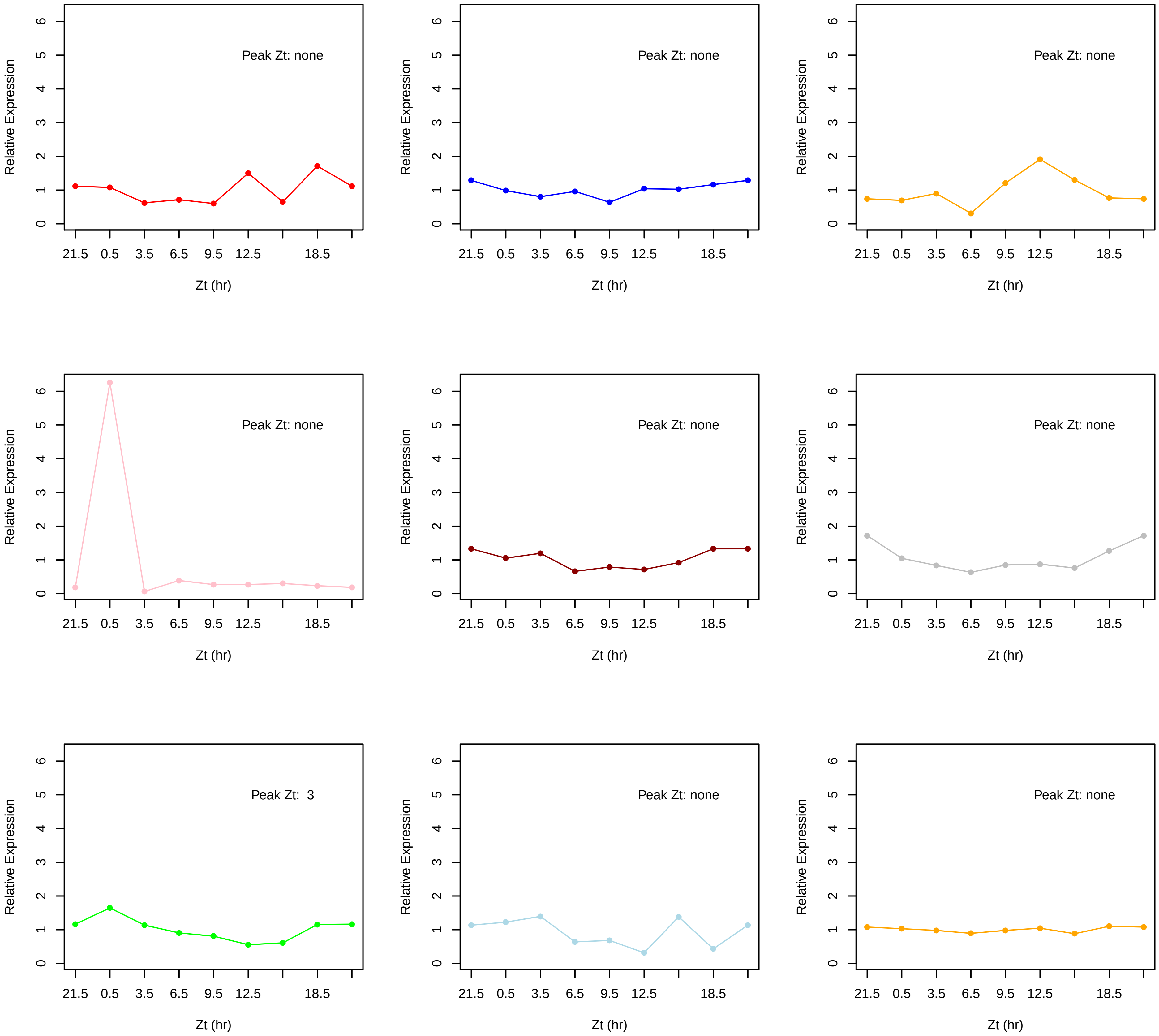

Singeleton: ENSXMAG00000001219 fitm2

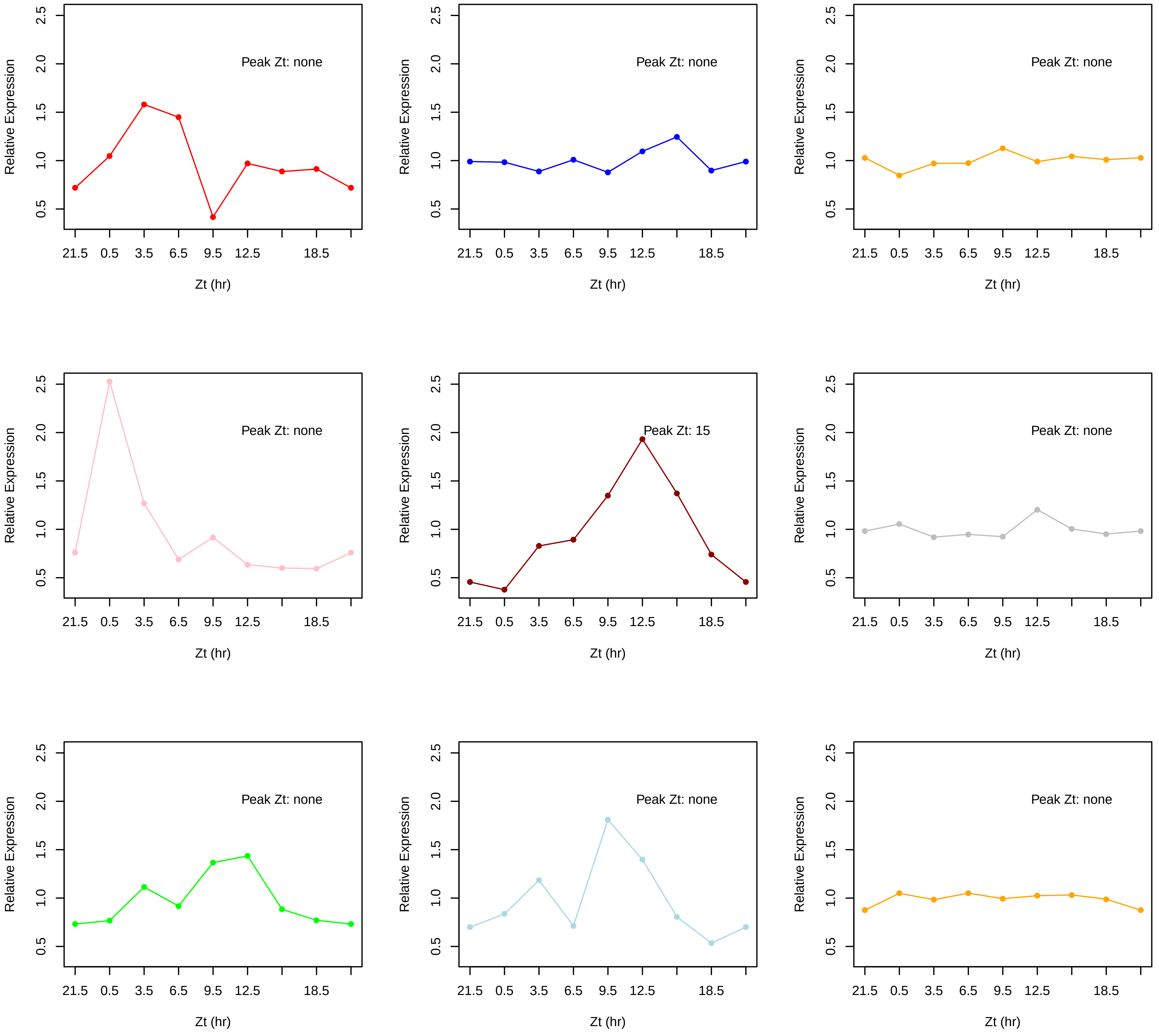

Singleton: ENSXMAG00000001444 aadac

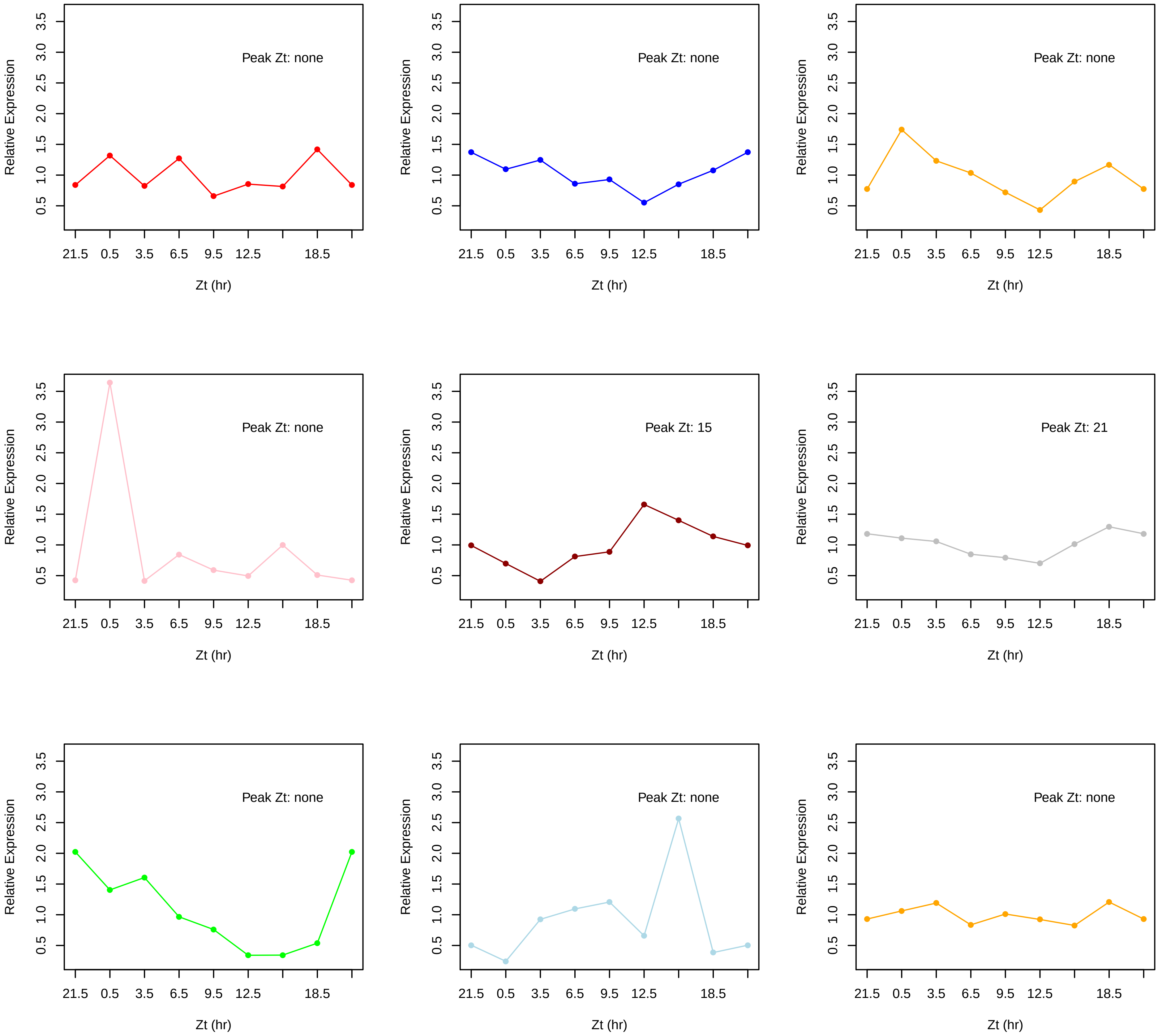

Singeleton: ENSXMAG00000023293 ralba

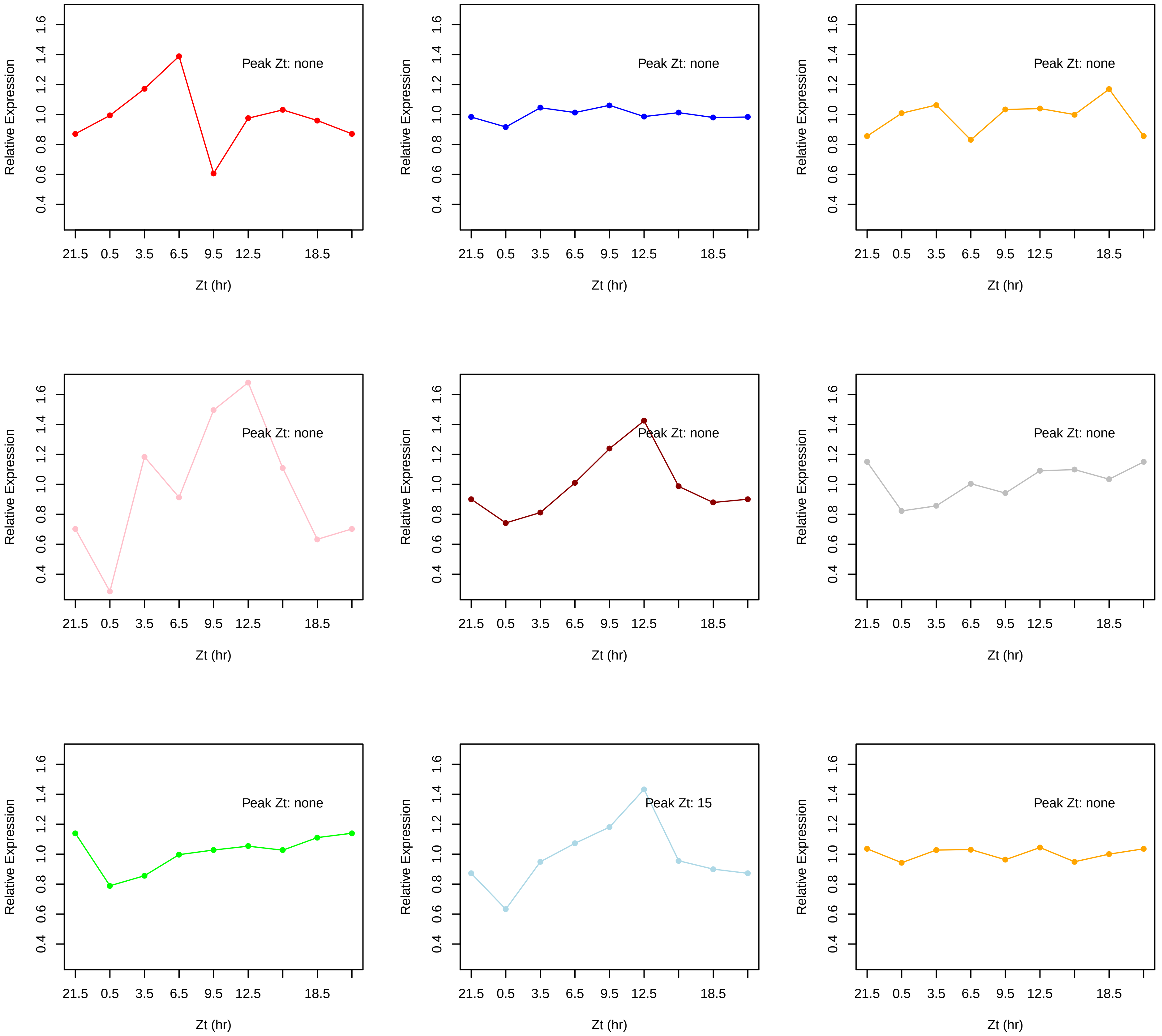

Singleton: ENSXMAG00000026280 doc2b

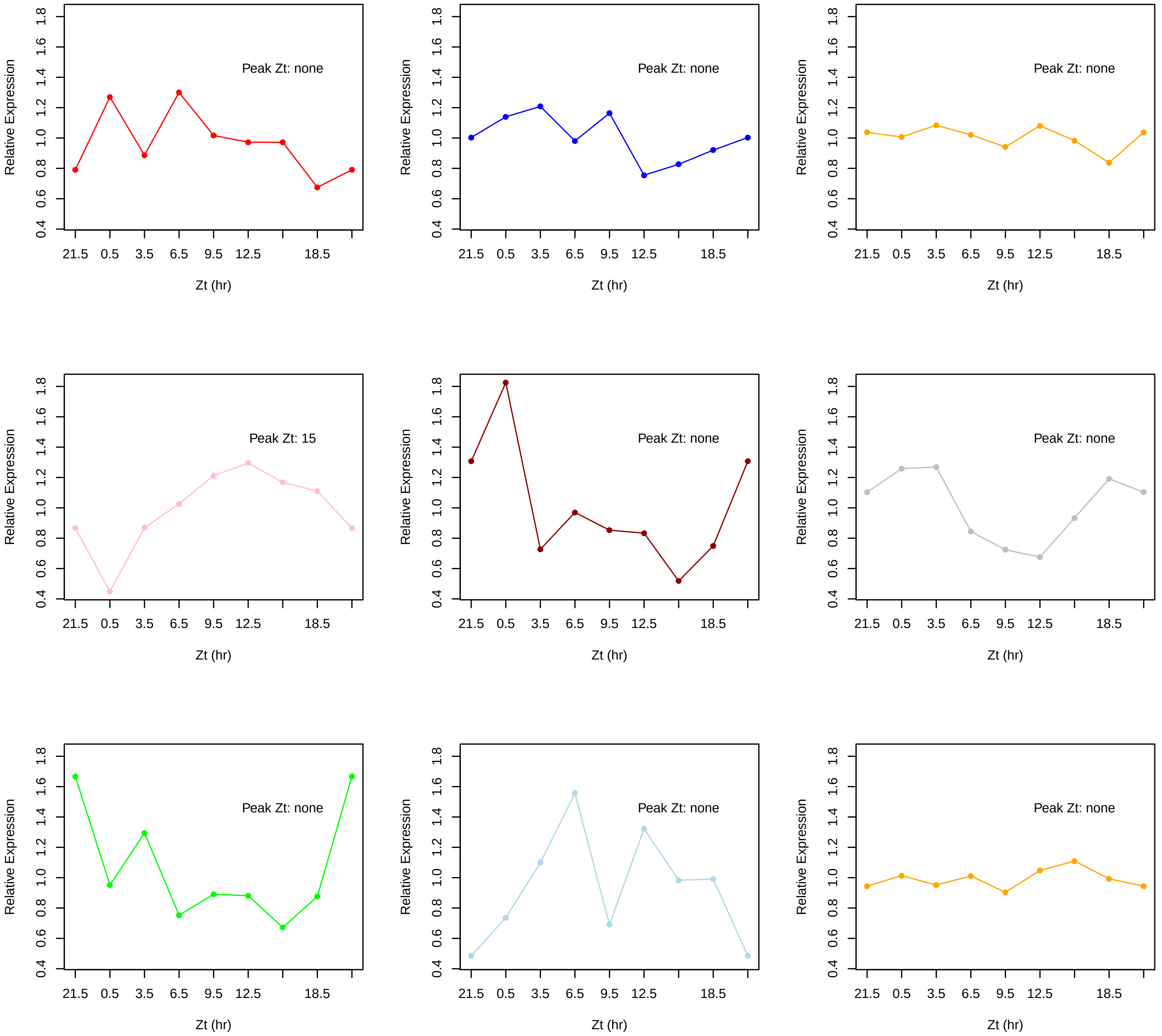

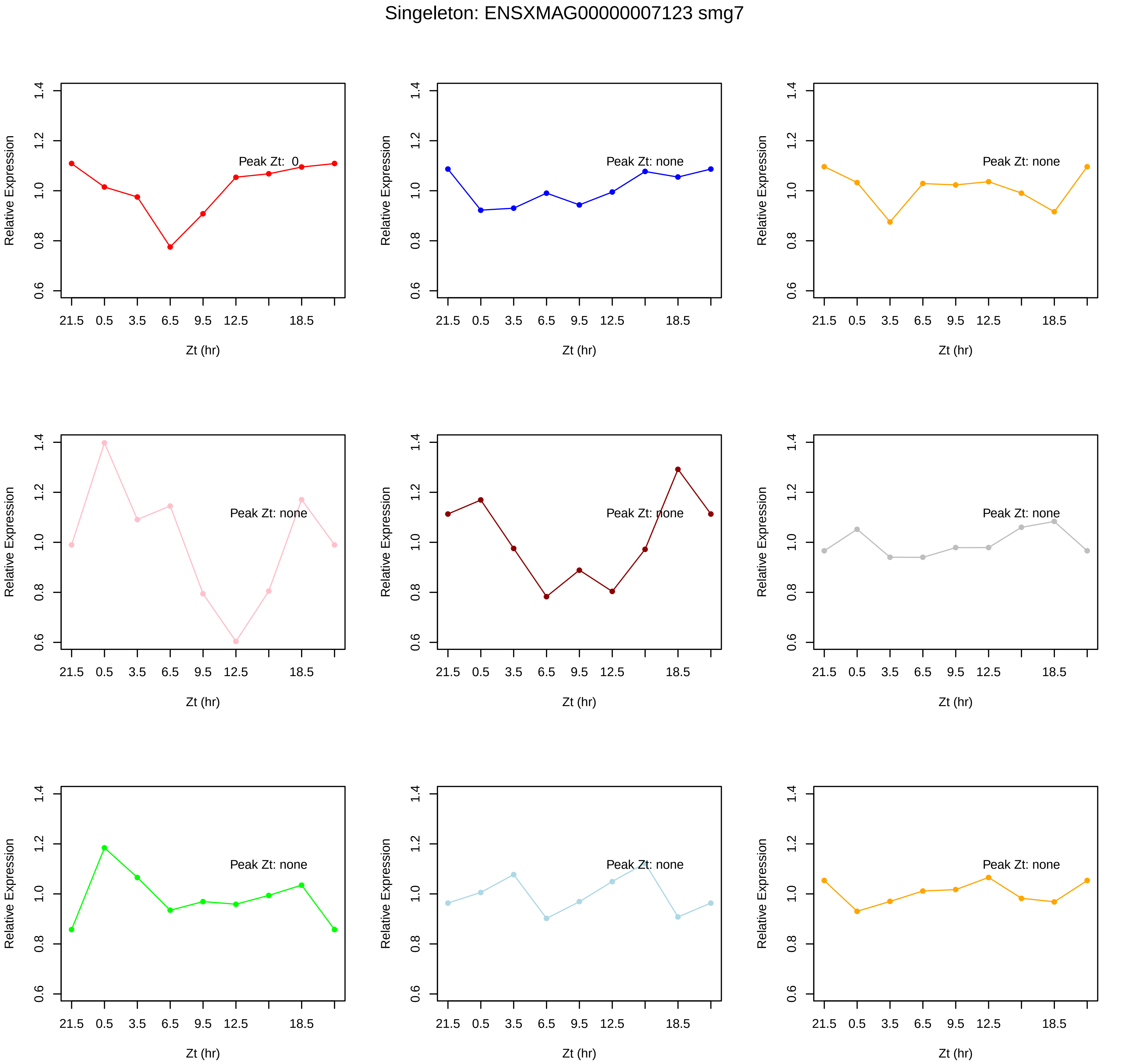
Singeleton: ENSXMAG00000007123 smg7
Peak Zt: 0
Peak Zt: none
Relative Expression
Zt (hr)
21.5
0.5
3.5
6.5
9.5
12.5
18.5
0.6
0.8
1.0
1.2
1.4

Singeleton: ENSXMAG00000006444 mapk1

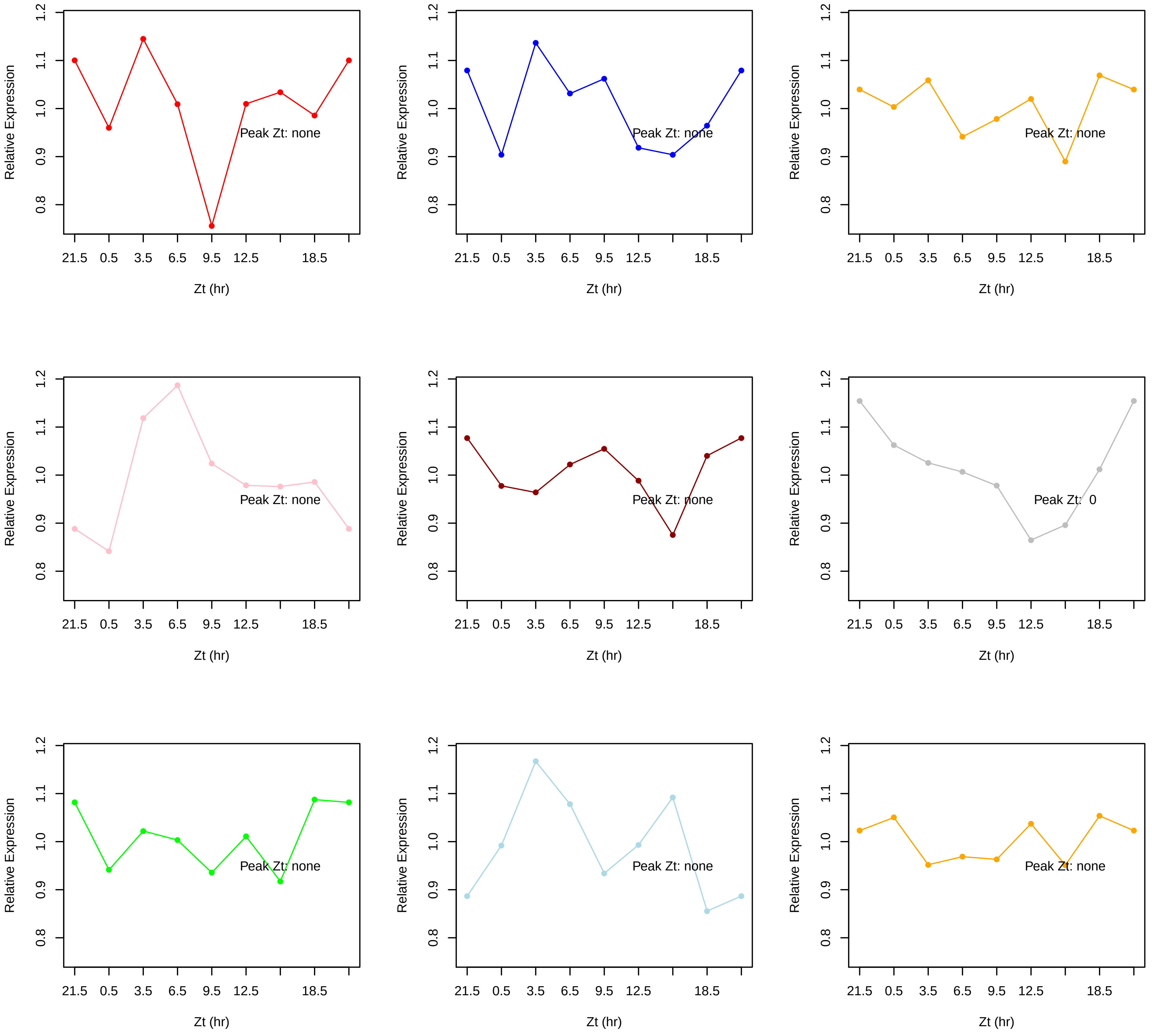

Singeleton: ENSXMAG00000026745 GLI2

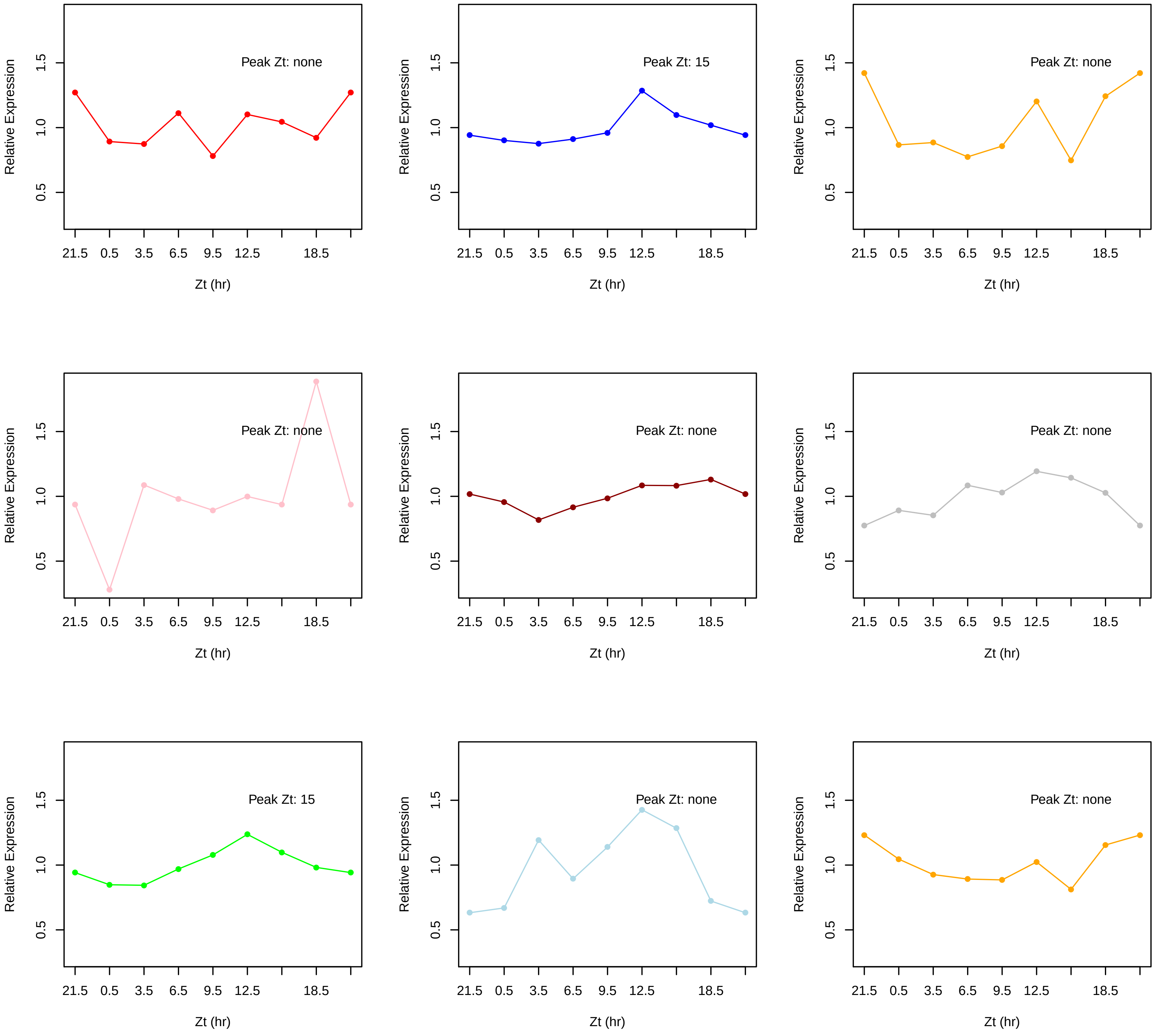

Singleton: ENSXMAG00000019243 abcc8

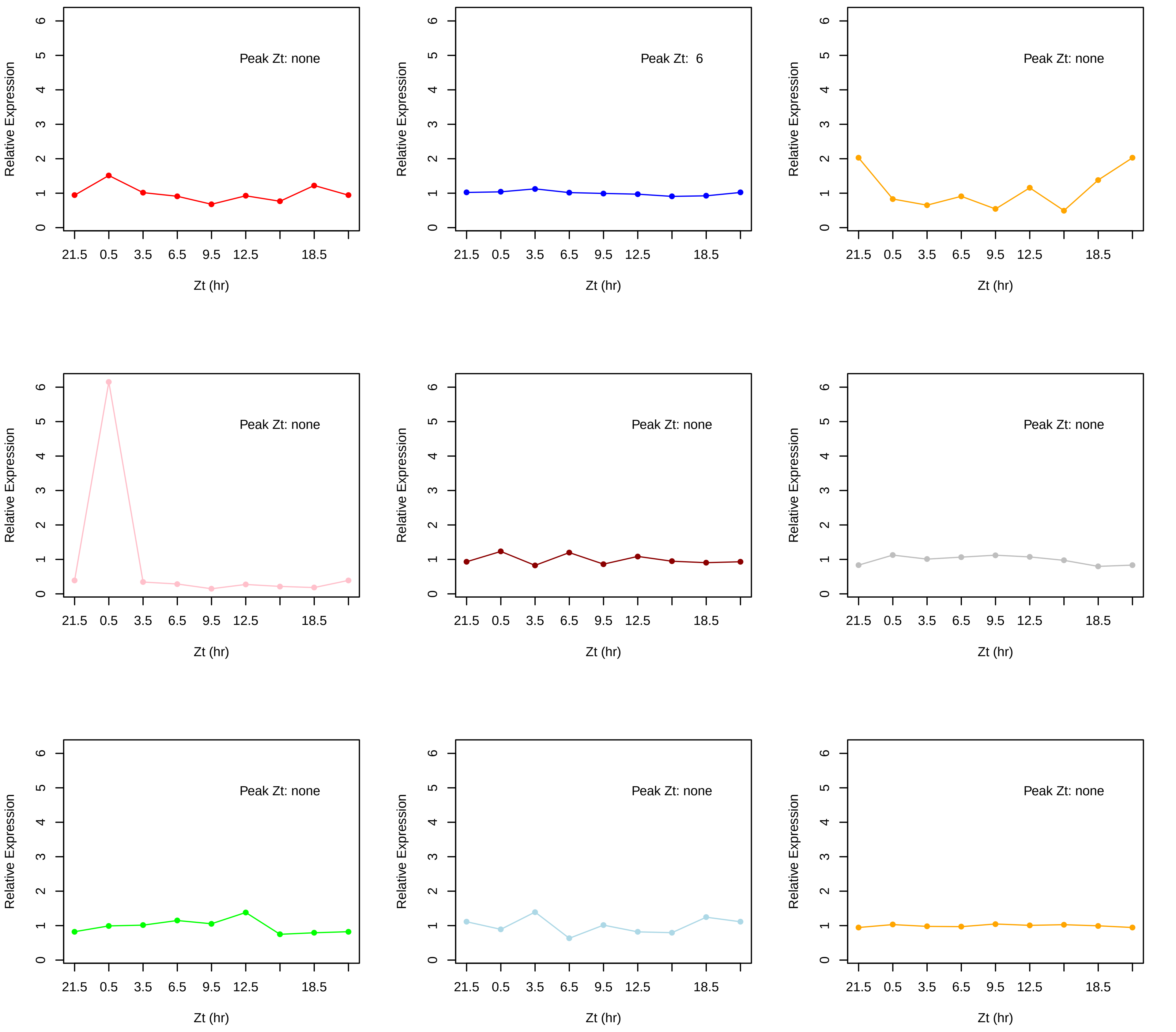

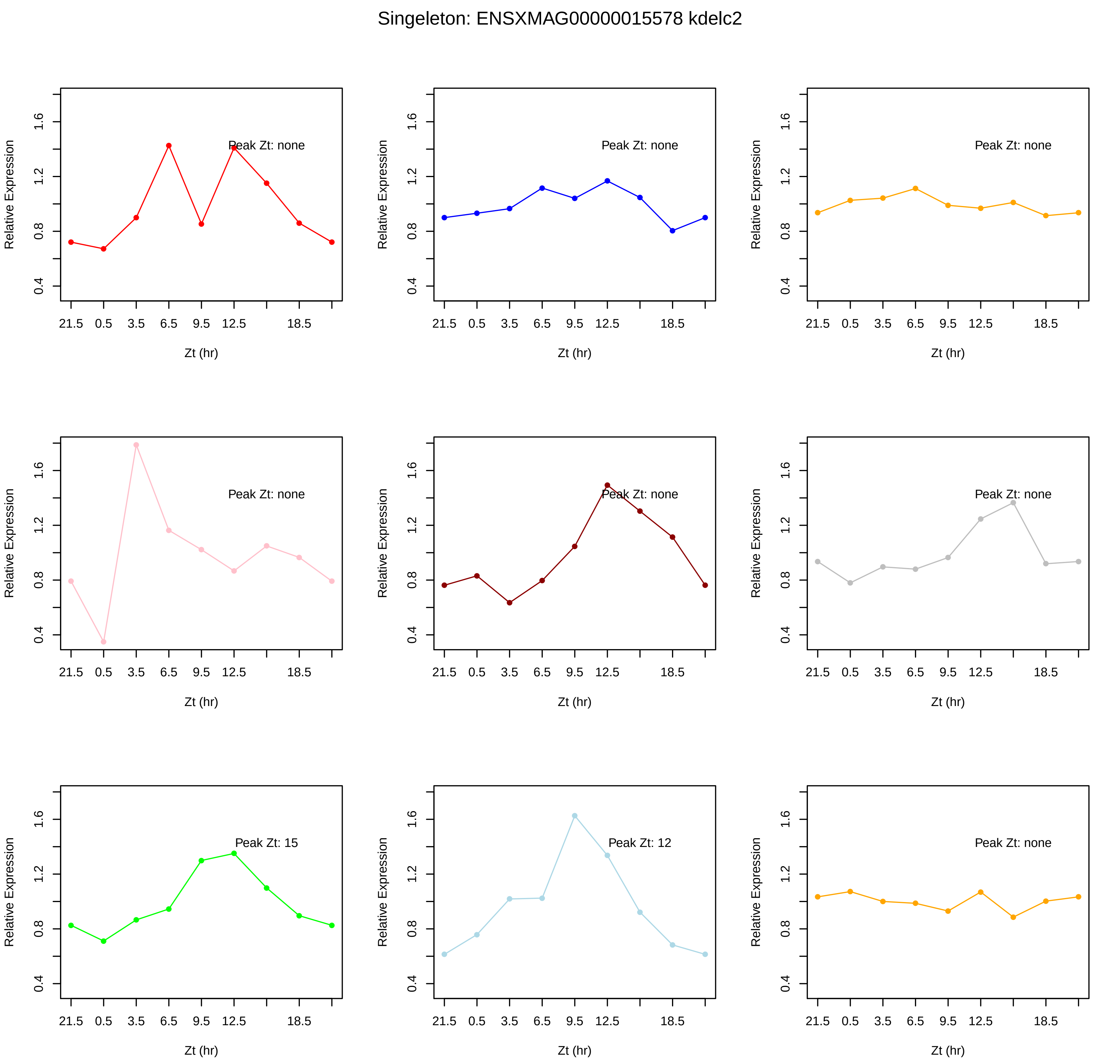
Singeleton: ENSXMAG00000015578 kdelc2
Relative Expression
Zt (hr)
21.5 0.5 3.5 6.5 9.5 12.5 18.5
0.4 0.8 1.2 1.6
Peak Zt: none
Peak Zt: none
Peak Zt: none
Peak Zt: none
Peak Zt: none
Peak Zt: none
Peak Zt: 15
Peak Zt: 12
Peak Zt: none

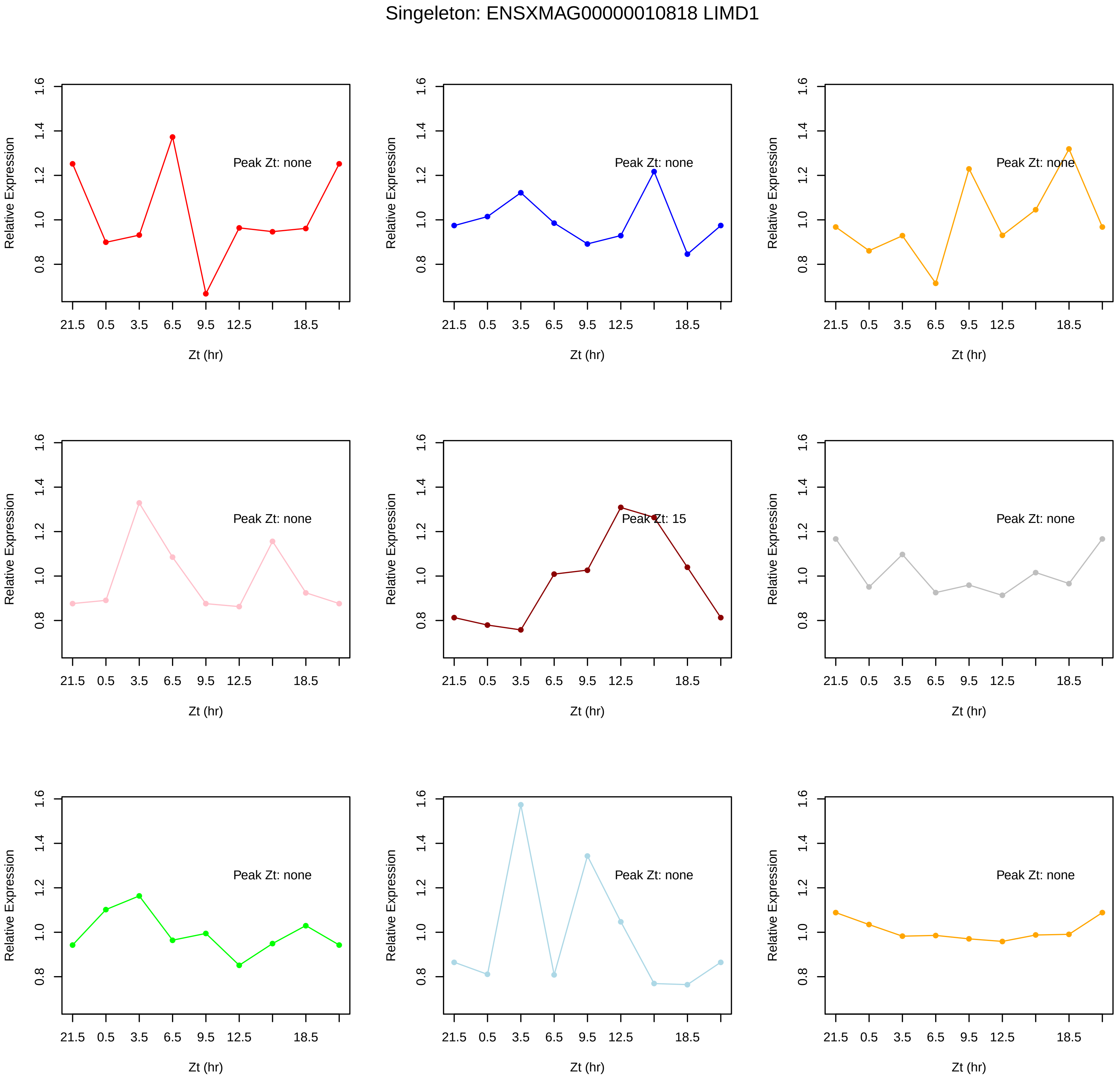
Singeleton: ENSXMAG00000010818 LIMD1
Peak Zt: none
Peak Zt: none
Peak Zt: none
Peak Zt: none
Peak Zt: 15
Peak Zt: none
Peak Zt: none
Peak Zt: none
Peak Zt: none
Relative Expression
Zt (hr)
21.5
0.5
3.5
6.5
9.5
12.5
18.5
0.8
1.0
1.2
1.4
1.6

Singeleton: ENSXMAG00000002532 PGPEP1

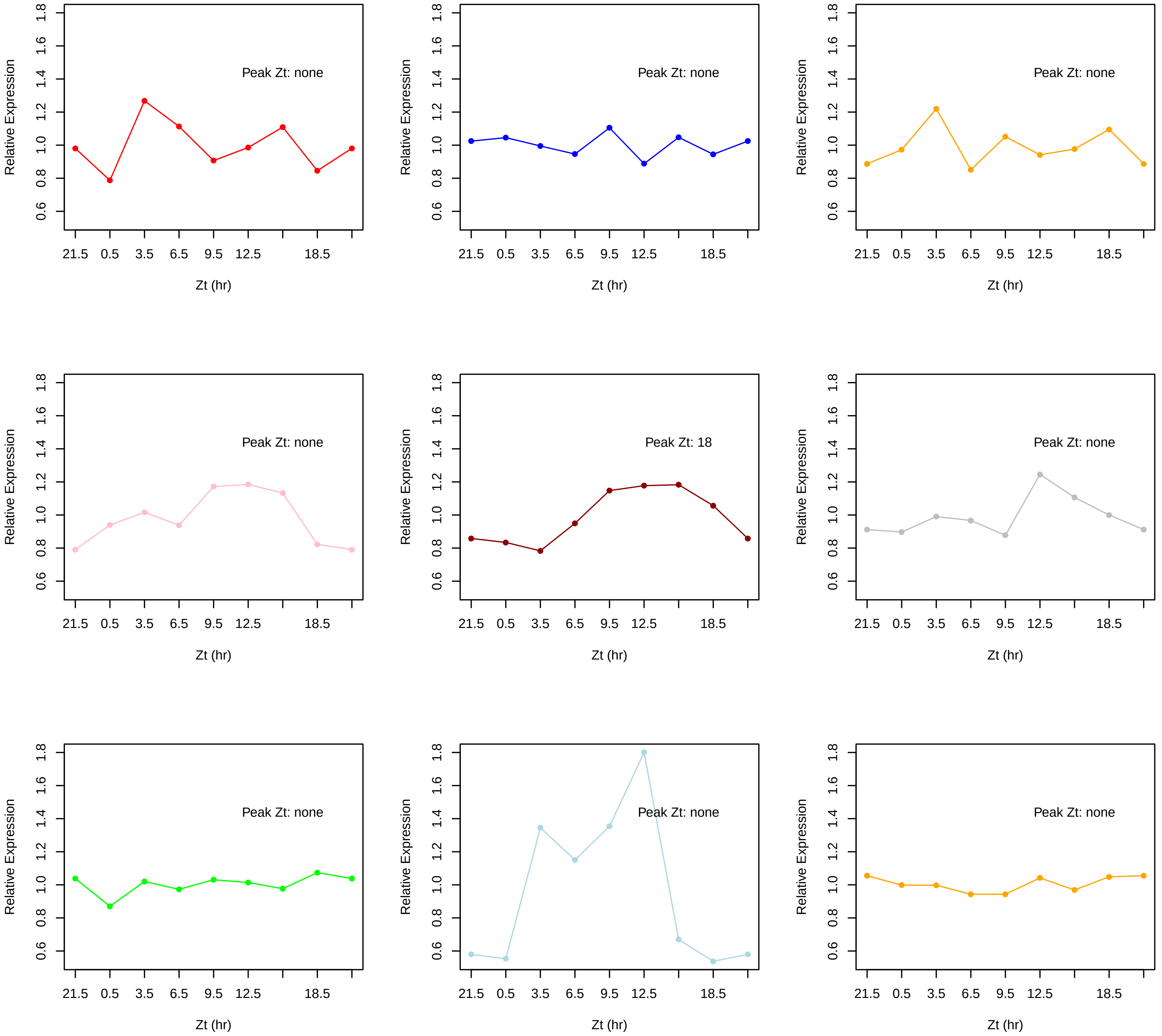

Singeleton: ENSXMAG00000011477 rad51d

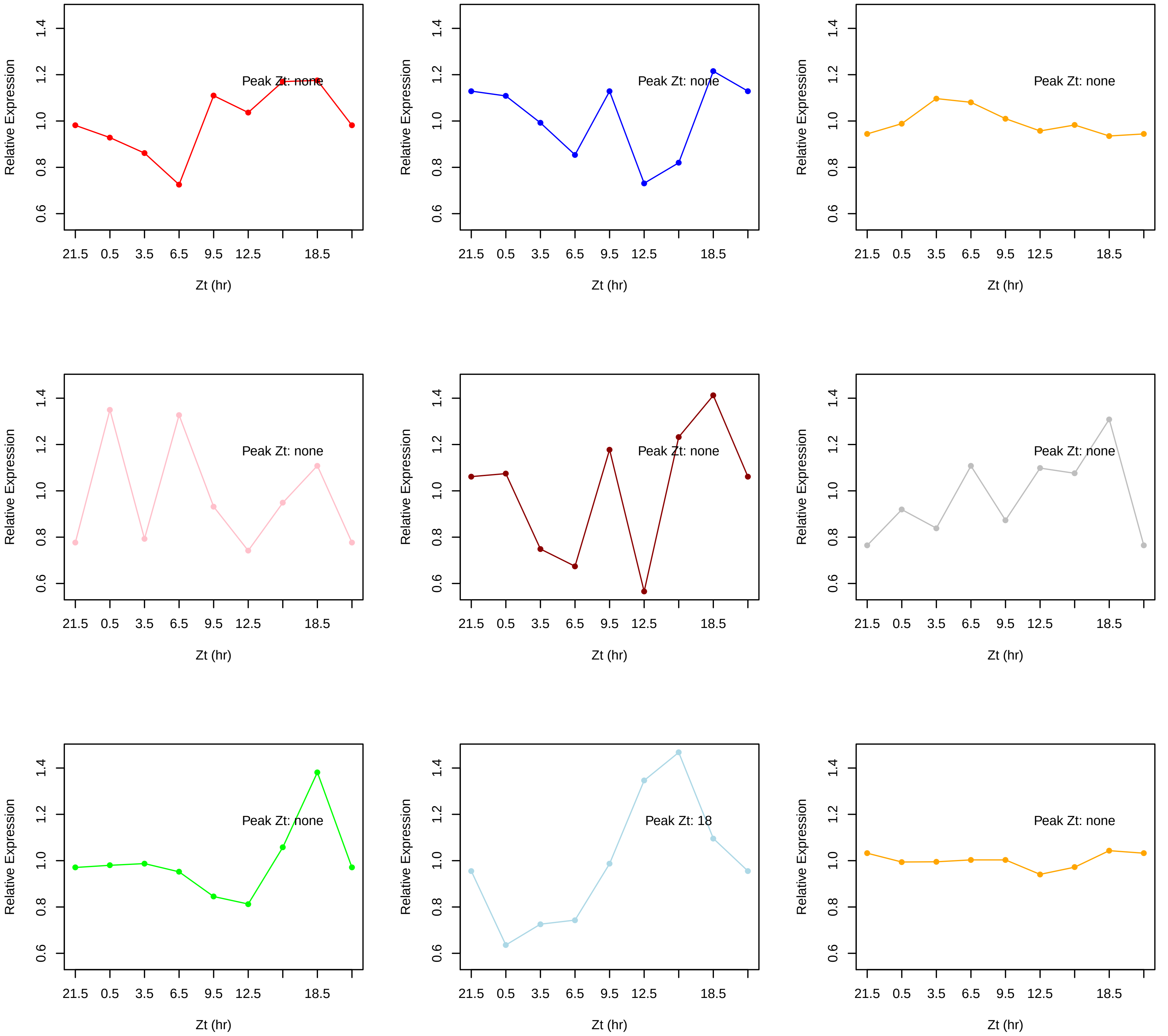

Singeleton: ENSXMAG00000004348 ppm1f

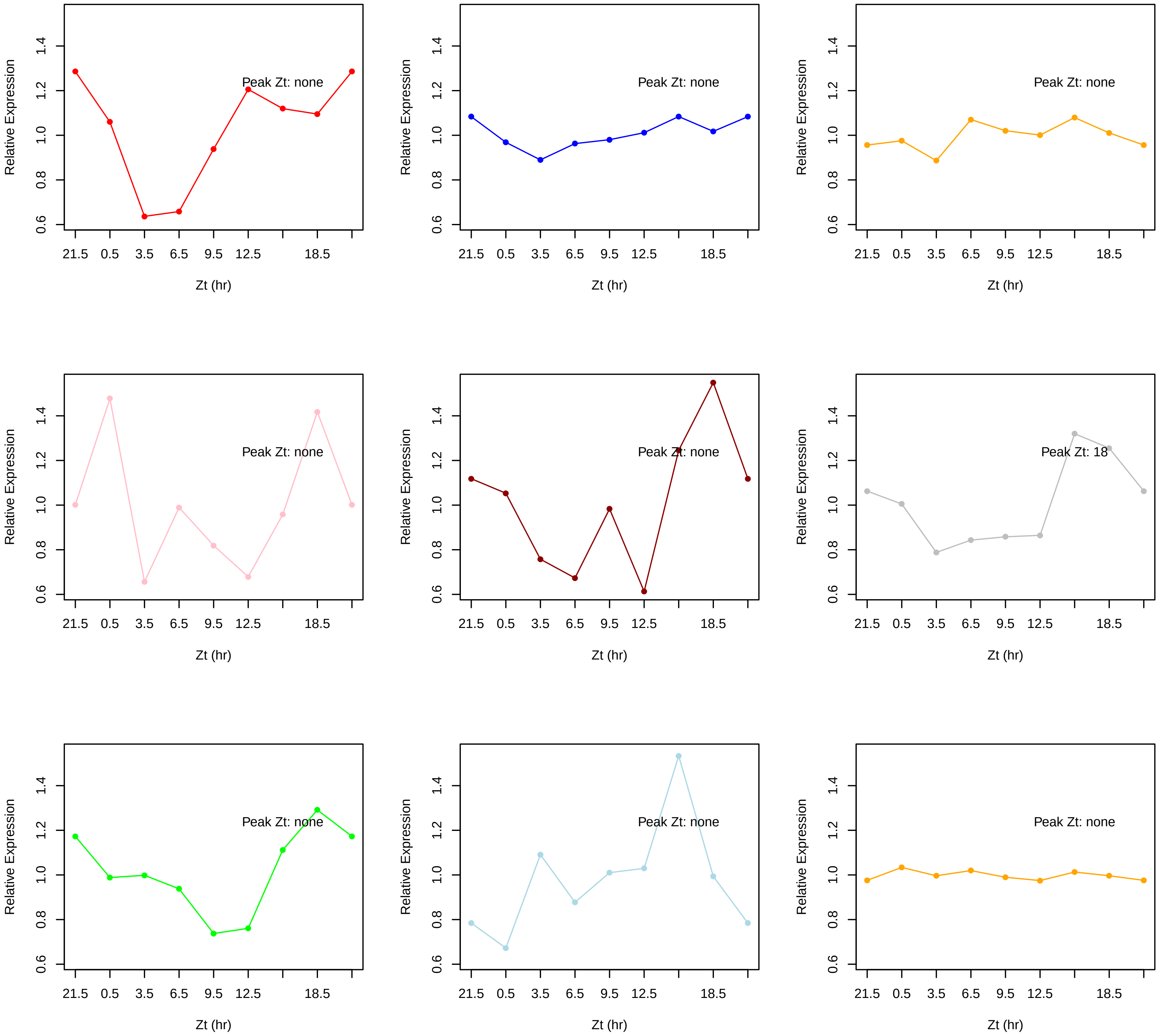

Singleton: ENSXMAG00000026959 usp14

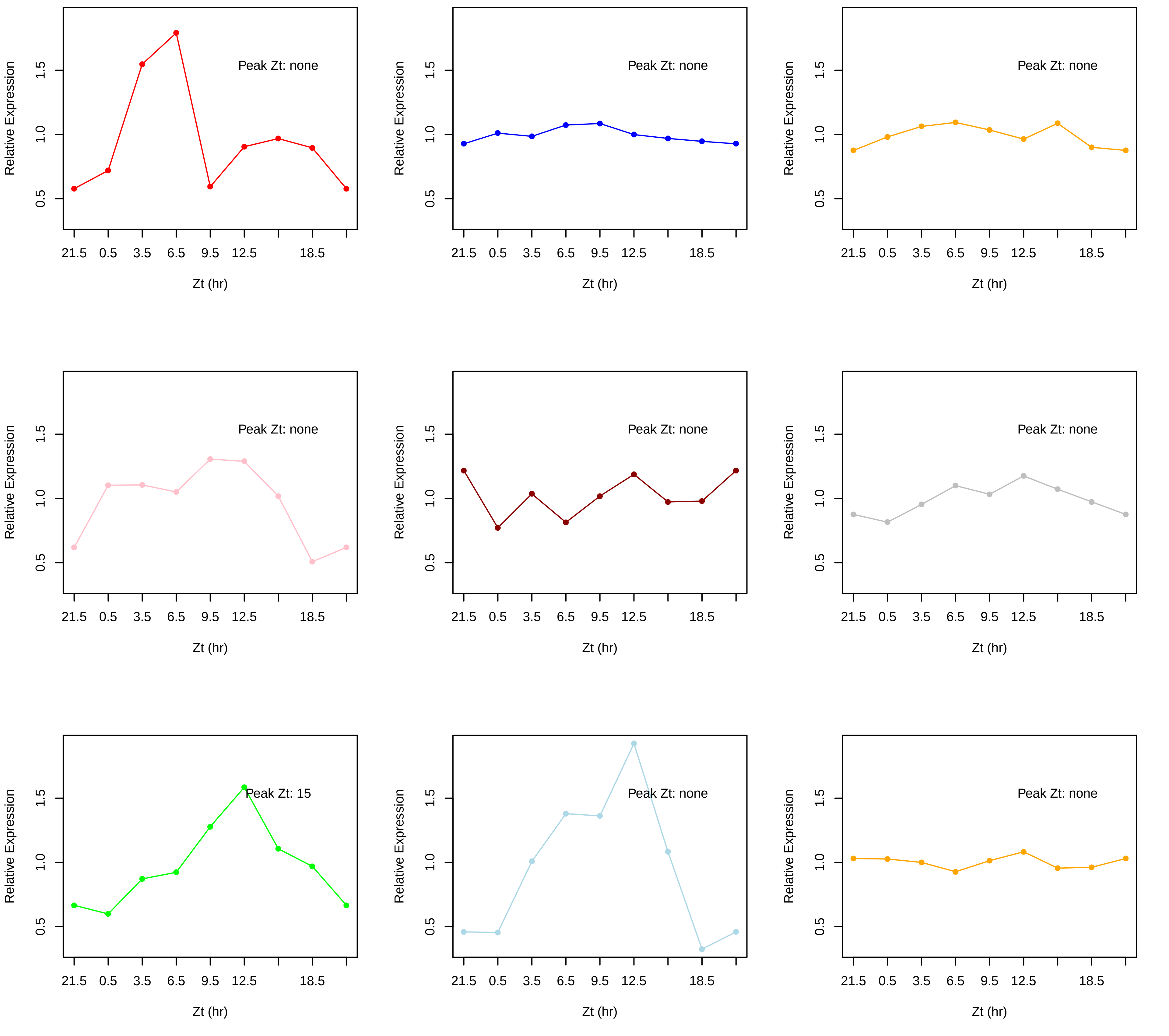

Singeleton: ENSXMAG00000029021 si:ch211−130m23.5

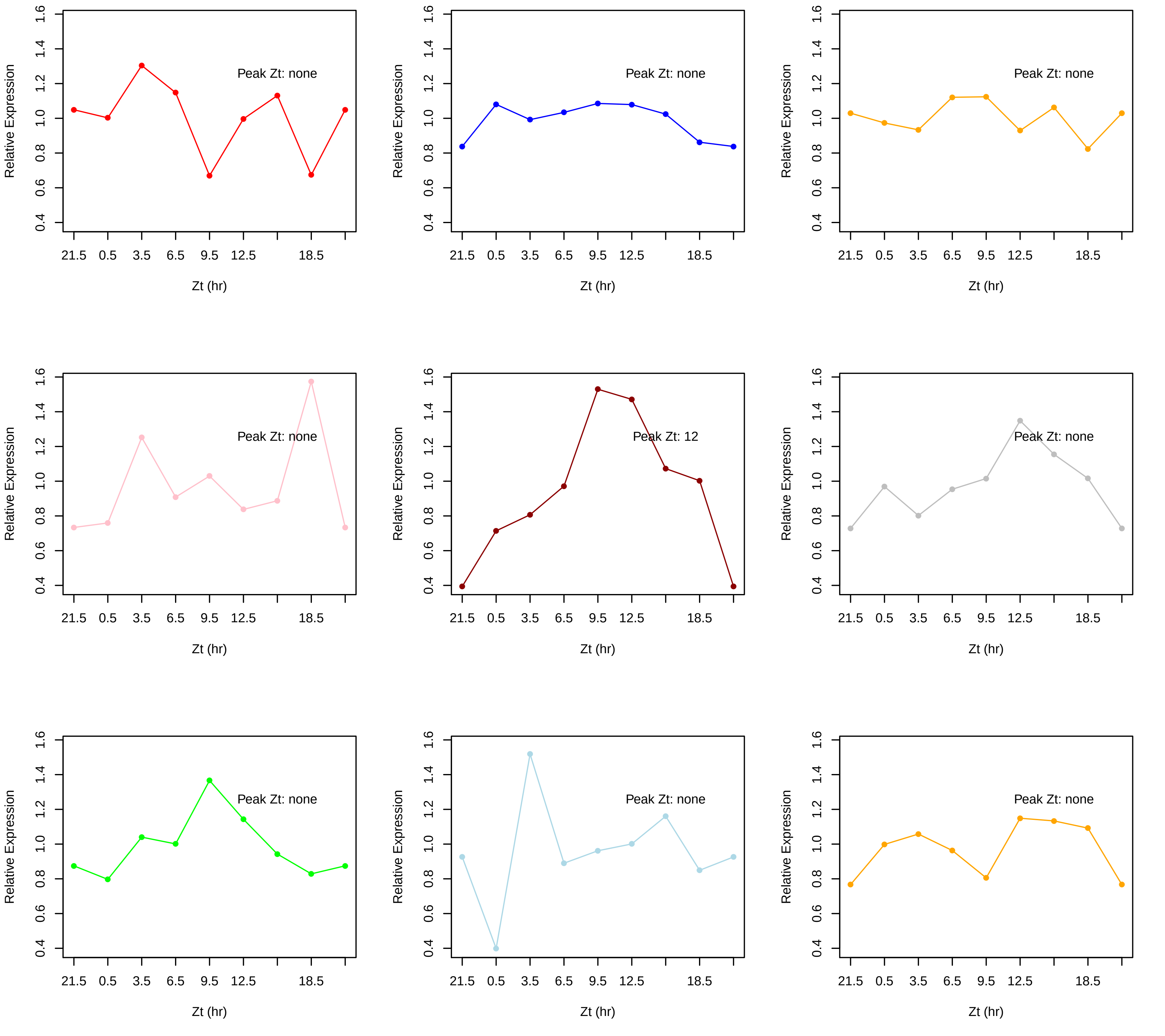

Singeleton: ENSXMAG00000027005 ss18l2

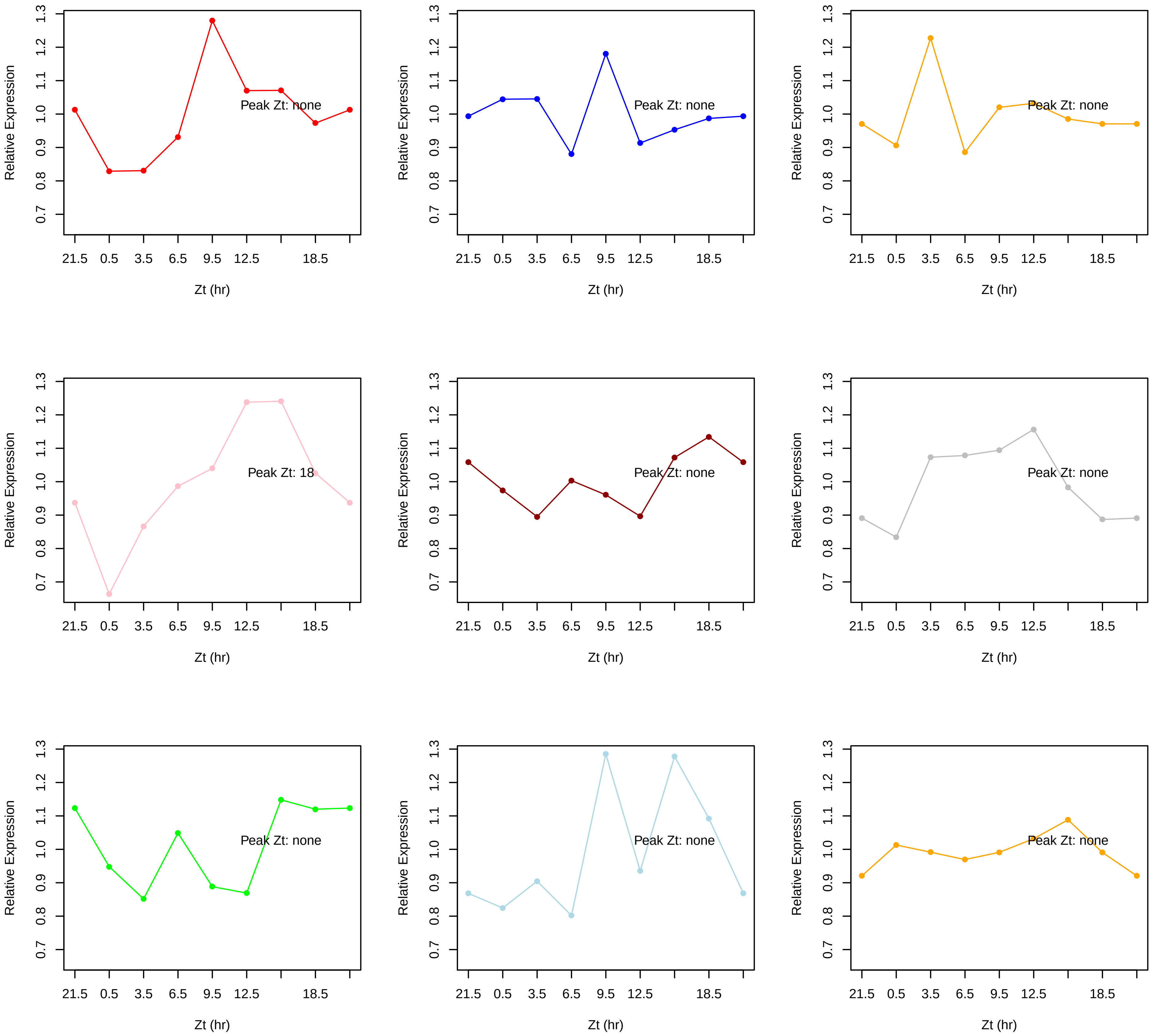

Singeleton: ENSXMAG00000001341 atg10

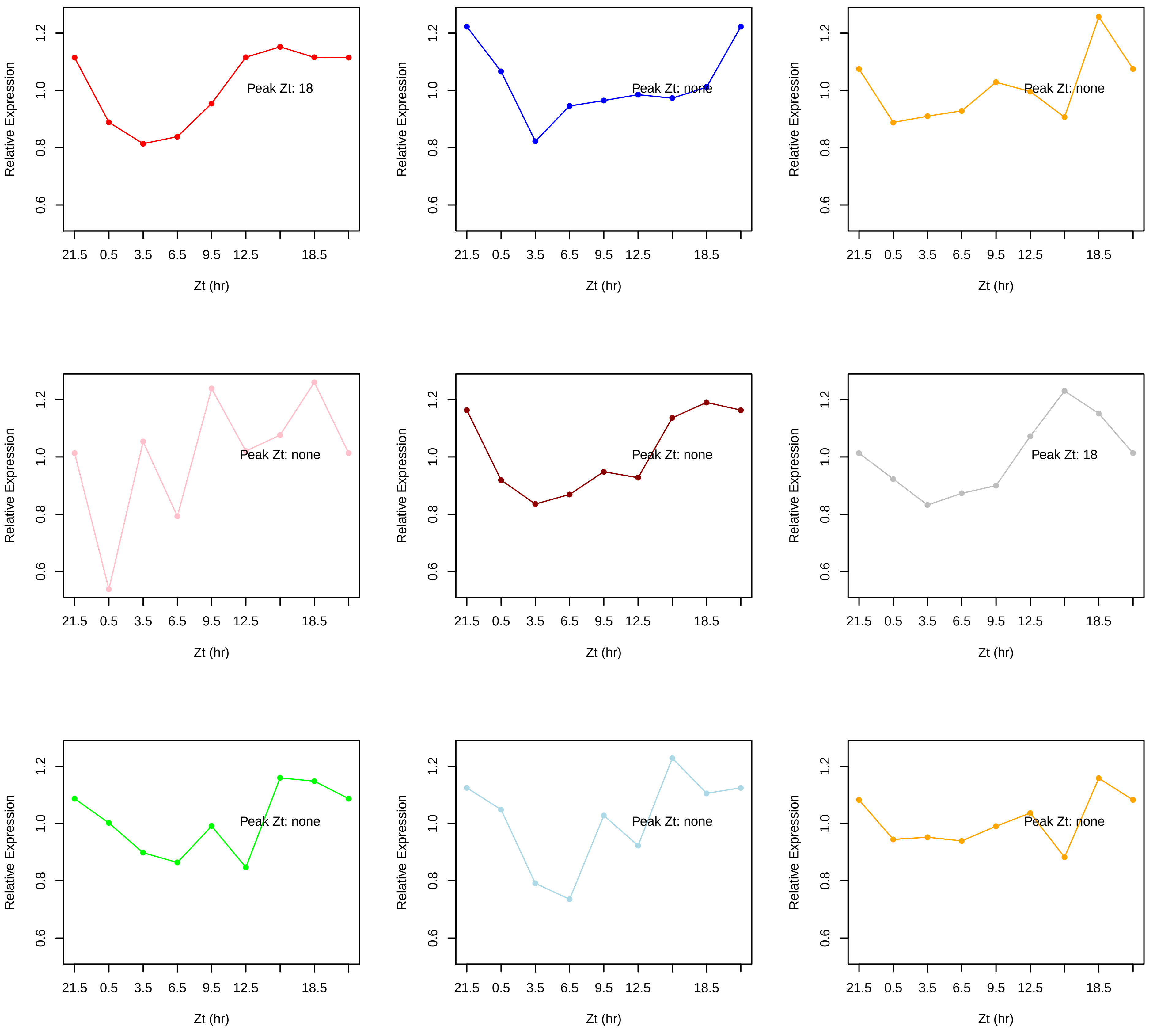

Singleton: ENSXMAG00000019258 ush1c

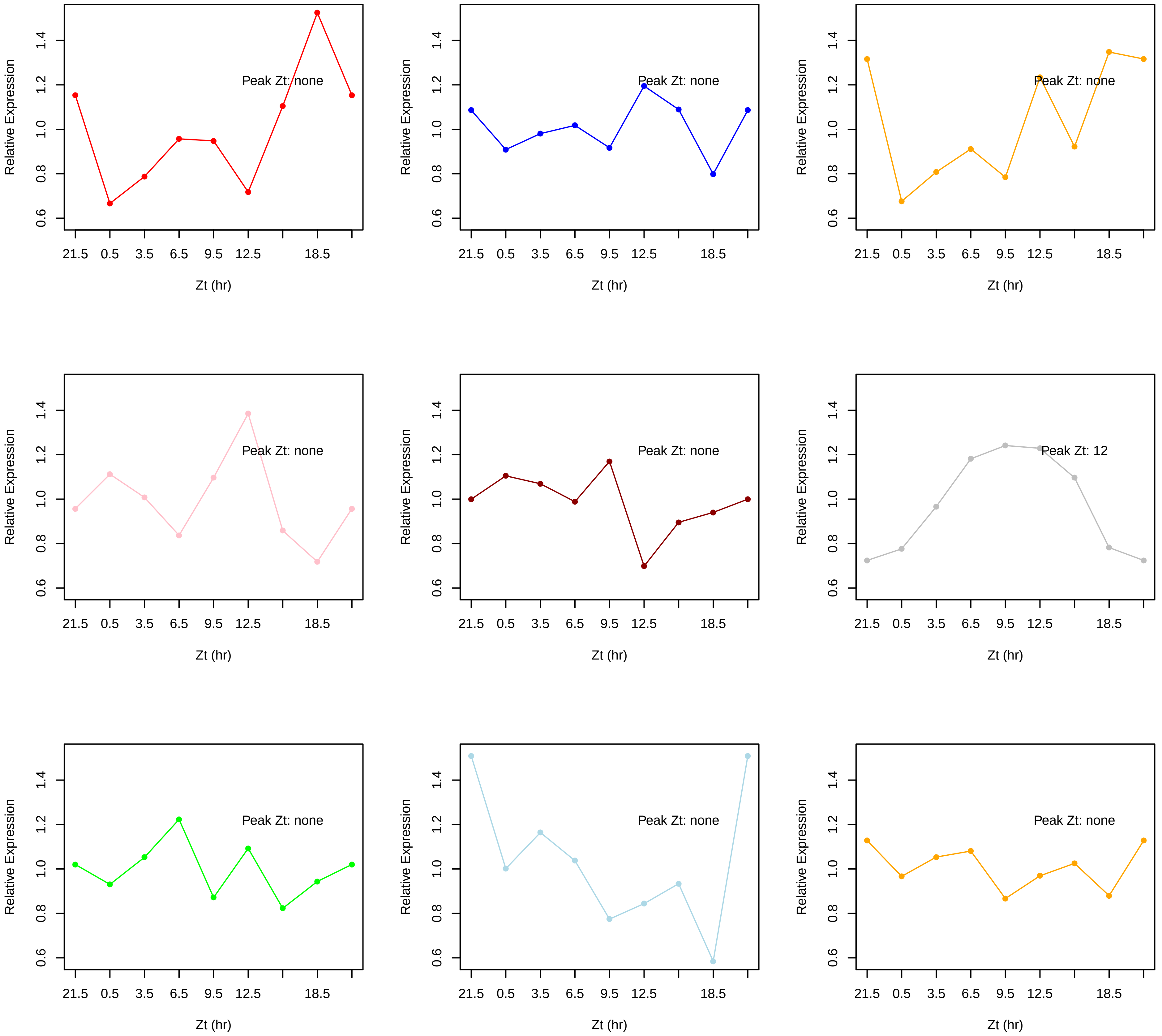

Singeleton: ENSXMAG00000006775 si:ch211−130m23.3

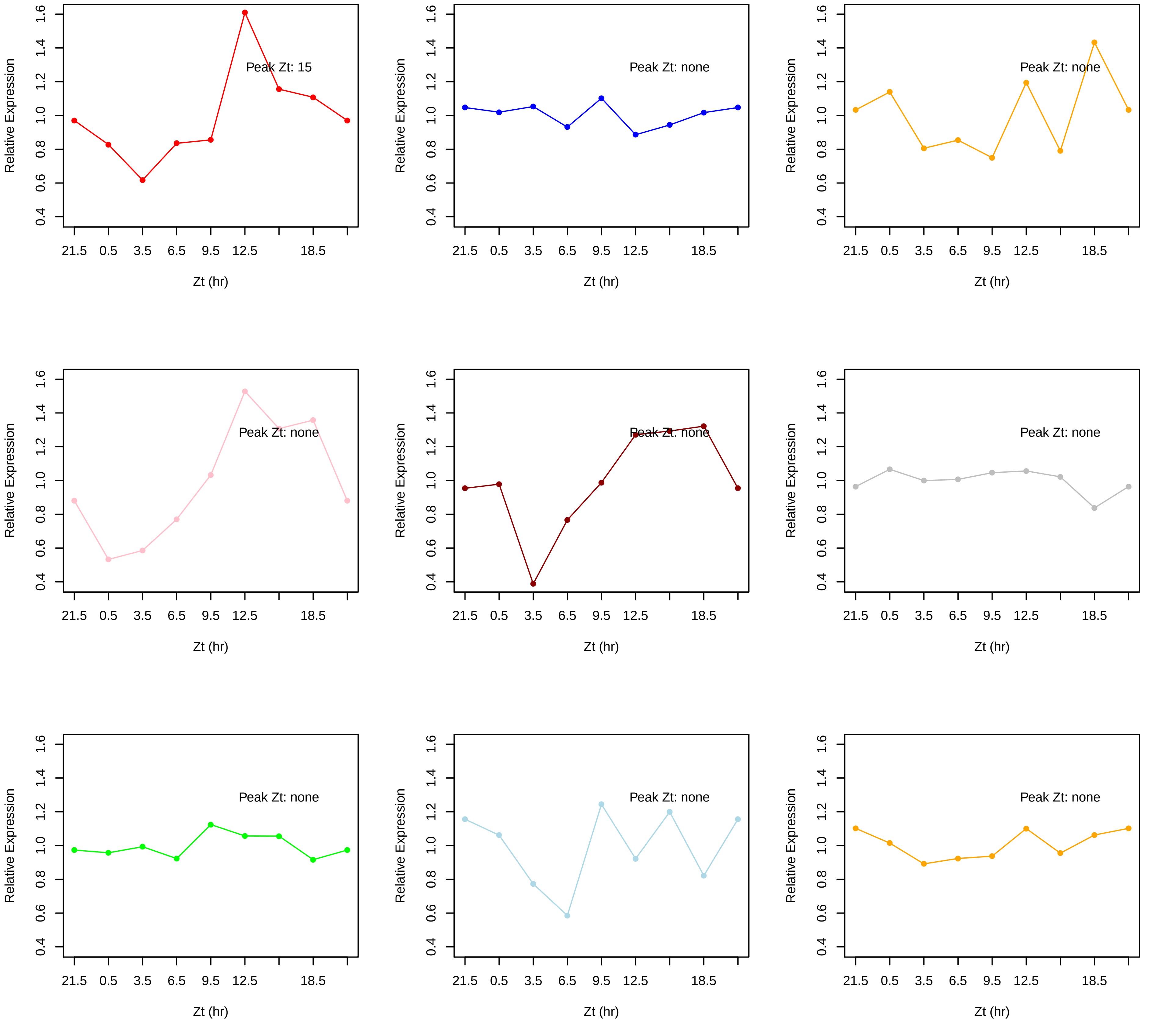

Singeleton: ENSXMAG00000014934 popdc2

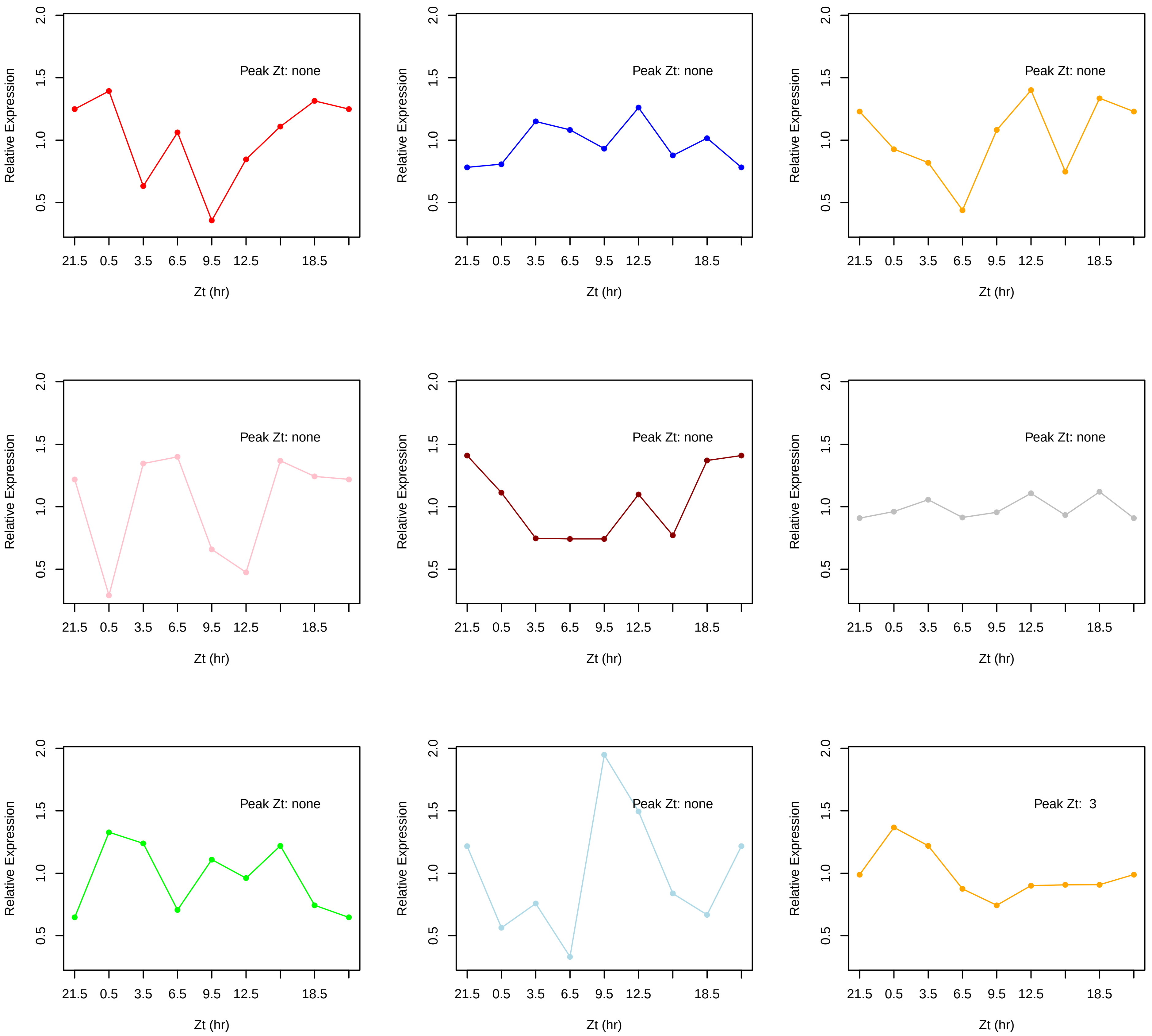

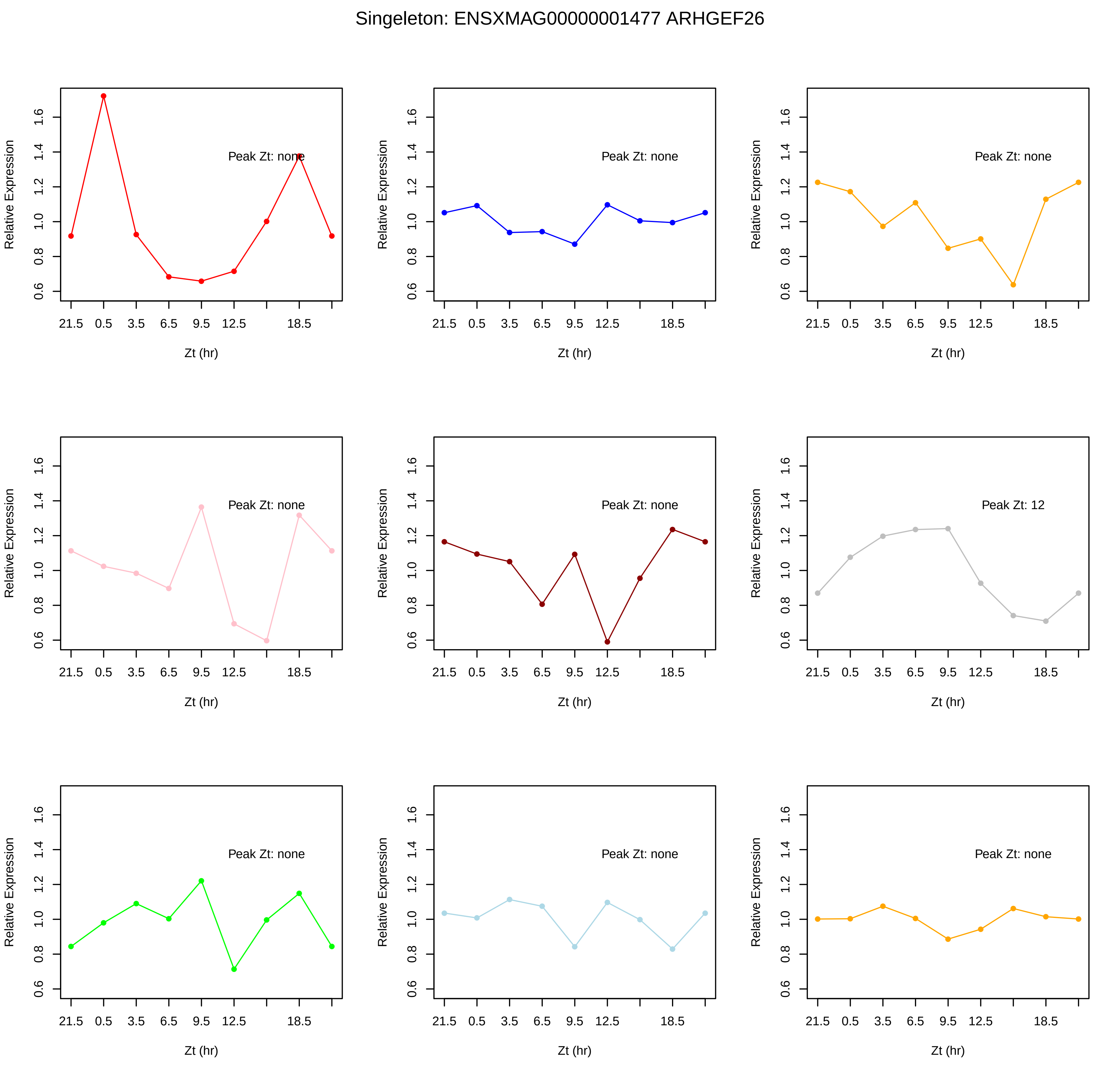
Singeleton: ENSXMAG00000001477 ARHGEF26
Relative Expression
Zt (hr)
Peak Zt: none
Peak Zt: 12

Singleton: ENSXMAG00000007961 men1

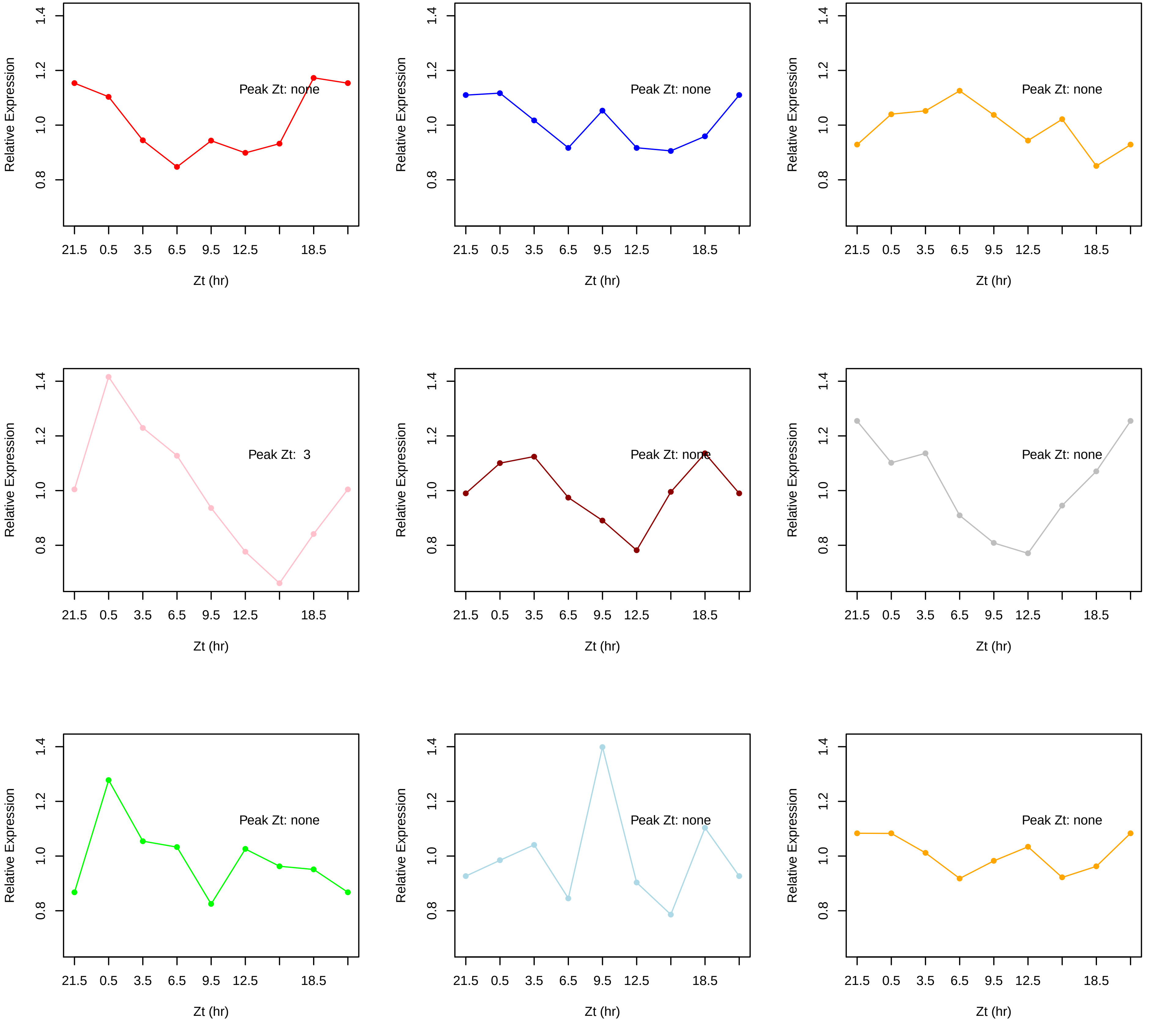

Singeleton: ENSXMAG00000004473 sdf2l1

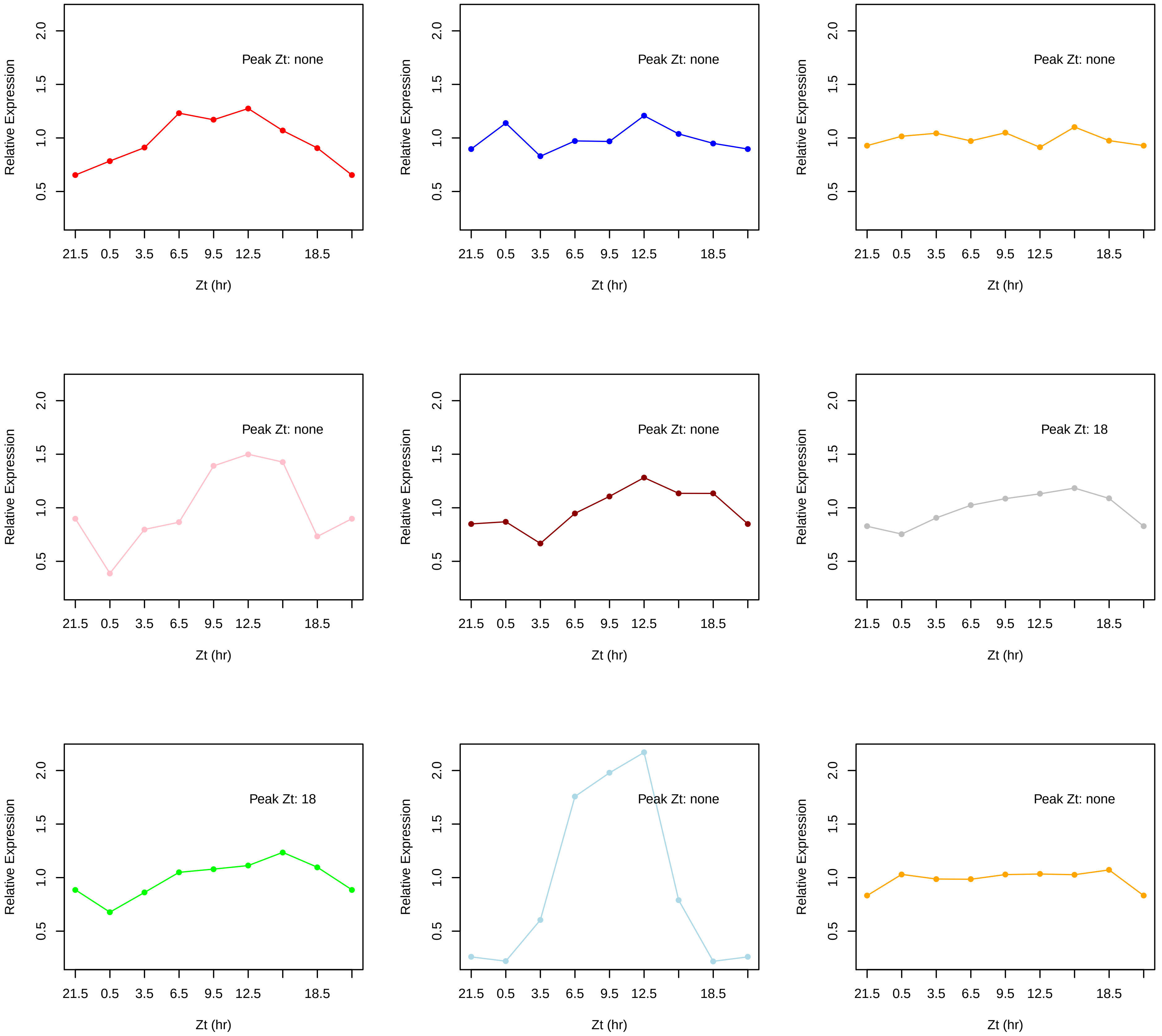

Singeleton: ENSXMAG00000006826 nyx

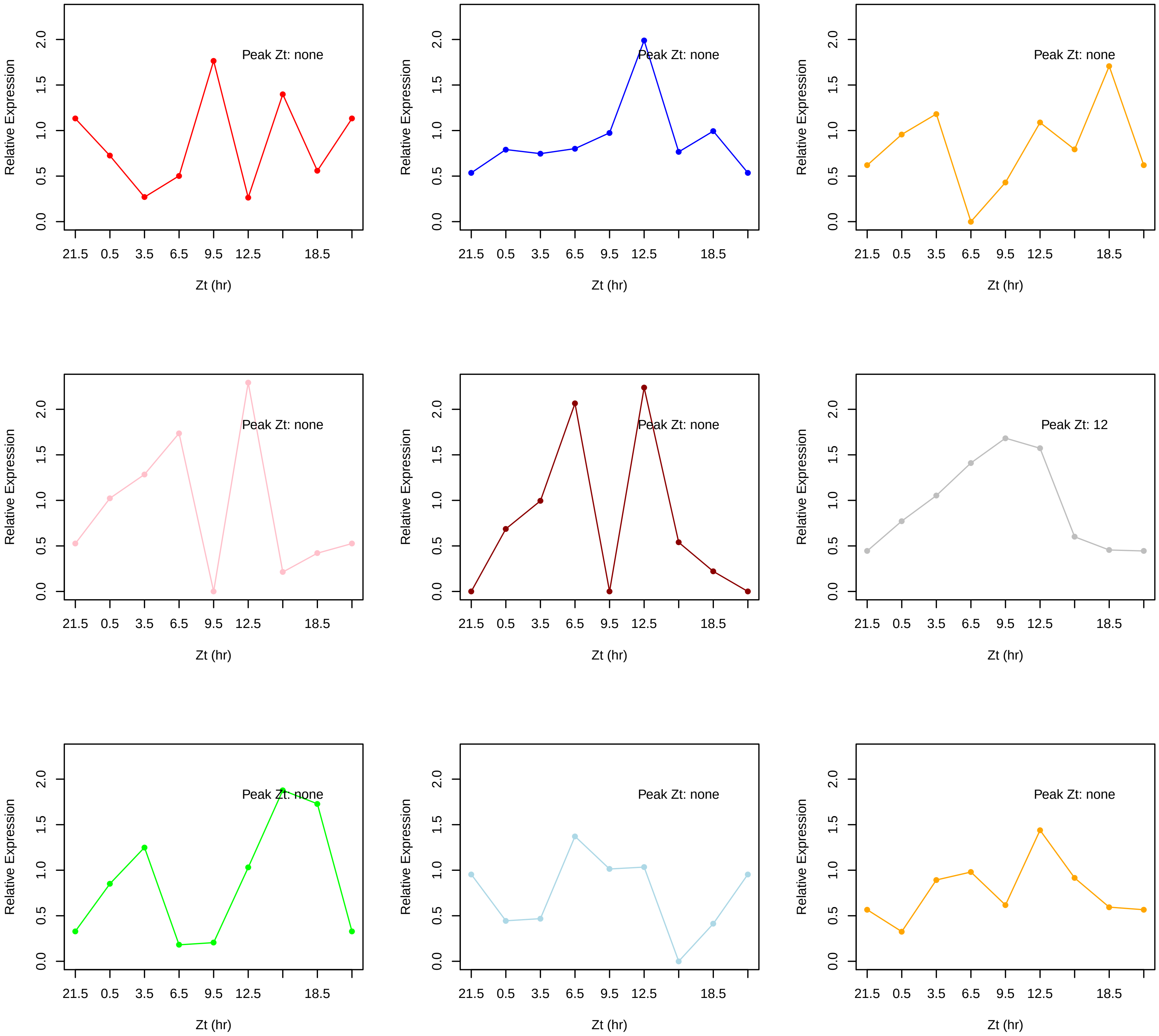

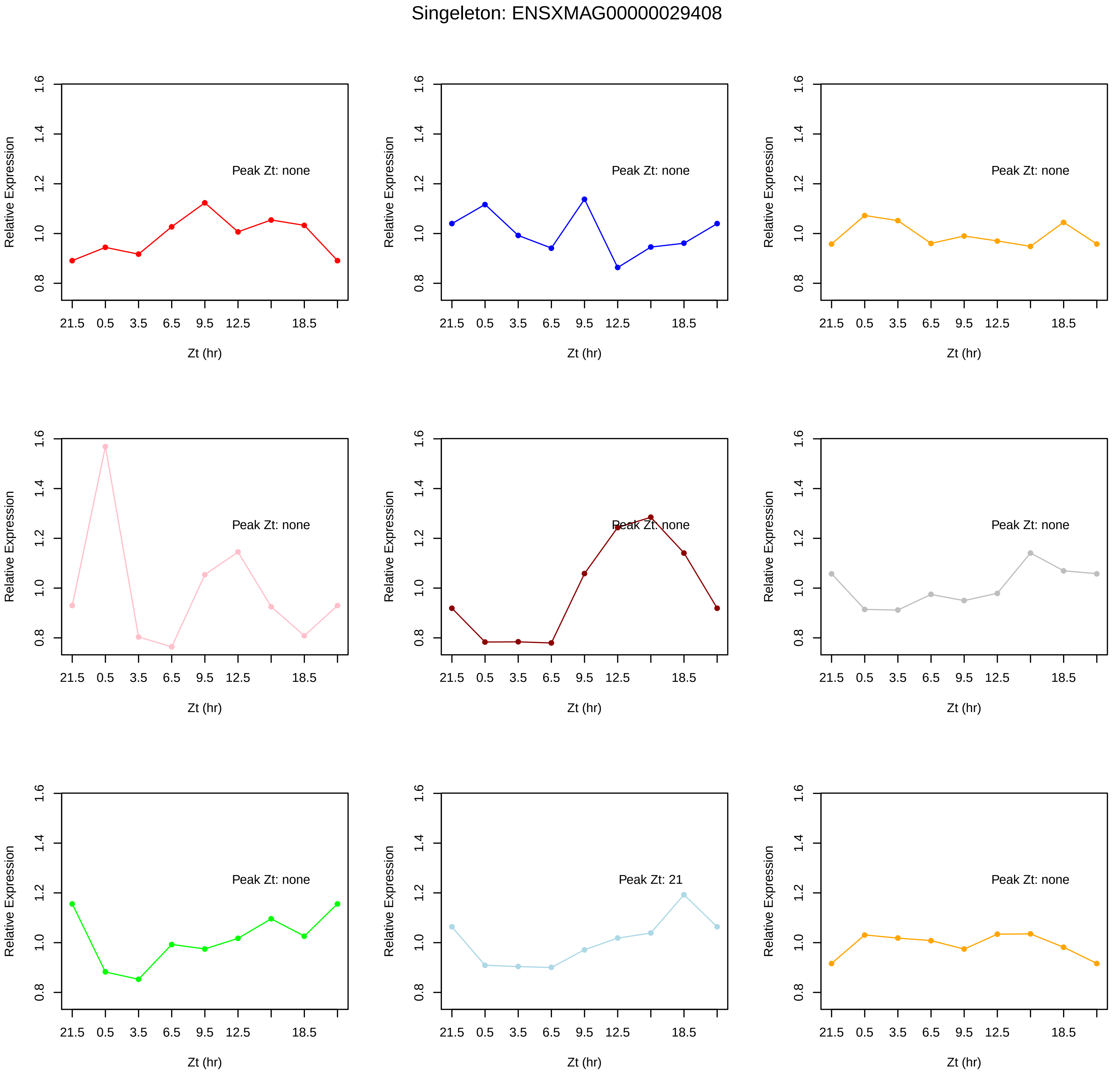

Singleton: ENSXMAG00000029408
Relative Expression
Zt (hr)
21.5 0.5 3.5 6.5 9.5 12.5 18.5
0.8 1.0 1.2 1.4 1.6
Peak Zt: none
Peak Zt: none
Peak Zt: none
Peak Zt: none
Peak Zt: none
Peak Zt: none
Peak Zt: none
Peak Zt: 21
Peak Zt: none

Singleton: ENSXMAG00000016543 pea15

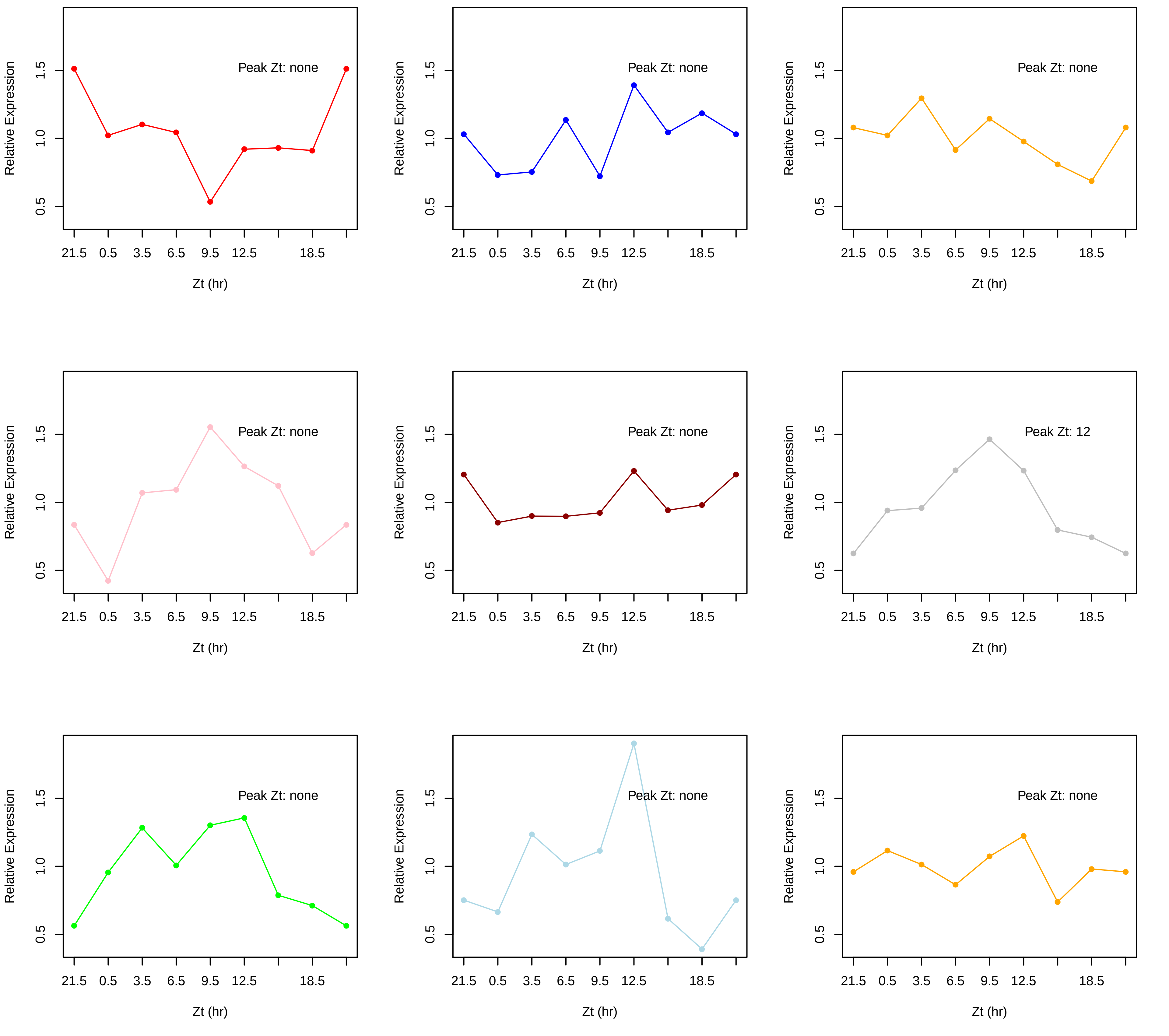

Singeleton: ENSXMAG00000007822

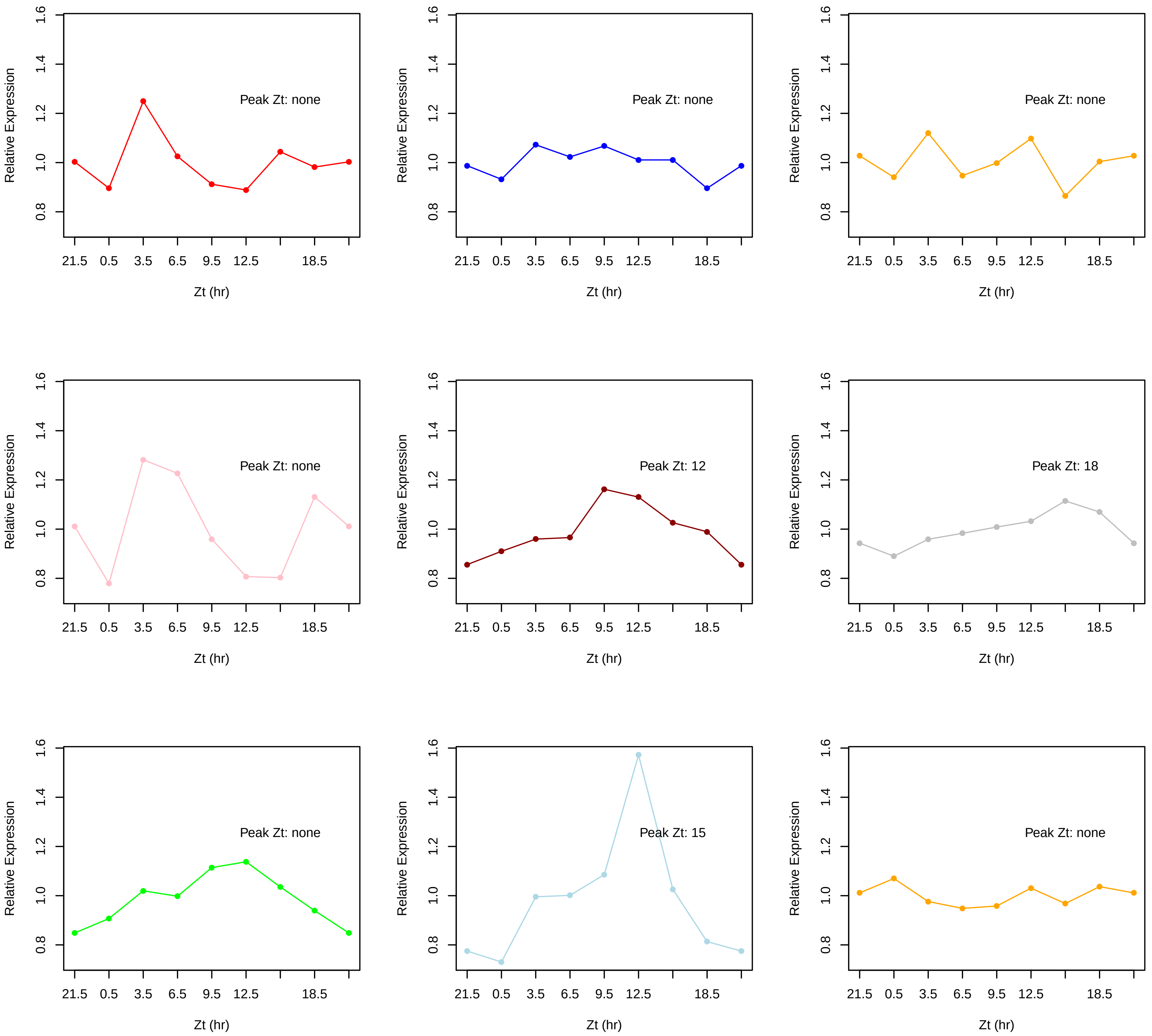

Singleton: ENSXMAG00000014199 abcg1

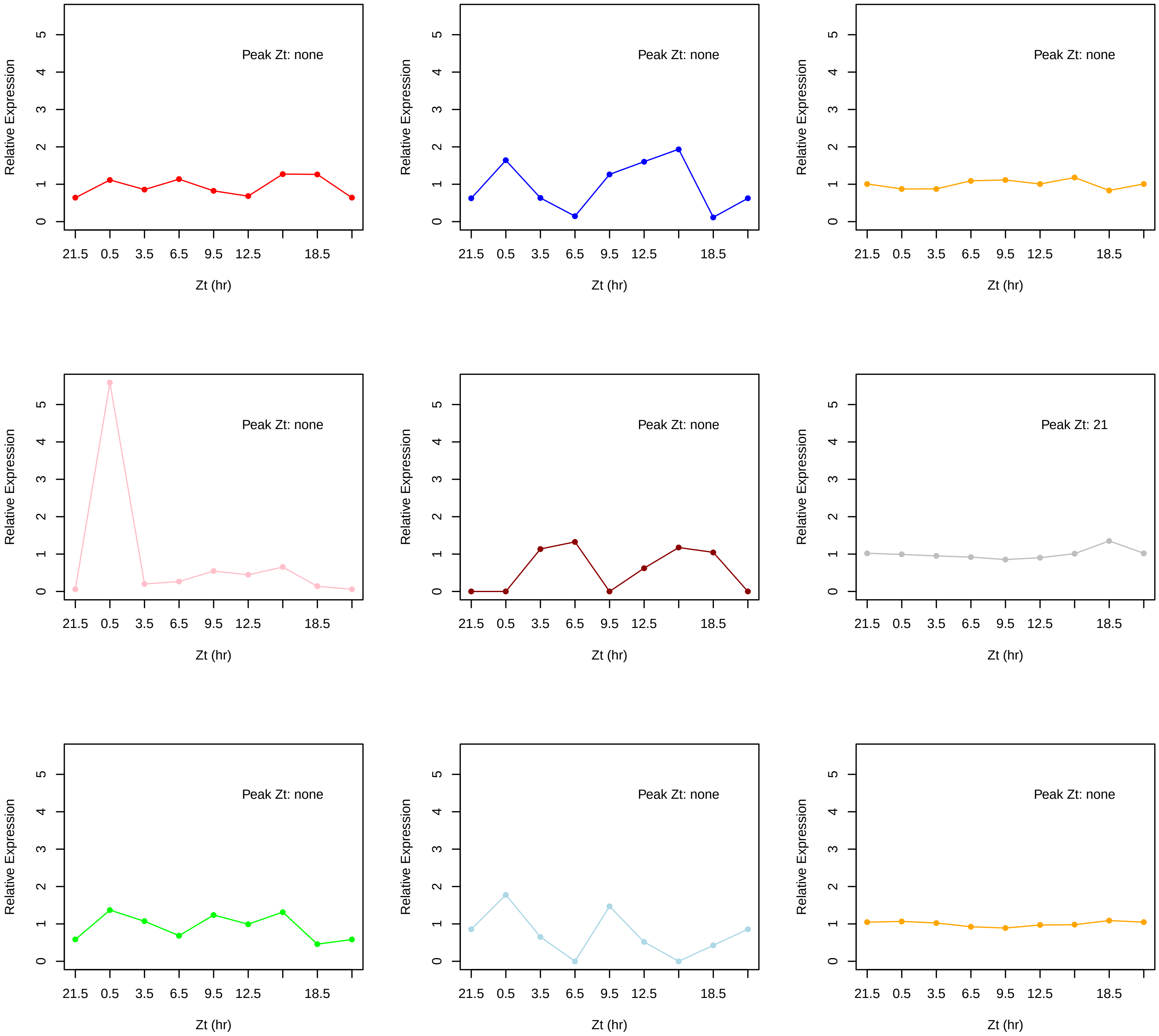

Singeleton: ENSXMAG00000025486 zdhhc3b

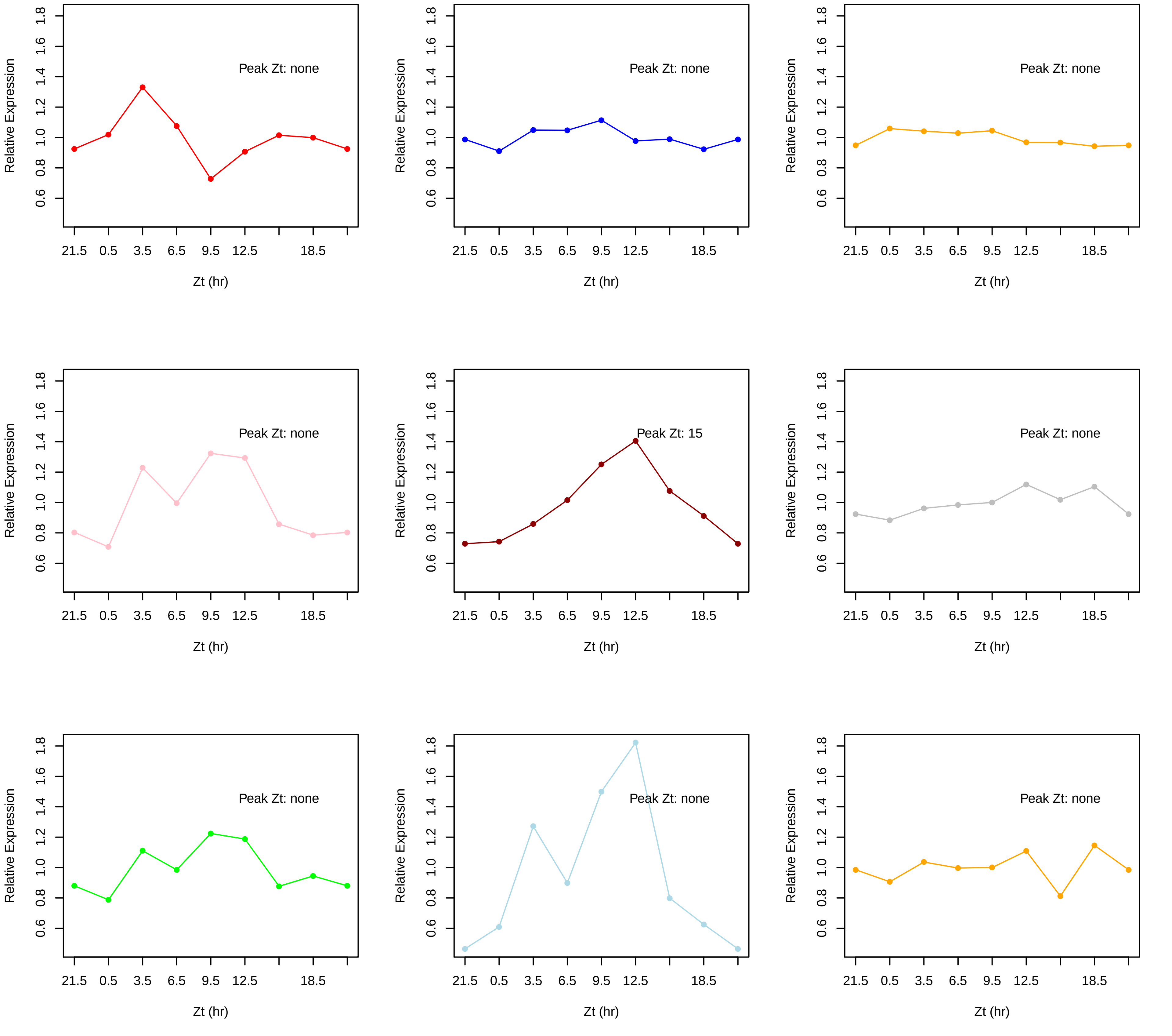

Singeleton: ENSXMAG00000011884 jpt2

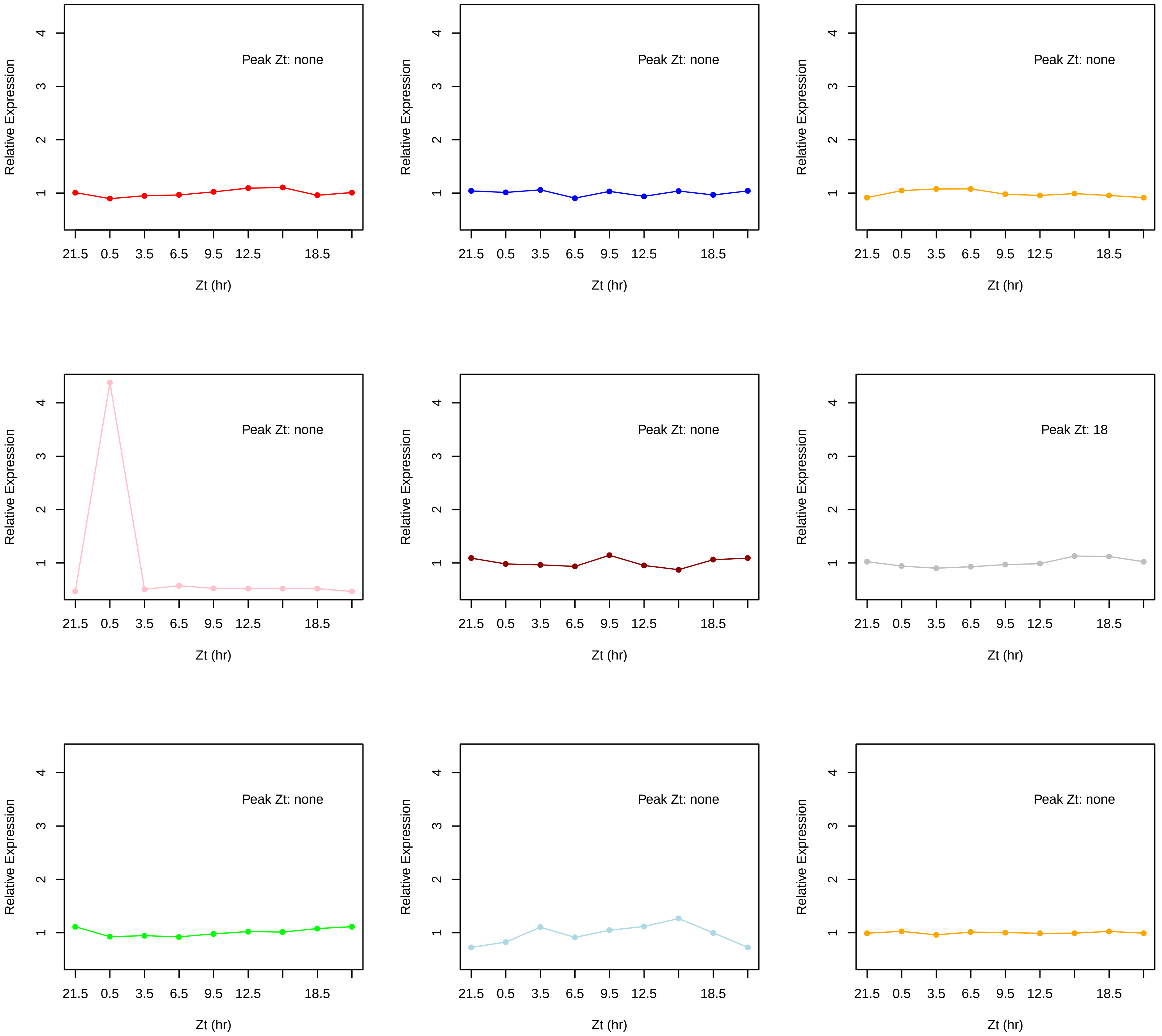

Singeleton: ENSXMAG00000024494 sdr42e1

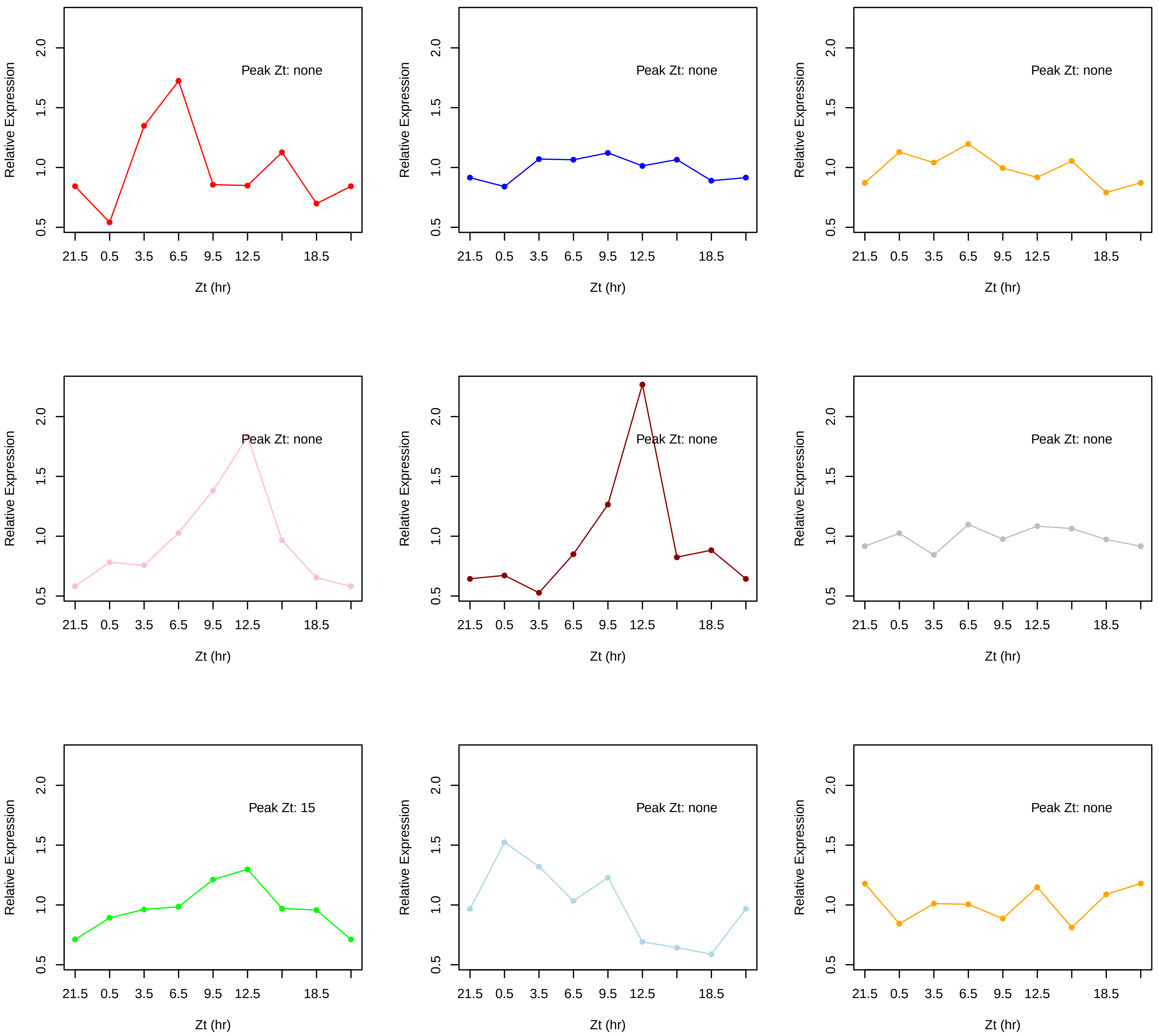

Singeleton: ENSXMAG00000021838 IDNK

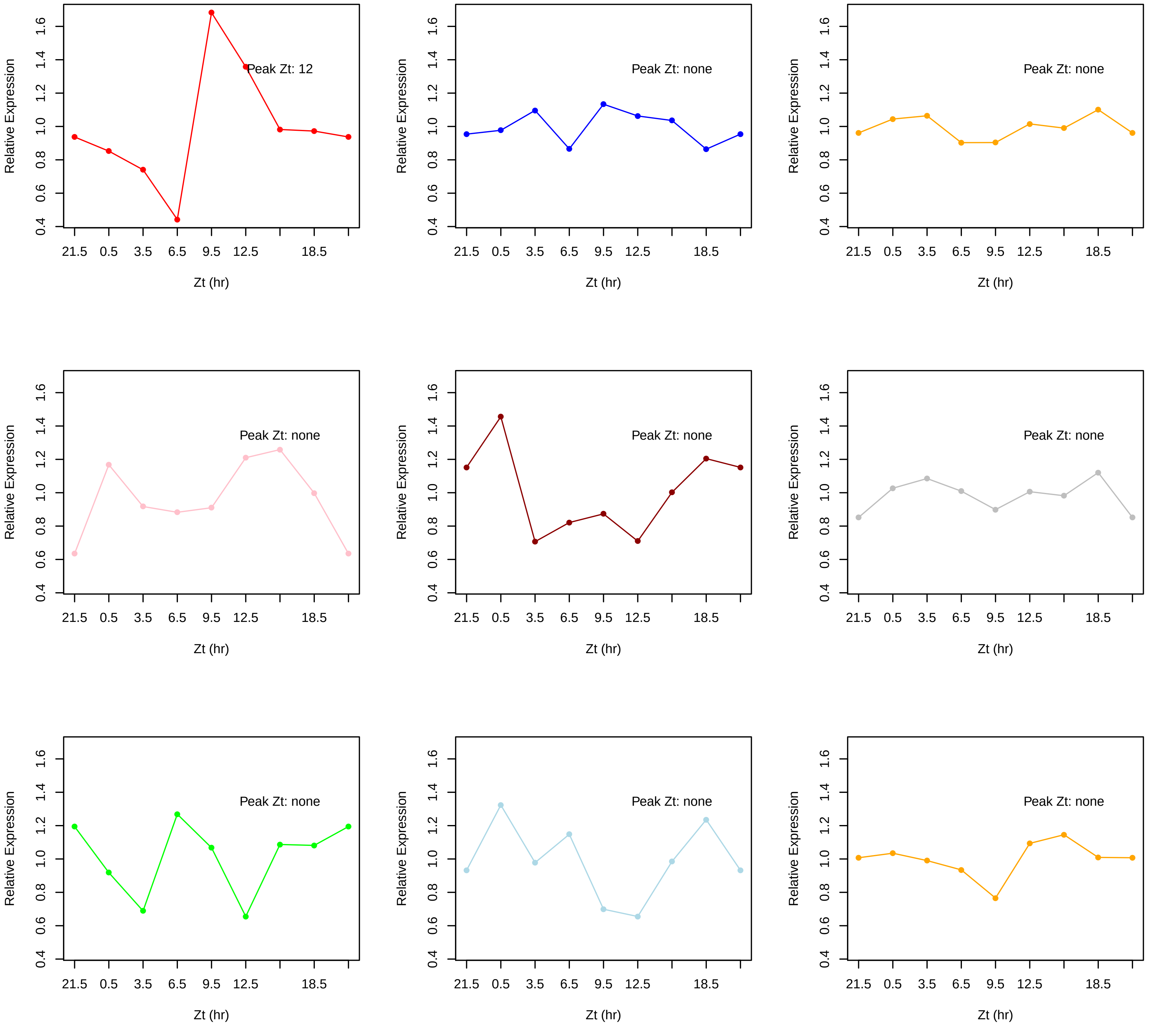

Singeleton: ENSXMAG00000023401 nifk

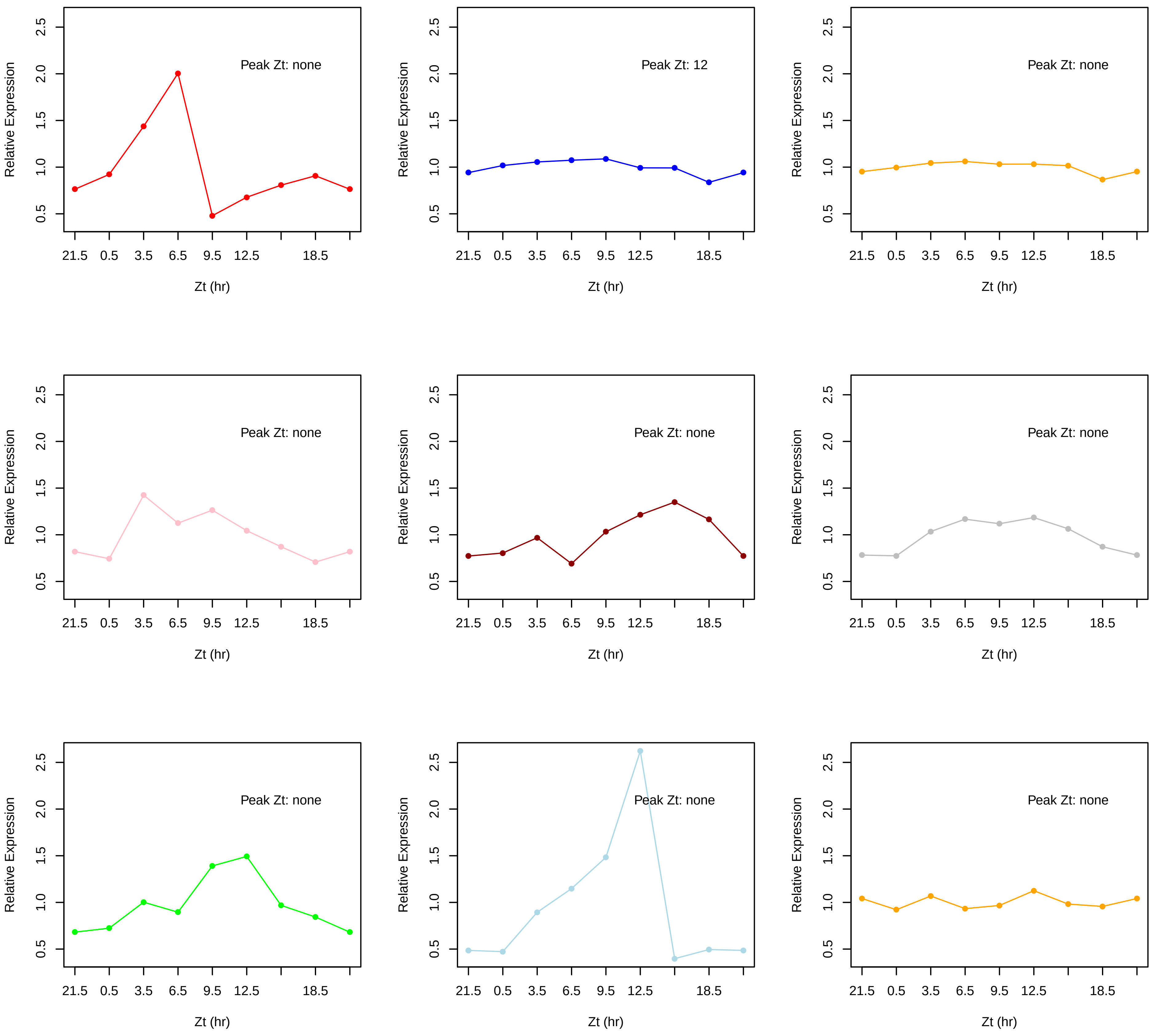

Singeleton: ENSXMAG00000007835 taf6l

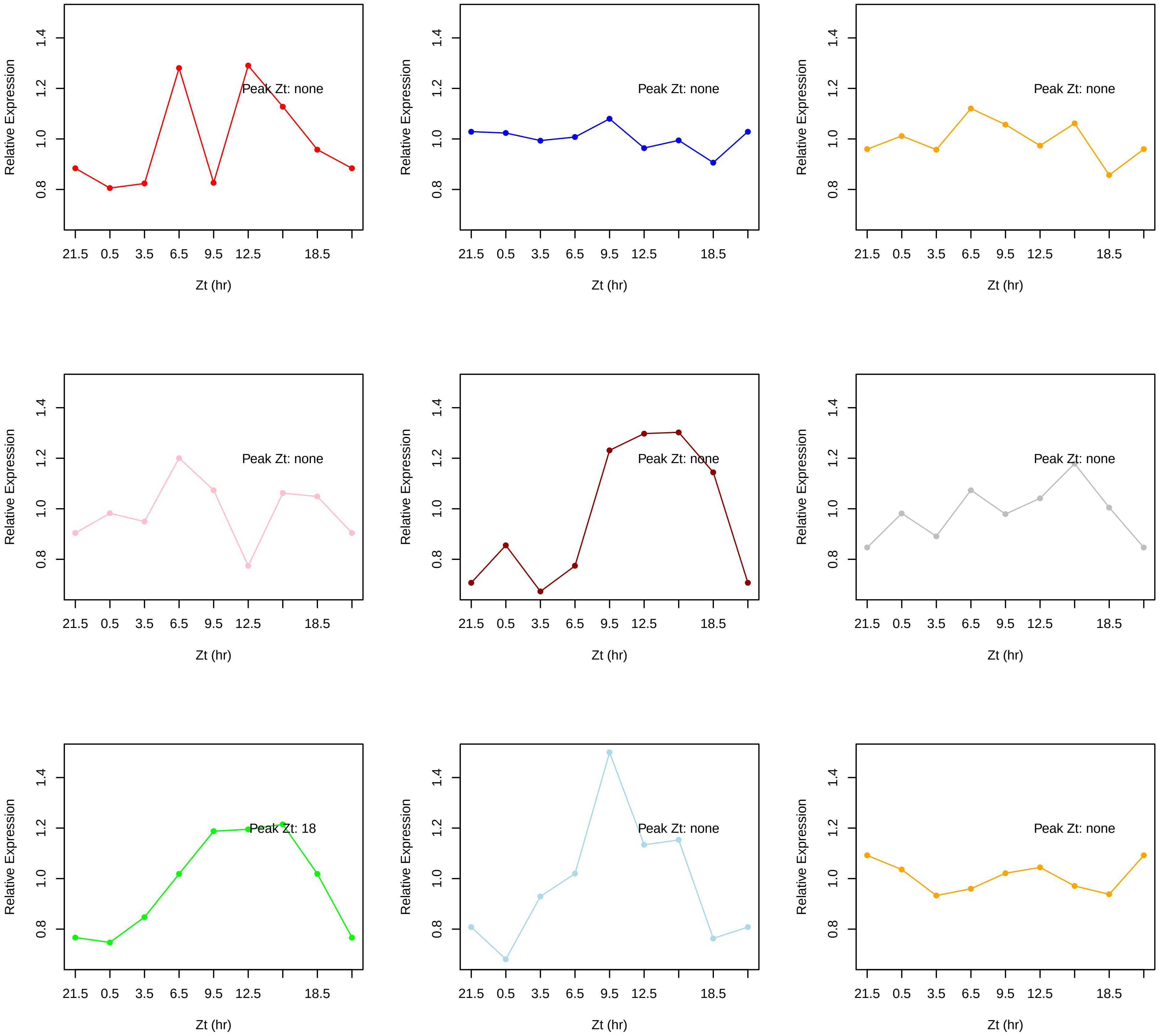

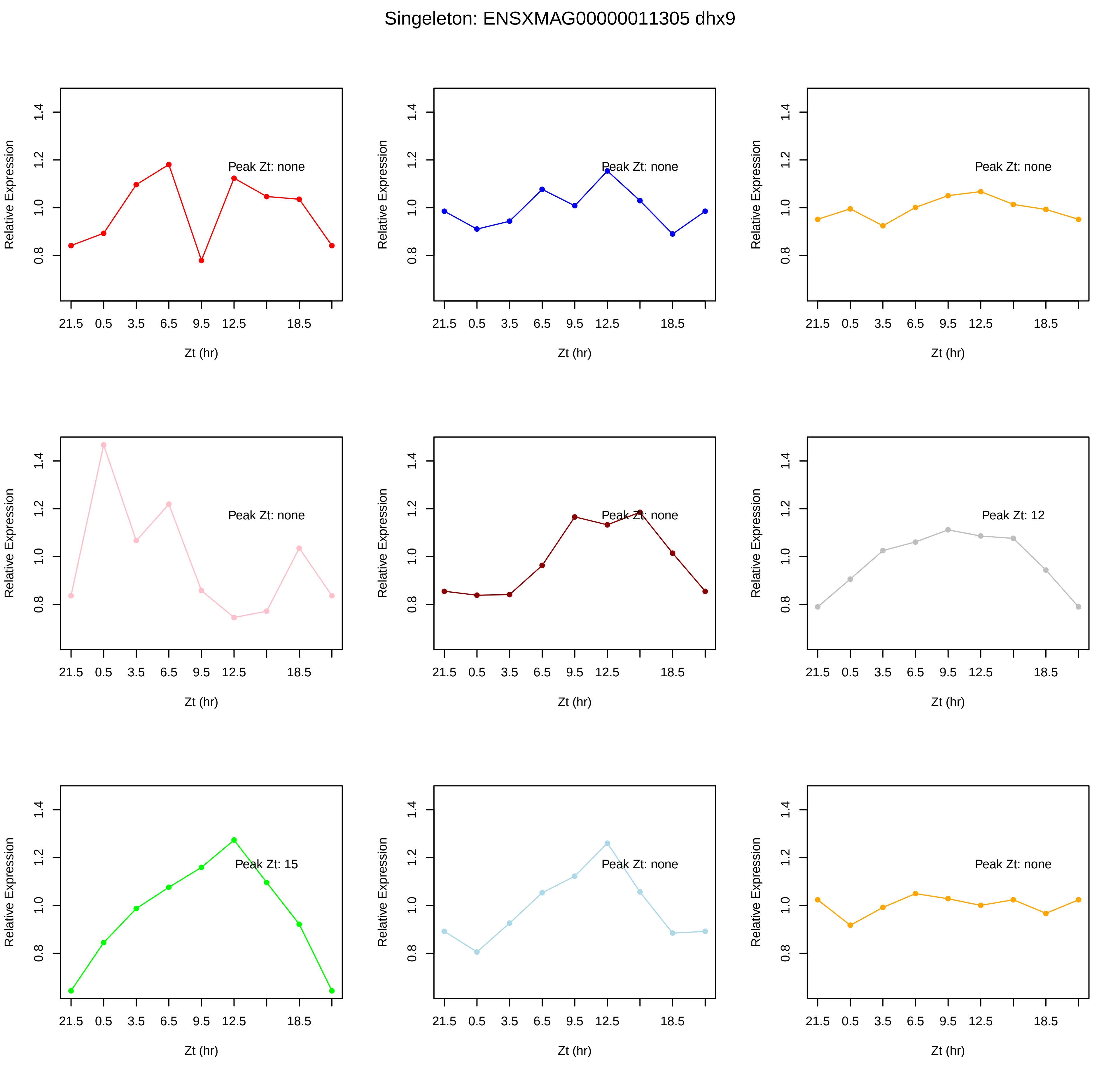
Singeleton: ENSXMAG00000011305 dhx9
Peak Zt: none
Peak Zt: none
Peak Zt: none
Peak Zt: none
Peak Zt: none
Peak Zt: 12
Peak Zt: 15
Peak Zt: none
Peak Zt: none
Relative Expression
Zt (hr)
21.5 0.5 3.5 6.5 9.5 12.5 18.5
0.8 1.0 1.2 1.4

Singeleton: ENSXMAG00000015329 clip2

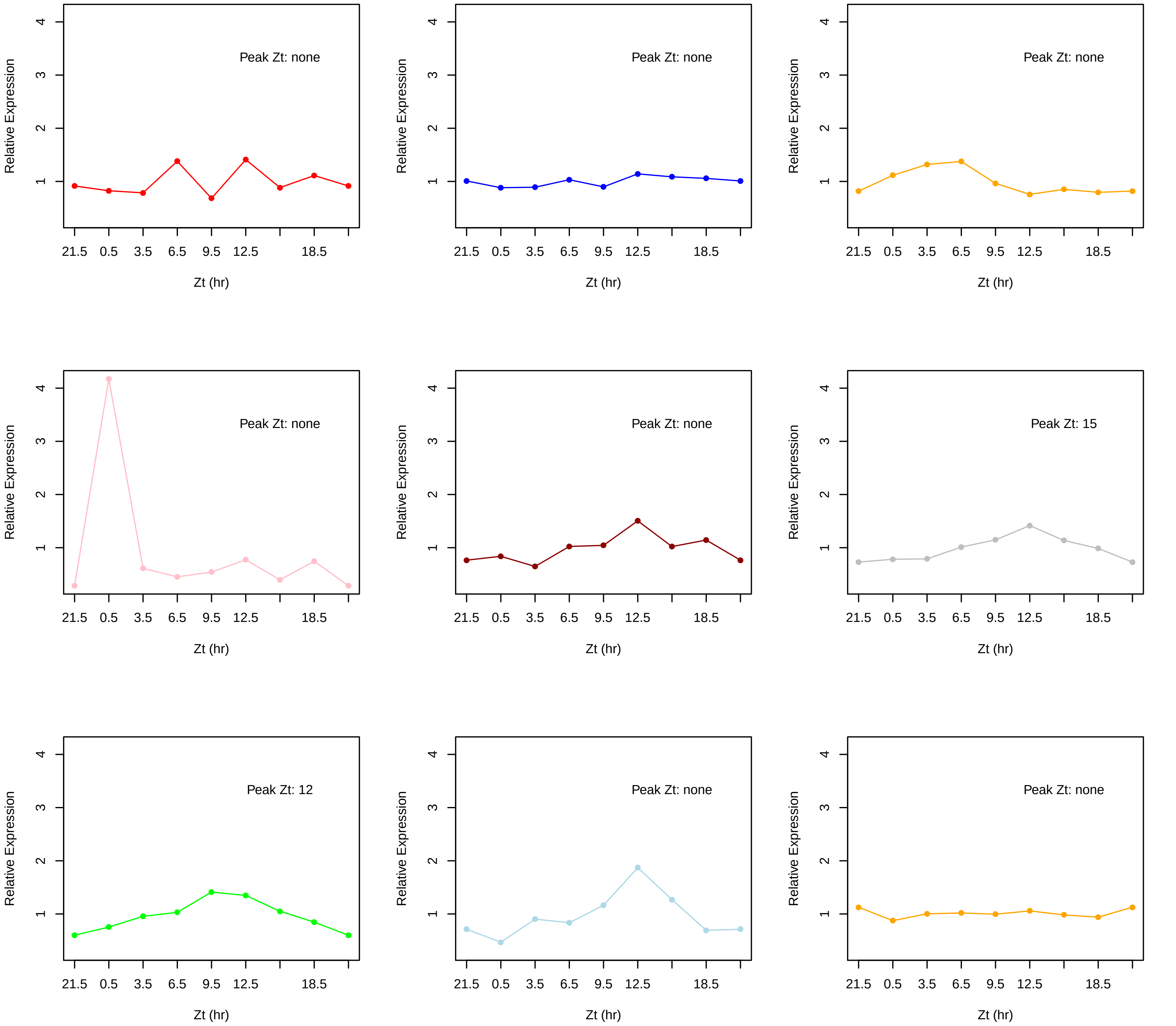

Singeleton: ENSXMAG00000000933

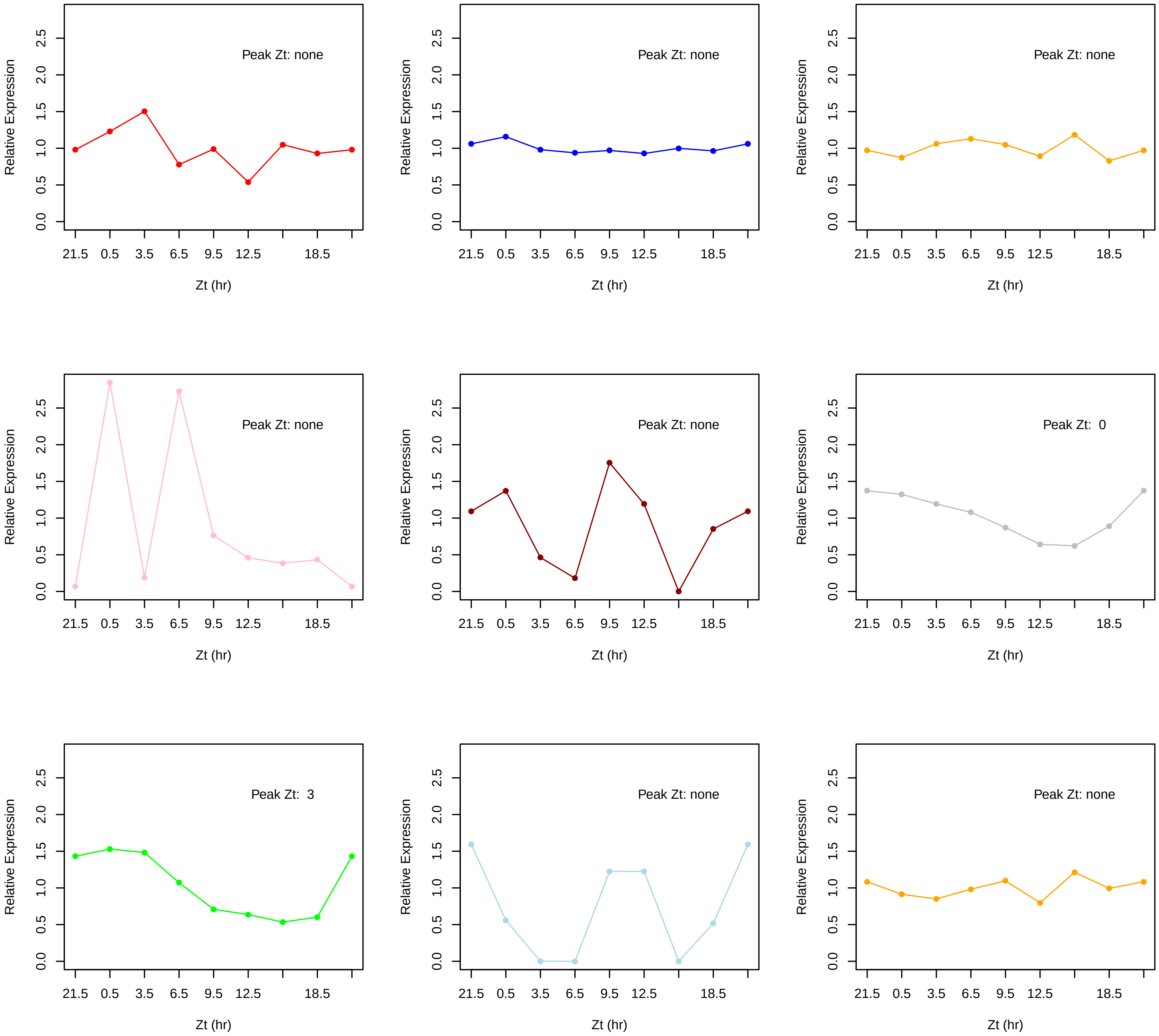

Singeleton: ENSXMAG00000012455 cptp

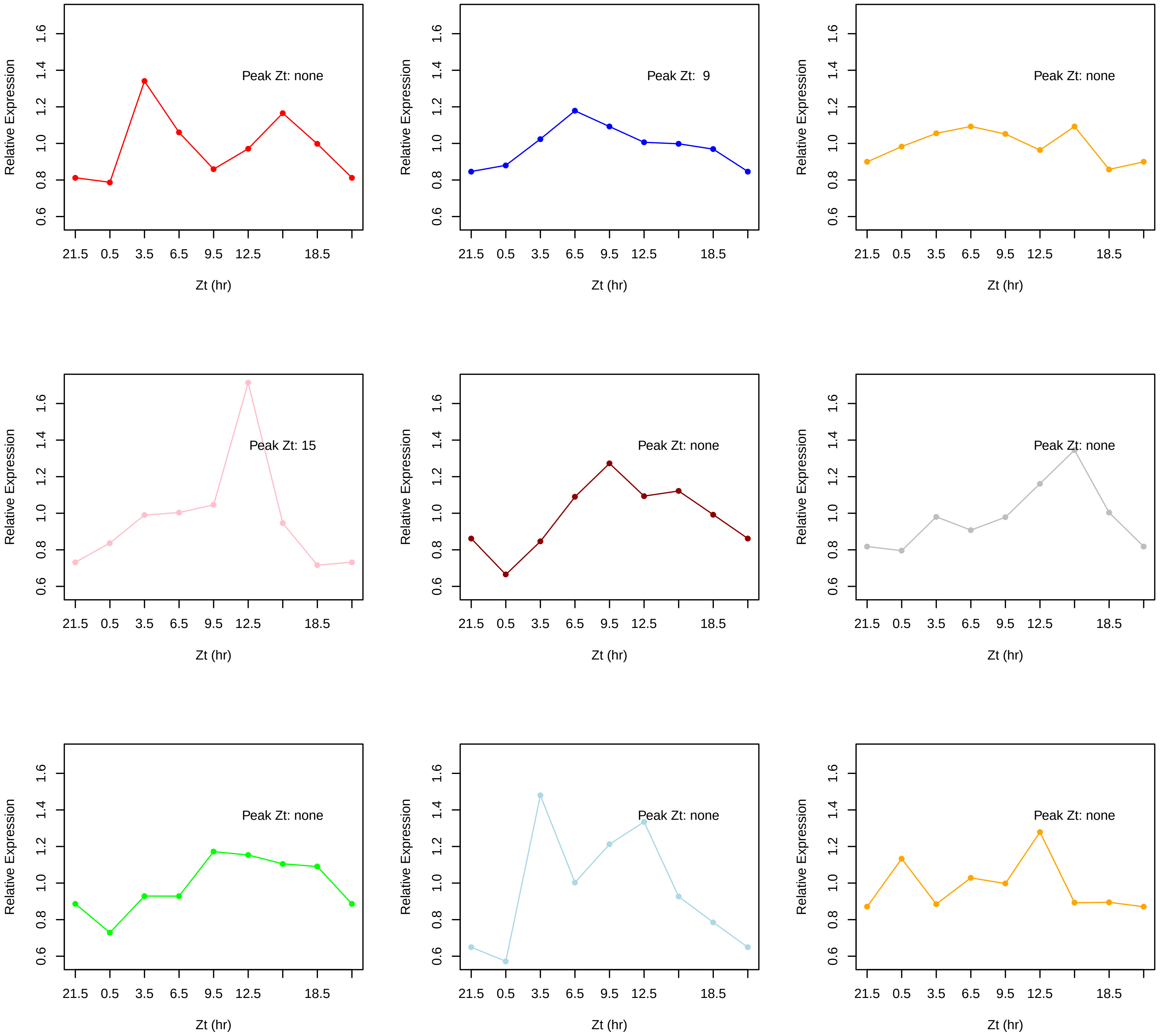

Singeleton: ENSXMAG00000016348 yars

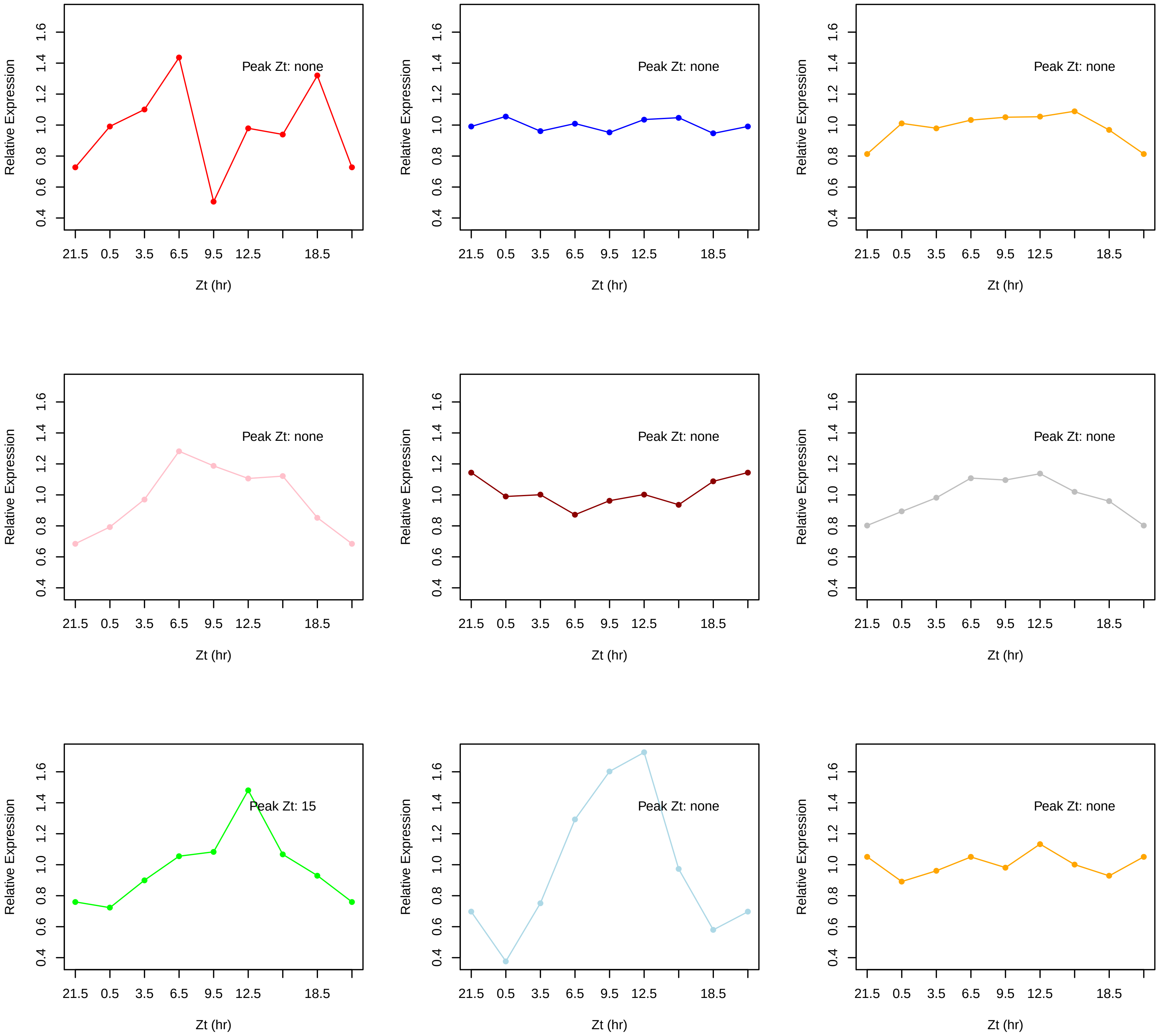

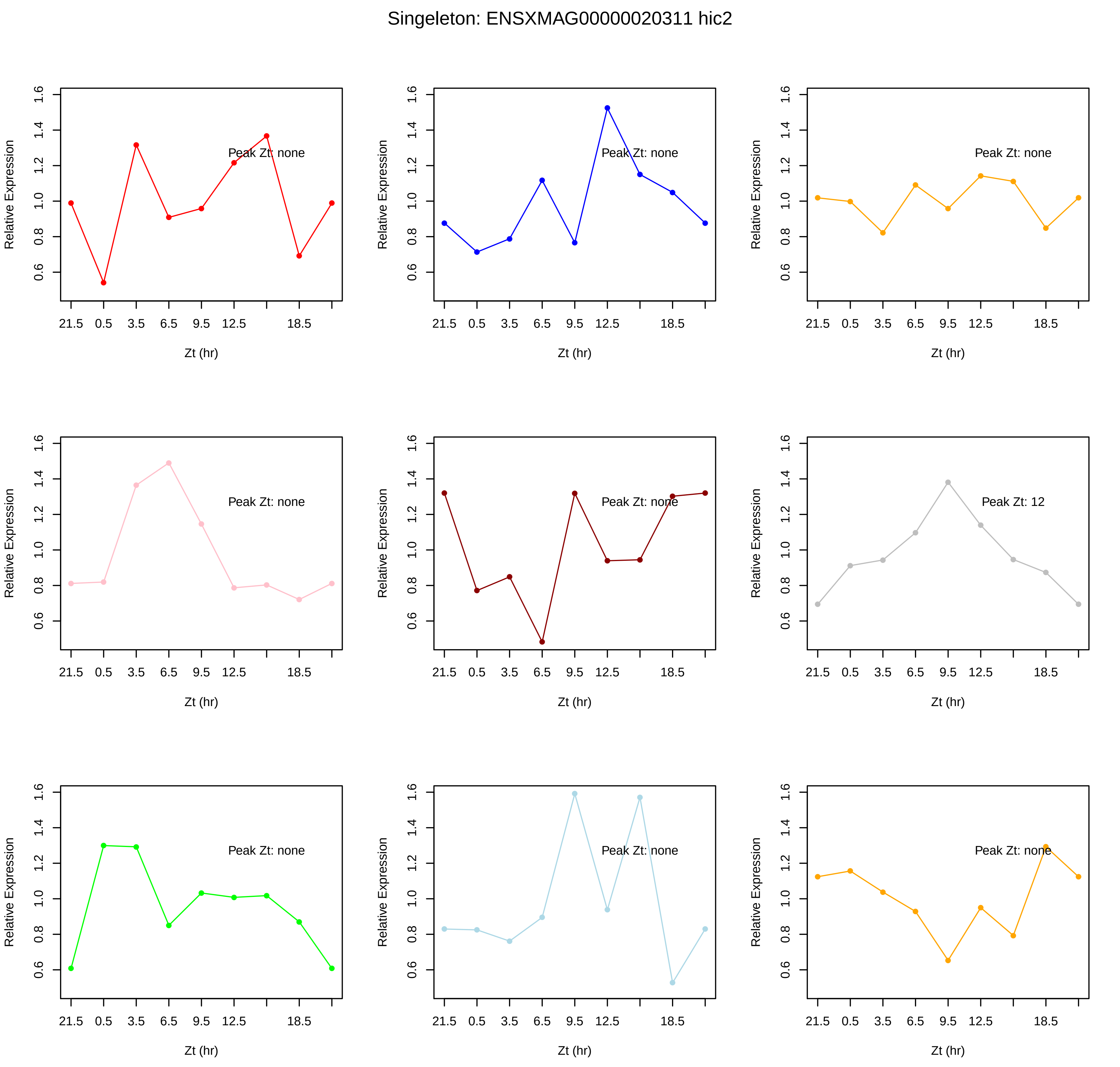
Singeleton: ENSXMAG00000020311 hic2
Relative Expression
Zt (hr)
21.5 0.5 3.5 6.5 9.5 12.5 18.5
0.6 0.8 1.0 1.2 1.4 1.6
Peak Zt: none
Peak Zt: none
Peak Zt: none
Peak Zt: none
Peak Zt: none
Peak Zt: 12
Peak Zt: none
Peak Zt: none
Peak Zt: none

Singeleton: ENSXMAG00000017060 arl2

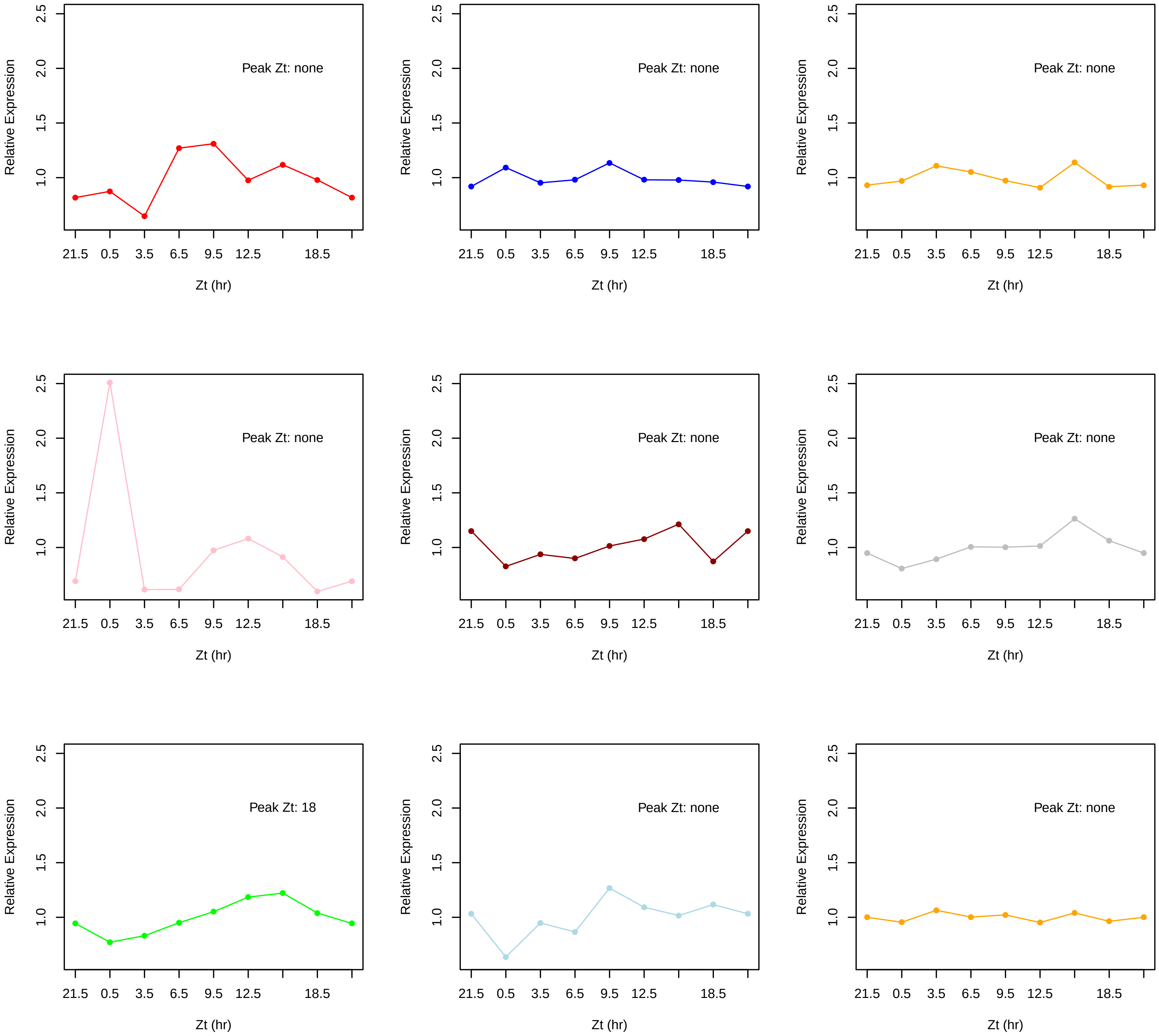

Singeleton: ENSXMAG00000000122 im:7160594

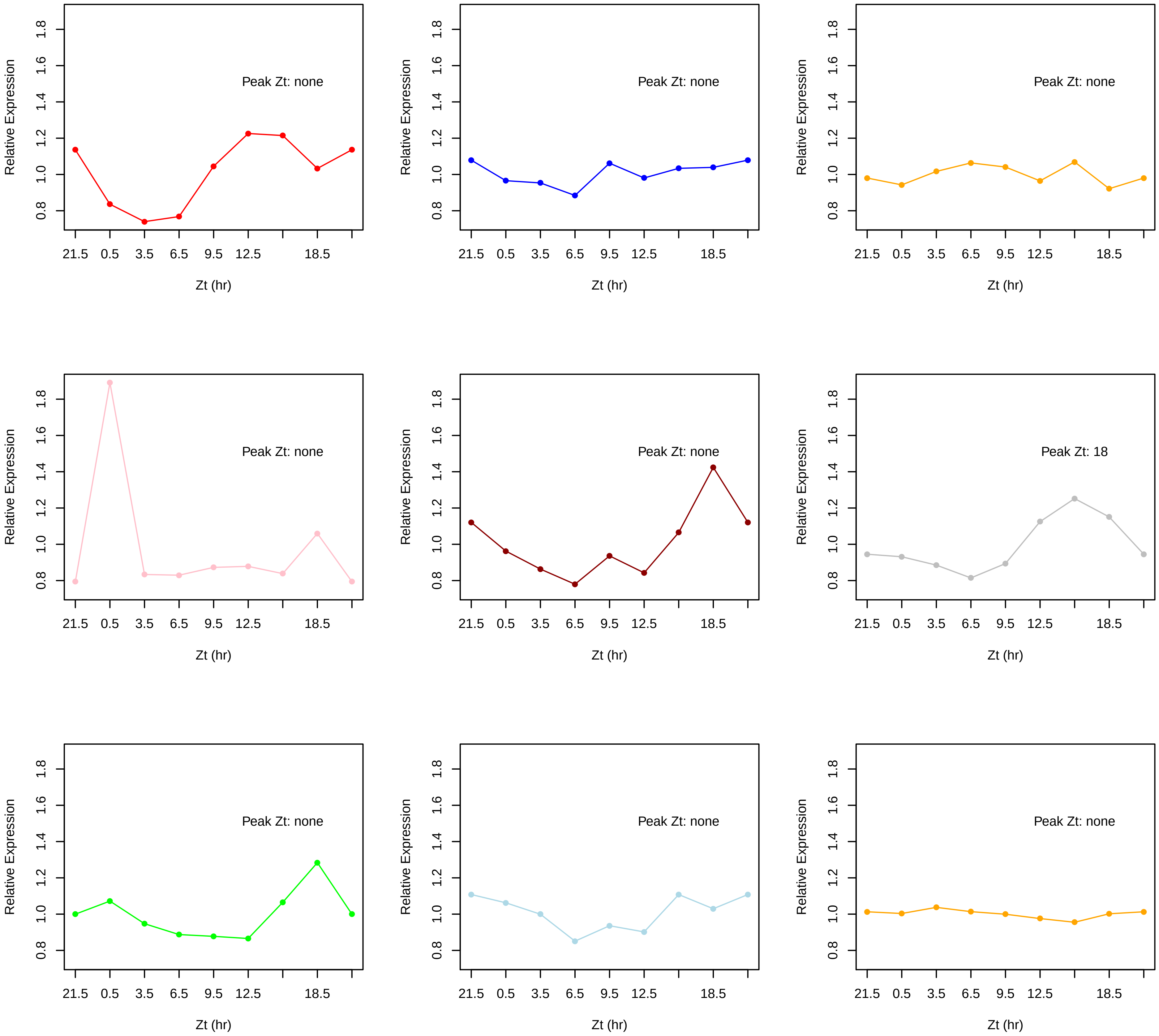

Singleton: ENSXMAG00000012456 ints11

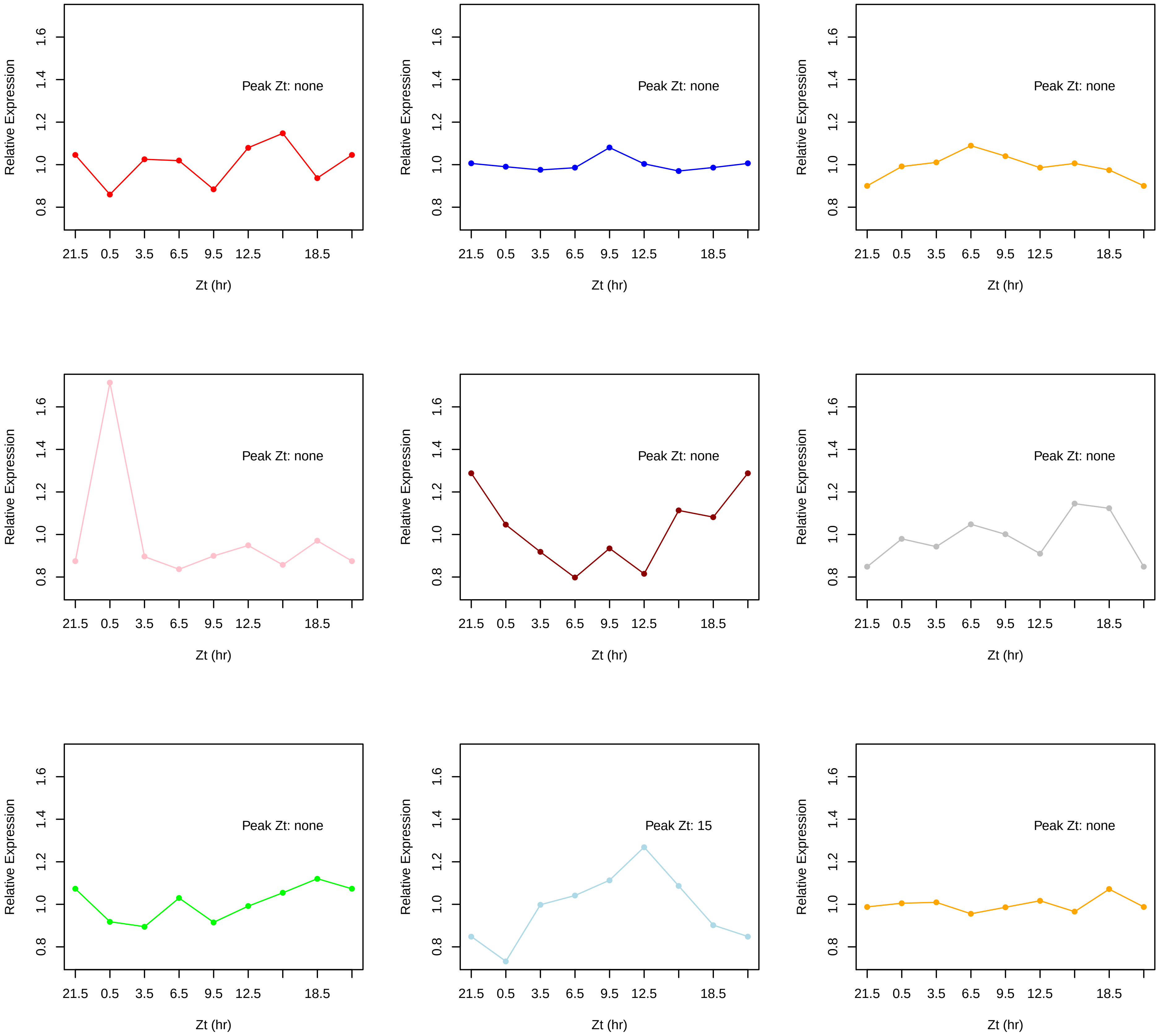

Singeleton: ENSXMAG00000022886 GPR149

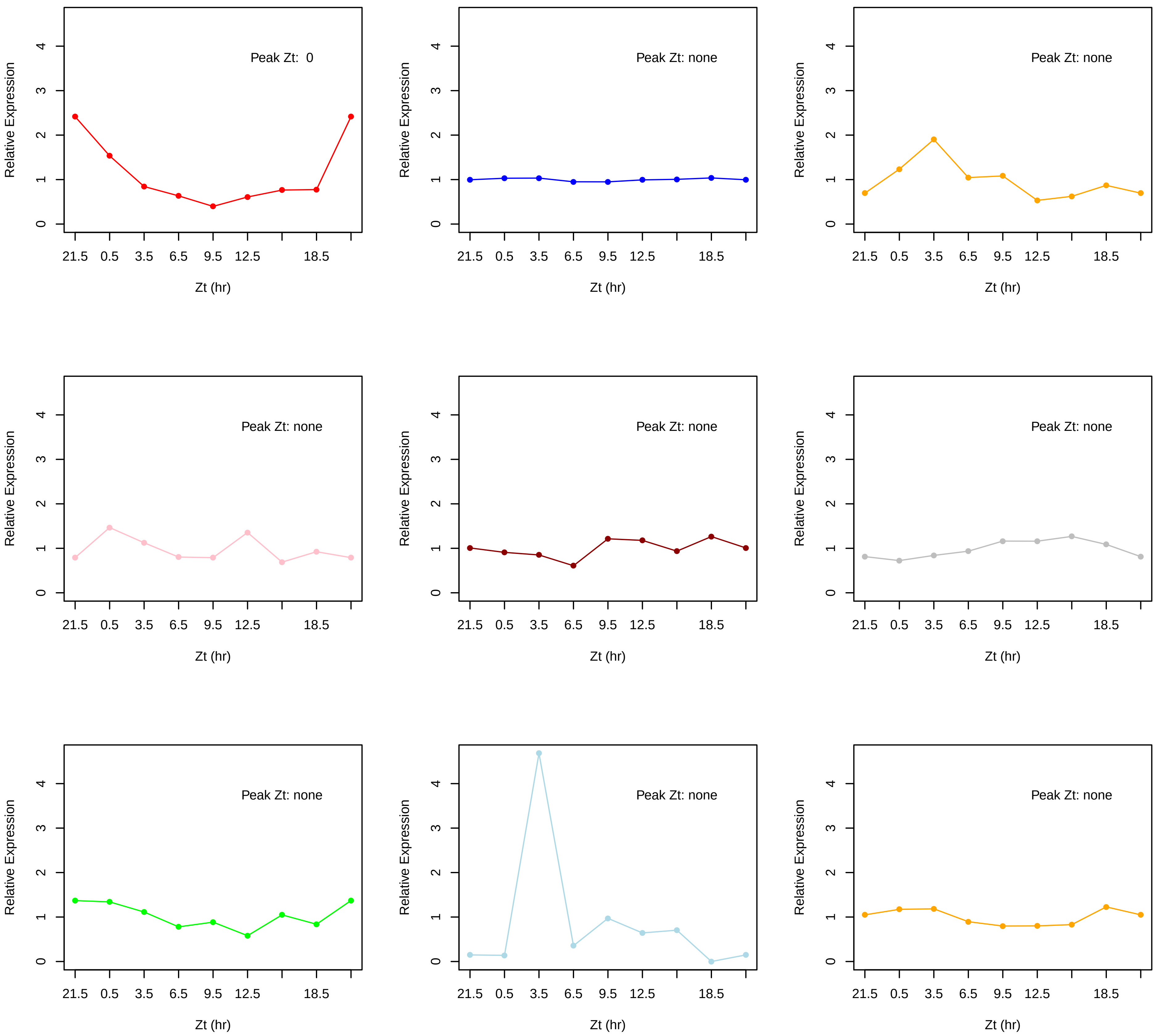

Singeleton: ENSXMAG00000015294 gtf2ird1

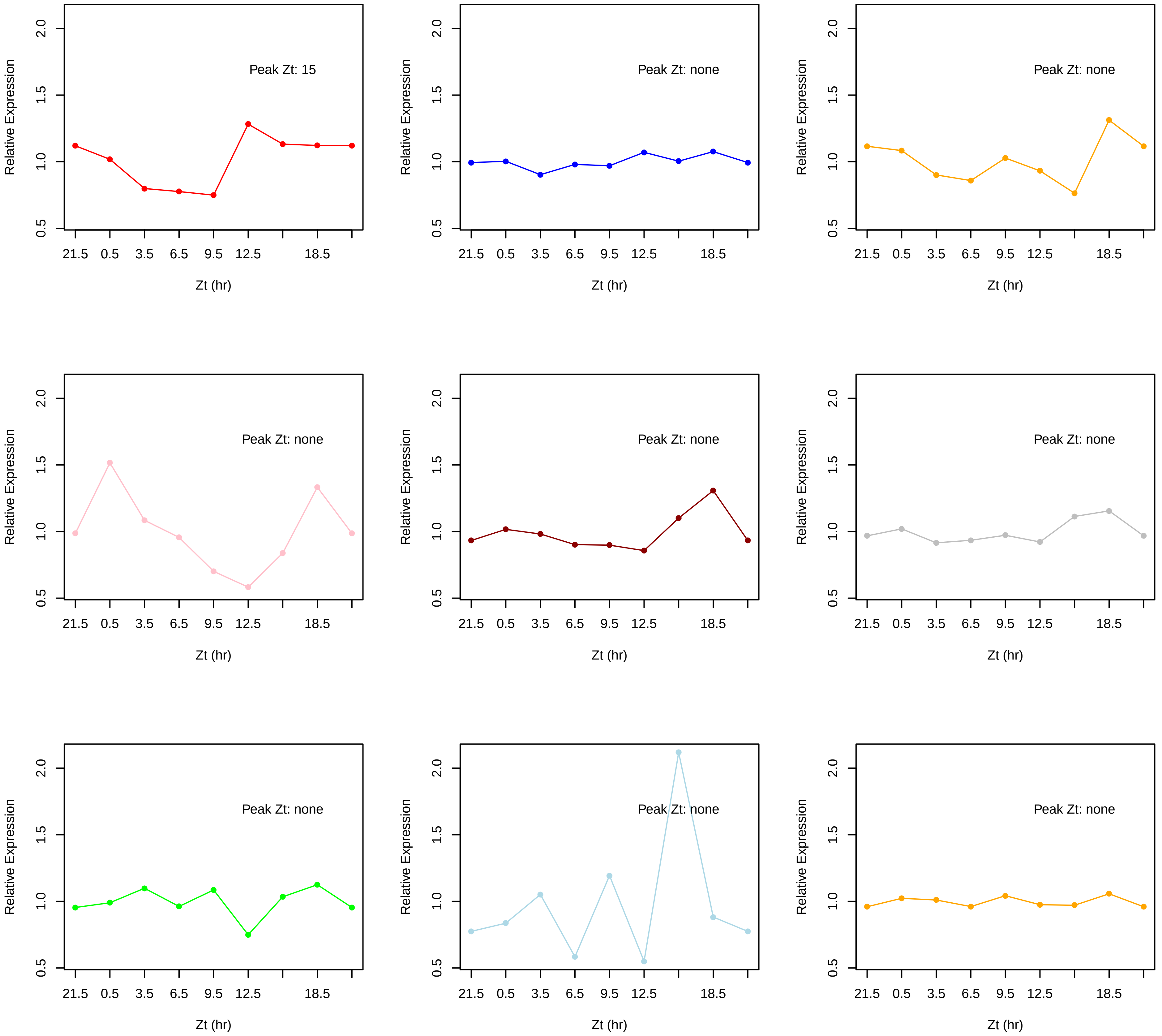

Singeleton: ENSXMAG00000022382 prox1b

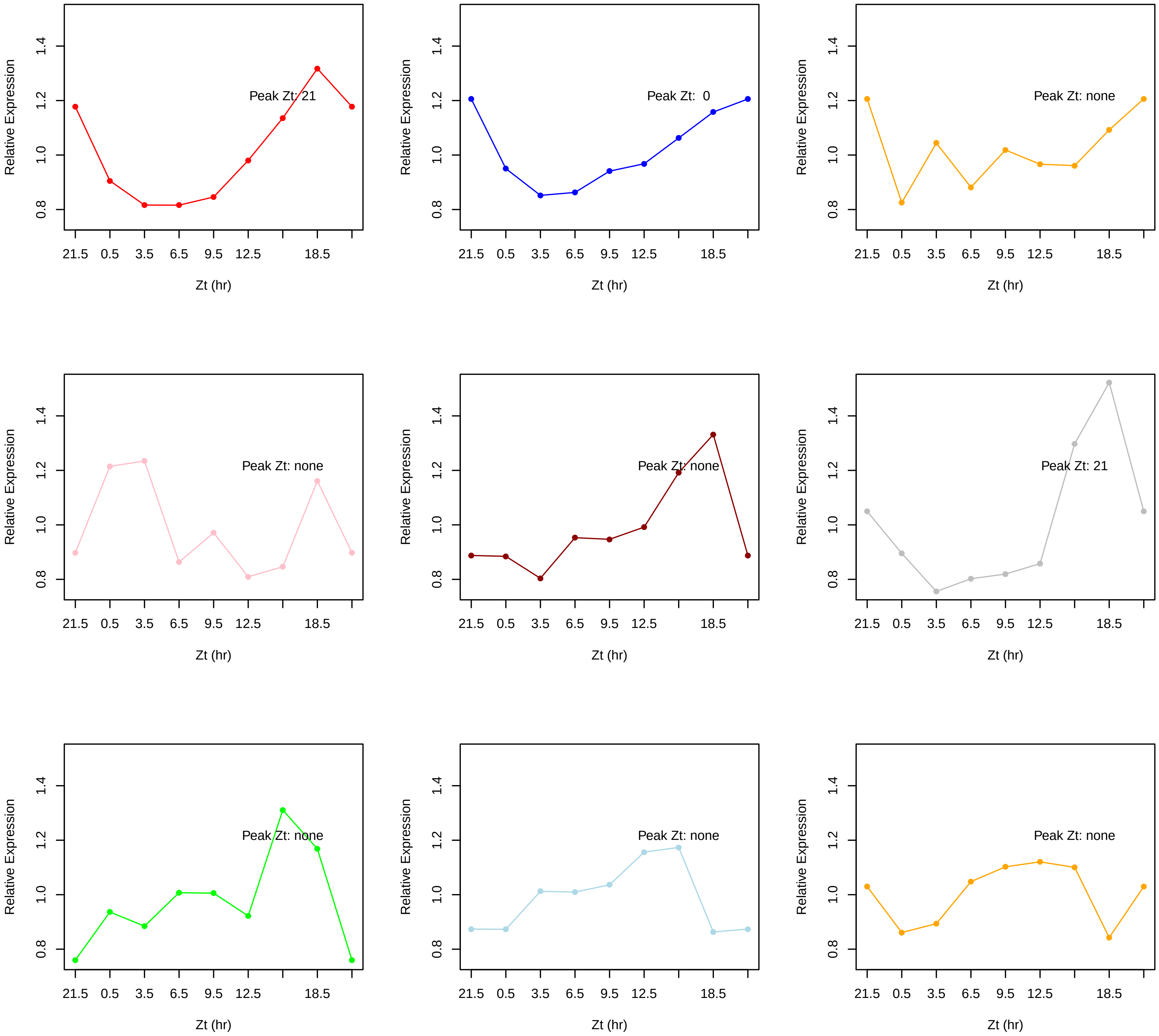

Singeleton: ENSXMAG00000007844 csnk2a4

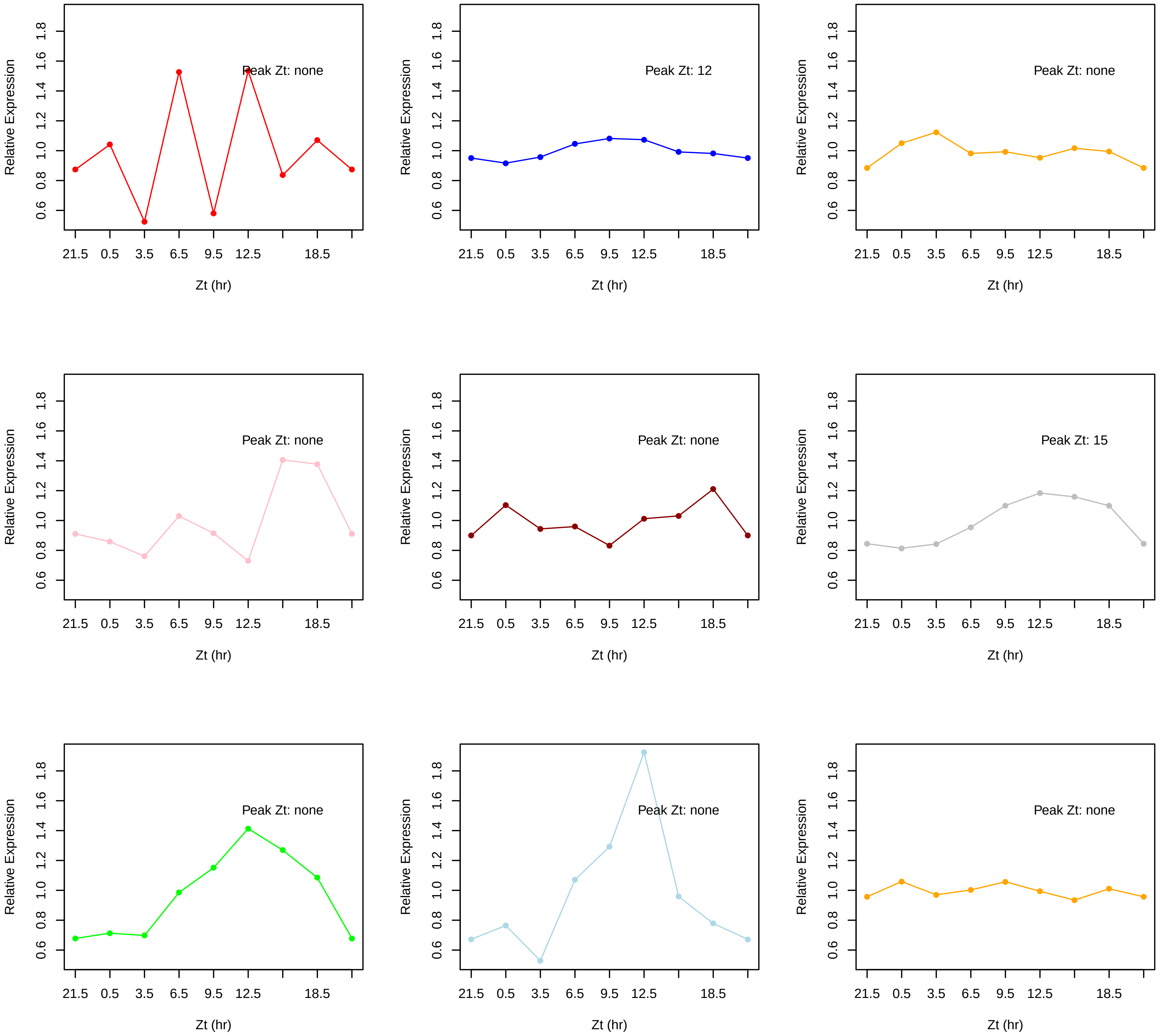

Singleton: ENSXMAG00000014237 spcs2

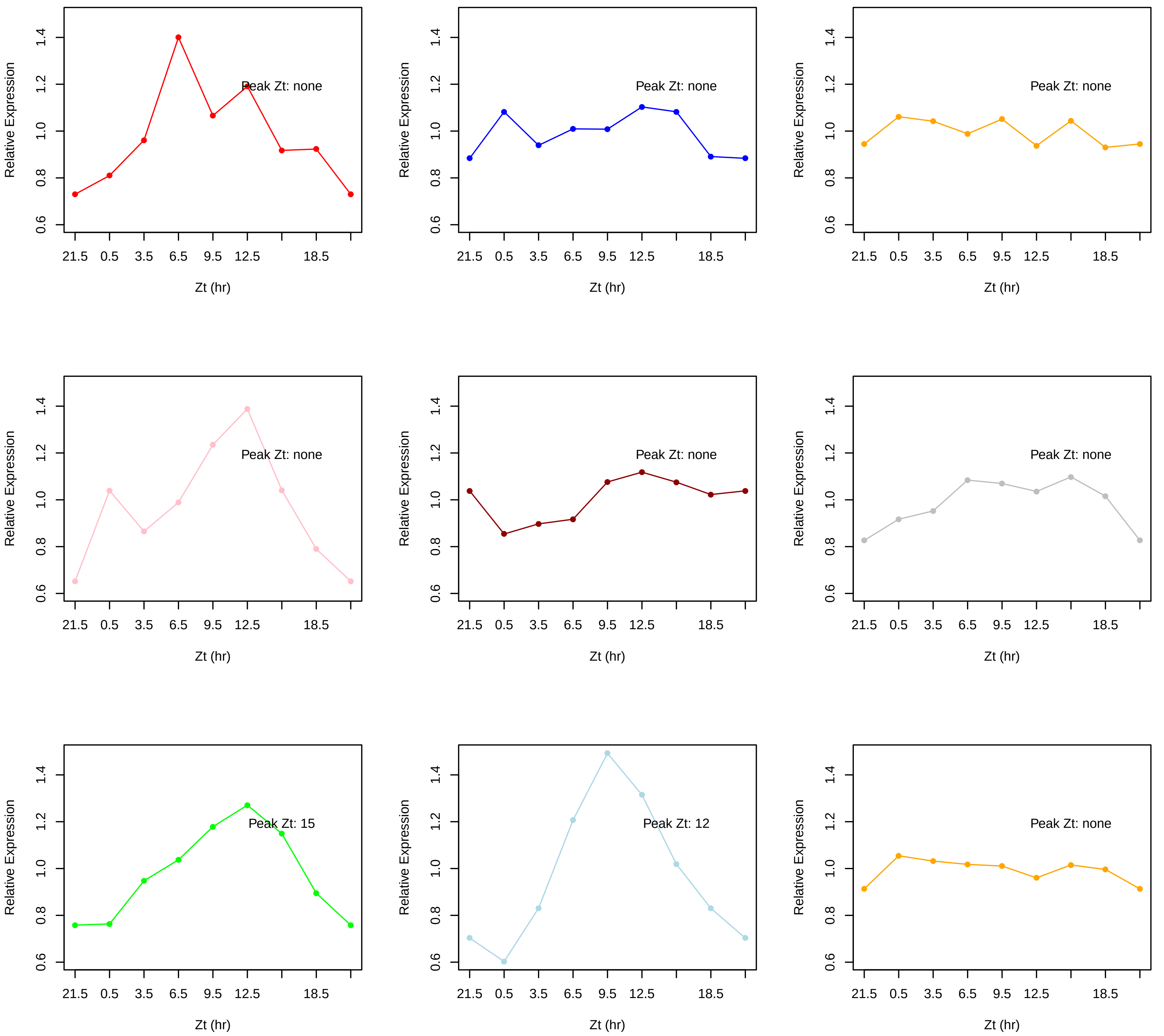

Singleton: ENSXMAG00000018846 pusl1

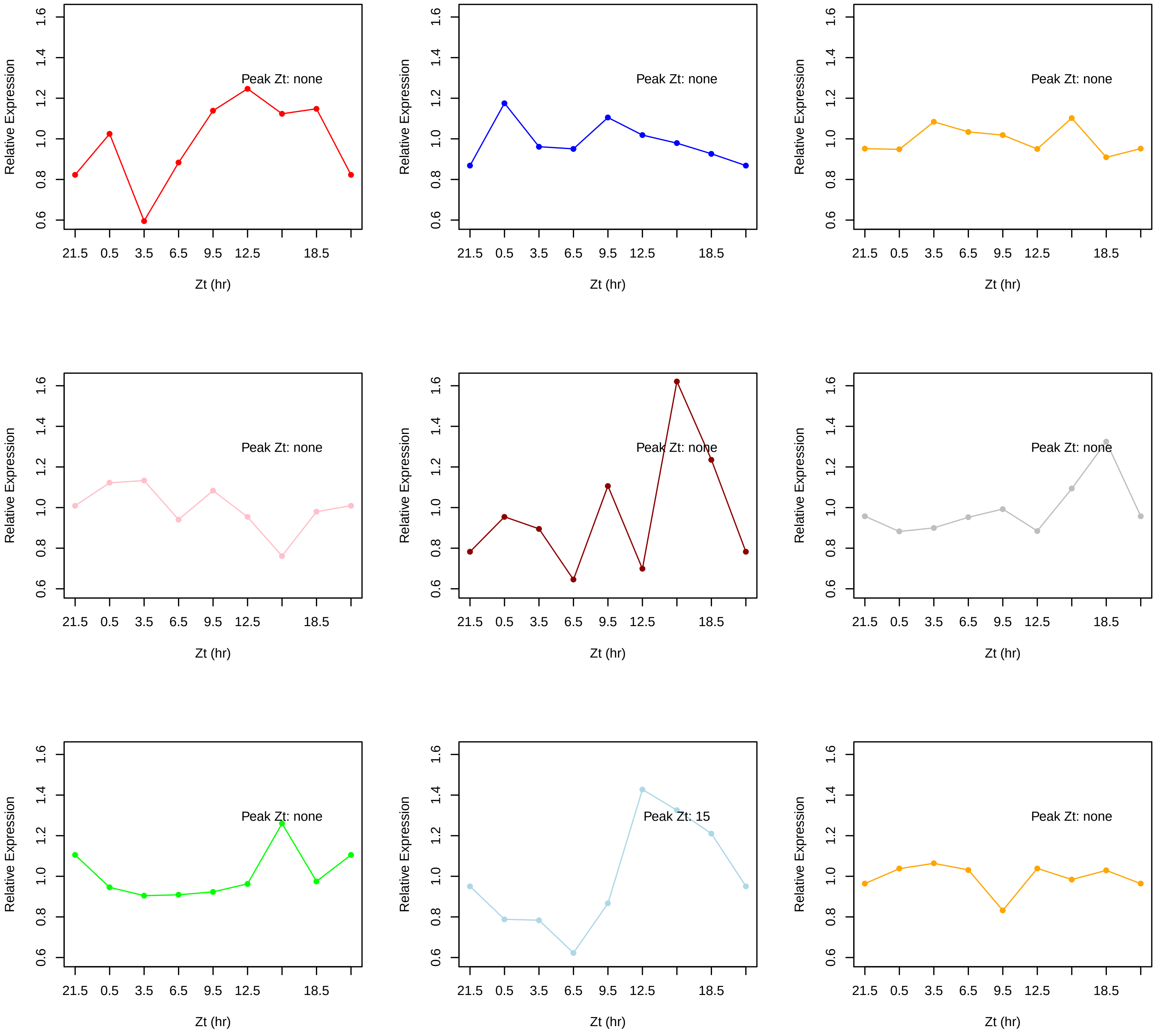

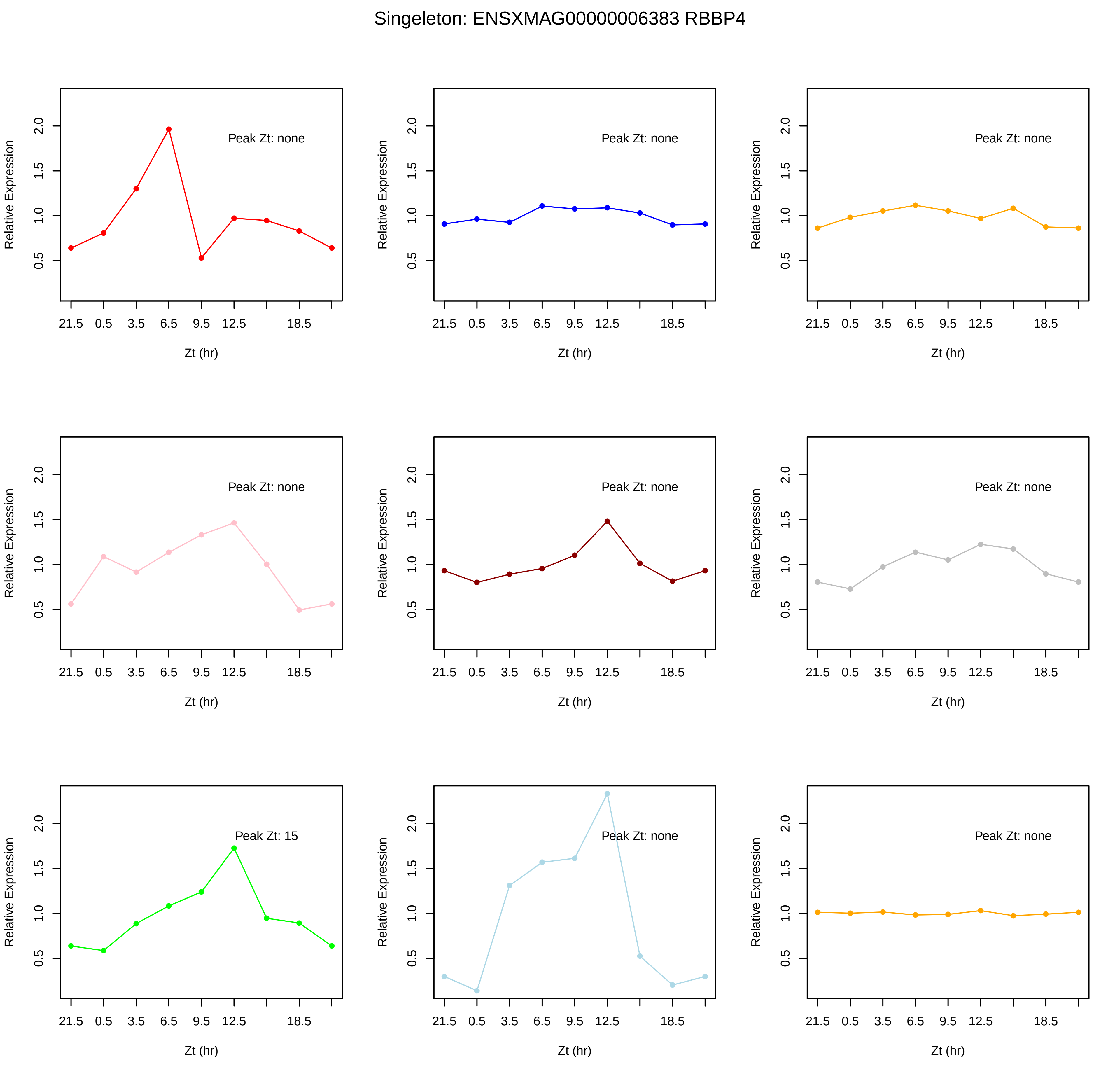
Singeleton: ENSXMAG00000006383 RBBP4
Peak Zt: none
Peak Zt: none
Peak Zt: none
Peak Zt: none
Peak Zt: none
Peak Zt: none
Peak Zt: 15
Peak Zt: none
Peak Zt: none
Relative Expression
Zt (hr)

Singleton: ENSXMAG00000009645 mlycd

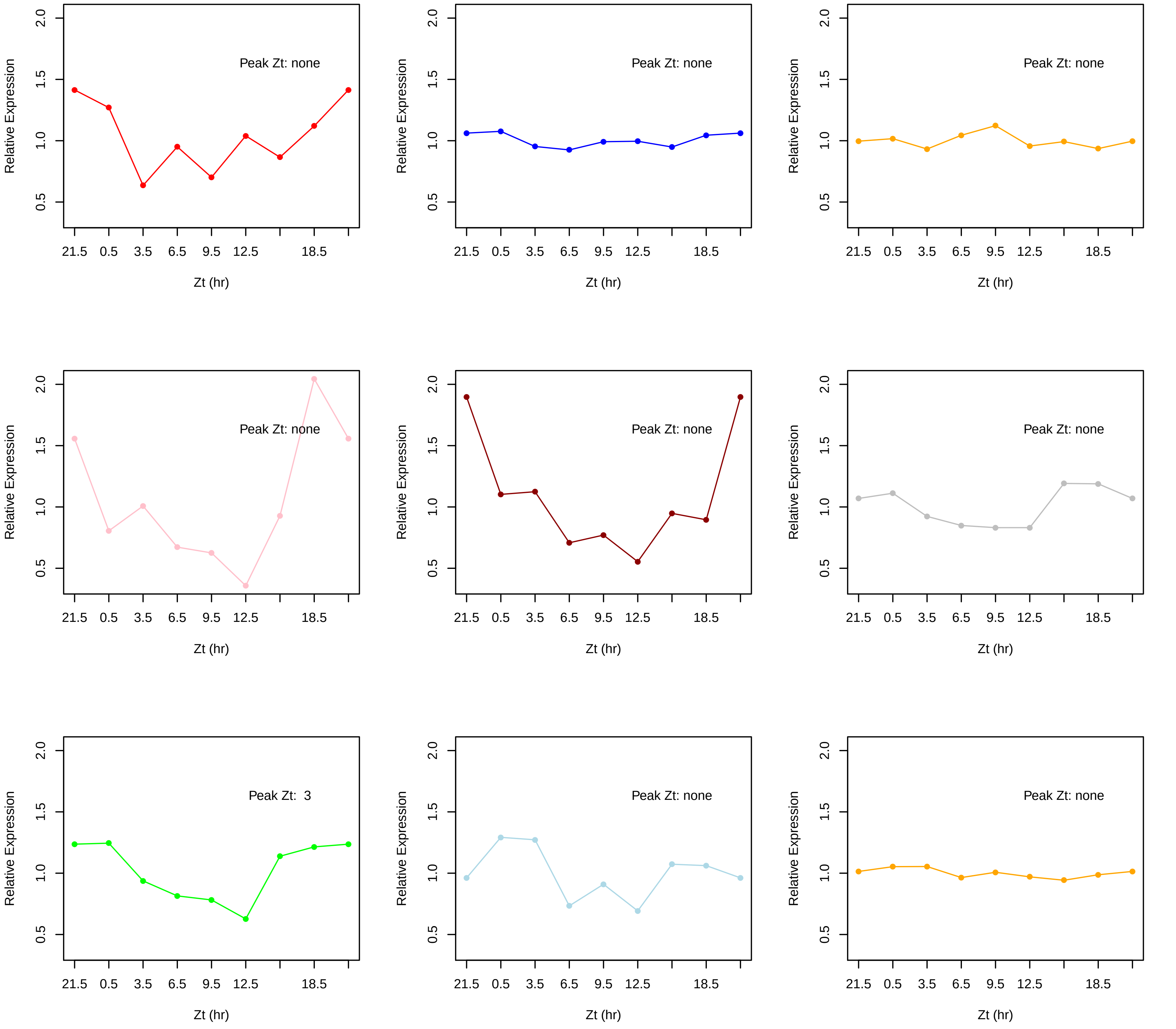

Singeleton: ENSXMAG00000003872 ptpa

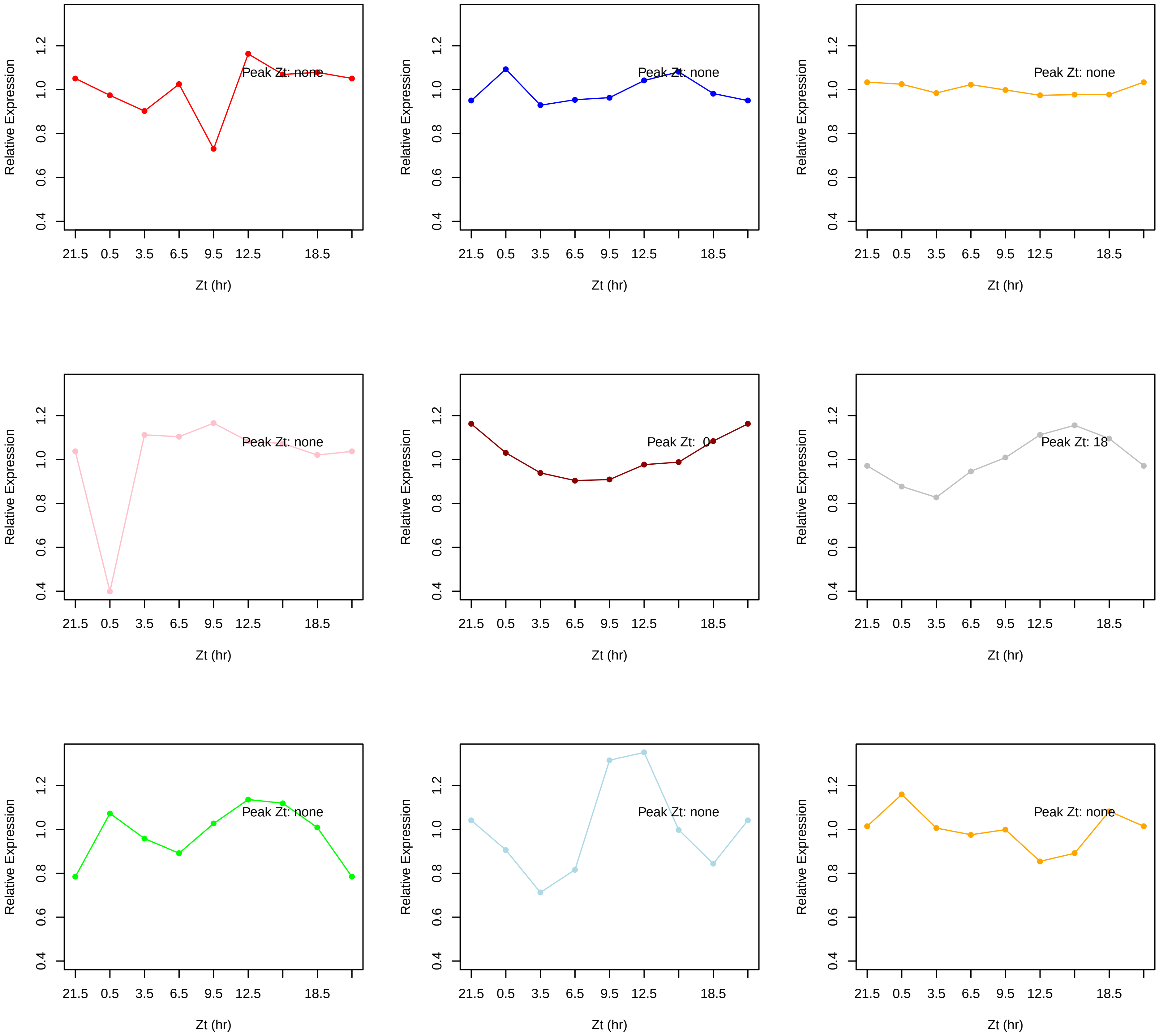

Singeleton: ENSXMAG00000029558 ccdc15

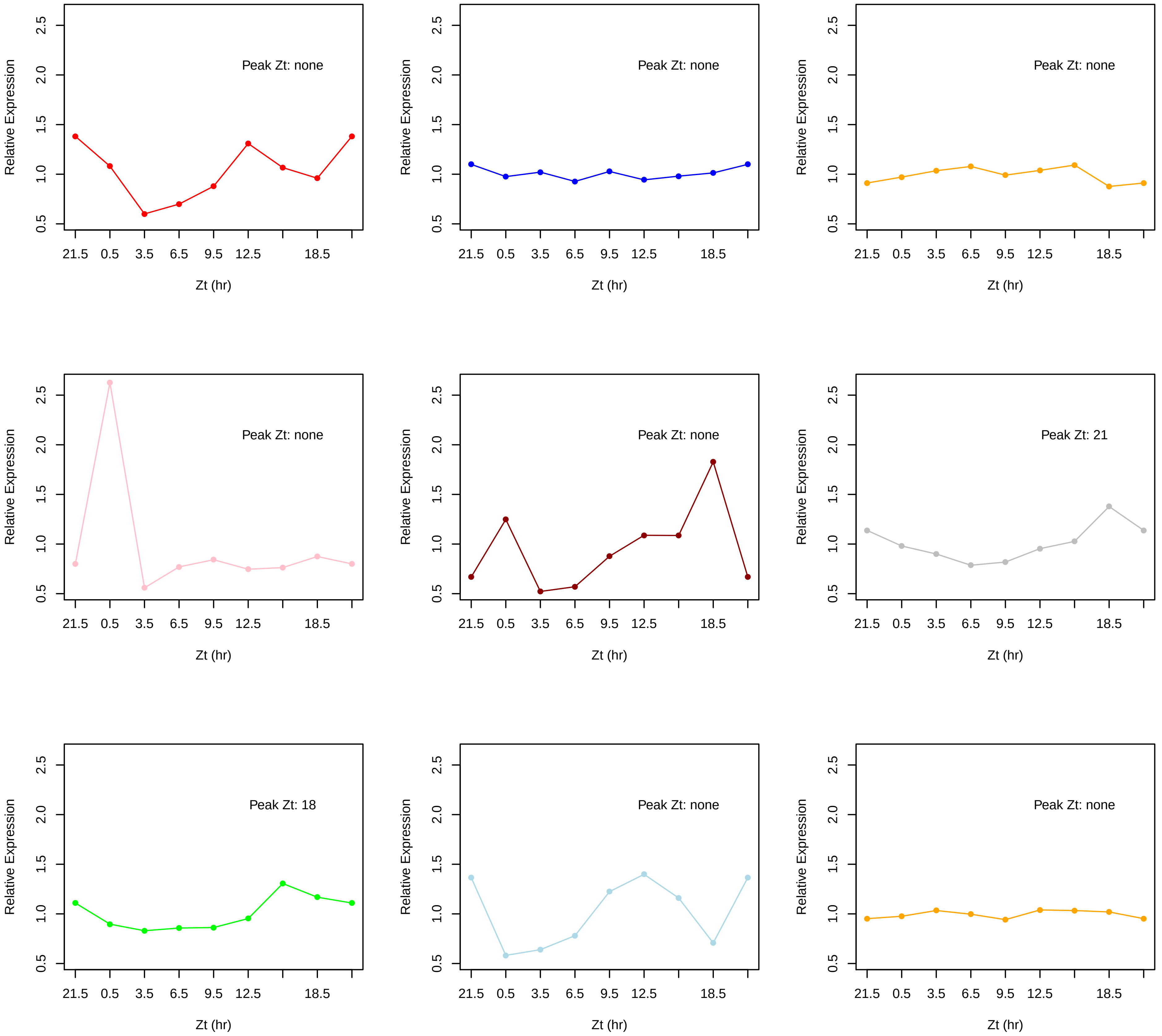

Singeleton: ENSXMAG00000018028 usp9

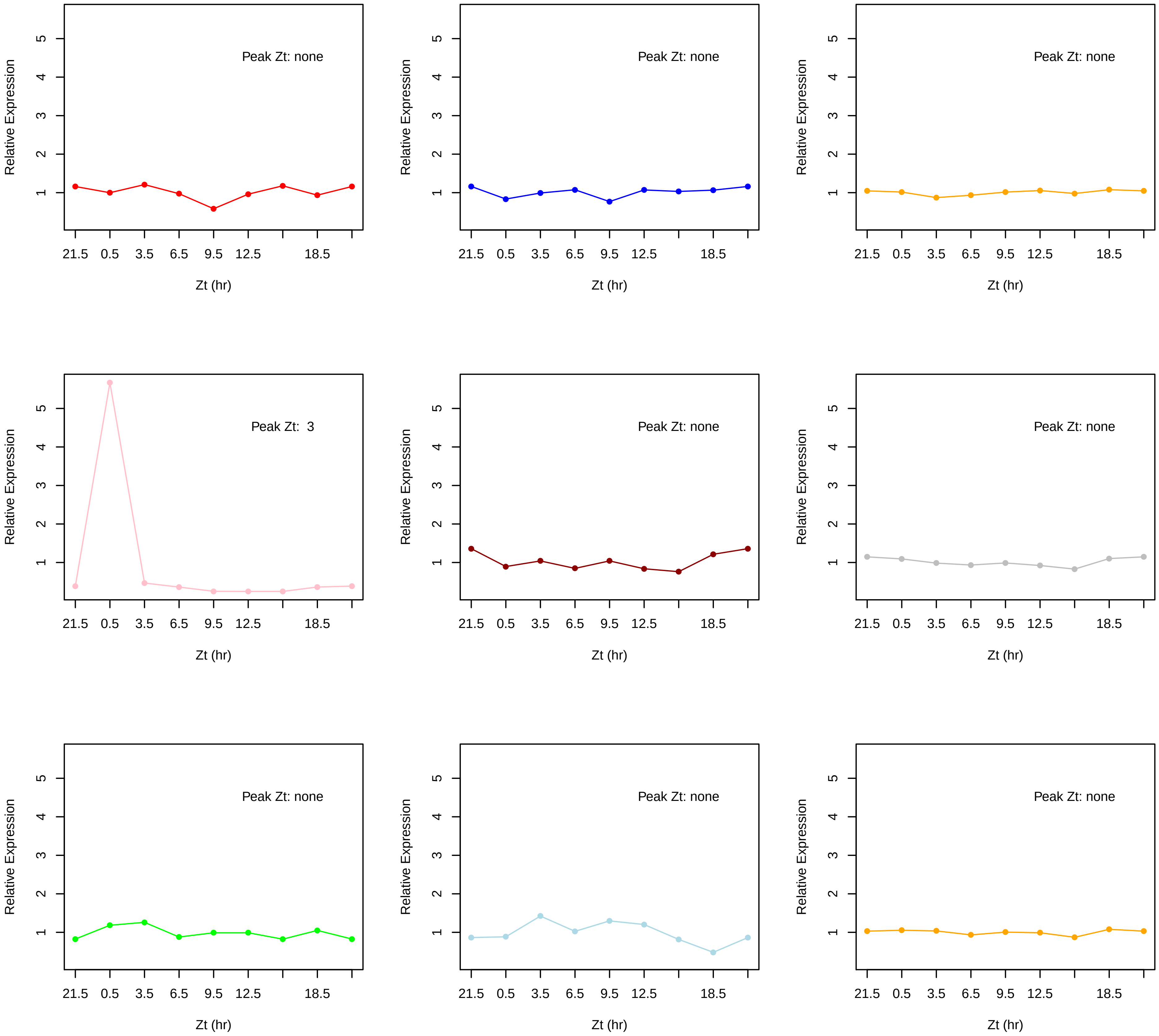

Singleton: ENSXMAG00000011927 nme3

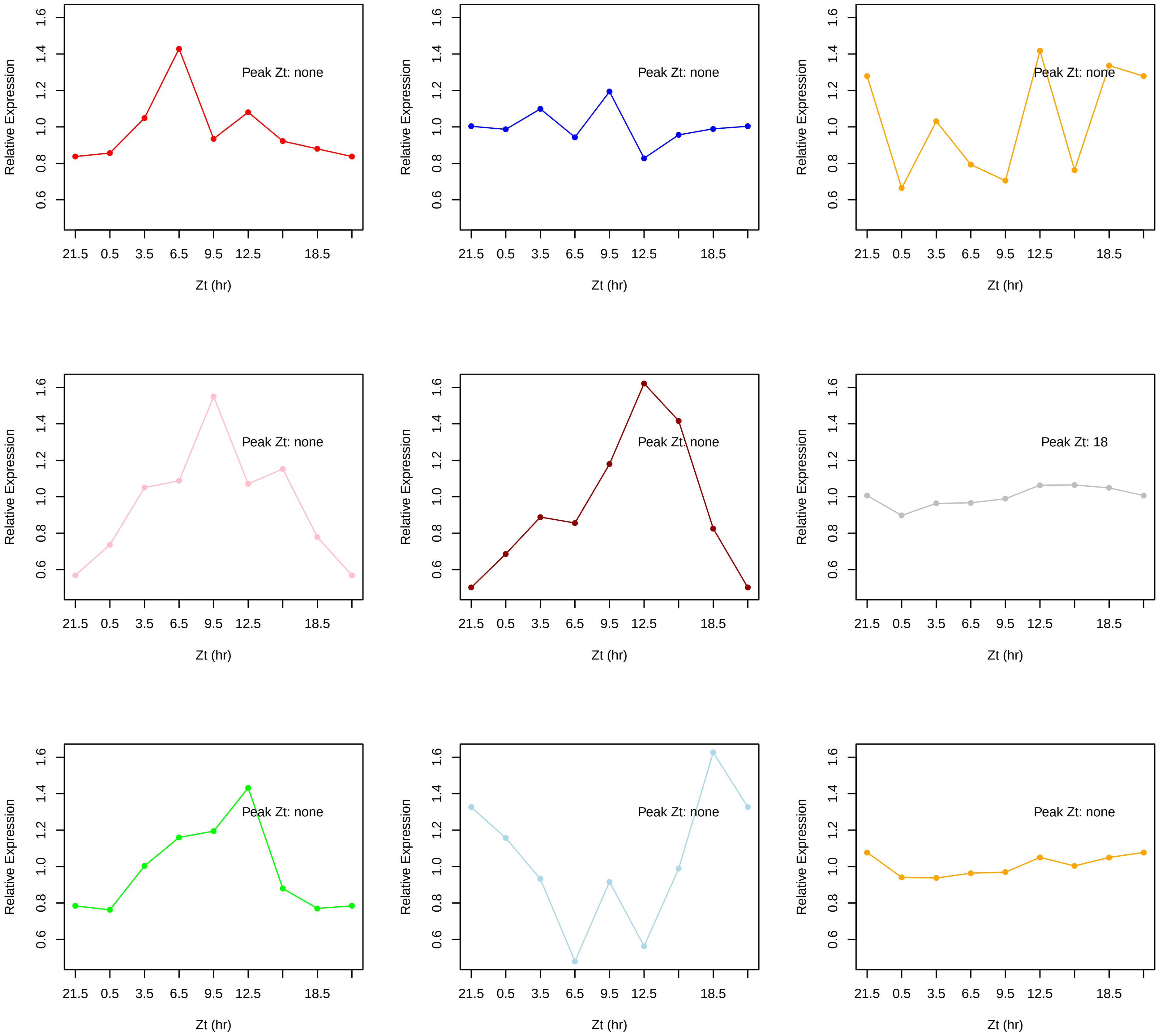

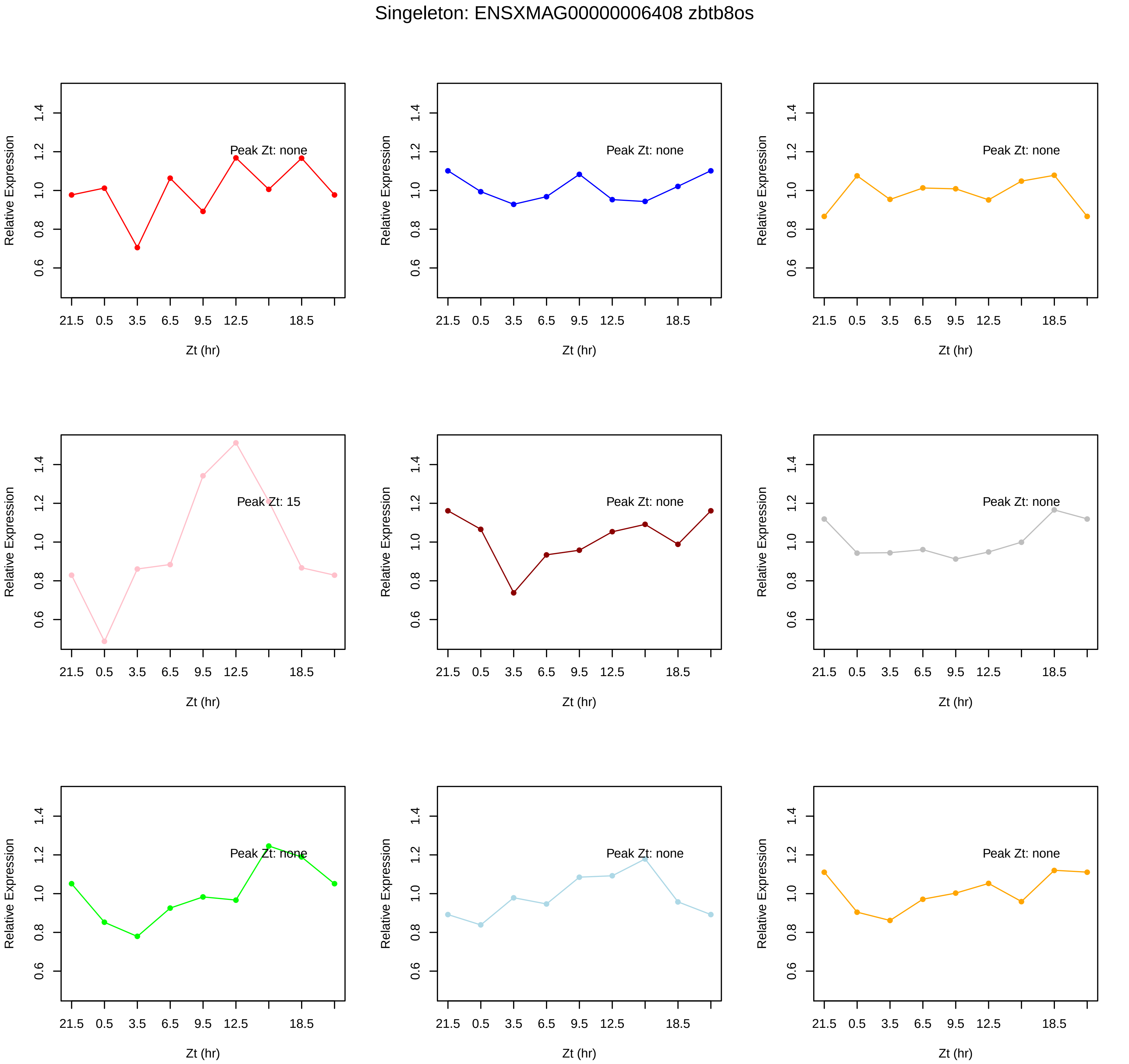
Singeleton: ENSXMAG00000006408 zbtb8os
Peak Zt: none
Peak Zt: none
Peak Zt: none
Peak Zt: 15
Peak Zt: none
Peak Zt: none
Peak Zt: none
Peak Zt: none
Peak Zt: none
Relative Expression
Zt (hr)
21.5
0.5
3.5
6.5
9.5
12.5
18.5
0.6
0.8
1.0
1.2
1.4

Singleton: ENSXMAG00000022547 srxn1

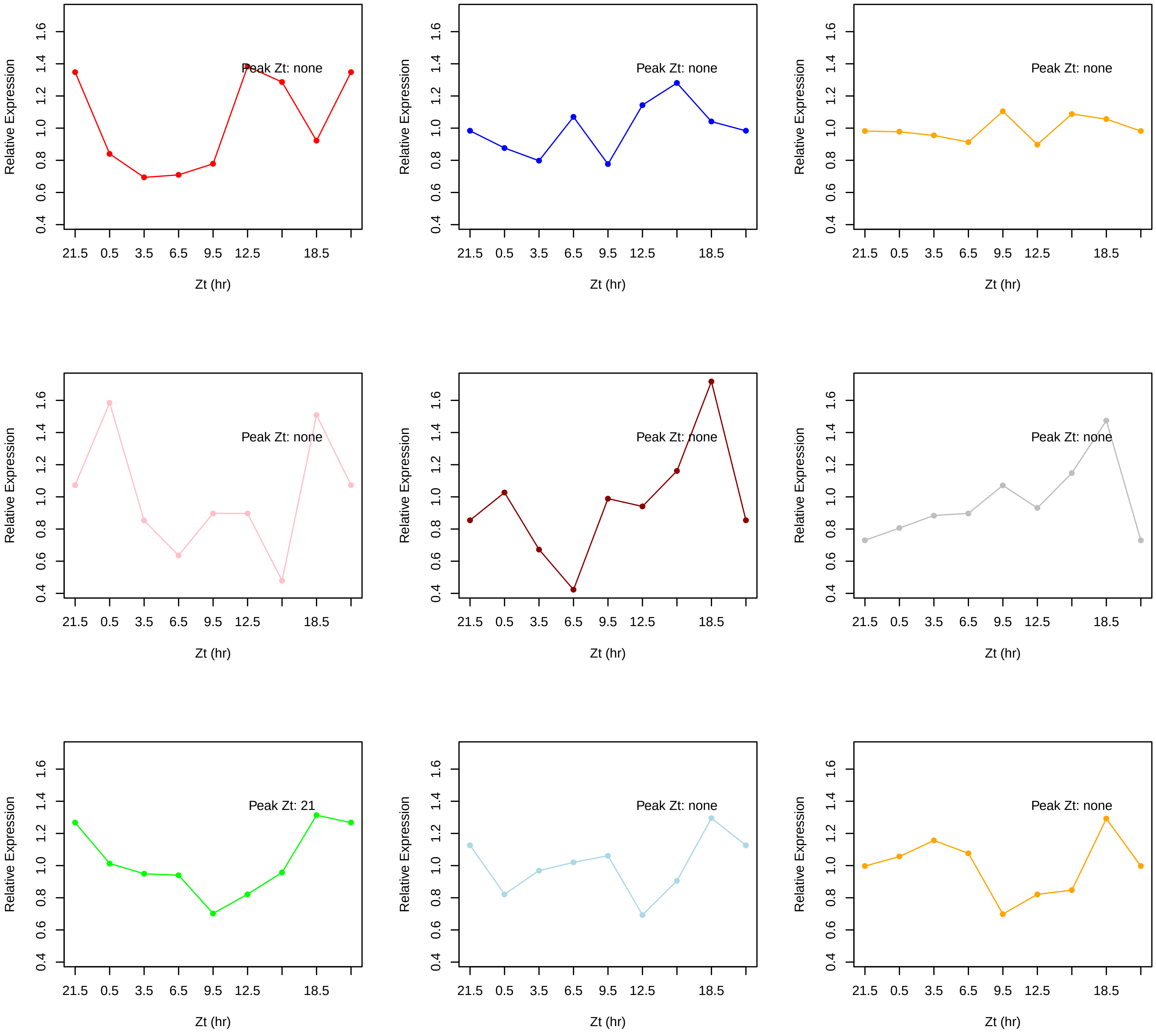

Singeleton: ENSXMAG00000011943 mrps34

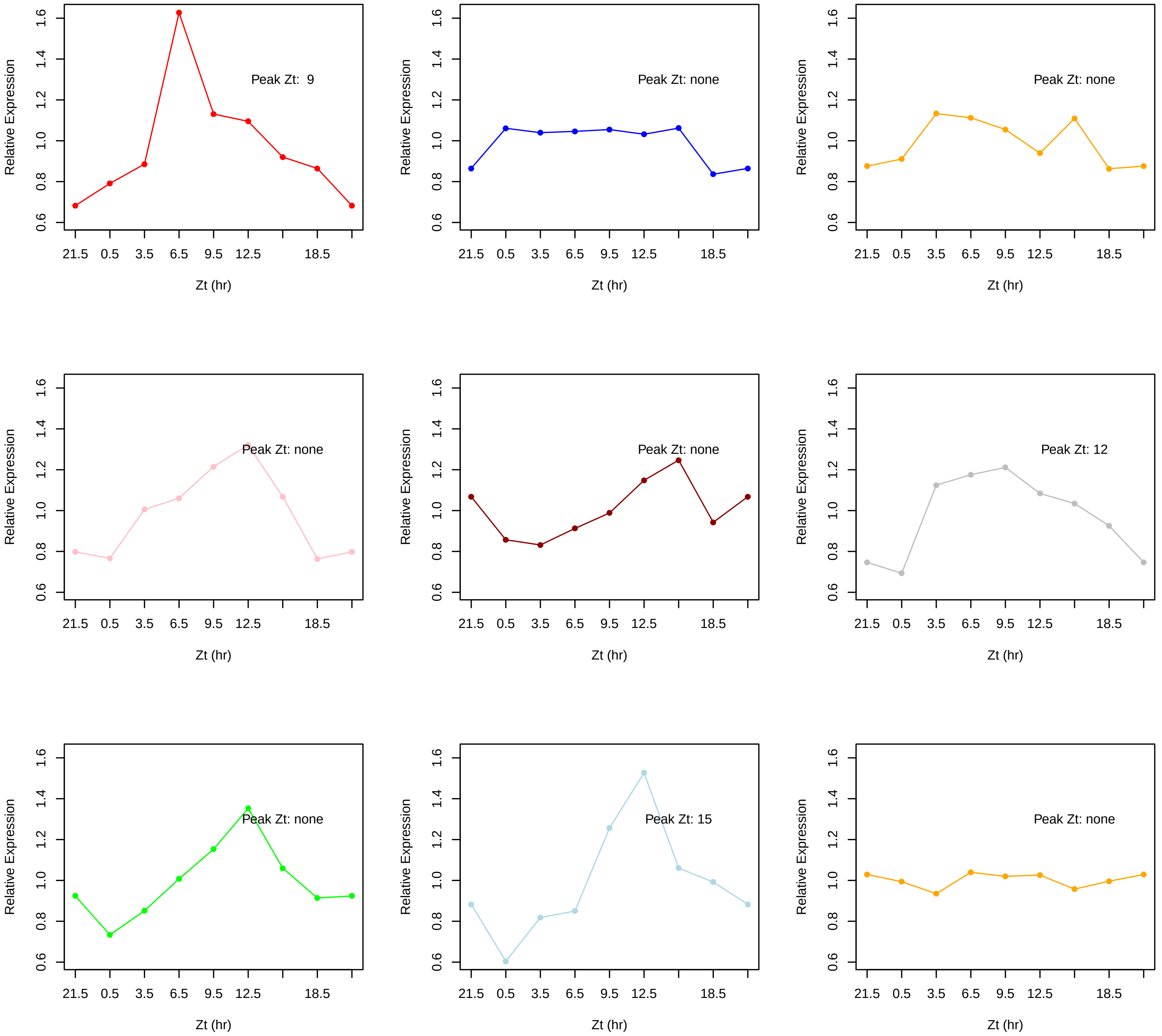

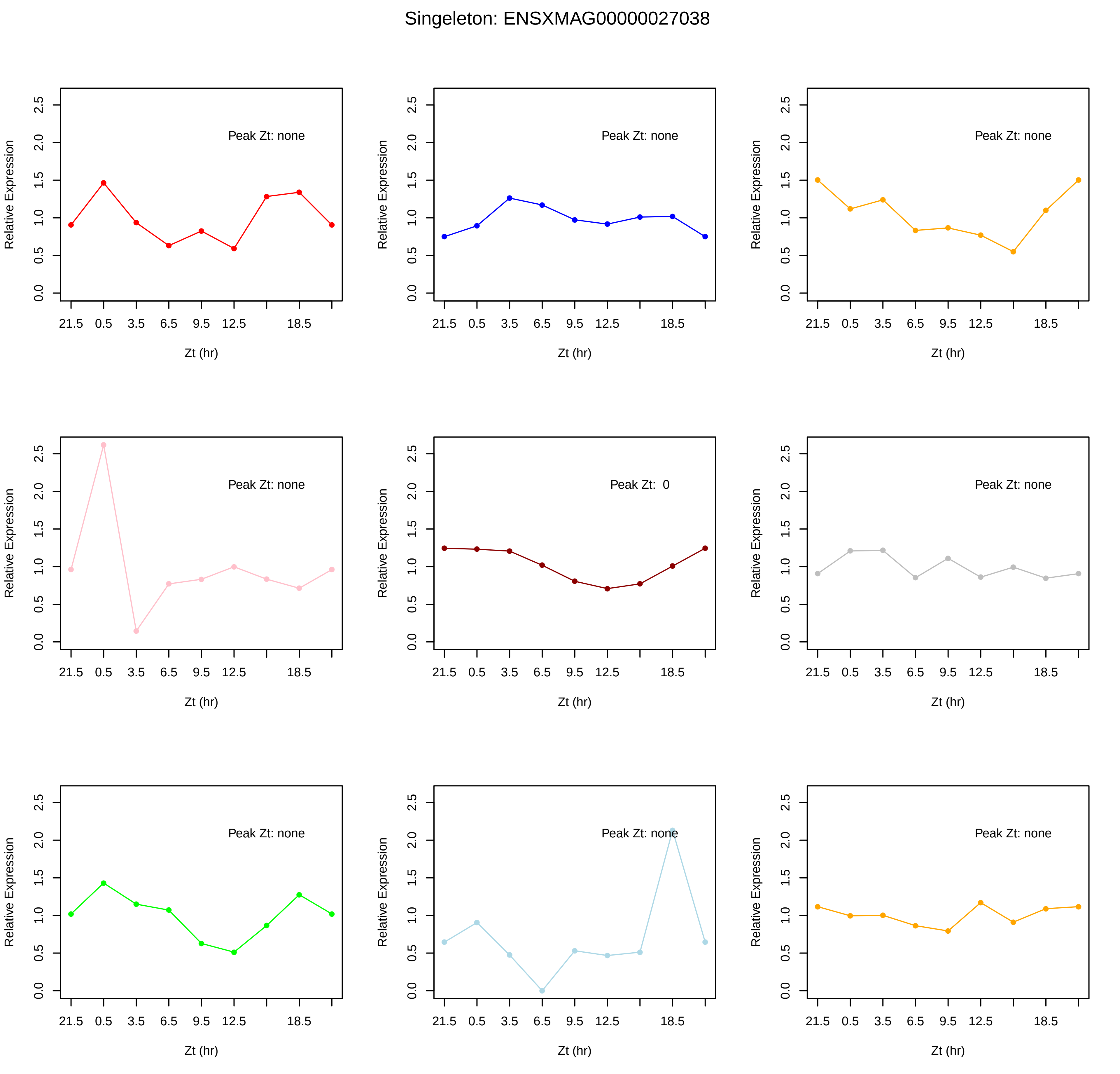
Singeleton: ENSXMAG00000027038
Peak Zt: none
Peak Zt: none
Peak Zt: none
Peak Zt: none
Peak Zt: 0
Peak Zt: none
Peak Zt: none
Peak Zt: none
Peak Zt: none
Relative Expression
Zt (hr)
21.5
0.5
3.5
6.5
9.5
12.5
18.5
0.0
0.5
1.0
1.5
2.0
2.5

Singeleton: ENSXMAG00000015055 zgc:136564

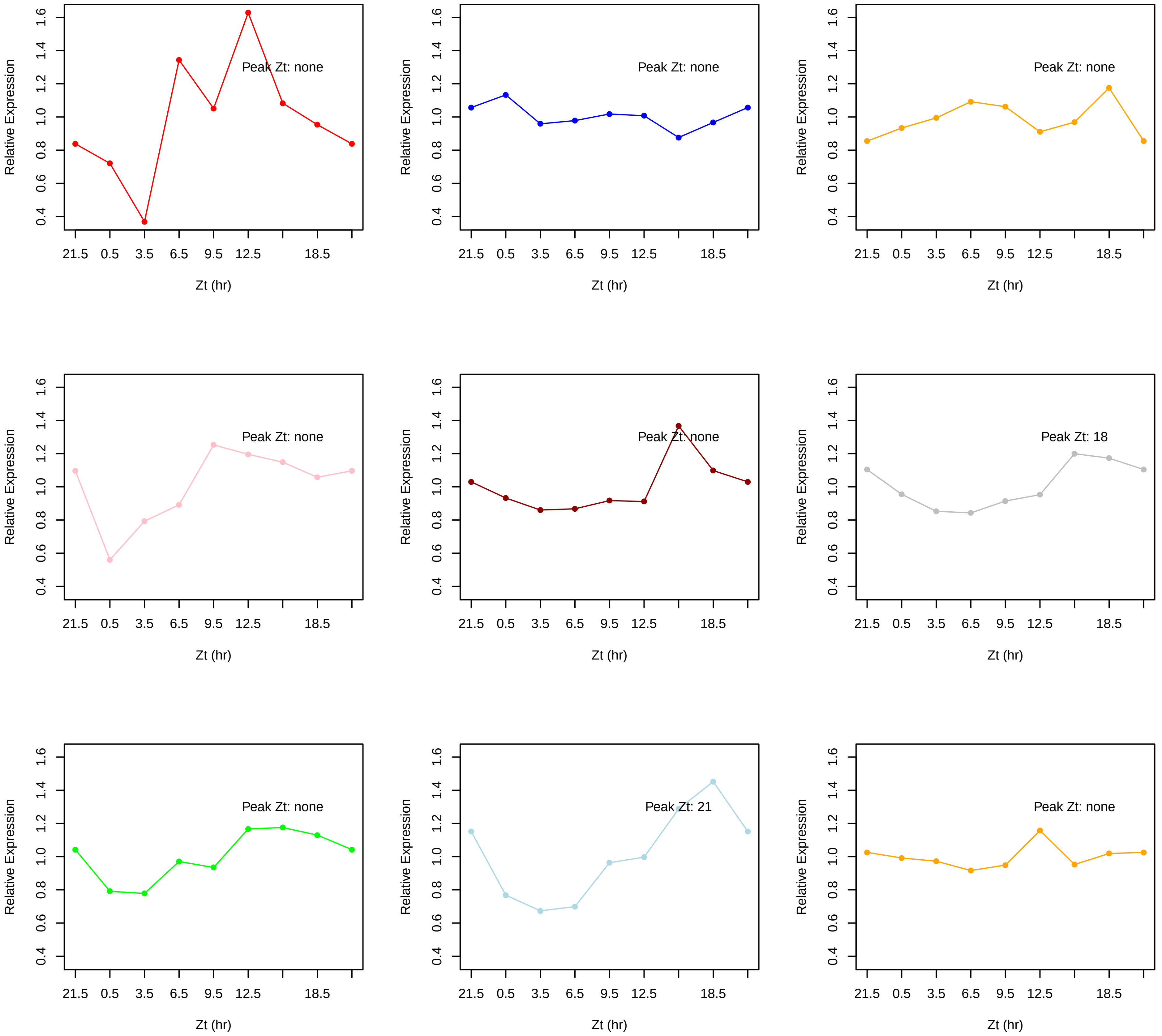

Singeleton: ENSXMAG00000026448 eaf2

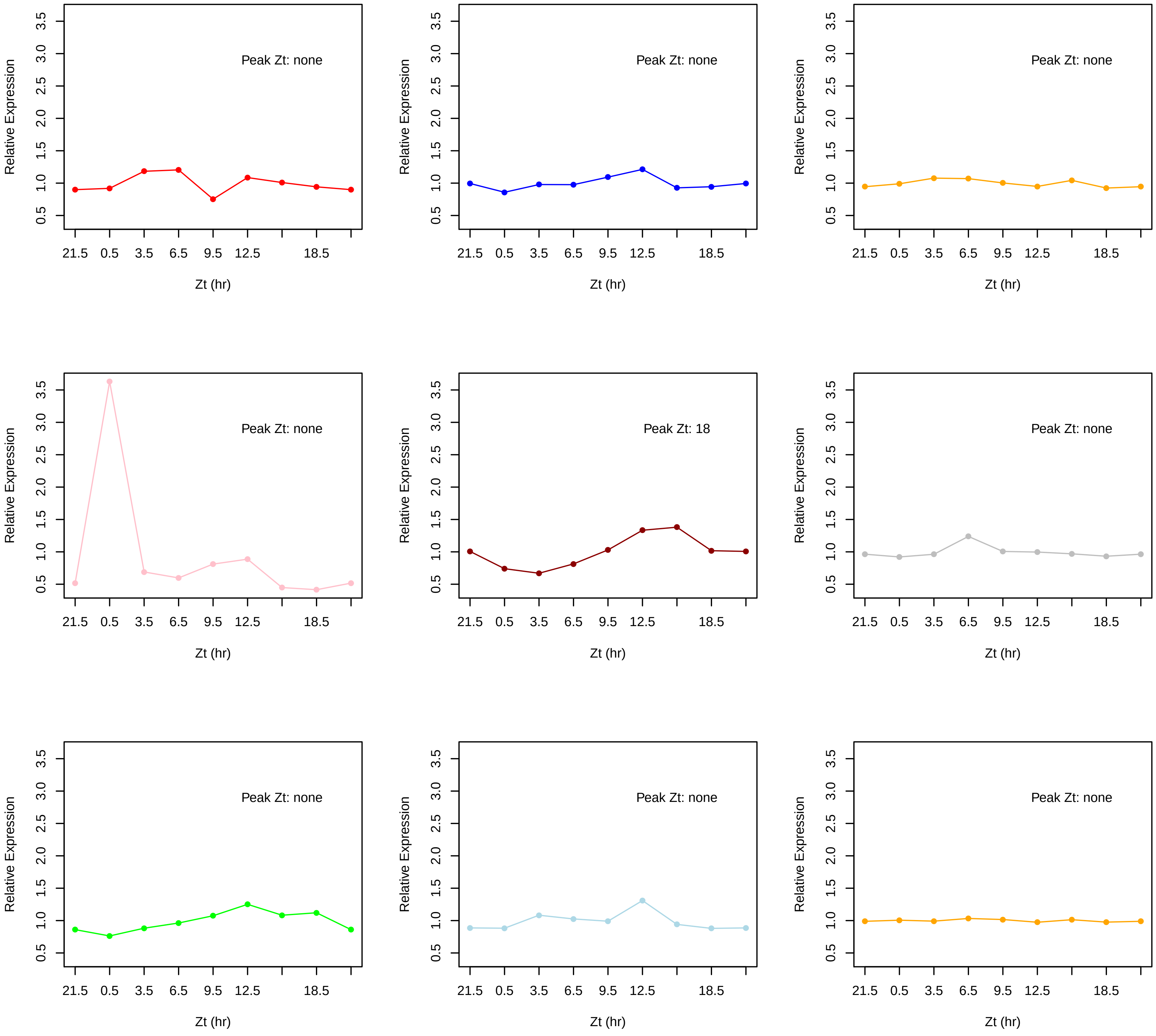

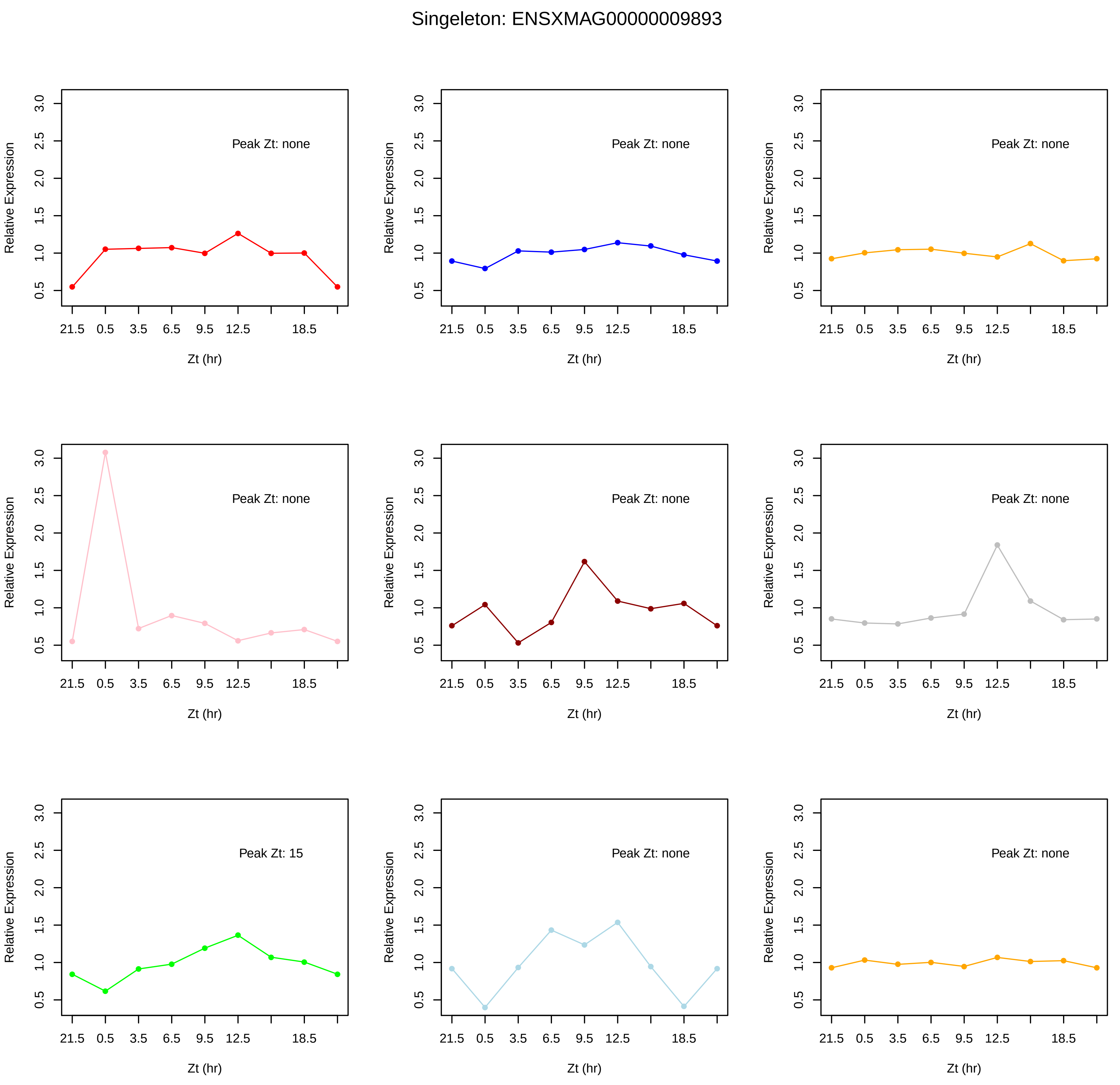

Singeleton: ENSXMAG00000009893
Peak Zt: none
Peak Zt: none
Peak Zt: none
Peak Zt: none
Peak Zt: none
Peak Zt: none
Peak Zt: 15
Peak Zt: none
Peak Zt: none
Relative Expression
Zt (hr)
21.5
0.5
3.5
6.5
9.5
12.5
18.5
0.5
1.0
1.5
2.0
2.5
3.0

Singeleton: ENSXMAG00000011578 slc52a3

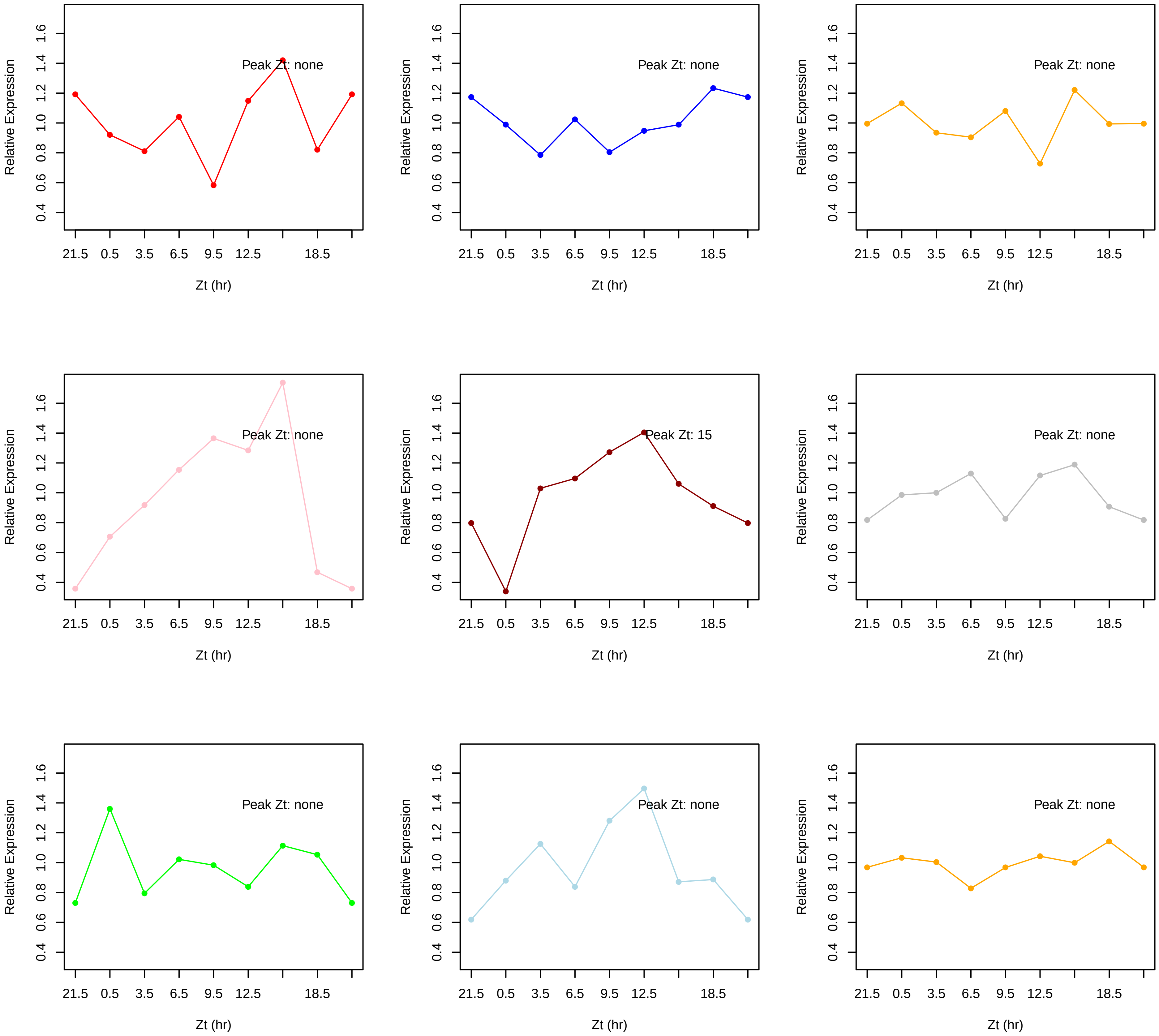

Singleton: ENSXMAG00000000891

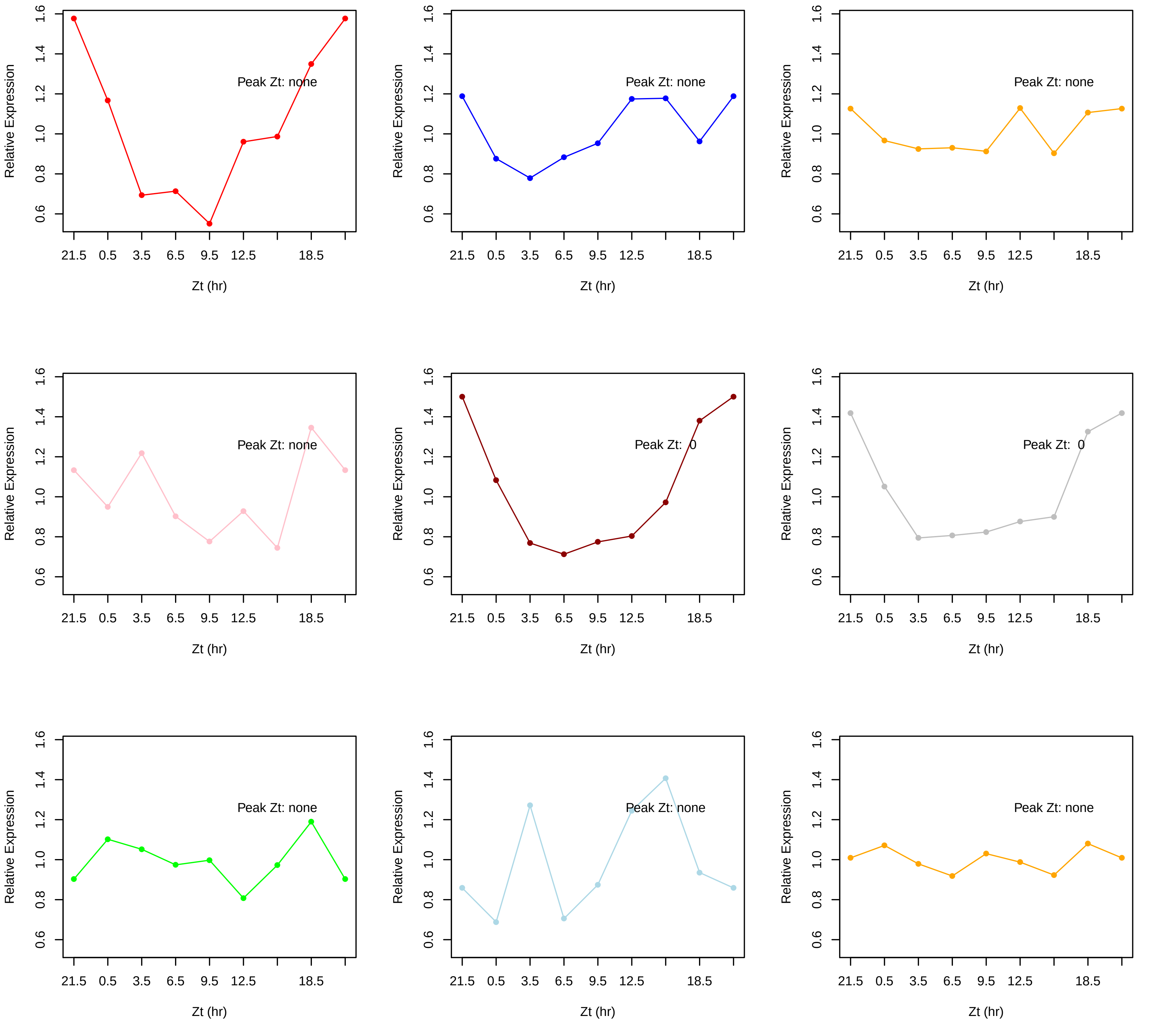

Singleton: ENSXMAG00000003862 ndor1

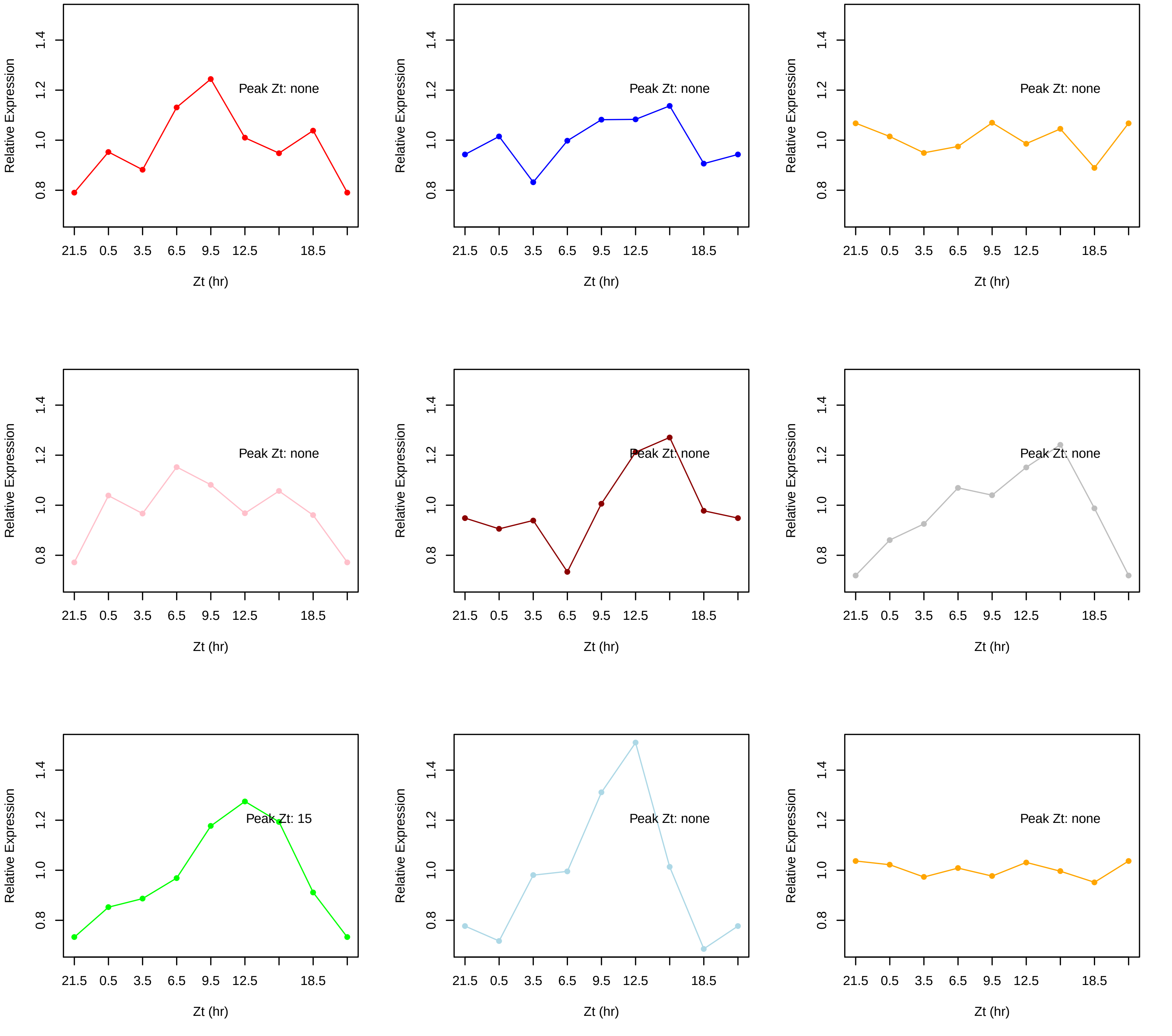

Singeleton: ENSXMAG00000000920 si:dkey−91i10.3

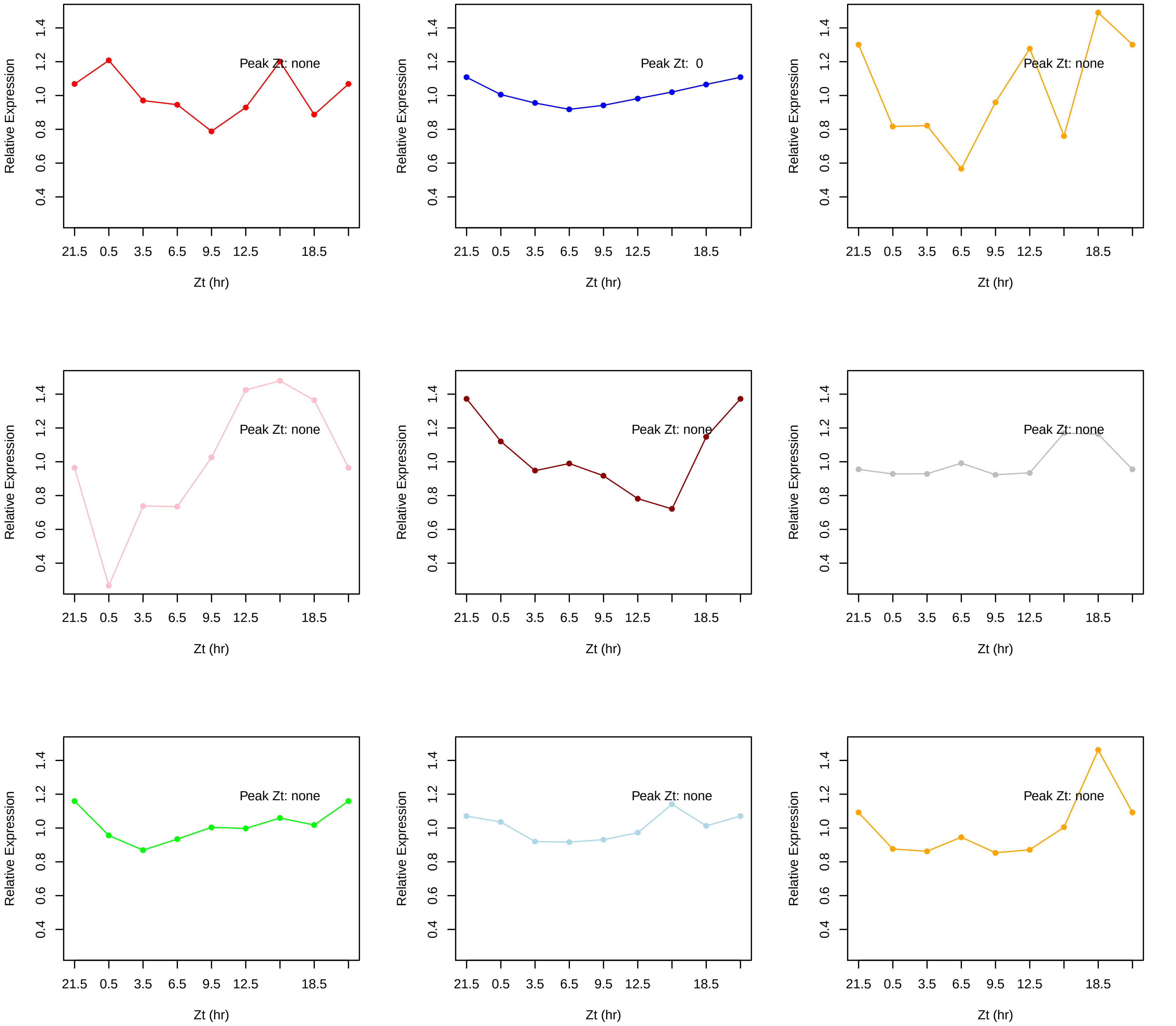

Singeleton: ENSXMAG00000005163 vps51

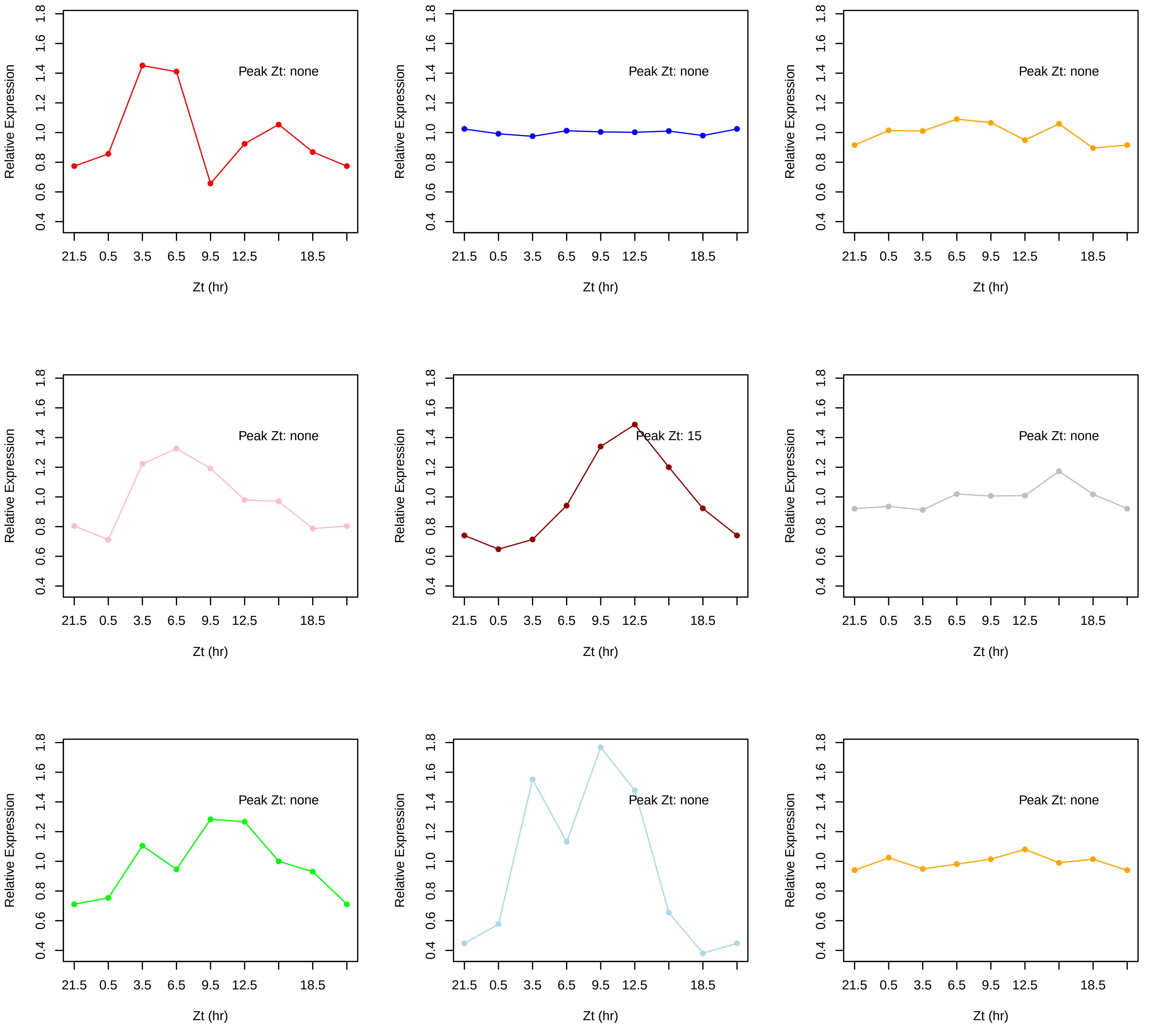

Singeleton: ENSXMAG00000020991 spsb3a

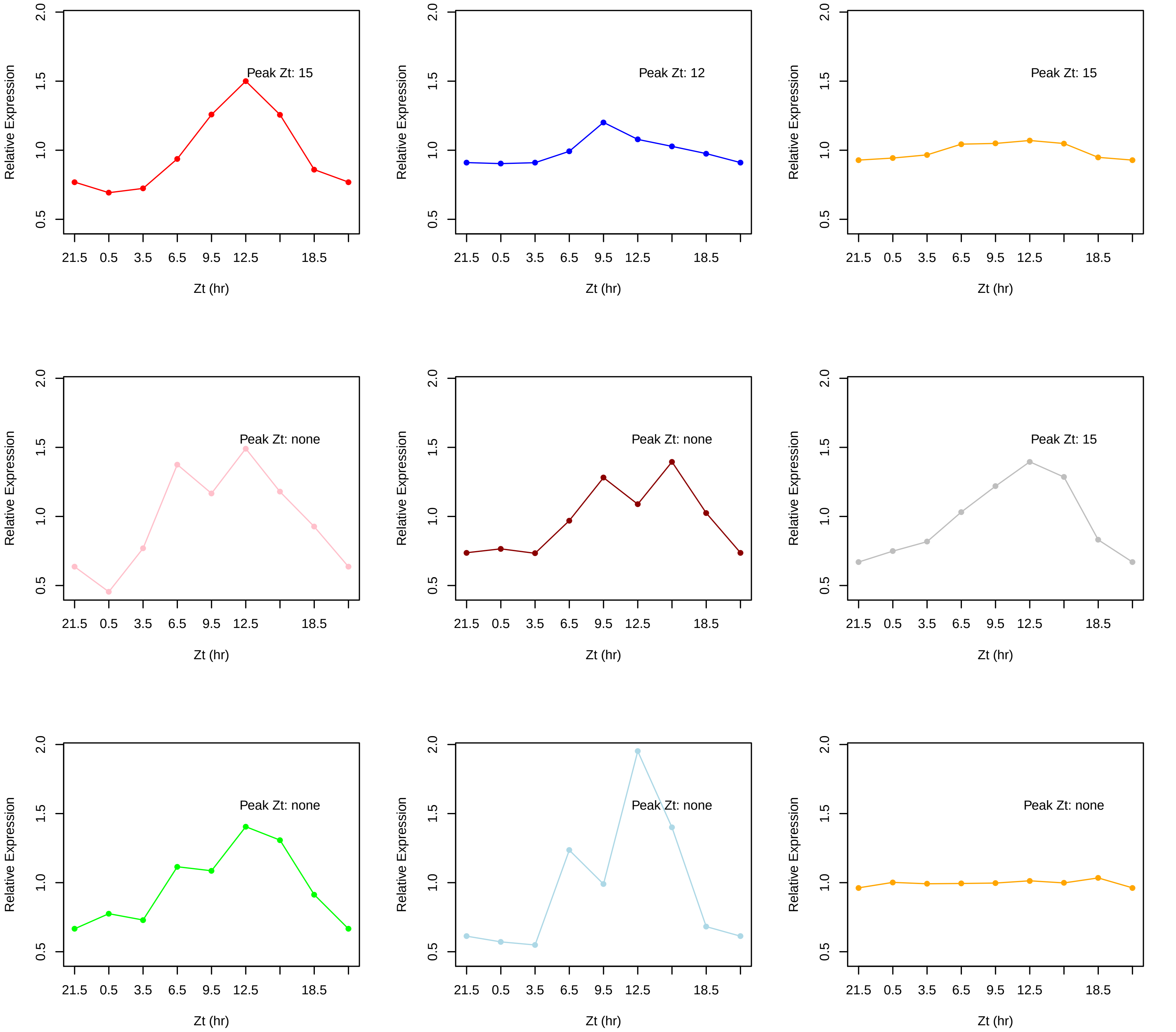

Singeleton: ENSXMAG00000000045 slc2a8

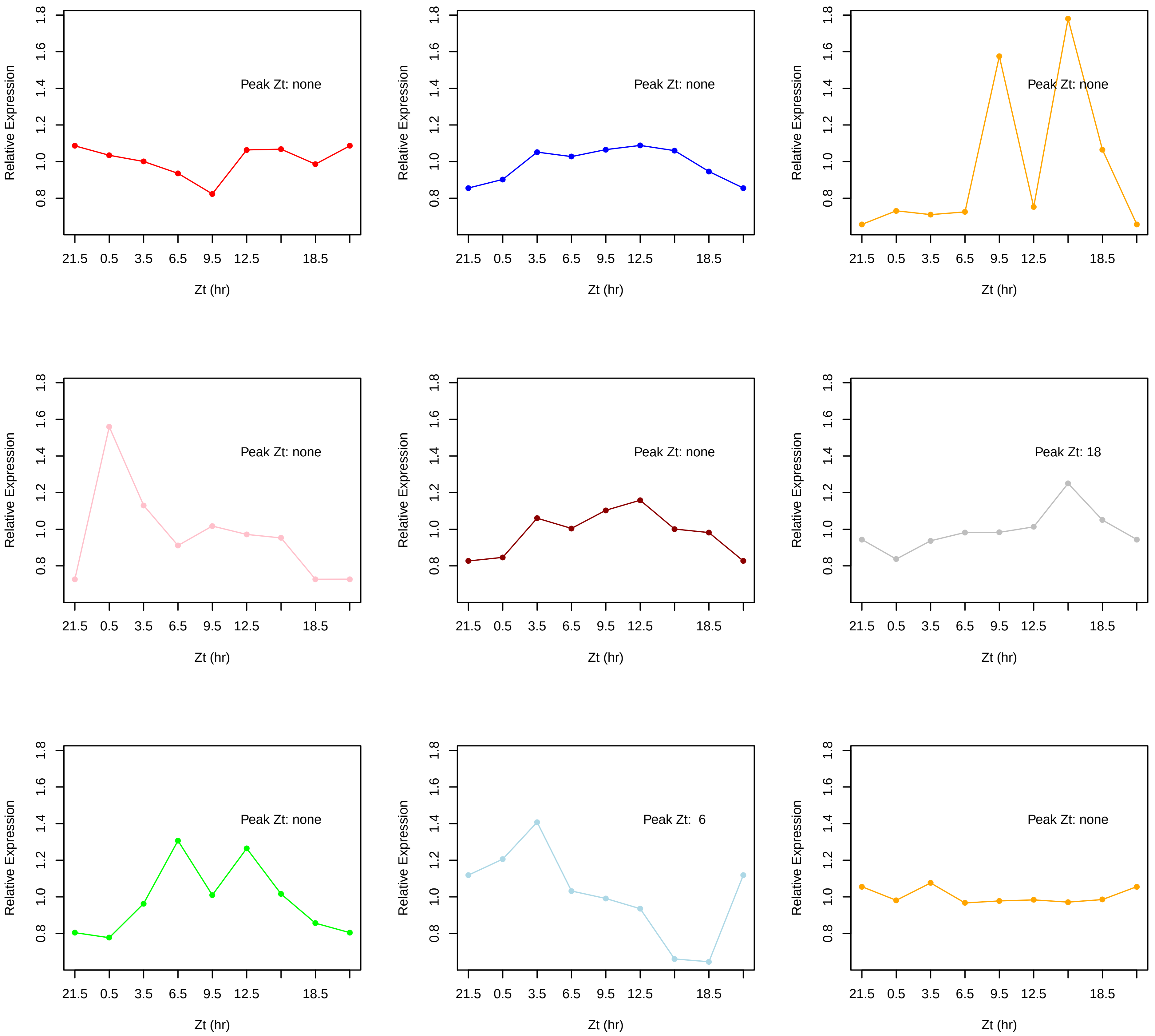

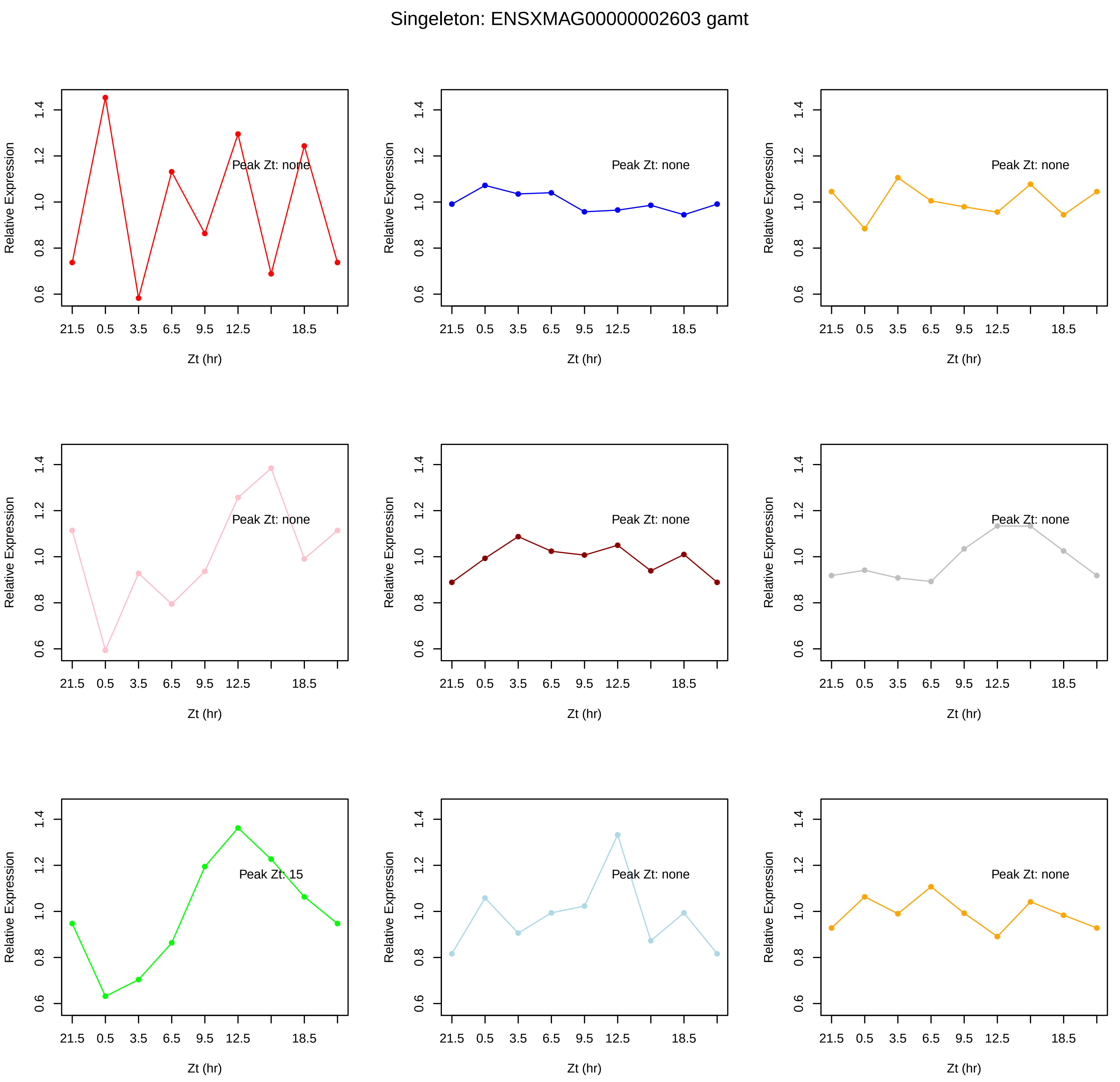
Singeleton: ENSXMAG00000002603 gamt
Peak Zt: none
Peak Zt: none
Peak Zt: none
Peak Zt: none
Peak Zt: none
Peak Zt: none
Peak Zt: 15
Peak Zt: none
Peak Zt: none
Relative Expression
Zt (hr)
21.5 0.5 3.5 6.5 9.5 12.5 18.5
0.6 0.8 1.0 1.2 1.4

Singeleton: ENSXMAG00000024236

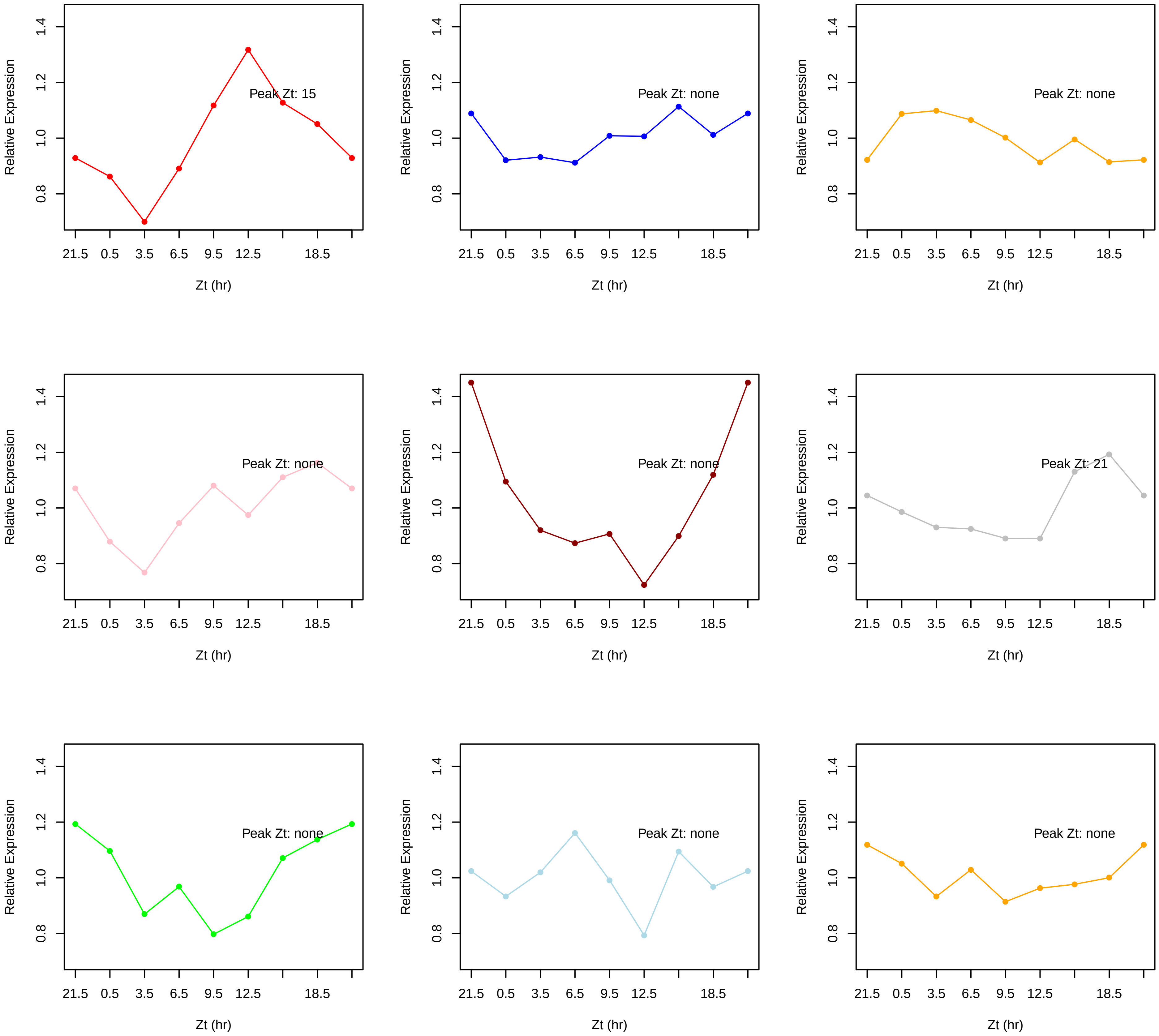

Singeleton: ENSXMAG00000014376 serpind1

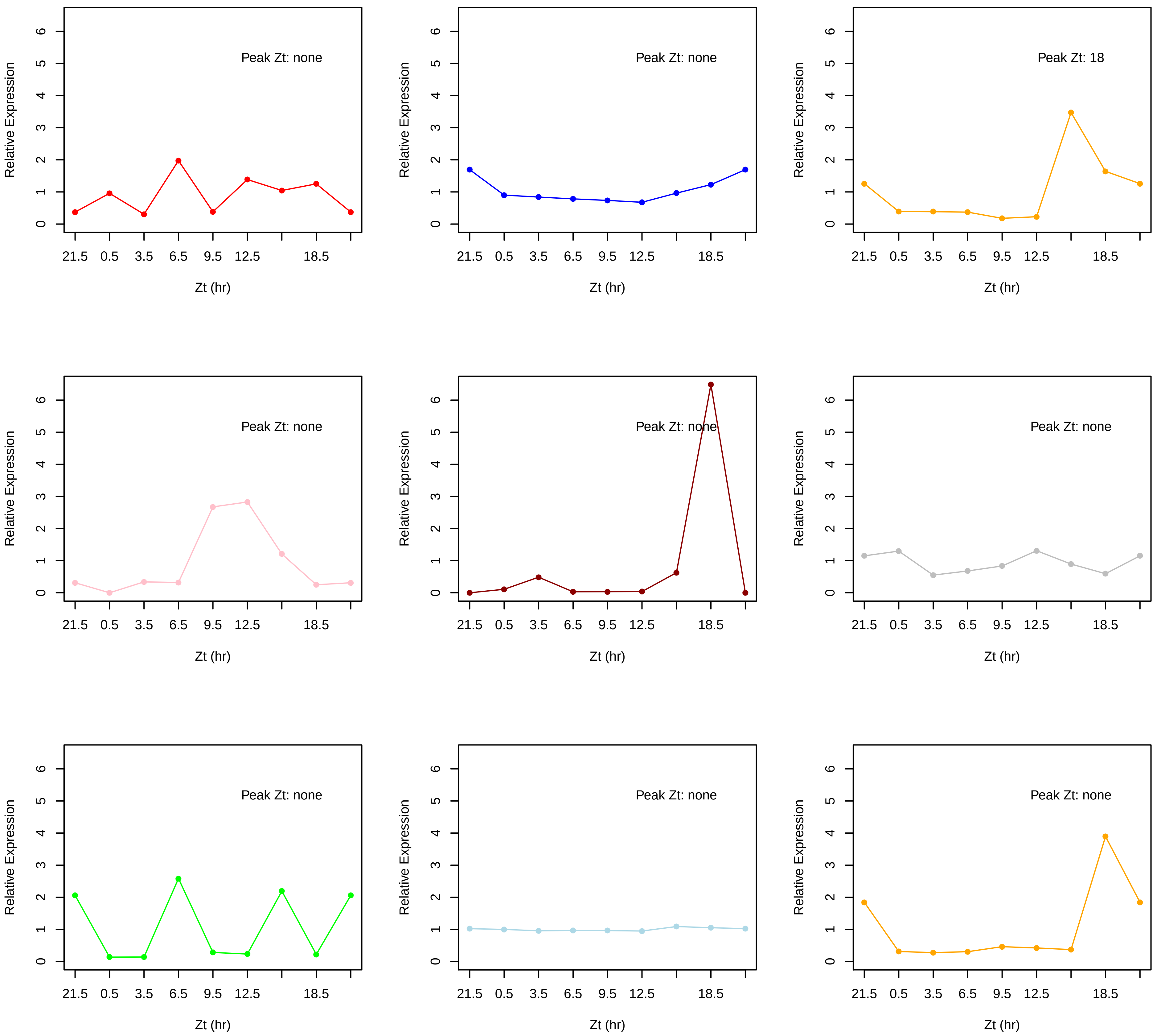

Singeleton: ENSXMAG00000011540 ptpn1

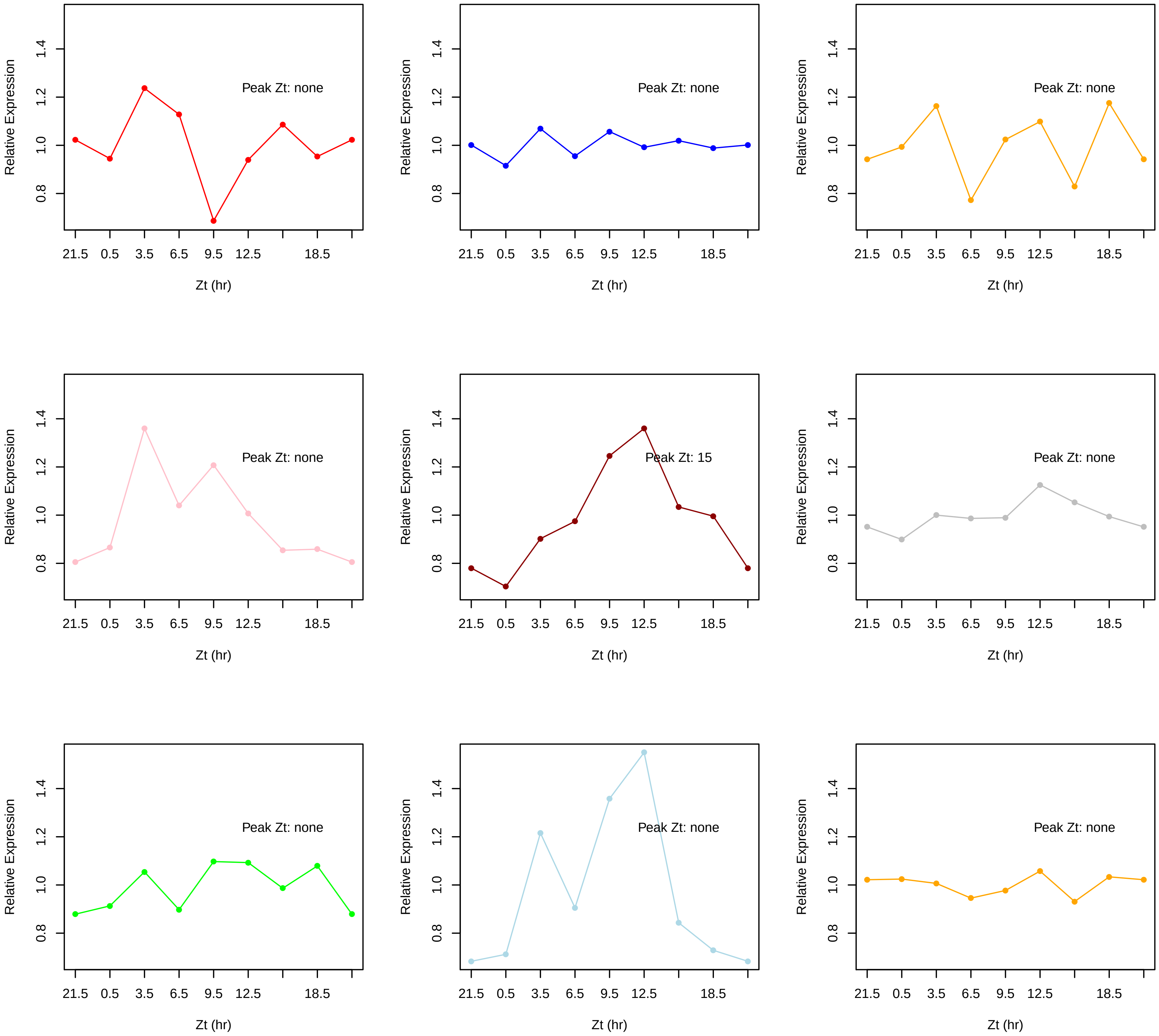

Singleton: ENSXMAG00000005374 si:dkey−246g23.4

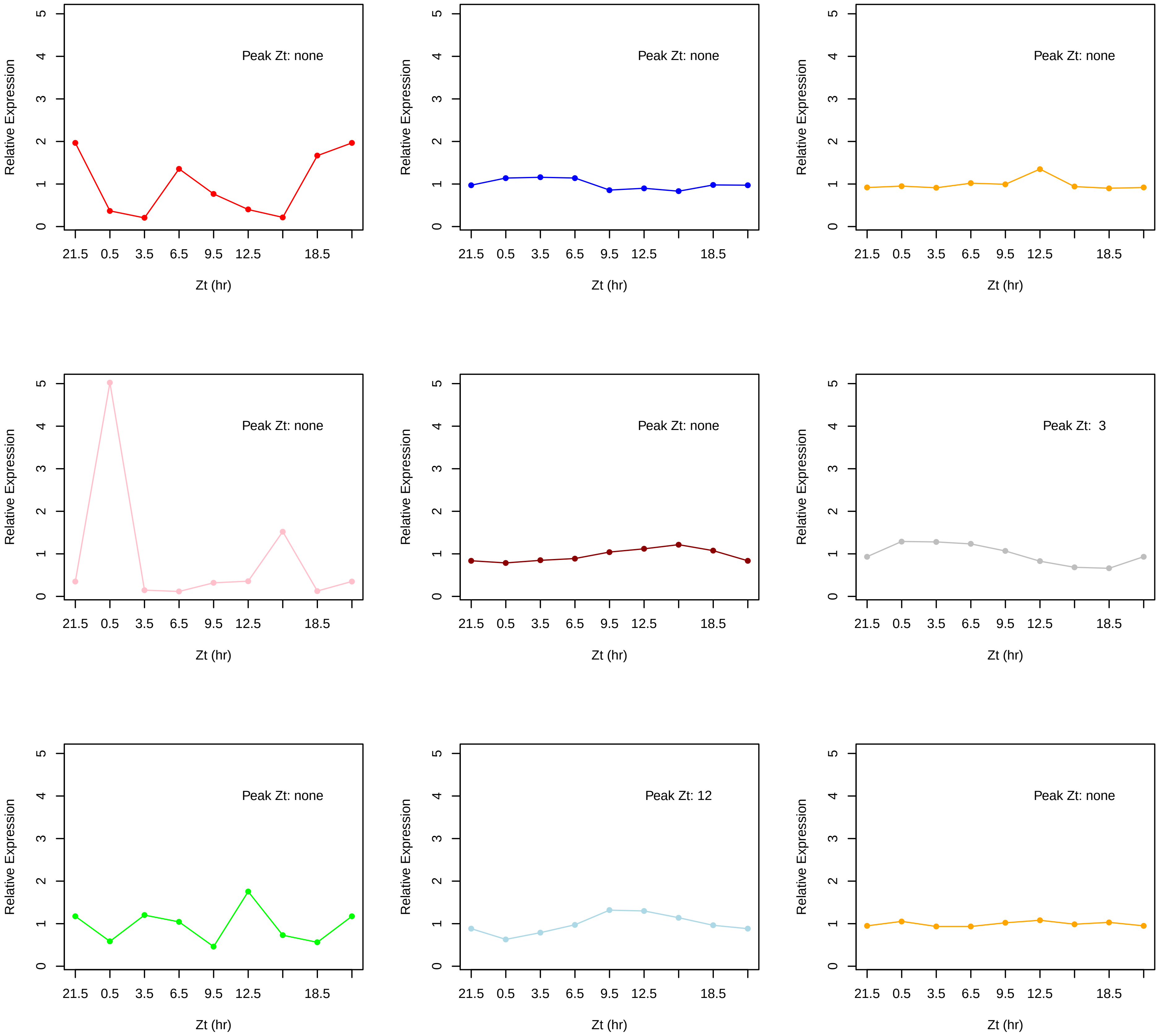

Singeleton: ENSXMAG00000018345 cop1

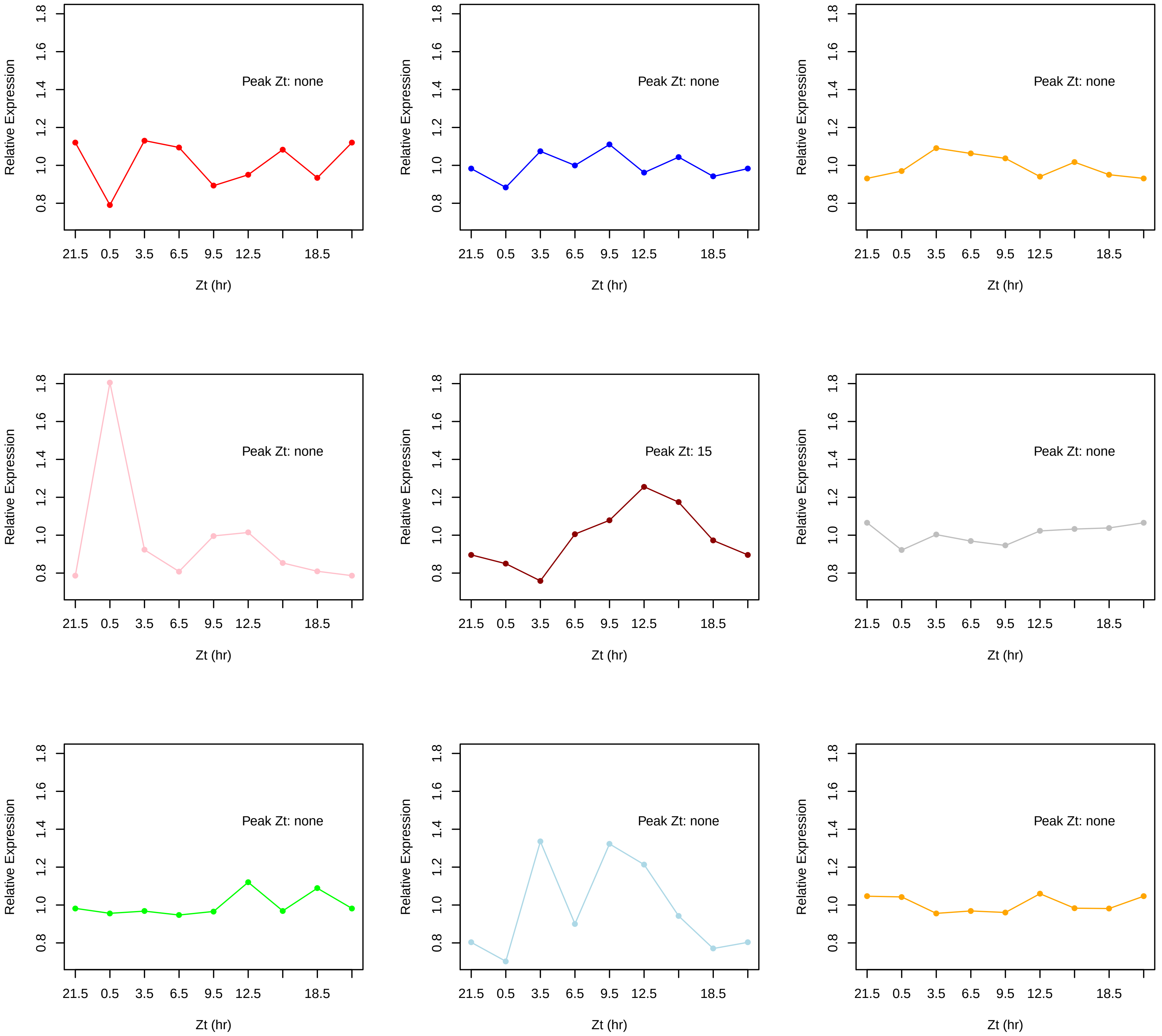

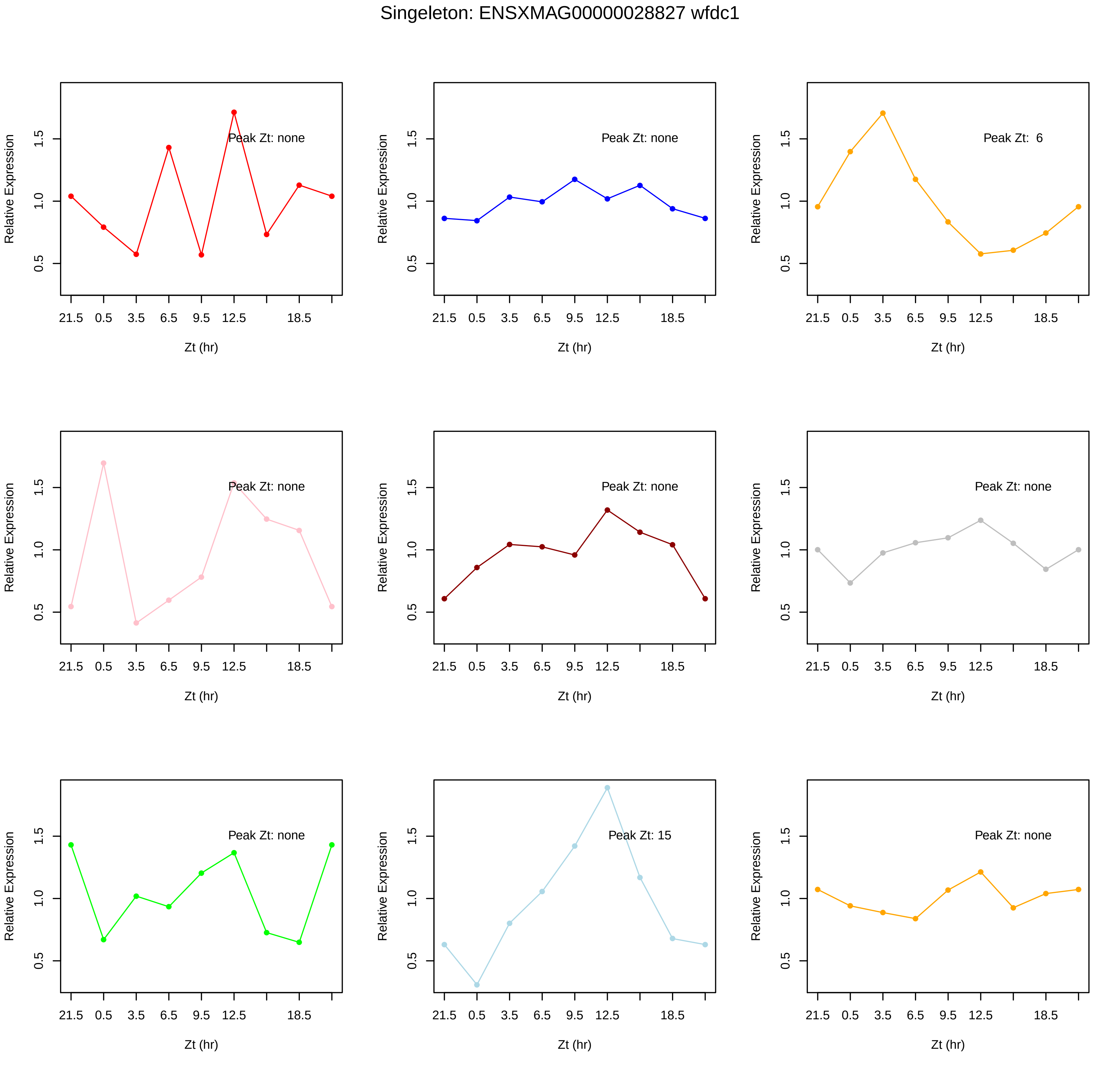
Singleton: ENSXMAG00000028827 wfdc1
Relative Expression
Zt (hr)
21.5
0.5
3.5
6.5
9.5
12.5
18.5
0.5
1.0
1.5
Peak Zt: none
Peak Zt: none
Peak Zt: 6
Peak Zt: none
Peak Zt: none
Peak Zt: none
Peak Zt: none
Peak Zt: 15
Peak Zt: none

Singeleton: ENSXMAG00000000861 slc15a2

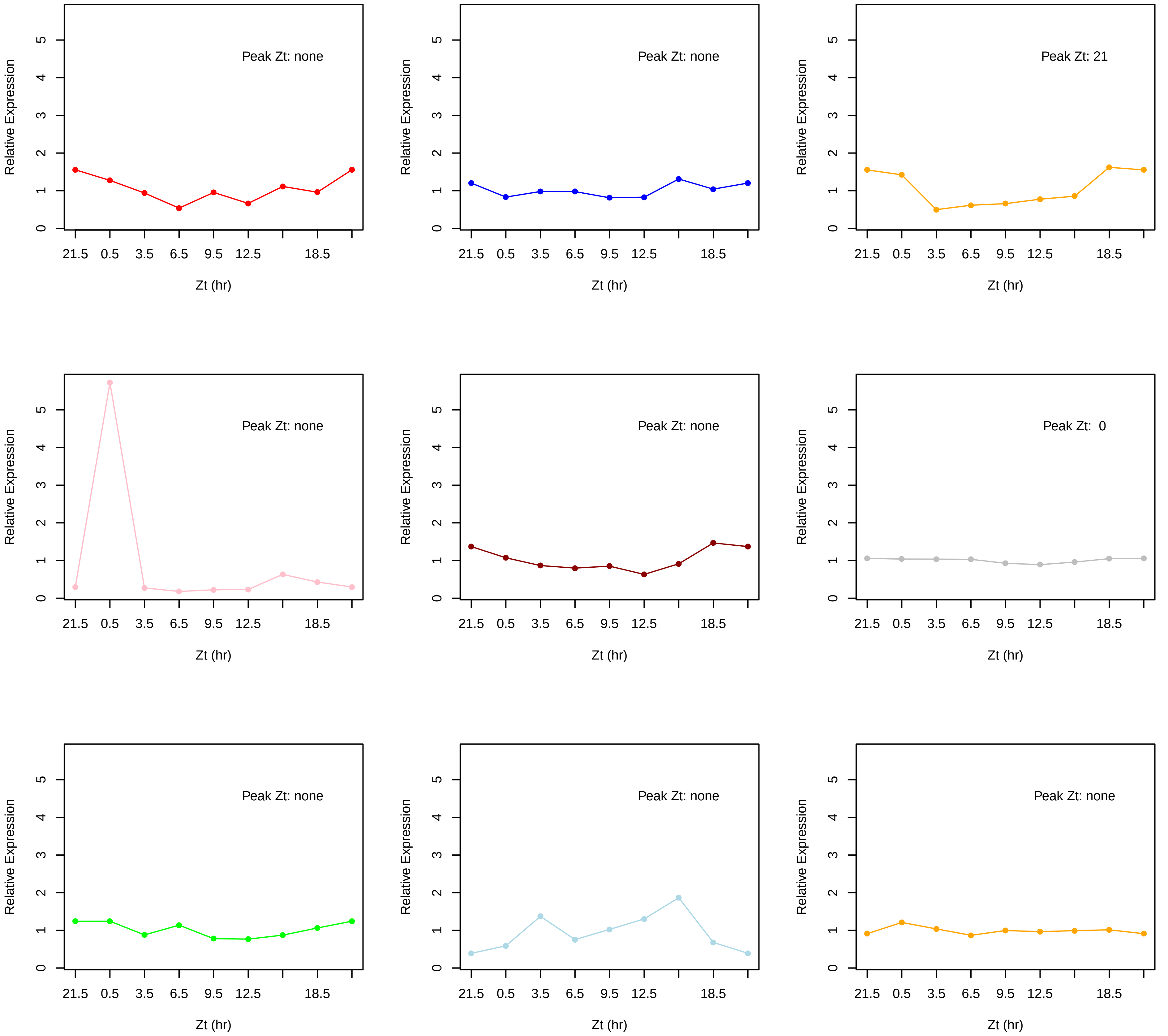

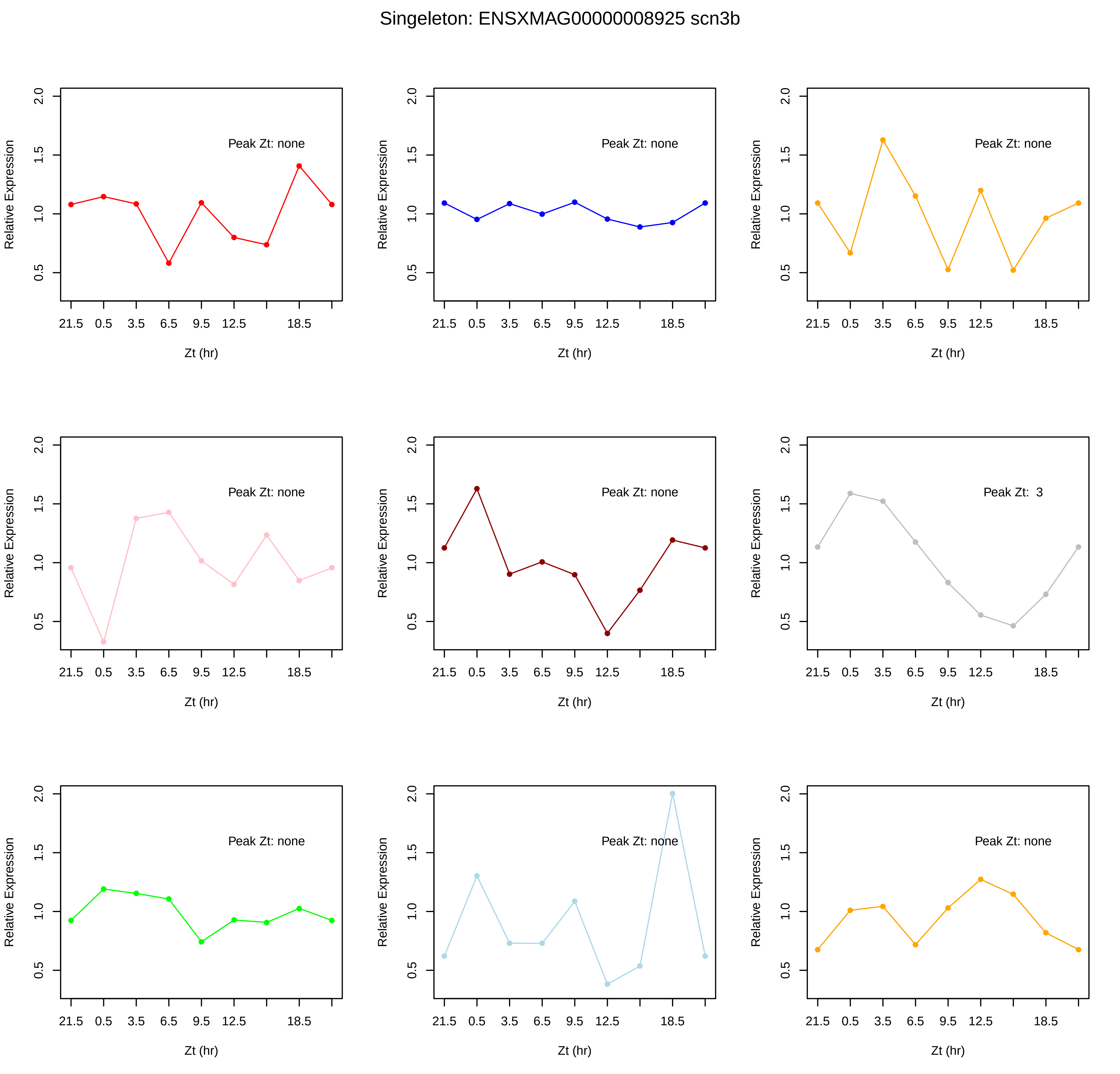
Singeleton: ENSXMAG00000008925 scn3b
Relative Expression
Zt (hr)
21.5
0.5
3.5
6.5
9.5
12.5
18.5
0.5
1.0
1.5
2.0
Peak Zt: none
Peak Zt: 3

Singeleton: ENSXMAG00000012587 c1qtnf12

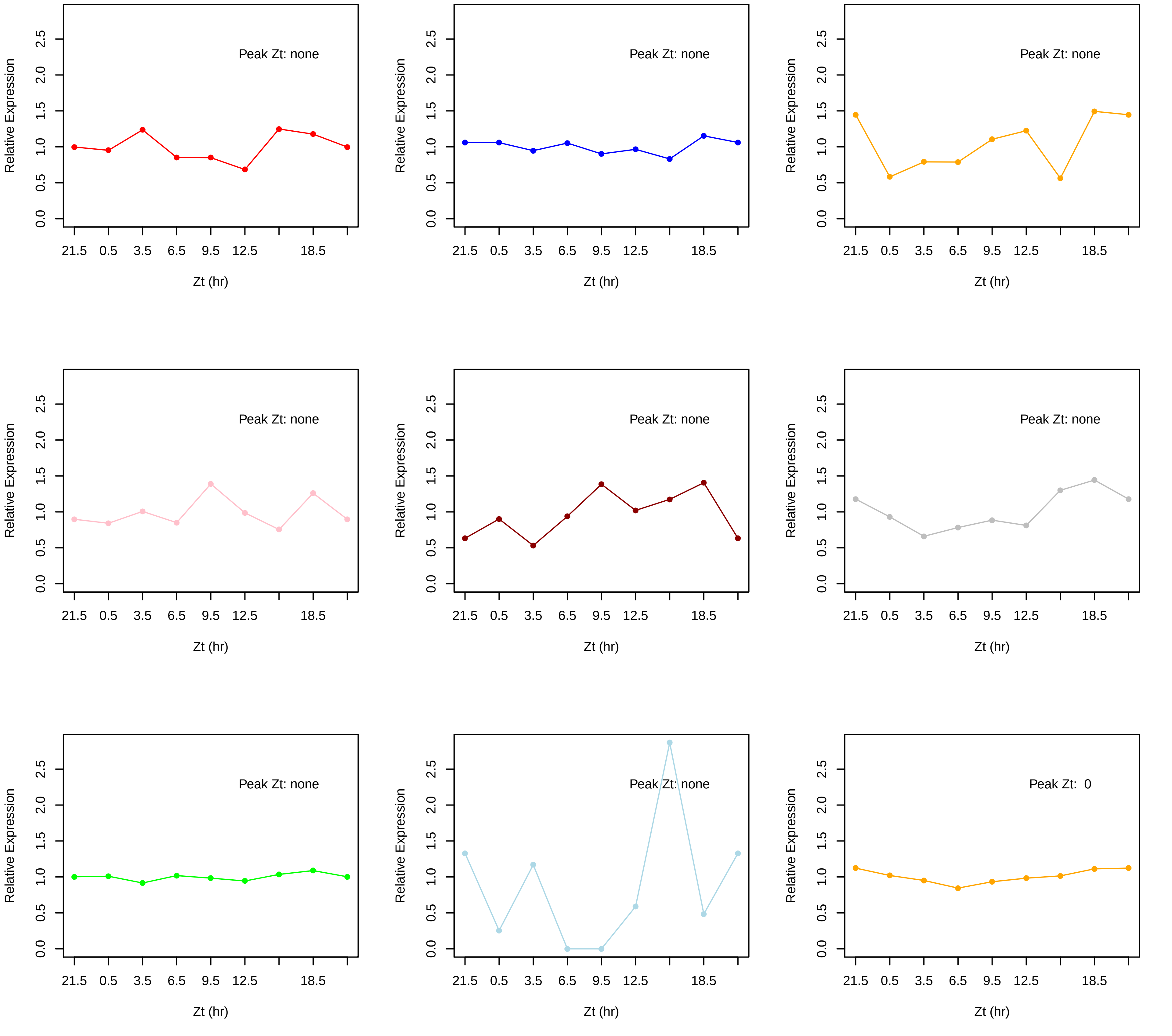

Singleton: ENSXMAG00000021783 rbms3

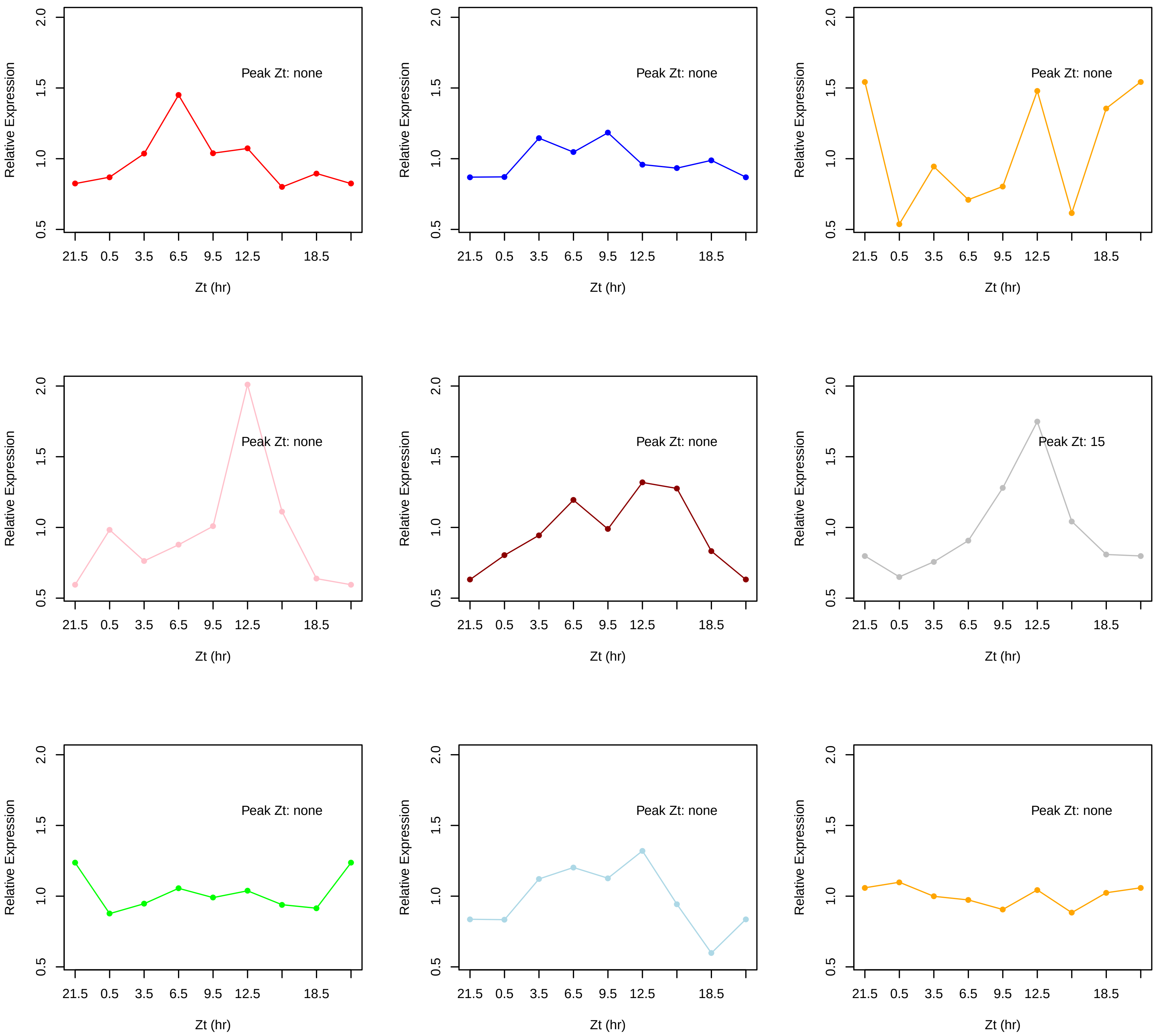

Singeleton: ENSXMAG00000011433 CXorf38

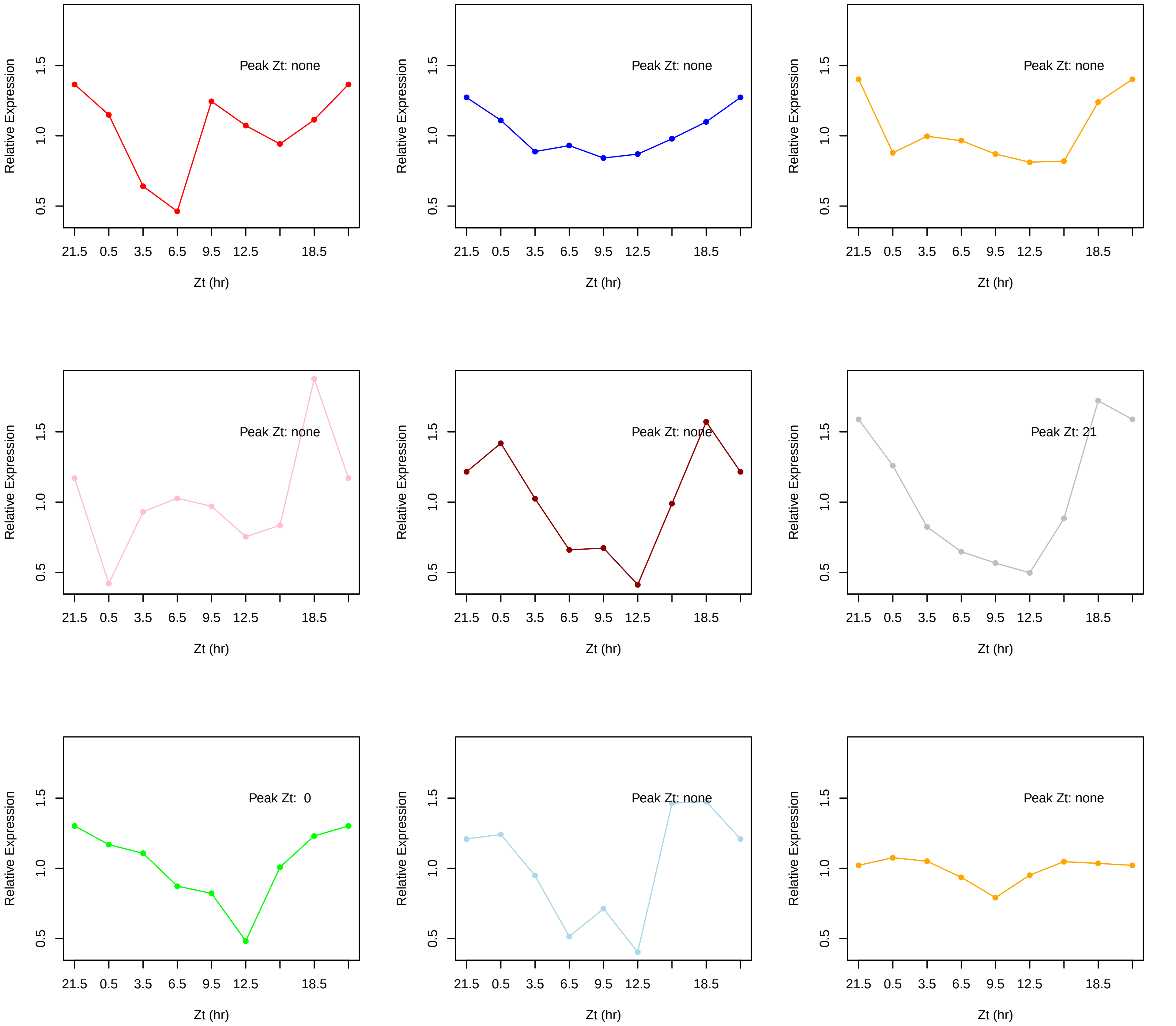

Singeleton: ENSXMAG00000012579 sdf4

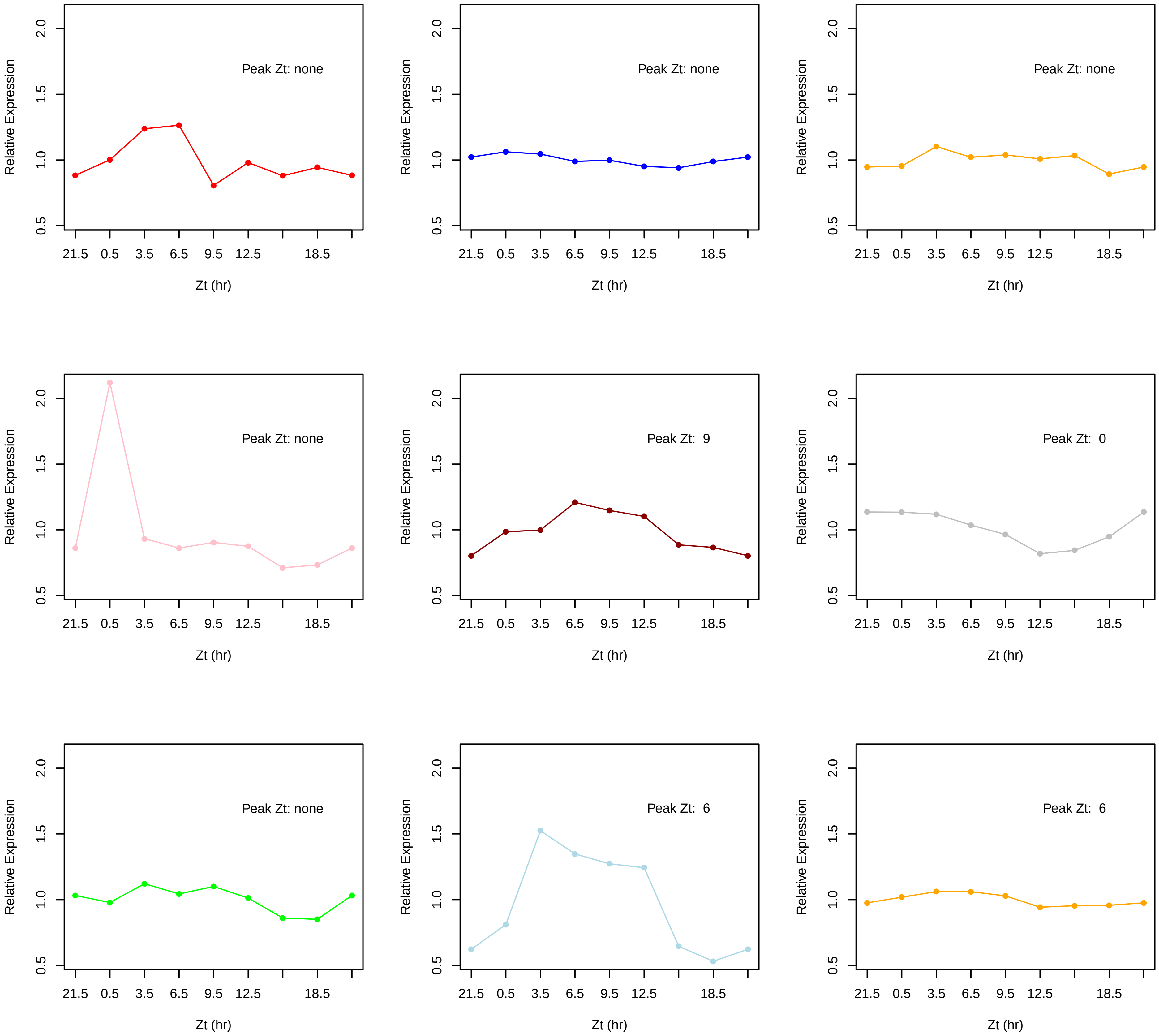

Singleton: ENSXMAG00000000977 gramd1bb

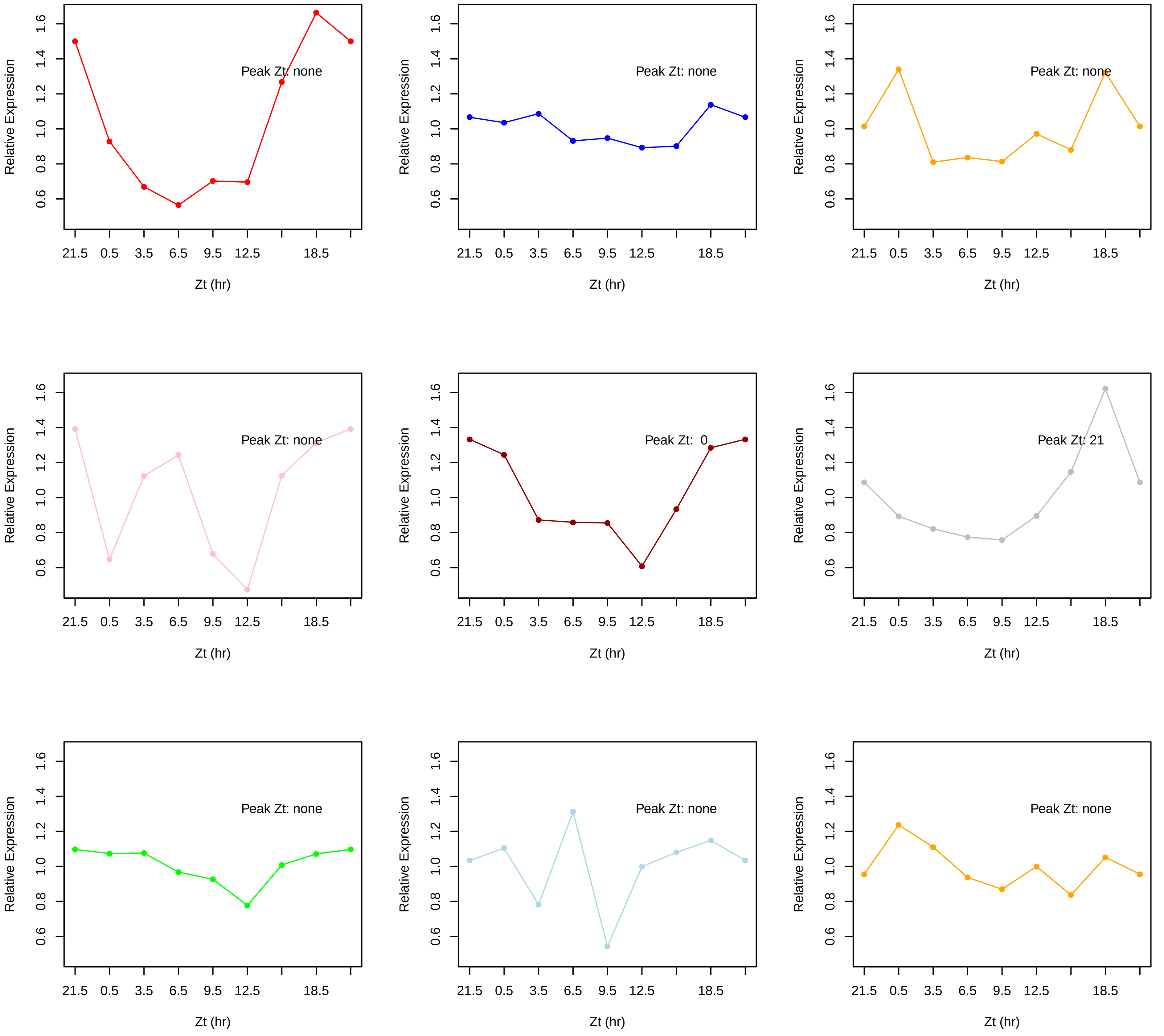

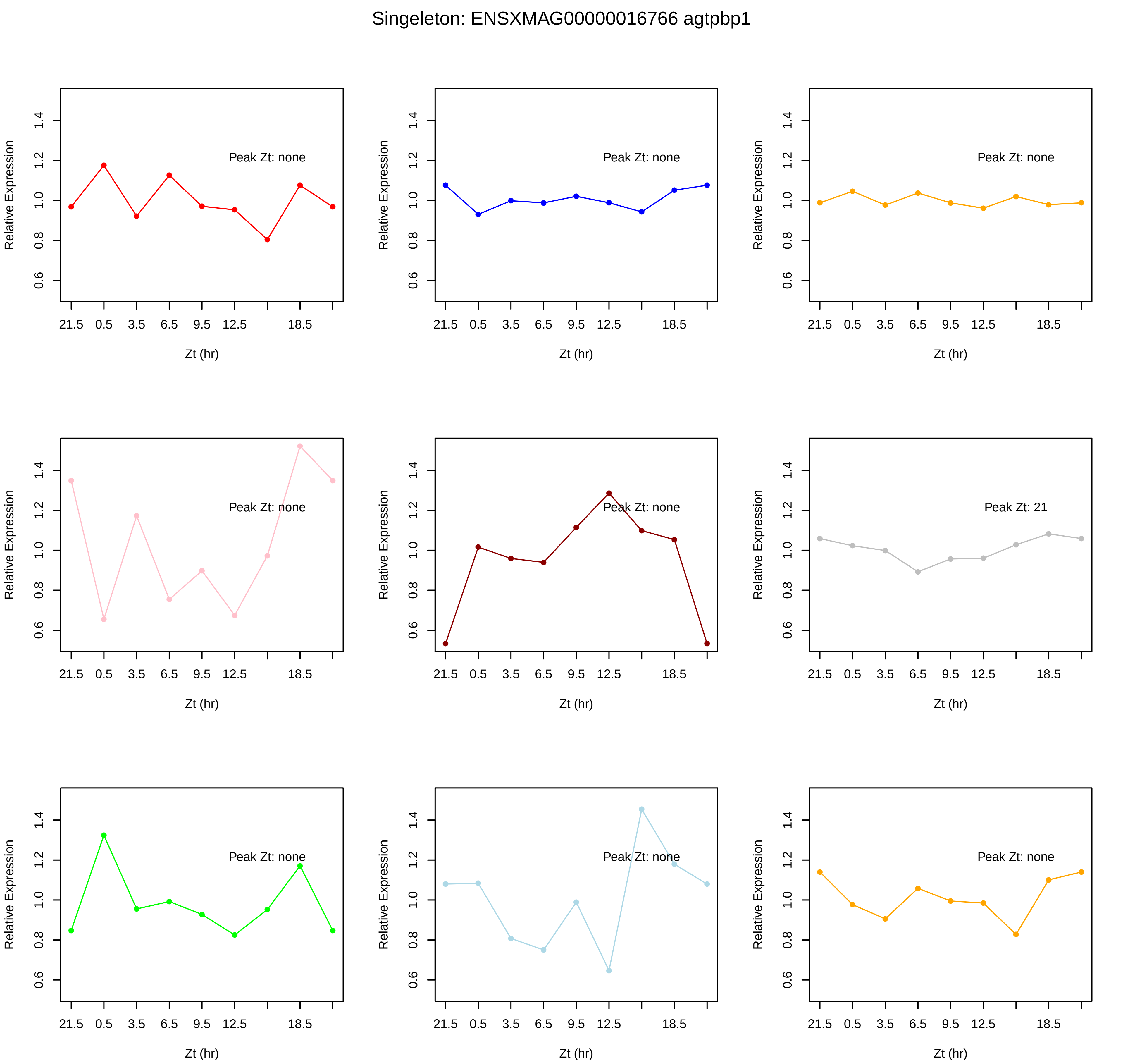
Singeleton: ENSXMAG00000016766 agtpbp1
Relative Expression
Zt (hr)
21.5 0.5 3.5 6.5 9.5 12.5 18.5
0.6 0.8 1.0 1.2 1.4
Peak Zt: none
Peak Zt: none
Peak Zt: none
Peak Zt: none
Peak Zt: none
Peak Zt: 21
Peak Zt: none
Peak Zt: none
Peak Zt: none

Singeleton: ENSXMAG00000002617 ctdspl3

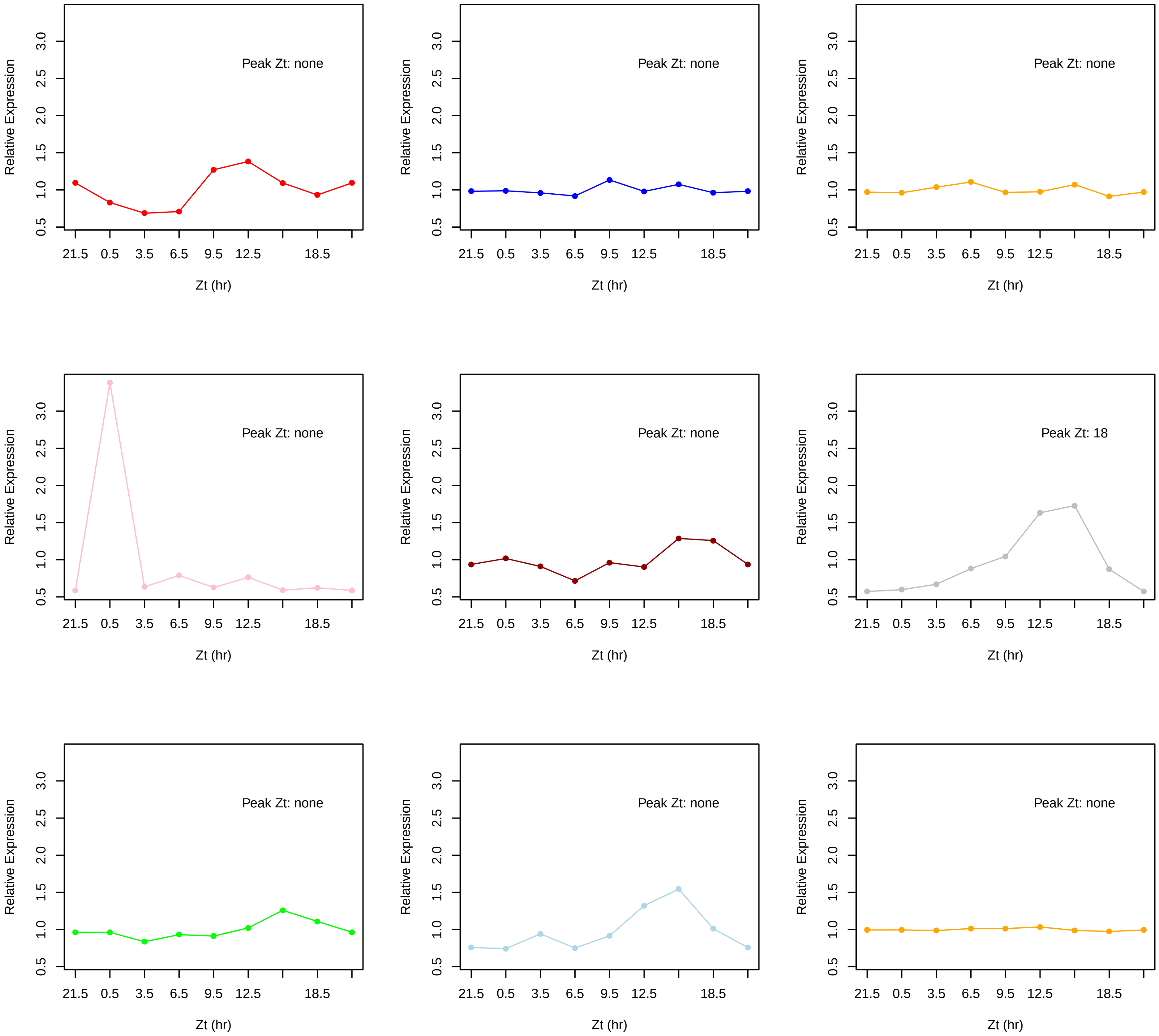

Singeleton: ENSXMAG00000014311 azi2

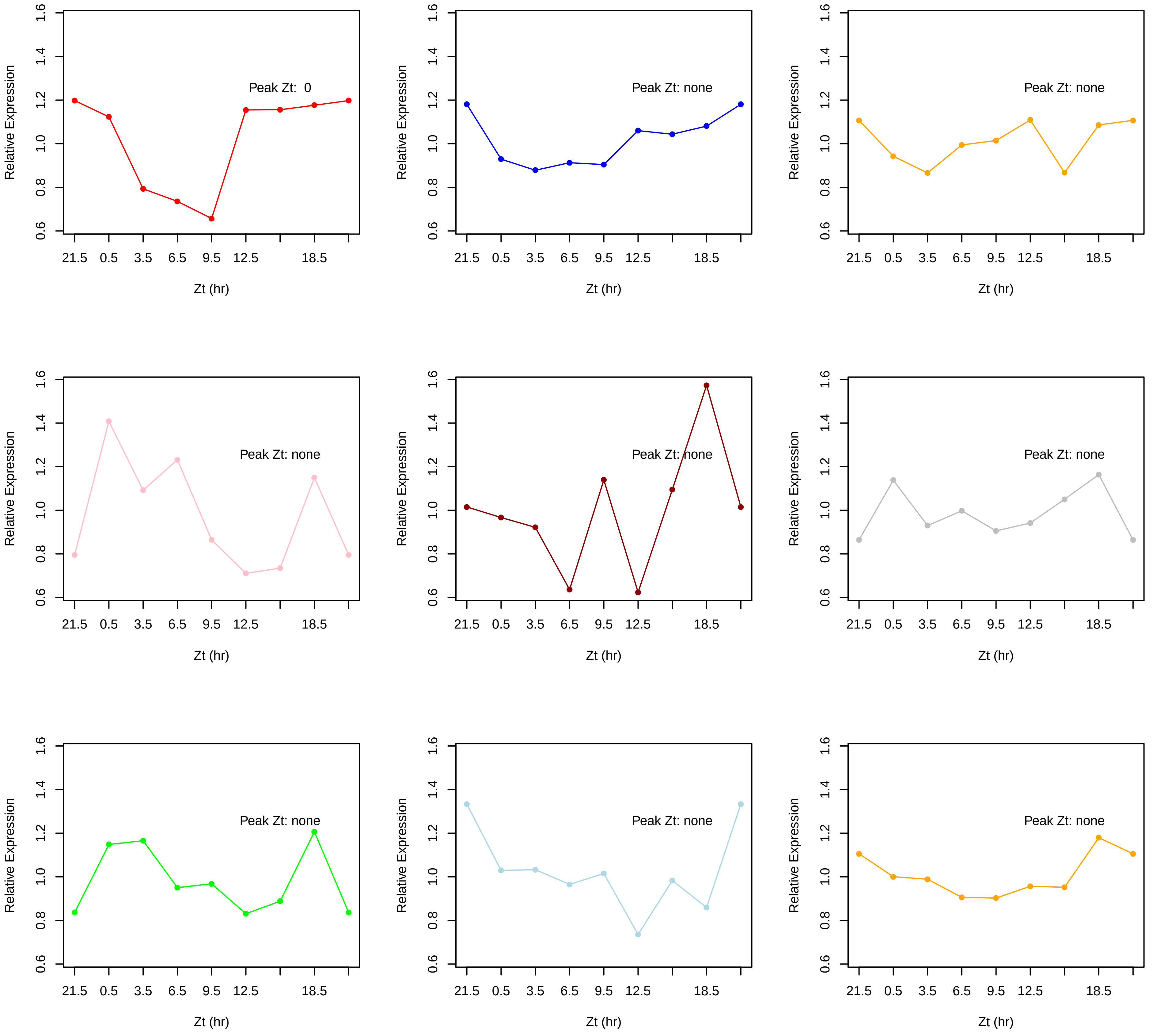

Singeleton: ENSXMAG00000014407

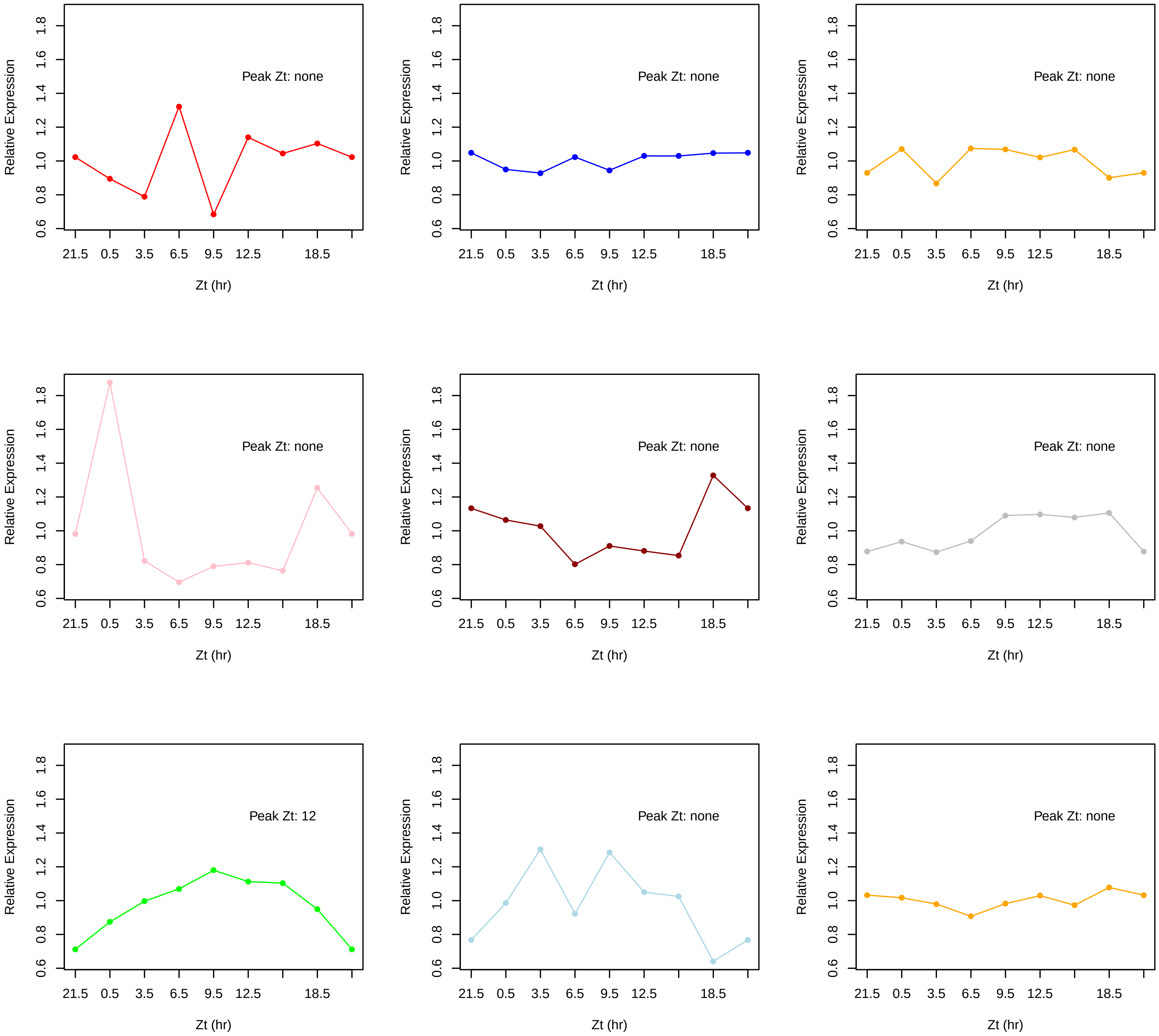

Singeleton: ENSXMAG00000015856 tnr

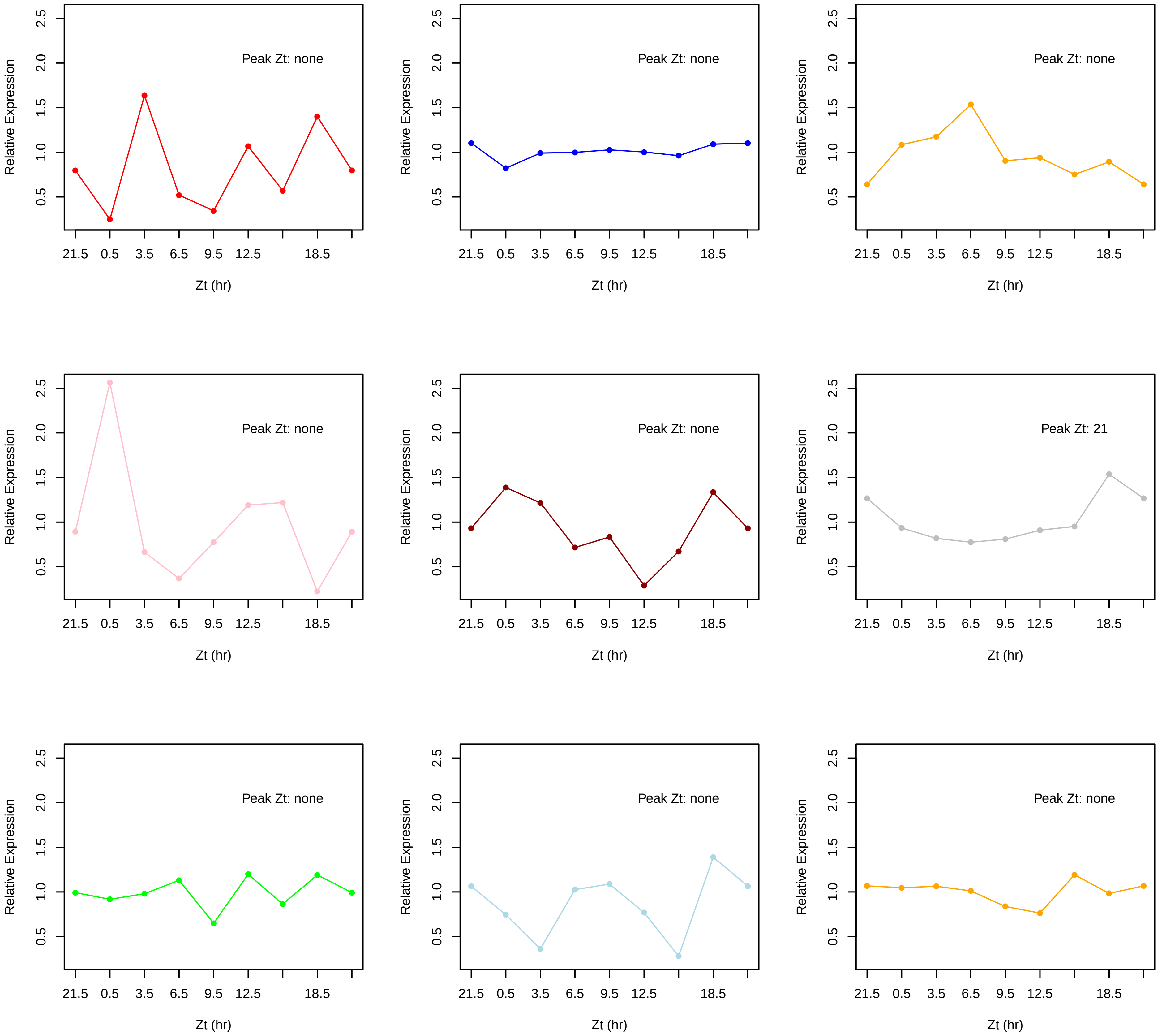

Singeleton: ENSXMAG00000002370 tbx1

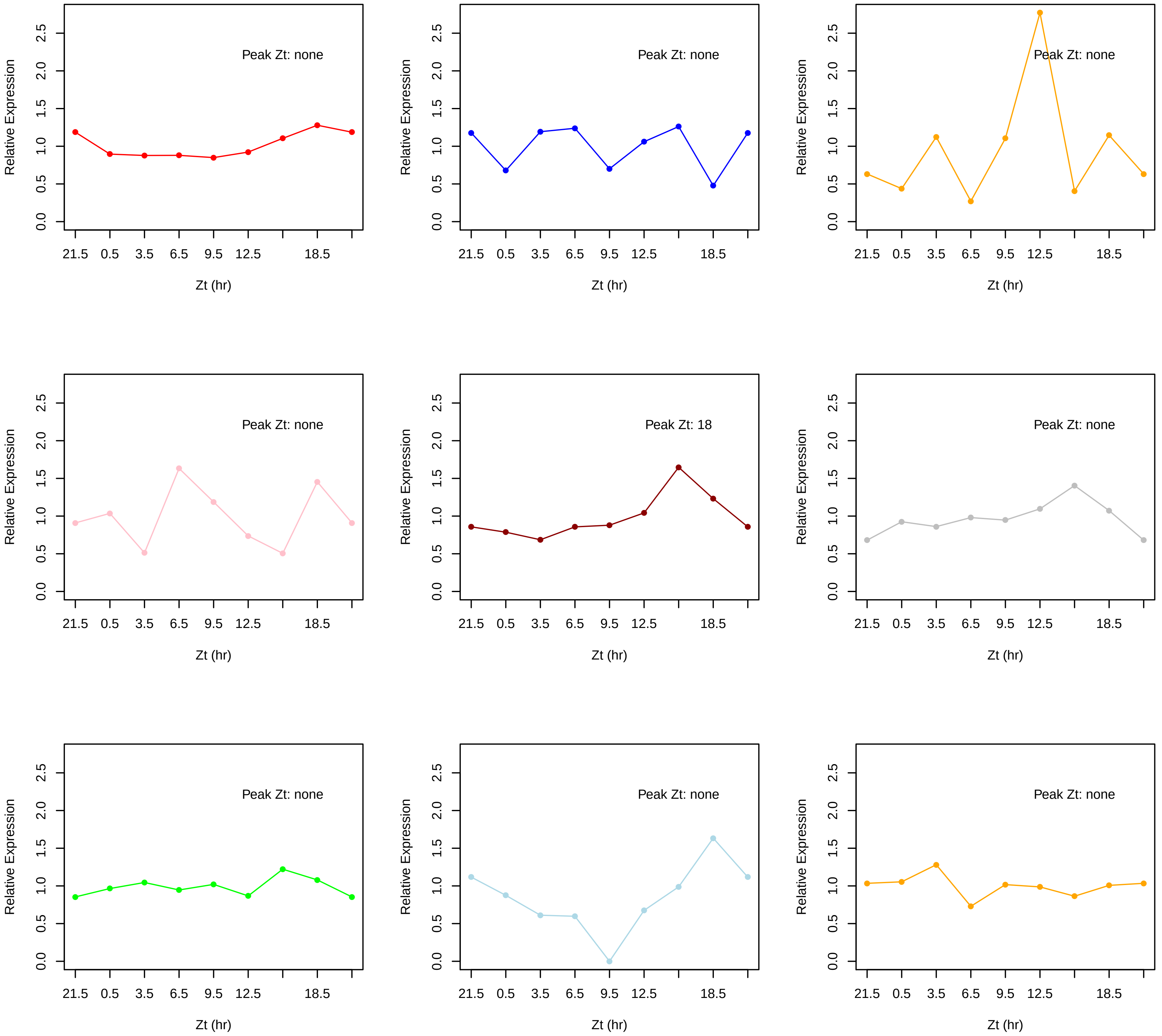

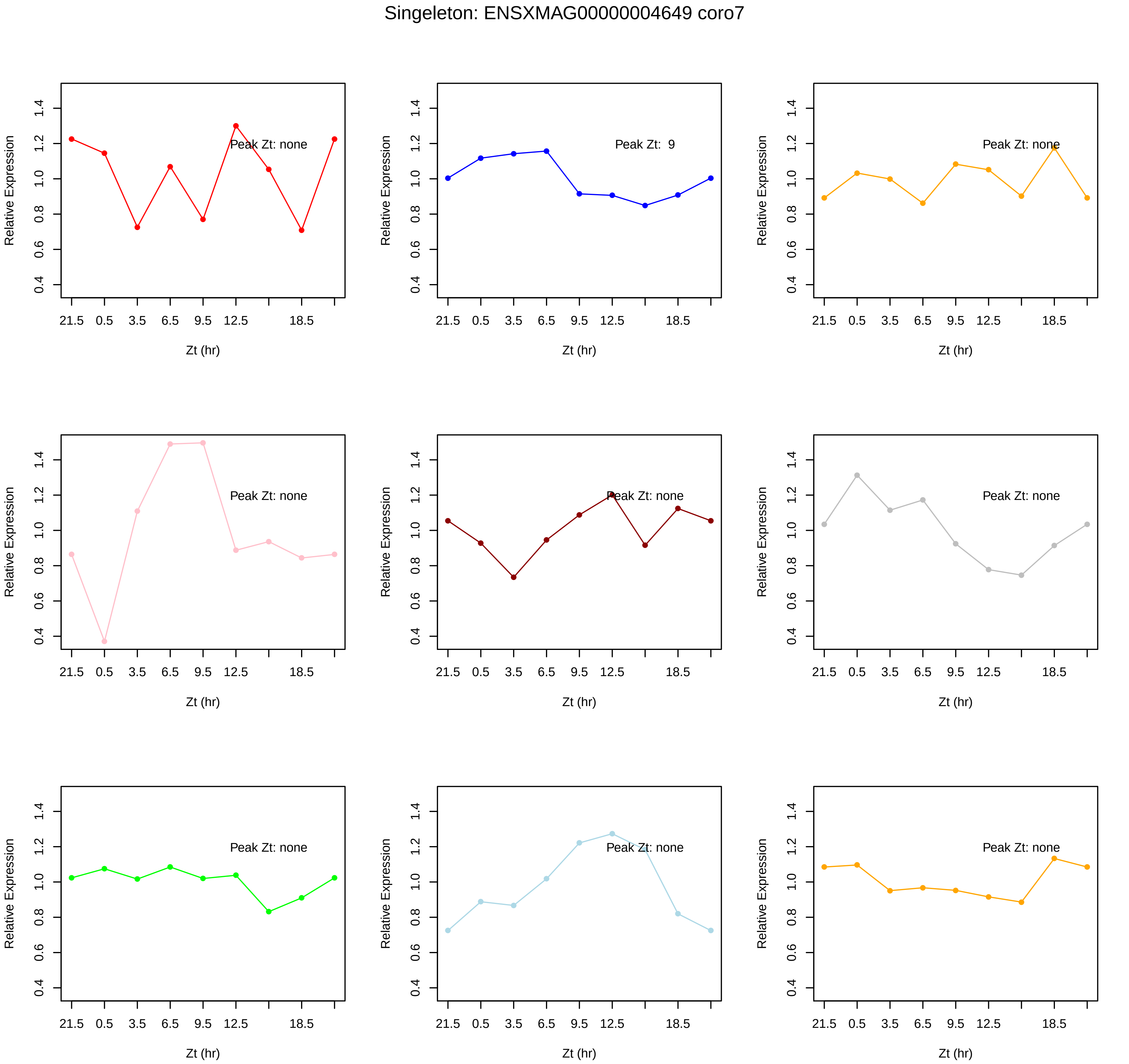
Singleton: ENSXMAG00000004649 coro7
Peak Zt: none
Peak Zt: 9
Peak Zt: none
Peak Zt: none
Peak Zt: none
Peak Zt: none
Peak Zt: none
Peak Zt: none
Peak Zt: none
Relative Expression
Zt (hr)
21.5 0.5 3.5 6.5 9.5 12.5 18.5
0.4 0.6 0.8 1.0 1.2 1.4

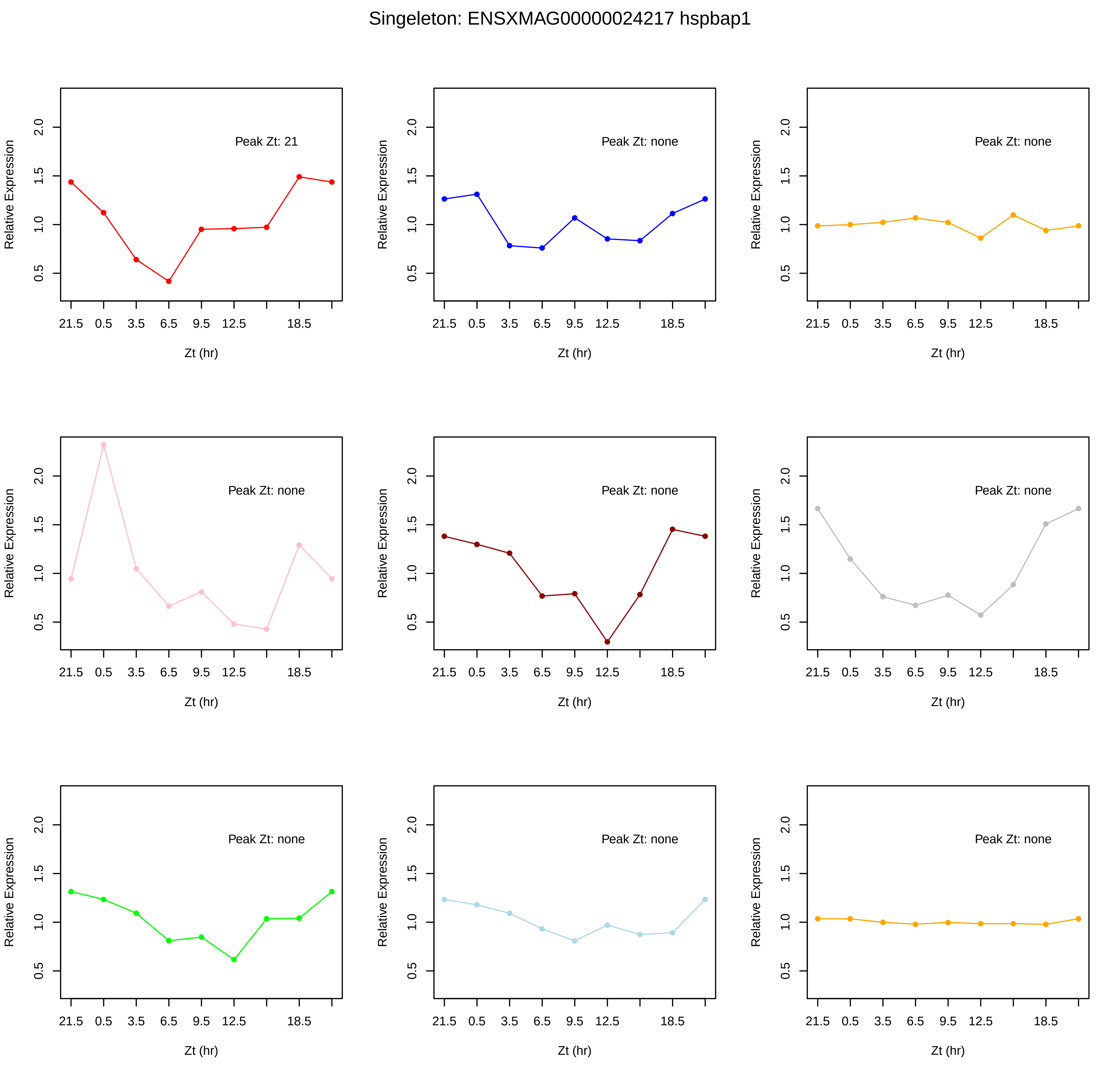
Singeleton: ENSXMAG00000024217 hspbap1
Peak Zt: 21
Peak Zt: none
Peak Zt: none
Peak Zt: none
Peak Zt: none
Peak Zt: none
Peak Zt: none
Peak Zt: none
Peak Zt: none
Relative Expression
Zt (hr)

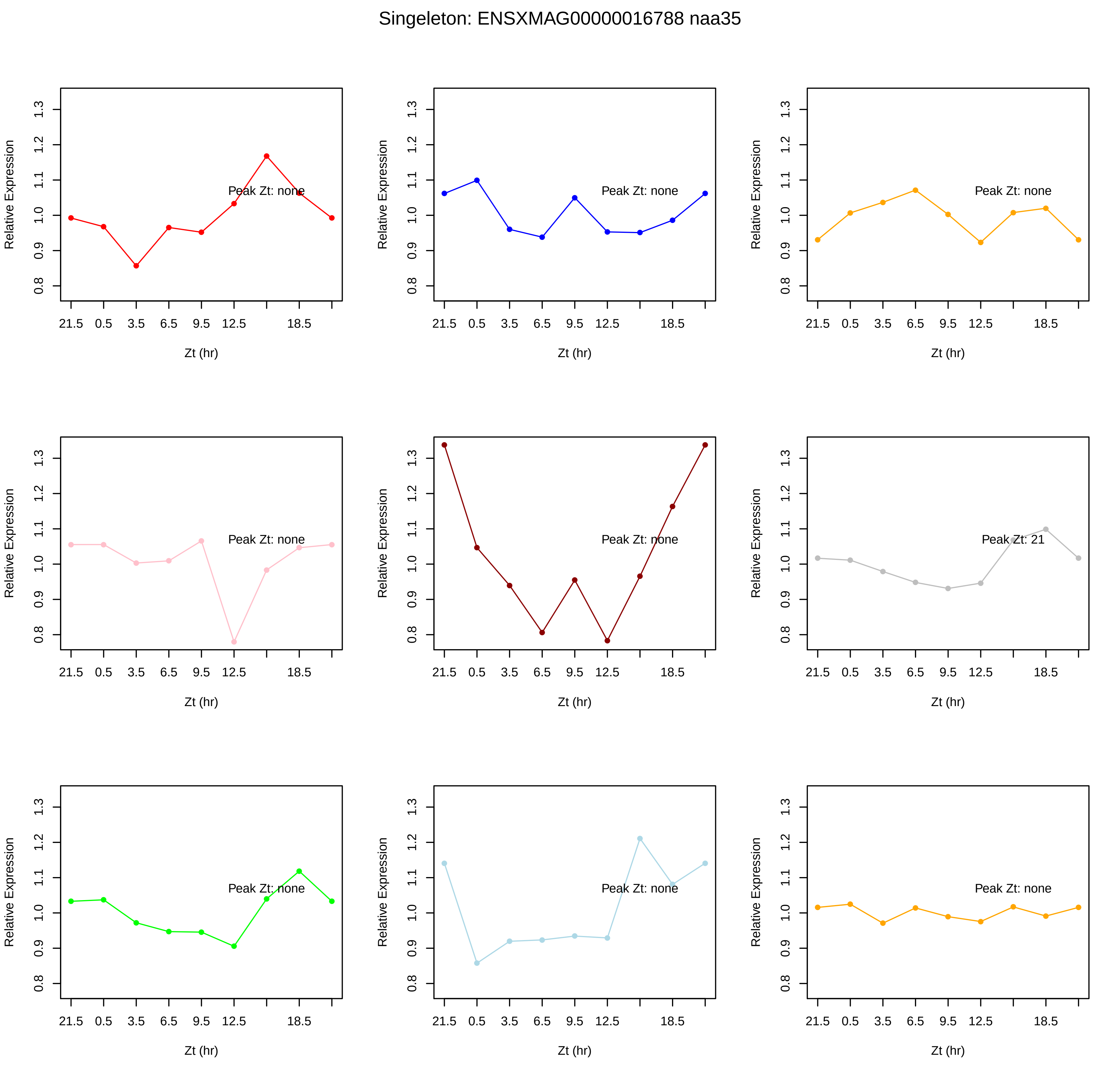
Singleton: ENSXMAG00000016788 naa35
Relative Expression
Zt (hr)
Peak Zt: none
Peak Zt: 21
21.5
0.5
3.5
6.5
9.5
12.5
18.5
0.8
0.9
1.0
1.1
1.2
1.3

Singeleton: ENSXMAG00000015195 ddr2l

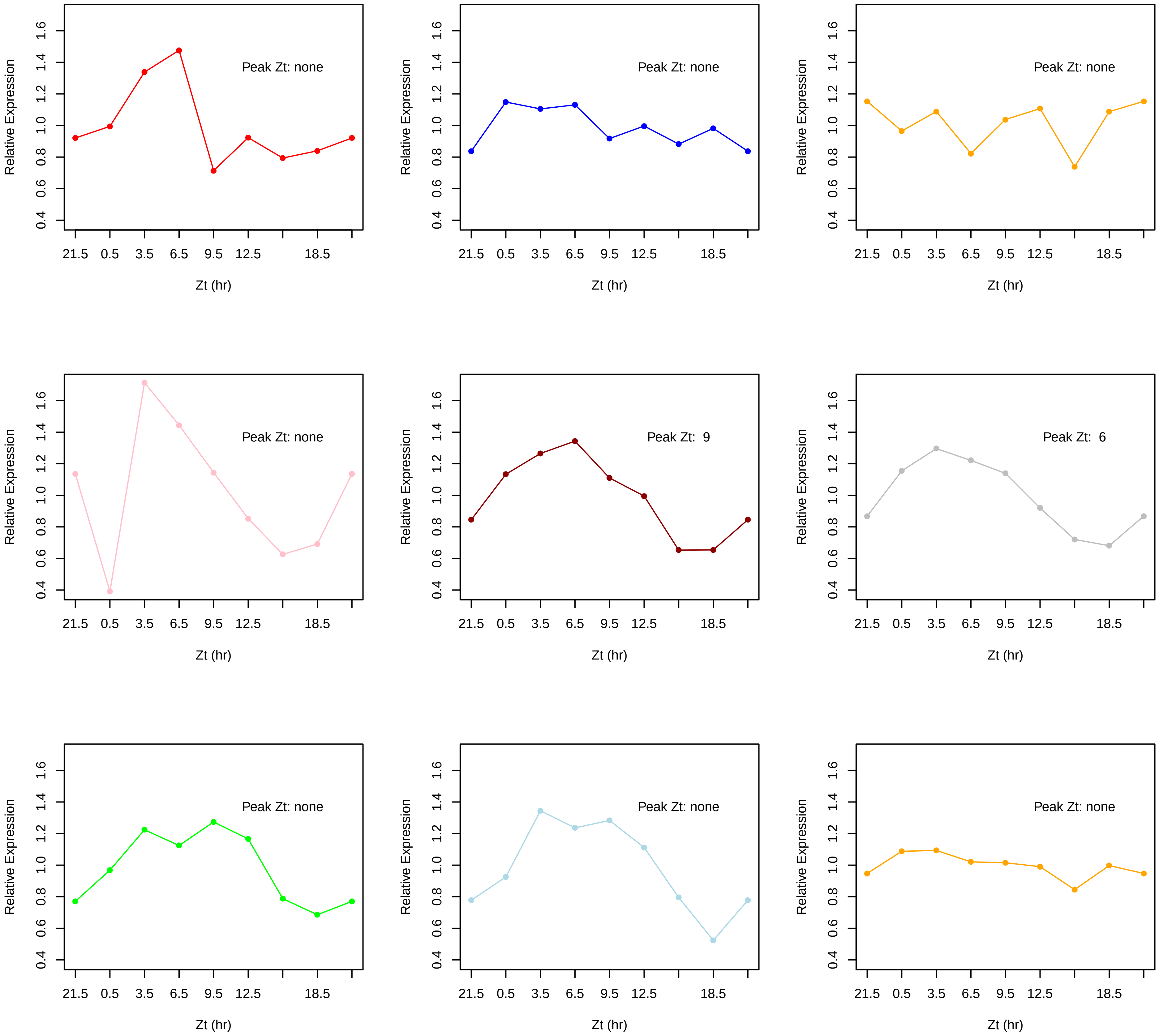

Singeleton: ENSXMAG00000002619 arid3a

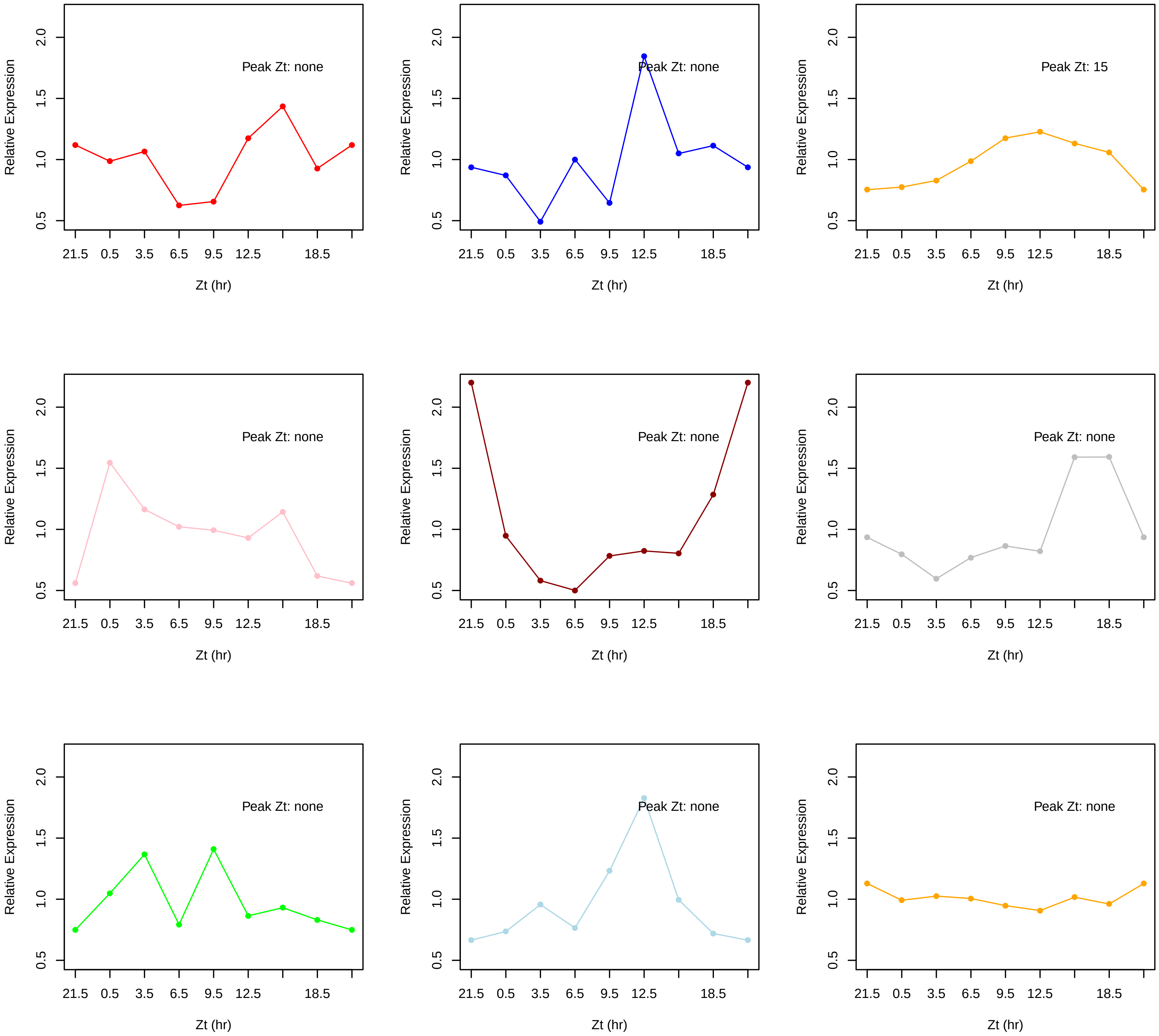

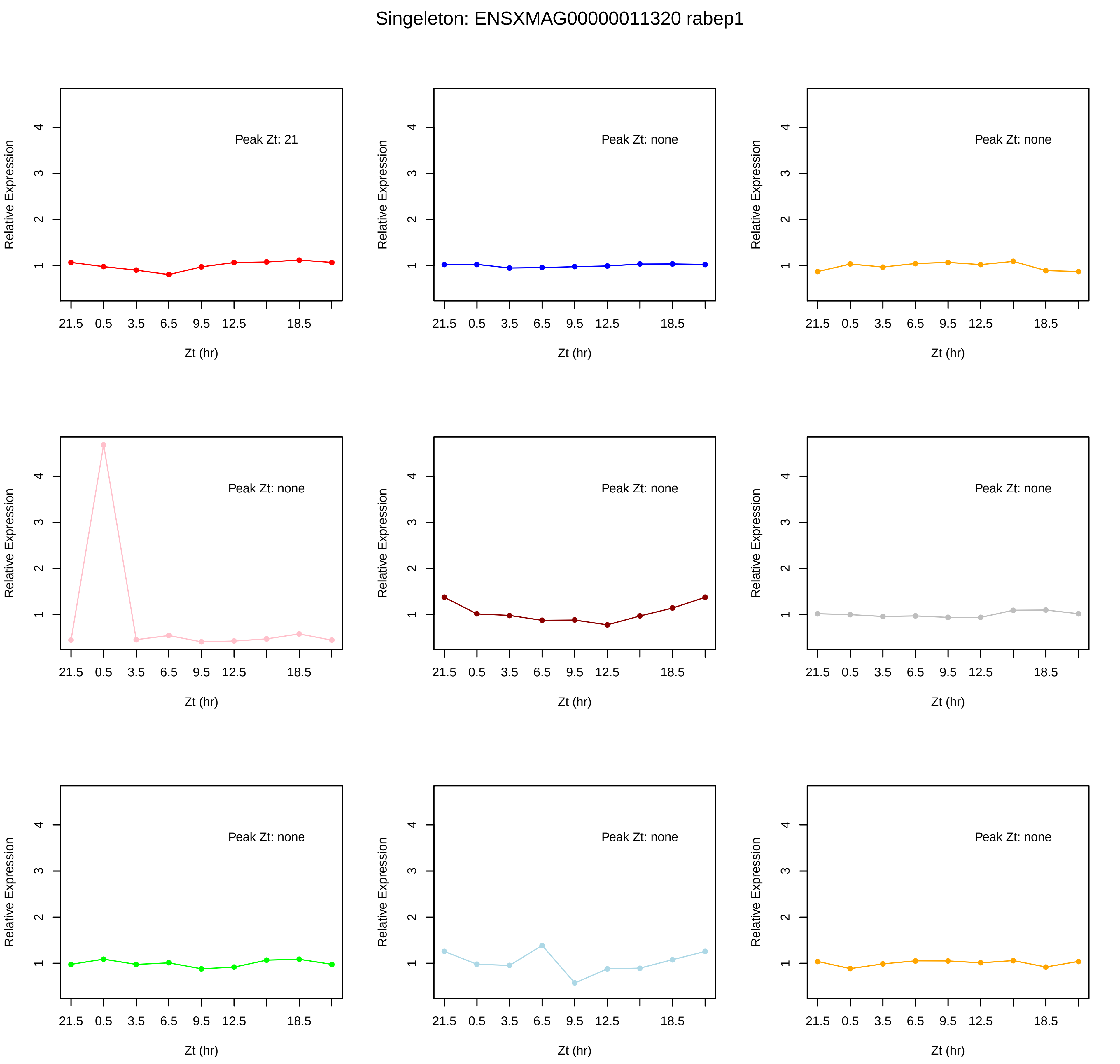
Singeleton: ENSXMAG00000011320 rabep1
Peak Zt: 21
Peak Zt: none
Peak Zt: none
Peak Zt: none
Peak Zt: none
Peak Zt: none
Peak Zt: none
Peak Zt: none
Peak Zt: none
Relative Expression
Zt (hr)
21.5
0.5
3.5
6.5
9.5
12.5
18.5

Singeleton: ENSXMAG00000021125 smfn

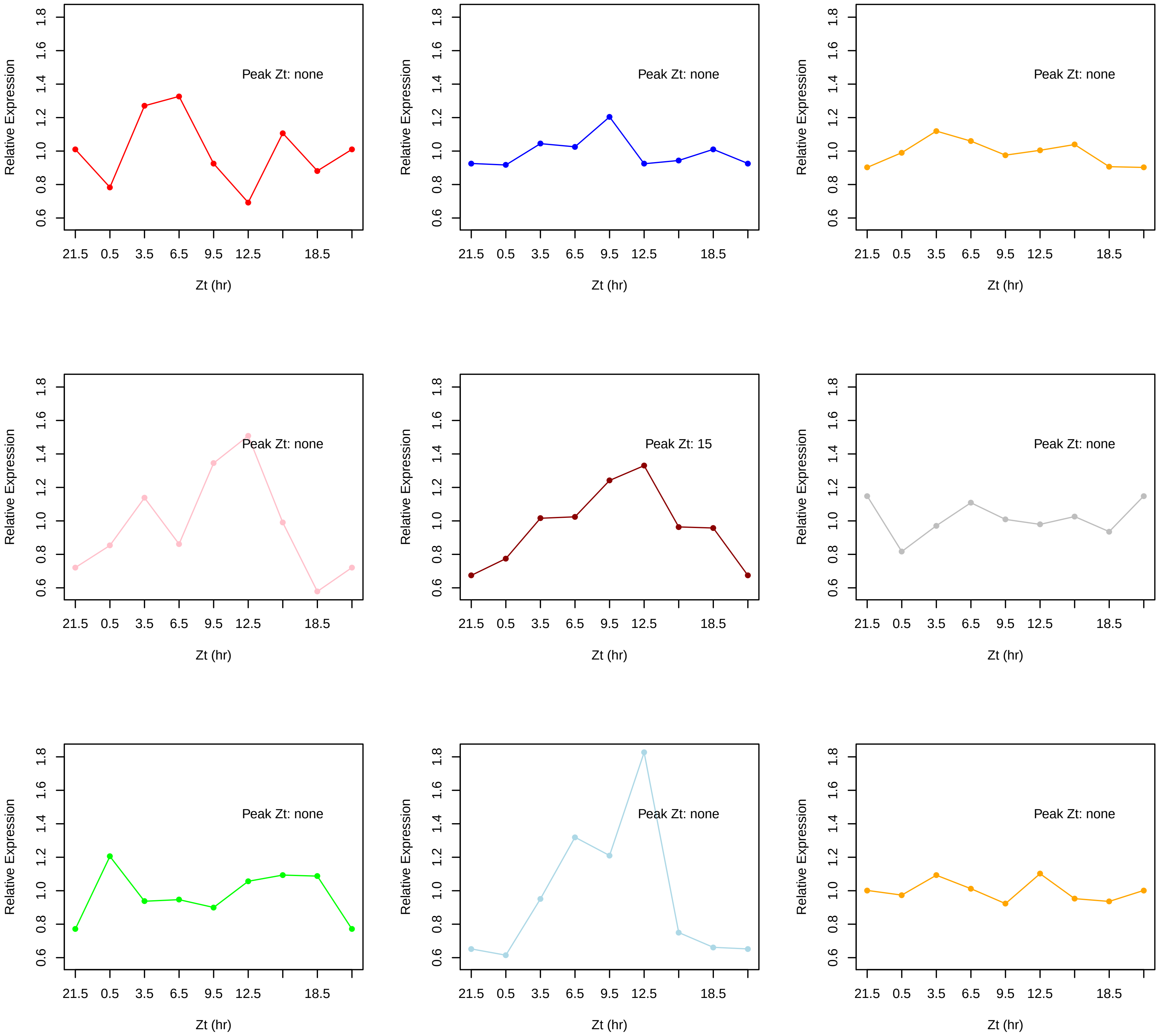

Singeleton: ENSXMAG00000024976 ATP9A

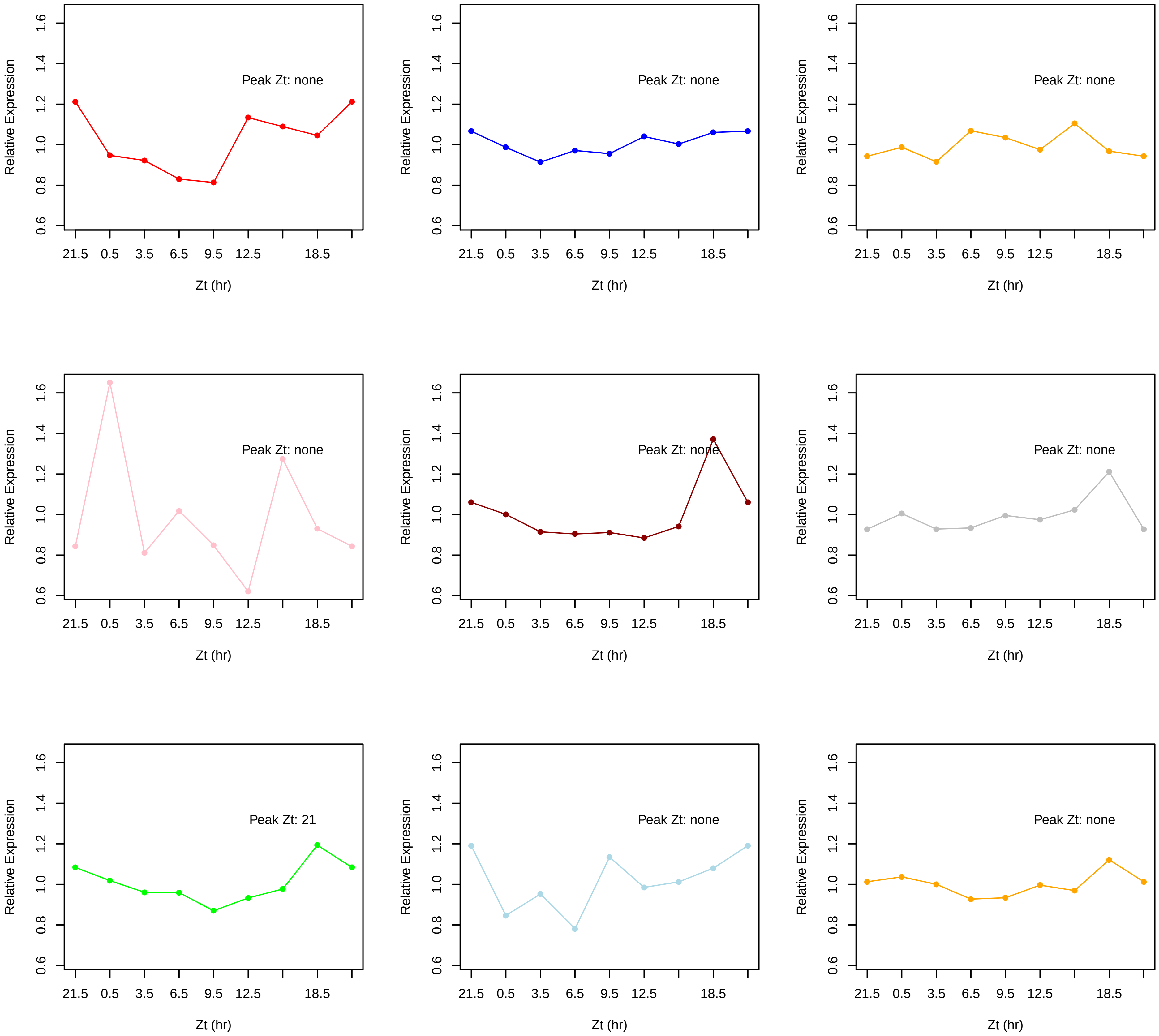

Singeleton: ENSXMAG00000006148 tango6

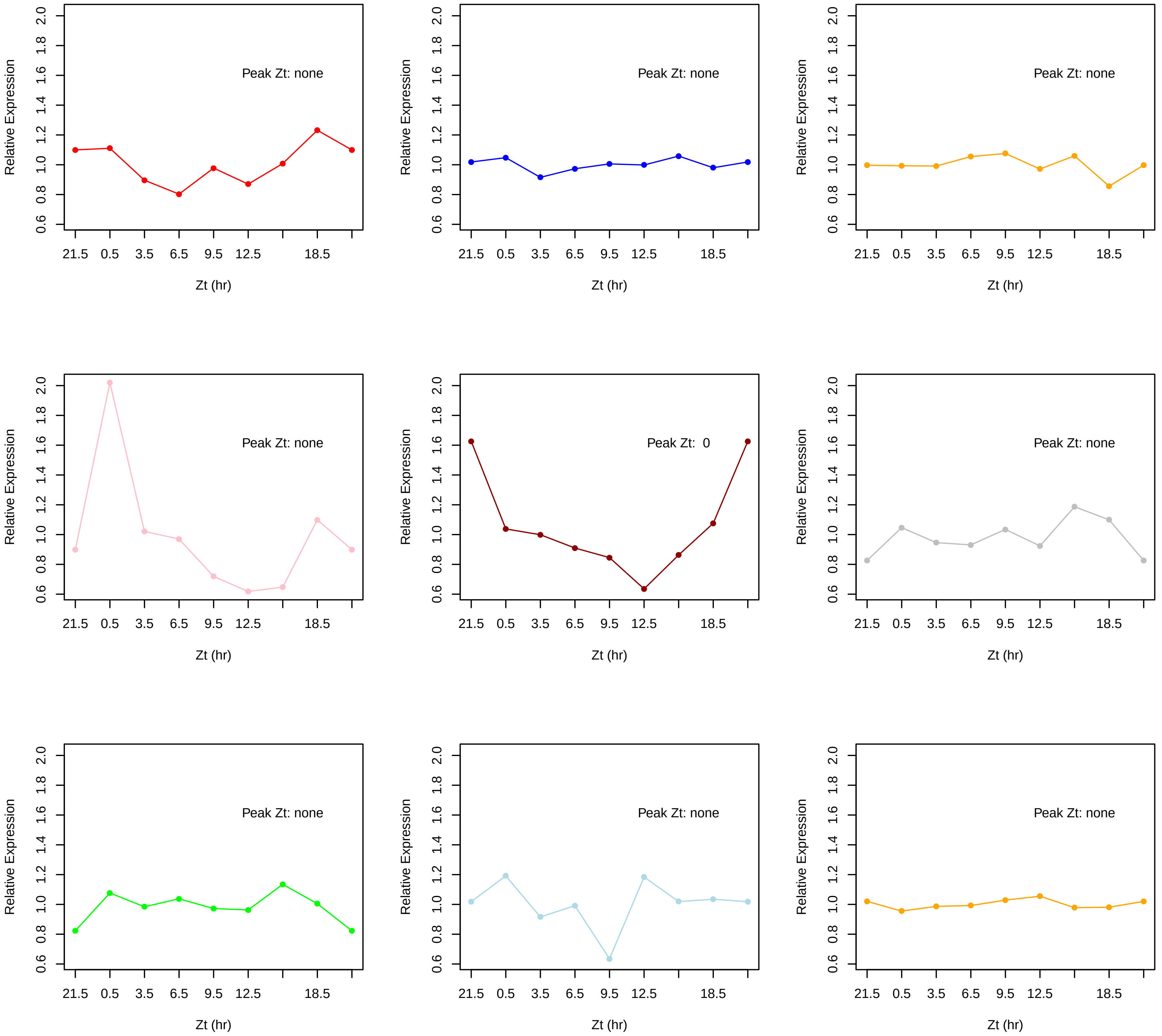

Singeleton: ENSXMAG00000005480 pdcd4a

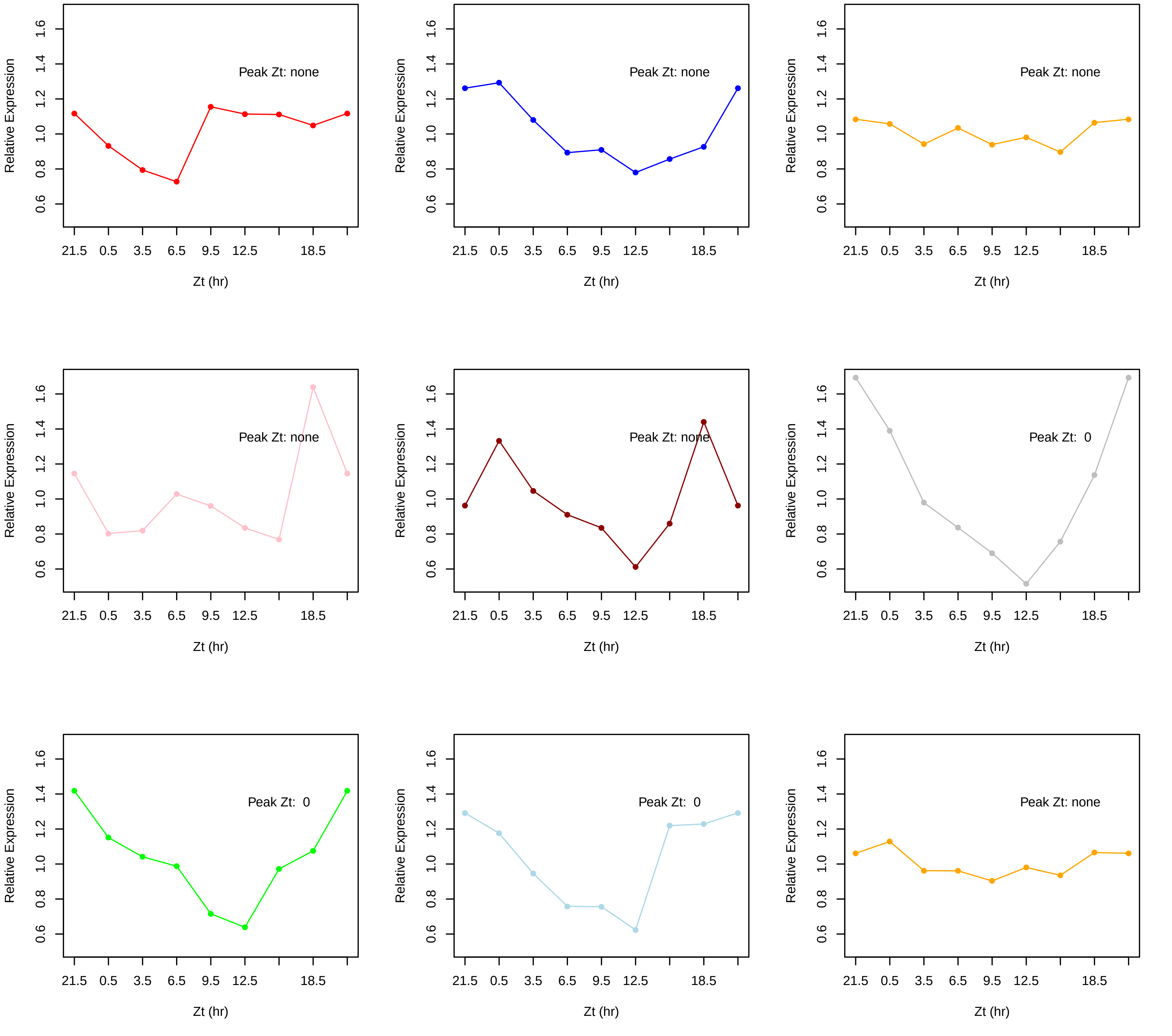

Singeleton: ENSXMAG00000000837 dirc2

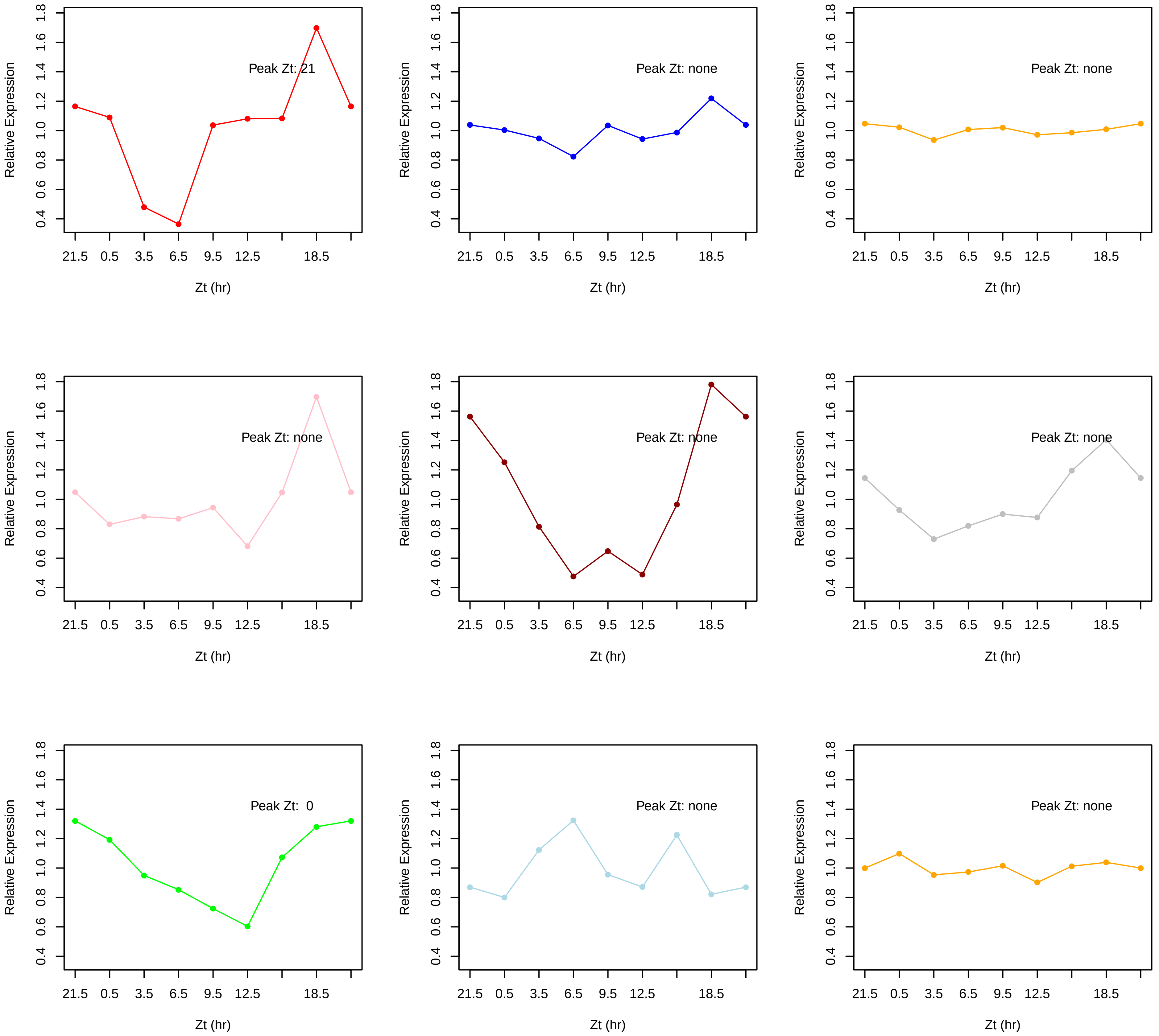

Singeleton: ENSXMAG00000016828 derl2

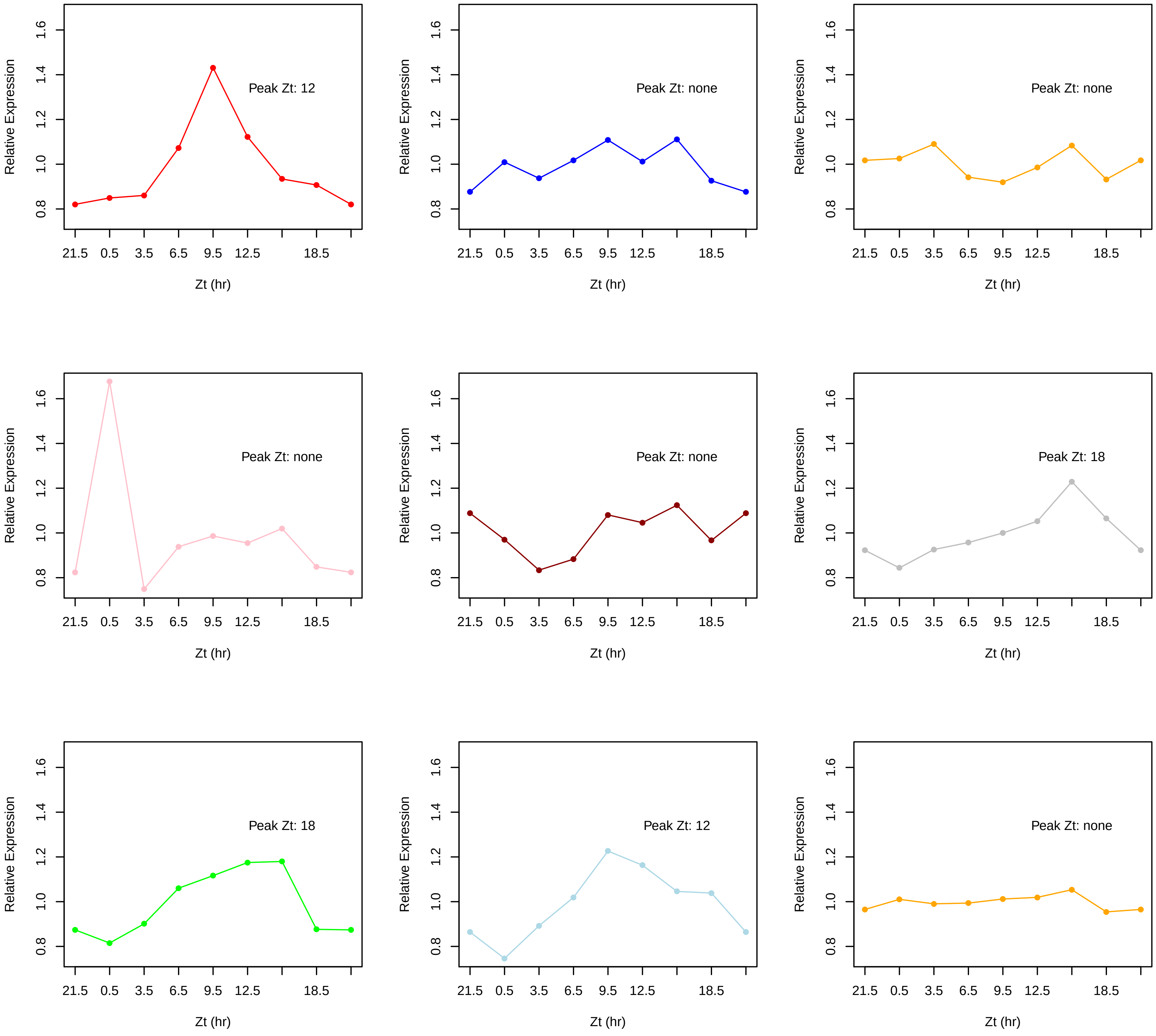

Singeleton: ENSXMAG00000002378 txnrd2.1

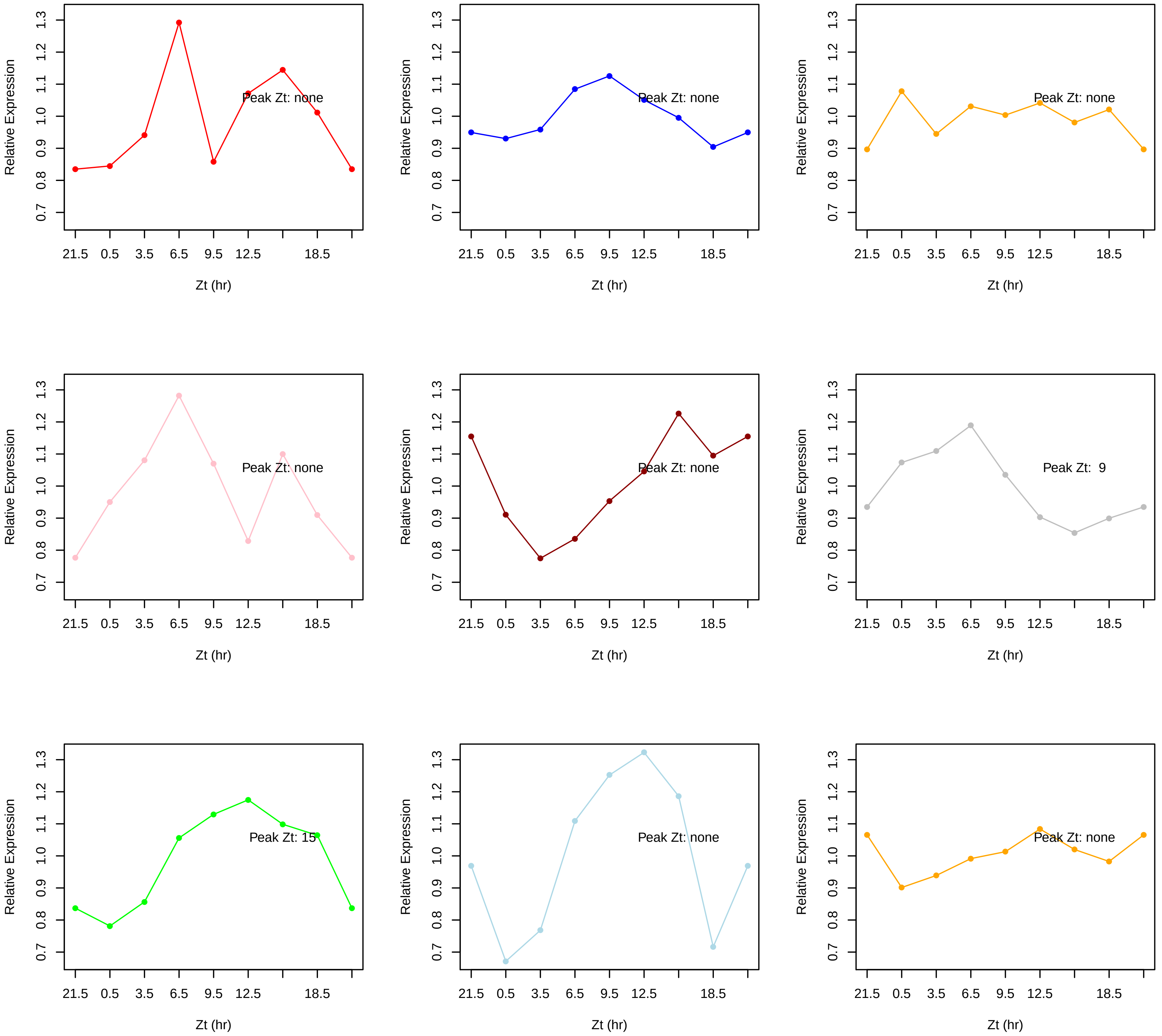

Singeleton: ENSXMAG00000016293 ric8a

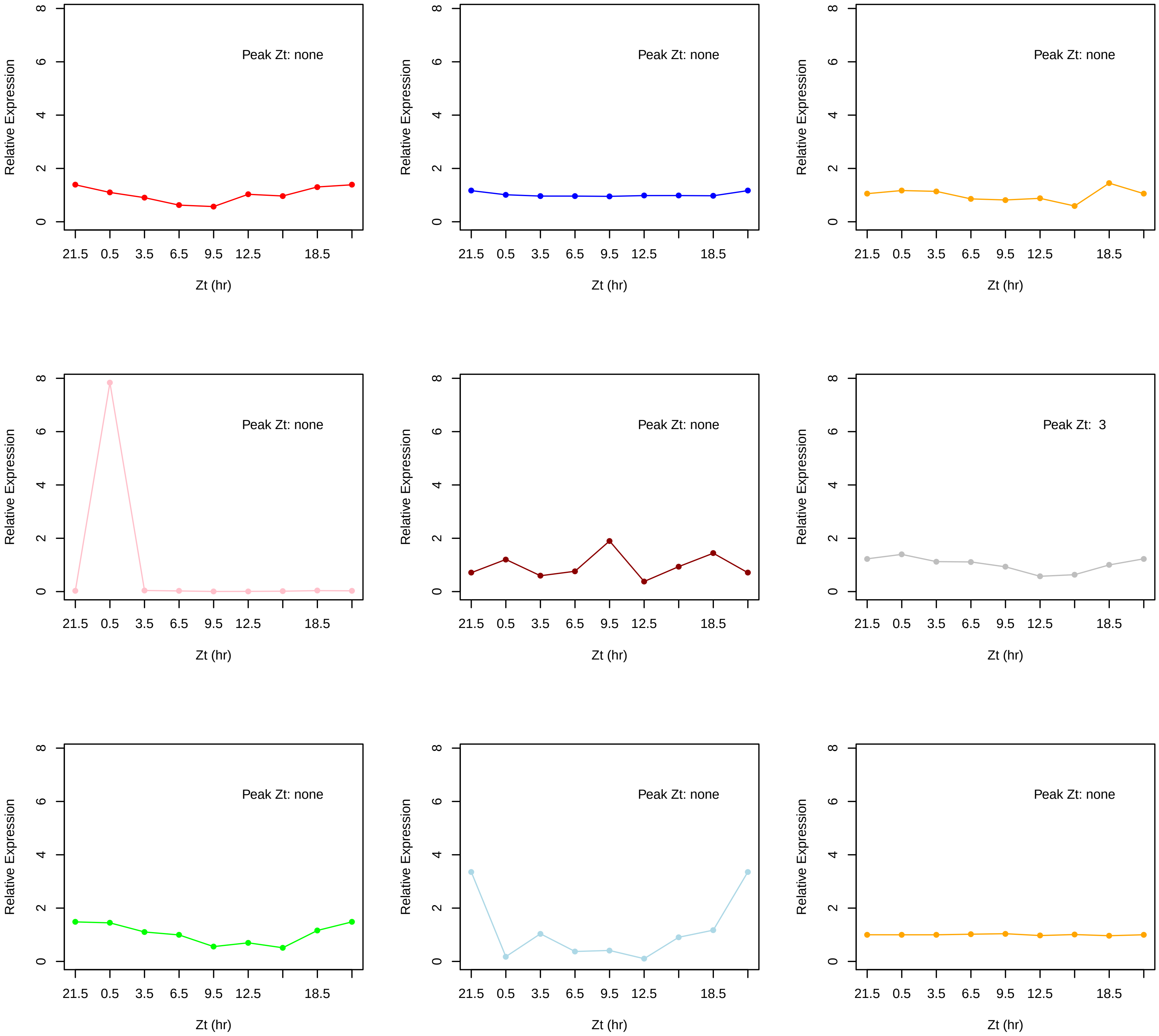

Singeleton: ENSXMAG00000003934

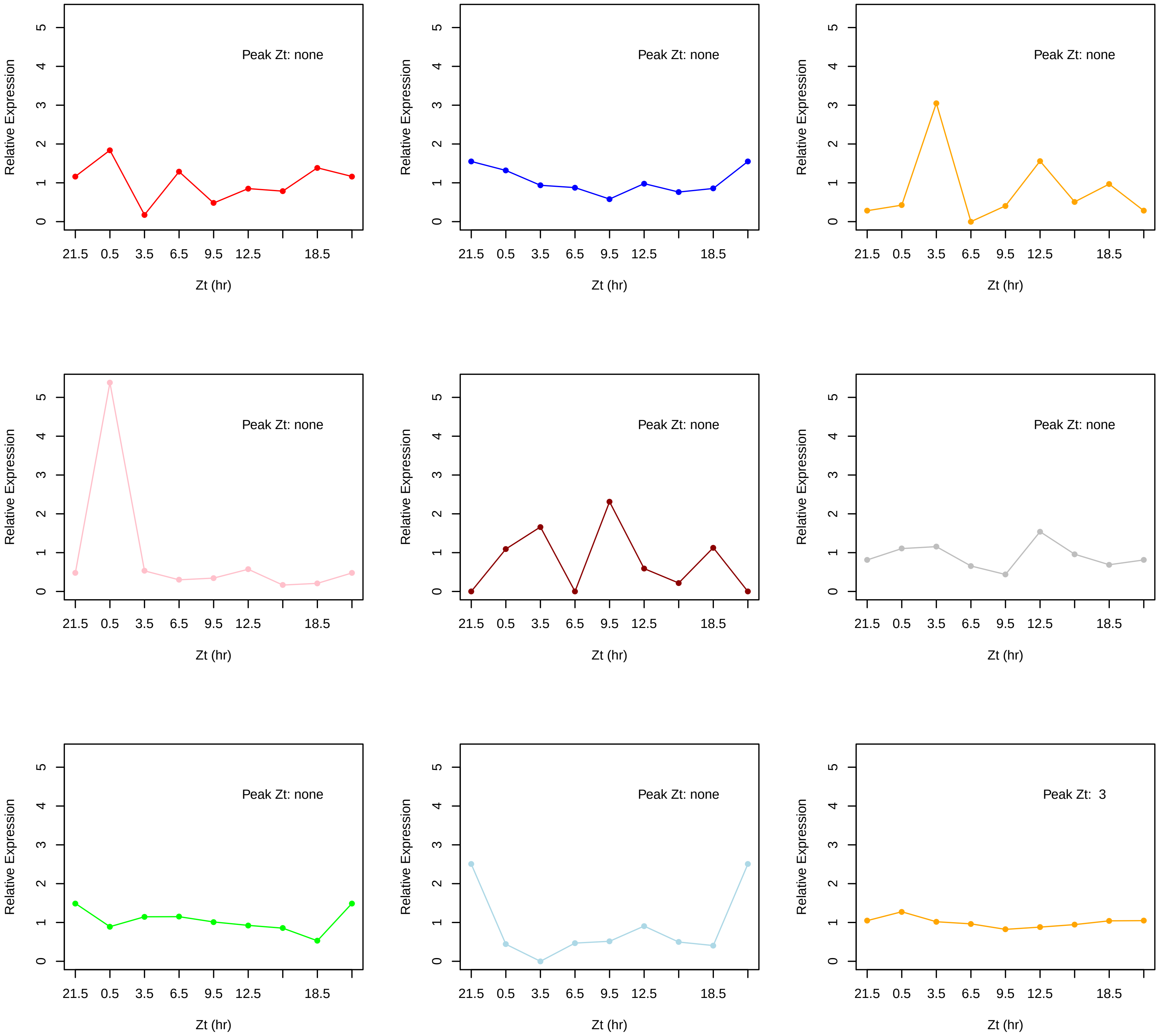

Singleton: ENSXMAG00000021337 mogs

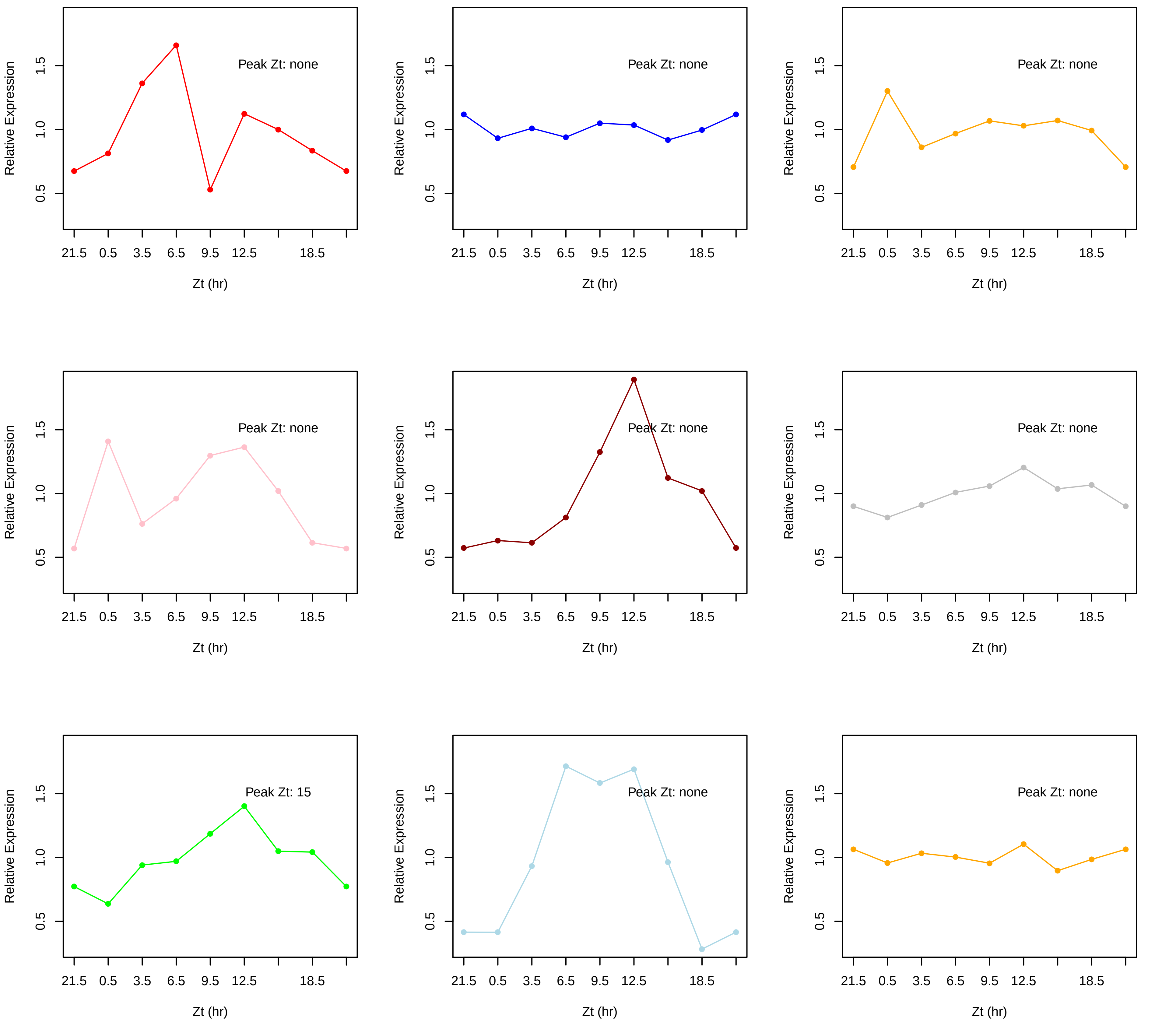

Singeleton: ENSXMAG00000000473

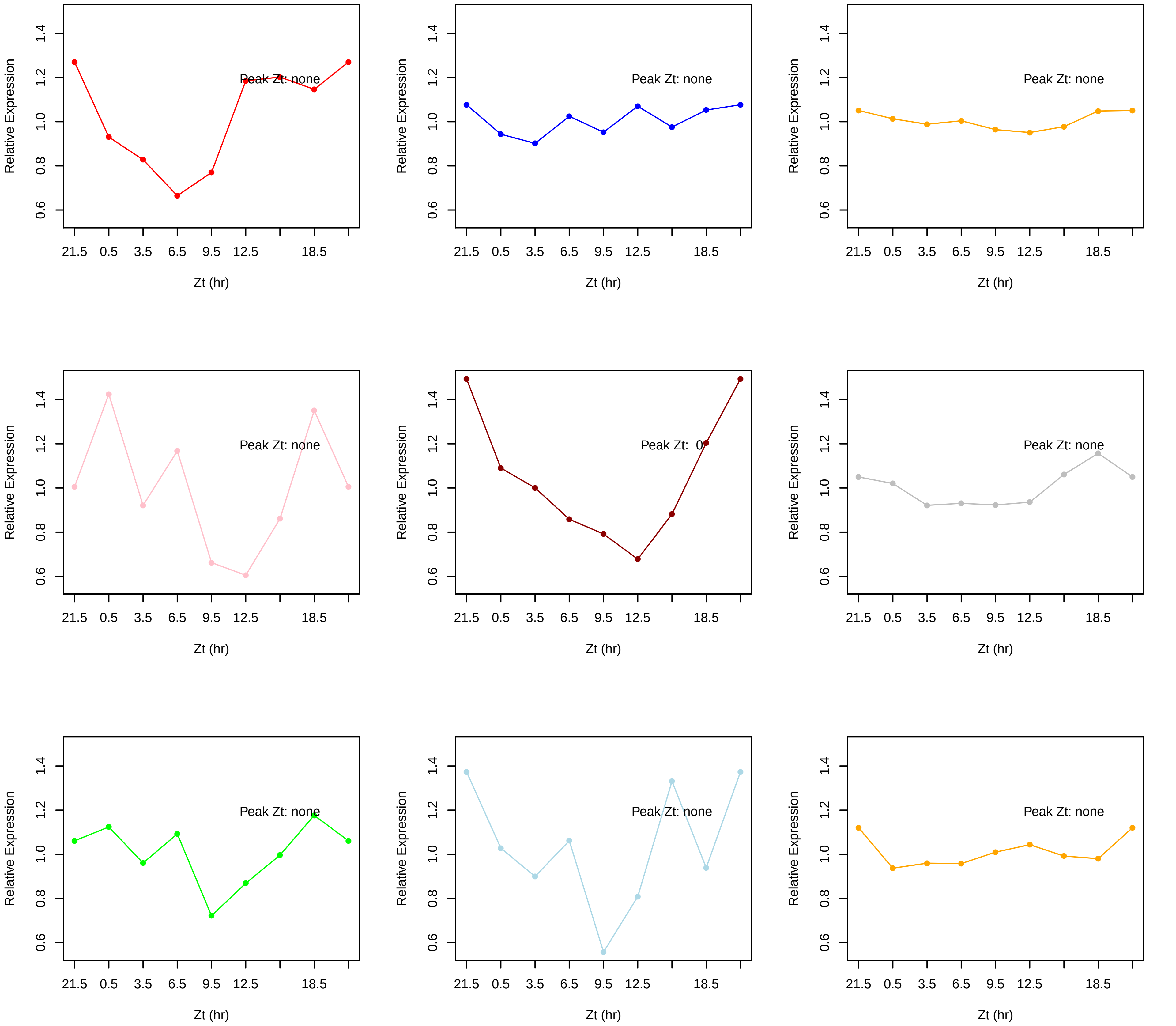

Singeleton: ENSXMAG00000022875 r3hdm4

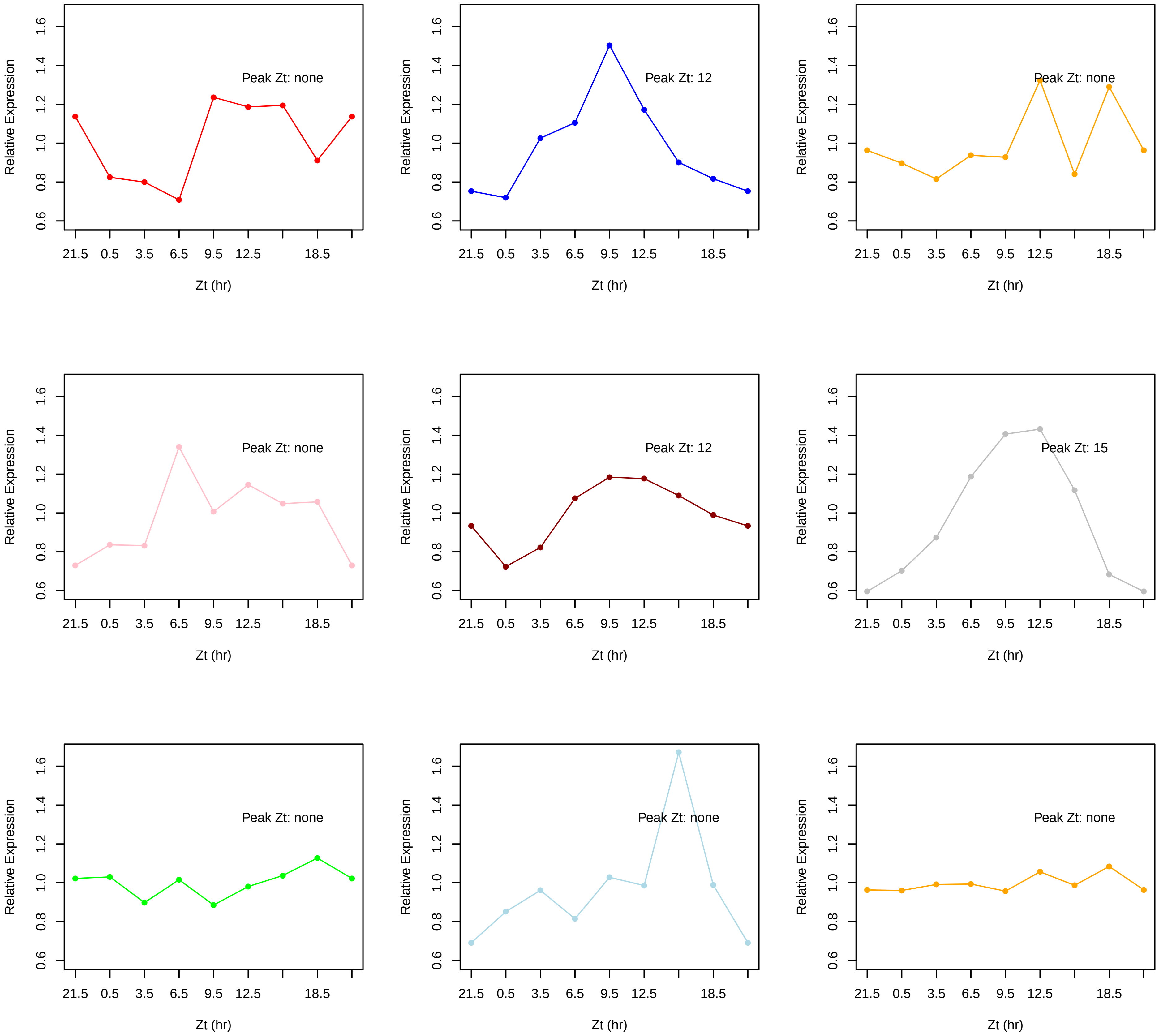

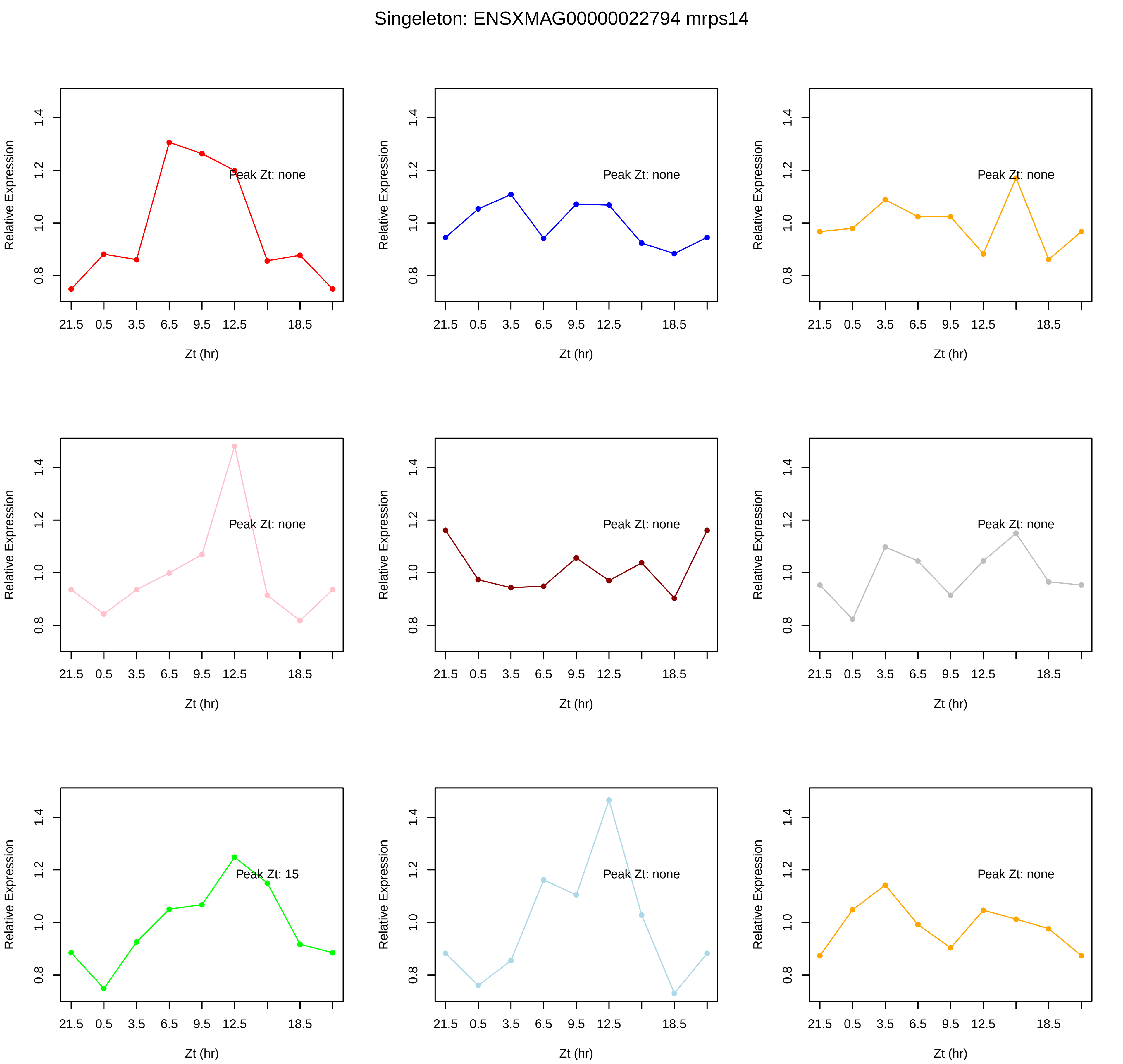

Singeleton: ENSXMAG00000022794 mrps14
Relative Expression
Zt (hr)
21.5
0.5
3.5
6.5
9.5
12.5
18.5
0.8
1.0
1.2
1.4
Peak Zt: none
Peak Zt: 15

Singleton: ENSXMAG00000028474 RAB31

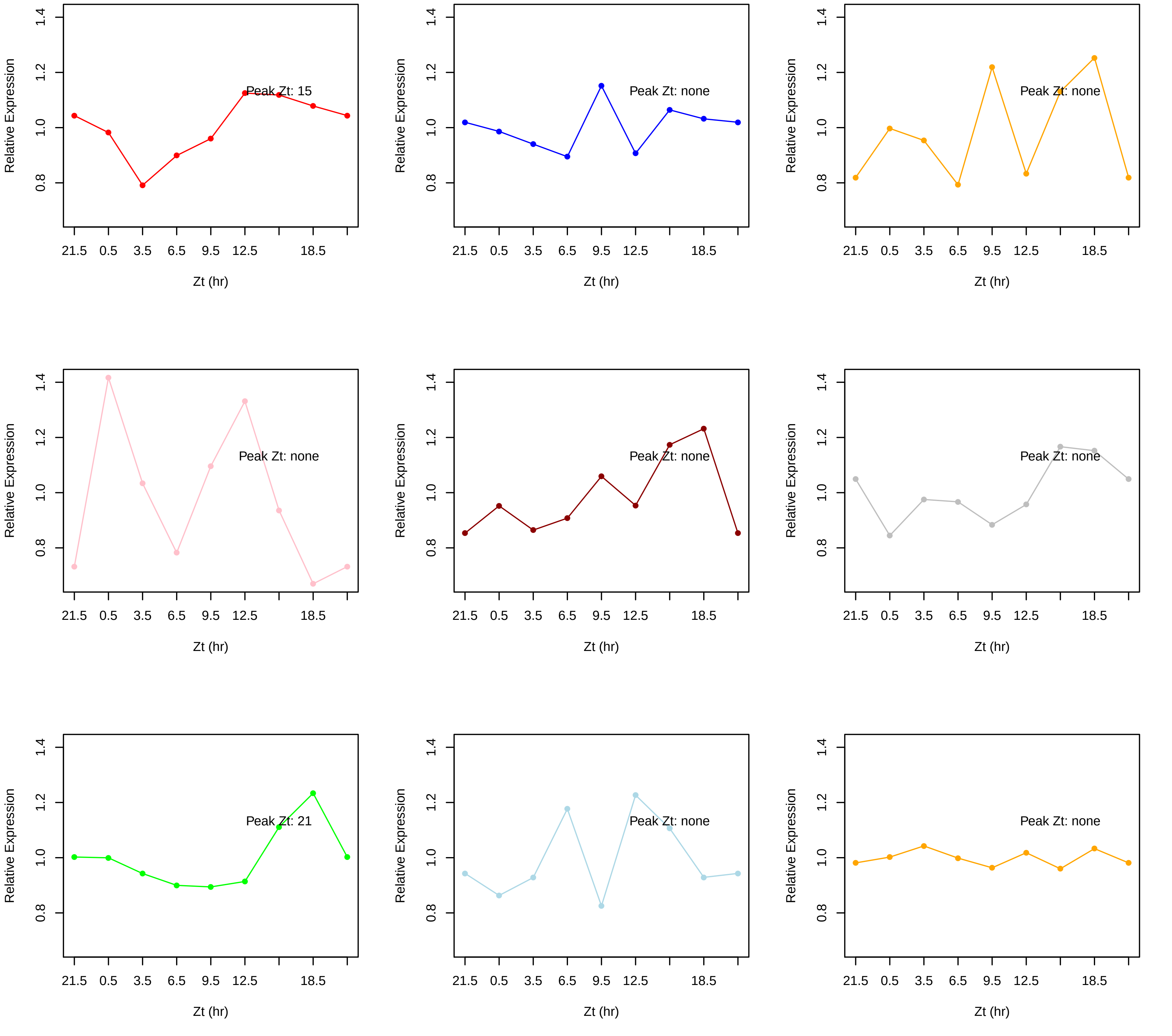

Singeleton: ENSXMAG00000015822 cacybp

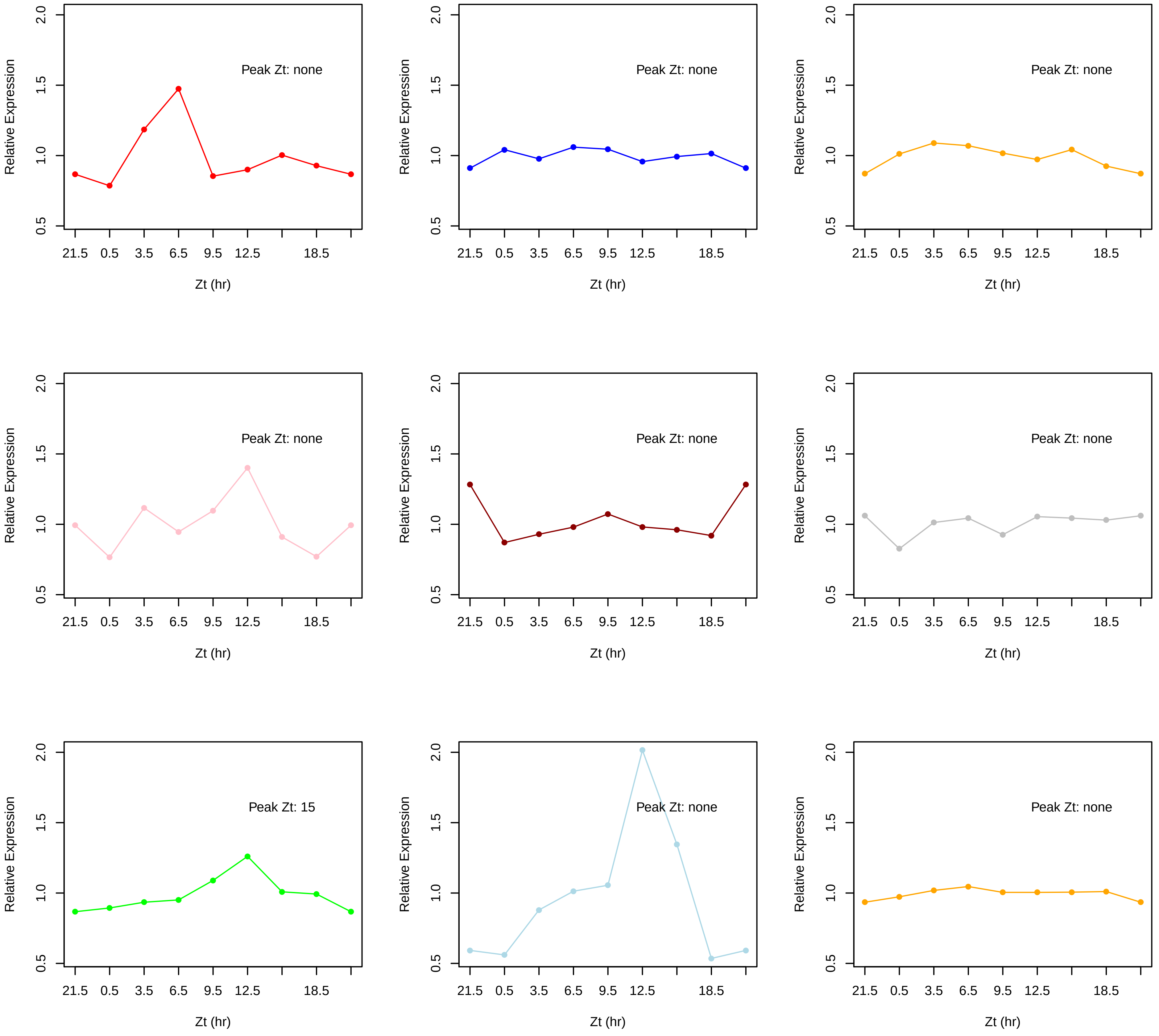

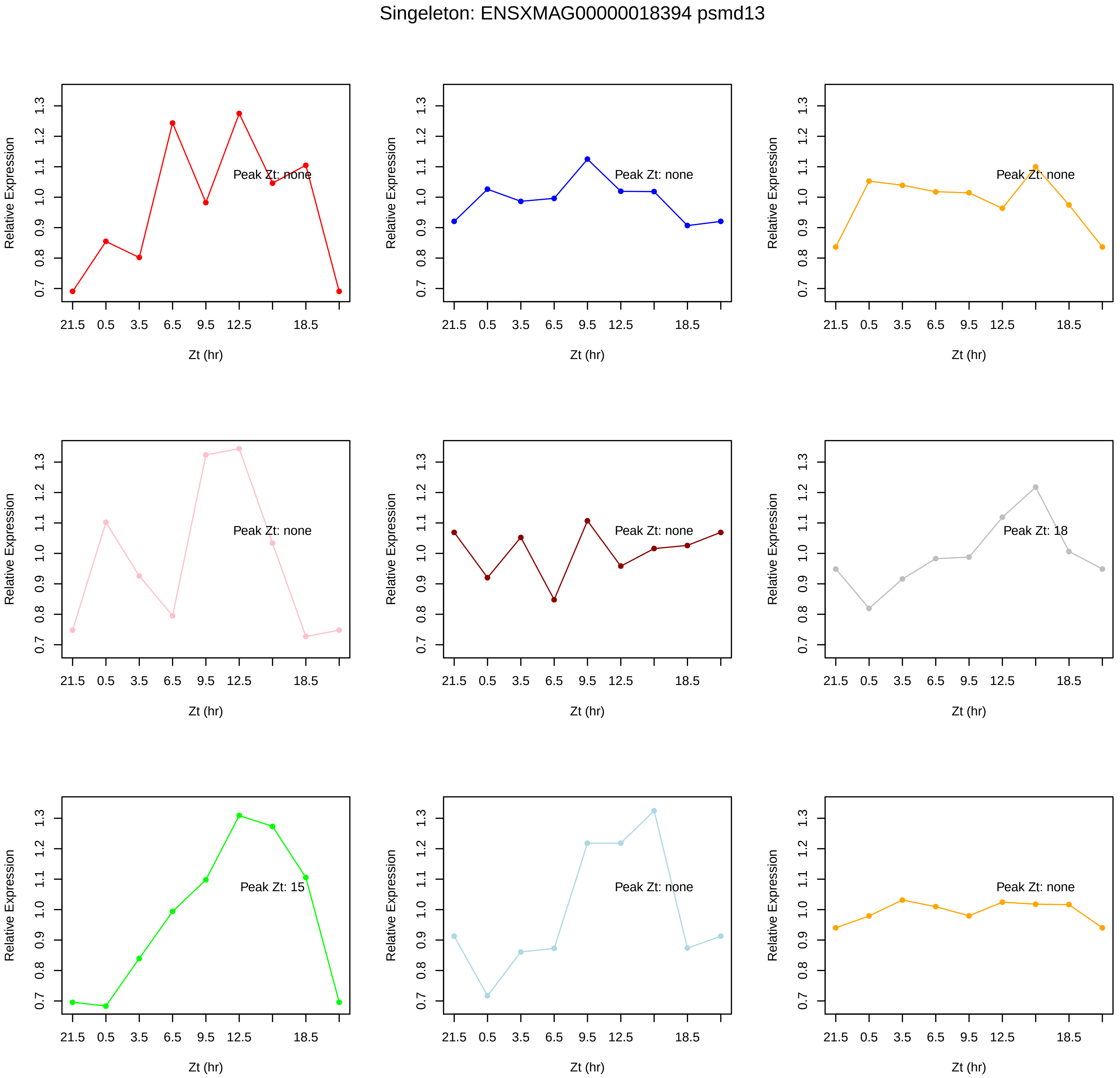

Singeleton: ENSXMAG00000018394 psmd13
Relative Expression
Zt (hr)
21.5
0.5
3.5
6.5
9.5
12.5
18.5
0.7
0.8
0.9
1.0
1.1
1.2
1.3
Peak Zt: none
Peak Zt: none
Peak Zt: none
Peak Zt: none
Peak Zt: none
Peak Zt: 18
Peak Zt: 15
Peak Zt: none
Peak Zt: none

Singeleton: ENSXMAG00000006239 zc3h18

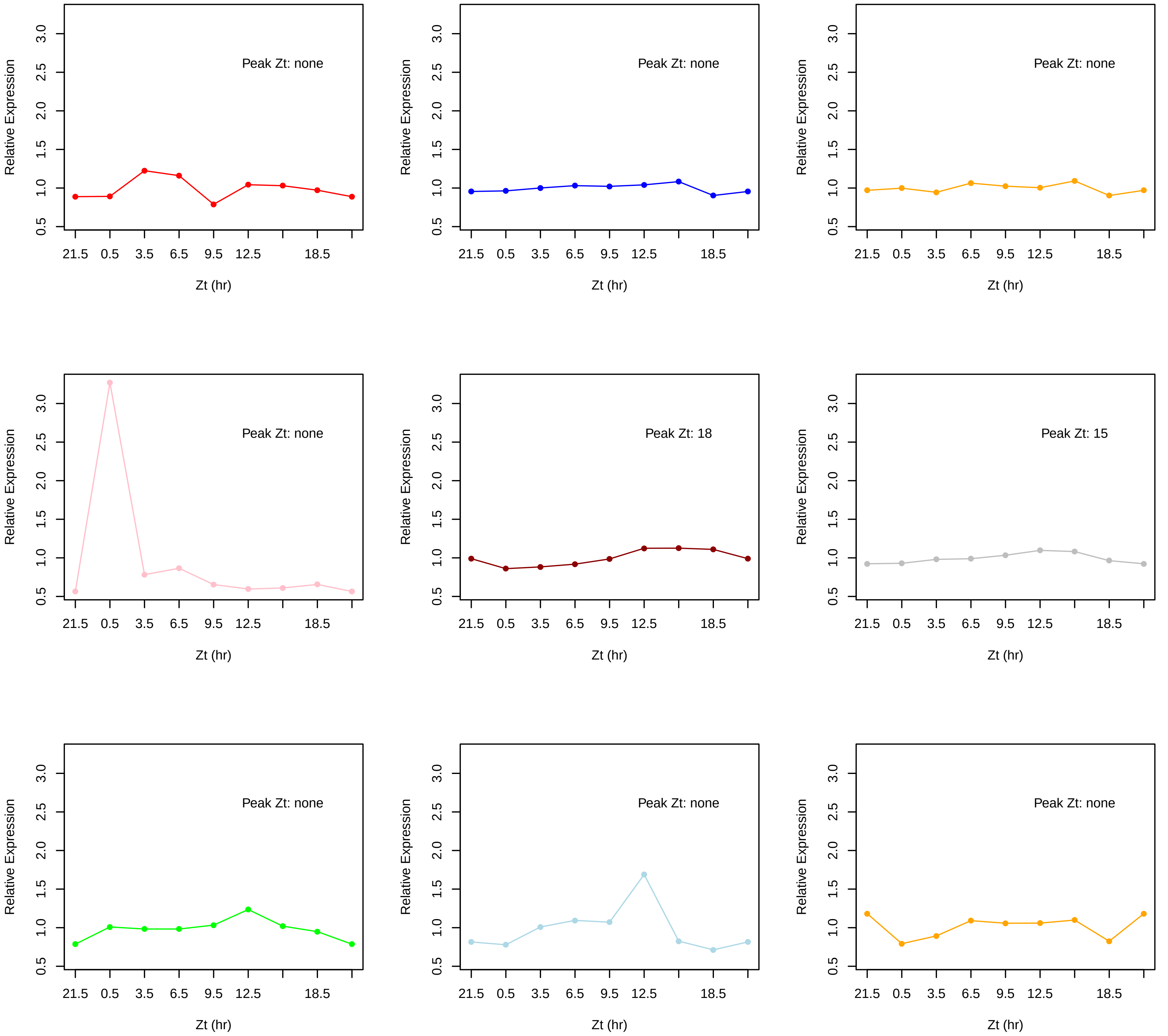

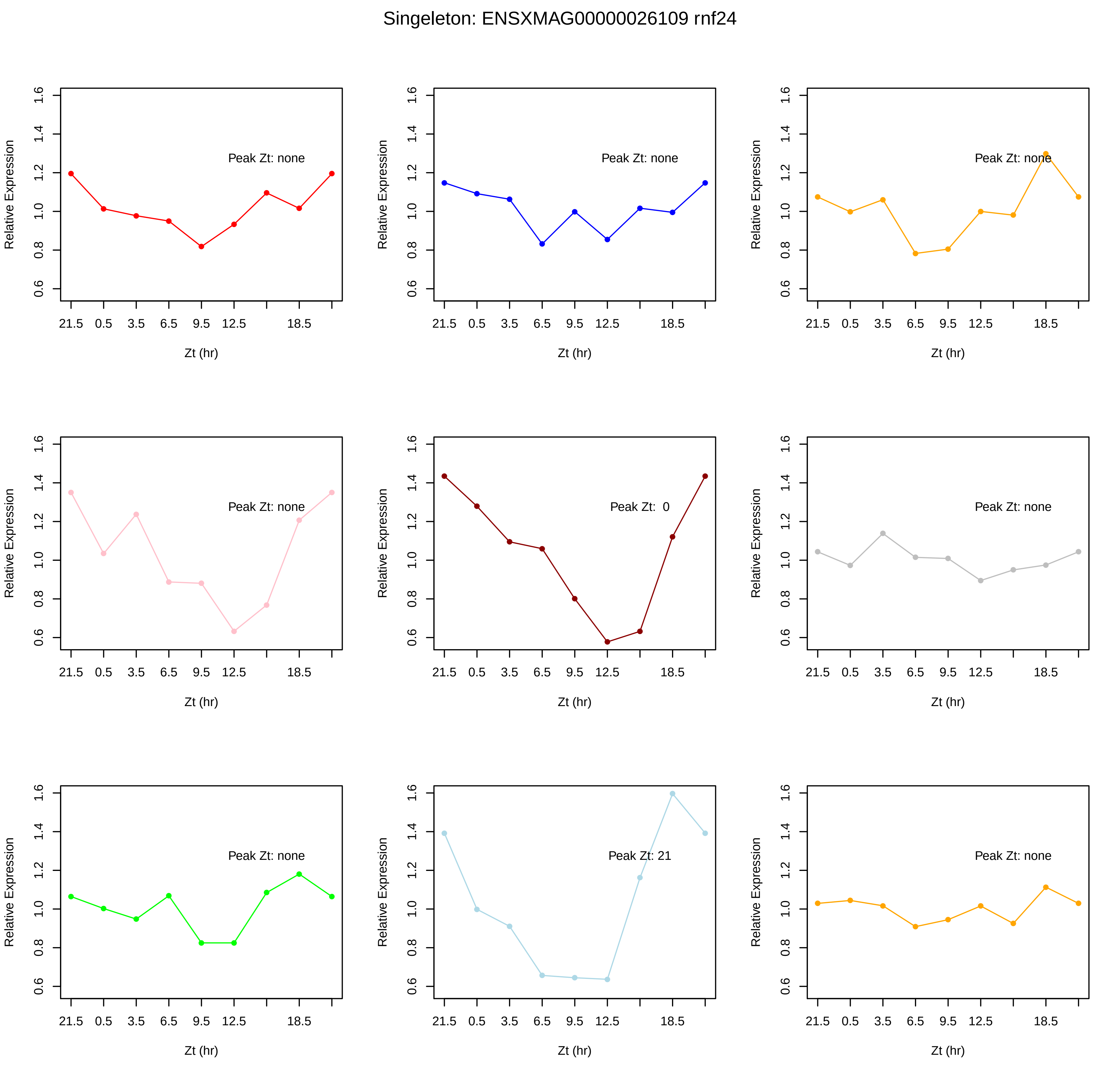
Singeleton: ENSXMAG00000026109 rnf24
Peak Zt: none
Peak Zt: none
Peak Zt: none
Peak Zt: none
Peak Zt: 0
Peak Zt: none
Peak Zt: none
Peak Zt: 21
Peak Zt: none
Relative Expression
Zt (hr)
21.5
0.5
3.5
6.5
9.5
12.5
18.5
0.6
0.8
1.0
1.2
1.4
1.6

Singeleton: ENSXMAG00000002782 isca1

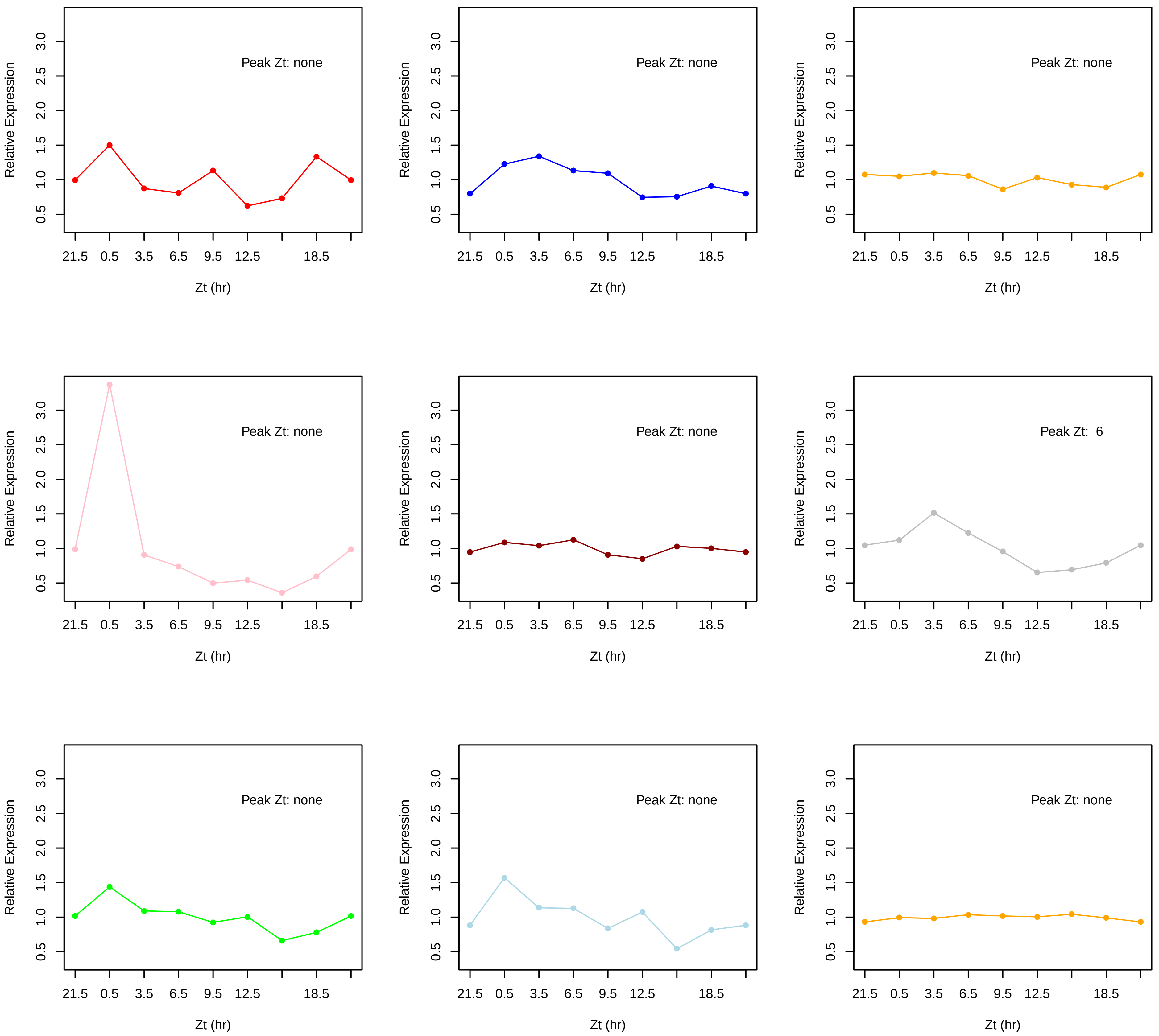

Singleton: ENSXMAG00000018906 zgc:154055

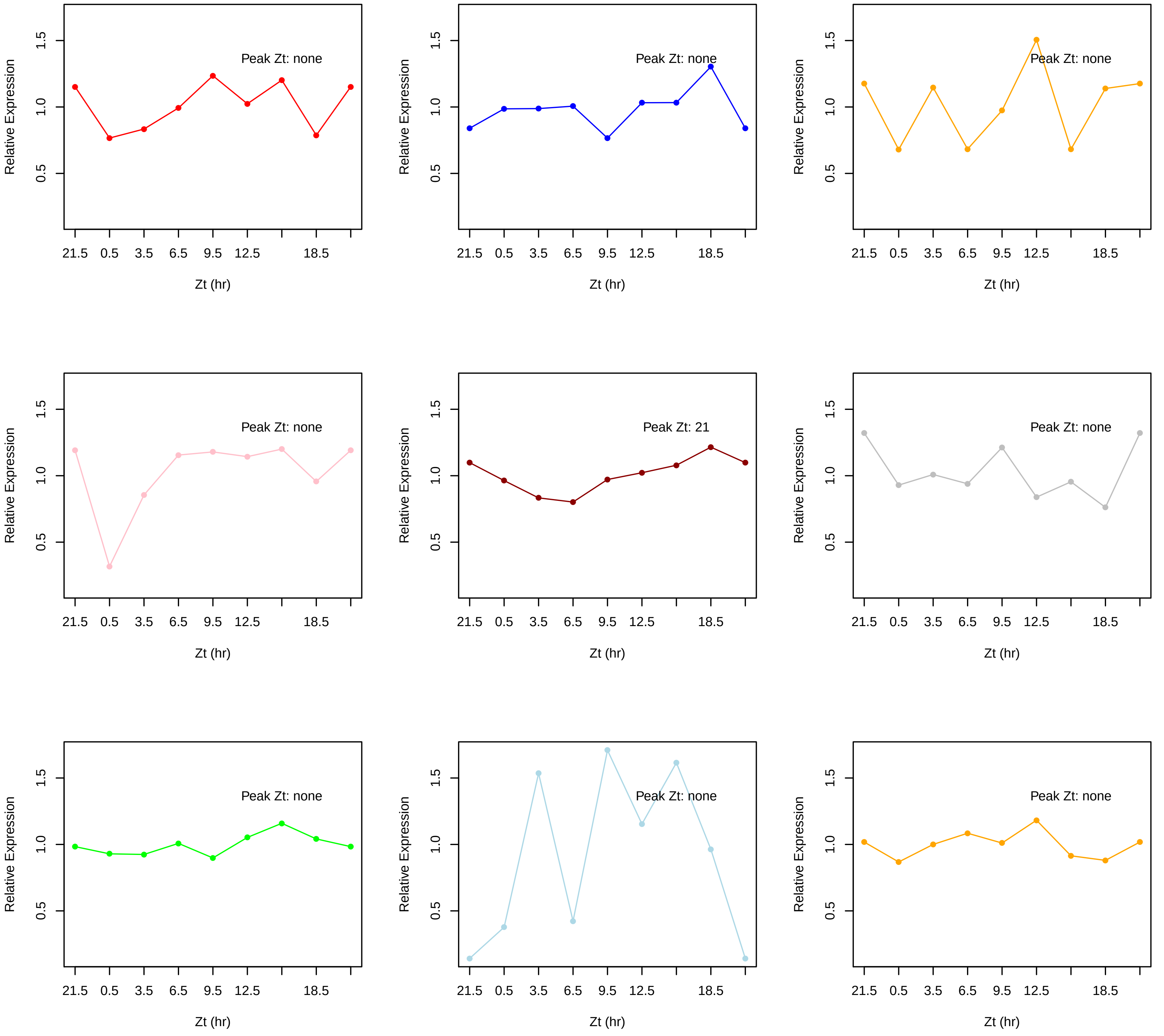

Singeleton: ENSXMAG00000005505

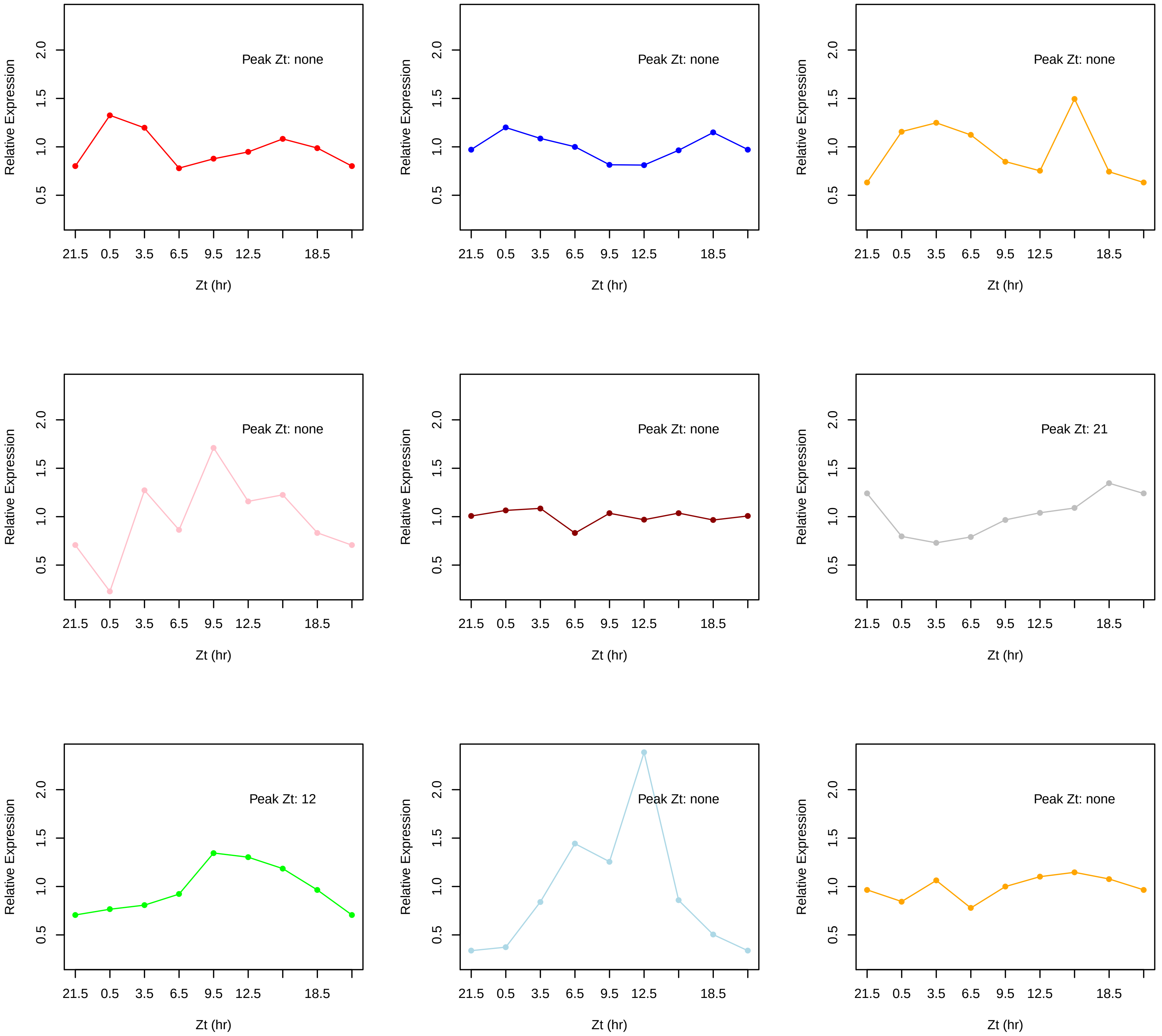

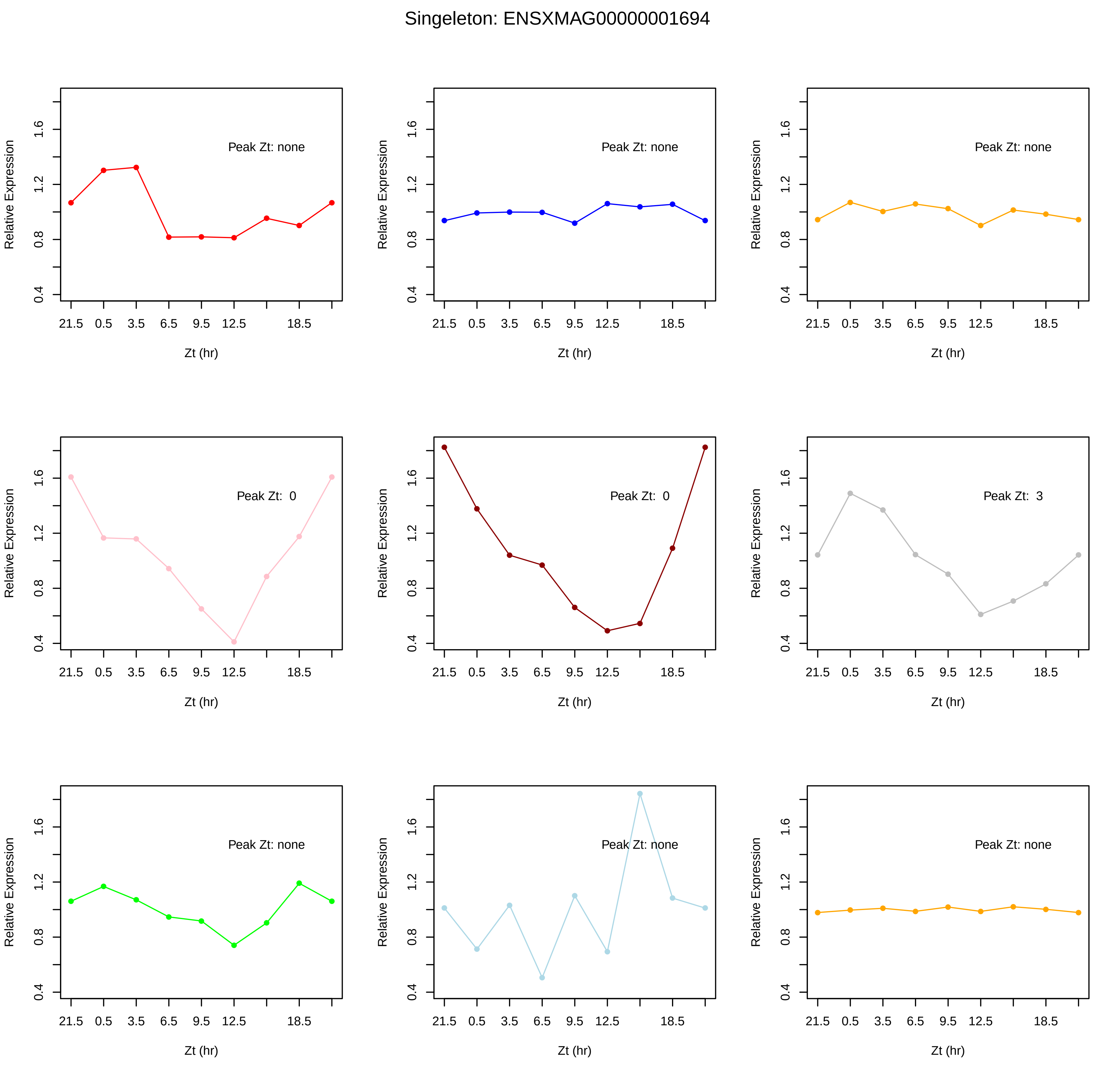
Singeleton: ENSXMAG00000001694
Peak Zt: none
Peak Zt: none
Peak Zt: none
Peak Zt: 0
Peak Zt: 0
Peak Zt: 3
Peak Zt: none
Peak Zt: none
Peak Zt: none
Relative Expression
Zt (hr)
0.4
0.8
1.2
1.6
21.5
0.5
3.5
6.5
9.5
12.5
18.5

Singleton: ENSXMAG00000005918 ruvbl2

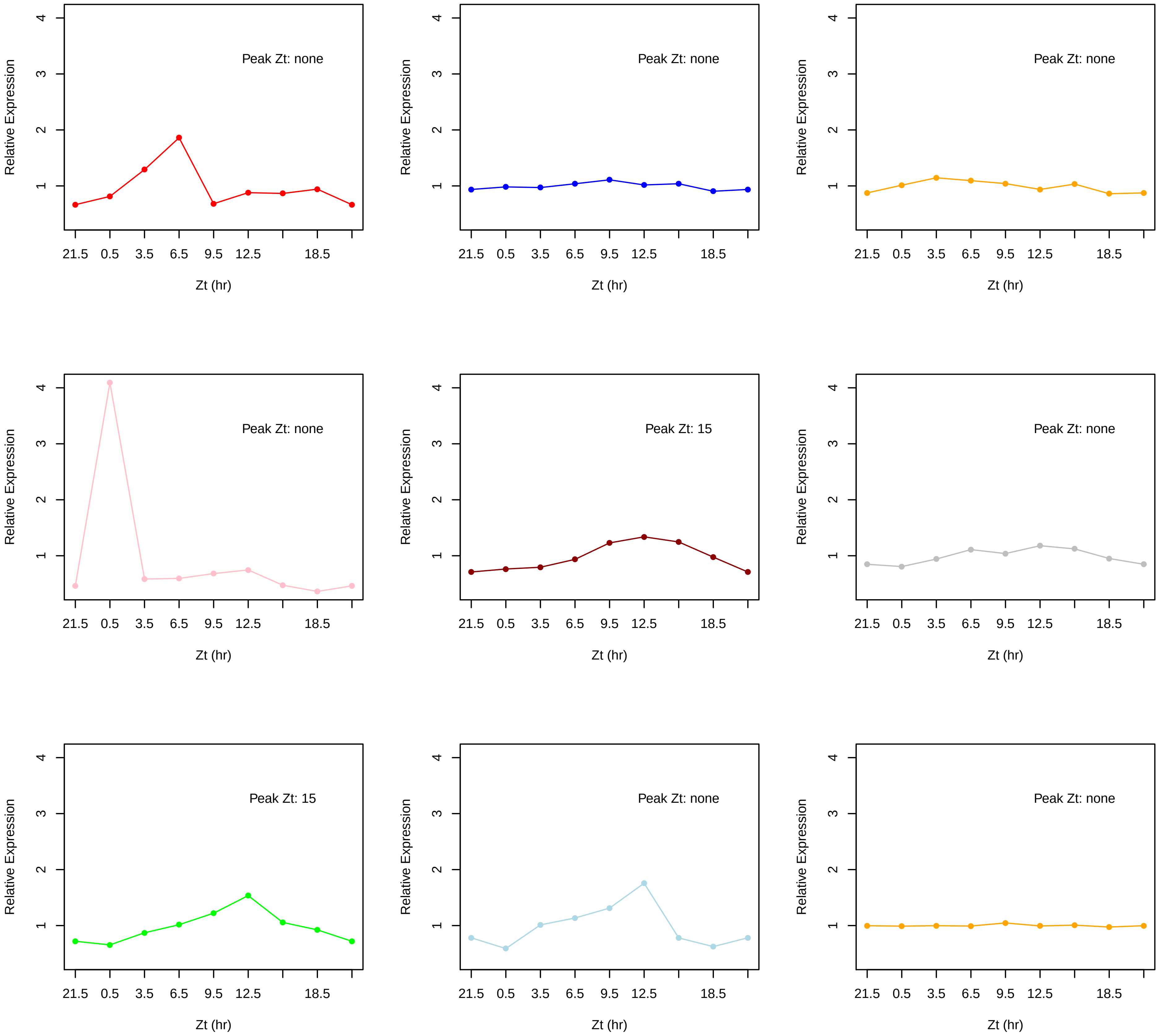

Singeleton: ENSXMAG00000002629 zgc:172302

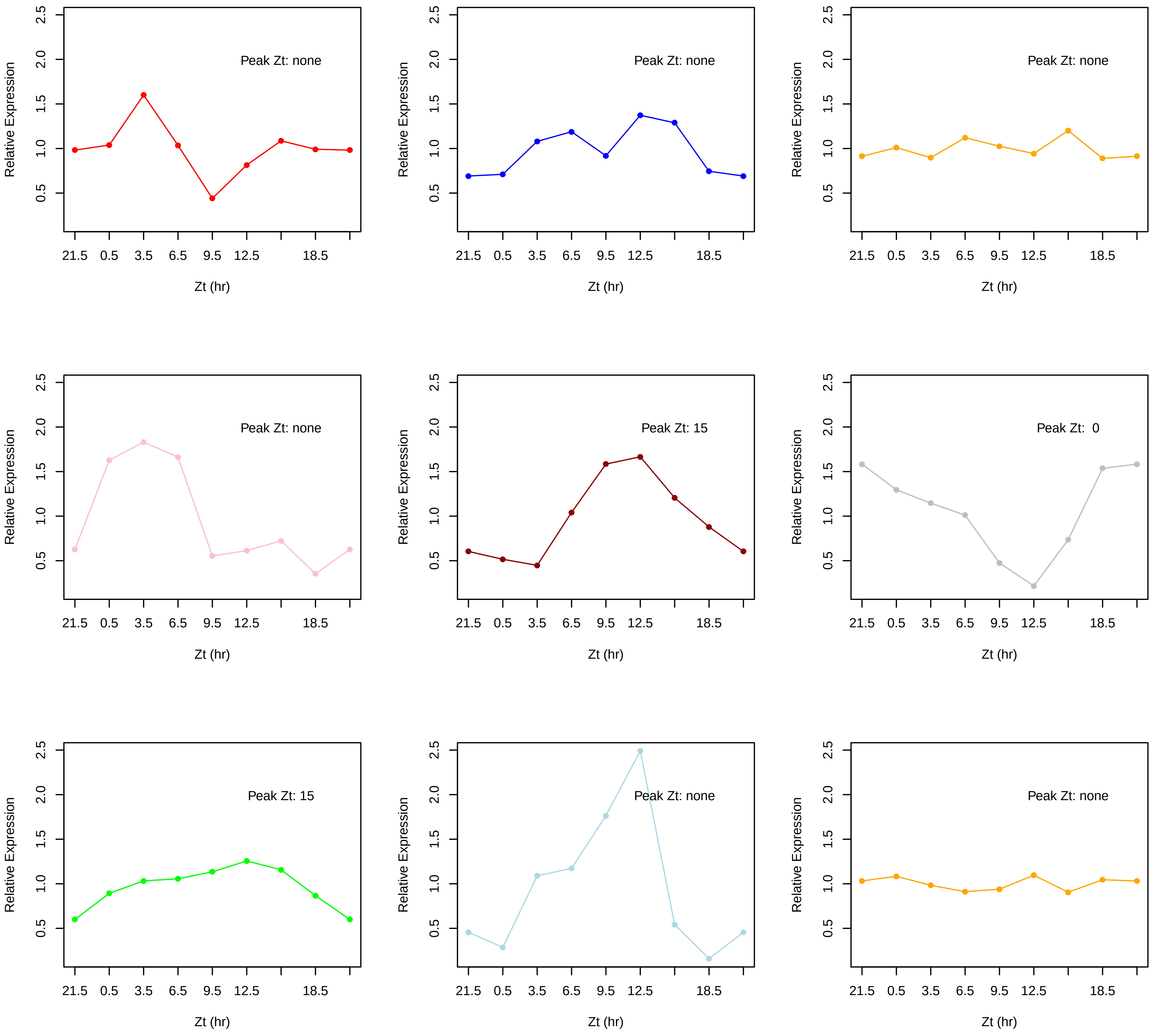

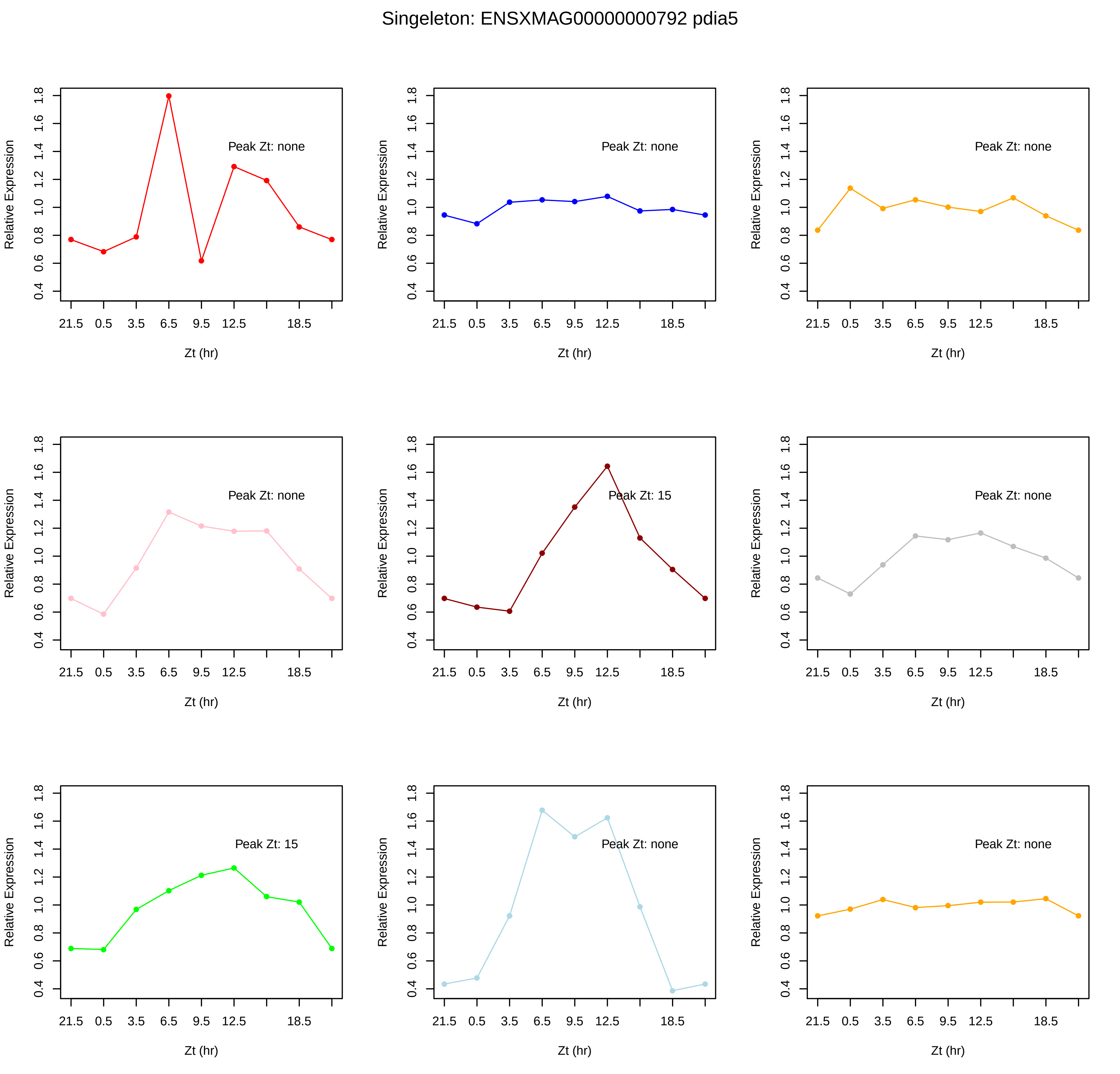

Singeleton: ENSXMAG00000000792 pdia5
Peak Zt: none
Peak Zt: none
Peak Zt: none
Peak Zt: none
Peak Zt: 15
Peak Zt: none
Peak Zt: 15
Peak Zt: none
Peak Zt: none
Relative Expression
Zt (hr)
21.5
0.5
3.5
6.5
9.5
12.5
18.5
0.4
0.6
0.8
1.0
1.2
1.4
1.6
1.8

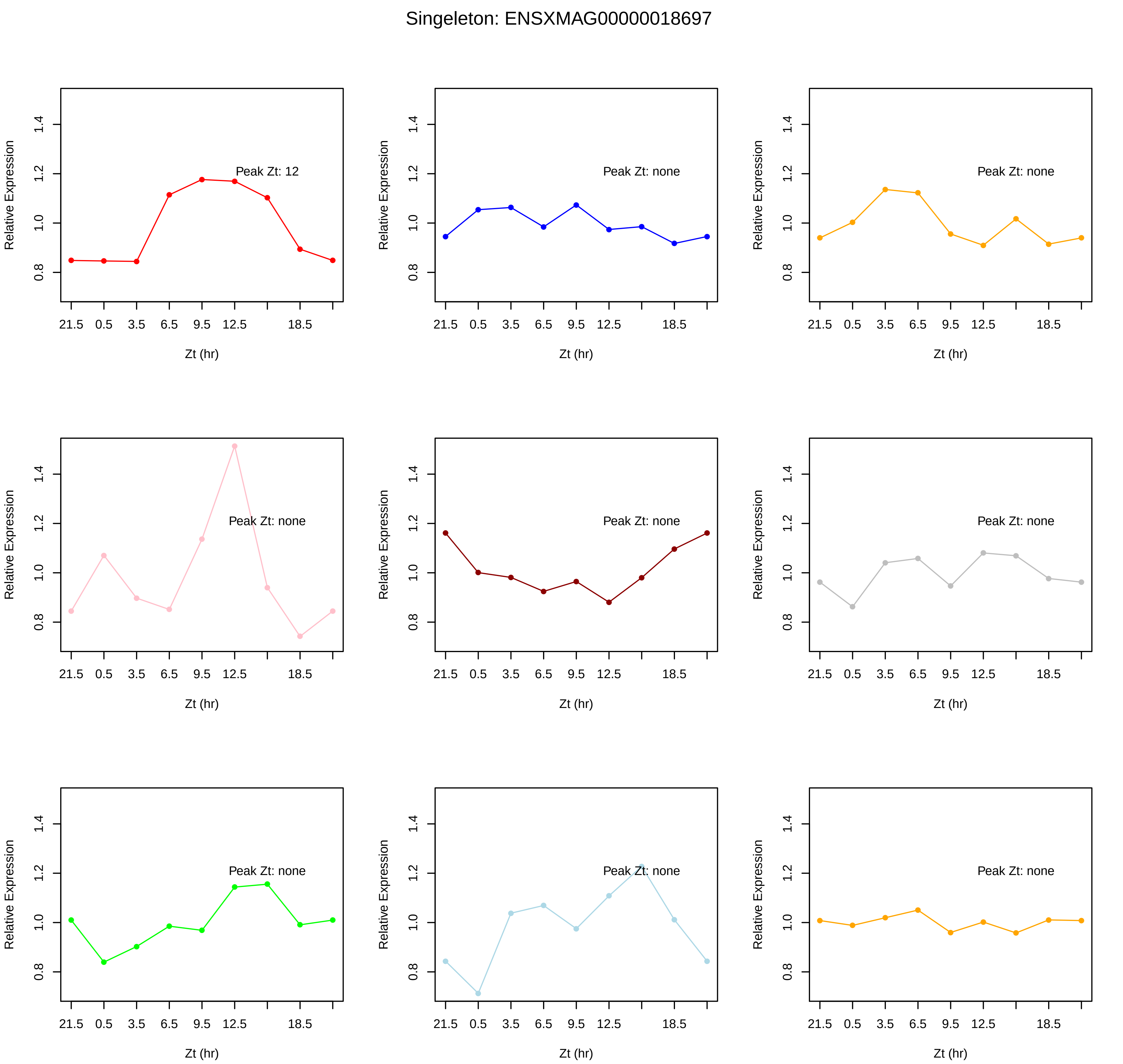
Singeleton: ENSXMAG00000018697
Peak Zt: 12
Peak Zt: none
Peak Zt: none
Peak Zt: none
Peak Zt: none
Peak Zt: none
Peak Zt: none
Peak Zt: none
Peak Zt: none
Relative Expression
Zt (hr)
21.5
0.5
3.5
6.5
9.5
12.5
18.5
0.8
1.0
1.2
1.4

Singleton: ENSXMAG00000024489 lmo4a

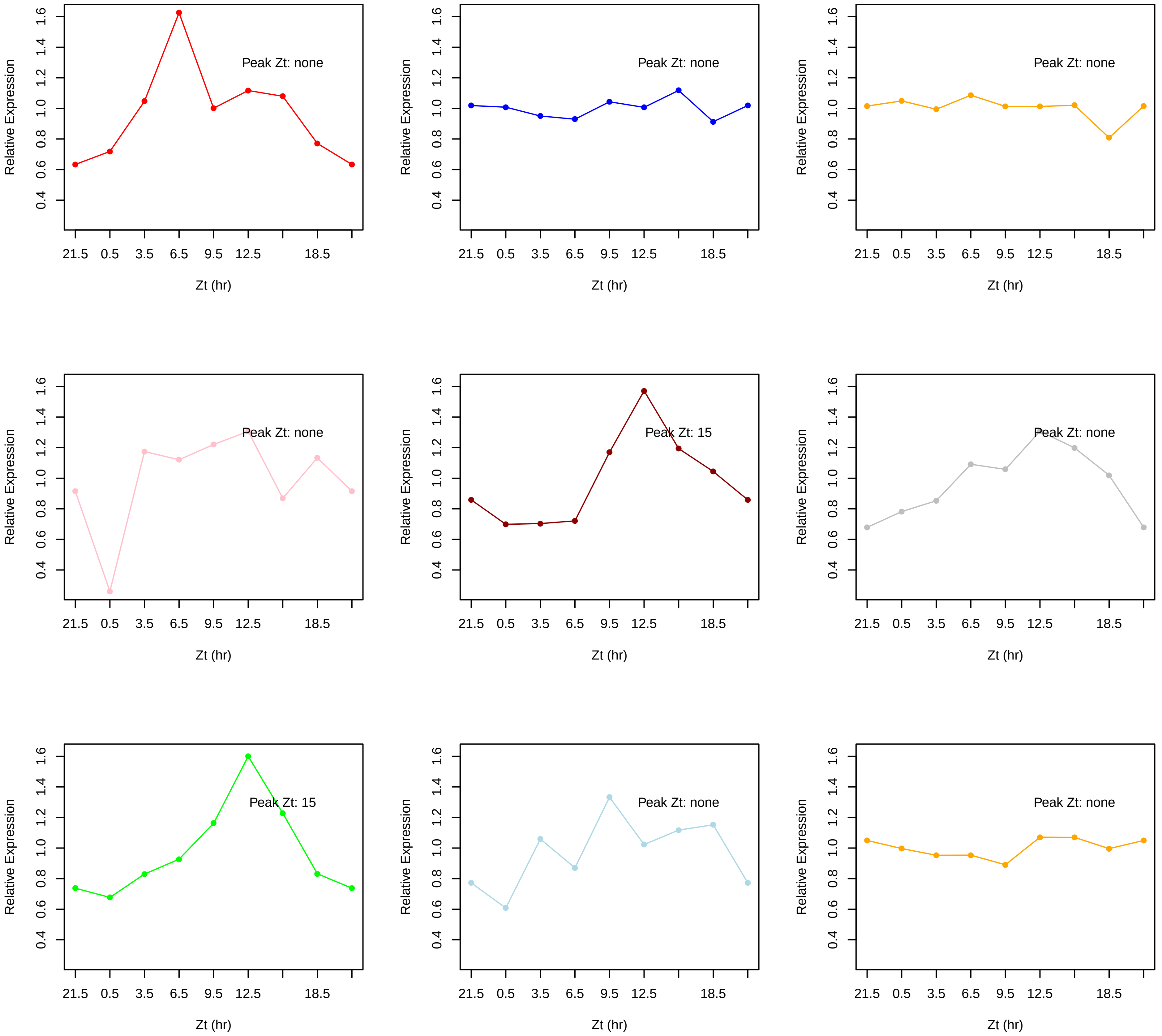

Singeleton: ENSXMAG00000008594 npr2

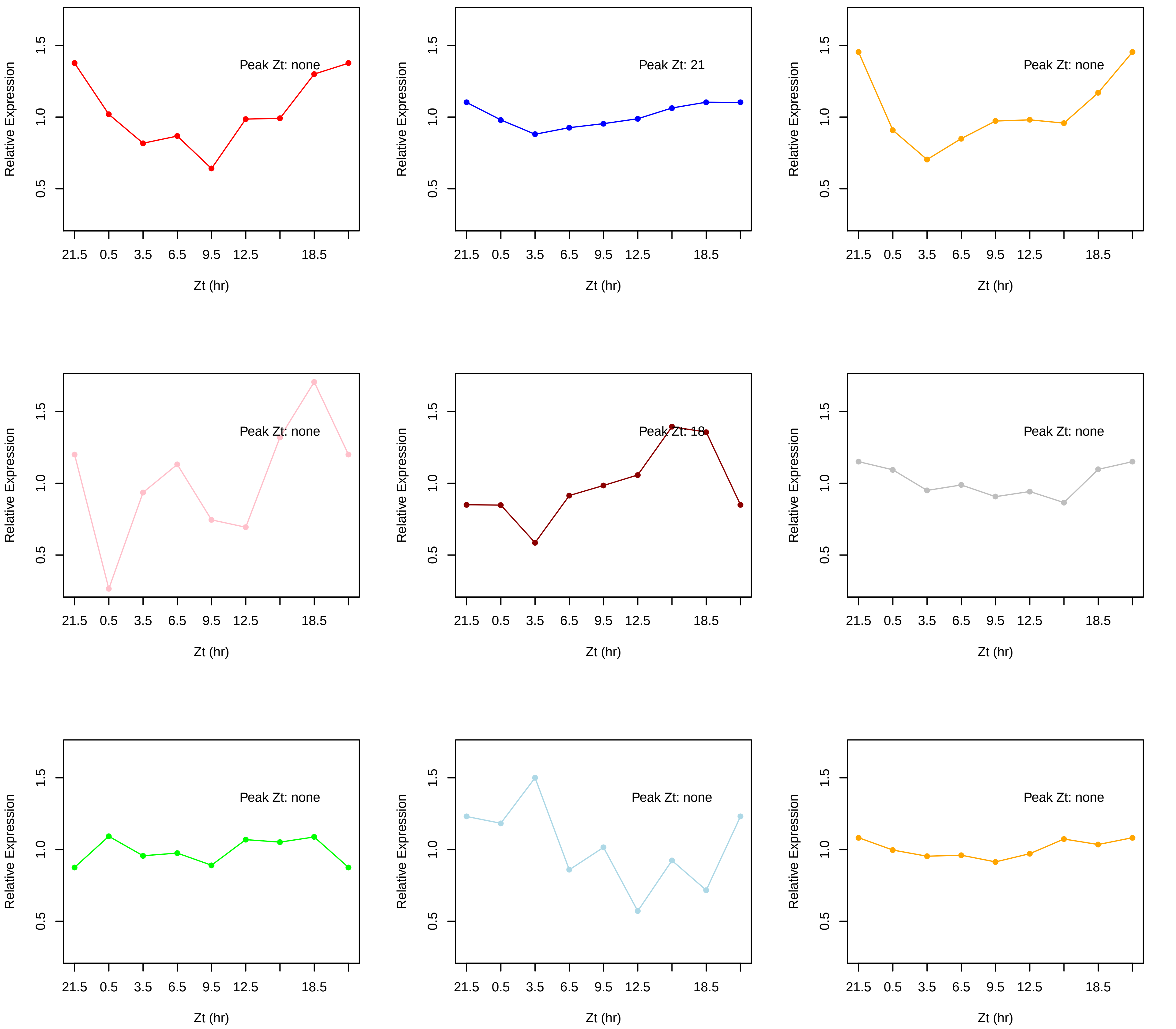

Singeleton: ENSXMAG00000002426 dgcr8

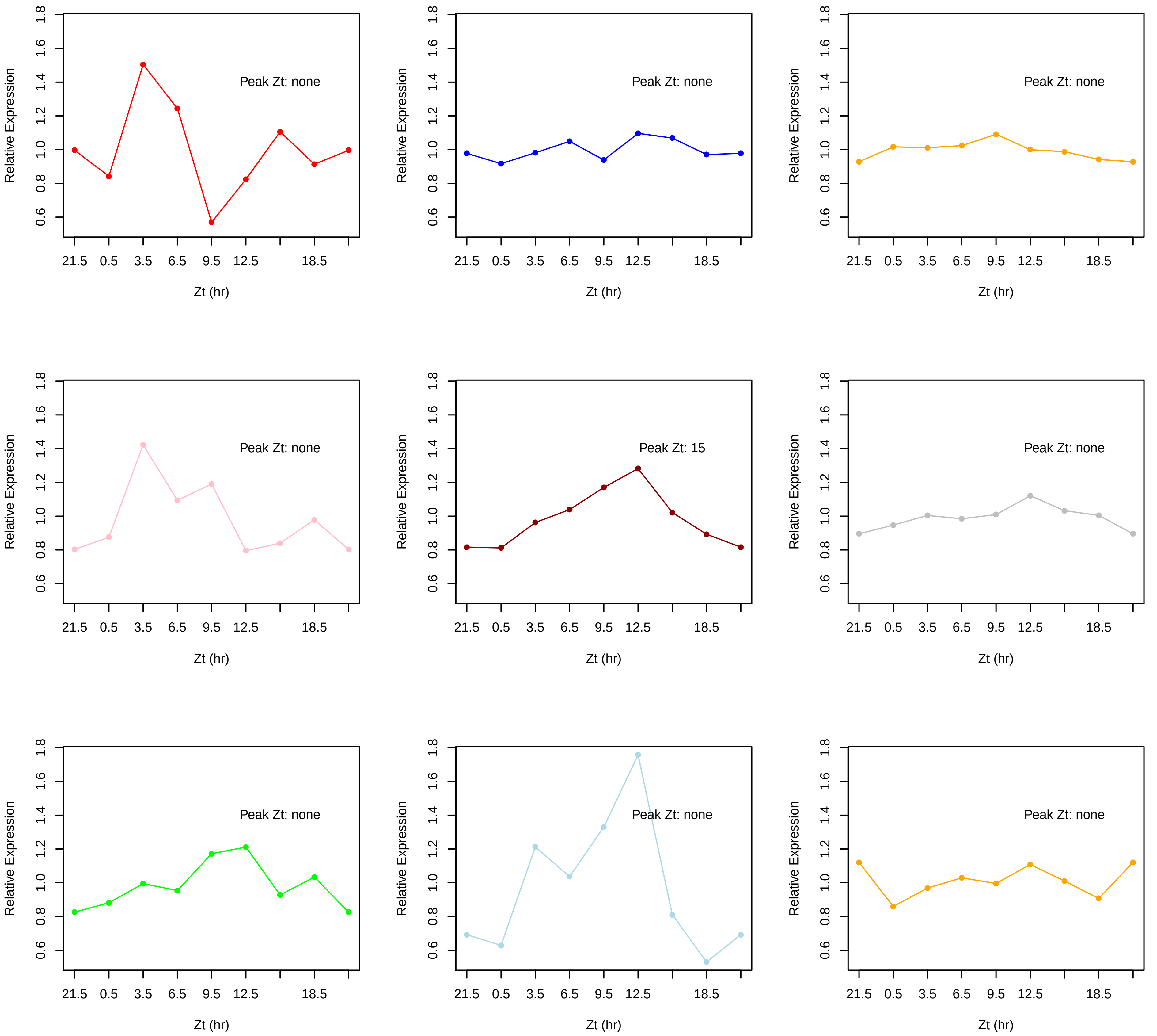

Singleton: ENSXMAG00000015759 eif2b3

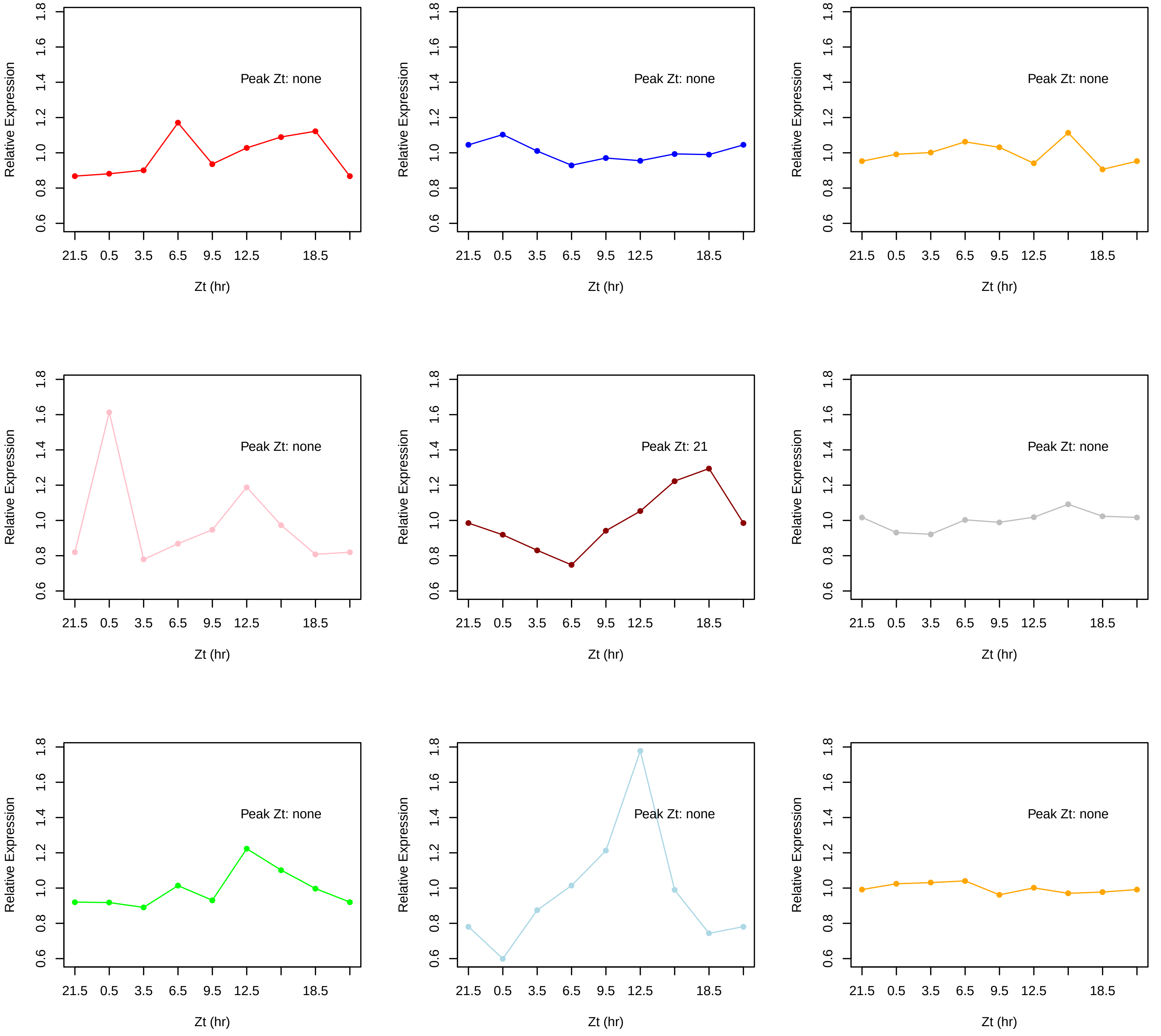

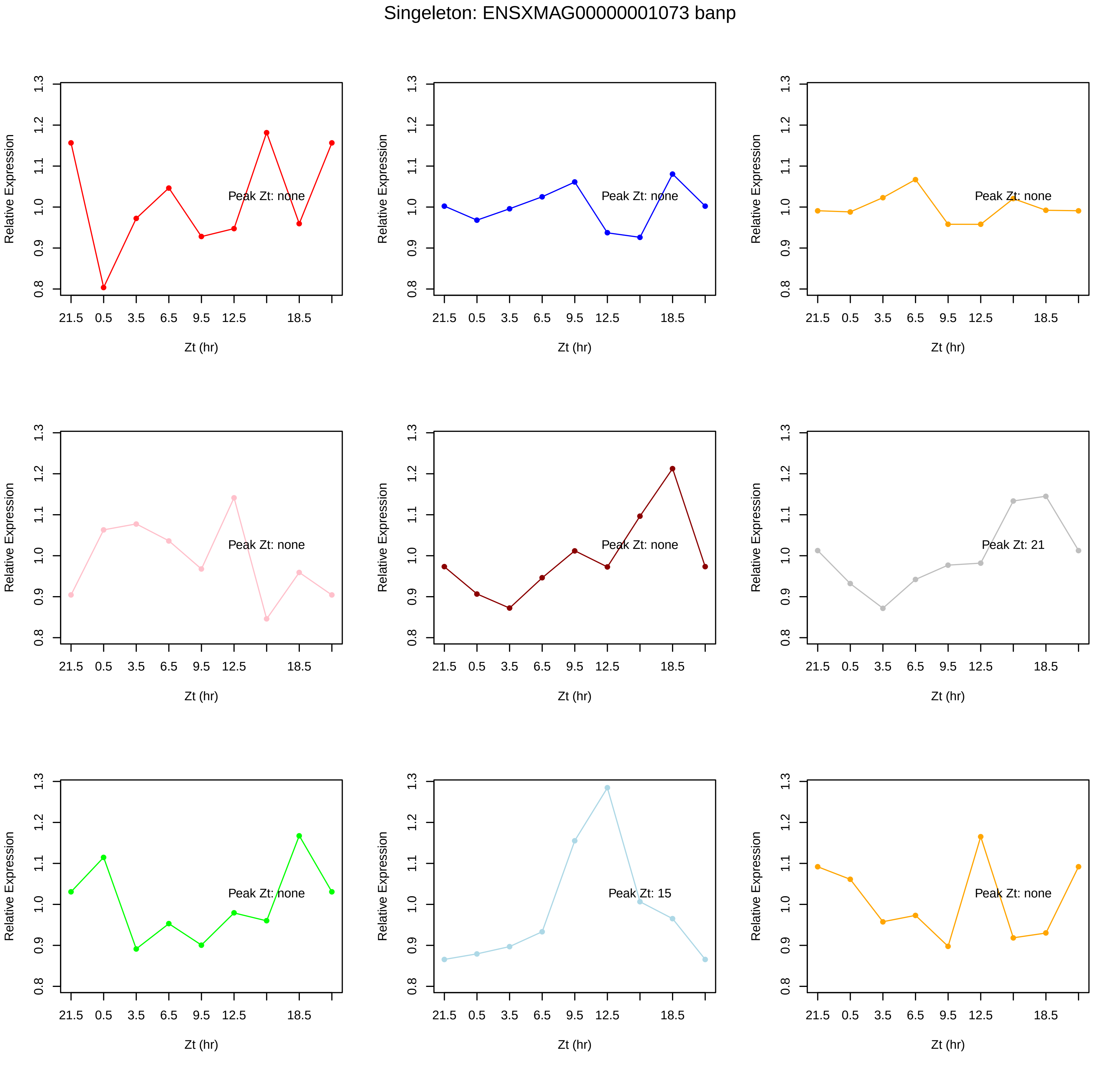
Singeleton: ENSXMAG00000001073 banp
Relative Expression
Zt (hr)
21.5
0.5
3.5
6.5
9.5
12.5
18.5
0.8
0.9
1.0
1.1
1.2
1.3
Peak Zt: none
Peak Zt: 21
Peak Zt: 15

Singeleton: ENSXMAG00000024576 ppih

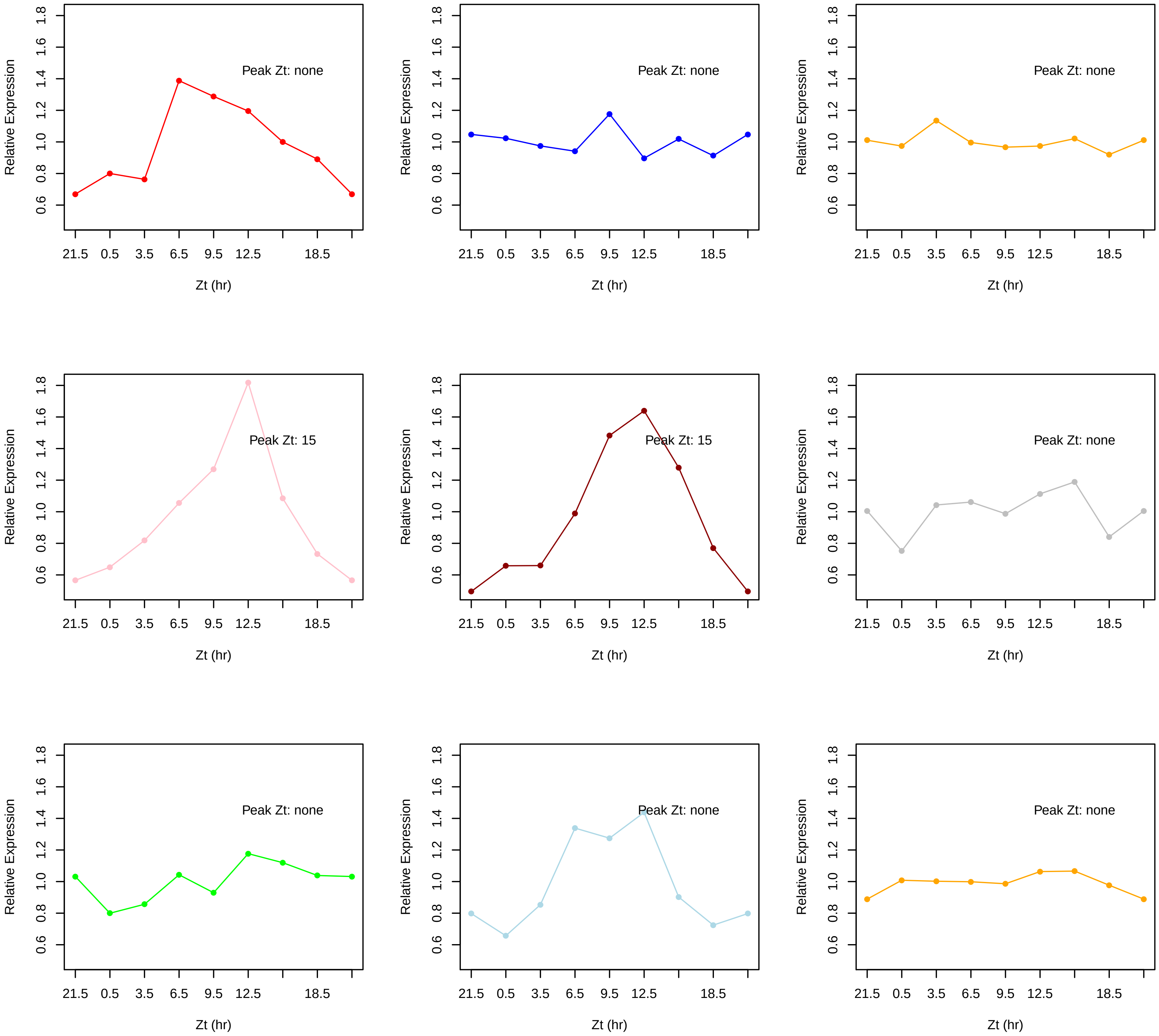

Singeleton: ENSXMAG00000023076 fam167b

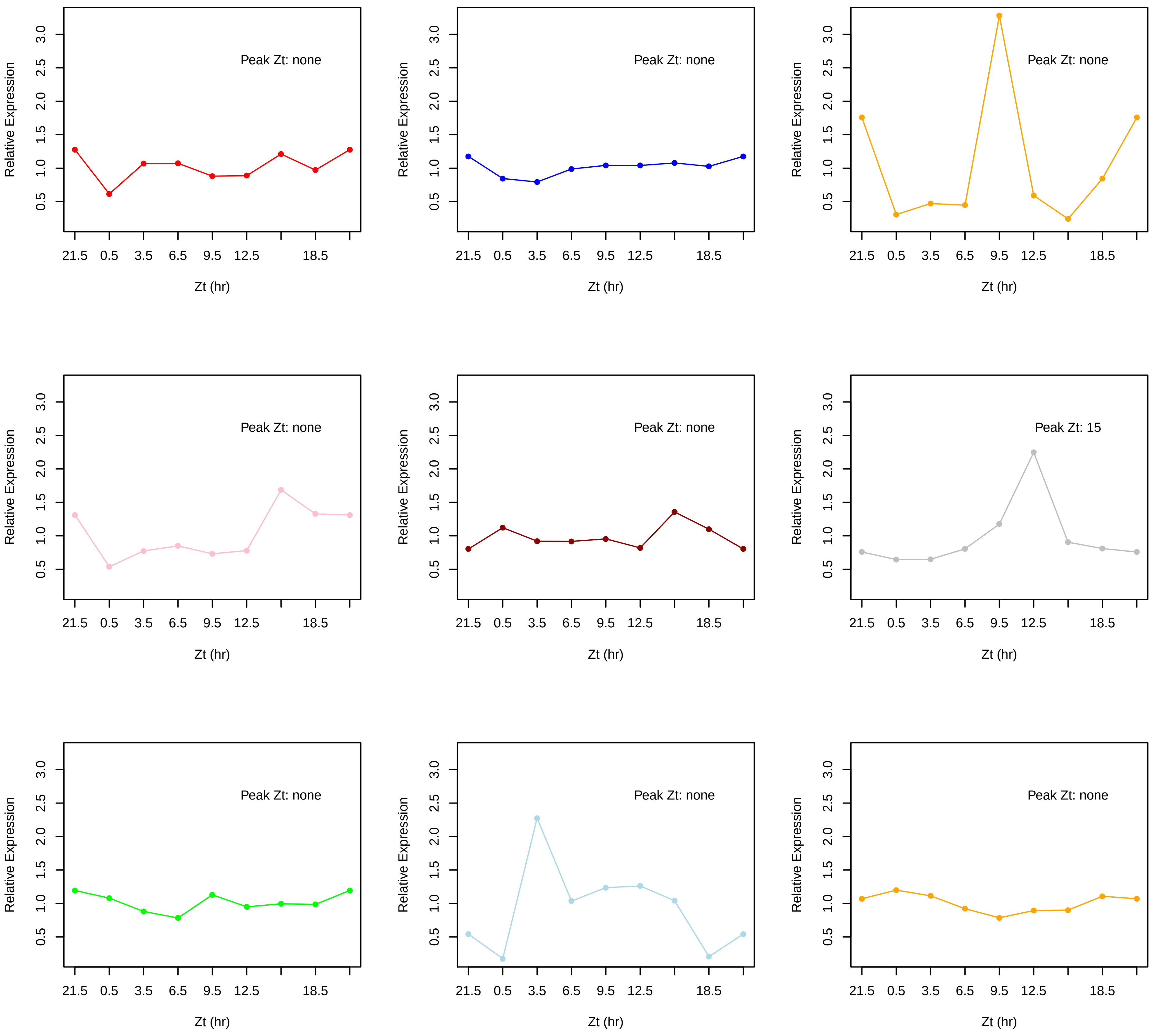

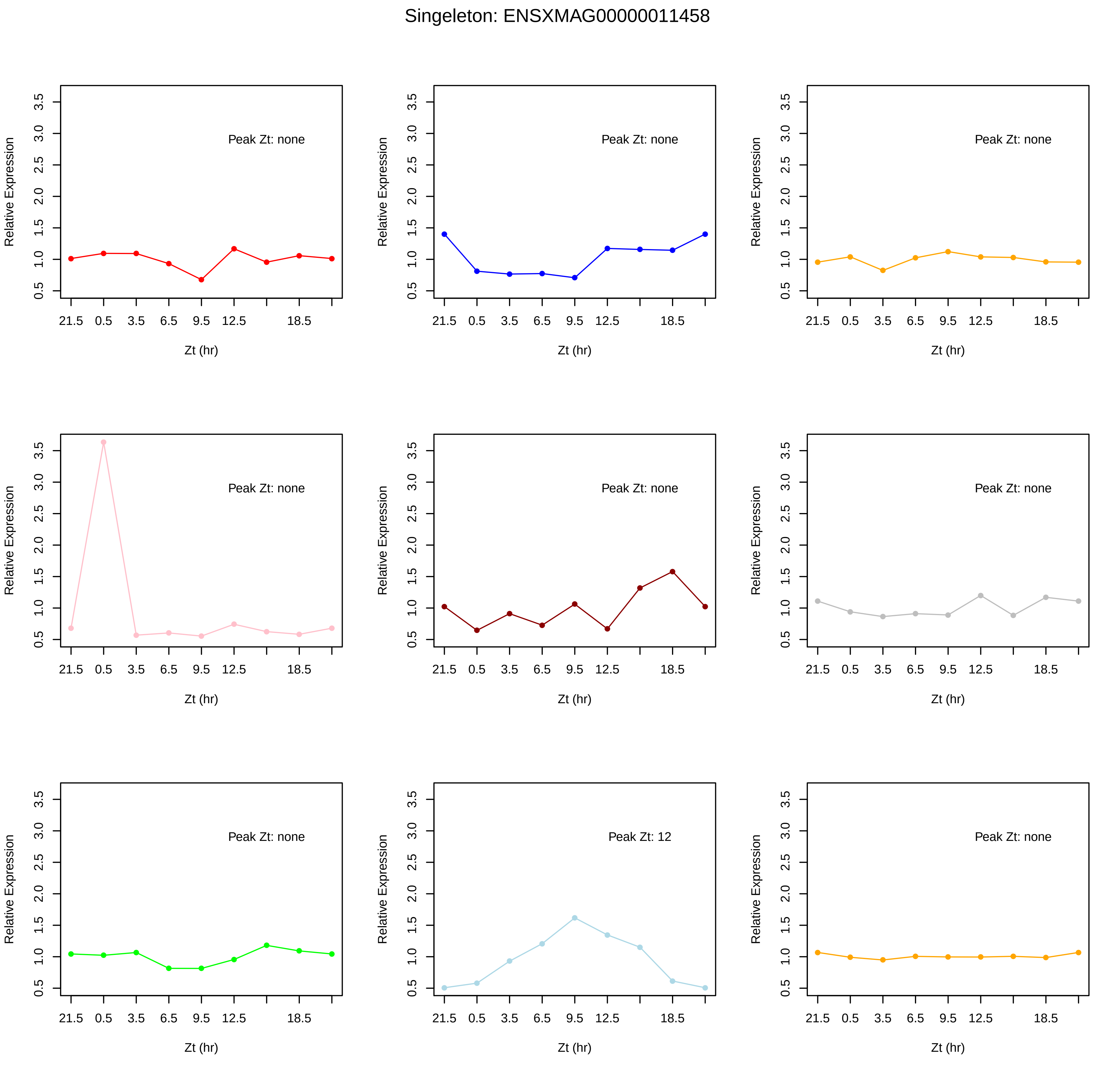

Singeleton: ENSXMAG00000011458
Peak Zt: none
Peak Zt: none
Peak Zt: none
Peak Zt: none
Peak Zt: none
Peak Zt: none
Peak Zt: none
Peak Zt: 12
Peak Zt: none
Relative Expression
Zt (hr)
21.5
0.5
3.5
6.5
9.5
12.5
18.5
0.5
1.0
1.5
2.0
2.5
3.0
3.5

Singeleton: ENSXMAG00000005907 SPNS2

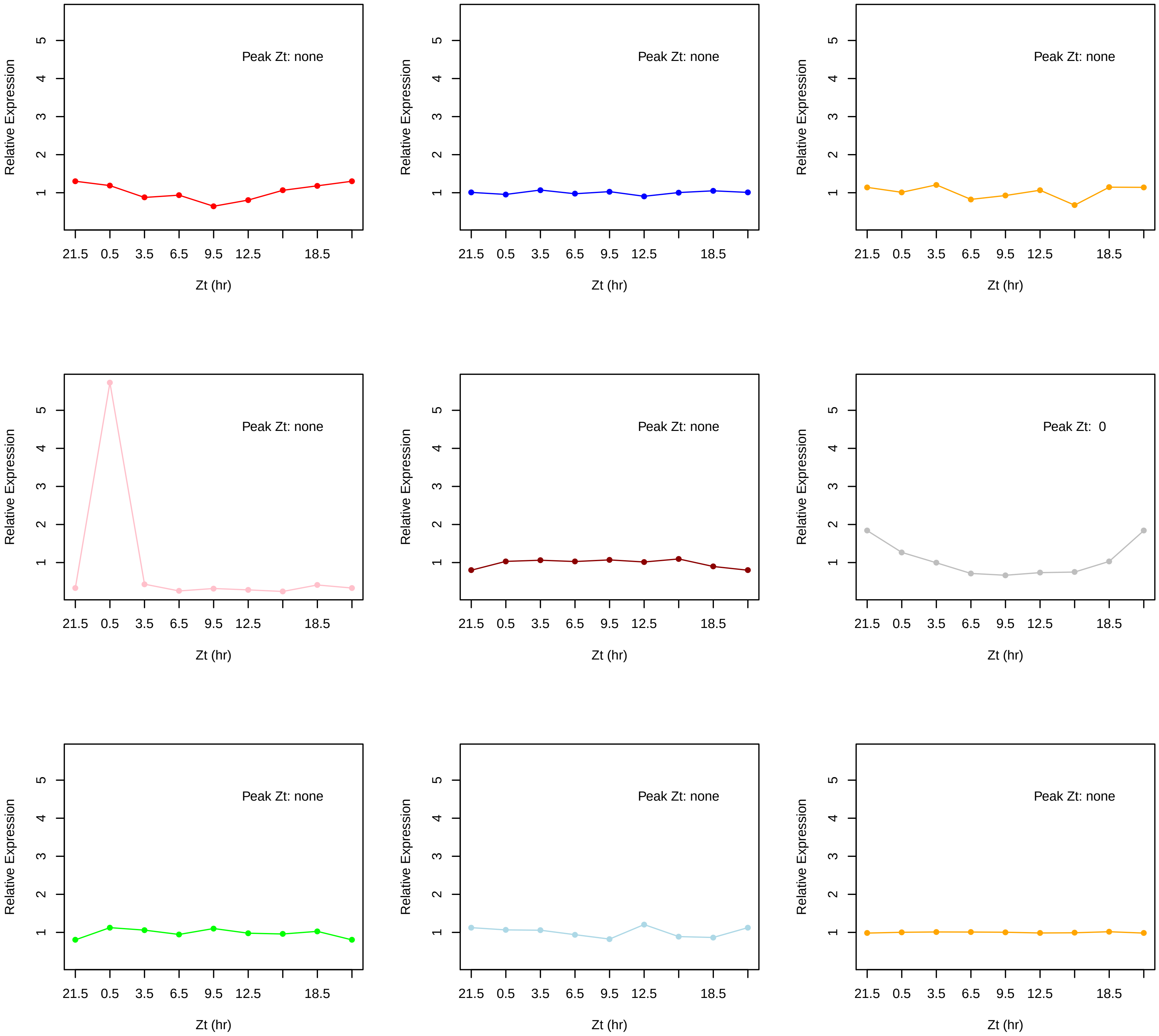

Singleton: ENSXMAG00000004797 adra1d

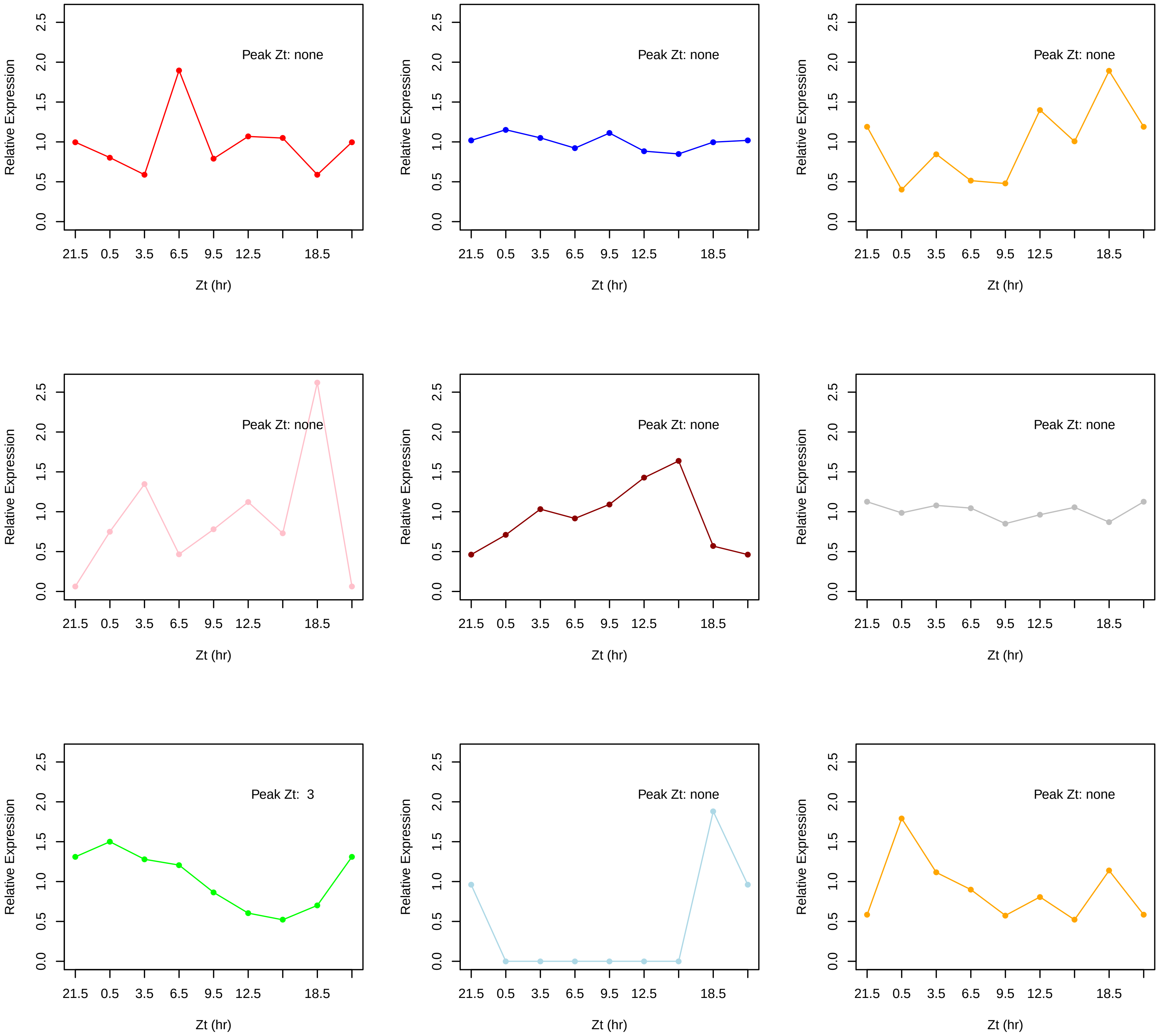

Singeleton: ENSXMAG00000018936 lck

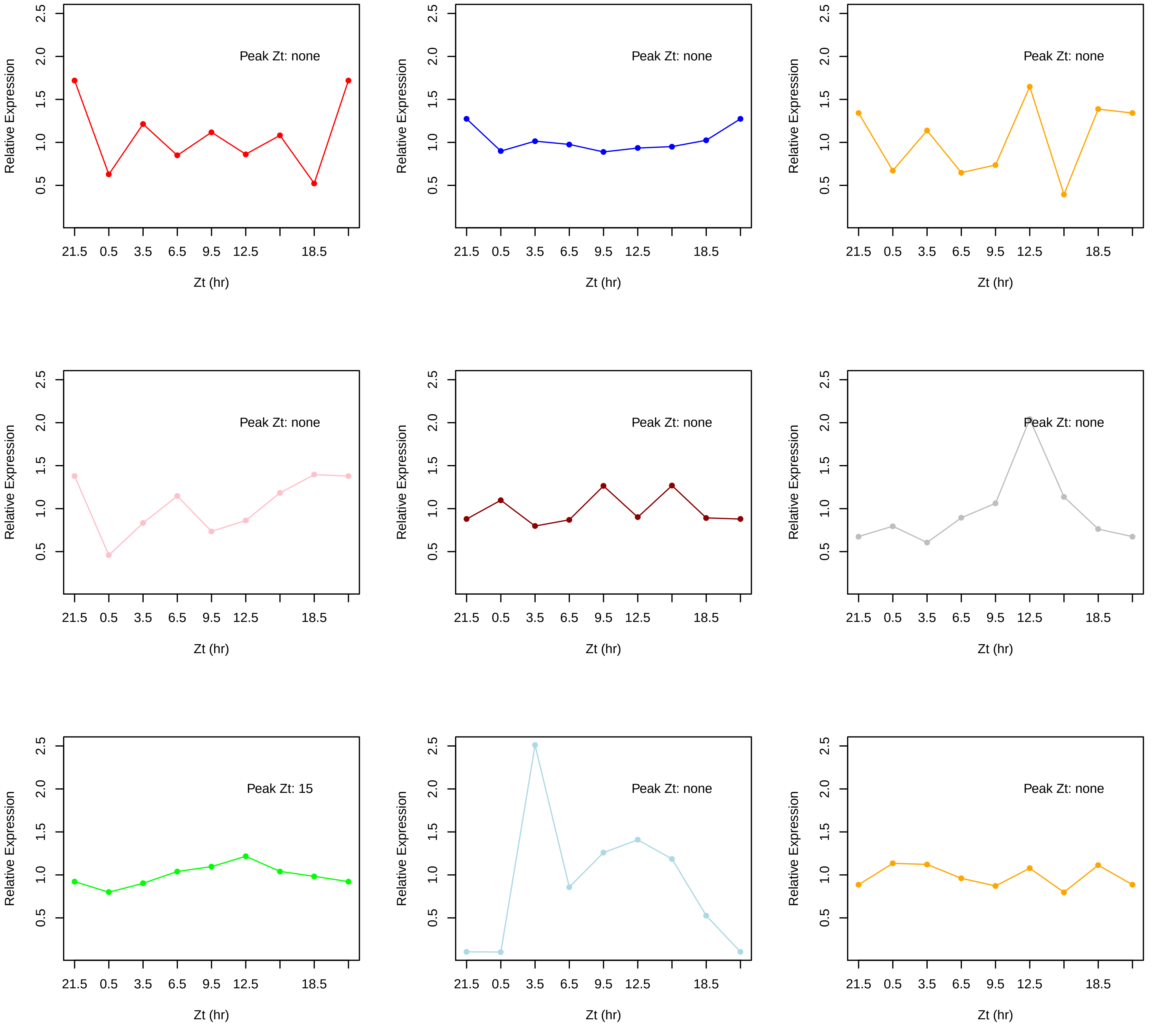

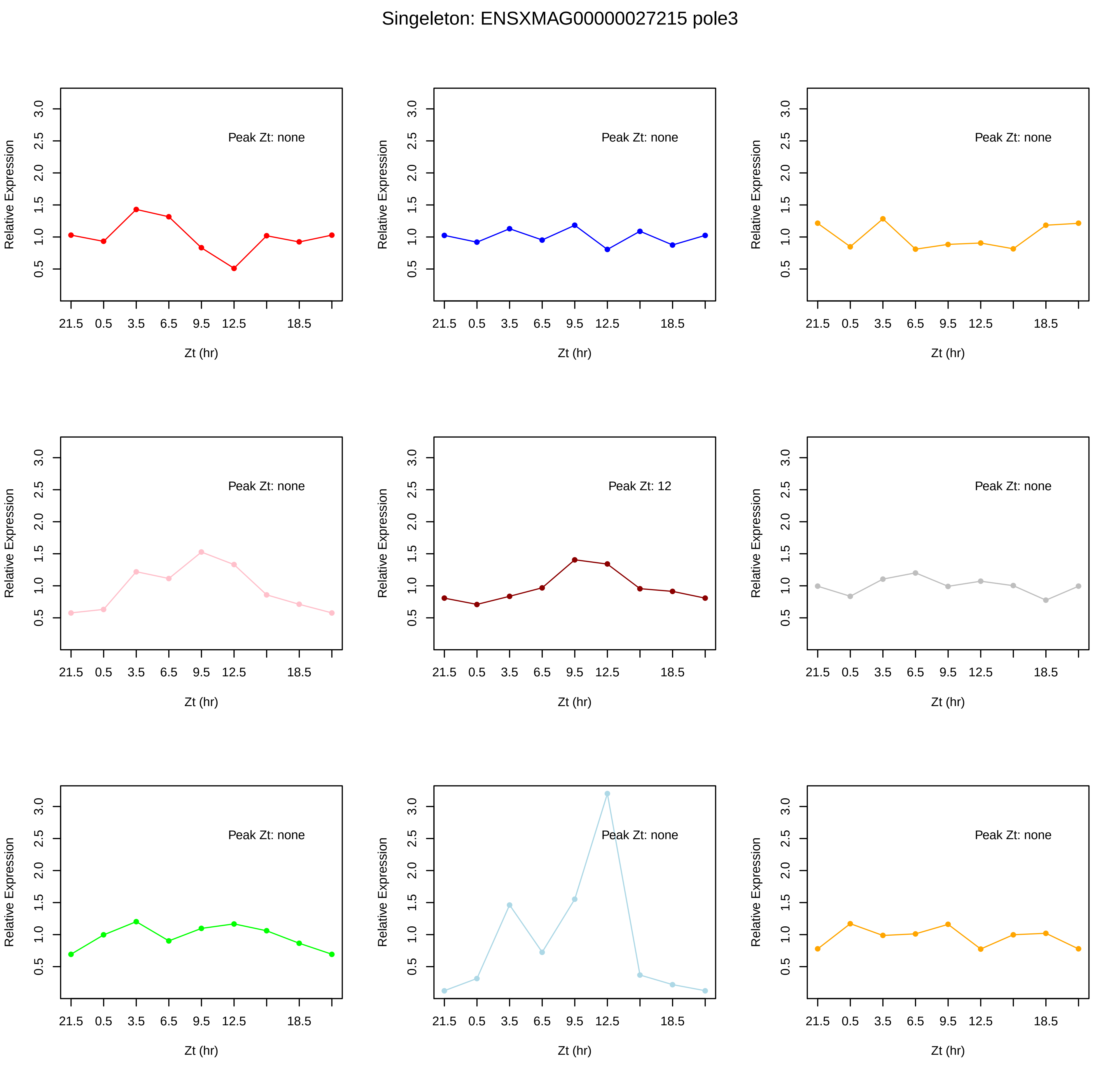
Singeleton: ENSXMAG00000027215 pole3
Peak Zt: none
Peak Zt: none
Peak Zt: none
Peak Zt: none
Peak Zt: 12
Peak Zt: none
Peak Zt: none
Peak Zt: none
Peak Zt: none
Relative Expression
Zt (hr)
21.5
0.5
3.5
6.5
9.5
12.5
18.5

Singleton: ENSXMAG00000010981 pcnx3

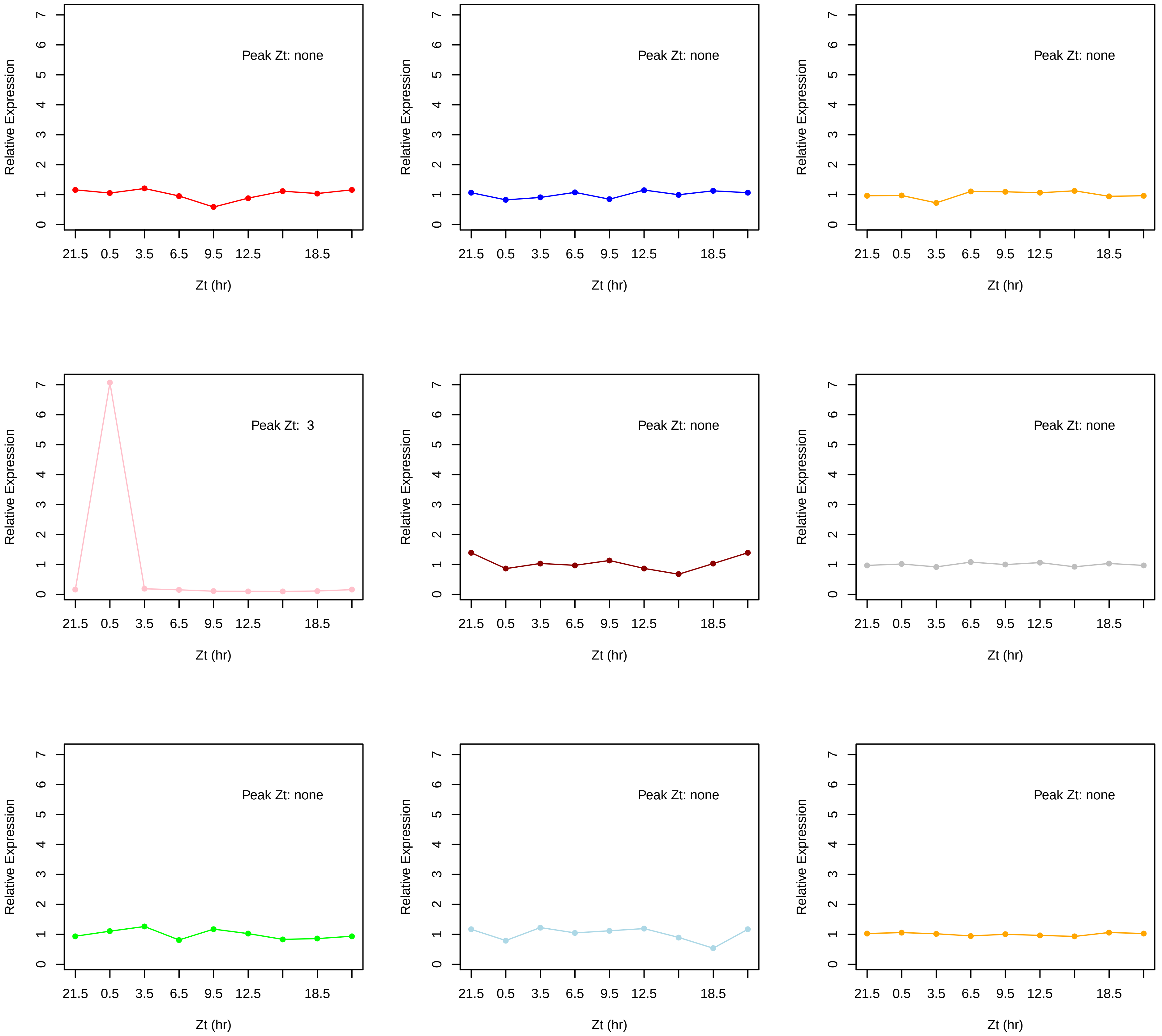

Singleton: ENSXMAG00000012243 hacd2

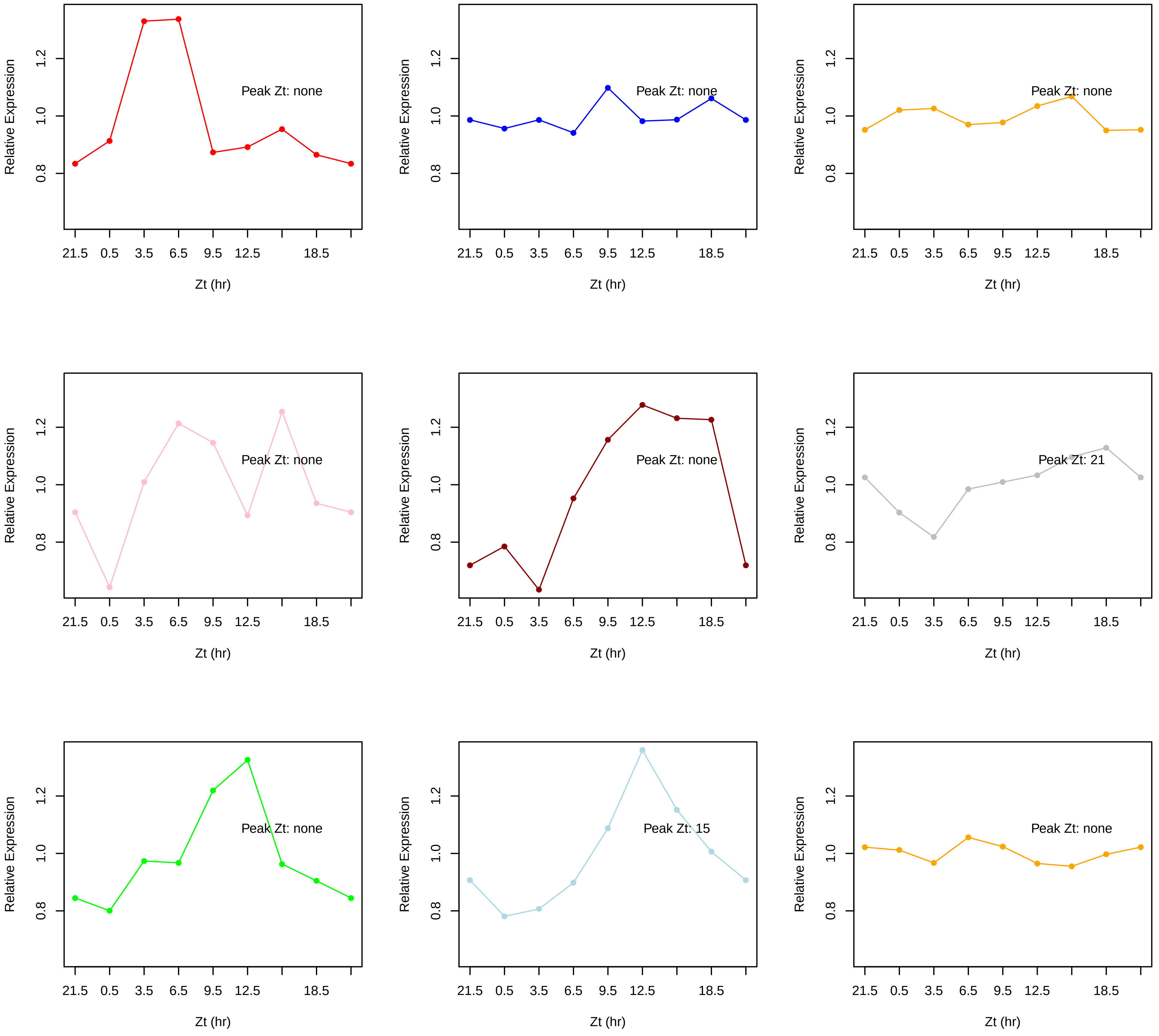

Singleton: ENSXMAG00000006268 zgc:91860

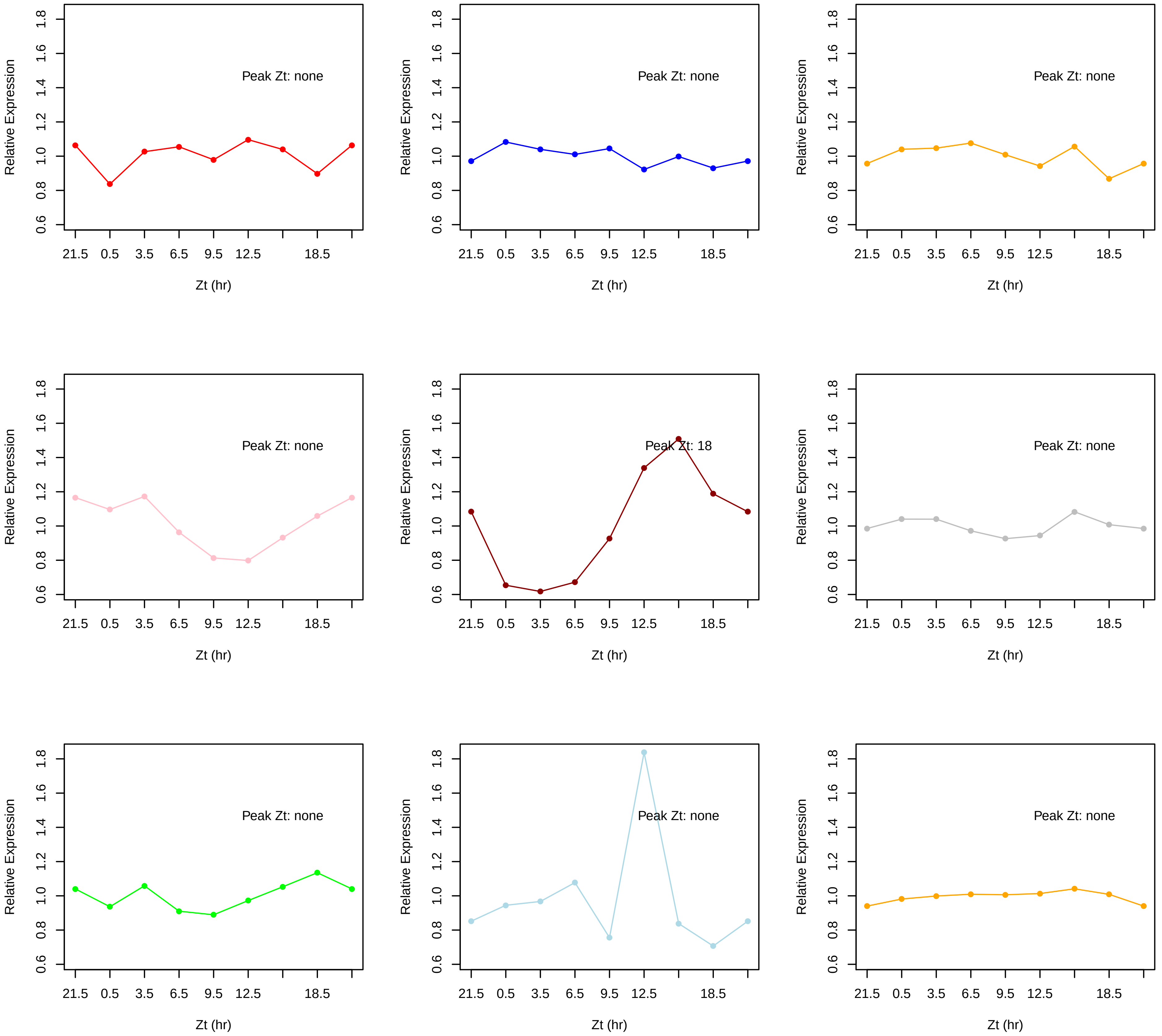

Singeleton: ENSXMAG00000013902

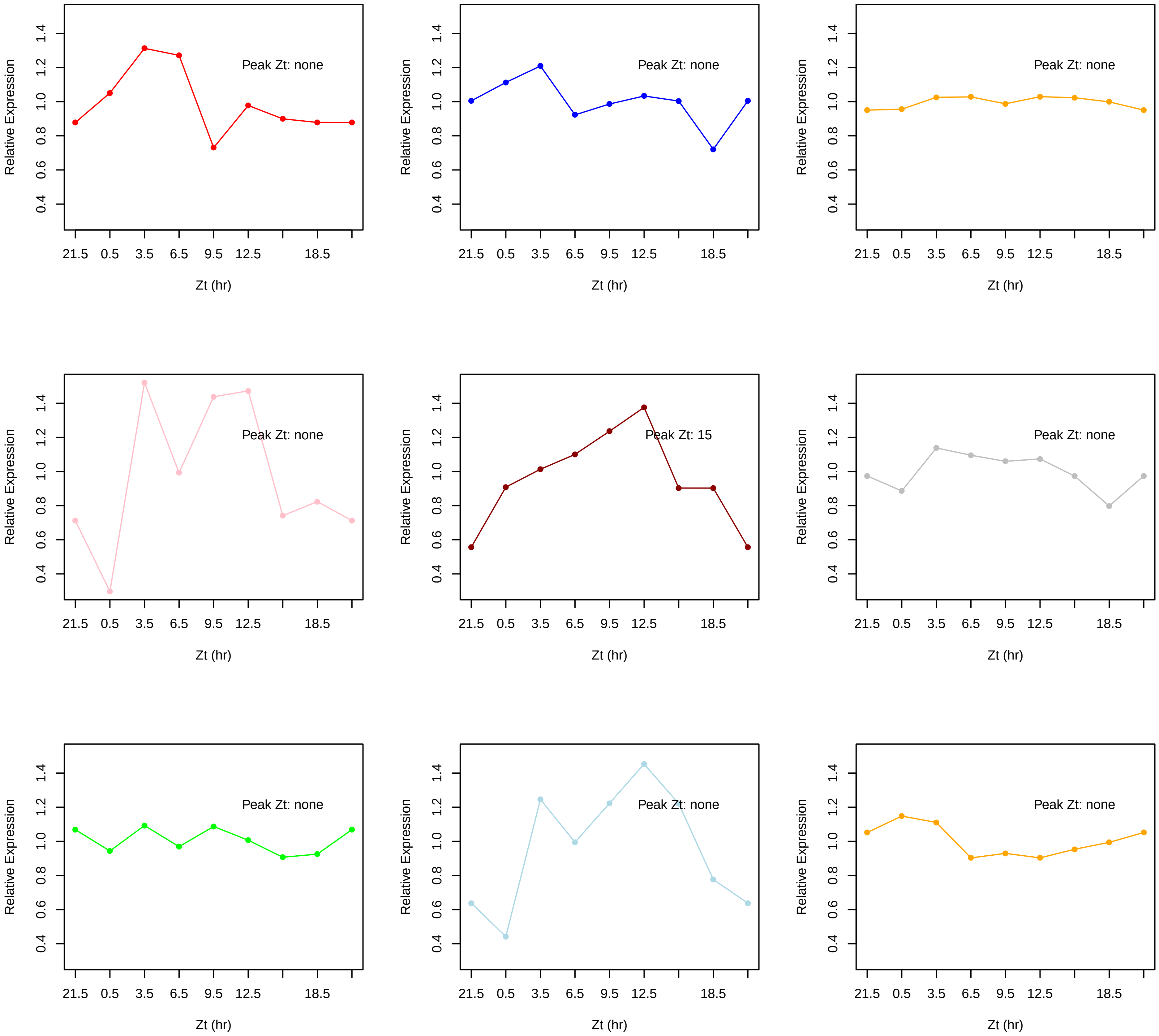

Singeleton: ENSXMAG00000012679 pfdn4

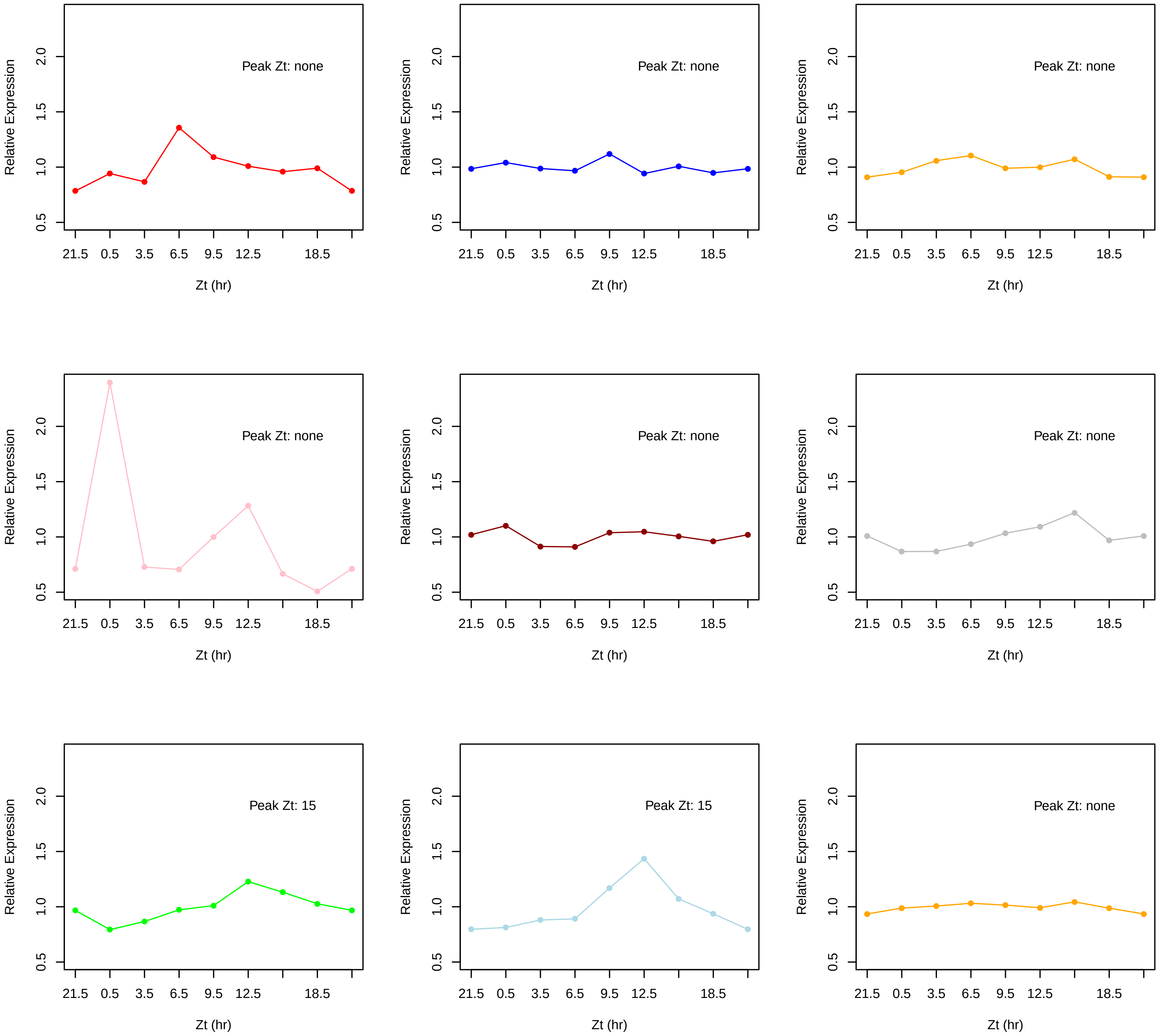

Singeleton: ENSXMAG00000017038 lrsam1

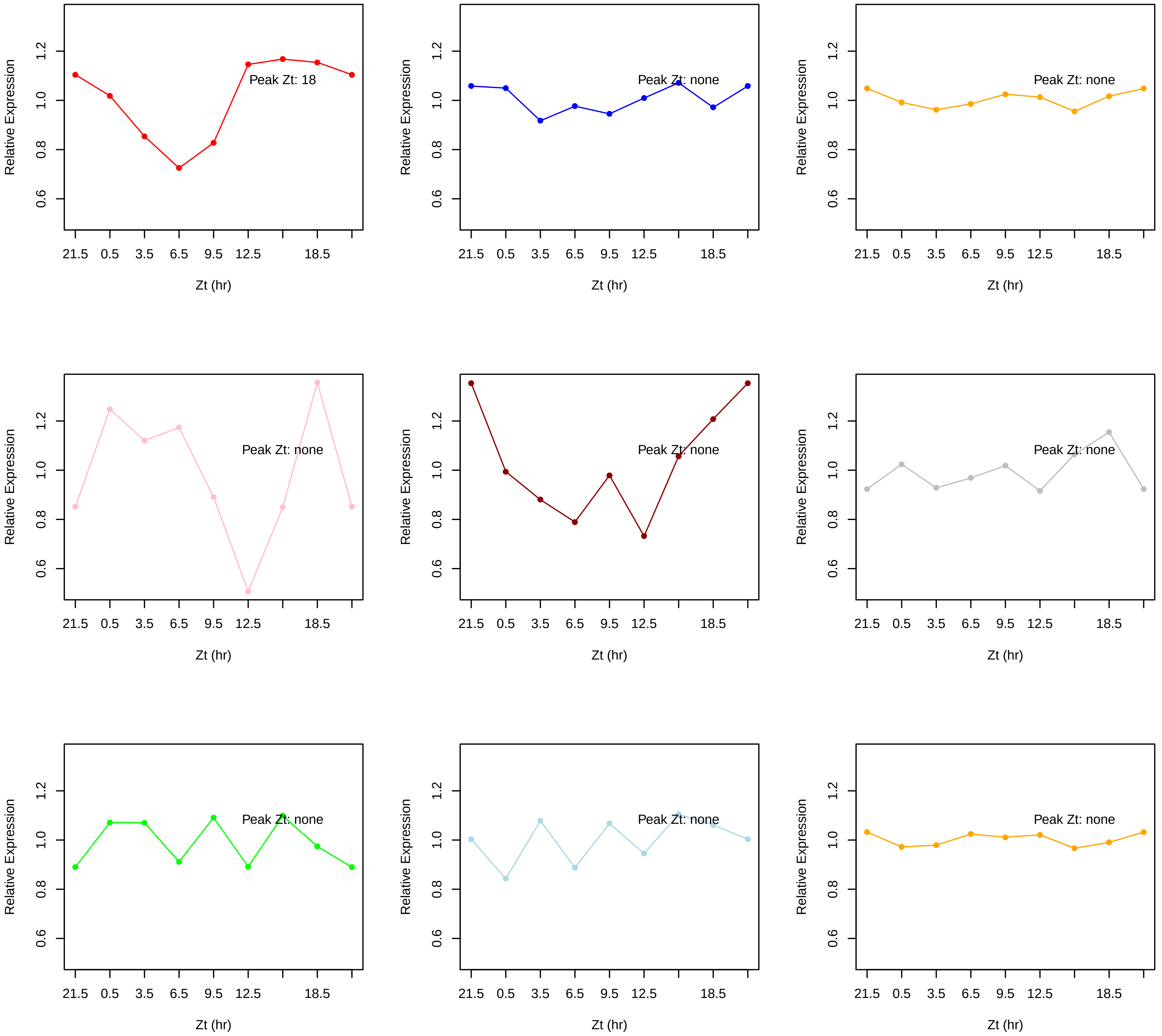

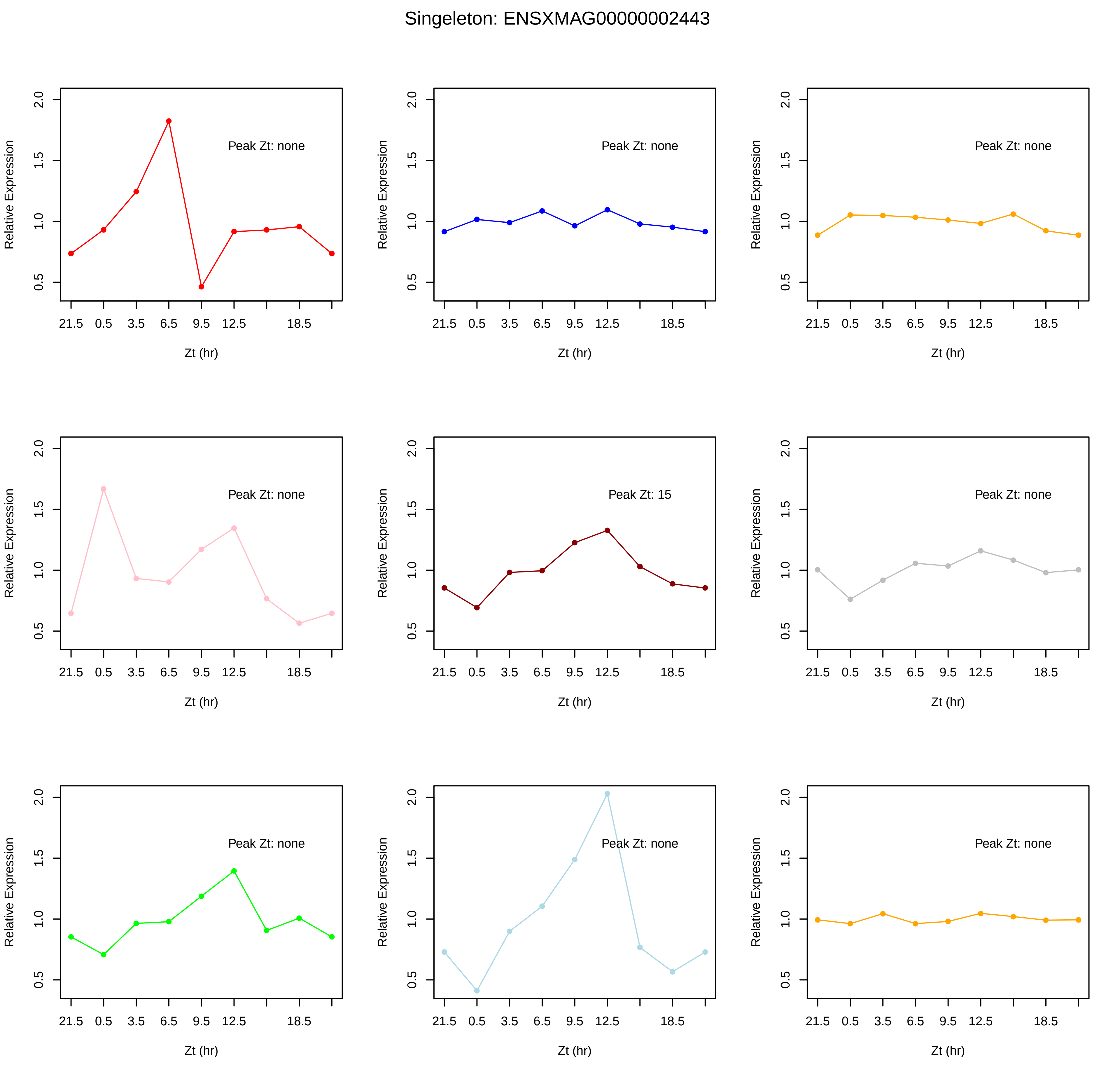
Singleton: ENSXMAG00000002443
Peak Zt: none
Peak Zt: none
Peak Zt: none
Peak Zt: none
Peak Zt: 15
Peak Zt: none
Peak Zt: none
Peak Zt: none
Peak Zt: none
Relative Expression
Zt (hr)
21.5
0.5
3.5
6.5
9.5
12.5
18.5
0.5
1.0
1.5
2.0

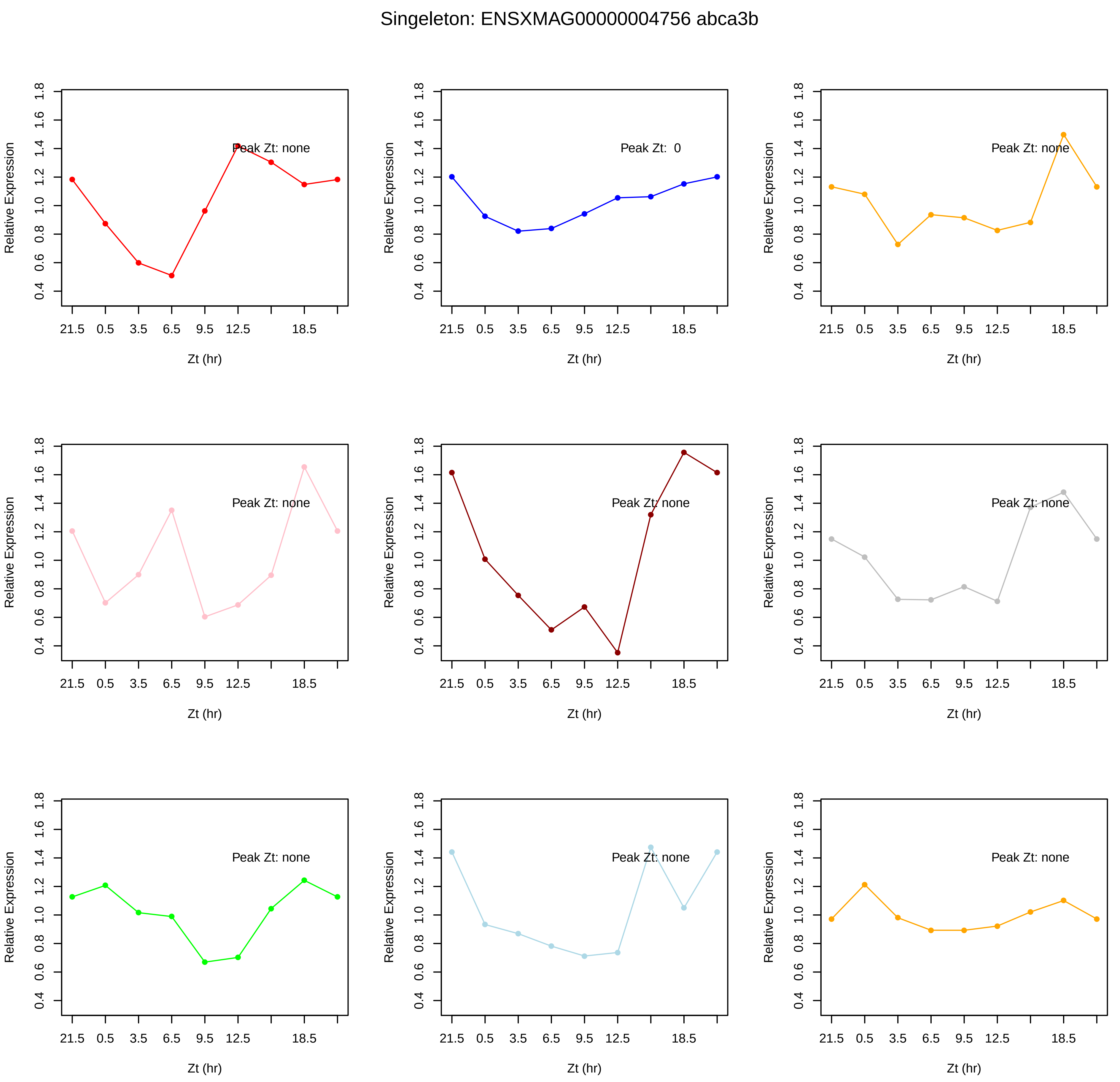

Singeleton: ENSXMAG00000004756 abca3b
Peak Zt: none
Peak Zt: 0
Peak Zt: none
Peak Zt: none
Peak Zt: none
Peak Zt: none
Peak Zt: none
Peak Zt: none
Peak Zt: none
Relative Expression
Zt (hr)
21.5 0.5 3.5 6.5 9.5 12.5 18.5
0.4 0.6 0.8 1.0 1.2 1.4 1.6 1.8

Singleton: ENSXMAG00000009325 si:ch211−63p21.1

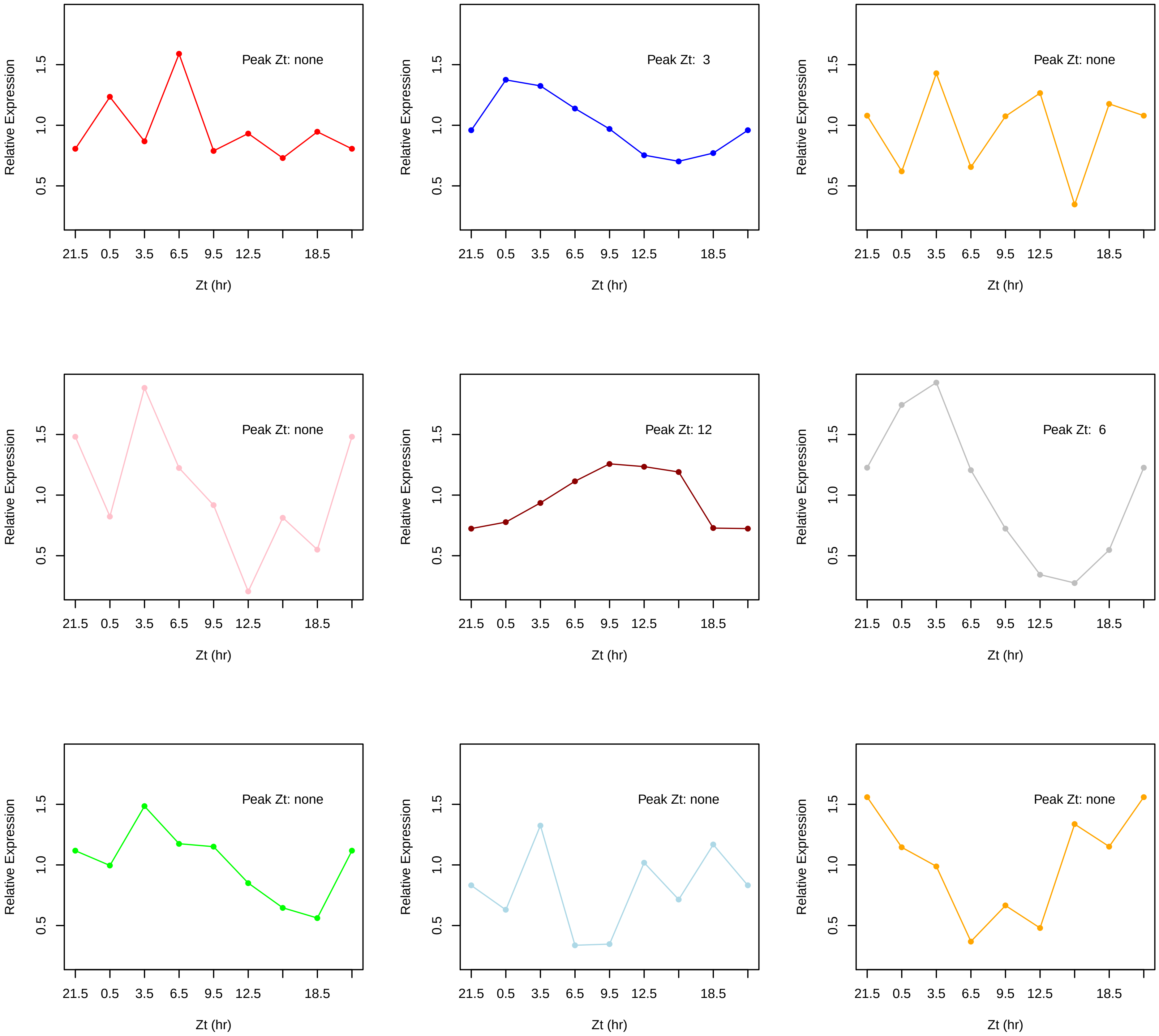

Singeleton: ENSXMAG00000016259 ppp6r3

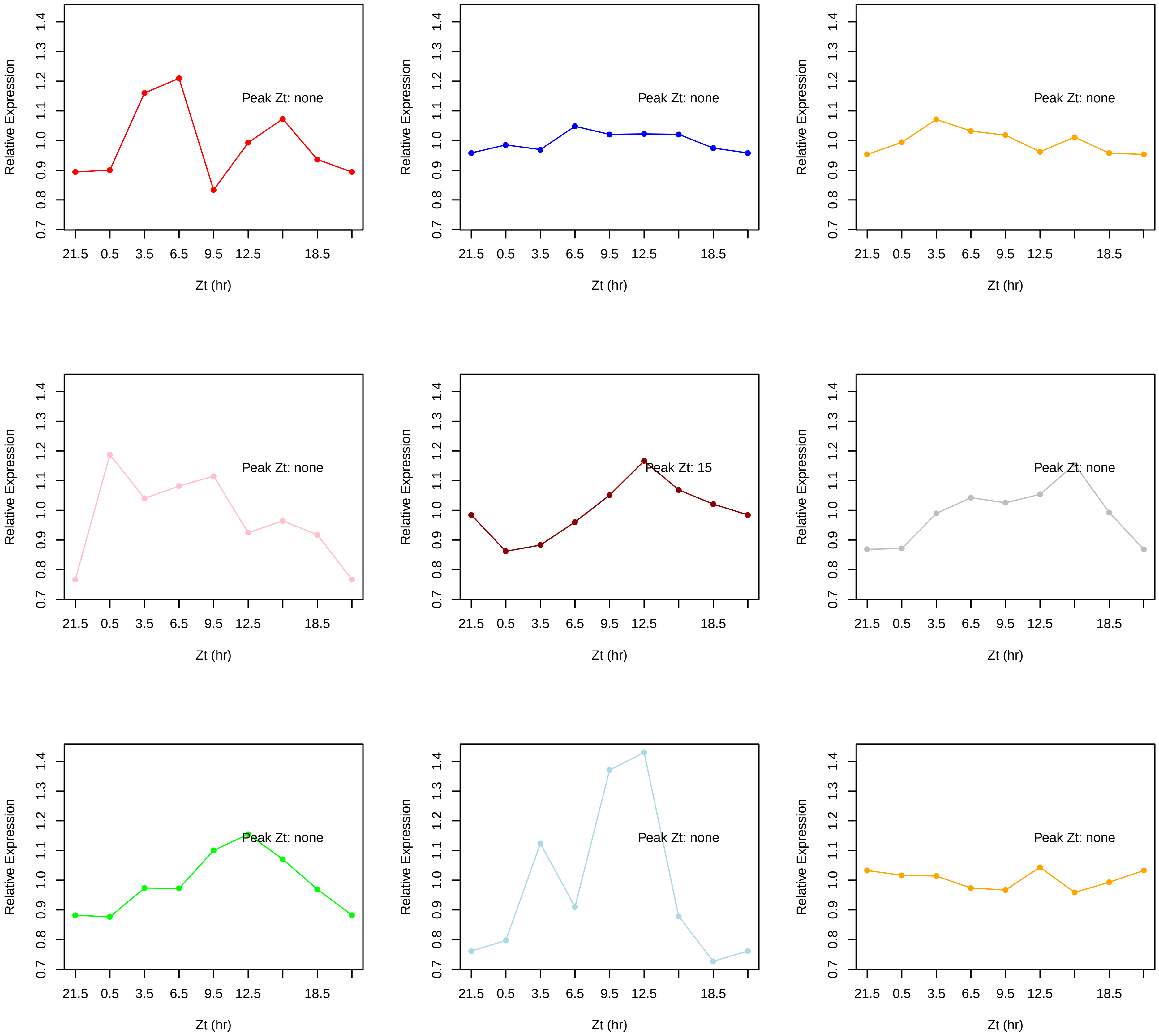

Singeleton: ENSXMAG00000002626 si:ch73−40i7.5

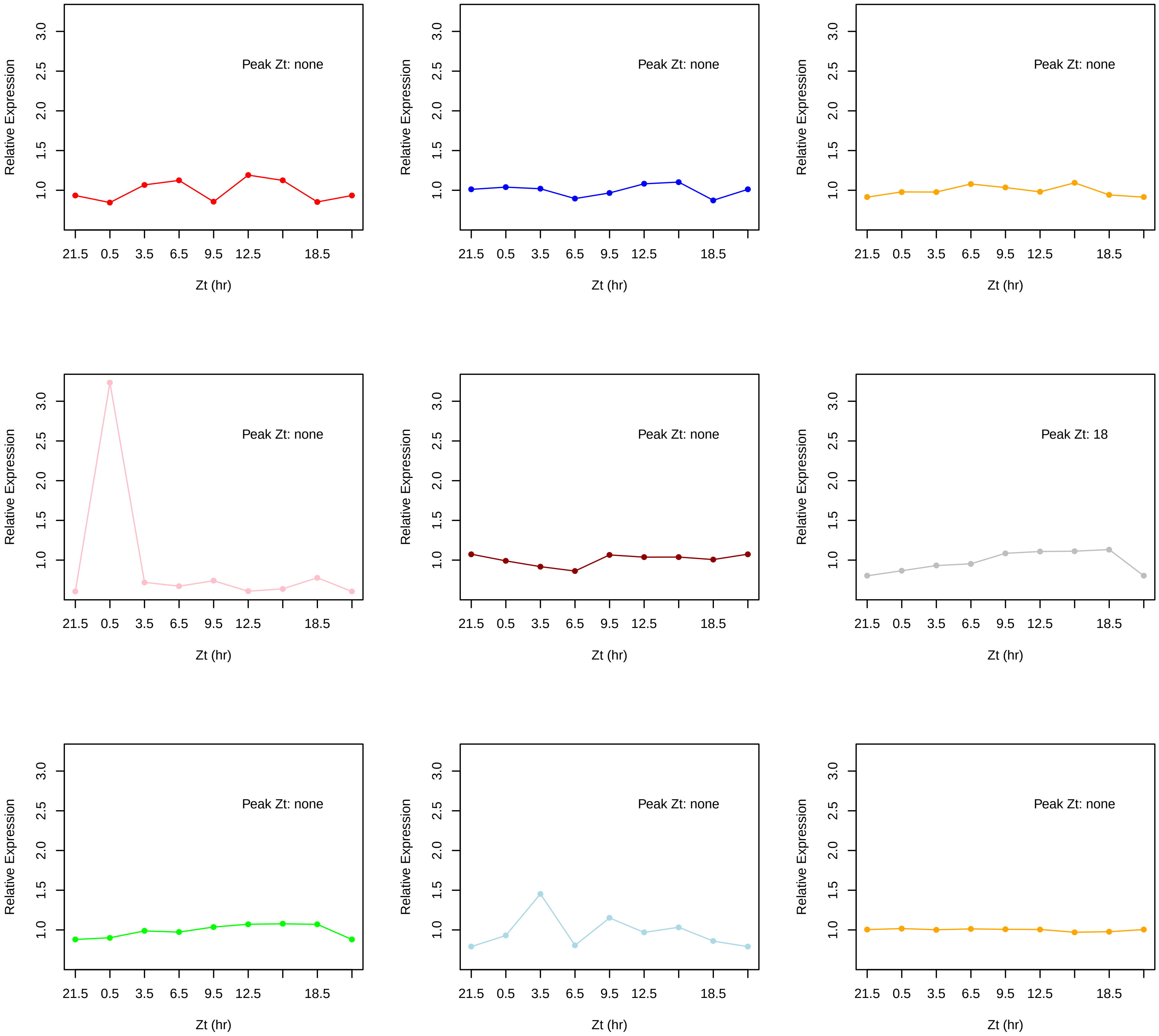

Singeleton: ENSXMAG00000004984

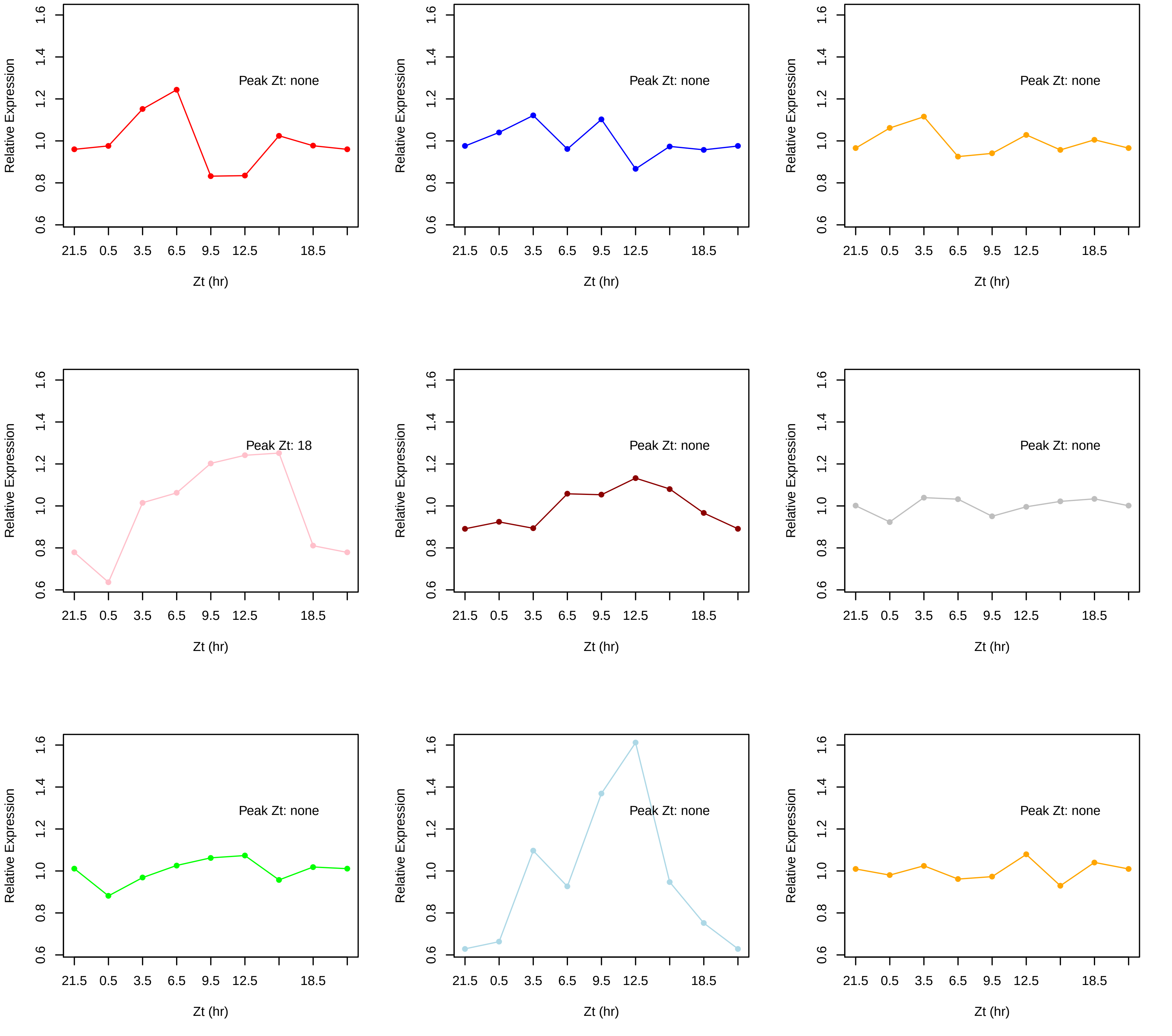

Singleton: ENSXMAG00000015704

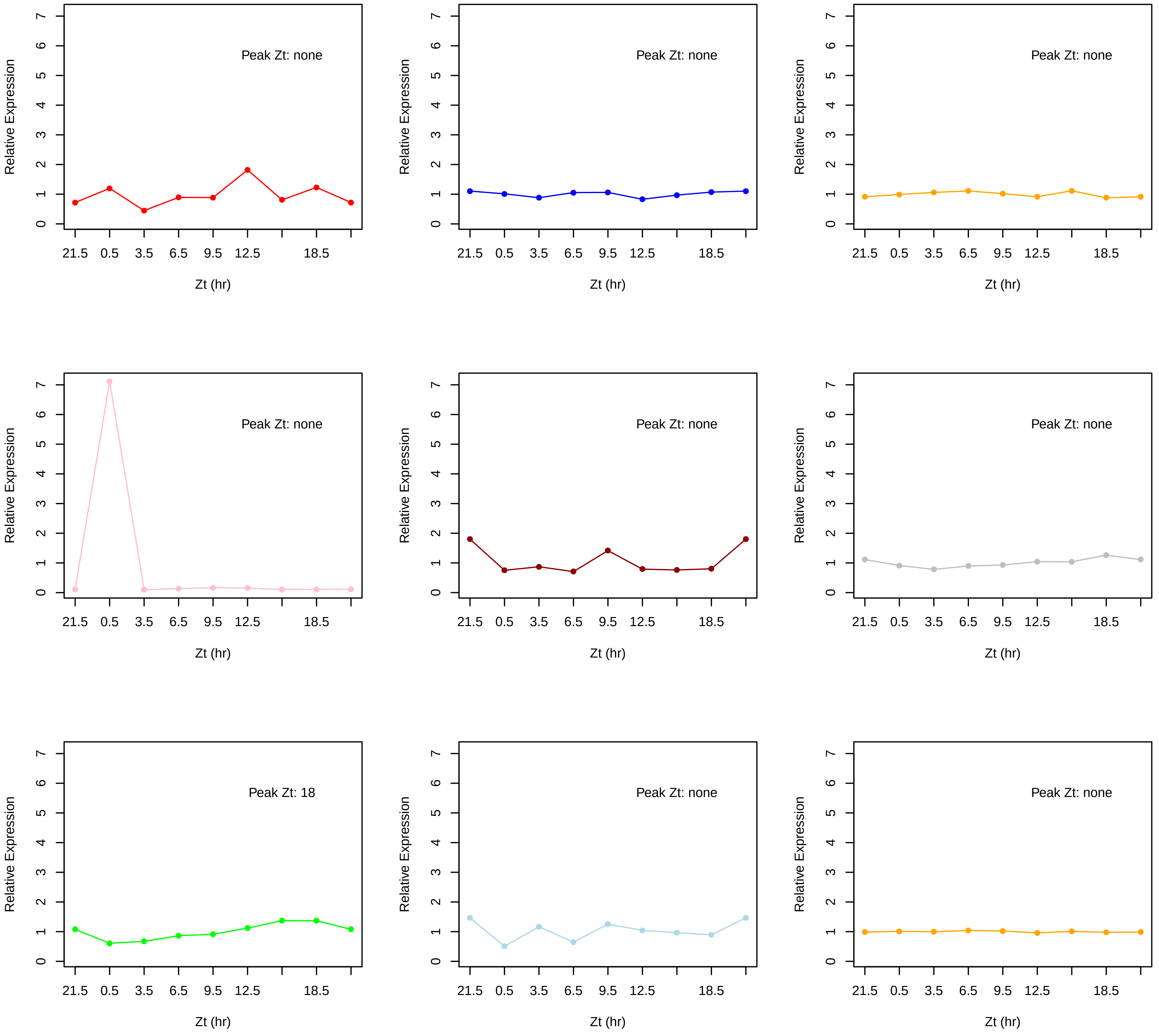

Singeleton: ENSXMAG00000013314 bbs9

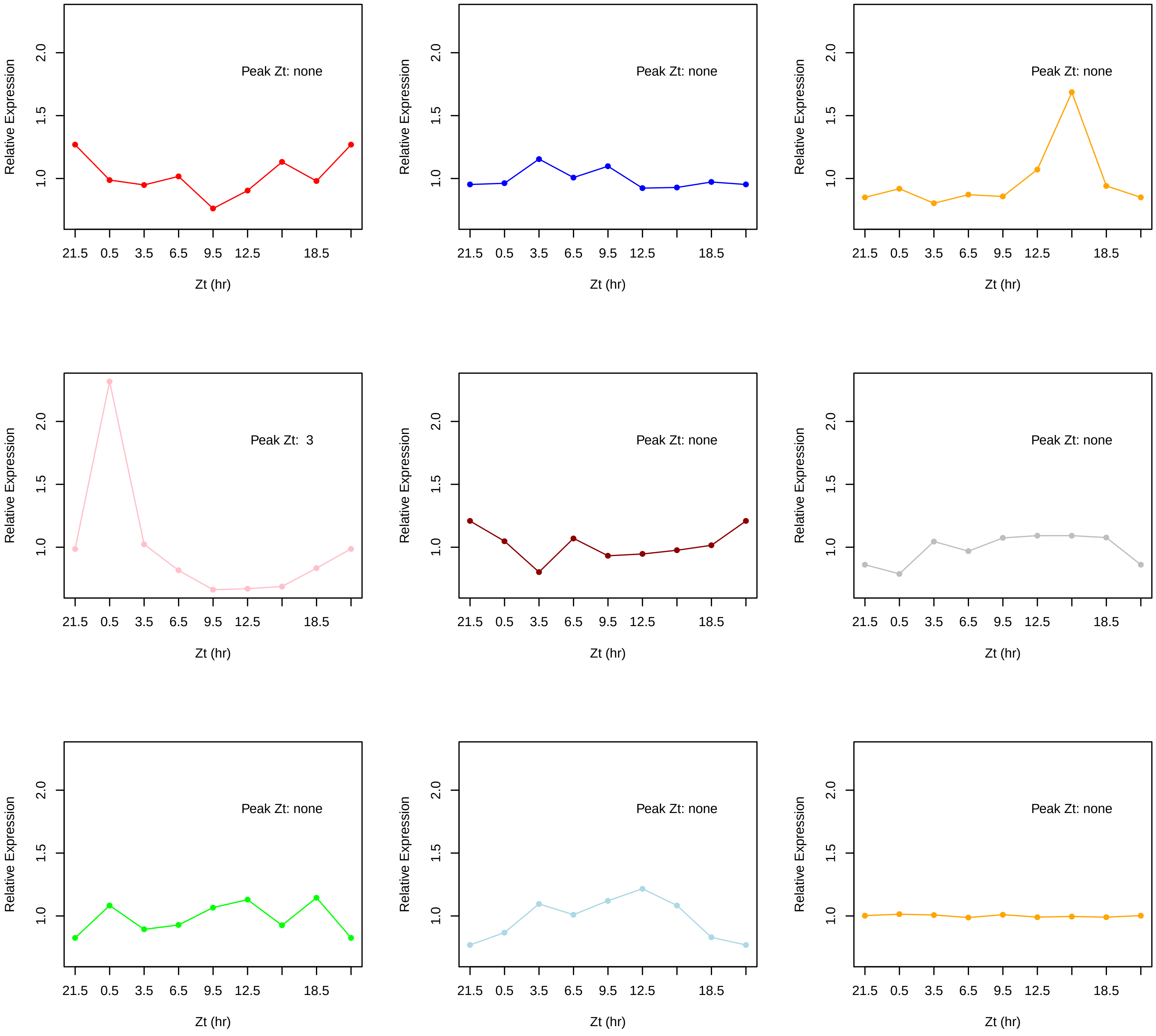

Singeleton: ENSXMAG00000001243 fli1a

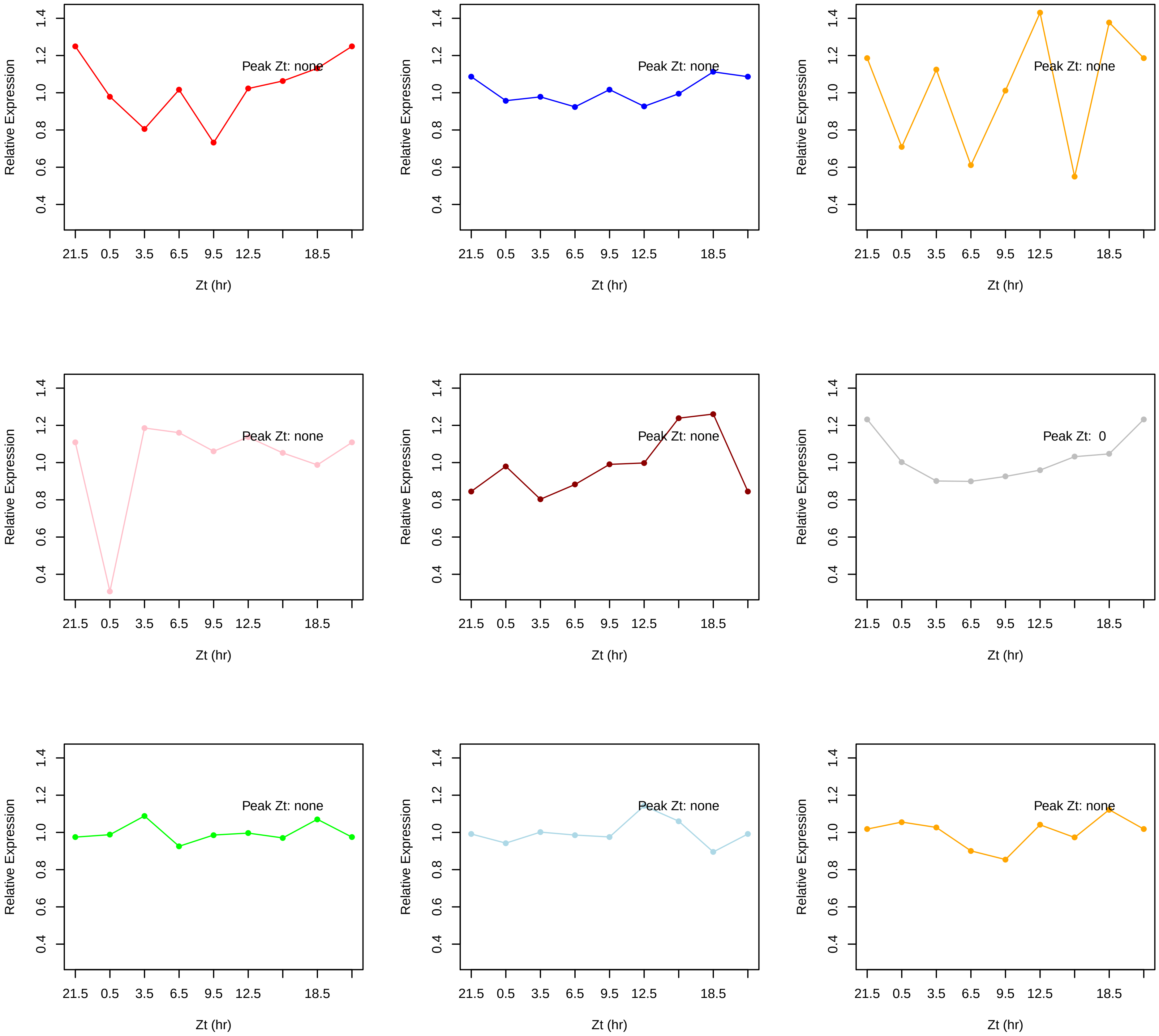

Singeleton: ENSXMAG00000024110 foxl1

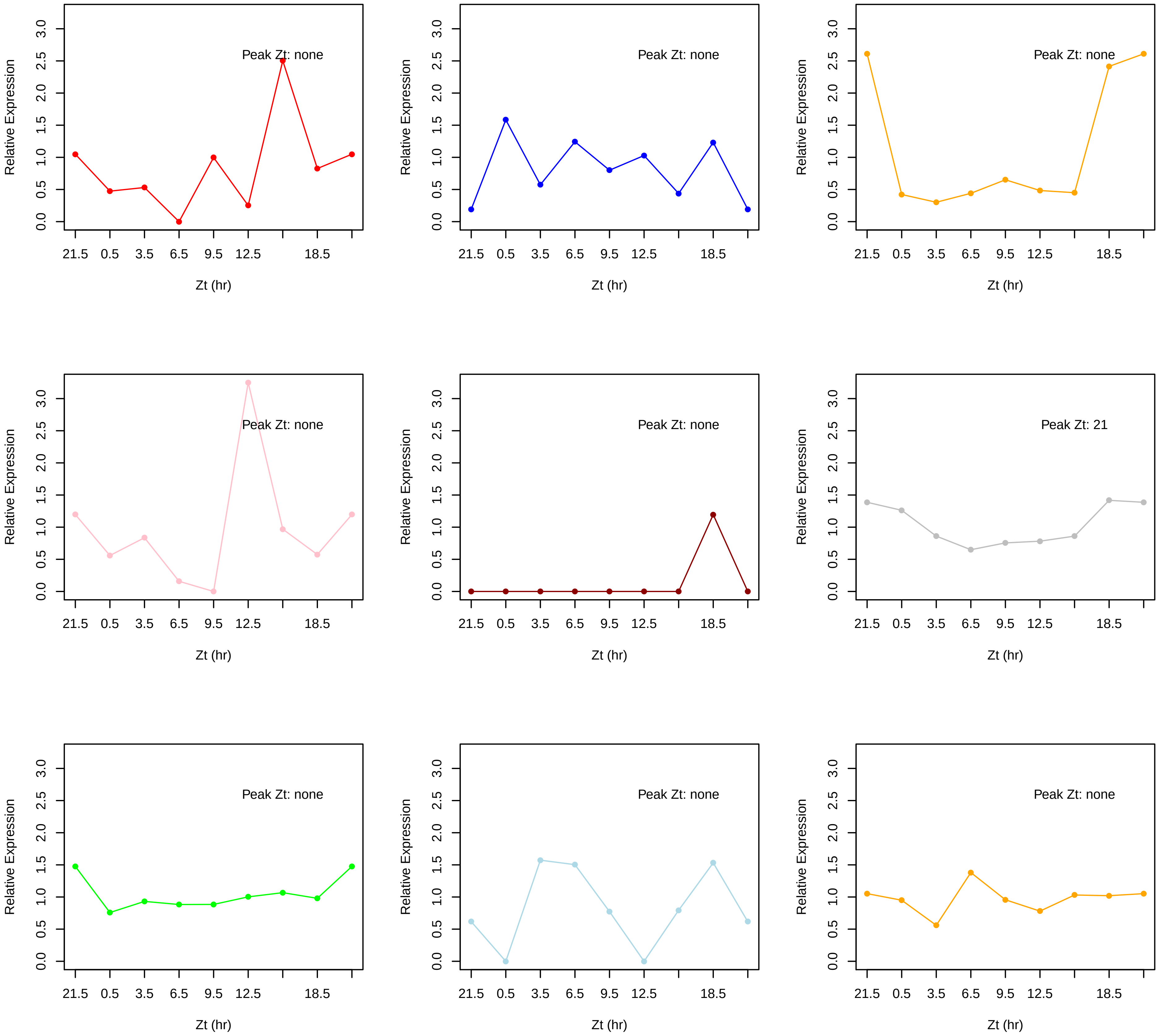

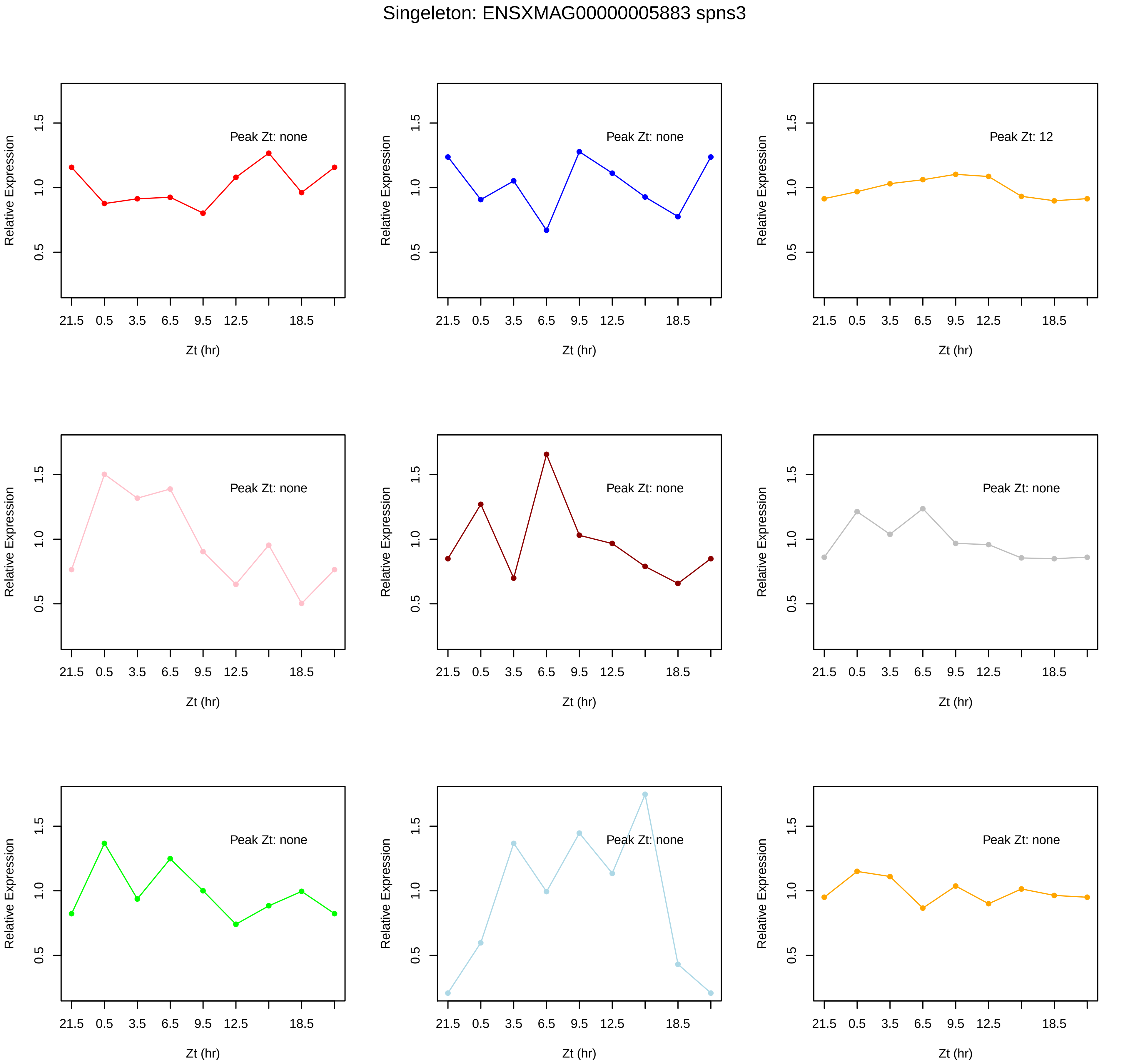
Singleton: ENSXMAG00000005883 spns3
Peak Zt: none
Peak Zt: none
Peak Zt: 12
Peak Zt: none
Peak Zt: none
Peak Zt: none
Peak Zt: none
Peak Zt: none
Peak Zt: none
Relative Expression
Zt (hr)
21.5
0.5
3.5
6.5
9.5
12.5
18.5
0.5
1.0
1.5

Singeleton: ENSXMAG00000003001 bbs1

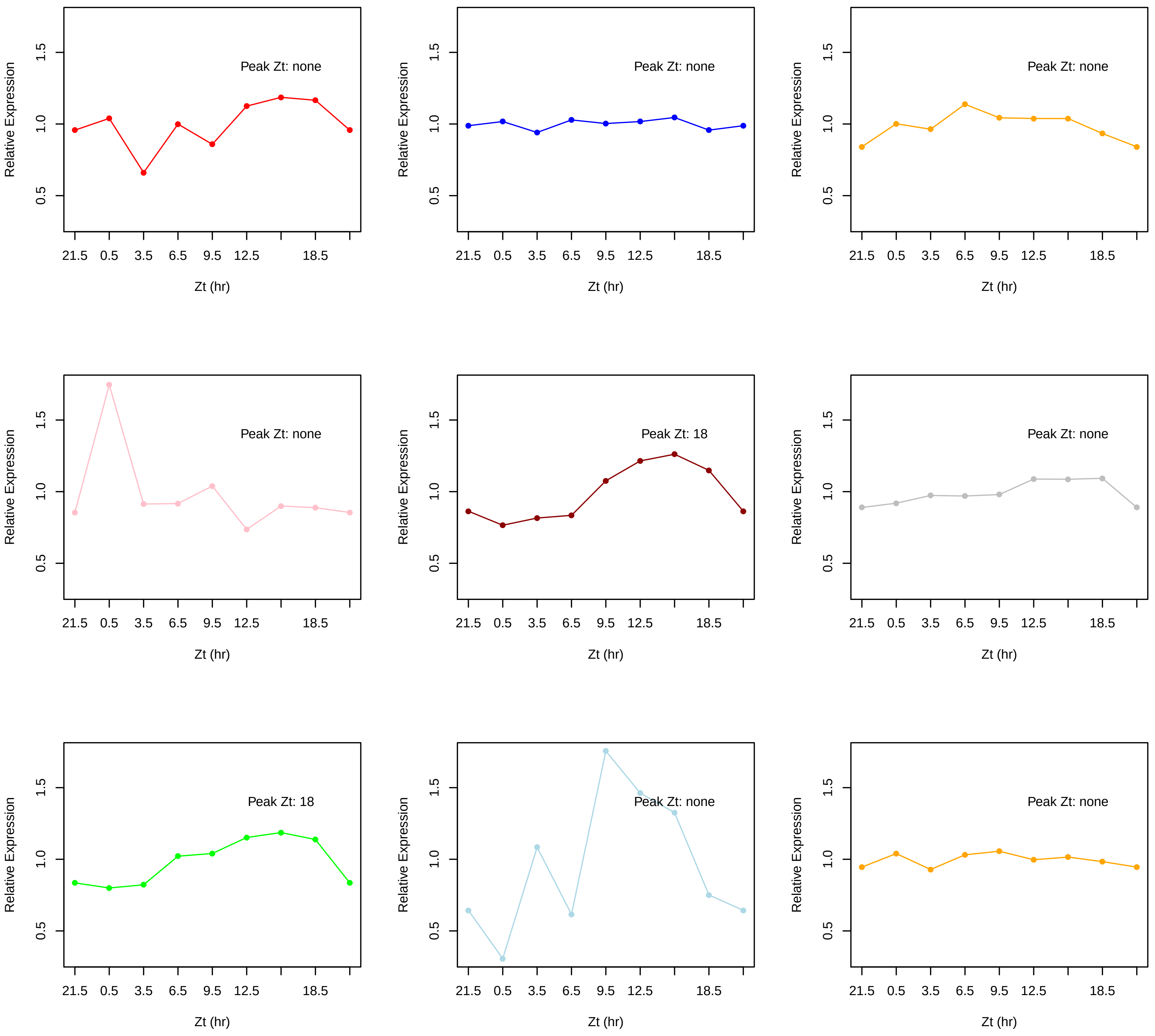

Singeleton: ENSXMAG00000002450 ZDHHC8

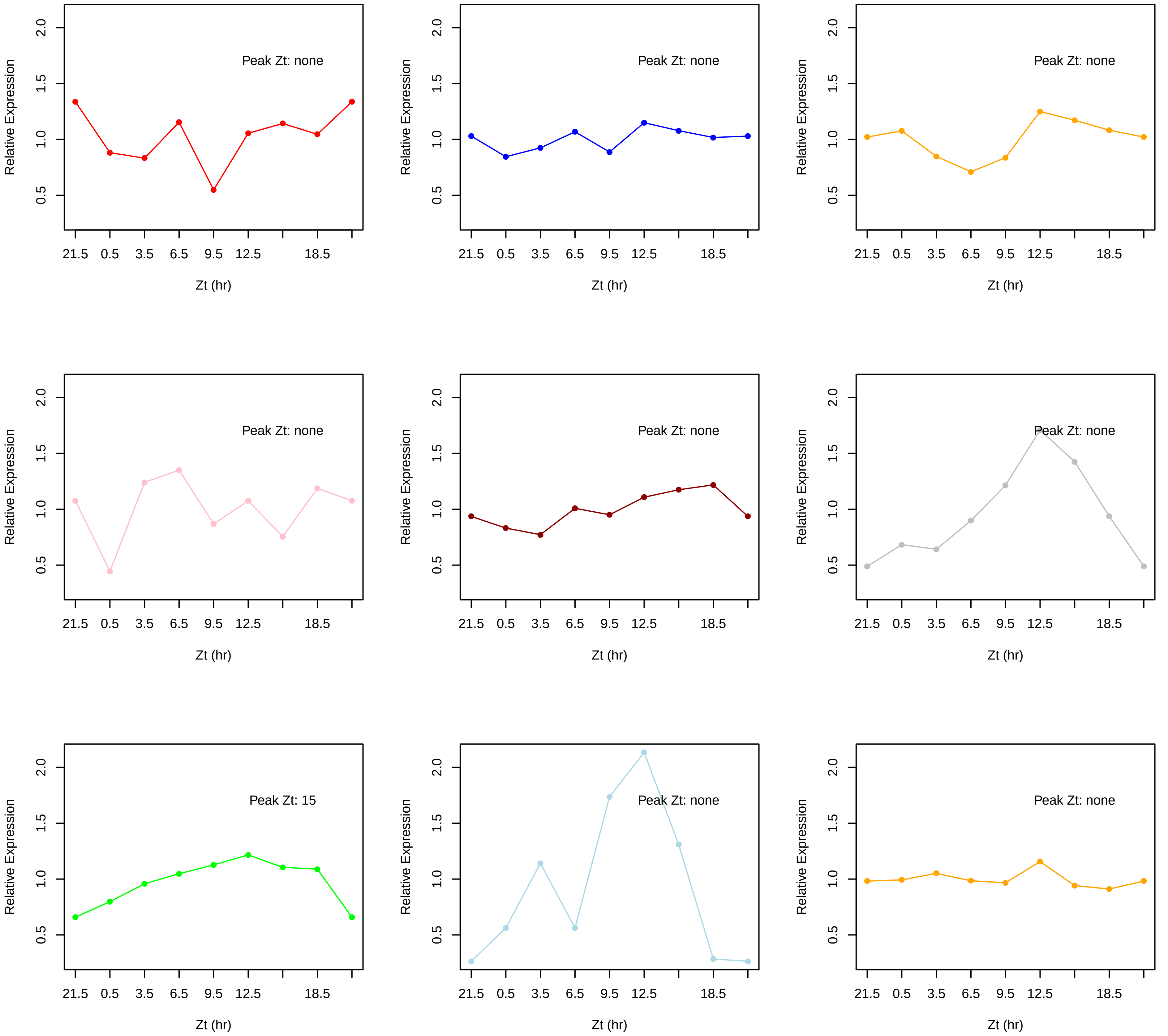

Singleton: ENSXMAG00000018748 bsdc1

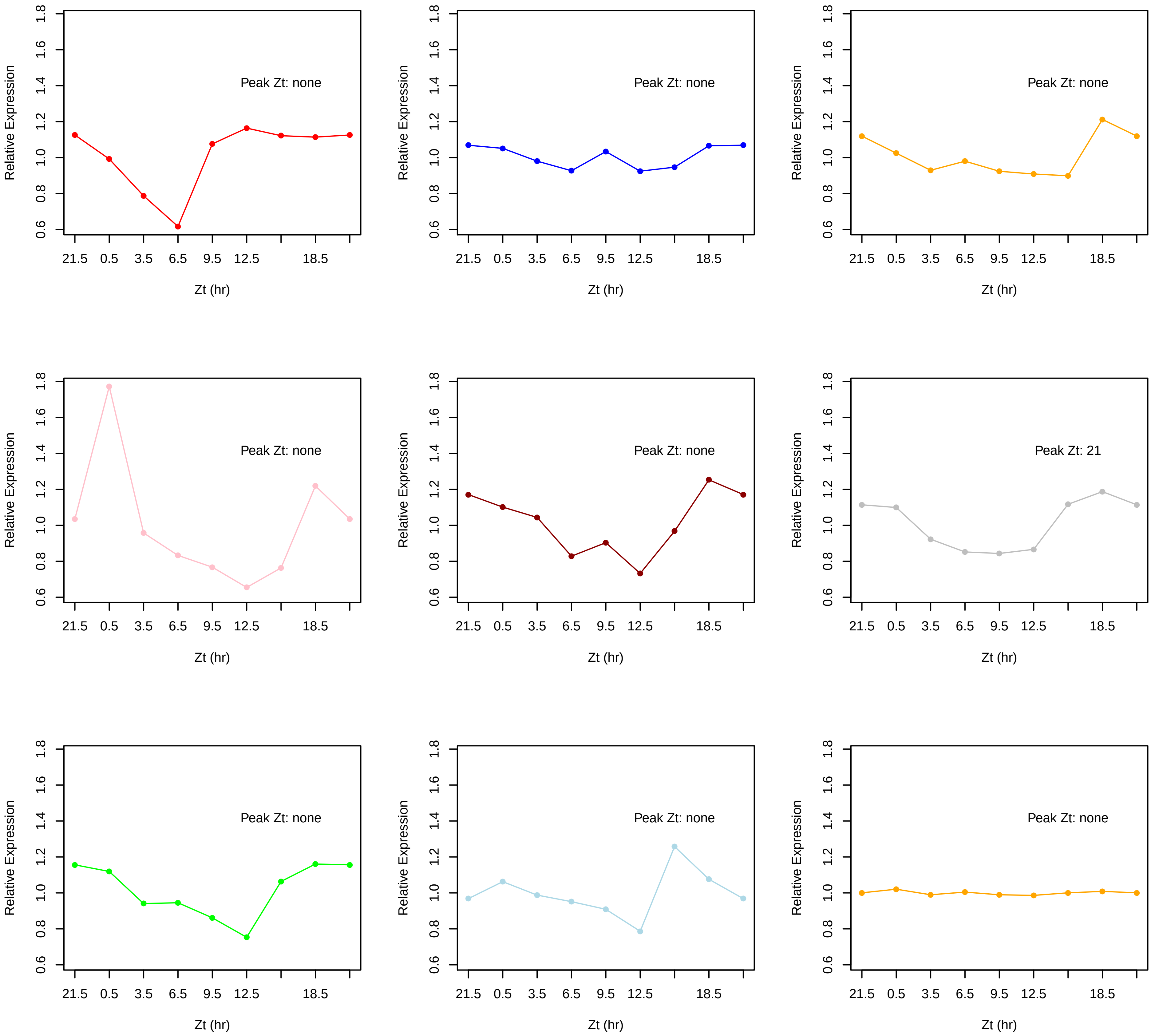

Singeleton: ENSXMAG00000002624 pex11g

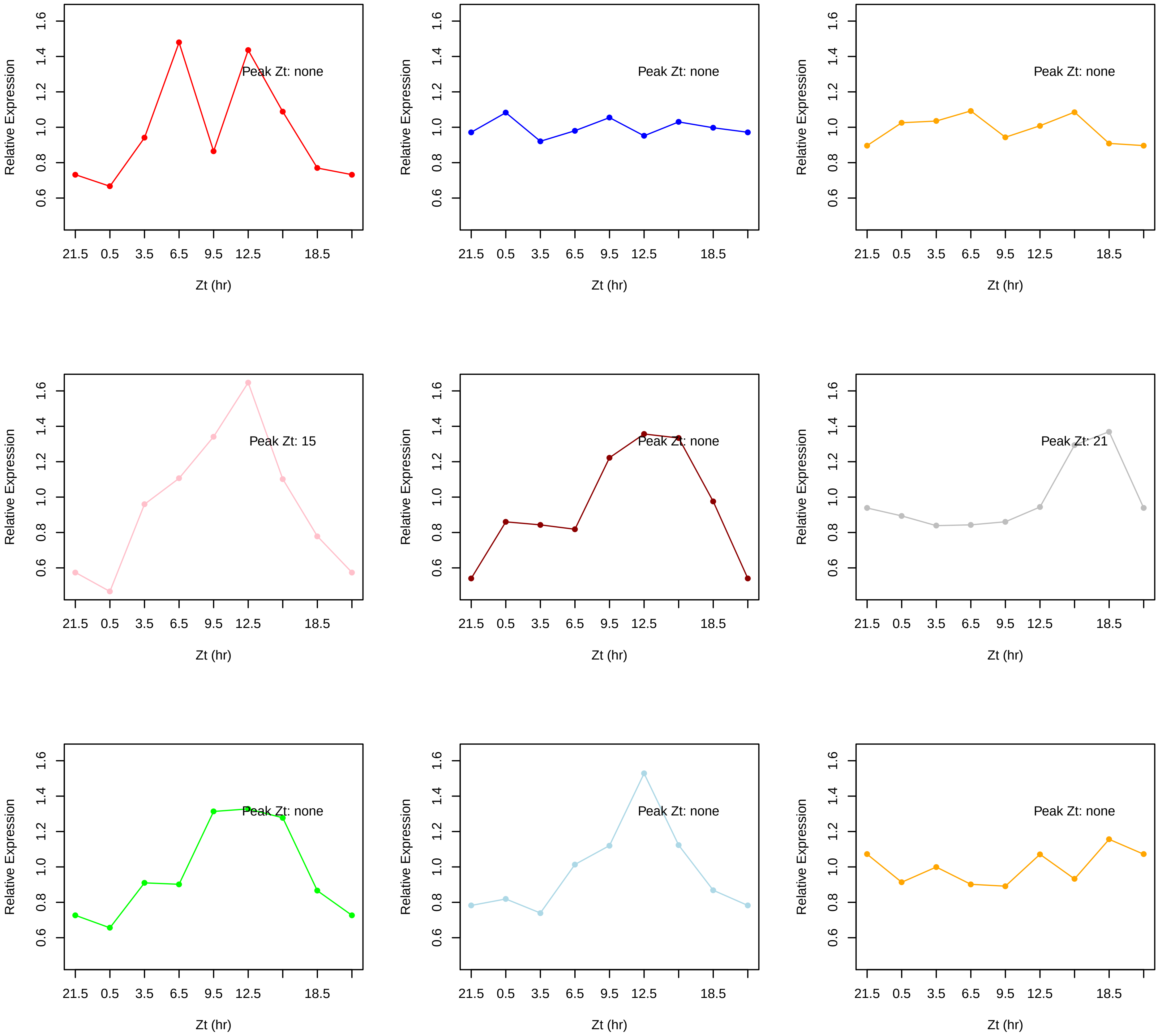

Singeleton: ENSXMAG00000006216 GAN

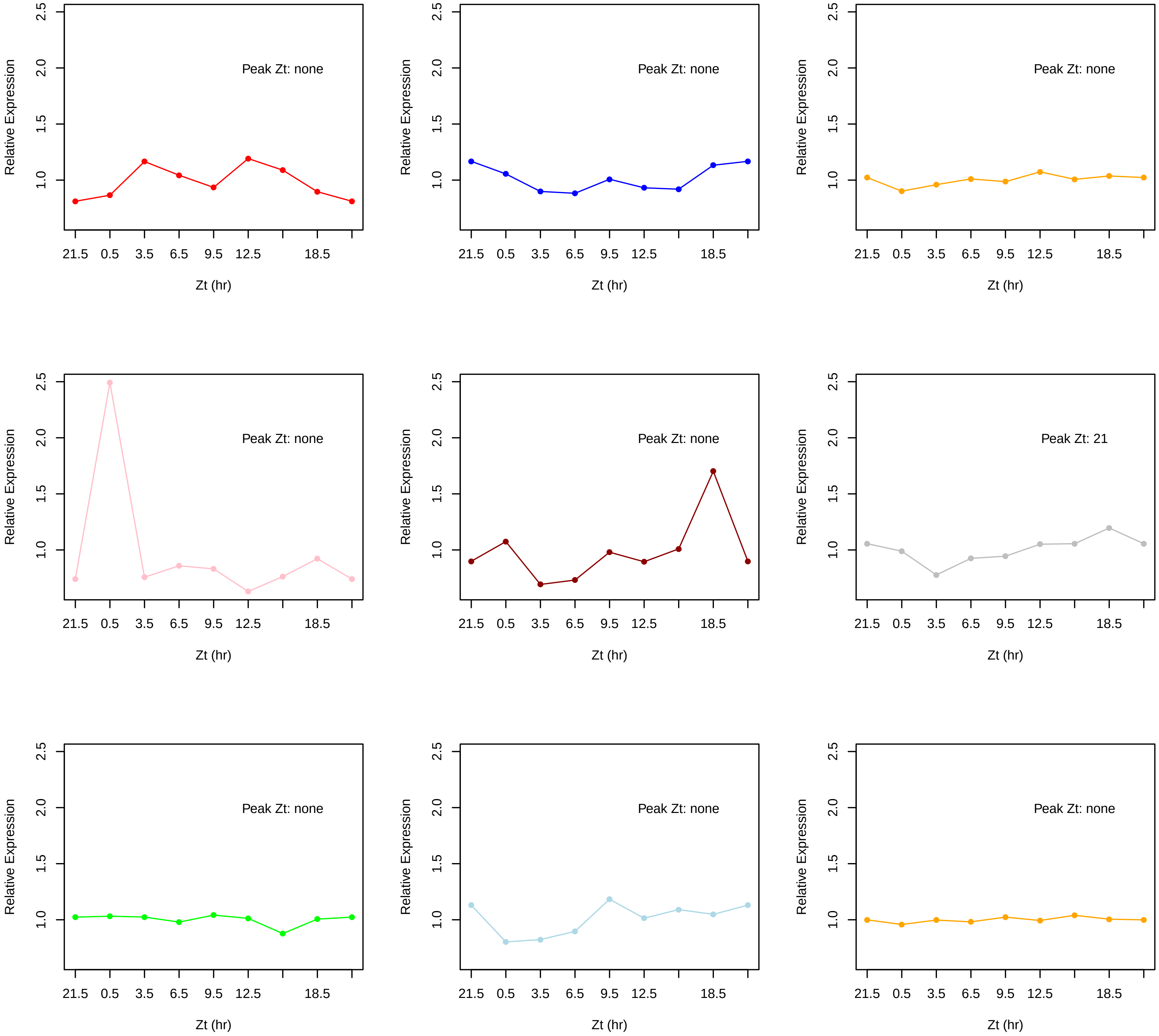

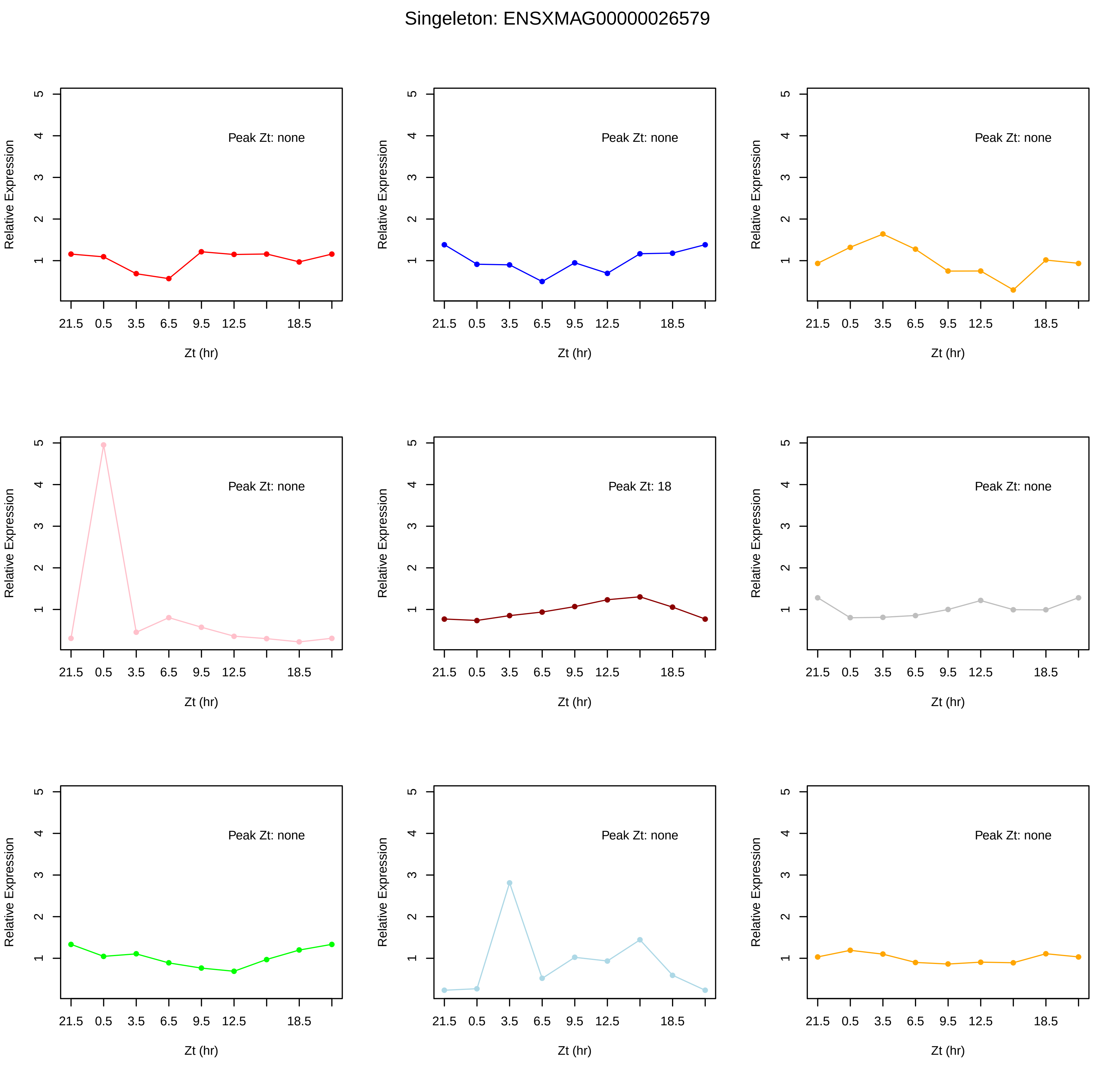

Singeleton: ENSXMAG00000026579
Peak Zt: none
Peak Zt: none
Peak Zt: none
Peak Zt: none
Peak Zt: 18
Peak Zt: none
Peak Zt: none
Peak Zt: none
Peak Zt: none
Relative Expression
Zt (hr)
21.5 0.5 3.5 6.5 9.5 12.5 18.5

Singeleton: ENSXMAG00000002459 pgam5

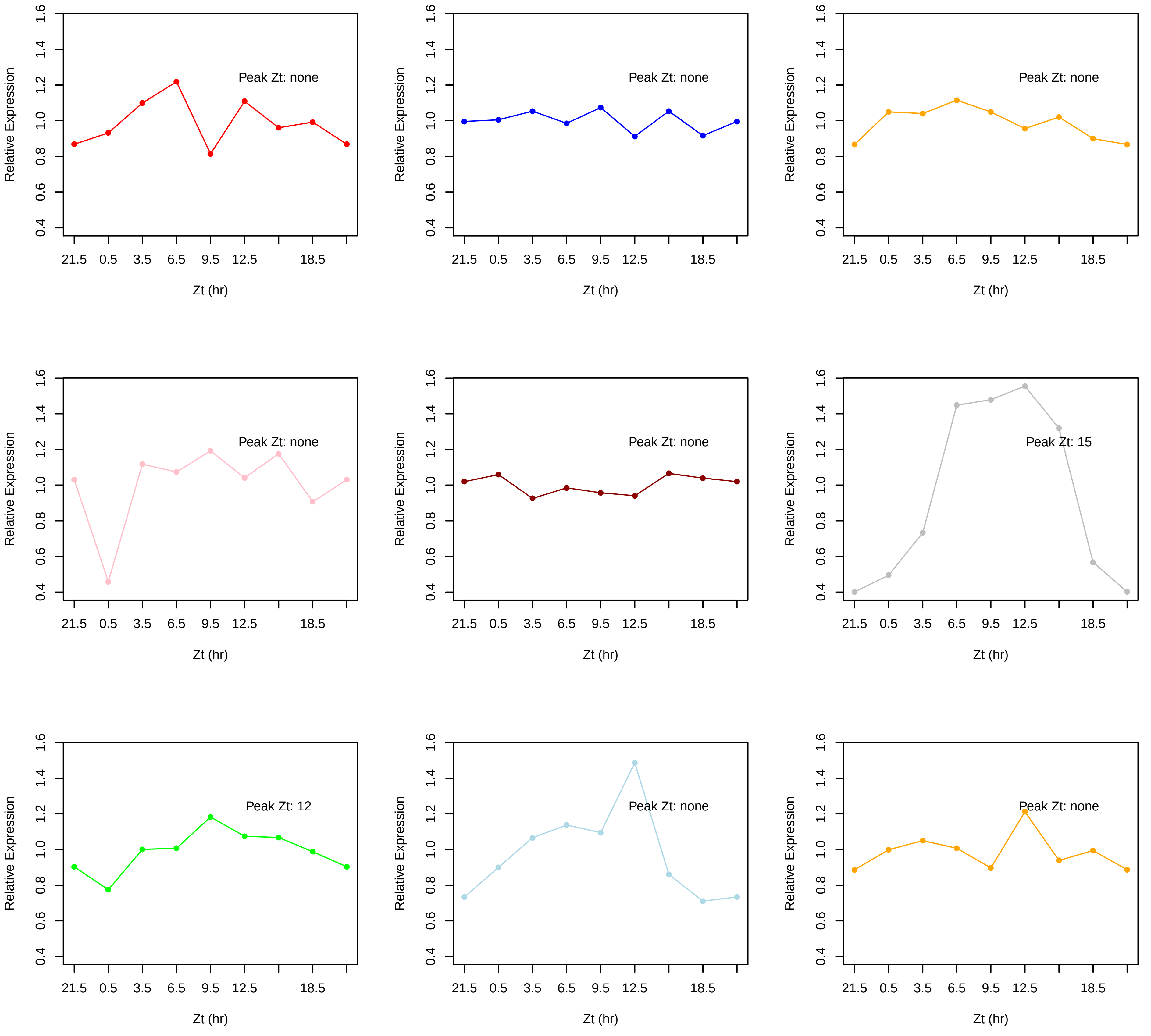

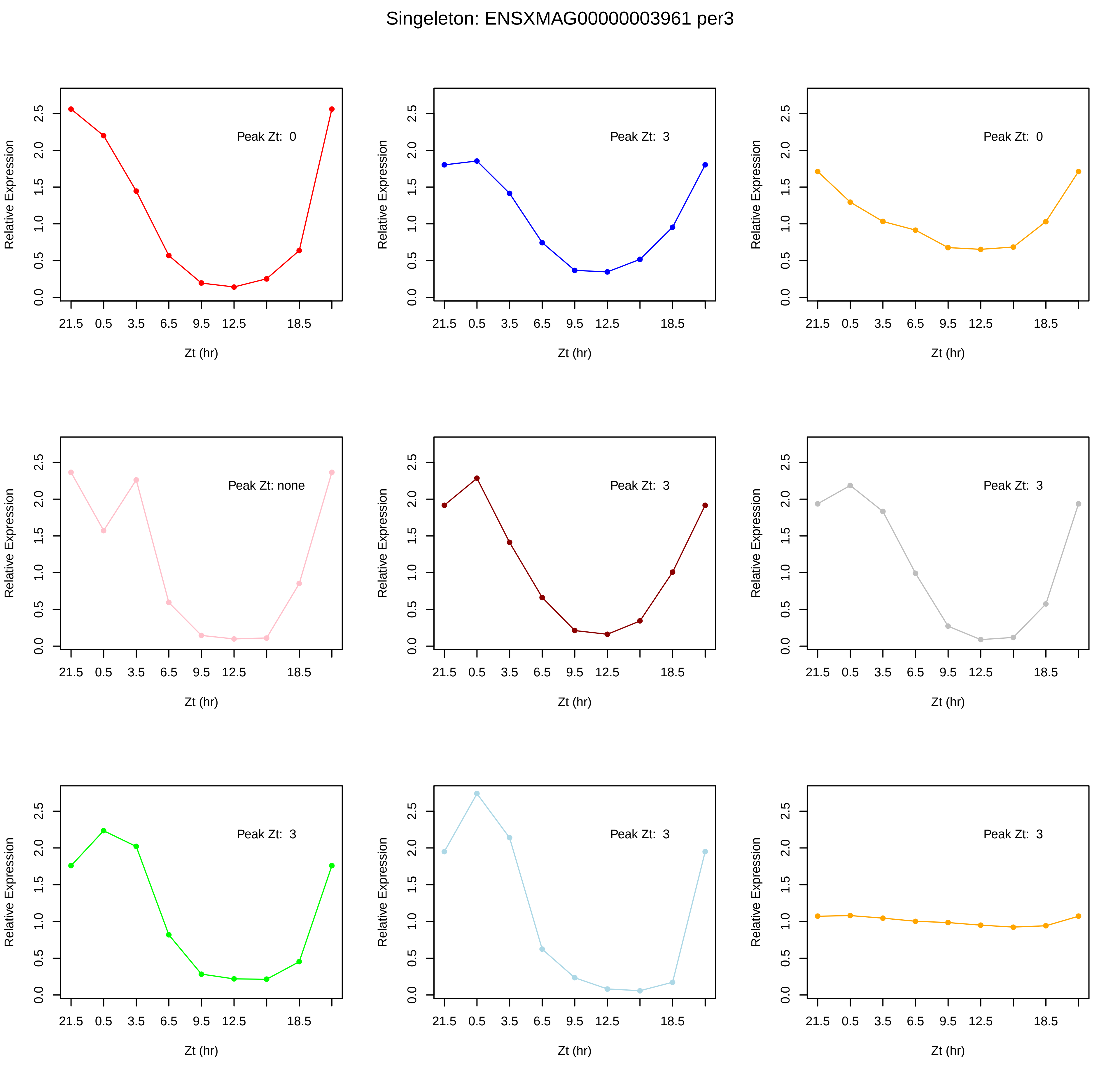

Singeleton: ENSXMAG00000003961 per3
Peak Zt: 0
Peak Zt: 3
Peak Zt: 0
Peak Zt: none
Peak Zt: 3
Peak Zt: 3
Peak Zt: 3
Peak Zt: 3
Peak Zt: 3
Relative Expression
Zt (hr)
0.0
0.5
1.0
1.5
2.0
2.5
21.5
0.5
3.5
6.5
9.5
12.5
18.5

Singeleton: ENSXMAG00000007983 arl5a

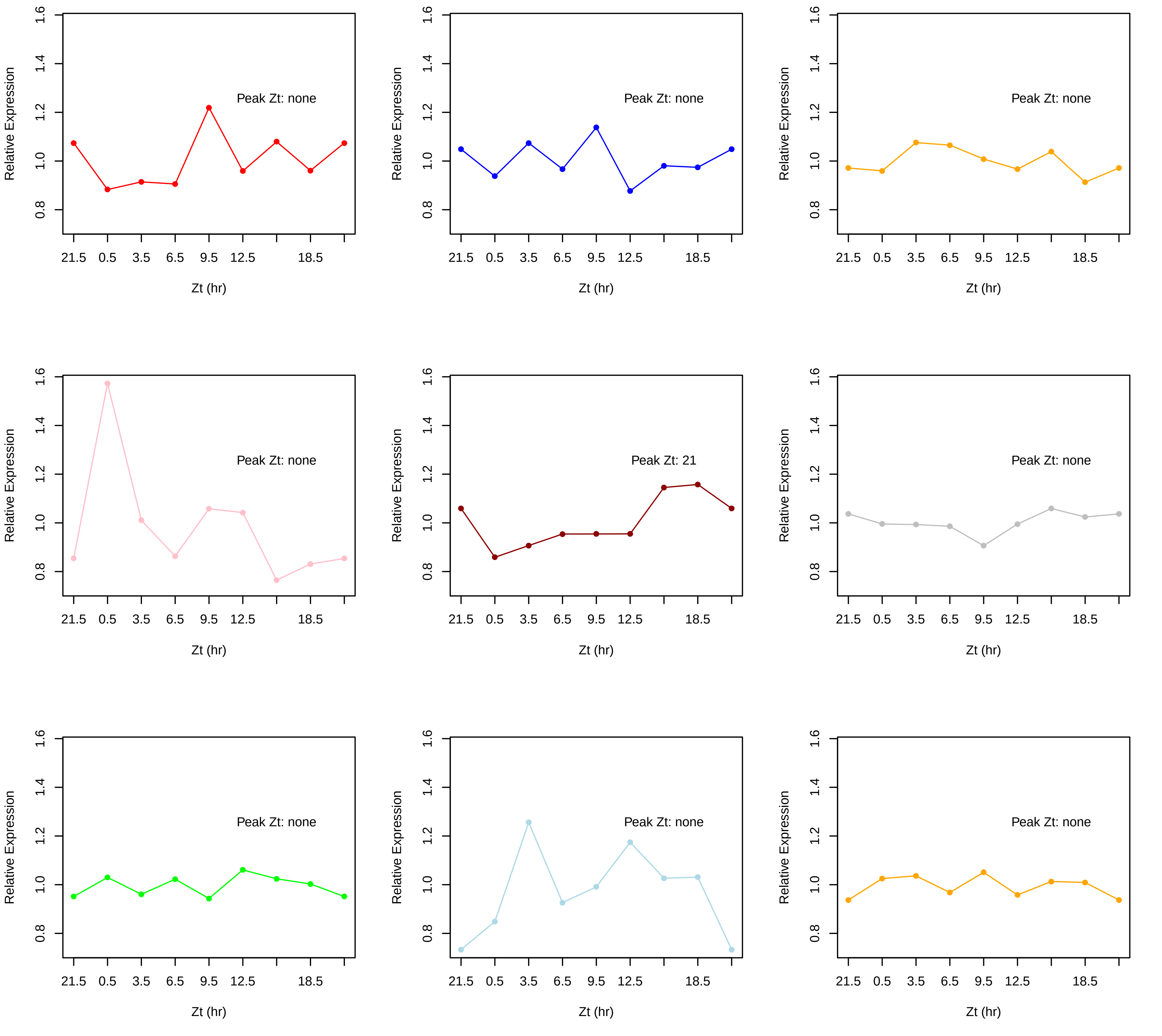

Singleton: ENSXMAG00000016233

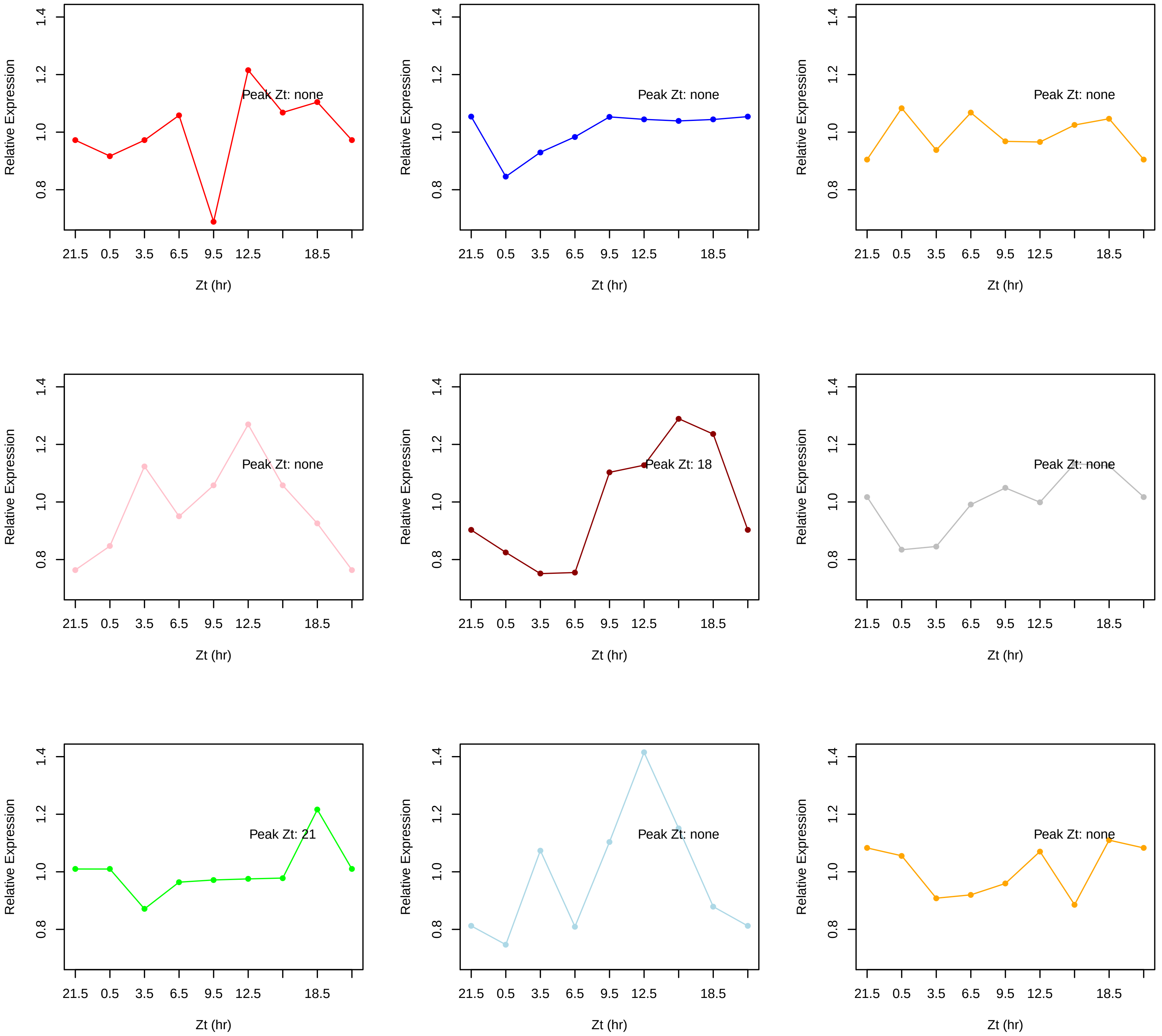

Singeleton: ENSXMAG00000021541 cyb5d2

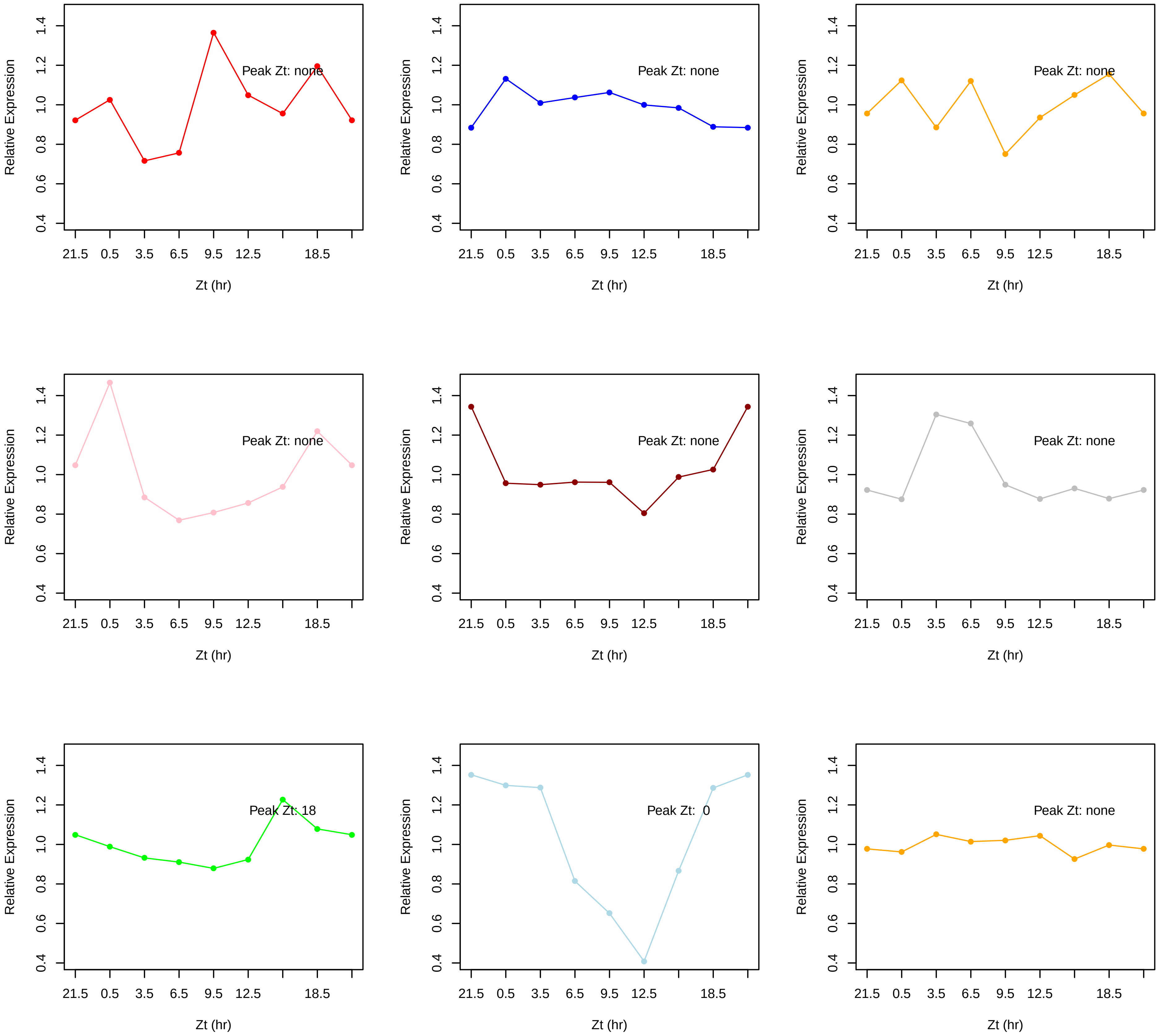

Singeleton: ENSXMAG00000002465 ankle2

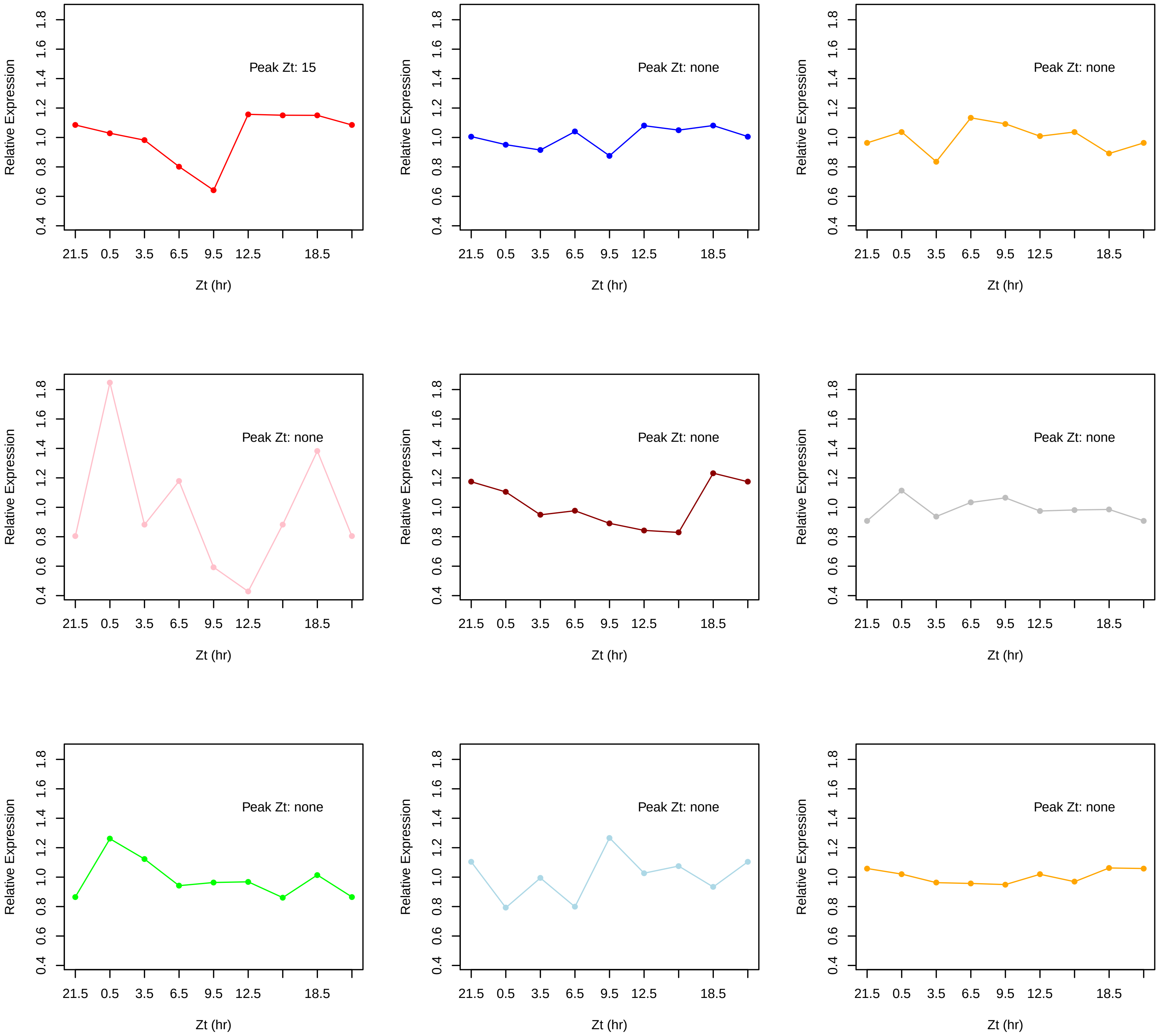

Singleton: ENSXMAG00000014305 atg16l2

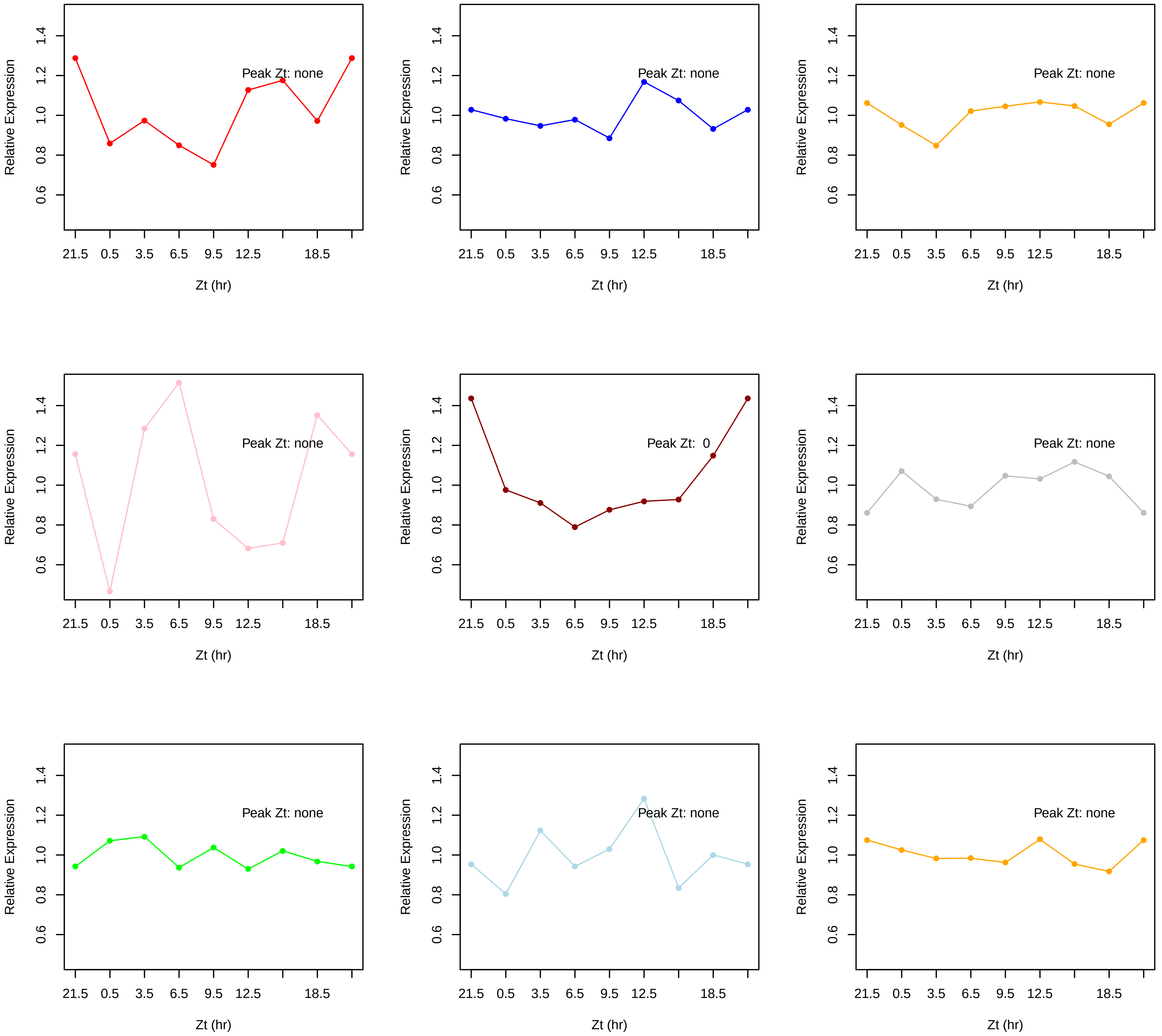

Singleton: ENSXMAG00000018000 srp72

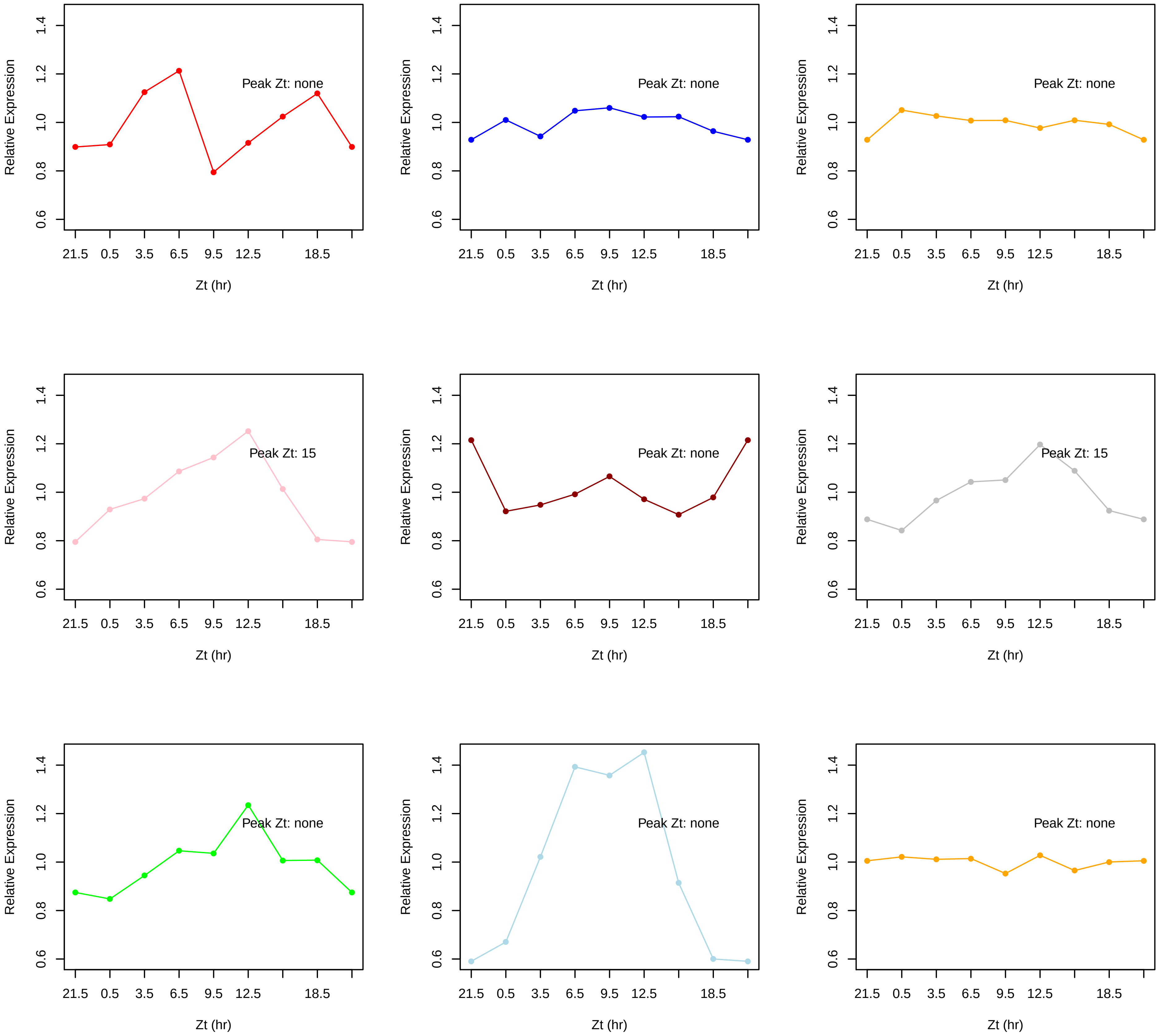

Singeleton: ENSXMAG00000003160 fkbp9

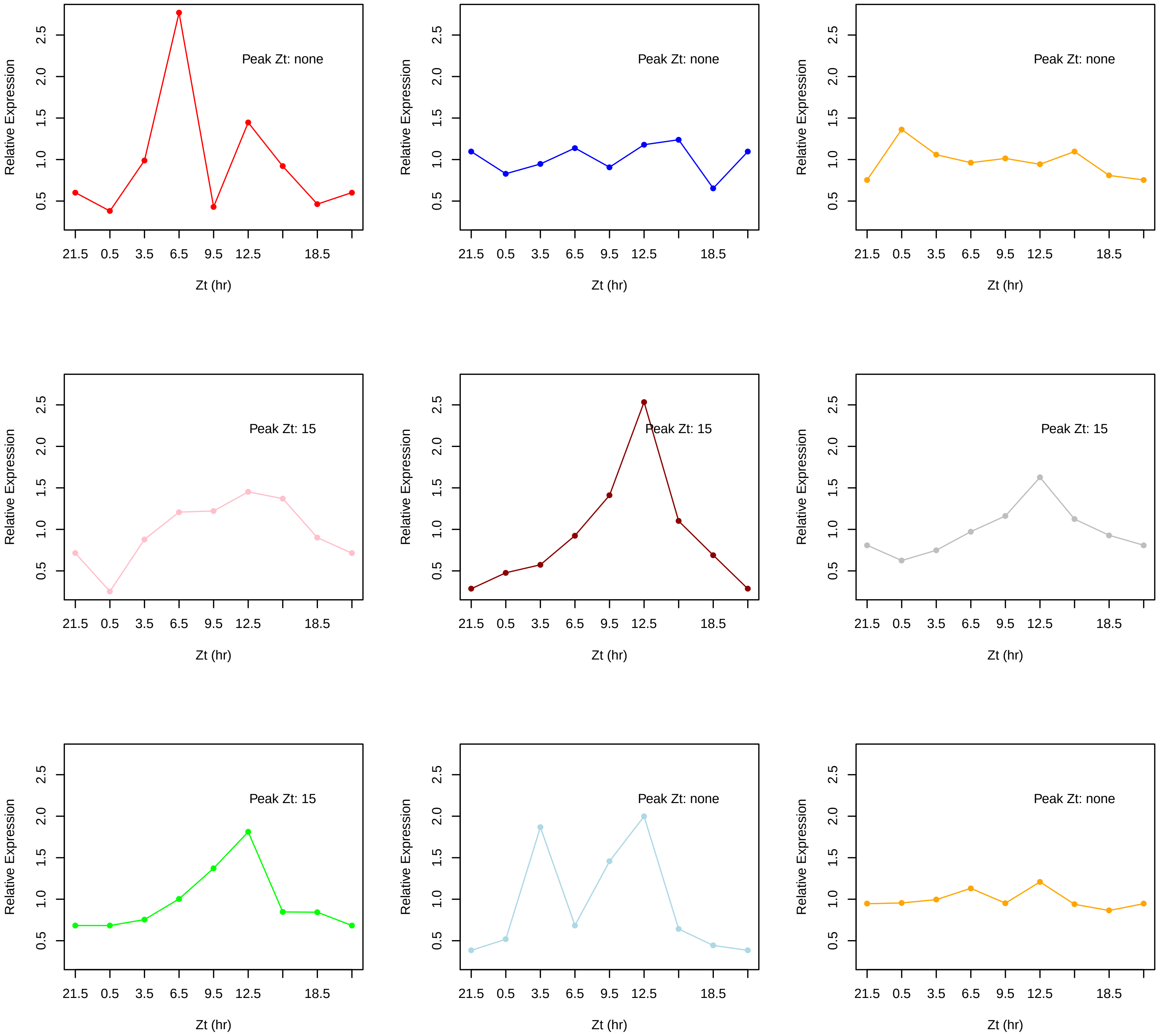

Singeleton: ENSXMAG00000013461 asmtl

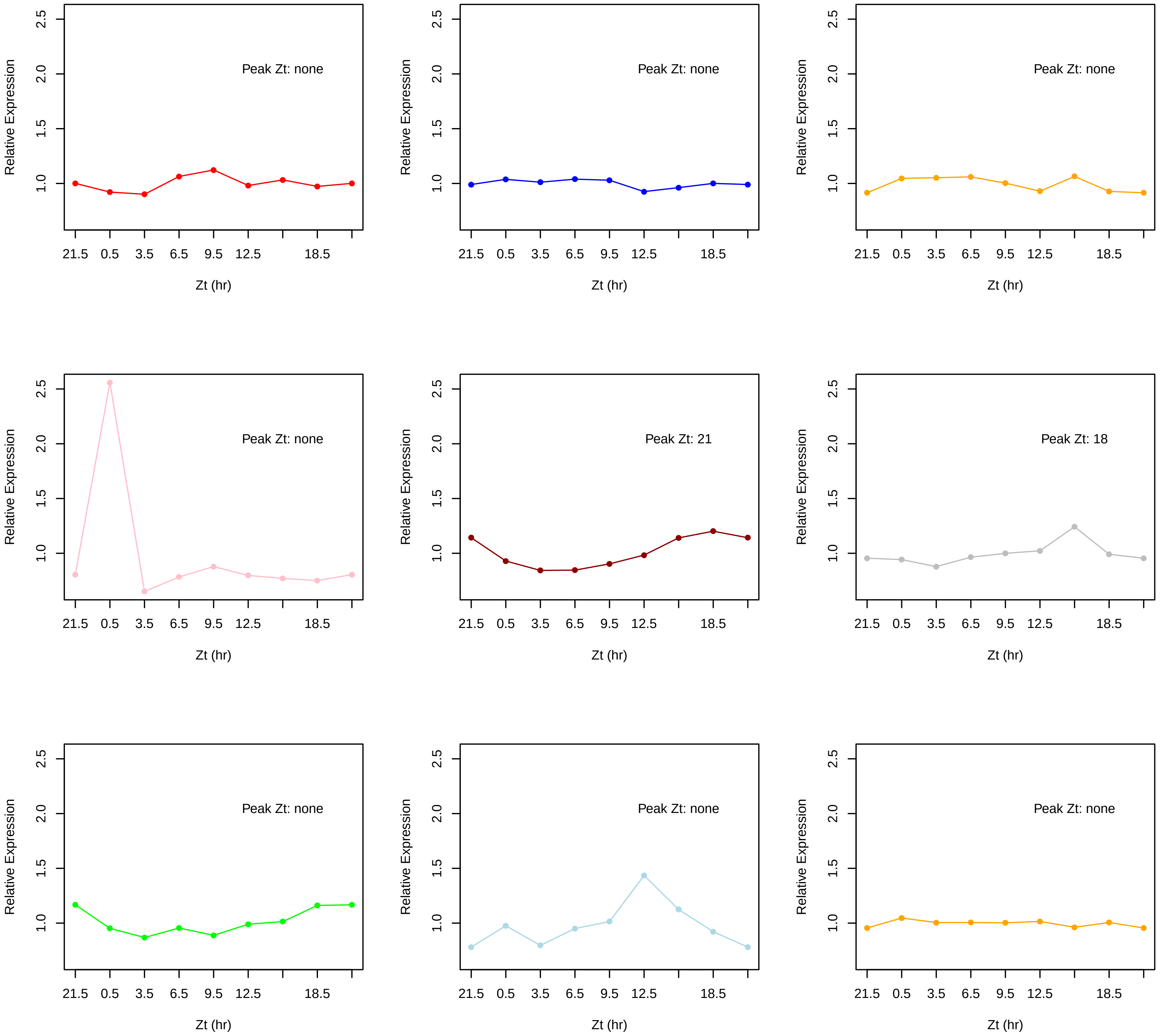

Singeleton: ENSXMAG00000005859 znfx1

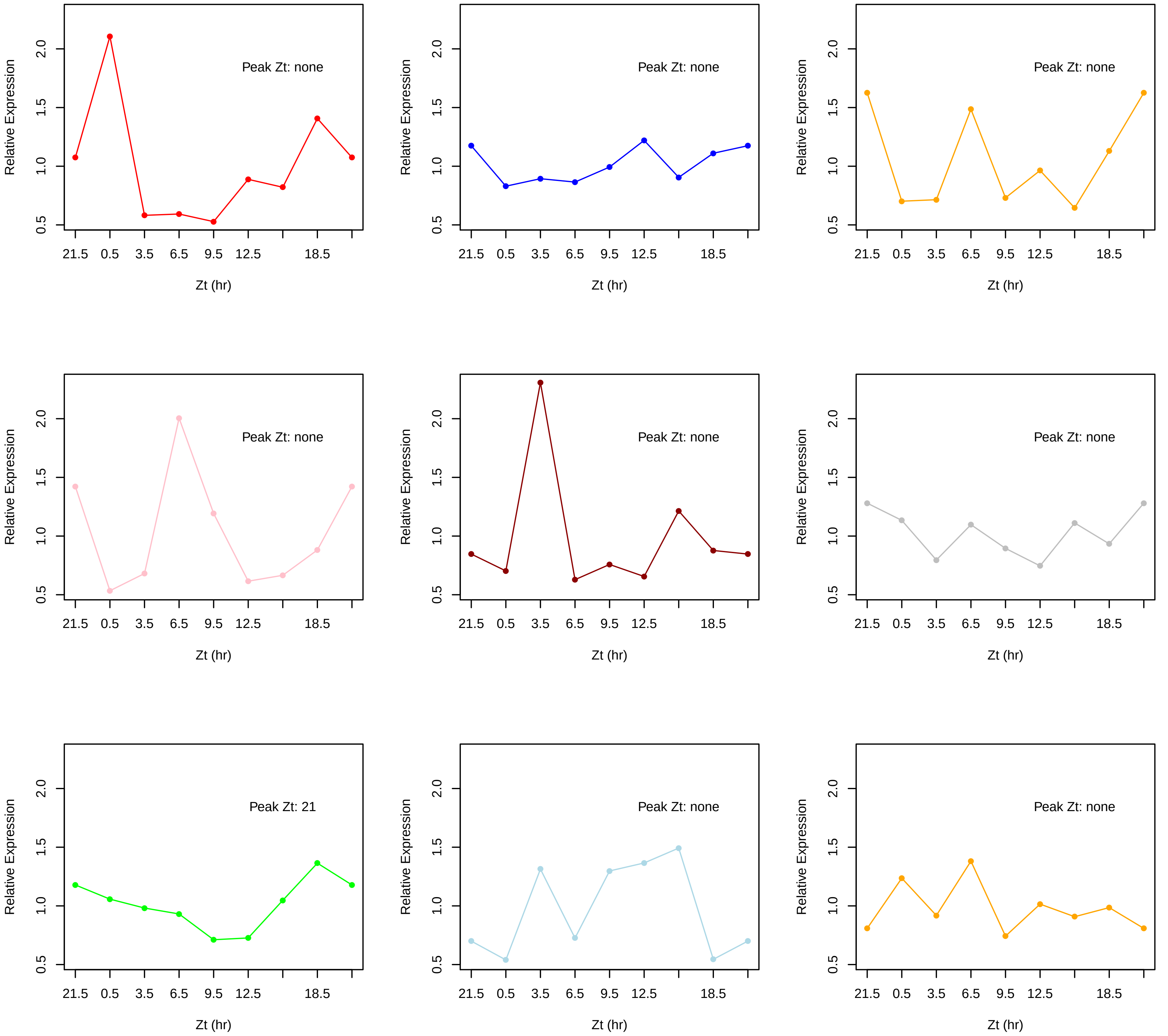

Singleton: ENSXMAG00000002470 golga3

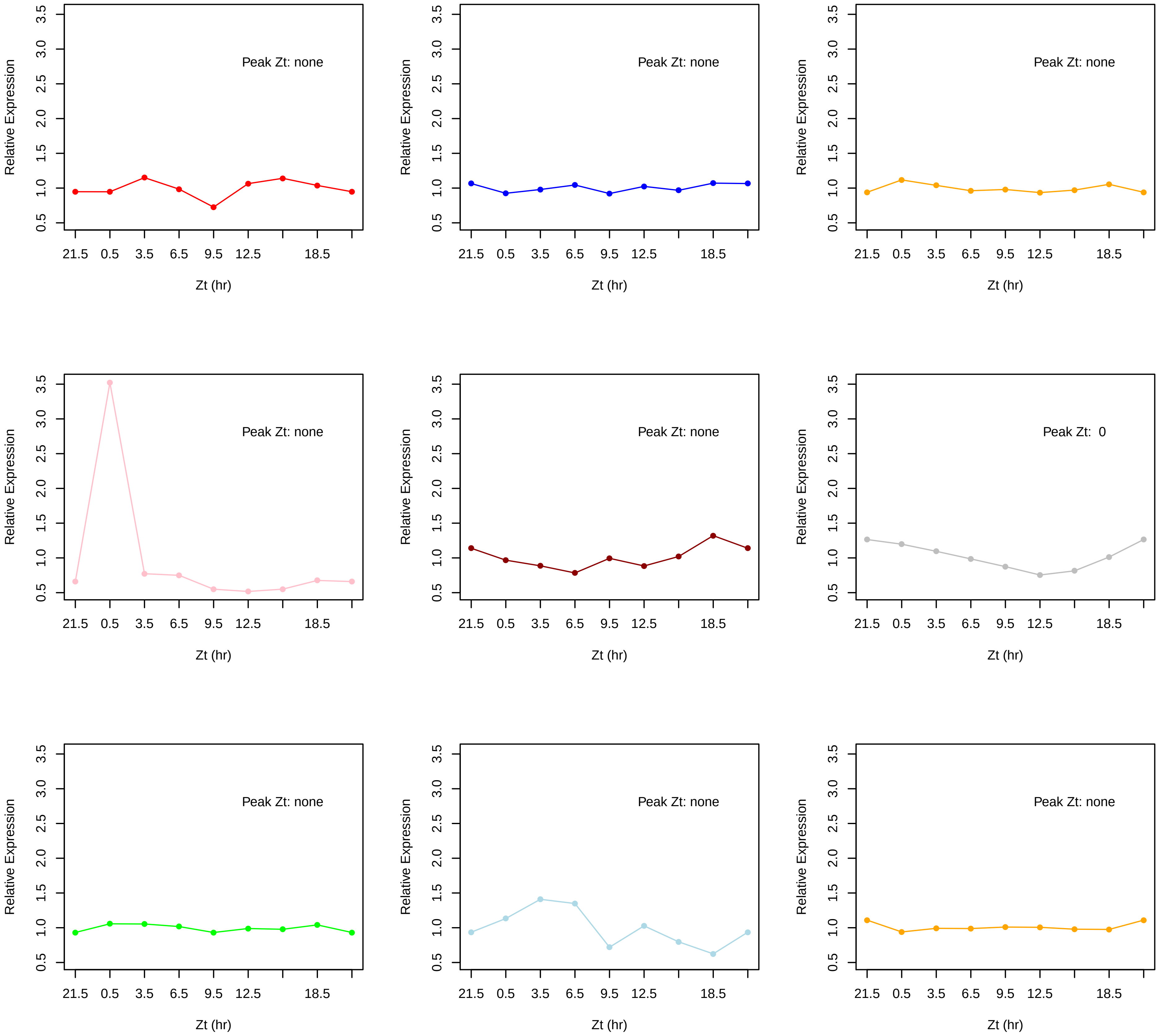

Singleton: ENSXMAG00000027105 kank3

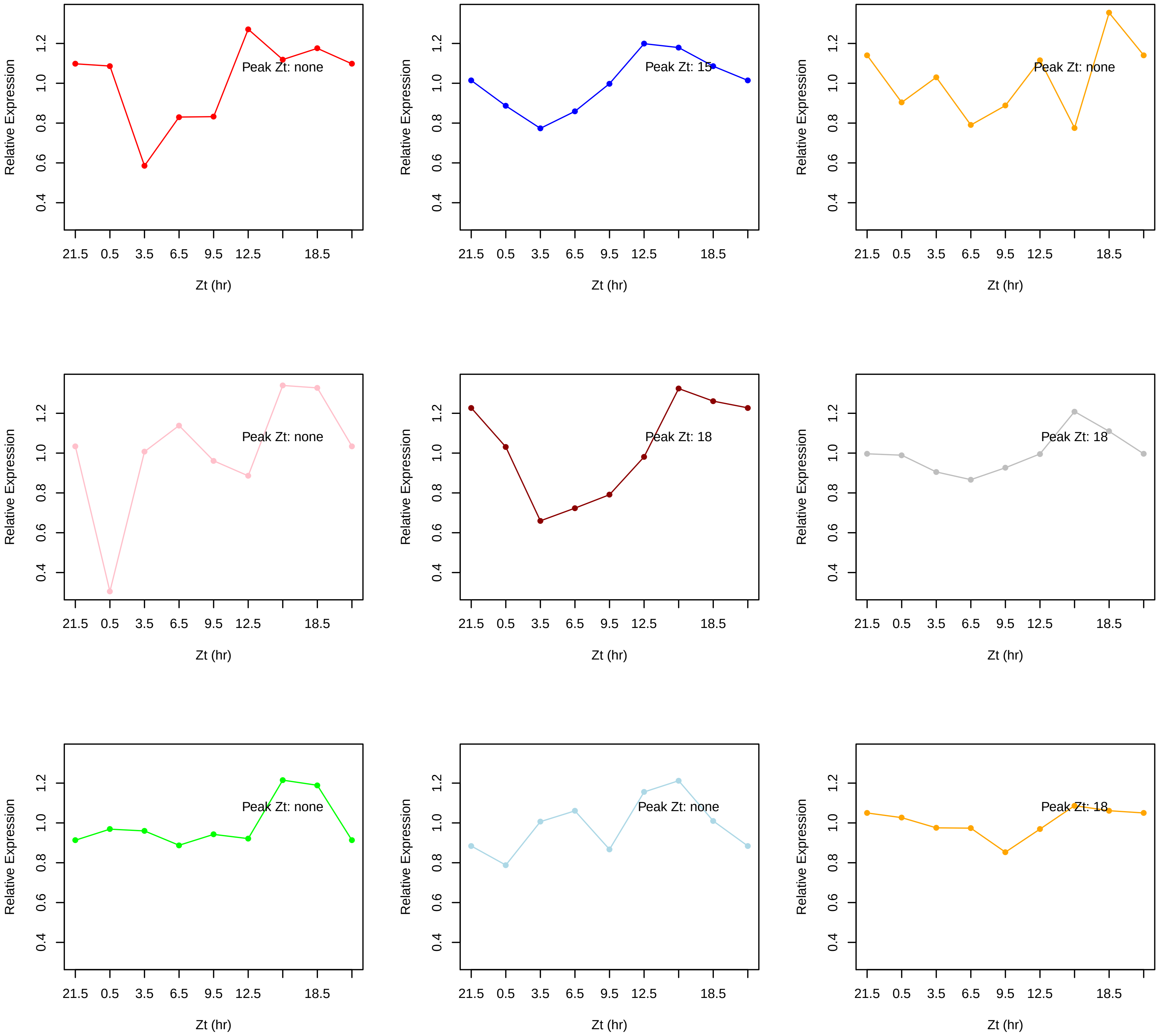

Singeleton: ENSXMAG00000029935 zgc:113531

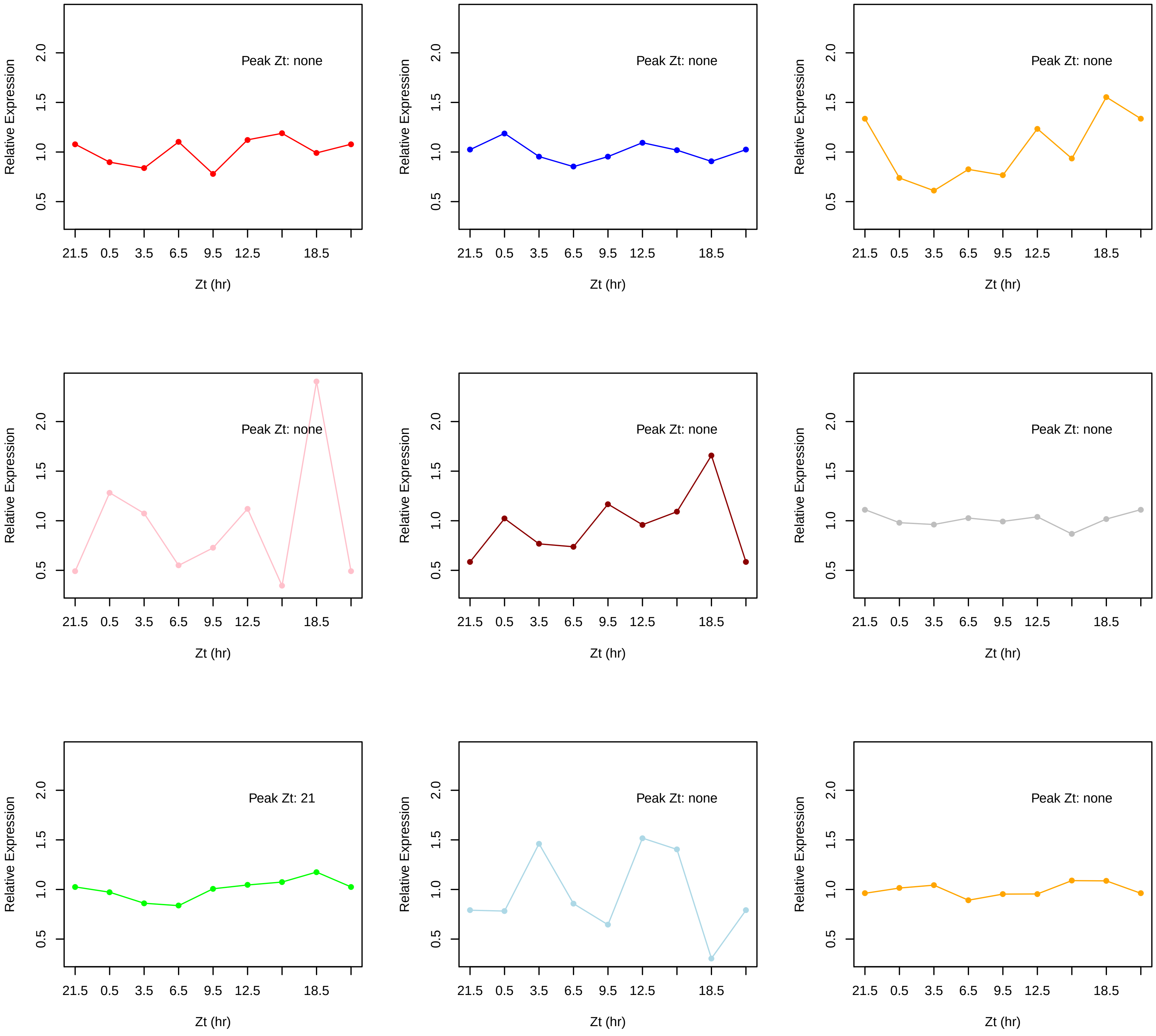

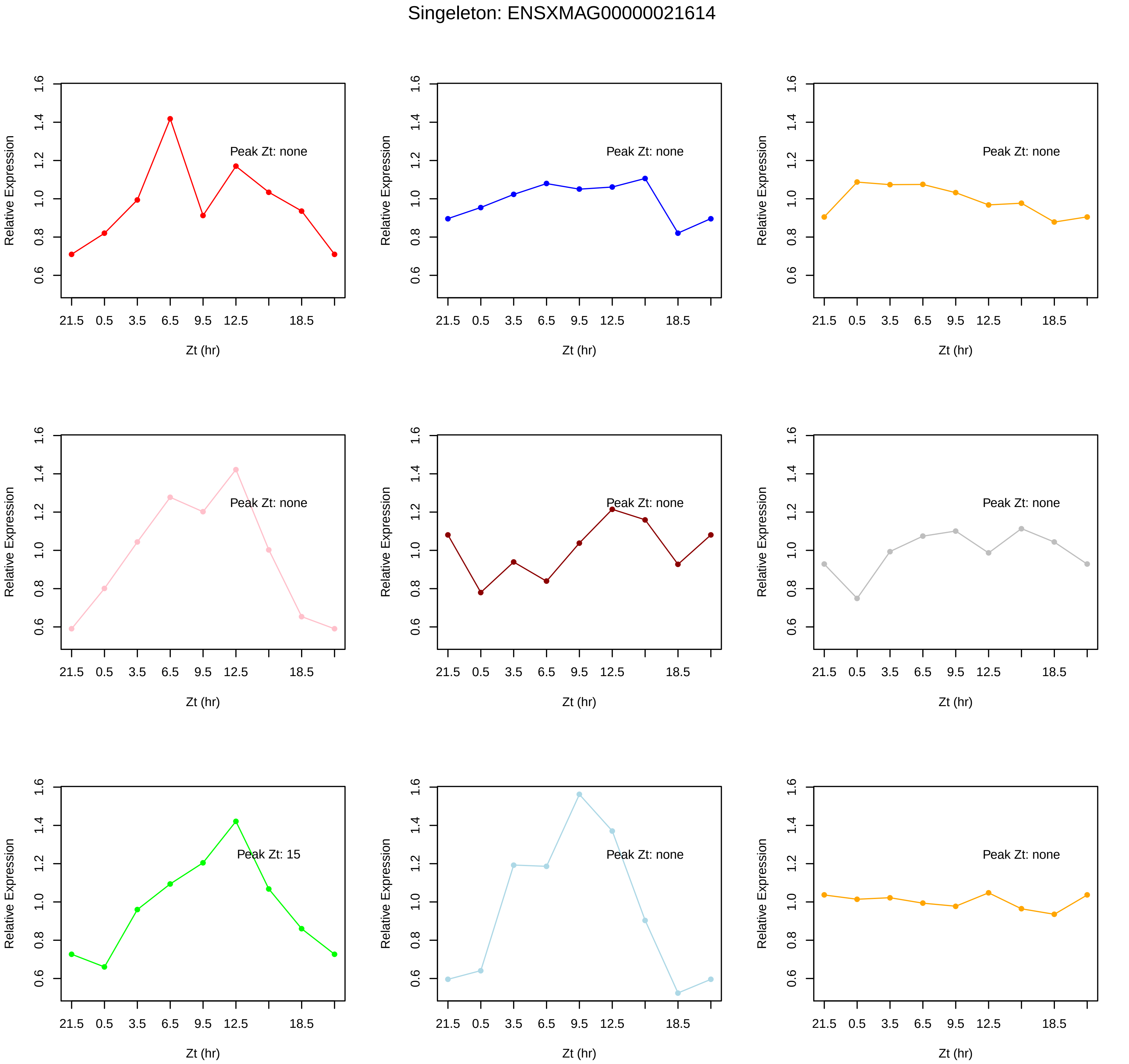

Singeleton: ENSXMAG00000021614
Peak Zt: none
Peak Zt: none
Peak Zt: none
Peak Zt: none
Peak Zt: none
Peak Zt: none
Peak Zt: 15
Peak Zt: none
Peak Zt: none
Relative Expression
Zt (hr)

Singeleton: ENSXMAG00000016167 camta1a

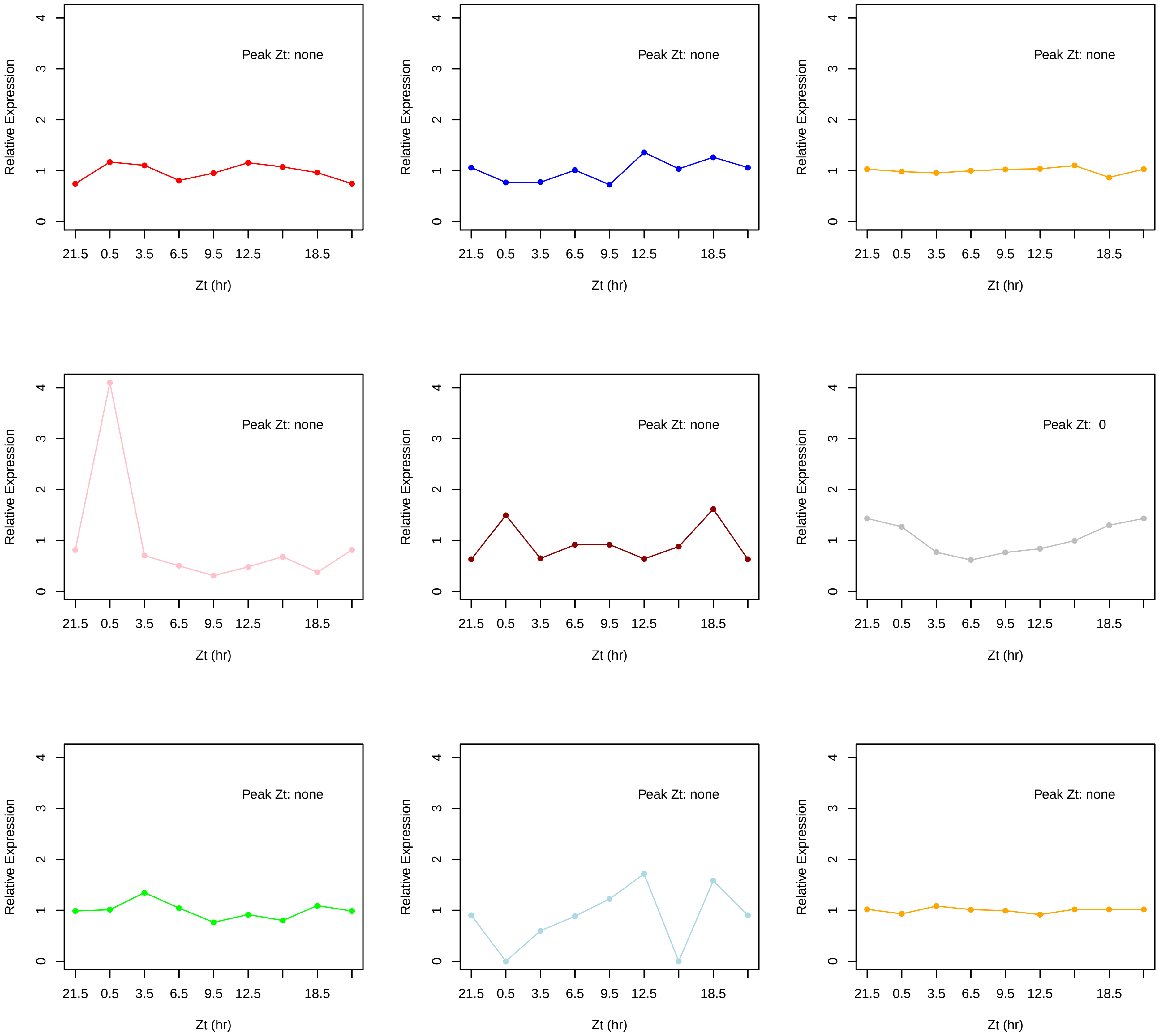

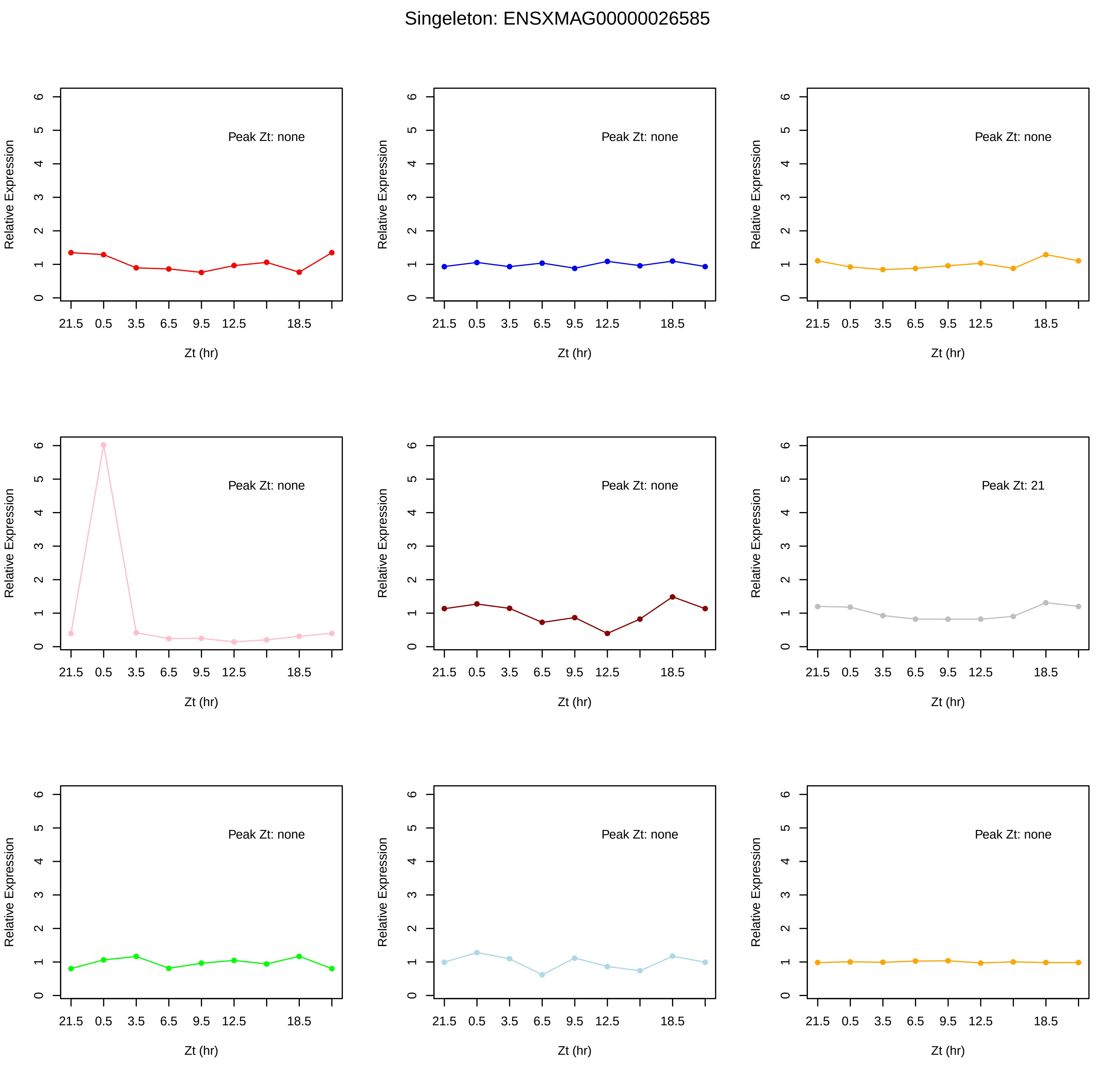

Singeleton: ENSXMAG00000026585
Peak Zt: none
Peak Zt: none
Peak Zt: none
Peak Zt: none
Peak Zt: none
Peak Zt: 21
Peak Zt: none
Peak Zt: none
Peak Zt: none
Relative Expression
Zt (hr)
21.5
0.5
3.5
6.5
9.5
12.5
18.5

Singleton: ENSXMAG00000006126 lpar1

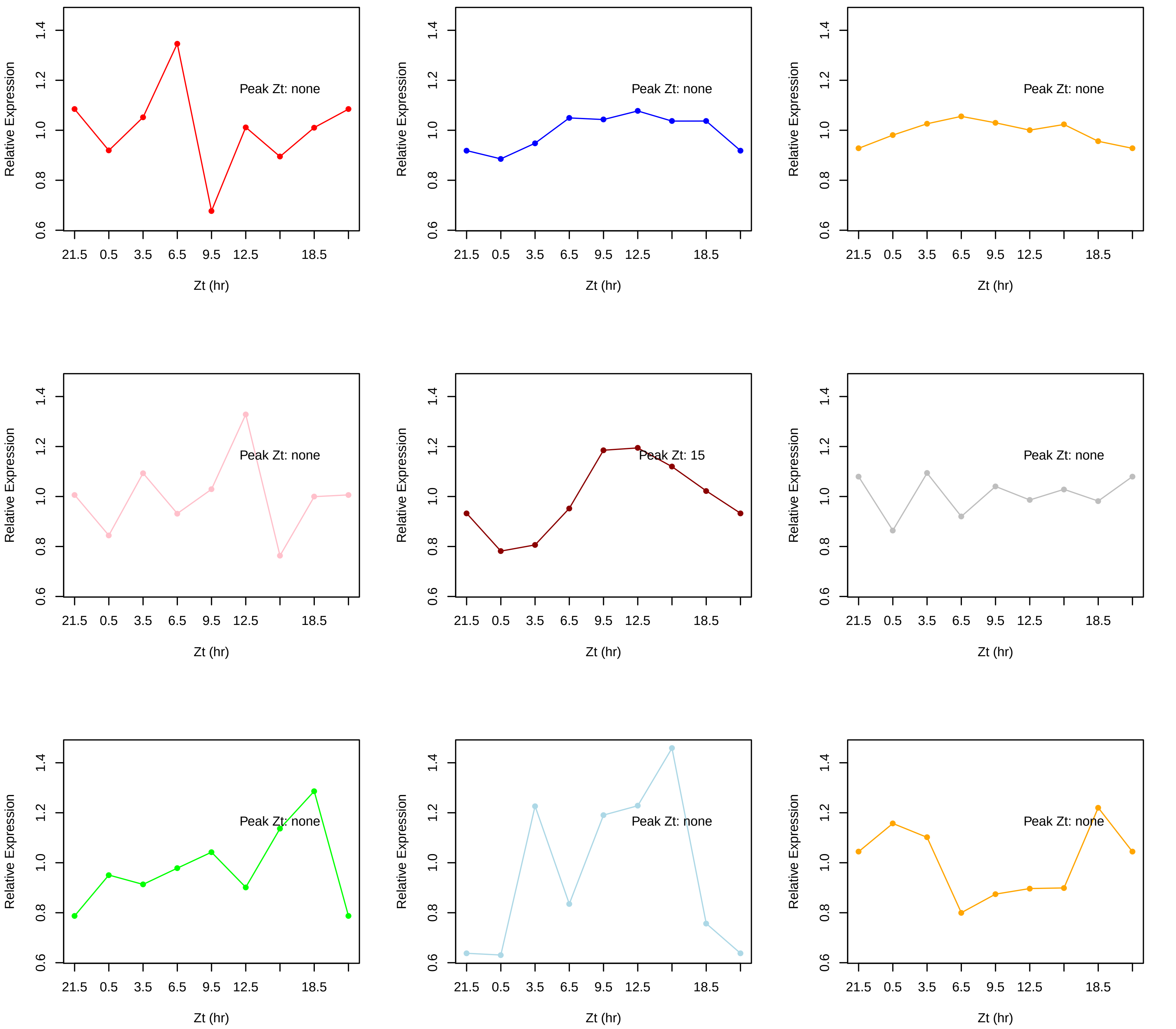

Singleton: ENSXMAG00000012435

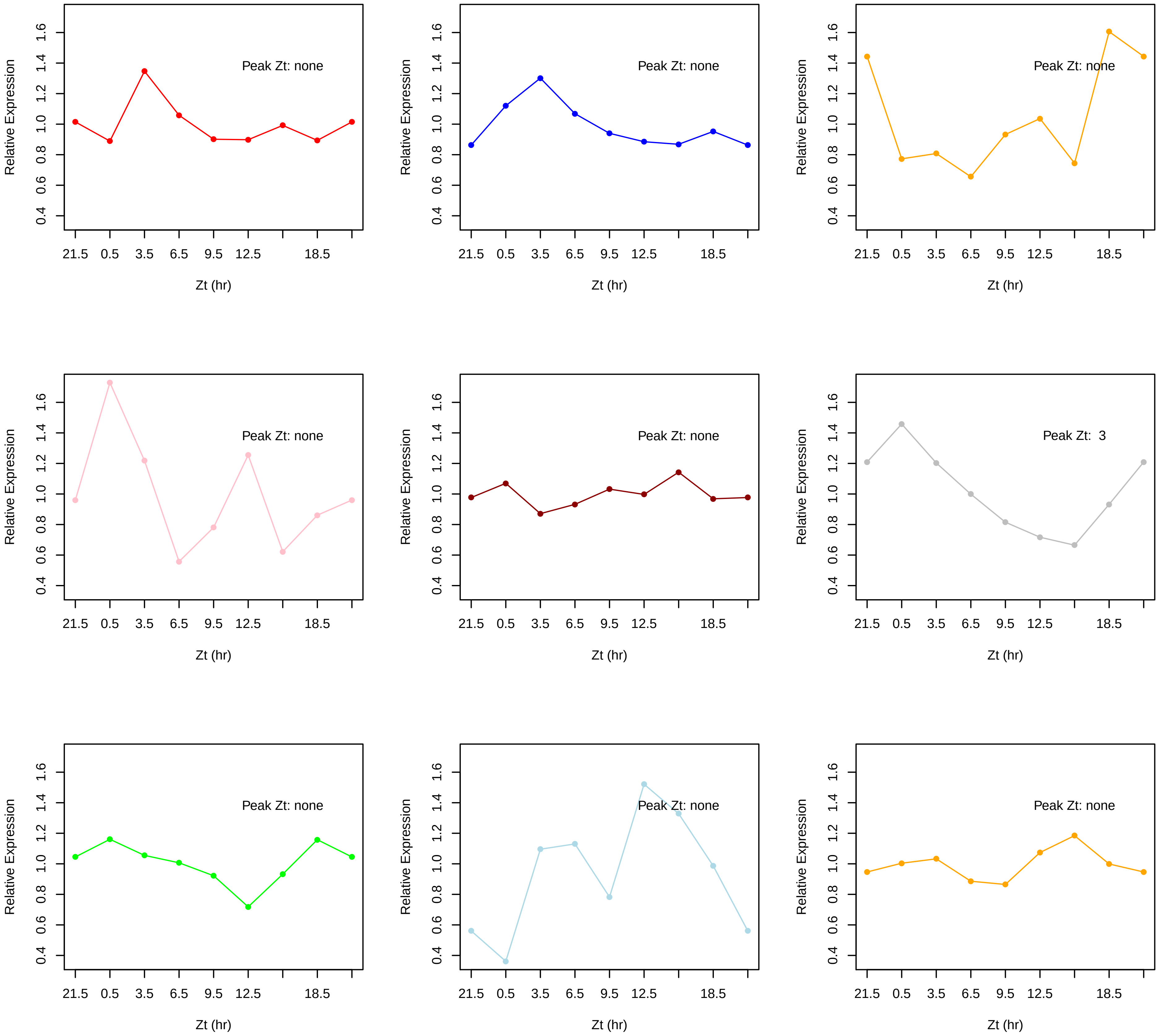

Singleton: ENSXMAG00000023617 rab12

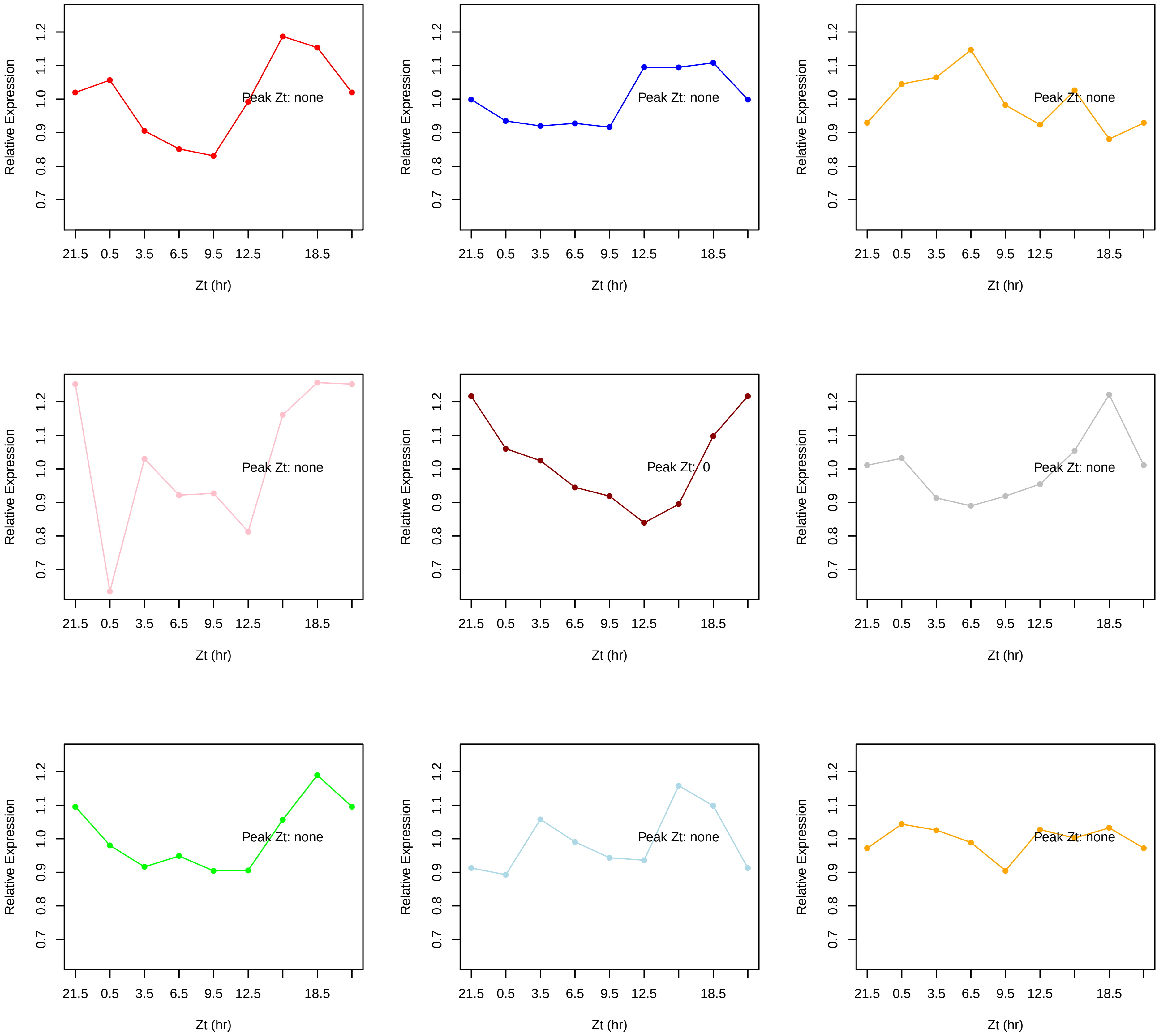

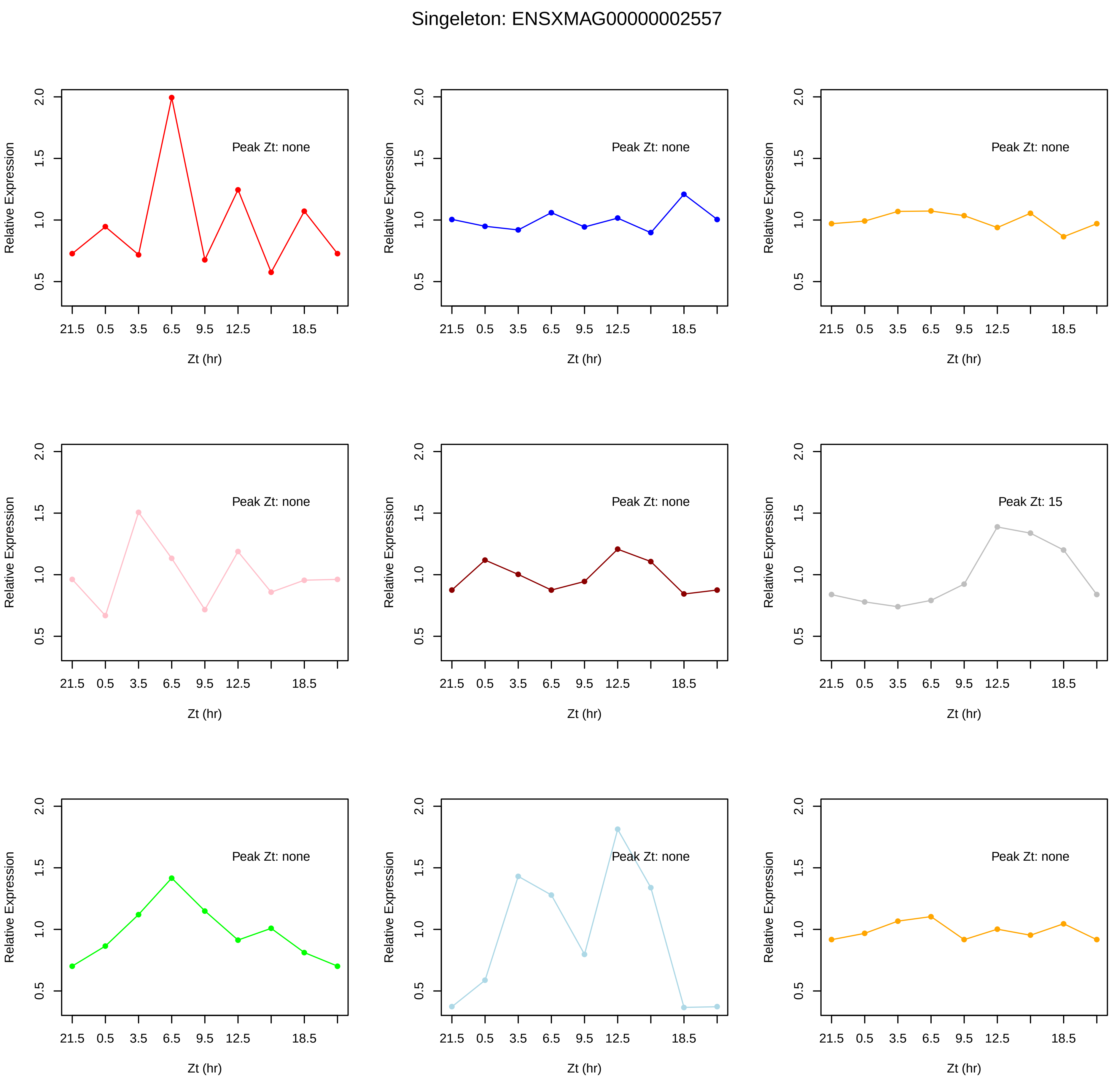
Singeleton: ENSXMAG00000002557
Peak Zt: none
Peak Zt: none
Peak Zt: none
Peak Zt: none
Peak Zt: none
Peak Zt: 15
Peak Zt: none
Peak Zt: none
Peak Zt: none
Relative Expression
Zt (hr)
0.5
1.0
1.5
2.0
21.5
0.5
3.5
6.5
9.5
12.5
18.5

Singleton: ENSXMAG00000000747 PTPRM

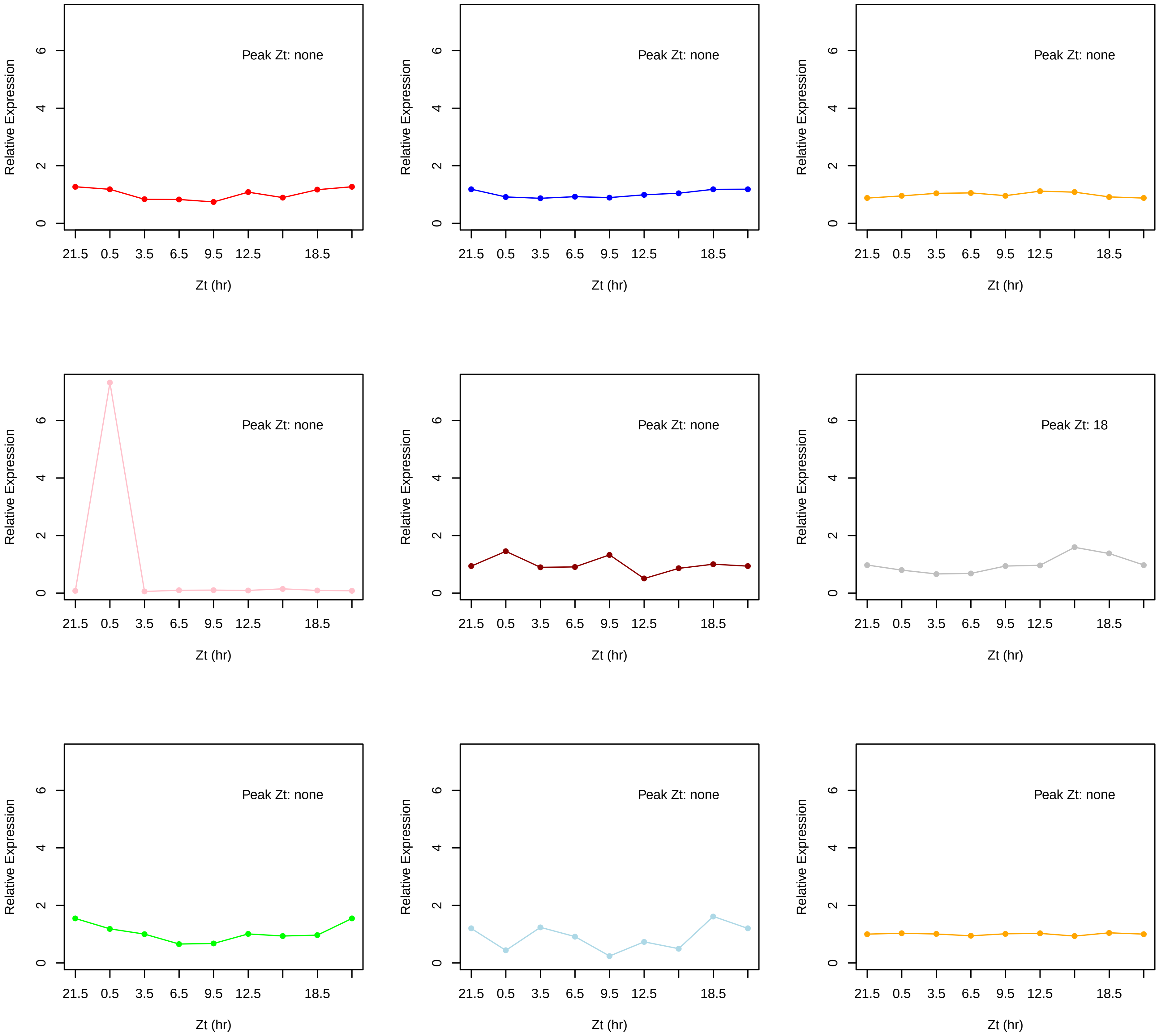

Singleton: ENSXMAG00000029488 susd5

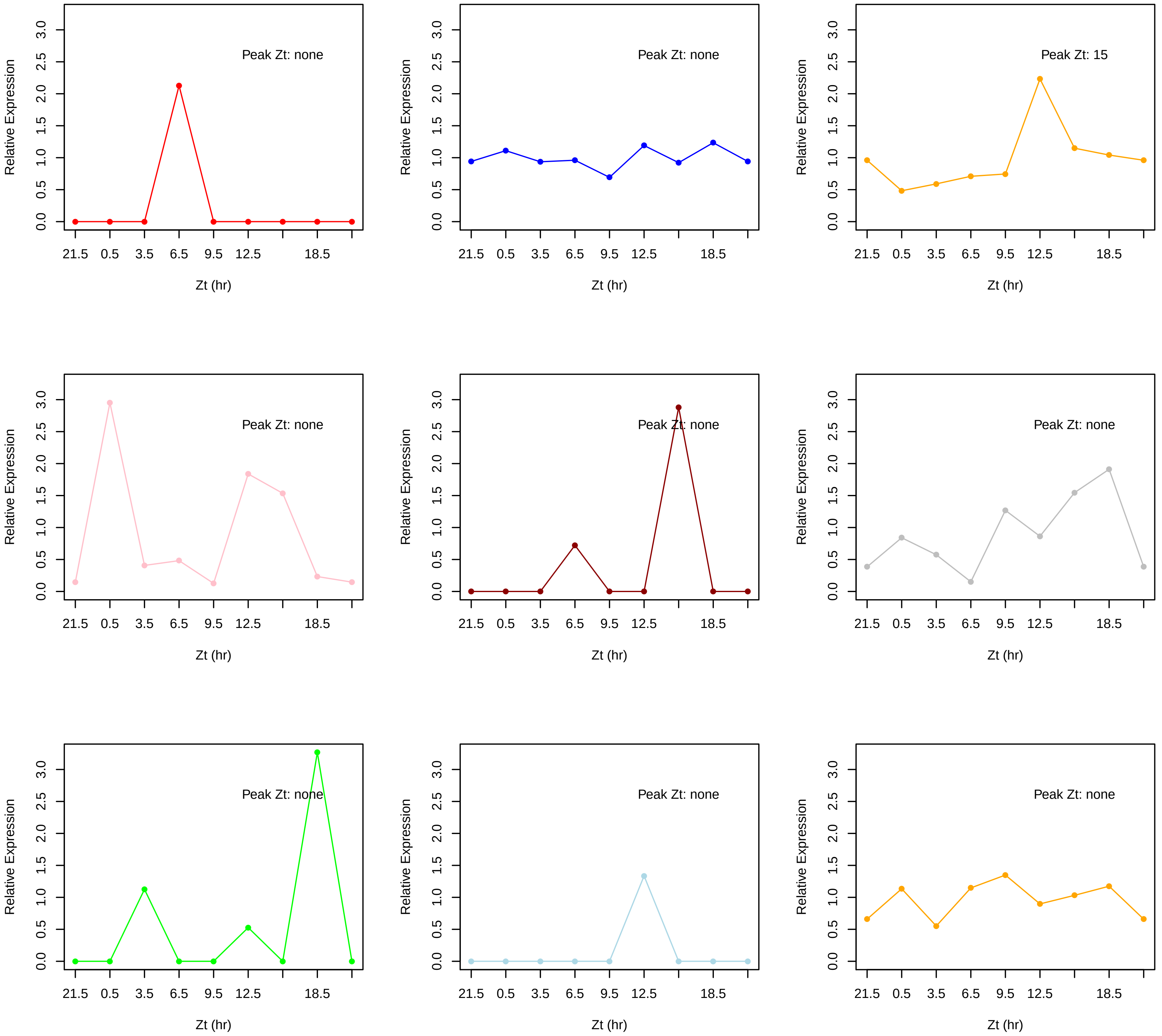

Singleton: ENSXMAG00000003040 tnfaip6

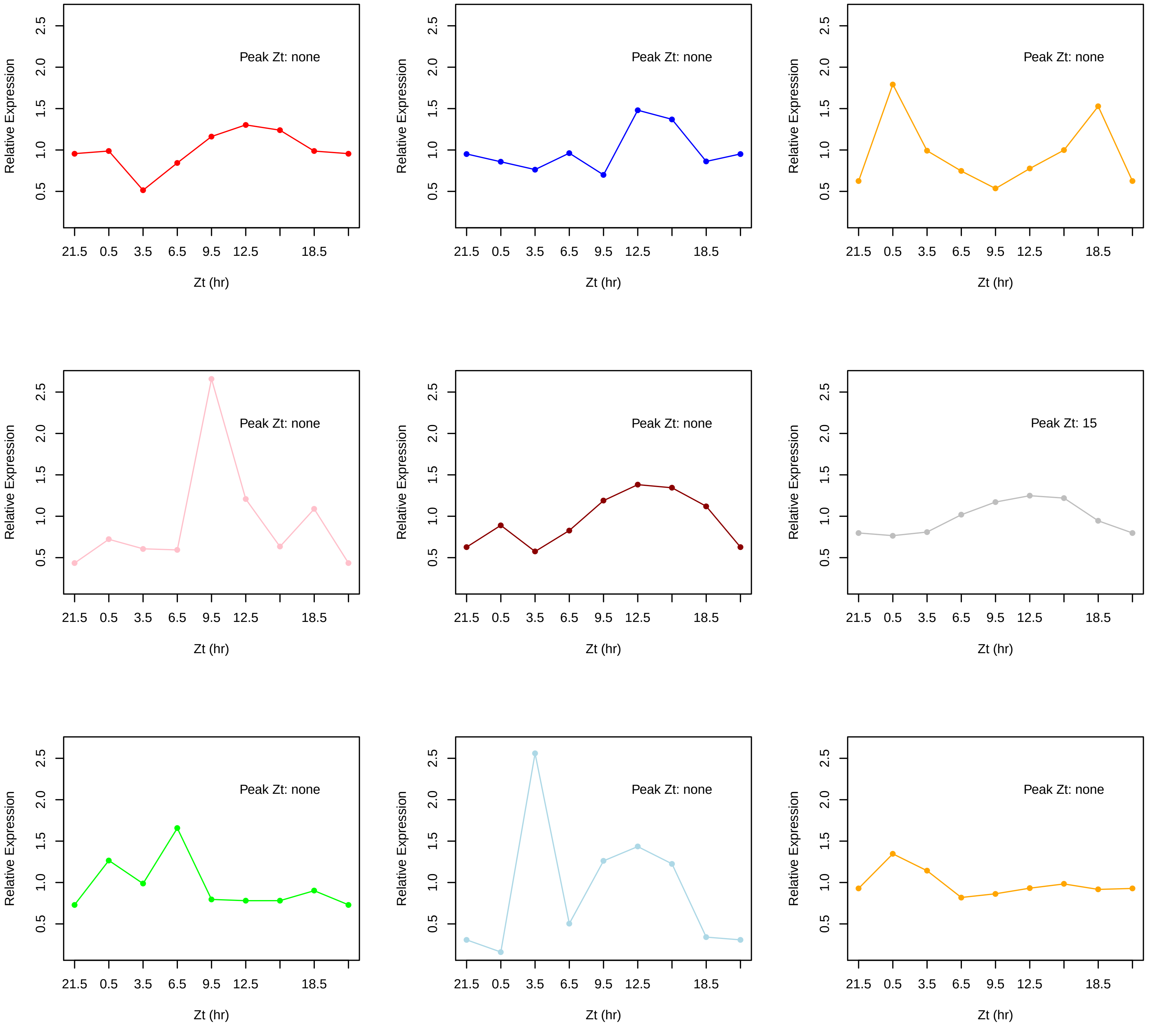

Singeleton: ENSXMAG00000002473 chfr

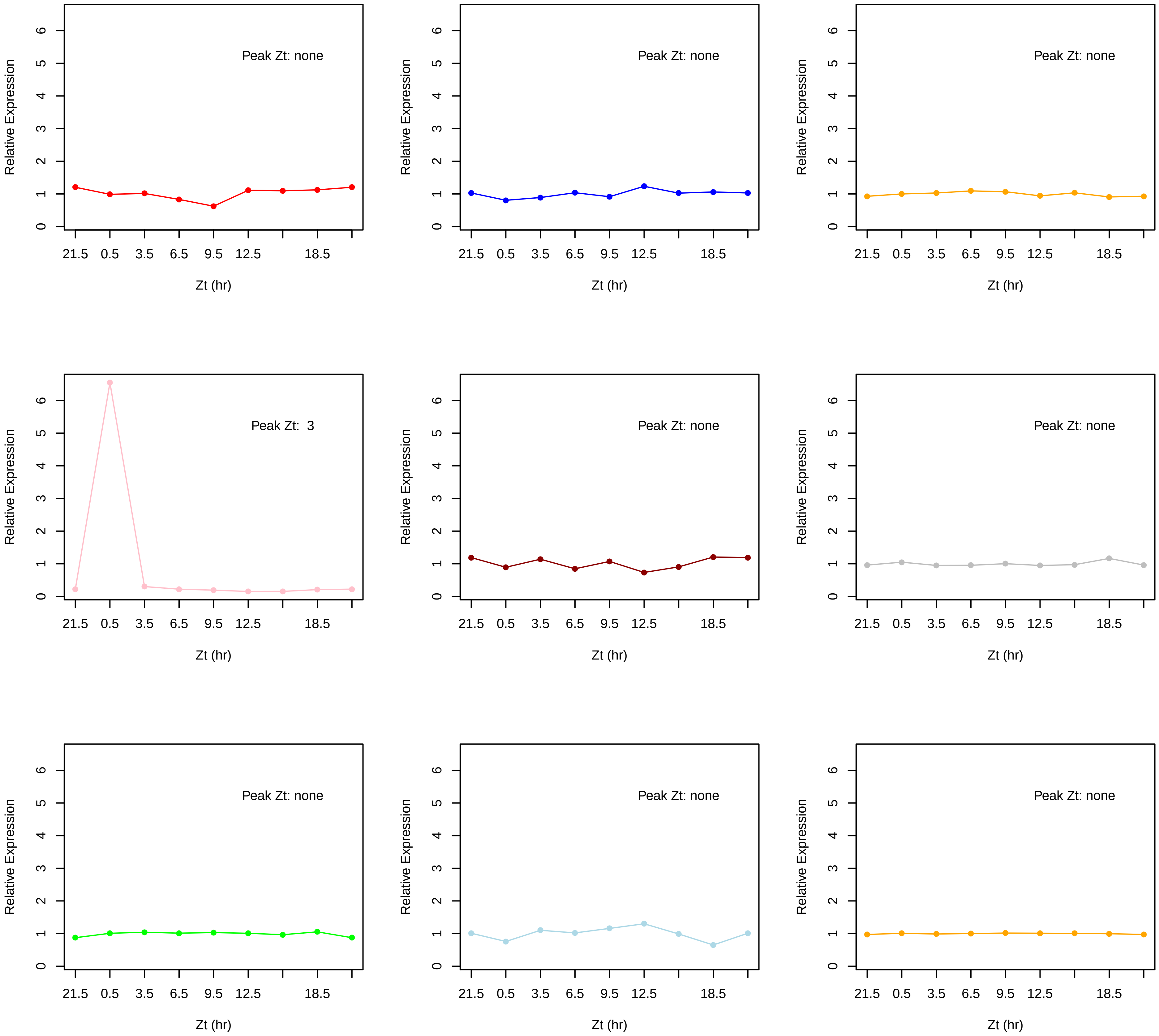

Singeleton: ENSXMAG00000003112 fbxl2

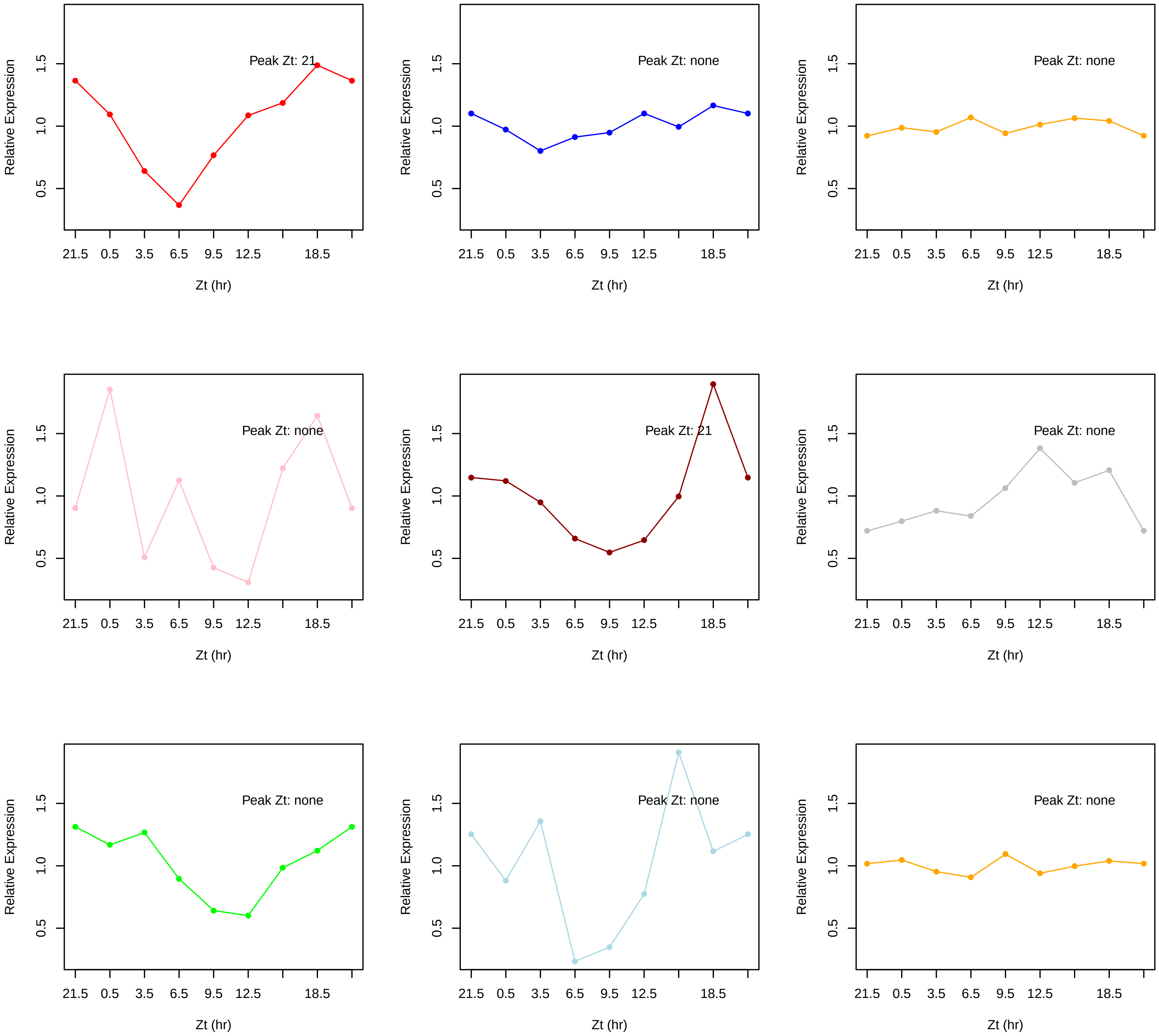

Singeleton: ENSXMAG00000005318 itgb5

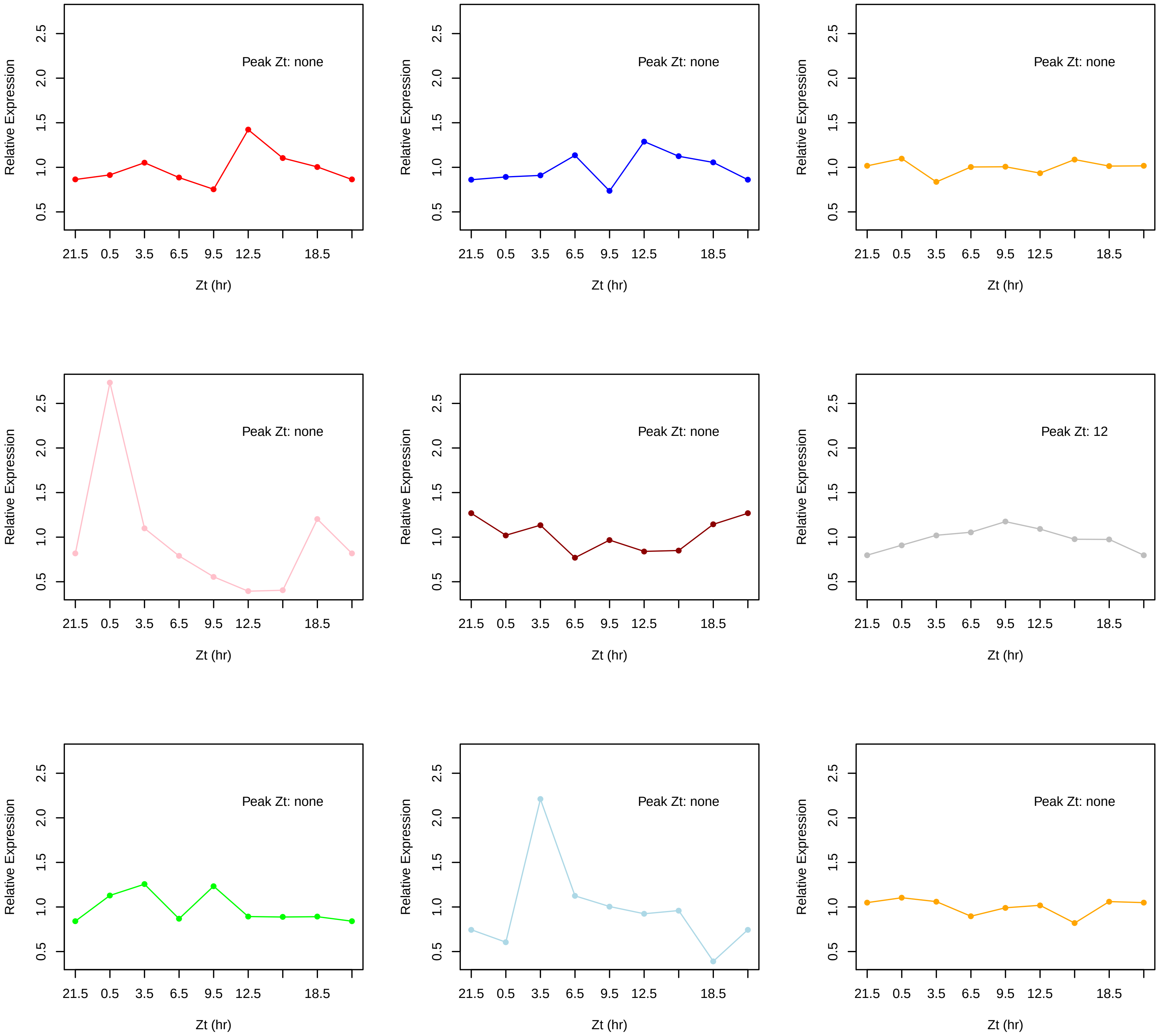

Singeleton: ENSXMAG00000018348 FAT4

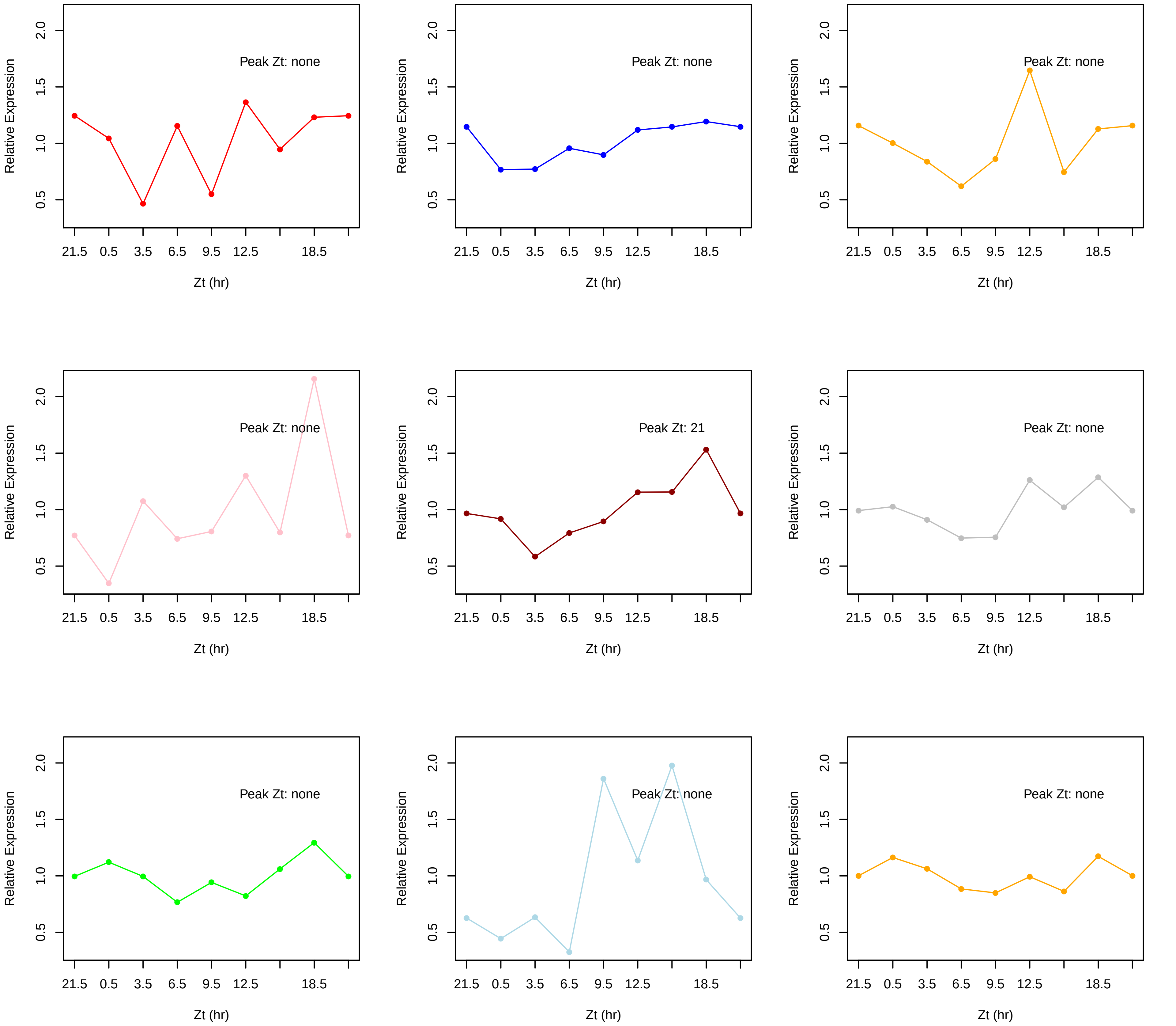

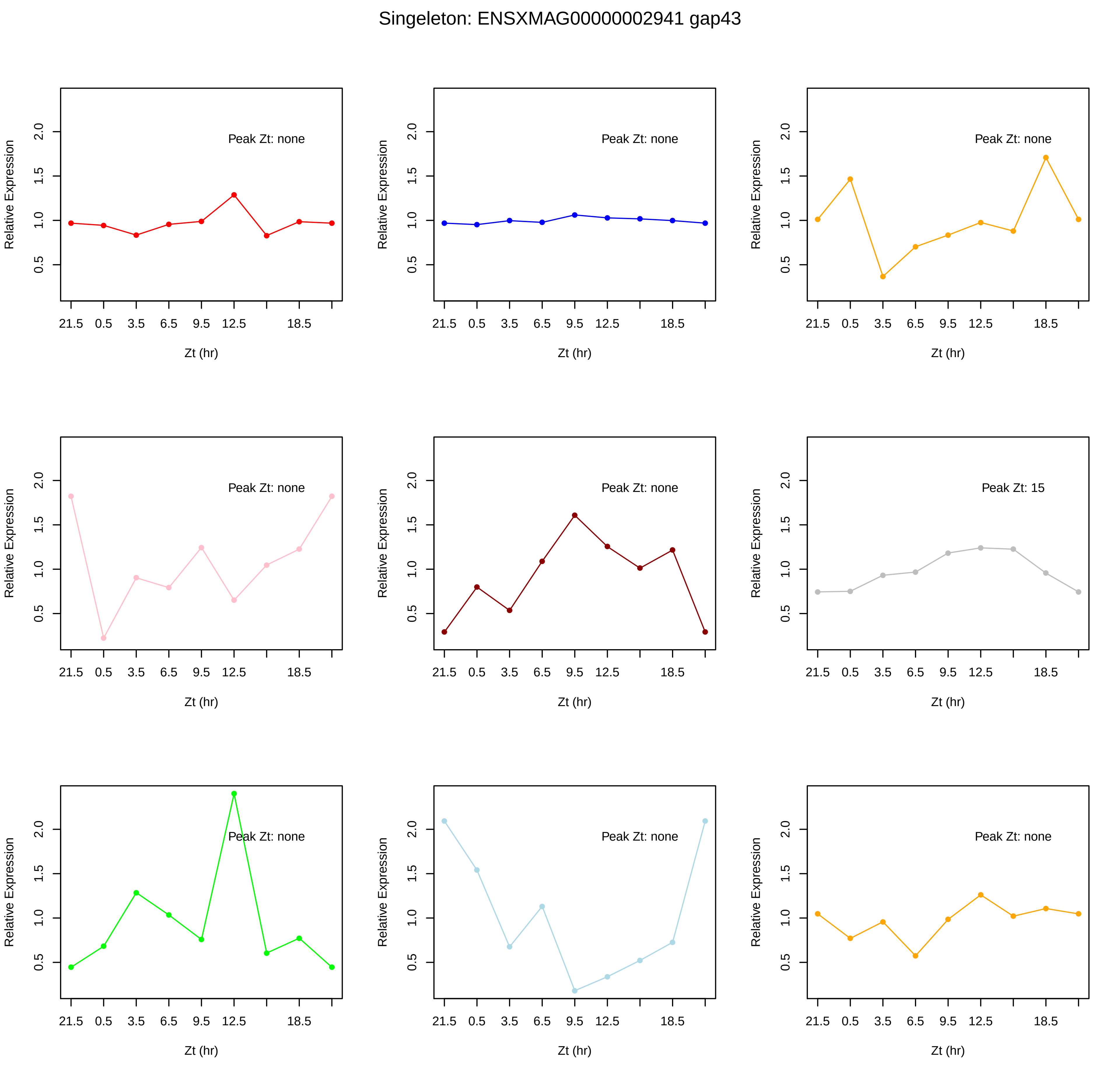
Singeleton: ENSXMAG00000002941 gap43
Peak Zt: none
Peak Zt: none
Peak Zt: none
Peak Zt: none
Peak Zt: none
Peak Zt: 15
Peak Zt: none
Peak Zt: none
Peak Zt: none
Relative Expression
Zt (hr)
21.5
0.5
3.5
6.5
9.5
12.5
18.5
0.5
1.0
1.5
2.0

Singeleton: ENSXMAG00000012014 tldc1

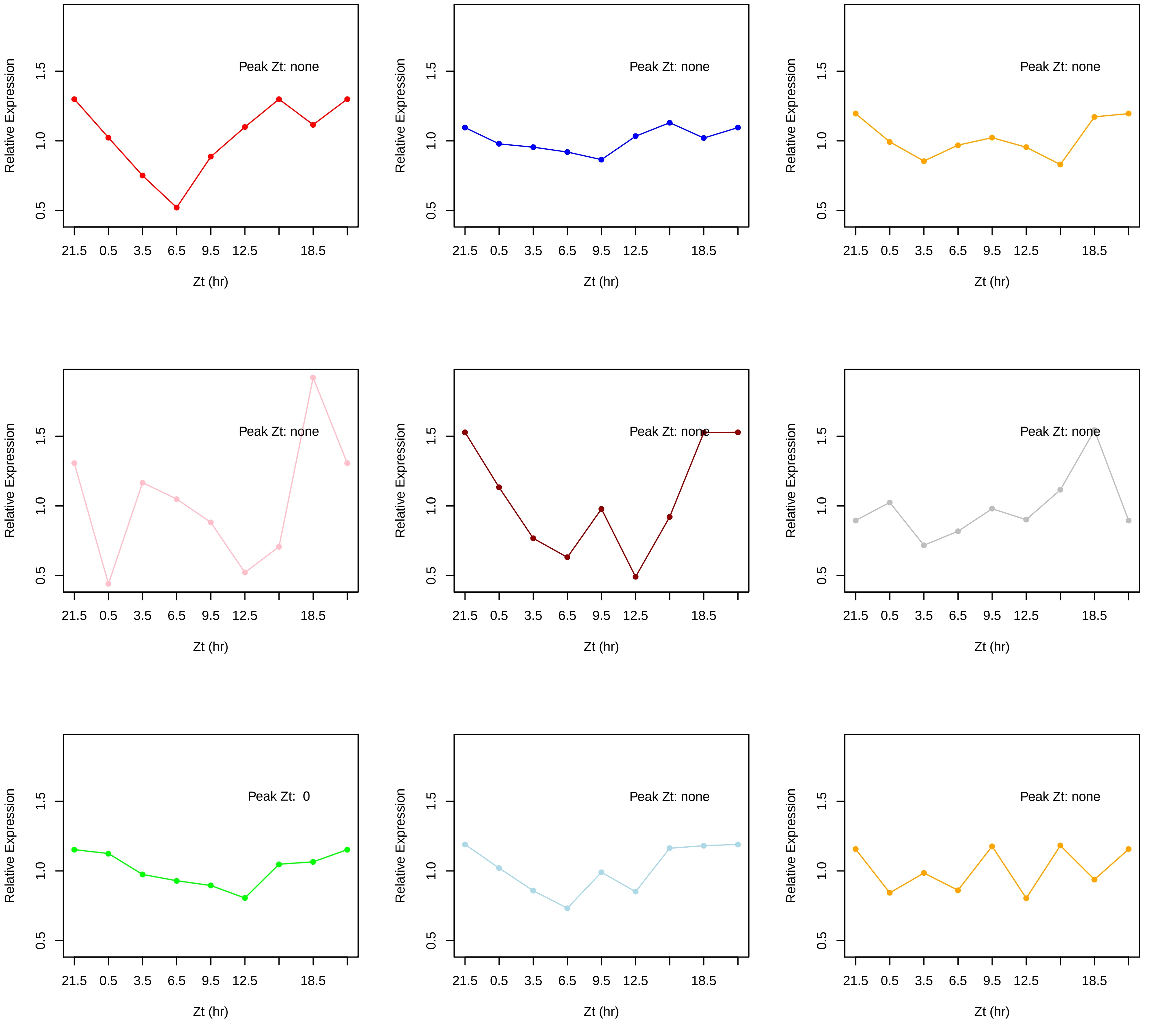

Singeleton: ENSXMAG00000022698 syf2

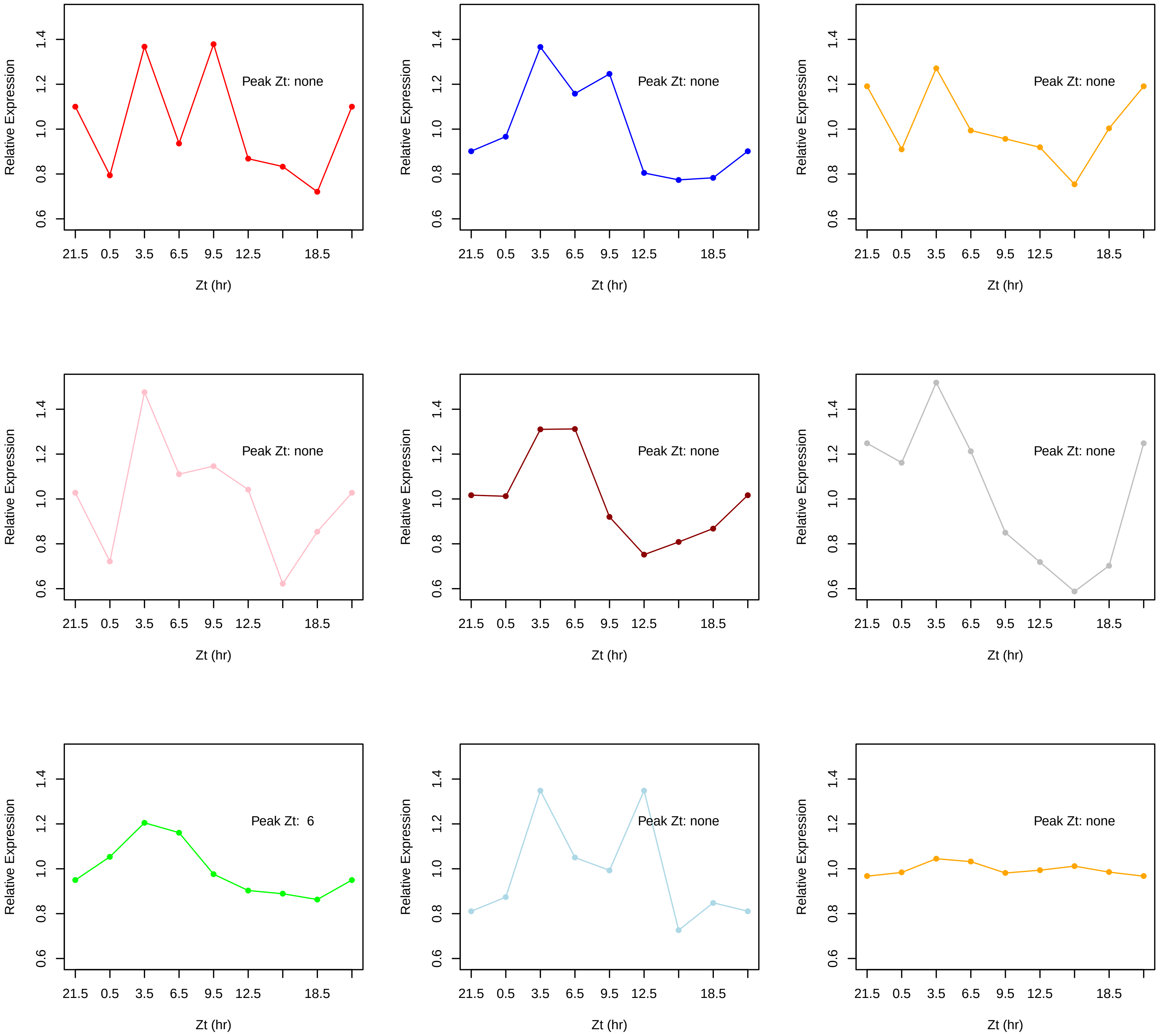

Singeleton: ENSXMAG00000016187 heg1

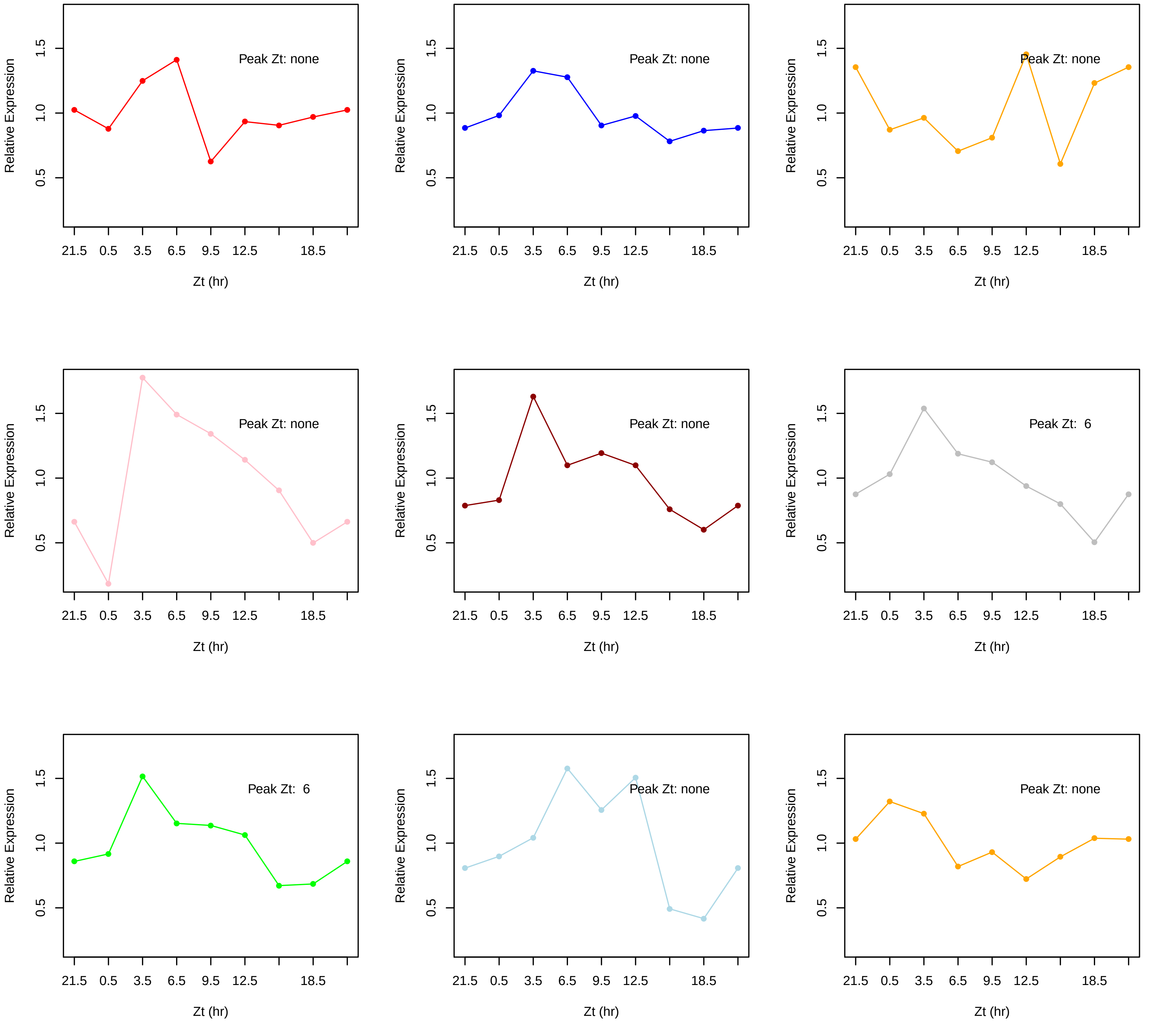

Singeleton: ENSXMAG00000024919 cotl1

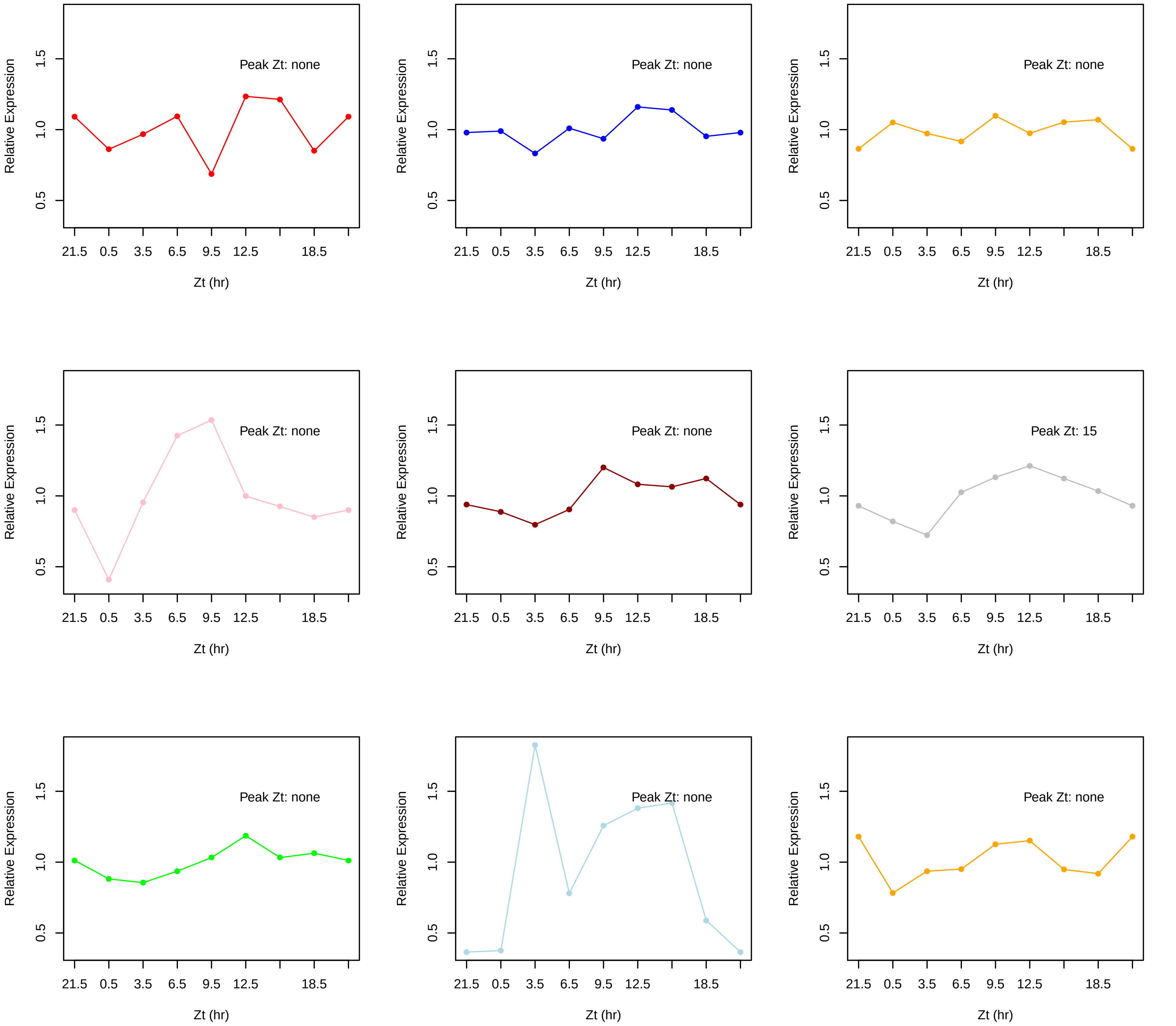

Singeleton: ENSXMAG00000004520 TRAP1

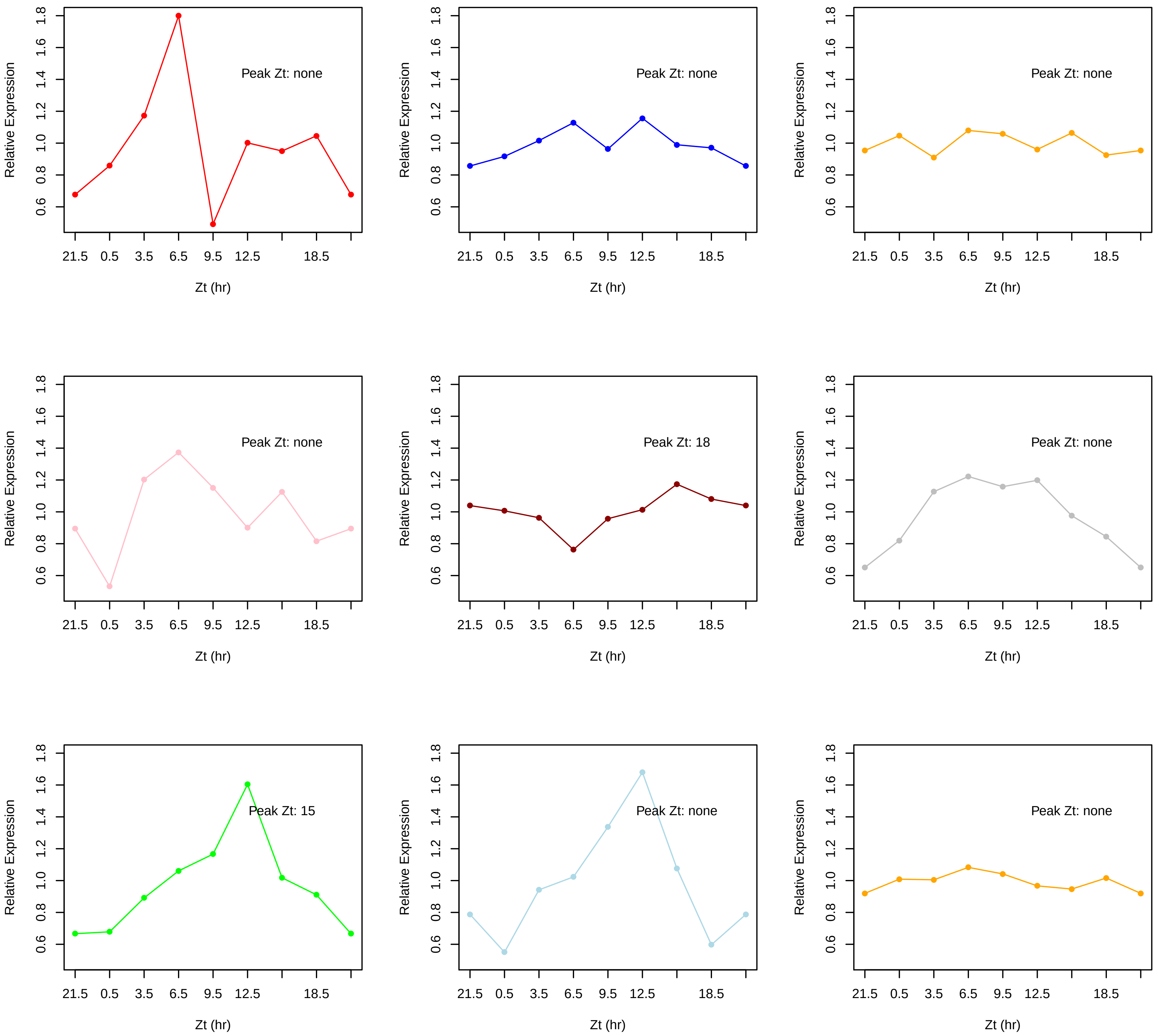

Singeleton: ENSXMAG00000018356 SPATA5

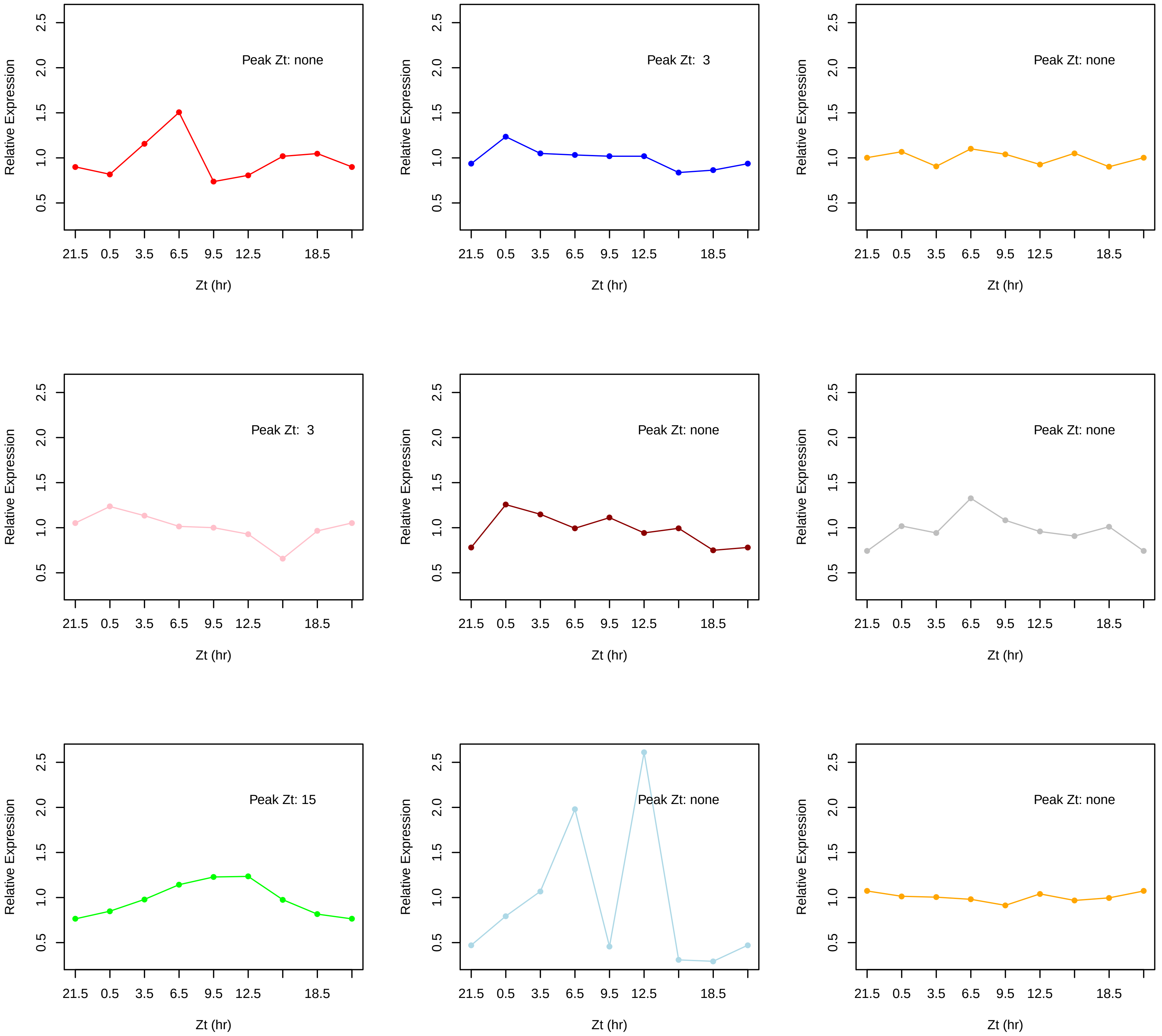

Singeleton: ENSXMAG00000001340 fam118b

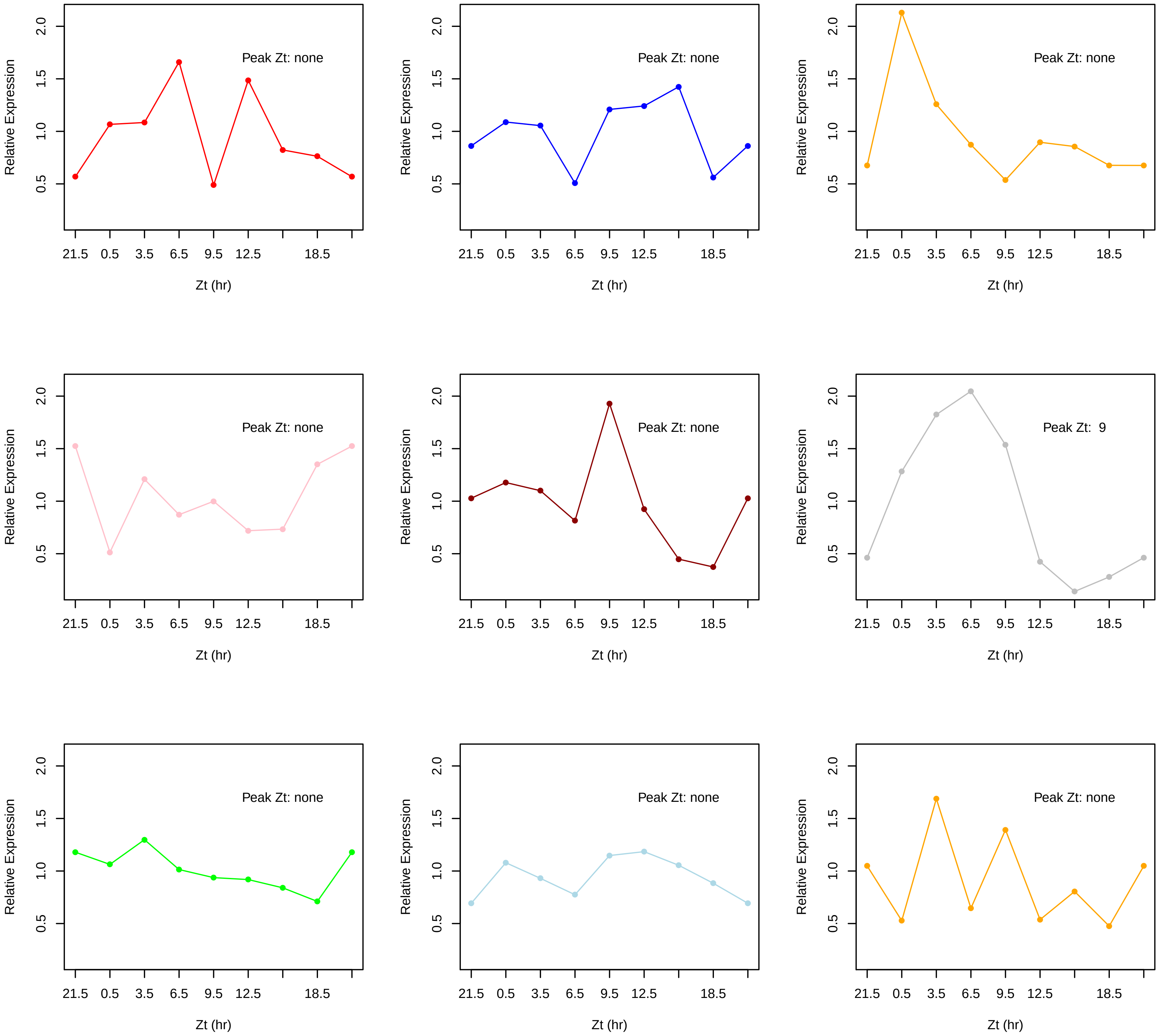

Singeleton: ENSXMAG00000021035 tp53i11b

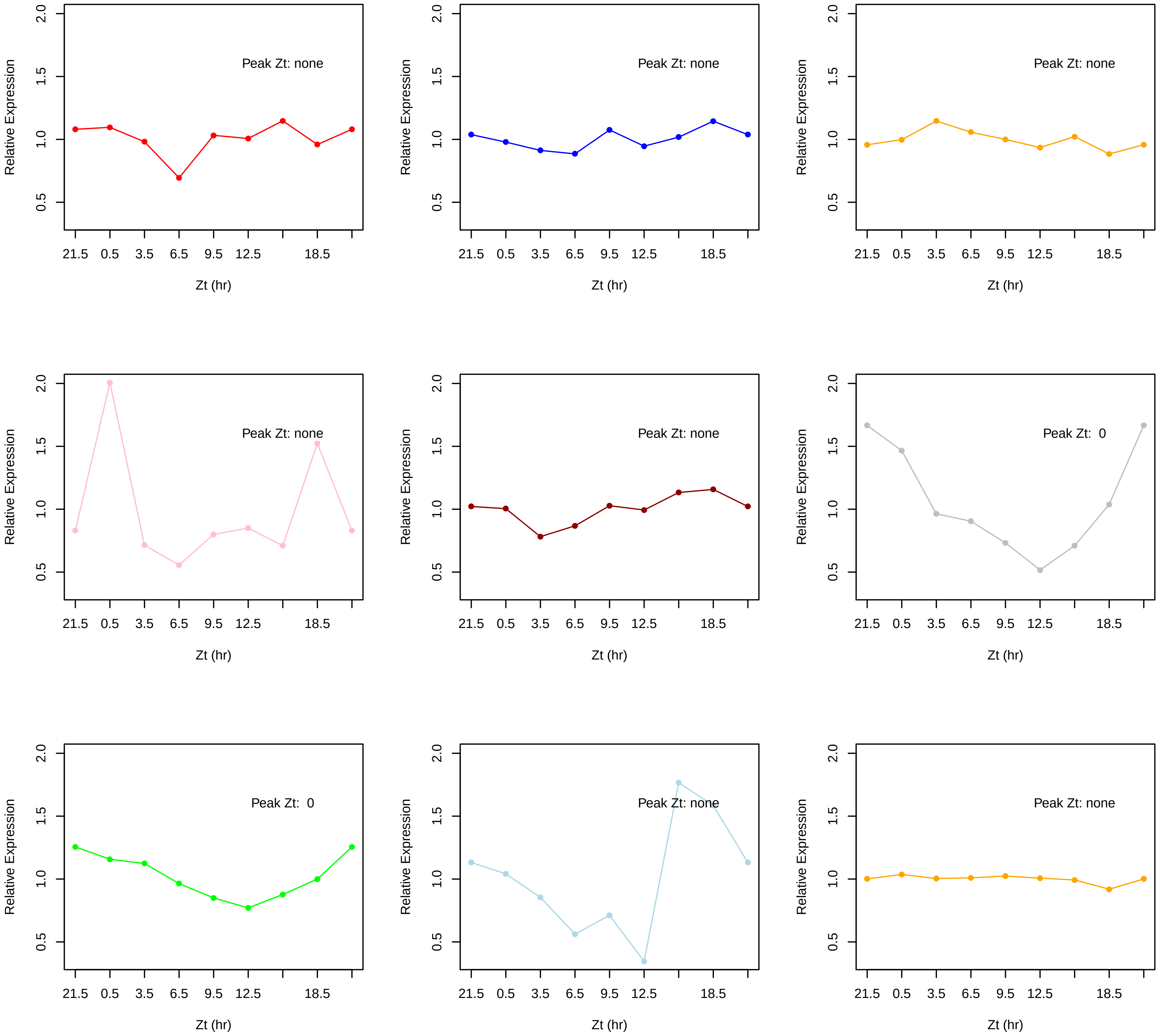

Singleton: ENSXMAG00000009101 klhl36

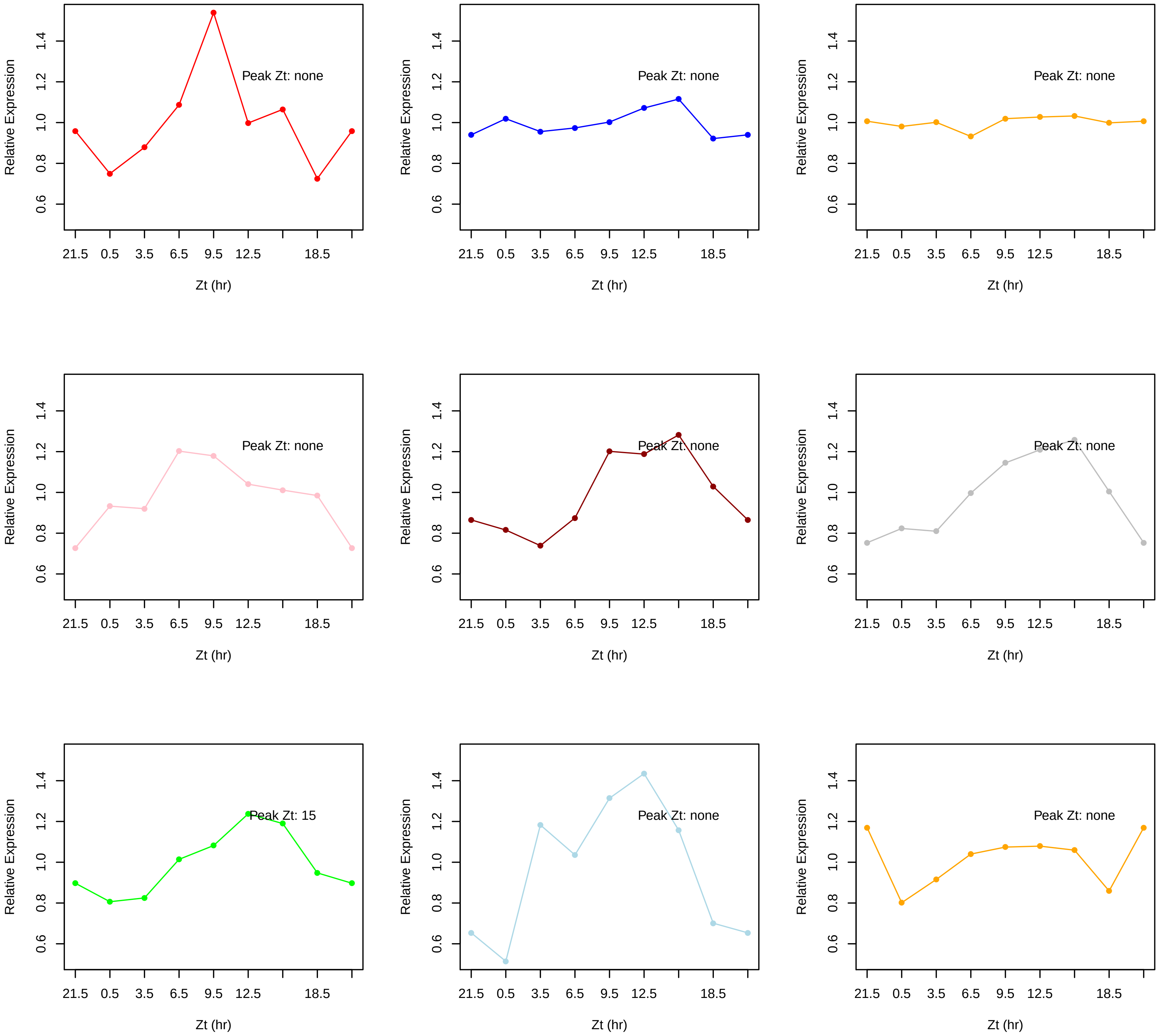

Singeleton: ENSXMAG00000001680 urm1

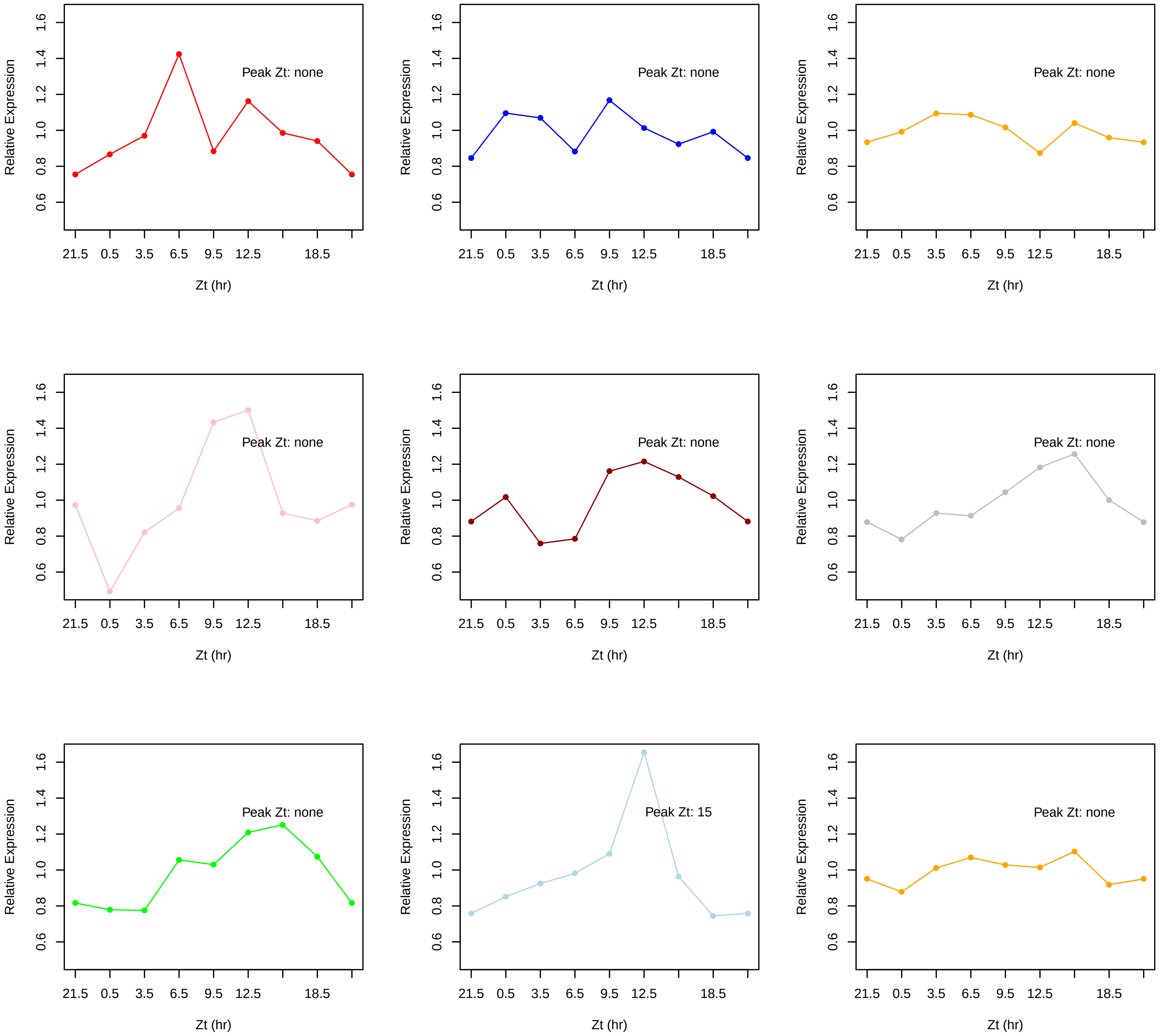

Singeleton: ENSXMAG00000012605 klhl21

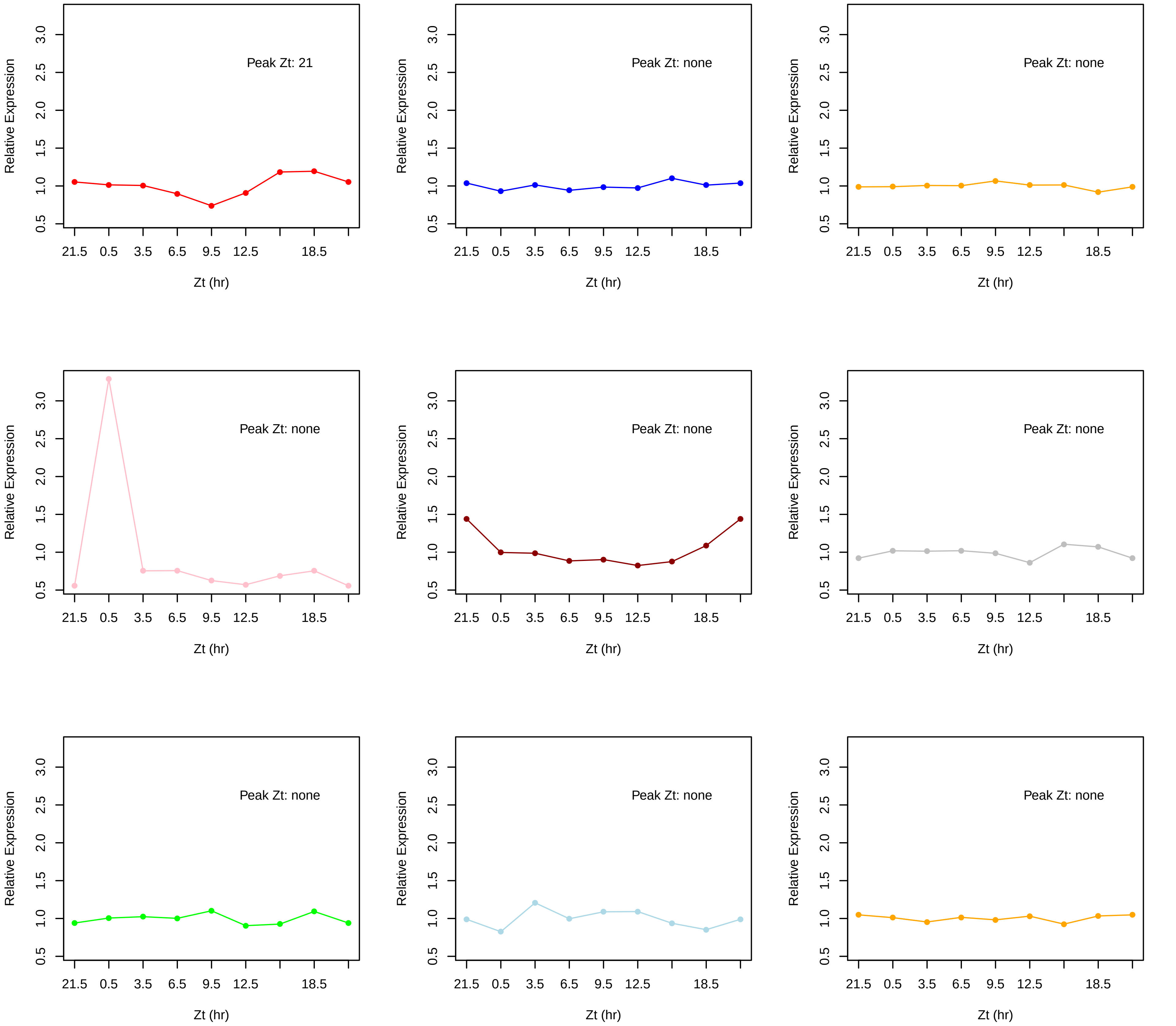

Singeleton: ENSXMAG00000013894 stt3a

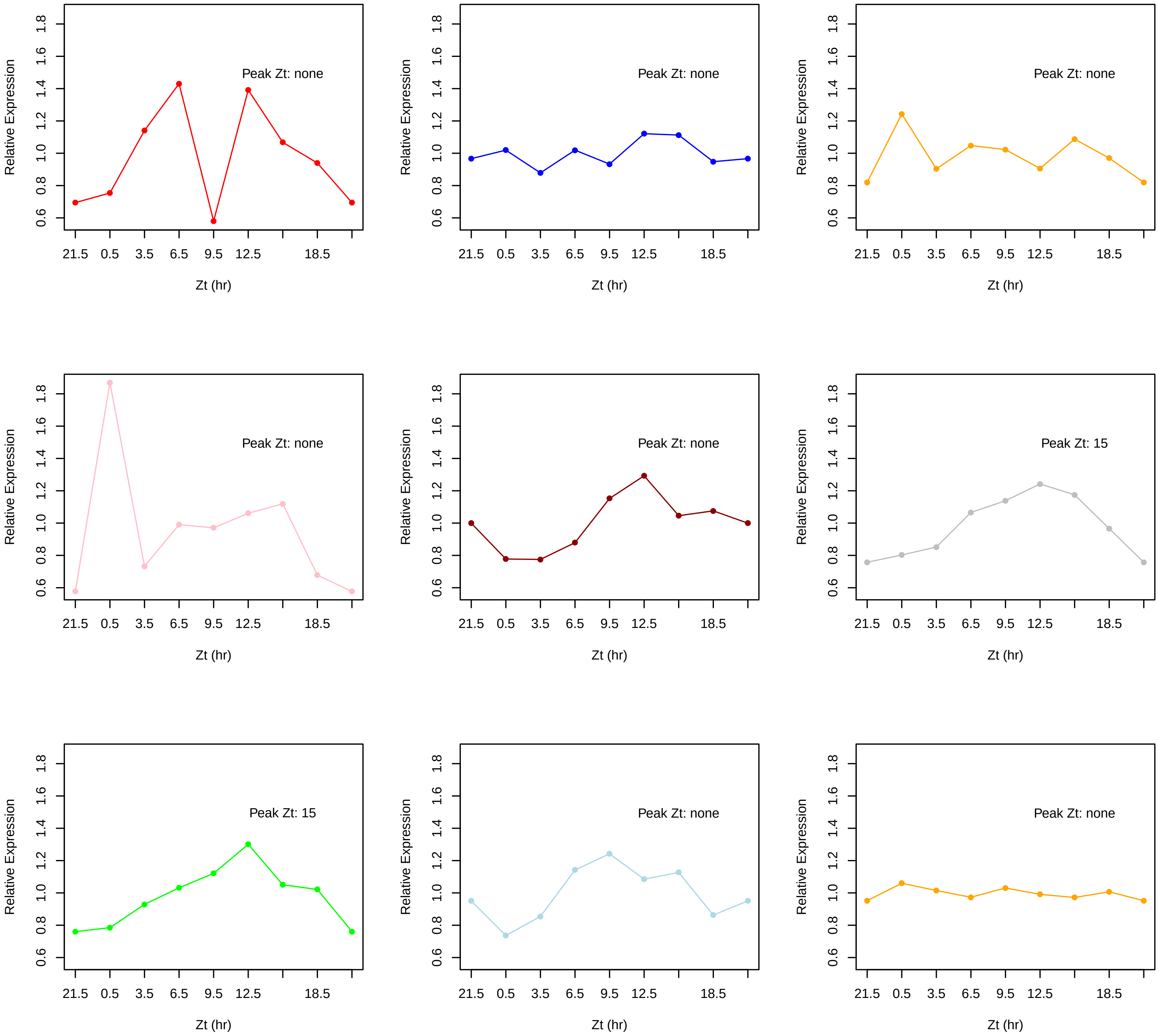

Singeleton: ENSXMAG00000022028 tor2a

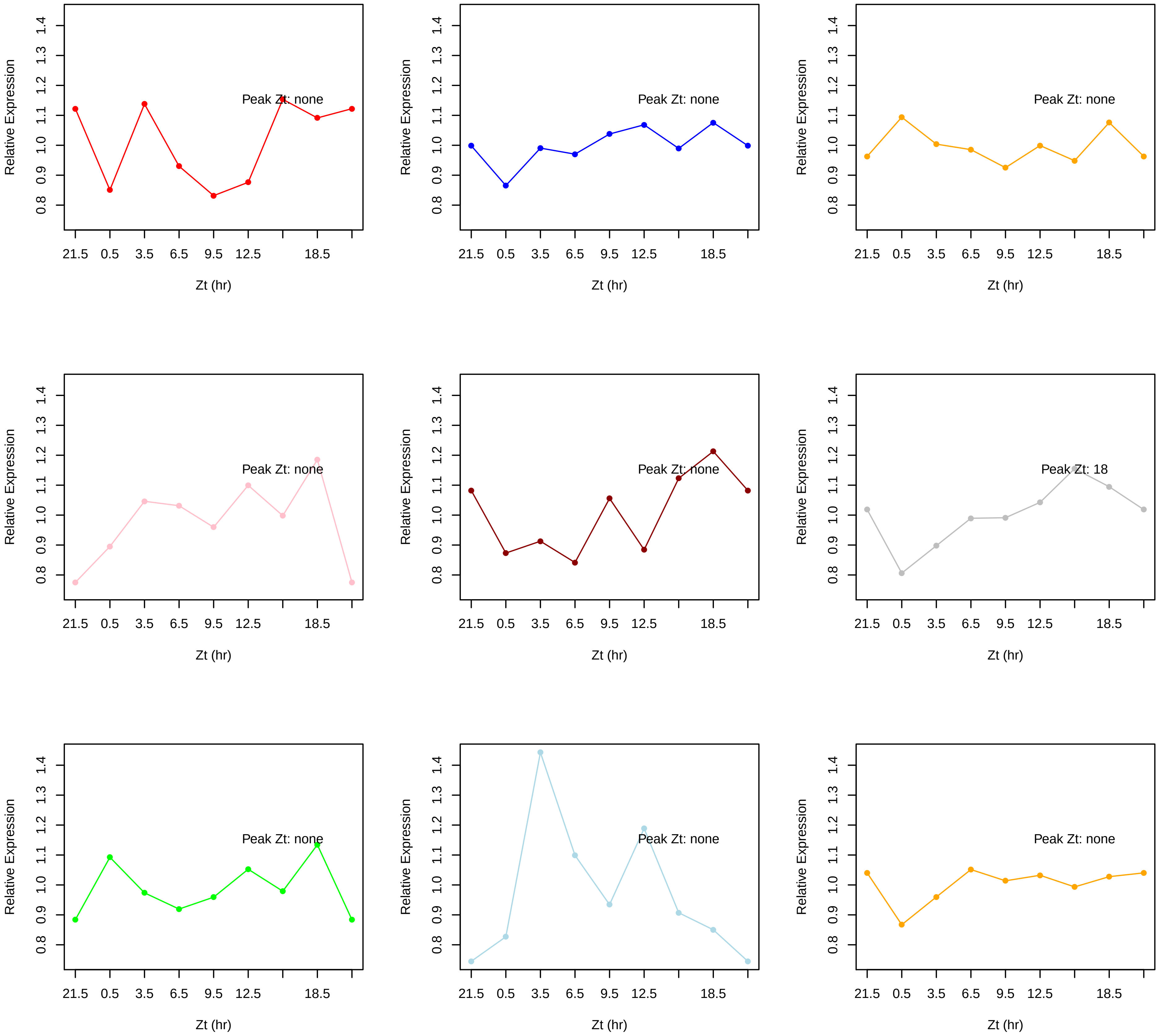

Singeleton: ENSXMAG00000005671

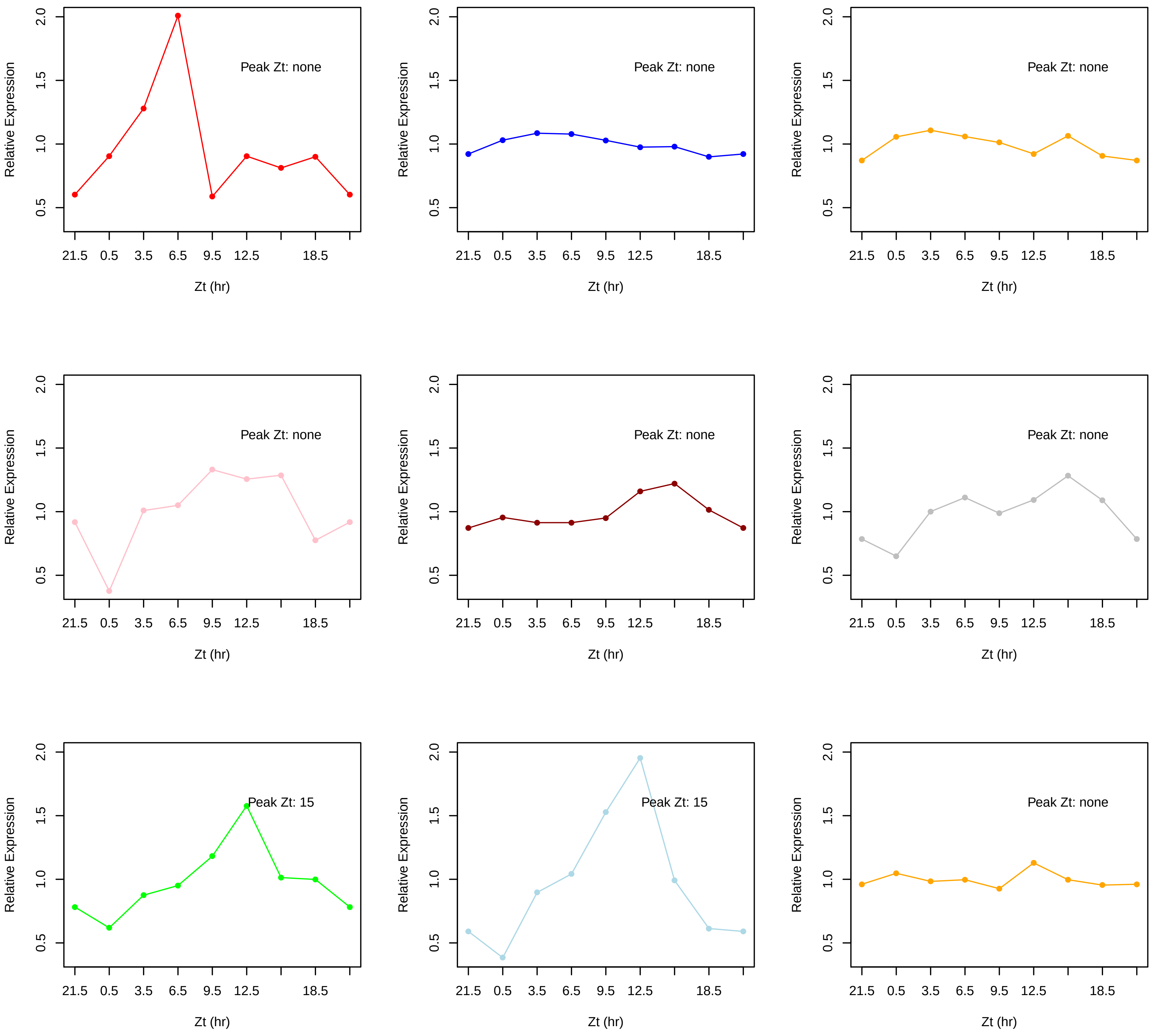

Singleton: ENSXMAG00000029886 nudt6

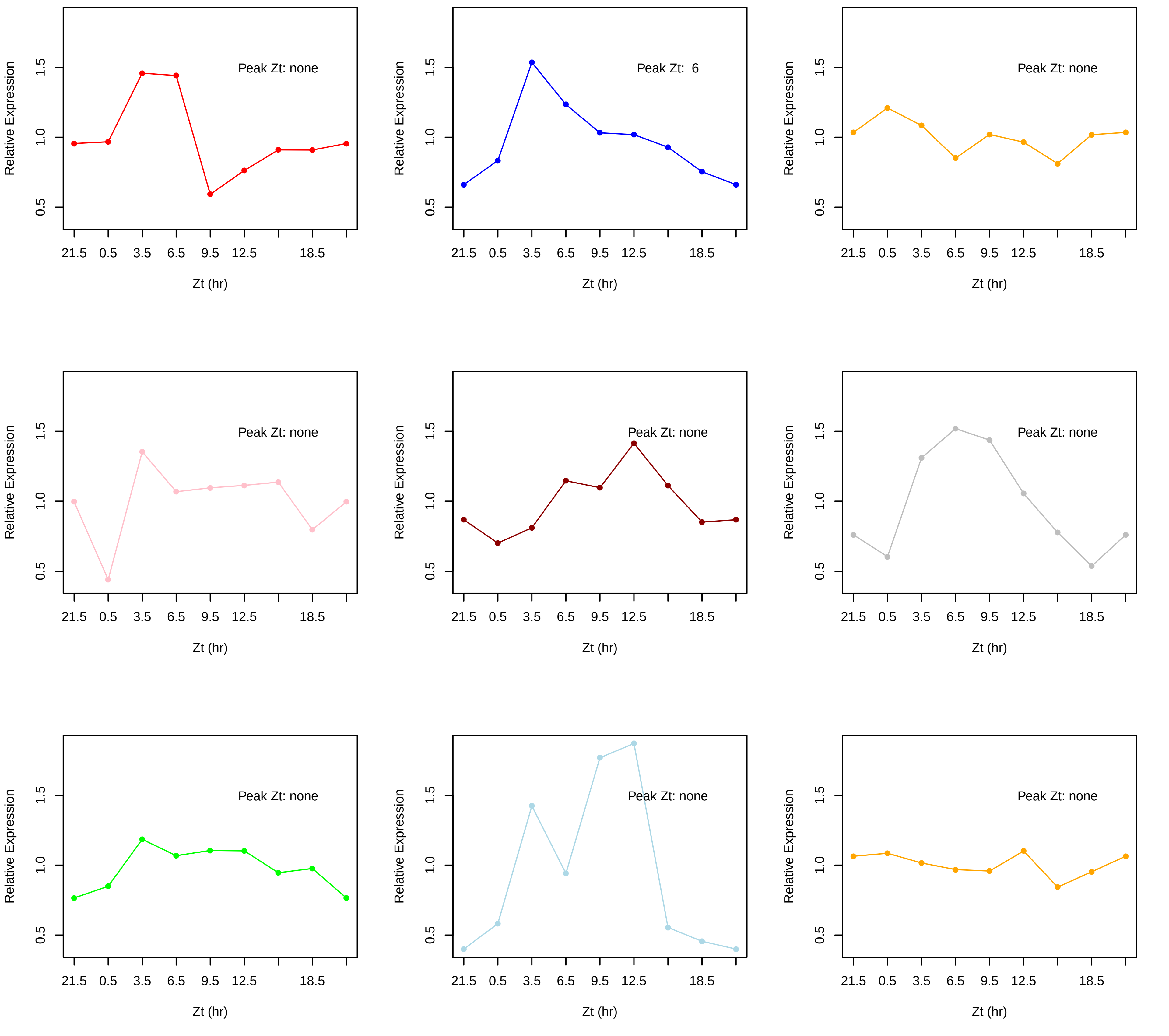

Singeleton: ENSXMAG00000008825 slc5a5

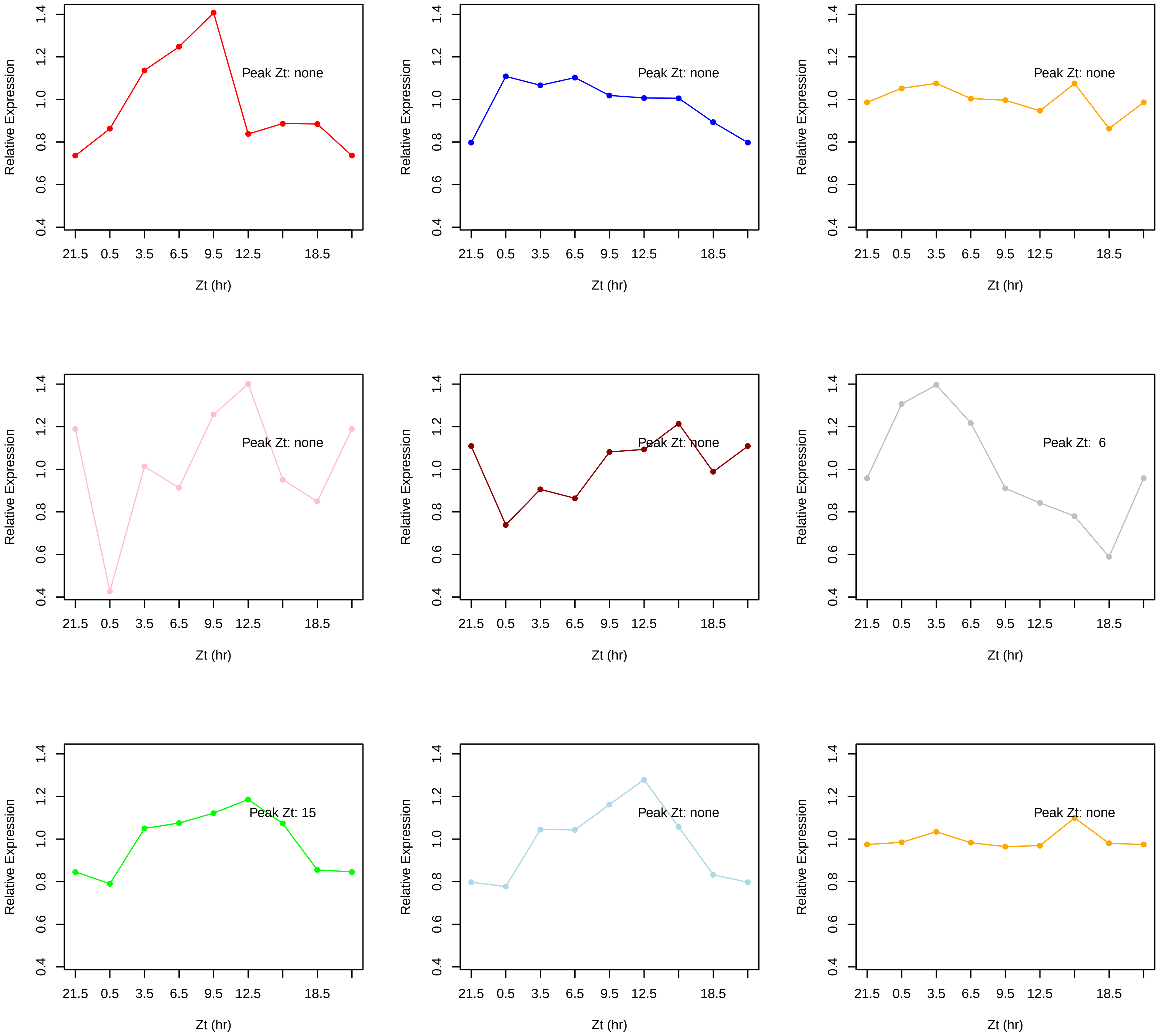

Singeleton: ENSXMAG00000018492 dmap1

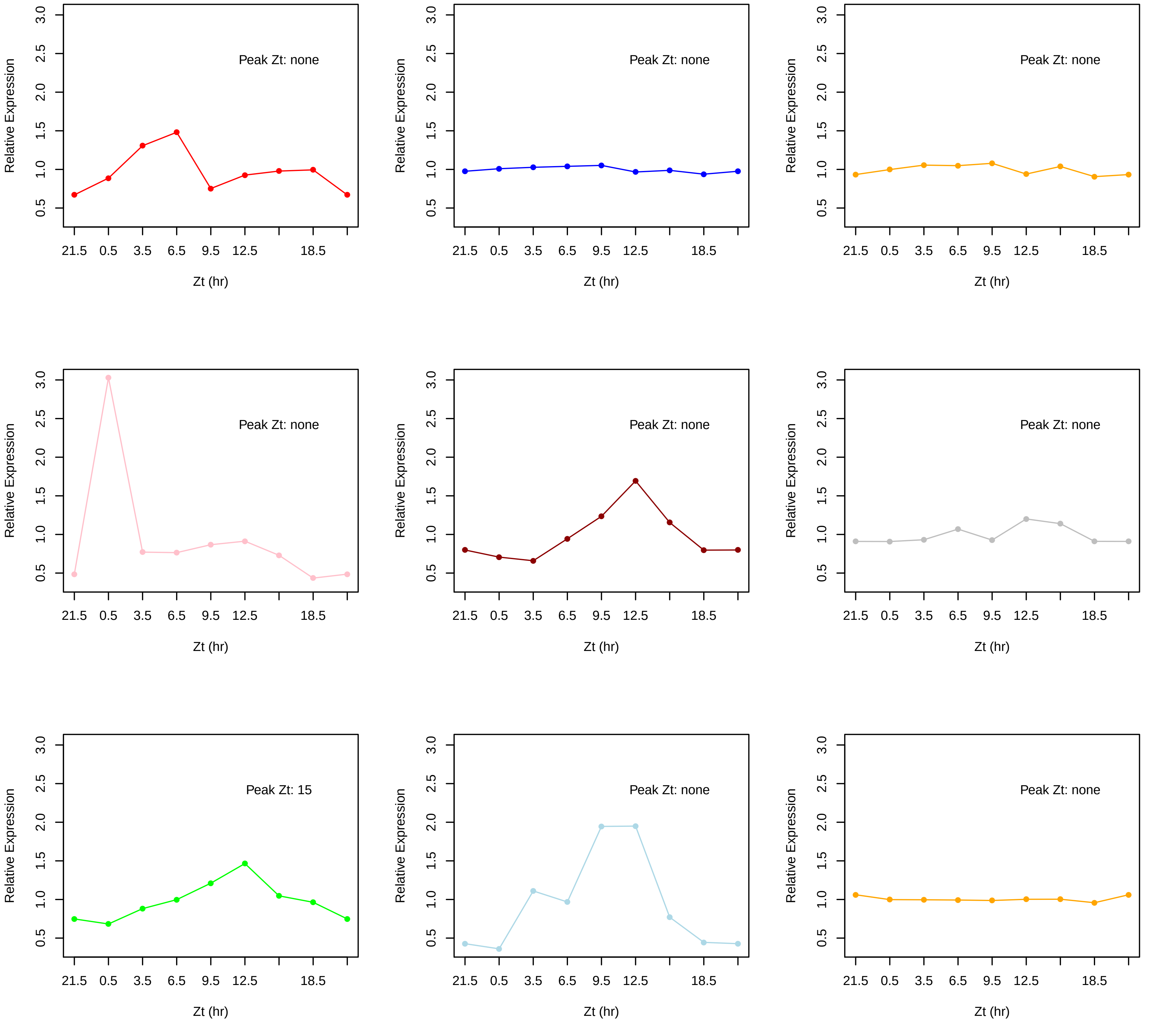

Singleton: ENSXMAG00000007692 rcan3

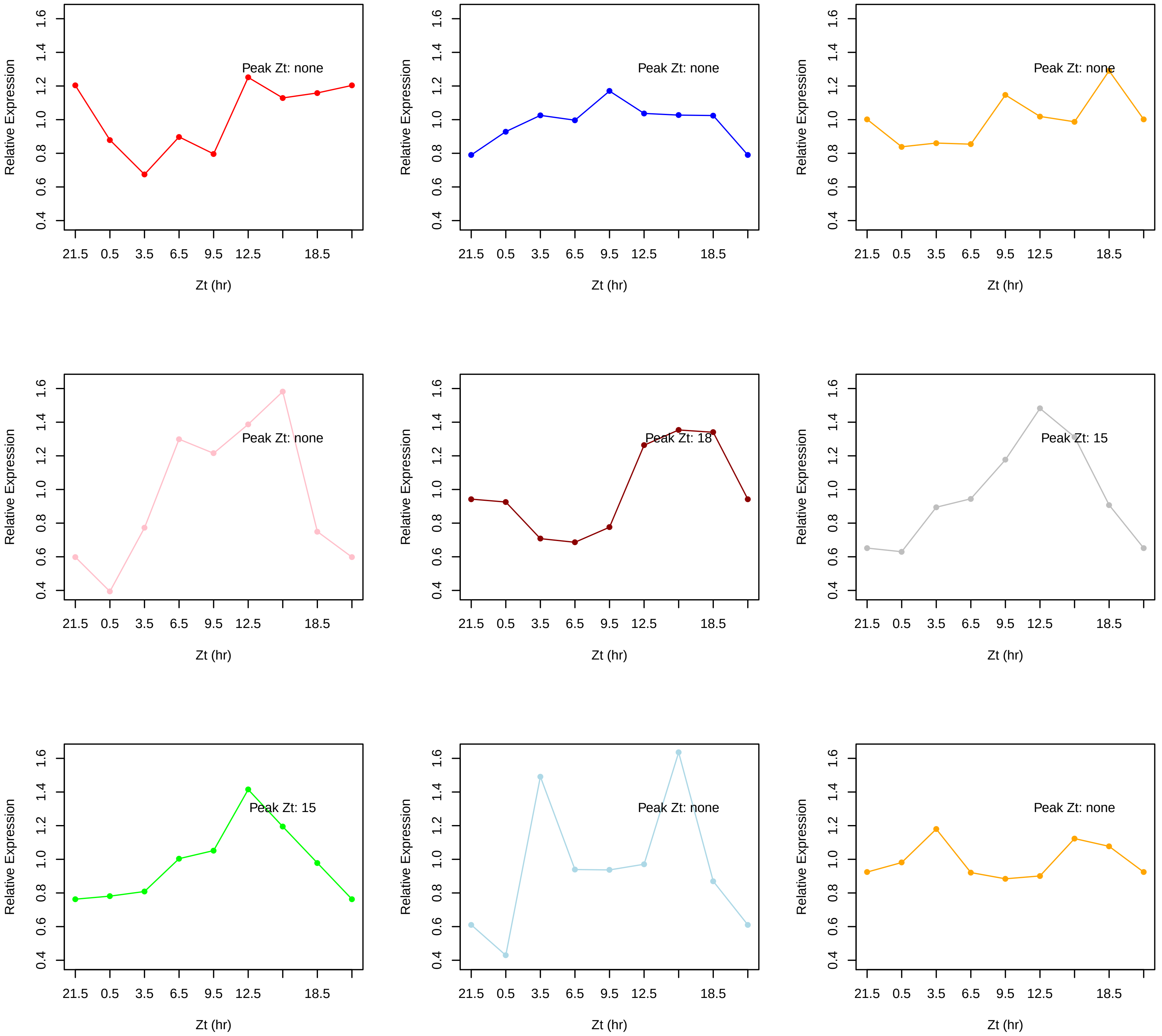

Singleton: ENSXMAG00000023623 tpd52

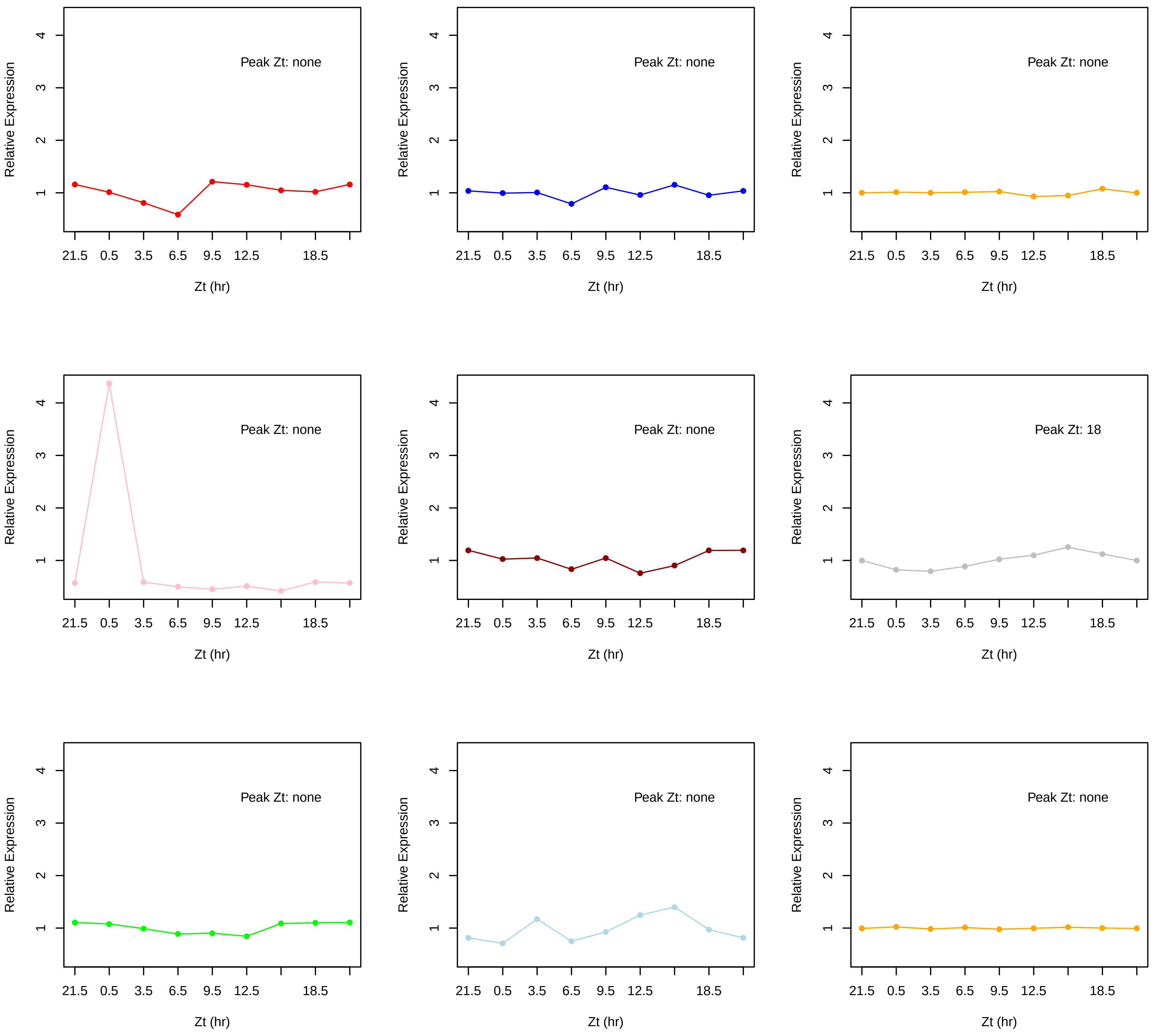

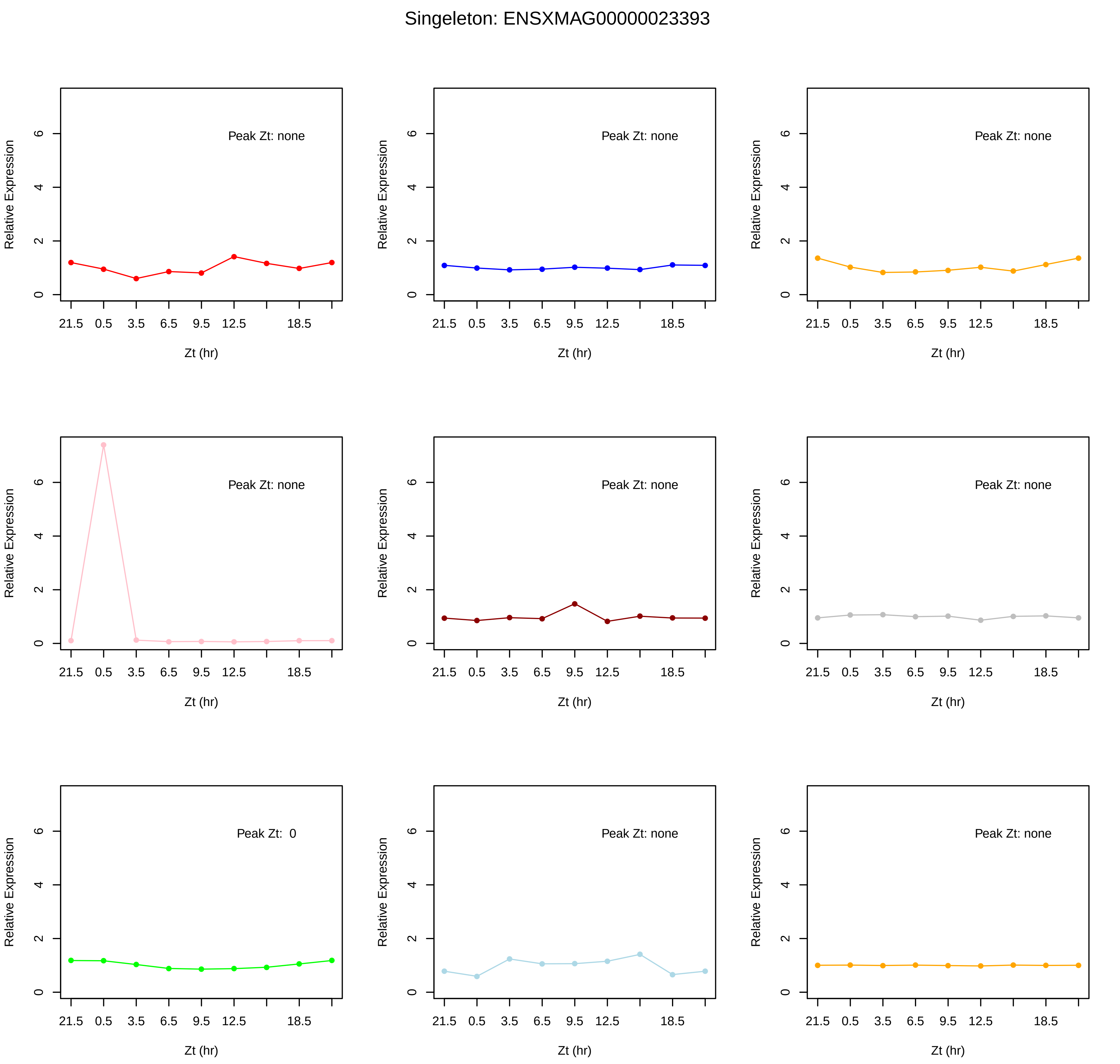
Singeleton: ENSXMAG00000023393
Peak Zt: none
Peak Zt: none
Peak Zt: none
Peak Zt: none
Peak Zt: none
Peak Zt: none
Peak Zt: 0
Peak Zt: none
Peak Zt: none
Relative Expression
Zt (hr)
21.5
0.5
3.5
6.5
9.5
12.5
18.5
0
2
4
6

Singeleton: ENSXMAG00000007194 ext2

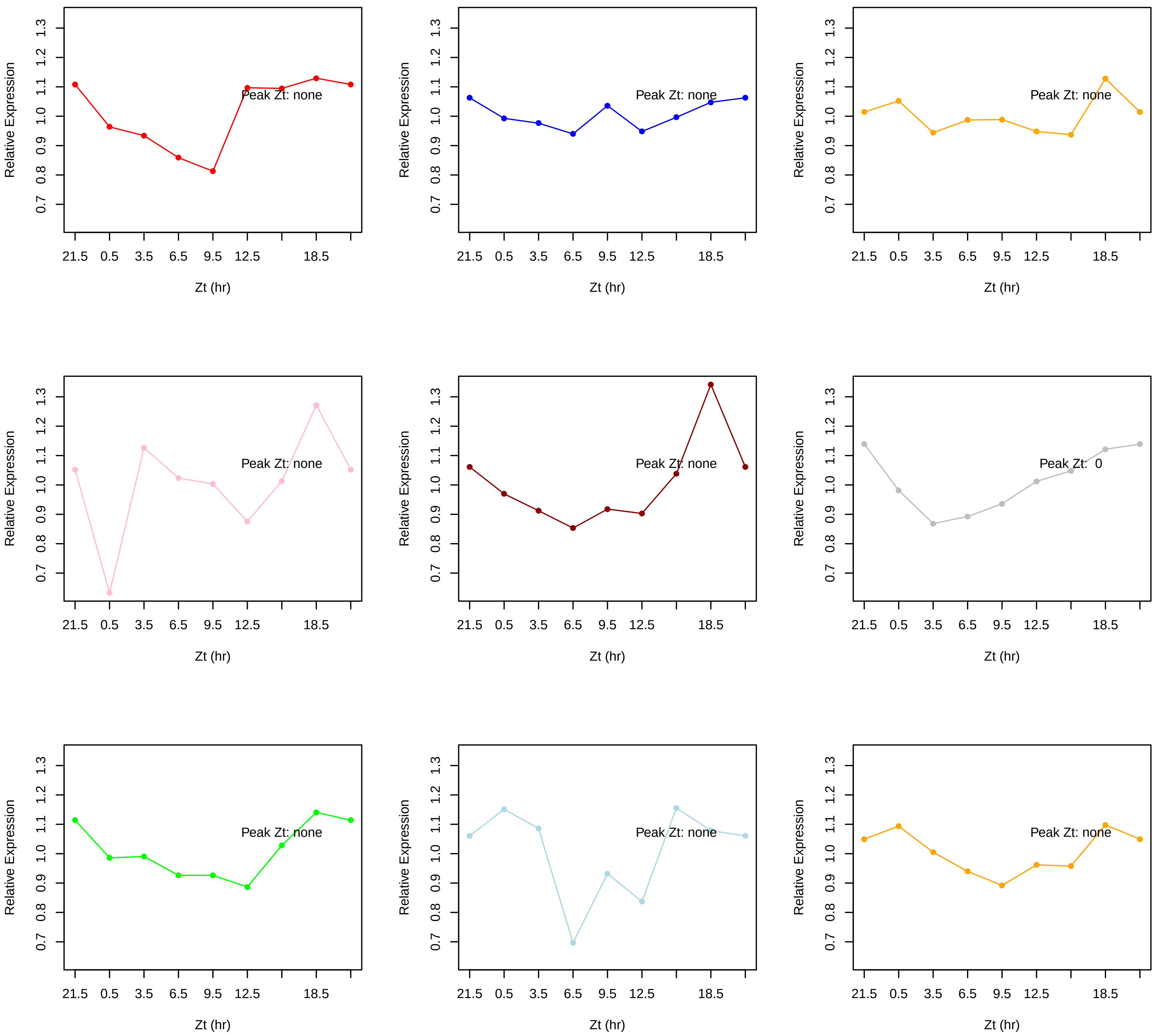

Singeleton: ENSXMAG00000028755 zgc:56622

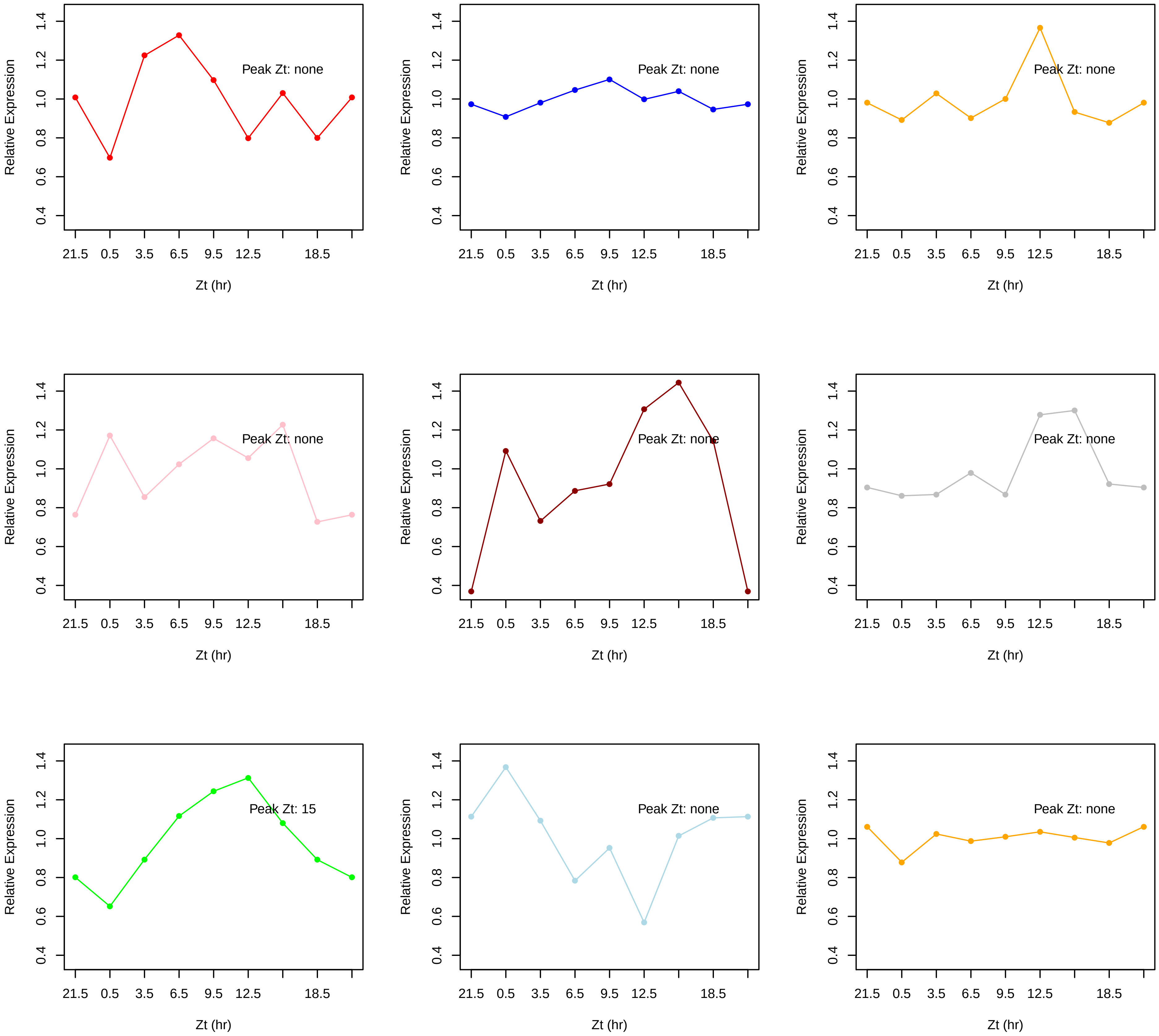

Singeleton: ENSXMAG00000013393 nalcn

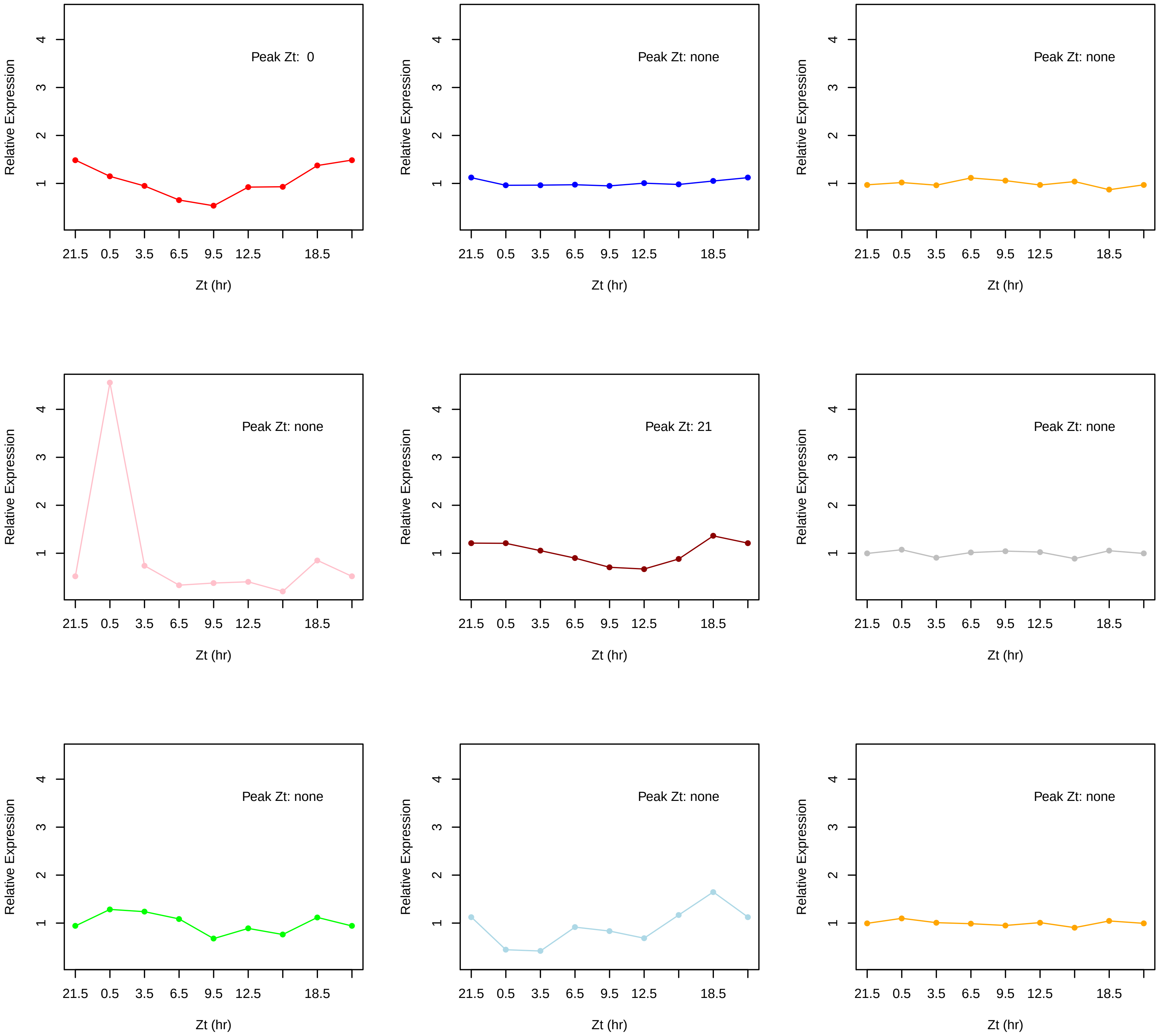

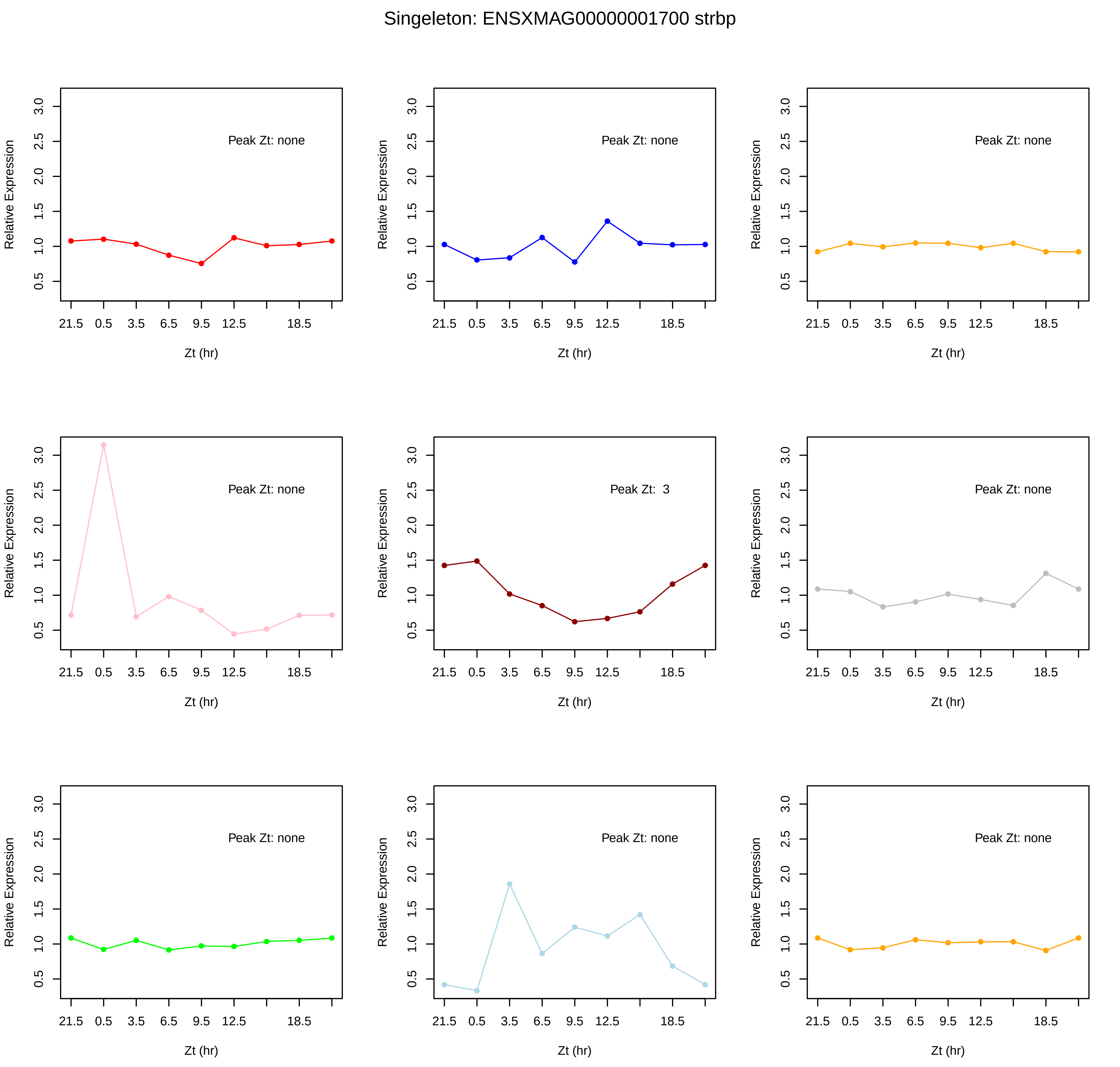

Singeleton: ENSXMAG00000001700 strbp
Peak Zt: none
Peak Zt: none
Peak Zt: none
Peak Zt: none
Peak Zt: 3
Peak Zt: none
Peak Zt: none
Peak Zt: none
Peak Zt: none
Relative Expression
Zt (hr)
21.5 0.5 3.5 6.5 9.5 12.5 18.5
0.5 1.0 1.5 2.0 2.5 3.0

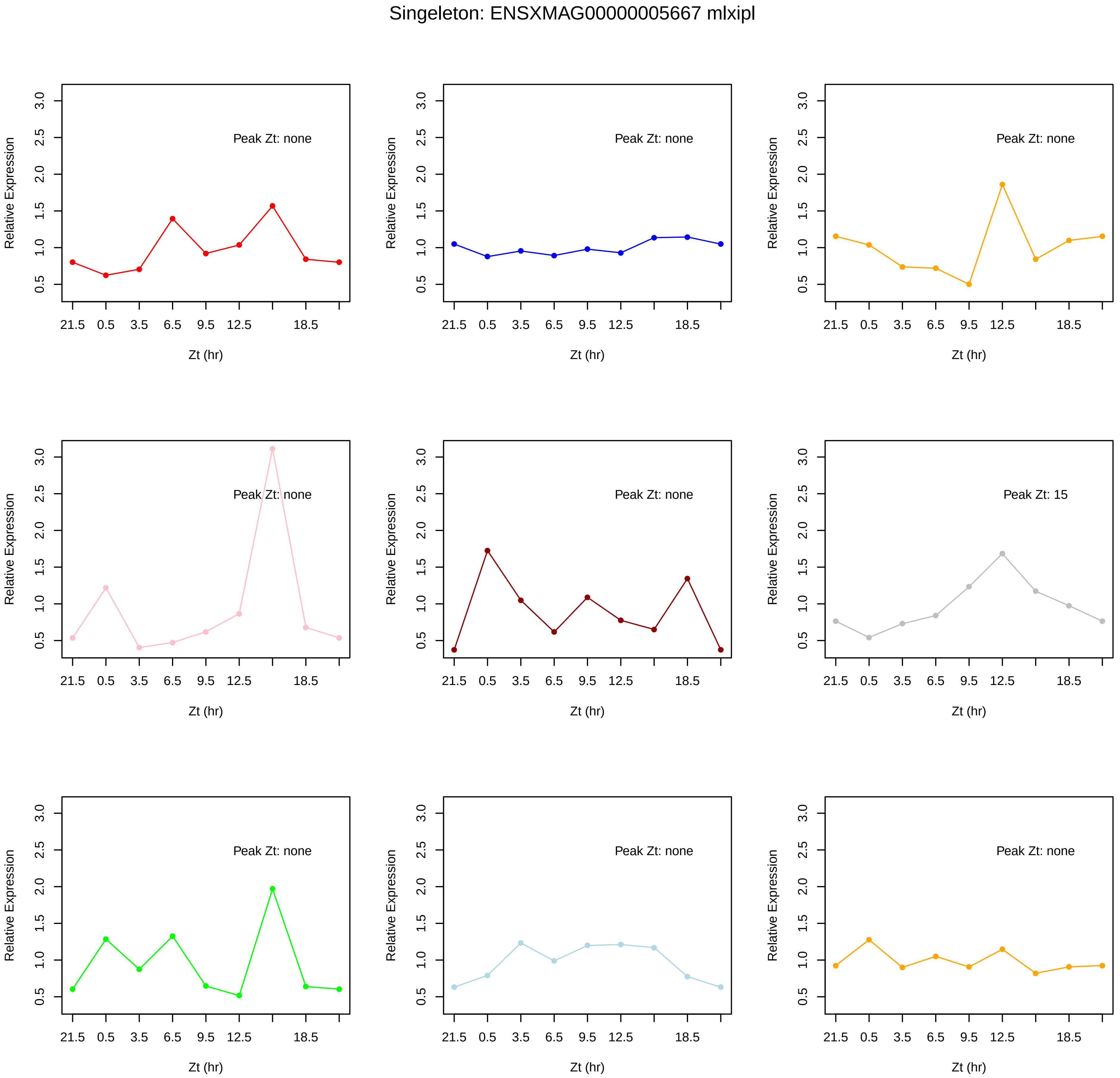
Singleton: ENSXMAG00000005667 mlxipl
Peak Zt: none
Peak Zt: none
Peak Zt: none
Peak Zt: none
Peak Zt: none
Peak Zt: 15
Peak Zt: none
Peak Zt: none
Peak Zt: none
Relative Expression
Zt (hr)
21.5
0.5
3.5
6.5
9.5
12.5
18.5
0.5
1.0
1.5
2.0
2.5
3.0

Singeleton: ENSXMAG00000001714 rabgap1

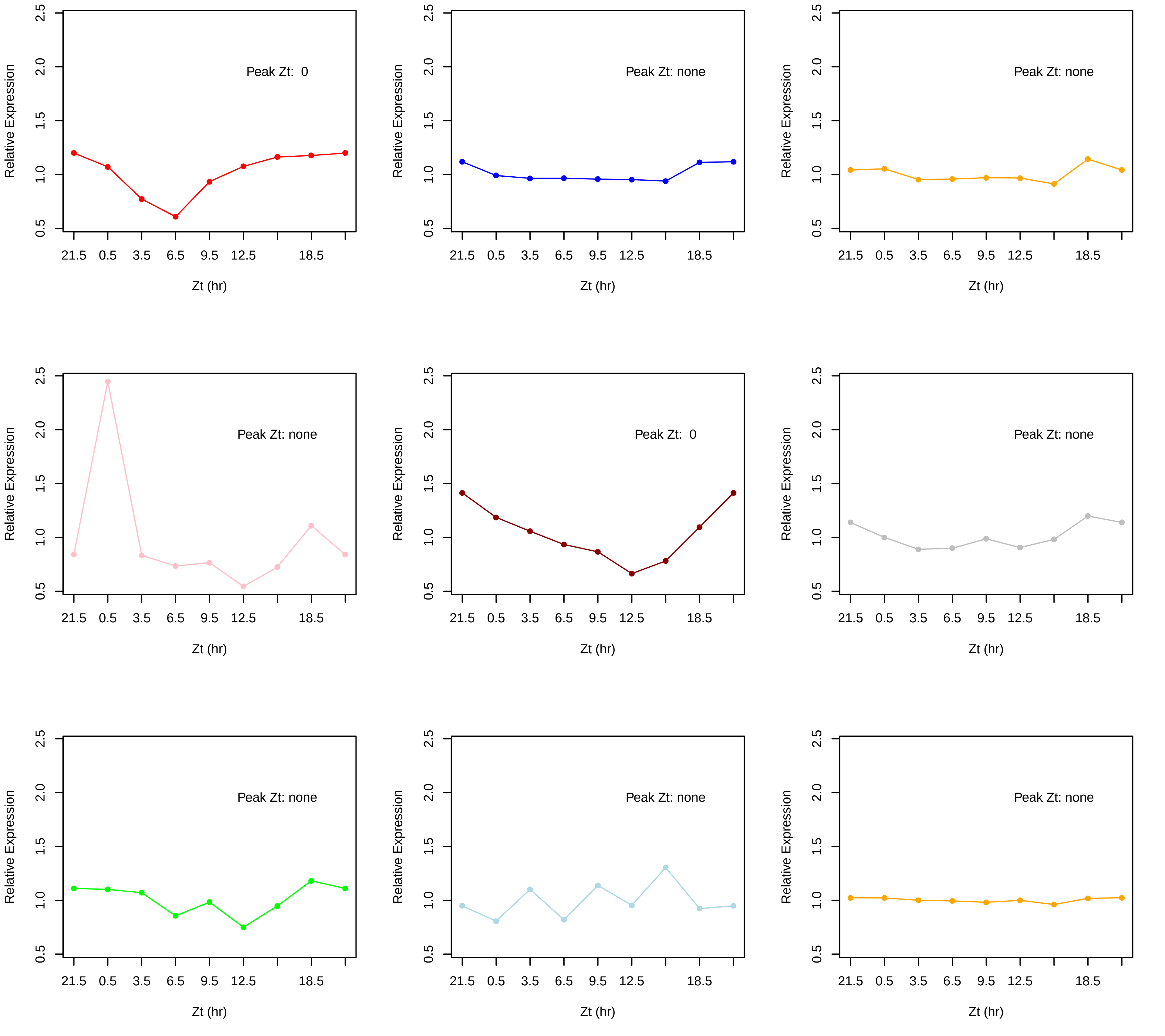

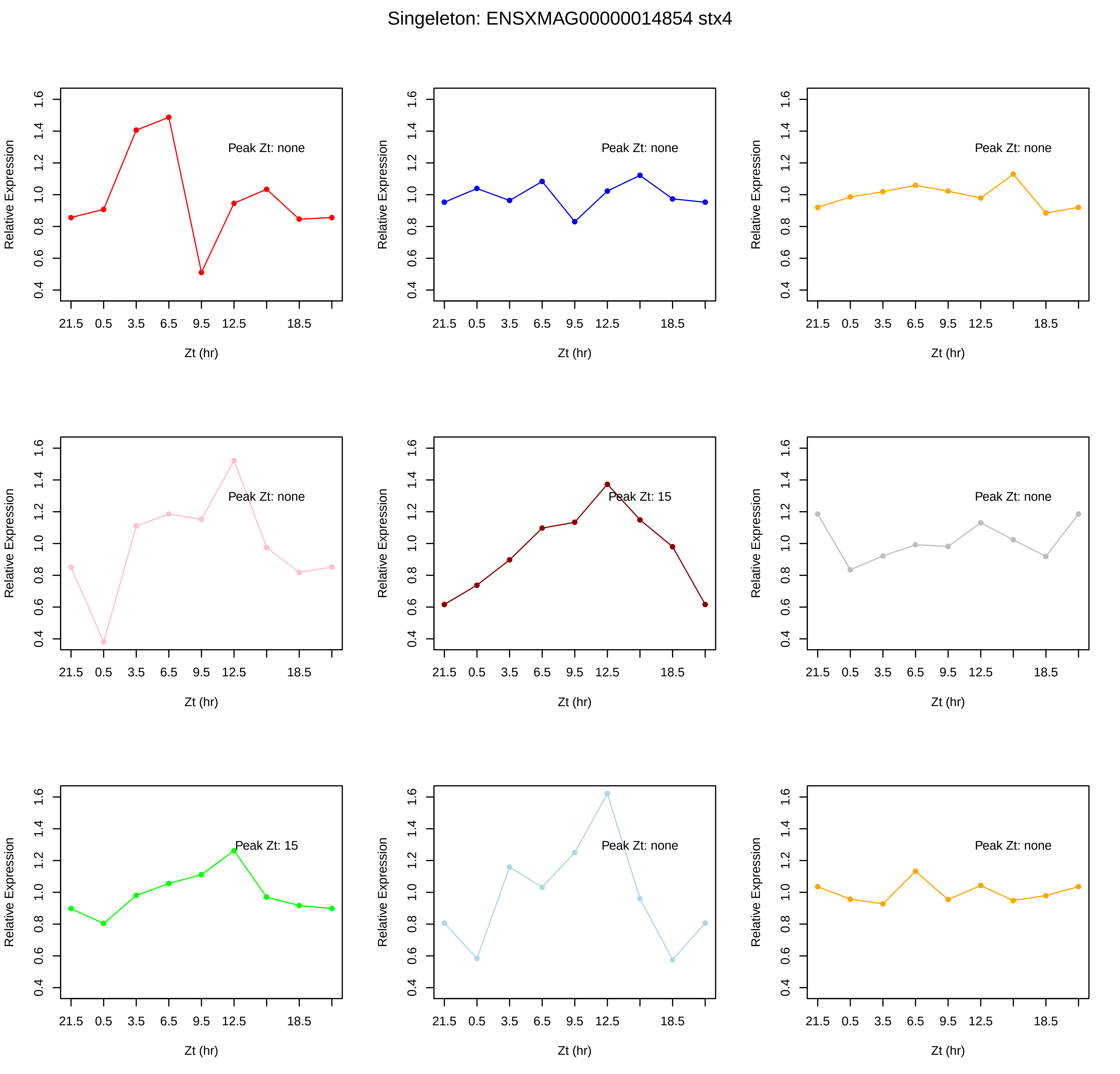
Singeleton: ENSXMAG00000014854 stx4
Peak Zt: none
Peak Zt: none
Peak Zt: none
Peak Zt: none
Peak Zt: 15
Peak Zt: none
Peak Zt: 15
Peak Zt: none
Peak Zt: none
Relative Expression
Zt (hr)

Singeleton: ENSXMAG00000003607 znf704

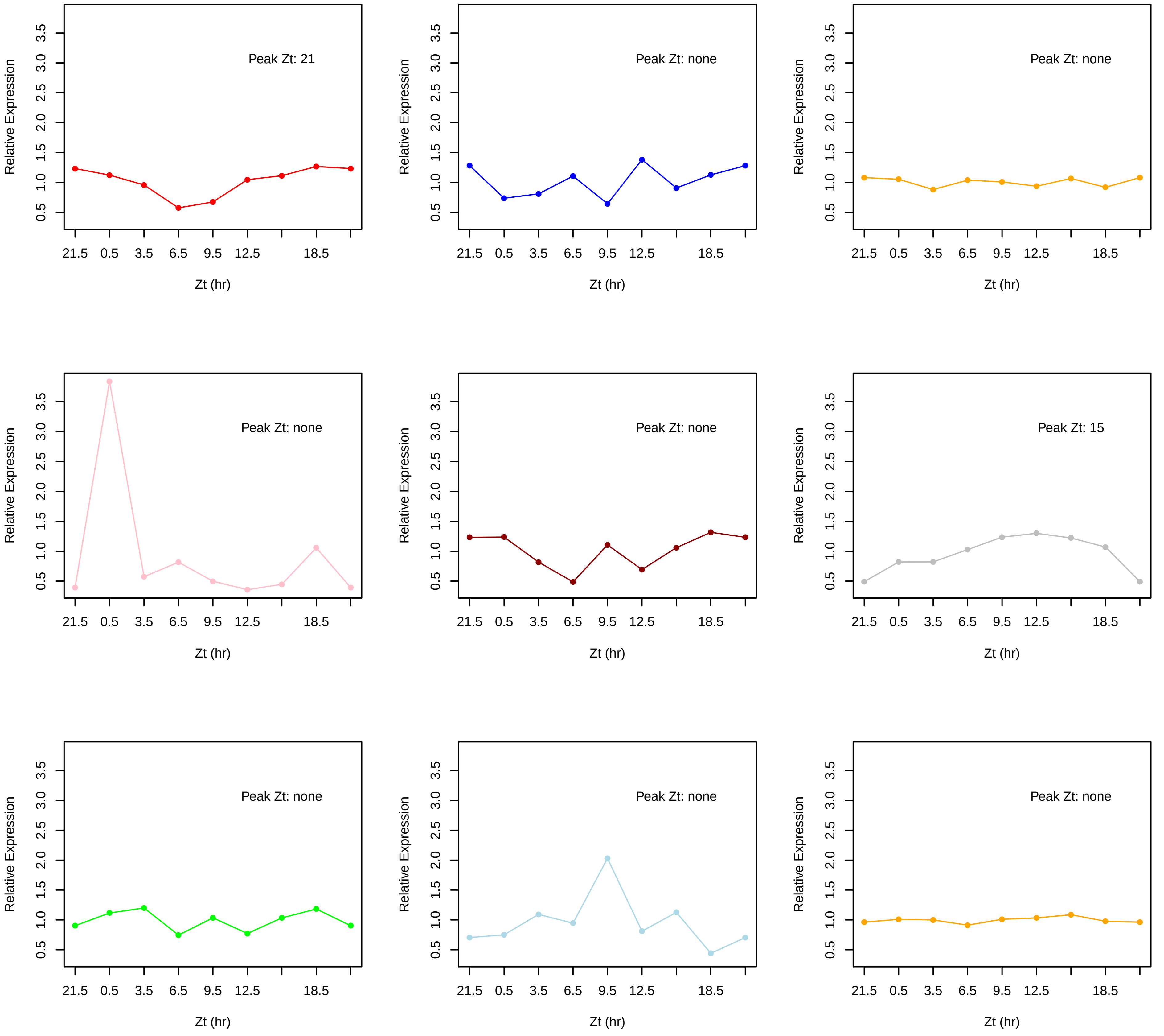

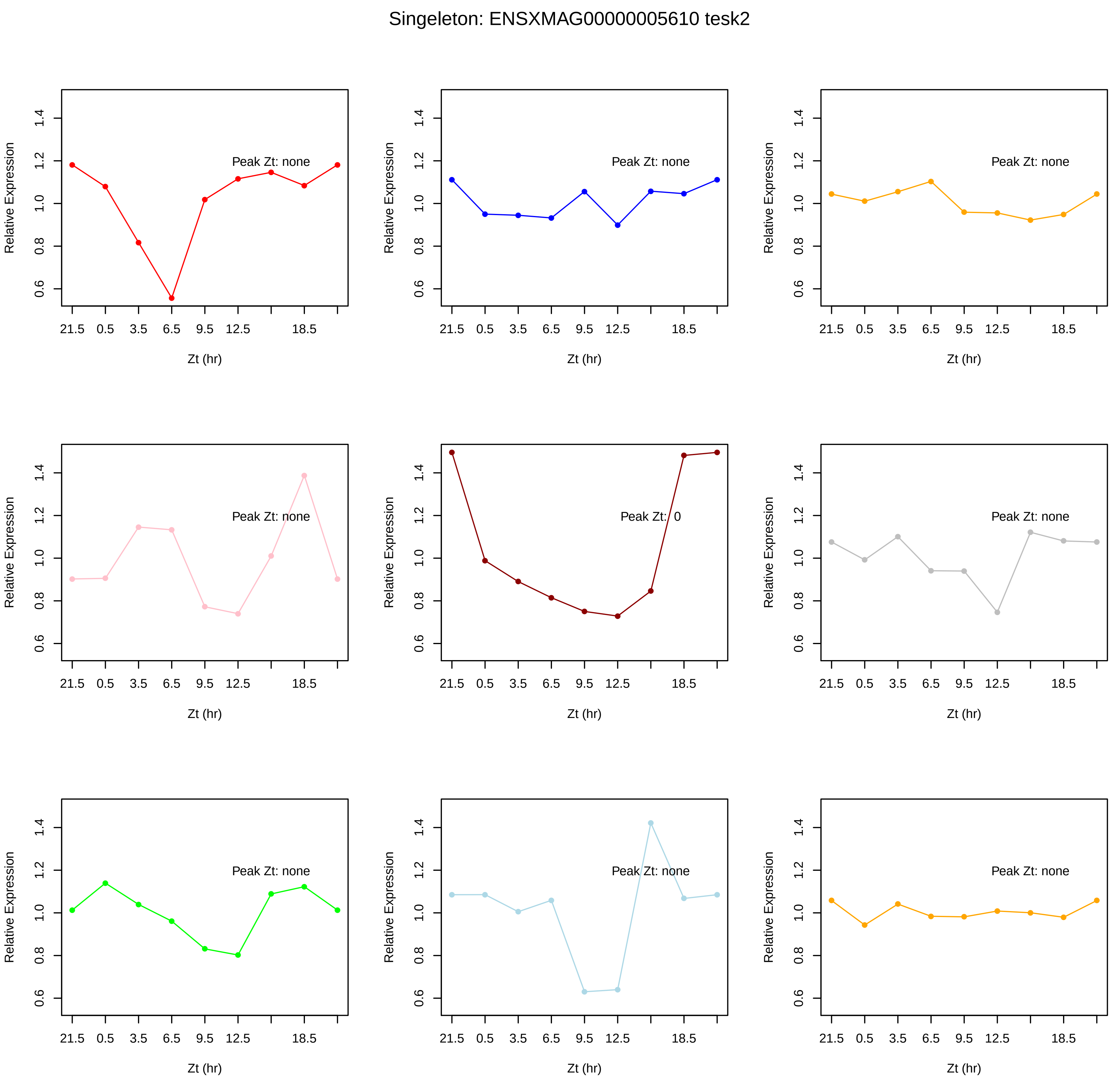
Singeleton: ENSXMAG00000005610 tesk2
Peak Zt: none
Peak Zt: none
Peak Zt: none
Peak Zt: none
Peak Zt: 0
Peak Zt: none
Peak Zt: none
Peak Zt: none
Peak Zt: none
Relative Expression
Zt (hr)
21.5
0.5
3.5
6.5
9.5
12.5
18.5
0.6
0.8
1.0
1.2
1.4

Singeleton: ENSXMAG00000009612 vps35l

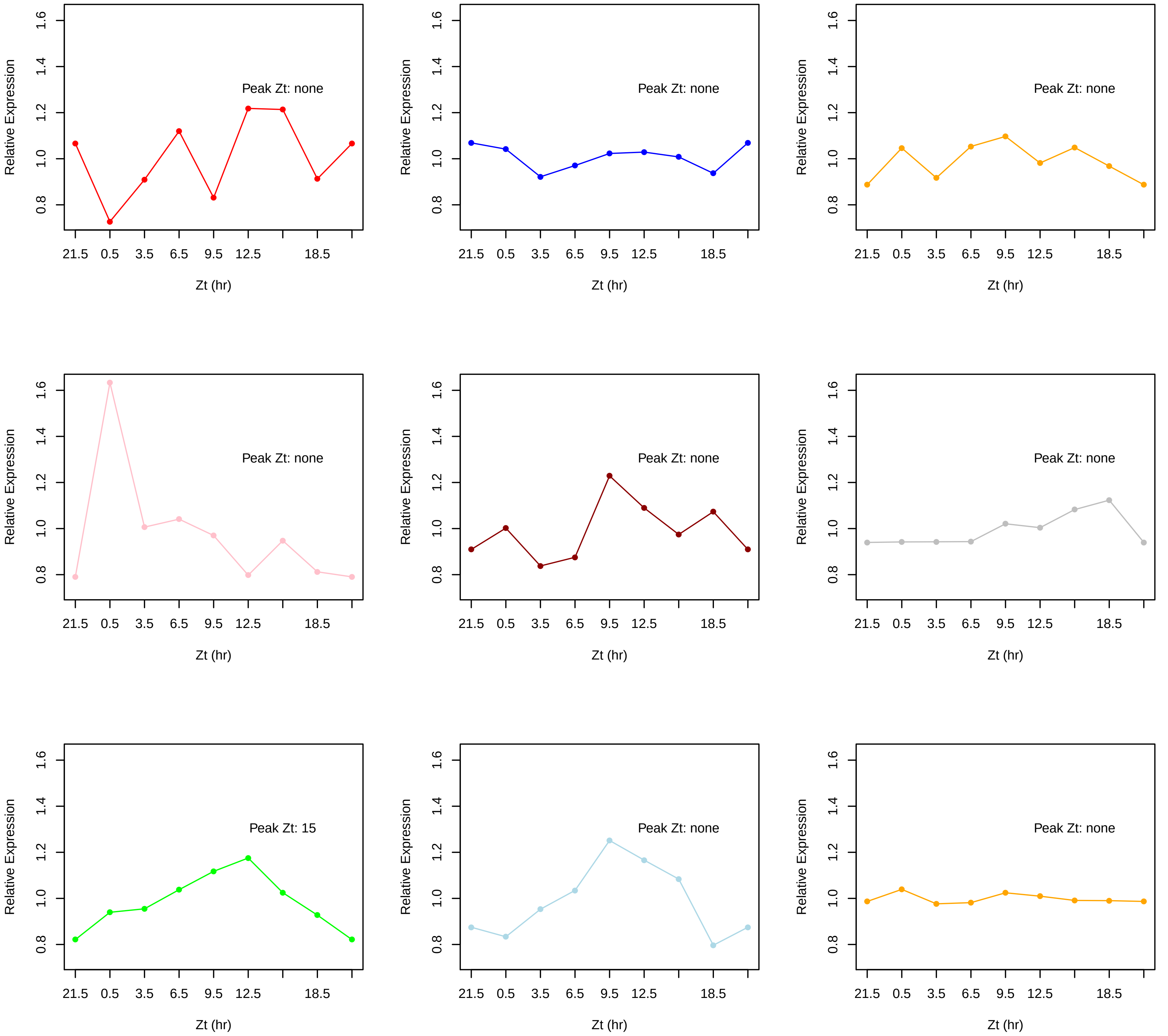

Singleton: ENSXMAG00000027516 acads

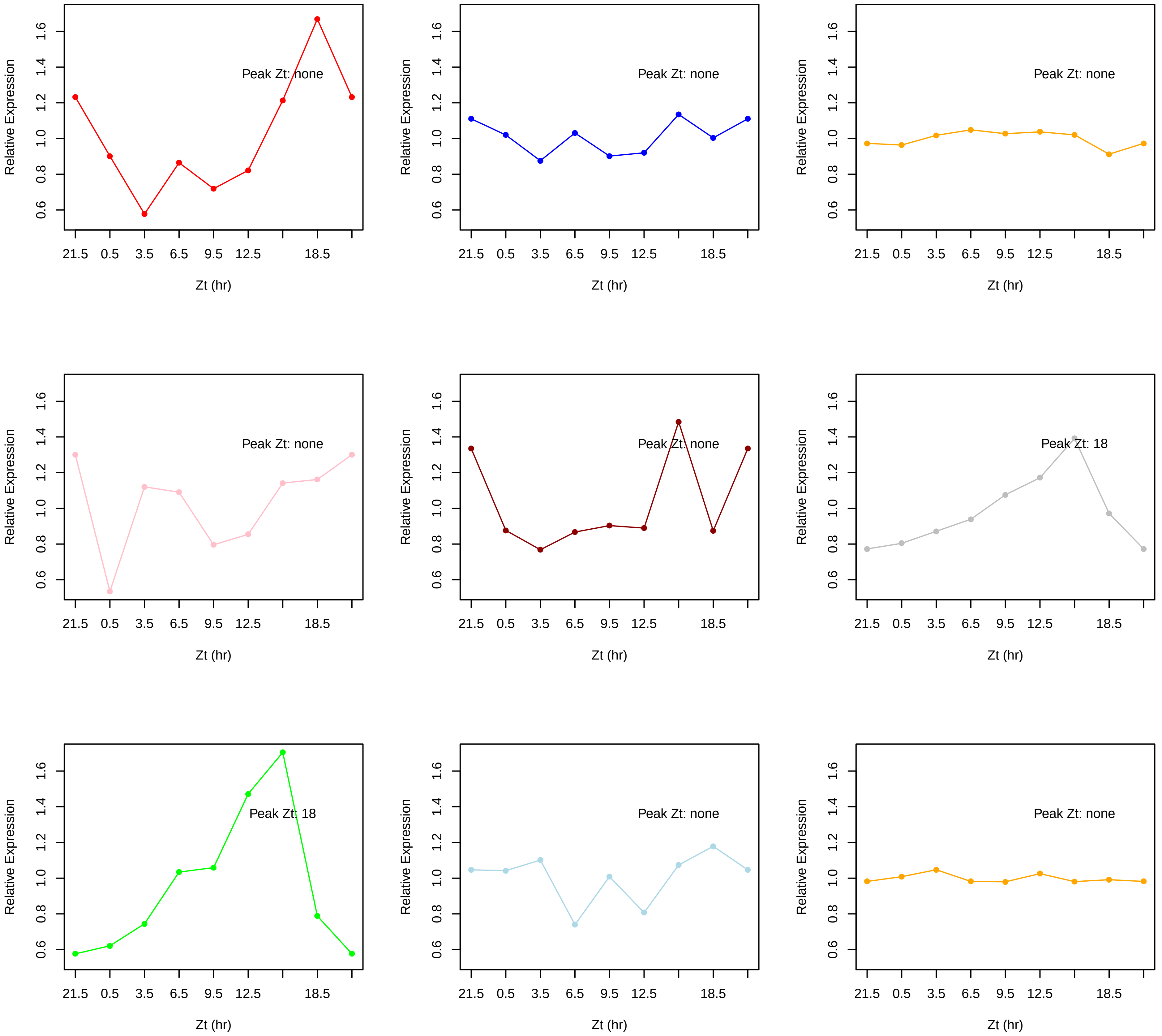

Singeleton: ENSXMAG00000025090 lmo2

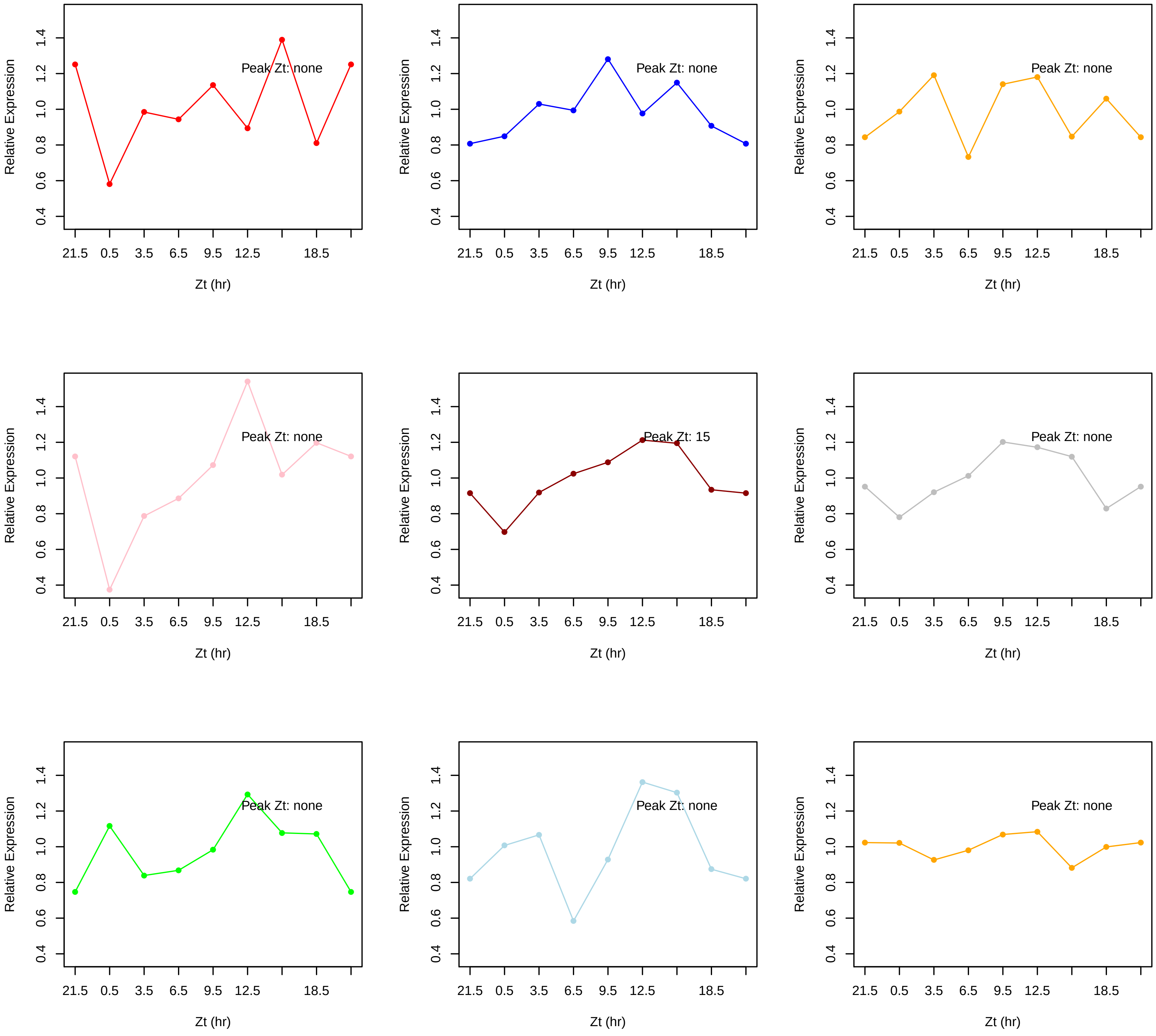

Singleton: ENSXMAG00000007949 gpd1c

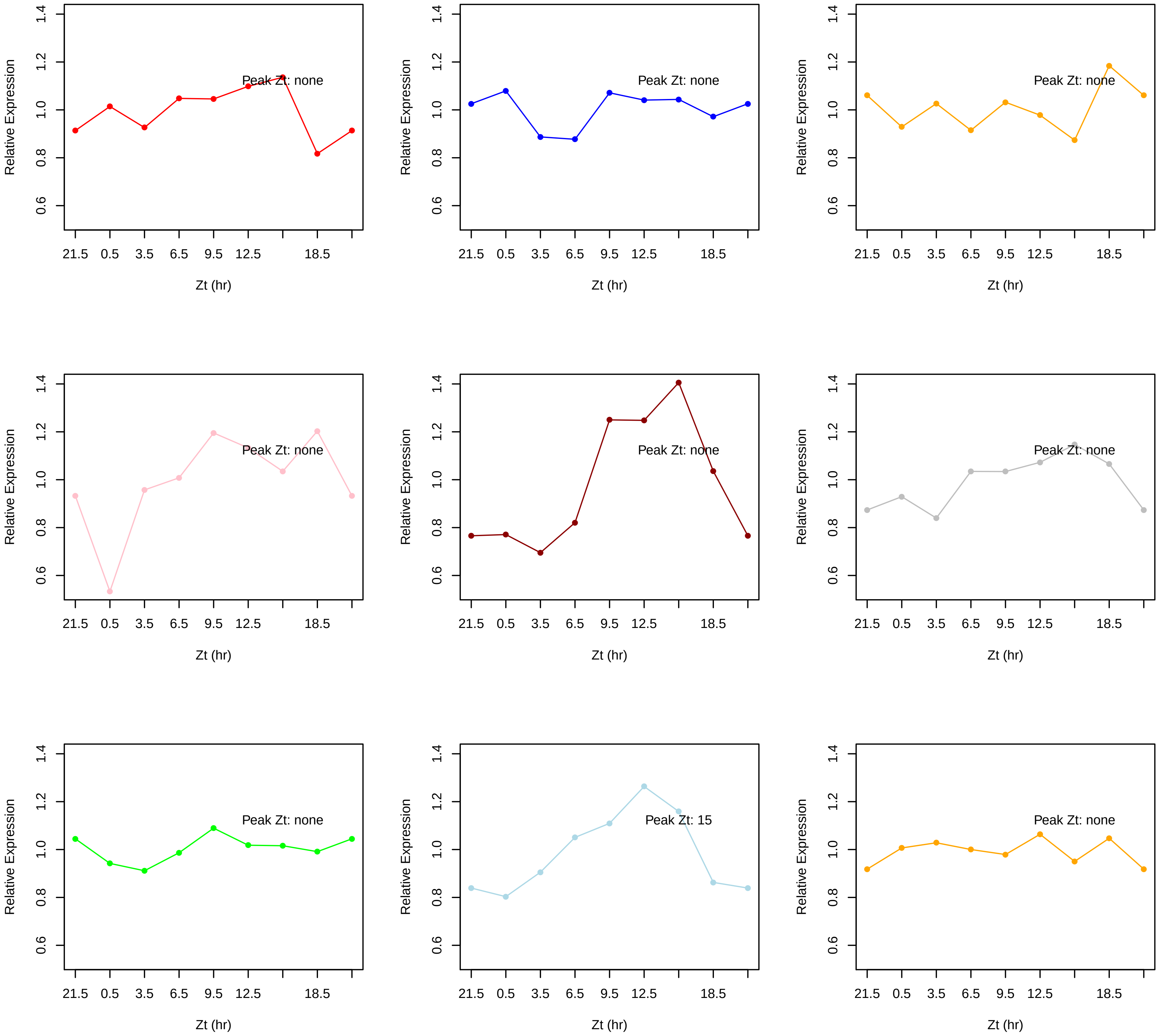

Singeleton: ENSXMAG00000011102 toe1

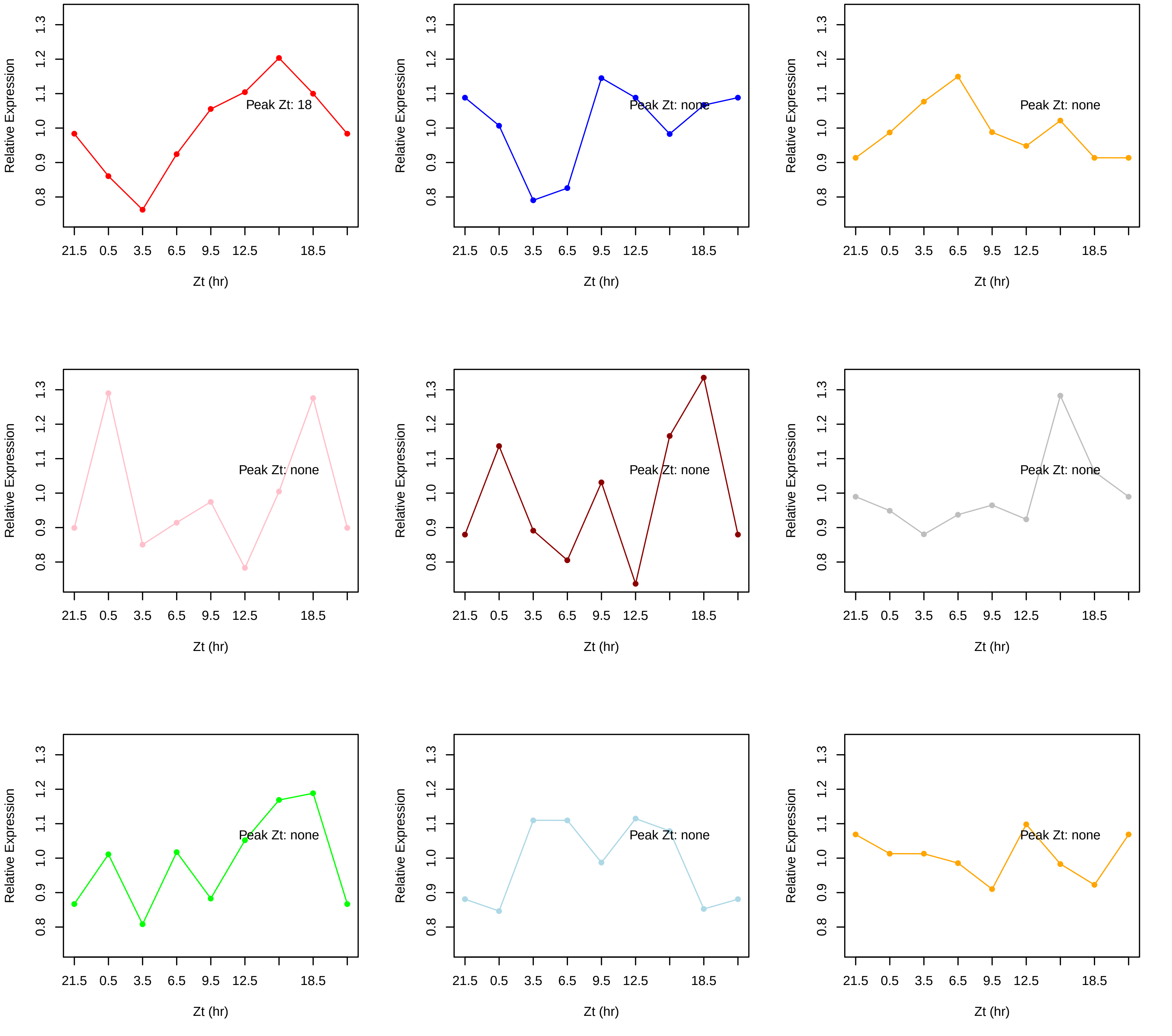

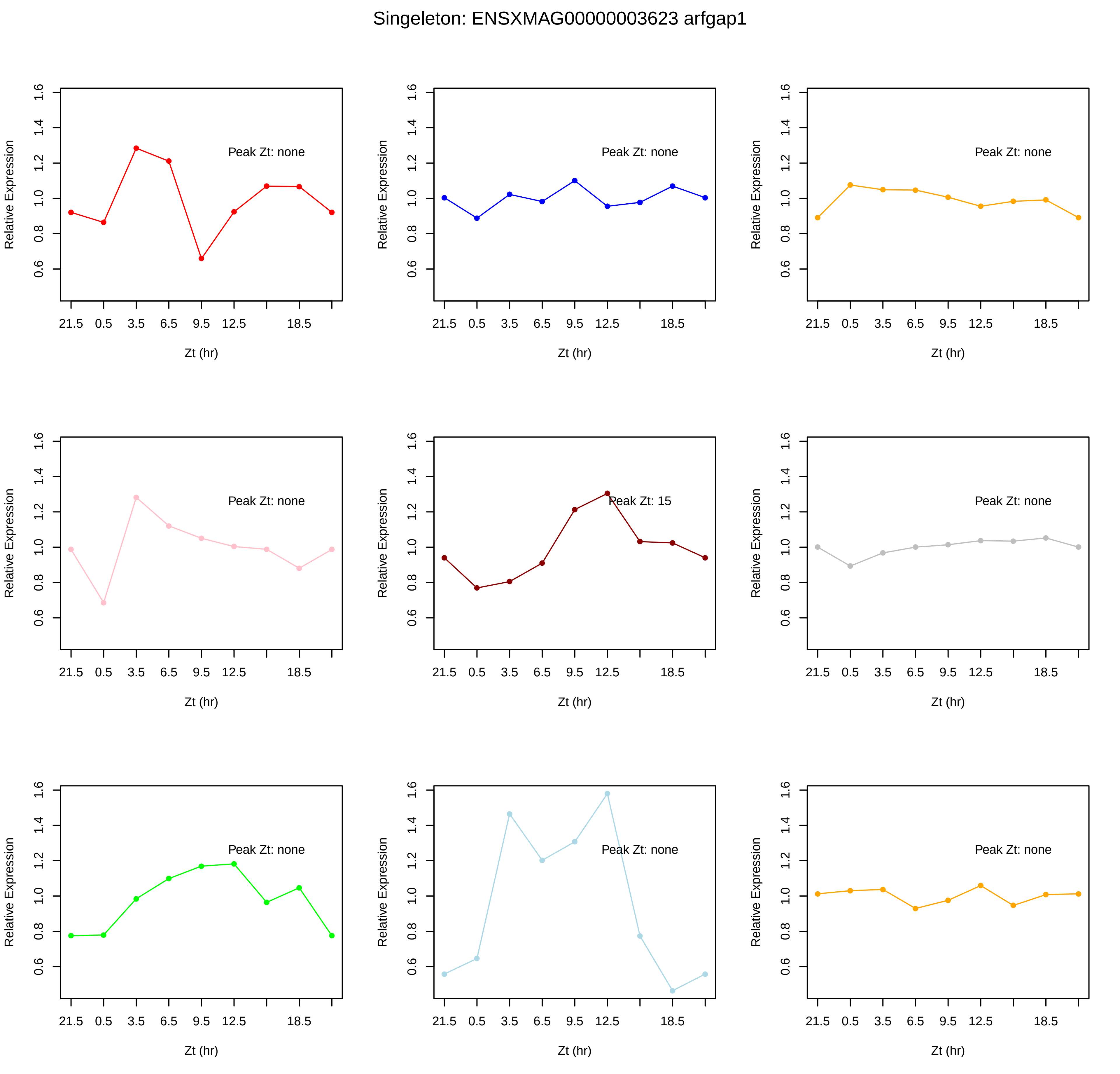
Singeleton: ENSXMAG00000003623 arfgap1
Relative Expression
Zt (hr)
Peak Zt: none
Peak Zt: none
Peak Zt: none
Peak Zt: none
Peak Zt: 15
Peak Zt: none
Peak Zt: none
Peak Zt: none
Peak Zt: none
21.5 0.5 3.5 6.5 9.5 12.5 18.5
0.6 0.8 1.0 1.2 1.4 1.6

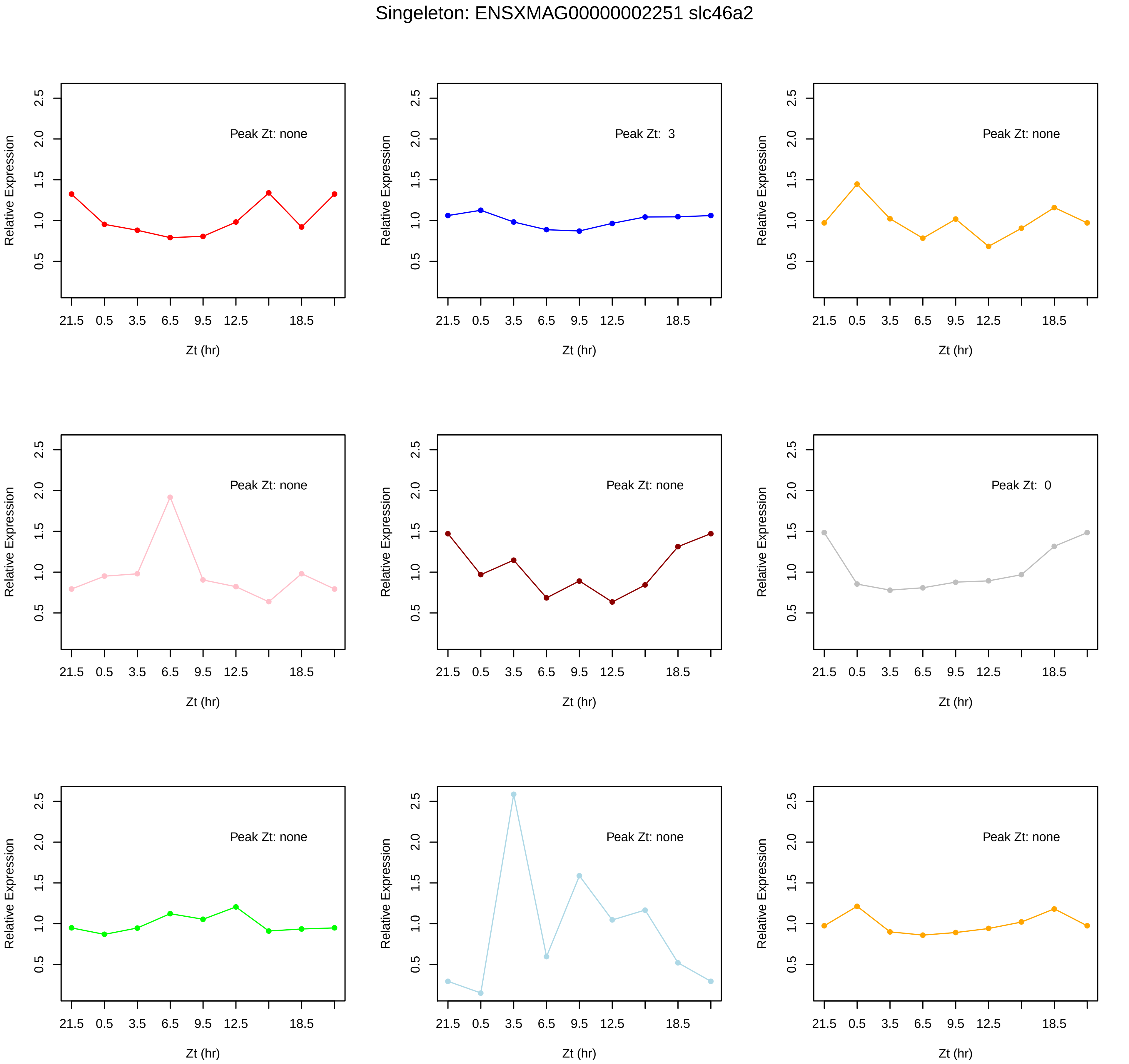
Singeleton: ENSXMAG00000002251 slc46a2
Peak Zt: none
Peak Zt: 3
Peak Zt: none
Peak Zt: none
Peak Zt: none
Peak Zt: 0
Peak Zt: none
Peak Zt: none
Peak Zt: none
Relative Expression
Zt (hr)
21.5
0.5
3.5
6.5
9.5
12.5
18.5
0.5
1.0
1.5
2.0
2.5

Singeleton: ENSXMAG00000013904 ei24

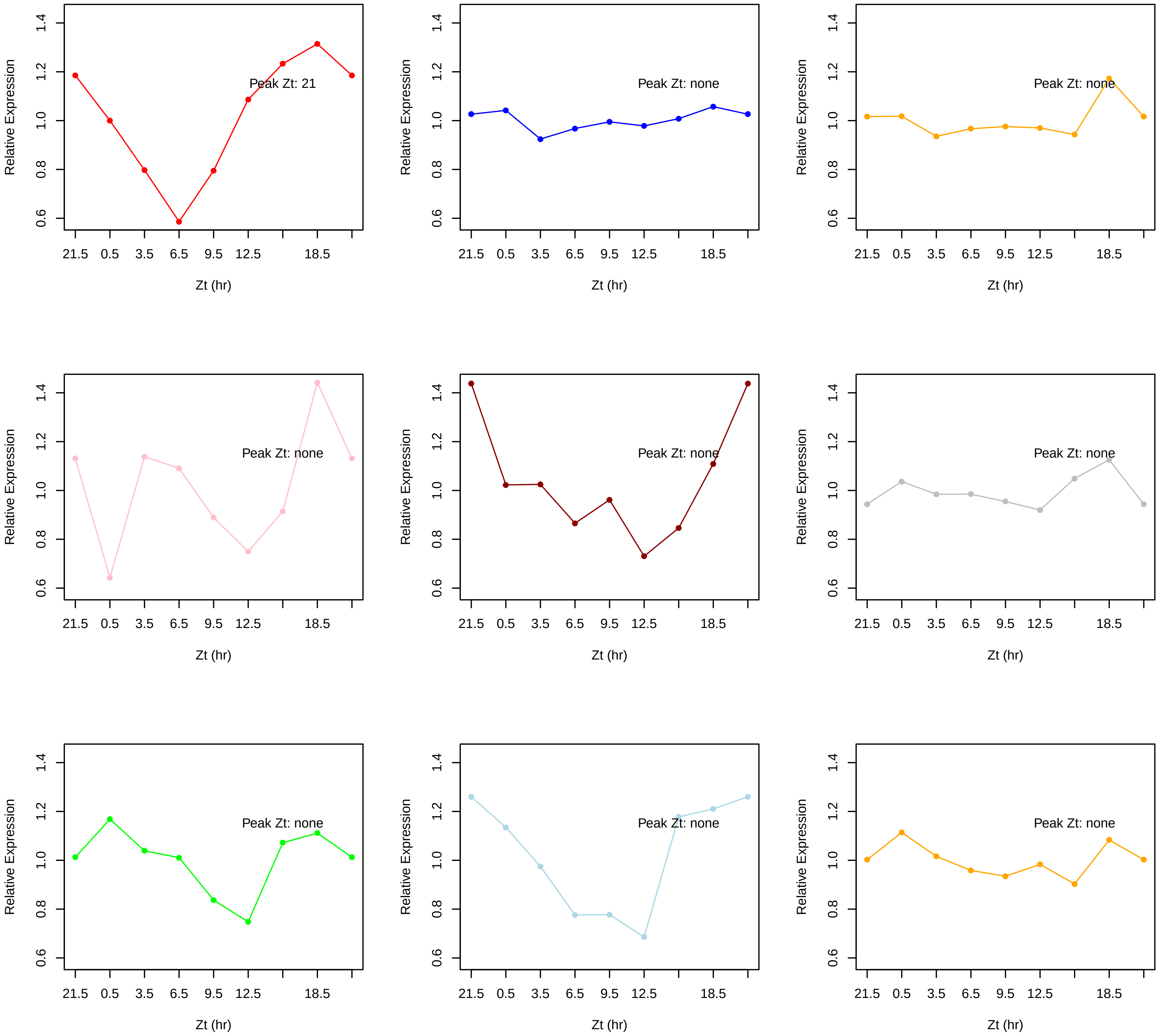

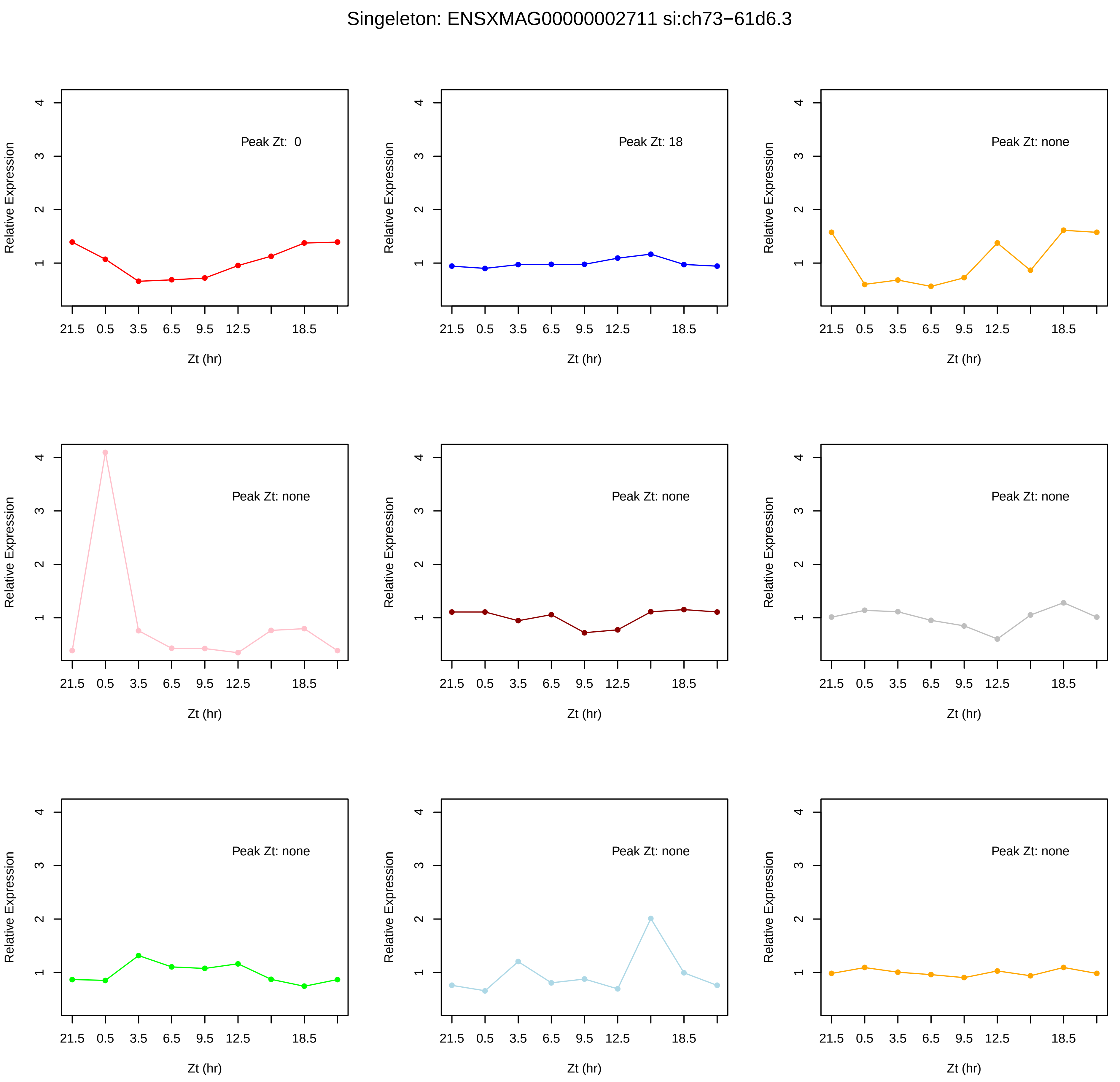
Singeleton: ENSXMAG00000002711 si:ch73−61d6.3
Peak Zt: 0
Peak Zt: 18
Peak Zt: none
Peak Zt: none
Peak Zt: none
Peak Zt: none
Peak Zt: none
Peak Zt: none
Peak Zt: none
Relative Expression
Zt (hr)
21.5 0.5 3.5 6.5 9.5 12.5 18.5

Singeleton: ENSXMAG00000003712 sppl3

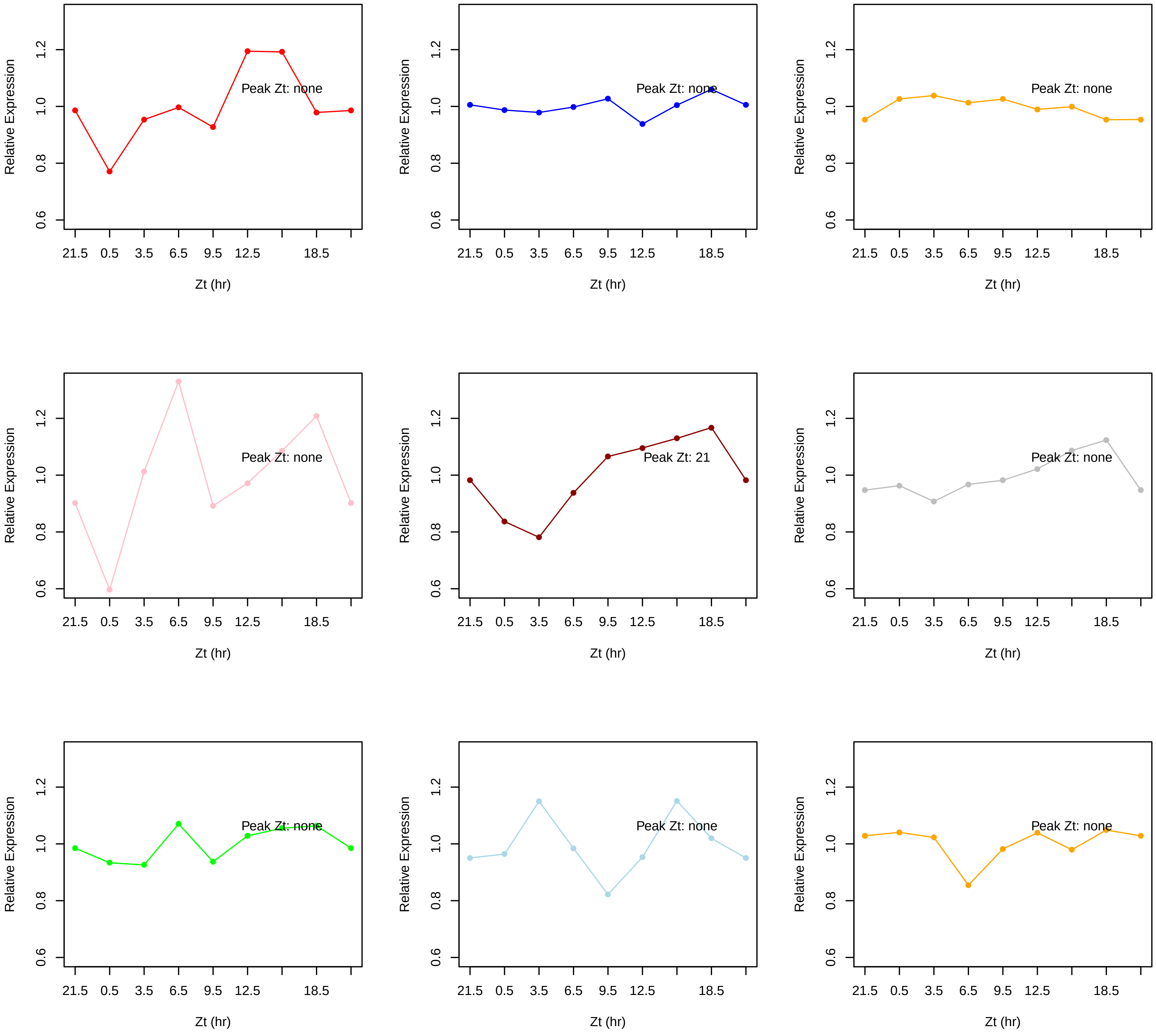

Singeleton: ENSXMAG00000025730 sntb2

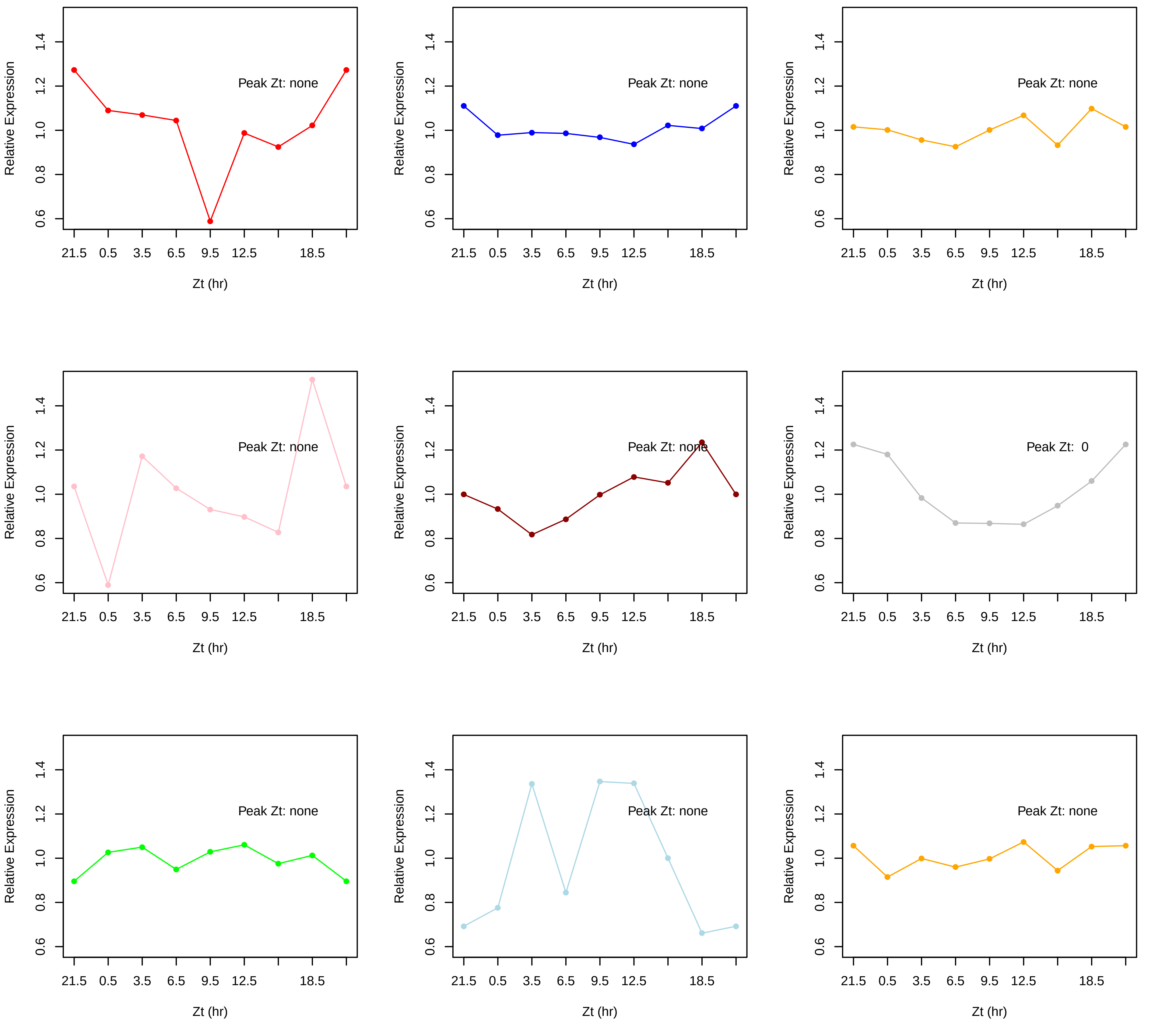

Singleton: ENSXMAG00000012720 knop1

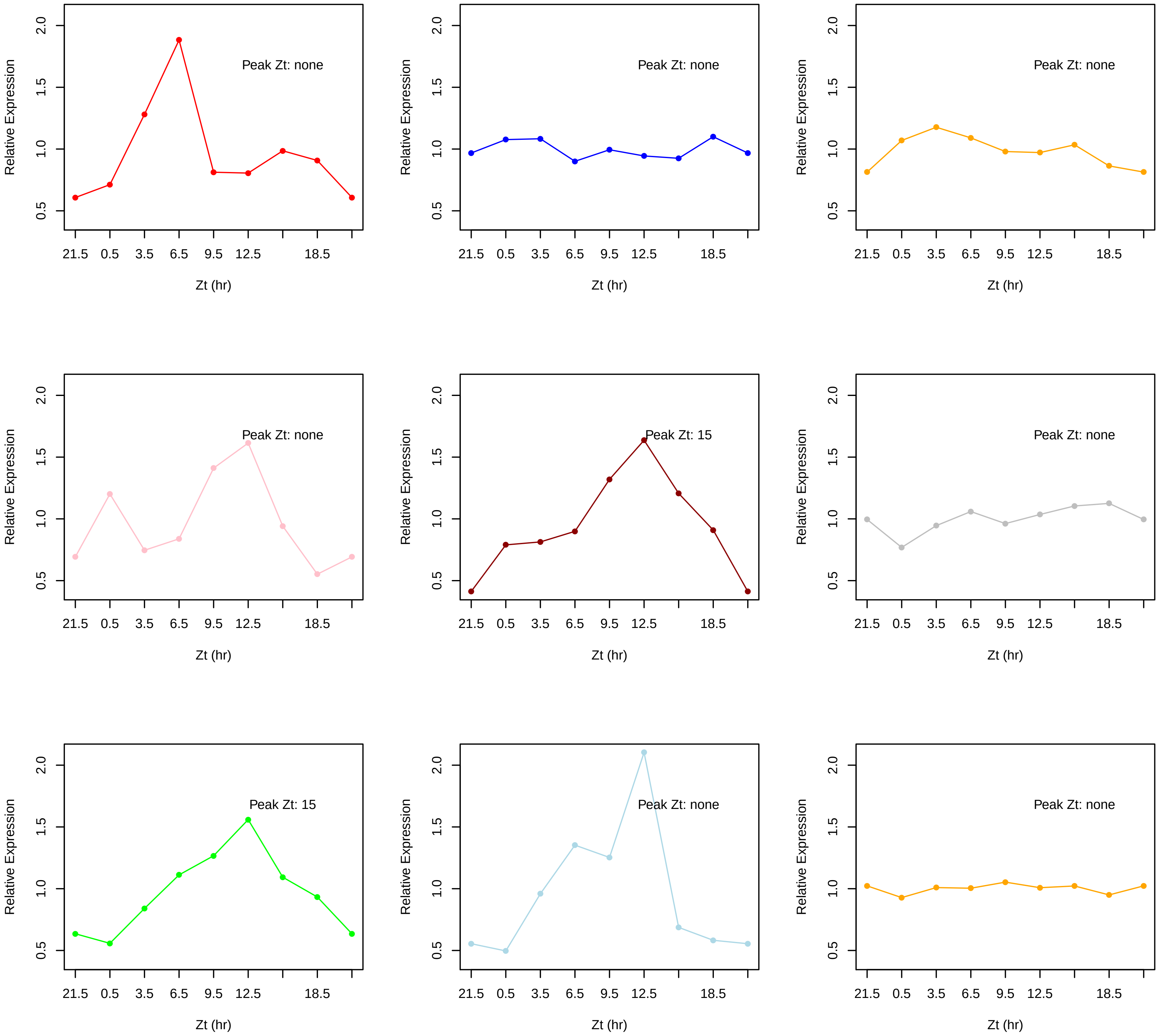

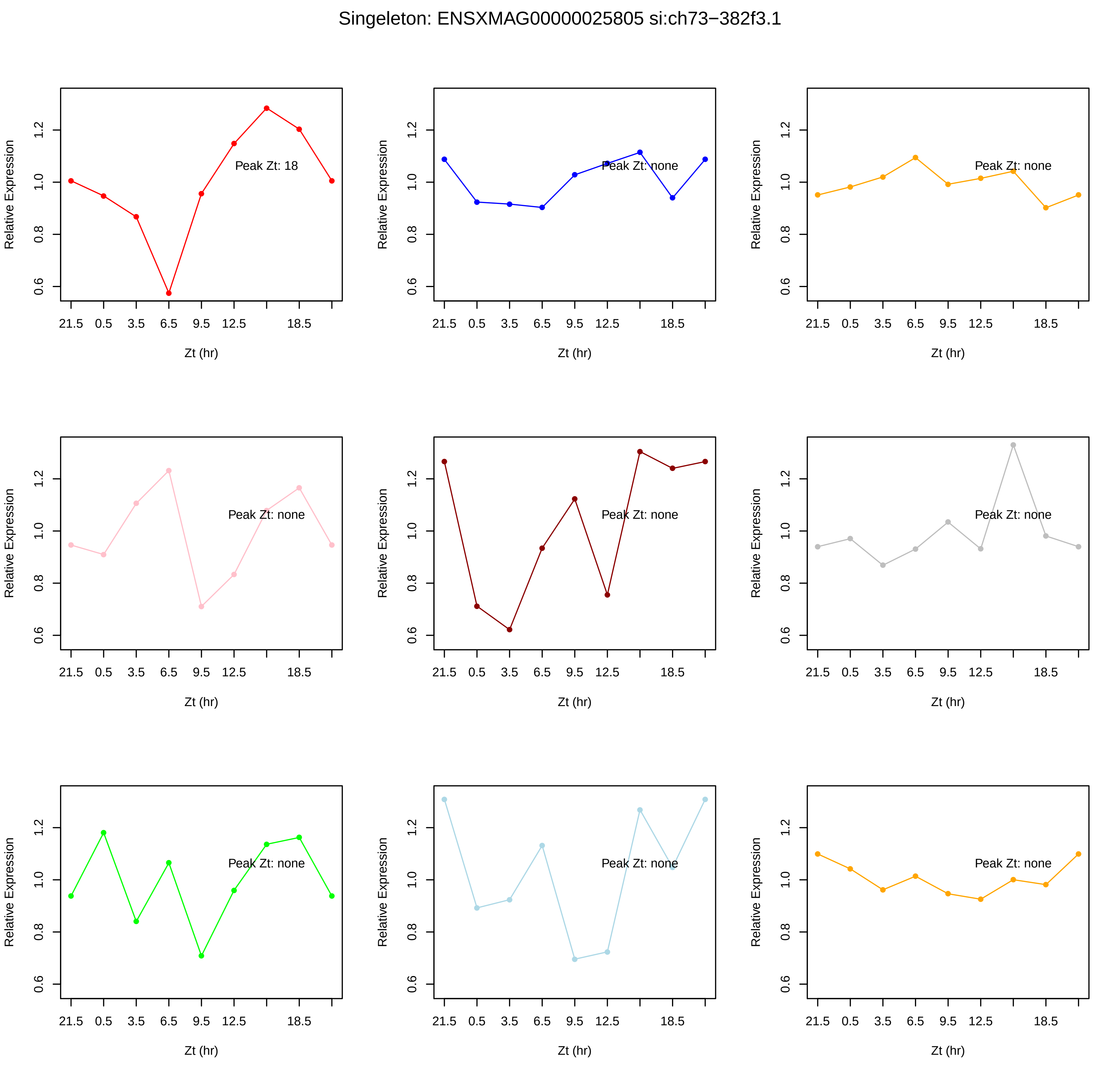
Singeleton: ENSXMAG00000025805 si:ch73−382f3.1
Peak Zt: 18
Peak Zt: none
Relative Expression
Zt (hr)
21.5 0.5 3.5 6.5 9.5 12.5 18.5
0.6 0.8 1.0 1.2

Singeleton: ENSXMAG00000019607 taf13

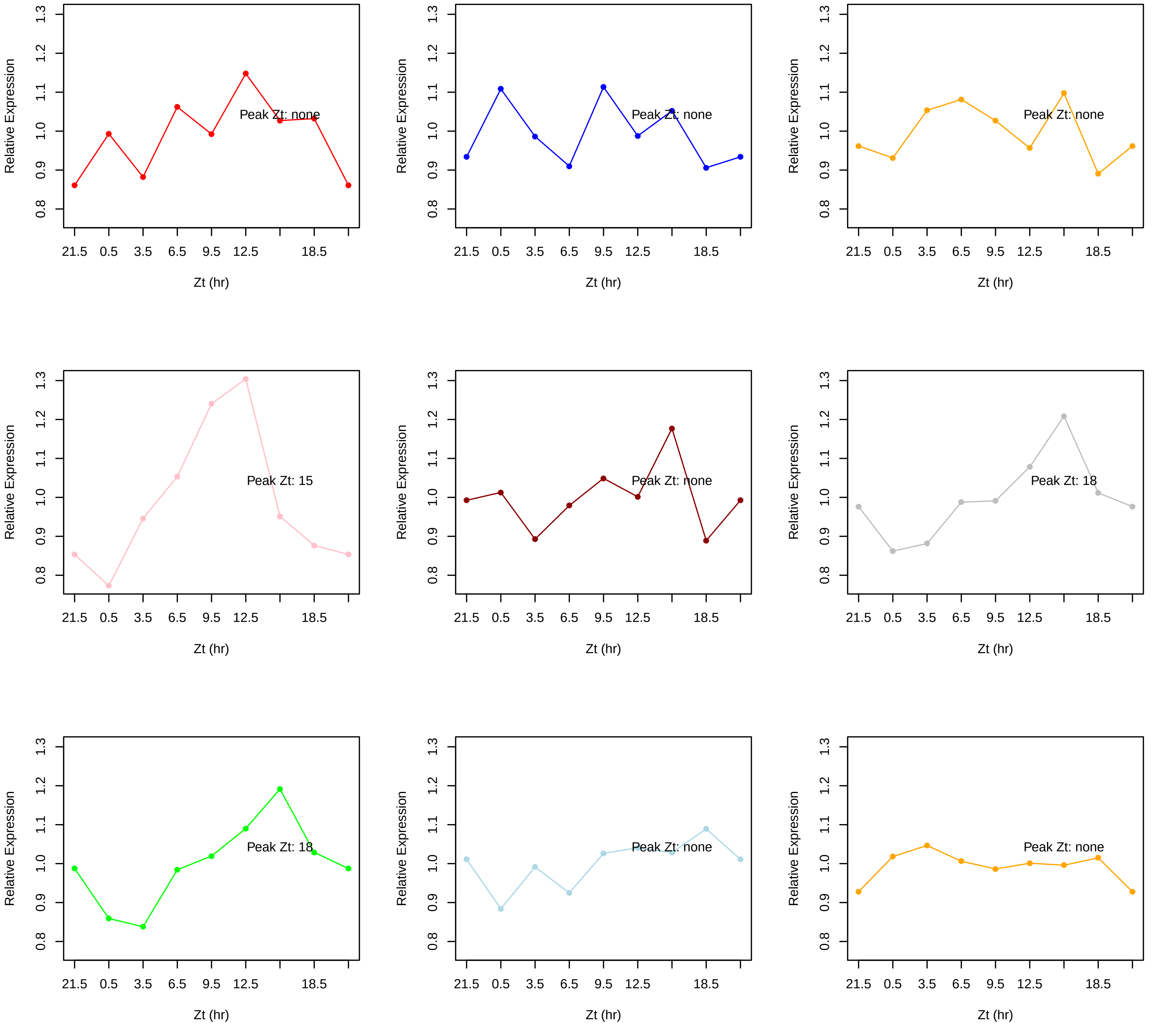

Singeleton: ENSXMAG00000025204

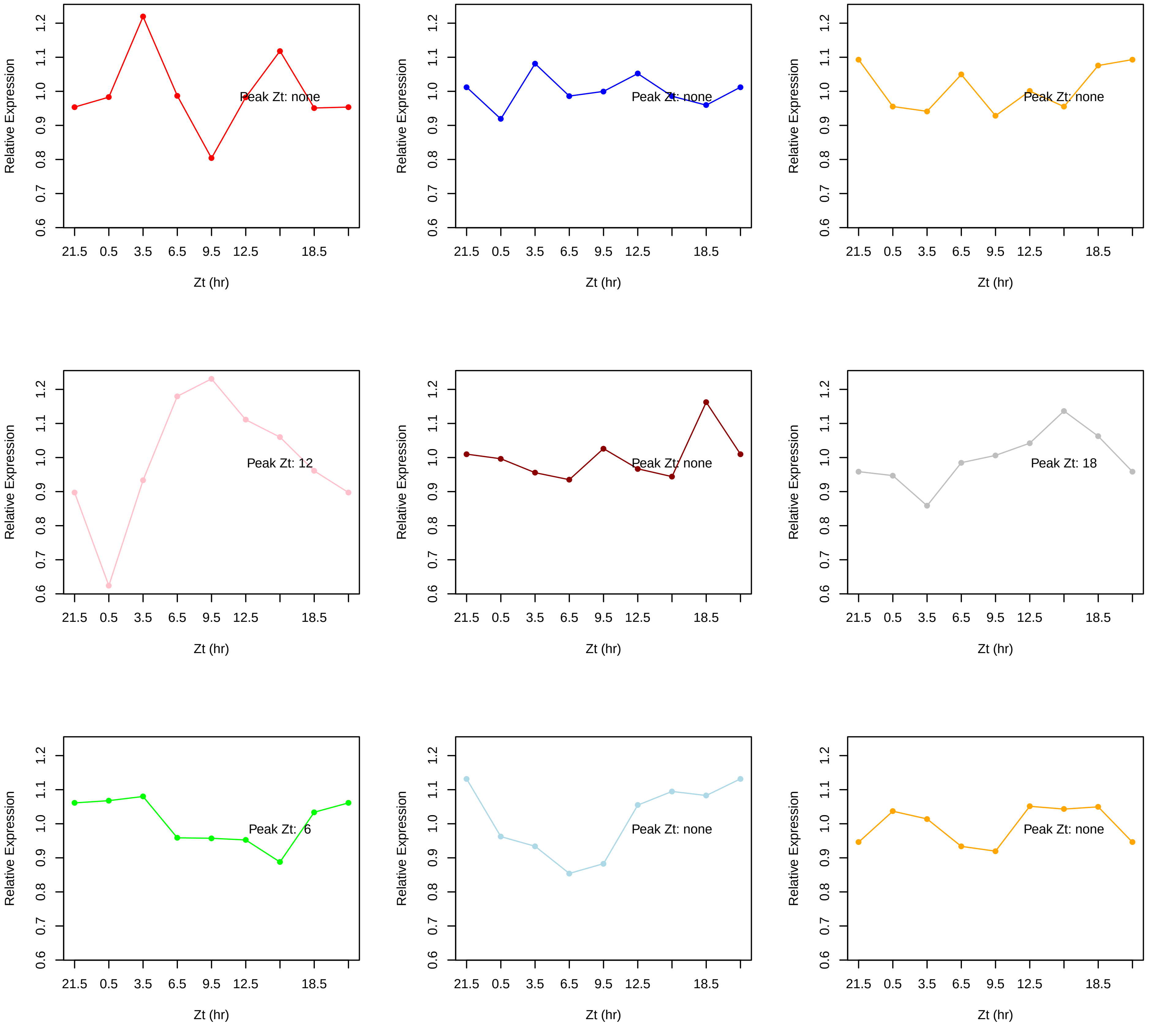

Singeleton: ENSXMAG00000001360 ilvbl

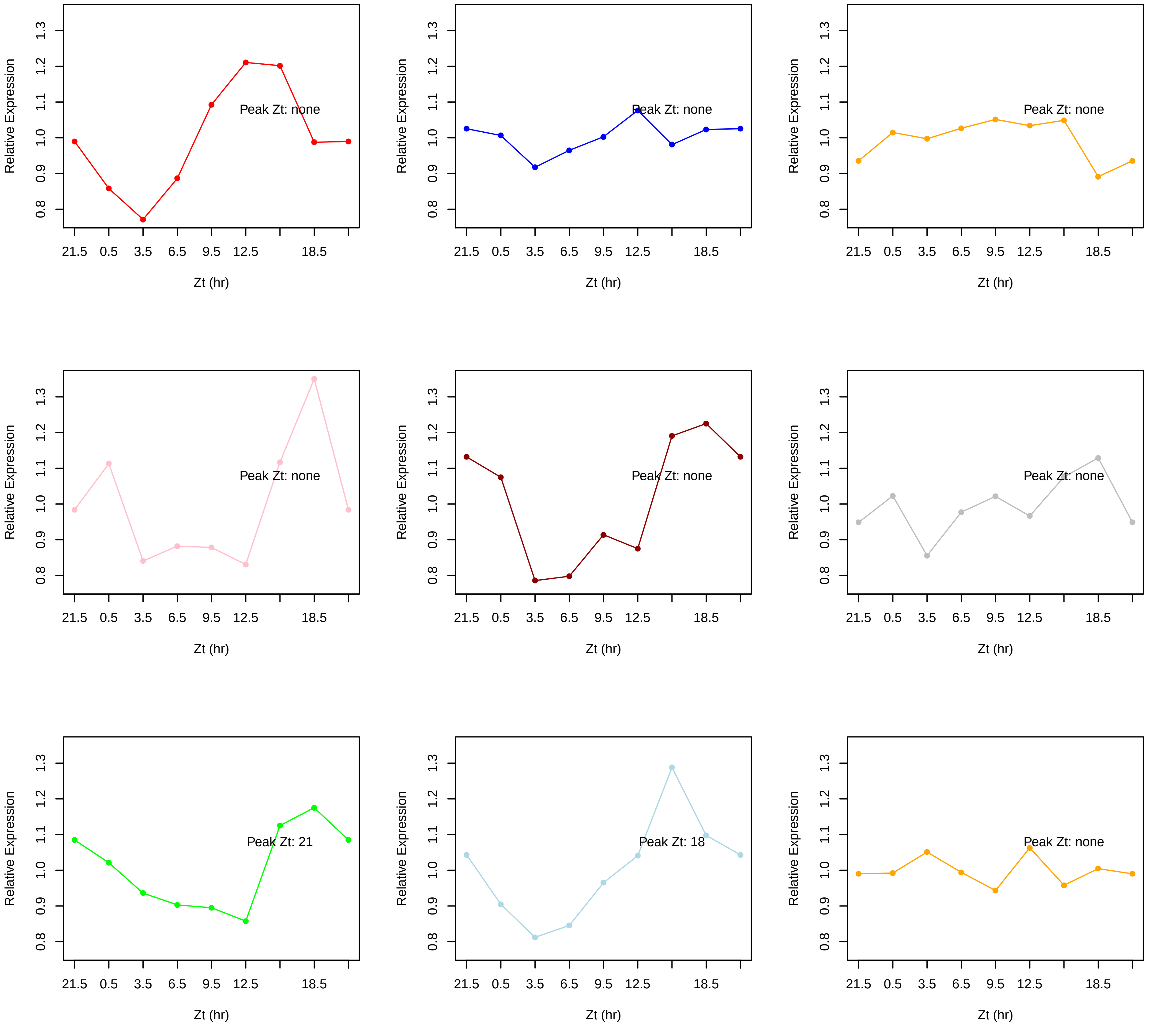

Singeleton: ENSXMAG00000019612

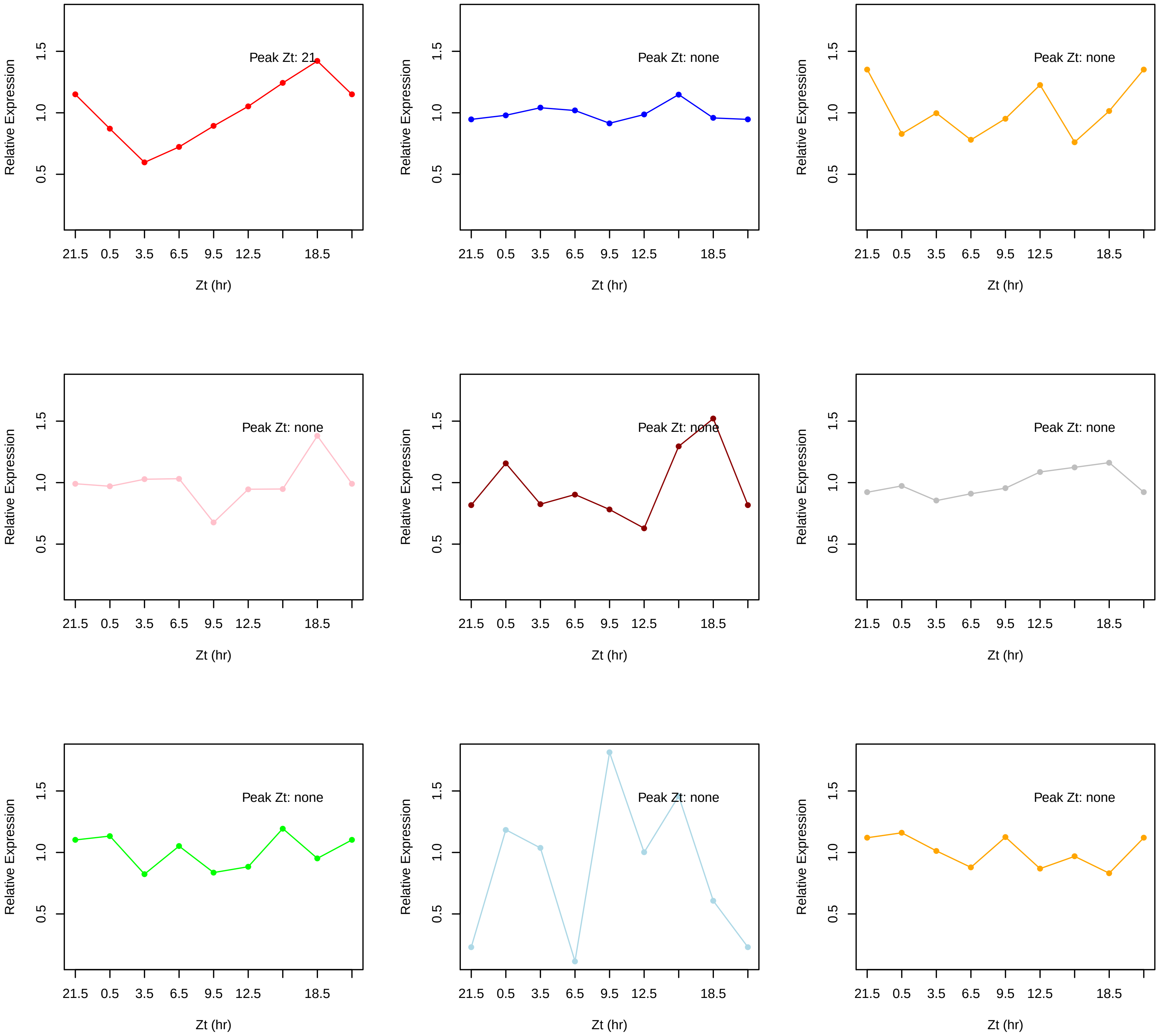

Singeleton: ENSXMAG00000001724 rc3h2

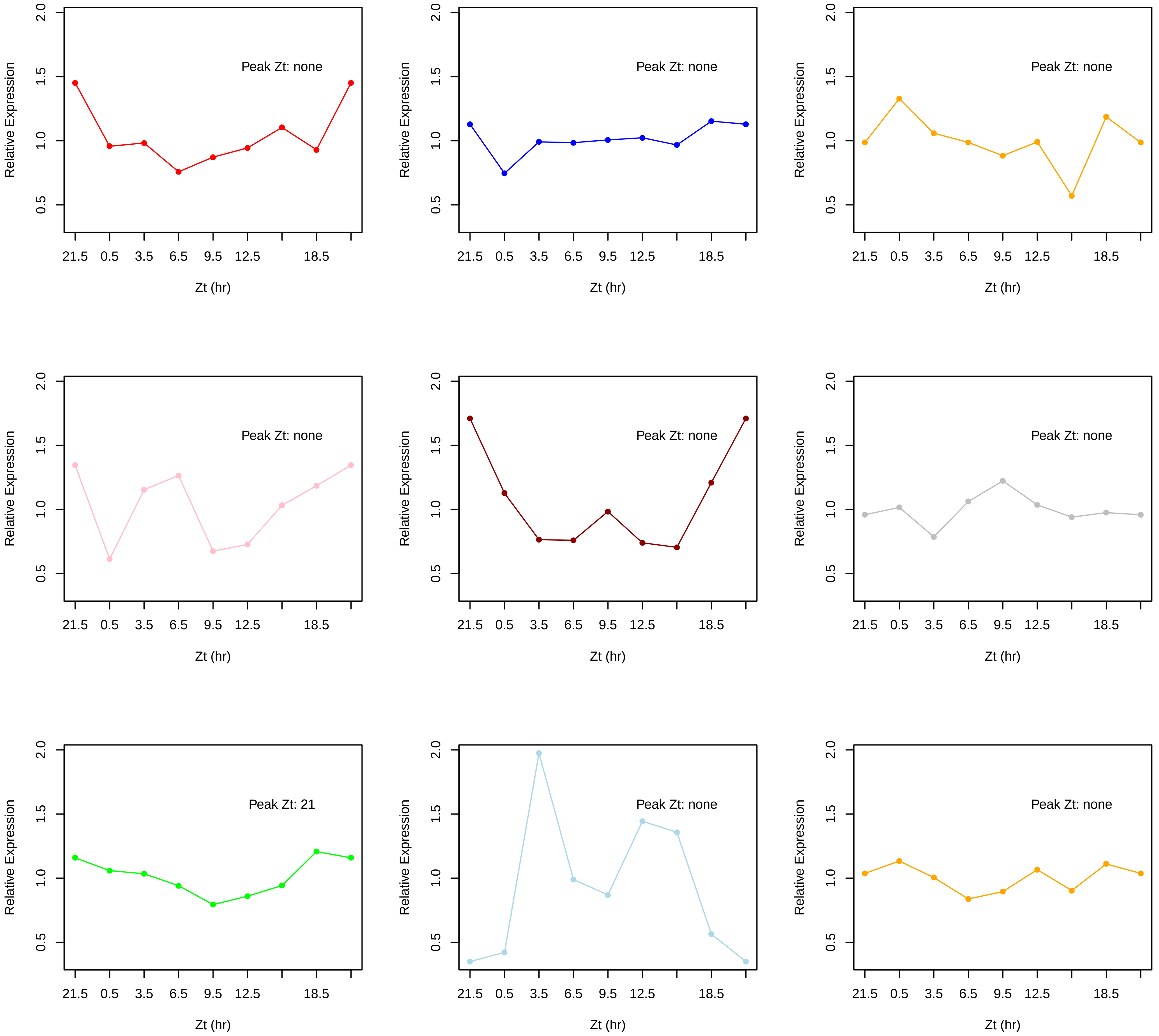

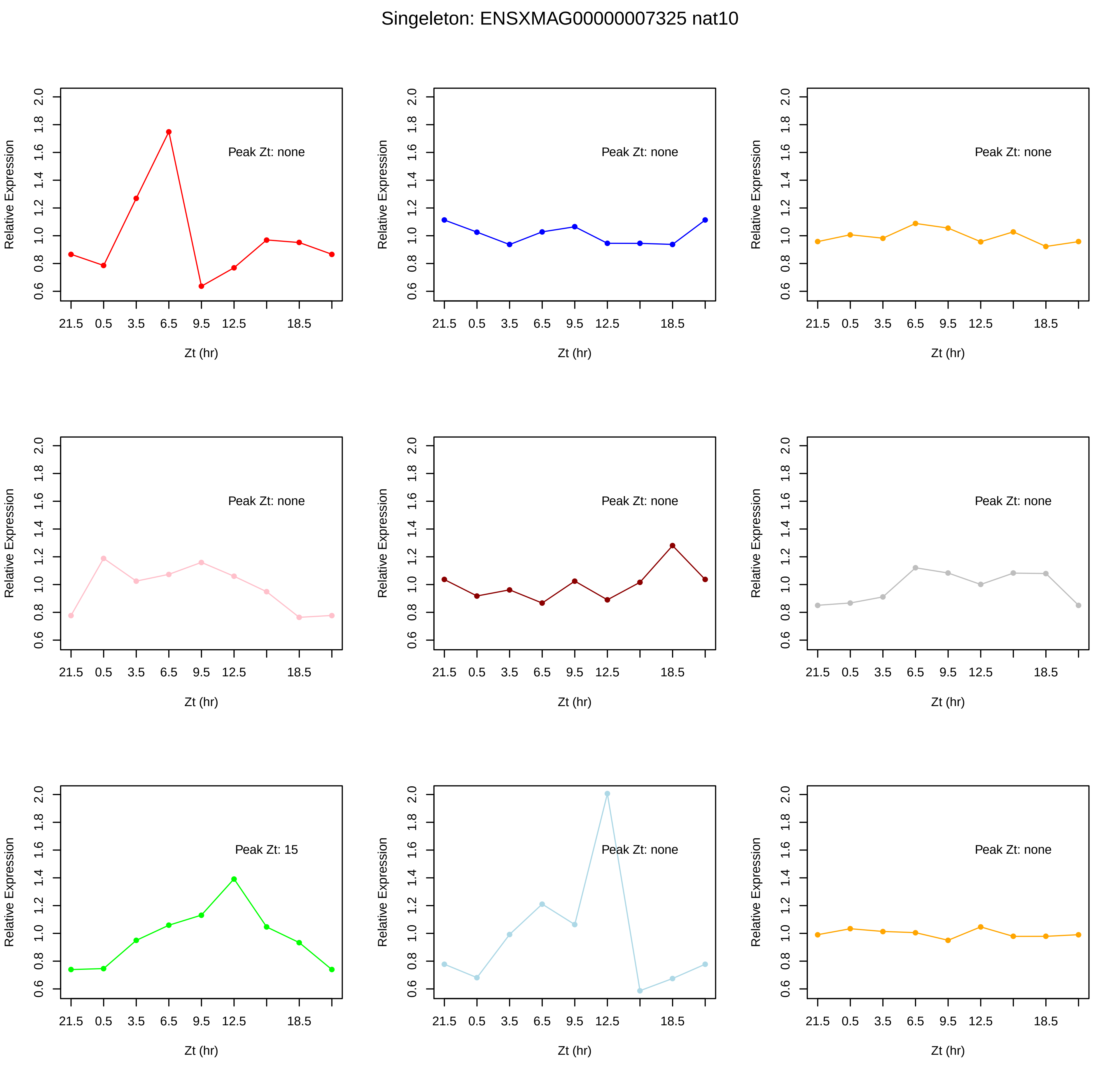
Singleton: ENSXMAG00000007325 nat10
Relative Expression
Zt (hr)
21.5
0.5
3.5
6.5
9.5
12.5
18.5
0.6
0.8
1.0
1.2
1.4
1.6
1.8
2.0
Peak Zt: none
Peak Zt: none
Peak Zt: none
Peak Zt: none
Peak Zt: none
Peak Zt: none
Peak Zt: 15
Peak Zt: none
Peak Zt: none

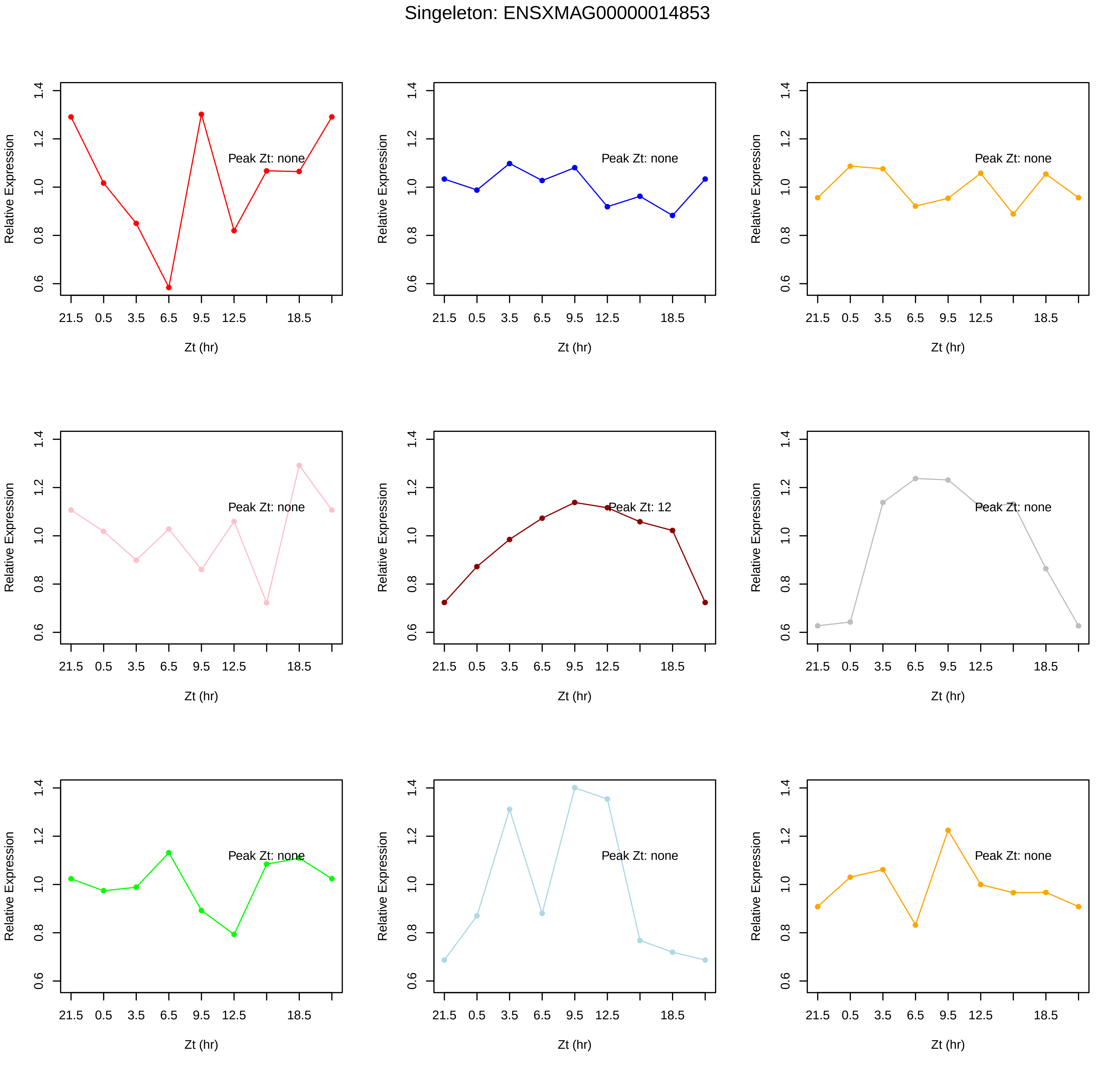

Singeleton: ENSXMAG00000014853
Peak Zt: none
Peak Zt: none
Peak Zt: none
Peak Zt: none
Peak Zt: 12
Peak Zt: none
Peak Zt: none
Peak Zt: none
Peak Zt: none
Relative Expression
Zt (hr)

Singeleton: ENSXMAG00000002971 mlec

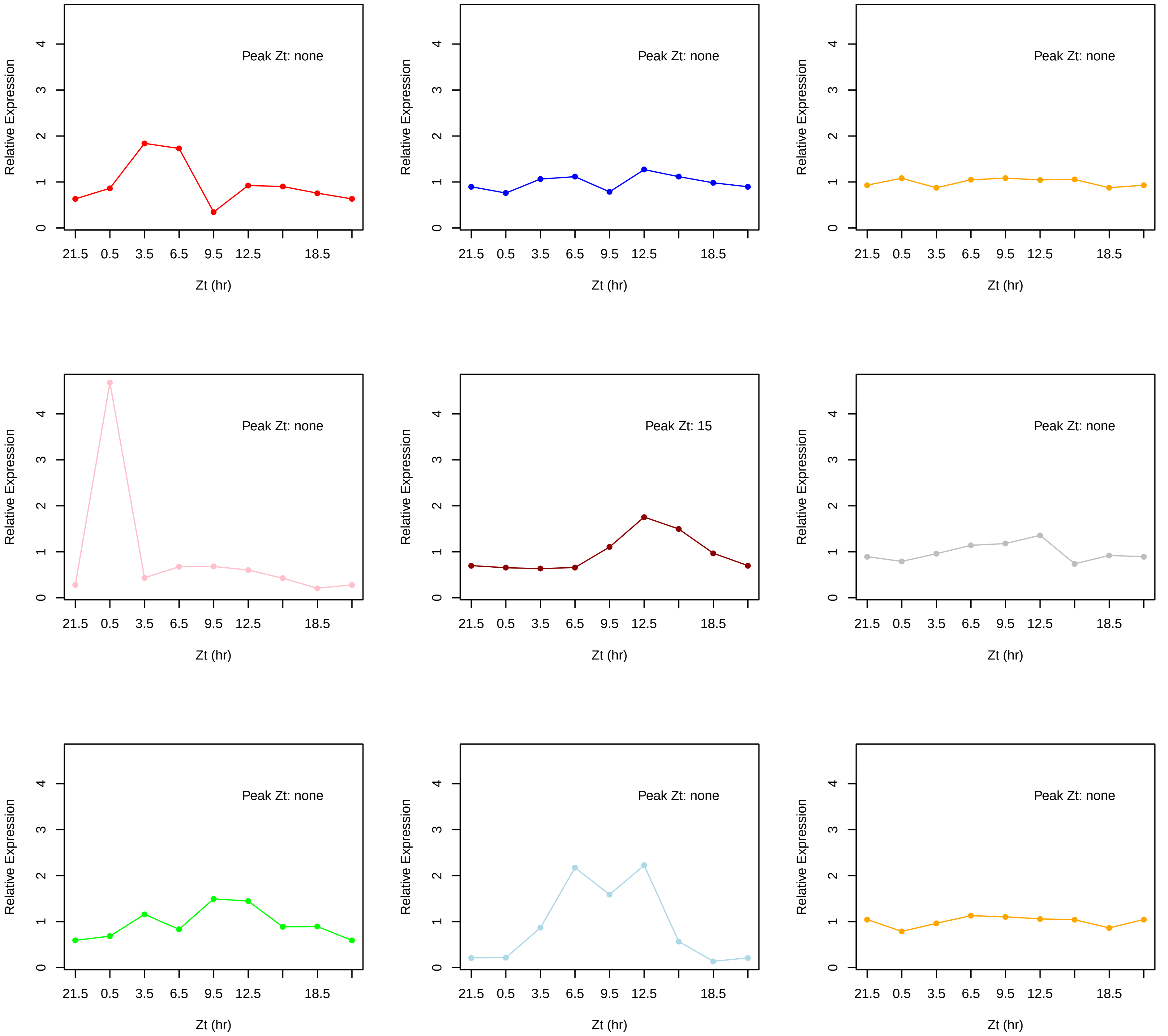

Singeleton: ENSXMAG00000002380 ushbp1

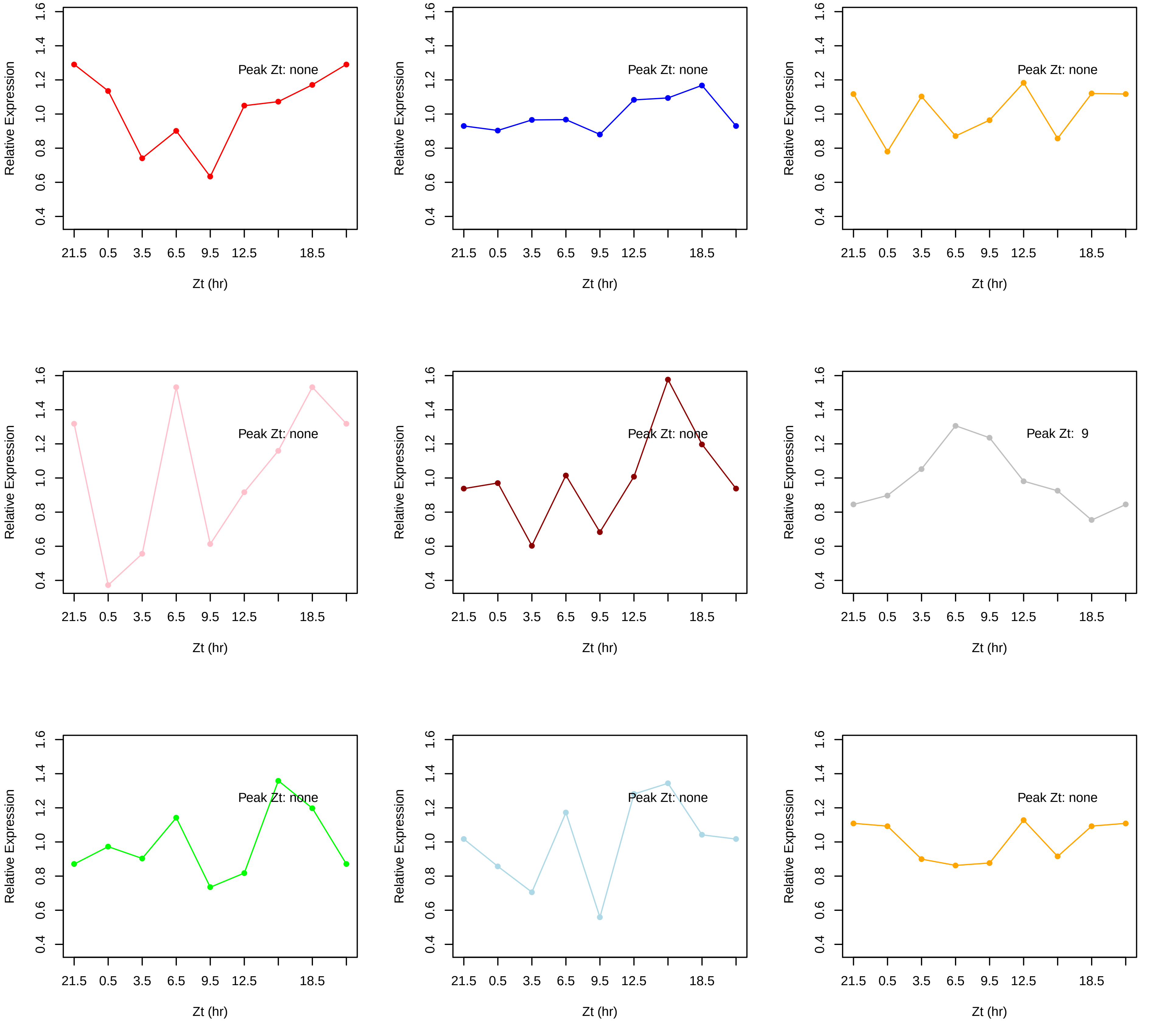

Singeleton: ENSXMAG00000003615 atxn1a

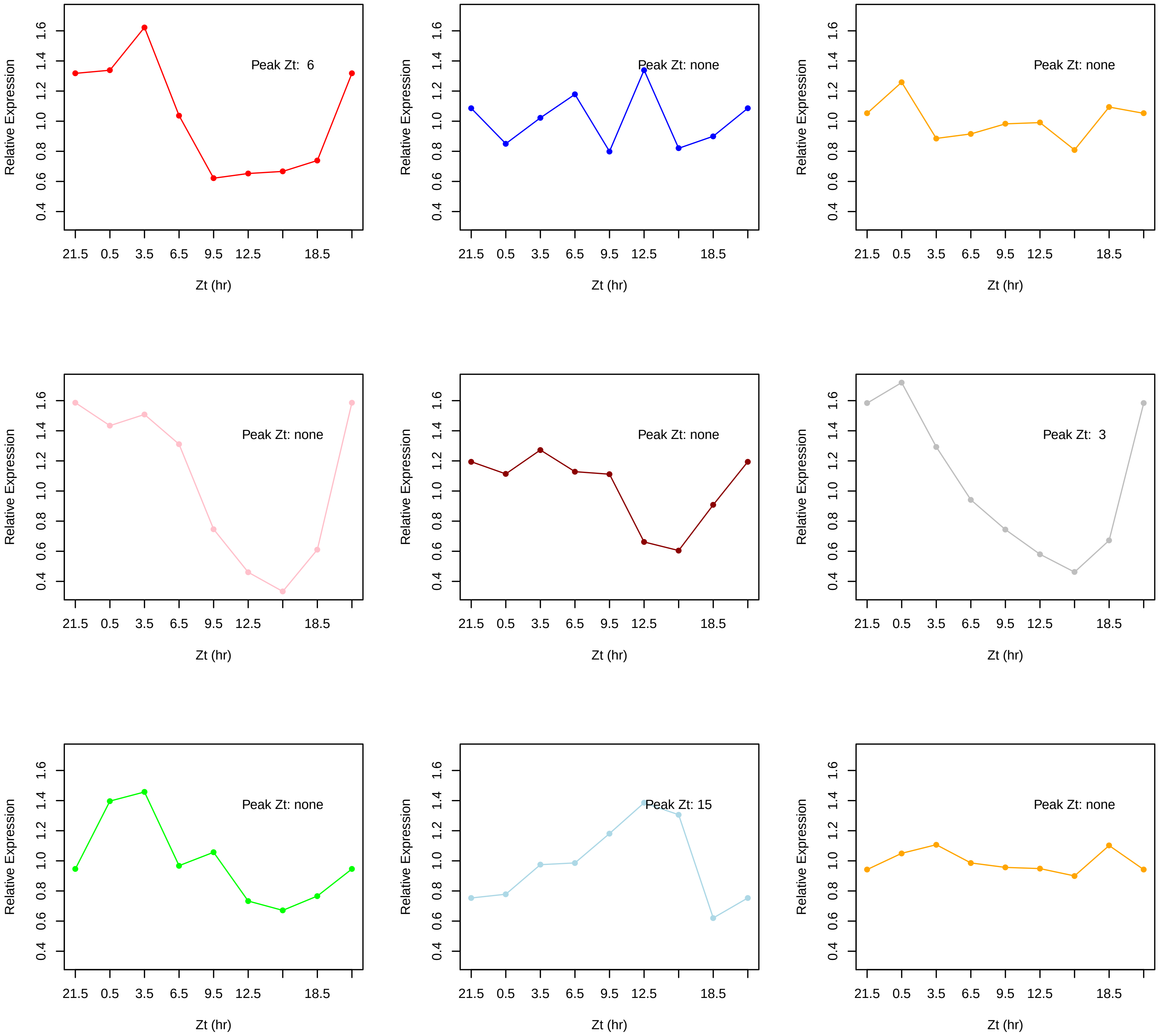

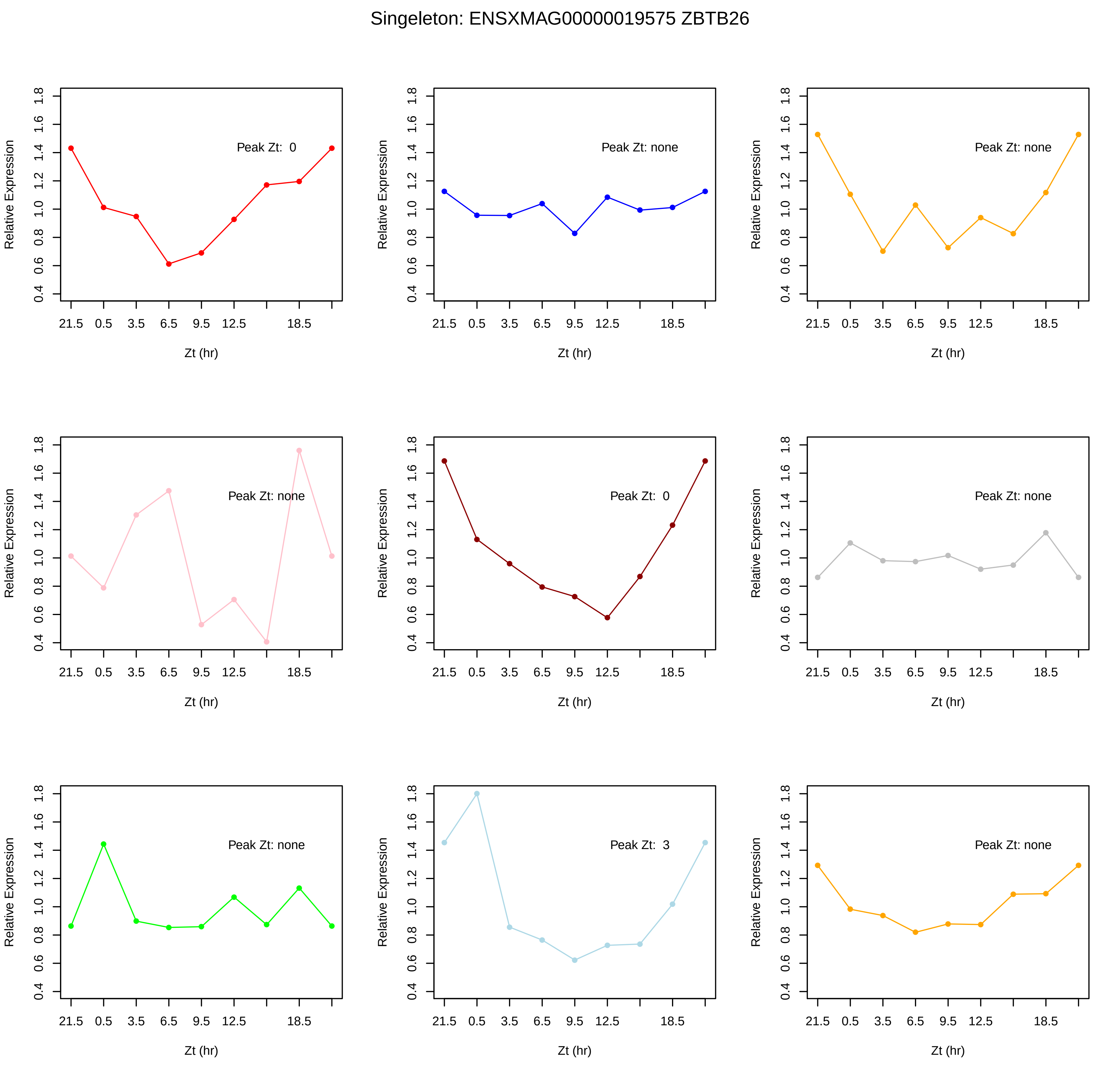
Singeleton: ENSXMAG00000019575 ZBTB26
Peak Zt: 0
Peak Zt: none
Peak Zt: none
Peak Zt: none
Peak Zt: 0
Peak Zt: none
Peak Zt: none
Peak Zt: 3
Peak Zt: none
Relative Expression
Zt (hr)
21.5 0.5 3.5 6.5 9.5 12.5 18.5
0.4 0.6 0.8 1.0 1.2 1.4 1.6 1.8

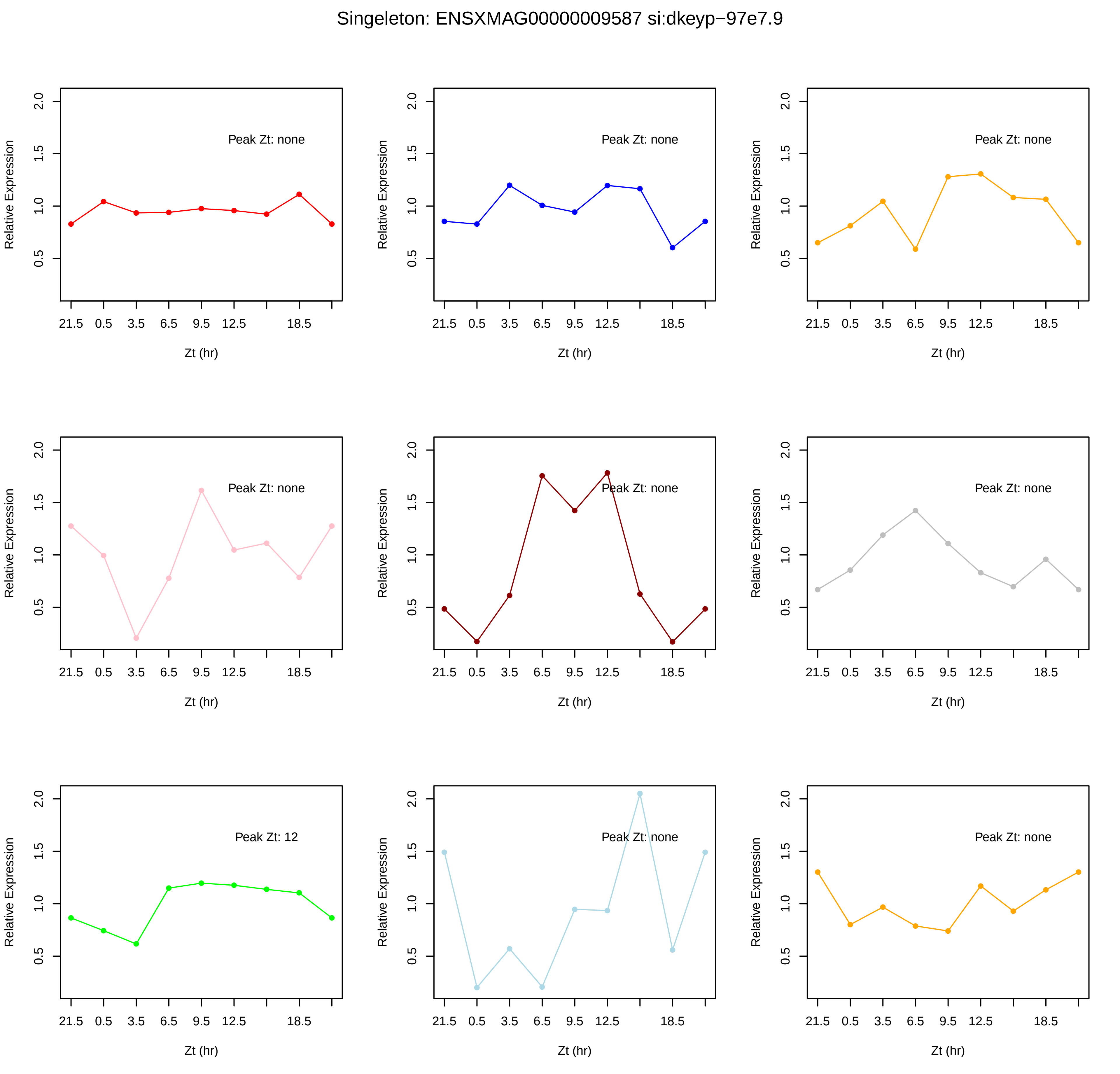
Singeleton: ENSXMAG00000009587 si:dkeyp−97e7.9
Peak Zt: none
Peak Zt: none
Peak Zt: none
Peak Zt: none
Peak Zt: none
Peak Zt: none
Peak Zt: 12
Peak Zt: none
Peak Zt: none
Relative Expression
Zt (hr)
21.5
0.5
3.5
6.5
9.5
12.5
18.5

Singeleton: ENSXMAG00000007119 nucb2a

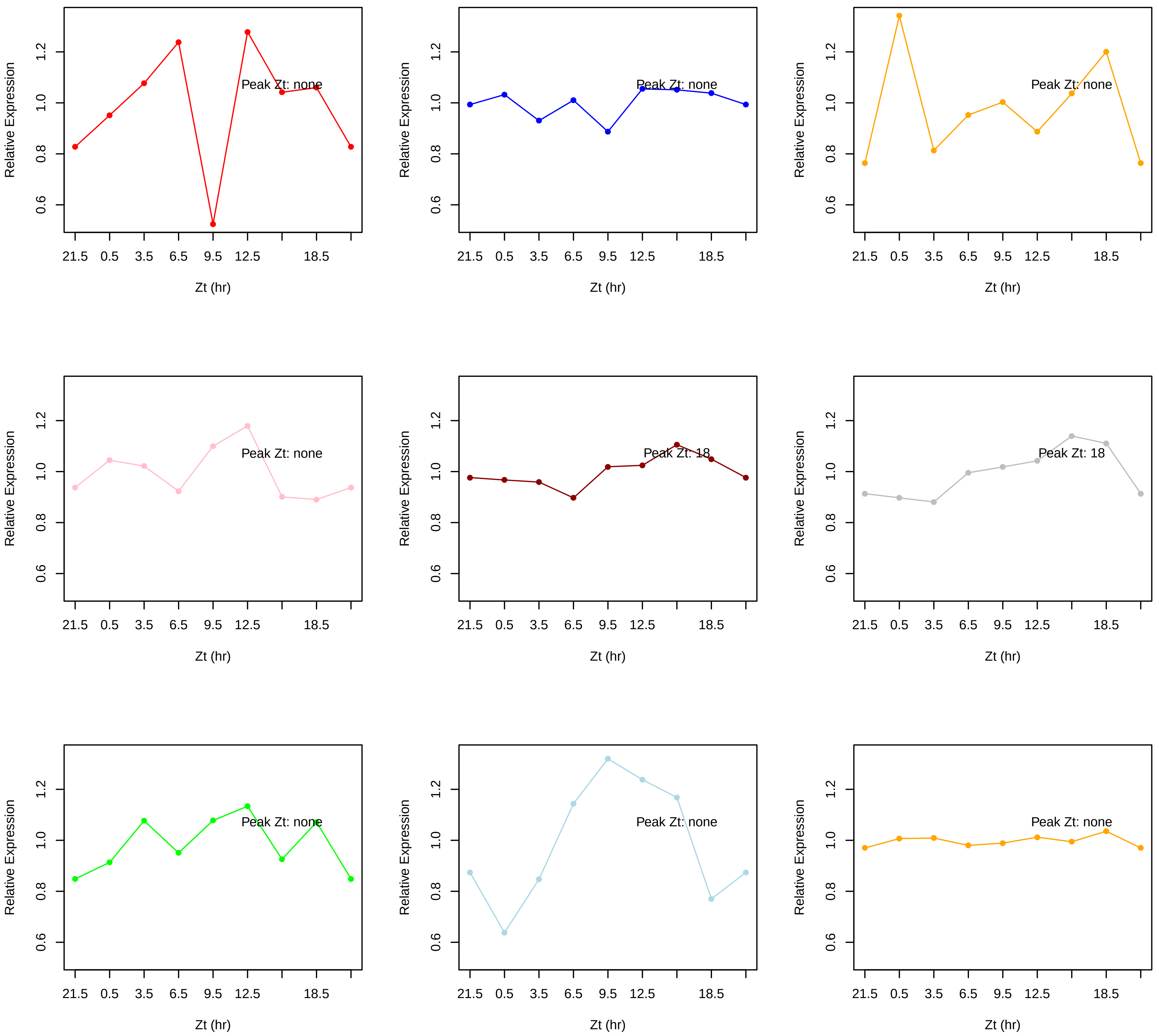

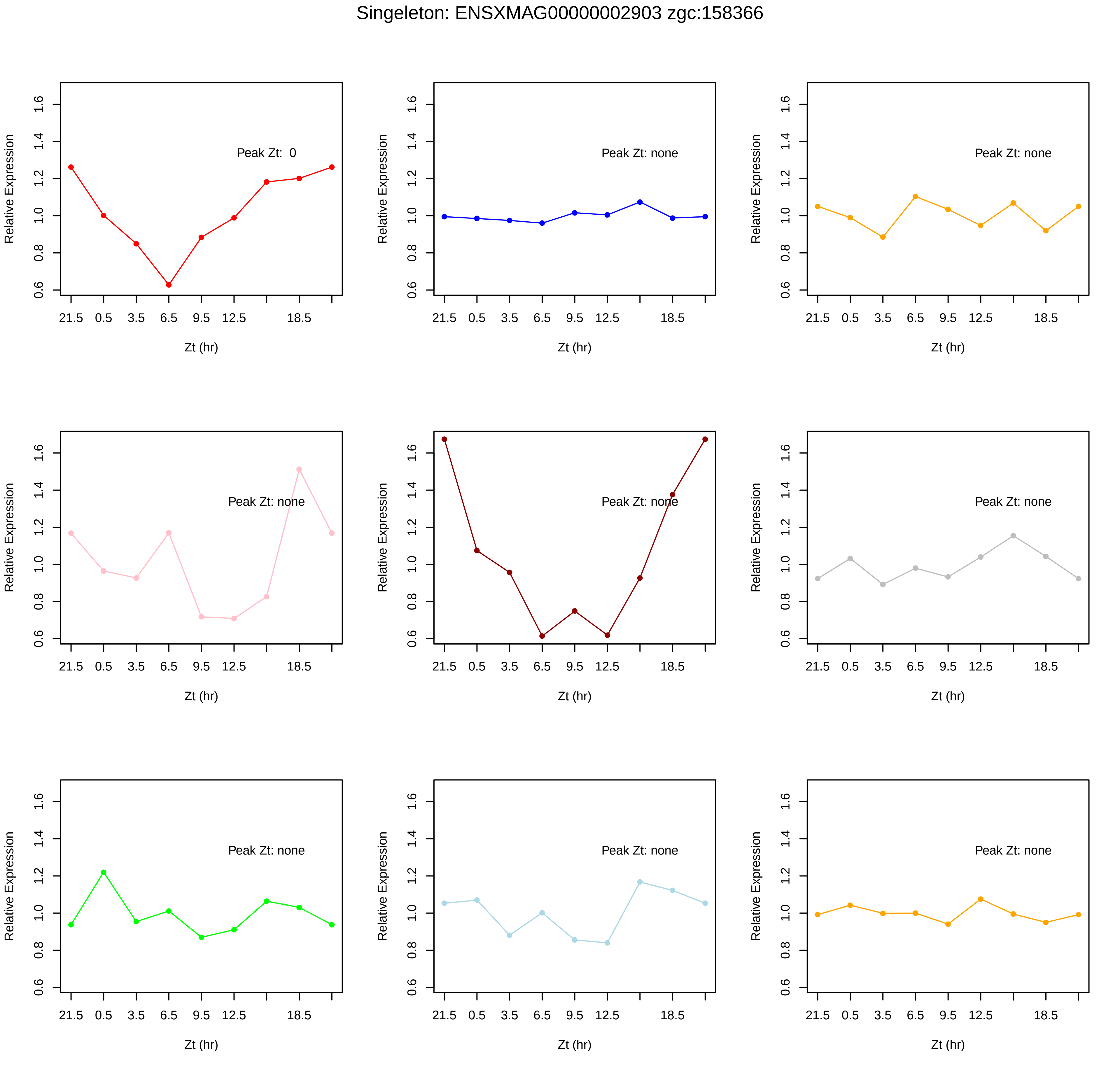
Singeleton: ENSXMAG00000002903 zgc:158366
Peak Zt: 0
Peak Zt: none
Relative Expression
Zt (hr)
0.6
0.8
1.0
1.2
1.4
1.6
21.5
0.5
3.5
6.5
9.5
12.5
18.5

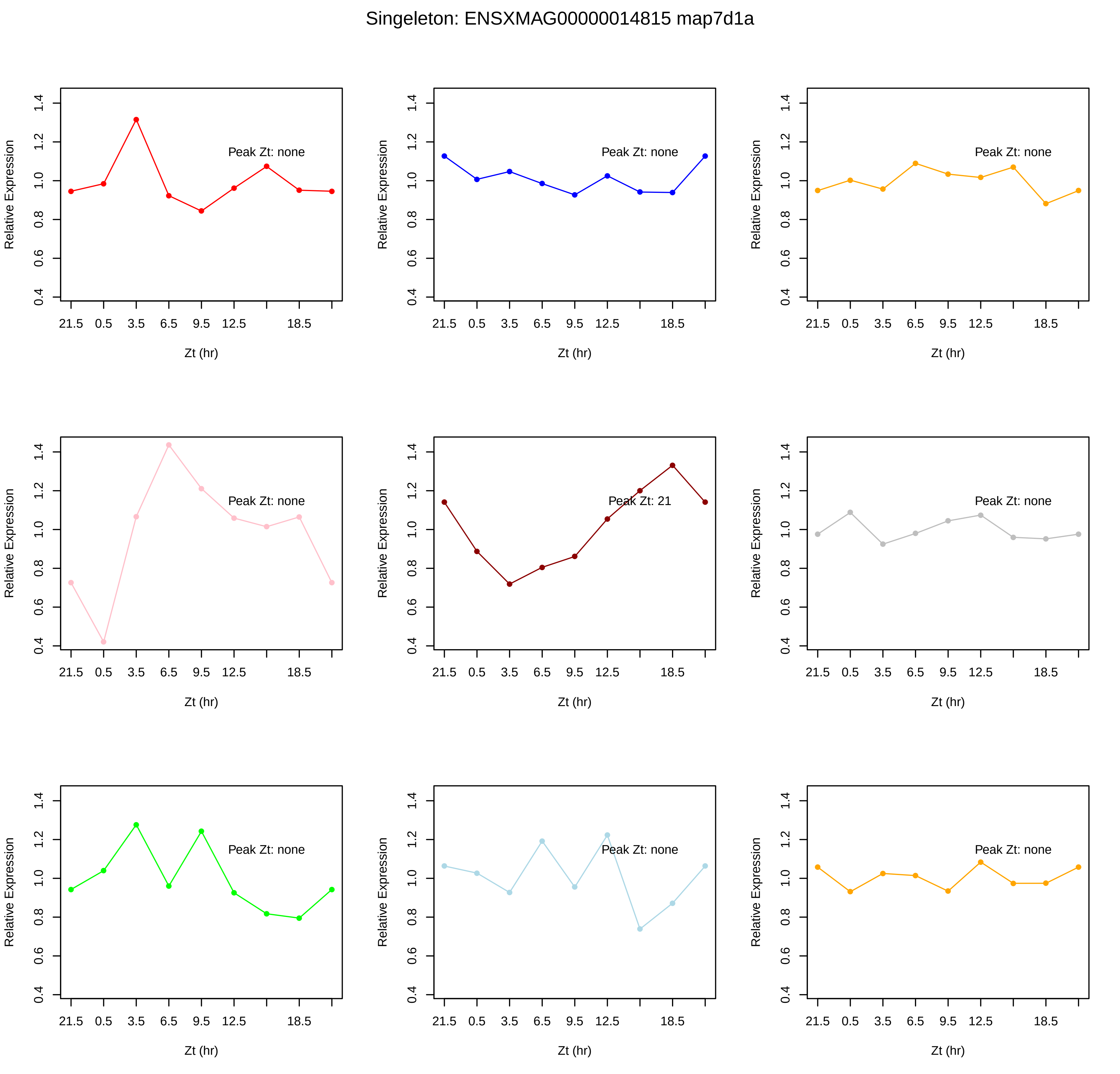
Singeleton: ENSXMAG00000014815 map7d1a
Peak Zt: none
Peak Zt: none
Peak Zt: none
Peak Zt: none
Peak Zt: 21
Peak Zt: none
Peak Zt: none
Peak Zt: none
Peak Zt: none
Relative Expression
Zt (hr)
21.5 0.5 3.5 6.5 9.5 12.5 18.5
0.4 0.6 0.8 1.0 1.2 1.4

Singeleton: ENSXMAG00000005653

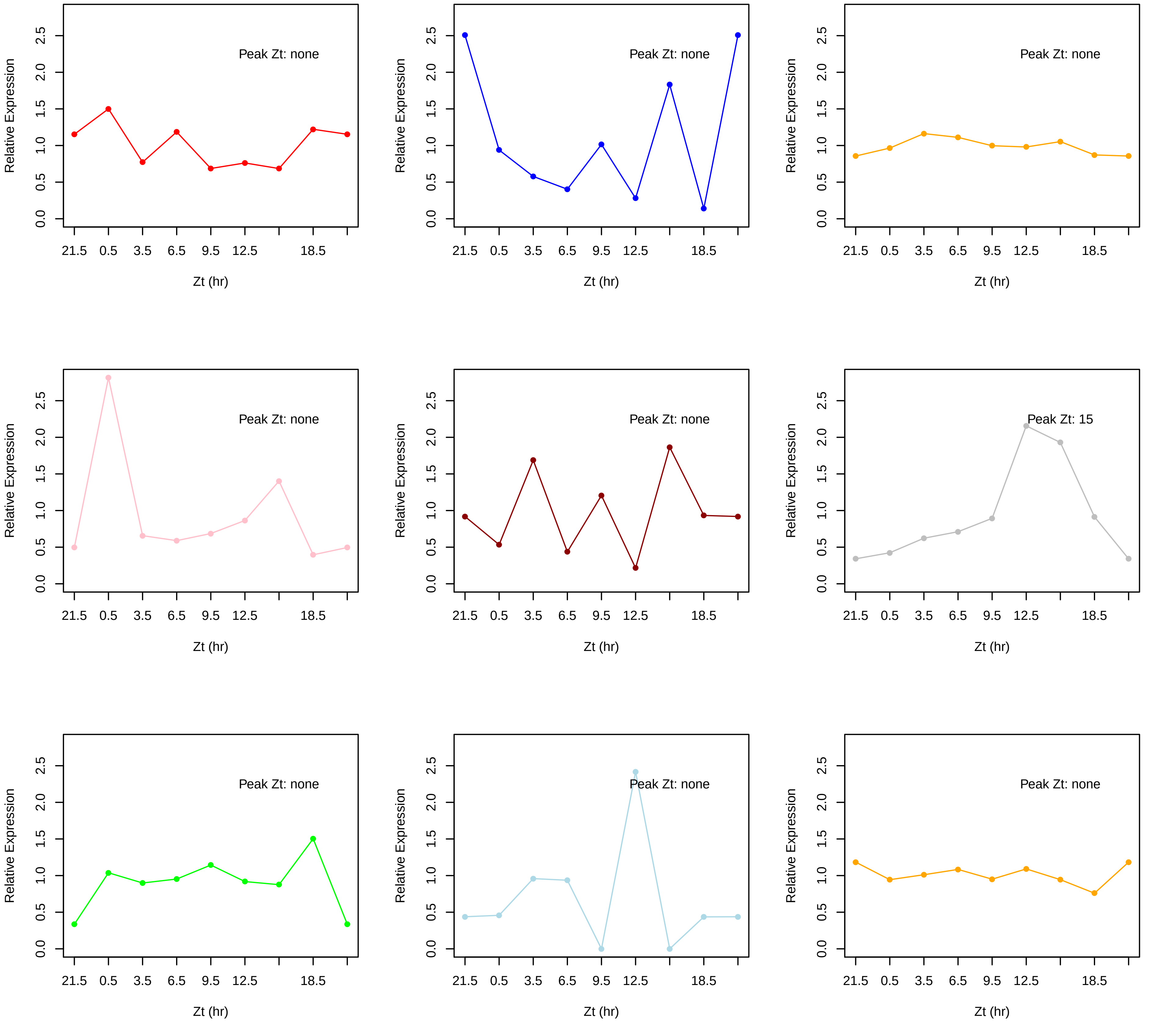

Singleton: ENSXMAG00000025135 rnf10

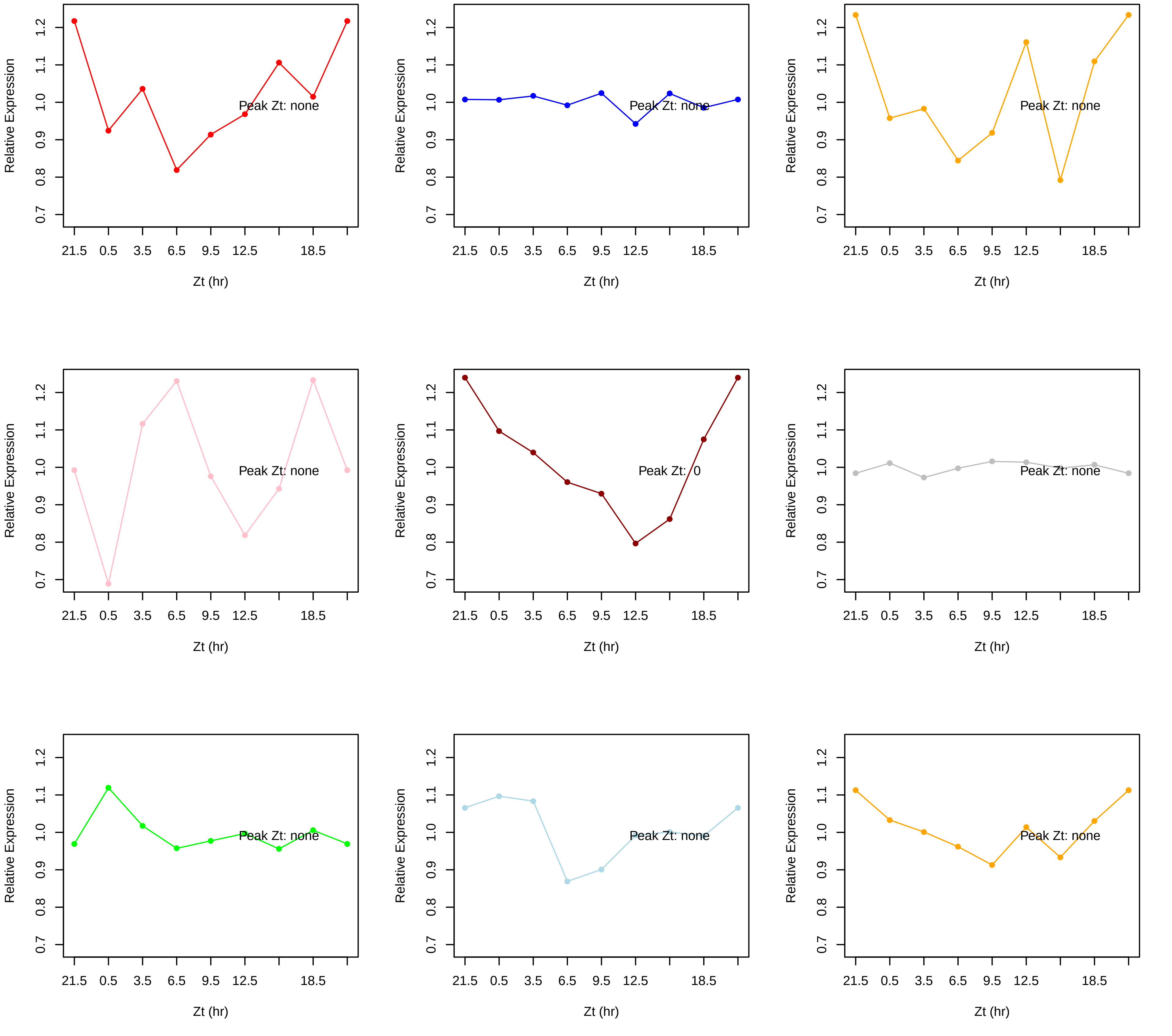

Singeleton: ENSXMAG00000029102 aurkaip1

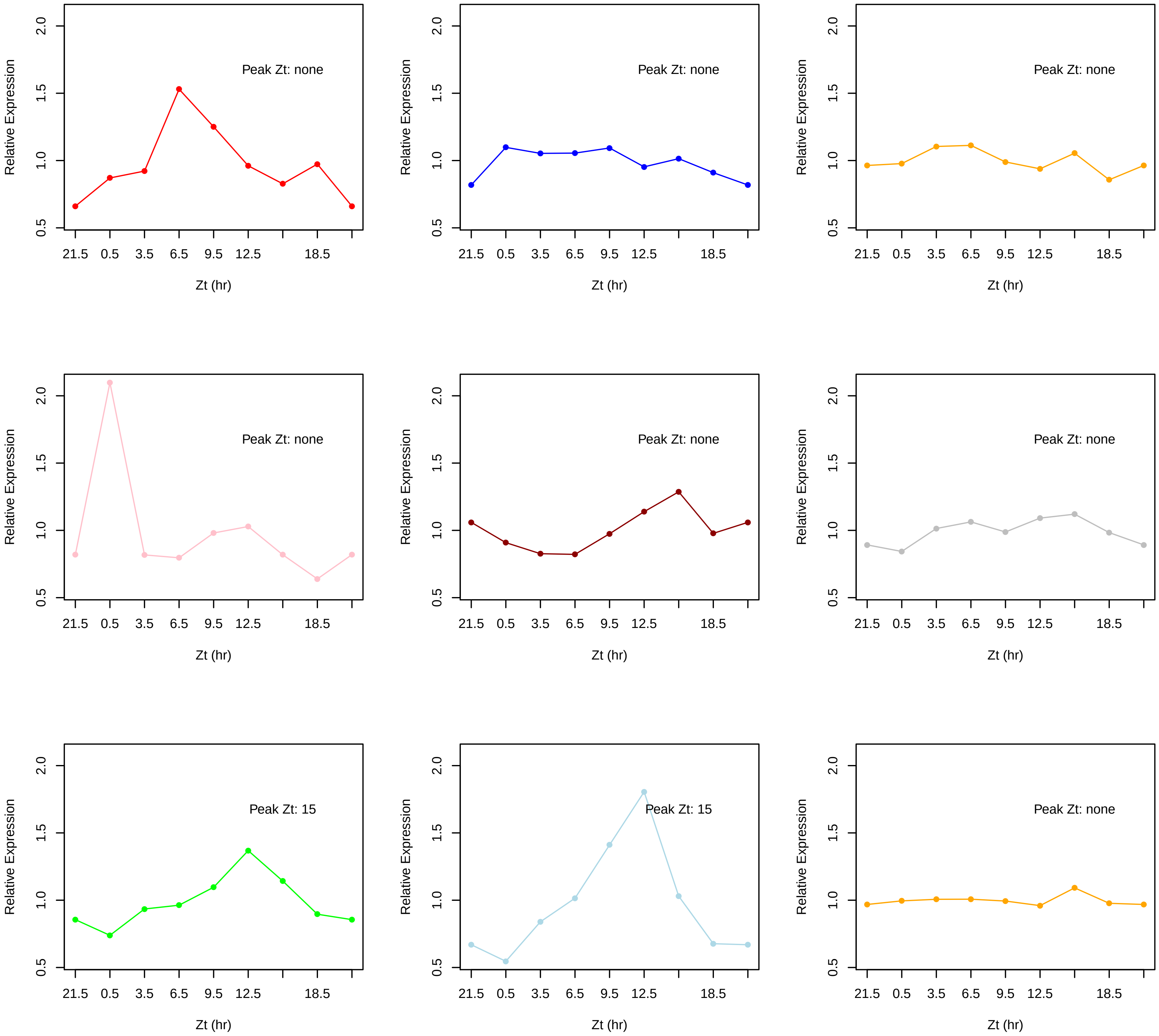

Singeleton: ENSXMAG00000026487 zswim5

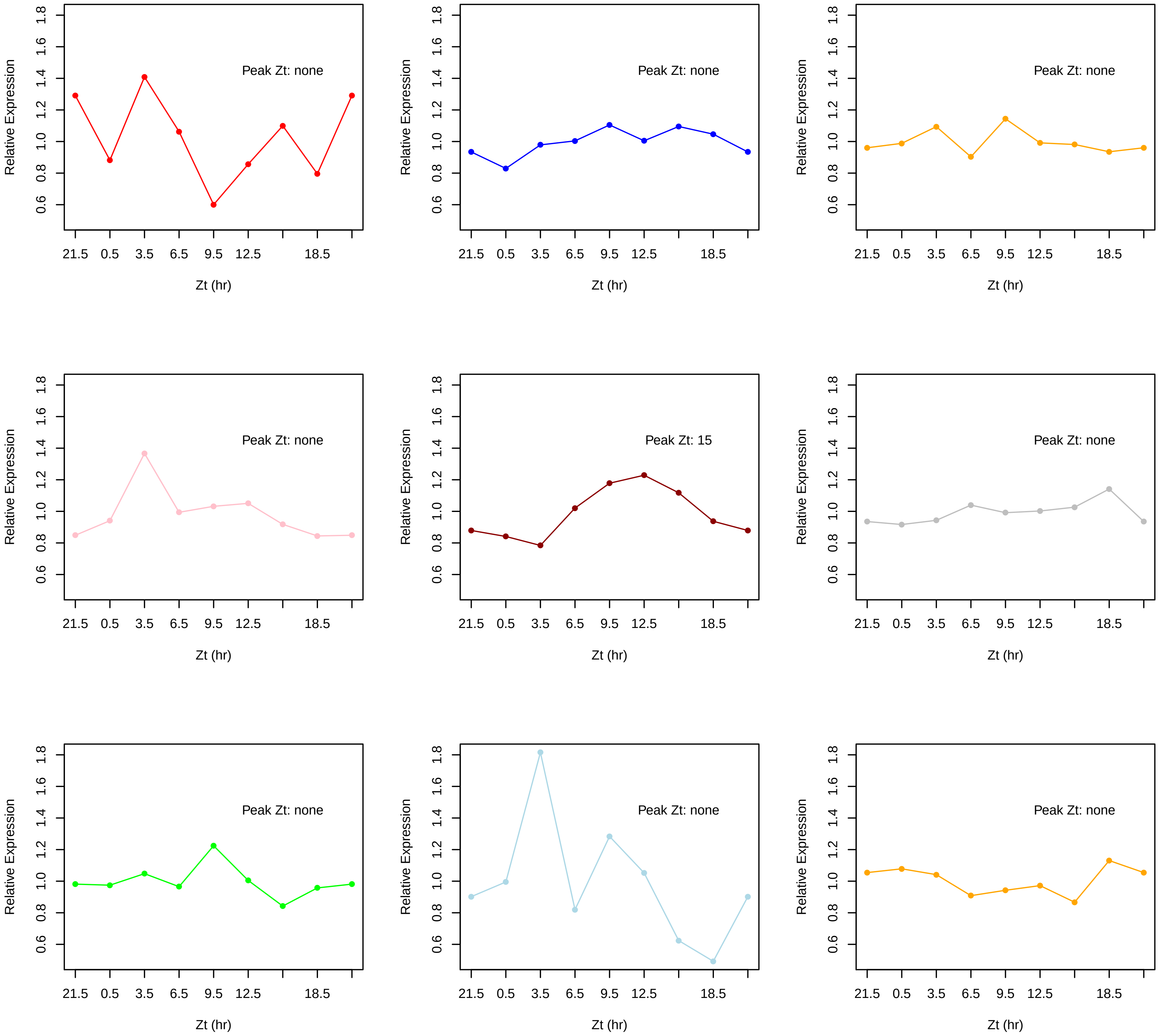

Singleton: ENSXMAG00000025830 ptbp3

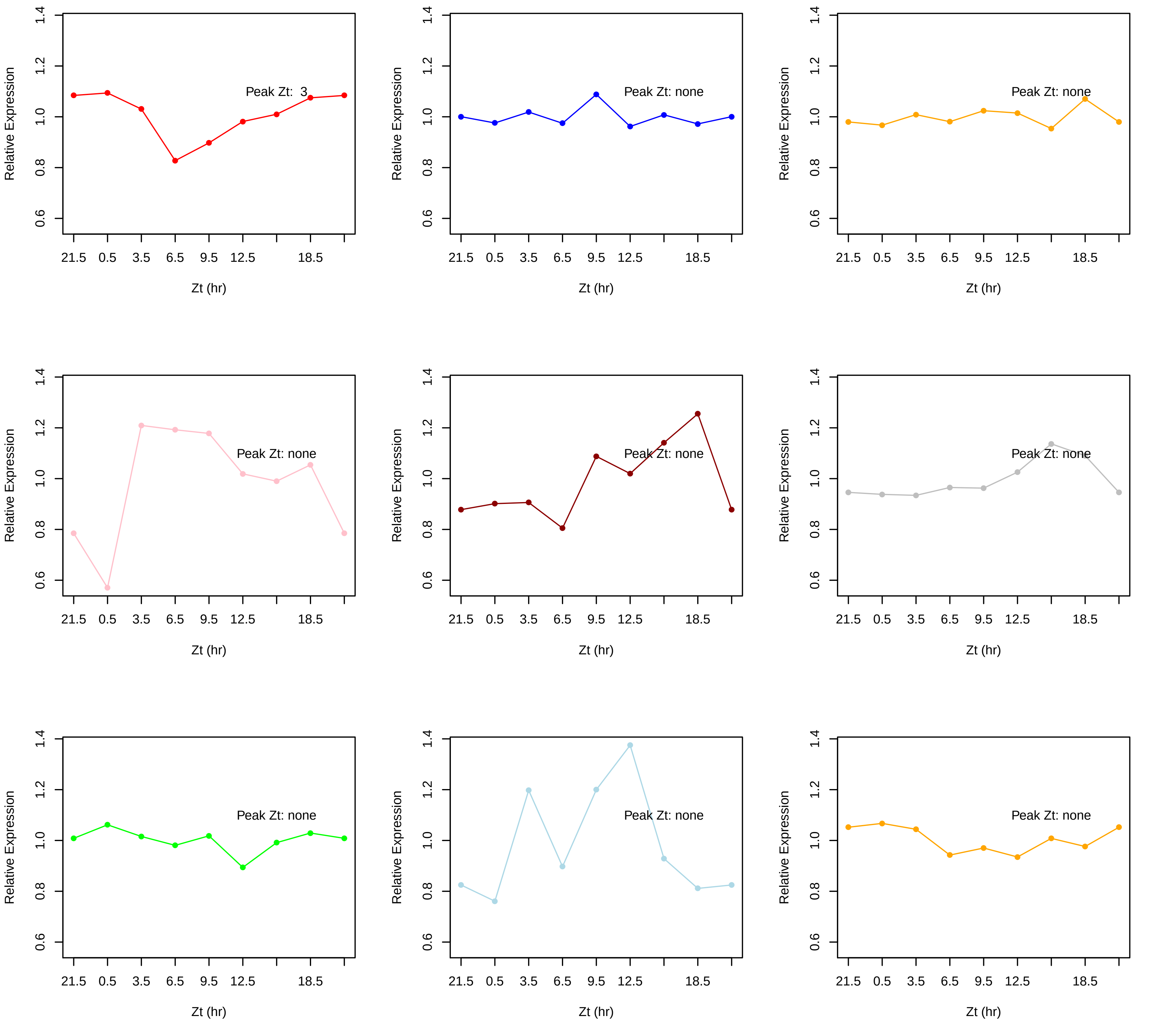

Singeleton: ENSXMAG00000011727 vps9d1

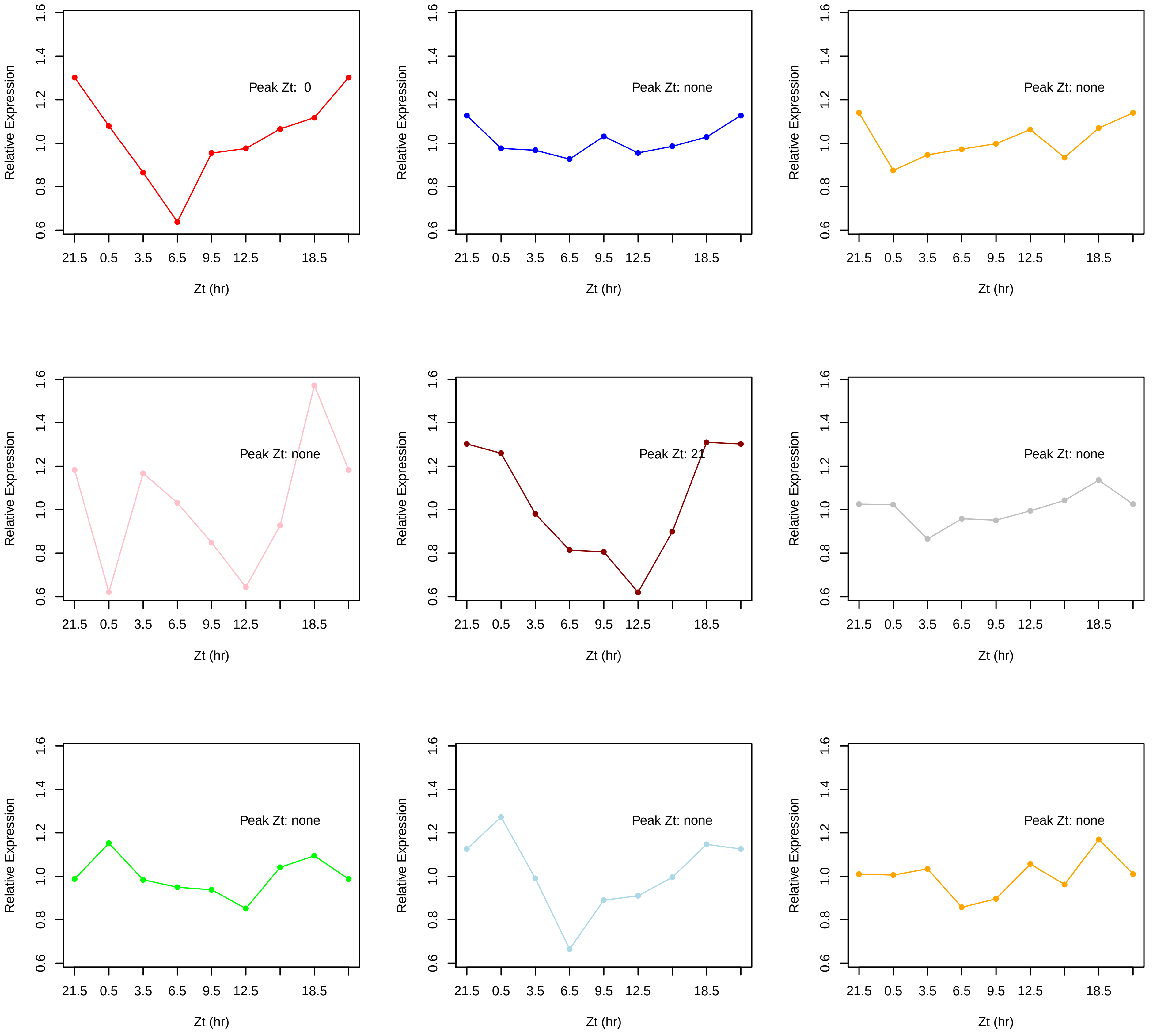

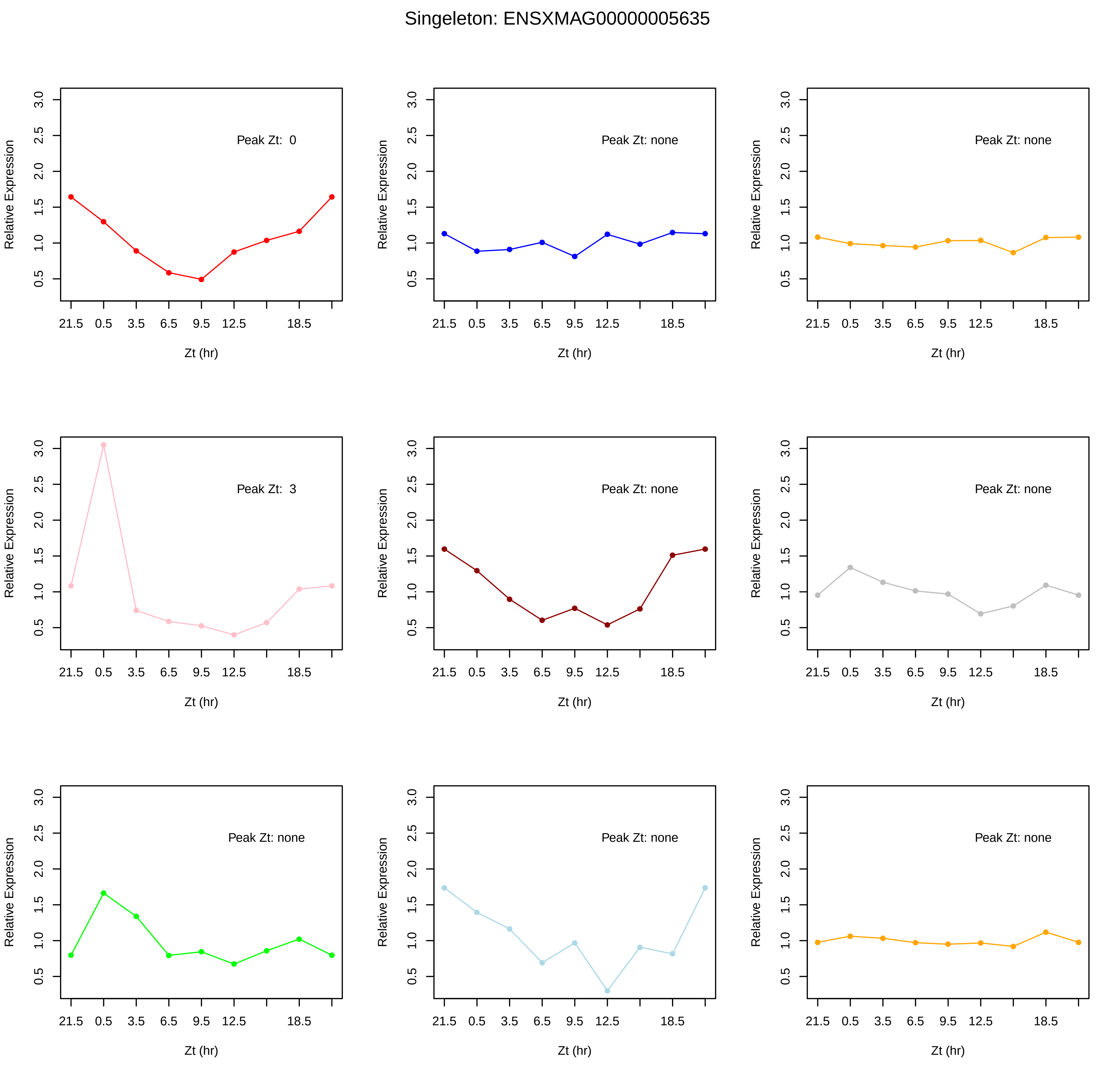

Singeleton: ENSXMAG00000005635
Peak Zt: 0
Peak Zt: none
Peak Zt: none
Peak Zt: 3
Peak Zt: none
Peak Zt: none
Peak Zt: none
Peak Zt: none
Peak Zt: none
Relative Expression
Zt (hr)
21.5
0.5
3.5
6.5
9.5
12.5
18.5
0.5
1.0
1.5
2.0
2.5
3.0

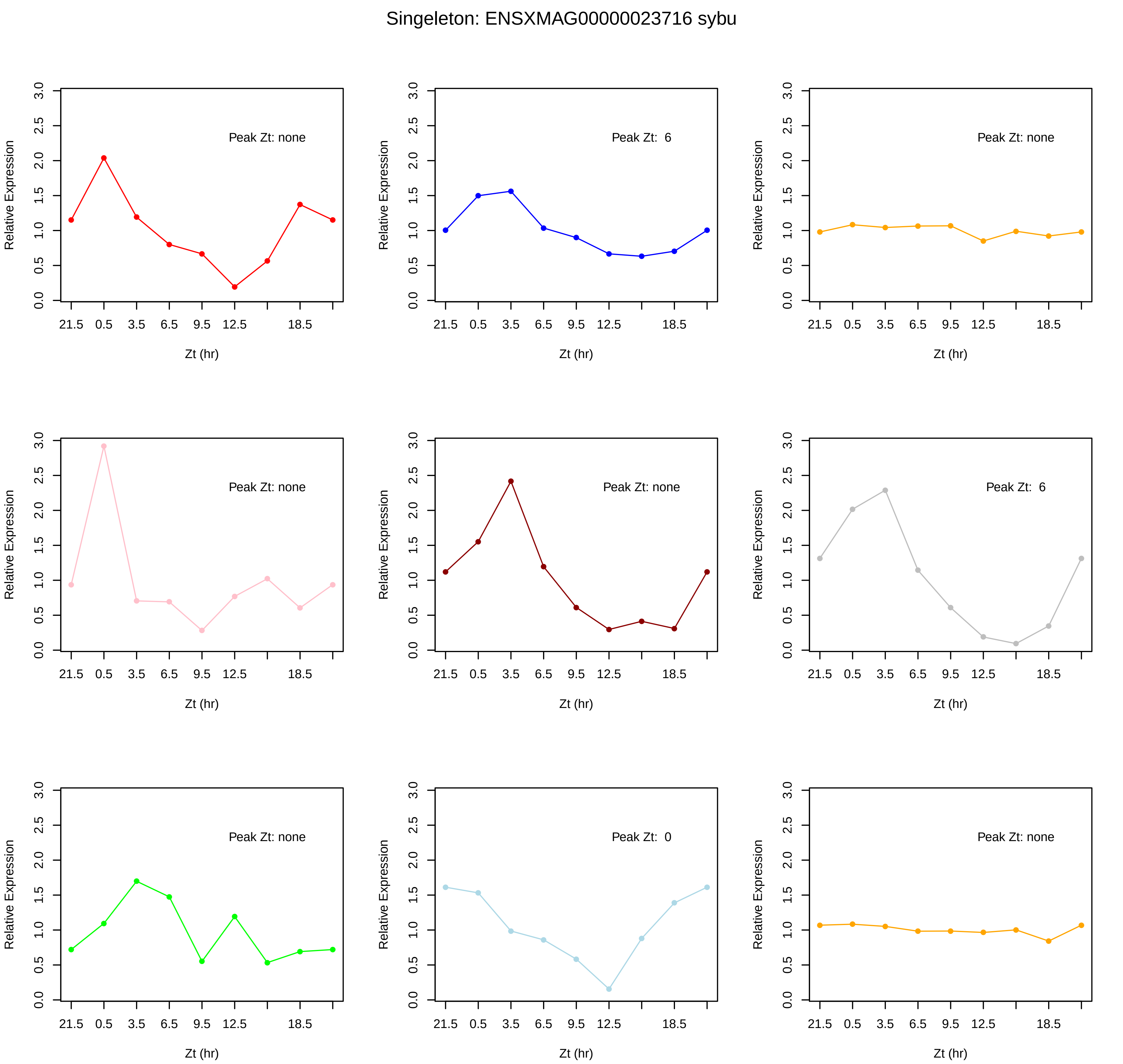
Singeleton: ENSXMAG00000023716 sybu
Peak Zt: none
Peak Zt: 6
Peak Zt: none
Peak Zt: none
Peak Zt: none
Peak Zt: 6
Peak Zt: none
Peak Zt: 0
Peak Zt: none
Relative Expression
Zt (hr)
21.5
0.5
3.5
6.5
9.5
12.5
18.5

Singleton: ENSXMAG00000007078 ebag9

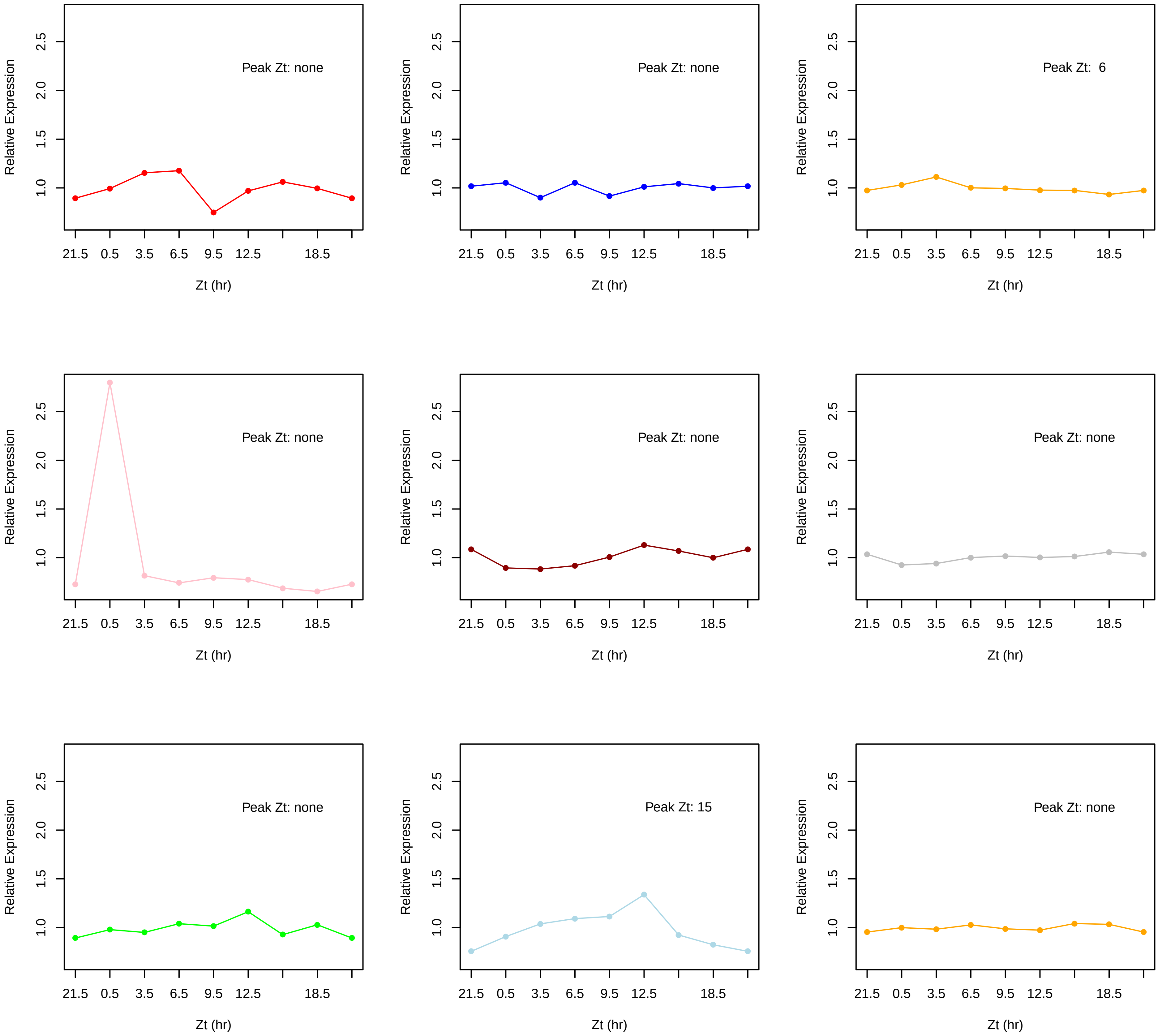

Singeleton: ENSXMAG00000012374 pik3c2a

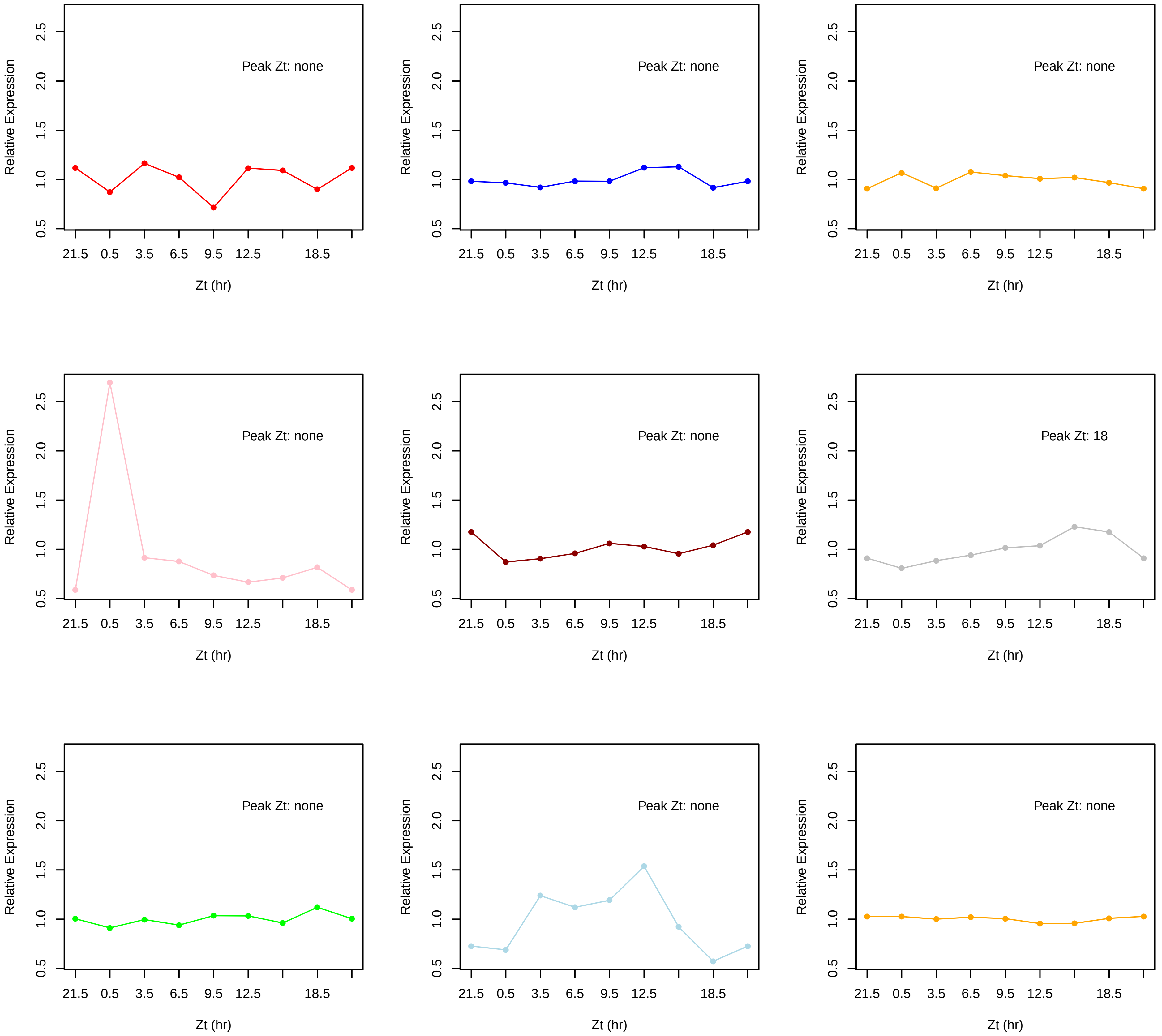

Singeleton: ENSXMAG00000005633 supt4h1

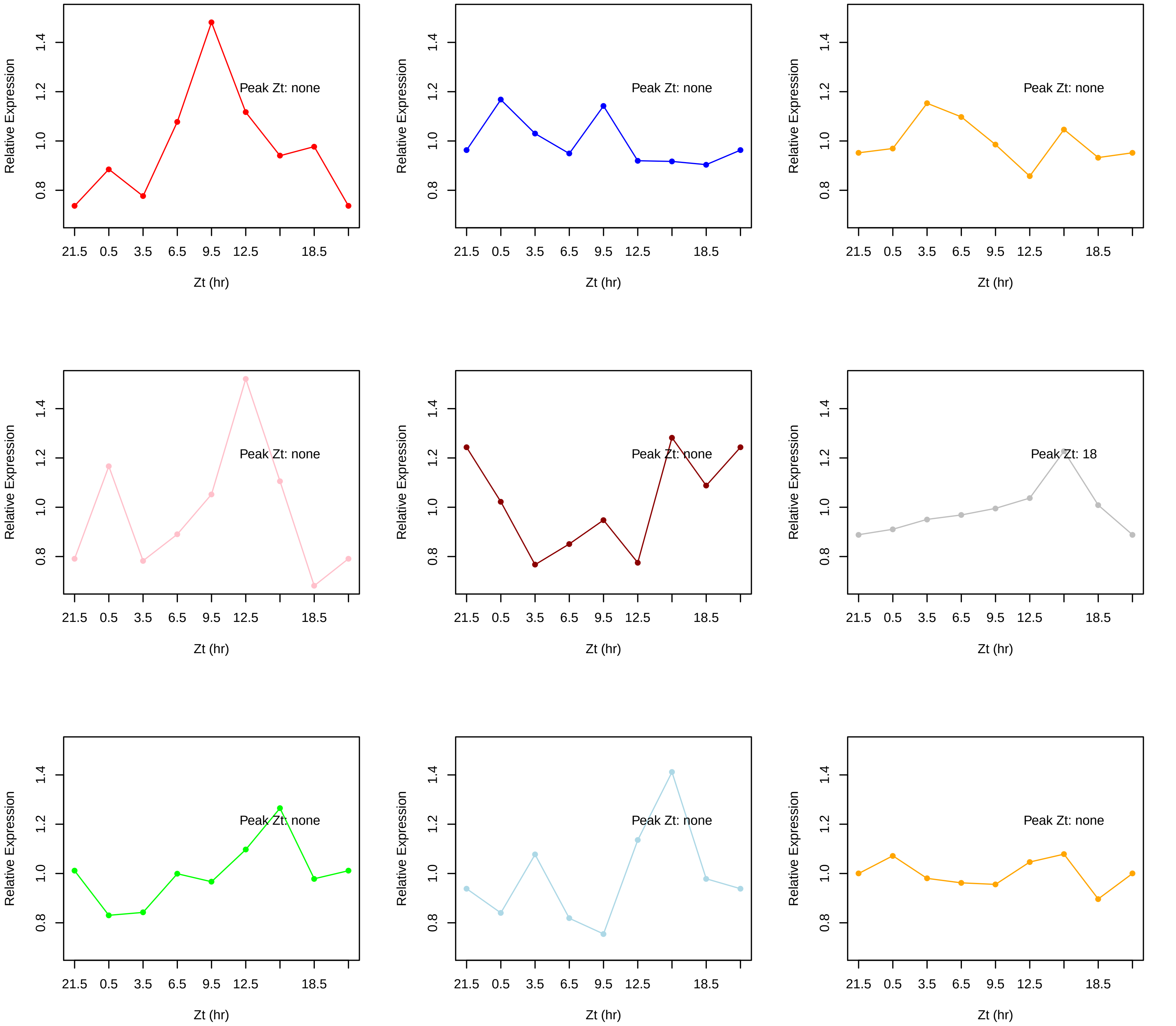

Singeleton: ENSXMAG00000005041 ugcg

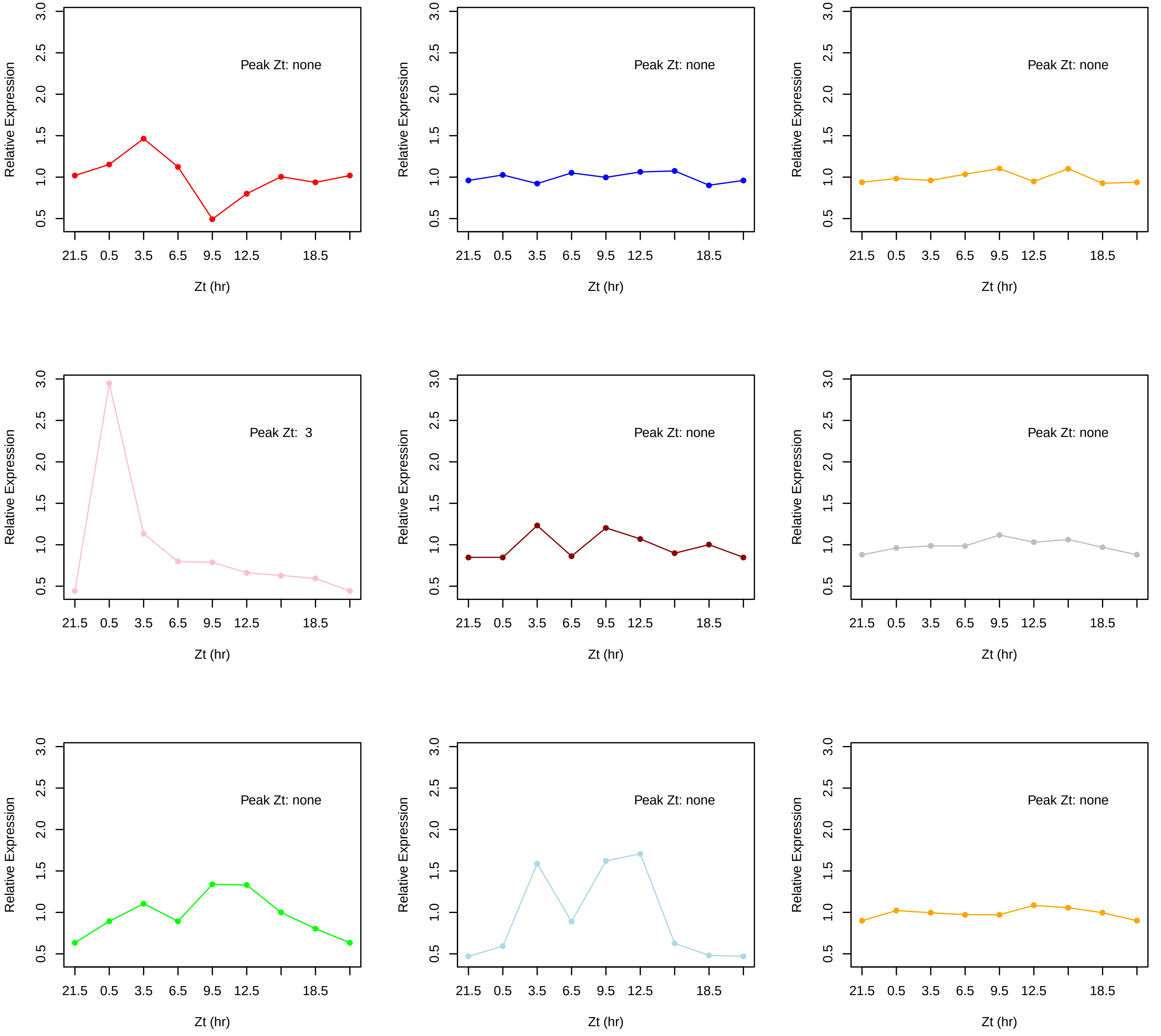

Singleton: ENSXMAG00000018018

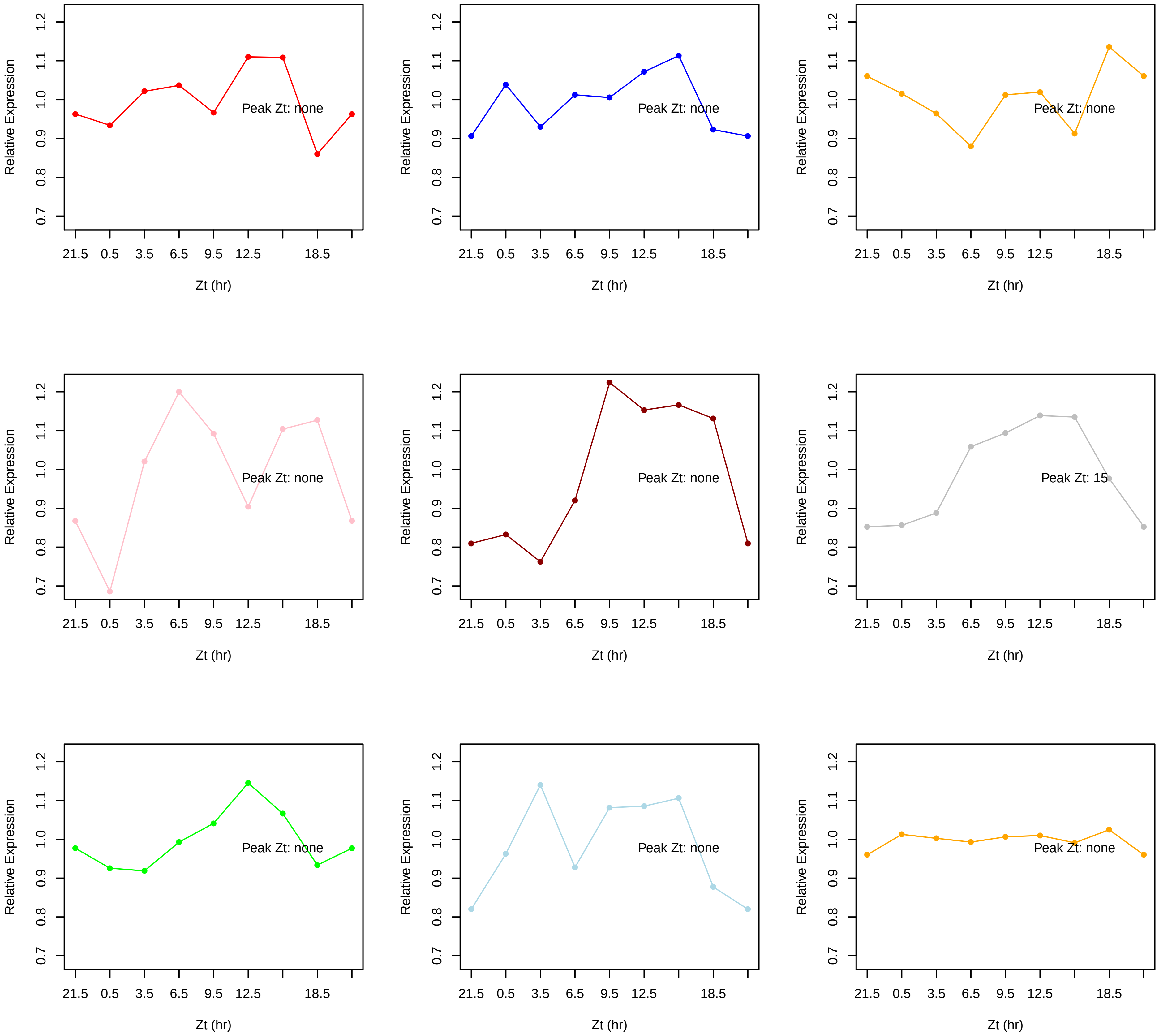

Singeleton: ENSXMAG00000001593 sh3d21

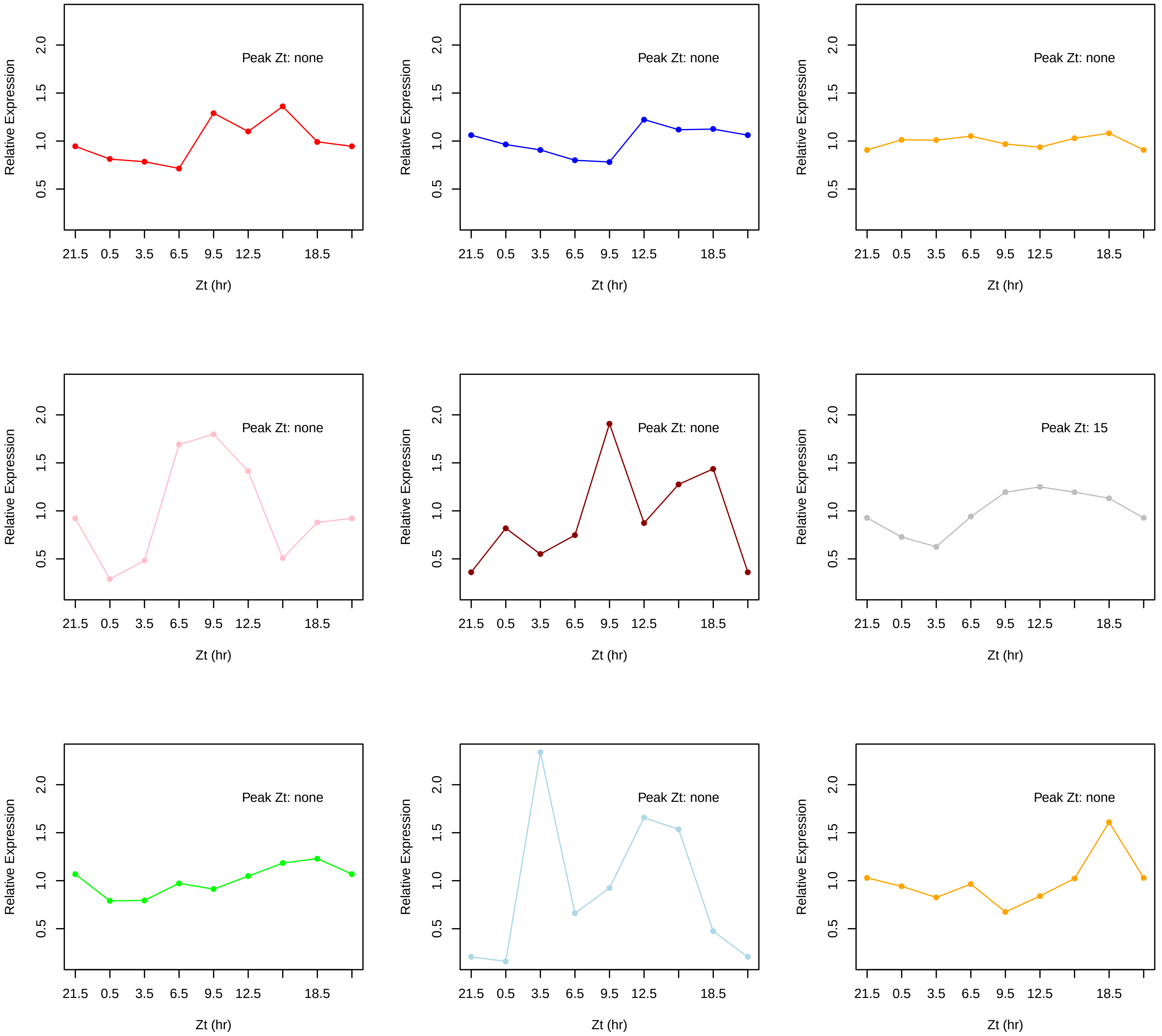

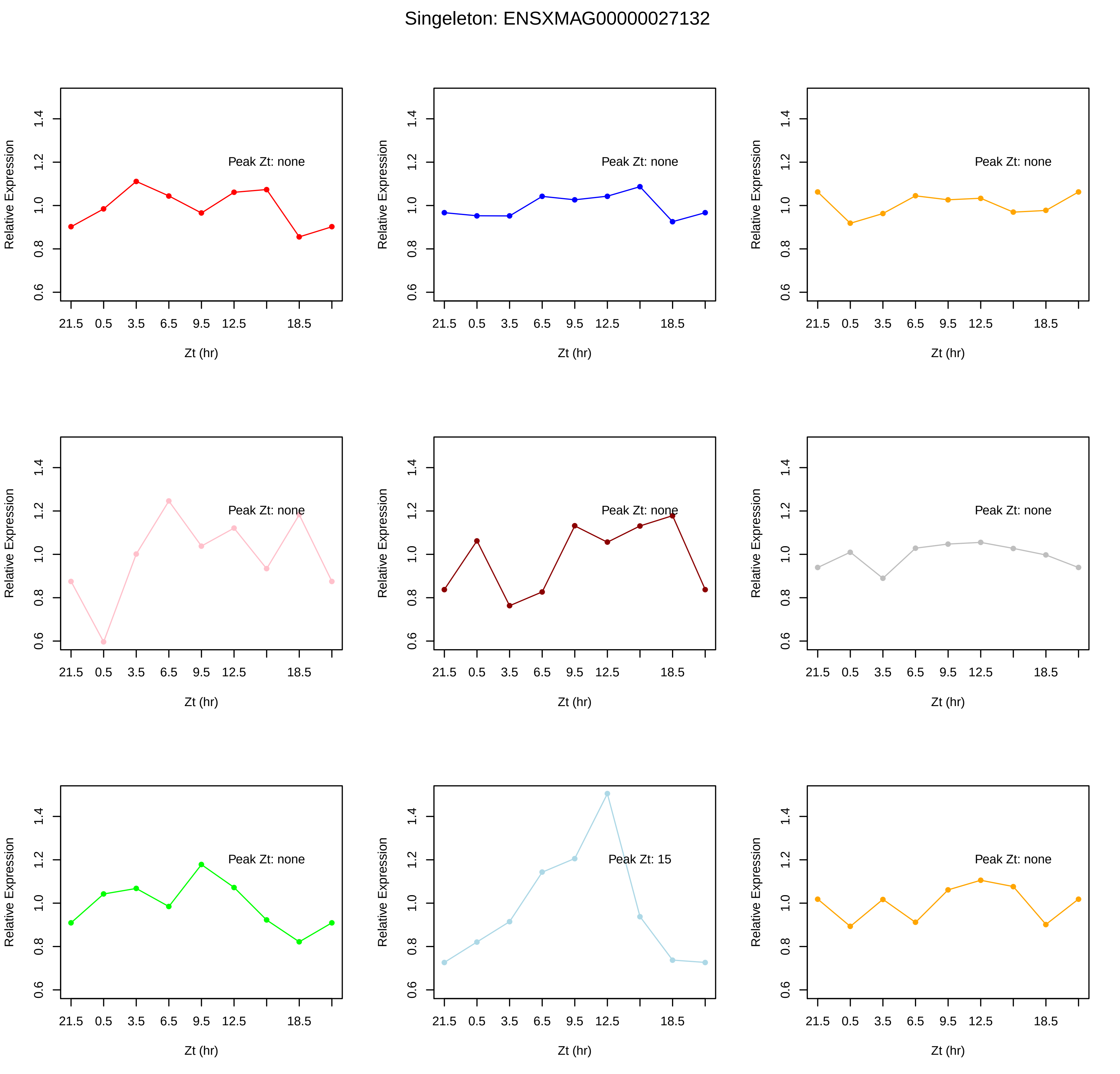
Singeleton: ENSXMAG00000027132
Peak Zt: none
Peak Zt: none
Peak Zt: none
Peak Zt: none
Peak Zt: none
Peak Zt: none
Peak Zt: none
Peak Zt: 15
Peak Zt: none
Relative Expression
Zt (hr)
21.5 0.5 3.5 6.5 9.5 12.5 18.5
0.6 0.8 1.0 1.2 1.4

Singeleton: ENSXMAG00000027768

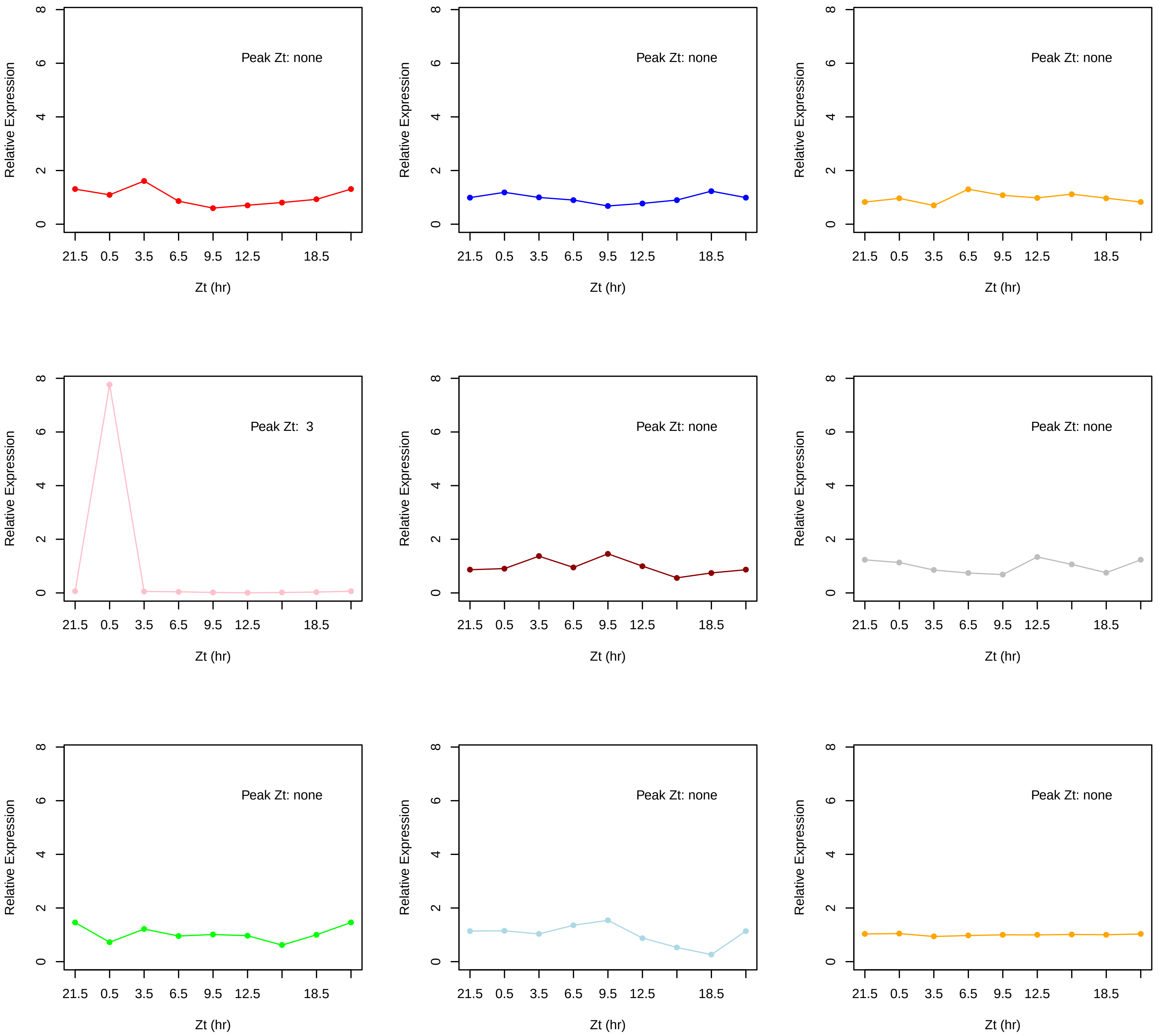

Singleton: ENSXMAG00000024934 ppp1r15b

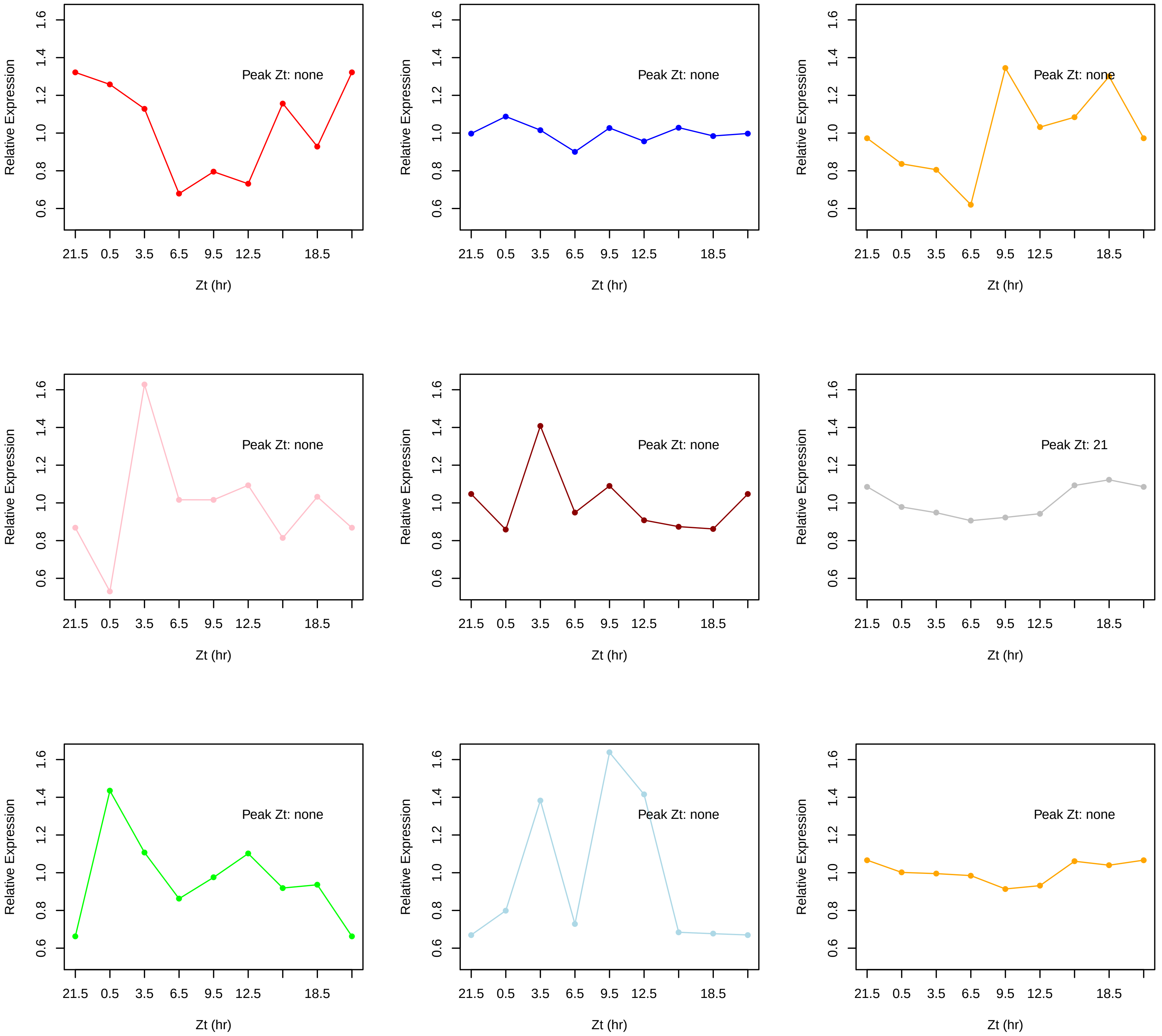

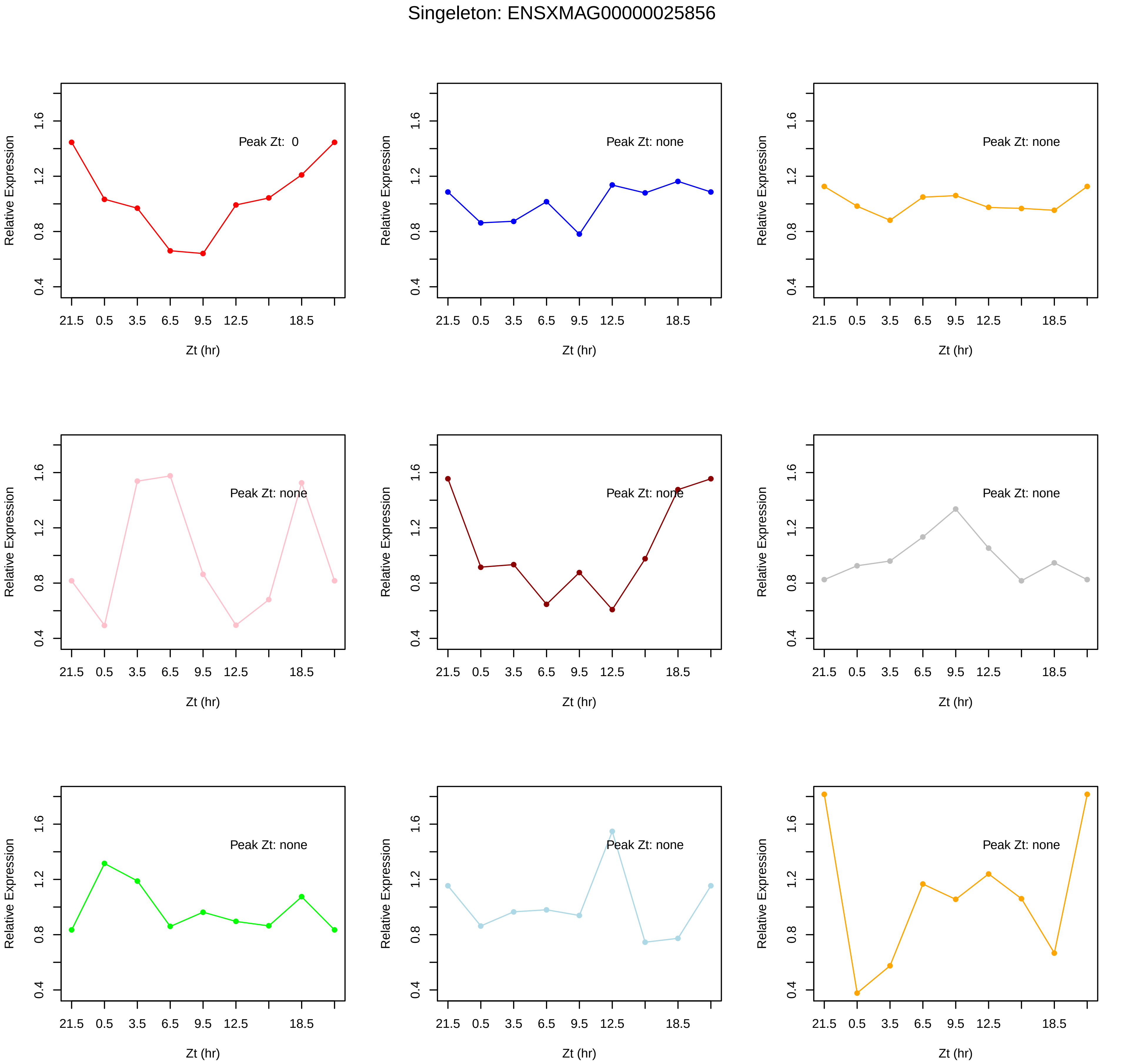

Singleton: ENSXMAG00000025856
Peak Zt: 0
Peak Zt: none
Peak Zt: none
Peak Zt: none
Peak Zt: none
Peak Zt: none
Peak Zt: none
Peak Zt: none
Peak Zt: none
Relative Expression
Zt (hr)
21.5
0.5
3.5
6.5
9.5
12.5
18.5
0.4
0.8
1.2
1.6

Singeleton: ENSXMAG00000021725 MRPL34

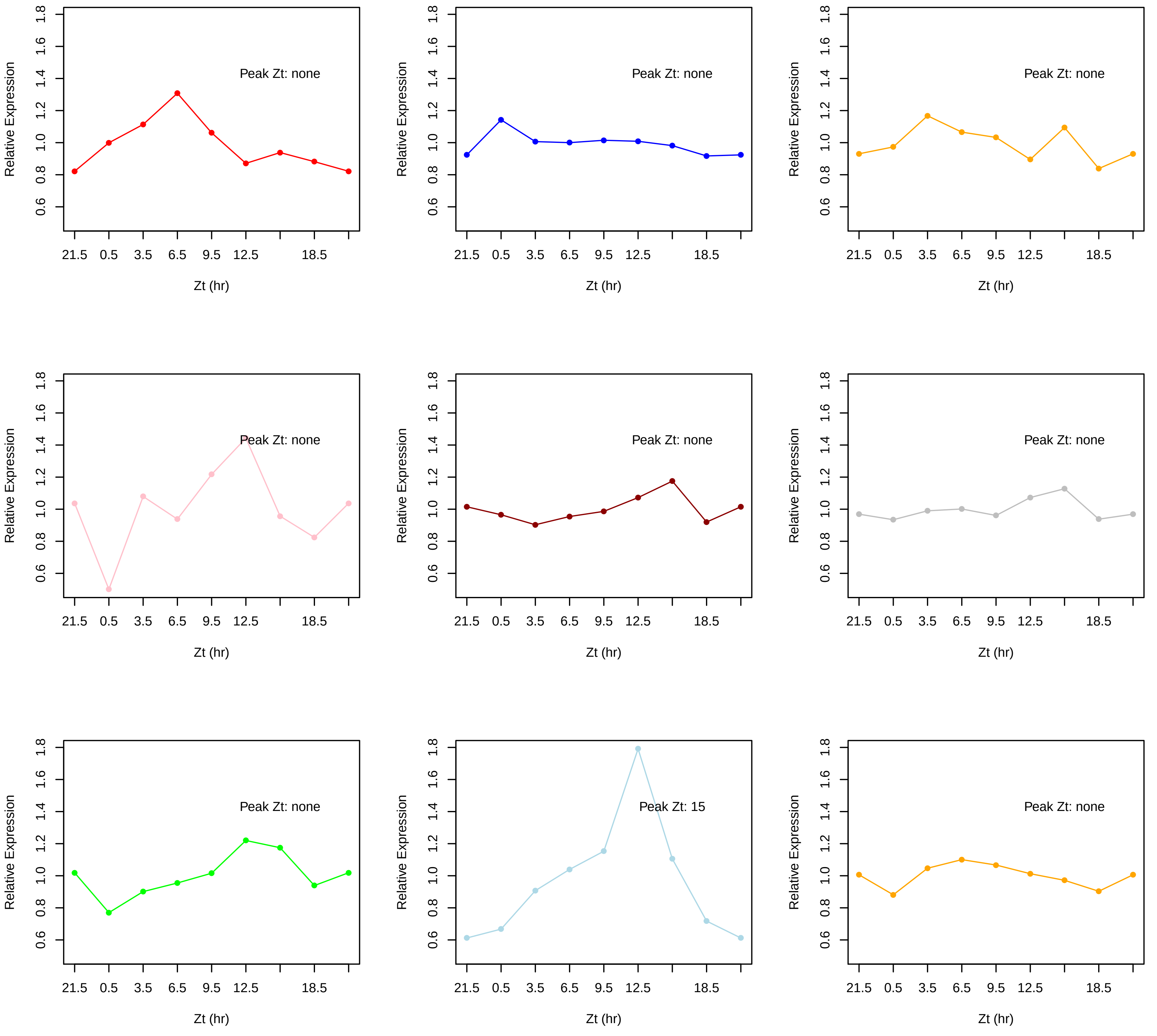

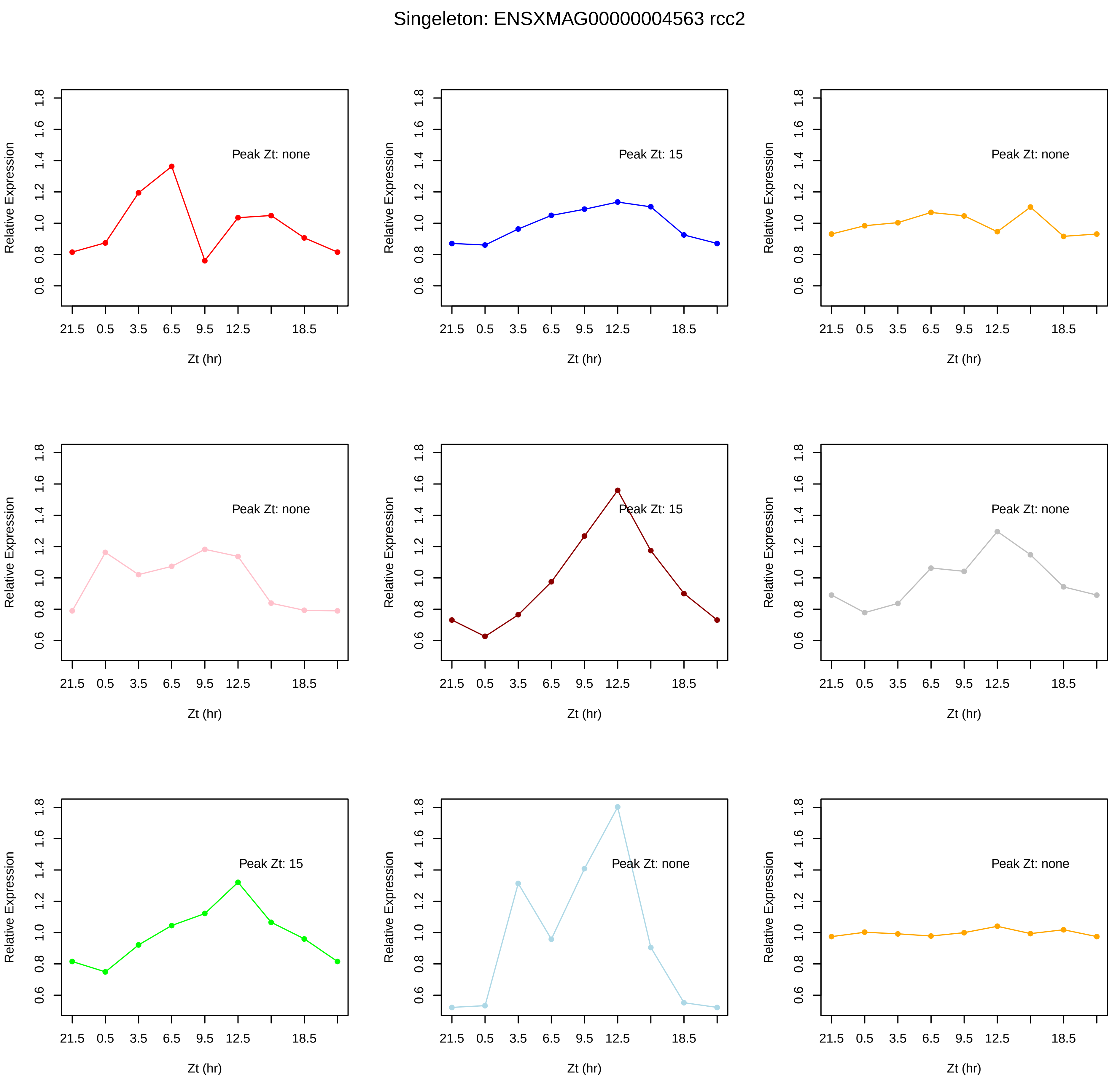
Singeleton: ENSXMAG00000004563 rcc2
Peak Zt: none
Peak Zt: 15
Peak Zt: none
Peak Zt: none
Peak Zt: 15
Peak Zt: none
Peak Zt: 15
Peak Zt: none
Peak Zt: none
Relative Expression
Zt (hr)
21.5
0.5
3.5
6.5
9.5
12.5
18.5
0.6
0.8
1.0
1.2
1.4
1.6
1.8

Singeleton: ENSXMAG00000014869 pcca

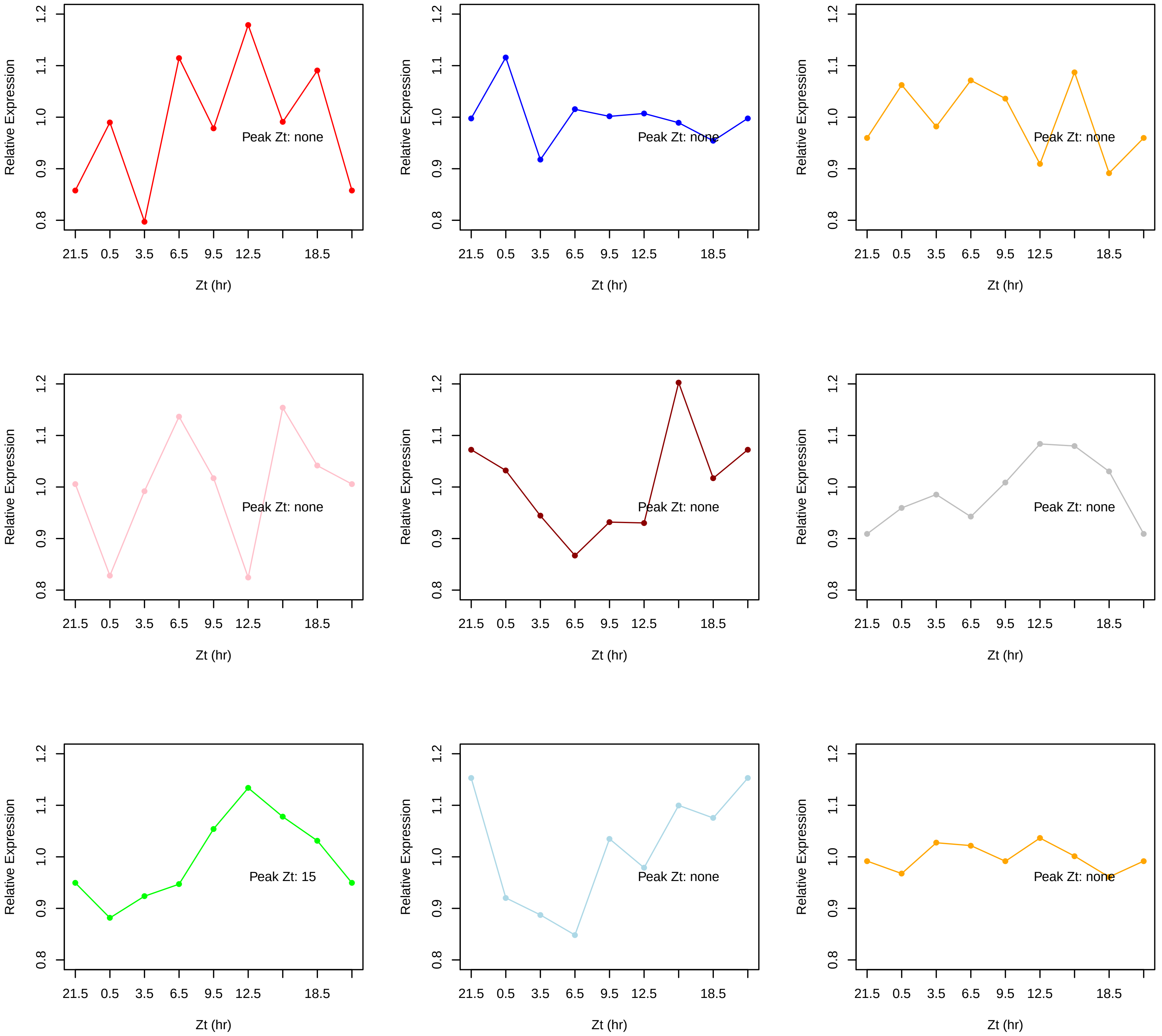

Singeleton: ENSXMAG00000005407 si:ch211−244a23.1

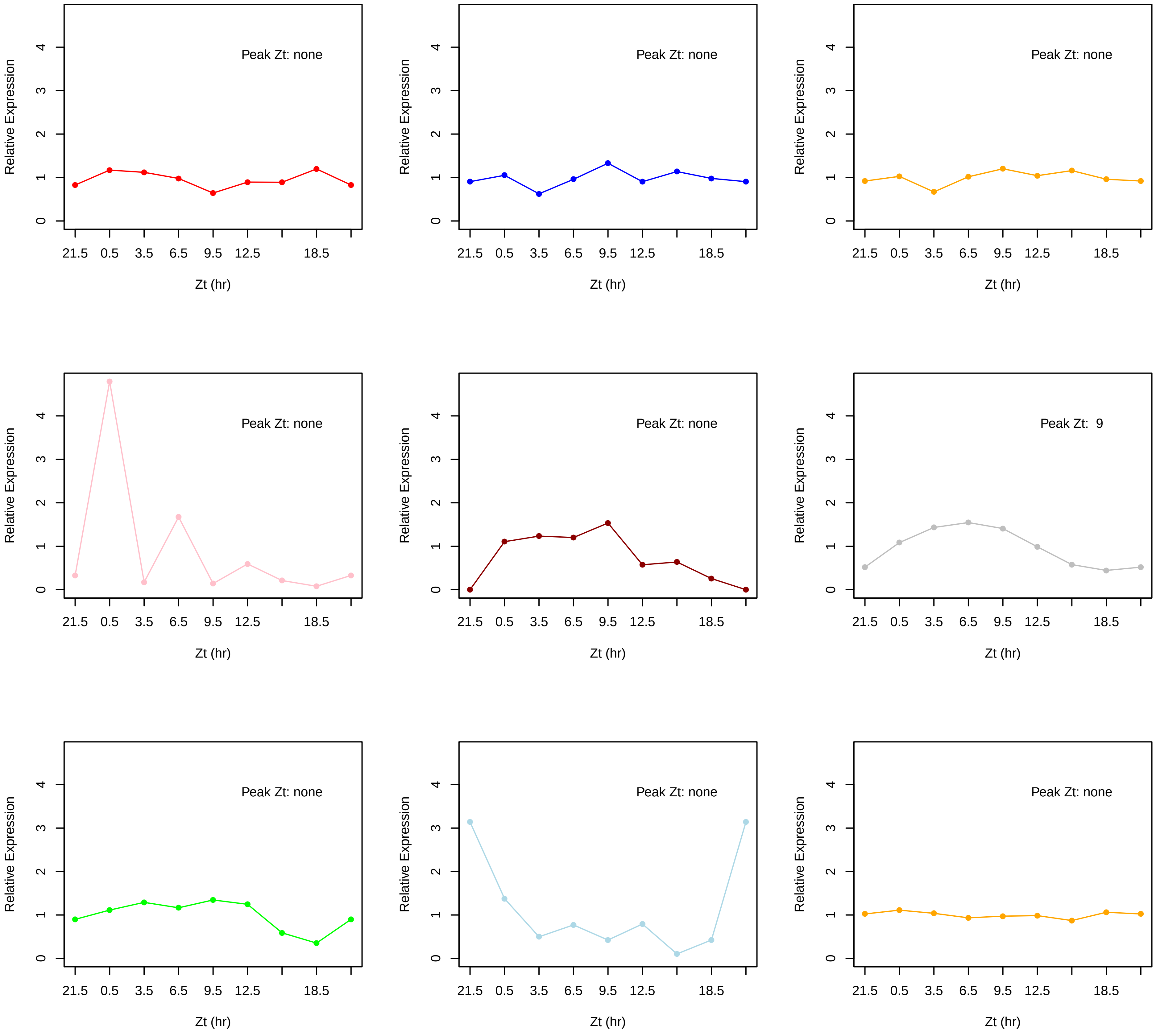

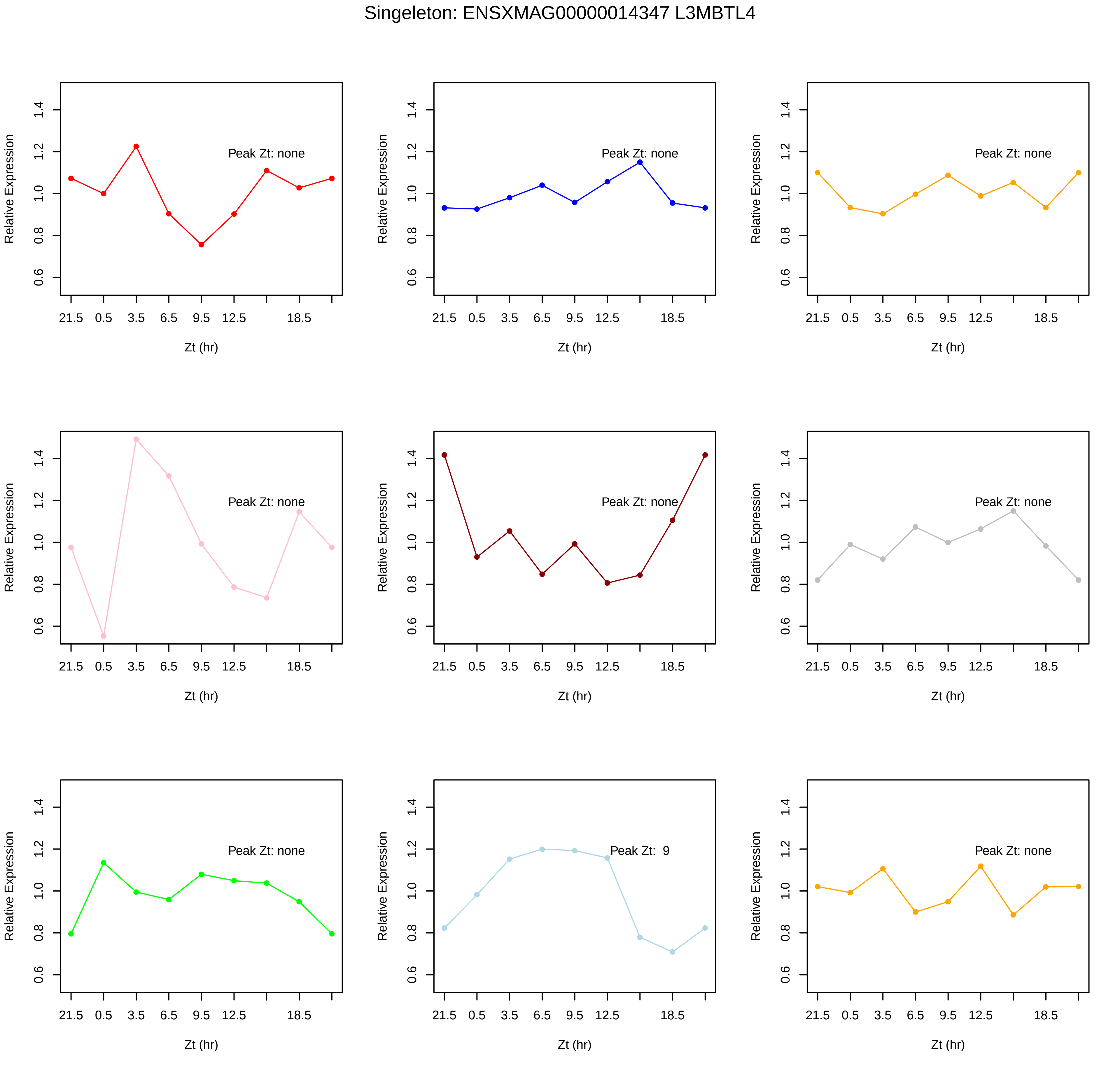

Singeleton: ENSXMAG00000014347 L3MBTL4
Relative Expression
Zt (hr)
Peak Zt: none
Peak Zt: 9
21.5
0.5
3.5
6.5
9.5
12.5
18.5
0.6
0.8
1.0
1.2
1.4

Singeleton: ENSXMAG00000014470 SCMH1

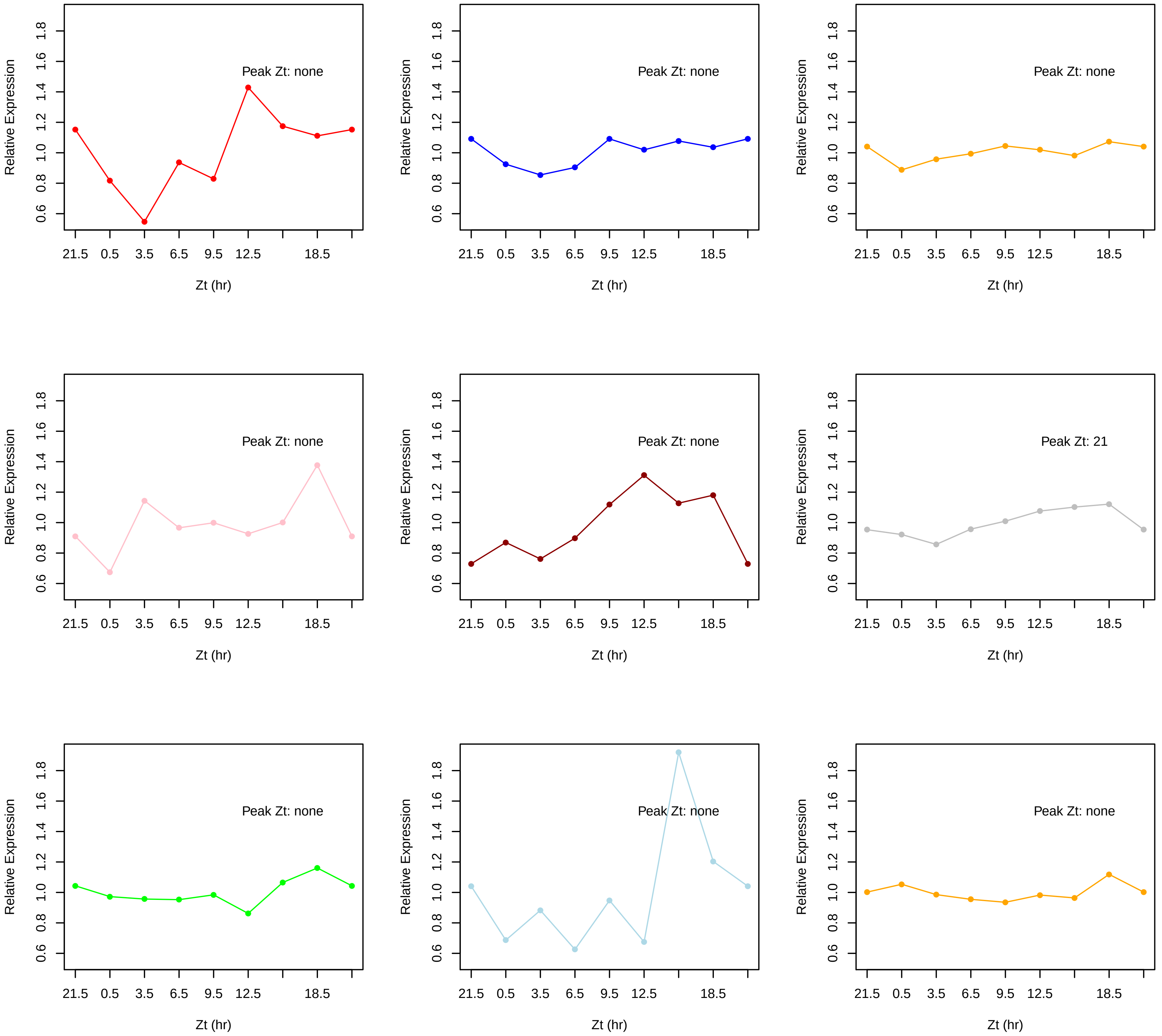

Singeleton: ENSXMAG00000015011 hectd3

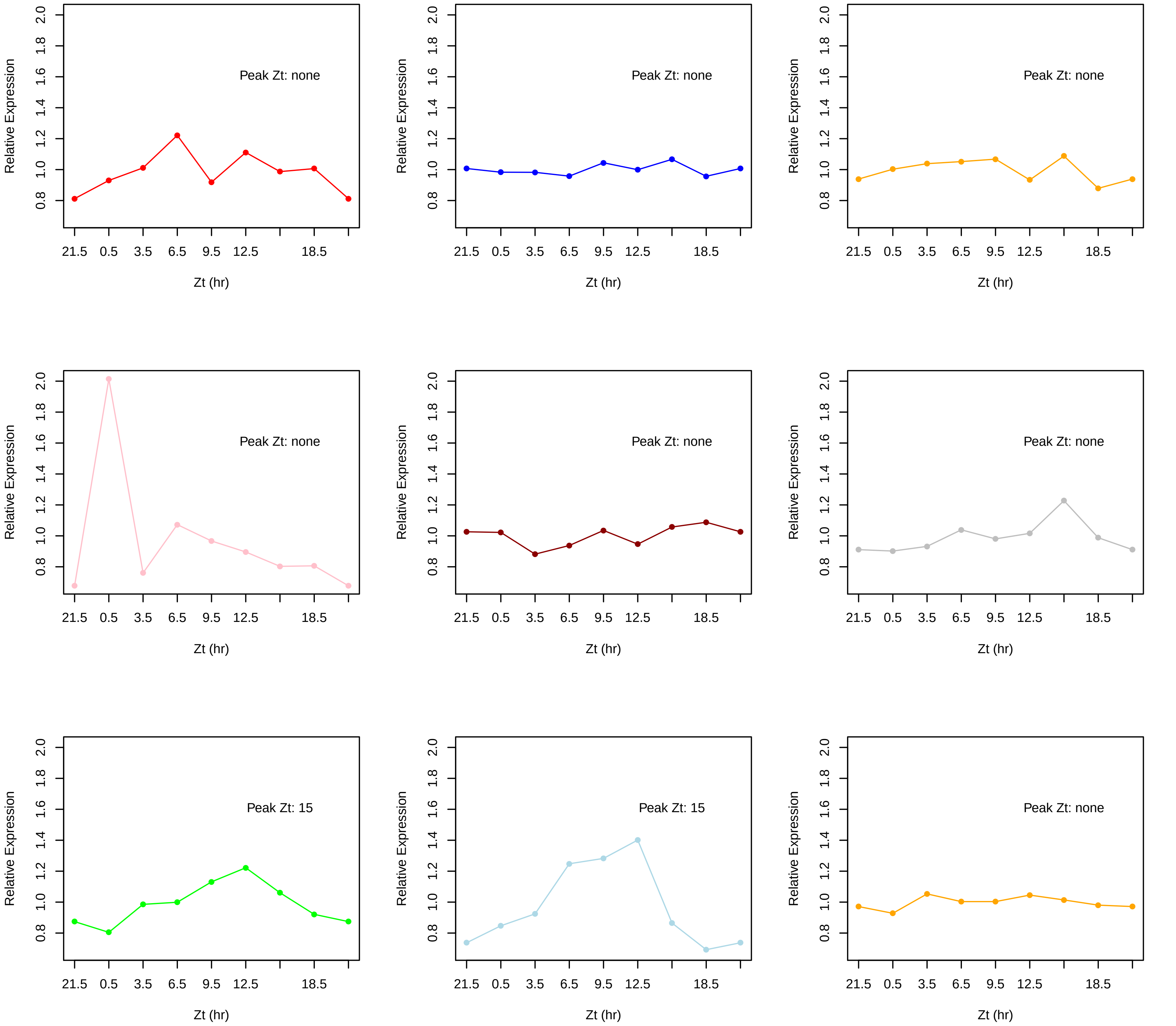

Singeleton: ENSXMAG00000025612 chmp1a

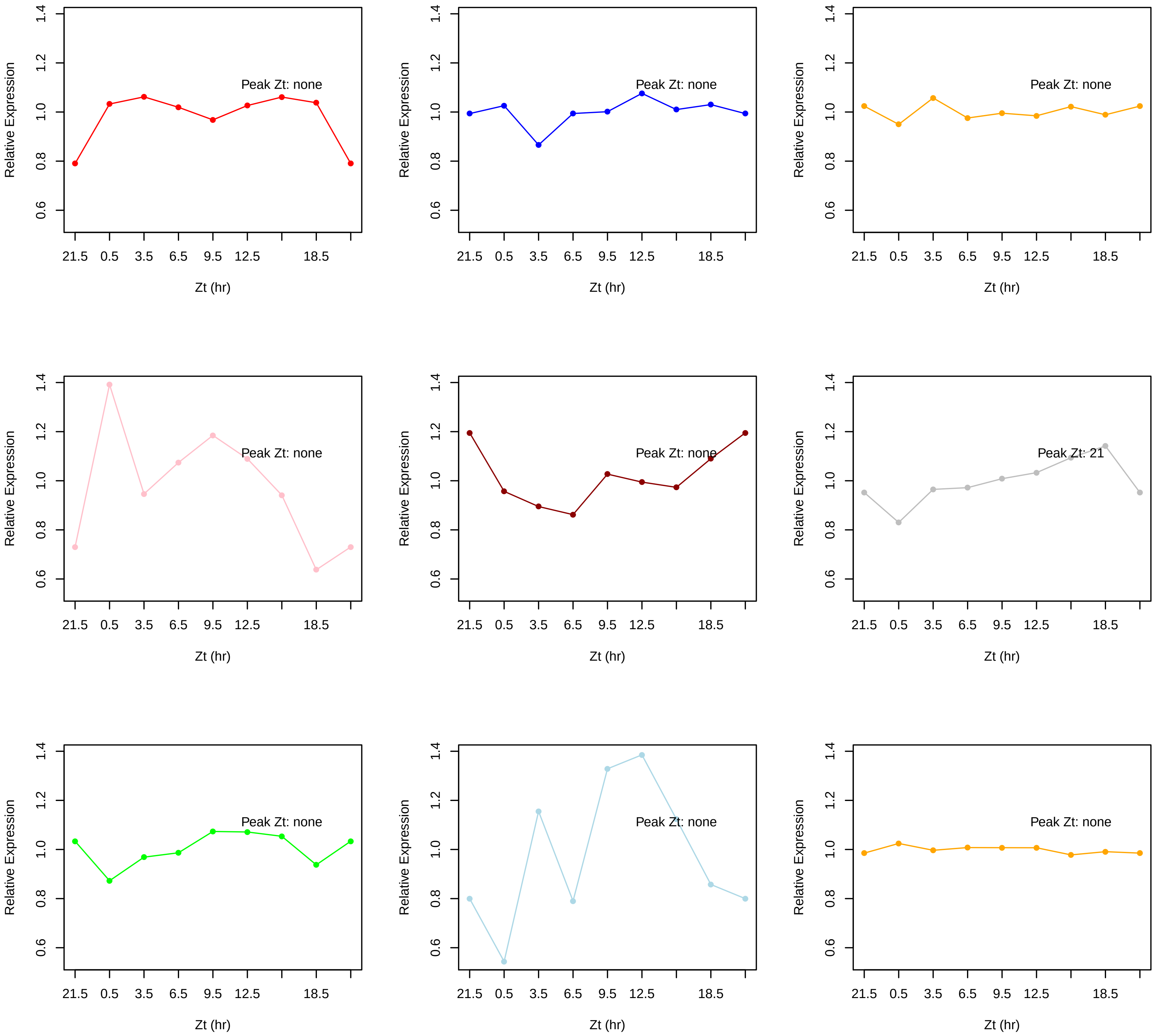

Singeleton: ENSXMAG00000005632 RNF43

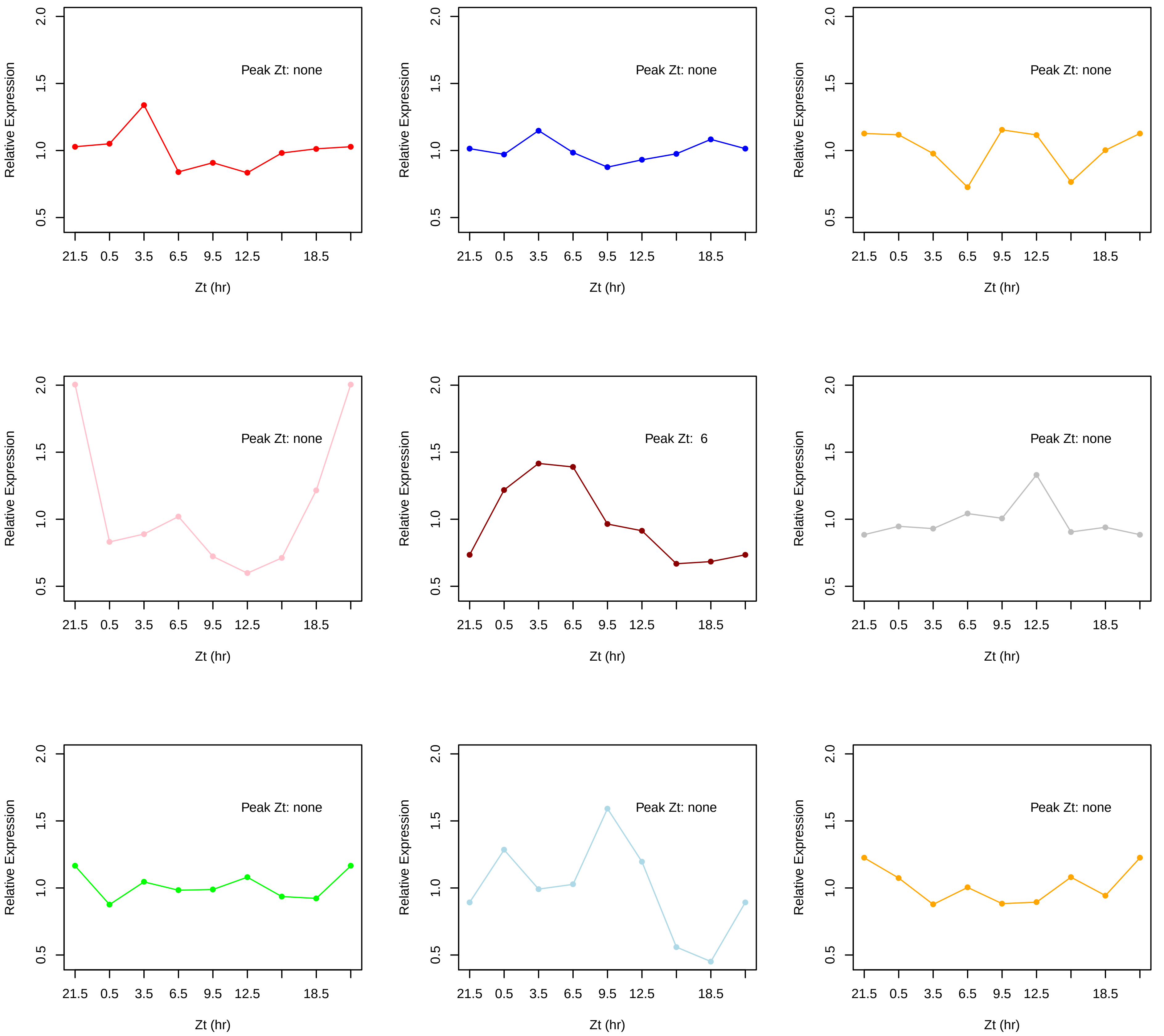

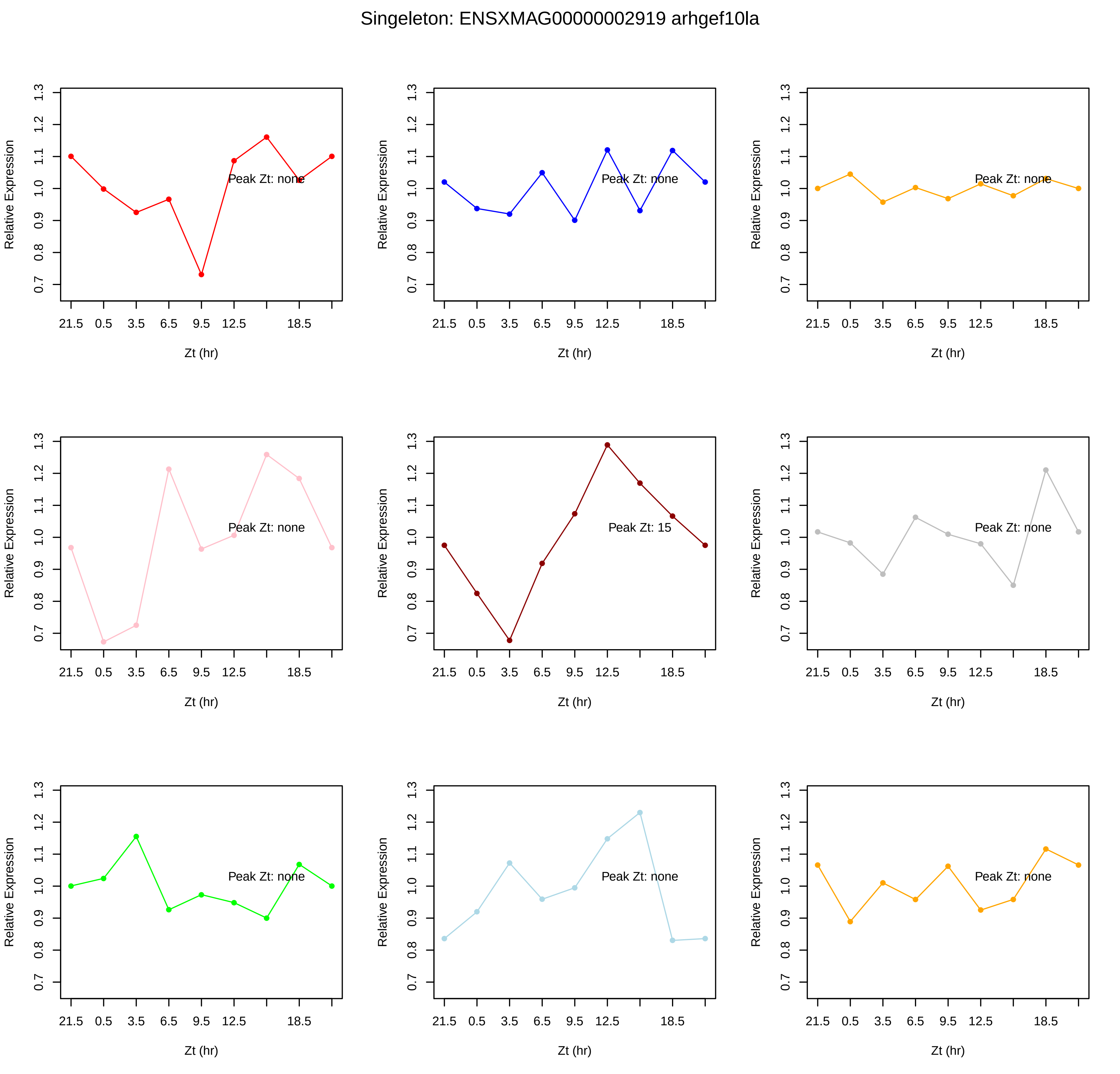
Singeleton: ENSXMAG00000002919 arhgef10la
Relative Expression
Zt (hr)
Peak Zt: none
Peak Zt: 15
21.5
0.5
3.5
6.5
9.5
12.5
18.5
0.7
0.8
0.9
1.0
1.1
1.2
1.3

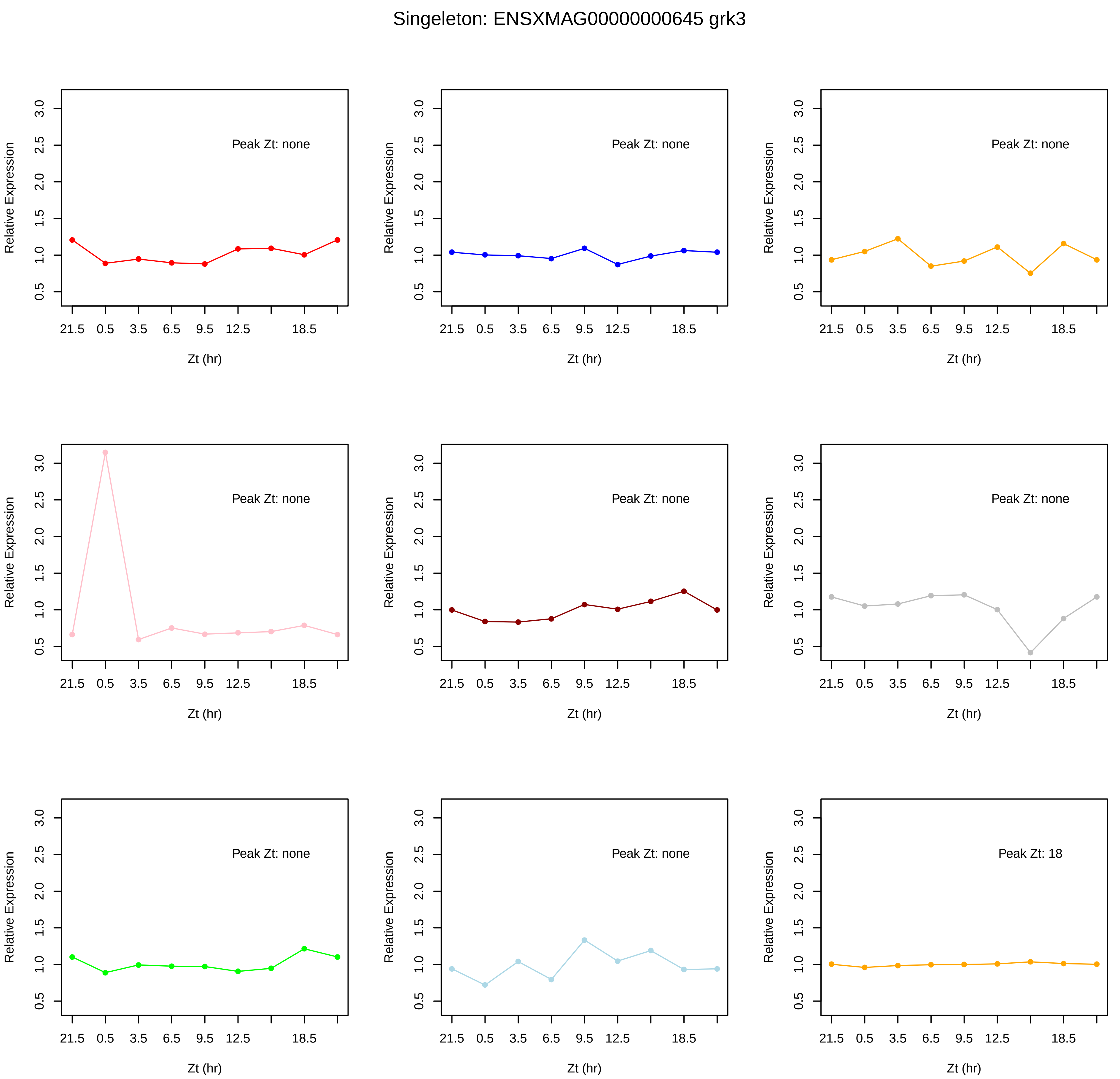
Singeleton: ENSXMAG00000000645 grk3
Peak Zt: none
Peak Zt: none
Peak Zt: none
Peak Zt: none
Peak Zt: none
Peak Zt: none
Peak Zt: none
Peak Zt: none
Peak Zt: 18
Relative Expression
Zt (hr)
21.5
0.5
3.5
6.5
9.5
12.5
18.5

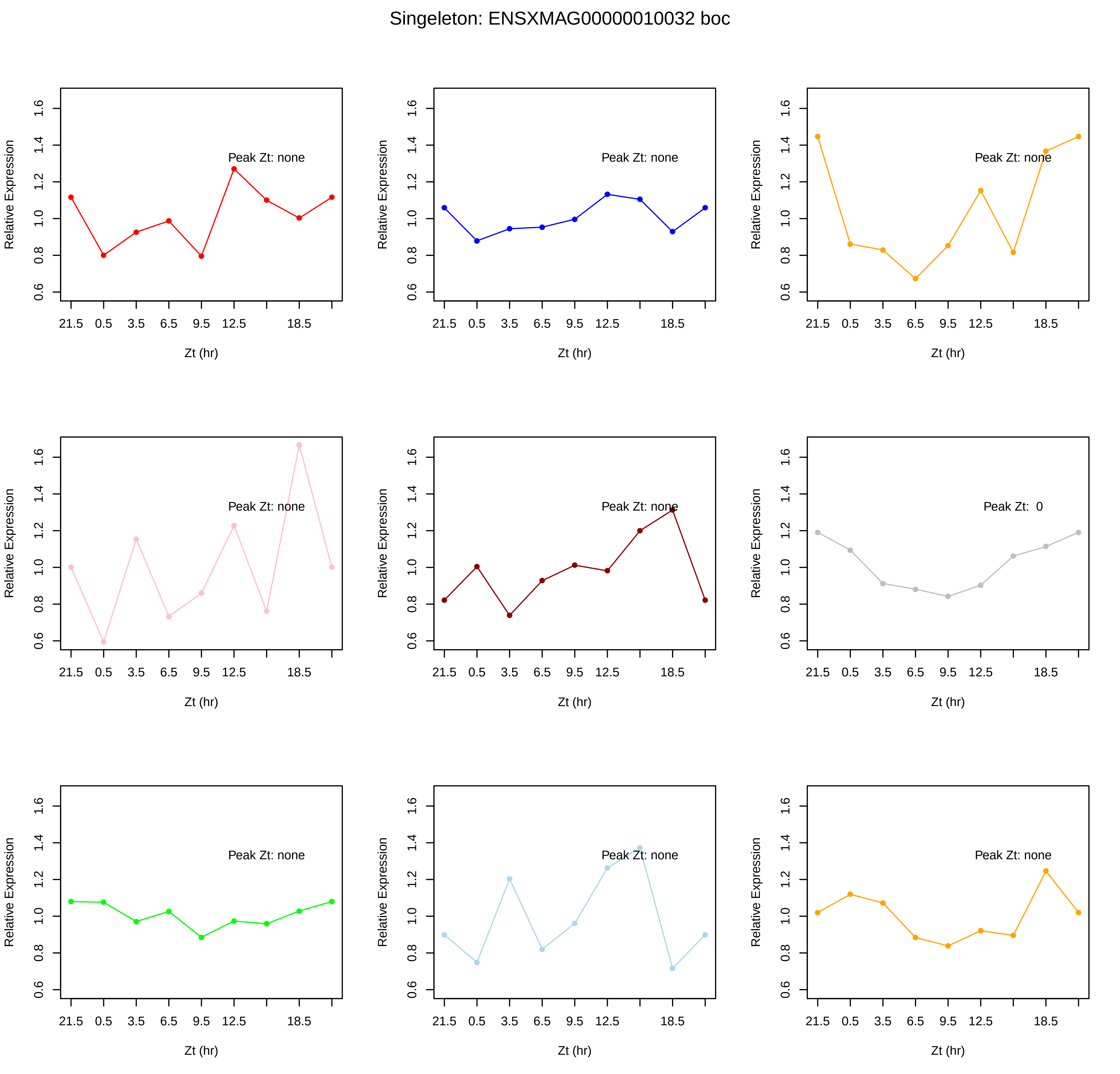

Singeleton: ENSXMAG00000010032 boc
Peak Zt: none
Peak Zt: none
Peak Zt: none
Peak Zt: none
Peak Zt: none
Peak Zt: 0
Peak Zt: none
Peak Zt: none
Peak Zt: none
Relative Expression
Zt (hr)
21.5 0.5 3.5 6.5 9.5 12.5 18.5
0.6 0.8 1.0 1.2 1.4 1.6

Singleton: ENSXMAG00000029804

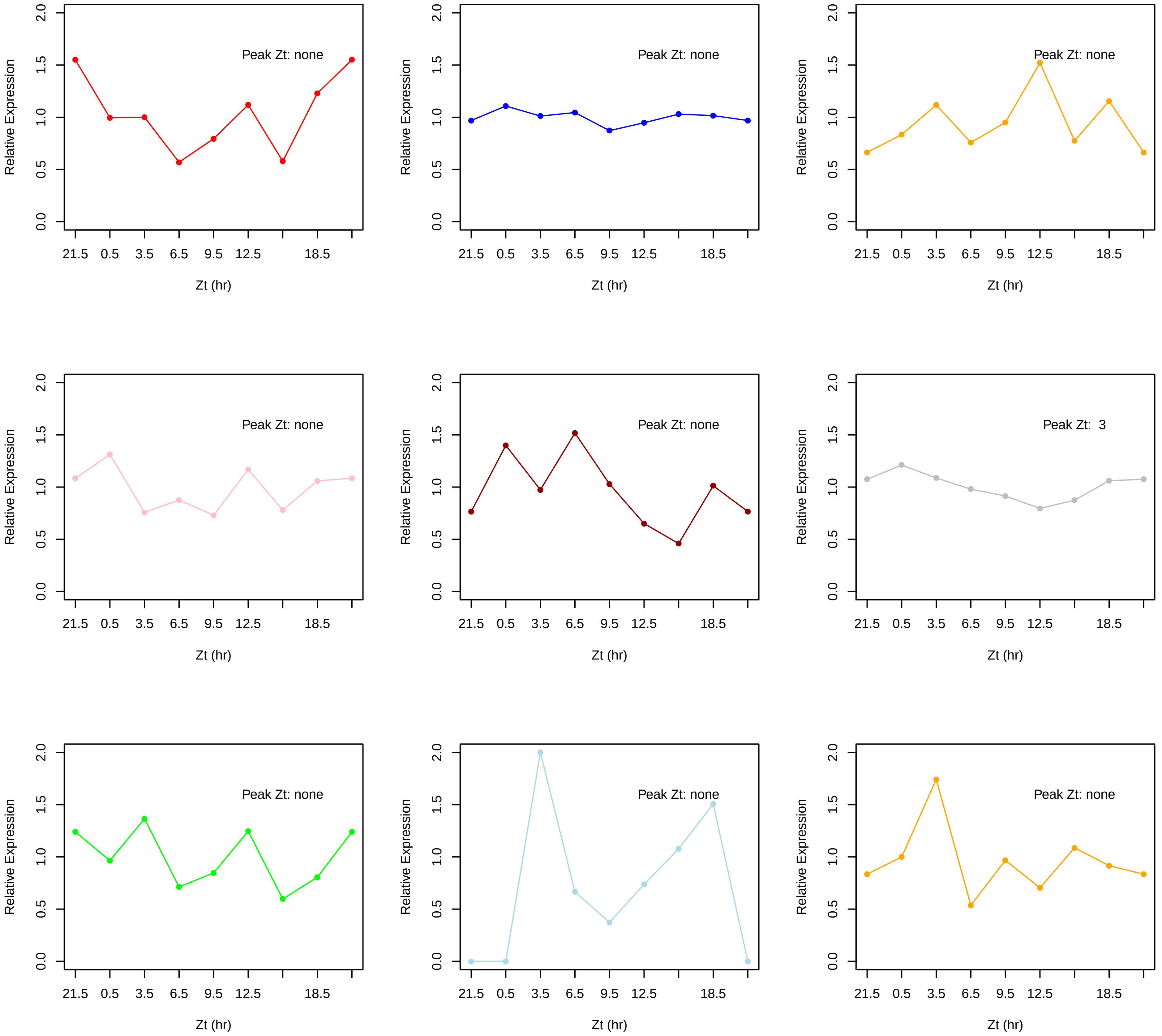

Singeleton: ENSXMAG00000001633 dnajc25

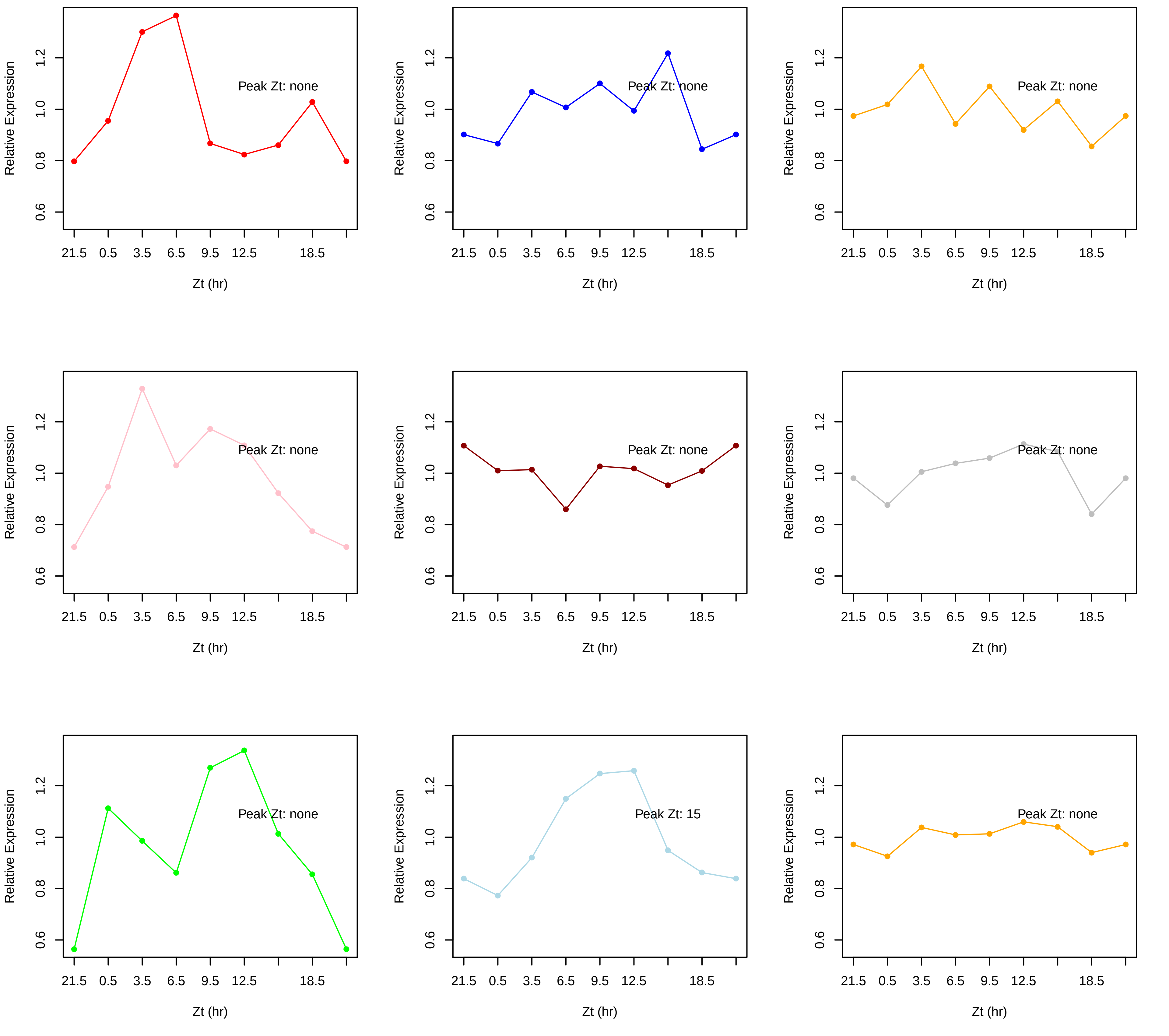

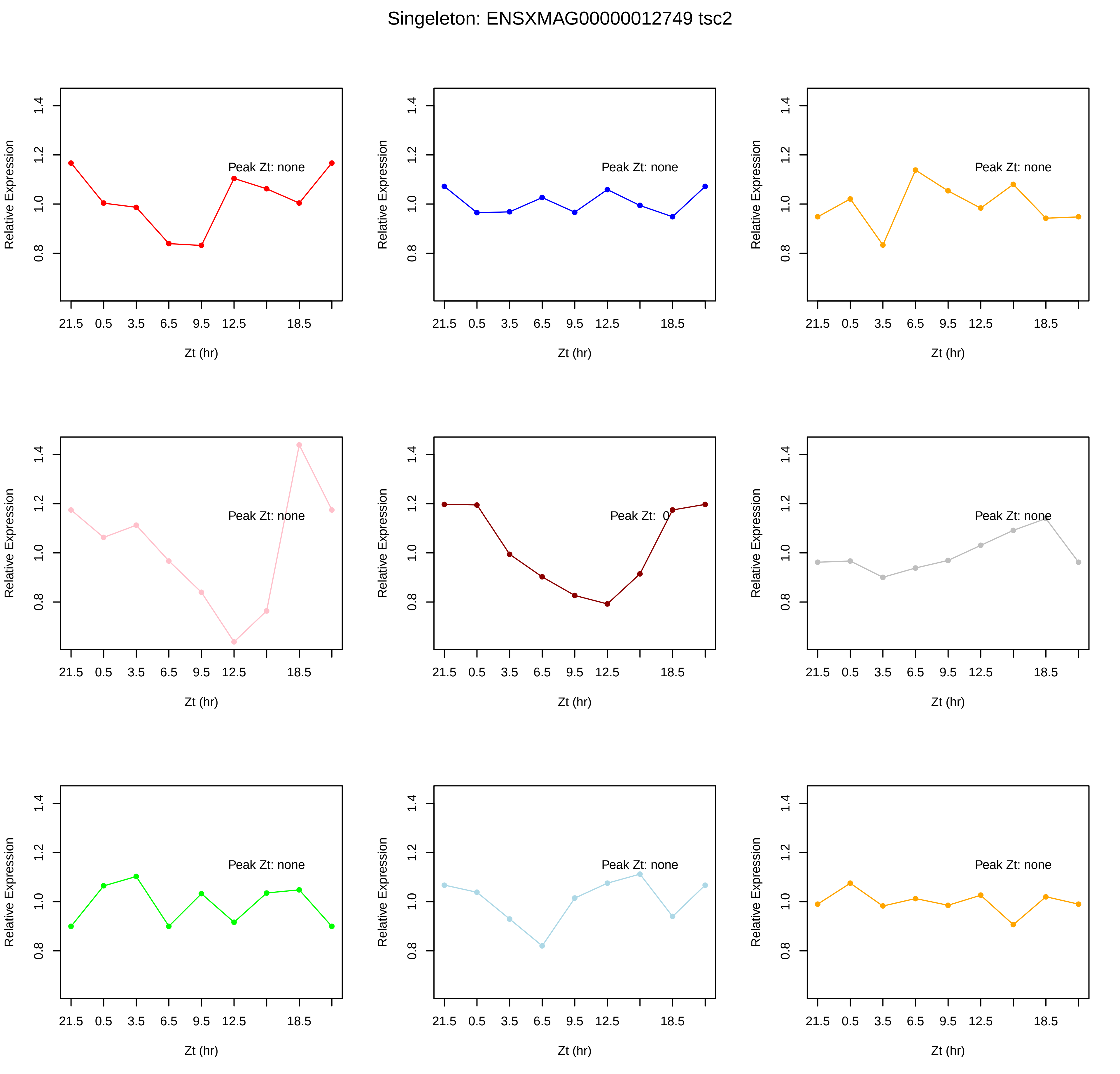

Singeleton: ENSXMAG00000012749 tsc2
Relative Expression
Zt (hr)
Peak Zt: none
Peak Zt: 0
21.5
0.5
3.5
6.5
9.5
12.5
18.5
0.8
1.0
1.2
1.4

Singeleton: ENSXMAG00000014481 med18

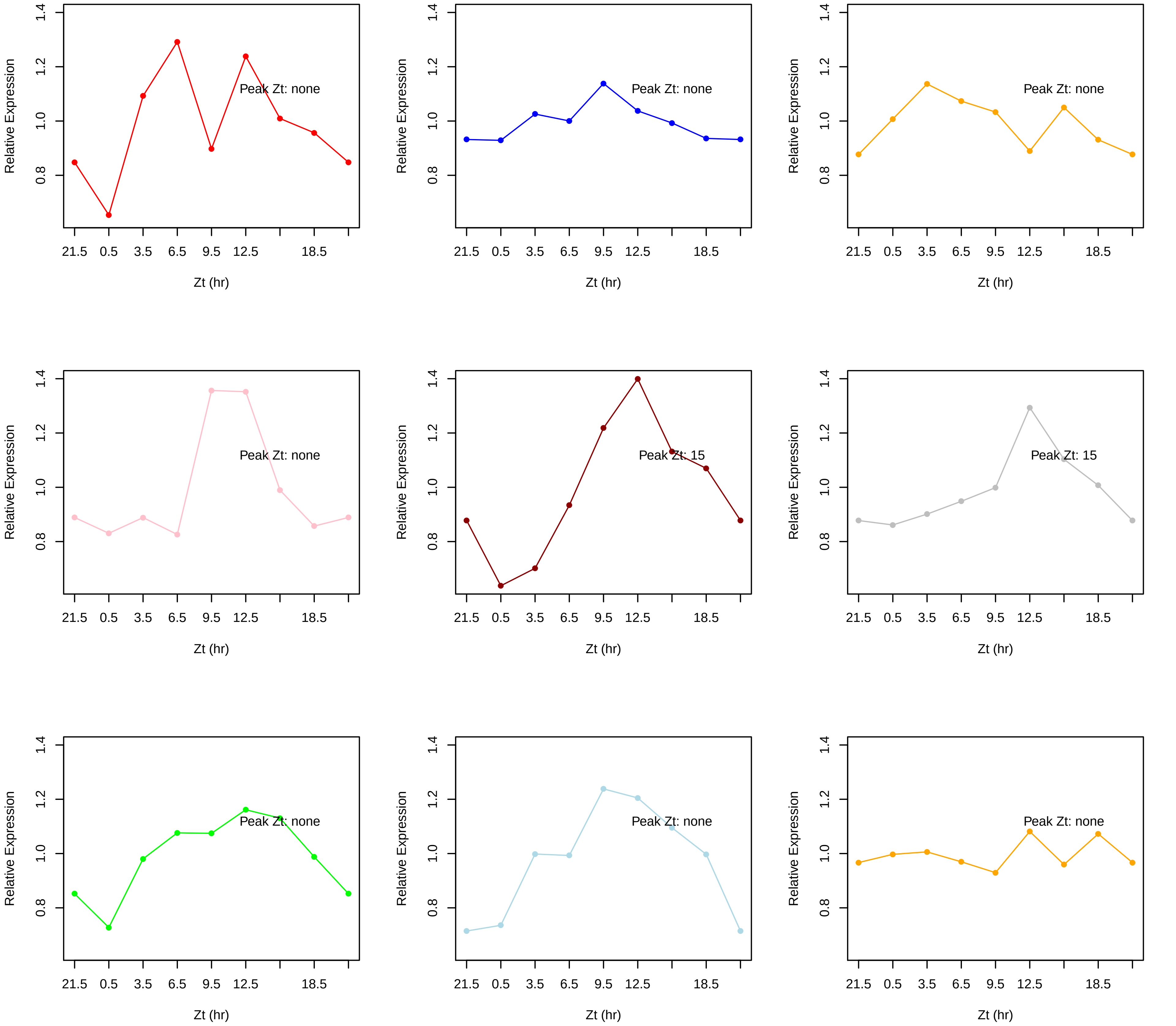

Singleton: ENSXMAG00000005196 ythdc1

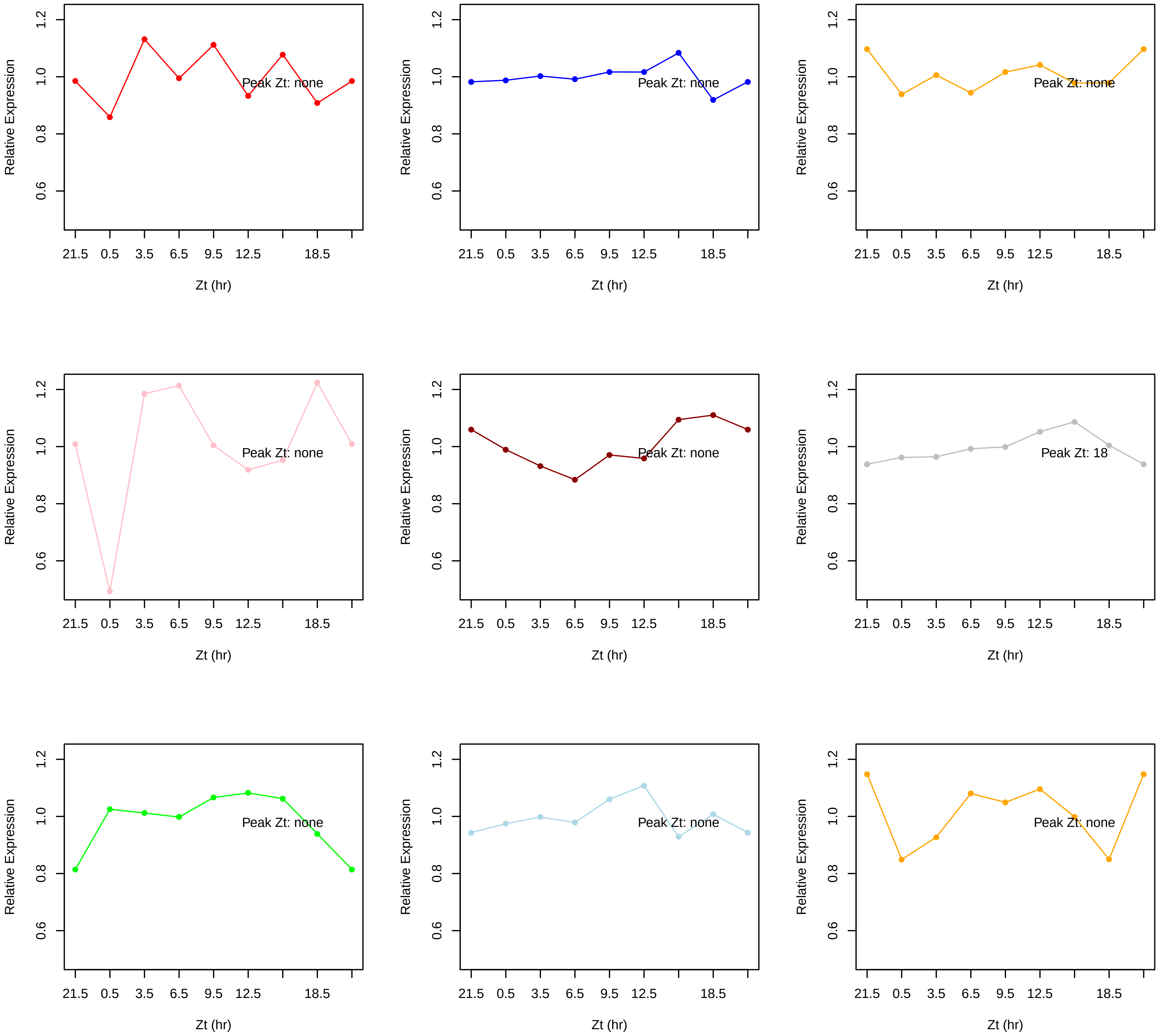

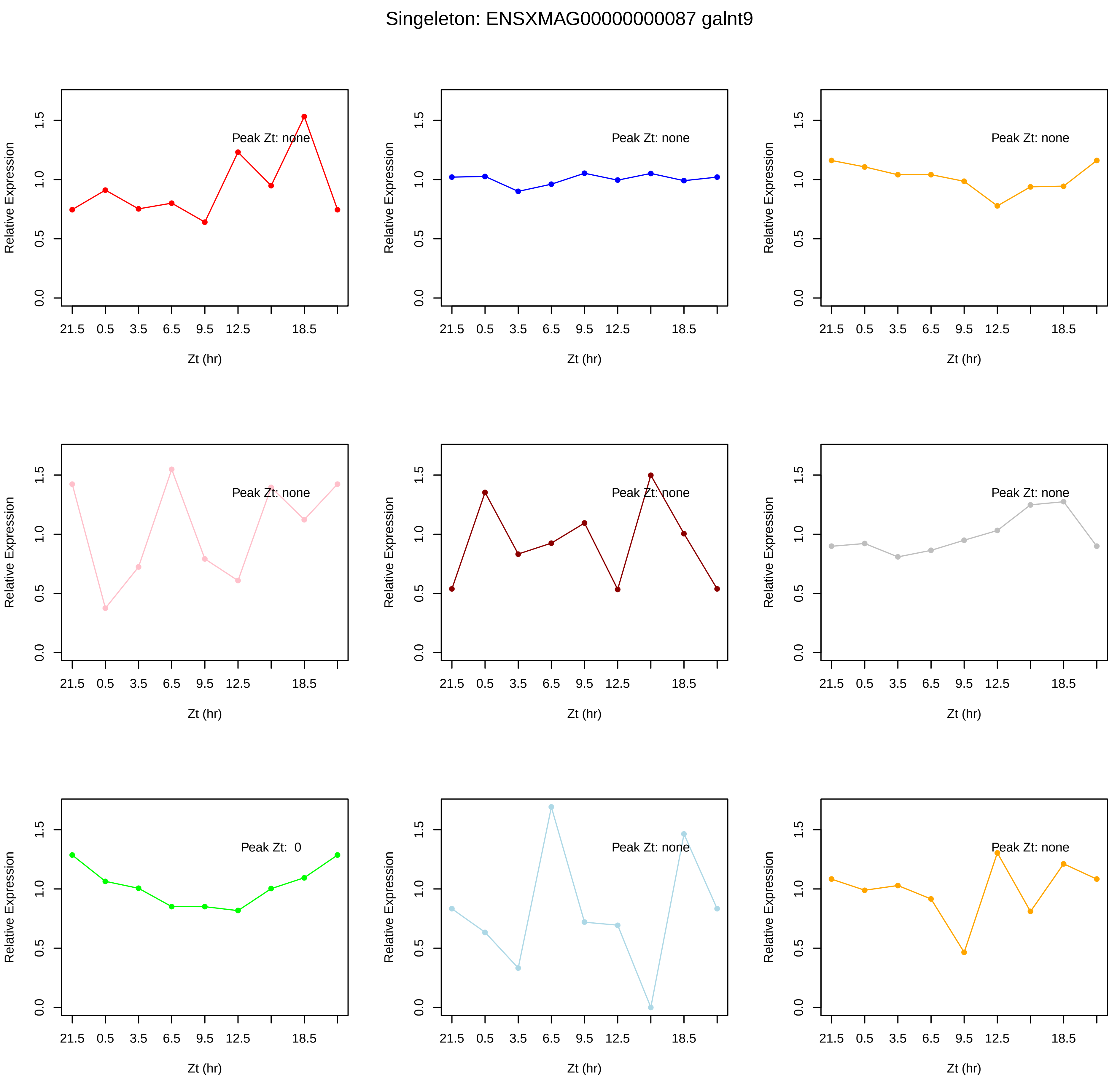

Singleton: ENSXMAG00000000087 galnt9
Peak Zt: none
Peak Zt: none
Peak Zt: none
Peak Zt: none
Peak Zt: none
Peak Zt: none
Peak Zt: 0
Peak Zt: none
Peak Zt: none
Relative Expression
Zt (hr)
21.5
0.5
3.5
6.5
9.5
12.5
18.5
0.0
0.5
1.0
1.5

Singeleton: ENSXMAG00000017079

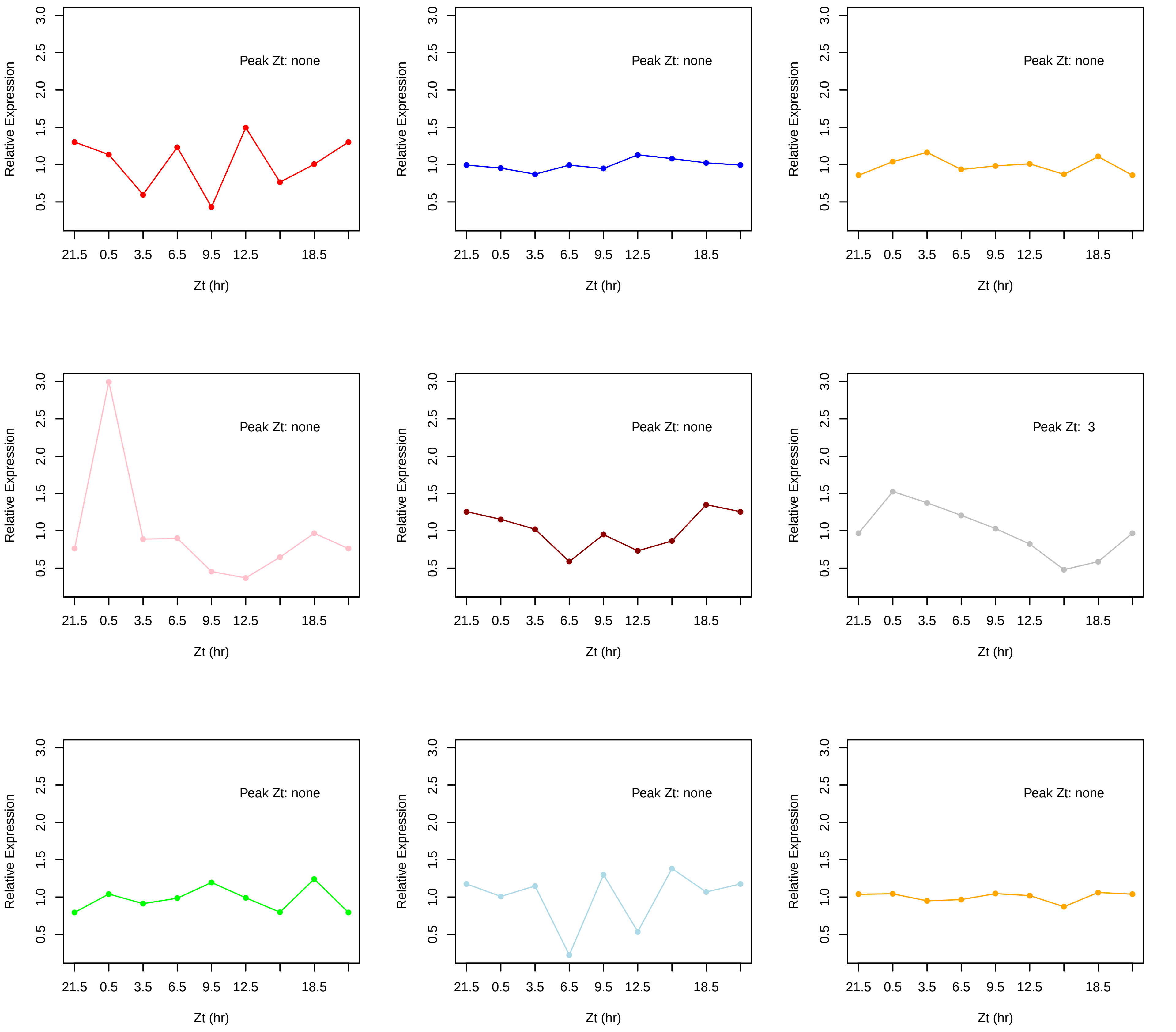

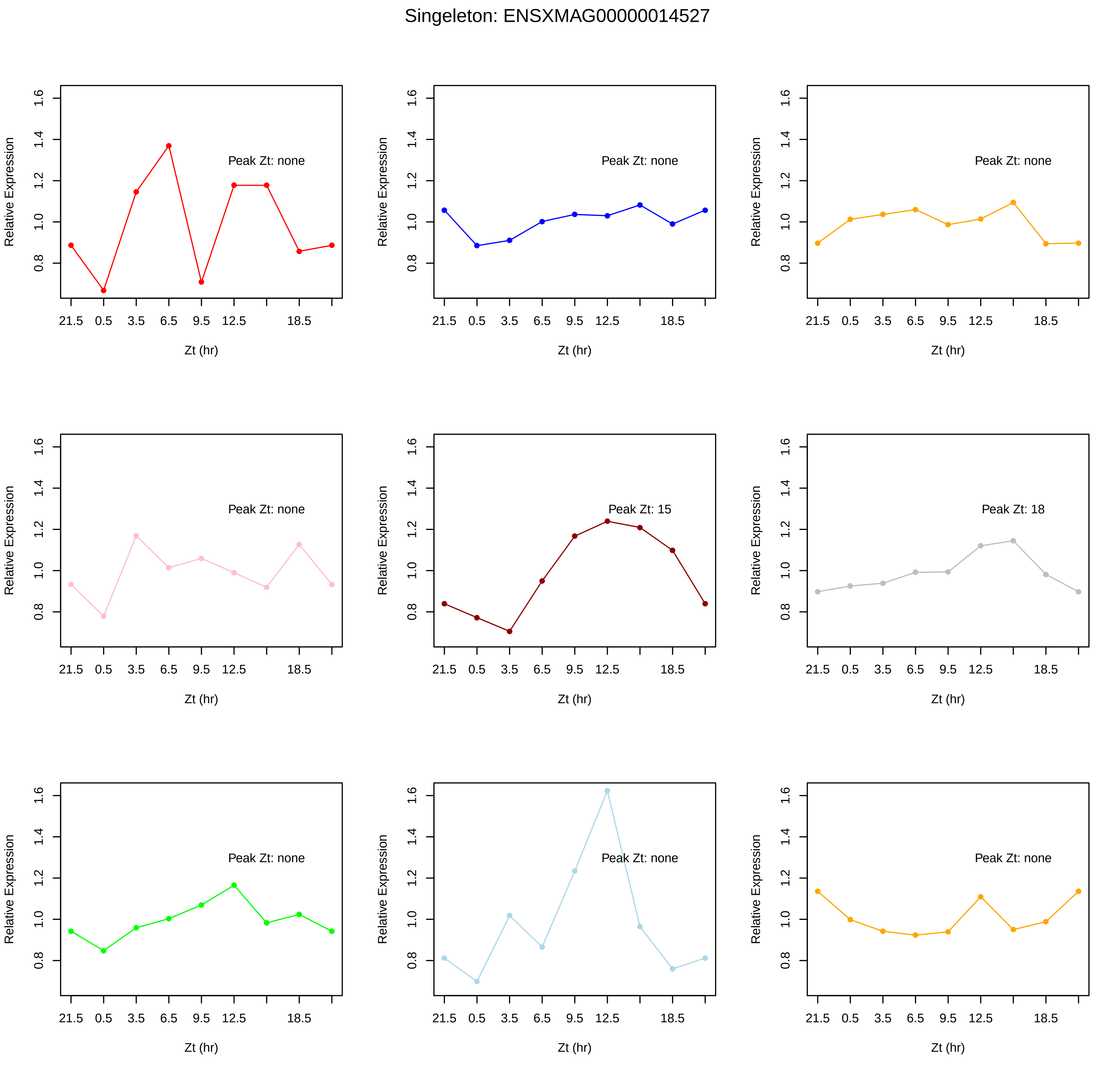

Singeleton: ENSXMAG00000014527
Peak Zt: none
Peak Zt: none
Peak Zt: none
Peak Zt: none
Peak Zt: 15
Peak Zt: 18
Peak Zt: none
Peak Zt: none
Peak Zt: none
Relative Expression
Zt (hr)
21.5
0.5
3.5
6.5
9.5
12.5
18.5
0.8
1.0
1.2
1.4
1.6

Singeleton: ENSXMAG00000007085 eny2

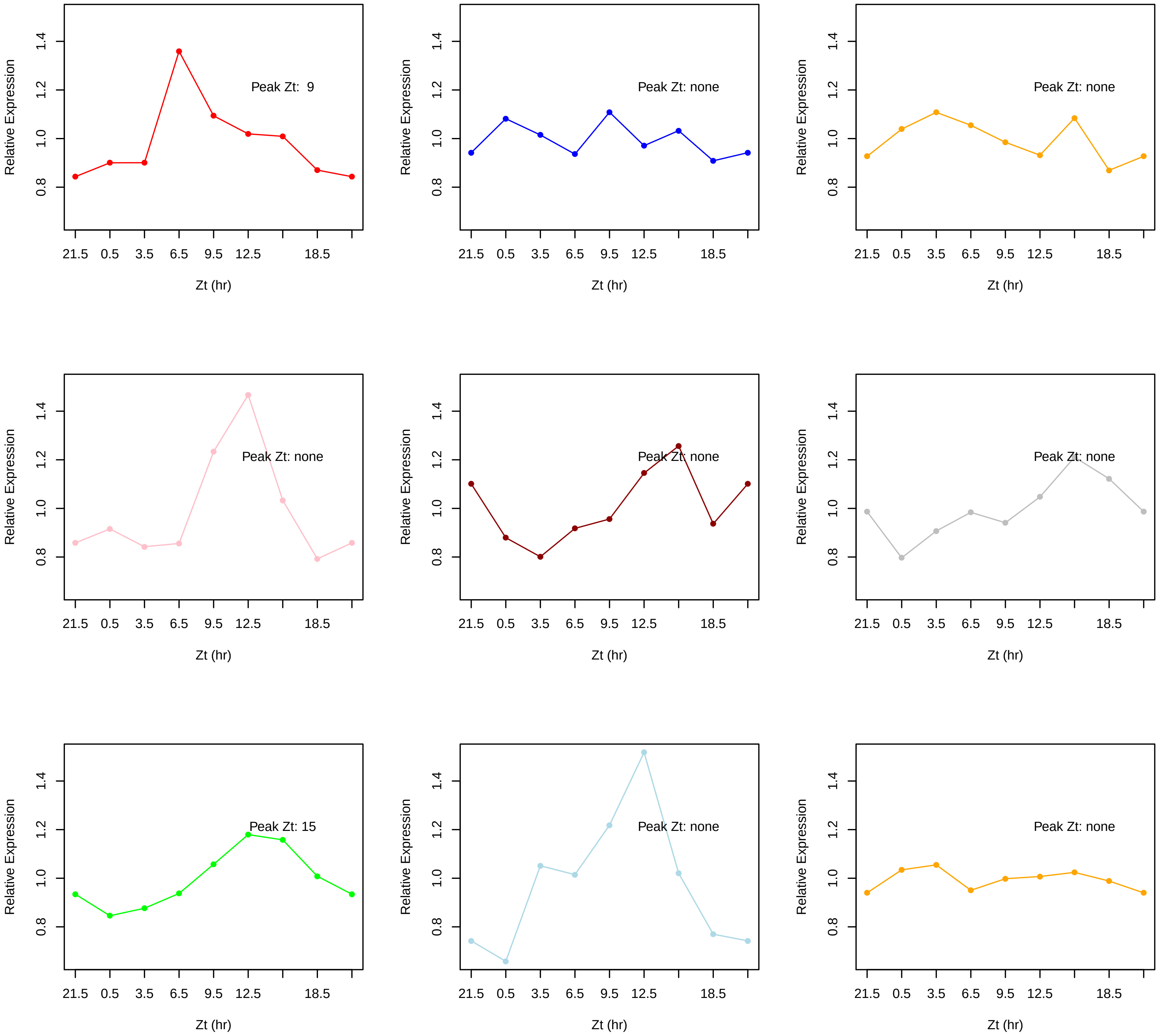

Singeleton: ENSXMAG00000012198 zgc:56106

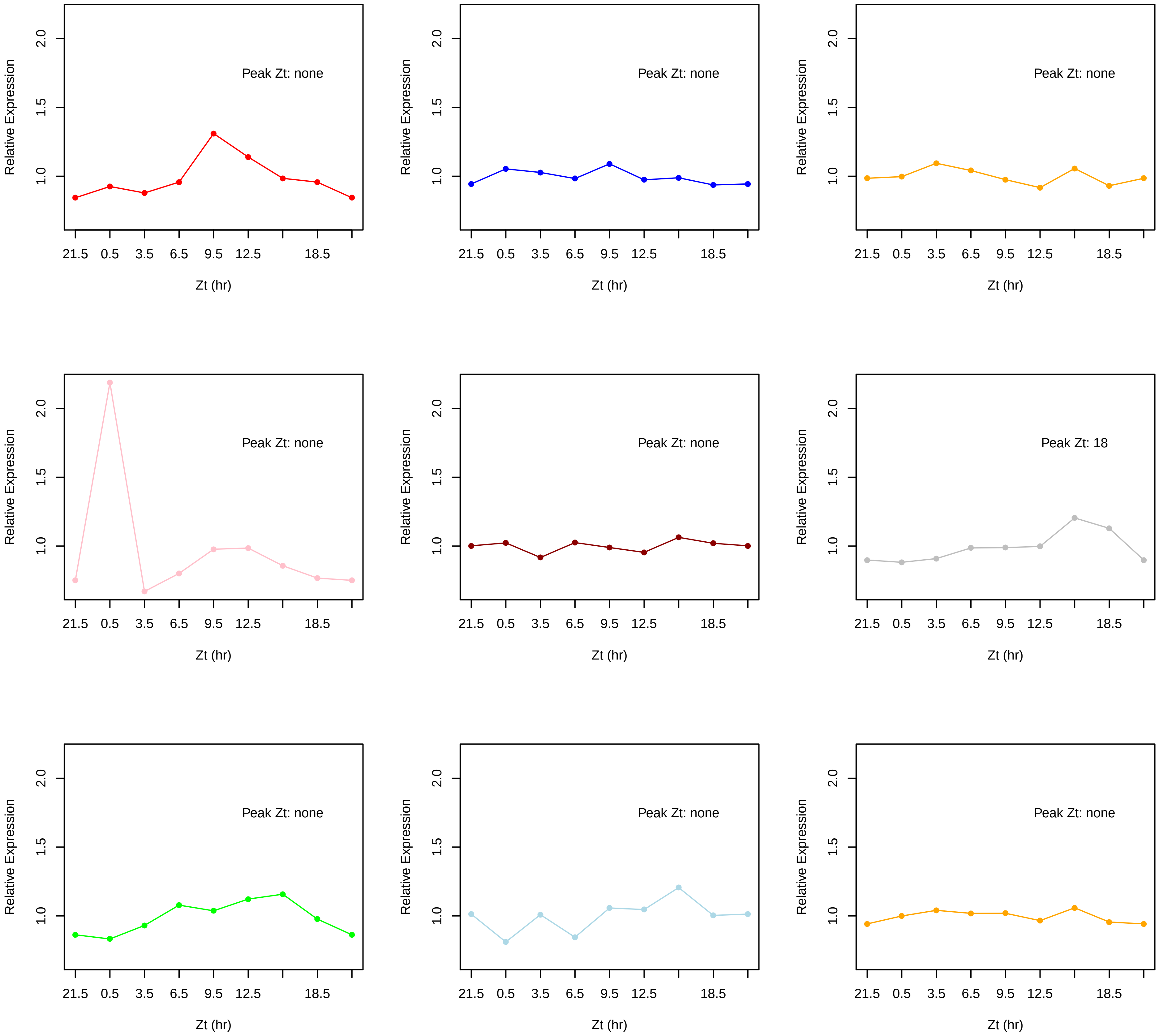

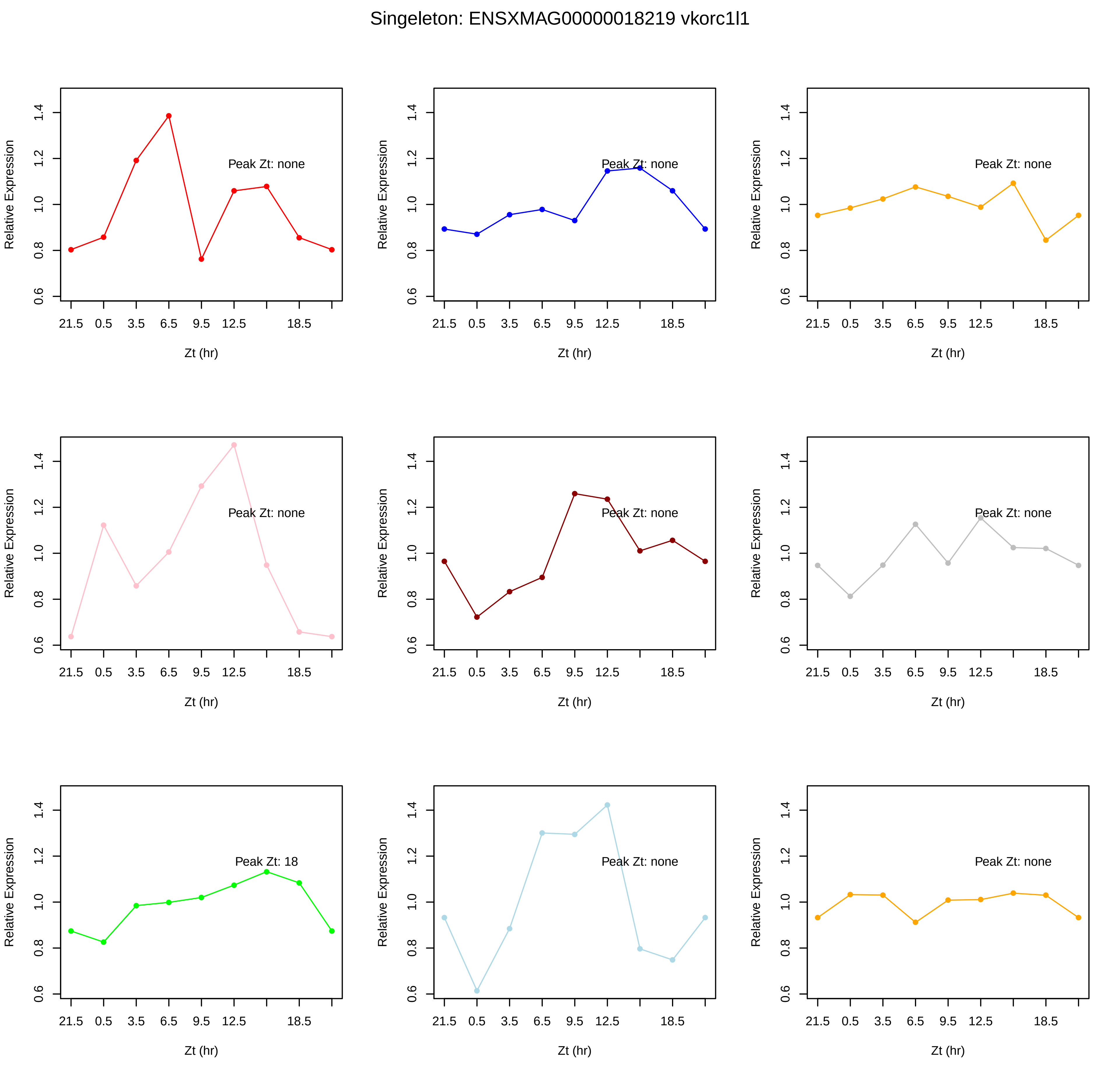

Singeleton: ENSXMAG00000018219 vkorc1l1
Relative Expression
Zt (hr)
Peak Zt: none
Peak Zt: 18
0.6
0.8
1.0
1.2
1.4
21.5
0.5
3.5
6.5
9.5
12.5
18.5

Singeleton: ENSXMAG00000005085 nudcd1

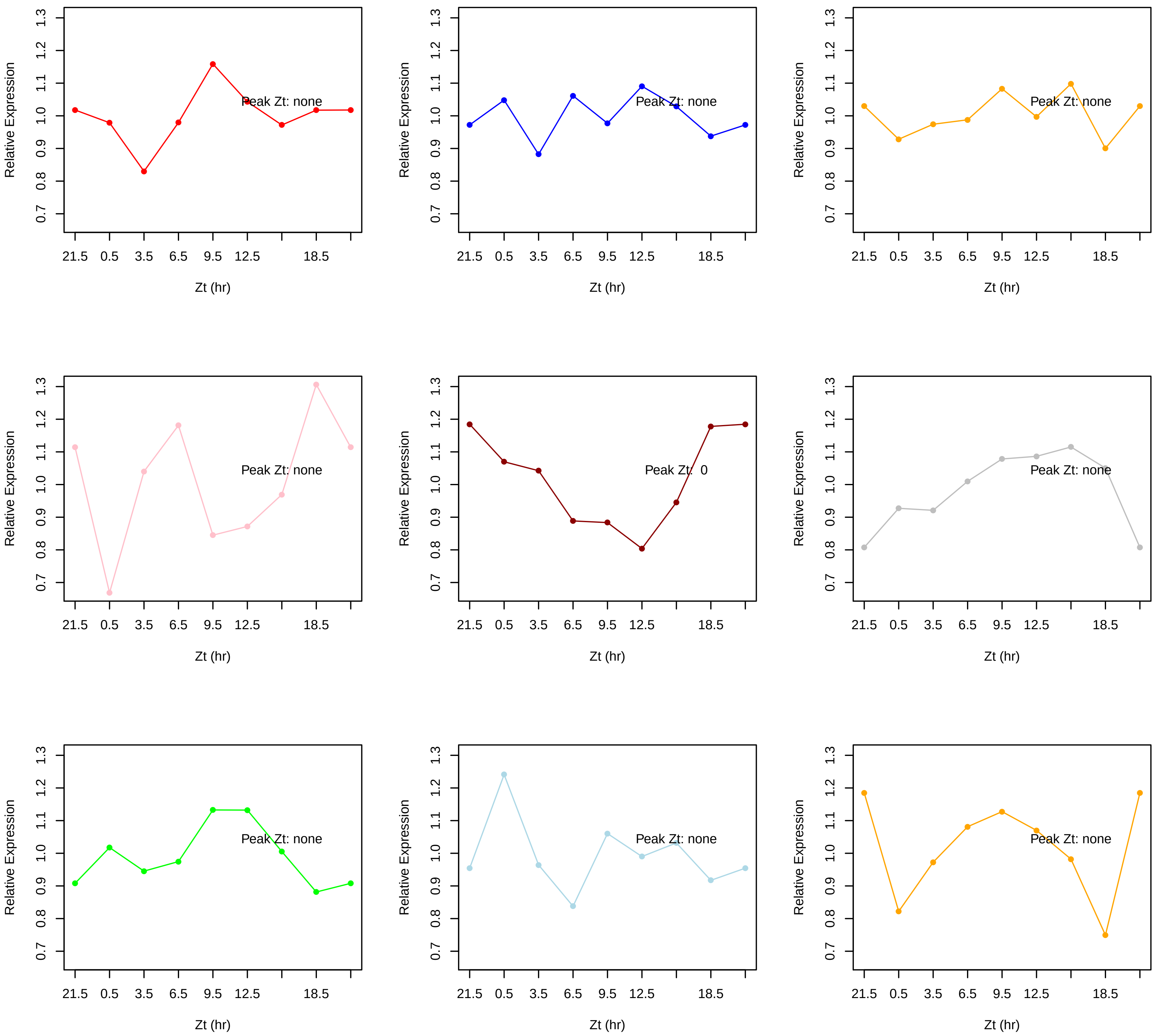

Singleton: ENSXMAG00000010052 naa50

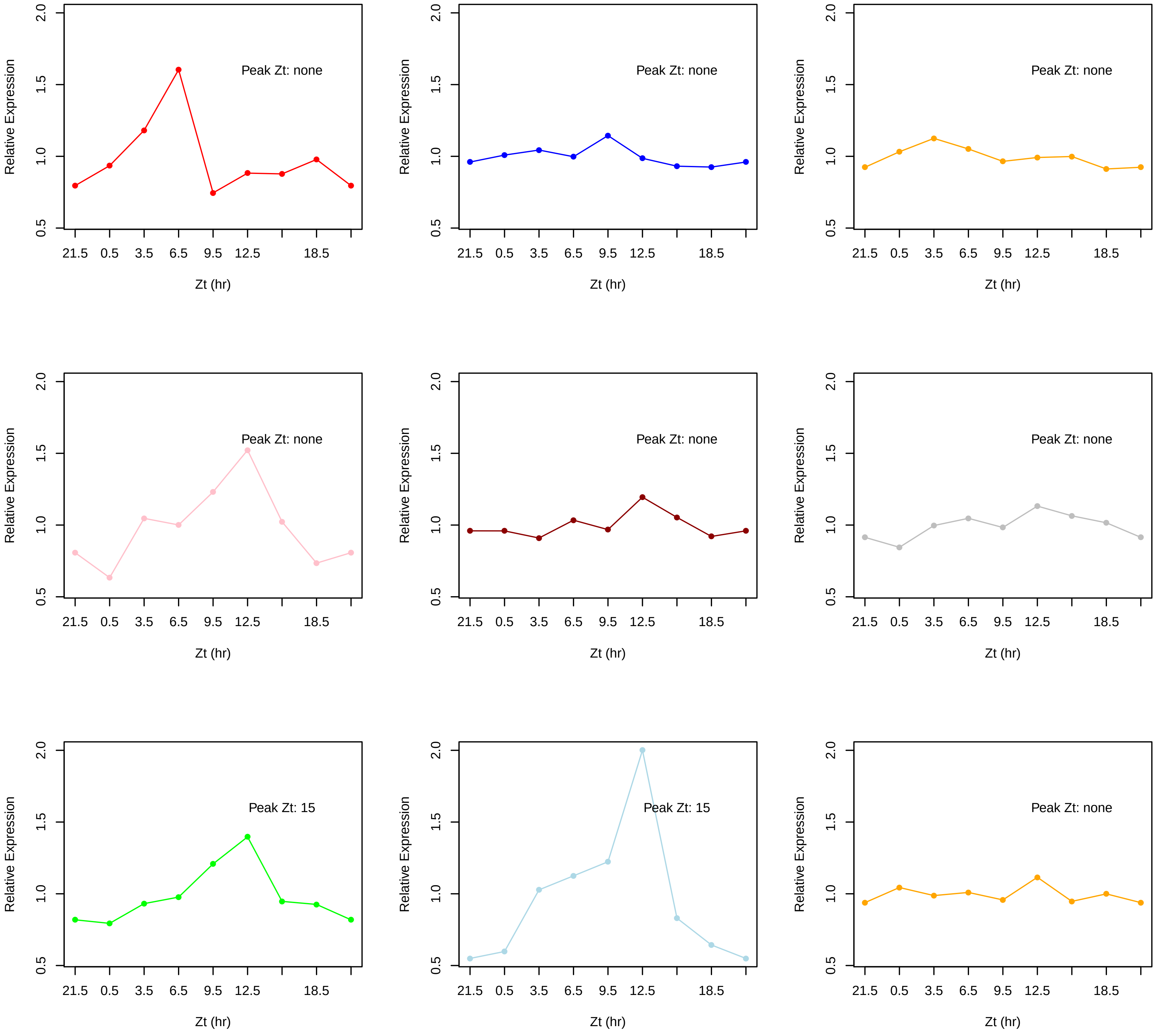

Singleton: ENSXMAG00000002684 si:dkey−174n20.1

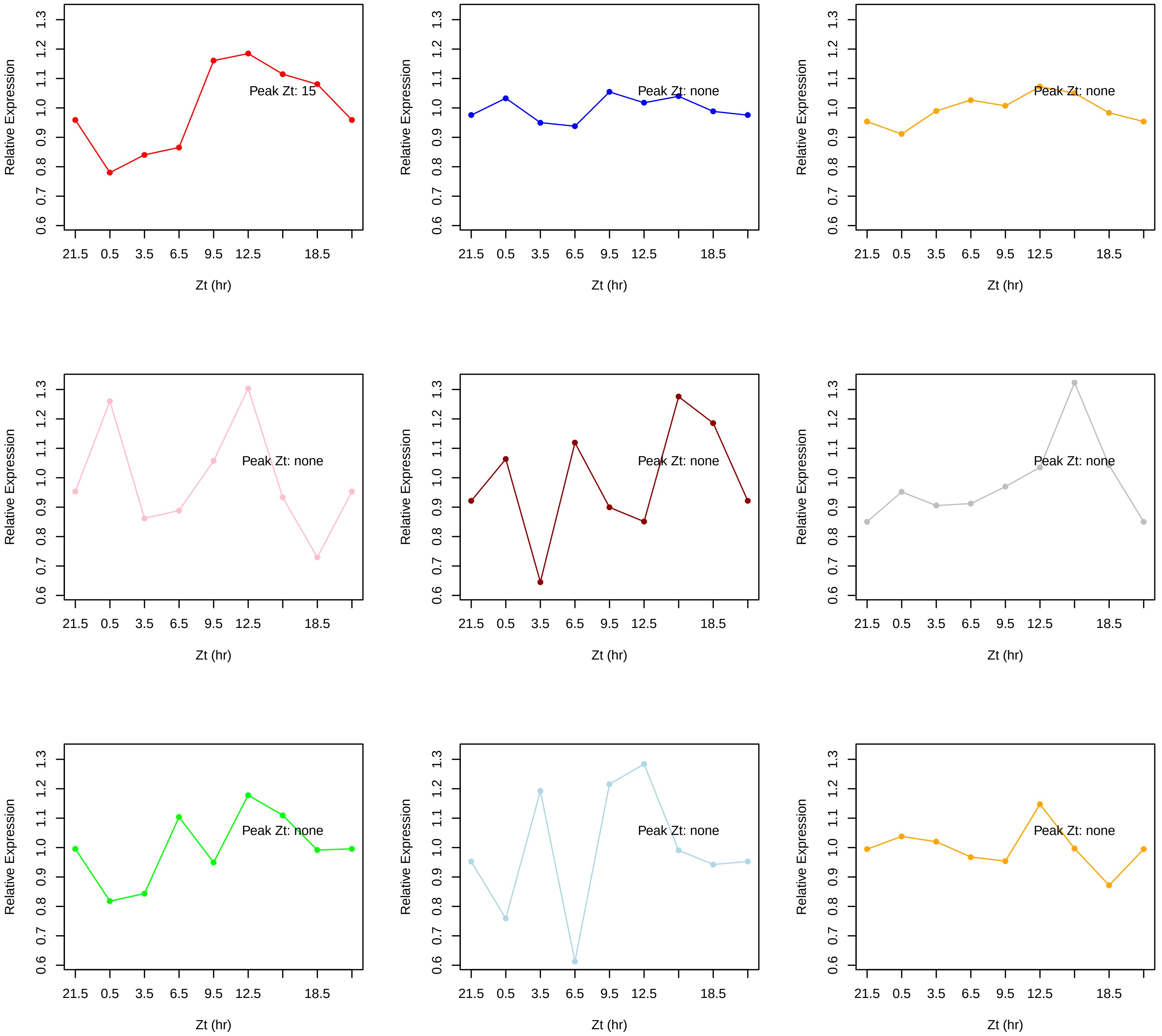

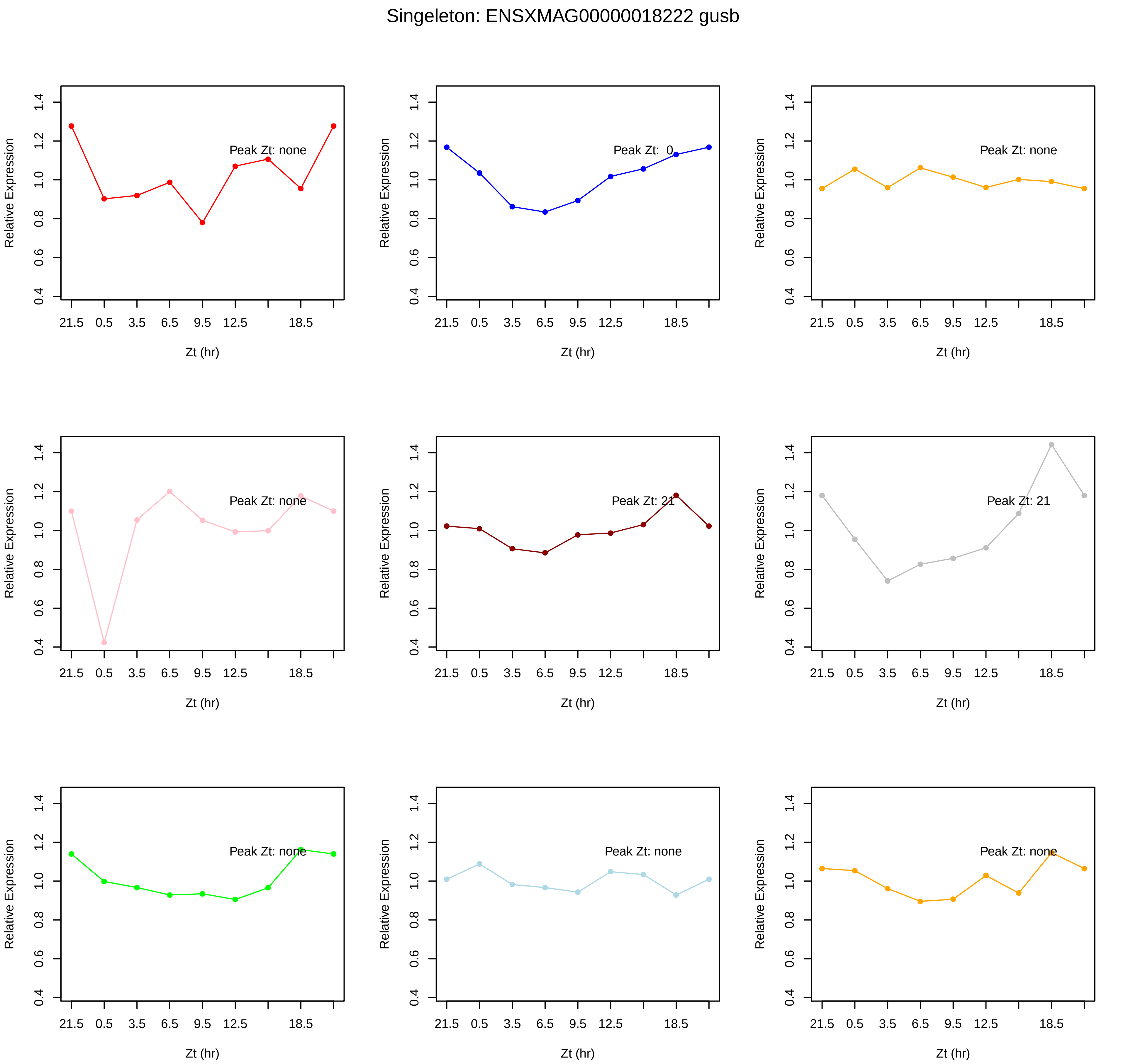
Singeleton: ENSXMAG00000018222 gusb
Relative Expression
Zt (hr)
21.5 0.5 3.5 6.5 9.5 12.5 18.5
0.4 0.6 0.8 1.0 1.2 1.4
Peak Zt: none
Peak Zt: 0
Peak Zt: none
Peak Zt: none
Peak Zt: 21
Peak Zt: 21
Peak Zt: none
Peak Zt: none
Peak Zt: none

Singleton: ENSXMAG00000007953 lmnb2

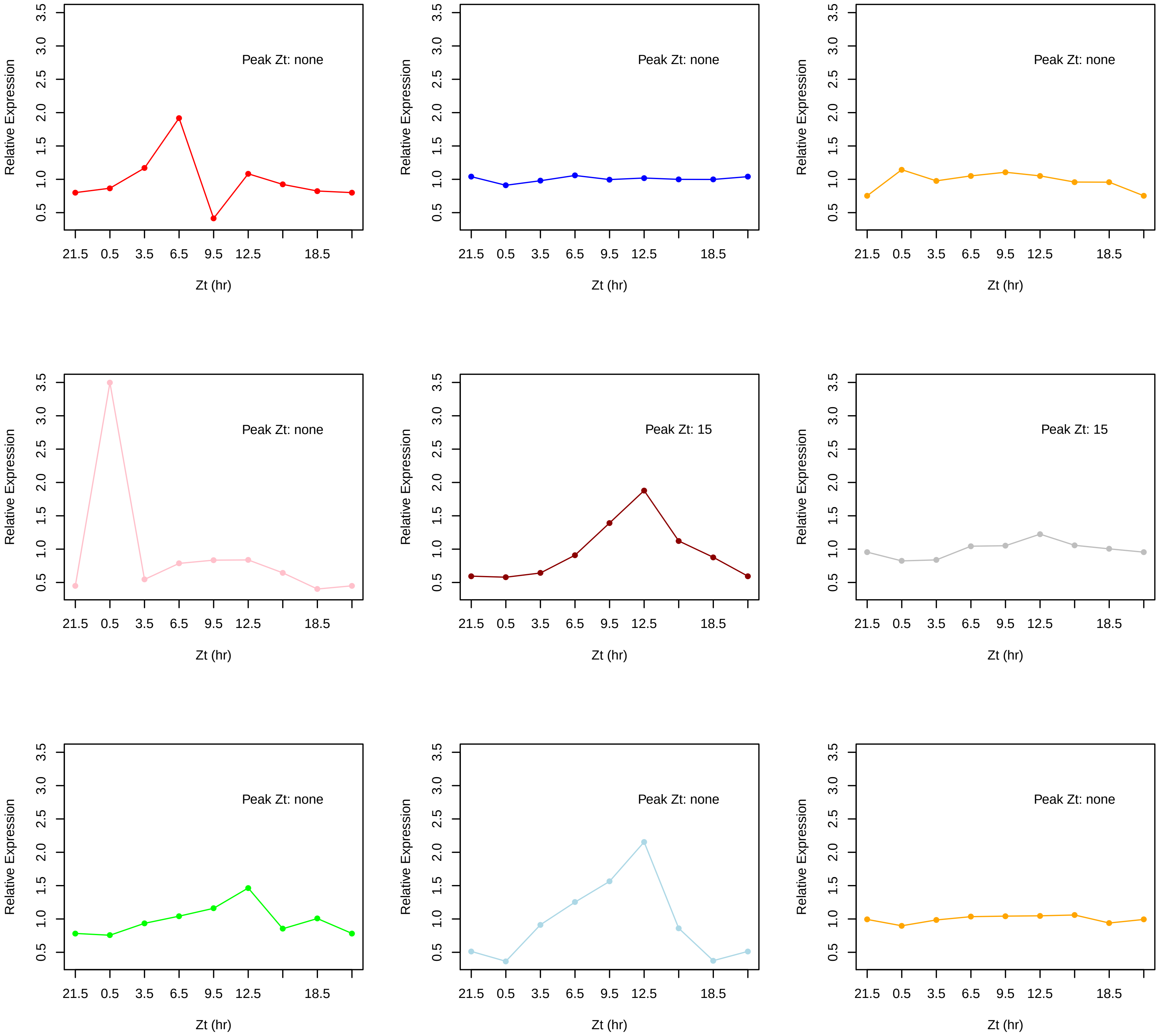

Singeleton: ENSXMAG00000001306 cse1l

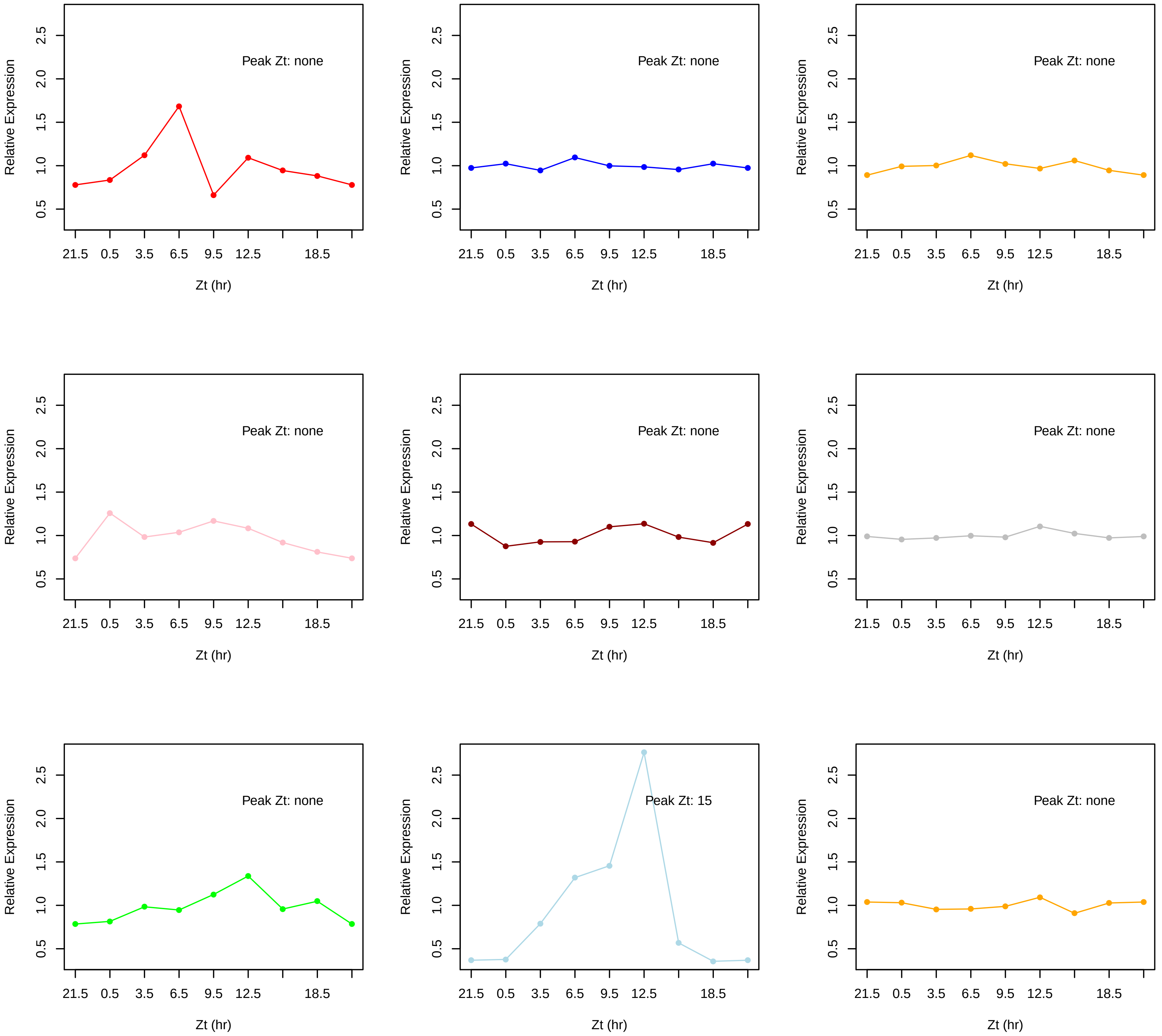

Singleton: ENSXMAG00000018003 ddx18

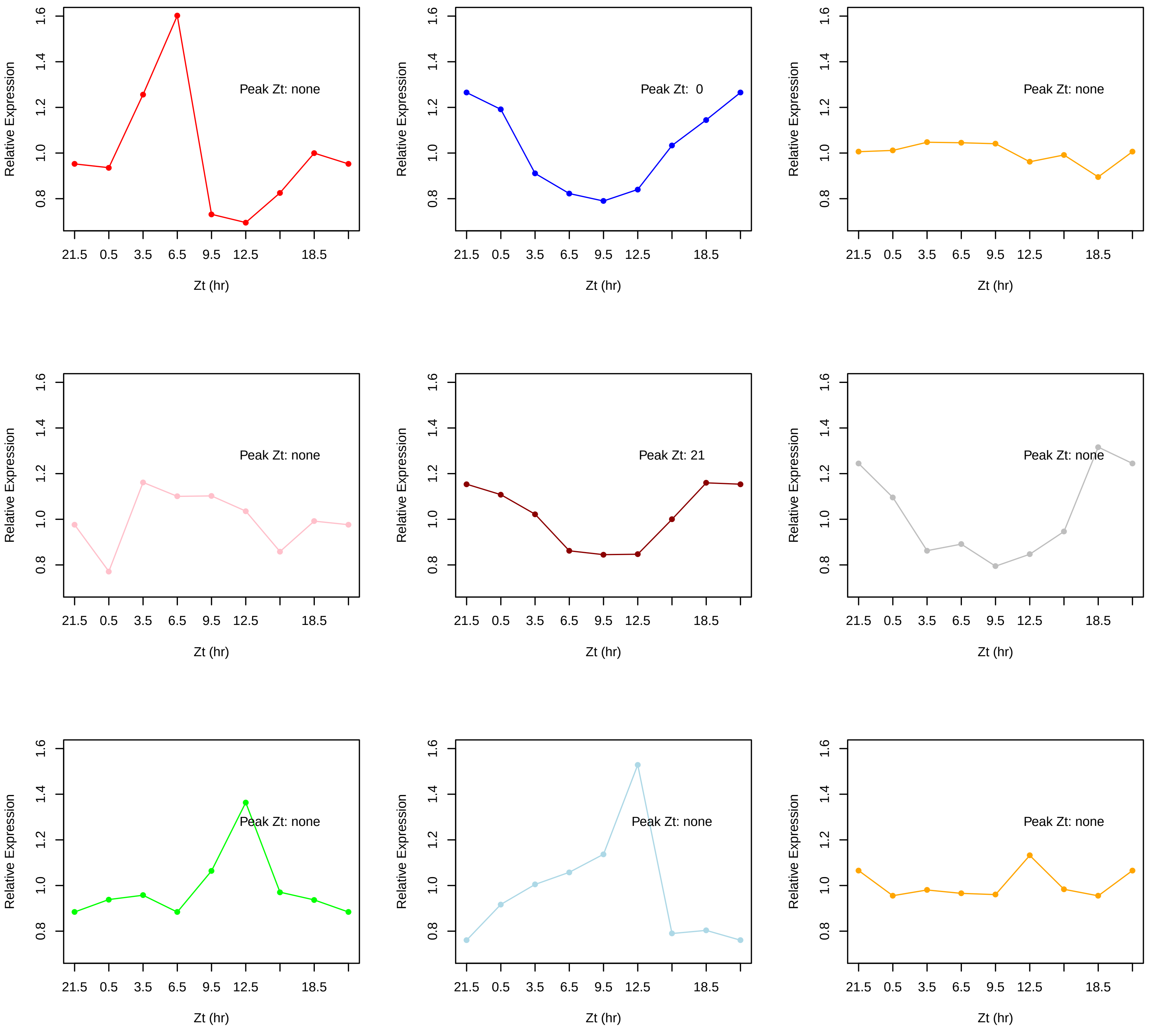

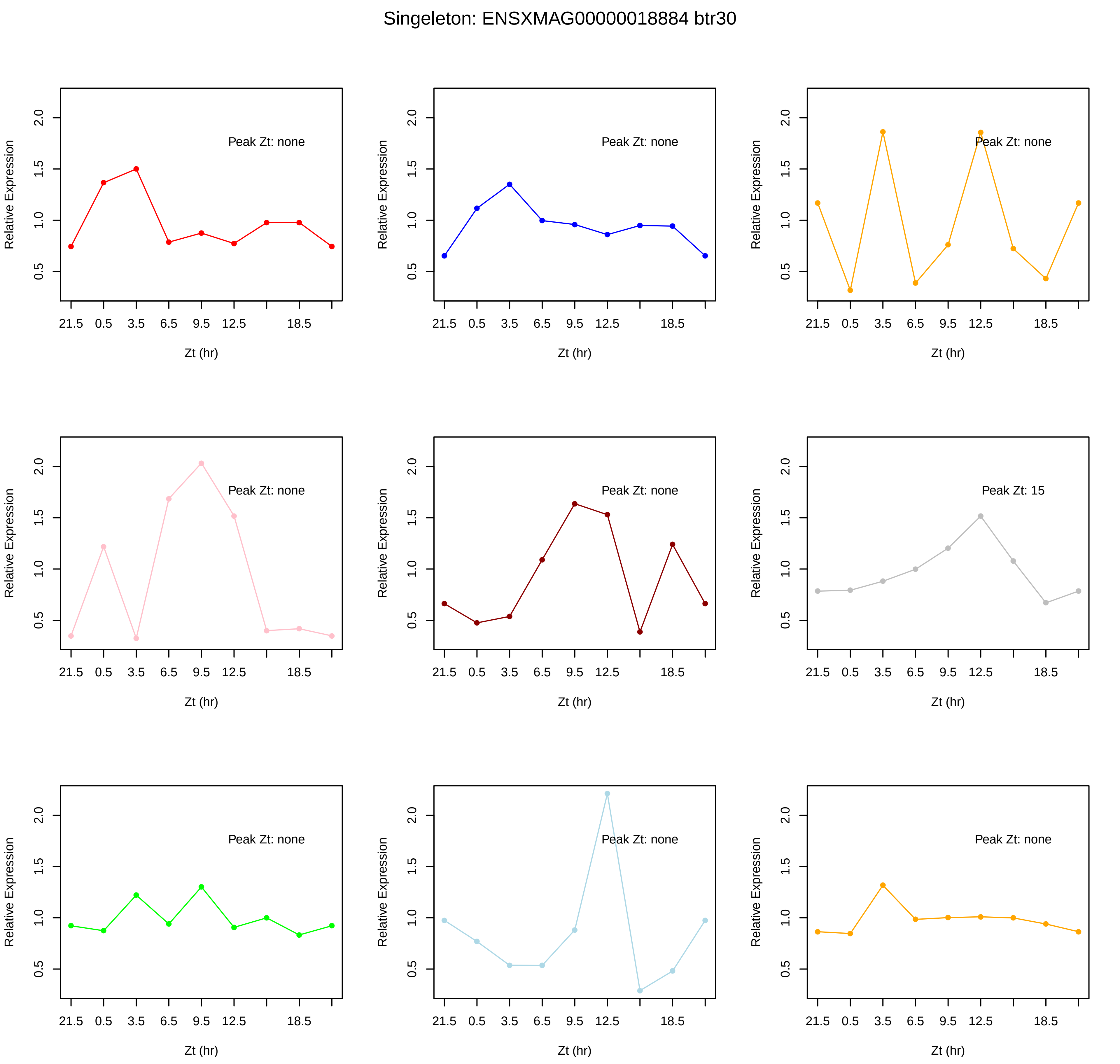
Singeleton: ENSXMAG00000018884 btr30
Peak Zt: none
Peak Zt: none
Peak Zt: none
Peak Zt: none
Peak Zt: none
Peak Zt: 15
Peak Zt: none
Peak Zt: none
Peak Zt: none
Relative Expression
Zt (hr)
21.5
0.5
3.5
6.5
9.5
12.5
18.5
0.5
1.0
1.5
2.0

Singeleton: ENSXMAG00000007990 LONP1

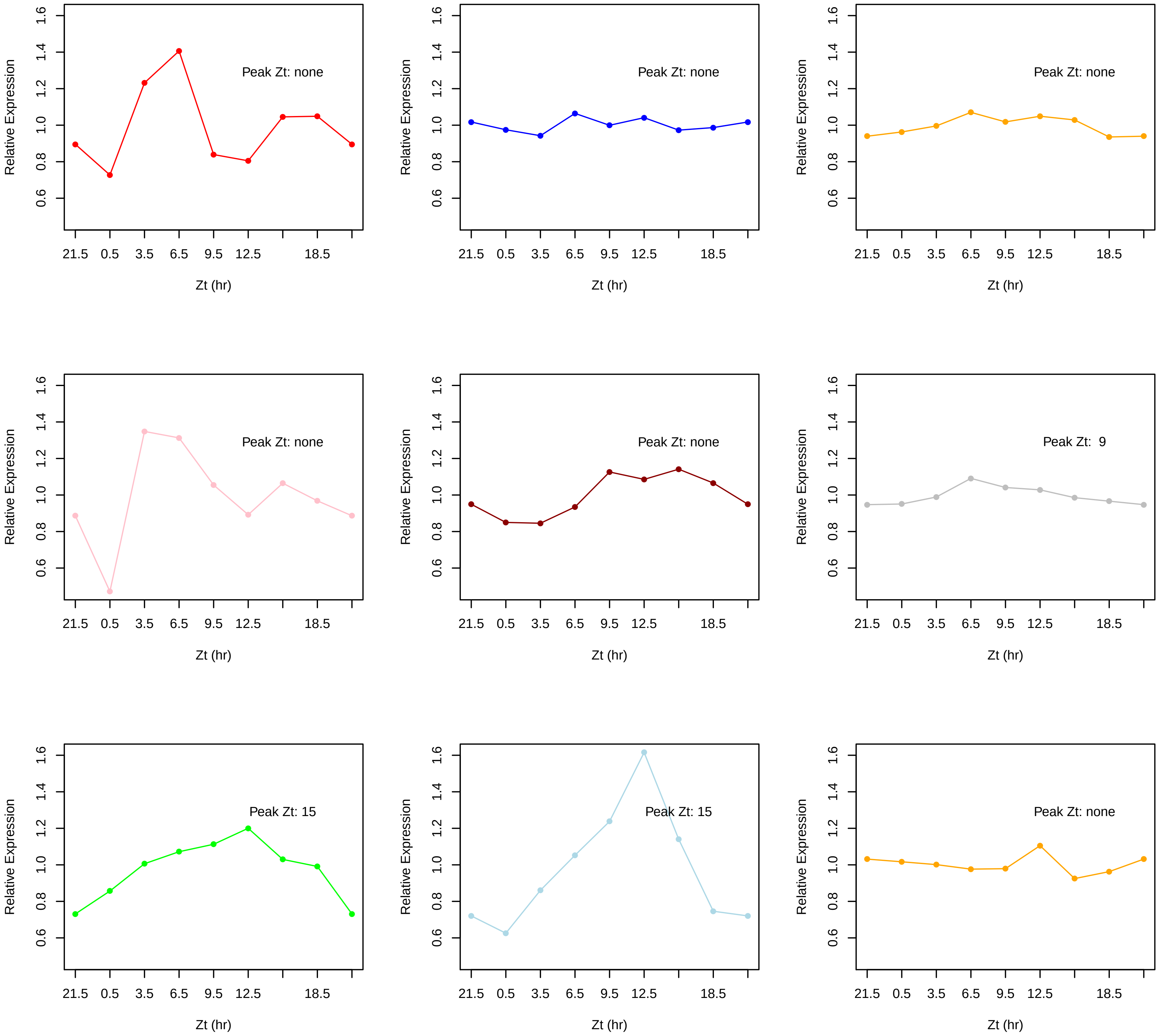

Singeleton: ENSXMAG00000001650 plch1

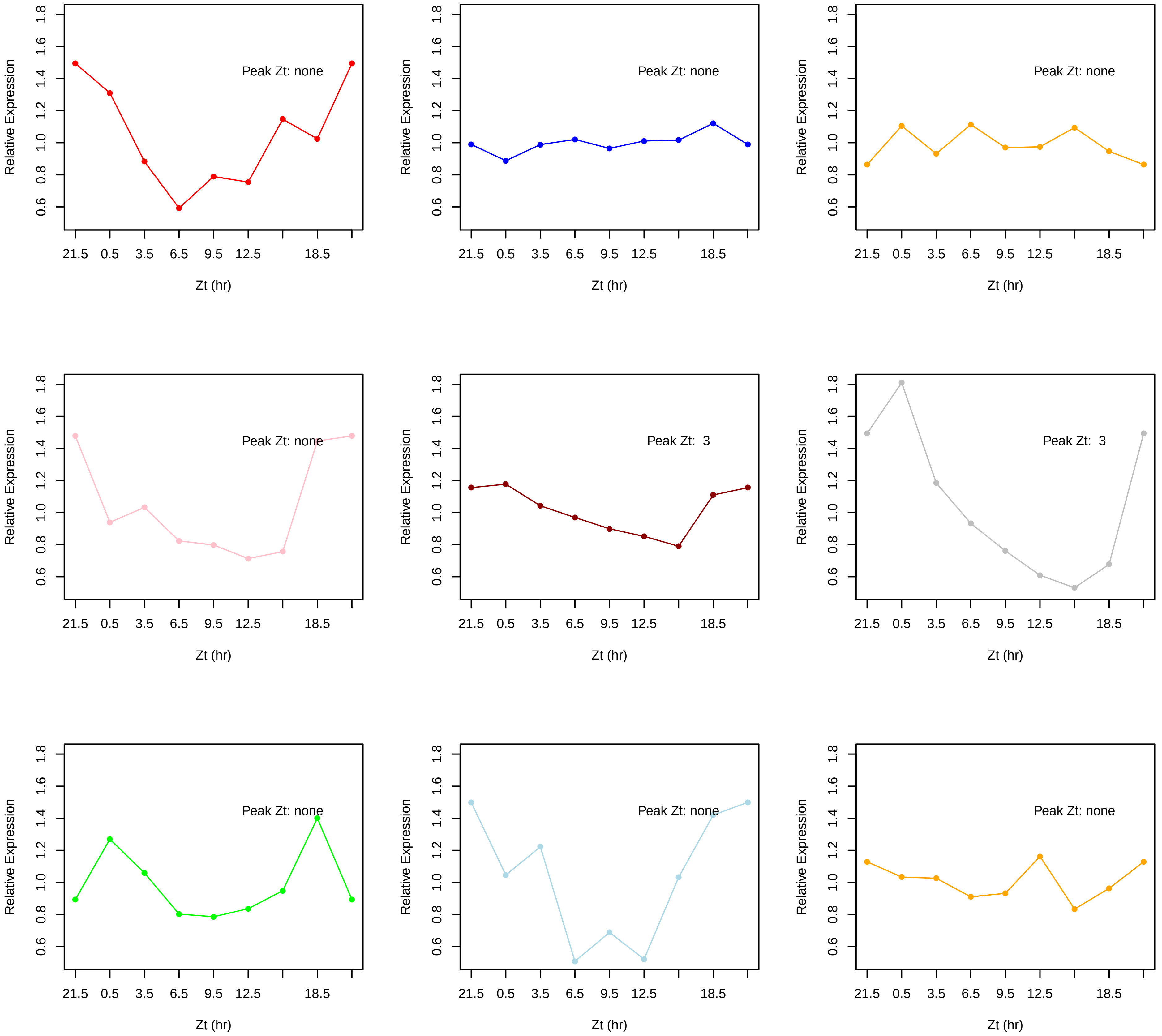

Singeleton: ENSXMAG00000016175 stau1

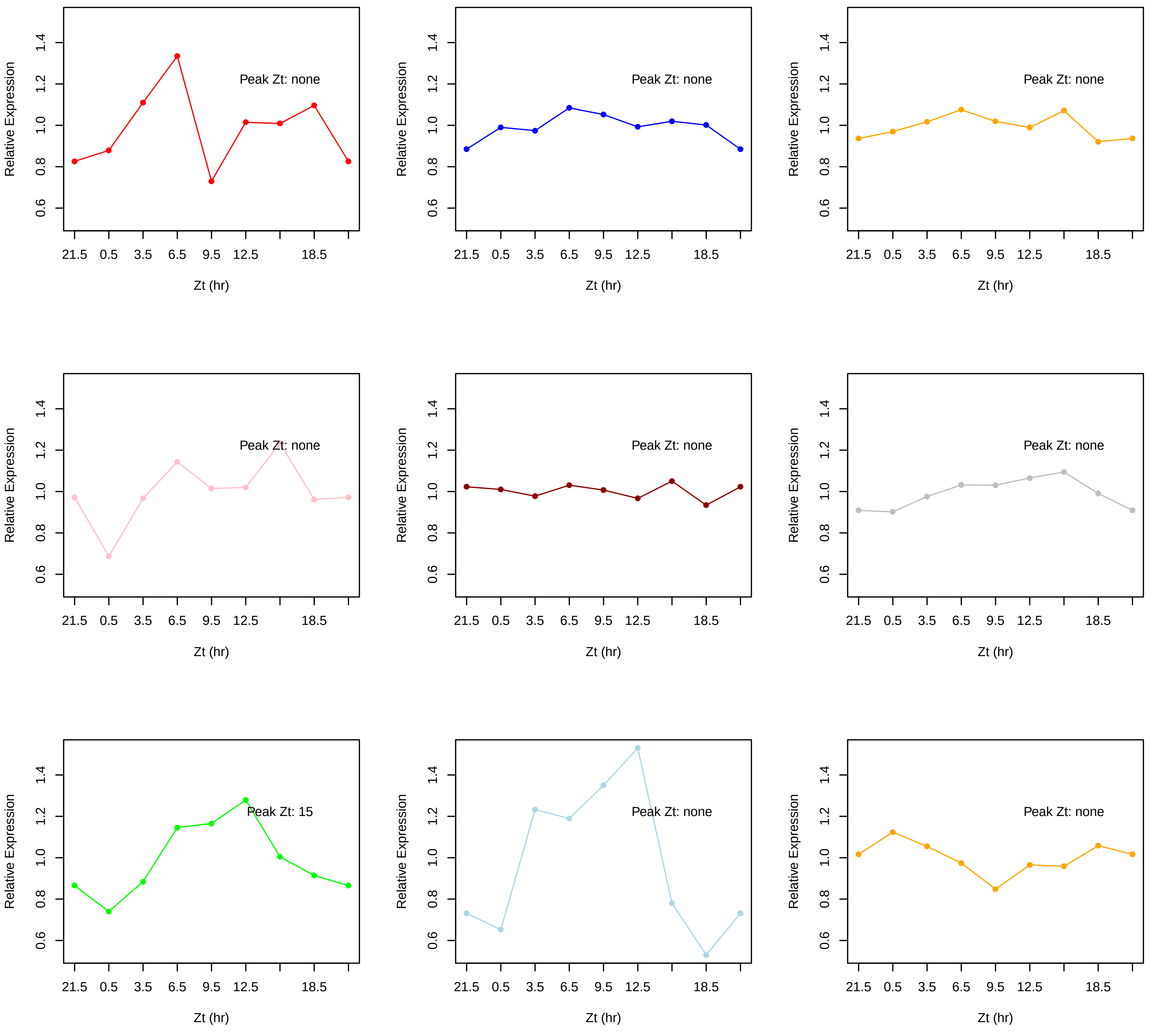

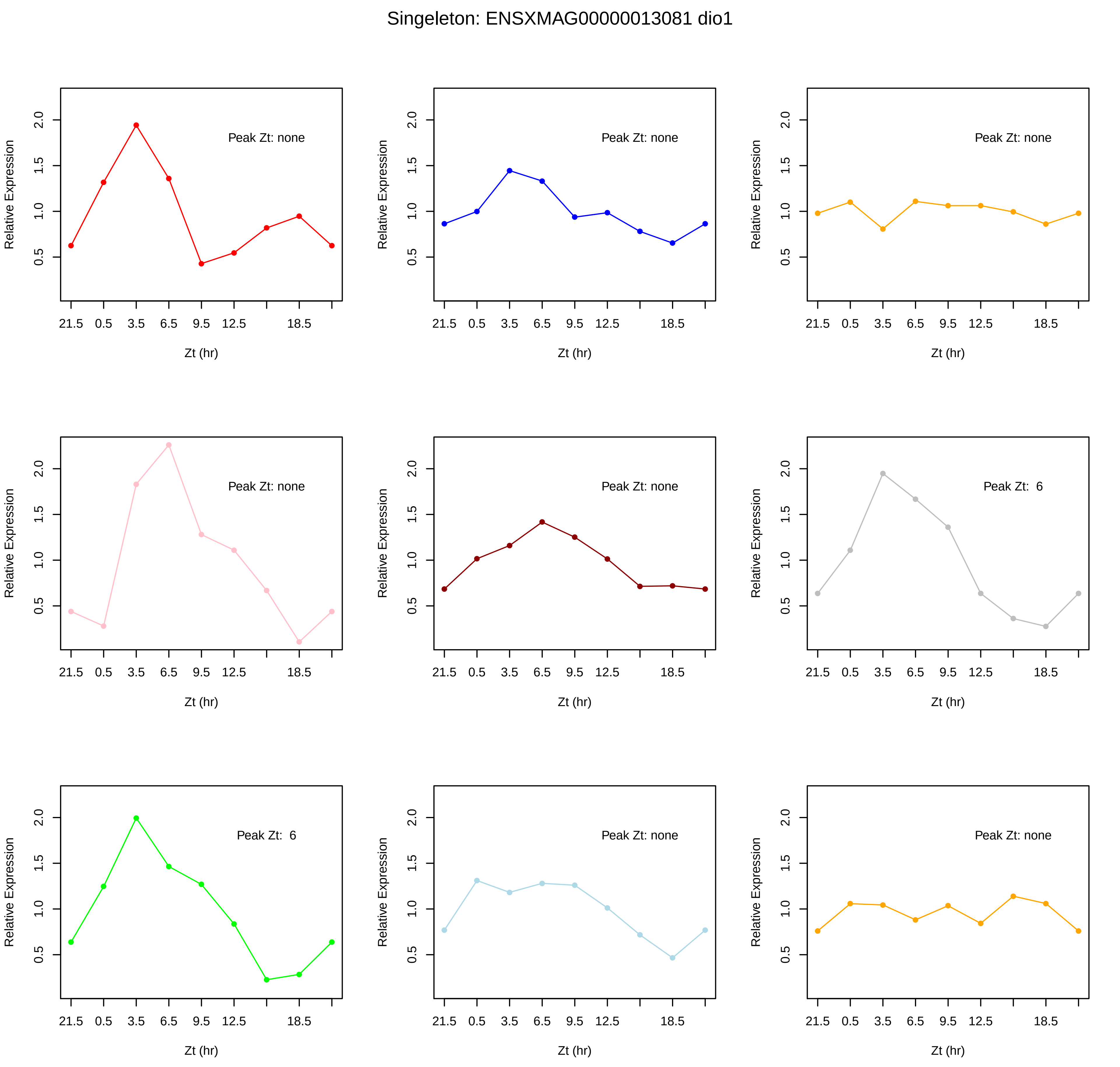
Singeleton: ENSXMAG00000013081 dio1
Peak Zt: none
Peak Zt: none
Peak Zt: none
Peak Zt: none
Peak Zt: none
Peak Zt: 6
Peak Zt: 6
Peak Zt: none
Peak Zt: none
Relative Expression
Zt (hr)
21.5
0.5
3.5
6.5
9.5
12.5
18.5

Singeleton: ENSXMAG00000002670 znrf3

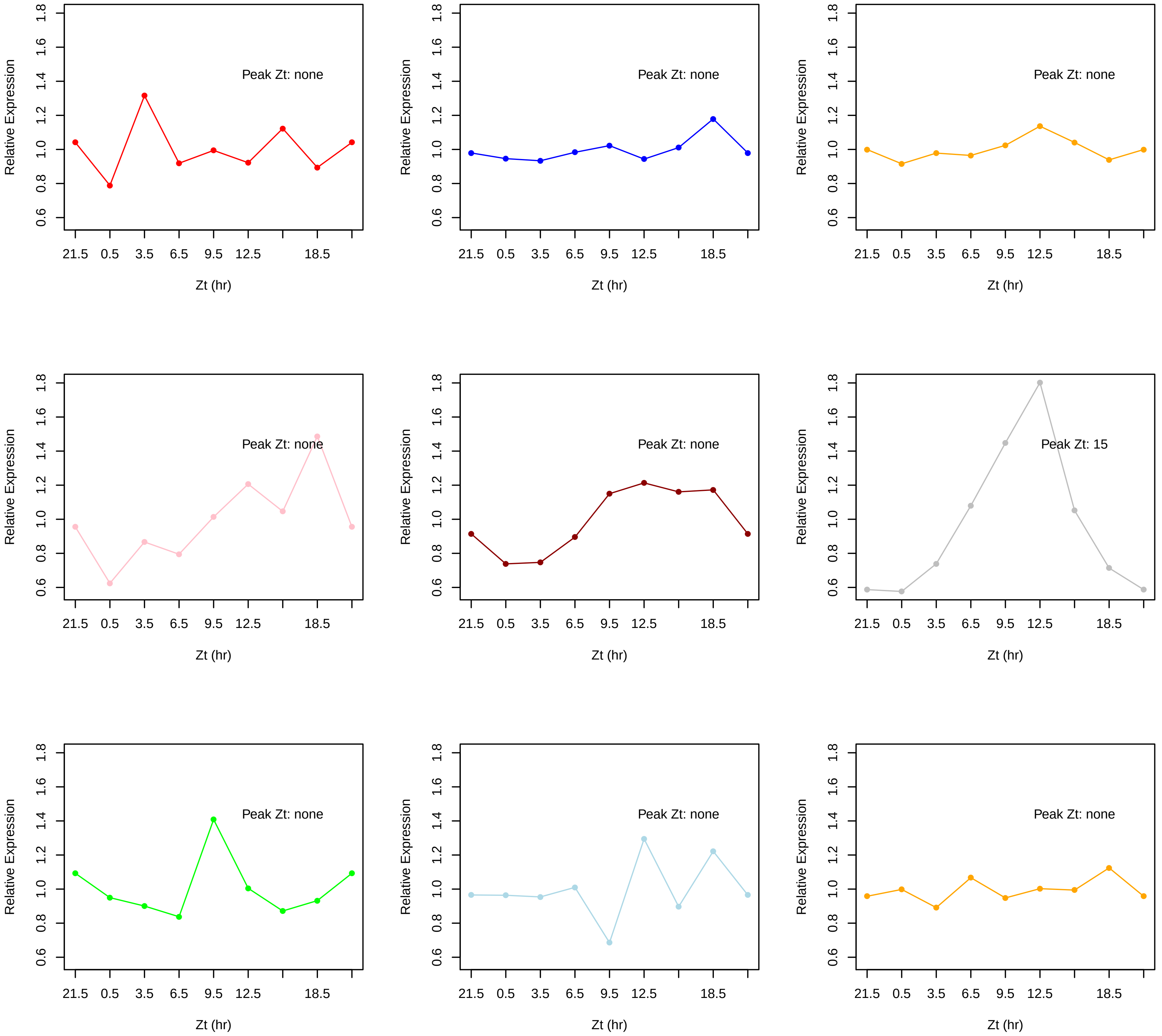

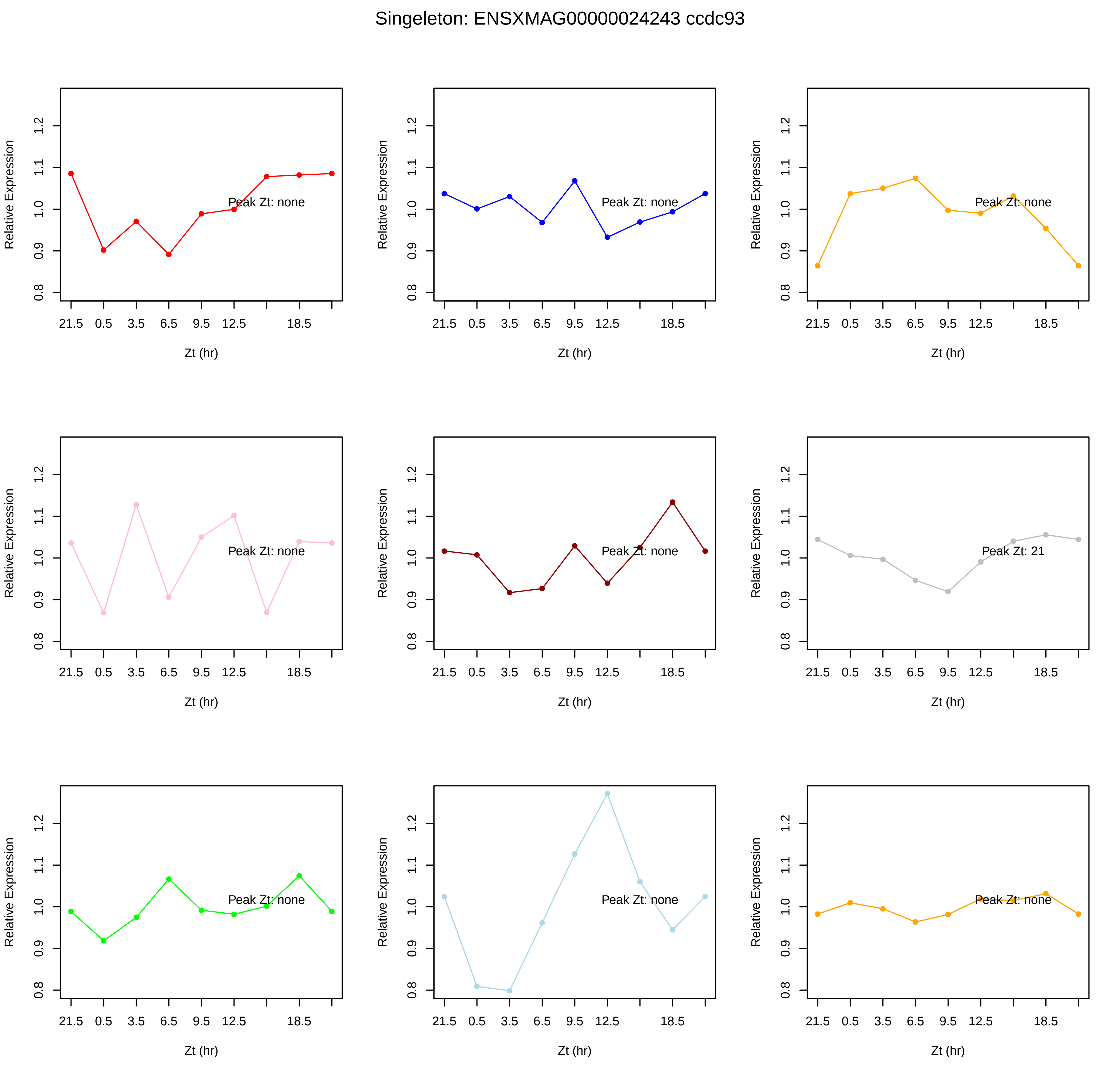
Singeleton: ENSXMAG00000024243 ccdc93
Relative Expression
Zt (hr)
21.5
0.5
3.5
6.5
9.5
12.5
18.5
0.8
0.9
1.0
1.1
1.2
Peak Zt: none
Peak Zt: 21

Singeleton: ENSXMAG00000011622 fam173a

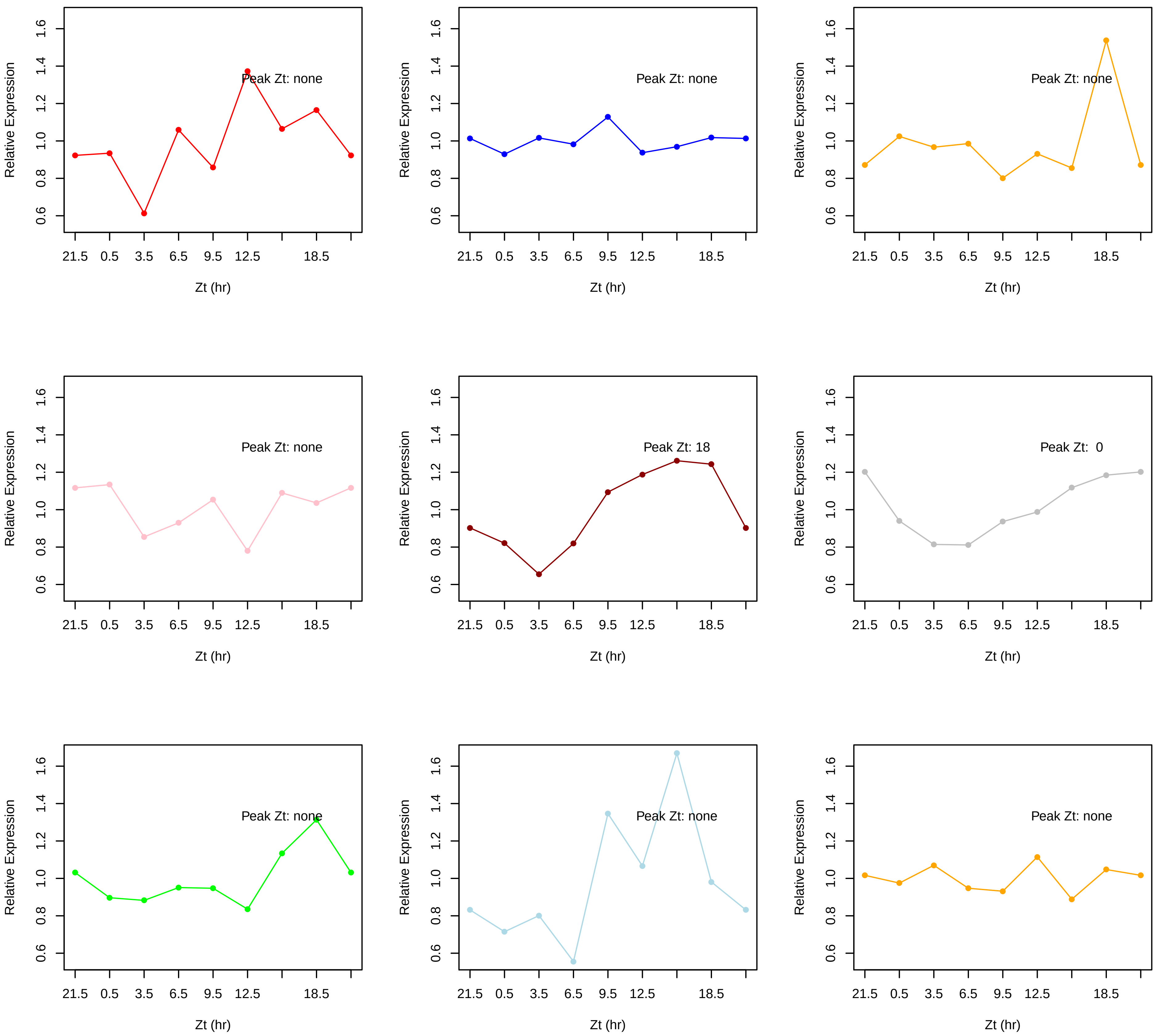

Singeleton: ENSXMAG00000002665 xbp1

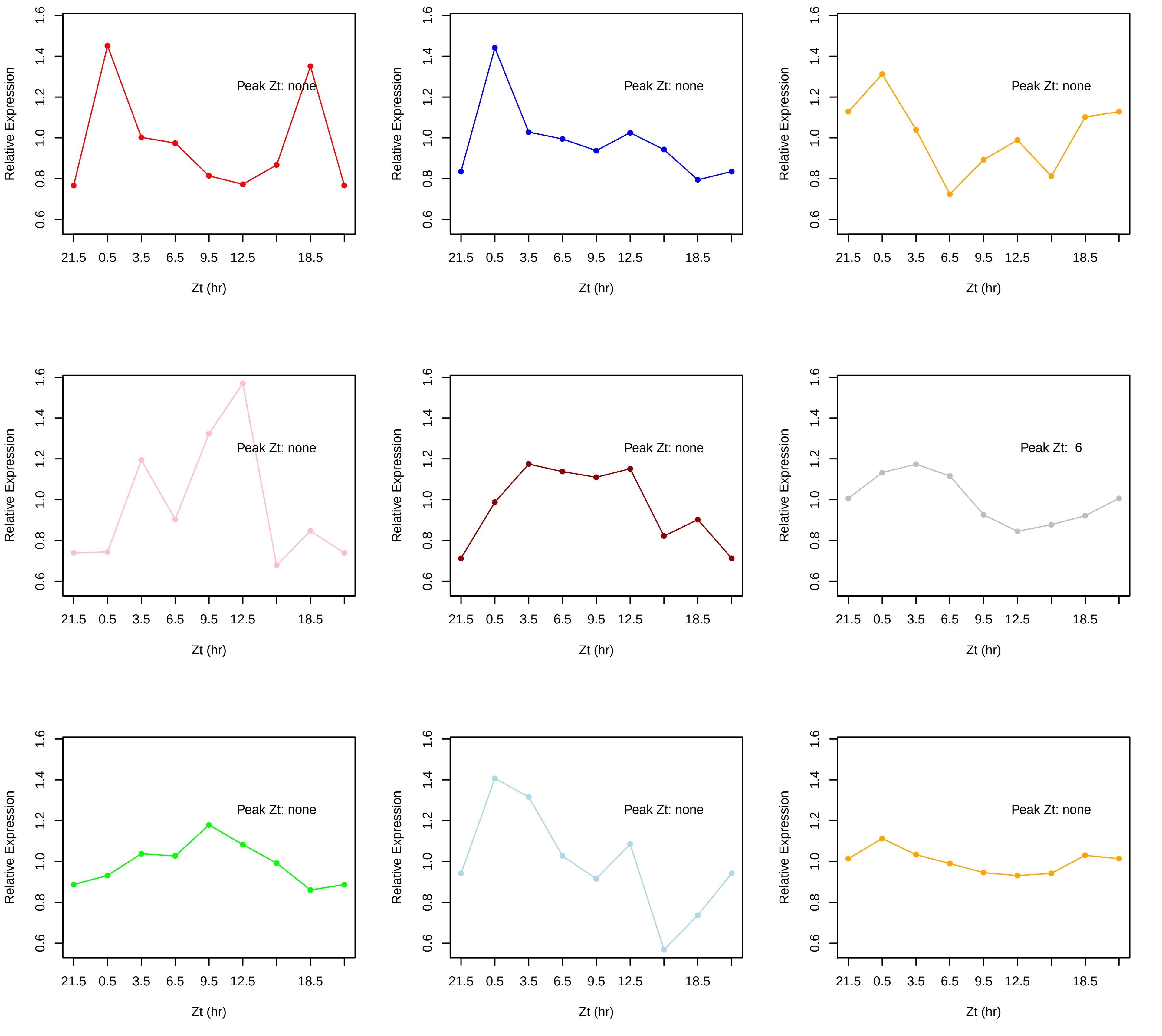

Singeleton: ENSXMAG00000029564 angpt1

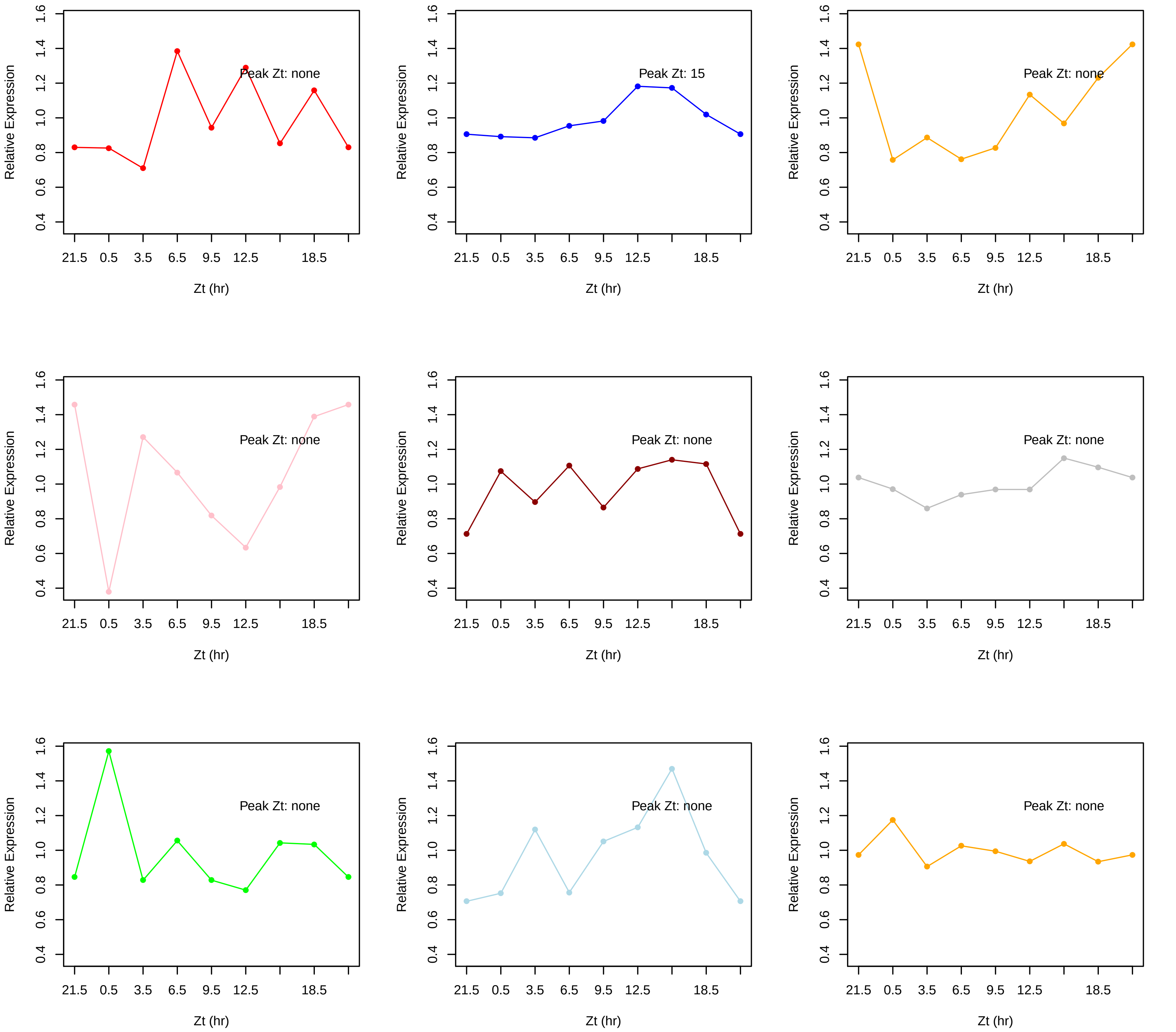

Singeleton: ENSXMAG00000025031 ldb2a

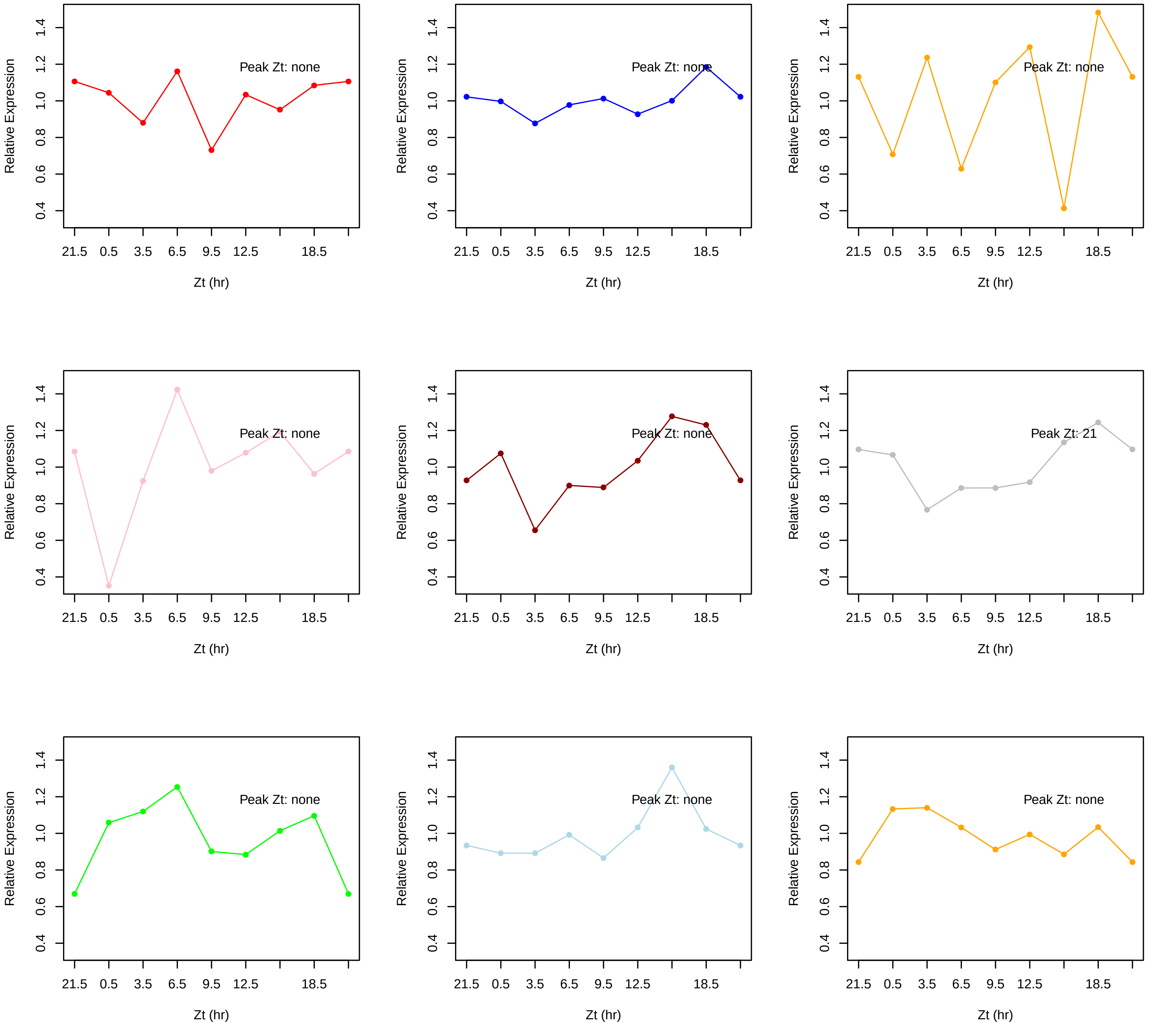

Singeleton: ENSXMAG00000025259 tgif1

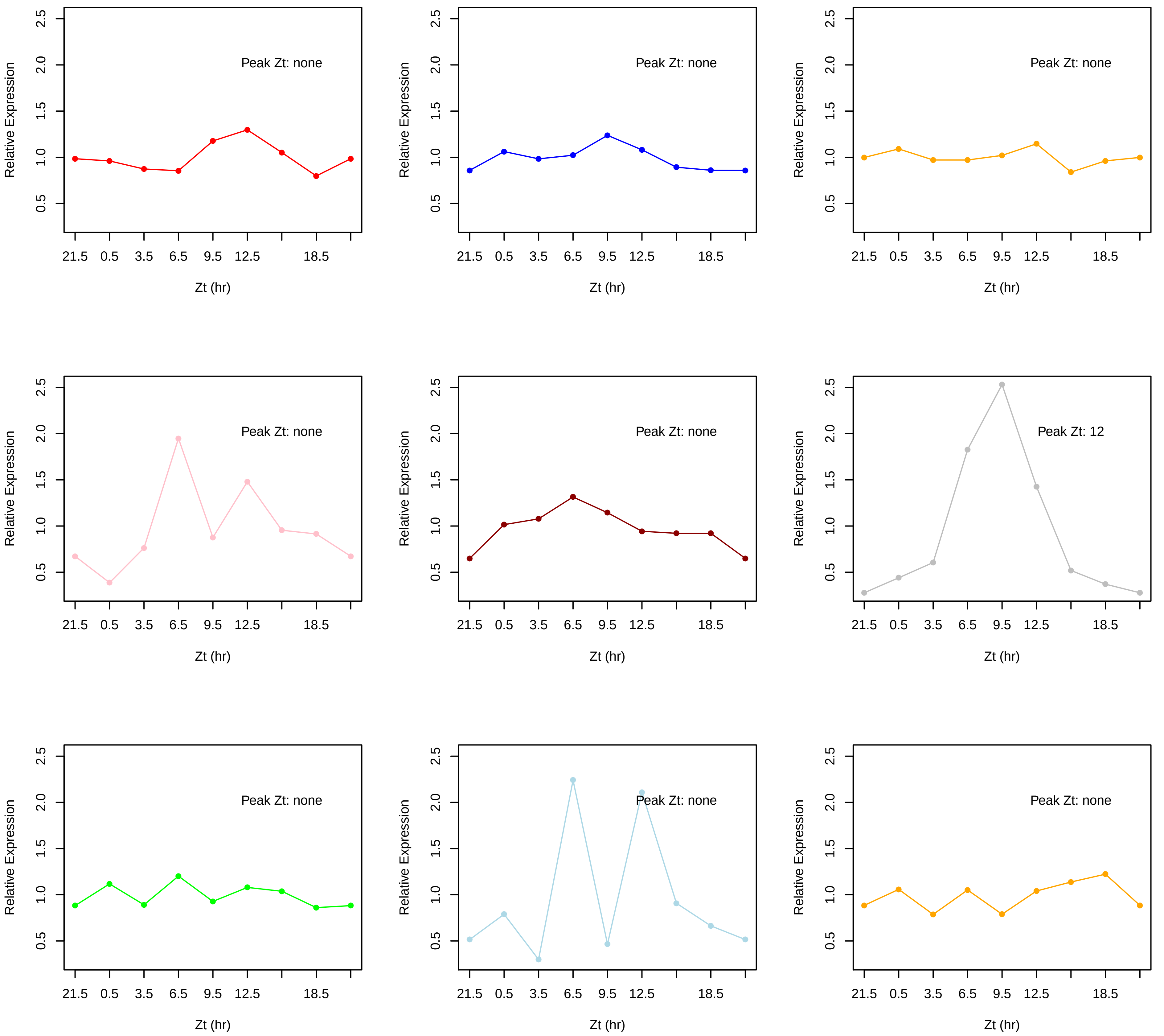

Singeleton: ENSXMAG00000025977 psma1

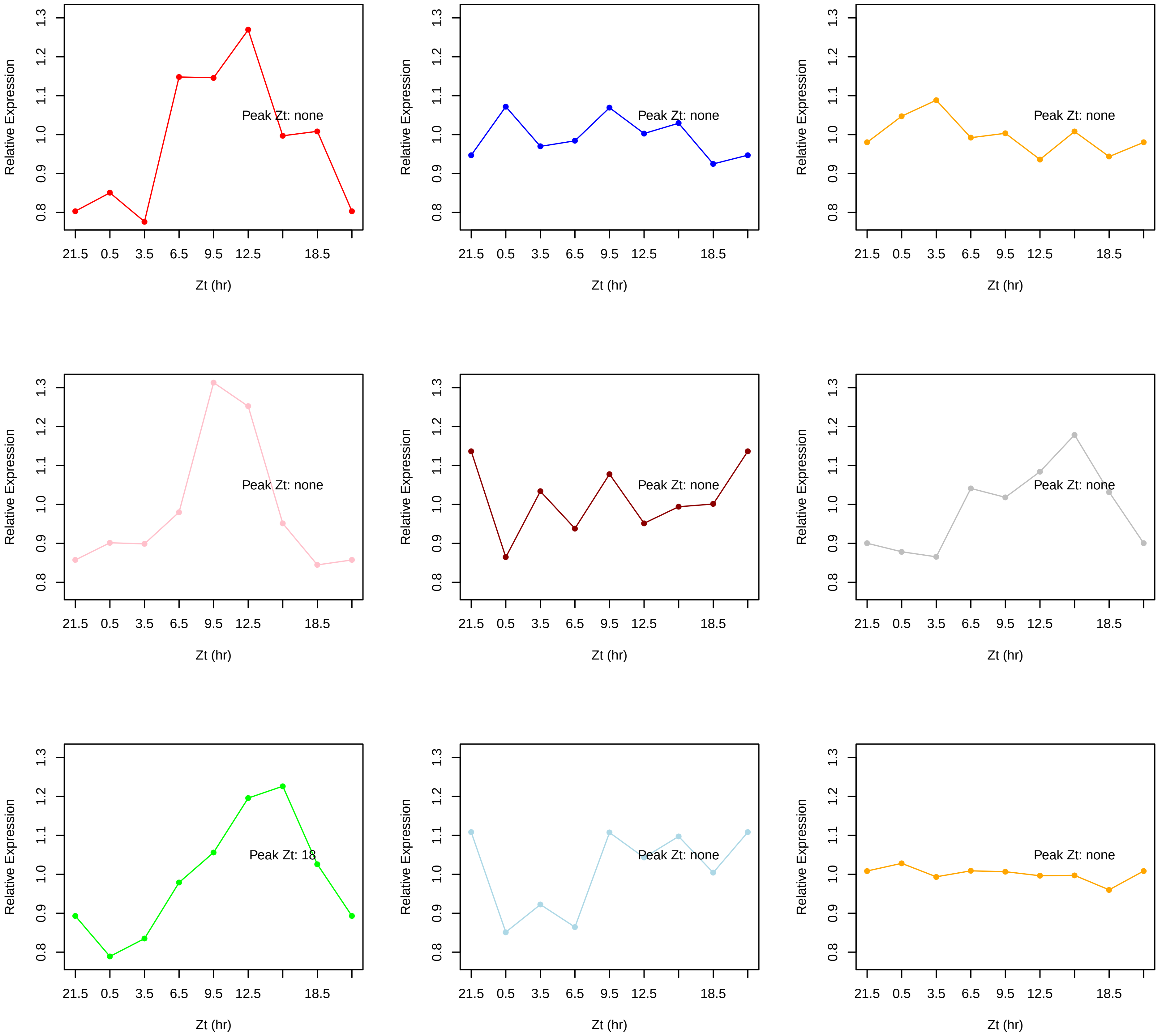

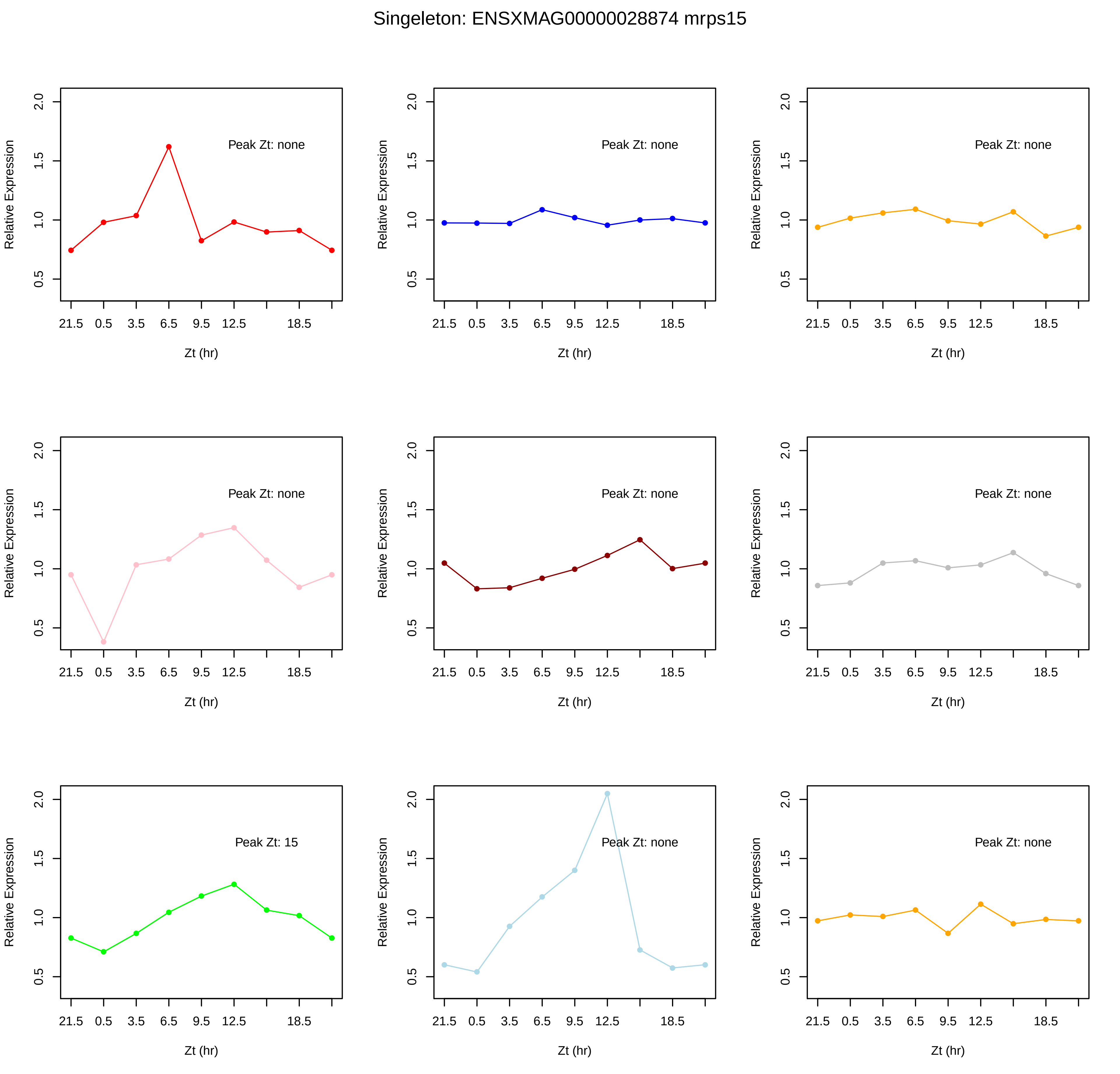
Singeleton: ENSXMAG00000028874 mrps15
Peak Zt: none
Peak Zt: none
Peak Zt: none
Peak Zt: none
Peak Zt: none
Peak Zt: none
Peak Zt: 15
Peak Zt: none
Peak Zt: none
Relative Expression
Zt (hr)
21.5
0.5
3.5
6.5
9.5
12.5
18.5
0.5
1.0
1.5
2.0

Singleton: ENSXMAG00000002901 insig2

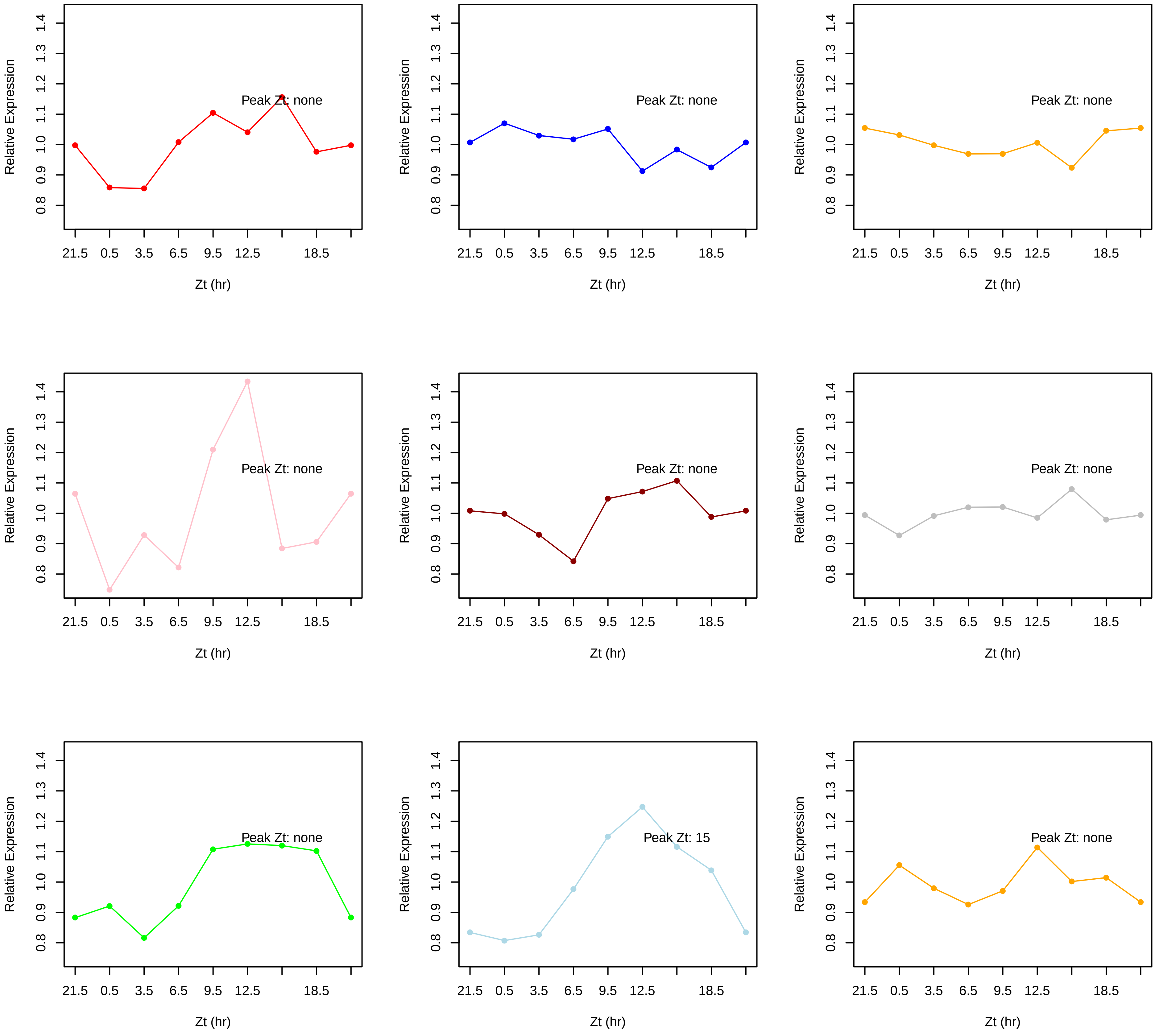

Singleton: ENSXMAG00000000464 lrrc42

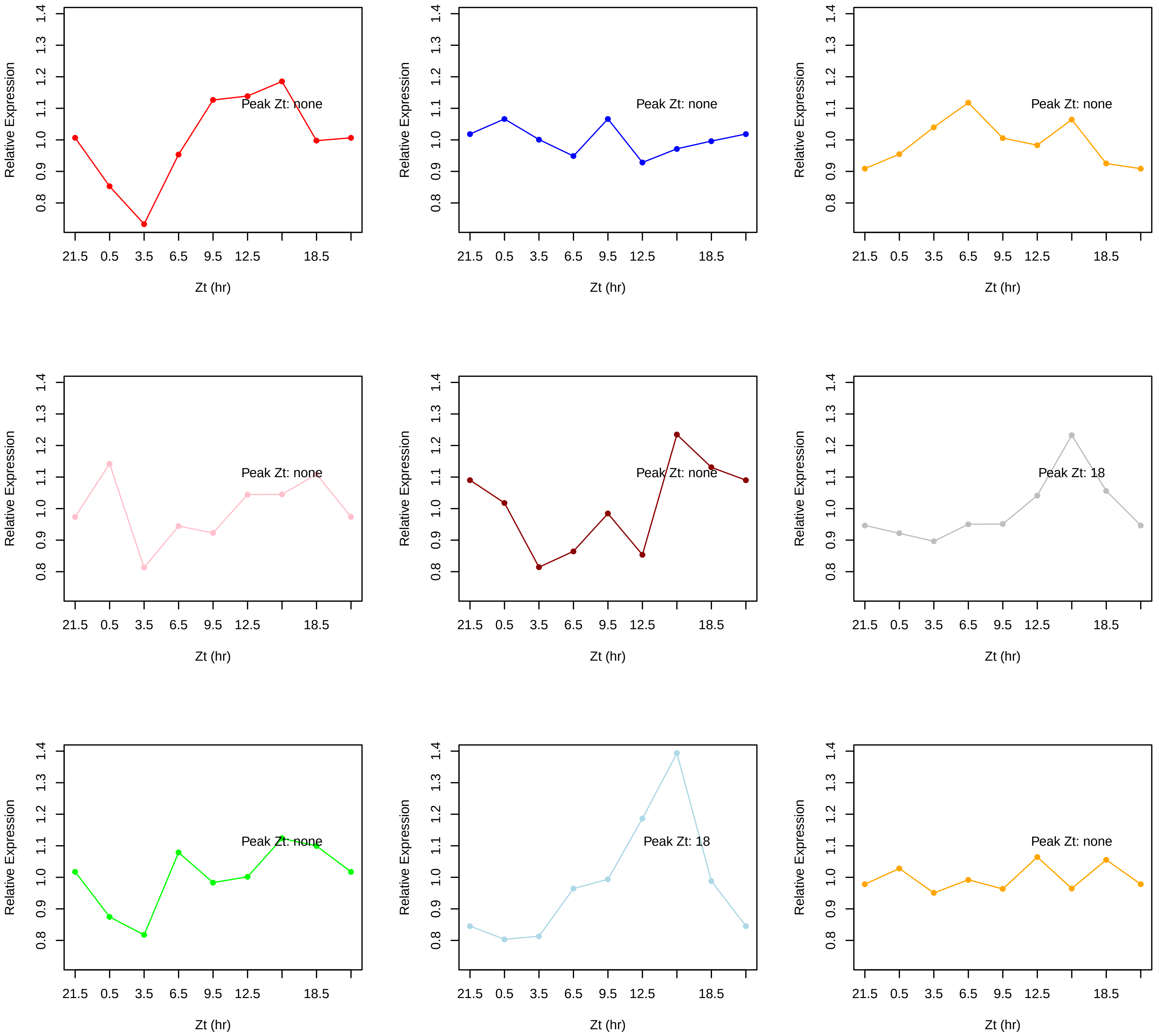

Singleton: ENSXMAG00000000684

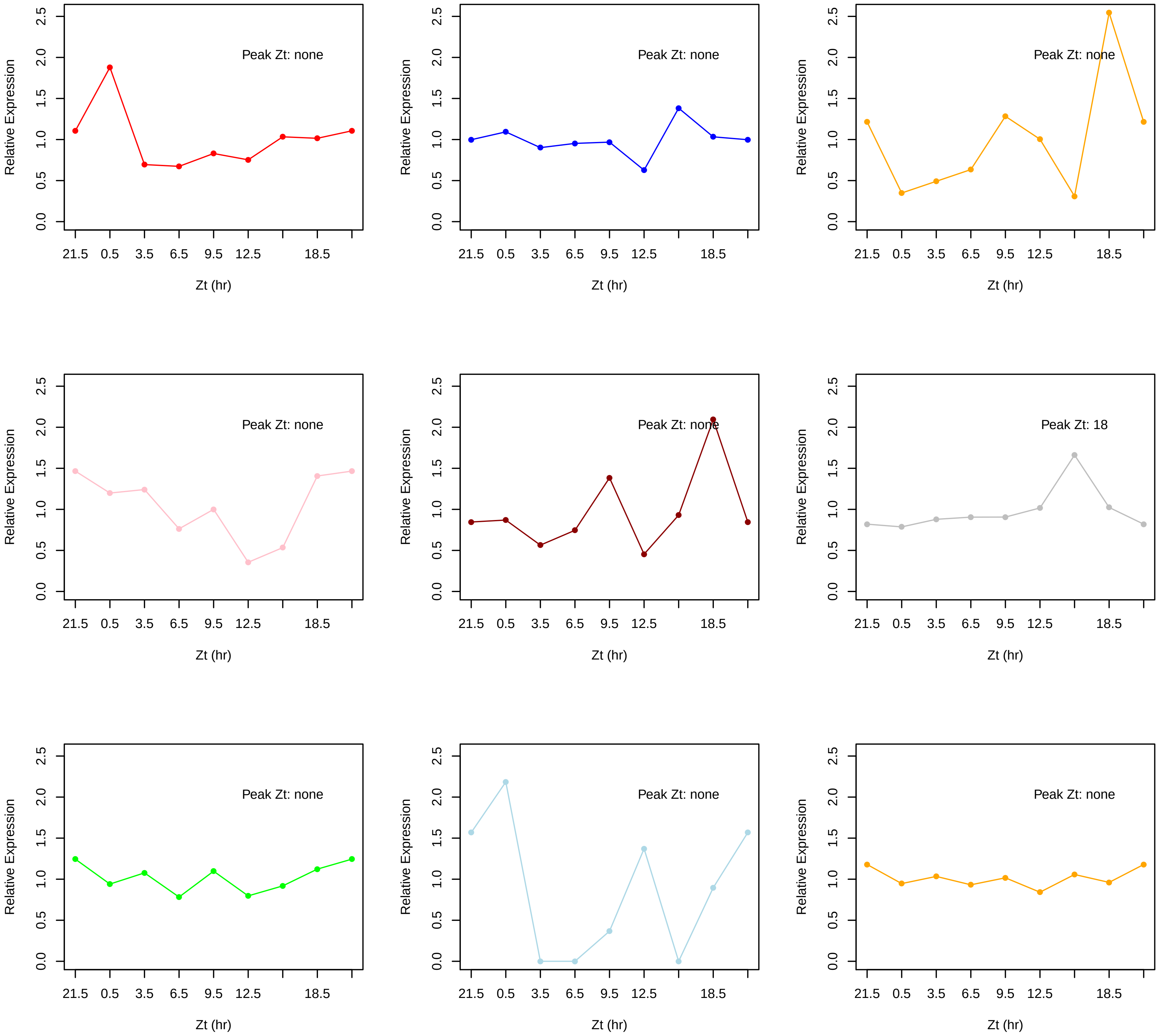

Singleton: ENSXMAG00000019061 ift140

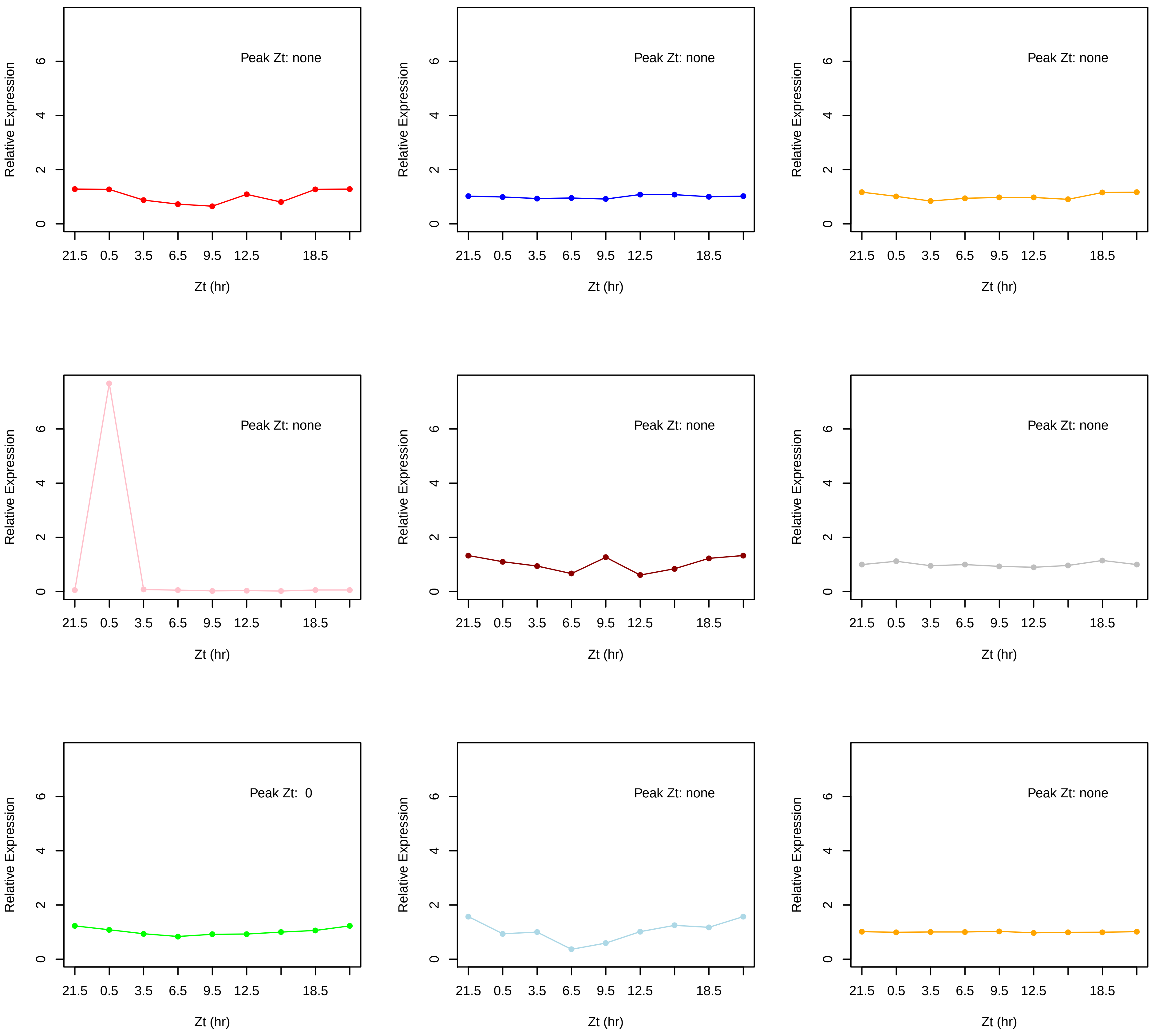

Singeleton: ENSXMAG00000014732 csf3r

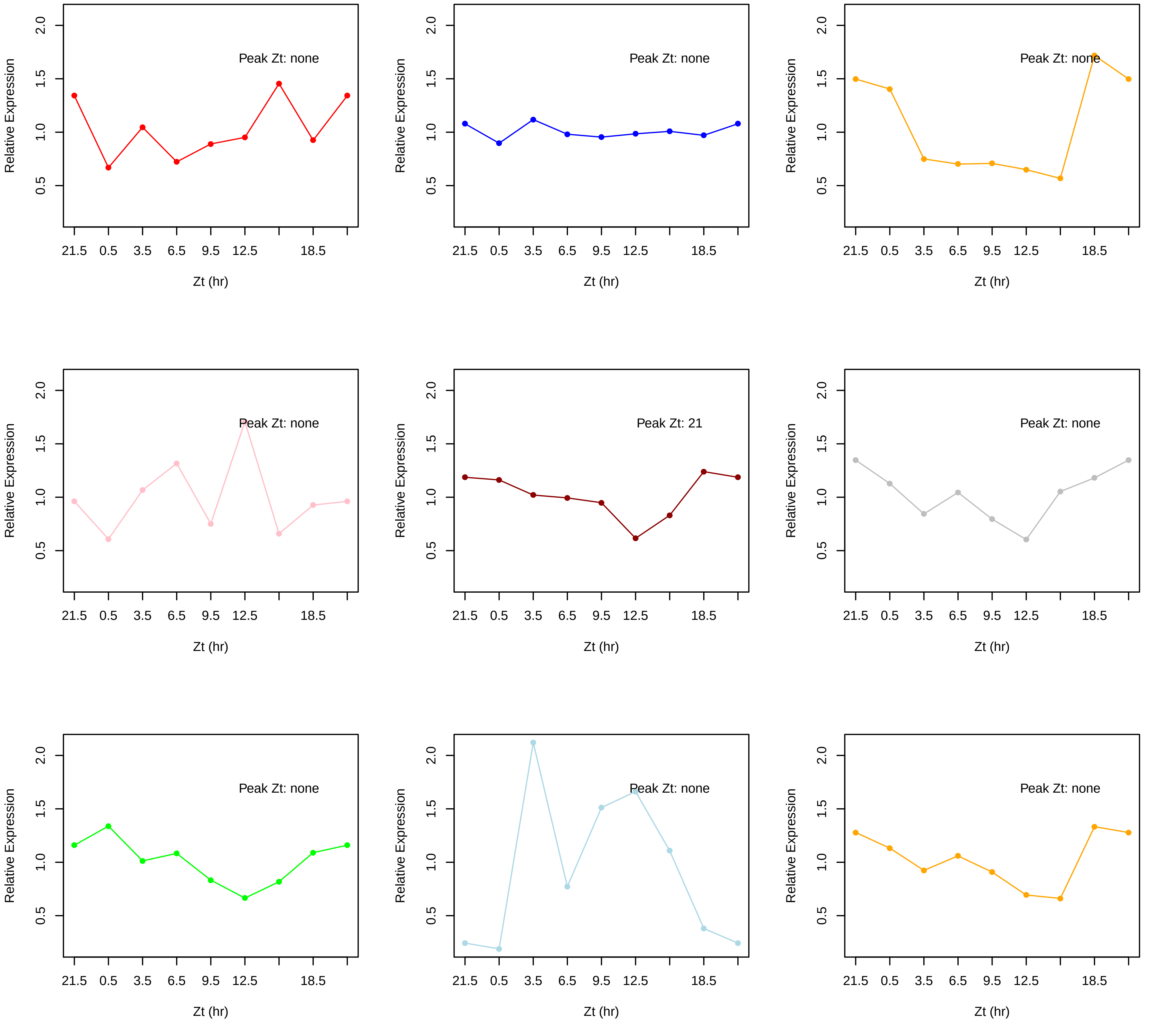

Singeleton: ENSXMAG00000021912 dhrs3b

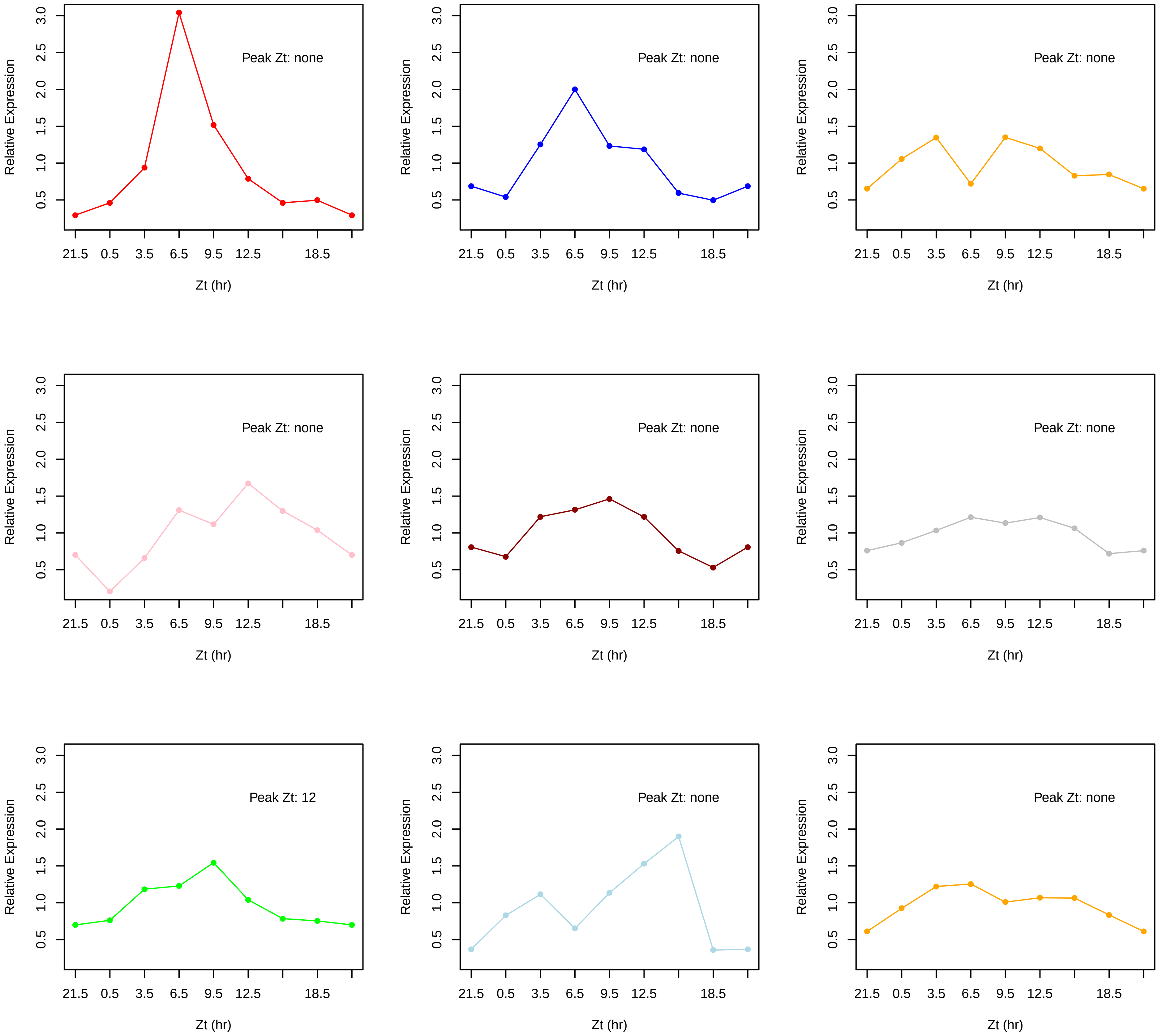

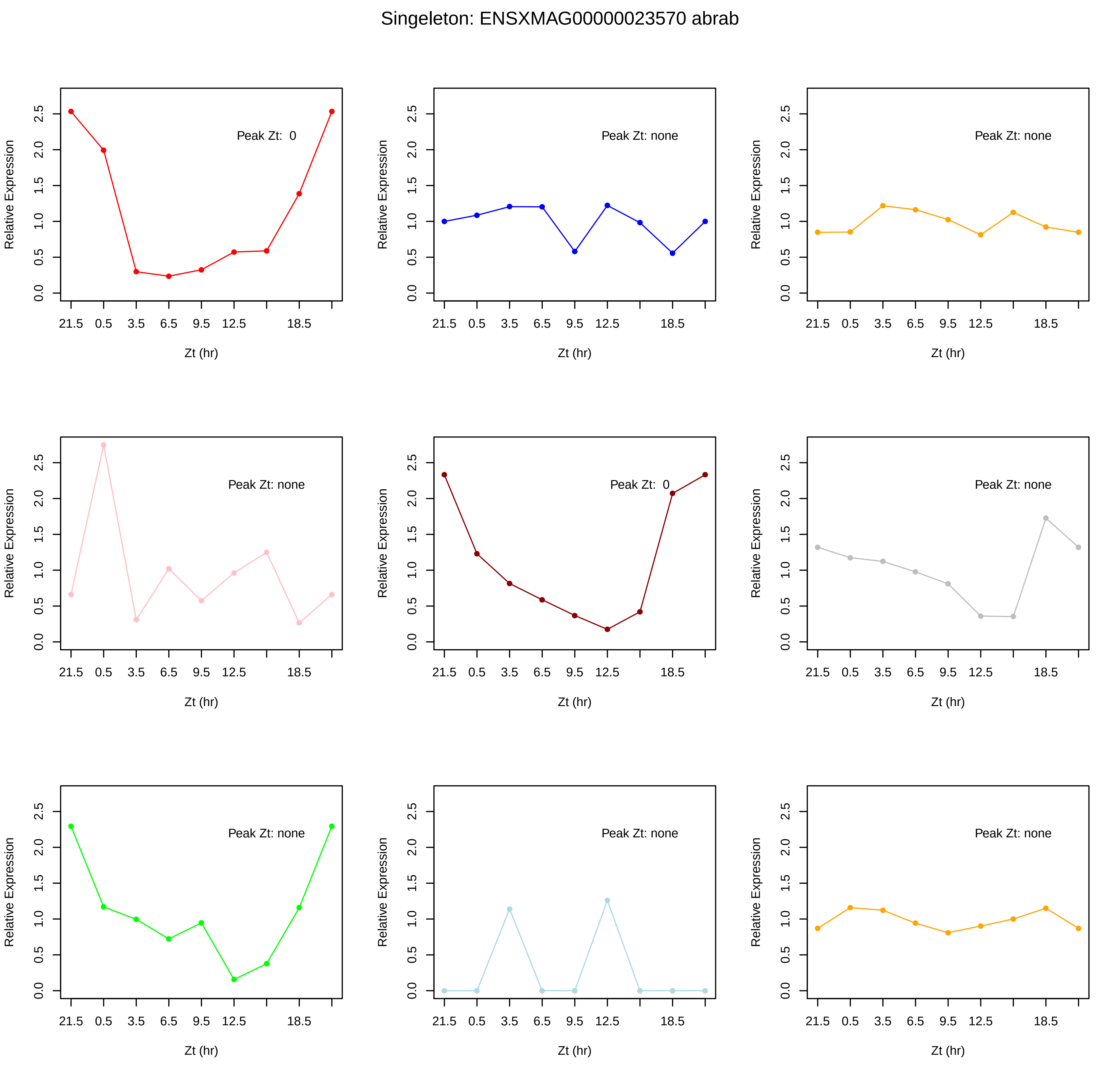

Singleton: ENSXMAG00000023570 abrab
Peak Zt: 0
Peak Zt: none
Peak Zt: none
Peak Zt: none
Peak Zt: 0
Peak Zt: none
Peak Zt: none
Peak Zt: none
Peak Zt: none
Relative Expression
Zt (hr)
21.5
0.5
3.5
6.5
9.5
12.5
18.5
0.0
0.5
1.0
1.5
2.0
2.5

Singleton: ENSXMAG00000007010 copb1

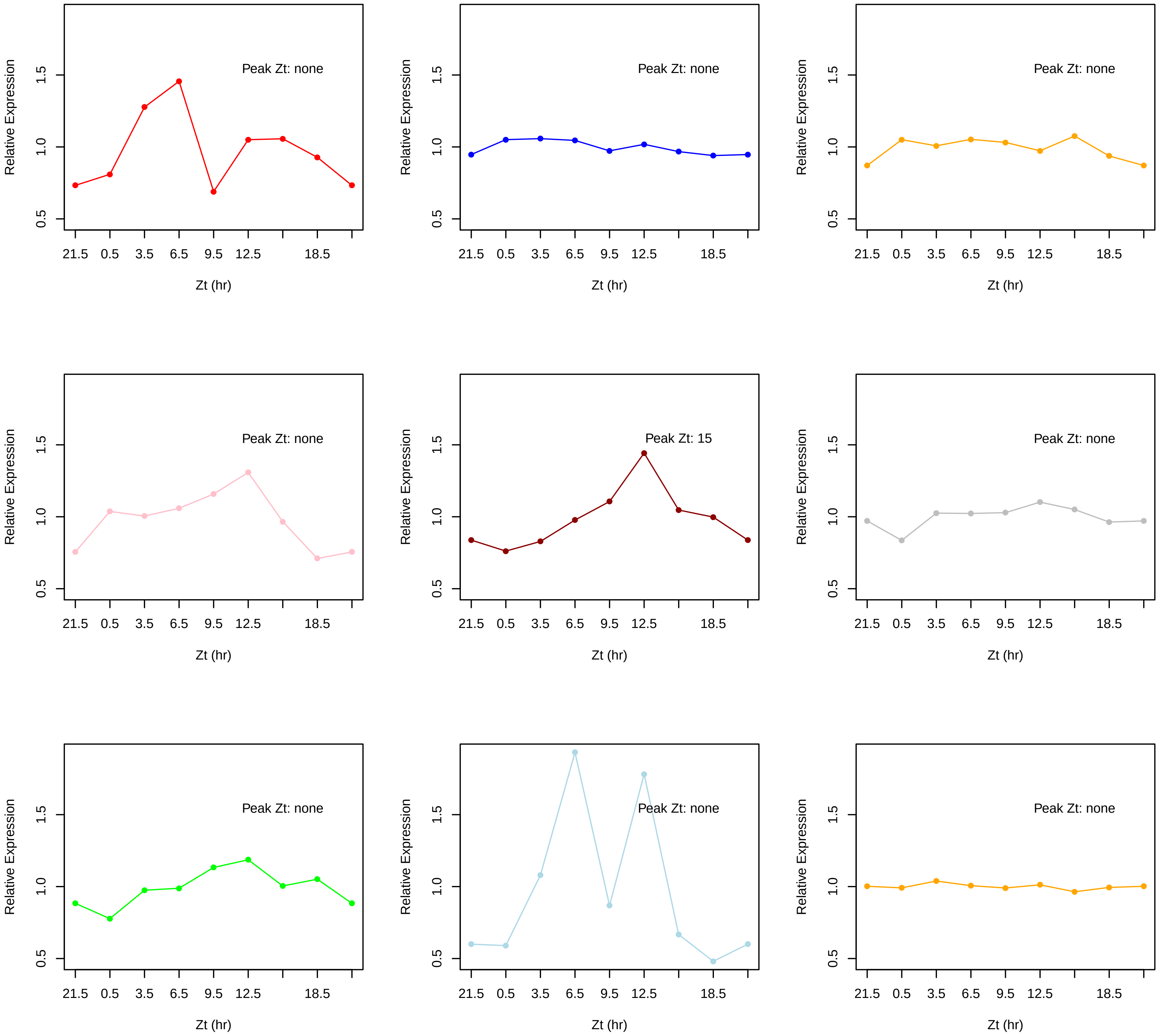

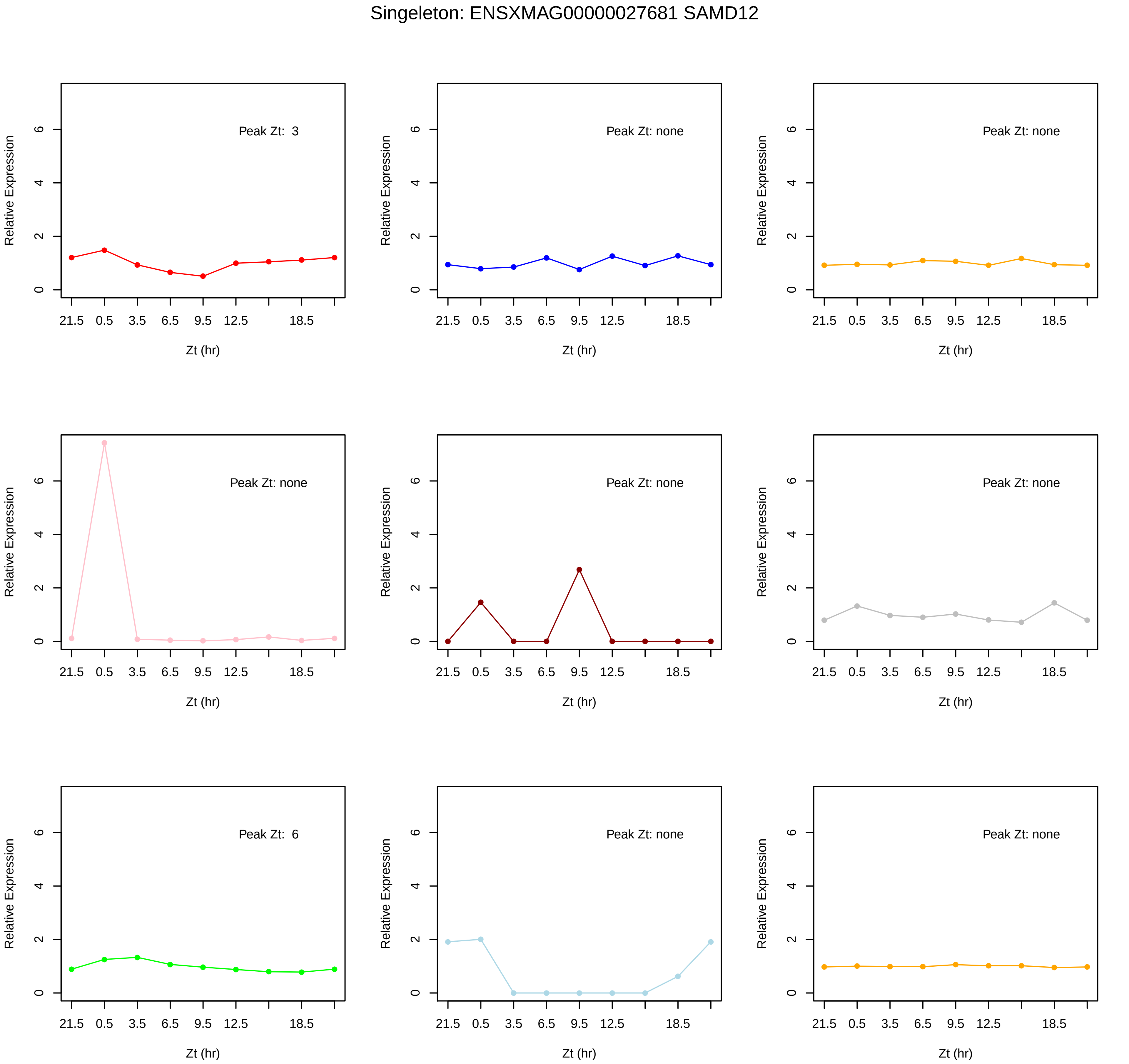

Singeleton: ENSXMAG00000027681 SAMD12
Peak Zt: 3
Peak Zt: none
Peak Zt: none
Peak Zt: none
Peak Zt: none
Peak Zt: none
Peak Zt: 6
Peak Zt: none
Peak Zt: none
Relative Expression
Zt (hr)
21.5
0.5
3.5
6.5
9.5
12.5
18.5

Singleton: ENSXMAG00000015984 polr2c

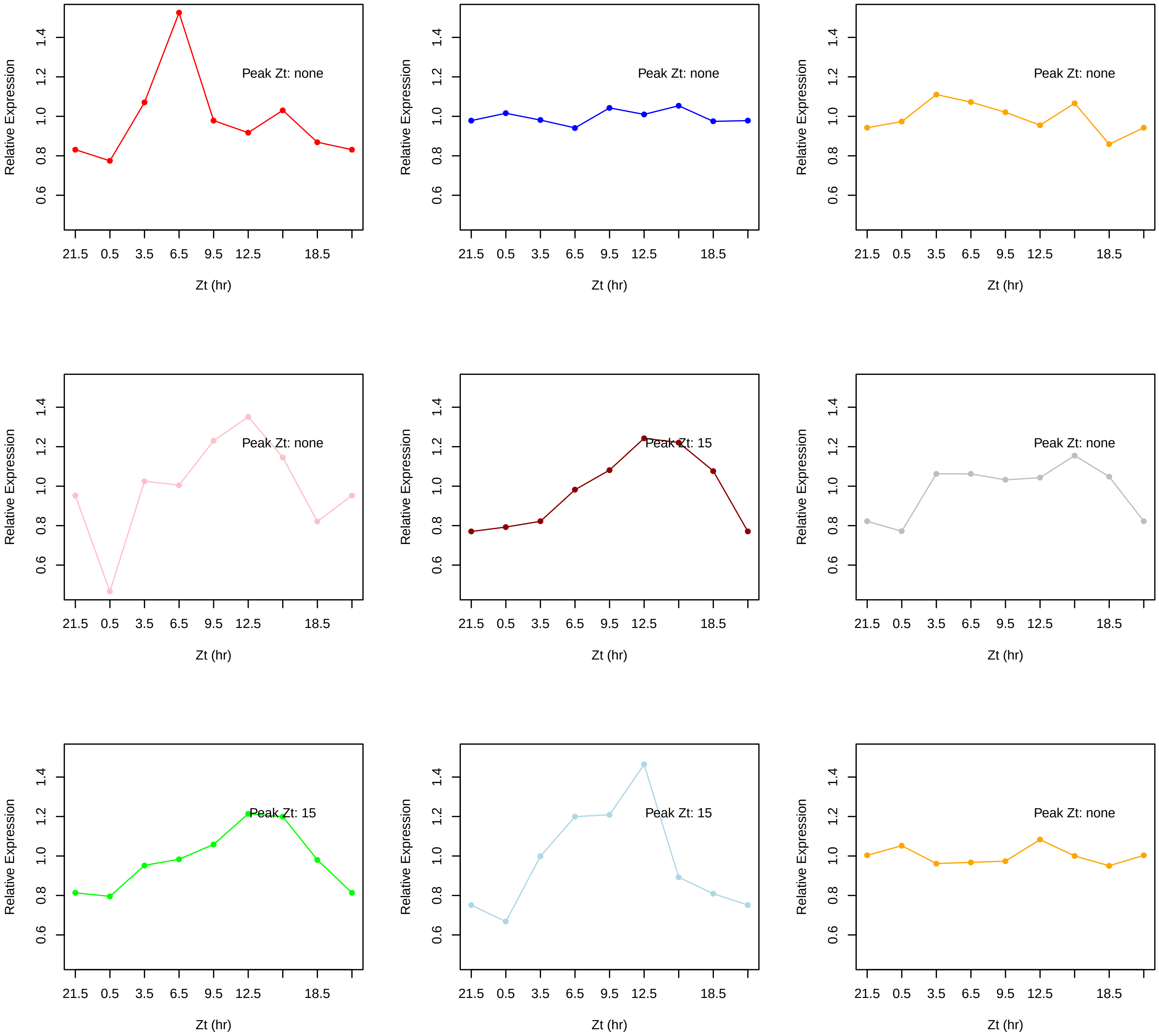

Singeleton: ENSXMAG00000010643

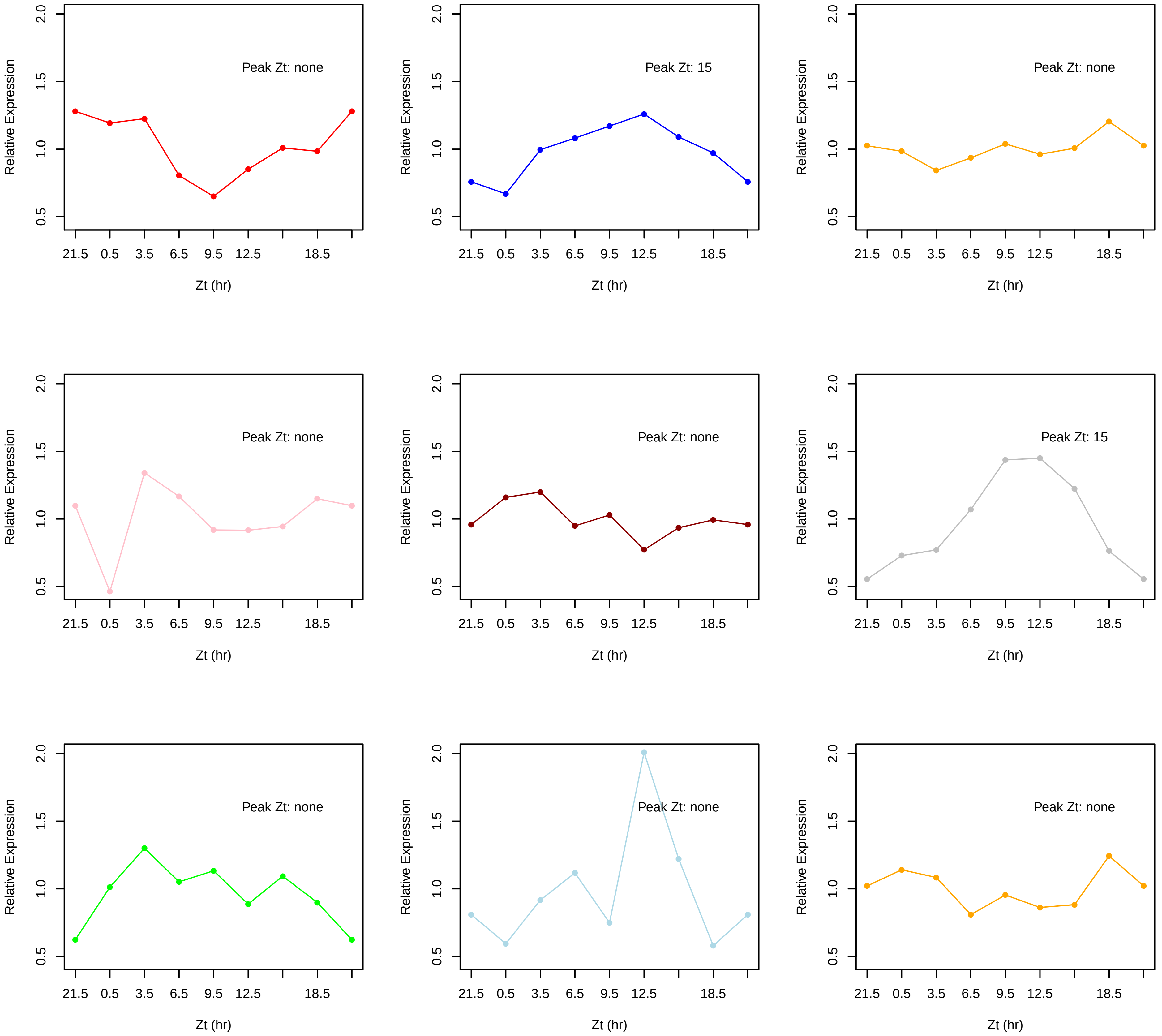

Singeleton: ENSXMAG00000007001 rras2

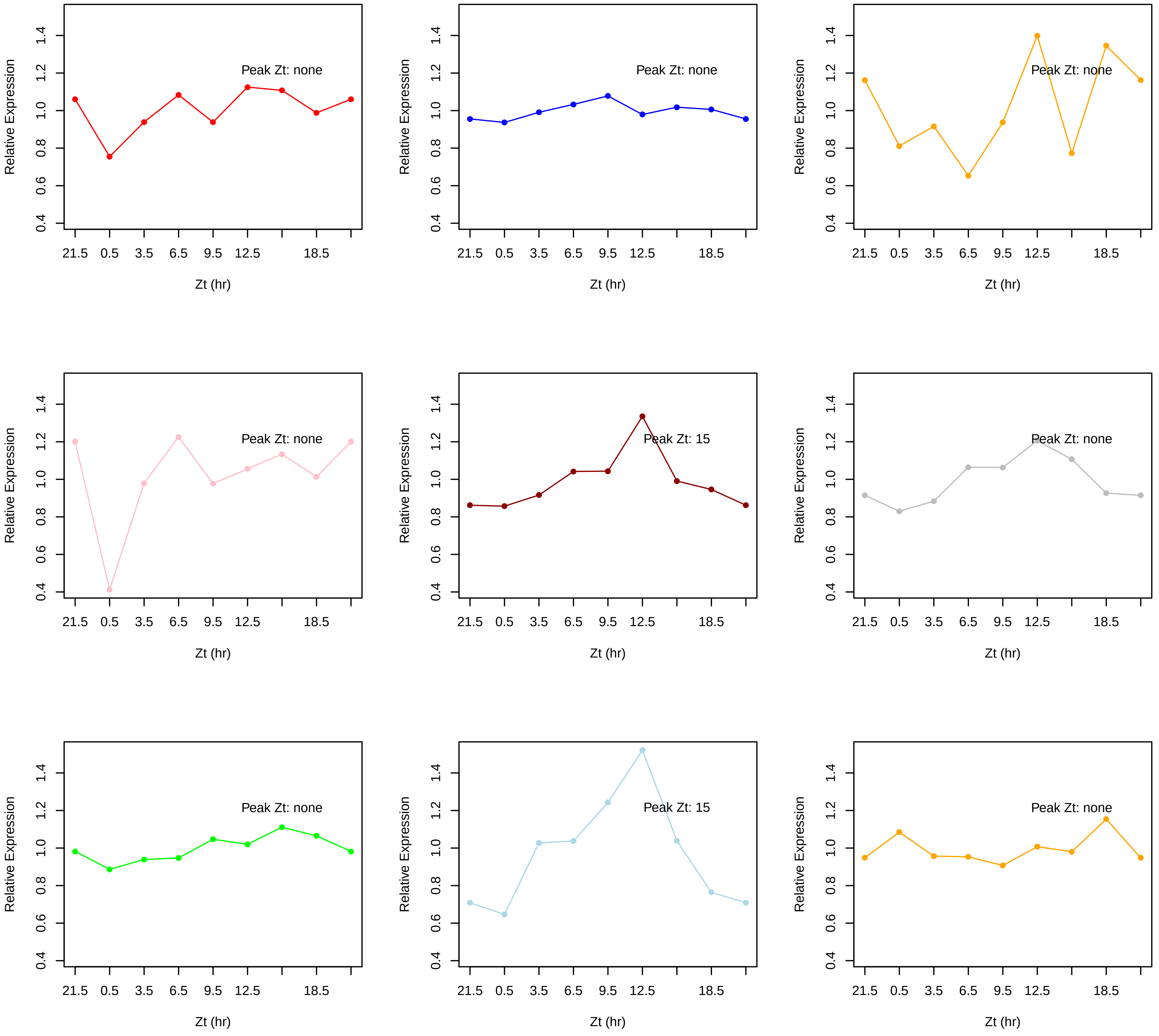

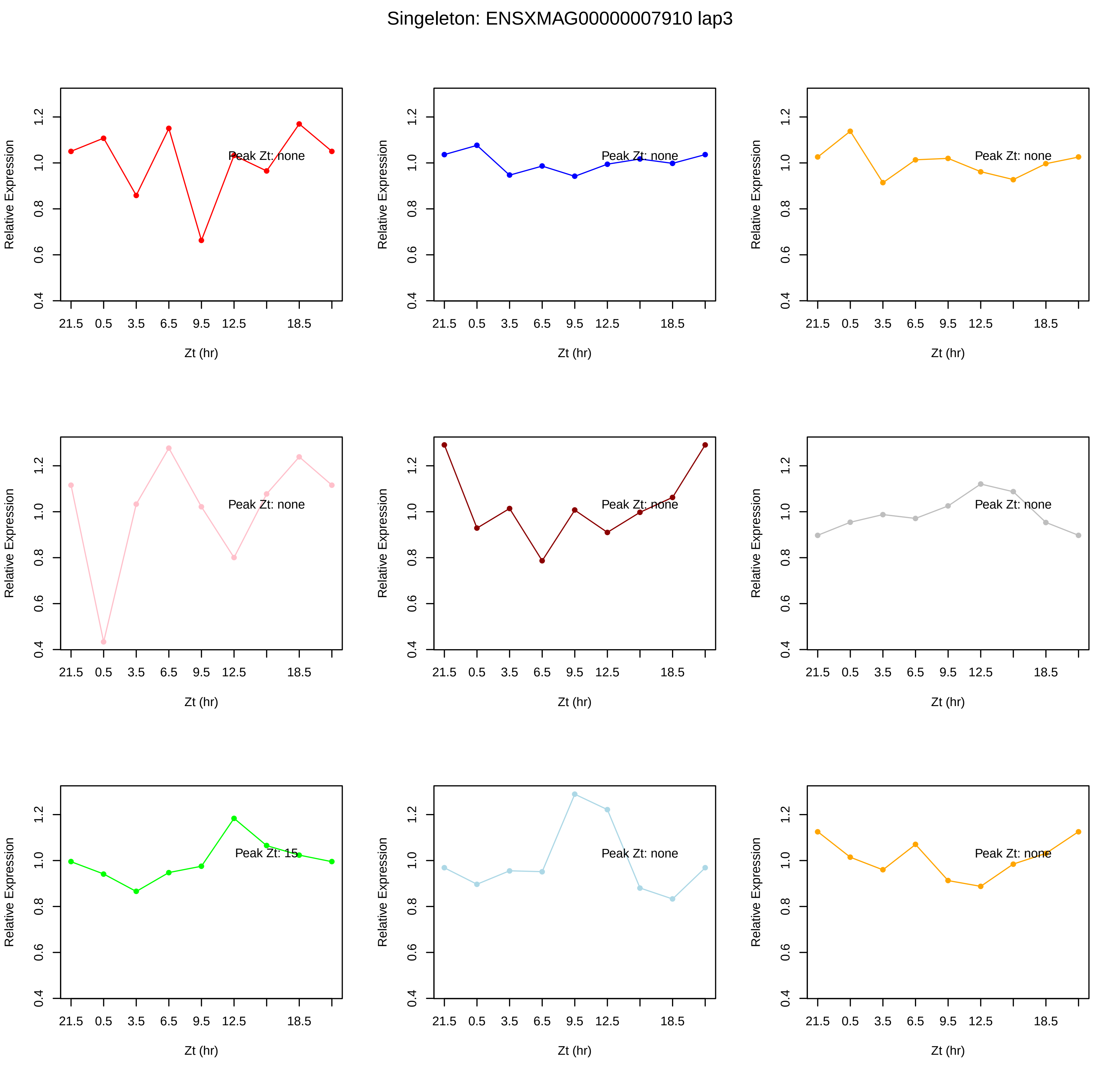
Singeleton: ENSXMAG00000007910 lap3
Relative Expression
Zt (hr)
21.5
0.5
3.5
6.5
9.5
12.5
18.5
0.4
0.6
0.8
1.0
1.2
Peak Zt: none
Peak Zt: 15

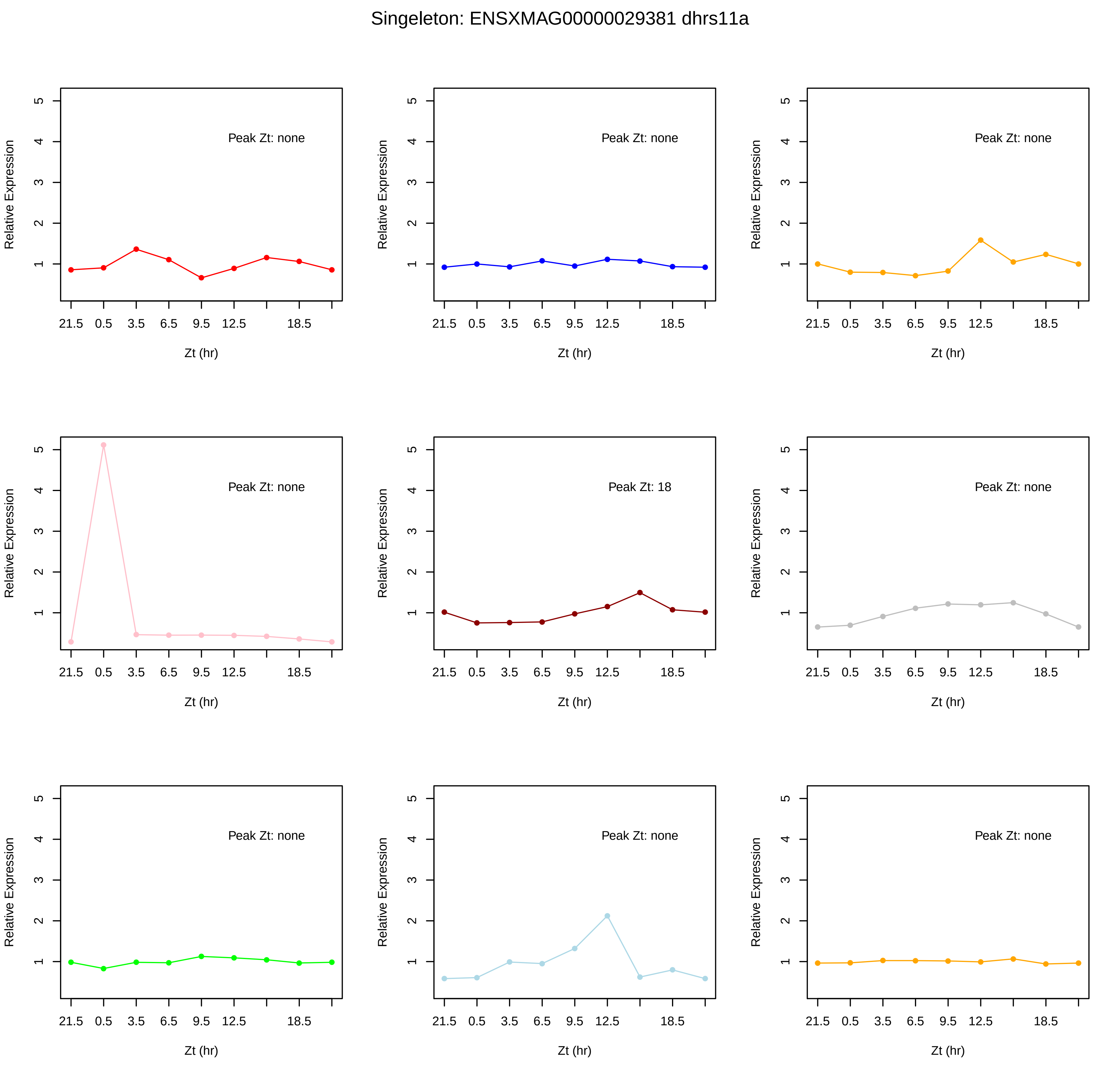
Singeleton: ENSXMAG00000029381 dhrs11a
Peak Zt: none
Peak Zt: none
Peak Zt: none
Peak Zt: none
Peak Zt: 18
Peak Zt: none
Peak Zt: none
Peak Zt: none
Peak Zt: none
Relative Expression
Zt (hr)
21.5 0.5 3.5 6.5 9.5 12.5 18.5
1 2 3 4 5

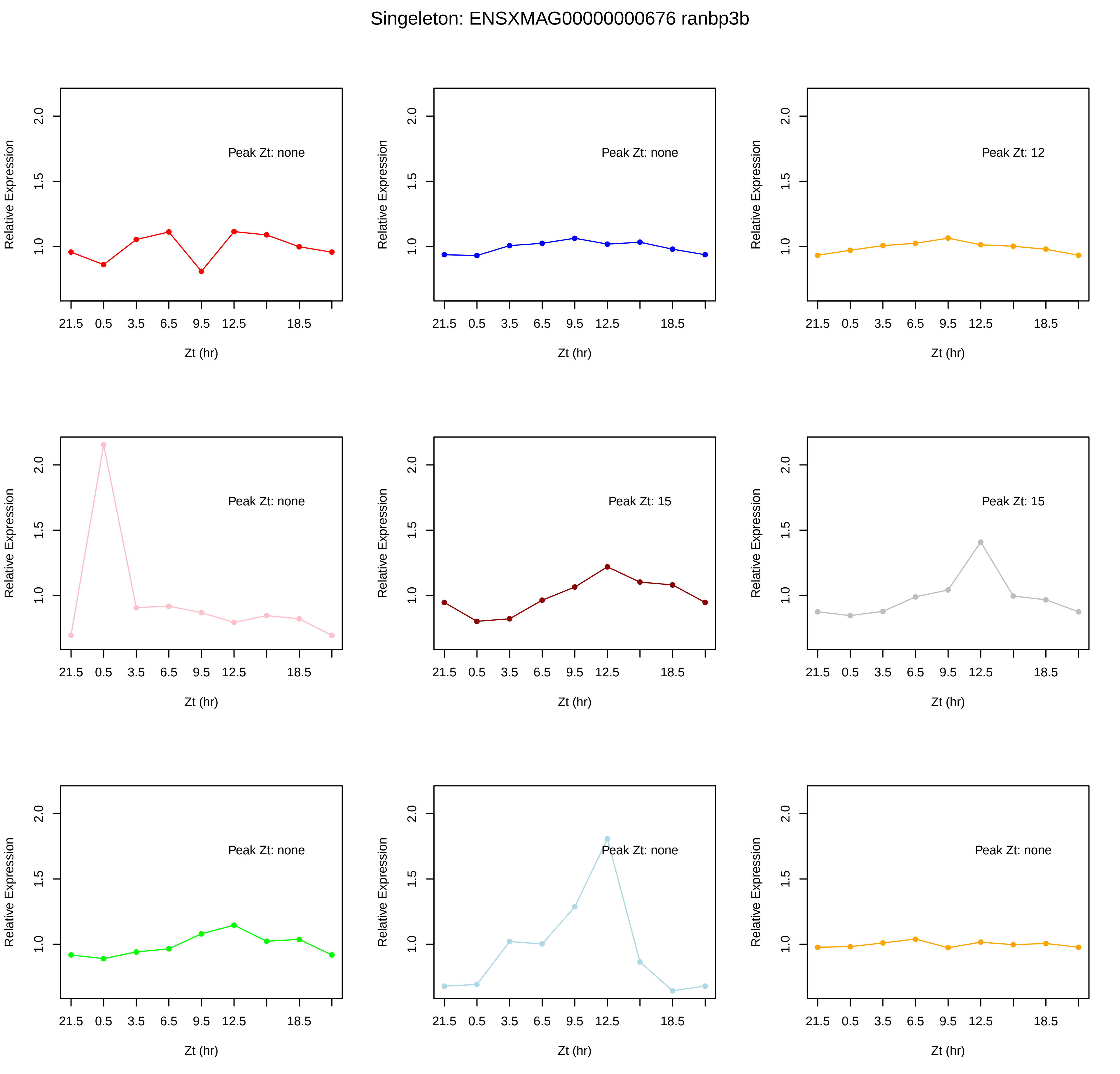
Singeleton: ENSXMAG00000000676 ranbp3b
Peak Zt: none
Peak Zt: none
Peak Zt: 12
Peak Zt: none
Peak Zt: 15
Peak Zt: 15
Peak Zt: none
Peak Zt: none
Peak Zt: none
Relative Expression
Zt (hr)
21.5 0.5 3.5 6.5 9.5 12.5 18.5
1.0 1.5 2.0

Singeleton: ENSXMAG00000019050 telo2

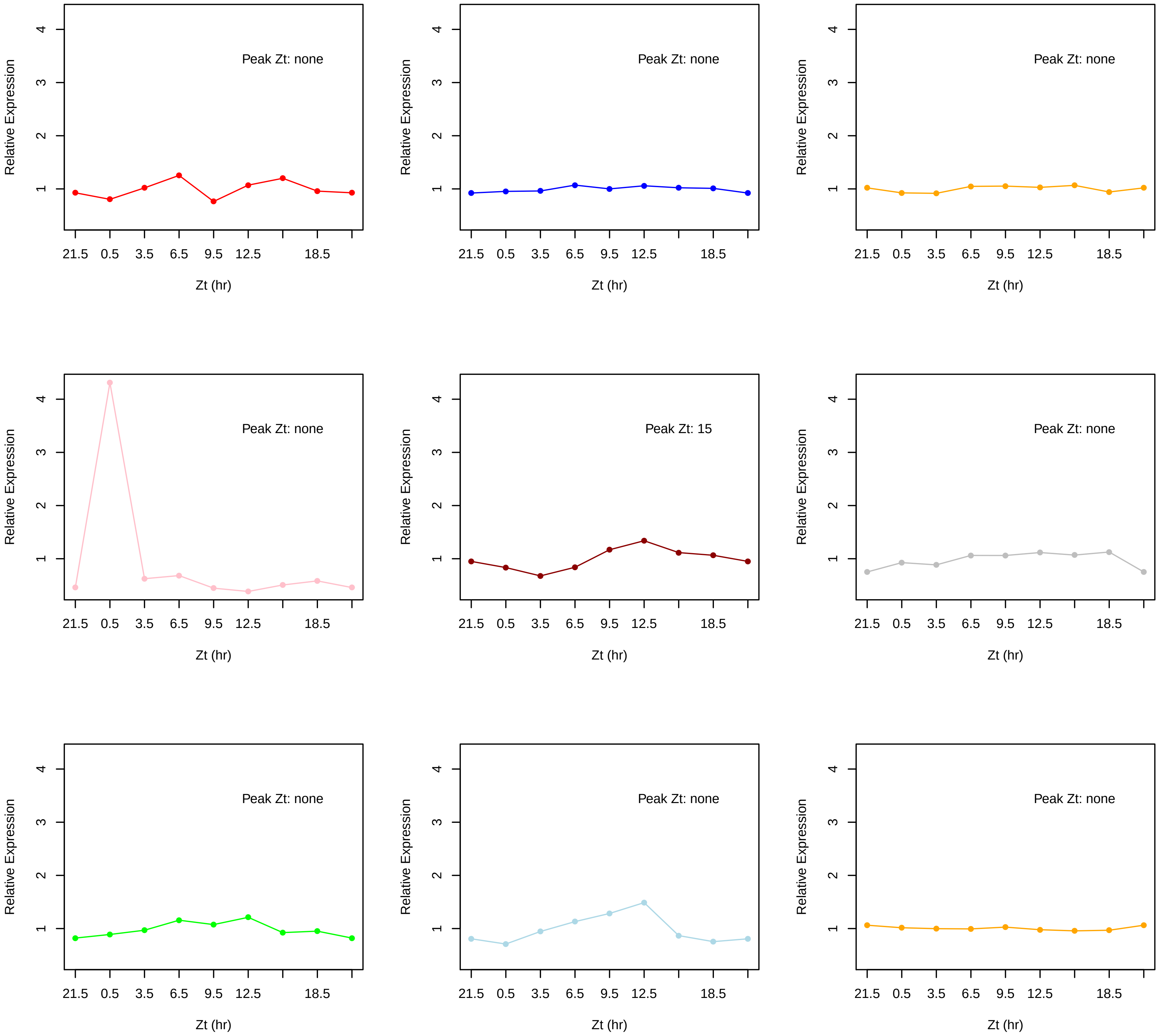

Singeleton: ENSXMAG00000022563 ccdc58

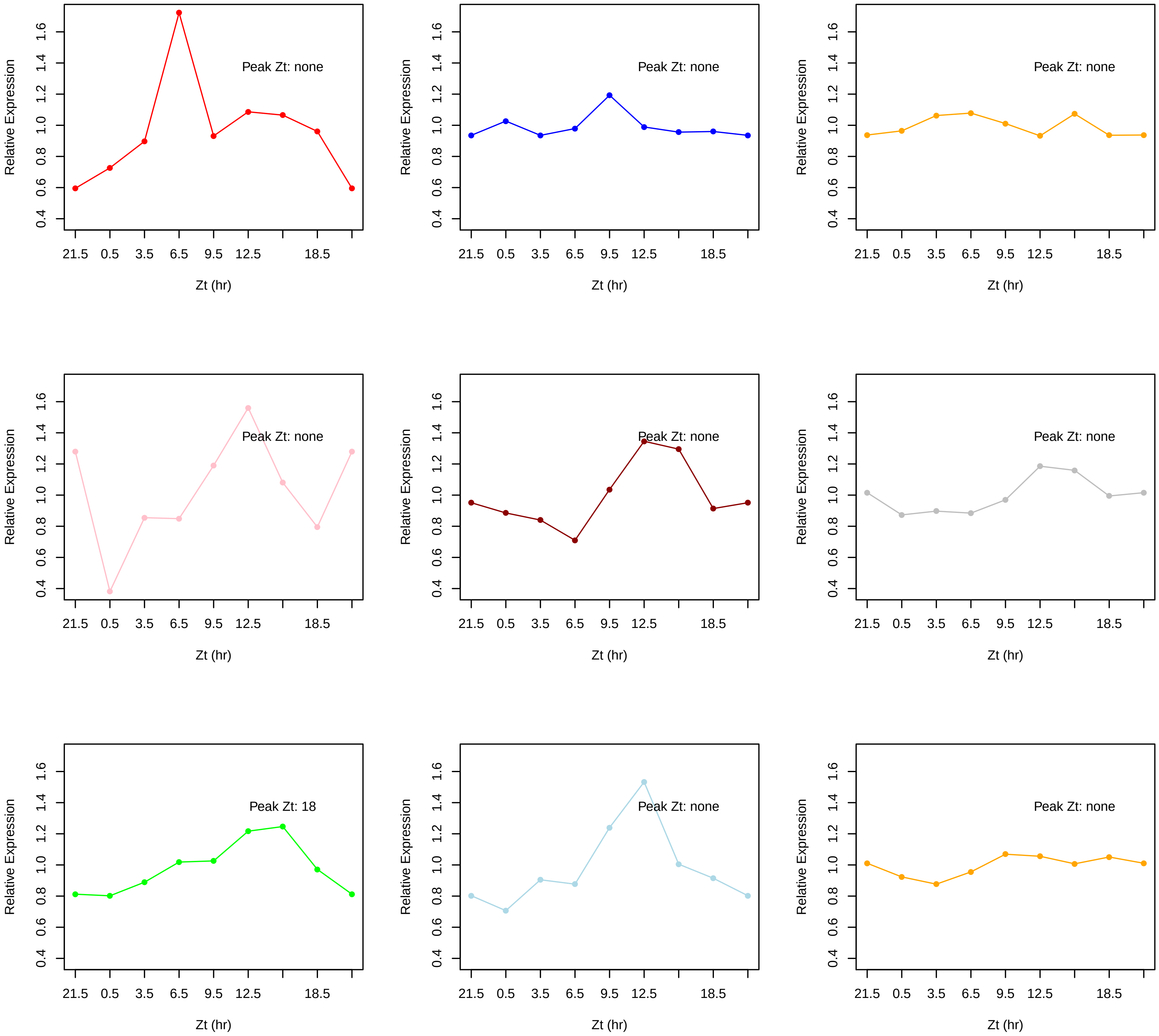

Singleton: ENSXMAG00000009510 acot8

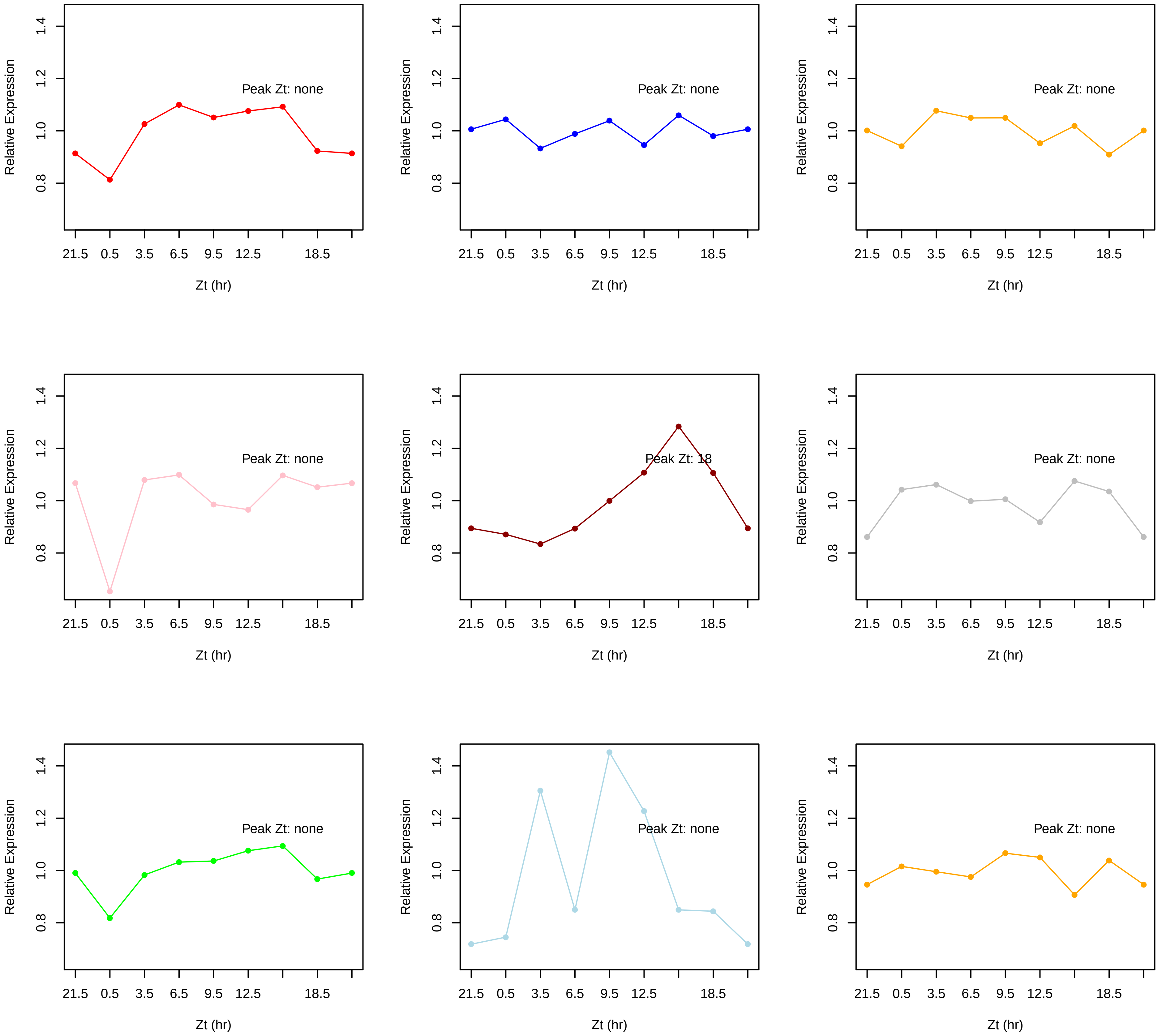

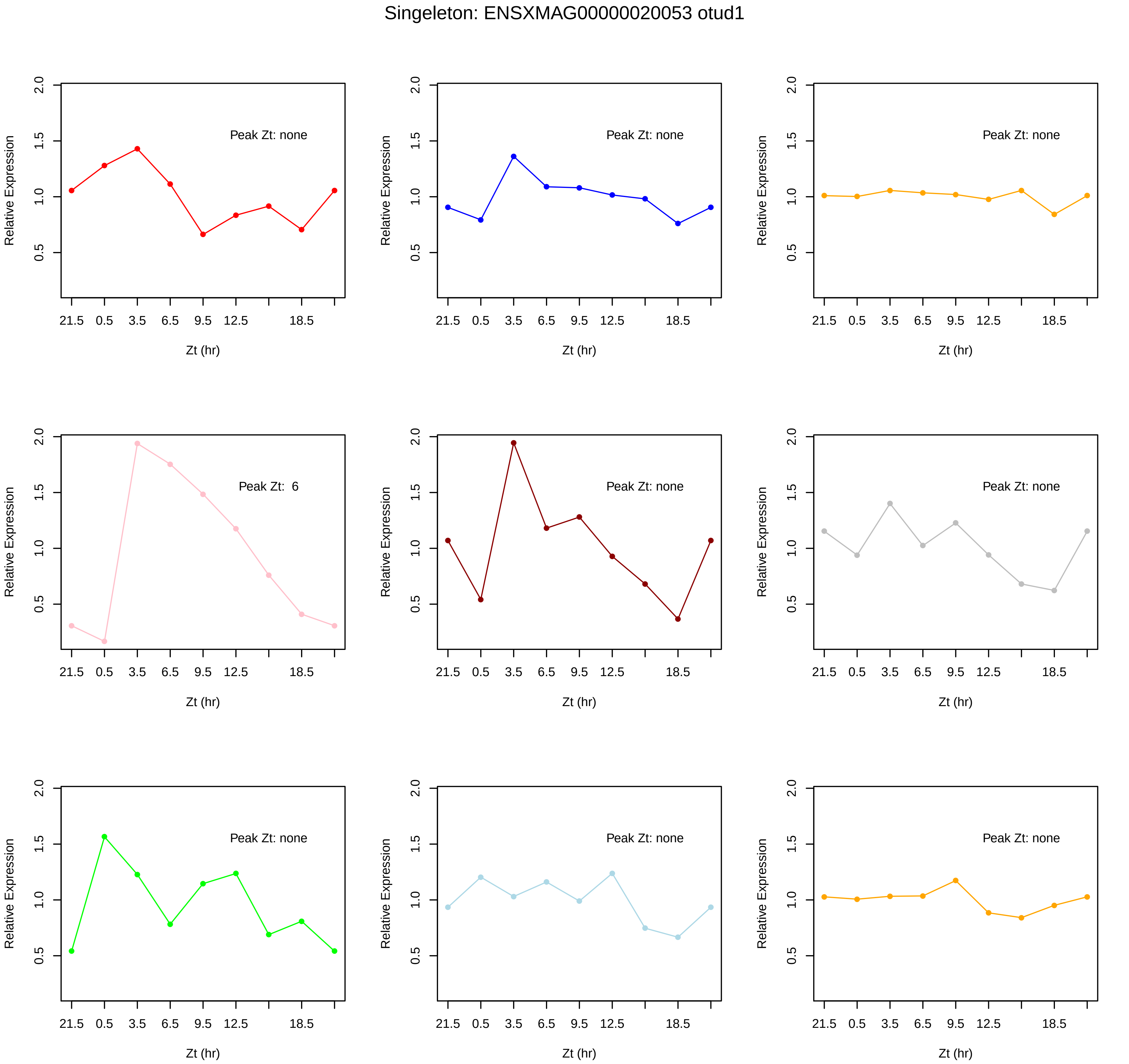
Singleton: ENSXMAG00000020053 otud1
Peak Zt: none
Peak Zt: none
Peak Zt: none
Peak Zt: 6
Peak Zt: none
Peak Zt: none
Peak Zt: none
Peak Zt: none
Peak Zt: none
Relative Expression
Zt (hr)
21.5
0.5
3.5
6.5
9.5
12.5
18.5
0.5
1.0
1.5
2.0

Singeleton: ENSXMAG00000023776 med28

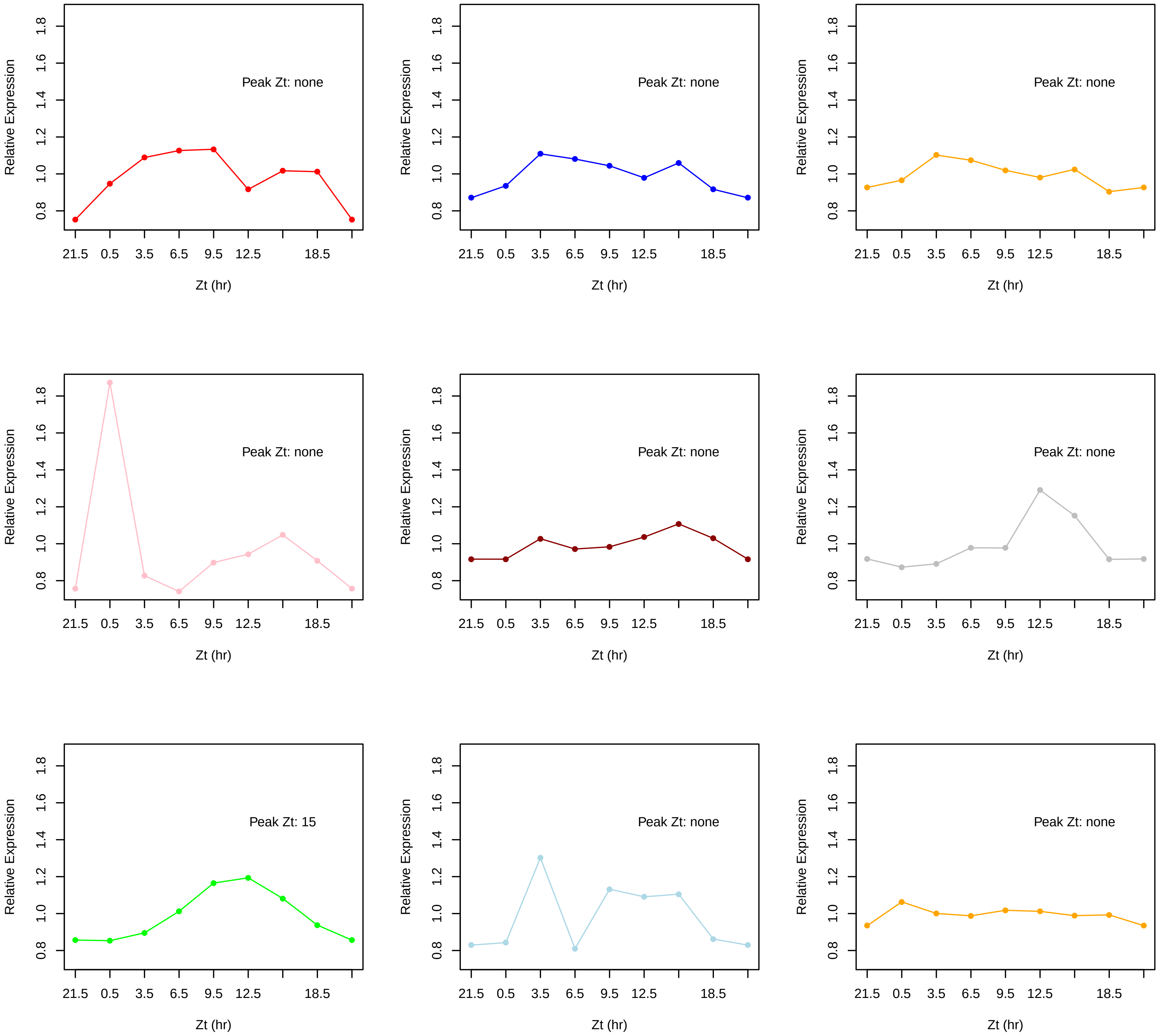

Singeleton: ENSXMAG00000021396 KCNAB1

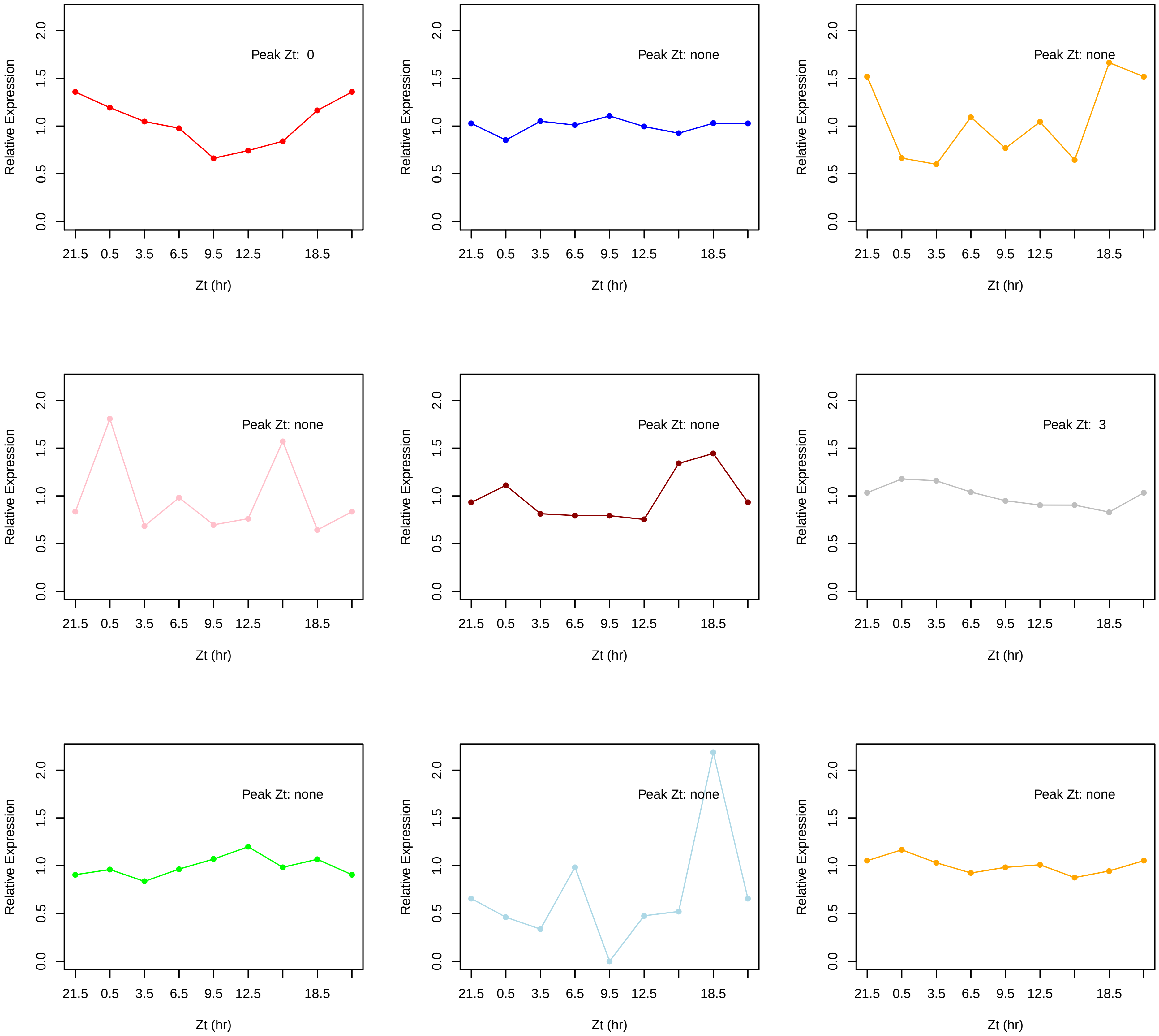

Singeleton: ENSXMAG00000009097 rnf170

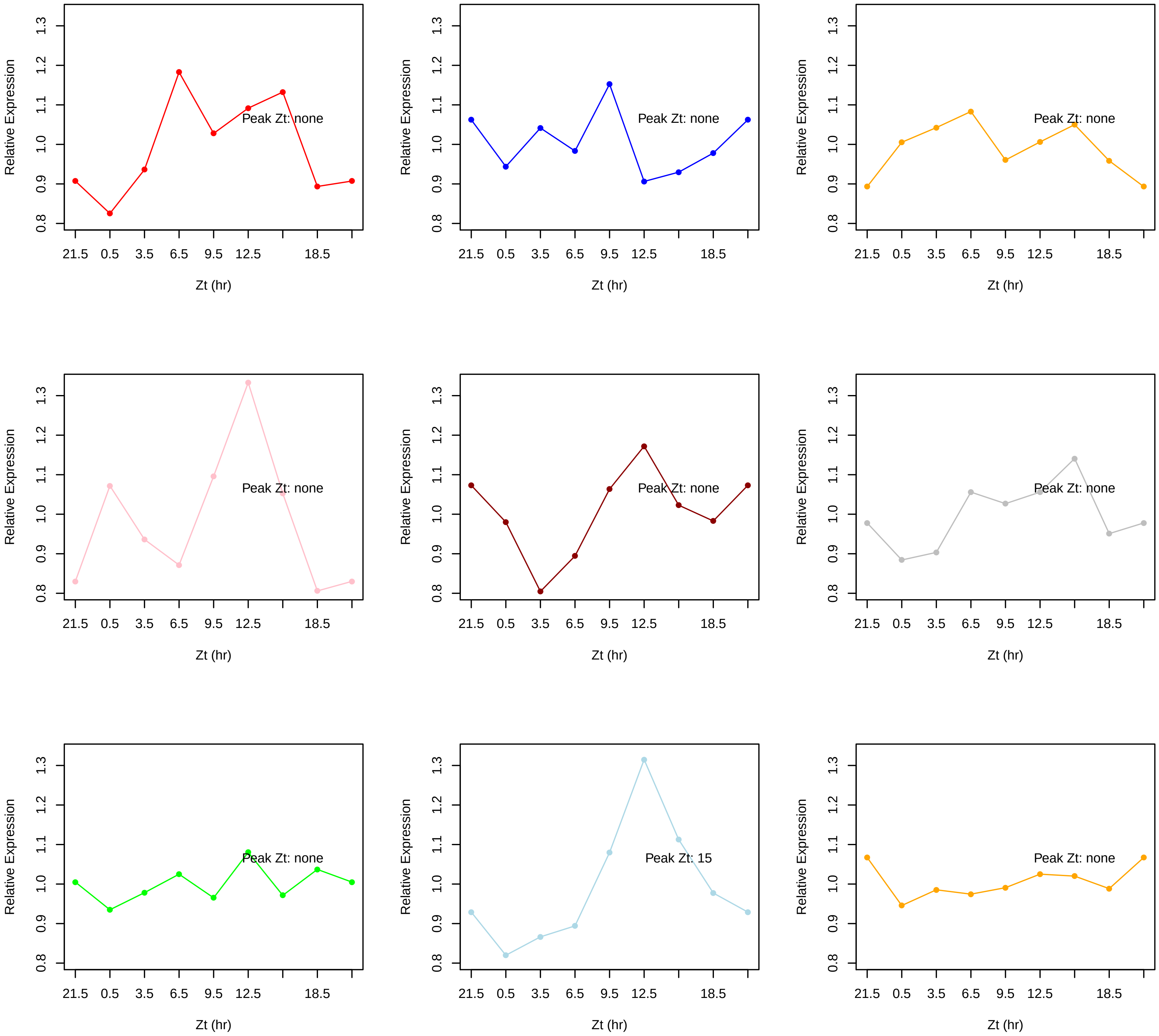

Singleton: ENSXMAG00000022713 suds3

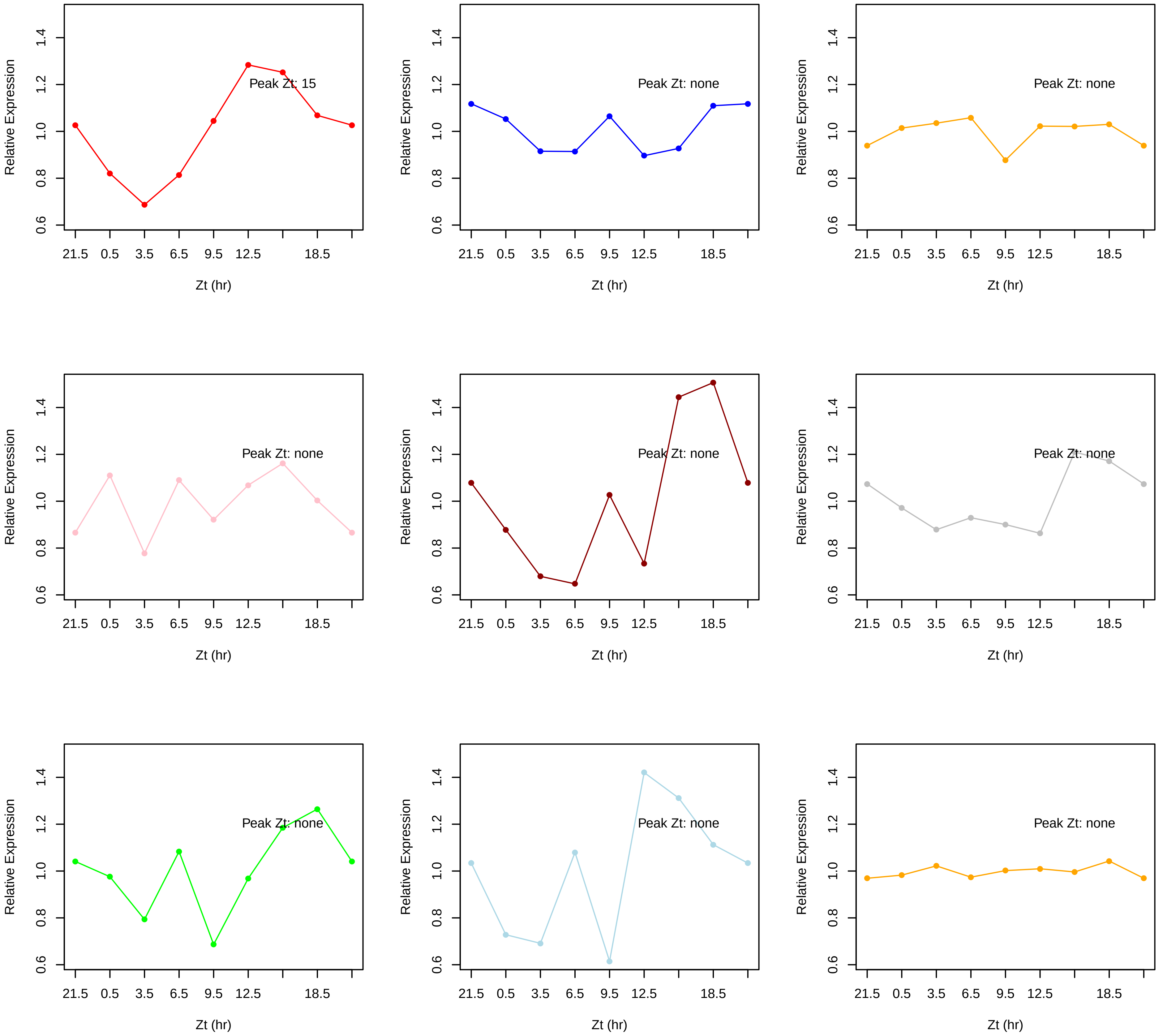

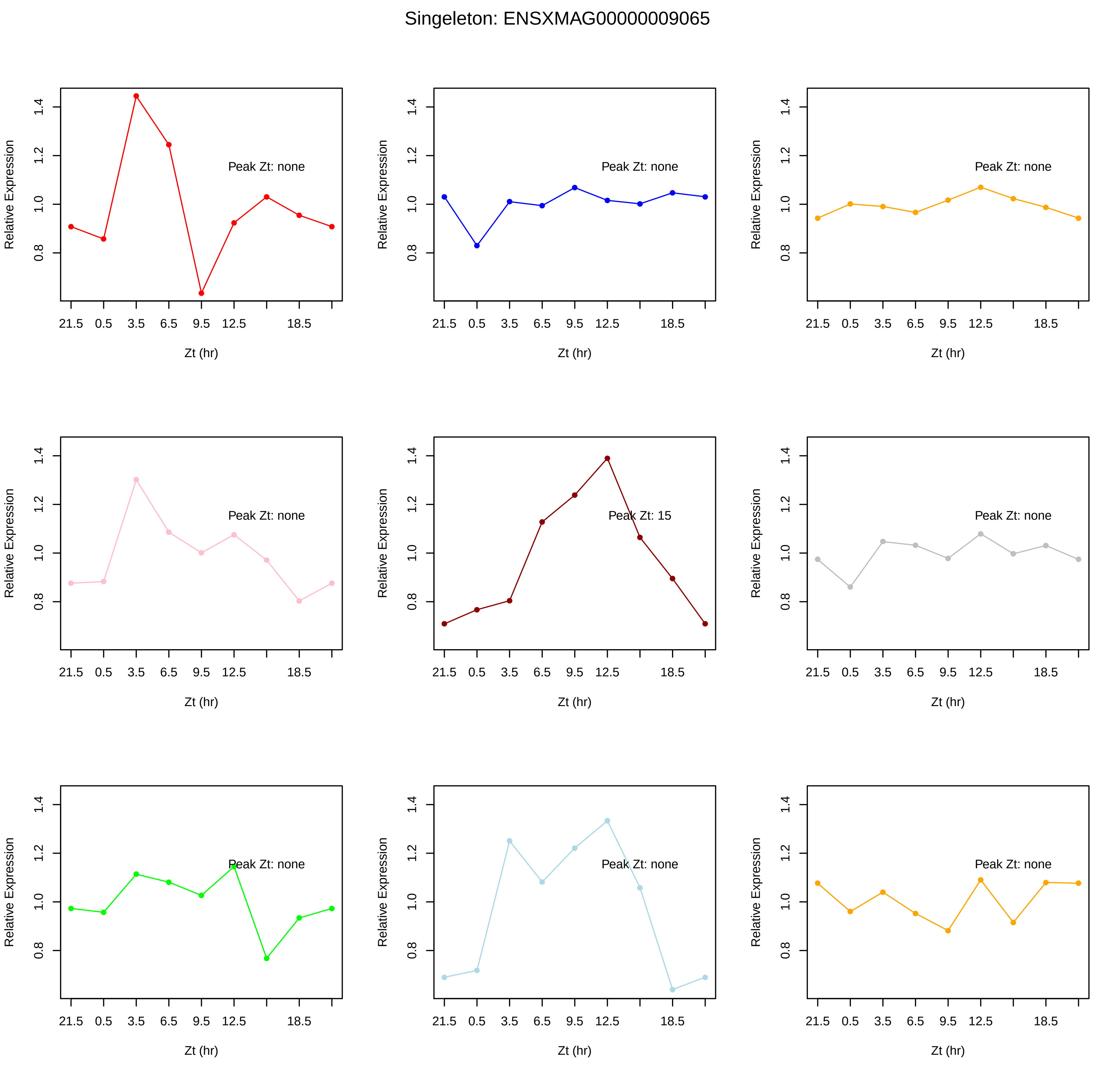
Singeleton: ENSXMAG00000009065
Peak Zt: none
Peak Zt: none
Peak Zt: none
Peak Zt: none
Peak Zt: 15
Peak Zt: none
Peak Zt: none
Peak Zt: none
Peak Zt: none
Relative Expression
Zt (hr)
21.5
0.5
3.5
6.5
9.5
12.5
18.5
0.8
1.0
1.2
1.4

Singleton: ENSXMAG00000022116

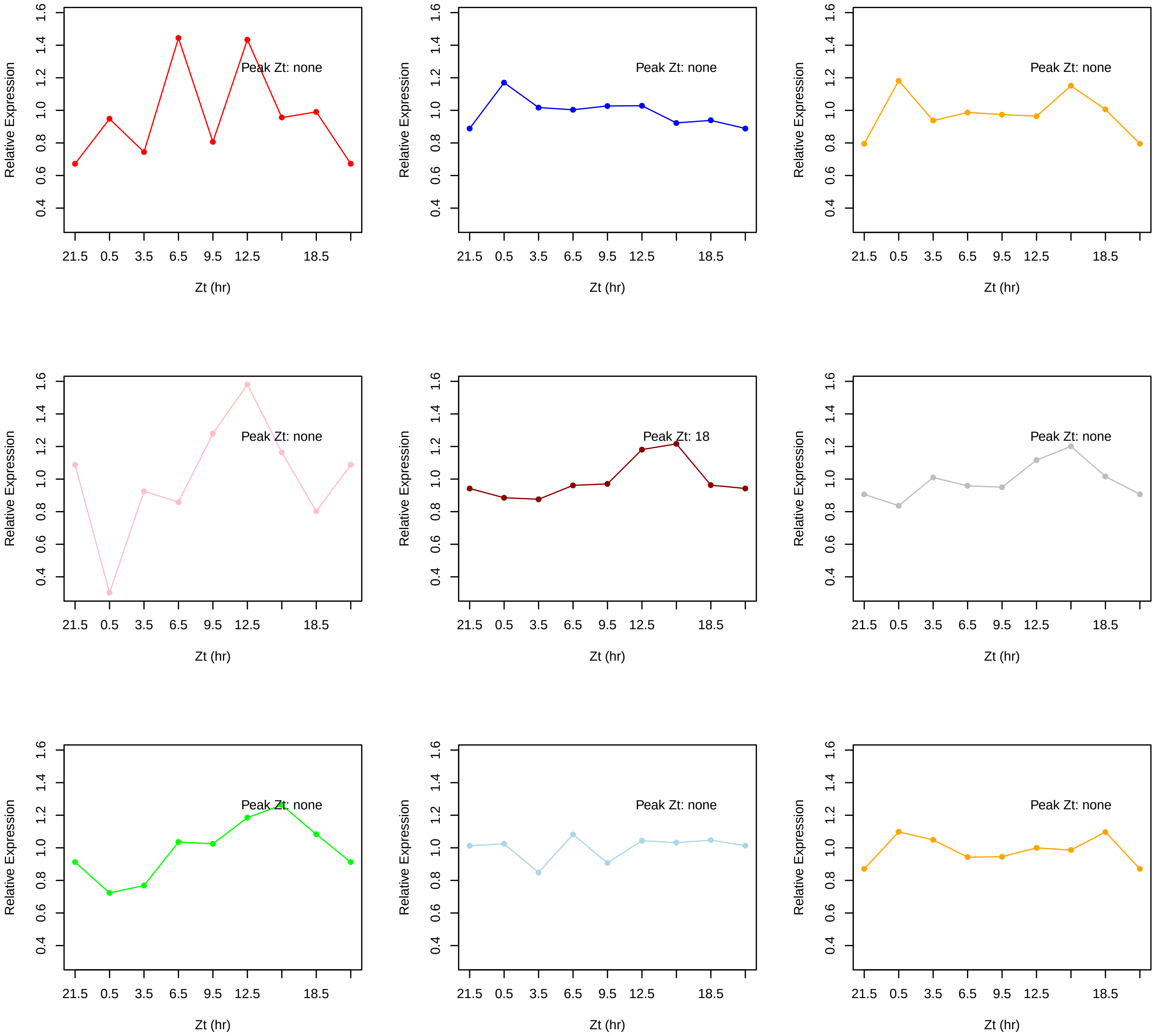

Singeleton: ENSXMAG00000015276 ano10b

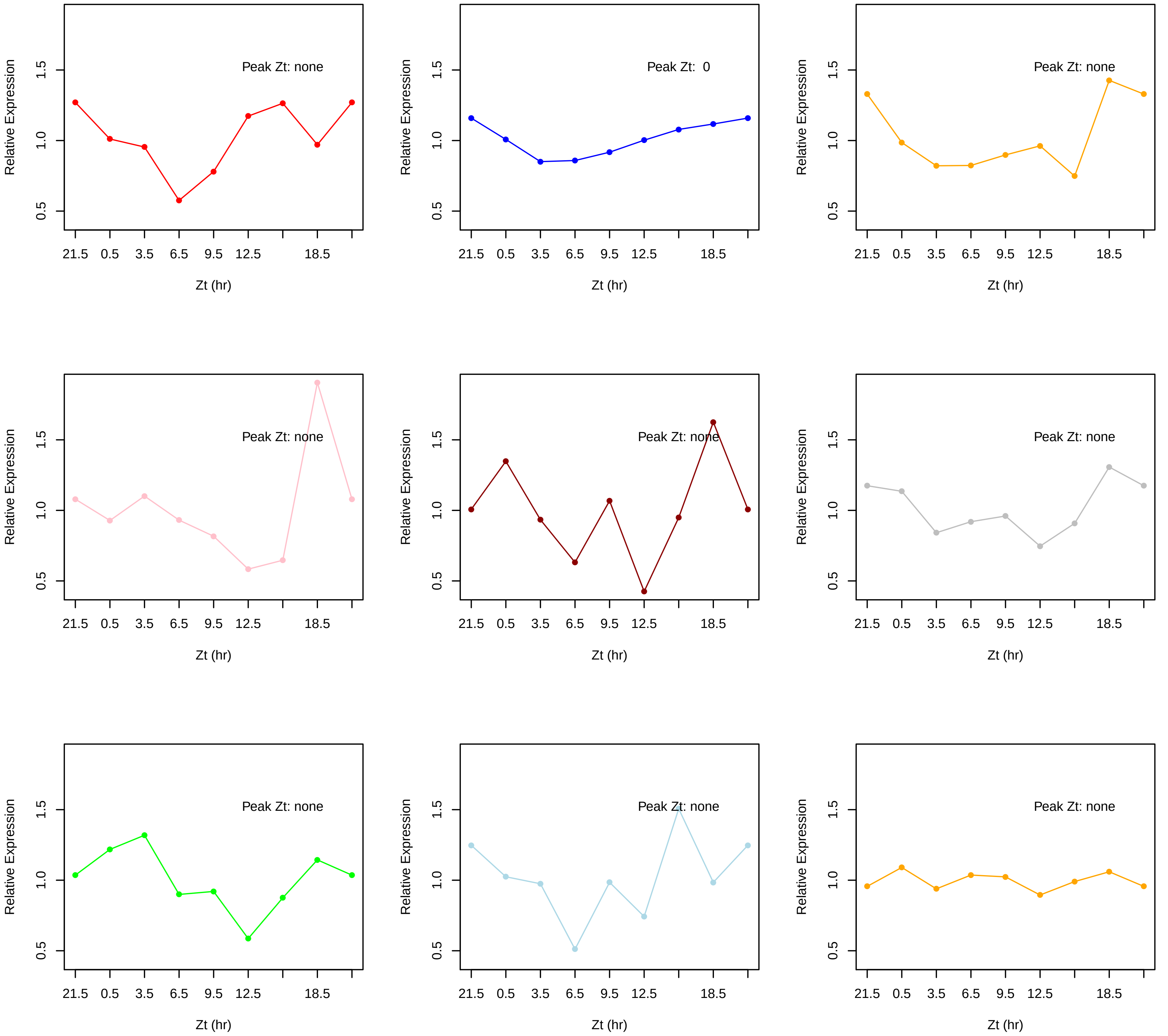

Singleton: ENSXMAG00000027948 kpna1

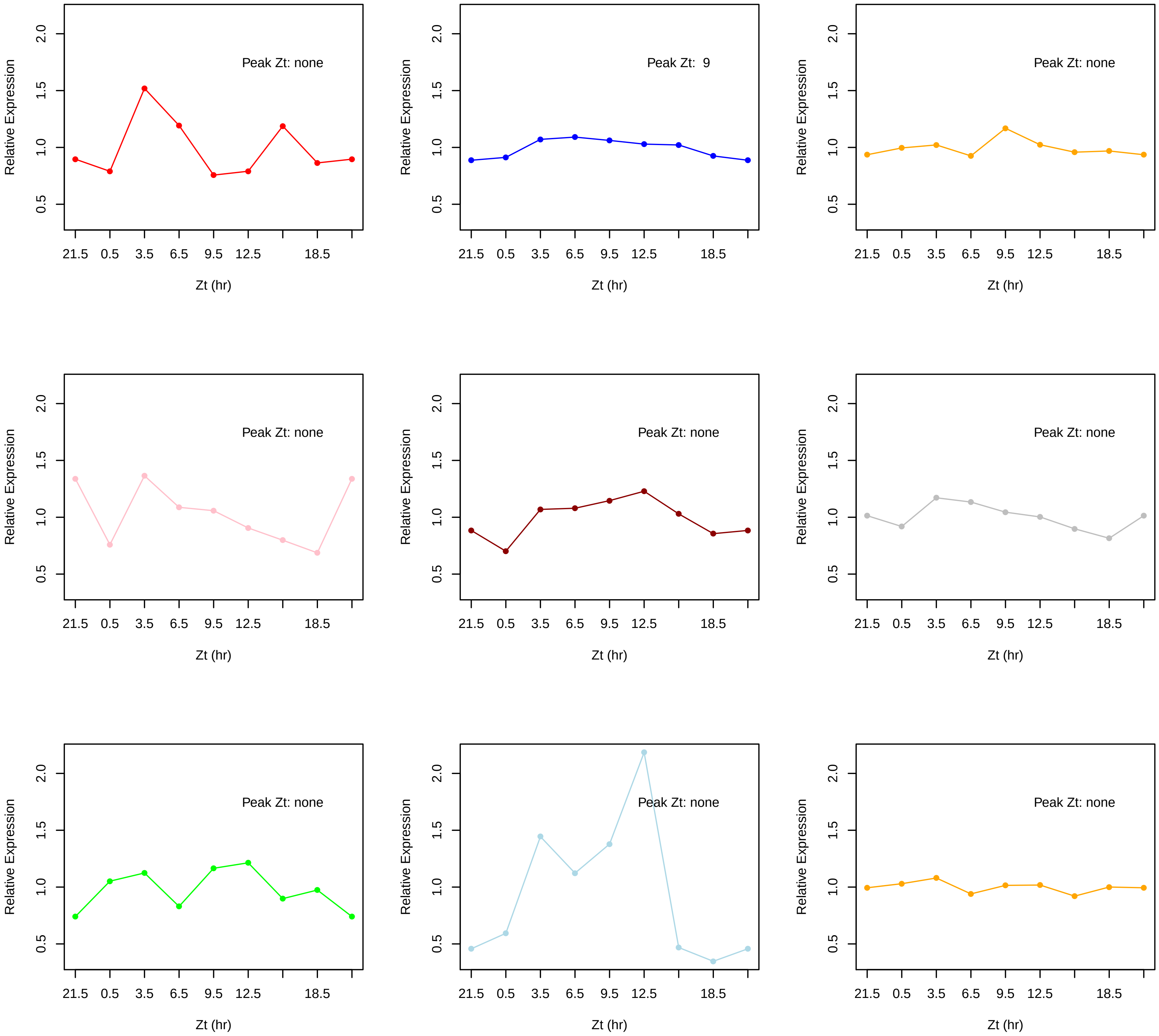

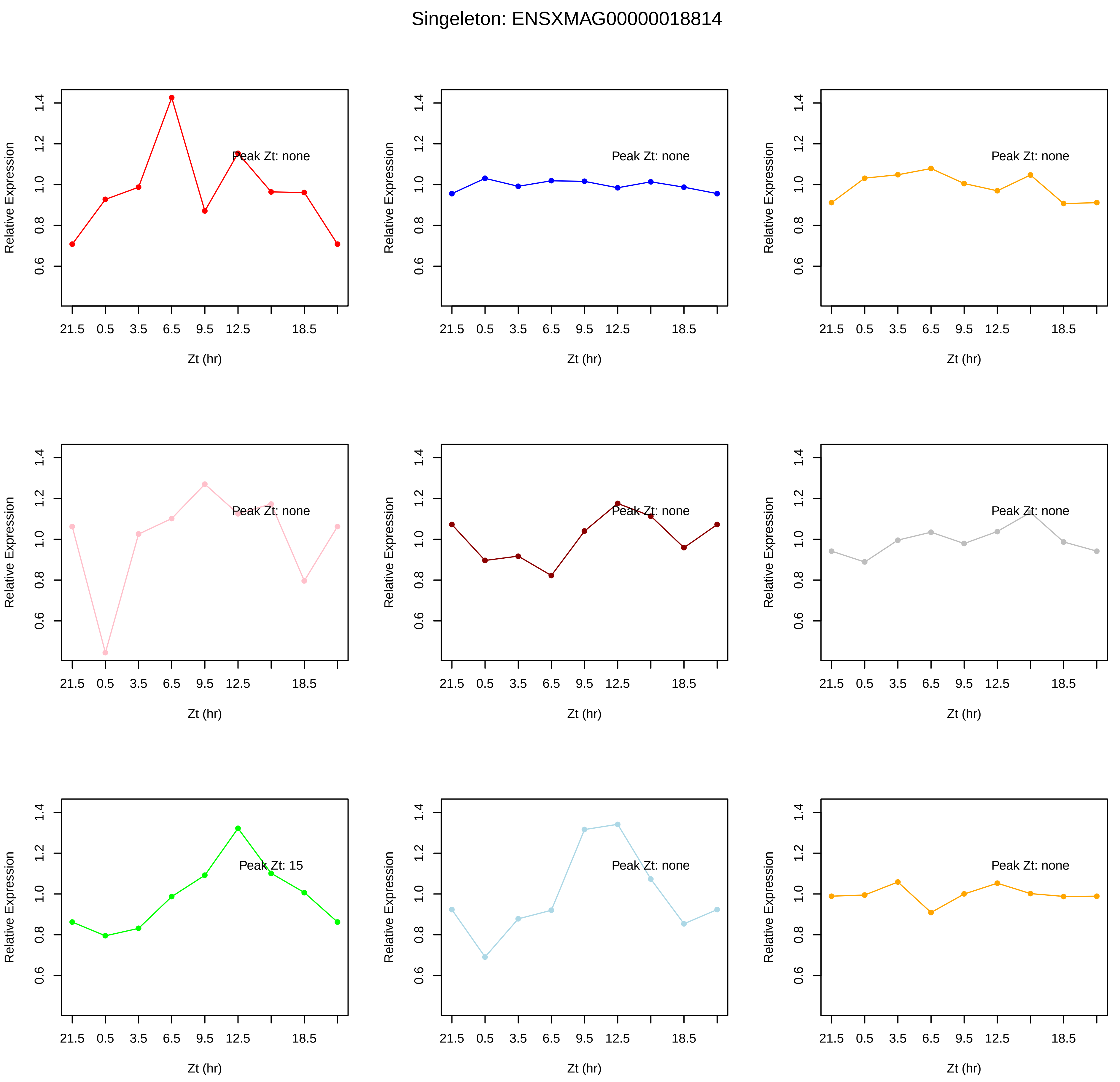

Singeleton: ENSXMAG00000018814
Relative Expression
0.6
0.8
1.0
1.2
1.4
Zt (hr)
21.5
0.5
3.5
6.5
9.5
12.5
18.5
Peak Zt: none
Peak Zt: none
Peak Zt: none
Peak Zt: none
Peak Zt: none
Peak Zt: none
Peak Zt: 15
Peak Zt: none
Peak Zt: none

Singeleton: ENSXMAG00000017141 ssr3

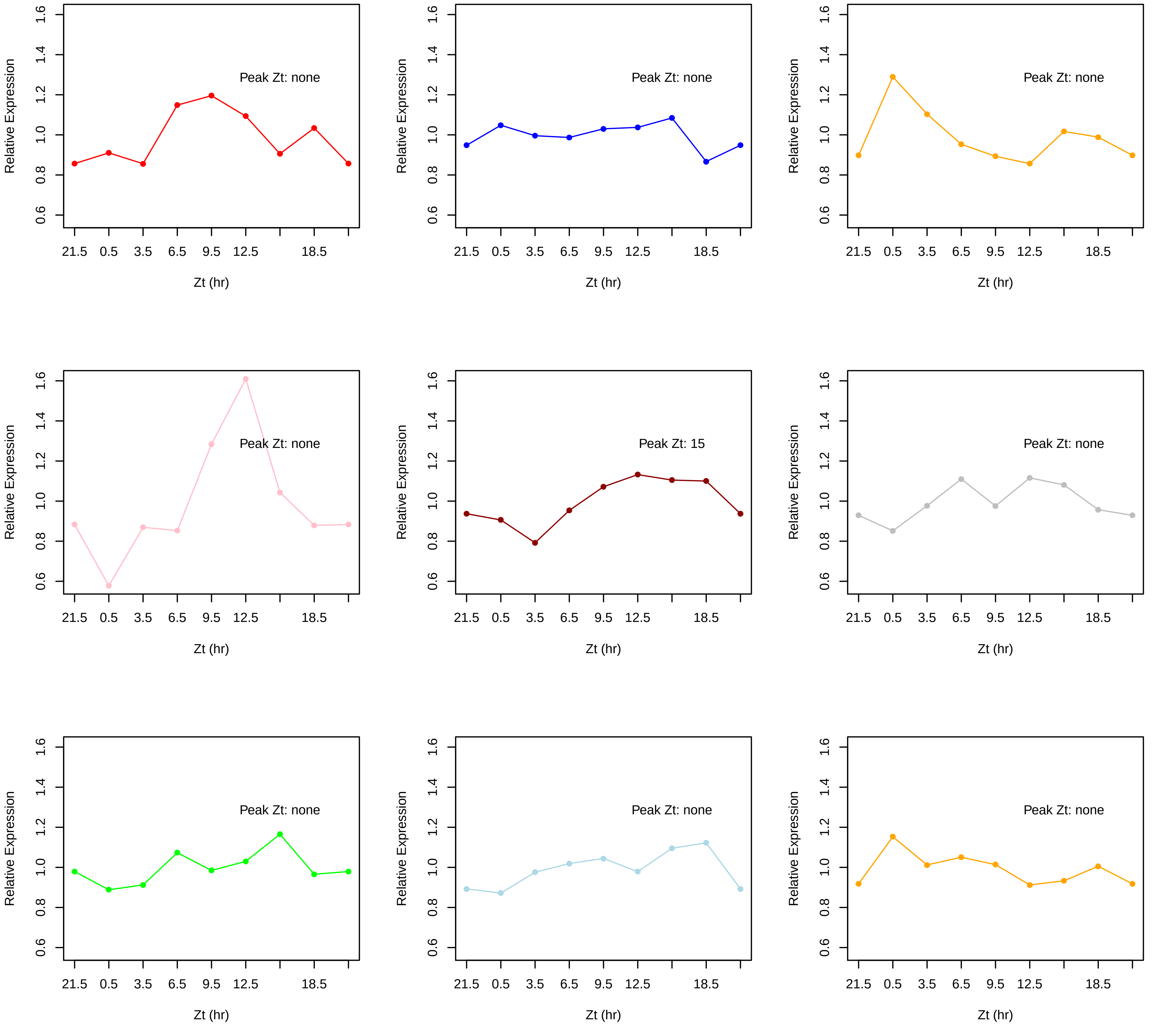

Singeleton: ENSXMAG00000014629 inpp5b

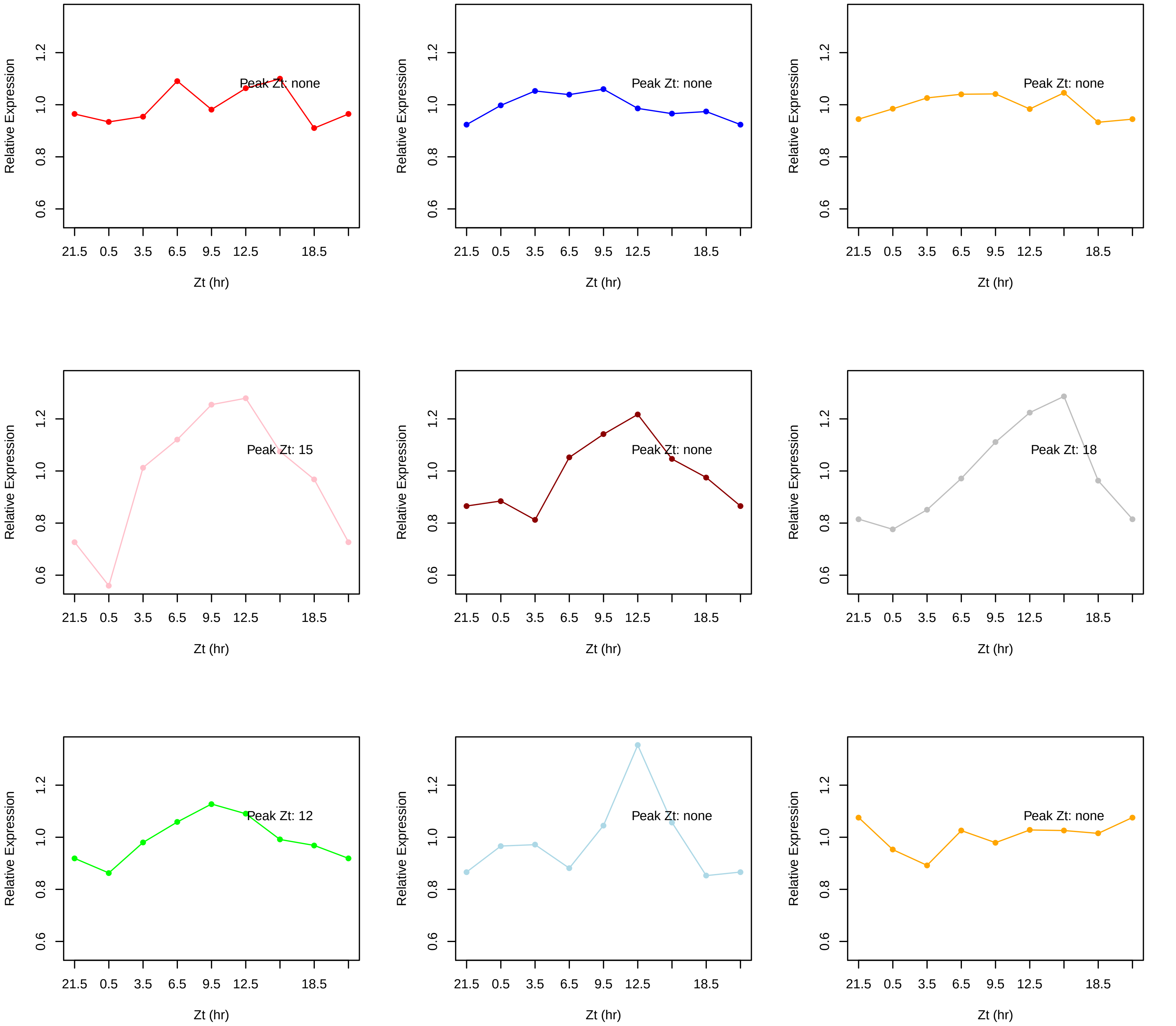

Singeleton: ENSXMAG00000015290 snrkb

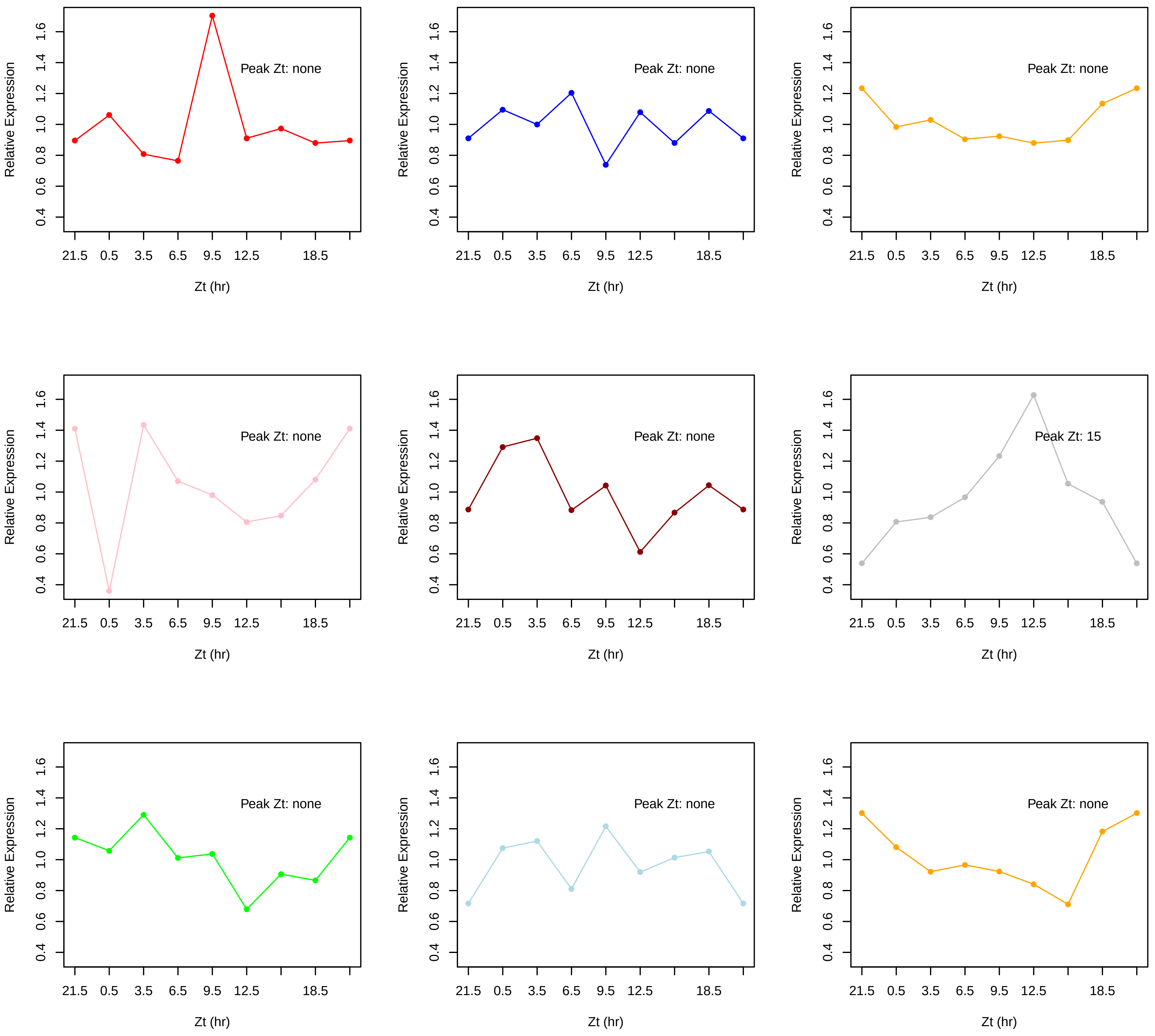

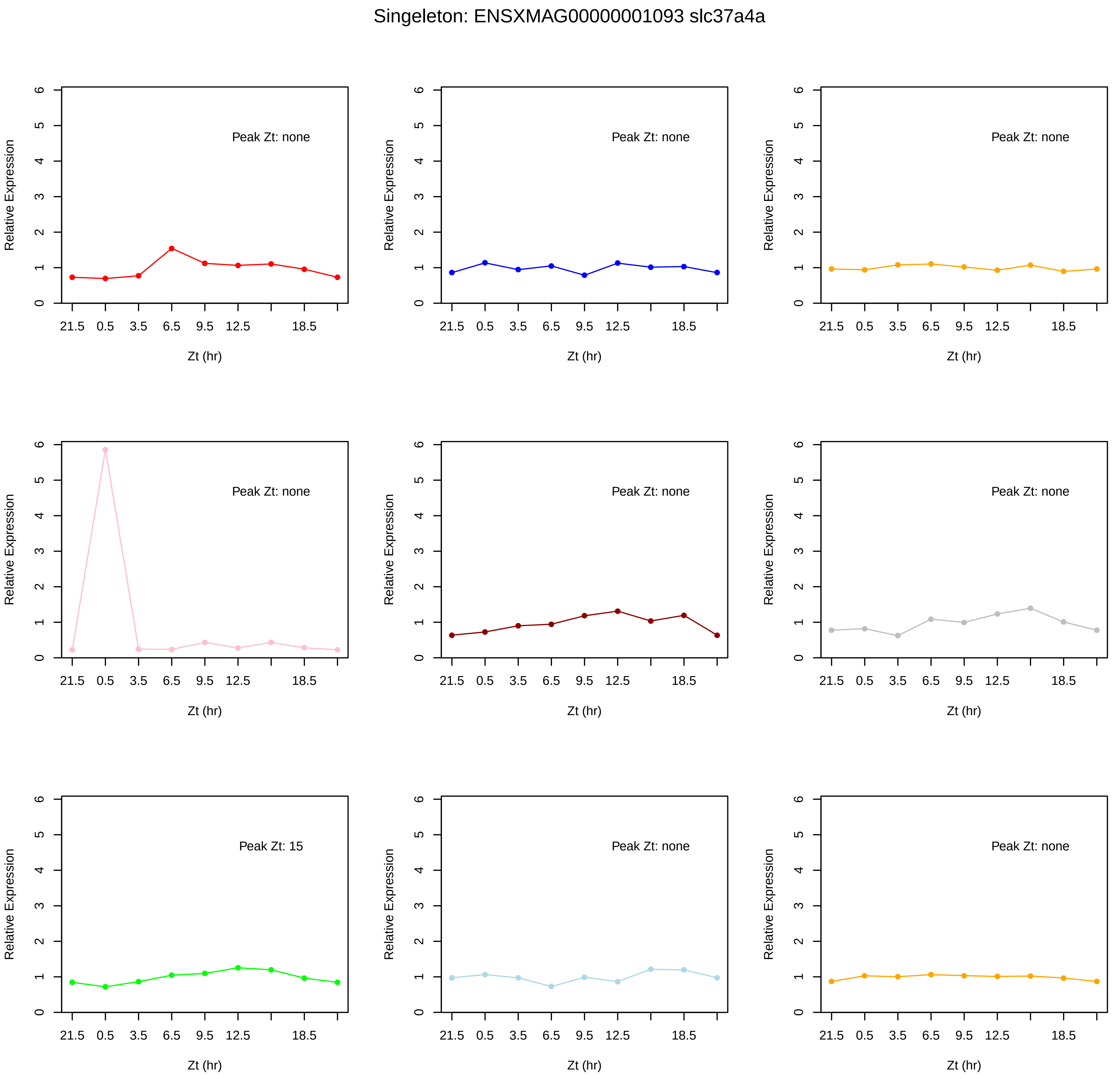
Singeleton: ENSXMAG00000001093 slc37a4a
Relative Expression
Zt (hr)
Peak Zt: none
Peak Zt: none
Peak Zt: none
Peak Zt: none
Peak Zt: none
Peak Zt: none
Peak Zt: 15
Peak Zt: none
Peak Zt: none
0
1
2
3
4
5
6
21.5
0.5
3.5
6.5
9.5
12.5
18.5

Singeleton: ENSXMAG00000025483 ccnl1a

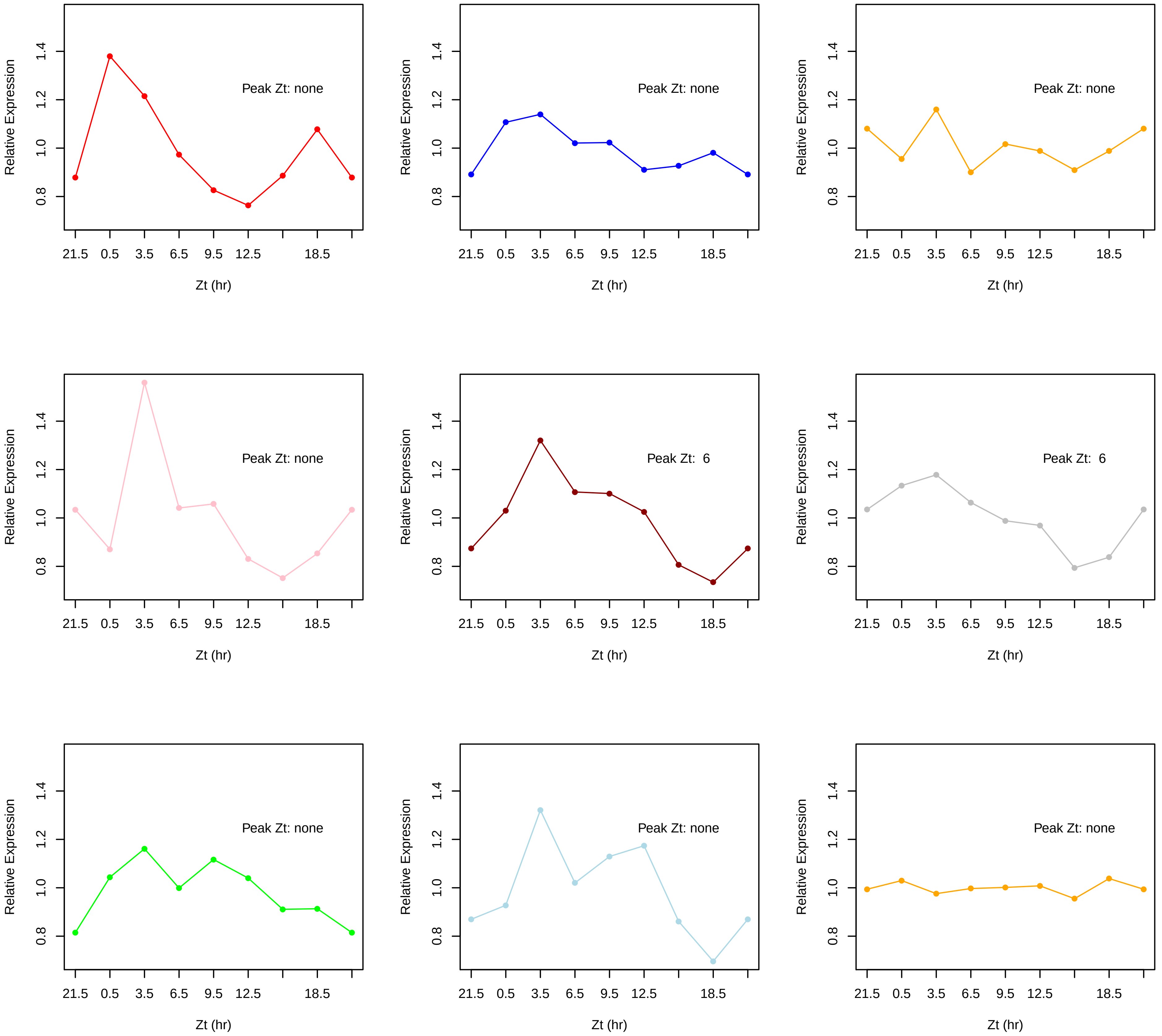

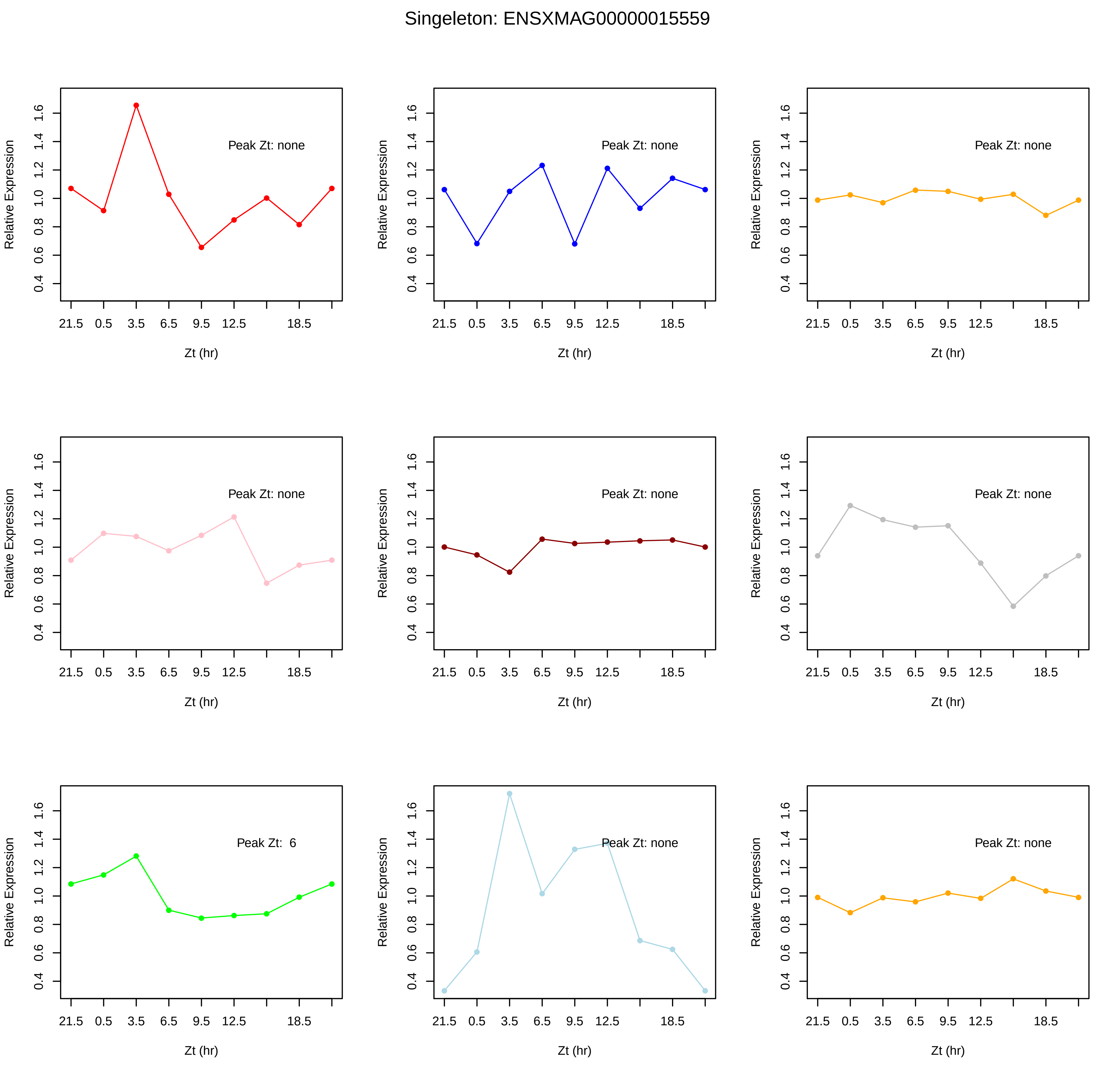
Singleton: ENSXMAG00000015559
Peak Zt: none
Peak Zt: none
Peak Zt: none
Peak Zt: none
Peak Zt: none
Peak Zt: none
Peak Zt: 6
Peak Zt: none
Peak Zt: none
Relative Expression
Zt (hr)
21.5 0.5 3.5 6.5 9.5 12.5 18.5
0.4 0.6 0.8 1.0 1.2 1.4 1.6

Singeleton: ENSXMAG00000002632 pebp1

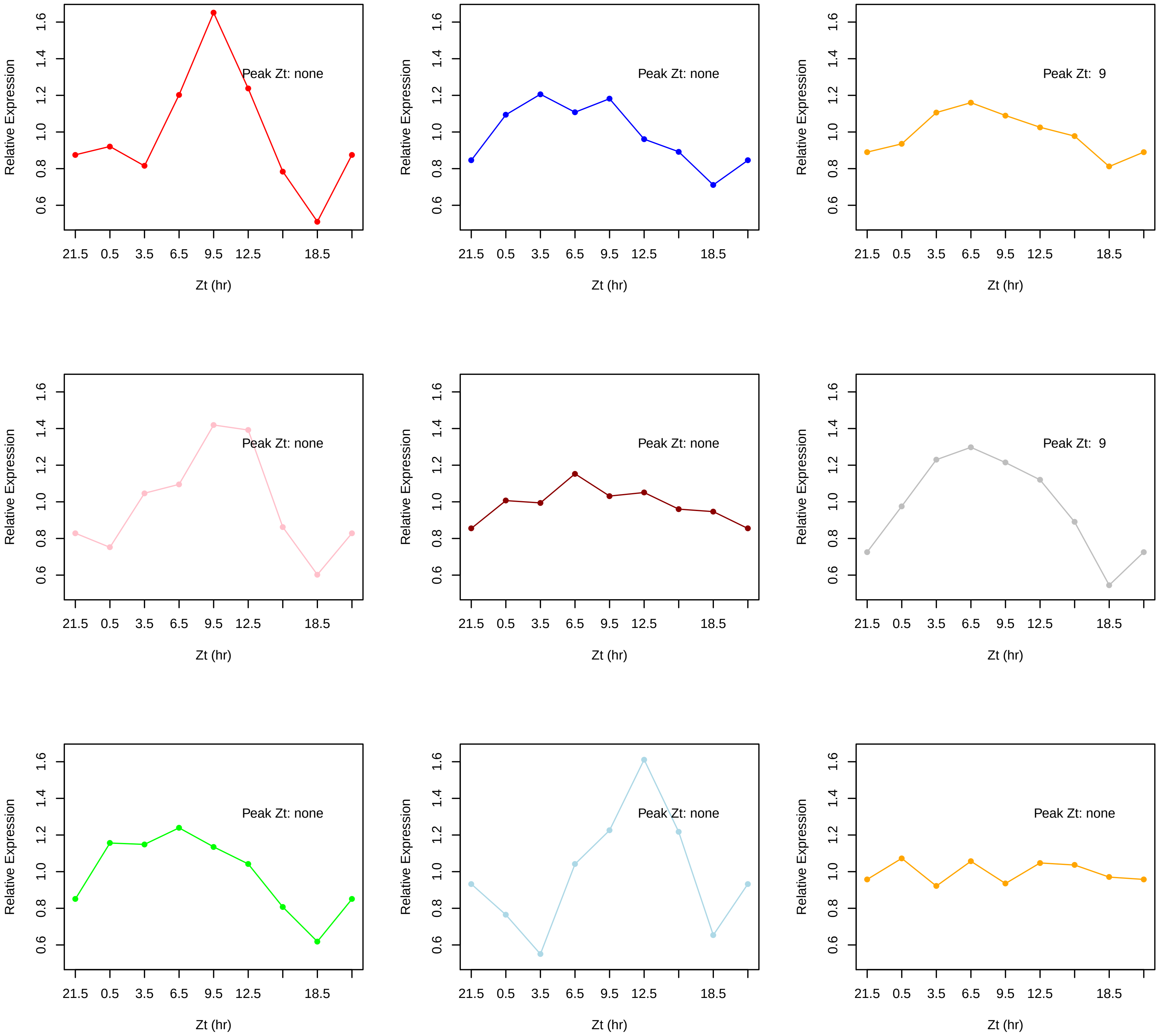

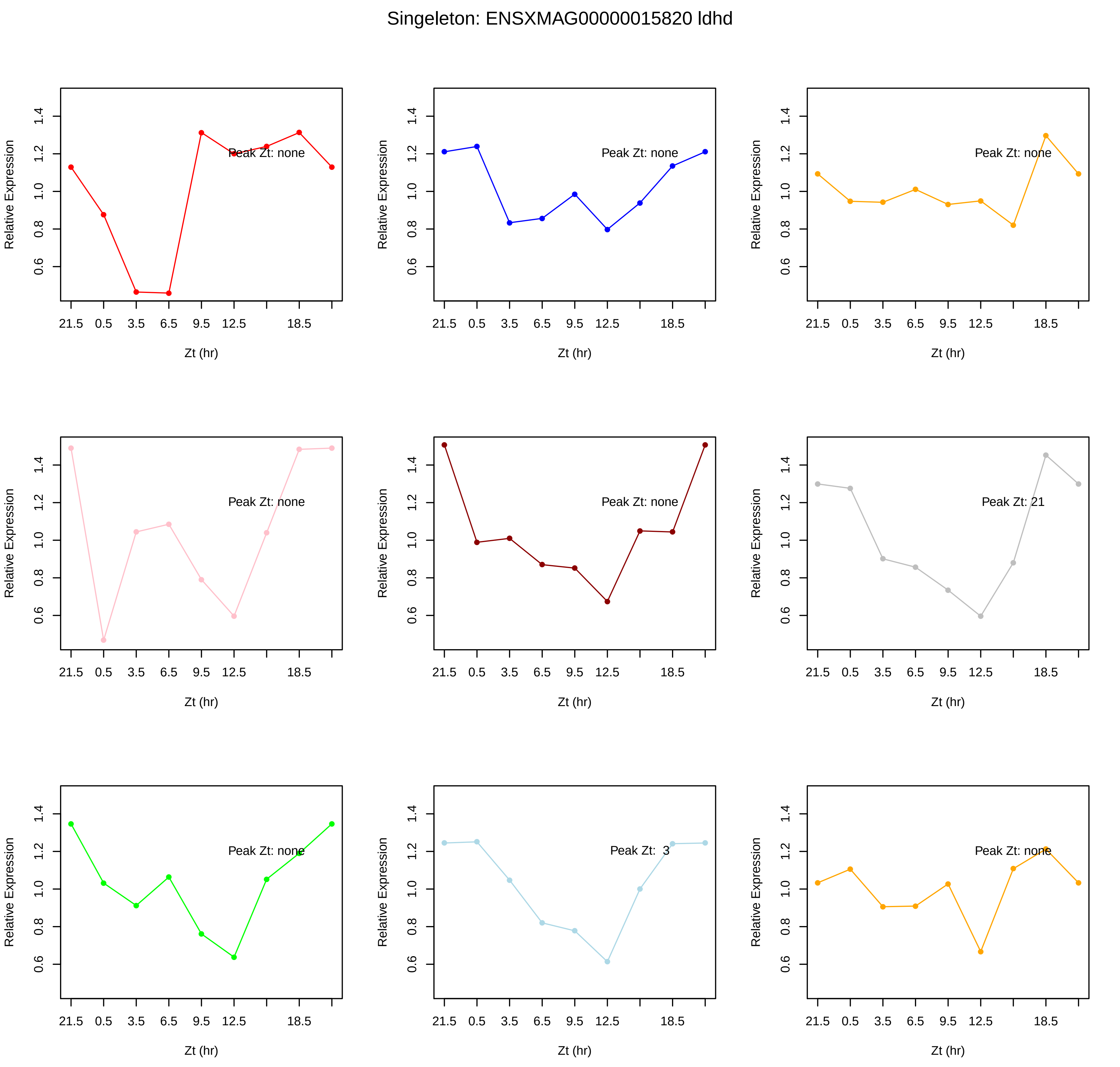
Singeleton: ENSXMAG00000015820 ldhd
Peak Zt: none
Peak Zt: none
Peak Zt: none
Peak Zt: none
Peak Zt: none
Peak Zt: 21
Peak Zt: none
Peak Zt: 3
Peak Zt: none
Relative Expression
Zt (hr)
21.5
0.5
3.5
6.5
9.5
12.5
18.5
0.6
0.8
1.0
1.2
1.4

Singeleton: ENSXMAG00000022255 lrrc38b

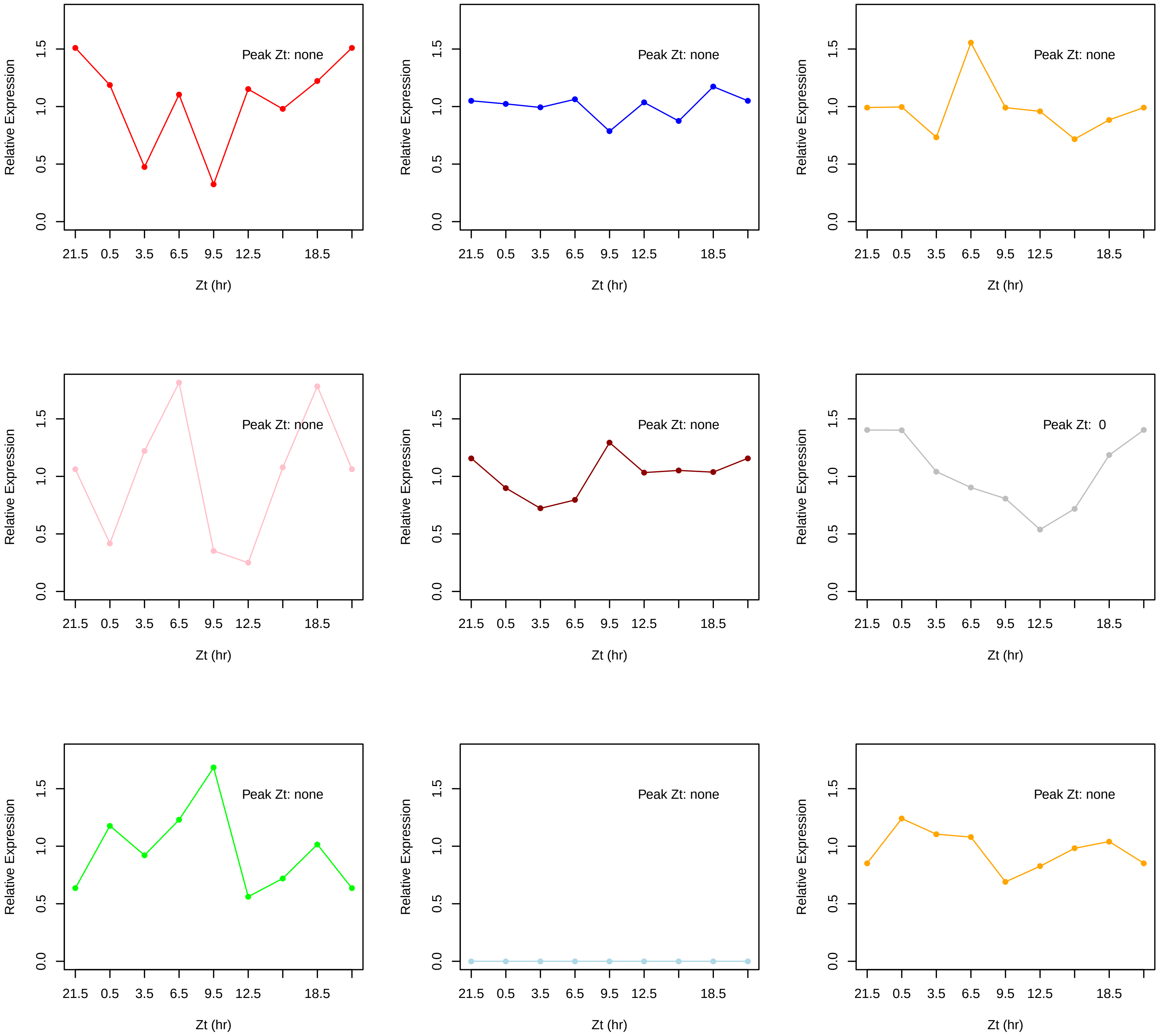

Singeleton: ENSXMAG00000014606 yrdc

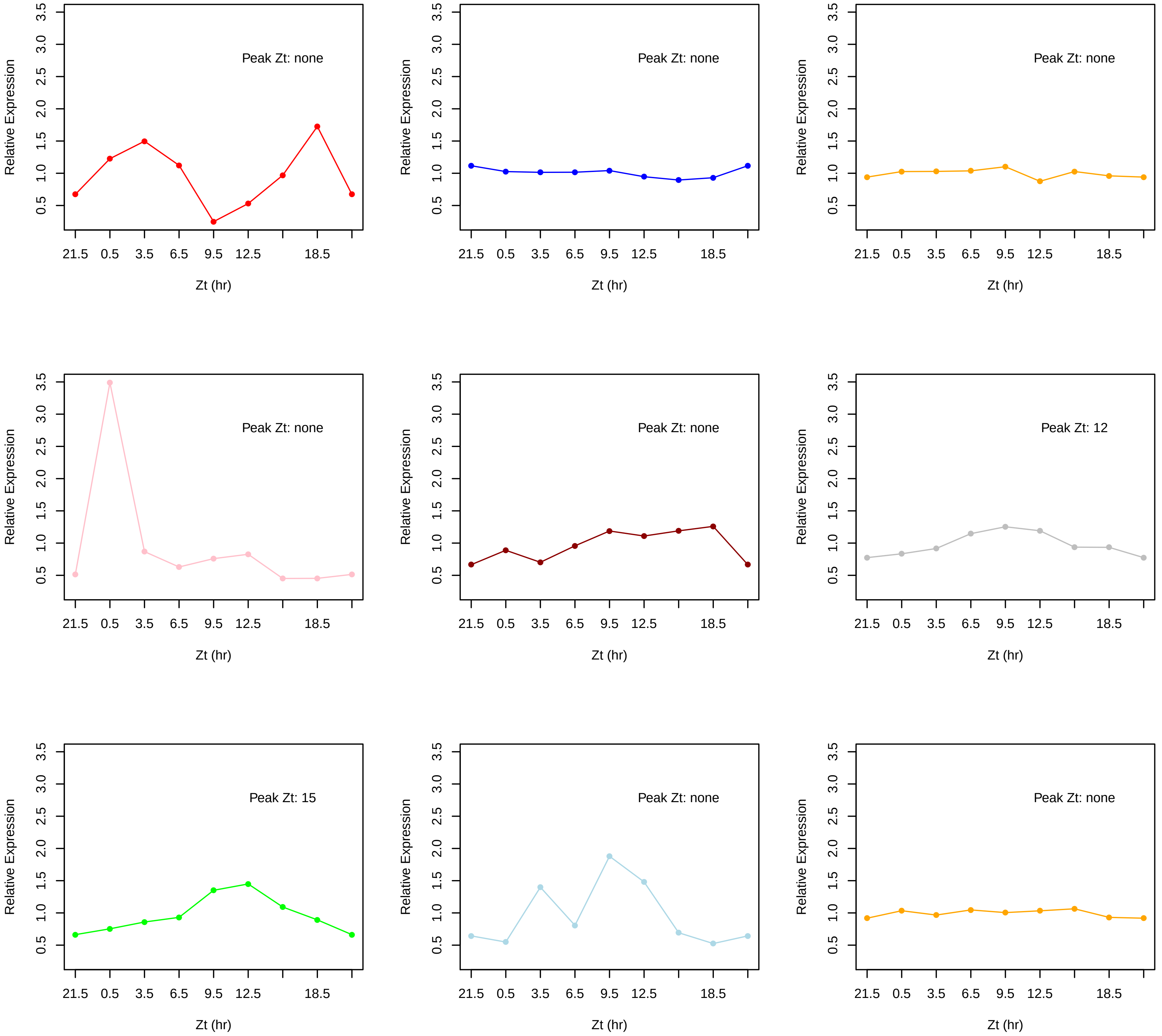

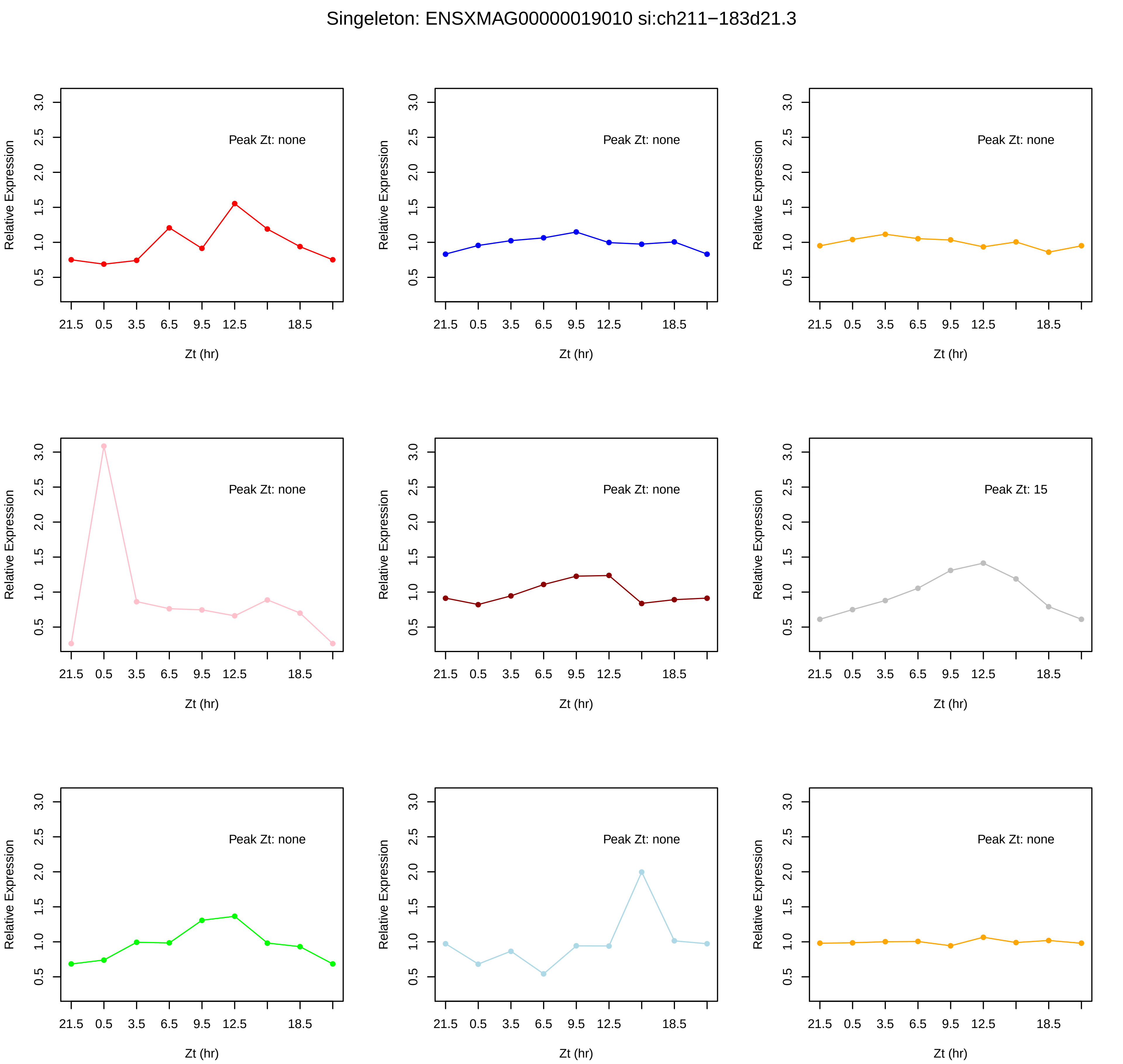

Singeleton: ENSXMAG00000019010 si:ch211−183d21.3
Peak Zt: none
Peak Zt: none
Peak Zt: none
Peak Zt: none
Peak Zt: none
Peak Zt: 15
Peak Zt: none
Peak Zt: none
Peak Zt: none
Relative Expression
Zt (hr)
21.5
0.5
3.5
6.5
9.5
12.5
18.5
0.5
1.0
1.5
2.0
2.5
3.0

Singleton: ENSXMAG00000024775

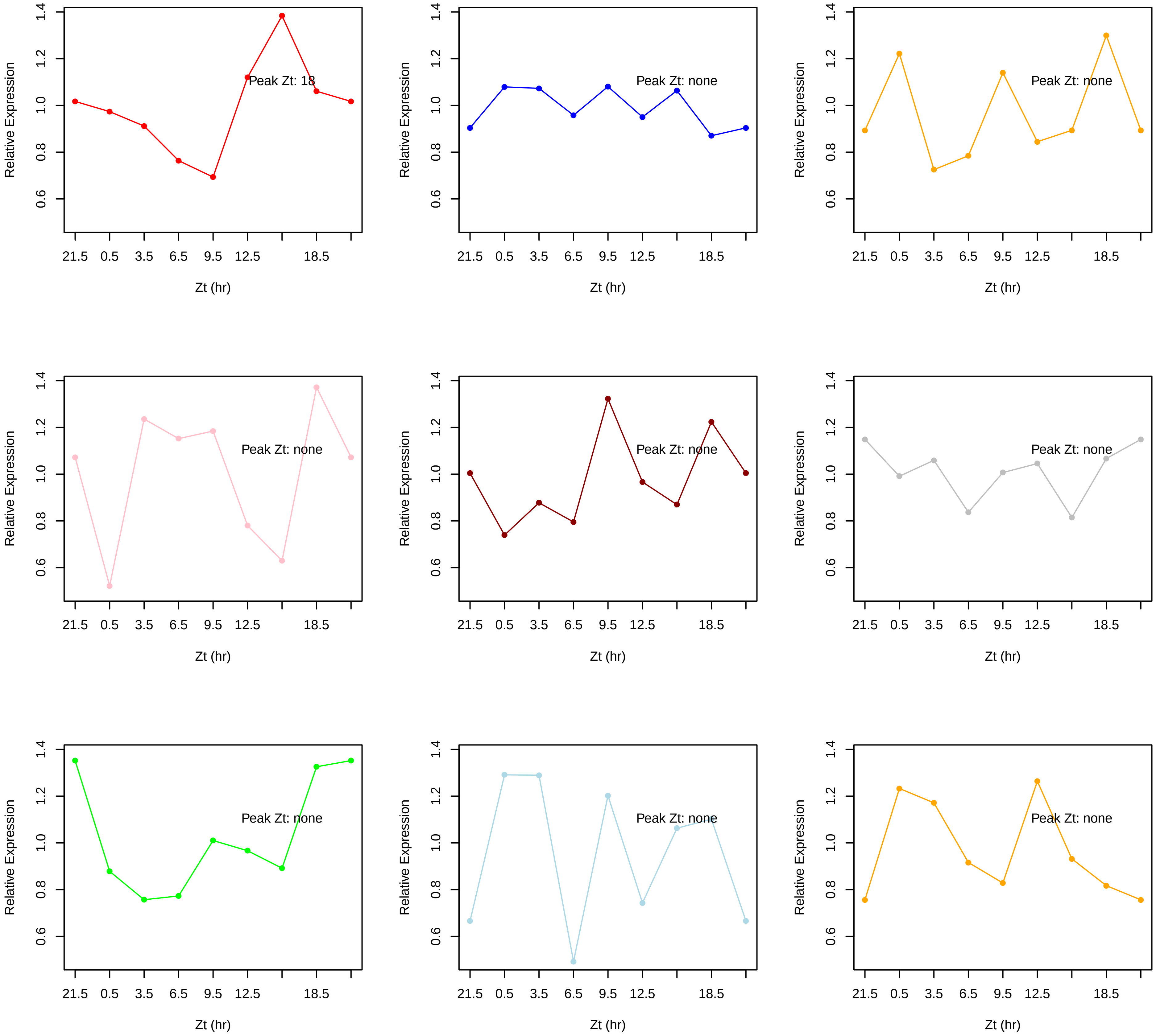

Singeleton: ENSXMAG00000023388 znrf1

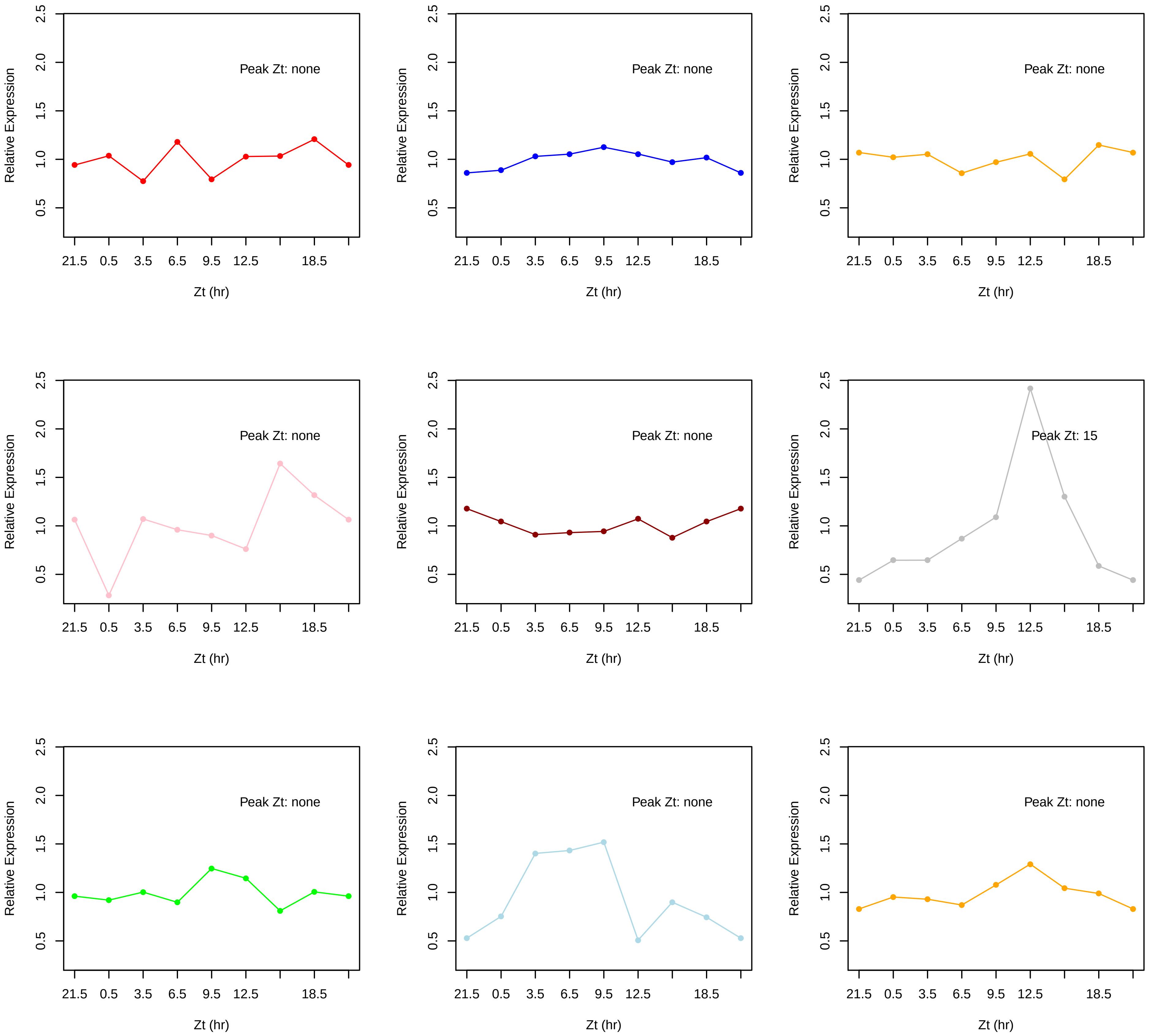

Singleton: ENSXMAG00000003328 camsap1b

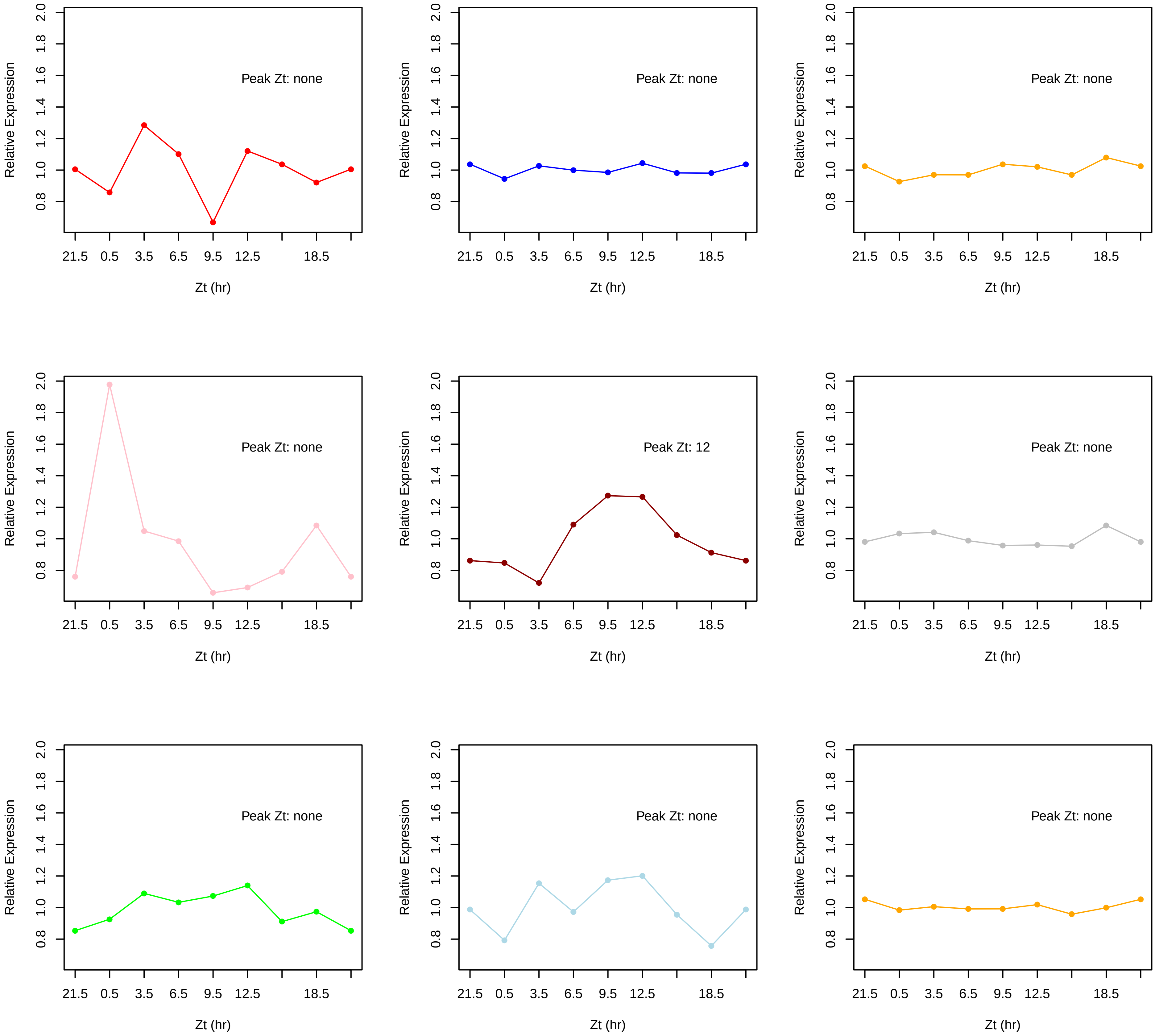

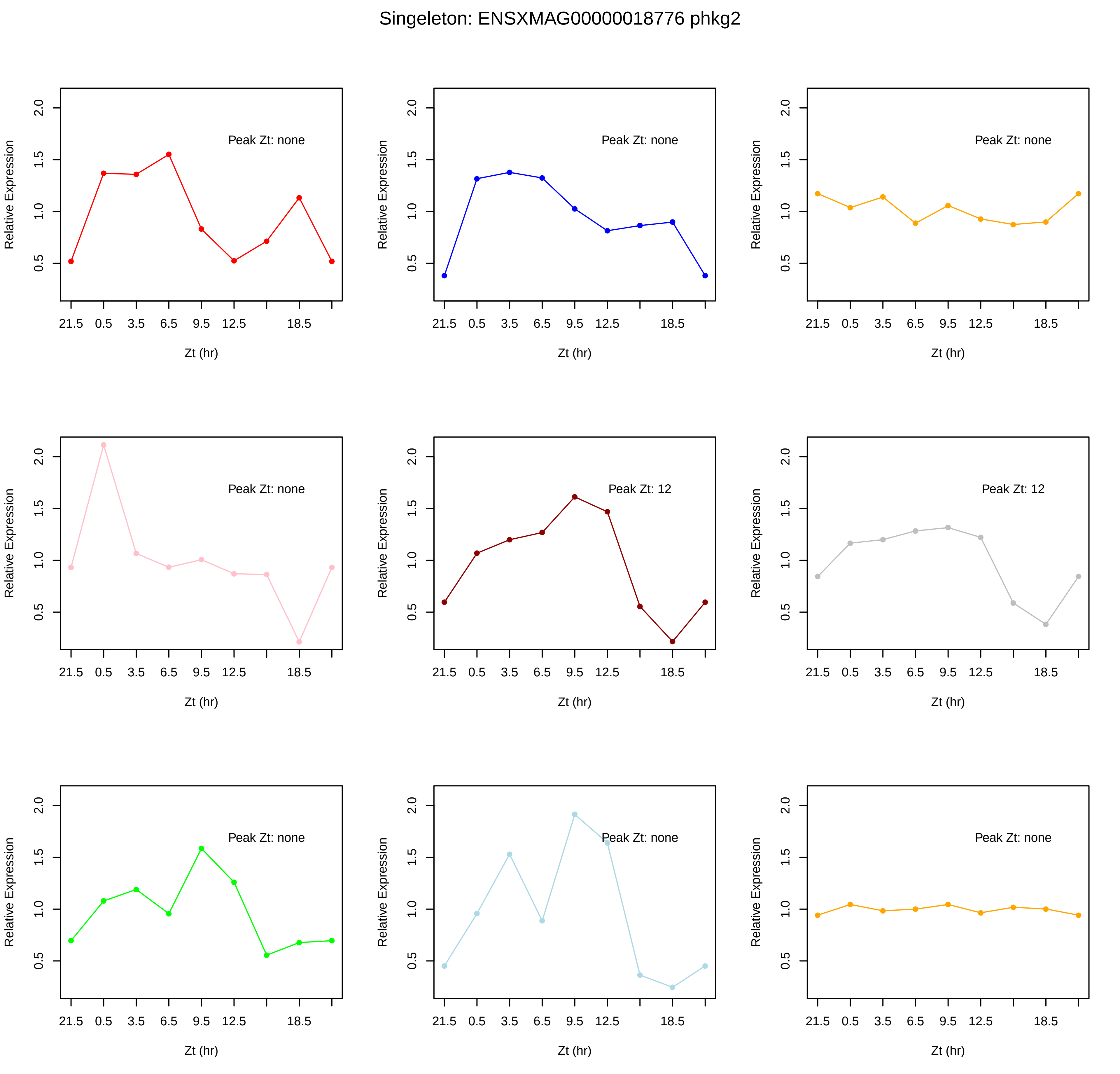
Singleton: ENSXMAG00000018776 phkg2
Peak Zt: none
Peak Zt: none
Peak Zt: none
Peak Zt: none
Peak Zt: 12
Peak Zt: 12
Peak Zt: none
Peak Zt: none
Peak Zt: none
Relative Expression
Zt (hr)
21.5
0.5
3.5
6.5
9.5
12.5
18.5
0.5
1.0
1.5
2.0

Singeleton: ENSXMAG00000018992 nlrc3

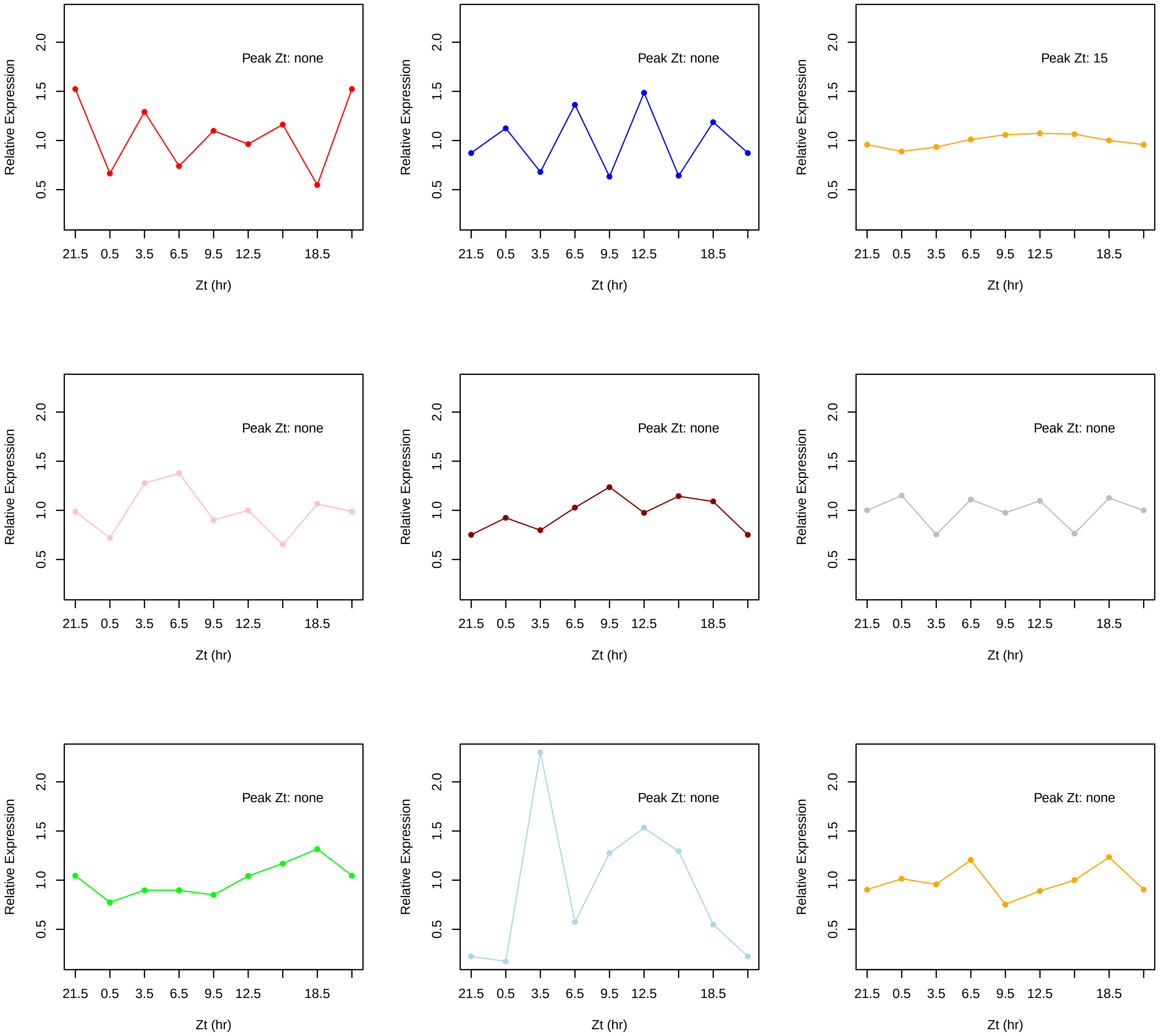

Singeleton: ENSXMAG00000016827 ttll9

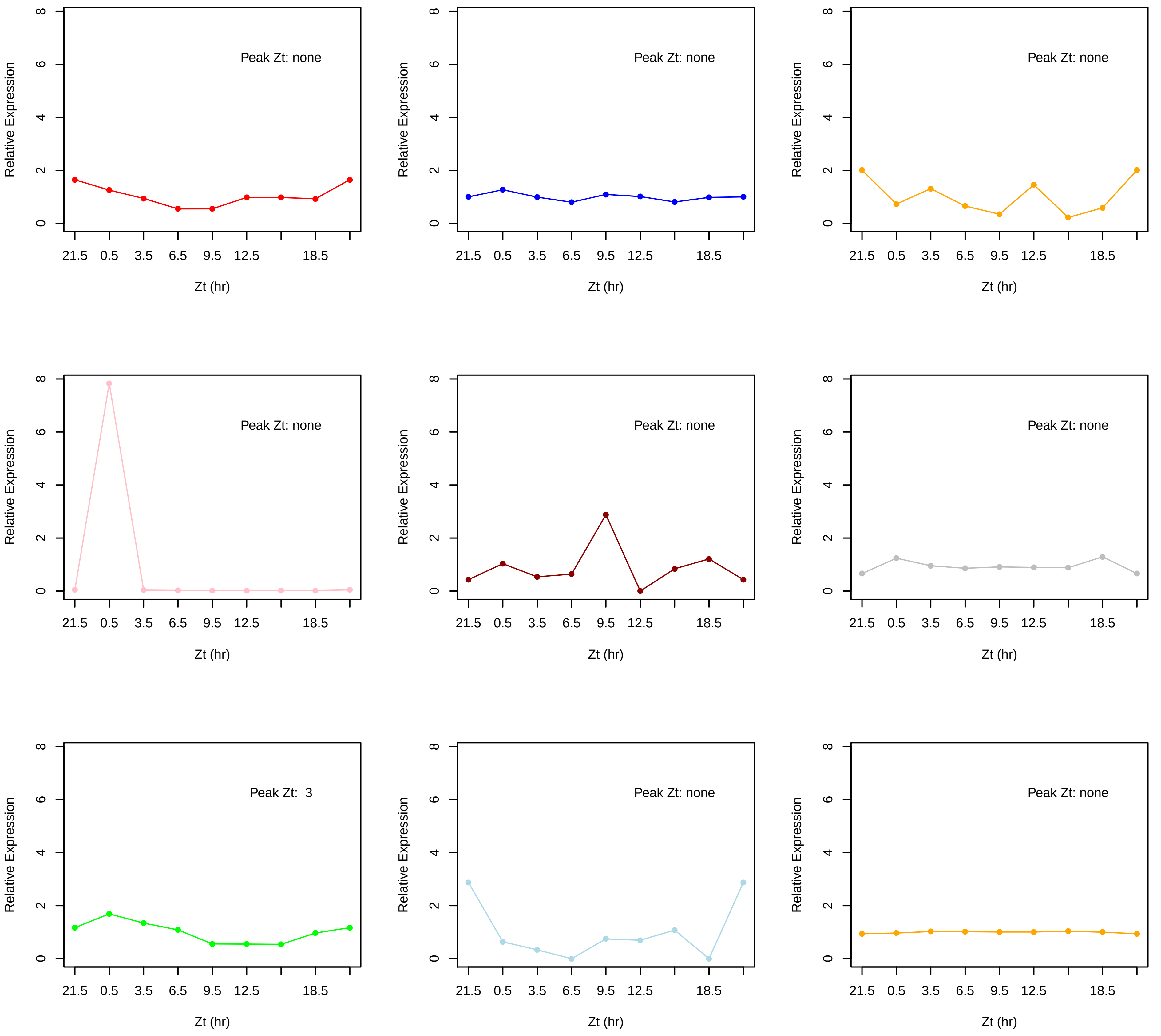

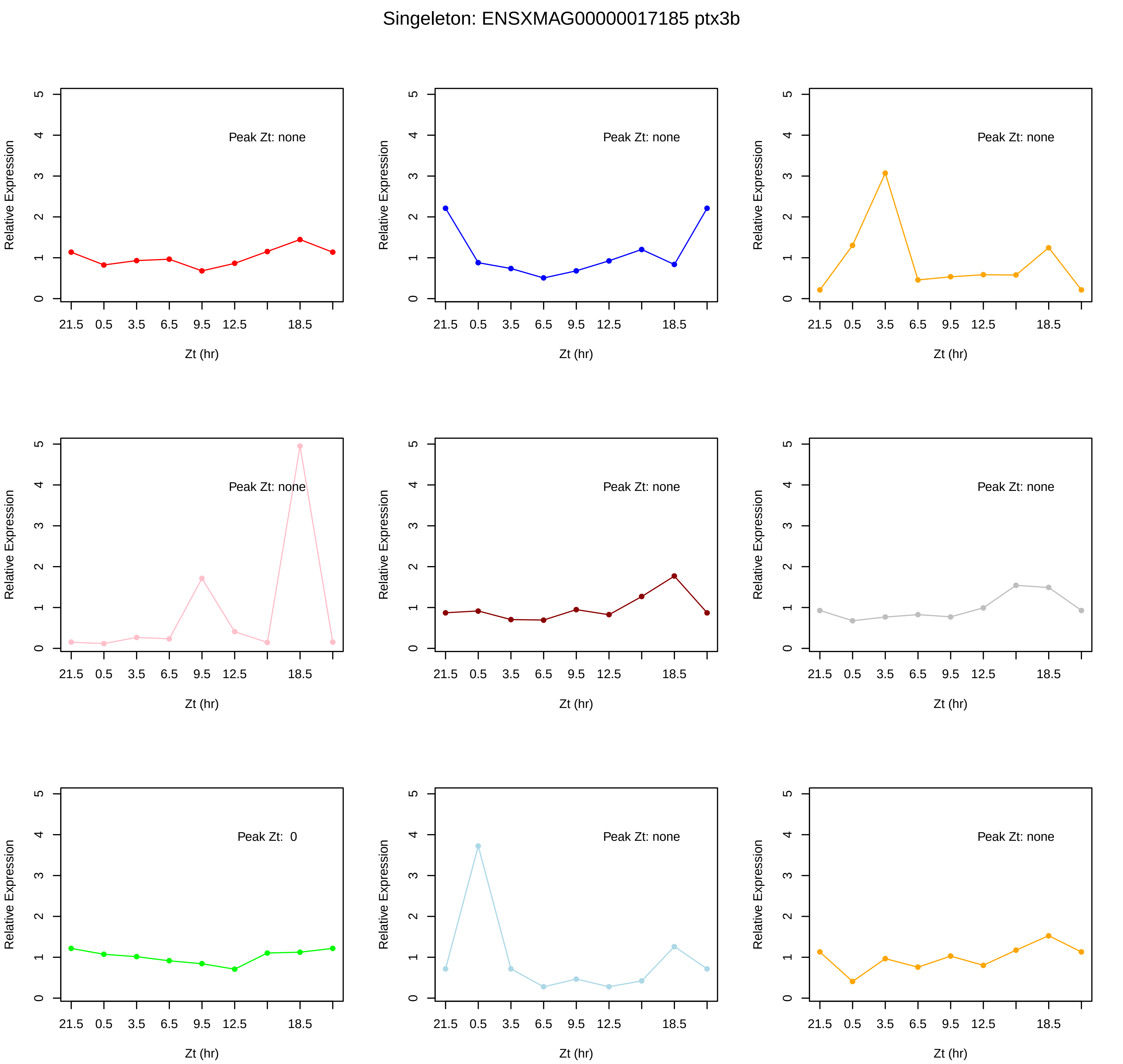
Singleton: ENSXMAG00000017185 ptx3b
Peak Zt: none
Peak Zt: none
Peak Zt: none
Peak Zt: none
Peak Zt: none
Peak Zt: none
Peak Zt: 0
Peak Zt: none
Peak Zt: none
Relative Expression
Zt (hr)
21.5
0.5
3.5
6.5
9.5
12.5
18.5
0
1
2
3
4
5

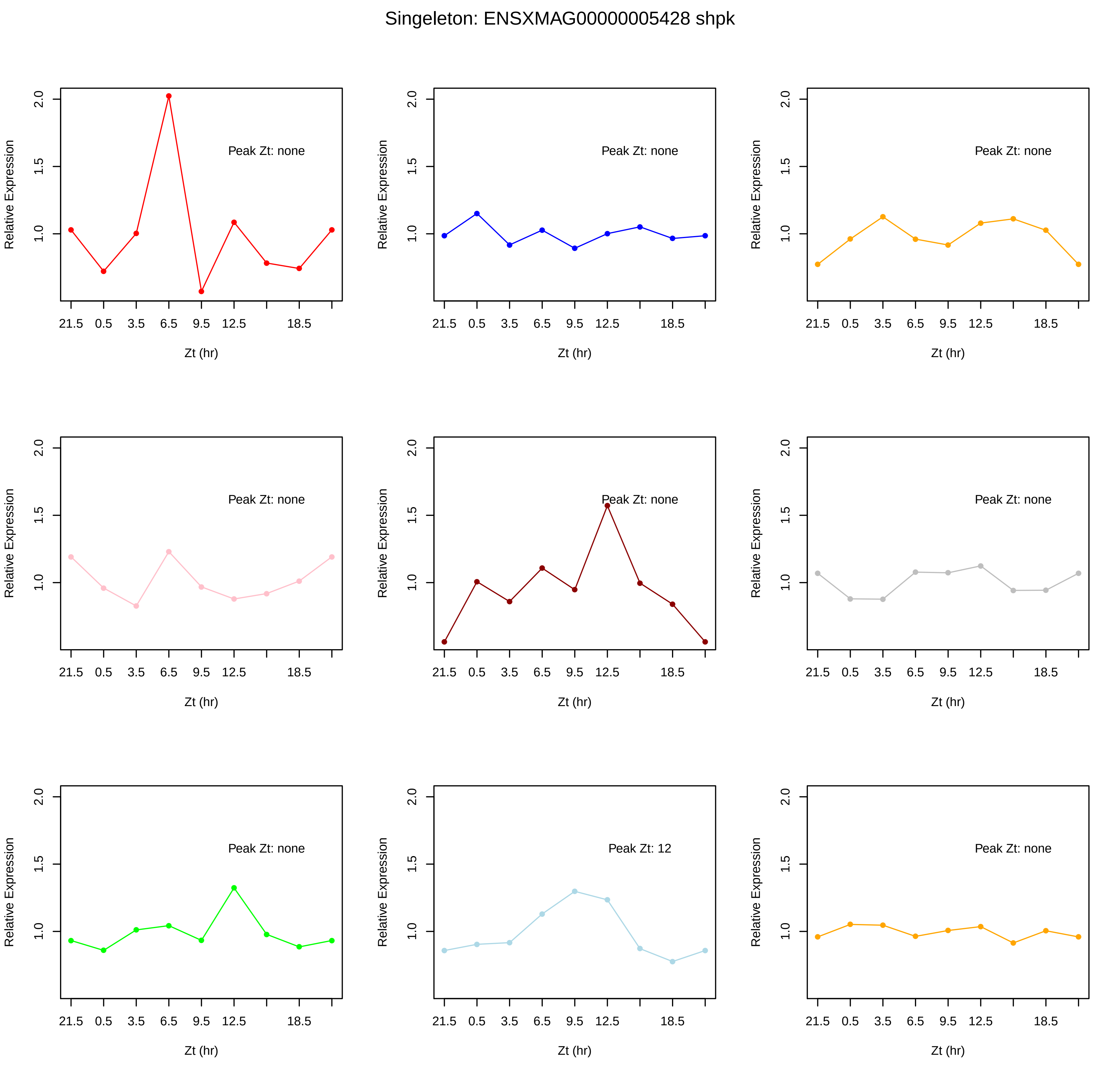
Singeleton: ENSXMAG00000005428 shpk
Peak Zt: none
Peak Zt: none
Peak Zt: none
Peak Zt: none
Peak Zt: none
Peak Zt: none
Peak Zt: none
Peak Zt: 12
Peak Zt: none
Relative Expression
Zt (hr)
21.5
0.5
3.5
6.5
9.5
12.5
18.5
1.0
1.5
2.0

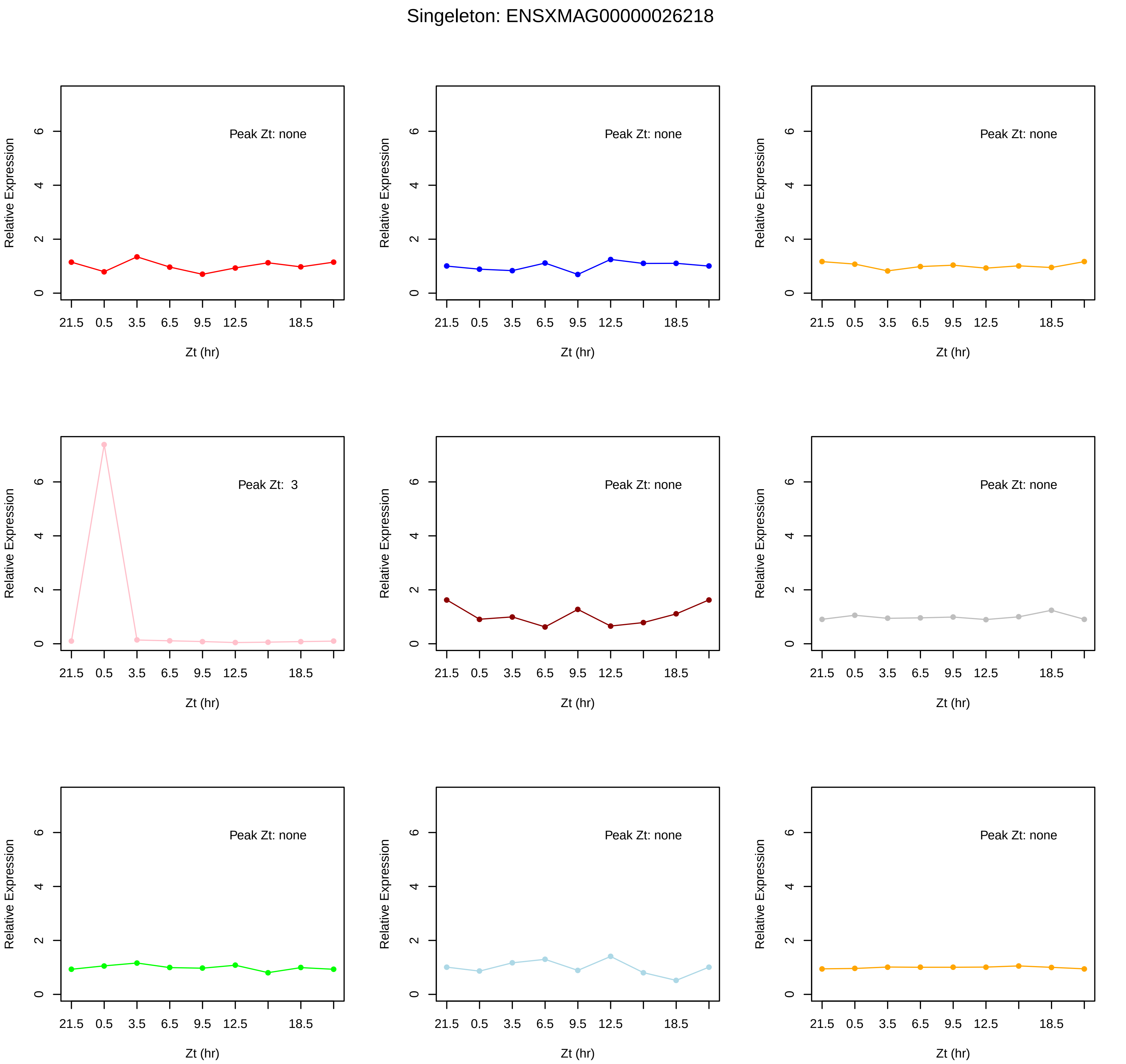
Singeleton: ENSXMAG00000026218
Peak Zt: none
Peak Zt: none
Peak Zt: none
Peak Zt: 3
Peak Zt: none
Peak Zt: none
Peak Zt: none
Peak Zt: none
Peak Zt: none
Relative Expression
Zt (hr)
21.5
0.5
3.5
6.5
9.5
12.5
18.5
0
2
4
6

Singeleton: ENSXMAG00000017835 prss12

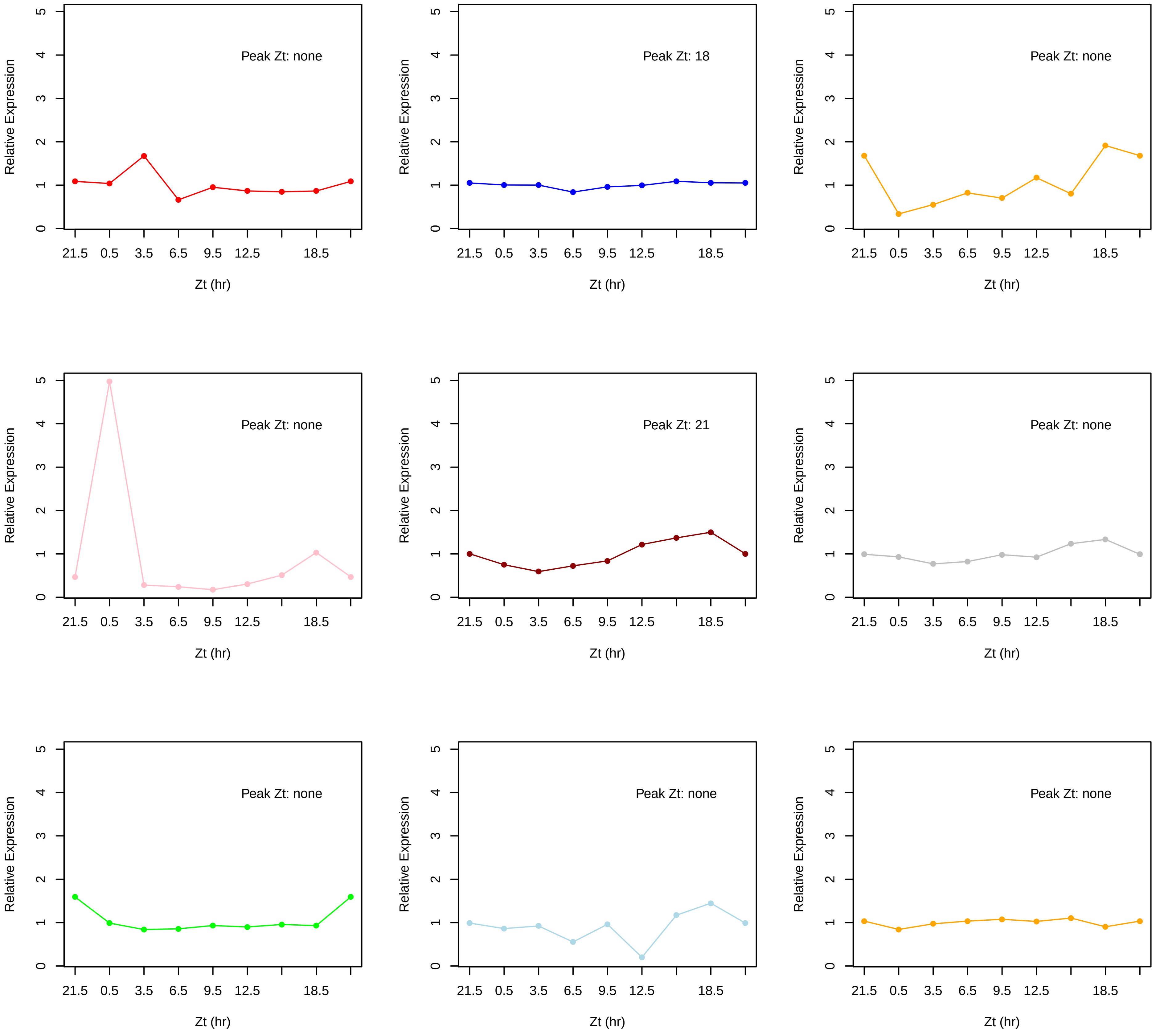

Singleton: ENSXMAG00000017189

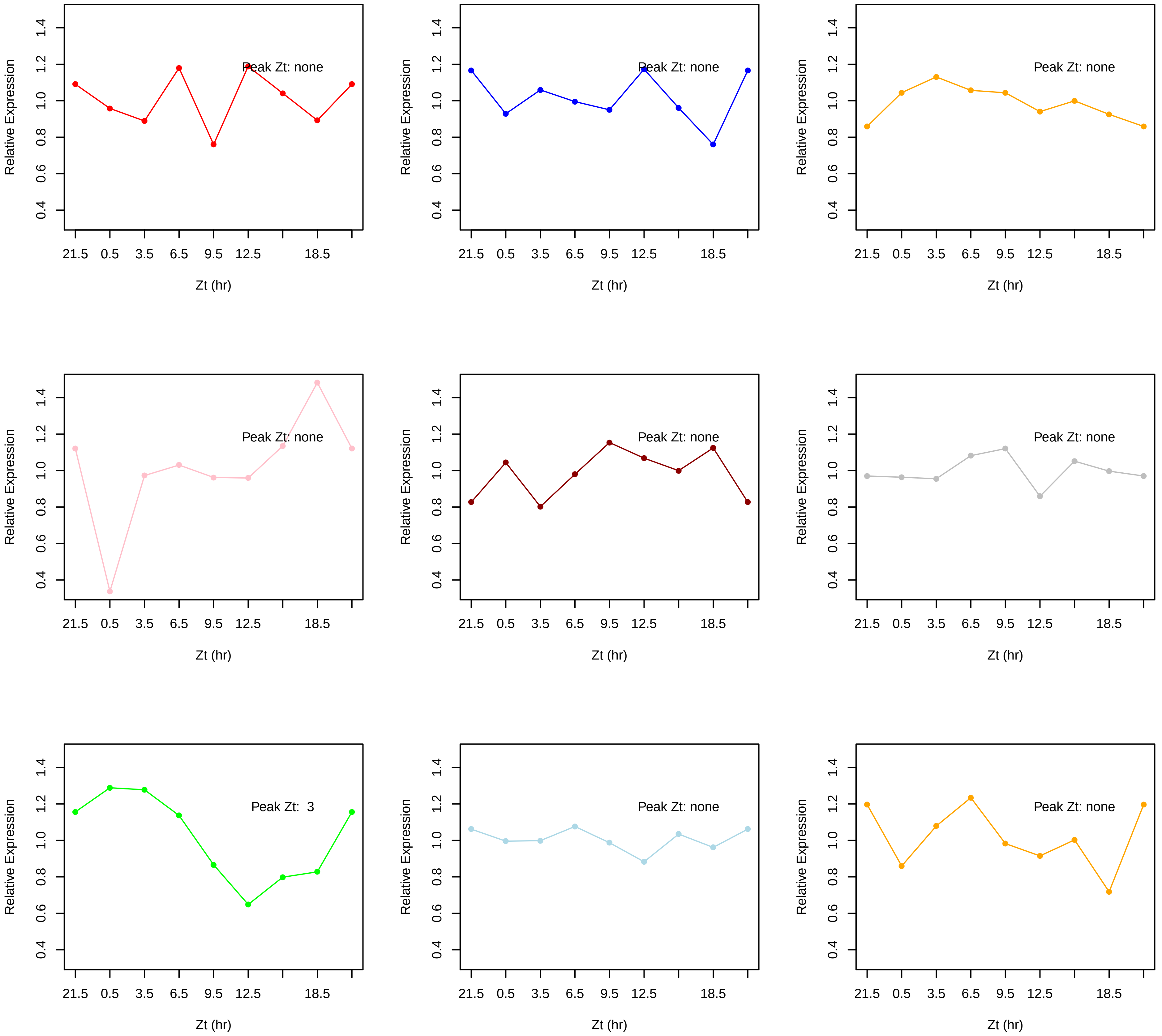

Singeleton: ENSXMAG00000029562 pdrg1

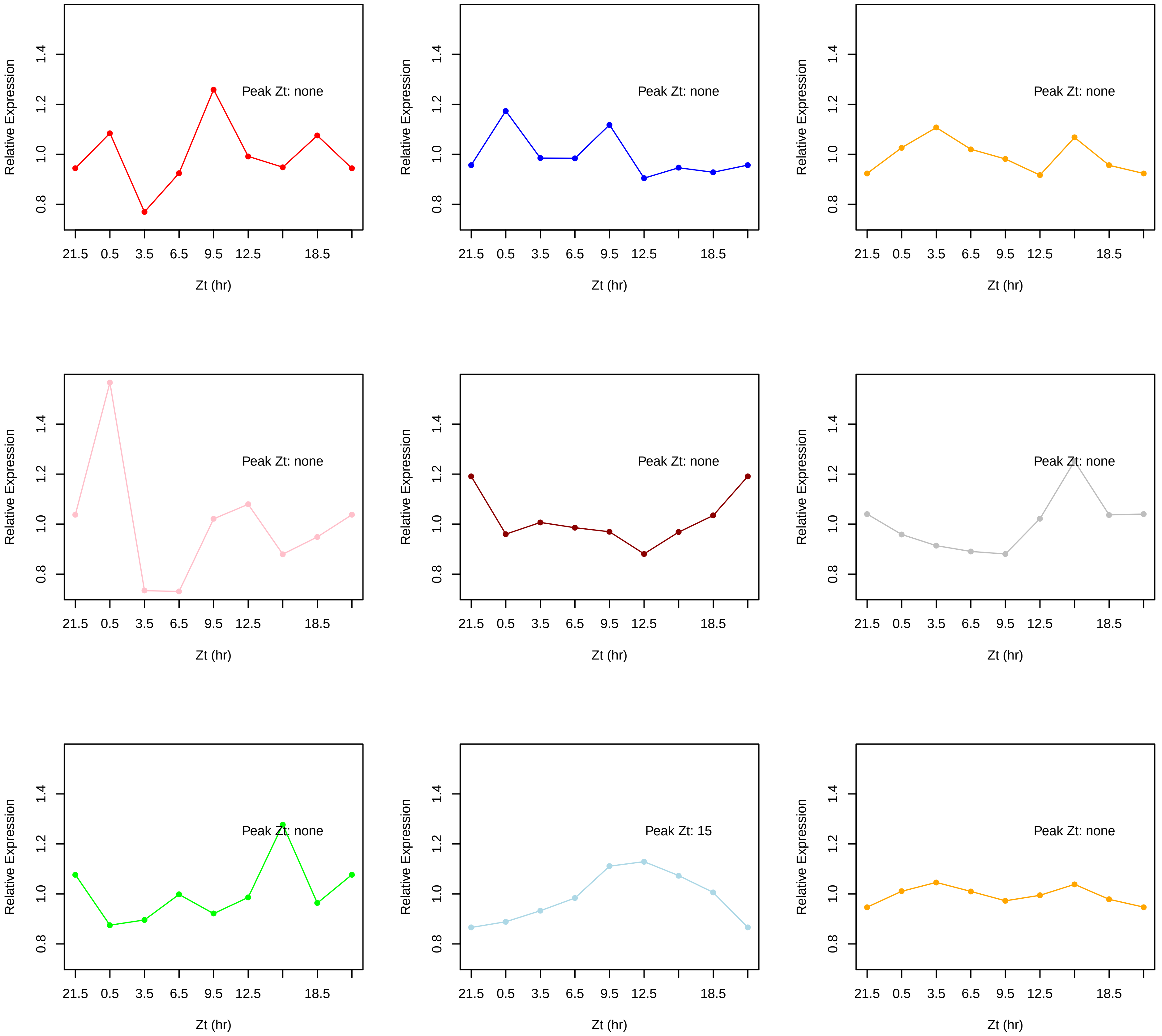

Singeleton: ENSXMAG00000026077 mecr

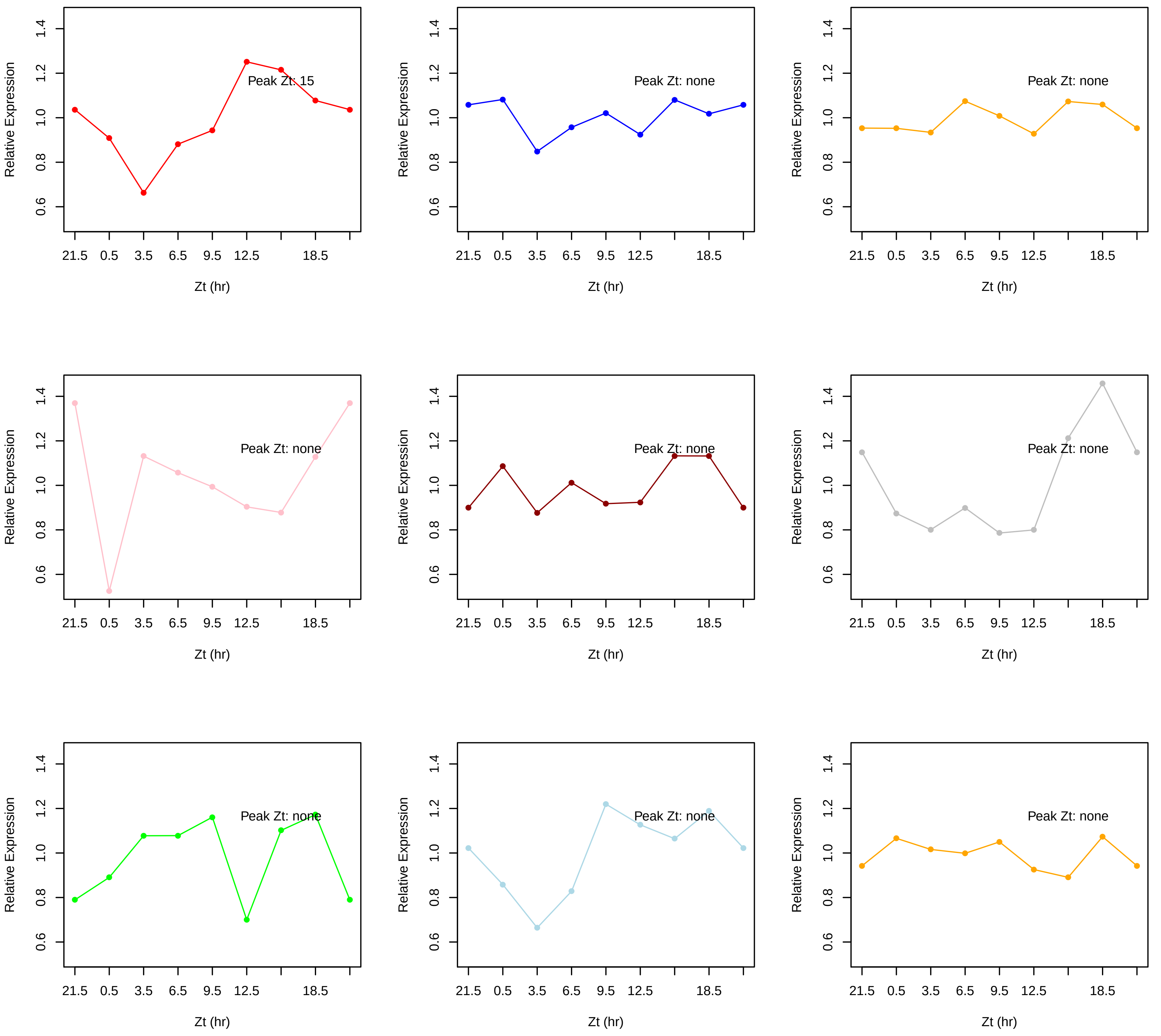

Singleton: ENSXMAG00000018852 cep70

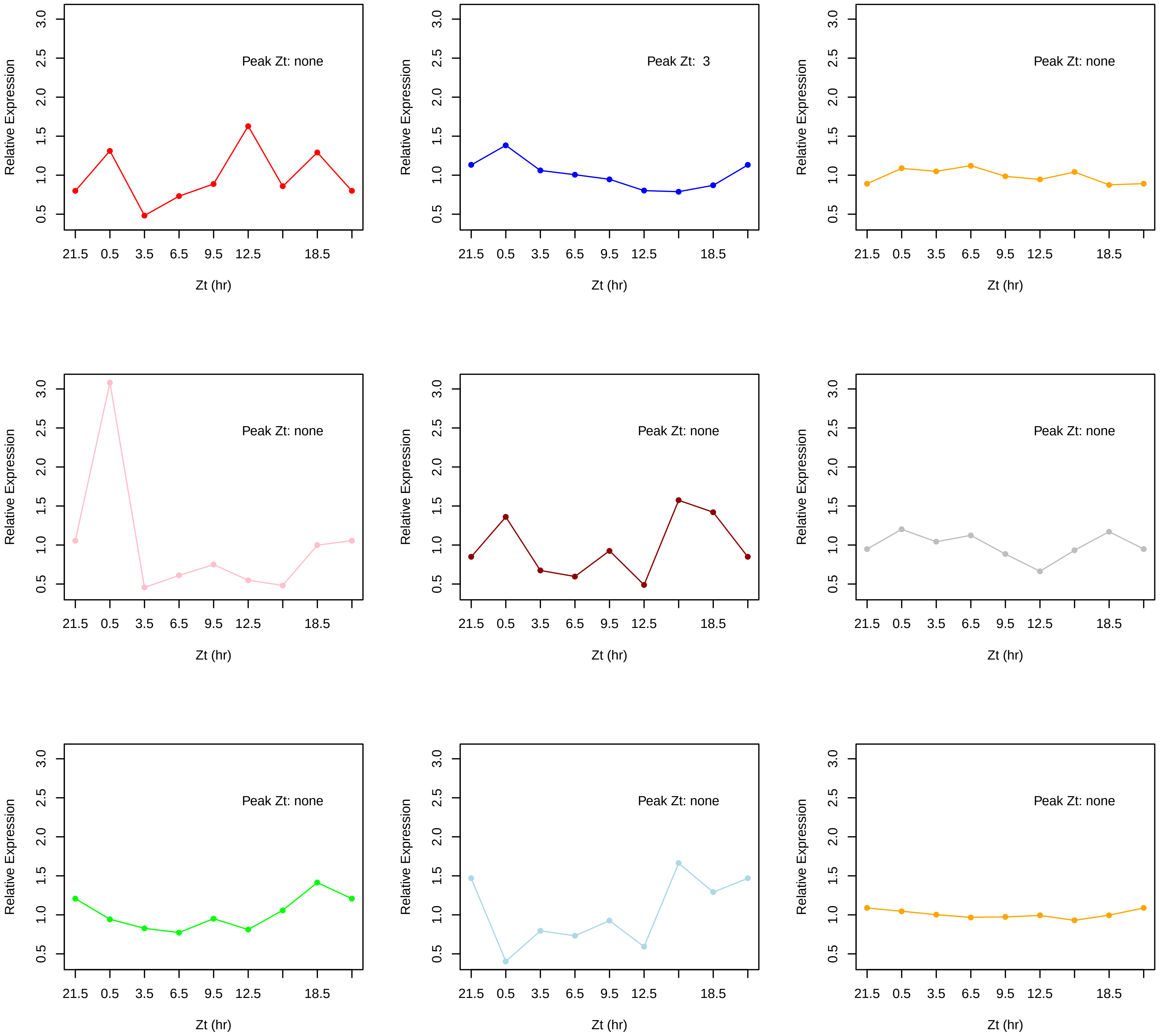

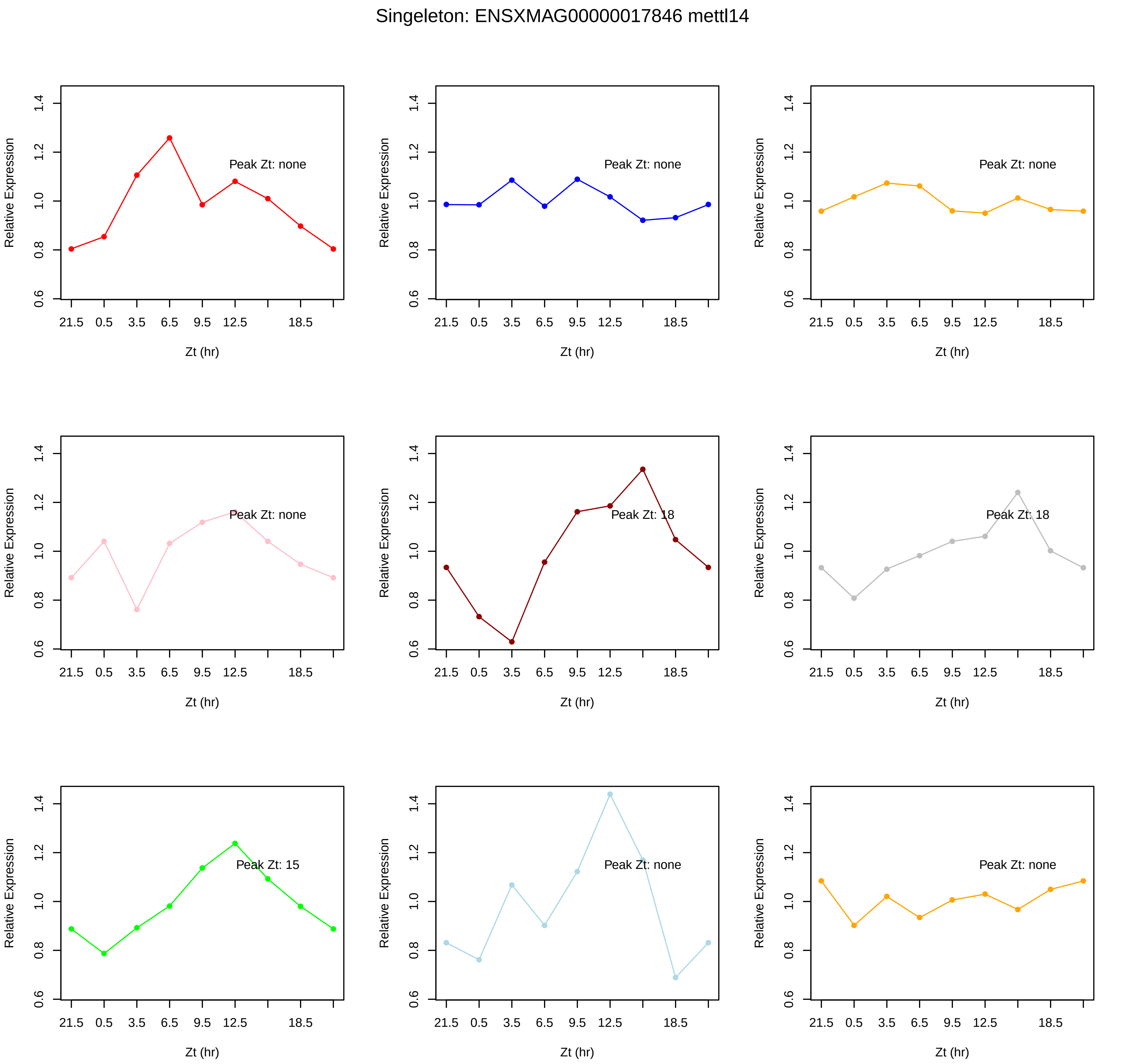
Singeleton: ENSXMAG00000017846 mettl14
Relative Expression
Zt (hr)
21.5
0.5
3.5
6.5
9.5
12.5
18.5
0.6
0.8
1.0
1.2
1.4
Peak Zt: none
Peak Zt: none
Peak Zt: none
Peak Zt: none
Peak Zt: 18
Peak Zt: 18
Peak Zt: 15
Peak Zt: none
Peak Zt: none

Singeleton: ENSXMAG00000017196 serpini1

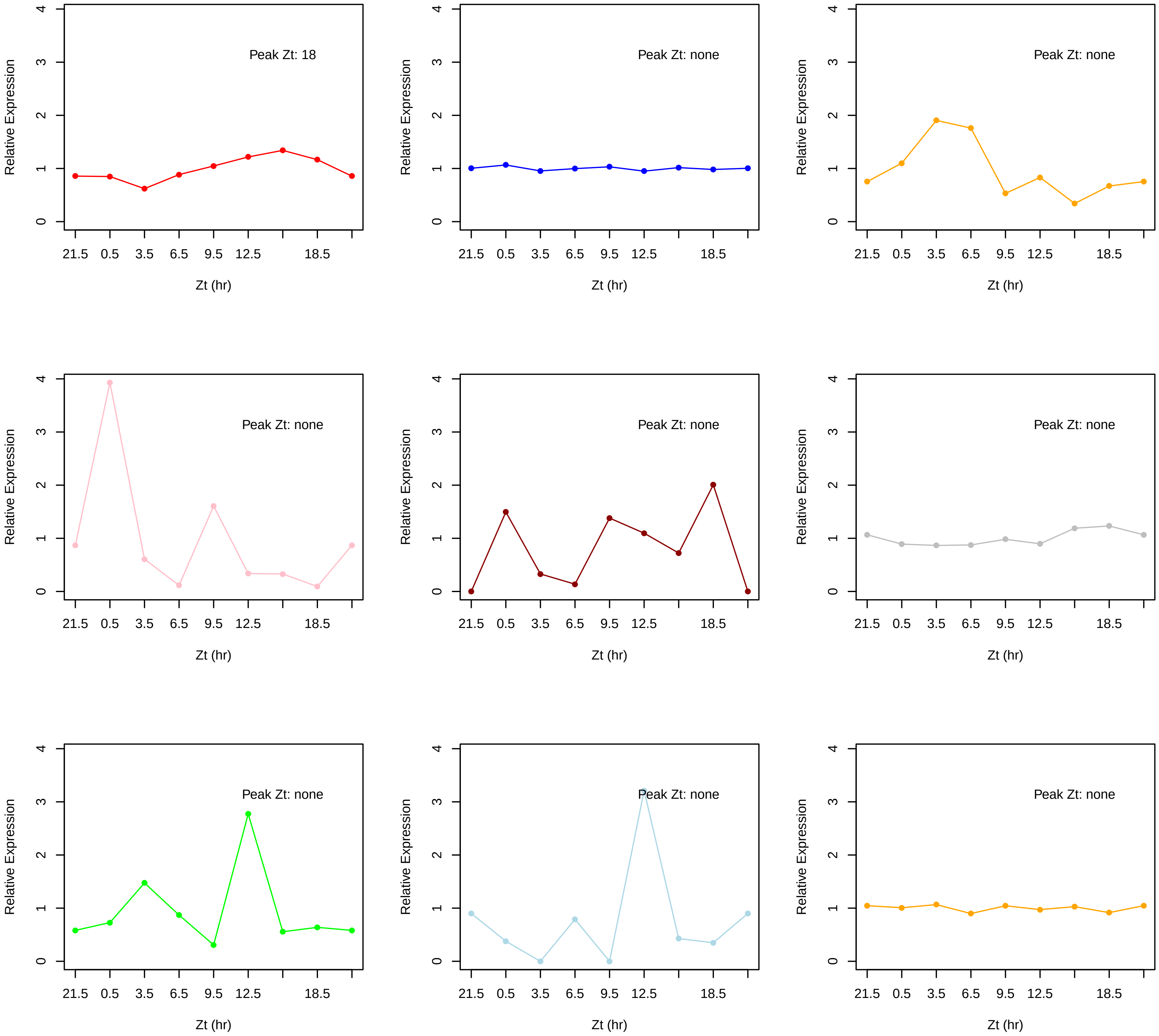

Singleton: ENSXMAG00000024906 faima

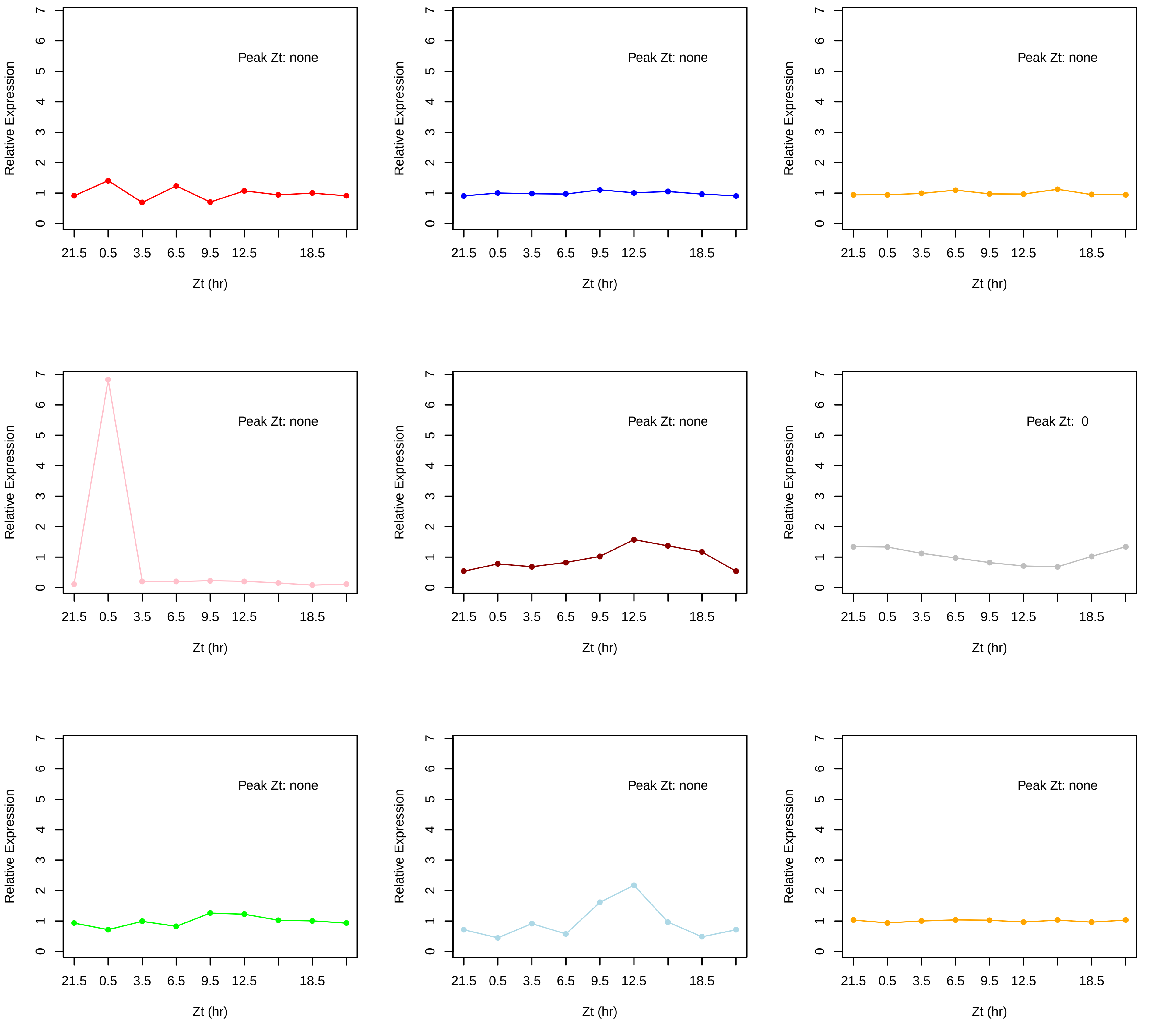

Singeleton: ENSXMAG00000013136 erich3

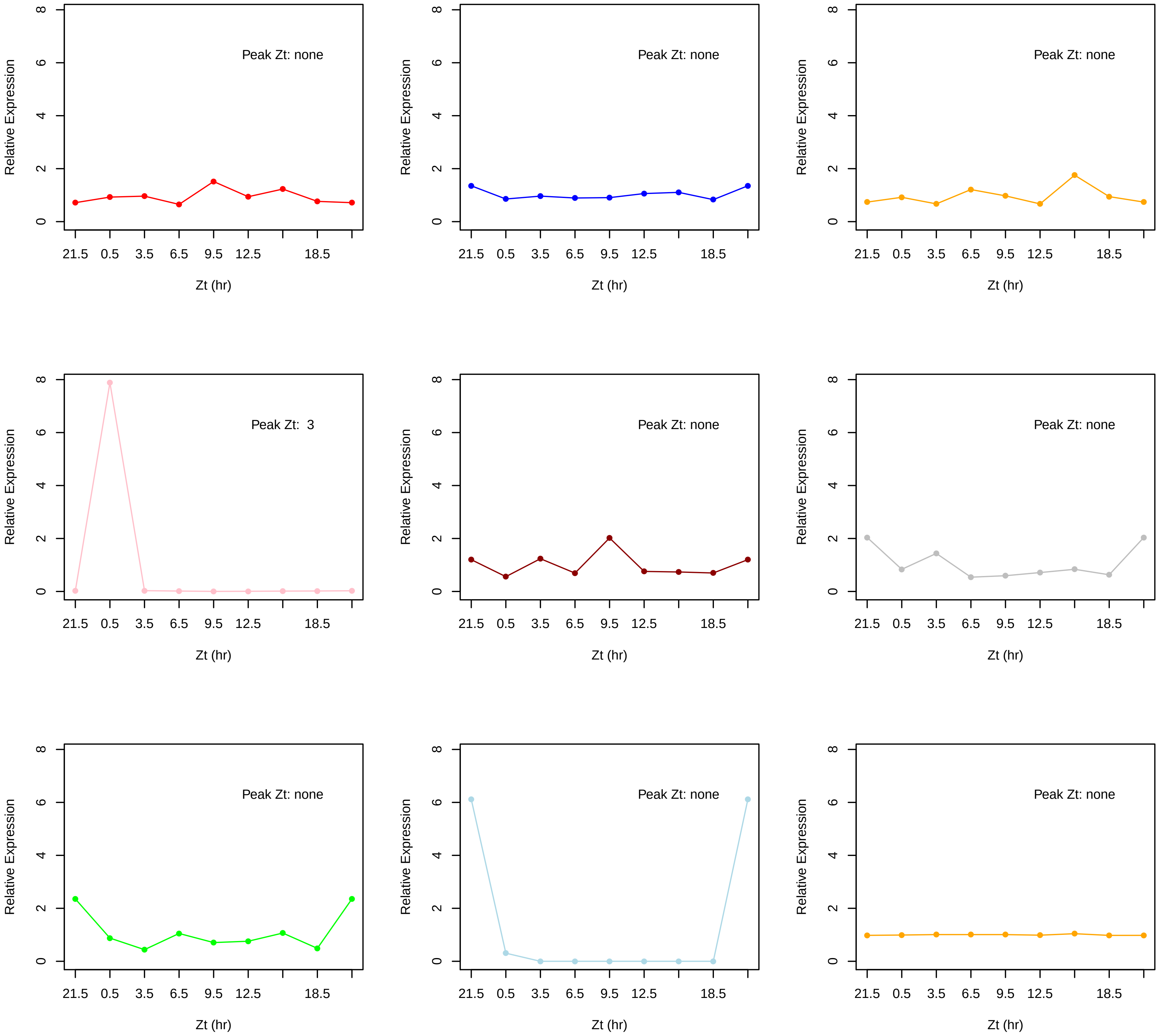

Singeleton: ENSXMAG00000002551 ksr2

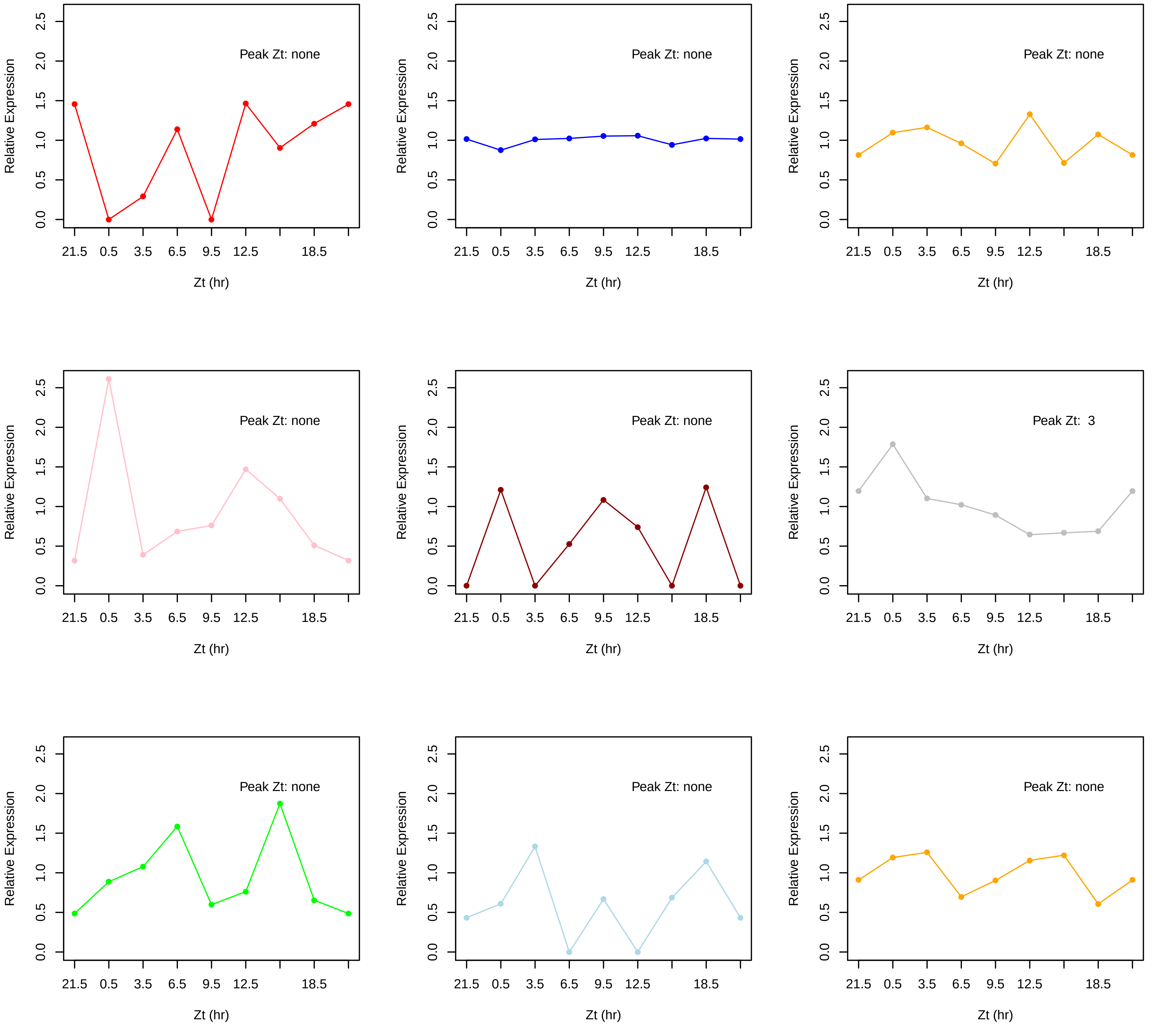

Singeleton: ENSXMAG00000012809 ctps1a

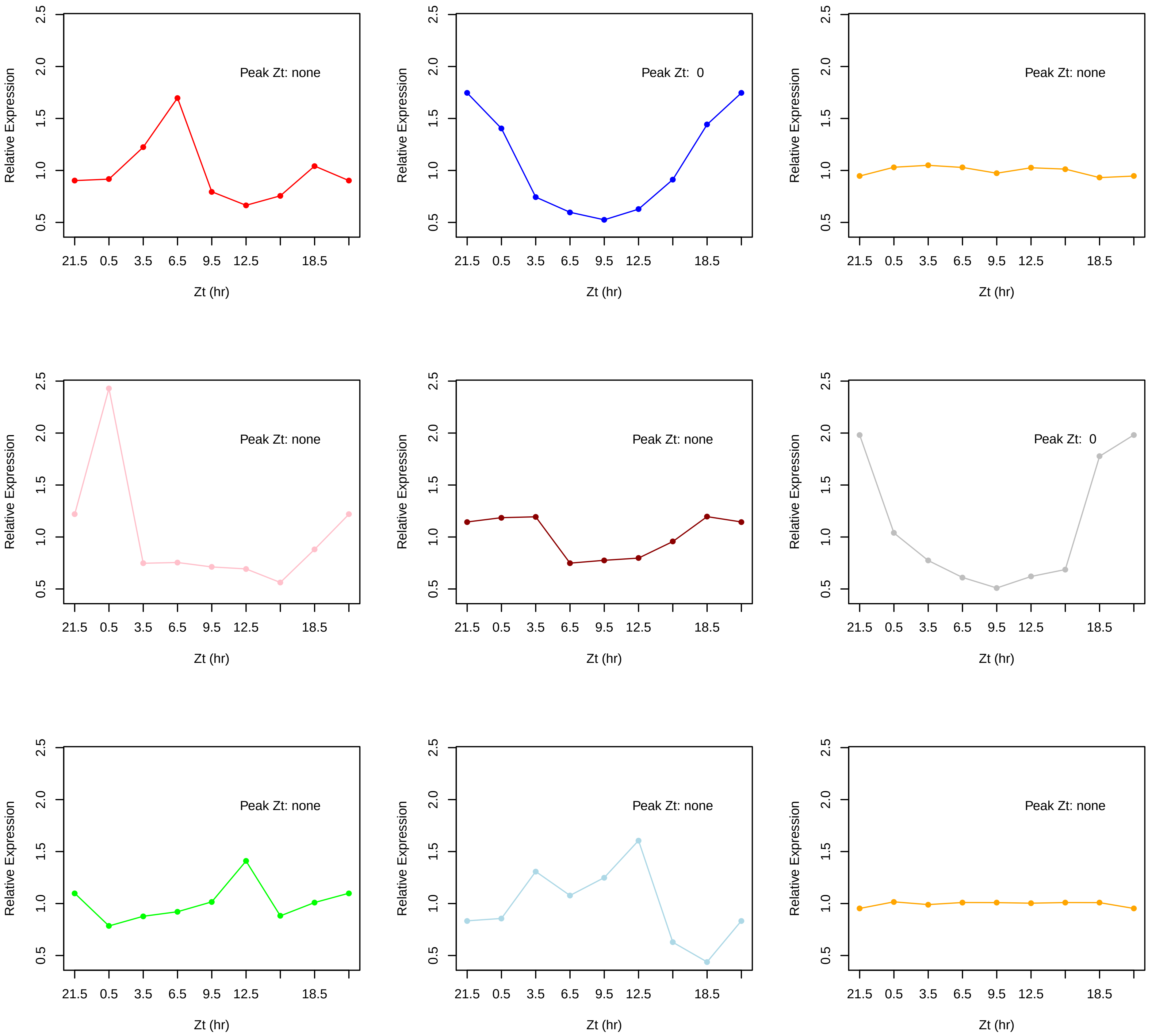

Singeleton: ENSXMAG00000018549 cryz

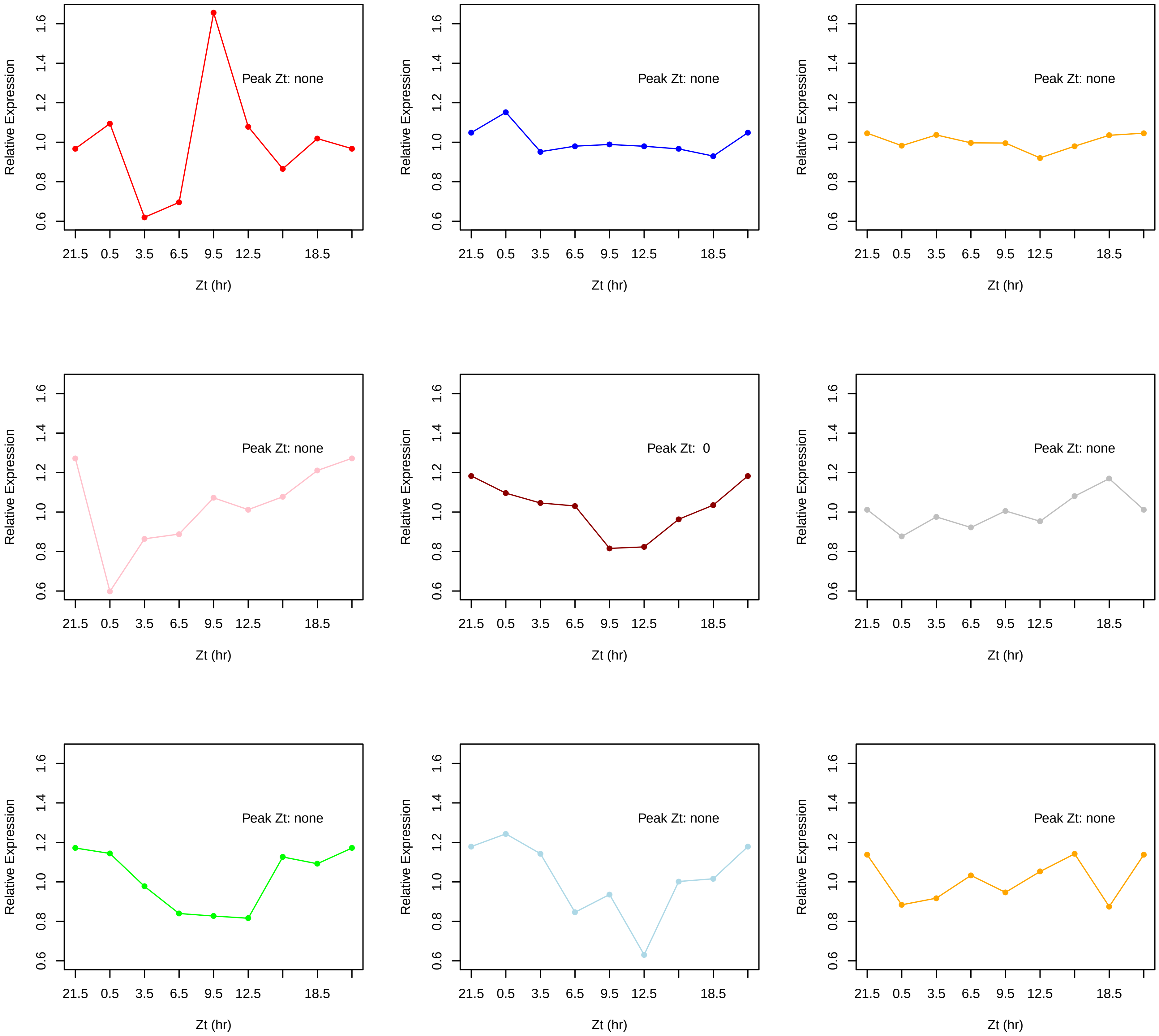

Singeleton: ENSXMAG00000011825 sat1b

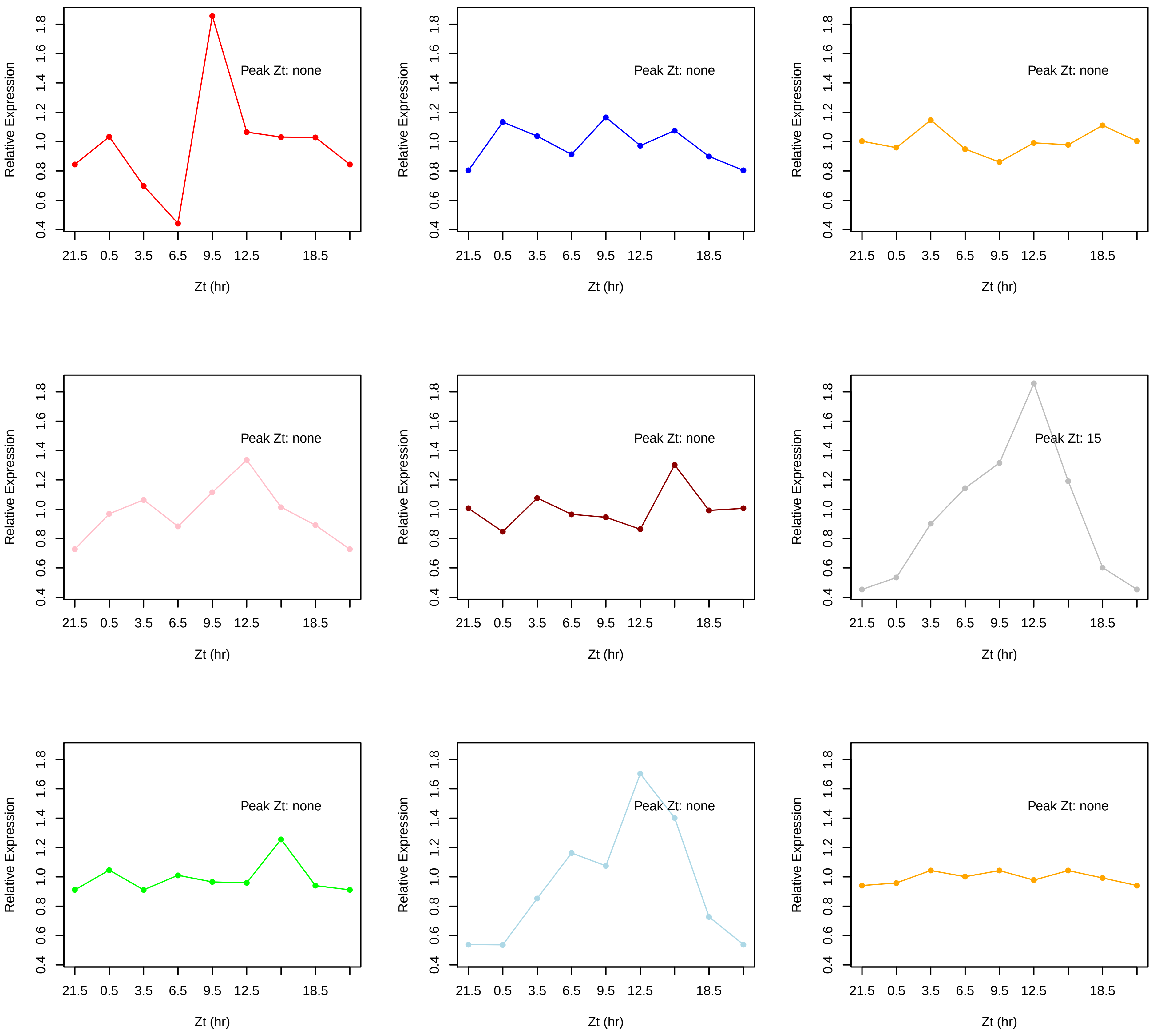

Singeleton: ENSXMAG00000028183 tax1bp3

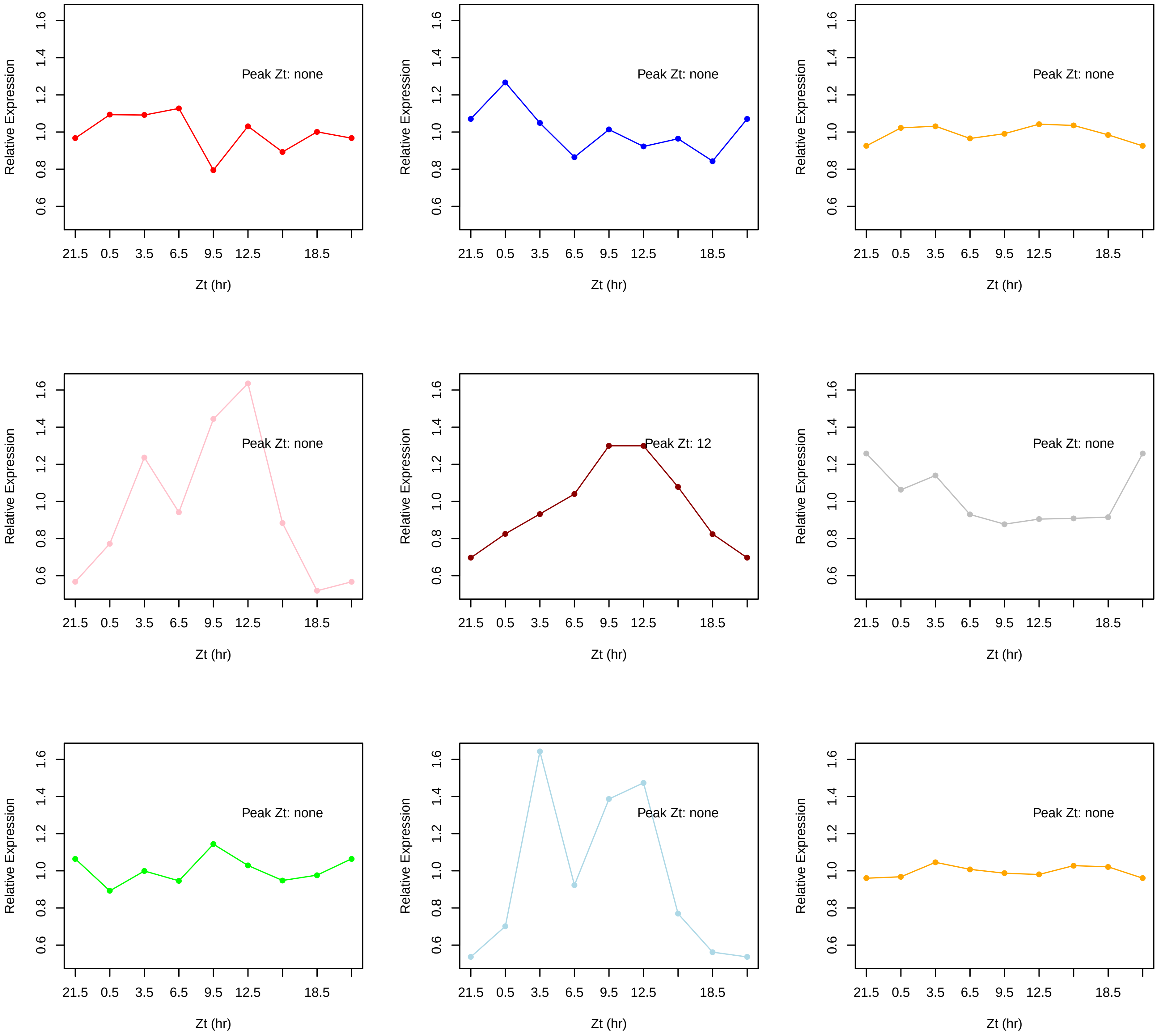

Singleton: ENSXMAG00000014005 cass4

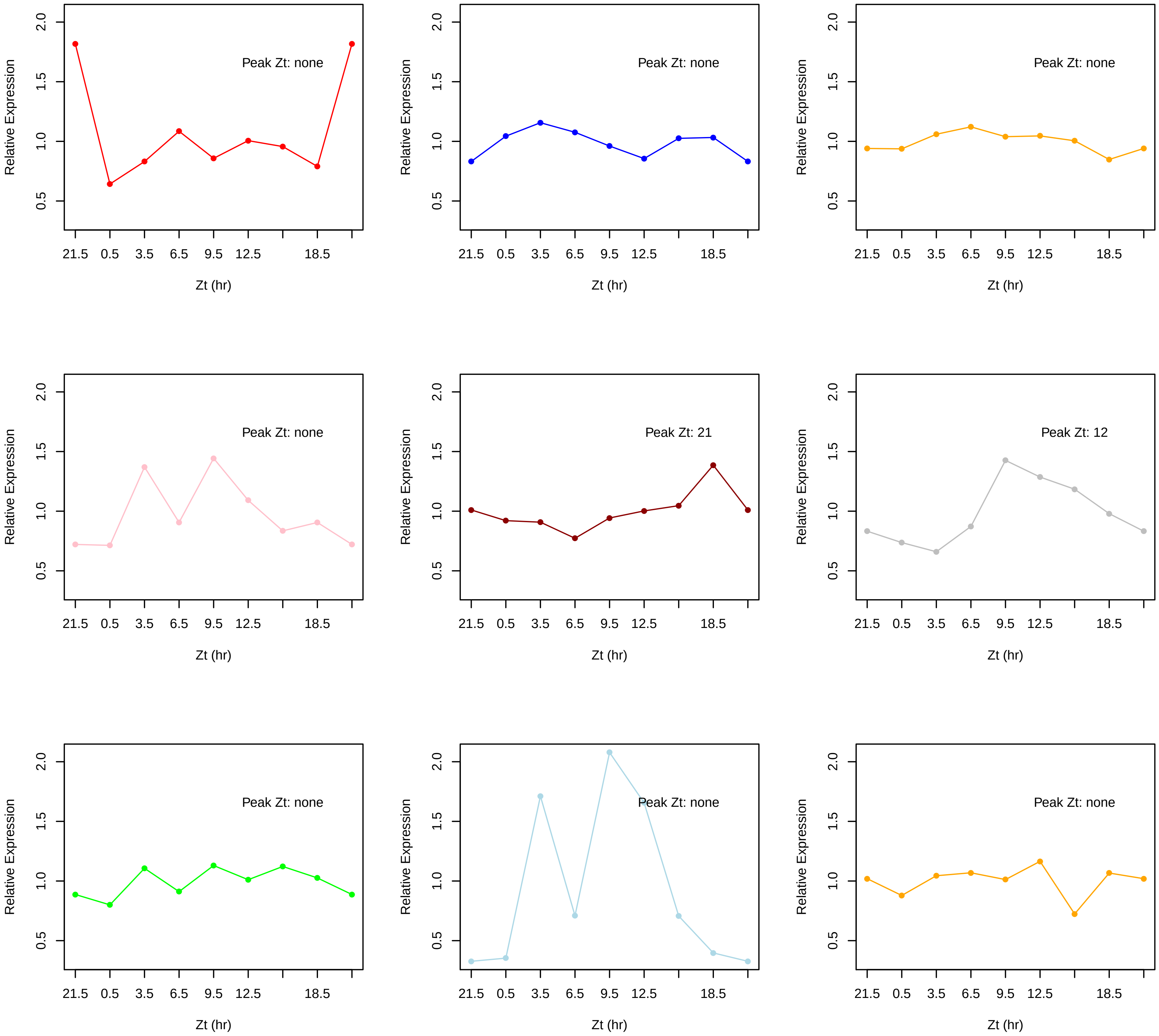

Singeleton: ENSXMAG00000026564 rnf26

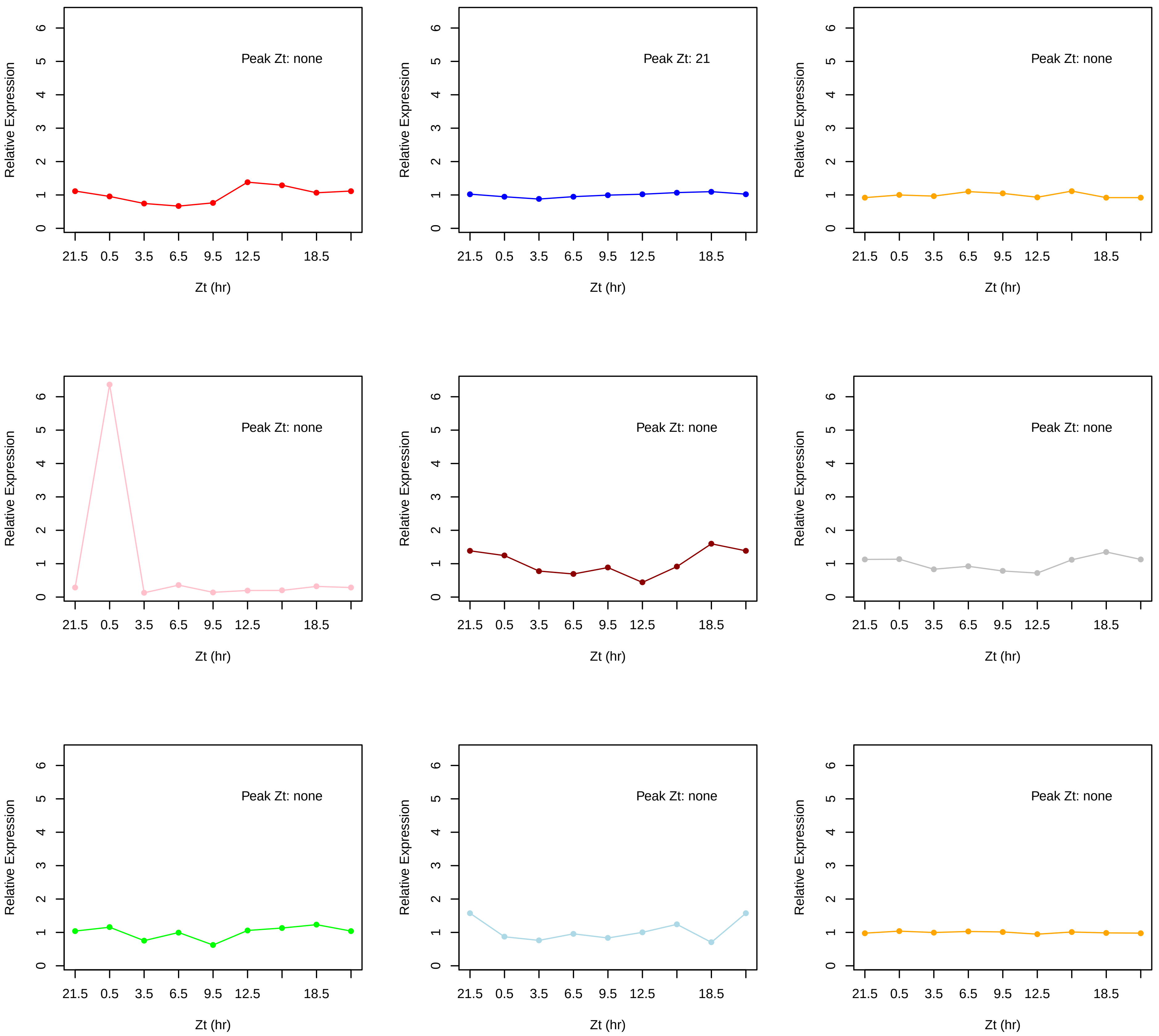

Singeleton: ENSXMAG00000008021 setd1a

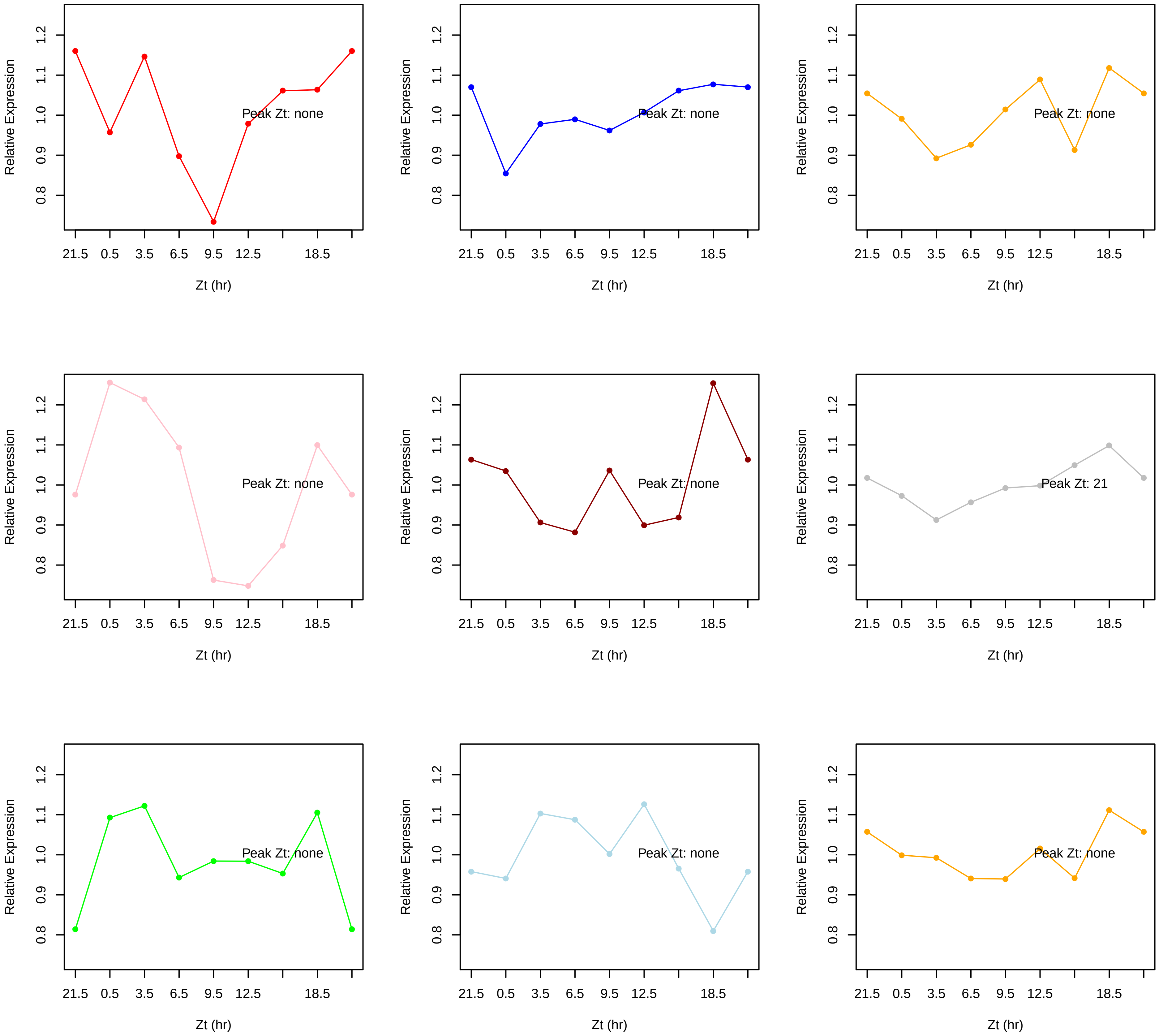

Singeleton: ENSXMAG00000017668 alpl

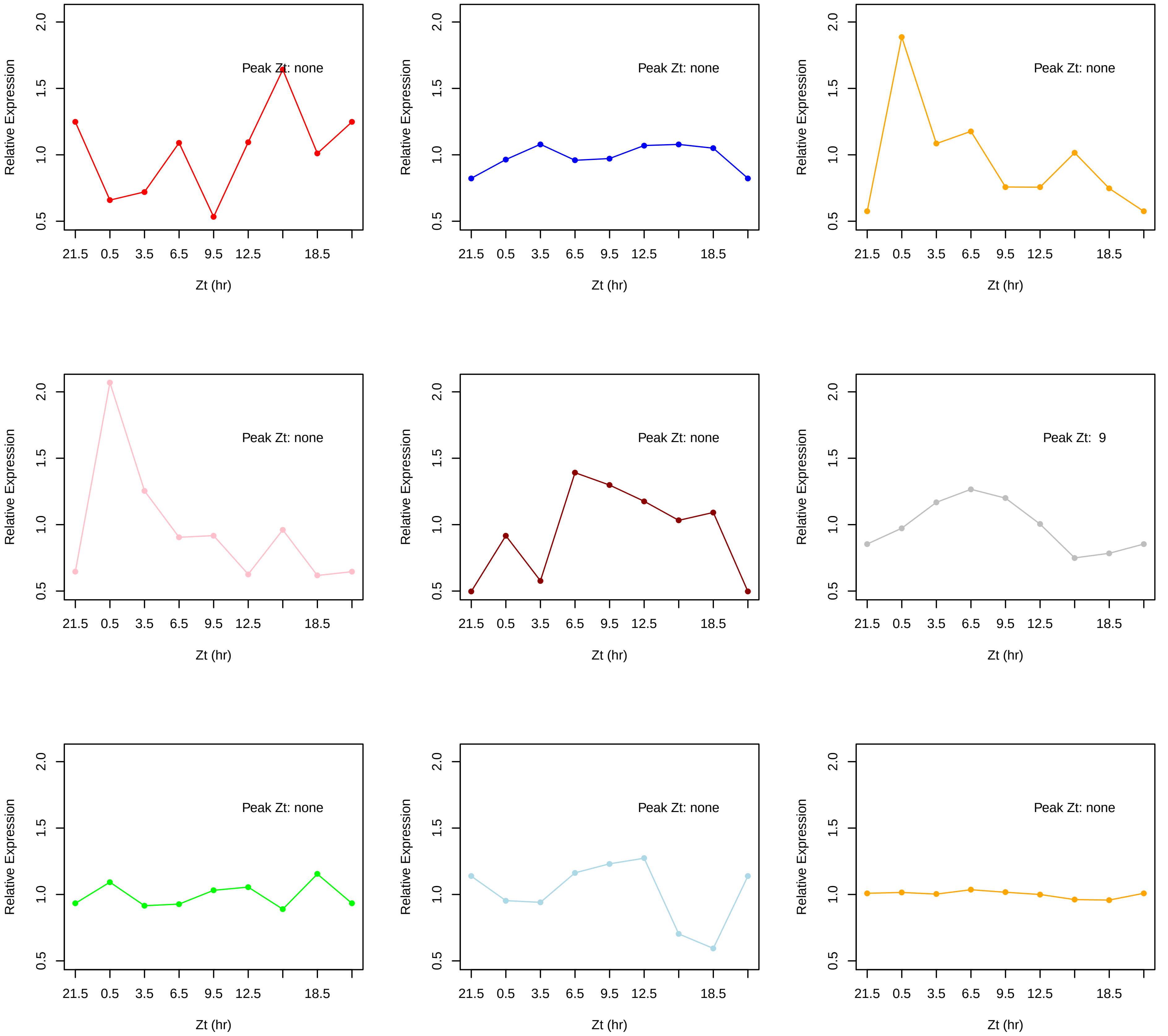

Singeleton: ENSXMAG00000005198

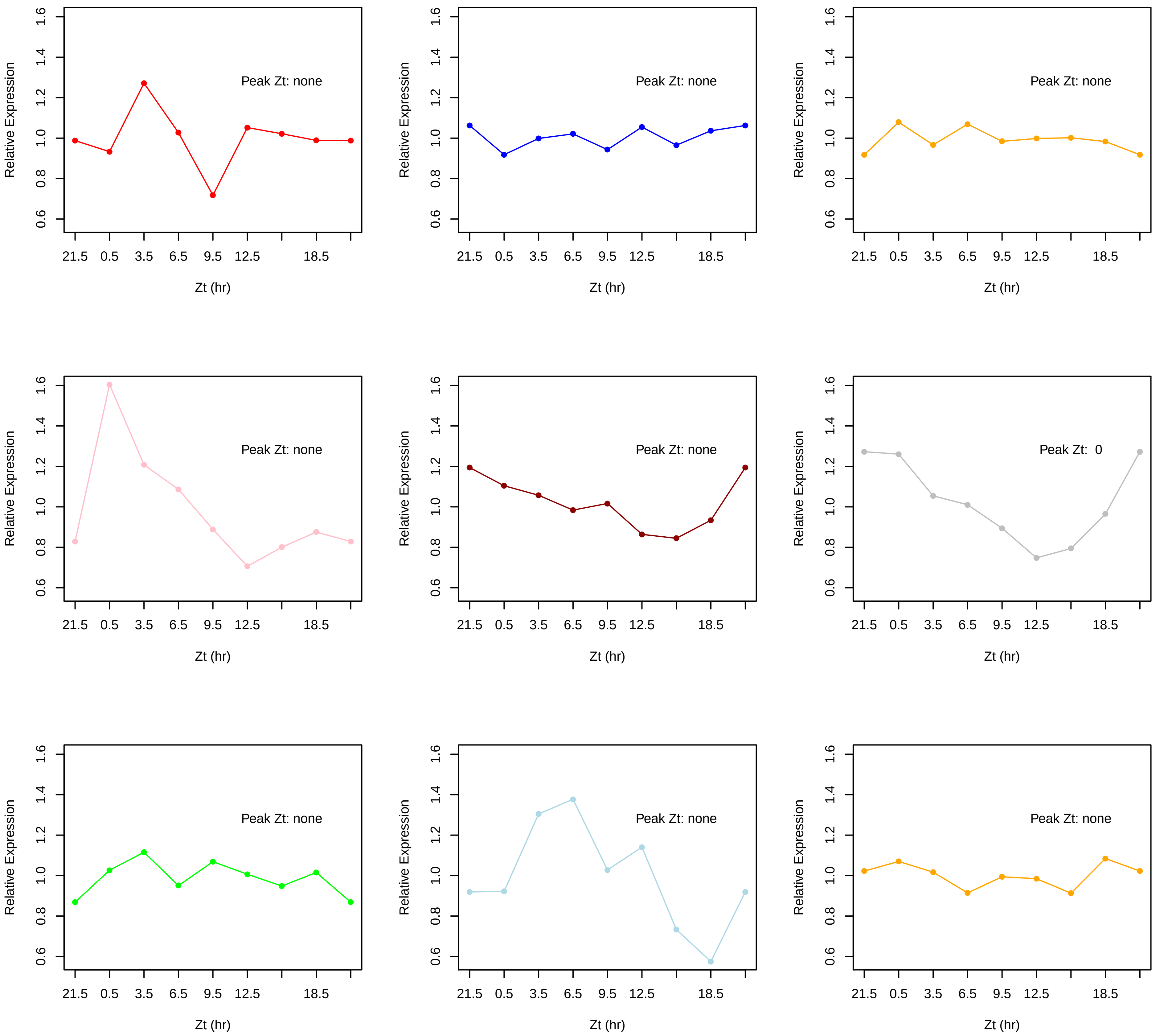

Singeleton: ENSXMAG00000010051 rae1

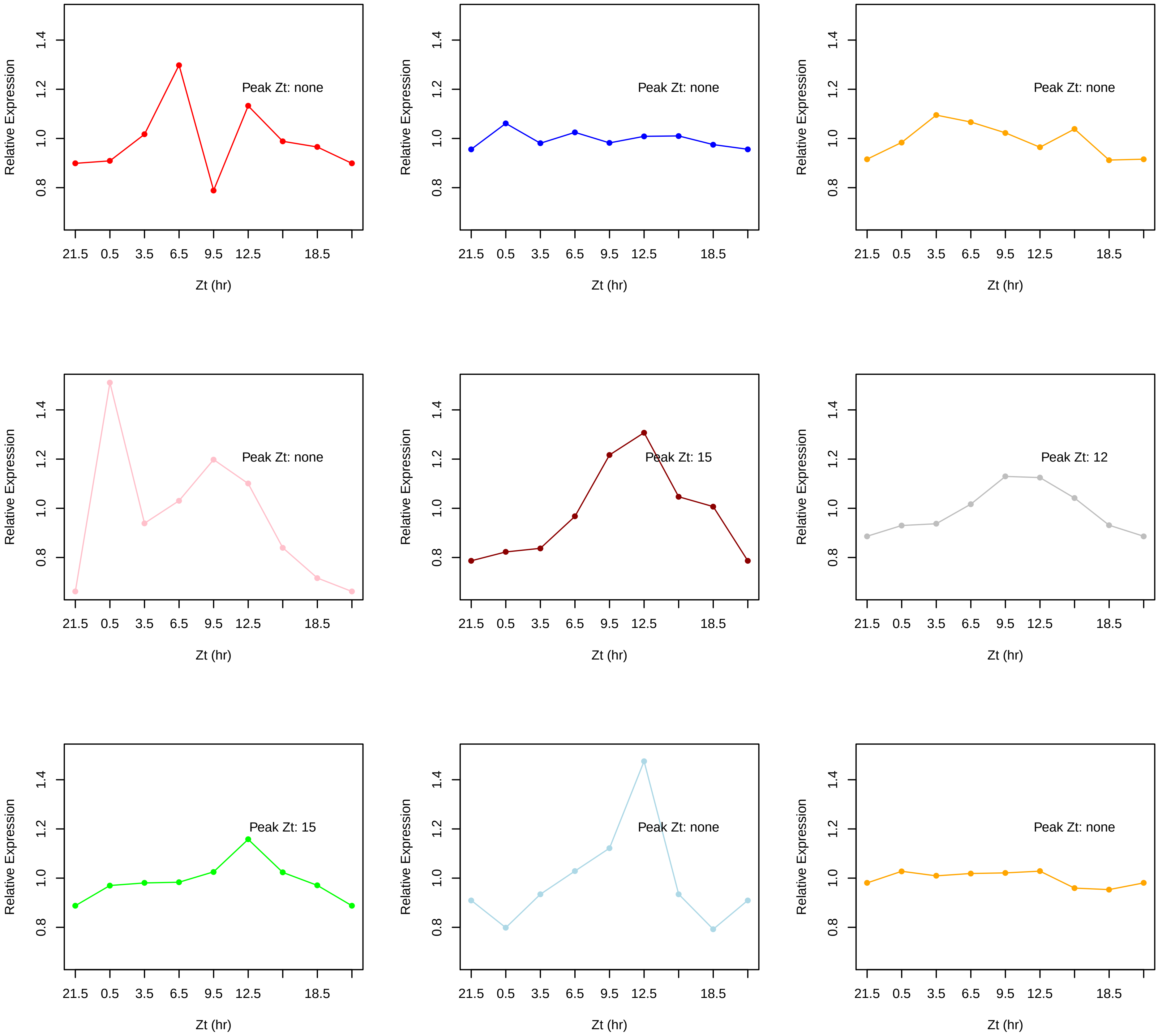

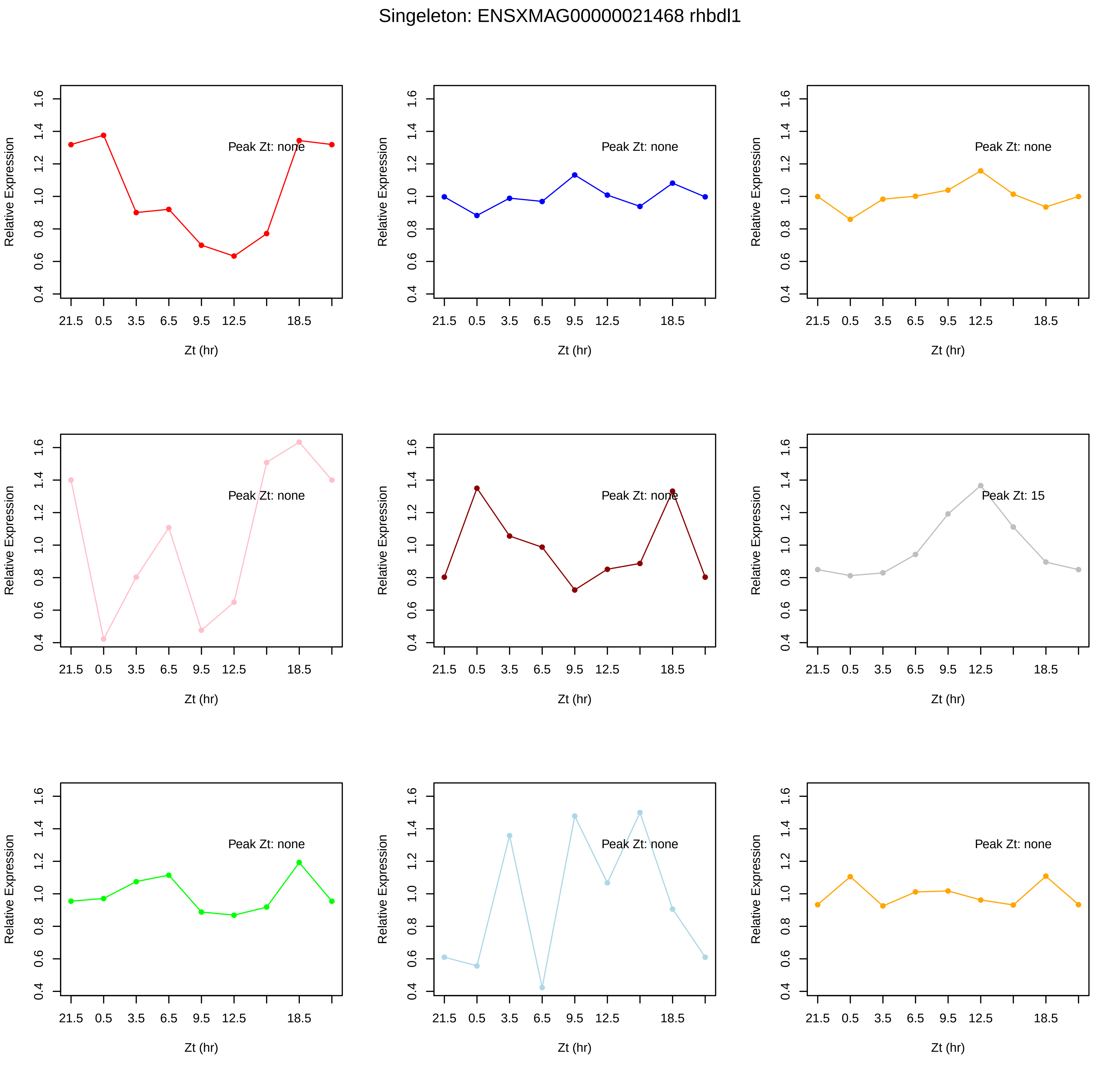
Singeleton: ENSXMAG00000021468 rhbdl1
Peak Zt: none
Peak Zt: none
Peak Zt: none
Peak Zt: none
Peak Zt: none
Peak Zt: 15
Peak Zt: none
Peak Zt: none
Peak Zt: none
Relative Expression
Zt (hr)
21.5
0.5
3.5
6.5
9.5
12.5
18.5
0.4
0.6
0.8
1.0
1.2
1.4
1.6

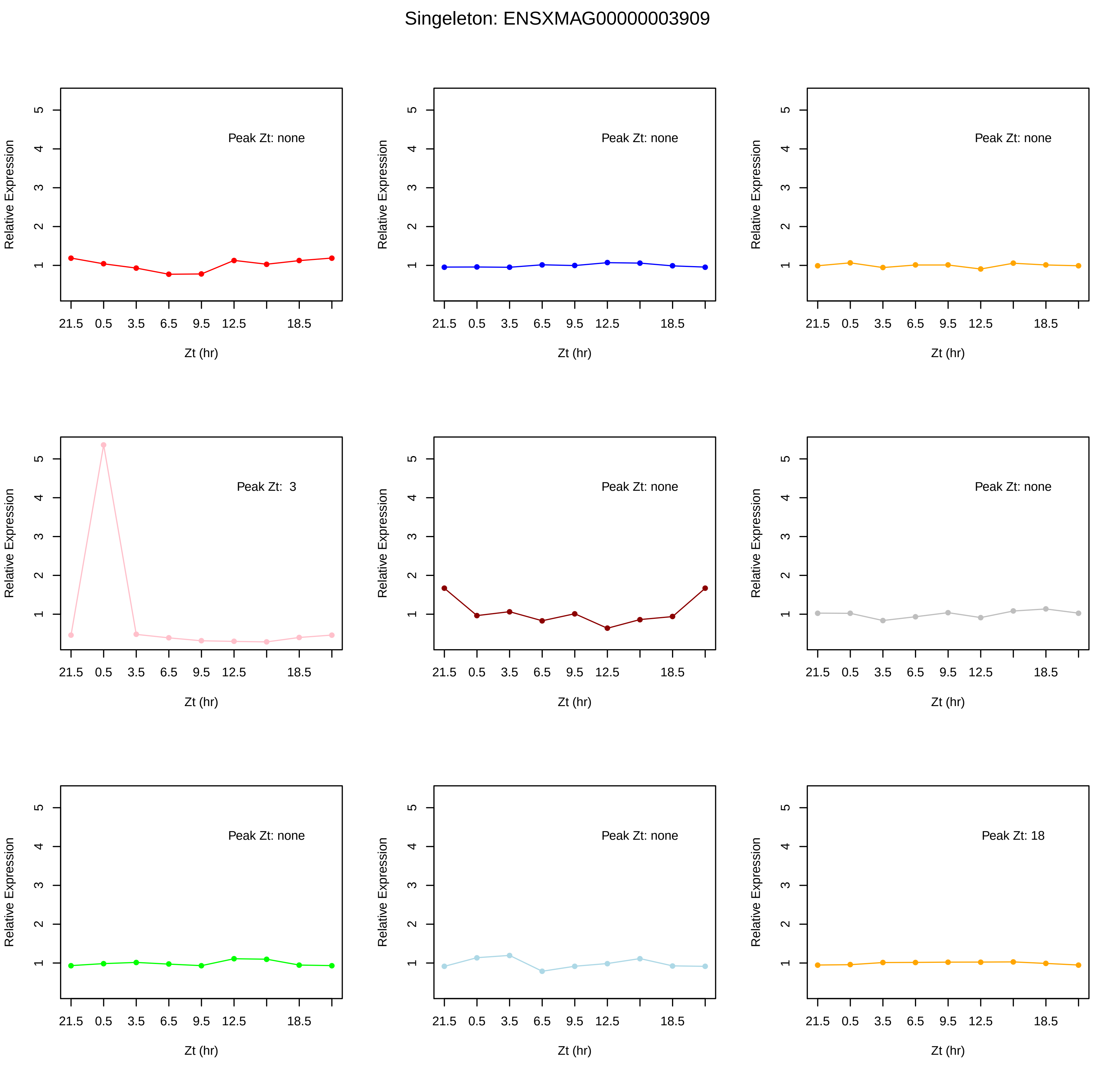
Singleton: ENSXMAG00000003909
Peak Zt: none
Peak Zt: none
Peak Zt: none
Peak Zt: 3
Peak Zt: none
Peak Zt: none
Peak Zt: none
Peak Zt: none
Peak Zt: 18
Relative Expression
Zt (hr)
21.5
0.5
3.5
6.5
9.5
12.5
18.5

Singleton: ENSXMAG00000020918 fxyd1

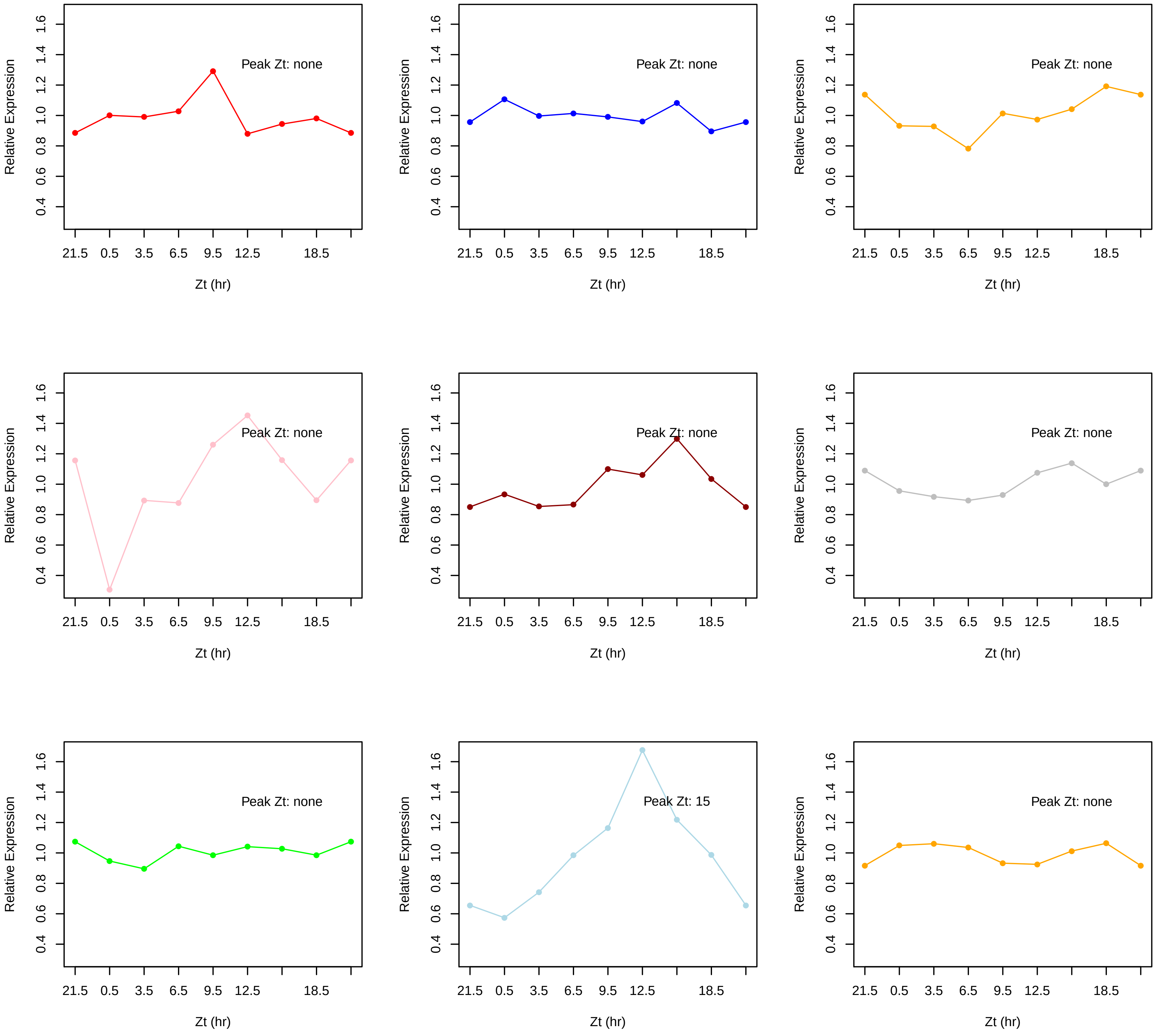

Singeleton: ENSXMAG00000017683 ece1

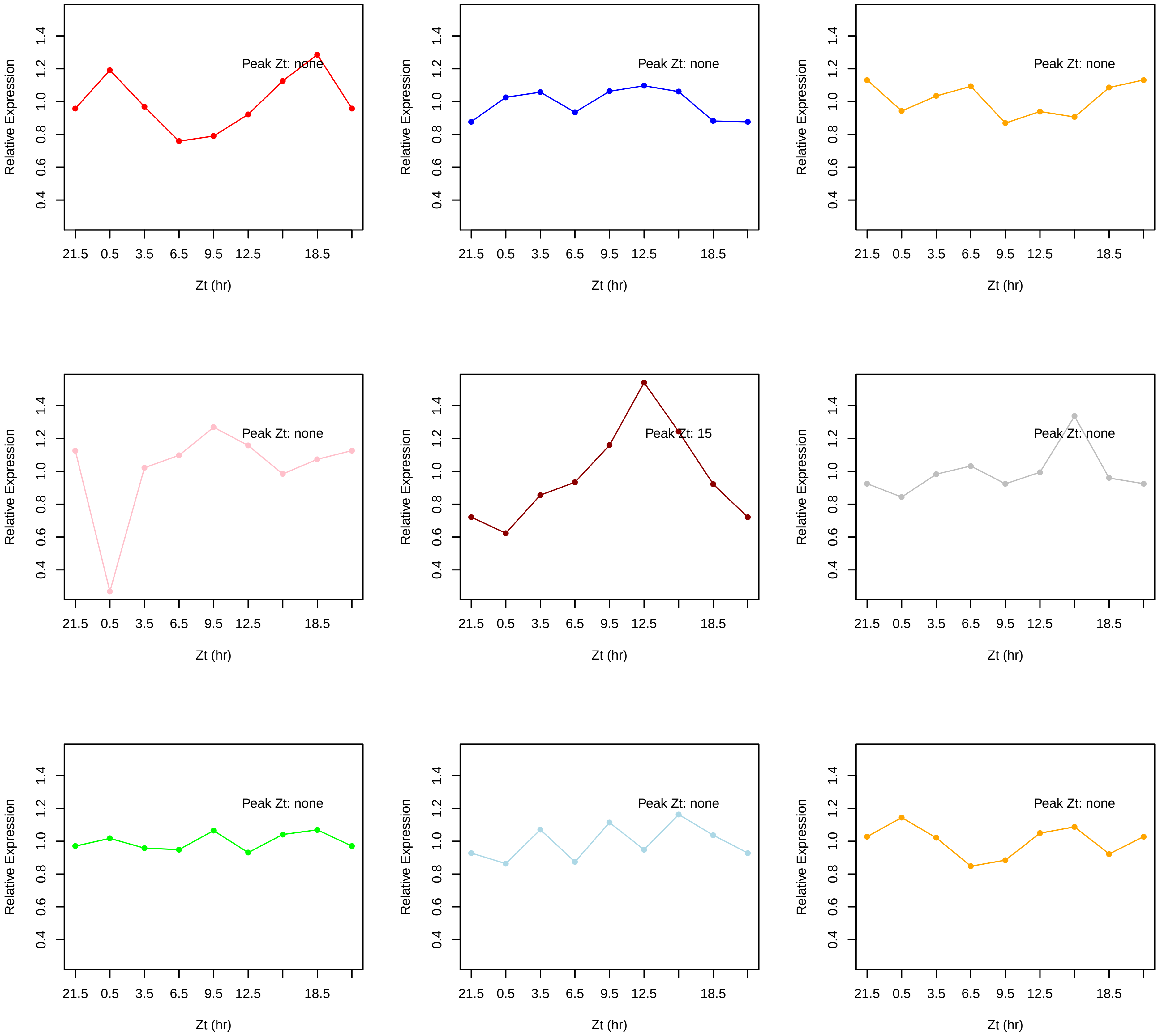

Singeleton: ENSXMAG00000003267 inpp5e

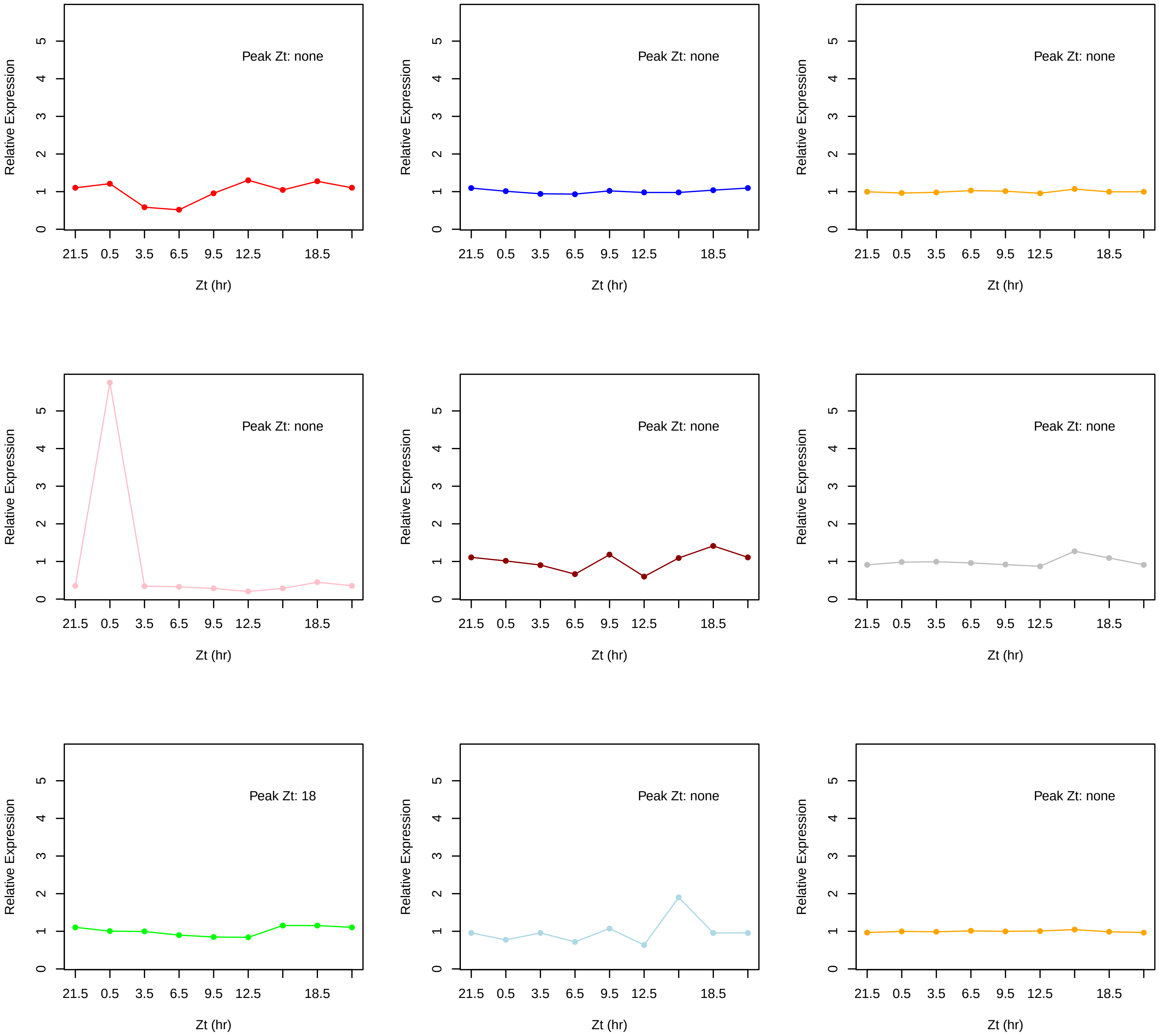

Singleton: ENSXMAG00000013013 prdx4

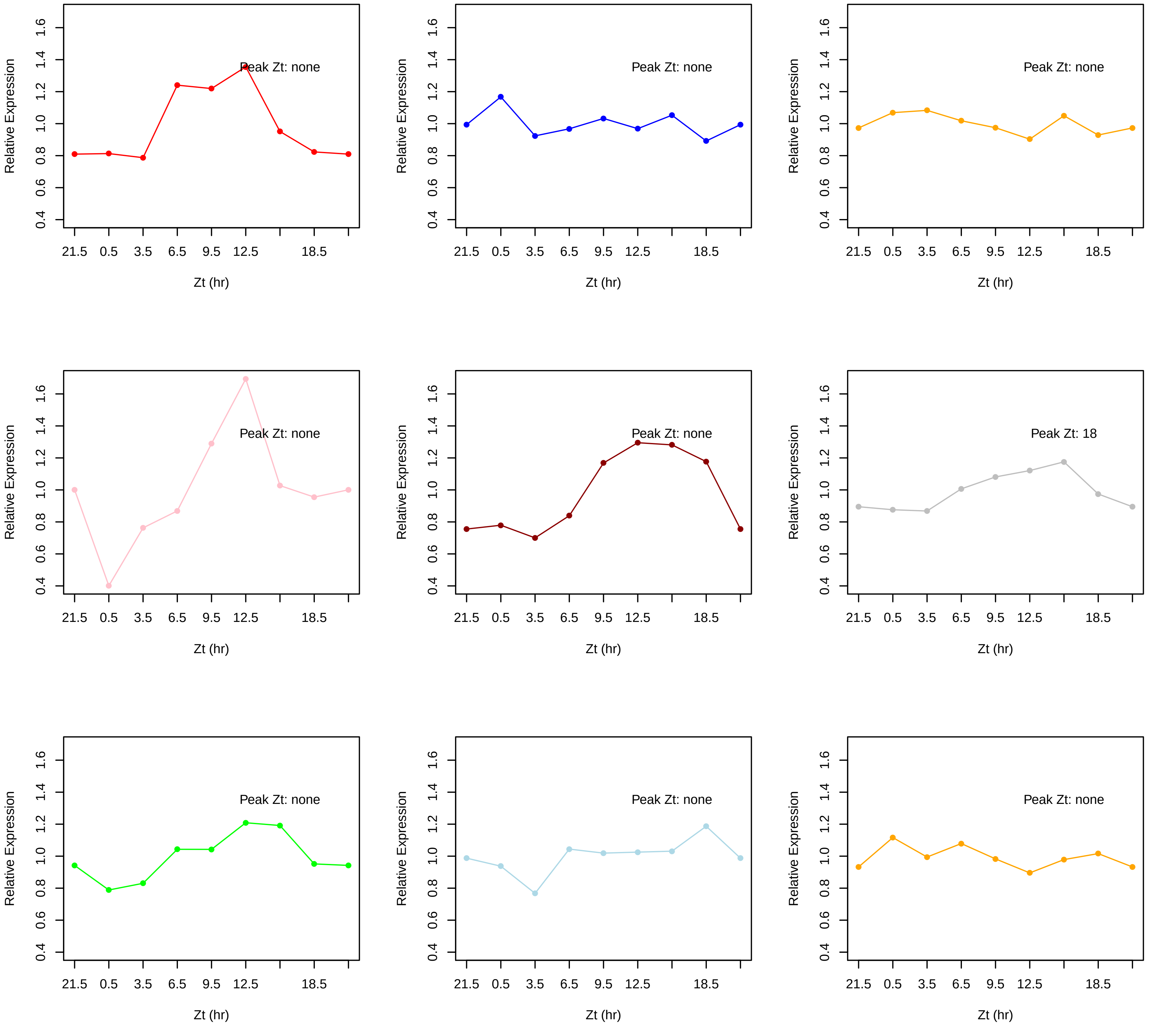

Singeleton: ENSXMAG00000024327 spock3

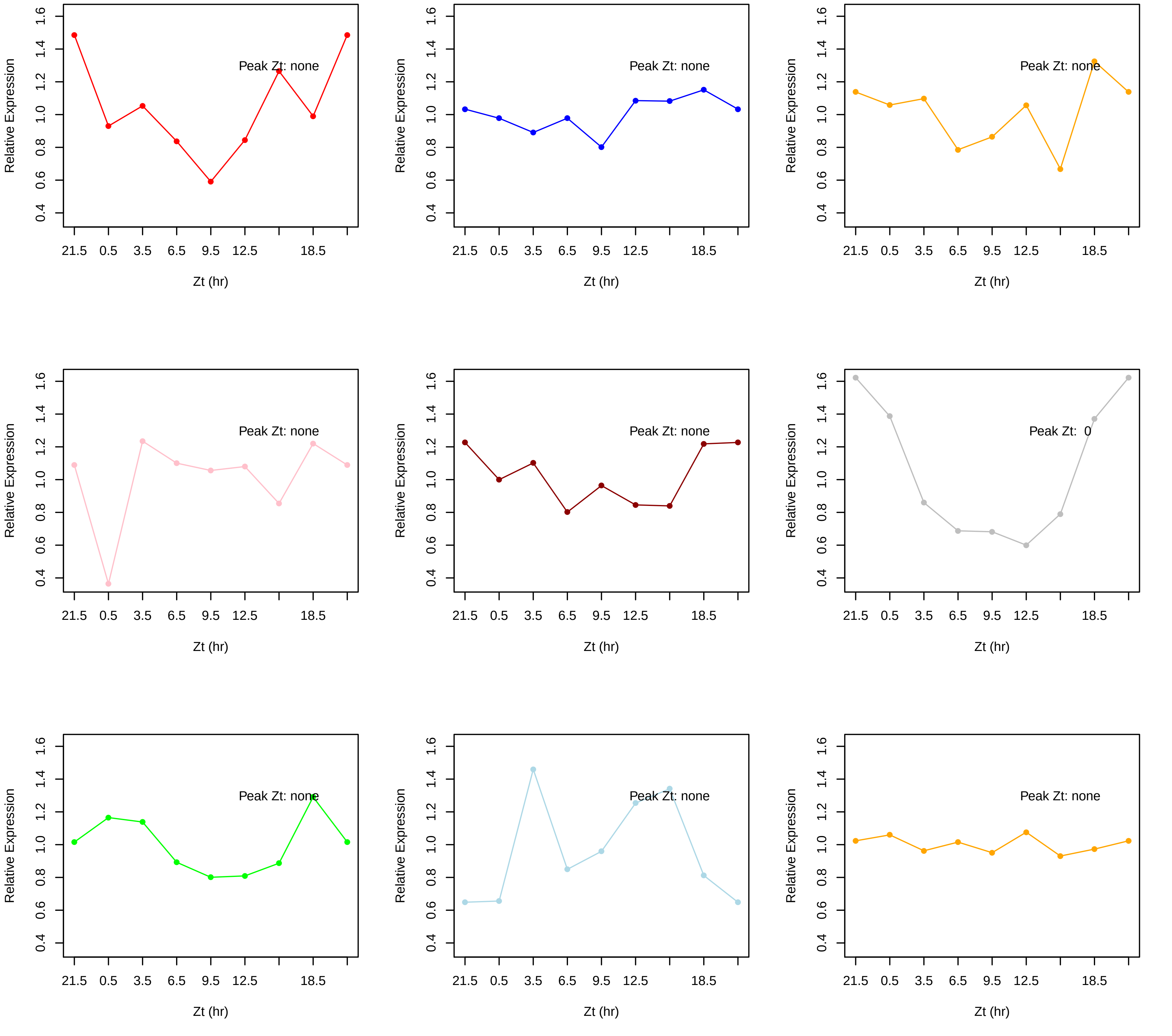

Singeleton: ENSXMAG00000014941 acadm

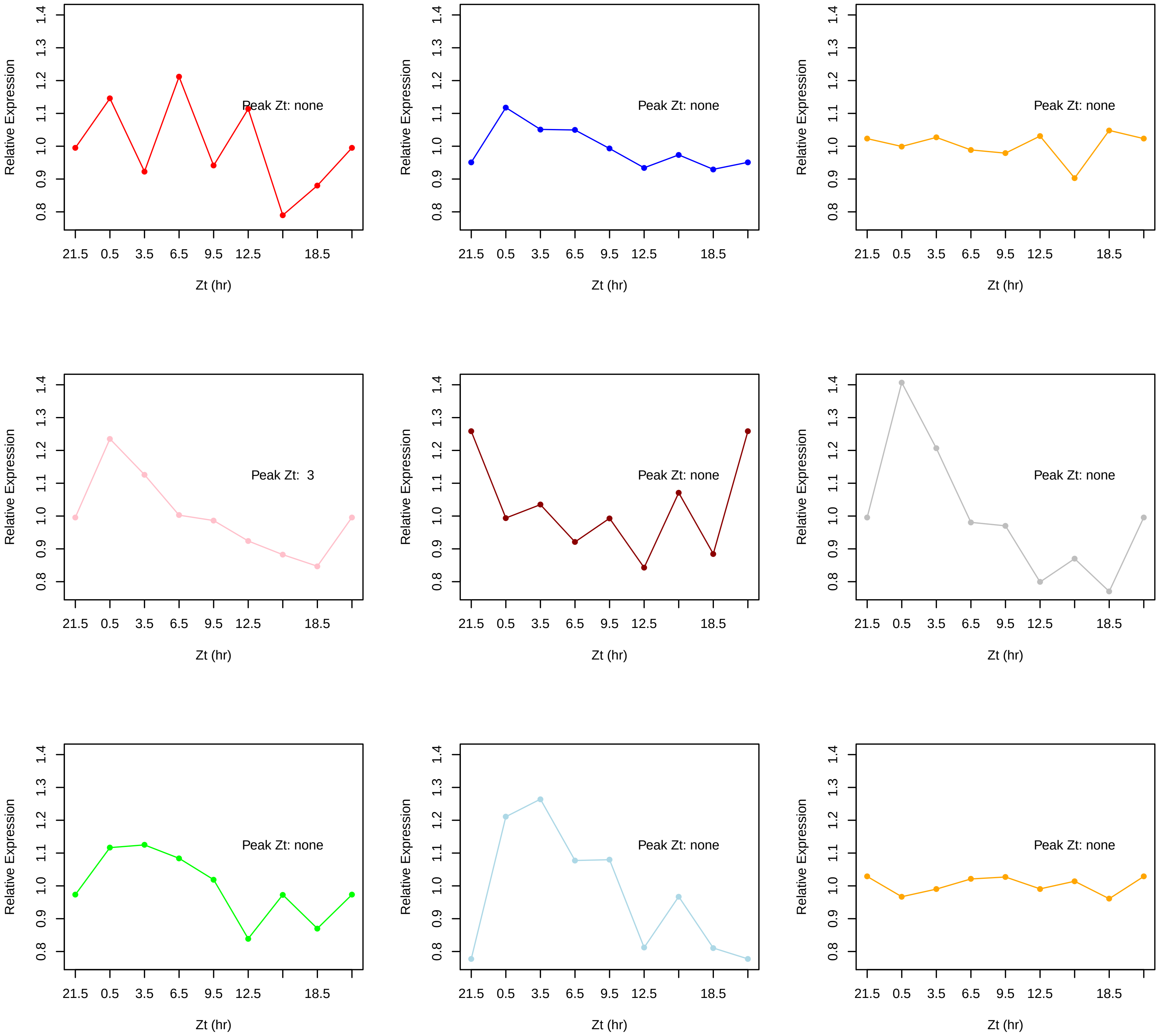

Singeleton: ENSXMAG00000003265

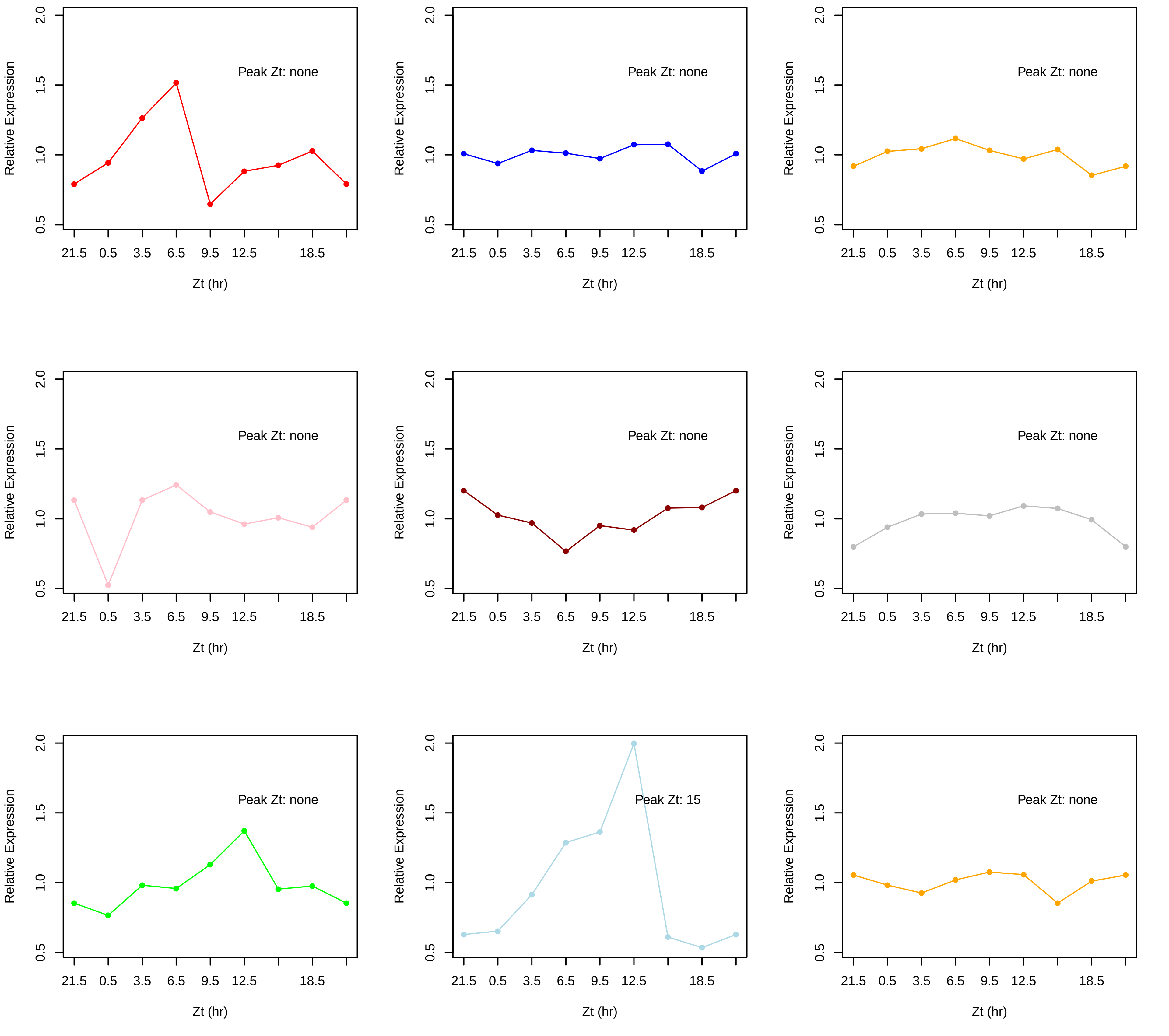

Singeleton: ENSXMAG00000025912

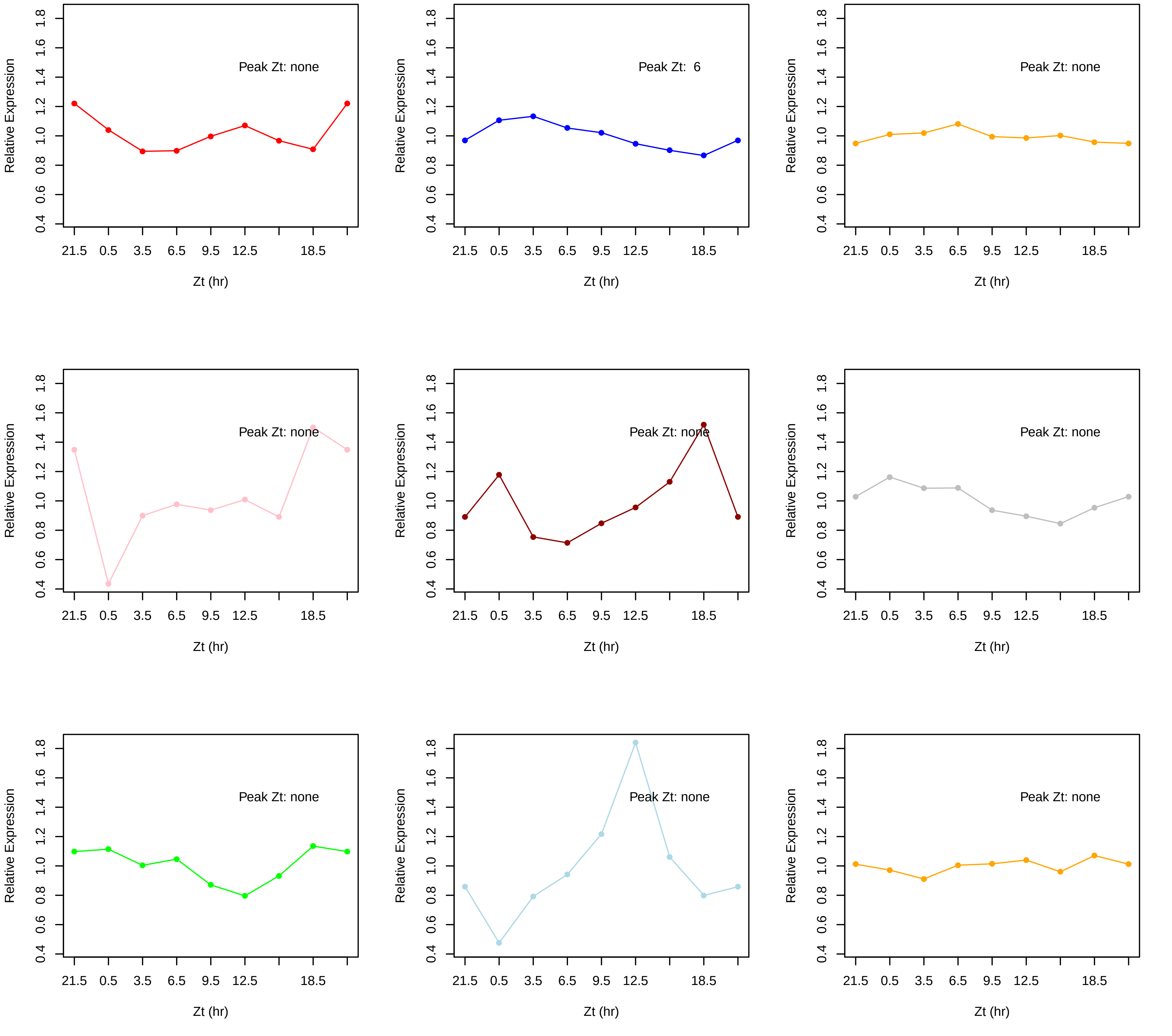

Singleton: ENSXMAG00000005388 ncor1

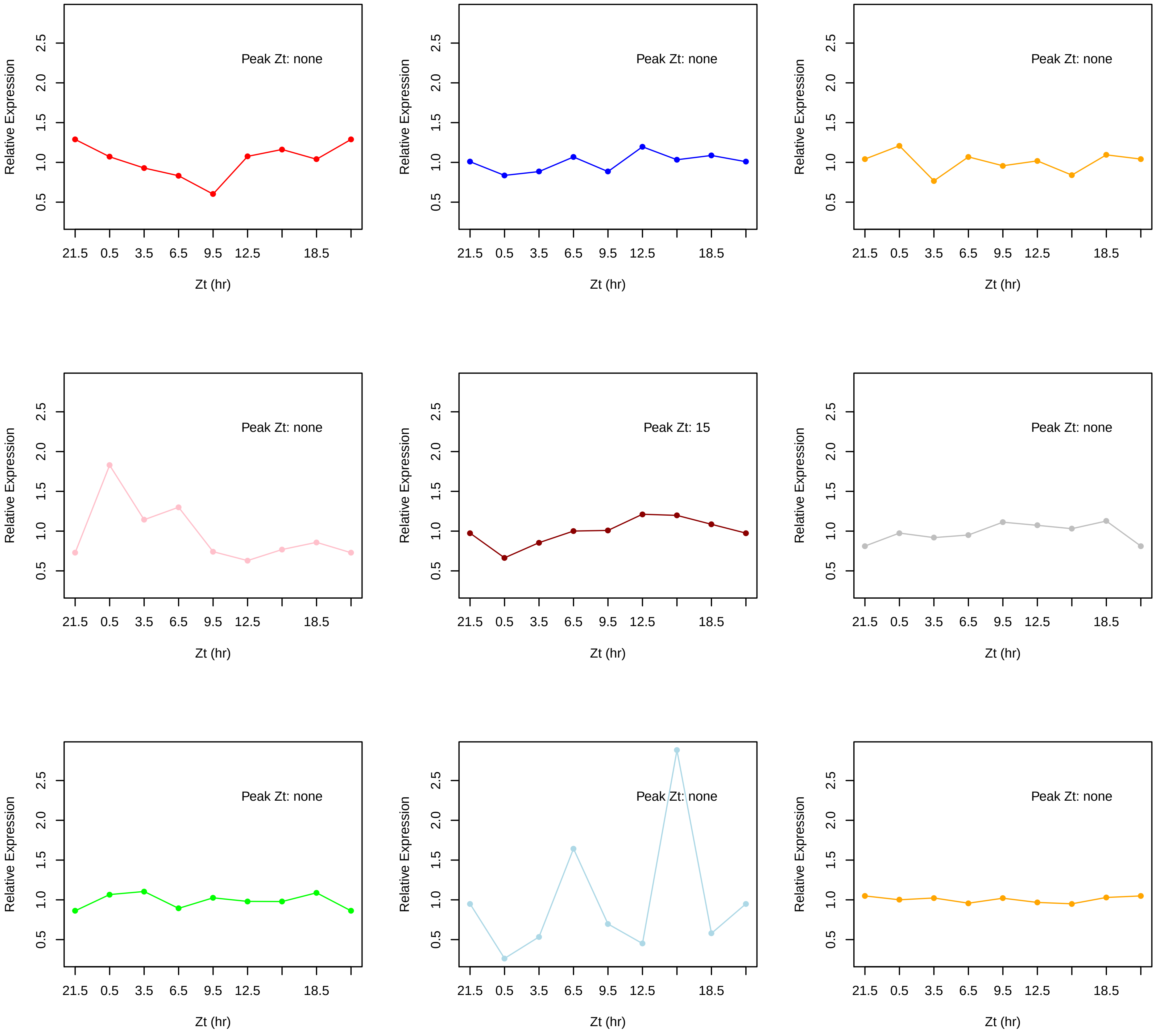

Singeleton: ENSXMAG00000017817 bmp7b

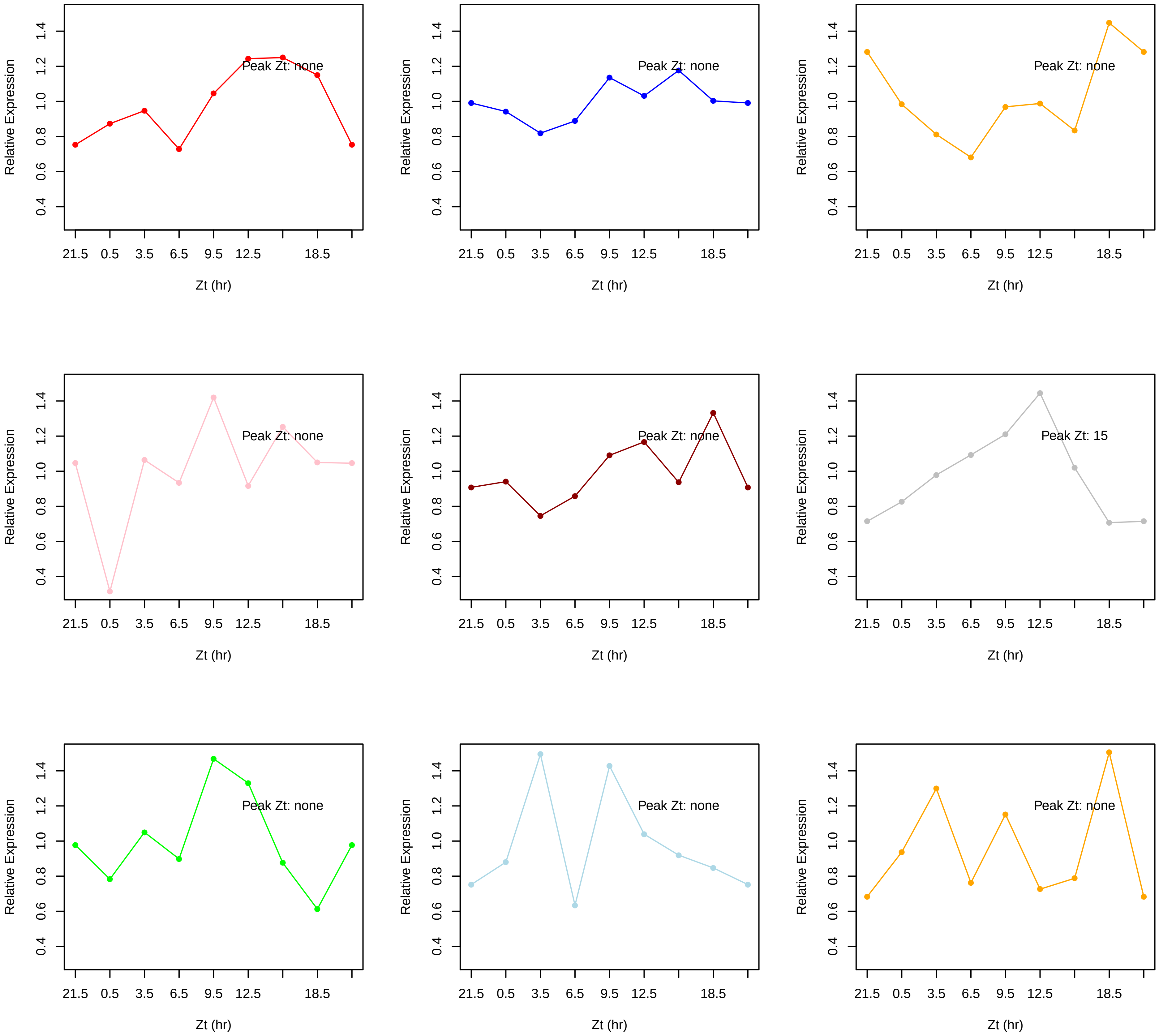

Singleton: ENSXMAG00000018970 wdr24

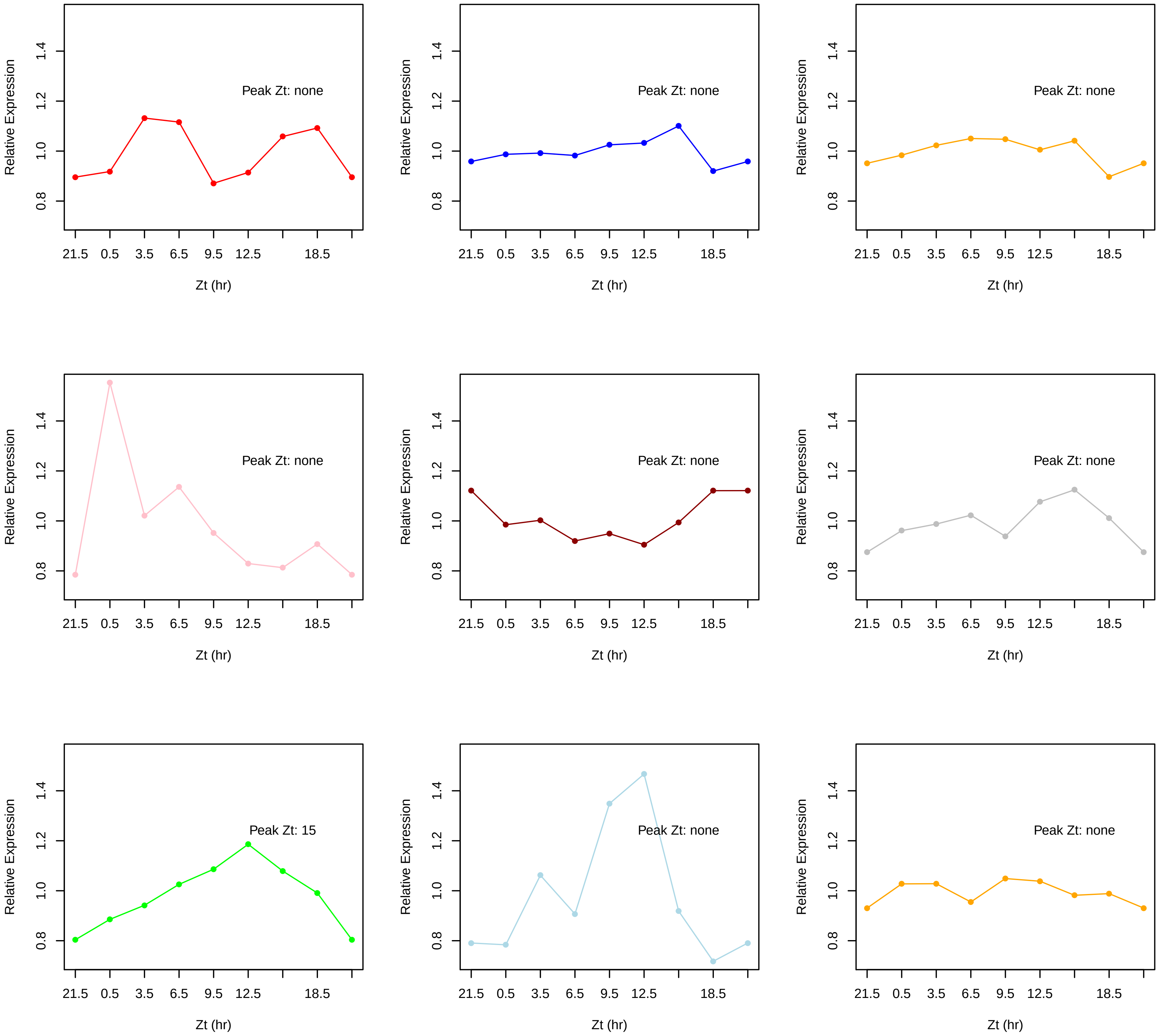

Singeleton: ENSXMAG00000012352 stk36

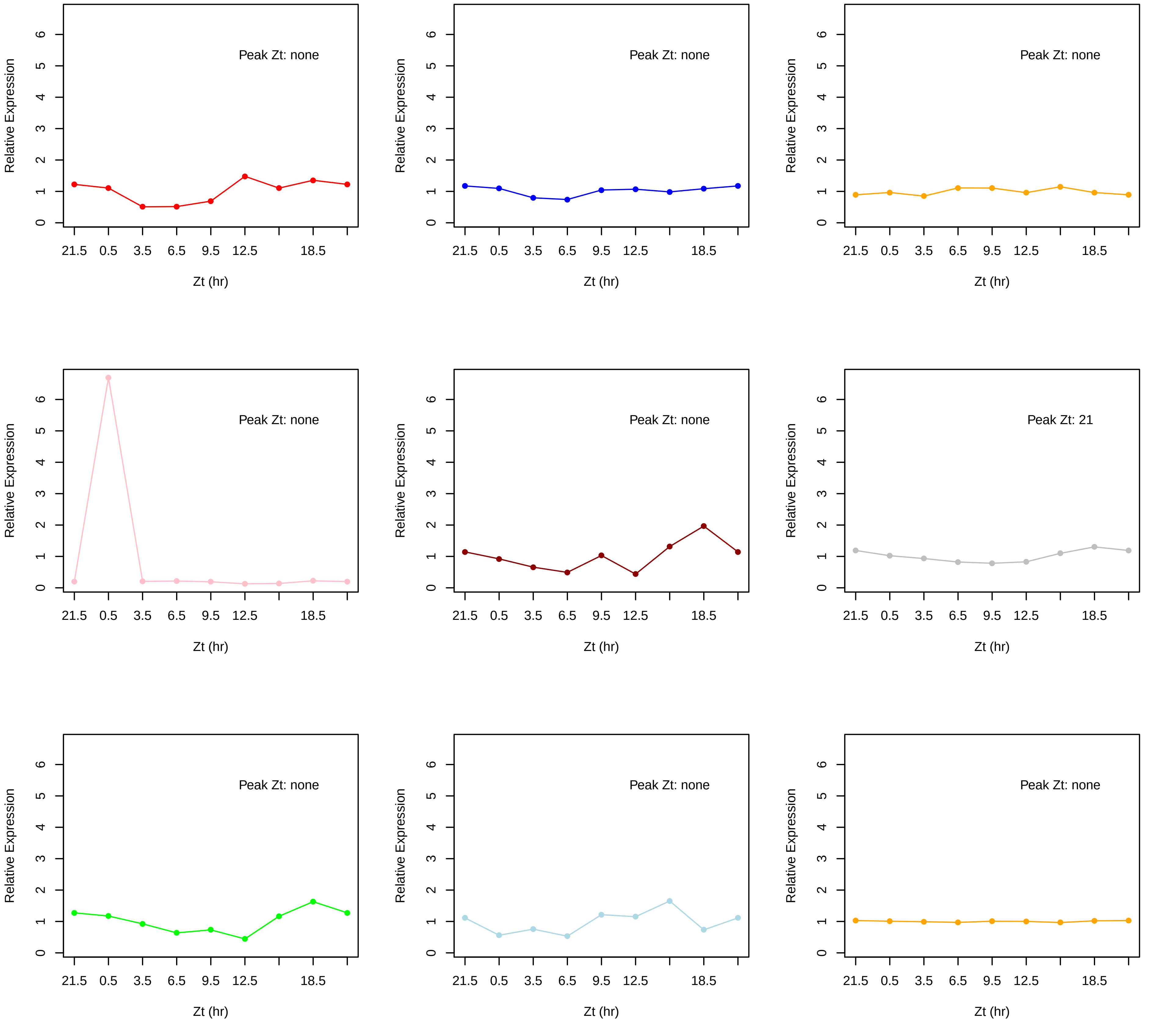

Singeleton: ENSXMAG00000021116 rabggtb

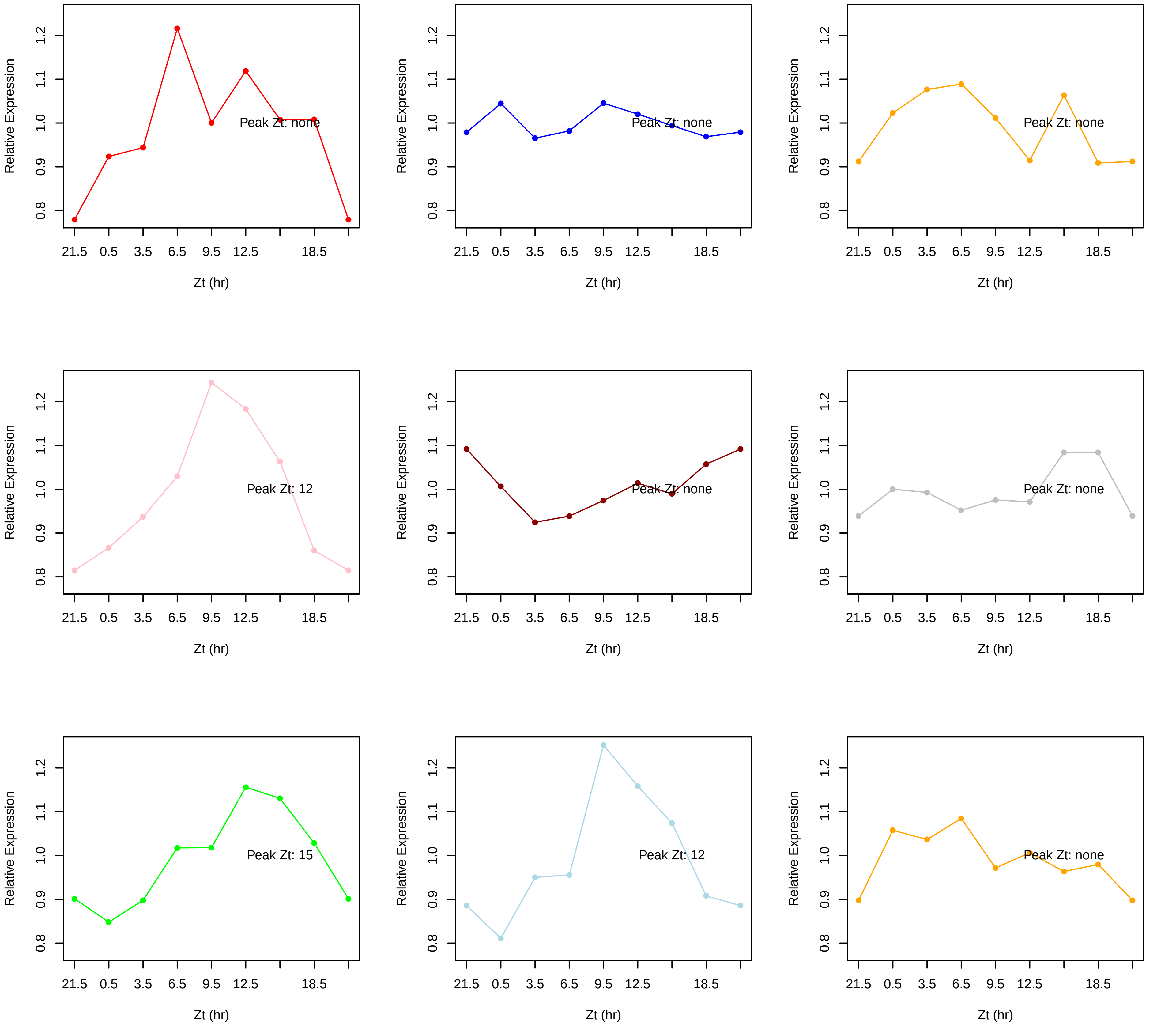

Singeleton: ENSXMAG00000027761

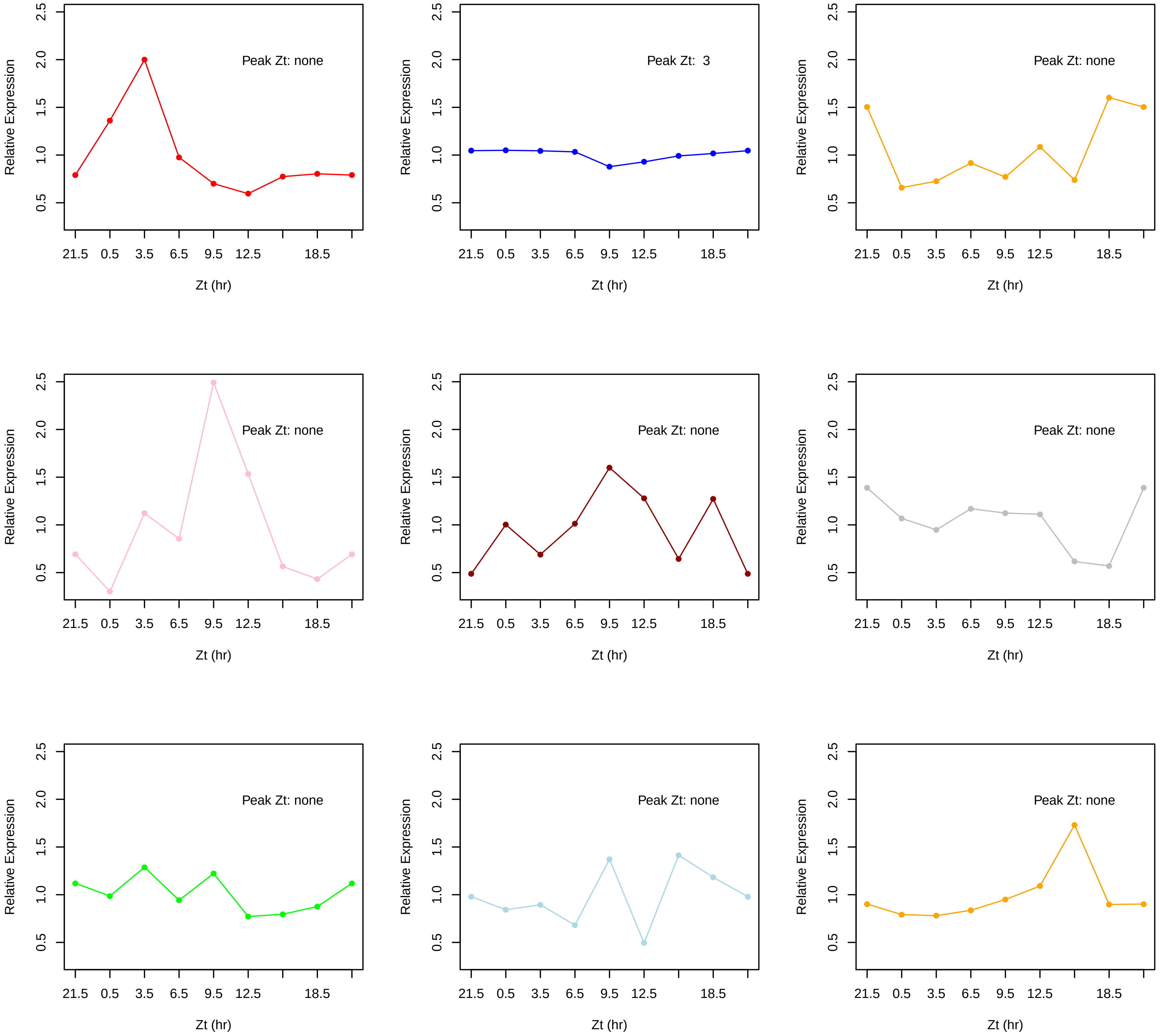

Singeleton: ENSXMAG00000005937 CACFD1

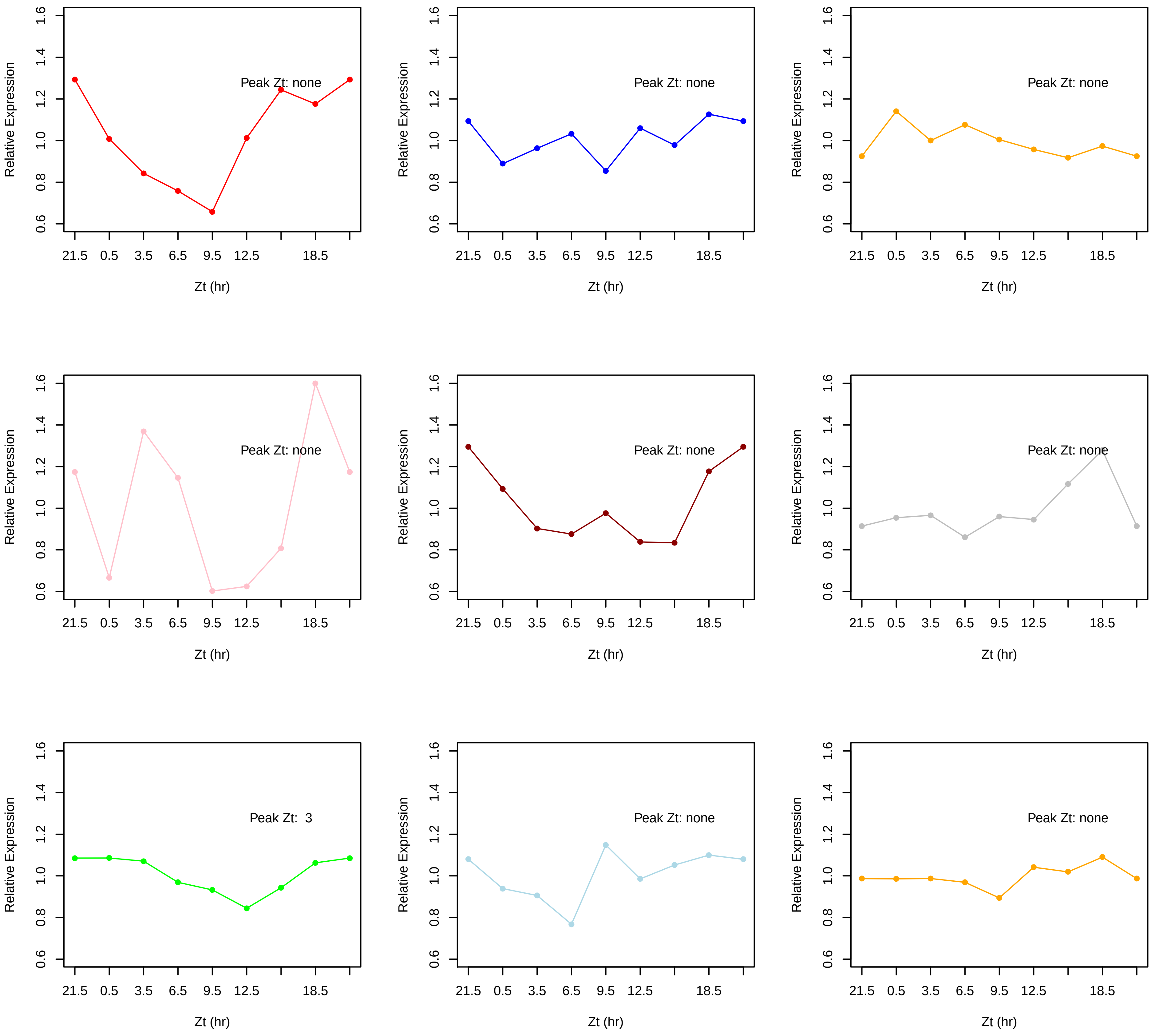

Singeleton: ENSXMAG00000008849

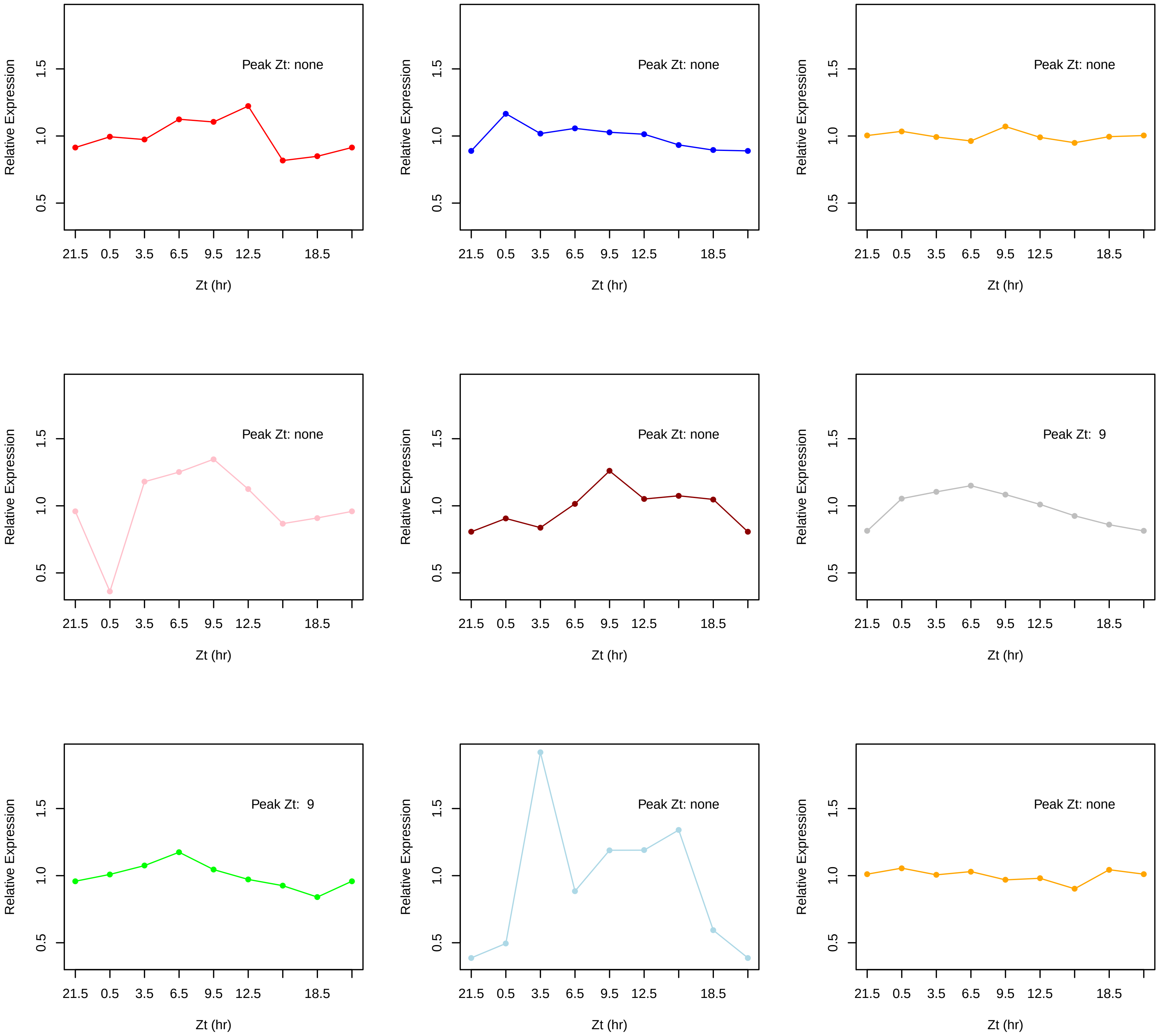

Singeleton: ENSXMAG00000003820

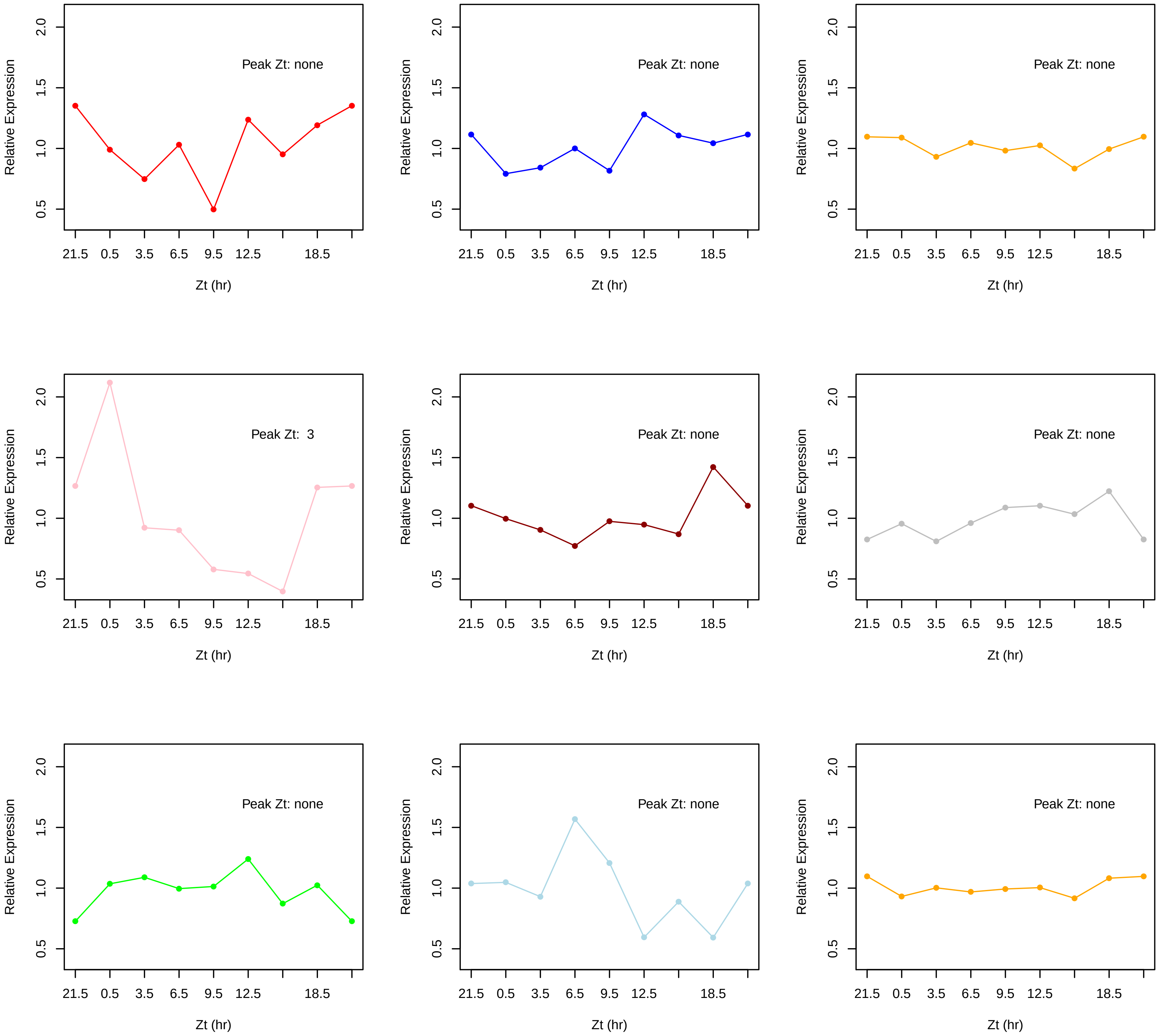

Singeleton: ENSXMAG00000005254 dgkg

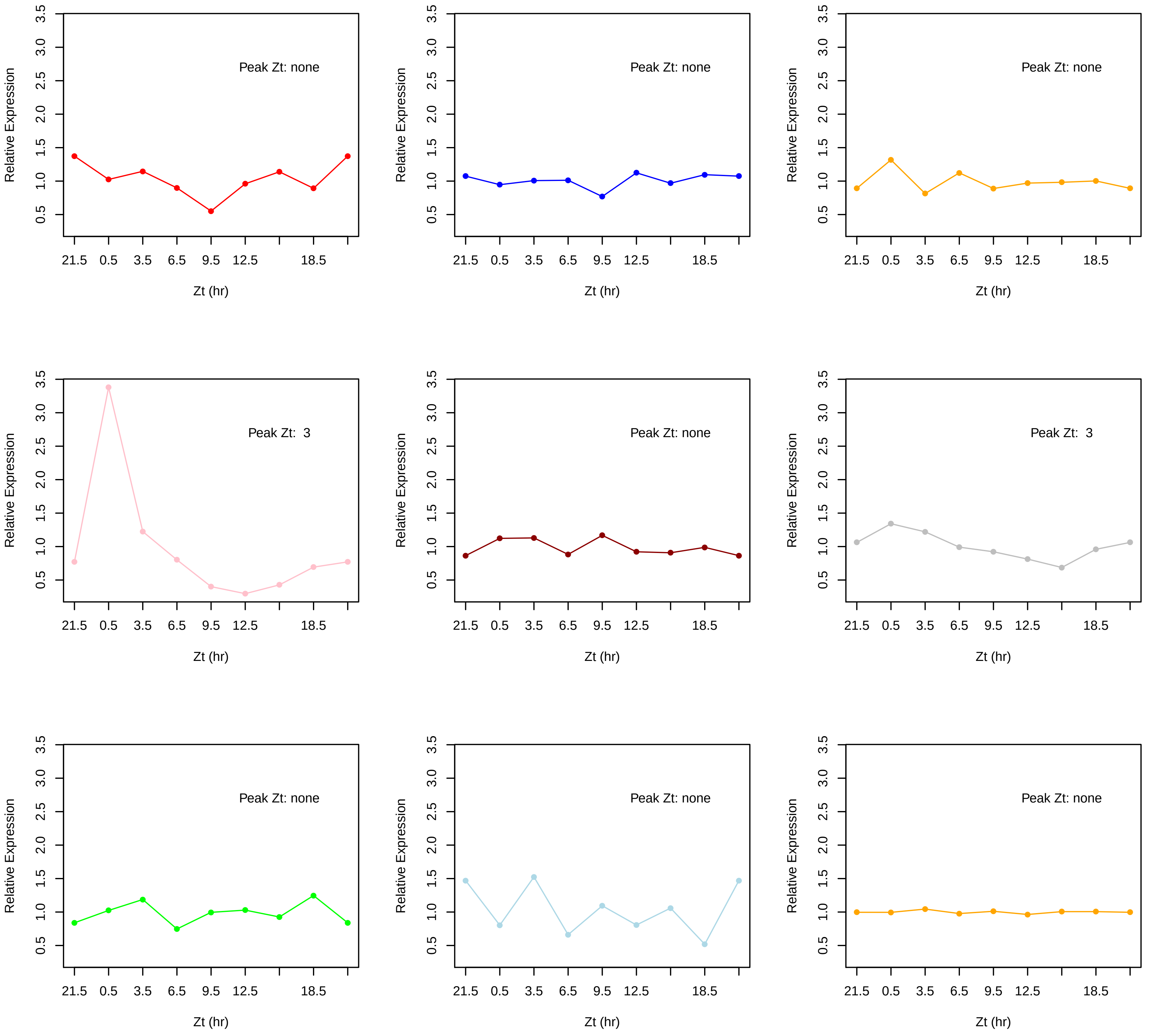

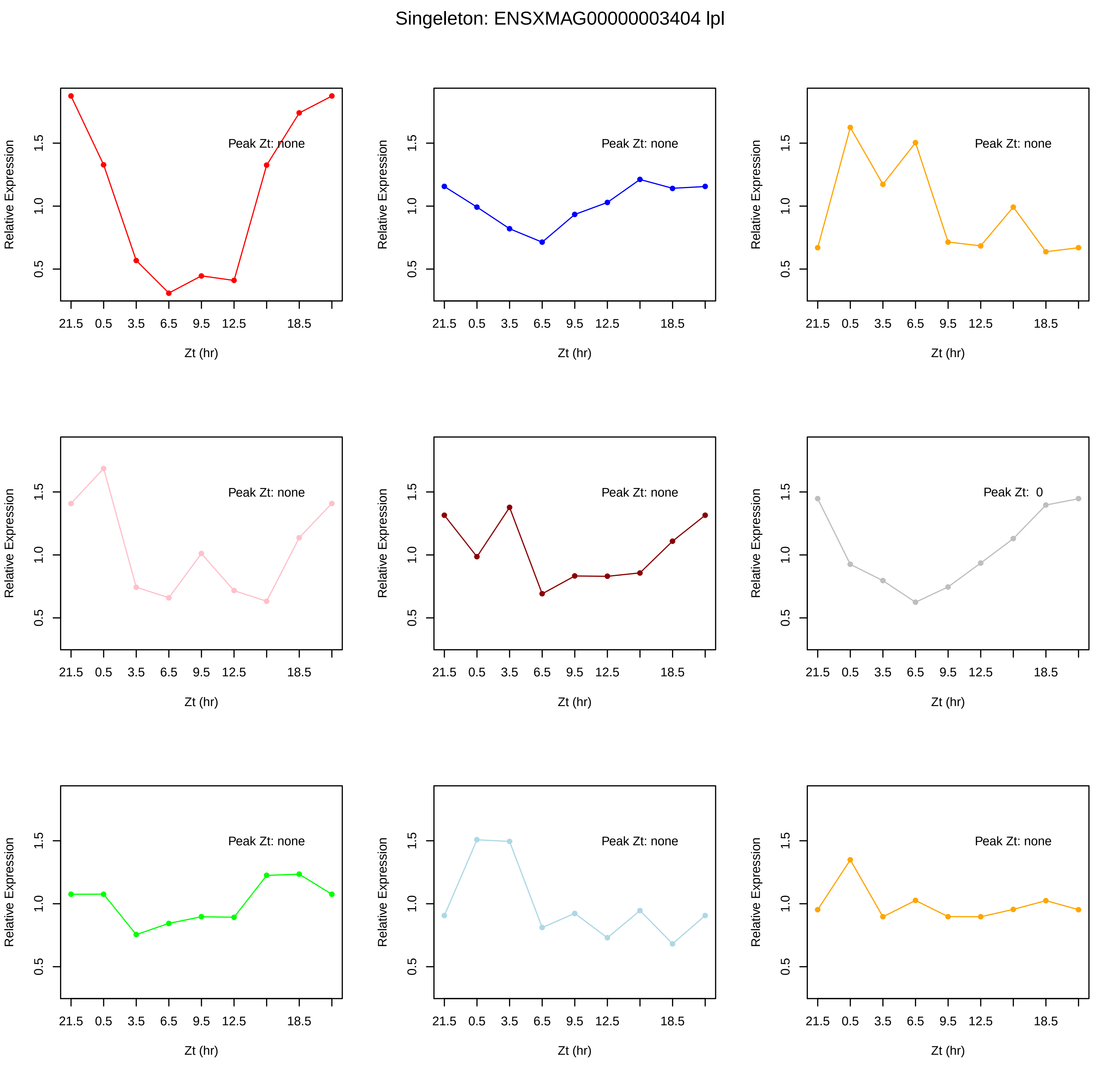

Singeleton: ENSXMAG00000003404 lpl
Peak Zt: none
Peak Zt: 0
Relative Expression
Zt (hr)
21.5
0.5
3.5
6.5
9.5
12.5
18.5
0.5
1.0
1.5

Singeleton: ENSXMAG00000005379 zswim7

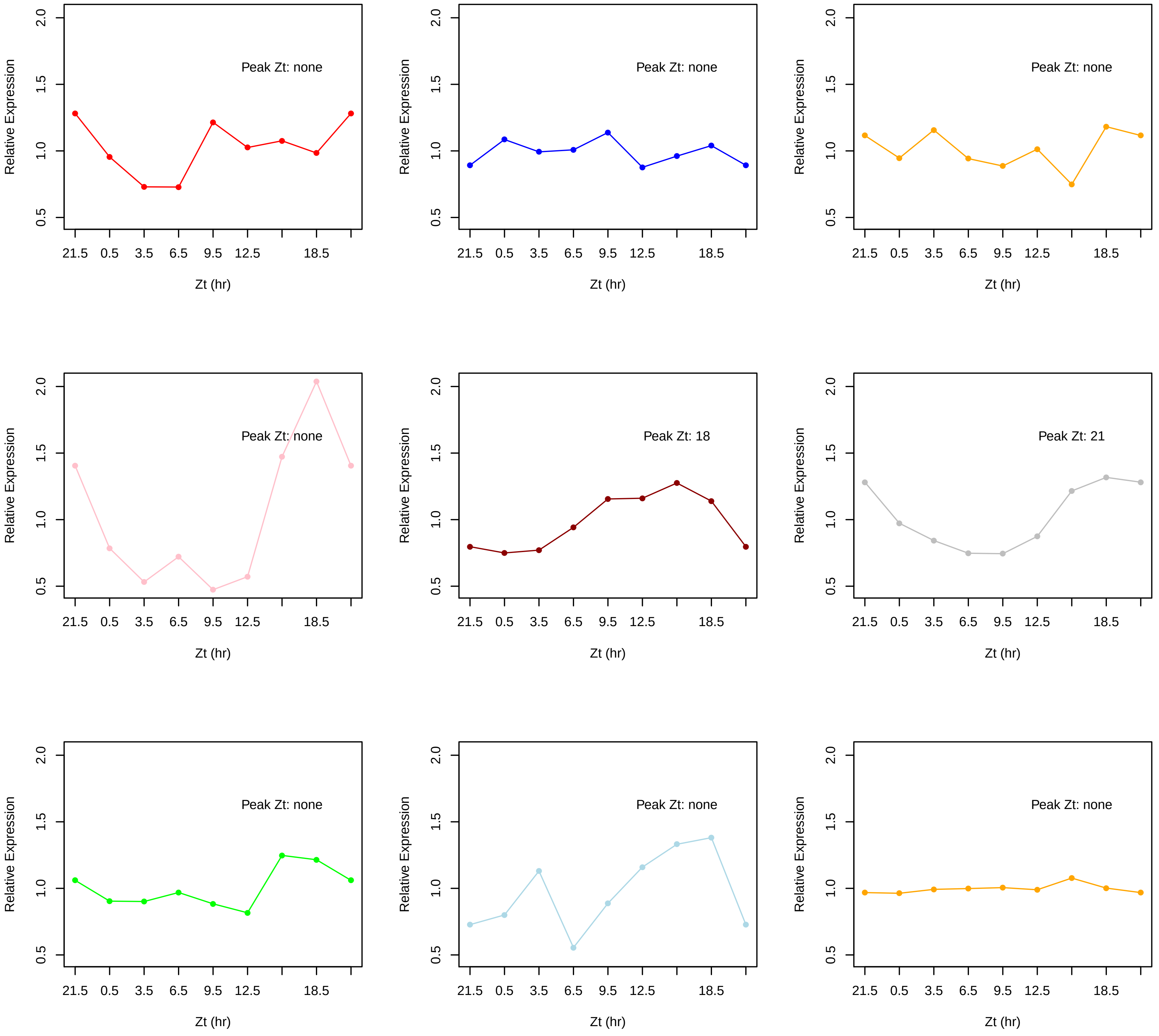

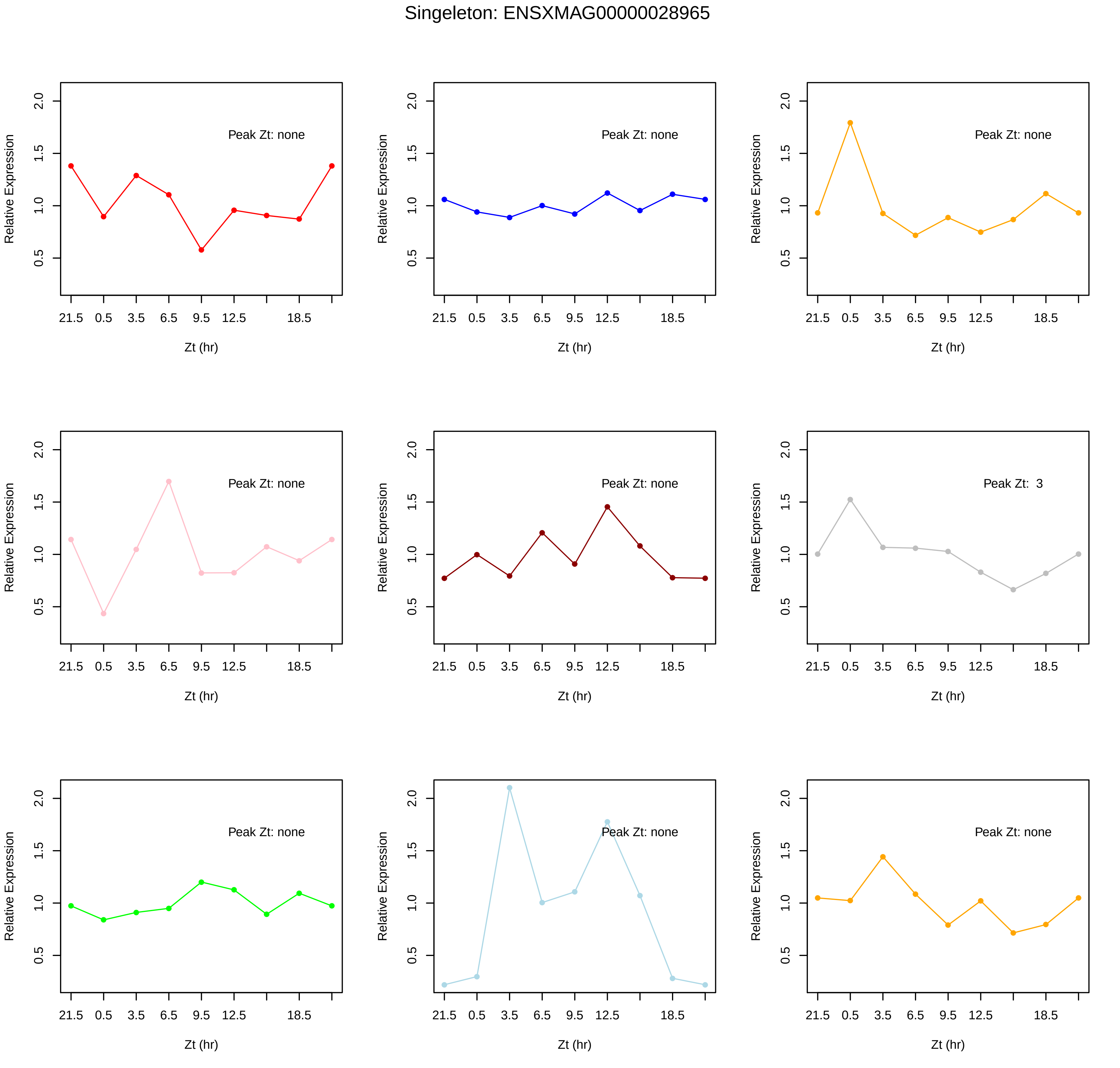
Singeleton: ENSXMAG00000028965
Peak Zt: none
Peak Zt: none
Peak Zt: none
Peak Zt: none
Peak Zt: none
Peak Zt: 3
Peak Zt: none
Peak Zt: none
Peak Zt: none
Relative Expression
Zt (hr)
21.5
0.5
3.5
6.5
9.5
12.5
18.5
0.5
1.0
1.5
2.0

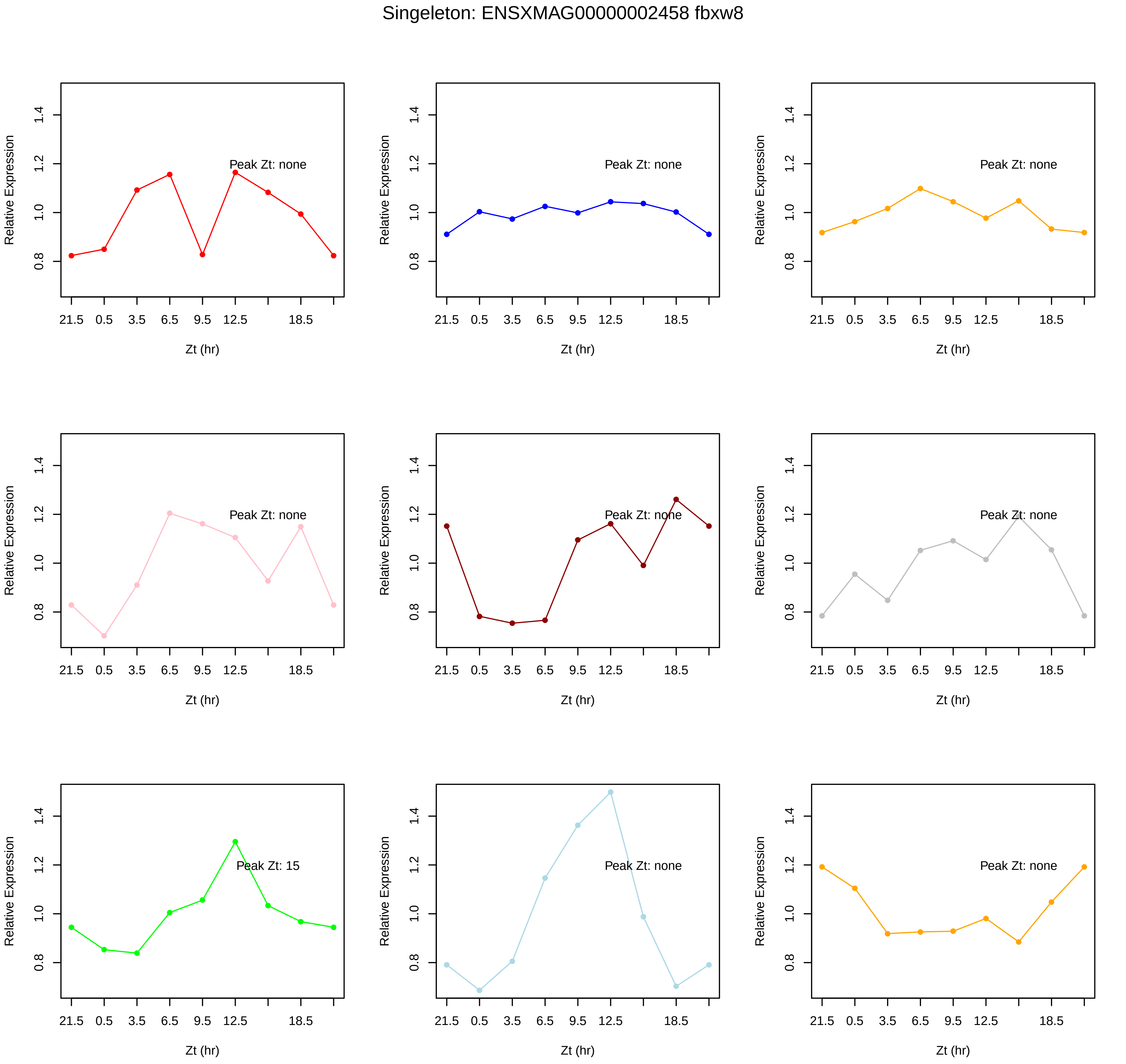

Singleton: ENSXMAG00000002458 fbxw8
Peak Zt: none
Peak Zt: none
Peak Zt: none
Peak Zt: none
Peak Zt: none
Peak Zt: none
Peak Zt: 15
Peak Zt: none
Peak Zt: none
Relative Expression
Zt (hr)
21.5
0.5
3.5
6.5
9.5
12.5
18.5
0.8
1.0
1.2
1.4

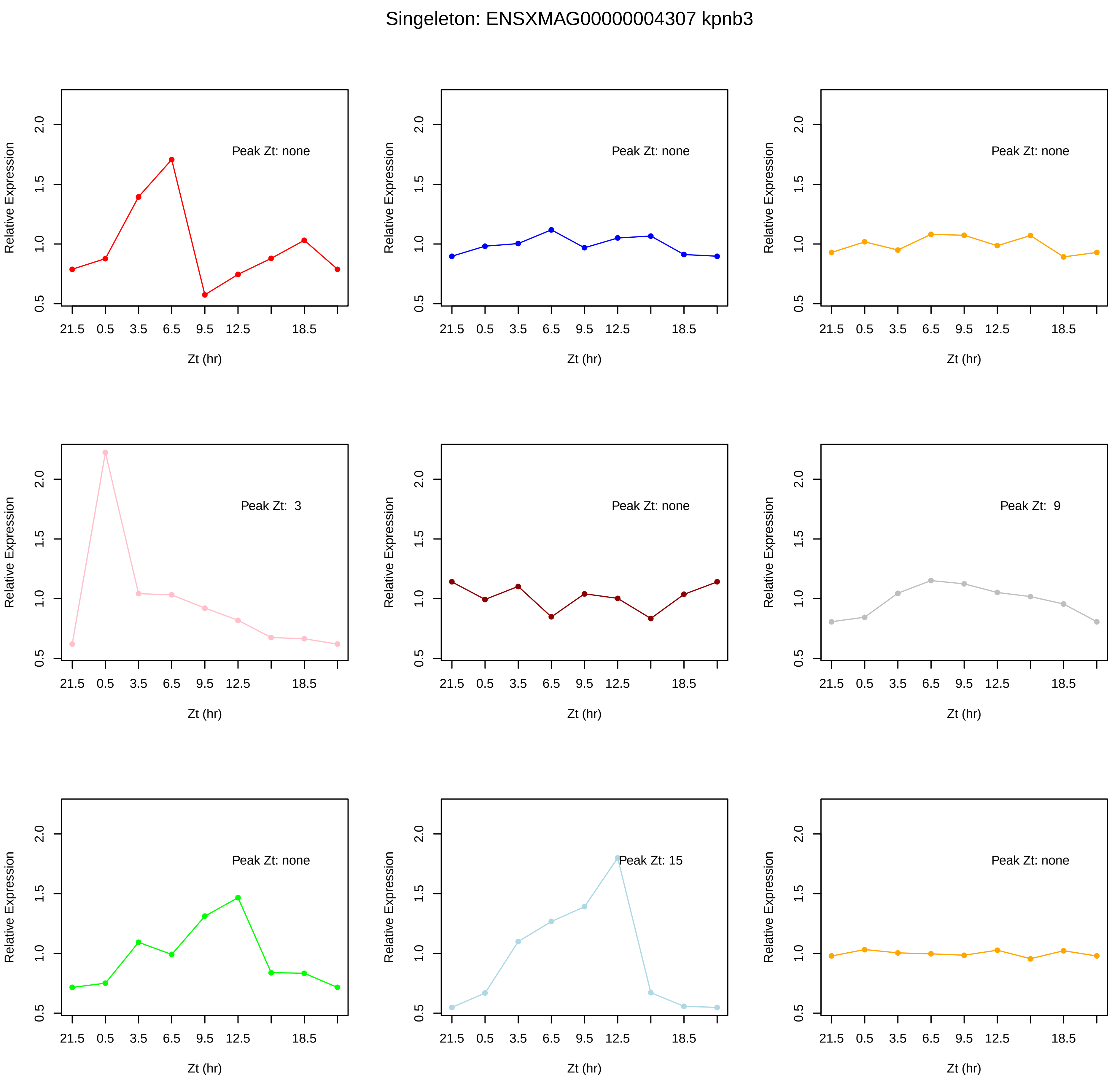

Singleton: ENSXMAG00000004307 kpnb3
Peak Zt: none
Peak Zt: none
Peak Zt: none
Peak Zt: 3
Peak Zt: none
Peak Zt: 9
Peak Zt: none
Peak Zt: 15
Peak Zt: none
Relative Expression
Zt (hr)
21.5
0.5
3.5
6.5
9.5
12.5
18.5
0.5
1.0
1.5
2.0

Singeleton: ENSXMAG00000009460 dennd5a

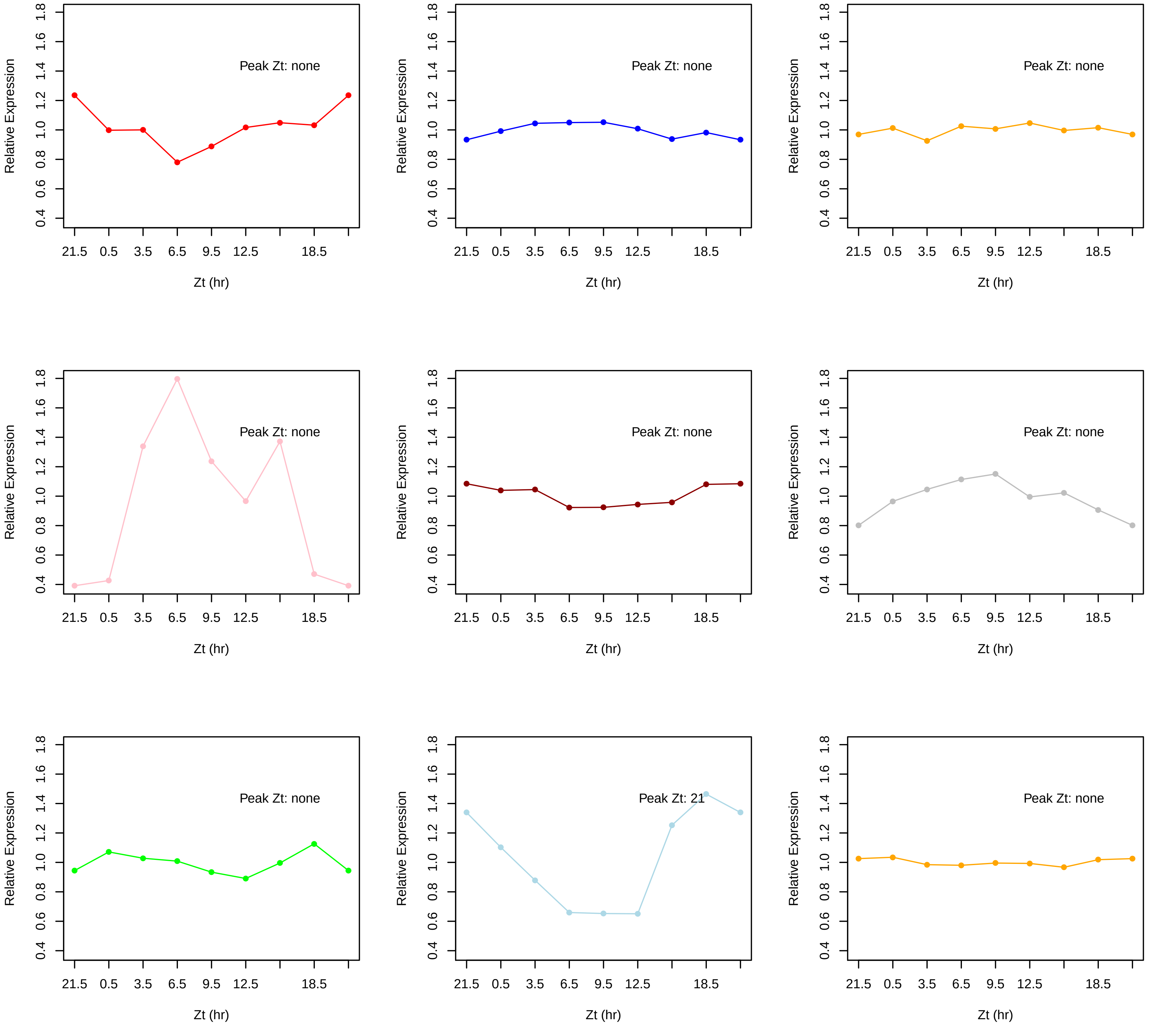

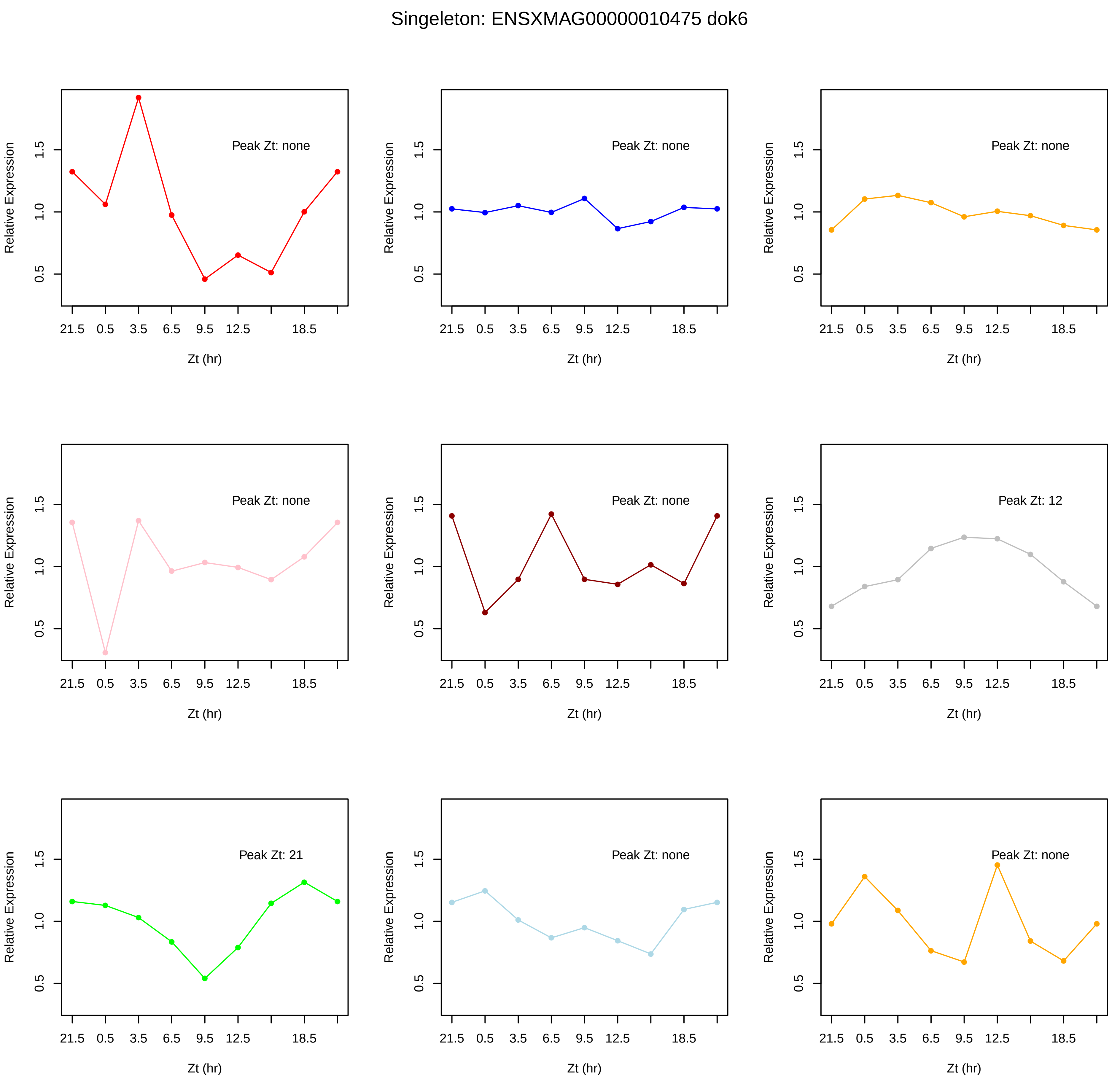

Singeleton: ENSXMAG00000010475 dok6
Peak Zt: none
Peak Zt: none
Peak Zt: none
Peak Zt: none
Peak Zt: none
Peak Zt: 12
Peak Zt: 21
Peak Zt: none
Peak Zt: none
Relative Expression
Zt (hr)
0.5
1.0
1.5
21.5
0.5
3.5
6.5
9.5
12.5
18.5

Singeleton: ENSXMAG00000016047 slc7a5

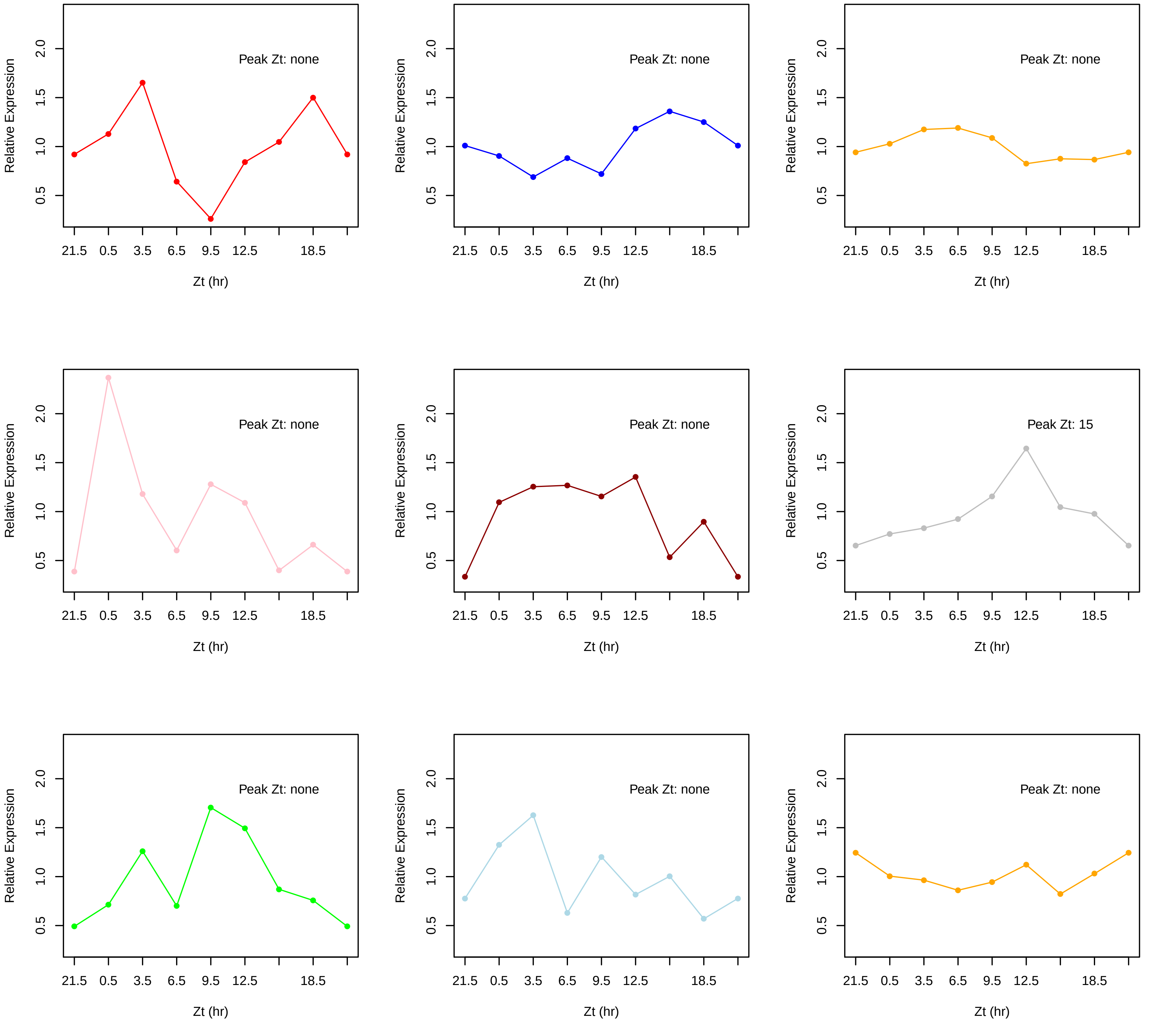

Singeleton: ENSXMAG00000003401

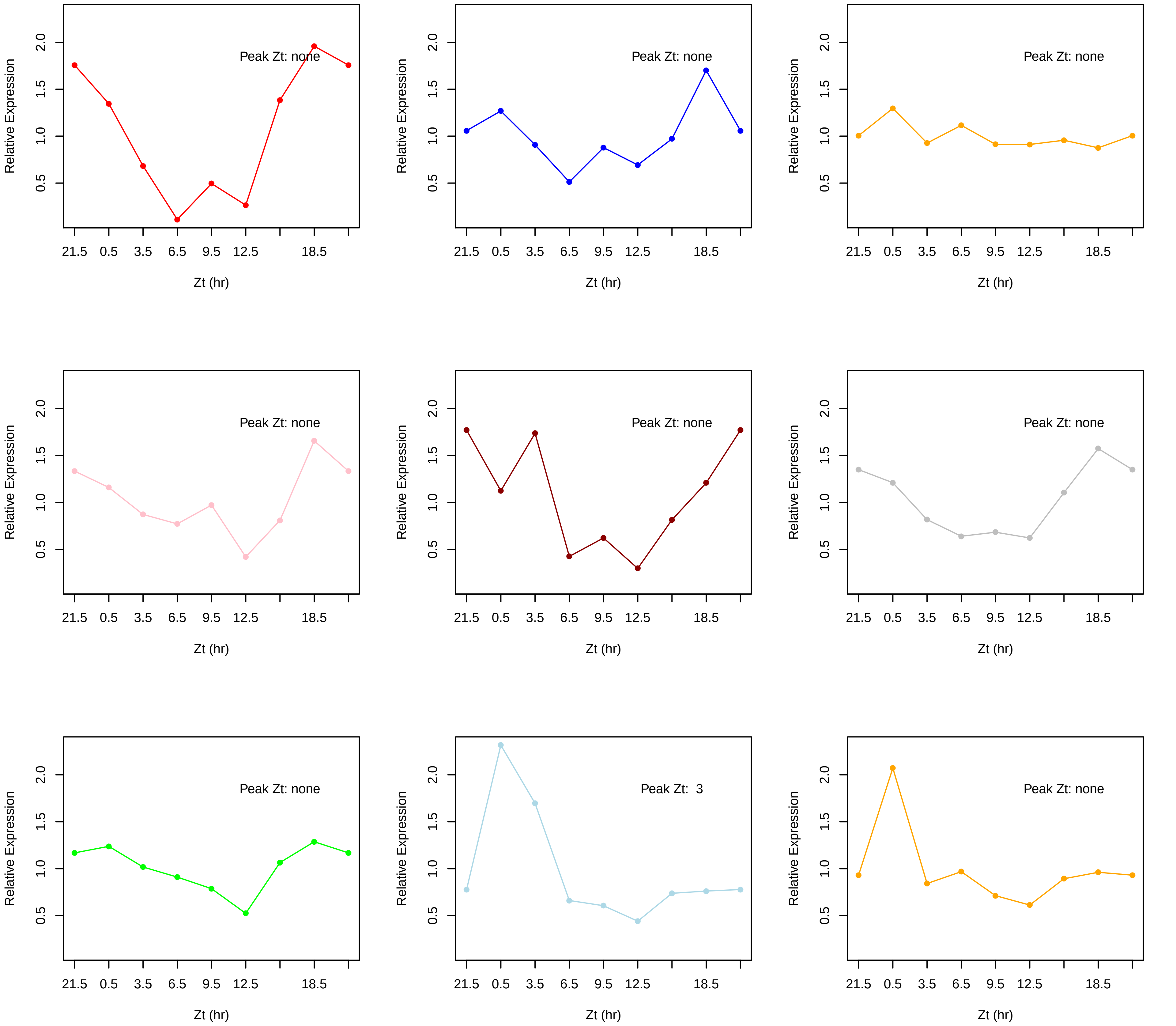

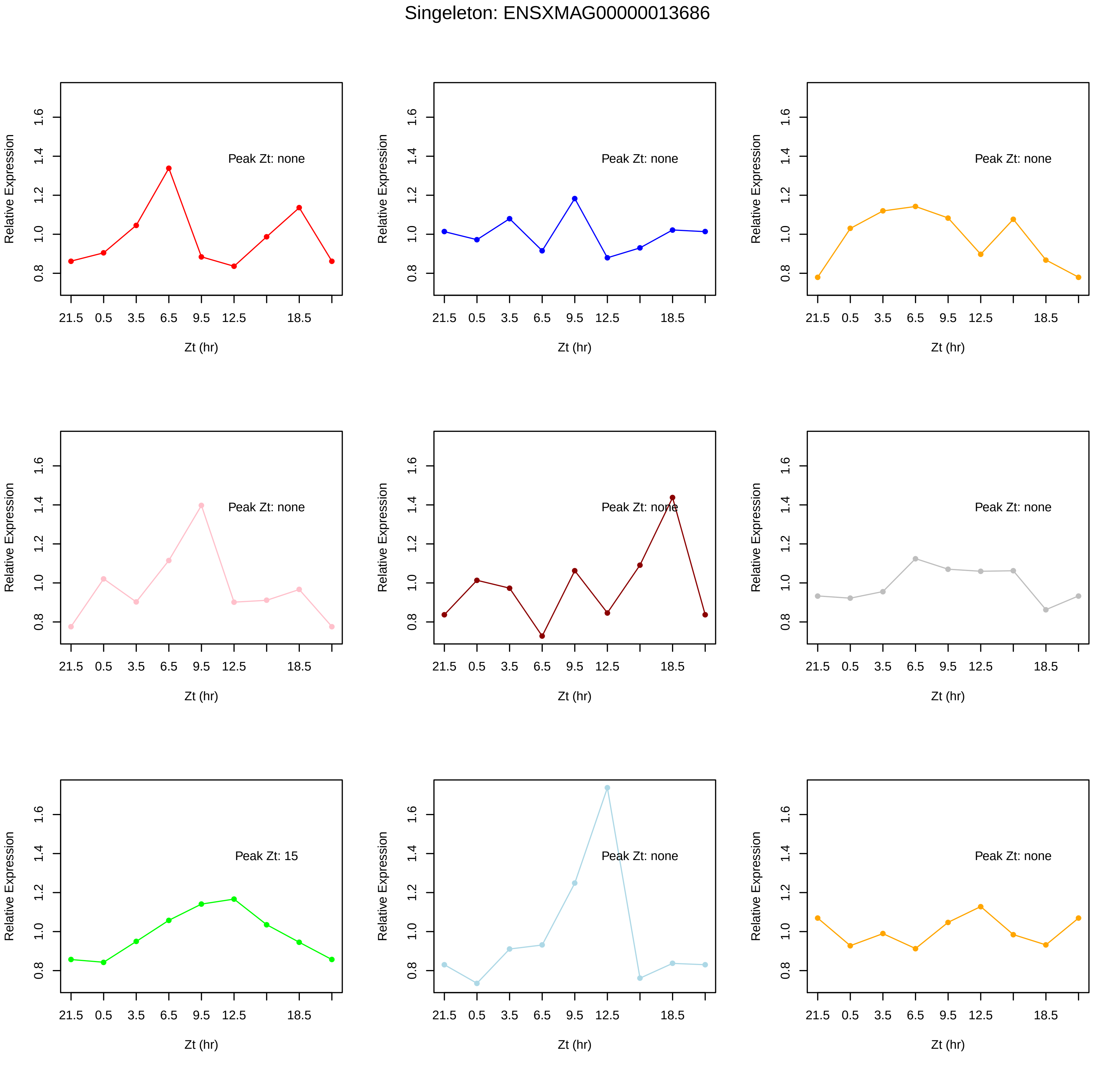
Singeleton: ENSXMAG00000013686
Peak Zt: none
Peak Zt: none
Peak Zt: none
Peak Zt: none
Peak Zt: none
Peak Zt: none
Peak Zt: 15
Peak Zt: none
Peak Zt: none
Relative Expression
Zt (hr)
21.5
0.5
3.5
6.5
9.5
12.5
18.5
0.8
1.0
1.2
1.4
1.6

Singleton: ENSXMAG00000024119 zgc:112185

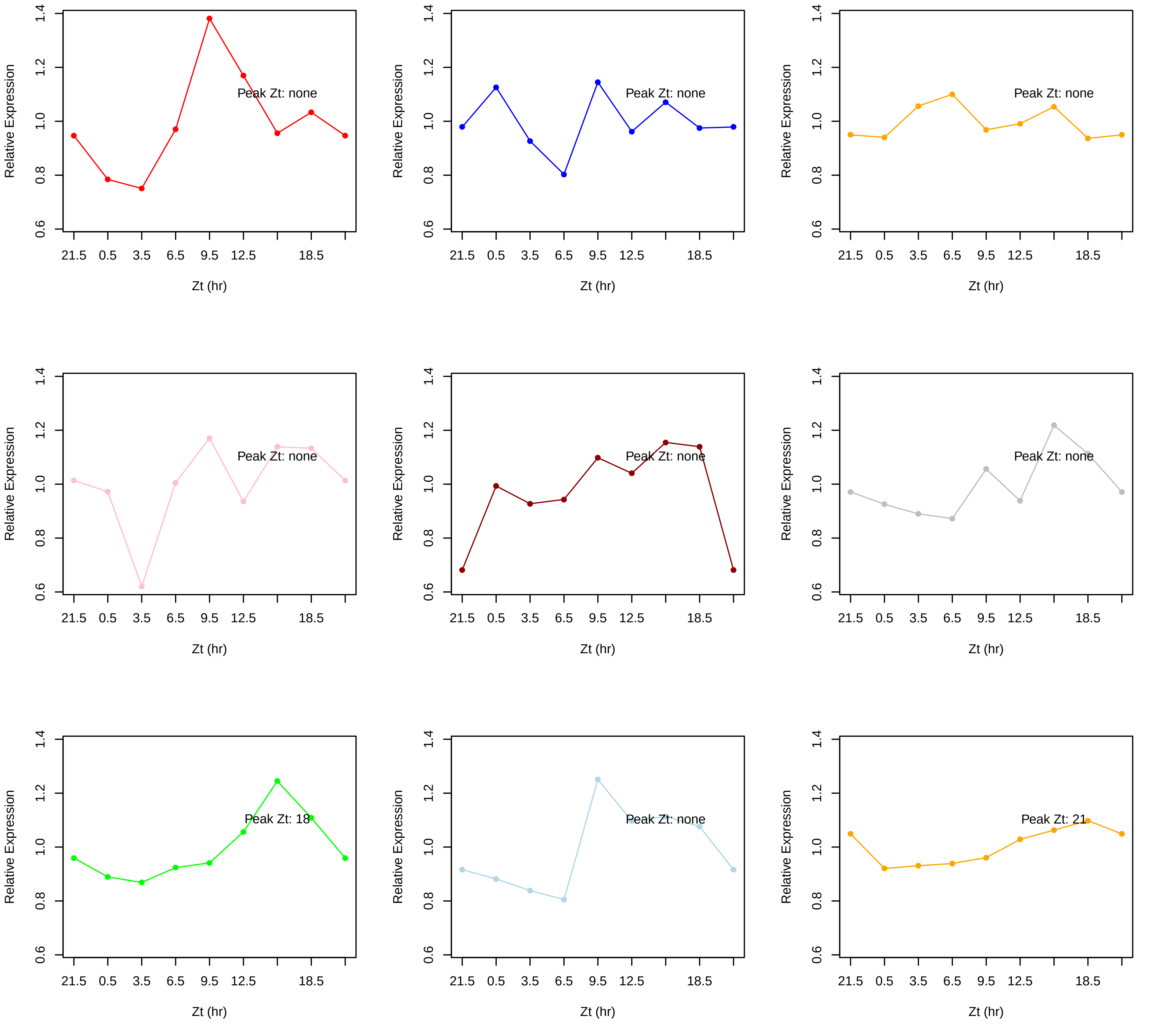

Singeleton: ENSXMAG00000009373 nckap5l

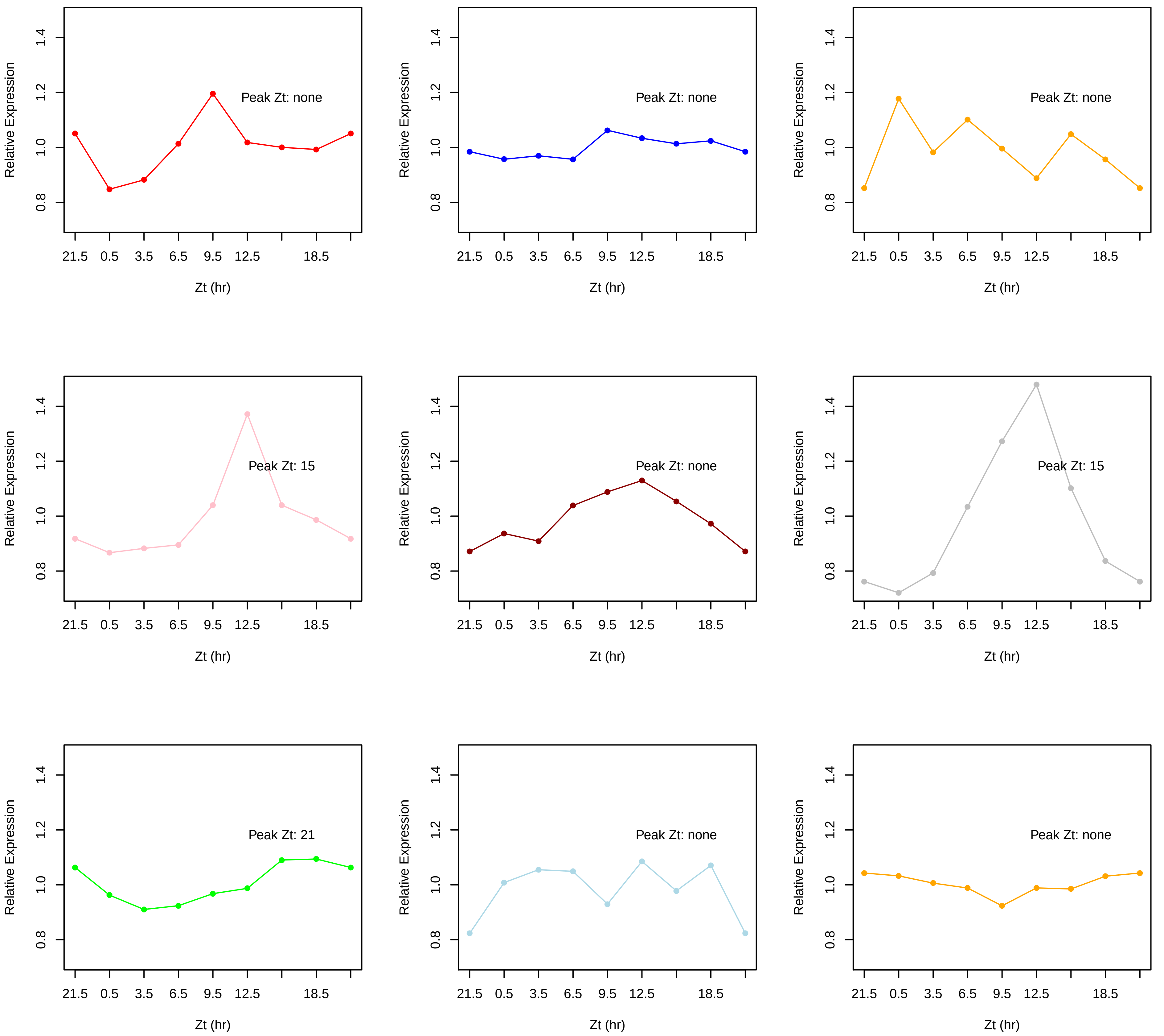

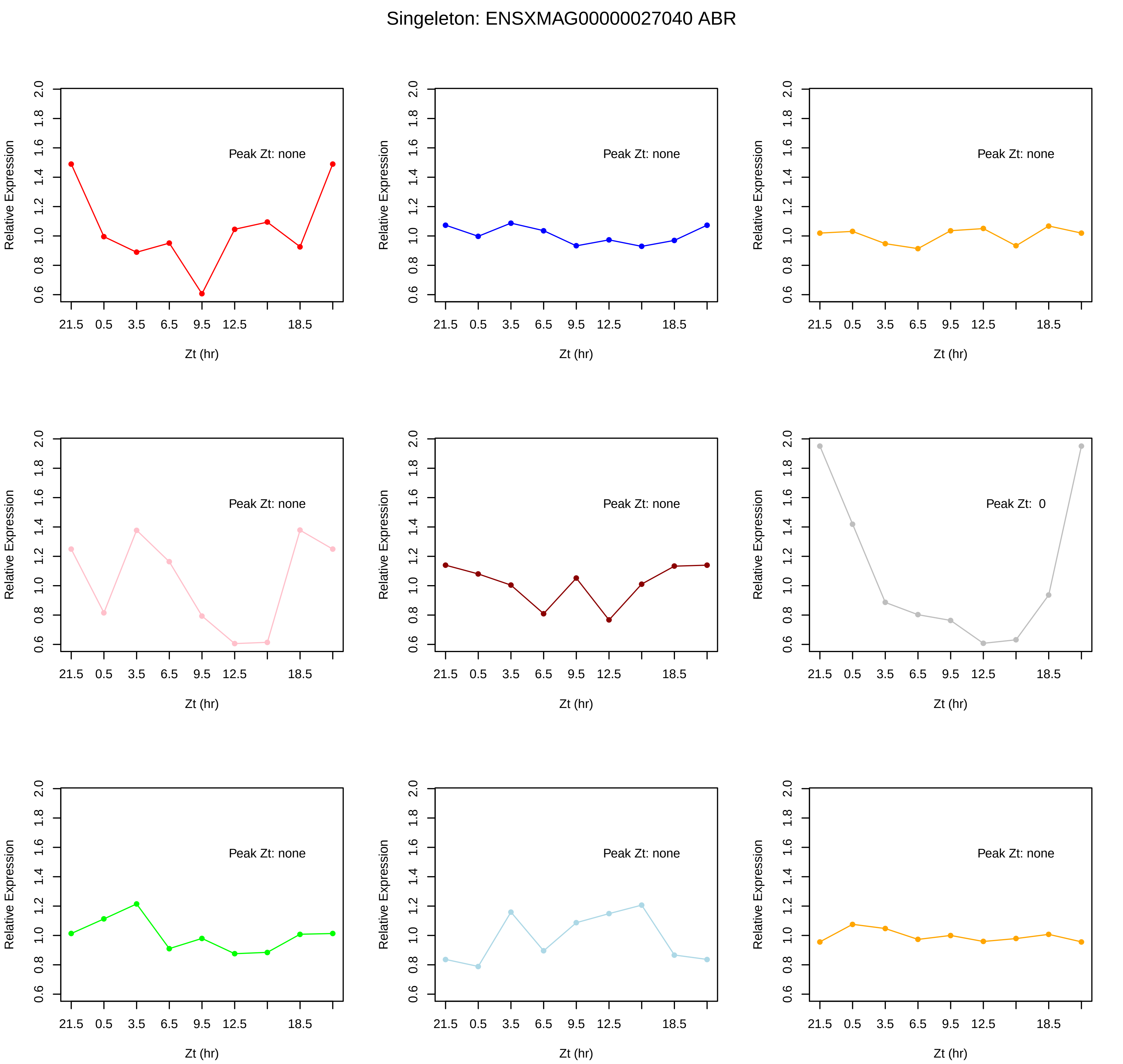
Singeleton: ENSXMAG00000027040 ABR
Peak Zt: none
Peak Zt: none
Peak Zt: none
Peak Zt: none
Peak Zt: none
Peak Zt: 0
Peak Zt: none
Peak Zt: none
Peak Zt: none
Relative Expression
Zt (hr)
21.5 0.5 3.5 6.5 9.5 12.5 18.5
0.6 0.8 1.0 1.2 1.4 1.6 1.8 2.0

Singeleton: ENSXMAG00000002436 fbxo21

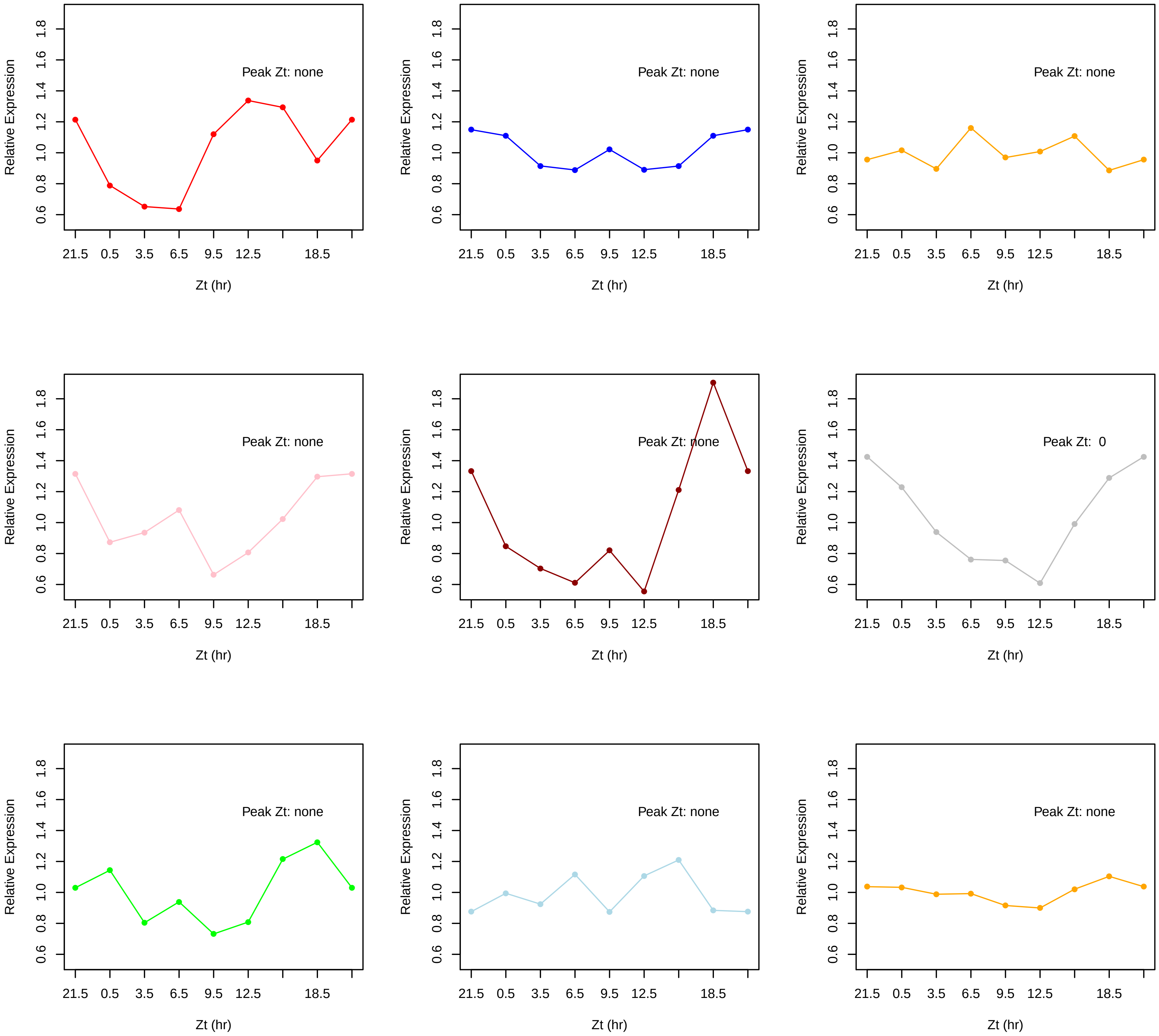

Singeleton: ENSXMAG00000011755 NARS

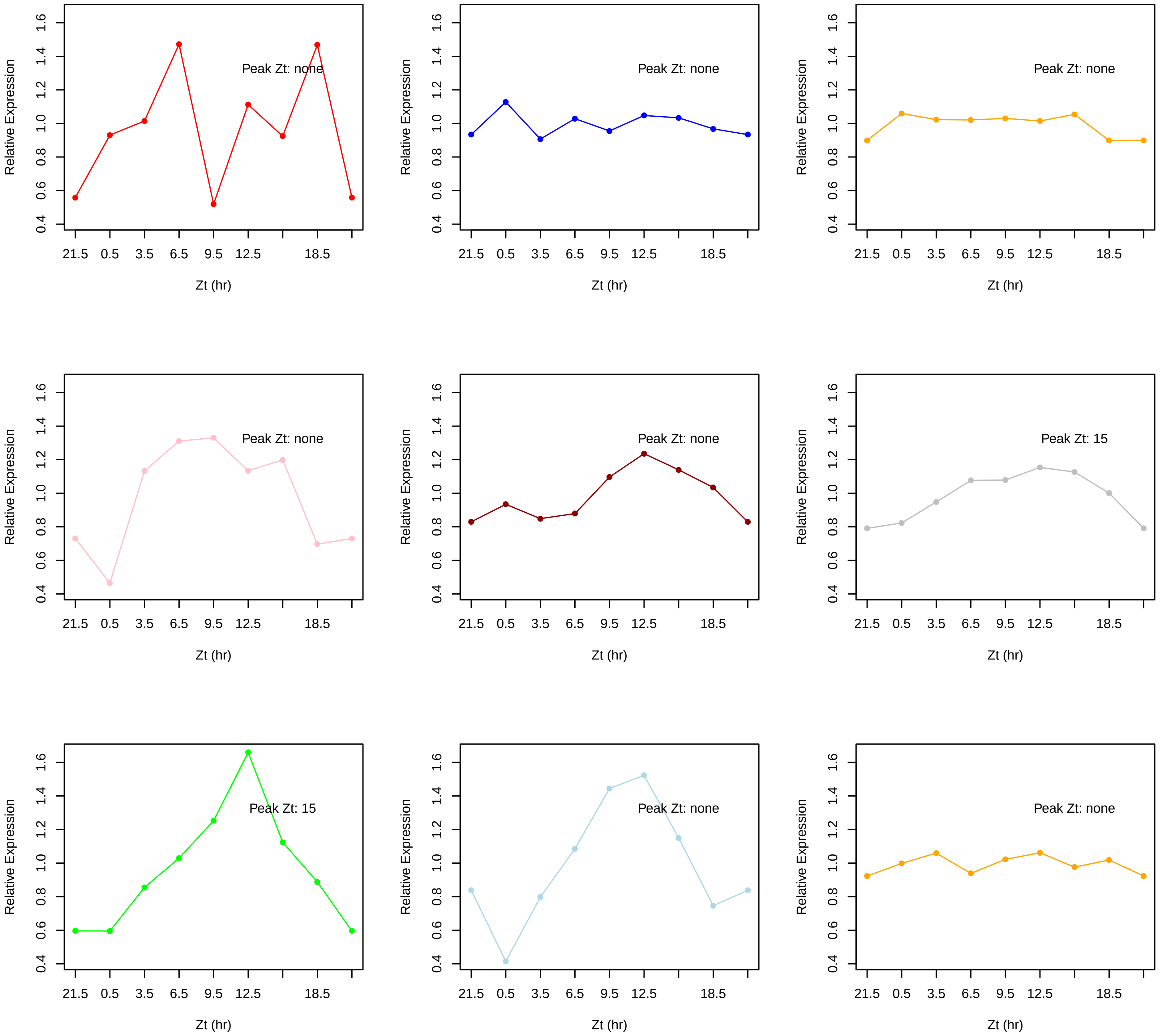

Singleton: ENSXMAG00000018950 zmp:0000000624

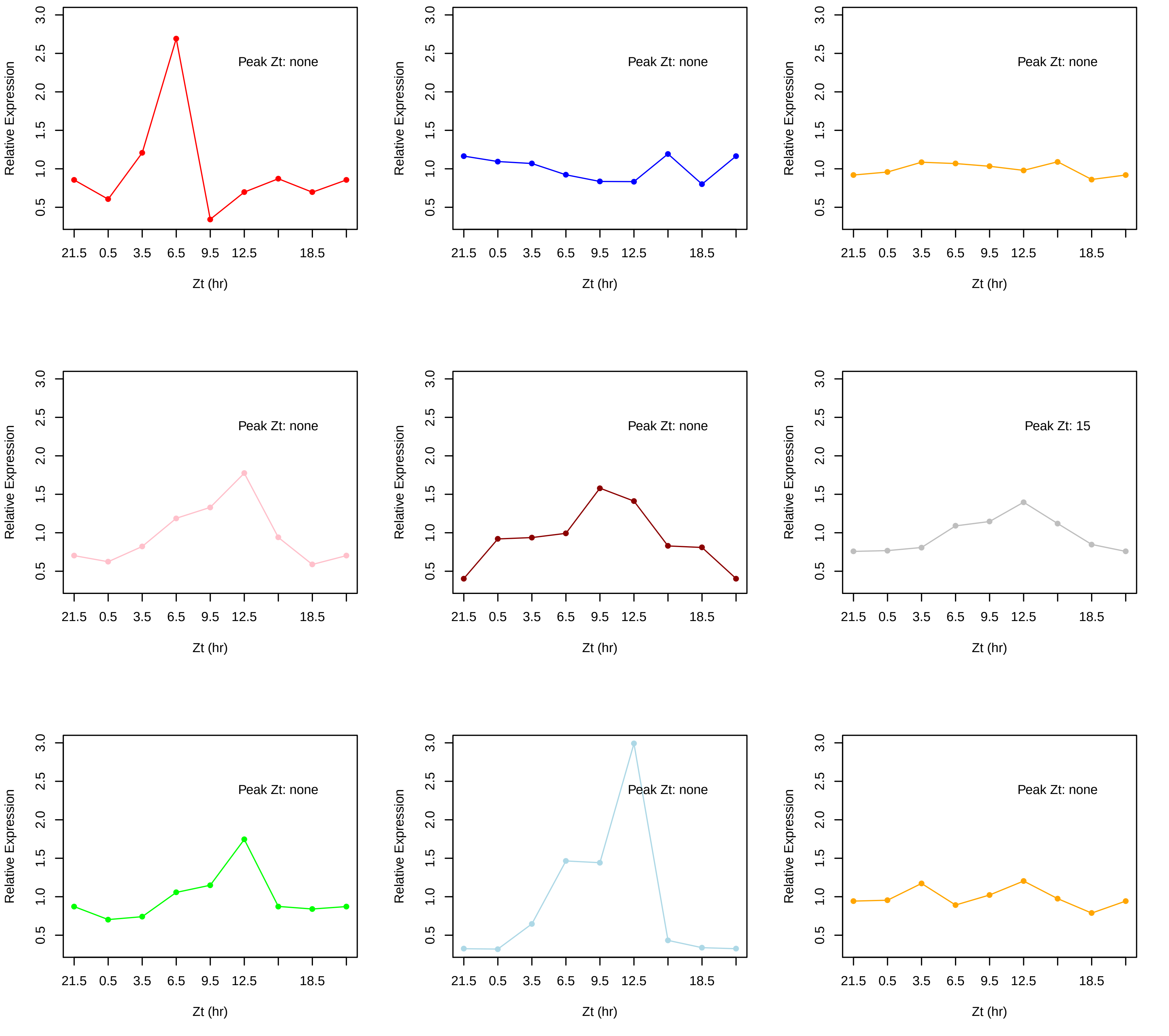

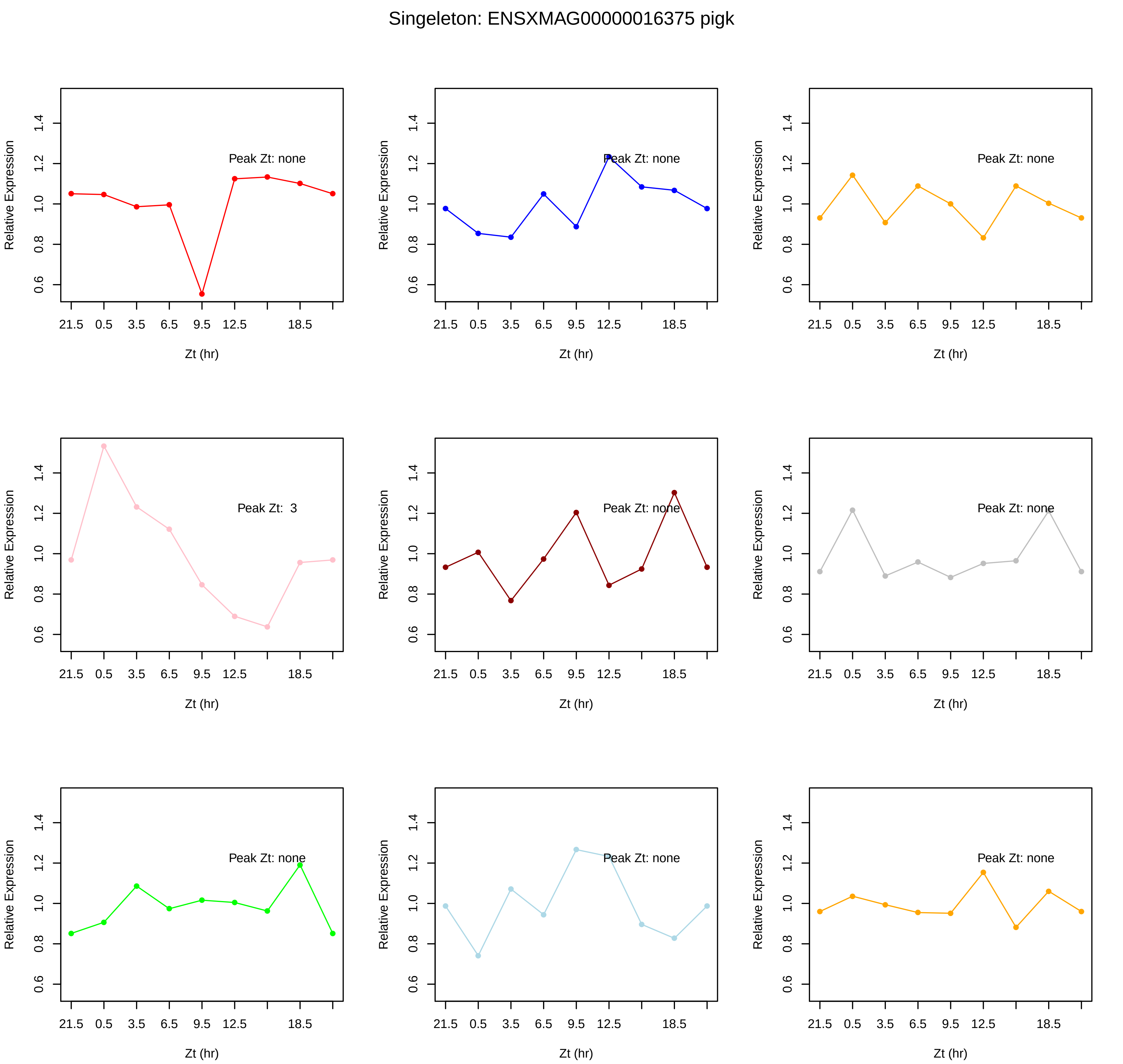
Singeleton: ENSXMAG00000016375 pigk
Relative Expression
Zt (hr)
Peak Zt: none
Peak Zt: none
Peak Zt: none
Peak Zt: 3
Peak Zt: none
Peak Zt: none
Peak Zt: none
Peak Zt: none
Peak Zt: none

Singeleton: ENSXMAG00000003458 safb

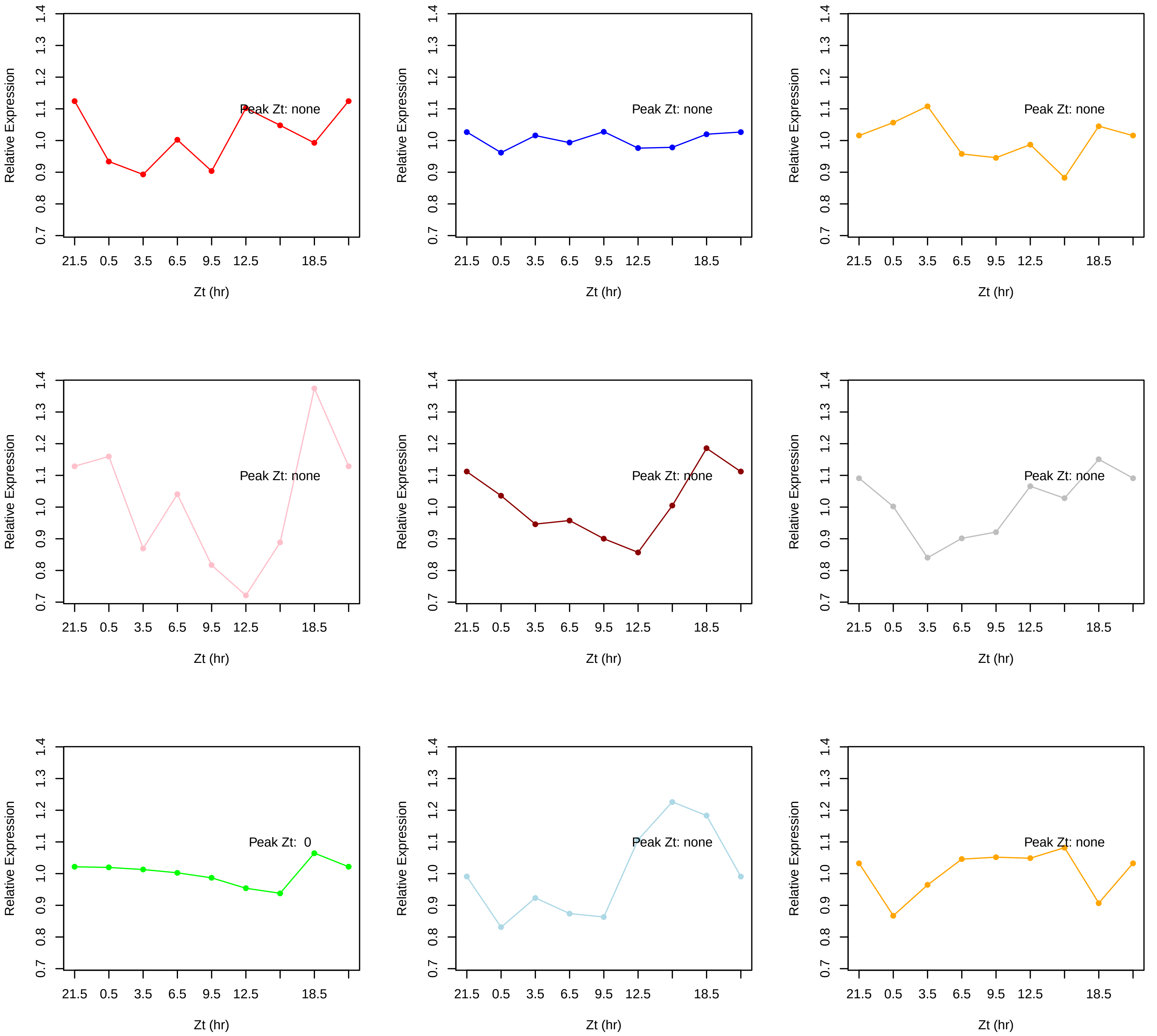

Singeleton: ENSXMAG00000001624 fech

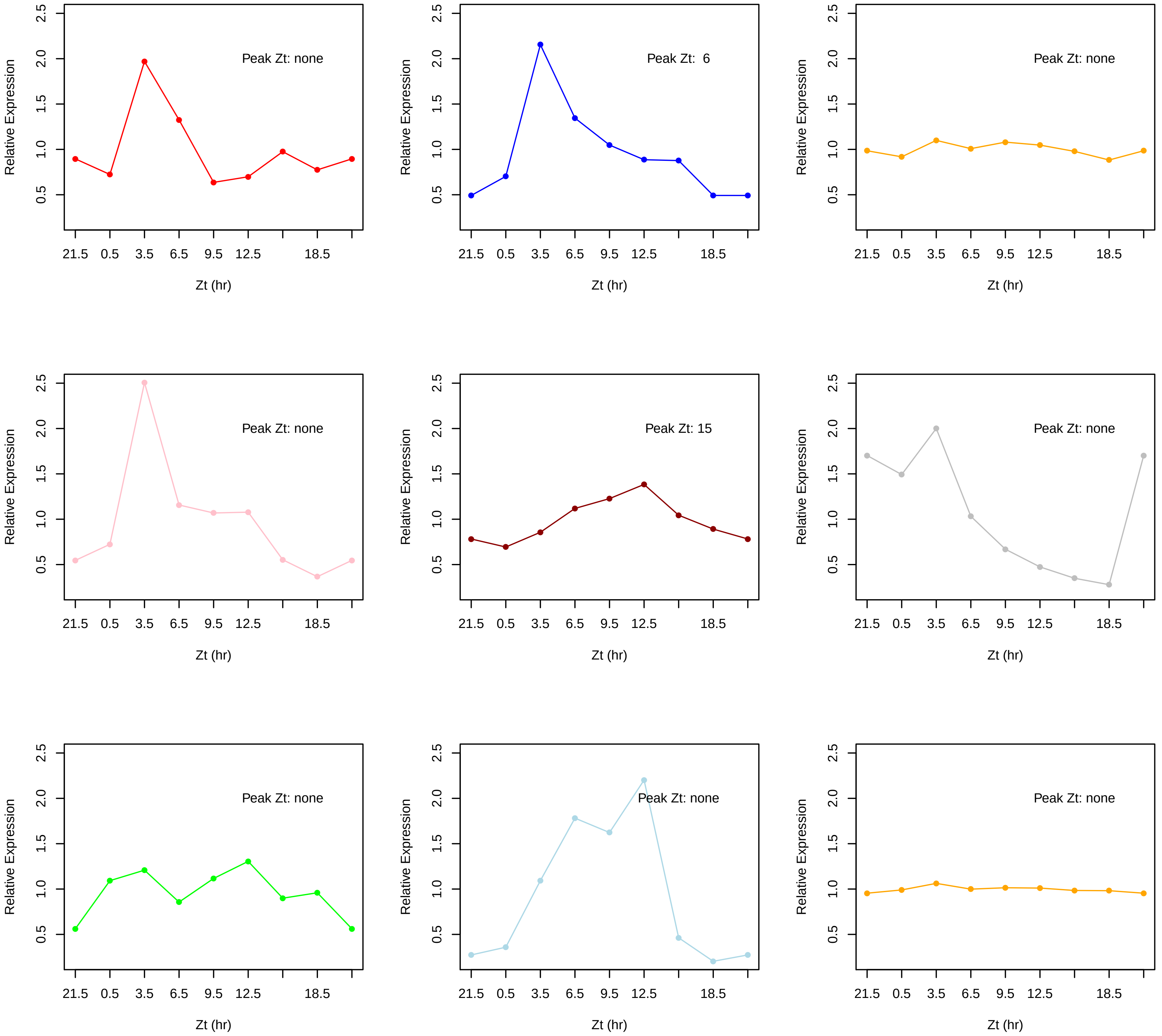

Singeleton: ENSXMAG00000016073 uba2

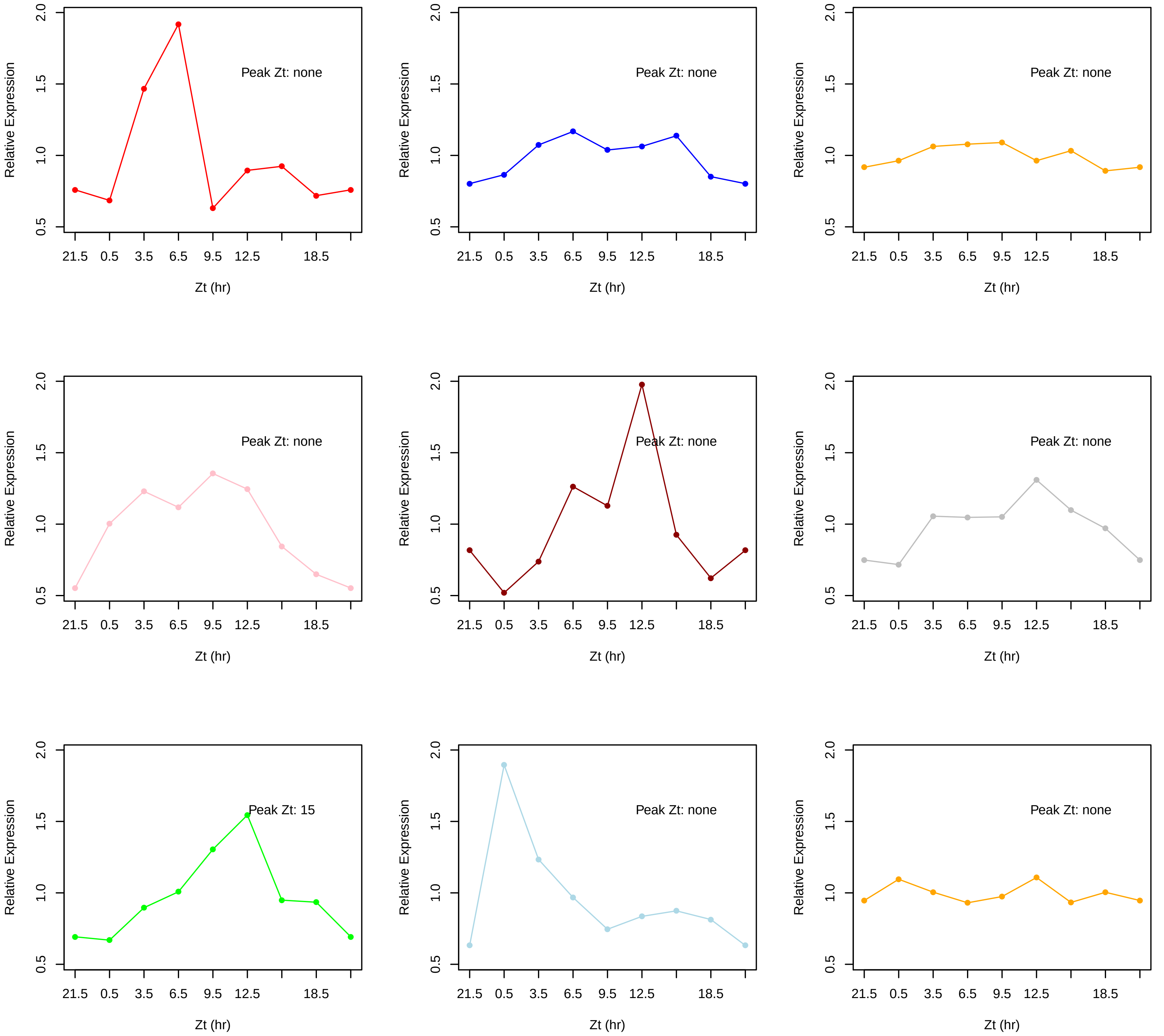

Singeleton: ENSXMAG00000009356 sarnp

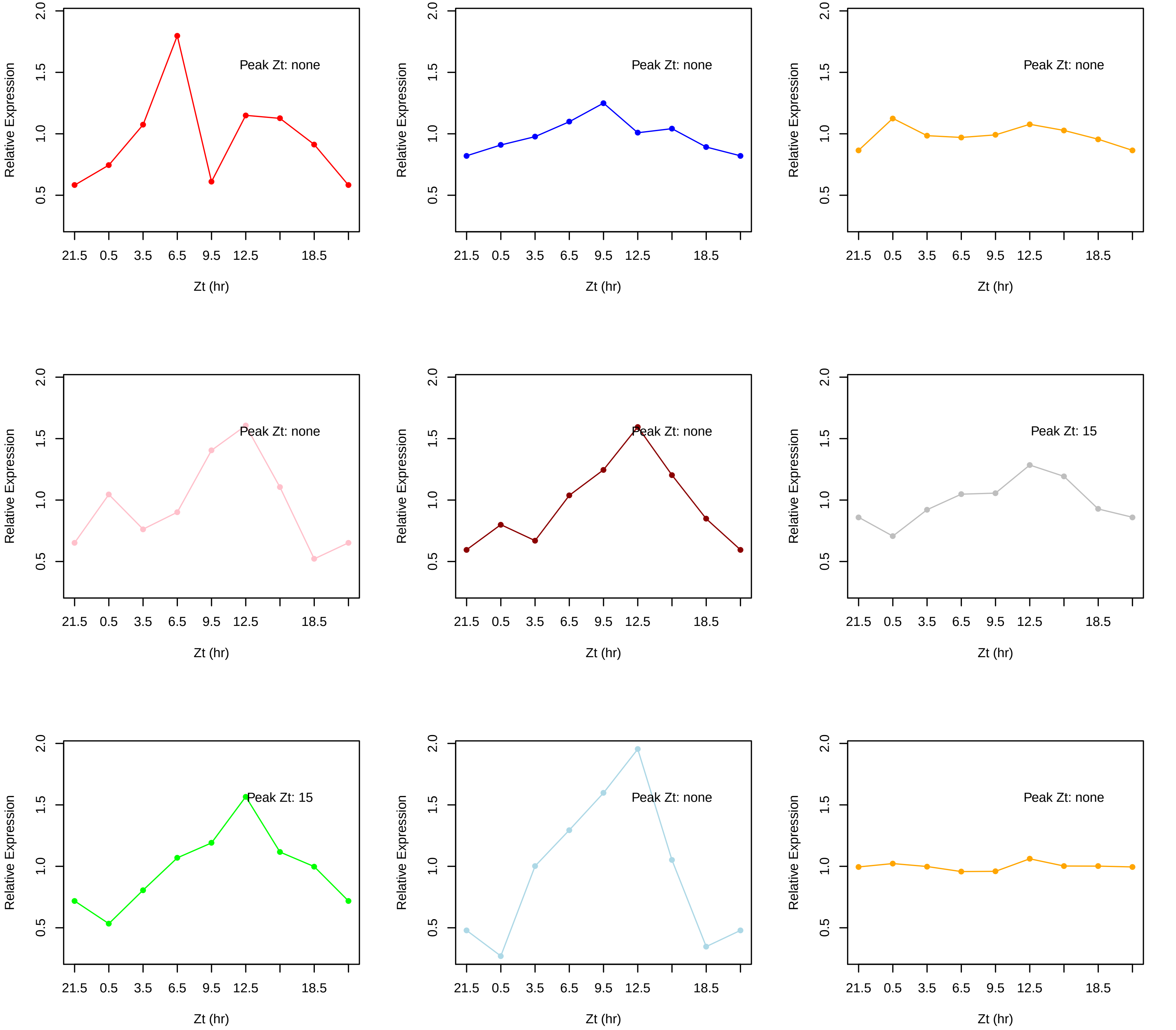

Singeleton: ENSXMAG00000021594 EIF1AY

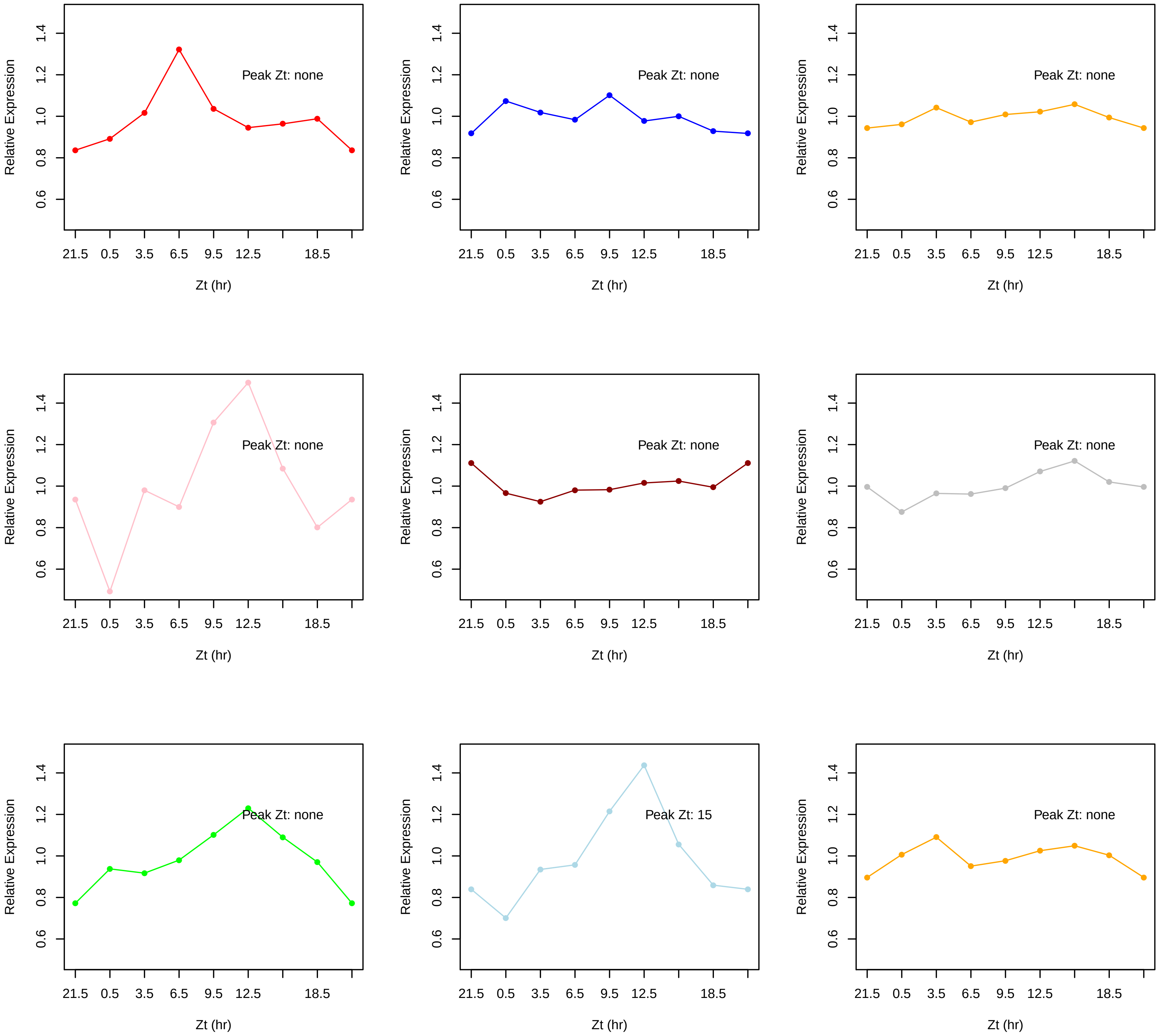

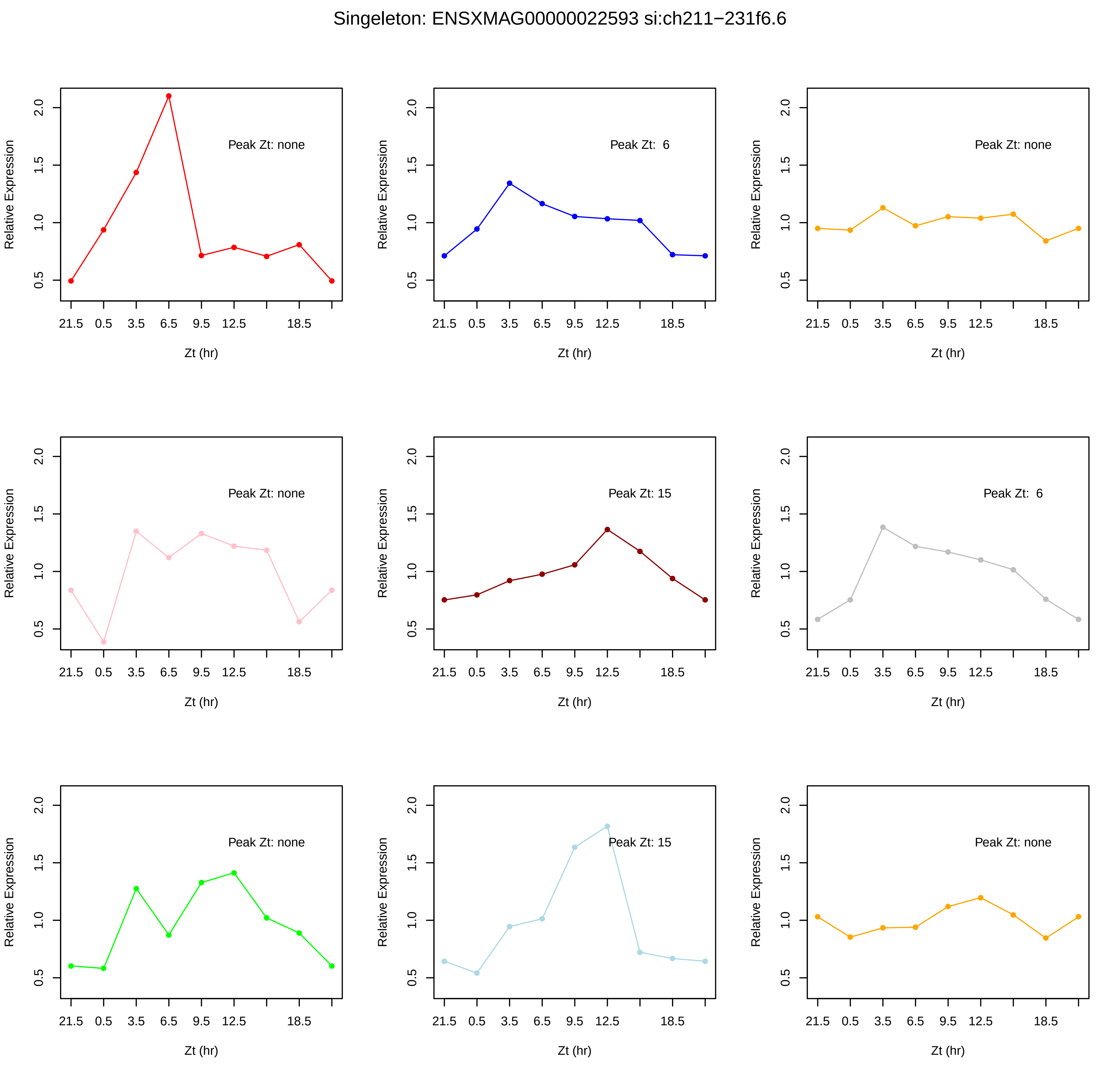

Singeleton: ENSXMAG00000022593 si:ch211−231f6.6
Peak Zt: none
Peak Zt: 6
Peak Zt: none
Peak Zt: none
Peak Zt: 15
Peak Zt: 6
Peak Zt: none
Peak Zt: 15
Peak Zt: none
Relative Expression
Zt (hr)
21.5
0.5
3.5
6.5
9.5
12.5
18.5
0.5
1.0
1.5
2.0

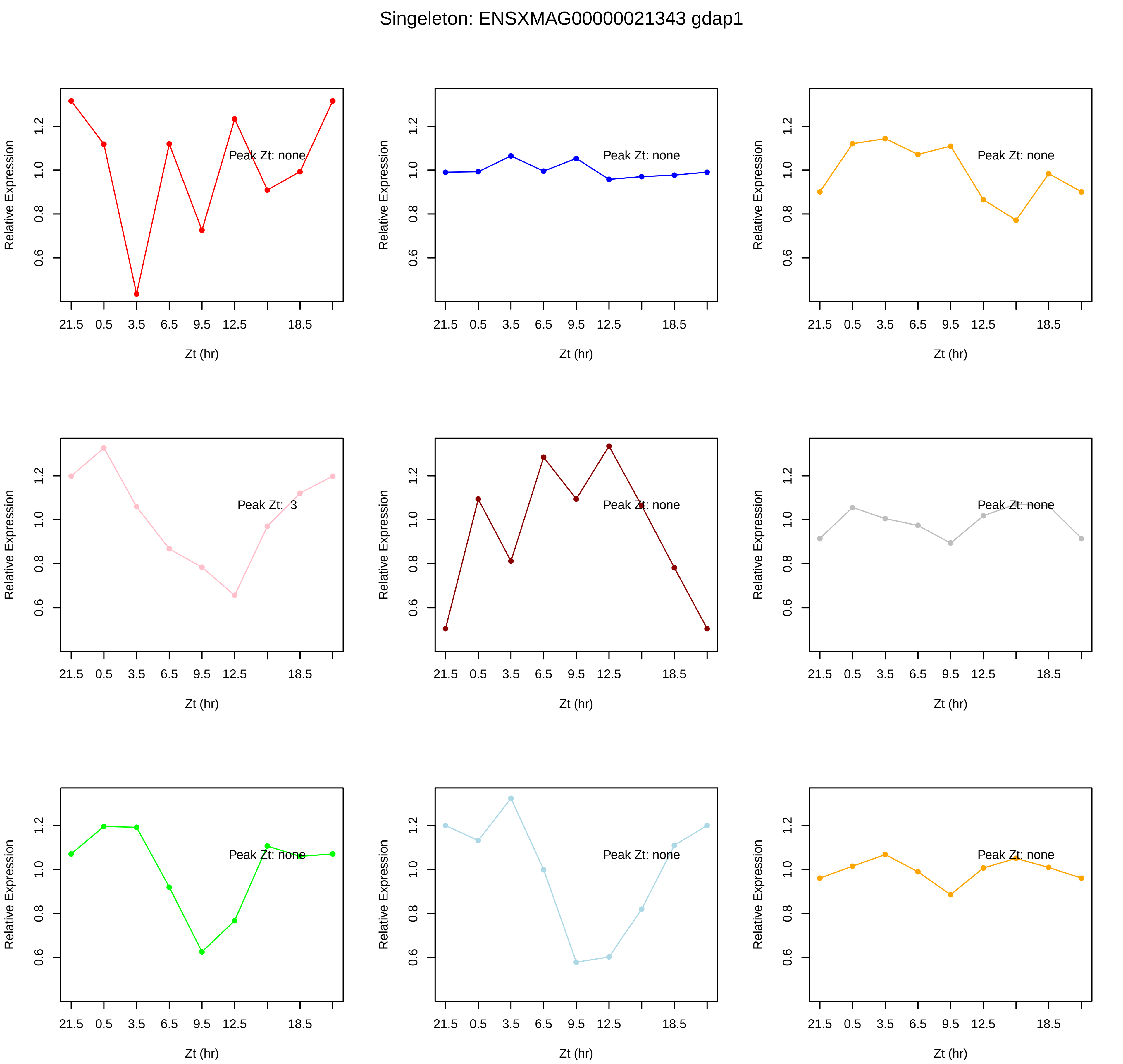
Singeleton: ENSXMAG00000021343 gdap1
Relative Expression
Zt (hr)
Peak Zt: none
Peak Zt: 3
21.5
0.5
3.5
6.5
9.5
12.5
18.5
0.6
0.8
1.0
1.2

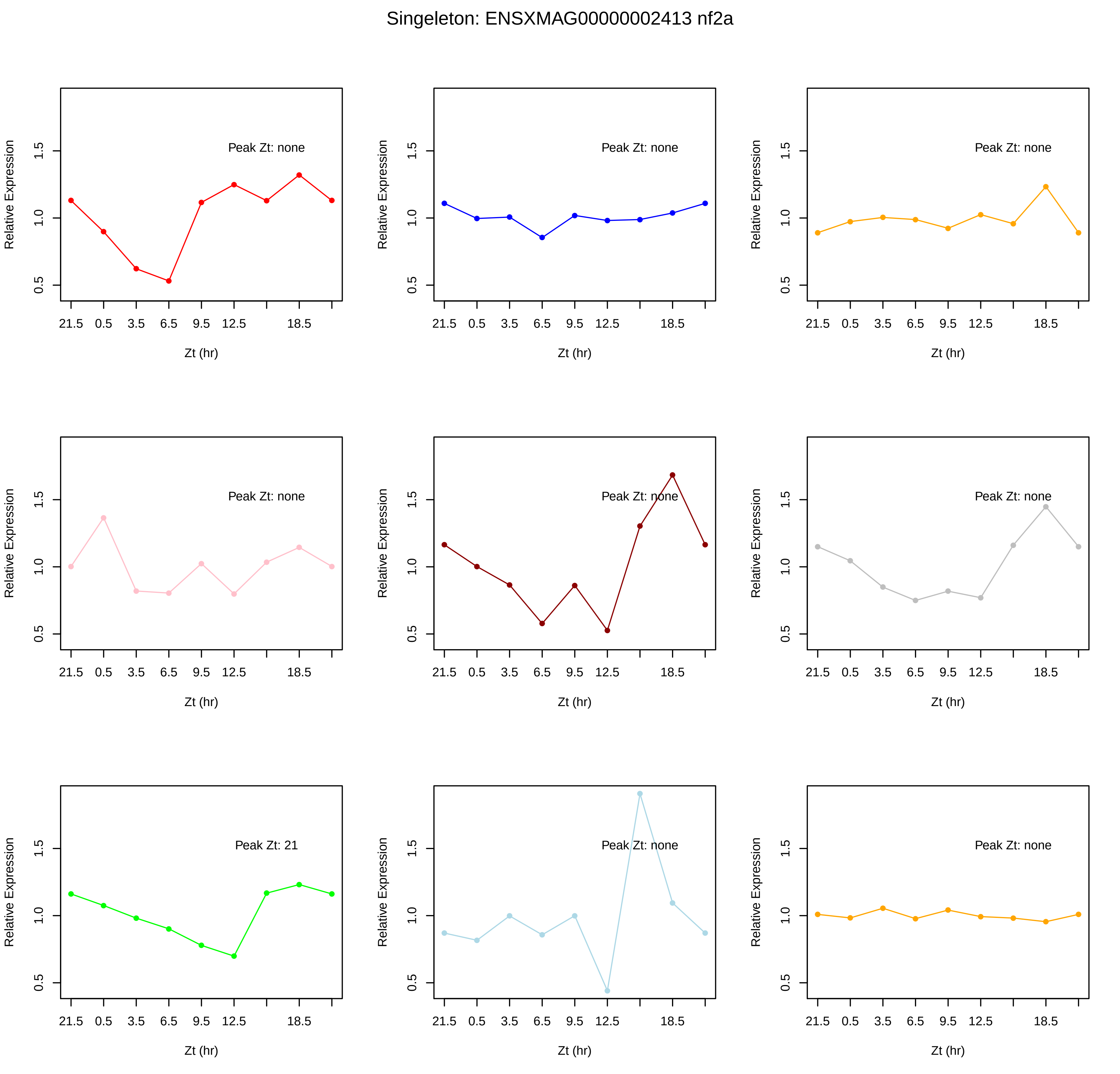

Singeleton: ENSXMAG00000002413 nf2a
Peak Zt: none
Peak Zt: none
Peak Zt: none
Peak Zt: none
Peak Zt: none
Peak Zt: none
Peak Zt: 21
Peak Zt: none
Peak Zt: none
Relative Expression
Zt (hr)
21.5
0.5
3.5
6.5
9.5
12.5
18.5
0.5
1.0
1.5

Singeleton: ENSXMAG00000005326 aspa

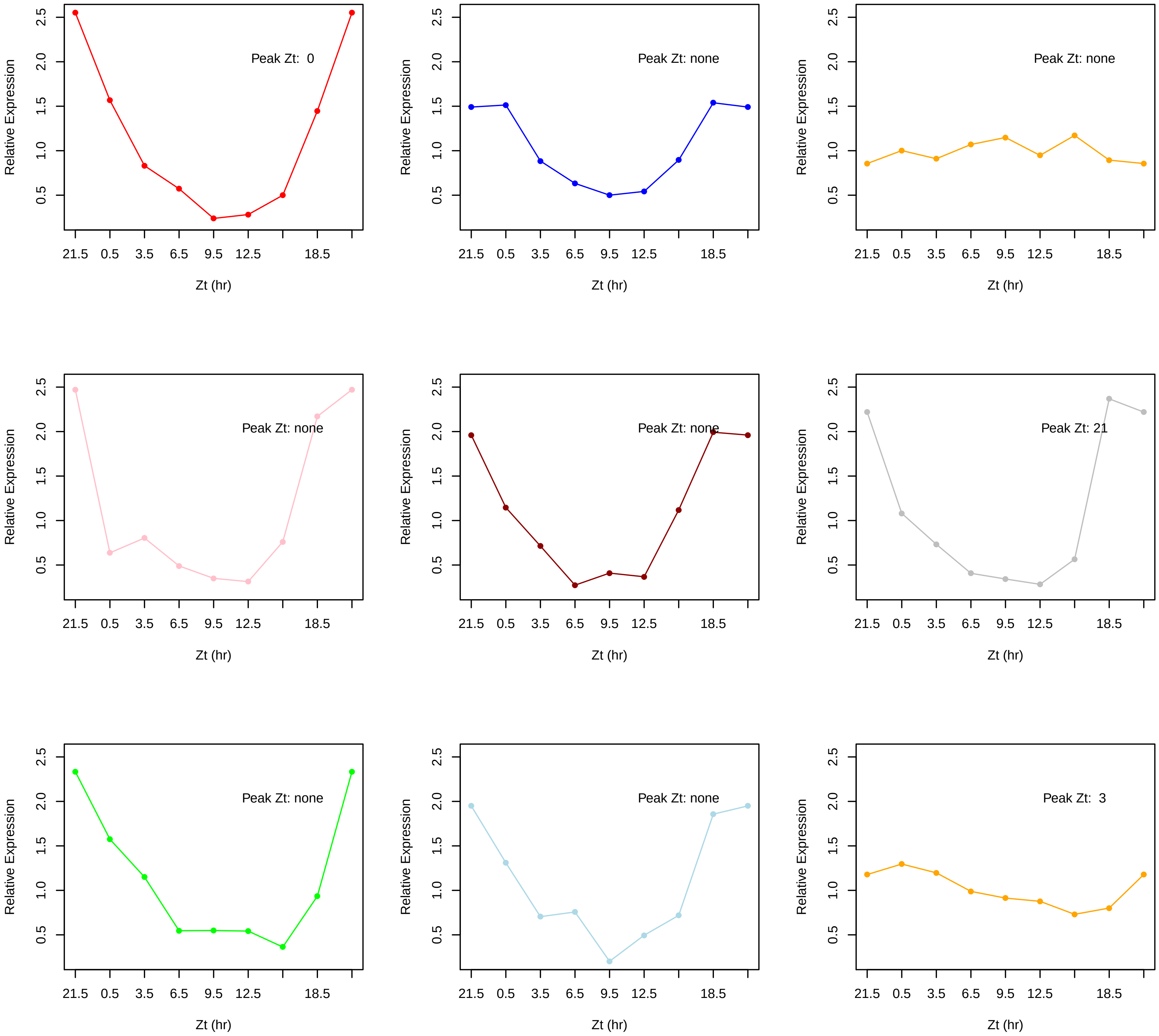

Singeleton: ENSXMAG00000027063 gdf11

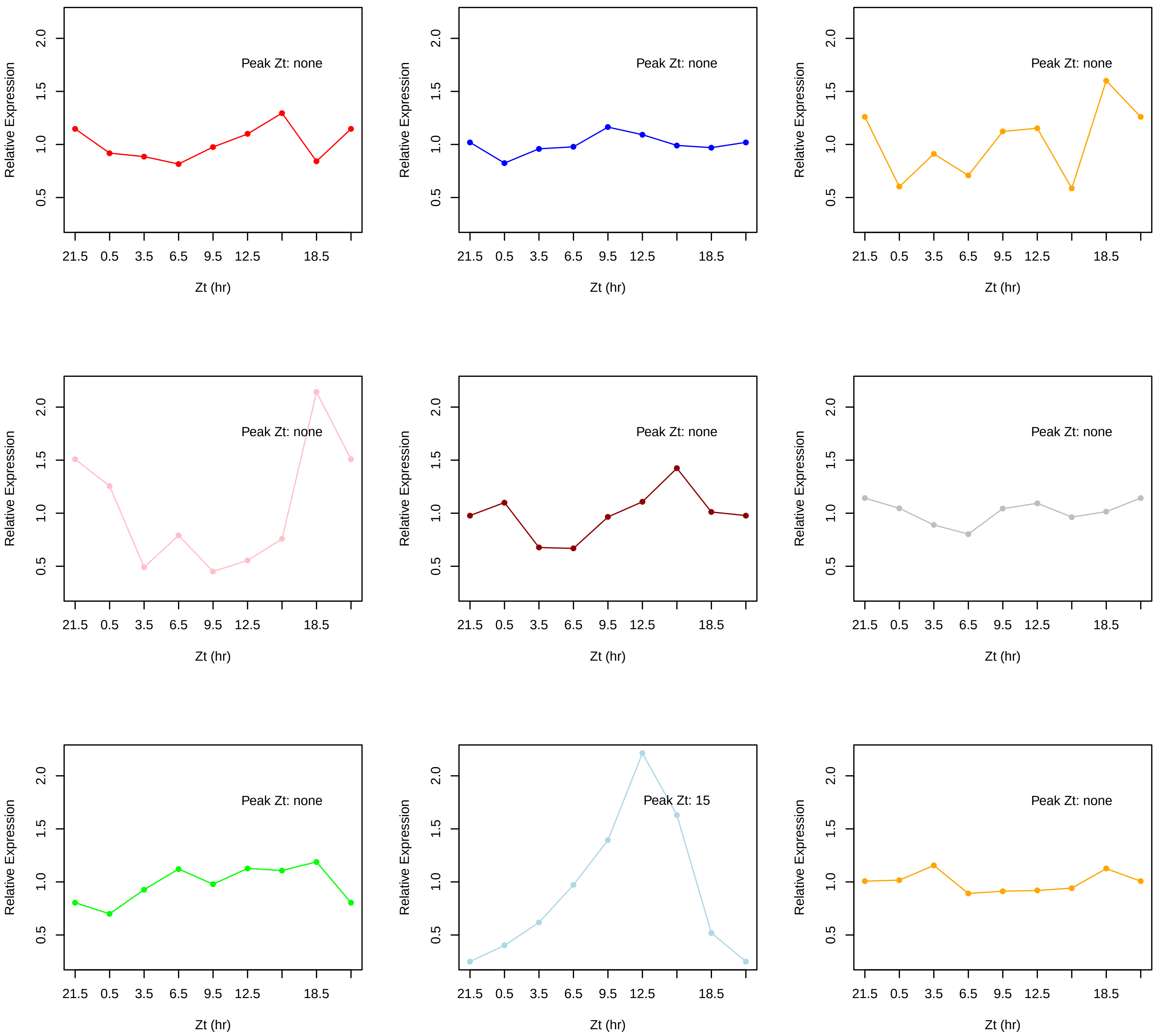

Singeleton: ENSXMAG00000012766 wee1

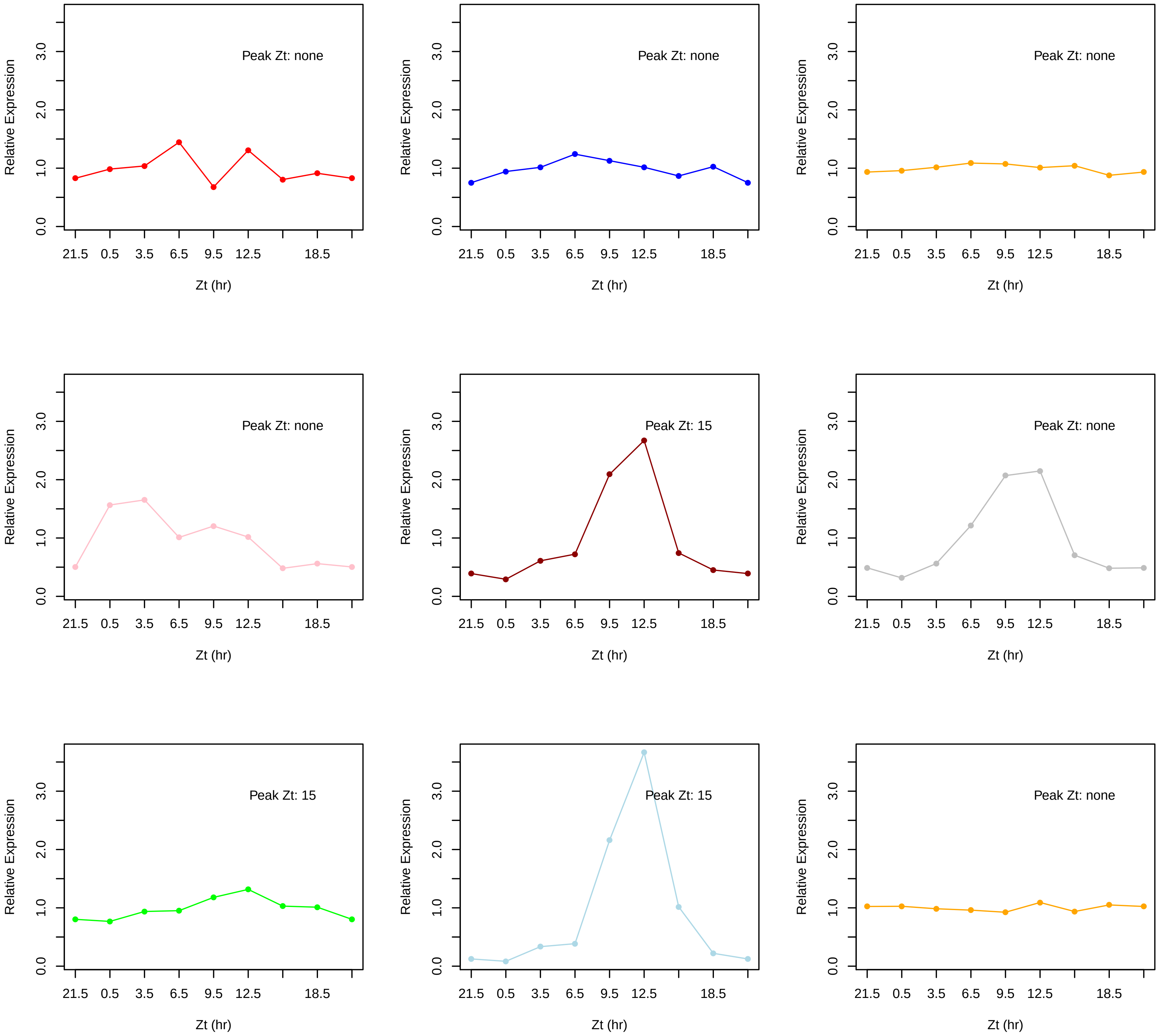

Singeleton: ENSXMAG00000028108 zzz3

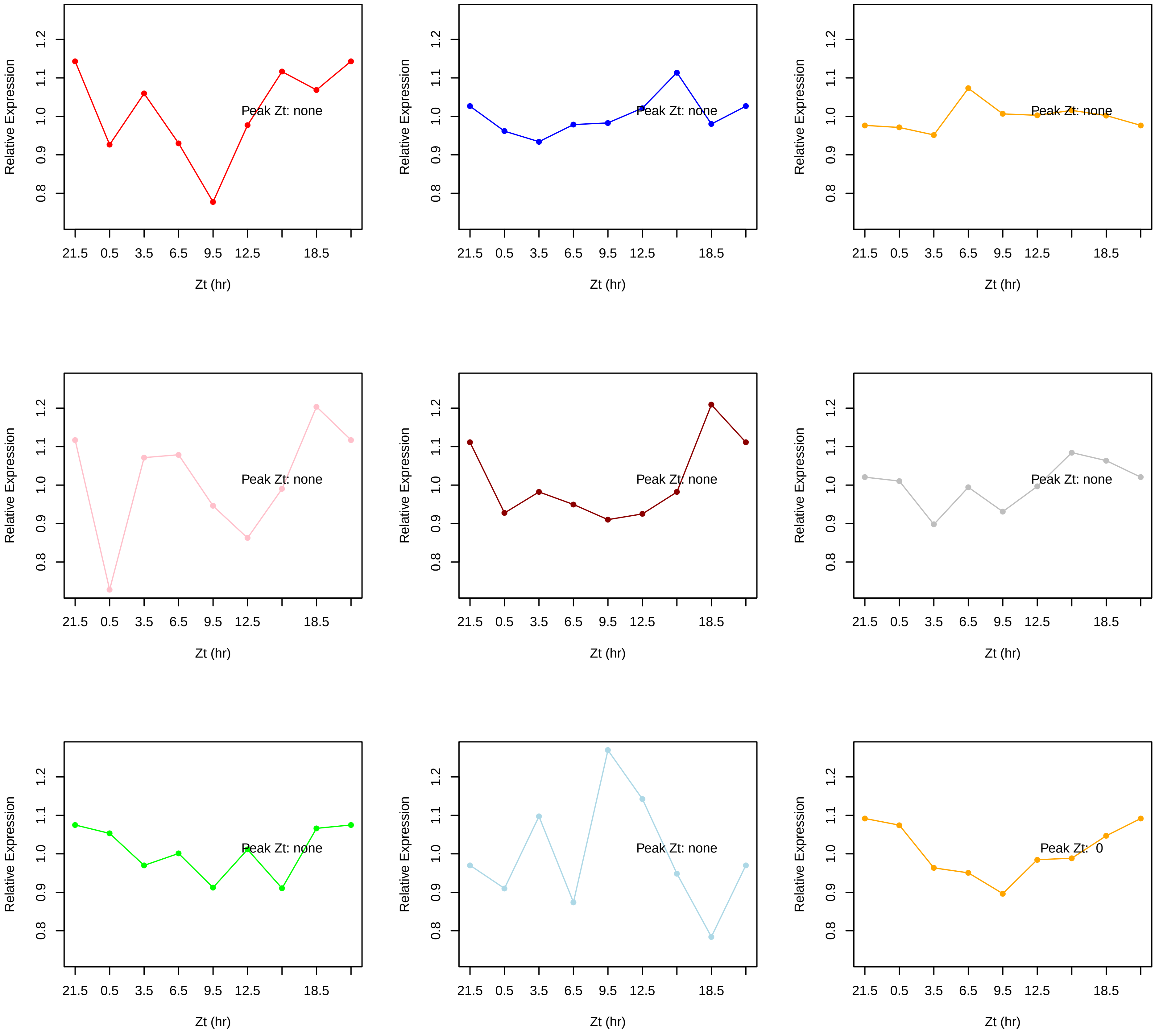

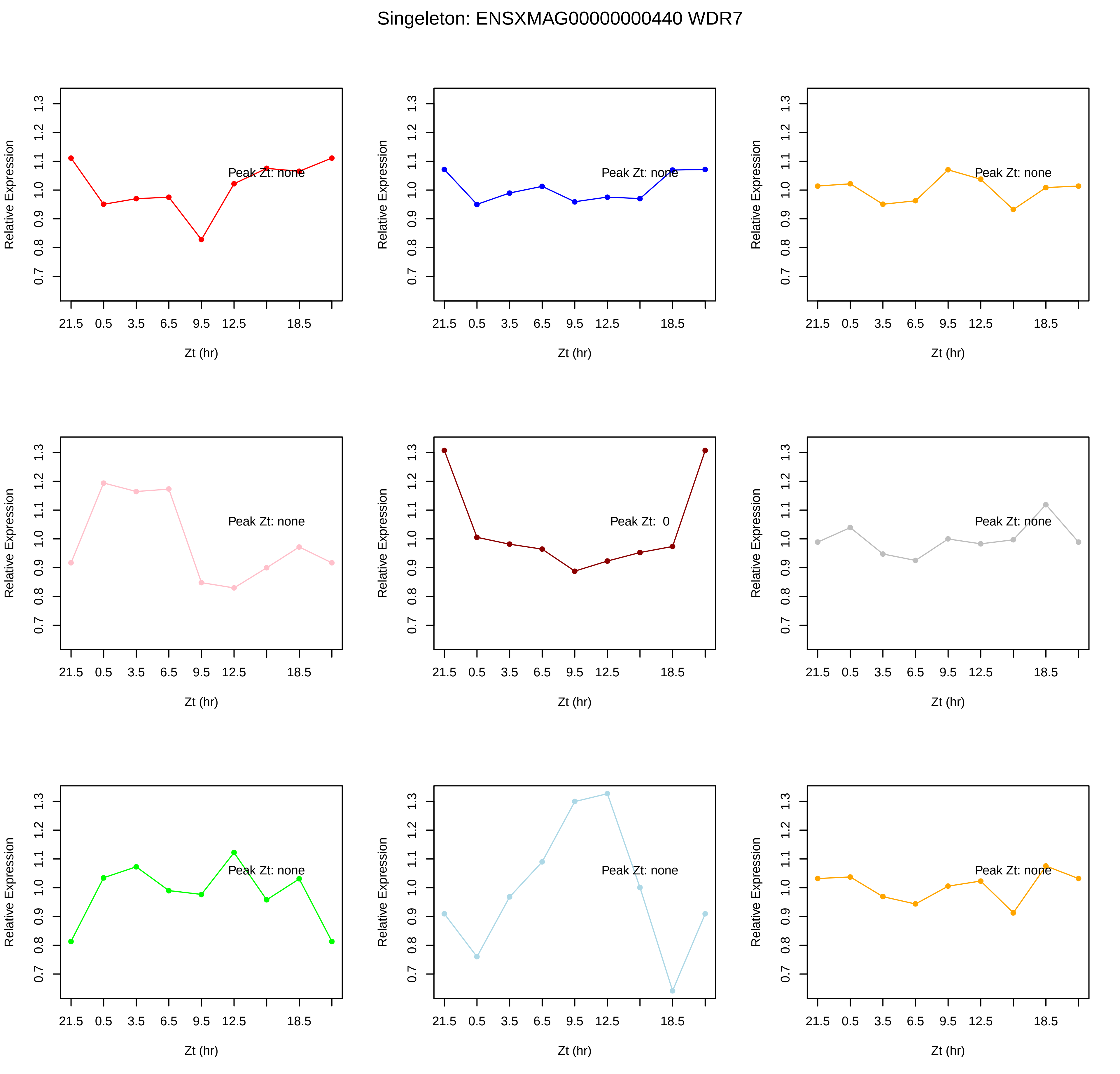
Singeleton: ENSXMAG00000000440 WDR7
Relative Expression
Zt (hr)
Peak Zt: none
Peak Zt: 0
21.5
0.5
3.5
6.5
9.5
12.5
18.5
0.7
0.8
0.9
1.0
1.1
1.2
1.3

Singeleton: ENSXMAG00000010657 rcc1

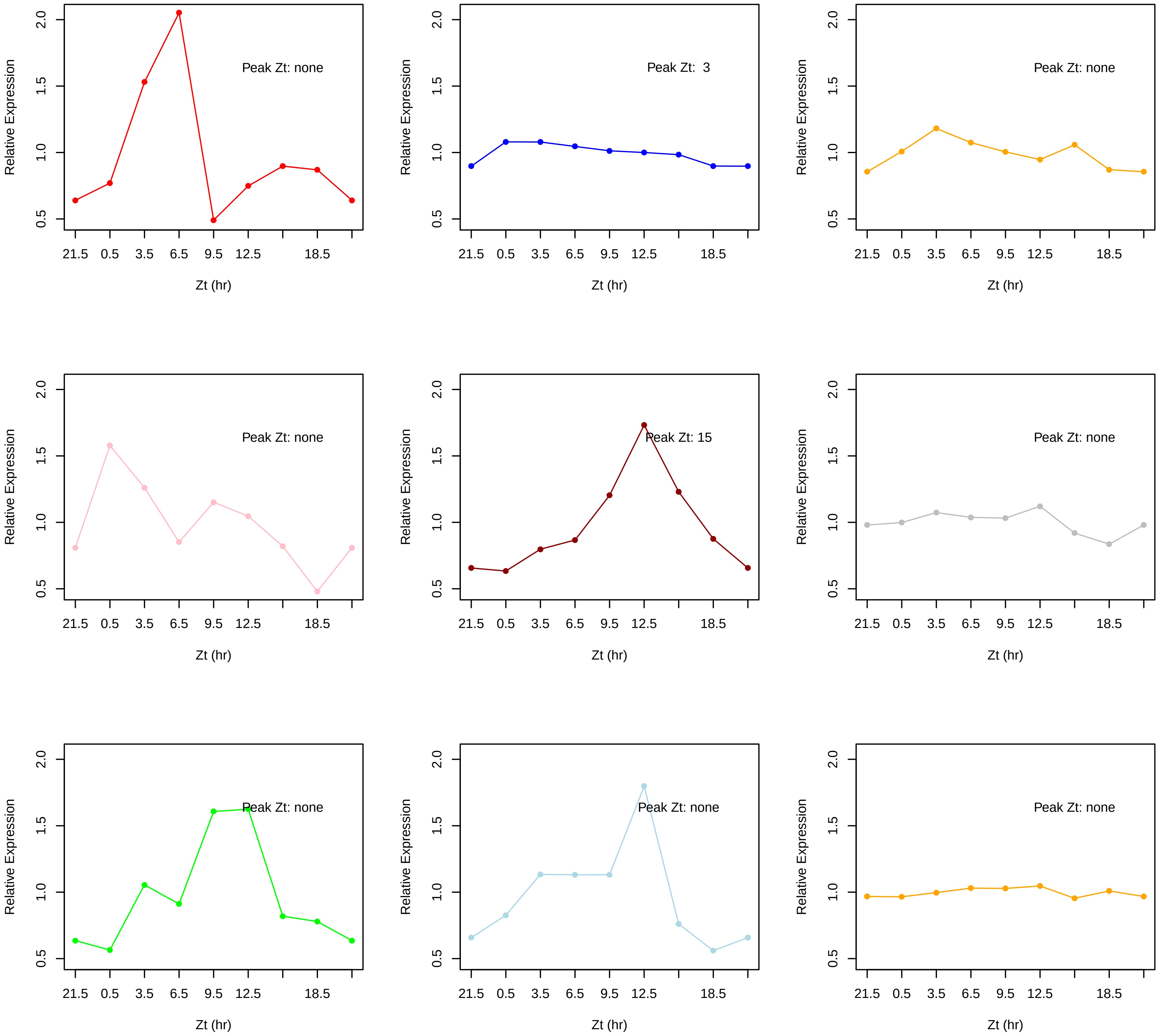

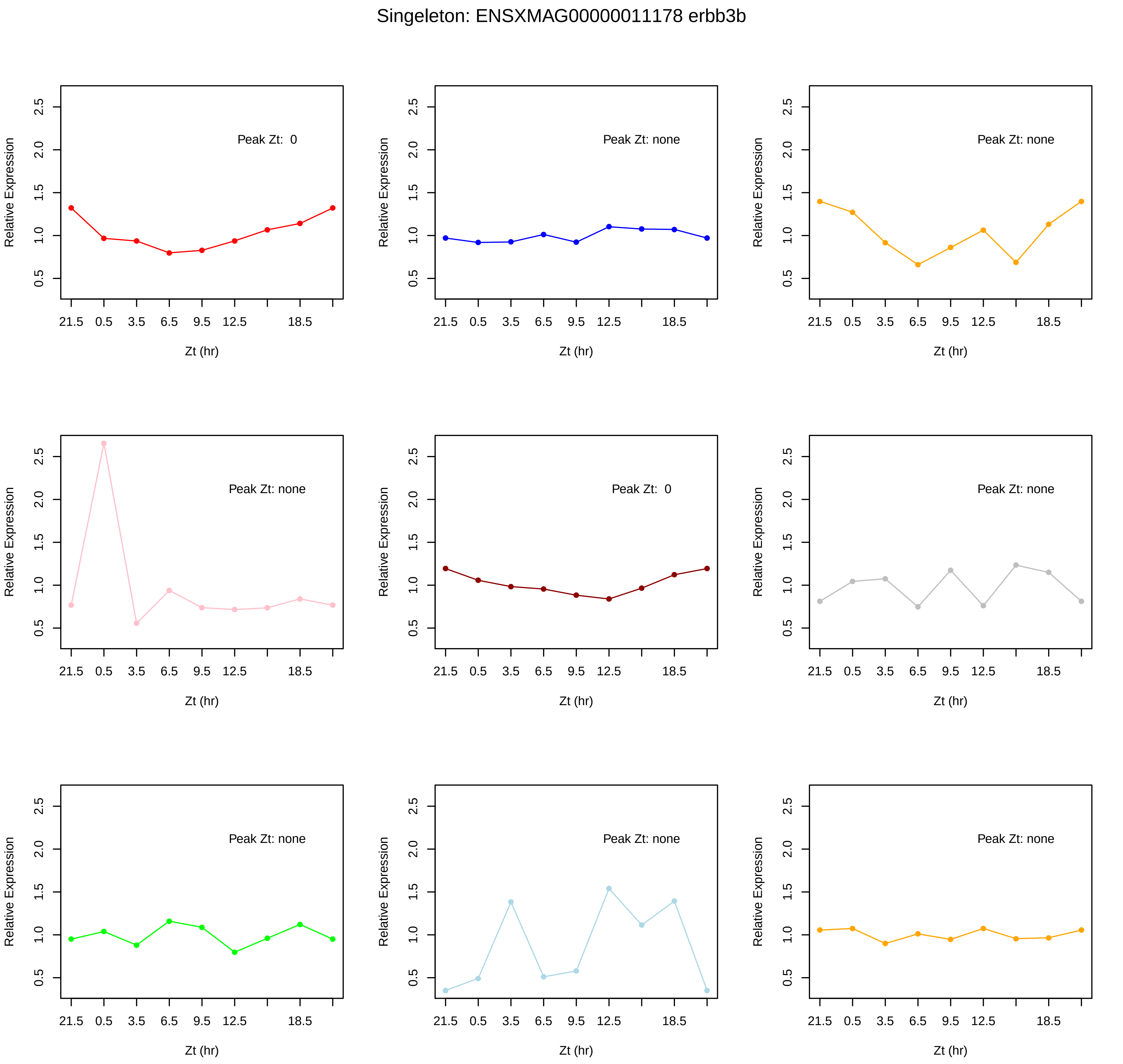

Singeleton: ENSXMAG00000011178 erbb3b
Relative Expression
Zt (hr)
0.5
1.0
1.5
2.0
2.5
21.5
0.5
3.5
6.5
9.5
12.5
18.5
Peak Zt: 0
Peak Zt: none
Peak Zt: none
Peak Zt: none
Peak Zt: 0
Peak Zt: none
Peak Zt: none
Peak Zt: none
Peak Zt: none

Singleton: ENSXMAG00000004561 phc2a

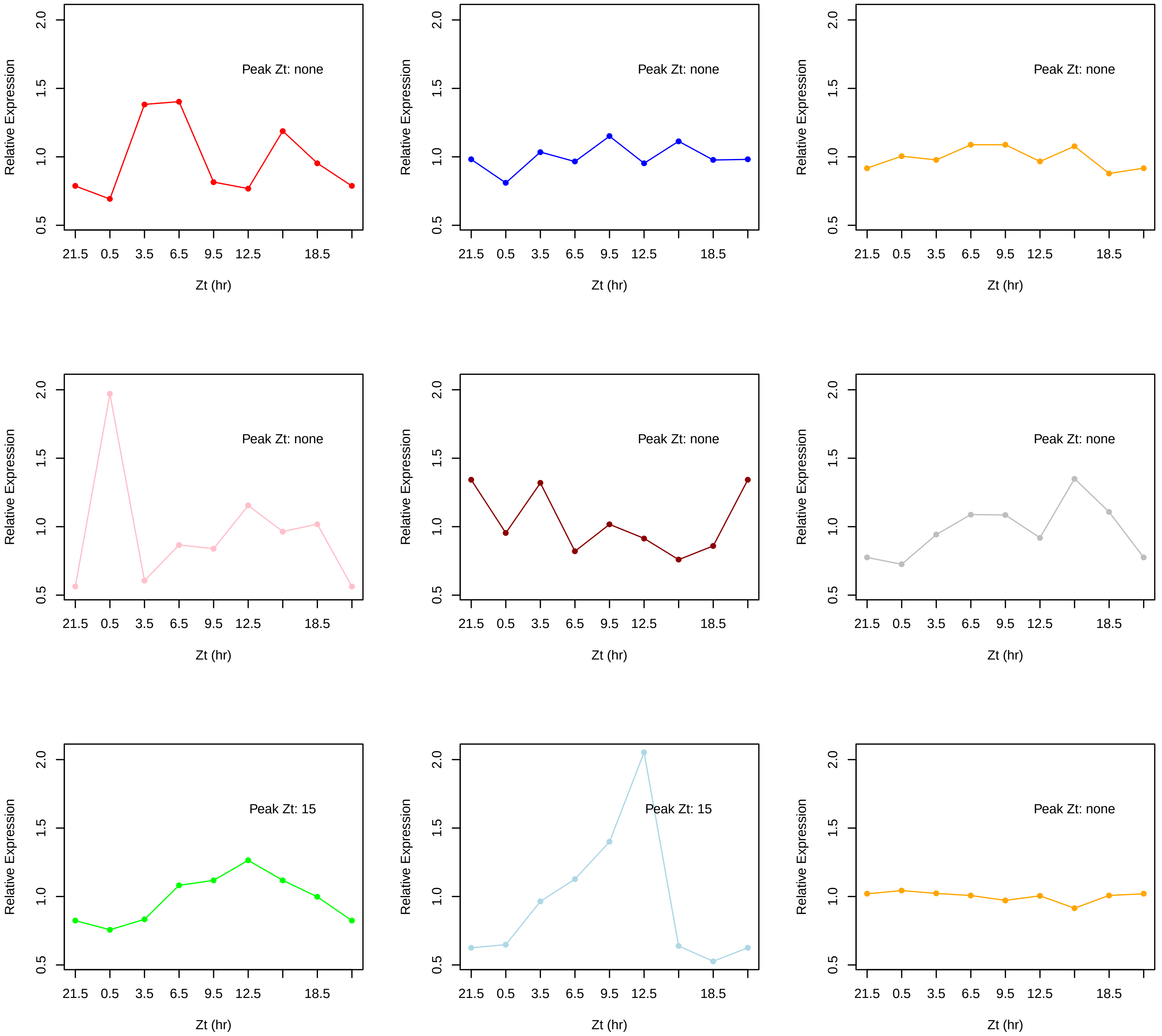

Singeleton: ENSXMAG00000018928 gnptg

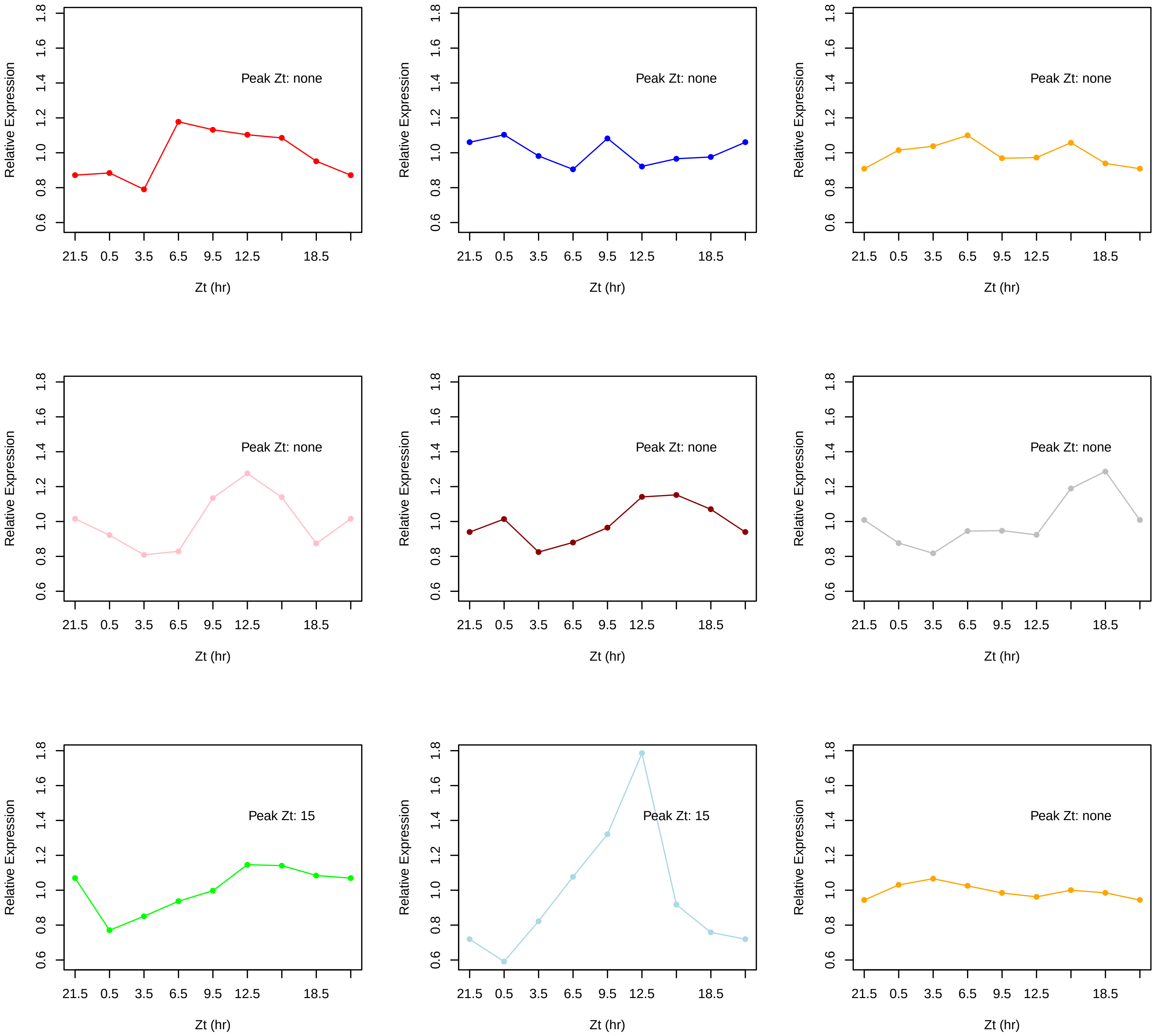

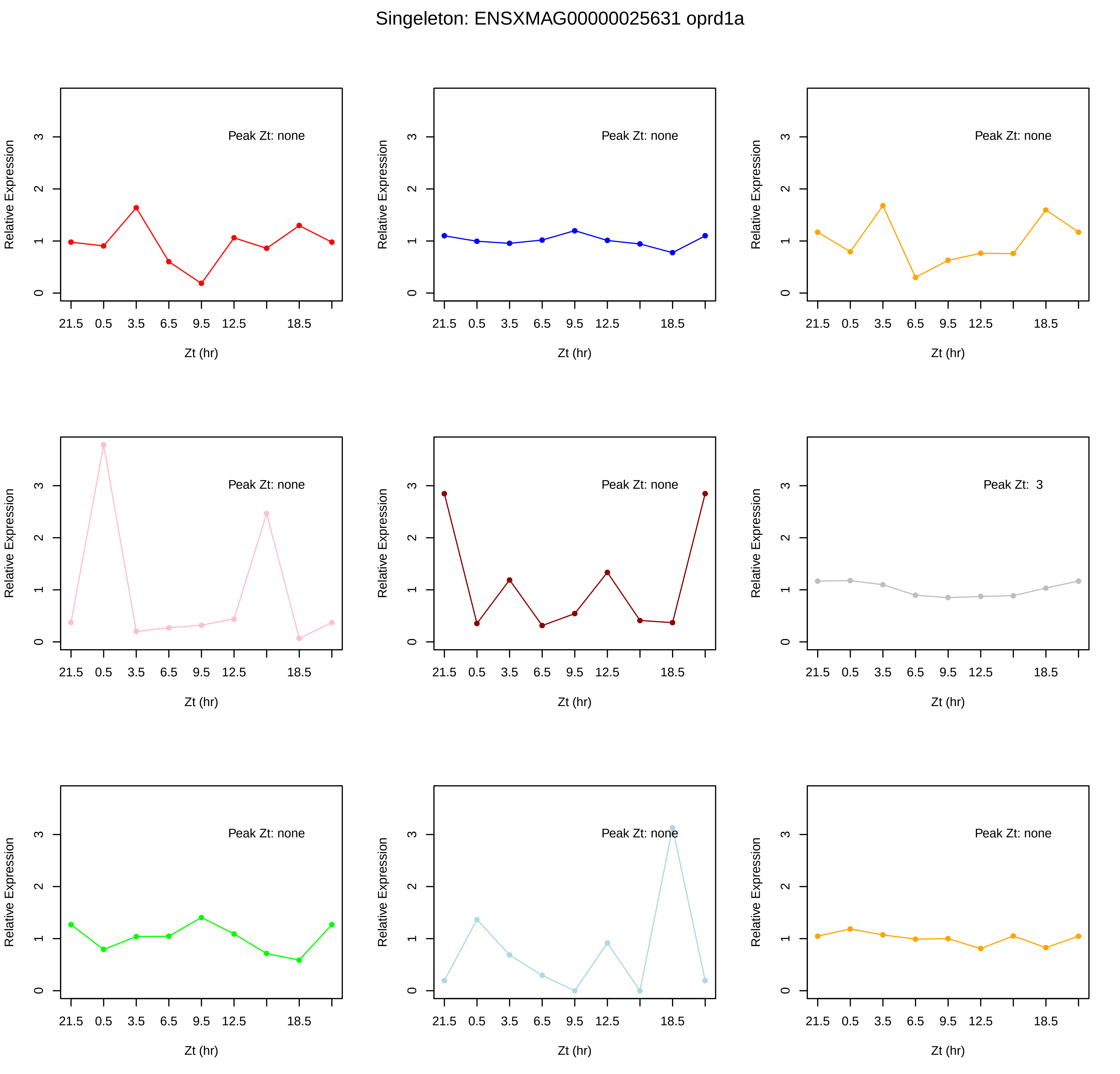
Singeleton: ENSXMAG00000025631 oprd1a
Peak Zt: none
Peak Zt: none
Peak Zt: none
Peak Zt: none
Peak Zt: none
Peak Zt: 3
Peak Zt: none
Peak Zt: none
Peak Zt: none
Relative Expression
Zt (hr)
21.5
0.5
3.5
6.5
9.5
12.5
18.5

Singeleton: ENSXMAG00000018921 tsr3

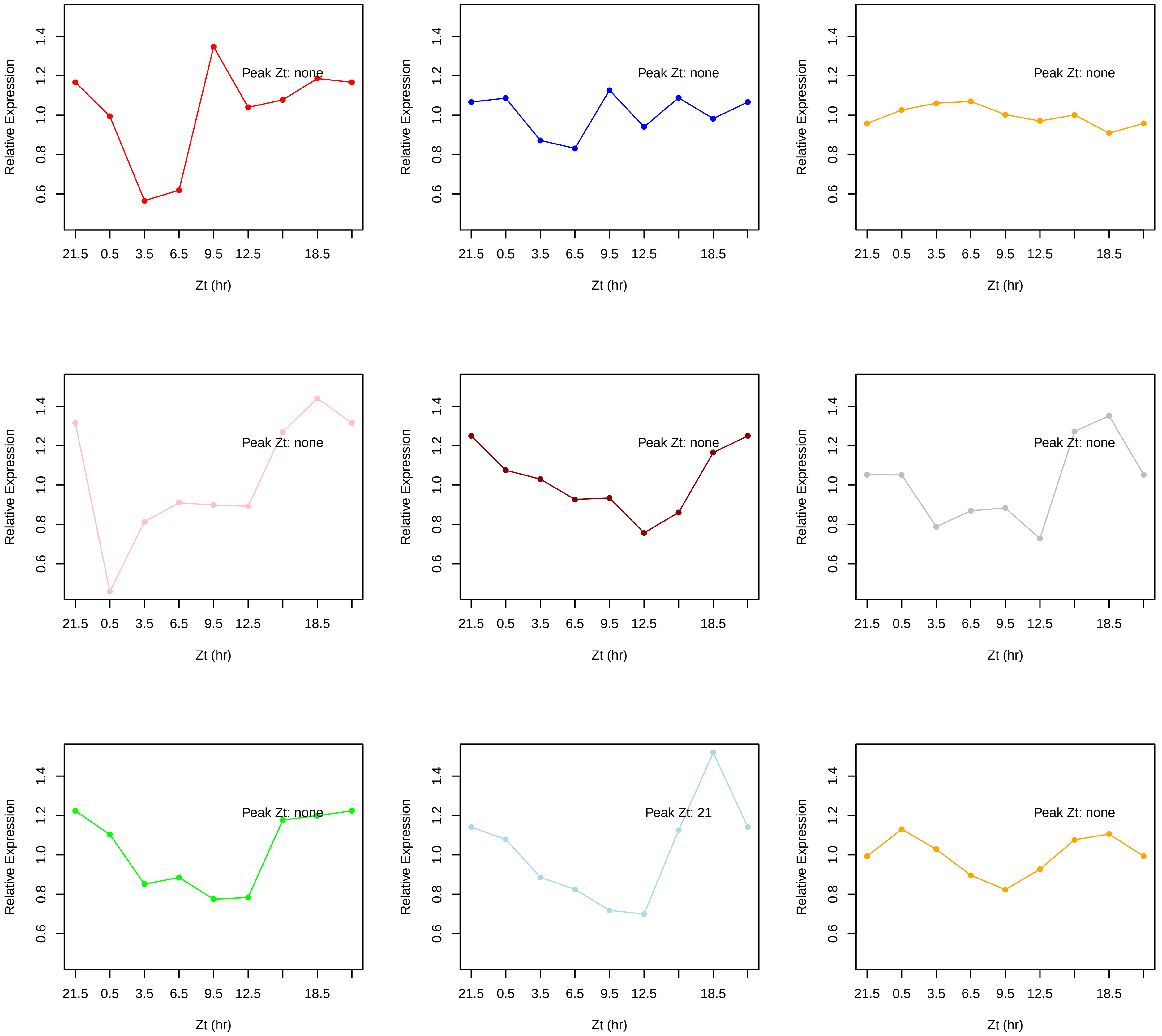

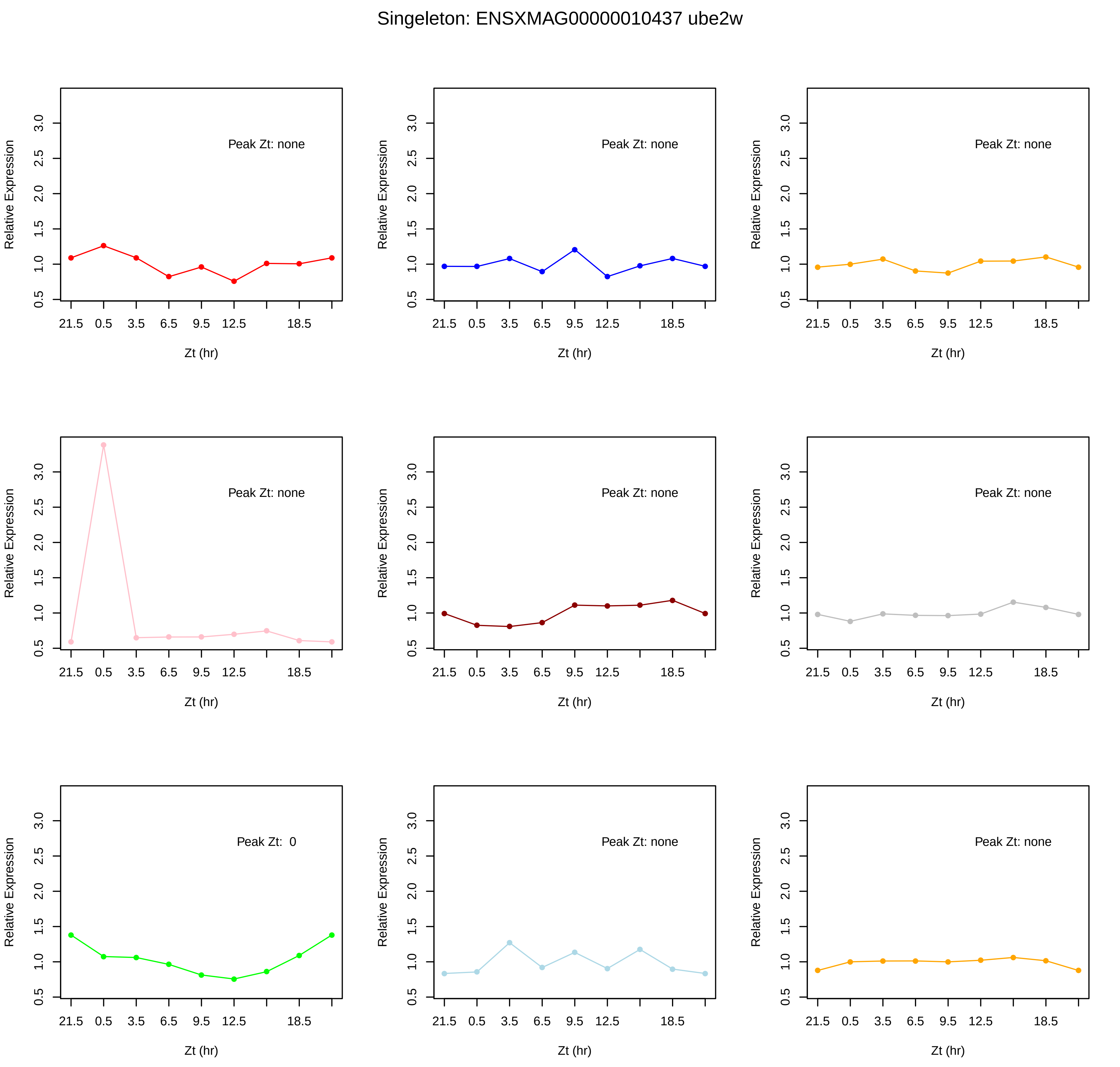
Singeleton: ENSXMAG00000010437 ube2w
Peak Zt: none
Peak Zt: none
Peak Zt: none
Peak Zt: none
Peak Zt: none
Peak Zt: none
Peak Zt: 0
Peak Zt: none
Peak Zt: none
Relative Expression
Zt (hr)
21.5
0.5
3.5
6.5
9.5
12.5
18.5
0.5
1.0
1.5
2.0
2.5
3.0

Singeleton: ENSXMAG00000010993 zfat

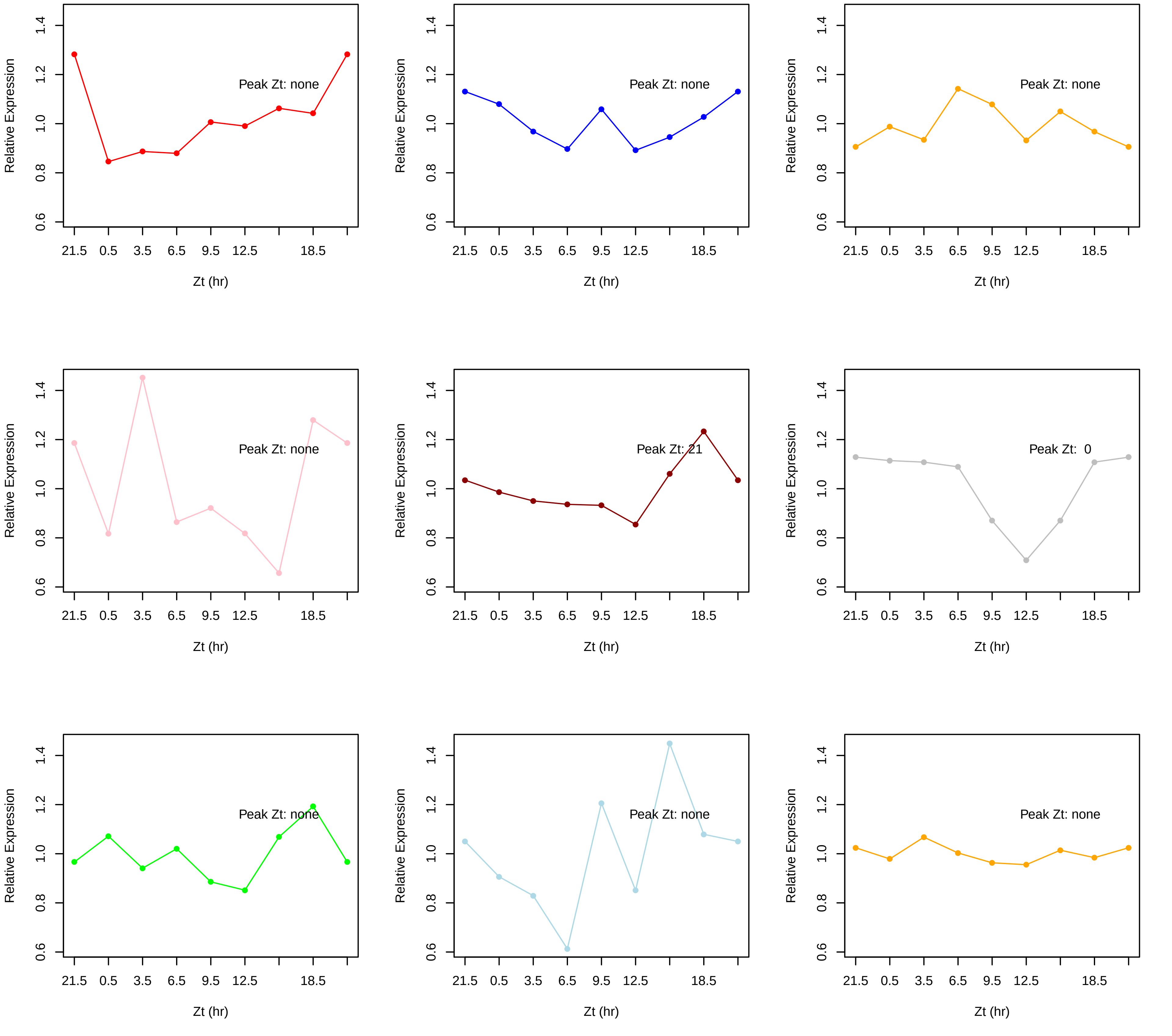

Singeleton: ENSXMAG00000000085 ints2

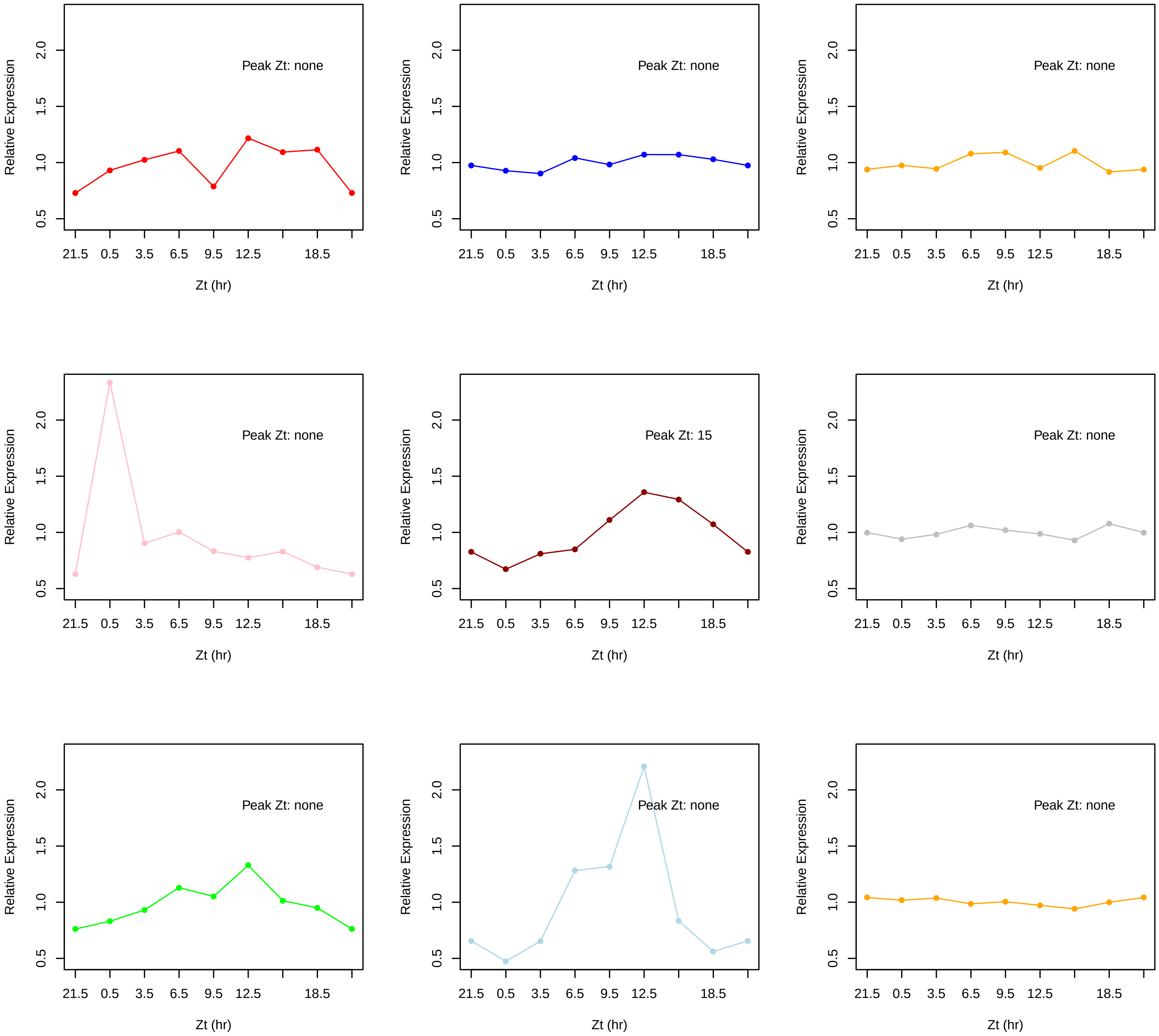

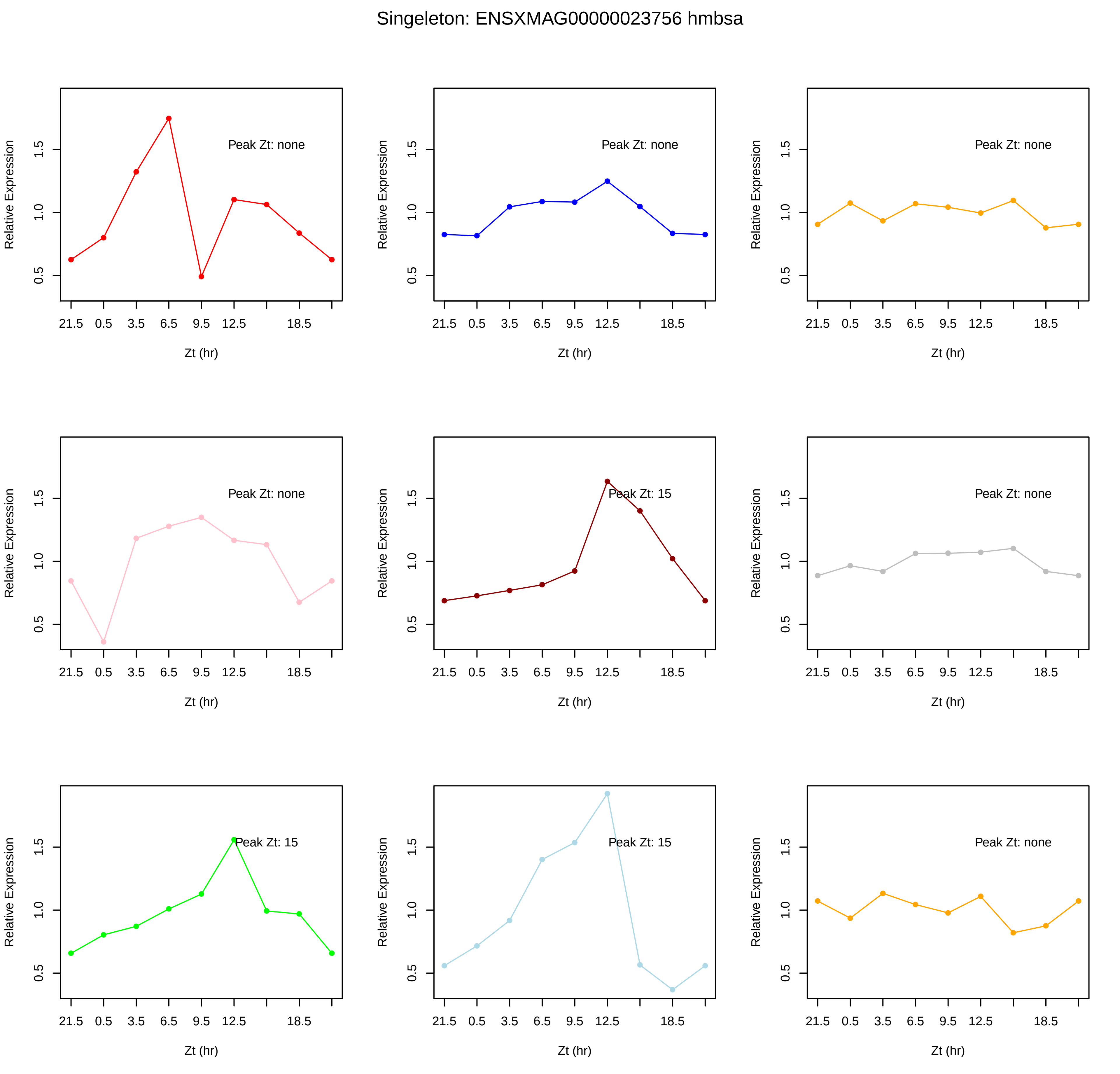
Singeleton: ENSXMAG00000023756 hmbsa
Peak Zt: none
Peak Zt: none
Peak Zt: none
Peak Zt: none
Peak Zt: 15
Peak Zt: none
Peak Zt: 15
Peak Zt: 15
Peak Zt: none
Relative Expression
Zt (hr)
21.5
0.5
3.5
6.5
9.5
12.5
18.5
0.5
1.0
1.5

Singleton: ENSXMAG00000001913 pa2g4a

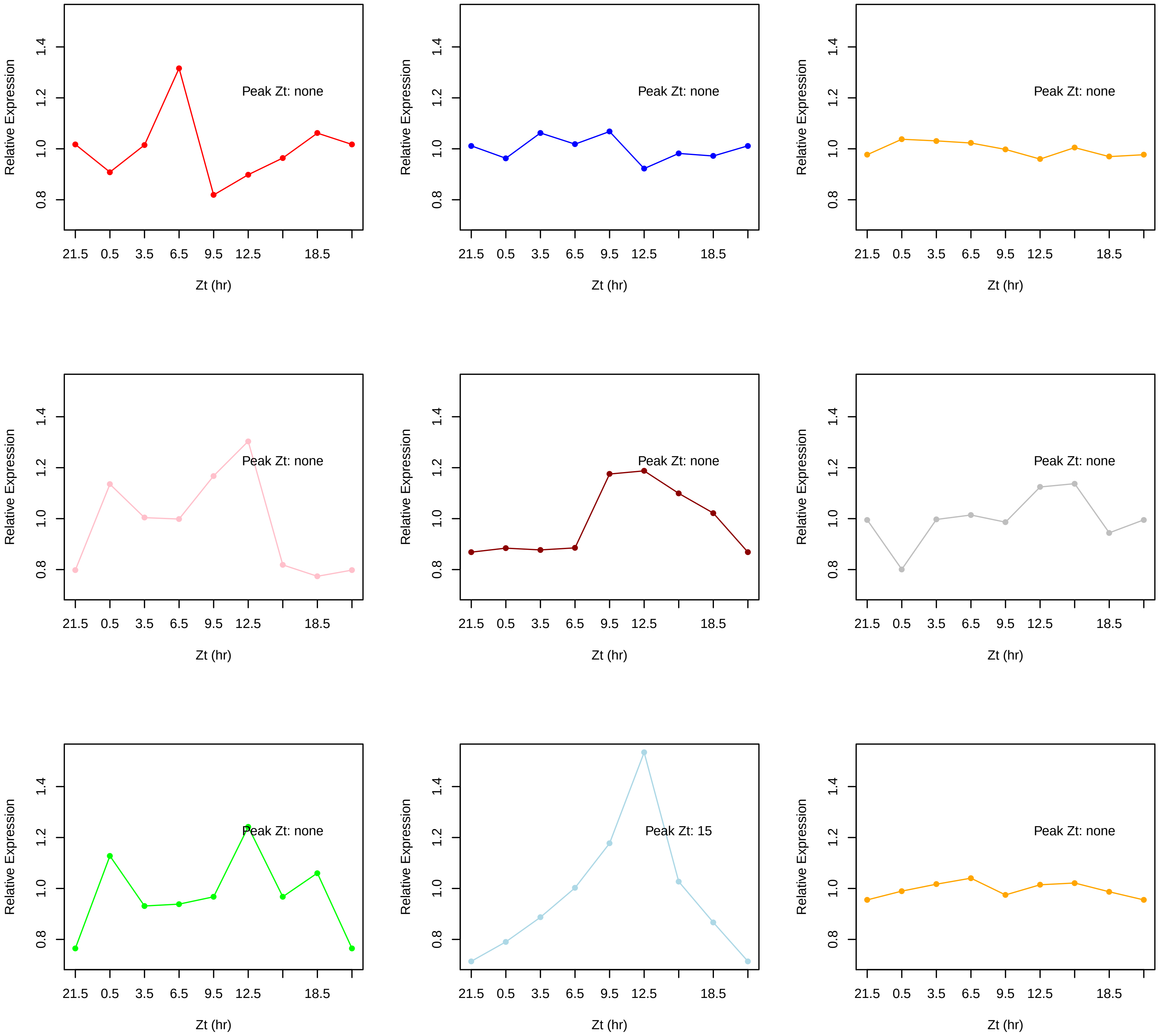

Singleton: ENSXMAG00000007120 miga1

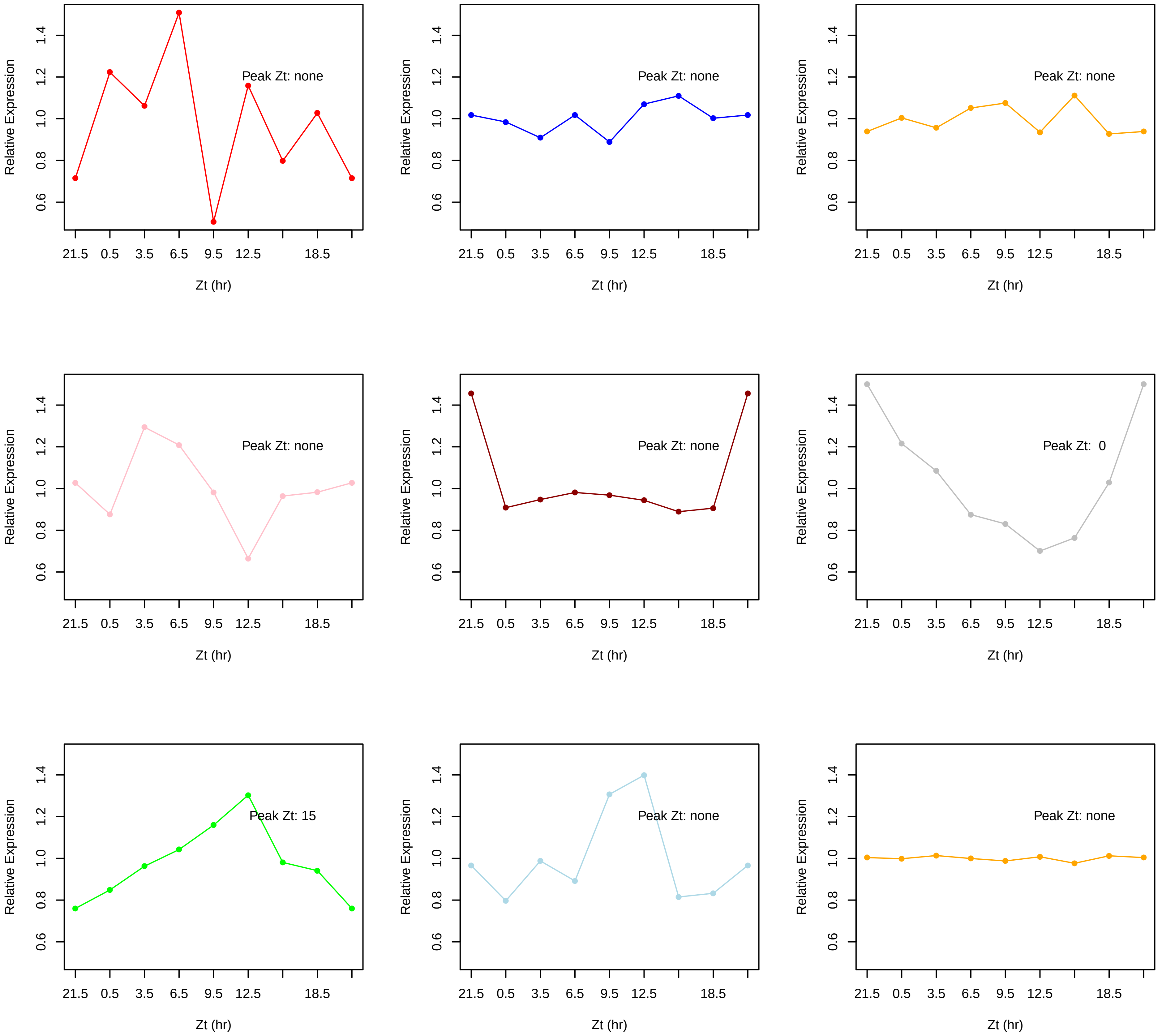

Singleton: ENSXMAG00000026007 calr3b

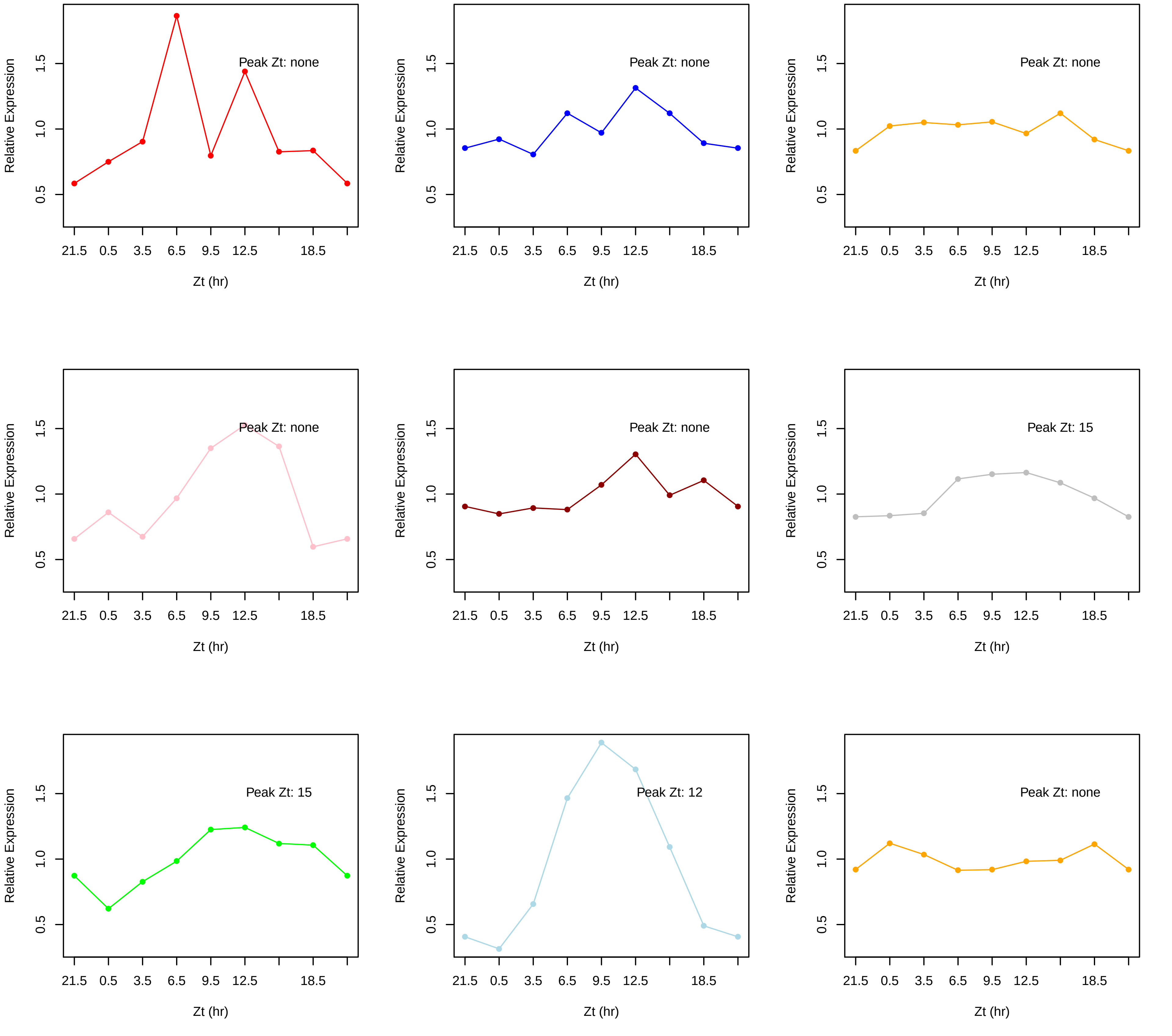

Singeleton: ENSXMAG00000026188

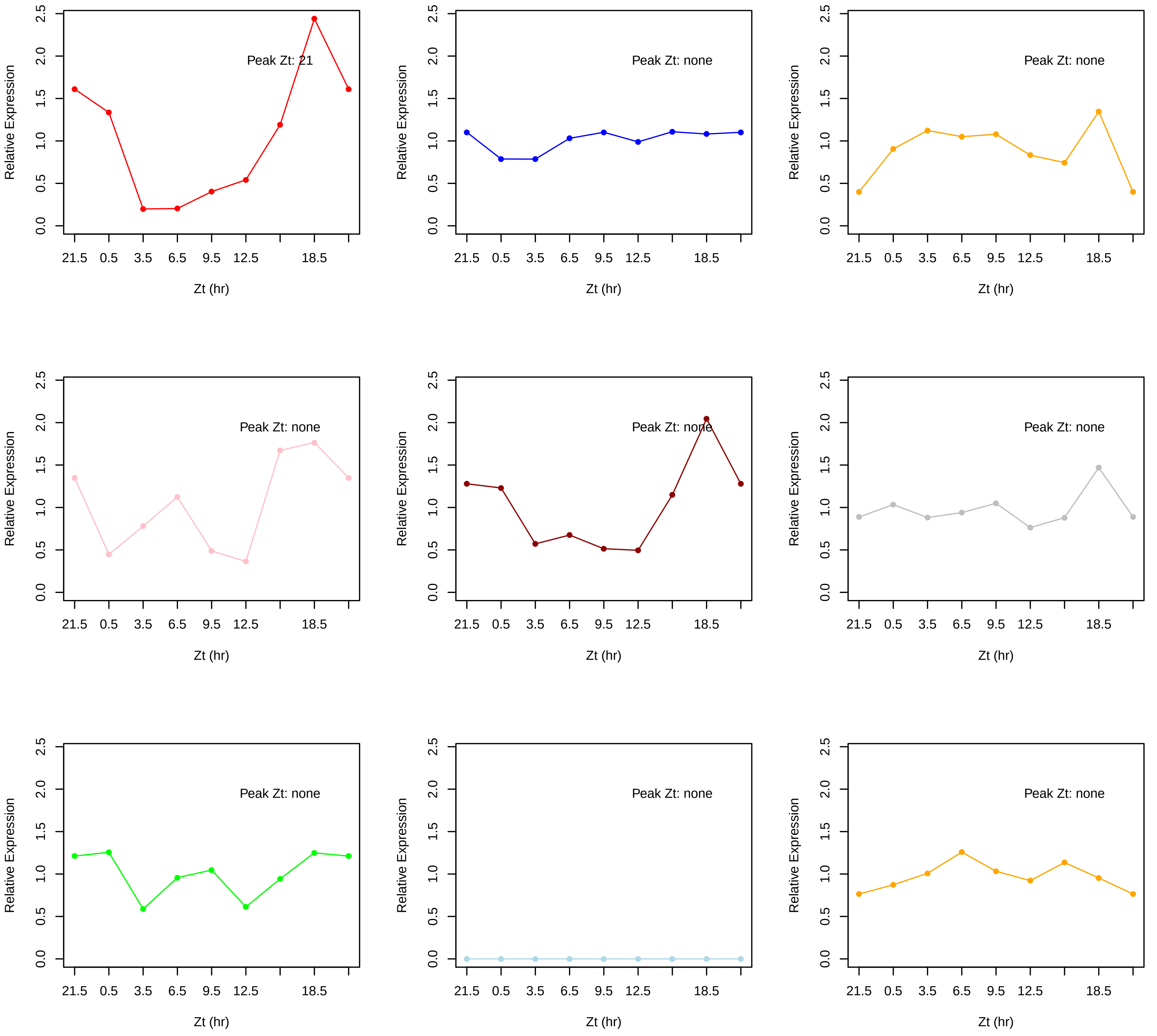

Singeleton: ENSXMAG00000018131

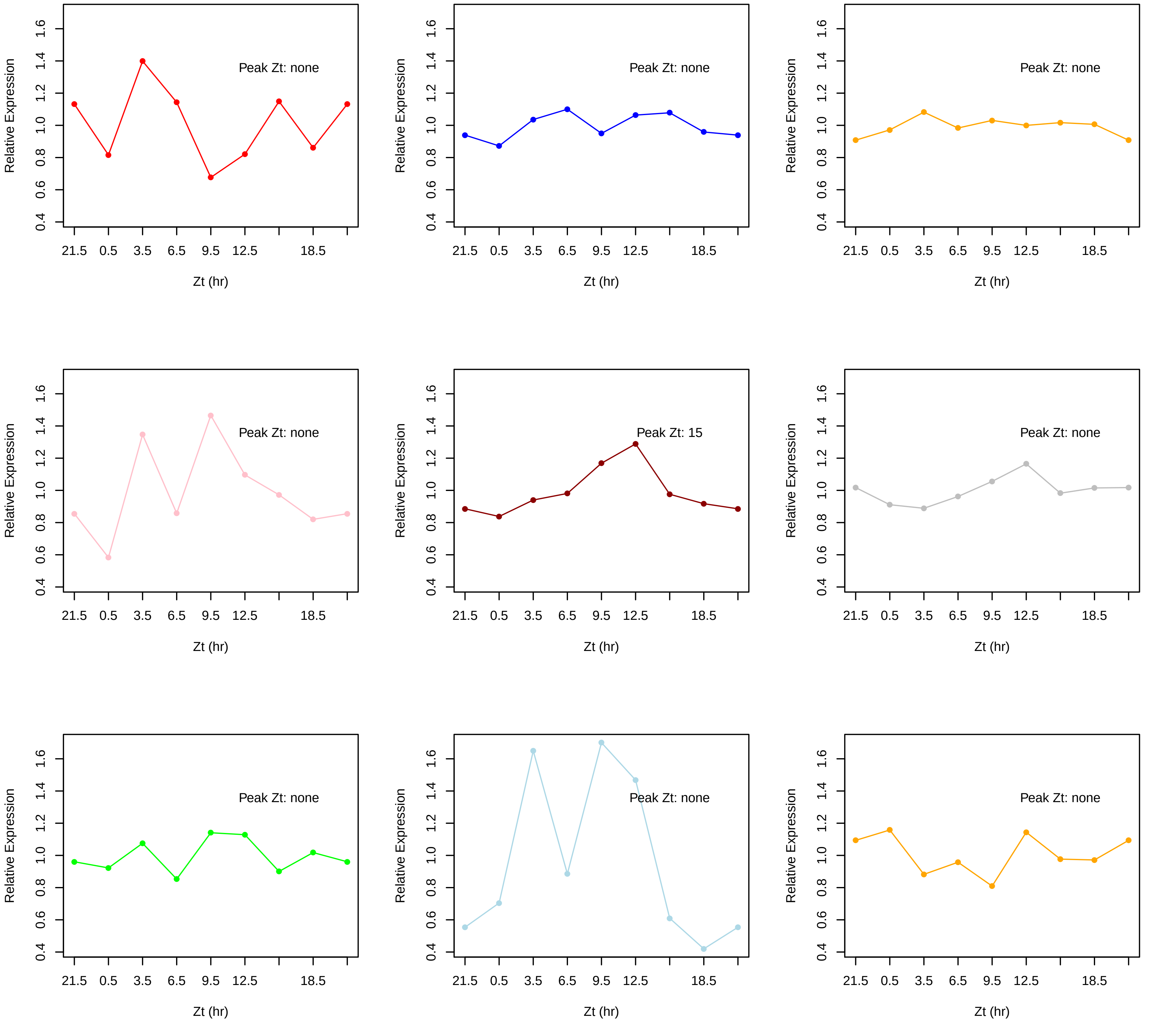

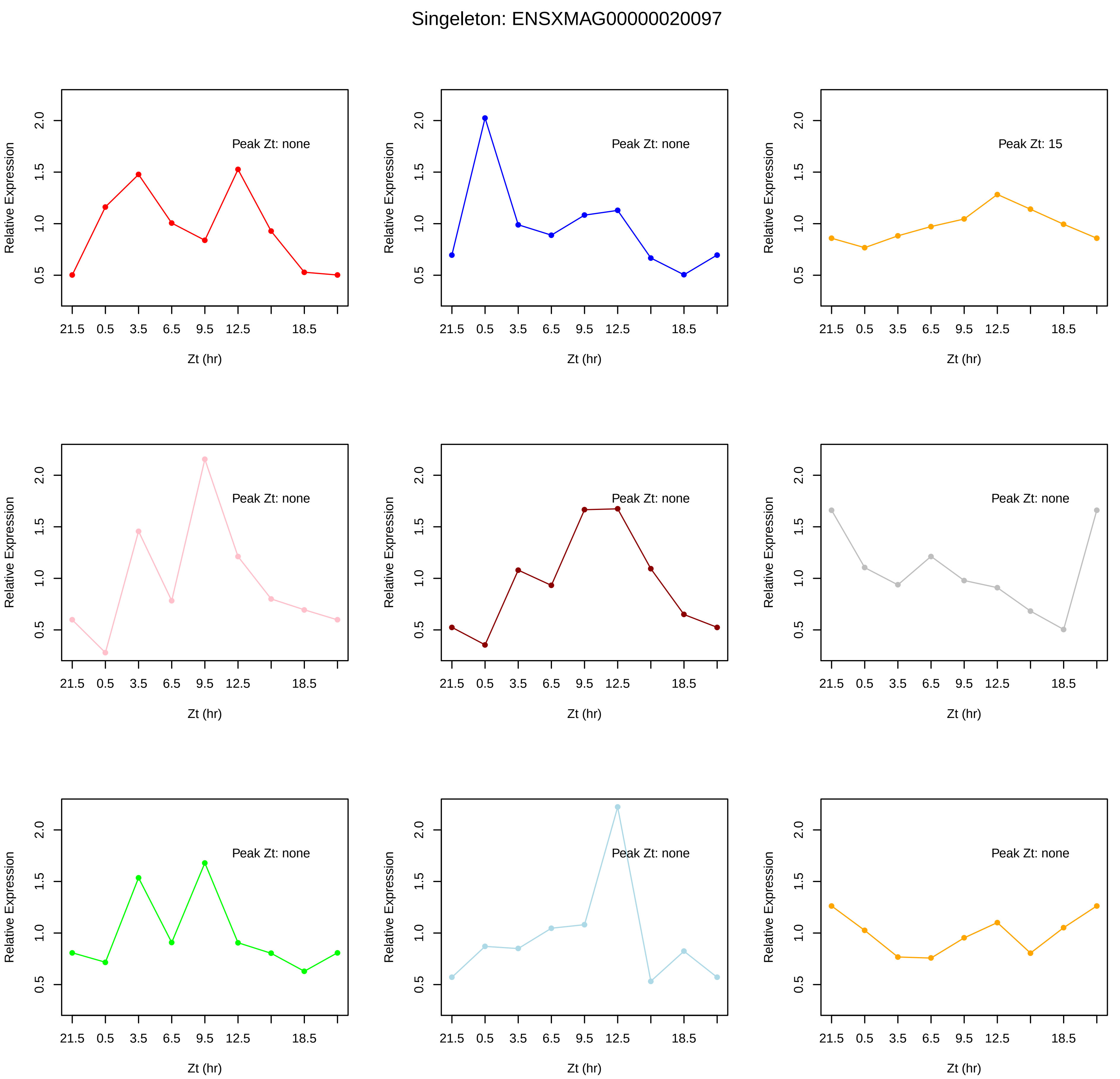

Singeleton: ENSXMAG00000020097
Peak Zt: none
Peak Zt: none
Peak Zt: 15
Peak Zt: none
Peak Zt: none
Peak Zt: none
Peak Zt: none
Peak Zt: none
Peak Zt: none
Relative Expression
Zt (hr)
21.5 0.5 3.5 6.5 9.5 12.5 18.5
0.5 1.0 1.5 2.0

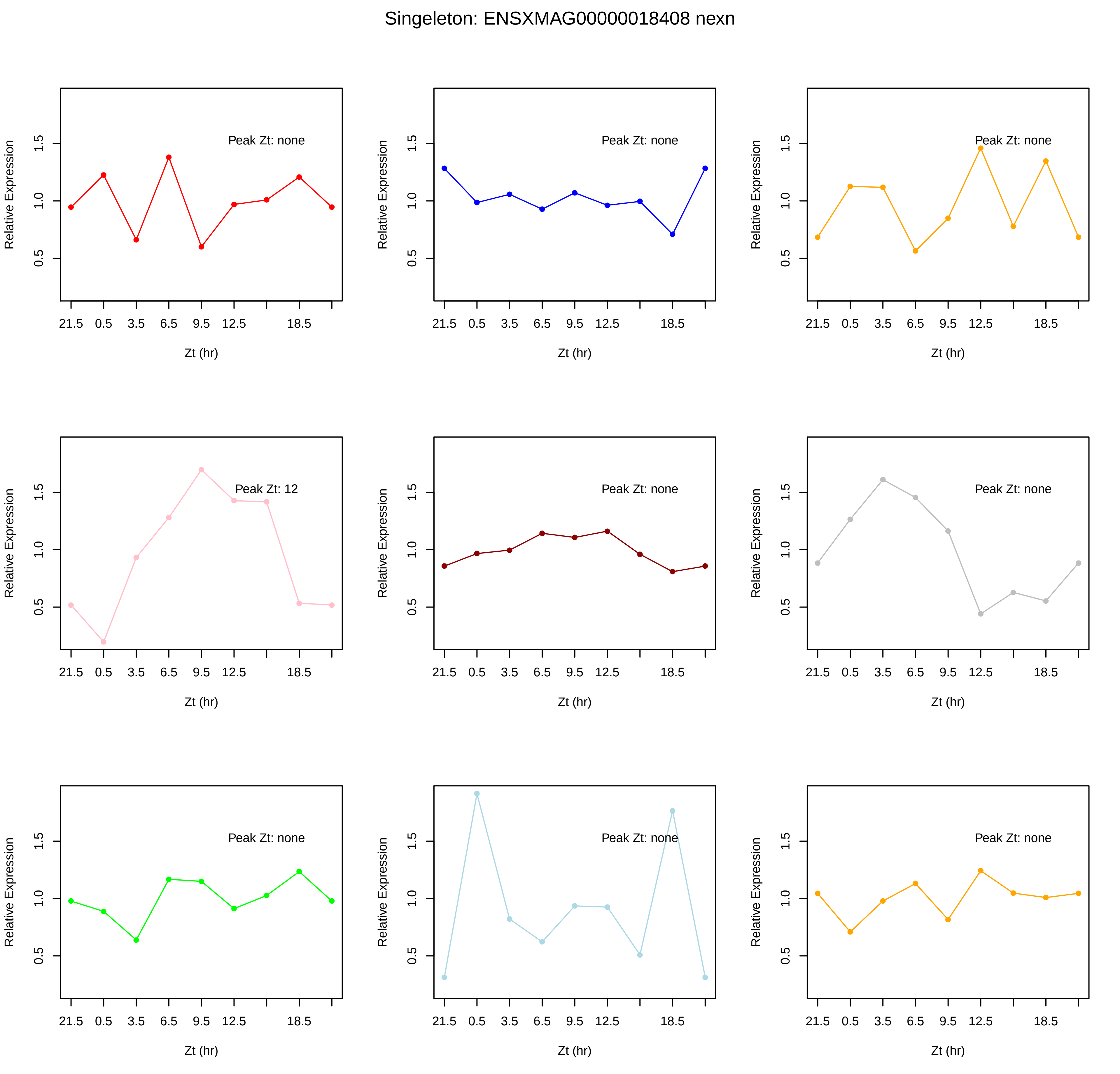

Singeleton: ENSXMAG00000018408 nexn
Peak Zt: none
Peak Zt: none
Peak Zt: none
Peak Zt: 12
Peak Zt: none
Peak Zt: none
Peak Zt: none
Peak Zt: none
Peak Zt: none
Relative Expression
Zt (hr)
0.5
1.0
1.5
21.5
0.5
3.5
6.5
9.5
12.5
18.5

Singeleton: ENSXMAG00000003675 mbtps2

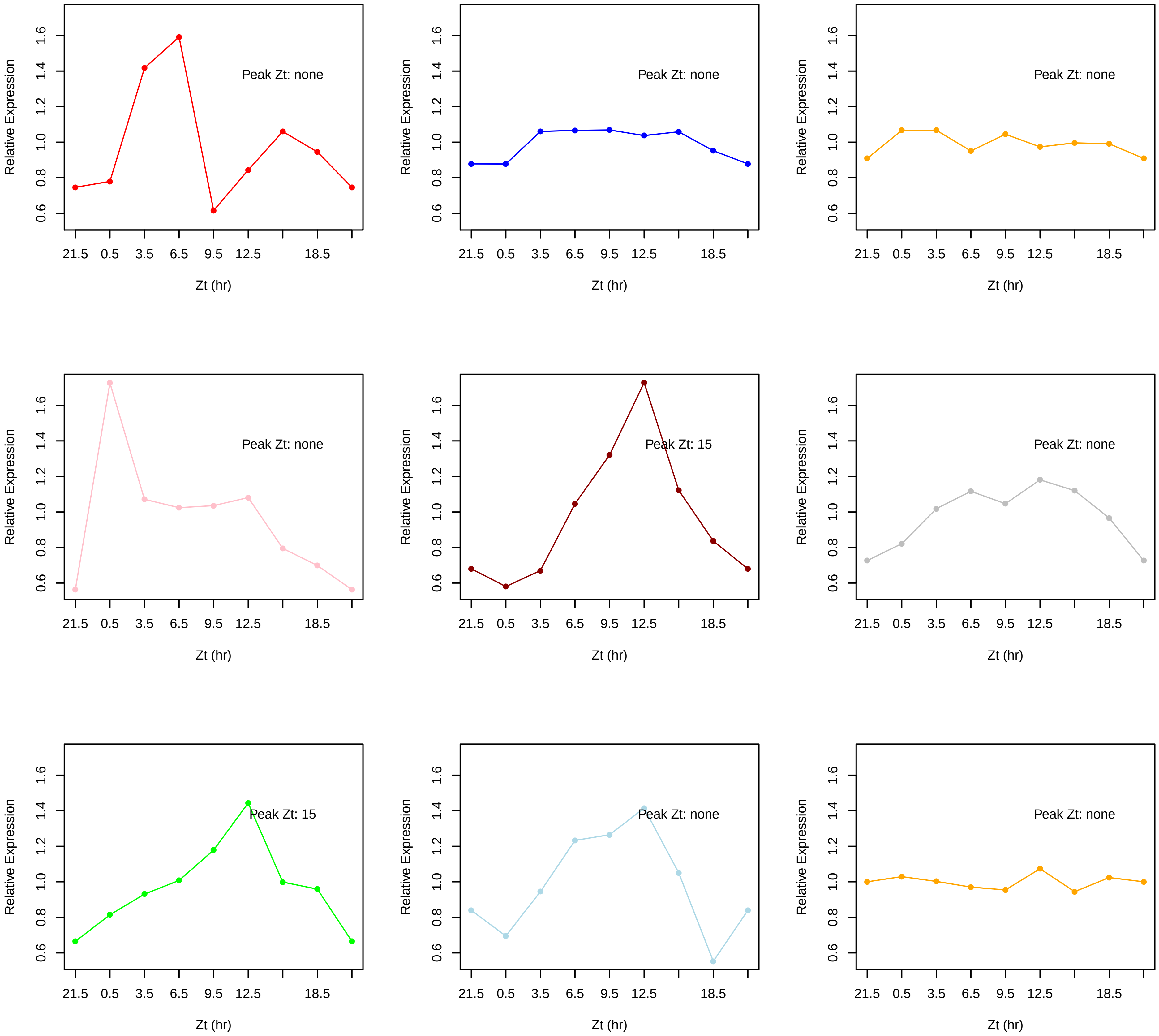

Singeleton: ENSXMAG00000018837 ube2ia

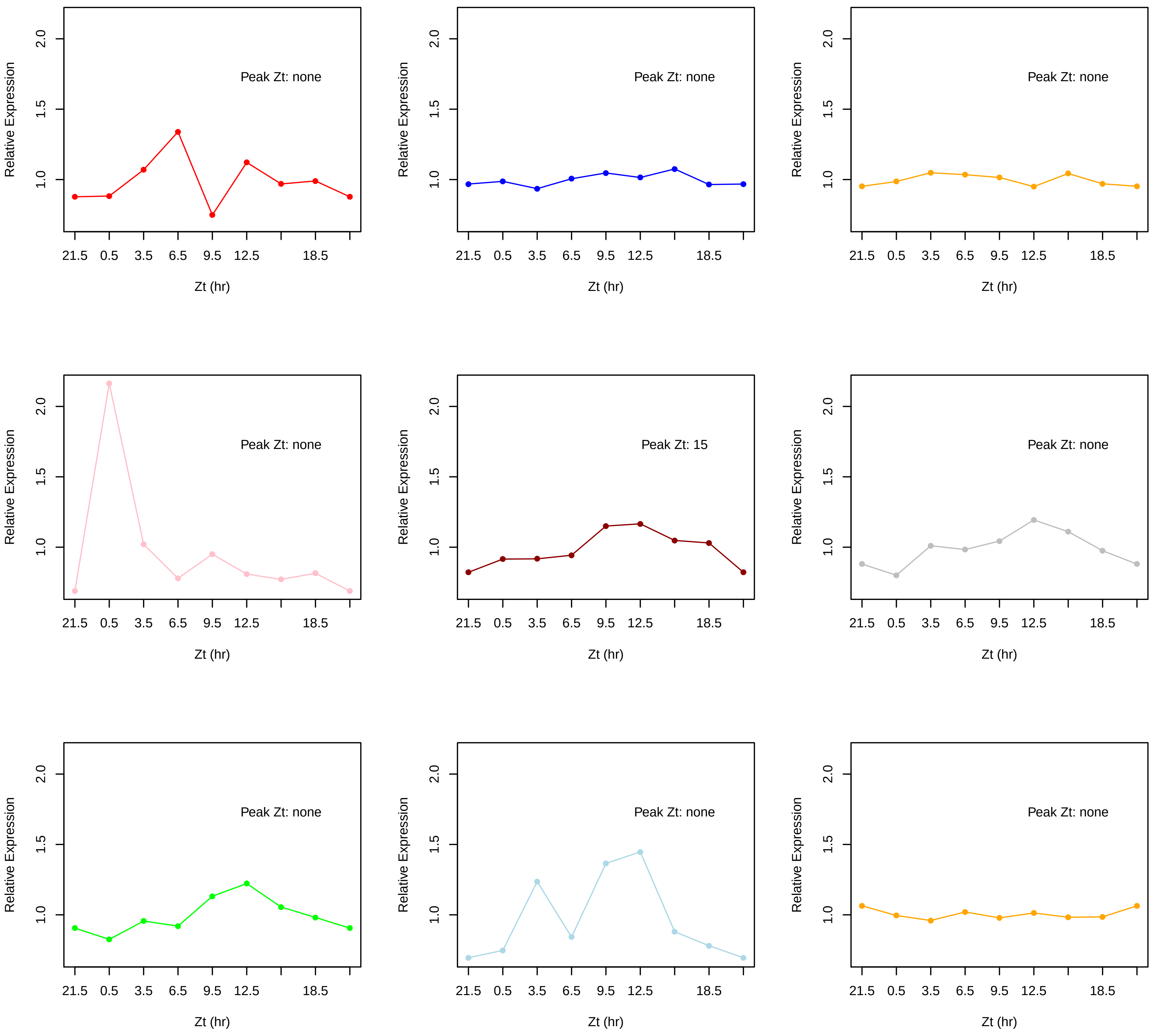

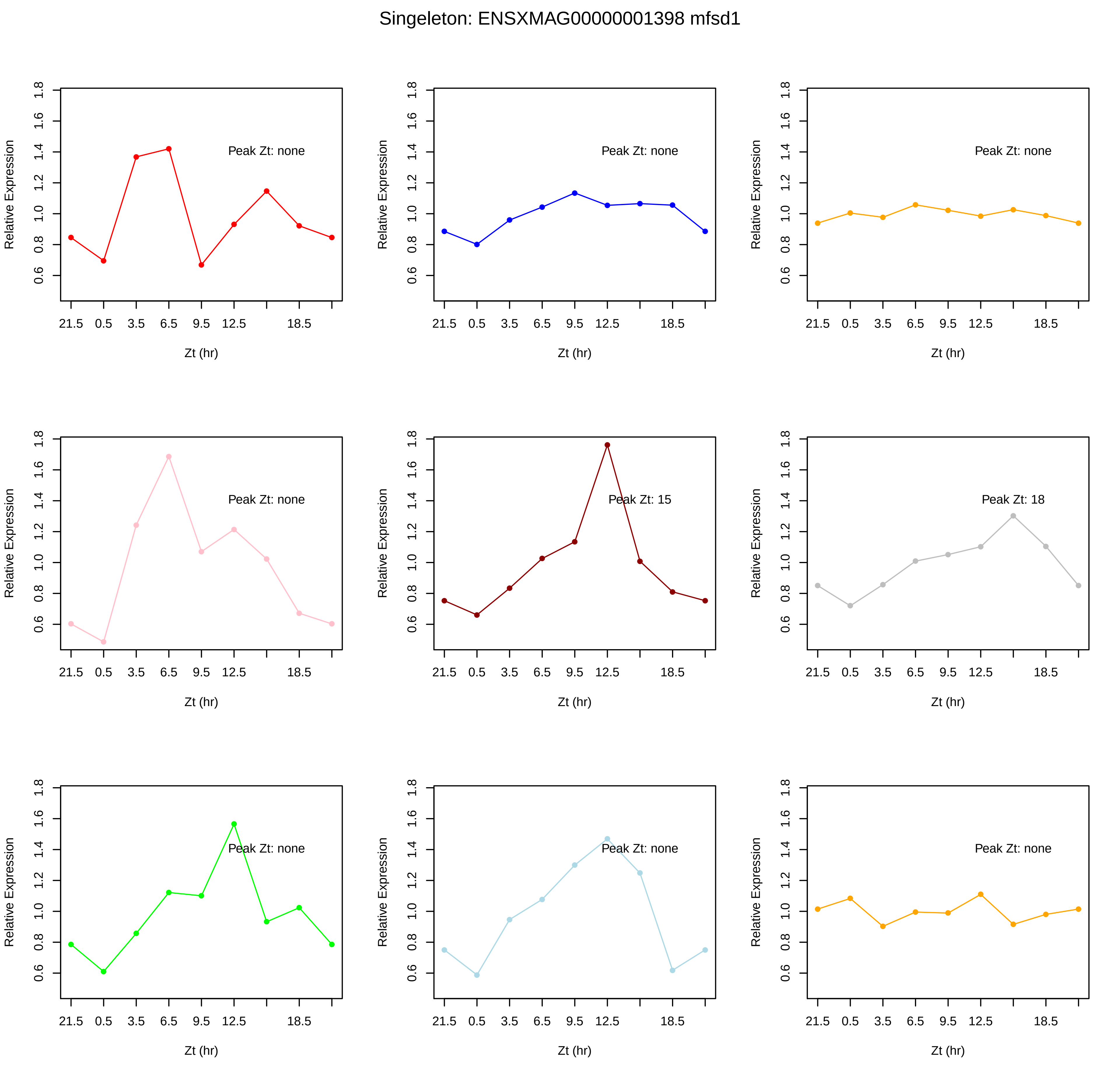
Singleton: ENSXMAG00000001398 mfsd1
Peak Zt: none
Peak Zt: none
Peak Zt: none
Peak Zt: none
Peak Zt: 15
Peak Zt: 18
Peak Zt: none
Peak Zt: none
Peak Zt: none
Relative Expression
Zt (hr)
21.5 0.5 3.5 6.5 9.5 12.5 18.5
0.6 0.8 1.0 1.2 1.4 1.6 1.8

Singleton: ENSXMAG00000005747 FAM135B

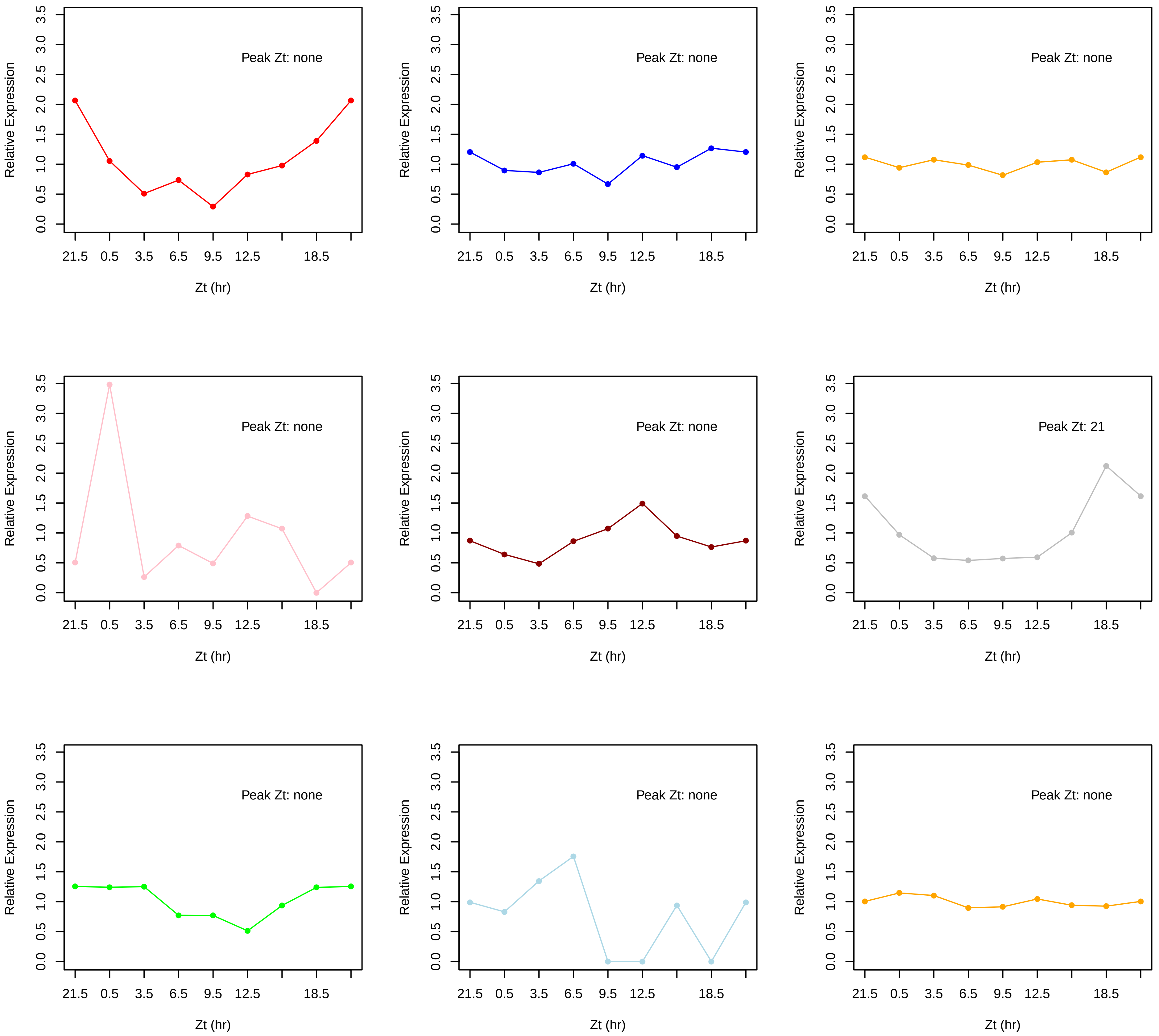

Singleton: ENSXMAG00000003600 agpat3

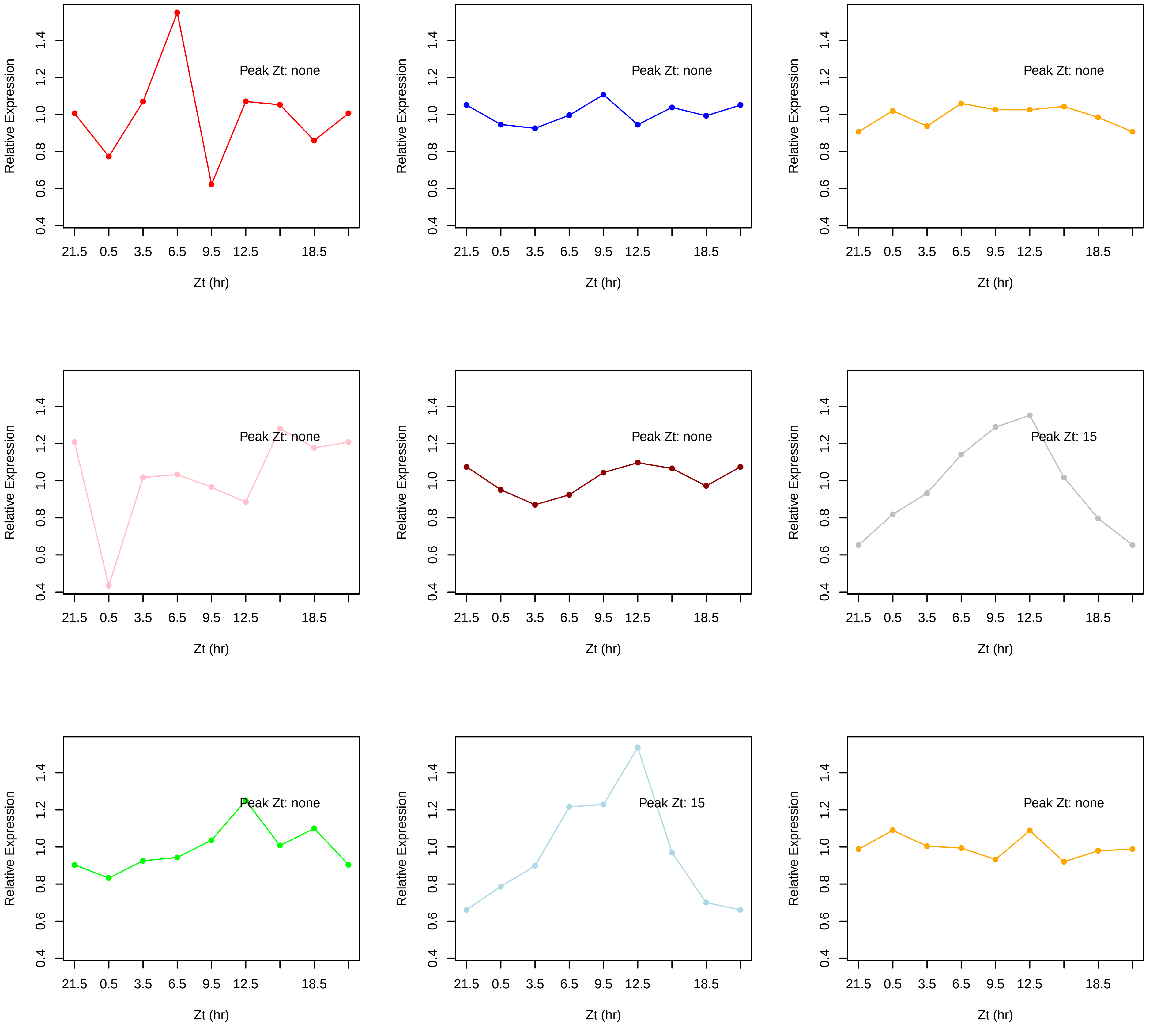

Singleton: ENSXMAG00000026003 si:ch211−207k7.4

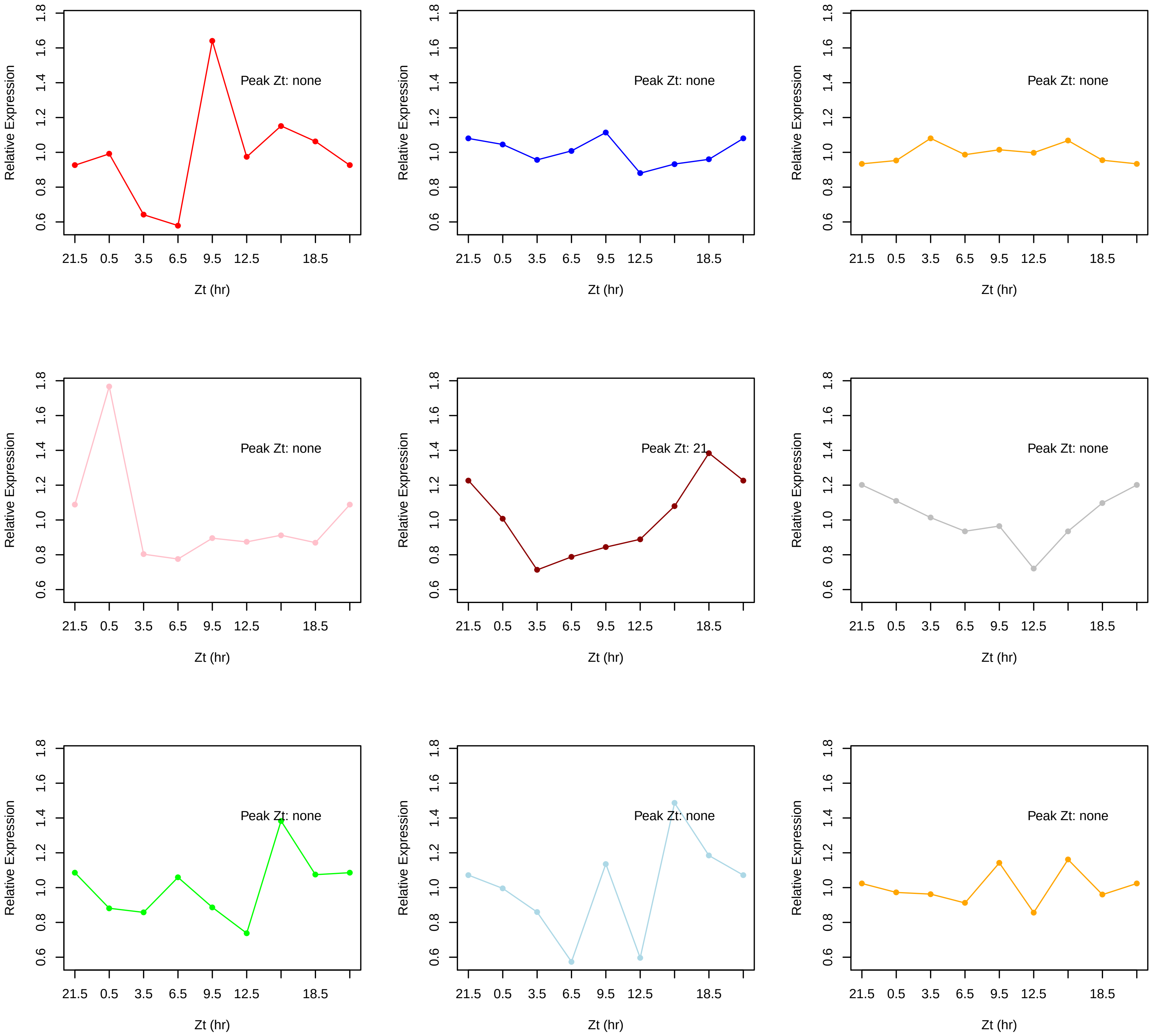

Singeleton: ENSXMAG00000010405 SBSPON

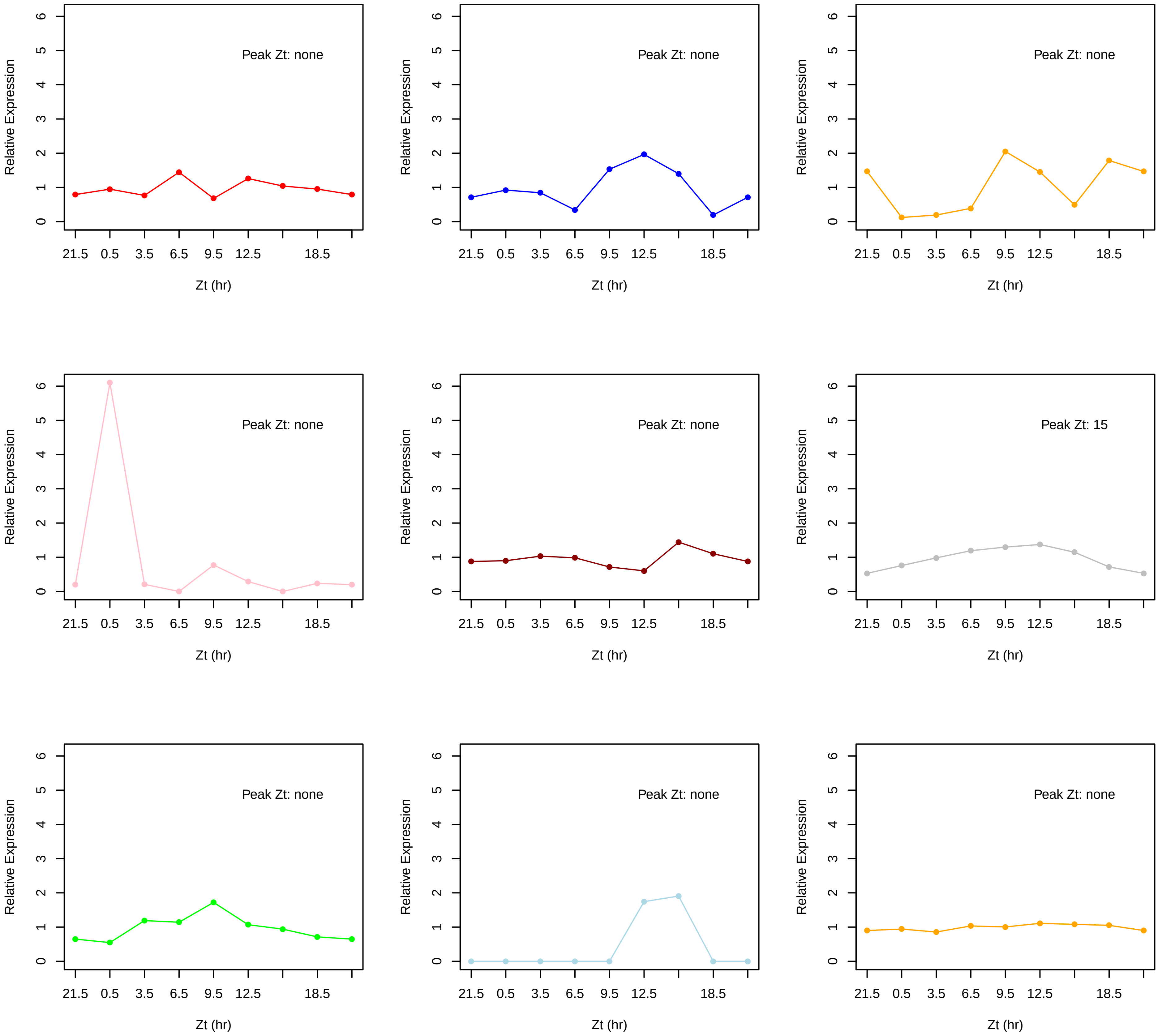

Singleton: ENSXMAG00000023382 exosc1

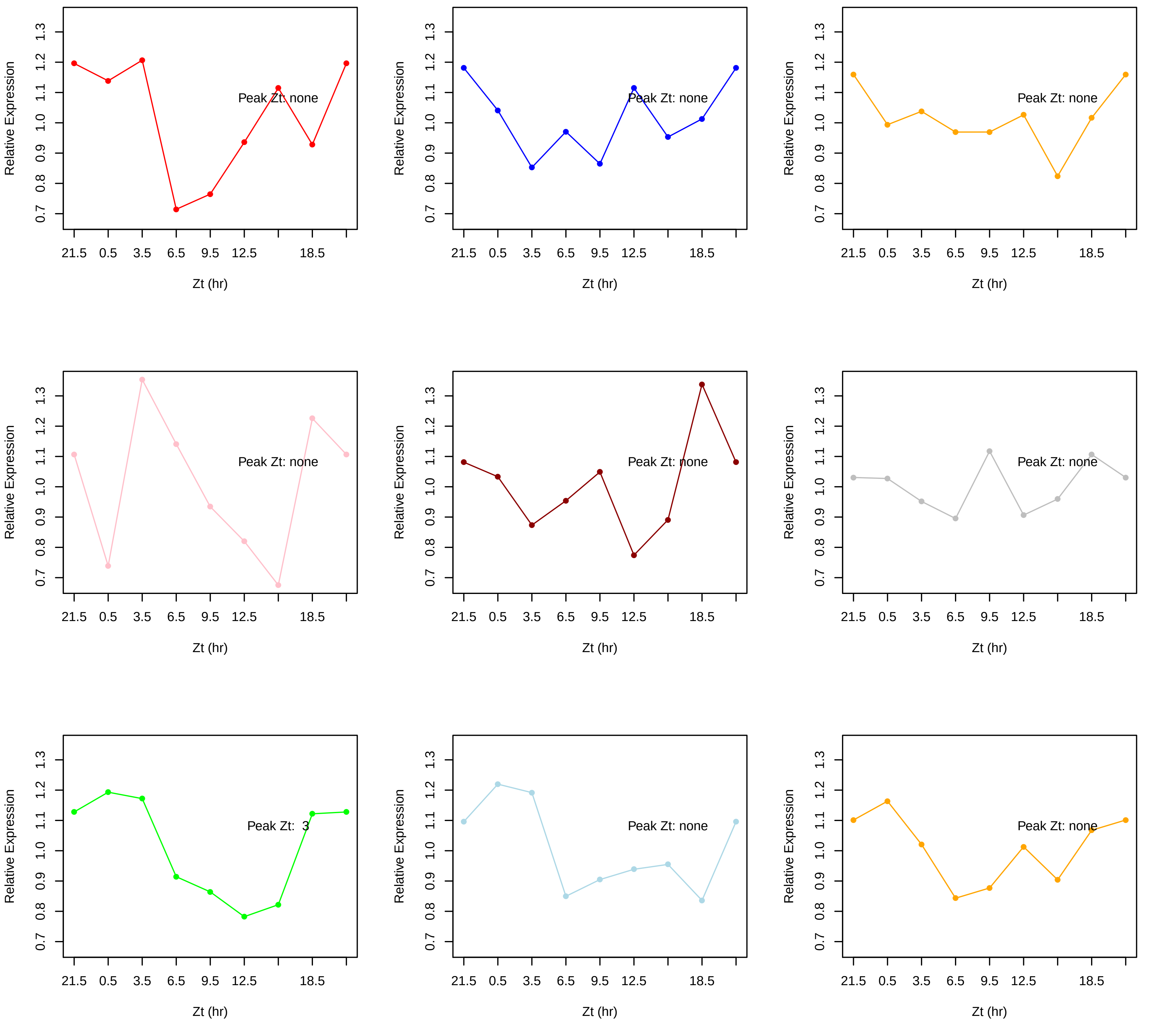

Singleton: ENSXMAG00000000261 GFM1

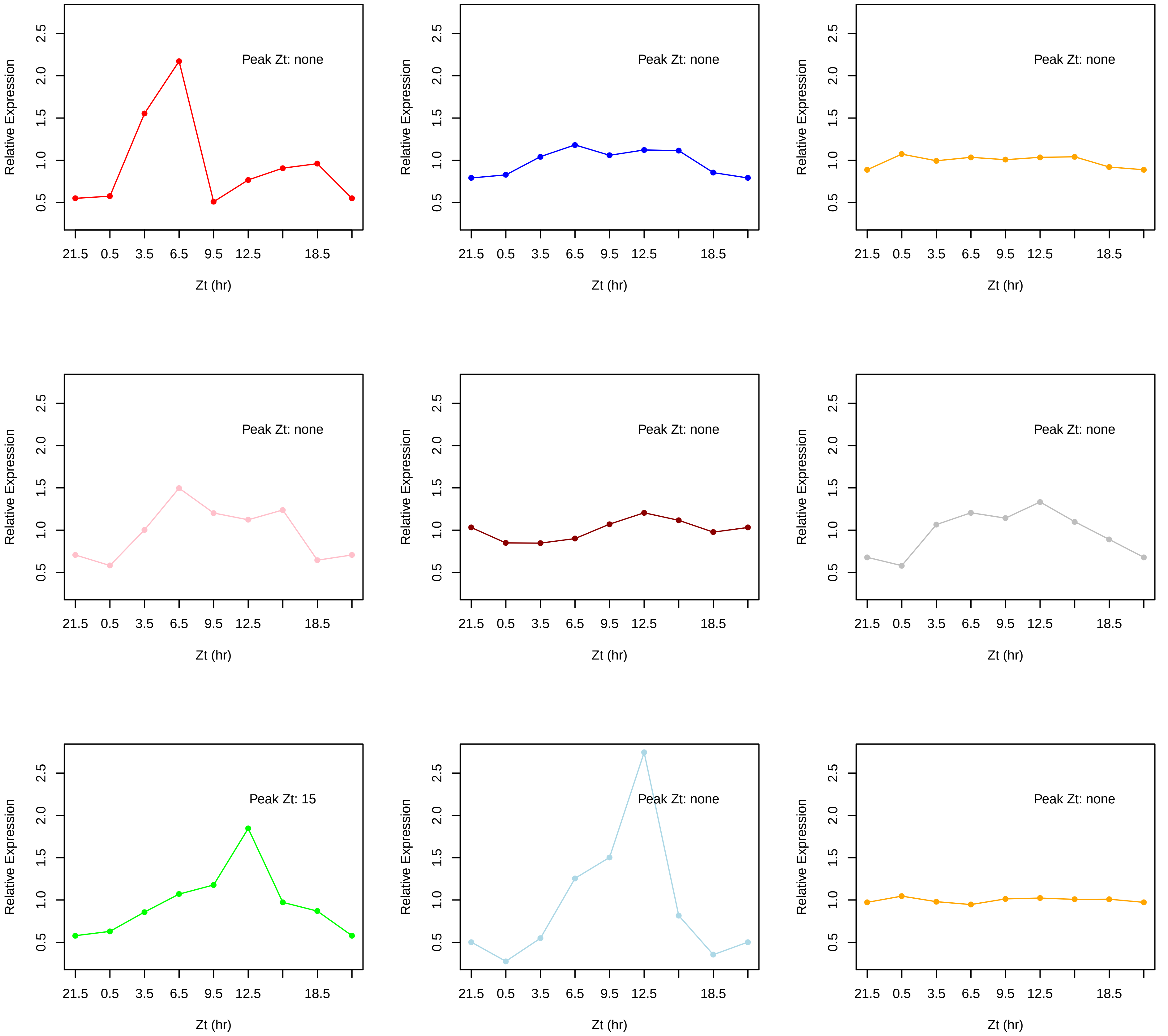

Singeleton: ENSXMAG00000003680 phex

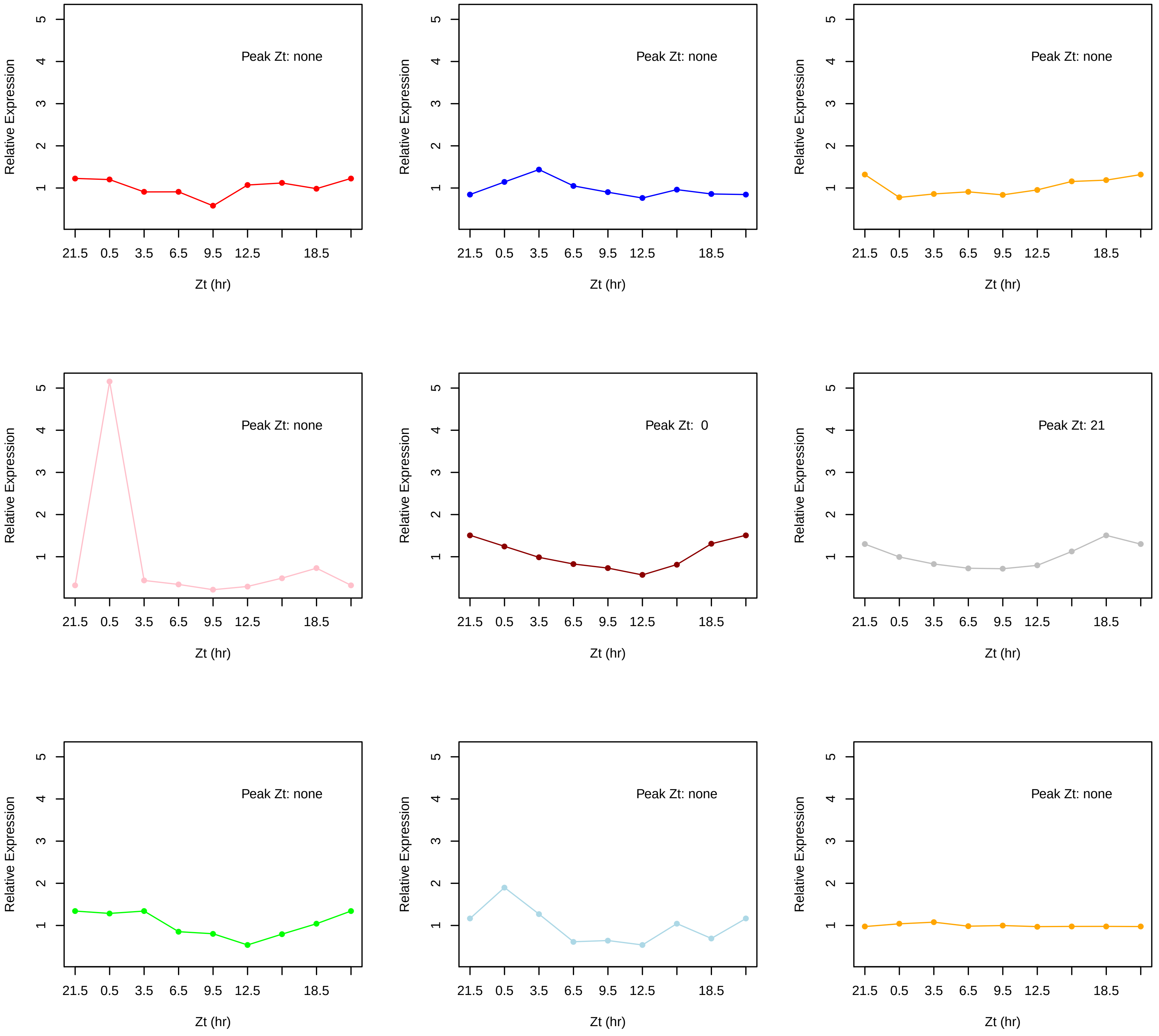

Singeleton: ENSXMAG00000005959 dpagt1

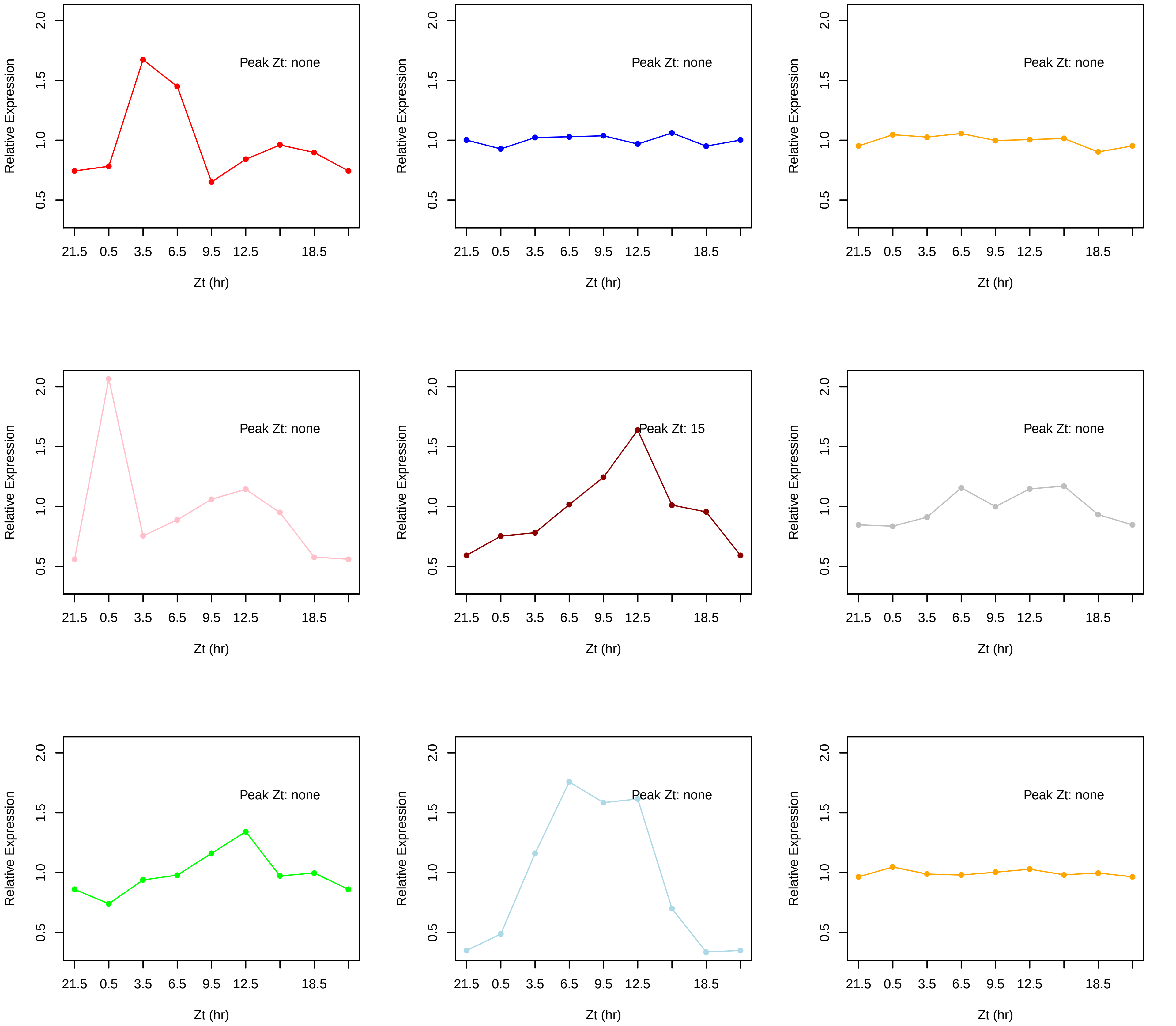

Singeleton: ENSXMAG00000010711 pigv

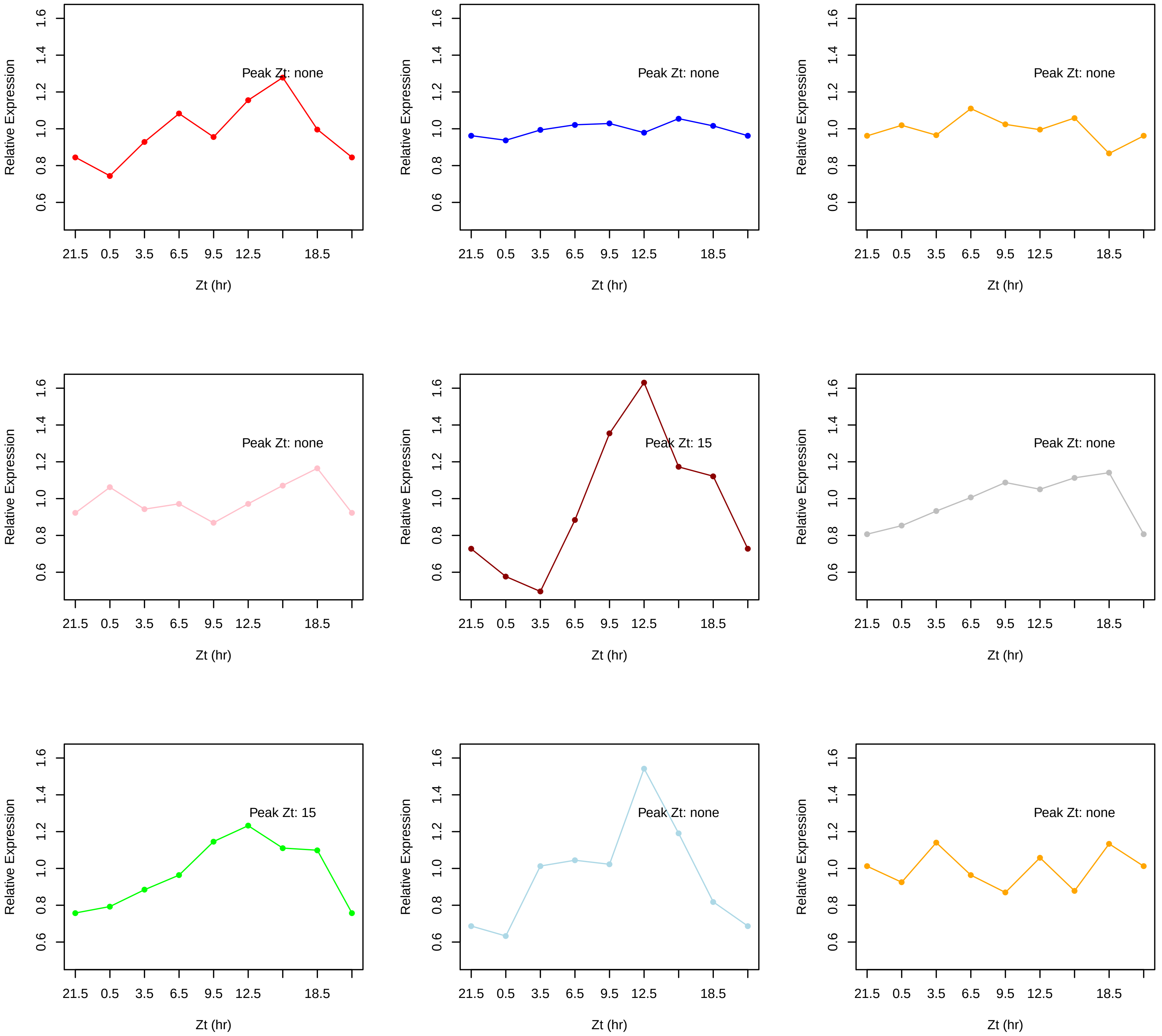

Singeleton: ENSXMAG00000008063 gstt2

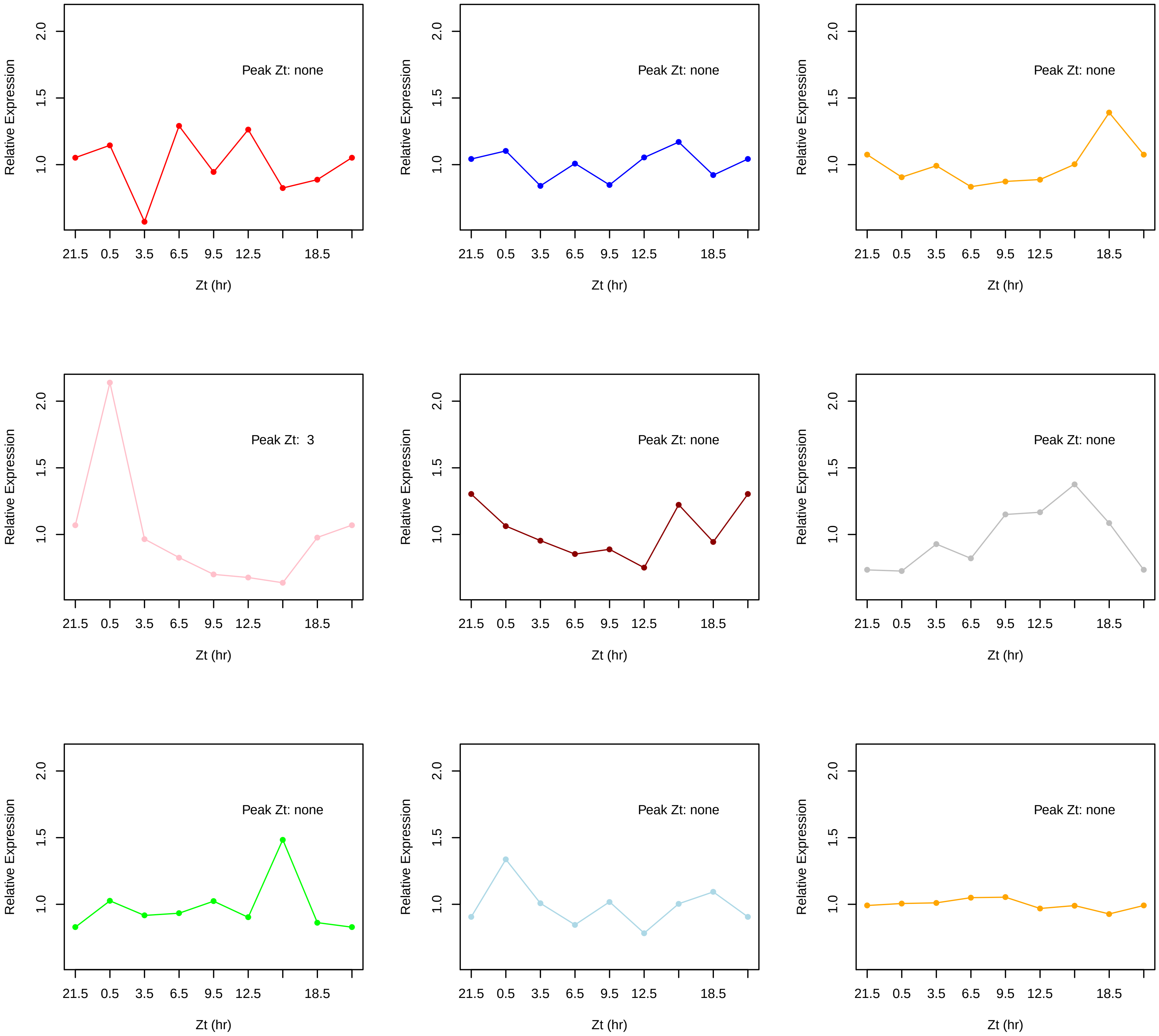

Singeleton: ENSXMAG00000005943 hinfp

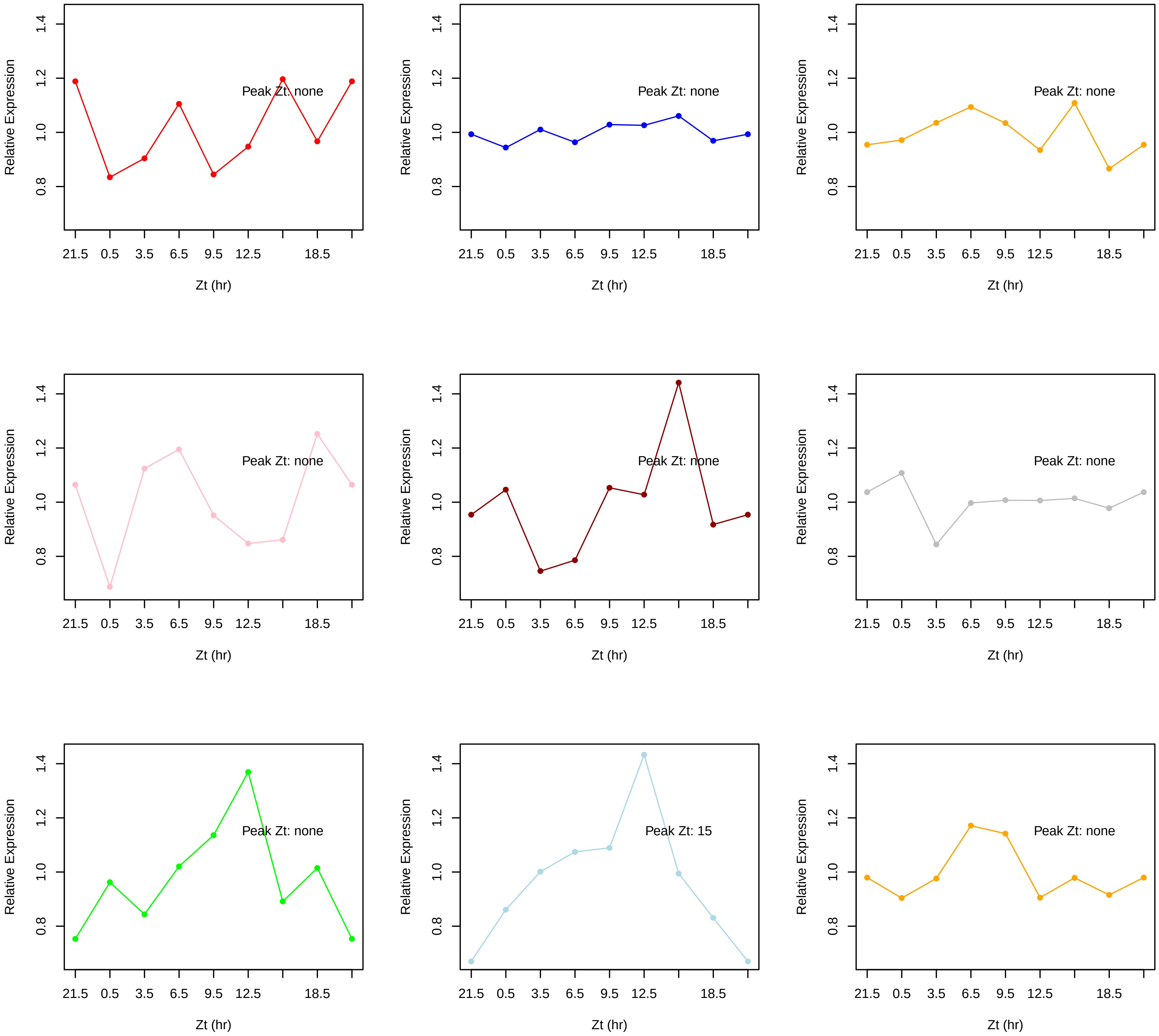

Singeleton: ENSXMAG00000012290 shroom3

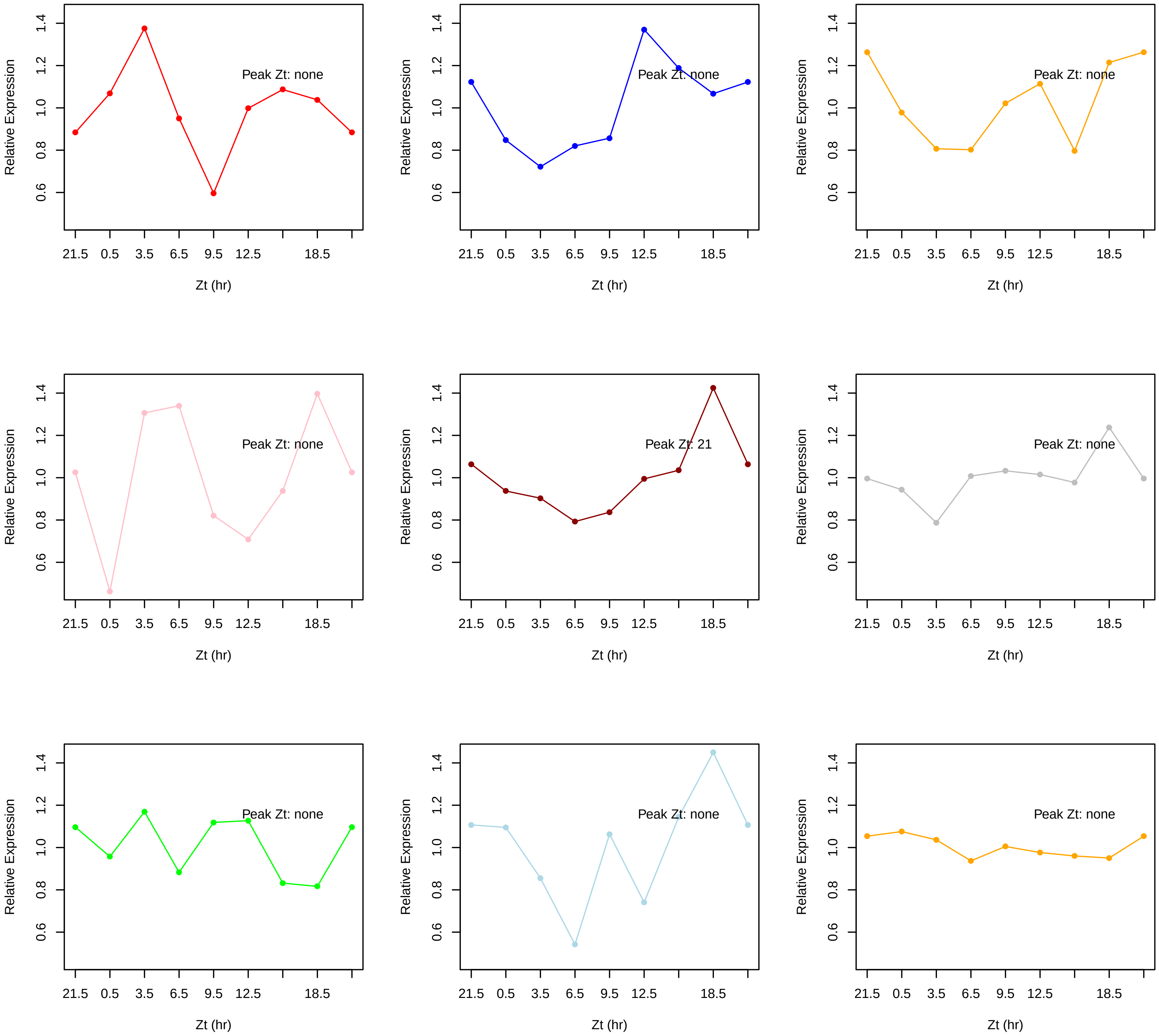

Singeleton: ENSXMAG00000003728 ptchd1

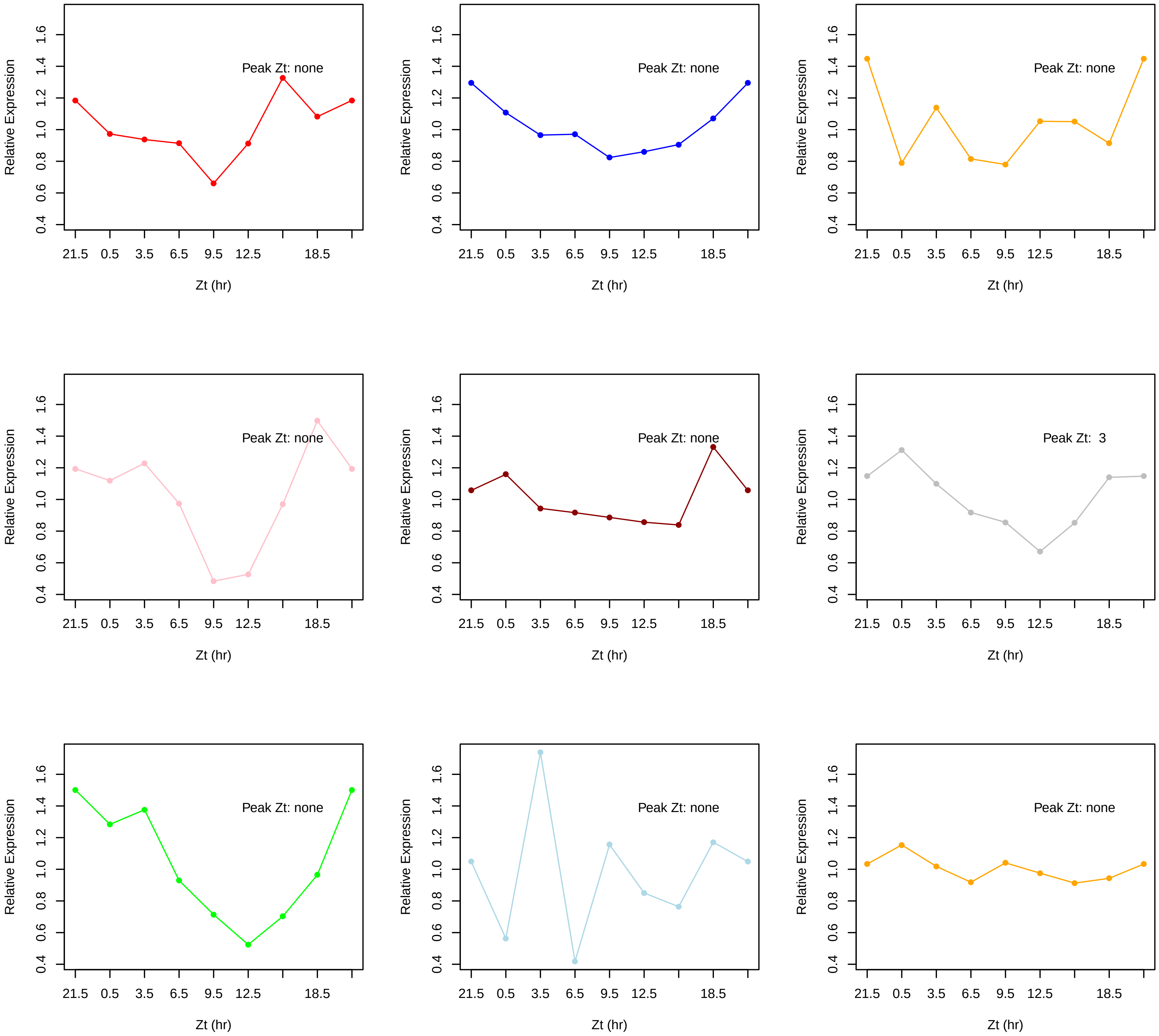

Singeleton: ENSXMAG00000009316

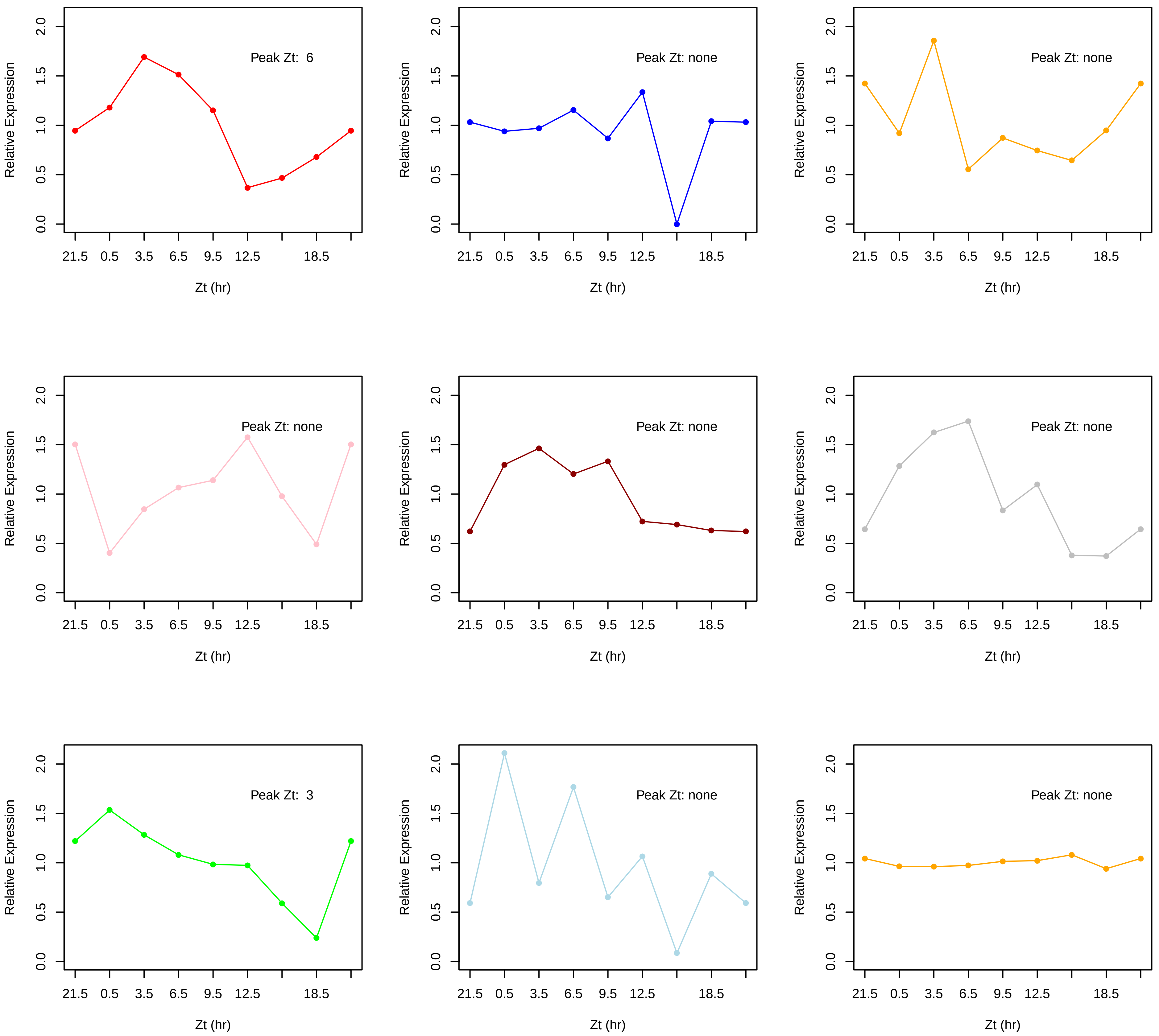

Singleton: ENSXMAG00000000642 ap1m1

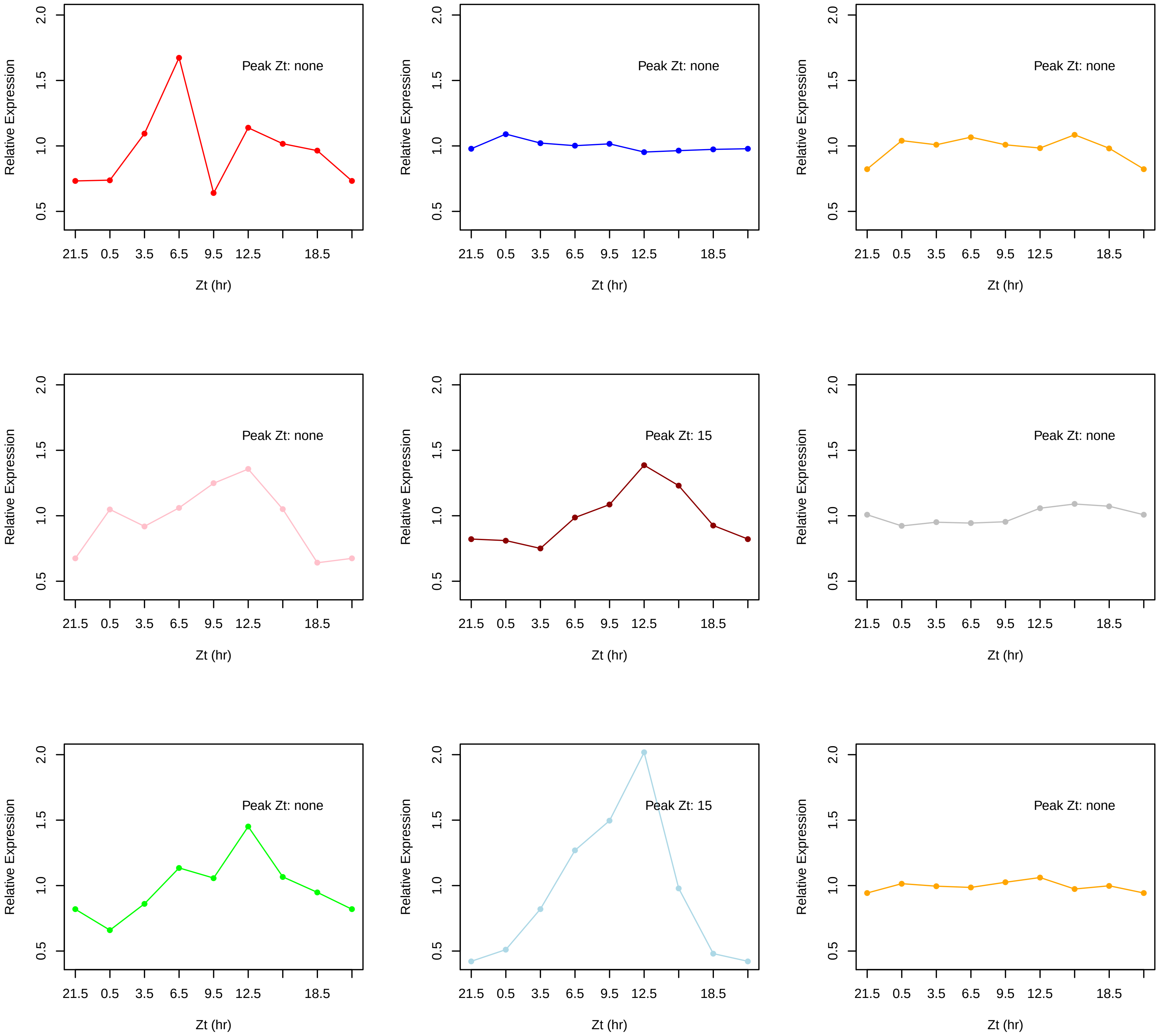

Singeleton: ENSXMAG00000010742 gpn2

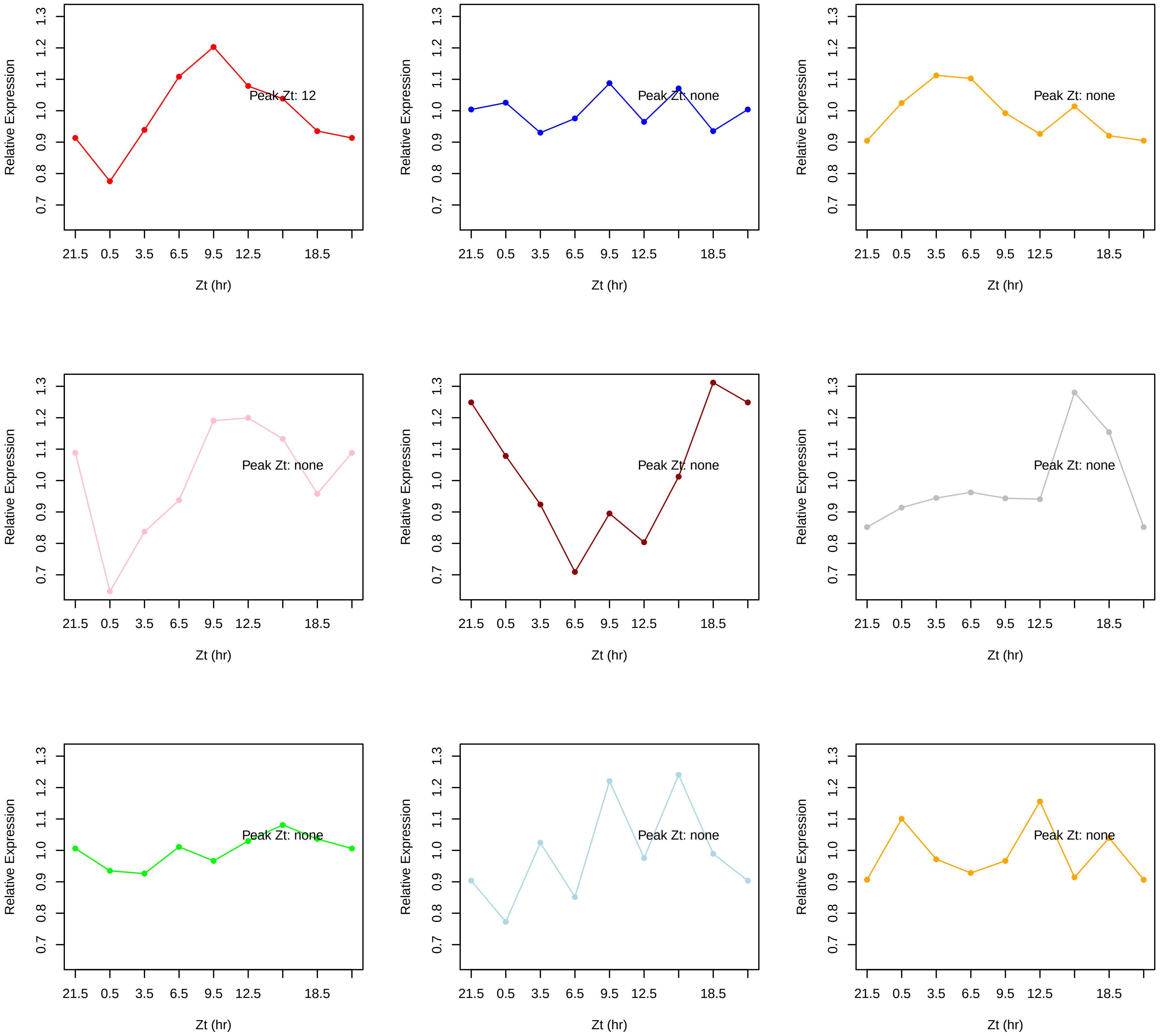

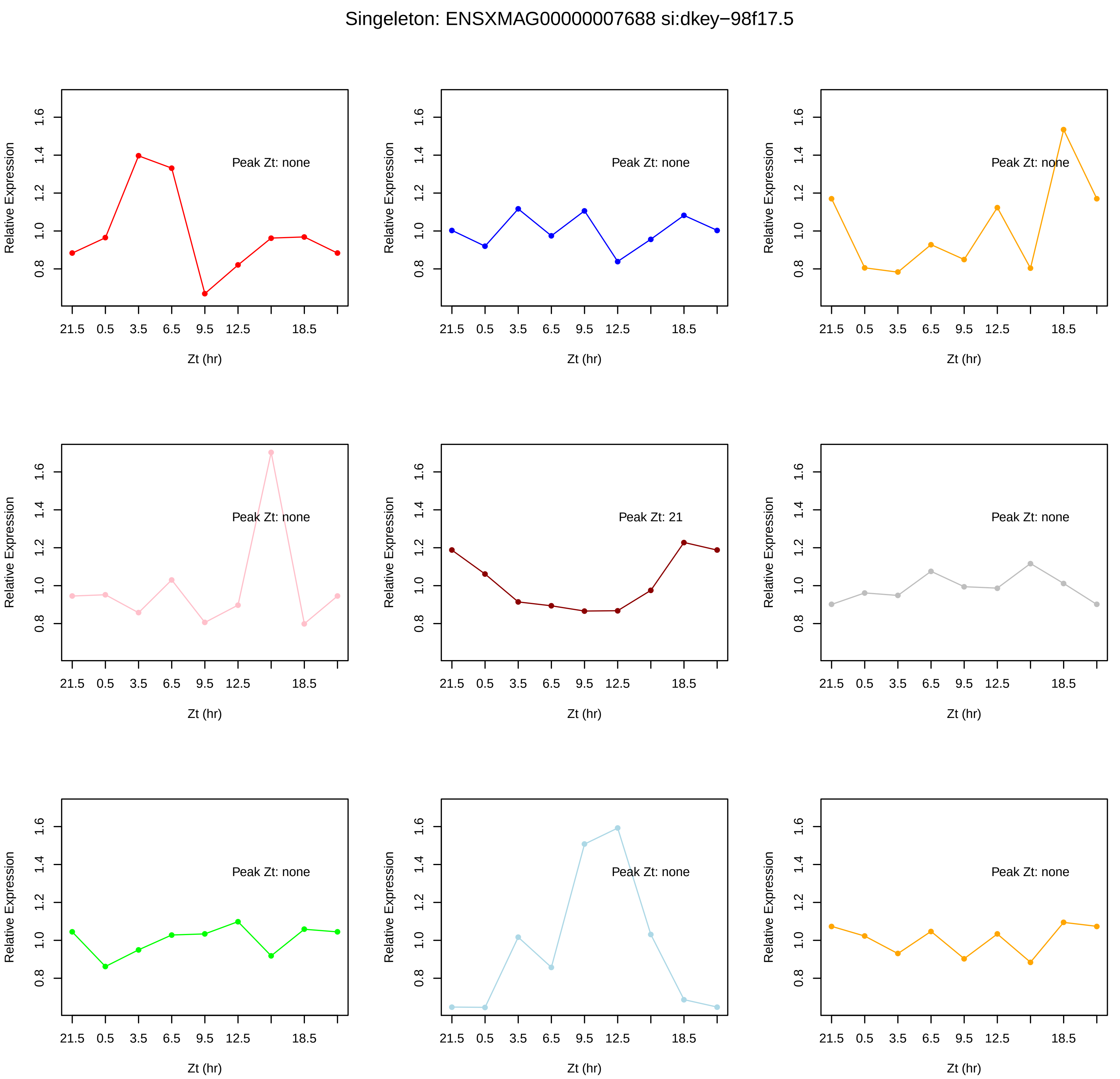

Singeleton: ENSXMAG00000007688 si:dkey−98f17.5
Peak Zt: none
Peak Zt: none
Peak Zt: none
Peak Zt: none
Peak Zt: 21
Peak Zt: none
Peak Zt: none
Peak Zt: none
Peak Zt: none
Relative Expression
Zt (hr)
21.5
0.5
3.5
6.5
9.5
12.5
18.5
0.8
1.0
1.2
1.4
1.6

Singeleton: ENSXMAG00000029199 RSRC1

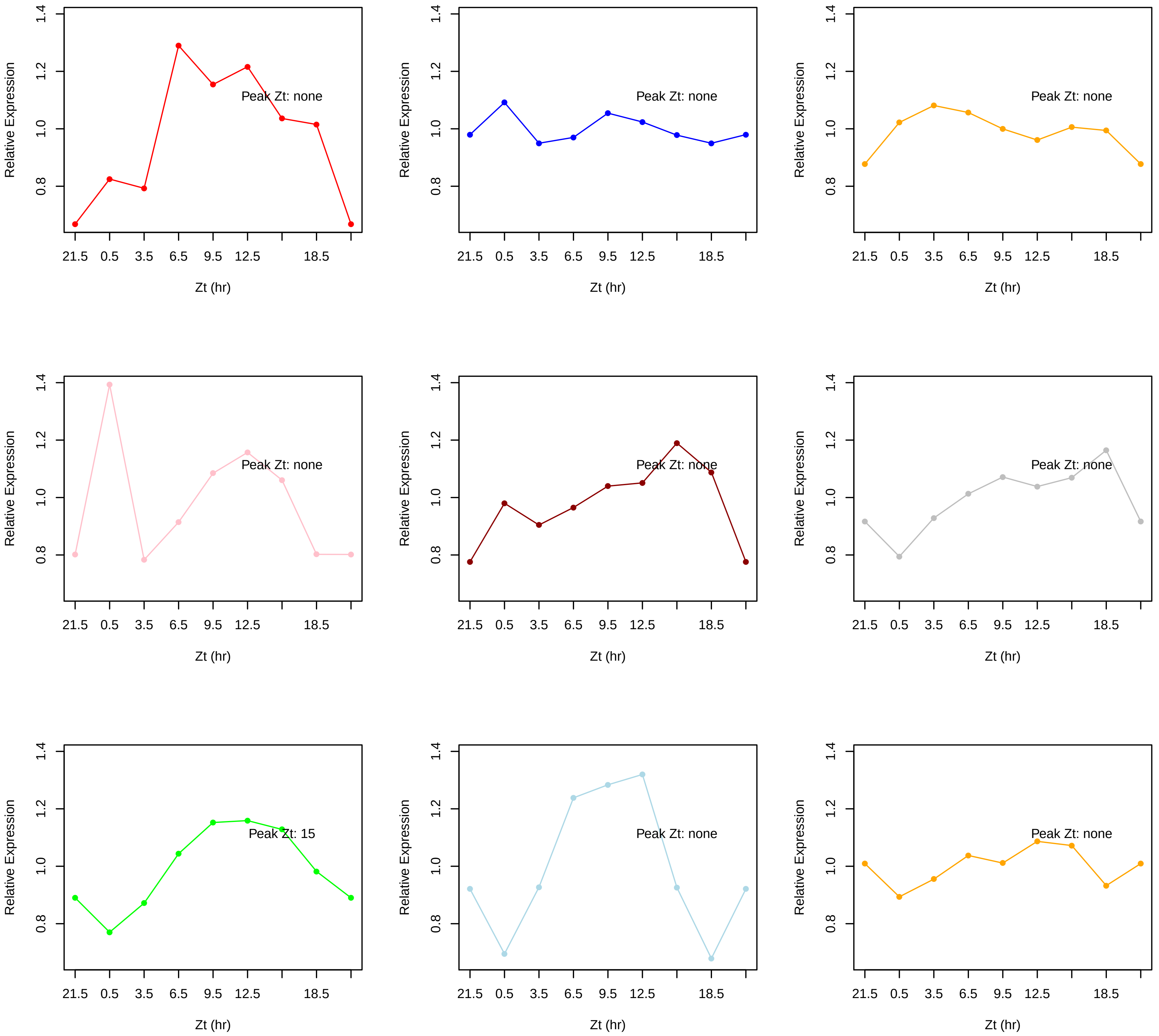

Singeleton: ENSXMAG00000011298 gpsm1b

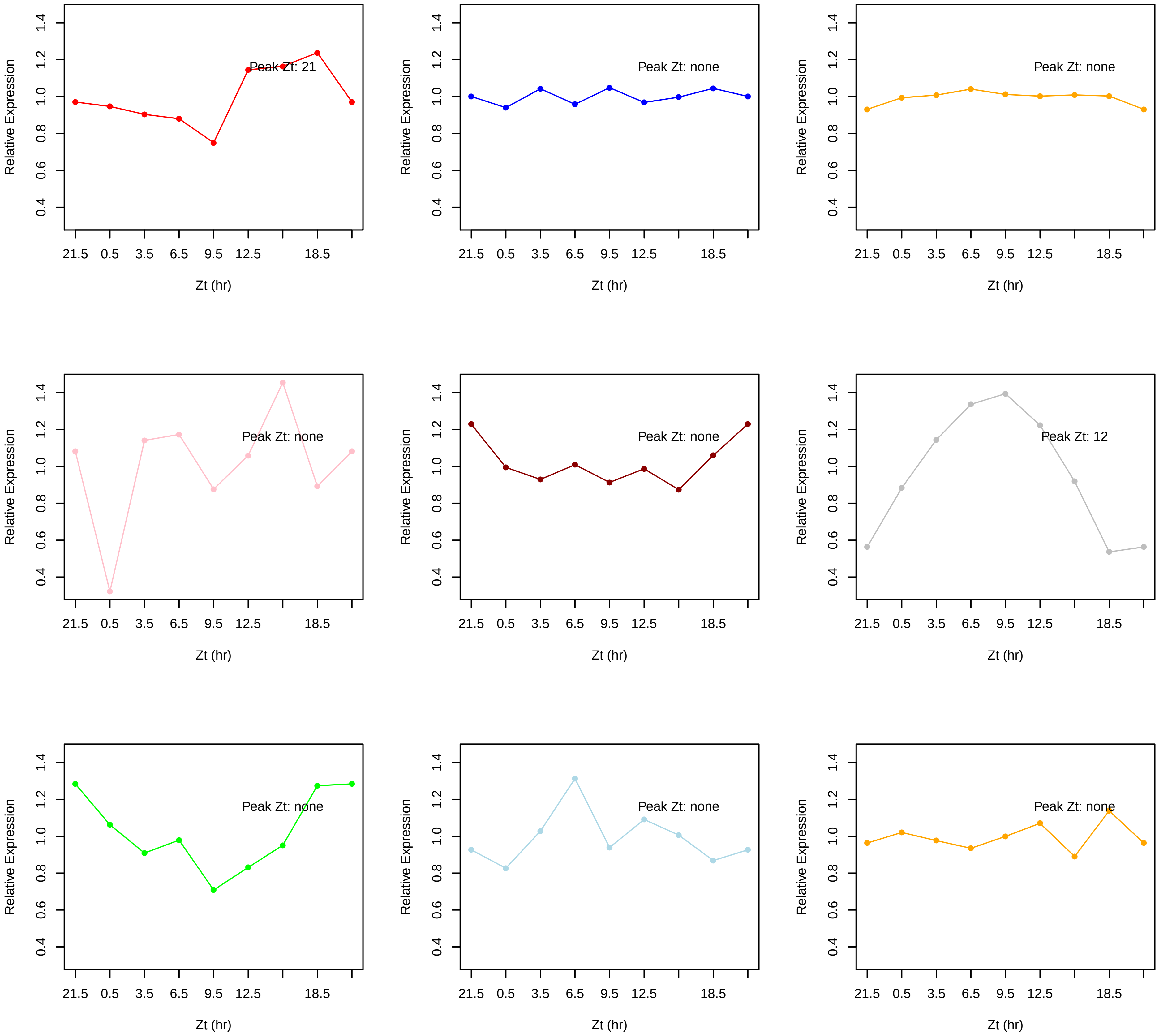

Singleton: ENSXMAG00000010334 trpa1b

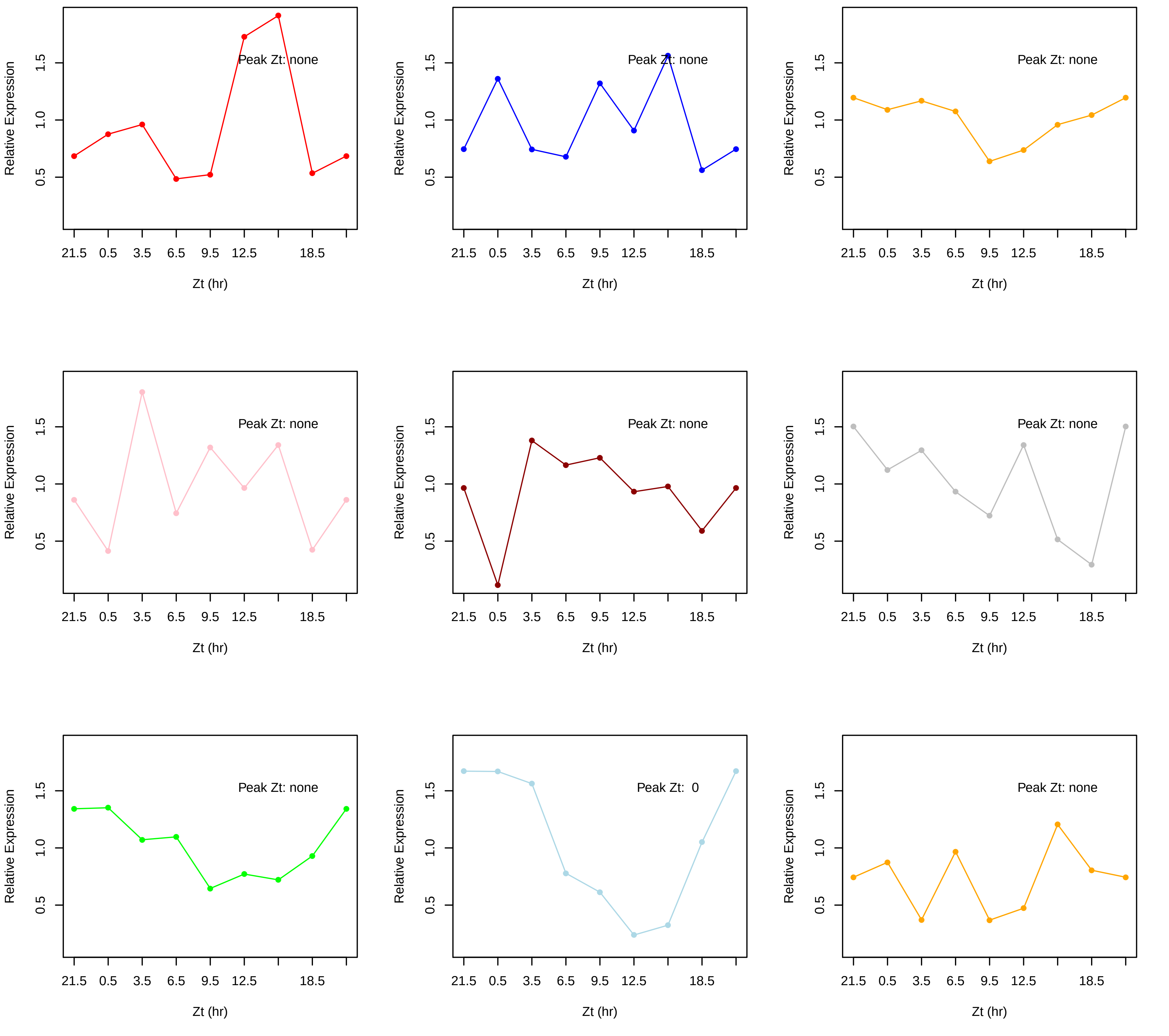

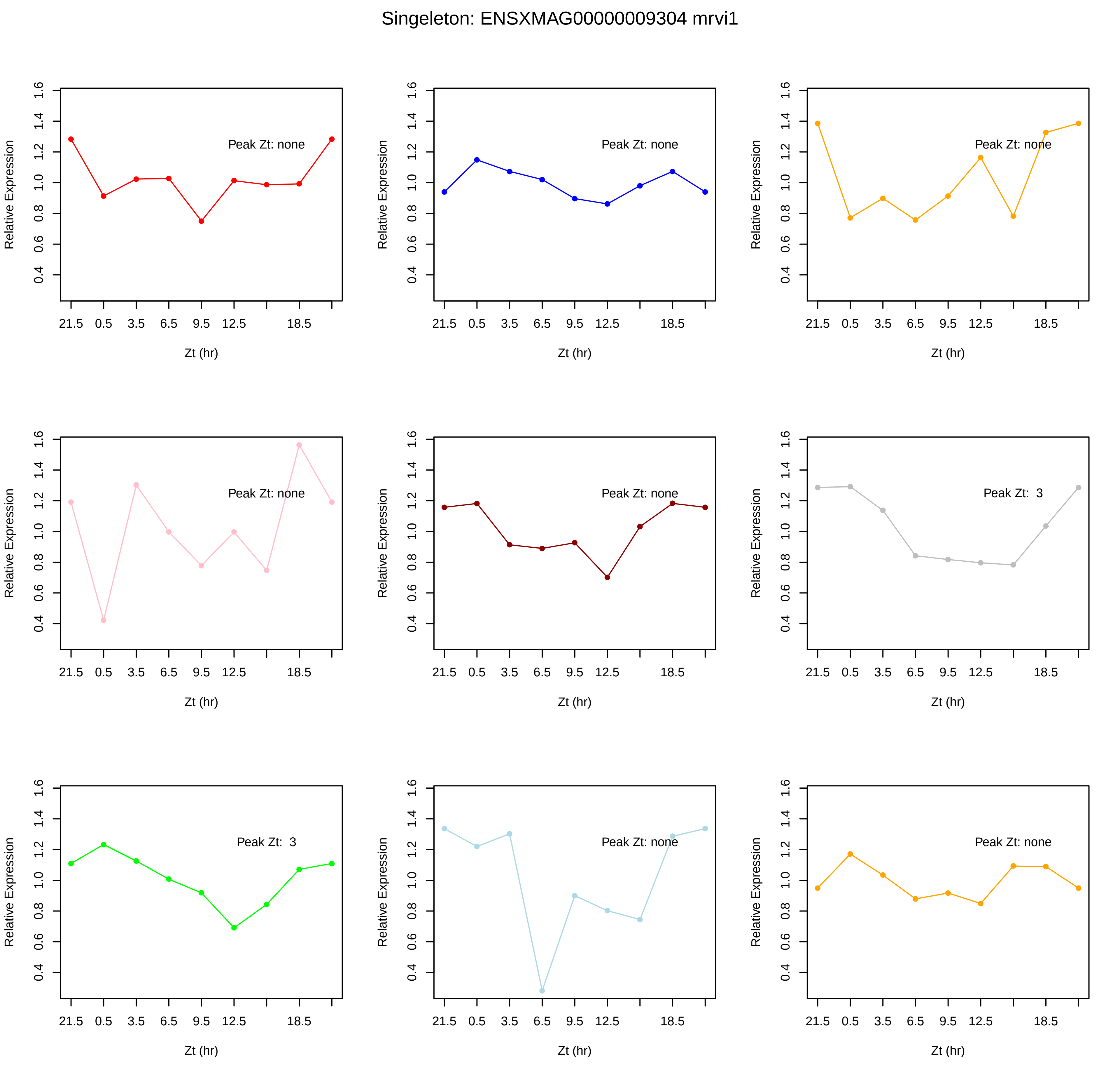

Singleton: ENSXMAG00000009304 mrvi1
Peak Zt: none
Peak Zt: none
Peak Zt: none
Peak Zt: none
Peak Zt: none
Peak Zt: 3
Peak Zt: 3
Peak Zt: none
Peak Zt: none
Relative Expression
Zt (hr)
21.5
0.5
3.5
6.5
9.5
12.5
18.5

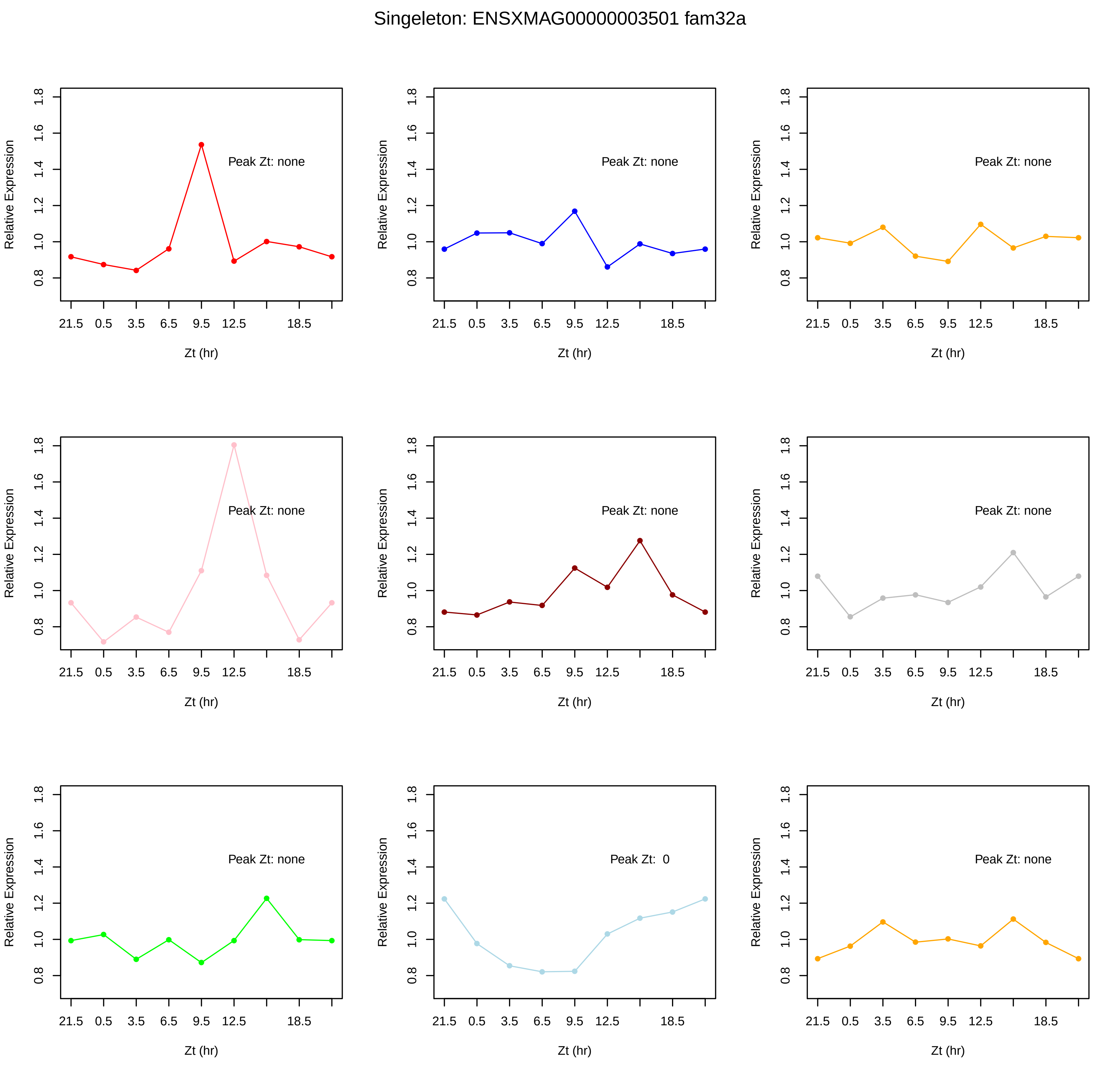
Singeleton: ENSXMAG00000003501 fam32a
Peak Zt: none
Peak Zt: none
Peak Zt: none
Peak Zt: none
Peak Zt: none
Peak Zt: none
Peak Zt: none
Peak Zt: 0
Peak Zt: none
Relative Expression
Zt (hr)

Singleton: ENSXMAG00000016973 tubd1

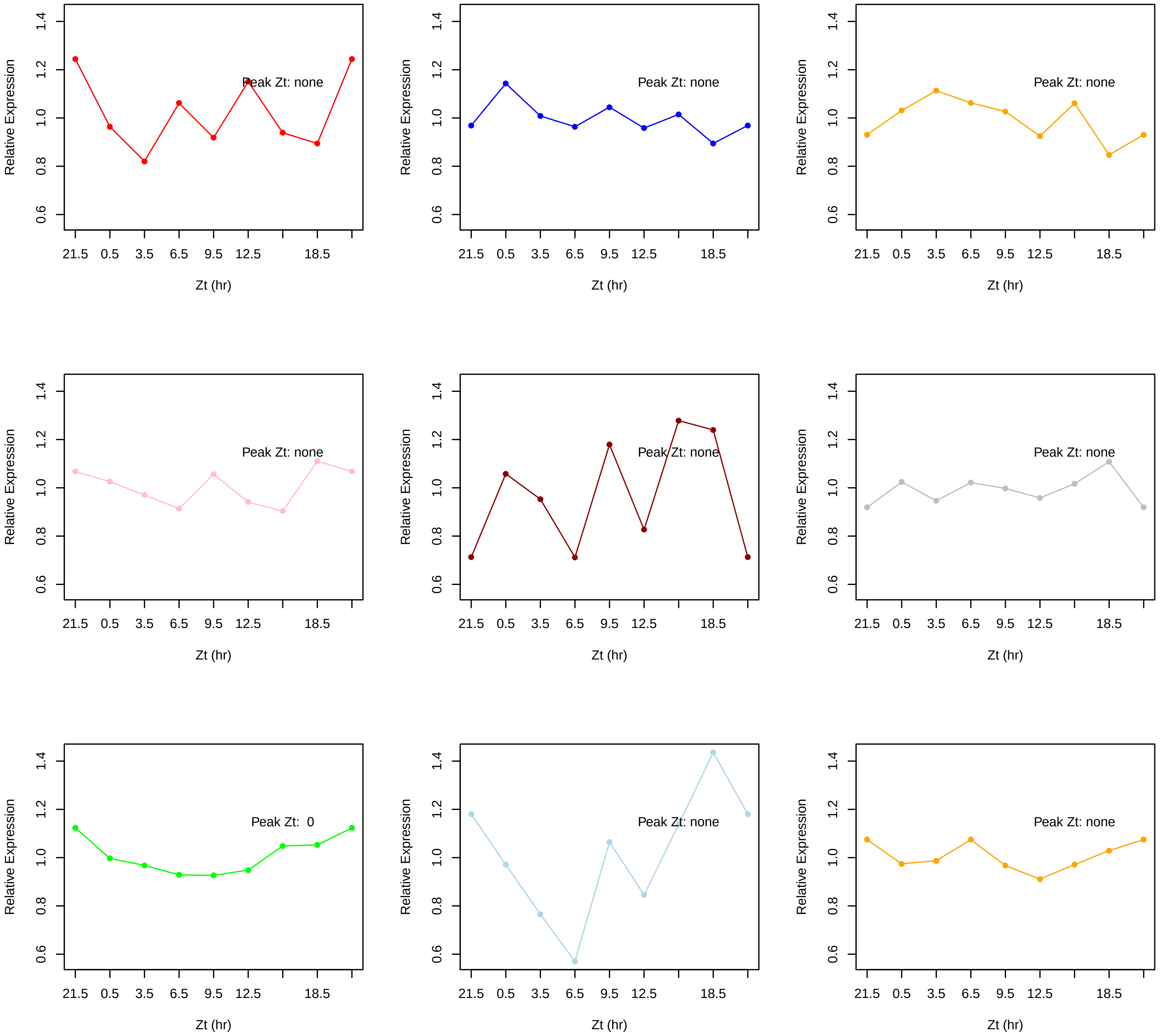

Singleton: ENSXMAG00000025022 qsox2

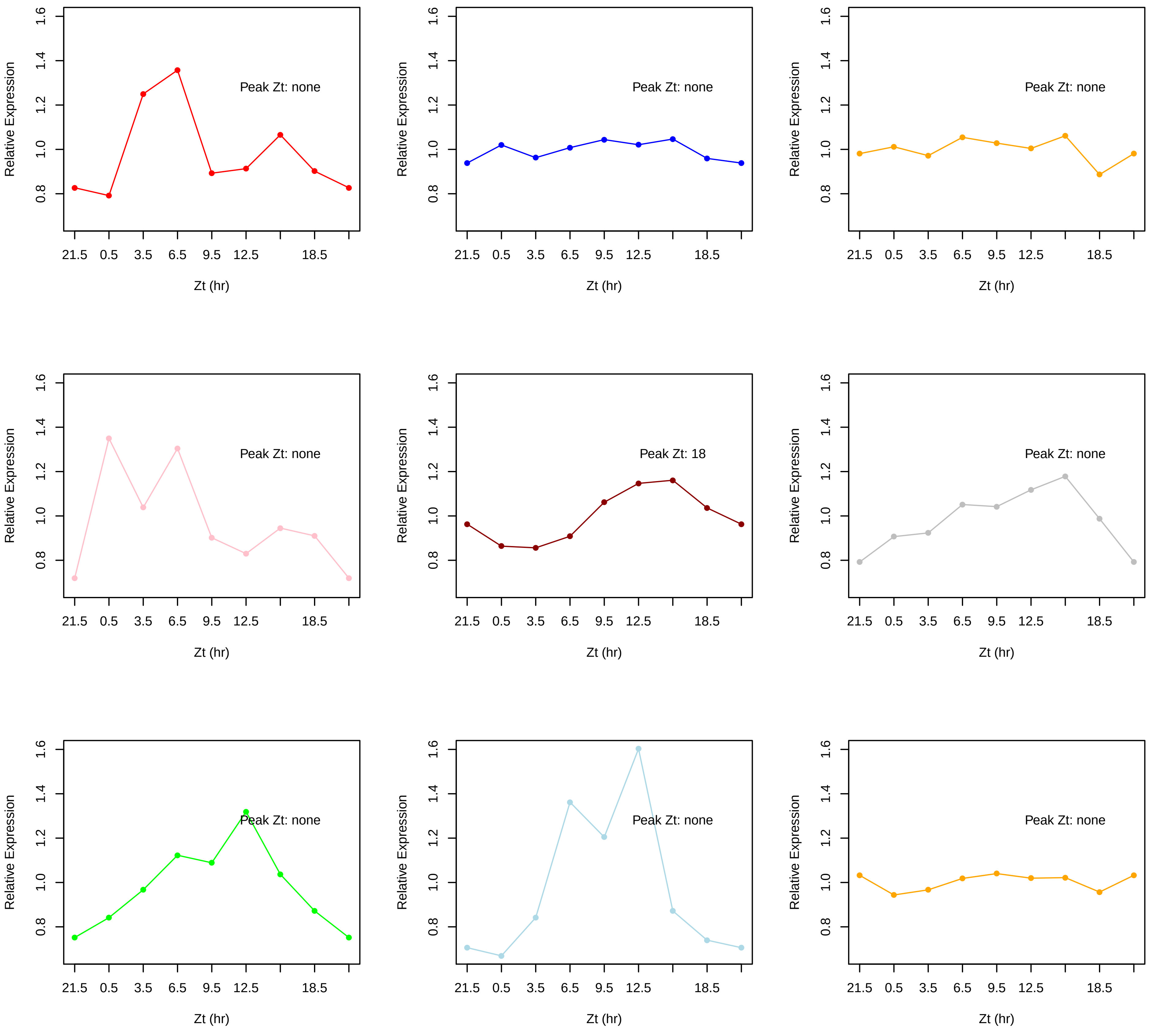

Singeleton: ENSXMAG00000003500 mrpl54

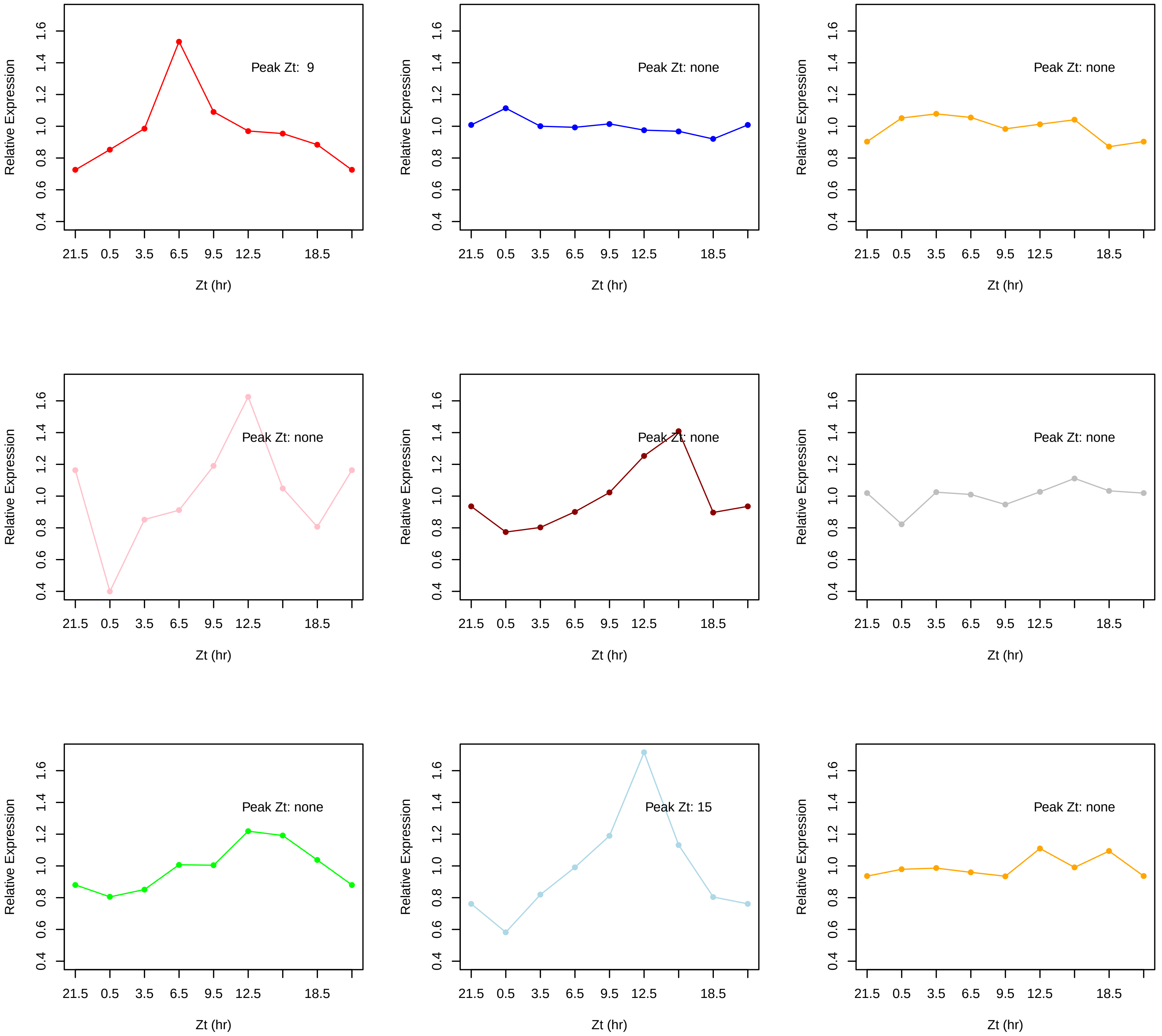

Singeleton: ENSXMAG00000003167 vat1l

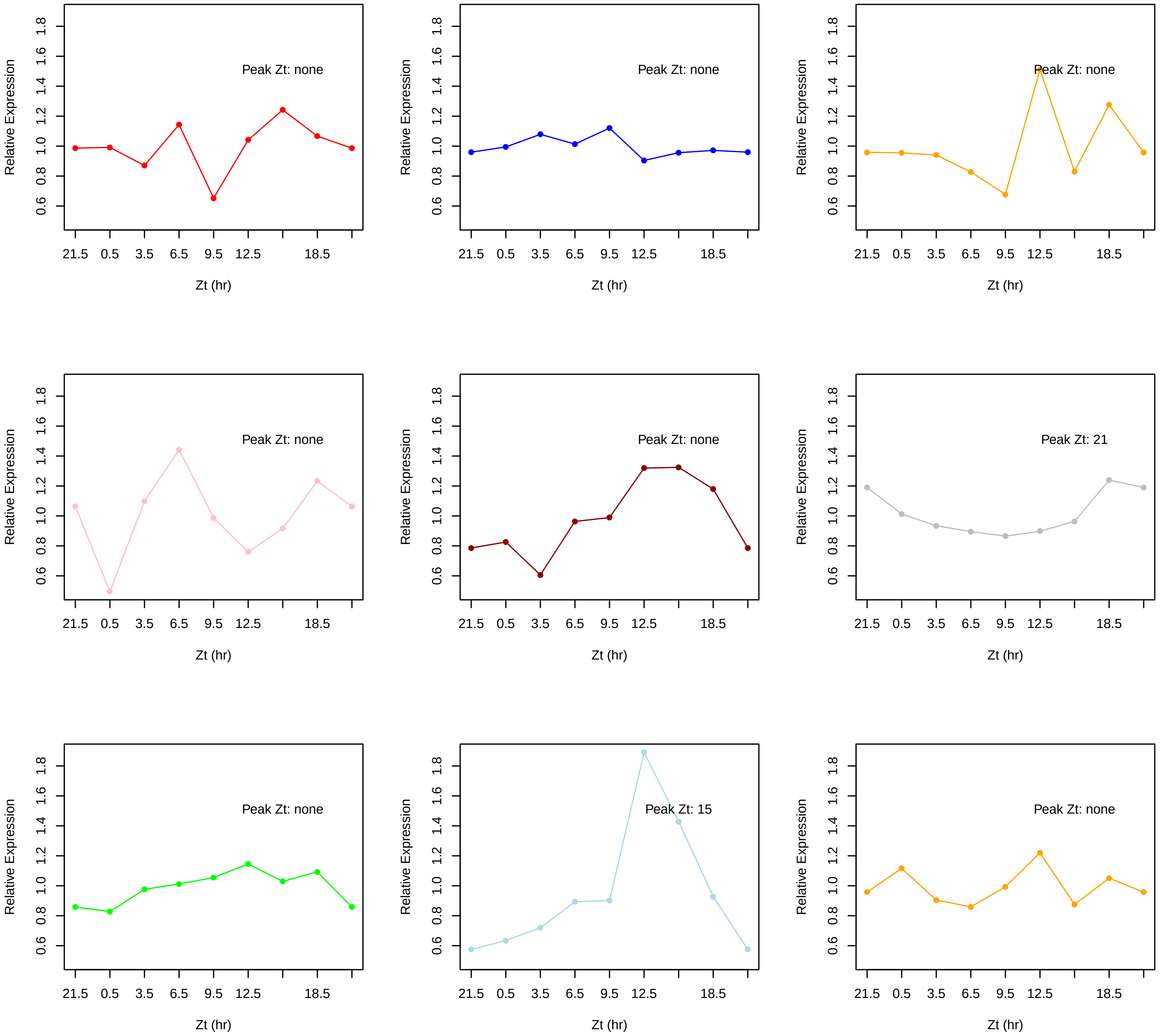

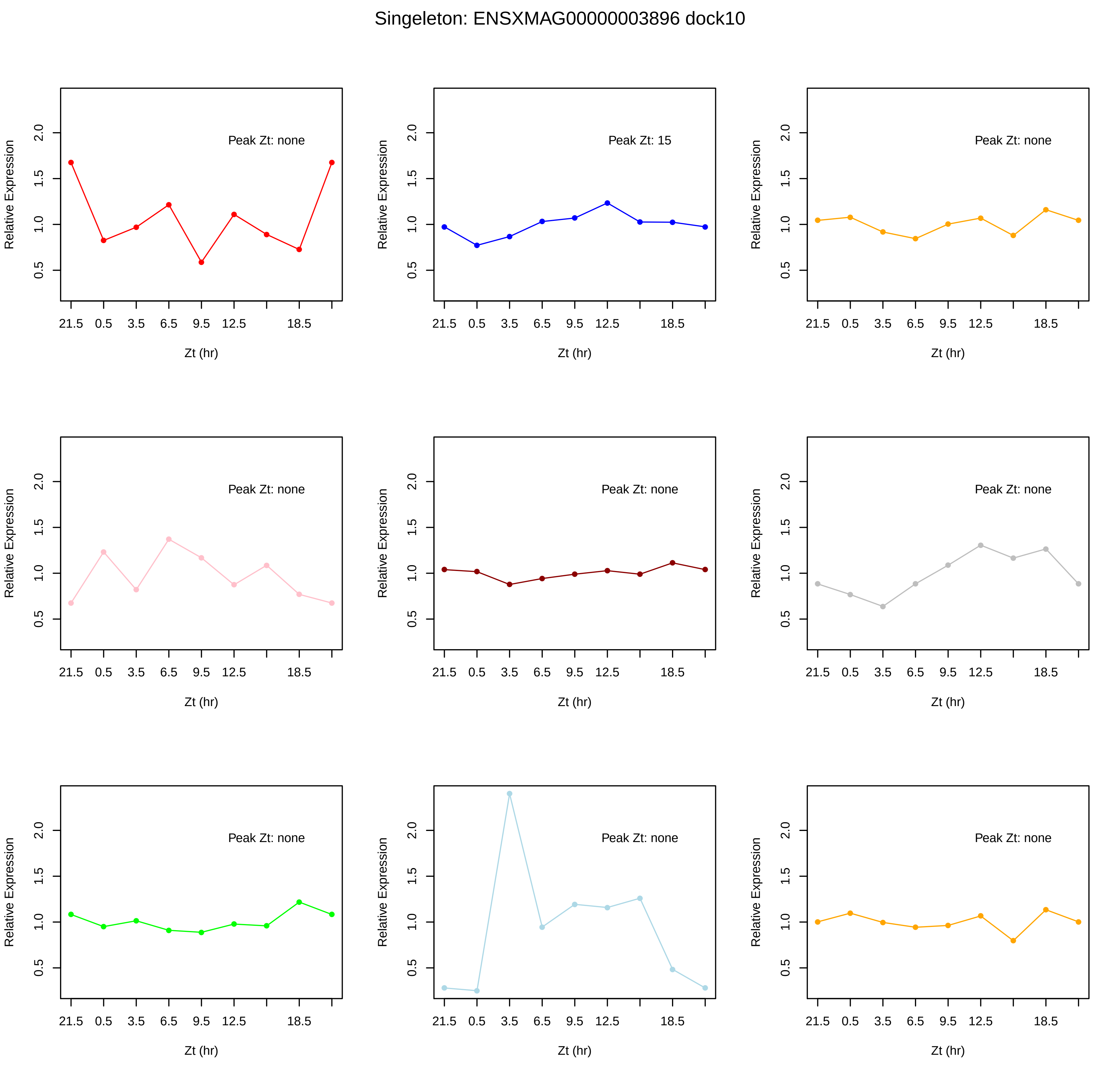

Singeleton: ENSXMAG00000003896 dock10
Peak Zt: none
Peak Zt: 15
Peak Zt: none
Peak Zt: none
Peak Zt: none
Peak Zt: none
Peak Zt: none
Peak Zt: none
Peak Zt: none
Relative Expression
Zt (hr)
21.5
0.5
3.5
6.5
9.5
12.5
18.5
0.5
1.0
1.5
2.0

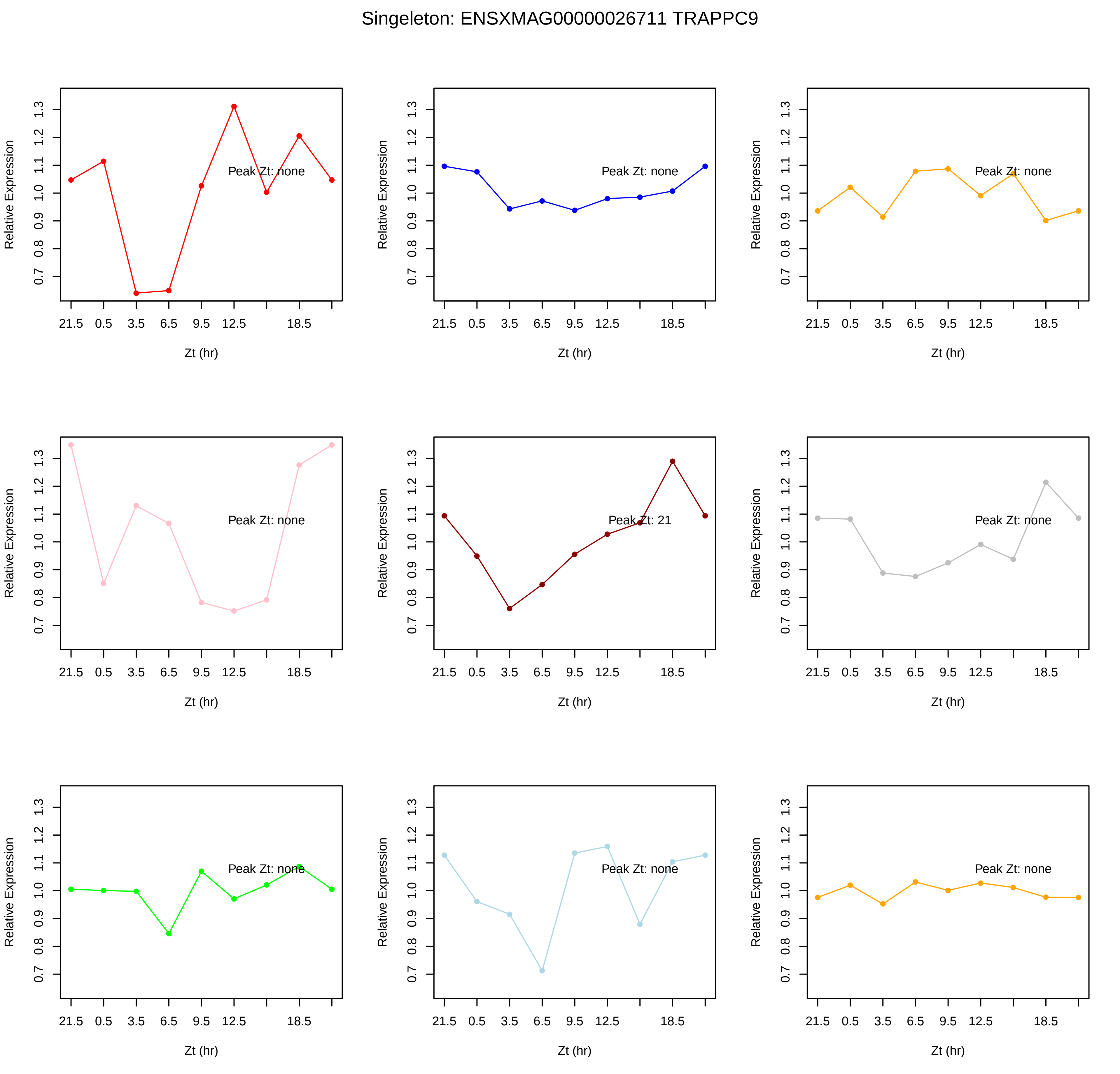
Singeleton: ENSXMAG00000026711 TRAPPC9
Relative Expression
Zt (hr)
Peak Zt: none
Peak Zt: 21
21.5
0.5
3.5
6.5
9.5
12.5
18.5
0.7
0.8
0.9
1.0
1.1
1.2
1.3

Singeleton: ENSXMAG00000003535

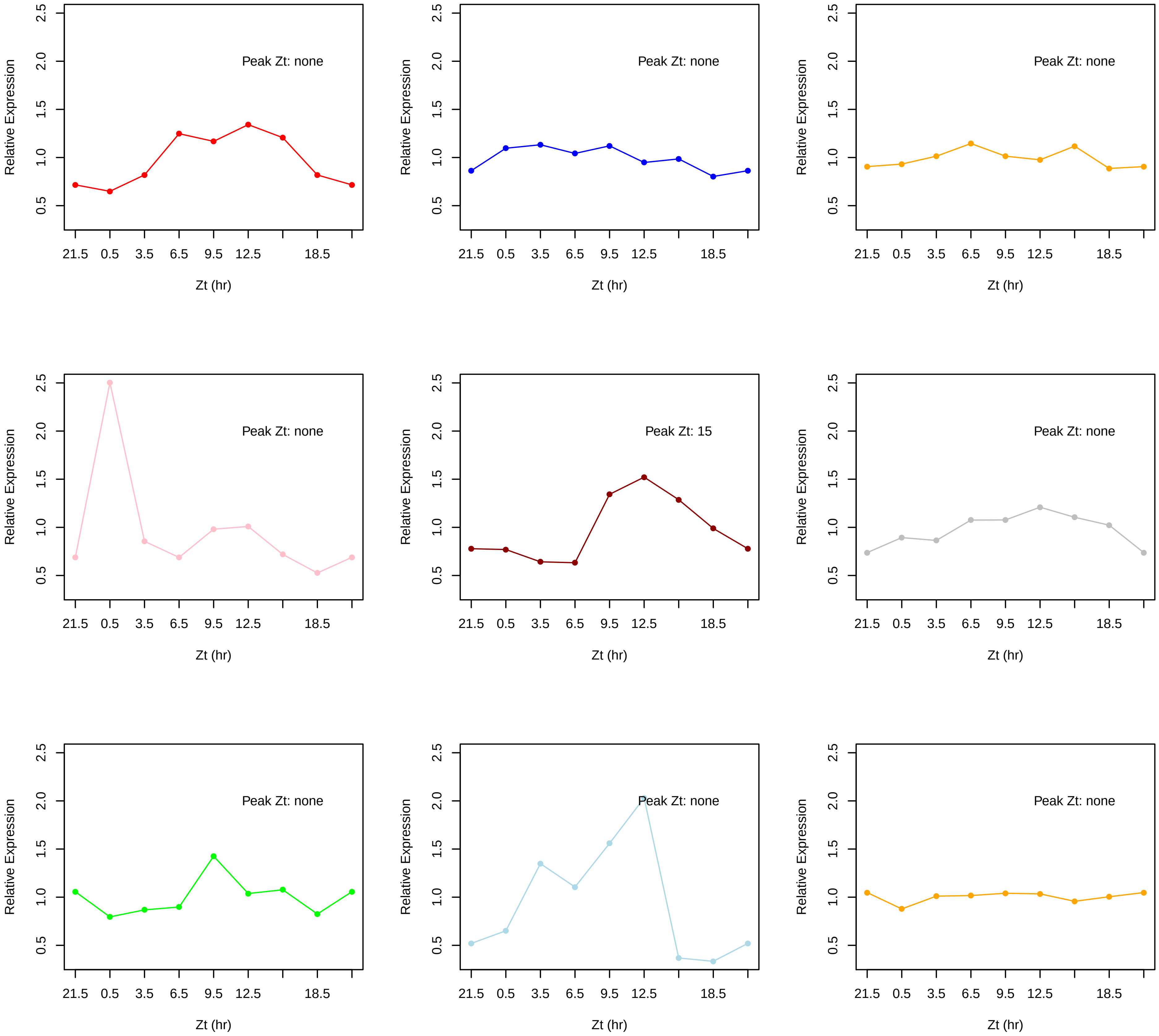

Singleton: ENSXMAG00000013542 antxr1d

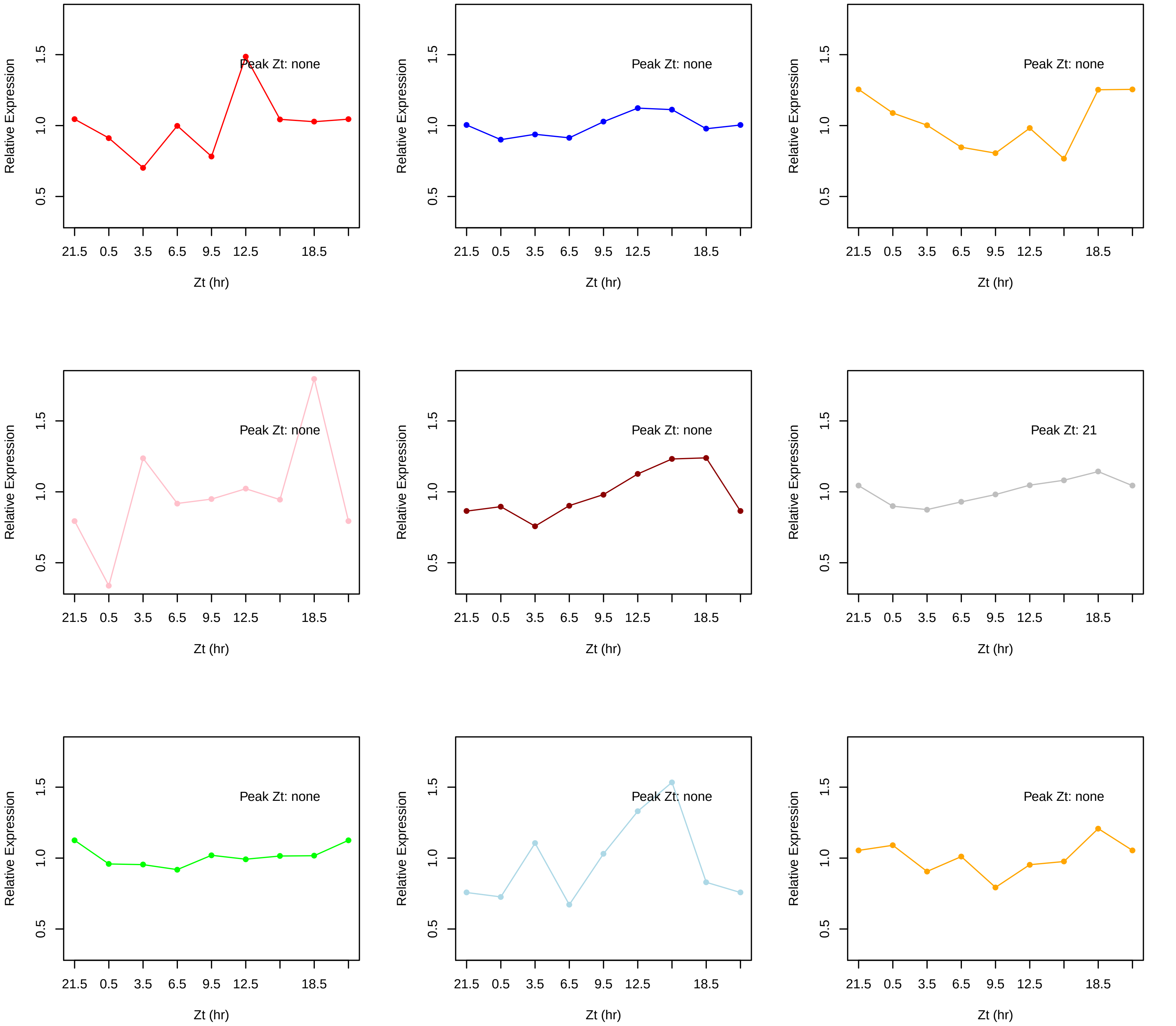

Singeleton: ENSXMAG00000019008 cog7

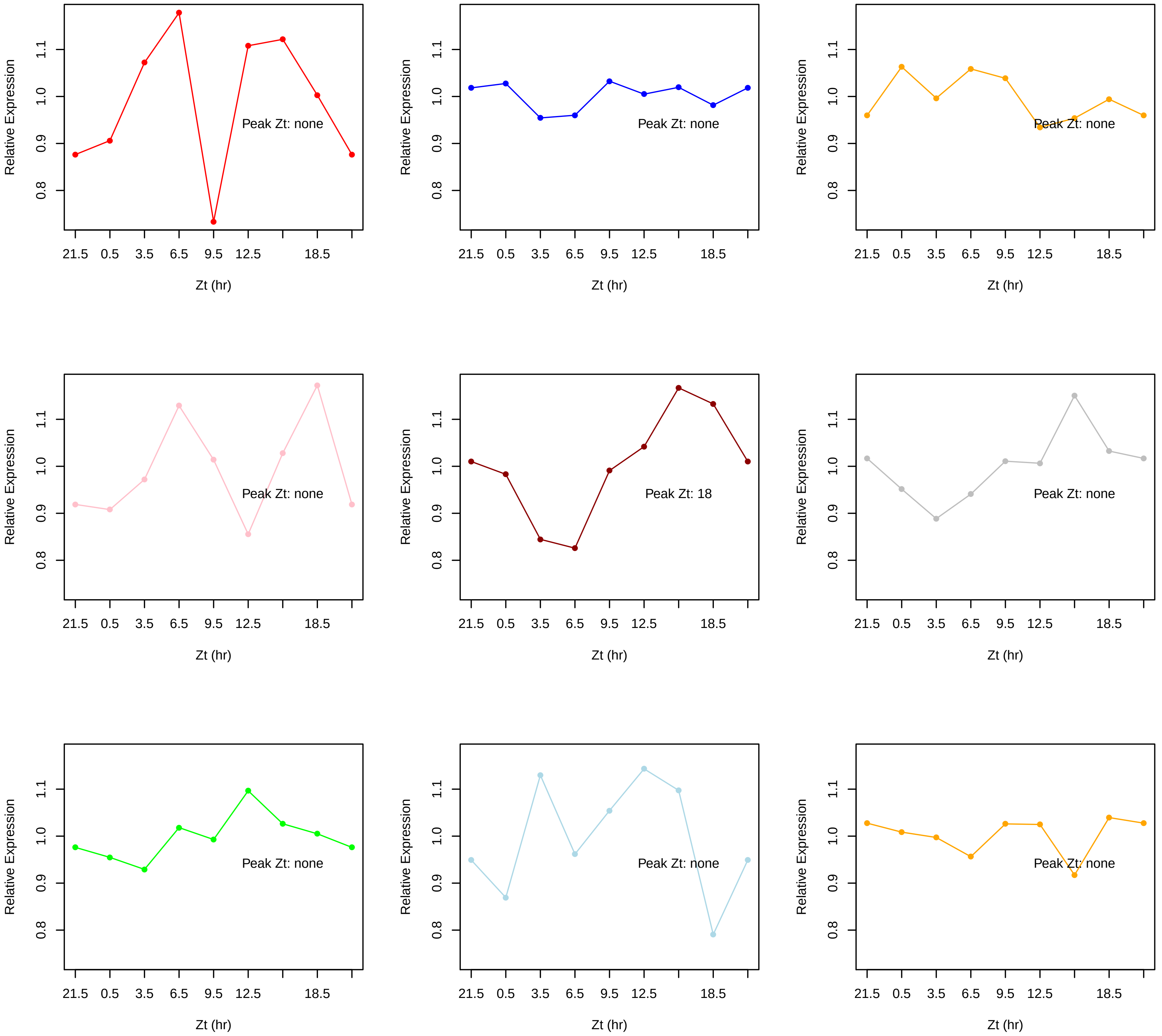

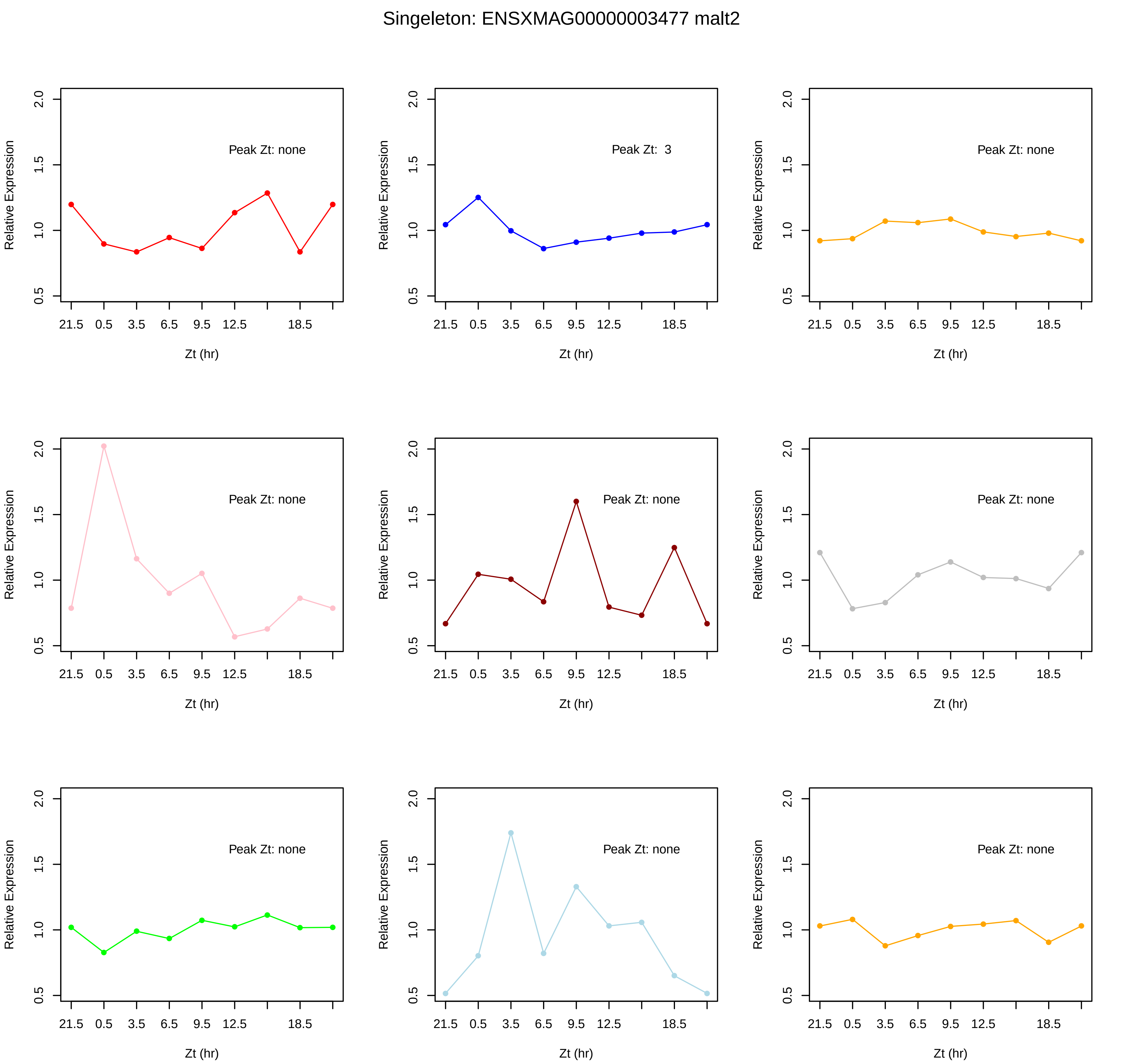

Singeleton: ENSXMAG00000003477 malt2
Peak Zt: none
Peak Zt: 3
Peak Zt: none
Peak Zt: none
Peak Zt: none
Peak Zt: none
Peak Zt: none
Peak Zt: none
Peak Zt: none
Relative Expression
Zt (hr)
21.5
0.5
3.5
6.5
9.5
12.5
18.5
0.5
1.0
1.5
2.0

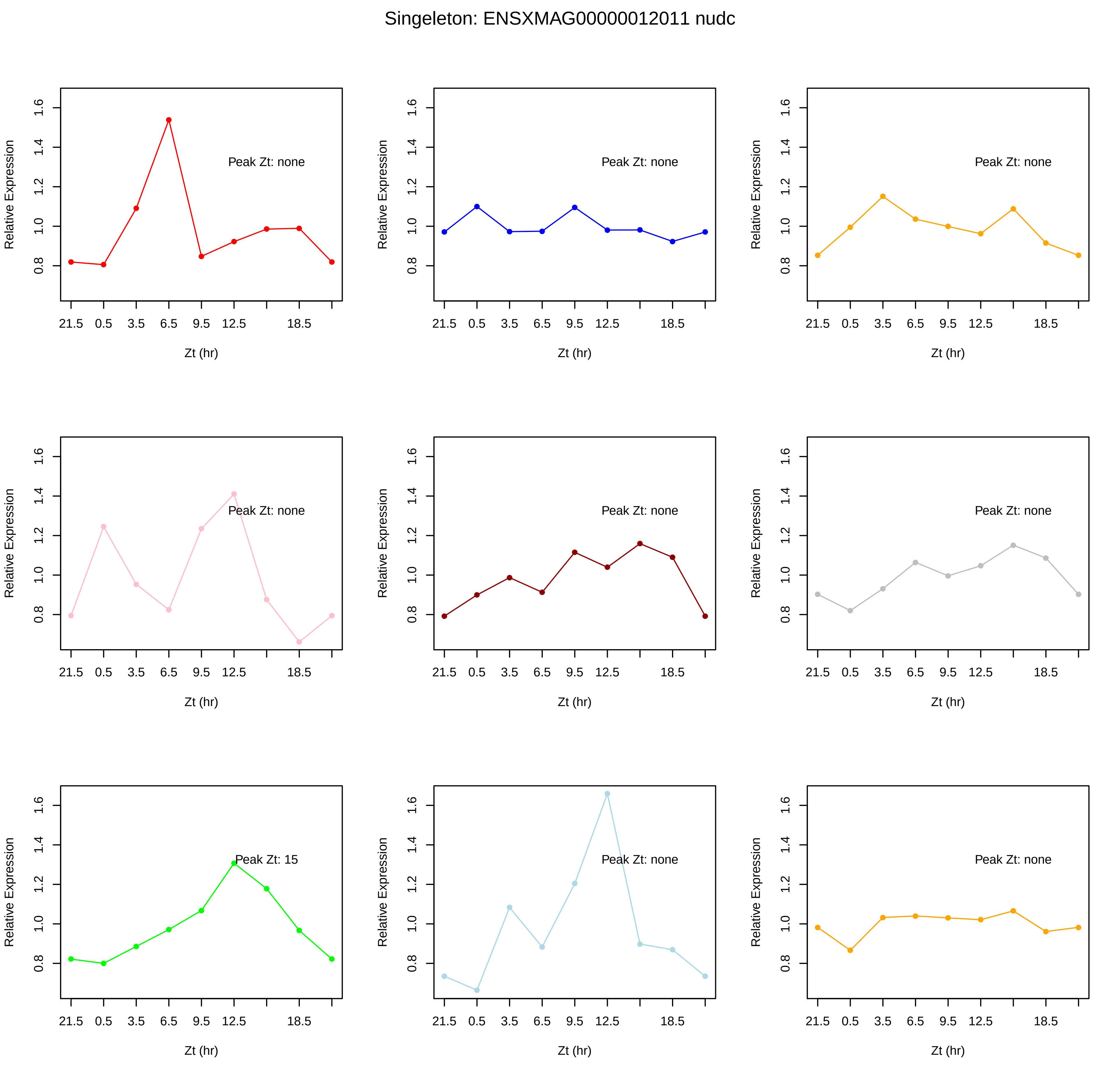

Singeleton: ENSXMAG00000012011 nudc
Peak Zt: none
Peak Zt: none
Peak Zt: none
Peak Zt: none
Peak Zt: none
Peak Zt: none
Peak Zt: 15
Peak Zt: none
Peak Zt: none
Relative Expression
Zt (hr)
21.5
0.5
3.5
6.5
9.5
12.5
18.5
0.8
1.0
1.2
1.4
1.6

Singeleton: ENSXMAG00000011308 LHX3

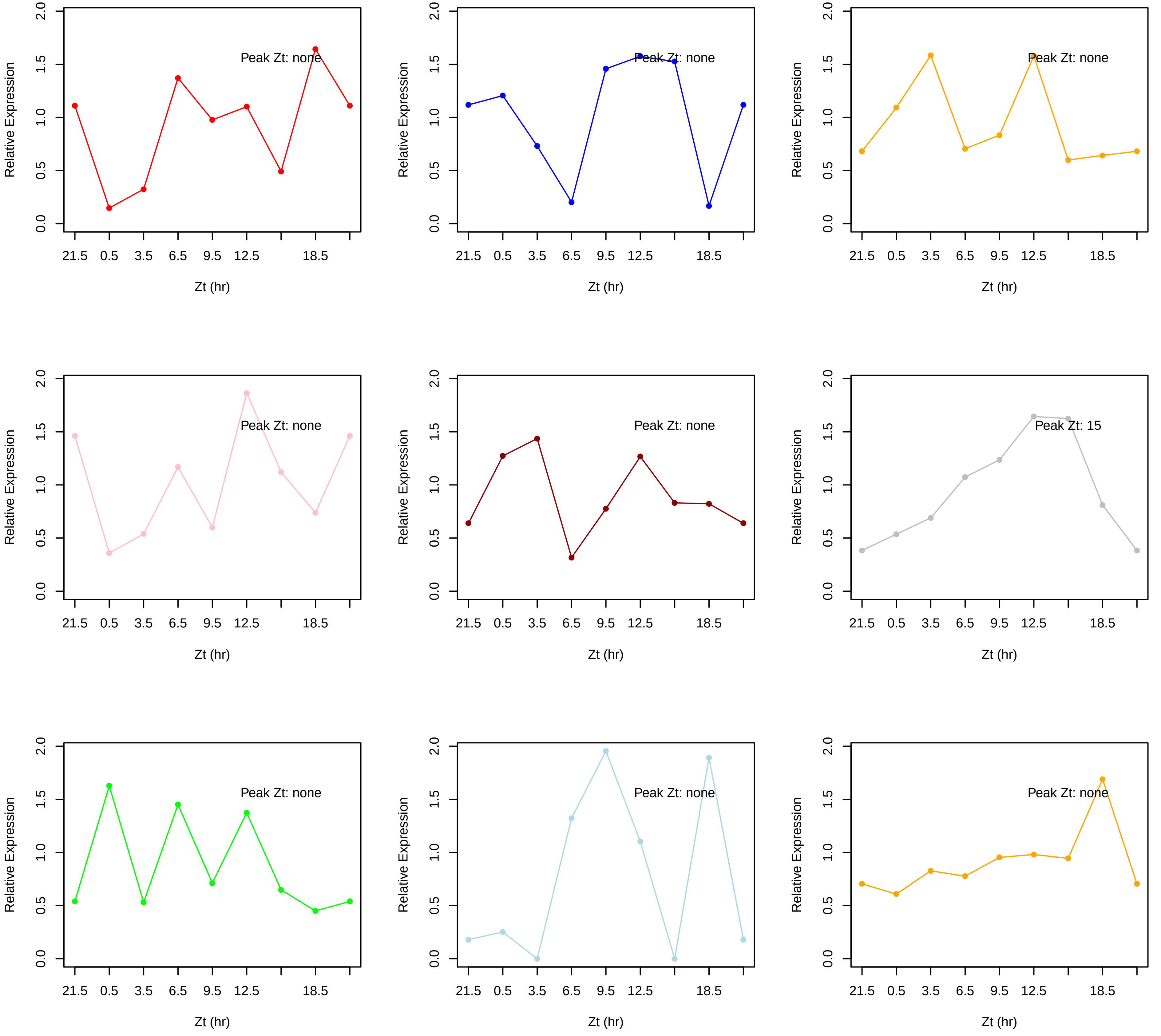

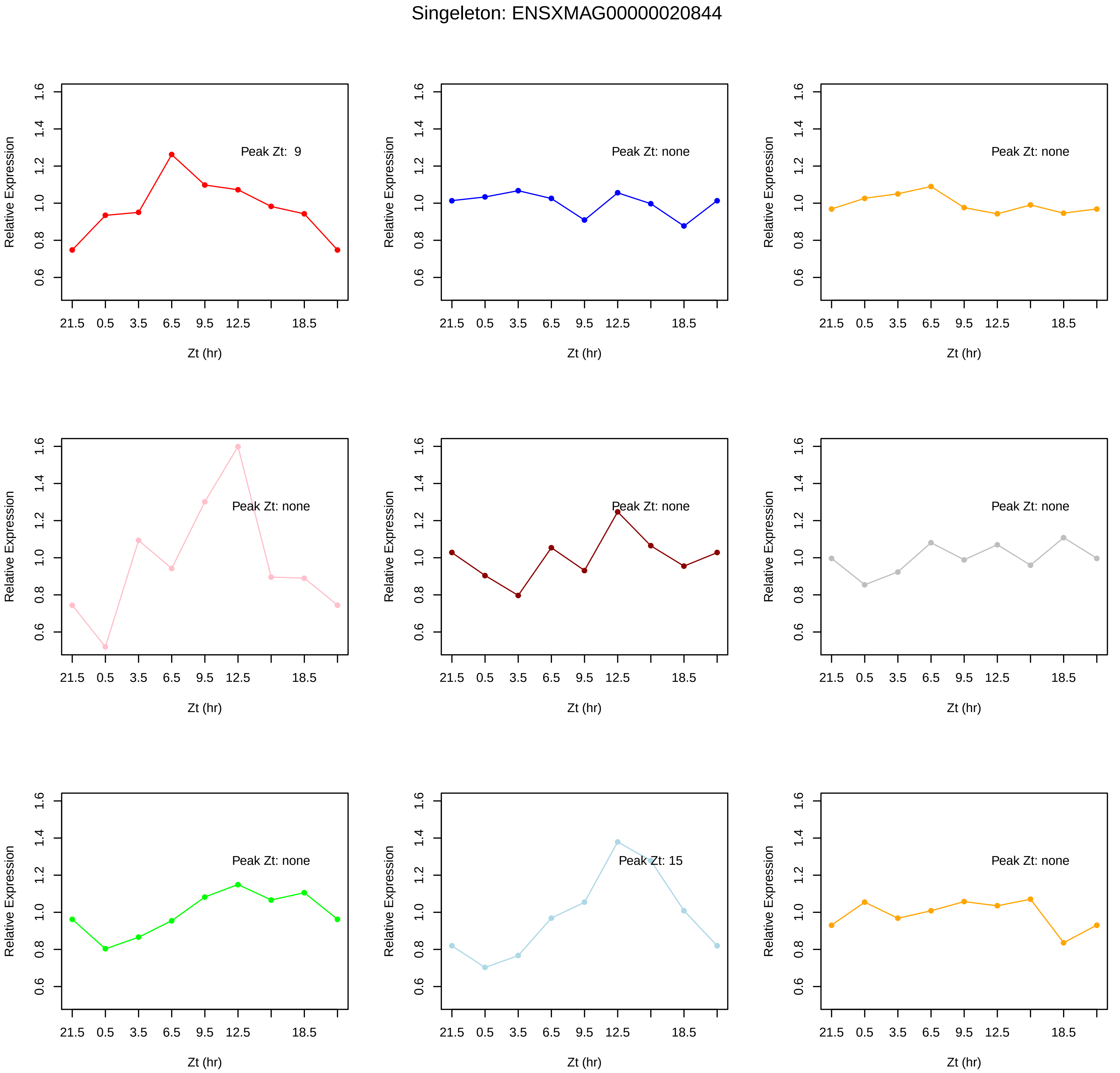
Singeleton: ENSXMAG00000020844
Peak Zt: 9
Peak Zt: none
Peak Zt: none
Peak Zt: none
Peak Zt: none
Peak Zt: none
Peak Zt: none
Peak Zt: 15
Peak Zt: none
Relative Expression
Zt (hr)
0.6
0.8
1.0
1.2
1.4
1.6
21.5
0.5
3.5
6.5
9.5
12.5
18.5

Singleton: ENSXMAG00000007369 sf3b1

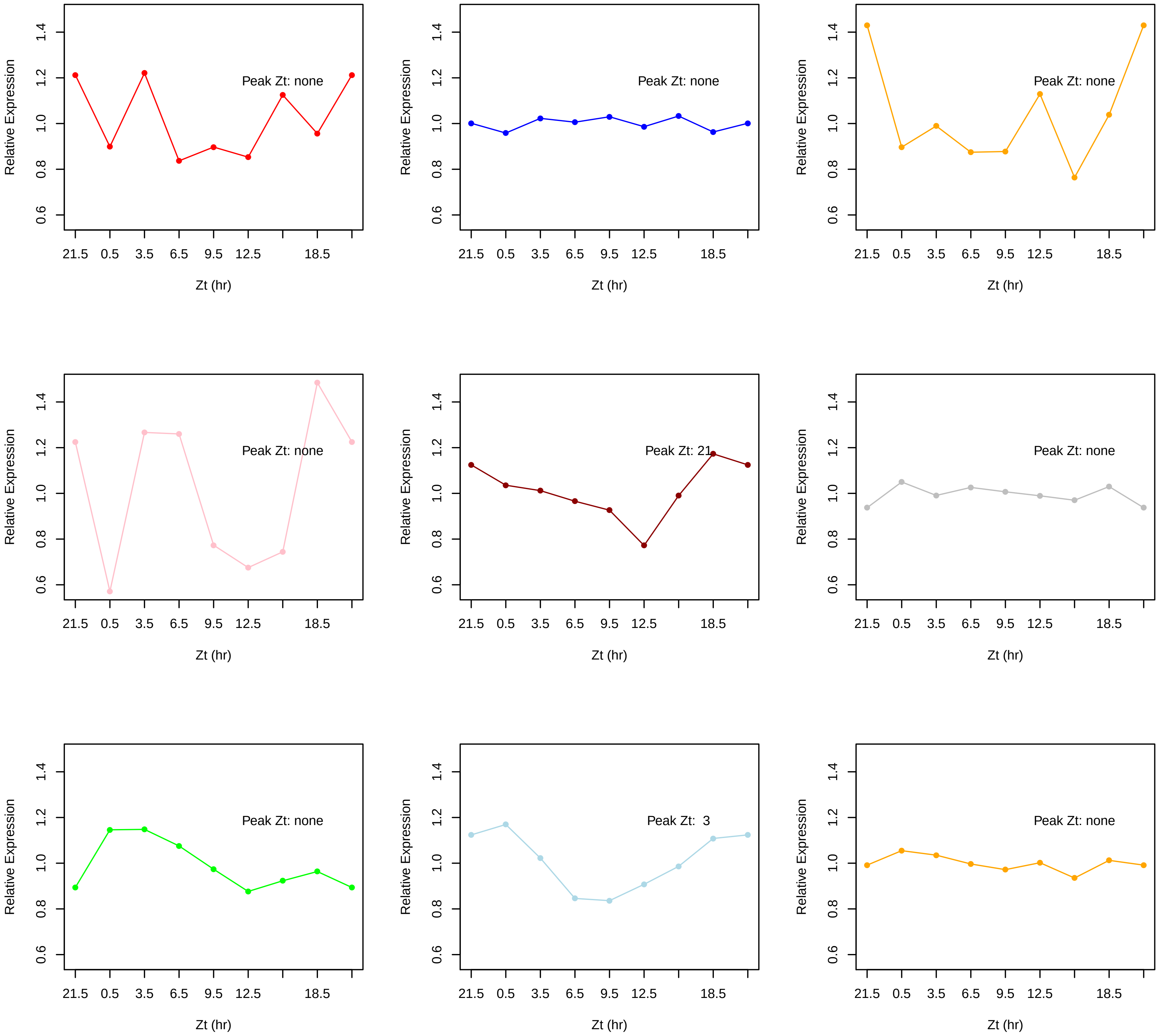

Singeleton: ENSXMAG00000009053 mbpb

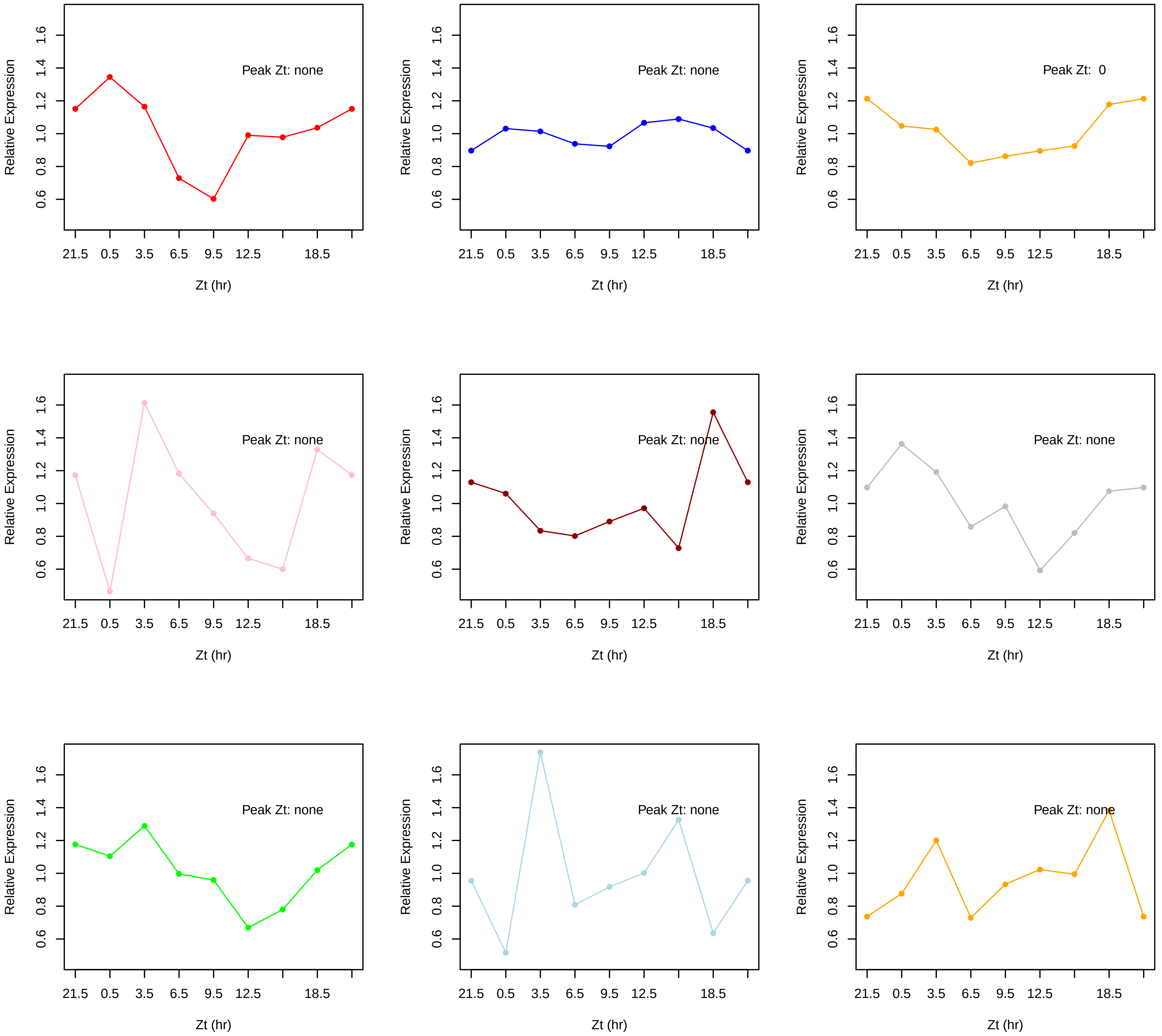

Singeleton: ENSXMAG00000001022 cc2d1b

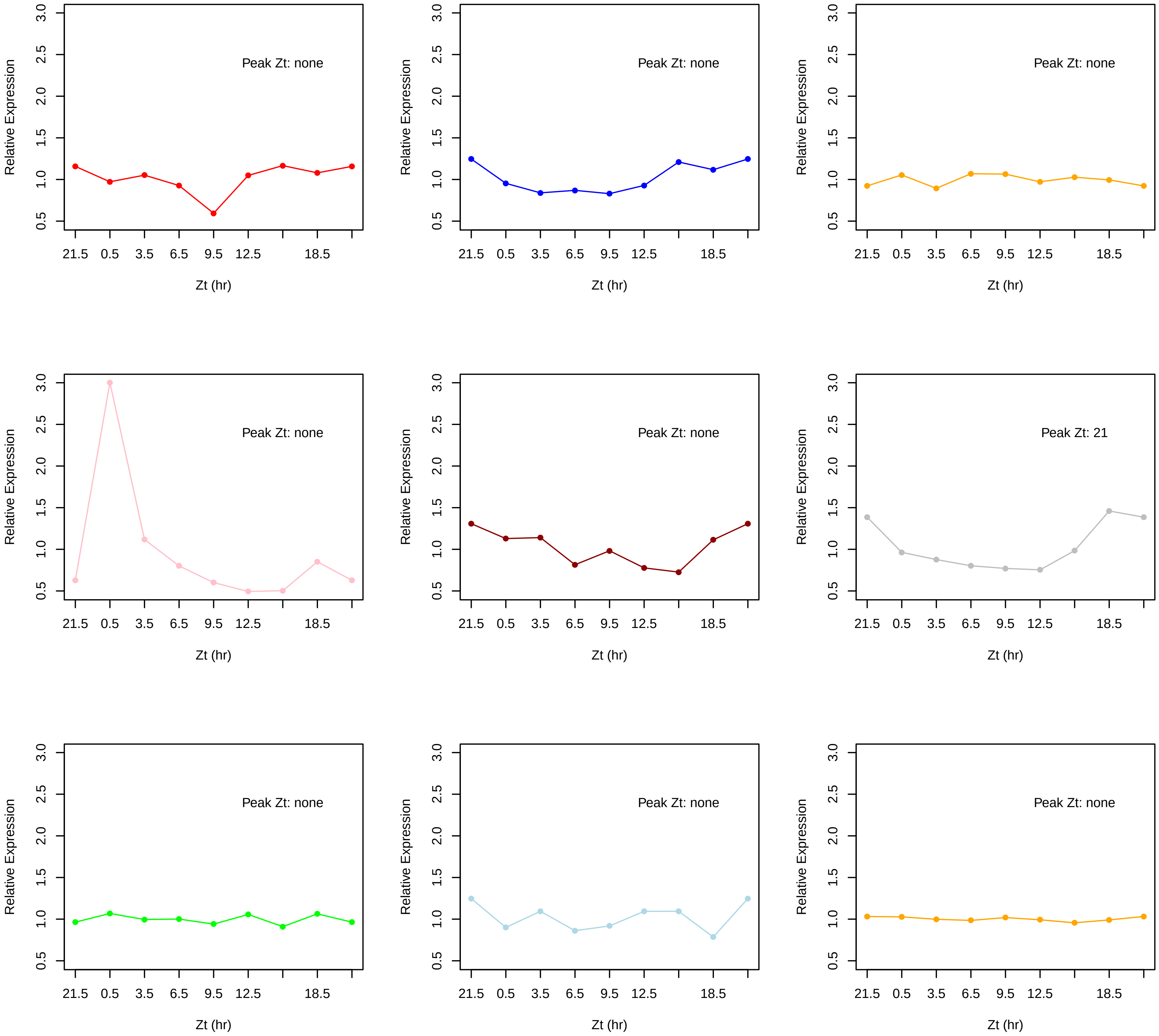

Singeleton: ENSXMAG00000020858 cplx4c

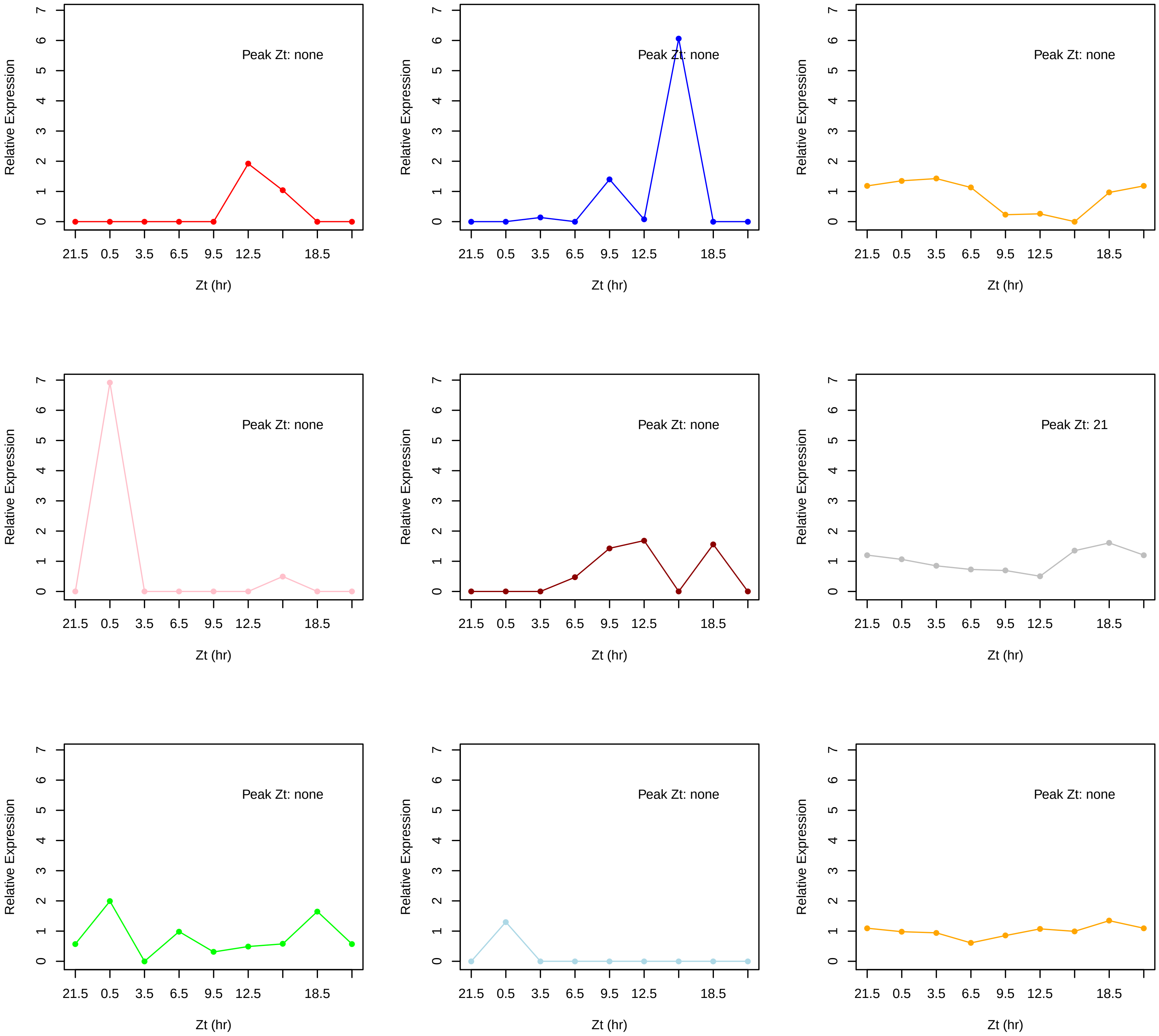

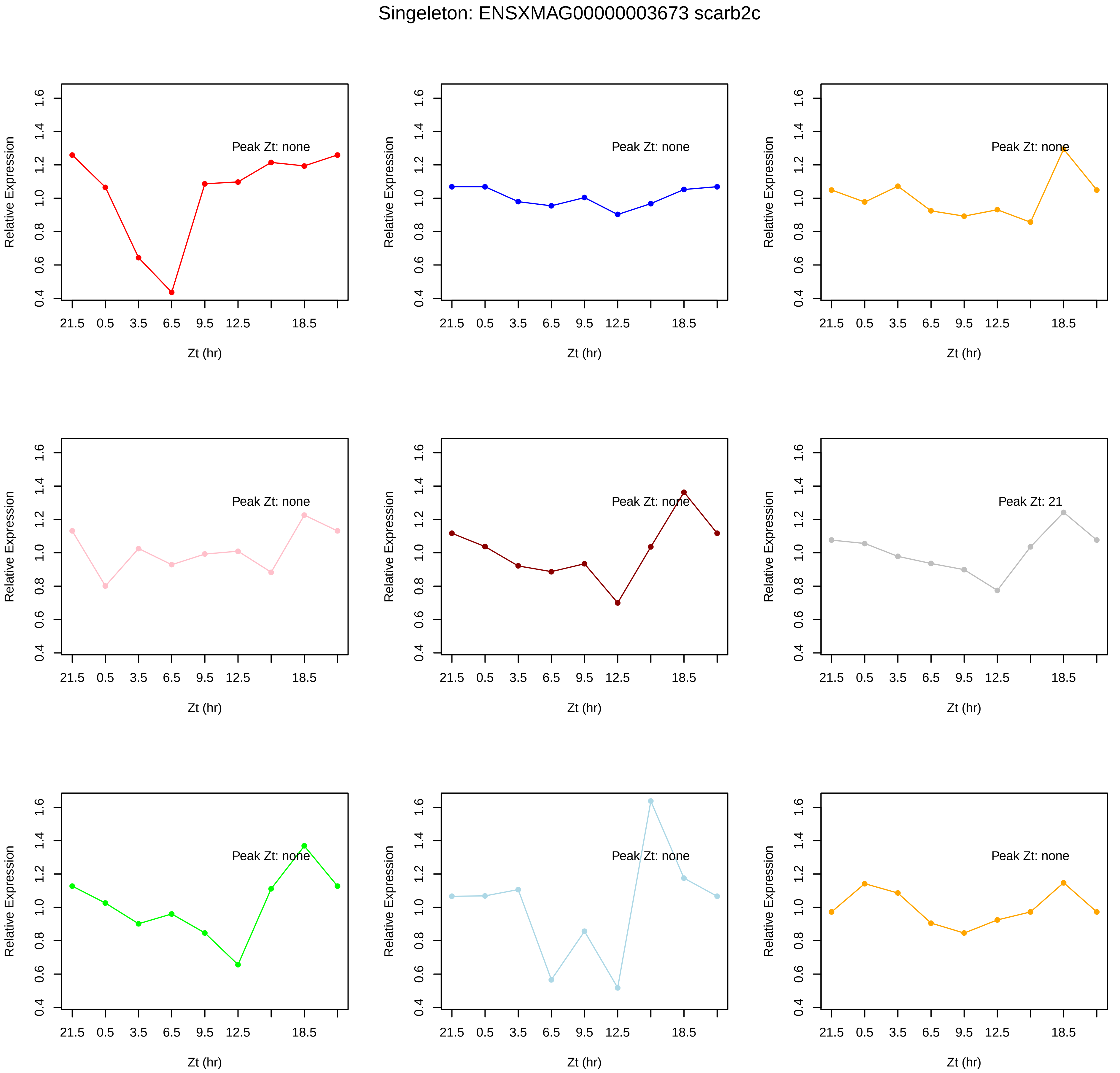
Singeleton: ENSXMAG00000003673 scarb2c
Peak Zt: none
Peak Zt: none
Peak Zt: none
Peak Zt: none
Peak Zt: none
Peak Zt: 21
Peak Zt: none
Peak Zt: none
Peak Zt: none
Relative Expression
Zt (hr)
21.5 0.5 3.5 6.5 9.5 12.5 18.5
0.4 0.6 0.8 1.0 1.2 1.4 1.6

Singleton: ENSXMAG00000015991 cdc20

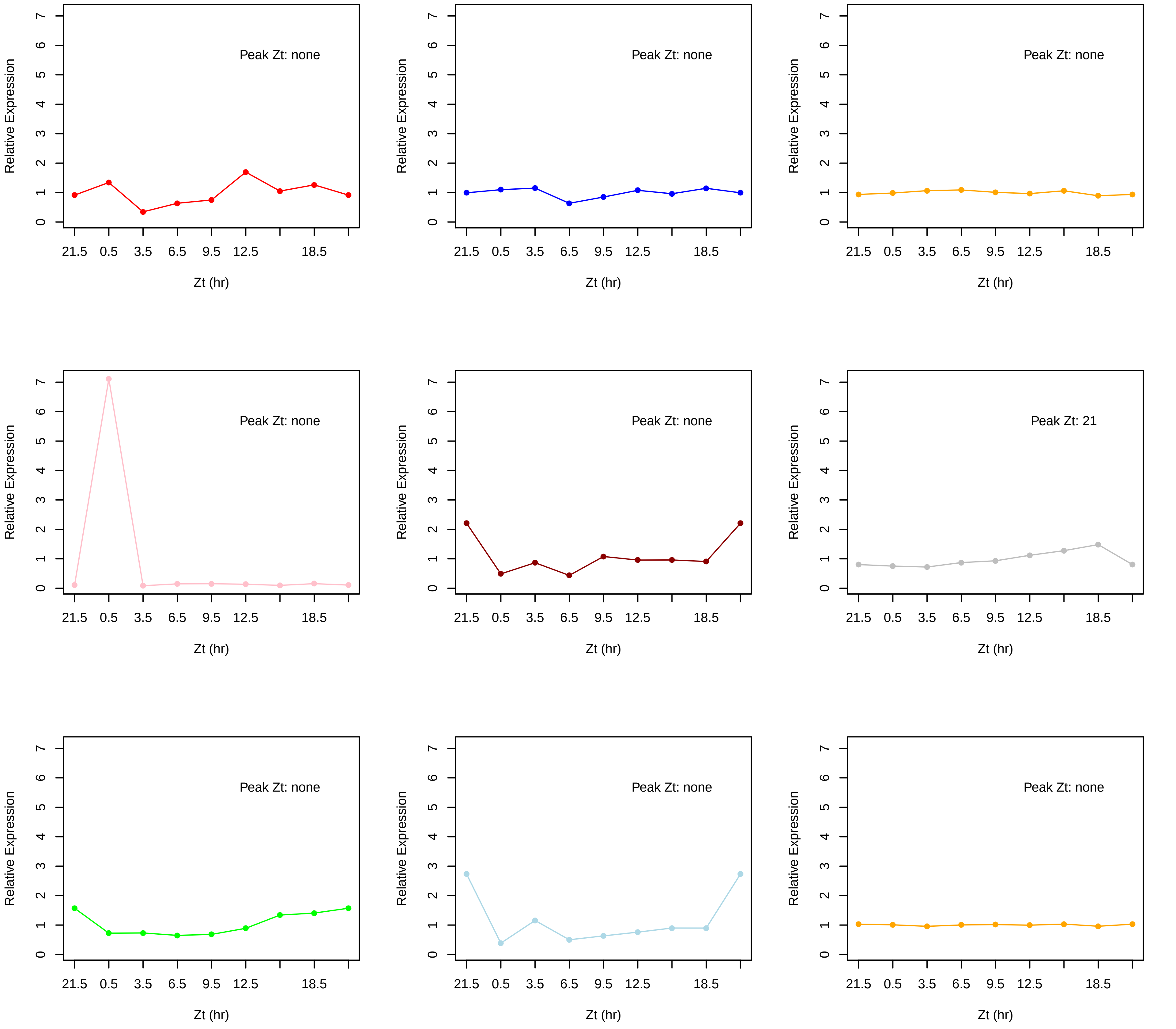

Singeleton: ENSXMAG00000000961 bco1l

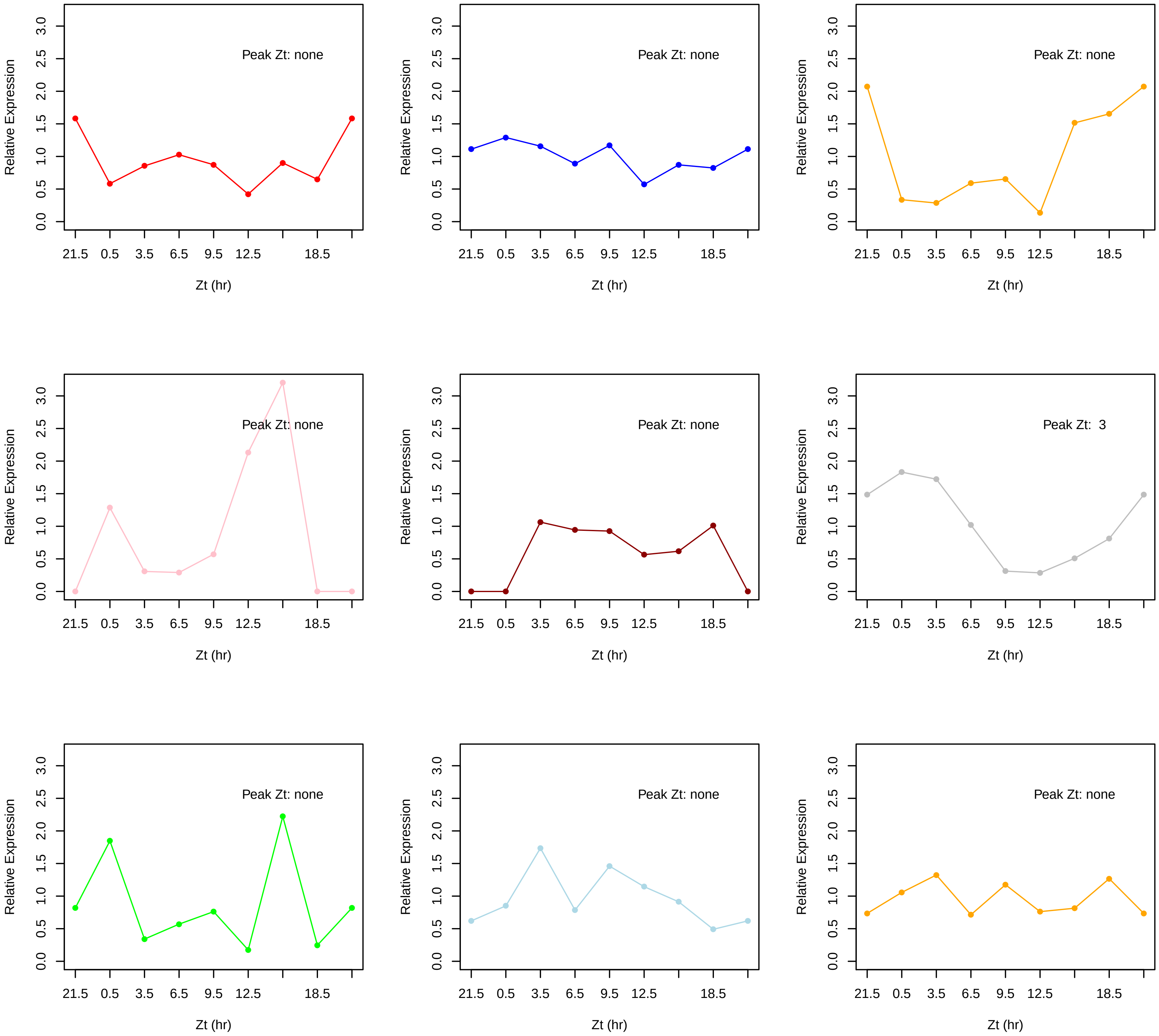

Singeleton: ENSXMAG00000000052 mrps16

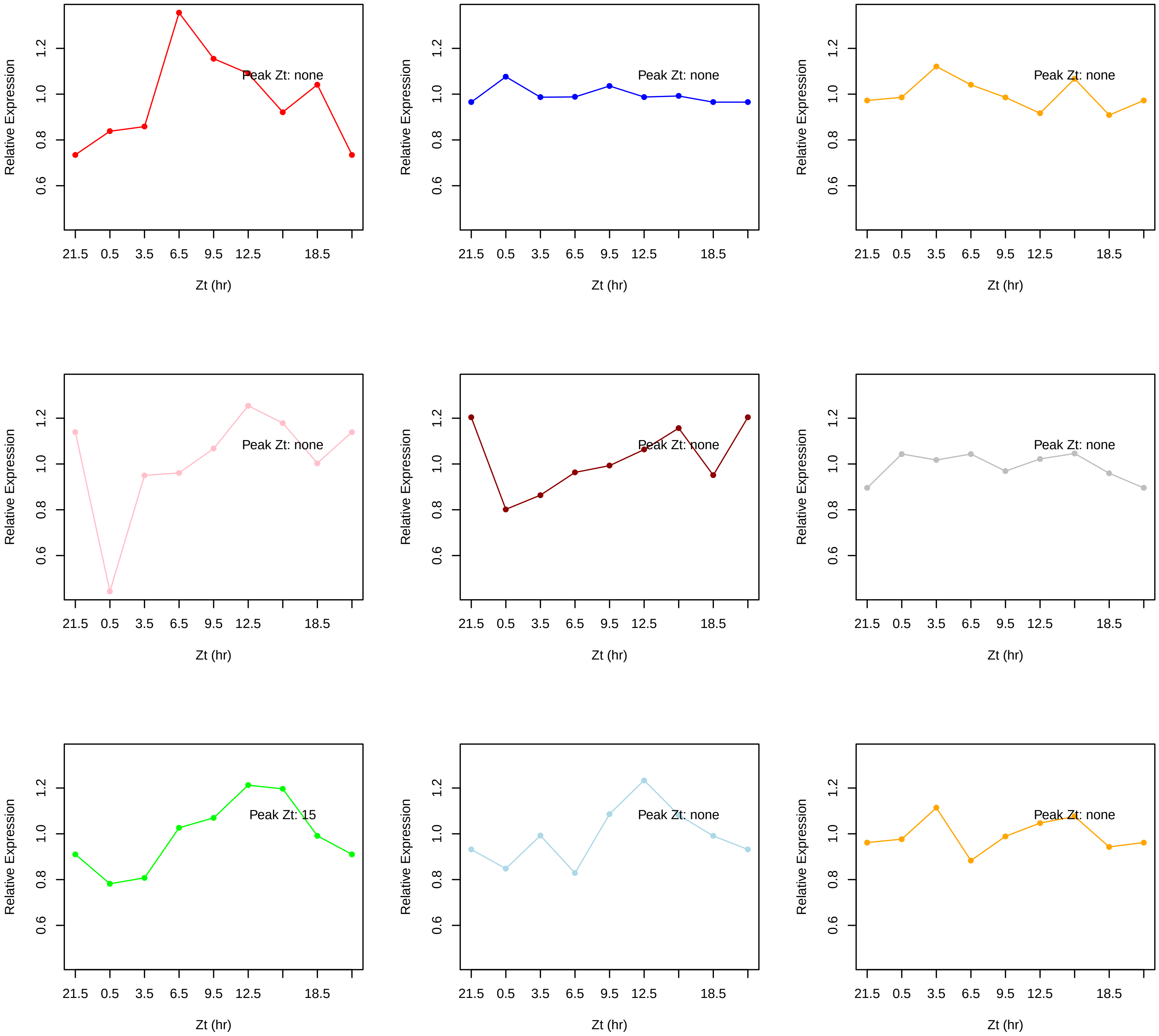

Singeleton: ENSXMAG00000008366 NLRX1

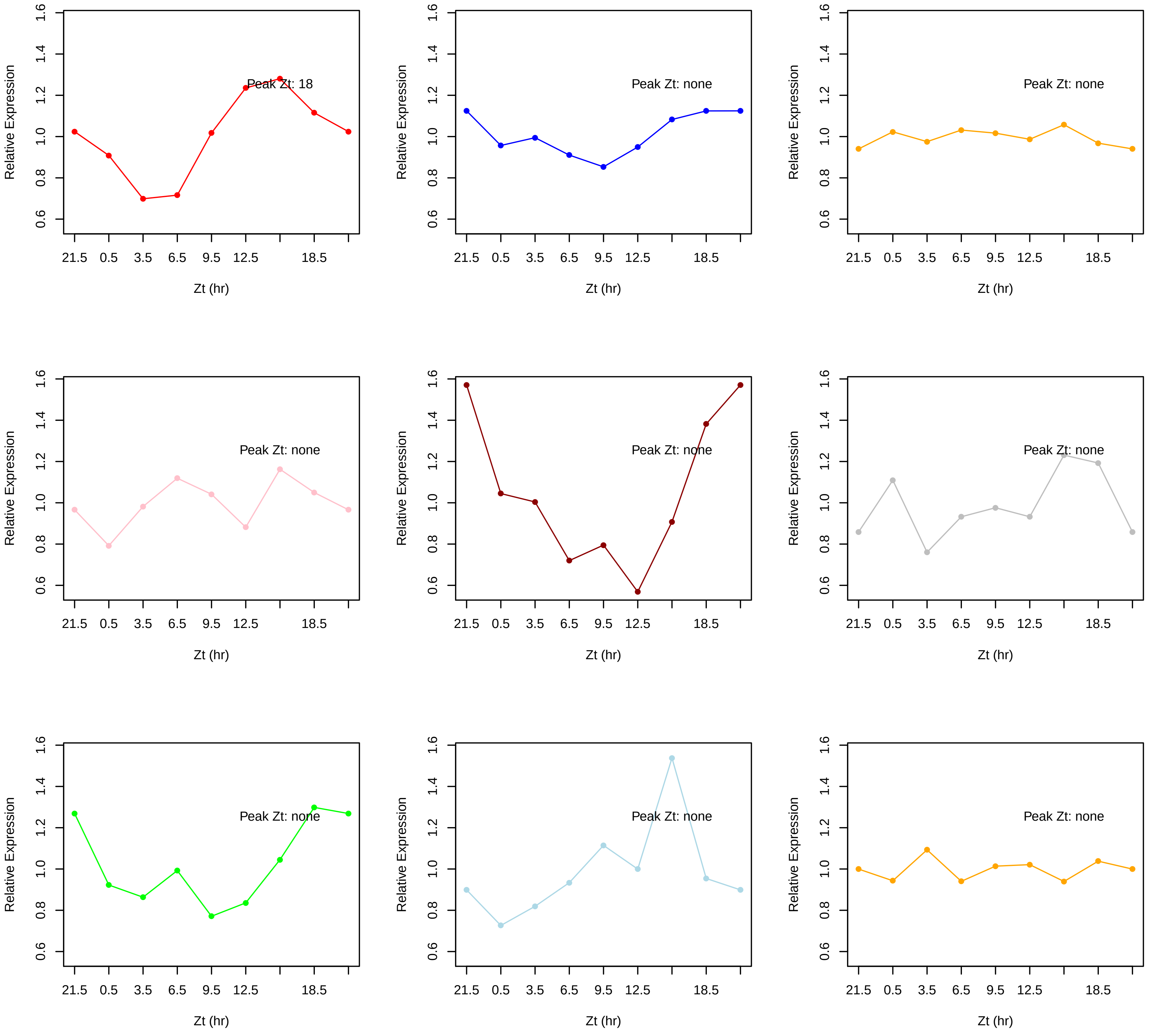

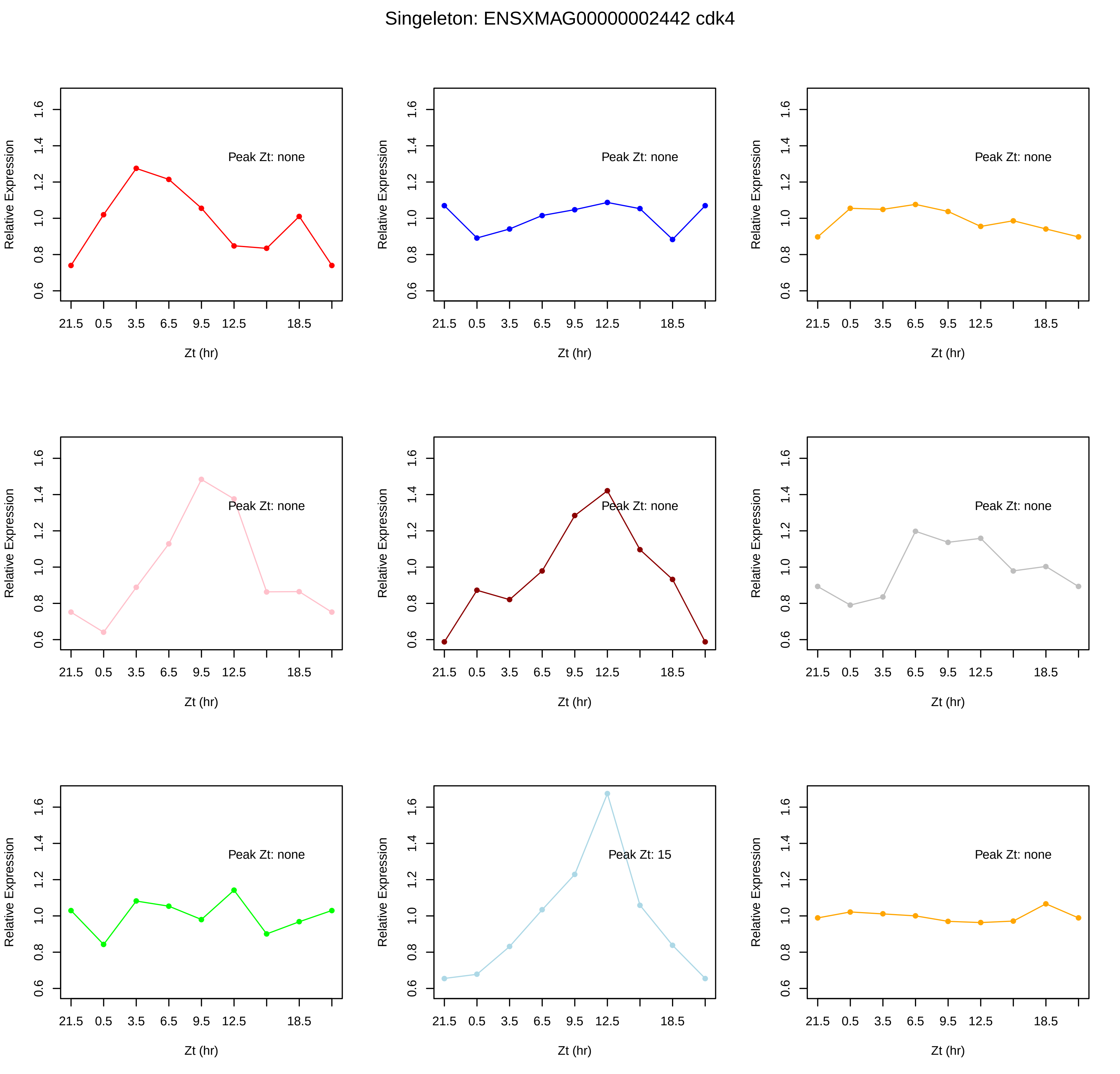
Singeleton: ENSXMAG00000002442 cdk4
Relative Expression
Zt (hr)
21.5
0.5
3.5
6.5
9.5
12.5
18.5
0.6
0.8
1.0
1.2
1.4
1.6
Peak Zt: none
Peak Zt: 15

Singeleton: ENSXMAG00000007708 pes

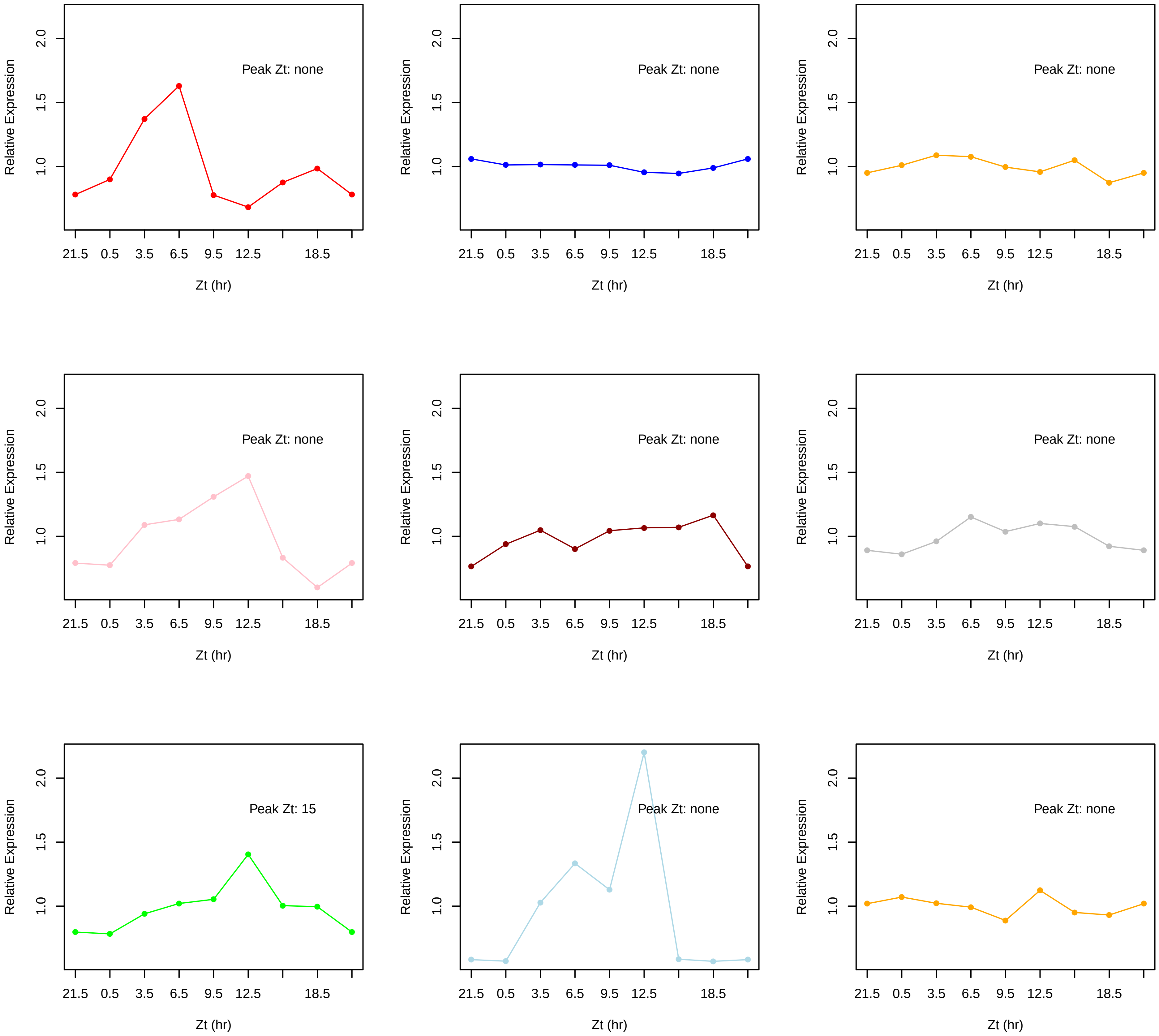

Singleton: ENSXMAG00000005891 hspd1

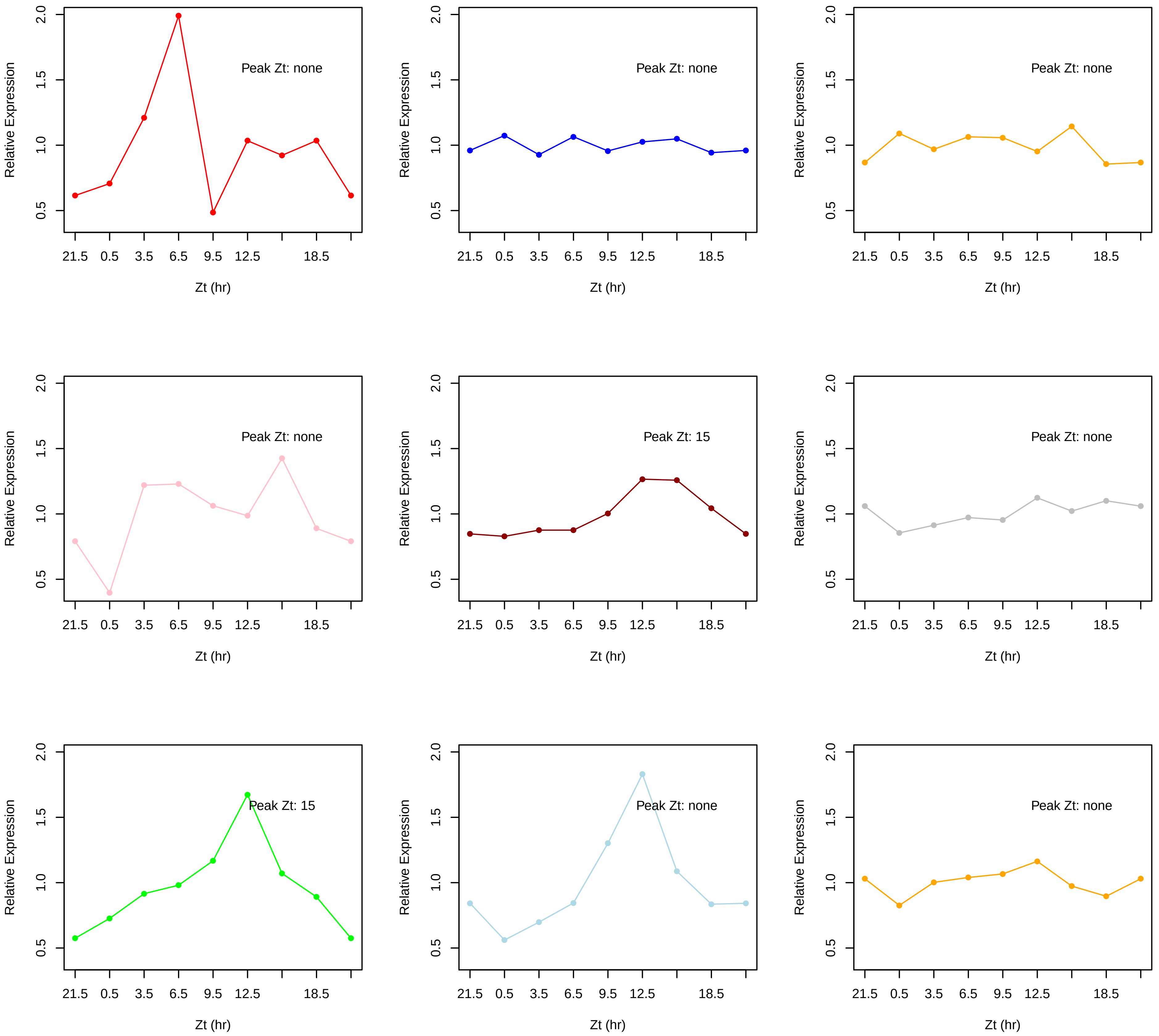

Singeleton: ENSXMAG00000010271 eya1

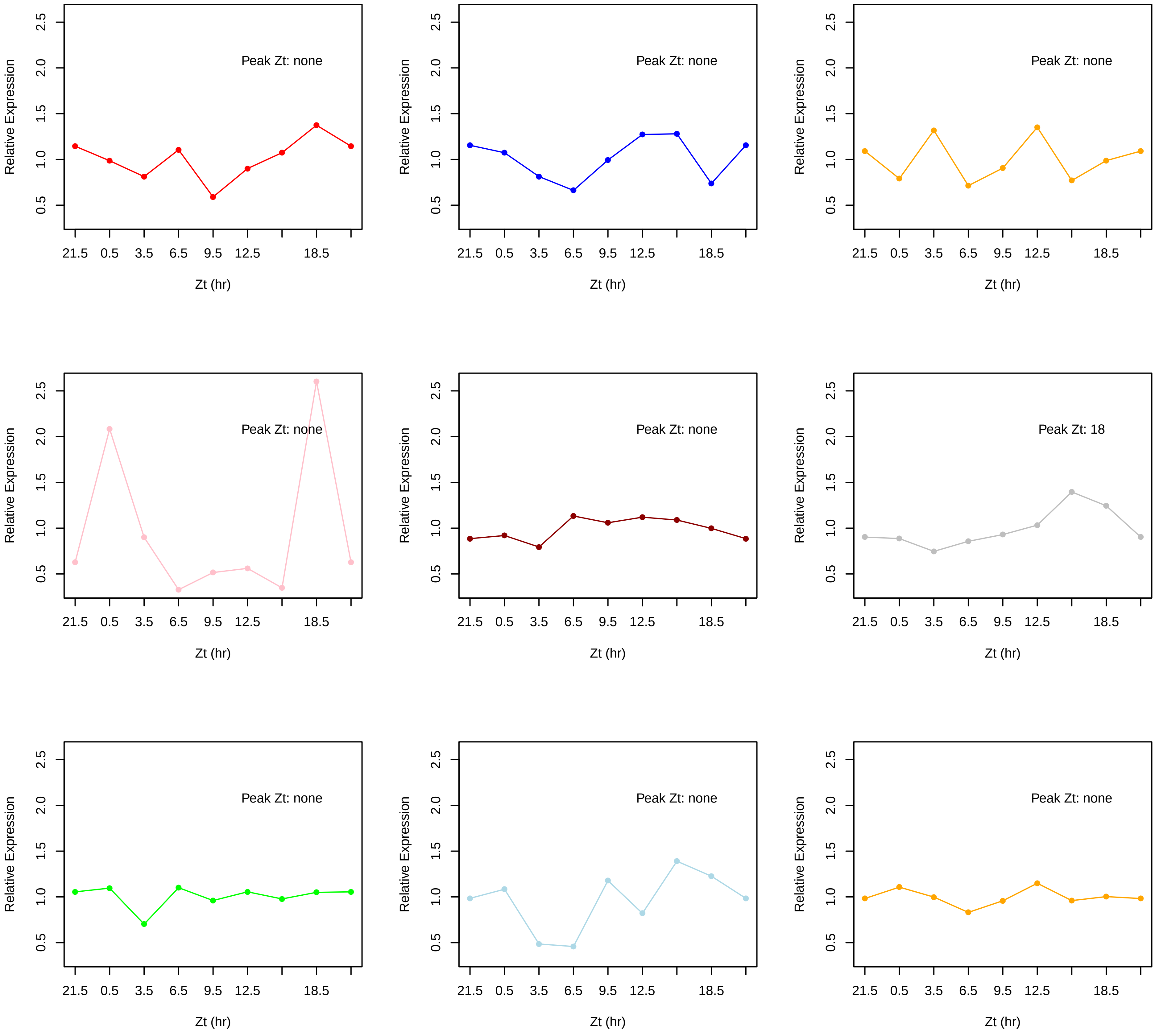

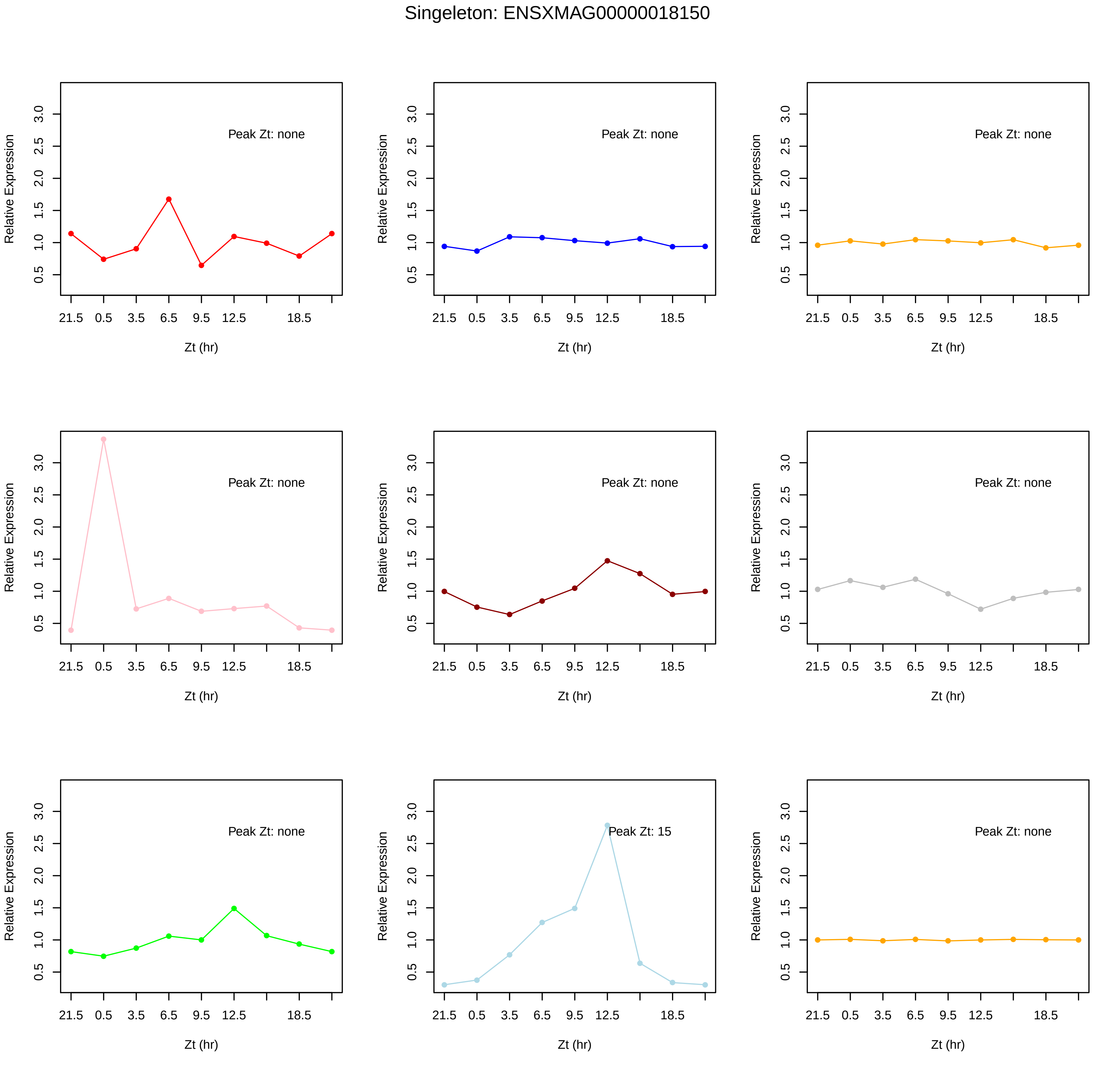
Singeleton: ENSXMAG00000018150
Peak Zt: none
Peak Zt: none
Peak Zt: none
Peak Zt: none
Peak Zt: none
Peak Zt: none
Peak Zt: none
Peak Zt: 15
Peak Zt: none
Relative Expression
Zt (hr)
21.5
0.5
3.5
6.5
9.5
12.5
18.5

Singeleton: ENSXMAG00000024833

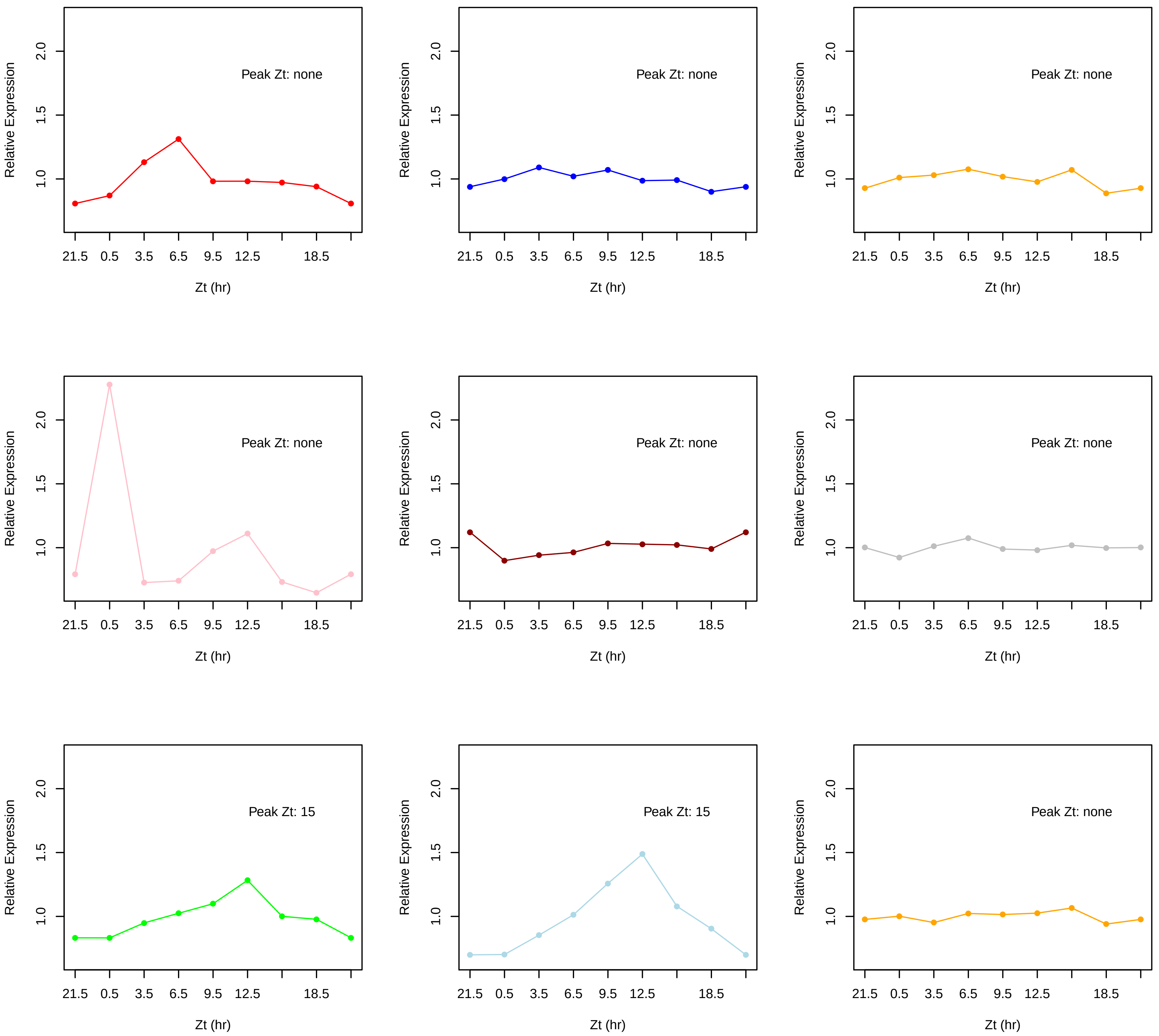

Singleton: ENSXMAG00000005320 zgc:85789

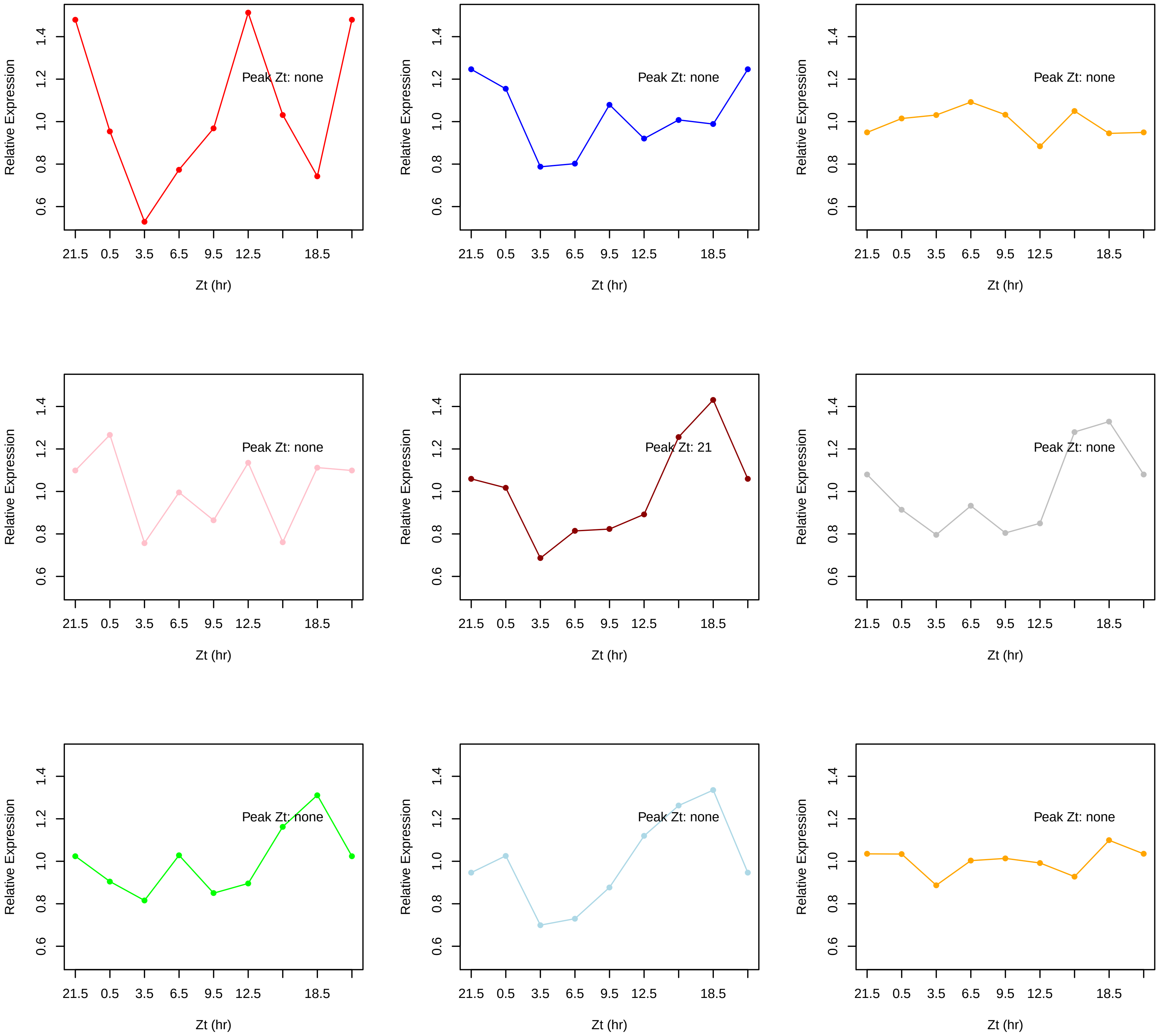

Singeleton: ENSXMAG00000028435 PTPN20

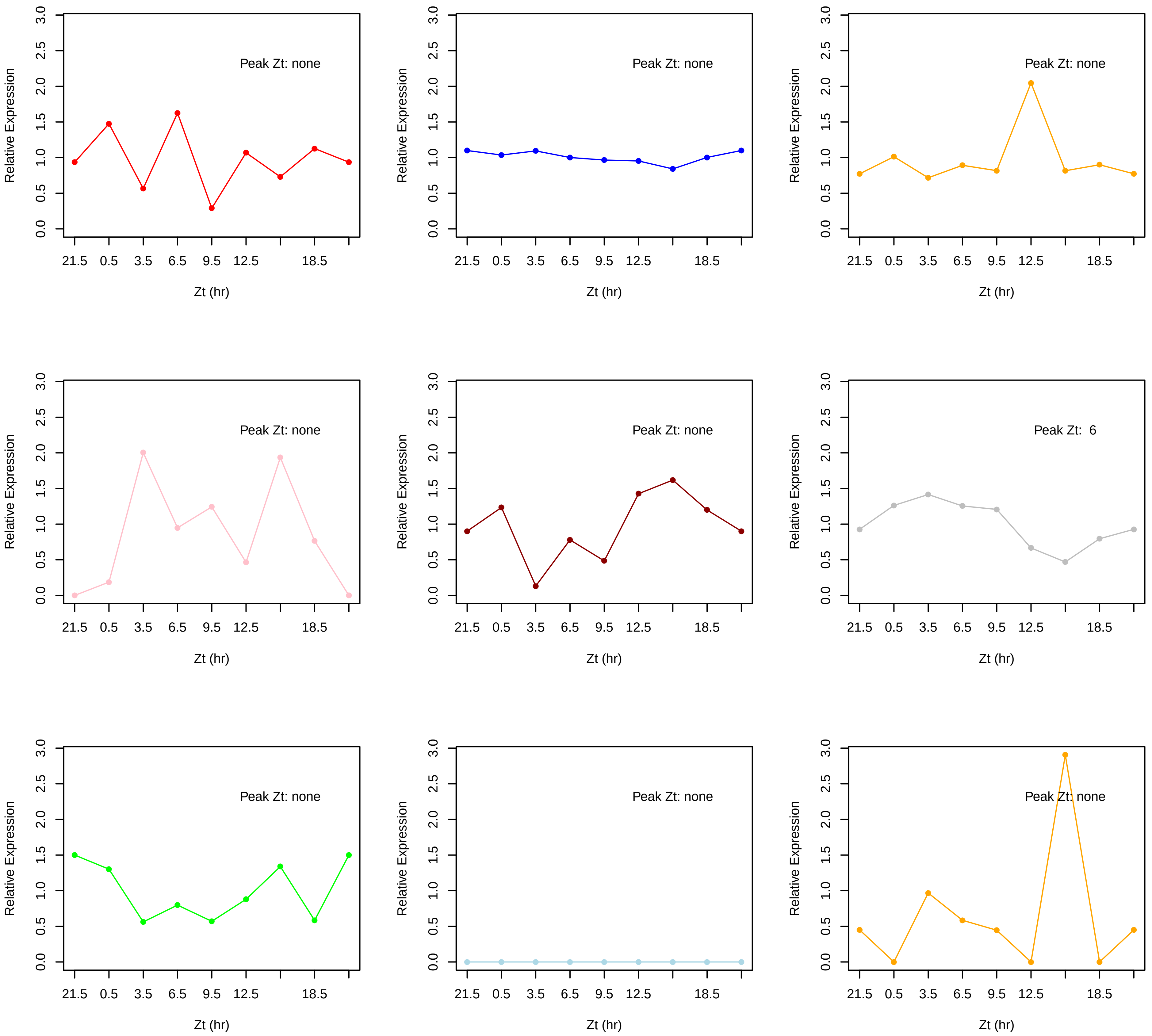

Singleton: ENSXMAG00000005889 hspe1

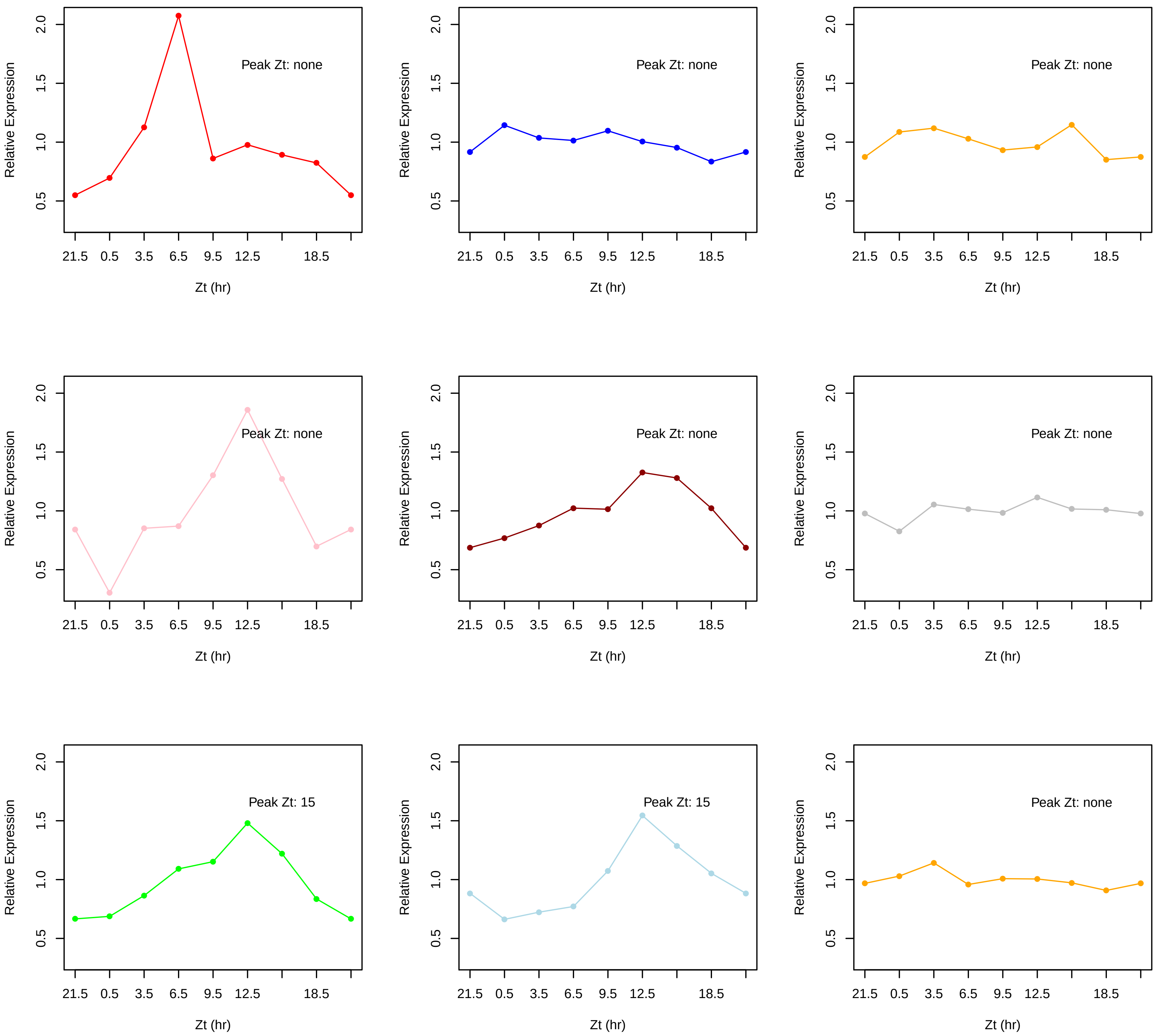

Singleton: ENSXMAG00000010763 wdtc1

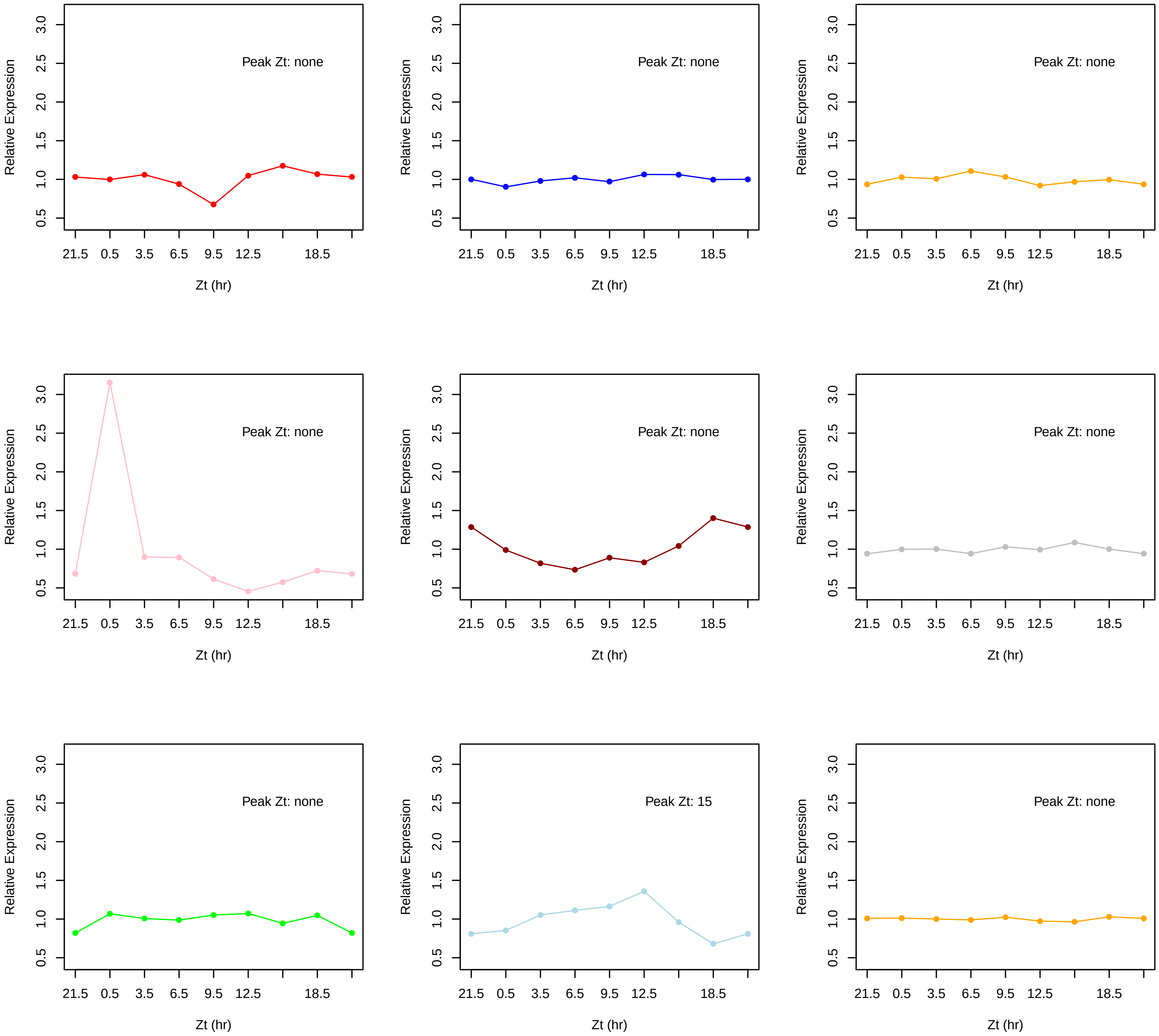

Singeleton: ENSXMAG00000009432 kdm8

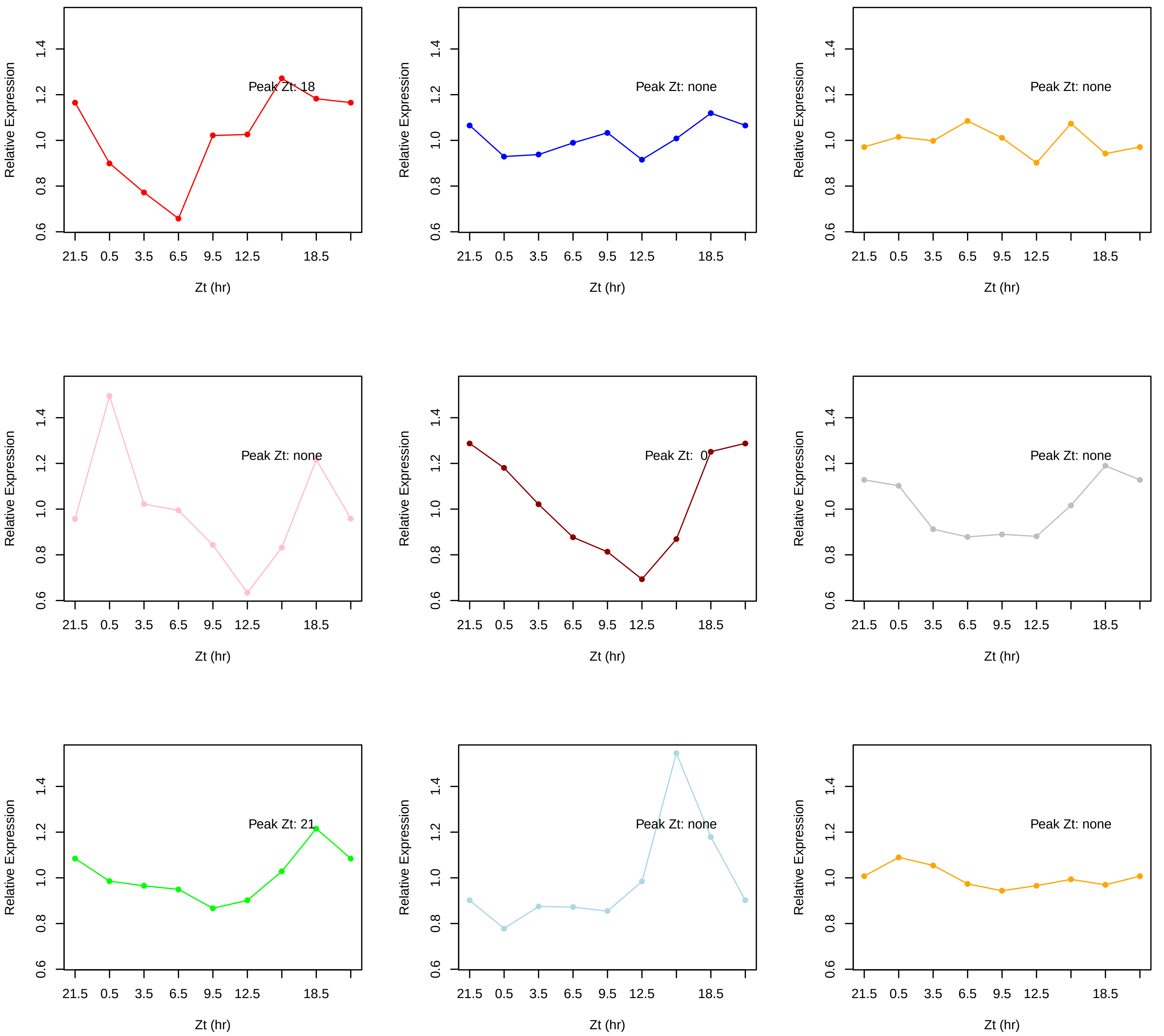

Singeleton: ENSXMAG00000009503 mapk8b

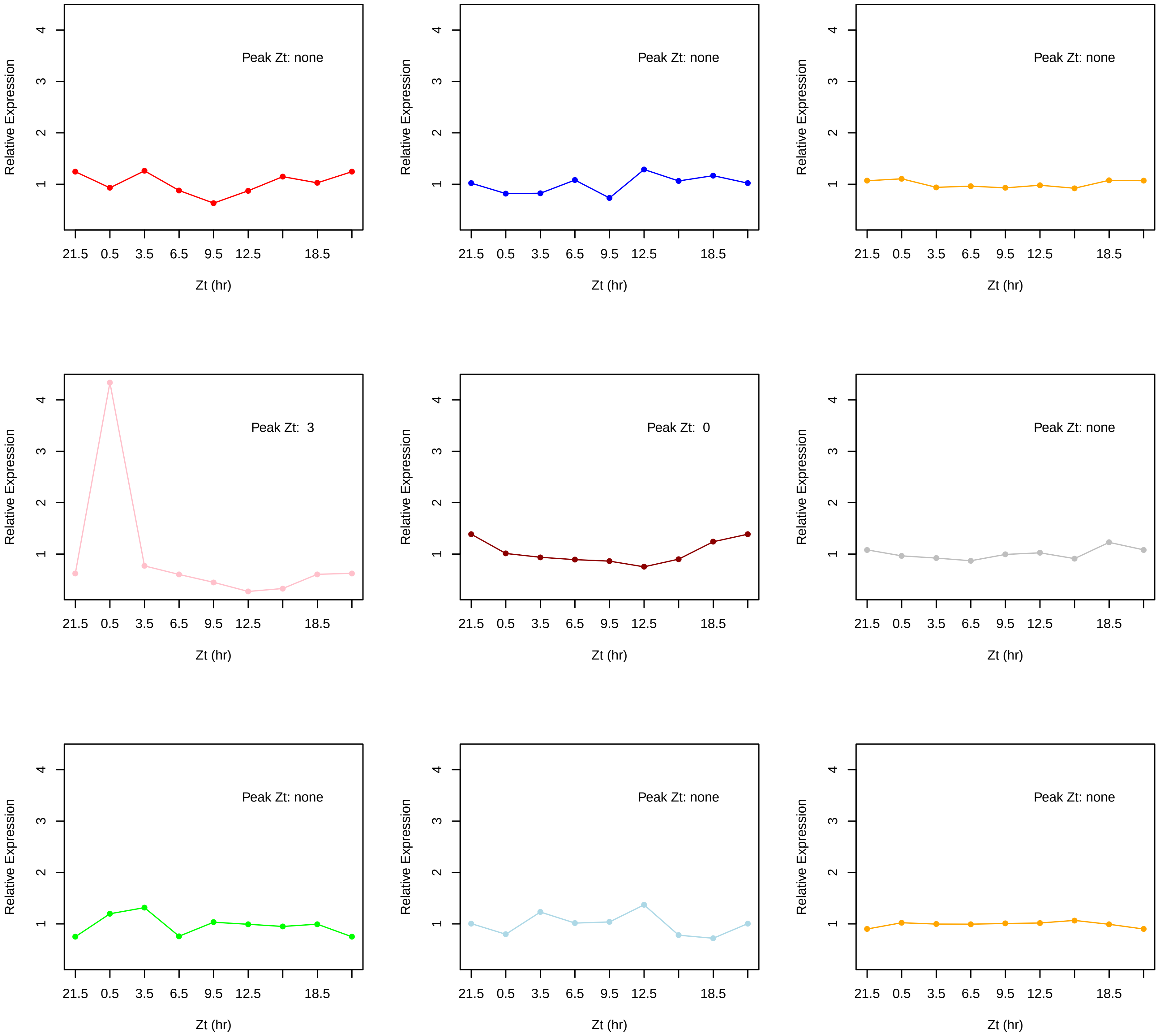

Singeleton: ENSXMAG00000009179 psmd7

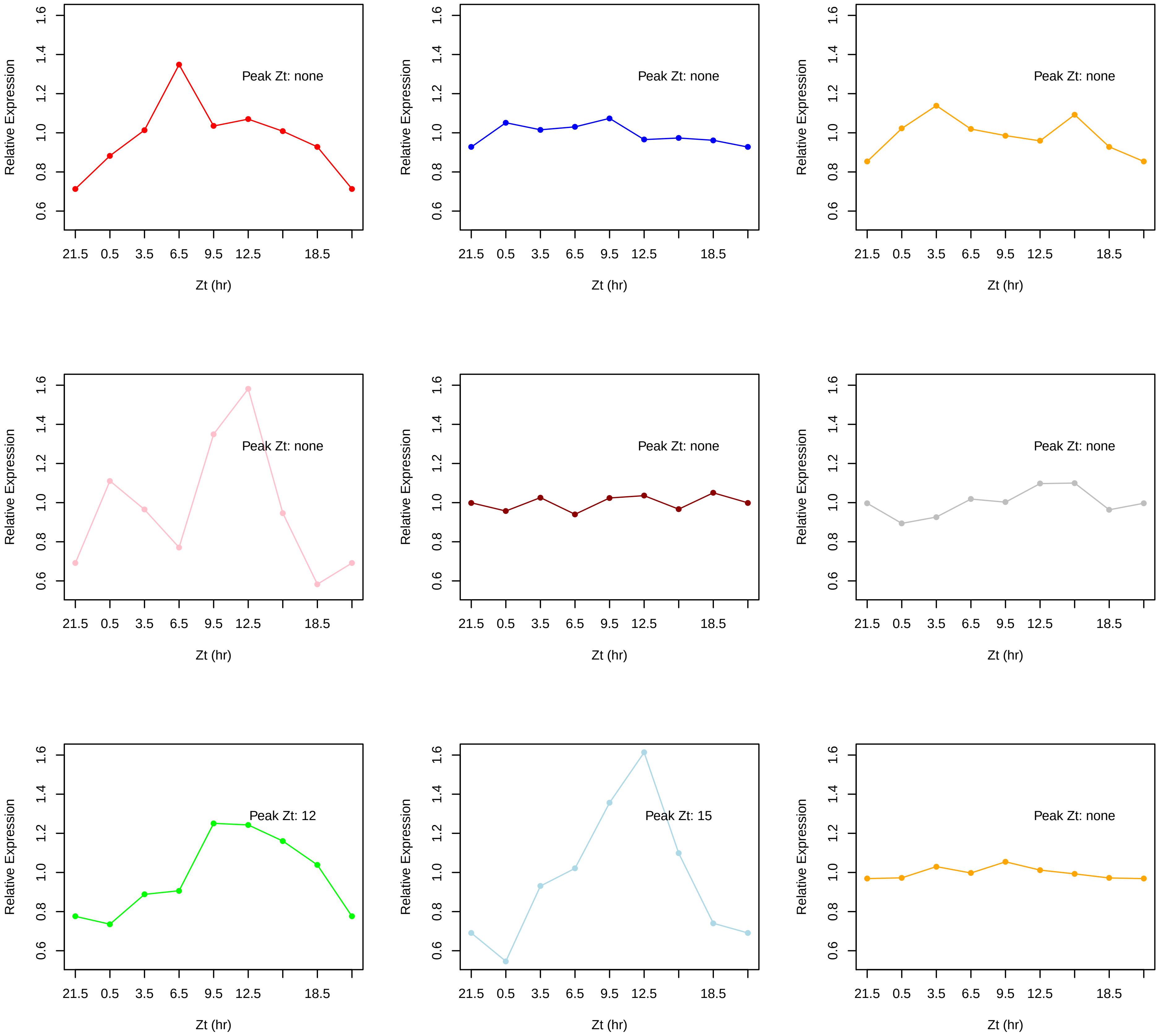

Singeleton: ENSXMAG00000010499 aplp1

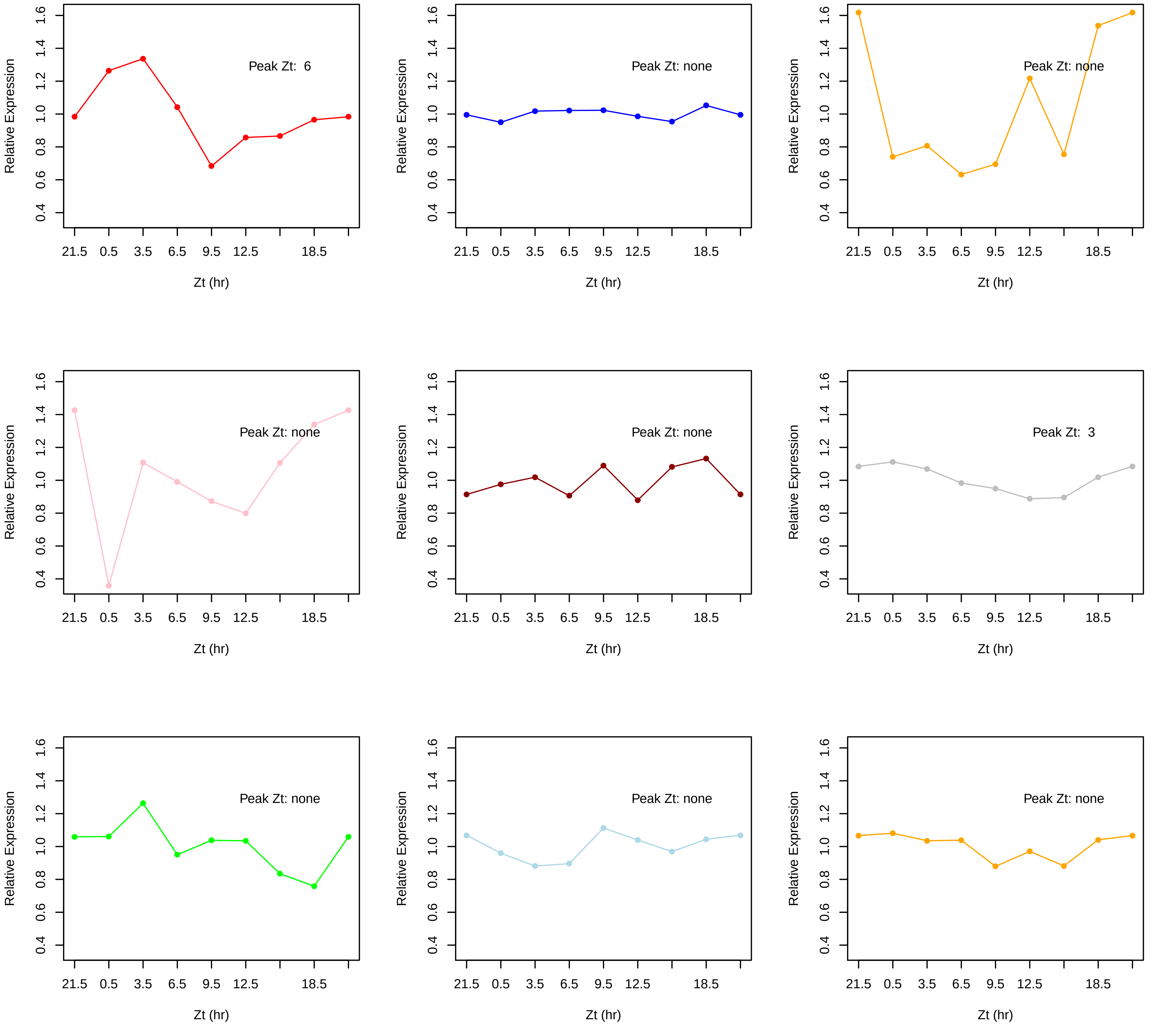

Singeleton: ENSXMAG00000009031 ZNF516

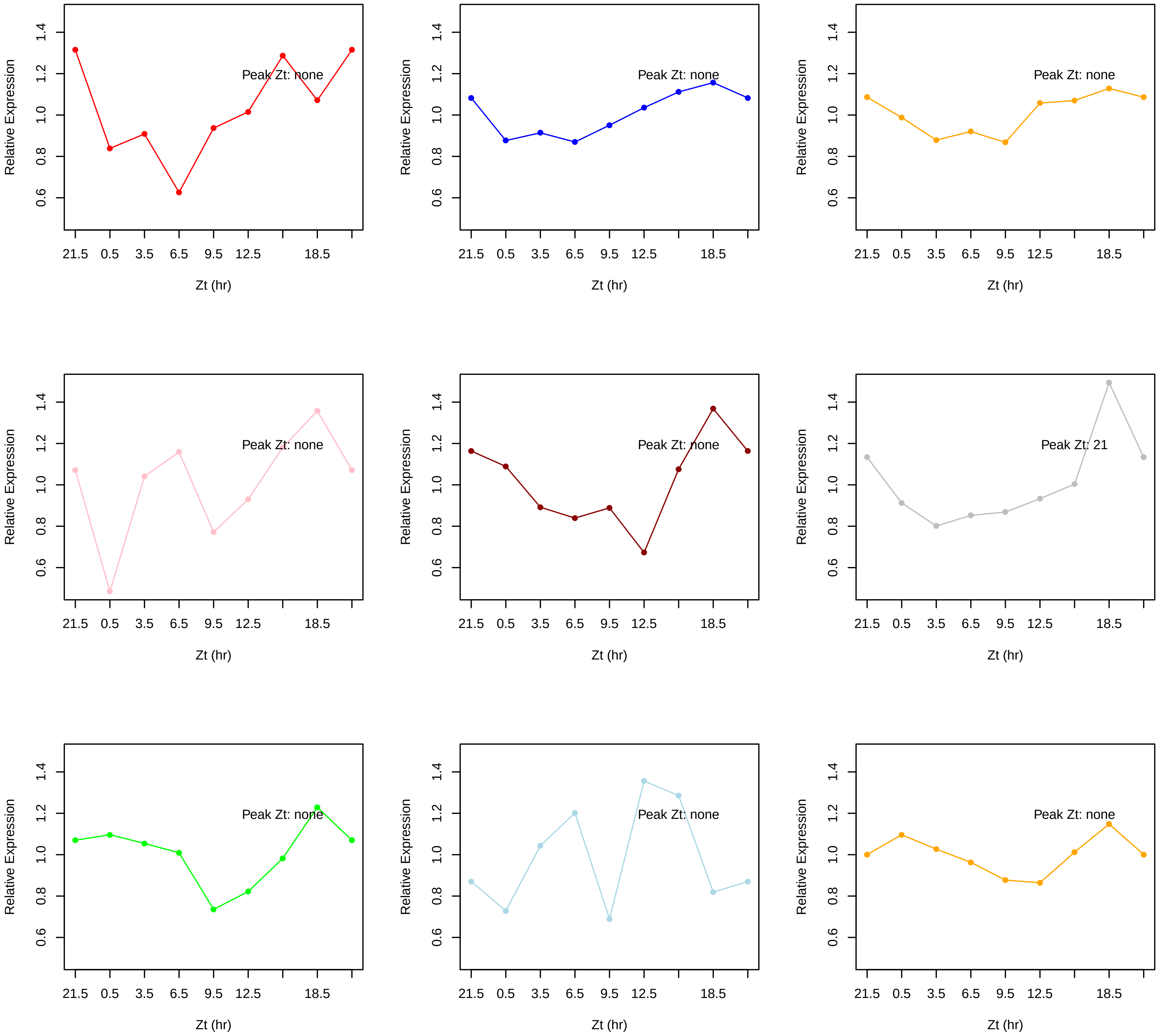

Singeleton: ENSXMAG00000007745 pole

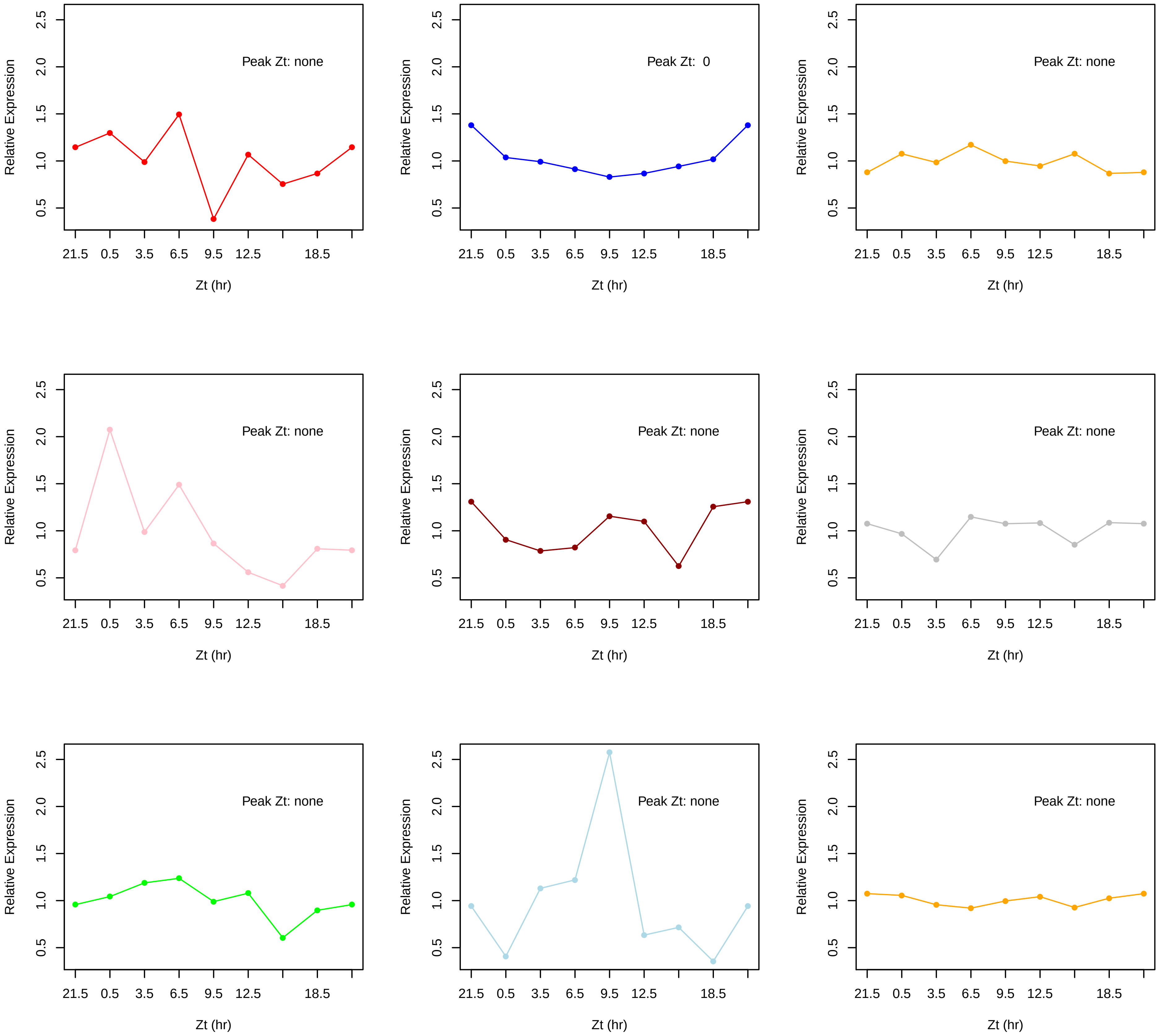

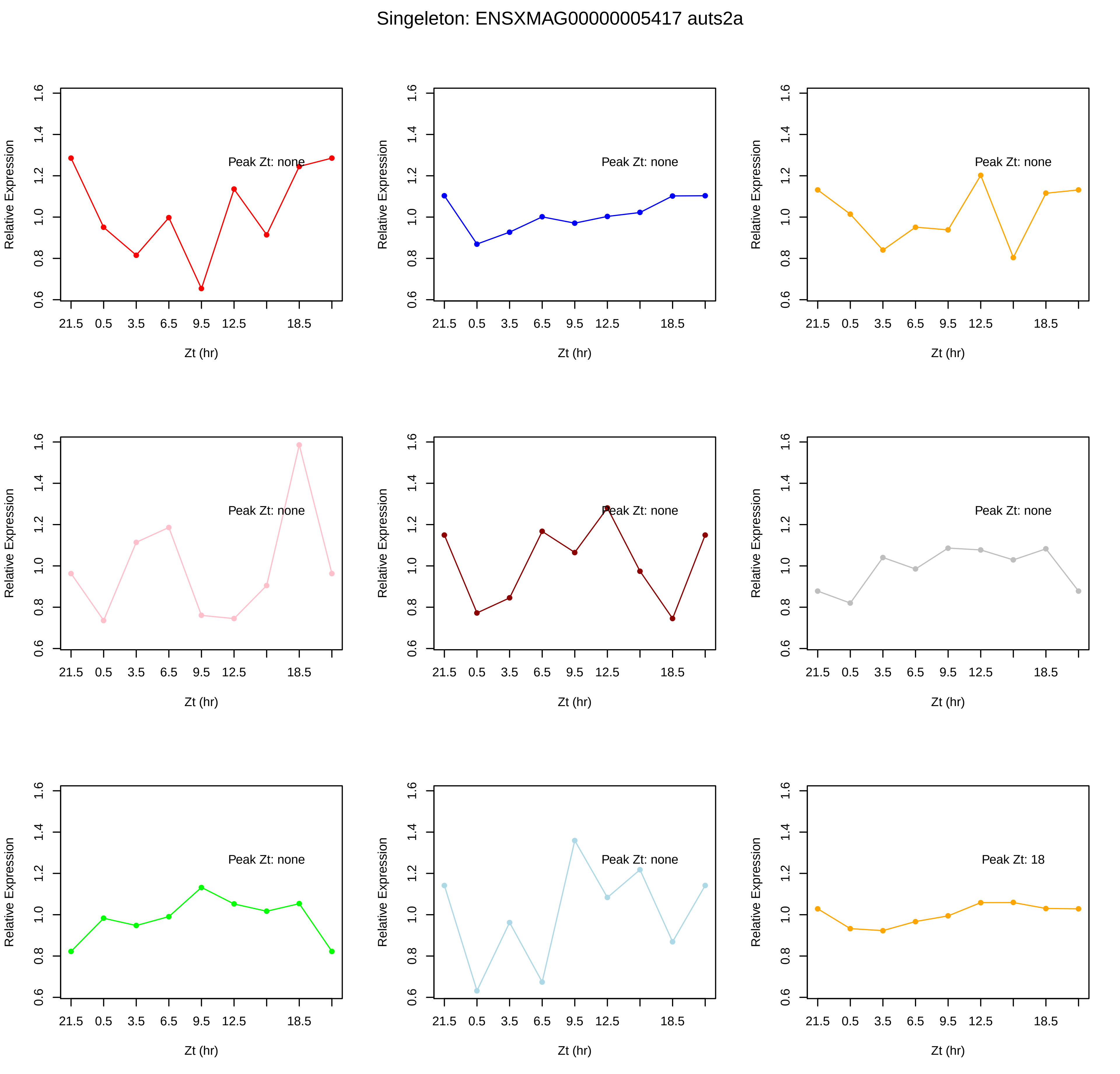
Singeleton: ENSXMAG00000005417 auts2a
Relative Expression
Zt (hr)
21.5
0.5
3.5
6.5
9.5
12.5
18.5
0.6
0.8
1.0
1.2
1.4
1.6
Peak Zt: none
Peak Zt: none
Peak Zt: none
Peak Zt: none
Peak Zt: none
Peak Zt: none
Peak Zt: none
Peak Zt: none
Peak Zt: 18

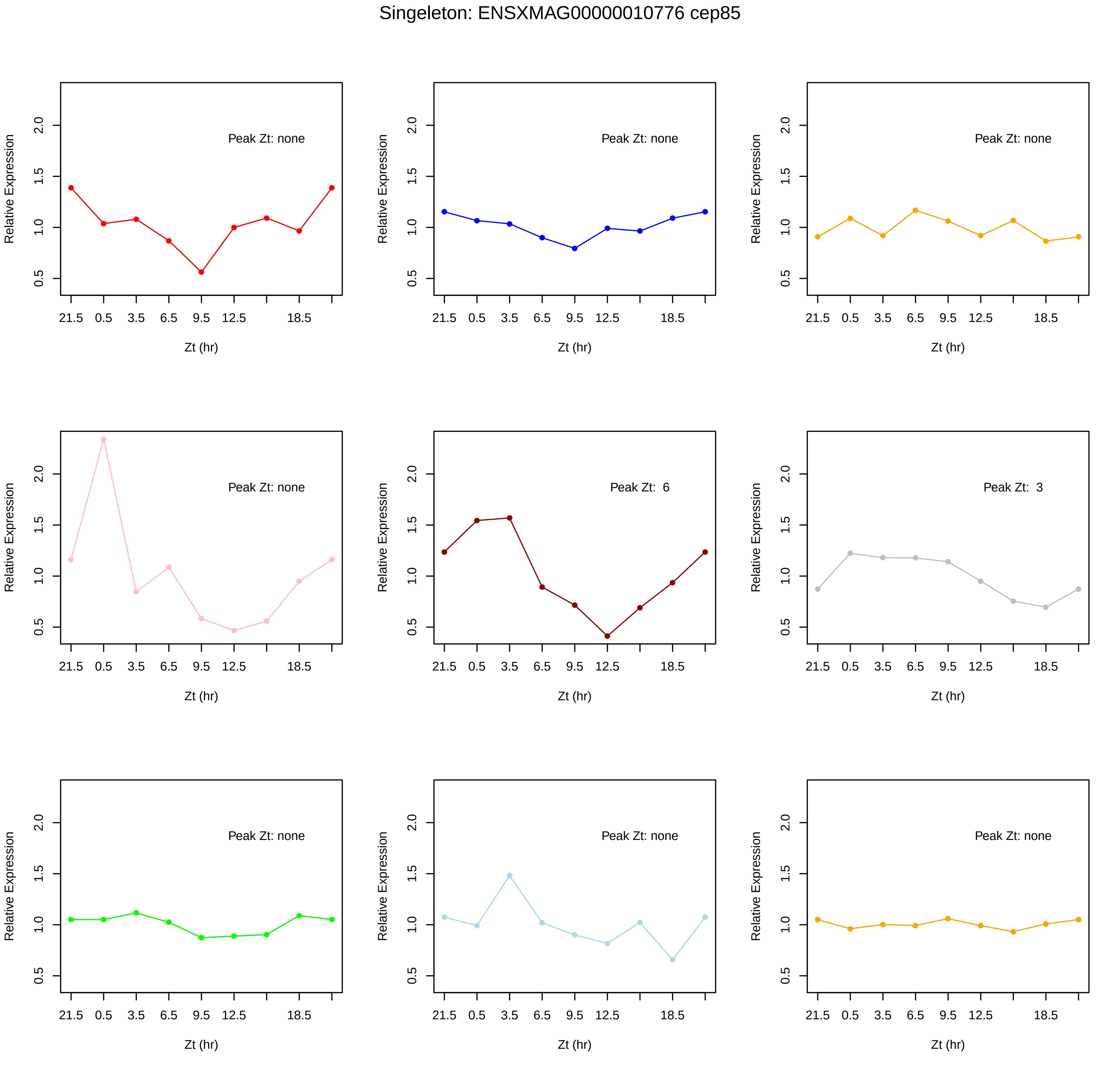
Singleton: ENSXMAG00000010776 cep85
Peak Zt: none
Peak Zt: none
Peak Zt: none
Peak Zt: none
Peak Zt: 6
Peak Zt: 3
Peak Zt: none
Peak Zt: none
Peak Zt: none
Relative Expression
Zt (hr)
21.5
0.5
3.5
6.5
9.5
12.5
18.5
0.5
1.0
1.5
2.0

Singeleton: ENSXMAG00000010989 fam124b

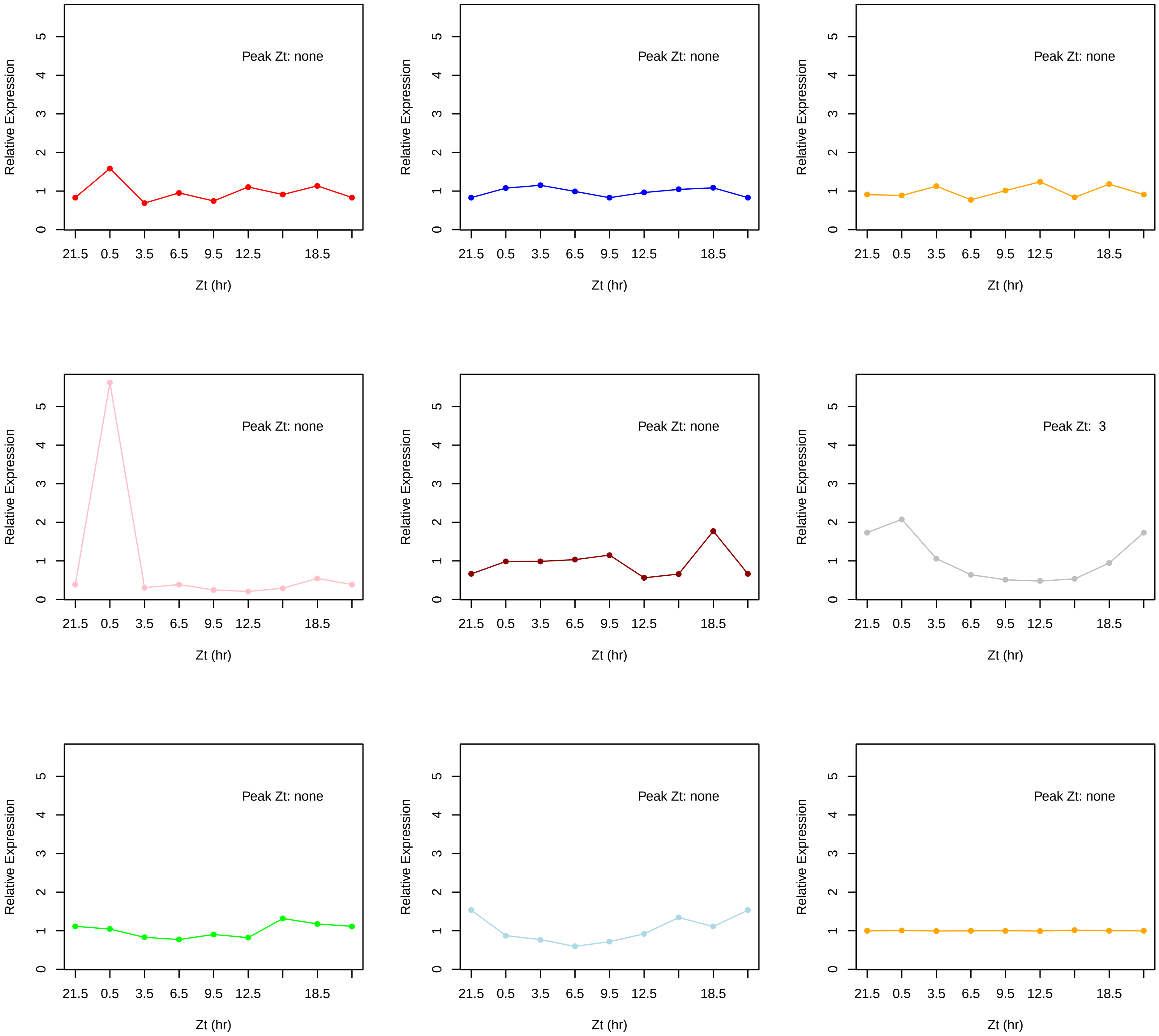

Singeleton: ENSXMAG00000006652 slc37a1

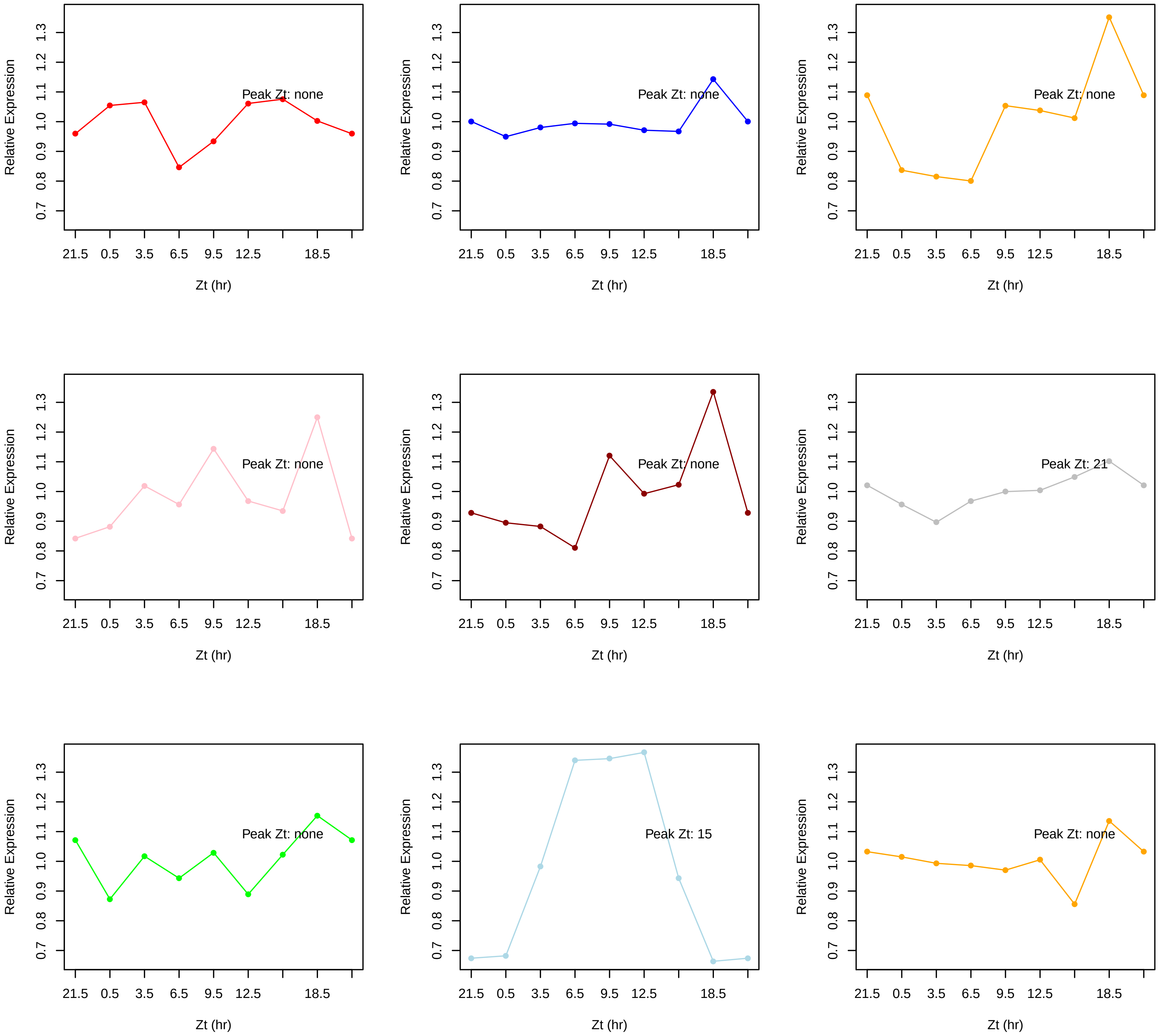

Singeleton: ENSXMAG00000008194 prss16

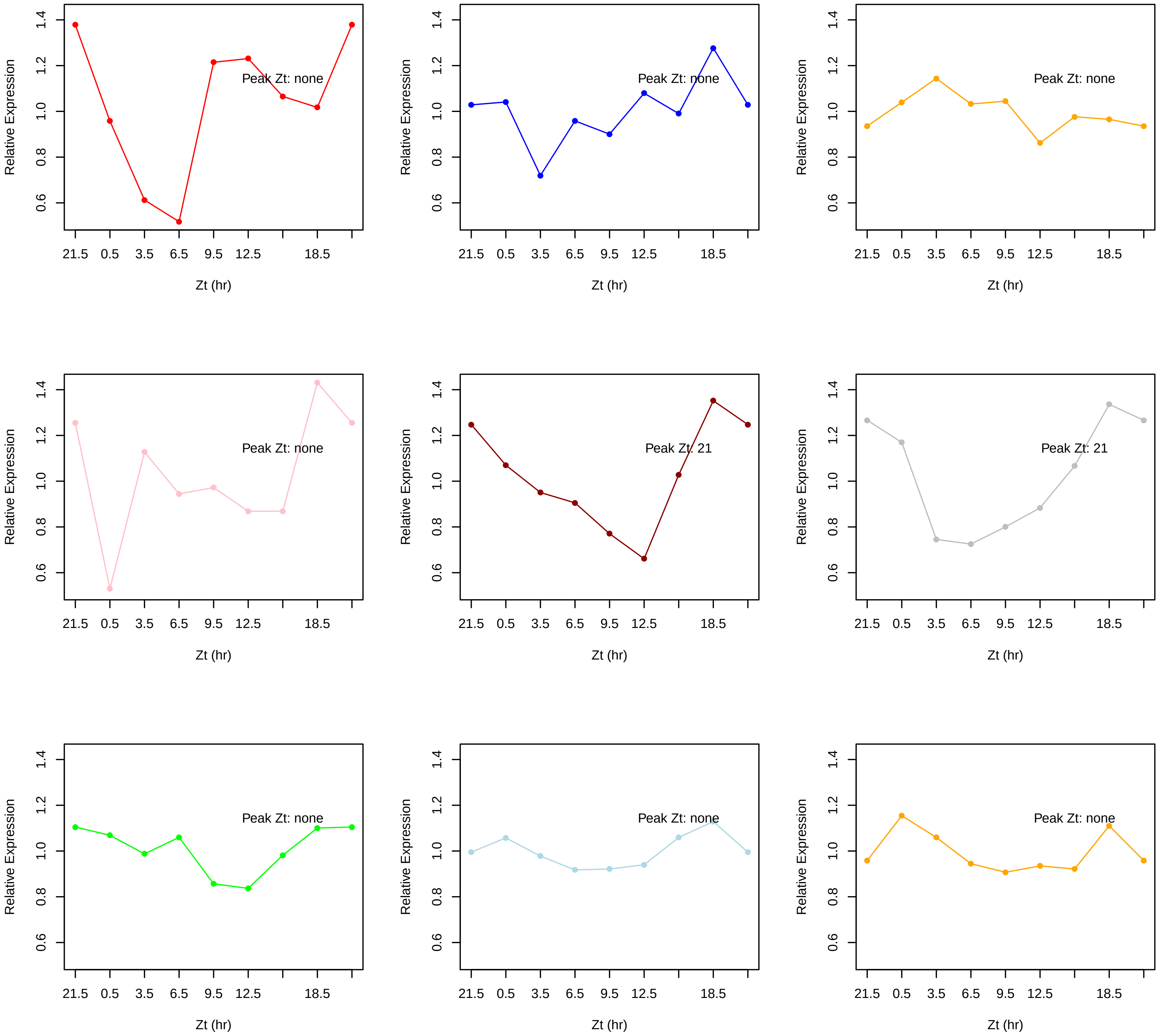

Singeleton: ENSXMAG00000013185 mknk1

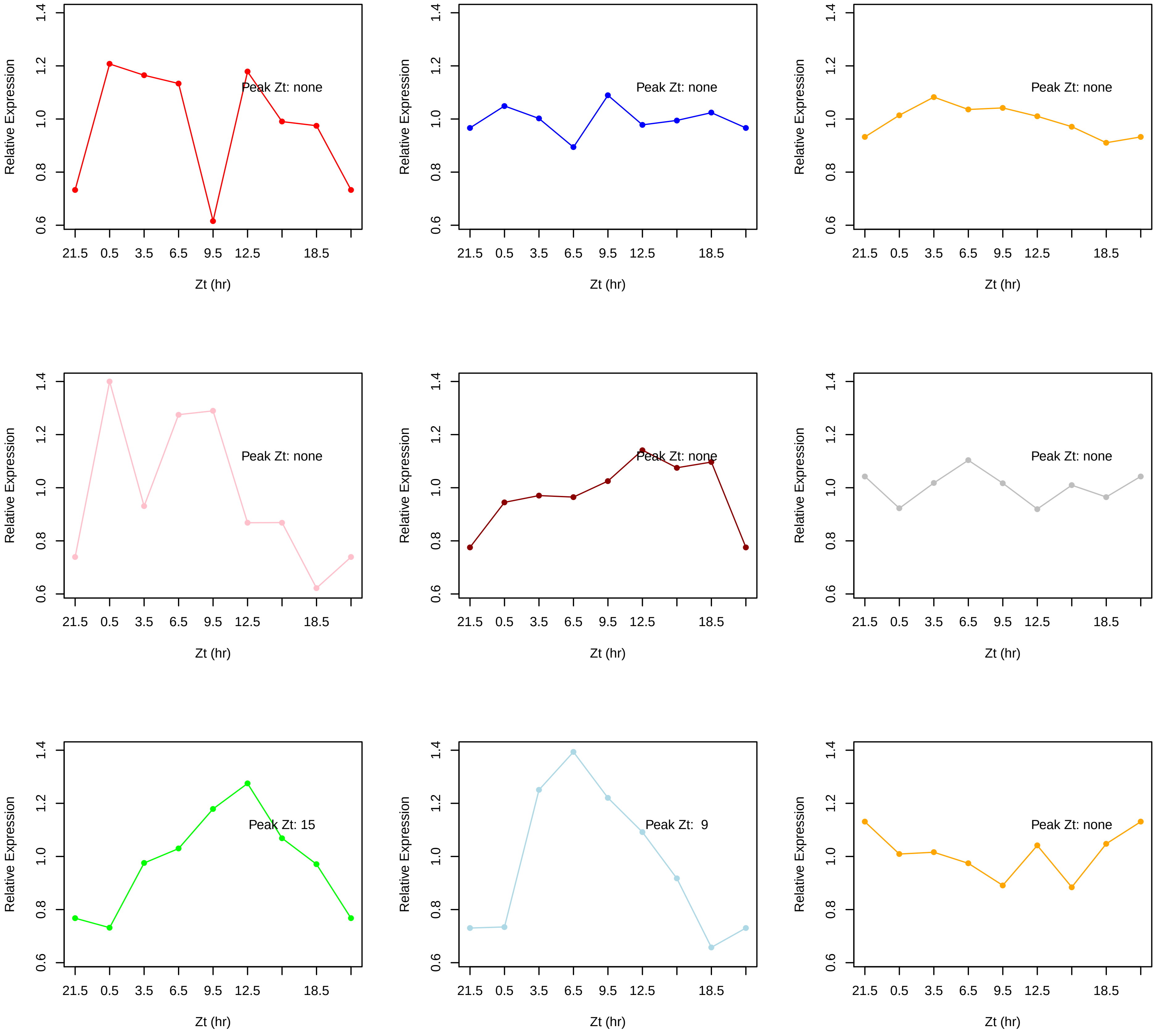

Singeleton: ENSXMAG00000015619 arntl1a

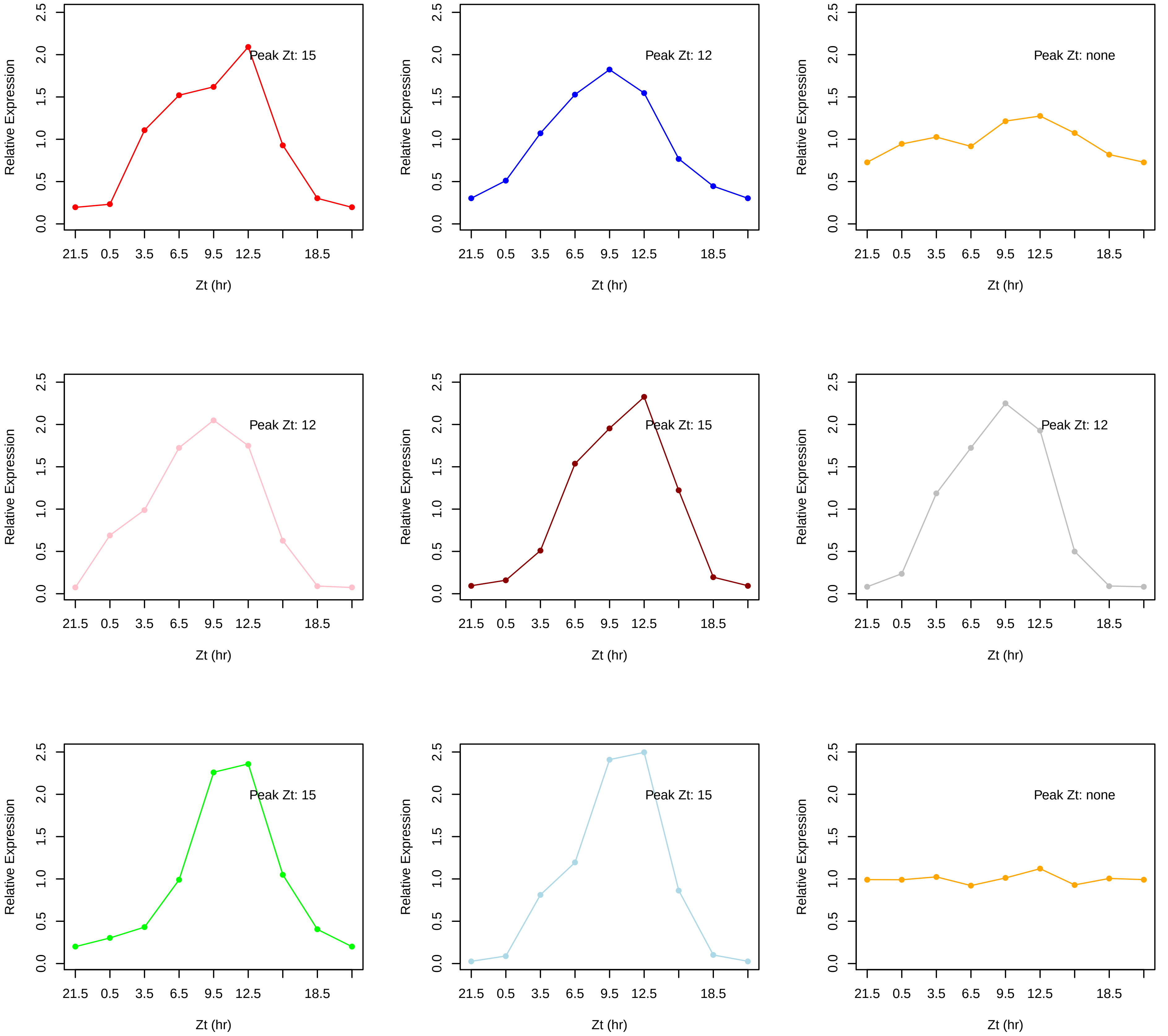

Singleton: ENSXMAG00000010530 si:ch211−113j13.2

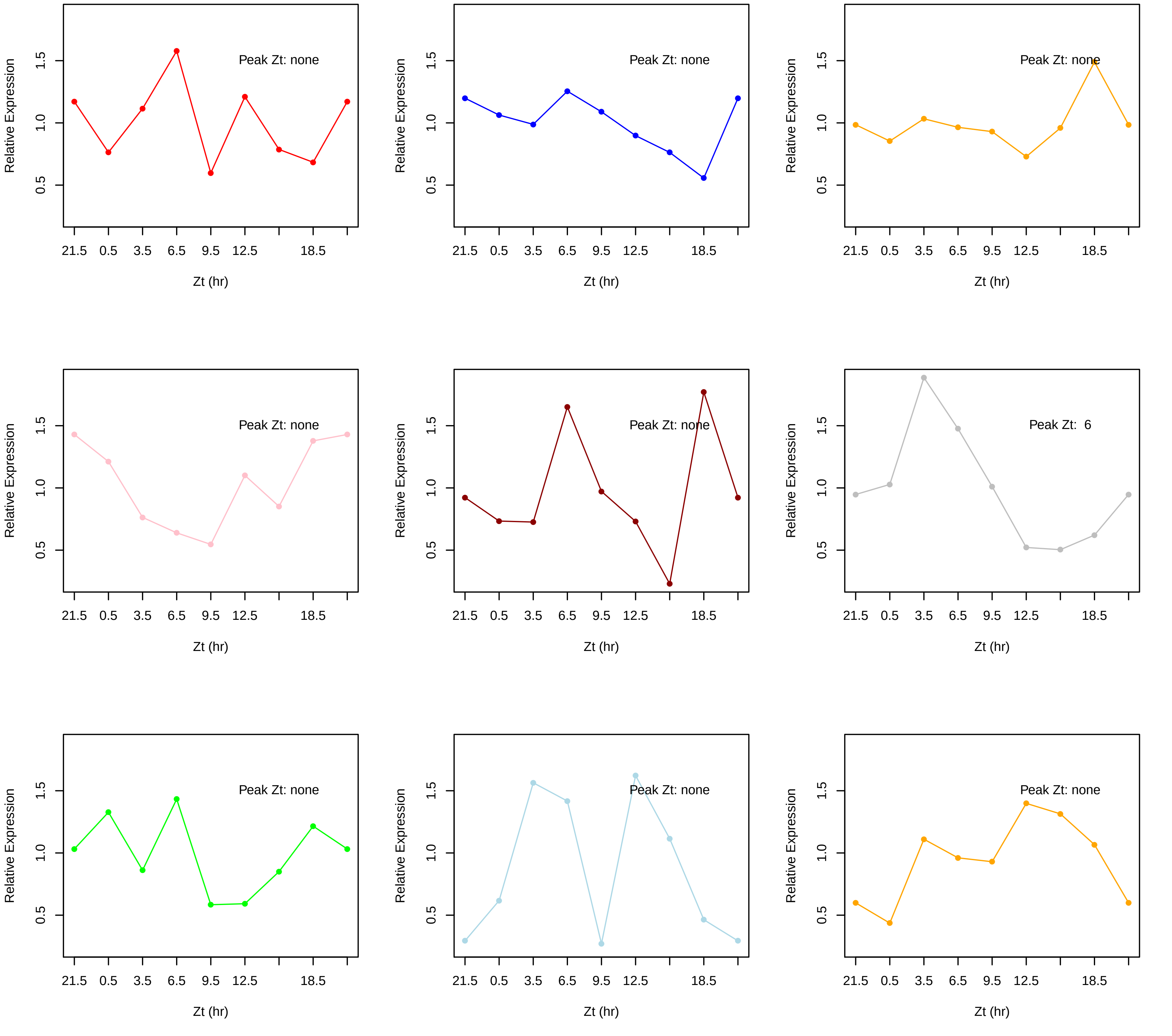

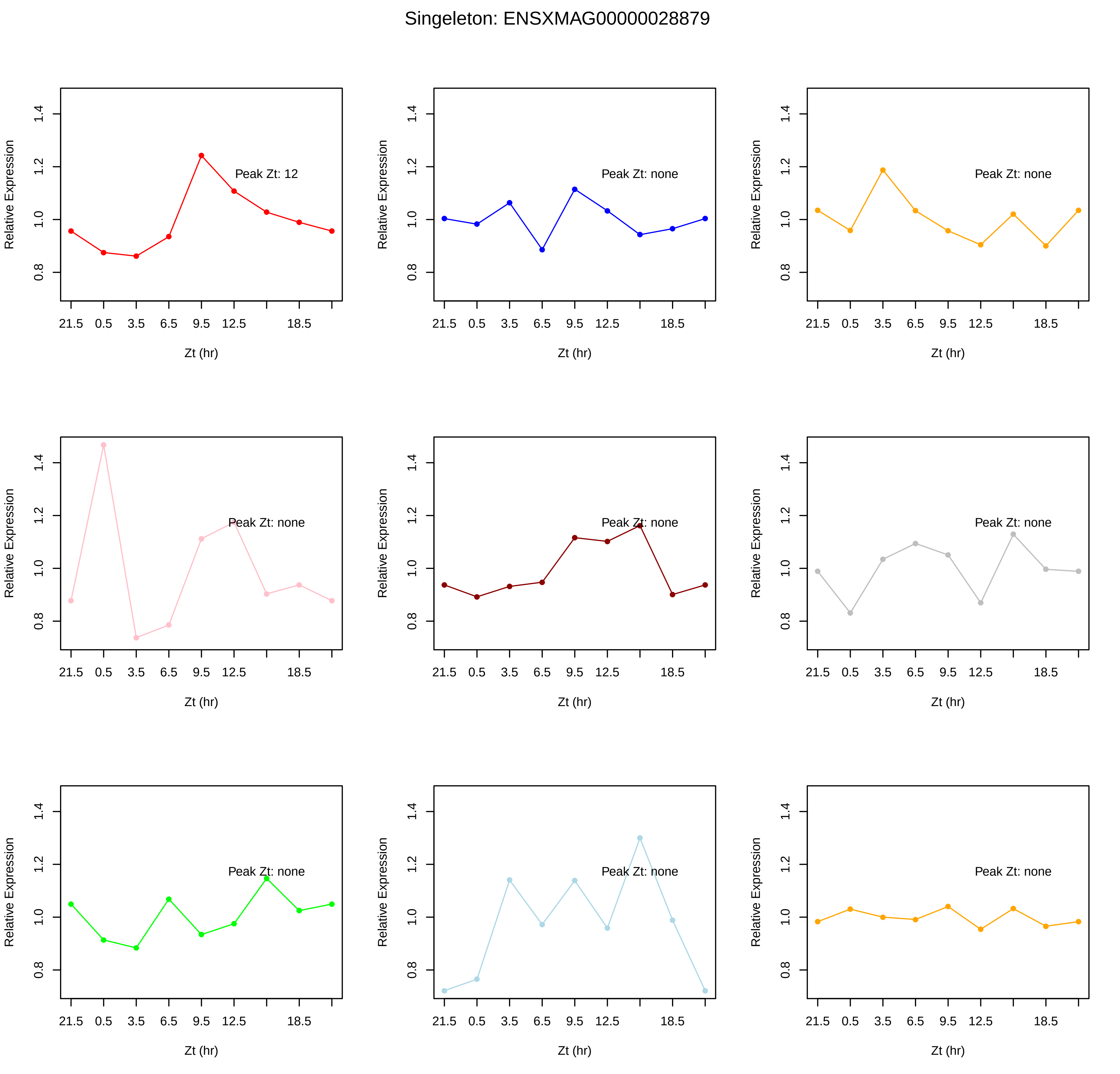

Singeleton: ENSXMAG00000028879
Peak Zt: 12
Peak Zt: none
Peak Zt: none
Peak Zt: none
Peak Zt: none
Peak Zt: none
Peak Zt: none
Peak Zt: none
Peak Zt: none
Relative Expression
Zt (hr)
0.8
1.0
1.2
1.4
21.5
0.5
3.5
6.5
9.5
12.5
18.5

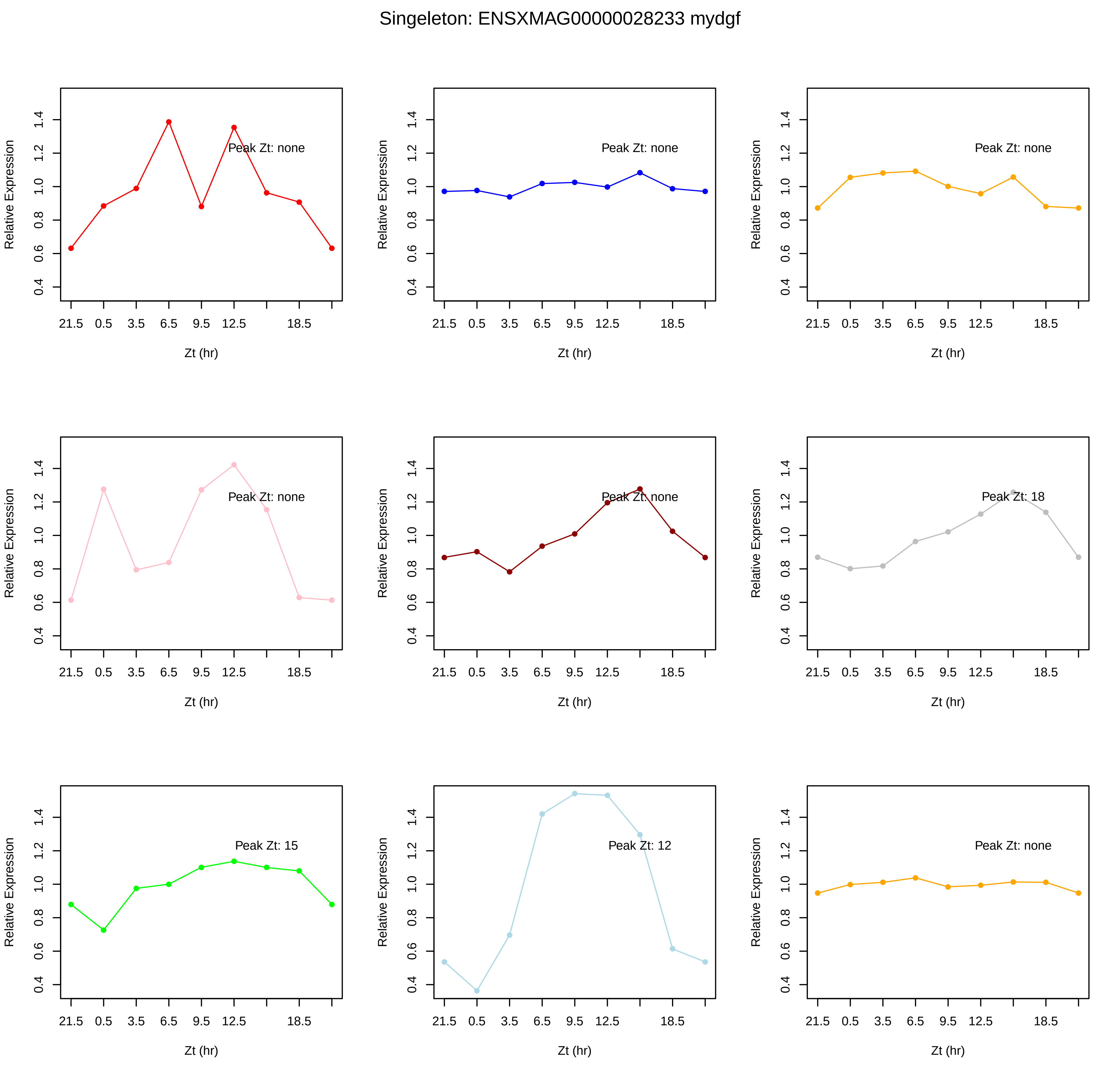

Singleton: ENSXMAG00000028233 mydgf
Relative Expression
Zt (hr)
21.5
0.5
3.5
6.5
9.5
12.5
18.5
0.4
0.6
0.8
1.0
1.2
1.4
Peak Zt: none
Peak Zt: none
Peak Zt: none
Peak Zt: none
Peak Zt: none
Peak Zt: 18
Peak Zt: 15
Peak Zt: 12
Peak Zt: none

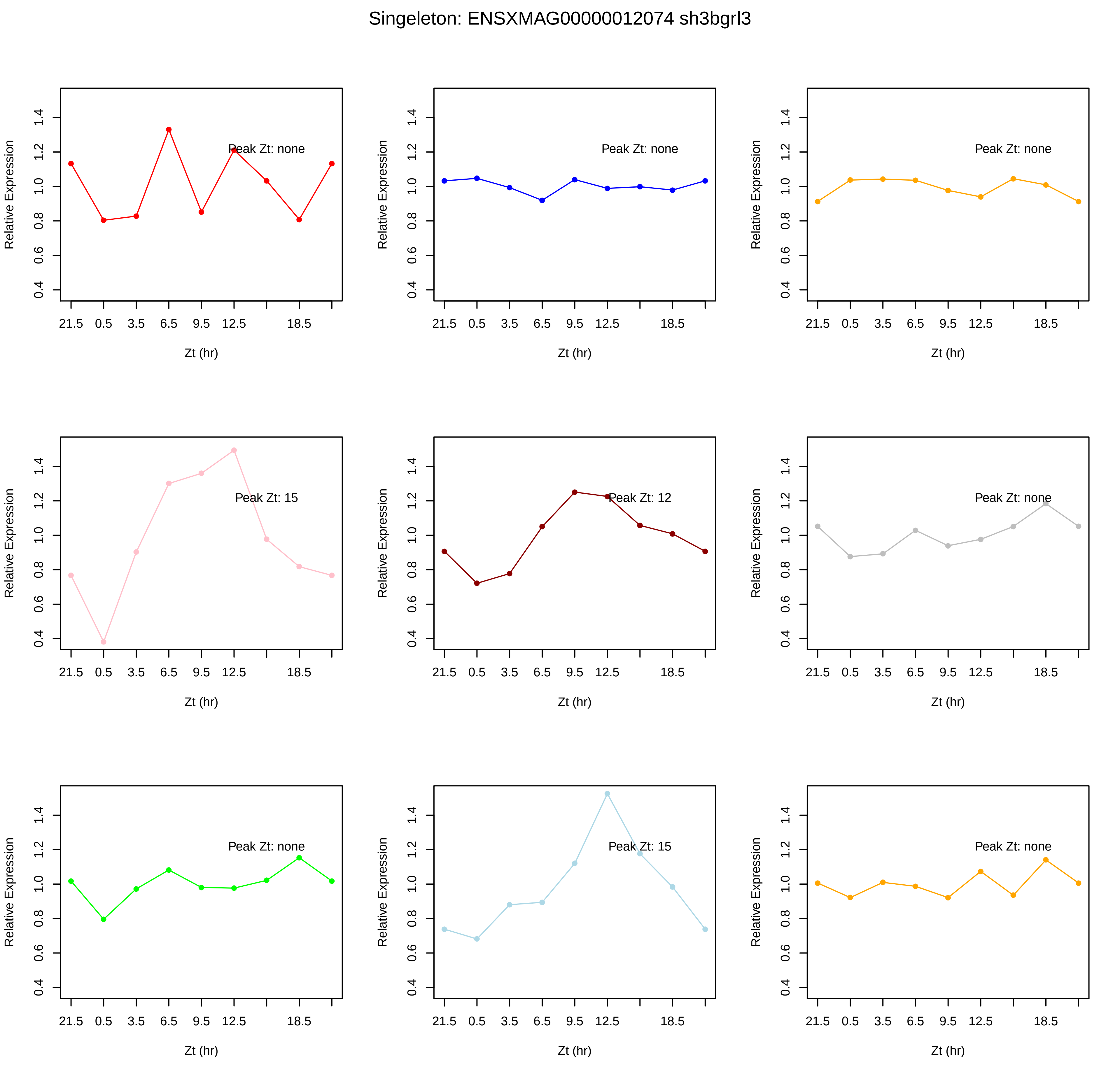
Singeleton: ENSXMAG00000012074 sh3bgrl3
Peak Zt: none
Peak Zt: none
Peak Zt: none
Peak Zt: 15
Peak Zt: 12
Peak Zt: none
Peak Zt: none
Peak Zt: 15
Peak Zt: none
Relative Expression
Zt (hr)
21.5
0.5
3.5
6.5
9.5
12.5
18.5
0.4
0.6
0.8
1.0
1.2
1.4

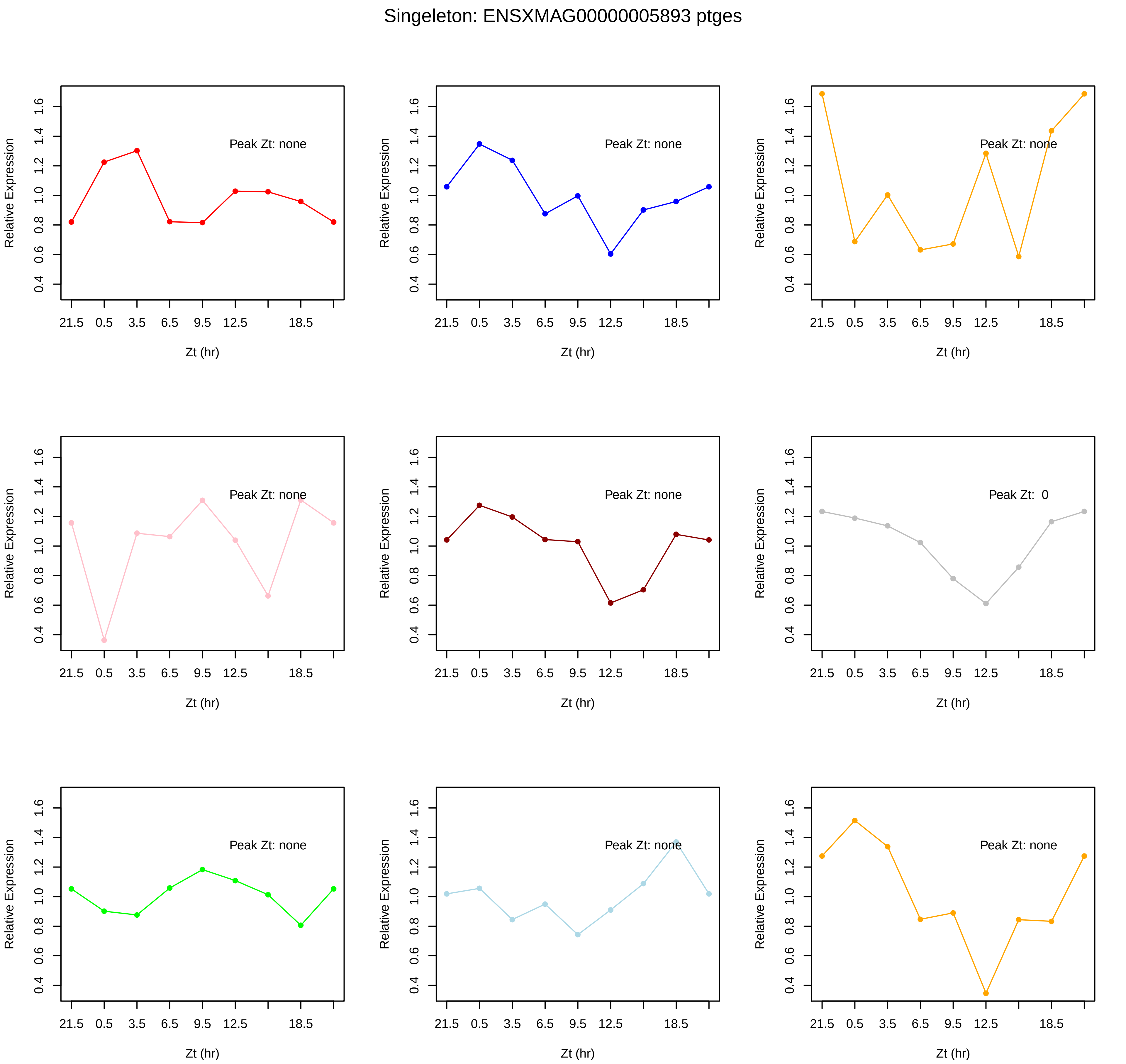
Singleton: ENSXMAG00000005893 ptges
Peak Zt: none
Peak Zt: none
Peak Zt: none
Peak Zt: none
Peak Zt: none
Peak Zt: 0
Peak Zt: none
Peak Zt: none
Peak Zt: none
Relative Expression
Zt (hr)
21.5
0.5
3.5
6.5
9.5
12.5
18.5
0.4
0.6
0.8
1.0
1.2
1.4
1.6

Singeleton: ENSXMAG00000025965 LACTB2

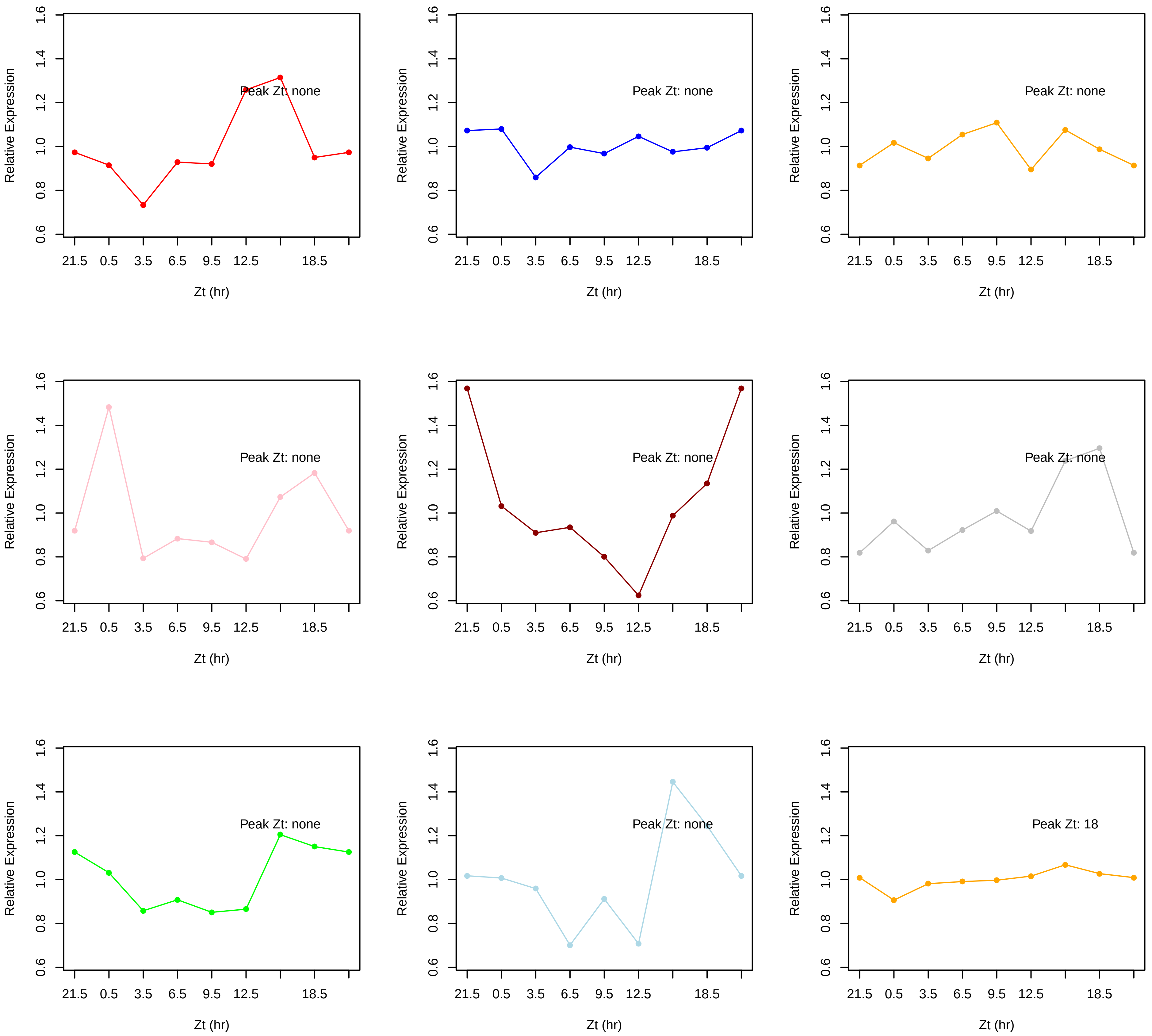

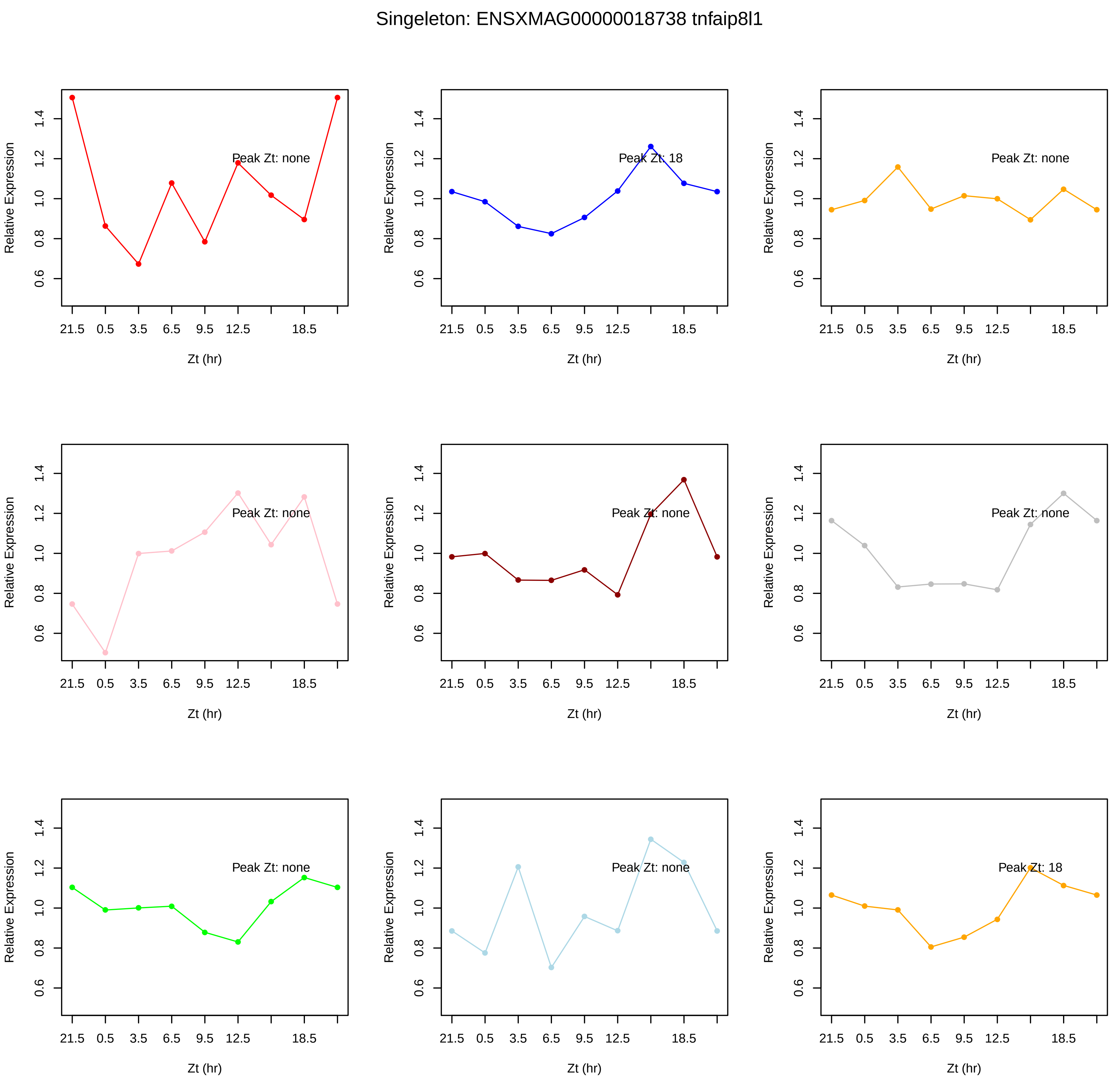
Singeleton: ENSXMAG00000018738 tnfaip8l1
Relative Expression
Zt (hr)
21.5
0.5
3.5
6.5
9.5
12.5
18.5
0.6
0.8
1.0
1.2
1.4
Peak Zt: none
Peak Zt: 18
Peak Zt: none
Peak Zt: none
Peak Zt: none
Peak Zt: none
Peak Zt: none
Peak Zt: none
Peak Zt: 18

Singleton: ENSXMAG00000005842 usp20

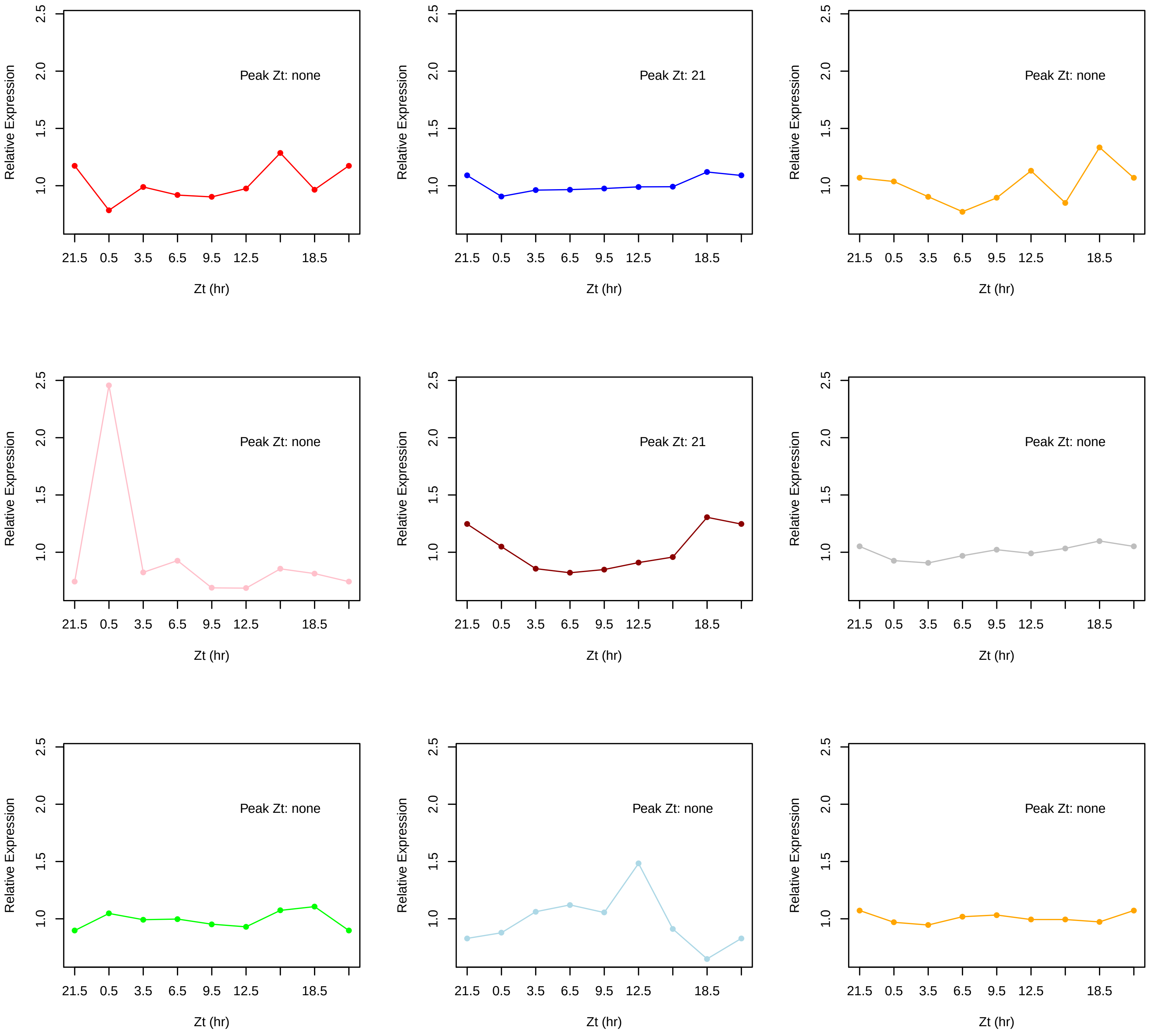

Singeleton: ENSXMAG00000018262 pxmp2

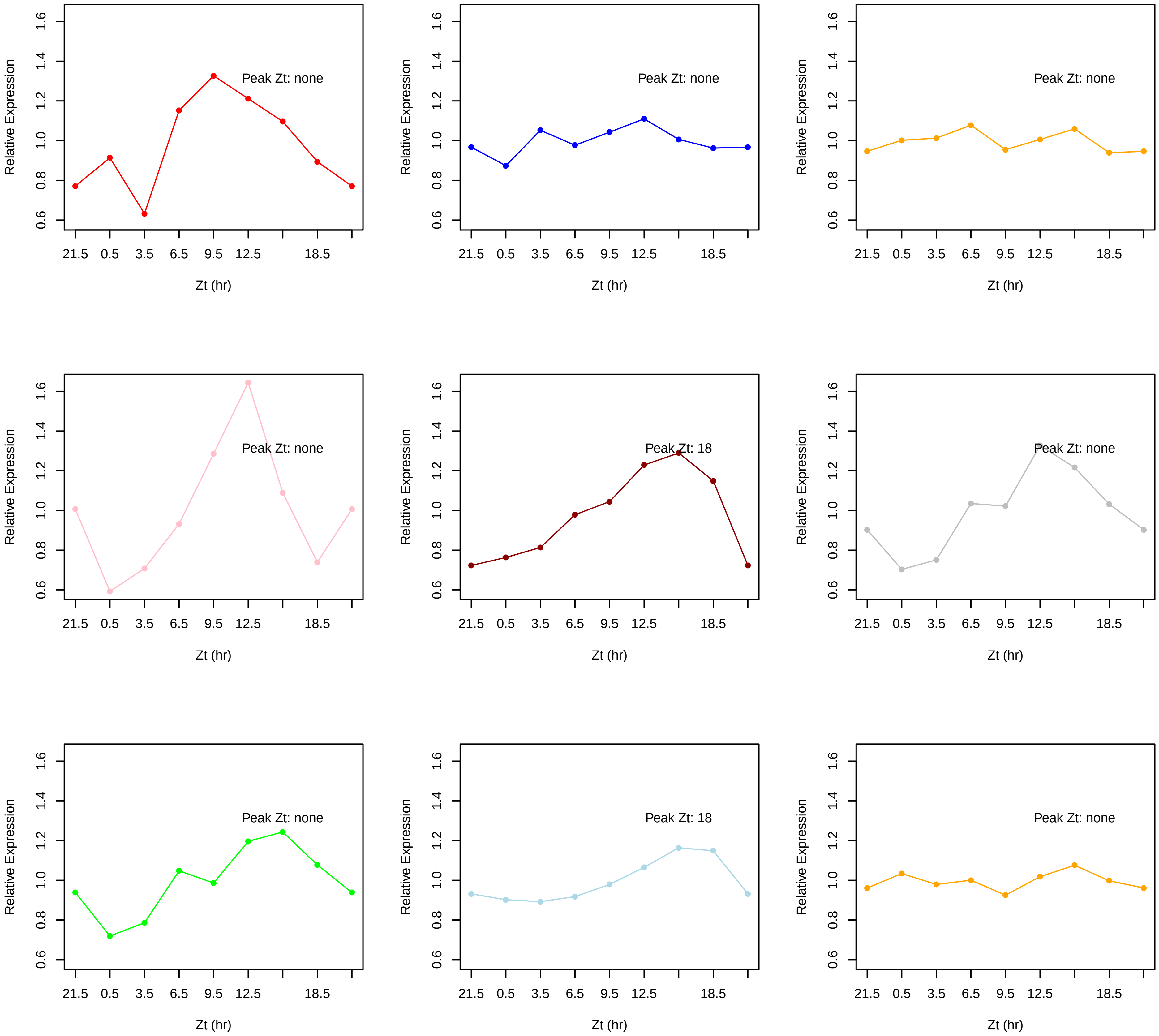

Singeleton: ENSXMAG00000018730 cope

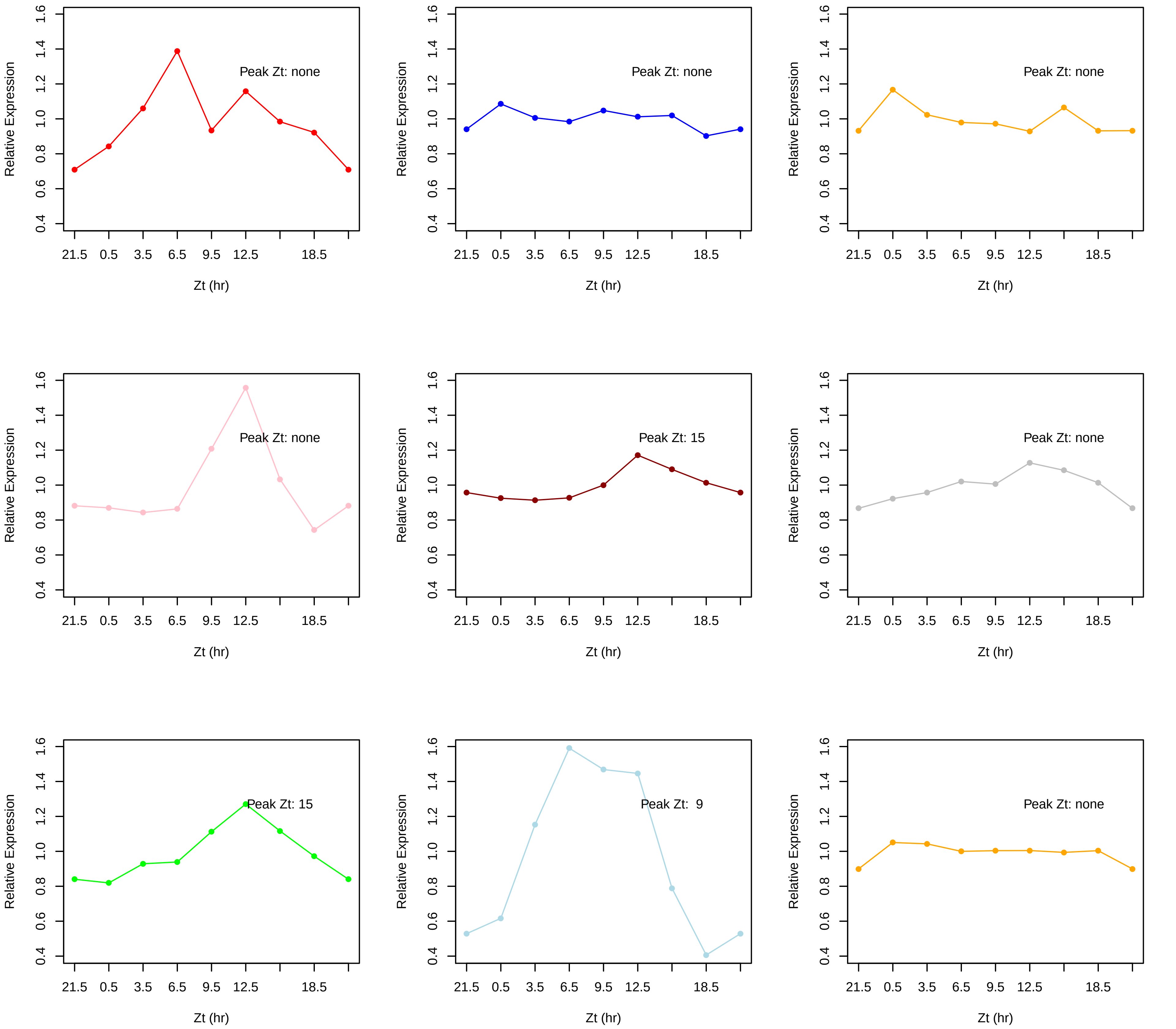

Singleton: ENSXMAG00000005275

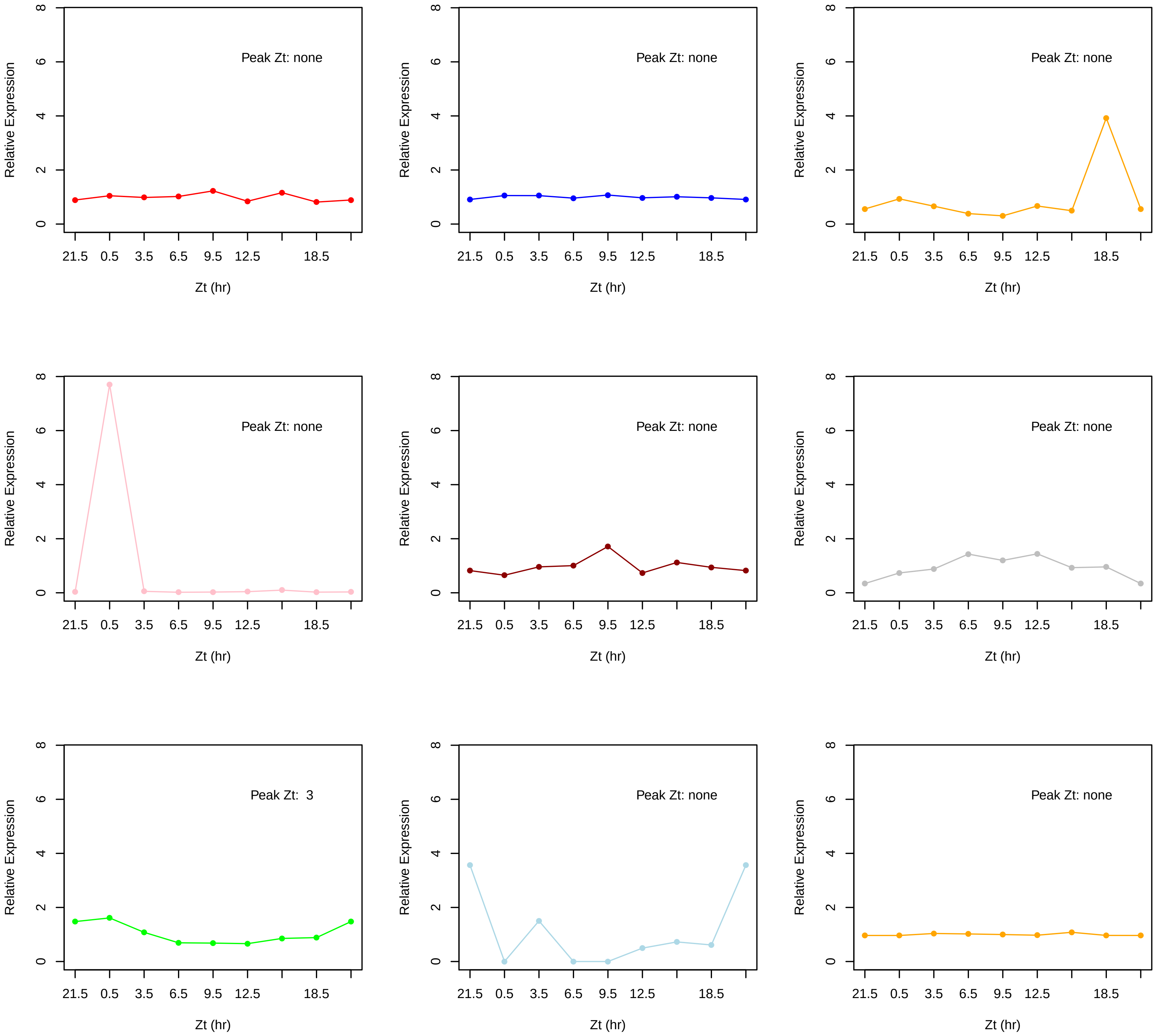

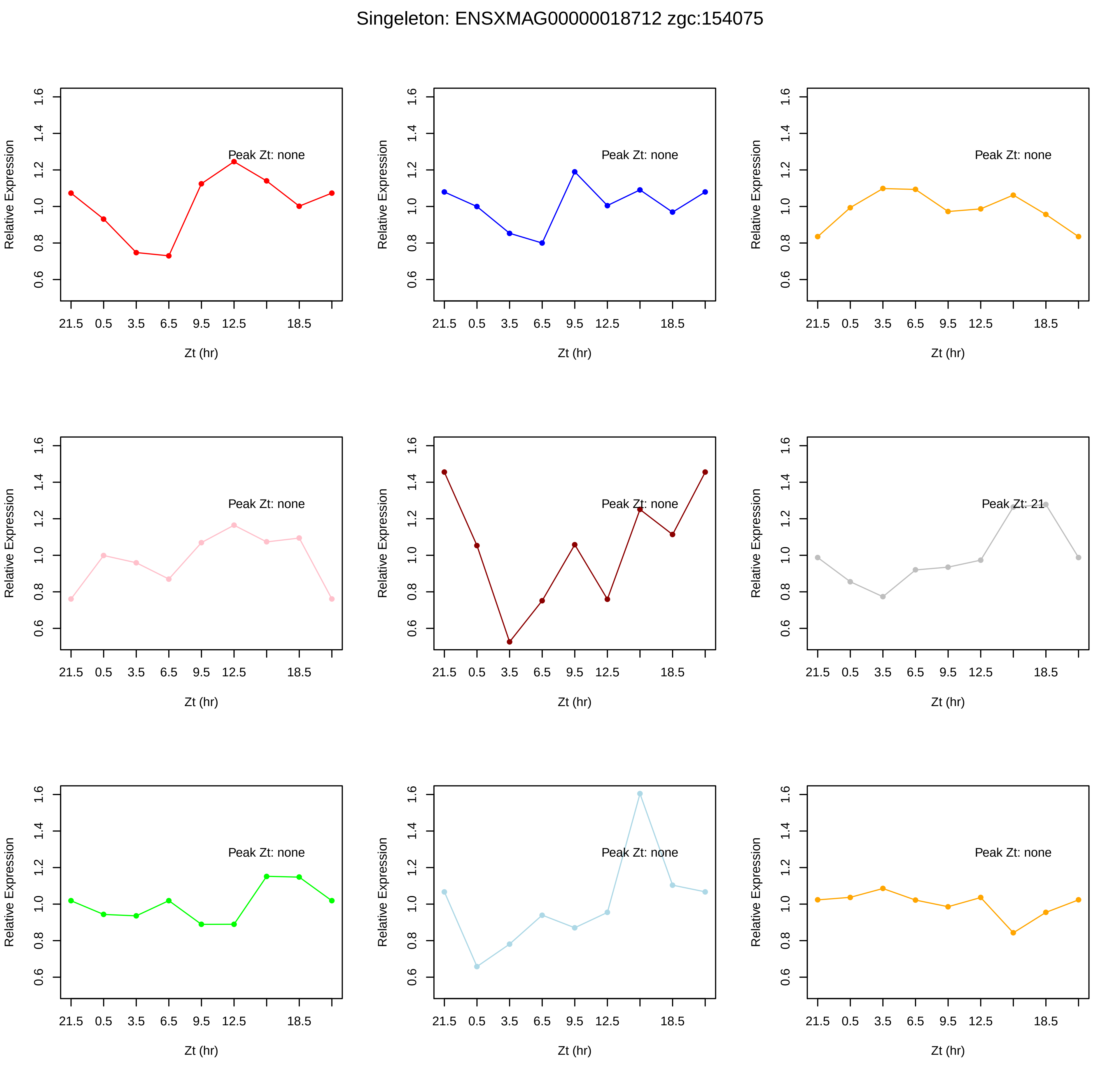
Singeleton: ENSXMAG00000018712 zgc:154075
Peak Zt: none
Peak Zt: none
Peak Zt: none
Peak Zt: none
Peak Zt: none
Peak Zt: 21
Peak Zt: none
Peak Zt: none
Peak Zt: none
Relative Expression
Zt (hr)
21.5
0.5
3.5
6.5
9.5
12.5
18.5

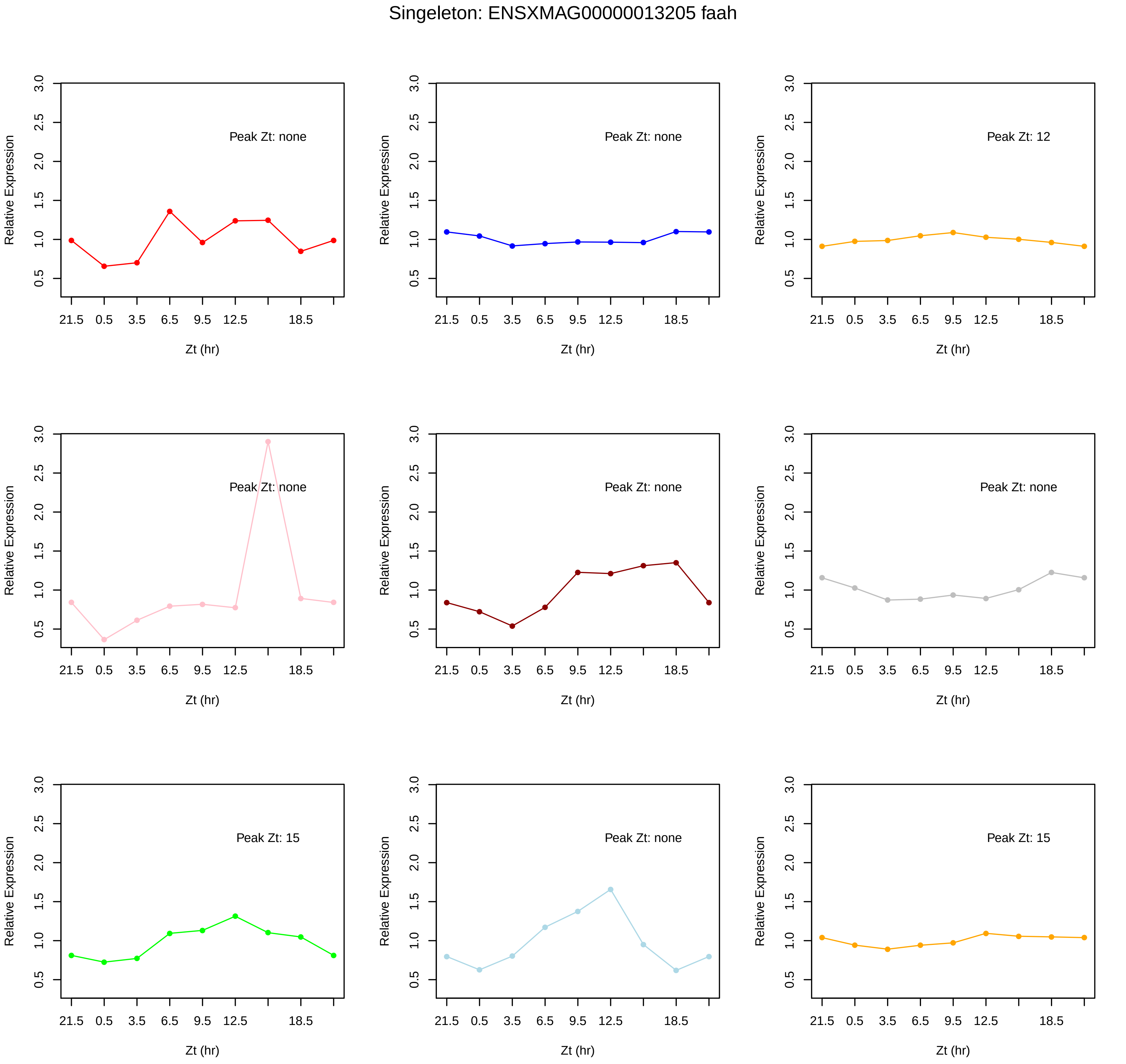

Singeleton: ENSXMAG00000013205 faah
Peak Zt: none
Peak Zt: none
Peak Zt: 12
Peak Zt: none
Peak Zt: none
Peak Zt: none
Peak Zt: 15
Peak Zt: none
Peak Zt: 15
Relative Expression
Zt (hr)
21.5 0.5 3.5 6.5 9.5 12.5 18.5
0.5 1.0 1.5 2.0 2.5 3.0

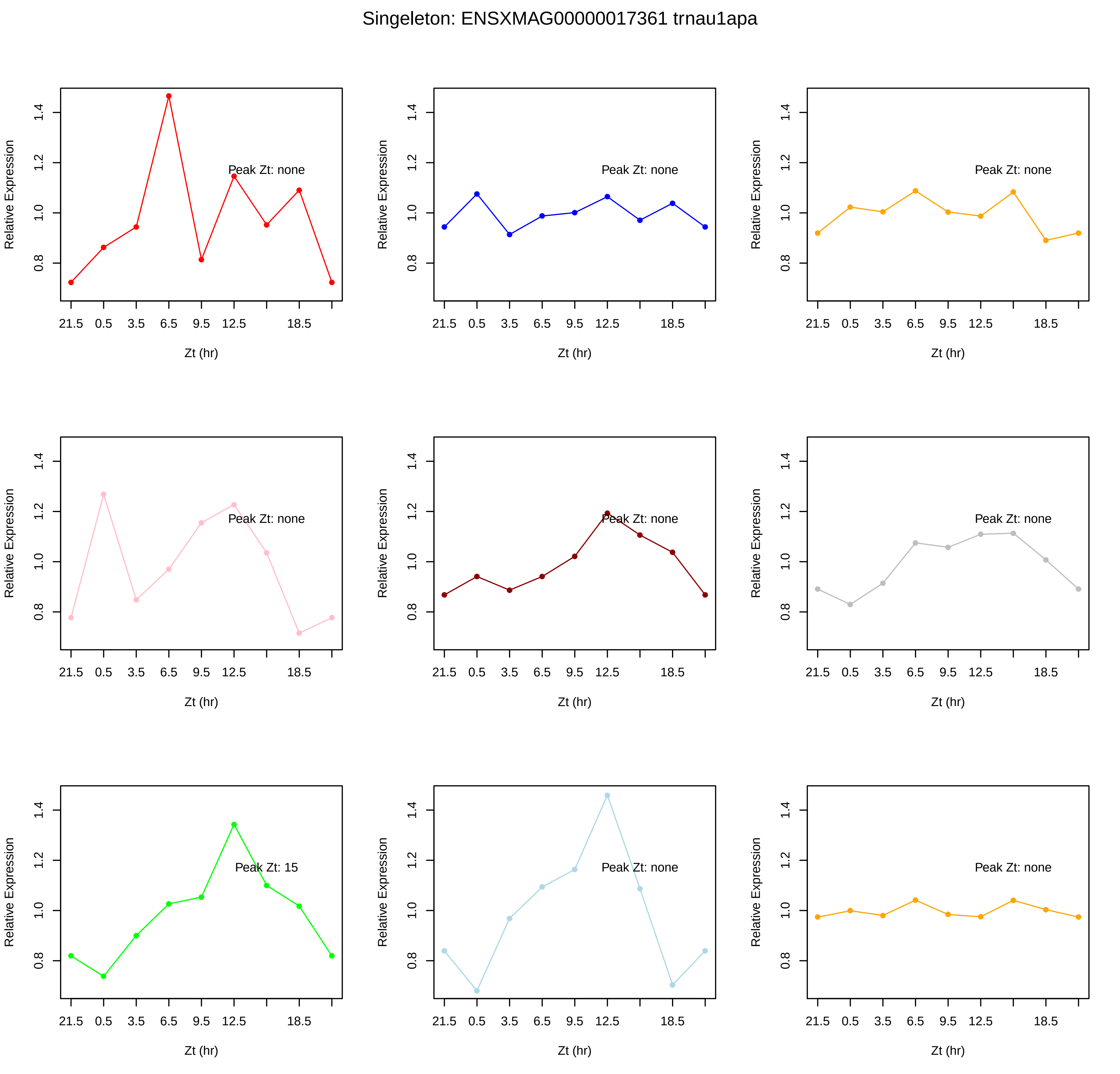
Singeleton: ENSXMAG00000017361 trnau1apa
Relative Expression
Zt (hr)
Peak Zt: none
Peak Zt: 15
21.5
0.5
3.5
6.5
9.5
12.5
18.5
0.8
1.0
1.2
1.4

Singeleton: ENSXMAG00000027759 znf407

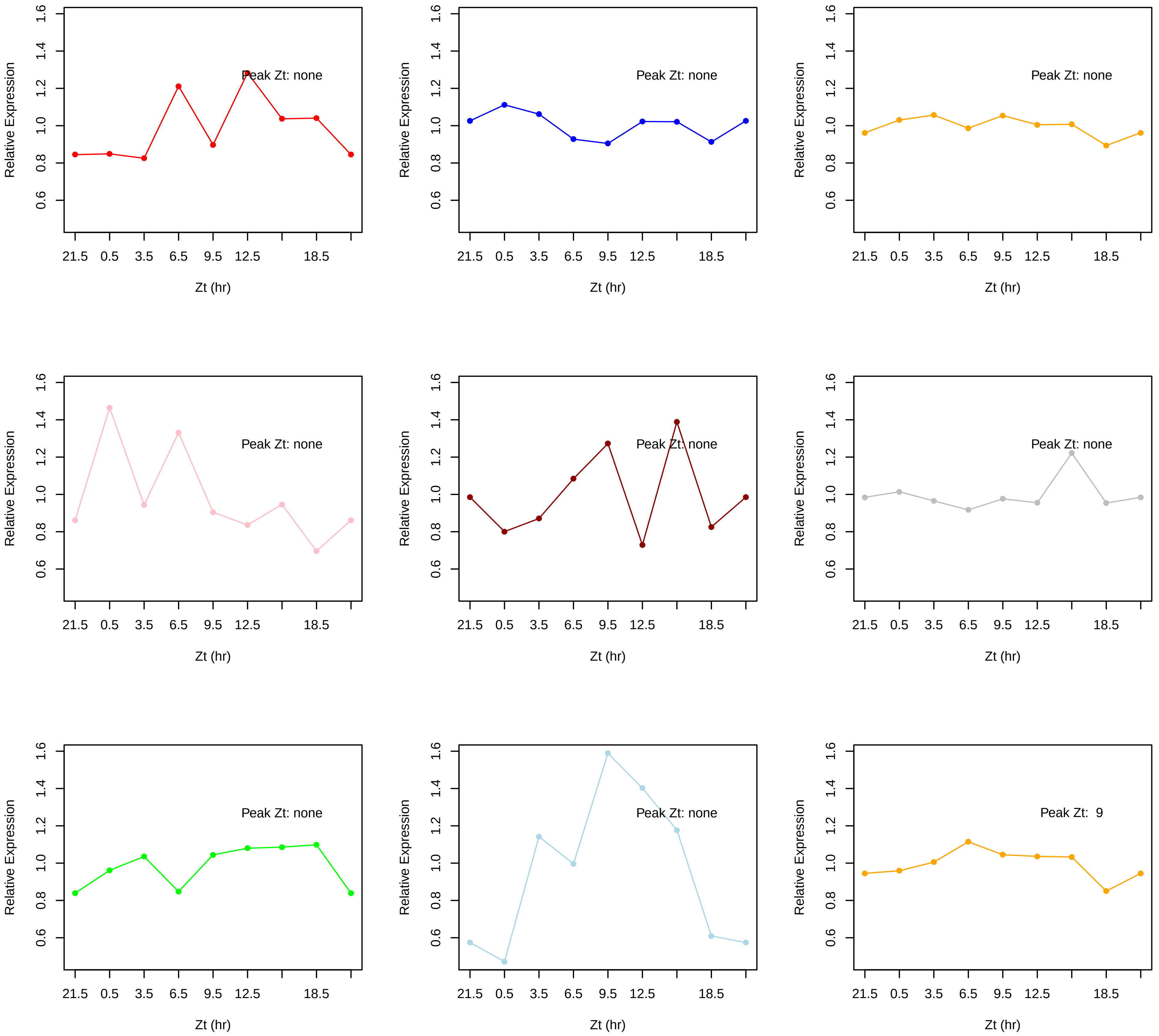

Singeleton: ENSXMAG00000018726 cers1

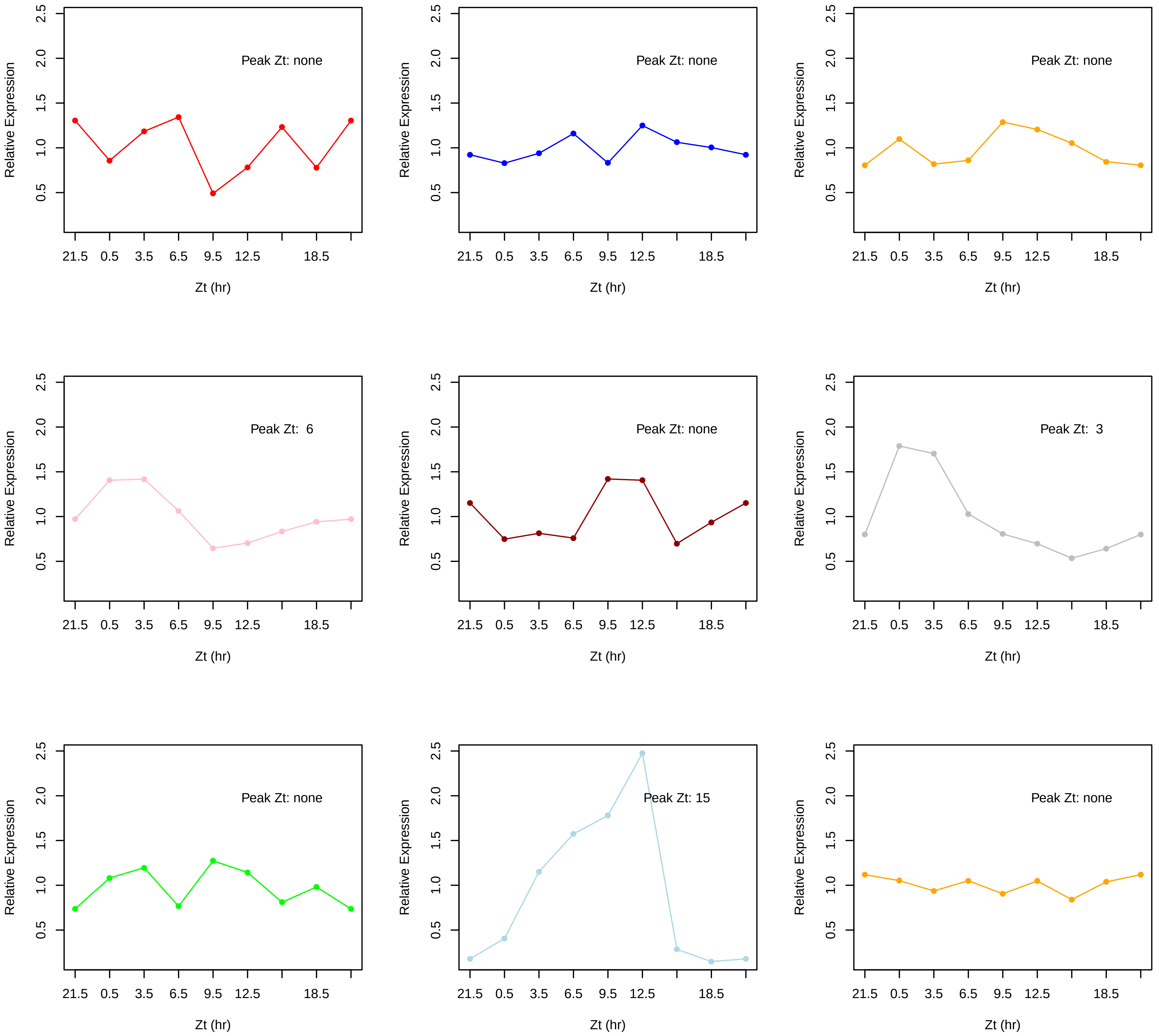

Singeleton: ENSXMAG00000004170 si:ch1073−390k14.1

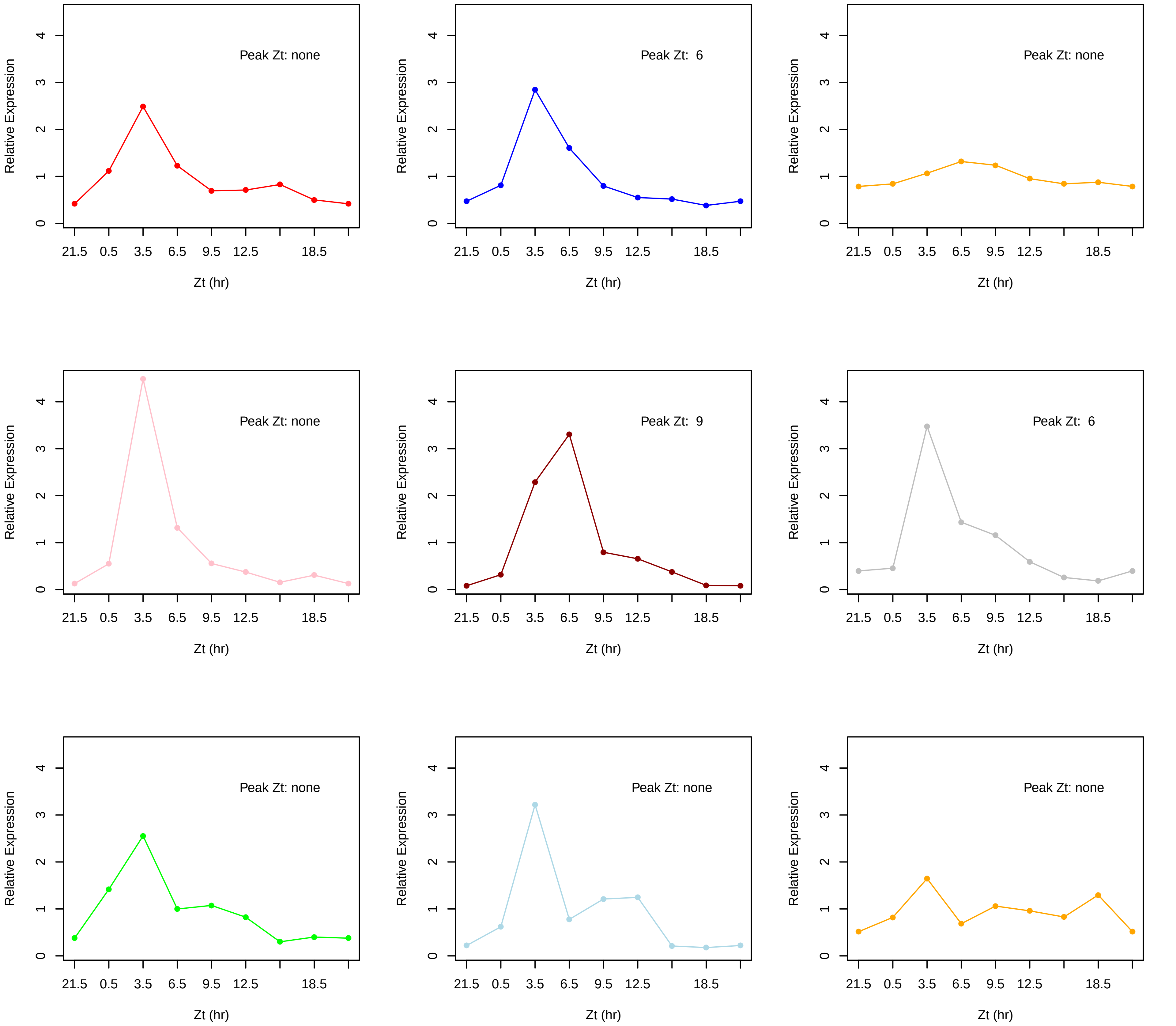

Singeleton: ENSXMAG00000010342 dnajb11

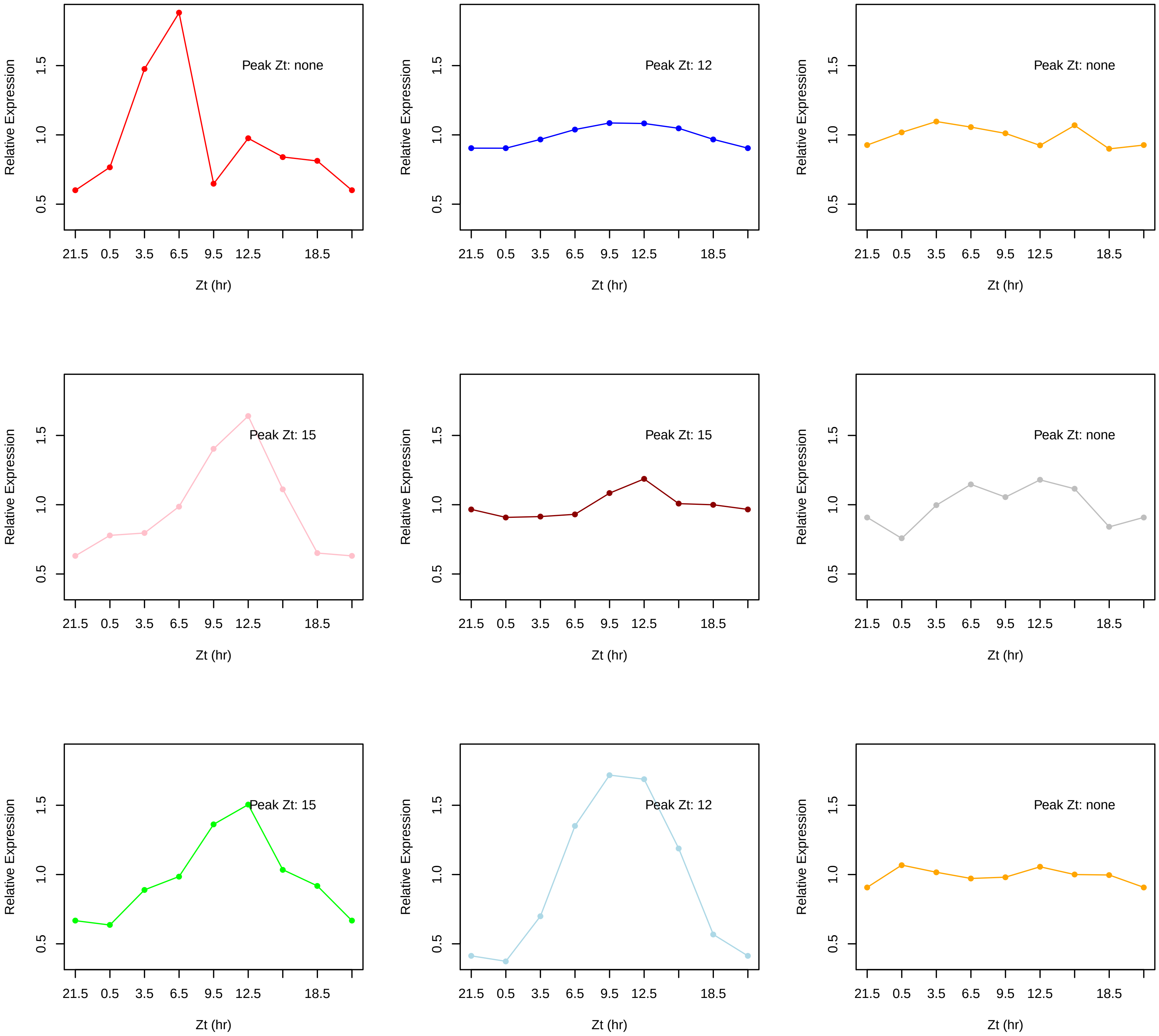

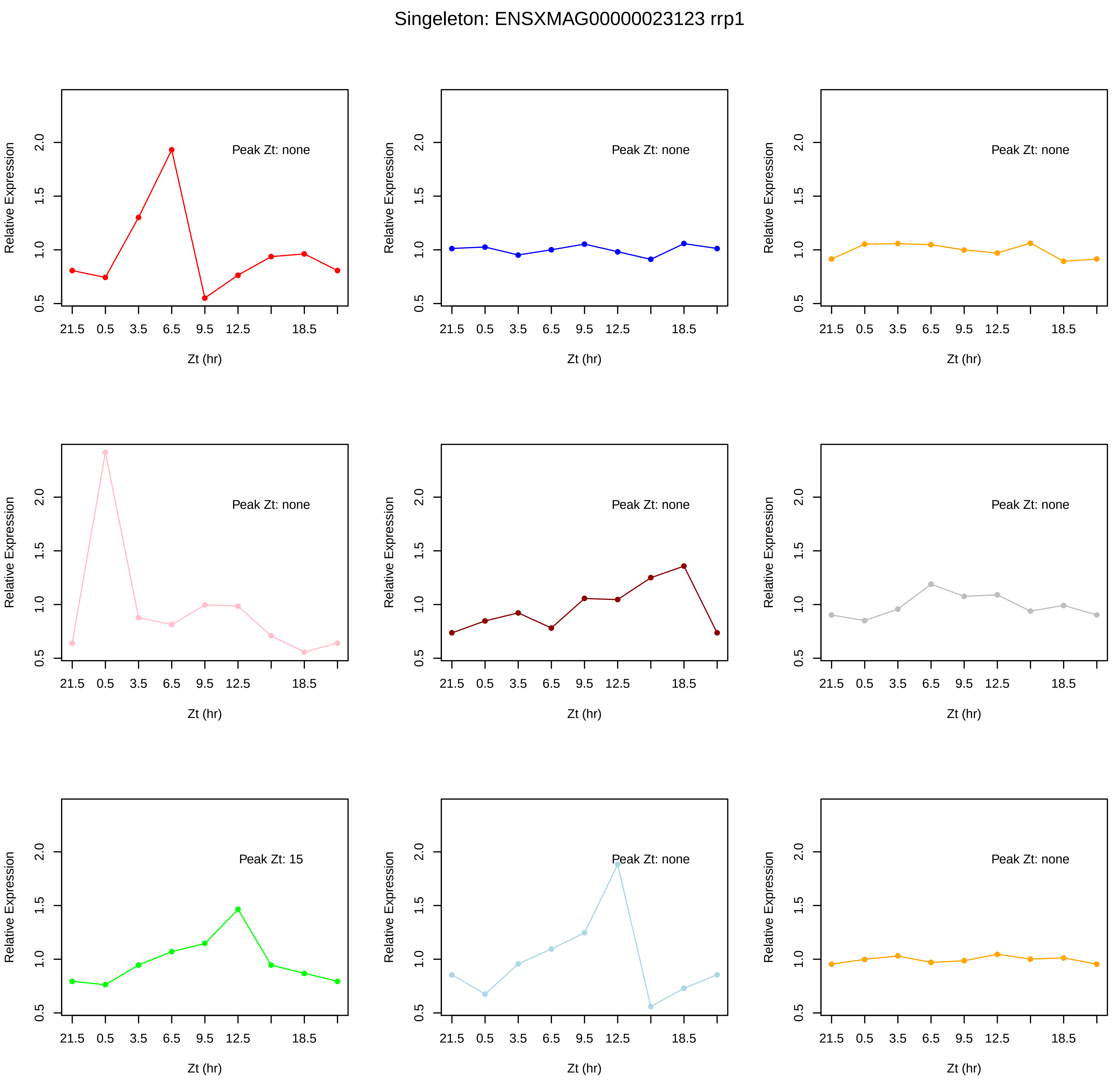
Singeleton: ENSXMAG00000023123 rrp1
Peak Zt: none
Peak Zt: none
Peak Zt: none
Peak Zt: none
Peak Zt: none
Peak Zt: none
Peak Zt: 15
Peak Zt: none
Peak Zt: none
Relative Expression
Zt (hr)
21.5
0.5
3.5
6.5
9.5
12.5
18.5
0.5
1.0
1.5
2.0

Singeleton: ENSXMAG00000010029 shisa2b

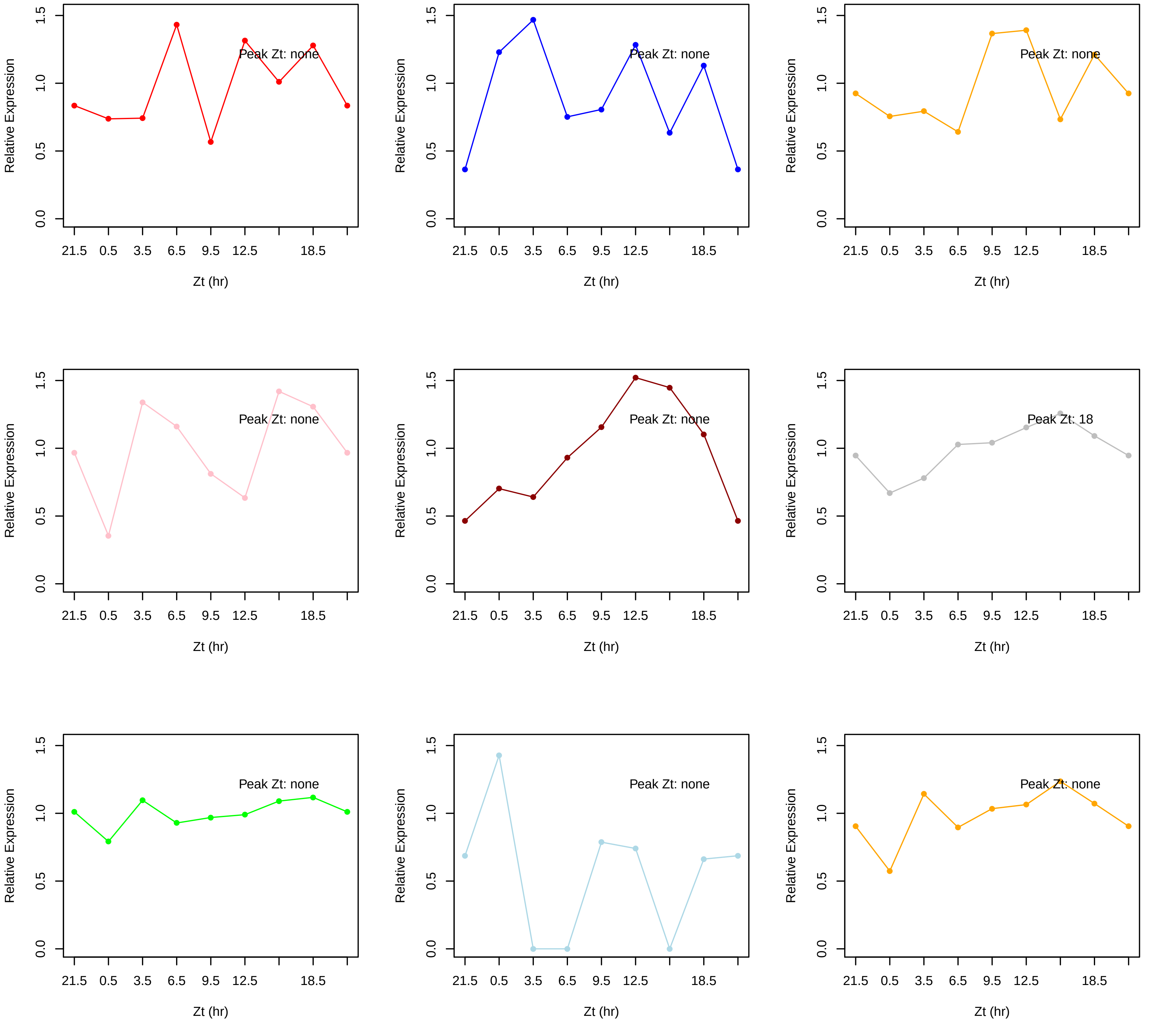

Singleton: ENSXMAG00000019585 triap1

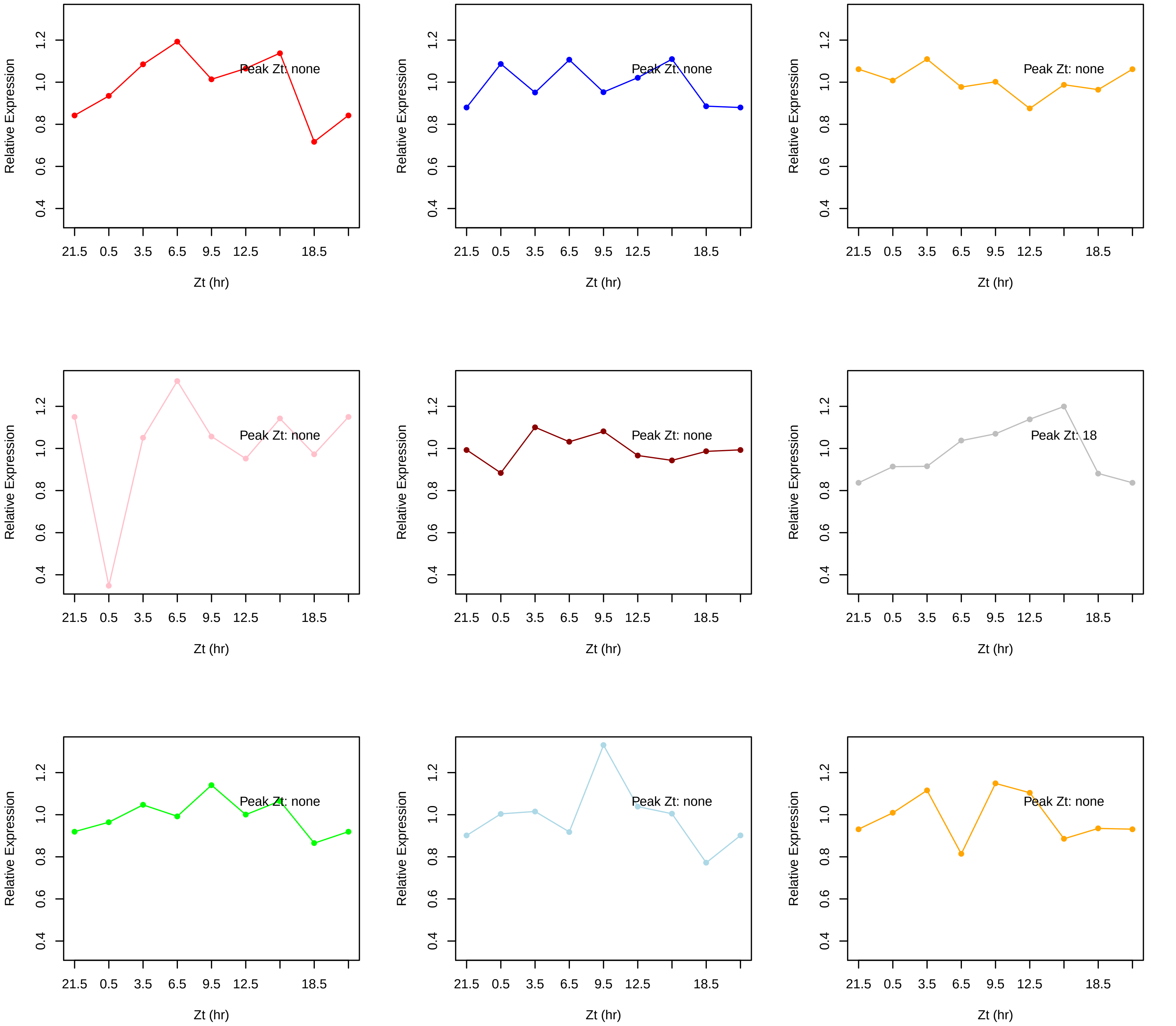

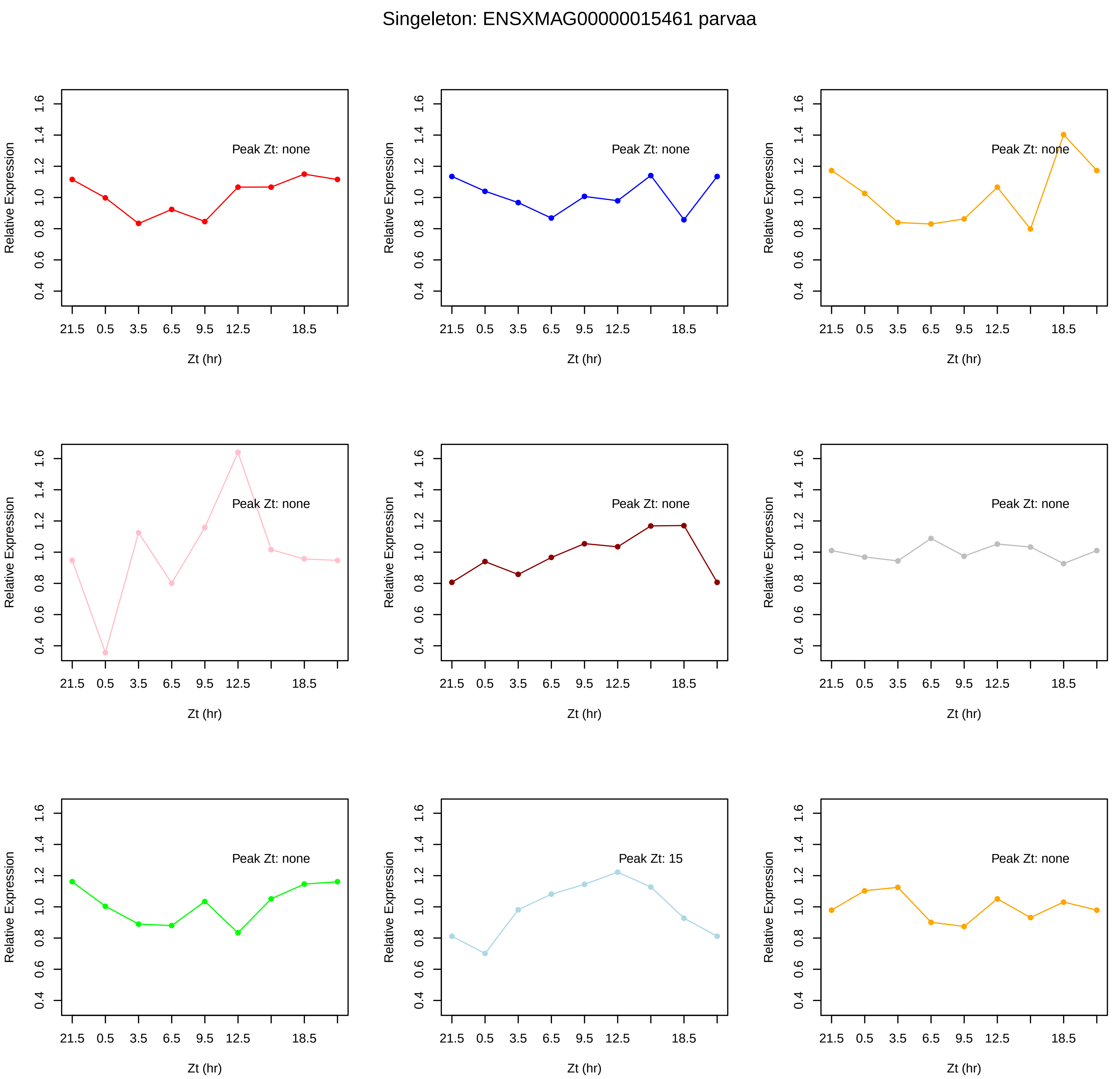

Singeleton: ENSXMAG00000015461 parvaa
Relative Expression
Zt (hr)
Peak Zt: none
Peak Zt: none
Peak Zt: none
Peak Zt: none
Peak Zt: none
Peak Zt: none
Peak Zt: none
Peak Zt: 15
Peak Zt: none
21.5 0.5 3.5 6.5 9.5 12.5 18.5
0.4 0.6 0.8 1.0 1.2 1.4 1.6

Singeleton: ENSXMAG00000015184 tbccd1

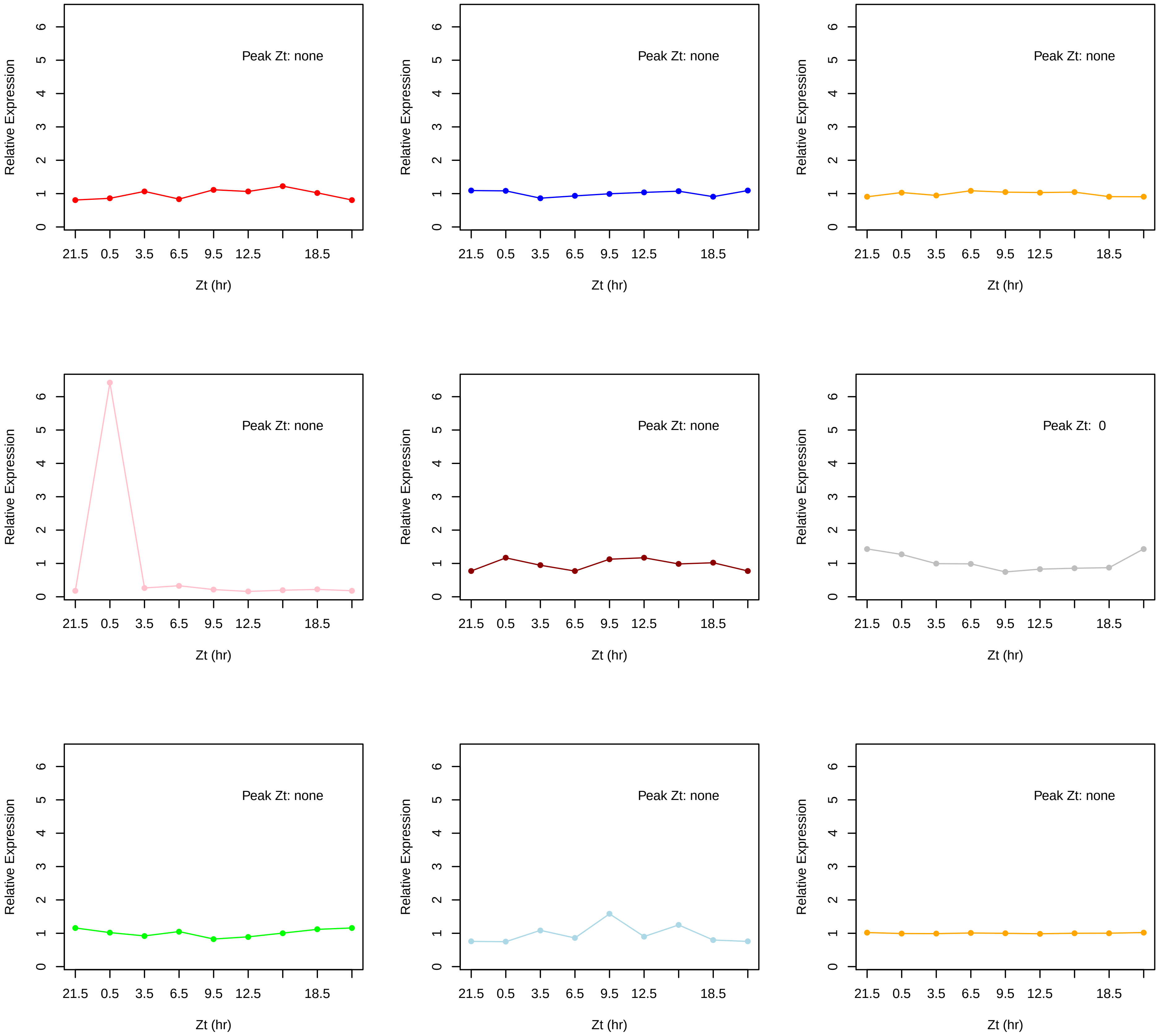

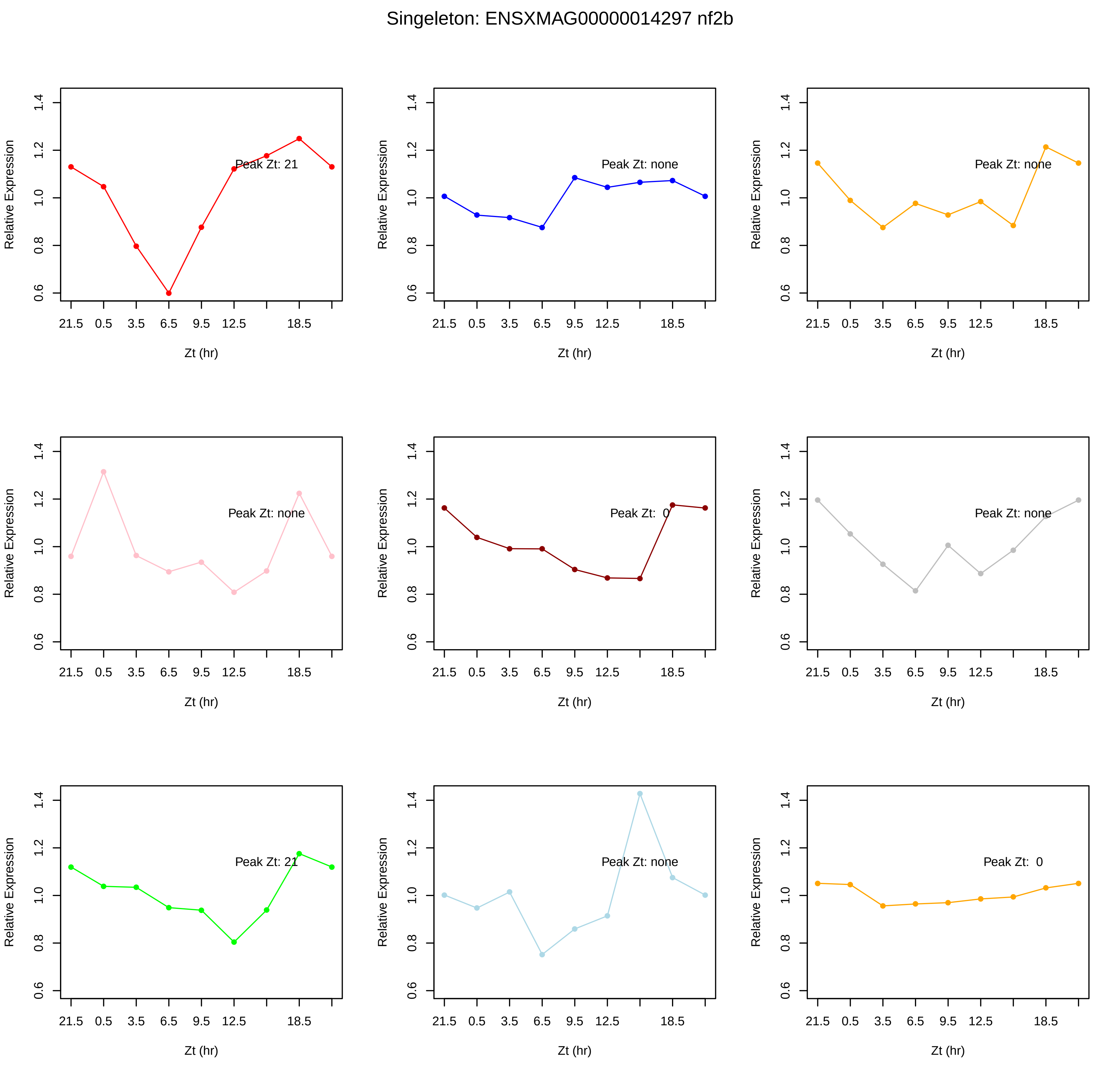

Singeleton: ENSXMAG00000014297 nf2b
Relative Expression
Zt (hr)
21.5 0.5 3.5 6.5 9.5 12.5 18.5
0.6 0.8 1.0 1.2 1.4
Peak Zt: 21
Peak Zt: none
Peak Zt: none
Peak Zt: none
Peak Zt: 0
Peak Zt: none
Peak Zt: 21
Peak Zt: none
Peak Zt: 0

Singeleton: ENSXMAG00000028344 RS1

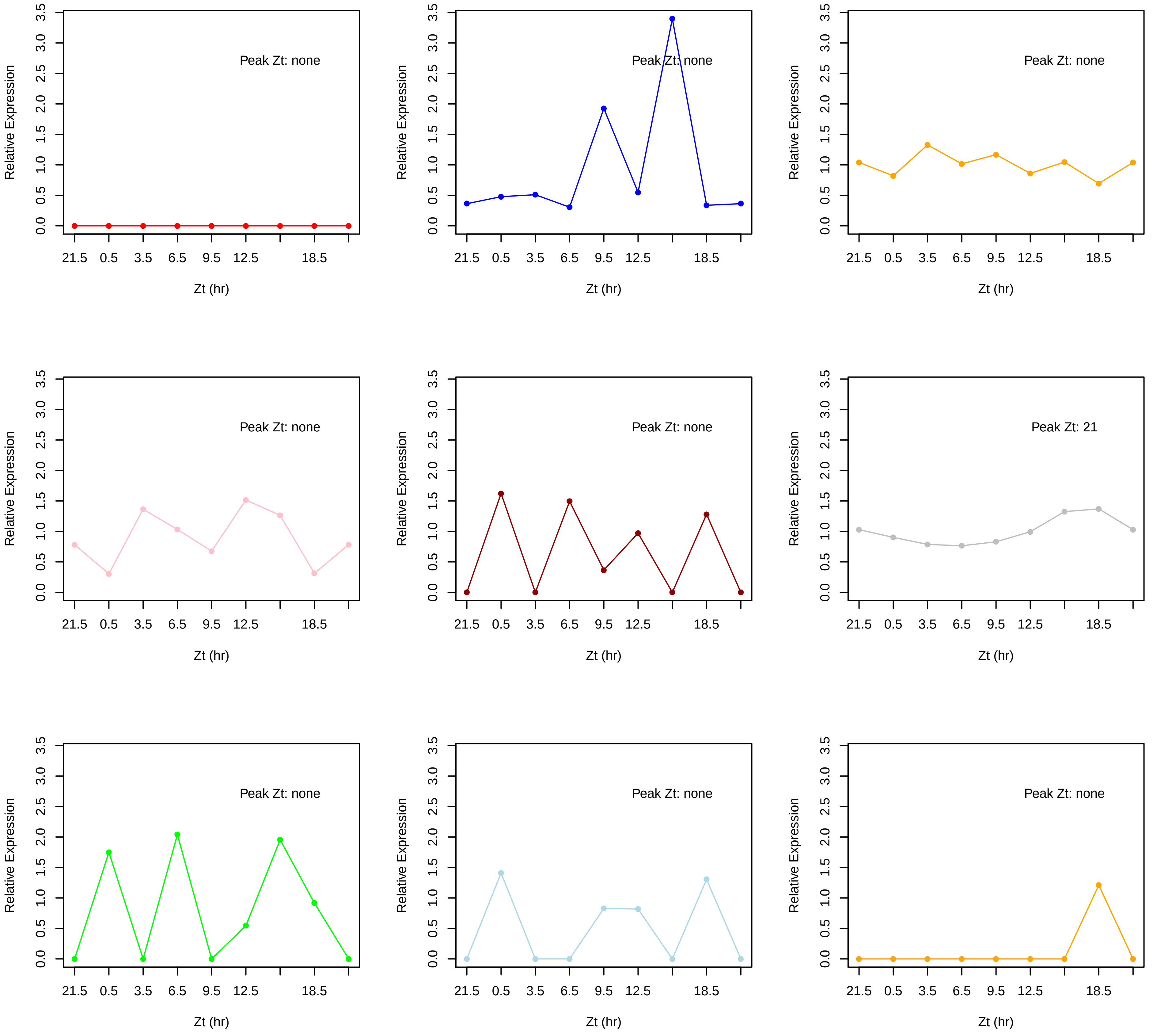

Singleton: ENSXMAG00000003296 hm13

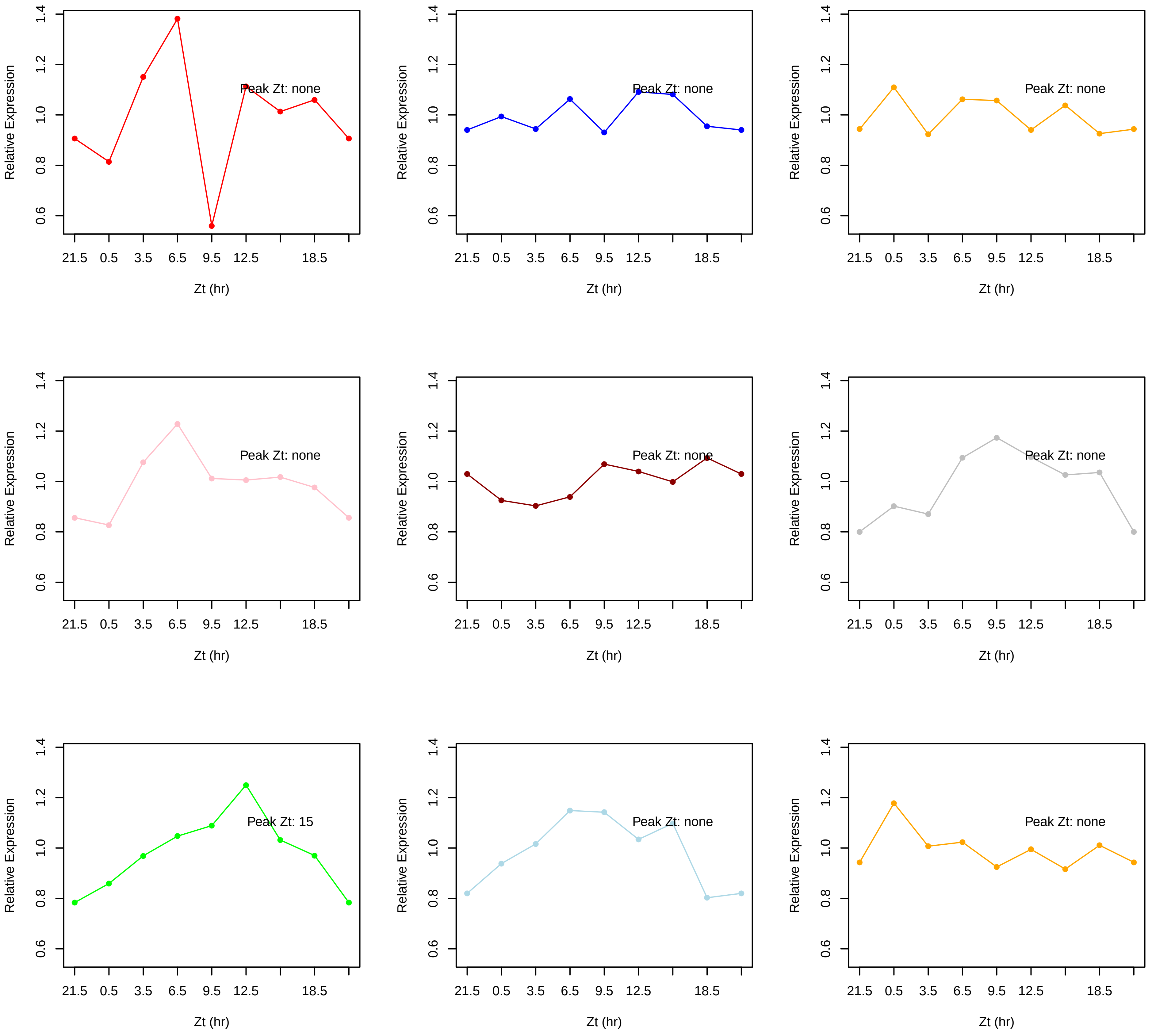

Singleton: ENSXMAG00000010572 hsc70

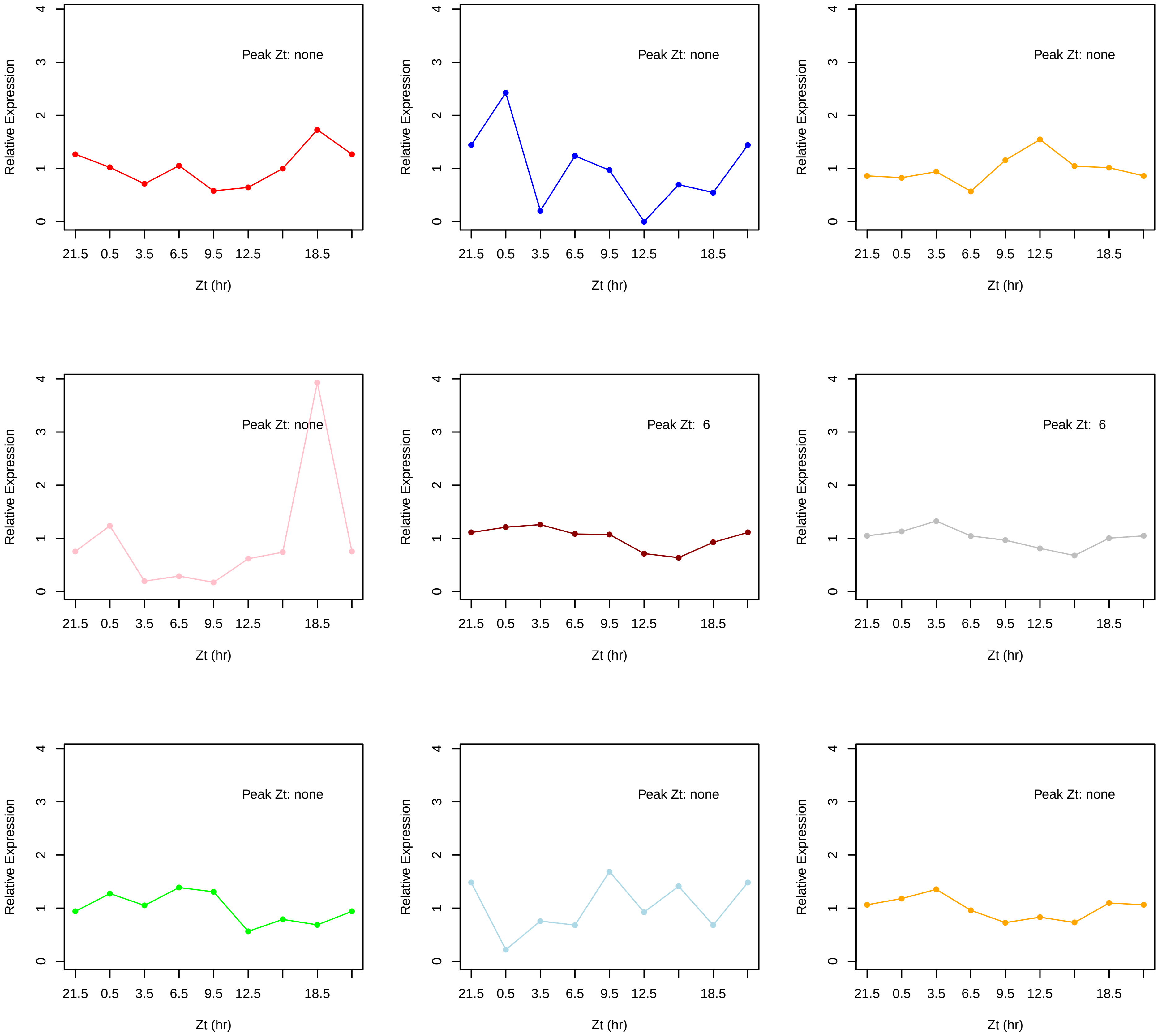

Singleton: ENSXMAG00000000578 hiat1a

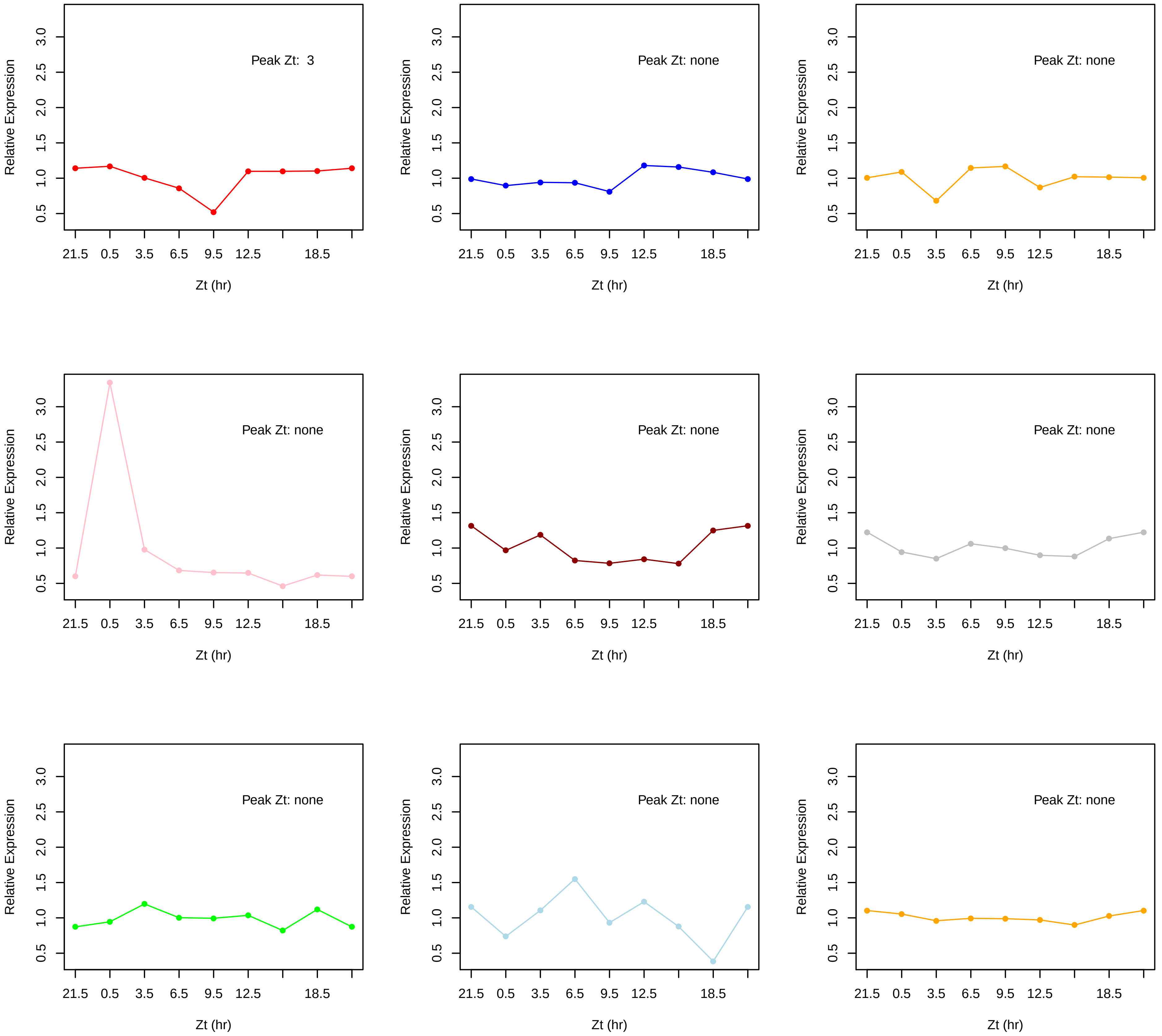

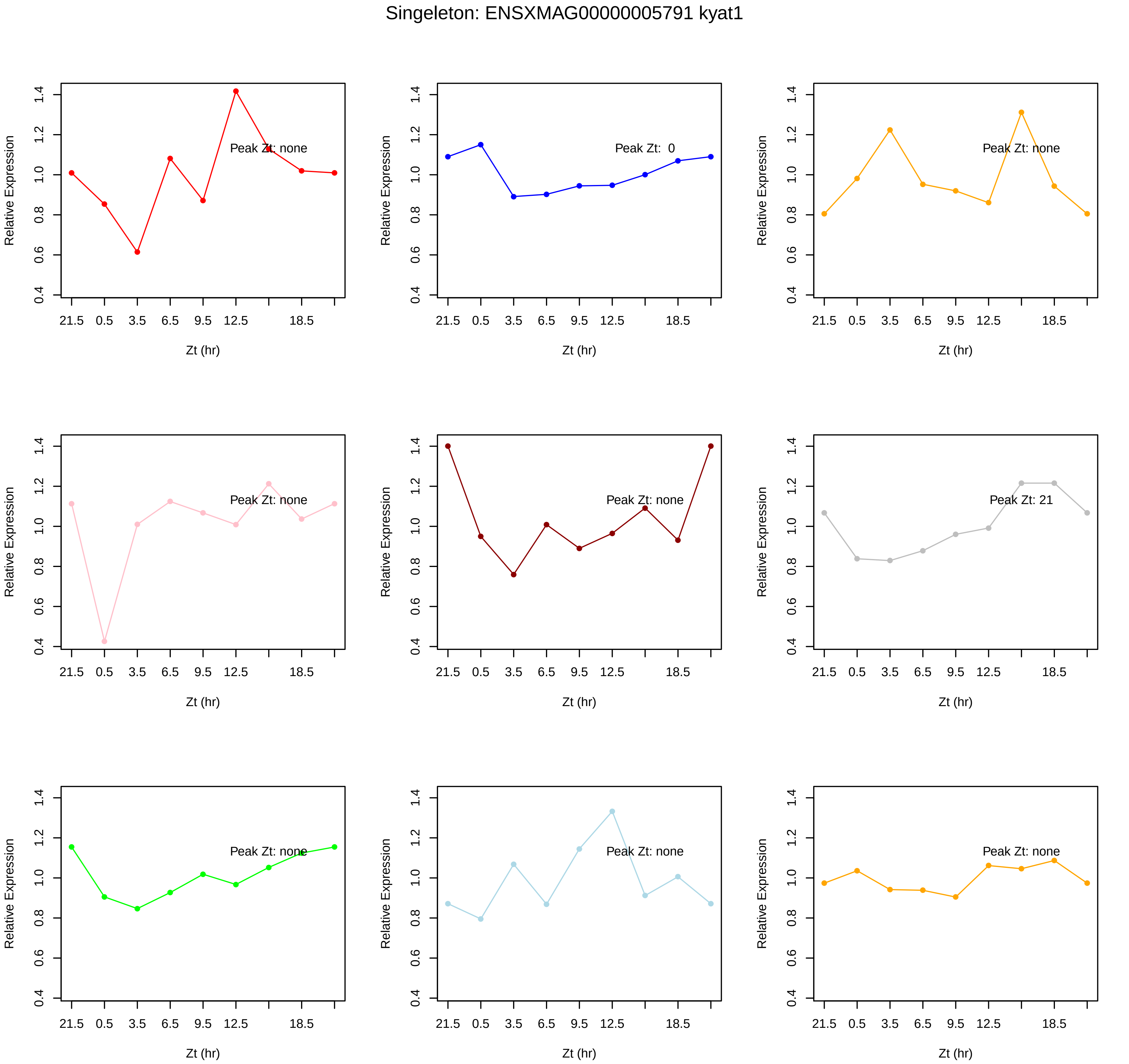
Singeleton: ENSXMAG00000005791 kyat1
Peak Zt: none
Peak Zt: 0
Peak Zt: none
Peak Zt: none
Peak Zt: none
Peak Zt: 21
Peak Zt: none
Peak Zt: none
Peak Zt: none
Relative Expression
Zt (hr)
21.5
0.5
3.5
6.5
9.5
12.5
18.5
0.4
0.6
0.8
1.0
1.2
1.4

Singleton: ENSXMAG00000007328 lcmt2

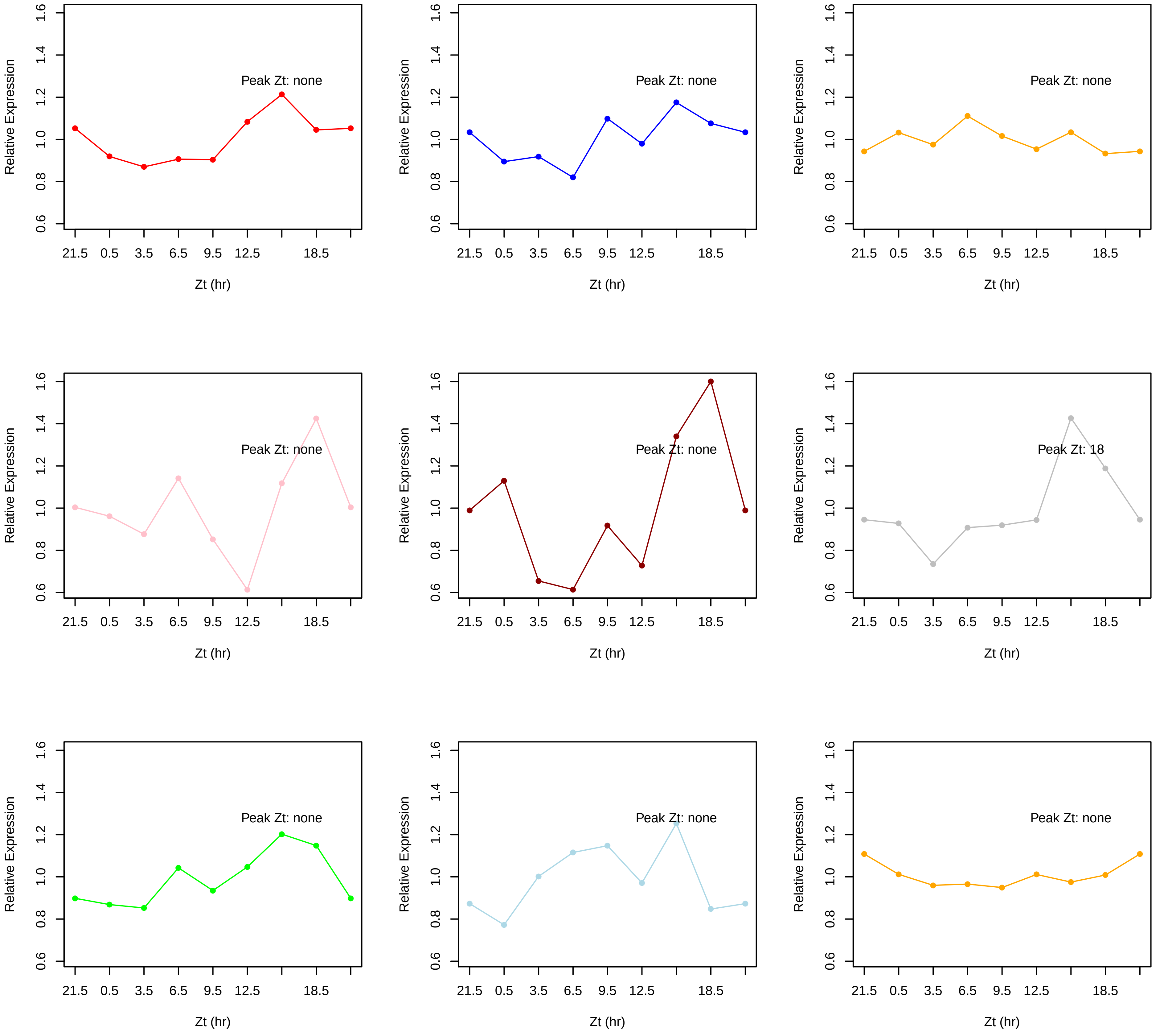

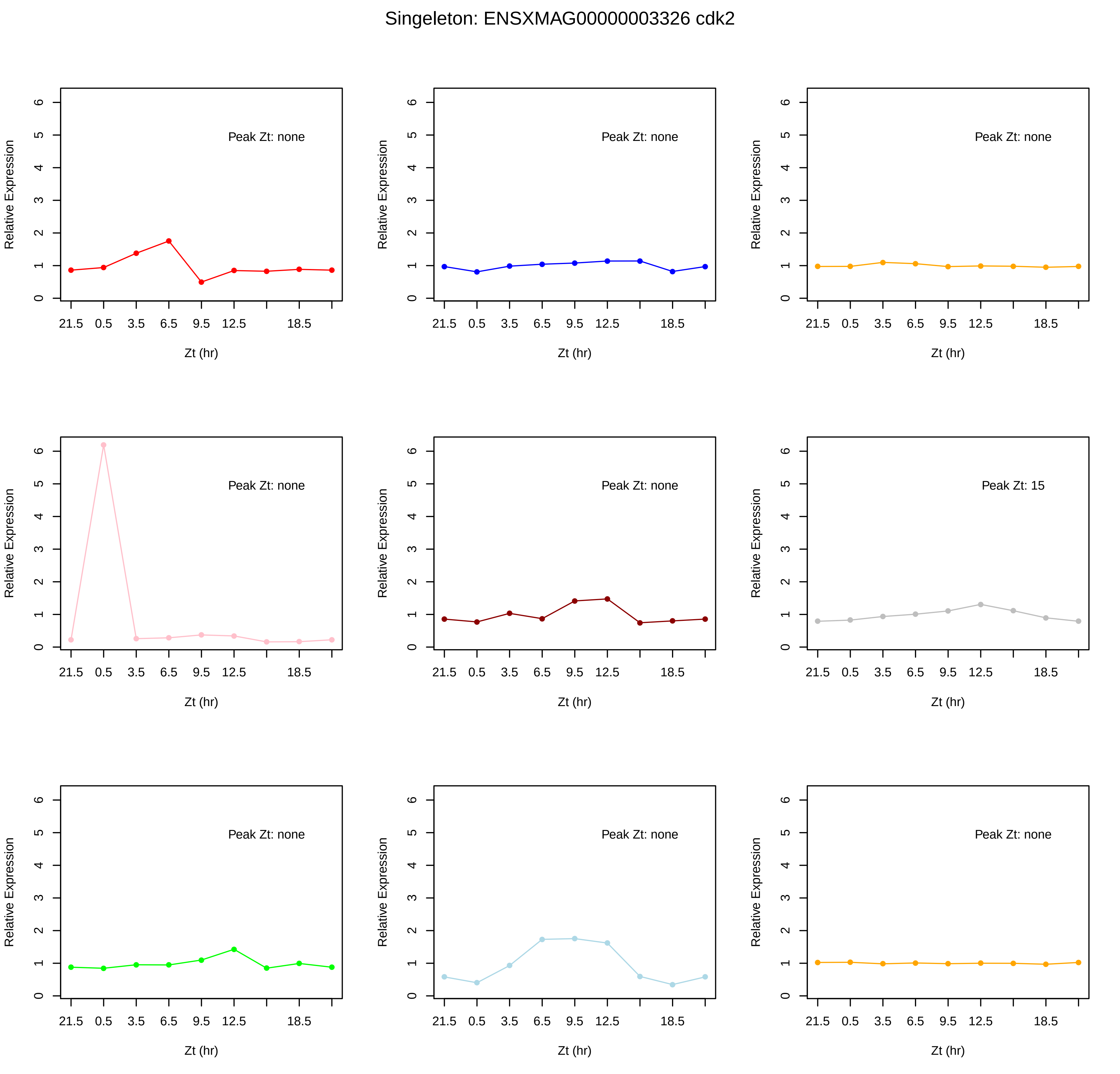
Singeleton: ENSXMAG00000003326 cdk2
Peak Zt: none
Peak Zt: none
Peak Zt: none
Peak Zt: none
Peak Zt: none
Peak Zt: 15
Peak Zt: none
Peak Zt: none
Peak Zt: none
Relative Expression
Zt (hr)
21.5
0.5
3.5
6.5
9.5
12.5
18.5

Singleton: ENSXMAG00000015266 cndp2

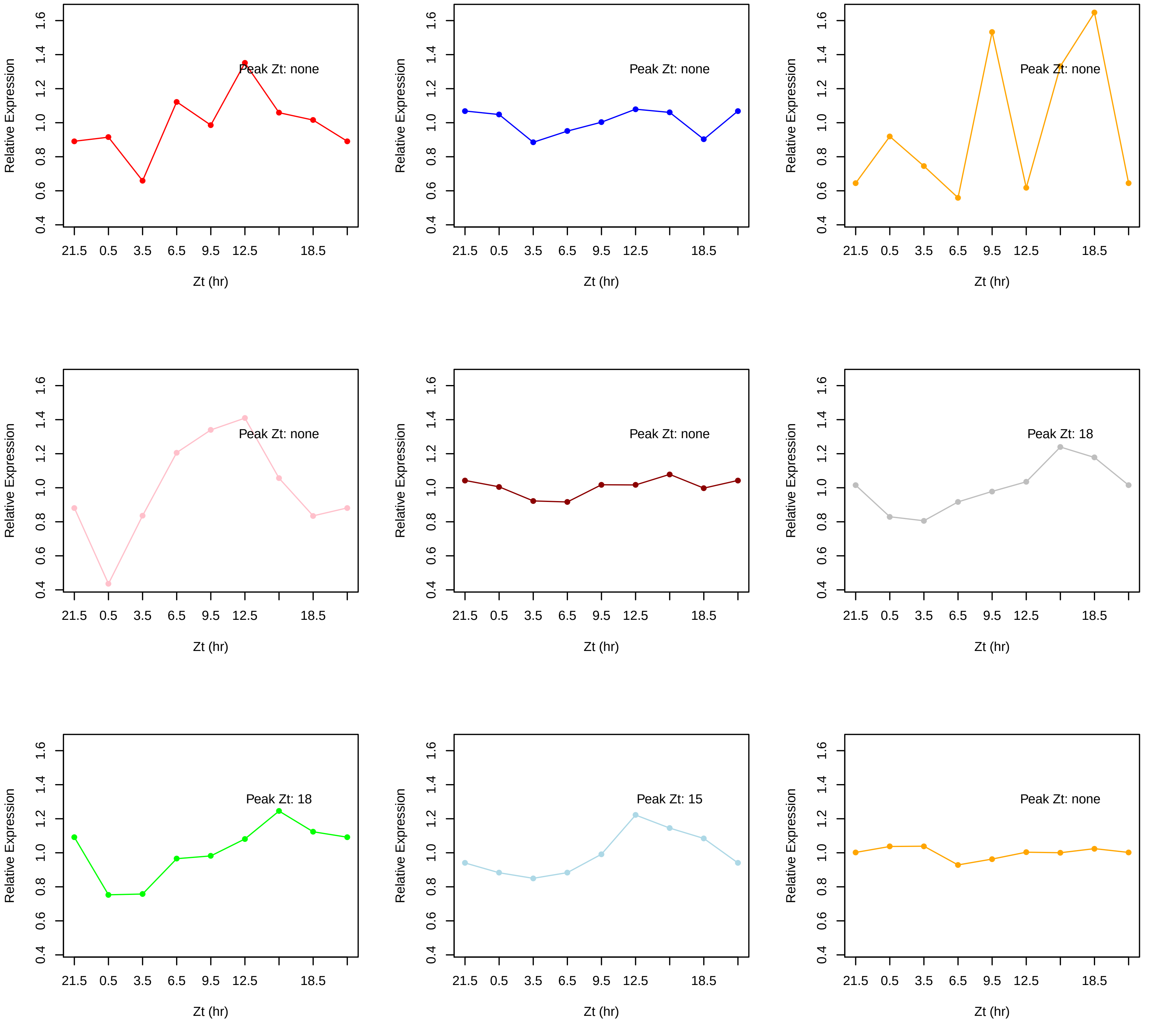

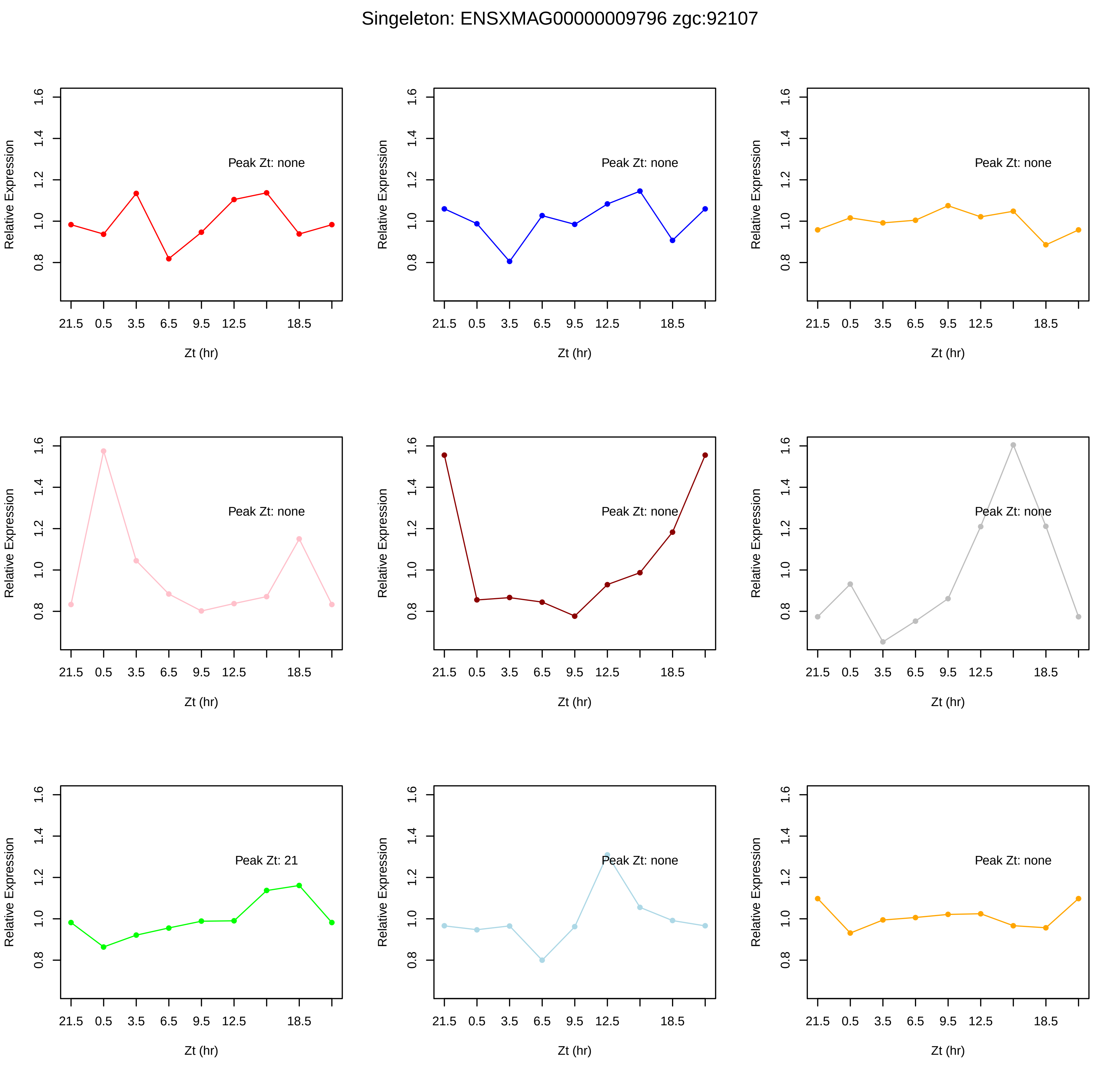
Singeleton: ENSXMAG00000009796 zgc:92107
Peak Zt: none
Peak Zt: none
Peak Zt: none
Peak Zt: none
Peak Zt: none
Peak Zt: none
Peak Zt: 21
Peak Zt: none
Peak Zt: none
Relative Expression
Zt (hr)
21.5
0.5
3.5
6.5
9.5
12.5
18.5
0.8
1.0
1.2
1.4
1.6

Singleton: ENSXMAG00000003148 usp47

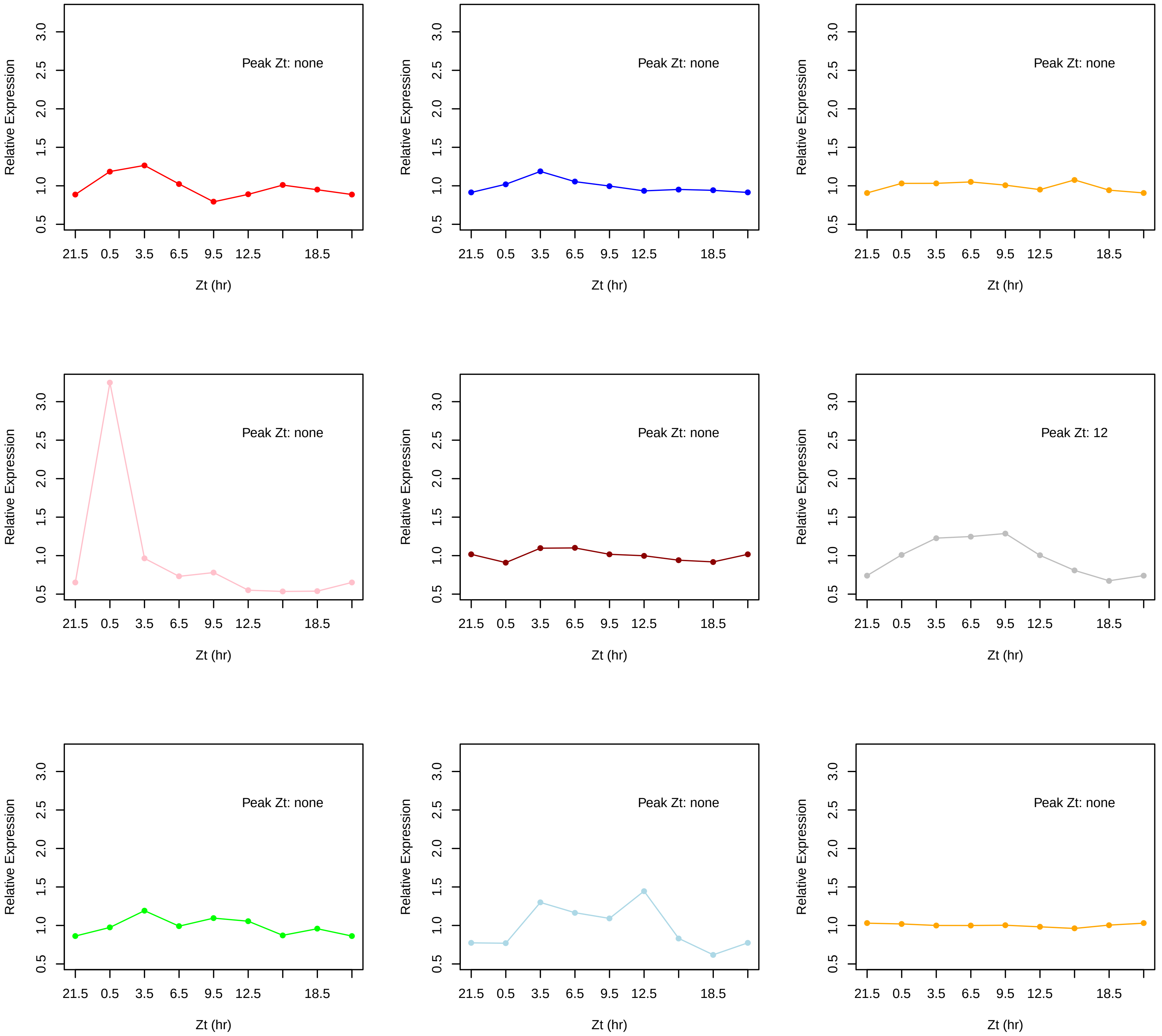

Singeleton: ENSXMAG00000008280 farsb

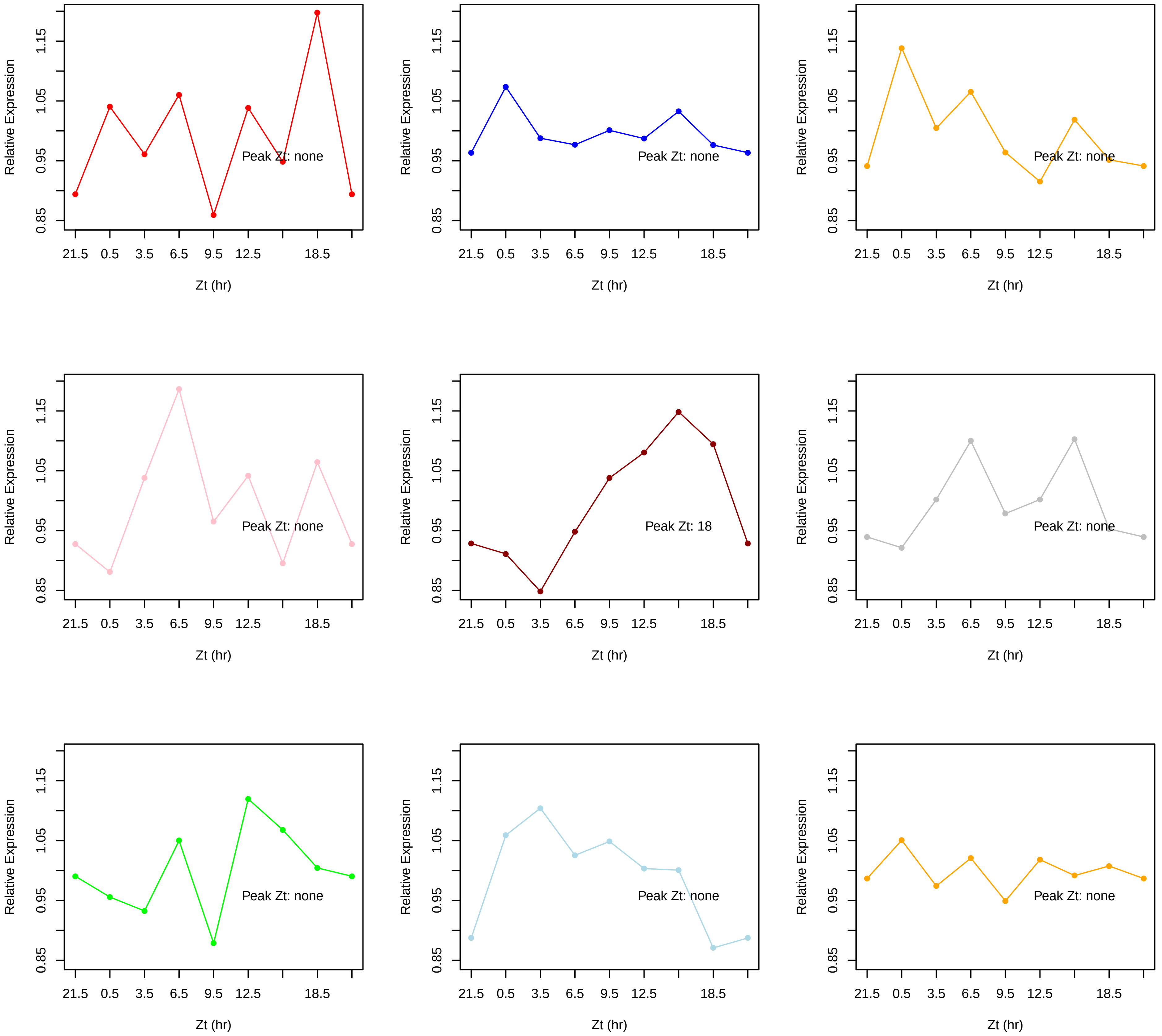

Singeleton: ENSXMAG00000005778 spout1

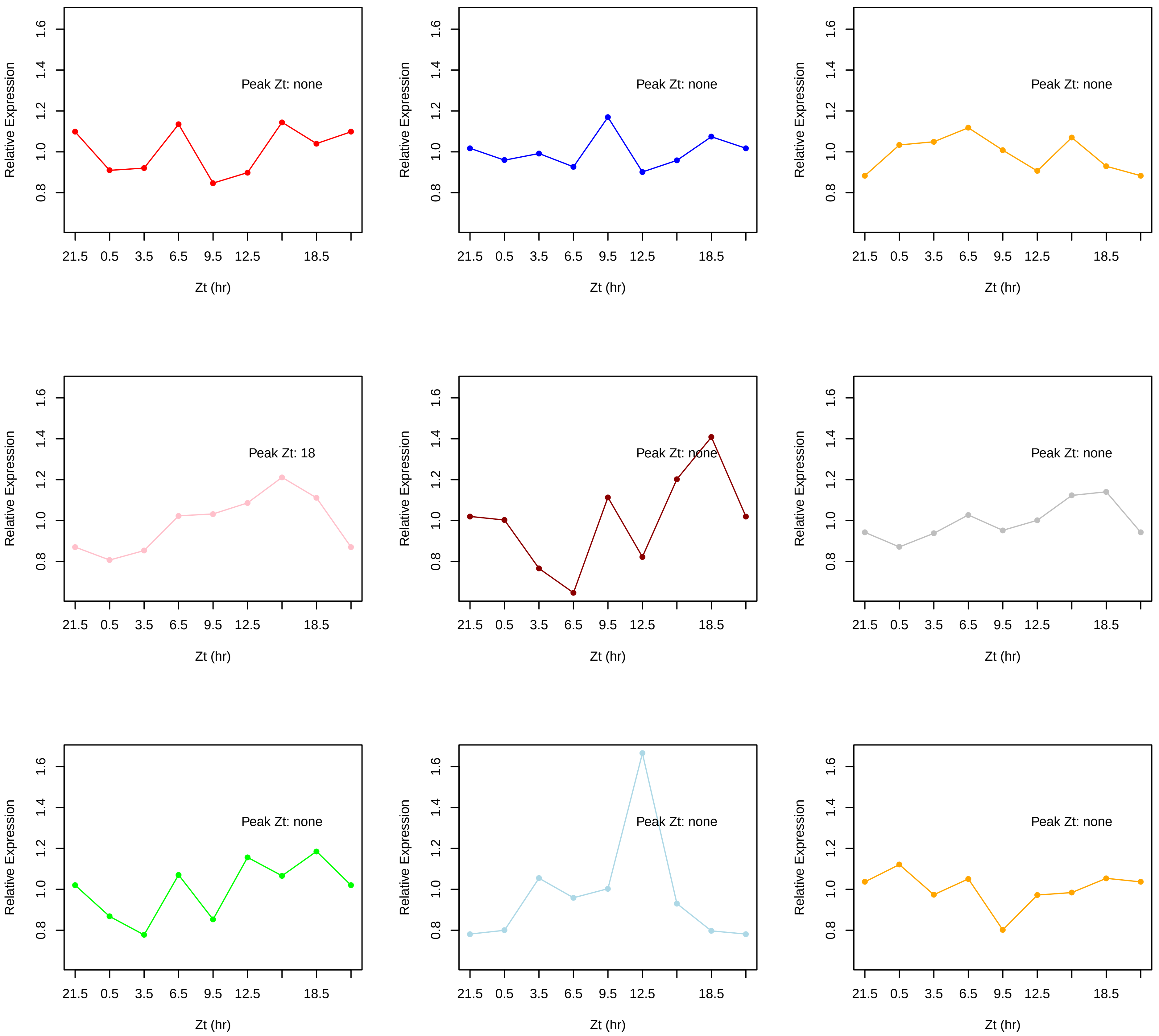

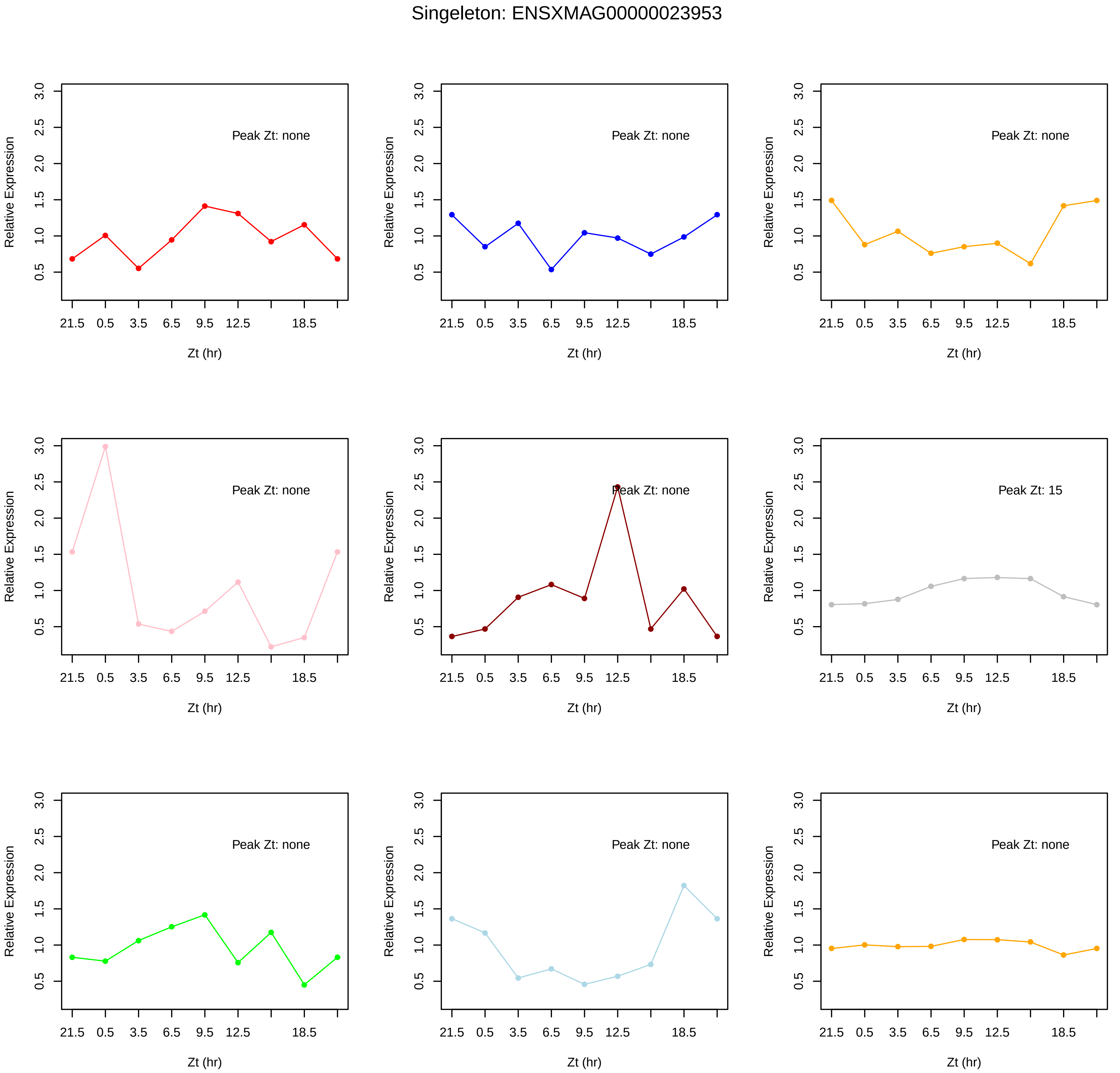
Singeleton: ENSXMAG00000023953
Peak Zt: none
Peak Zt: 15
Relative Expression
Zt (hr)
21.5
0.5
3.5
6.5
9.5
12.5
18.5
0.5
1.0
1.5
2.0
2.5
3.0

Singeleton: ENSXMAG00000009886 usp12a

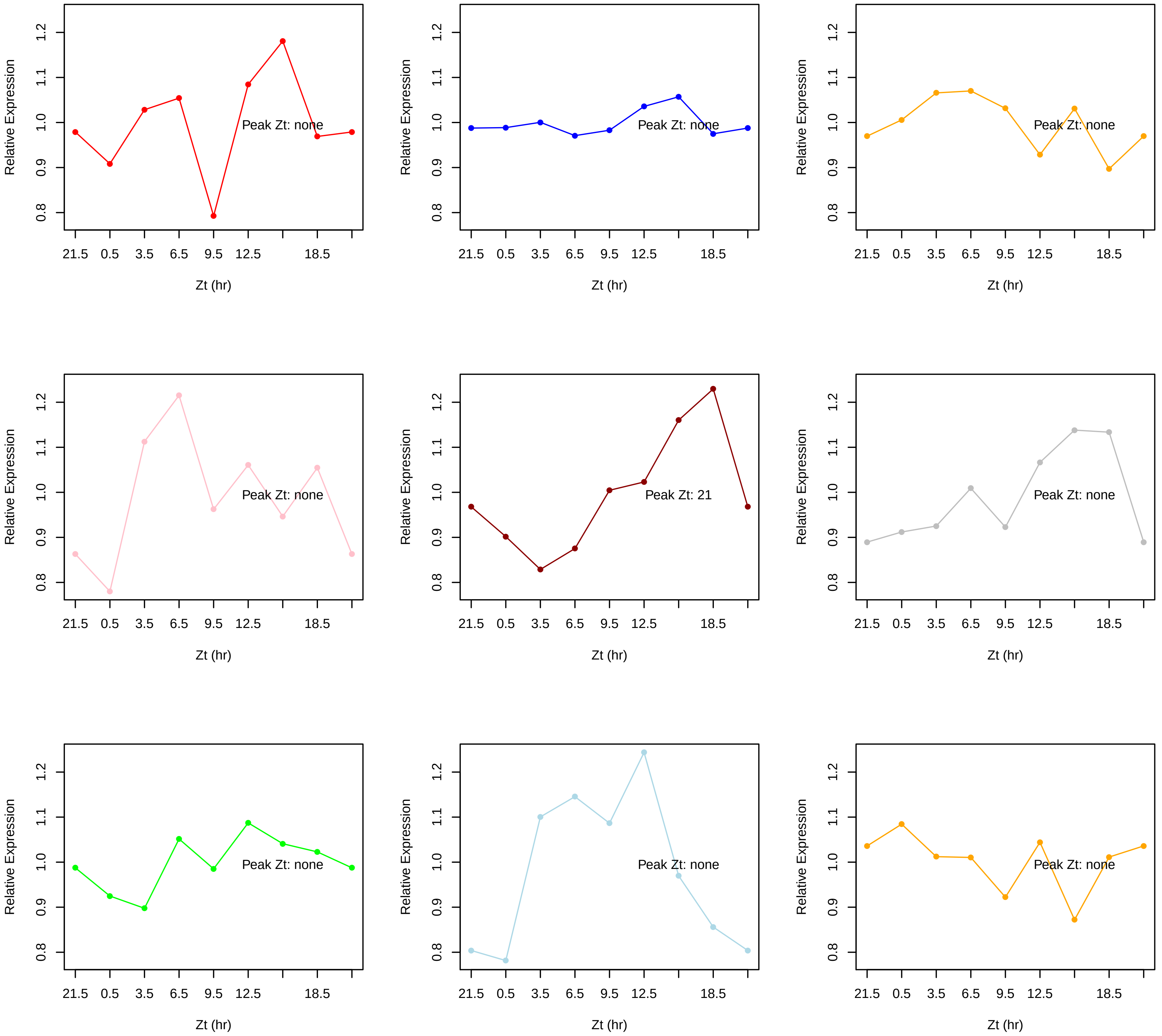

Singeleton: ENSXMAG00000003155

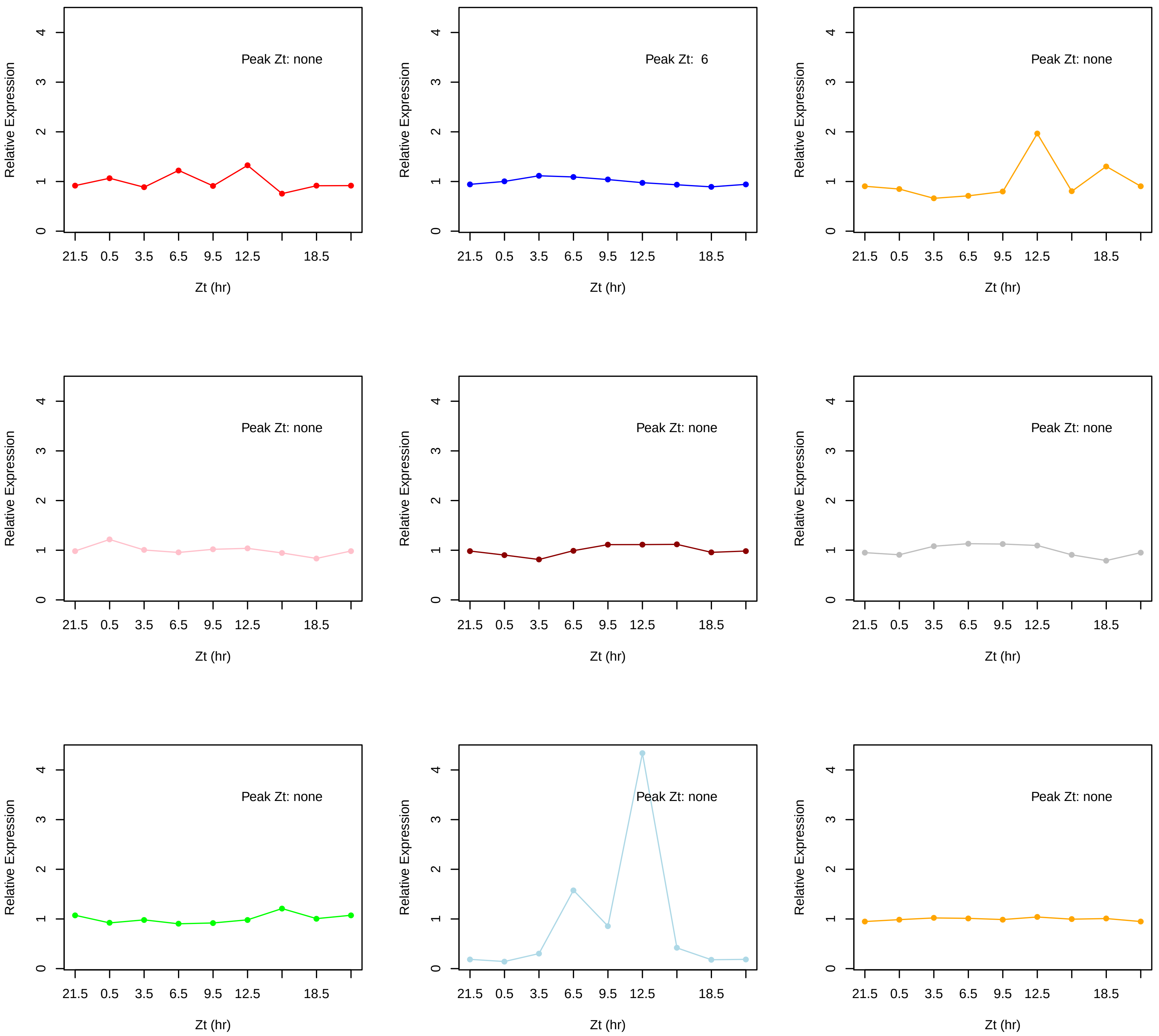

Singleton: ENSXMAG00000003284 cyb5b

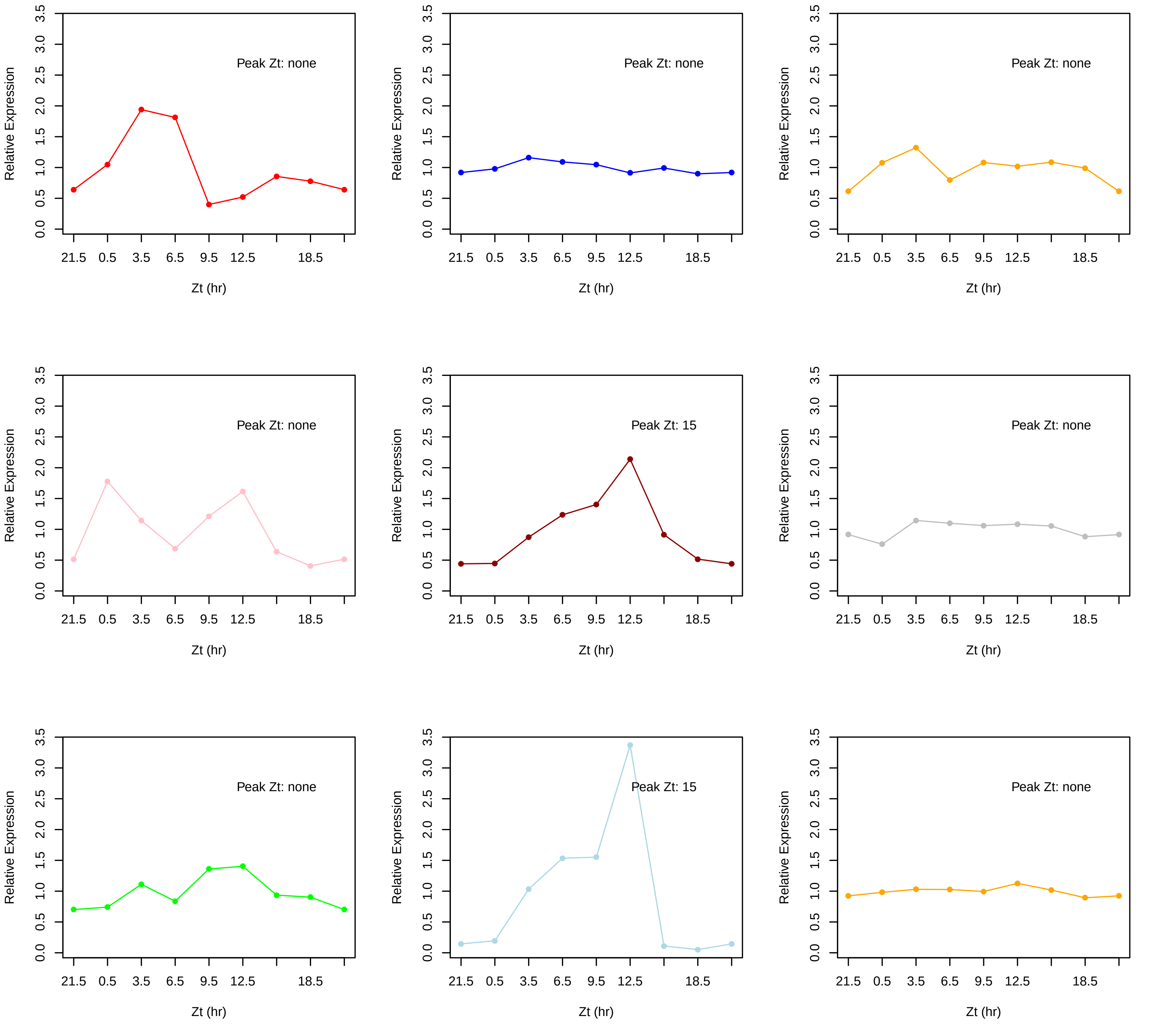

Singeleton: ENSXMAG00000026757 tuba1c

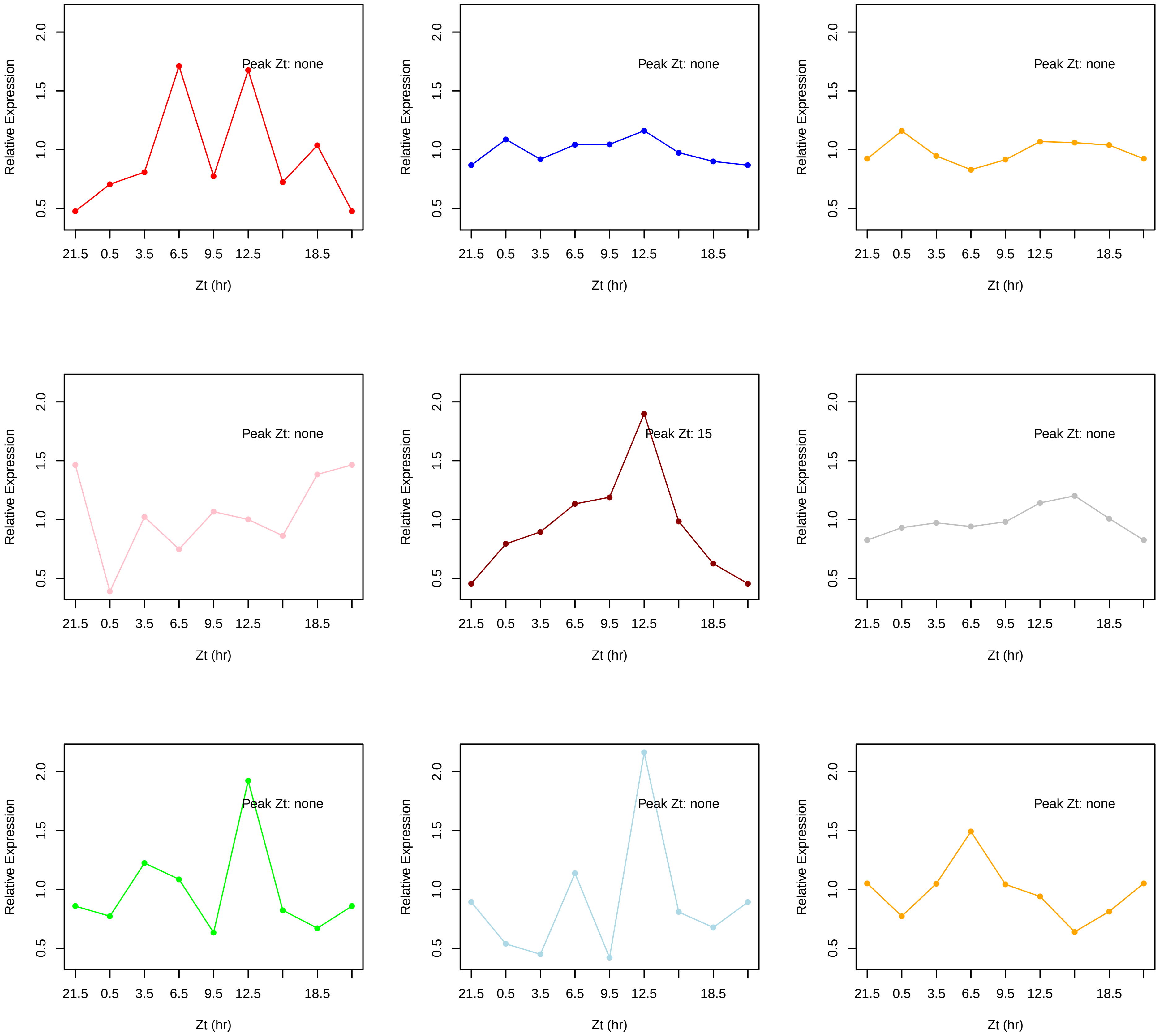

Singeleton: ENSXMAG00000007397

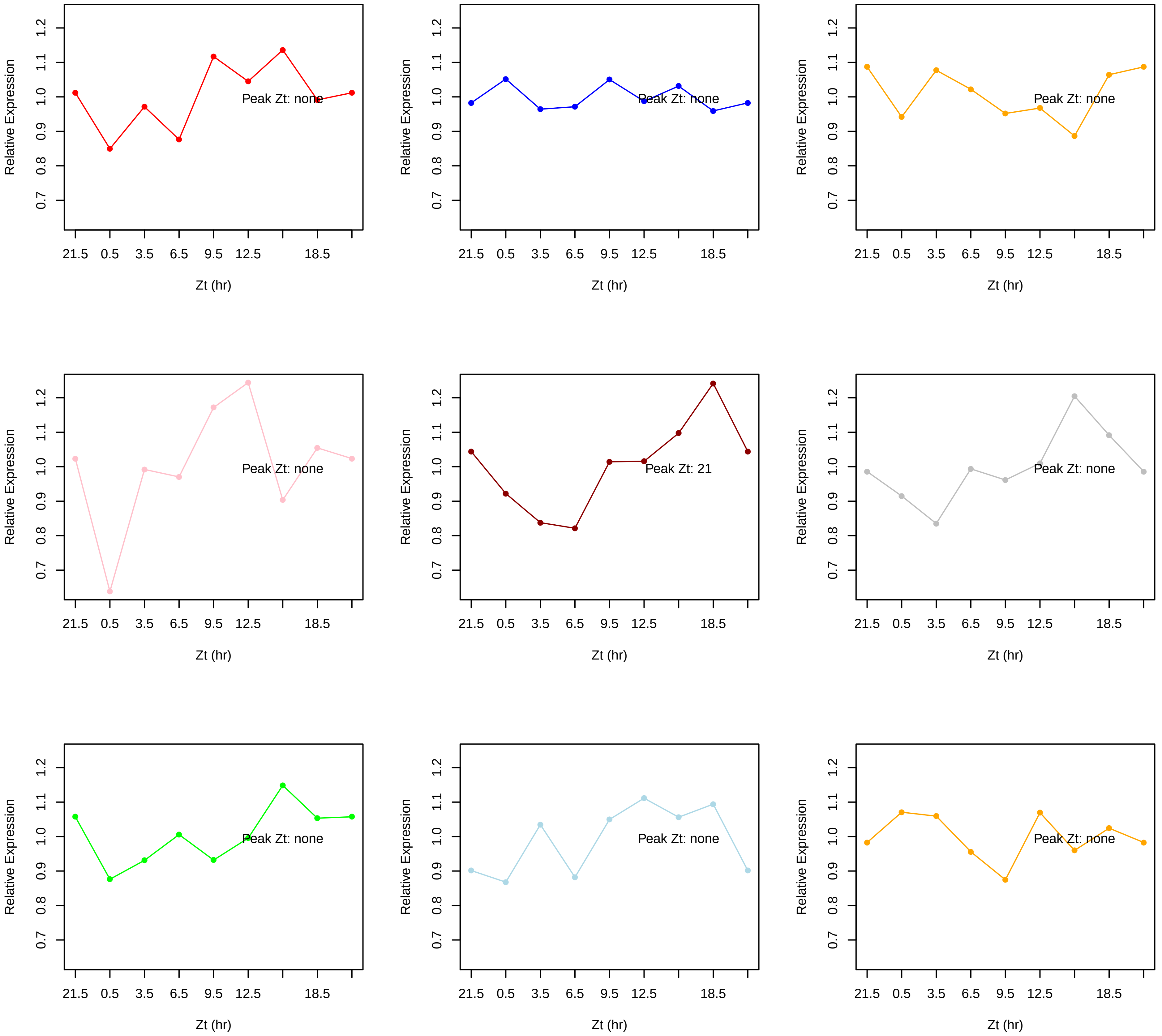

Singeleton: ENSXMAG00000007311 dbr1

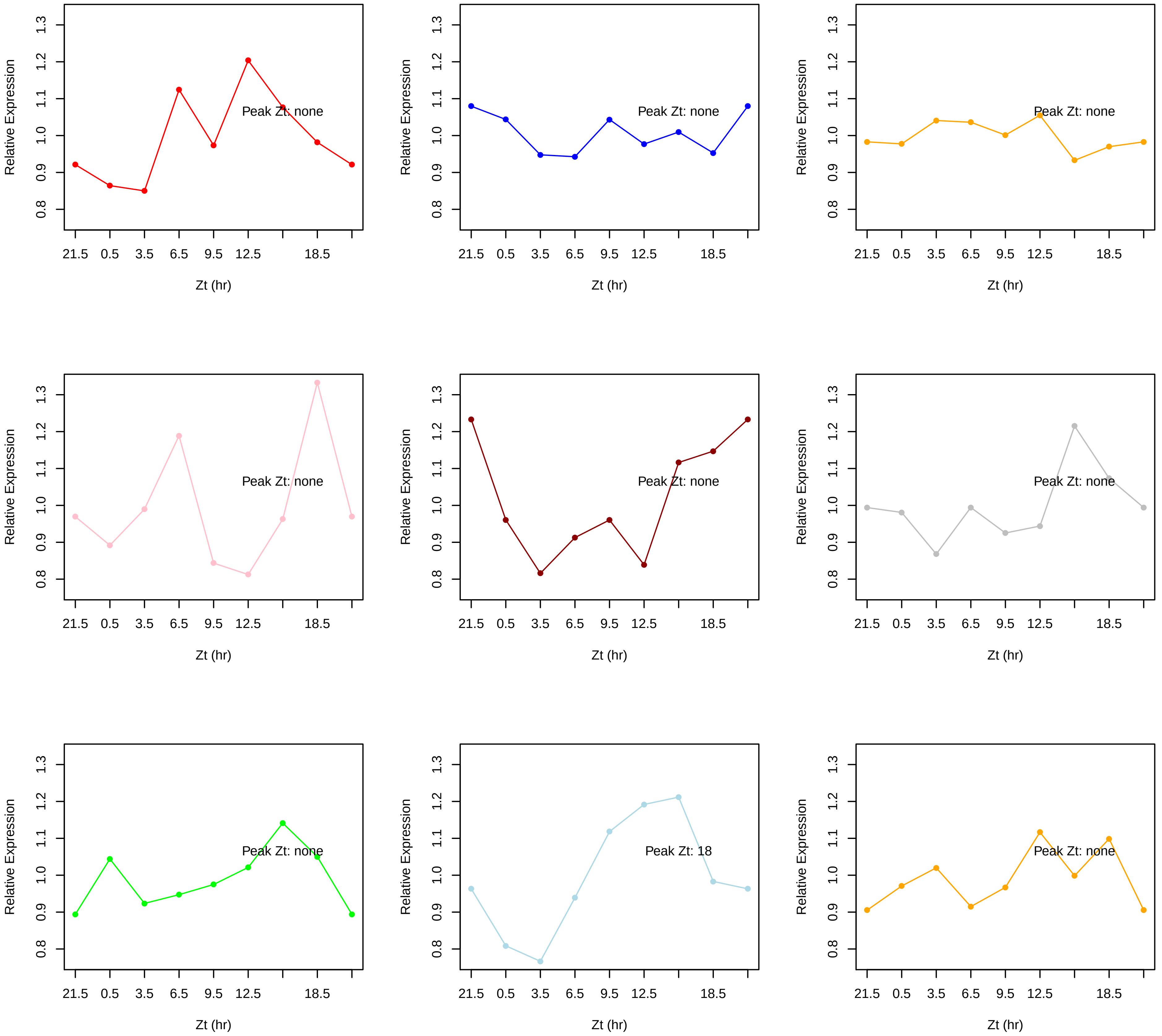

Singeleton: ENSXMAG00000008381 zbtb44

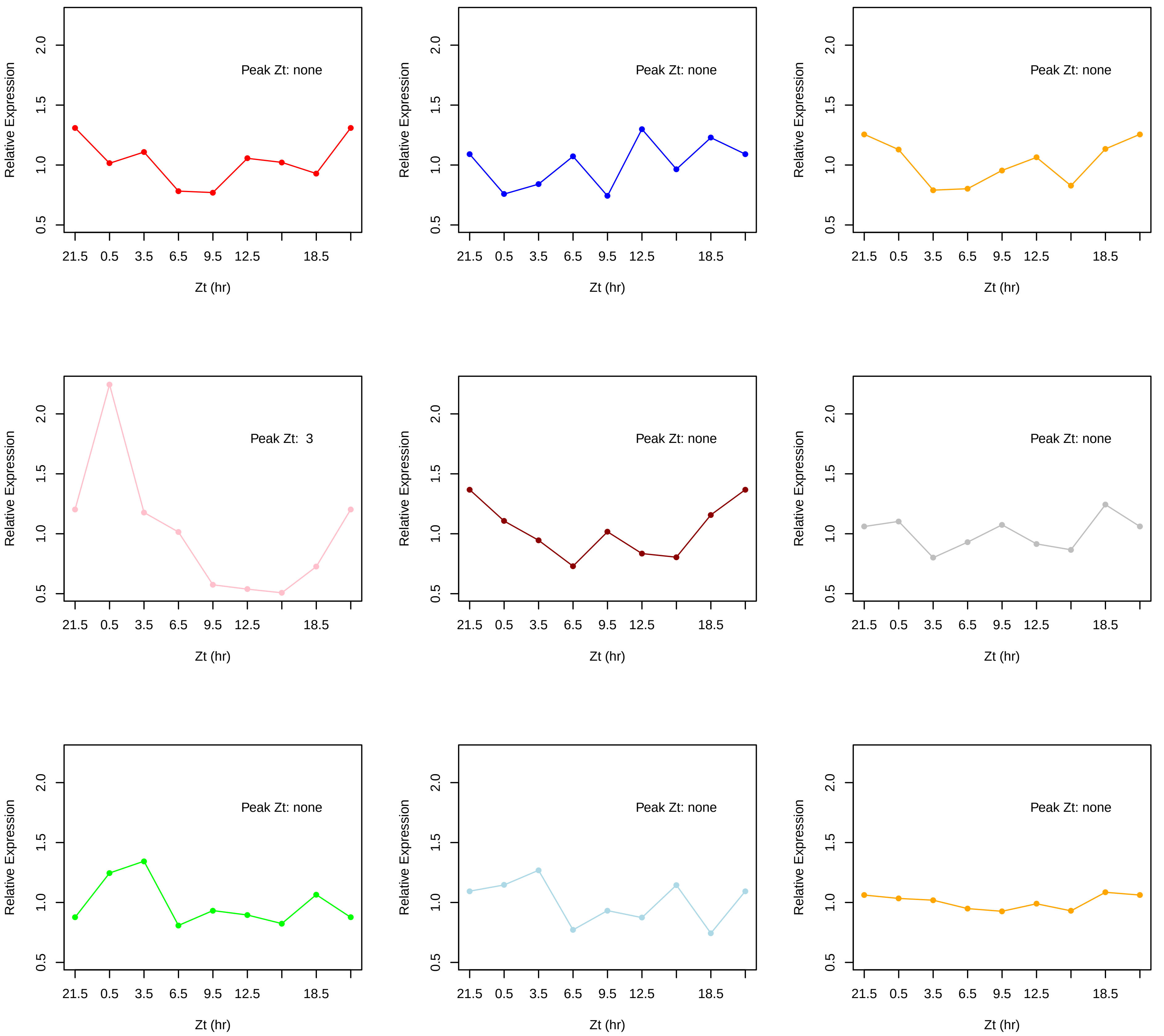

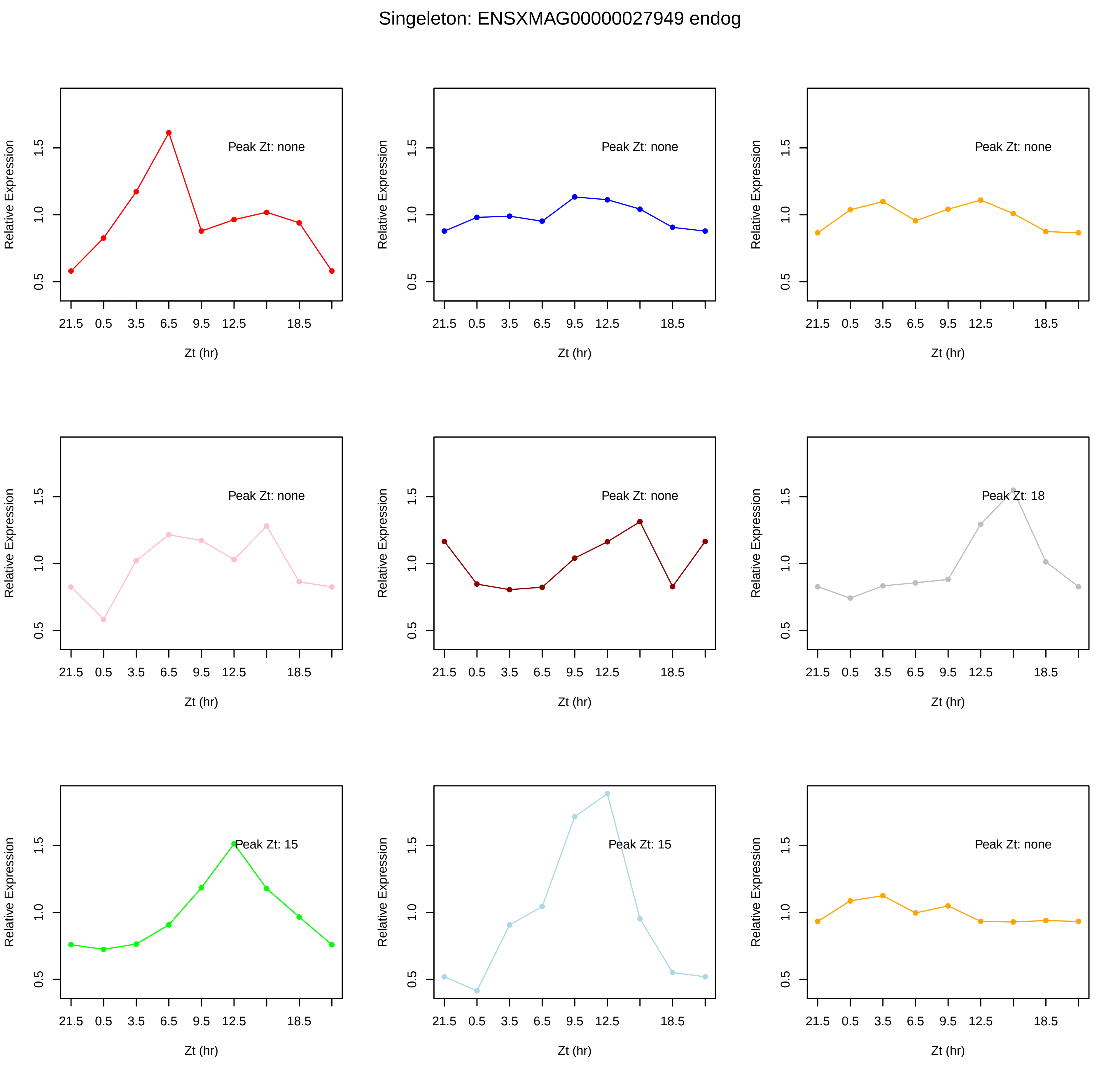
Singeleton: ENSXMAG00000027949 endog
Peak Zt: none
Peak Zt: none
Peak Zt: none
Peak Zt: none
Peak Zt: none
Peak Zt: 18
Peak Zt: 15
Peak Zt: 15
Peak Zt: none
Relative Expression
Zt (hr)
21.5
0.5
3.5
6.5
9.5
12.5
18.5
0.5
1.0
1.5

Singeleton: ENSXMAG00000003304 prph

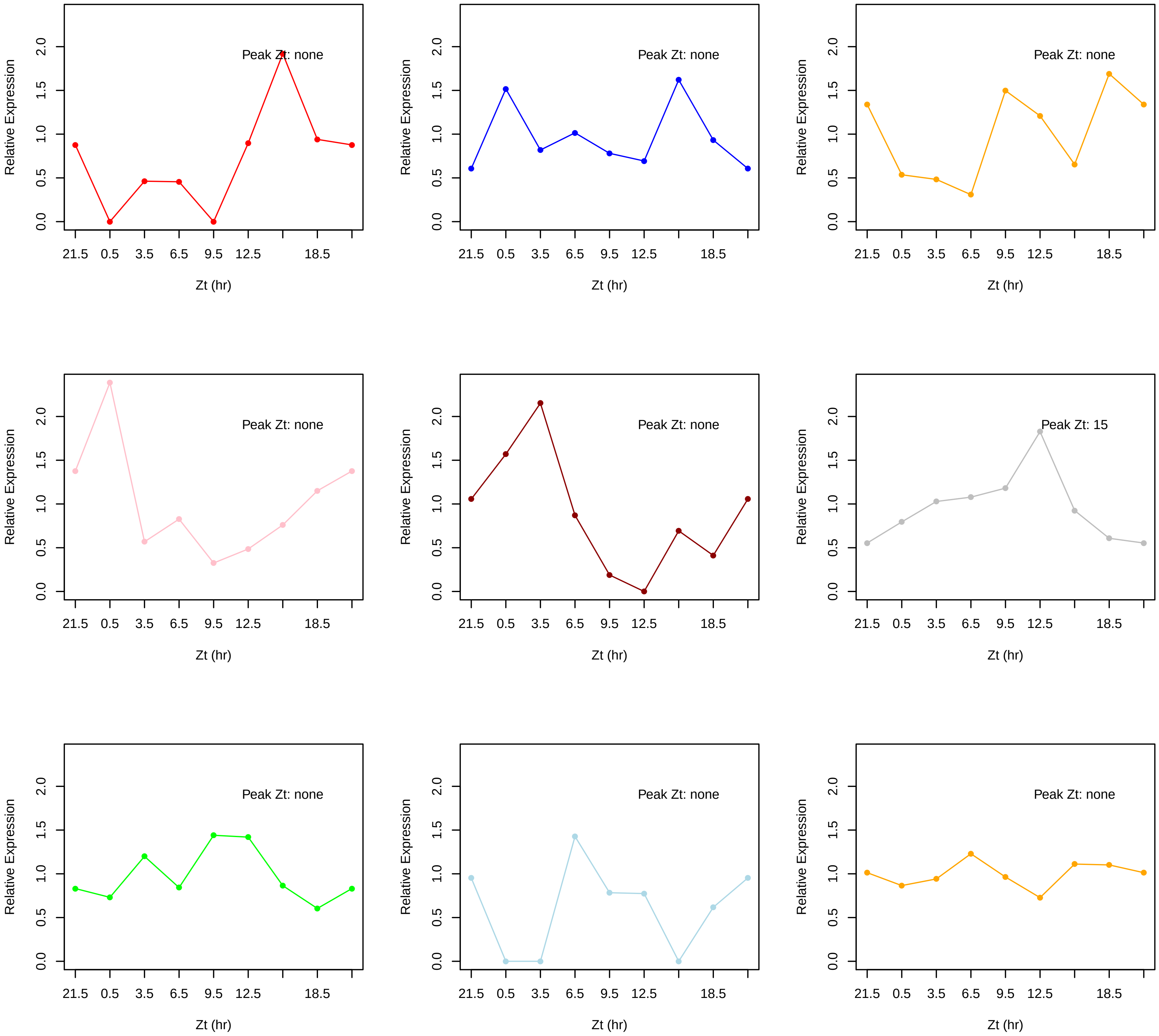

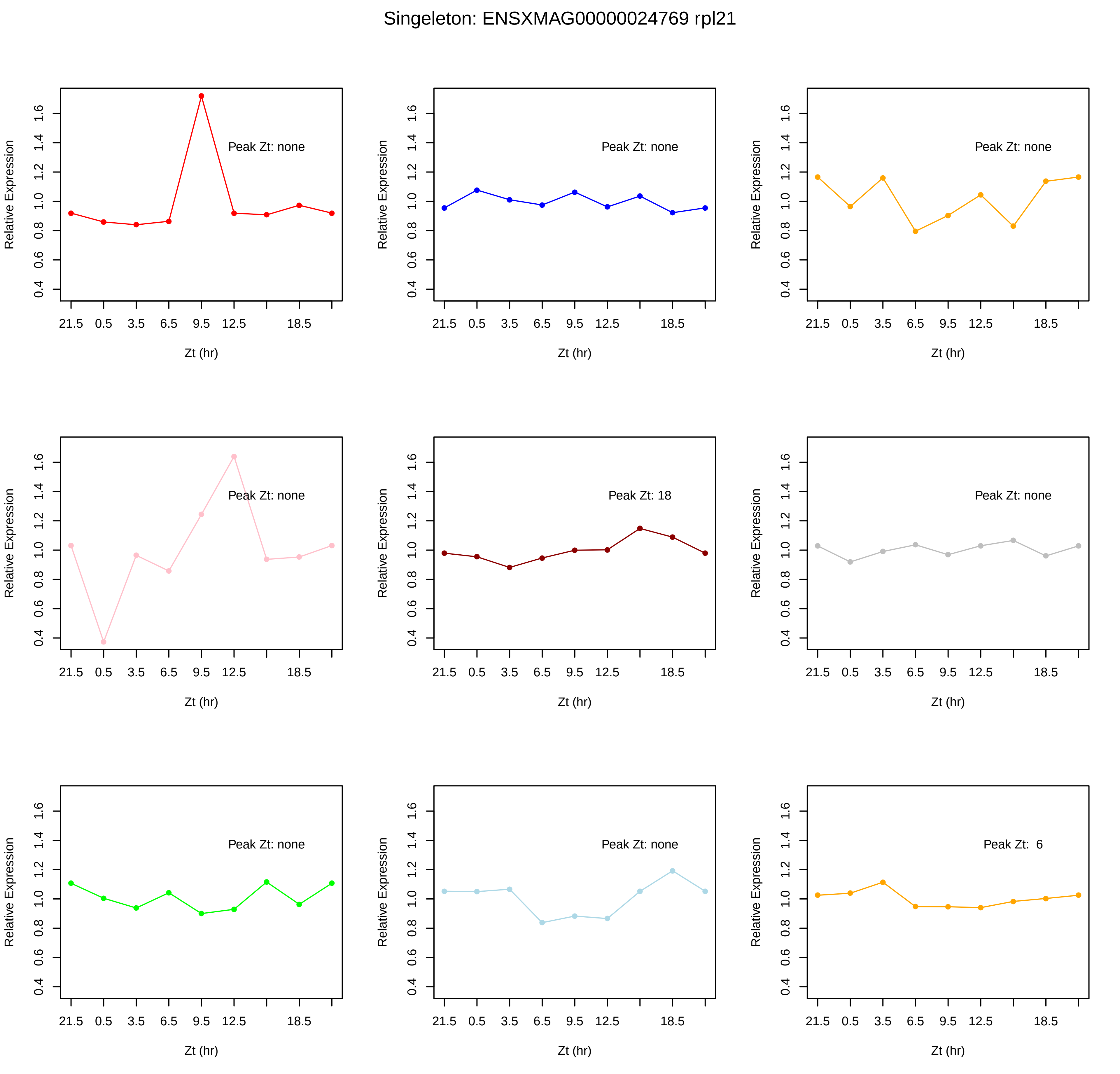
Singeleton: ENSXMAG00000024769 rpl21
Relative Expression
Zt (hr)
21.5
0.5
3.5
6.5
9.5
12.5
18.5
0.4
0.6
0.8
1.0
1.2
1.4
1.6
Peak Zt: none
Peak Zt: none
Peak Zt: none
Peak Zt: none
Peak Zt: 18
Peak Zt: none
Peak Zt: none
Peak Zt: none
Peak Zt: 6

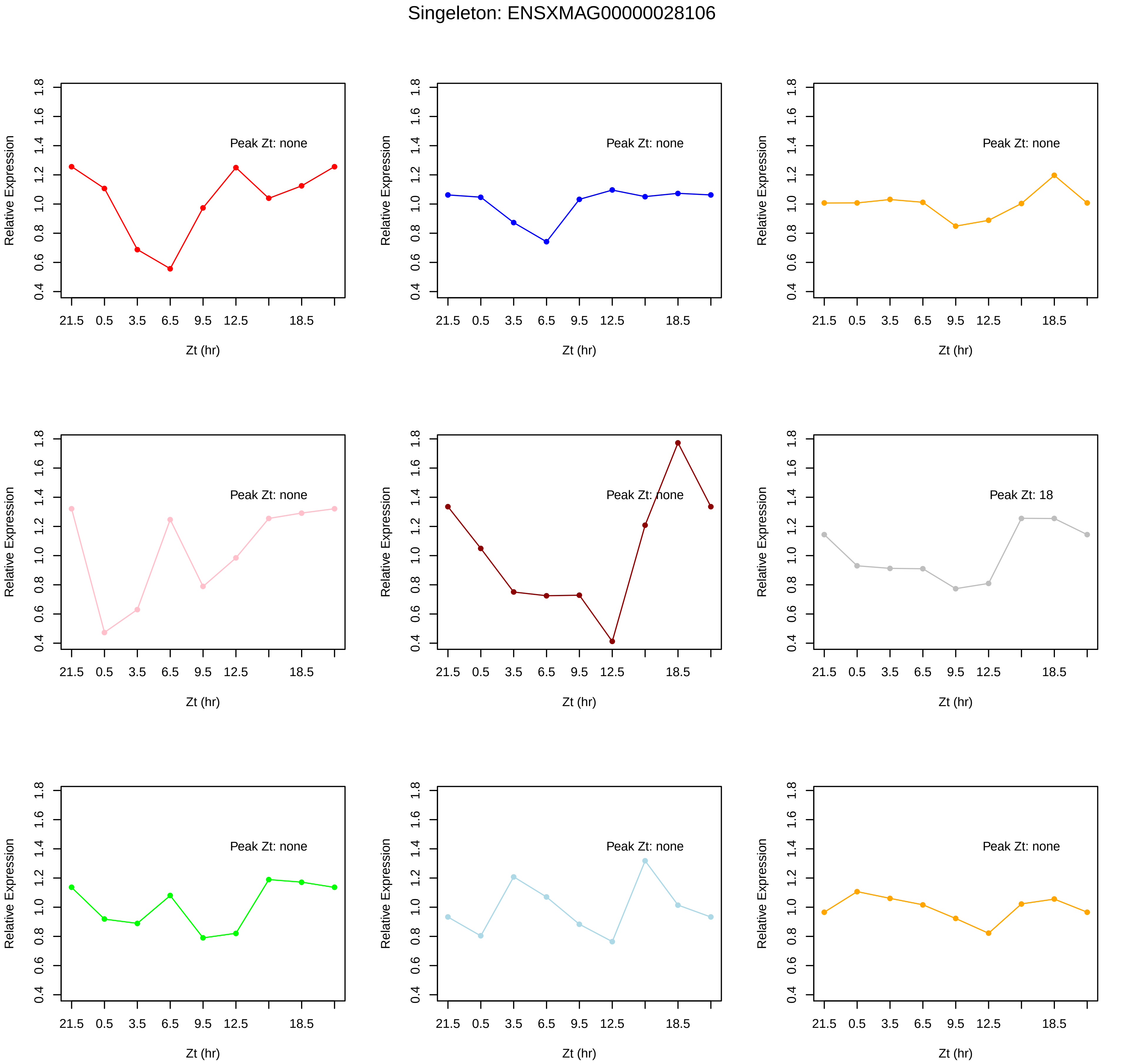
Singeleton: ENSXMAG00000028106
Peak Zt: none
Peak Zt: none
Peak Zt: none
Peak Zt: none
Peak Zt: none
Peak Zt: 18
Peak Zt: none
Peak Zt: none
Peak Zt: none
Relative Expression
Zt (hr)
0.4
0.6
0.8
1.0
1.2
1.4
1.6
1.8
21.5
0.5
3.5
6.5
9.5
12.5
18.5

Singeleton: ENSXMAG00000027058 adamts15b

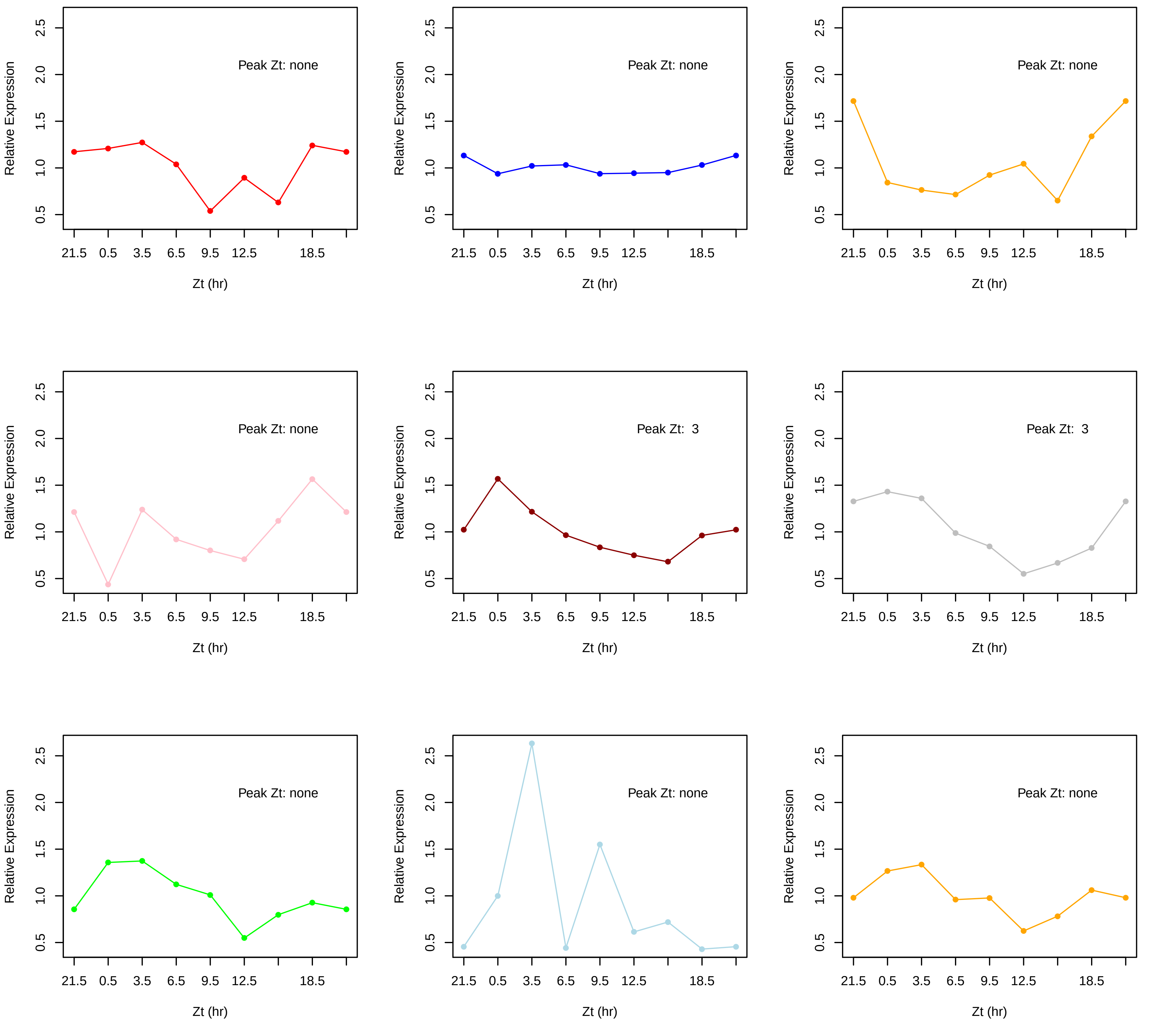

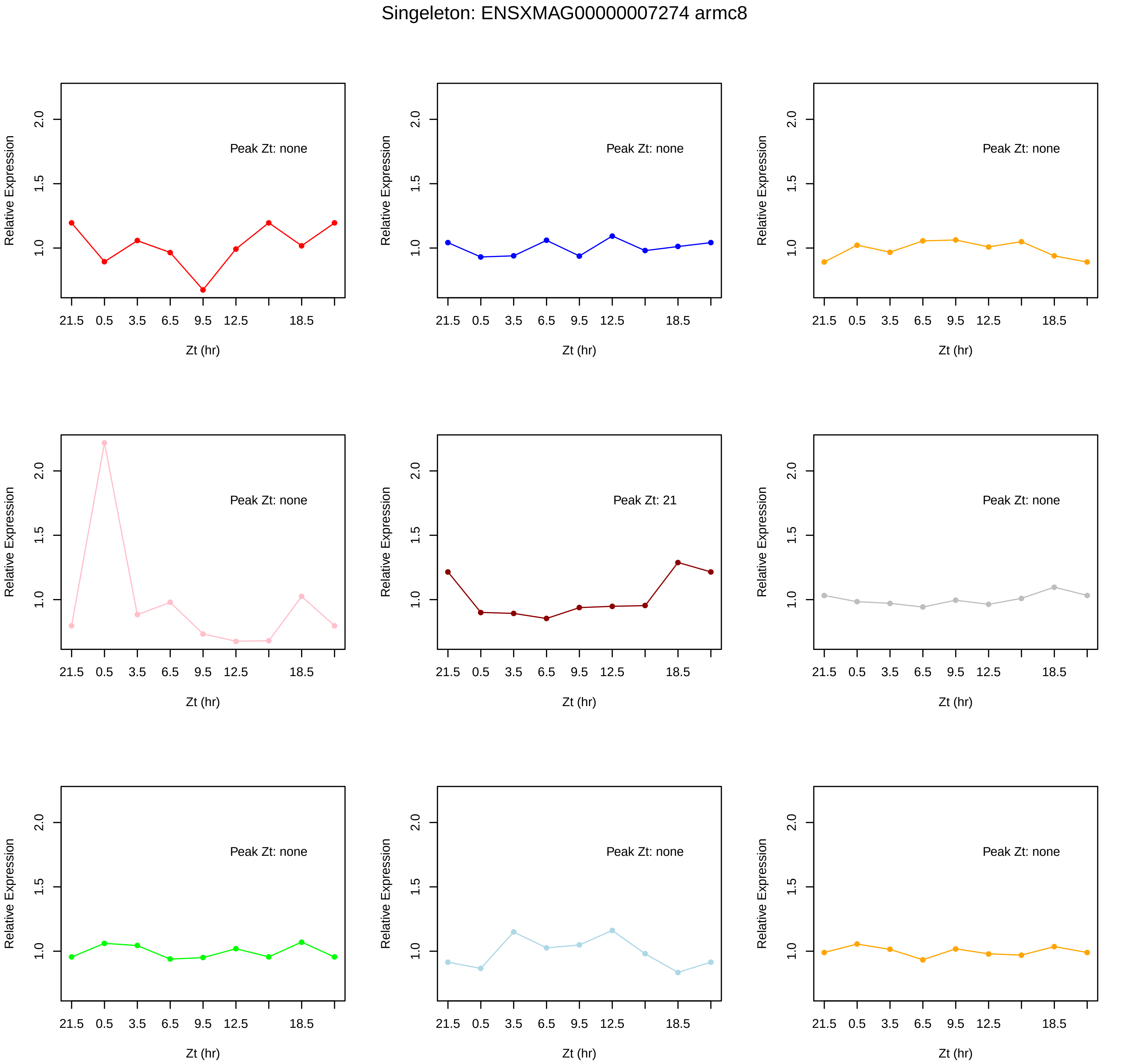
Singeleton: ENSXMAG00000007274 armc8
Relative Expression
Zt (hr)
21.5 0.5 3.5 6.5 9.5 12.5 18.5
1.0 1.5 2.0
Peak Zt: none
Peak Zt: none
Peak Zt: none
Peak Zt: none
Peak Zt: 21
Peak Zt: none
Peak Zt: none
Peak Zt: none
Peak Zt: none

Singeleton: ENSXMAG00000013595 snrnp48

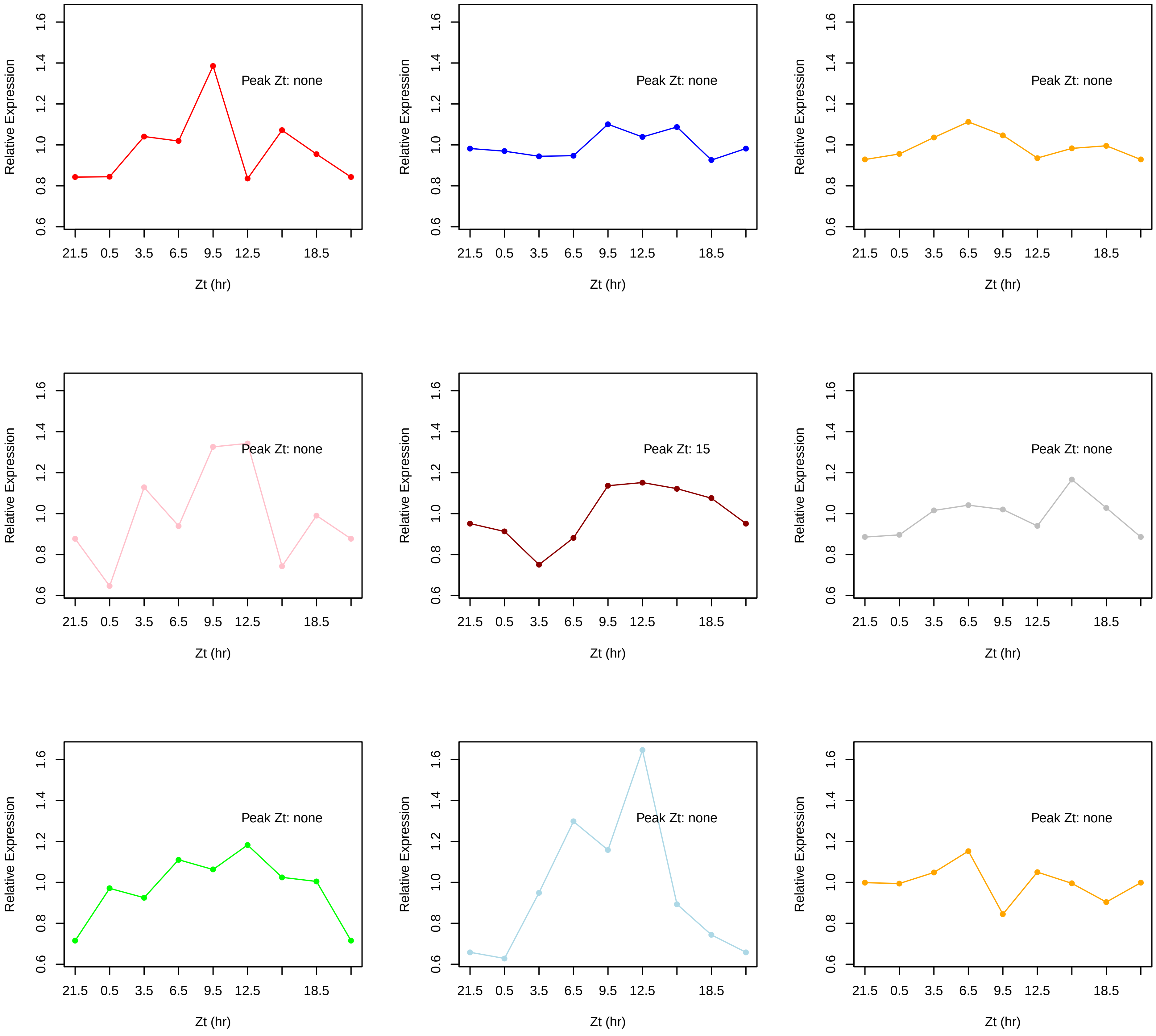

Singeleton: ENSXMAG00000008306 epha4a

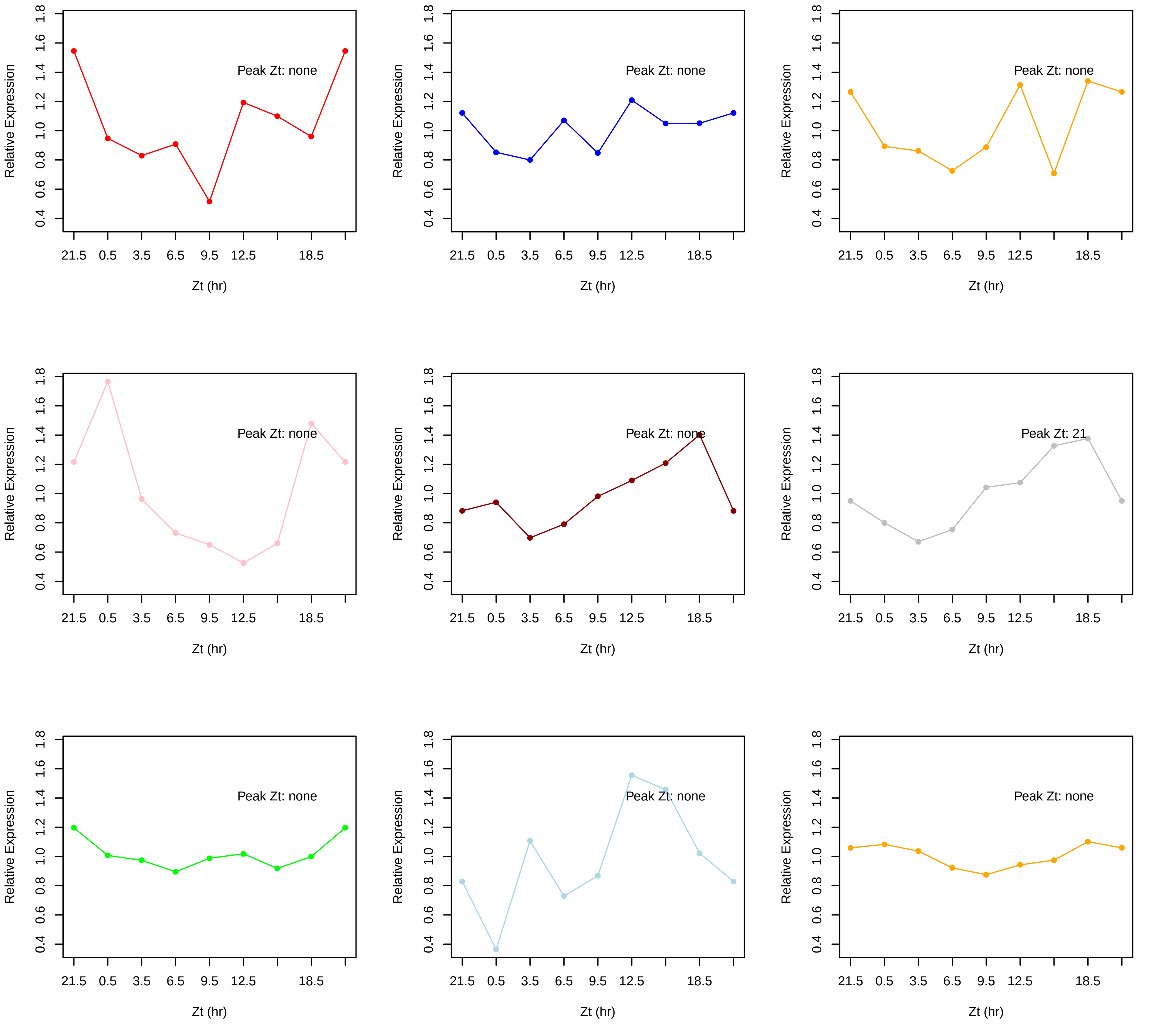

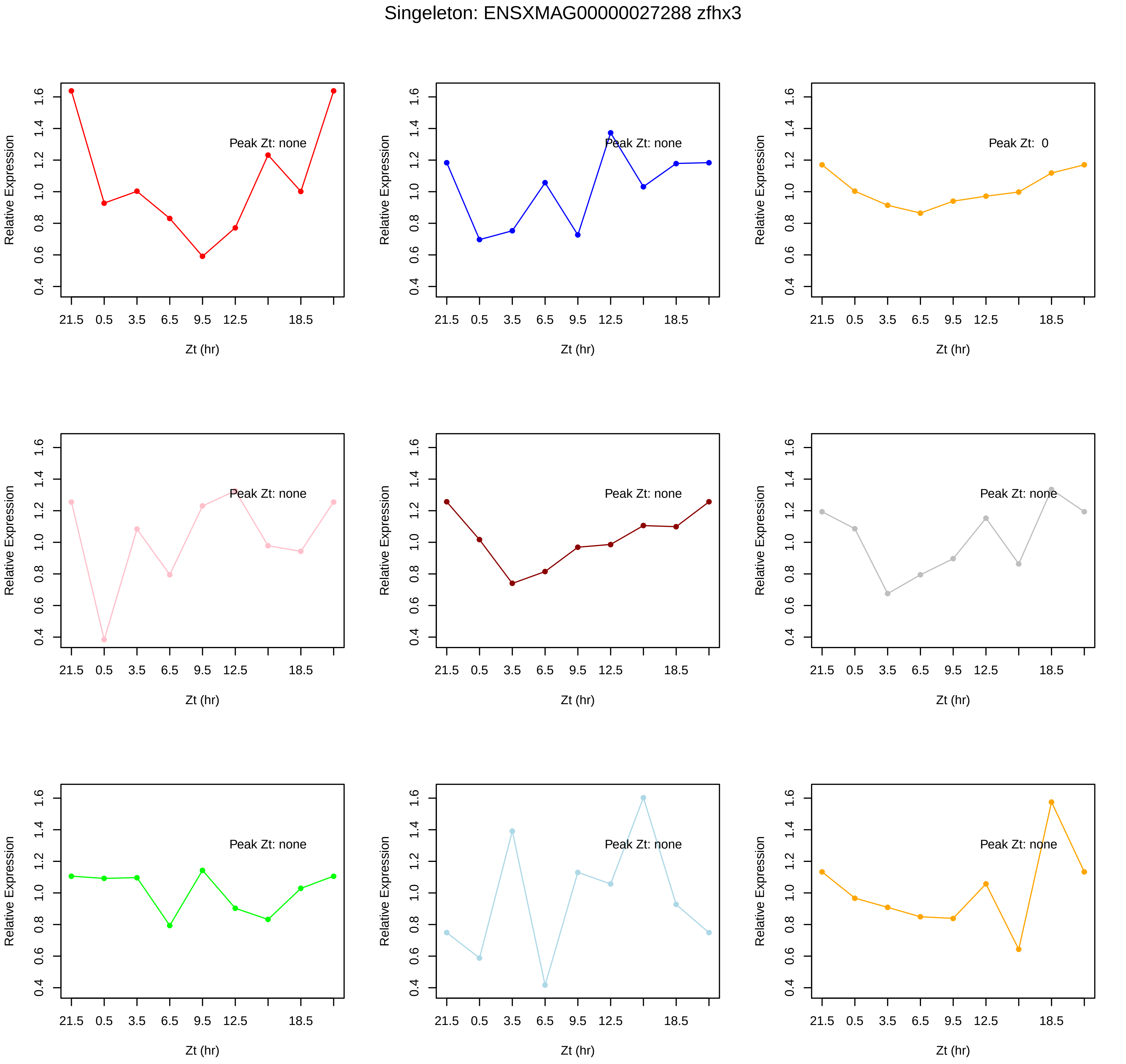
Singeleton: ENSXMAG00000027288 zfhx3
Peak Zt: none
Peak Zt: none
Peak Zt: 0
Peak Zt: none
Peak Zt: none
Peak Zt: none
Peak Zt: none
Peak Zt: none
Peak Zt: none
Relative Expression
Zt (hr)
21.5
0.5
3.5
6.5
9.5
12.5
18.5
0.4
0.6
0.8
1.0
1.2
1.4
1.6

Singeleton: ENSXMAG00000024057 PHKG1

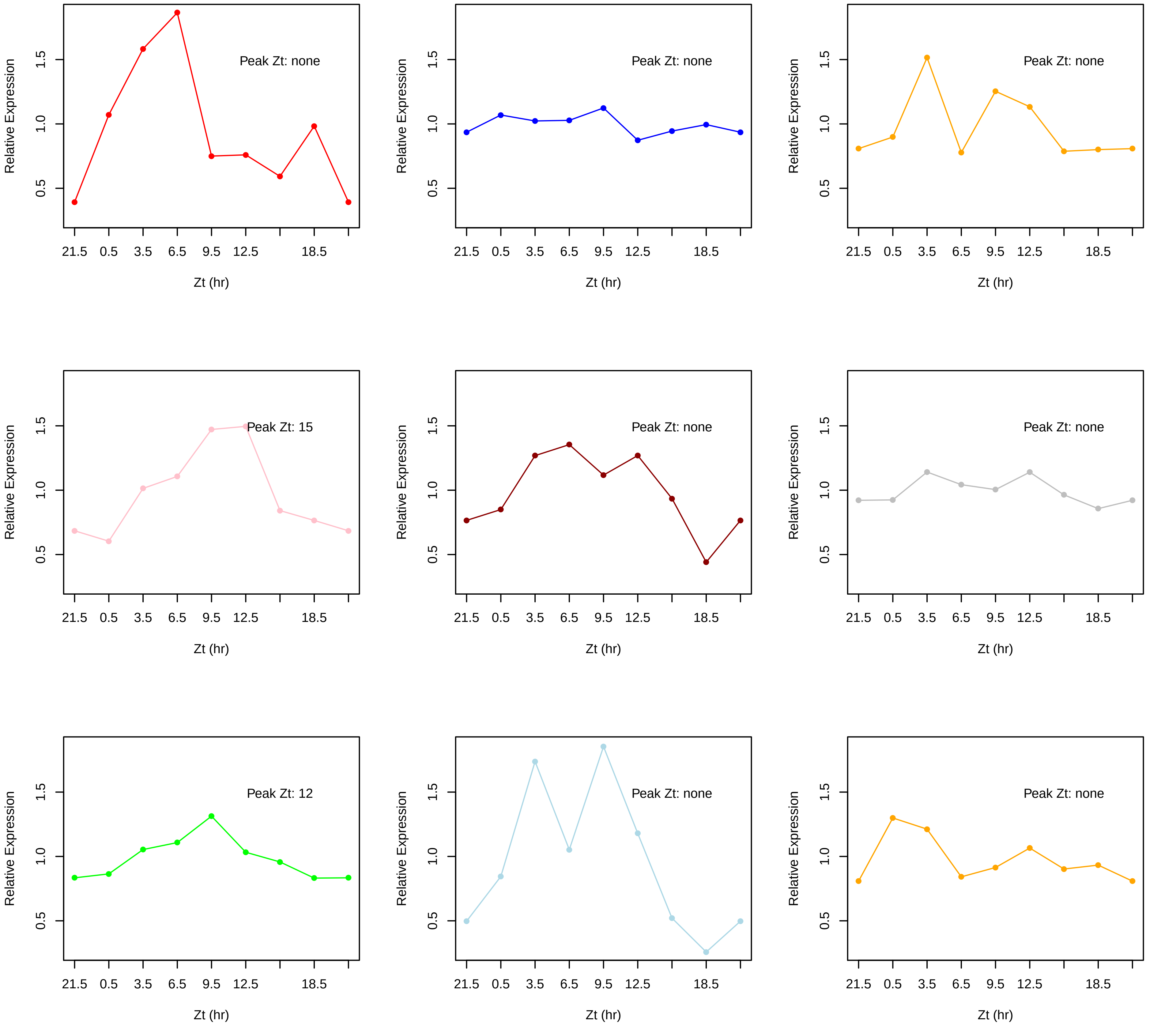

Singeleton: ENSXMAG00000028478

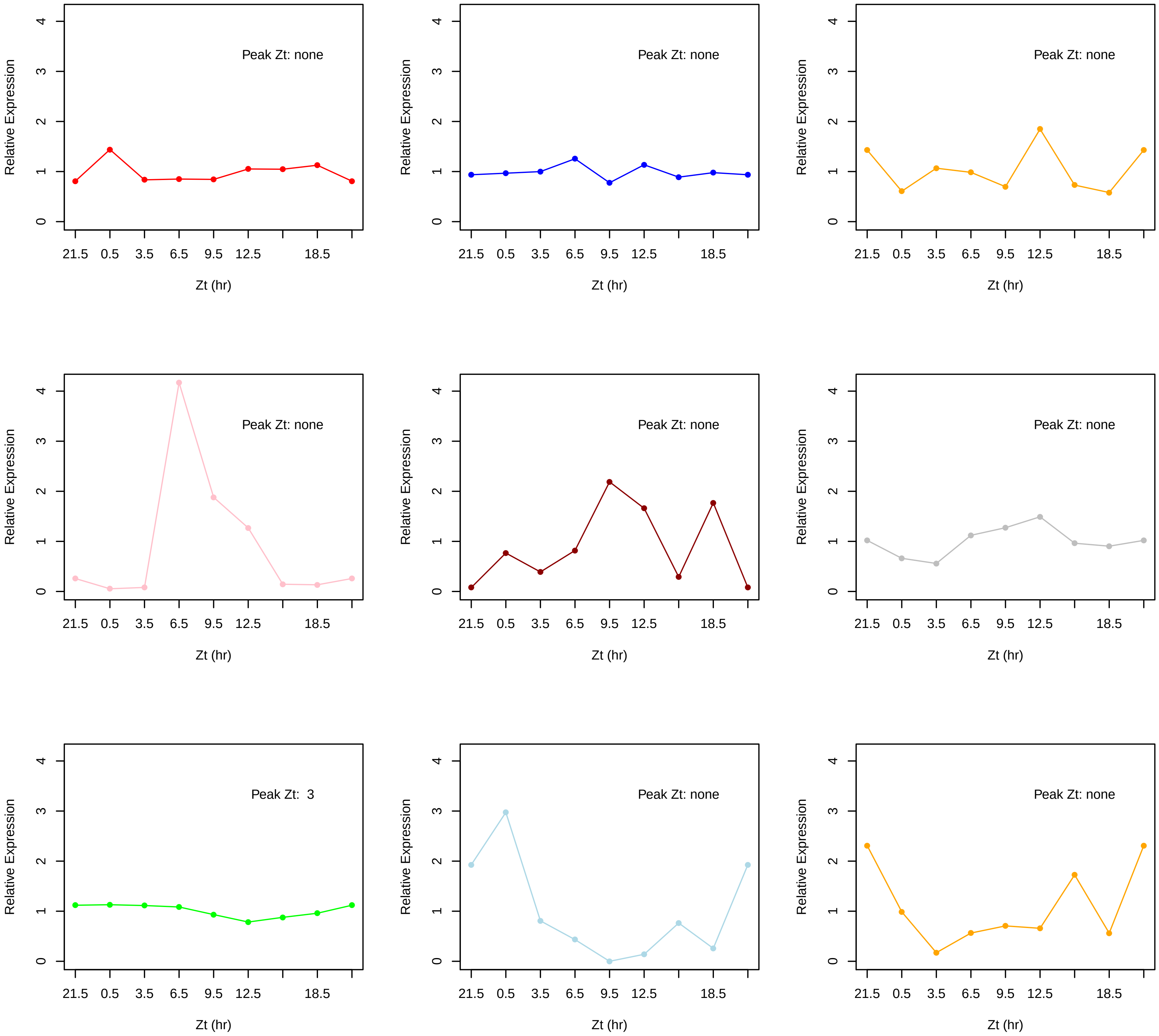

Singeleton: ENSXMAG00000008348 pxylp1

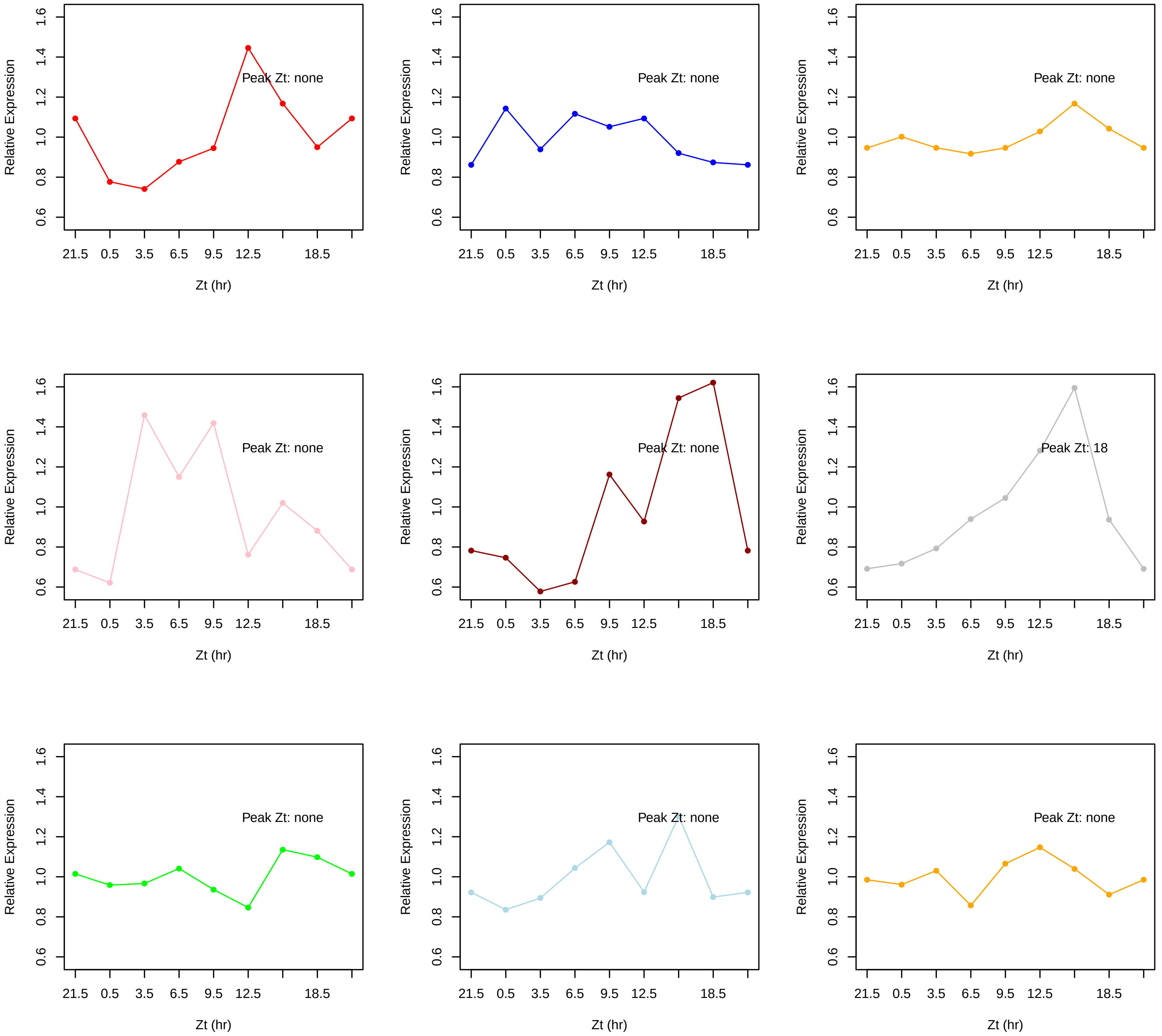

Singeleton: ENSXMAG00000017972 eya3

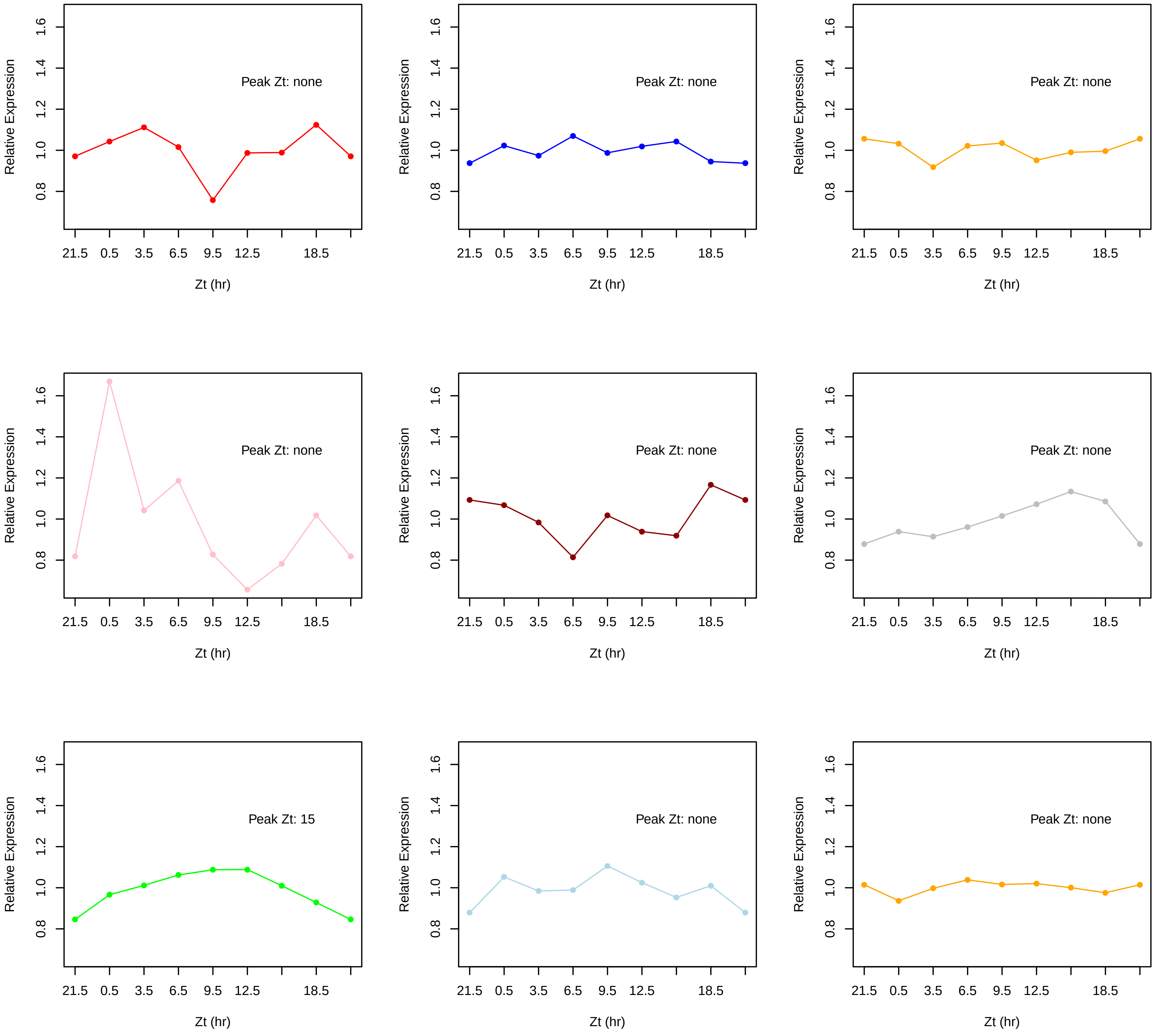

Singeleton: ENSXMAG00000015548 far1

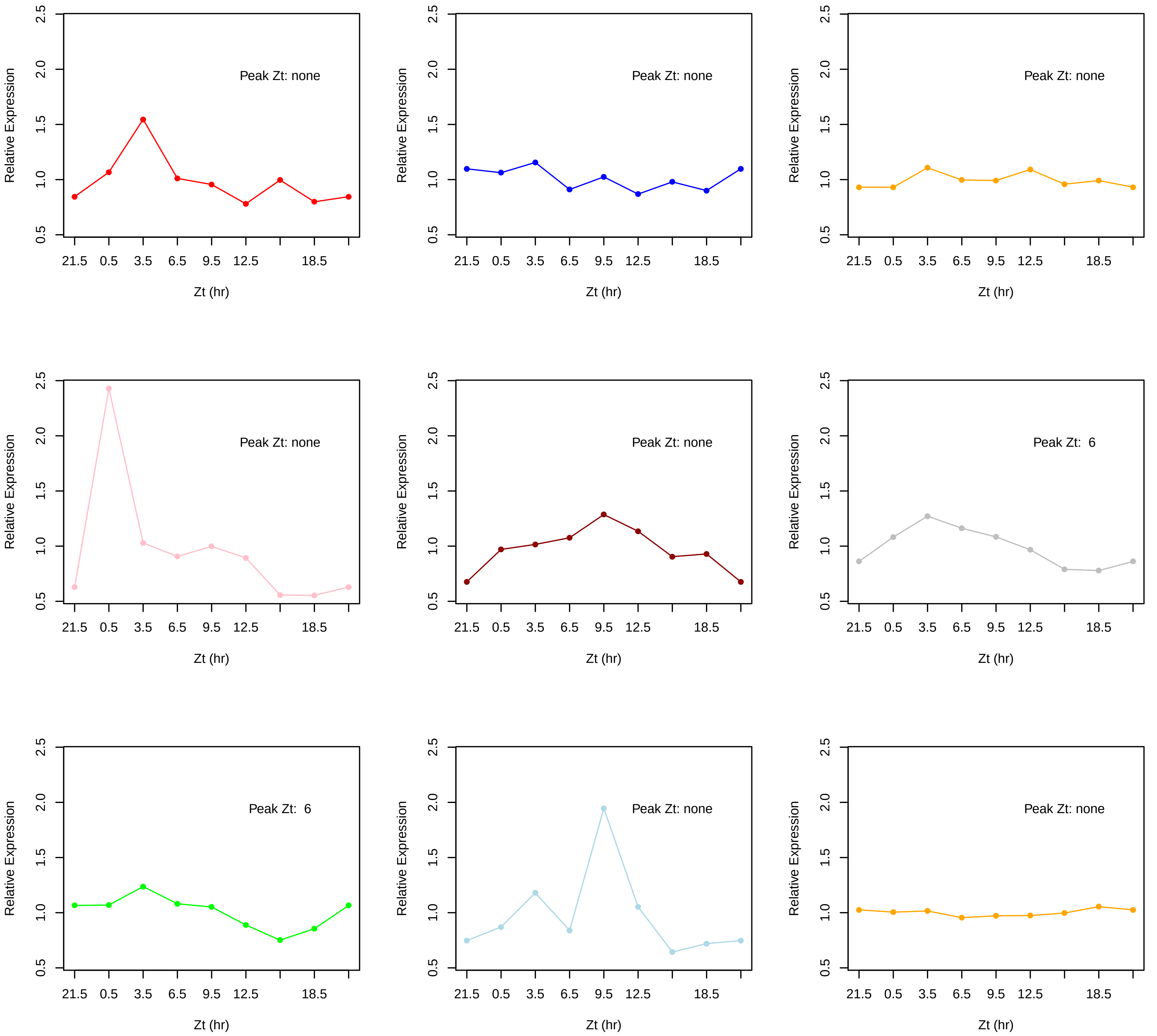

Singeleton: ENSXMAG00000004700 brat1

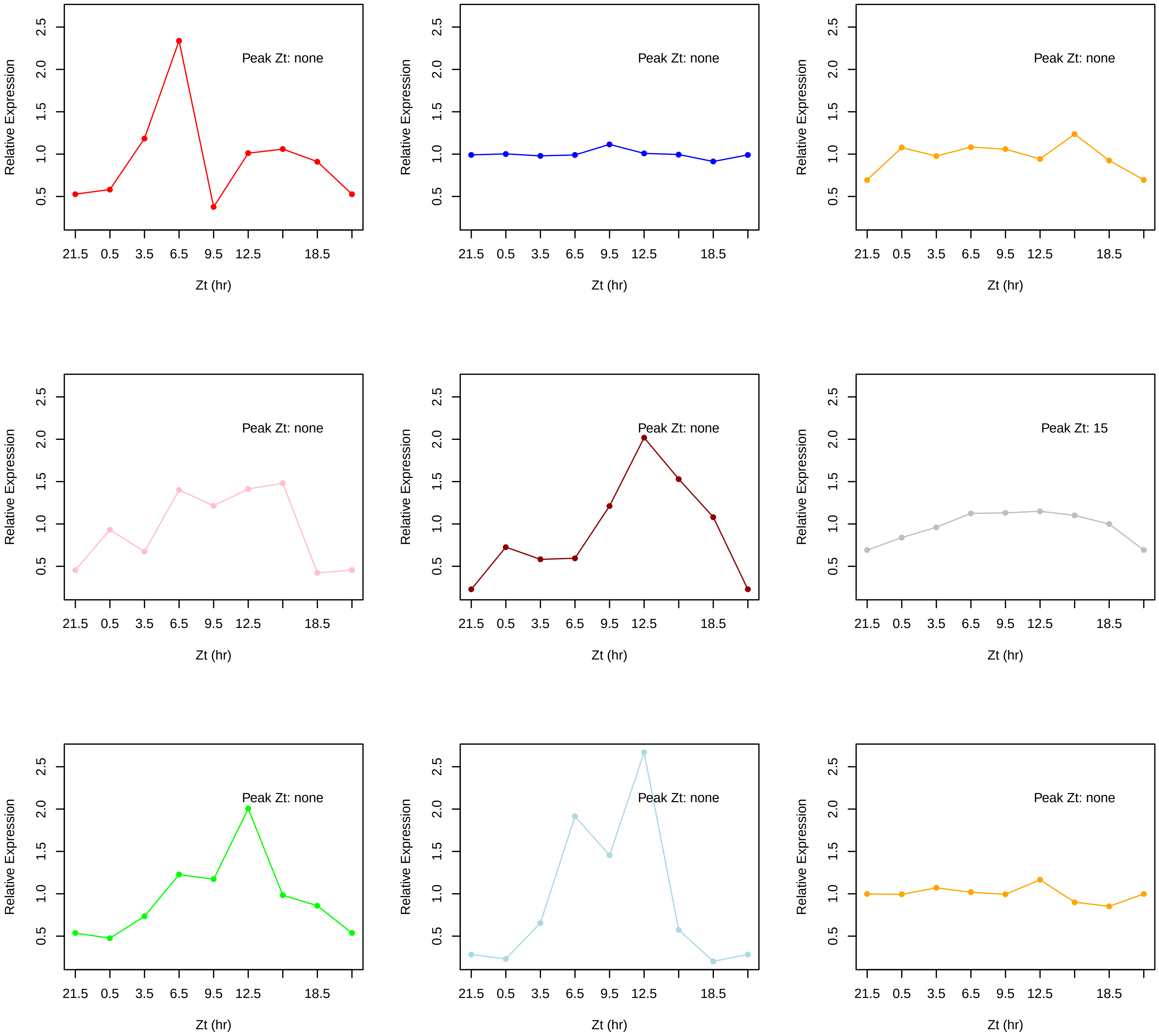

Singeleton: ENSXMAG00000005734 dolpp1

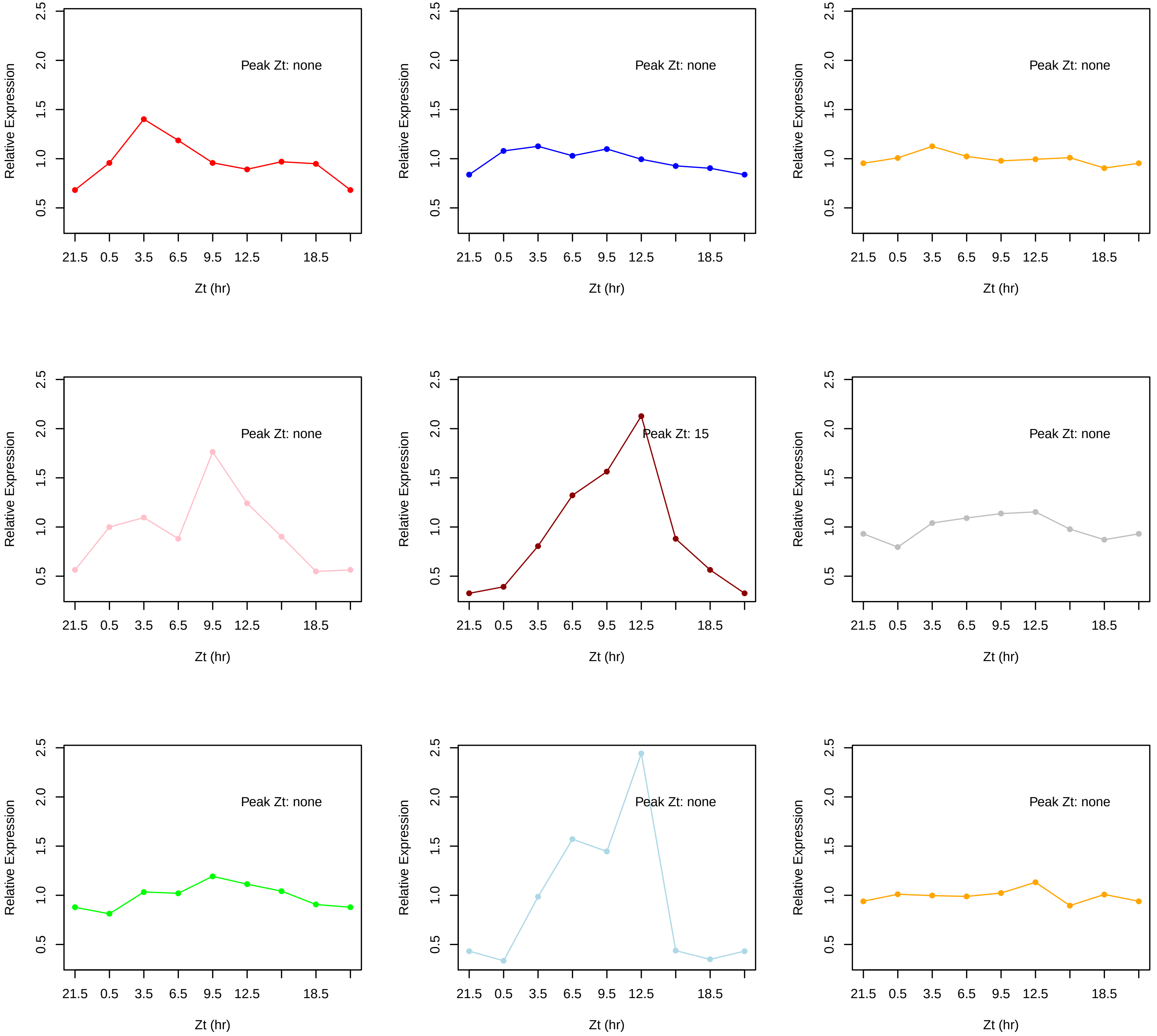

Singeleton: ENSXMAG00000029949 st3gal3b

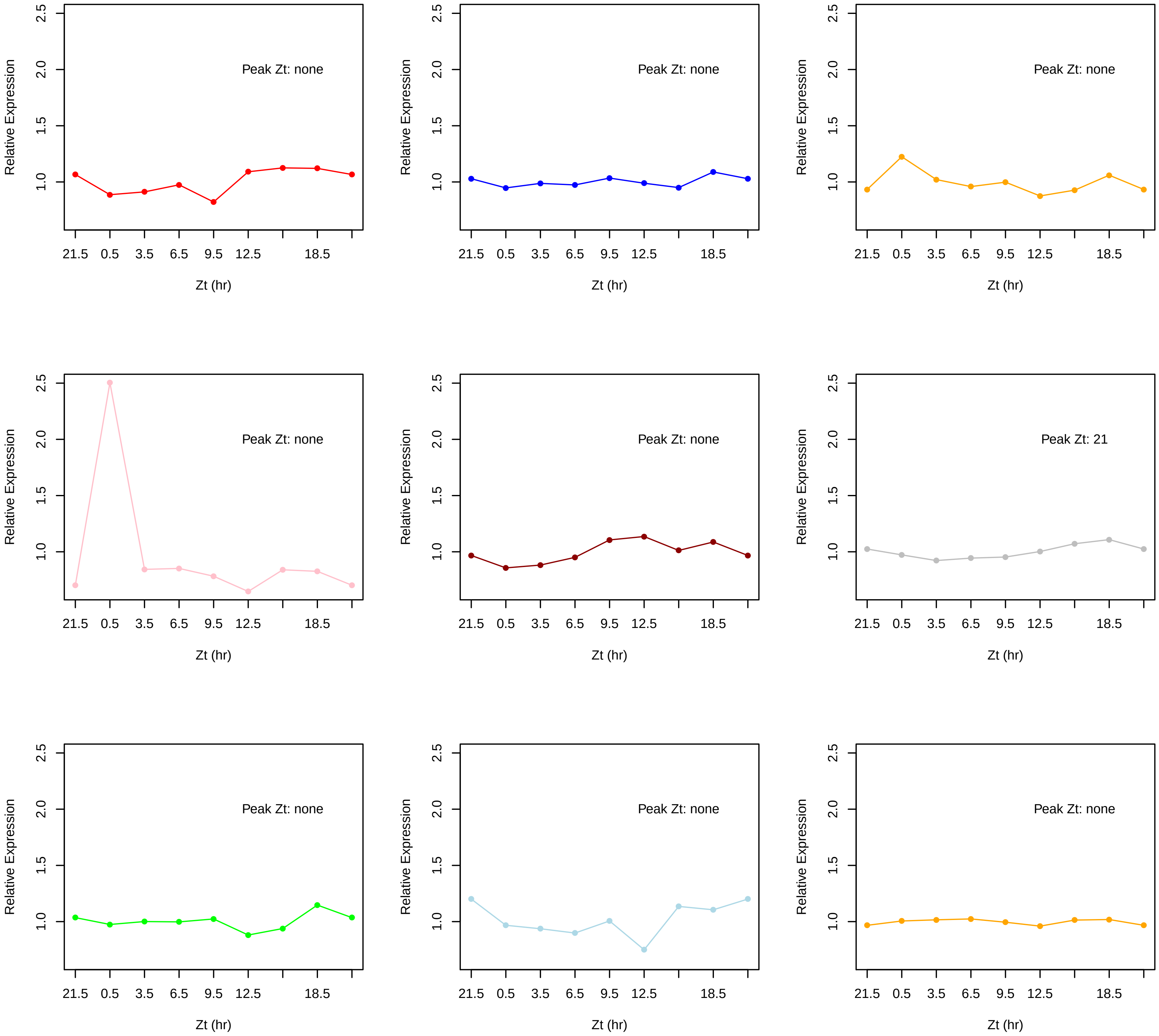

Singleton: ENSXMAG00000005219 mcrs1

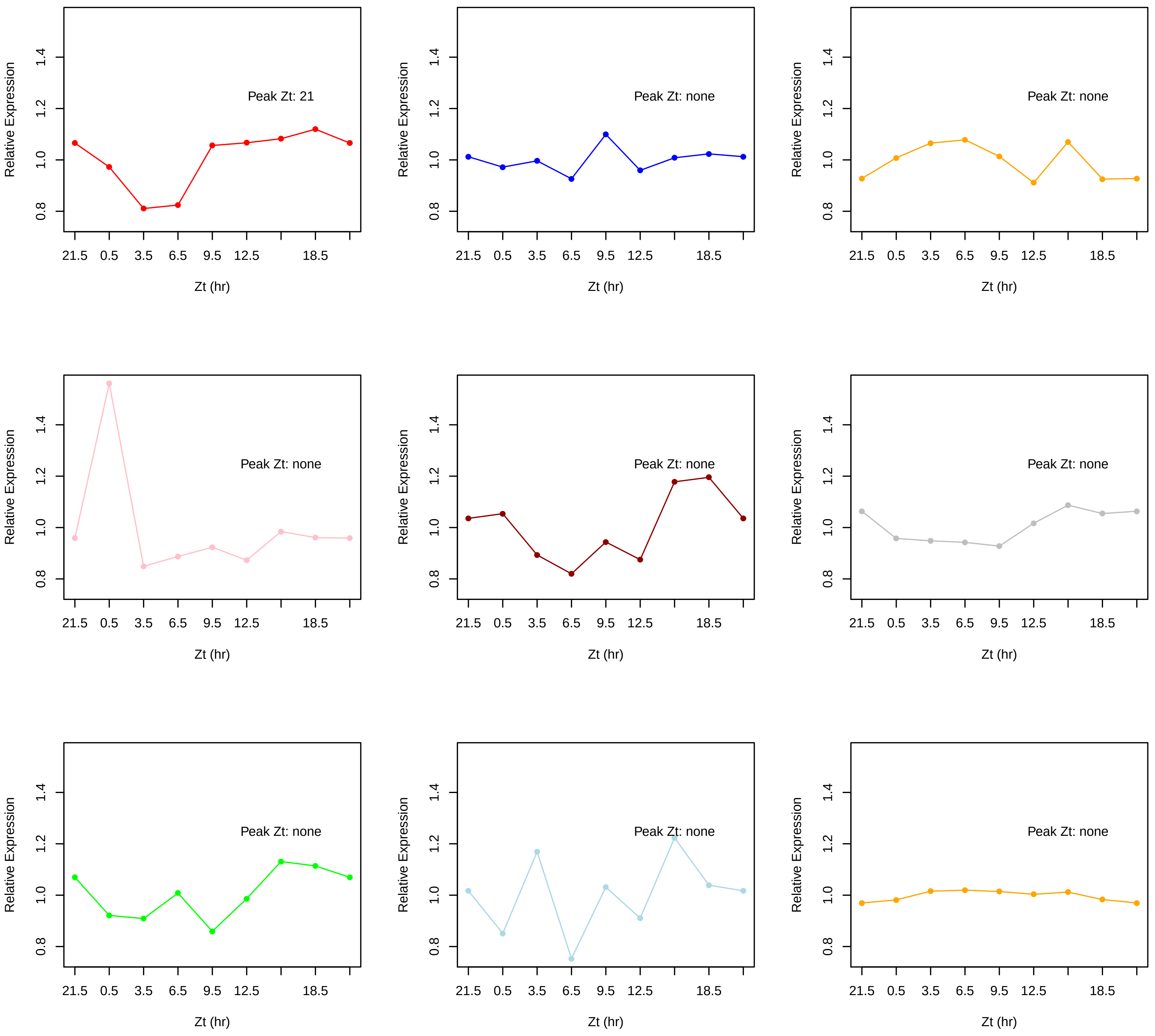

Singeleton: ENSXMAG00000025069

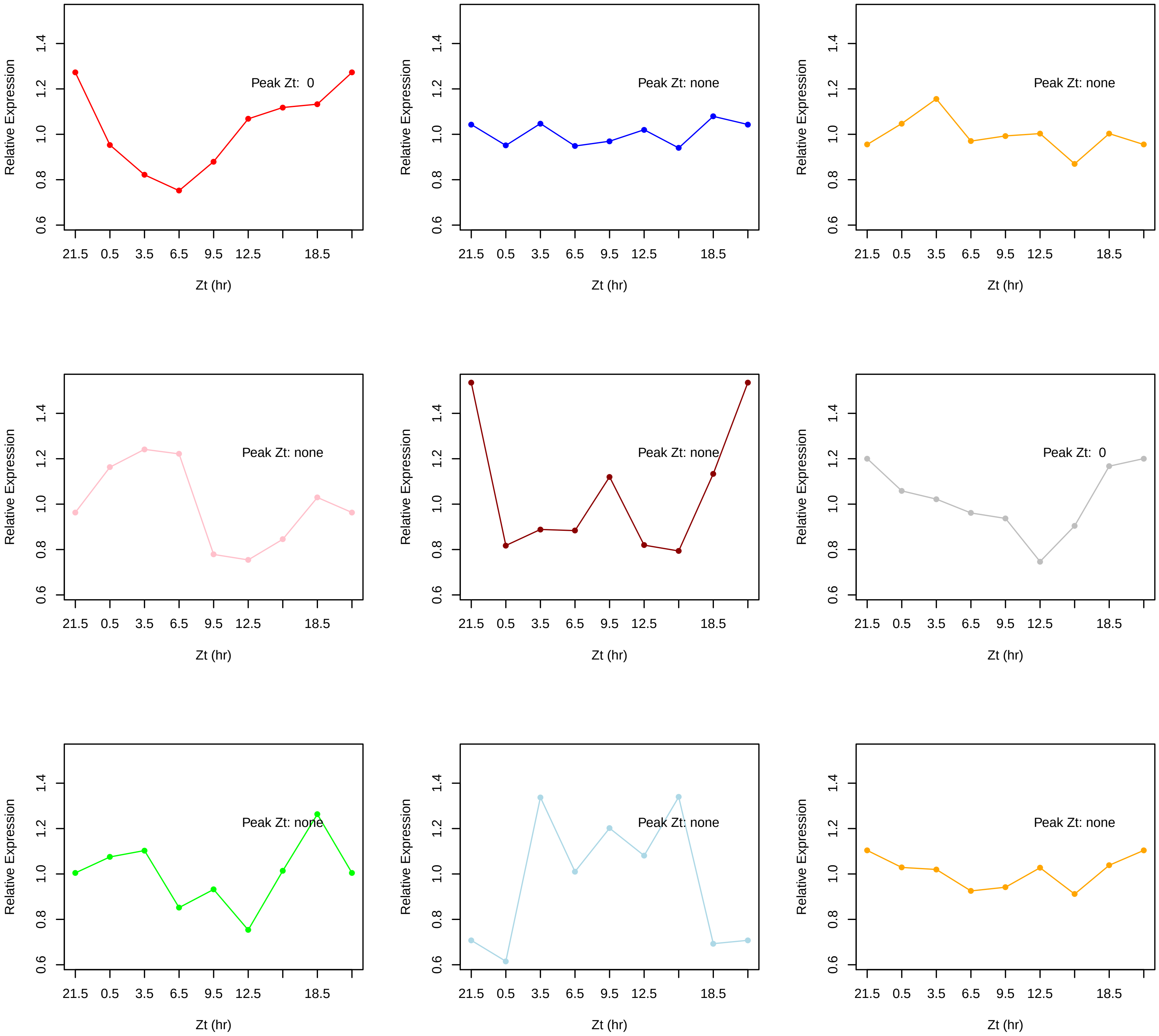

Singeleton: ENSXMAG00000017150 miip

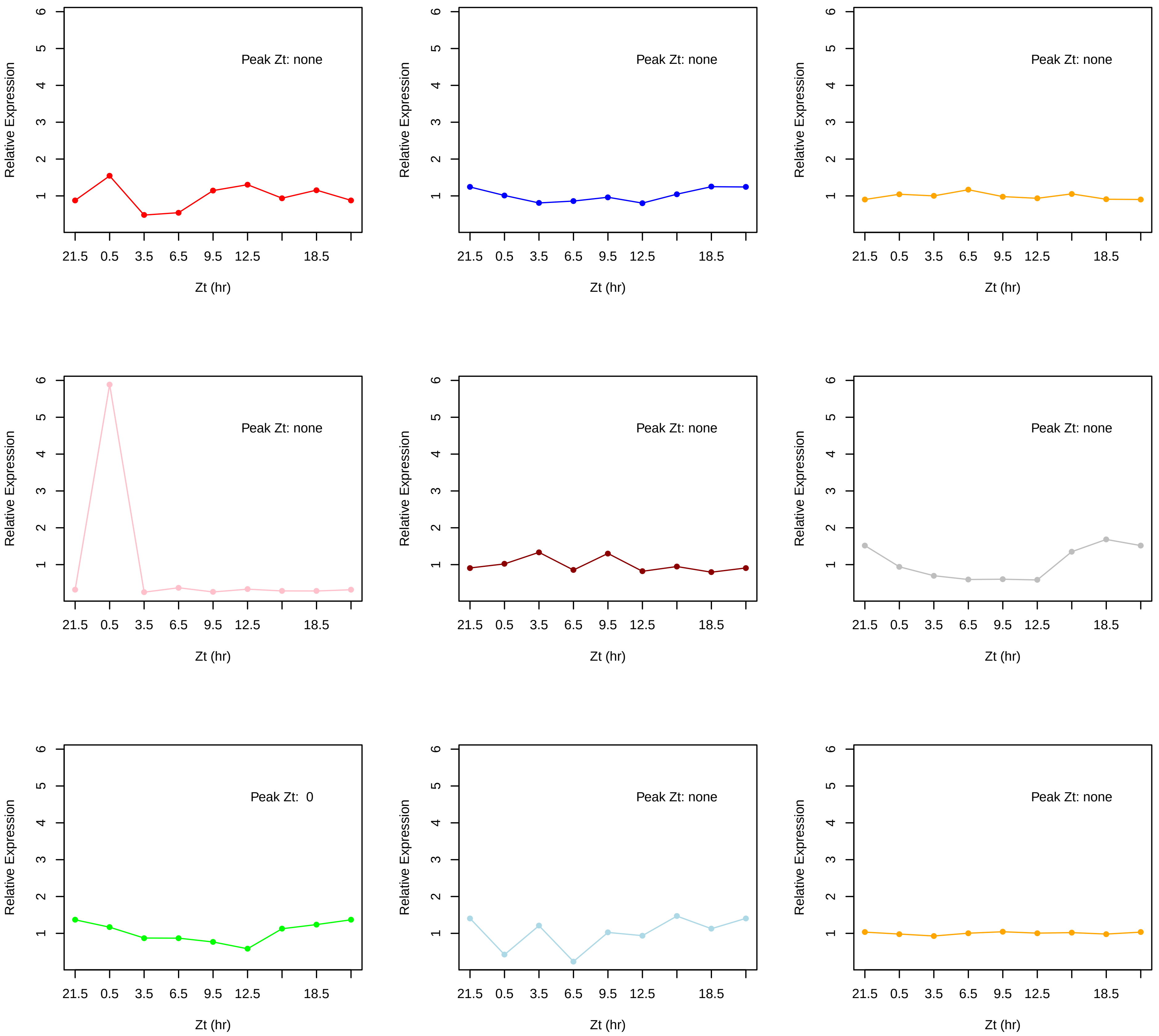

Singeleton: ENSXMAG00000015561 cyb5r2

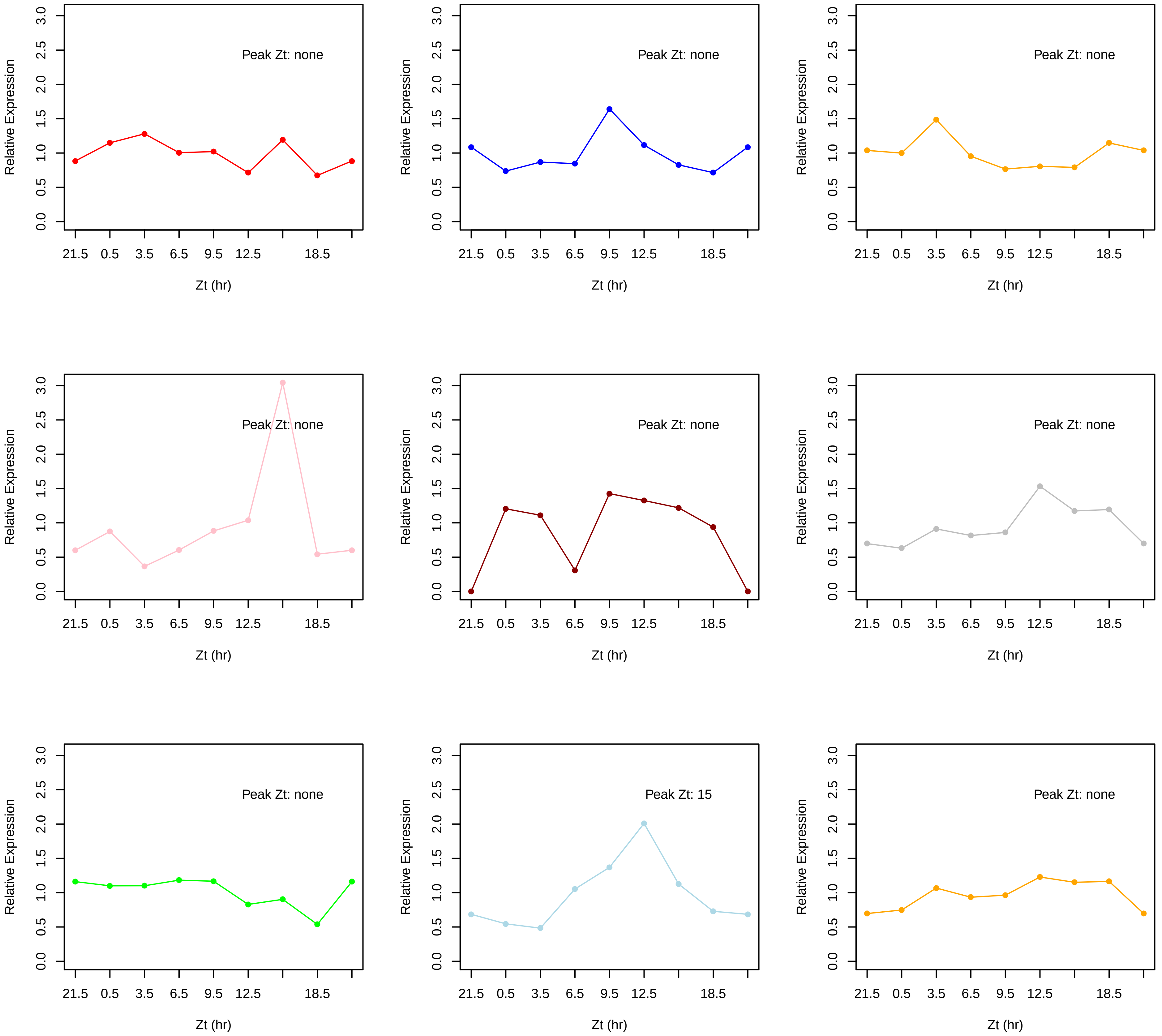

Singeleton: ENSXMAG00000010123 prex2

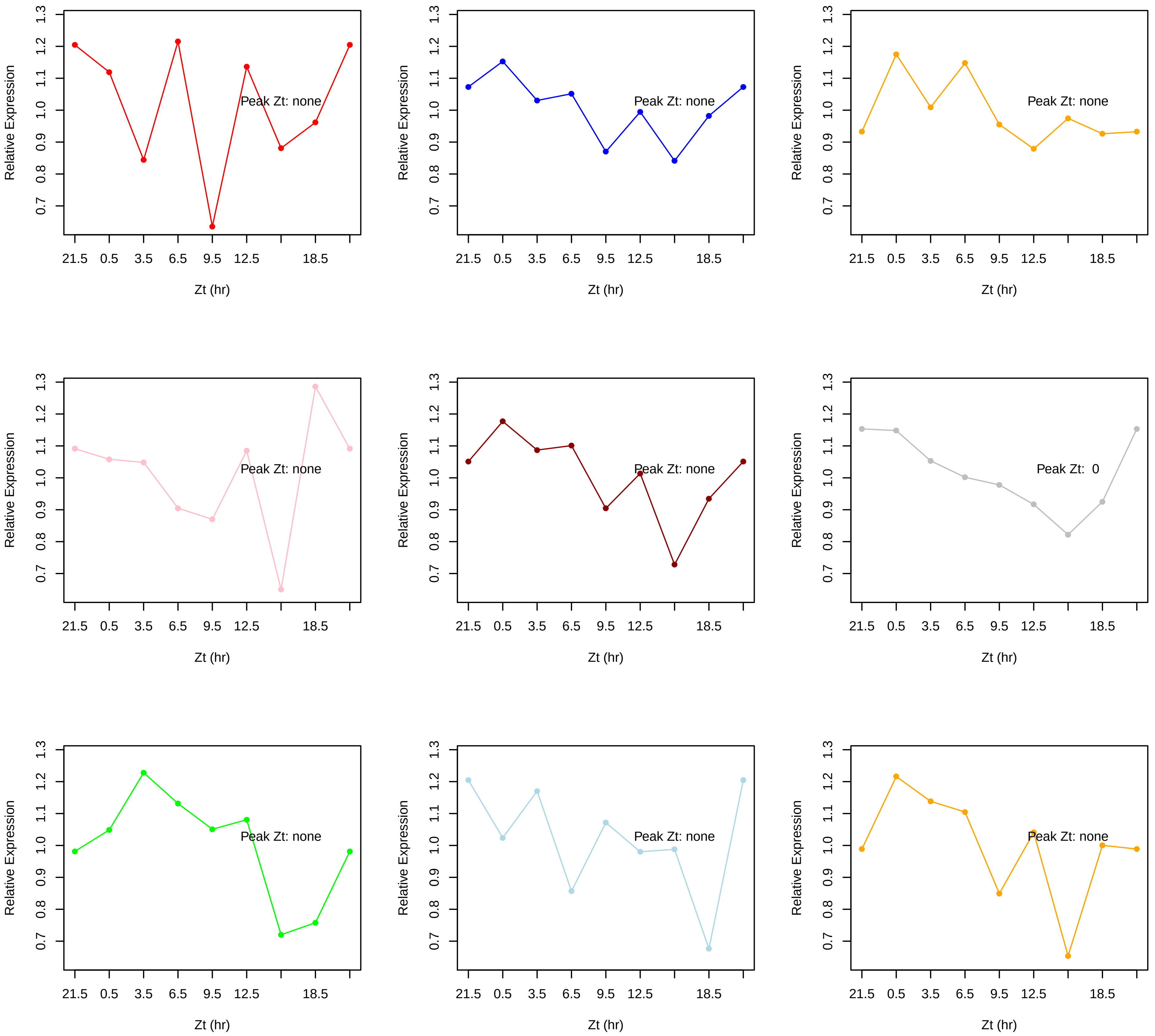

Singeleton: ENSXMAG00000007916 crispld2

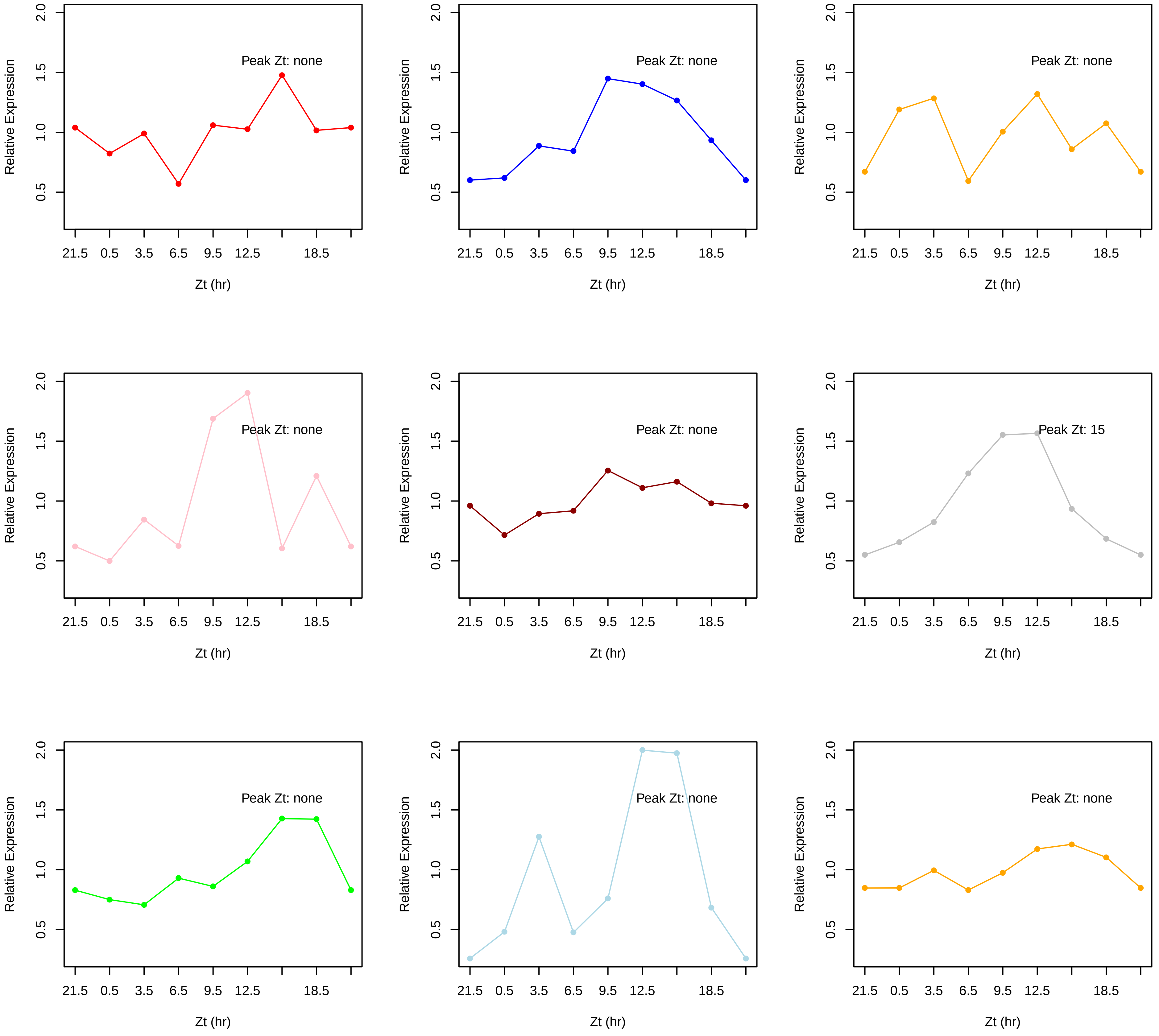

Singeleton: ENSXMAG00000013710 fuom

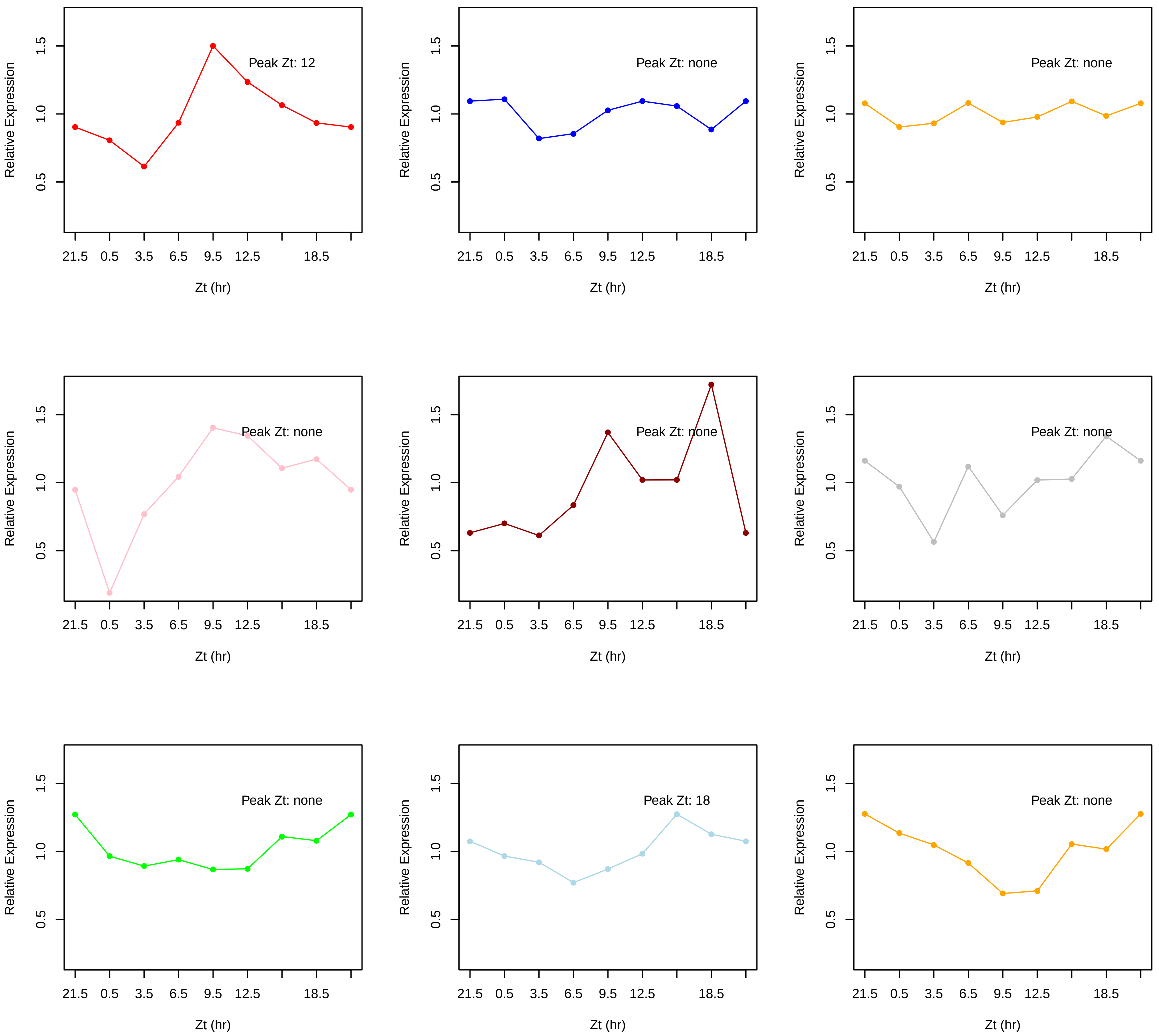

Singeleton: ENSXMAG00000009644 trpc4apa

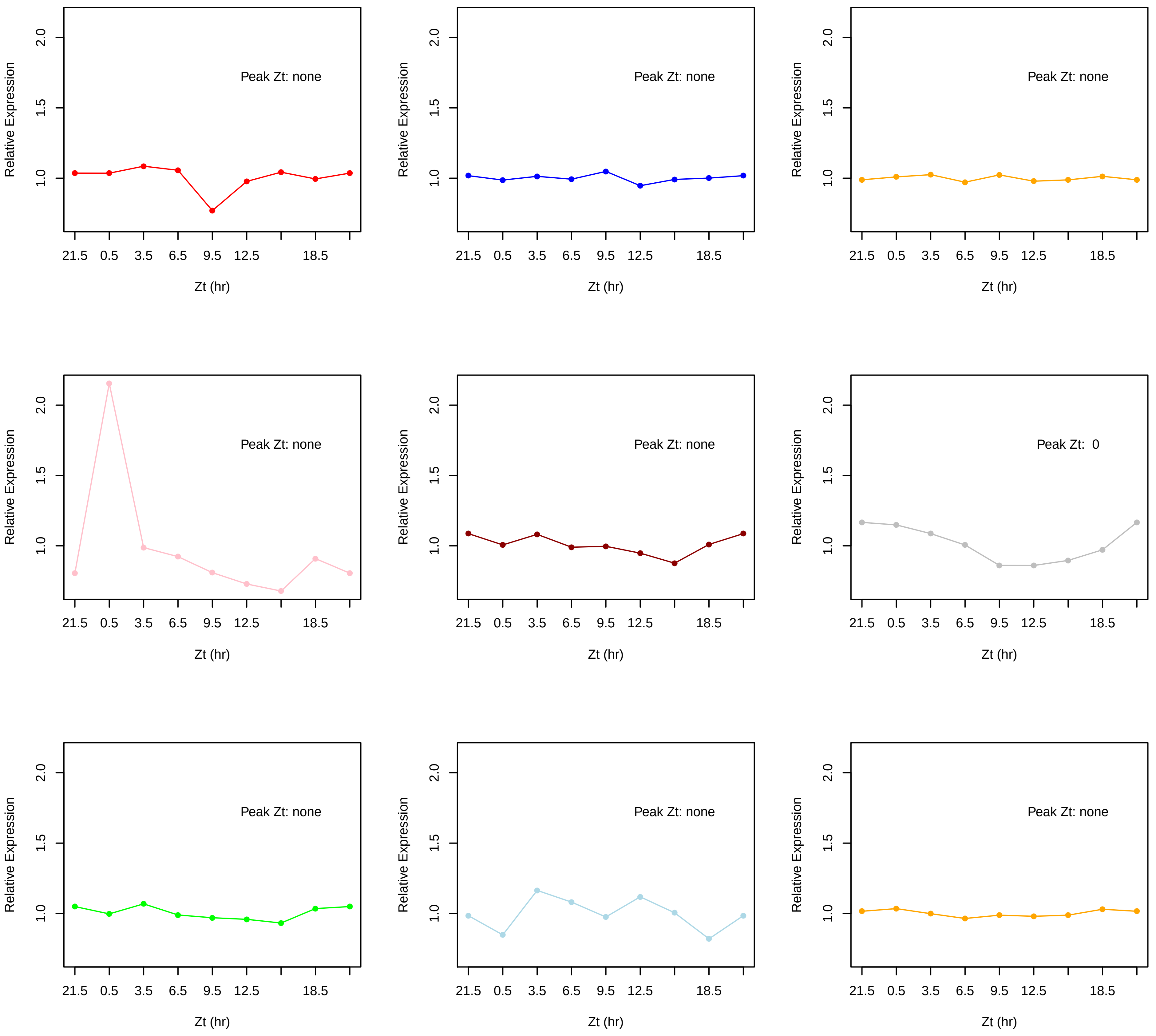

Singeleton: ENSXMAG00000017144

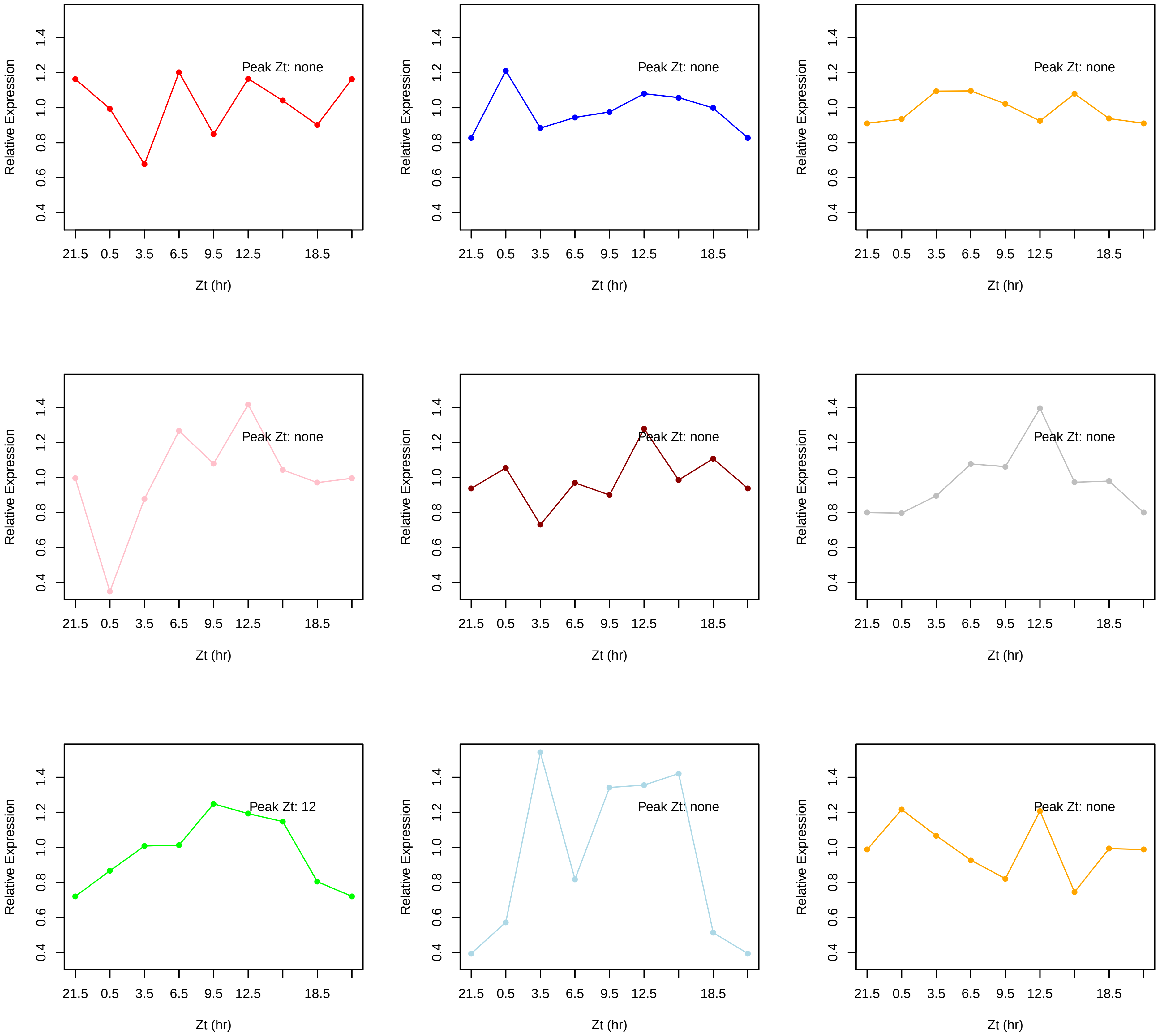

Singeleton: ENSXMAG00000008019 fam222a

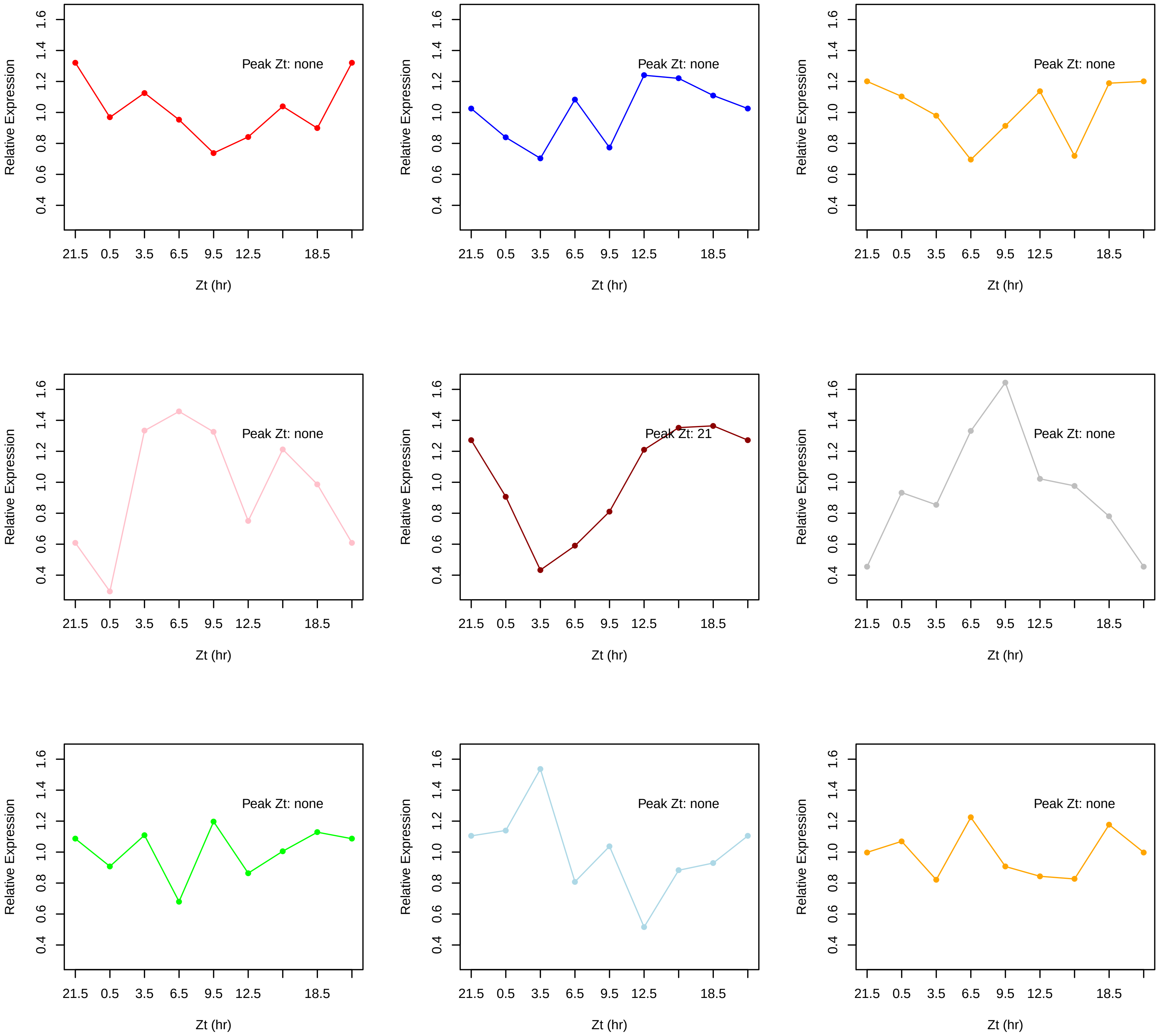

Singeleton: ENSXMAG00000007321 abhd11

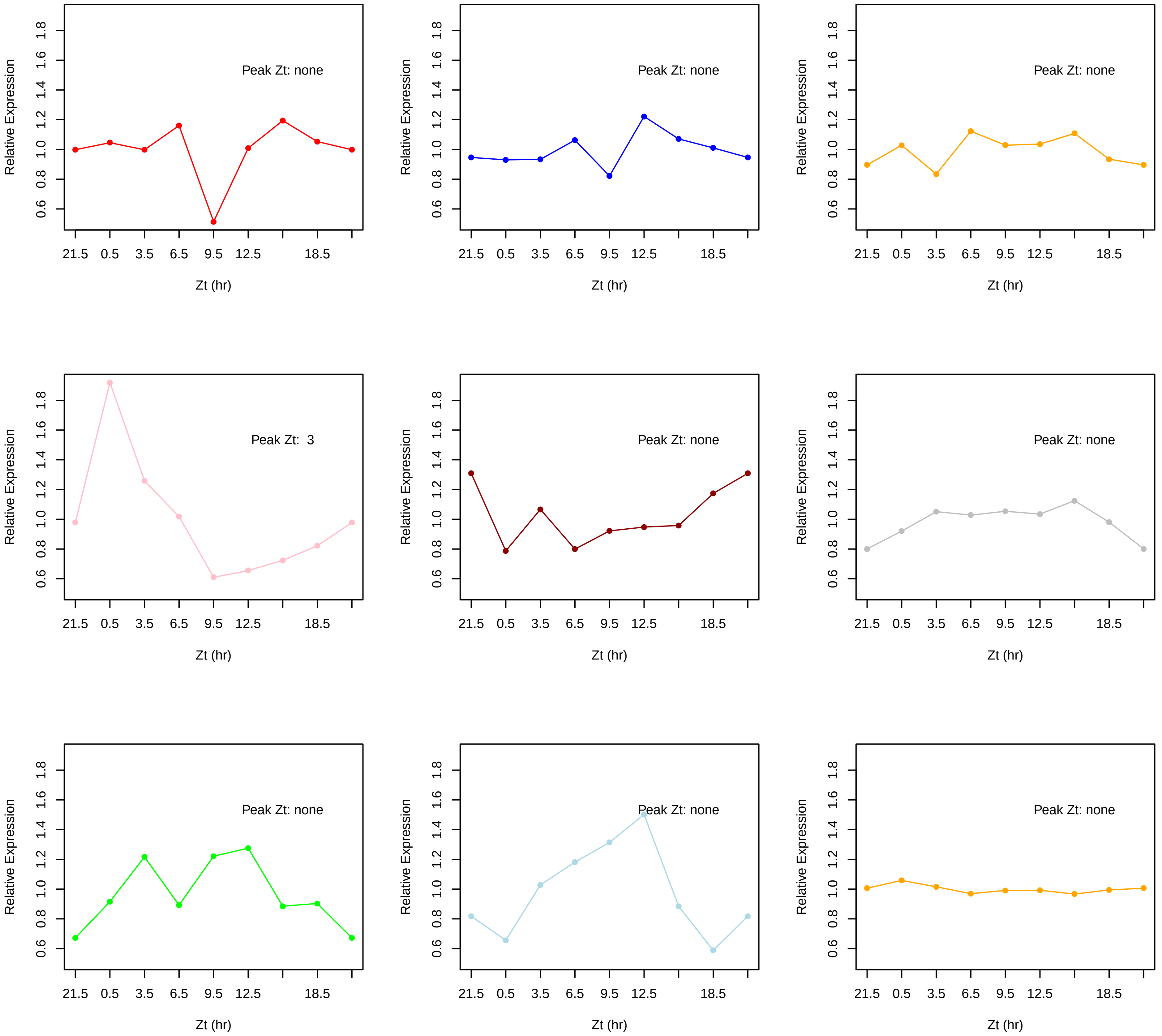

Singleton: ENSXMAG00000026179 usp10

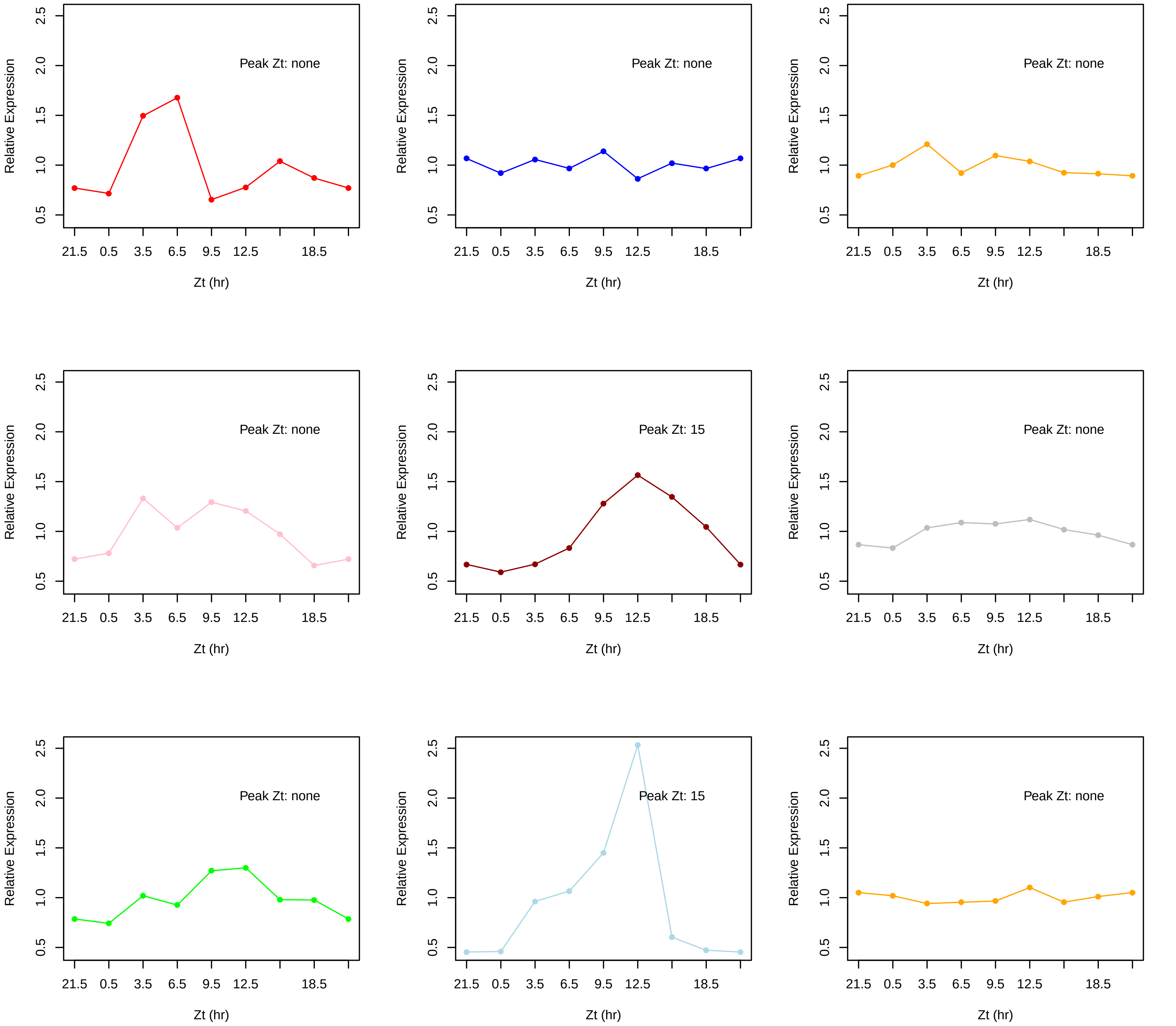

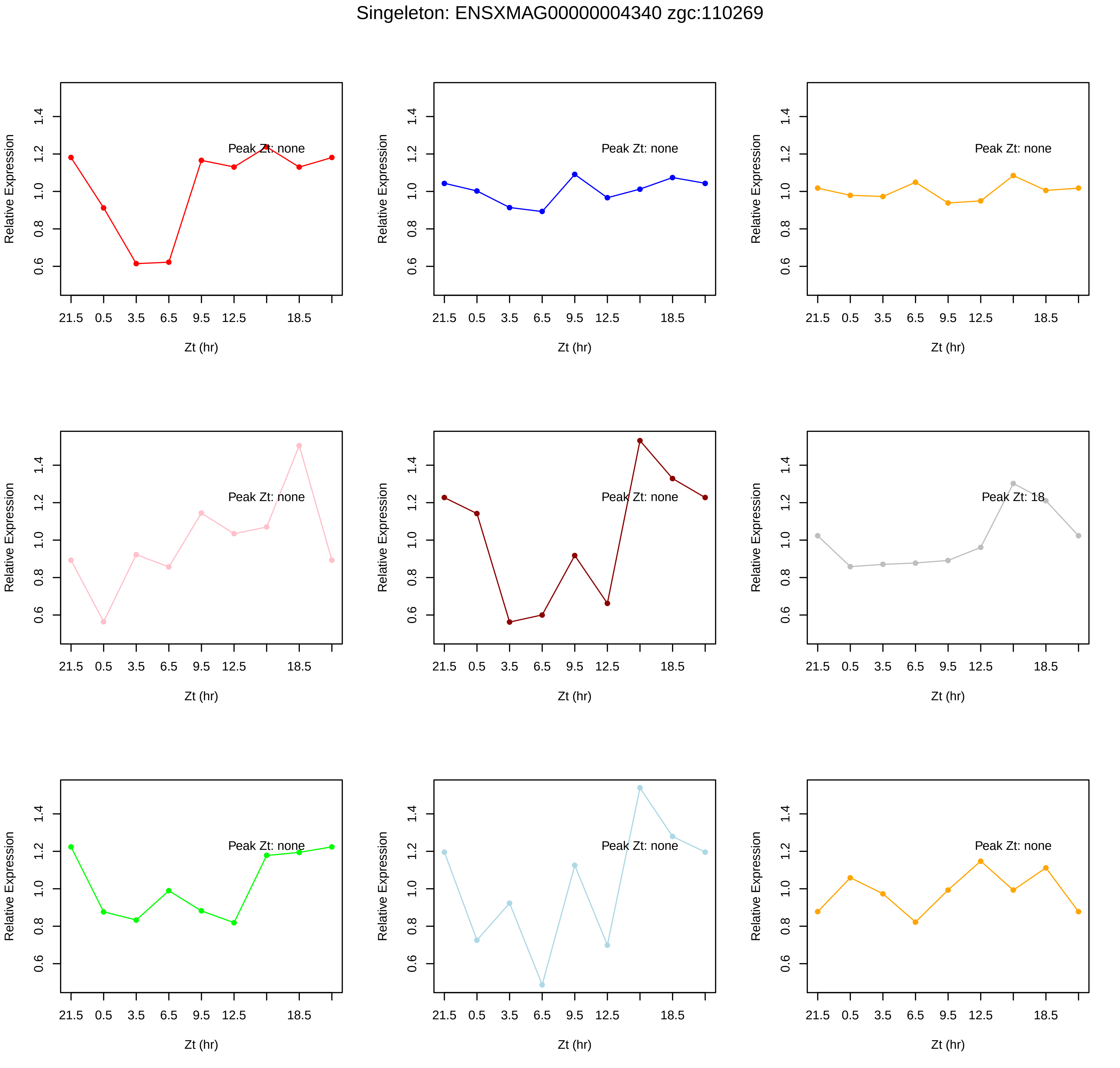

Singeleton: ENSXMAG00000004340 zgc:110269
Peak Zt: none
Peak Zt: none
Peak Zt: none
Peak Zt: none
Peak Zt: none
Peak Zt: 18
Peak Zt: none
Peak Zt: none
Peak Zt: none
Relative Expression
Zt (hr)

Singeleton: ENSXMAG00000014459 cadm4

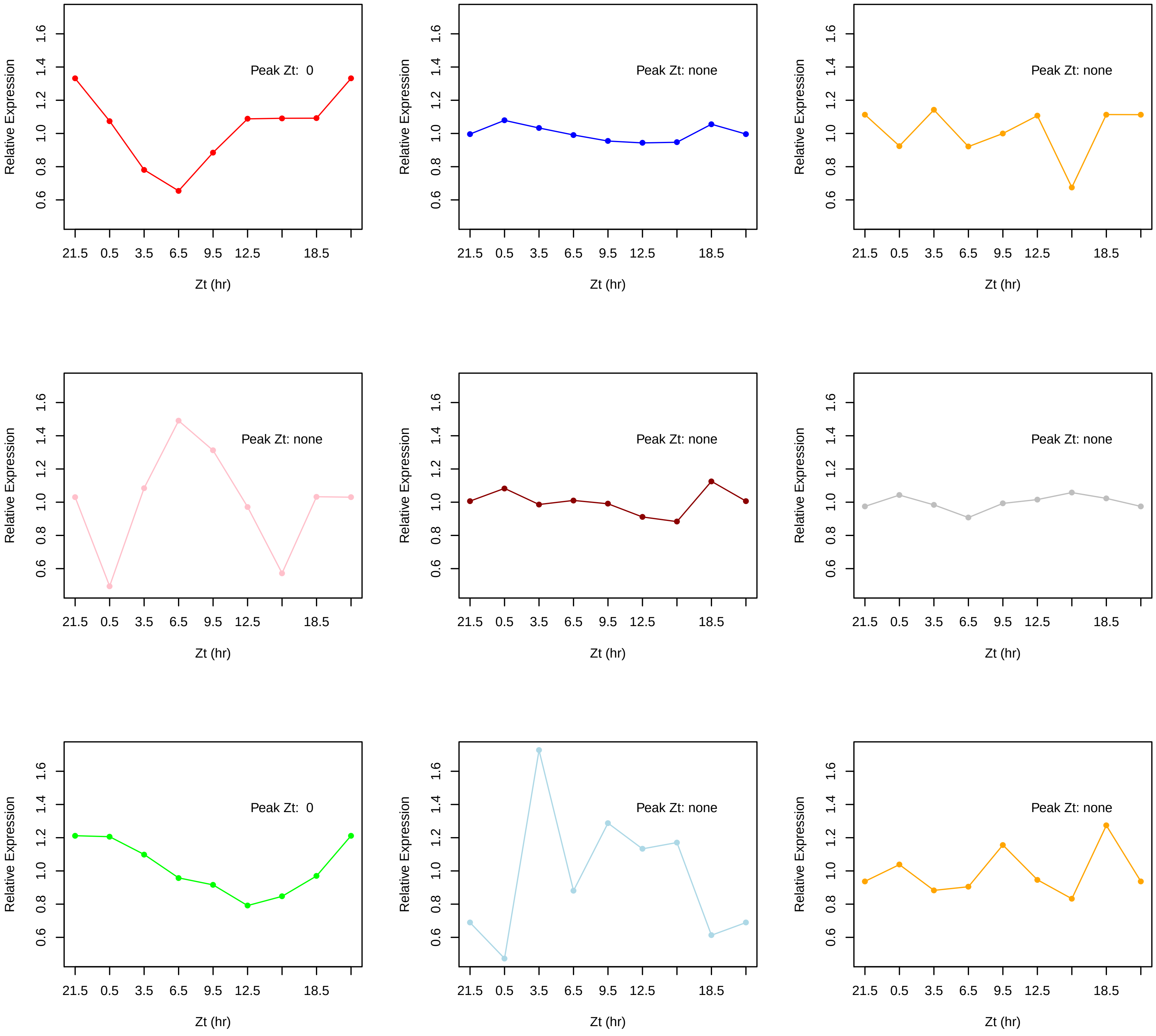

Singeleton: ENSXMAG00000022411 timm13

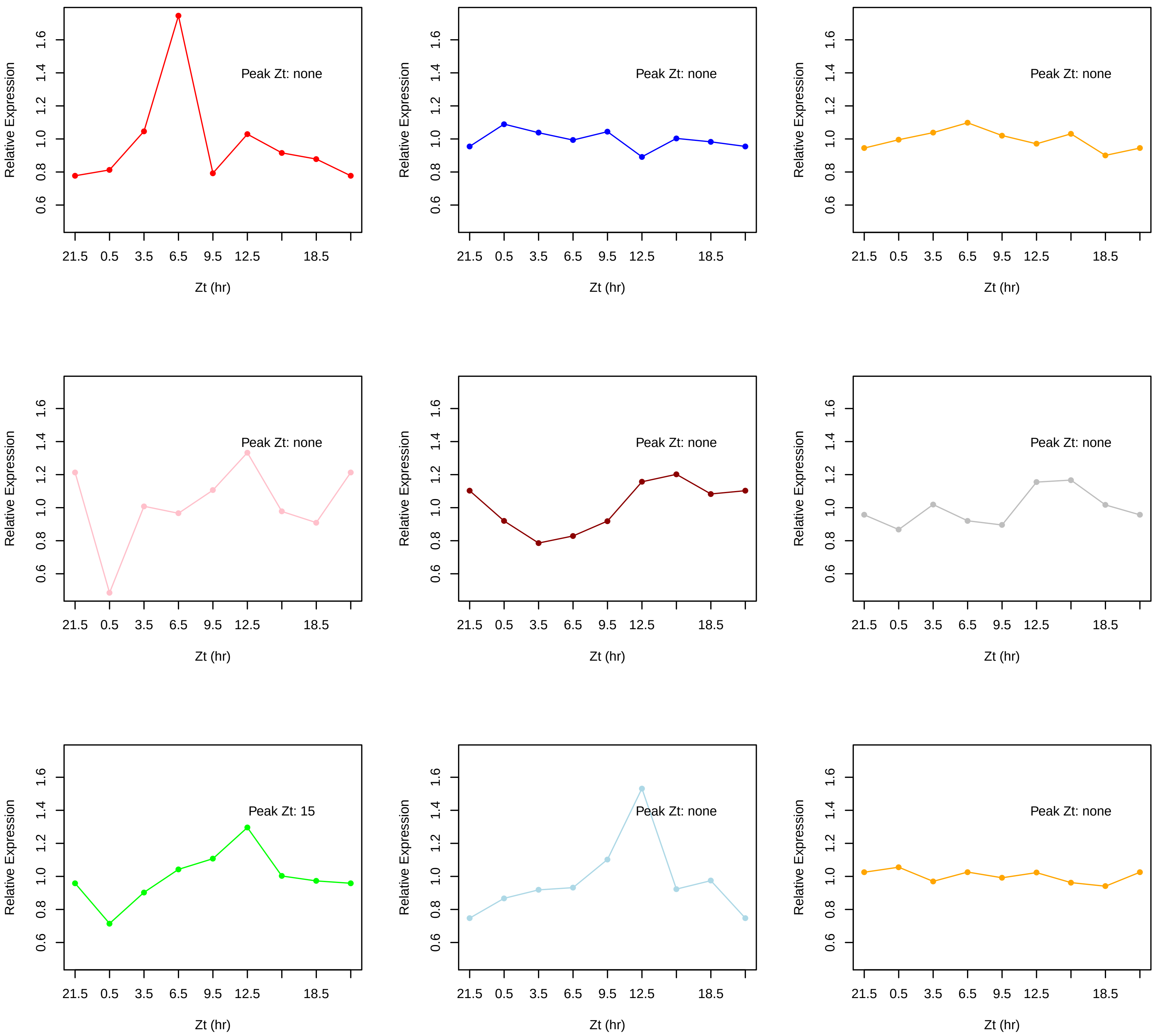

Singleton: ENSXMAG00000000386 ptprfb

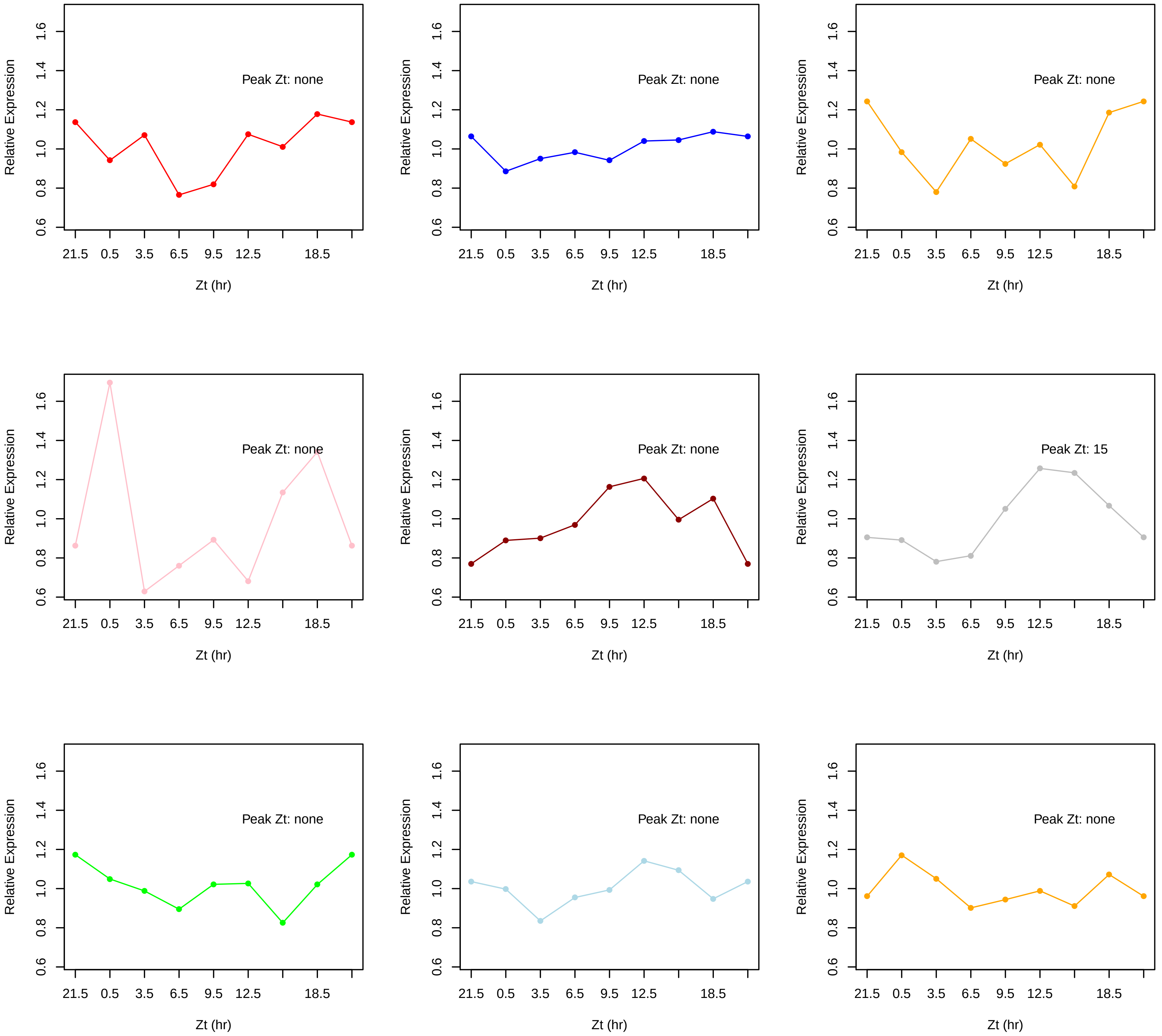

Singeleton: ENSXMAG00000019136 ptpn23a

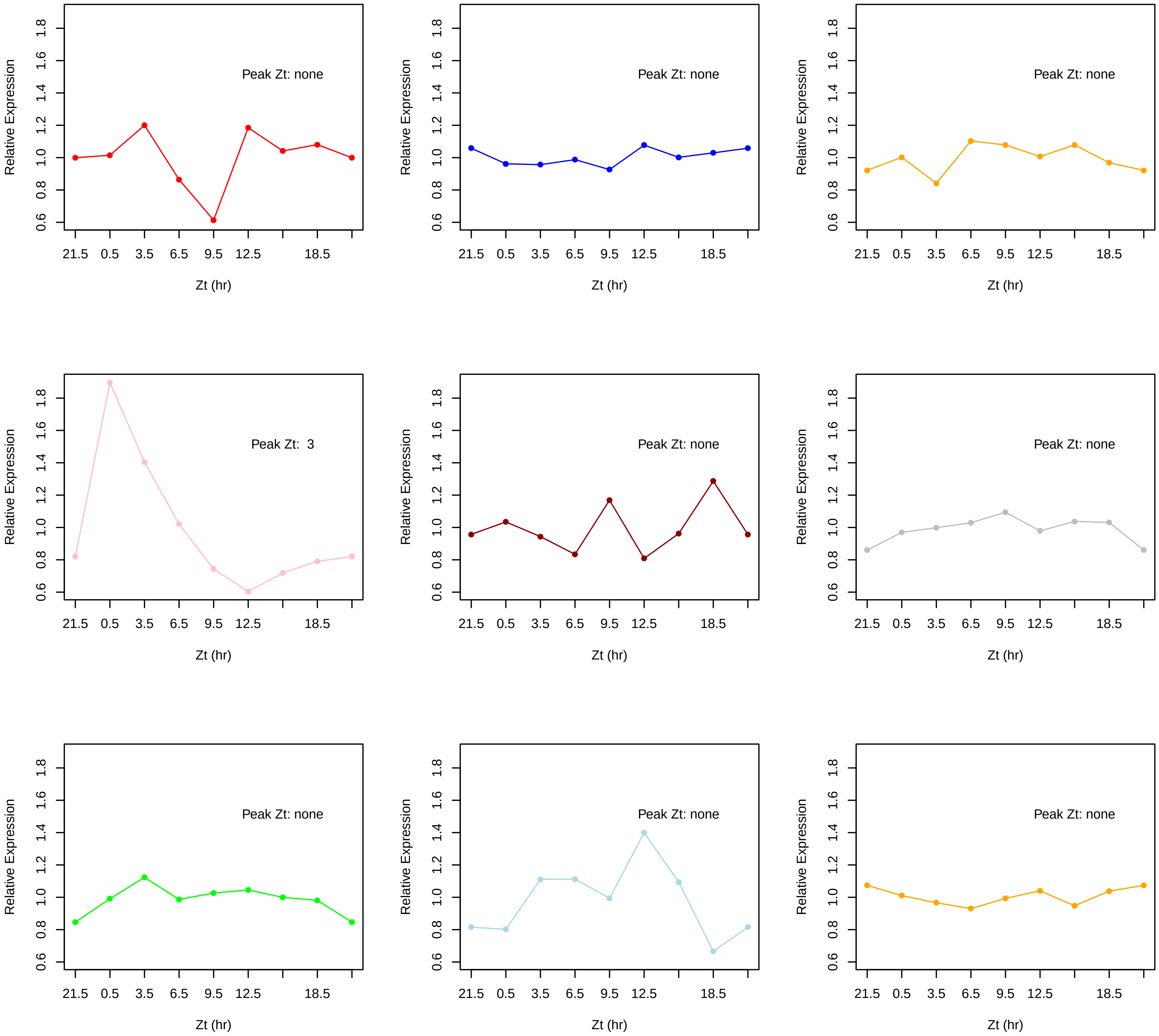

Singeleton: ENSXMAG00000007954 marveld3

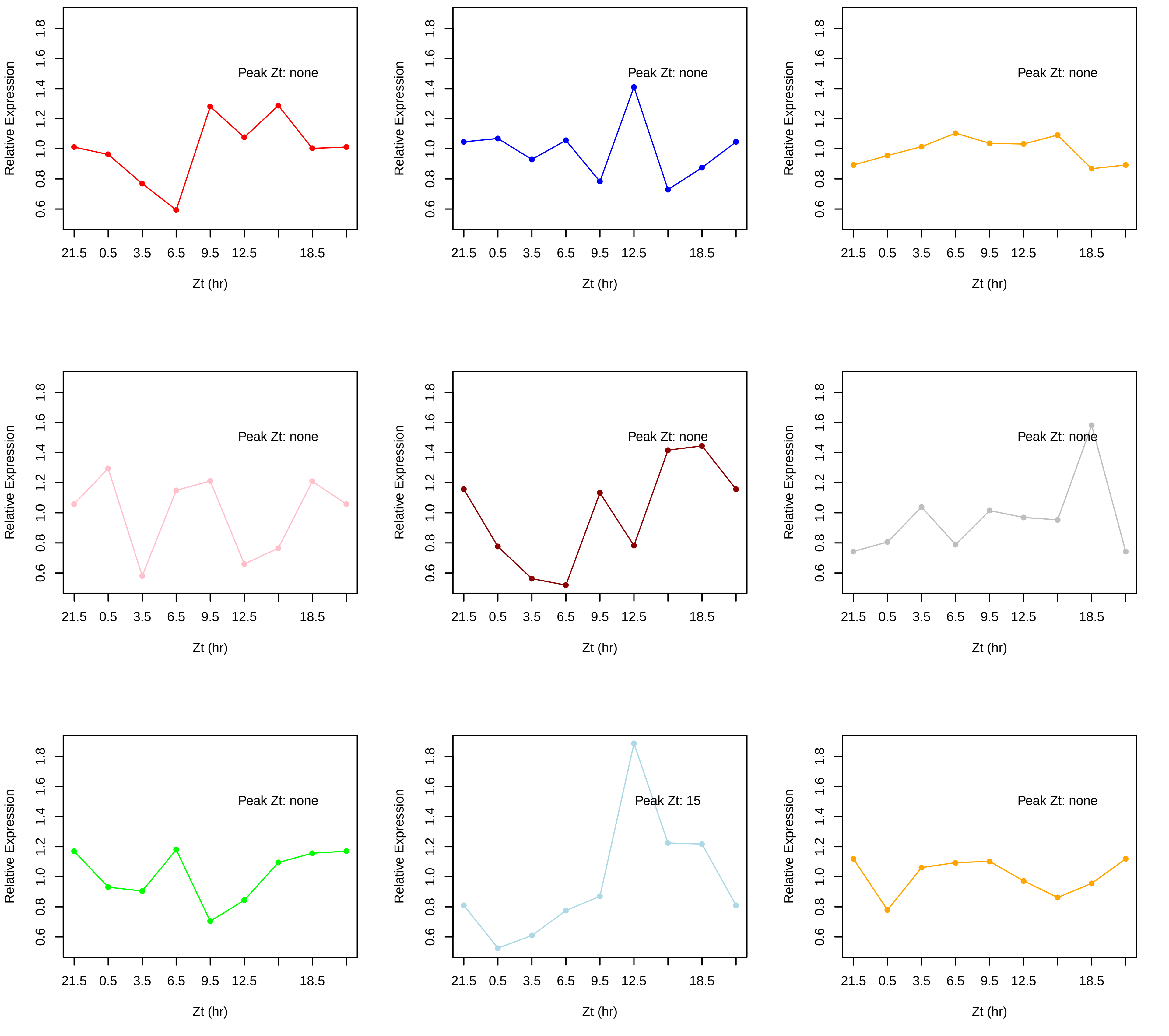

Singeleton: ENSXMAG00000005703 rapgef1b

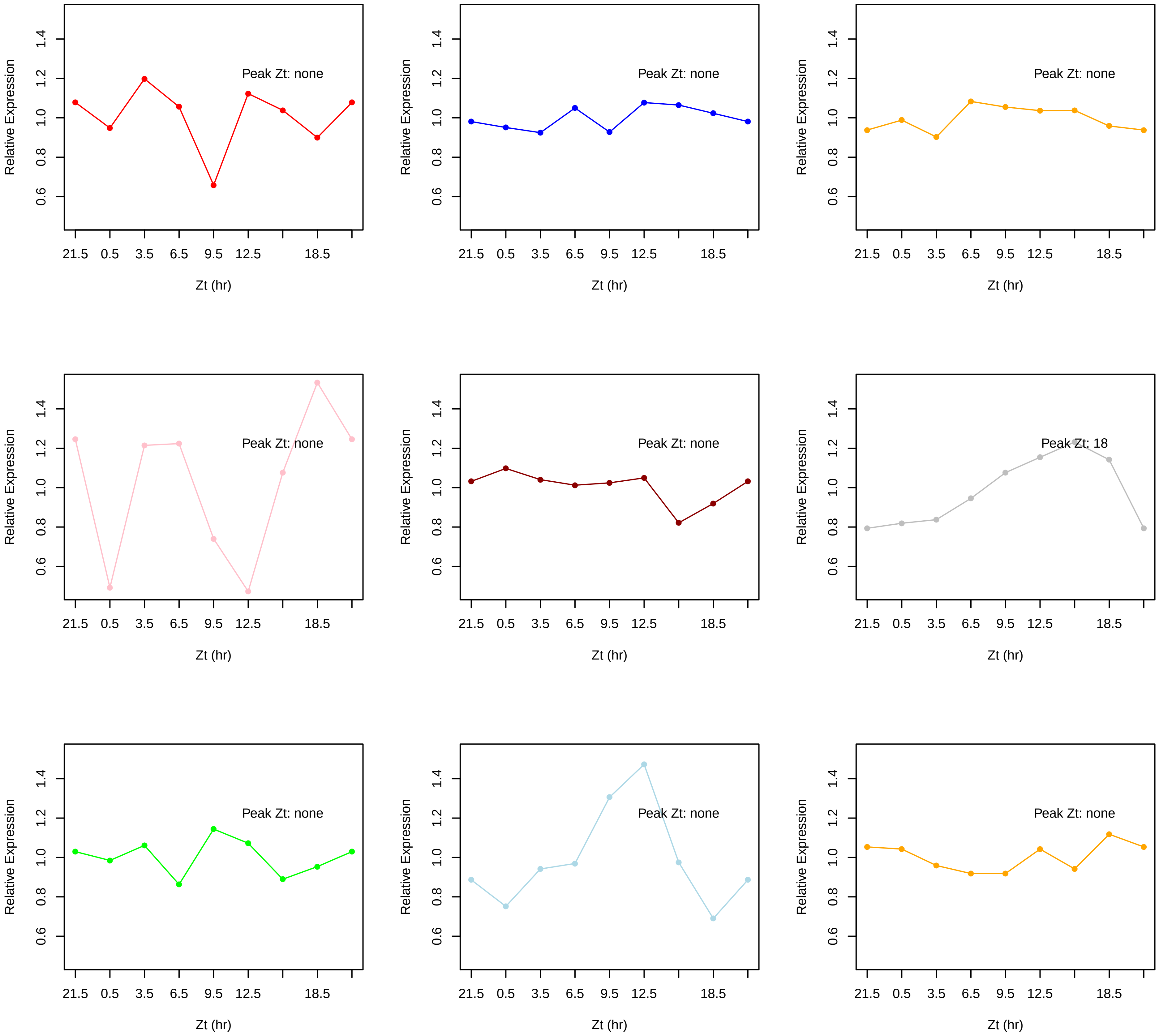

Singeleton: ENSXMAG00000017270 megf6b

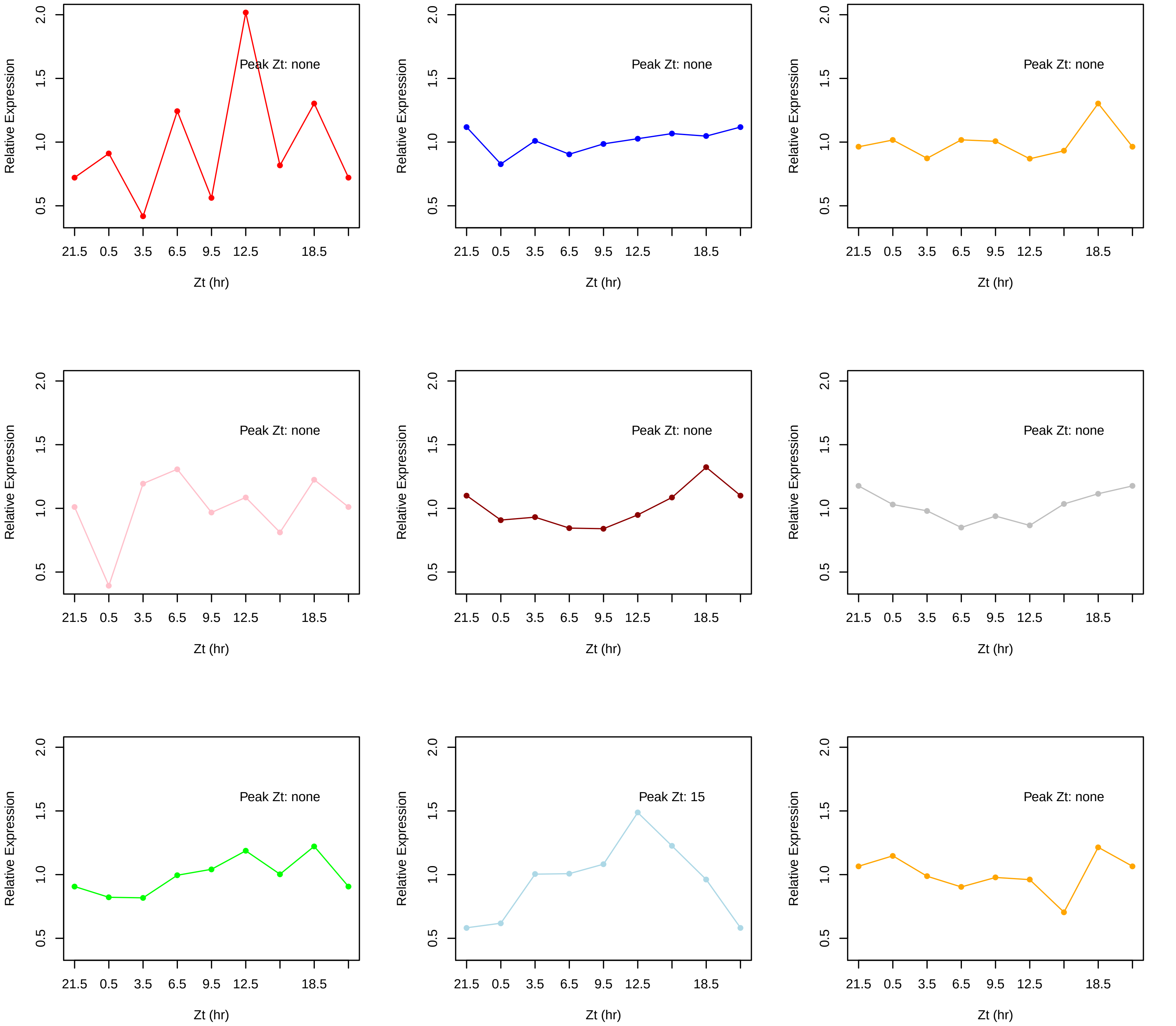

Singeleton: ENSXMAG00000014330 mettl27

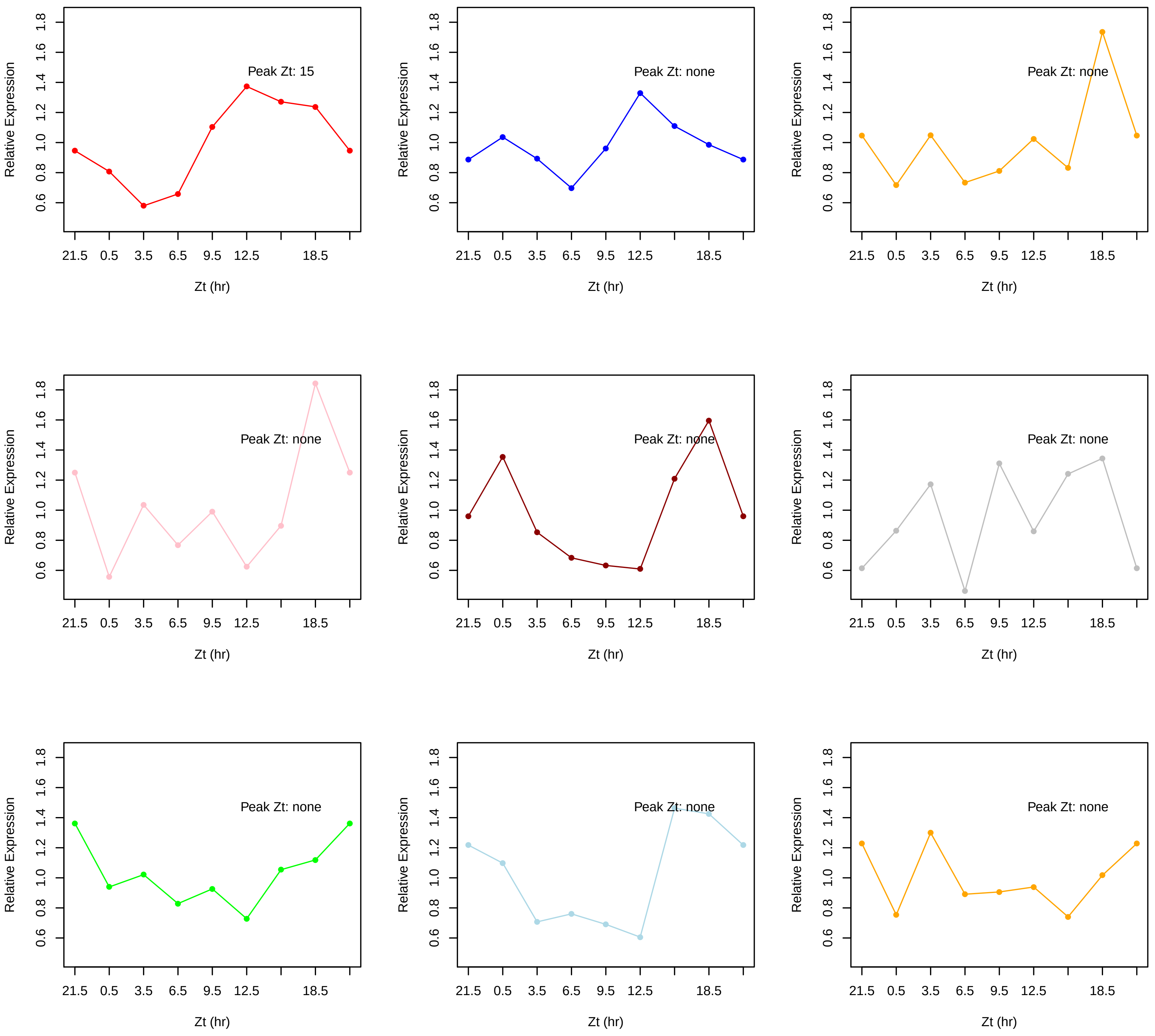

Singeleton: ENSXMAG00000029315 PHLPP2

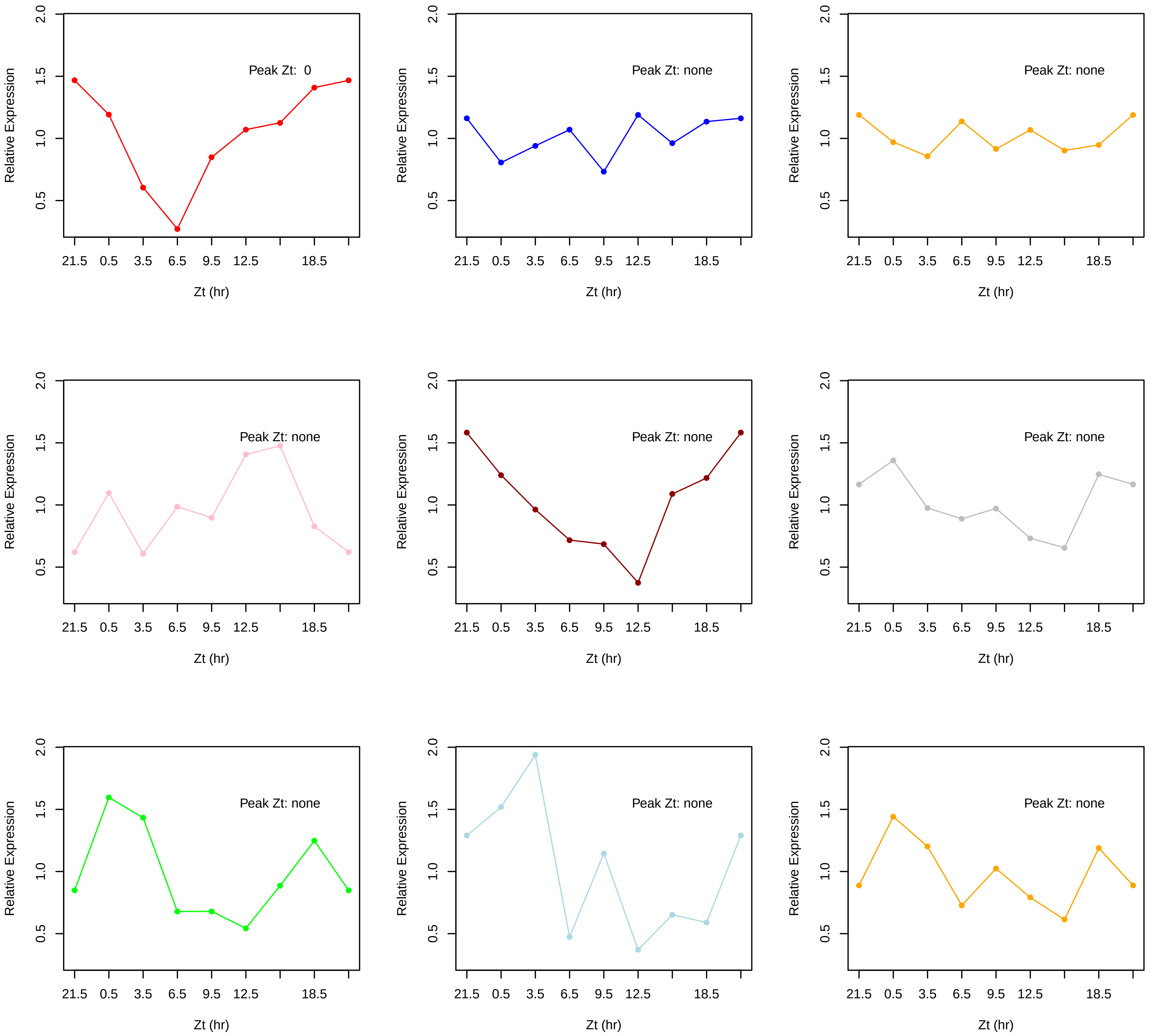

Singeleton: ENSXMAG00000000521 sppl2

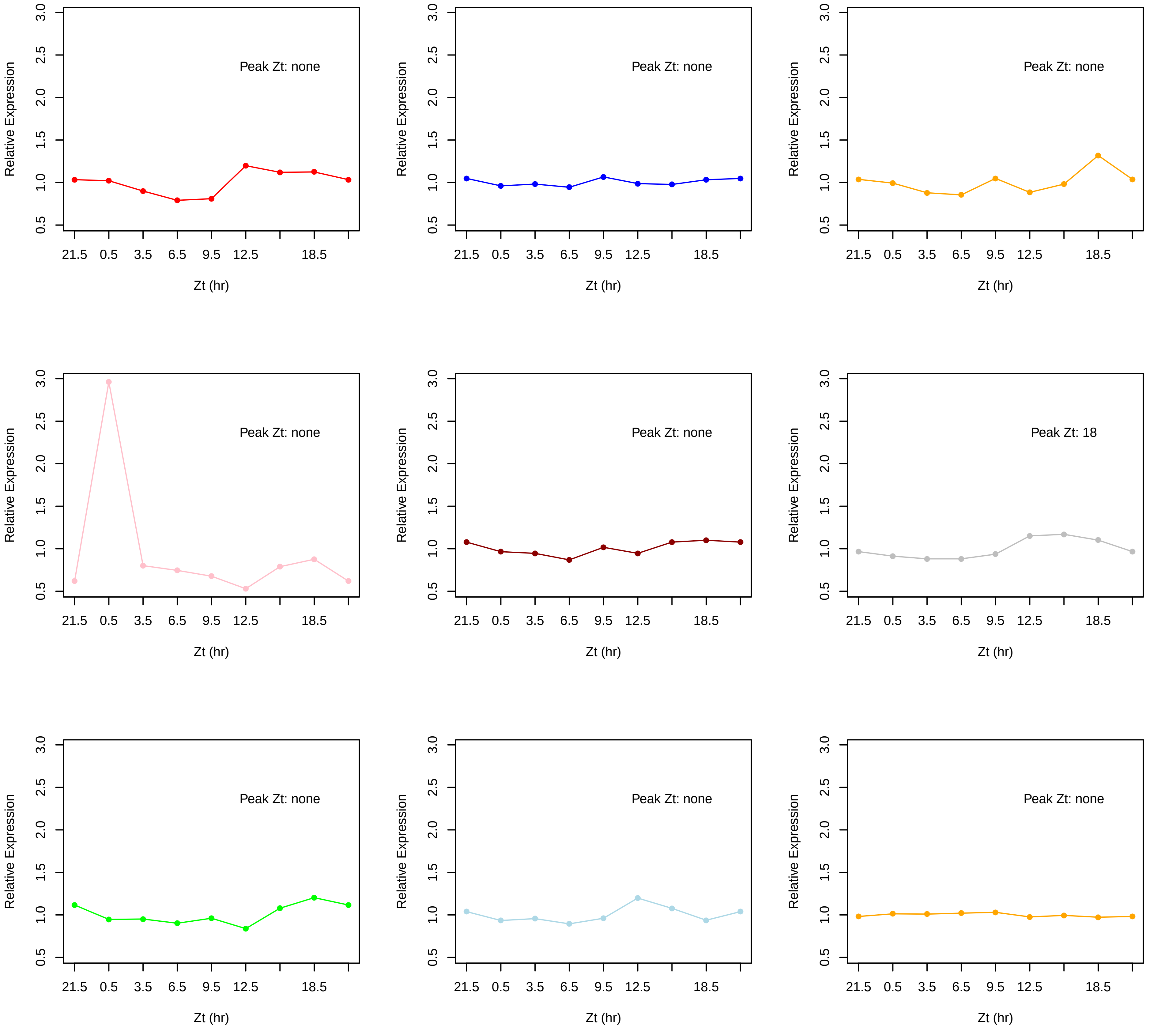

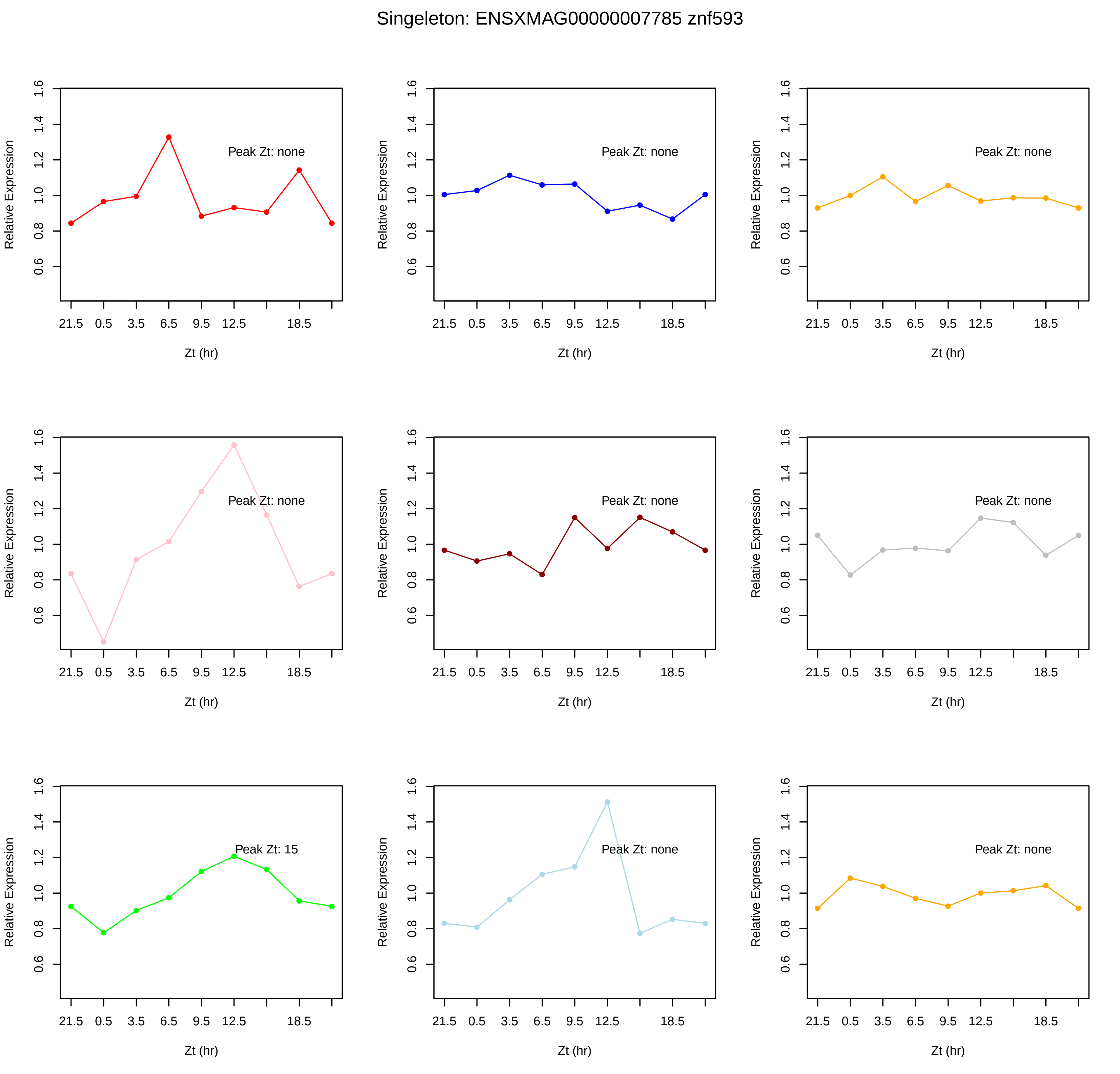
Singeleton: ENSXMAG00000007785 znf593
Peak Zt: none
Peak Zt: none
Peak Zt: none
Peak Zt: none
Peak Zt: none
Peak Zt: none
Peak Zt: 15
Peak Zt: none
Peak Zt: none
Relative Expression
Zt (hr)
21.5
0.5
3.5
6.5
9.5
12.5
18.5
0.6
0.8
1.0
1.2
1.4
1.6

Singleton: ENSXMAG00000006320 sdk1a

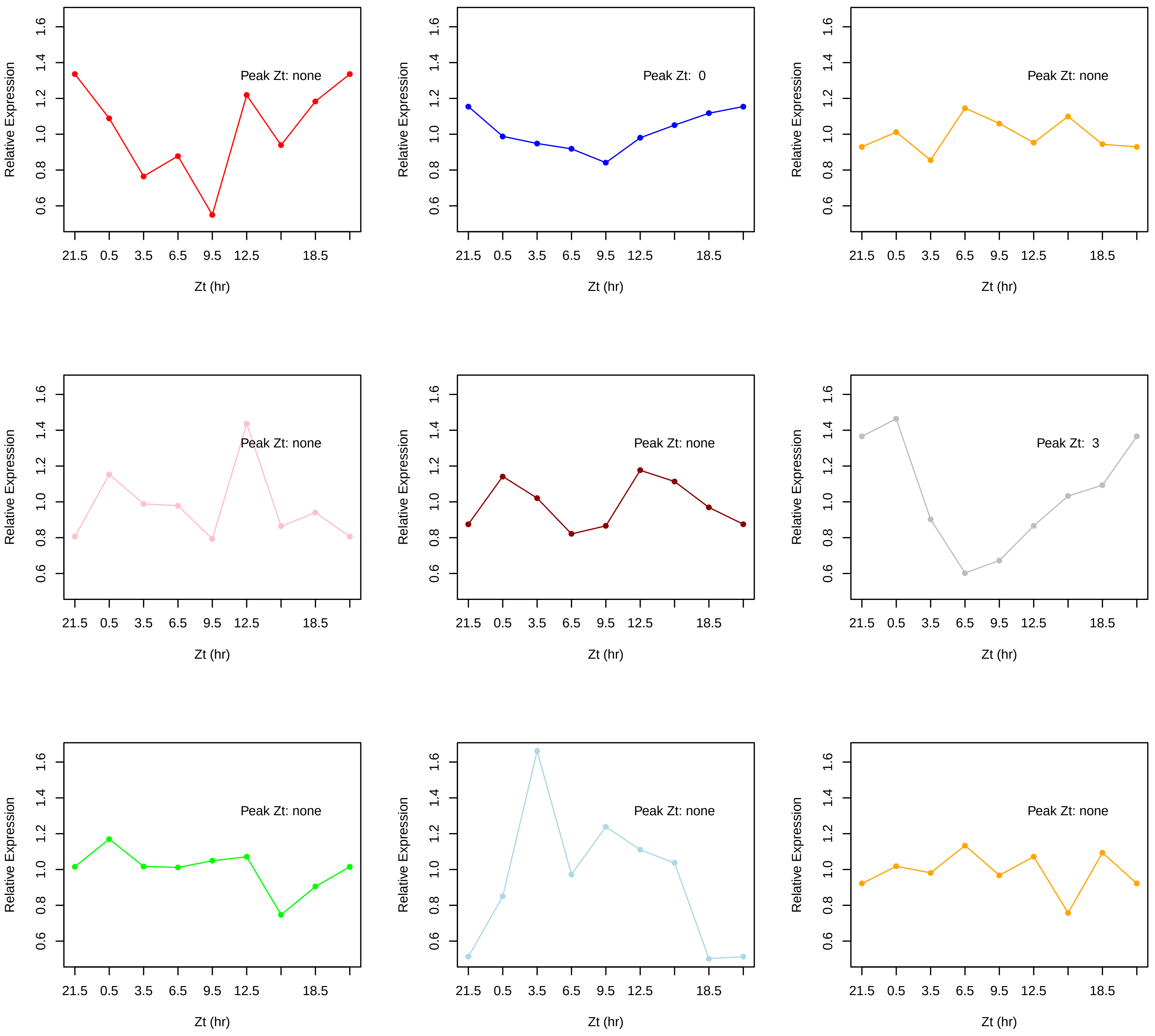

Singeleton: ENSXMAG00000025488 znf511

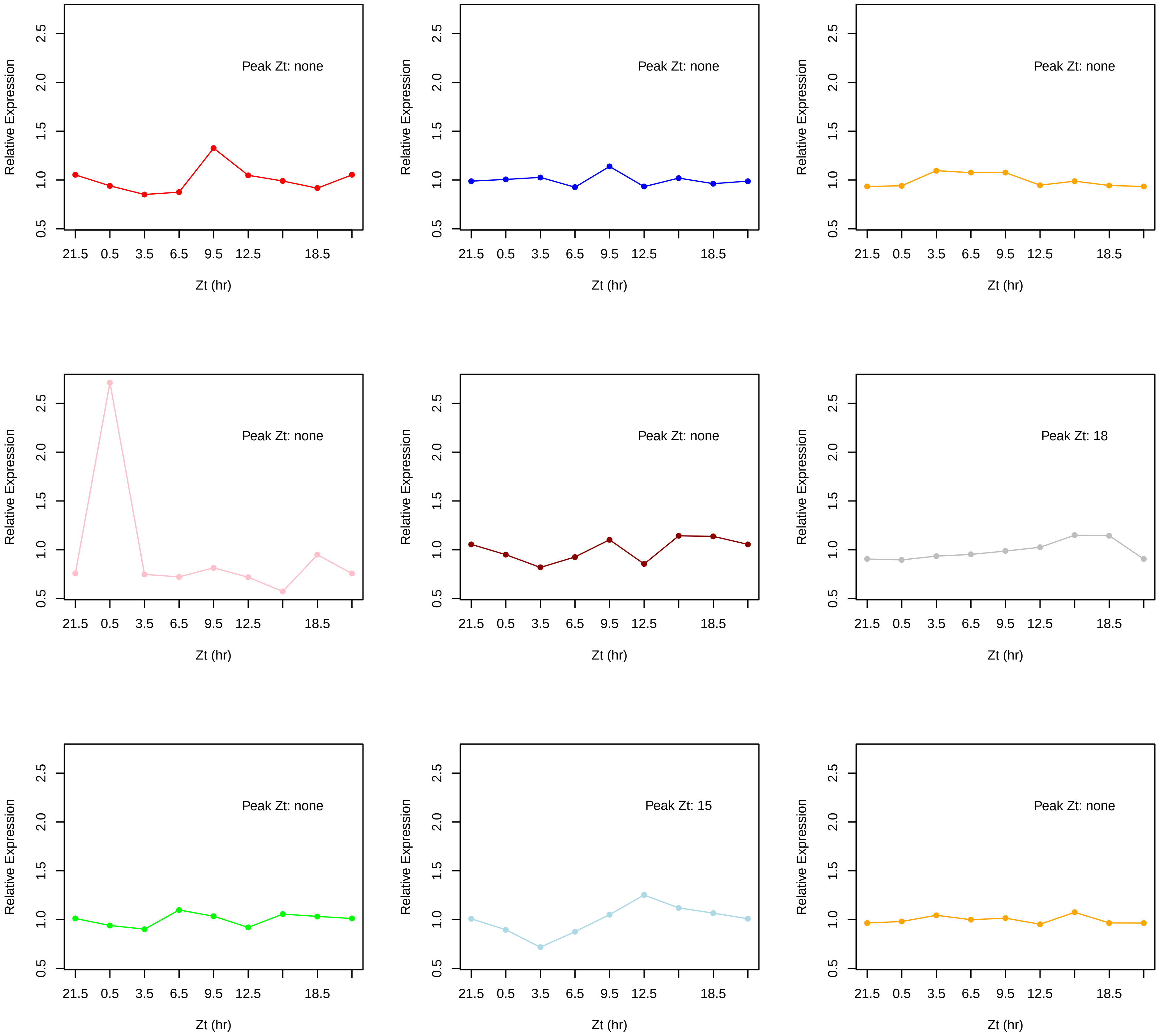

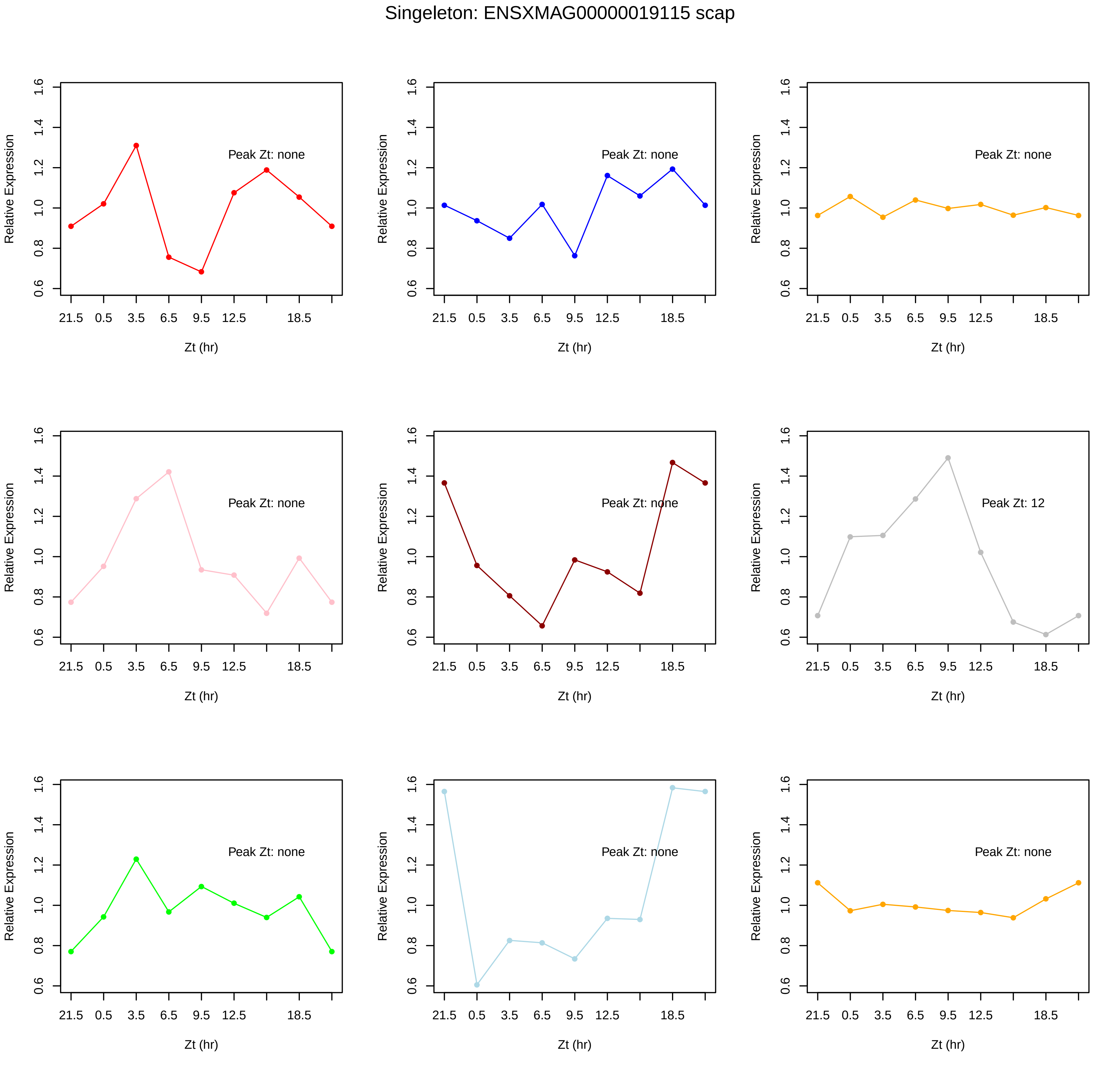

Singeleton: ENSXMAG00000019115 scap
Relative Expression
Zt (hr)
0.6
0.8
1.0
1.2
1.4
1.6
21.5
0.5
3.5
6.5
9.5
12.5
18.5
Peak Zt: none
Peak Zt: none
Peak Zt: none
Peak Zt: none
Peak Zt: none
Peak Zt: 12
Peak Zt: none
Peak Zt: none
Peak Zt: none

Singleton: ENSXMAG00000005983 ap1g1

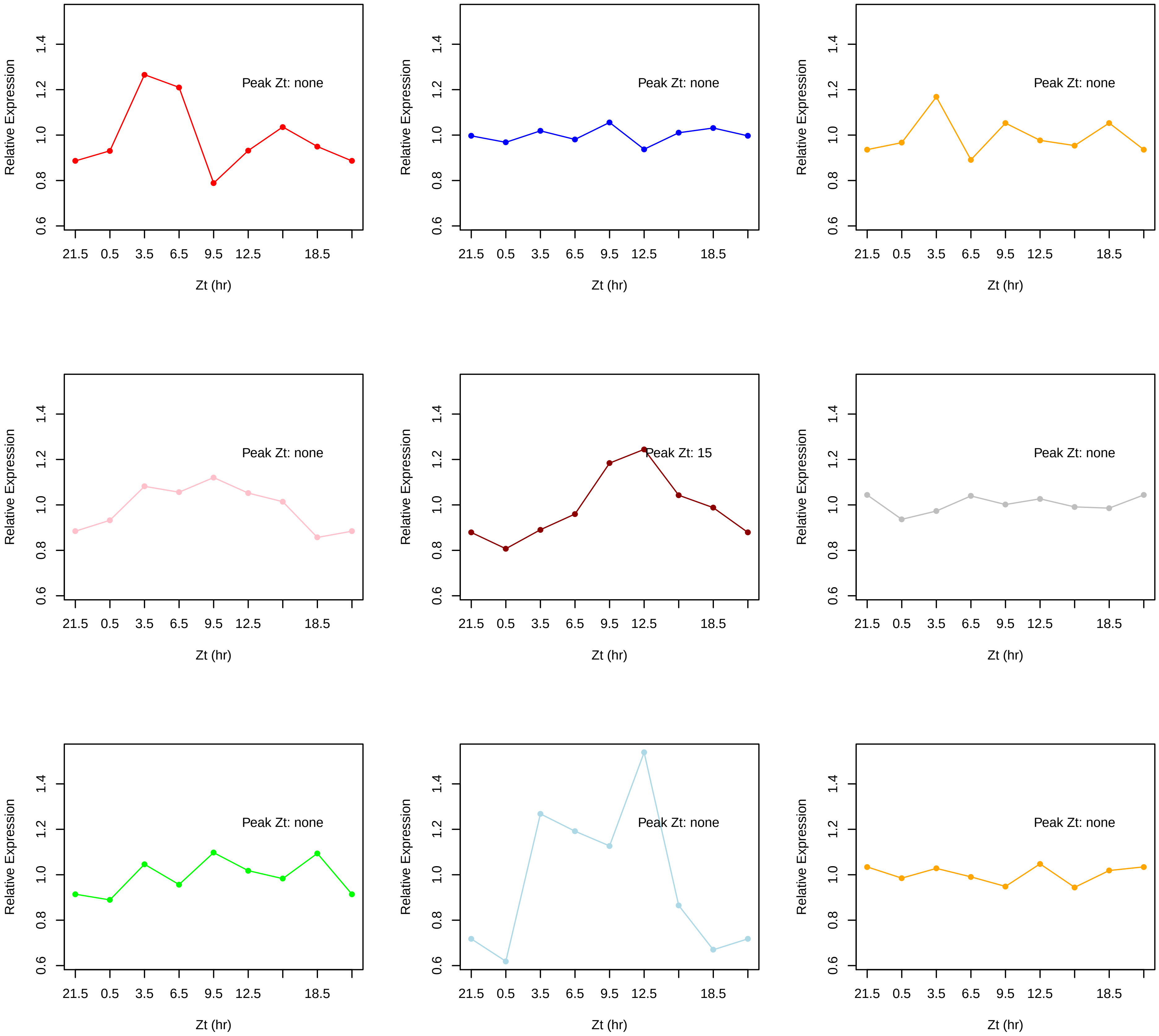

Singeleton: ENSXMAG00000007776 rlf

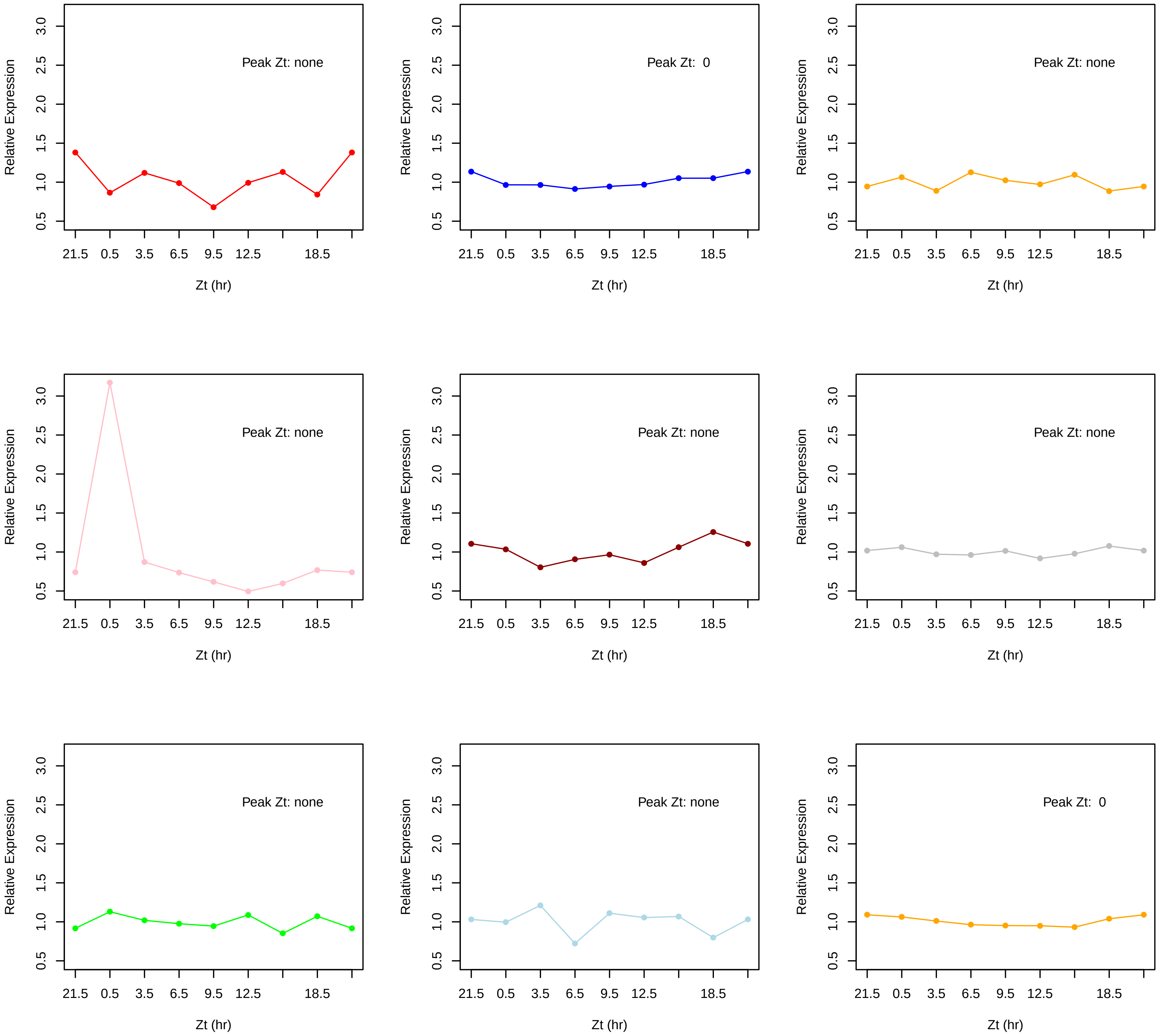

Singeleton: ENSXMAG00000005642 nup188

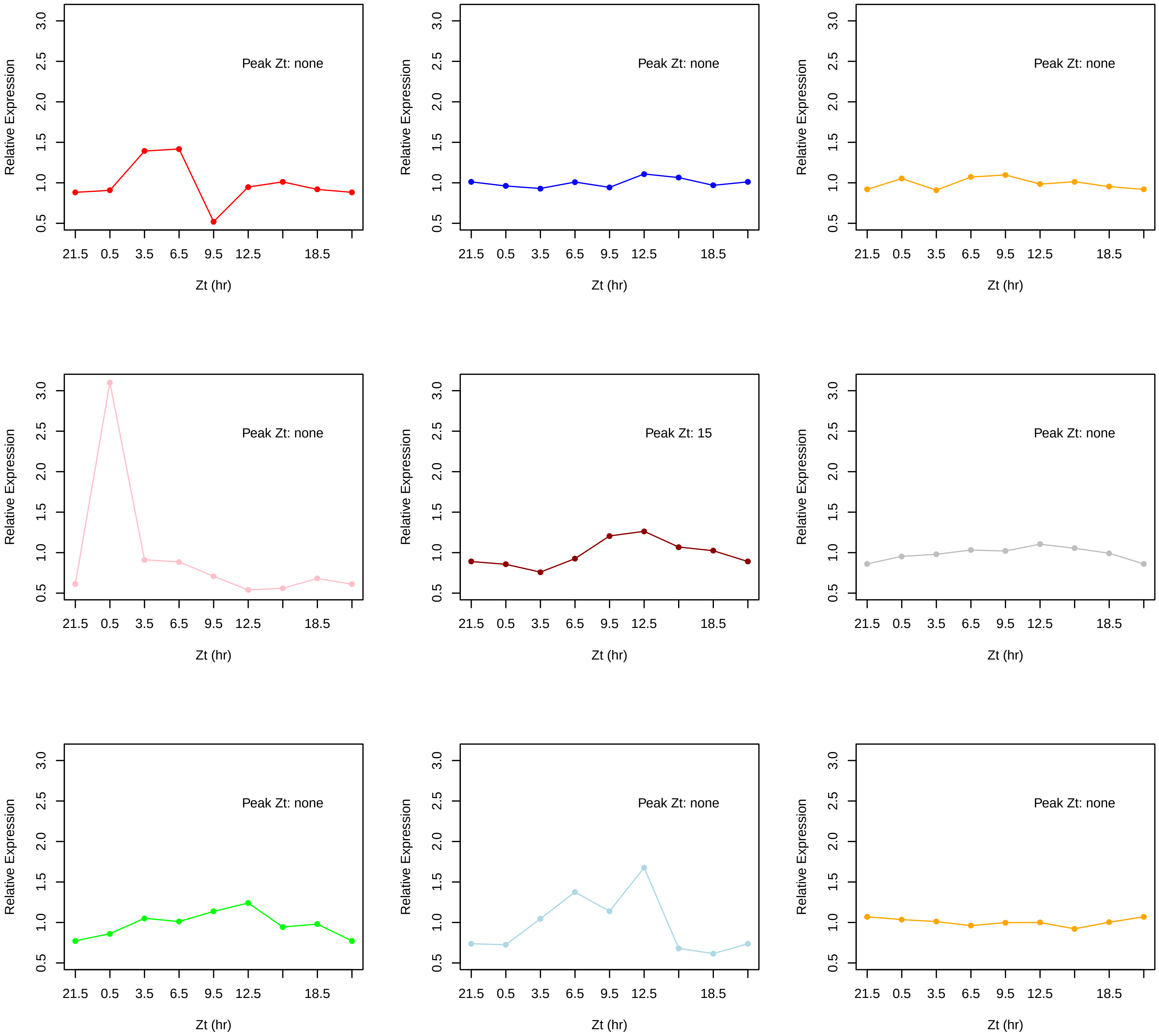

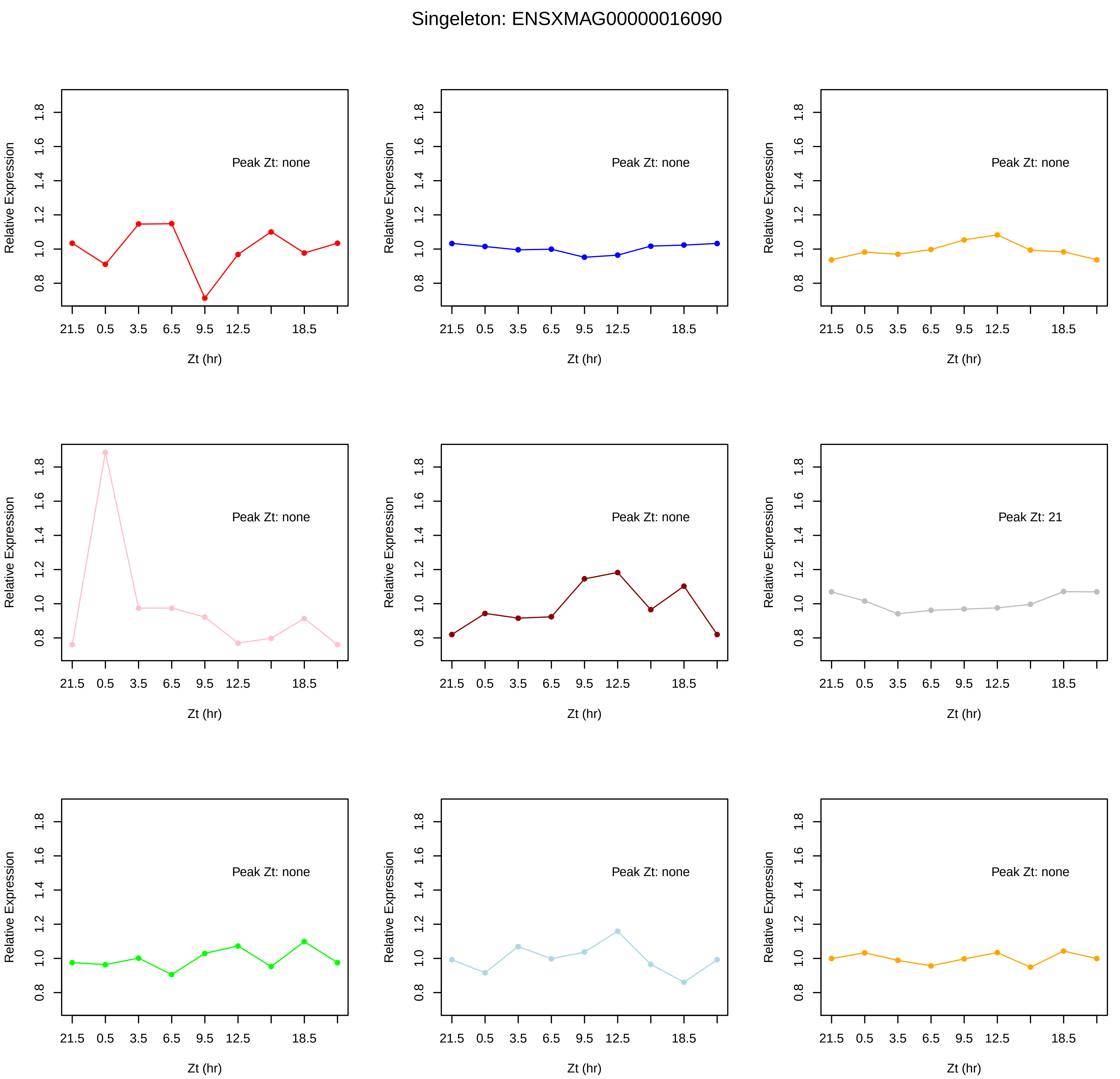
Singeleton: ENSXMAG00000016090
Peak Zt: none
Peak Zt: none
Peak Zt: none
Peak Zt: none
Peak Zt: none
Peak Zt: 21
Peak Zt: none
Peak Zt: none
Peak Zt: none
Relative Expression
Zt (hr)
21.5 0.5 3.5 6.5 9.5 12.5 18.5
0.8 1.0 1.2 1.4 1.6 1.8

Singeleton: ENSXMAG00000018630 lsm7

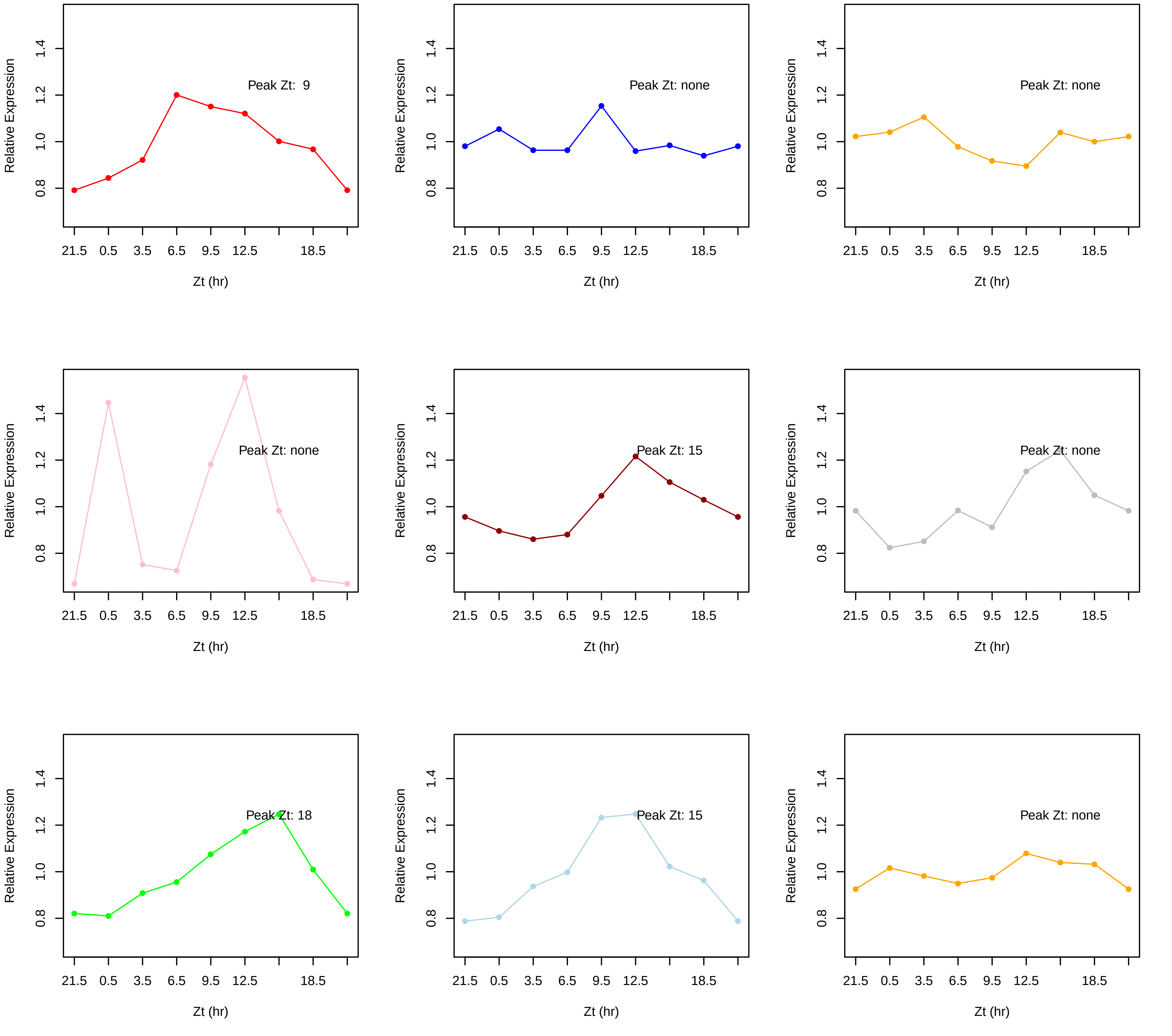

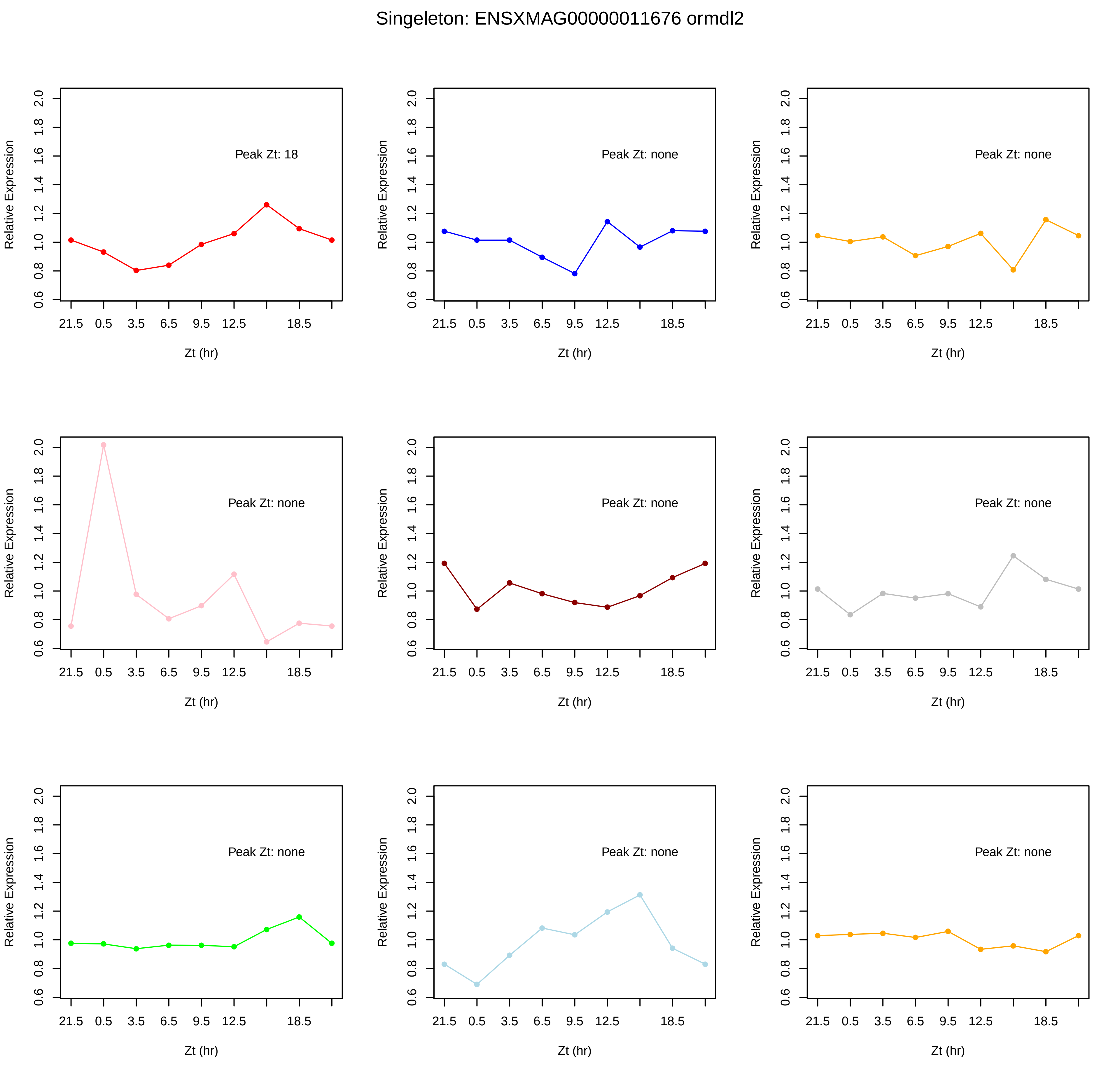
Singeleton: ENSXMAG00000011676 ormdl2
Peak Zt: 18
Peak Zt: none
Relative Expression
Zt (hr)
21.5
0.5
3.5
6.5
9.5
12.5
18.5
0.6
0.8
1.0
1.2
1.4
1.6
1.8
2.0

Singeleton: ENSXMAG00000019110 elp6

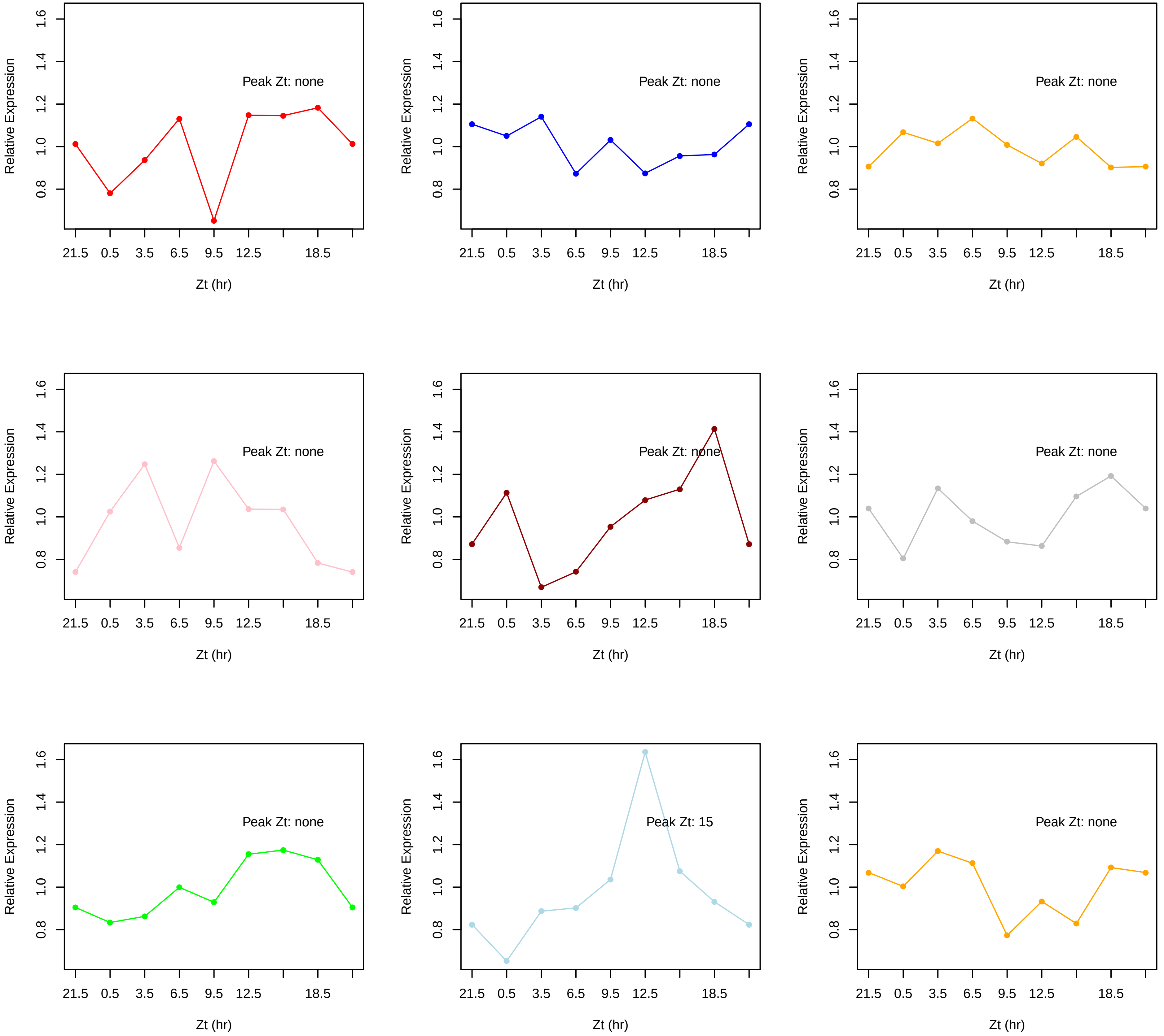

Singeleton: ENSXMAG00000000394 erap1b

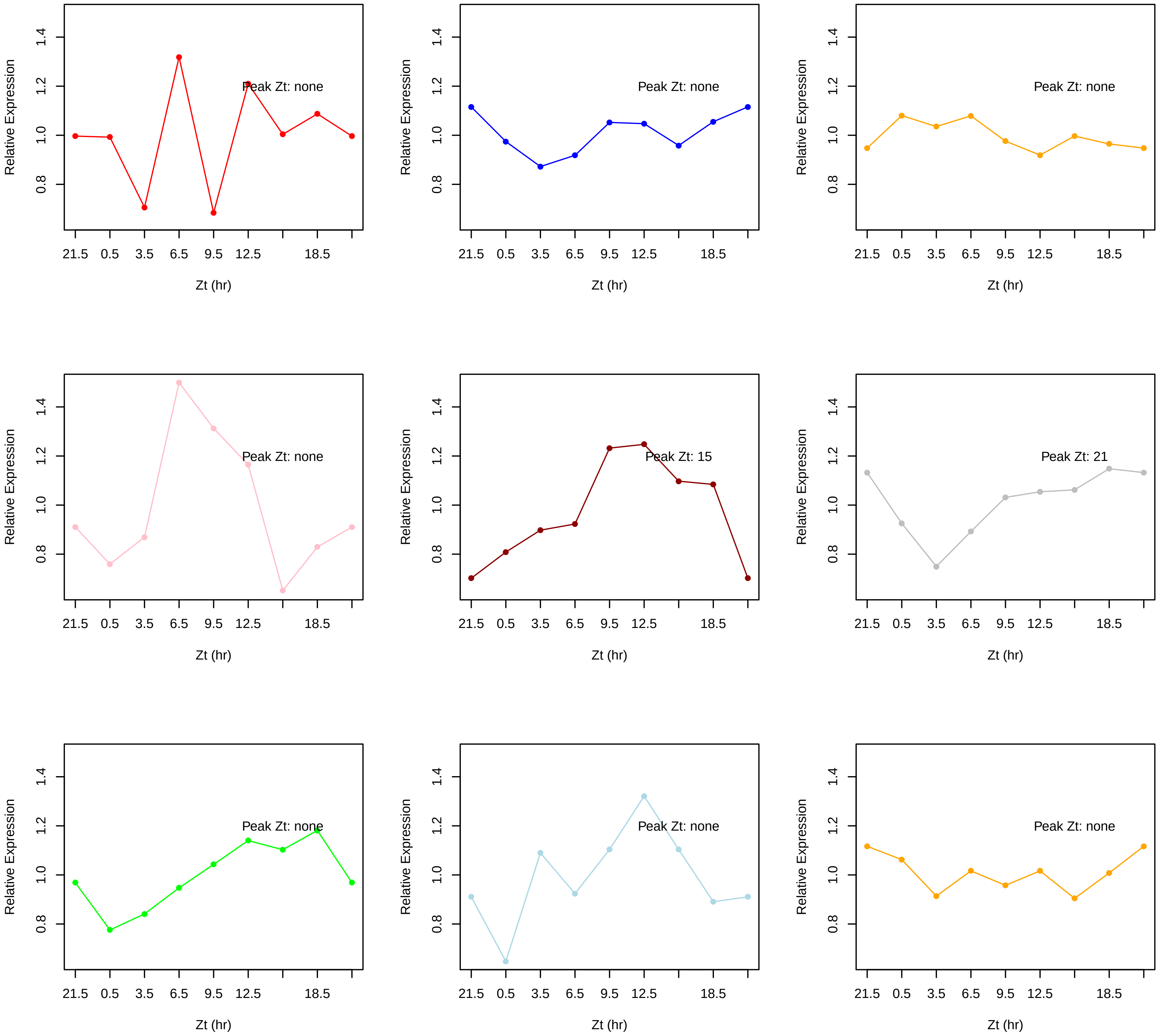

Singleton: ENSXMAG00000007768 zmpste24

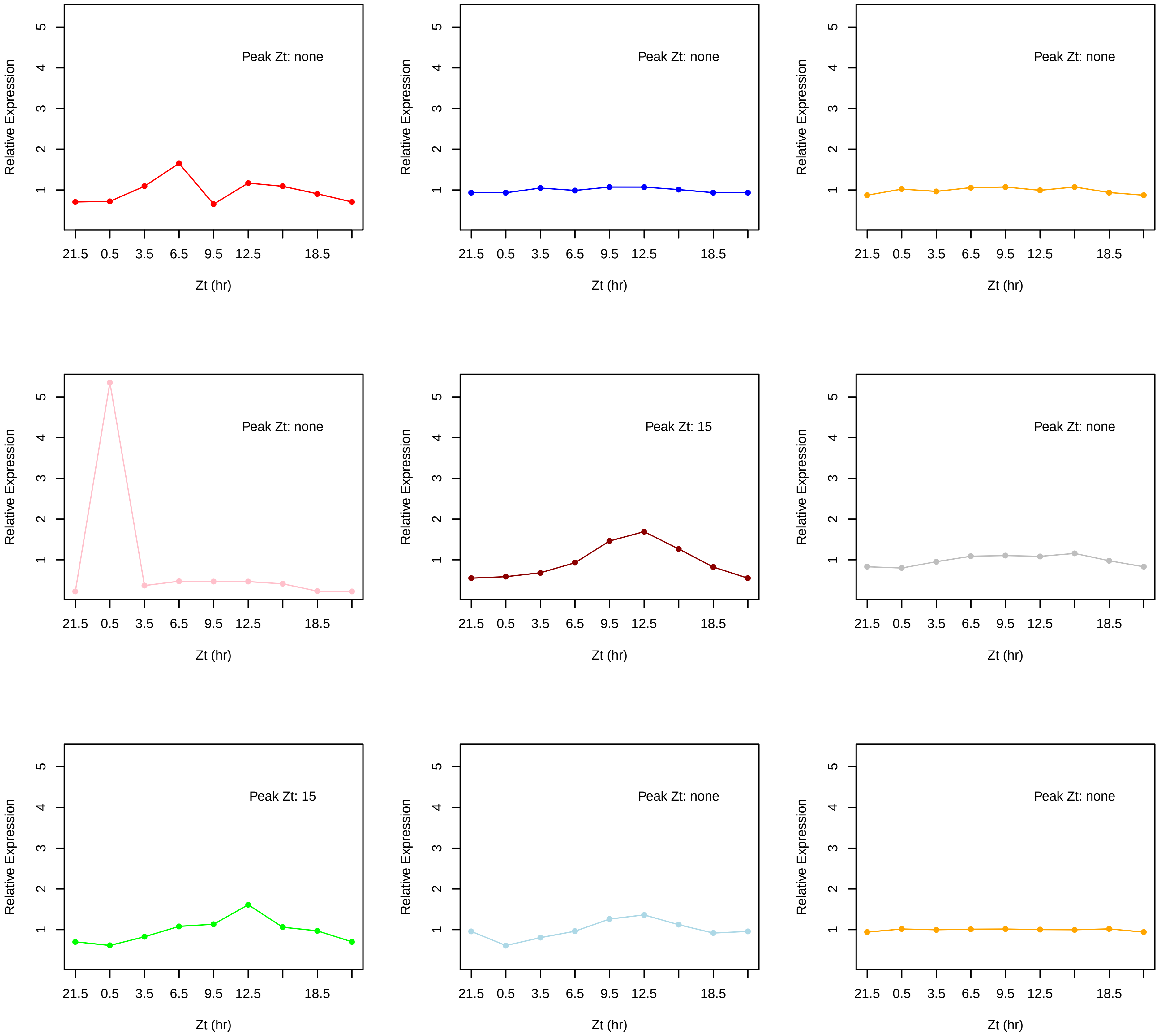

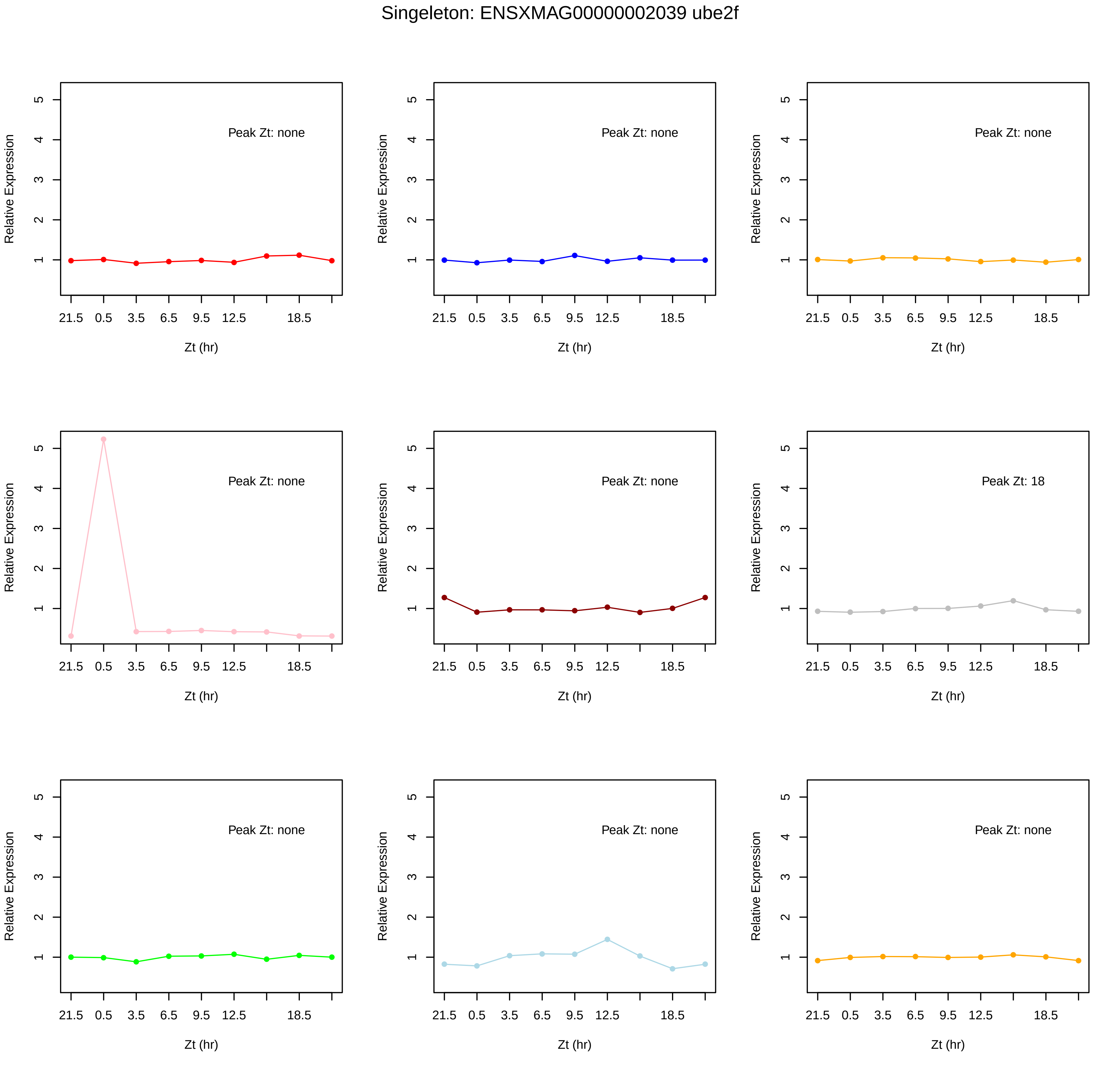
Singleton: ENSXMAG00000002039 ube2f
Peak Zt: none
Peak Zt: none
Peak Zt: none
Peak Zt: none
Peak Zt: none
Peak Zt: 18
Peak Zt: none
Peak Zt: none
Peak Zt: none
Relative Expression
Zt (hr)
21.5
0.5
3.5
6.5
9.5
12.5
18.5
1
2
3
4
5

Singleton: ENSXMAG00000018937 copb2

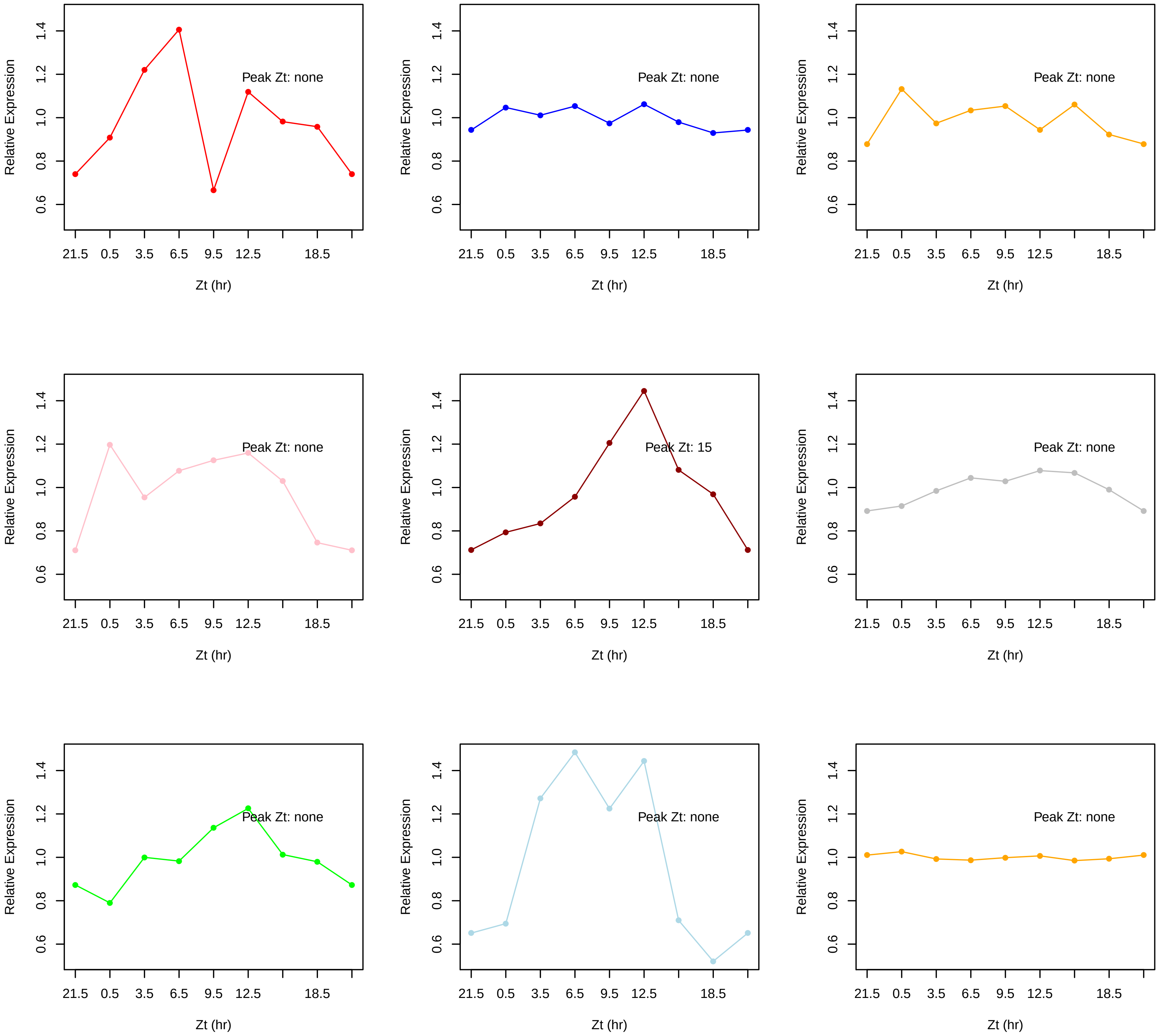

Singleton: ENSXMAG00000026279

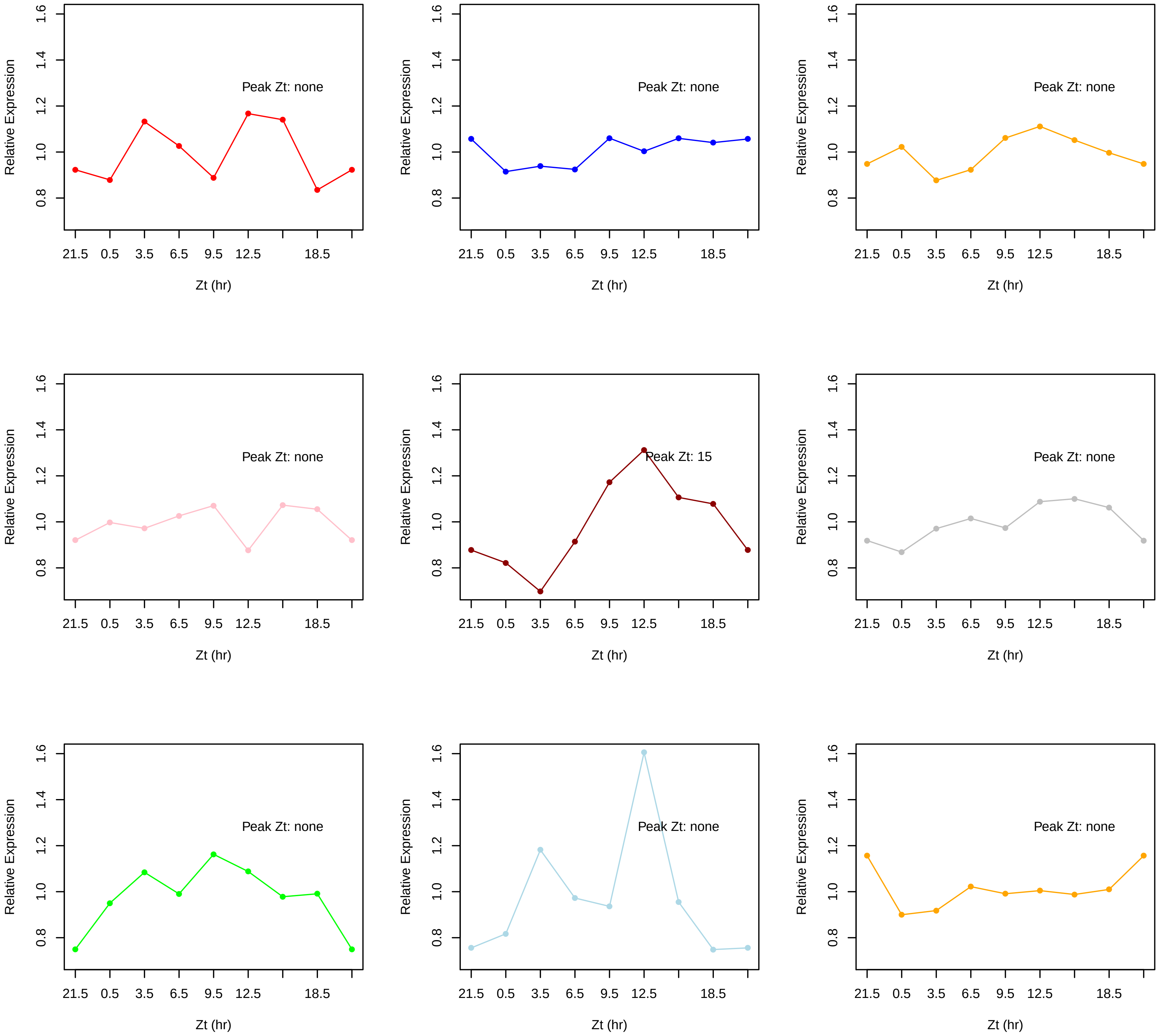

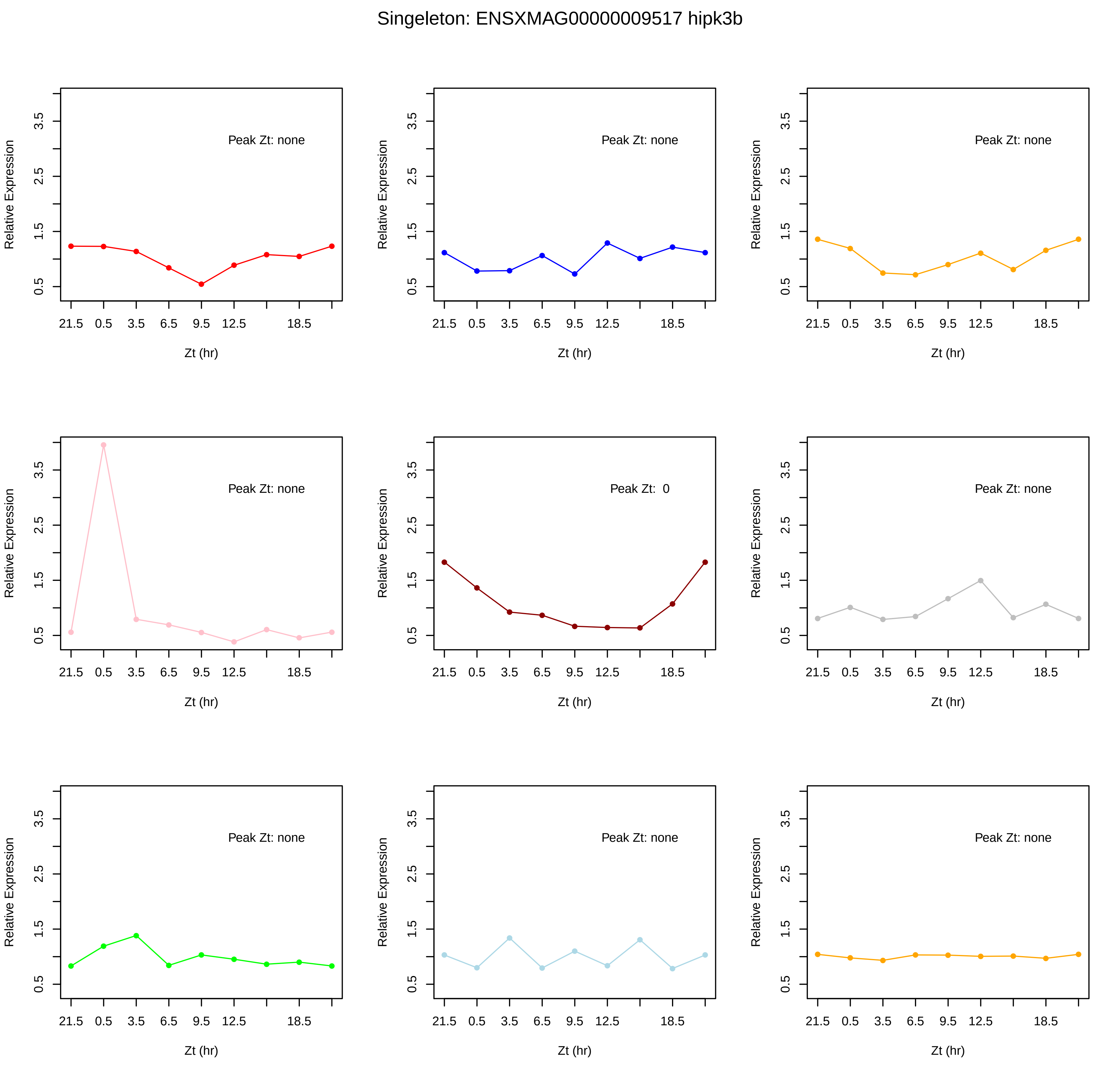
Singeleton: ENSXMAG00000009517 hipk3b
Peak Zt: none
Peak Zt: none
Peak Zt: none
Peak Zt: none
Peak Zt: 0
Peak Zt: none
Peak Zt: none
Peak Zt: none
Peak Zt: none
Relative Expression
Zt (hr)
0.5
1.5
2.5
3.5
21.5
0.5
3.5
6.5
9.5
12.5
18.5

Singeleton: ENSXMAG00000005677 phyhd1

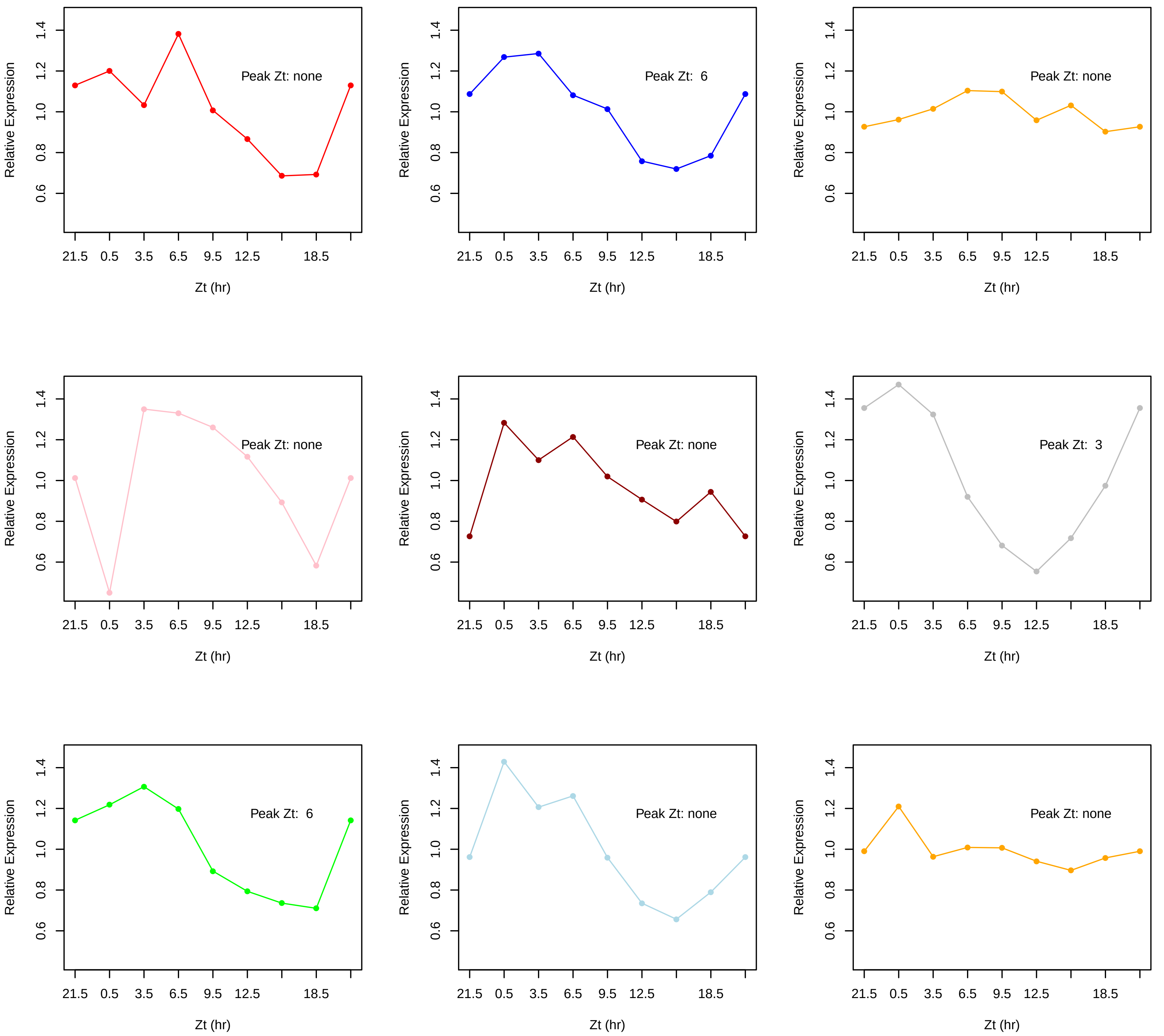

Singeleton: ENSXMAG00000003442 lrrc8ab

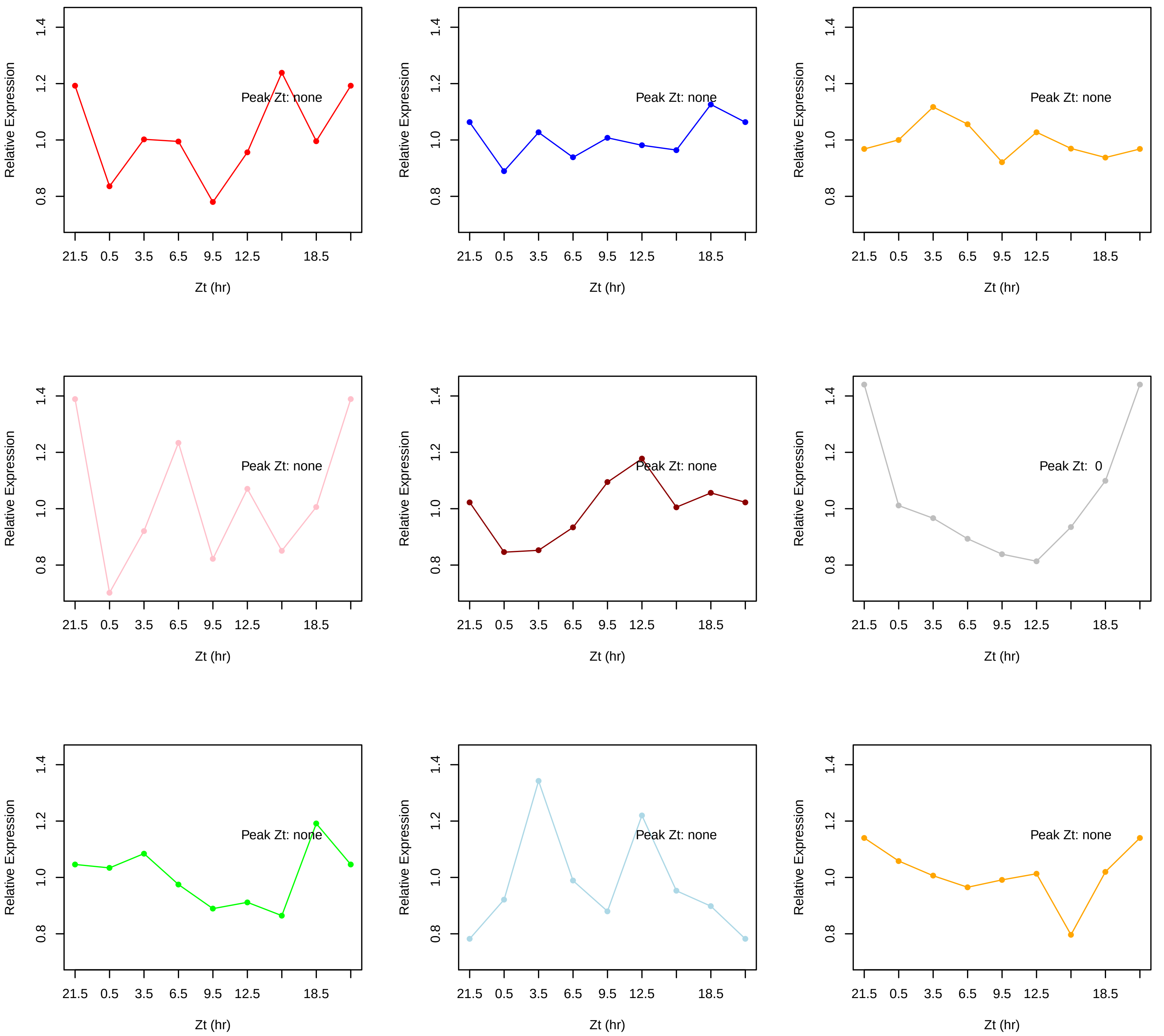

Singeleton: ENSXMAG00000014480 hnrnpr

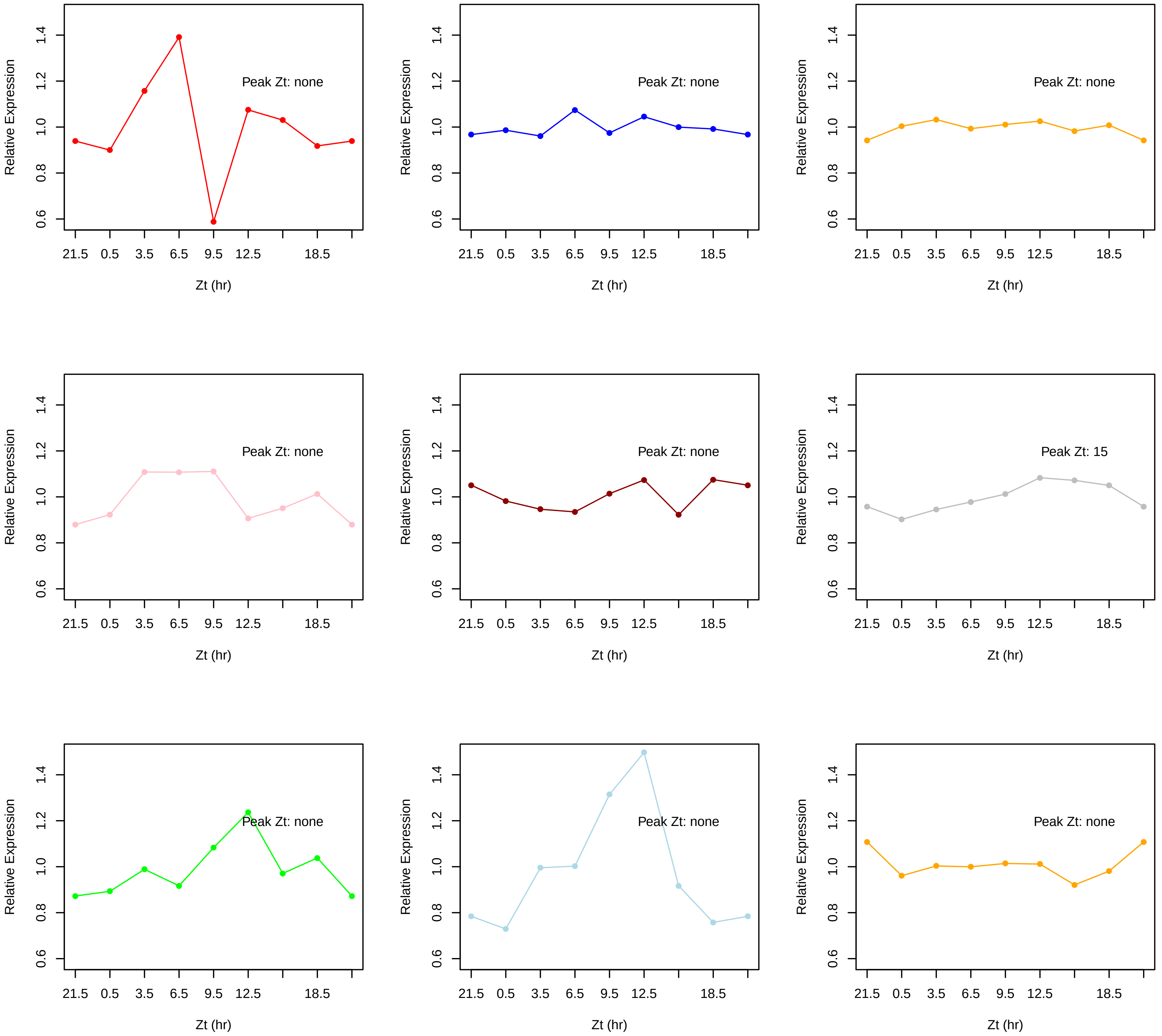

Singleton: ENSXMAG00000006328 med8

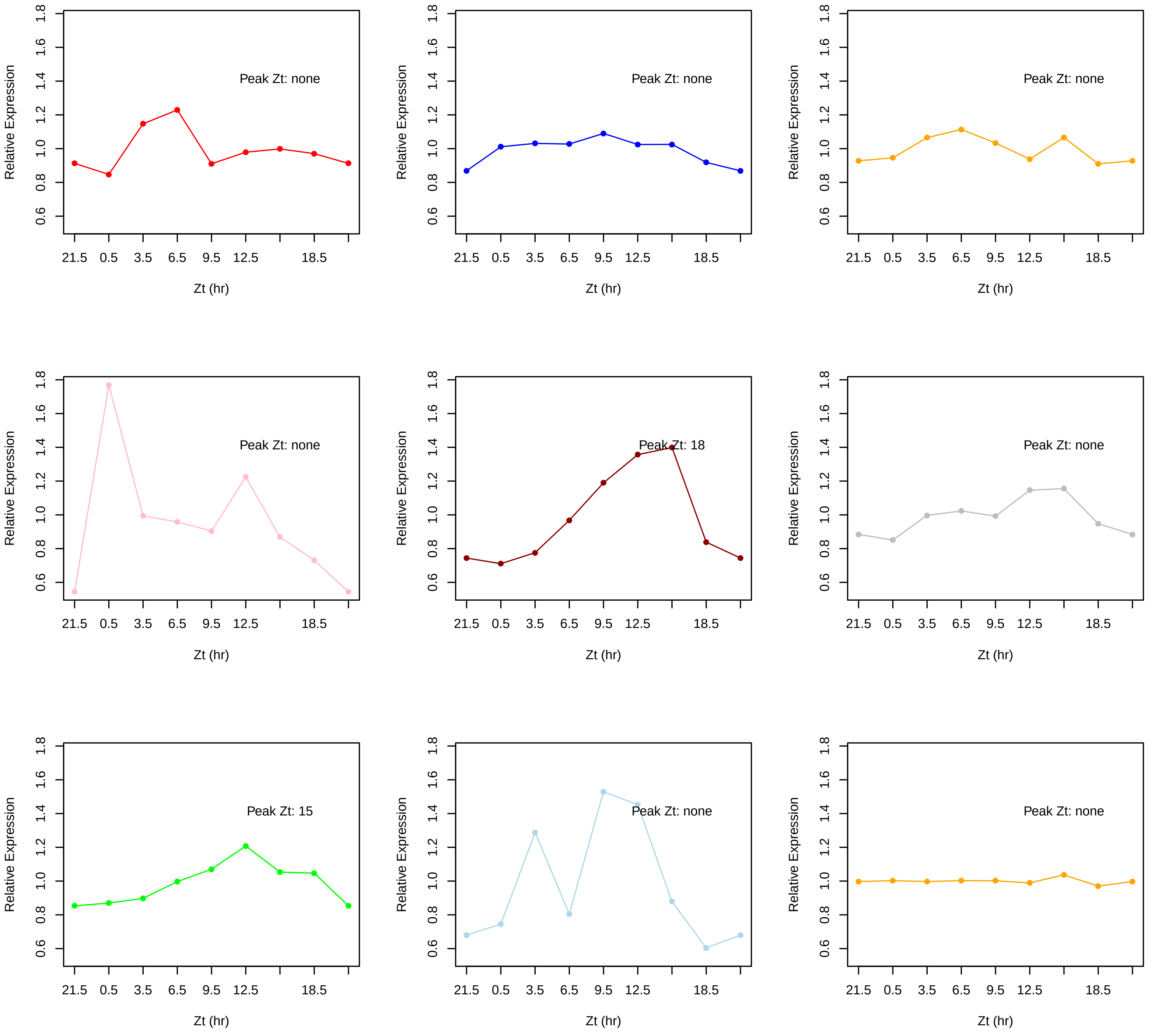

Singeleton: ENSXMAG00000015956 pofut2

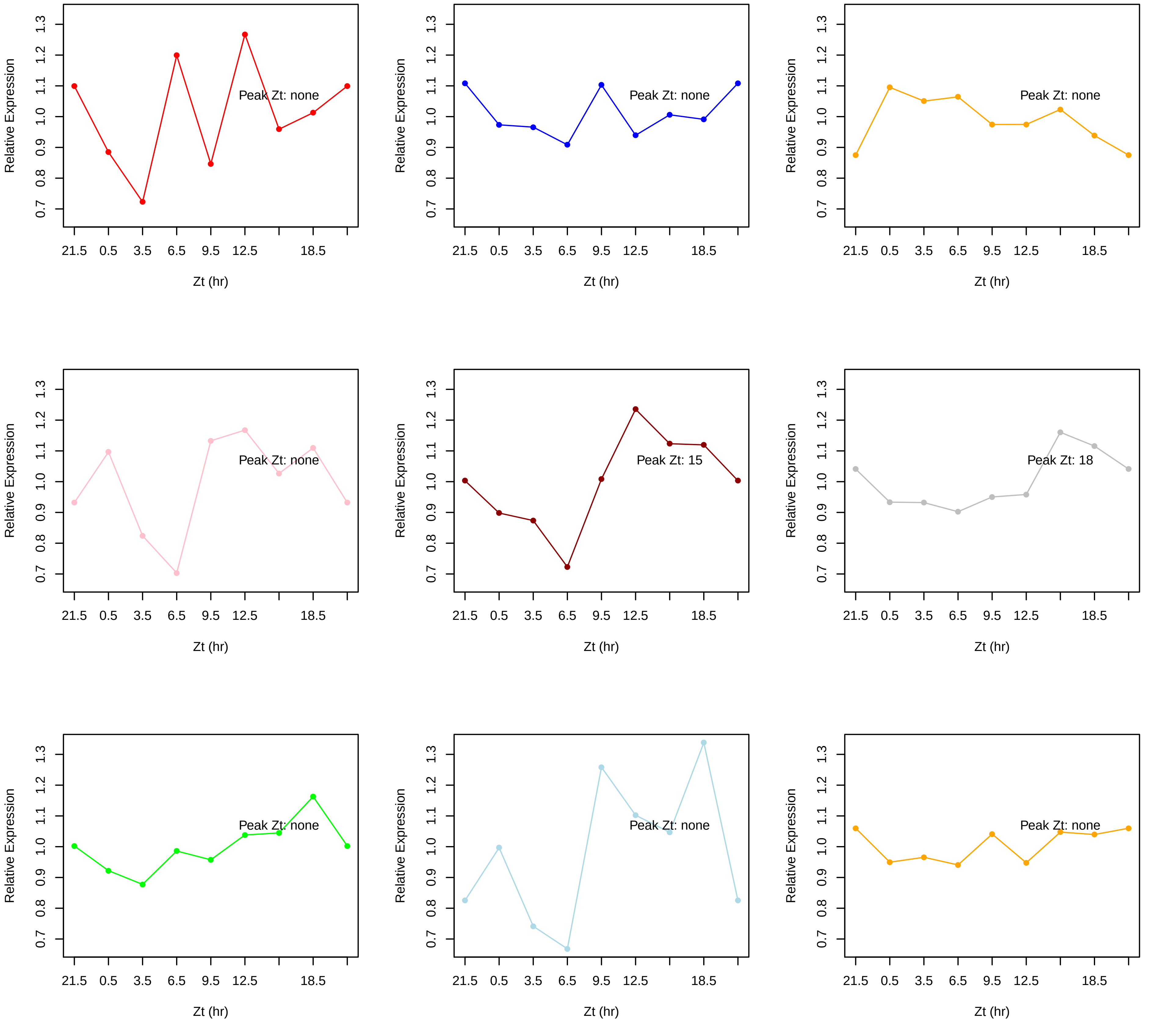

Singeleton: ENSXMAG00000004993 pigo

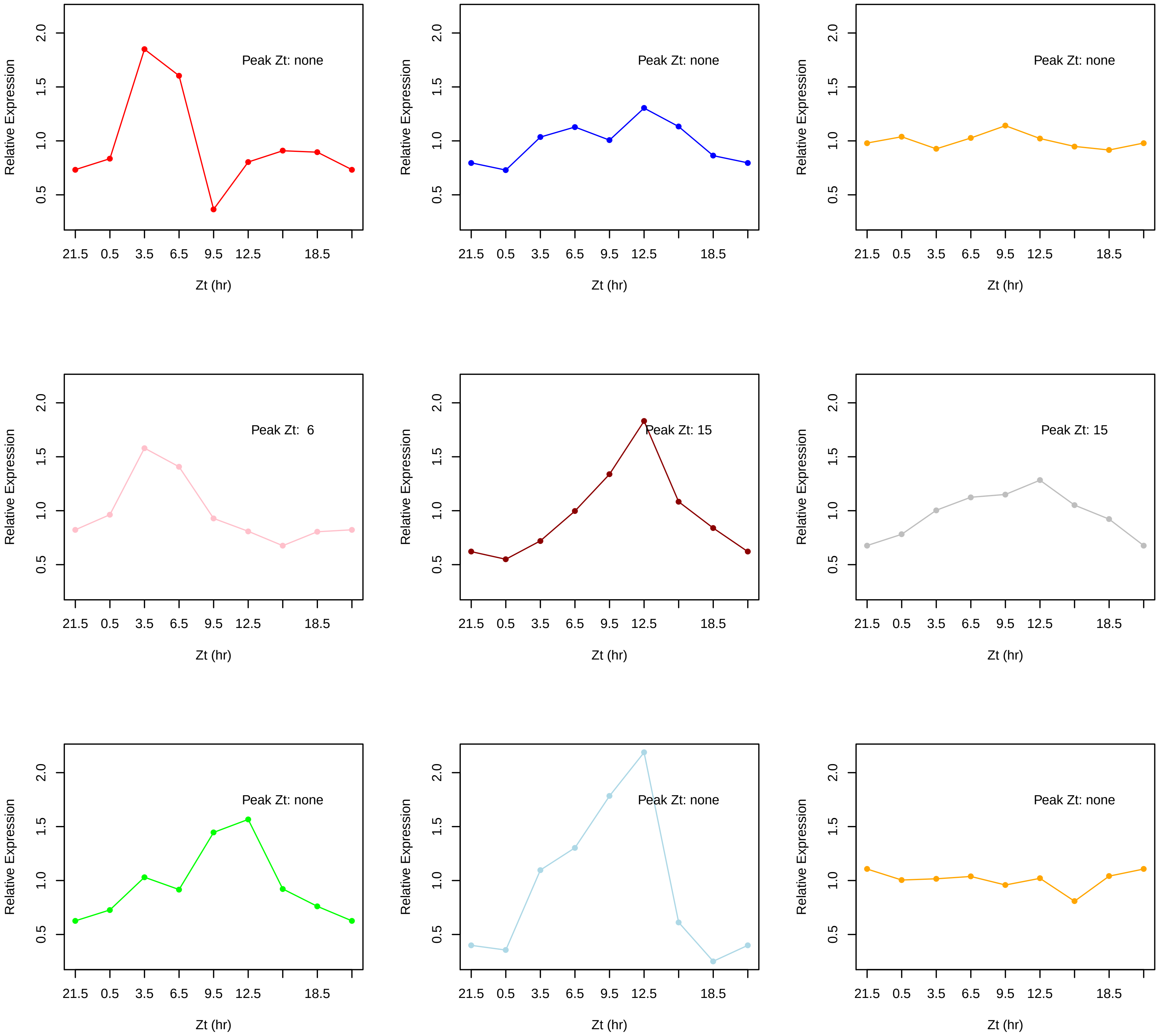

Singleton: ENSXMAG00000016513 myo1b

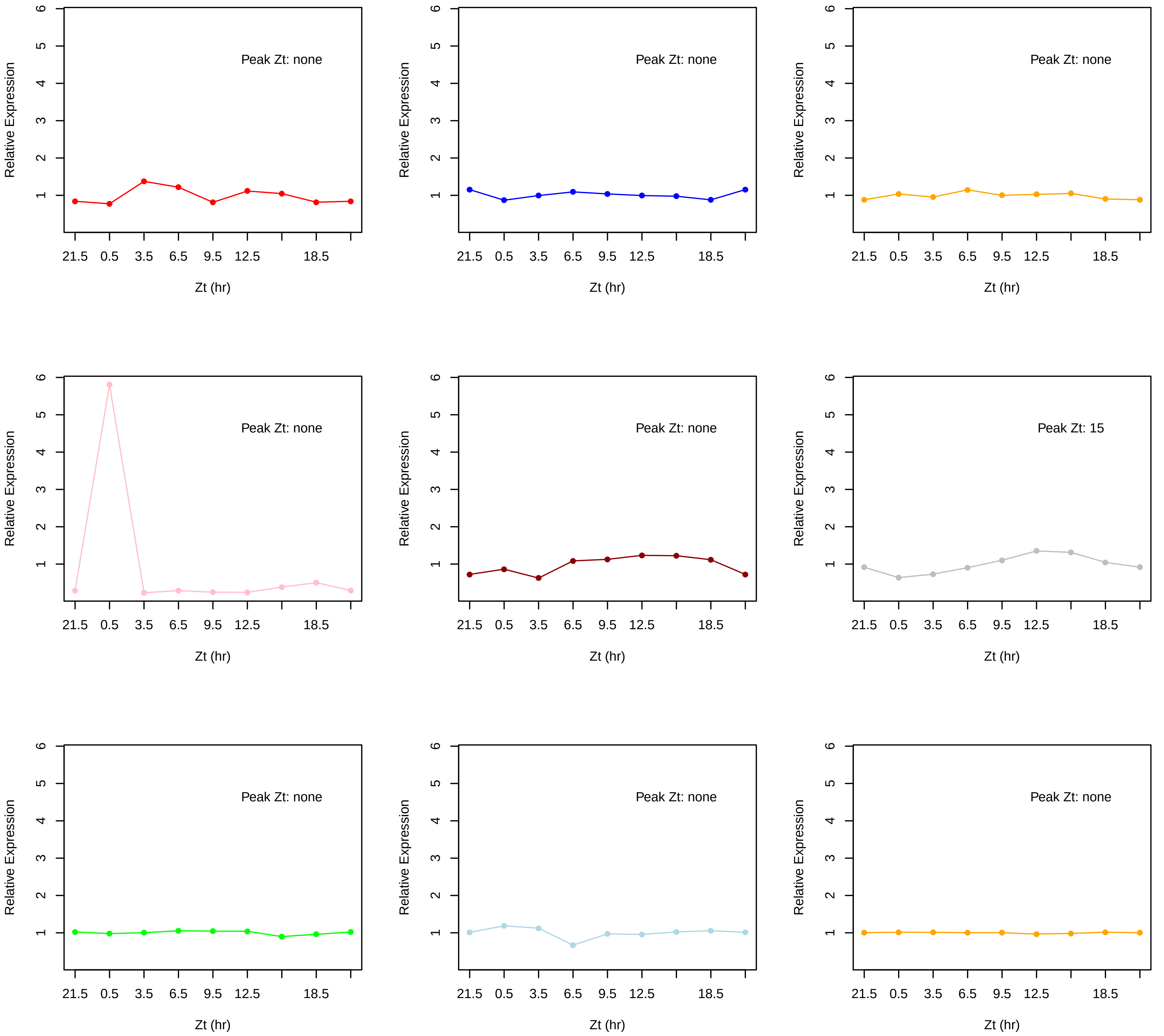

Singeleton: ENSXMAG00000008615 mettl16

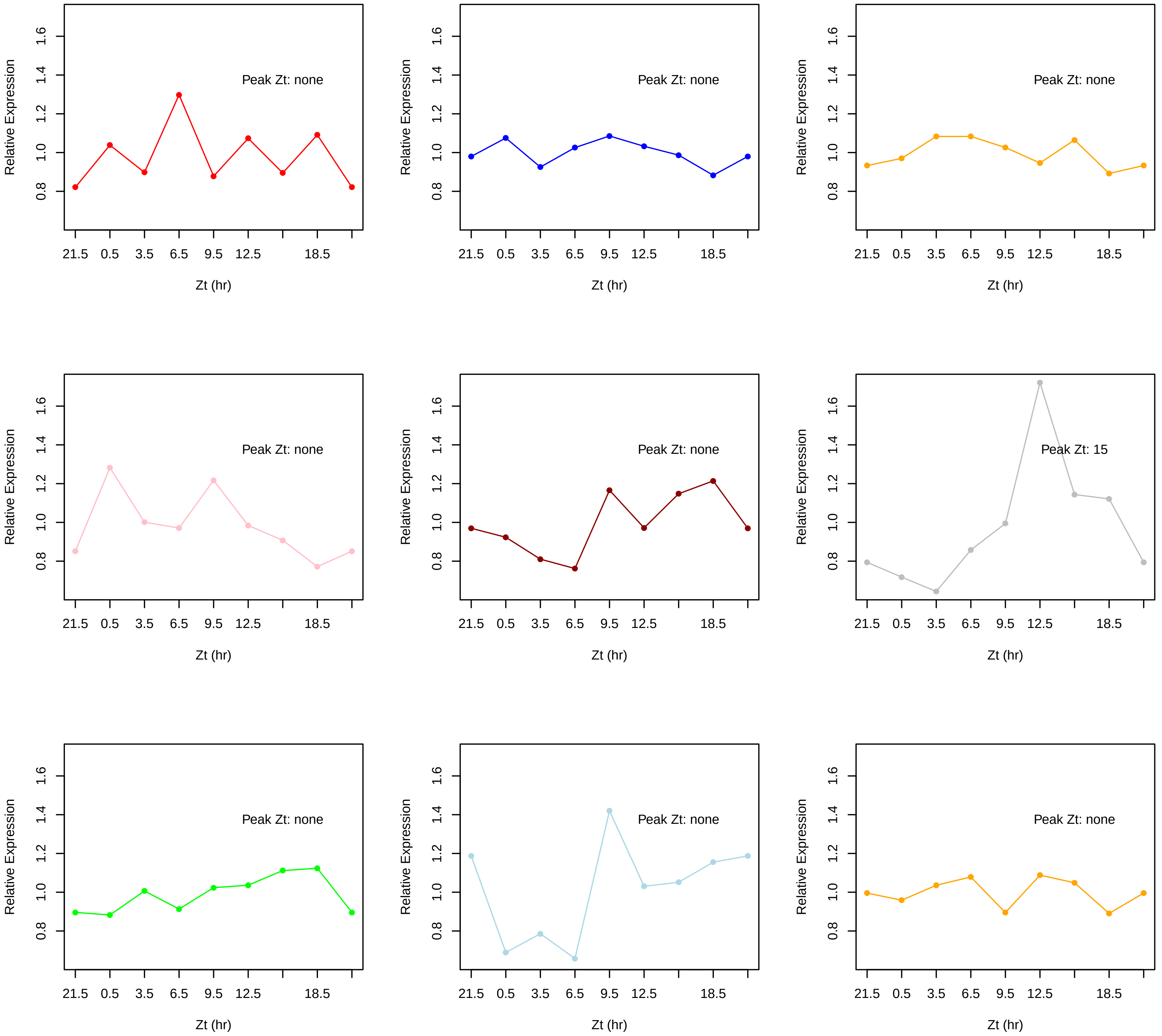

Singeleton: ENSXMAG00000004975 stoml2

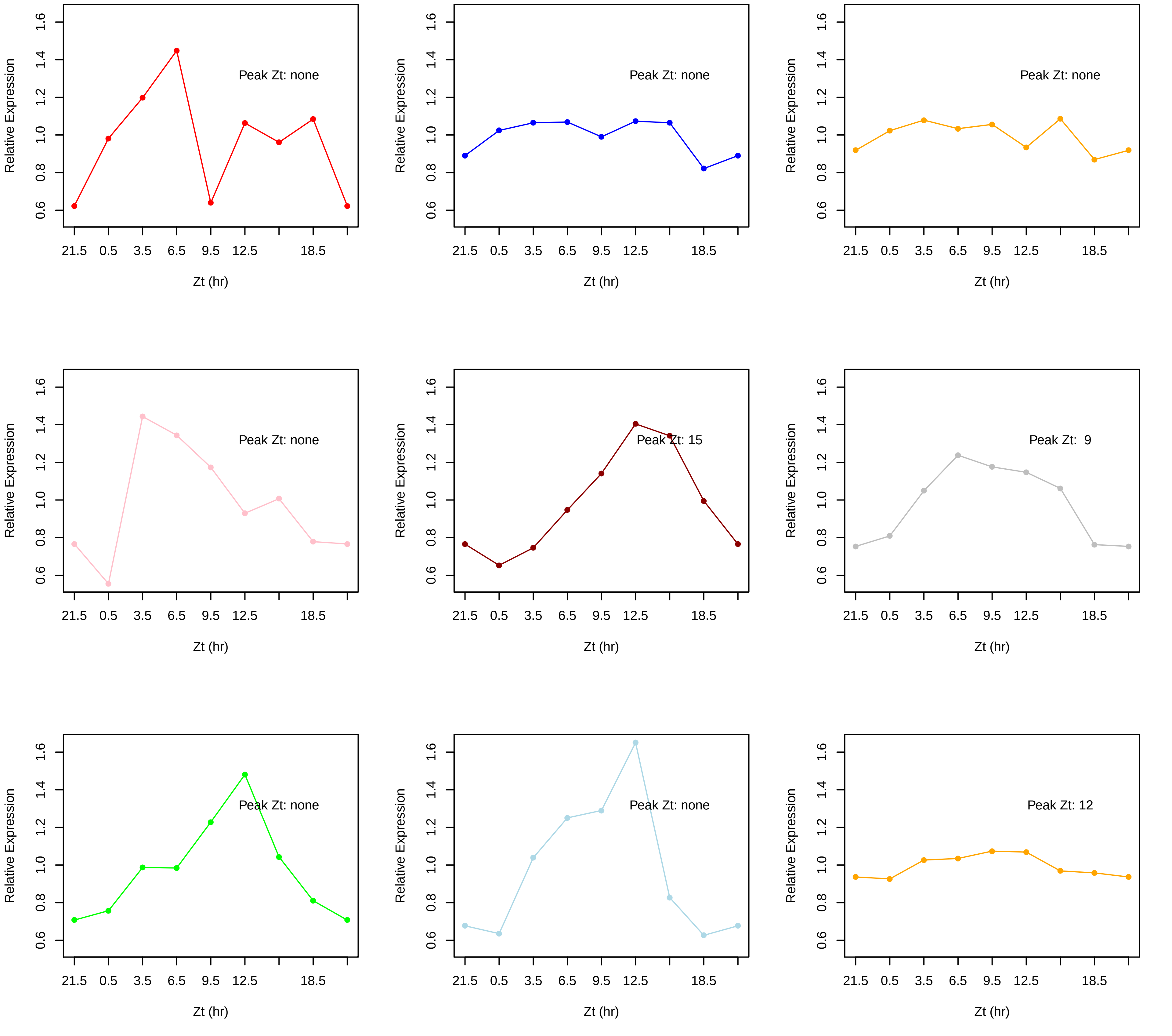

Singeleton: ENSXMAG00000006107 luzp1

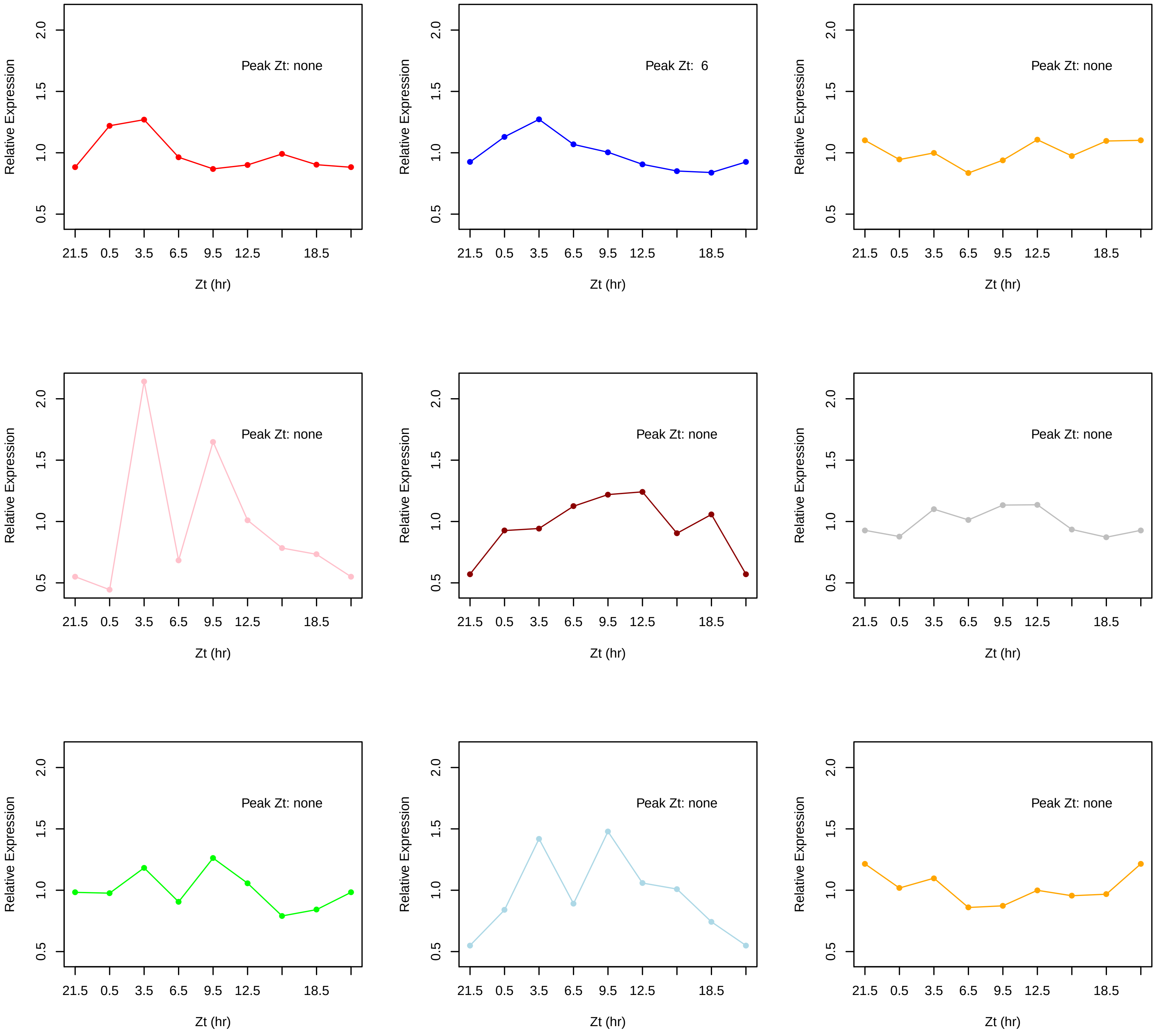

Singeleton: ENSXMAG00000015354 tcp11l1

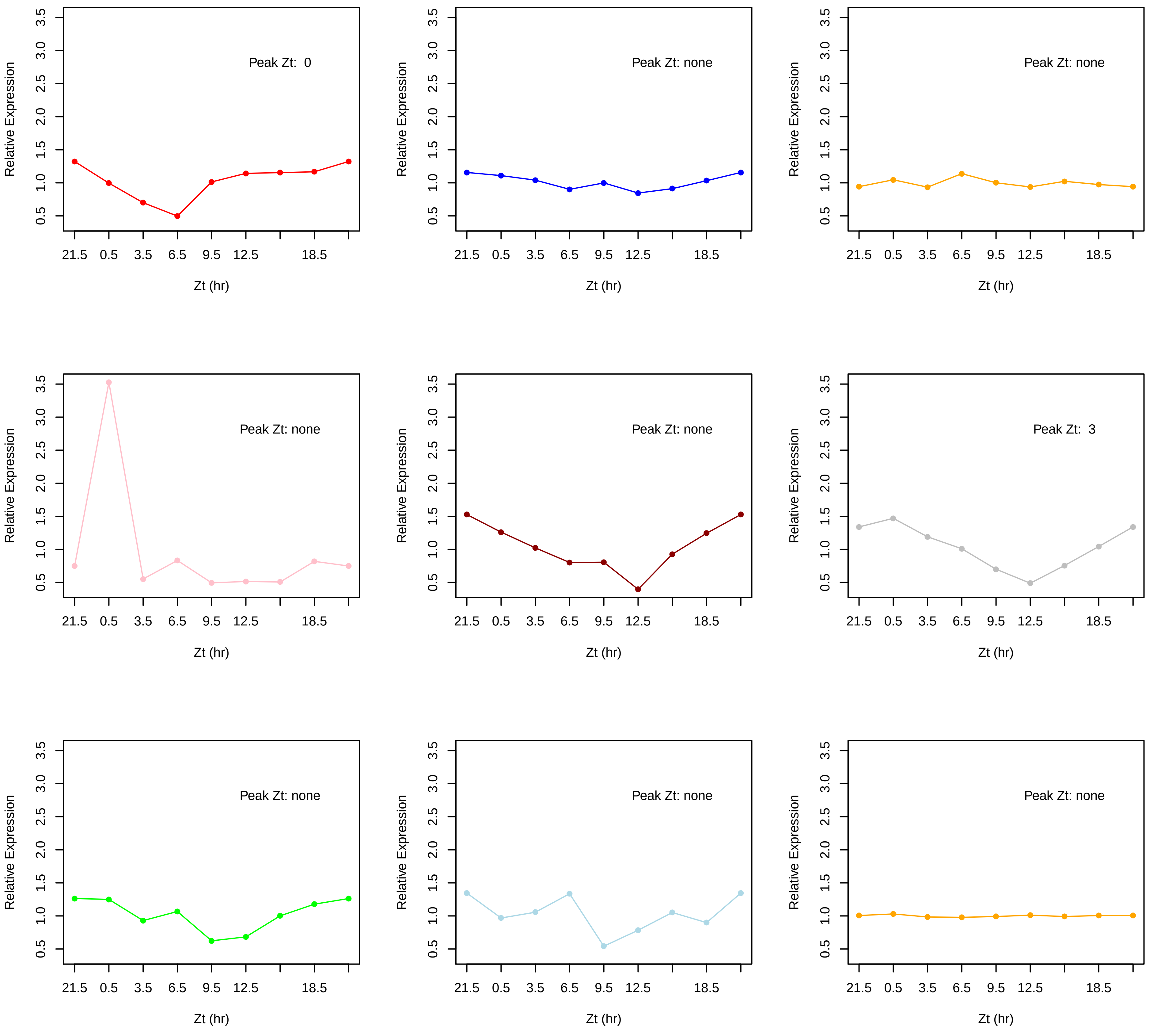

Singeleton: ENSXMAG00000006741 mrpl17

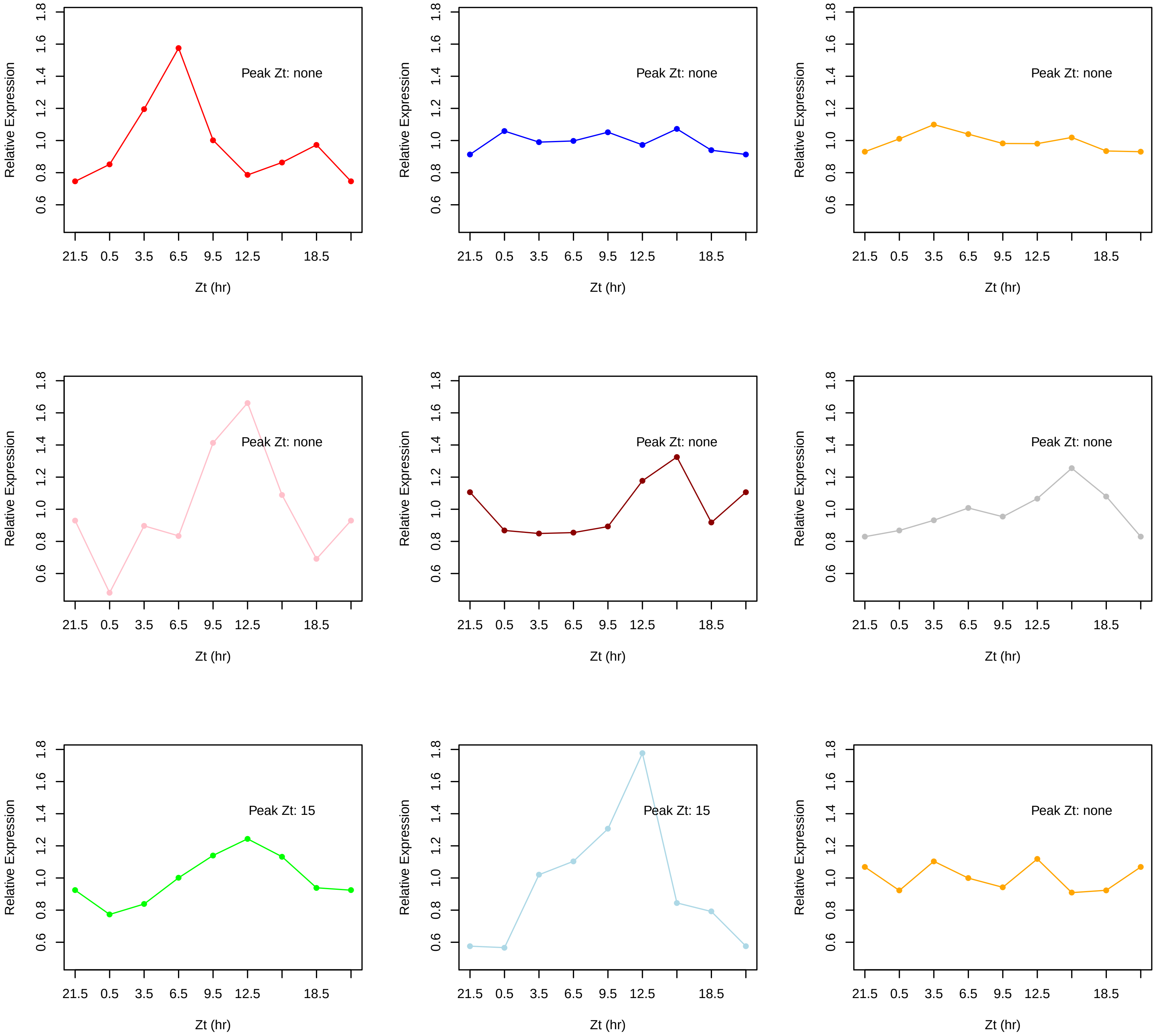

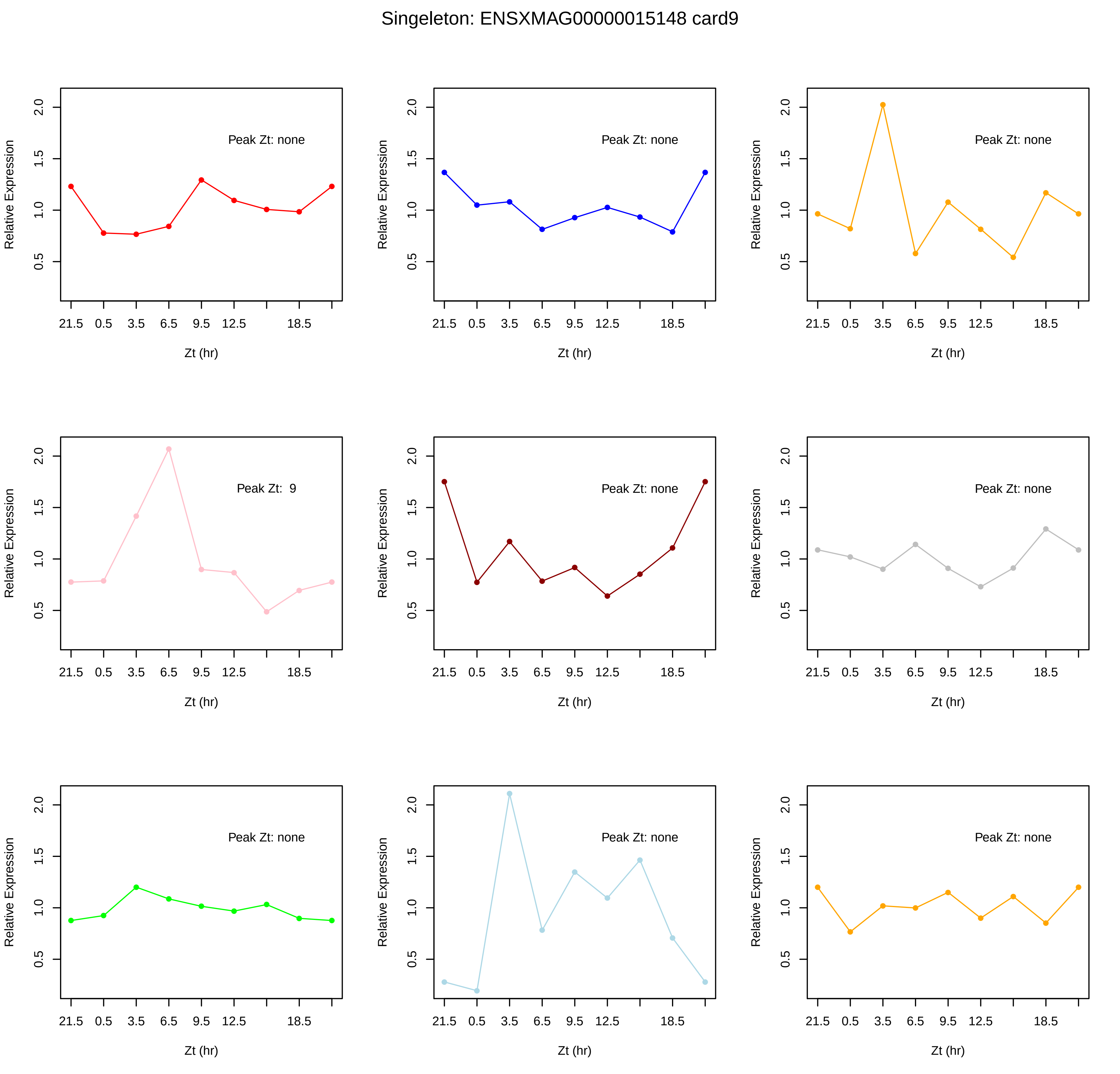

Singleton: ENSXMAG00000015148 card9
Peak Zt: none
Peak Zt: none
Peak Zt: none
Peak Zt: 9
Peak Zt: none
Peak Zt: none
Peak Zt: none
Peak Zt: none
Peak Zt: none
Relative Expression
Zt (hr)
21.5
0.5
3.5
6.5
9.5
12.5
18.5
0.5
1.0
1.5
2.0

Singleton: ENSXMAG00000007821 acacb

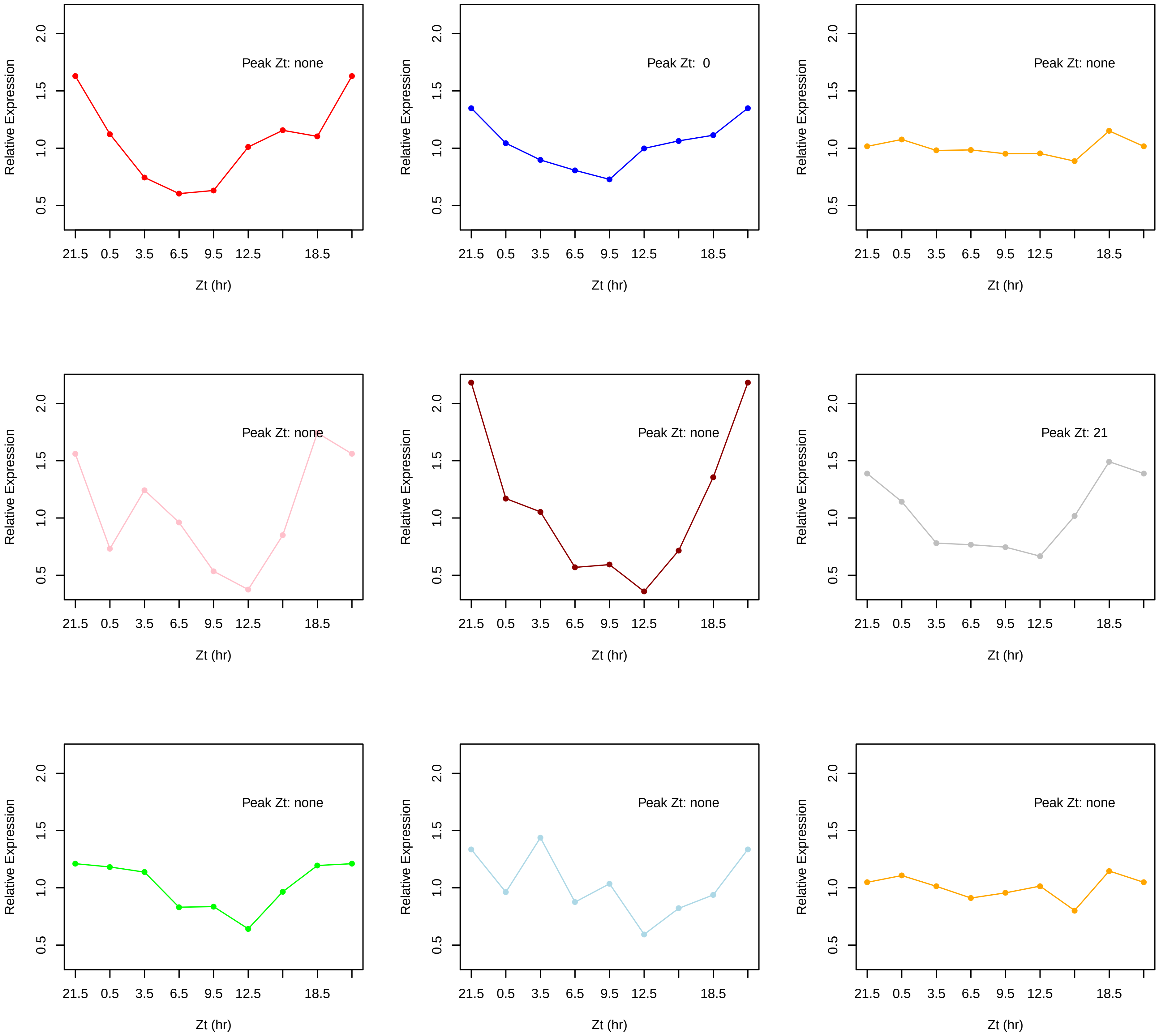

Singeleton: ENSXMAG00000009711 tagln3a

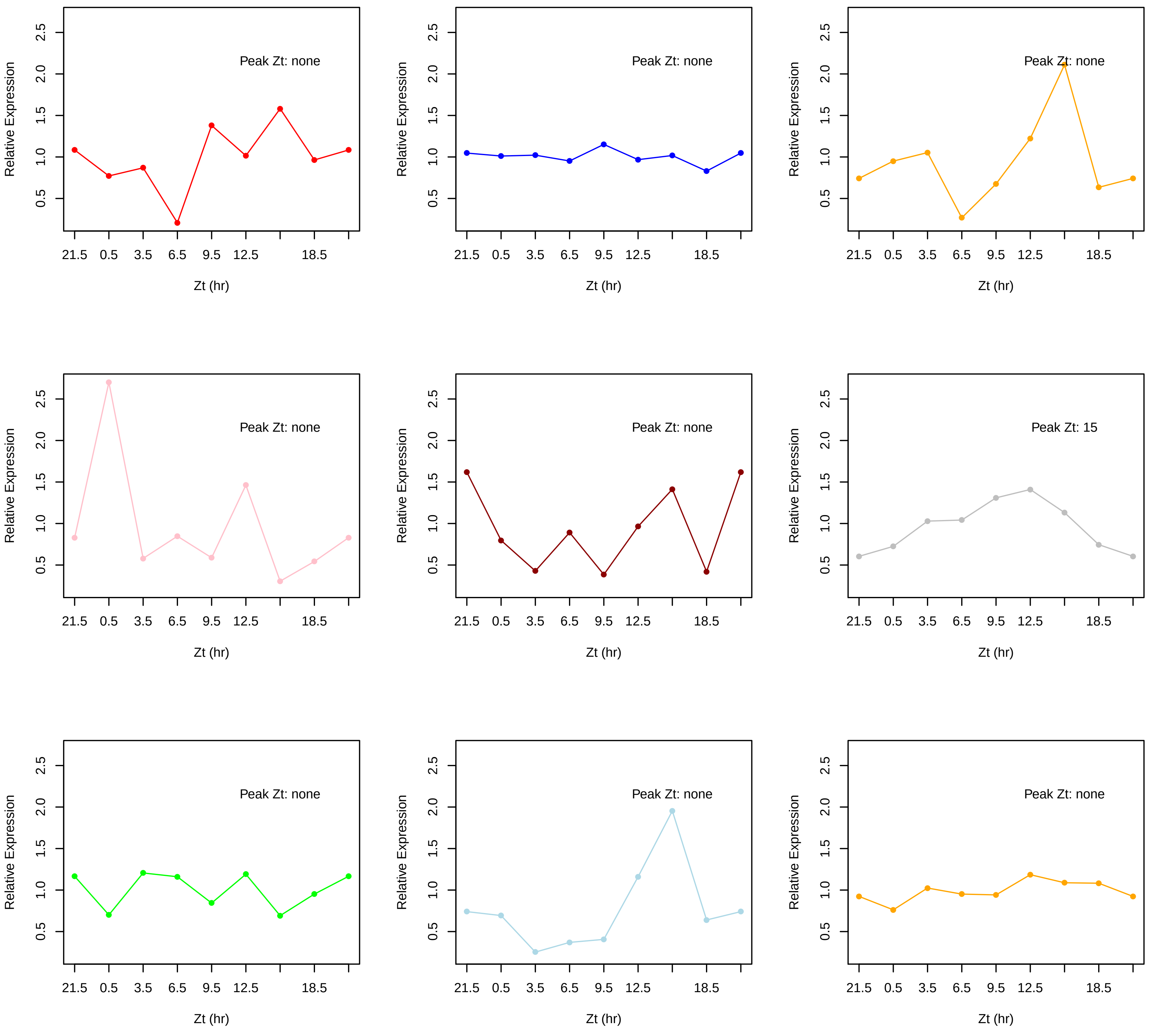

Singeleton: ENSXMAG00000026878 fam214b

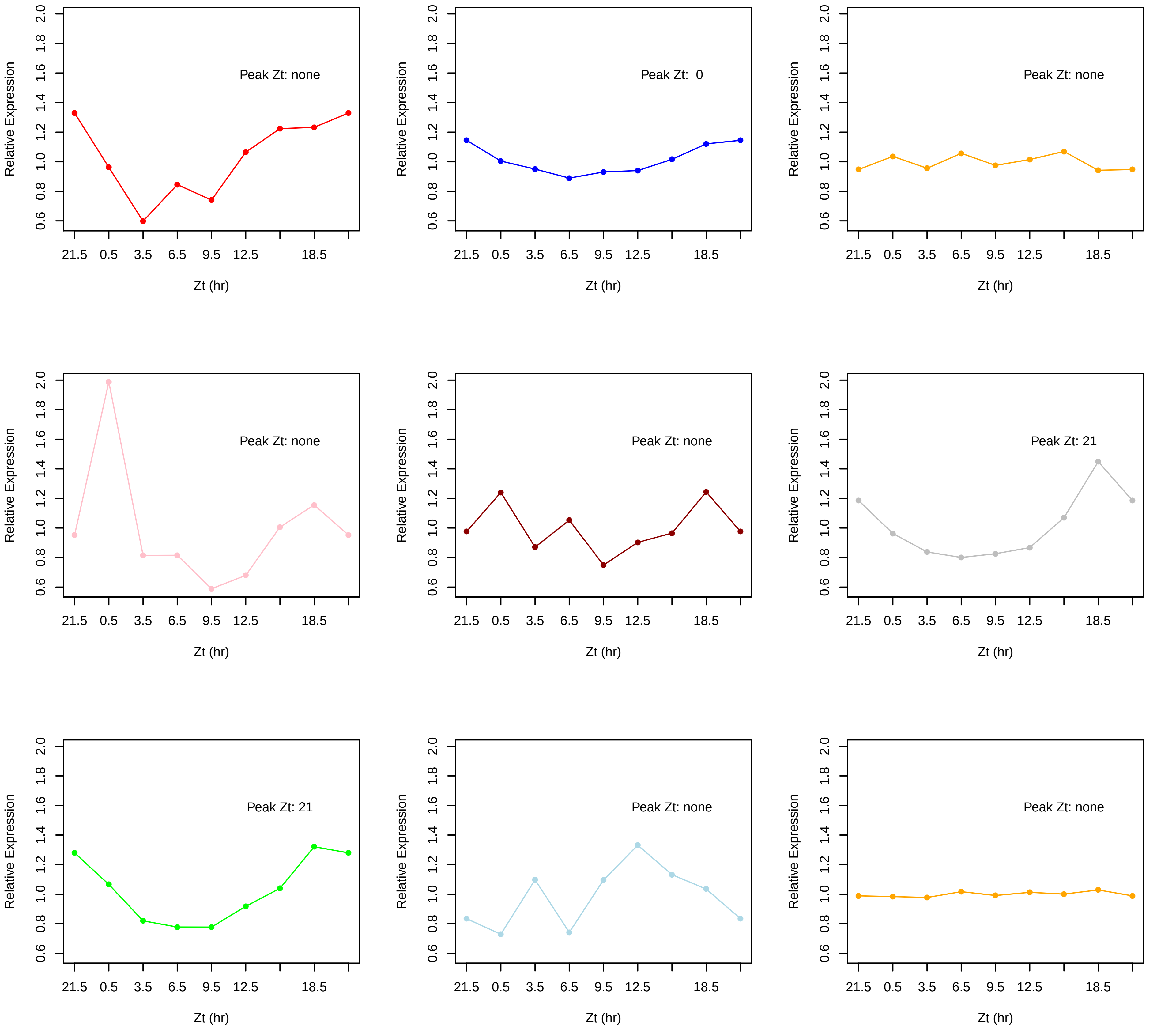

Singleton: ENSXMAG00000029684 pbx1a

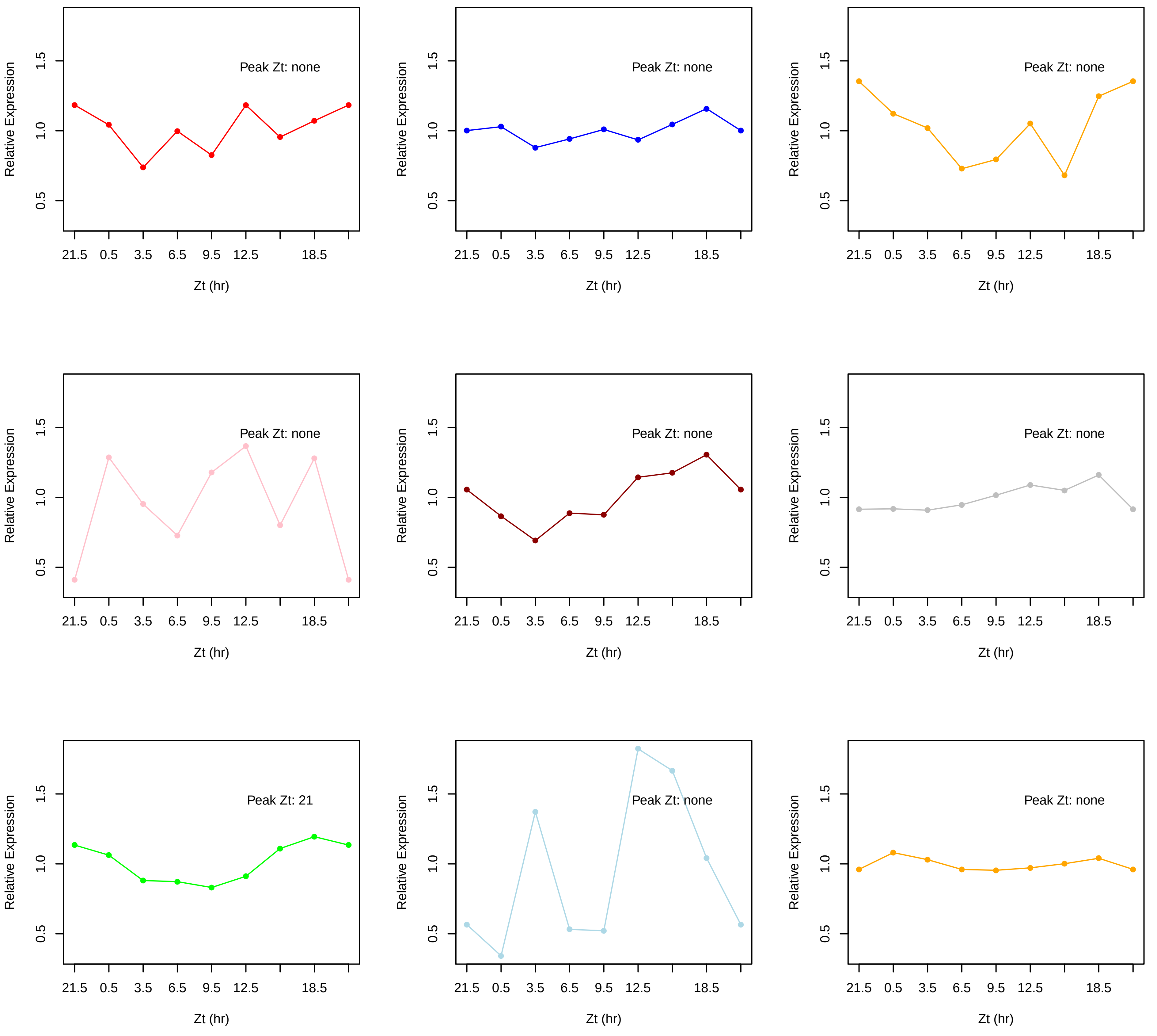

Singleton: ENSXMAG00000011684 letmd1

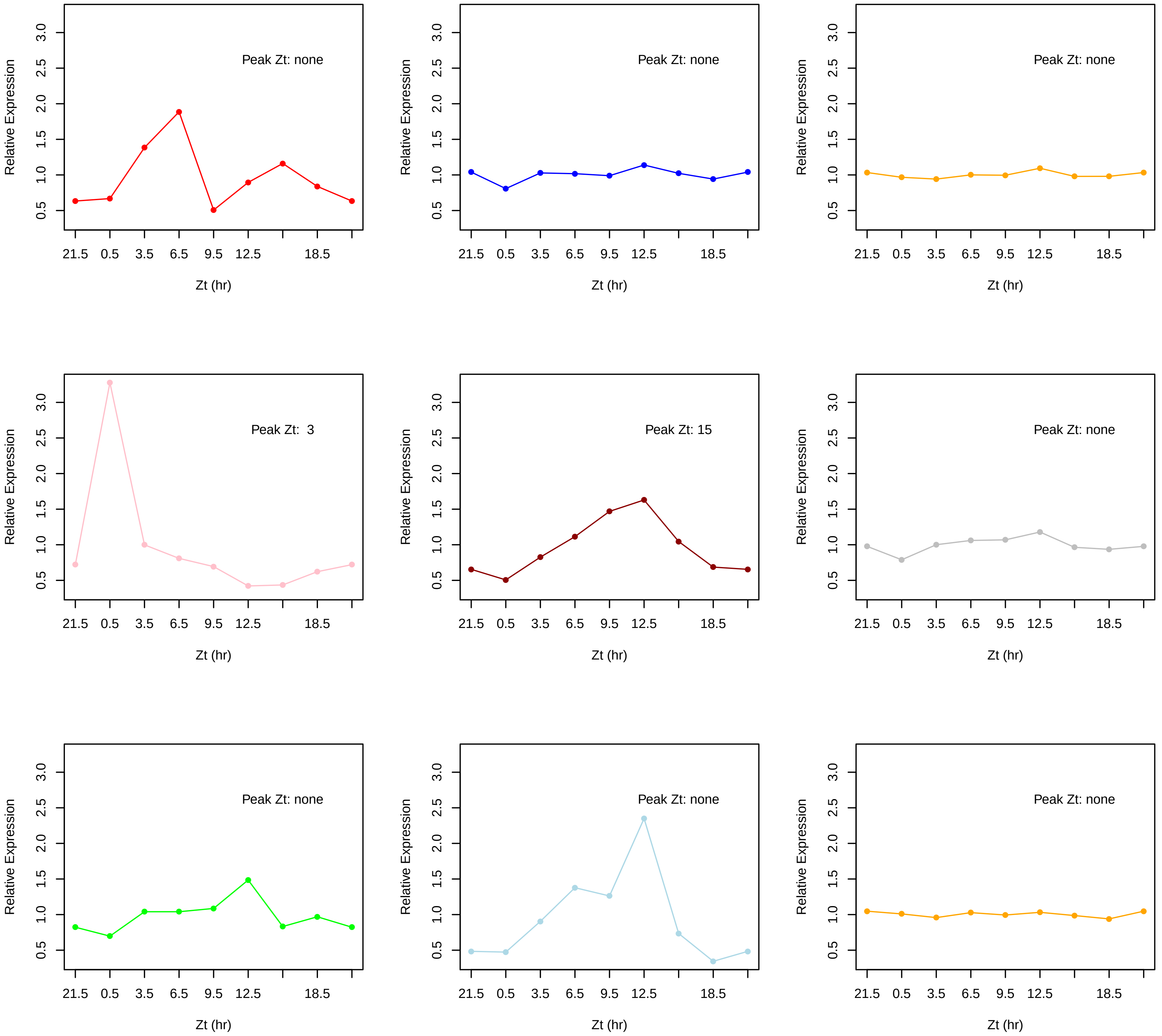

Singeleton: ENSXMAG00000023061

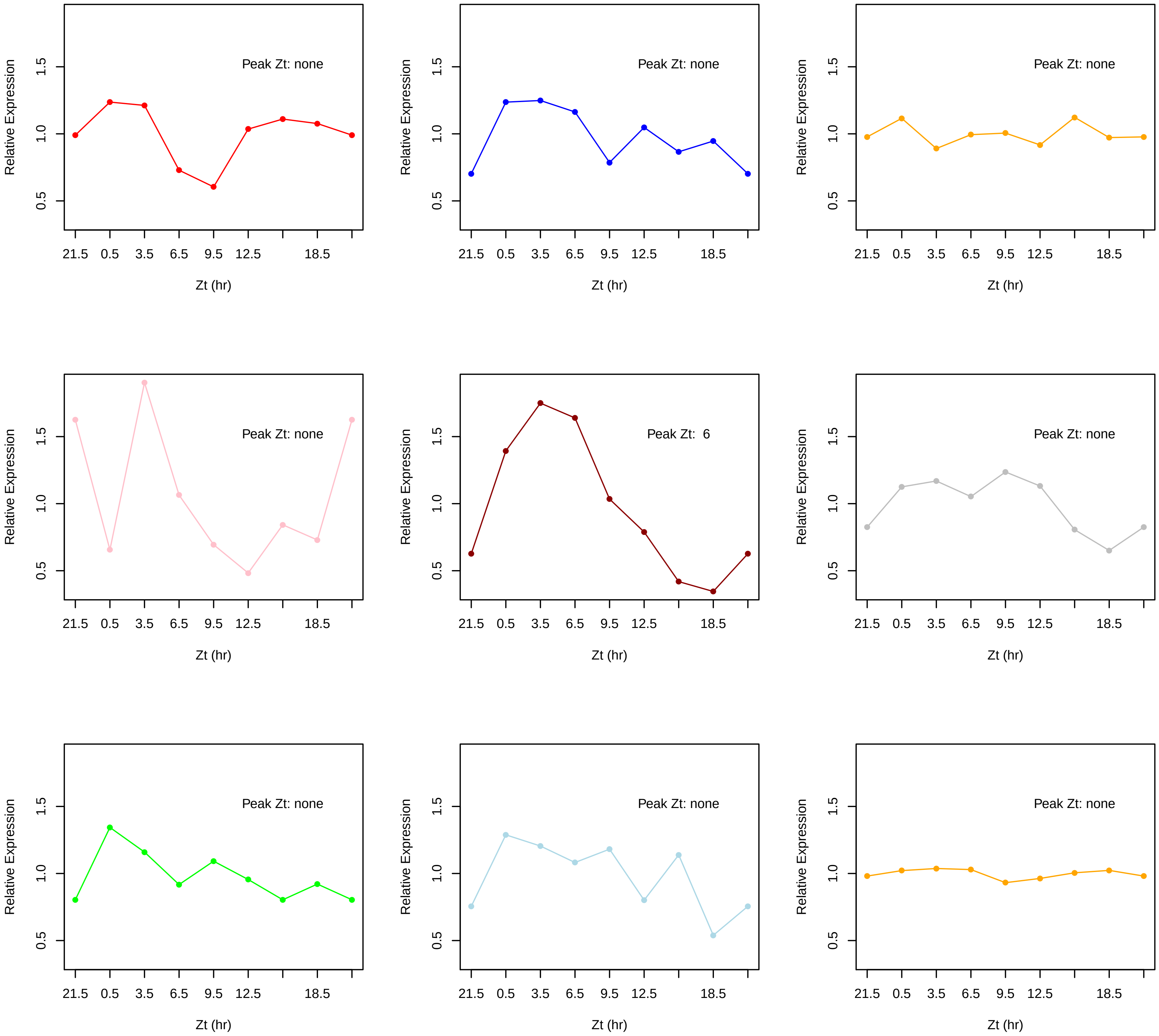

Singeleton: ENSXMAG00000018502 hnrnpm

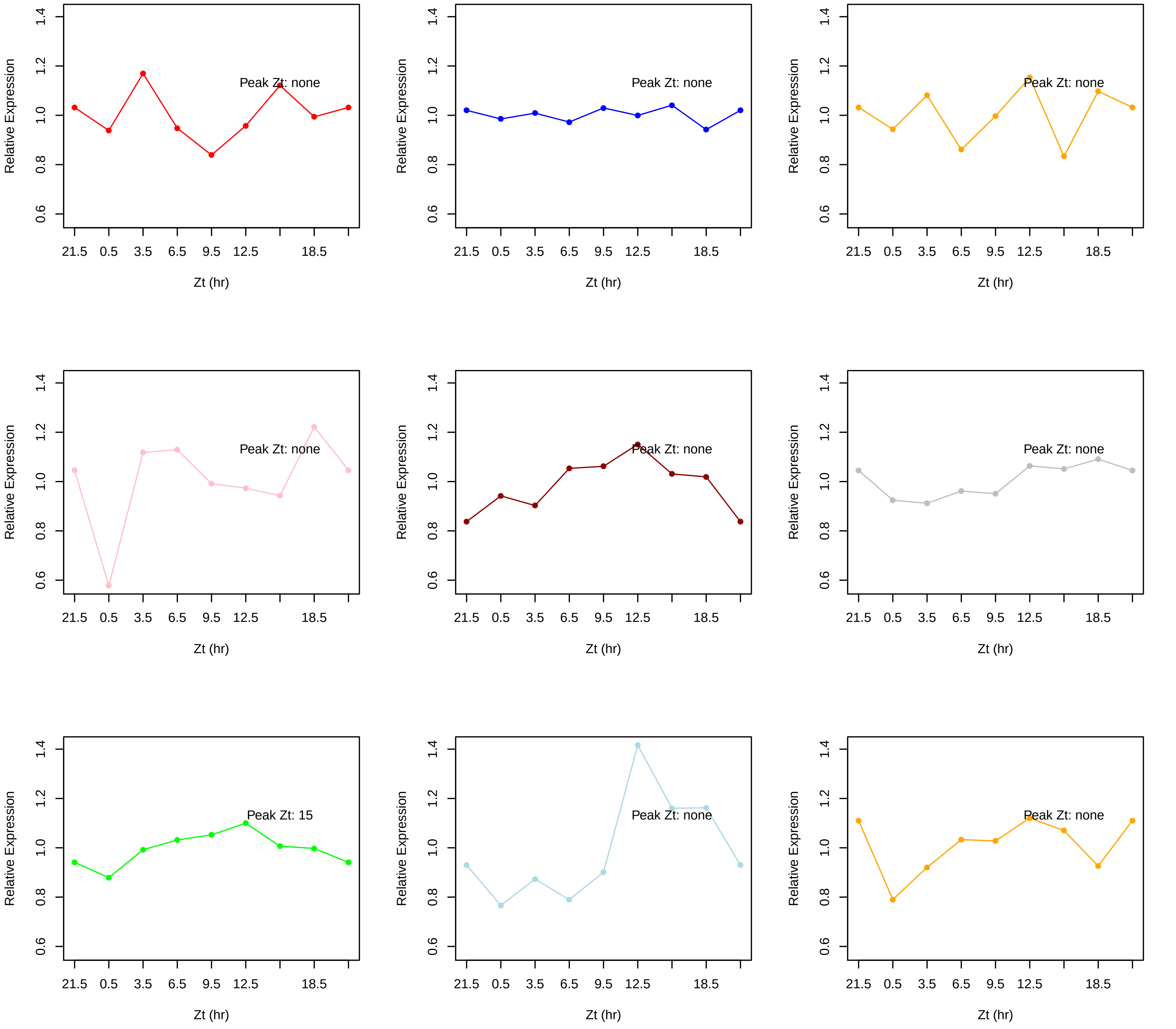

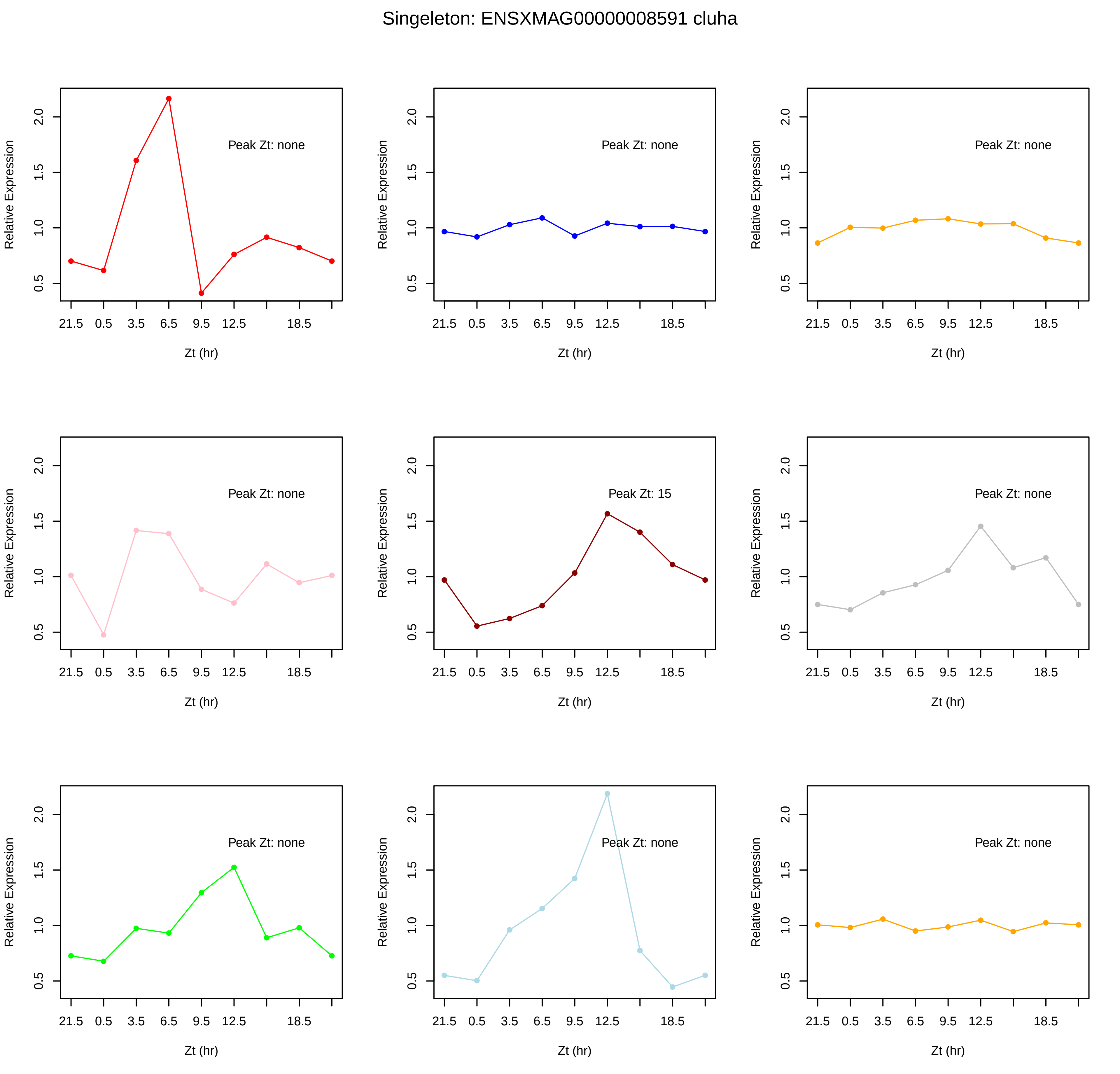
Singeleton: ENSXMAG00000008591 cluha
Peak Zt: none
Peak Zt: none
Peak Zt: none
Peak Zt: none
Peak Zt: 15
Peak Zt: none
Peak Zt: none
Peak Zt: none
Peak Zt: none
Relative Expression
Zt (hr)
21.5
0.5
3.5
6.5
9.5
12.5
18.5
0.5
1.0
1.5
2.0

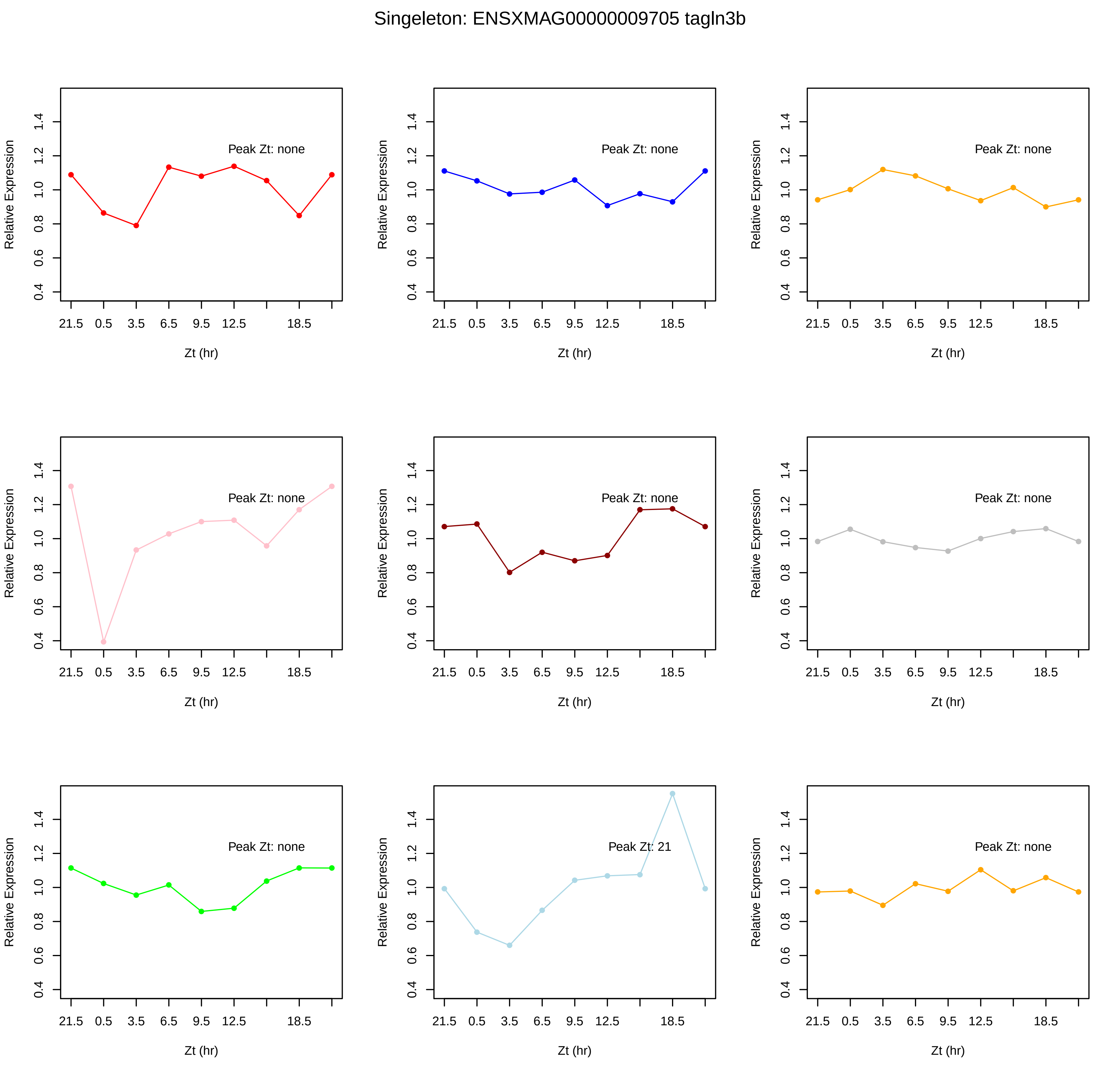
Singeleton: ENSXMAG00000009705 tagln3b
Peak Zt: none
Peak Zt: none
Peak Zt: none
Peak Zt: none
Peak Zt: none
Peak Zt: none
Peak Zt: none
Peak Zt: 21
Peak Zt: none
Relative Expression
Zt (hr)
21.5
0.5
3.5
6.5
9.5
12.5
18.5
0.4
0.6
0.8
1.0
1.2
1.4

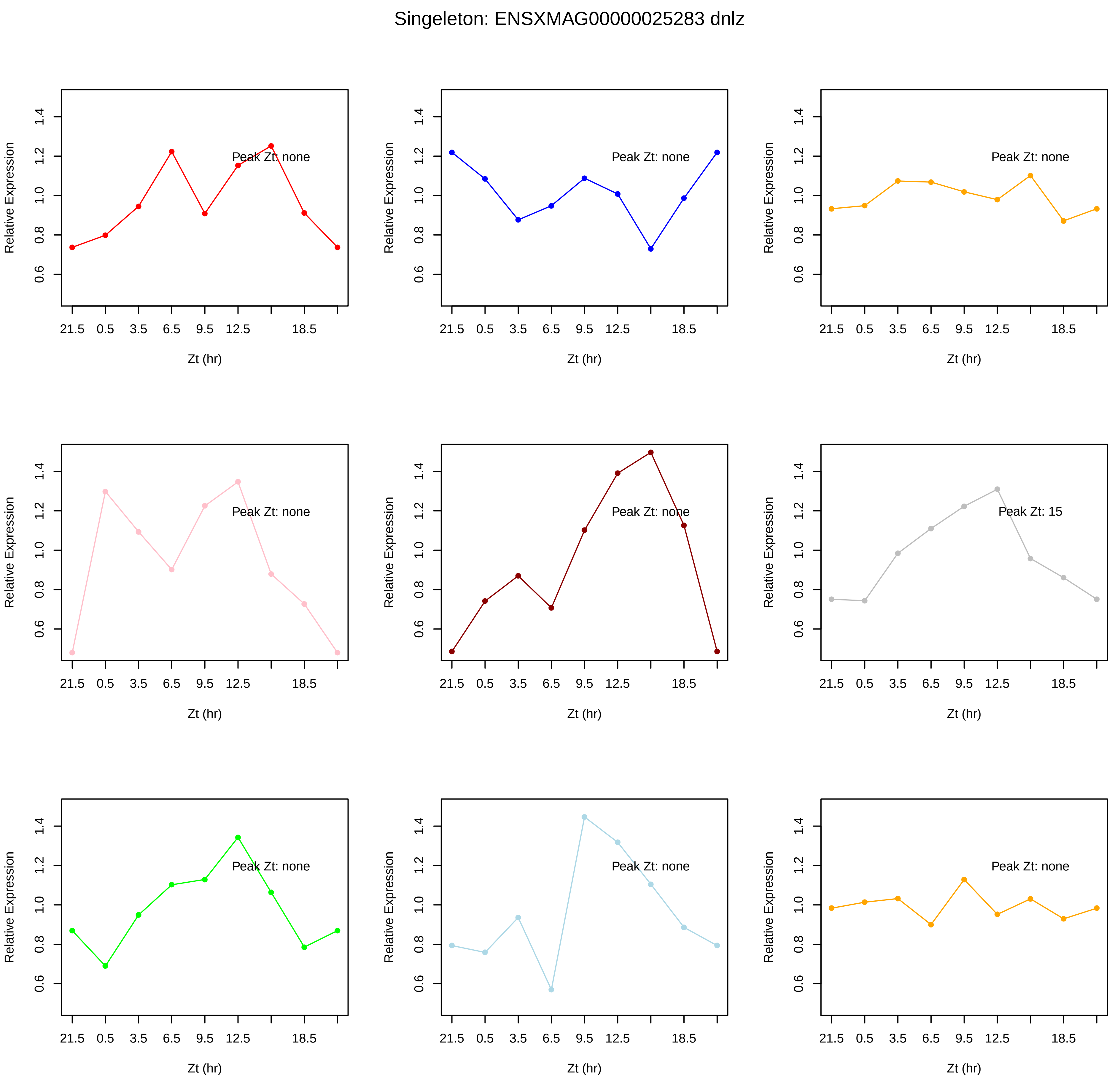
Singeleton: ENSXMAG00000025283 dnlz
Peak Zt: none
Peak Zt: none
Peak Zt: none
Peak Zt: none
Peak Zt: none
Peak Zt: 15
Peak Zt: none
Peak Zt: none
Peak Zt: none
Relative Expression
Zt (hr)
21.5 0.5 3.5 6.5 9.5 12.5 18.5
0.6 0.8 1.0 1.2 1.4

Singeleton: ENSXMAG00000015558 vclb

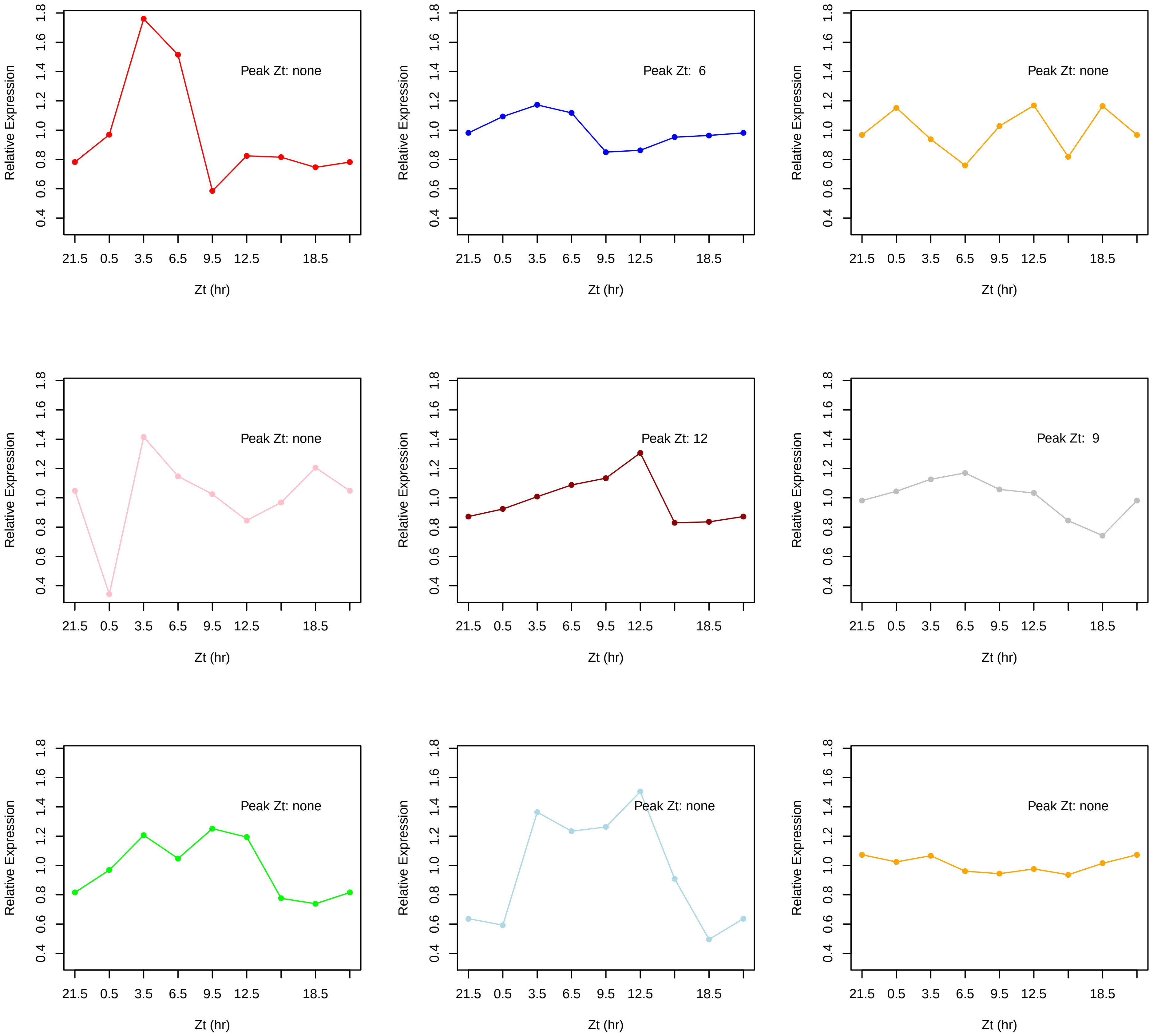

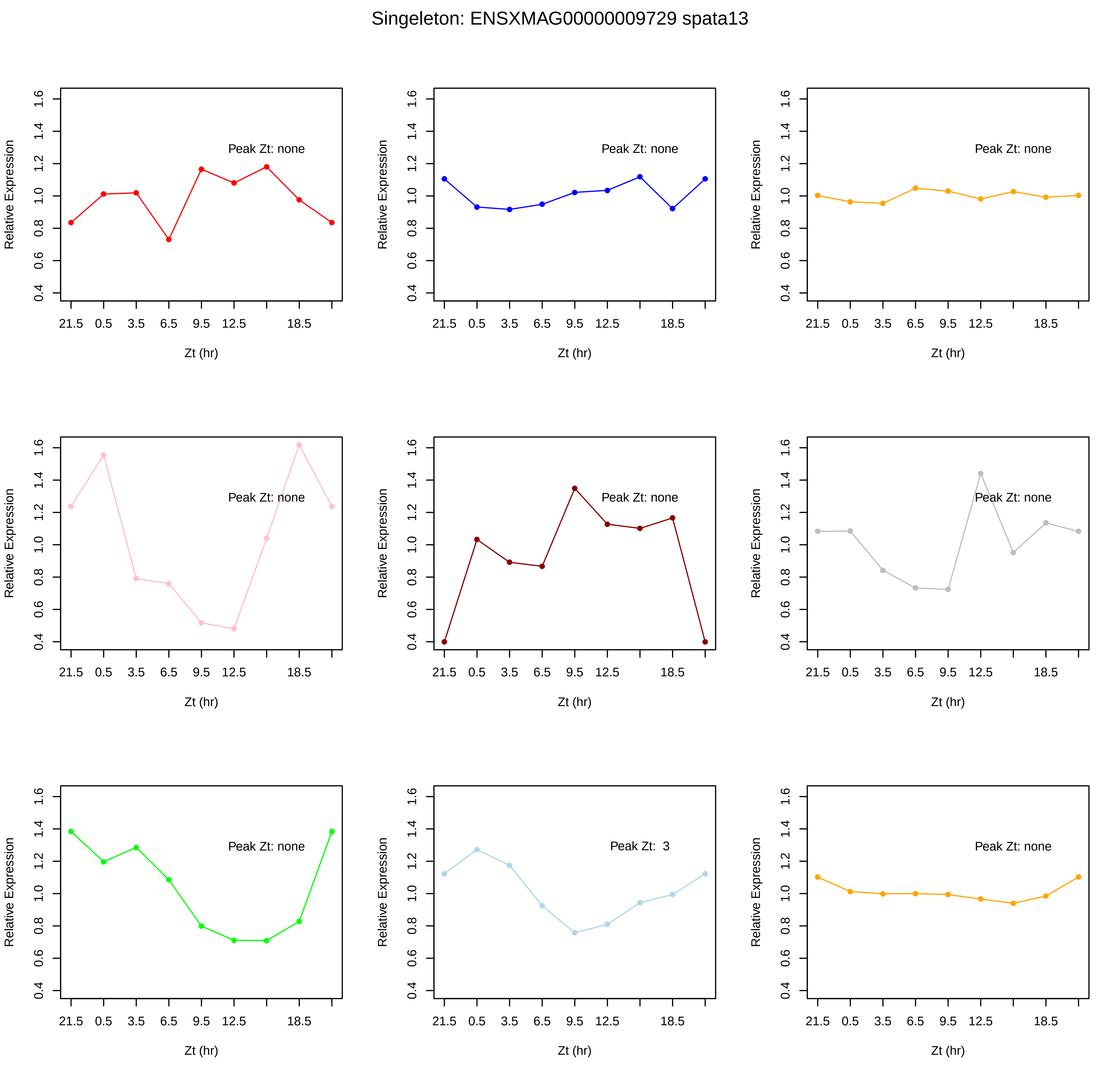

Singeleton: ENSXMAG00000009729 spata13
Relative Expression
Zt (hr)
Peak Zt: none
Peak Zt: 3

Singeleton: ENSXMAG00000018254 unga

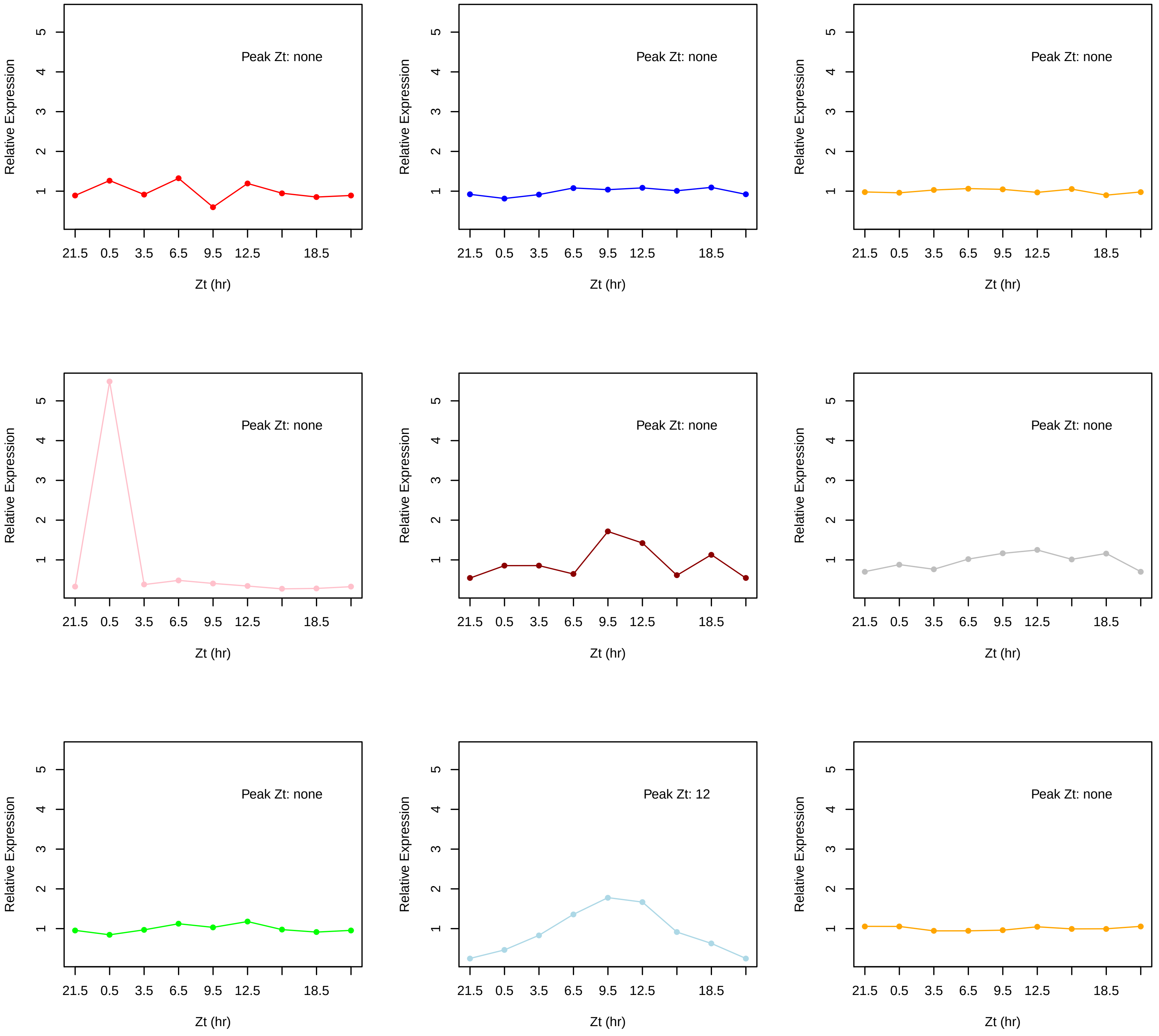

Singleton: ENSXMAG00000018715 emc1

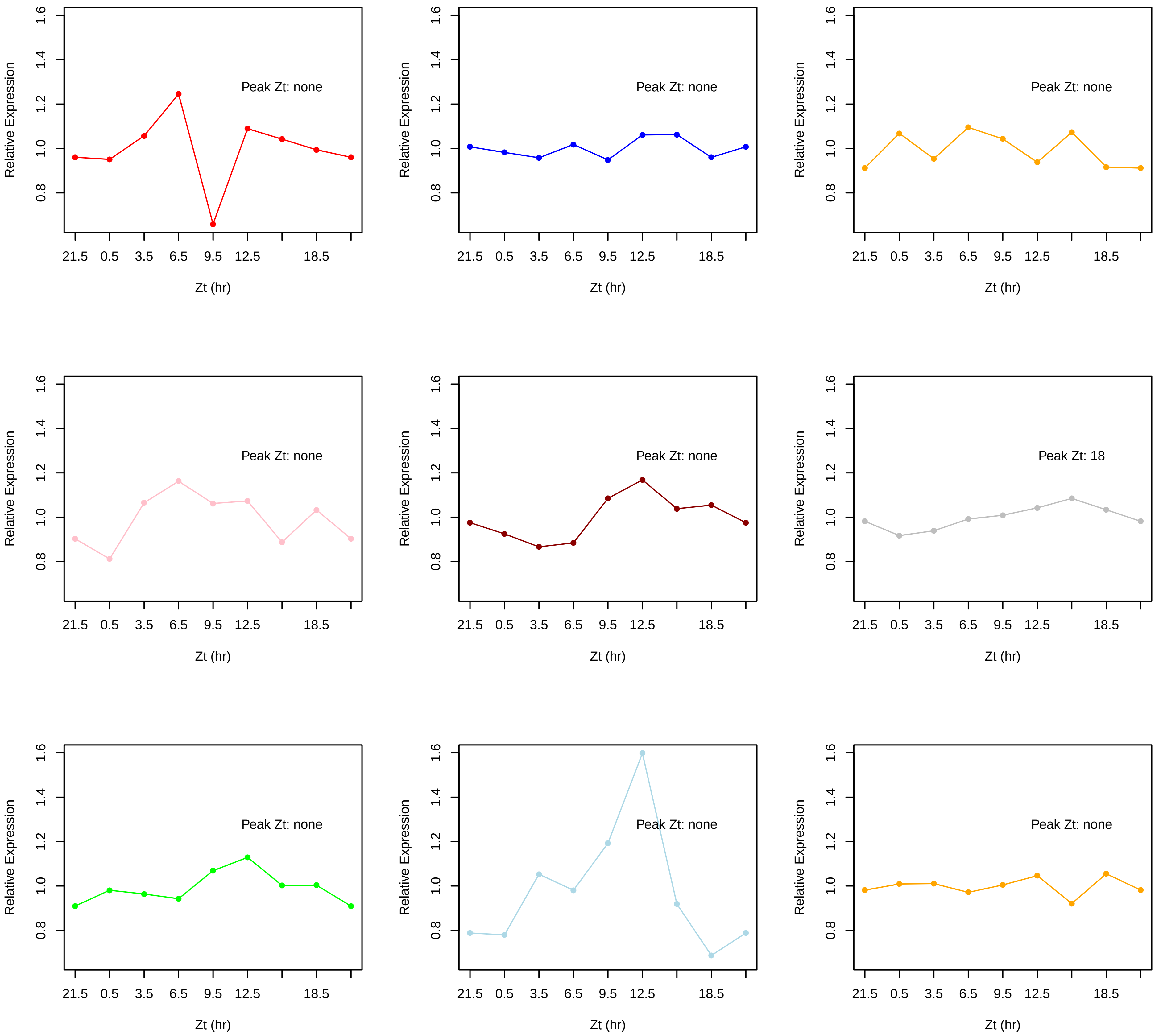

Singleton: ENSXMAG00000001756 VAV2

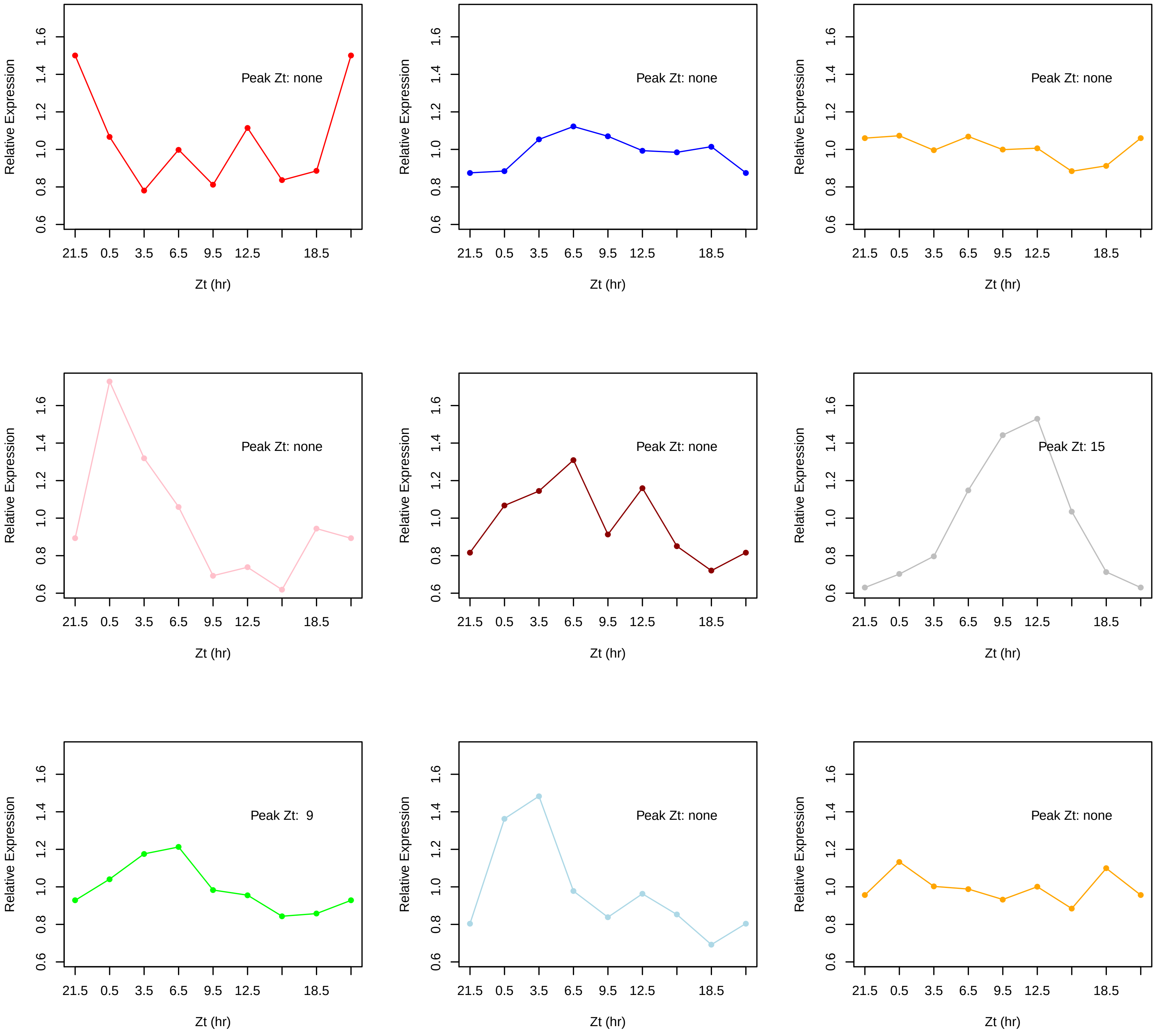

Singleton: ENSXMAG00000015291 GGT7

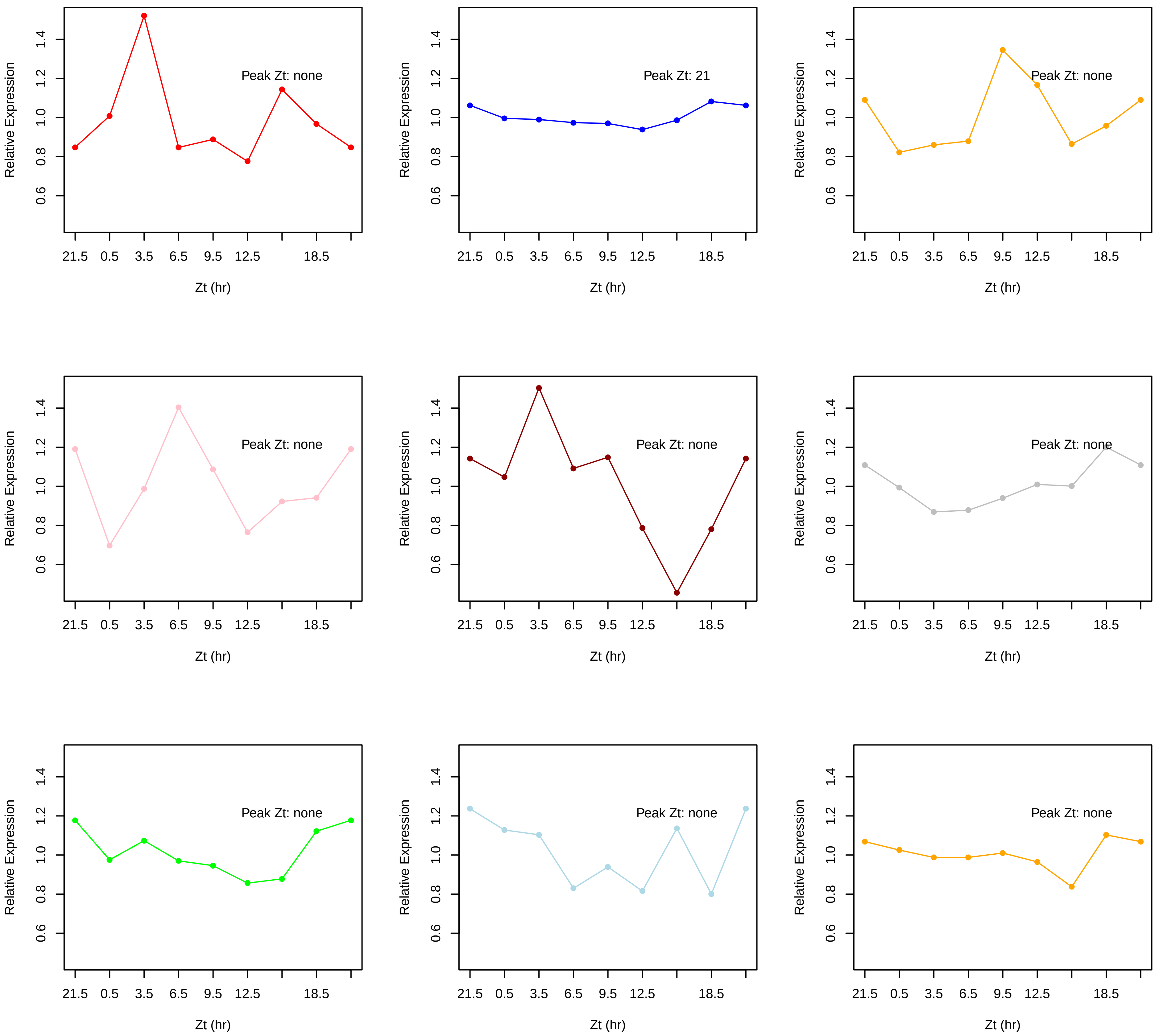

Singeleton: ENSXMAG00000010431 ap3m1

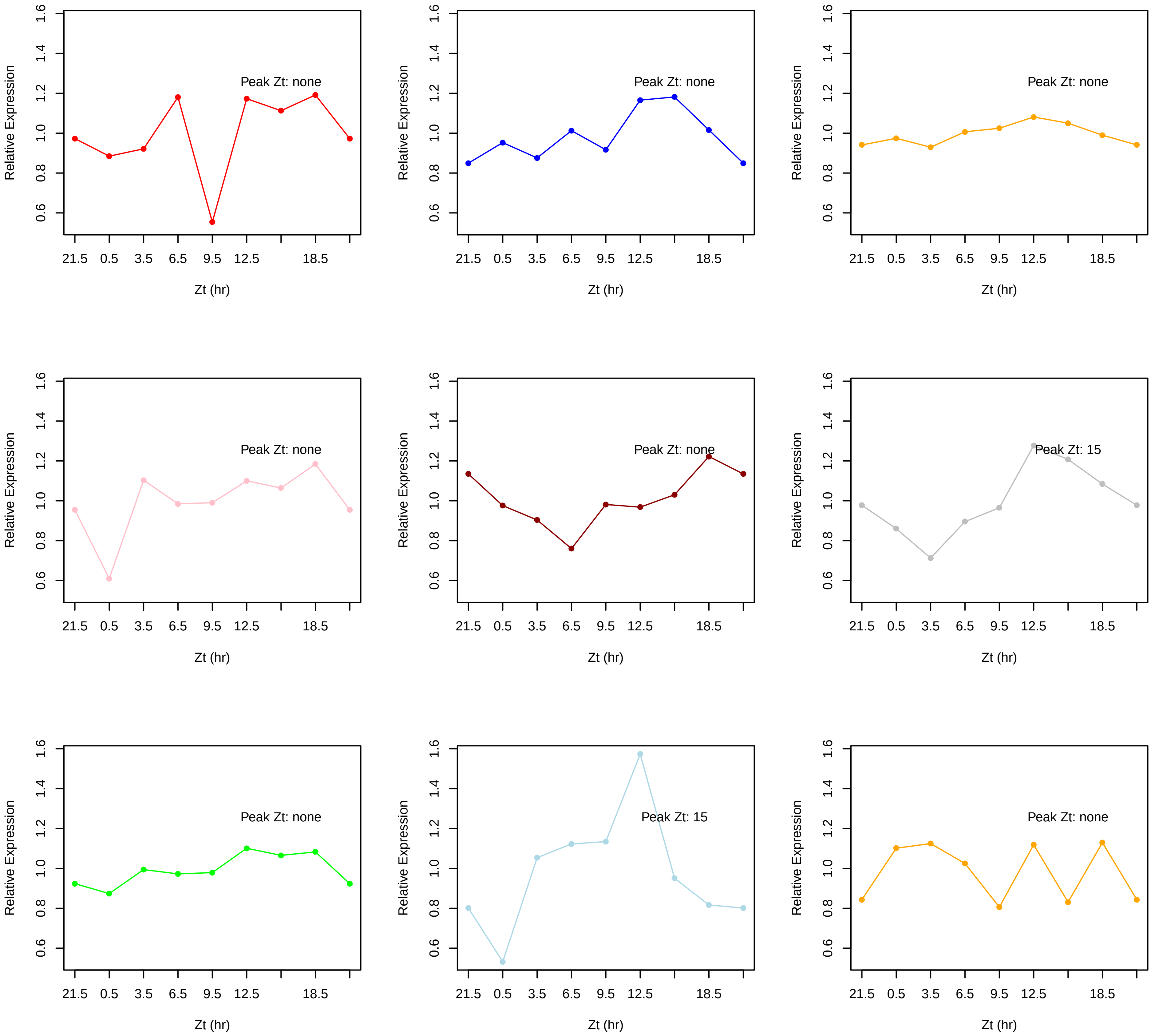

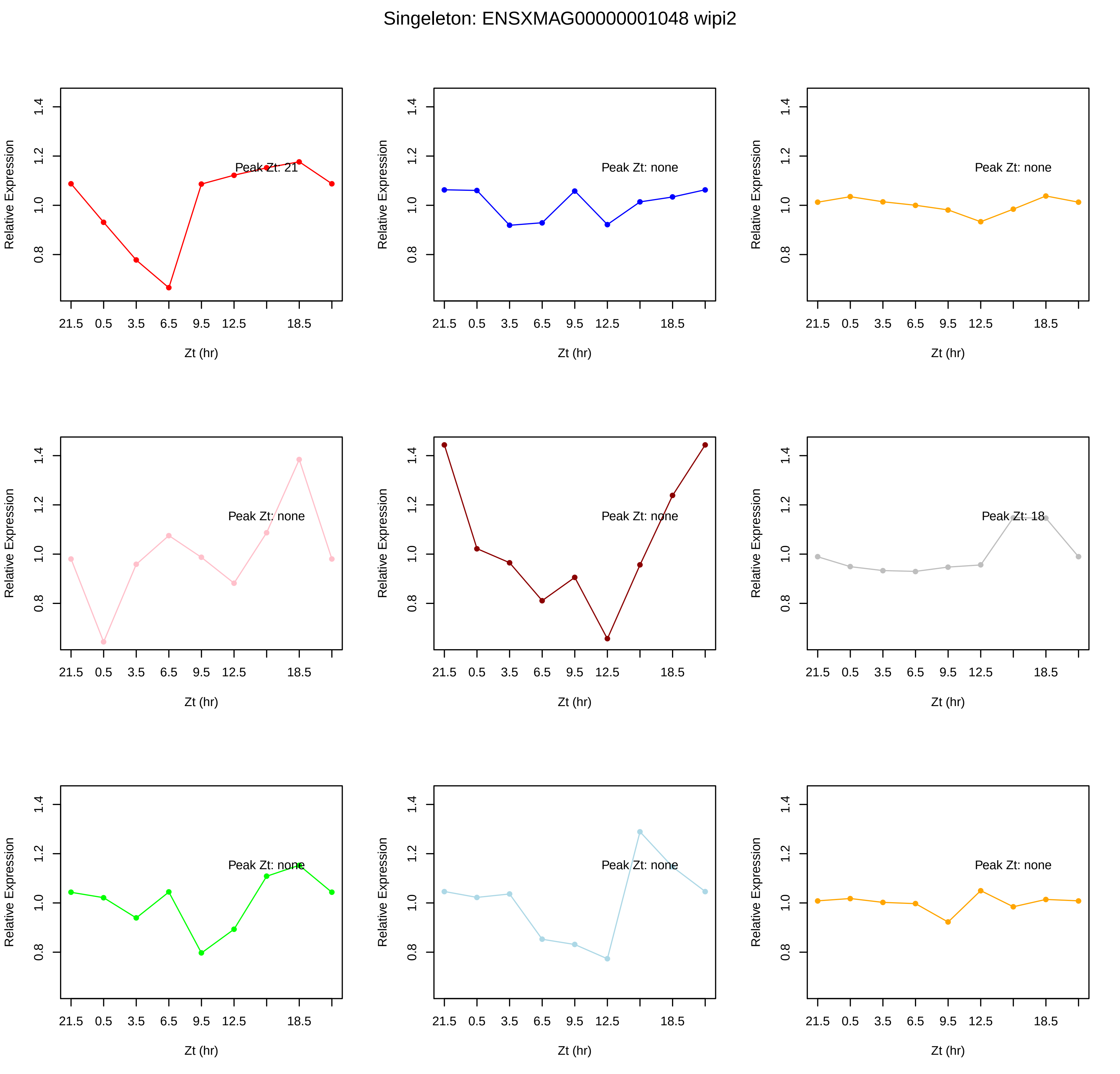

Singeleton: ENSXMAG00000001048 wipi2
Relative Expression
Zt (hr)
1.4
1.2
1.0
0.8
21.5
0.5
3.5
6.5
9.5
12.5
18.5
Peak Zt: 21
Peak Zt: none
Peak Zt: none
Peak Zt: none
Peak Zt: none
Peak Zt: 18
Peak Zt: none
Peak Zt: none
Peak Zt: none

Singleton: ENSXMAG00000030048 cxadr

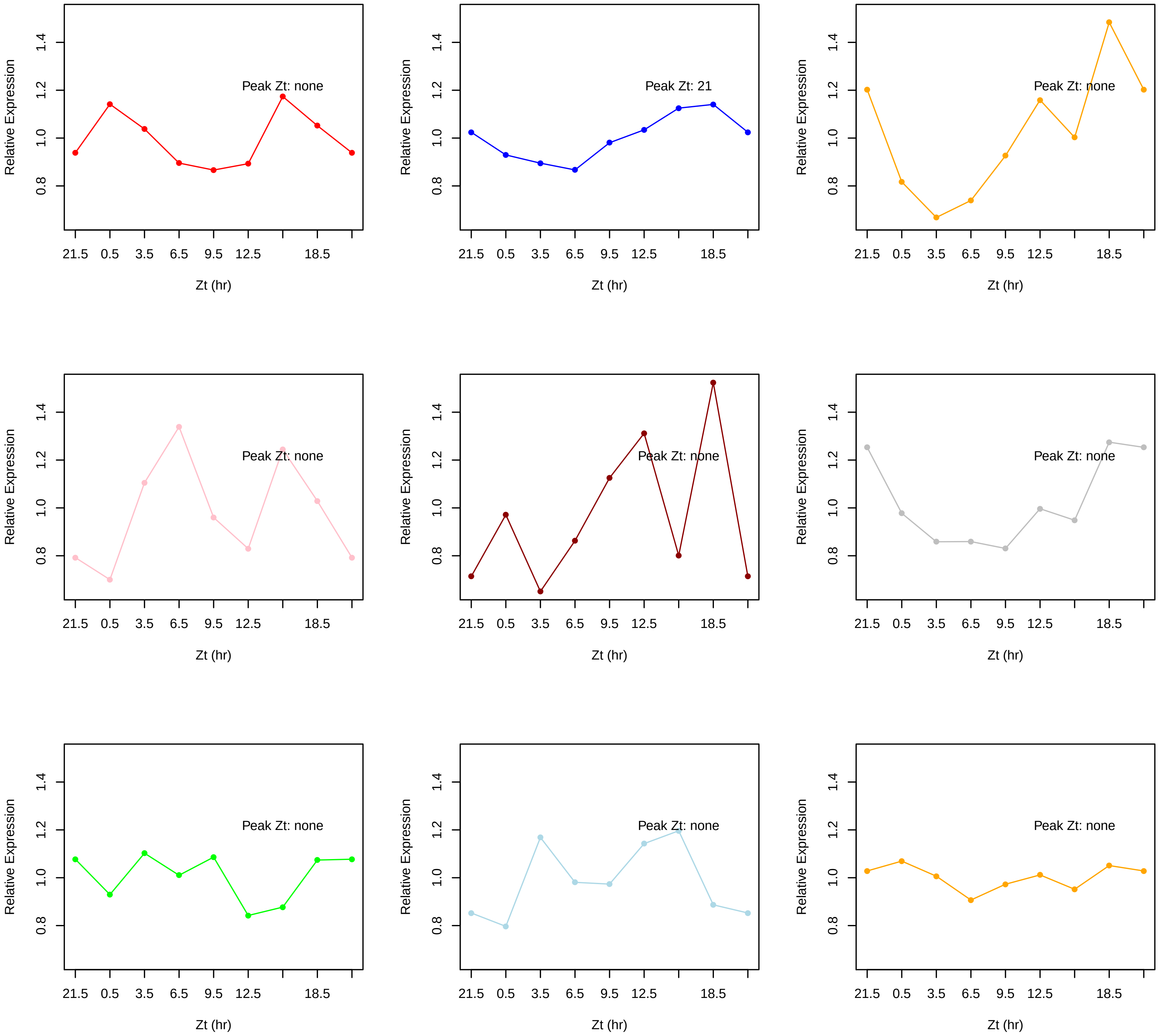

Singleton: ENSXMAG00000018244 usp30

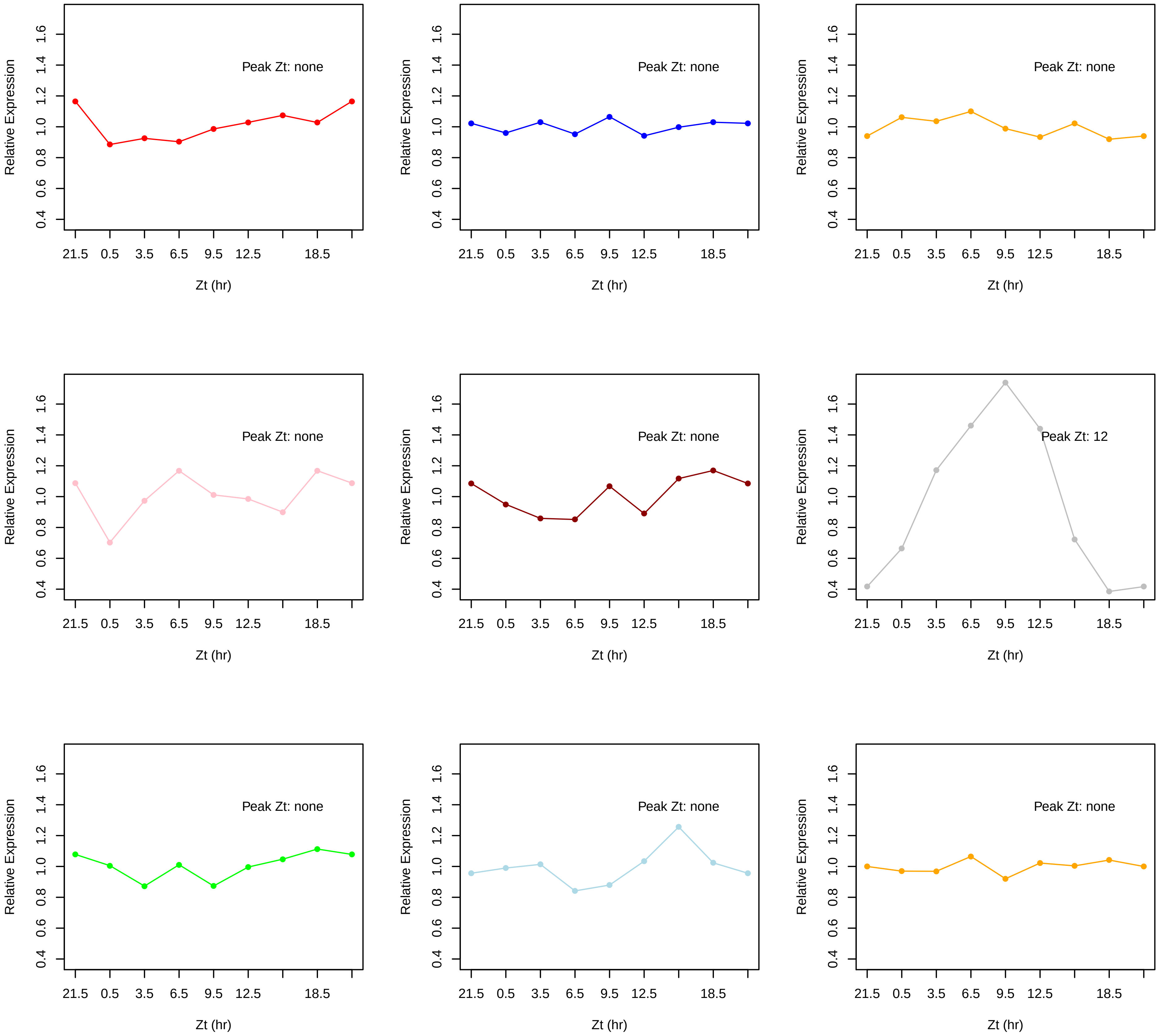

Singeleton: ENSXMAG00000008576 aifm4

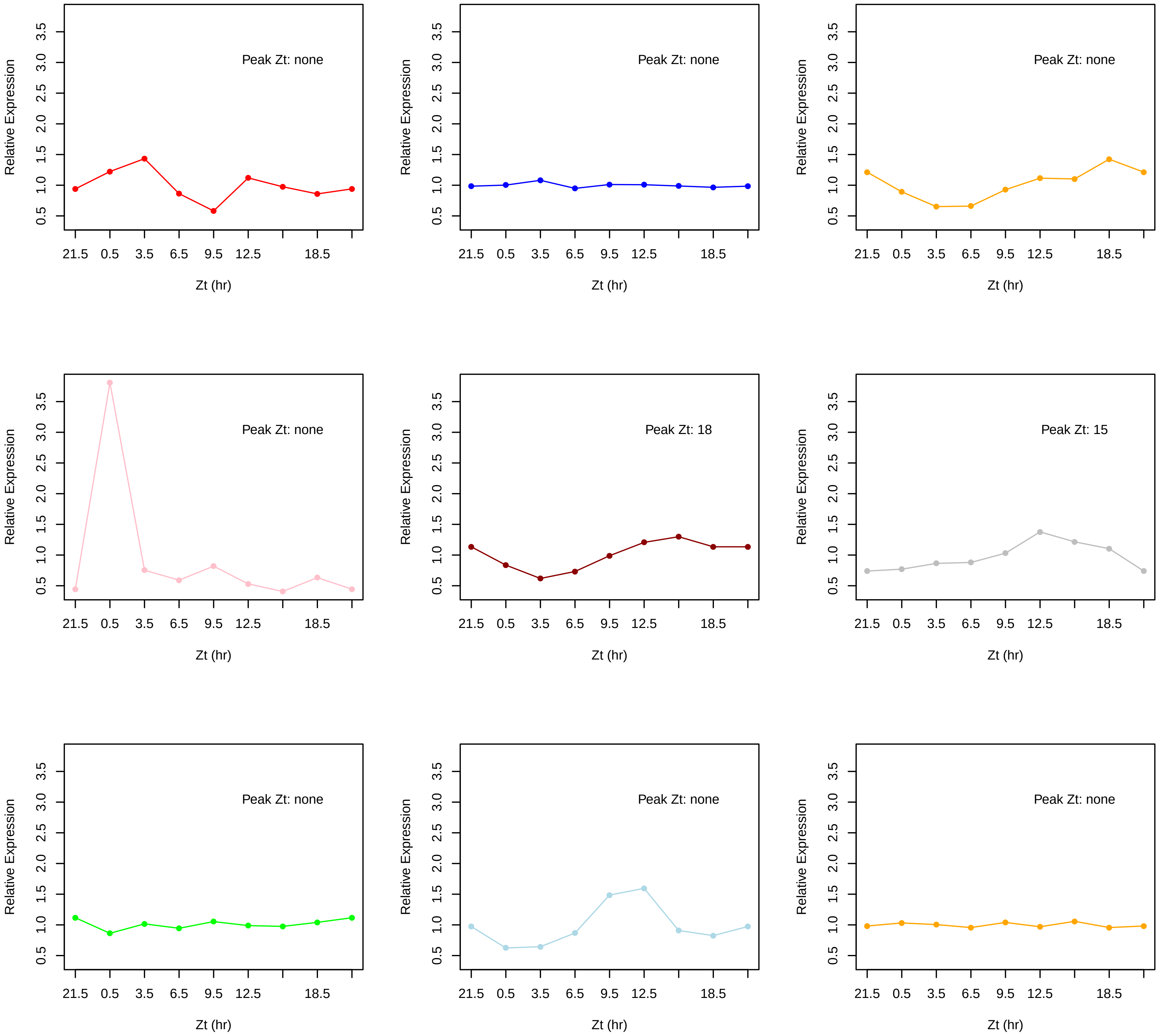

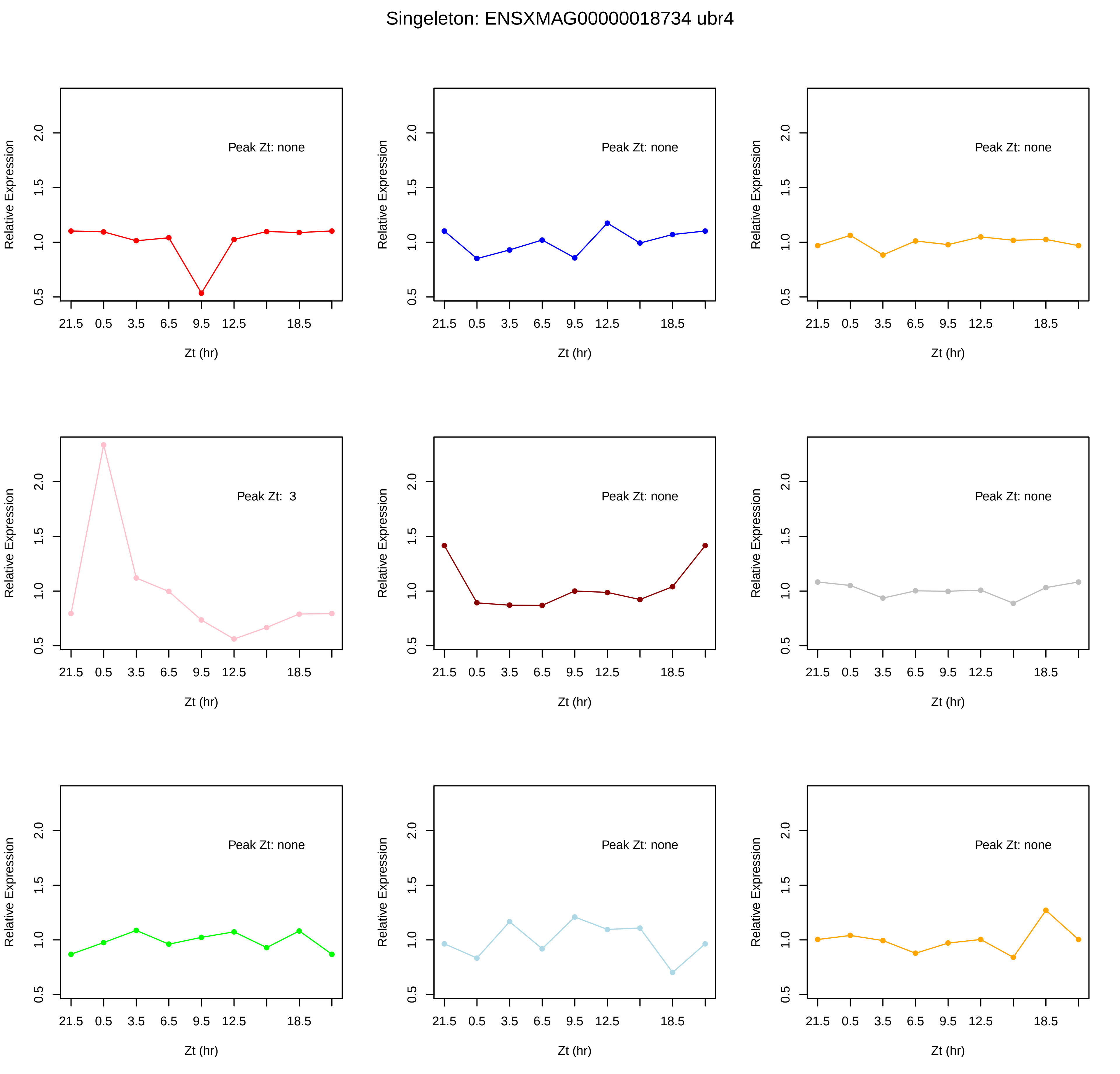

Singeleton: ENSXMAG00000018734 ubr4
Peak Zt: none
Peak Zt: none
Peak Zt: none
Peak Zt: 3
Peak Zt: none
Peak Zt: none
Peak Zt: none
Peak Zt: none
Peak Zt: none
Relative Expression
Zt (hr)
21.5
0.5
3.5
6.5
9.5
12.5
18.5
0.5
1.0
1.5
2.0

Singleton: ENSXMAG00000015523 kat6b

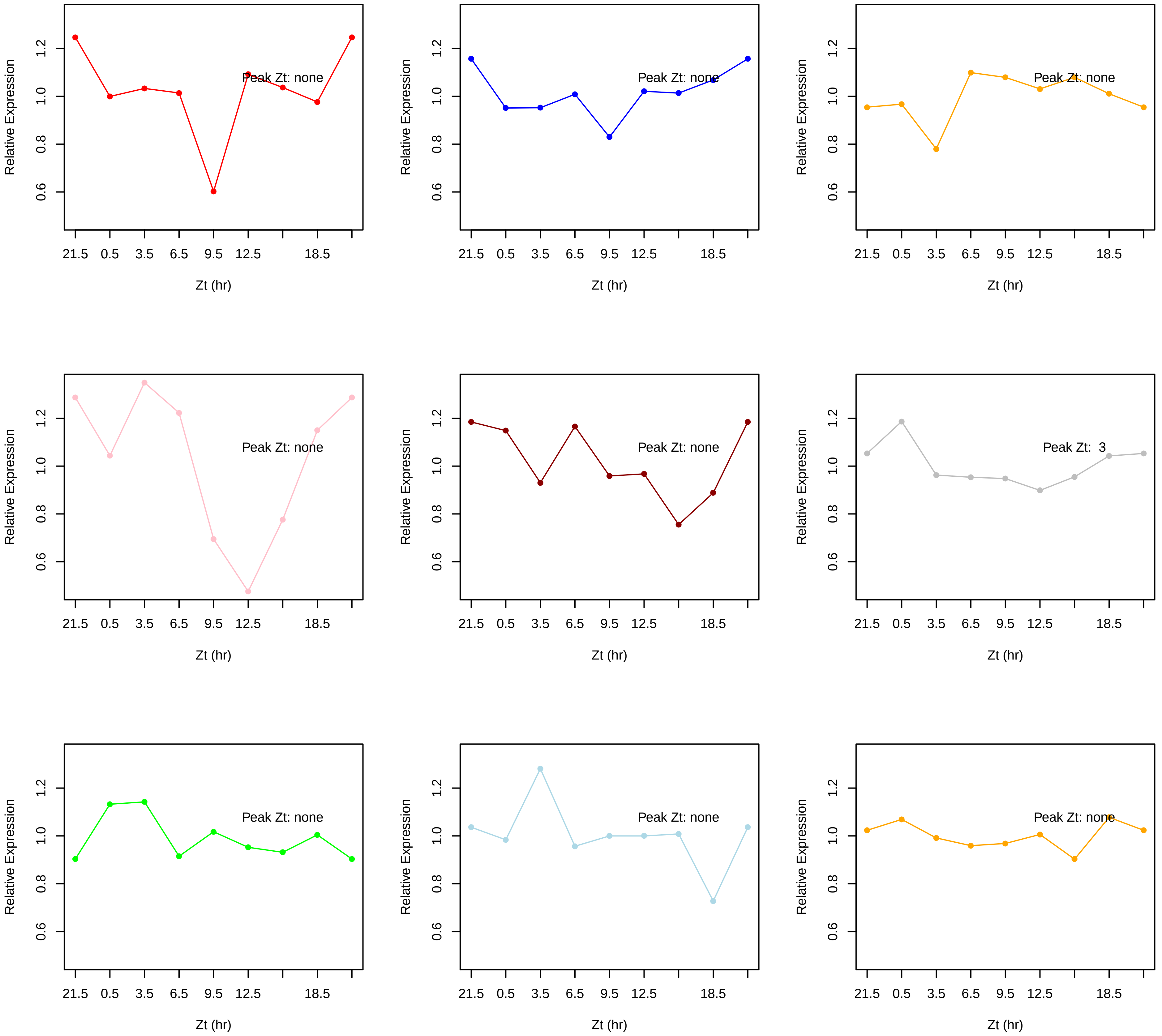

Singleton: ENSXMAG00000003471 arpc1b

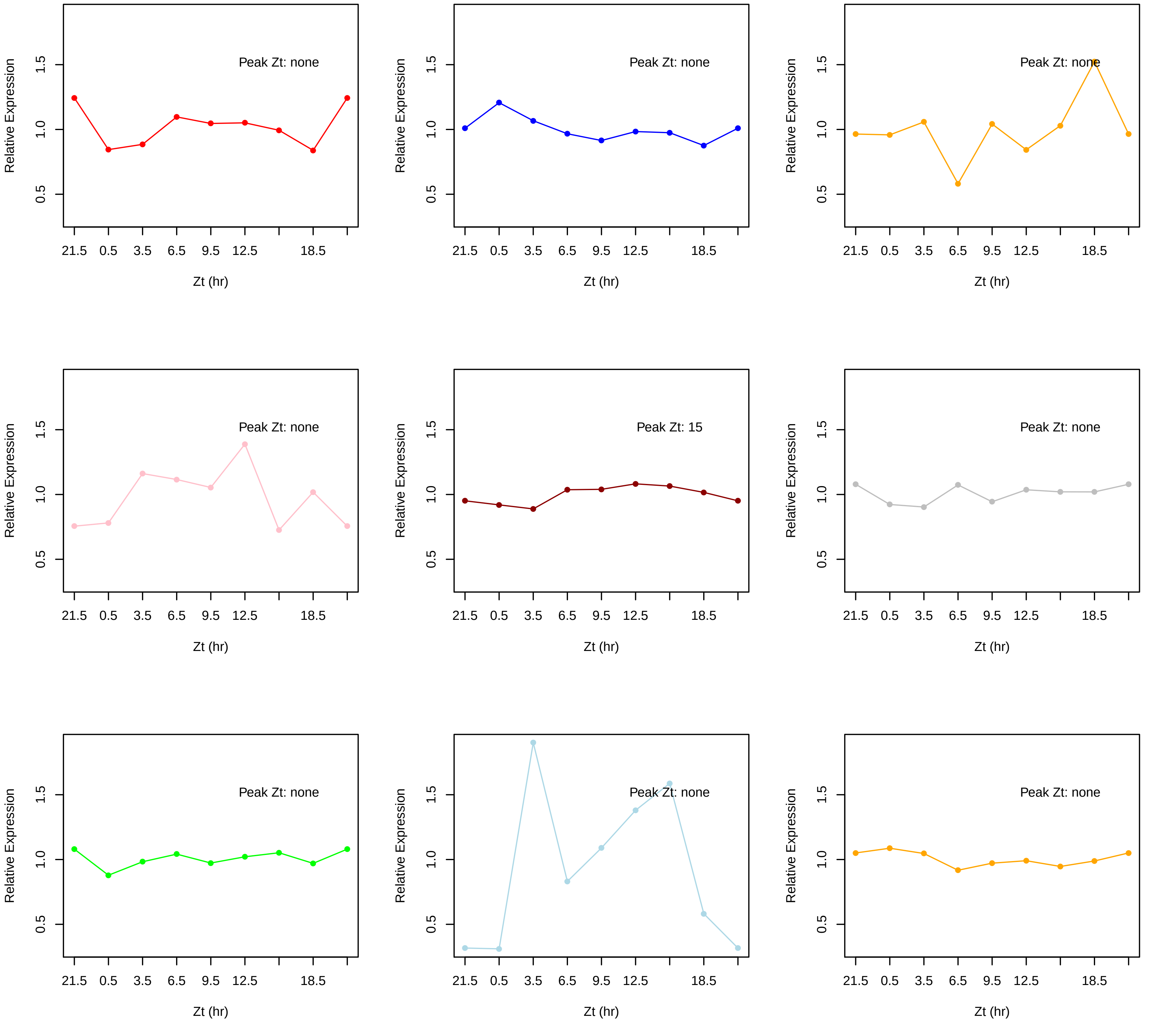

Singeleton: ENSXMAG00000024037 zgc:110006

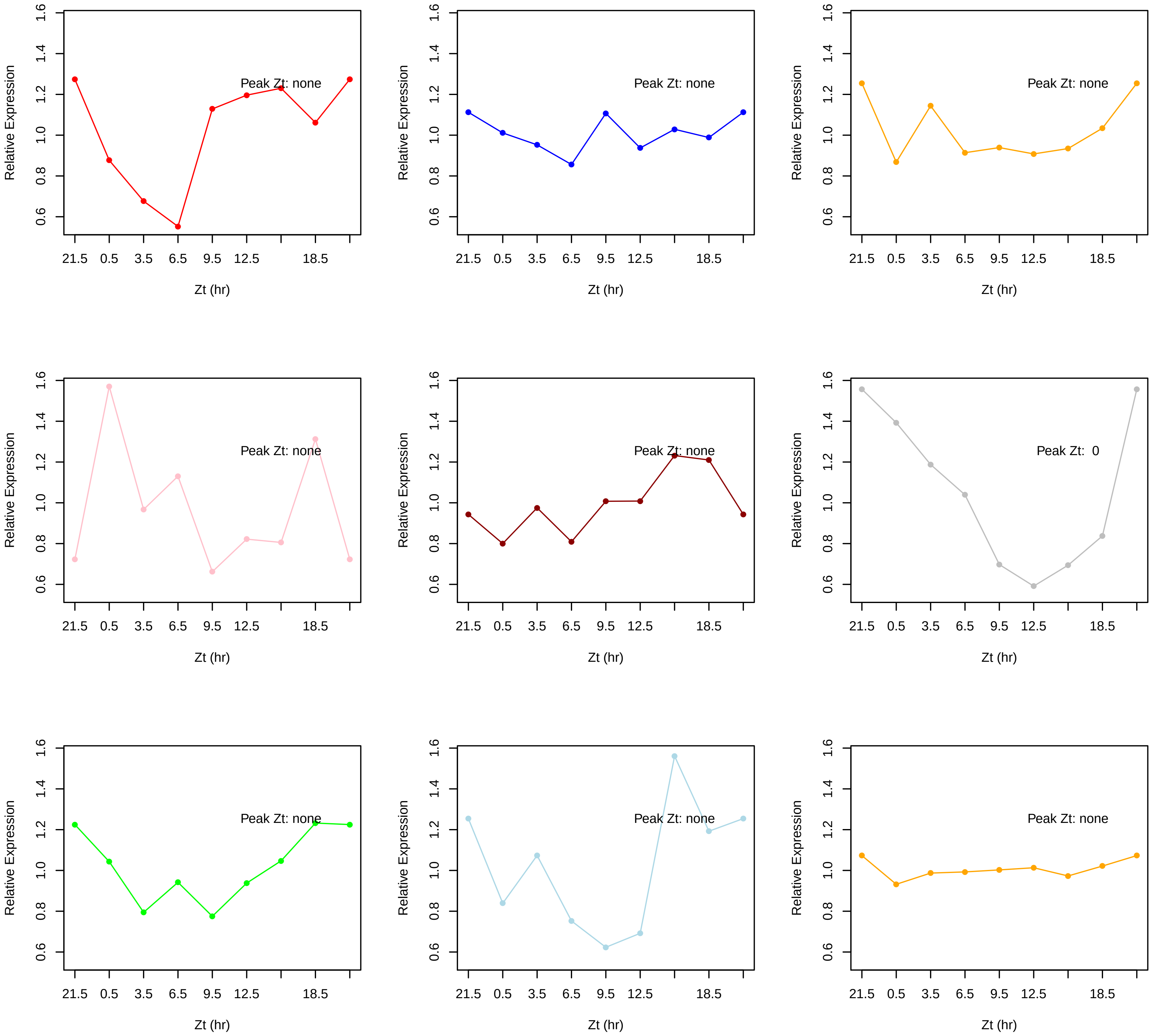

Singeleton: ENSXMAG00000027978 dnajc8

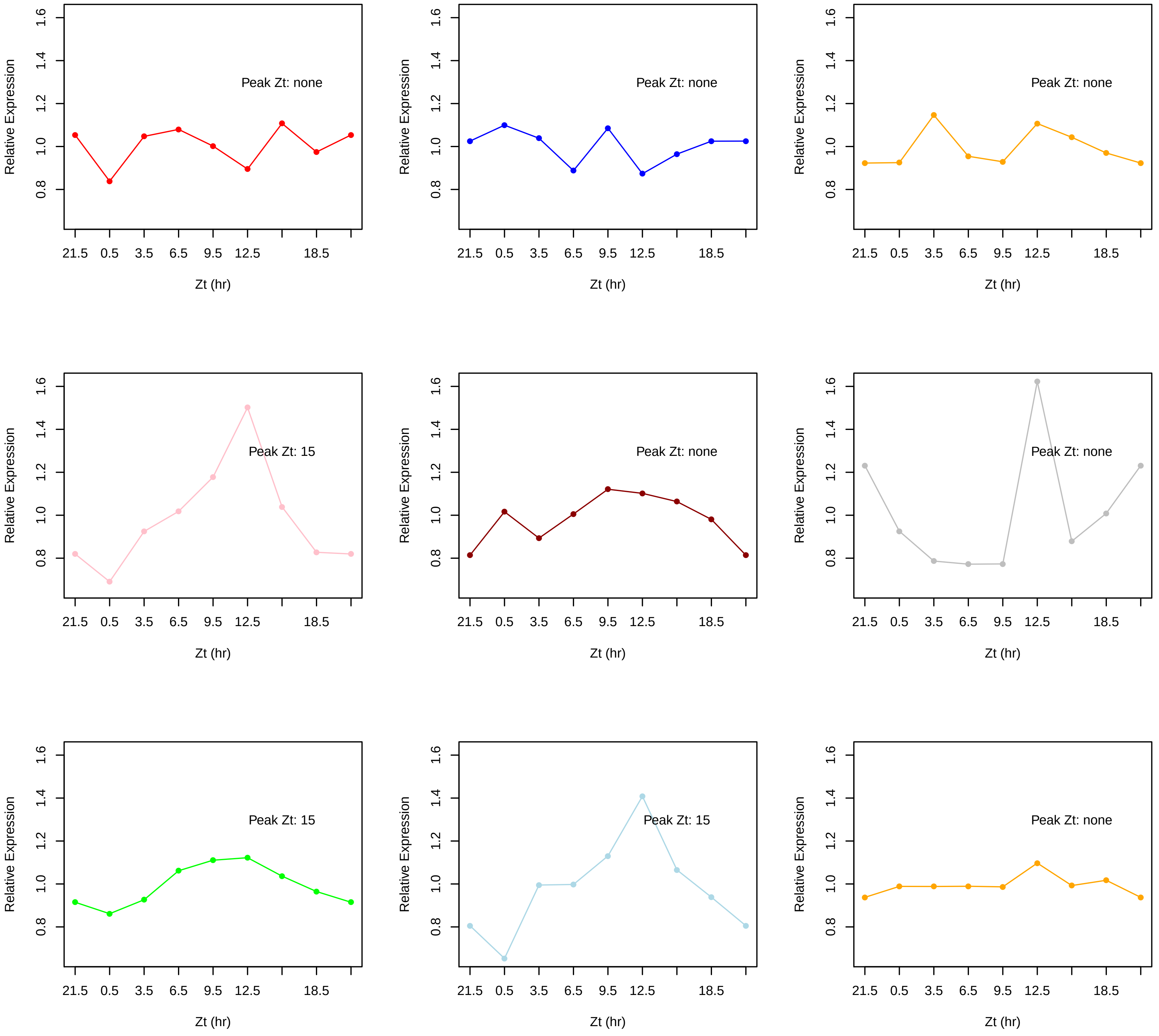

Singleton: ENSXMAG00000008431 vps53

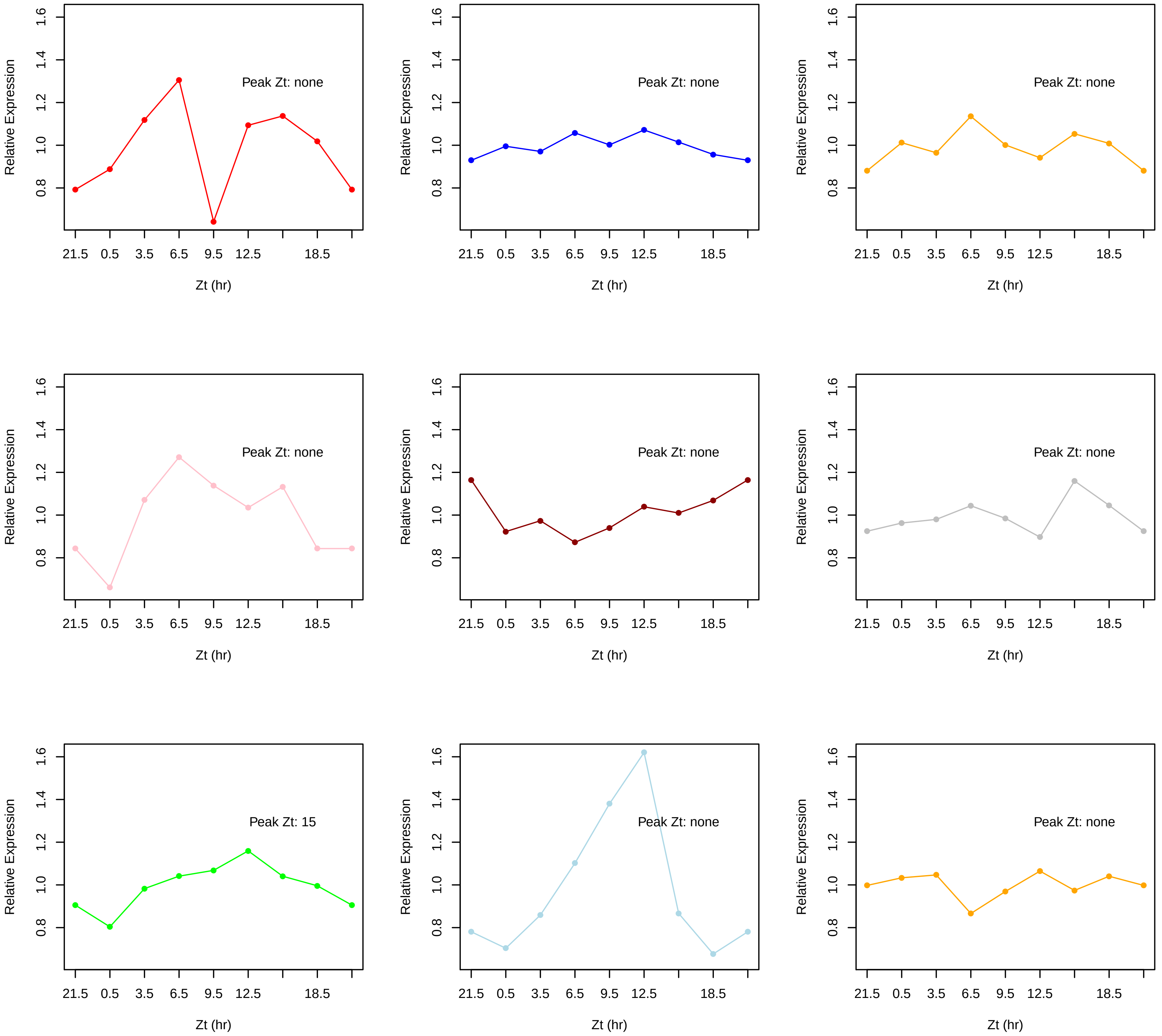

Singeleton: ENSXMAG00000003993 wls

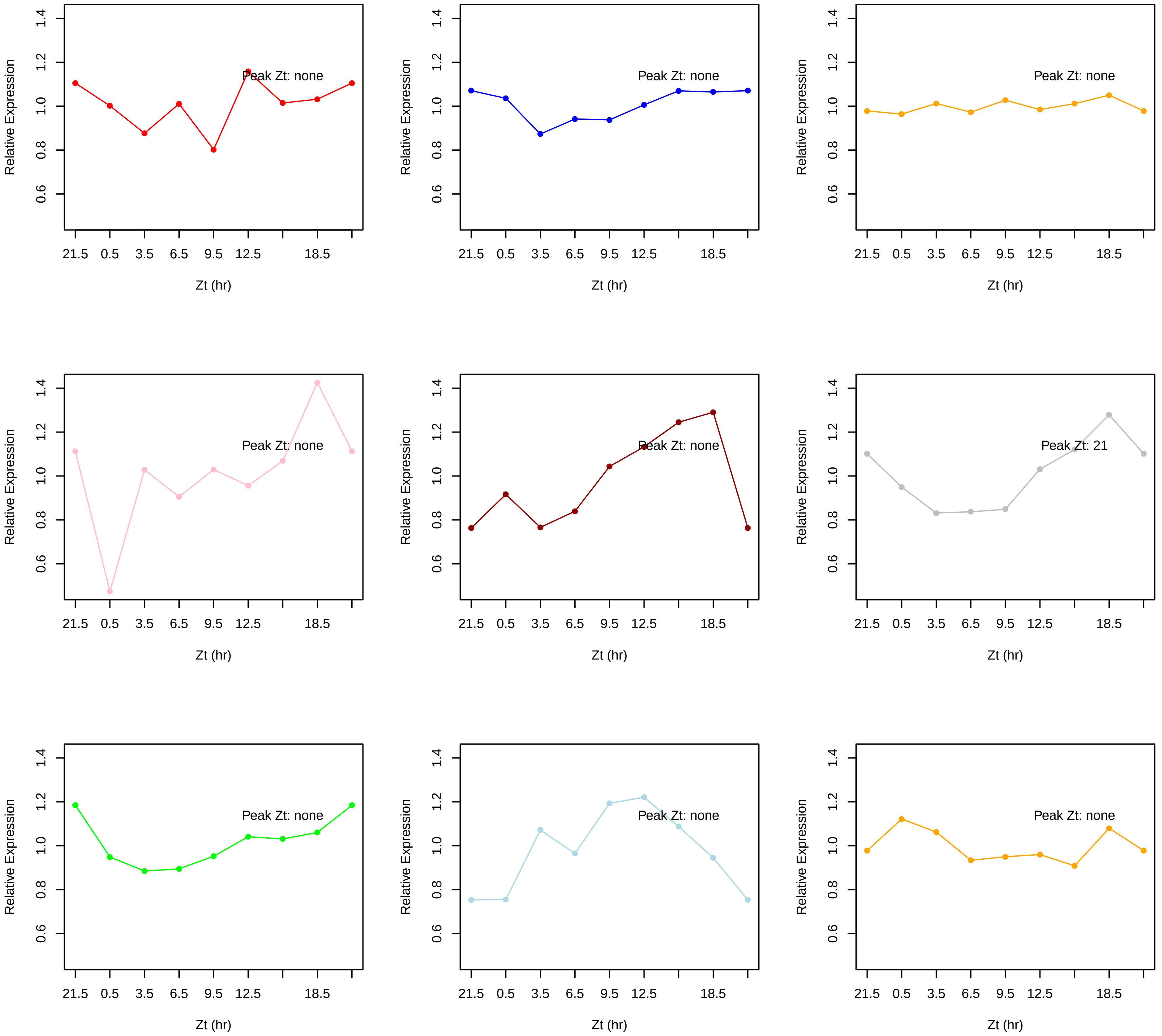

Singeleton: ENSXMAG00000012059 tjp3

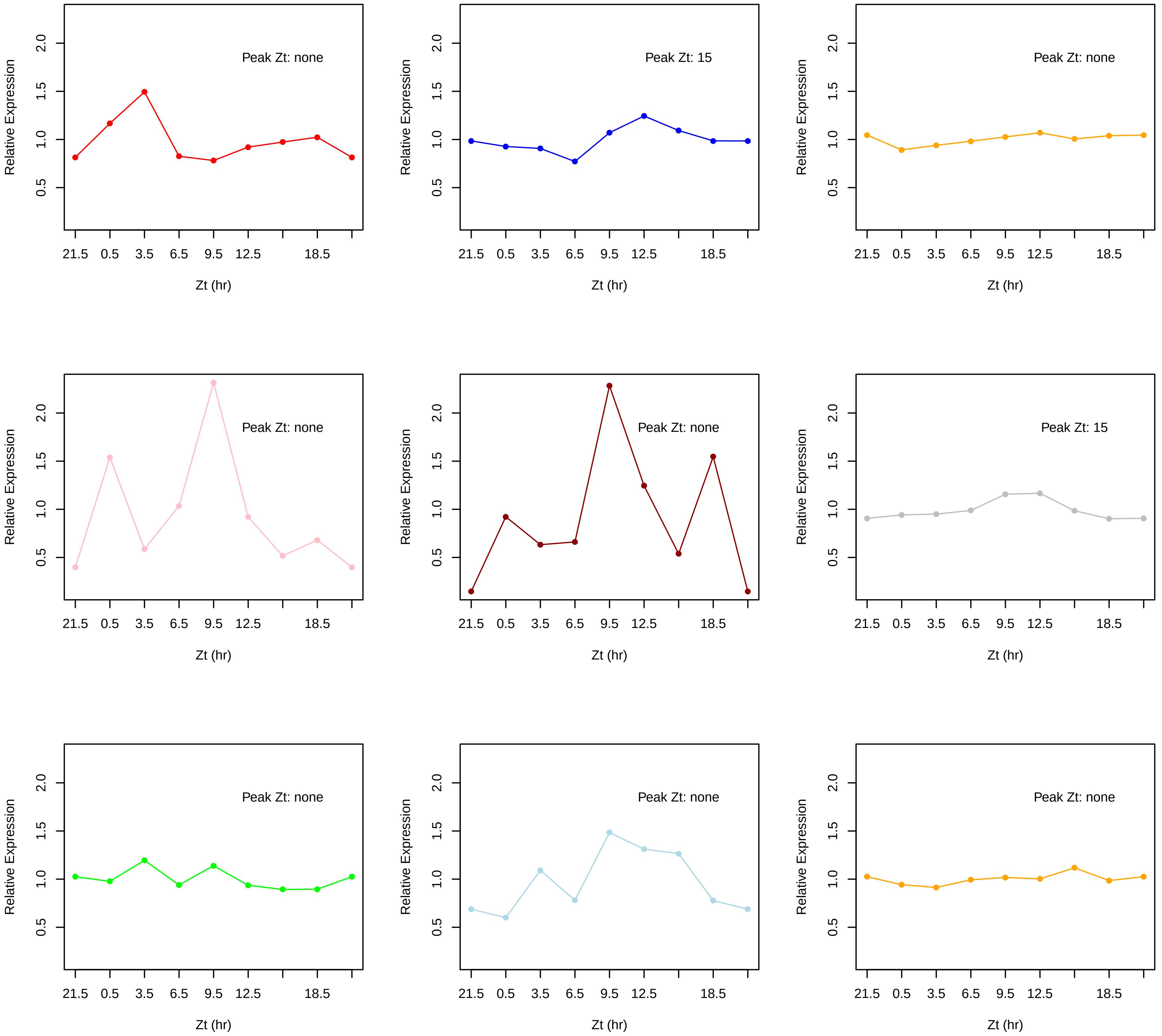

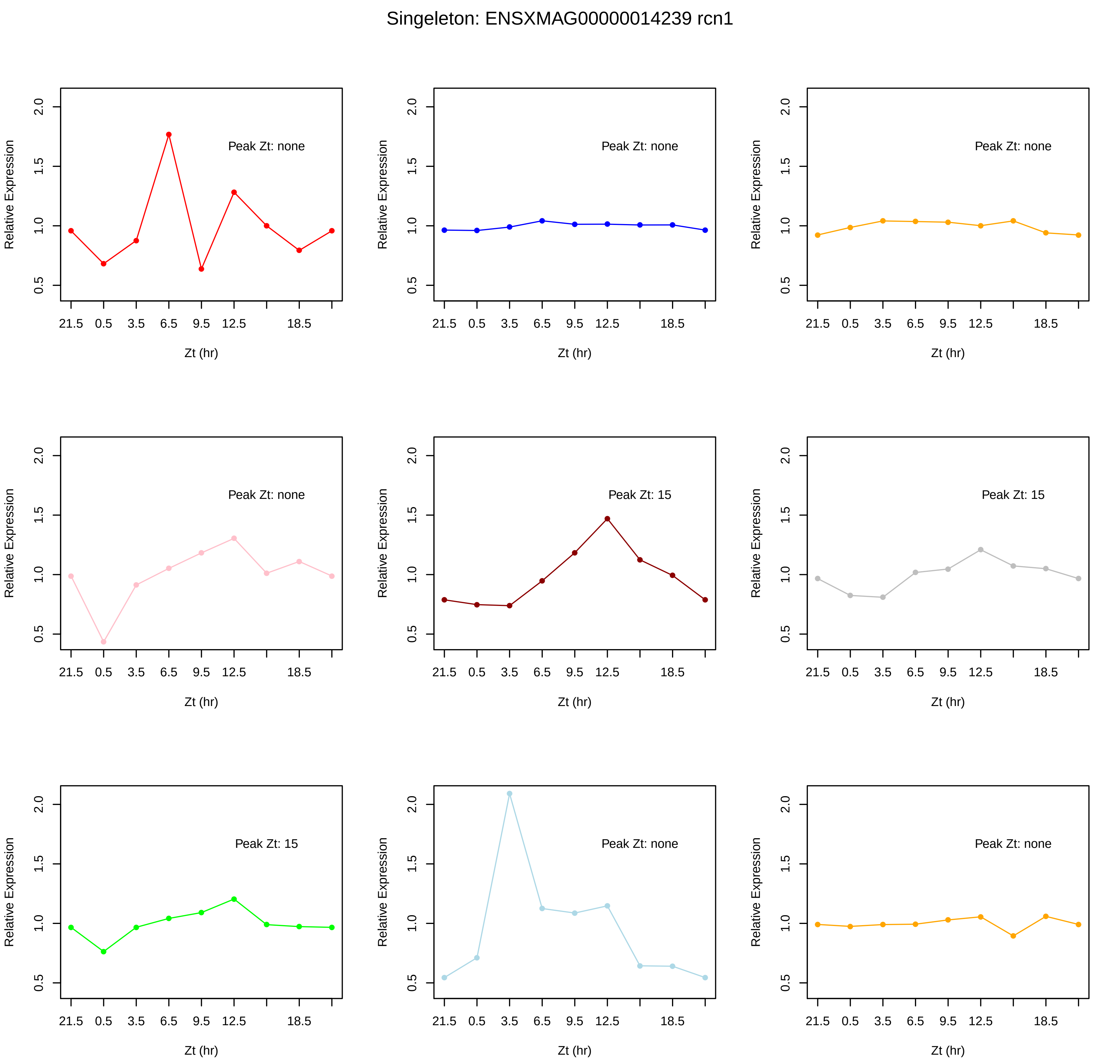
Singeleton: ENSXMAG00000014239 rcn1
Peak Zt: none
Peak Zt: none
Peak Zt: none
Peak Zt: none
Peak Zt: 15
Peak Zt: 15
Peak Zt: 15
Peak Zt: none
Peak Zt: none
Relative Expression
Zt (hr)
21.5 0.5 3.5 6.5 9.5 12.5 18.5
0.5 1.0 1.5 2.0

Singeleton: ENSXMAG00000018217 dao.2

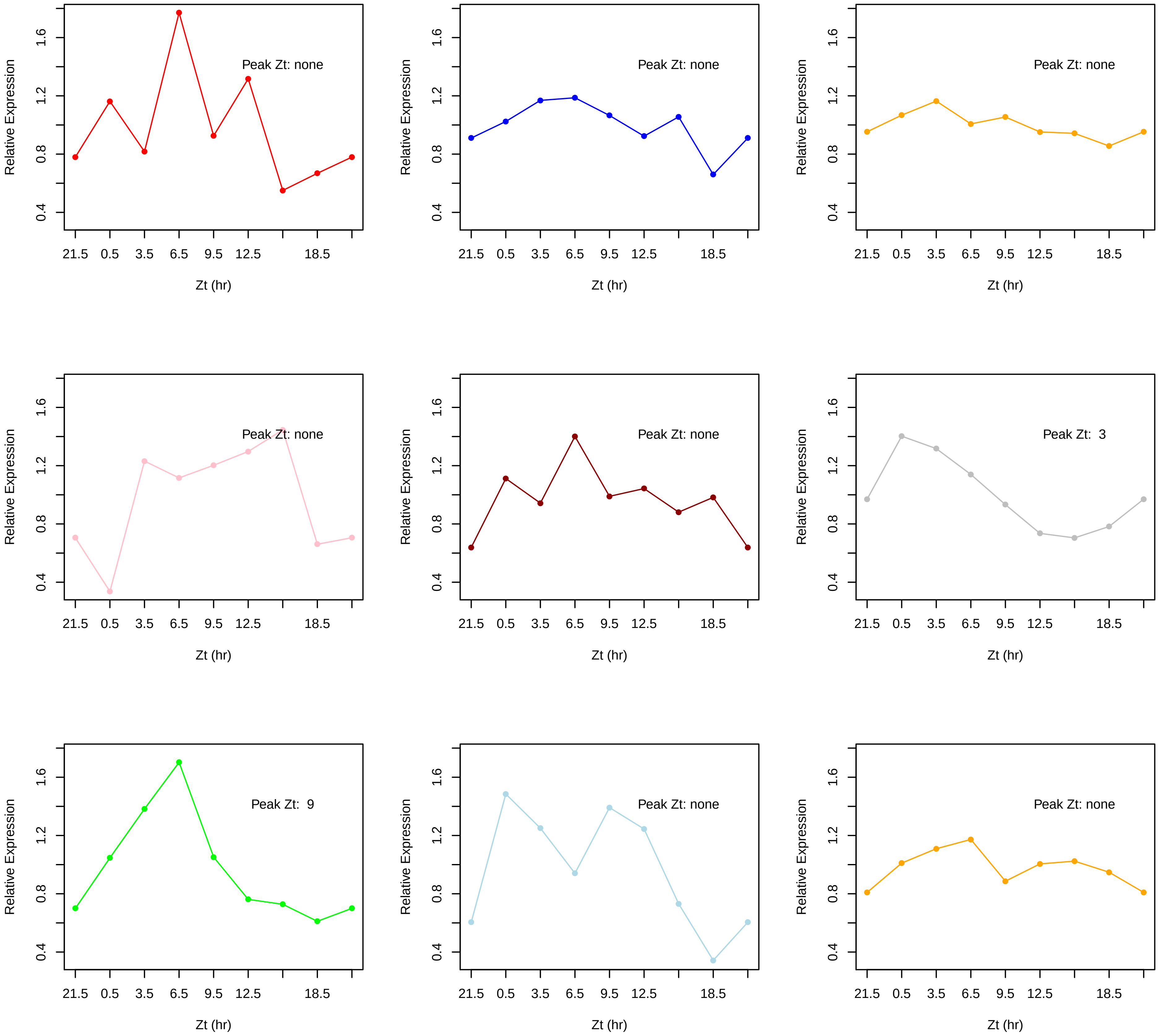

Singeleton: ENSXMAG00000003101 dr1

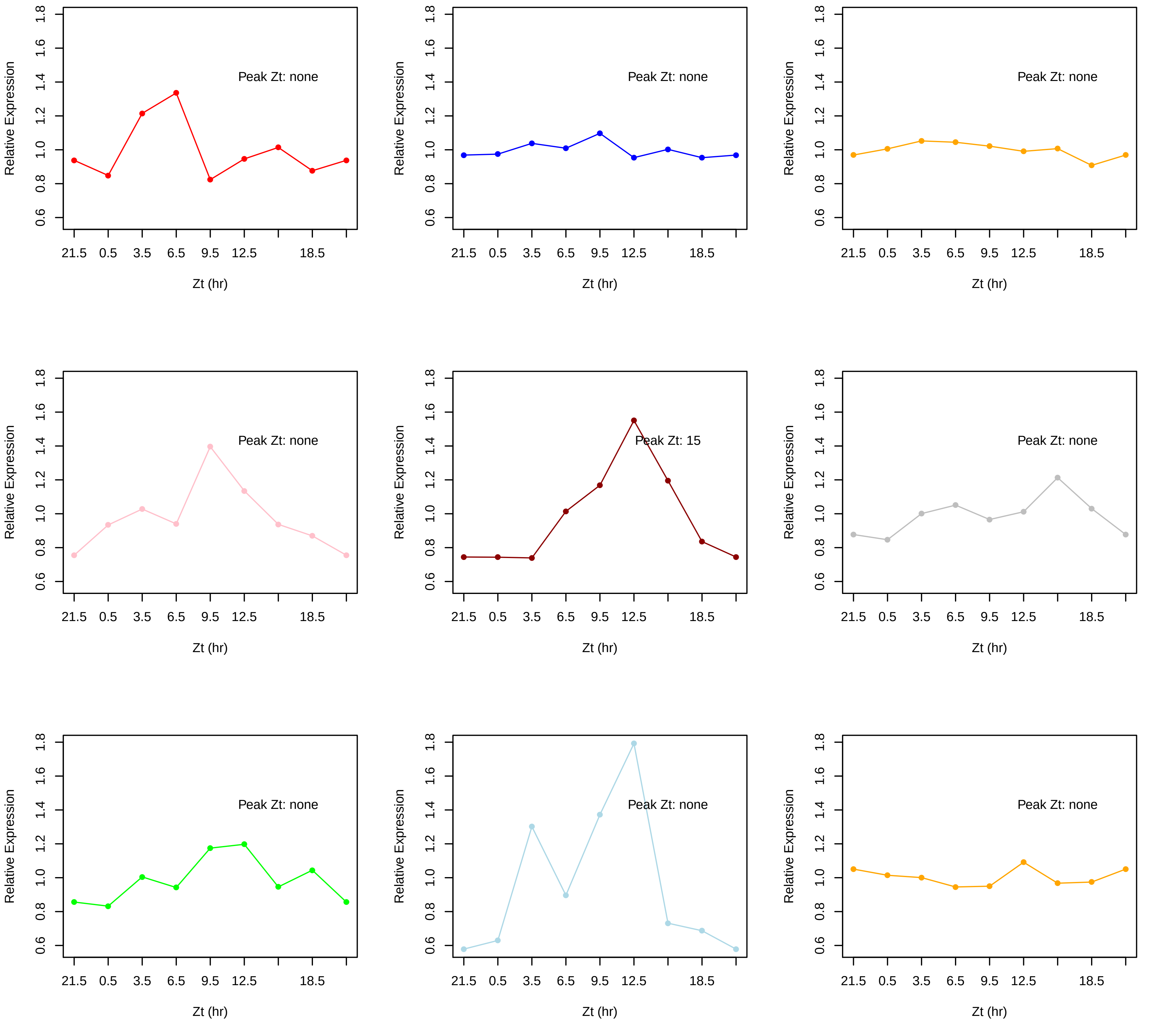

Singeleton: ENSXMAG00000008453 rflnb

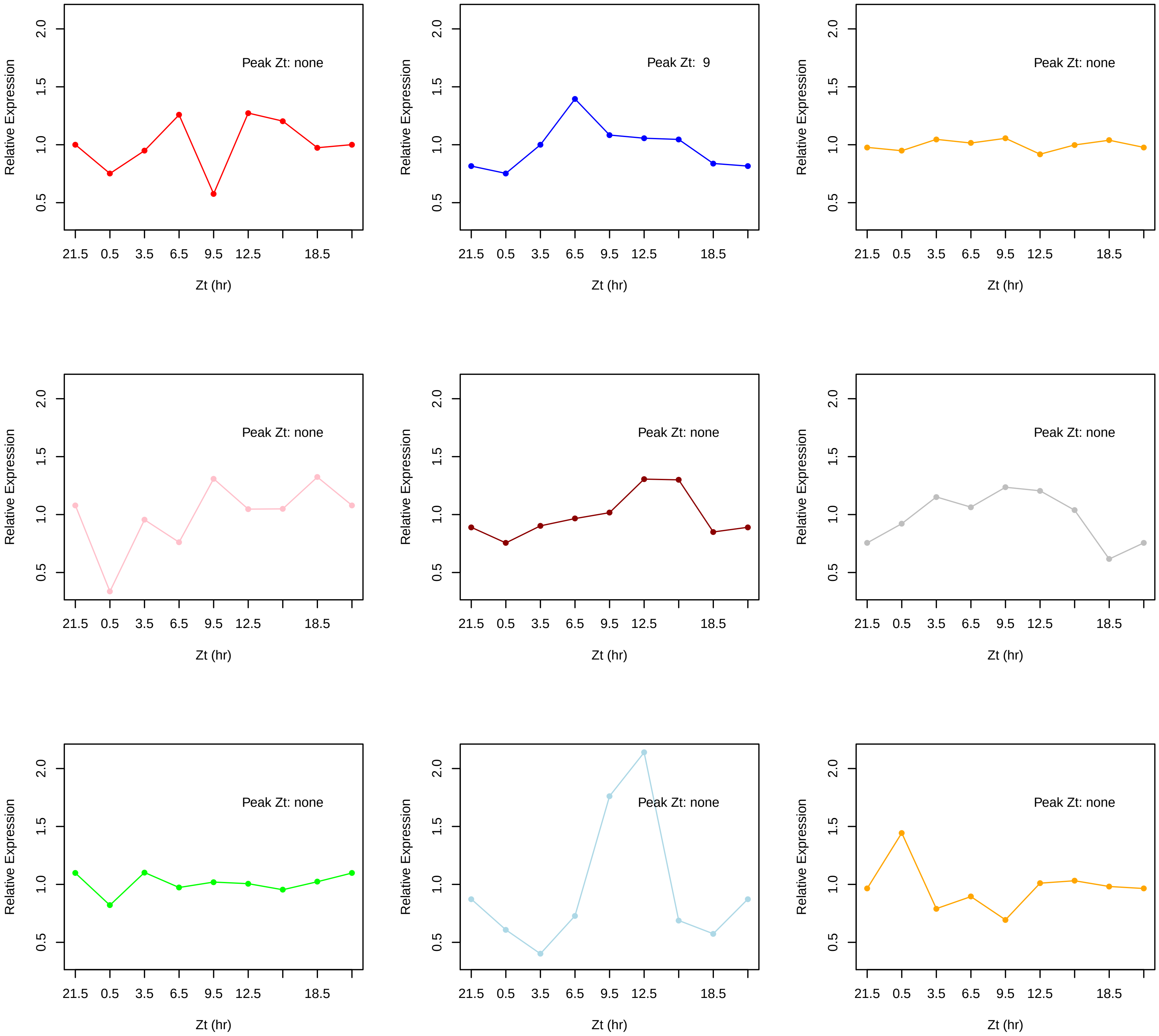

Singeleton: ENSXMAG00000003529 acot13

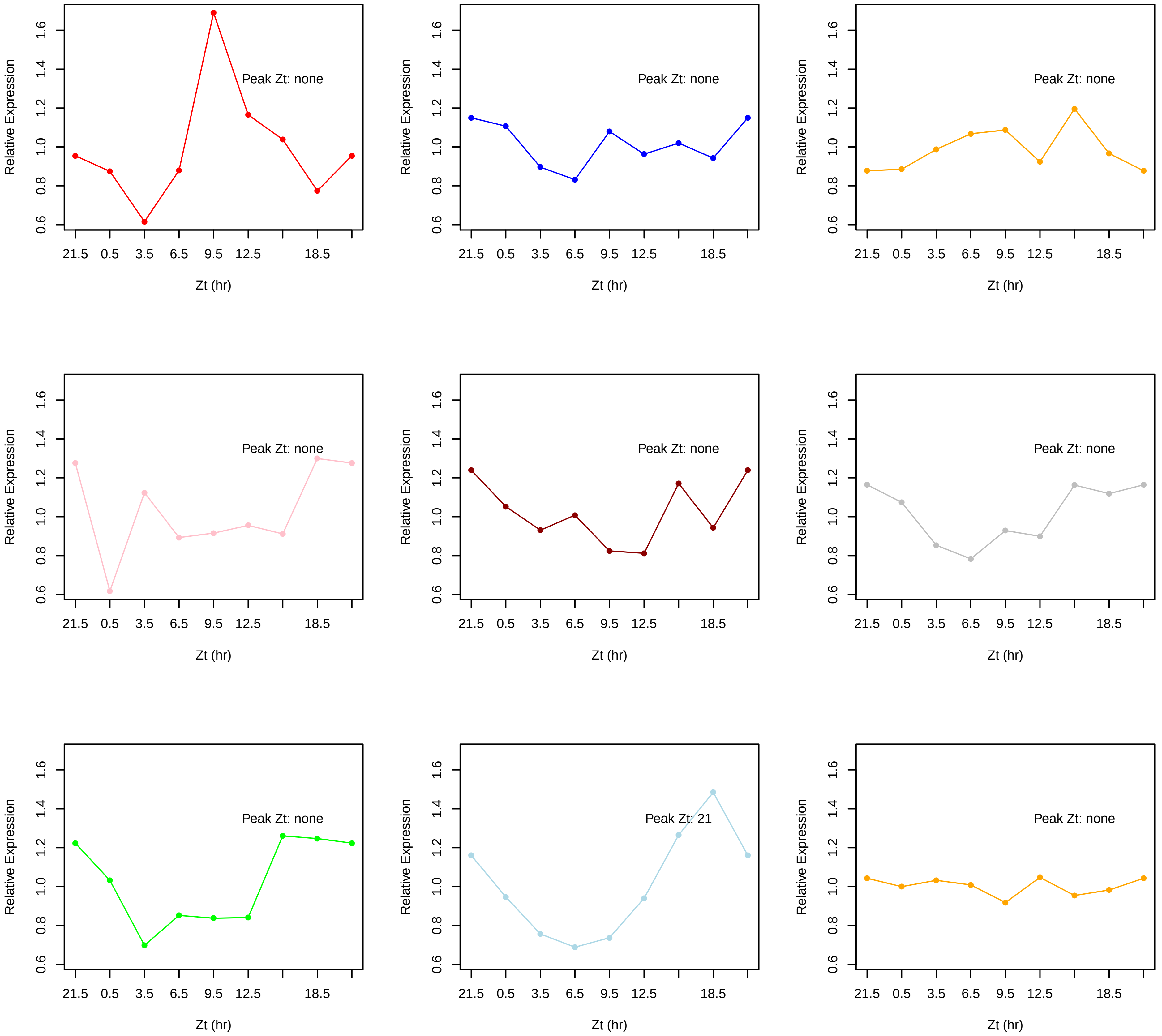

Singeleton: ENSXMAG00000001735 rxraa

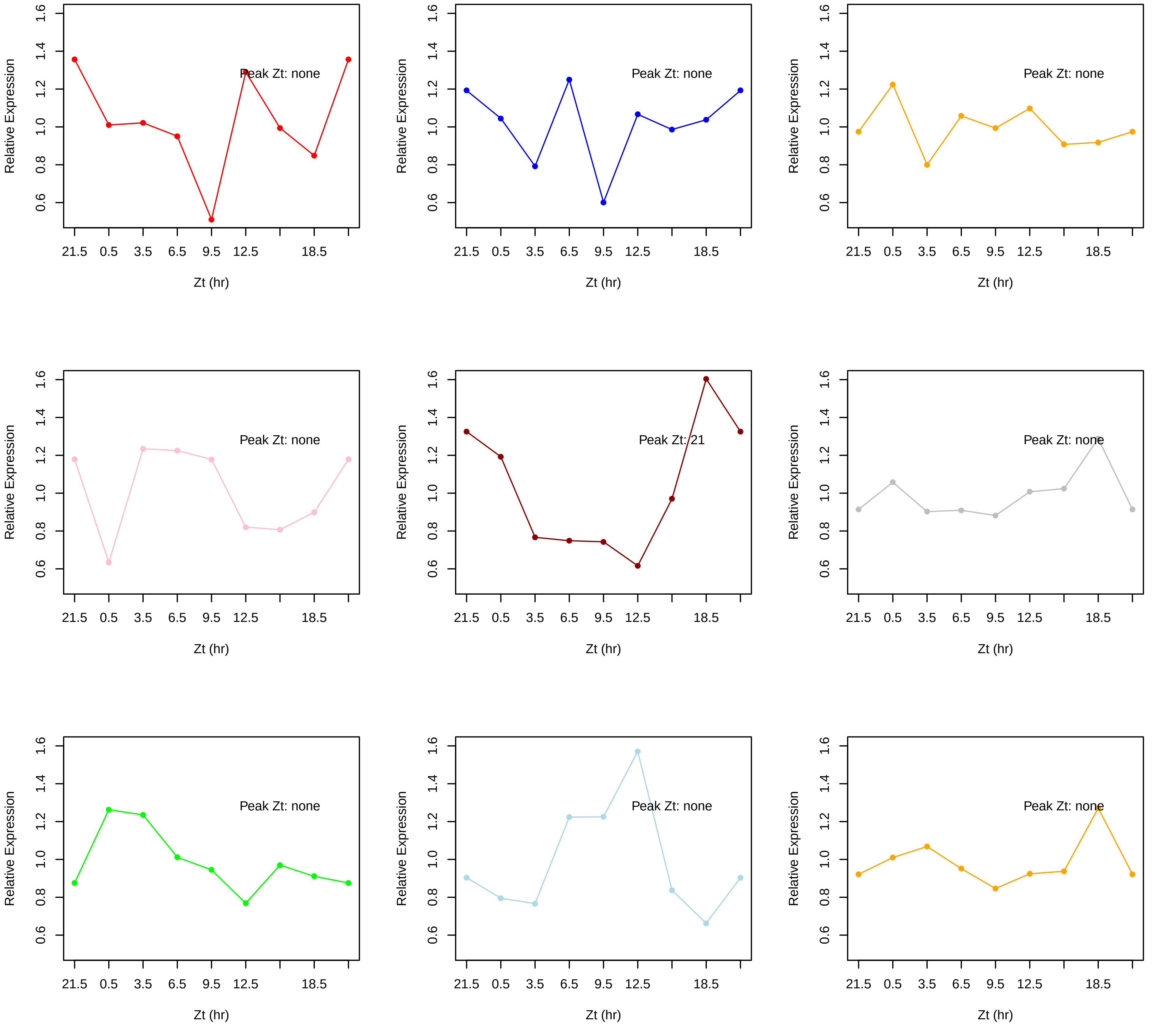

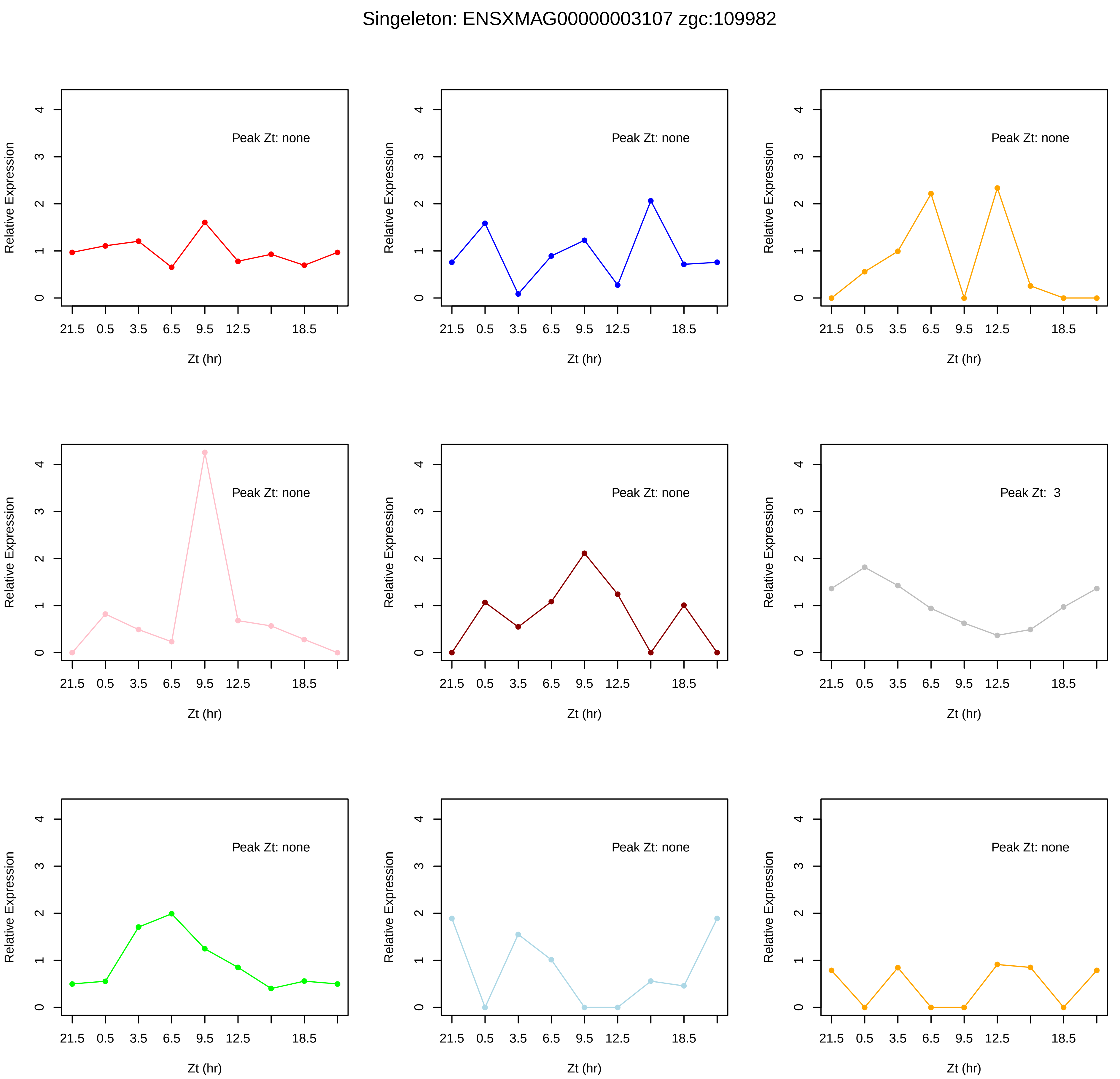

Singeleton: ENSXMAG00000003107 zgc:109982
Peak Zt: none
Peak Zt: none
Peak Zt: none
Peak Zt: none
Peak Zt: none
Peak Zt: 3
Peak Zt: none
Peak Zt: none
Peak Zt: none
Relative Expression
Zt (hr)
21.5
0.5
3.5
6.5
9.5
12.5
18.5

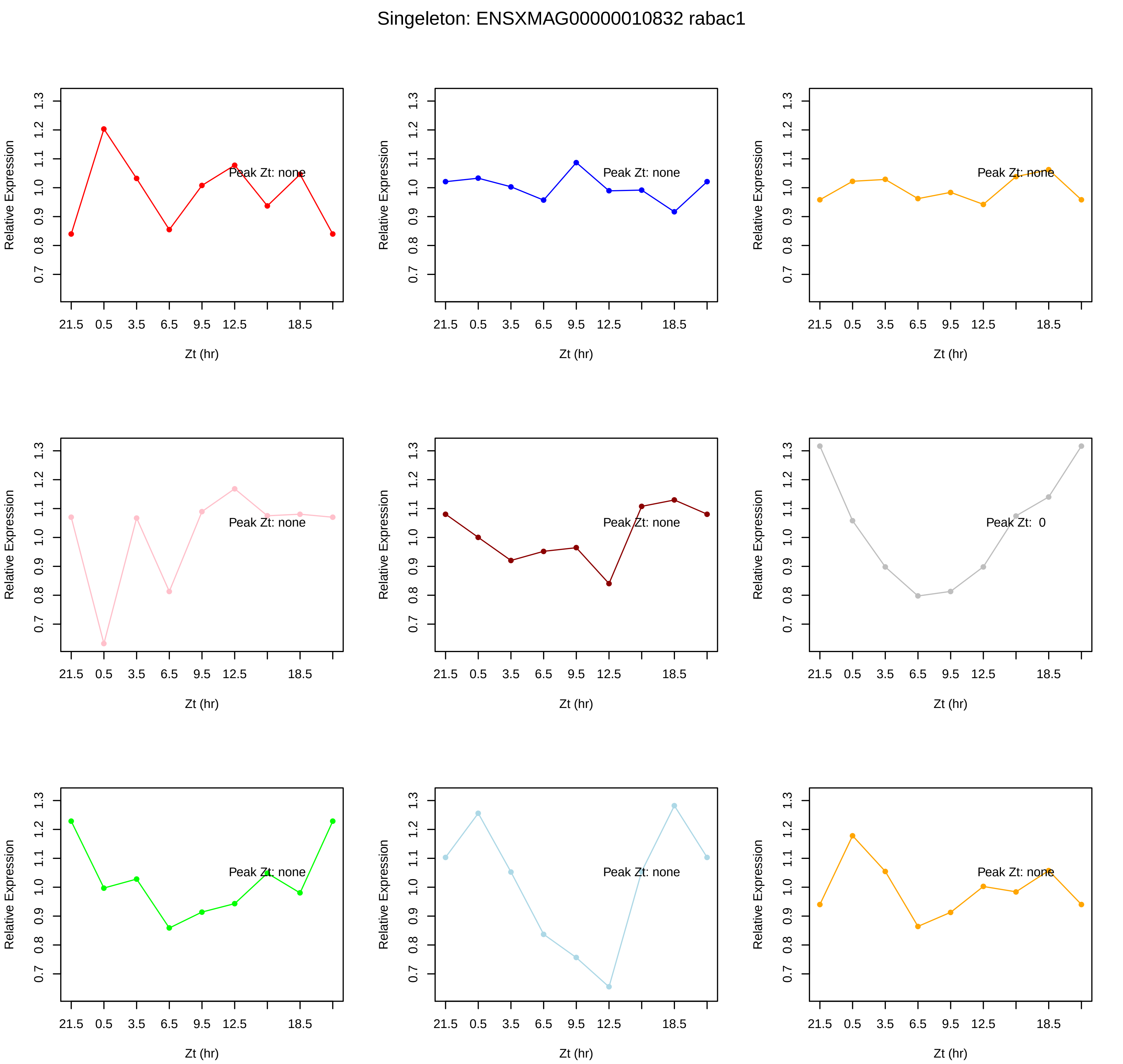

Singeleton: ENSXMAG00000010832 rabac1
Peak Zt: none
Peak Zt: none
Peak Zt: none
Peak Zt: none
Peak Zt: none
Peak Zt: 0
Peak Zt: none
Peak Zt: none
Peak Zt: none
Relative Expression
Zt (hr)
21.5
0.5
3.5
6.5
9.5
12.5
18.5
0.7
0.8
0.9
1.0
1.1
1.2
1.3

Singeleton: ENSXMAG00000008472 abhd15a

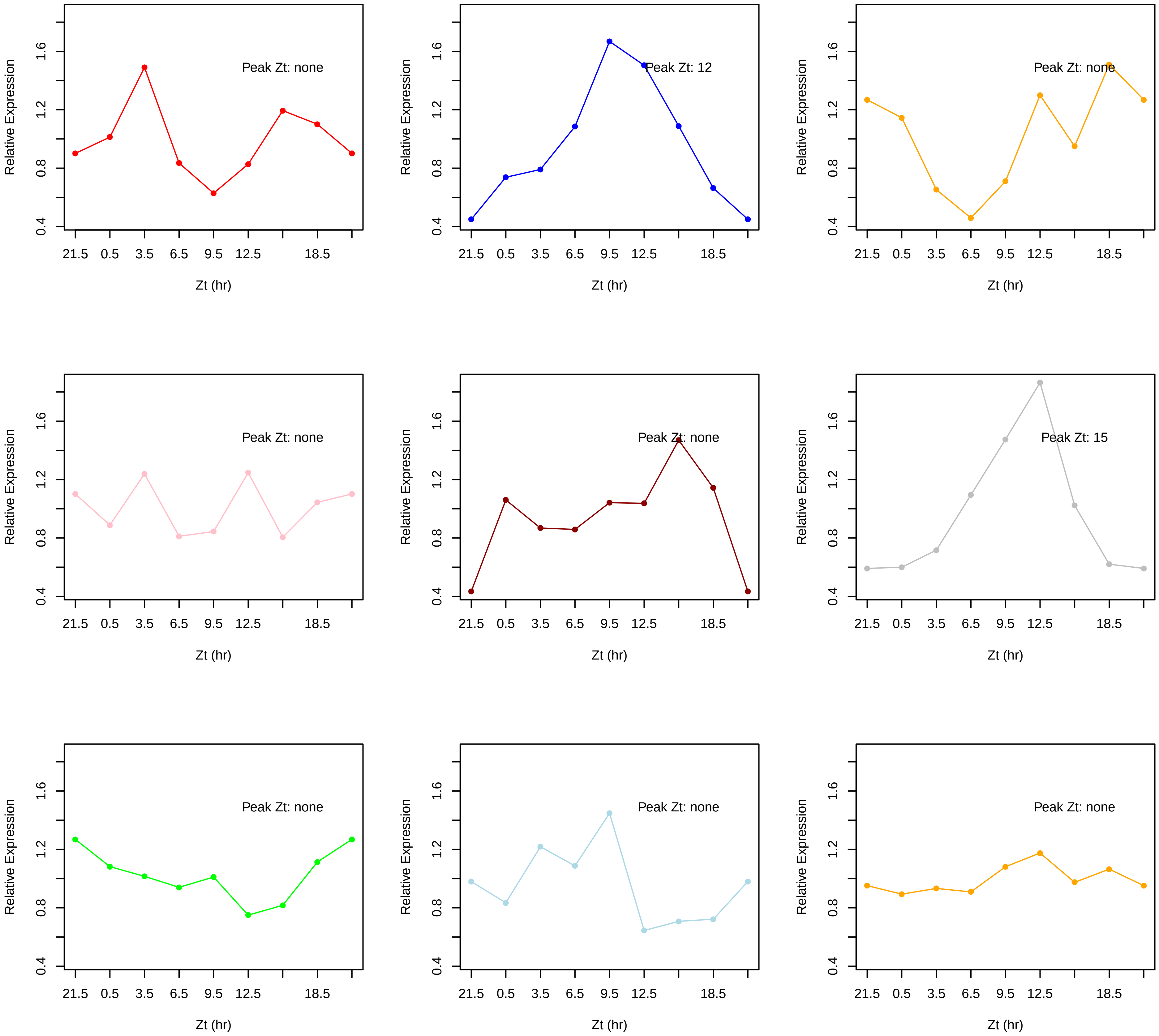

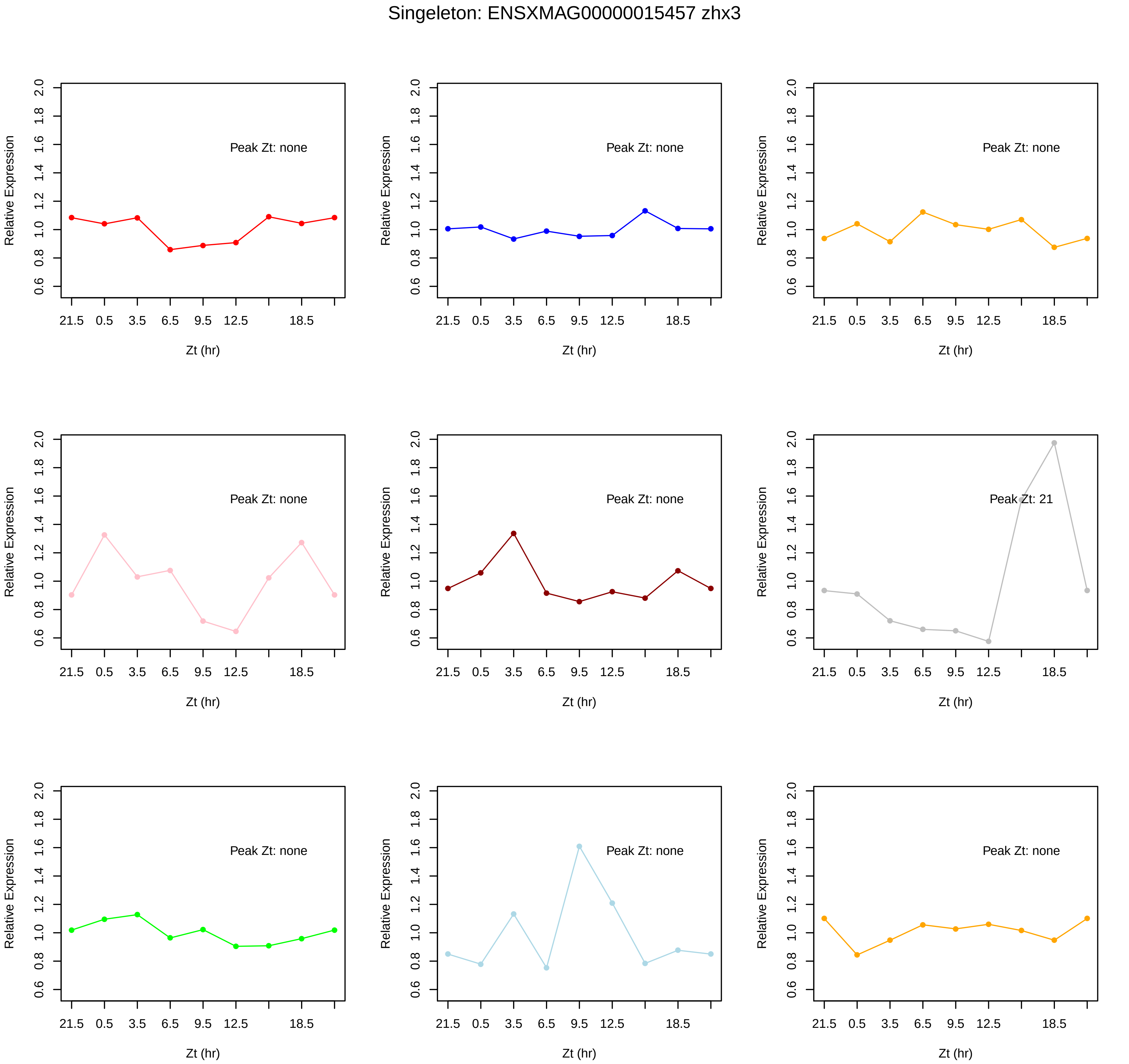

Singeleton: ENSXMAG00000015457 zhx3
Peak Zt: none
Peak Zt: none
Peak Zt: none
Peak Zt: none
Peak Zt: none
Peak Zt: 21
Peak Zt: none
Peak Zt: none
Peak Zt: none
Relative Expression
Zt (hr)

Singeleton: ENSXMAG00000013849 PRDM15

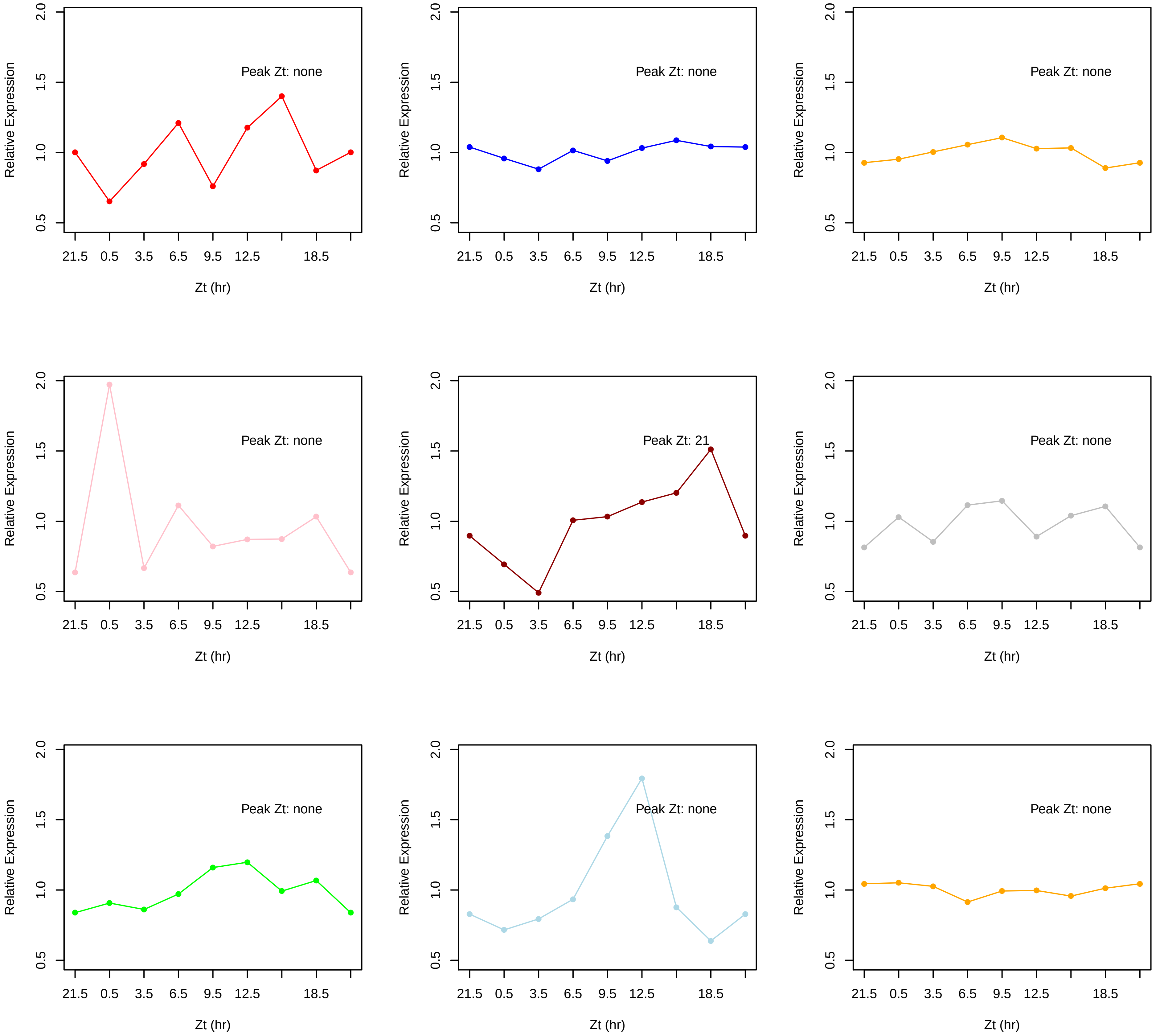

Singleton: ENSXMAG00000010827 dedd1

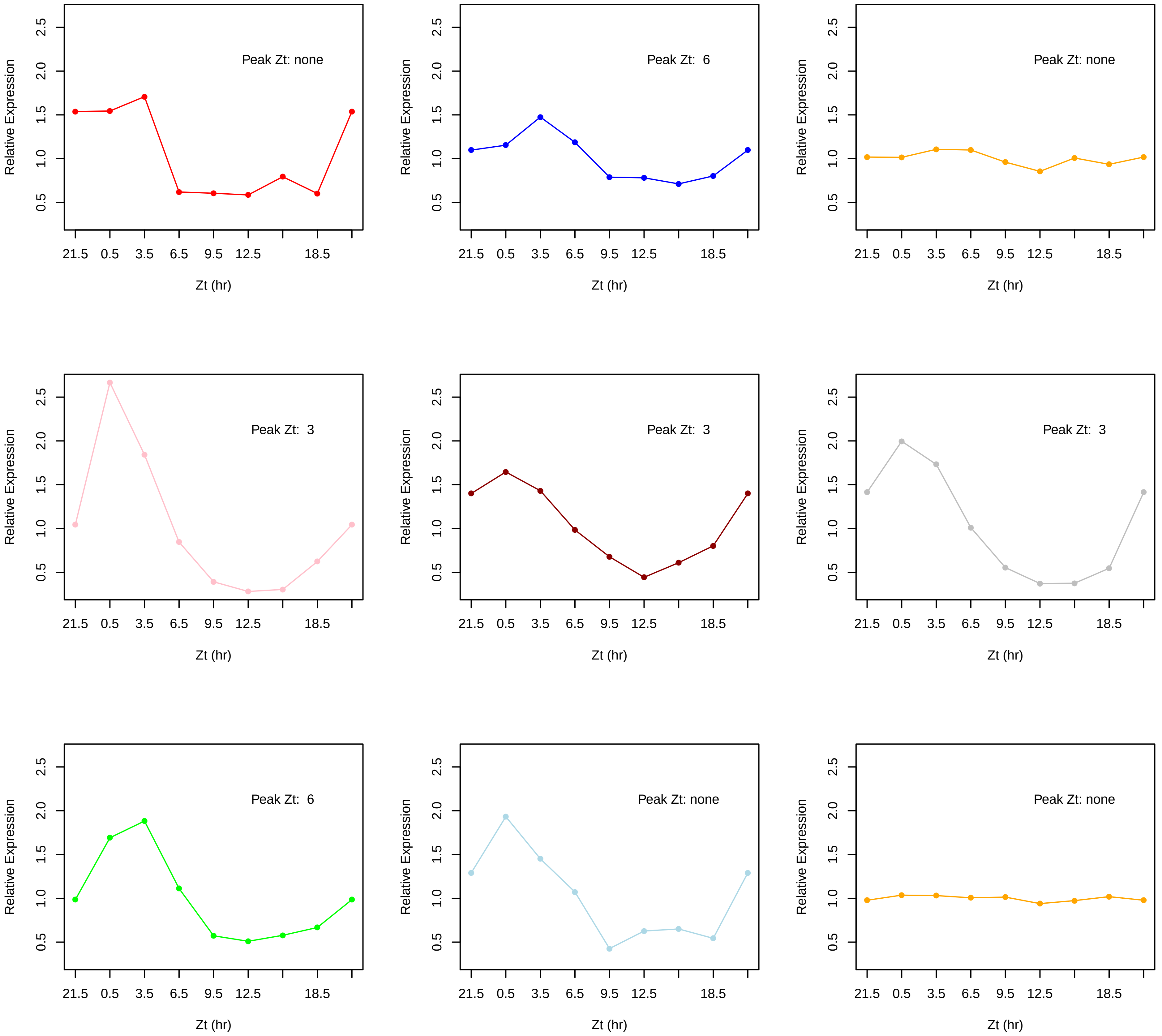

Singeleton: ENSXMAG00000008474 aipl1

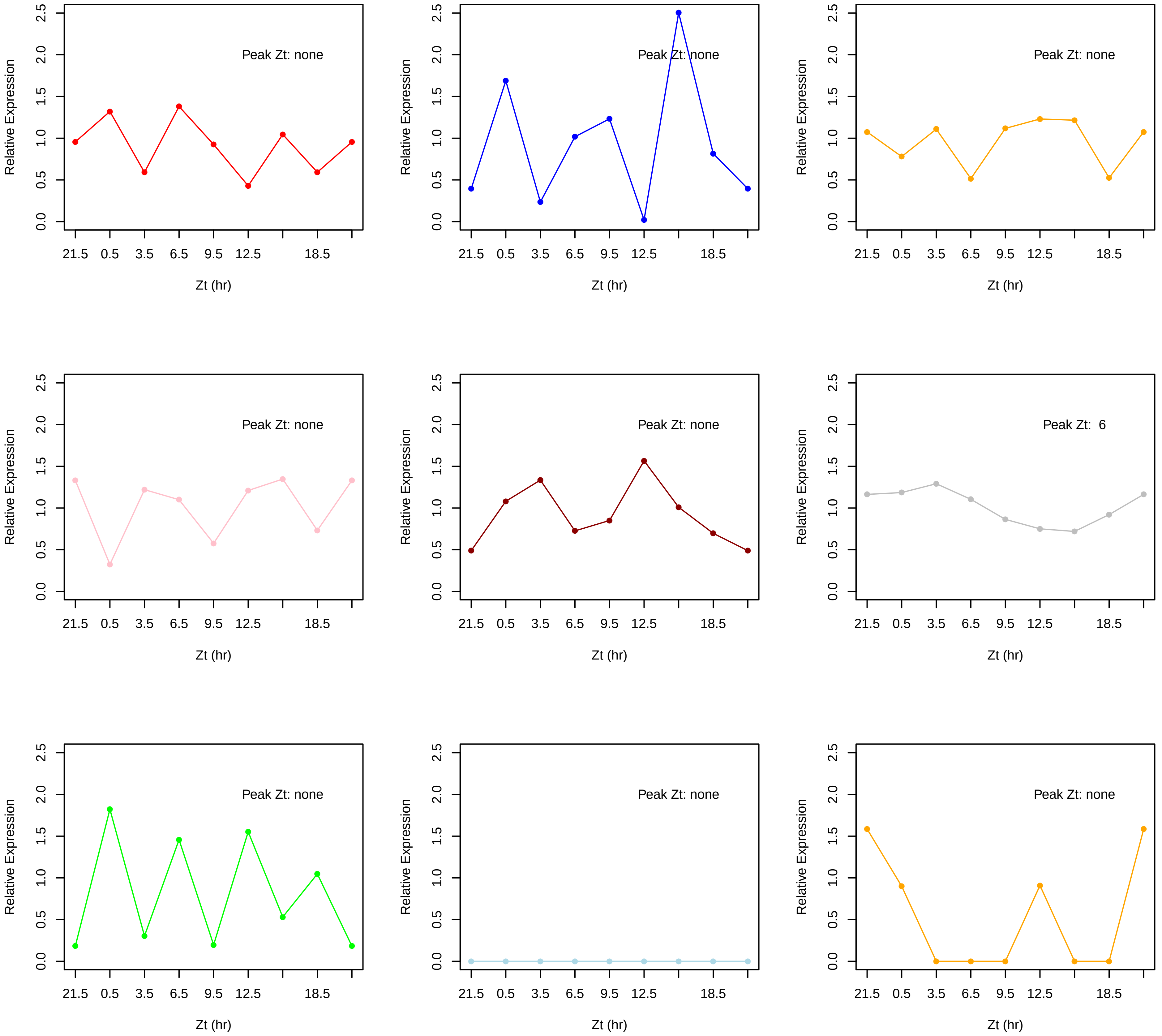

Singeleton: ENSXMAG00000013253 qkia

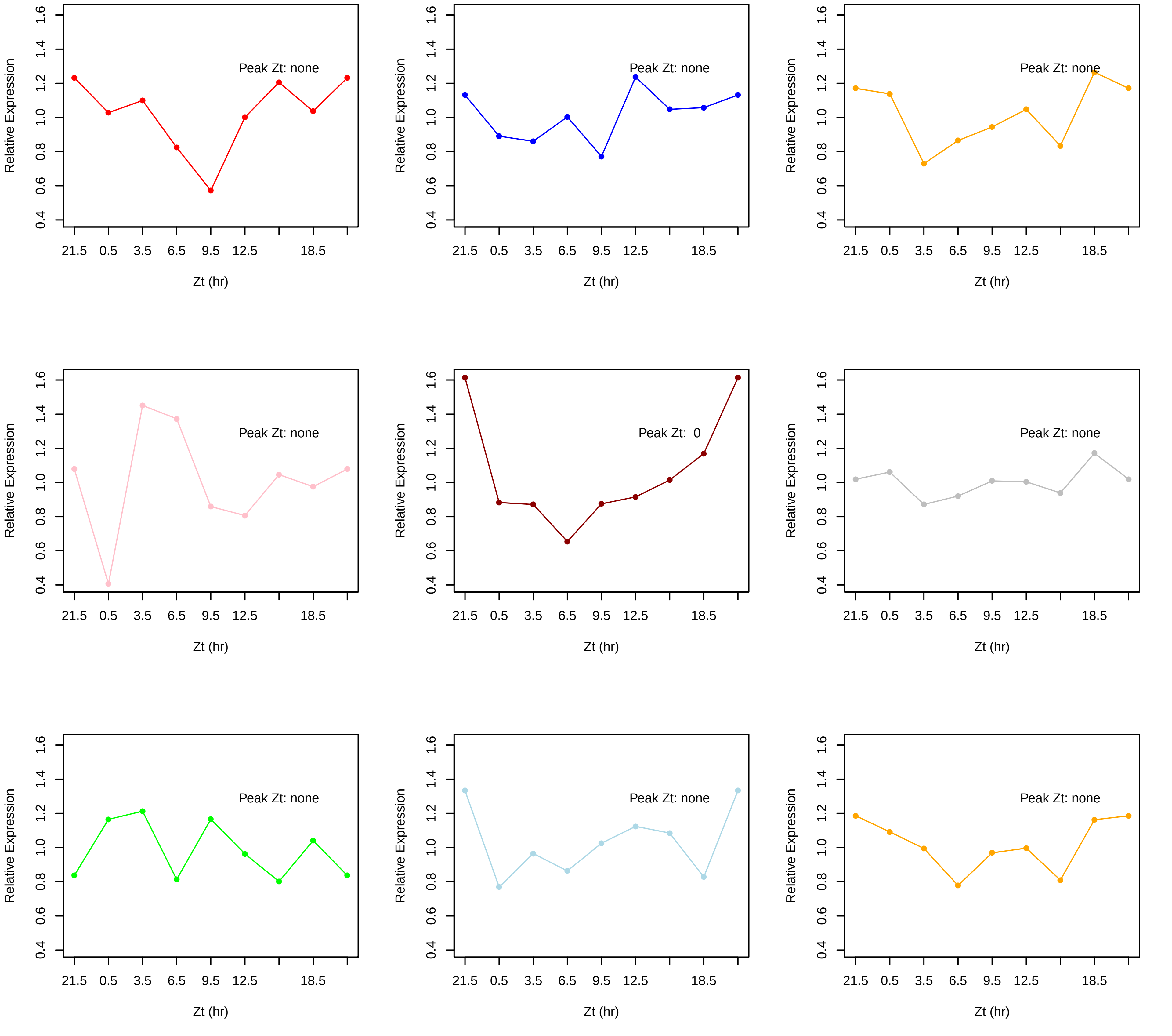

Singeleton: ENSXMAG00000019267

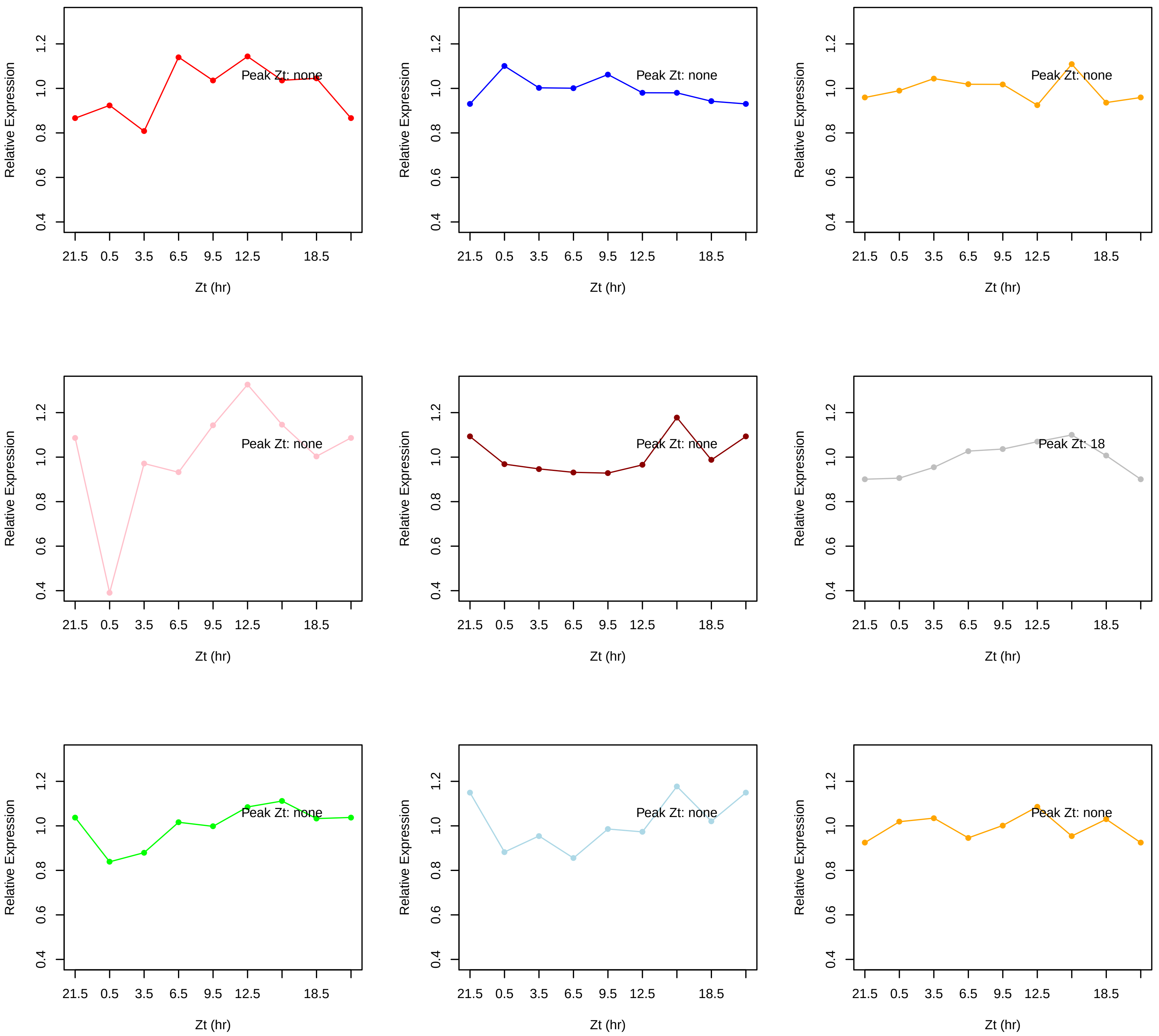

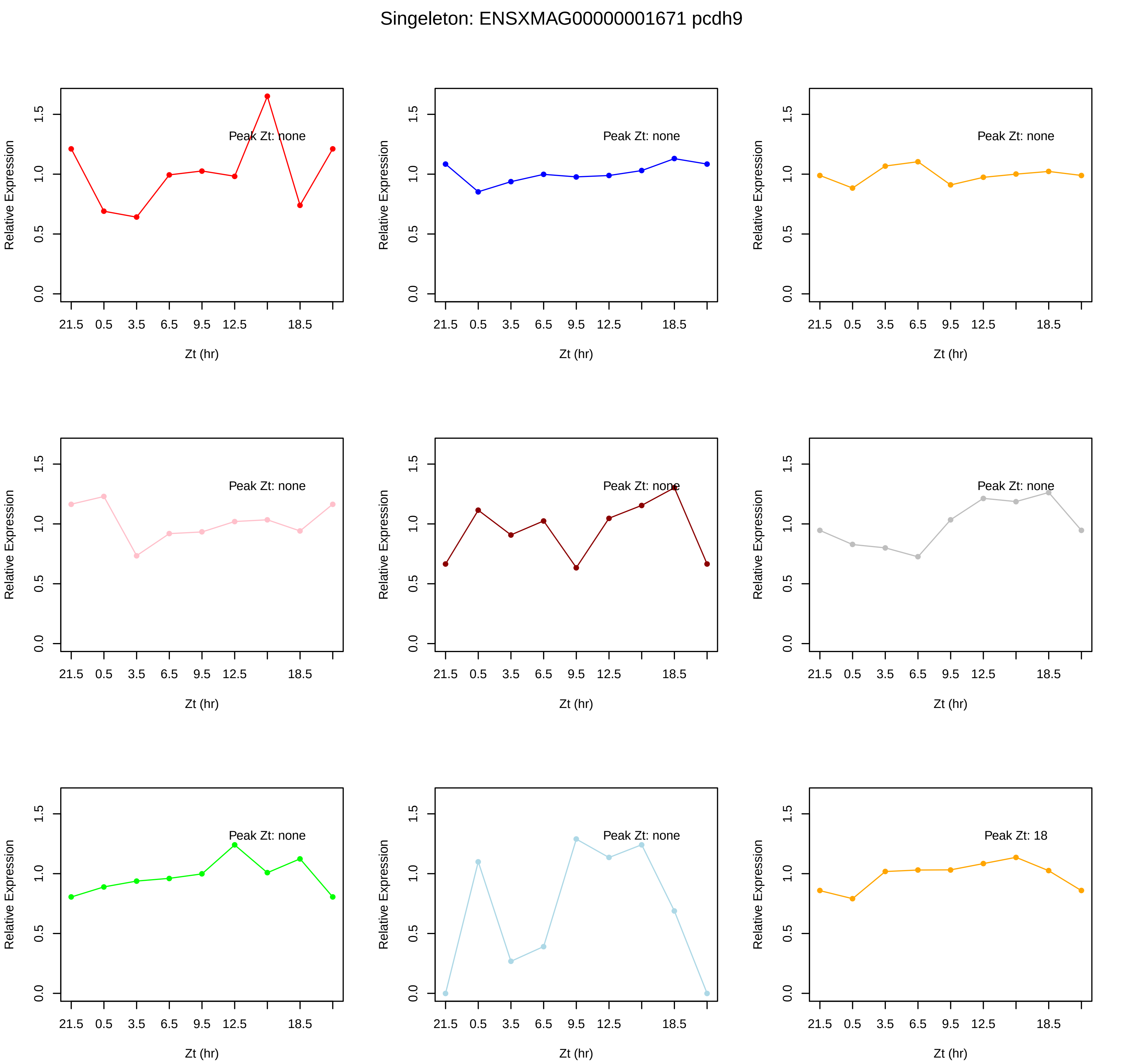
Singleton: ENSXMAG00000001671 pcdh9
Peak Zt: none
Peak Zt: none
Peak Zt: none
Peak Zt: none
Peak Zt: none
Peak Zt: none
Peak Zt: none
Peak Zt: none
Peak Zt: 18
Relative Expression
Zt (hr)
0.0
0.5
1.0
1.5
21.5
0.5
3.5
6.5
9.5
12.5
18.5

Singeleton: ENSXMAG00000004056 ccdc18

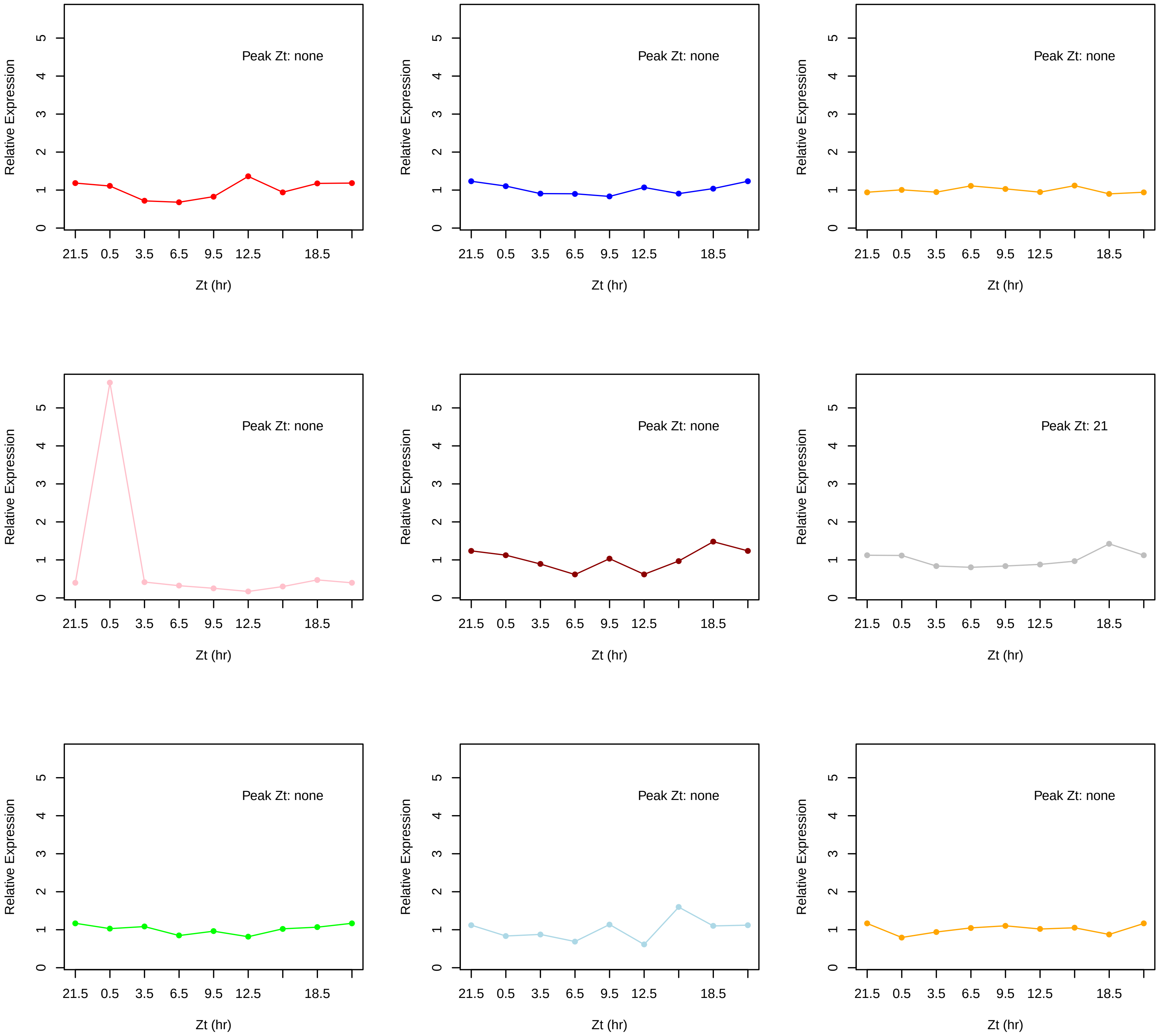

Singeleton: ENSXMAG00000014310 immp1l

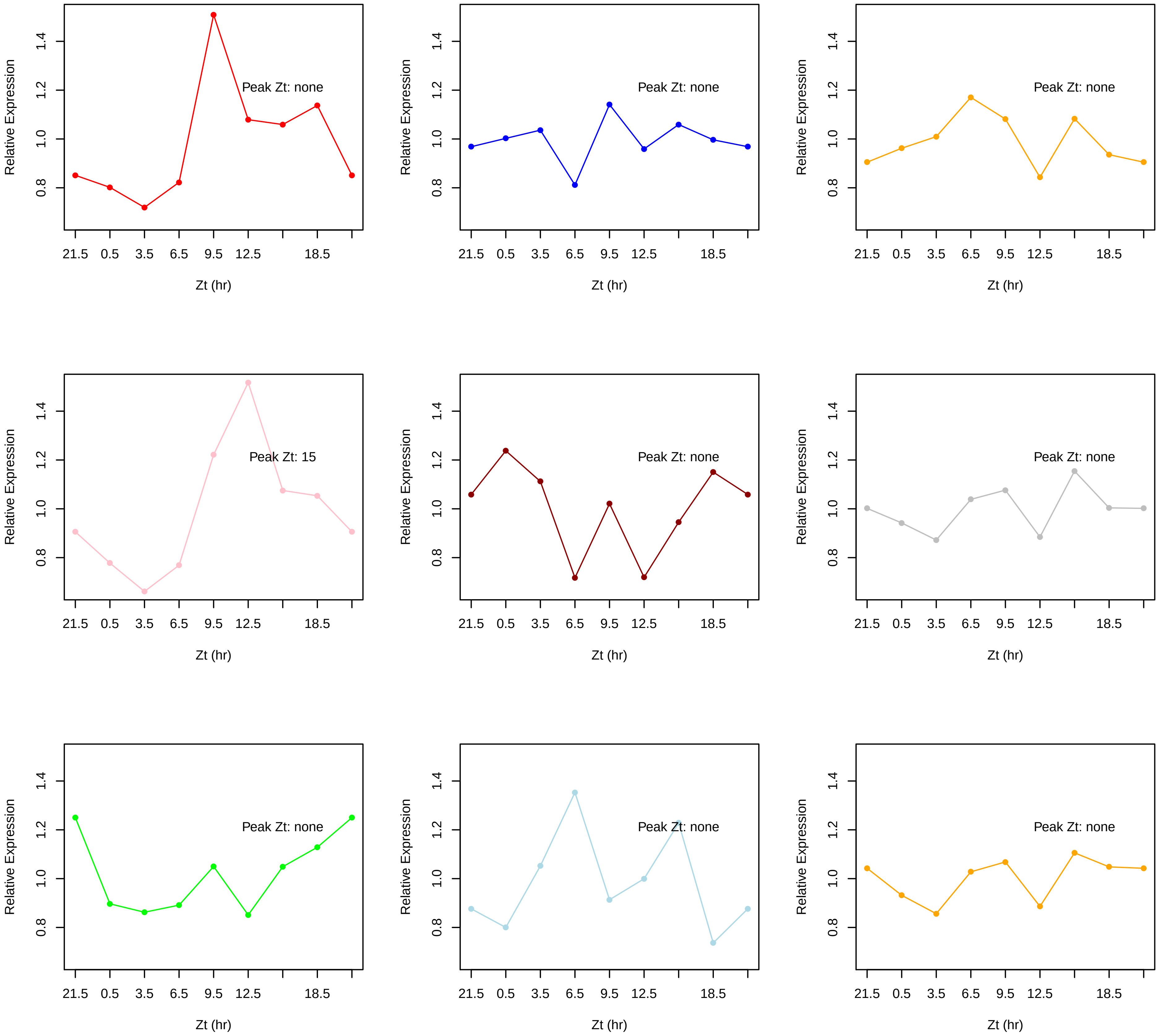

Singleton: ENSXMAG00000007902 lancl1

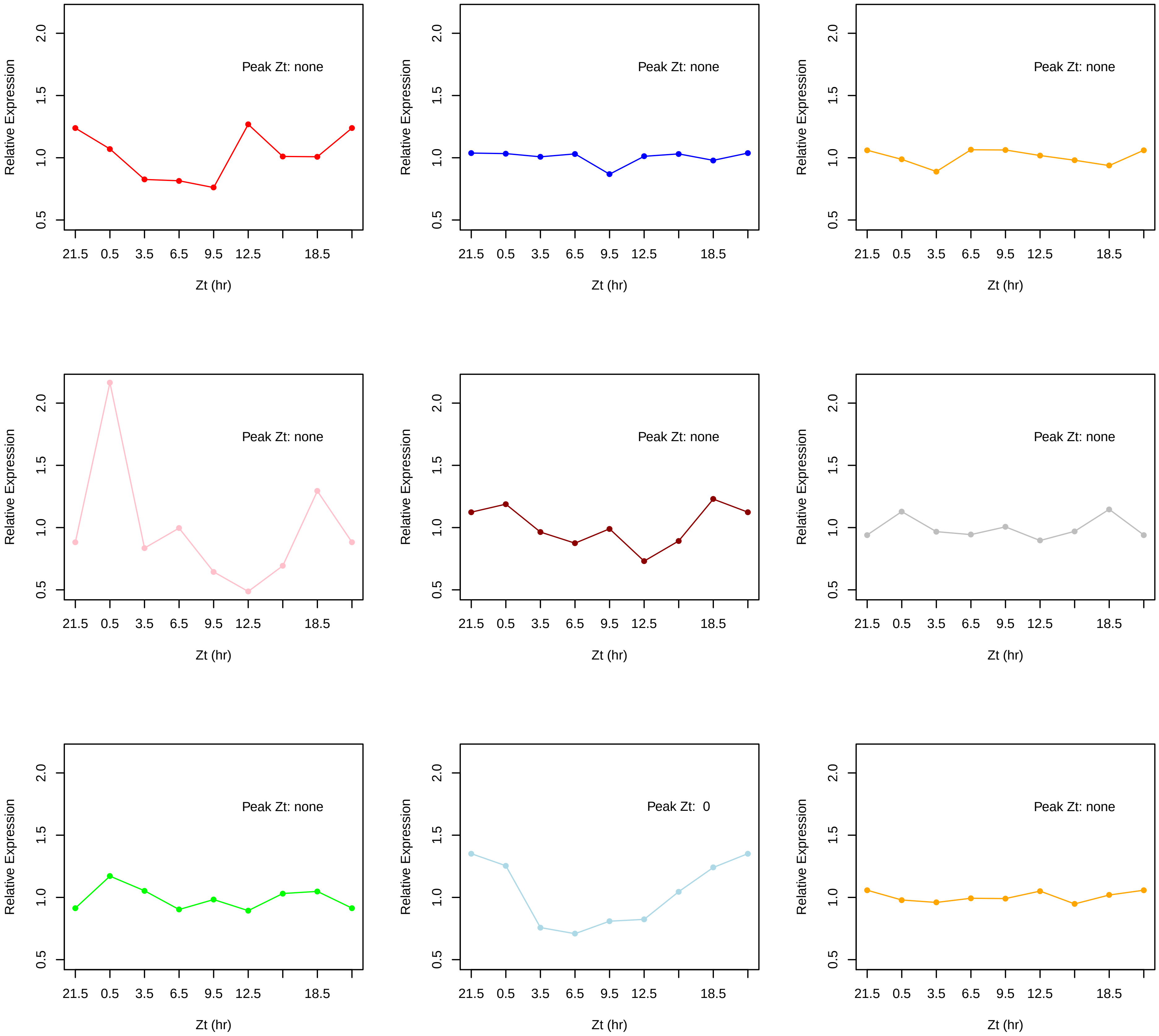

Singeleton: ENSXMAG00000008853 zbtb16a

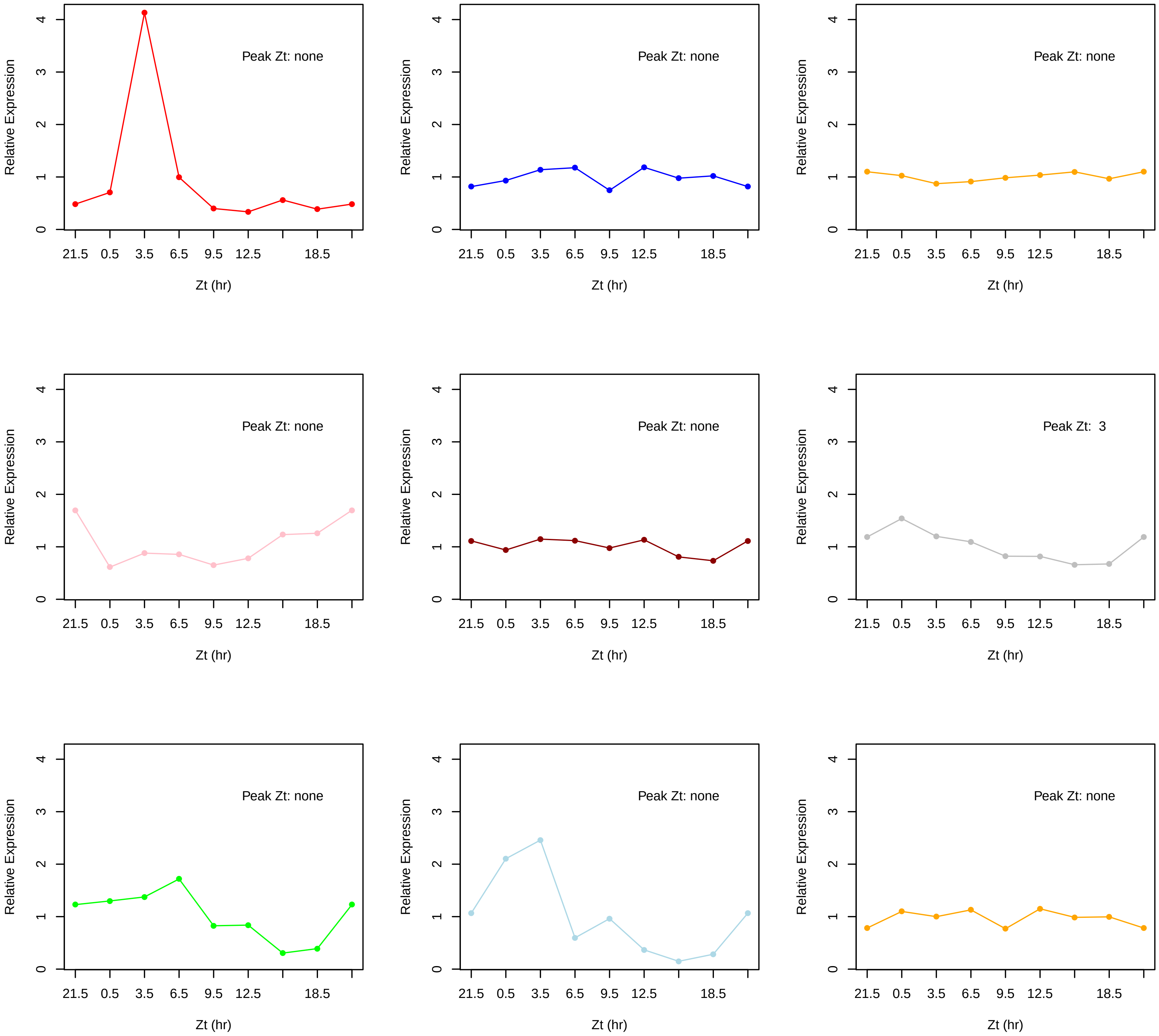

Singeleton: ENSXMAG00000015468 DHX35

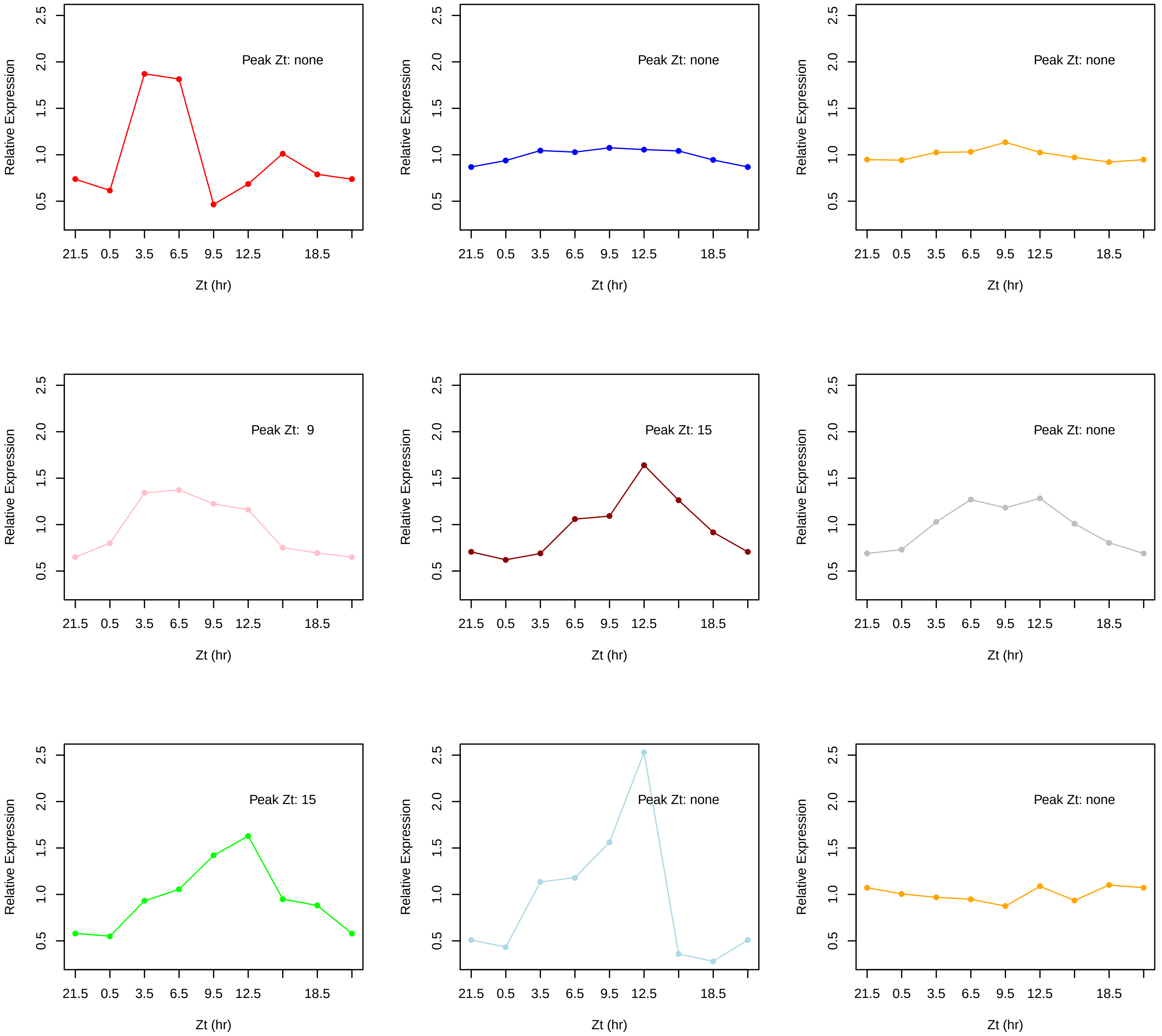

Singleton: ENSXMAG00000029670 ahdc1

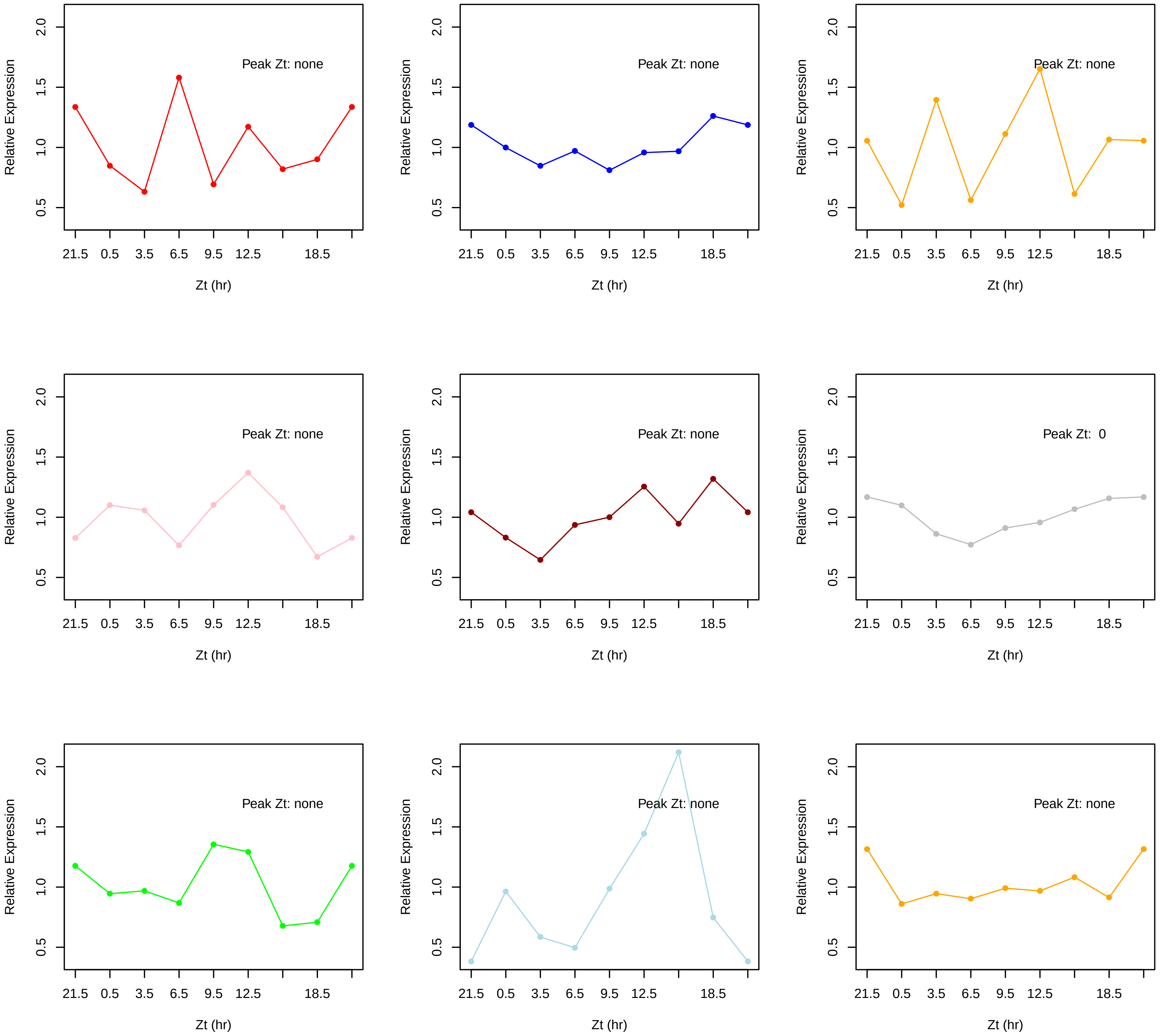

Singeleton: ENSXMAG00000028913 gosr1

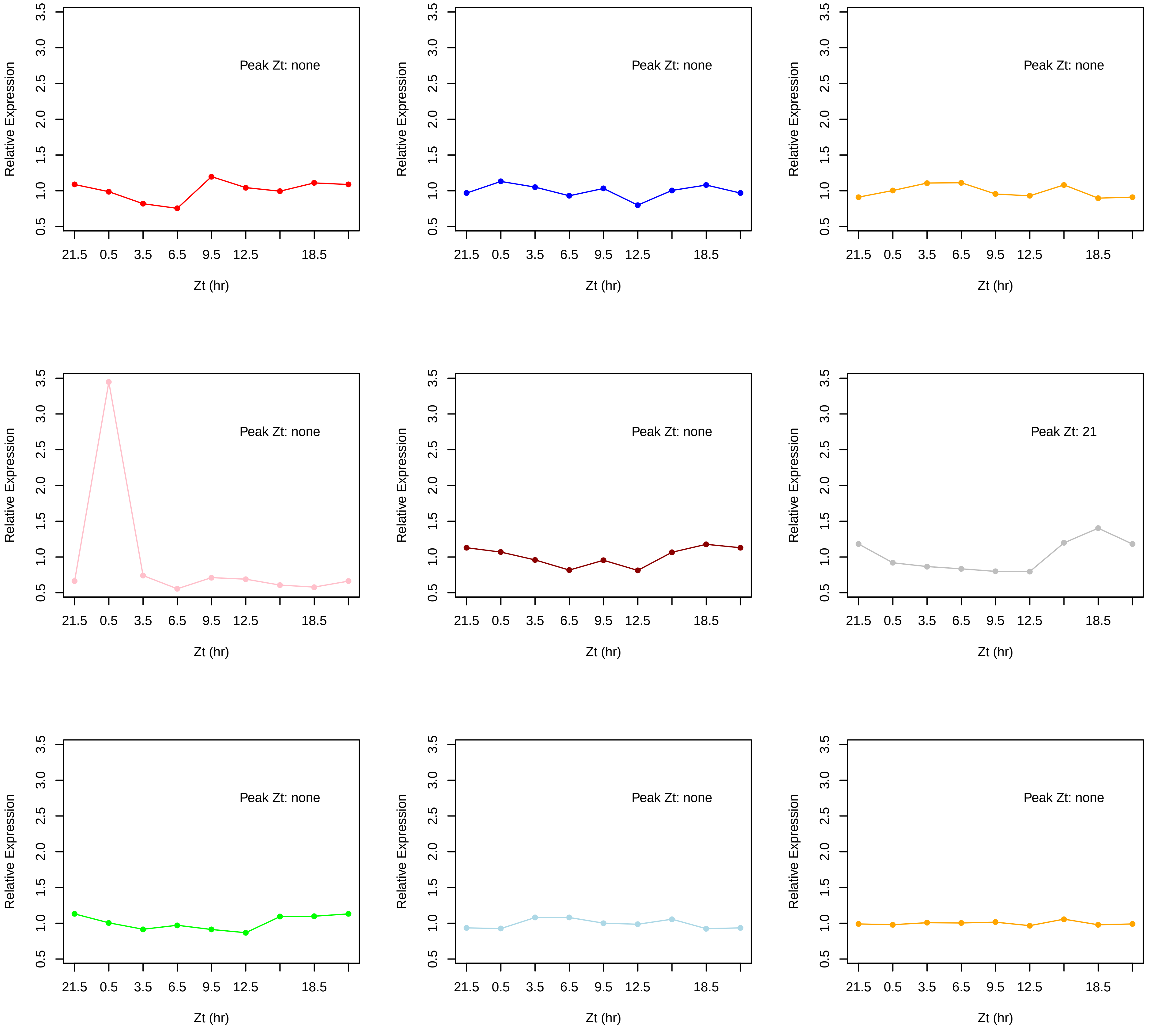

Singeleton: ENSXMAG00000010559 si:dkey−27m7.4

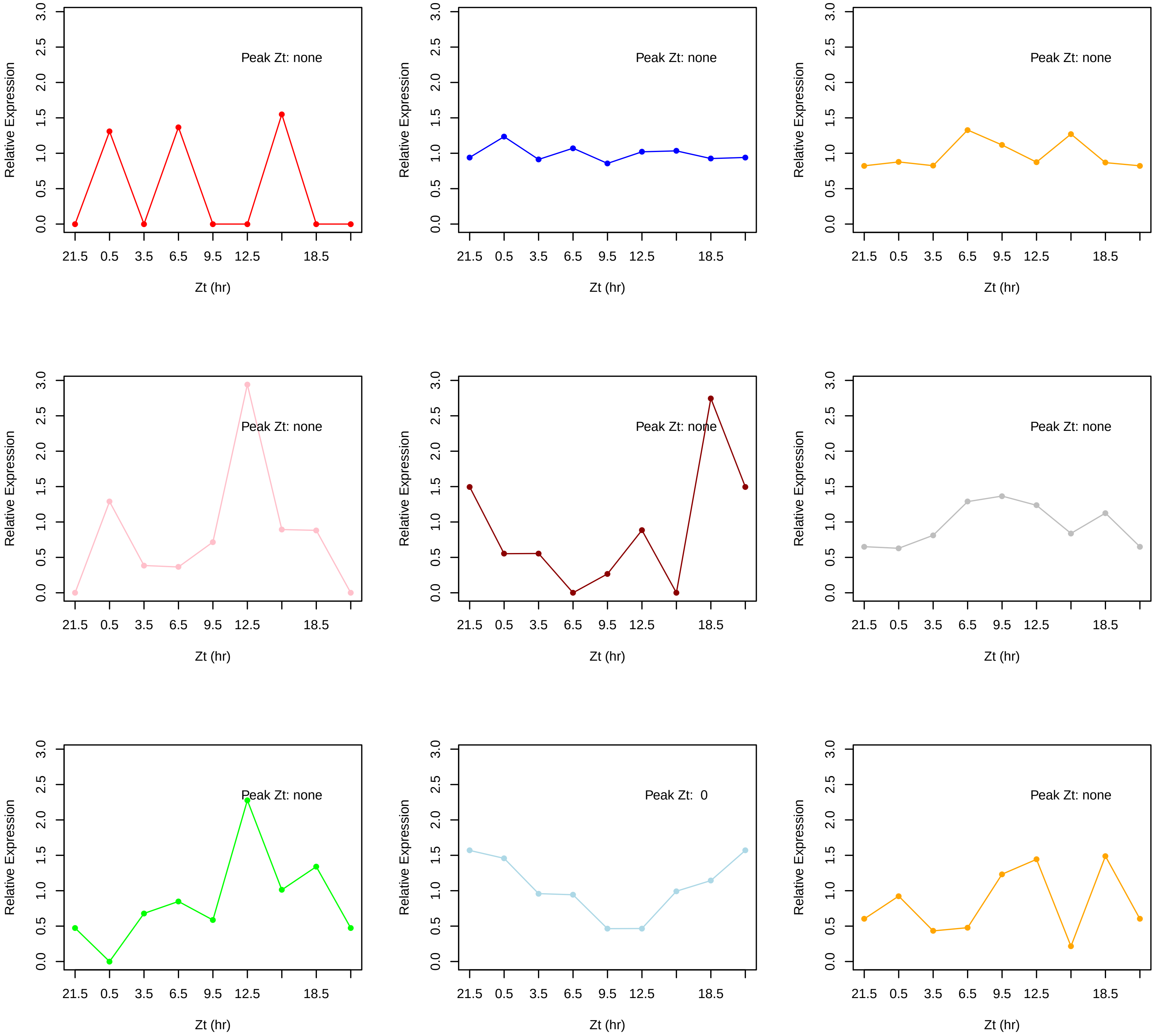

Singleton: ENSXMAG00000000316 eif2b5

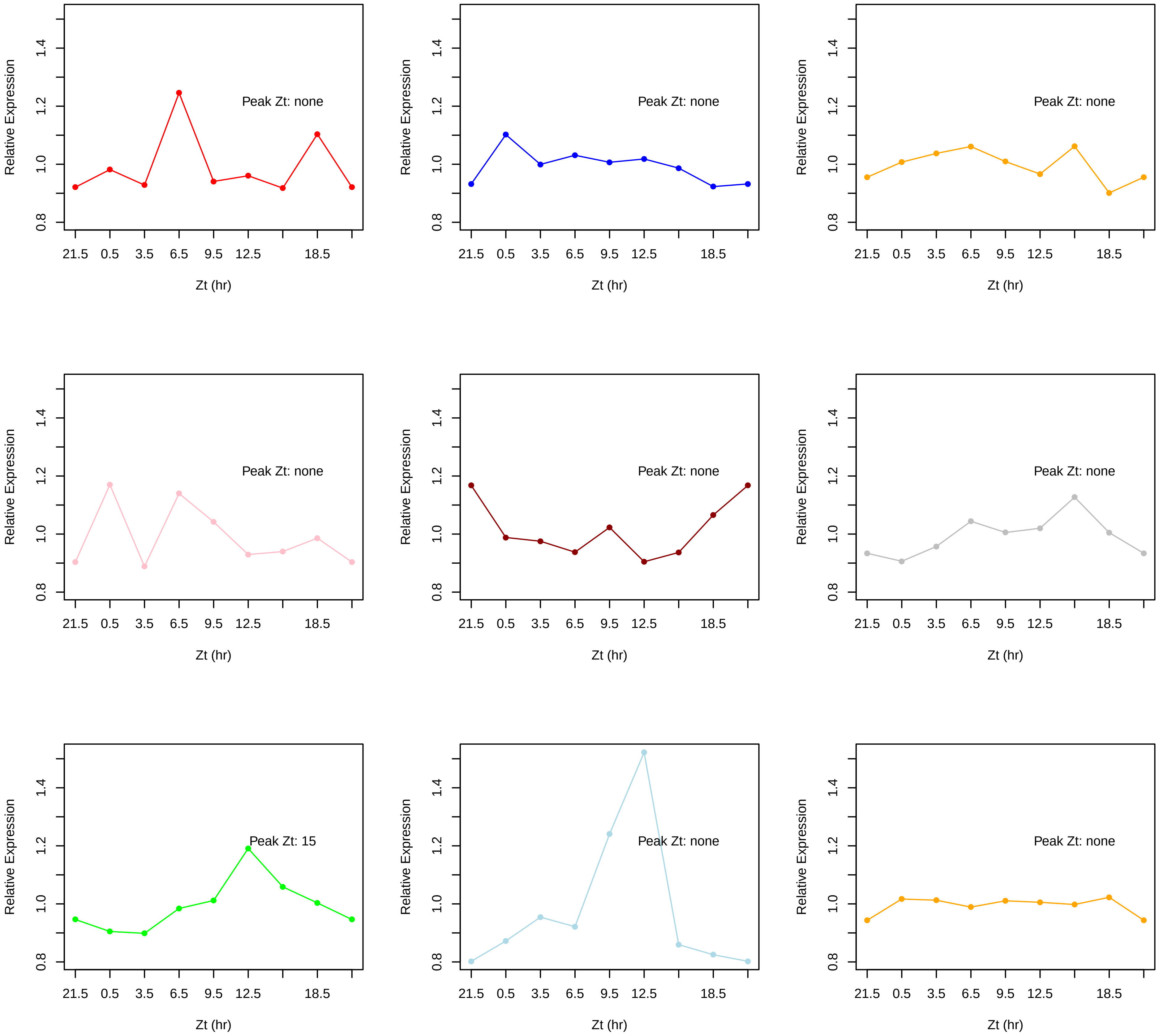

Singleton: ENSXMAG00000003539 ripor2

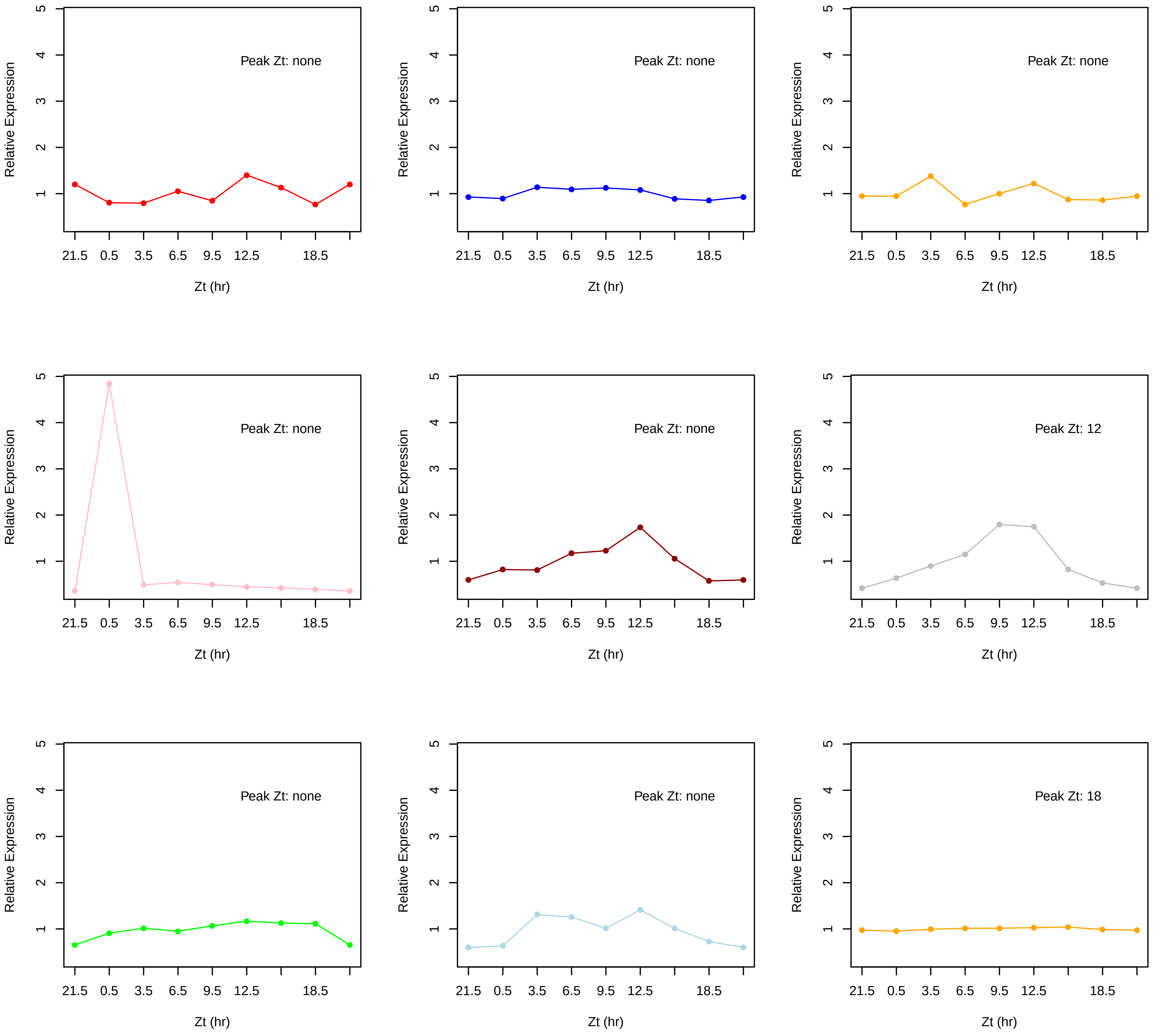

Singeleton: ENSXMAG00000004060 mtf2

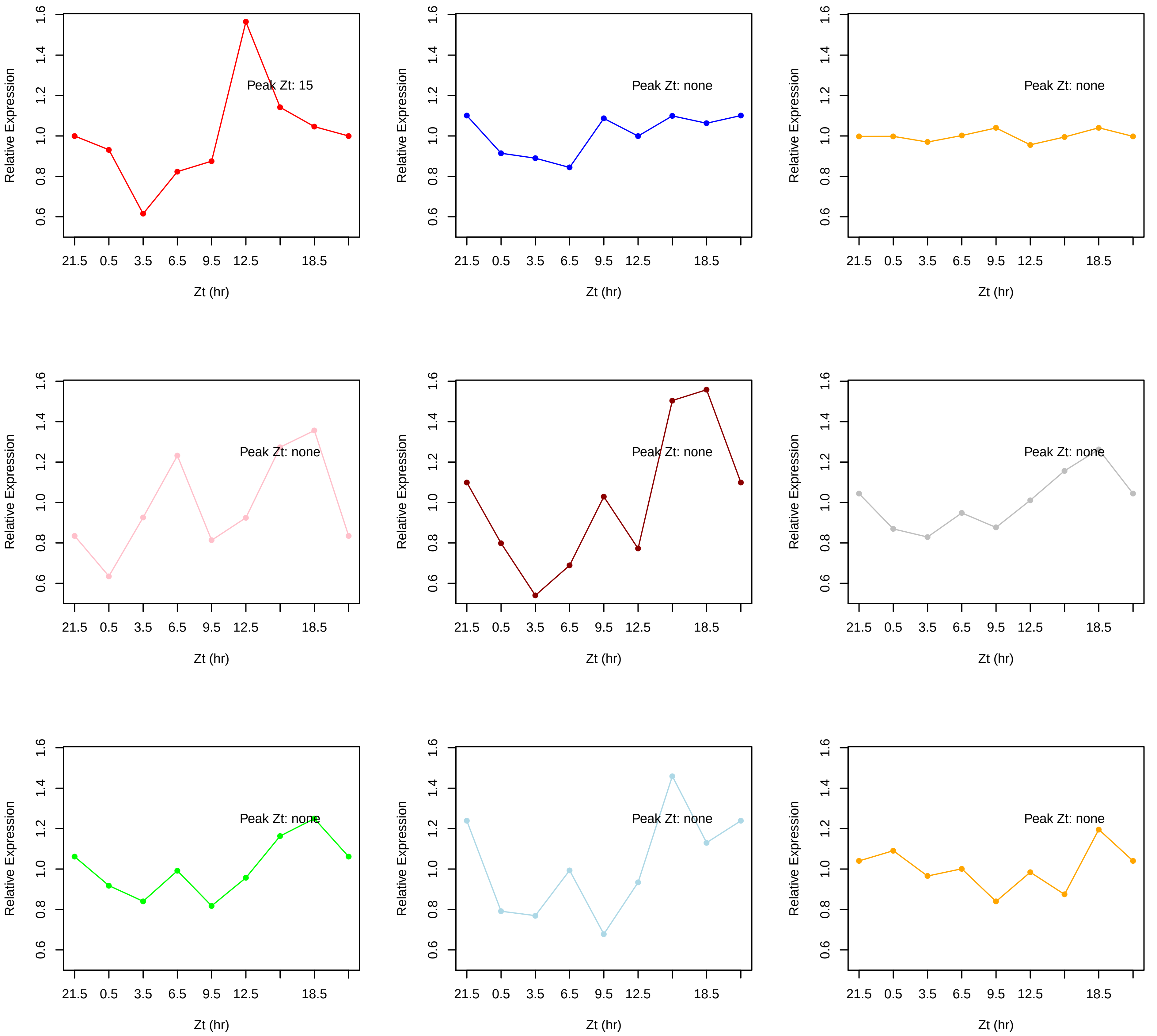

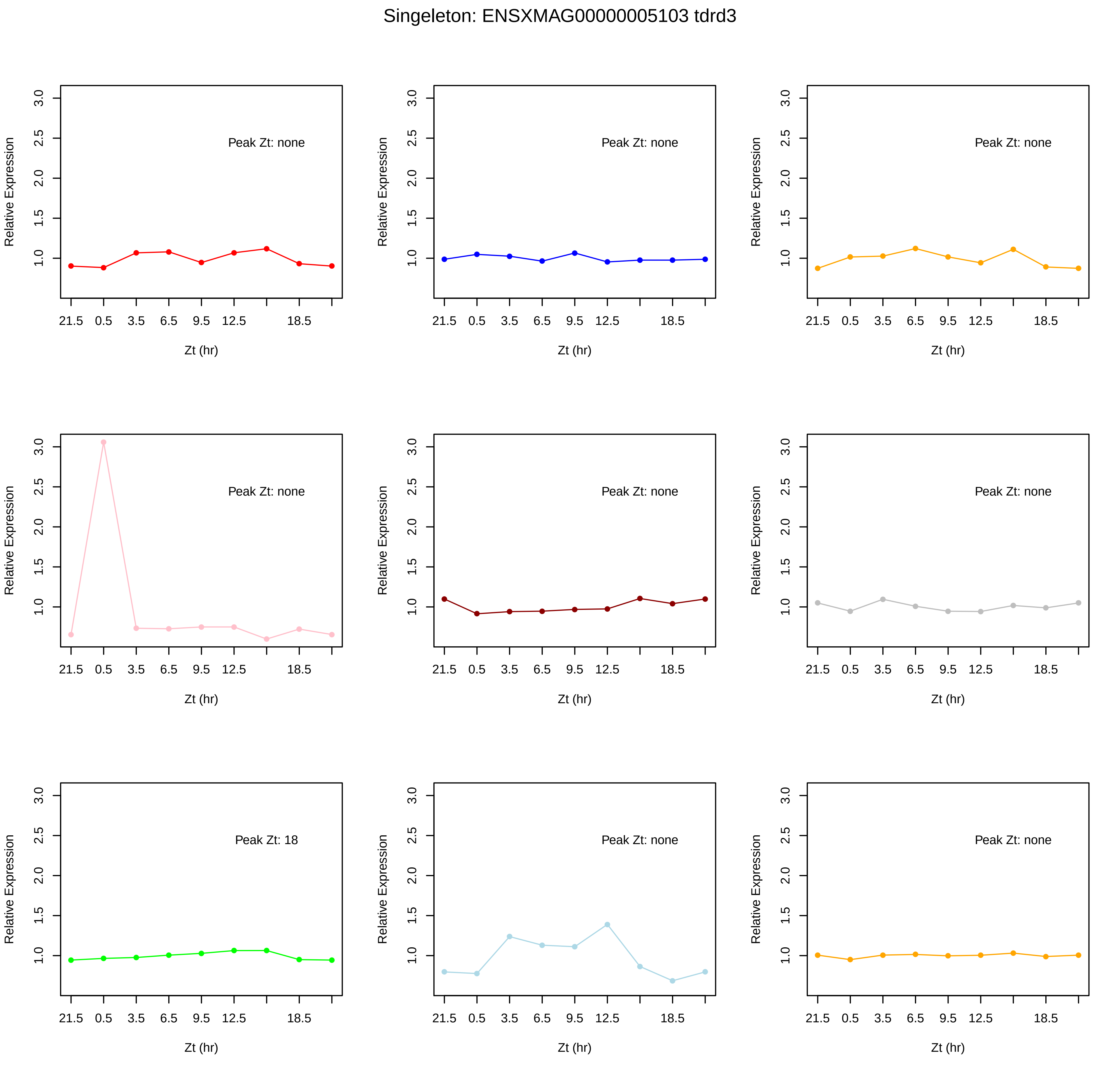
Singeleton: ENSXMAG00000005103 tdrd3
Relative Expression
Zt (hr)
Peak Zt: none
Peak Zt: 18
21.5 0.5 3.5 6.5 9.5 12.5 18.5
1.0 1.5 2.0 2.5 3.0

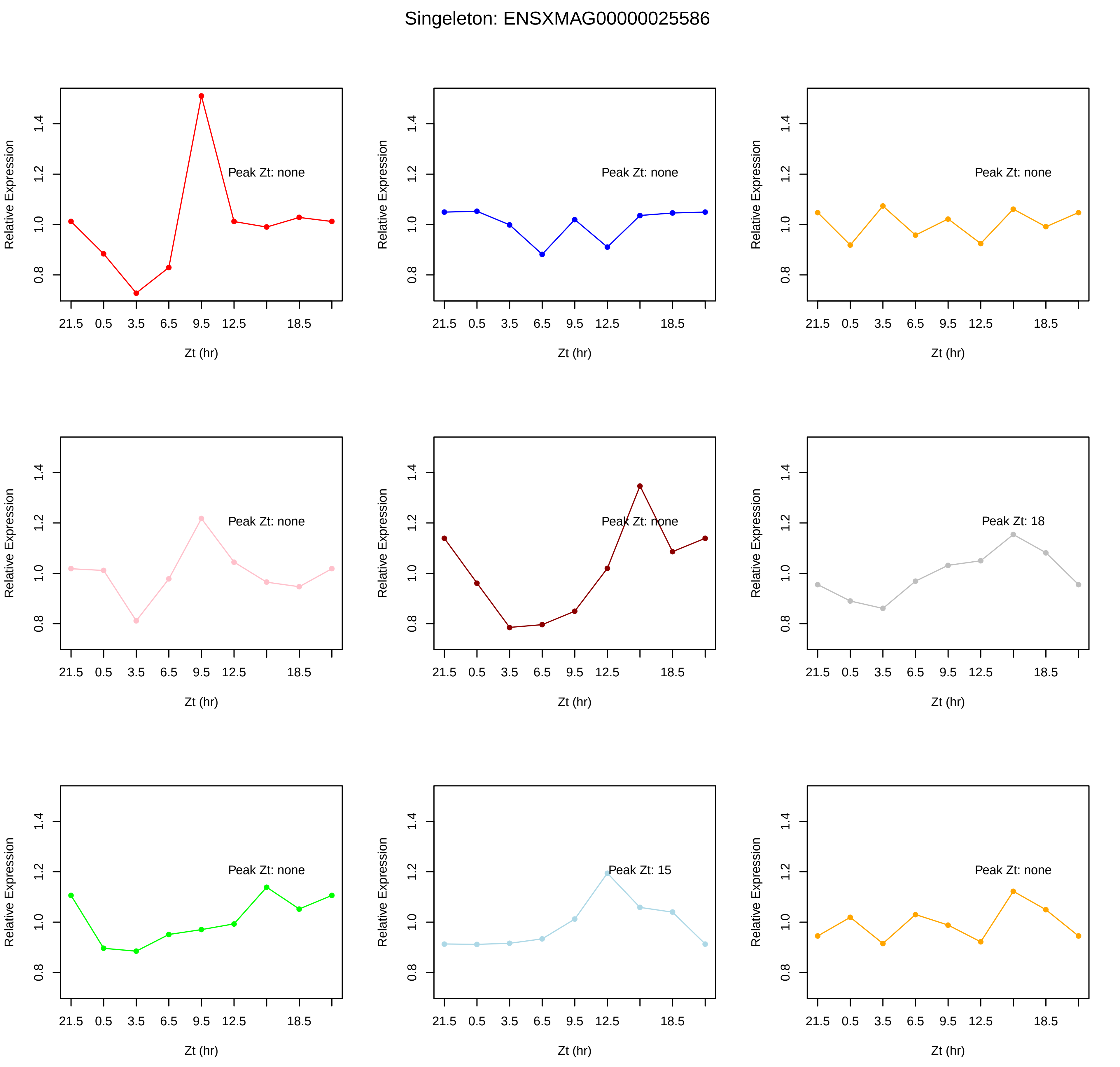
Singeleton: ENSXMAG00000025586
Peak Zt: none
Peak Zt: none
Peak Zt: none
Peak Zt: none
Peak Zt: none
Peak Zt: 18
Peak Zt: none
Peak Zt: 15
Peak Zt: none
Relative Expression
Zt (hr)
21.5
0.5
3.5
6.5
9.5
12.5
18.5
0.8
1.0
1.2
1.4

Singeleton: ENSXMAG00000013870 ZBTB21

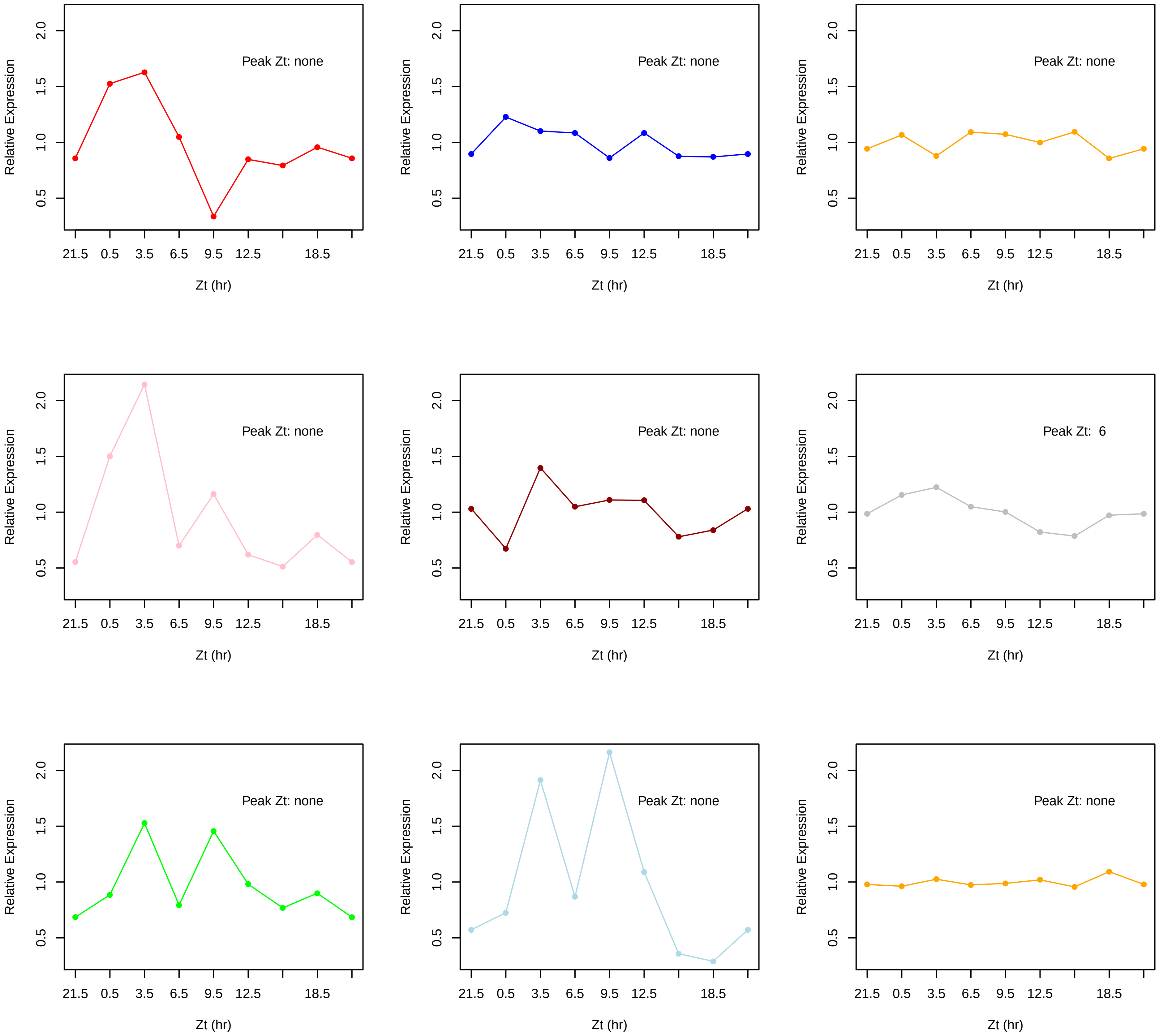

Singeleton: ENSXMAG00000001006 rnf216

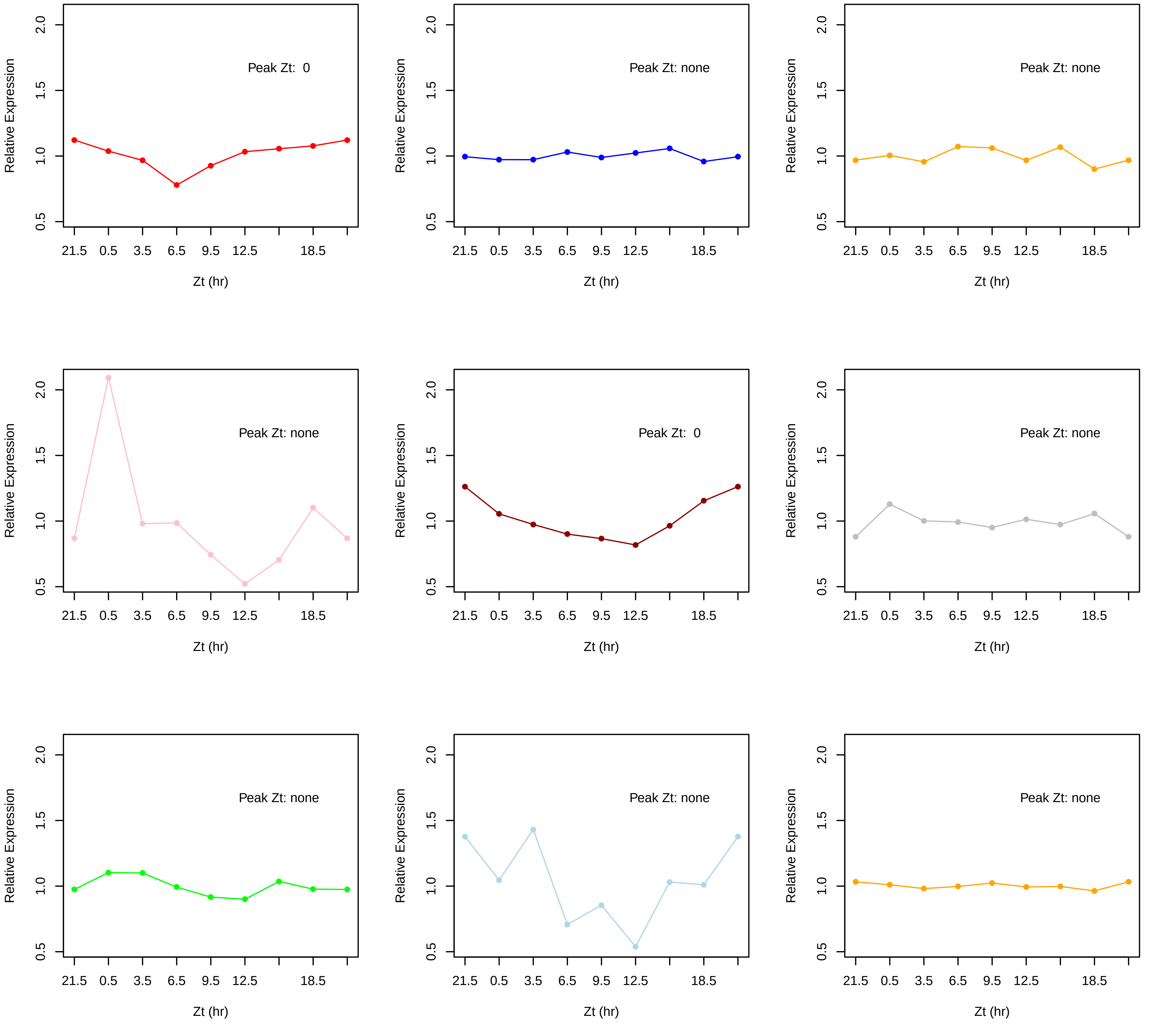

Singeleton: ENSXMAG00000023420 cbfa2t3

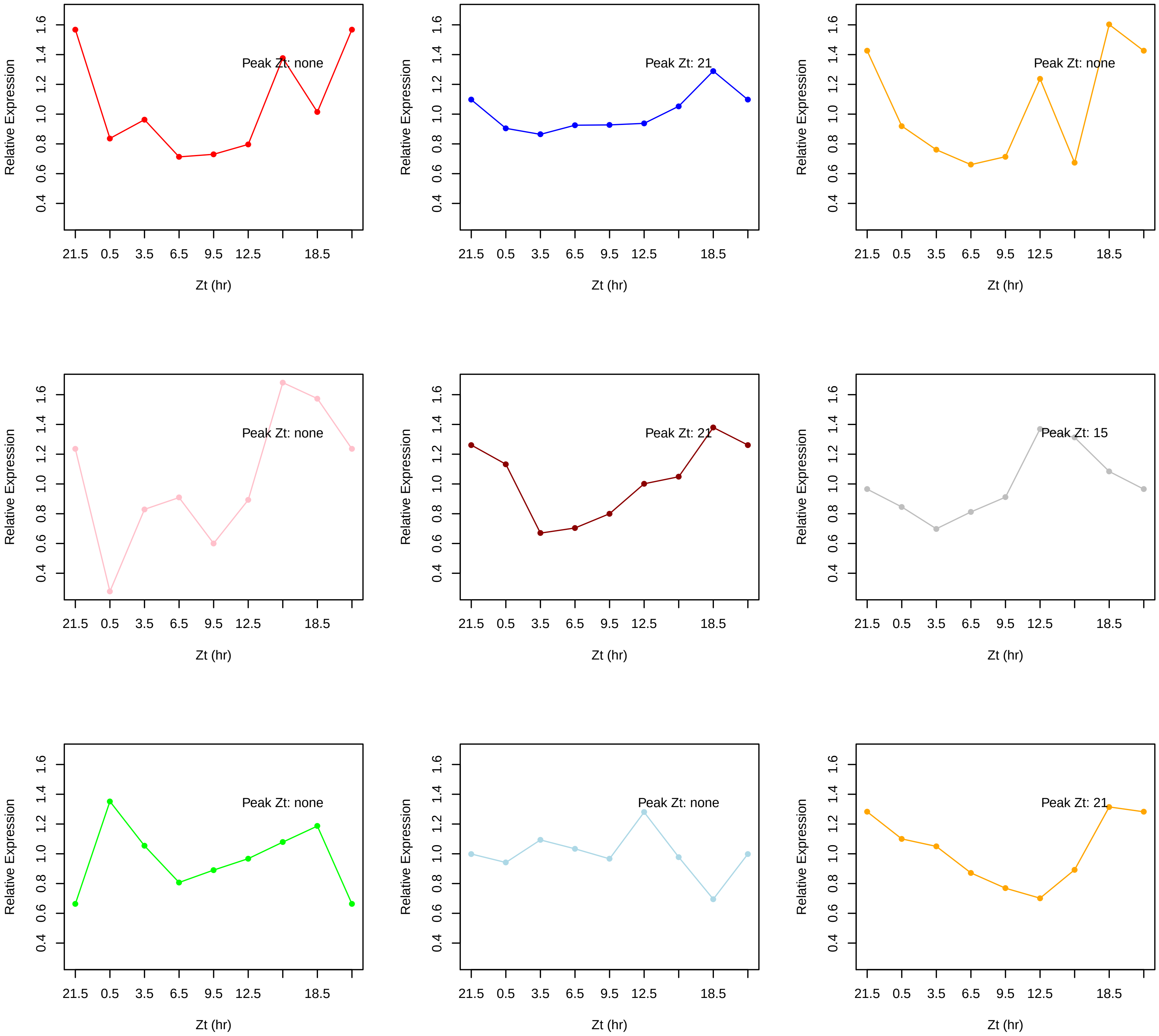

Singleton: ENSXMAG00000013871 umodl1

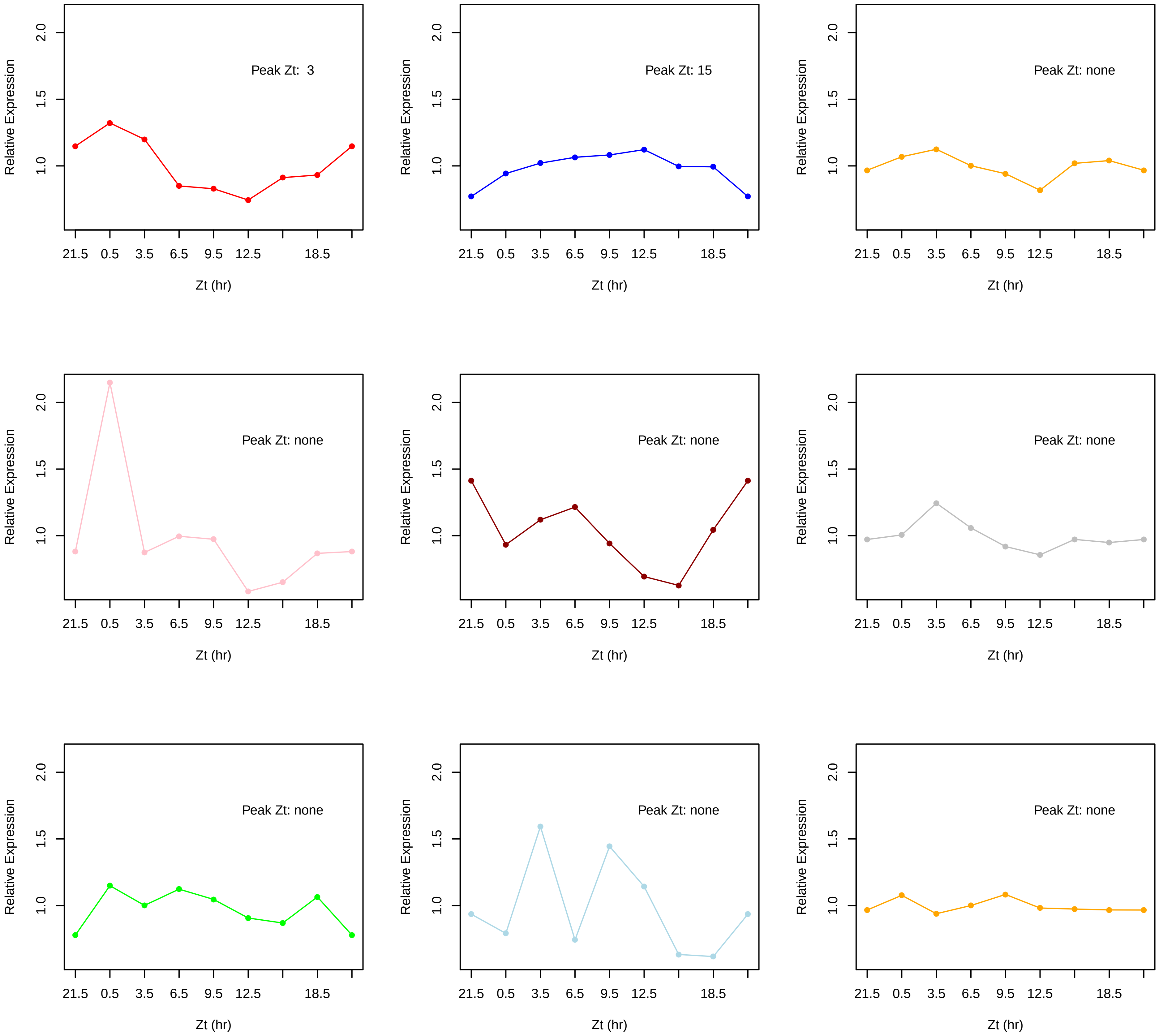

Singleton: ENSXMAG00000024768 cnpy2

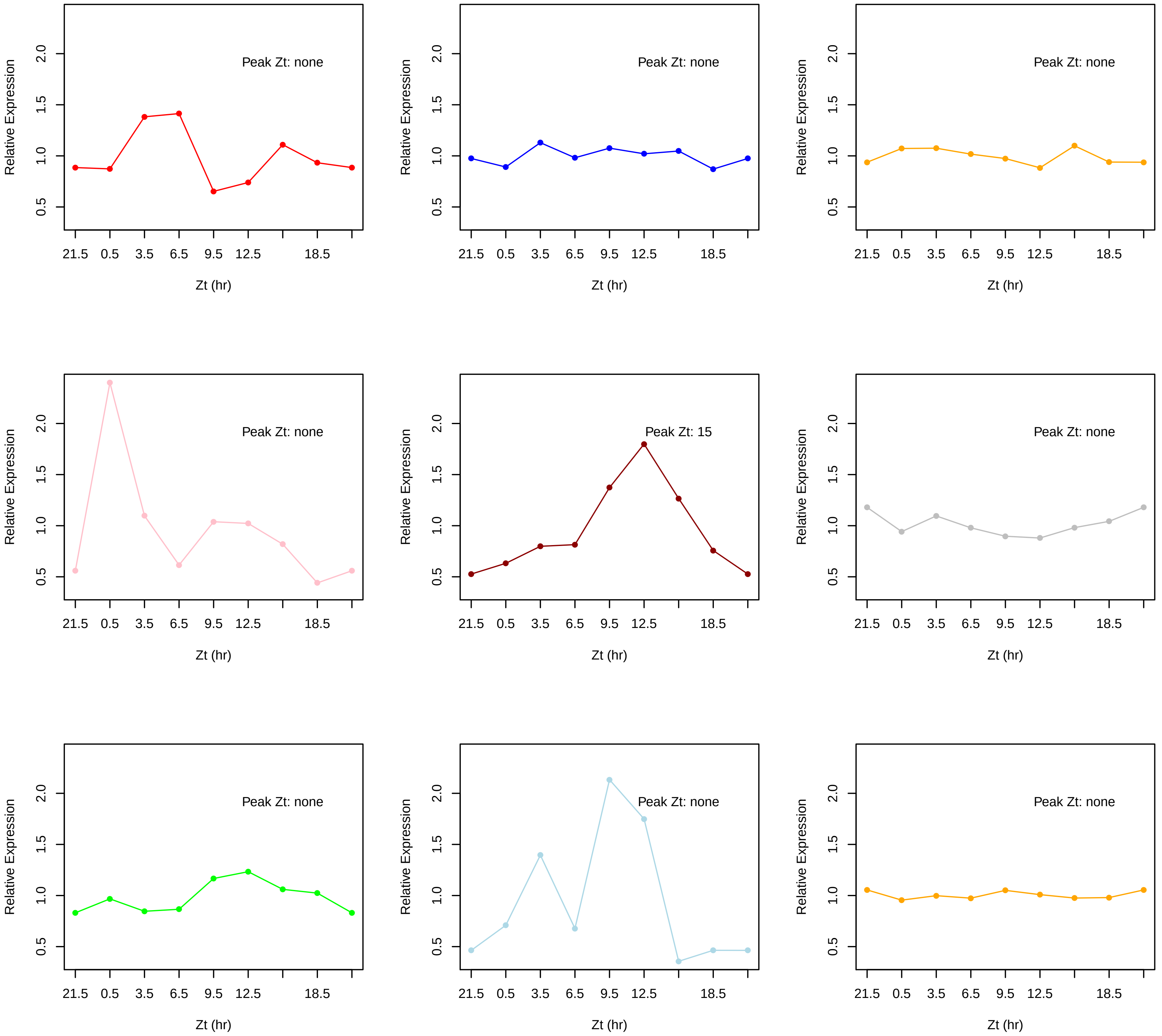

Singleton: ENSXMAG00000013297 si:dkeyp−97a10.2

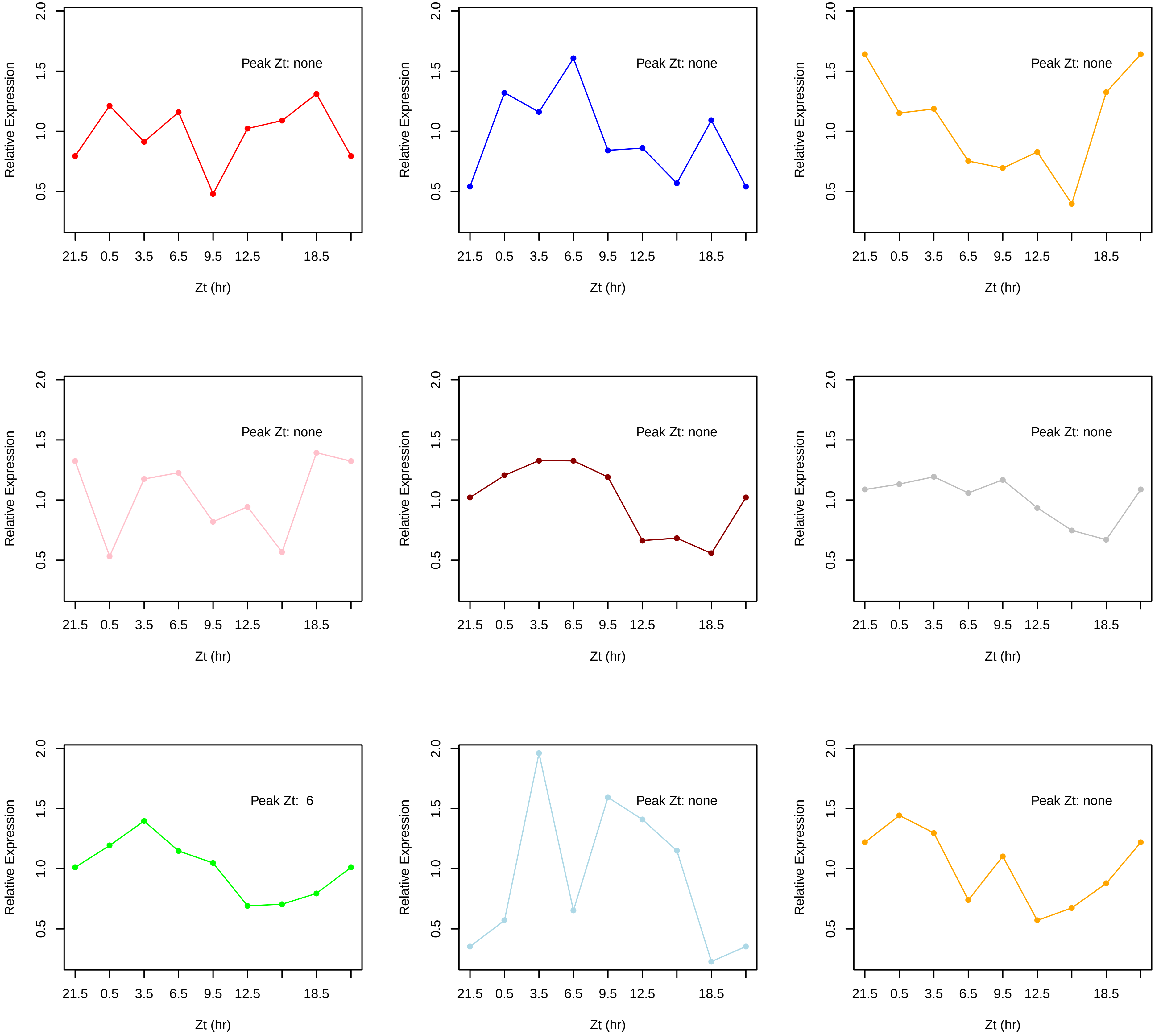

Singleton: ENSXMAG00000000608 msi2a

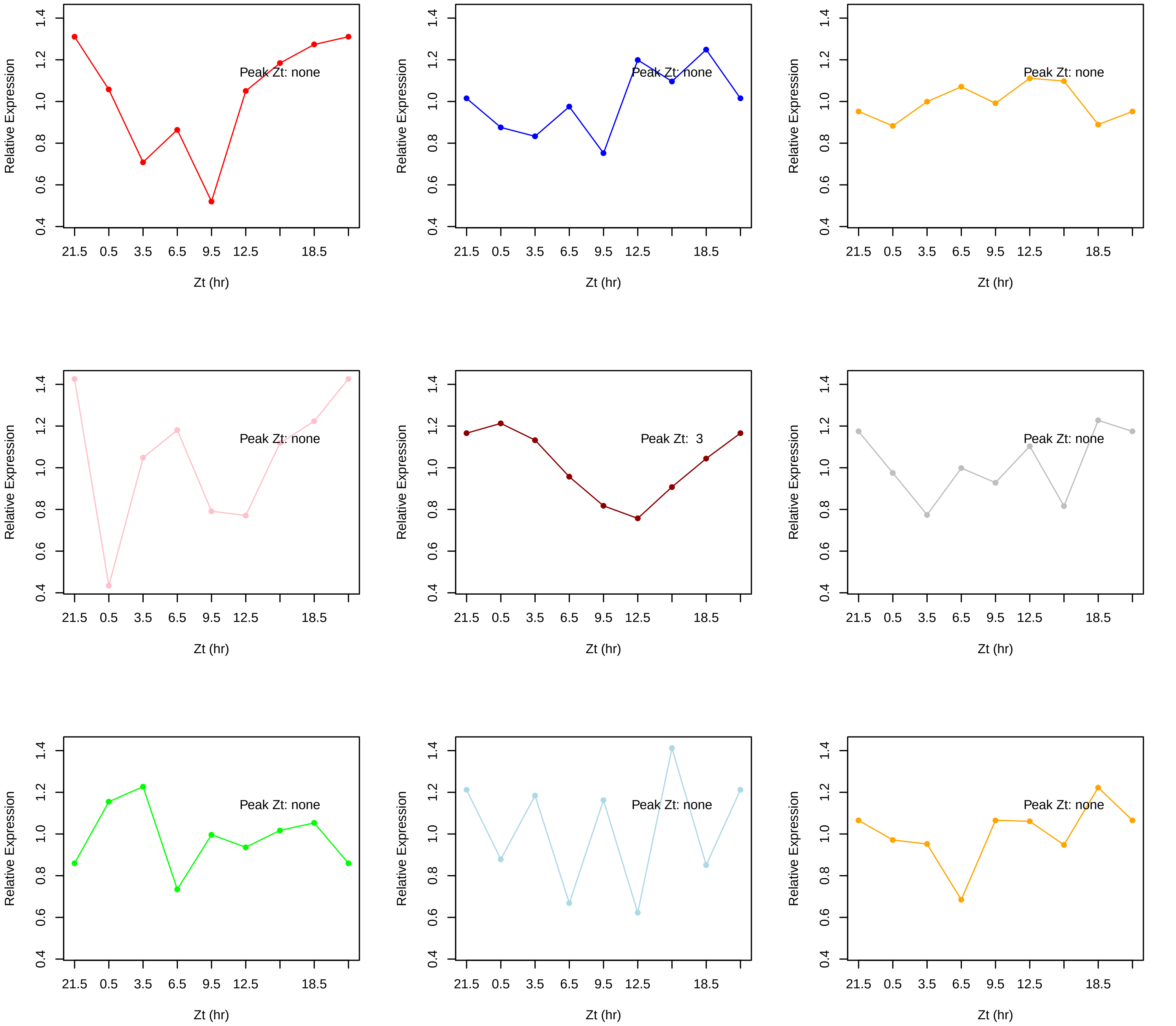

Singeleton: ENSXMAG00000005638 psmd1

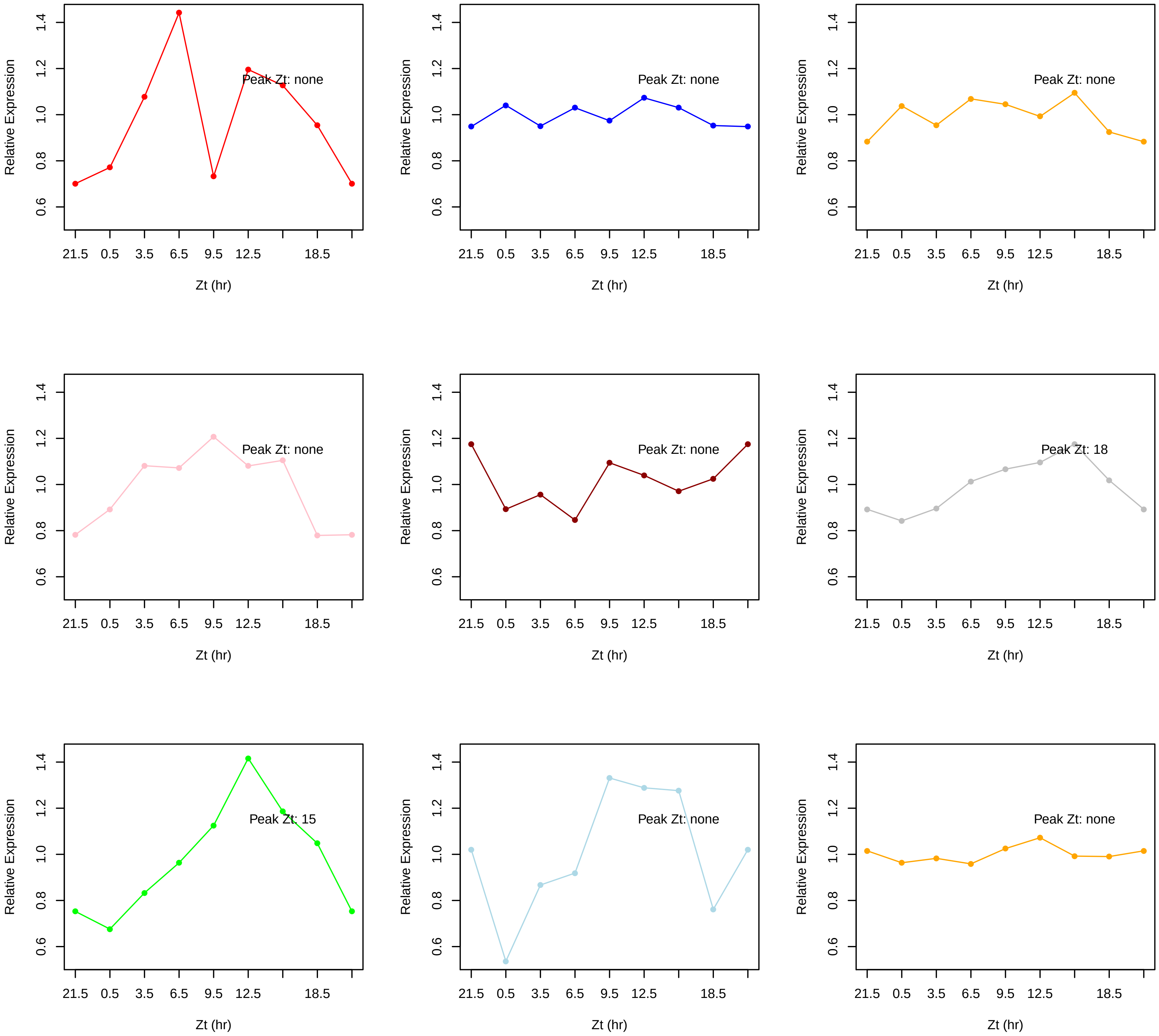

Singleton: ENSXMAG00000015512 rnf114

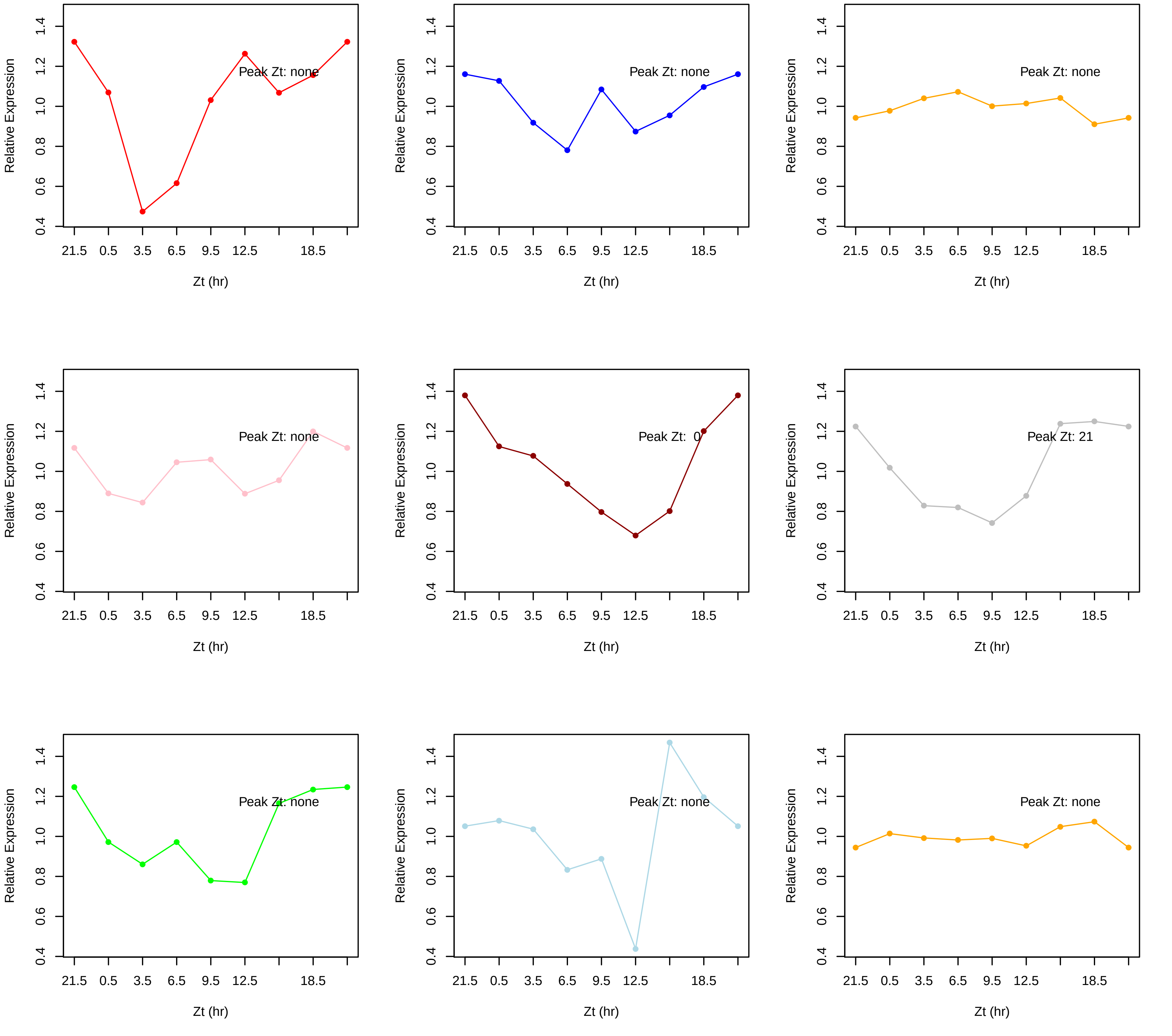

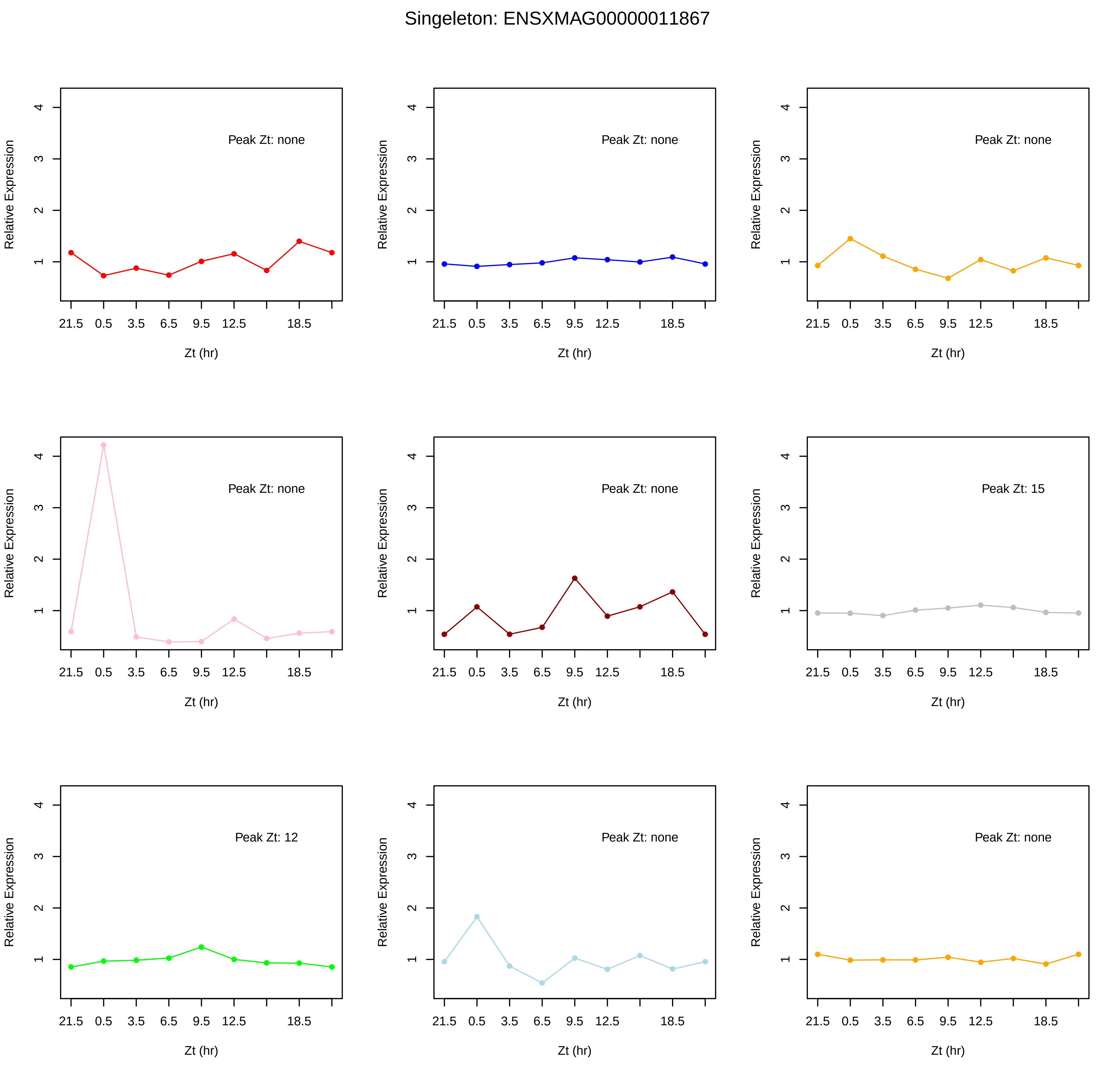

Singeleton: ENSXMAG00000011867
Peak Zt: none
Peak Zt: none
Peak Zt: none
Peak Zt: none
Peak Zt: none
Peak Zt: 15
Peak Zt: 12
Peak Zt: none
Peak Zt: none
Relative Expression
Zt (hr)
21.5
0.5
3.5
6.5
9.5
12.5
18.5

Singleton: ENSXMAG00000000999 fscn1a

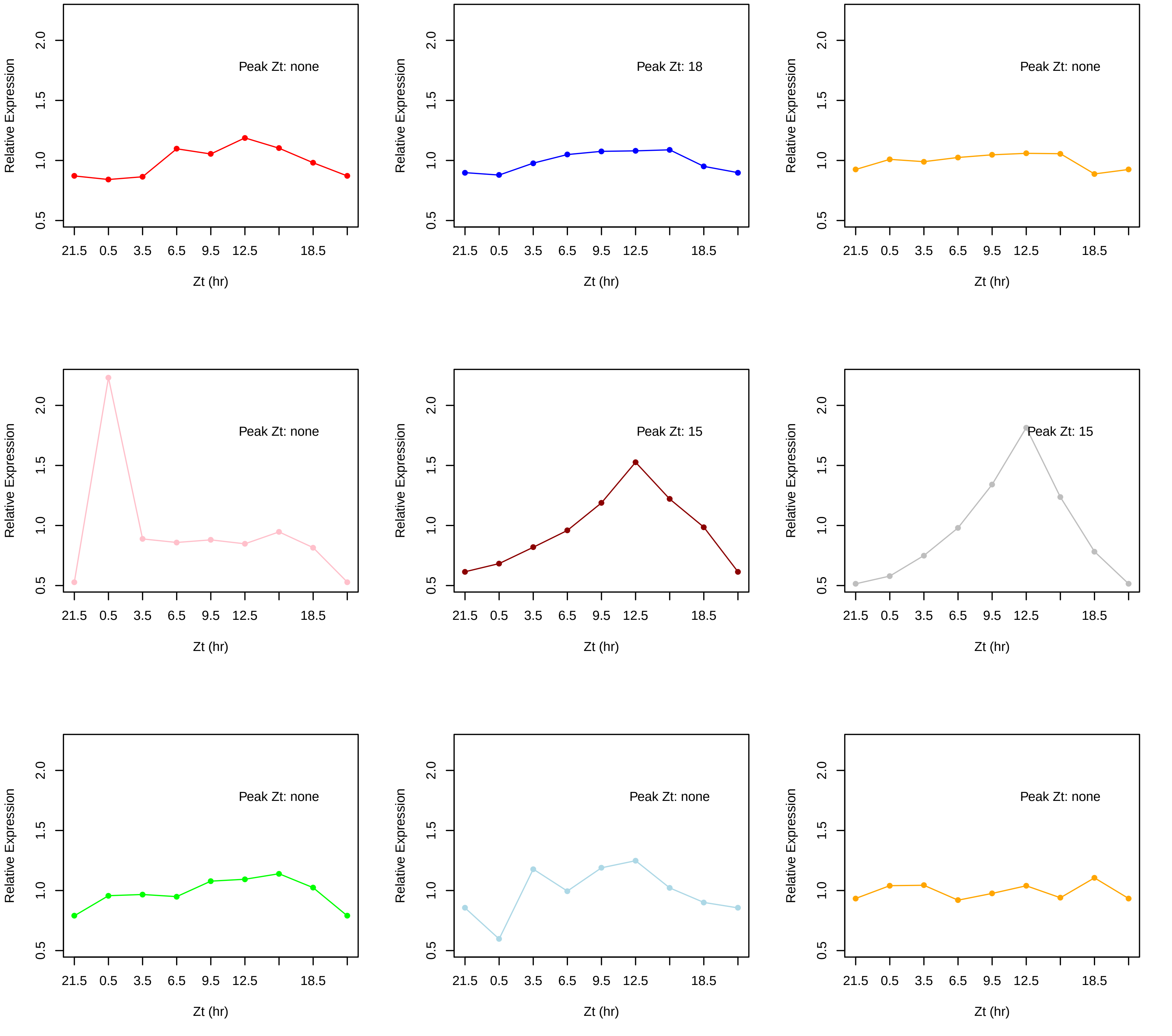

Singeleton: ENSXMAG00000003156 evi5b

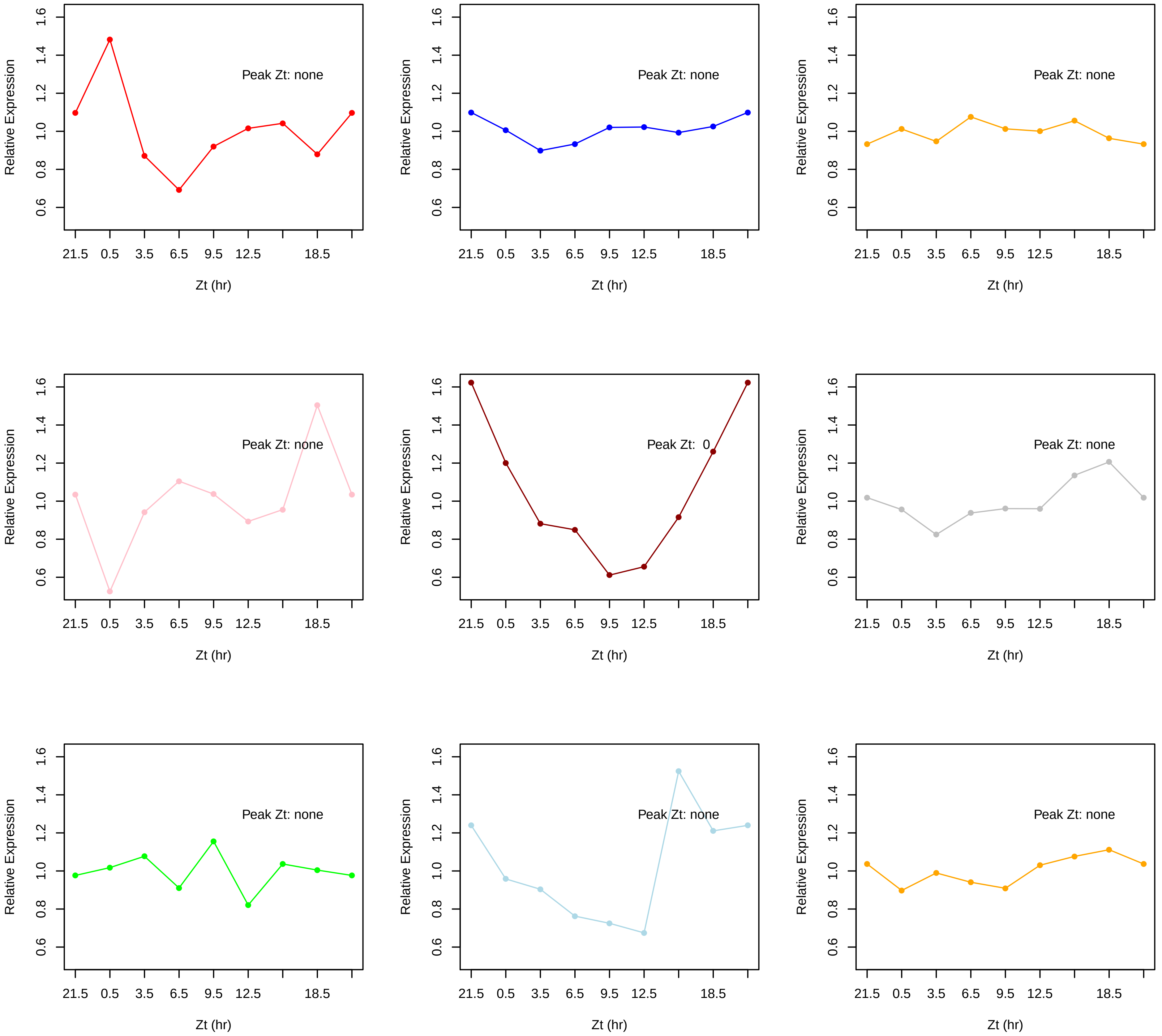

Singeleton: ENSXMAG00000003585 dyrk1b

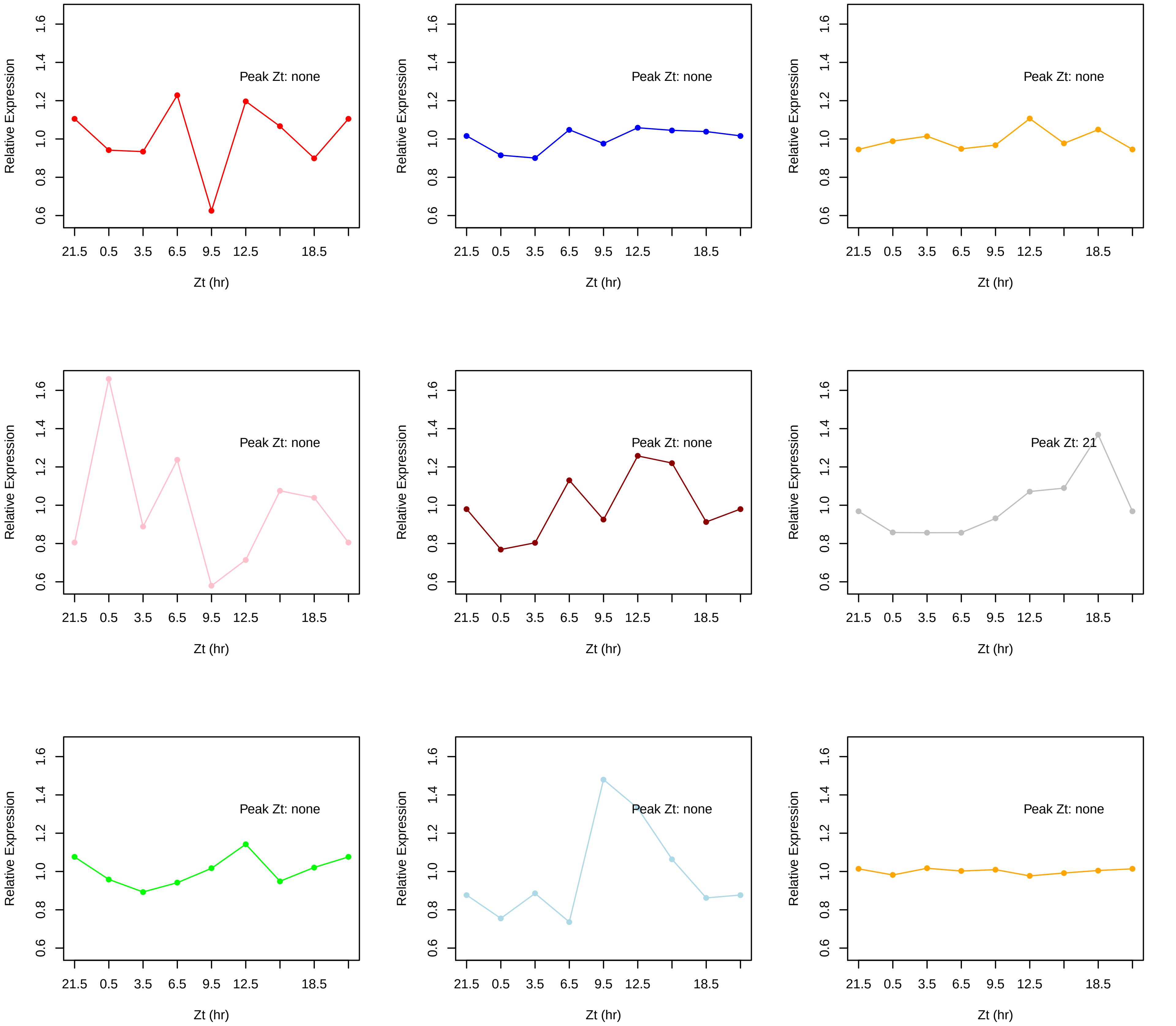

Singeleton: ENSXMAG00000012222

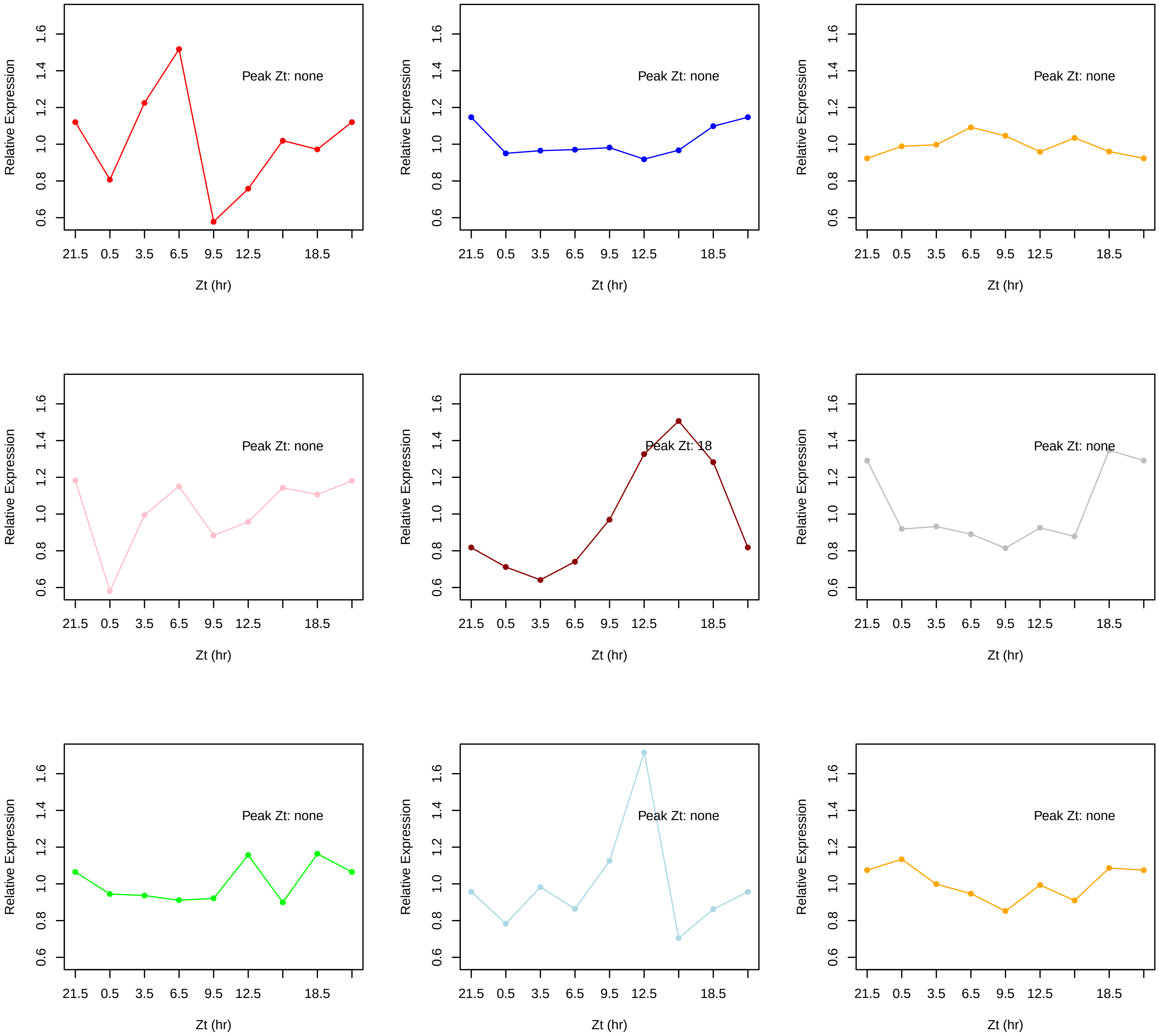

Singeleton: ENSXMAG00000015528 spata2

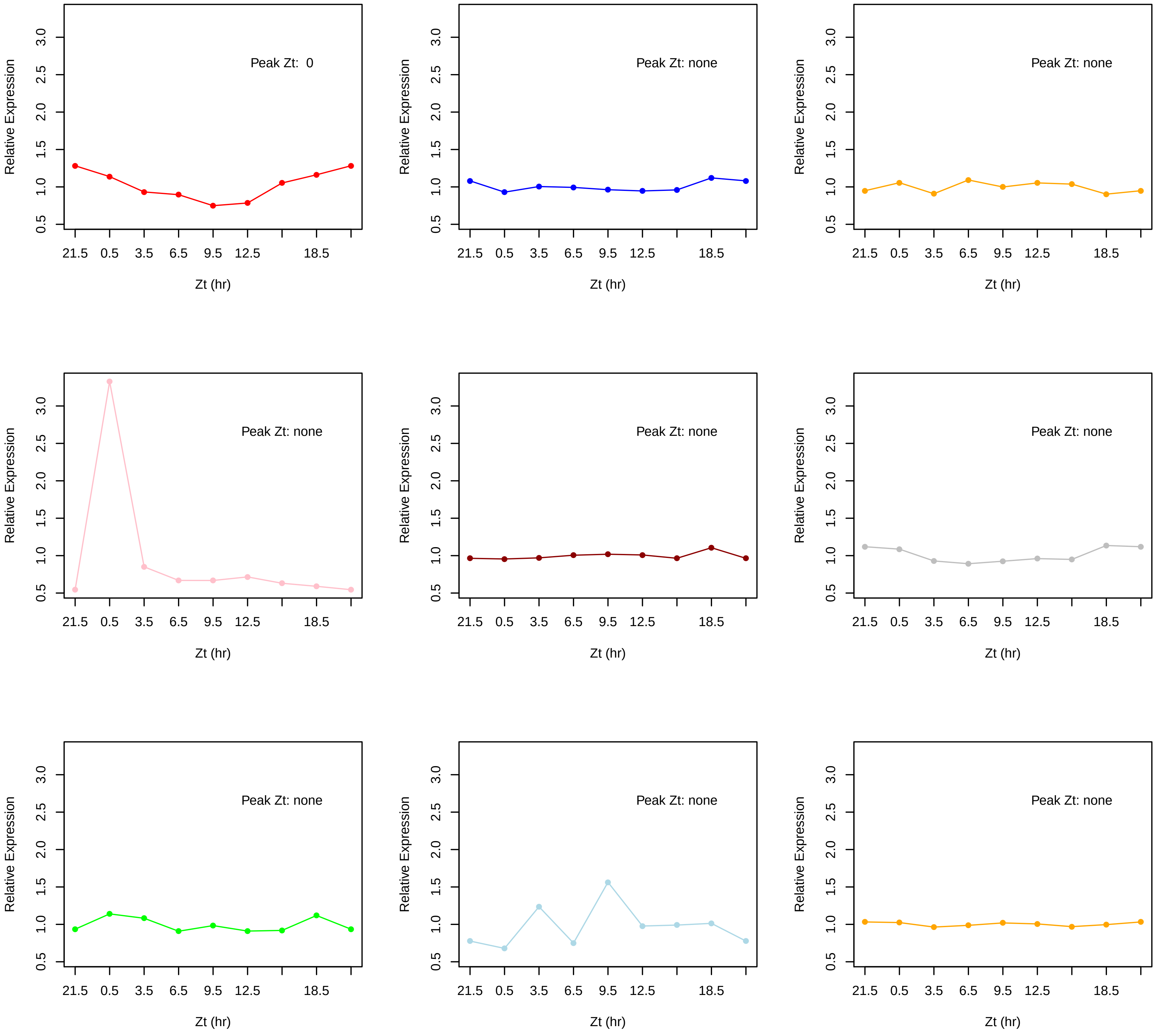

Singeleton: ENSXMAG00000018160 sart3

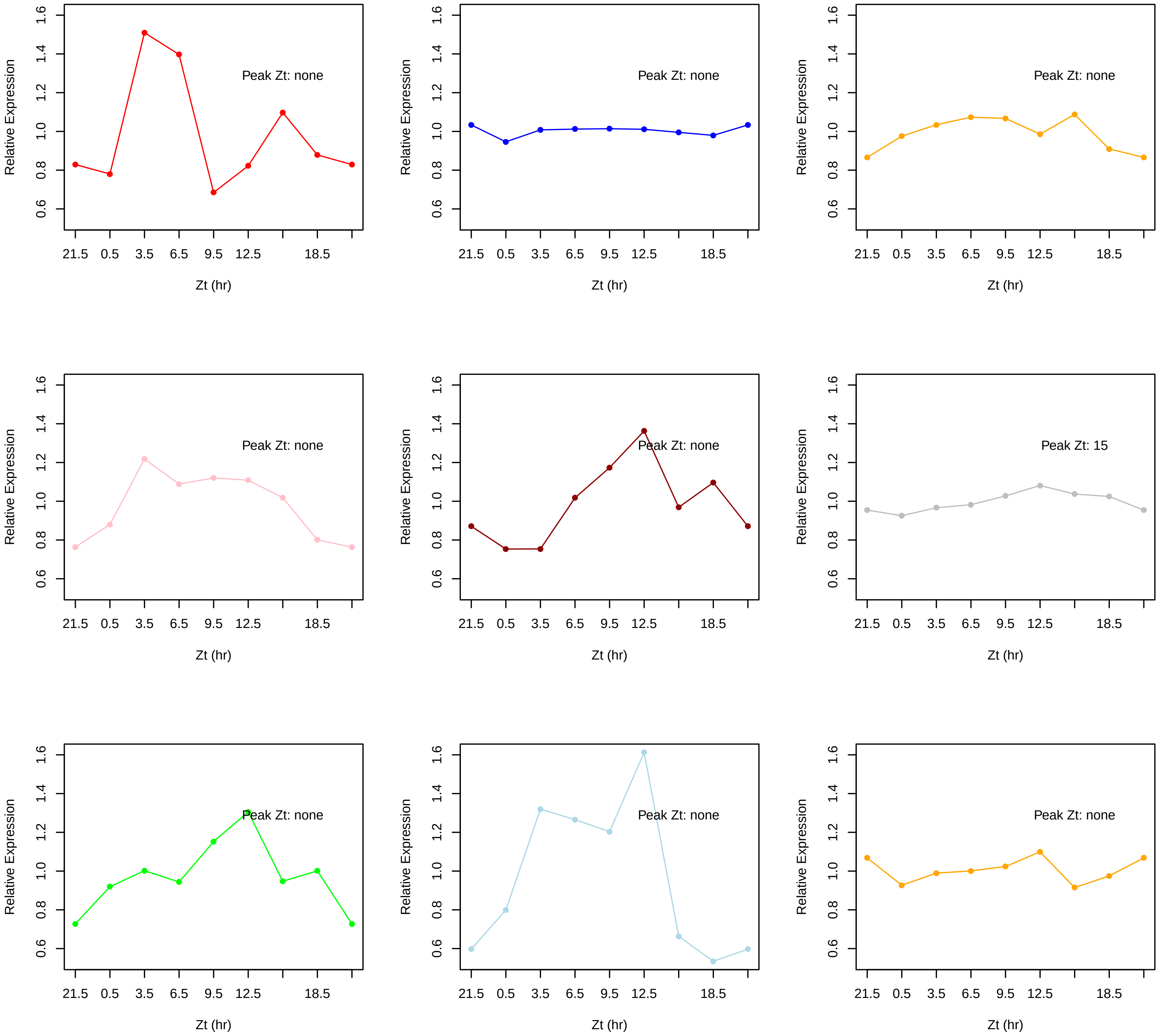

Singeleton: ENSXMAG00000008522

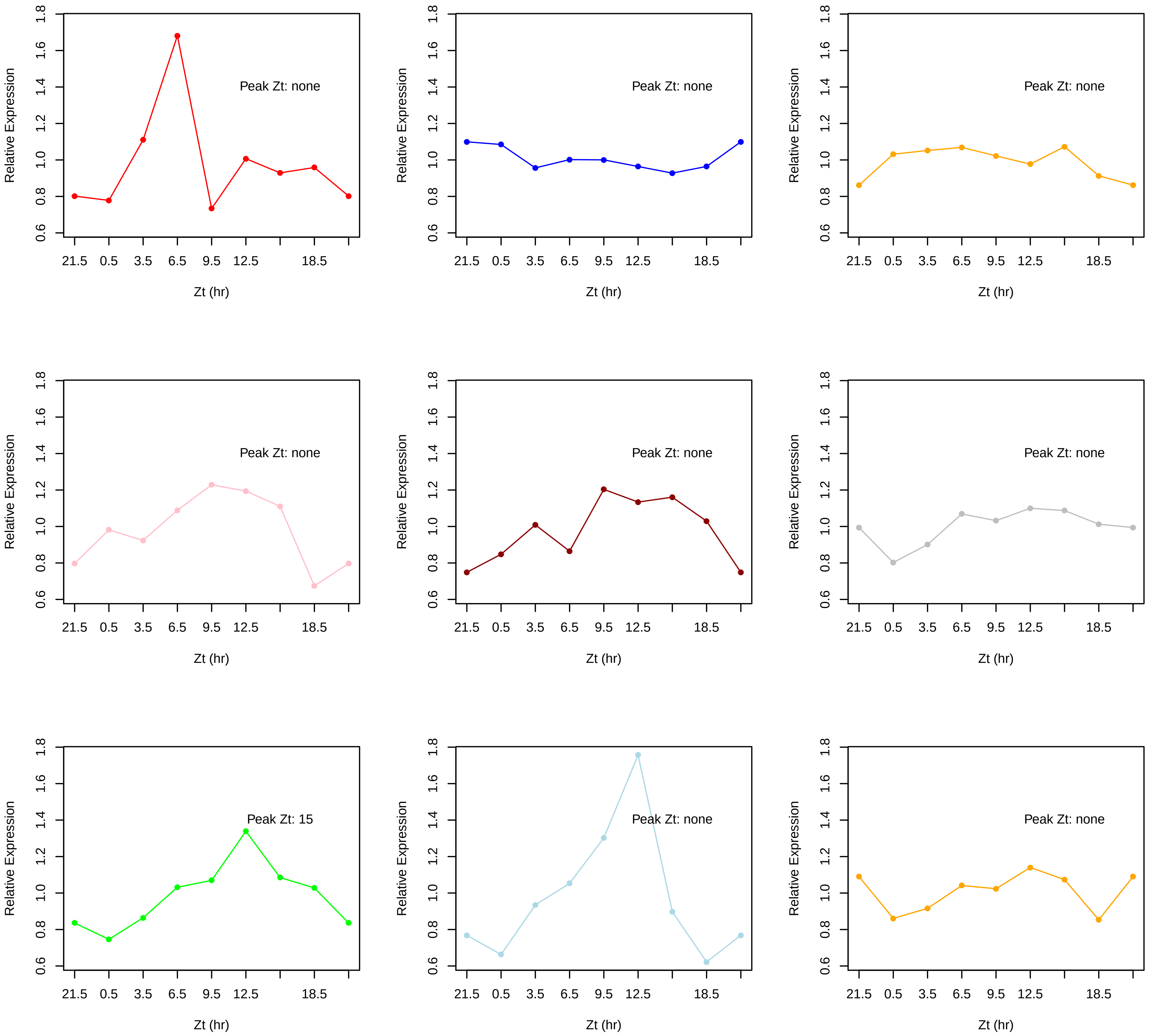

Singeleton: ENSXMAG00000005678 acap2

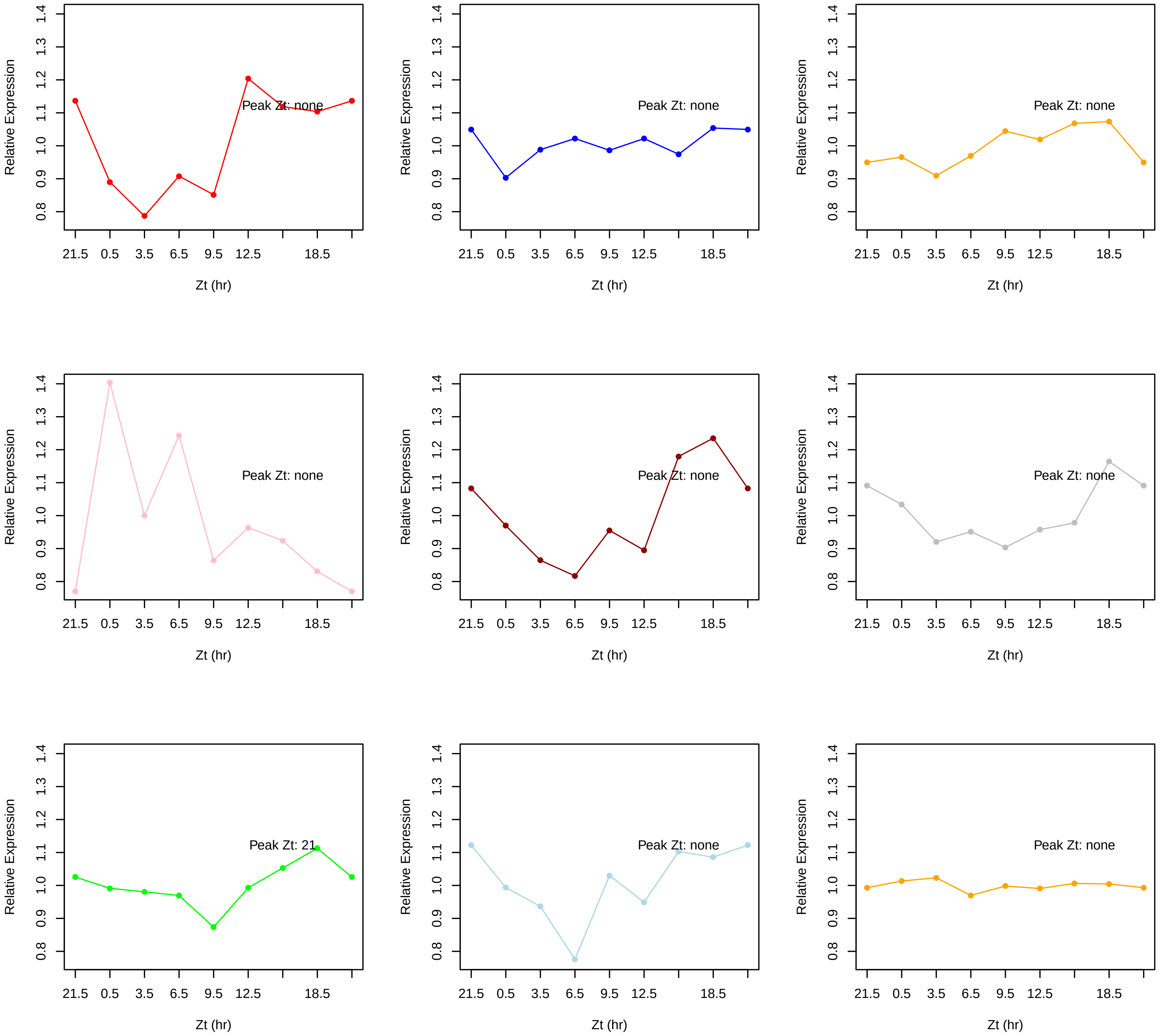

Singleton: ENSXMAG00000022200 dgat2

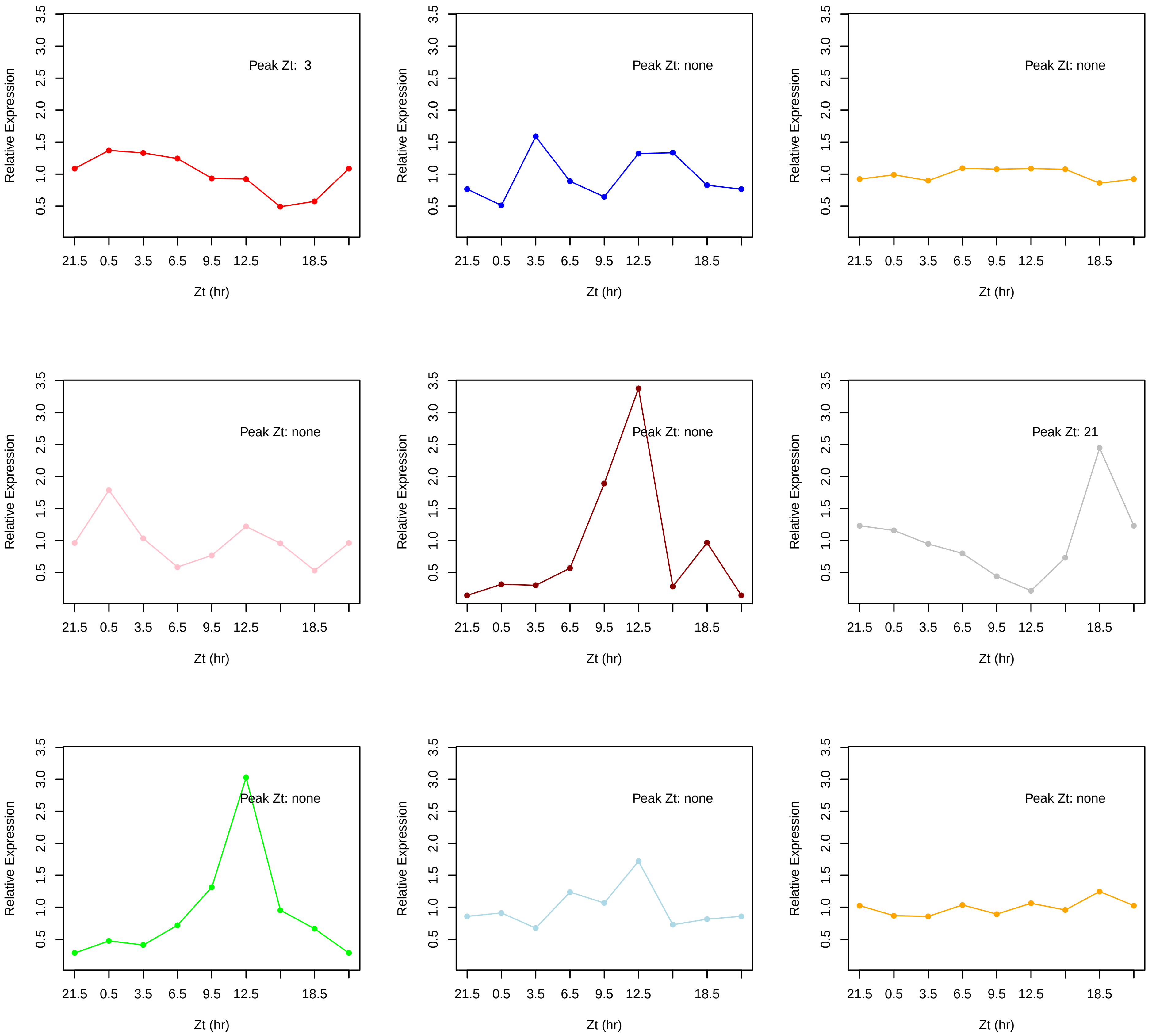

Singeleton: ENSXMAG00000005728 pappaa

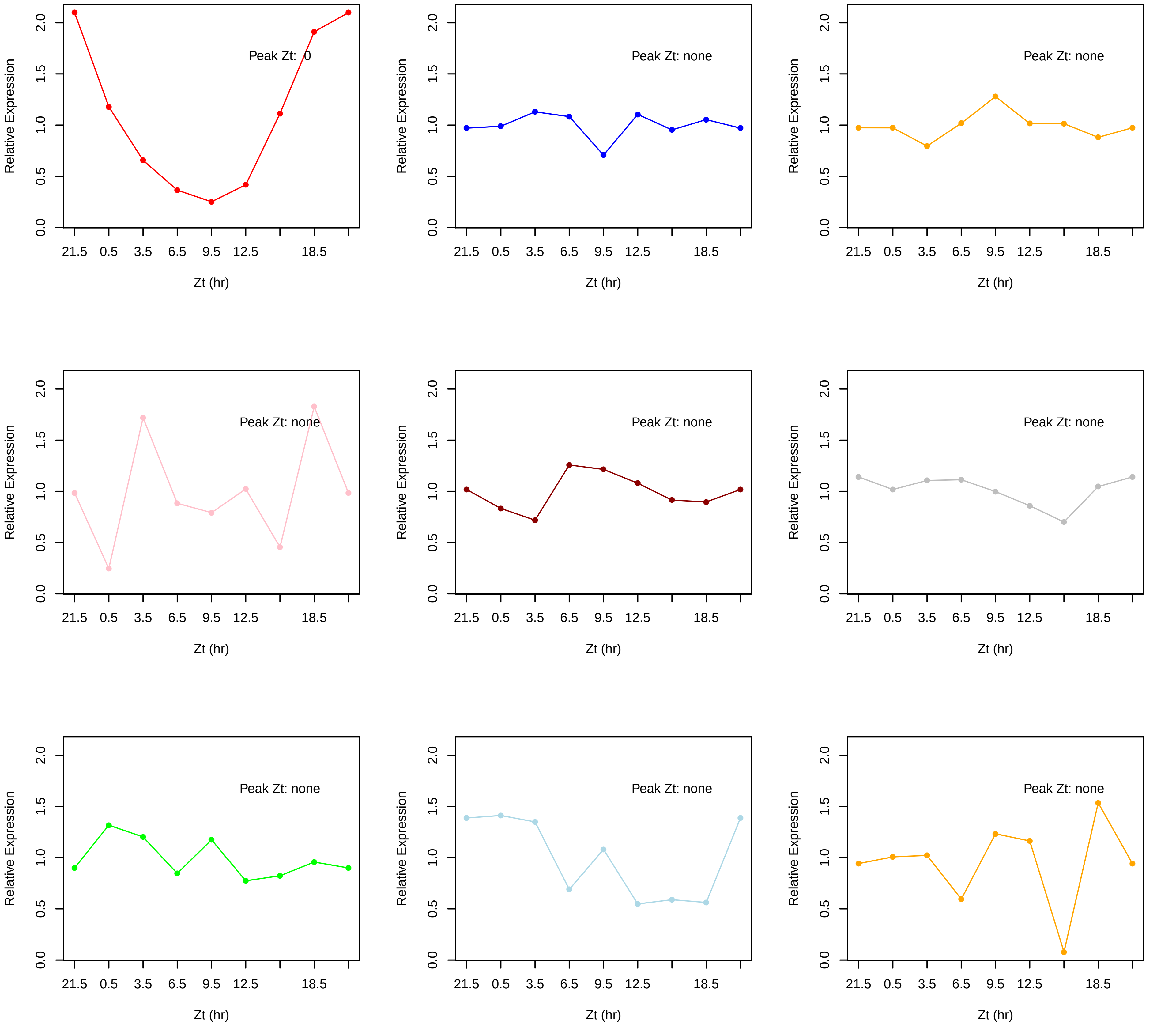

Singeleton: ENSXMAG00000018880 galns

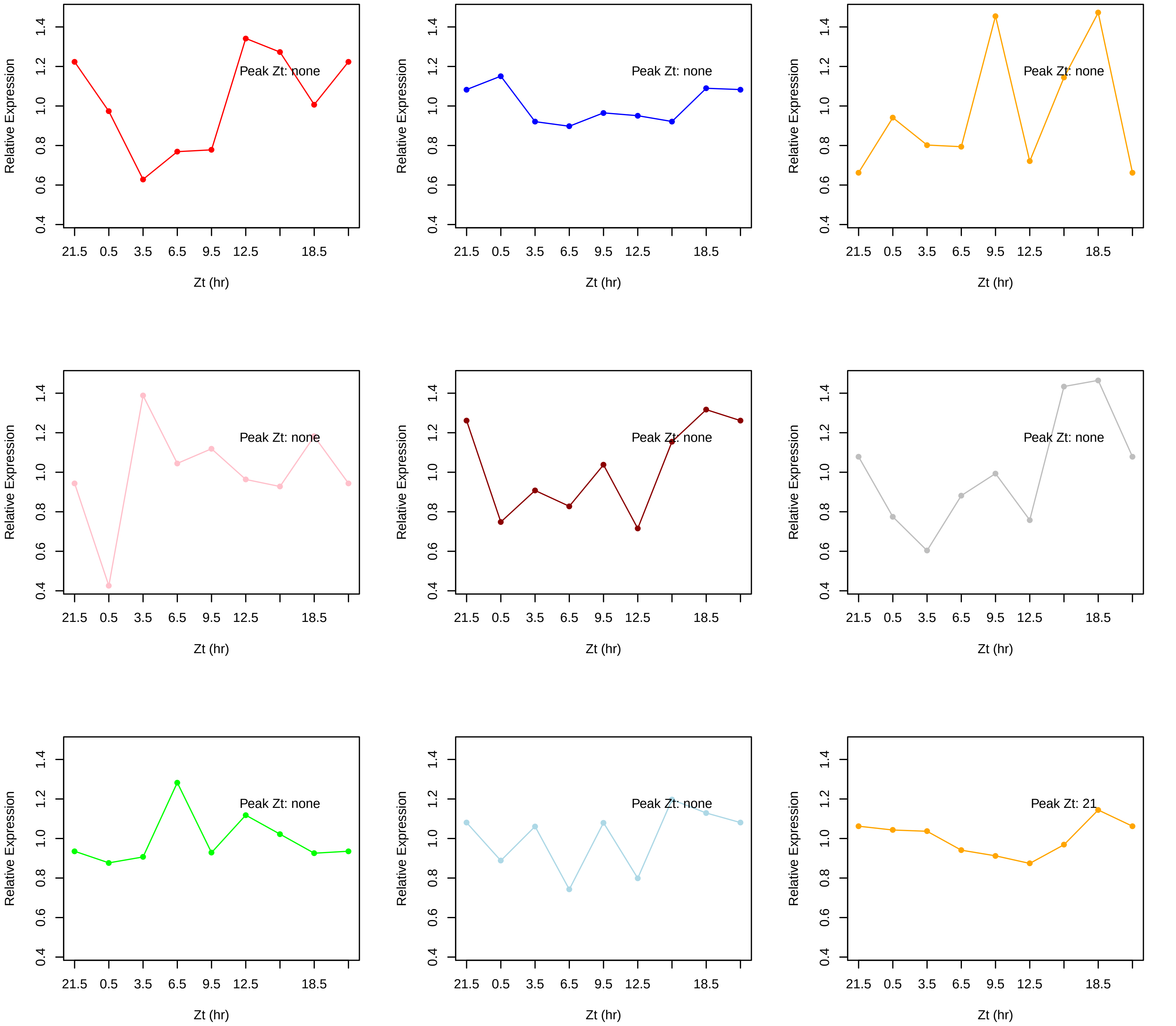

Singeleton: ENSXMAG00000023810 znf618

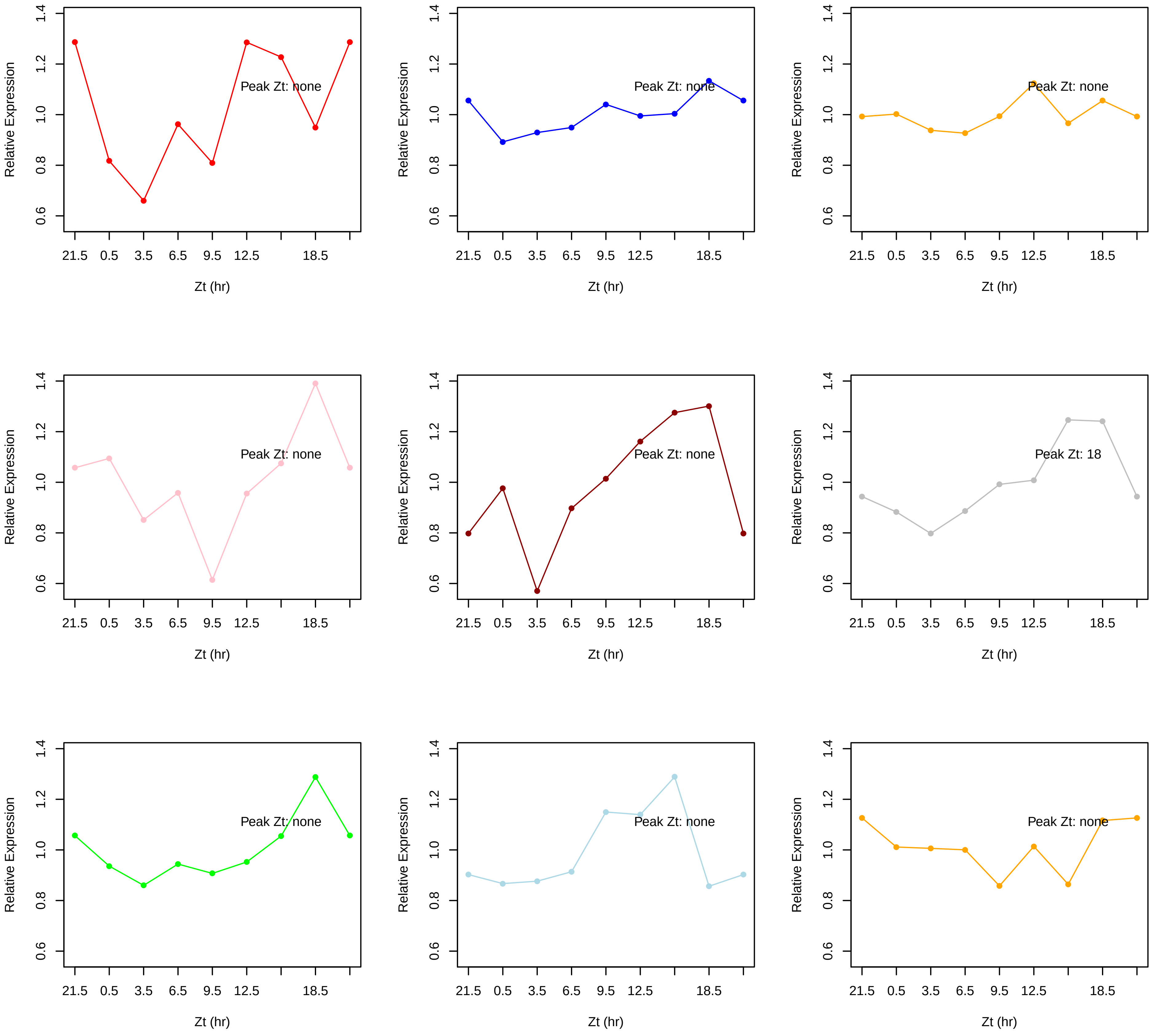

Singleton: ENSXMAG00000023943 si:ch211−210c8.6

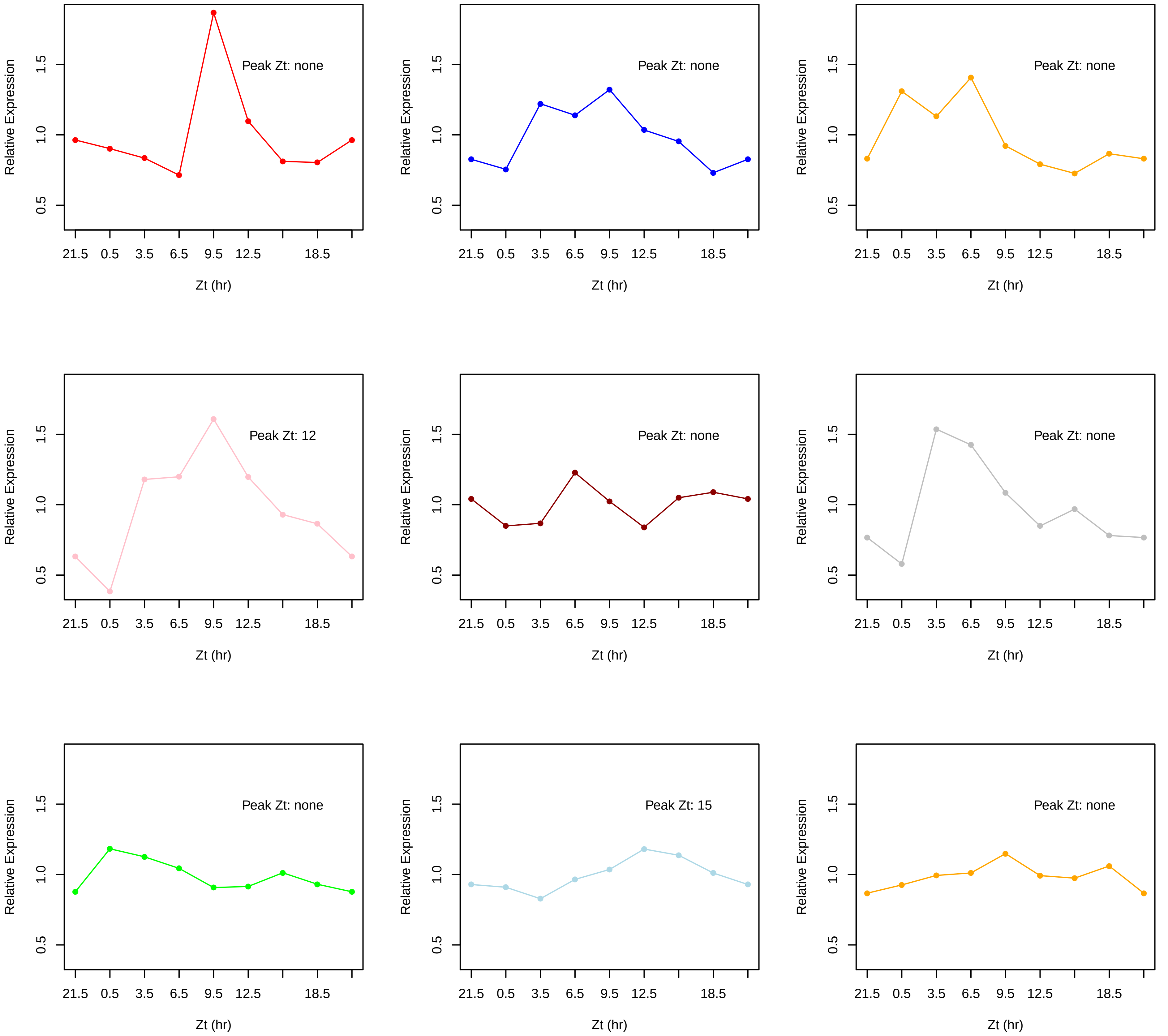

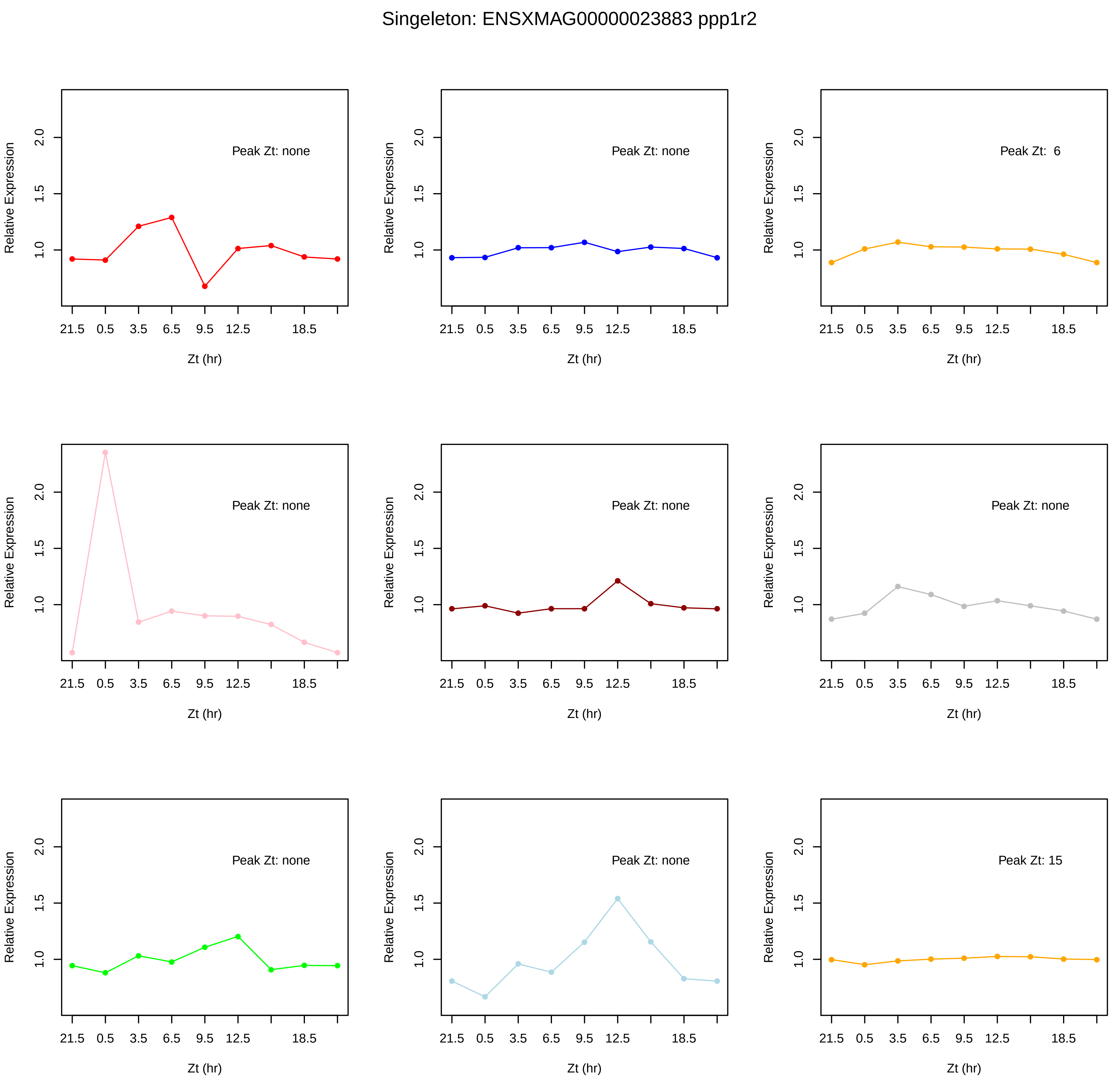
Singeleton: ENSXMAG00000023883 ppp1r2
Peak Zt: none
Peak Zt: none
Peak Zt: 6
Peak Zt: none
Peak Zt: none
Peak Zt: none
Peak Zt: none
Peak Zt: none
Peak Zt: 15
Relative Expression
Zt (hr)
21.5 0.5 3.5 6.5 9.5 12.5 18.5
1.0 1.5 2.0

Singeleton: ENSXMAG00000029314 sc5d

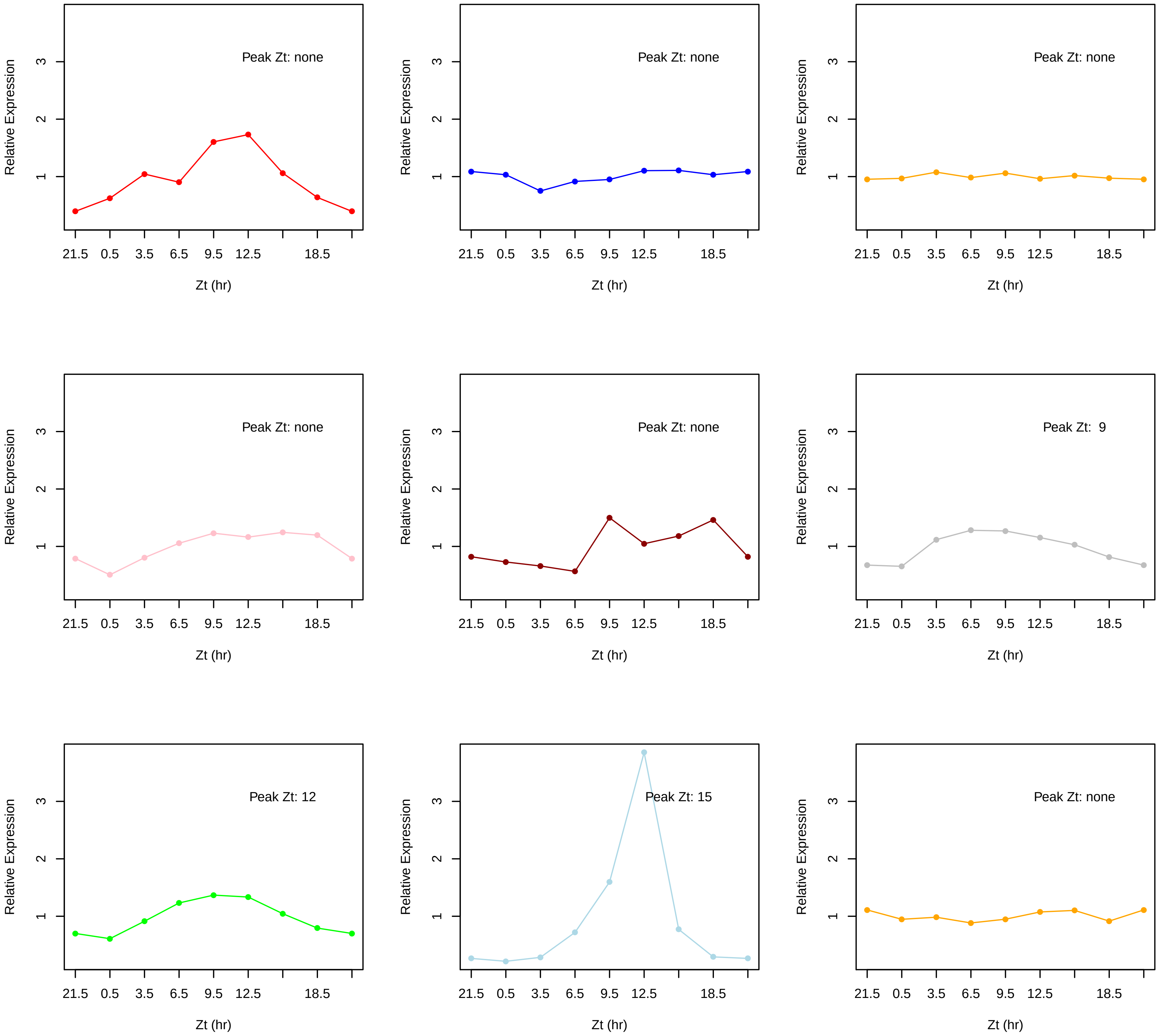

Singeleton: ENSXMAG00000026912 ppox

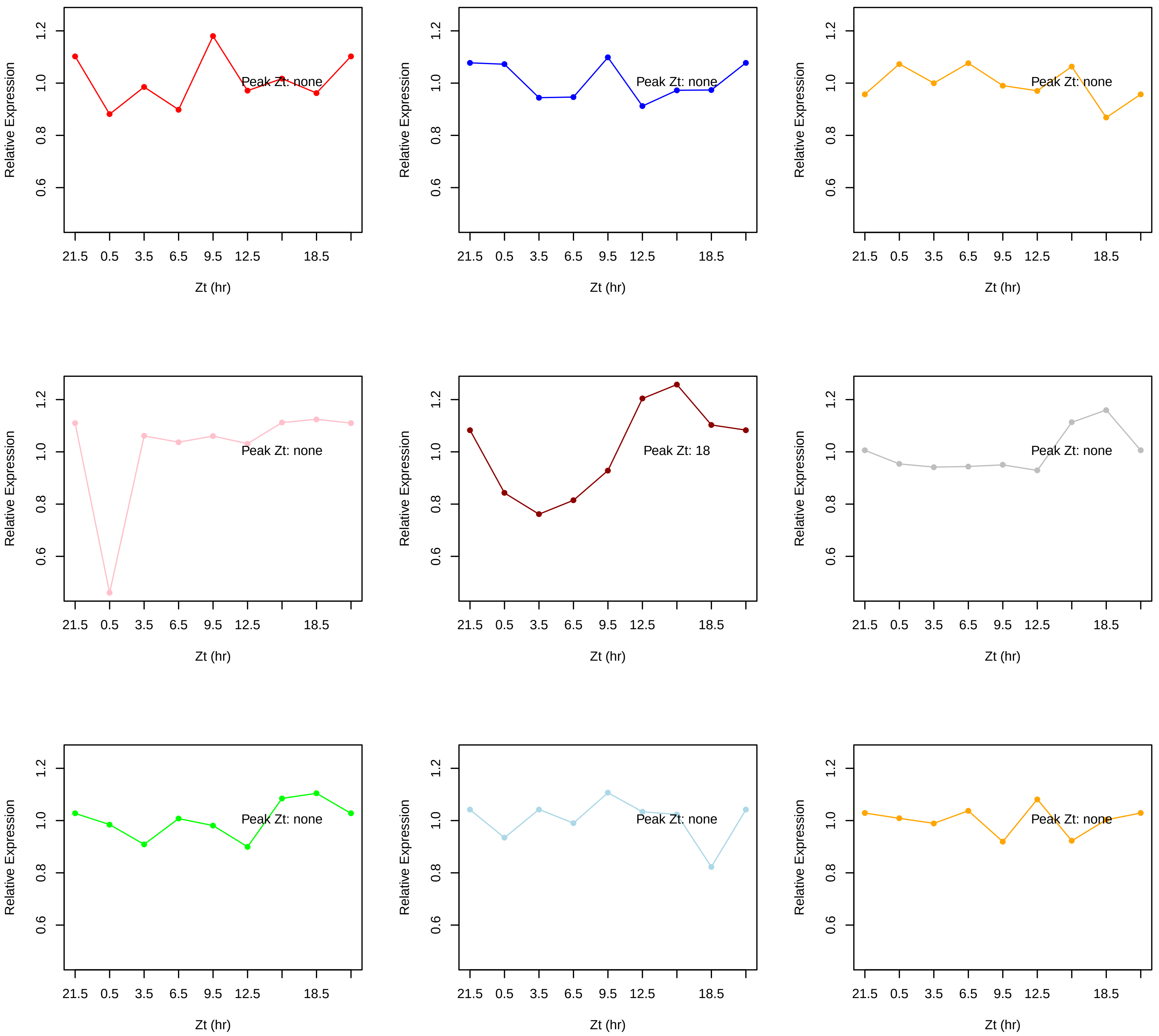

Singleton: ENSXMAG00000026740 wsb1

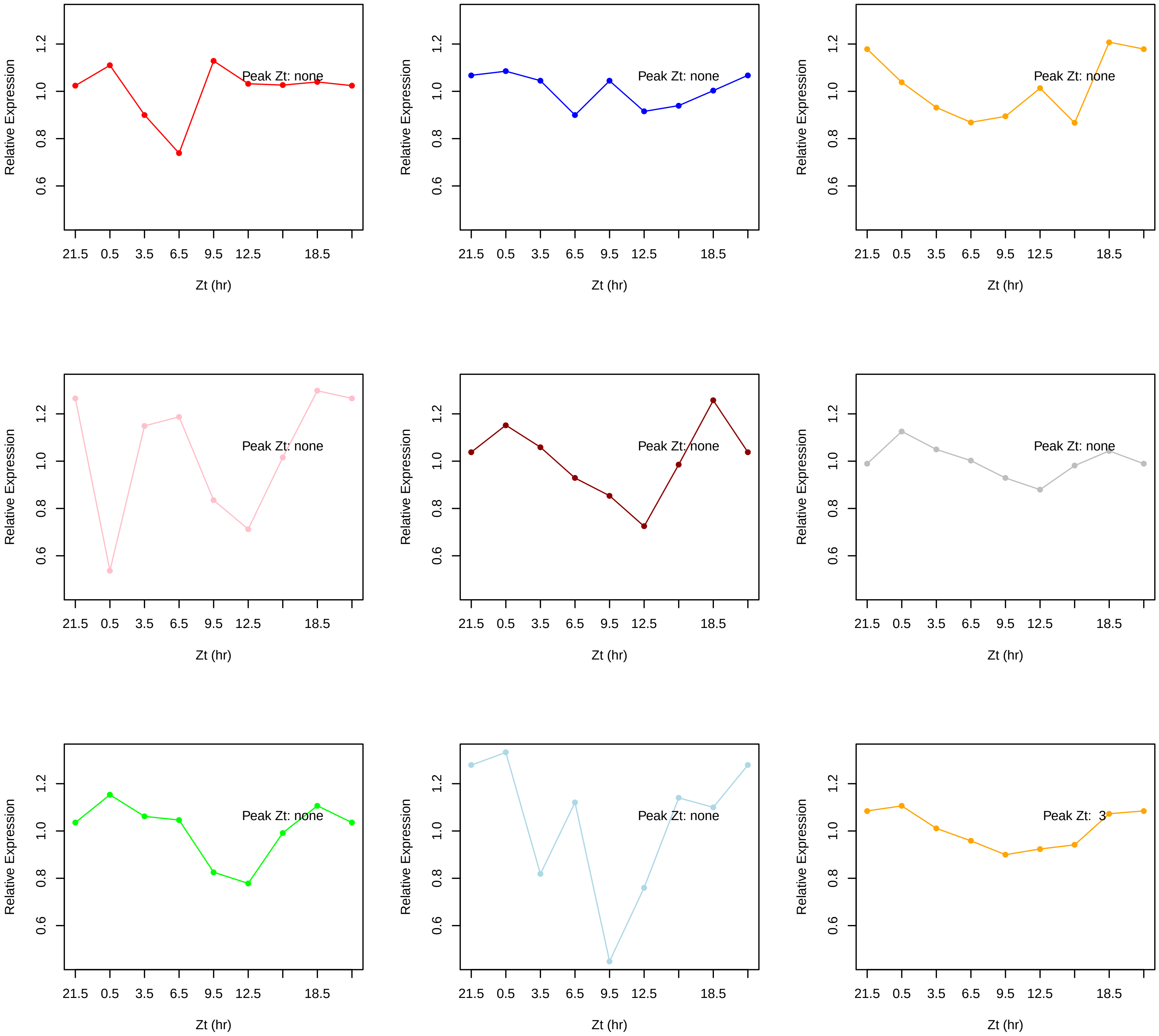

Singeleton: ENSXMAG00000026805 rgs3b

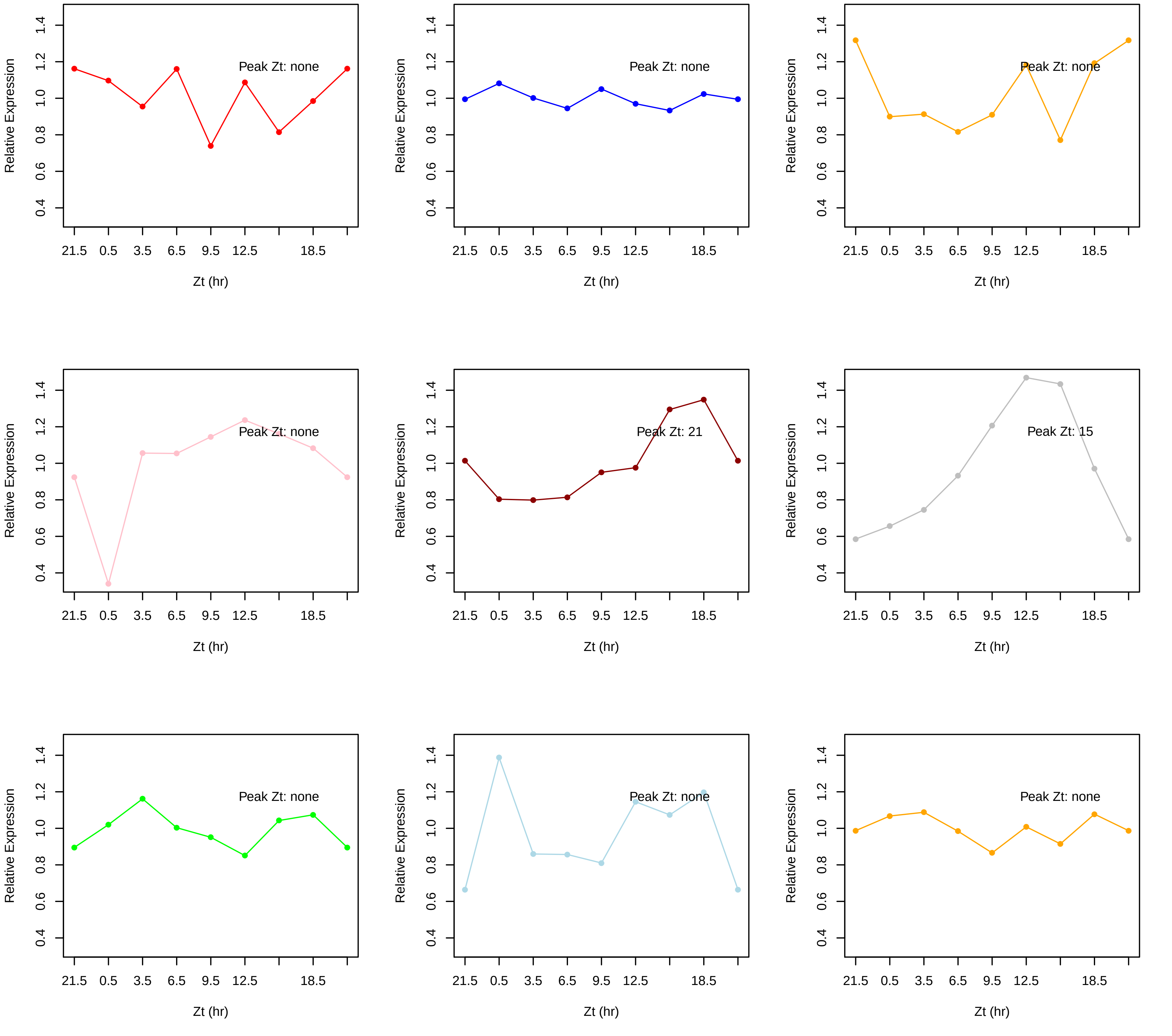

Singleton: ENSXMAG00000025235 rfxank

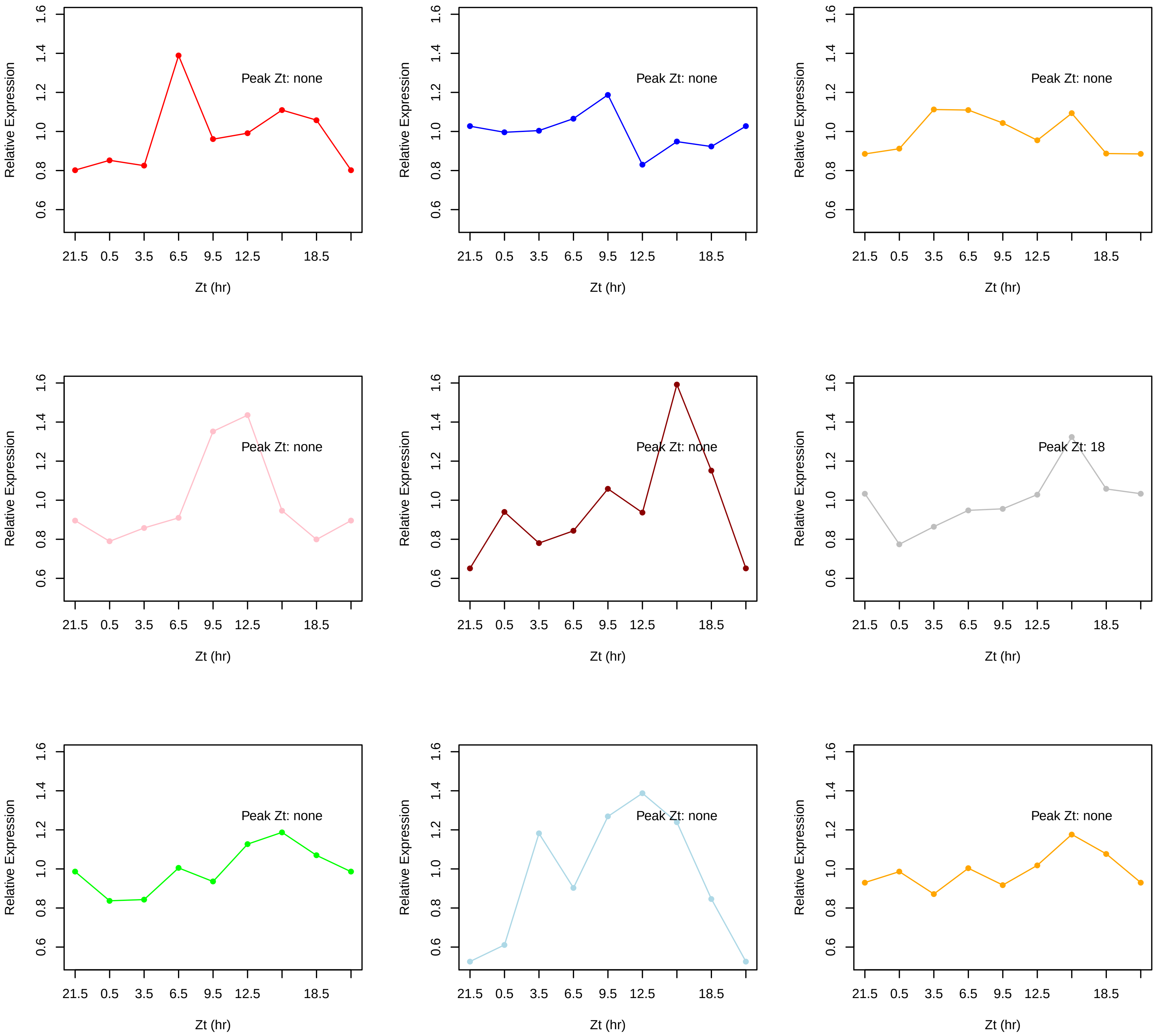

Singeleton: ENSXMAG00000021955 aprt

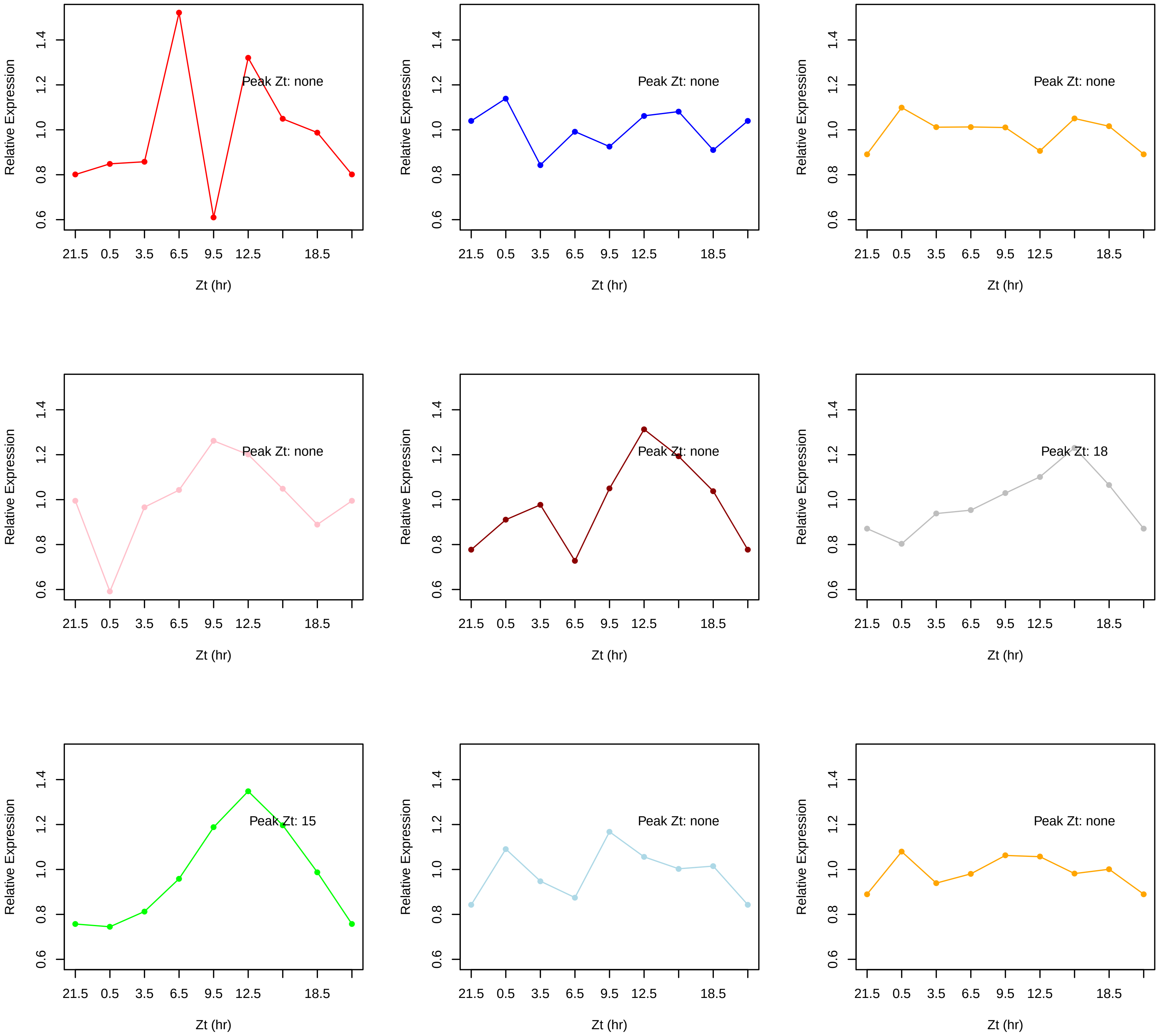

Singeleton: ENSXMAG00000013888 wnt11r

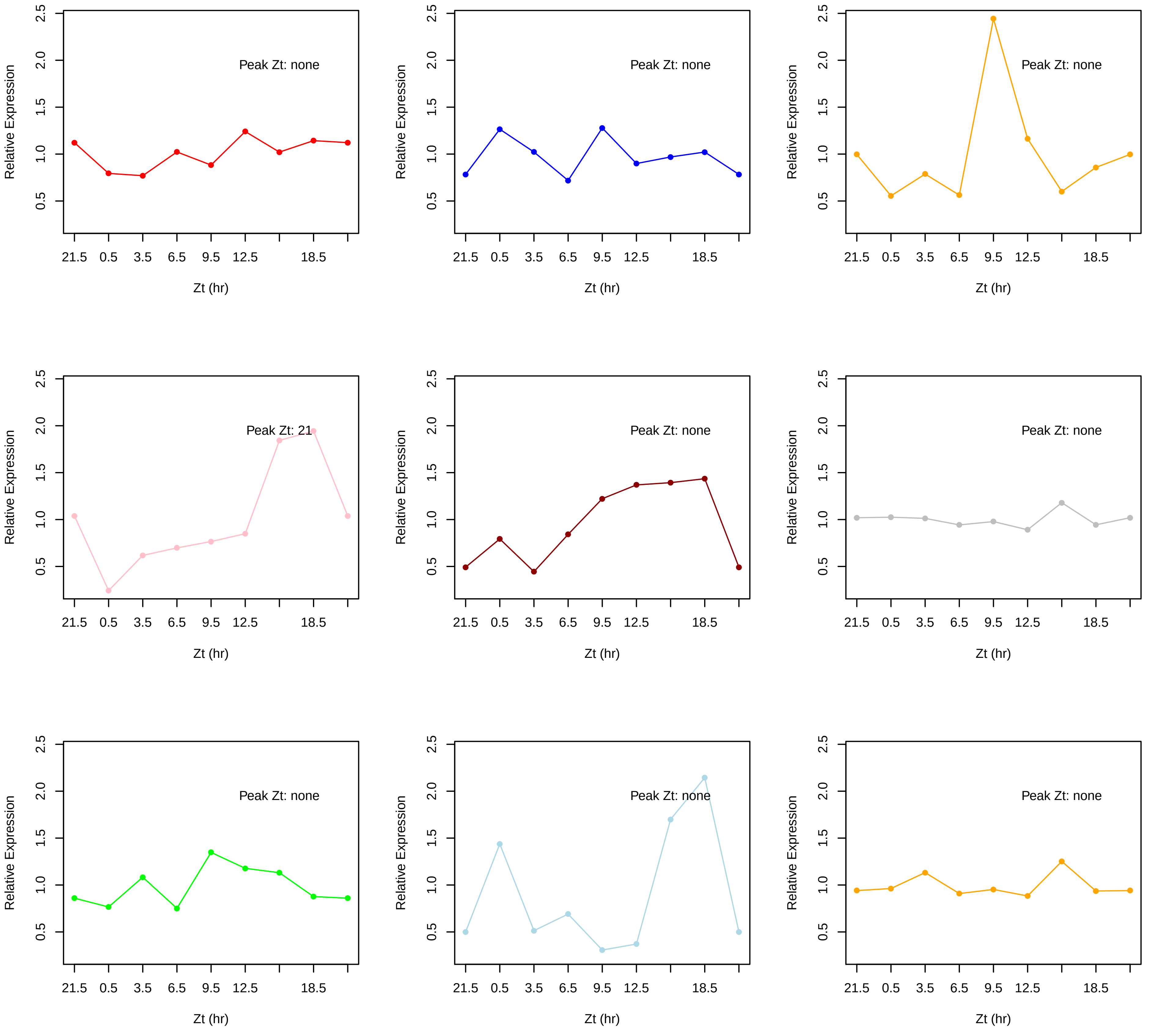

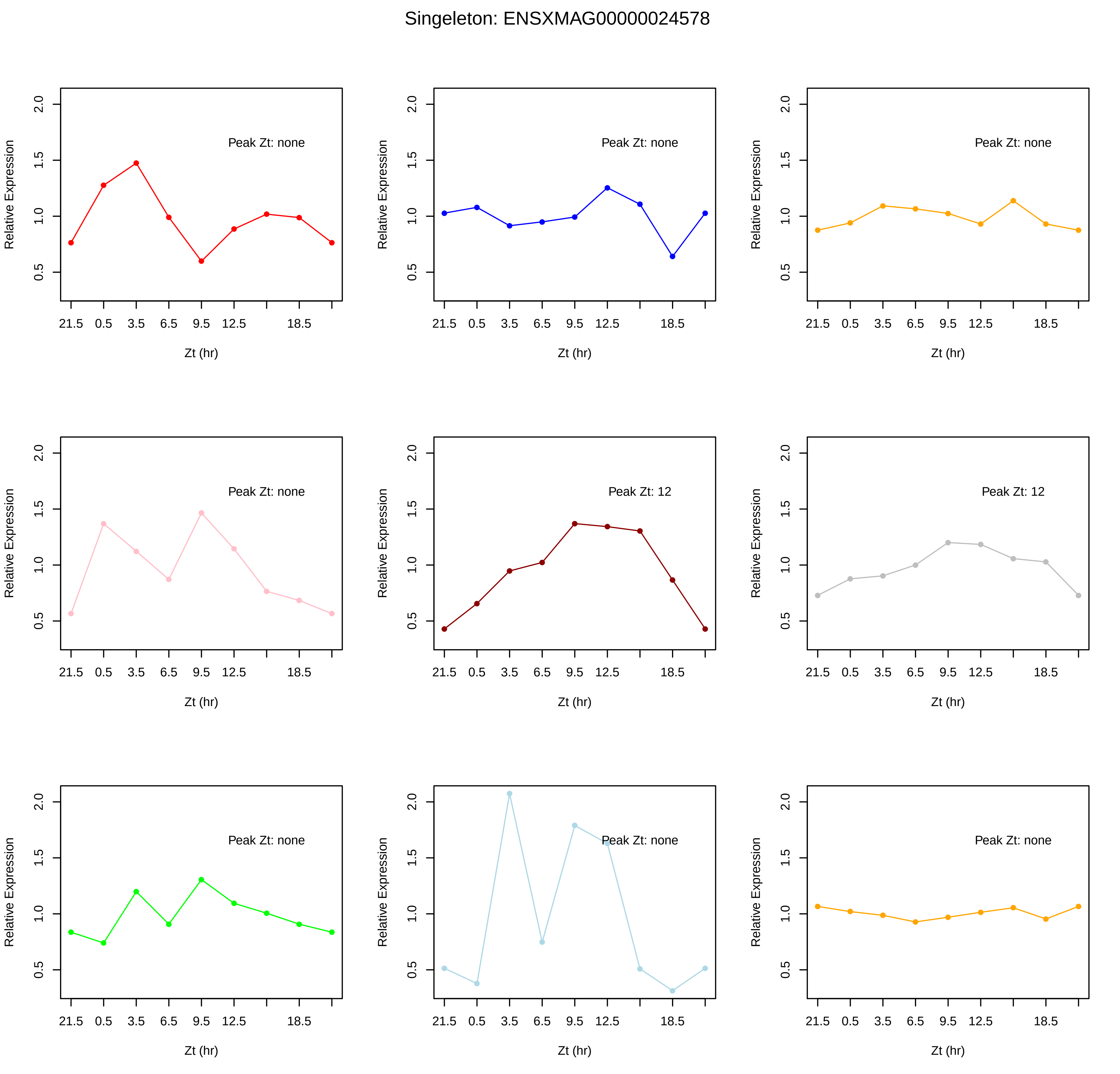
Singeleton: ENSXMAG00000024578
Peak Zt: none
Peak Zt: none
Peak Zt: none
Peak Zt: none
Peak Zt: 12
Peak Zt: 12
Peak Zt: none
Peak Zt: none
Peak Zt: none
Relative Expression
Zt (hr)
21.5 0.5 3.5 6.5 9.5 12.5 18.5
0.5 1.0 1.5 2.0

Singleton: ENSXMAG00000000598 ksr1a

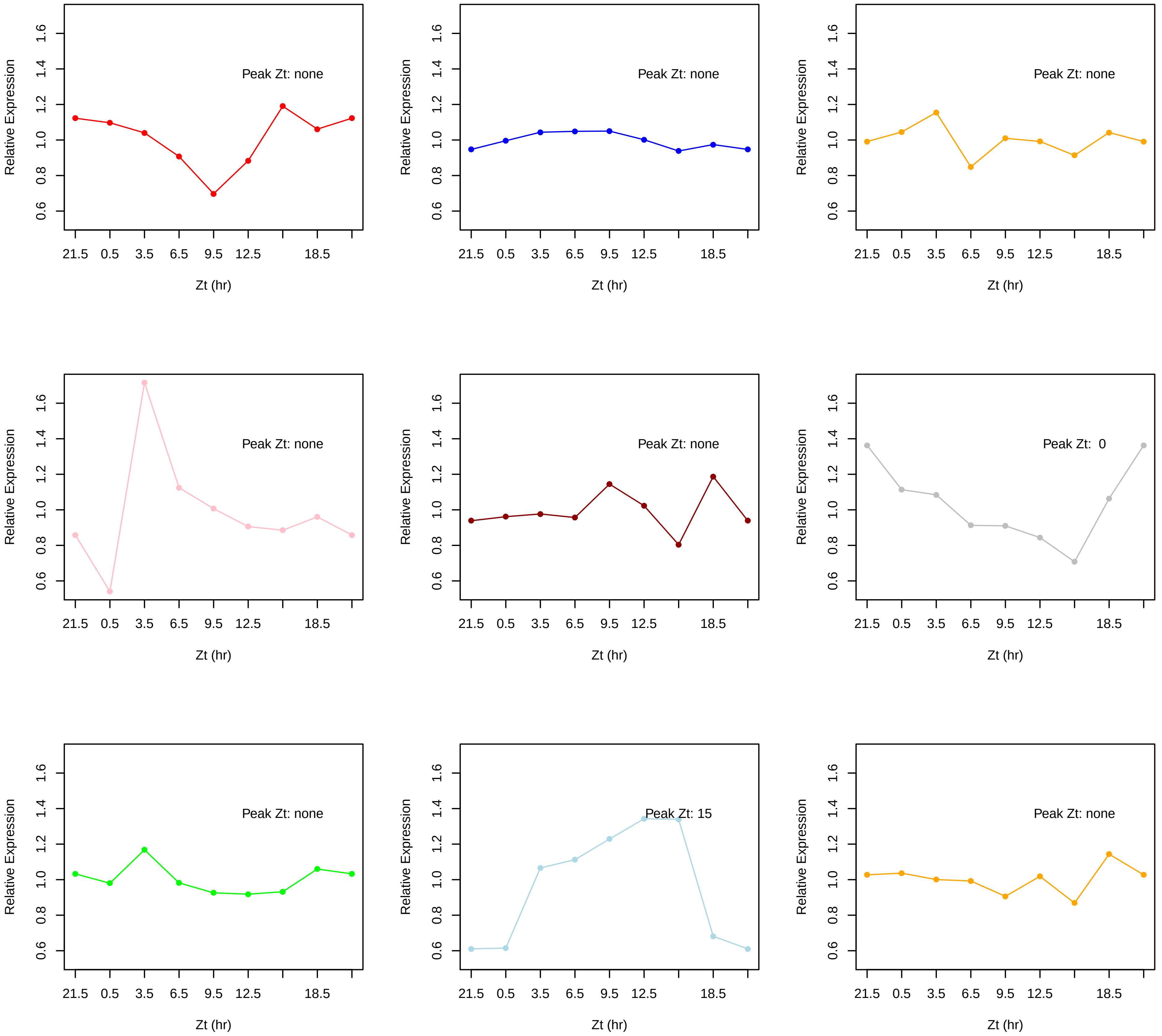

Singeleton: ENSXMAG00000002876 sytl1

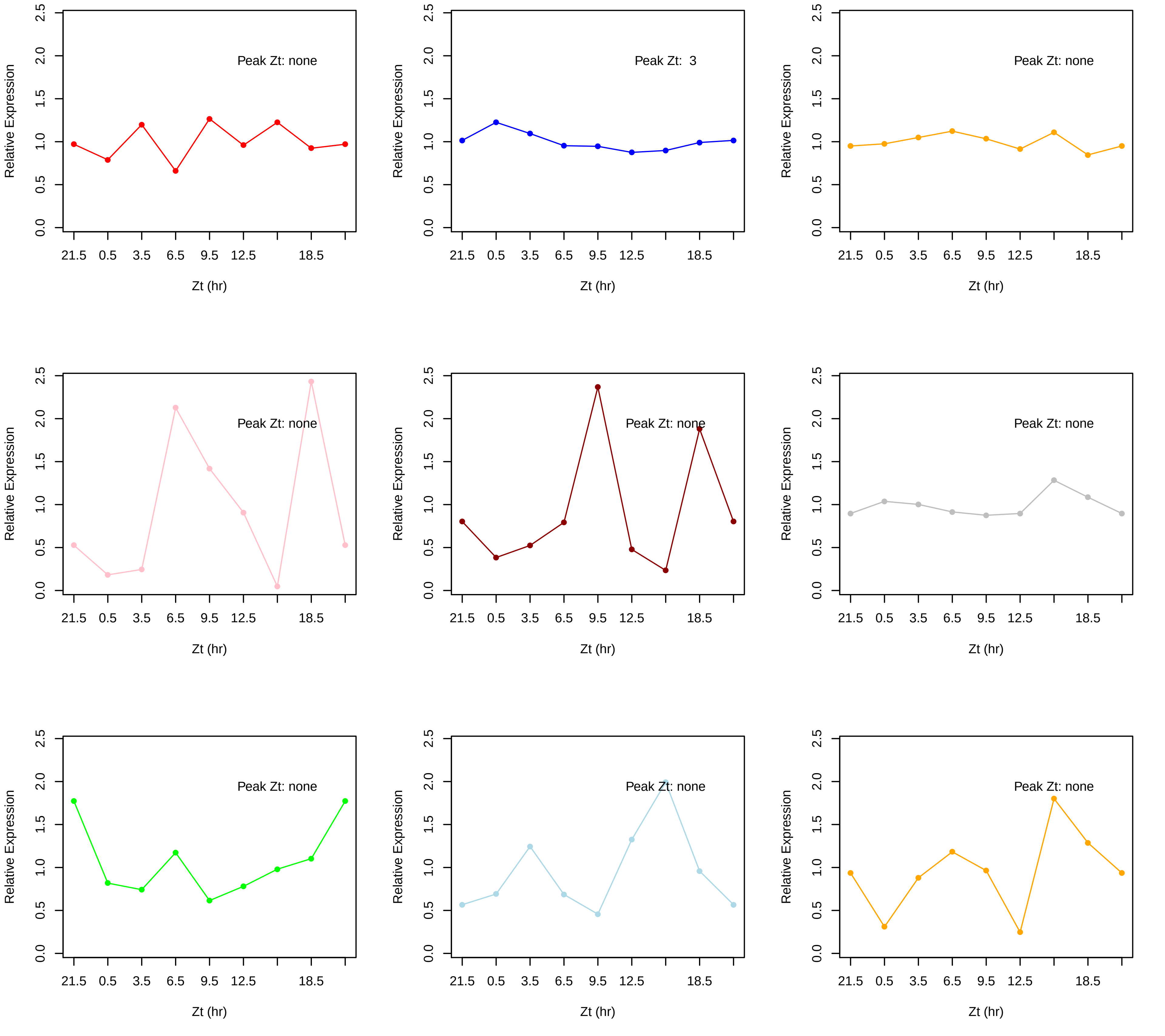

Singeleton: ENSXMAG00000000842 arhgap32b

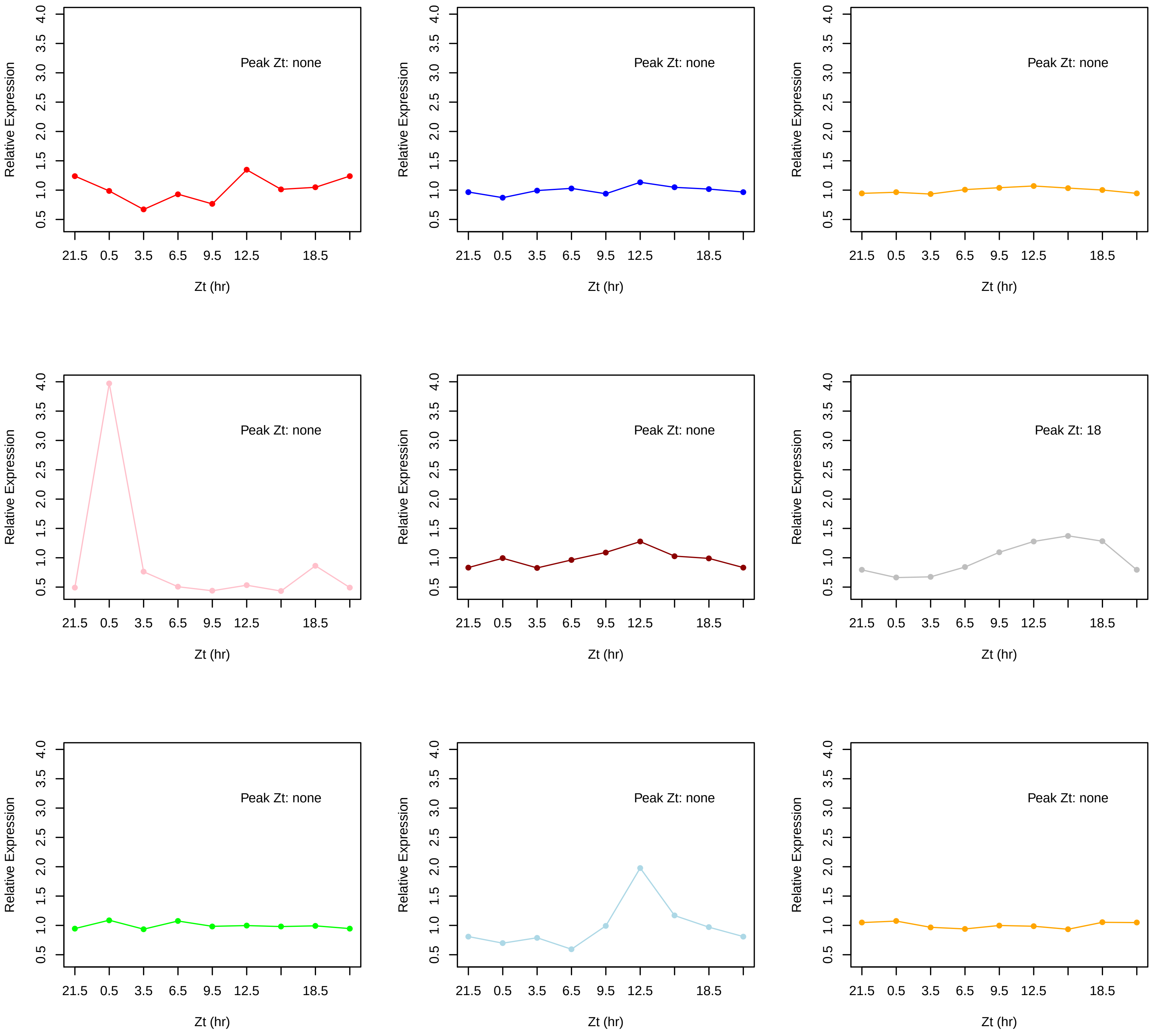

Singeleton: ENSXMAG00000013892

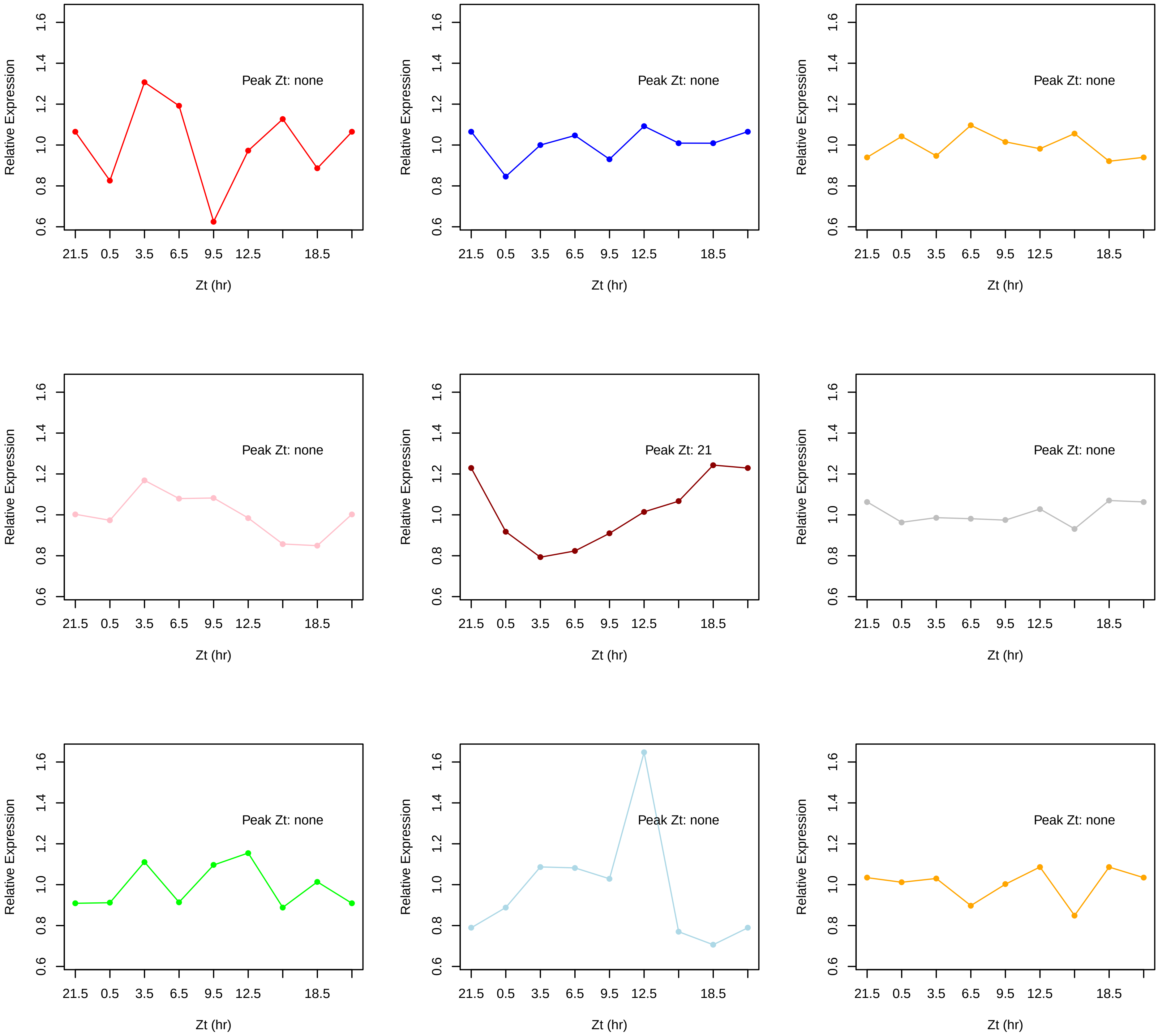

Singeleton: ENSXMAG00000020951

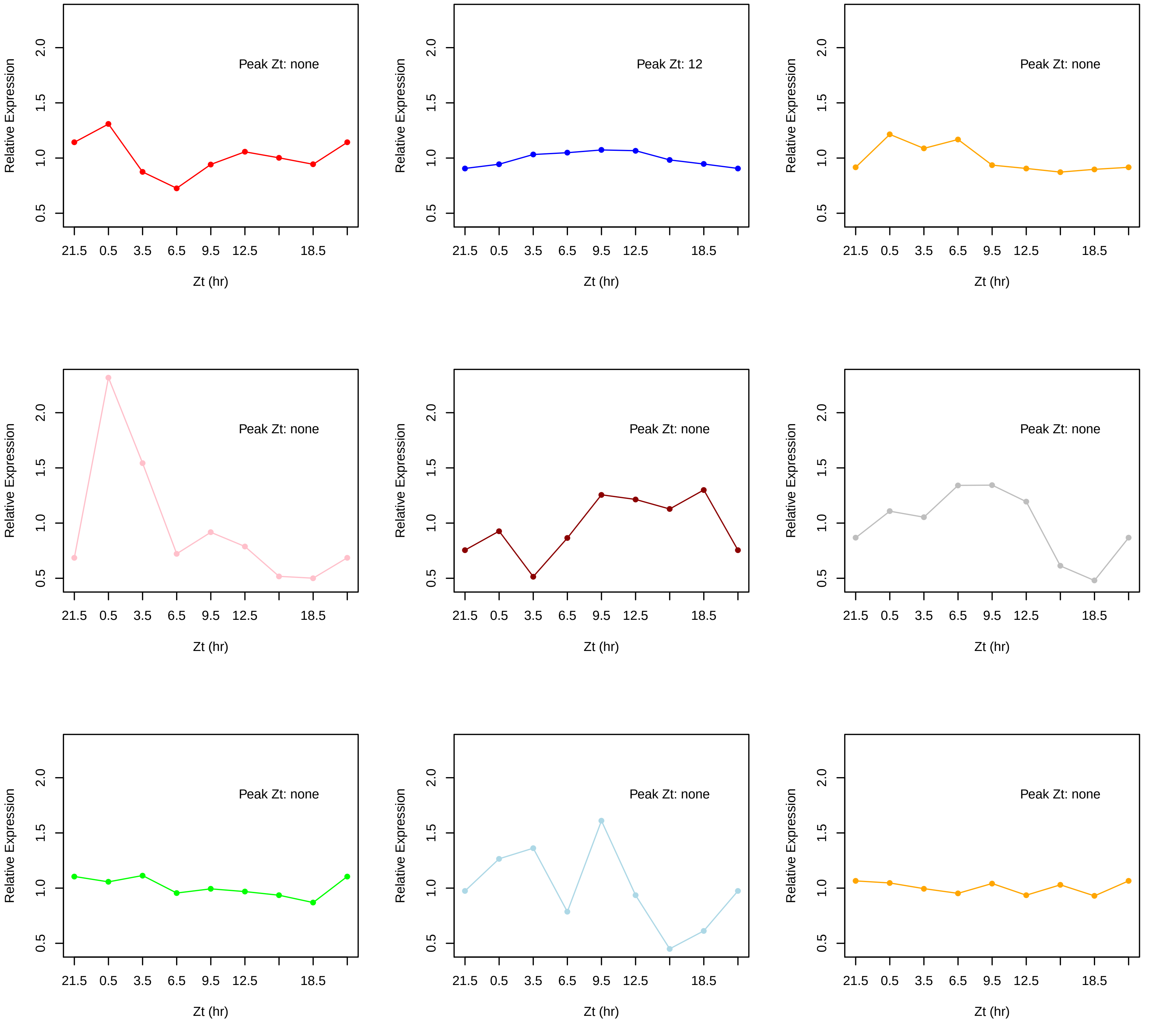

Singeleton: ENSXMAG00000012261 borcs8

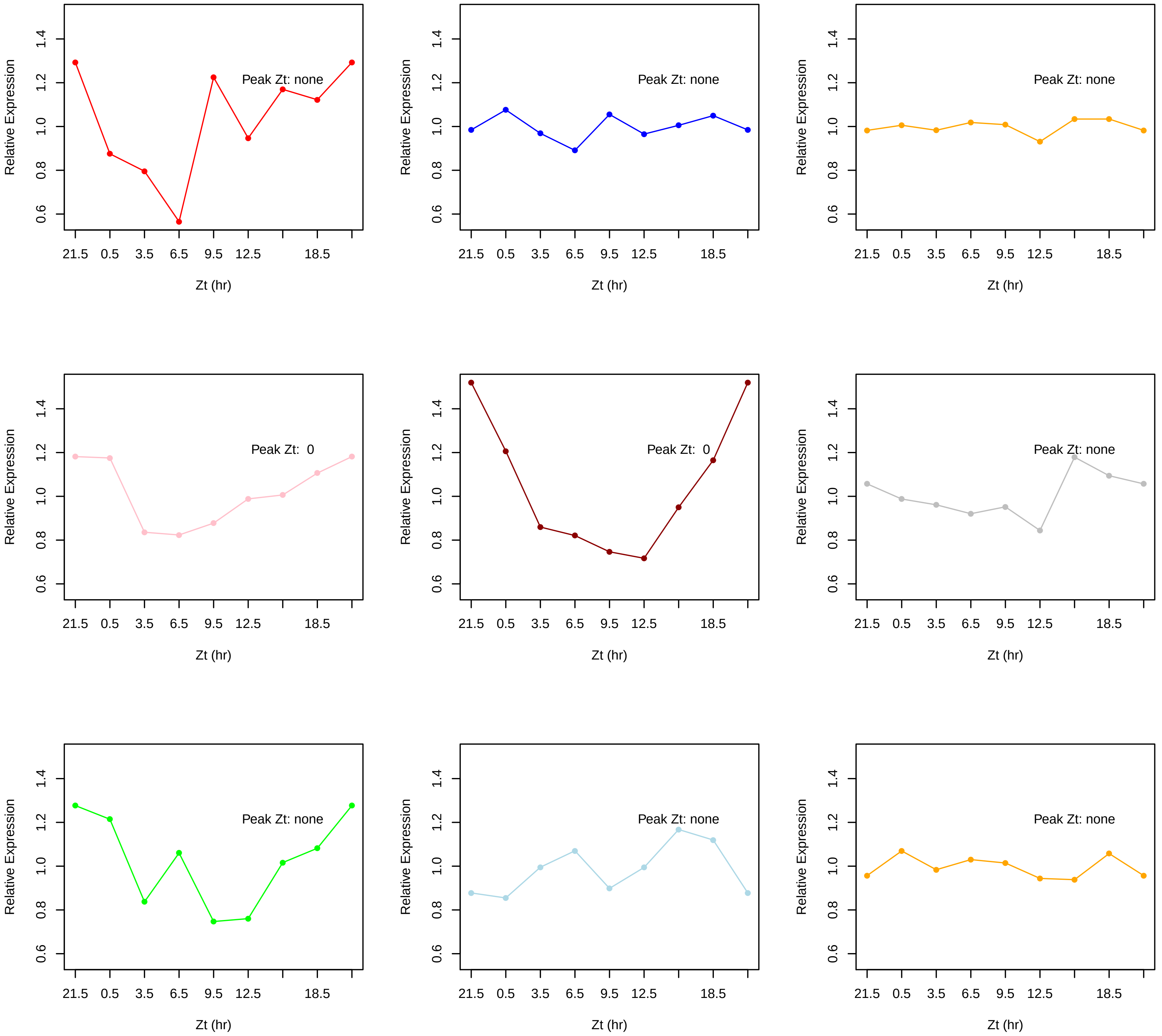

Singeleton: ENSXMAG00000026950 stac

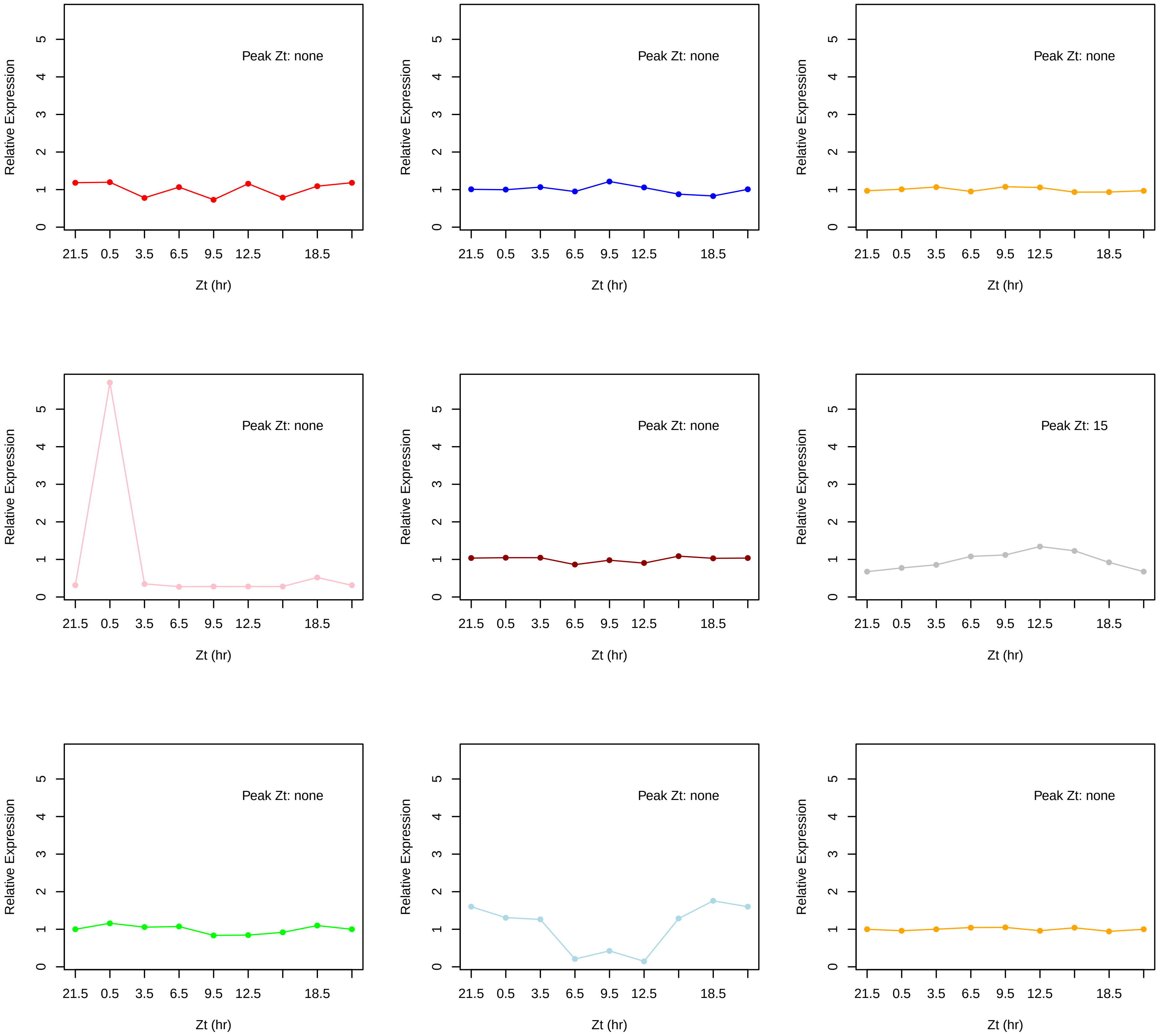

Singleton: ENSXMAG00000027708 gpc5a

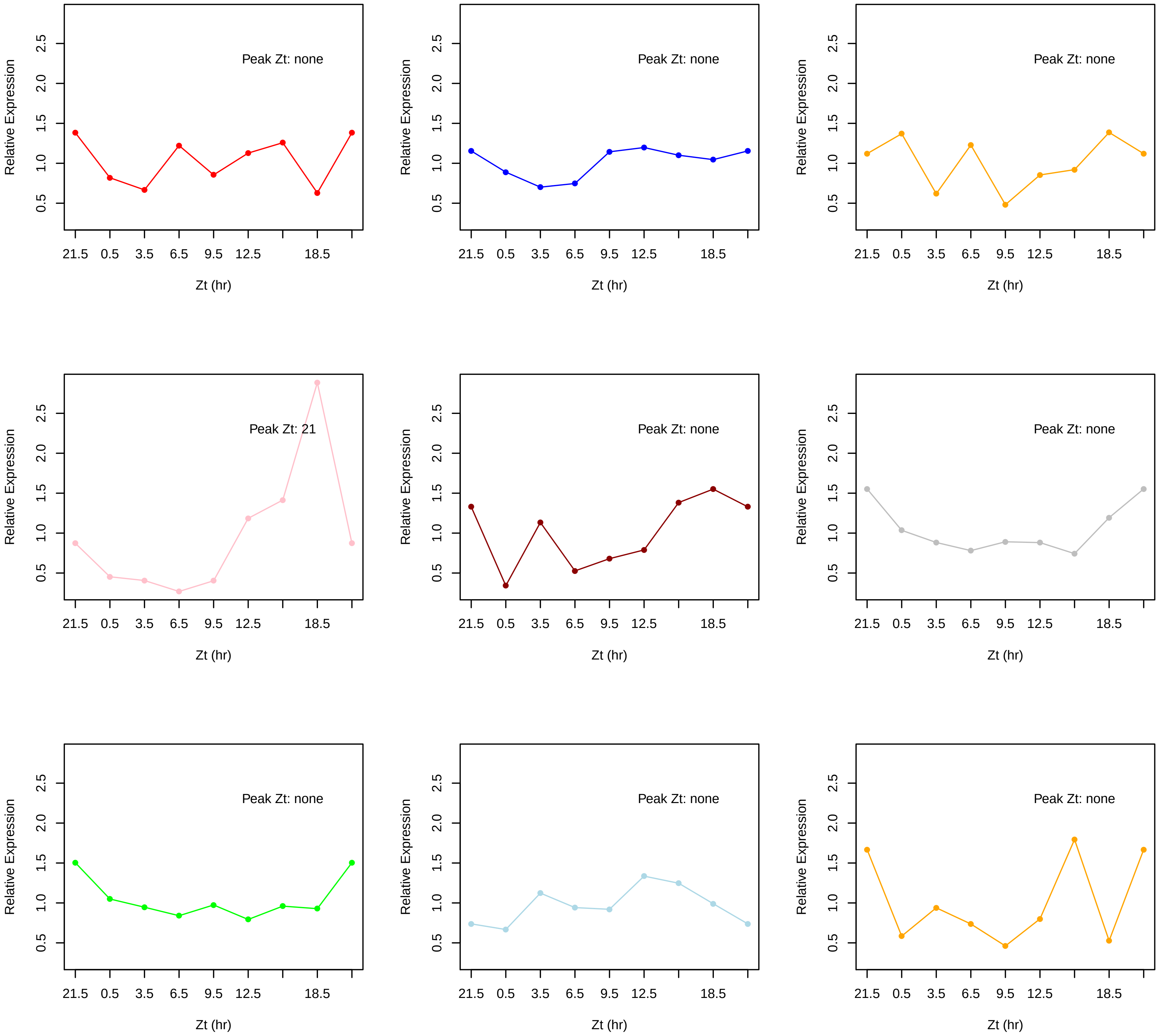

Singeleton: ENSXMAG00000022342 cdt1

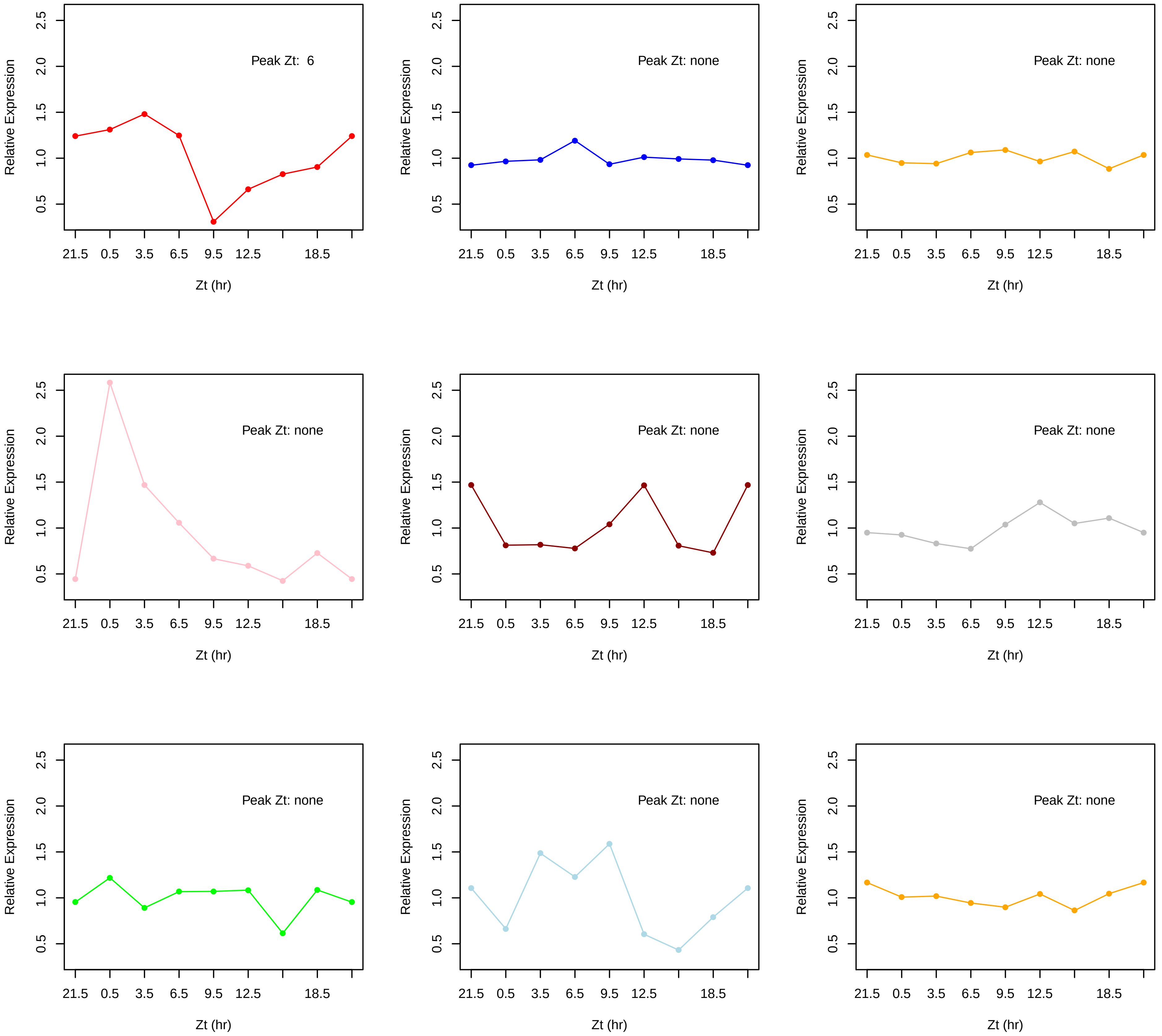

Singeleton: ENSXMAG00000005944 alpi.1

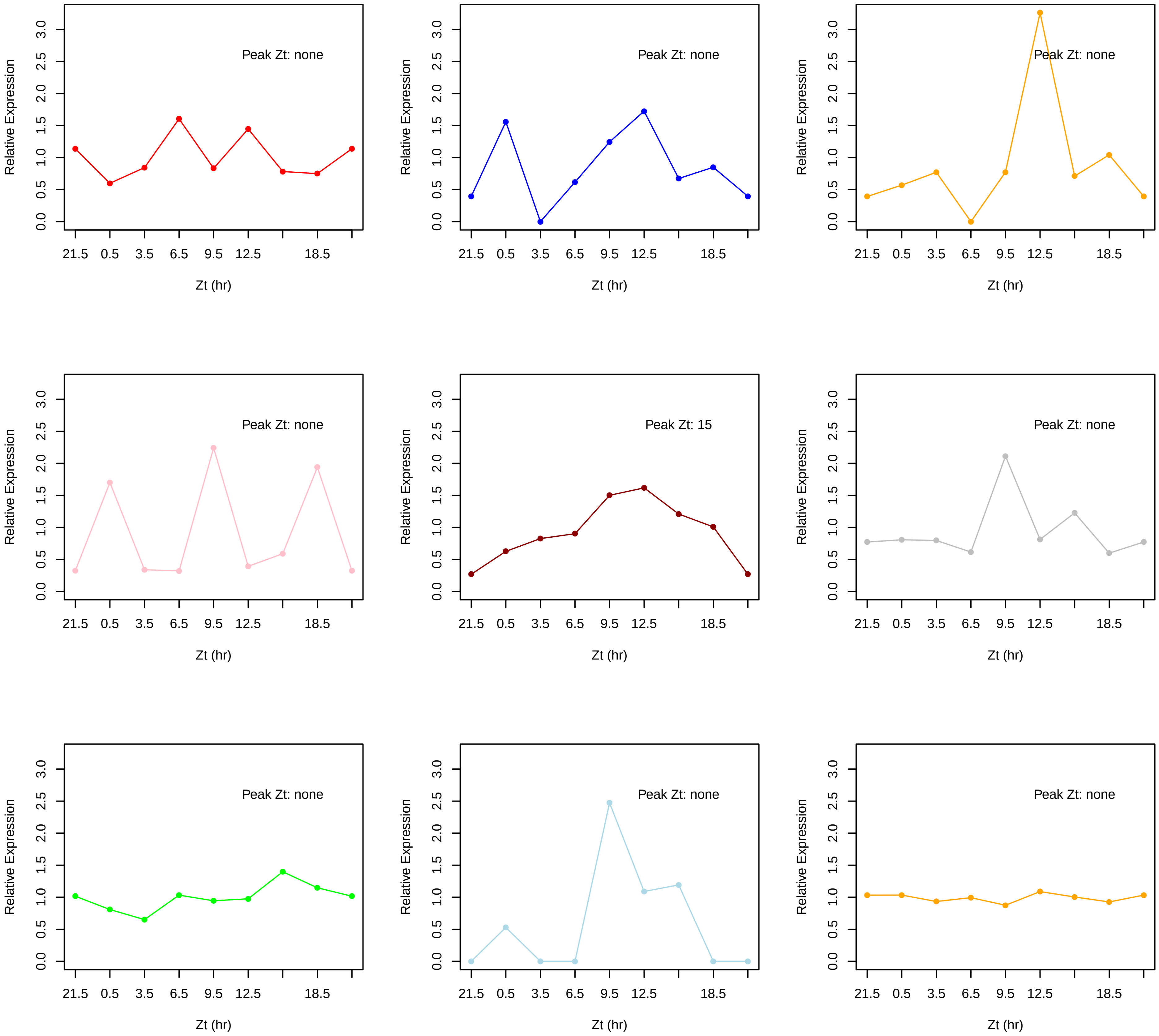

Singleton: ENSXMAG00000006201 pank4

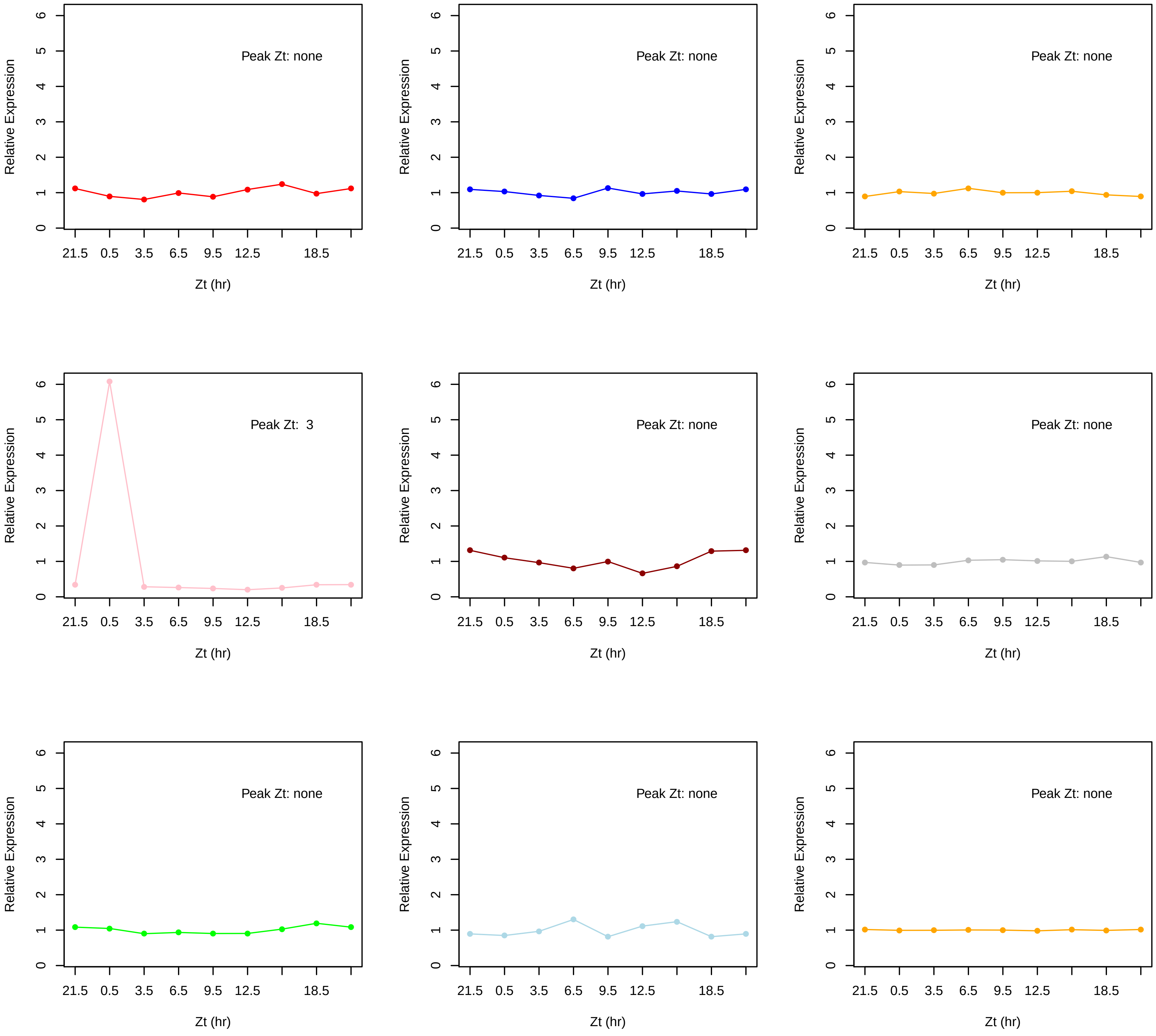

Singeleton: ENSXMAG00000005189 dazap2

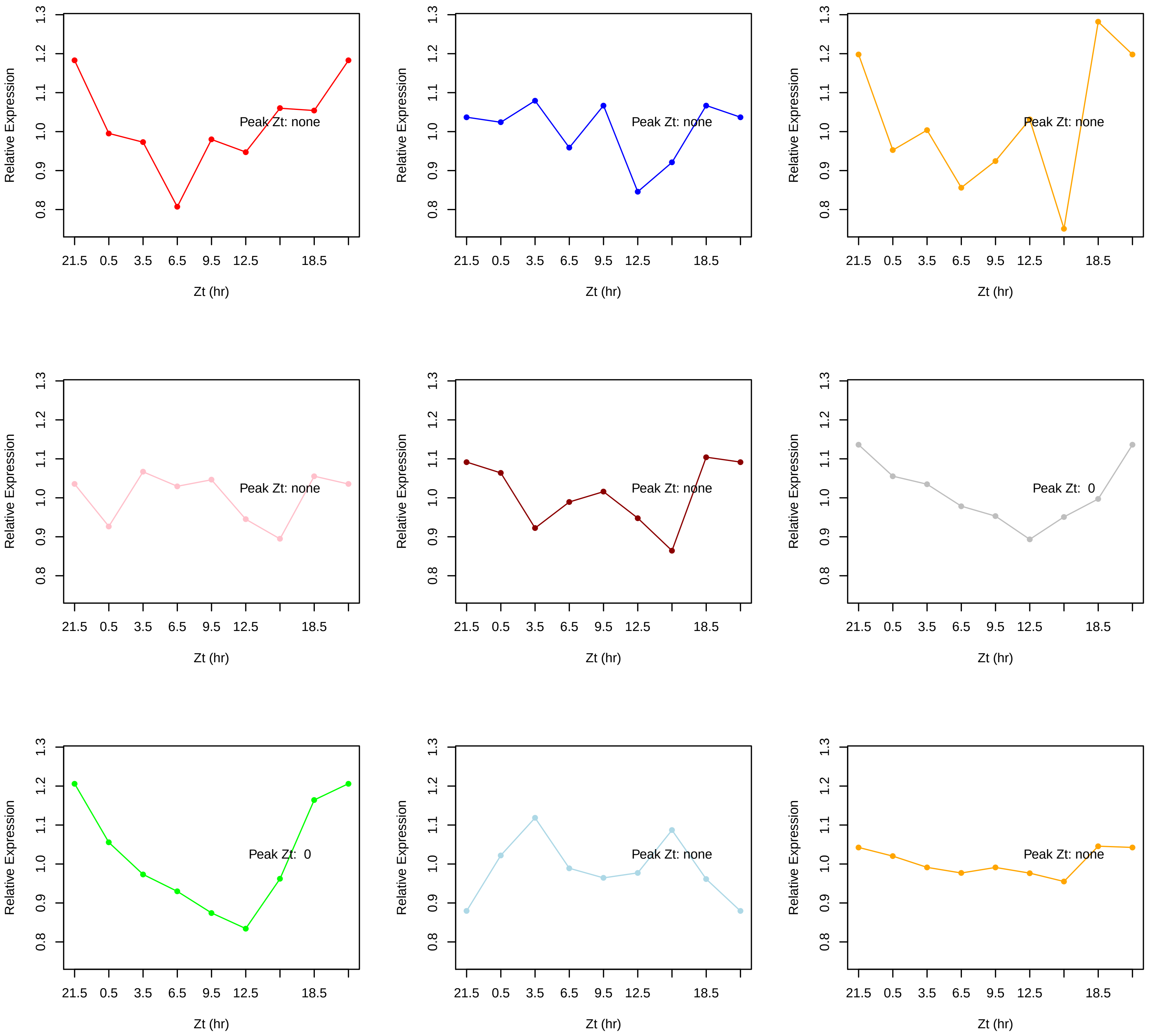

Singeleton: ENSXMAG00000010253 zdhhc13

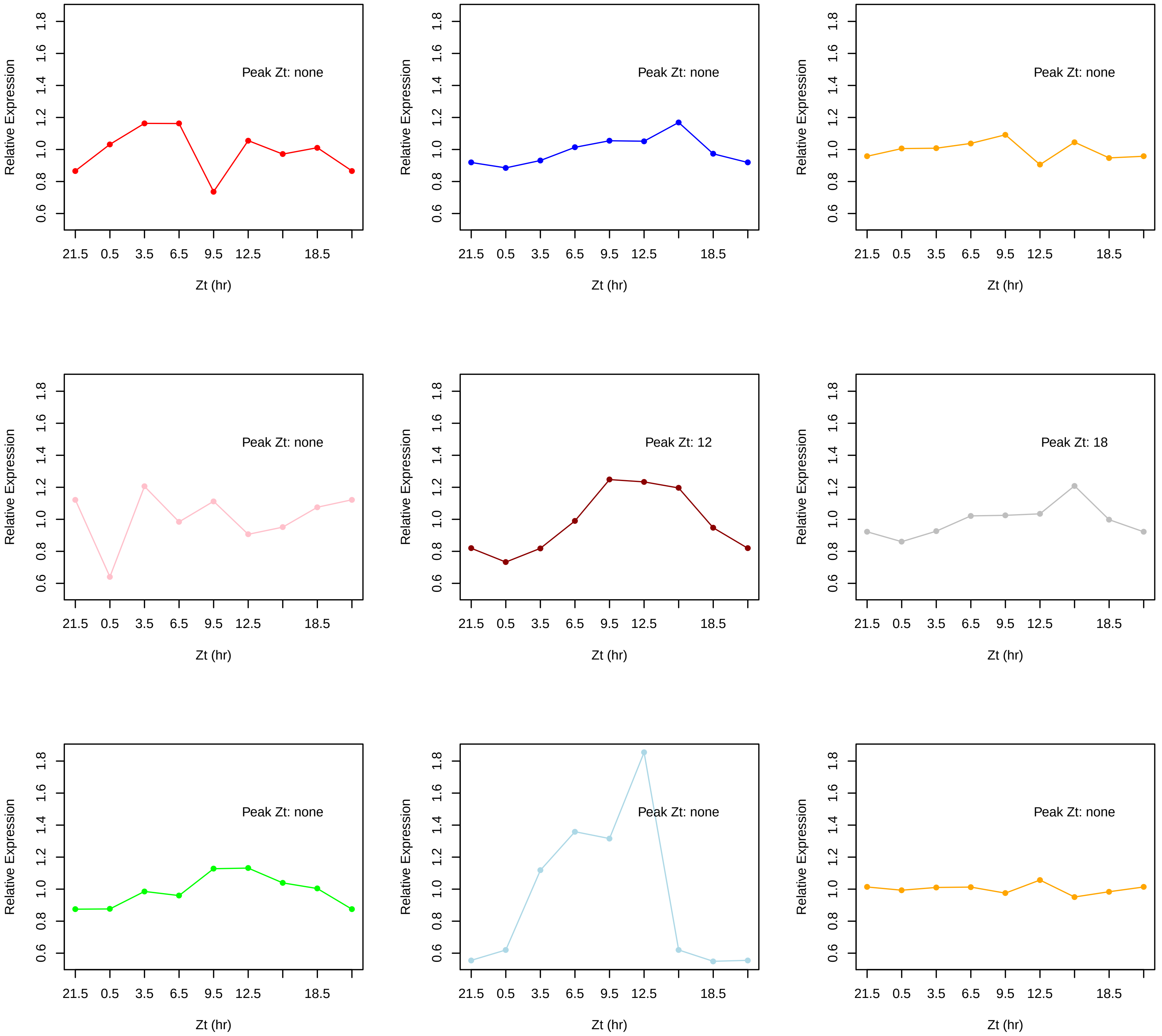

Singeleton: ENSXMAG00000011377 edf1

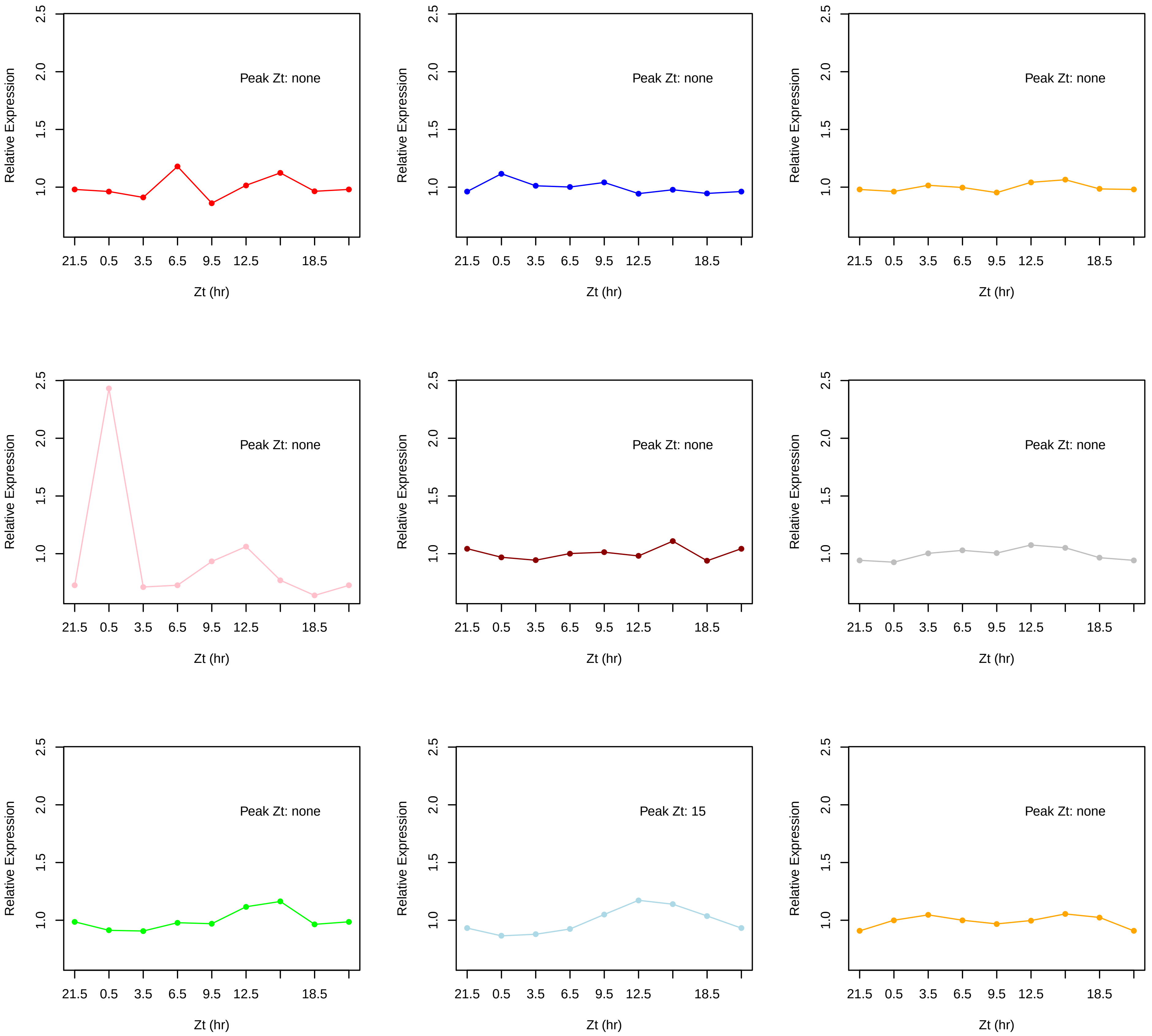

Singeleton: ENSXMAG00000012229 cuedc1b

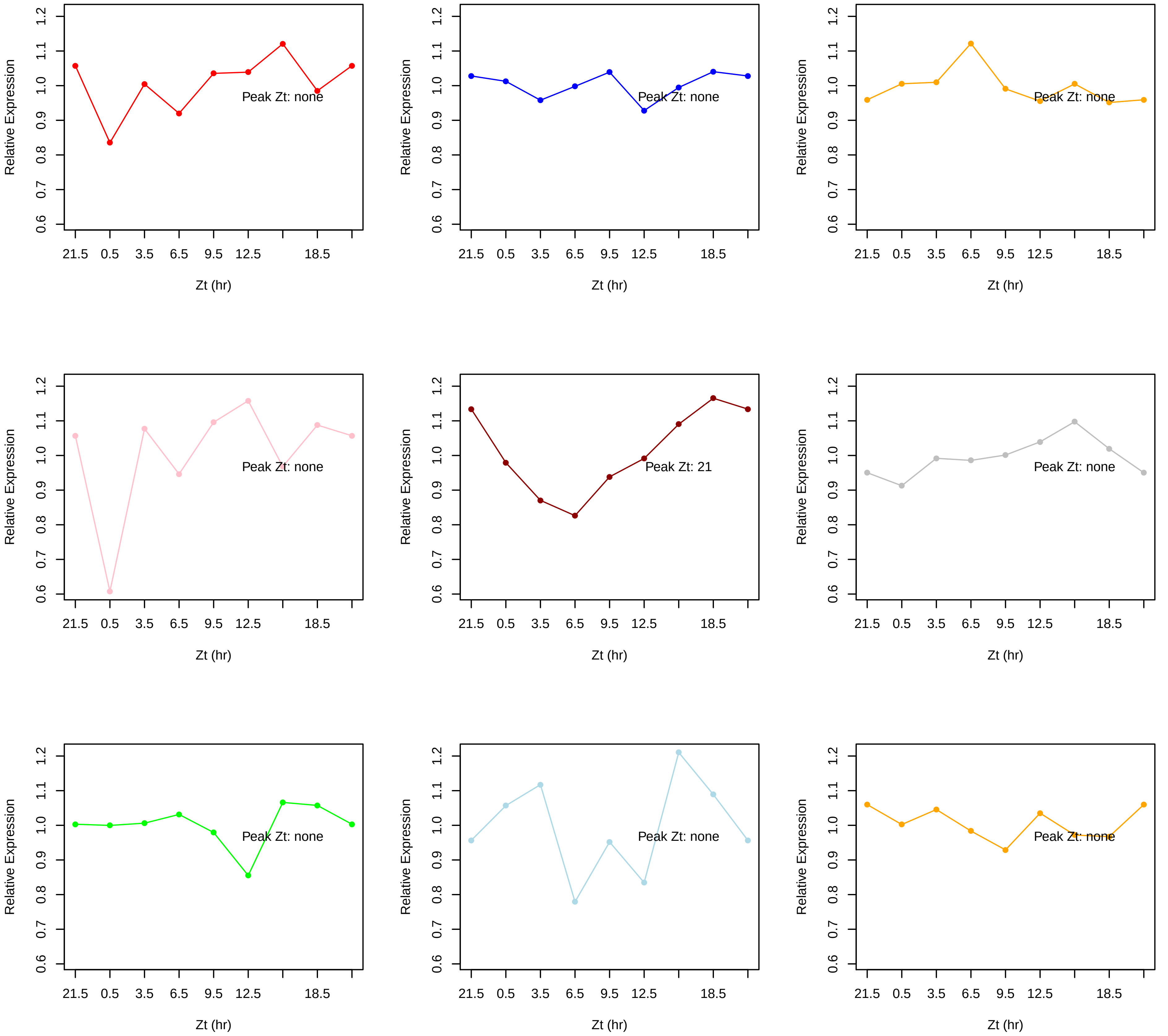

Singeleton: ENSXMAG00000018177 zgc:162344

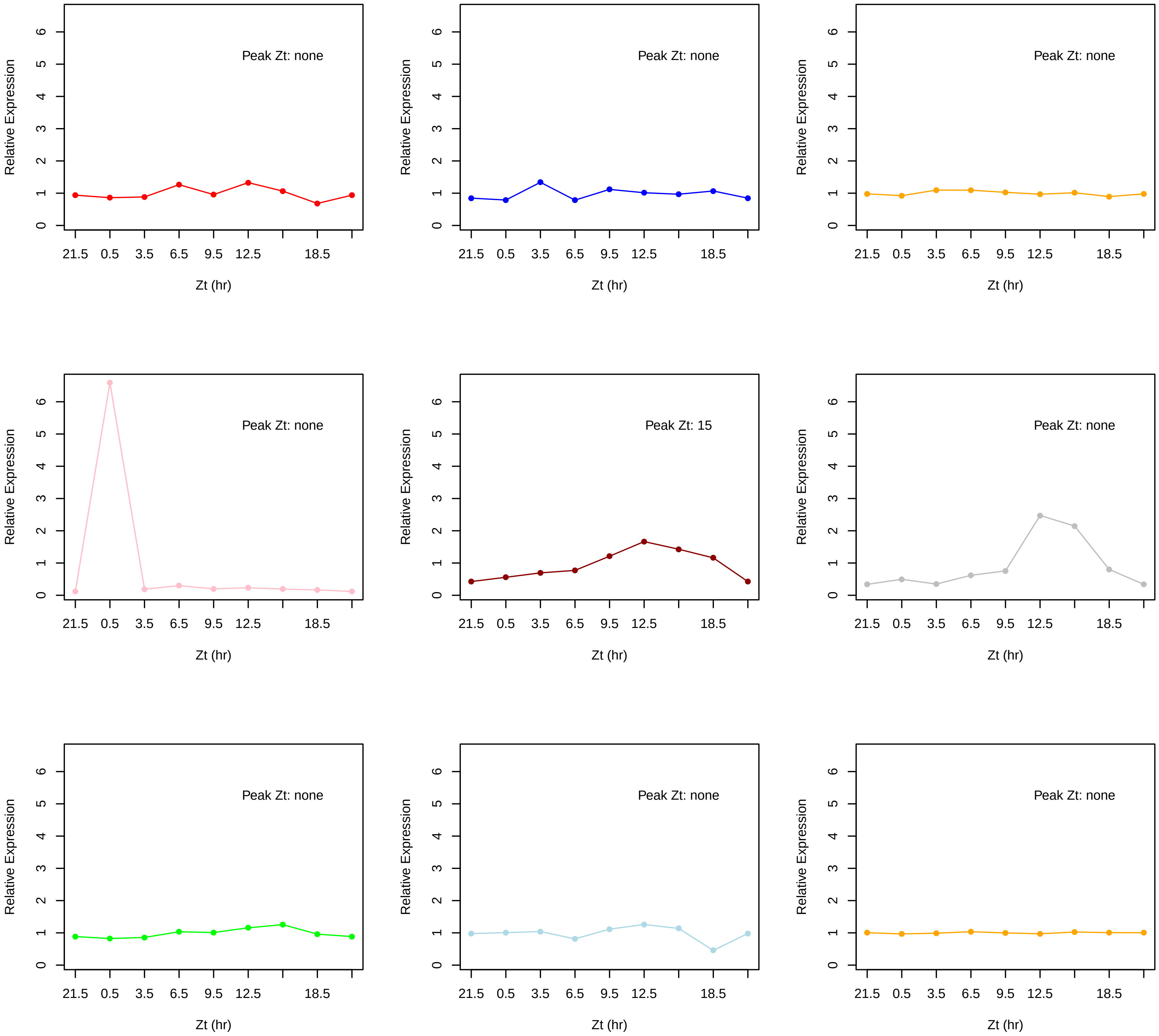

Singeleton: ENSXMAG00000015577 aar2

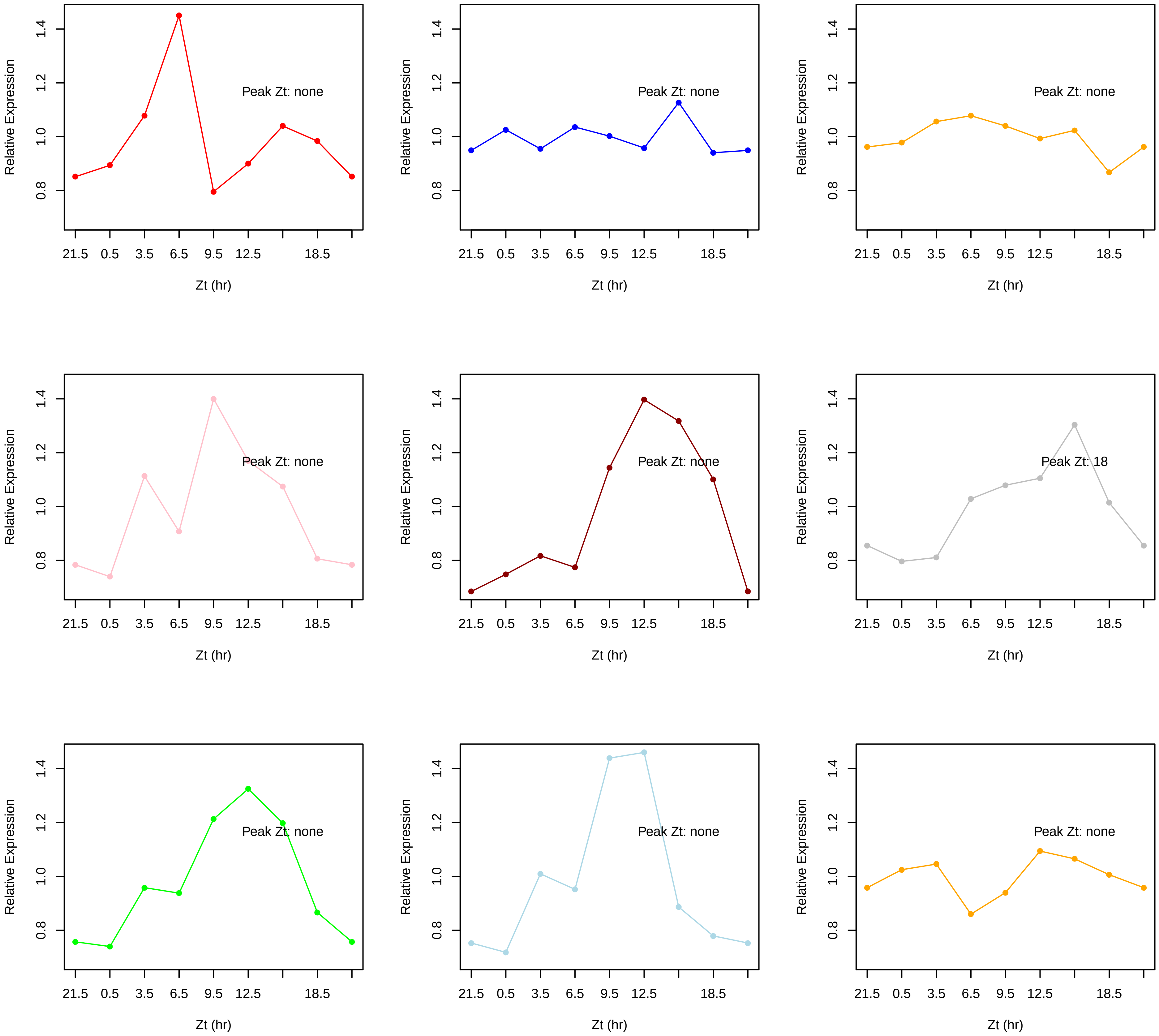

Singeleton: ENSXMAG00000000442 zgc:110239

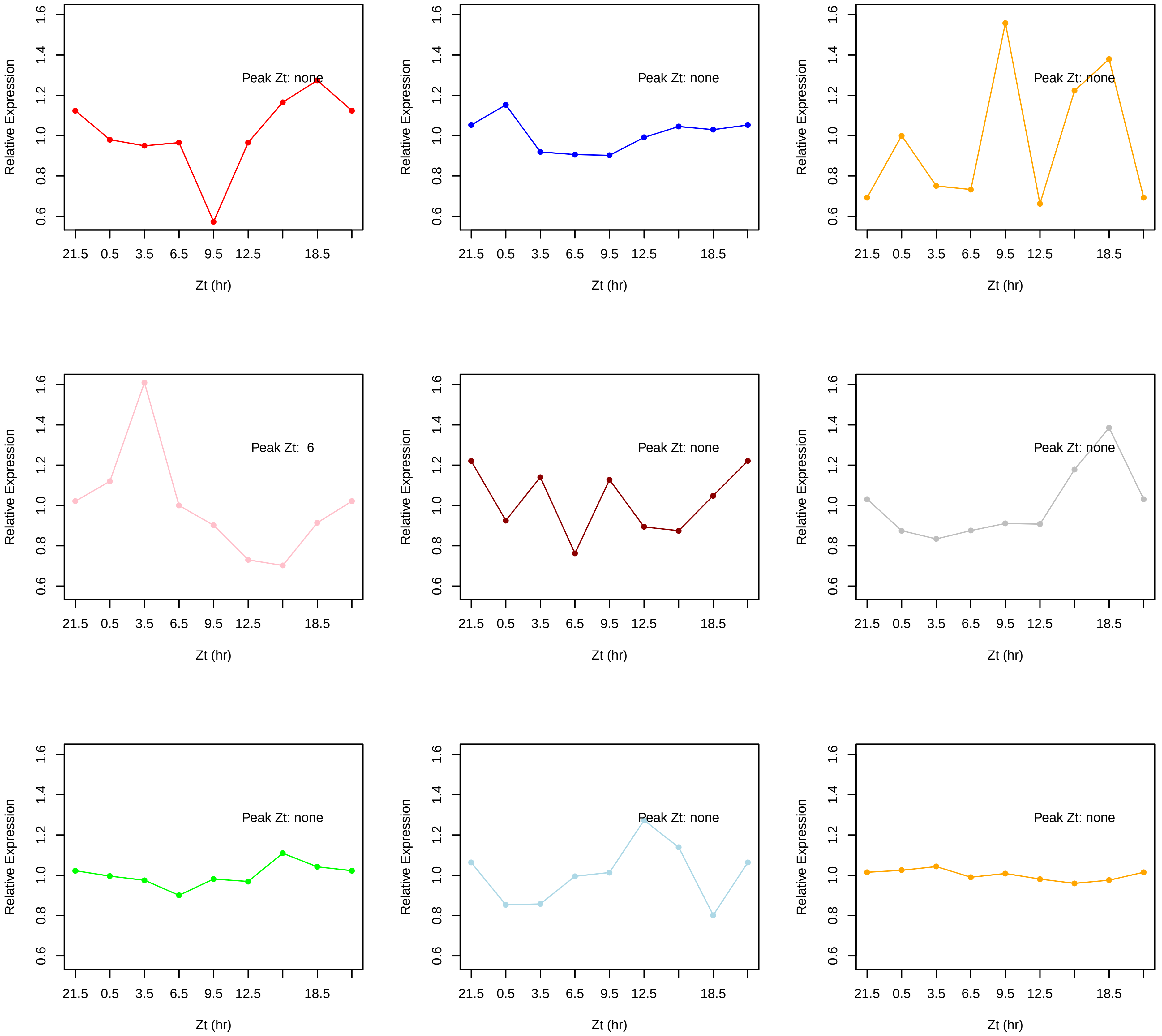

Singleton: ENSXMAG00000009670 pde7a

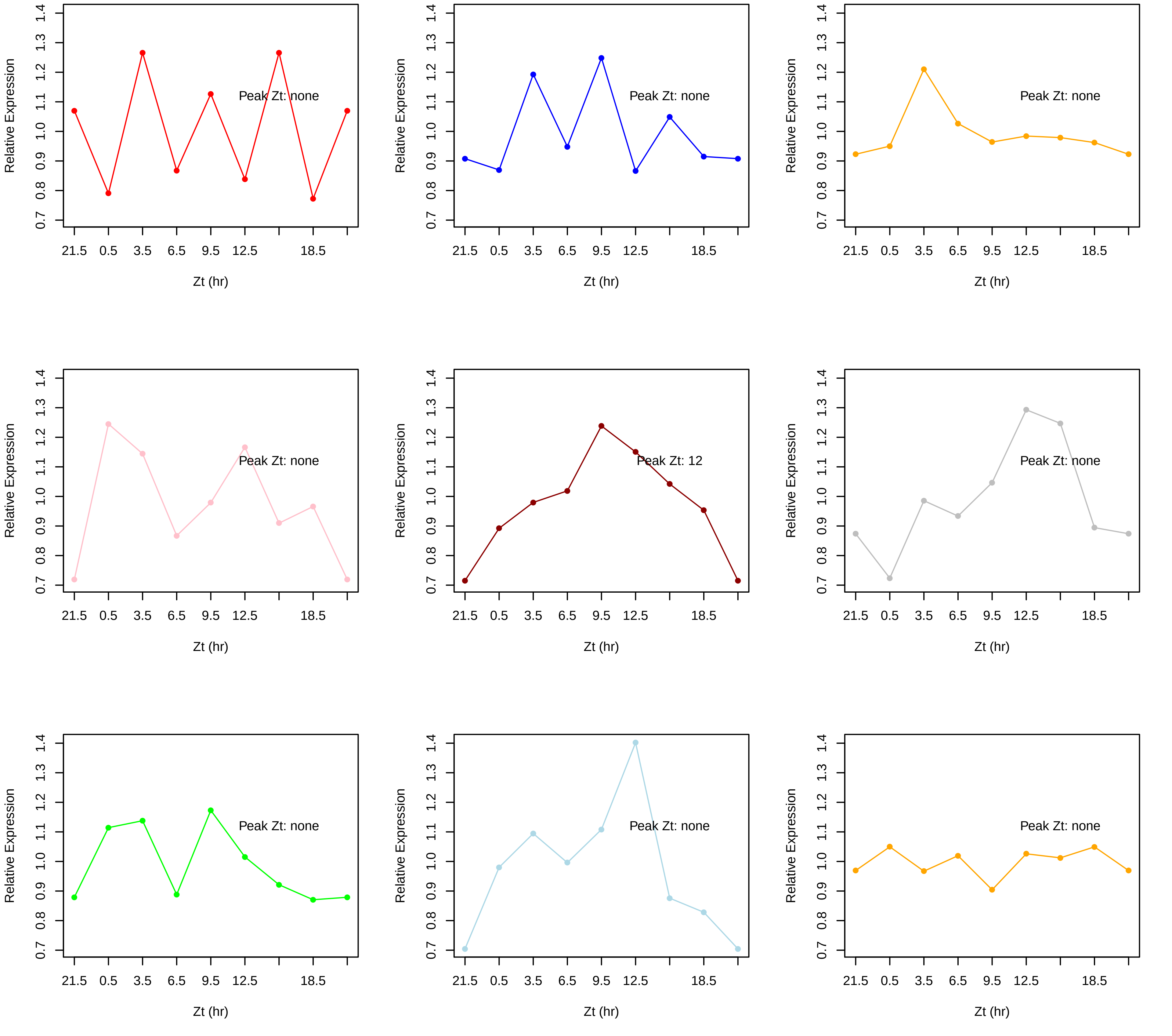

Singleton: ENSXMAG00000006235 draxin

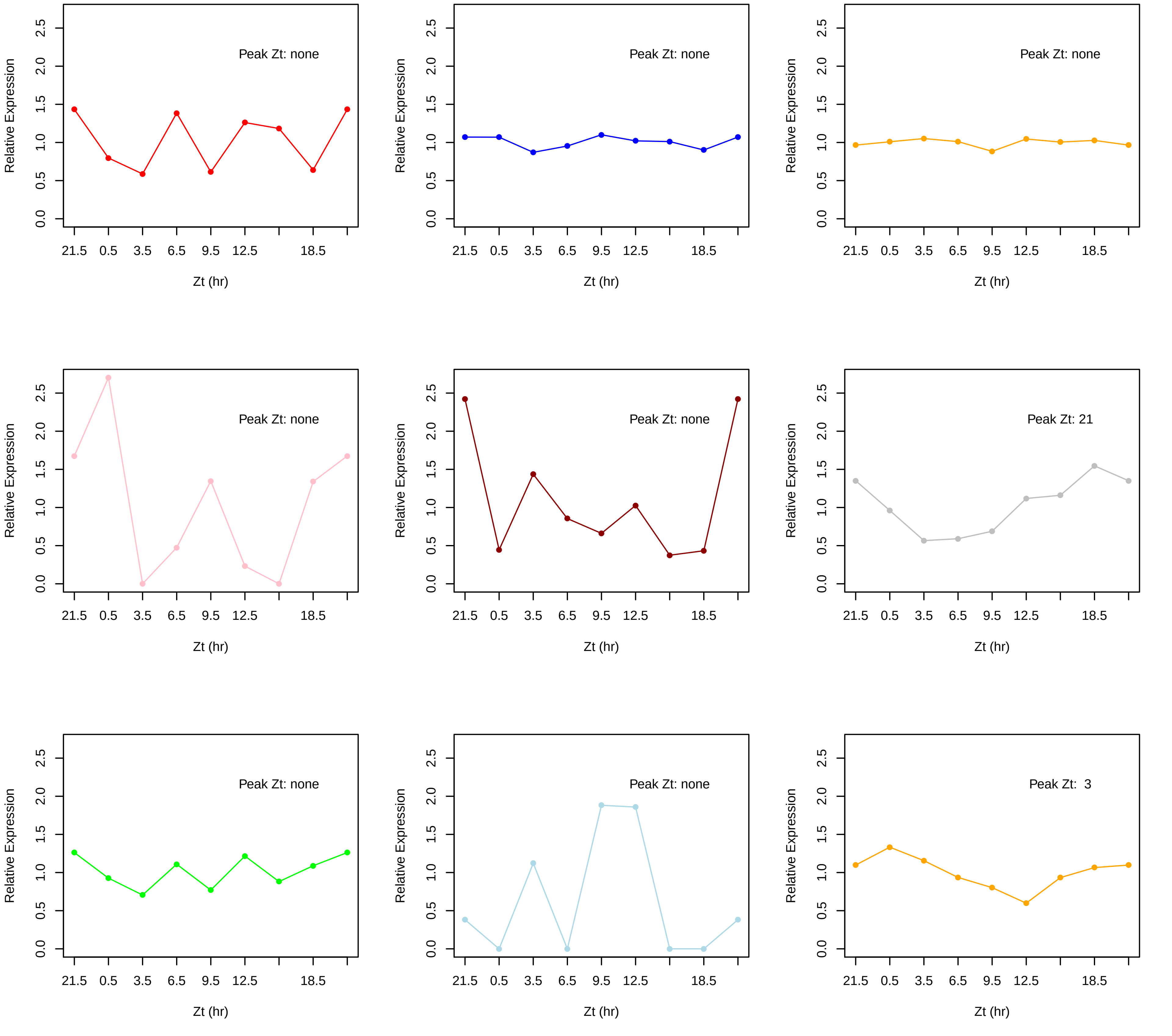

Singeleton: ENSXMAG00000018172 tpgs2

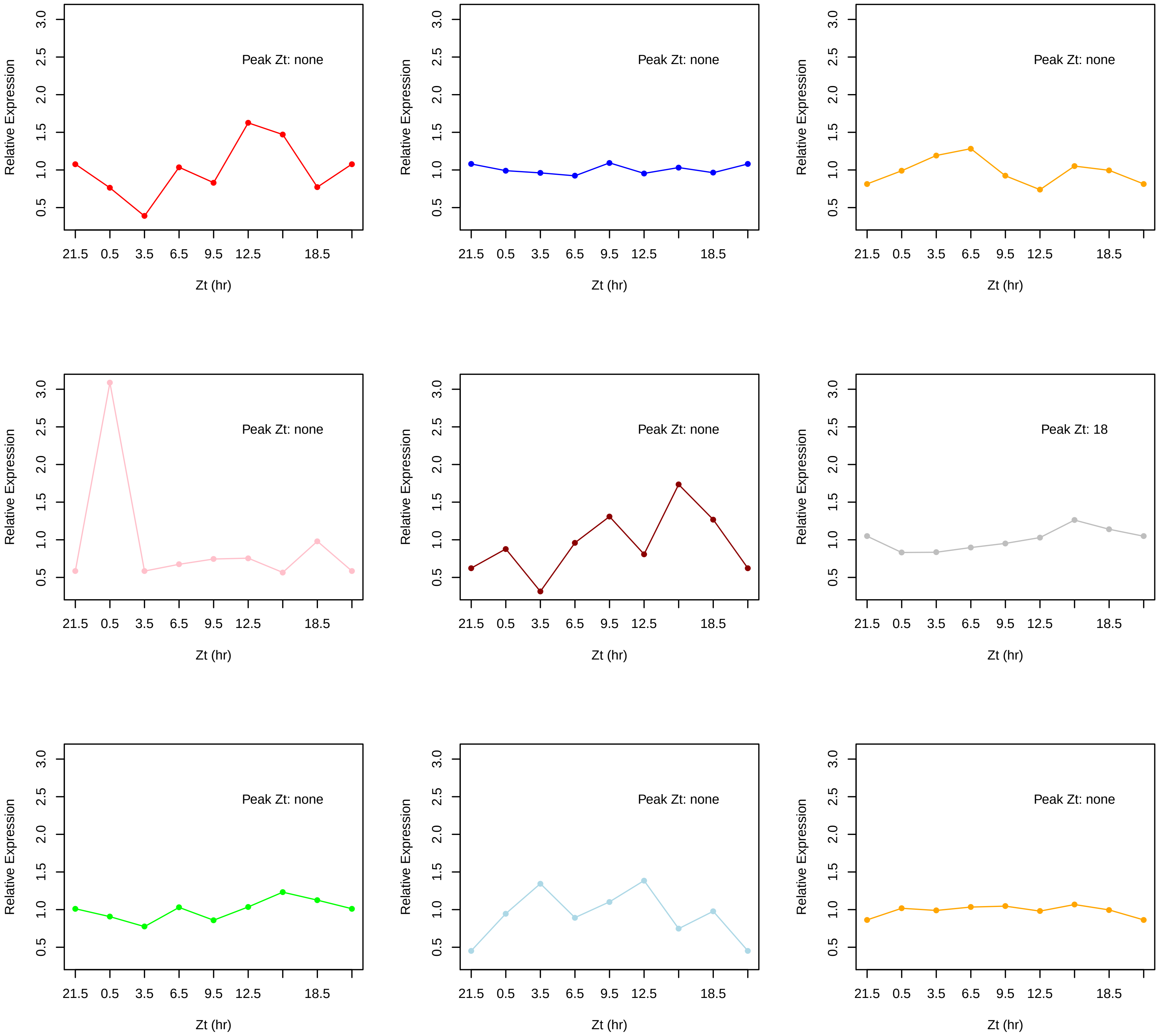

Singeleton: ENSXMAG00000018076 limk2

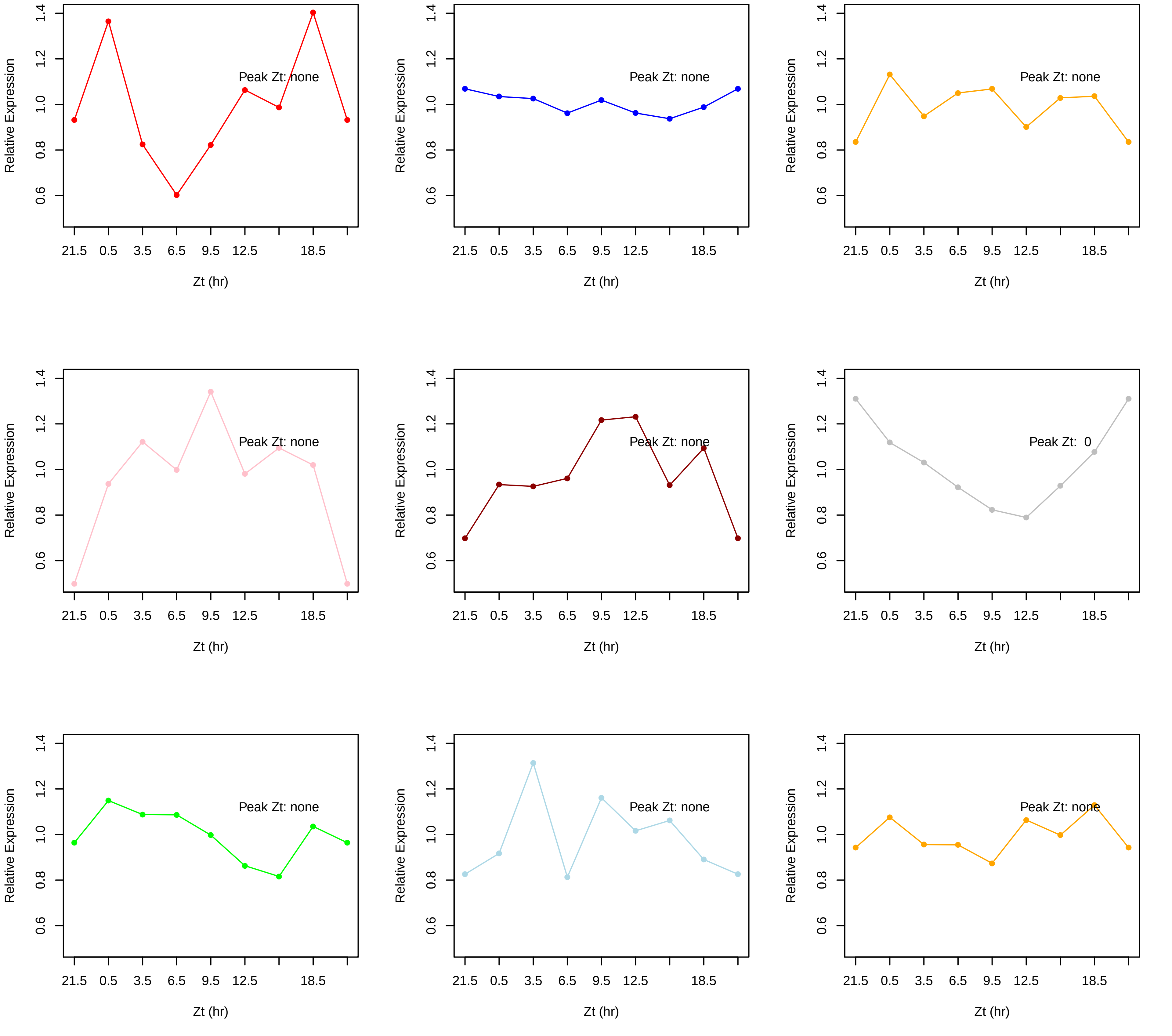

Singeleton: ENSXMAG00000015581 prpf6

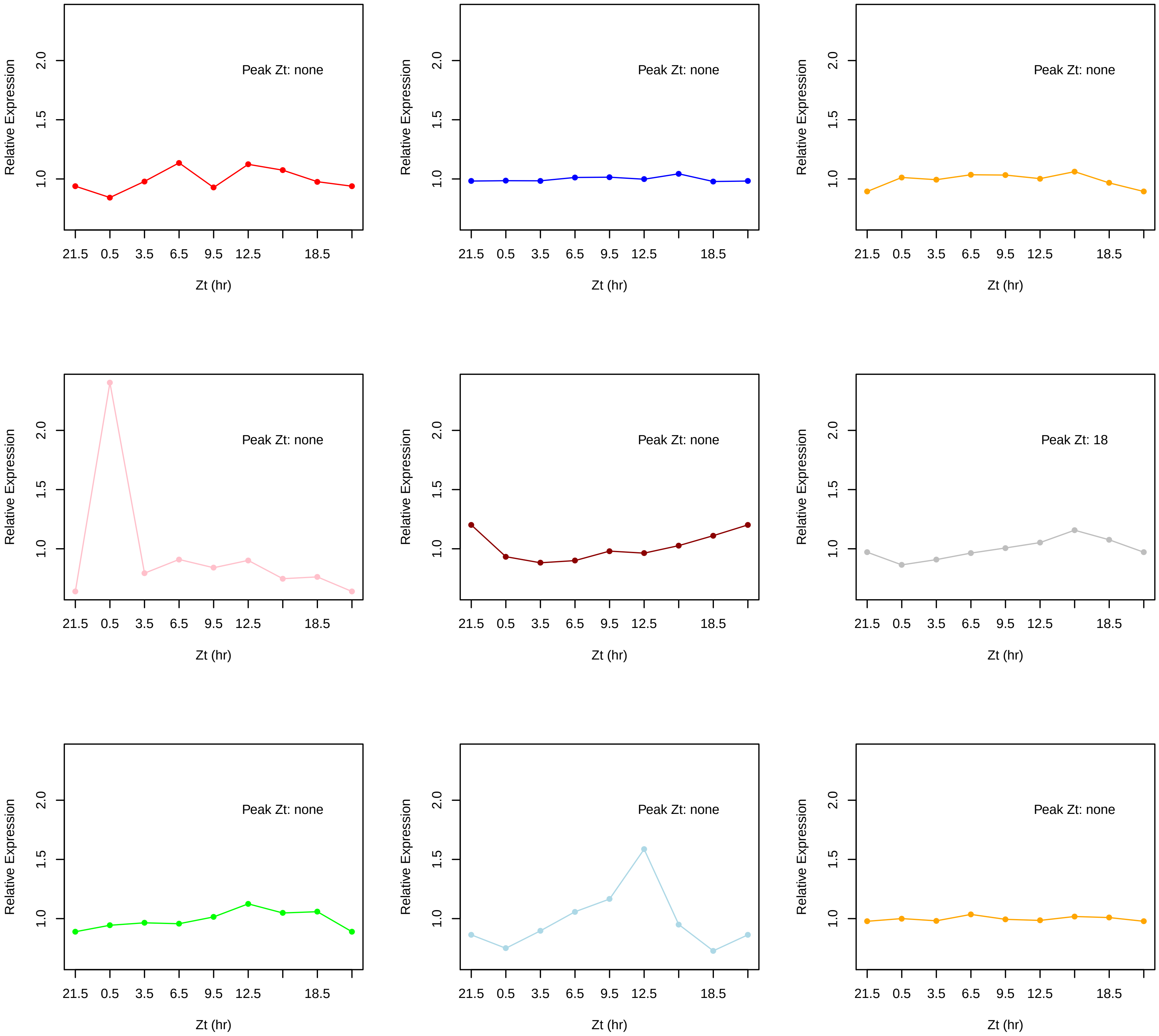

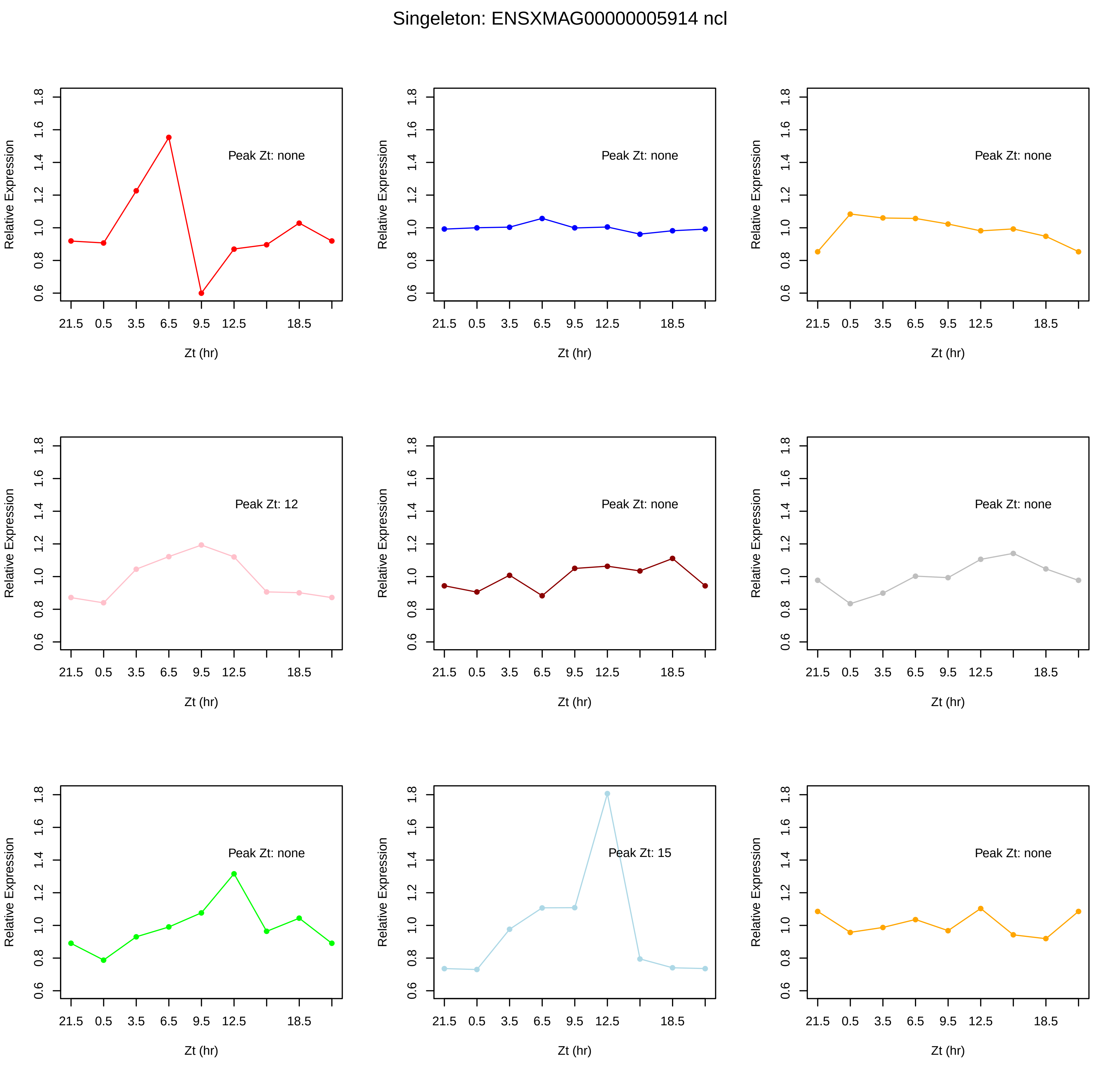

Singeleton: ENSXMAG00000005914 ncl
Peak Zt: none
Peak Zt: none
Peak Zt: none
Peak Zt: 12
Peak Zt: none
Peak Zt: none
Peak Zt: none
Peak Zt: 15
Peak Zt: none
Relative Expression
Zt (hr)
21.5
0.5
3.5
6.5
9.5
12.5
18.5
0.6
0.8
1.0
1.2
1.4
1.6
1.8

Singleton: ENSXMAG00000012265 tmem161a

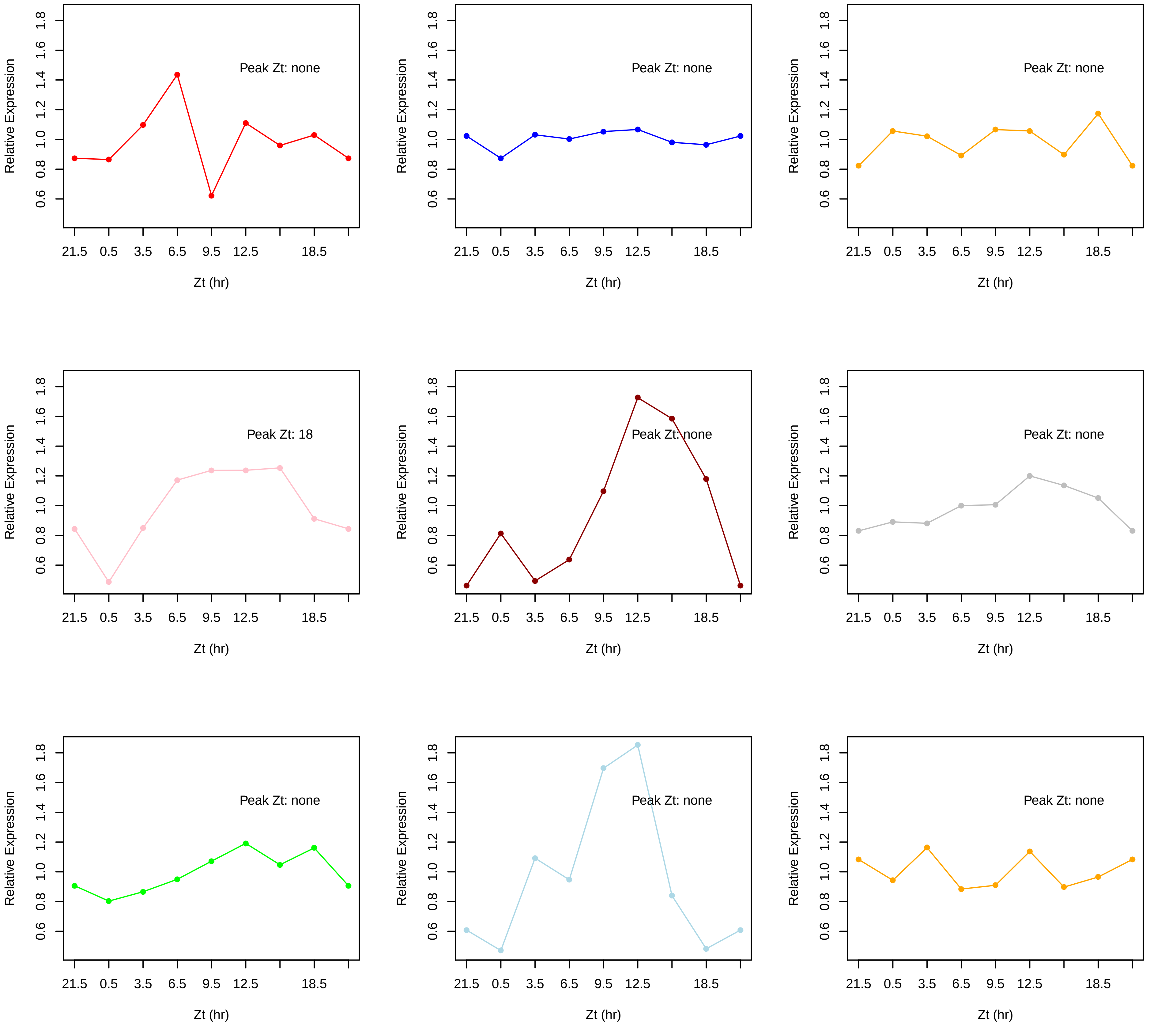

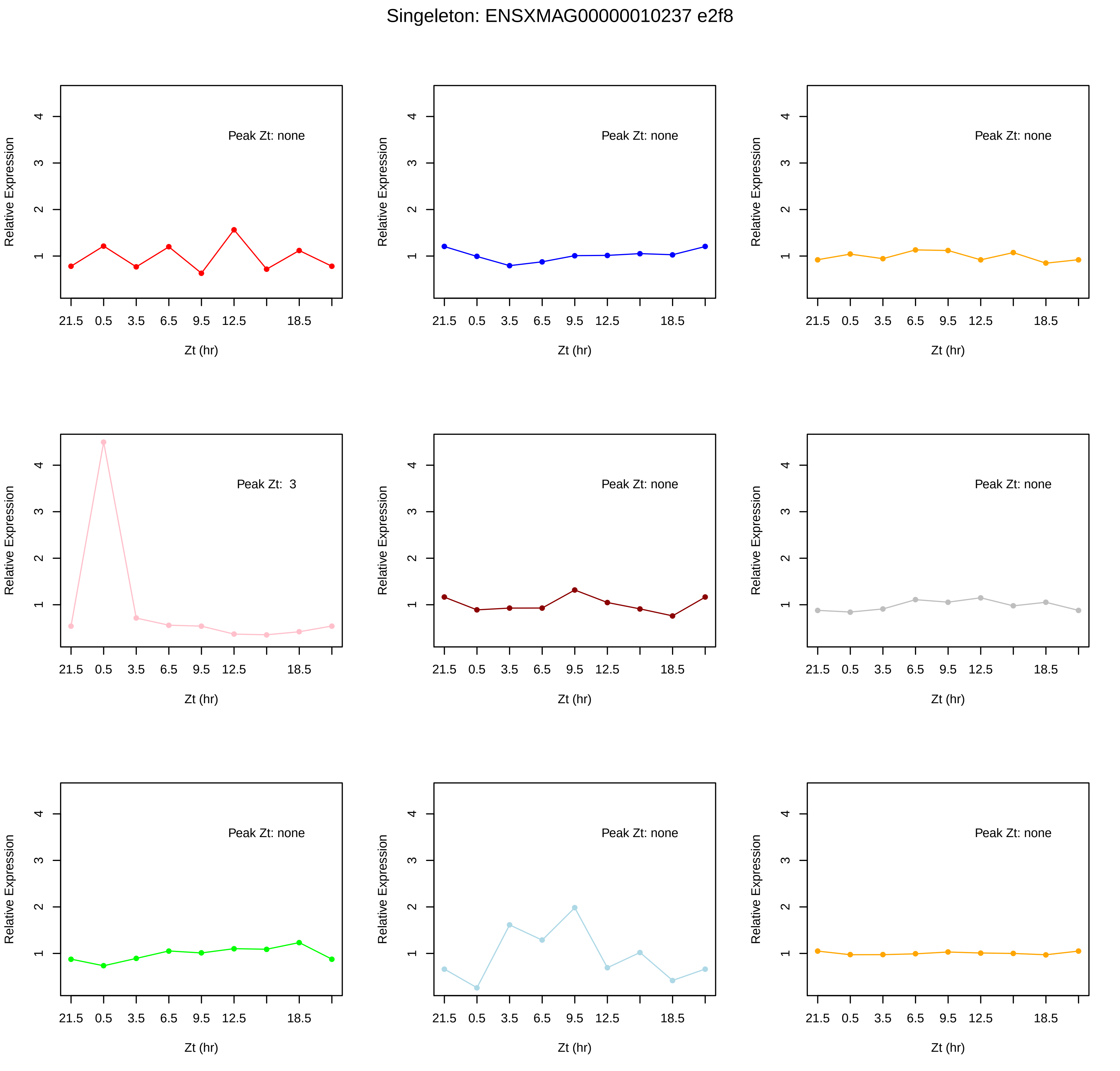
Singeleton: ENSXMAG00000010237 e2f8
Peak Zt: none
Peak Zt: none
Peak Zt: none
Peak Zt: 3
Peak Zt: none
Peak Zt: none
Peak Zt: none
Peak Zt: none
Peak Zt: none
Relative Expression
Zt (hr)
21.5
0.5
3.5
6.5
9.5
12.5
18.5
1
2
3
4

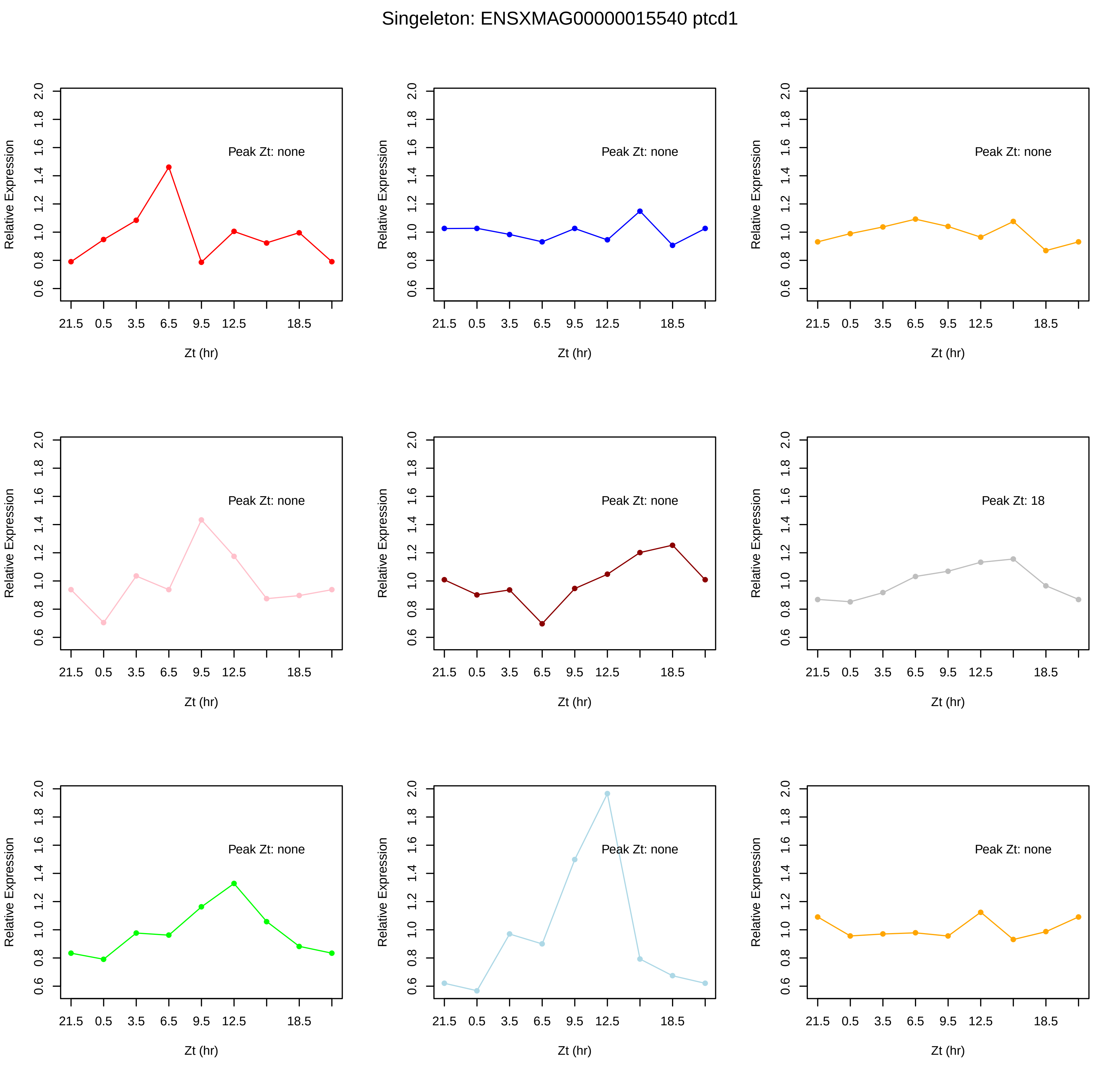
Singeleton: ENSXMAG00000015540 ptcd1
Peak Zt: none
Peak Zt: none
Peak Zt: none
Peak Zt: none
Peak Zt: none
Peak Zt: 18
Peak Zt: none
Peak Zt: none
Peak Zt: none
Relative Expression
Zt (hr)
21.5
0.5
3.5
6.5
9.5
12.5
18.5

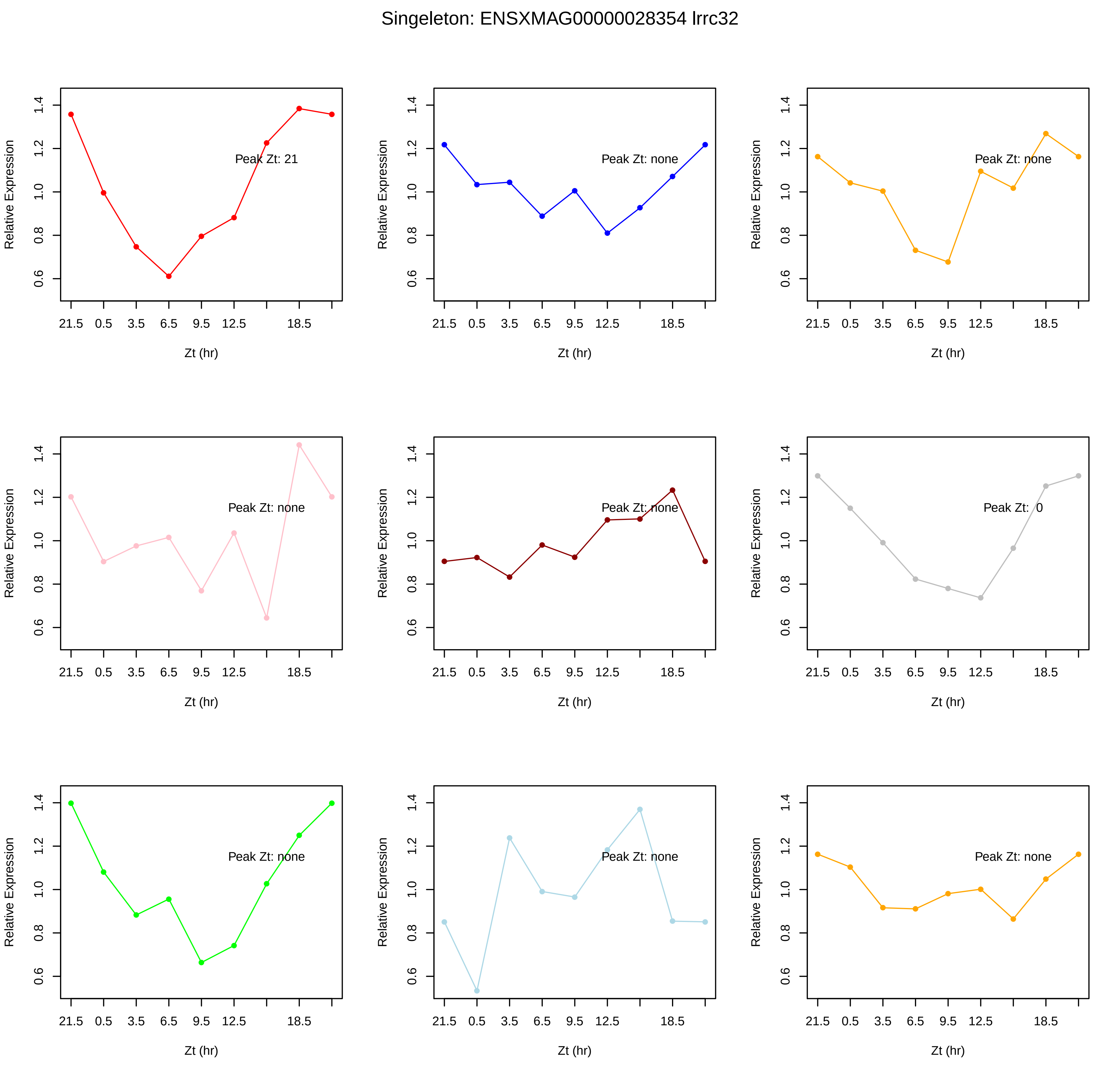
Singleton: ENSXMAG00000028354 lrrc32
Relative Expression
Zt (hr)
21.5 0.5 3.5 6.5 9.5 12.5 18.5
0.6 0.8 1.0 1.2 1.4
Peak Zt: 21
Peak Zt: none
Peak Zt: none
Peak Zt: none
Peak Zt: none
Peak Zt: 0
Peak Zt: none
Peak Zt: none
Peak Zt: none

Singeleton: ENSXMAG00000018071 pik3ip1

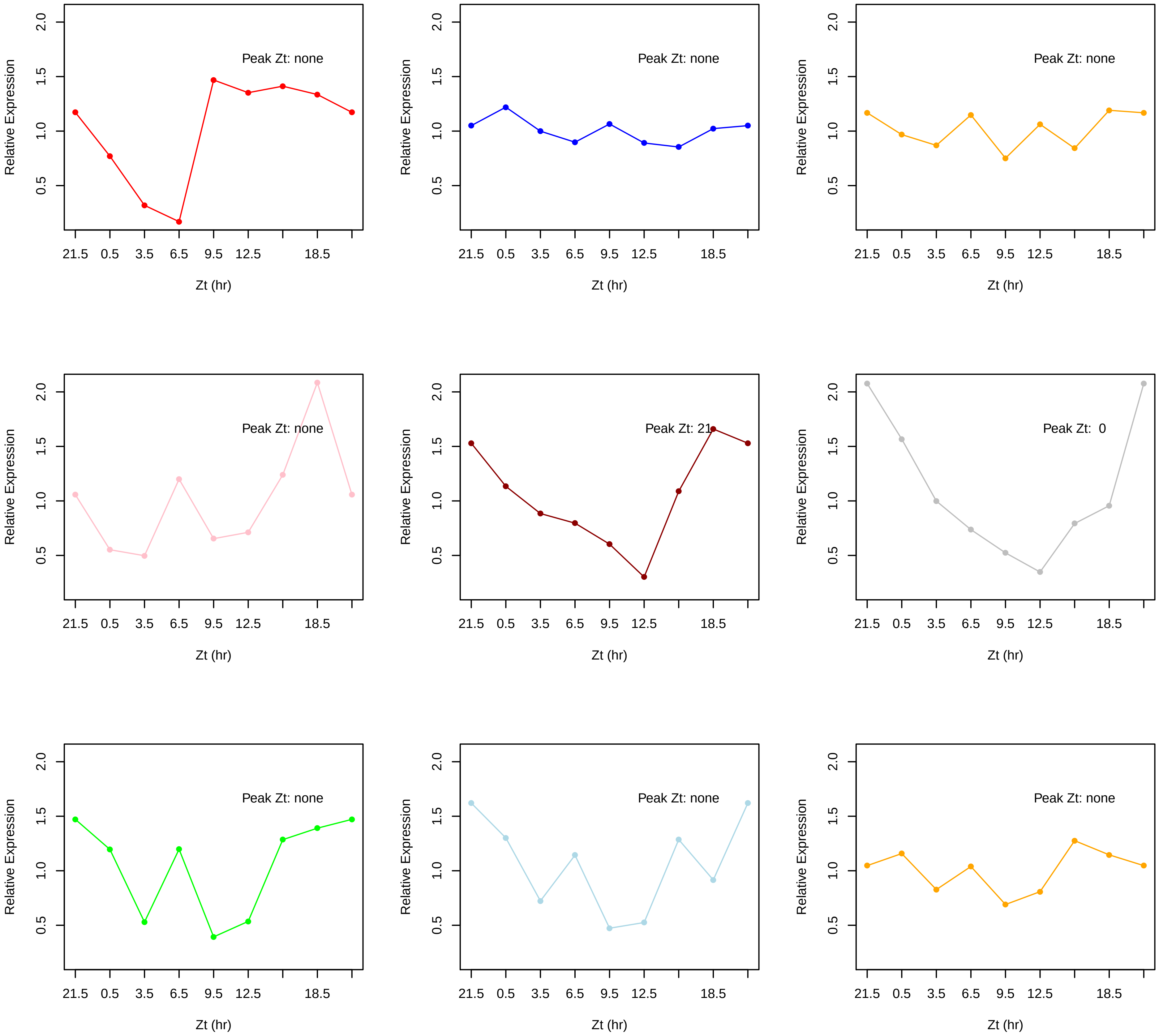

Singleton: ENSXMAG00000010189 nav2a

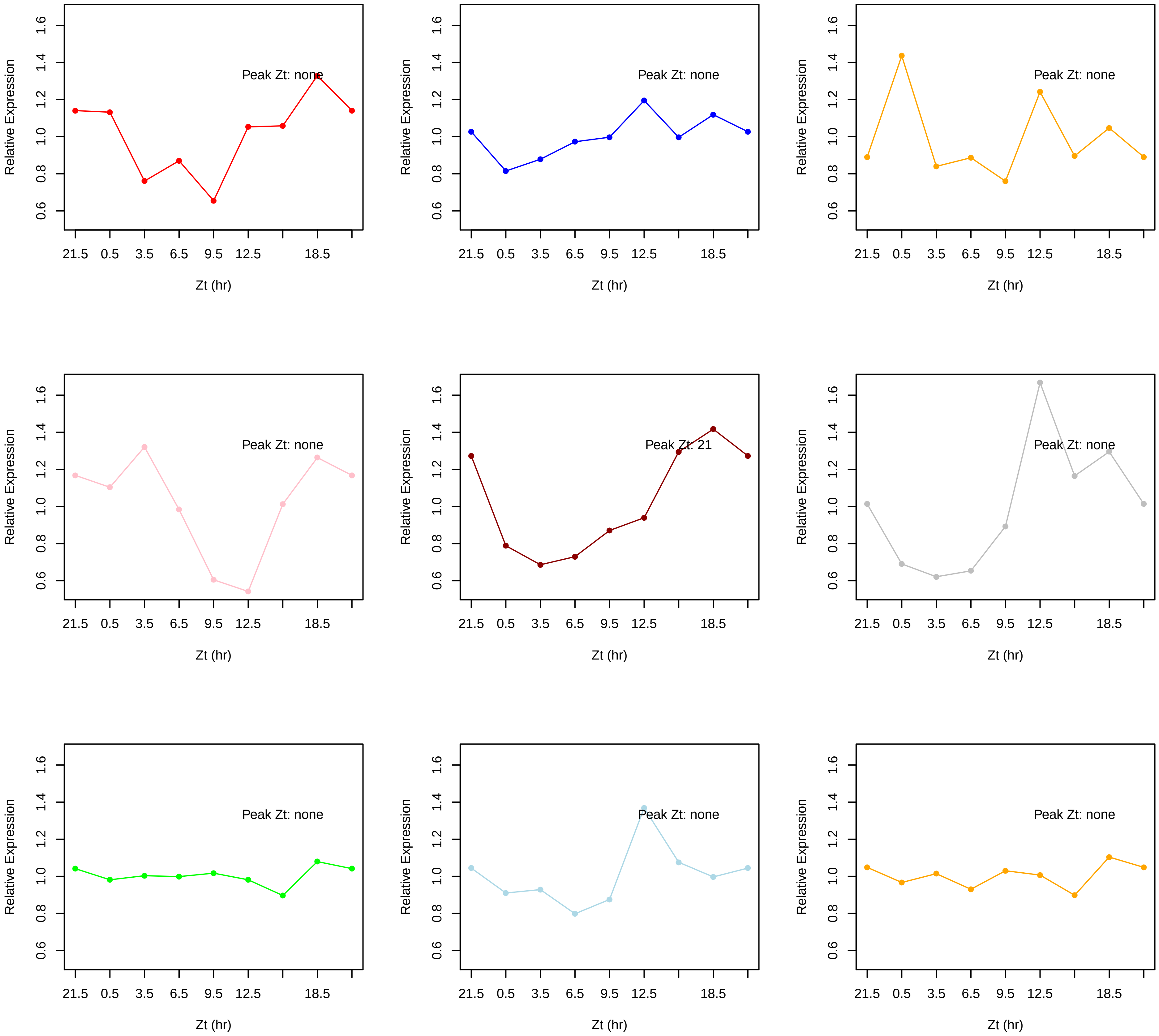

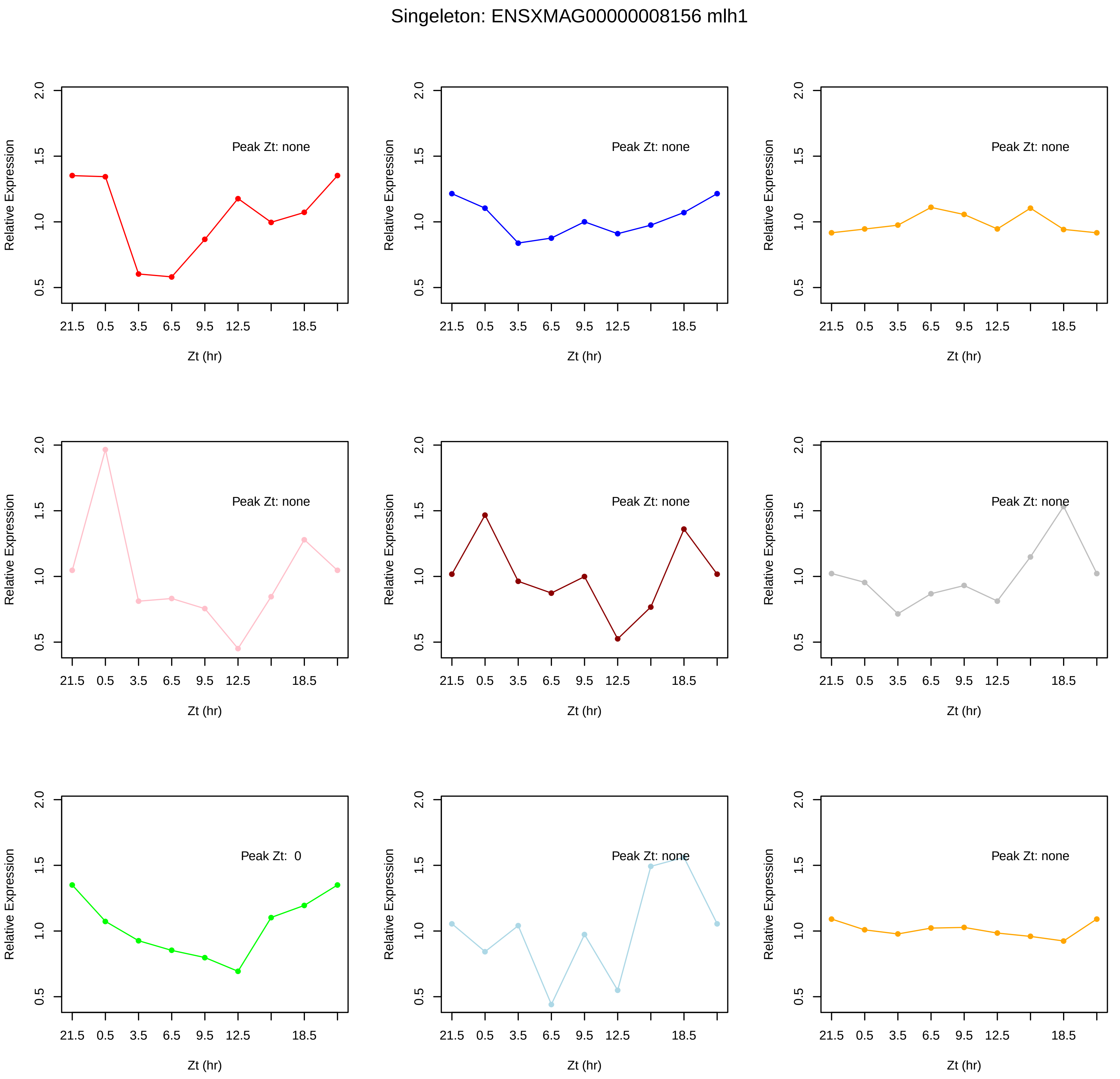
Singeleton: ENSXMAG00000008156 mlh1
Peak Zt: none
Peak Zt: 0
Relative Expression
Zt (hr)
21.5
0.5
3.5
6.5
9.5
12.5
18.5
0.5
1.0
1.5
2.0

Singeleton: ENSXMAG00000017105 fli1b

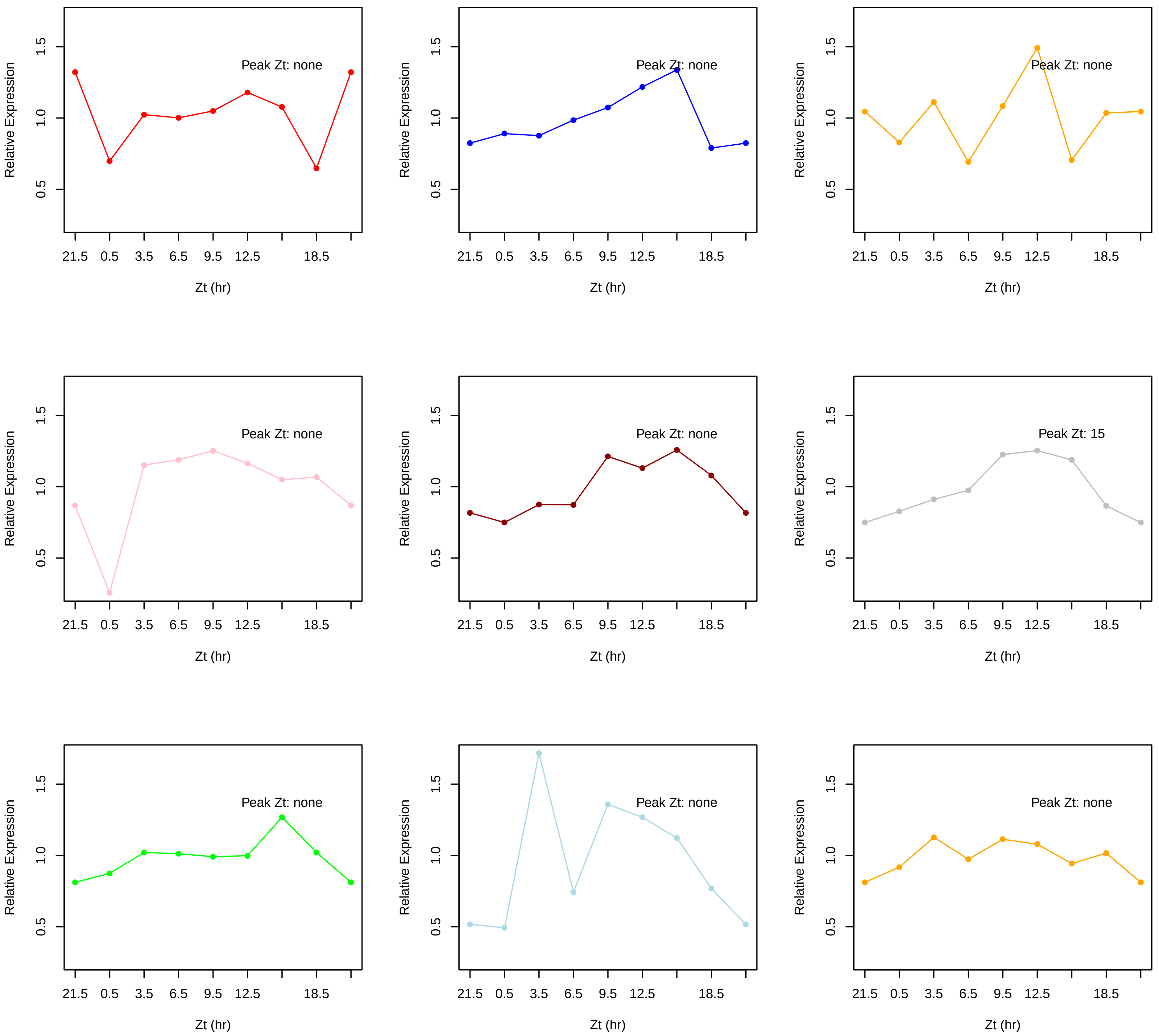

Singeleton: ENSXMAG00000023165 rnf166

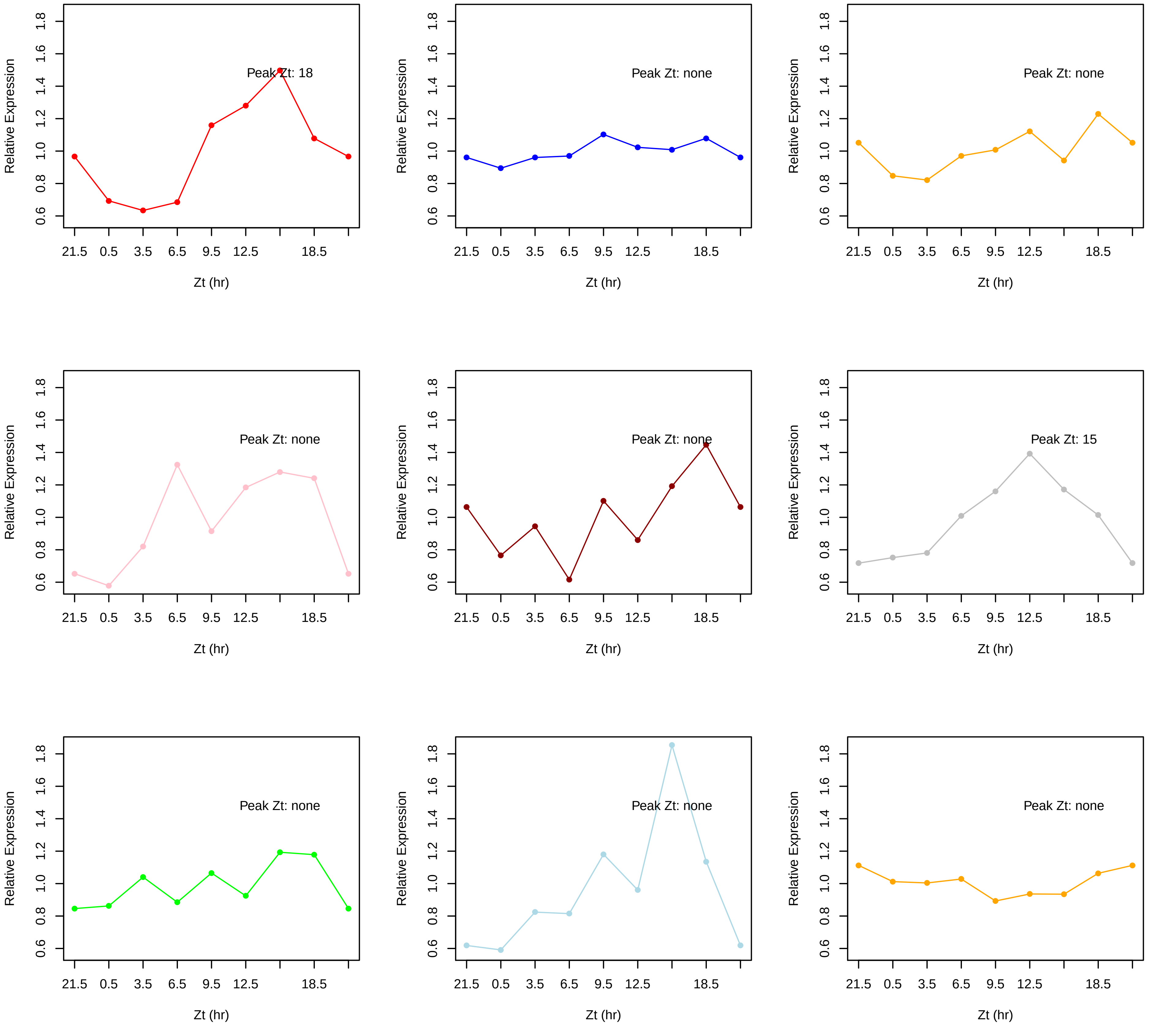

Singleton: ENSXMAG00000003664 bud31

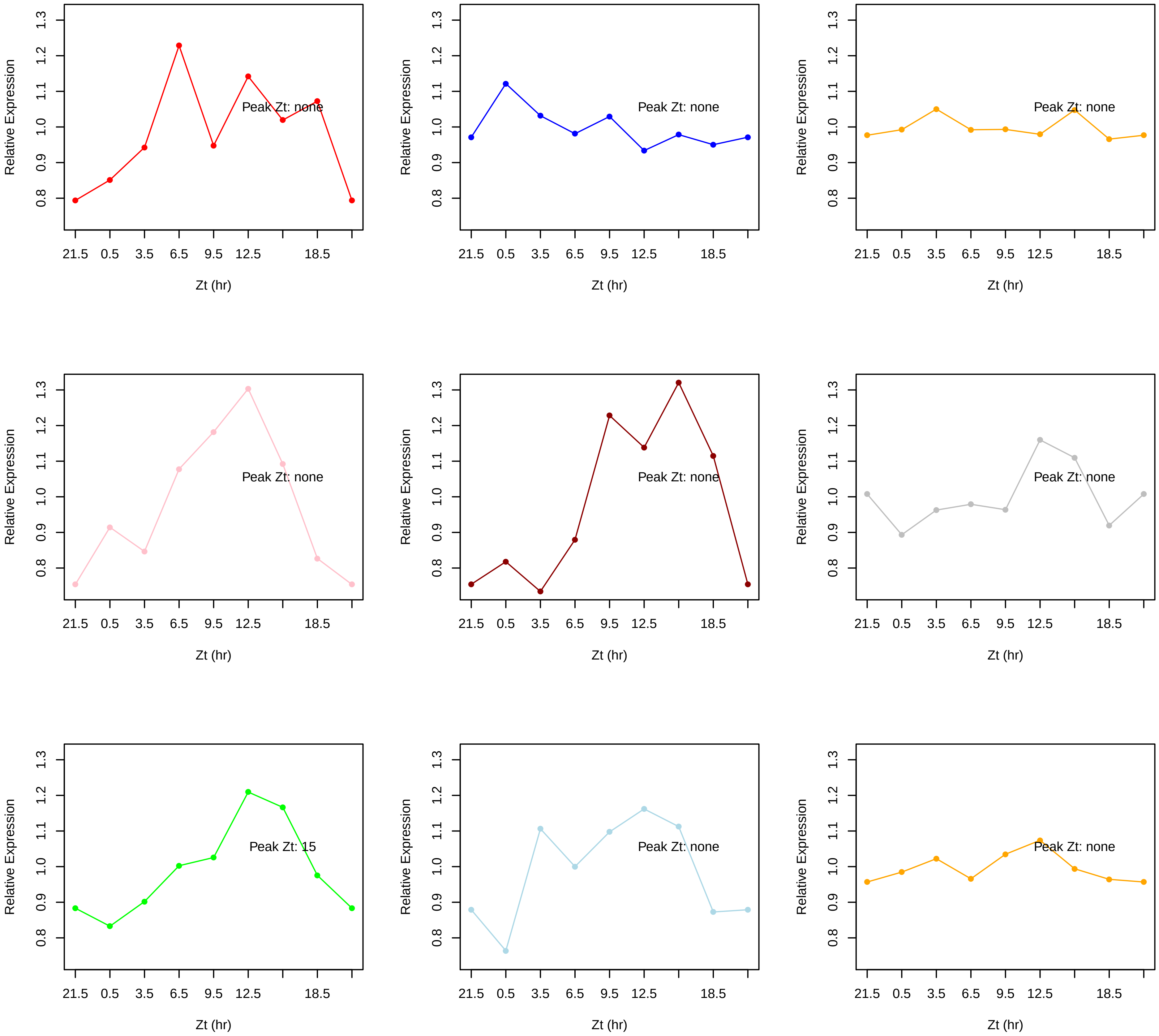

Singeleton: ENSXMAG00000019340 ARMC6

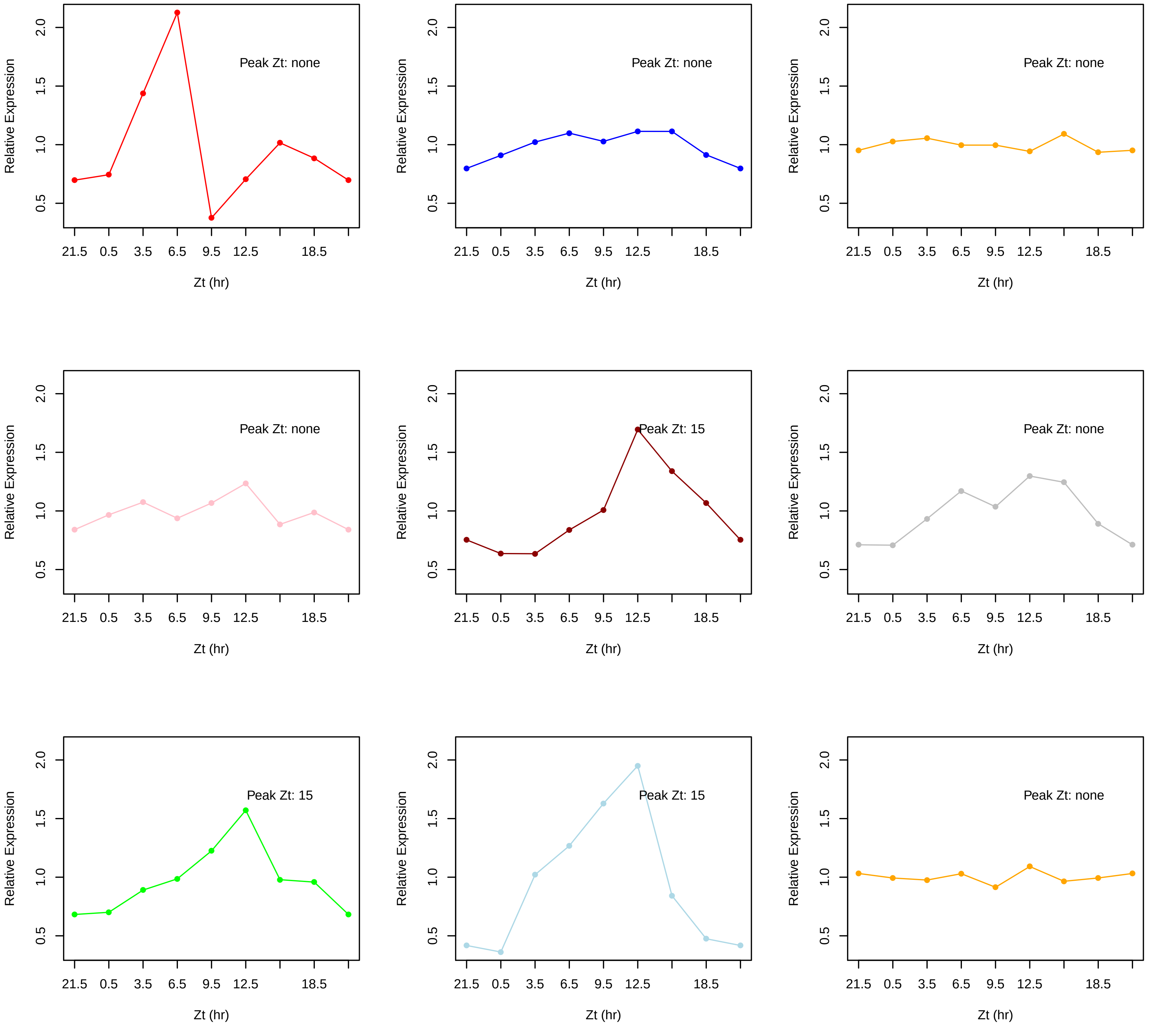

Singeleton: ENSXMAG00000021941 icmt

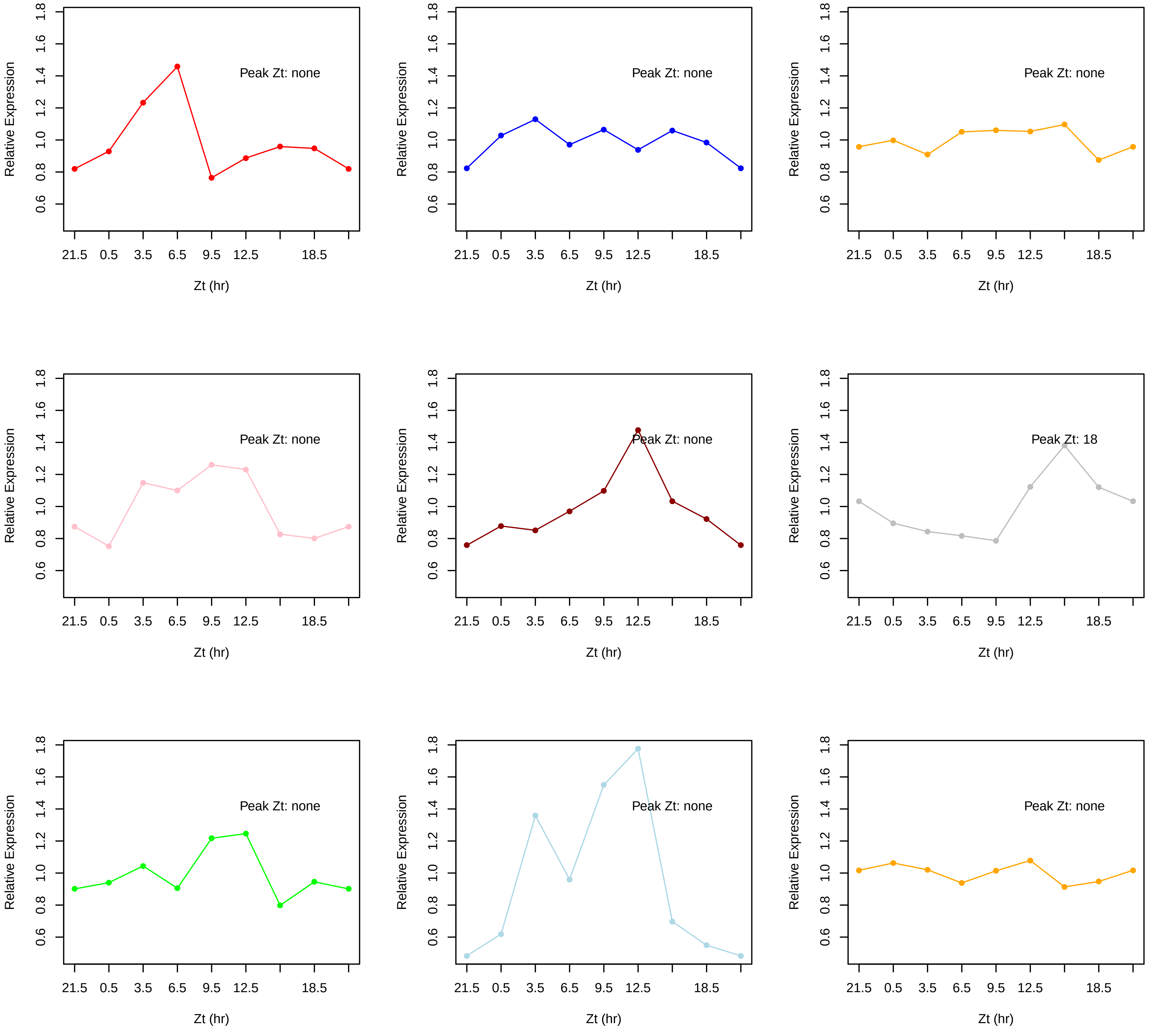

Singleton: ENSXMAG00000005876 armc9

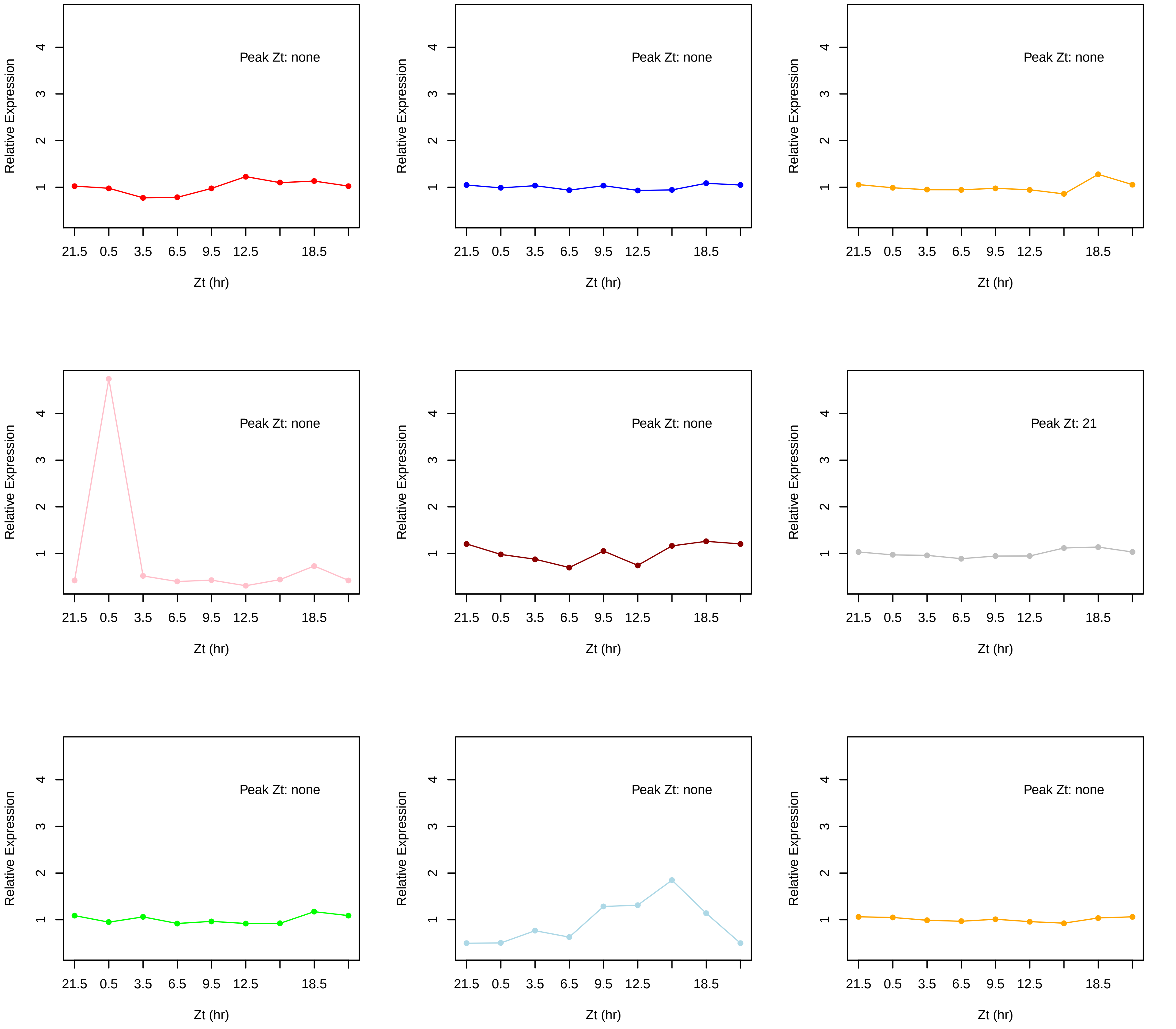

Singeleton: ENSXMAG00000012037 pipox

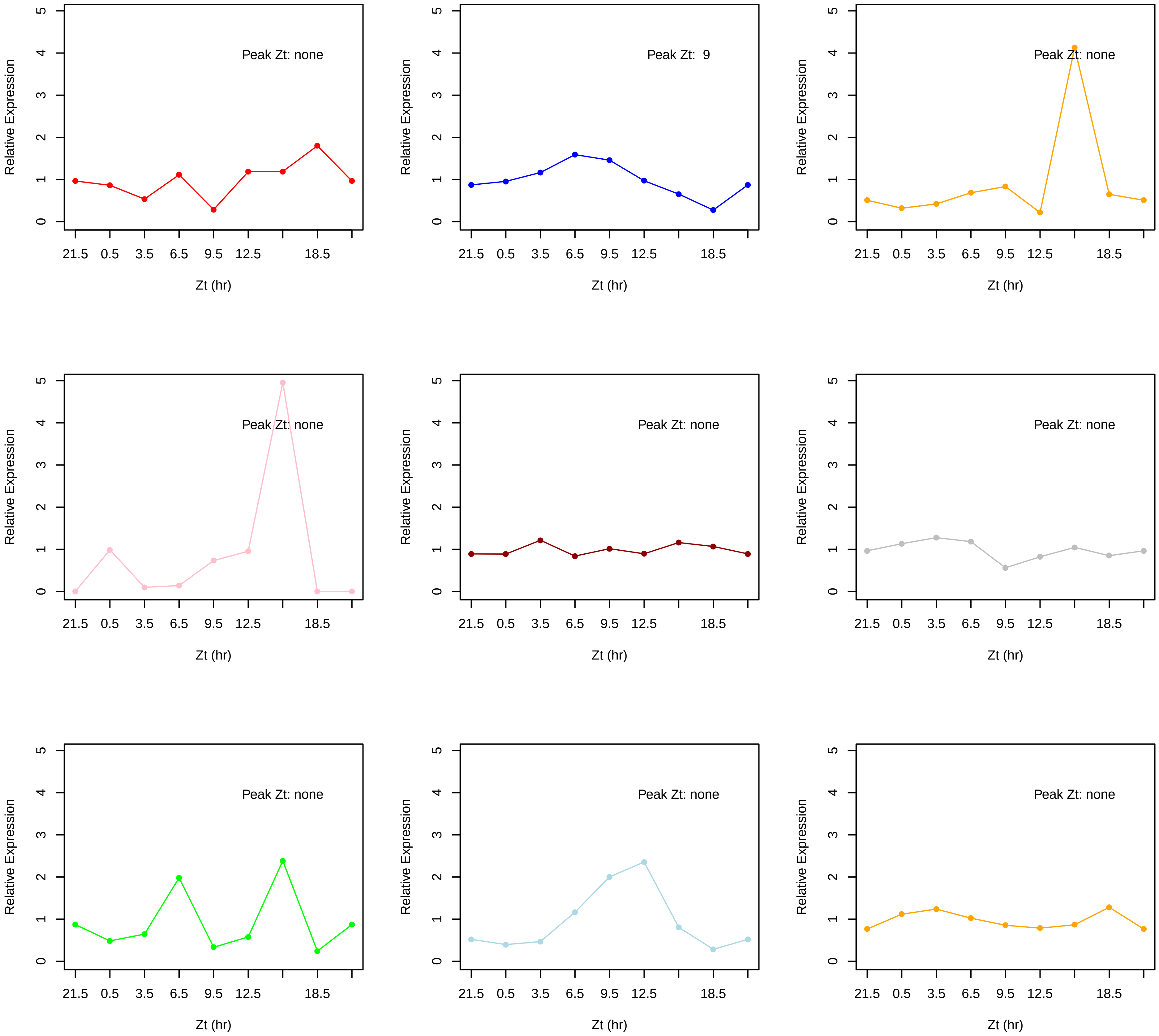

Singleton: ENSXMAG00000028787 tgfbr3

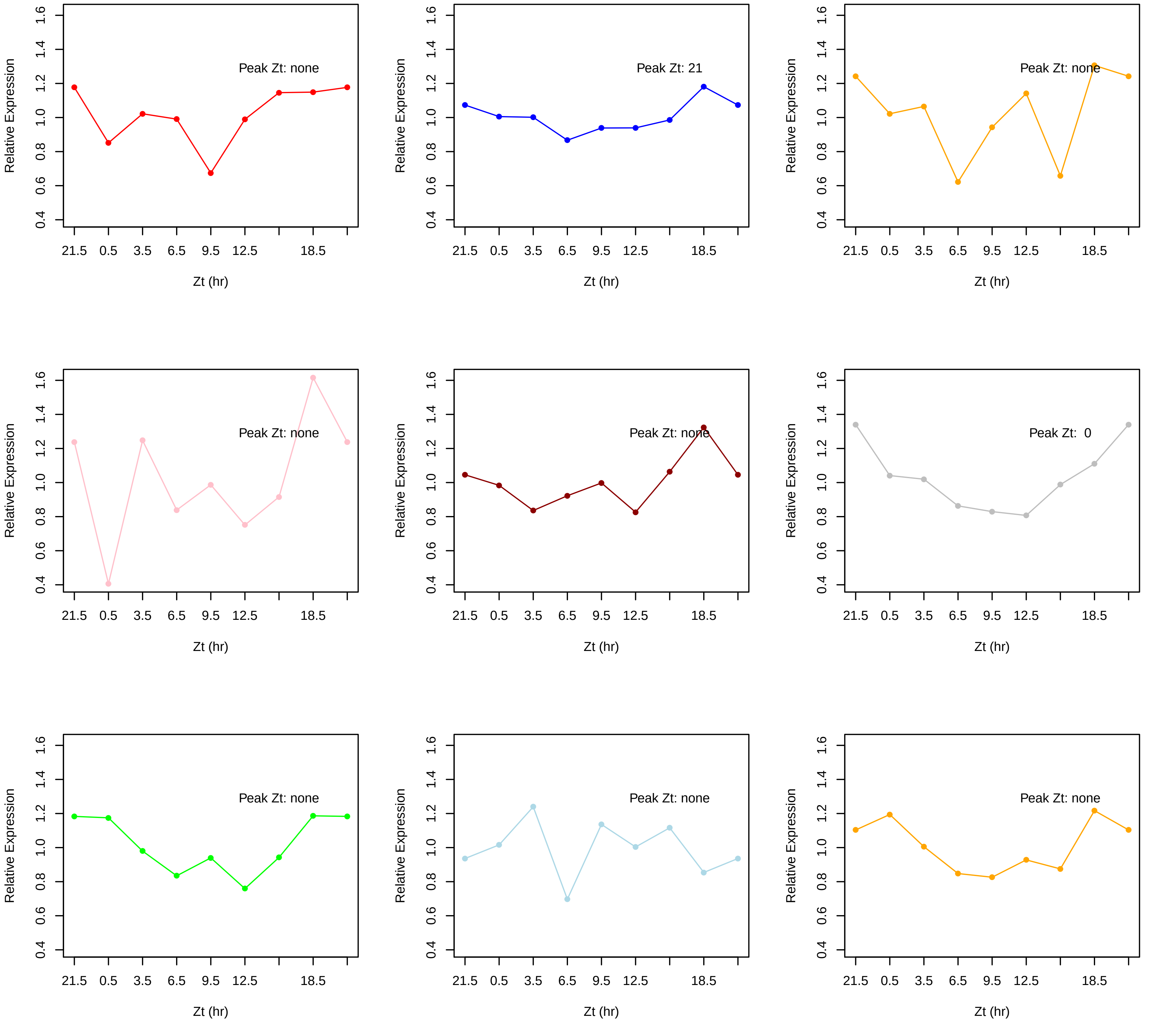

Singeleton: ENSXMAG00000003656 pdap1b

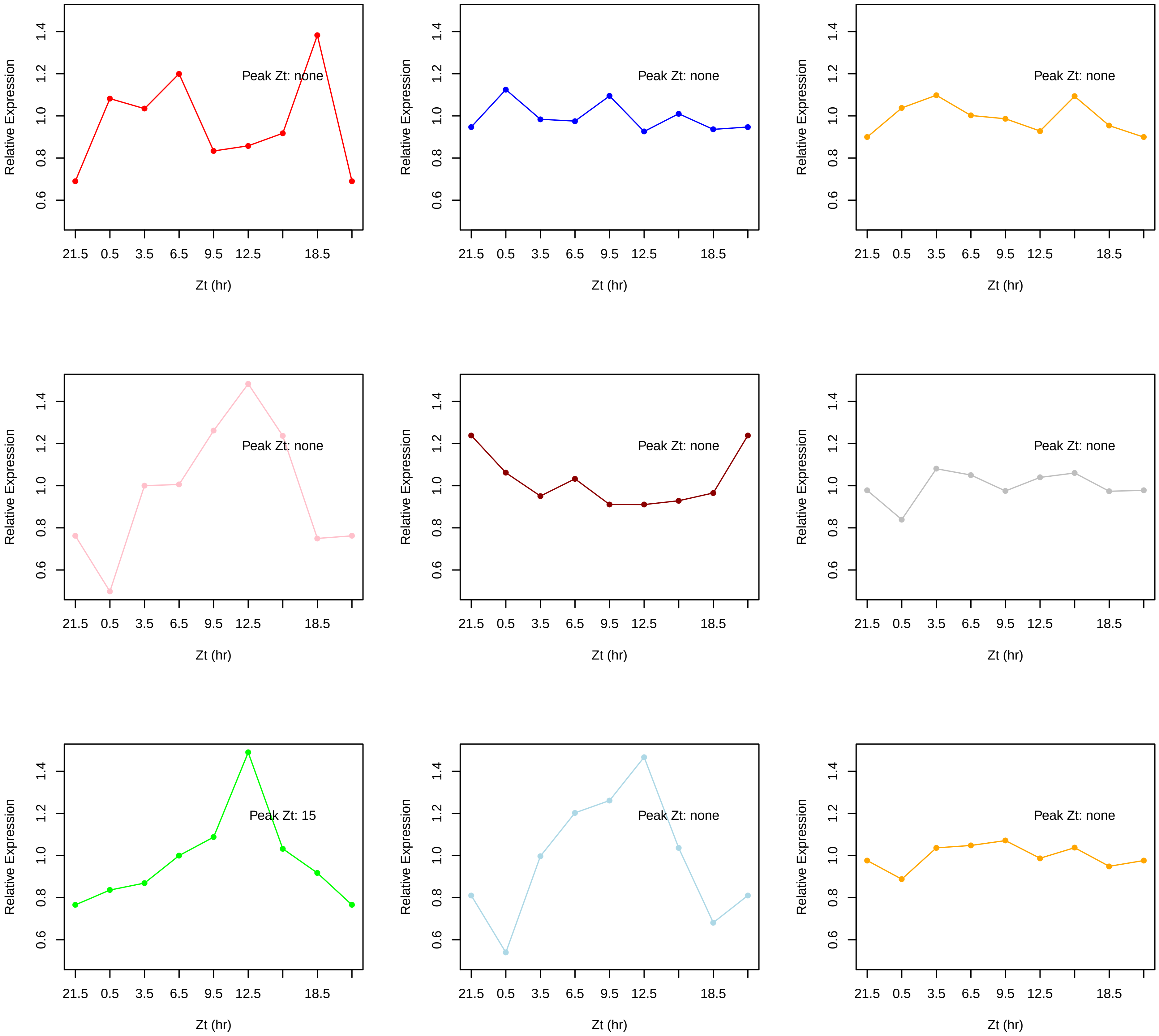

Singeleton: ENSXMAG00000021245 ppifb

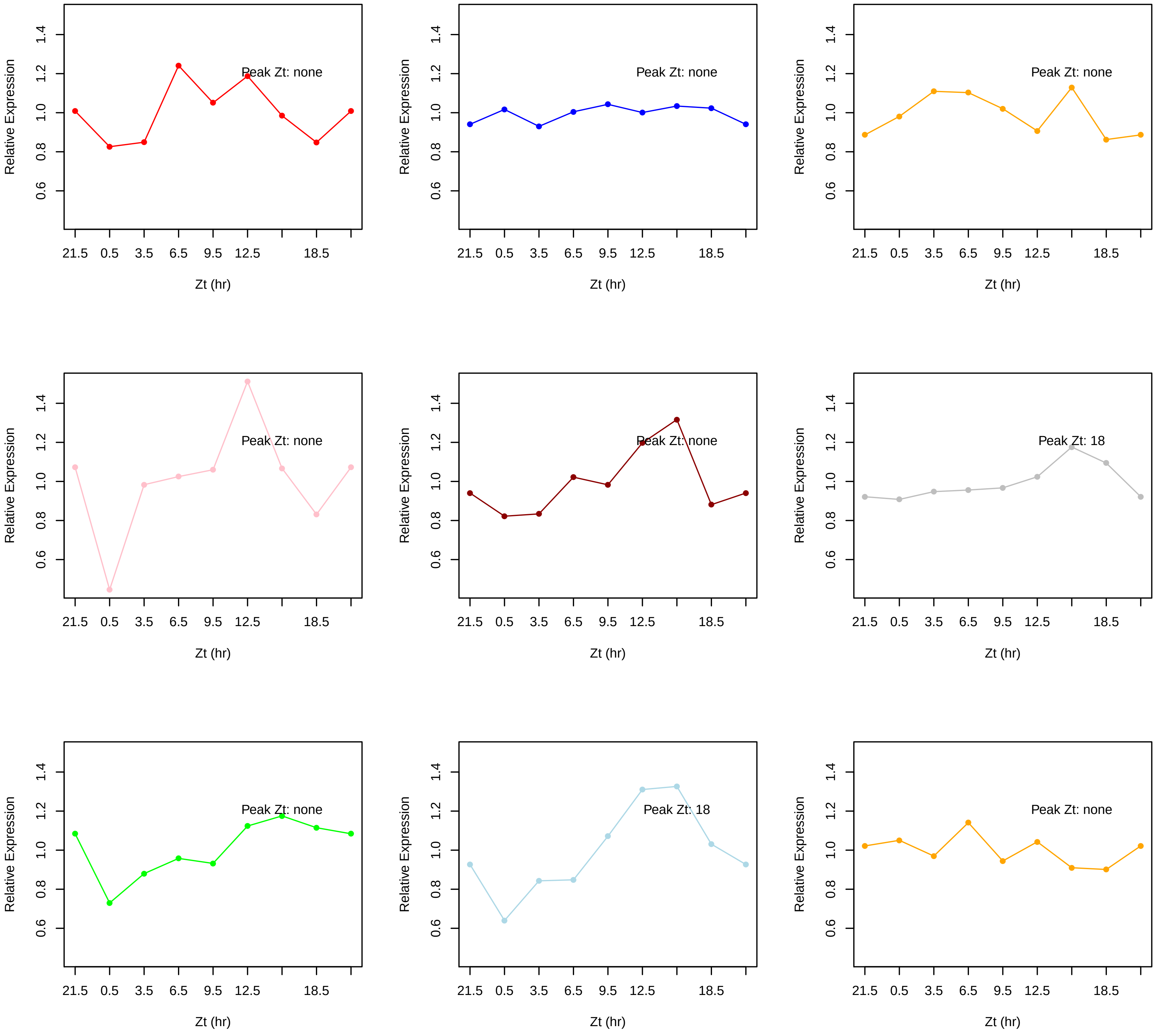

Singeleton: ENSXMAG00000011358 mpzl1l

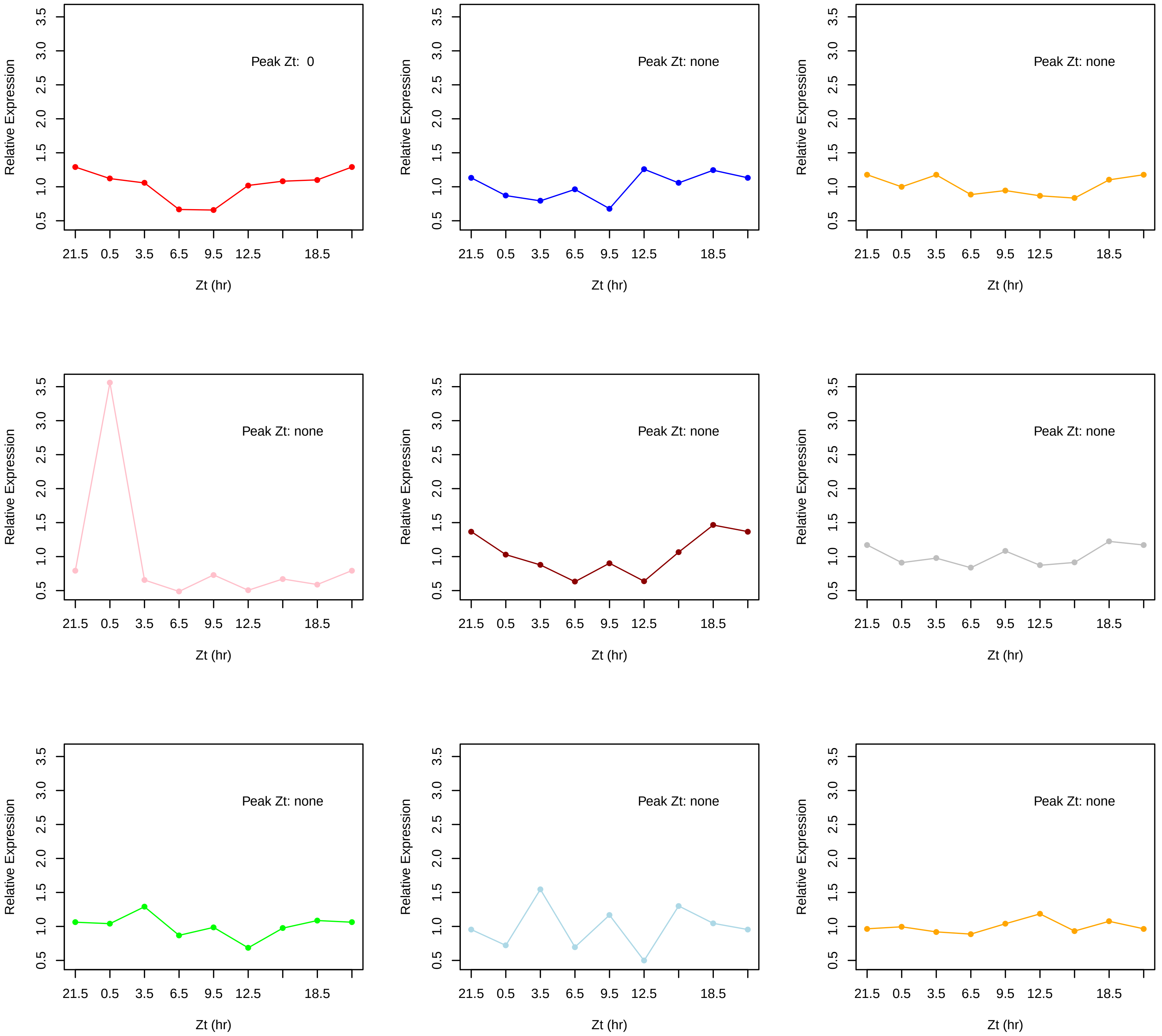

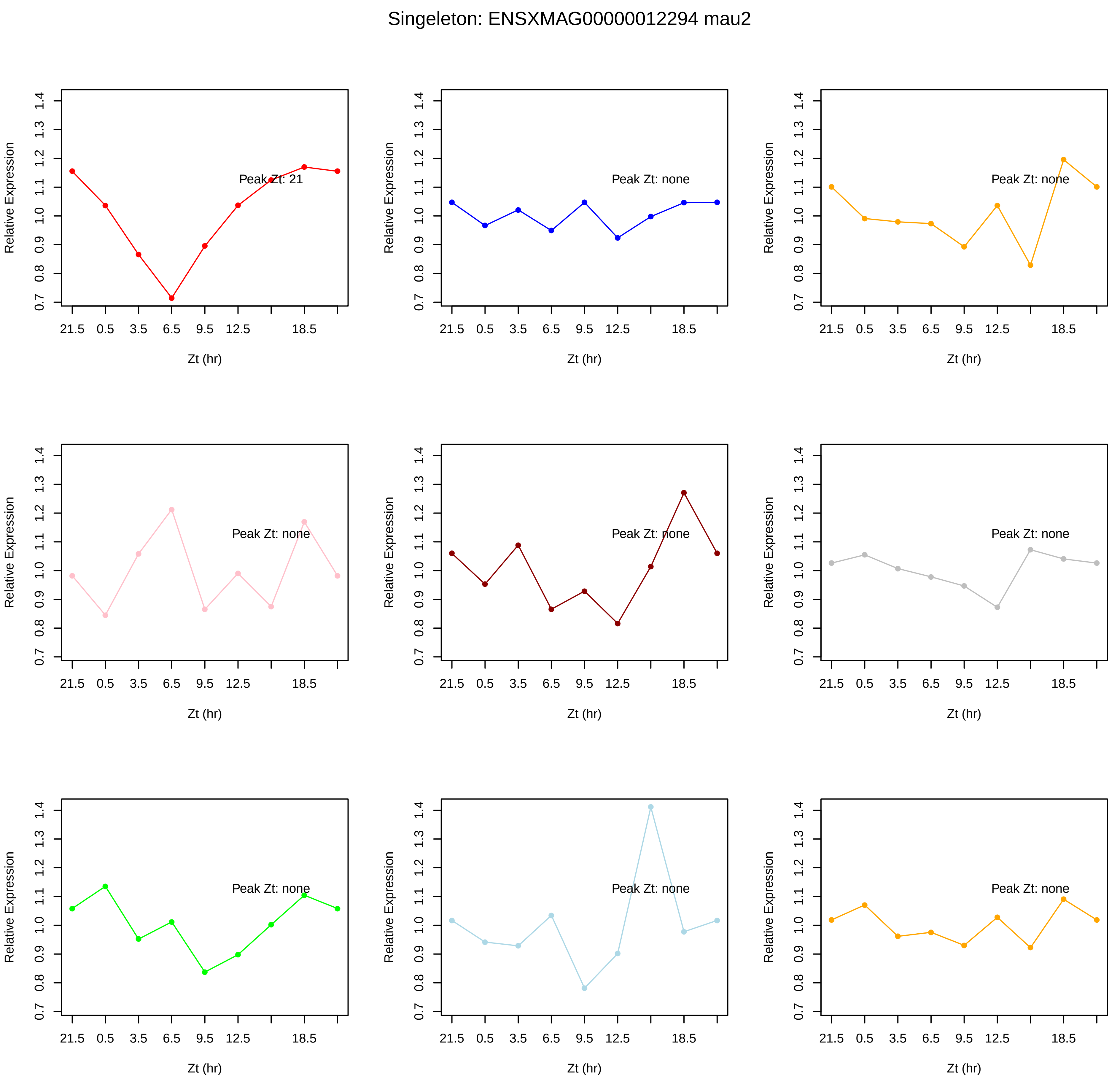
Singleton: ENSXMAG00000012294 mau2
Relative Expression
Zt (hr)
21.5
0.5
3.5
6.5
9.5
12.5
18.5
0.7
0.8
0.9
1.0
1.1
1.2
1.3
1.4
Peak Zt: 21
Peak Zt: none

Singeleton: ENSXMAG00000014726 RNF207

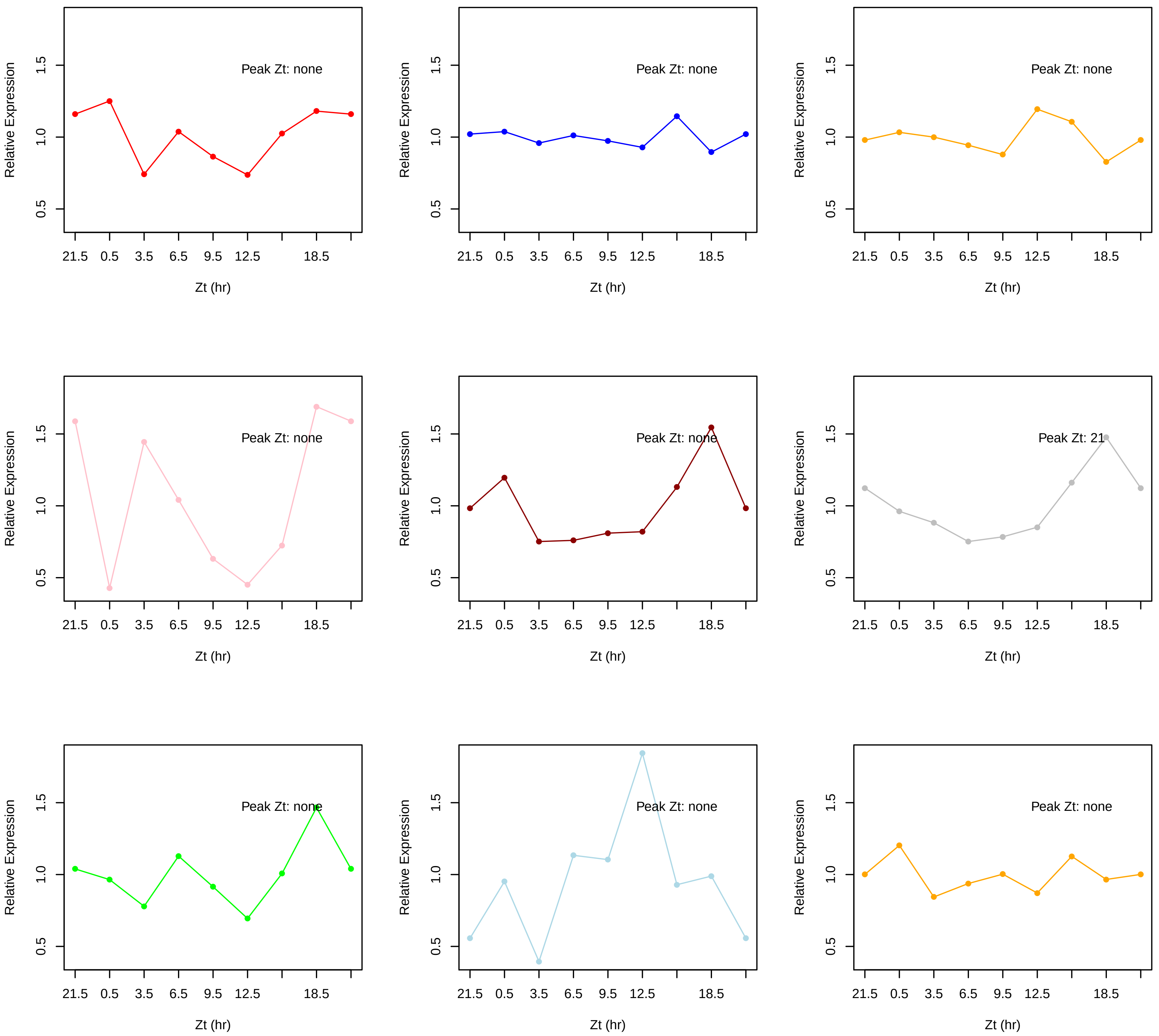

Singleton: ENSXMAG00000015609 ZNF512B

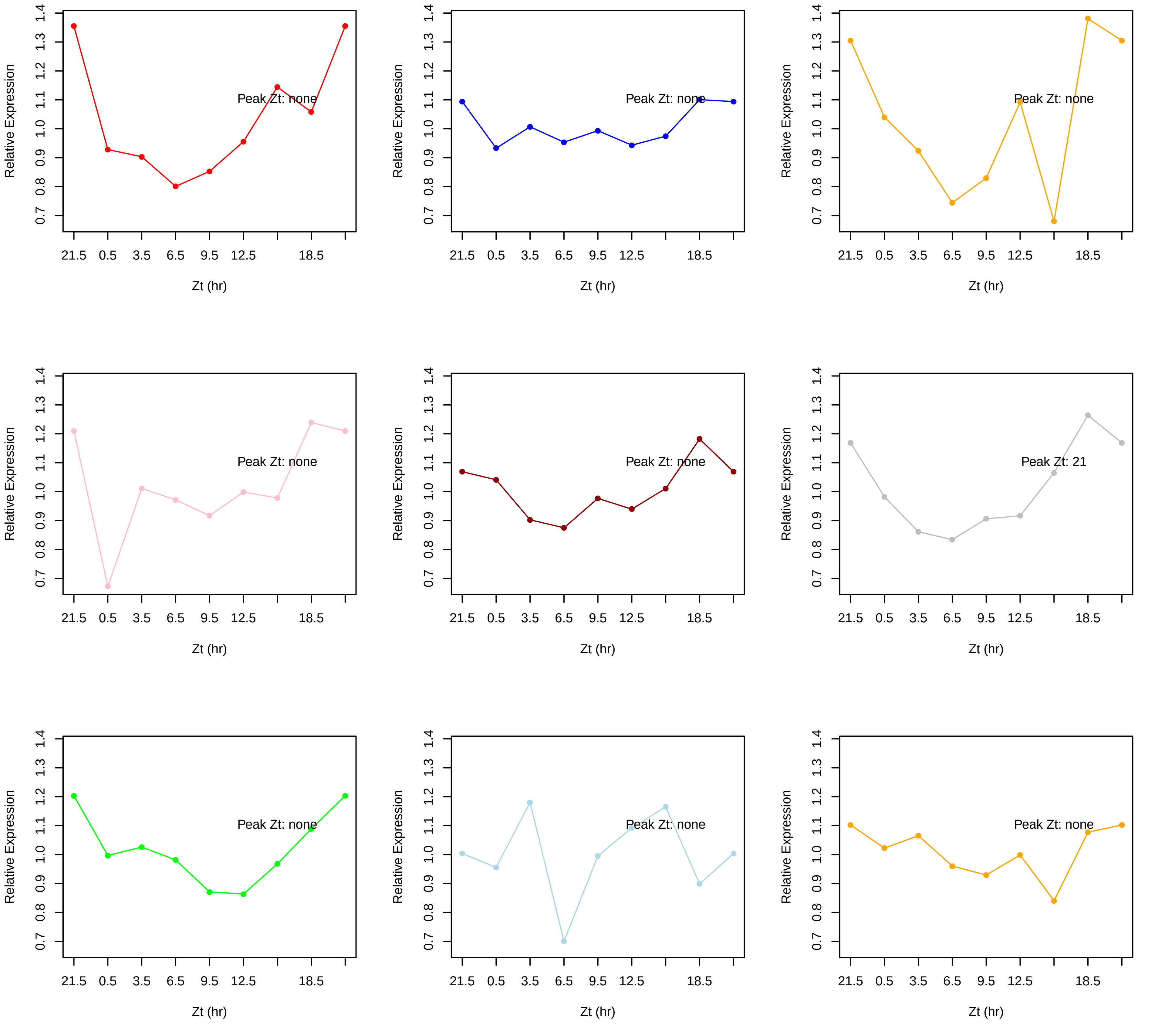

Singeleton: ENSXMAG00000027950 psmg1

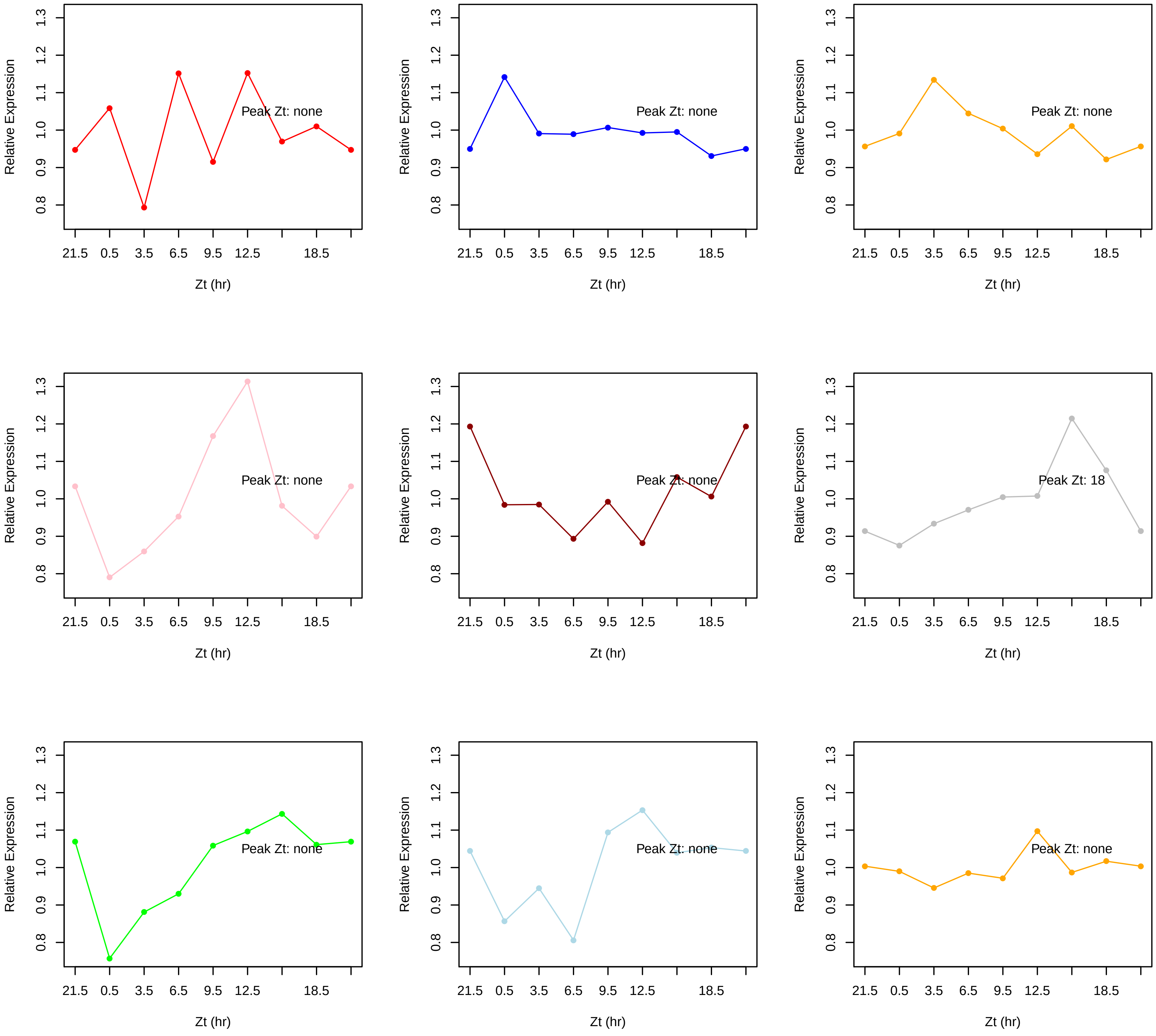

Singleton: ENSXMAG00000003644 shmt1

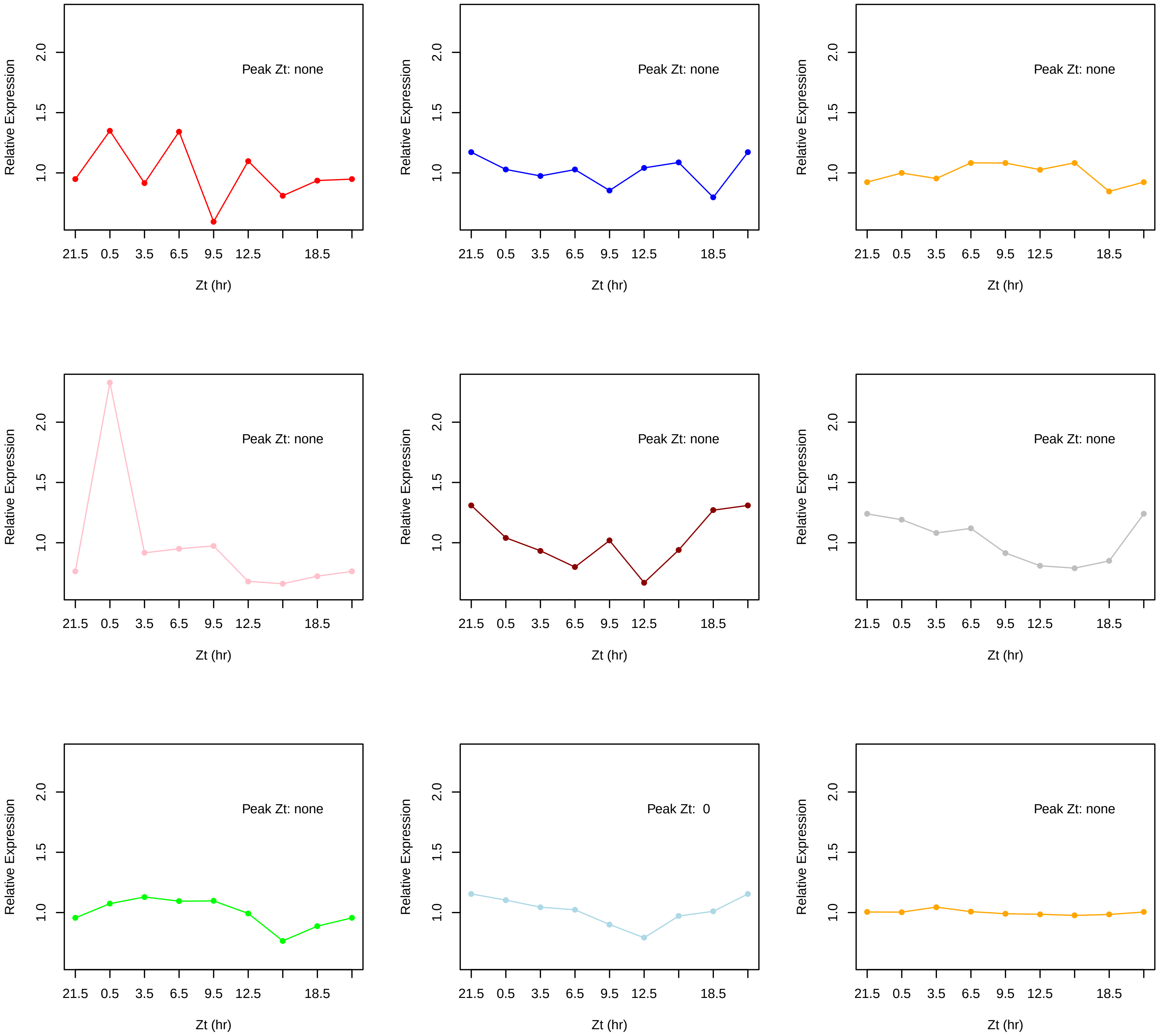

Singleton: ENSXMAG00000000571 sez6a

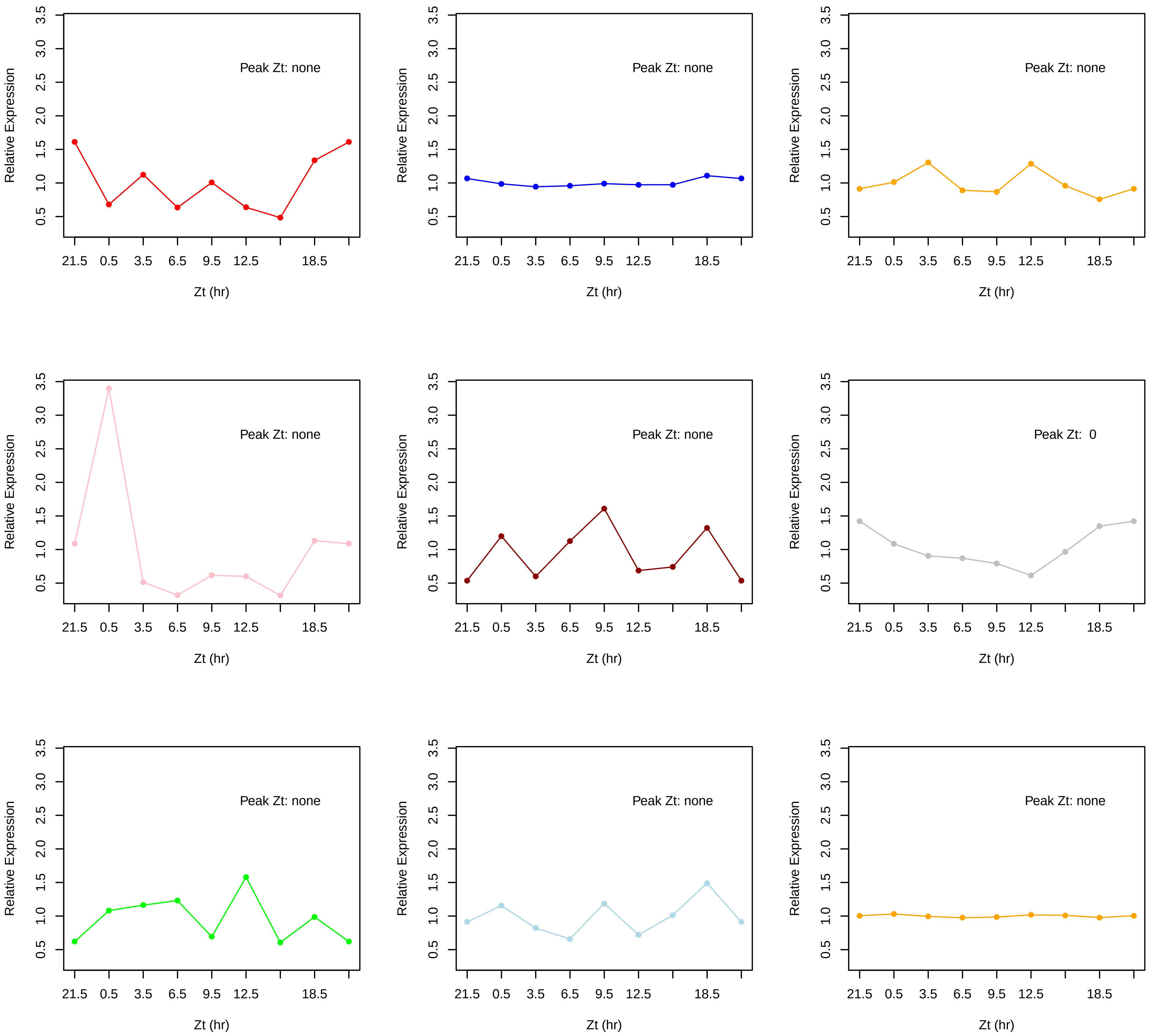

Singeleton: ENSXMAG00000015618 uckl1b

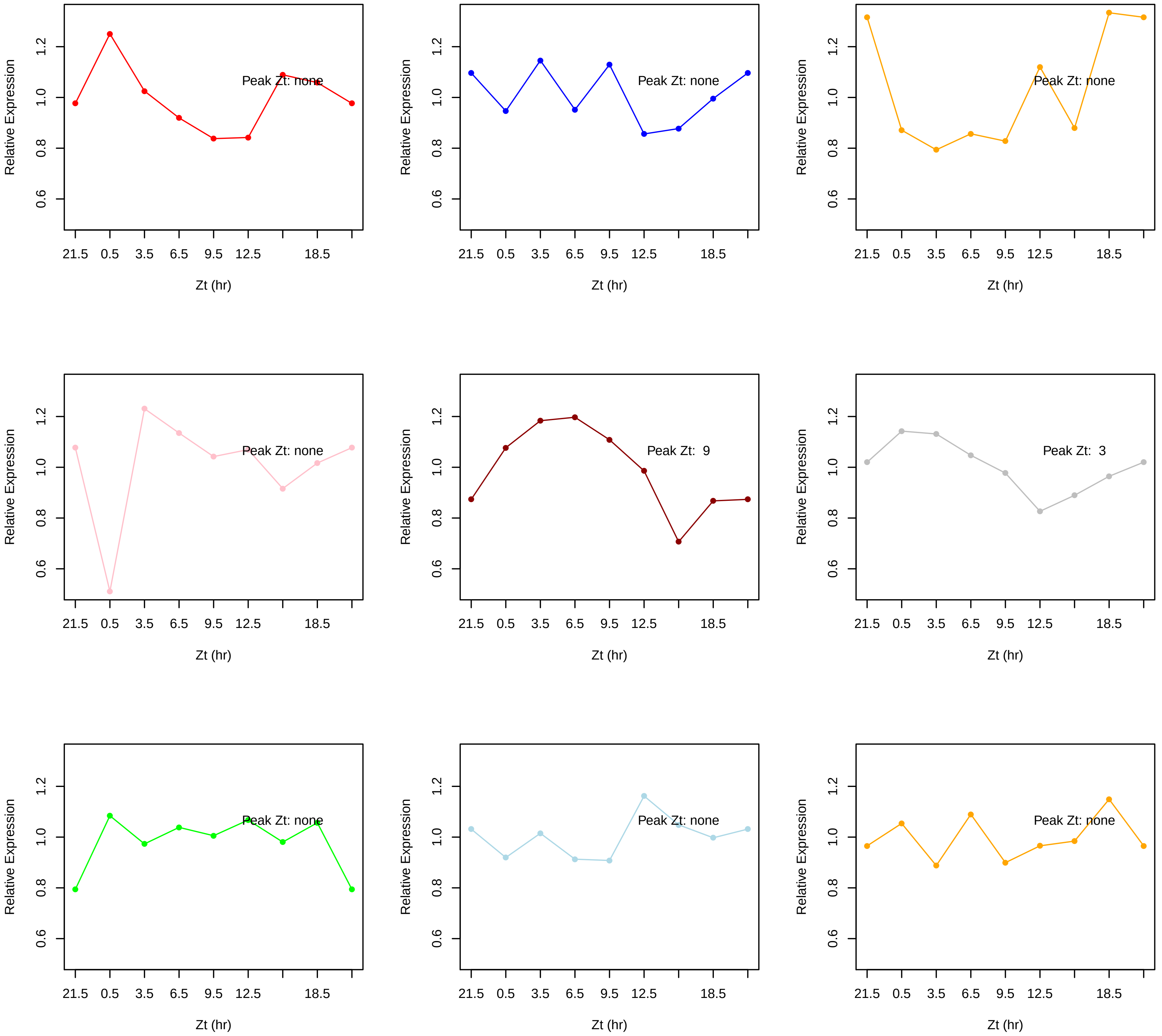

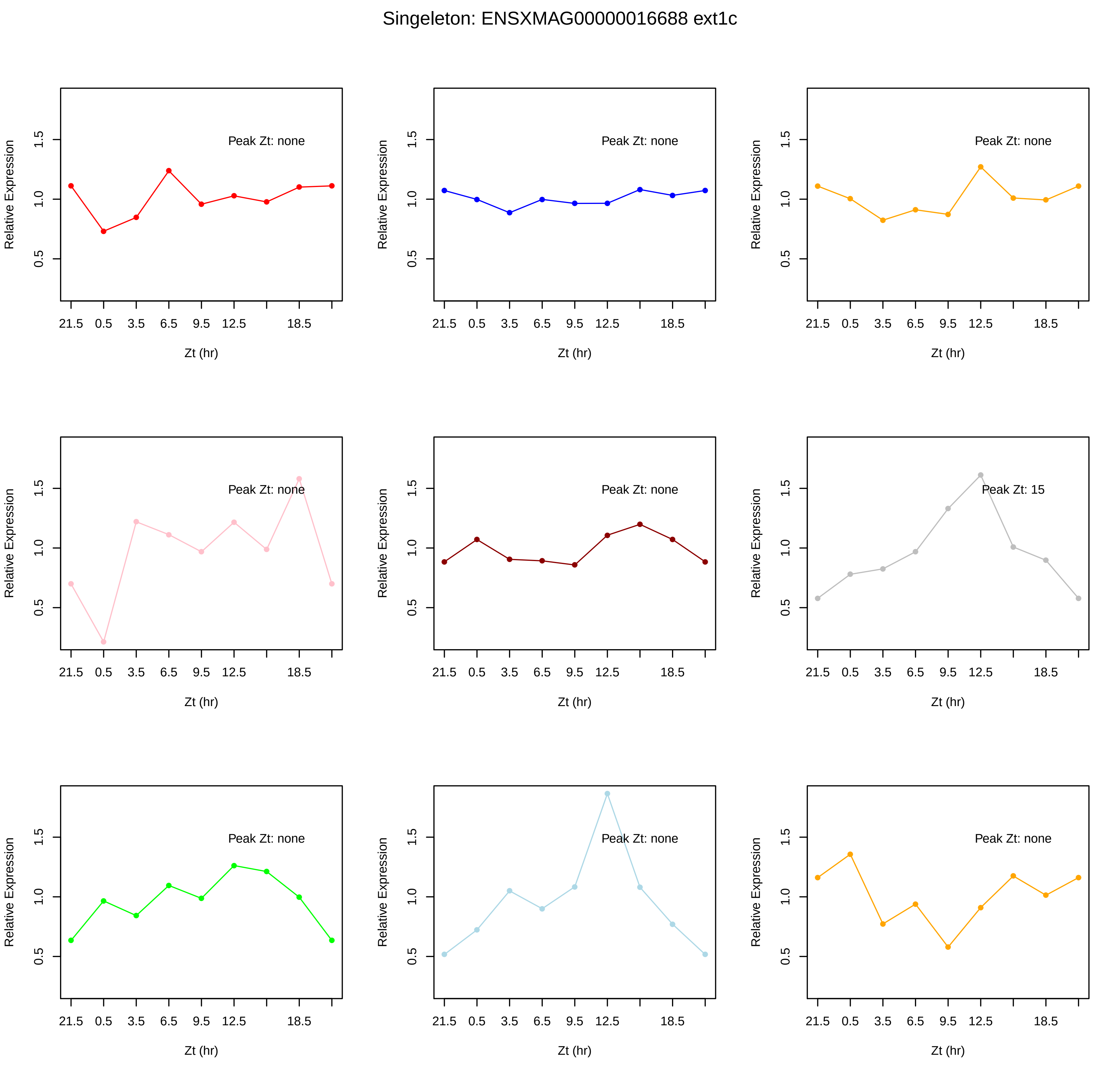
Singeleton: ENSXMAG00000016688 ext1c
Peak Zt: none
Peak Zt: none
Peak Zt: none
Peak Zt: none
Peak Zt: none
Peak Zt: 15
Peak Zt: none
Peak Zt: none
Peak Zt: none
Relative Expression
Zt (hr)
21.5 0.5 3.5 6.5 9.5 12.5 18.5
0.5 1.0 1.5

Singleton: ENSXMAG00000010154 prmt3

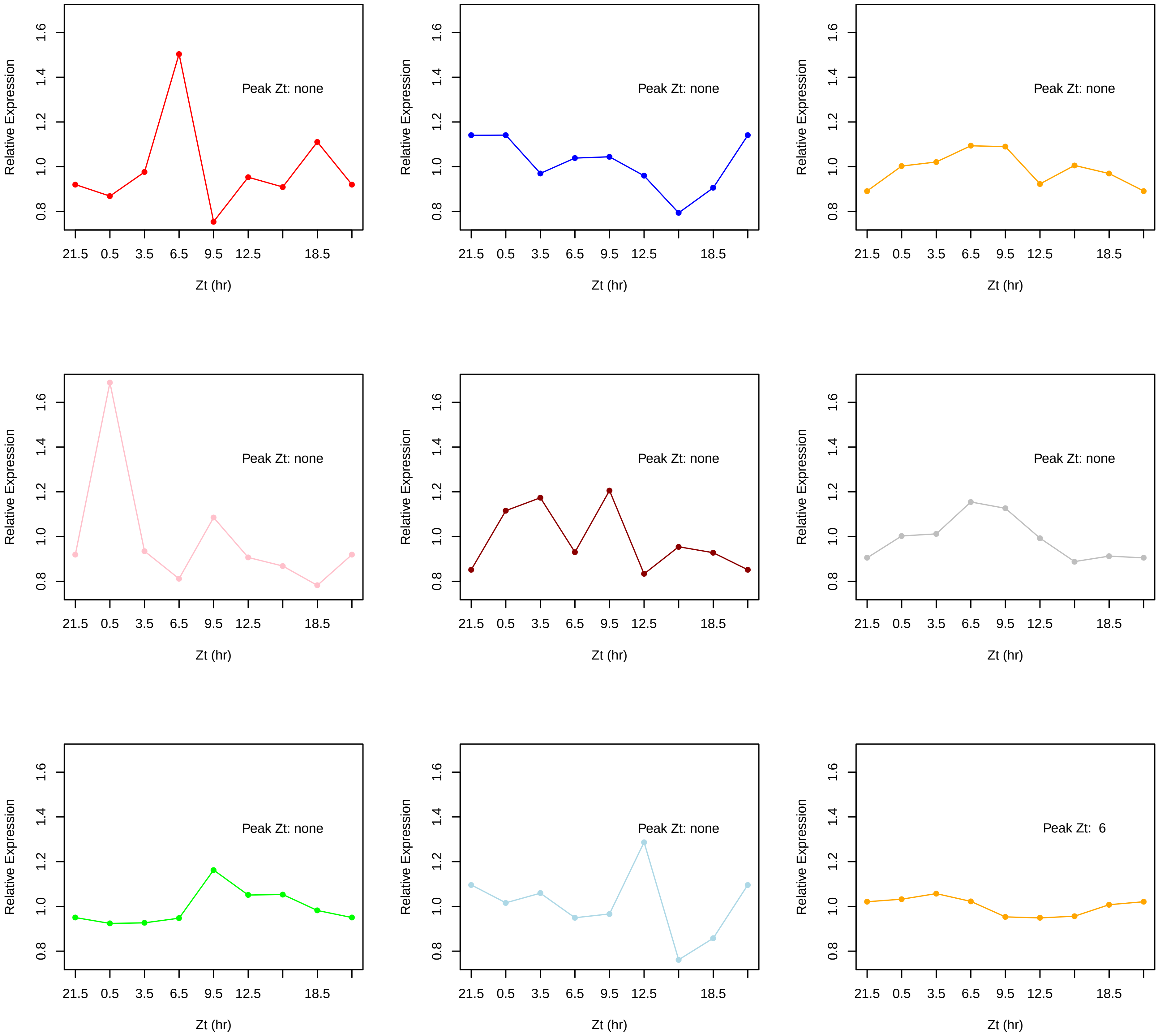

Singeleton: ENSXMAG00000003670 smcr8a

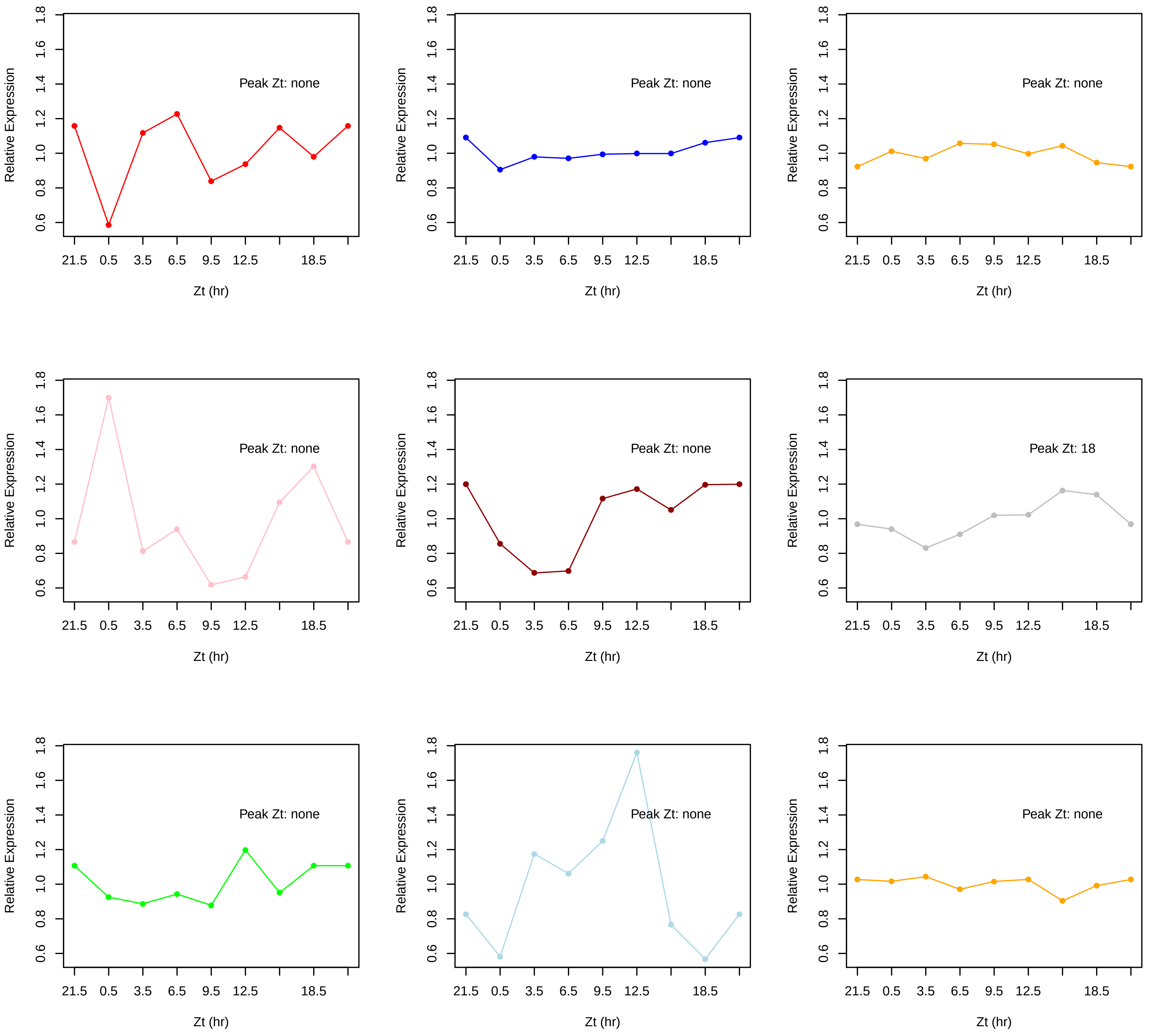

Singeleton: ENSXMAG00000024303 zgc:114119

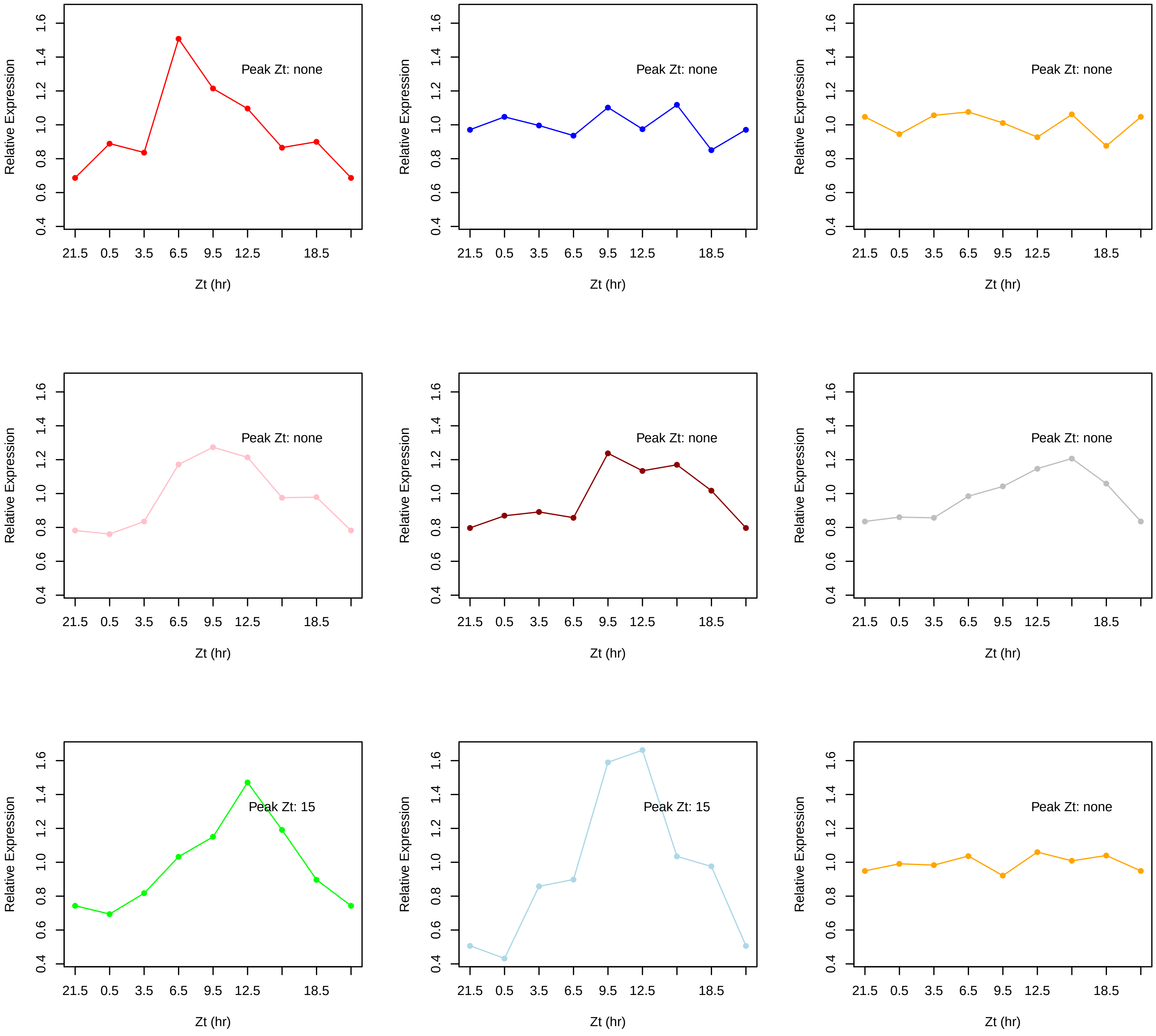

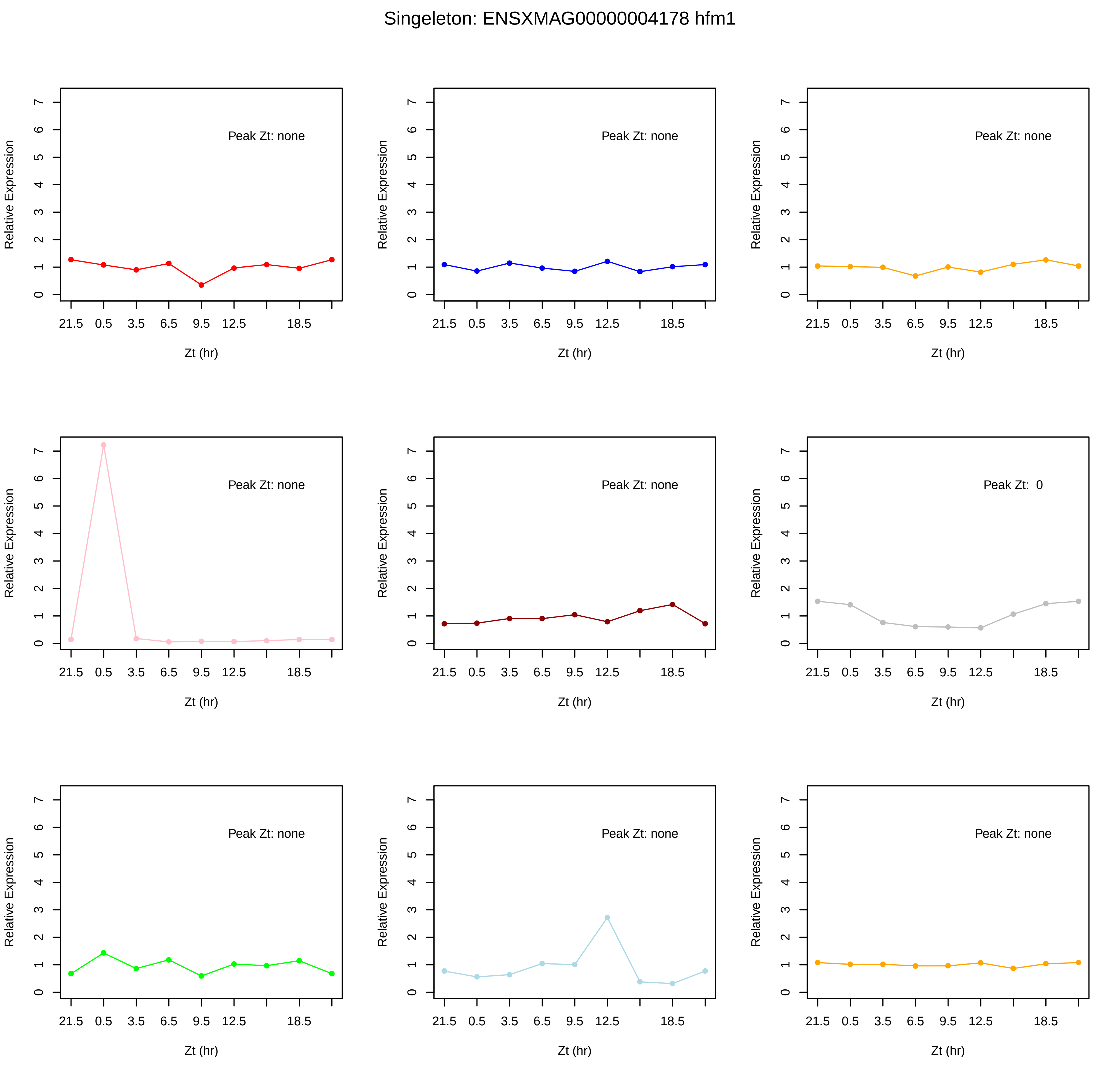
Singeleton: ENSXMAG00000004178 hfm1
Peak Zt: none
Peak Zt: none
Peak Zt: none
Peak Zt: none
Peak Zt: none
Peak Zt: 0
Peak Zt: none
Peak Zt: none
Peak Zt: none
Relative Expression
Zt (hr)
21.5
0.5
3.5
6.5
9.5
12.5
18.5

Singleton: ENSXMAG00000003671 top3a

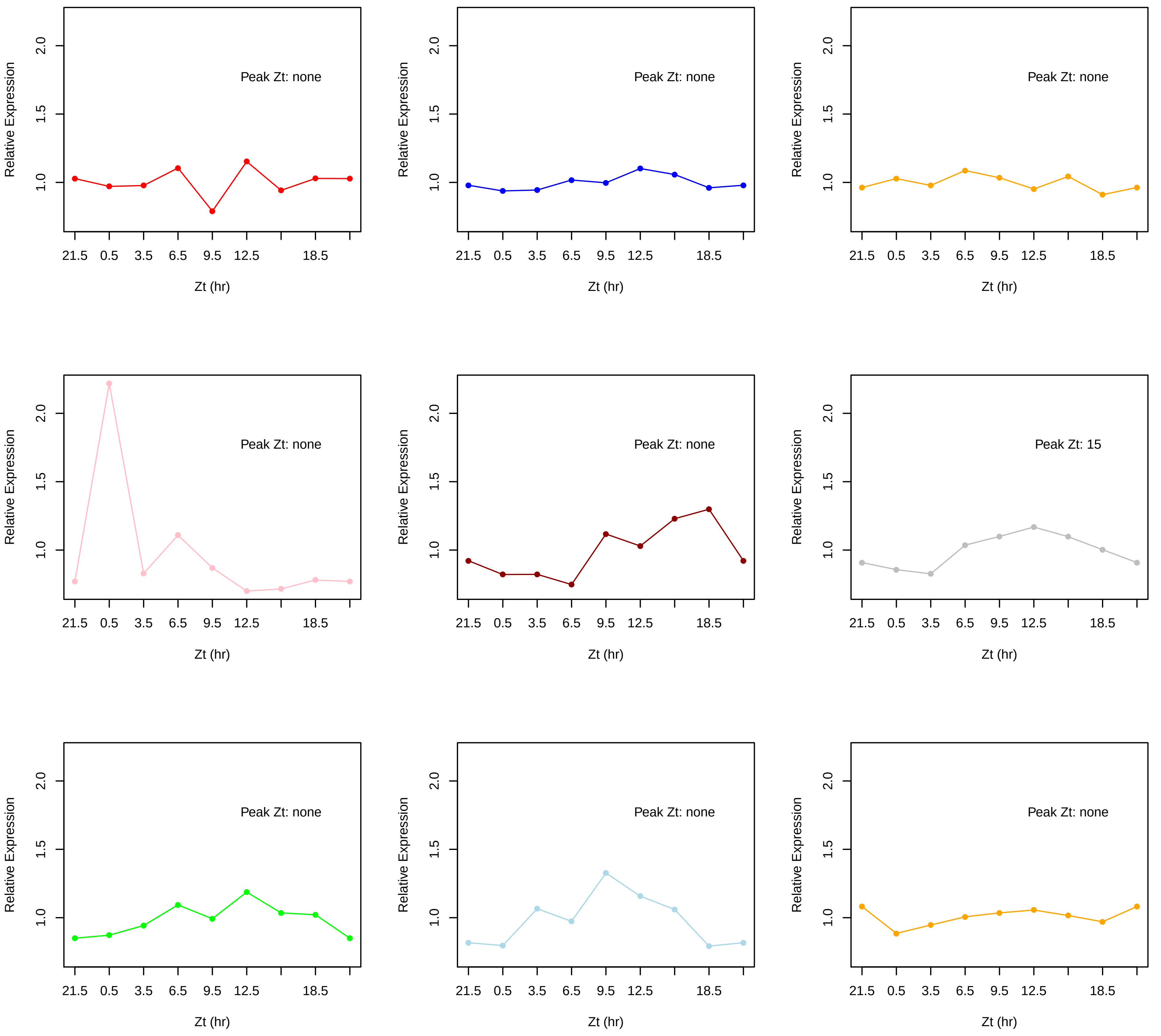

Singeleton: ENSXMAG00000024301 ubap1

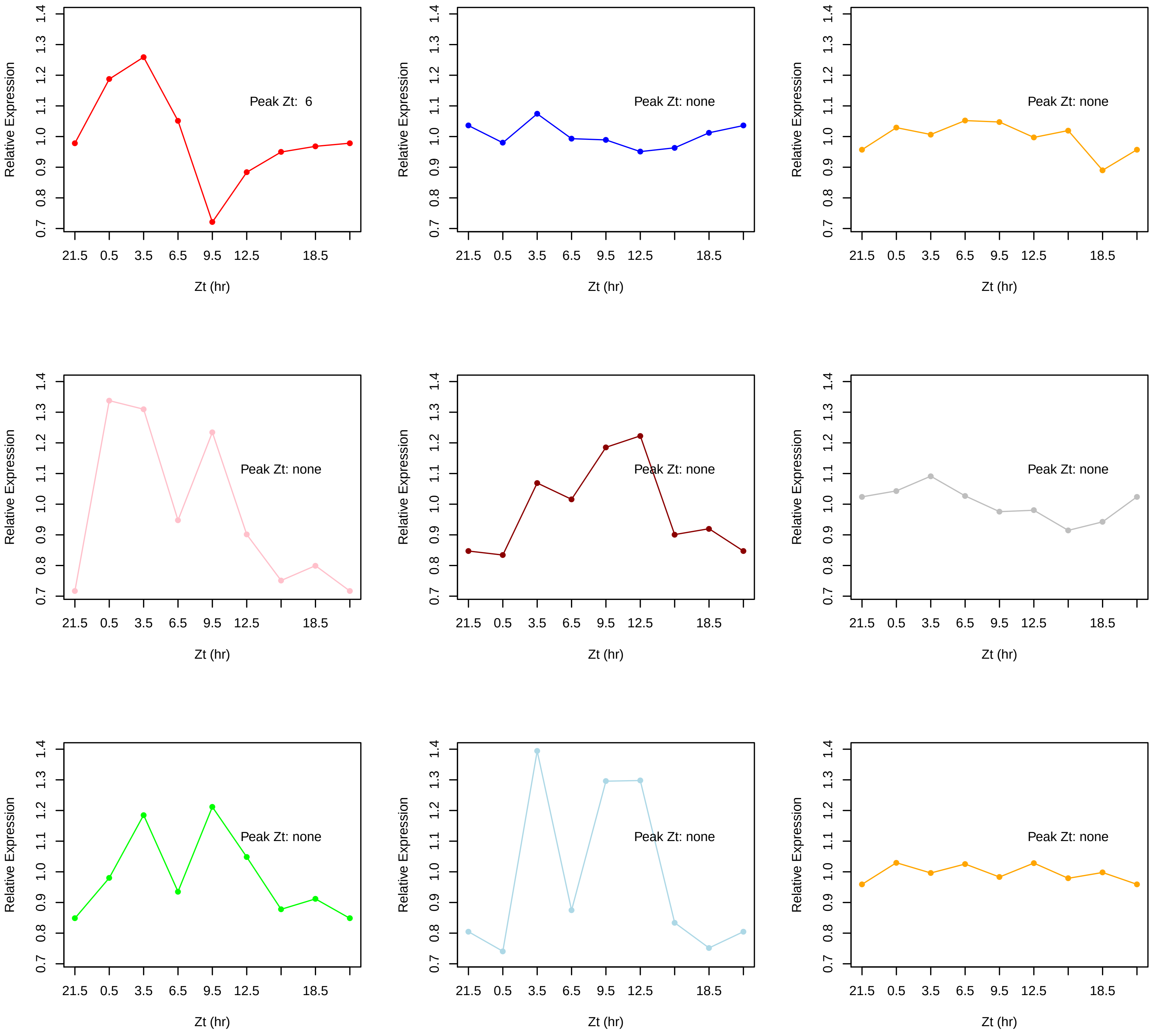

Singeleton: ENSXMAG00000005652 ciao2b

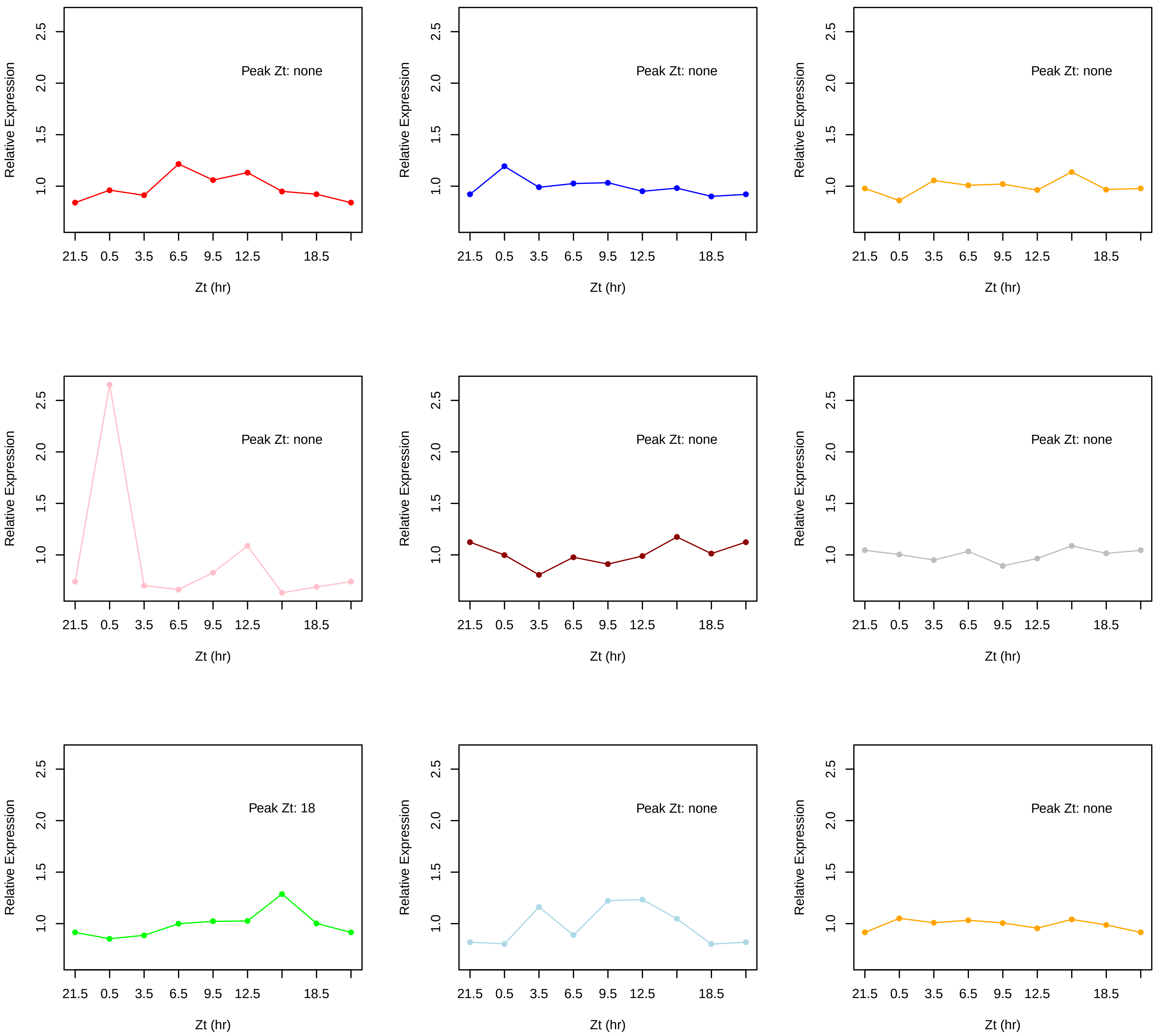

Singleton: ENSXMAG00000014890 mrm1

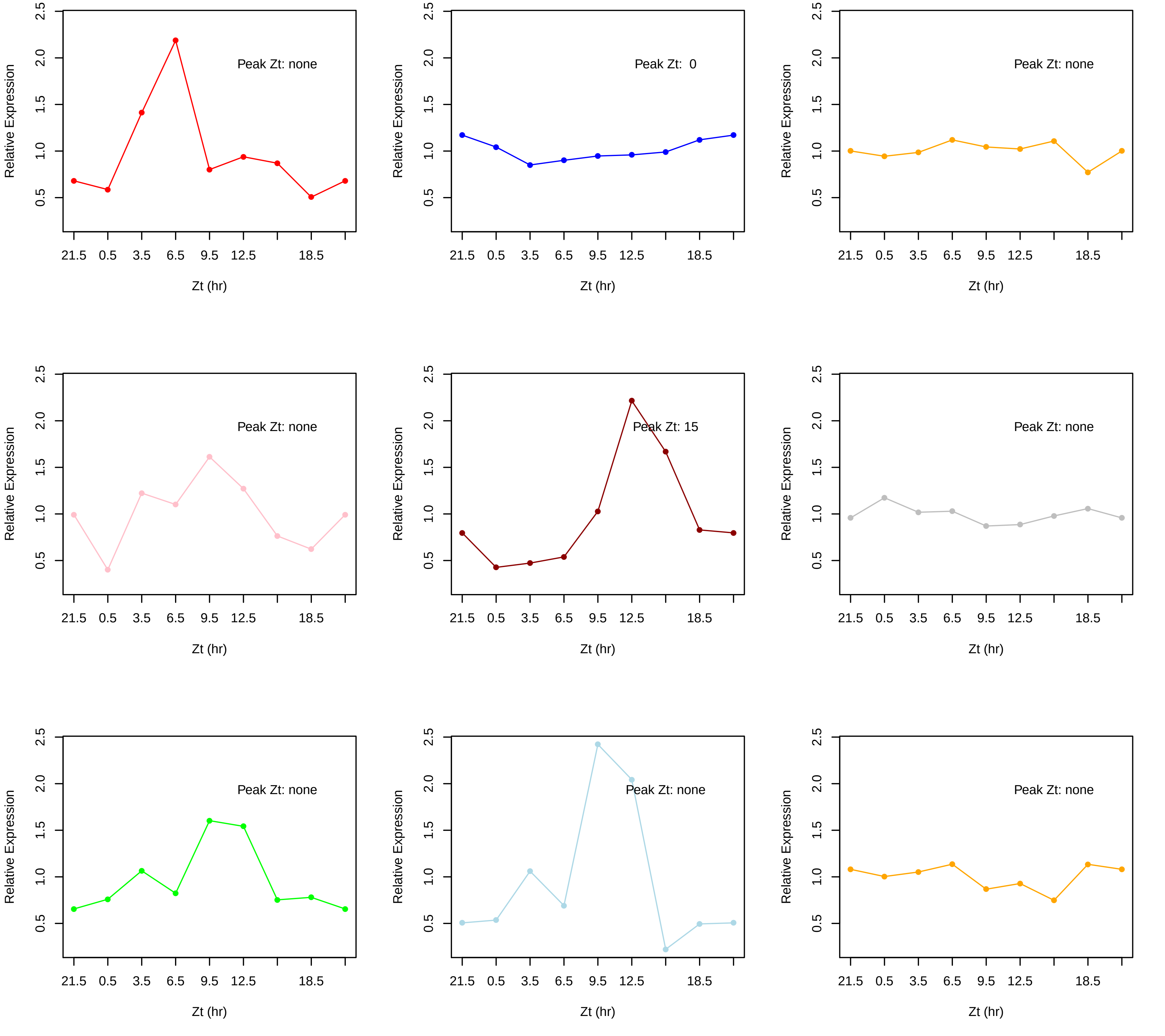

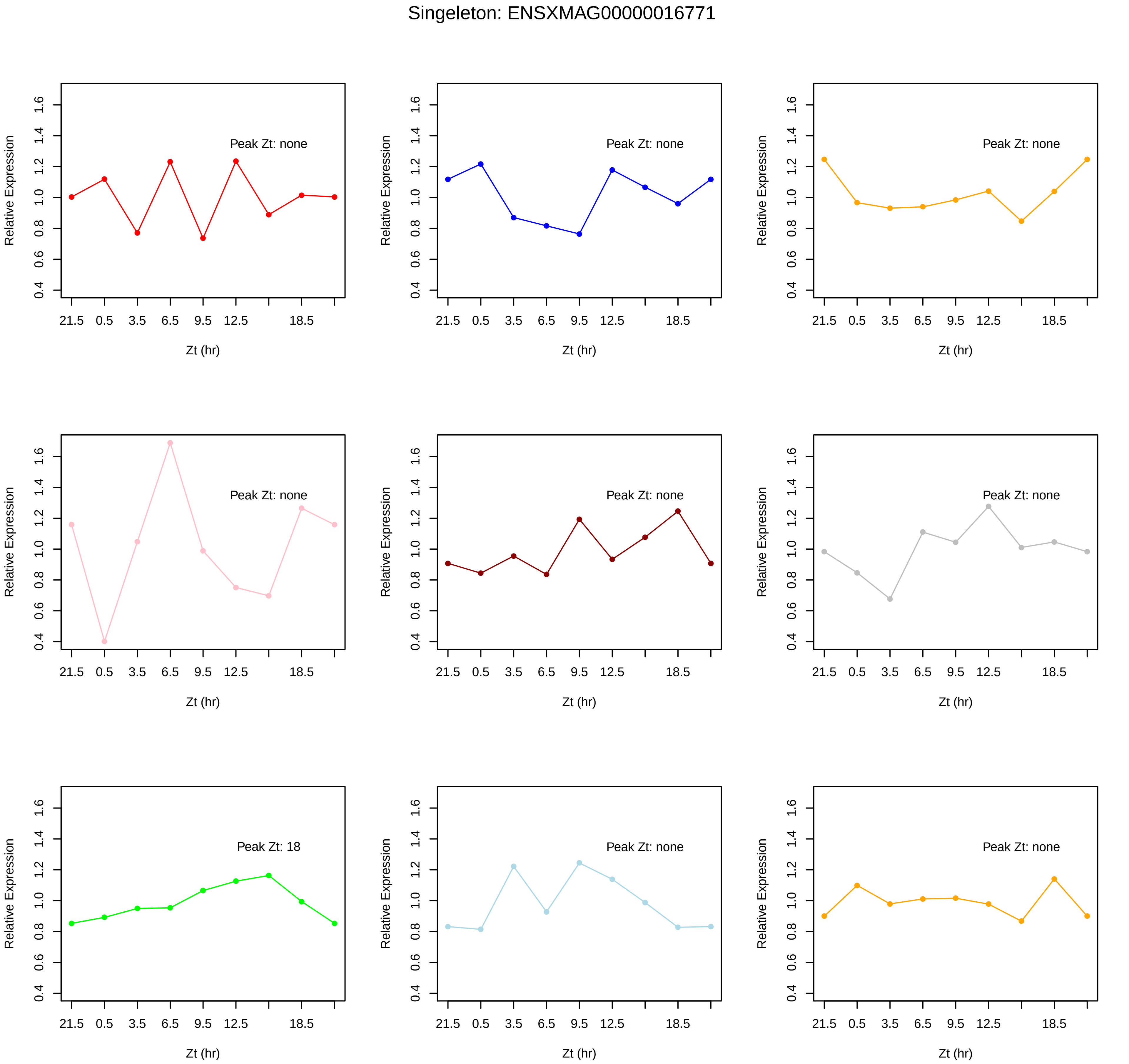
Singeleton: ENSXMAG00000016771
Peak Zt: none
Peak Zt: none
Peak Zt: none
Peak Zt: none
Peak Zt: none
Peak Zt: none
Peak Zt: 18
Peak Zt: none
Peak Zt: none
Relative Expression
Zt (hr)
21.5 0.5 3.5 6.5 9.5 12.5 18.5
0.4 0.6 0.8 1.0 1.2 1.4 1.6

Singeleton: ENSXMAG00000005554 atp13a3

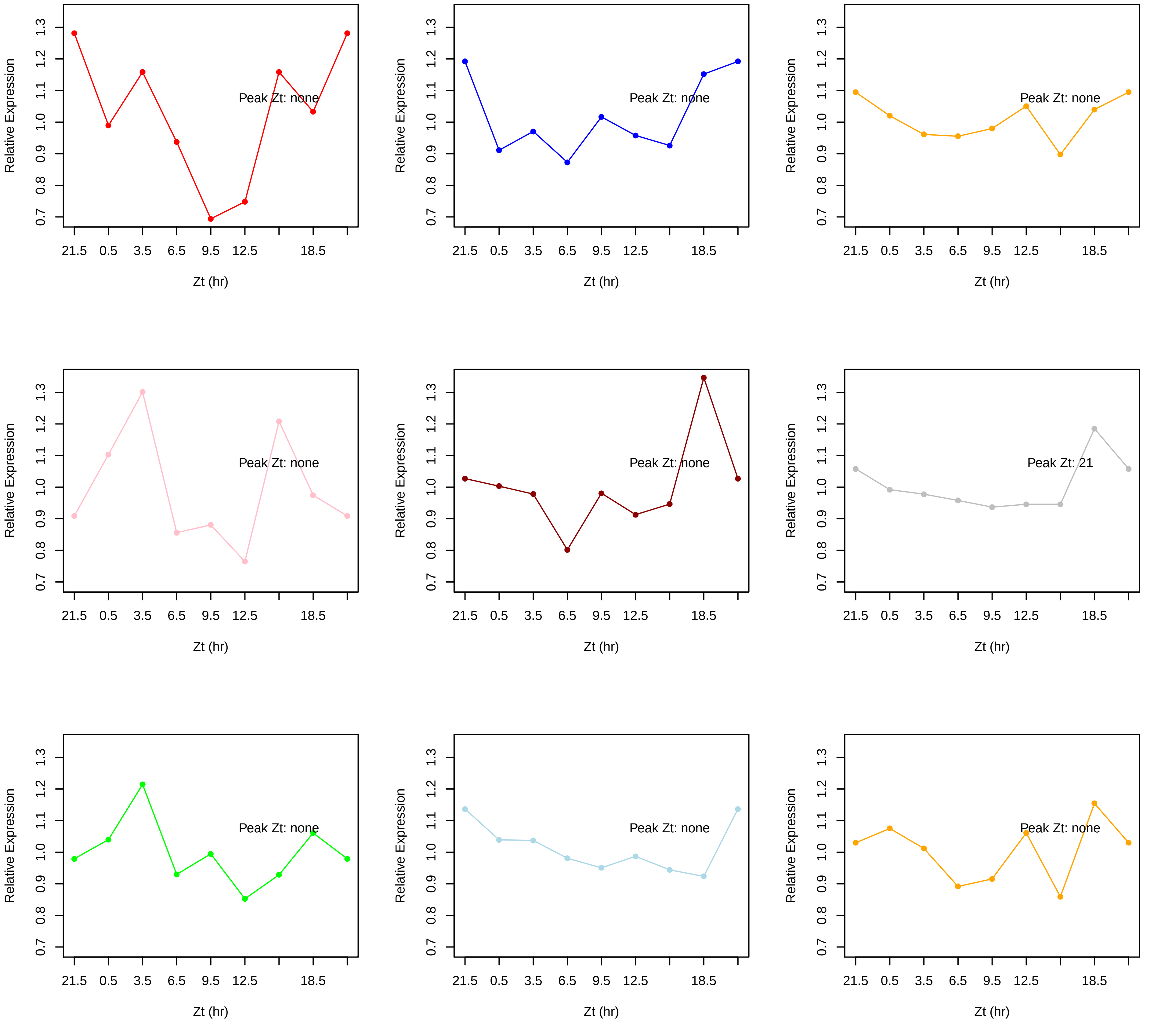

Singeleton: ENSXMAG00000010152 slc6a5

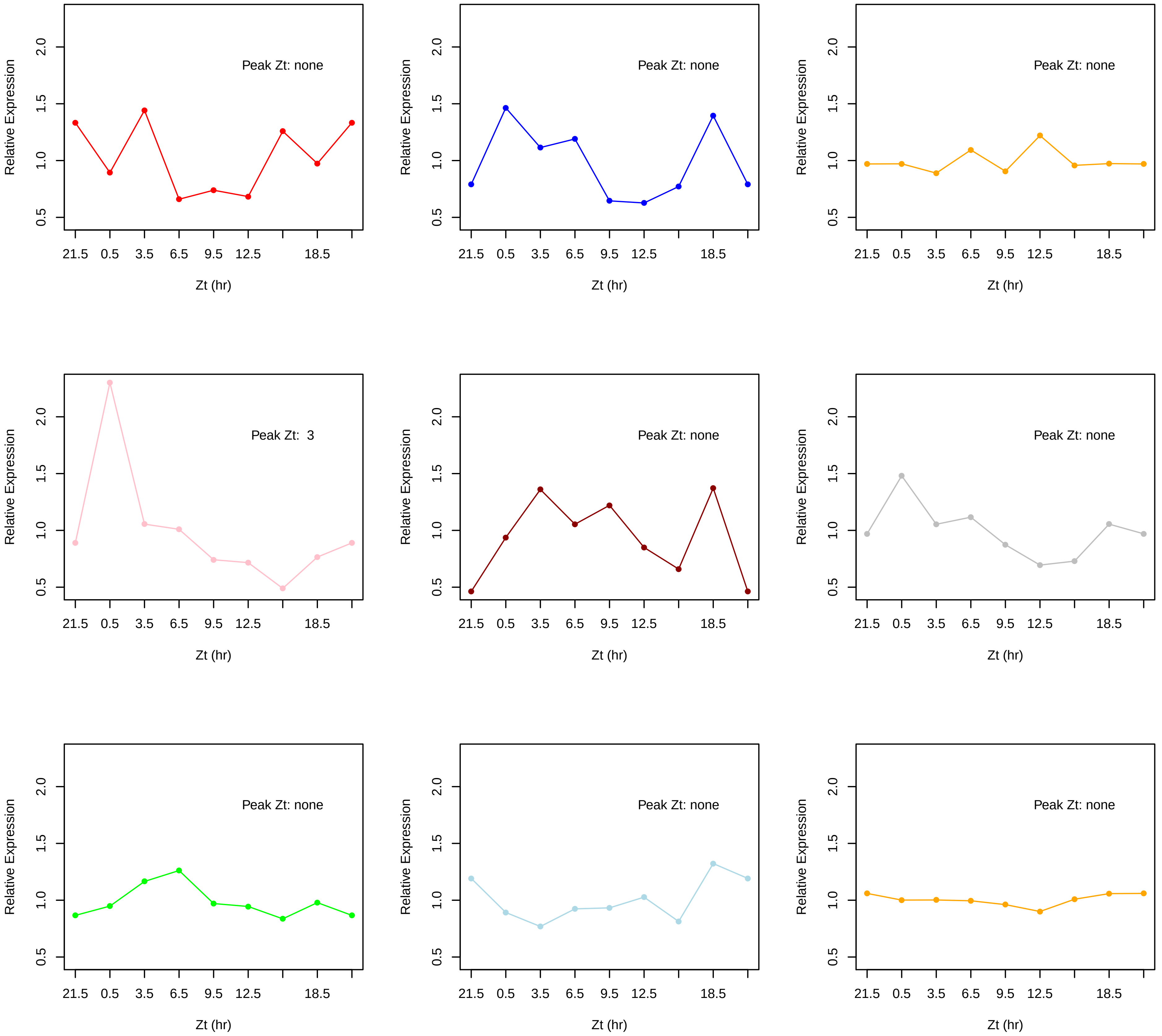

Singeleton: ENSXMAG00000001707 dyrk1ab

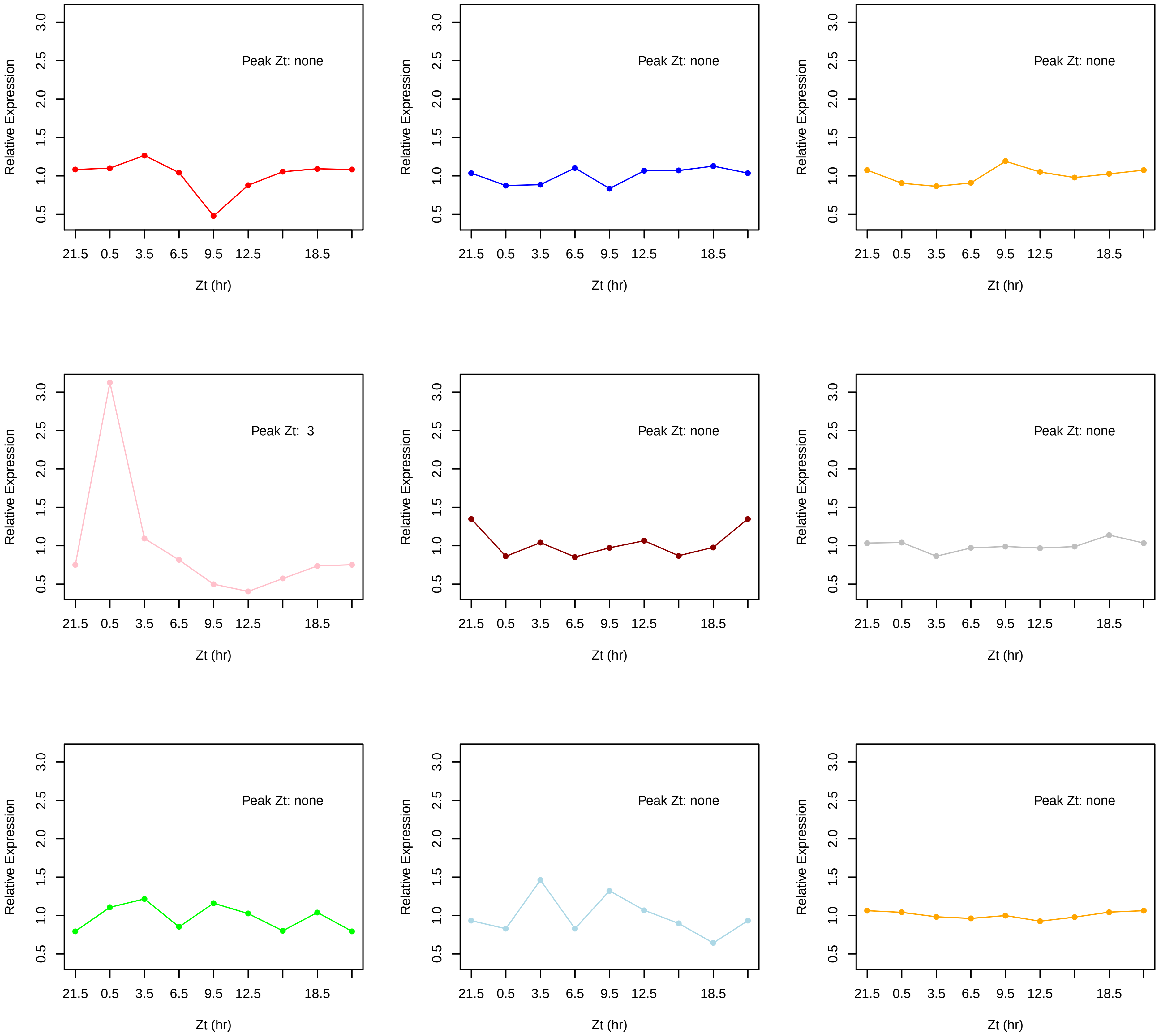

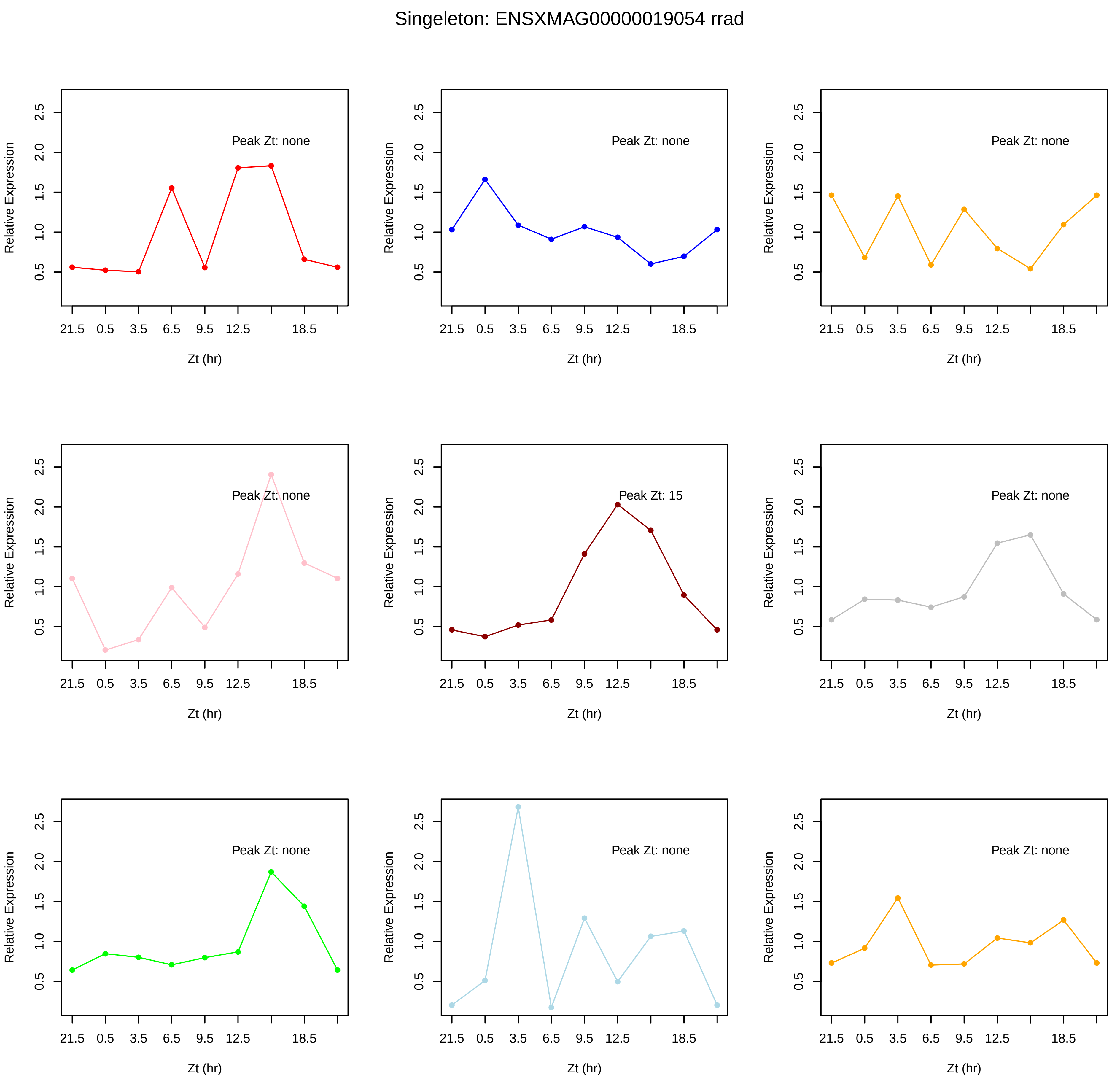
Singeleton: ENSXMAG00000019054 rrad
Peak Zt: none
Peak Zt: none
Peak Zt: none
Peak Zt: none
Peak Zt: 15
Peak Zt: none
Peak Zt: none
Peak Zt: none
Peak Zt: none
Relative Expression
Zt (hr)
21.5
0.5
3.5
6.5
9.5
12.5
18.5
0.5
1.0
1.5
2.0
2.5

Singleton: ENSXMAG00000017644 ITGA7

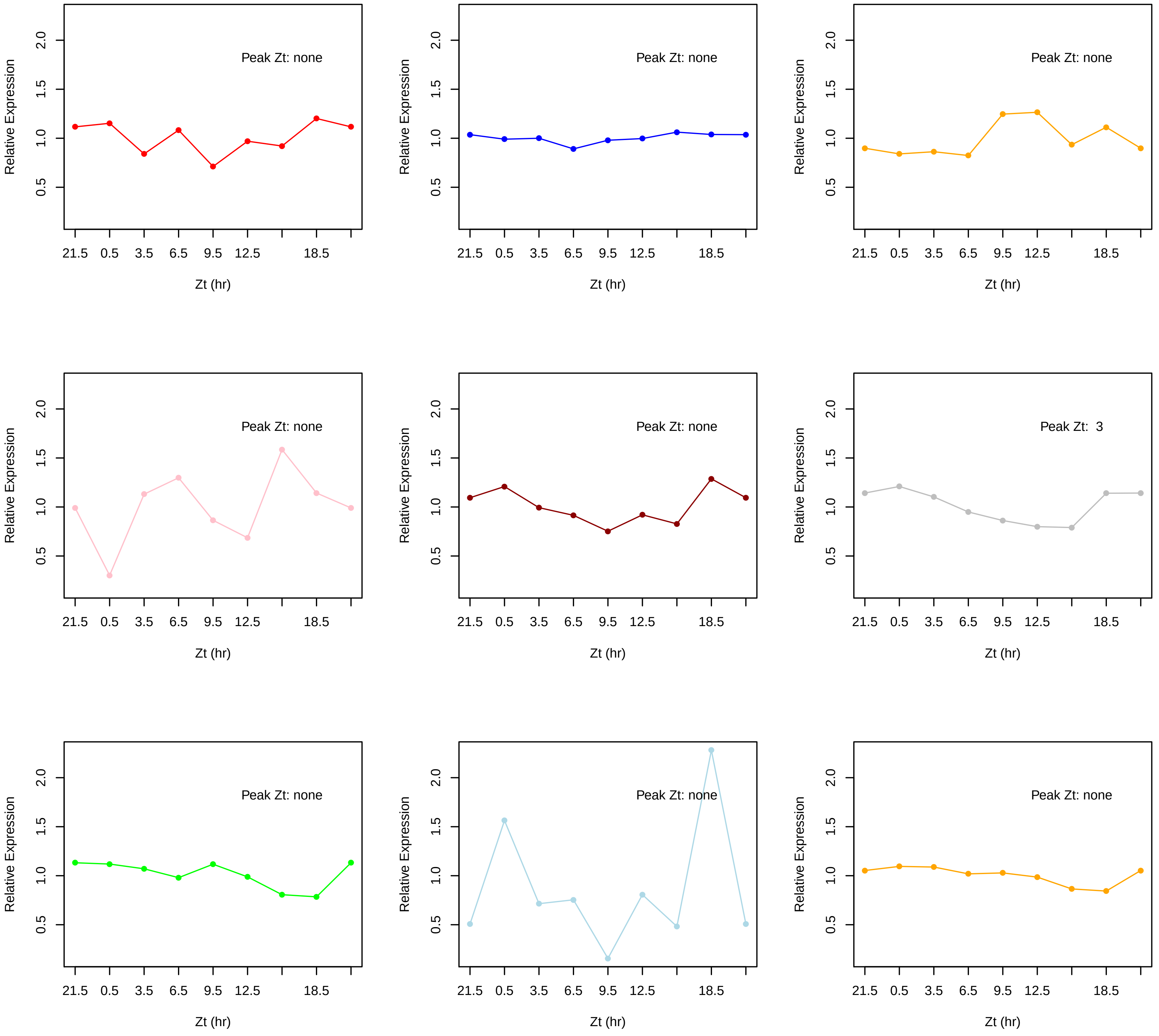

Singeleton: ENSXMAG00000028116 gramd1a

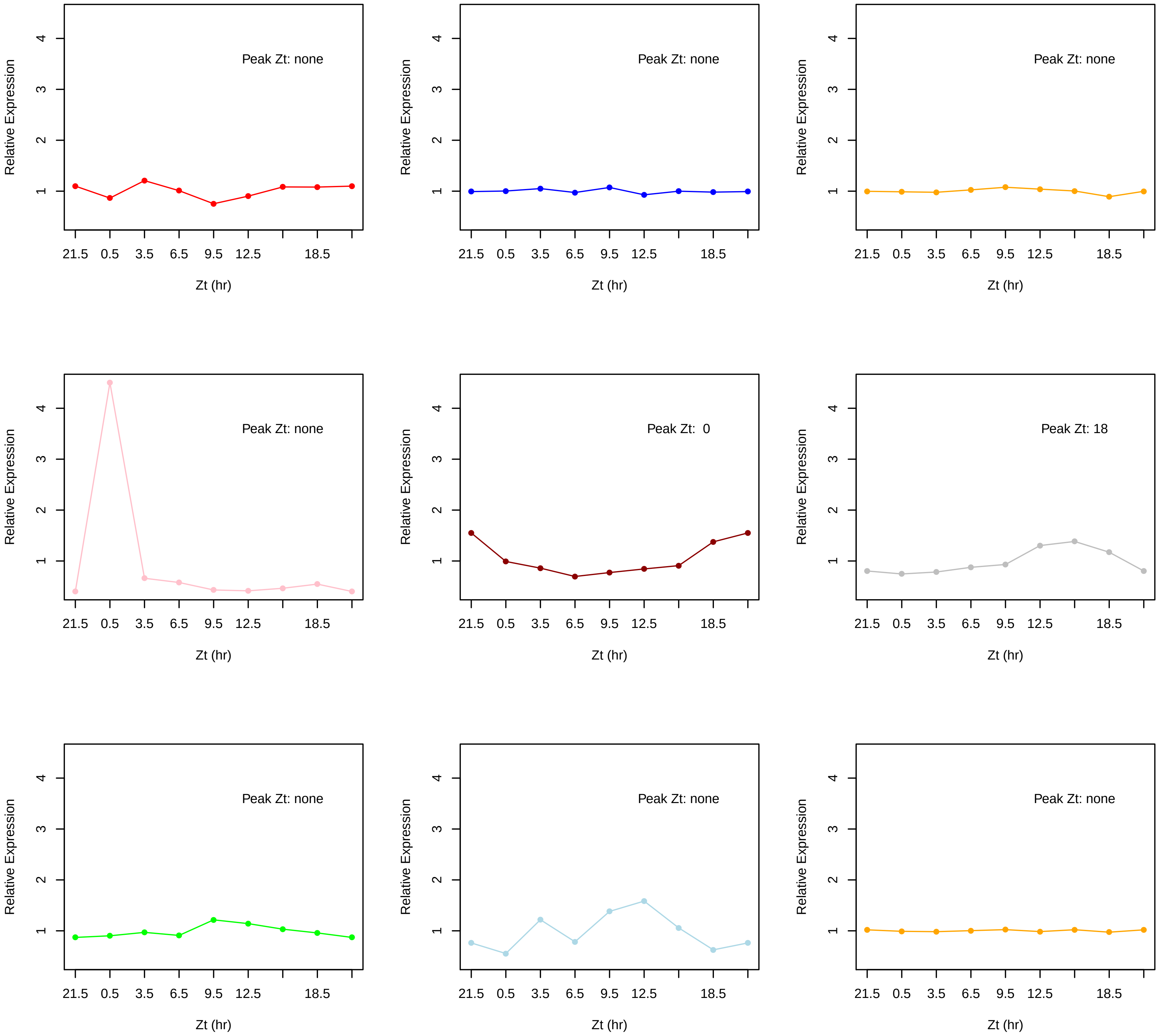

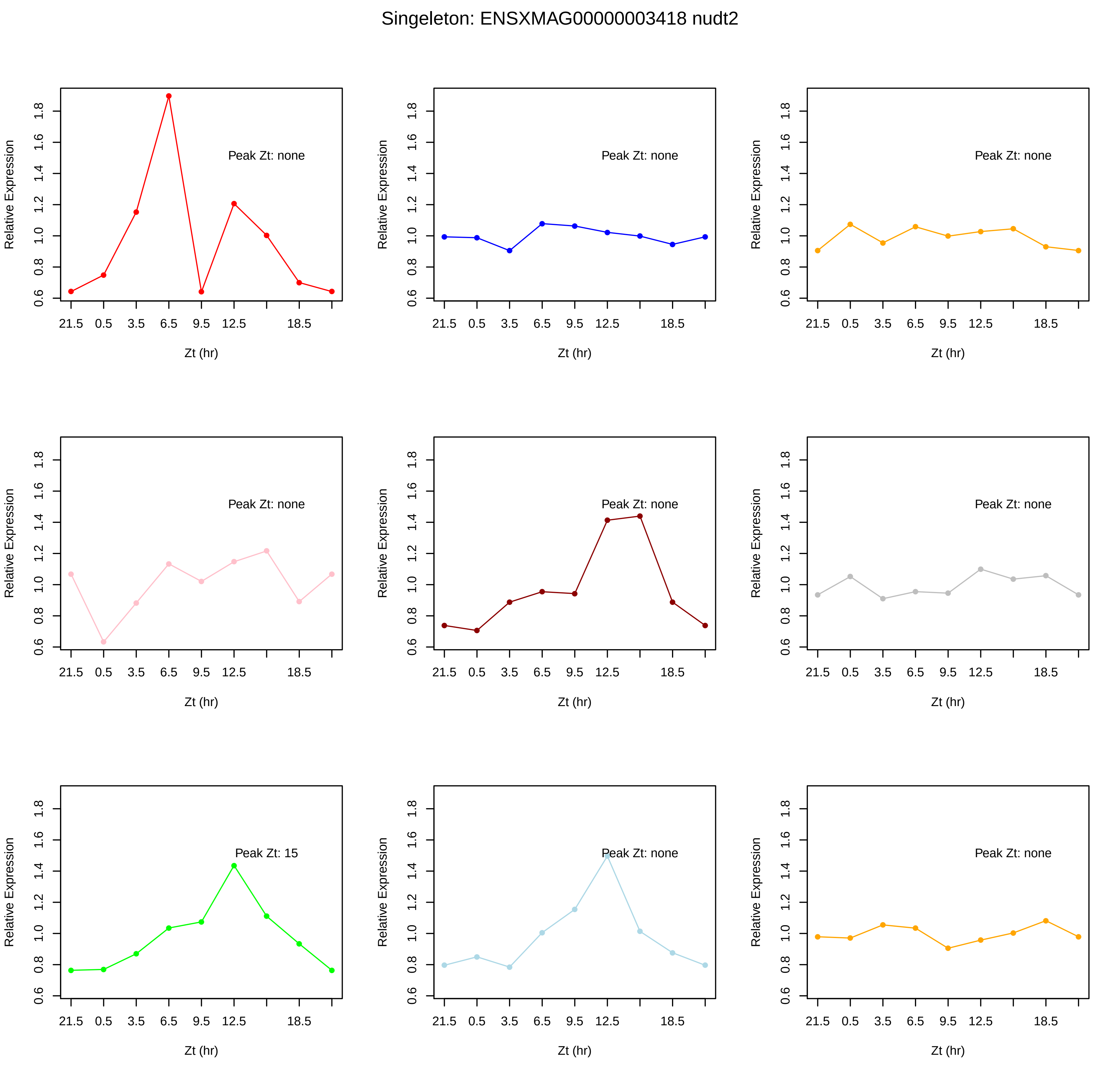
Singeleton: ENSXMAG00000003418 nudt2
Peak Zt: none
Peak Zt: none
Peak Zt: none
Peak Zt: none
Peak Zt: none
Peak Zt: none
Peak Zt: 15
Peak Zt: none
Peak Zt: none
Relative Expression
Zt (hr)
21.5
0.5
3.5
6.5
9.5
12.5
18.5
0.6
0.8
1.0
1.2
1.4
1.6
1.8

Singeleton: ENSXMAG00000002913 vps26c

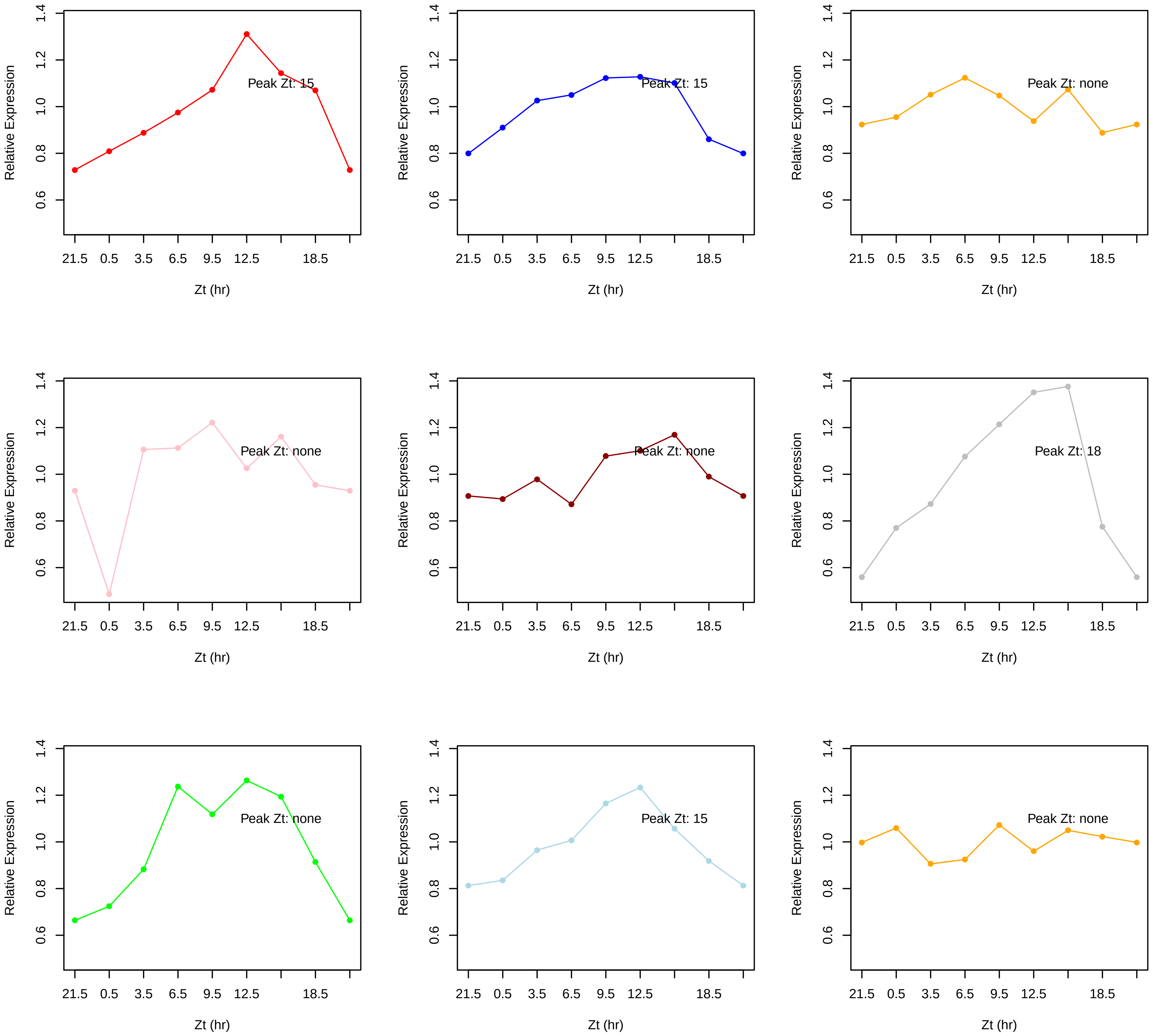

Singeleton: ENSXMAG00000001346 LLGL1

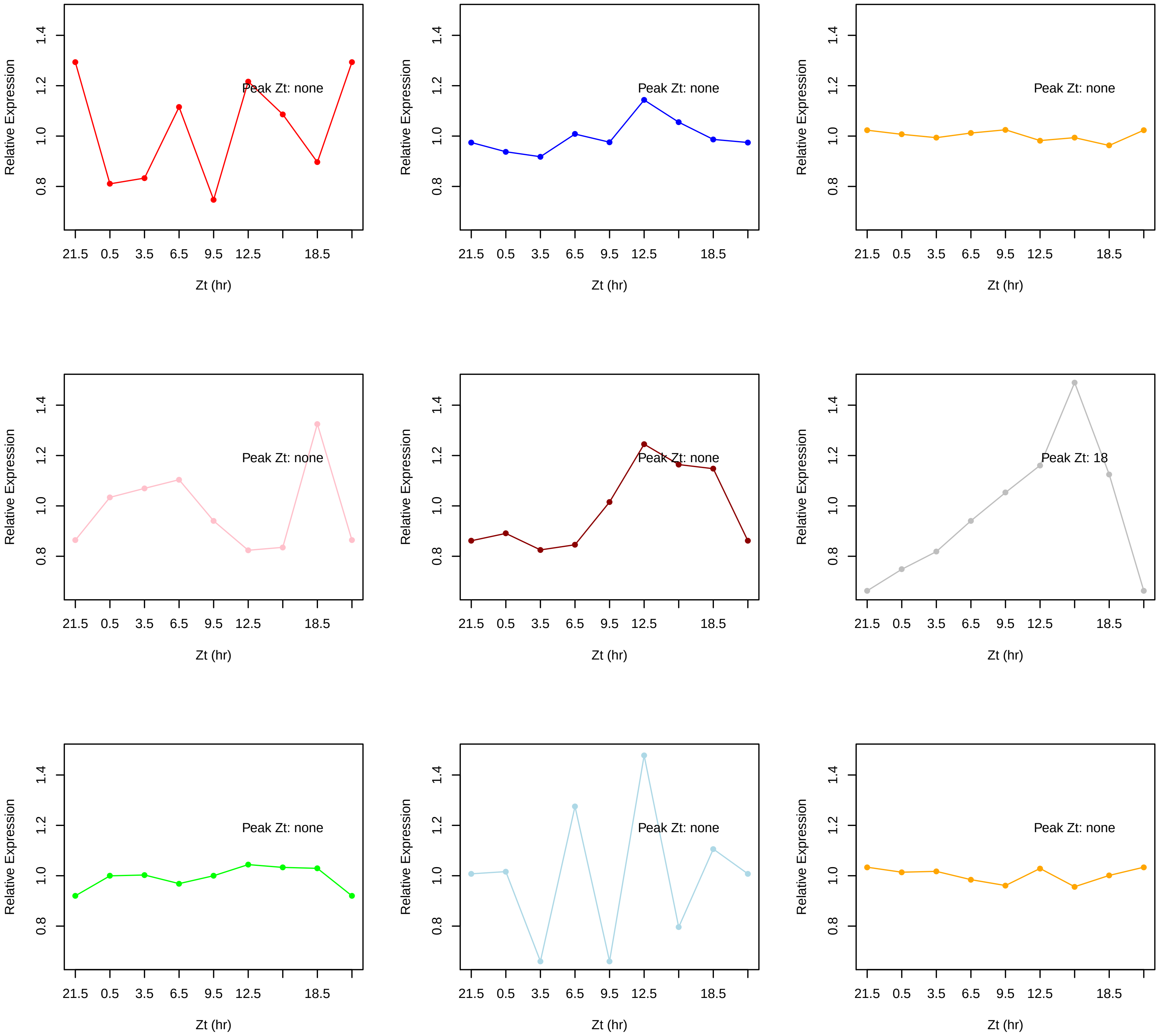

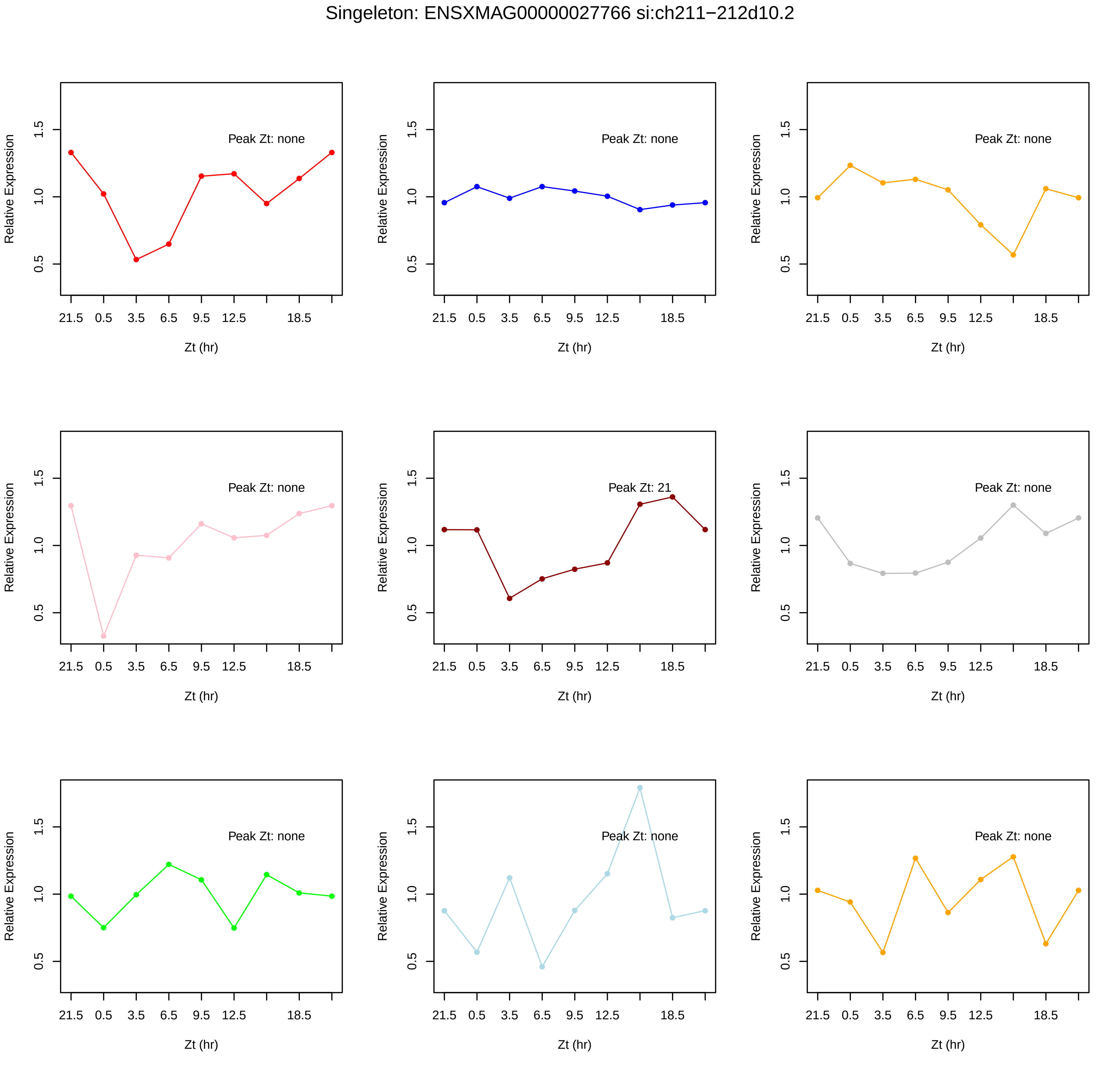
Singleton: ENSXMAG00000027766 si:ch211−212d10.2
Peak Zt: none
Peak Zt: none
Peak Zt: none
Peak Zt: none
Peak Zt: 21
Peak Zt: none
Peak Zt: none
Peak Zt: none
Peak Zt: none
Relative Expression
Zt (hr)
21.5
0.5
3.5
6.5
9.5
12.5
18.5
0.5
1.0
1.5

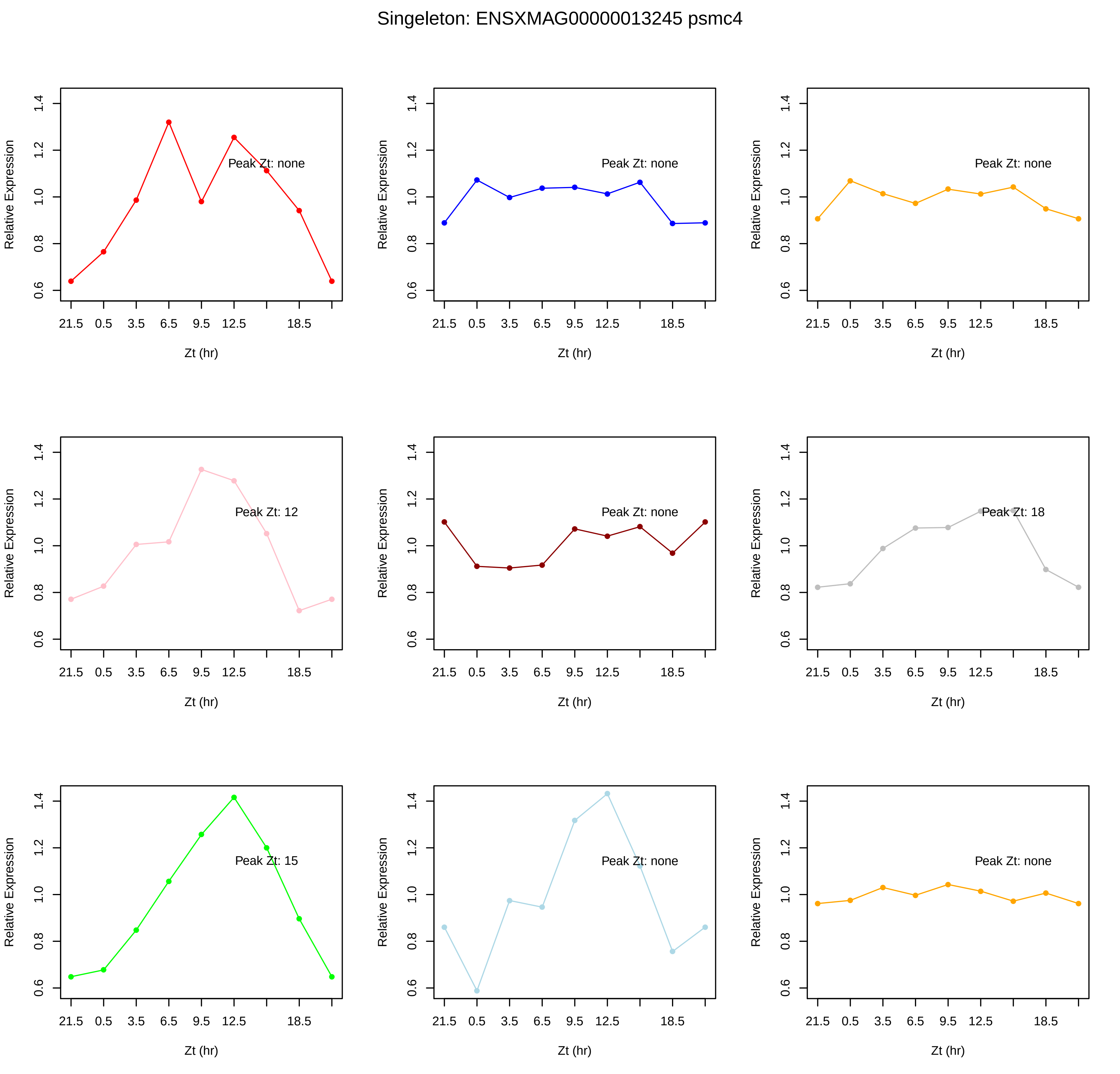

Singeleton: ENSXMAG00000013245 psmc4
Relative Expression
Zt (hr)
21.5 0.5 3.5 6.5 9.5 12.5 18.5
0.6 0.8 1.0 1.2 1.4
Peak Zt: none
Peak Zt: none
Peak Zt: none
Peak Zt: 12
Peak Zt: none
Peak Zt: 18
Peak Zt: 15
Peak Zt: none
Peak Zt: none

Singleton: ENSXMAG00000023590 barhl2

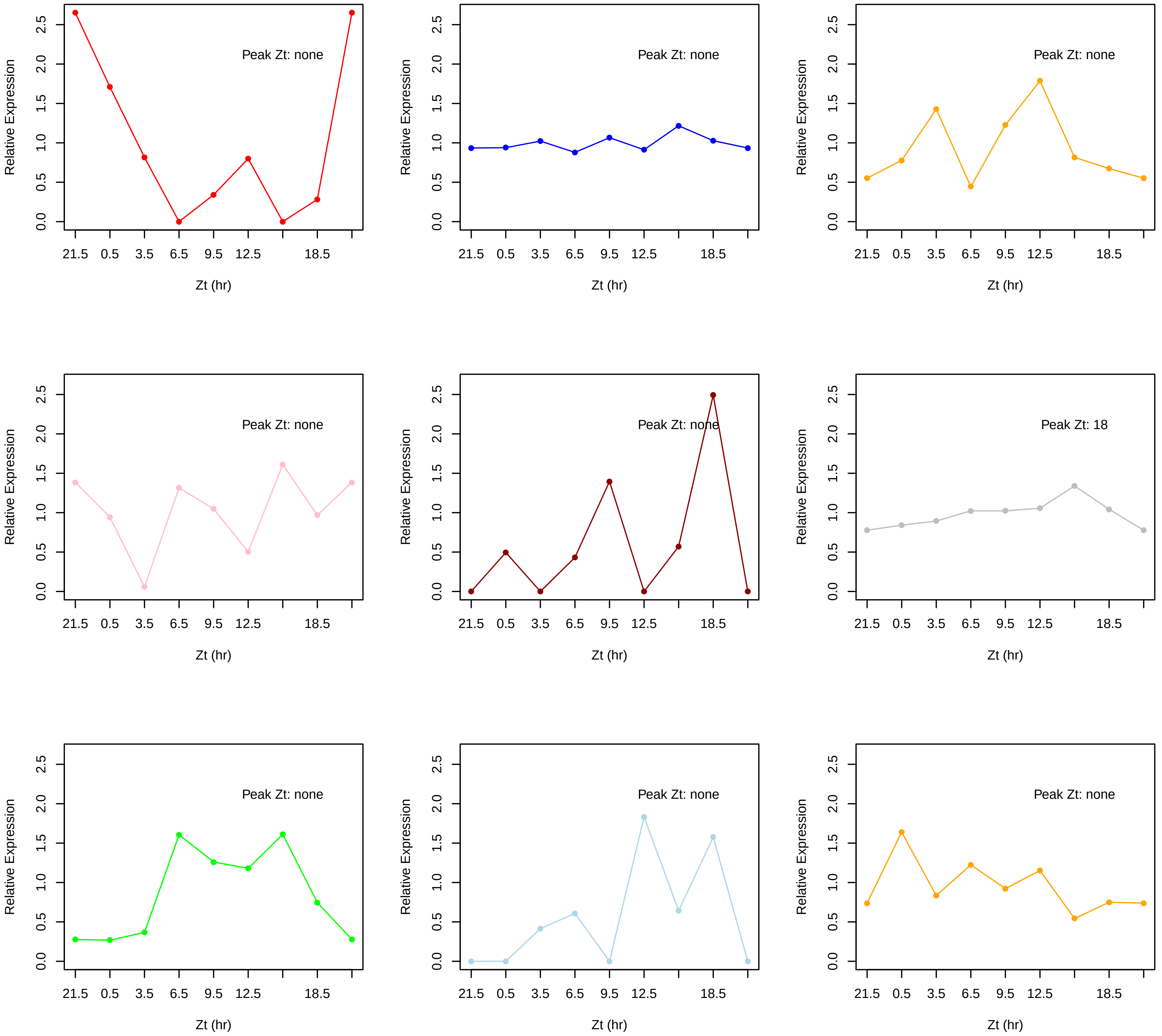

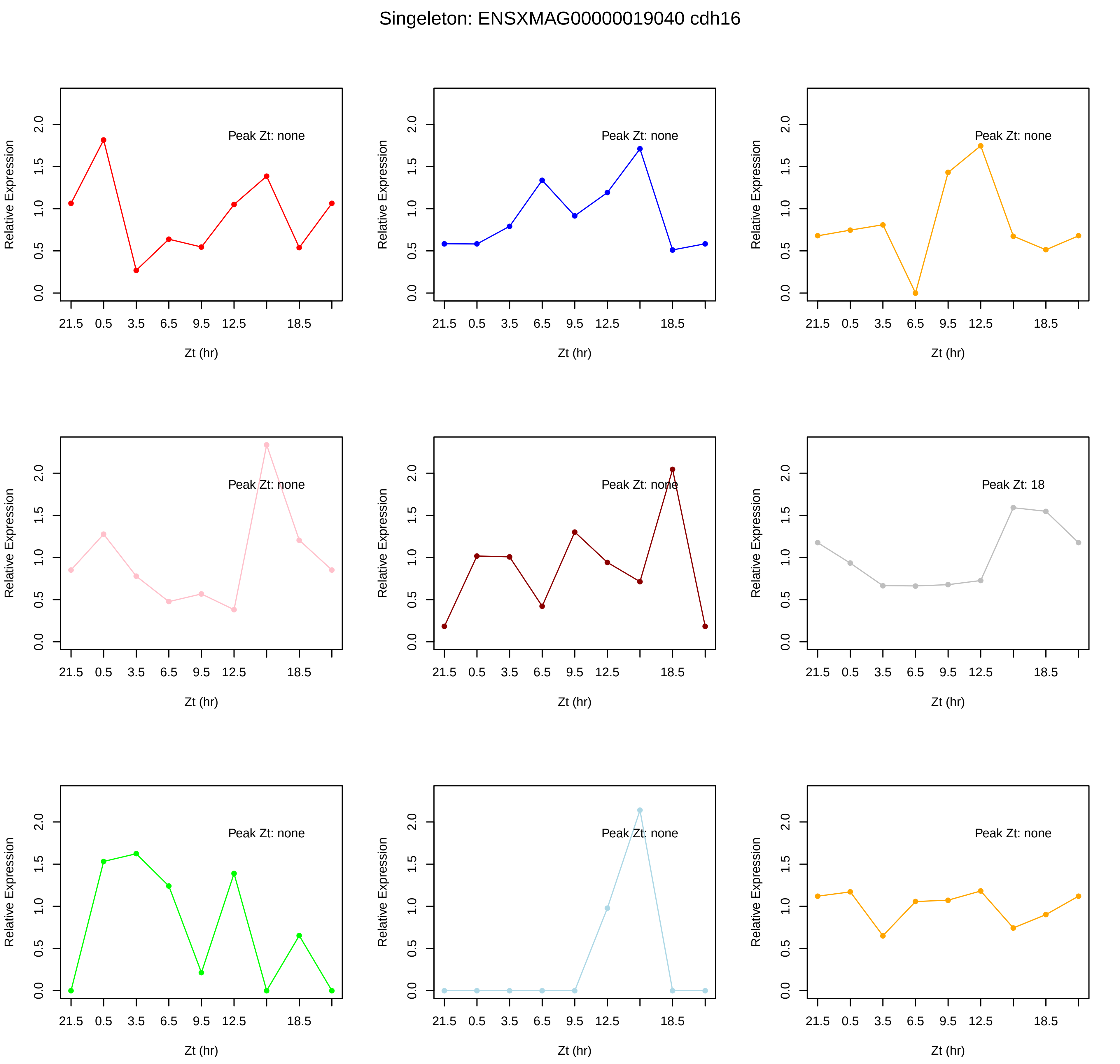
Singleton: ENSXMAG00000019040 cdh16
Peak Zt: none
Peak Zt: none
Peak Zt: none
Peak Zt: none
Peak Zt: none
Peak Zt: 18
Peak Zt: none
Peak Zt: none
Peak Zt: none
Relative Expression
Zt (hr)
21.5
0.5
3.5
6.5
9.5
12.5
18.5
0.0
0.5
1.0
1.5
2.0

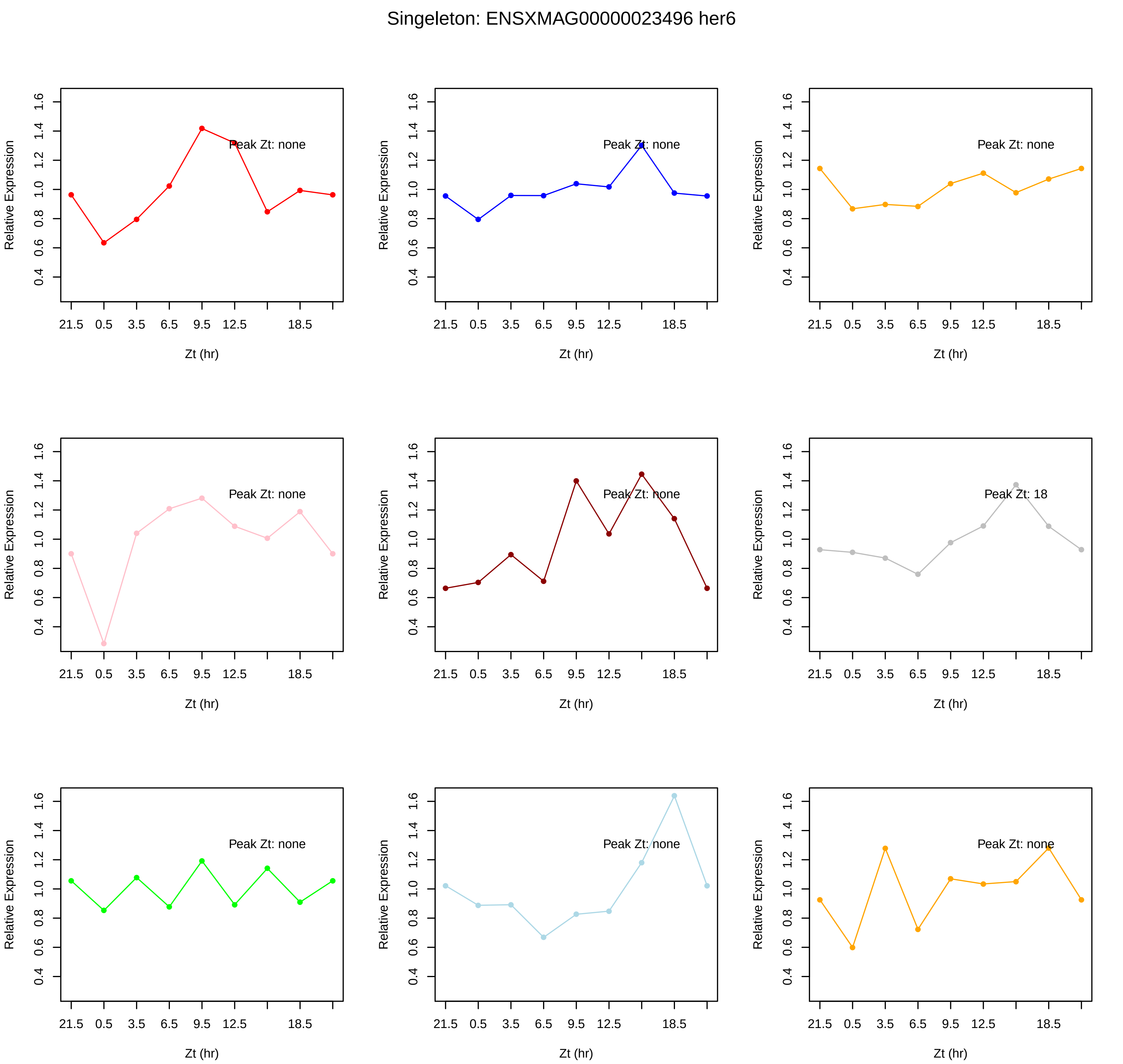

Singeleton: ENSXMAG00000023496 her6
Relative Expression
Zt (hr)
21.5
0.5
3.5
6.5
9.5
12.5
18.5
0.4
0.6
0.8
1.0
1.2
1.4
1.6
Peak Zt: none
Peak Zt: 18

Singeleton: ENSXMAG00000019408

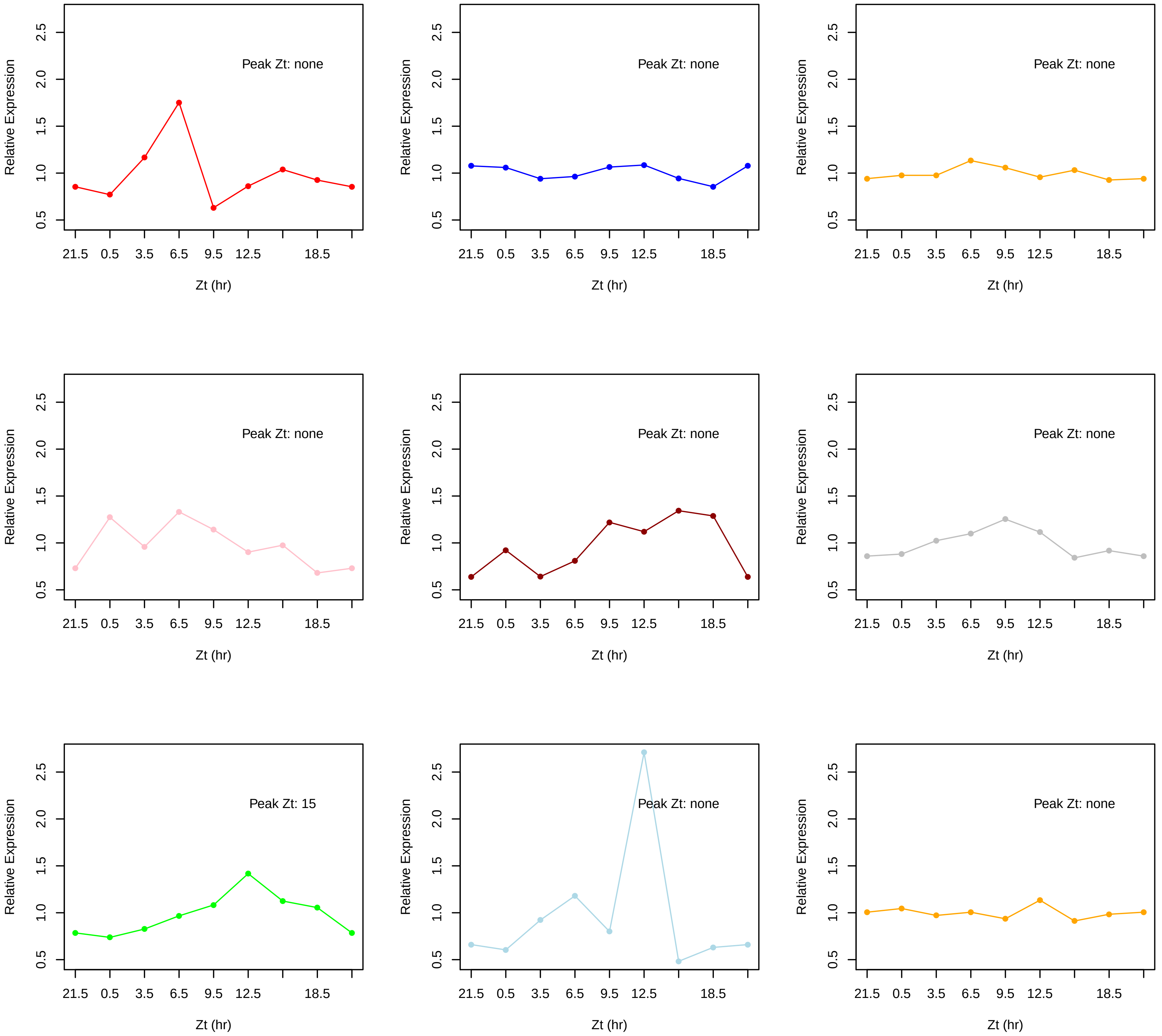

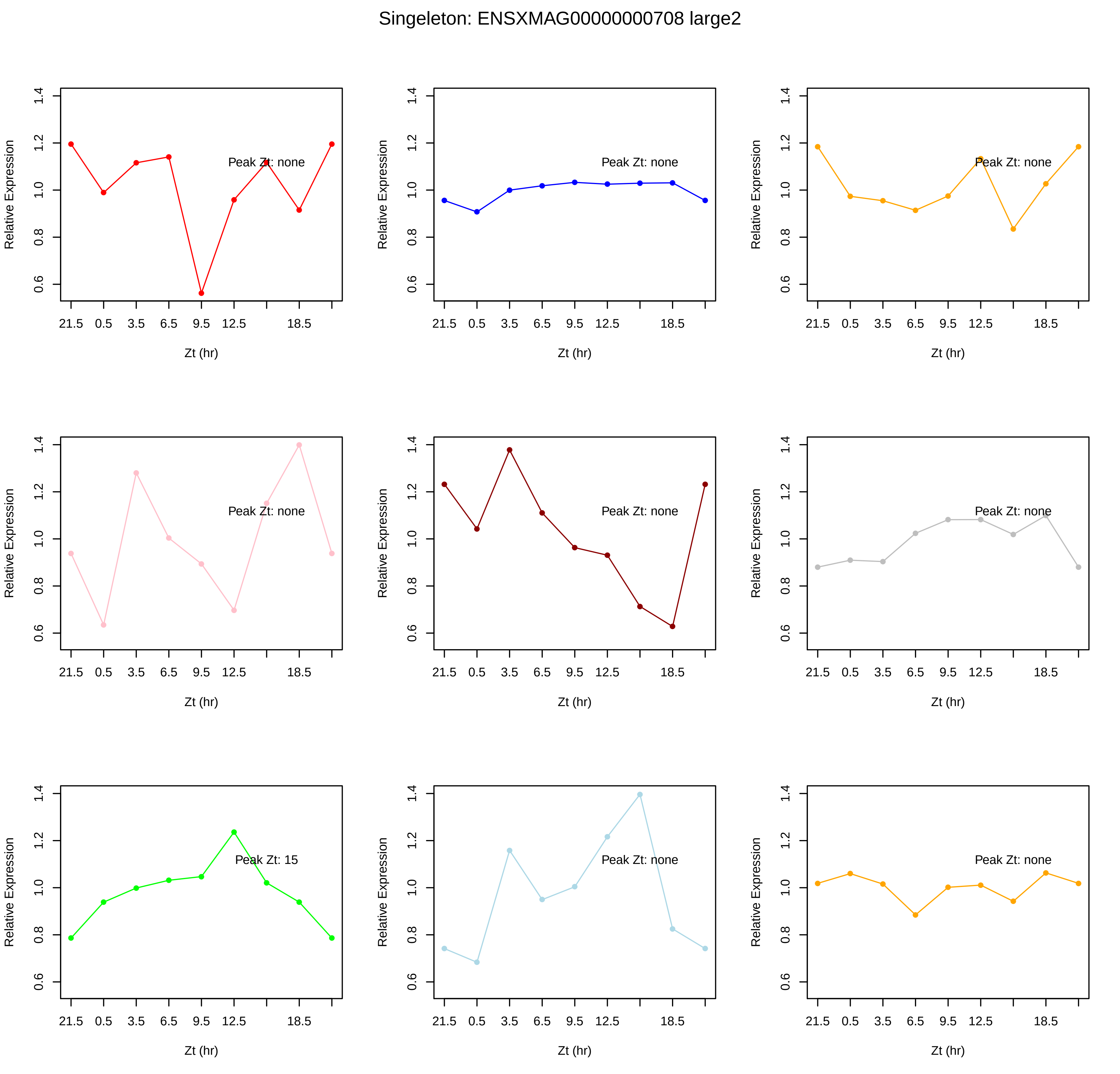
Singleton: ENSXMAG00000000708 large2
Relative Expression
Zt (hr)
21.5 0.5 3.5 6.5 9.5 12.5 18.5
0.6 0.8 1.0 1.2 1.4
Peak Zt: none
Peak Zt: 15

Singeleton: ENSXMAG00000013241 si:dkey−199f5.8

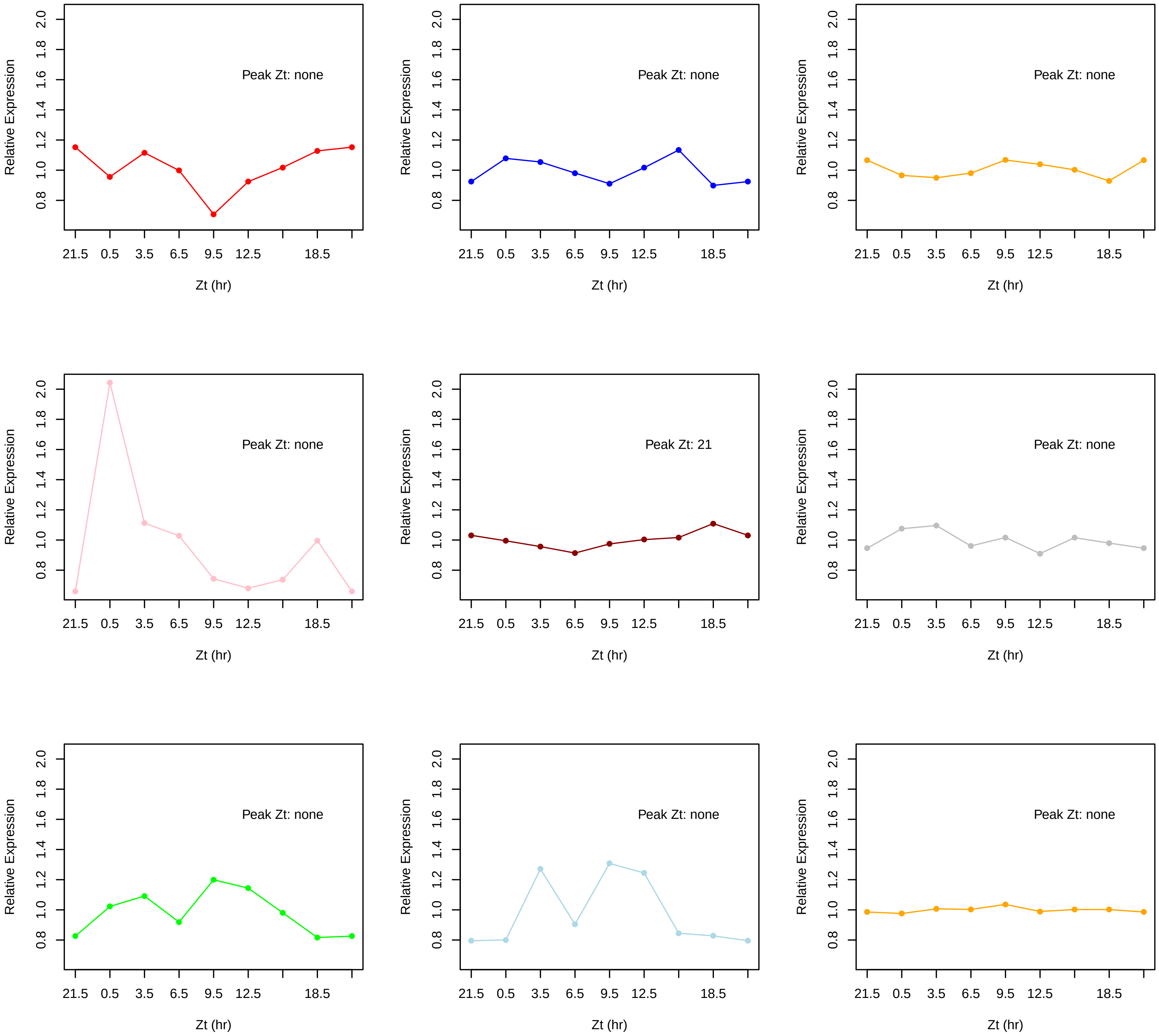

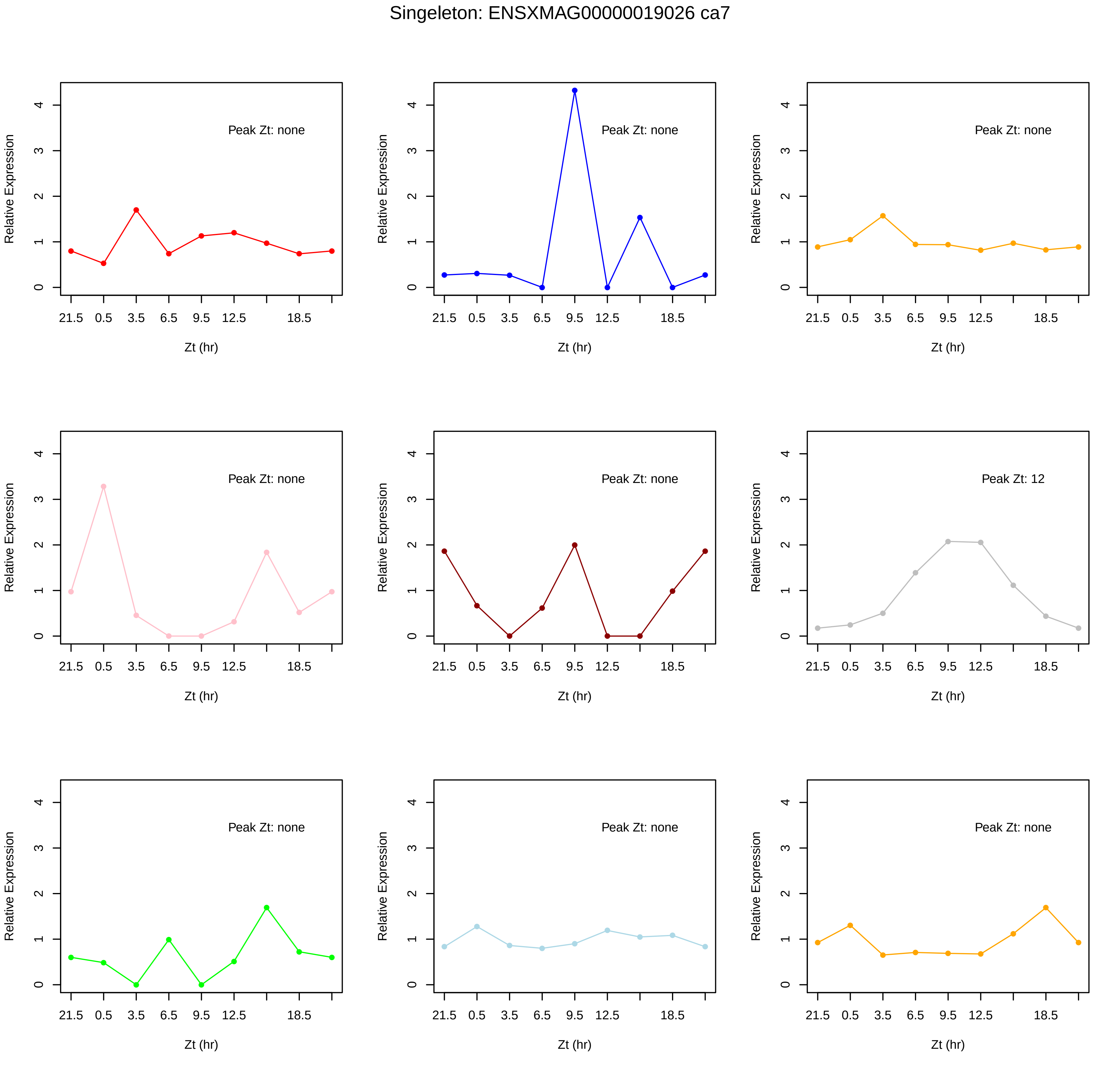
Singeleton: ENSXMAG00000019026 ca7
Peak Zt: none
Peak Zt: none
Peak Zt: none
Peak Zt: none
Peak Zt: none
Peak Zt: 12
Peak Zt: none
Peak Zt: none
Peak Zt: none
Relative Expression
Zt (hr)
21.5 0.5 3.5 6.5 9.5 12.5 18.5
0 1 2 3 4

Singleton: ENSXMAG00000021147 PPP1R1A

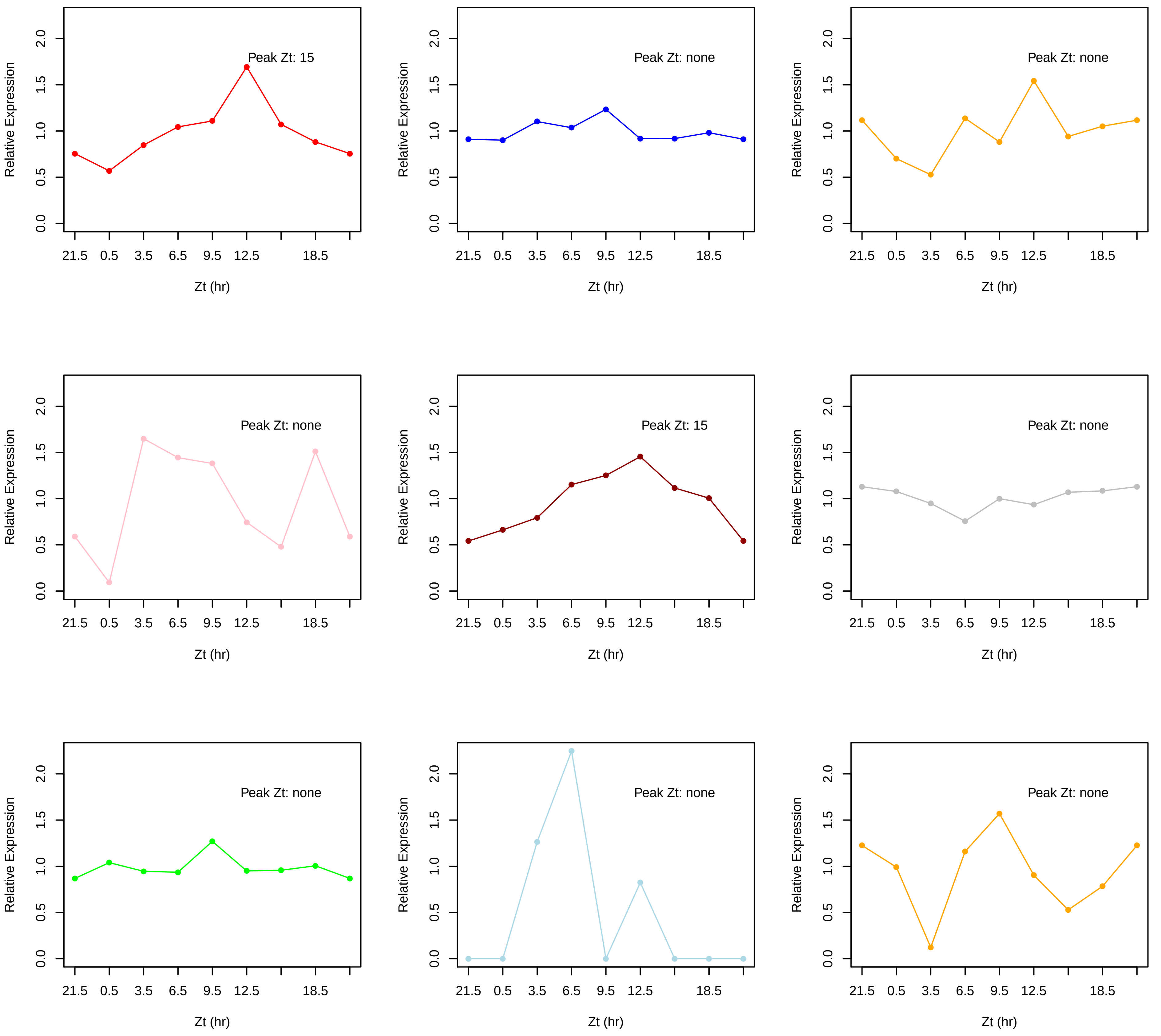

Singeleton: ENSXMAG00000005593 opa1

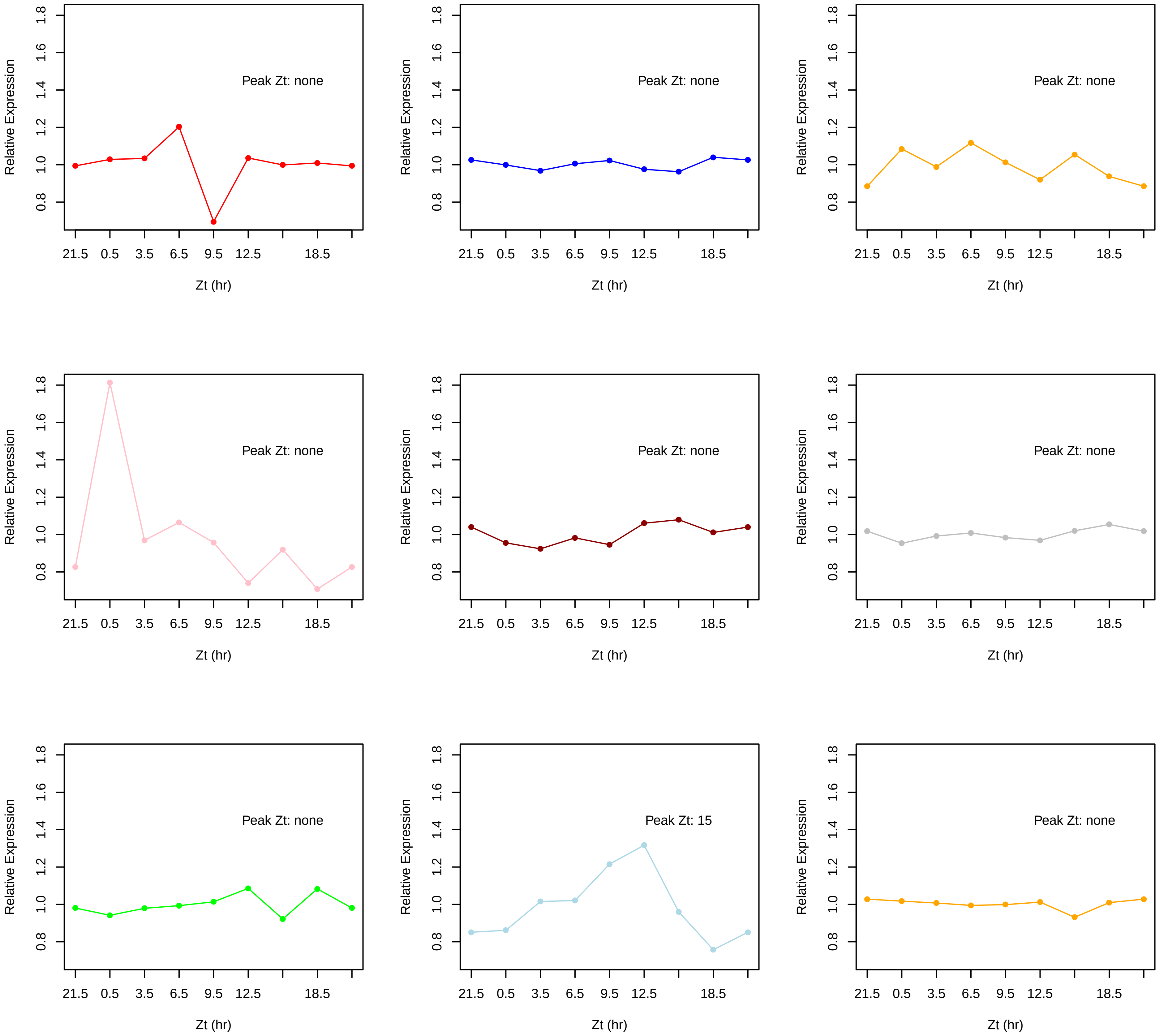

Singleton: ENSXMAG00000028573 dhcr7

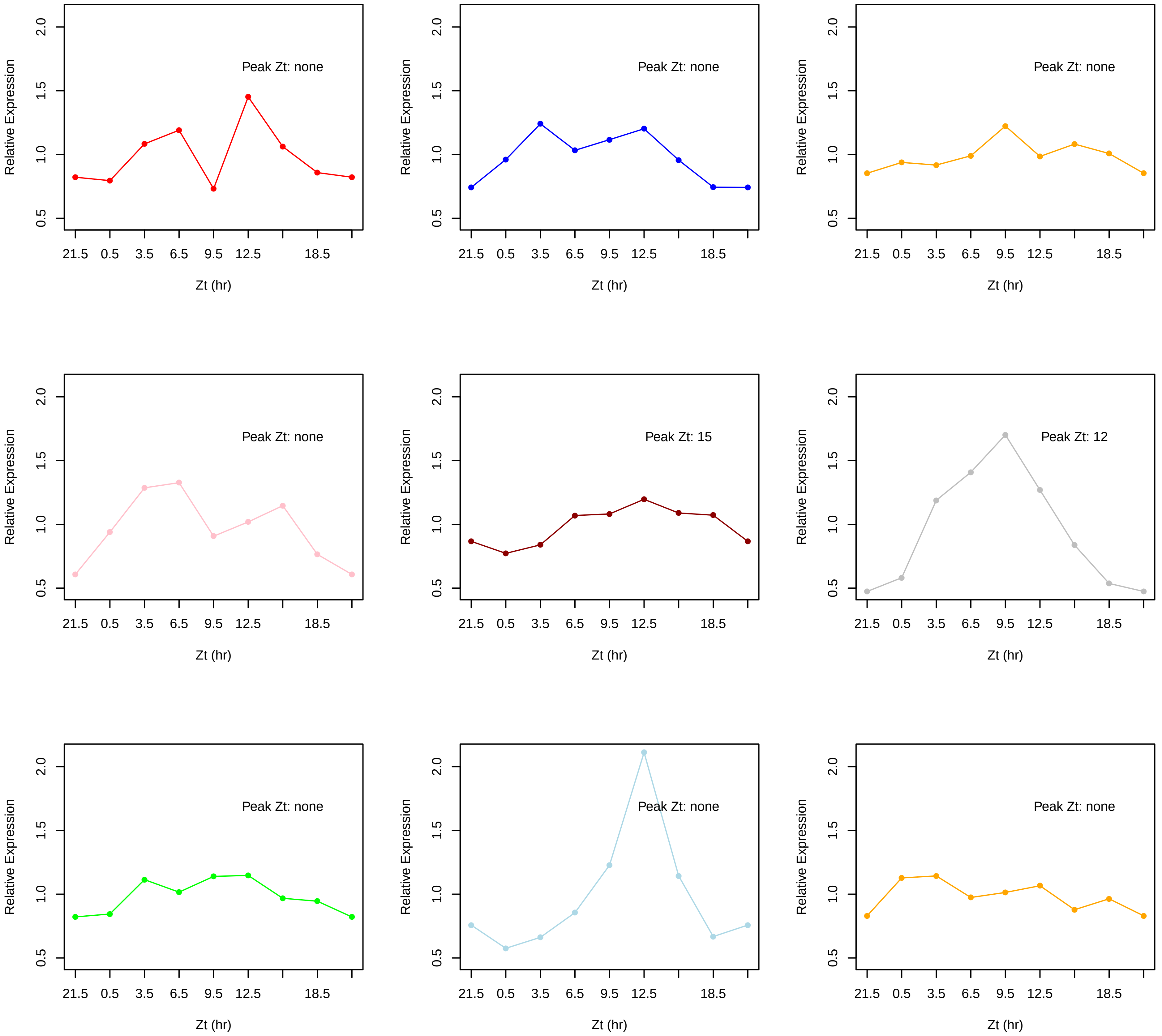

Singleton: ENSXMAG00000005273 lrrc8c

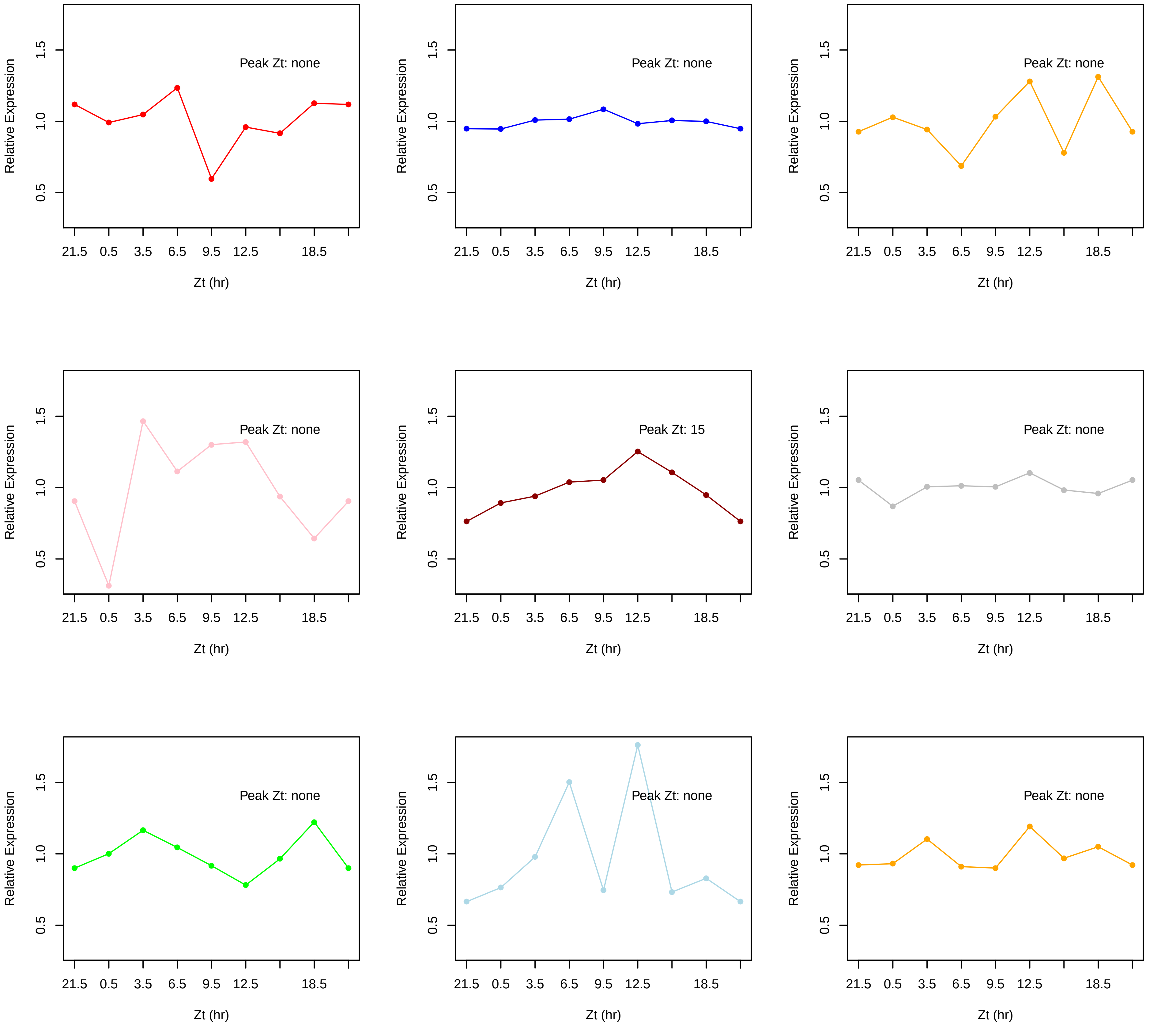

Singeleton: ENSXMAG00000021091 scai

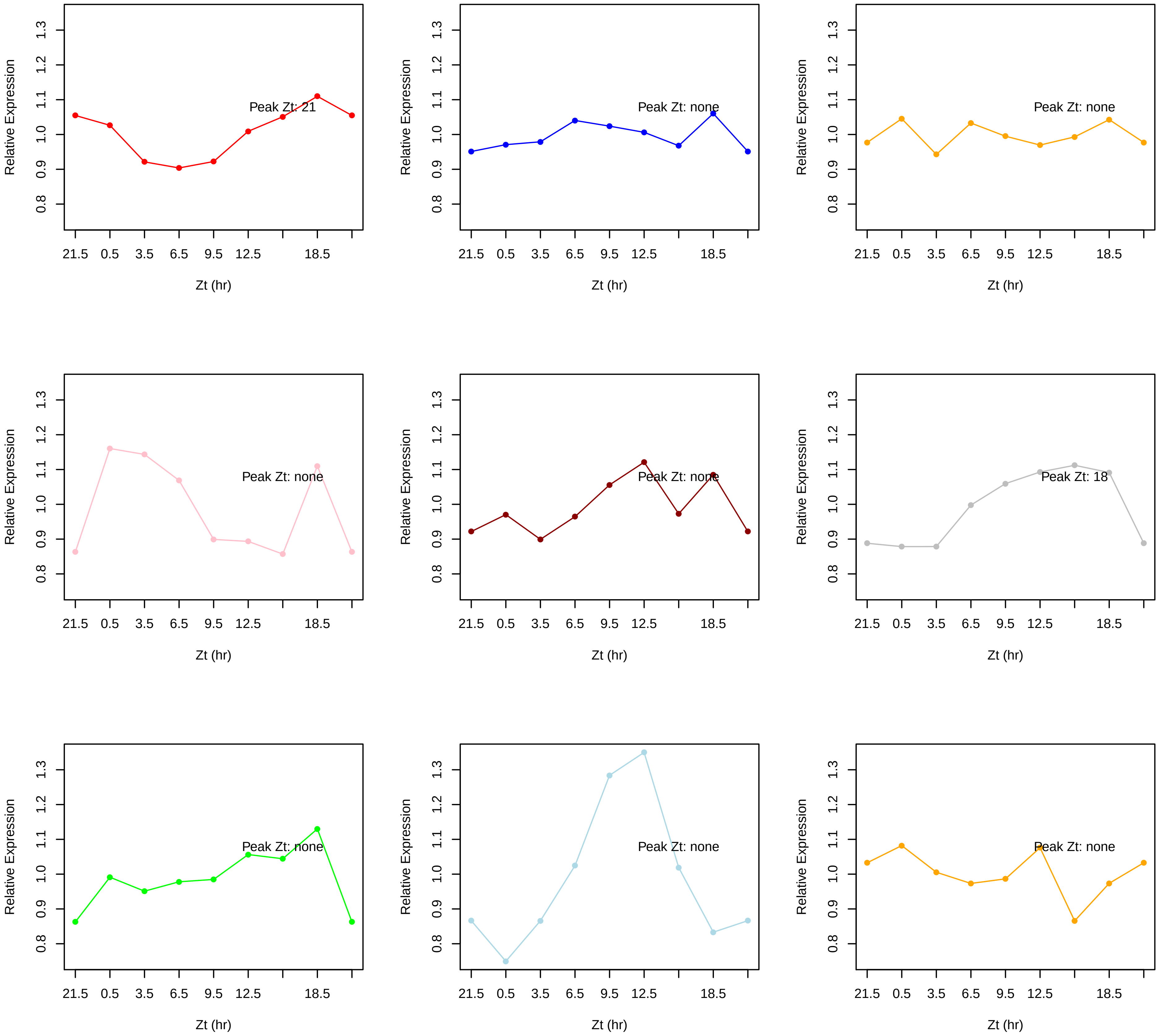

Singeleton: ENSXMAG00000009467

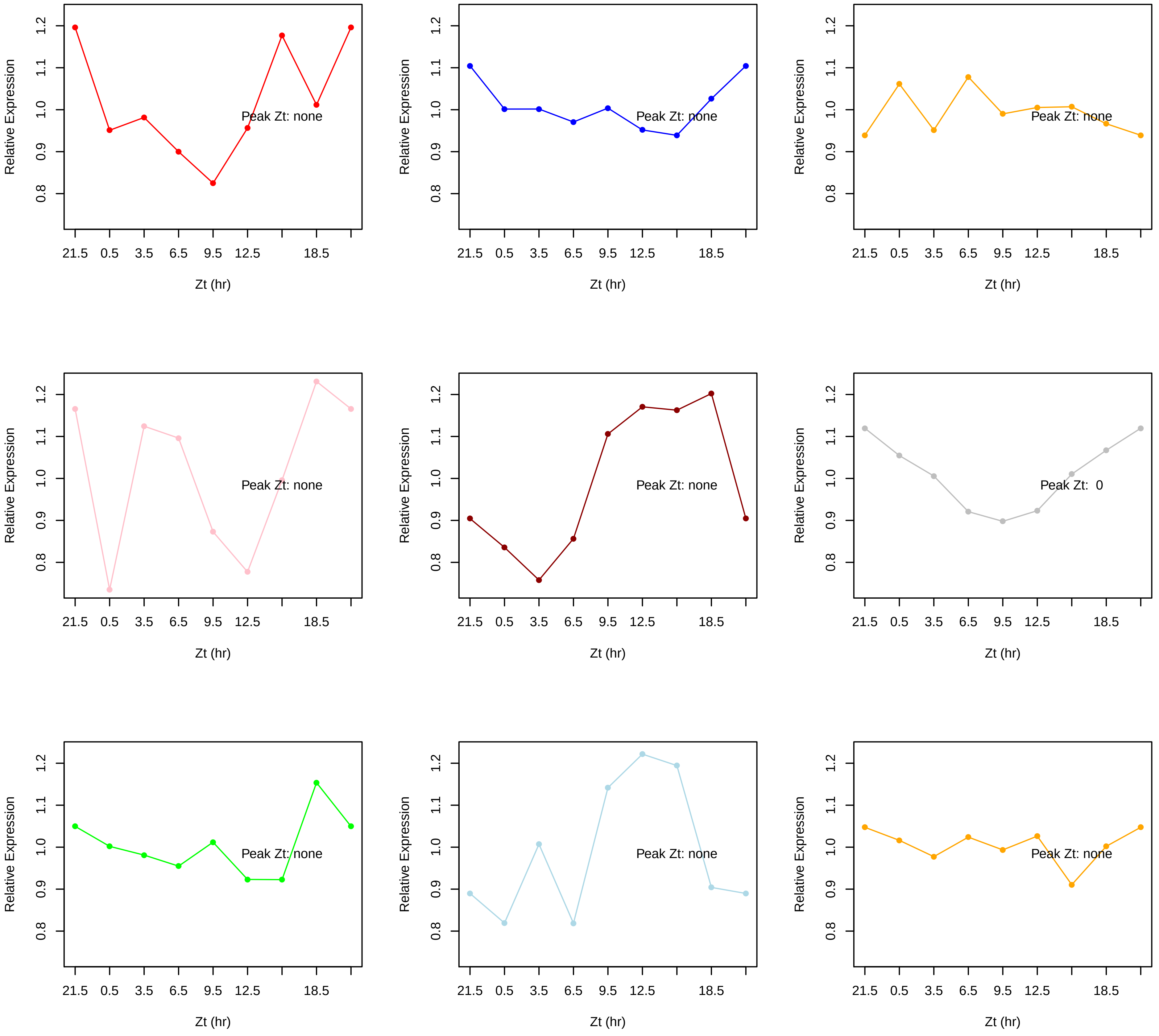

Singeleton: ENSXMAG00000005231 kyat3

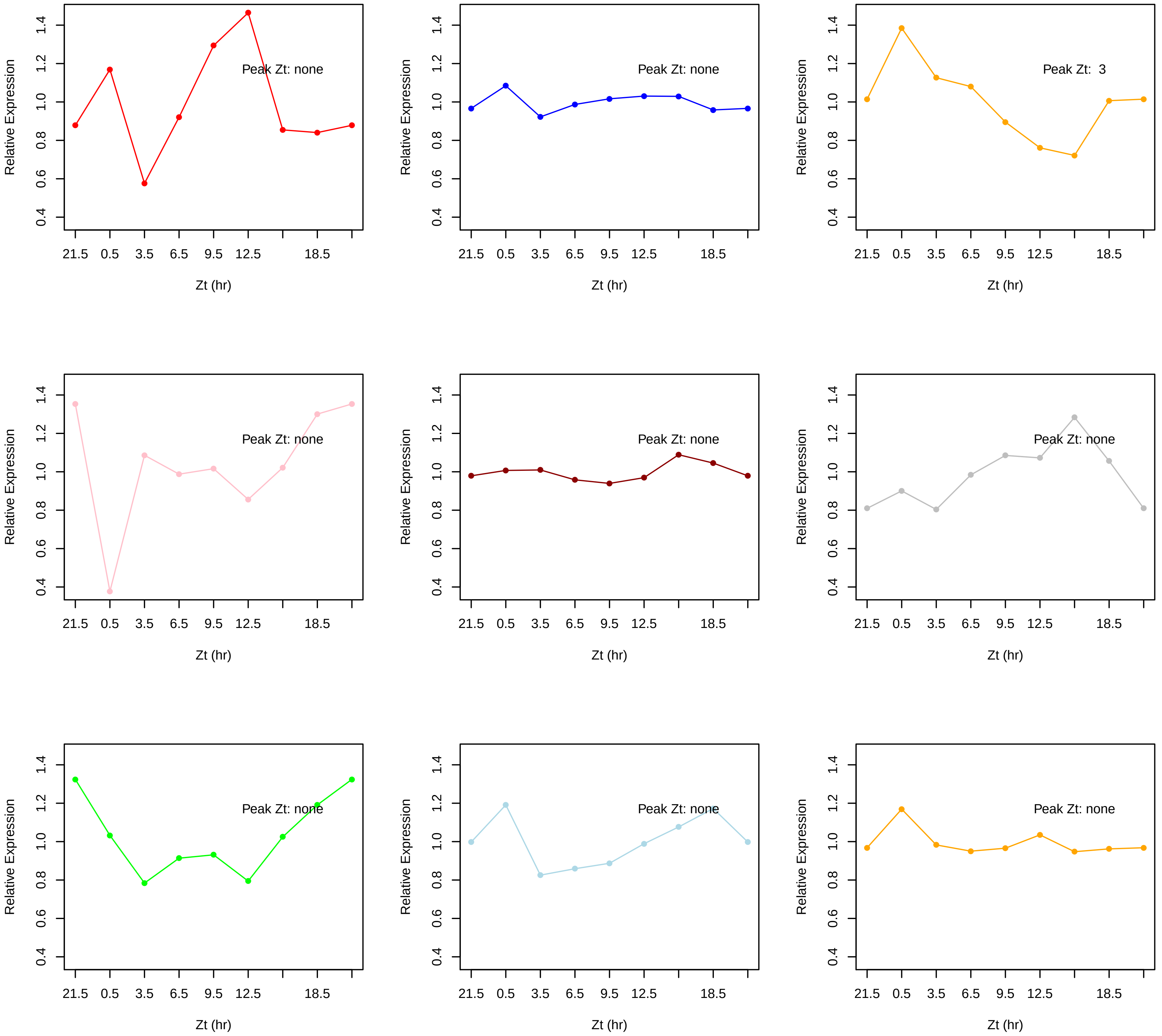

Singeleton: ENSXMAG00000018650 espn

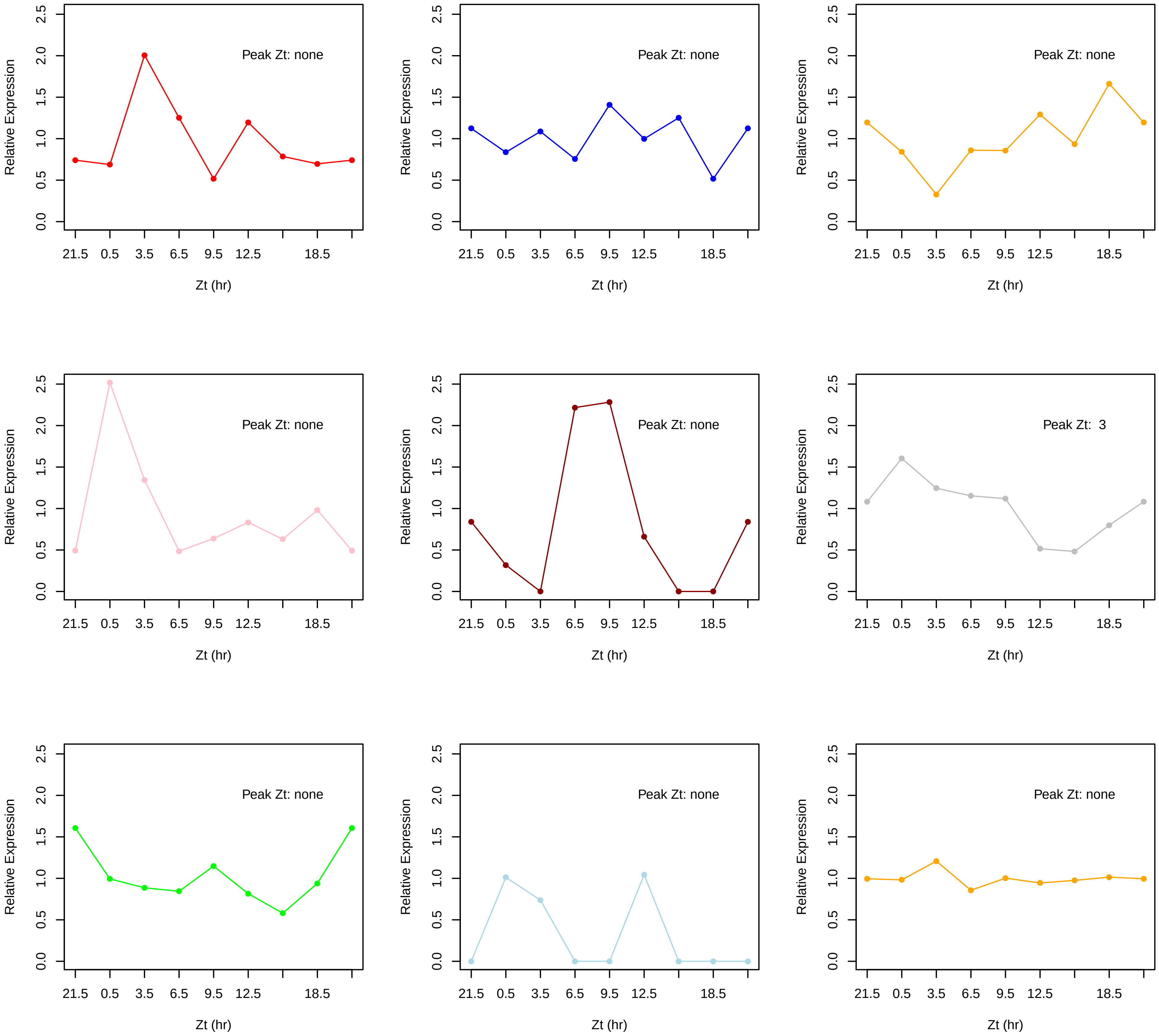

Singeleton: ENSXMAG00000012420 midn

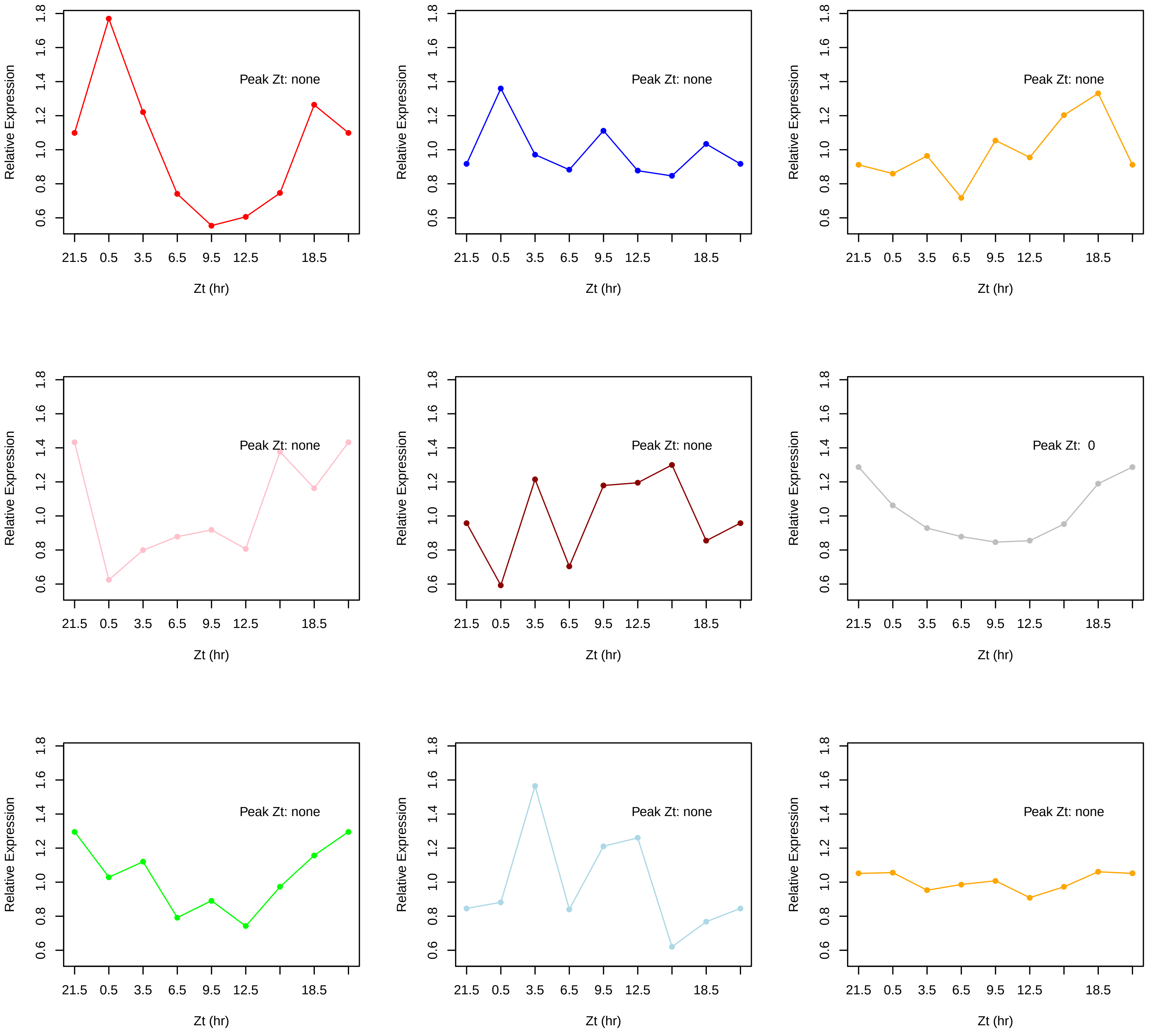

Singeleton: ENSXMAG00000002925 golga1

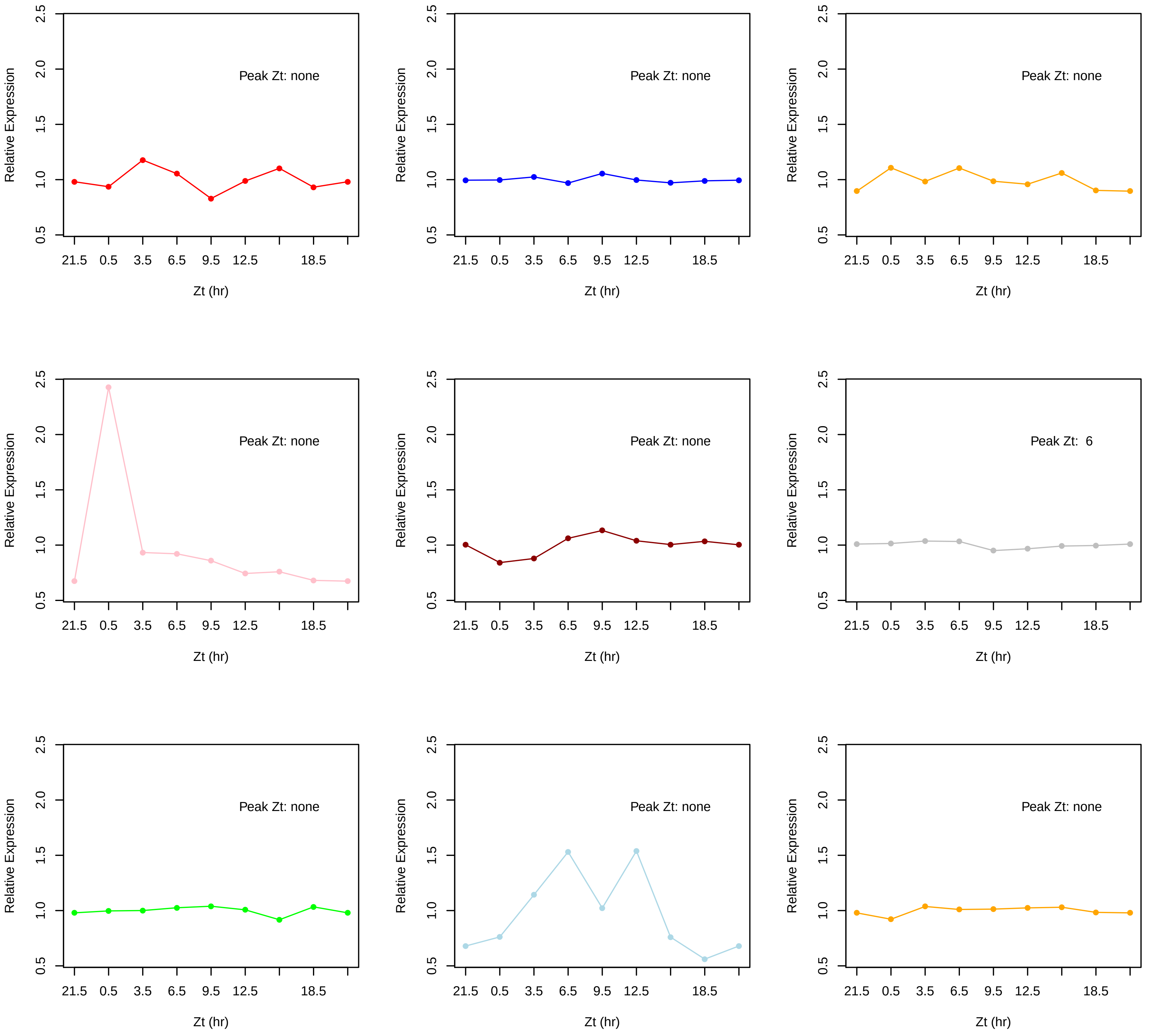

Singeleton: ENSXMAG00000015155 adamts14

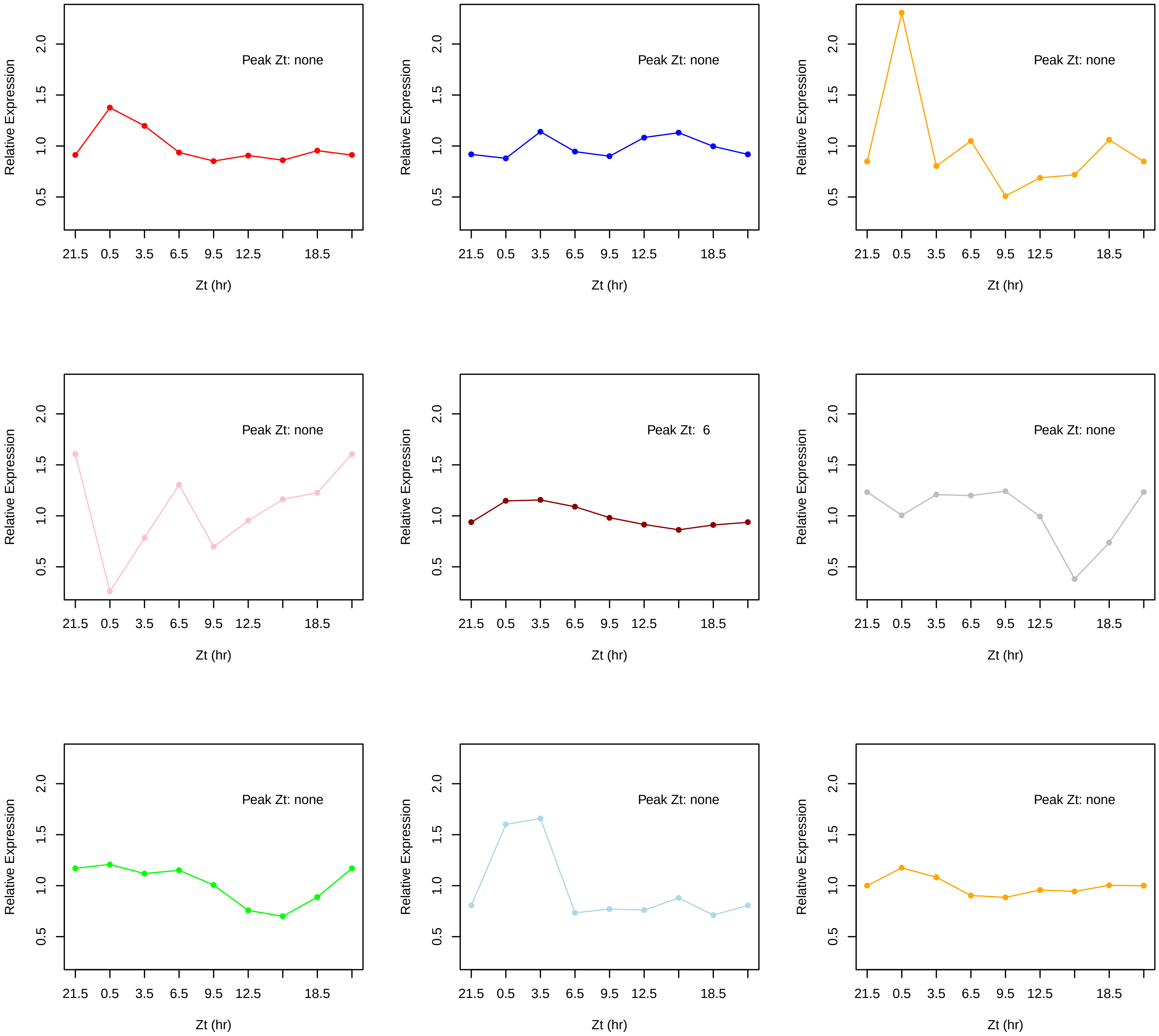

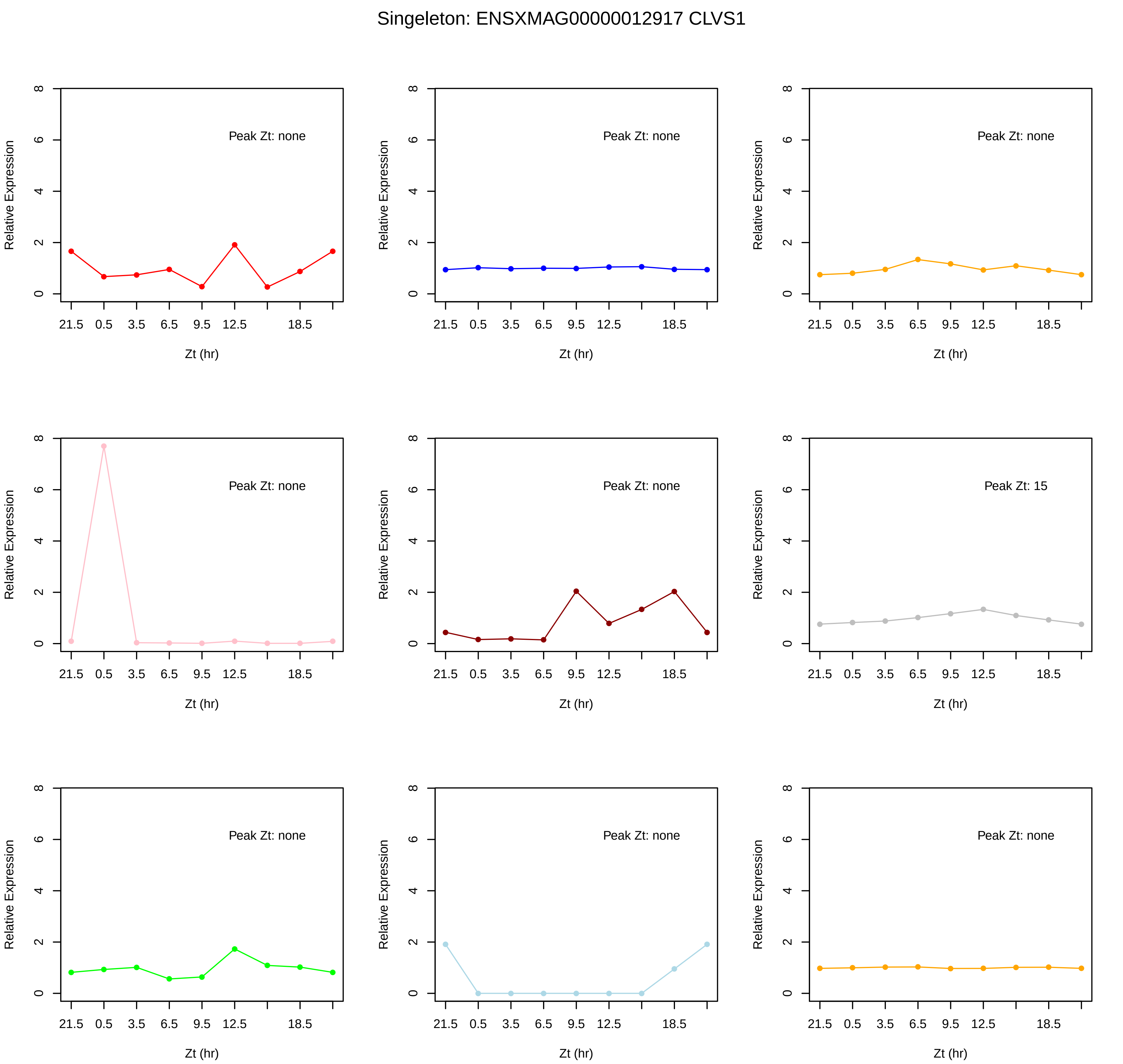
Singeleton: ENSXMAG00000012917 CLVS1
Peak Zt: none
Peak Zt: none
Peak Zt: none
Peak Zt: none
Peak Zt: none
Peak Zt: 15
Peak Zt: none
Peak Zt: none
Peak Zt: none
Relative Expression
Zt (hr)
21.5 0.5 3.5 6.5 9.5 12.5 18.5
0 2 4 6 8

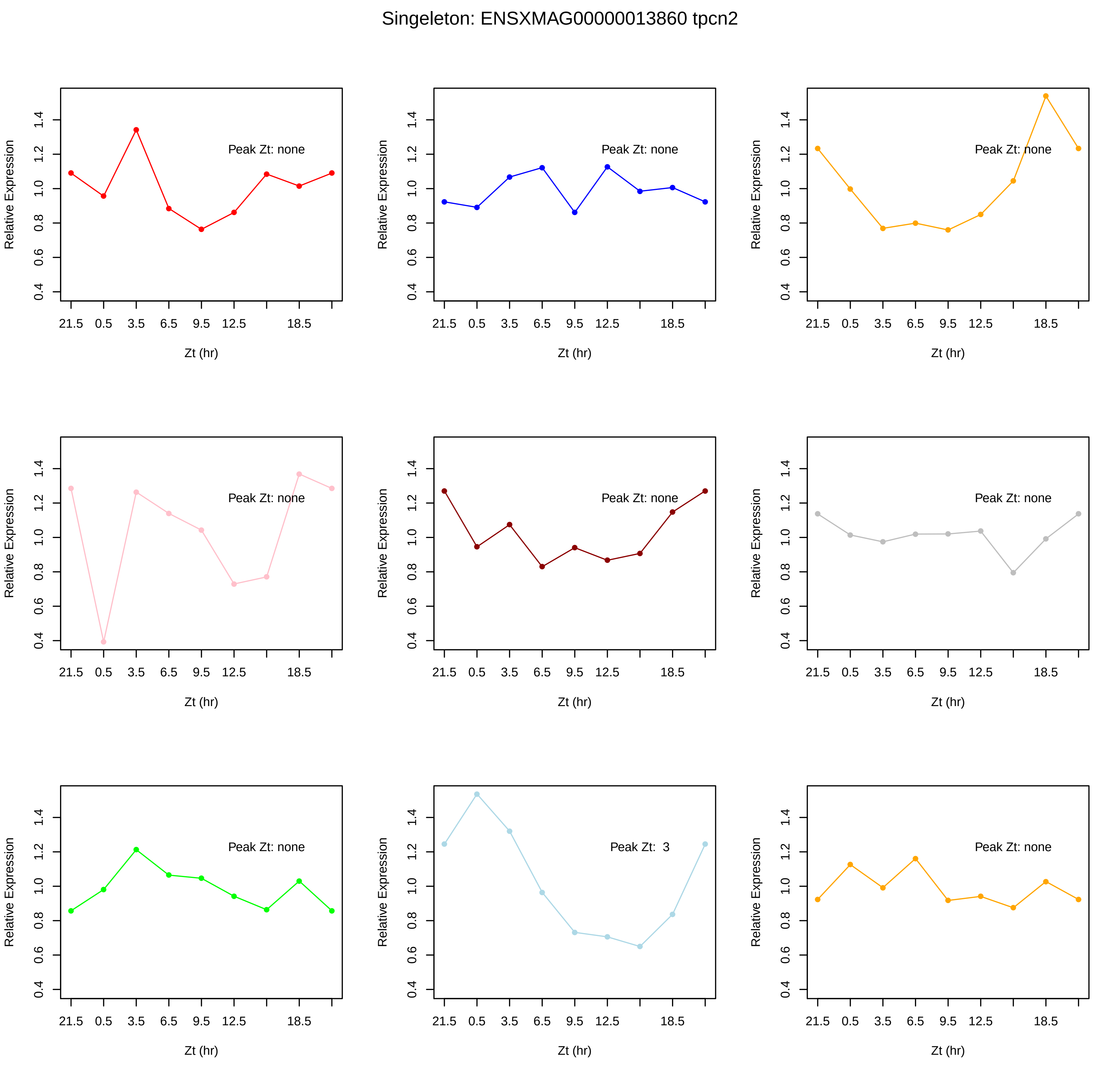
Singeleton: ENSXMAG00000013860 tpcn2
Peak Zt: none
Peak Zt: none
Peak Zt: none
Peak Zt: none
Peak Zt: none
Peak Zt: none
Peak Zt: none
Peak Zt: 3
Peak Zt: none
Relative Expression
Zt (hr)
21.5
0.5
3.5
6.5
9.5
12.5
18.5
0.4
0.6
0.8
1.0
1.2
1.4

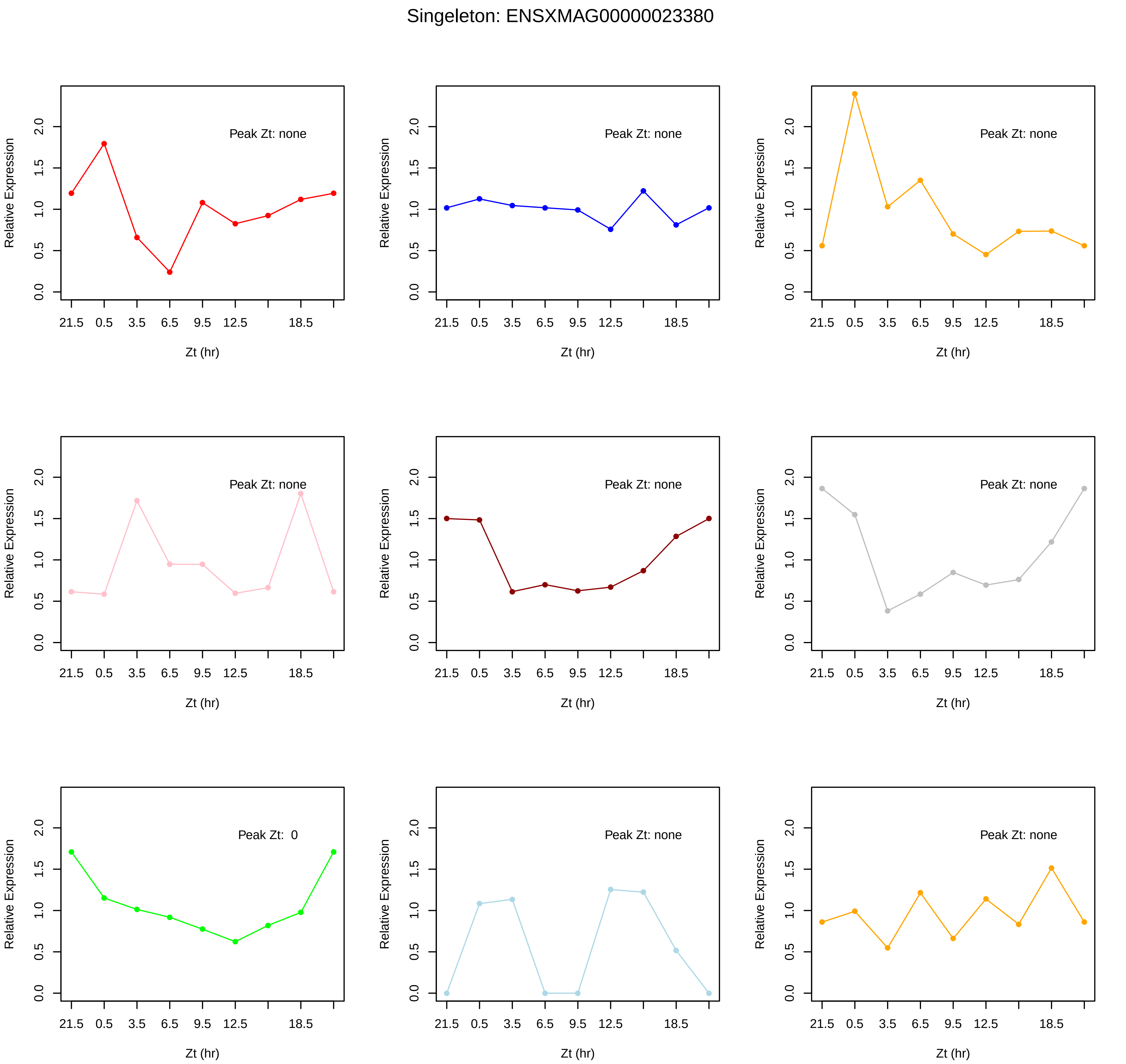

Singeleton: ENSXMAG00000023380
Peak Zt: none
Peak Zt: none
Peak Zt: none
Peak Zt: none
Peak Zt: none
Peak Zt: none
Peak Zt: 0
Peak Zt: none
Peak Zt: none
Relative Expression
Zt (hr)
21.5
0.5
3.5
6.5
9.5
12.5
18.5
0.0
0.5
1.0
1.5
2.0

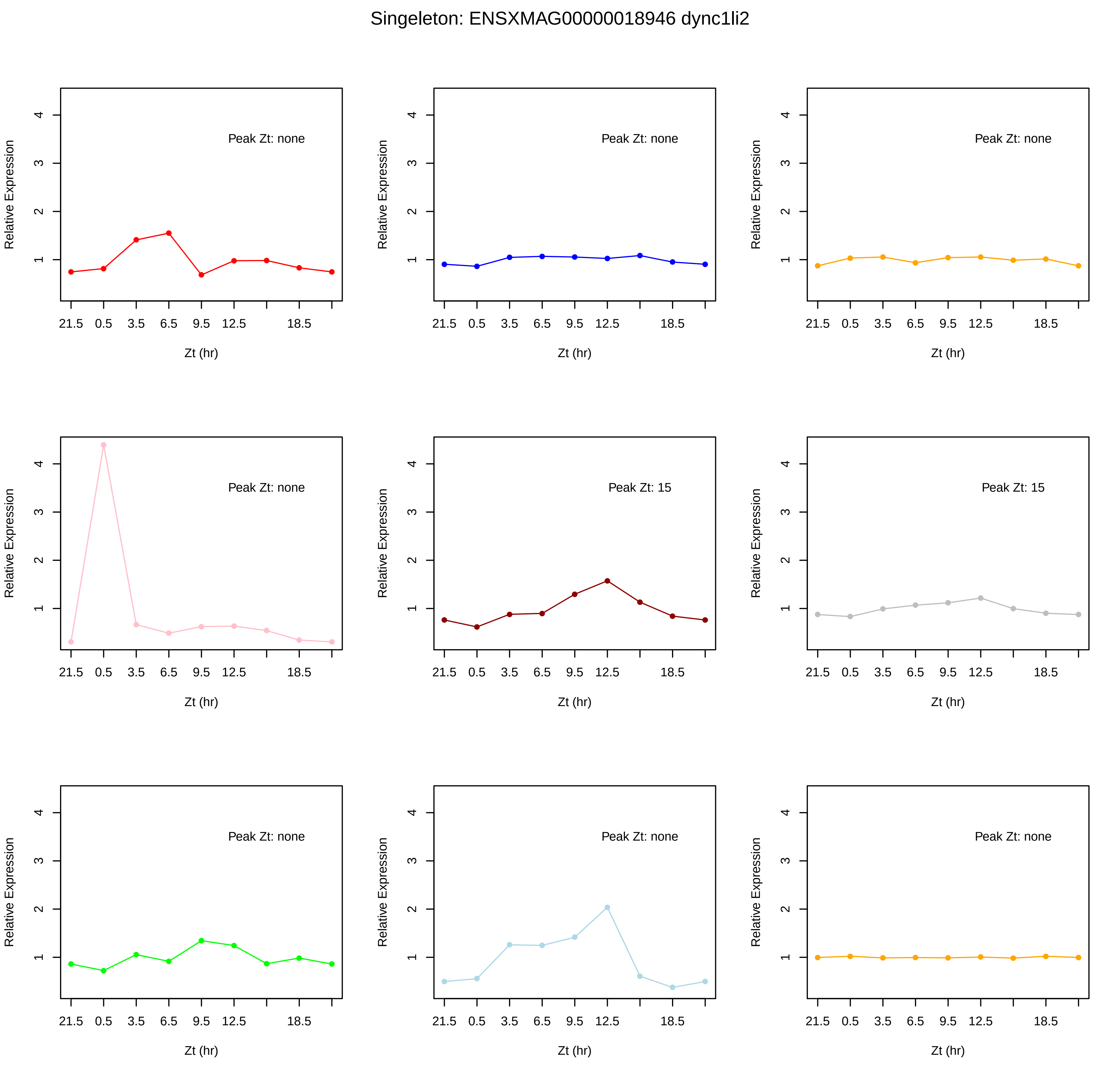
Singeleton: ENSXMAG00000018946 dync1li2
Peak Zt: none
Peak Zt: none
Peak Zt: none
Peak Zt: none
Peak Zt: 15
Peak Zt: 15
Peak Zt: none
Peak Zt: none
Peak Zt: none
Relative Expression
Zt (hr)
21.5 0.5 3.5 6.5 9.5 12.5 18.5
1 2 3 4

Singeleton: ENSXMAG00000004206 gtf2b

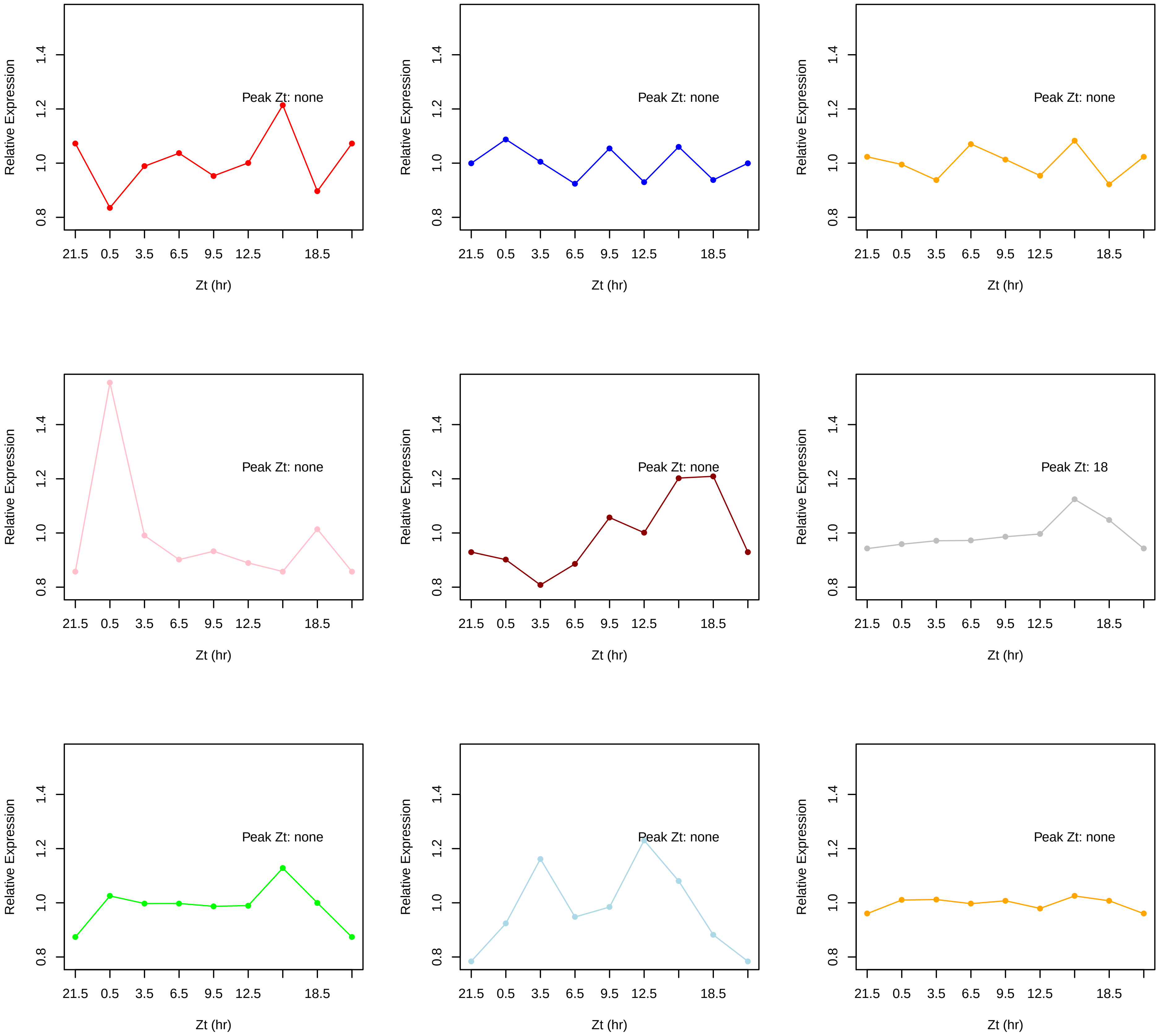

Singeleton: ENSXMAG00000004214 pkn2

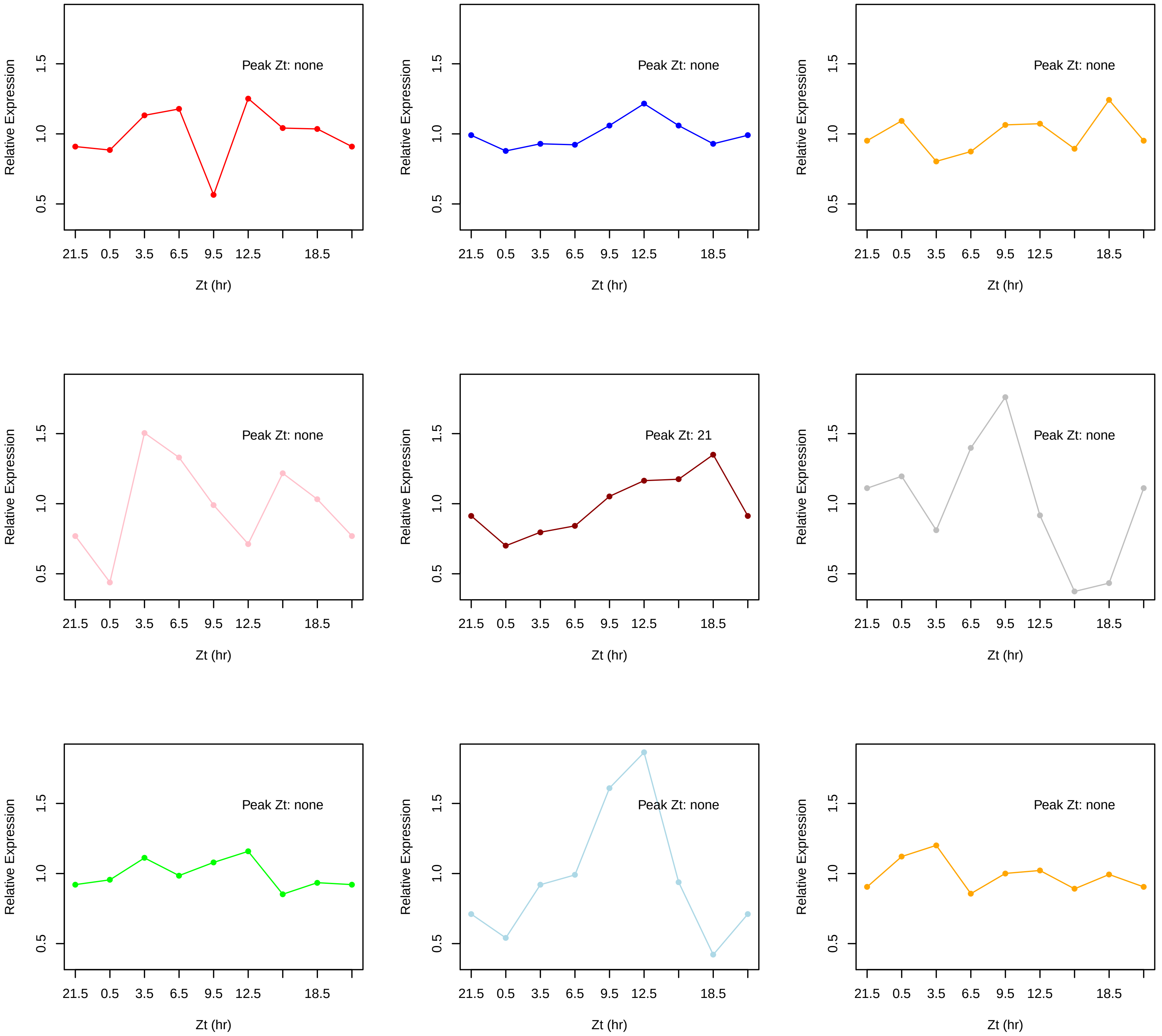

Singeleton: ENSXMAG00000029040 slc39a10

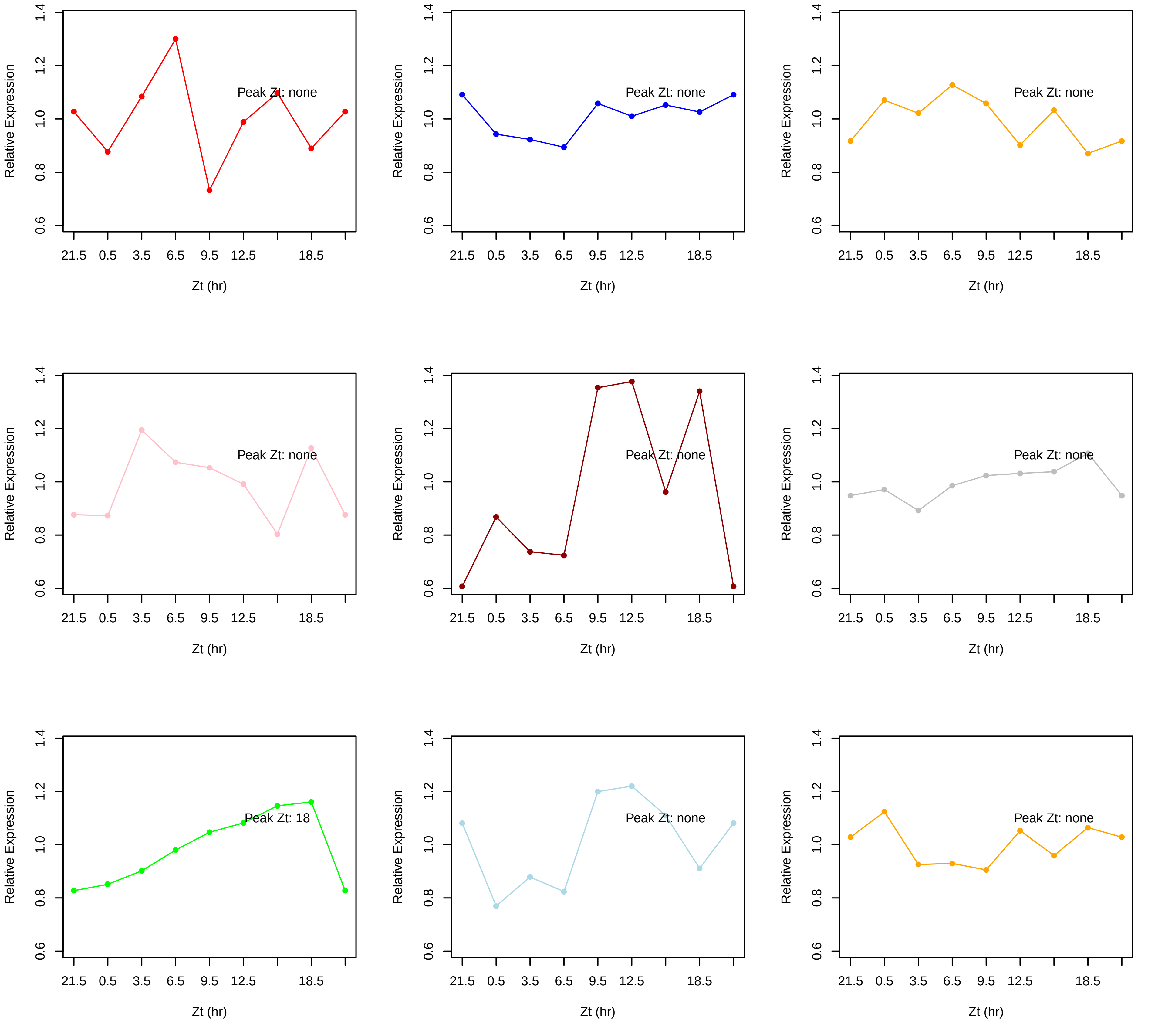

Singeleton: ENSXMAG00000019849 fahd1

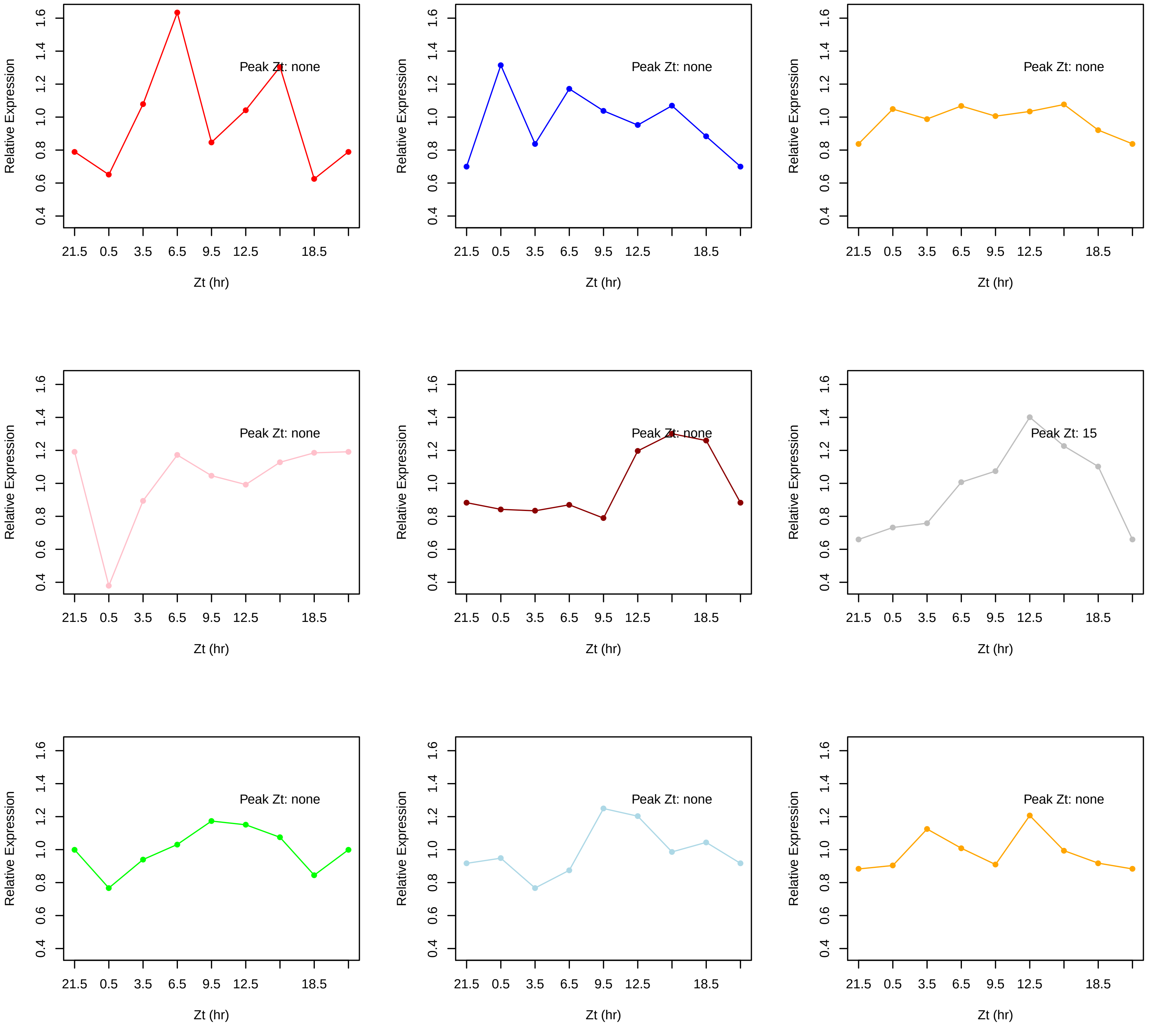

Singeleton: ENSXMAG00000028142 cmtm3

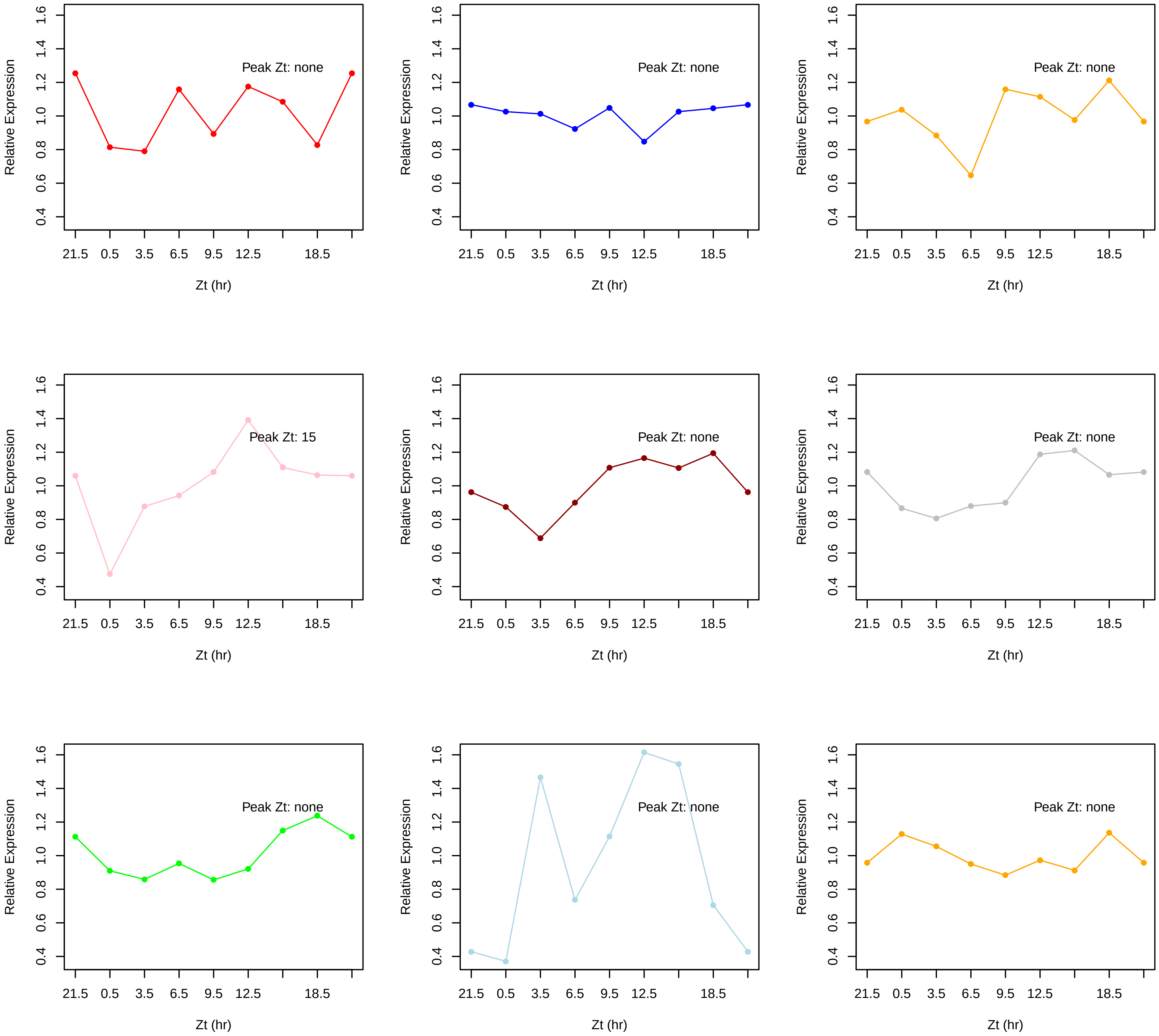

Singleton: ENSXMAG00000002371 bcas3

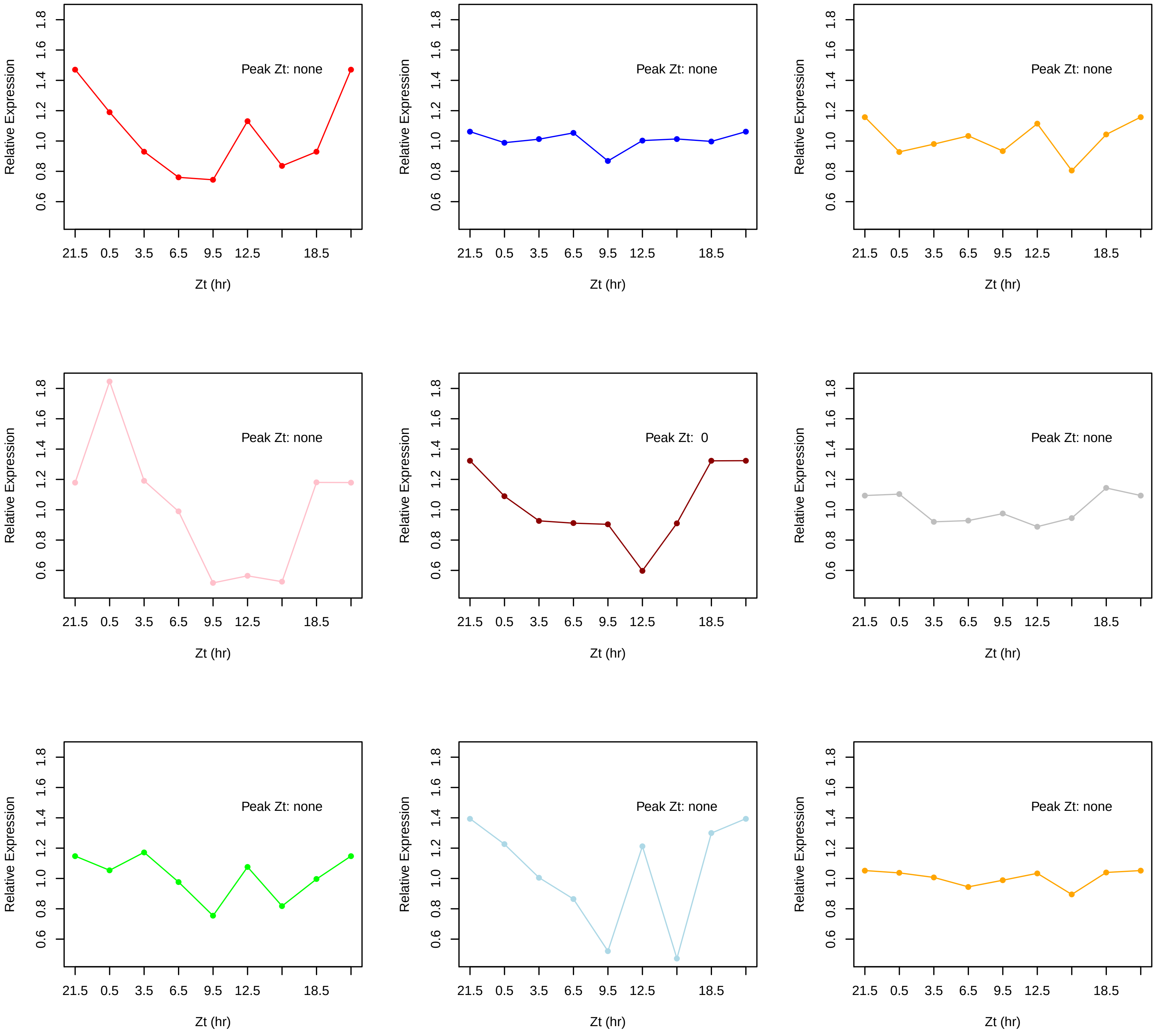

Singeleton: ENSXMAG00000015174 kdm2ba

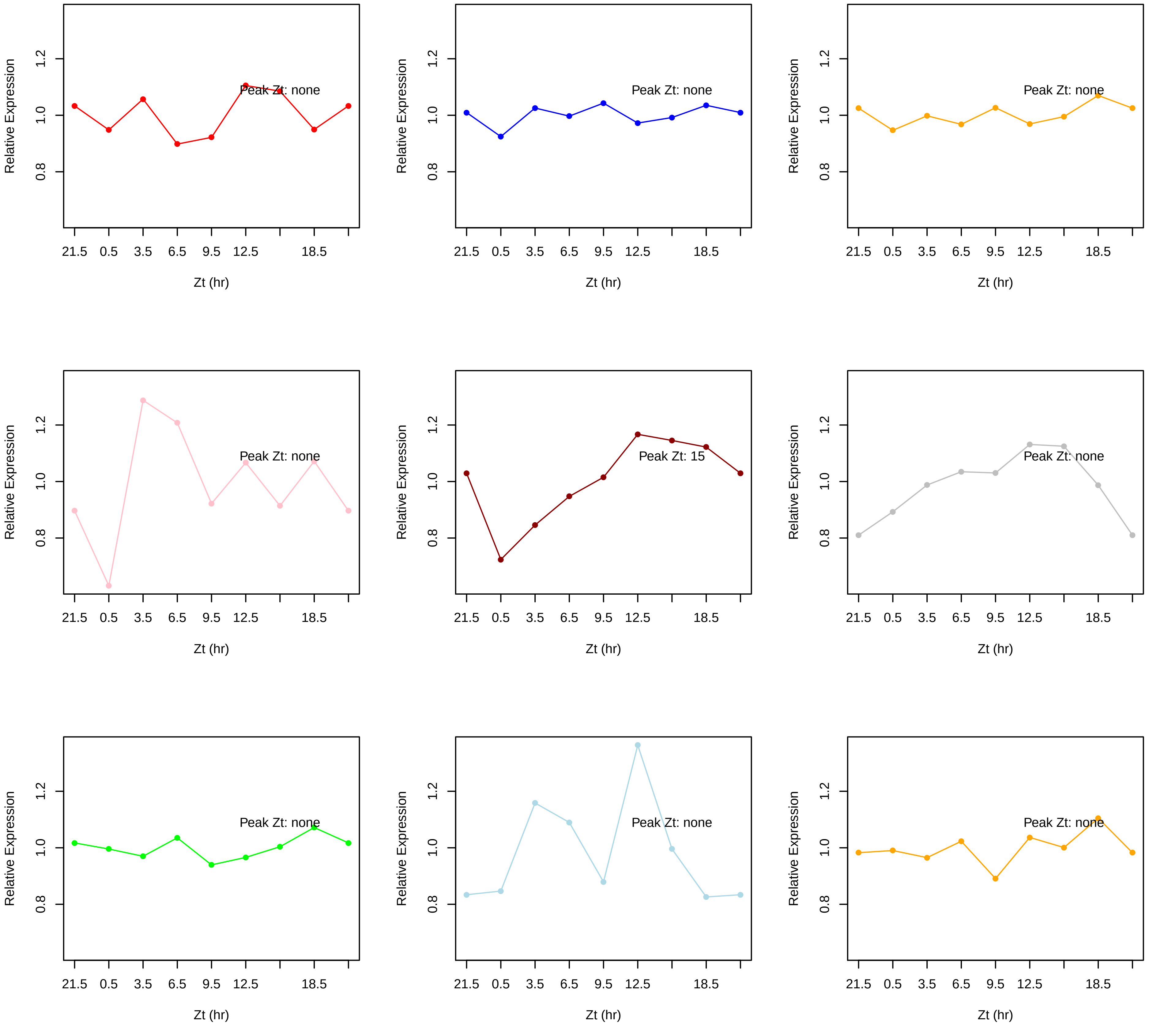

Singleton: ENSXMAG00000024267 rab2a

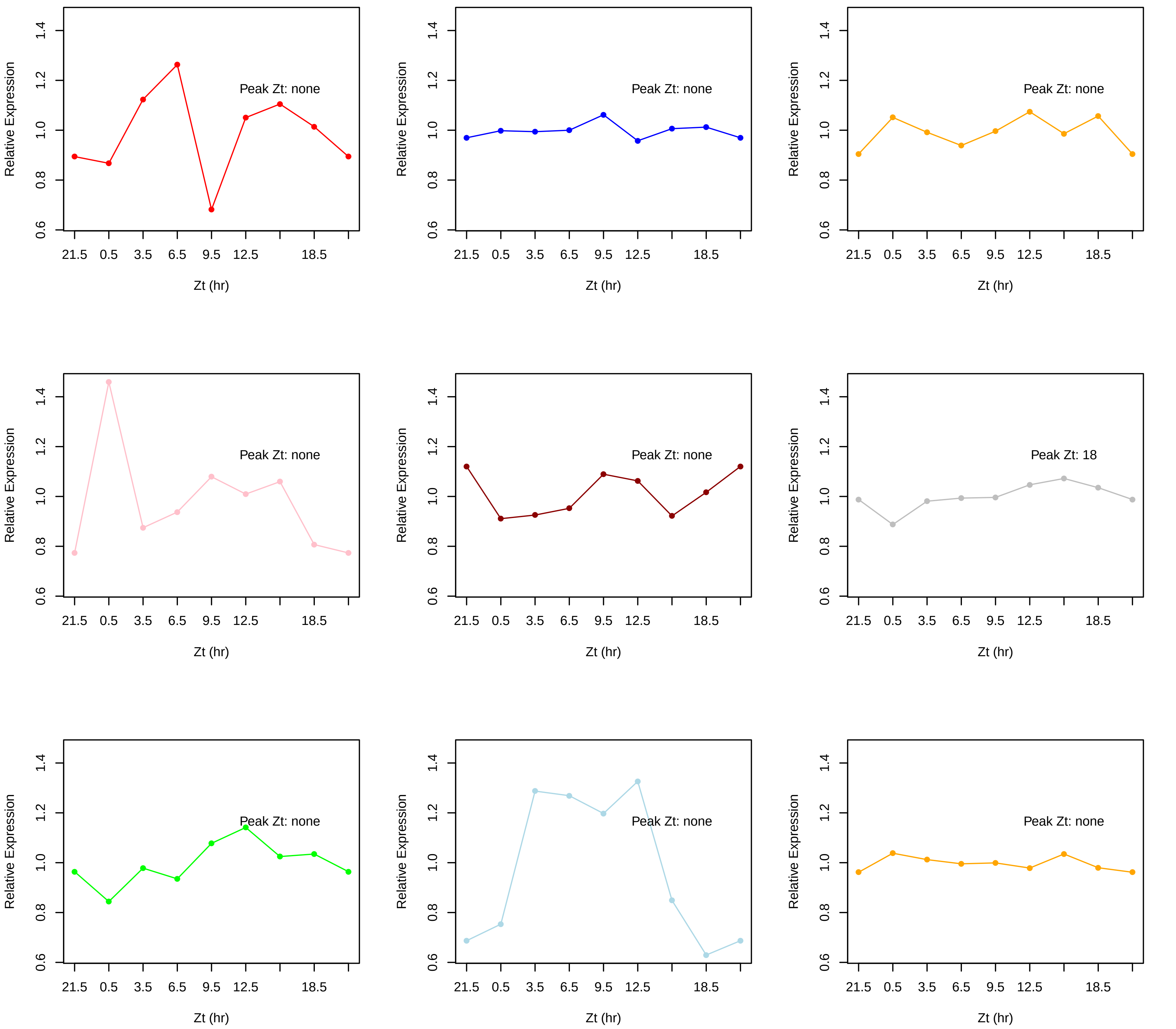

Singeleton: ENSXMAG00000026342 olfml2a

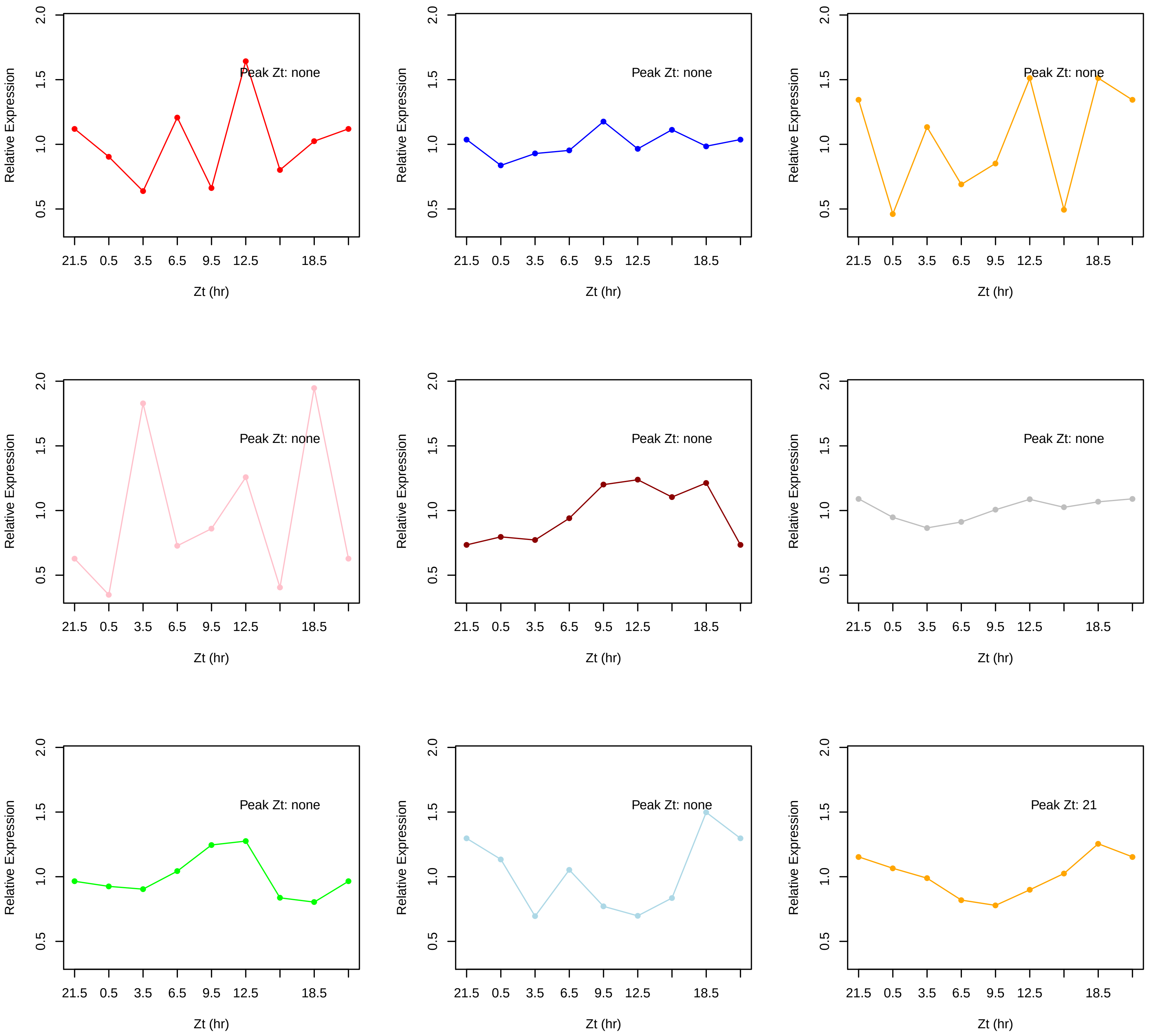

Singeleton: ENSXMAG00000015632

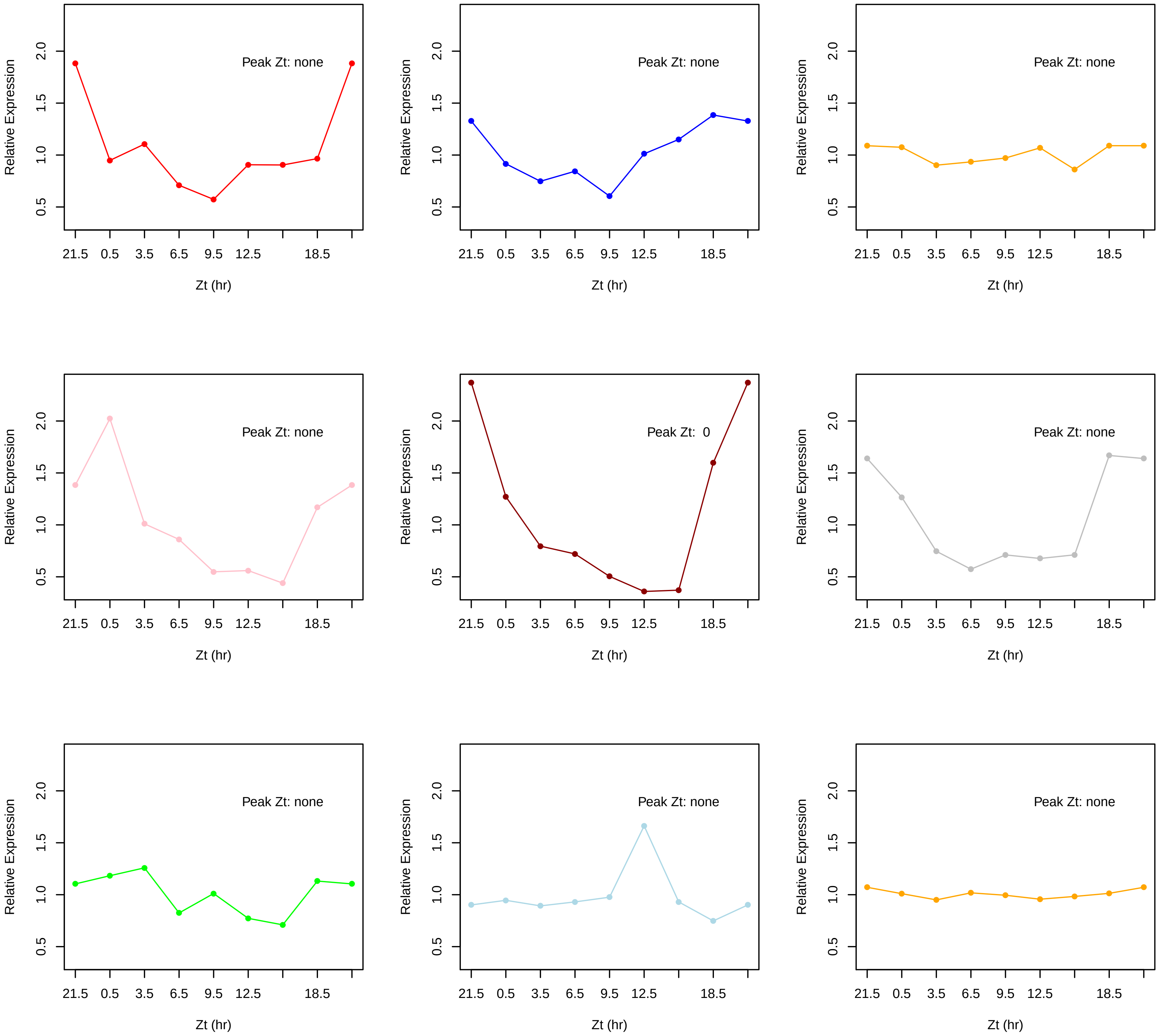

Singeleton: ENSXMAG00000019412 rhobtb4

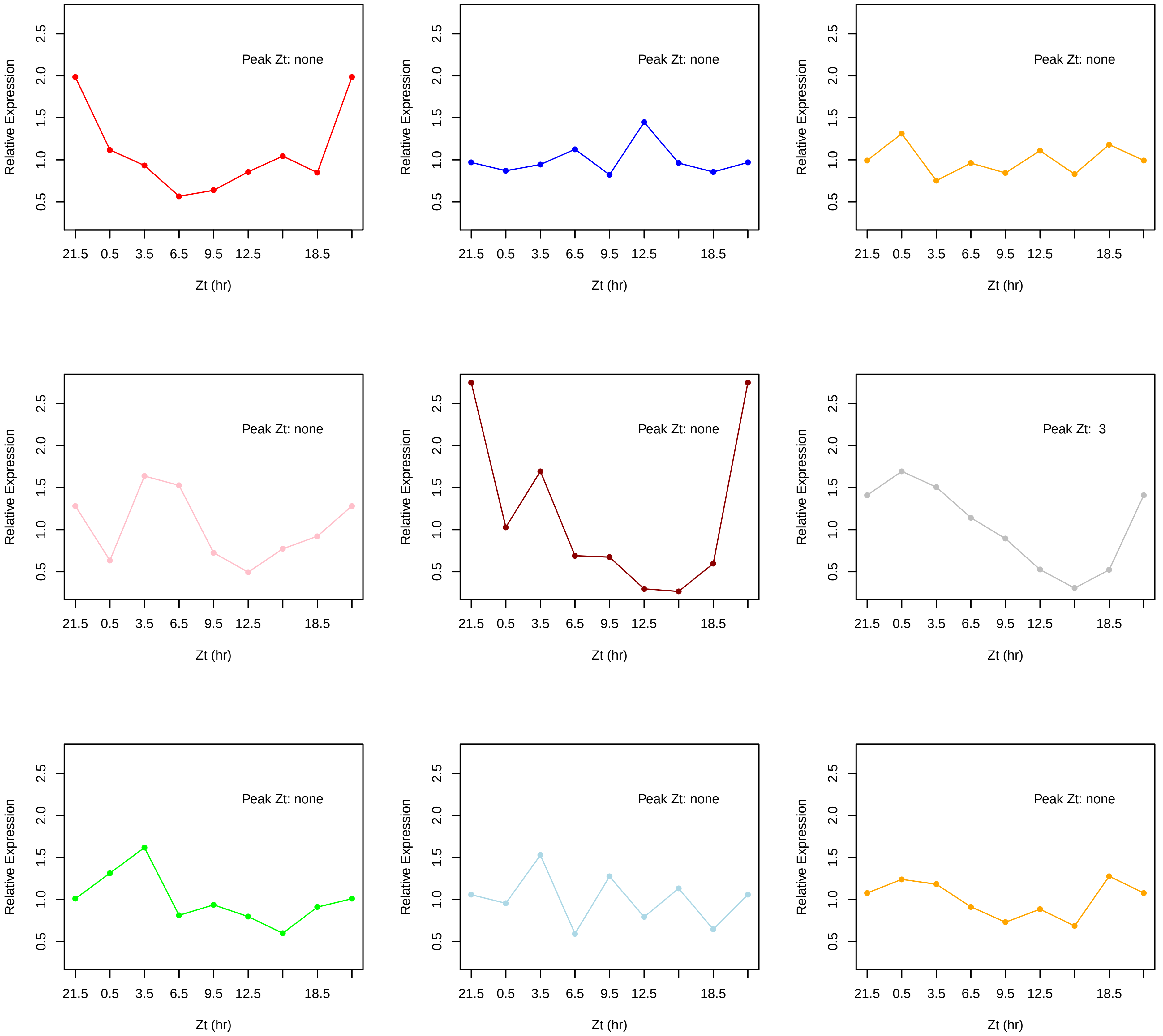

Singeleton: ENSXMAG00000004627 dnajc3a

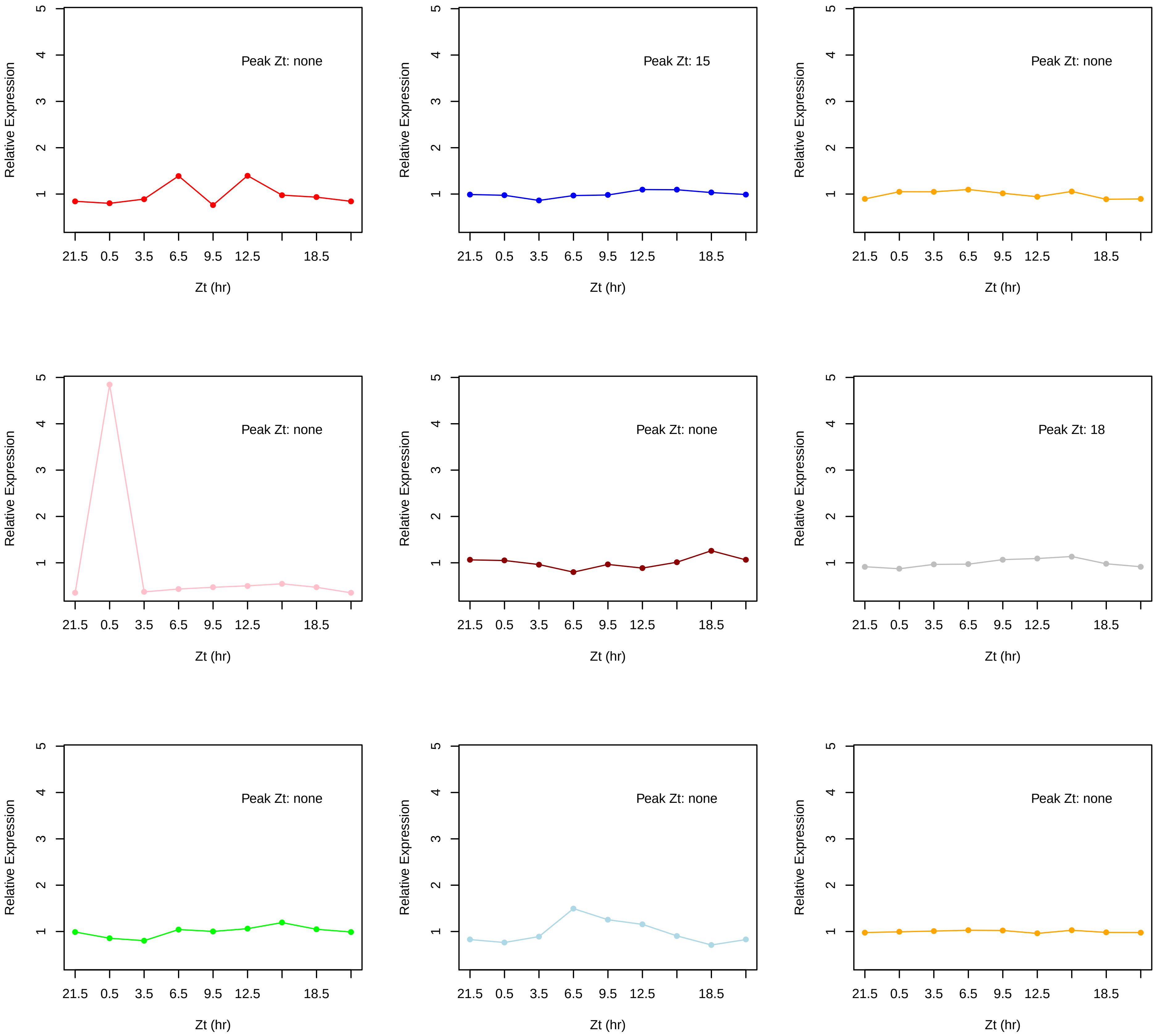

Singeleton: ENSXMAG00000003157 eif4ebp2

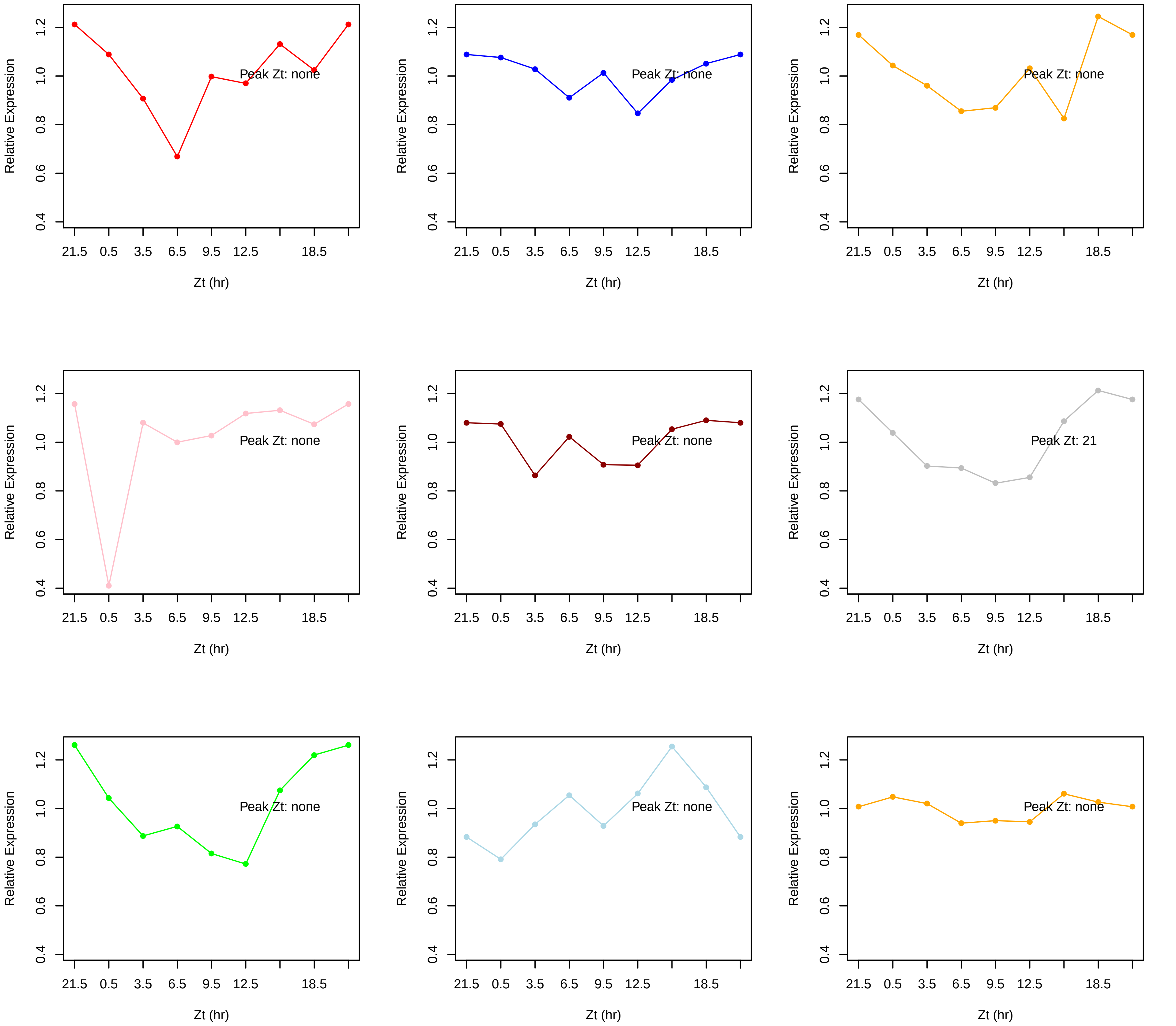

Singleton: ENSXMAG00000013103 ptpn3

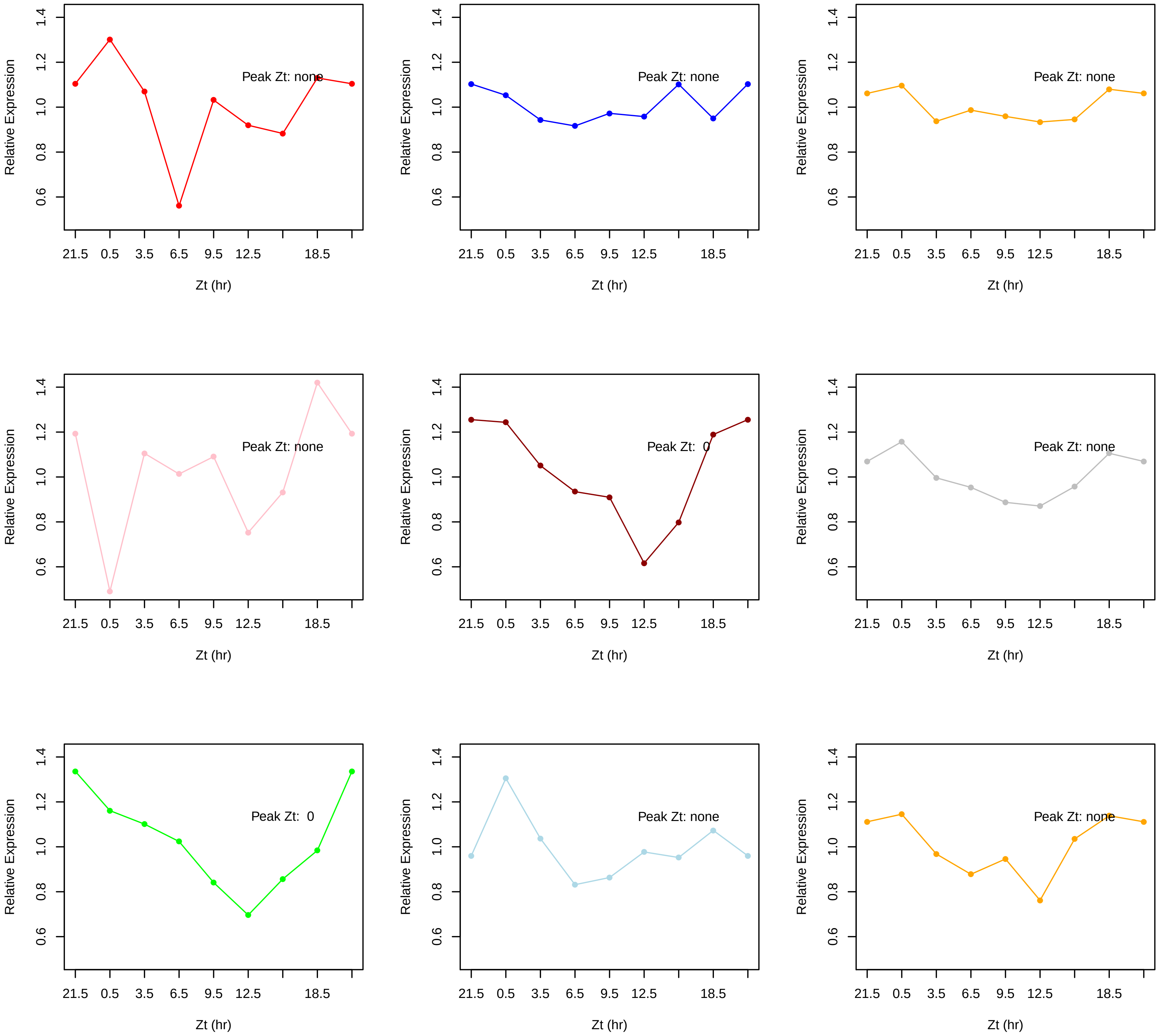

Singeleton: ENSXMAG00000018362 p3h2

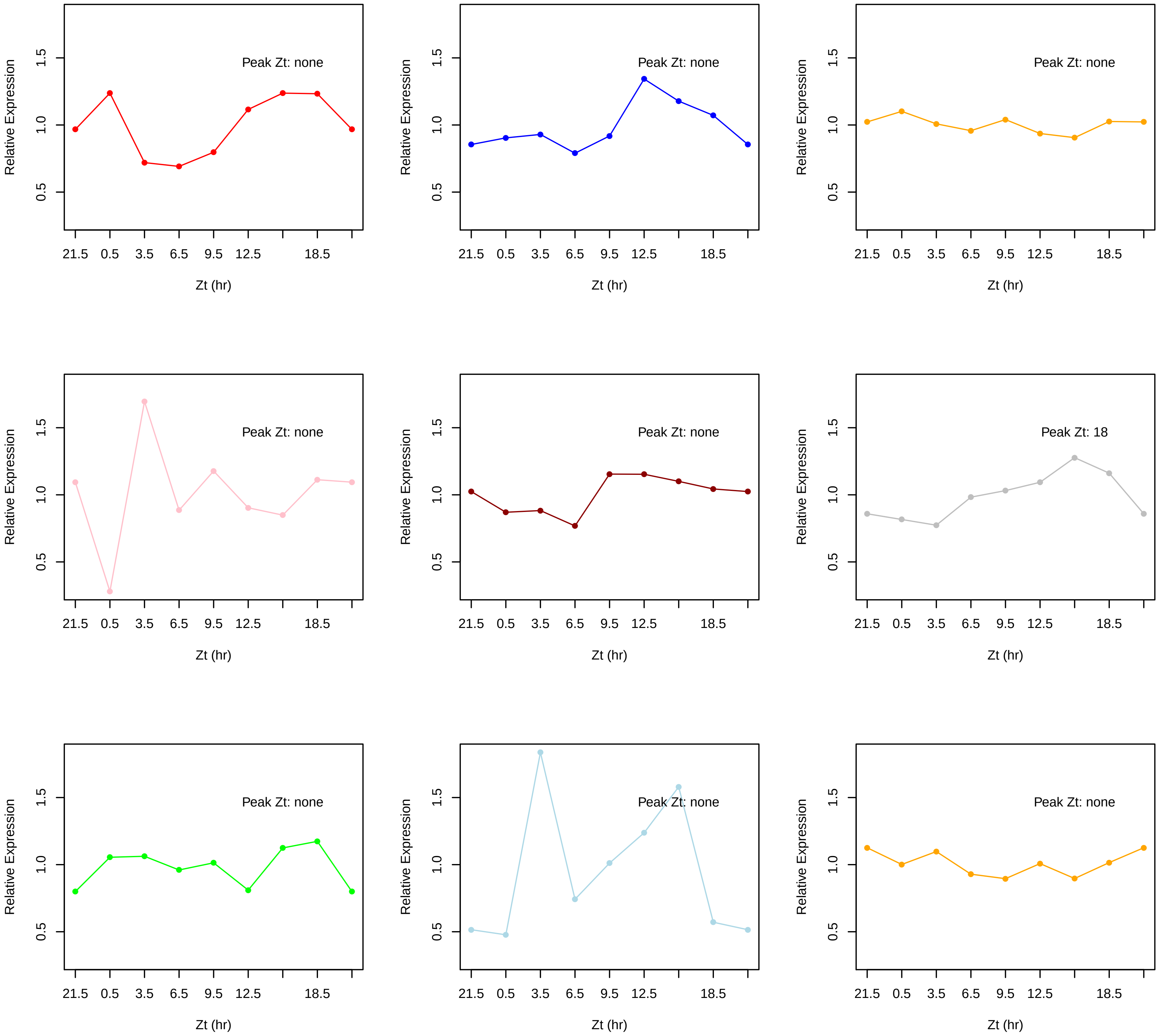

Singeleton: ENSXMAG00000015029 HAGHL

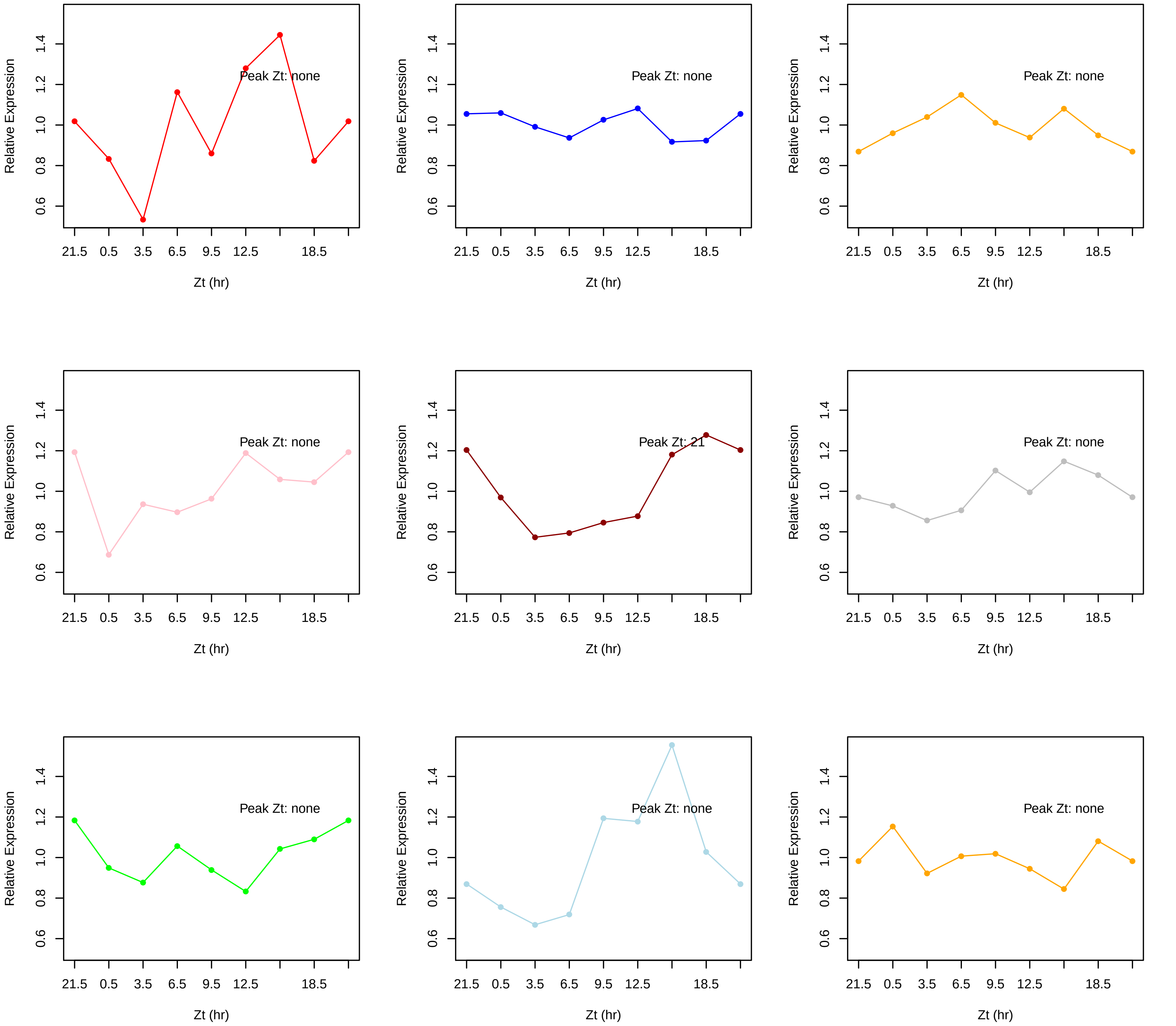

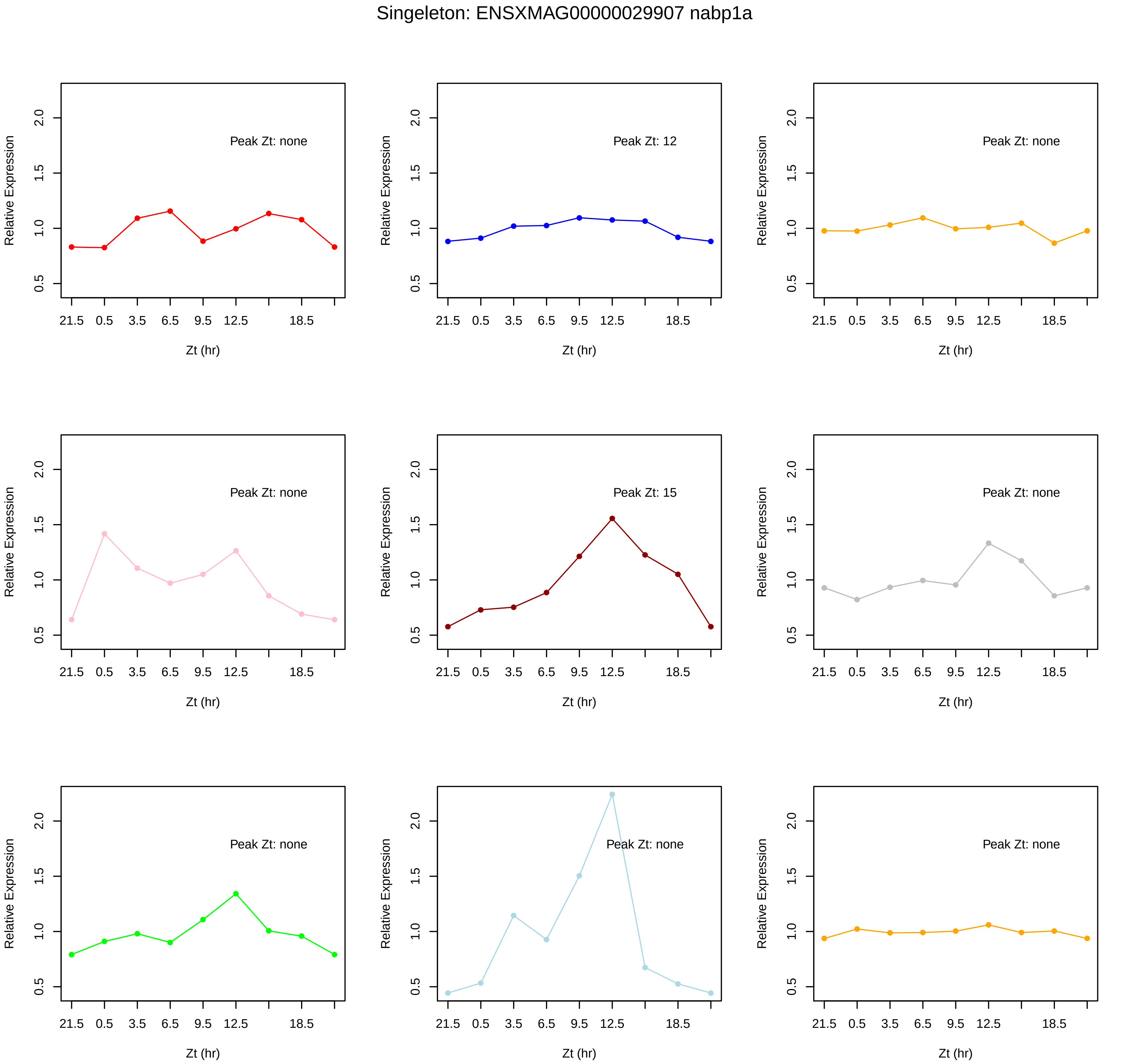
Singeleton: ENSXMAG00000029907 nabp1a
Peak Zt: none
Peak Zt: 12
Peak Zt: none
Peak Zt: none
Peak Zt: 15
Peak Zt: none
Peak Zt: none
Peak Zt: none
Peak Zt: none
Relative Expression
Zt (hr)
21.5
0.5
3.5
6.5
9.5
12.5
18.5
0.5
1.0
1.5
2.0

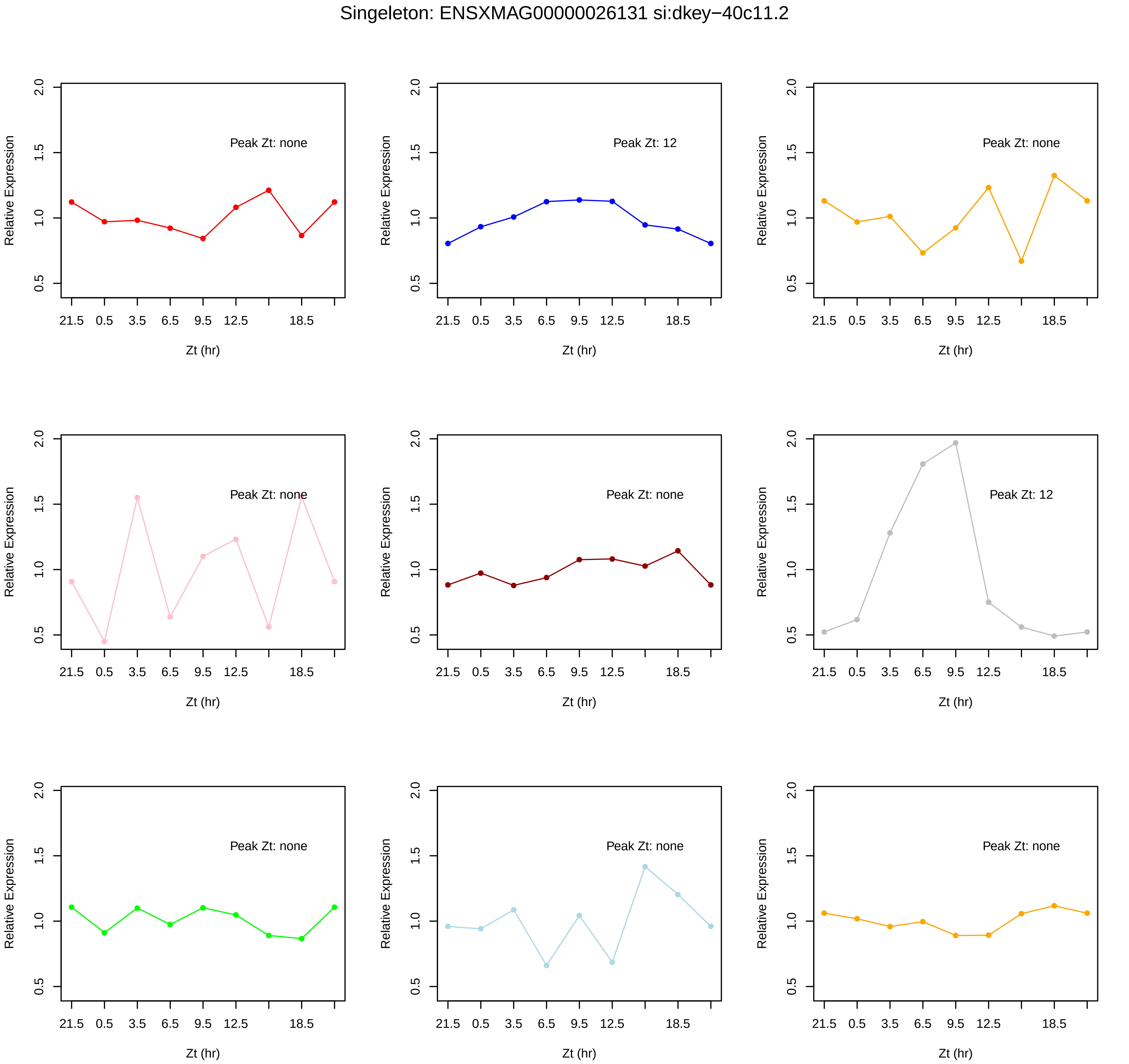
Singeleton: ENSXMAG00000026131 si:dkey−40c11.2
Peak Zt: none
Peak Zt: 12
Peak Zt: none
Peak Zt: none
Peak Zt: none
Peak Zt: 12
Peak Zt: none
Peak Zt: none
Peak Zt: none
Relative Expression
Zt (hr)
21.5
0.5
3.5
6.5
9.5
12.5
18.5
0.5
1.0
1.5
2.0

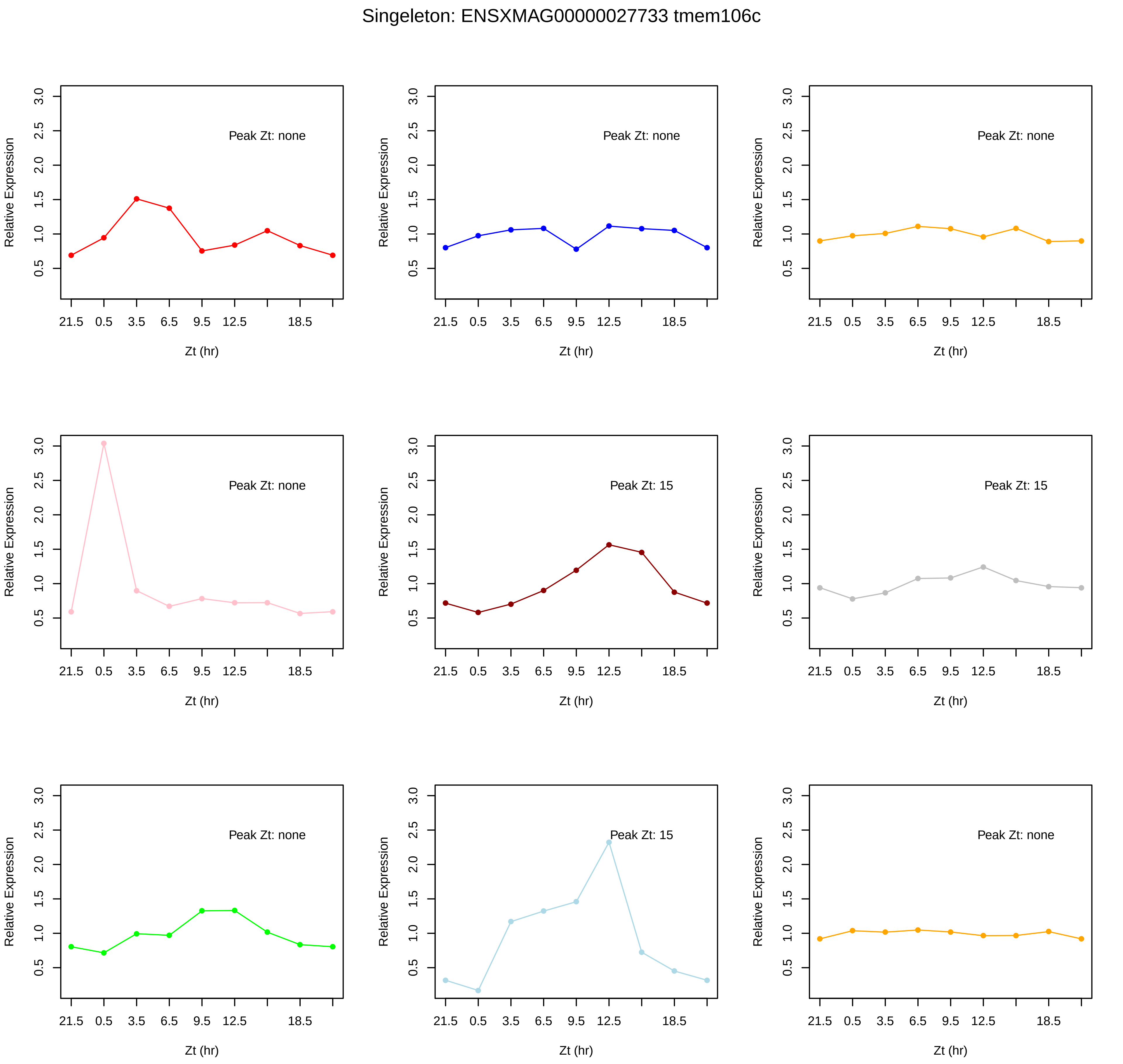
Singeleton: ENSXMAG00000027733 tmem106c
Peak Zt: none
Peak Zt: none
Peak Zt: none
Peak Zt: none
Peak Zt: 15
Peak Zt: 15
Peak Zt: none
Peak Zt: 15
Peak Zt: none
Relative Expression
Zt (hr)
21.5 0.5 3.5 6.5 9.5 12.5 18.5

Singleton: ENSXMAG00000015106 lrrc20

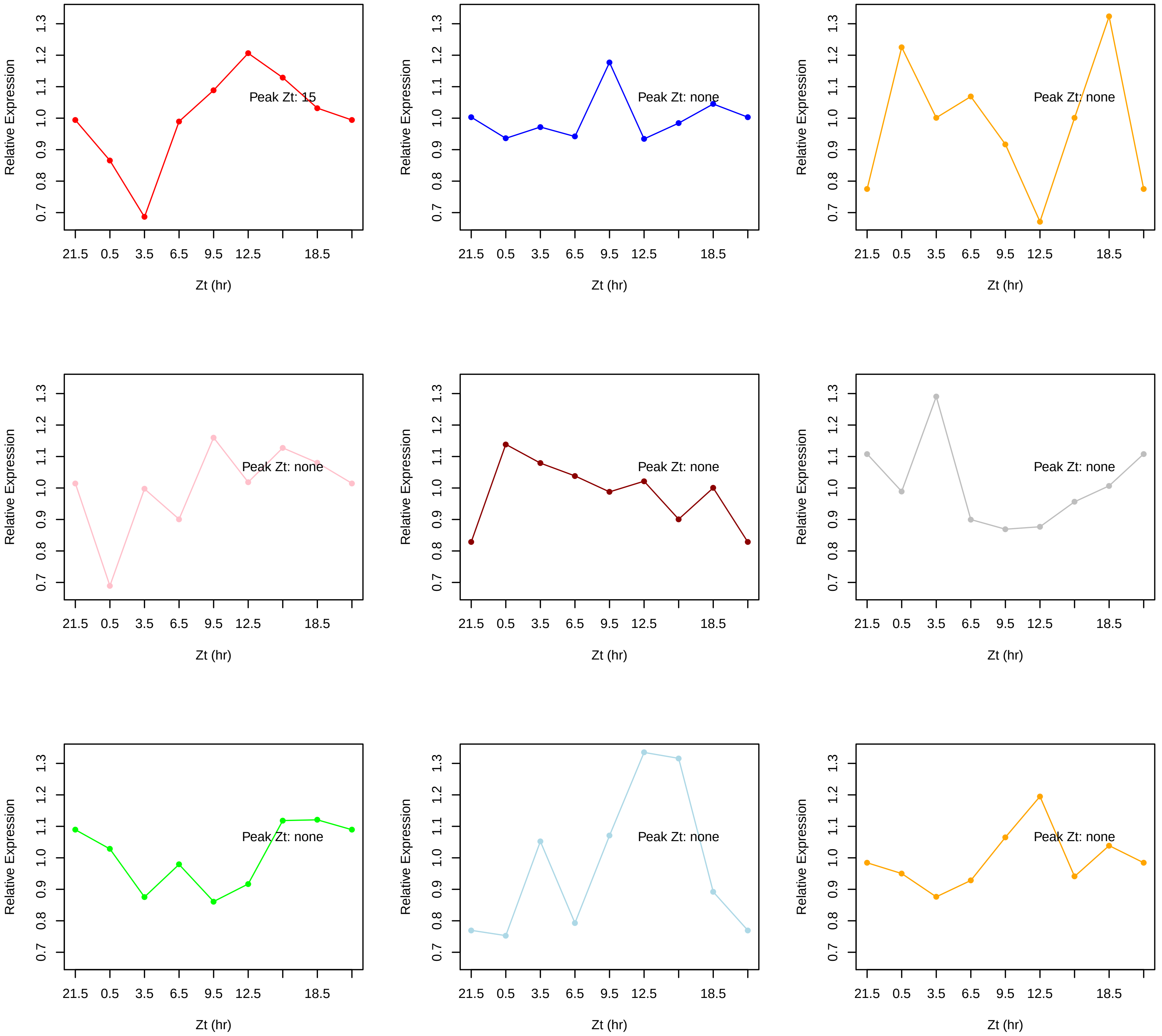

Singeleton: ENSXMAG00000018638 gpr153

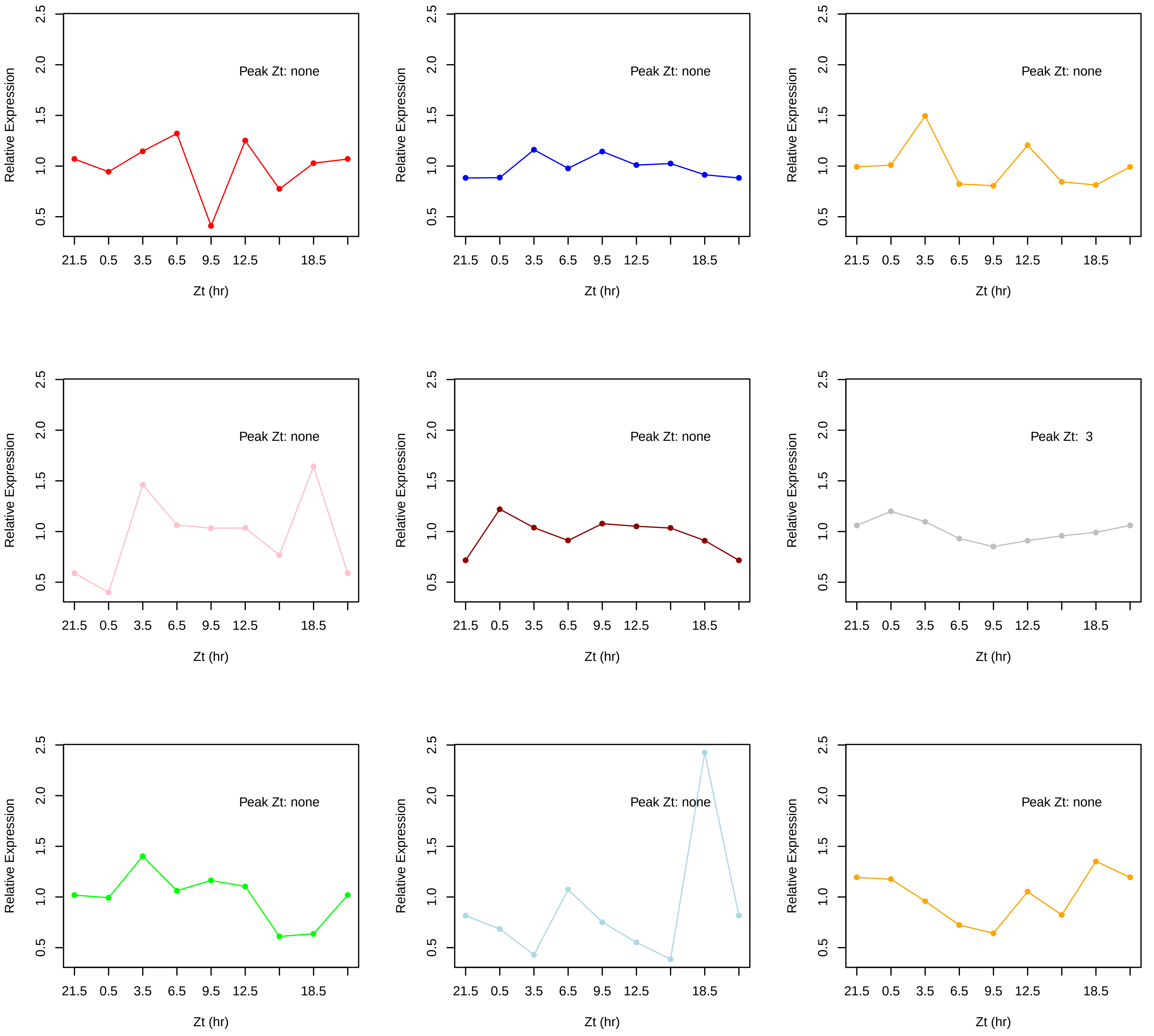

Singeleton: ENSXMAG00000028994 selenof

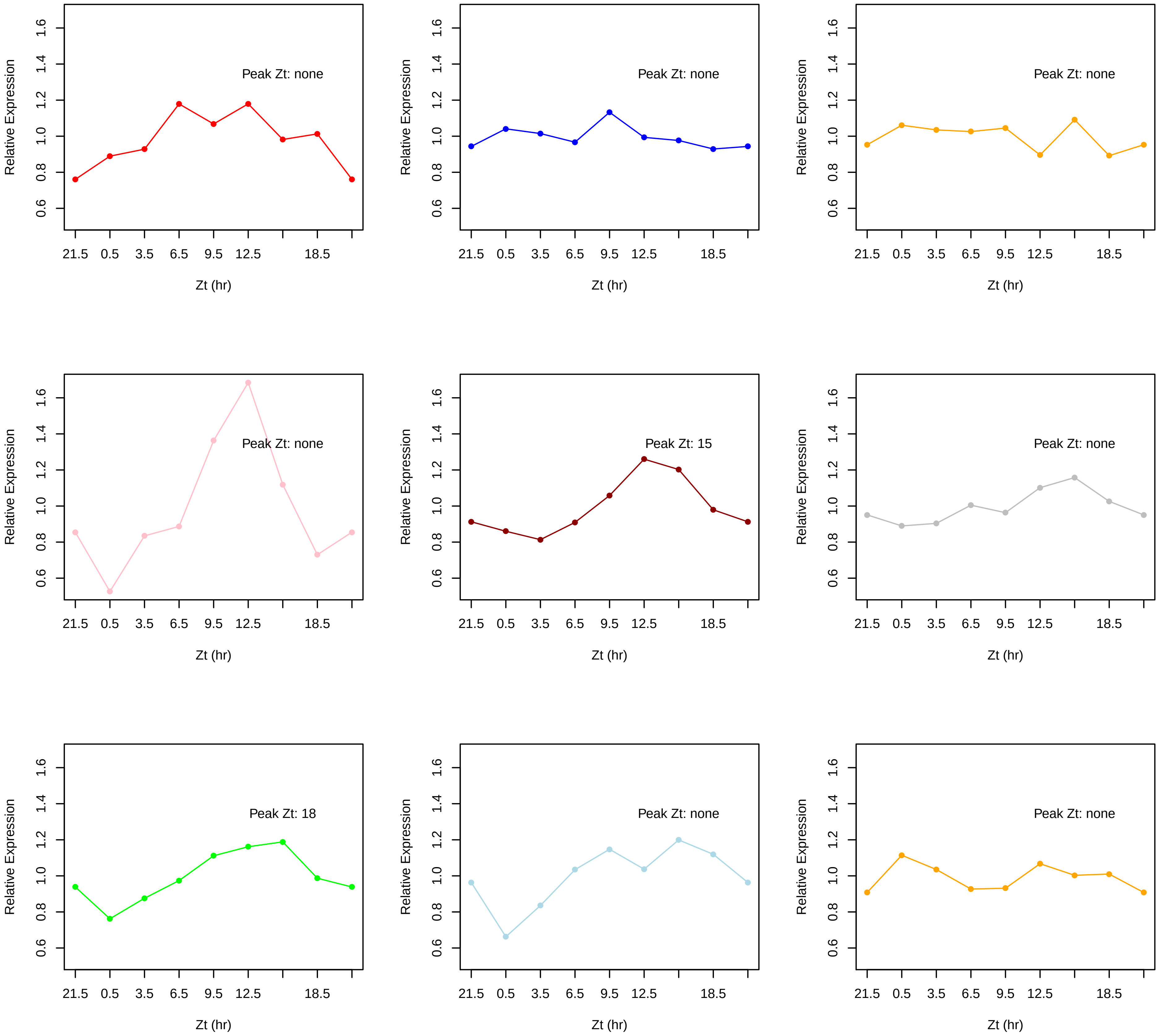

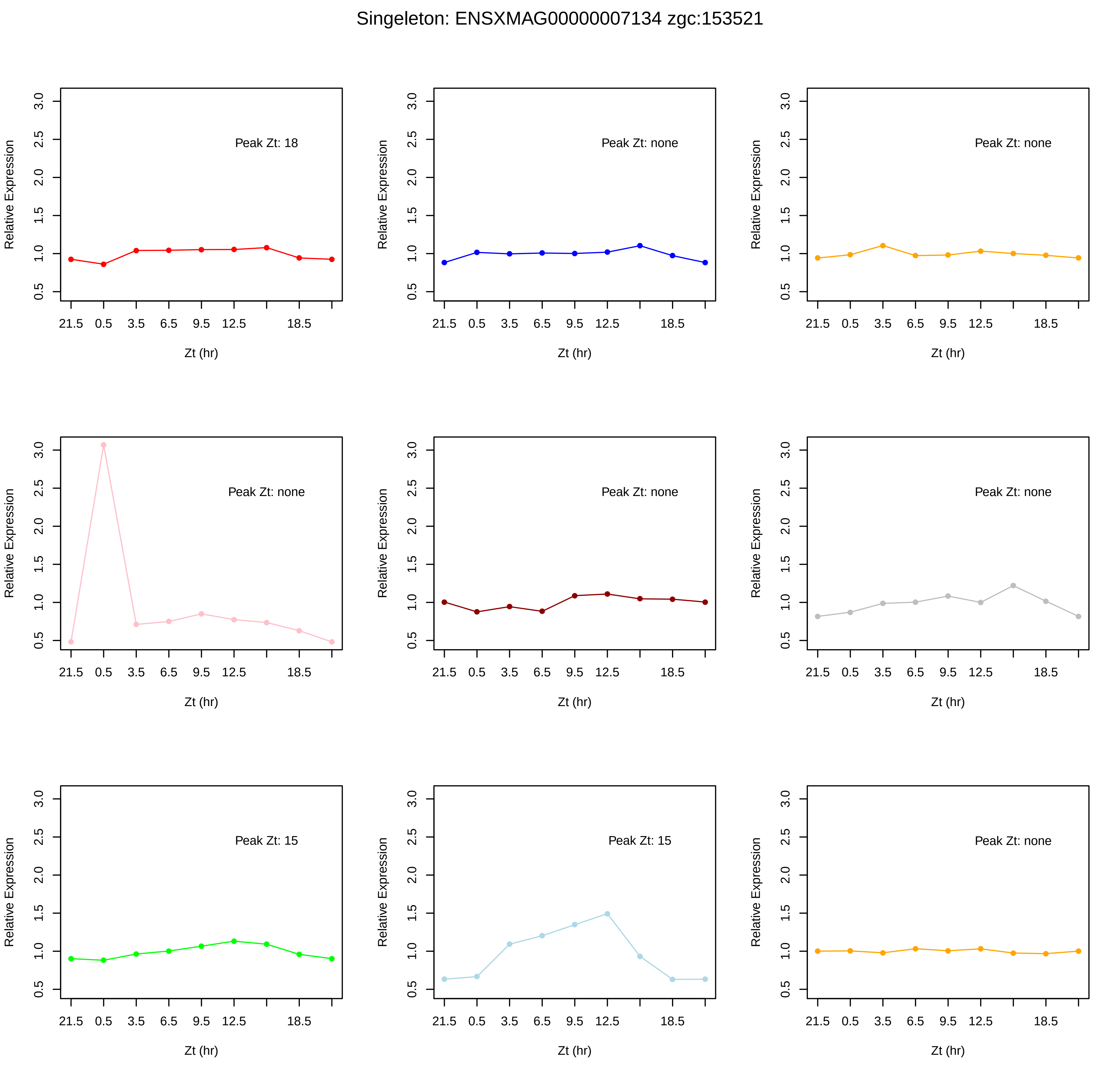
Singeleton: ENSXMAG00000007134 zgc:153521
Peak Zt: 18
Peak Zt: none
Peak Zt: none
Peak Zt: none
Peak Zt: none
Peak Zt: none
Peak Zt: 15
Peak Zt: 15
Peak Zt: none
Relative Expression
Zt (hr)
21.5
0.5
3.5
6.5
9.5
12.5
18.5
0.5
1.0
1.5
2.0
2.5
3.0

Singeleton: ENSXMAG00000014094 rhof

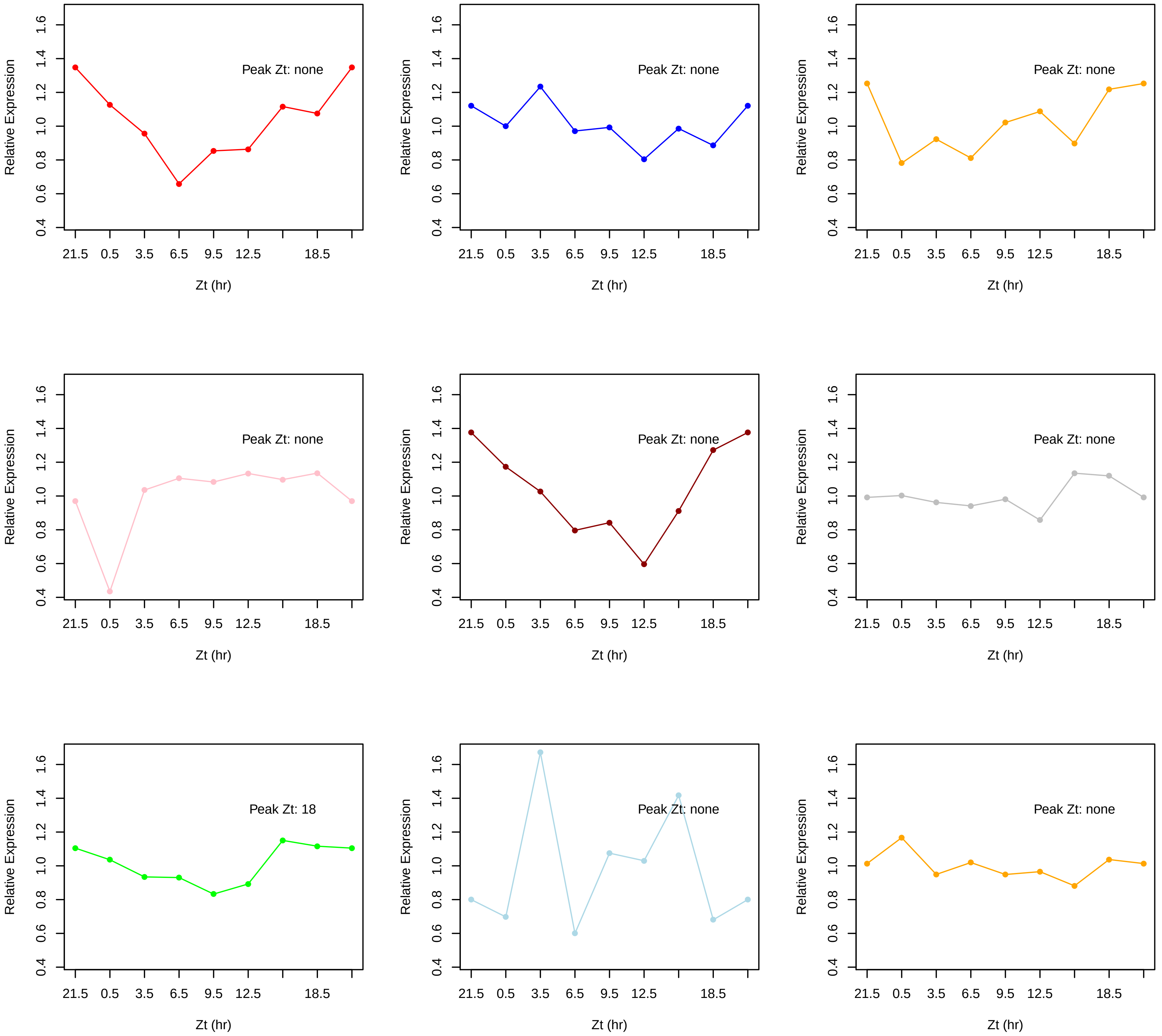

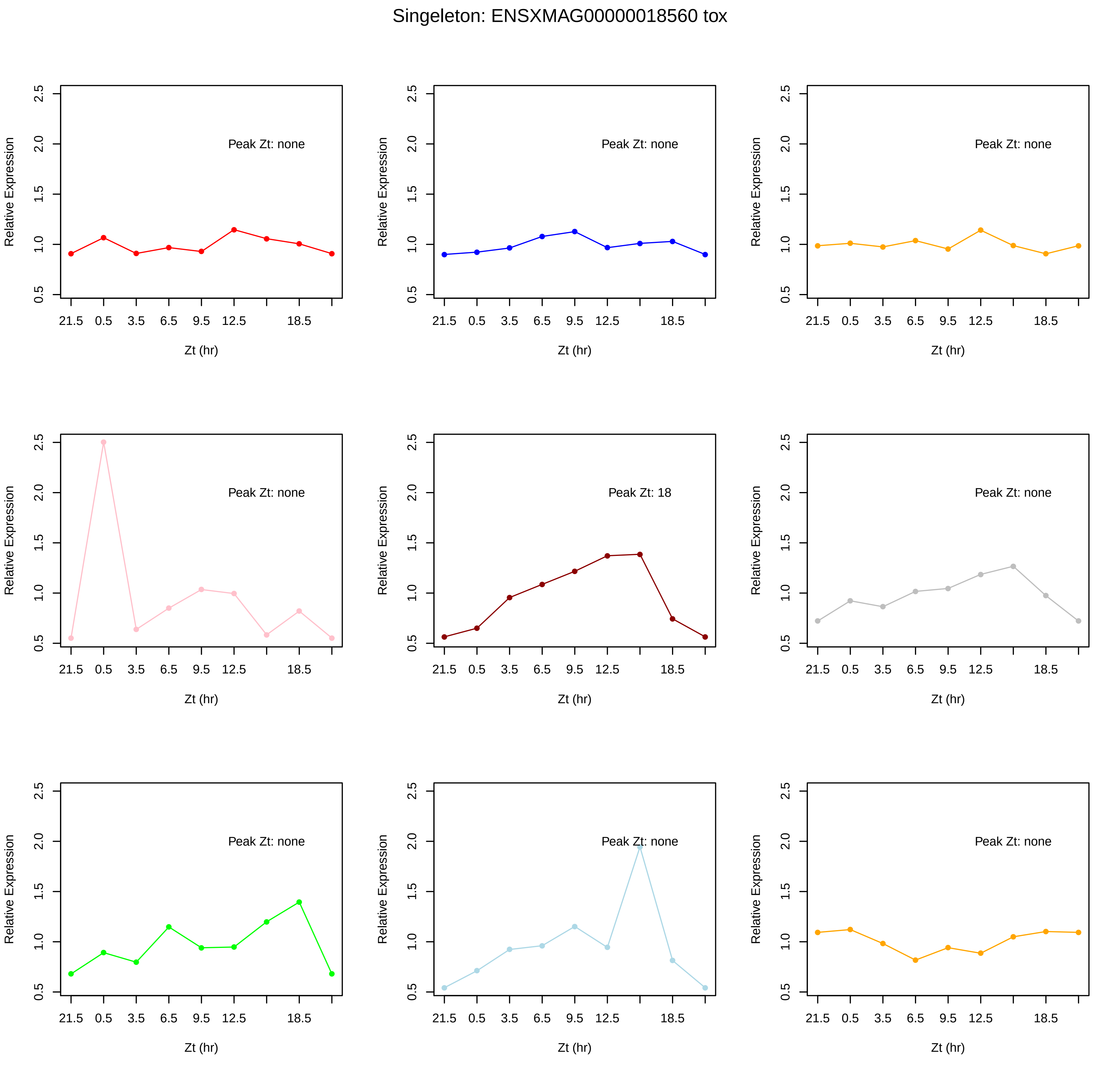
Singeleton: ENSXMAG00000018560 tox
Peak Zt: none
Peak Zt: none
Peak Zt: none
Peak Zt: none
Peak Zt: 18
Peak Zt: none
Peak Zt: none
Peak Zt: none
Peak Zt: none
Relative Expression
Zt (hr)
21.5 0.5 3.5 6.5 9.5 12.5 18.5
0.5 1.0 1.5 2.0 2.5

Singeleton: ENSXMAG00000004769 adgrd2

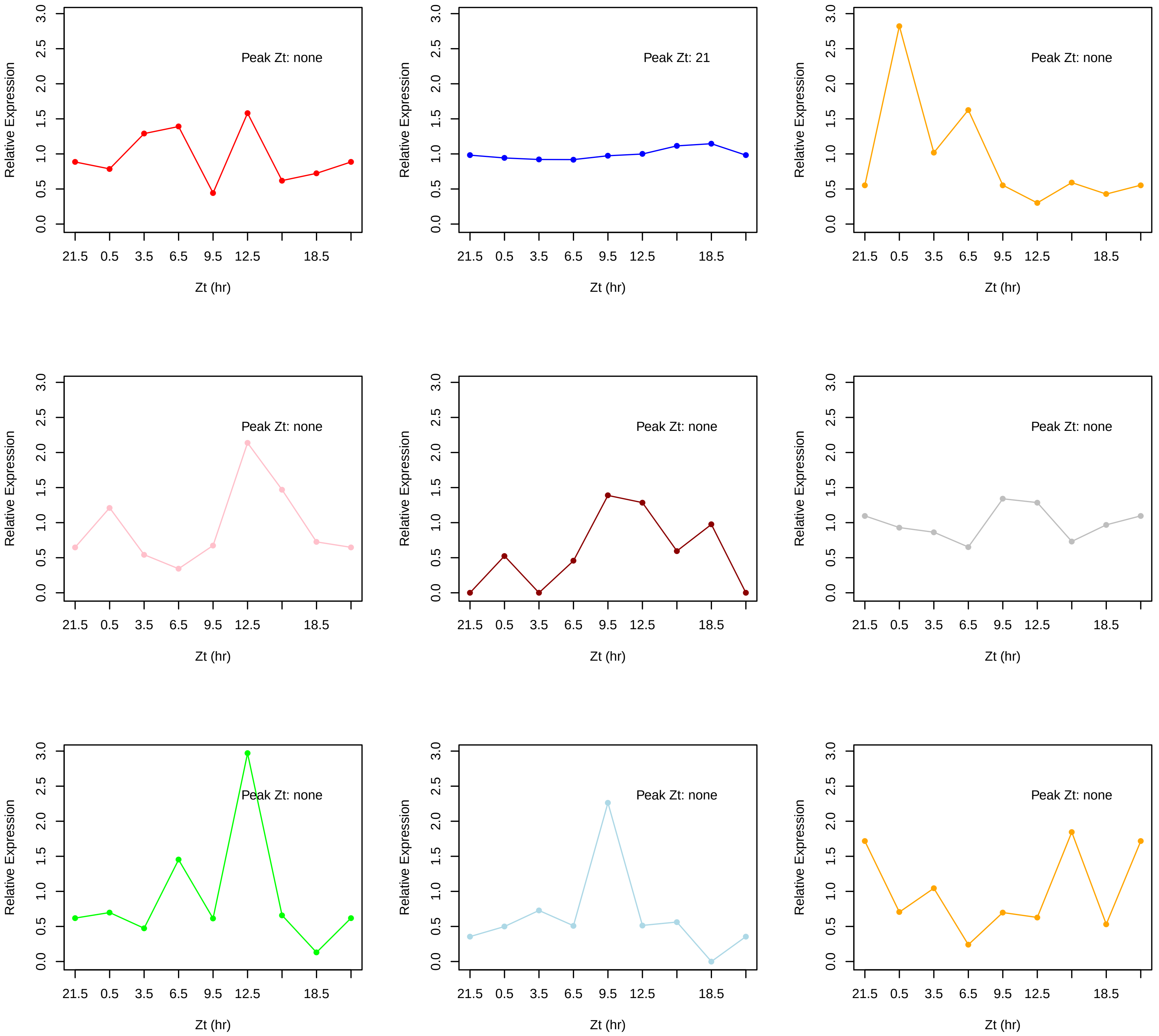

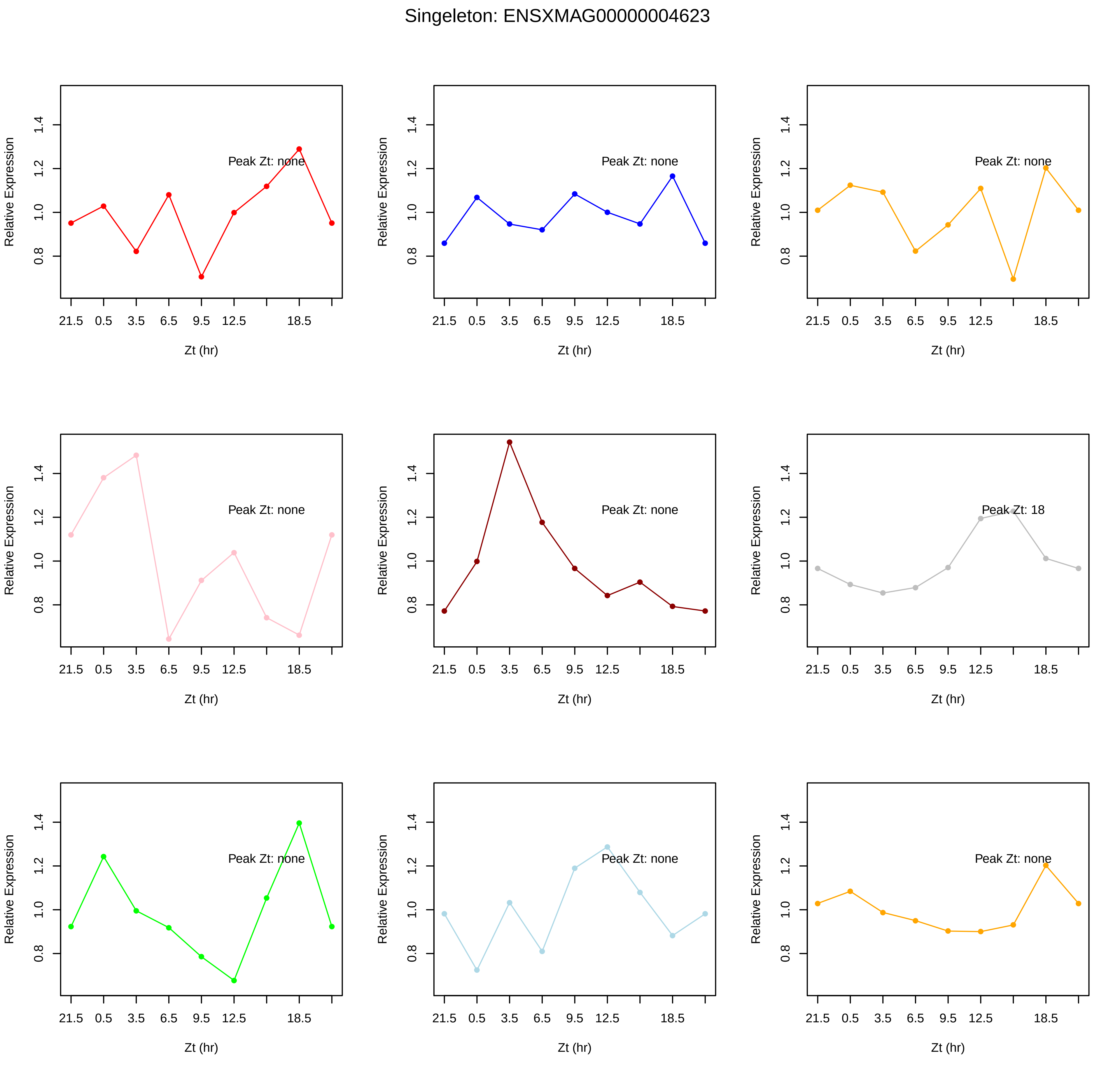
Singeleton: ENSXMAG00000004623
Peak Zt: none
Peak Zt: none
Peak Zt: none
Peak Zt: none
Peak Zt: none
Peak Zt: 18
Peak Zt: none
Peak Zt: none
Peak Zt: none
Relative Expression
Zt (hr)
21.5
0.5
3.5
6.5
9.5
12.5
18.5
0.8
1.0
1.2
1.4

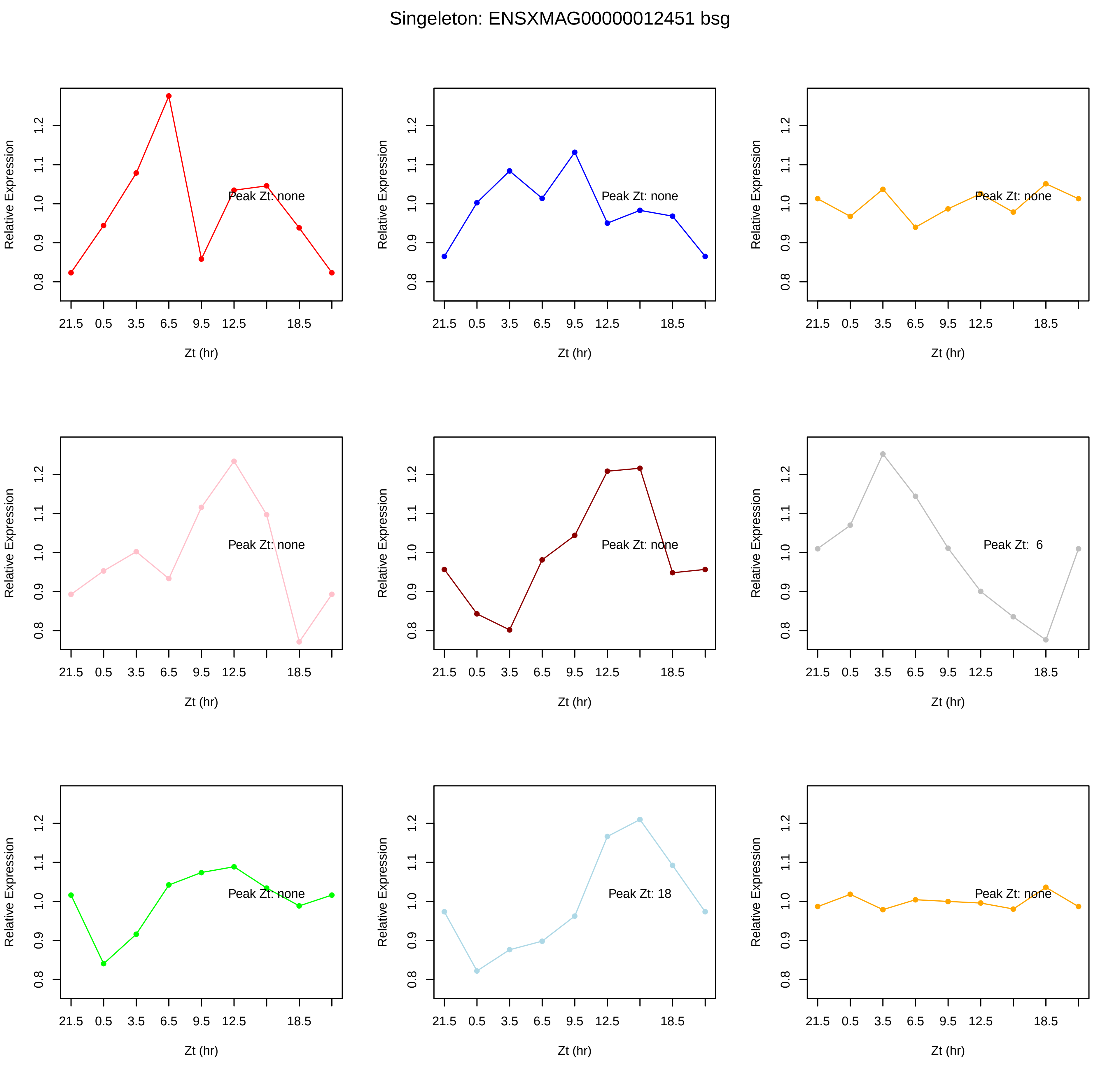
Singeleton: ENSXMAG00000012451 bsg
Relative Expression
Zt (hr)
21.5 0.5 3.5 6.5 9.5 12.5 18.5
0.8 0.9 1.0 1.1 1.2
Peak Zt: none
Peak Zt: none
Peak Zt: none
Peak Zt: none
Peak Zt: none
Peak Zt: 6
Peak Zt: none
Peak Zt: 18
Peak Zt: none

Singeleton: ENSXMAG00000012180 bean1

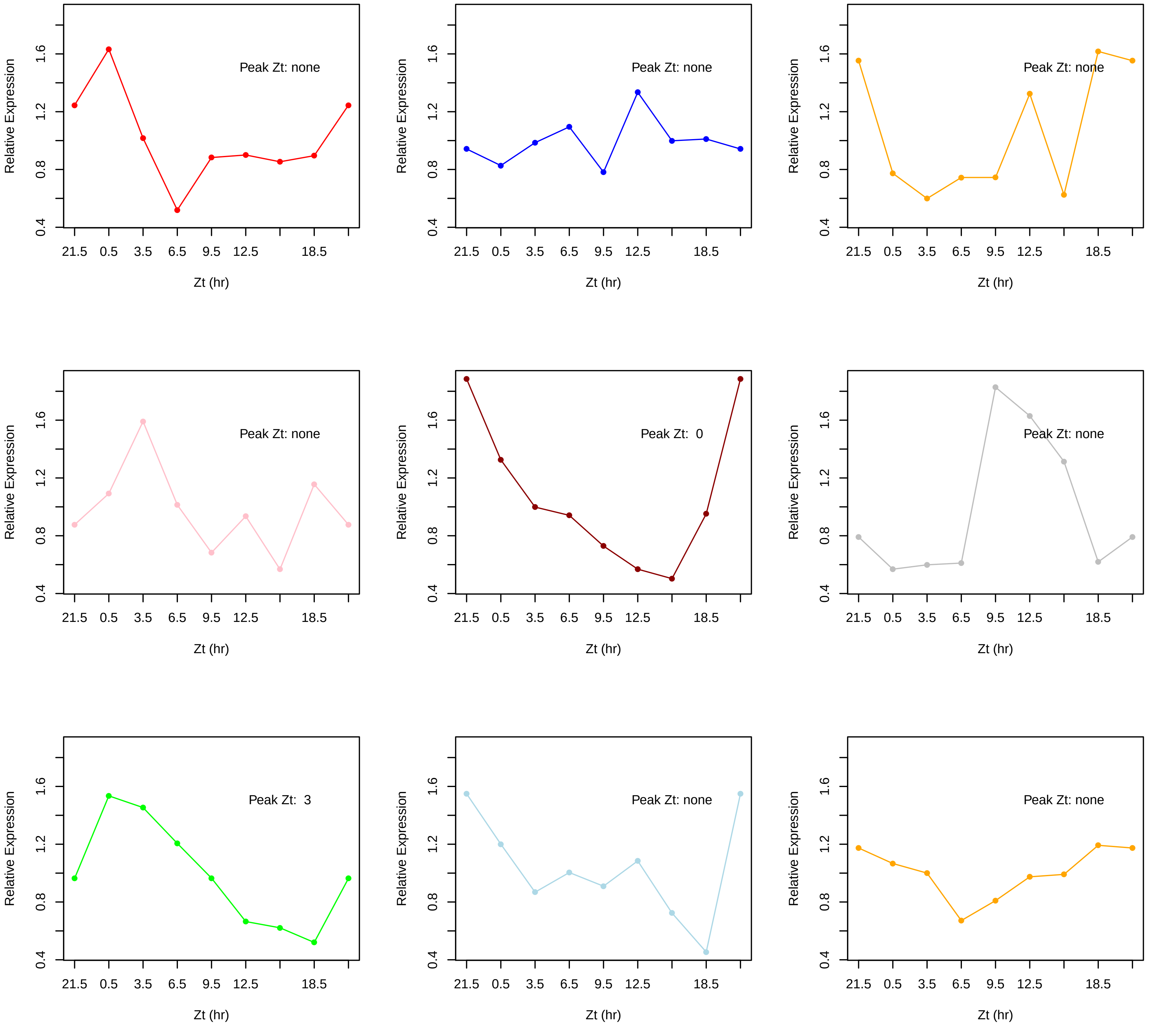

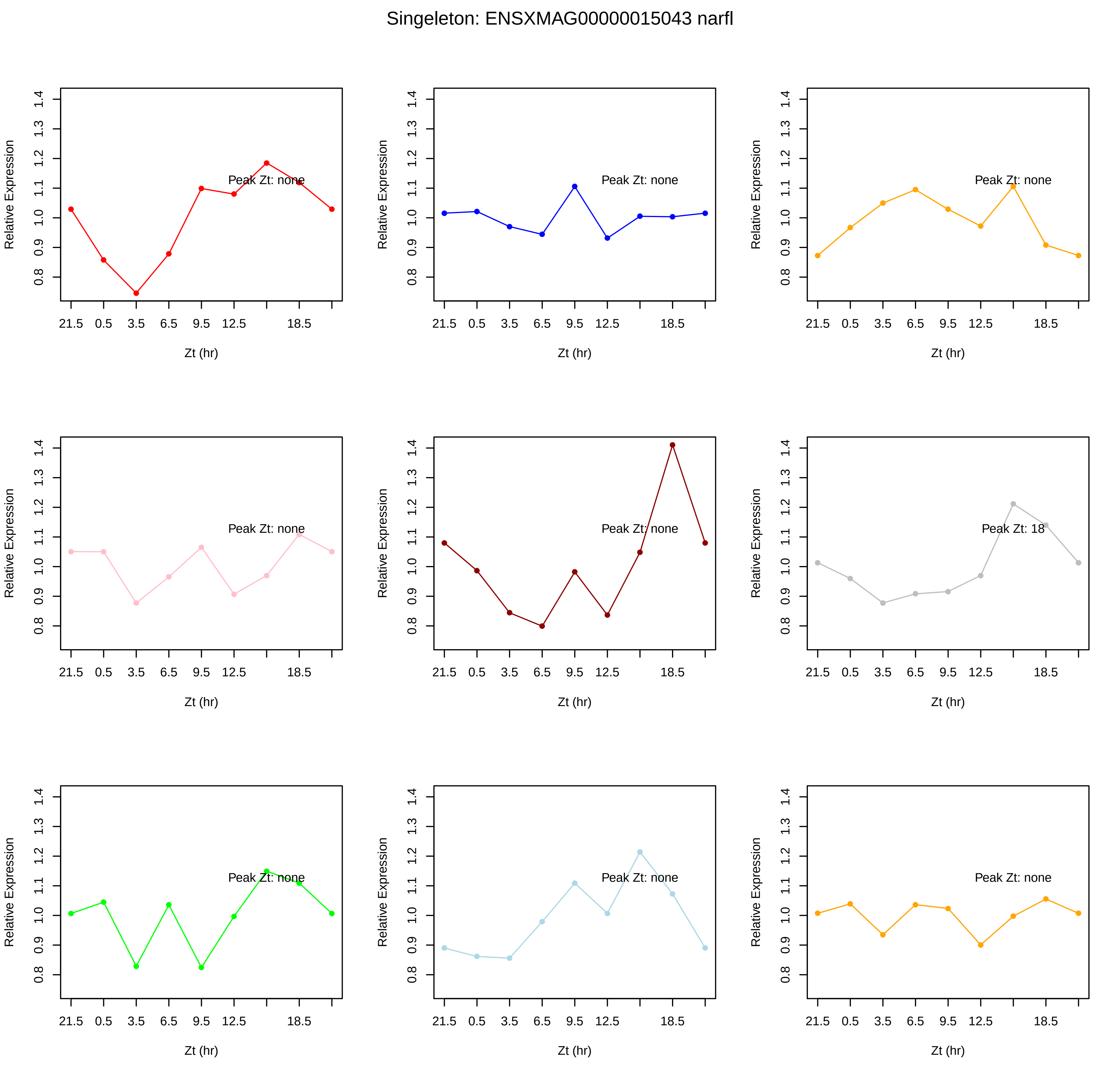

Singeleton: ENSXMAG00000015043 narfl
Relative Expression
Zt (hr)
21.5
0.5
3.5
6.5
9.5
12.5
18.5
0.8
0.9
1.0
1.1
1.2
1.3
1.4
Peak Zt: none
Peak Zt: 18

Singeleton: ENSXMAG00000024347 tprg1

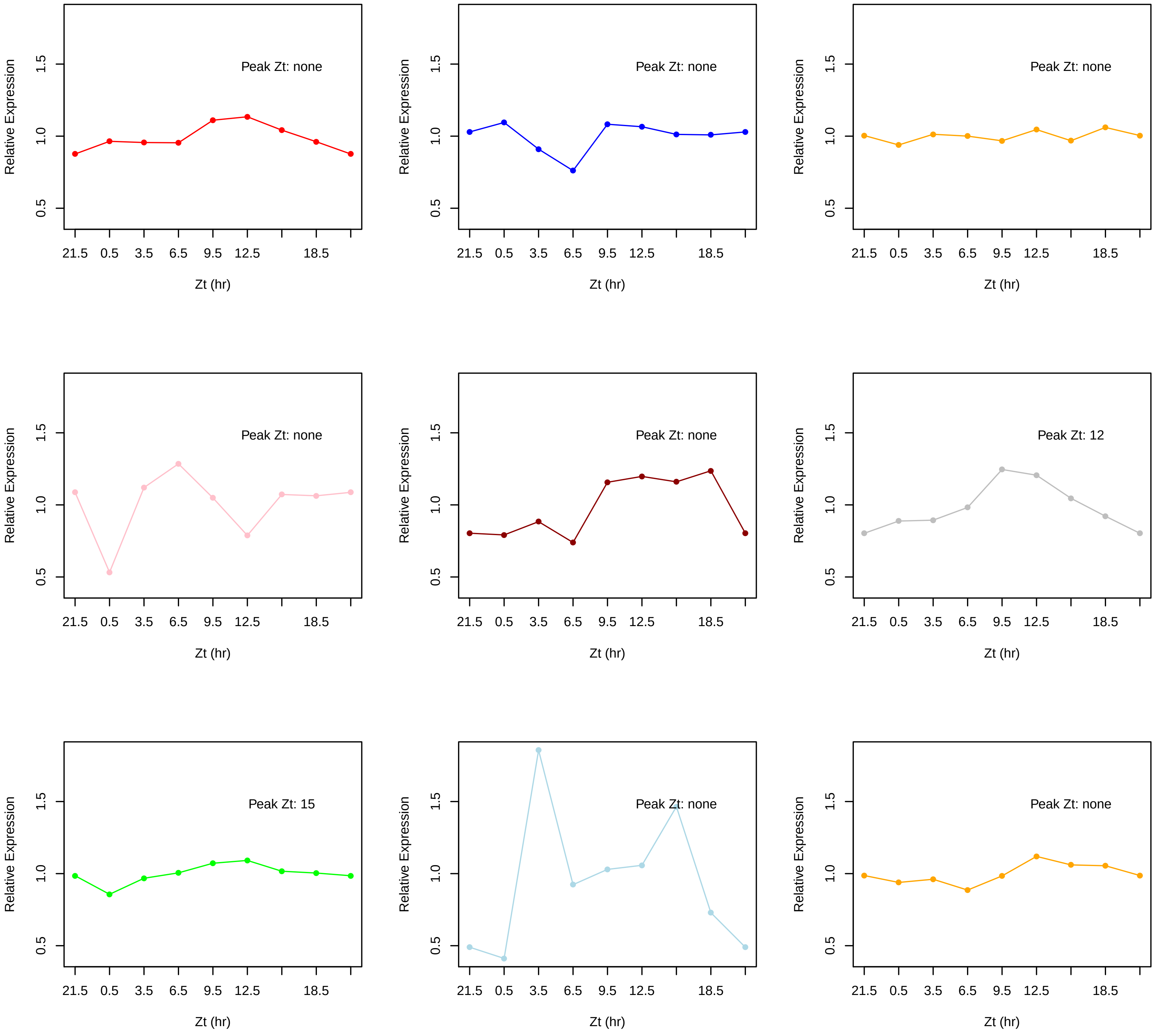

Singleton: ENSXMAG00000025493 stk17b

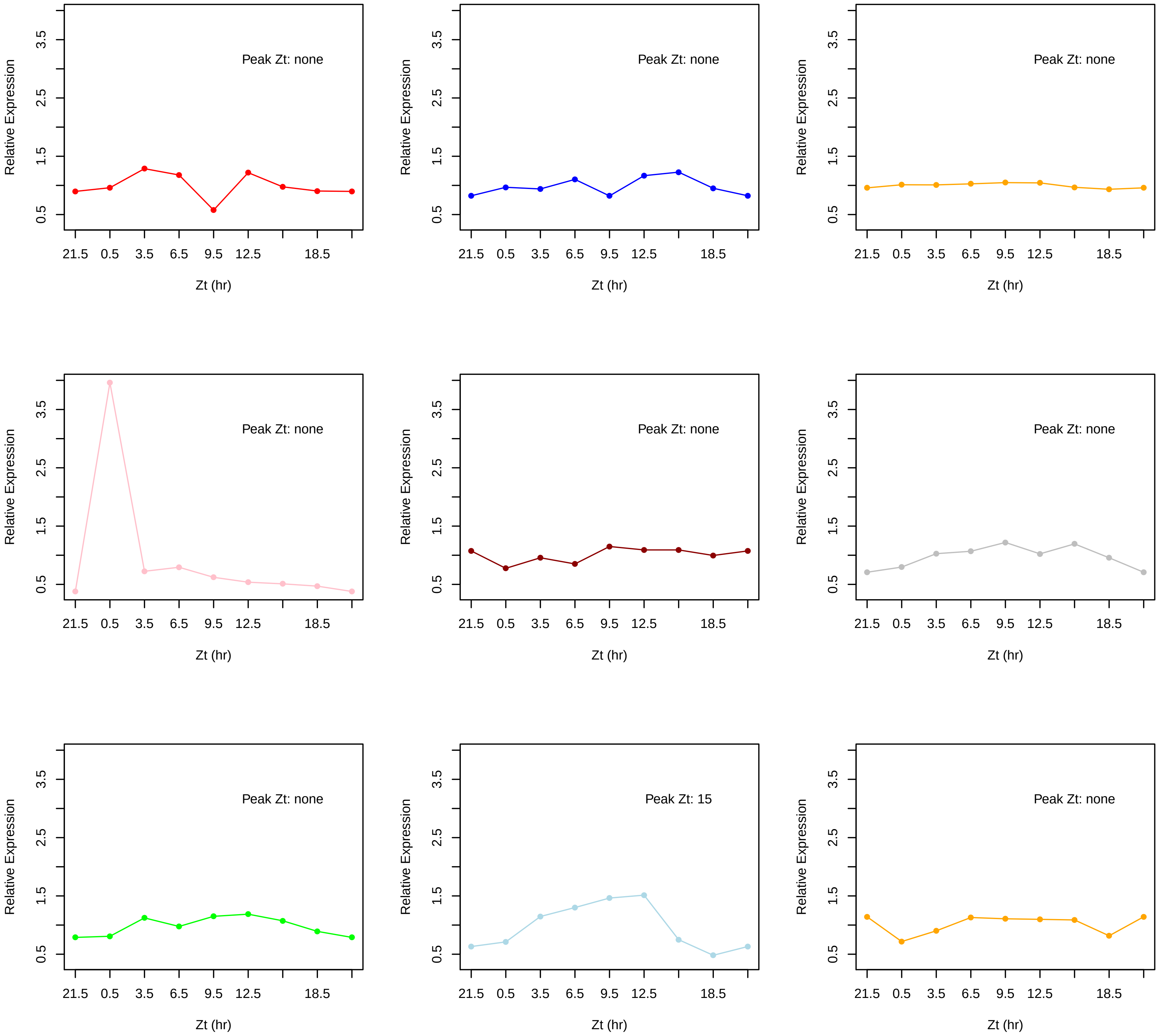

Singeleton: ENSXMAG00000006612

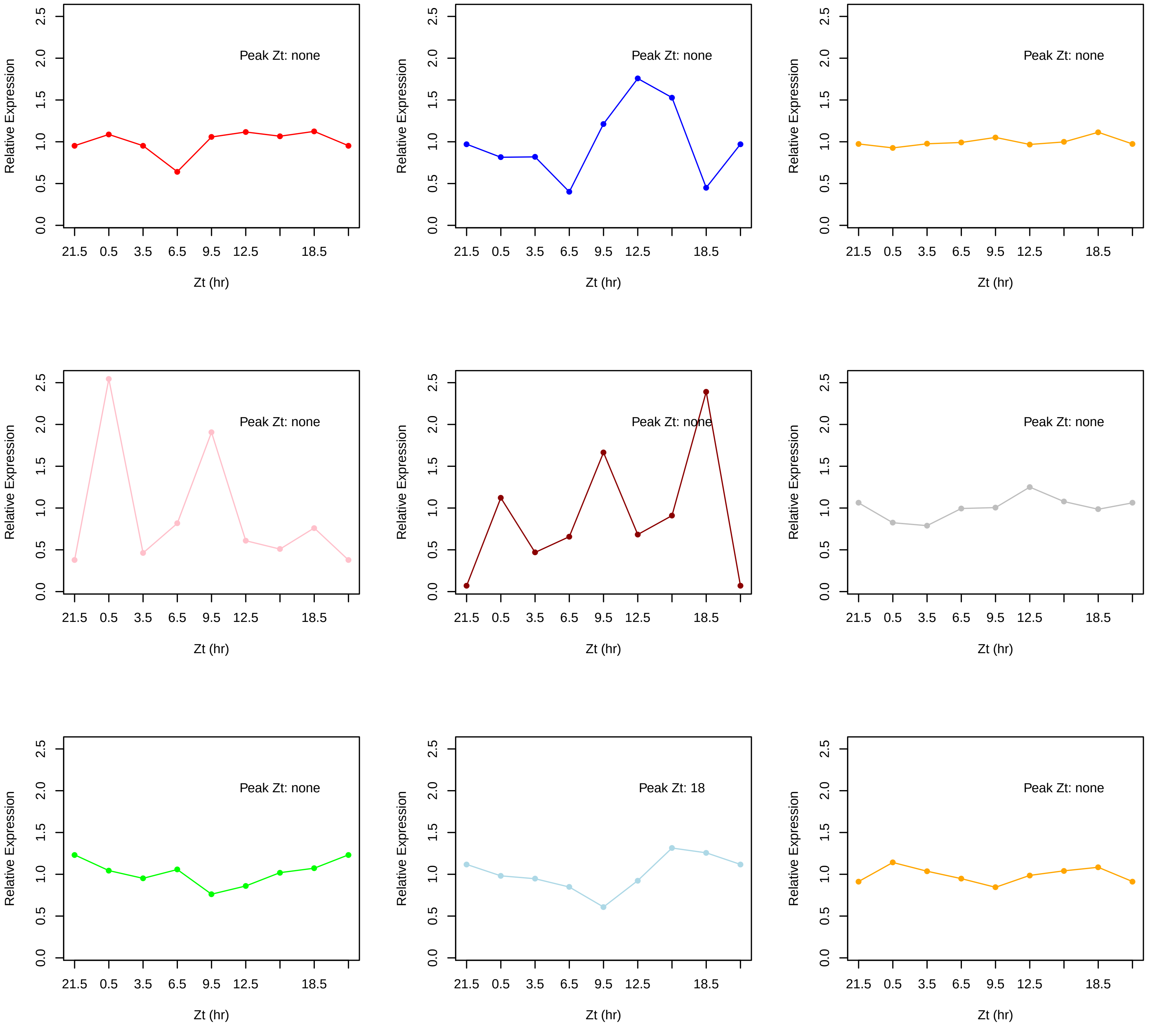

Singeleton: ENSXMAG00000014097 setd1ba

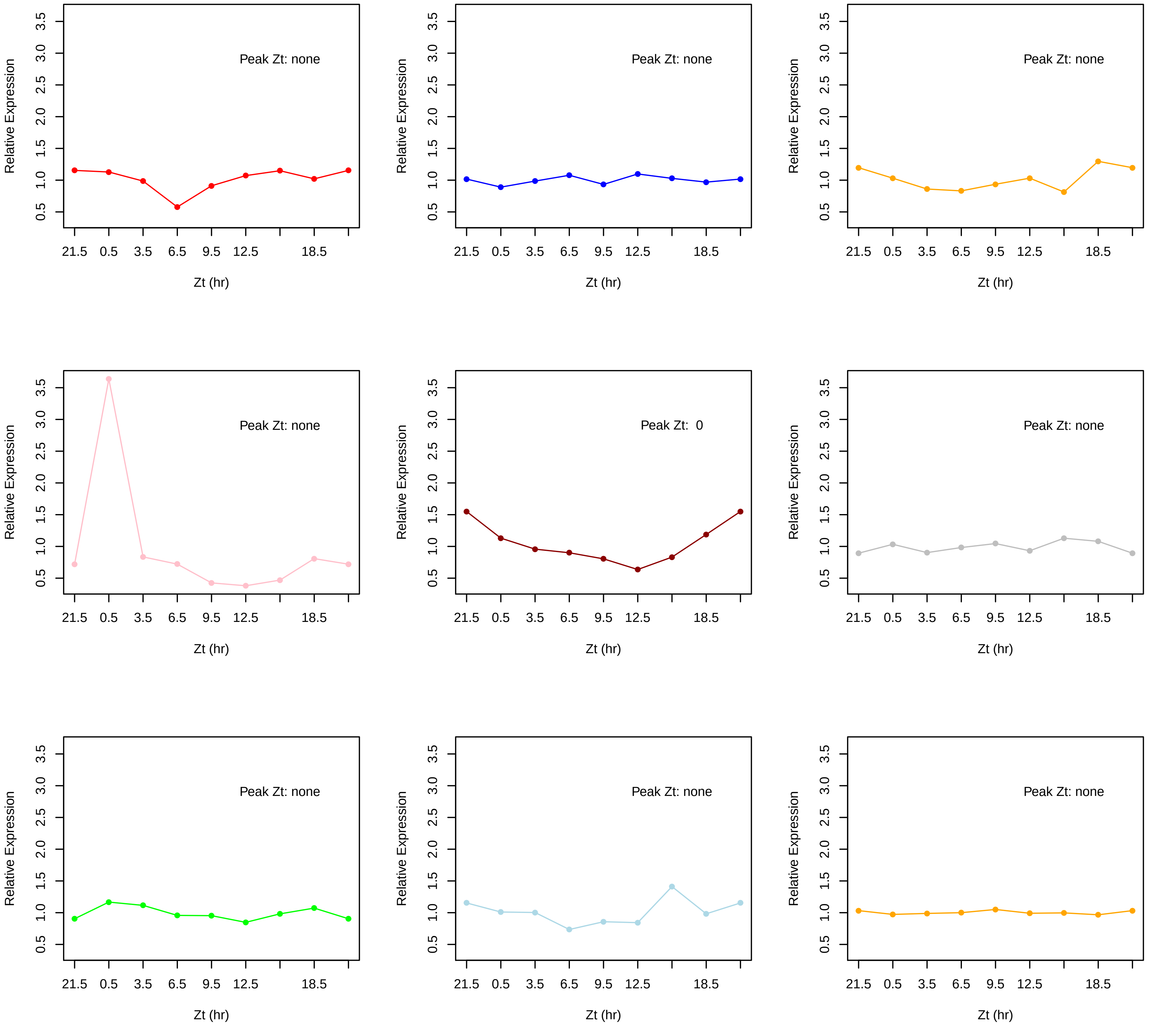

Singeleton: ENSXMAG00000004948 cdh5

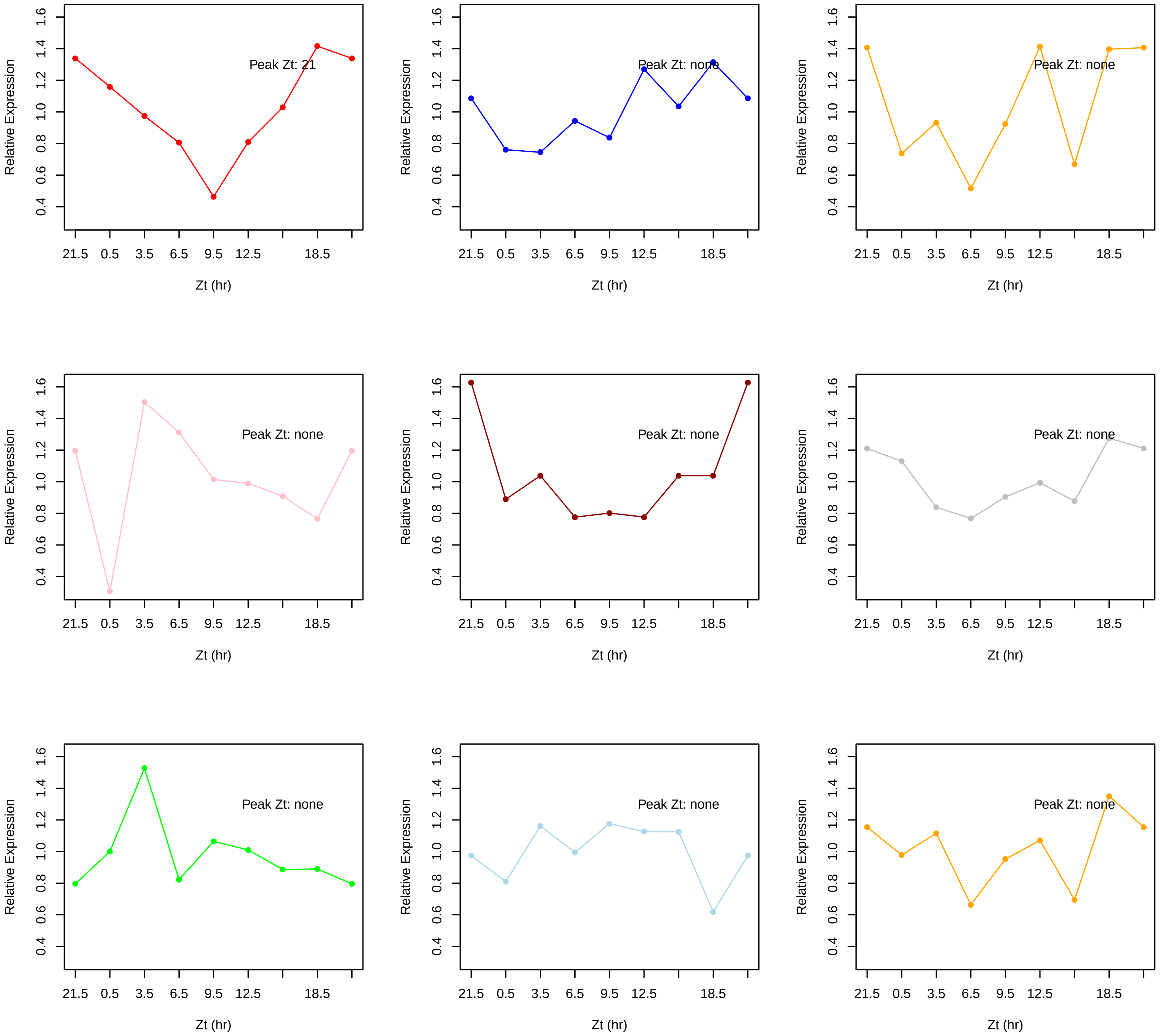

Singeleton: ENSXMAG00000013883 PPFIA1

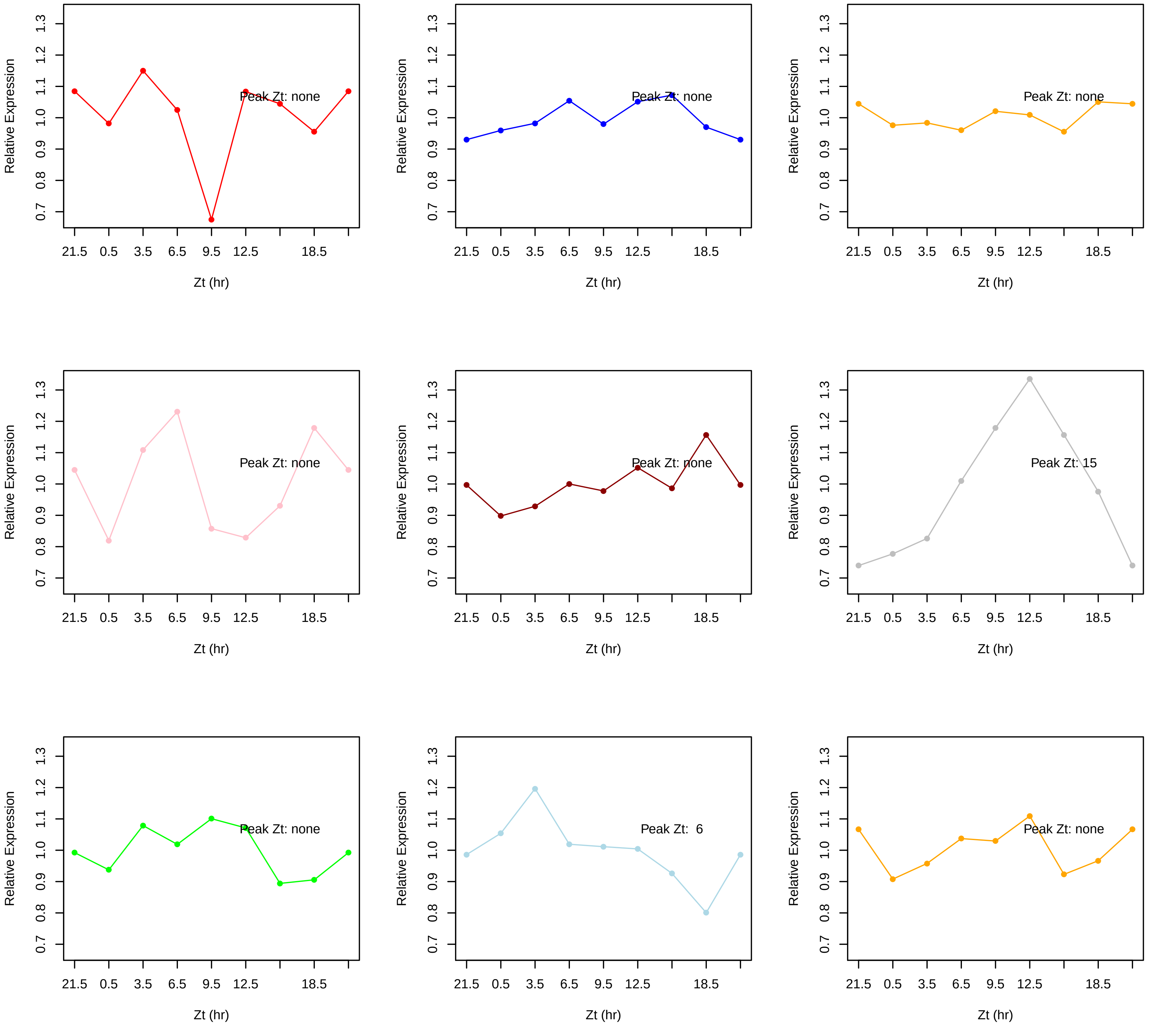

Singeleton: ENSXMAG00000007535 psmb7

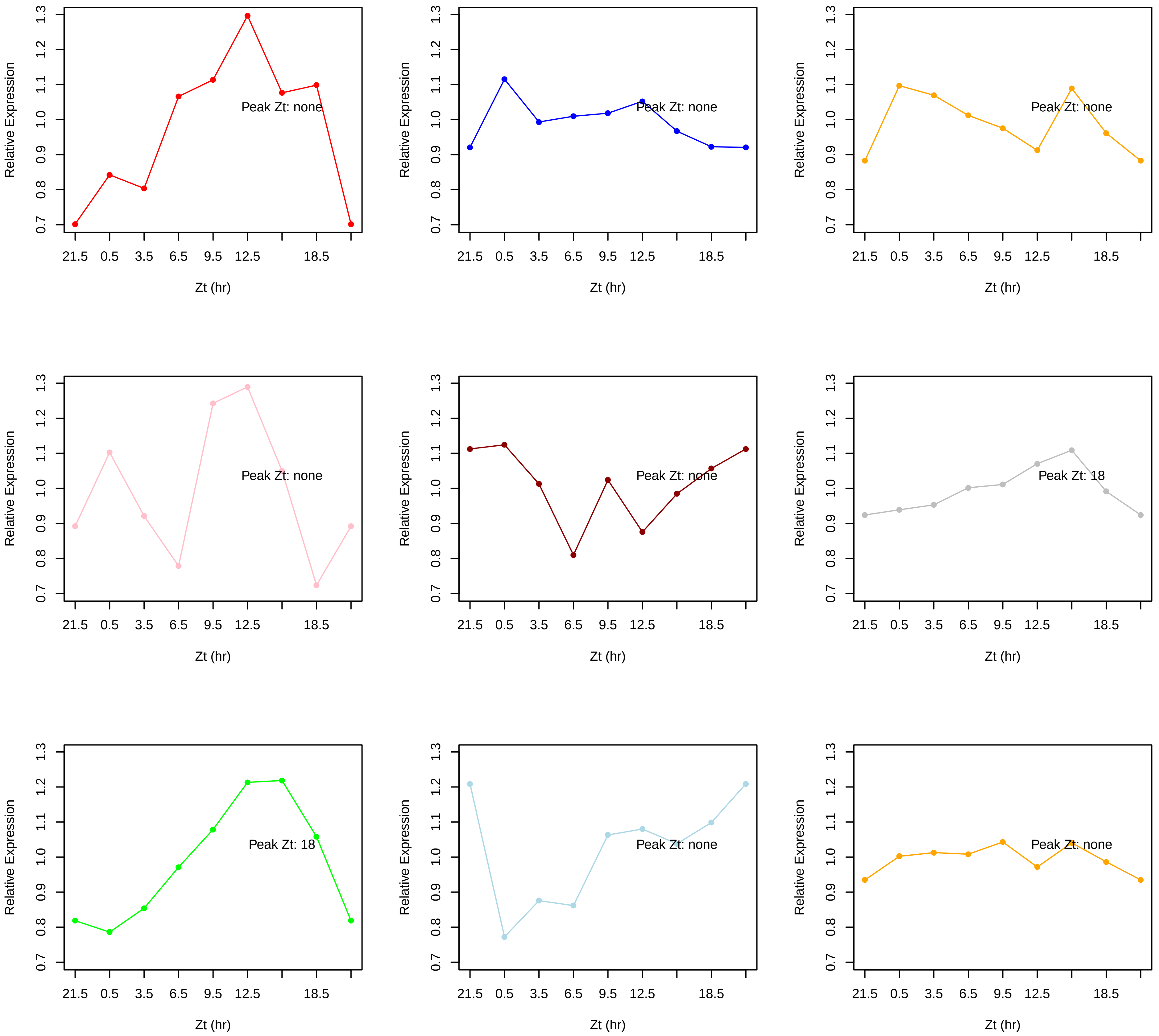

Singleton: ENSXMAG00000002945 pum1

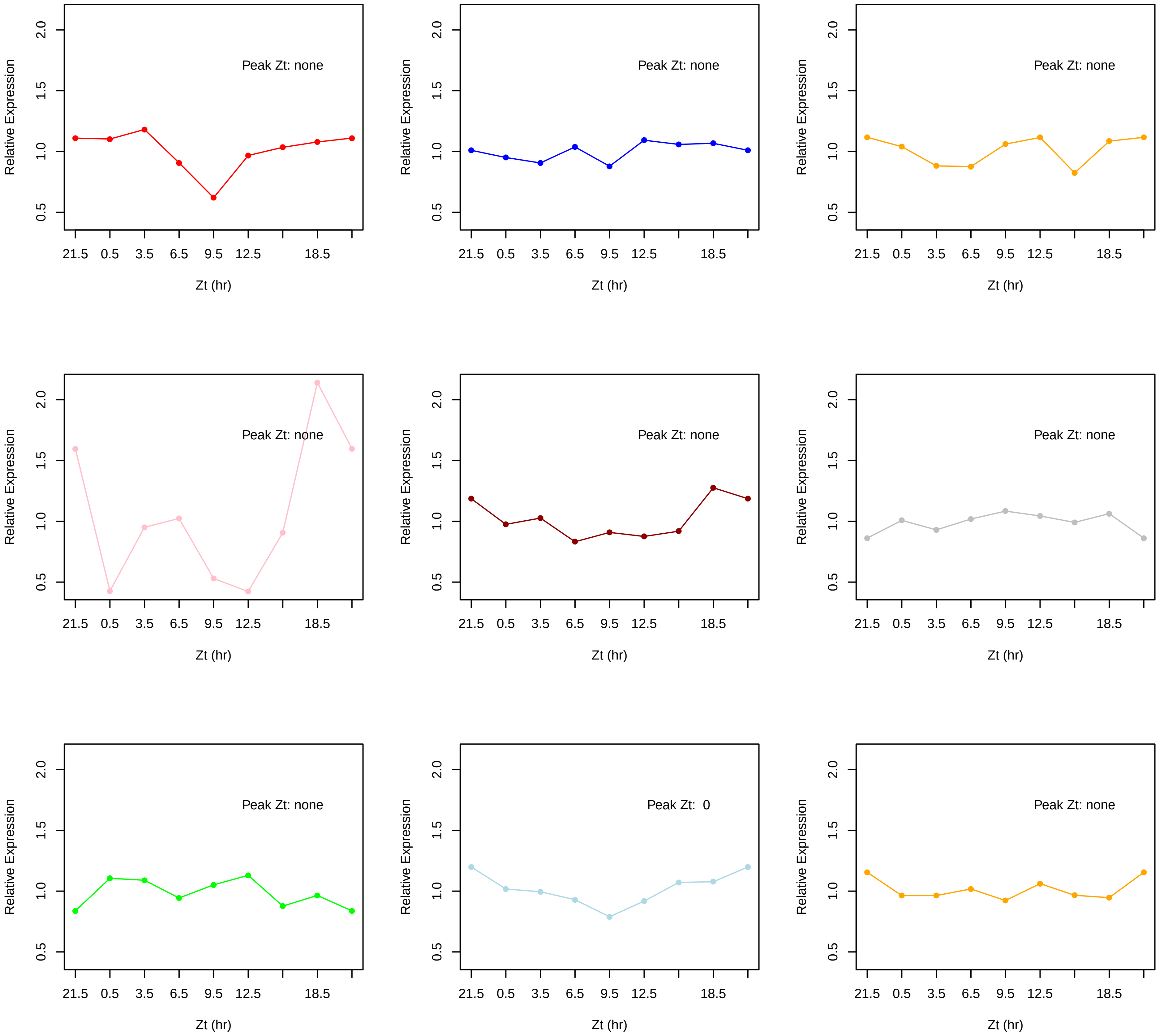

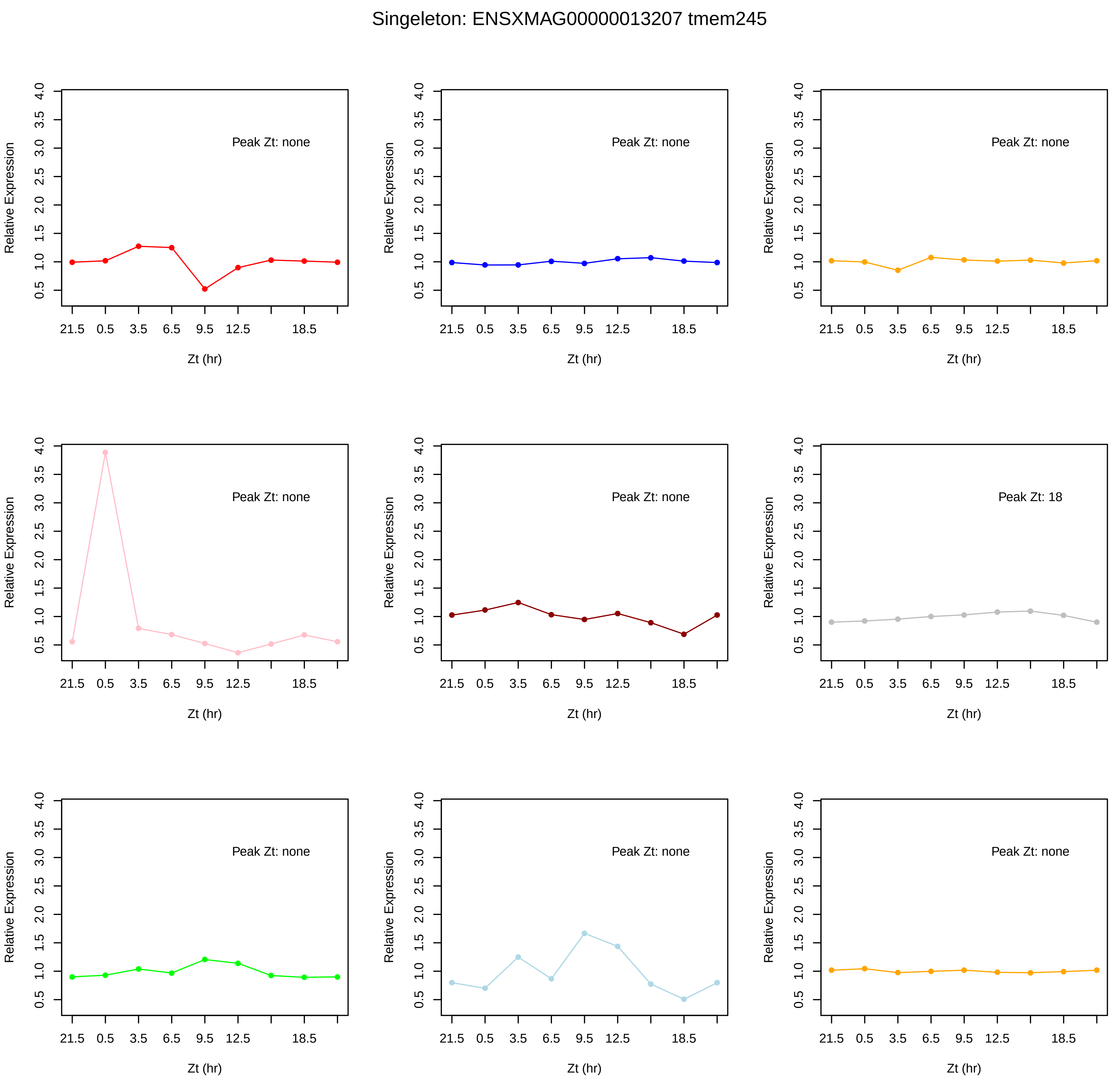

Singeleton: ENSXMAG00000013207 tmem245
Peak Zt: none
Peak Zt: none
Peak Zt: none
Peak Zt: none
Peak Zt: none
Peak Zt: 18
Peak Zt: none
Peak Zt: none
Peak Zt: none
Relative Expression
Zt (hr)
21.5 0.5 3.5 6.5 9.5 12.5 18.5
0.5 1.0 1.5 2.0 2.5 3.0 3.5 4.0

Singeleton: ENSXMAG00000004296 PCMTD2

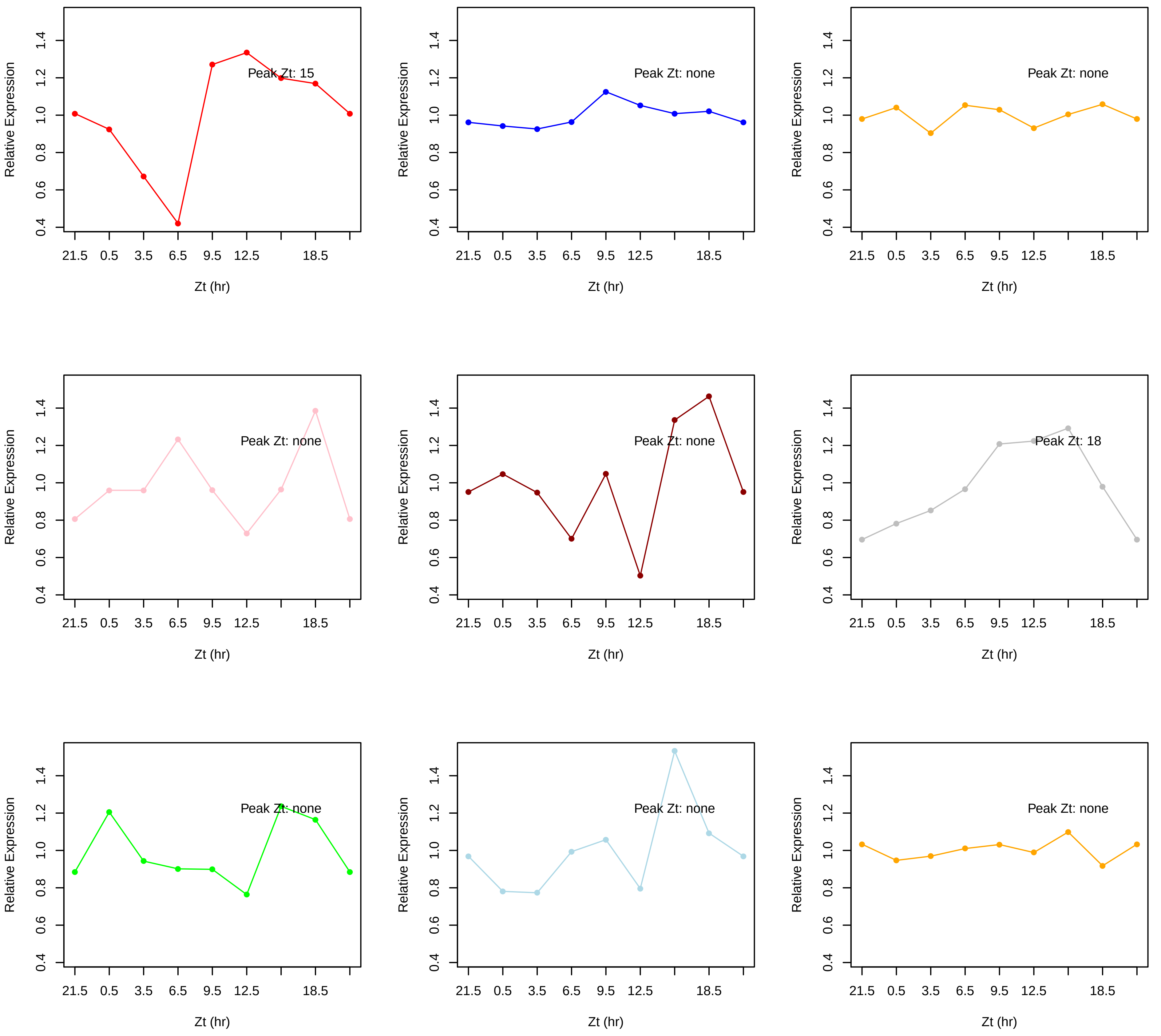

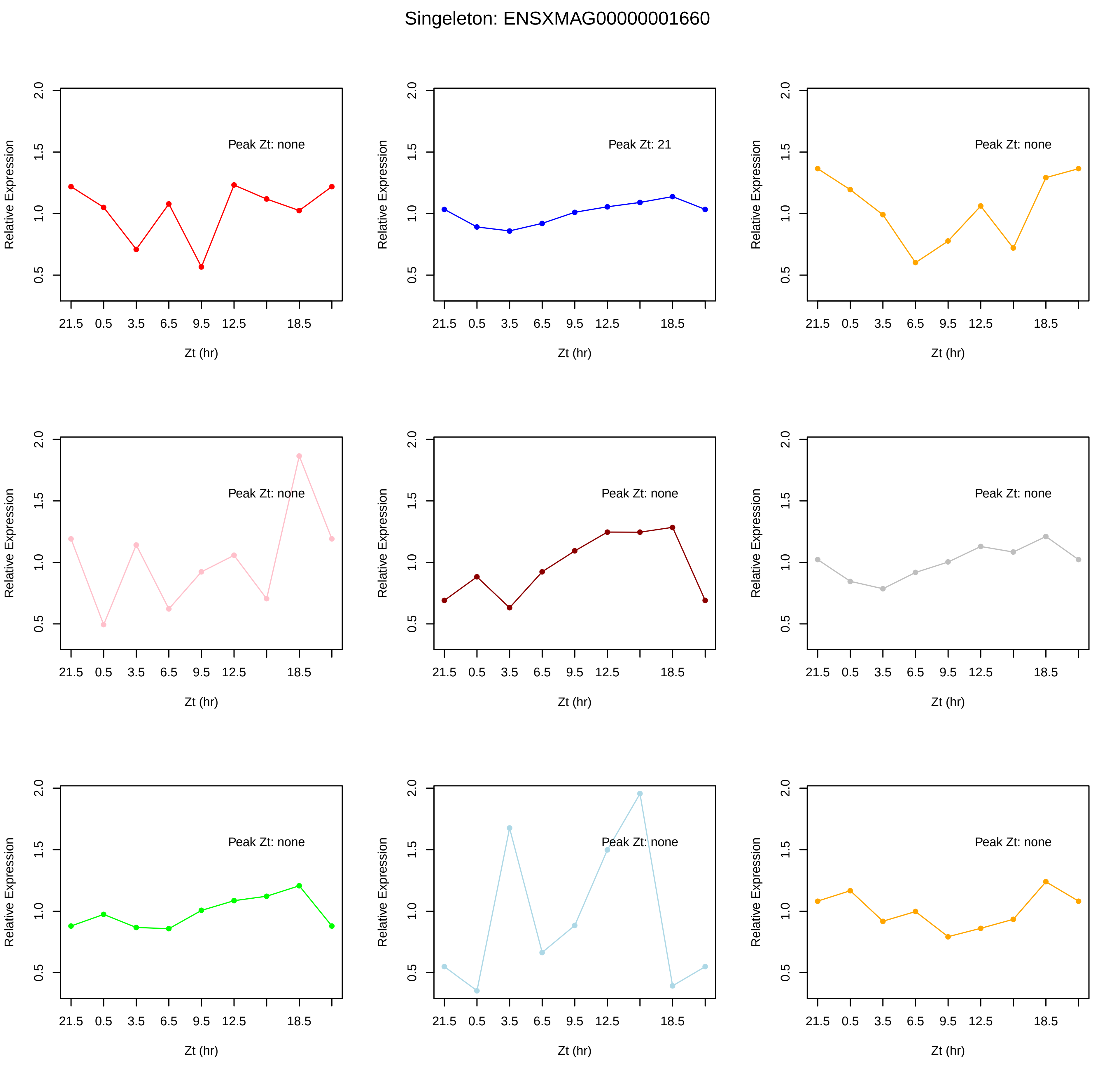
Singeleton: ENSXMAG00000001660
Peak Zt: none
Peak Zt: 21
Peak Zt: none
Peak Zt: none
Peak Zt: none
Peak Zt: none
Peak Zt: none
Peak Zt: none
Peak Zt: none
Relative Expression
Zt (hr)
0.5
1.0
1.5
2.0
21.5
0.5
3.5
6.5
9.5
12.5
18.5

Singleton: ENSXMAG00000028018 smx5

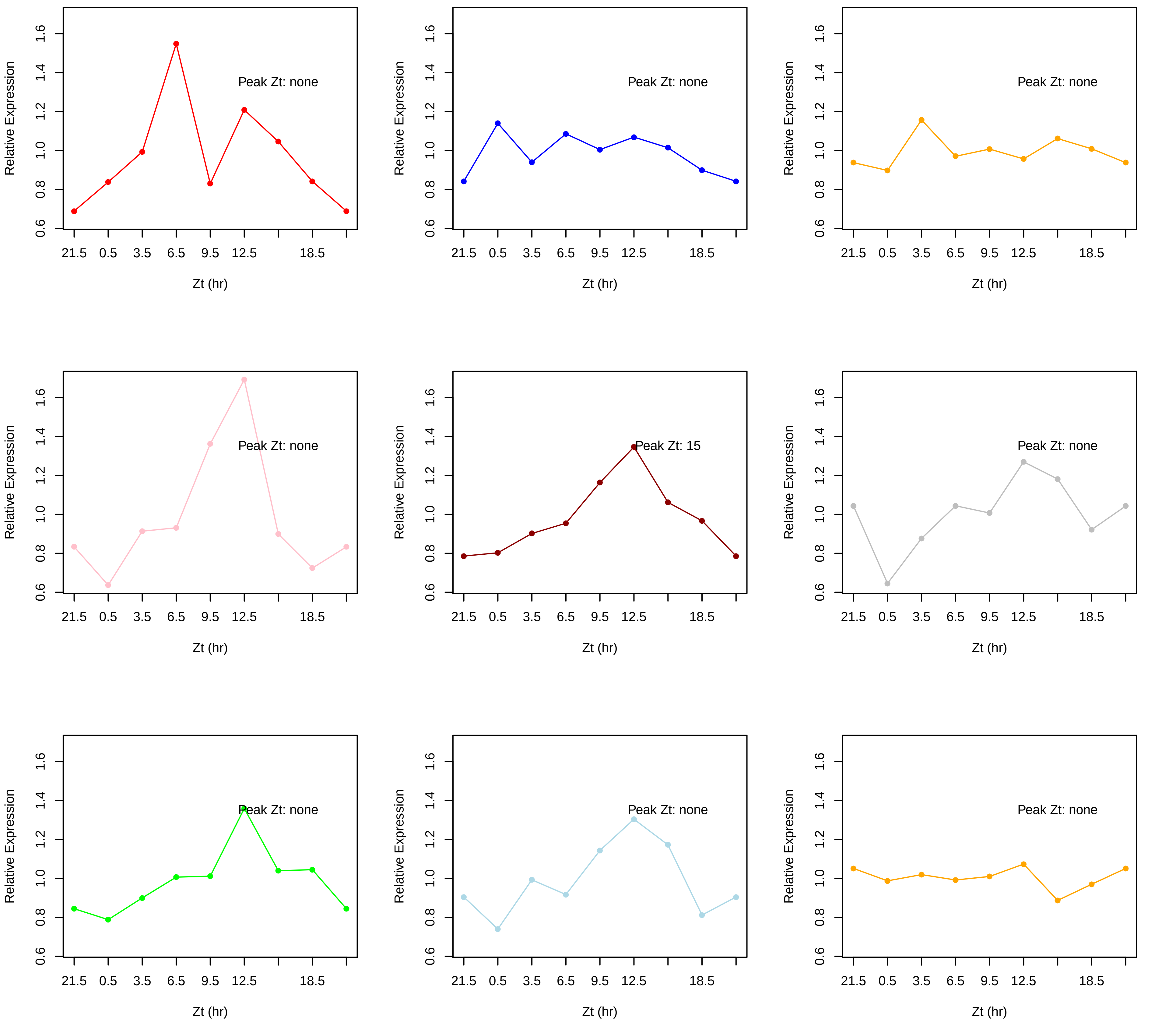

Singeleton: ENSXMAG00000020104 tysnd1

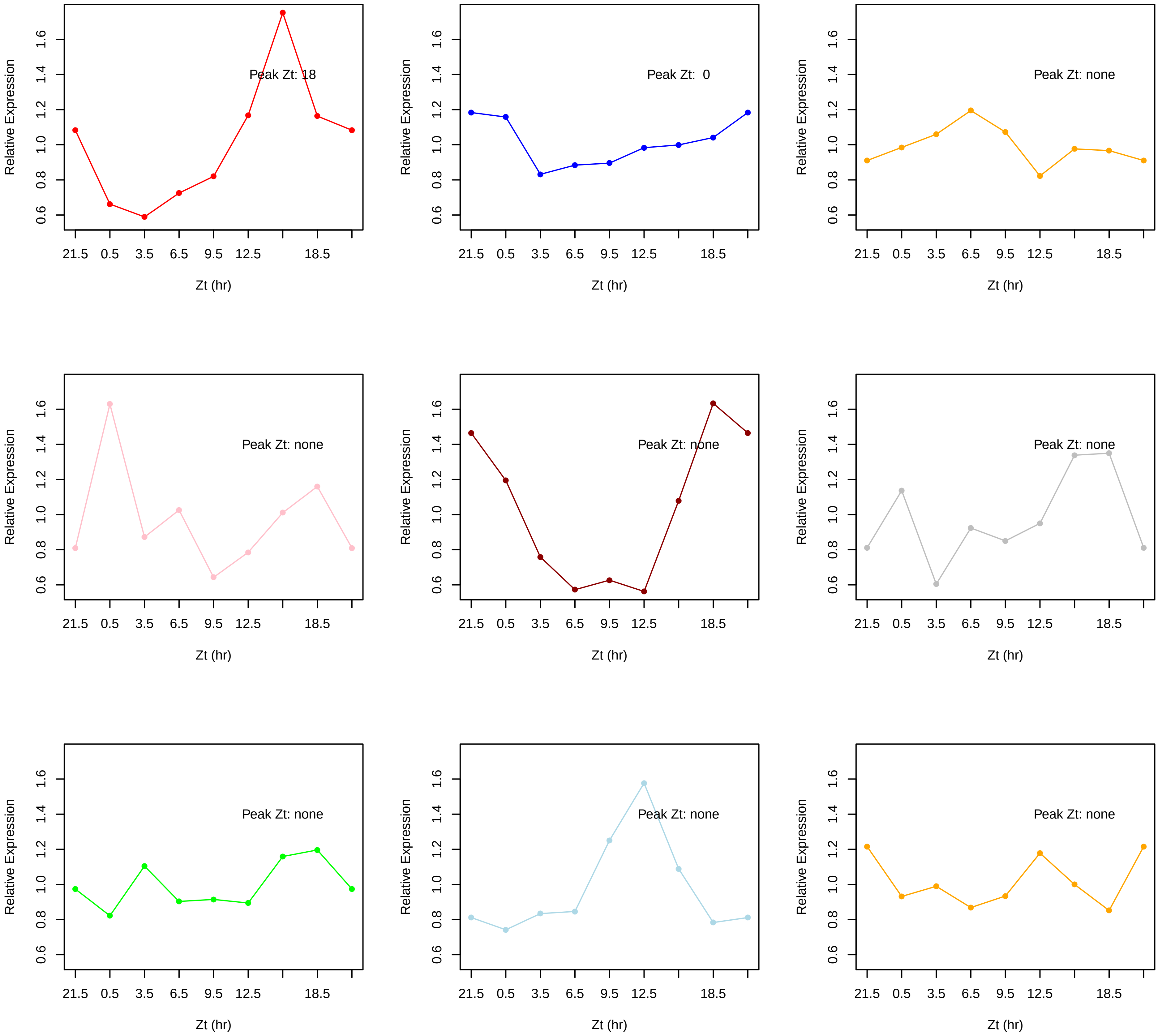

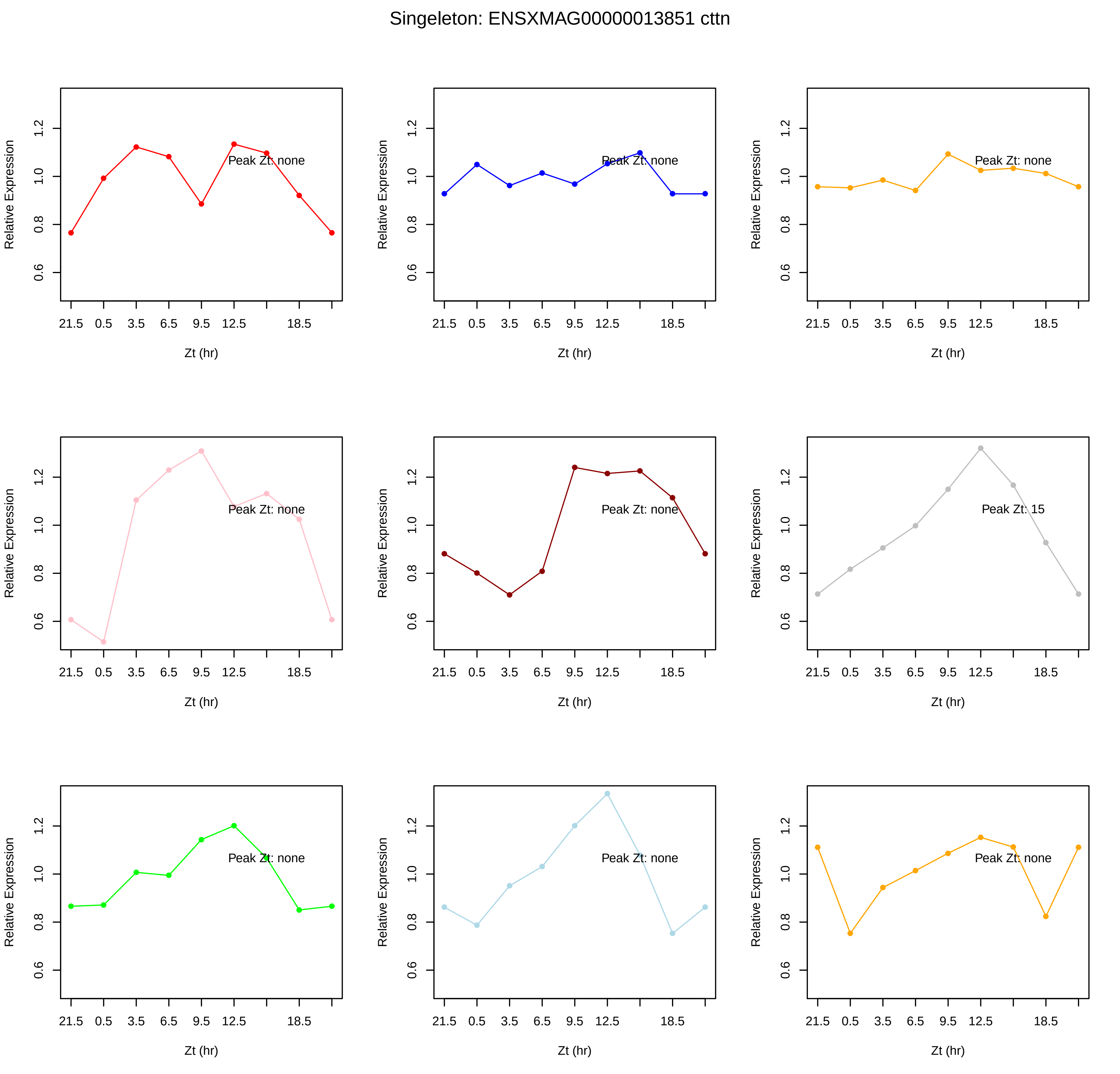
Singeleton: ENSXMAG00000013851 cttn
Peak Zt: none
Peak Zt: none
Peak Zt: none
Peak Zt: none
Peak Zt: none
Peak Zt: 15
Peak Zt: none
Peak Zt: none
Peak Zt: none
Relative Expression
Zt (hr)

Singeleton: ENSXMAG00000029375 pgp

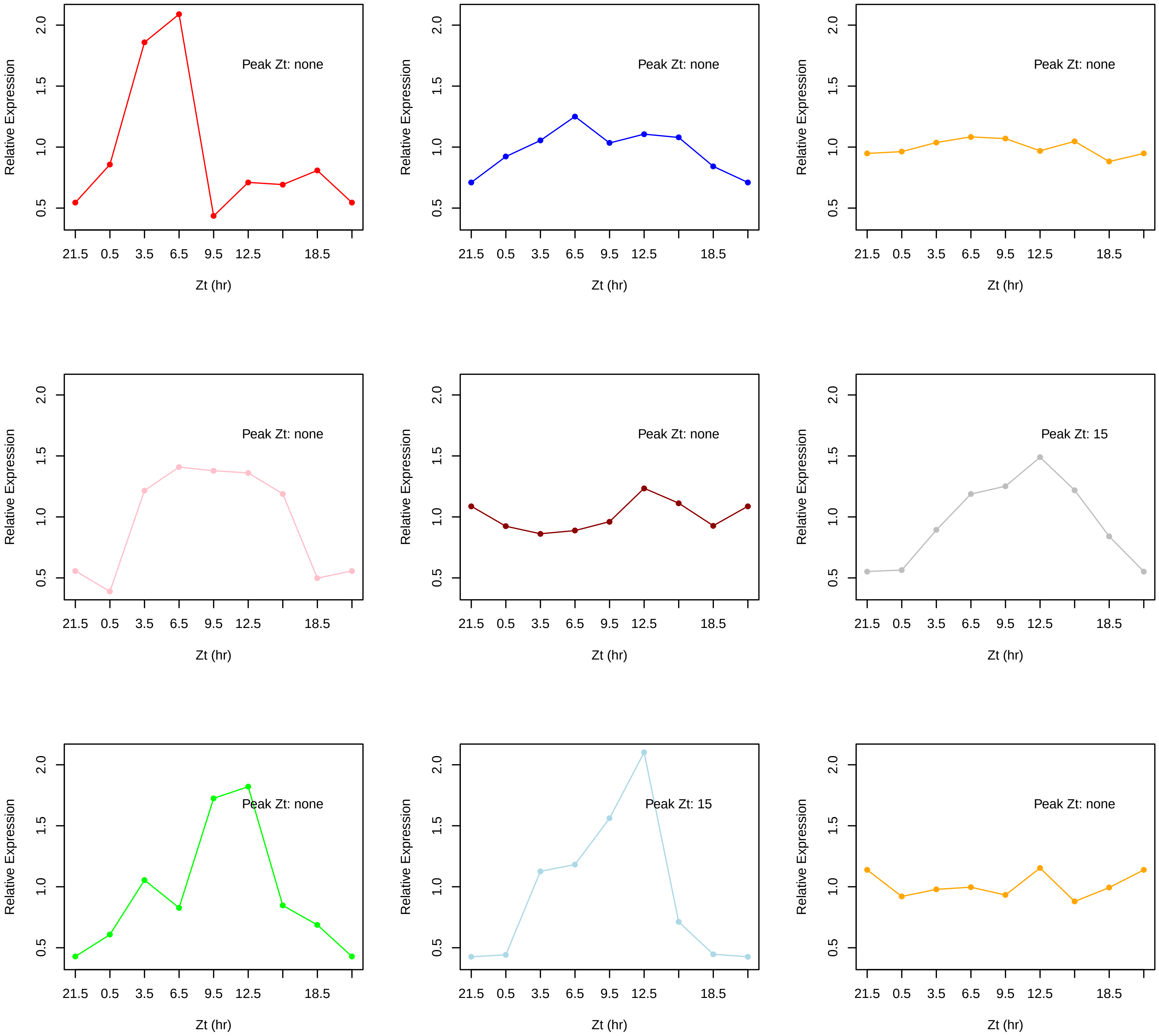

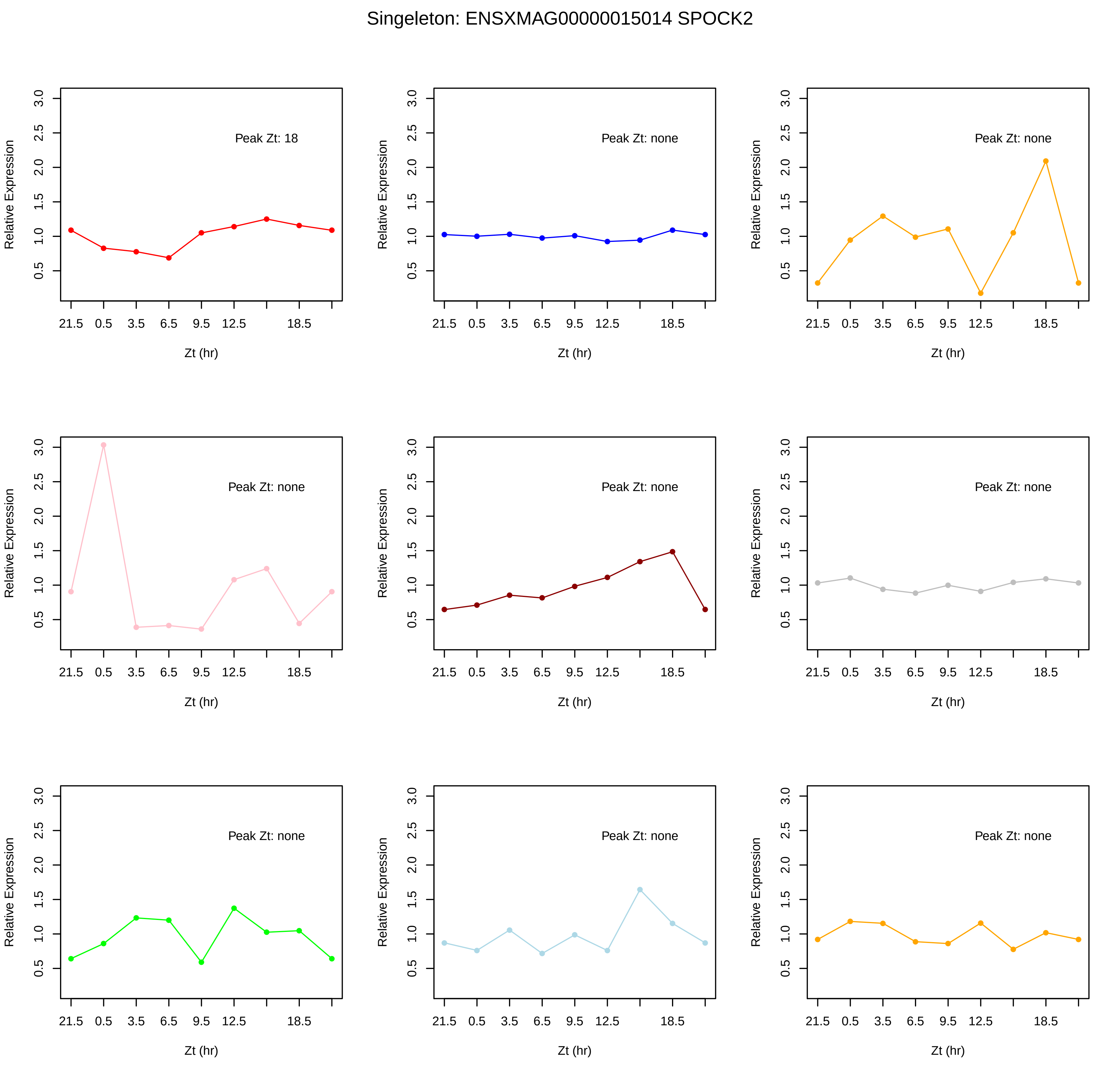
Singeleton: ENSXMAG00000015014 SPOCK2
Peak Zt: 18
Peak Zt: none
Peak Zt: none
Peak Zt: none
Peak Zt: none
Peak Zt: none
Peak Zt: none
Peak Zt: none
Peak Zt: none
Relative Expression
Zt (hr)
21.5
0.5
3.5
6.5
9.5
12.5
18.5
0.5
1.0
1.5
2.0
2.5
3.0

Singeleton: ENSXMAG00000017517 psmd9

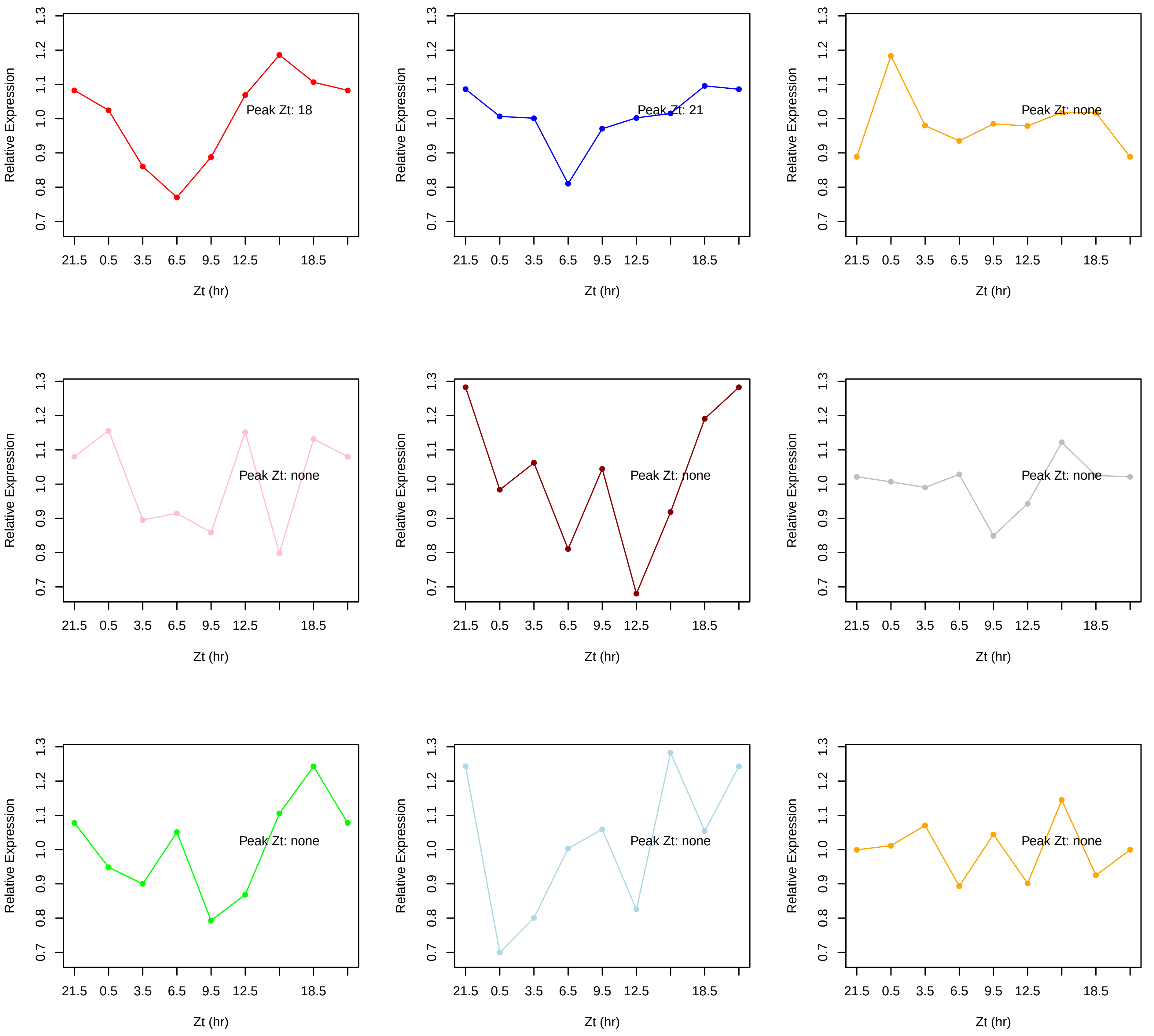

Singeleton: ENSXMAG00000013208 galnt1

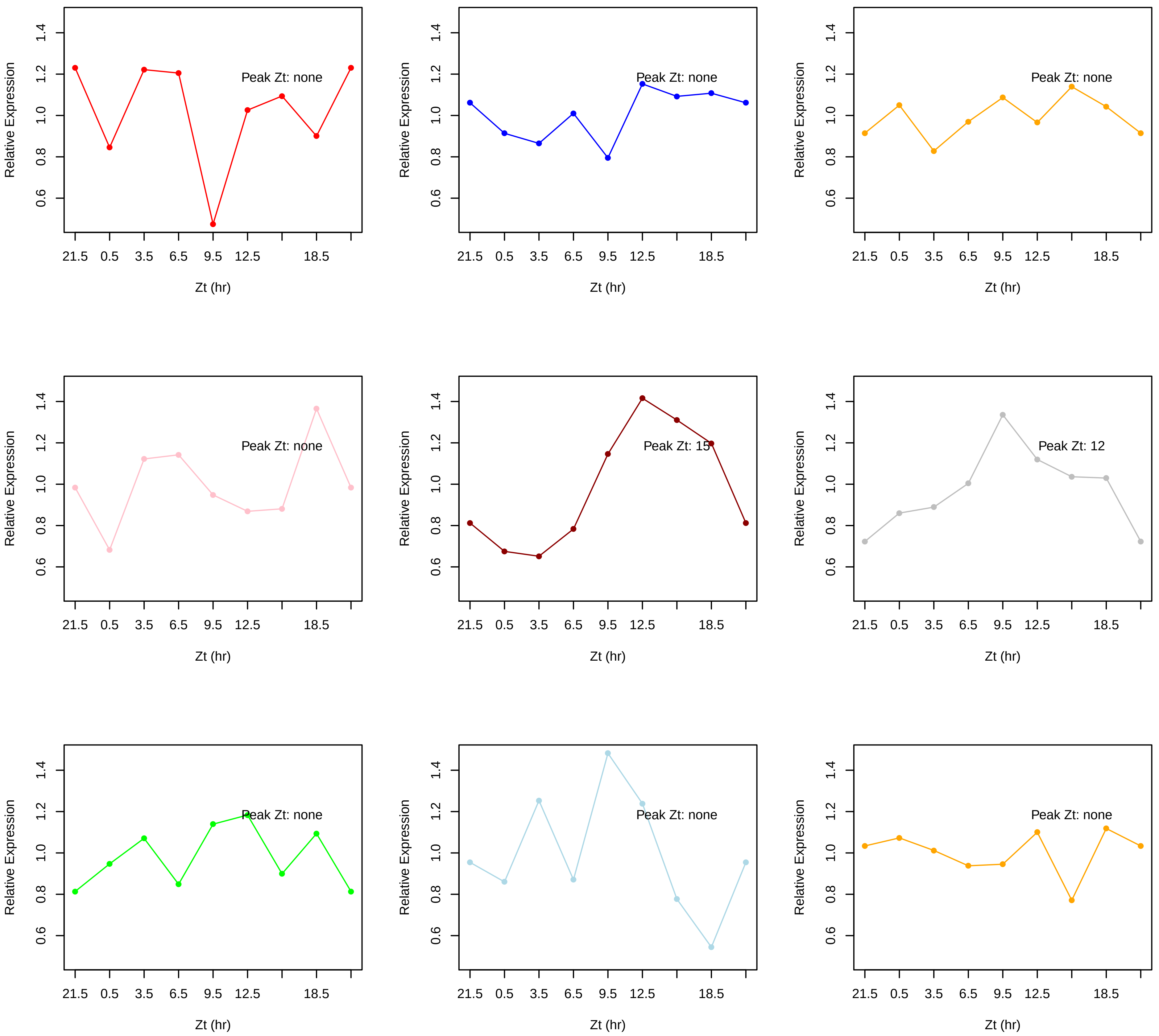

Singeleton: ENSXMAG00000012088 rapgef3

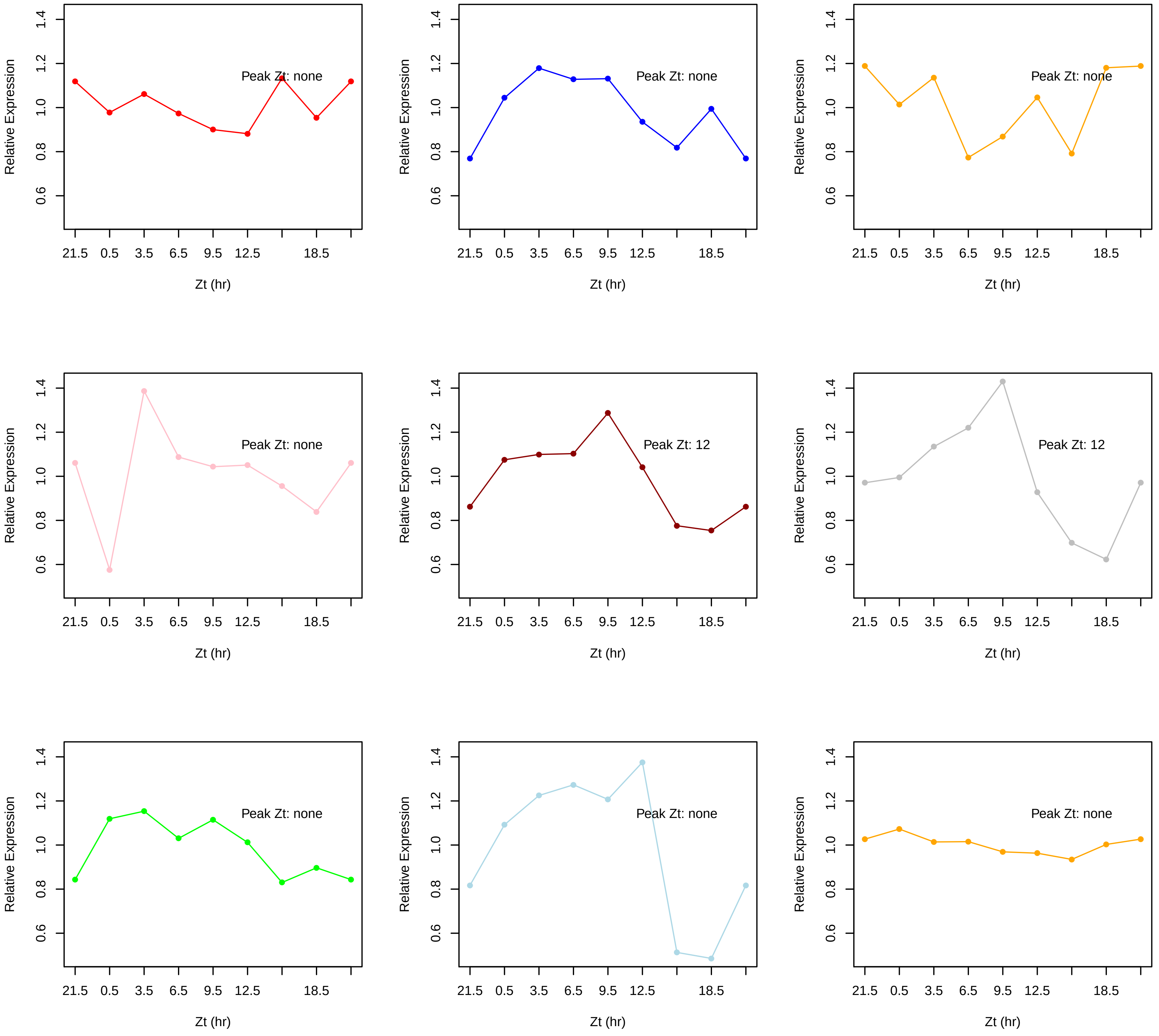

Singleton: ENSXMAG00000003908 lhx2b

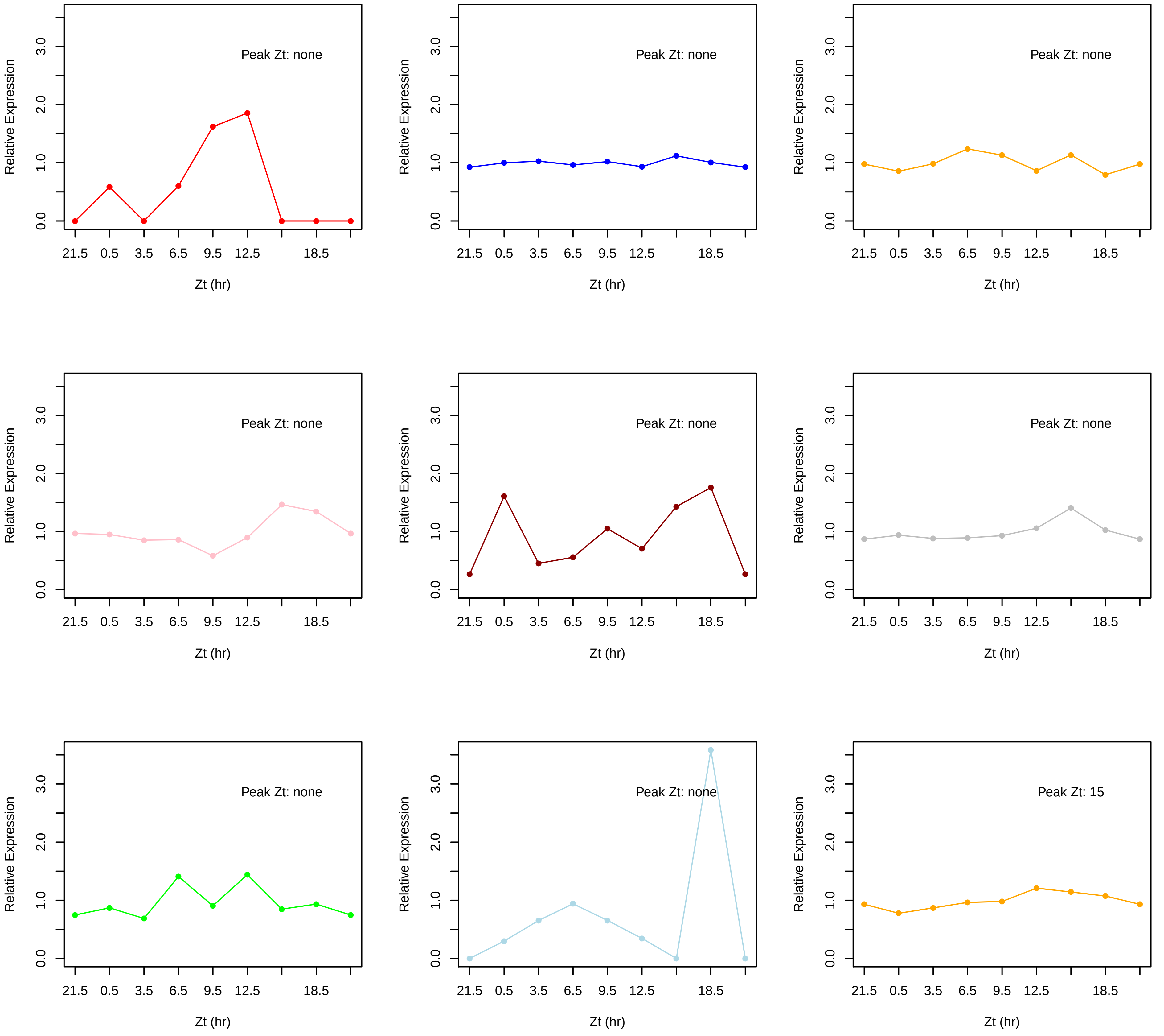

Singeleton: ENSXMAG00000002970 snrnp40

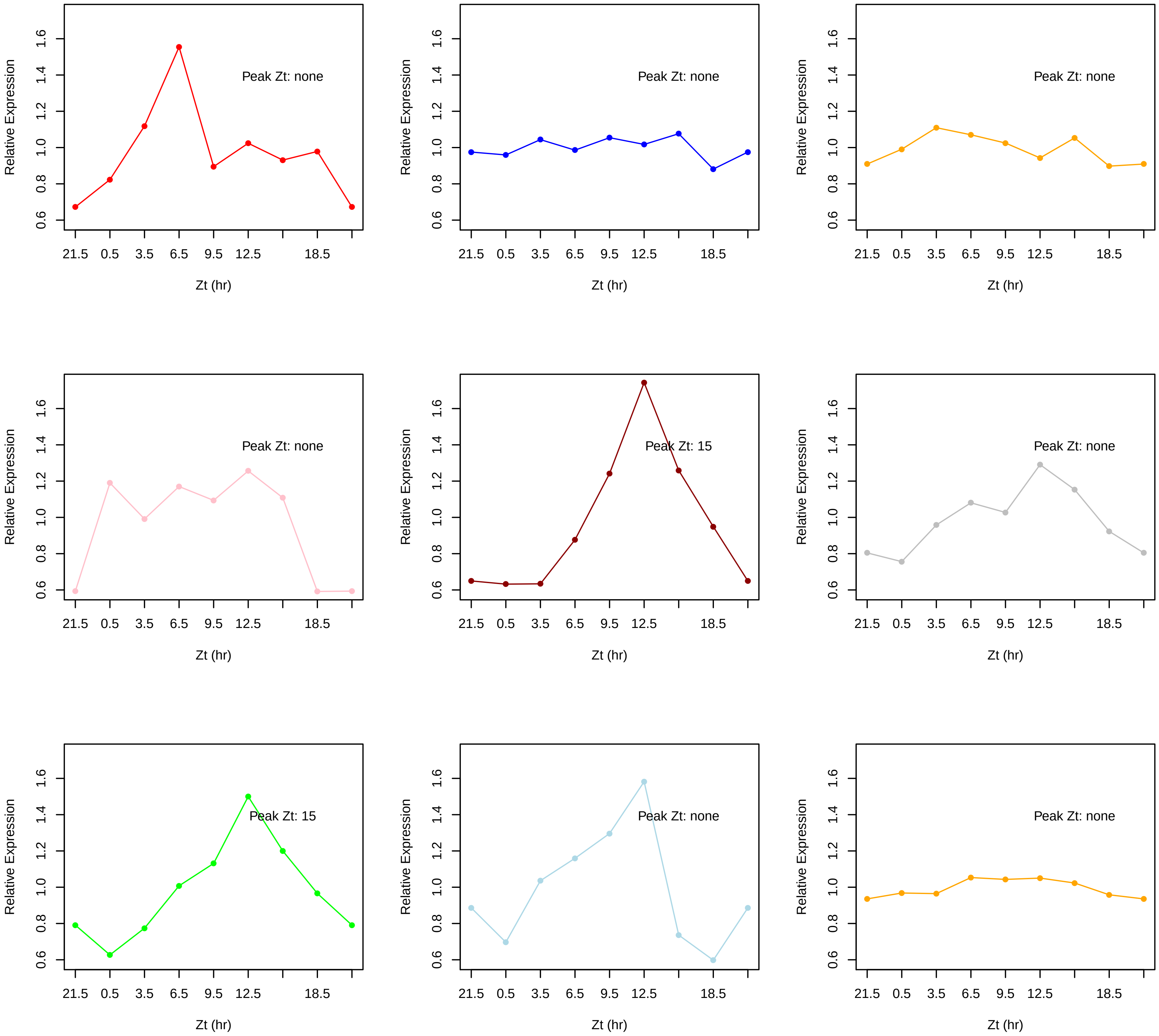

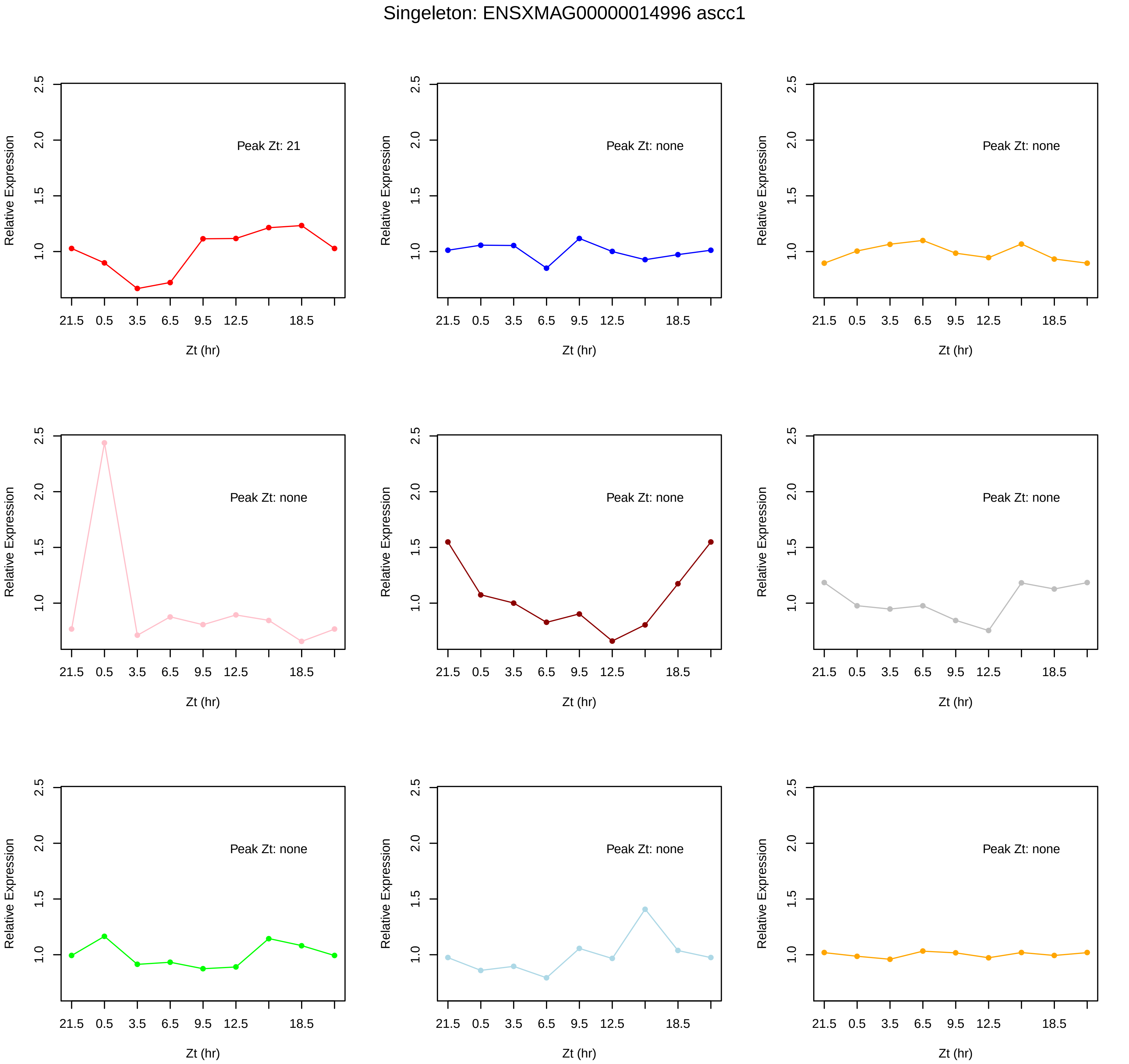
Singleton: ENSXMAG00000014996 ascc1
Peak Zt: 21
Peak Zt: none
Peak Zt: none
Peak Zt: none
Peak Zt: none
Peak Zt: none
Peak Zt: none
Peak Zt: none
Peak Zt: none
Relative Expression
Zt (hr)
21.5
0.5
3.5
6.5
9.5
12.5
18.5
1.0
1.5
2.0
2.5

Singeleton: ENSXMAG00000014496 ino80c

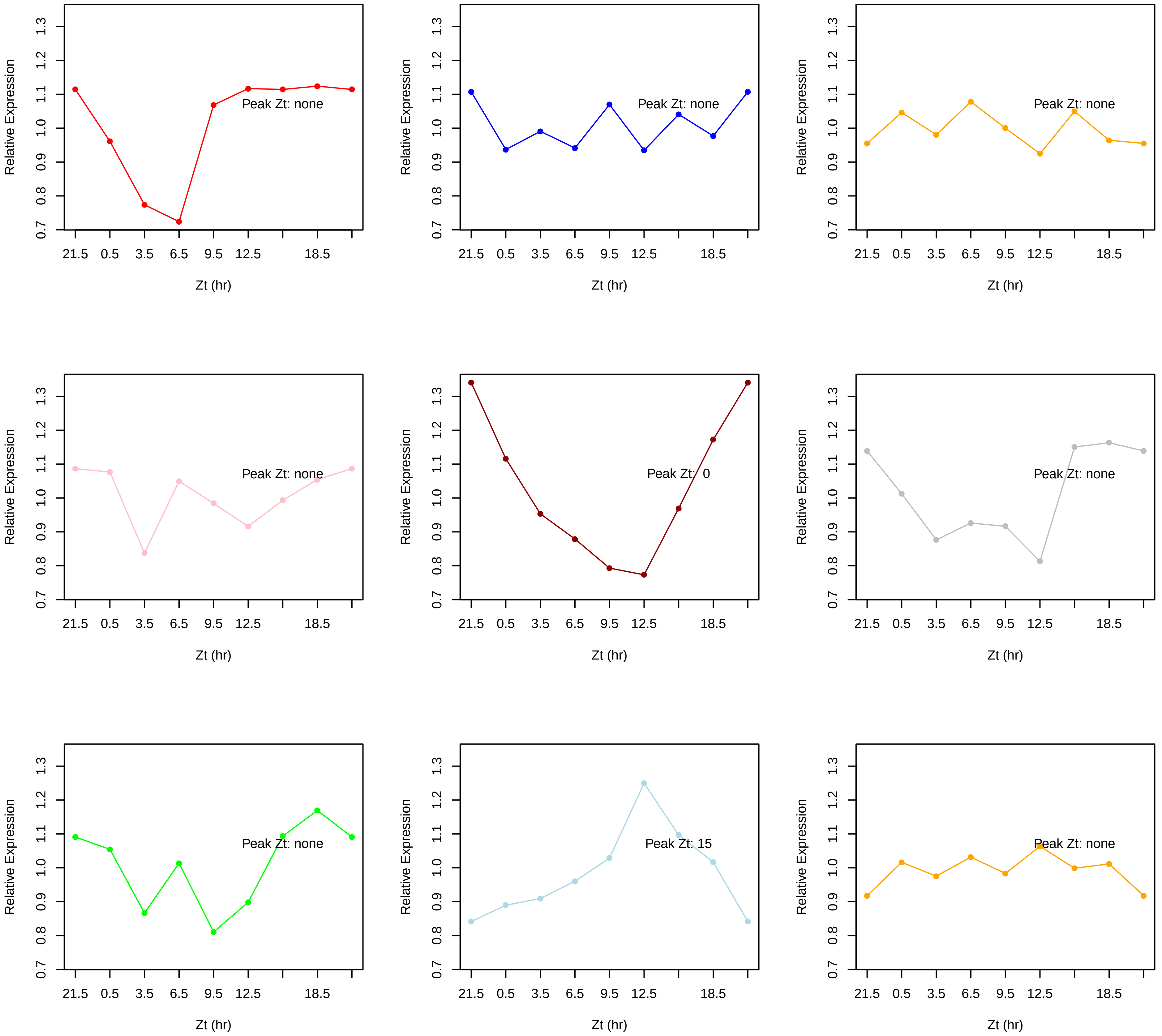

Singeleton: ENSXMAG00000026682 picalmb

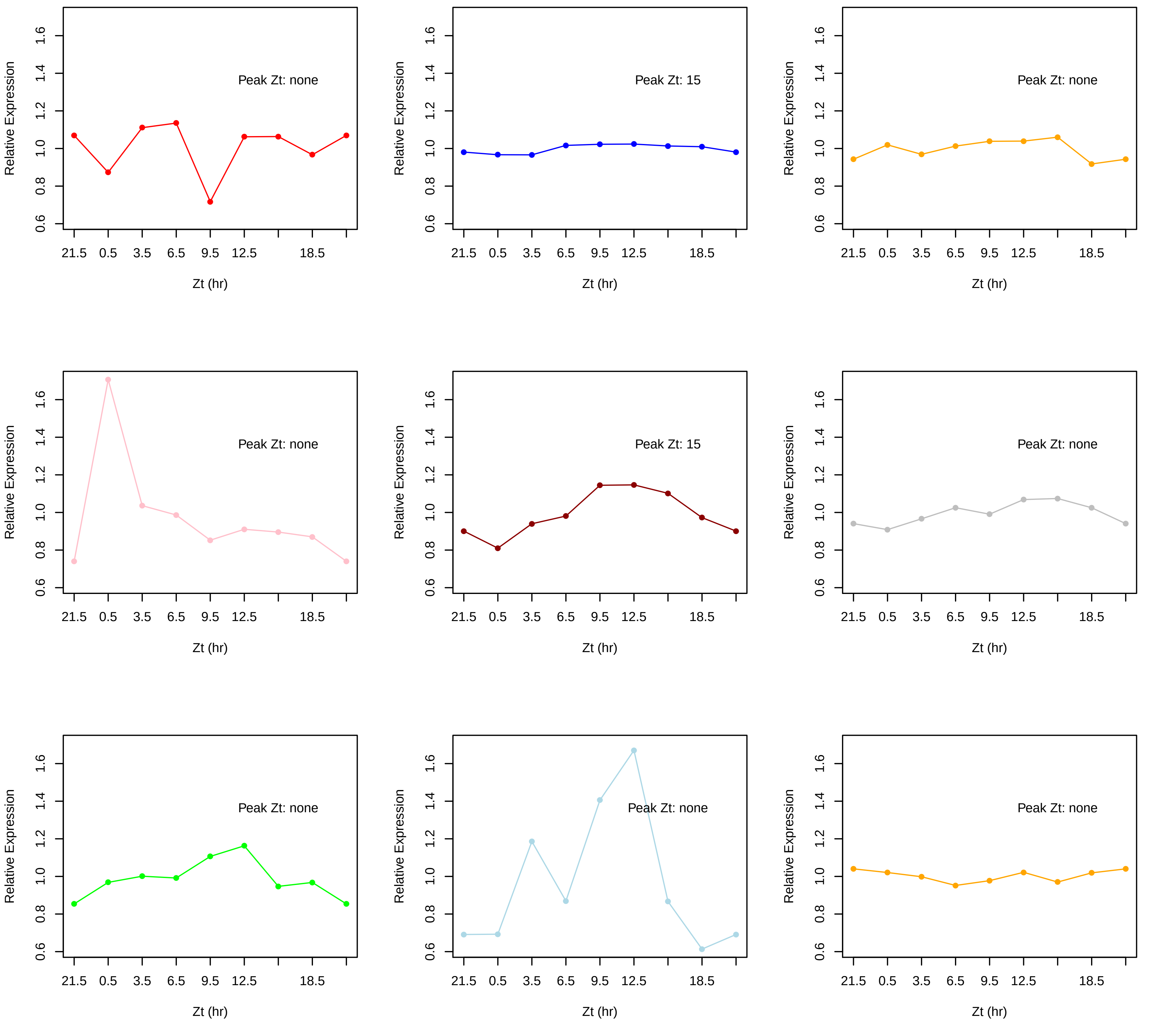

Singeleton: ENSXMAG00000009483 wrap73

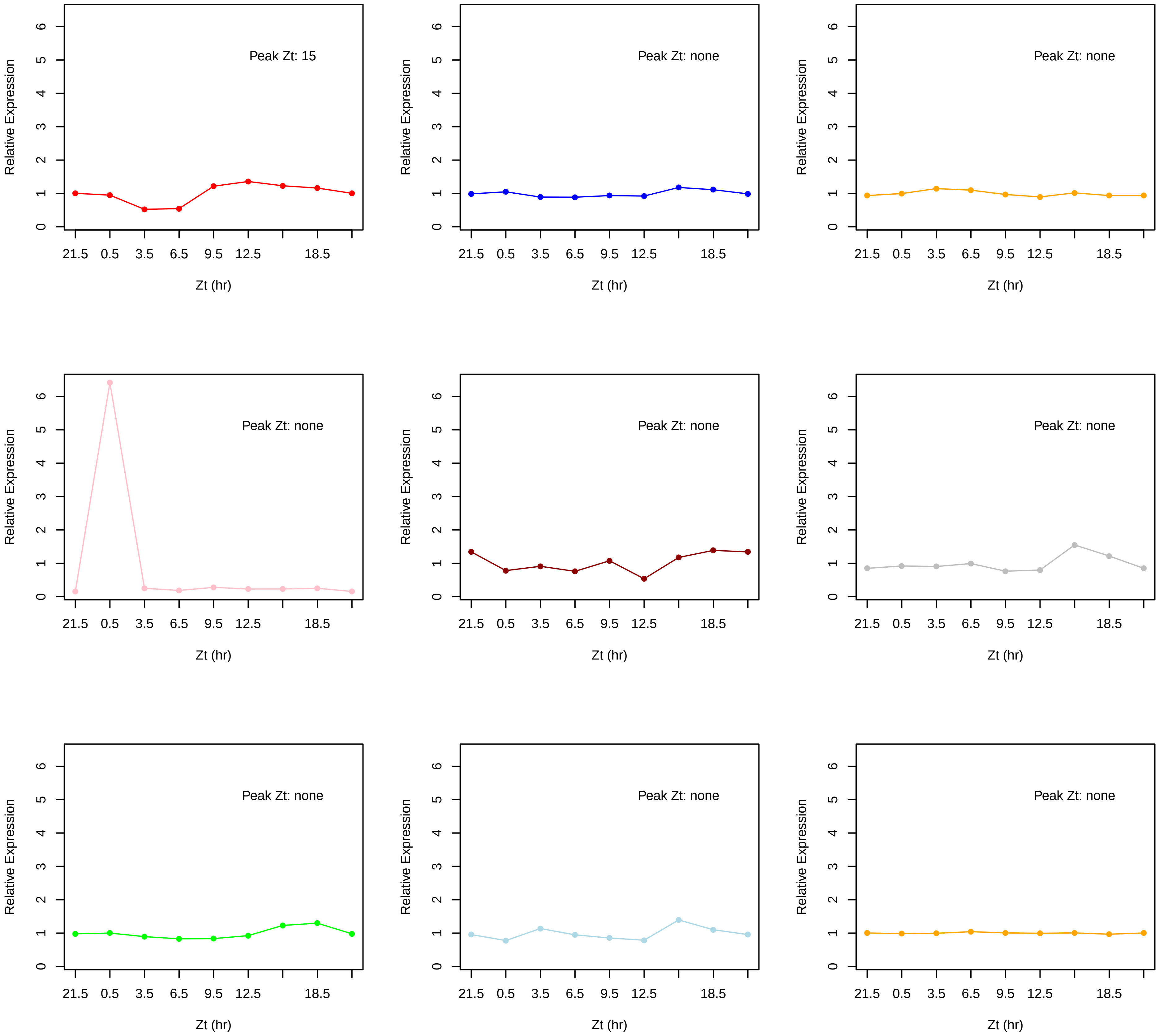

Singeleton: ENSXMAG00000020299 tpst1

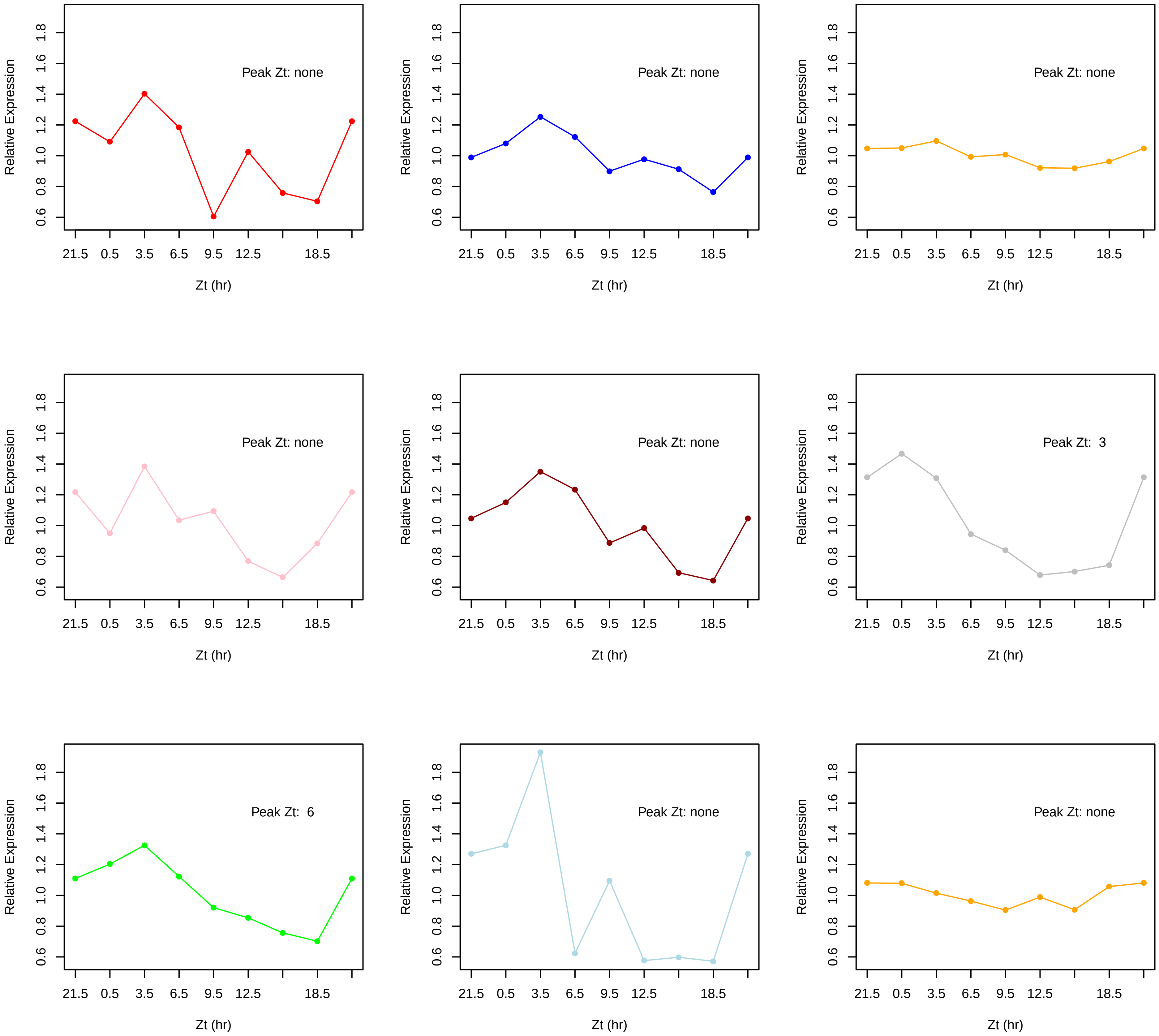

Singeleton: ENSXMAG00000011890 abcc12

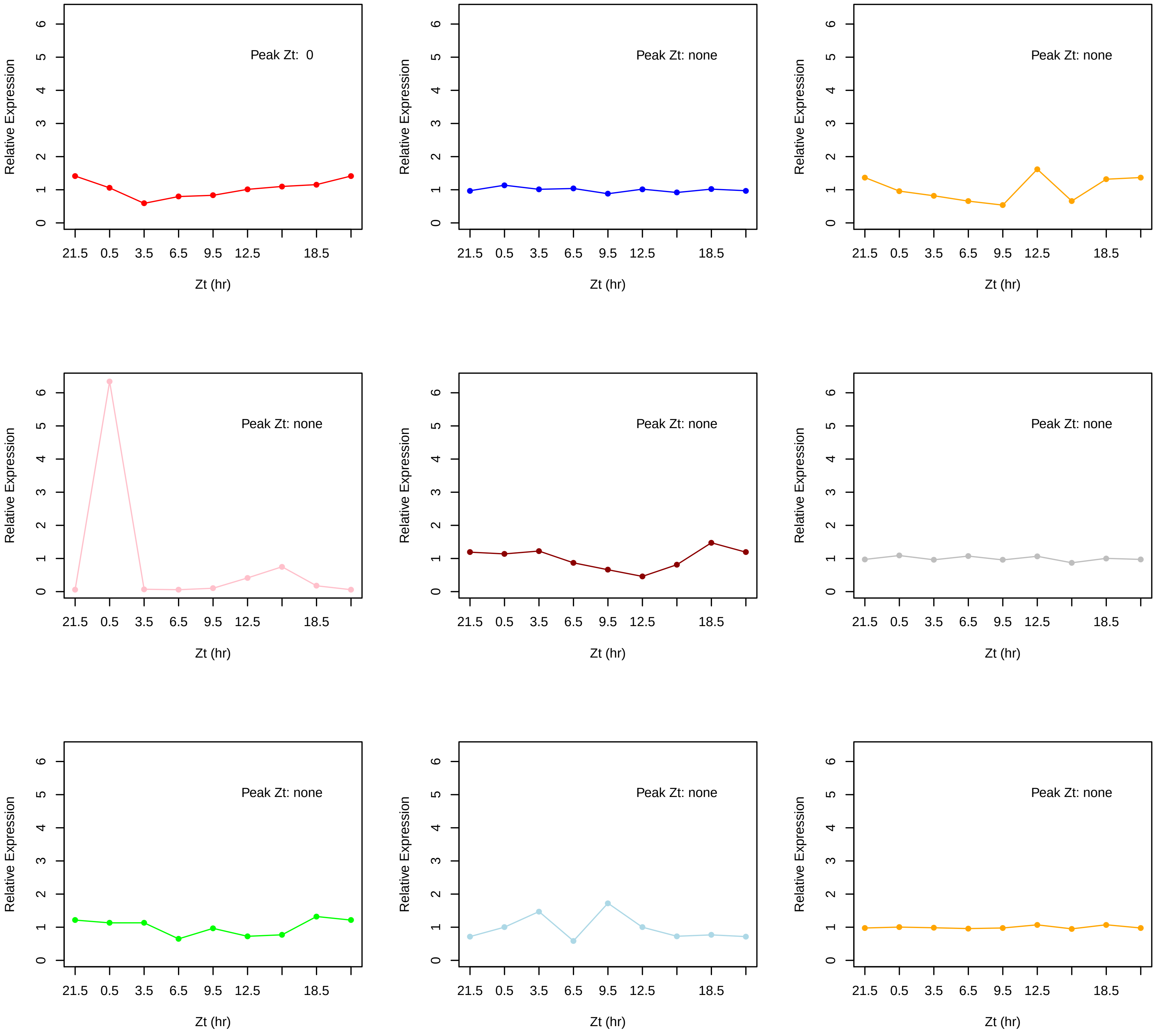

Singeleton: ENSXMAG00000022222 crcp

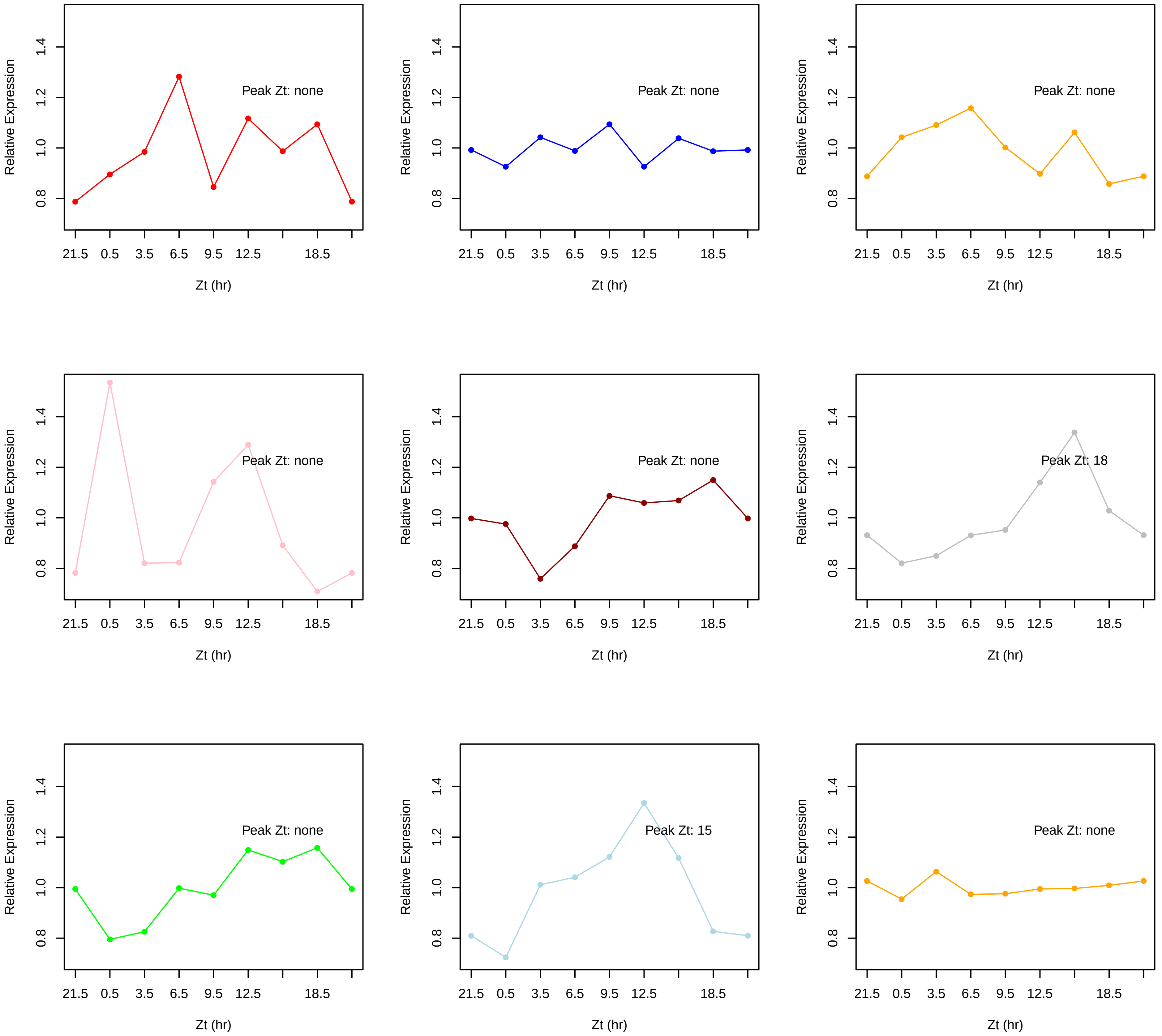

Singleton: ENSXMAG00000014422 glipr2l

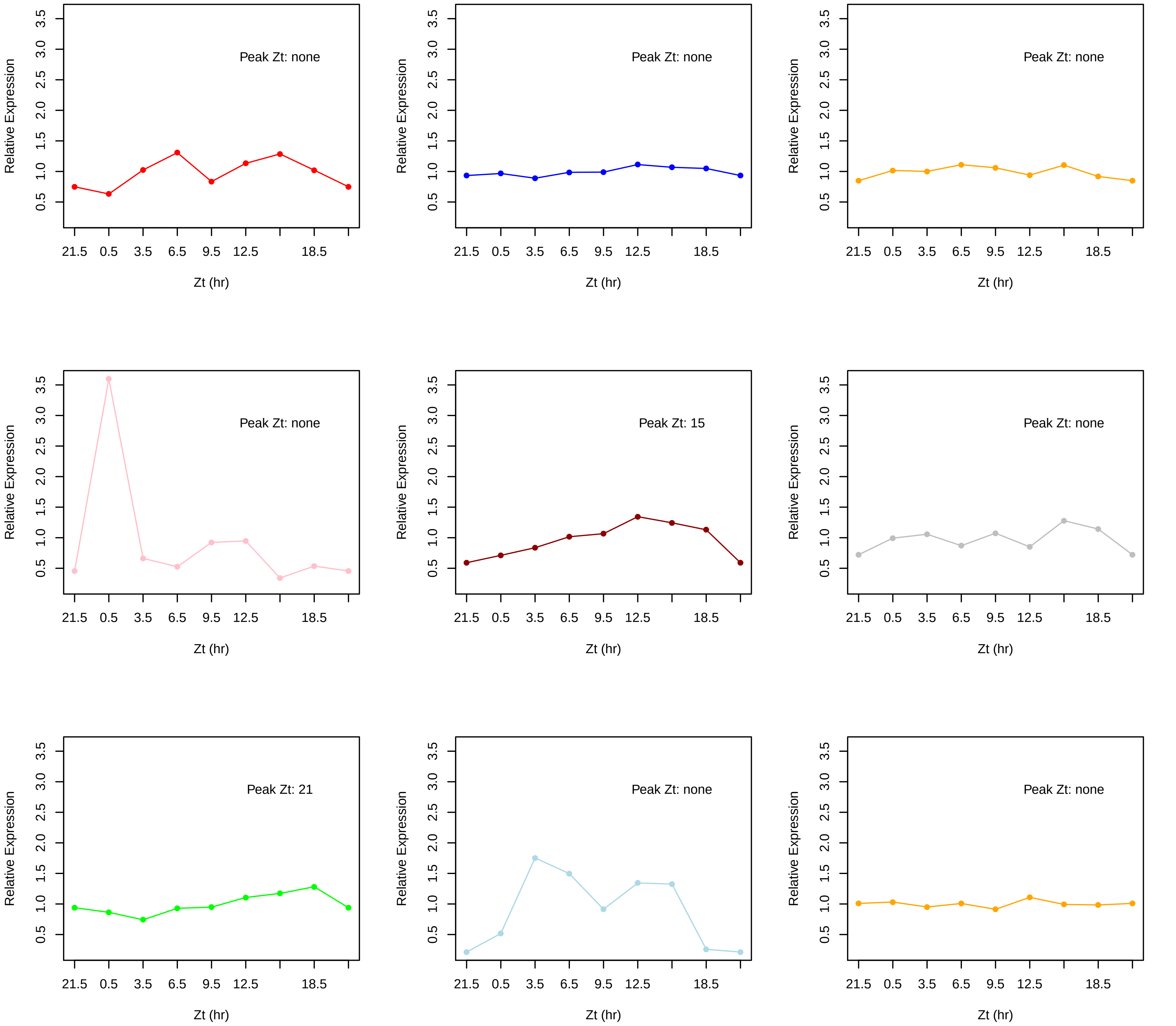

Singeleton: ENSXMAG00000024669 CNMD

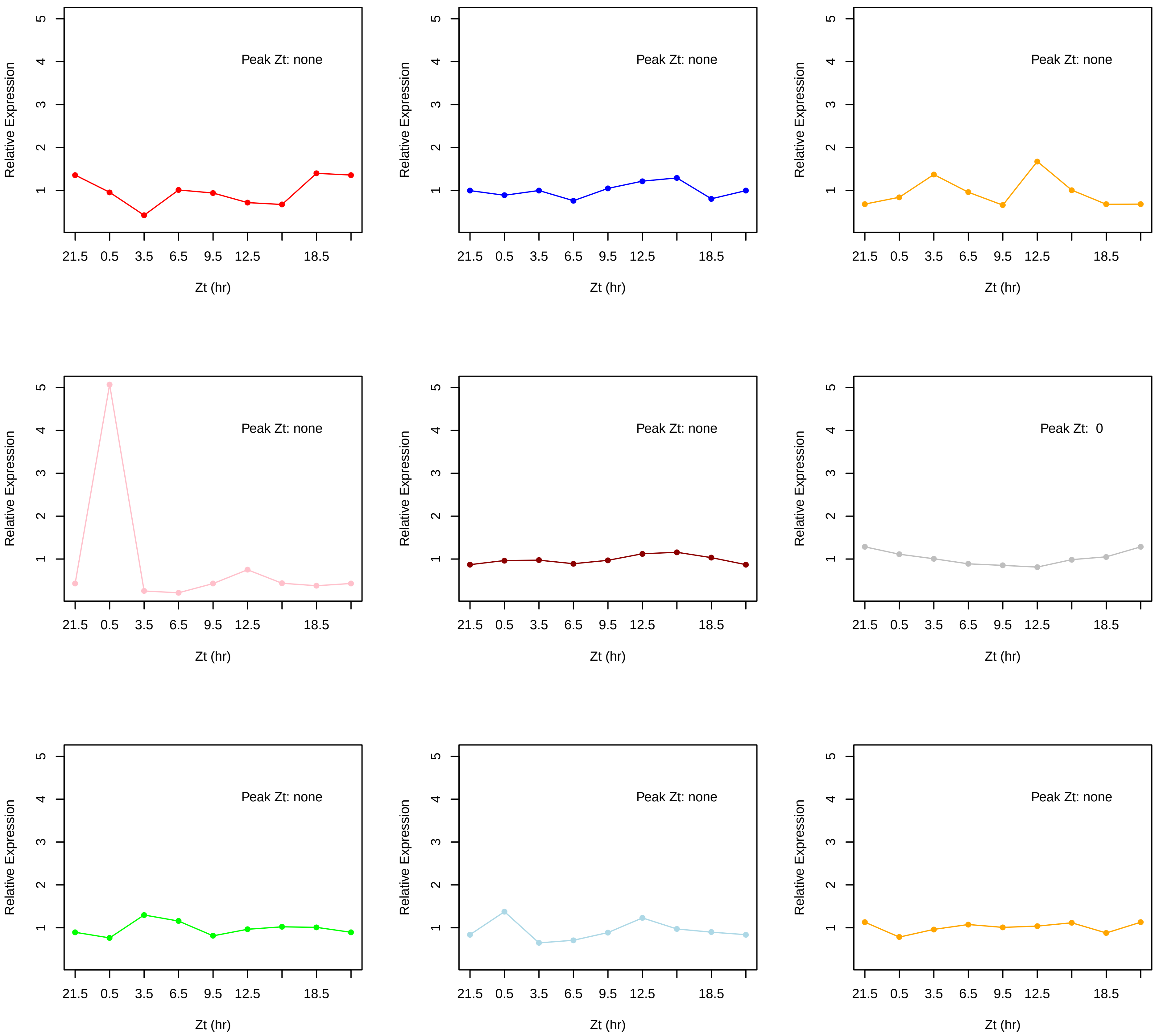

Singeleton: ENSXMAG00000024362 tmem218

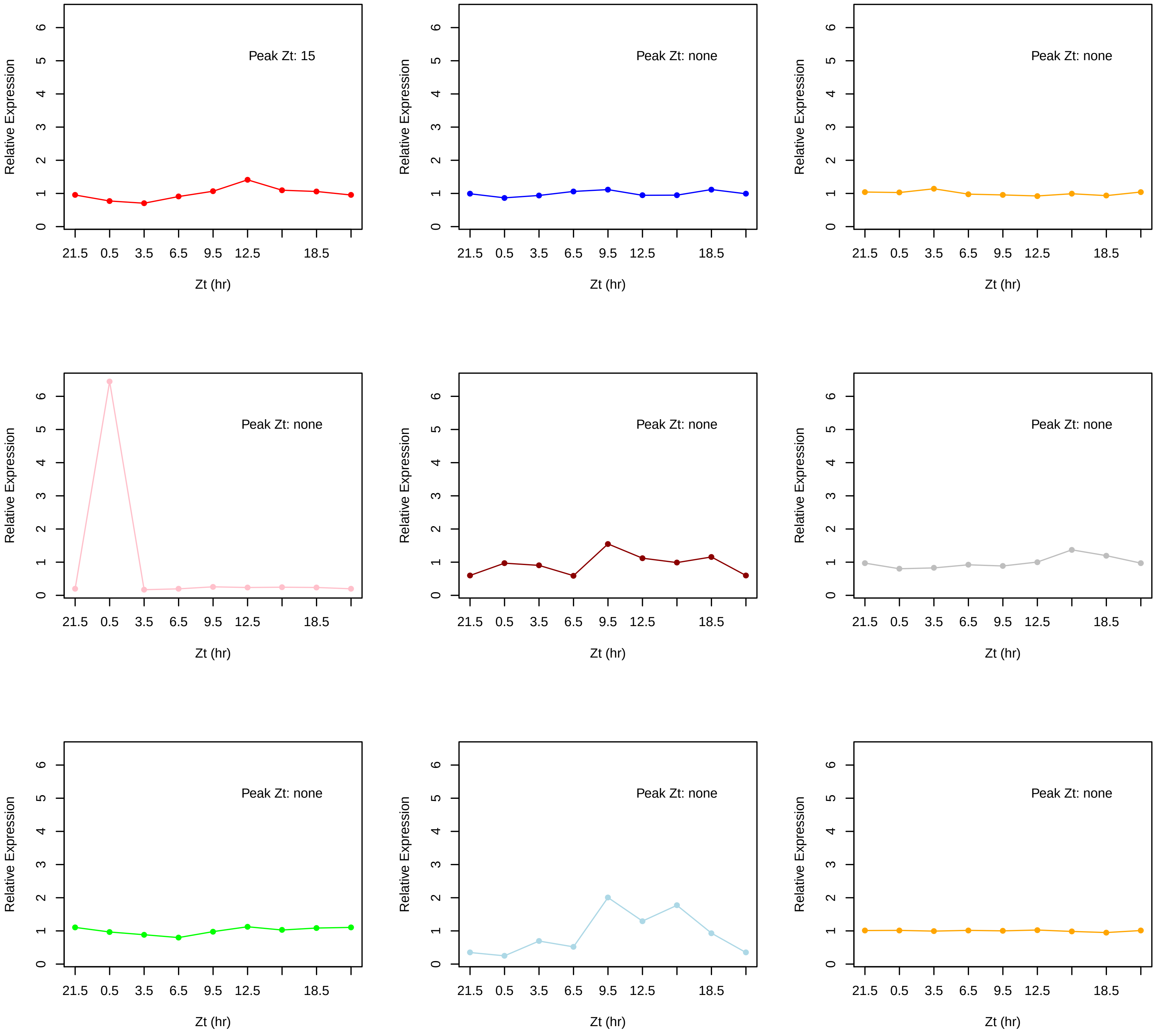

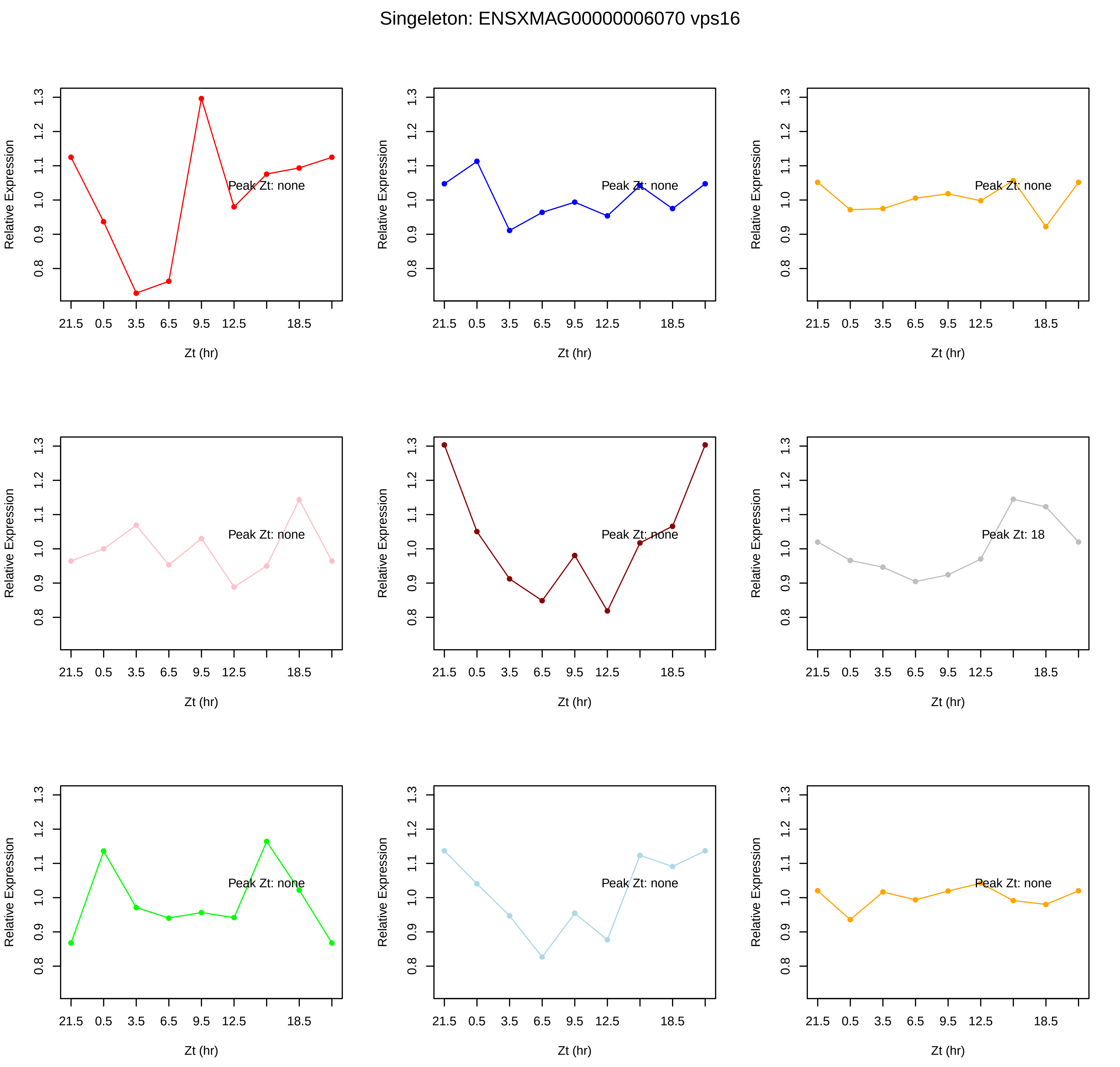
Singleton: ENSXMAG00000006070 vps16
Relative Expression
Zt (hr)
21.5
0.5
3.5
6.5
9.5
12.5
18.5
0.8
0.9
1.0
1.1
1.2
1.3
Peak Zt: none
Peak Zt: 18

Singeleton: ENSXMAG00000027398

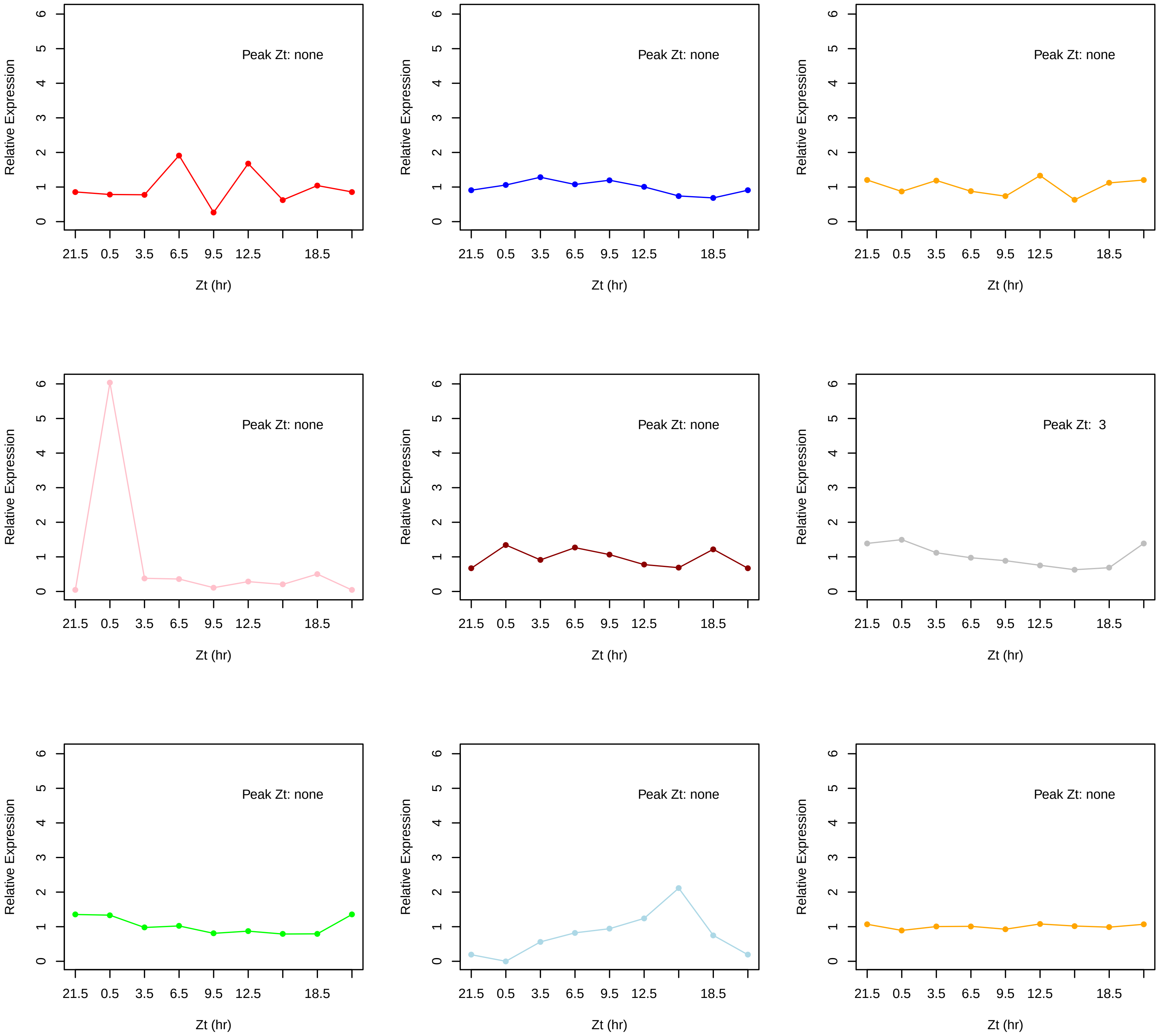

Singleton: ENSXMAG00000029424 tprg1l

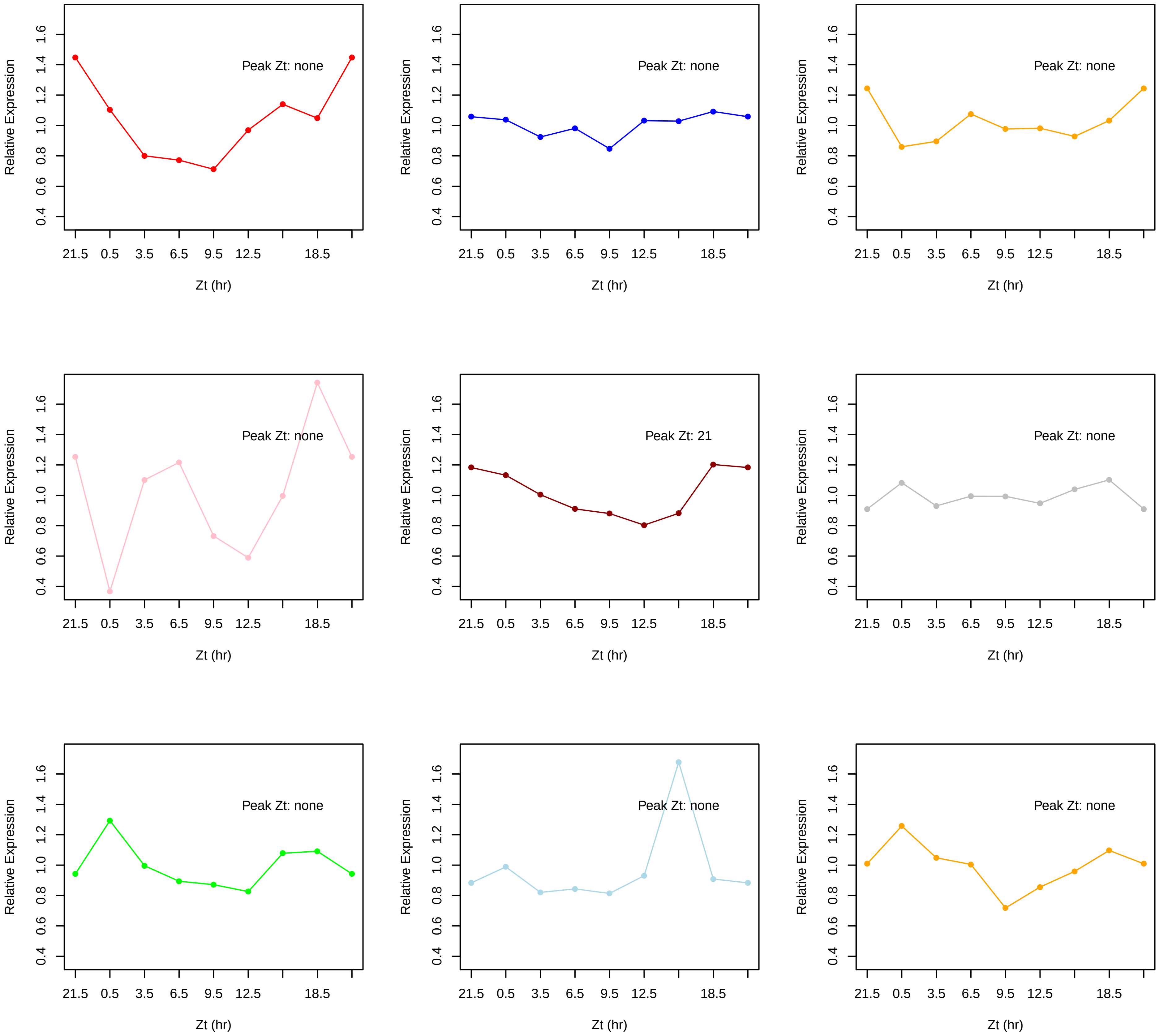

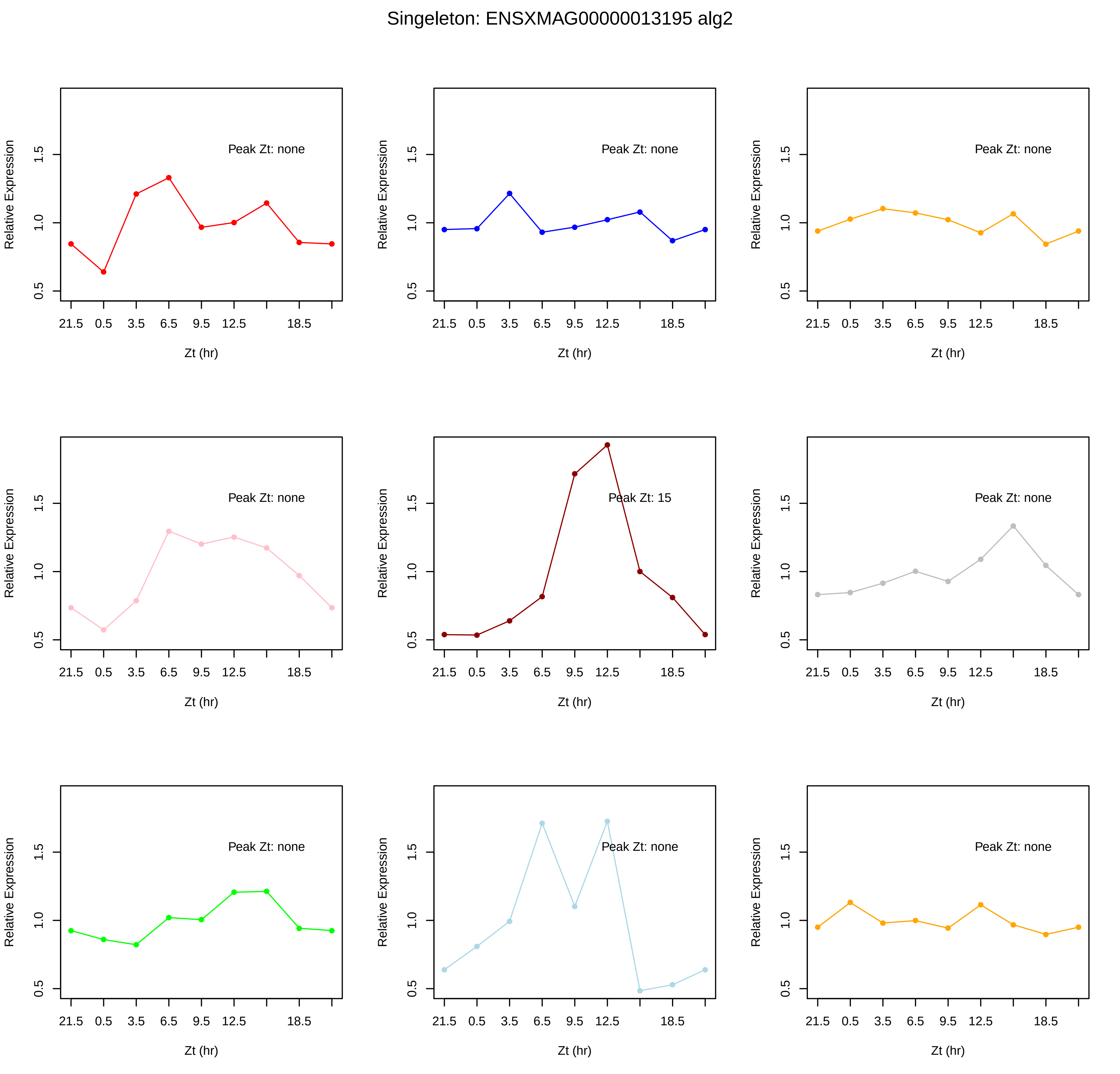
Singeleton: ENSXMAG00000013195 alg2
Peak Zt: none
Peak Zt: 15
Relative Expression
Zt (hr)
21.5
0.5
3.5
6.5
9.5
12.5
18.5
0.5
1.0
1.5

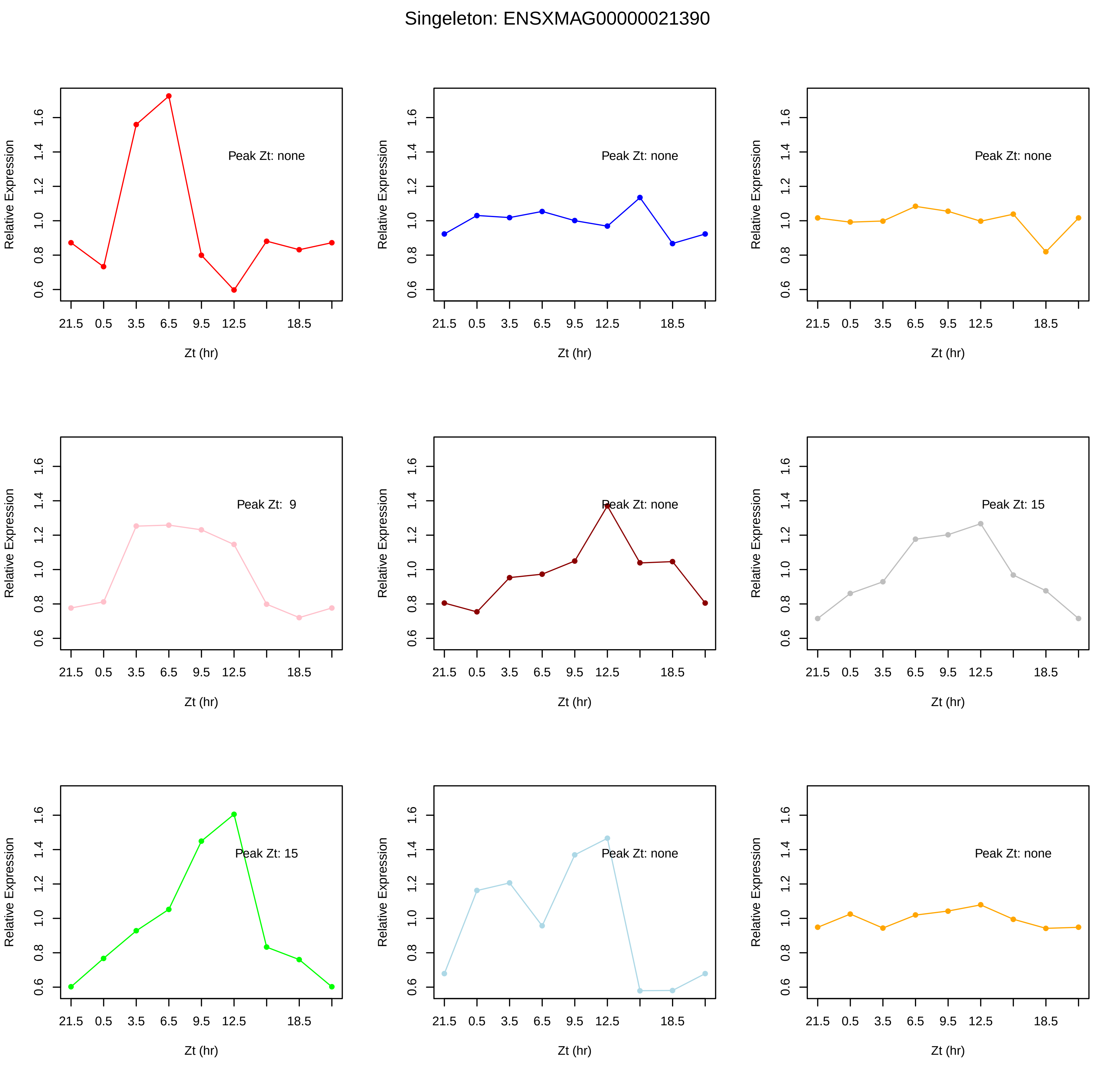

Singeleton: ENSXMAG00000021390
Peak Zt: none
Peak Zt: none
Peak Zt: none
Peak Zt: 9
Peak Zt: none
Peak Zt: 15
Peak Zt: 15
Peak Zt: none
Peak Zt: none
Relative Expression
Zt (hr)

Singeleton: ENSXMAG00000017557 mlxip

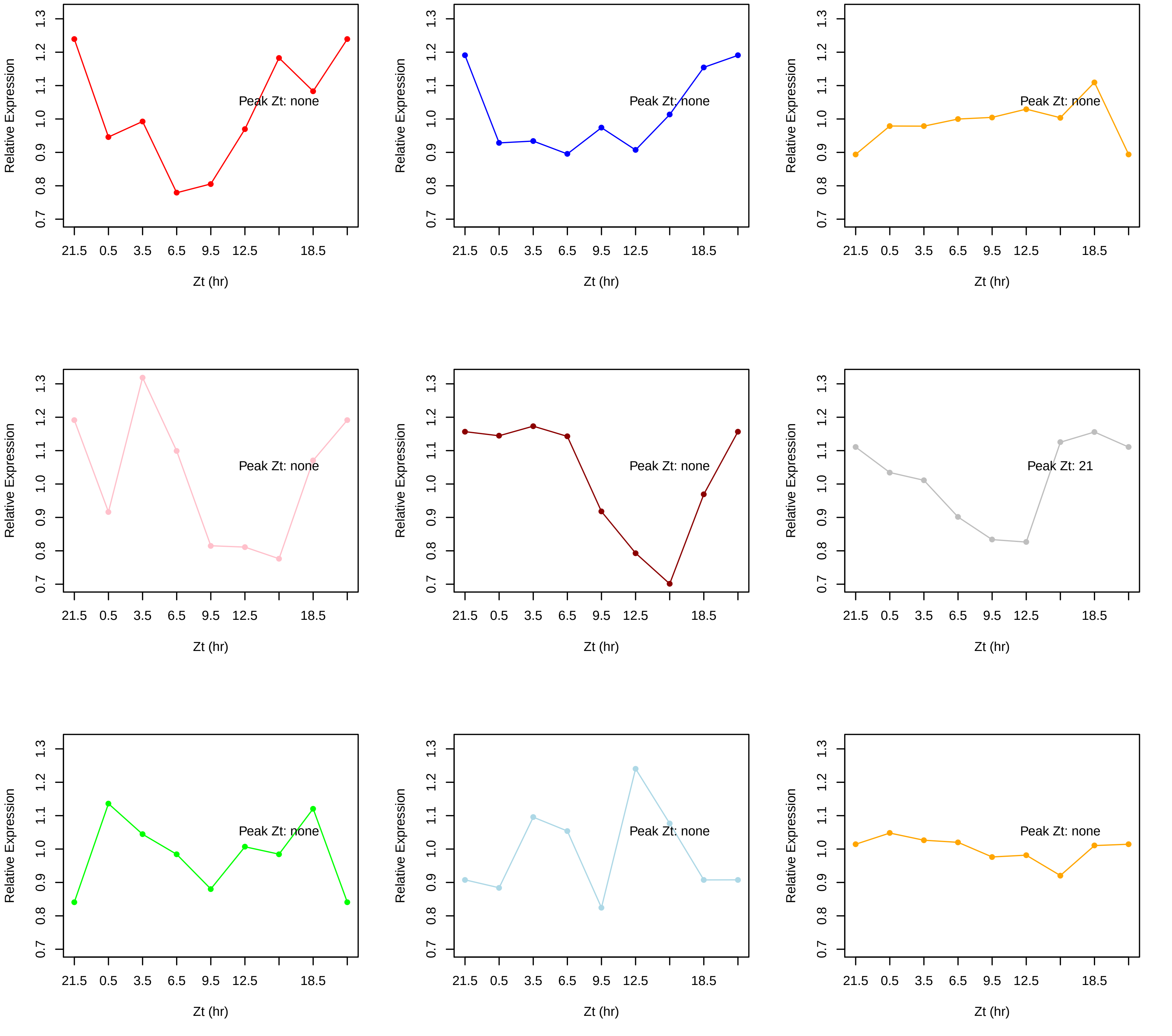

Singeleton: ENSXMAG00000013355 SUGT1

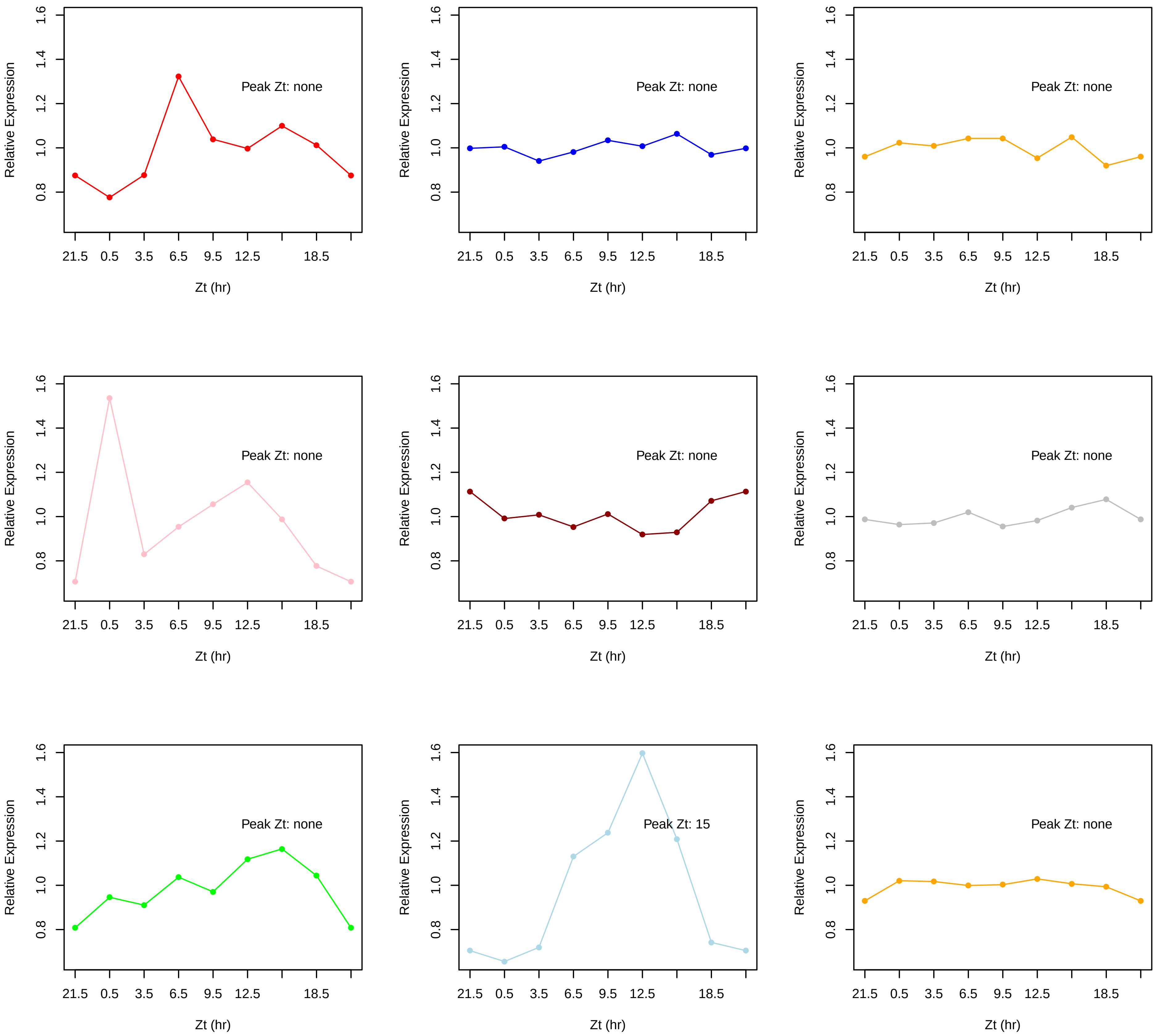

Singleton: ENSXMAG00000019072 si:ch211−263k4.2

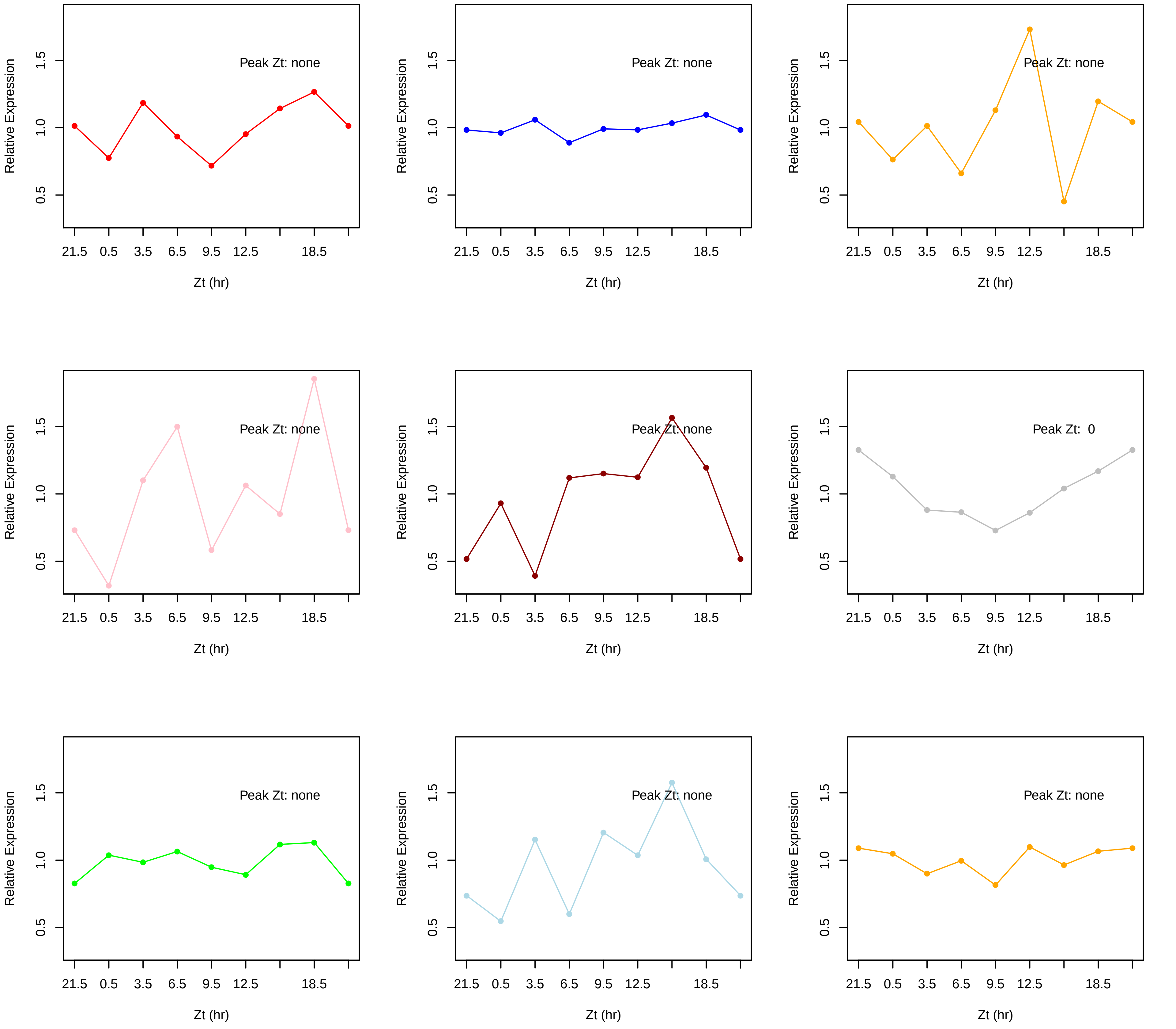

Singeleton: ENSXMAG00000010389 smarcd1

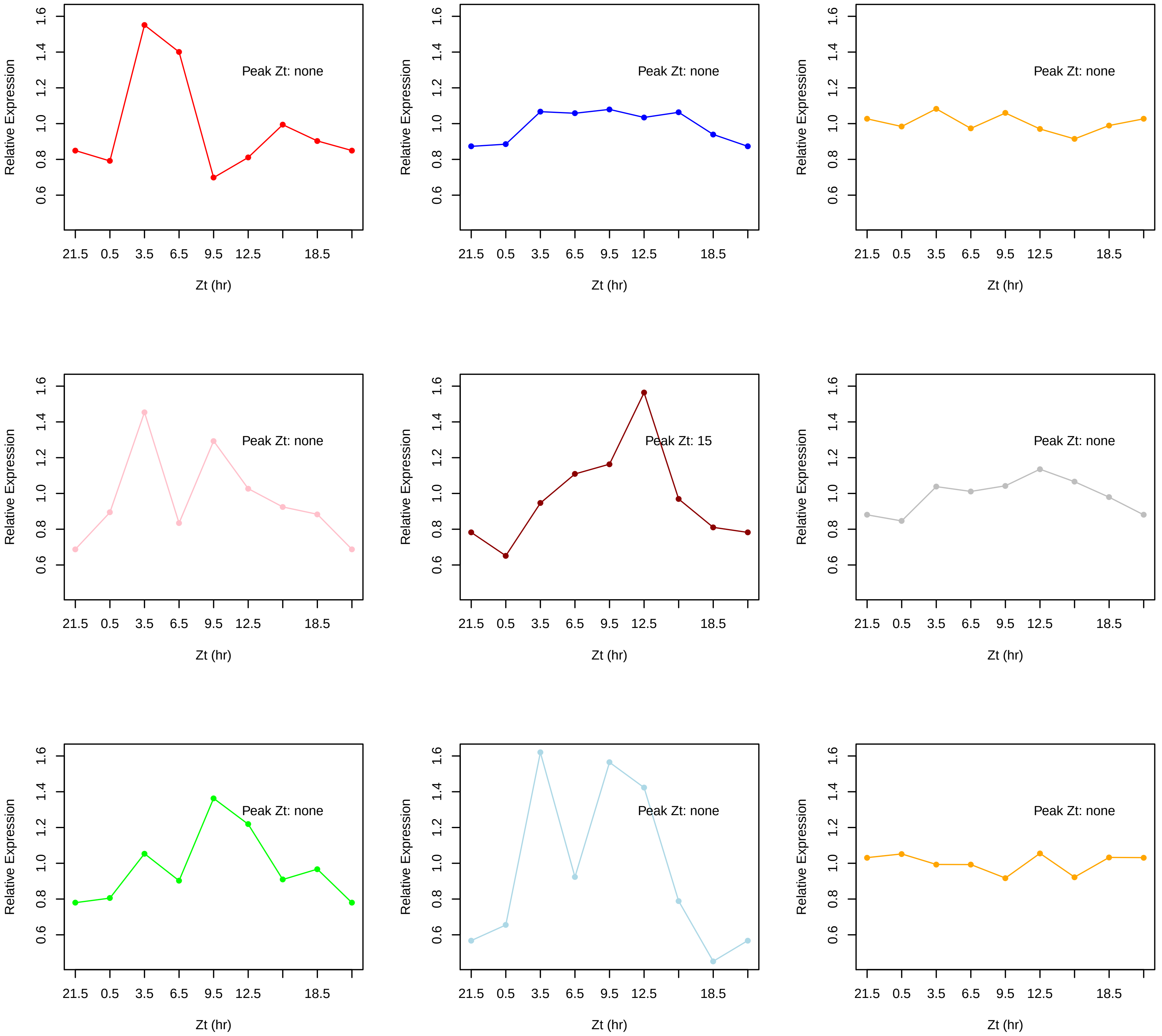

Singeleton: ENSXMAG00000027051 etfb

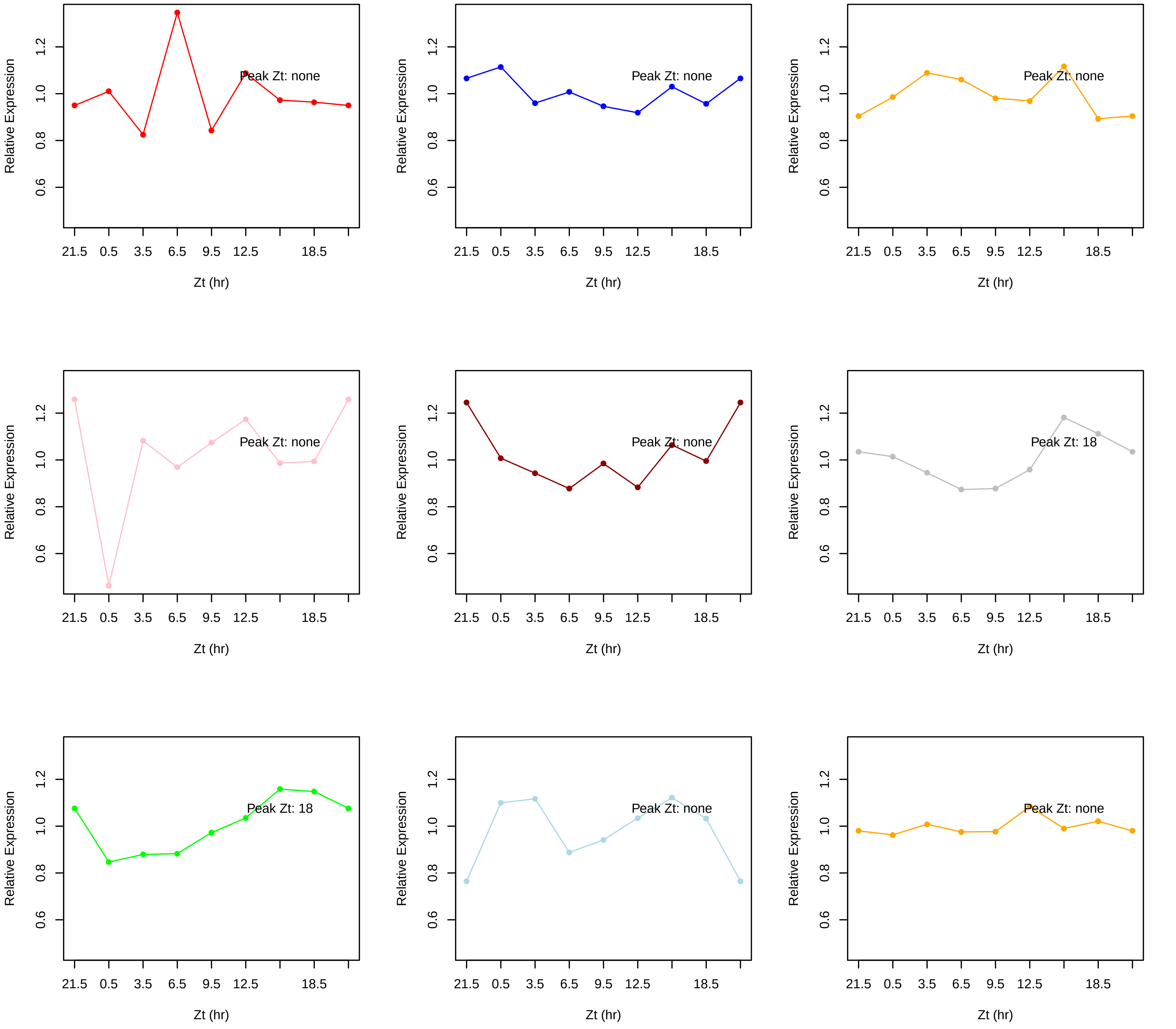

Singeleton: ENSXMAG00000027138

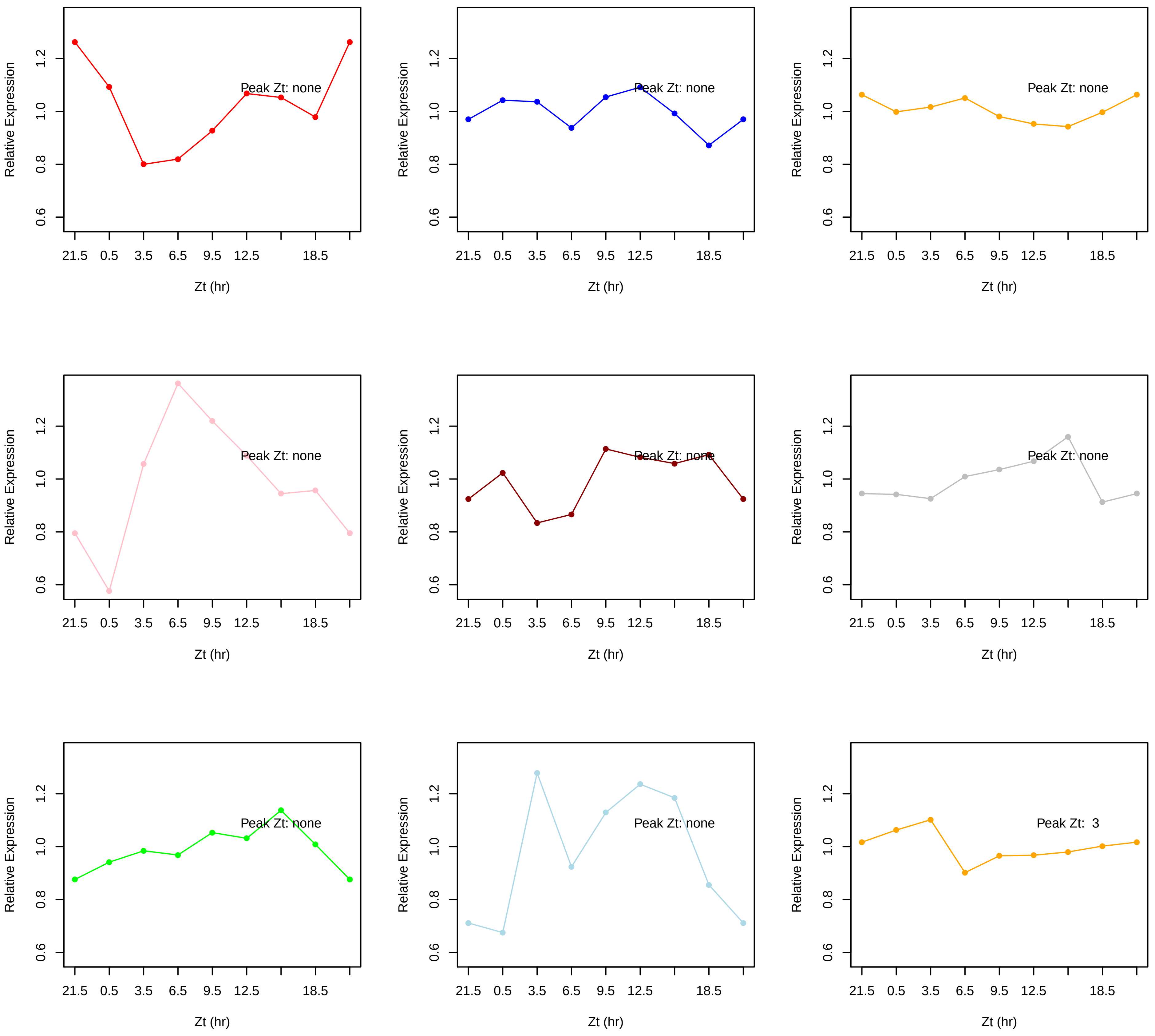

Singeleton: ENSXMAG00000021269 surf4

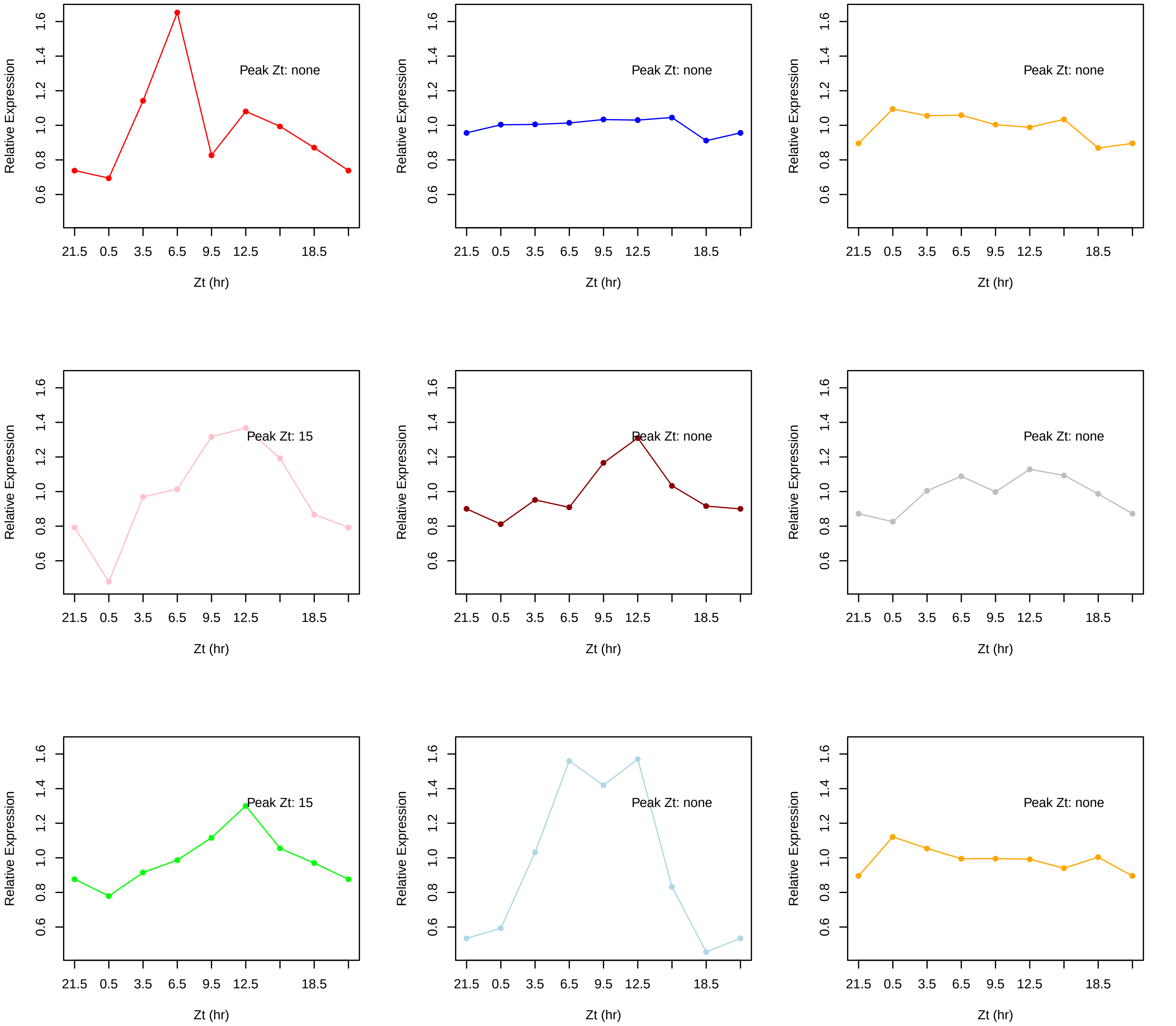

Singleton: ENSXMAG00000018758 ythdf2

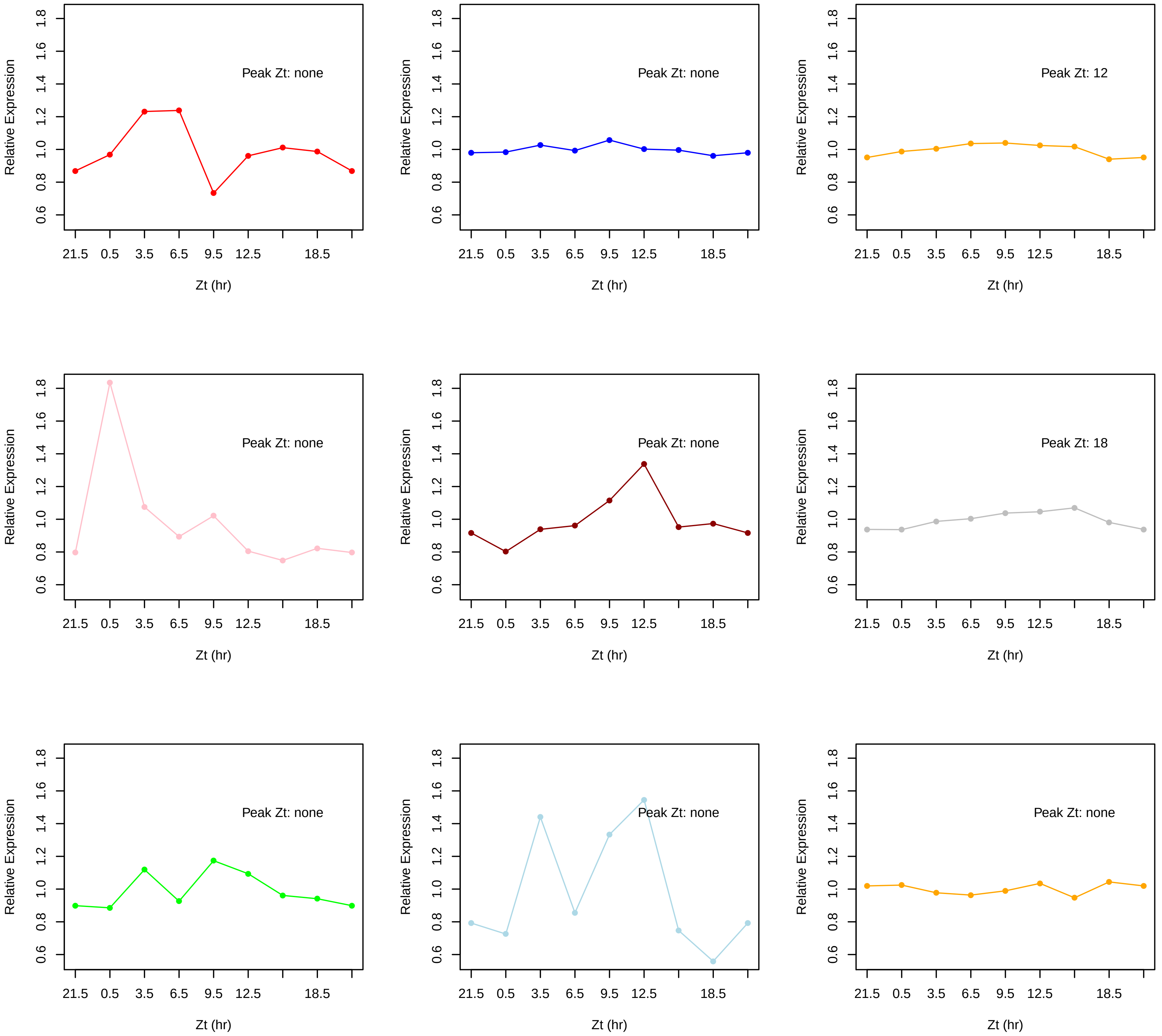

Singleton: ENSXMAG00000009323 tcea2

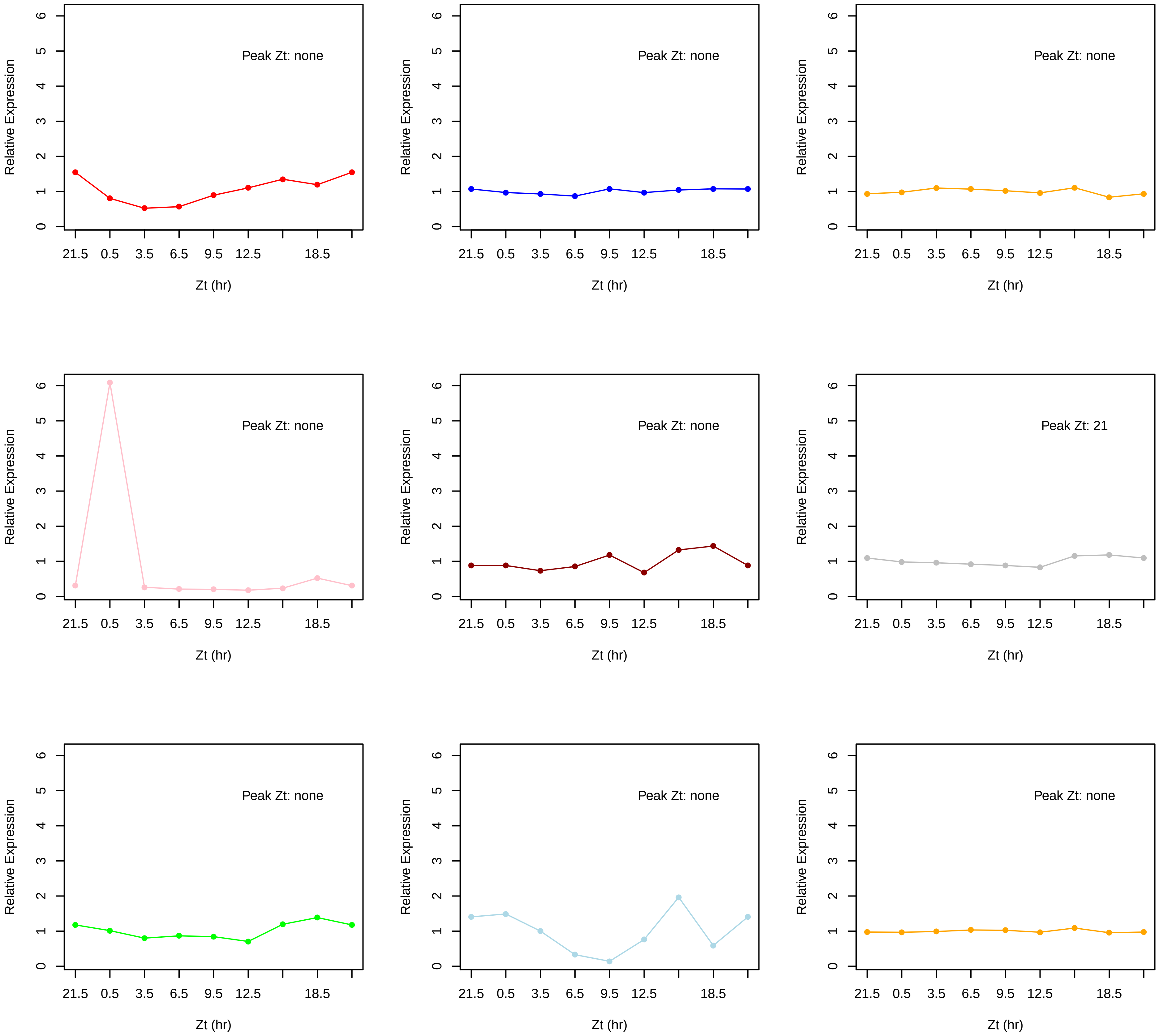

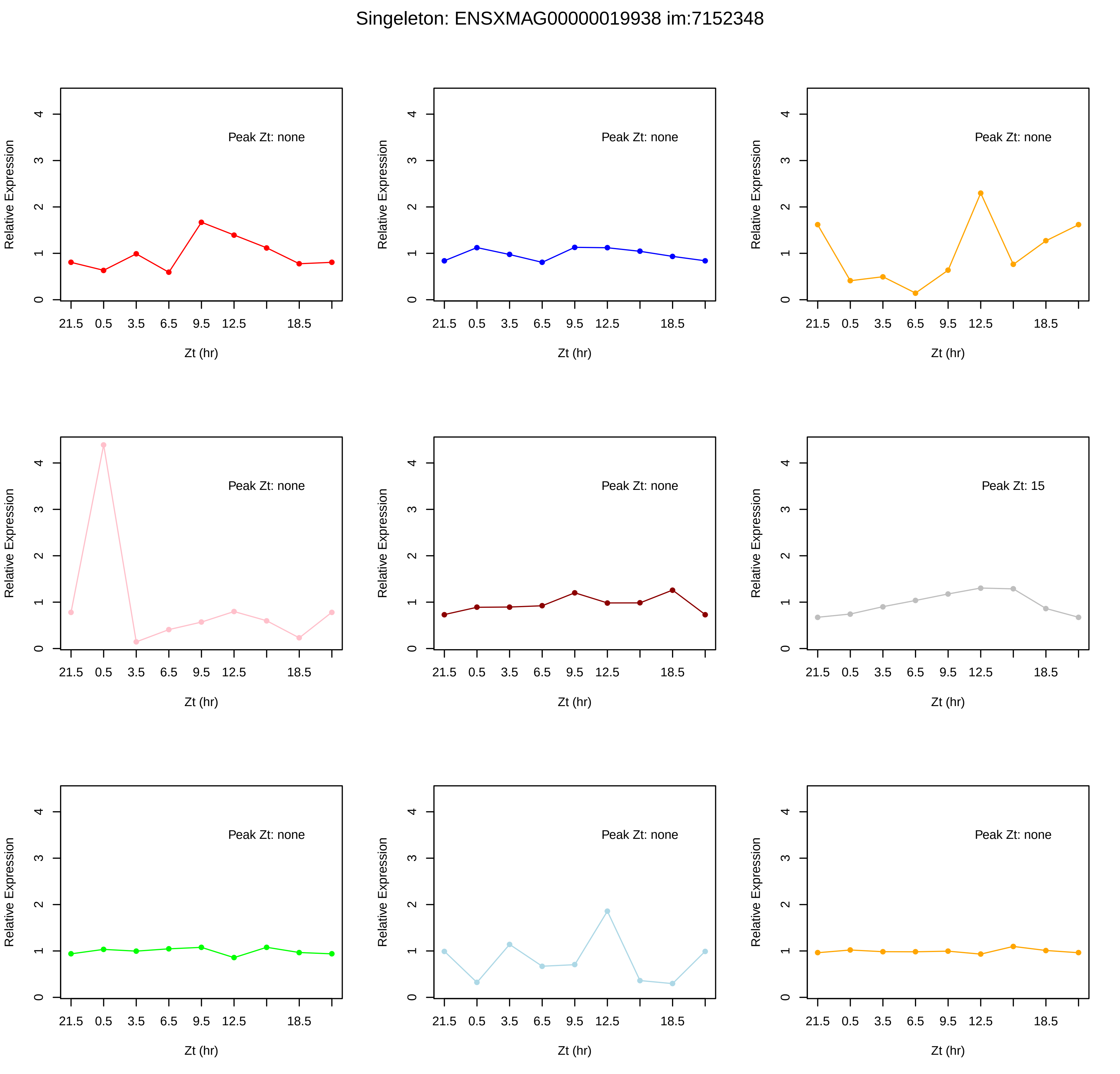

Singeleton: ENSXMAG00000019938 im:7152348
Peak Zt: none
Peak Zt: none
Peak Zt: none
Peak Zt: none
Peak Zt: none
Peak Zt: 15
Peak Zt: none
Peak Zt: none
Peak Zt: none
Relative Expression
Zt (hr)
21.5 0.5 3.5 6.5 9.5 12.5 18.5

Singeleton: ENSXMAG00000005775 ulk2

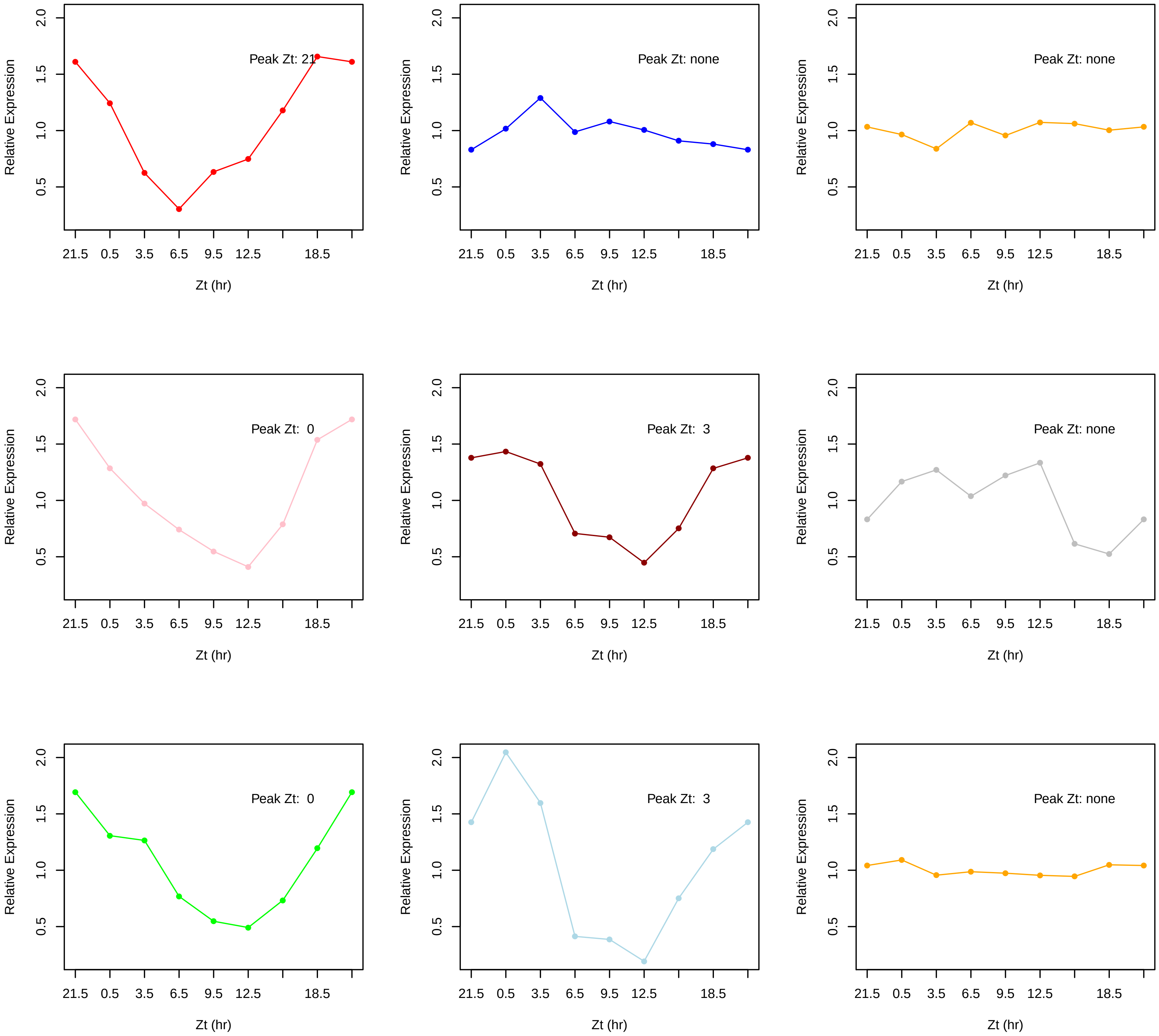

Singeleton: ENSXMAG00000004637 mfn2

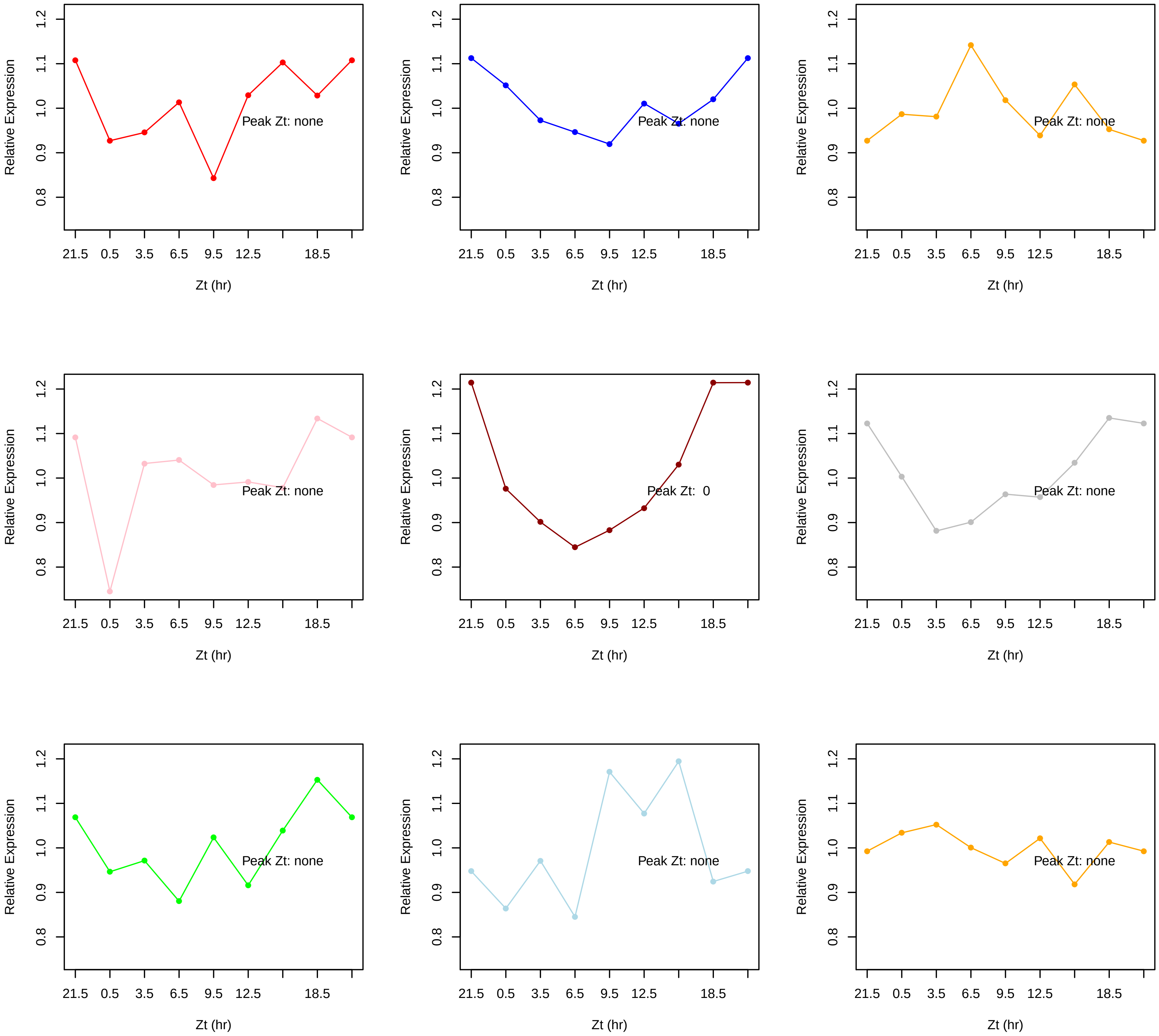

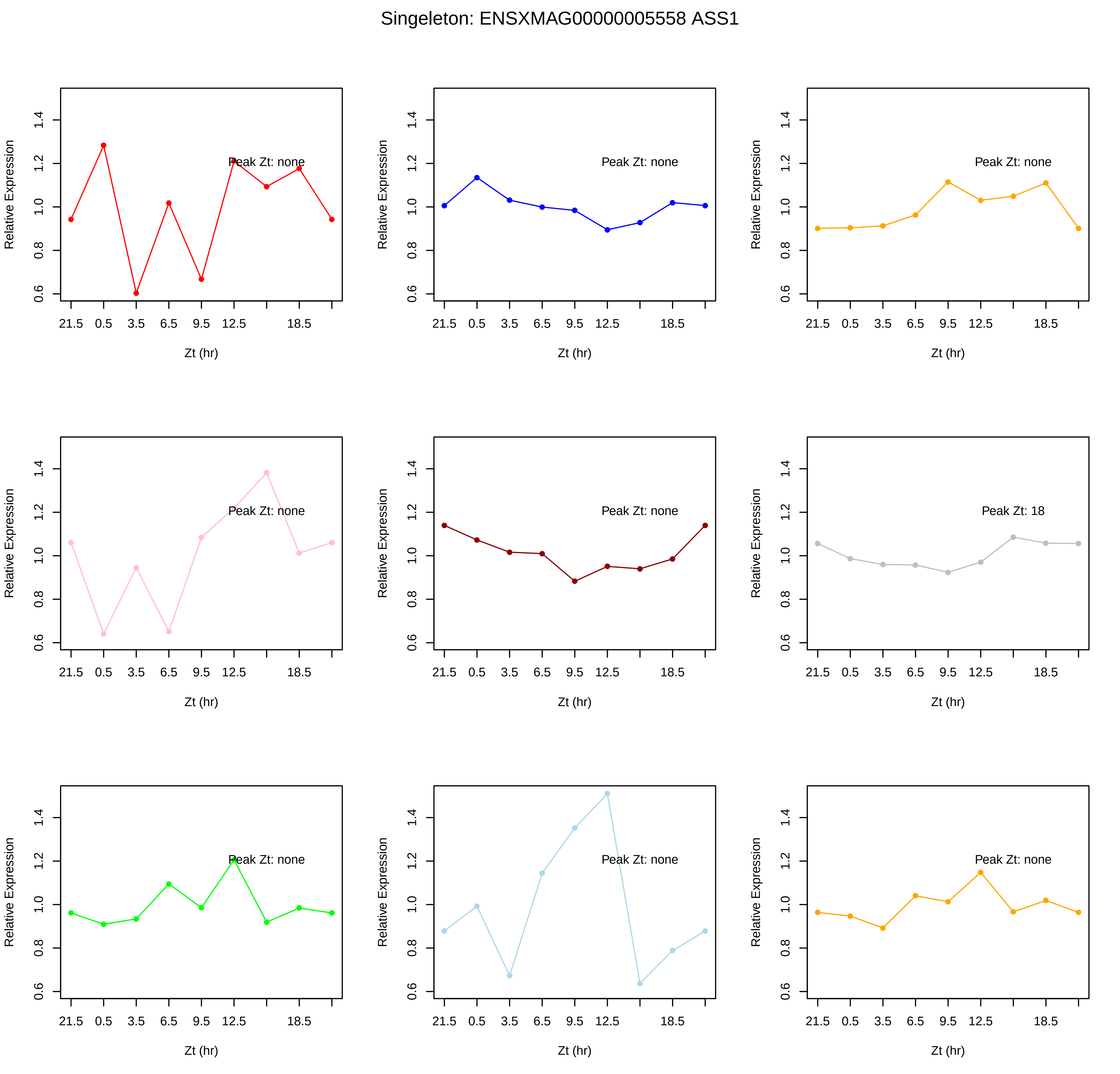
Singleton: ENSXMAG00000005558 ASS1
Peak Zt: none
Peak Zt: none
Peak Zt: none
Peak Zt: none
Peak Zt: none
Peak Zt: 18
Peak Zt: none
Peak Zt: none
Peak Zt: none
Relative Expression
Zt (hr)
21.5
0.5
3.5
6.5
9.5
12.5
18.5
0.6
0.8
1.0
1.2
1.4

Singeleton: ENSXMAG00000017430 lcmt1

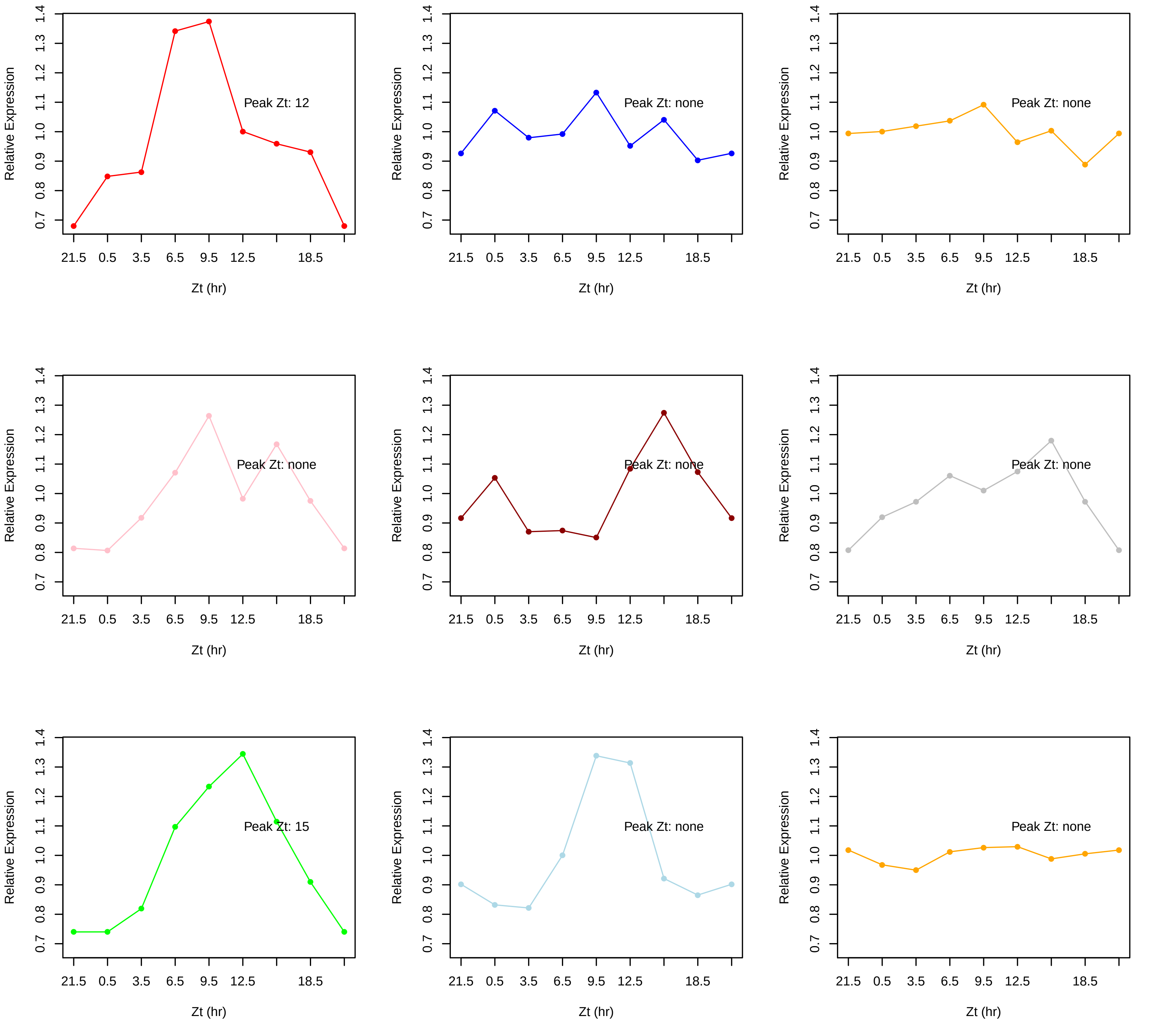

Singeleton: ENSXMAG00000013186 stx17

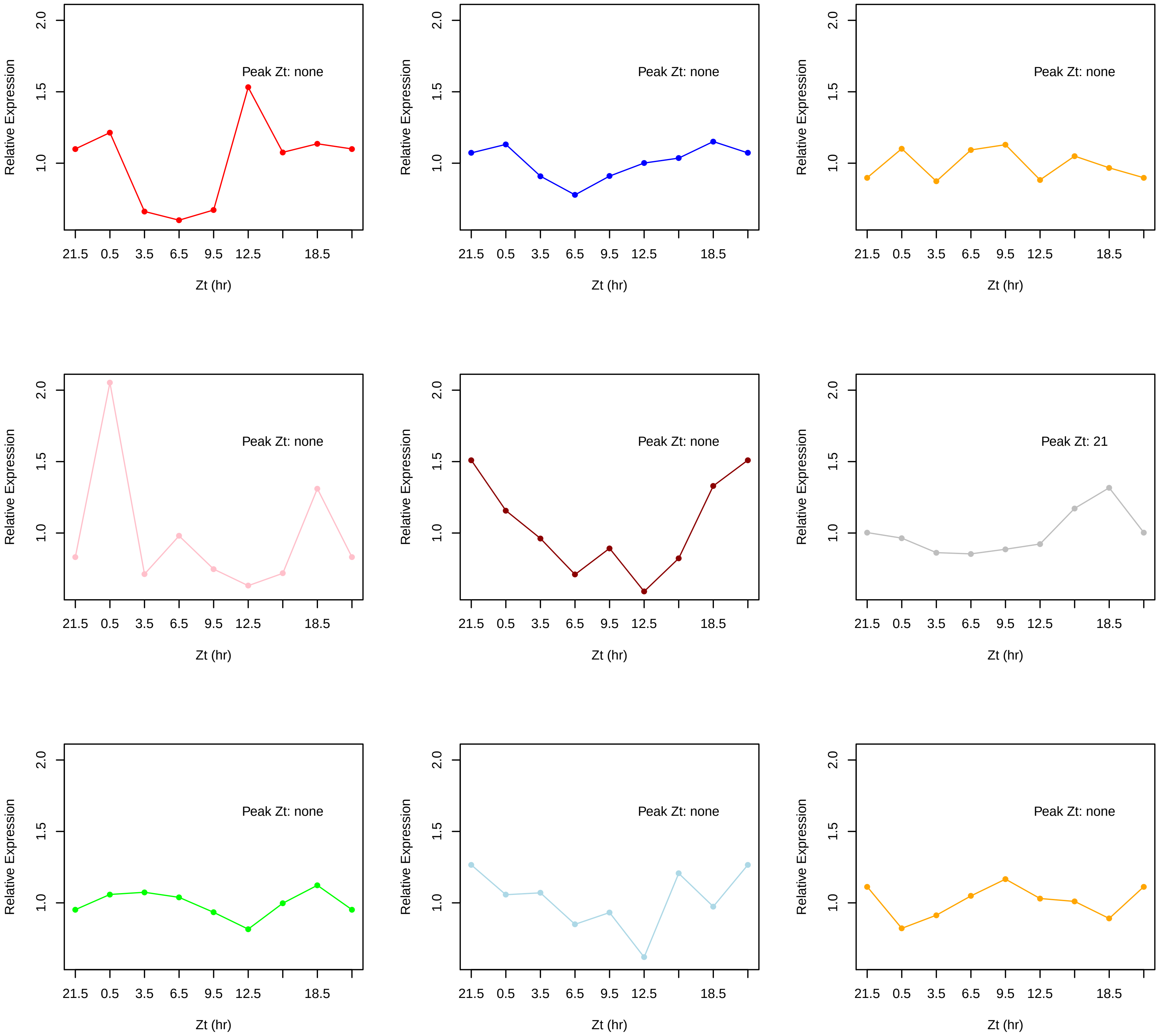

Singeleton: ENSXMAG00000024951 opcml

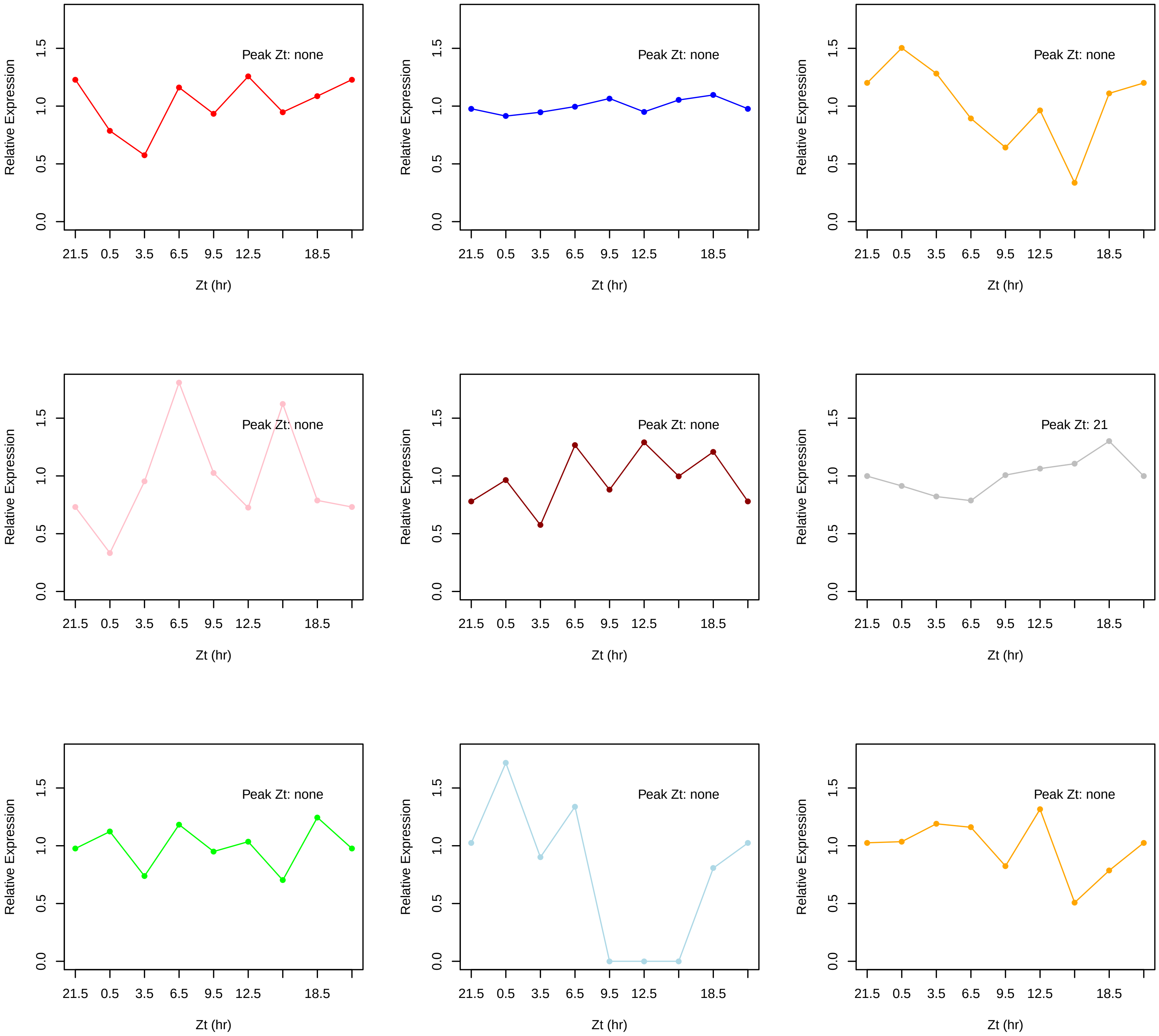

Singleton: ENSXMAG00000004420 chchd7

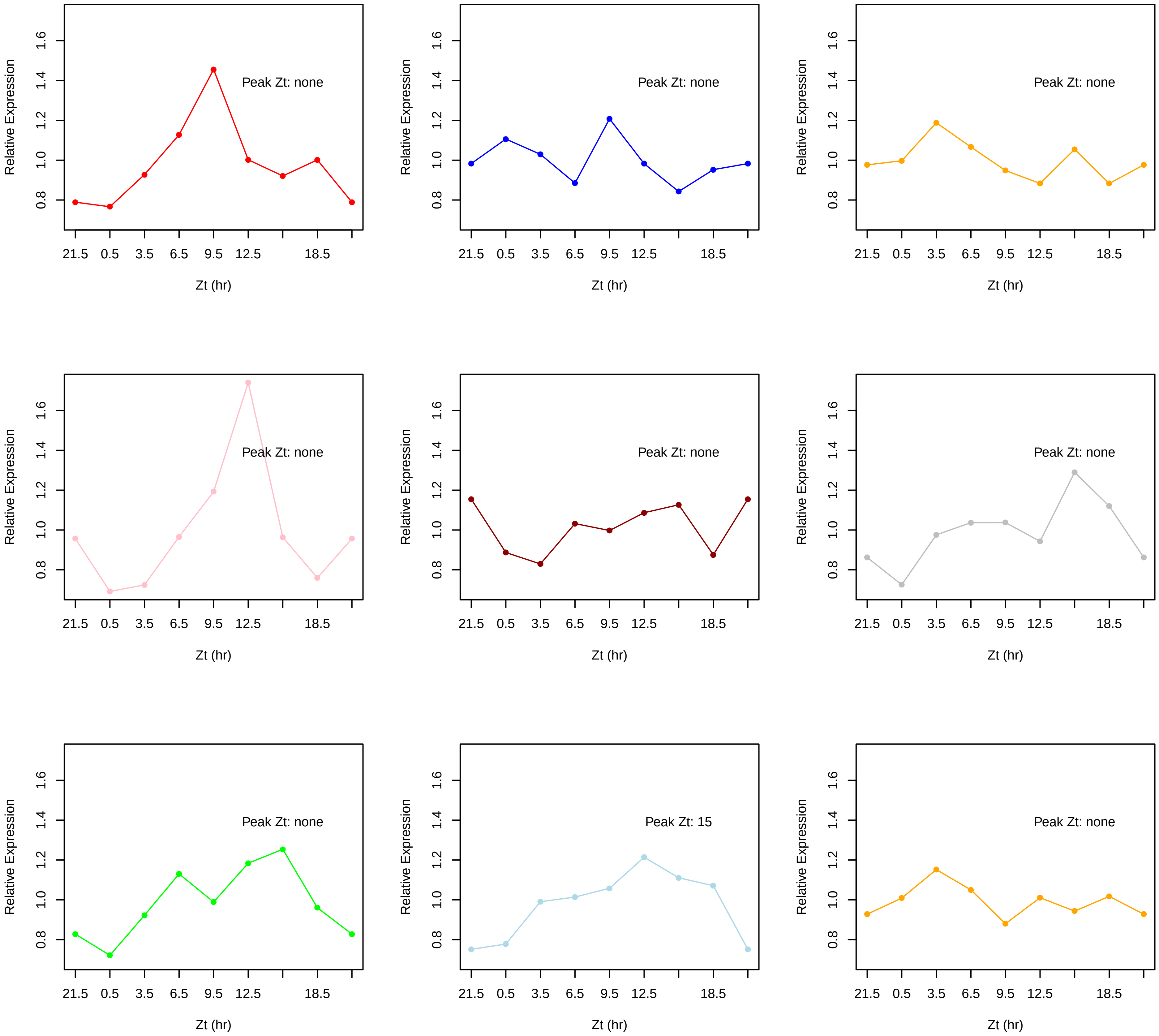

Singleton: ENSXMAG00000026085 erp44

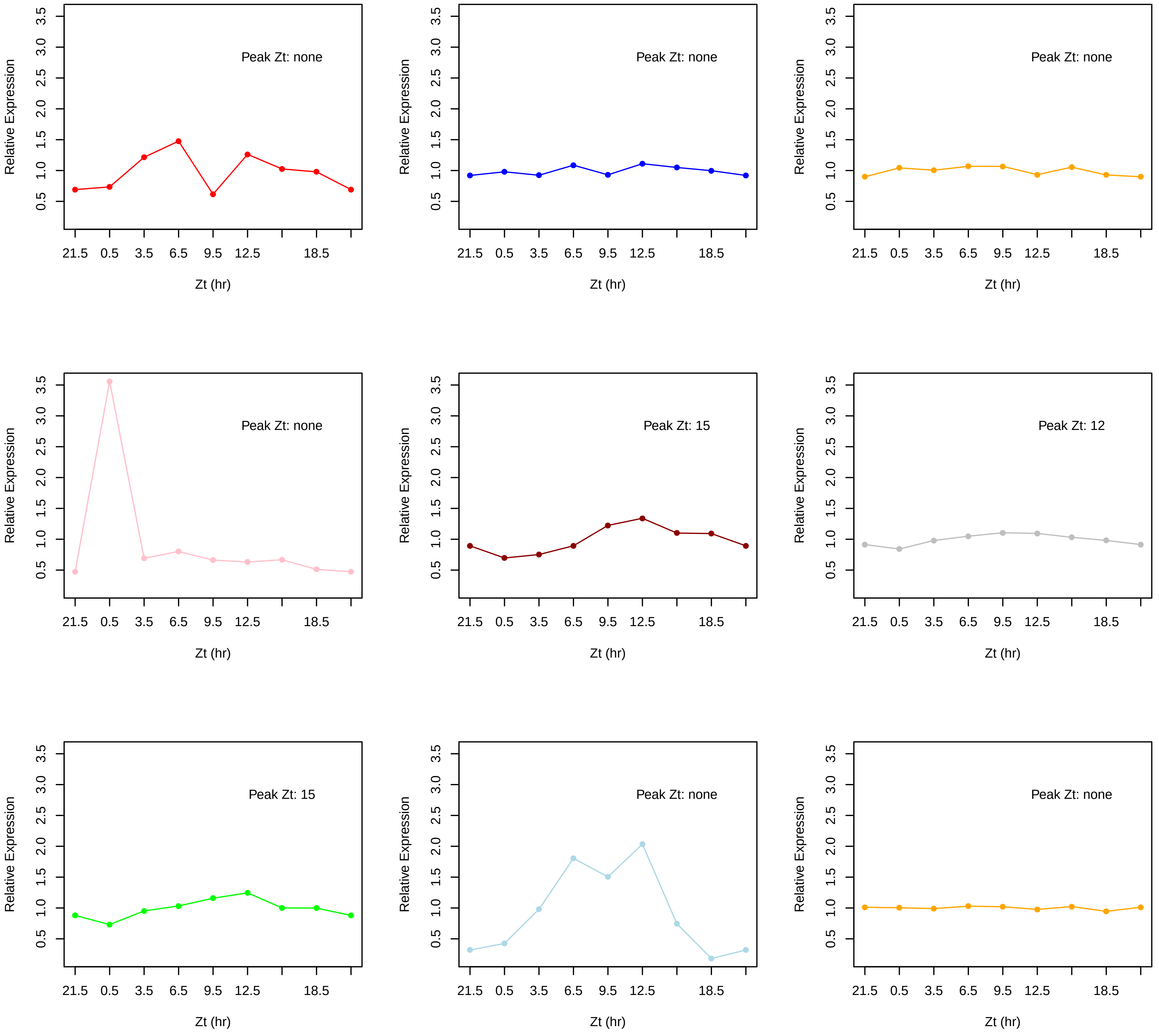

Singeleton: ENSXMAG00000016647 slco2a1

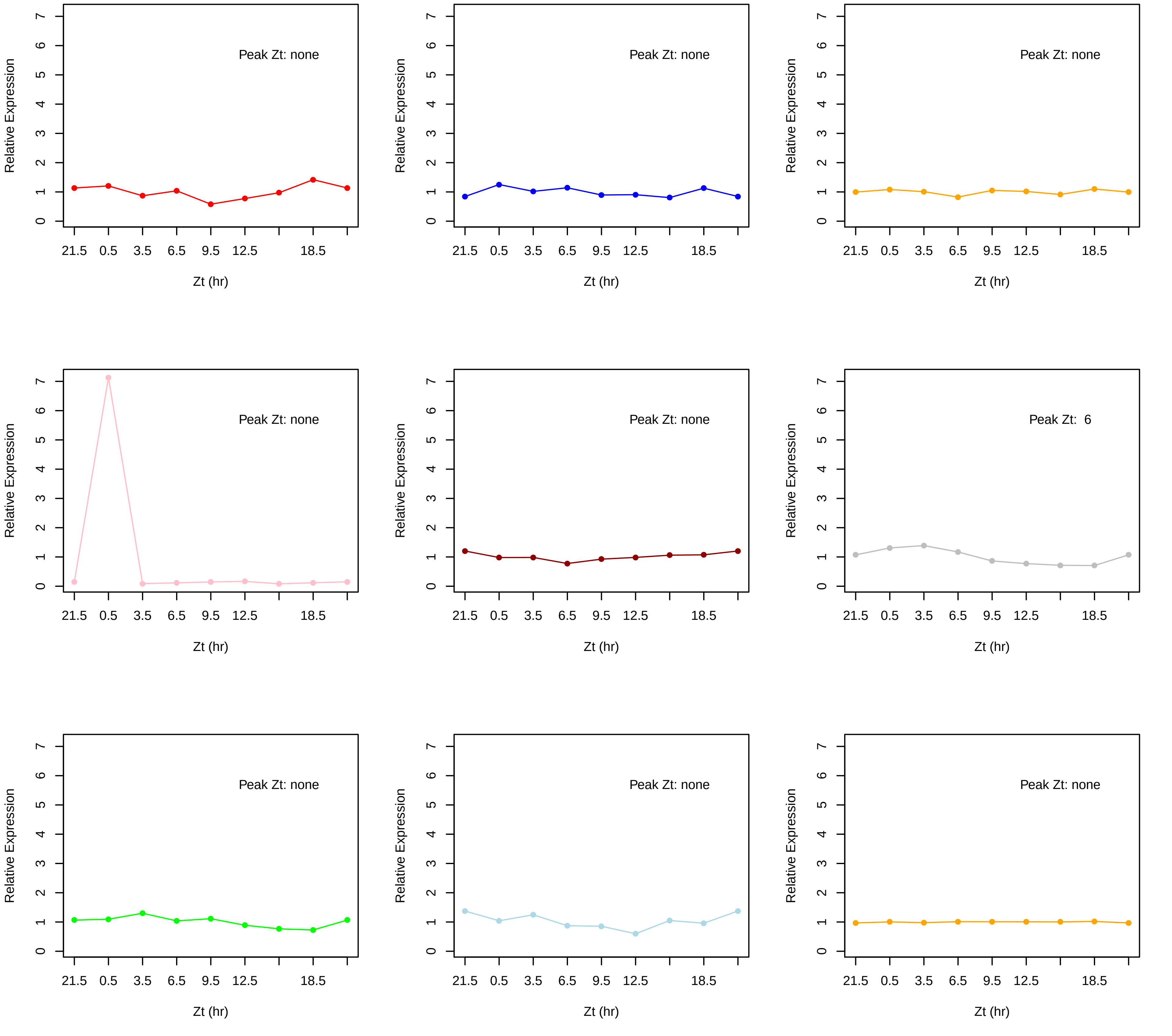

Singeleton: ENSXMAG00000027318 si:ch211−71n6.4

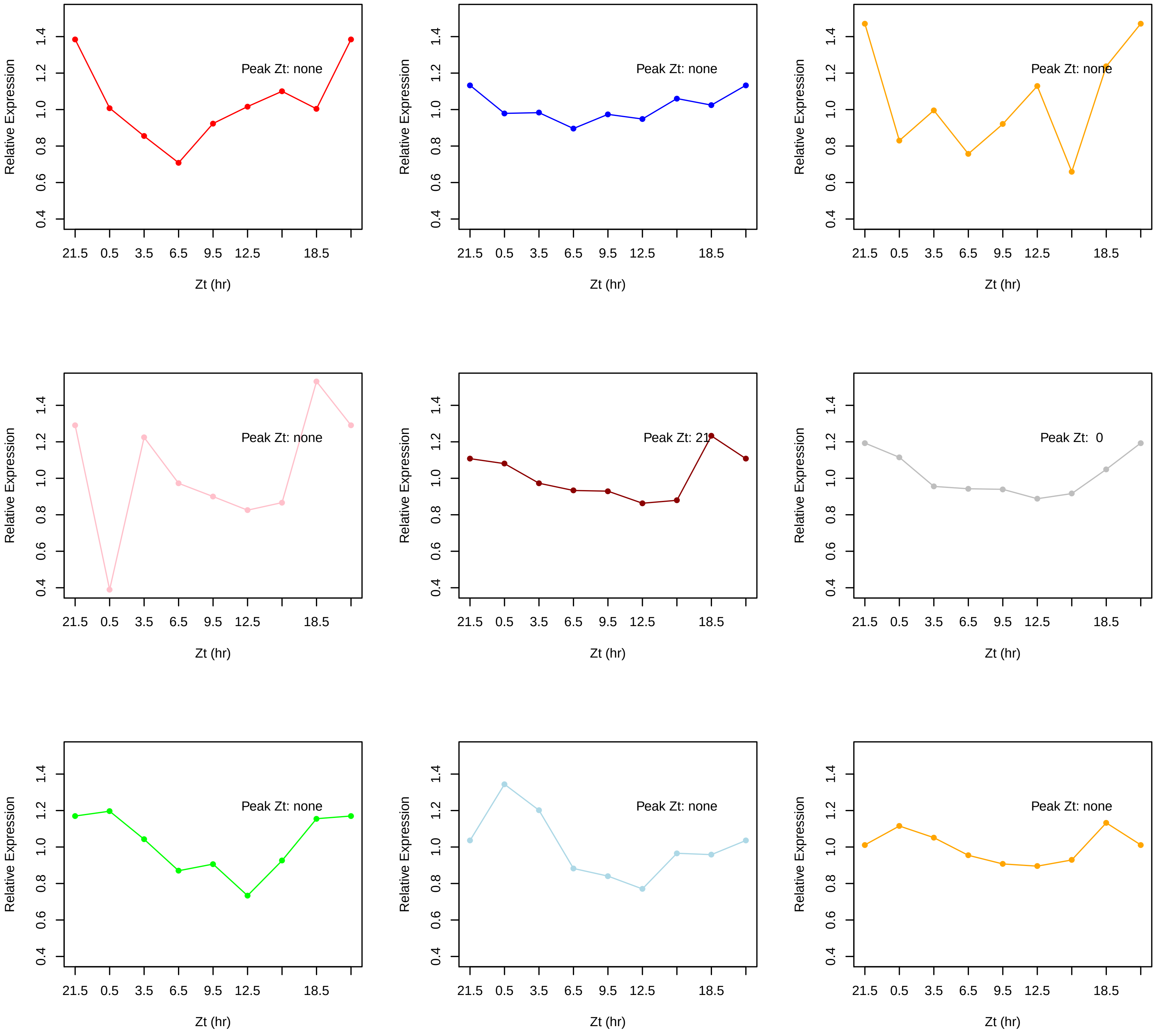

Singeleton: ENSXMAG00000017062

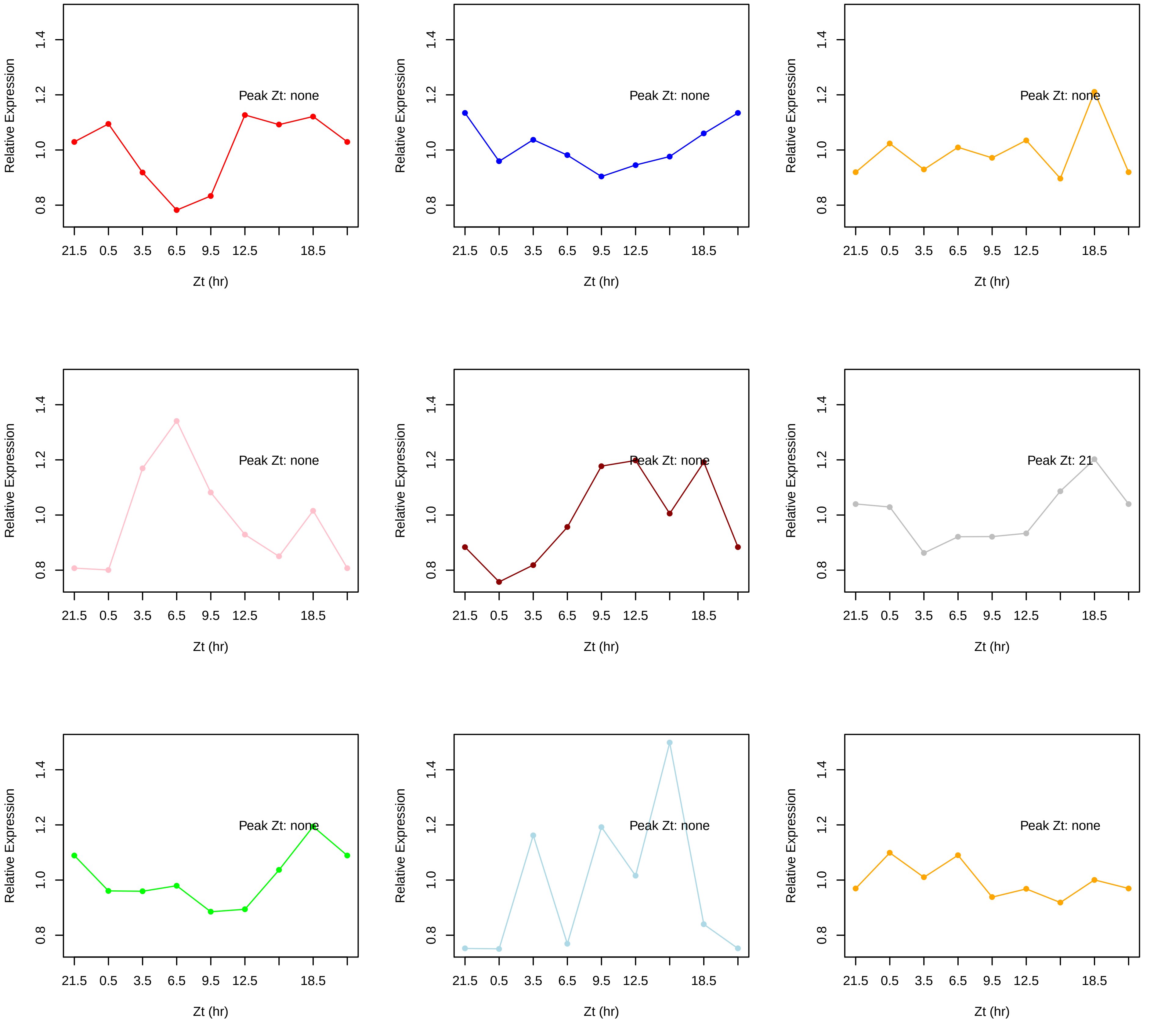

Singeleton: ENSXMAG00000014012 arfgap2

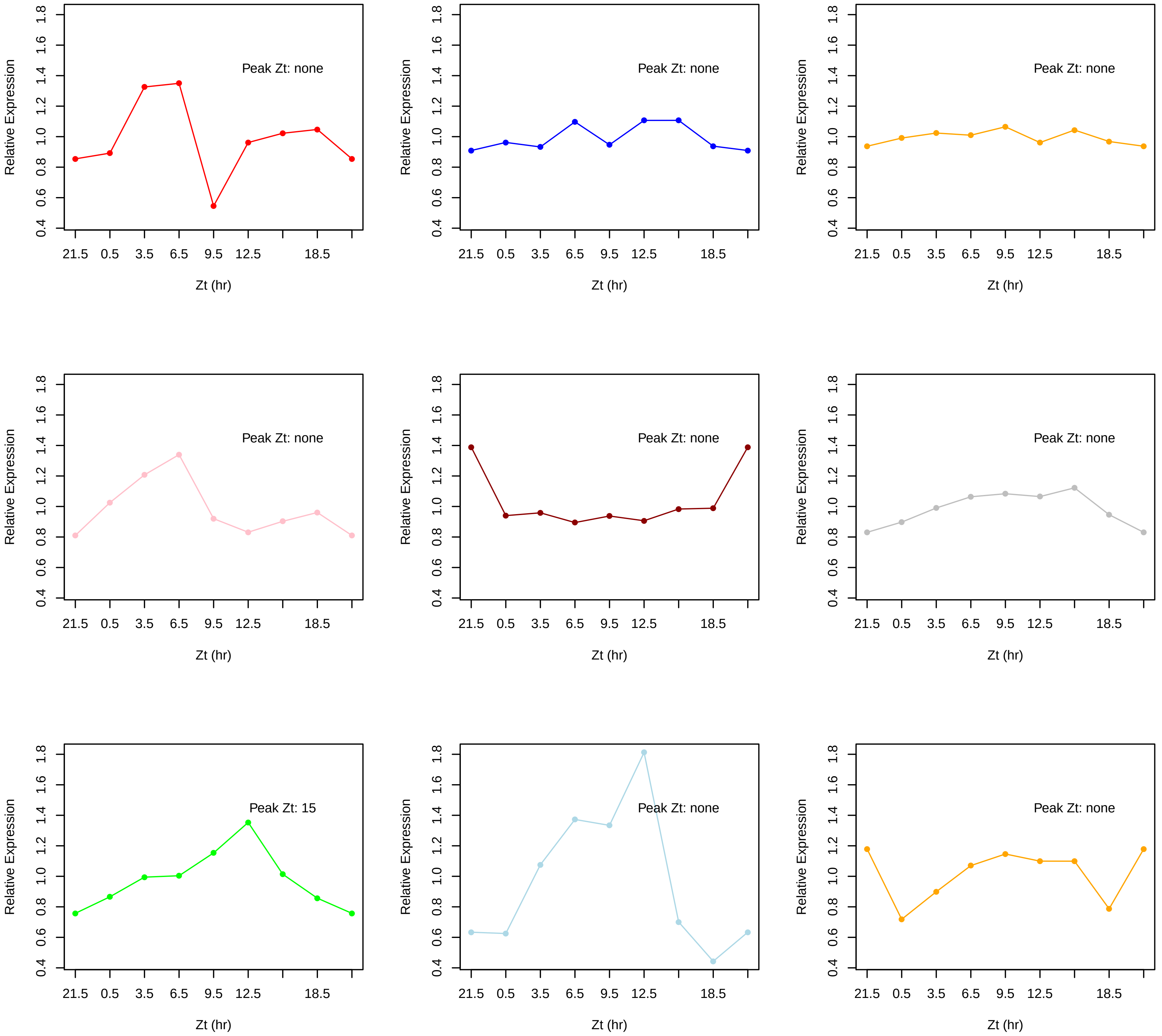

Singeleton: ENSXMAG00000027033 si:dkey−78l4.14

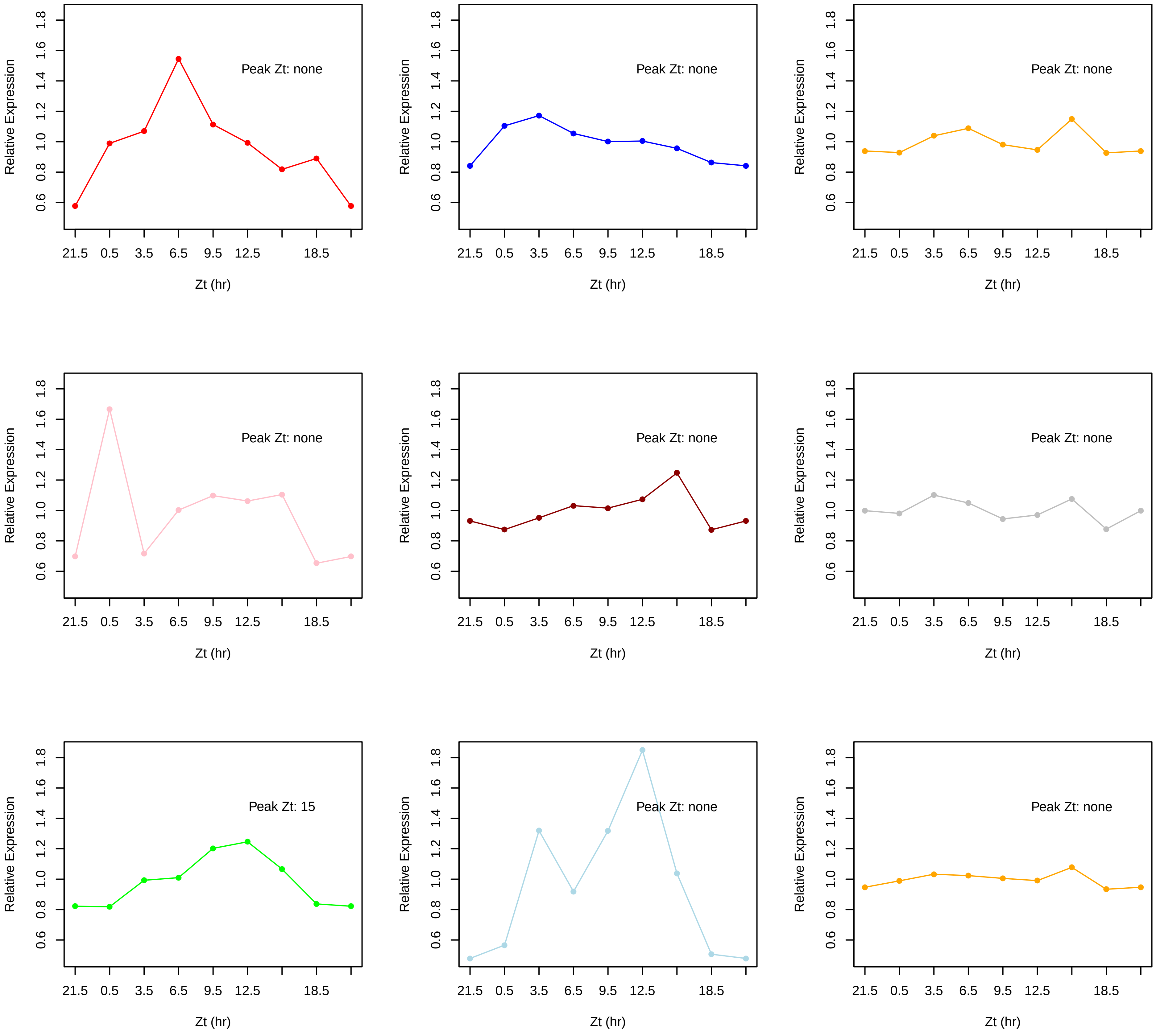

Singeleton: ENSXMAG00000003064 hif1an

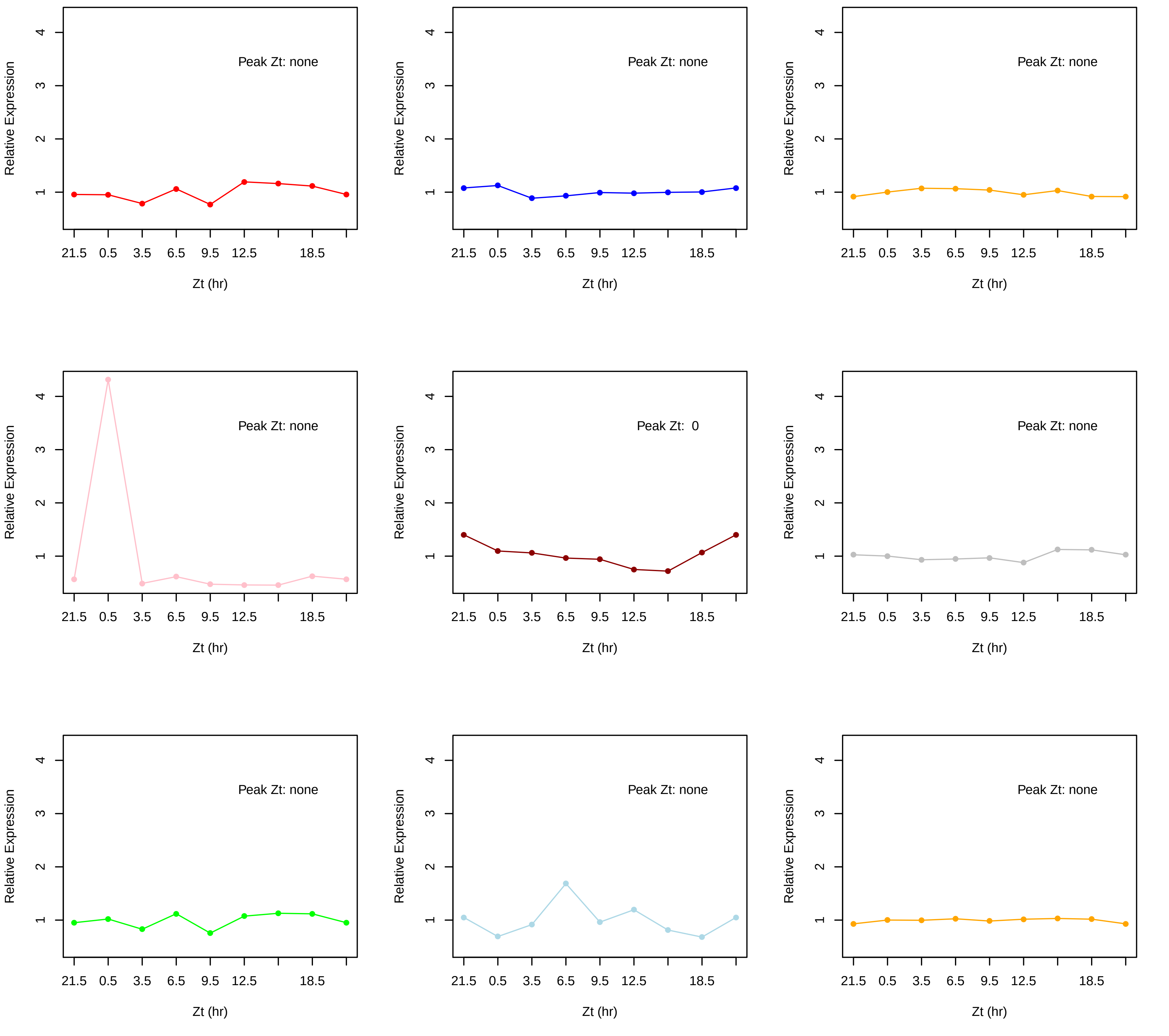

Singeleton: ENSXMAG00000009448

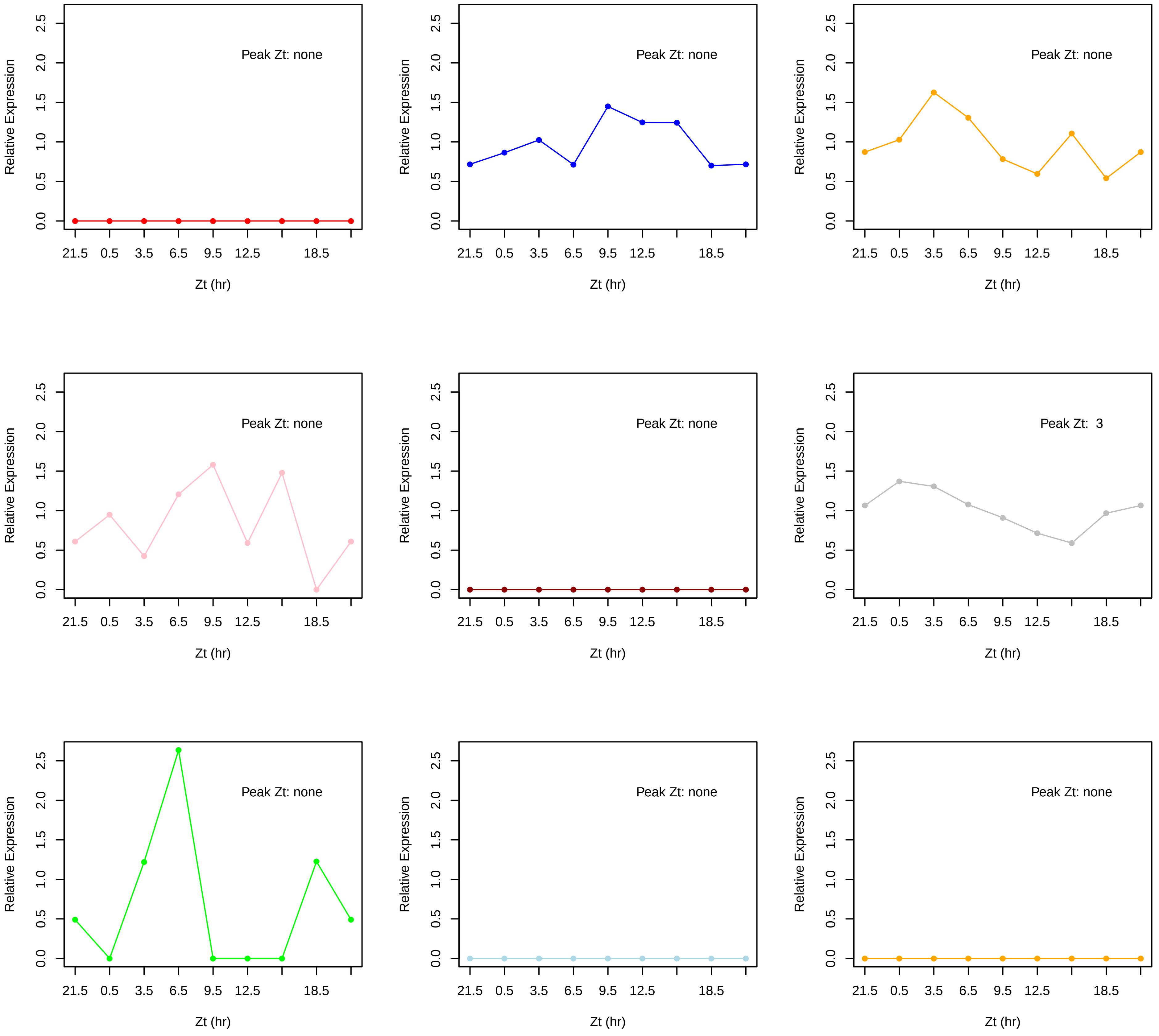

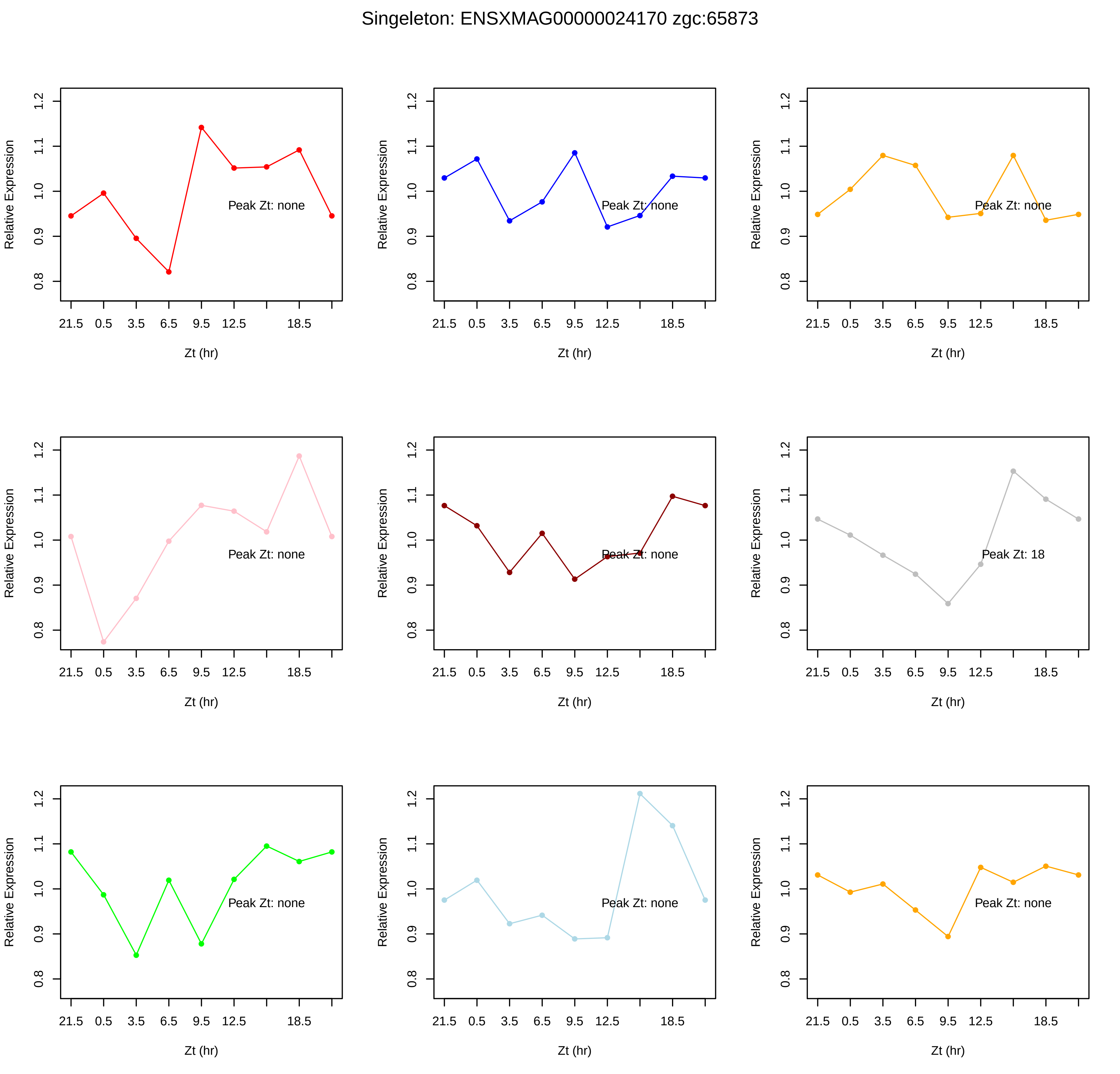
Singleton: ENSXMAG00000024170 zgc:65873
Relative Expression
Zt (hr)
21.5
0.5
3.5
6.5
9.5
12.5
18.5
0.8
0.9
1.0
1.1
1.2
Peak Zt: none
Peak Zt: none
Peak Zt: none
Peak Zt: none
Peak Zt: none
Peak Zt: 18
Peak Zt: none
Peak Zt: none
Peak Zt: none

Singleton: ENSXMAG00000001814 umps

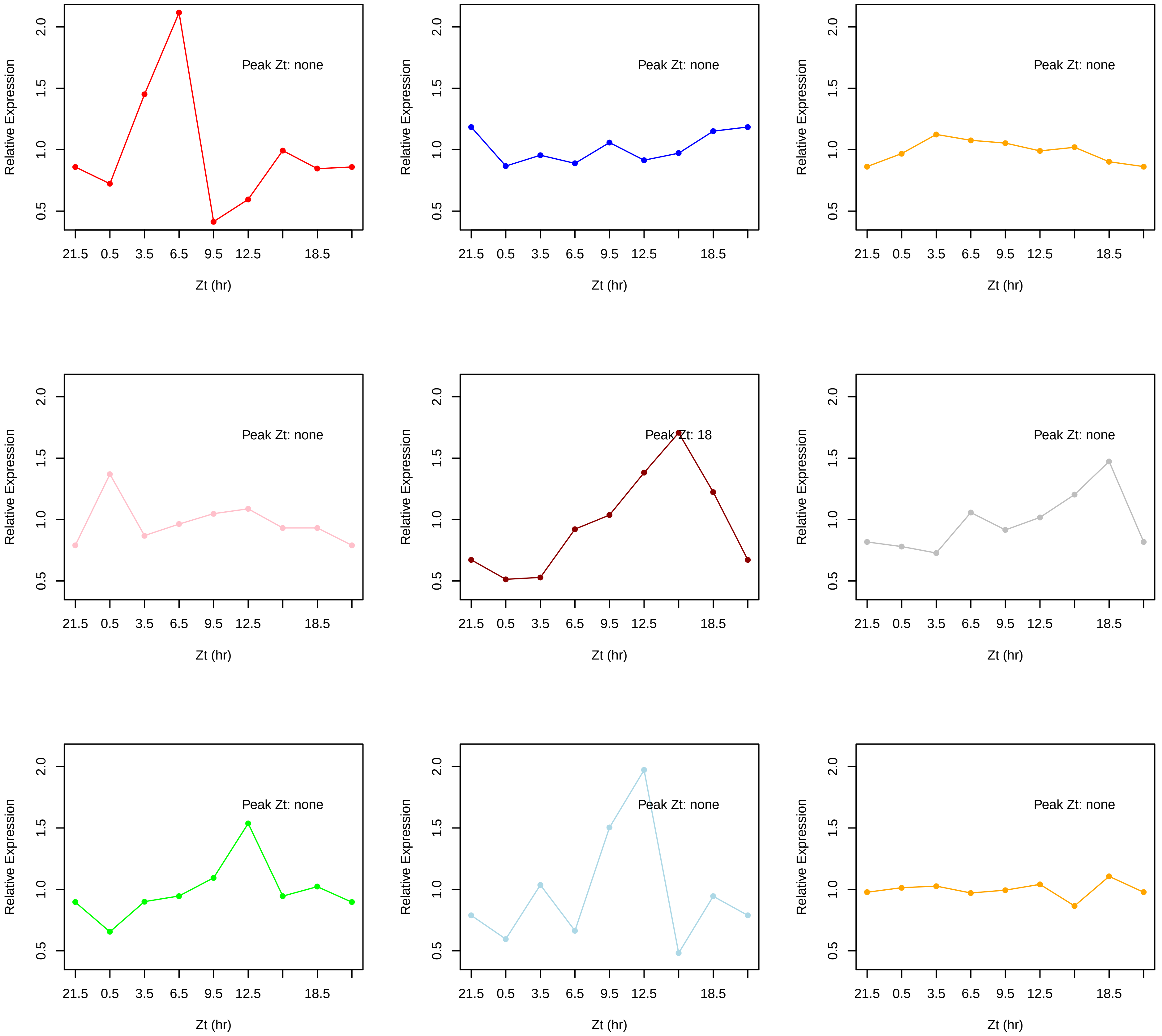

Singeleton: ENSXMAG00000024463 rsrc2

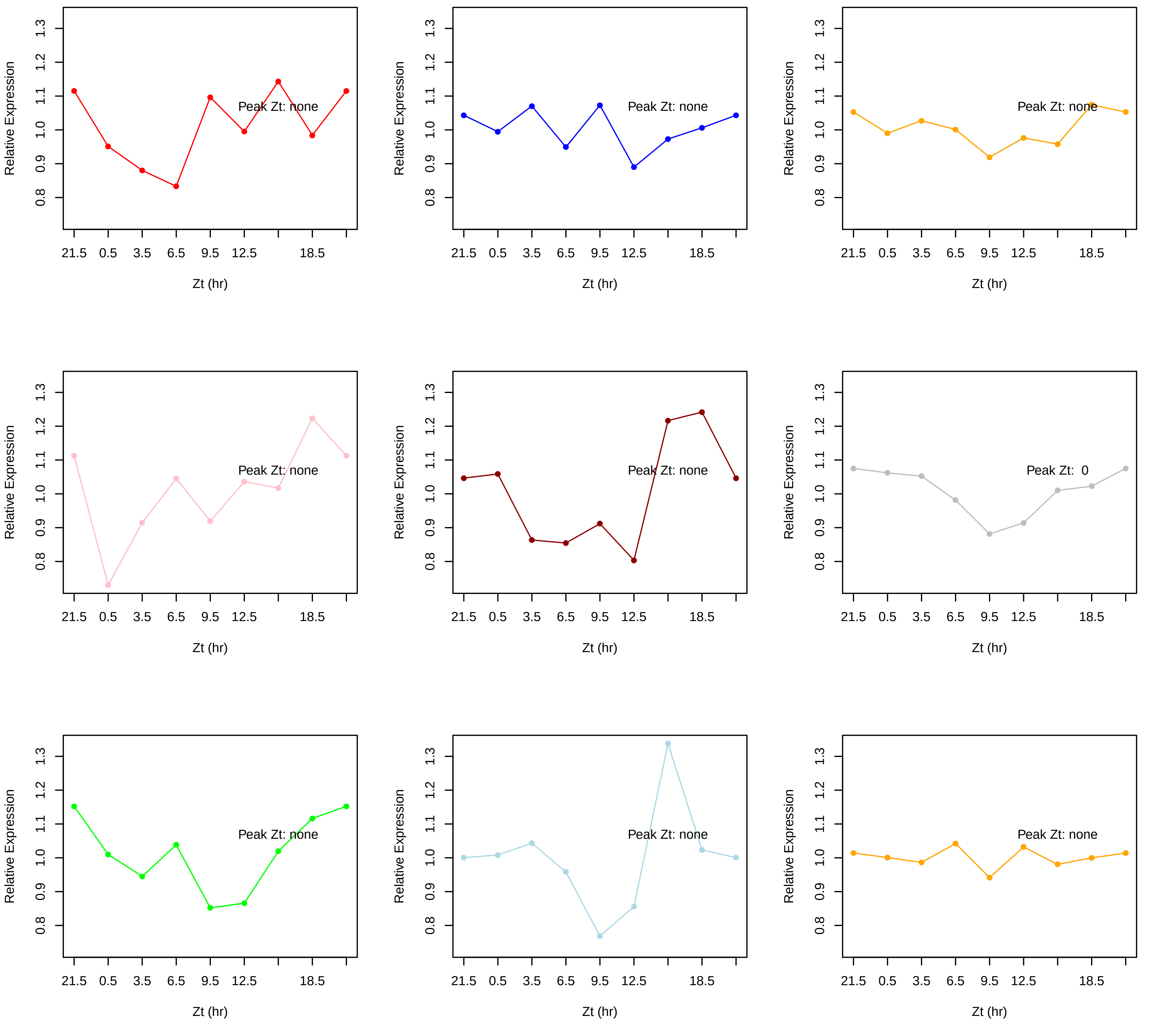

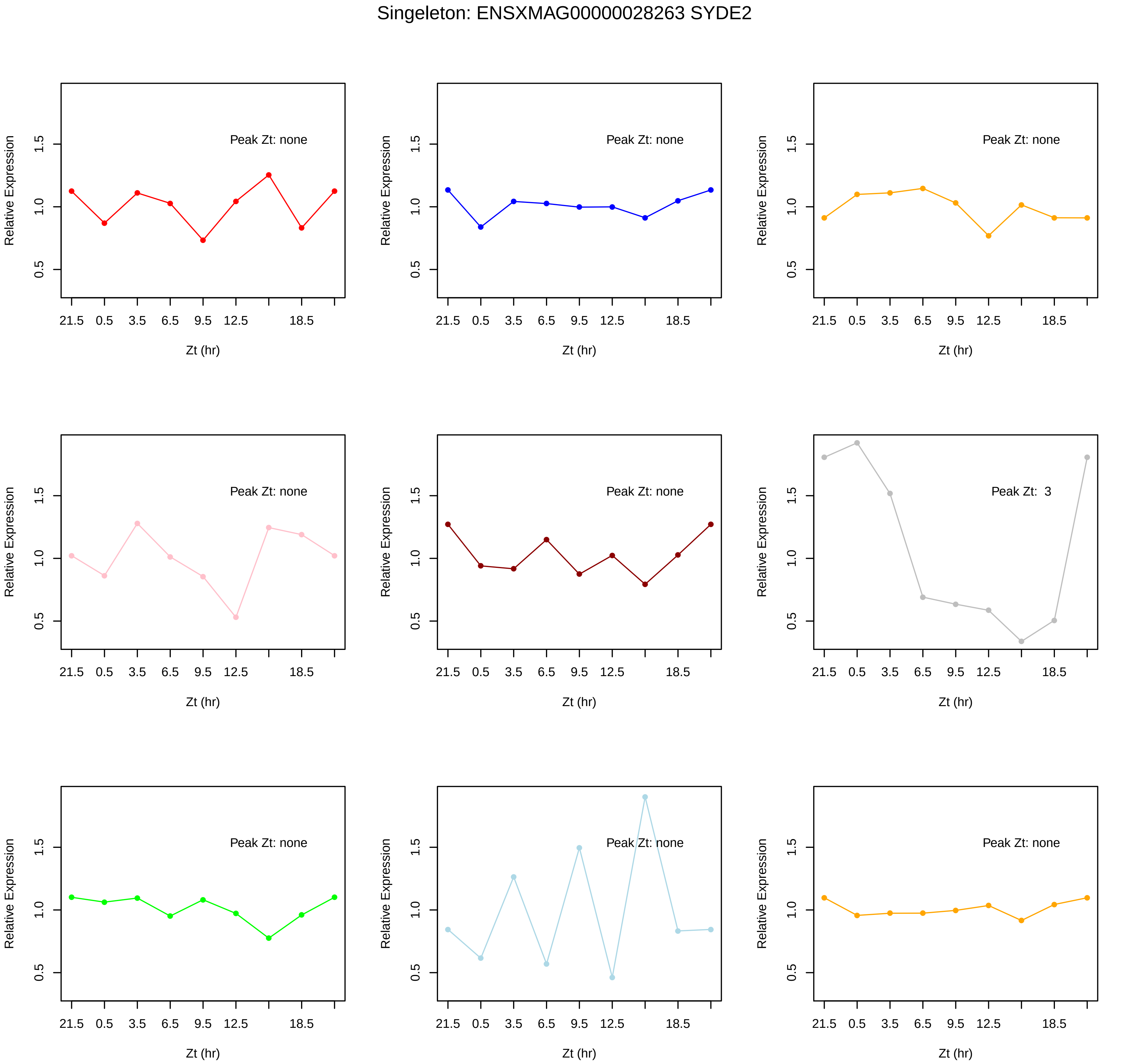
Singeleton: ENSXMAG00000028263 SYDE2
Peak Zt: none
Peak Zt: none
Peak Zt: none
Peak Zt: none
Peak Zt: none
Peak Zt: 3
Peak Zt: none
Peak Zt: none
Peak Zt: none
Relative Expression
Zt (hr)
21.5
0.5
3.5
6.5
9.5
12.5
18.5
0.5
1.0
1.5

Singeleton: ENSXMAG00000022122 pacsin3

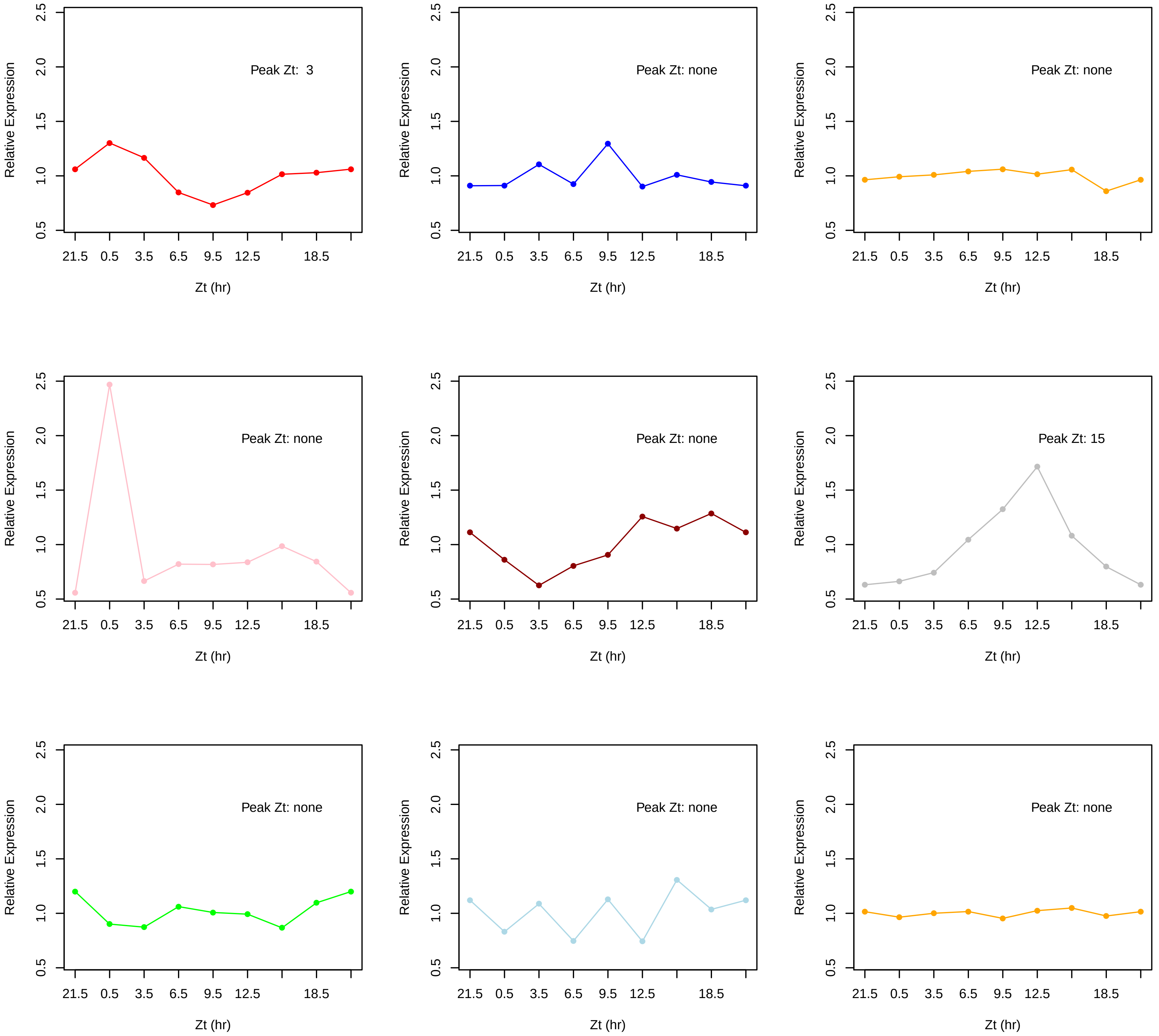

Singleton: ENSXMAG00000029455 si:ch211−197h24.6

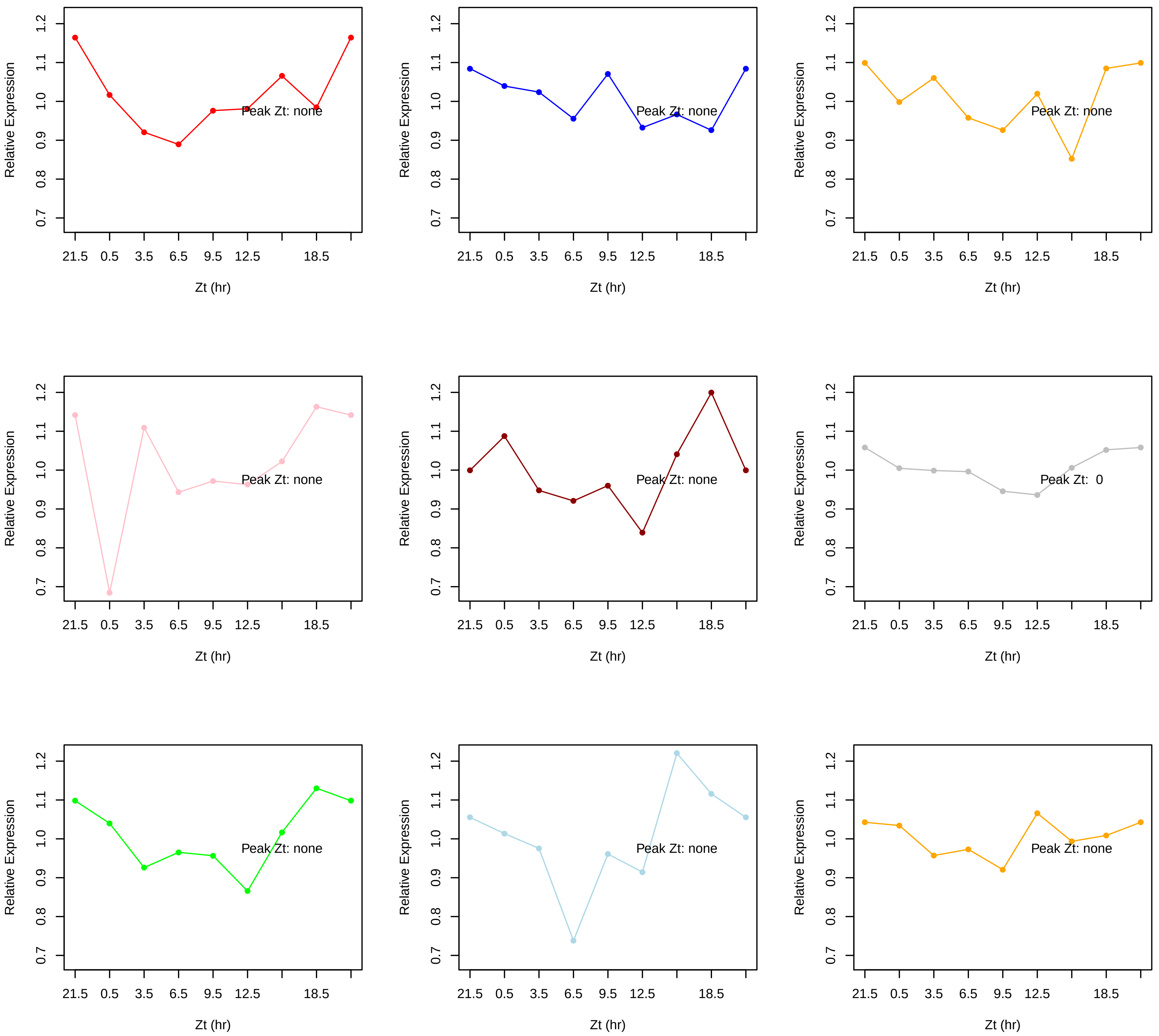

Singeleton: ENSXMAG00000018844 rps6ka1

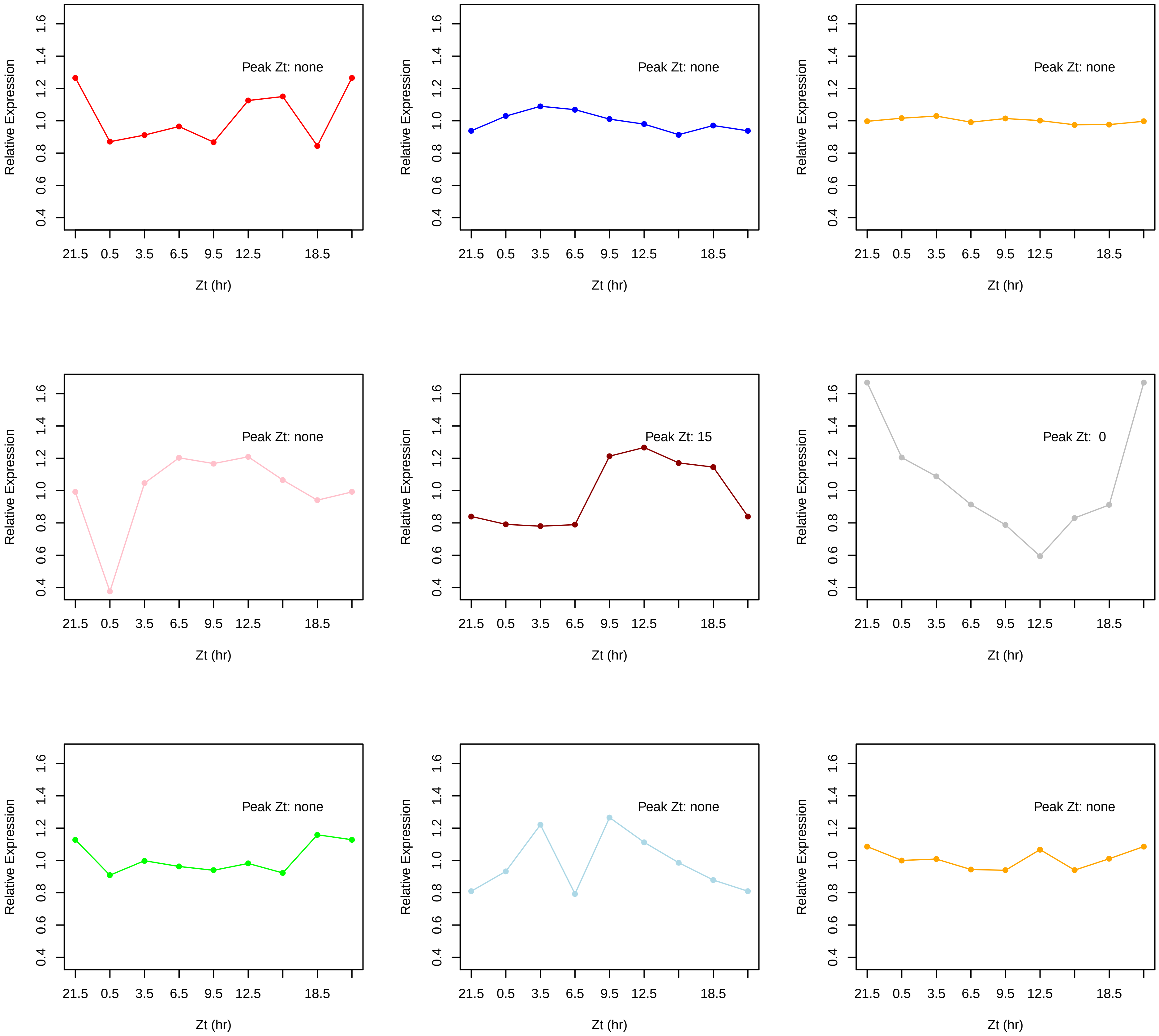

Singleton: ENSXMAG00000021359 sned1

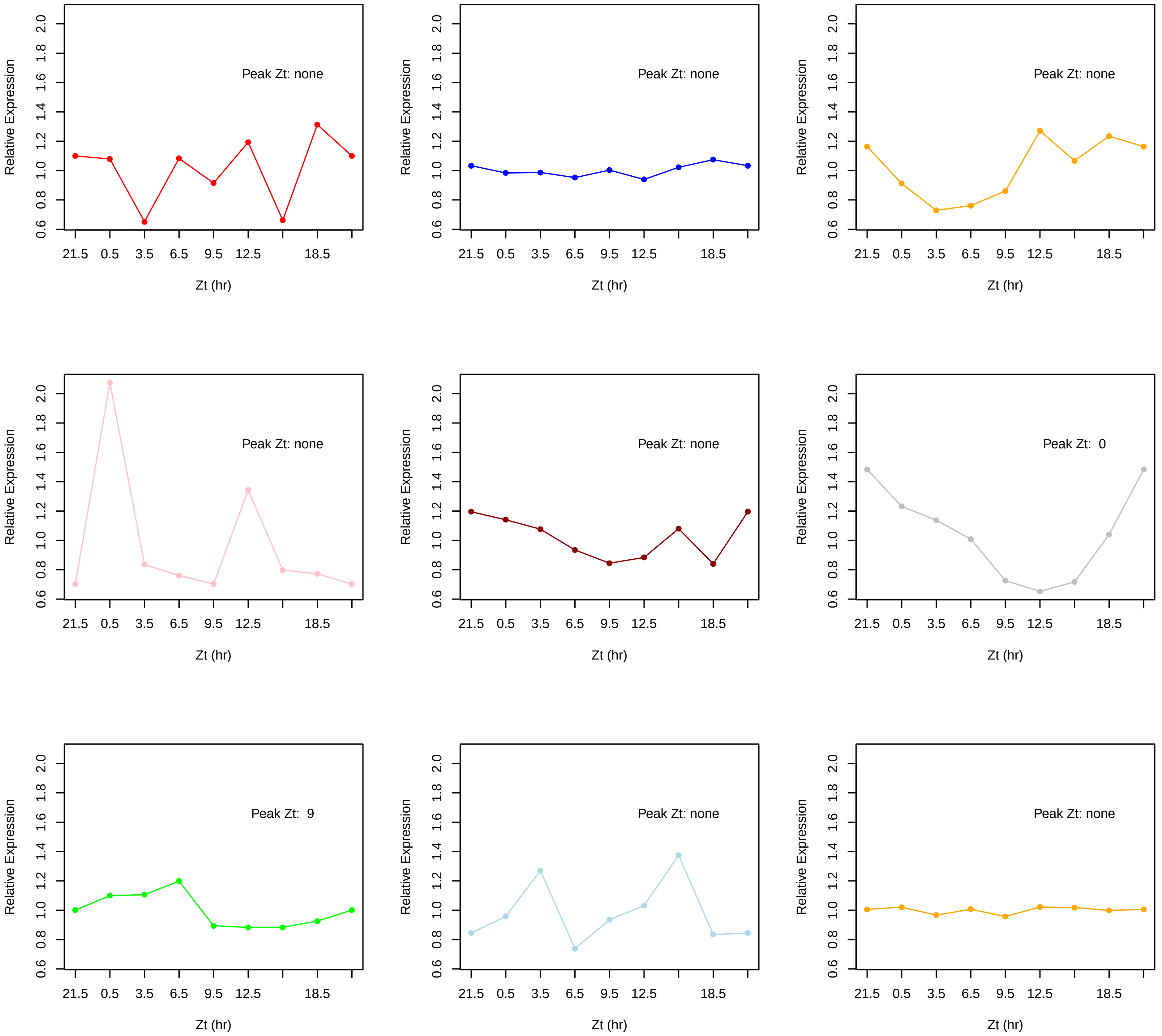

Singleton: ENSXMAG00000005081 kalrna

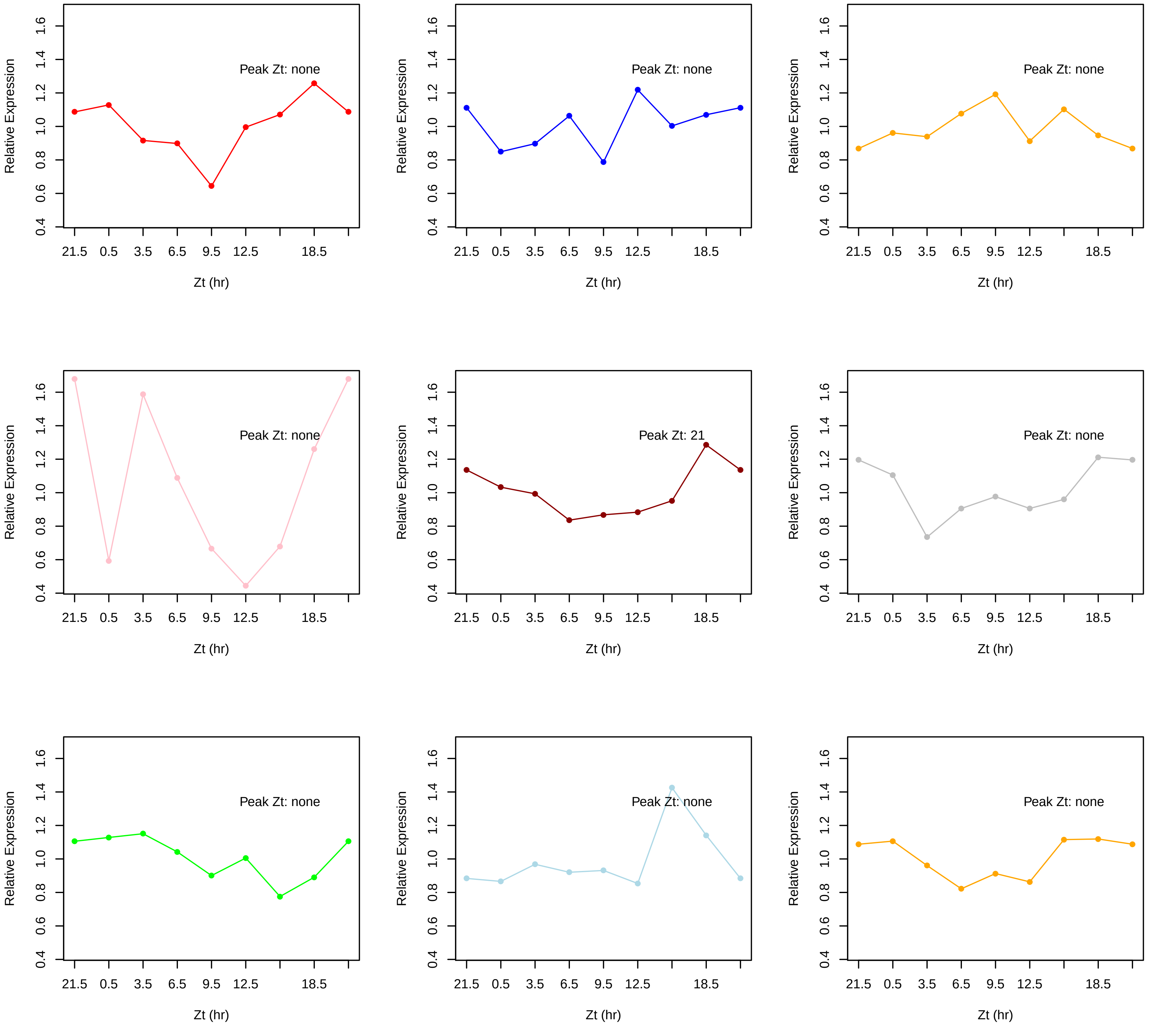

Singeleton: ENSXMAG00000012585 rgmd

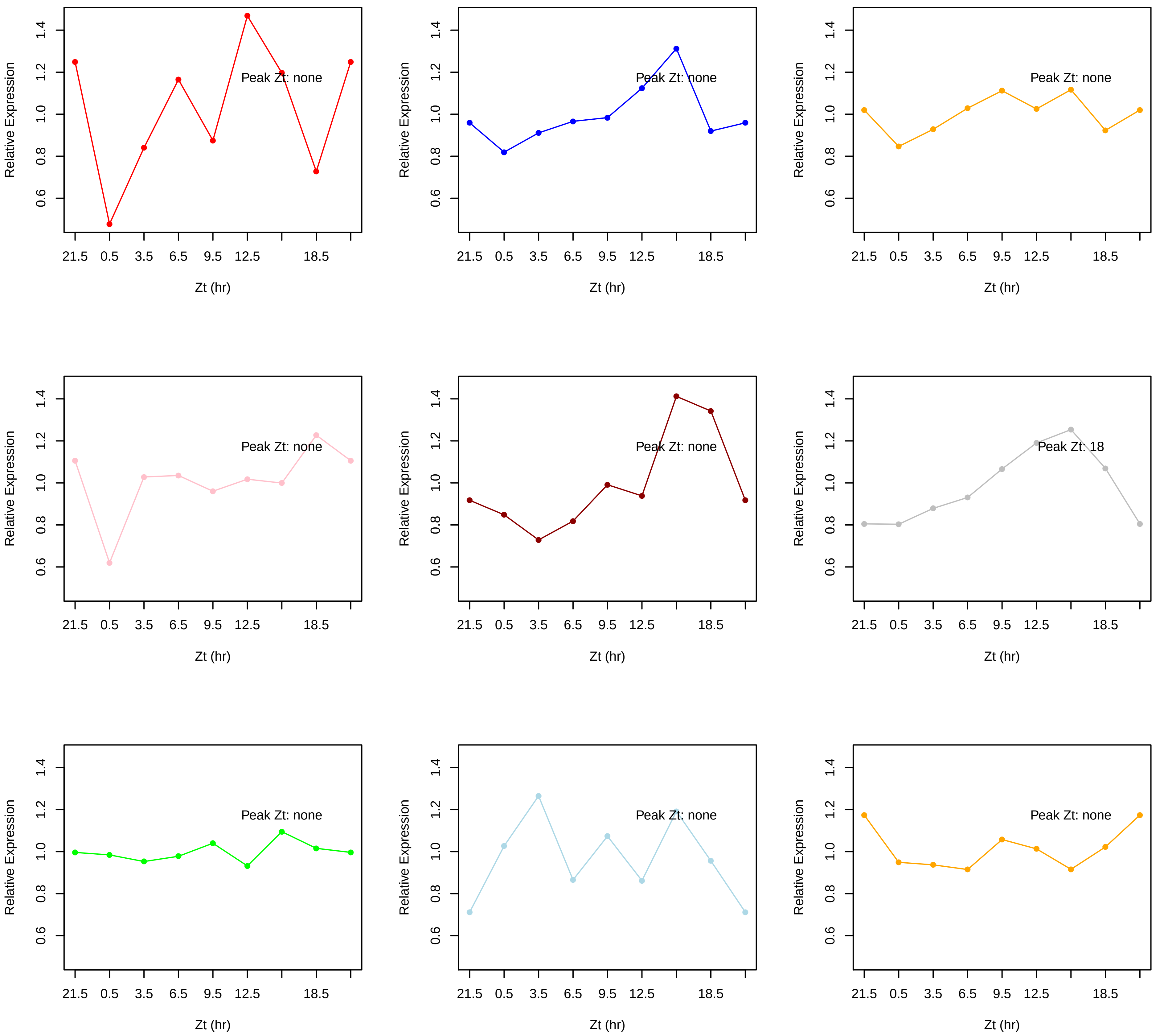

Singleton: ENSXMAG00000017627 kntc1

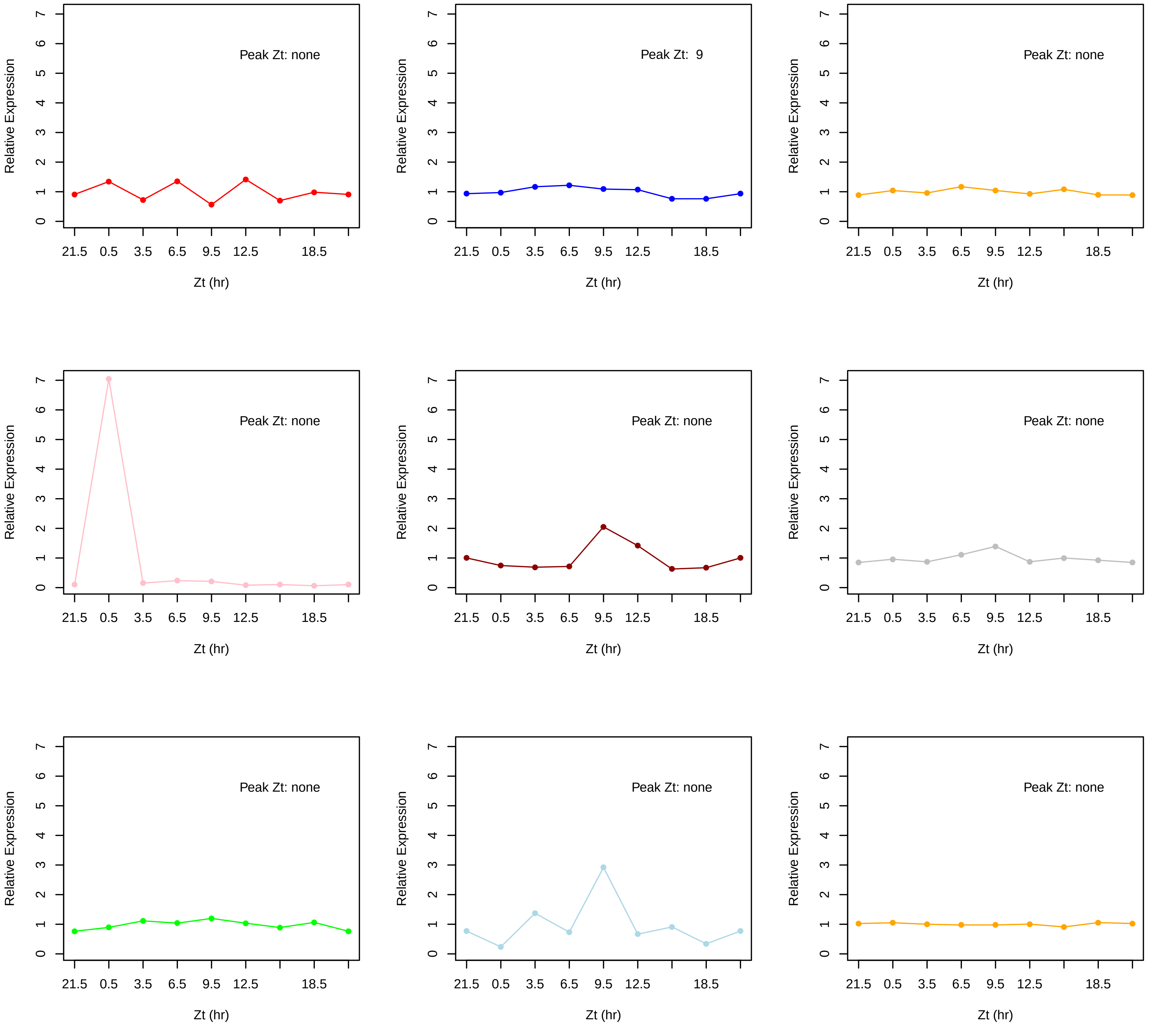

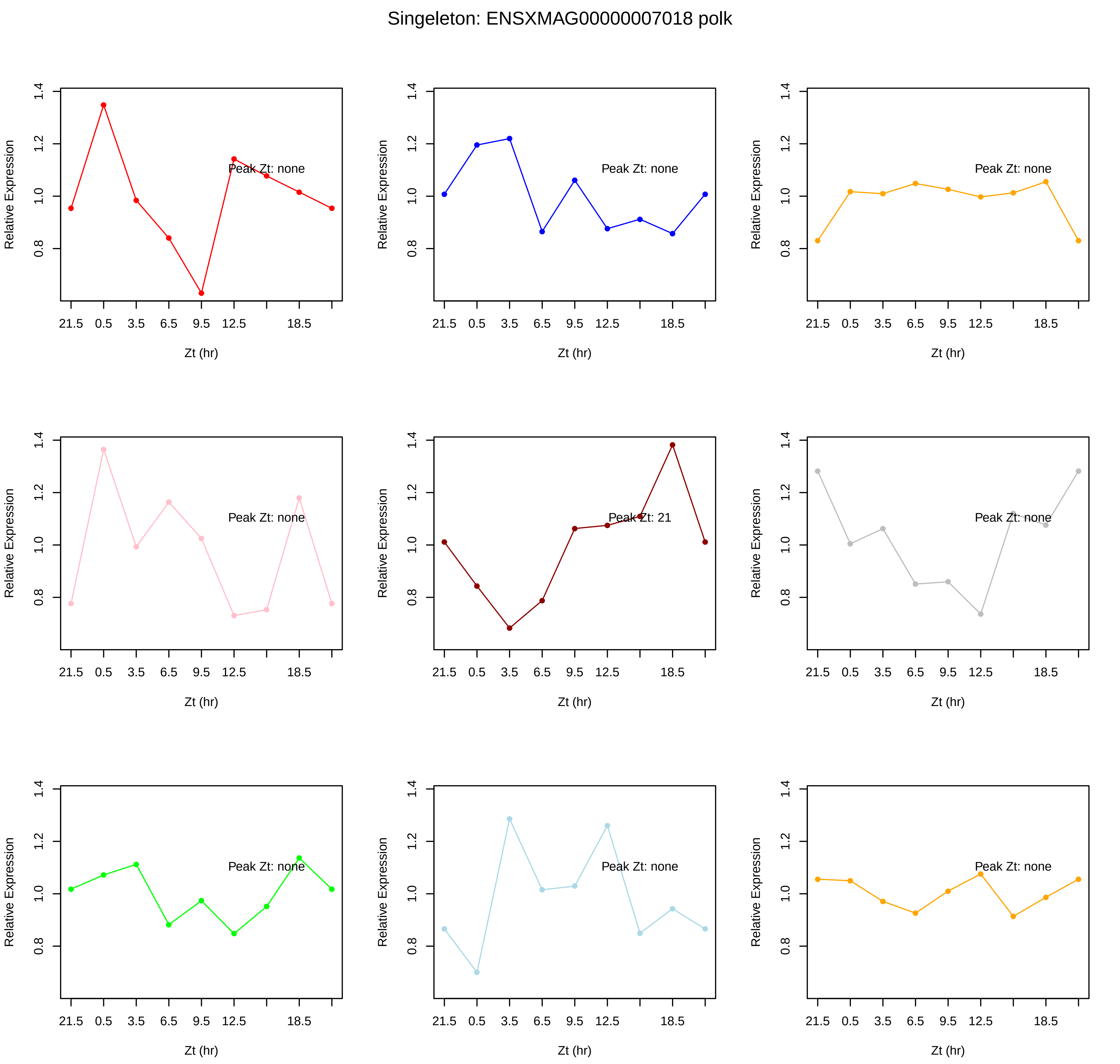
Singeleton: ENSXMAG00000007018 polk
Relative Expression
Zt (hr)
Peak Zt: none
Peak Zt: 21
1.4
1.2
1.0
0.8
21.5
0.5
3.5
6.5
9.5
12.5
18.5

Singeleton: ENSXMAG00000003694 ddb2

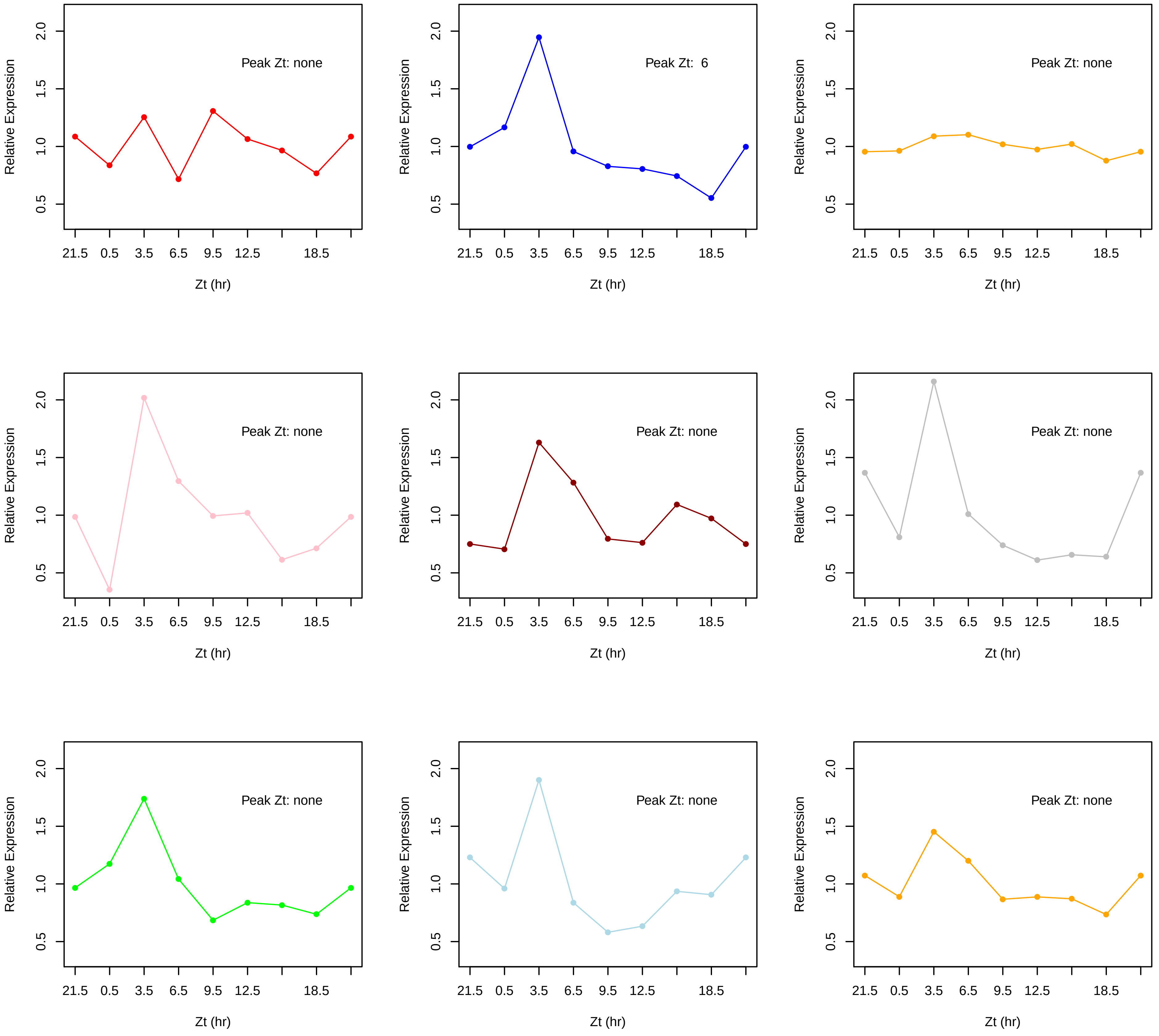

Singeleton: ENSXMAG00000013641 cnksr1

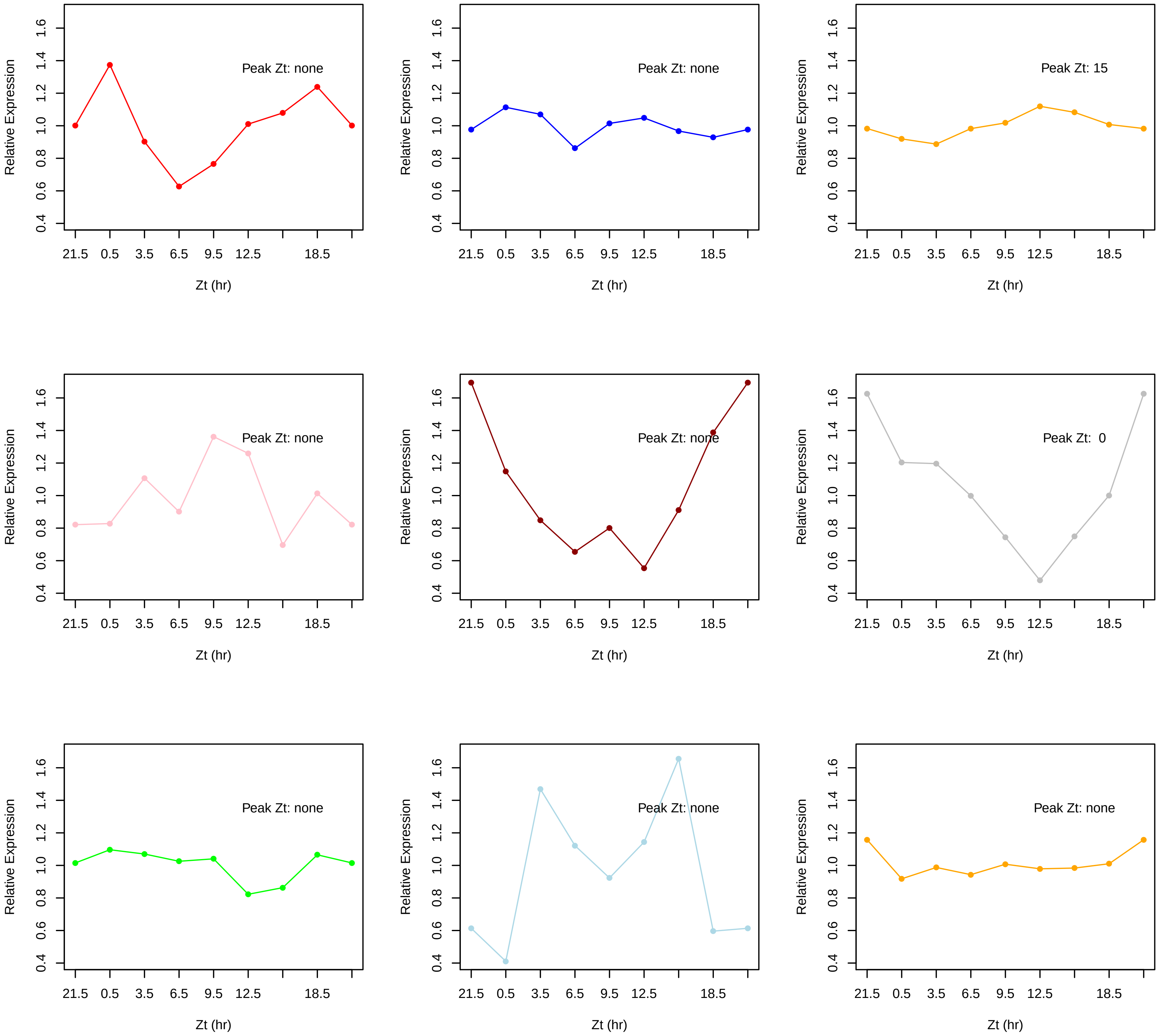

Singleton: ENSXMAG00000018673 eif4bb

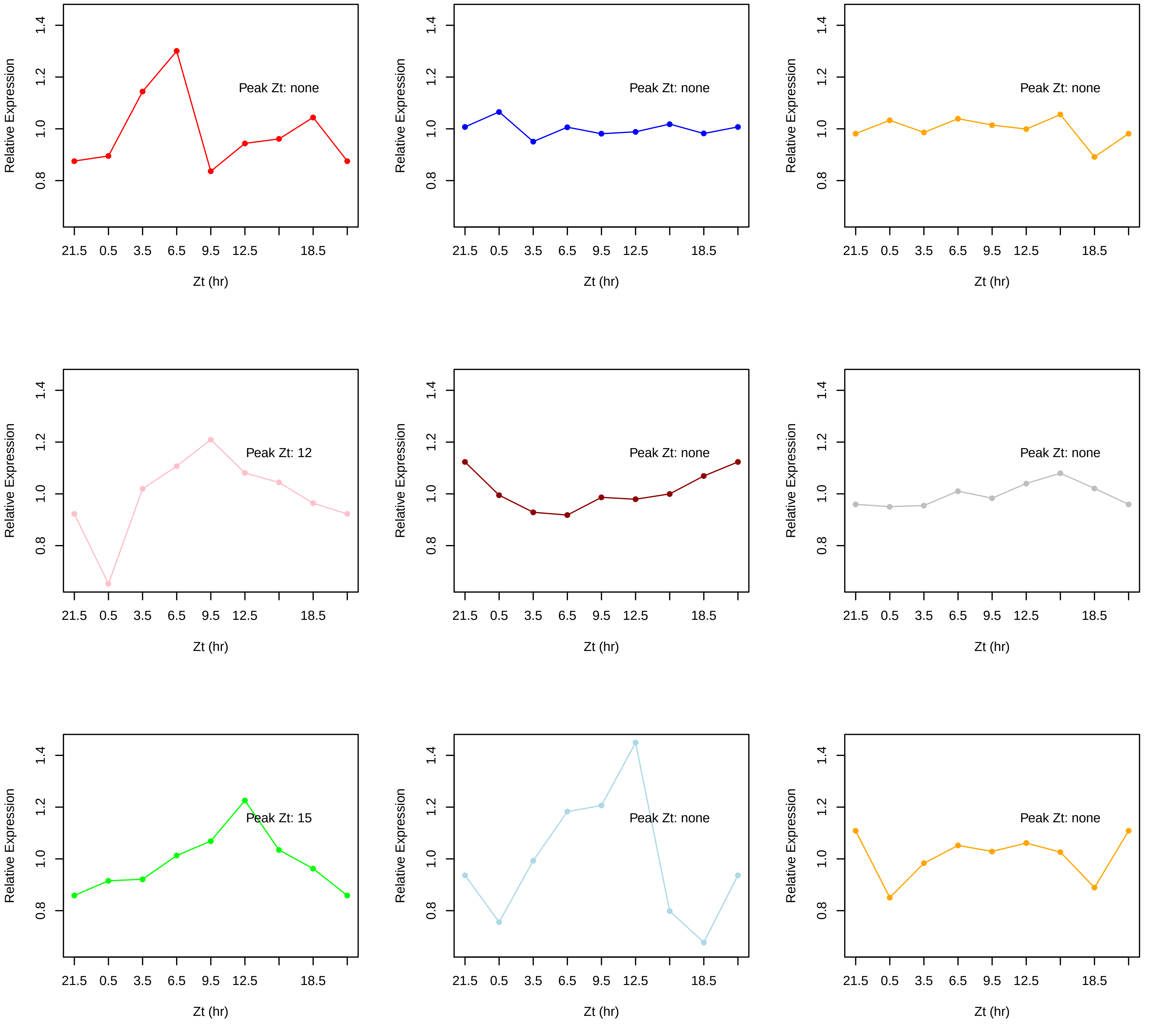

Singeleton: ENSXMAG00000019247 denr

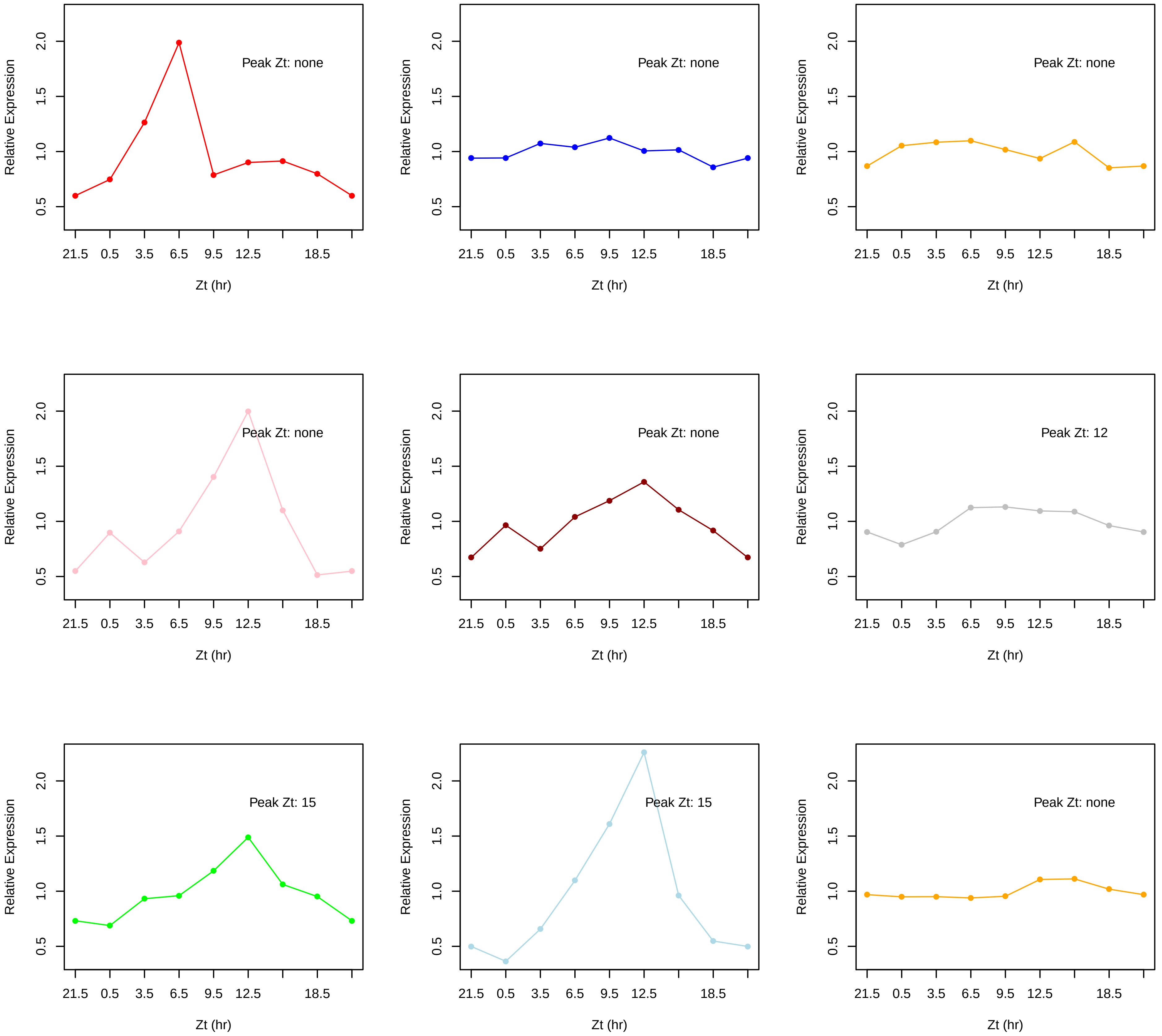

Singleton: ENSXMAG00000018438

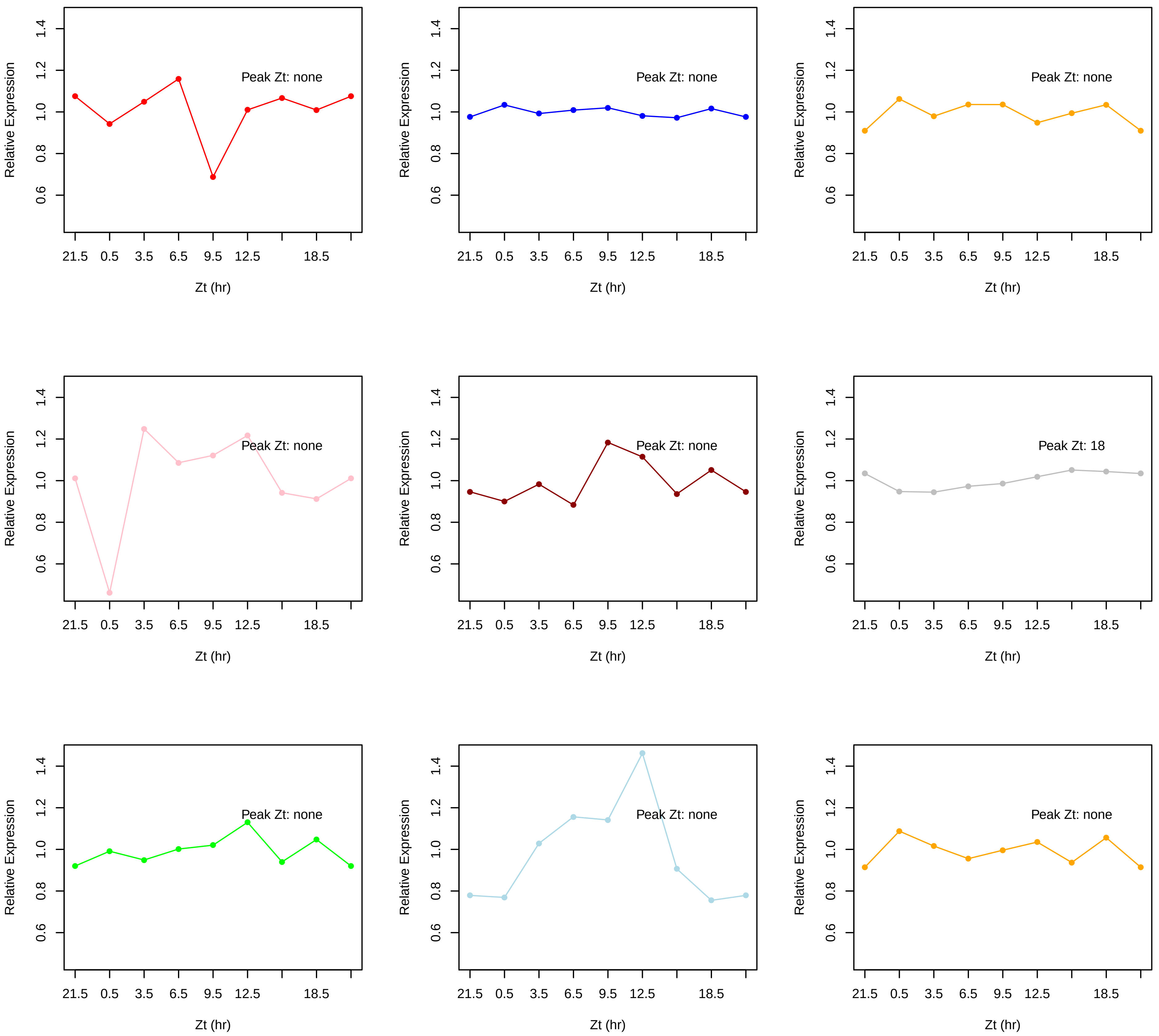

Singleton: ENSXMAG00000003669 klhl30

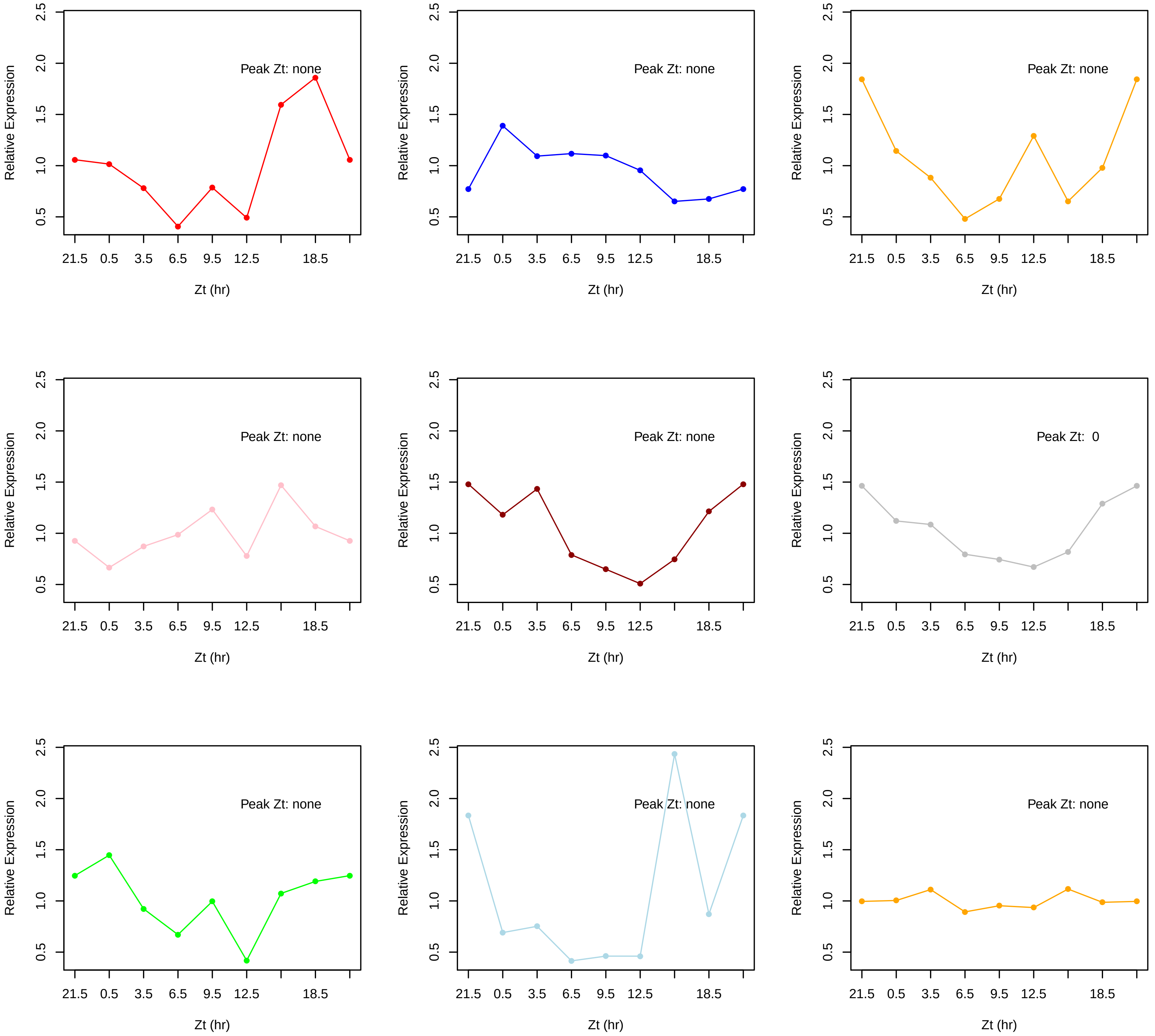

Singeleton: ENSXMAG00000028249 unc119b

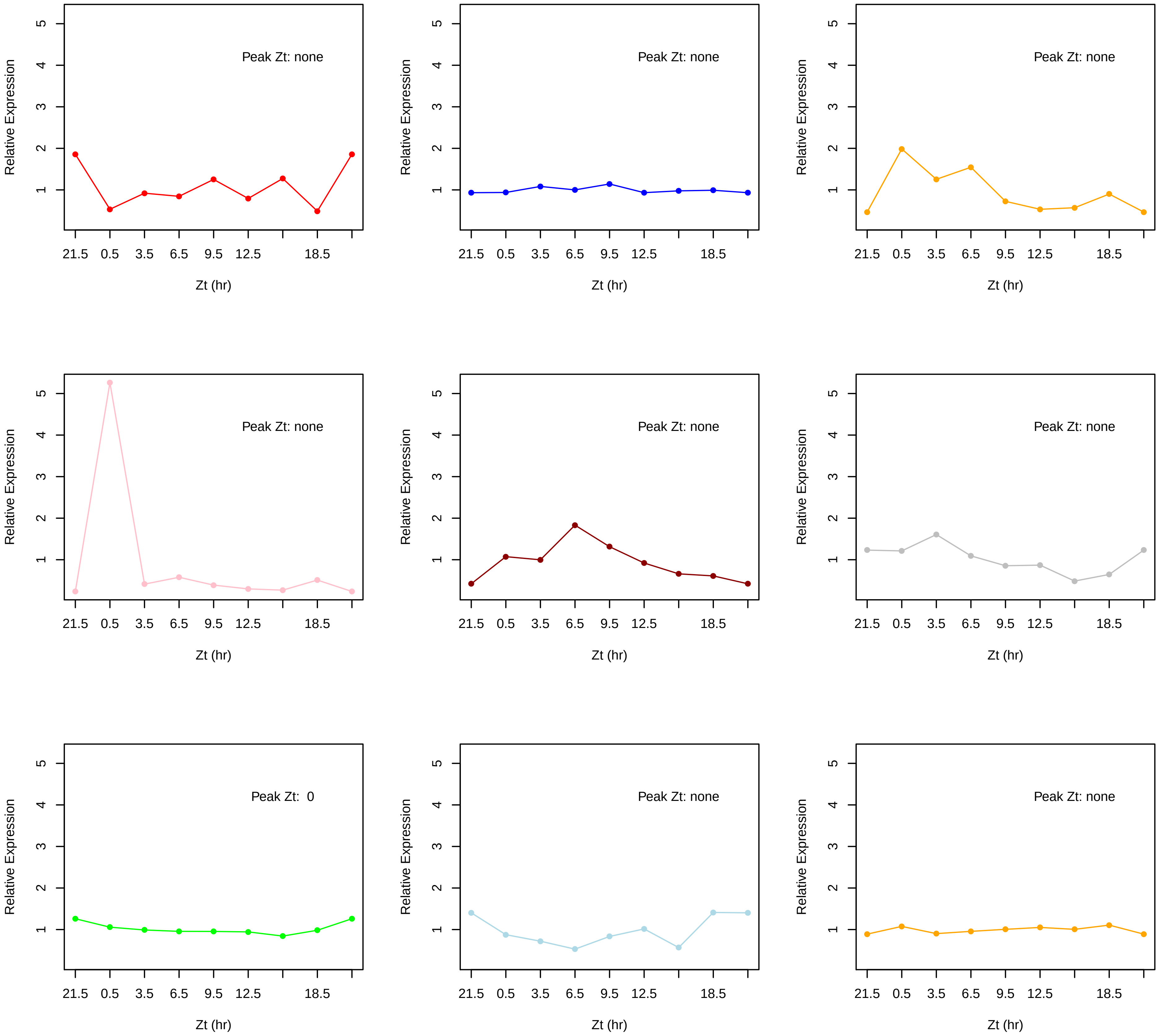

Singeleton: ENSXMAG00000007002 poc5

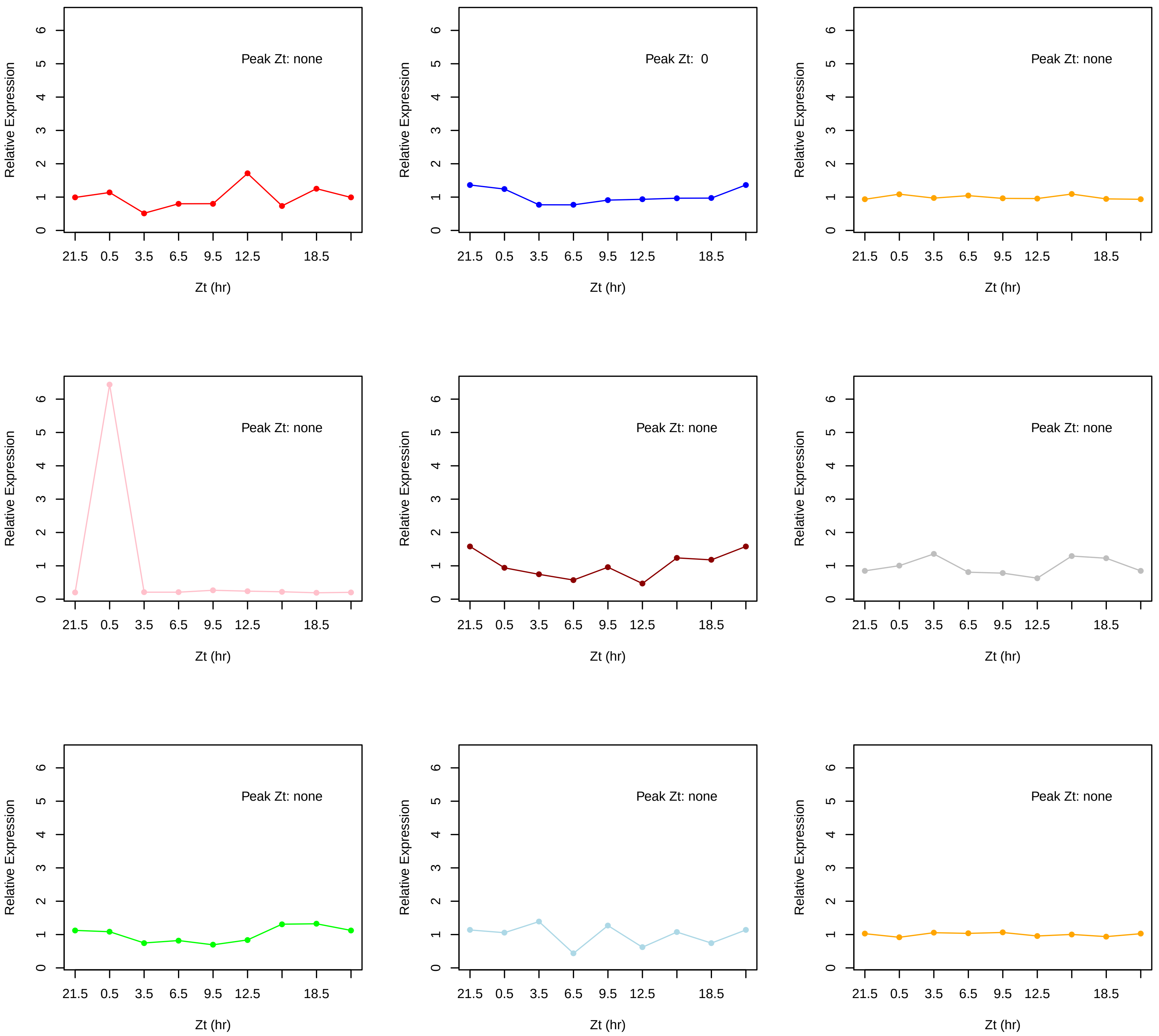

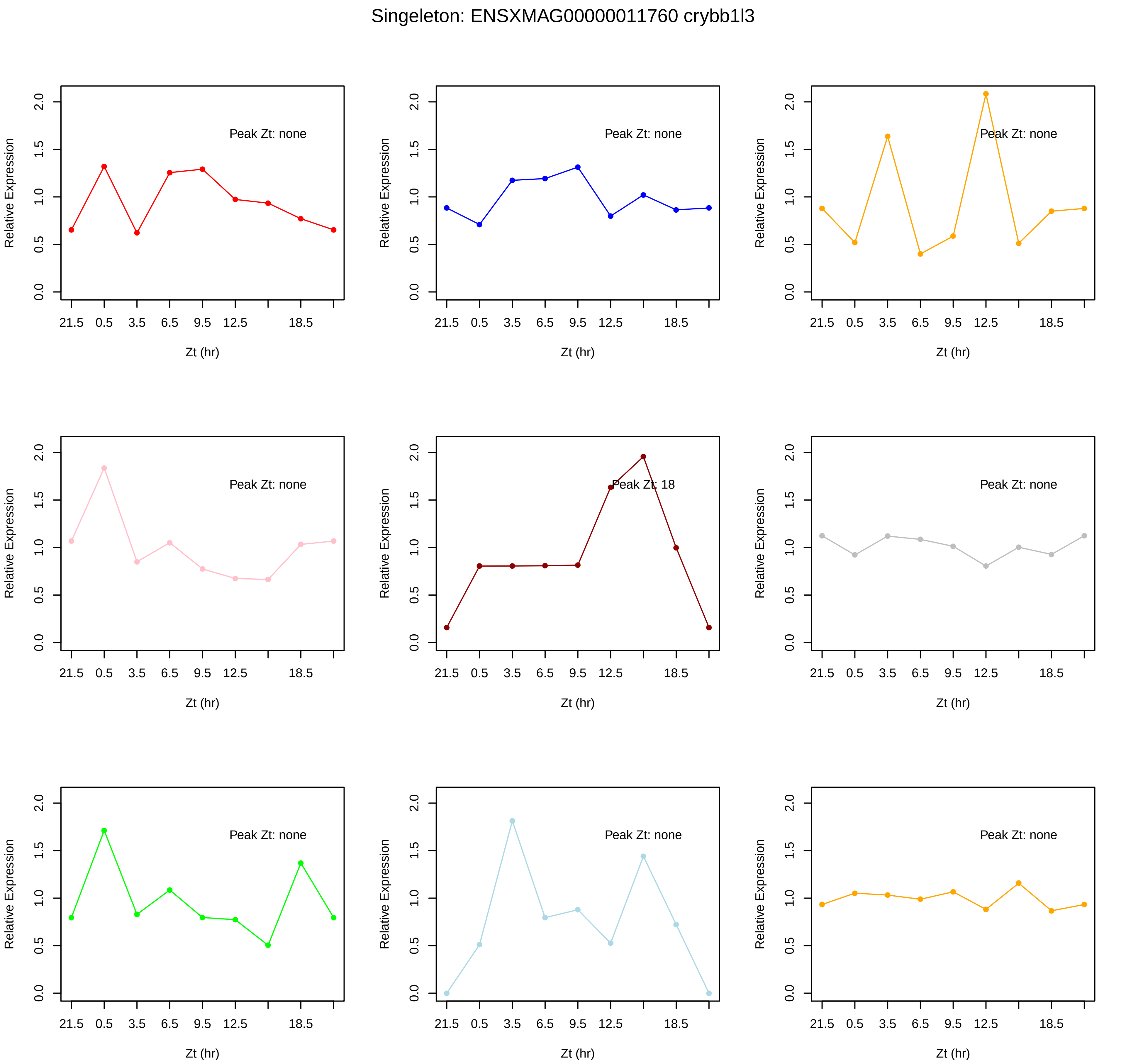

Singeleton: ENSXMAG00000011760 crybb1l3
Peak Zt: none
Peak Zt: none
Peak Zt: none
Peak Zt: none
Peak Zt: 18
Peak Zt: none
Peak Zt: none
Peak Zt: none
Peak Zt: none
Relative Expression
Zt (hr)
21.5 0.5 3.5 6.5 9.5 12.5 18.5
0.0 0.5 1.0 1.5 2.0

Singeleton: ENSXMAG00000001712 gpalpp1

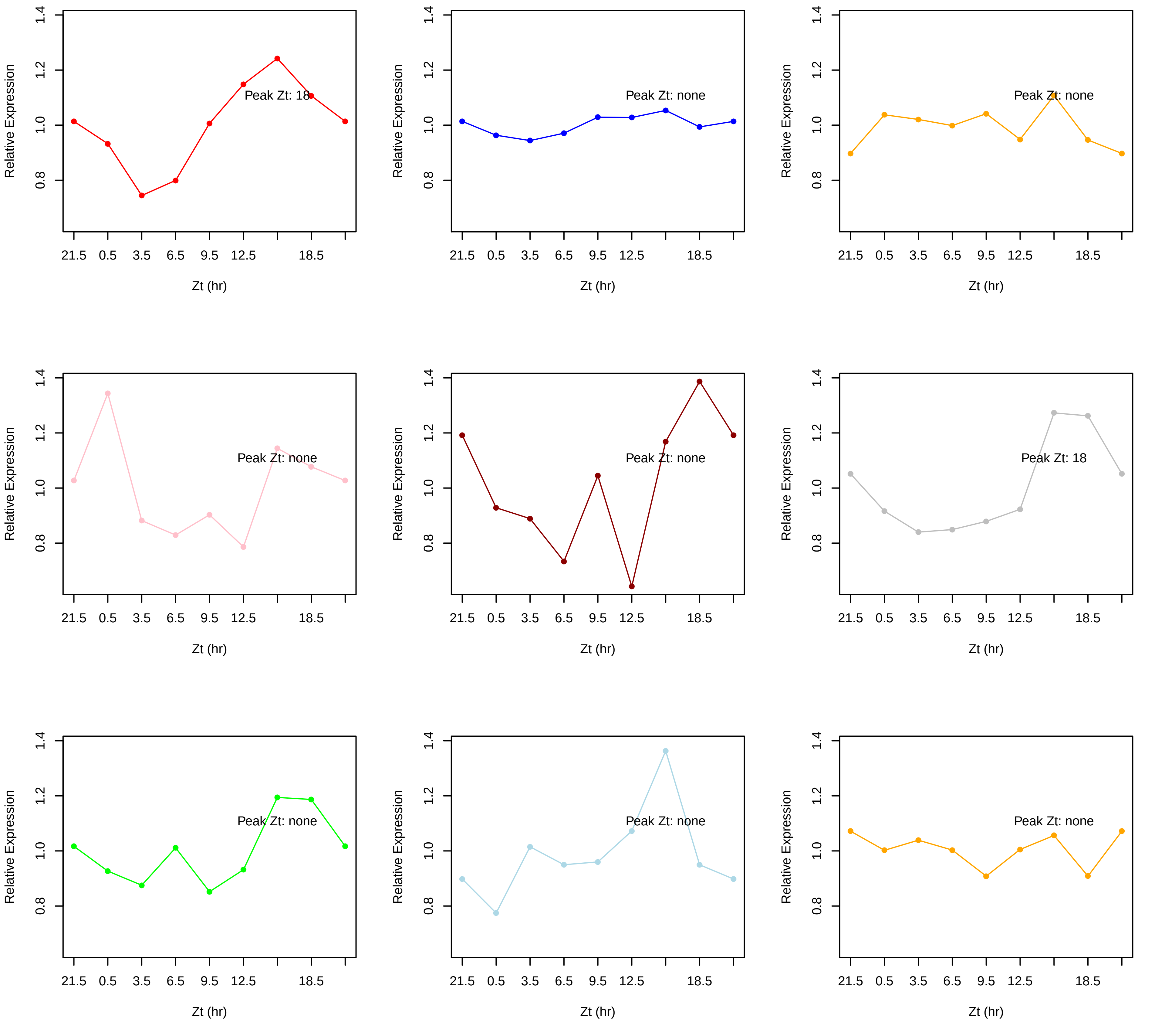

Singeleton: ENSXMAG00000007553 pgr

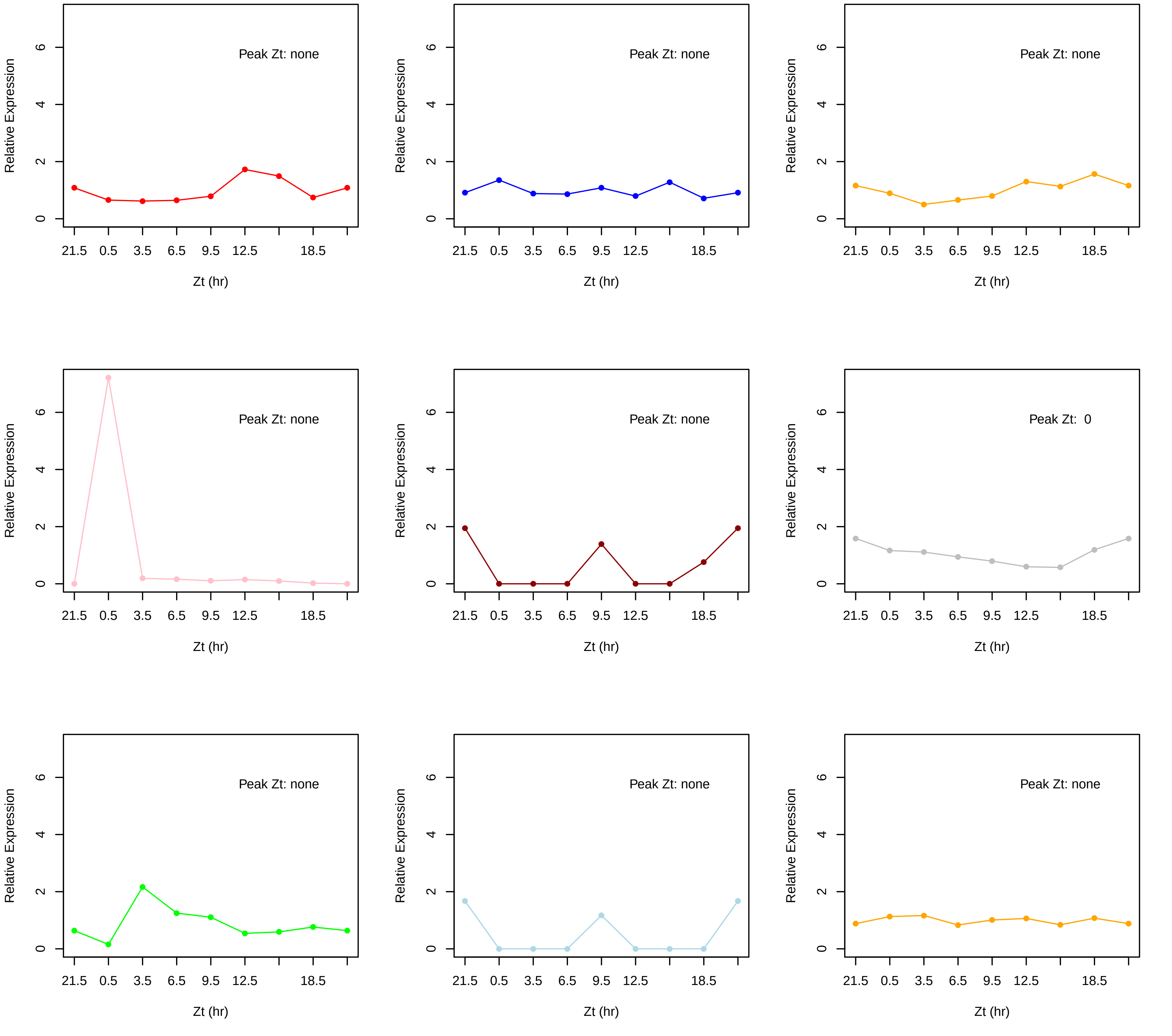

Singeleton: ENSXMAG00000006794 iqgap2

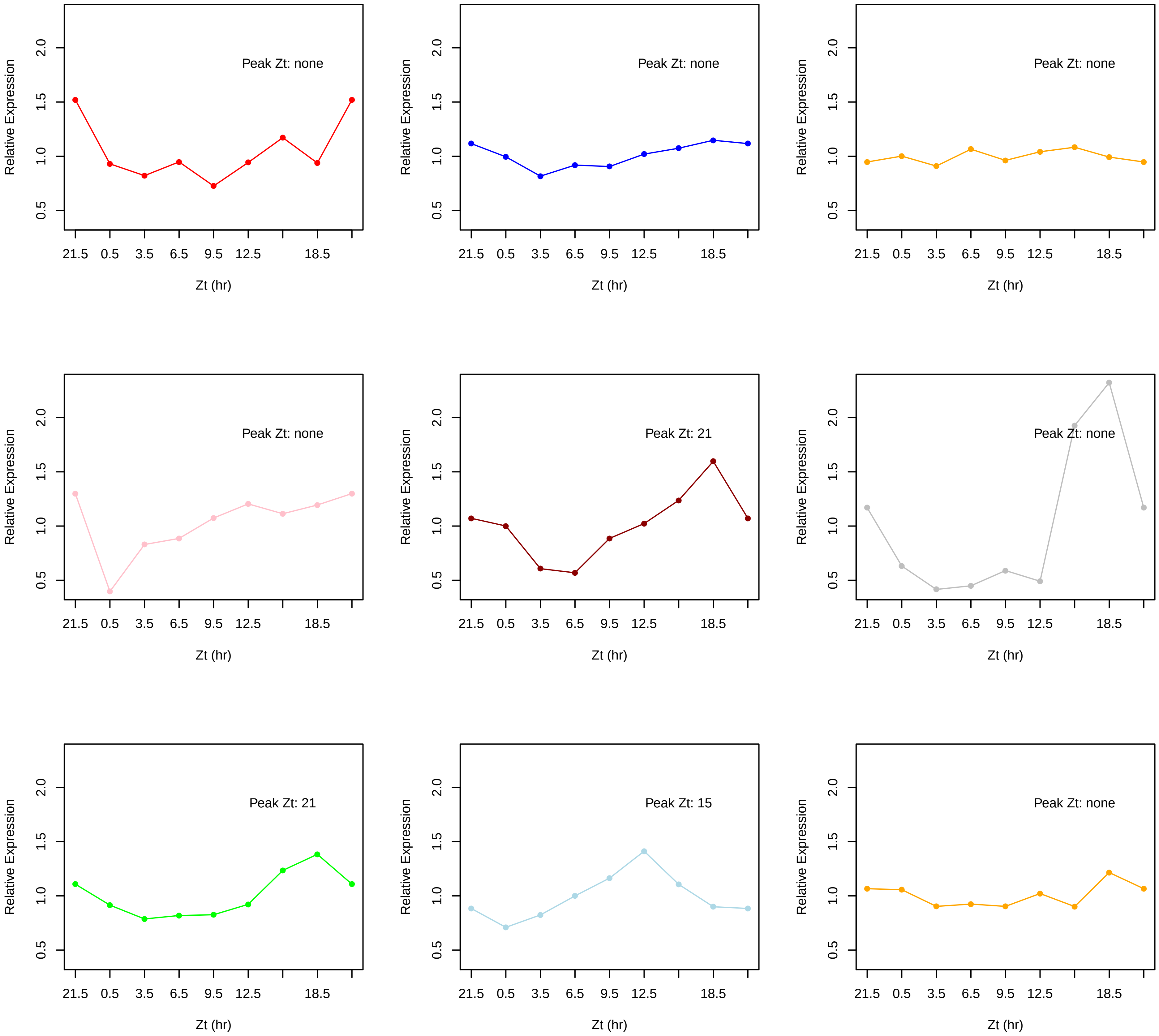

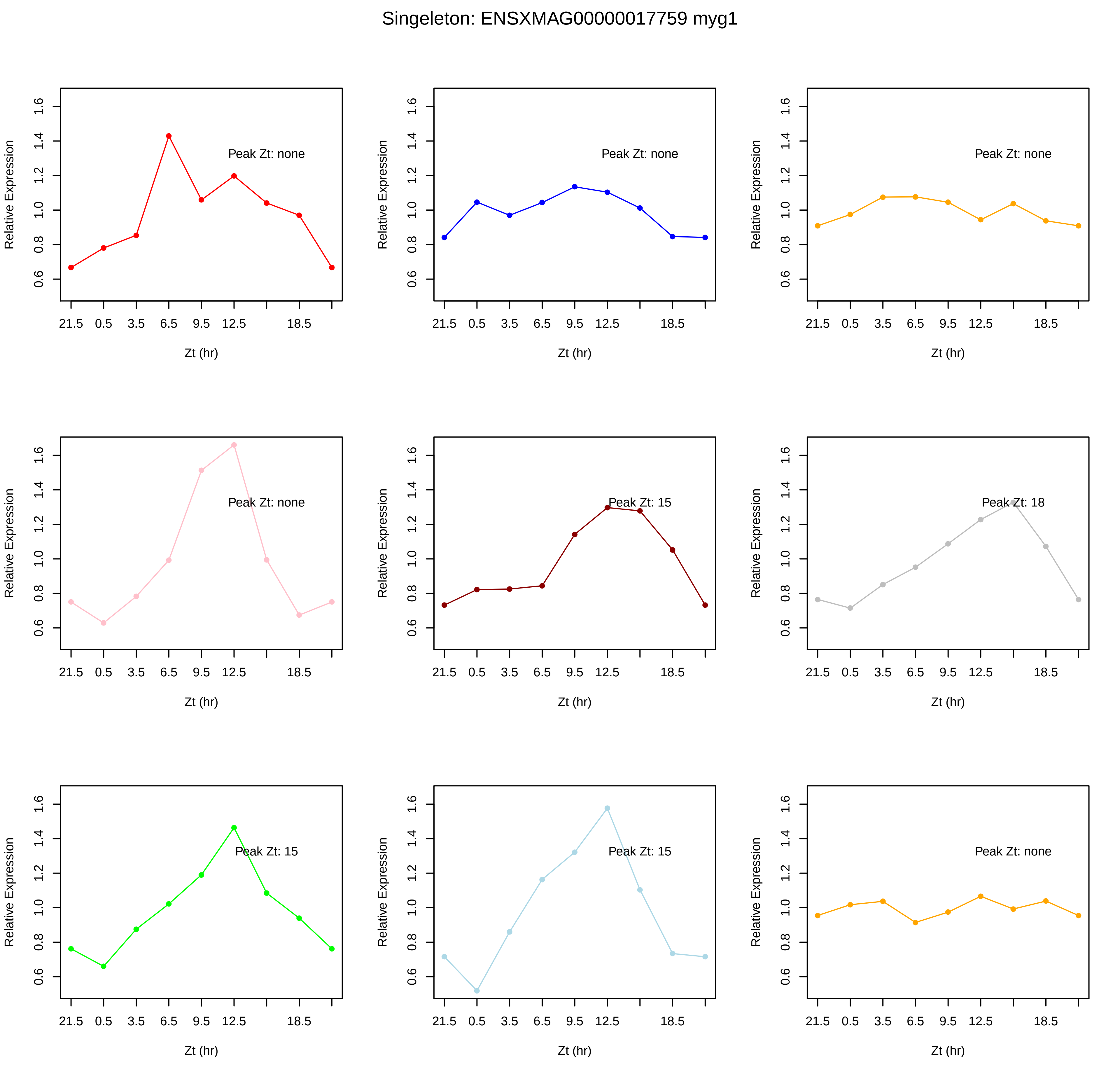

Singeleton: ENSXMAG00000017759 myg1
Peak Zt: none
Peak Zt: none
Peak Zt: none
Peak Zt: none
Peak Zt: 15
Peak Zt: 18
Peak Zt: 15
Peak Zt: 15
Peak Zt: none
Relative Expression
Zt (hr)
21.5
0.5
3.5
6.5
9.5
12.5
18.5
0.6
0.8
1.0
1.2
1.4
1.6

Singleton: ENSXMAG00000013272 dhdds

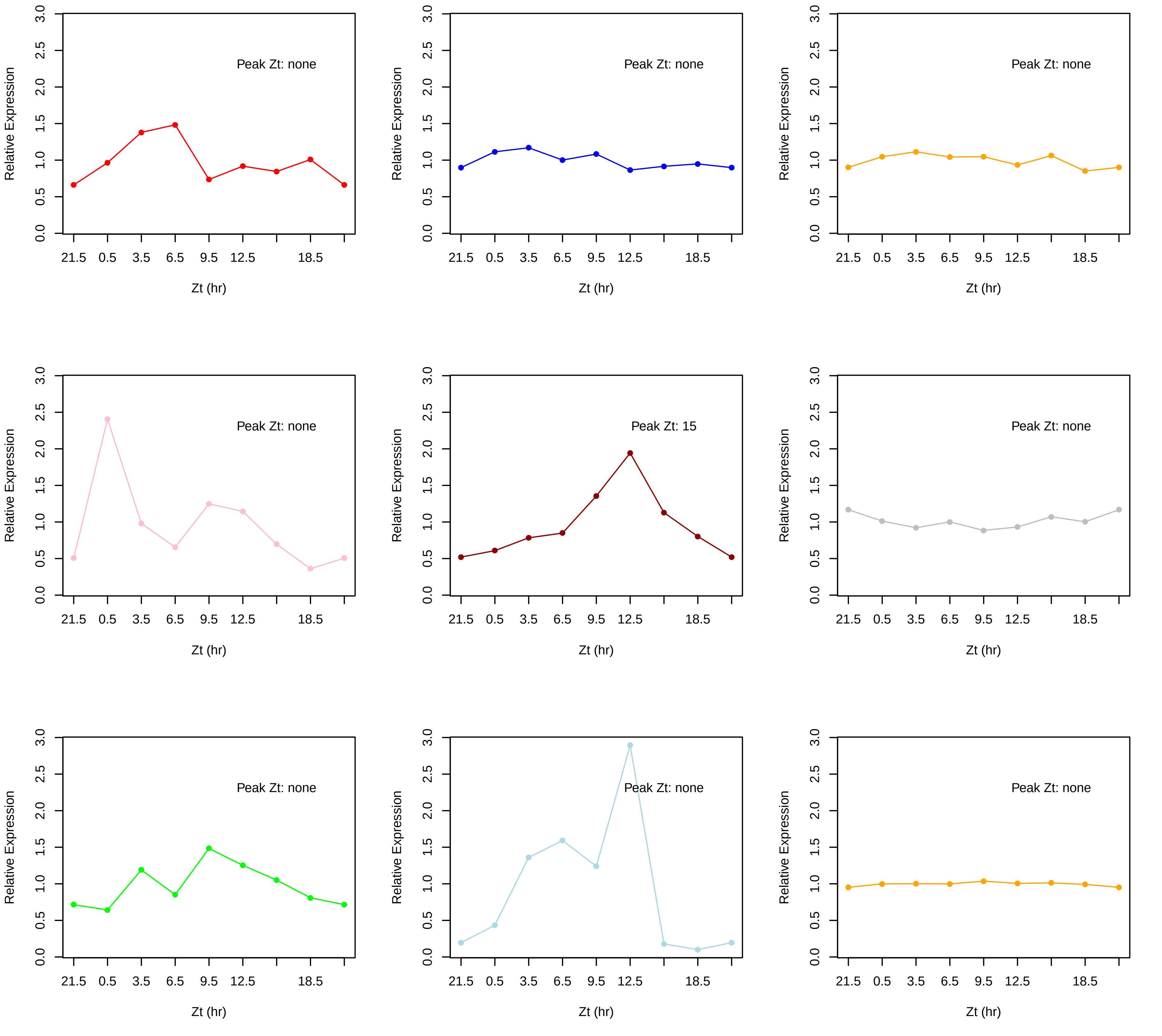

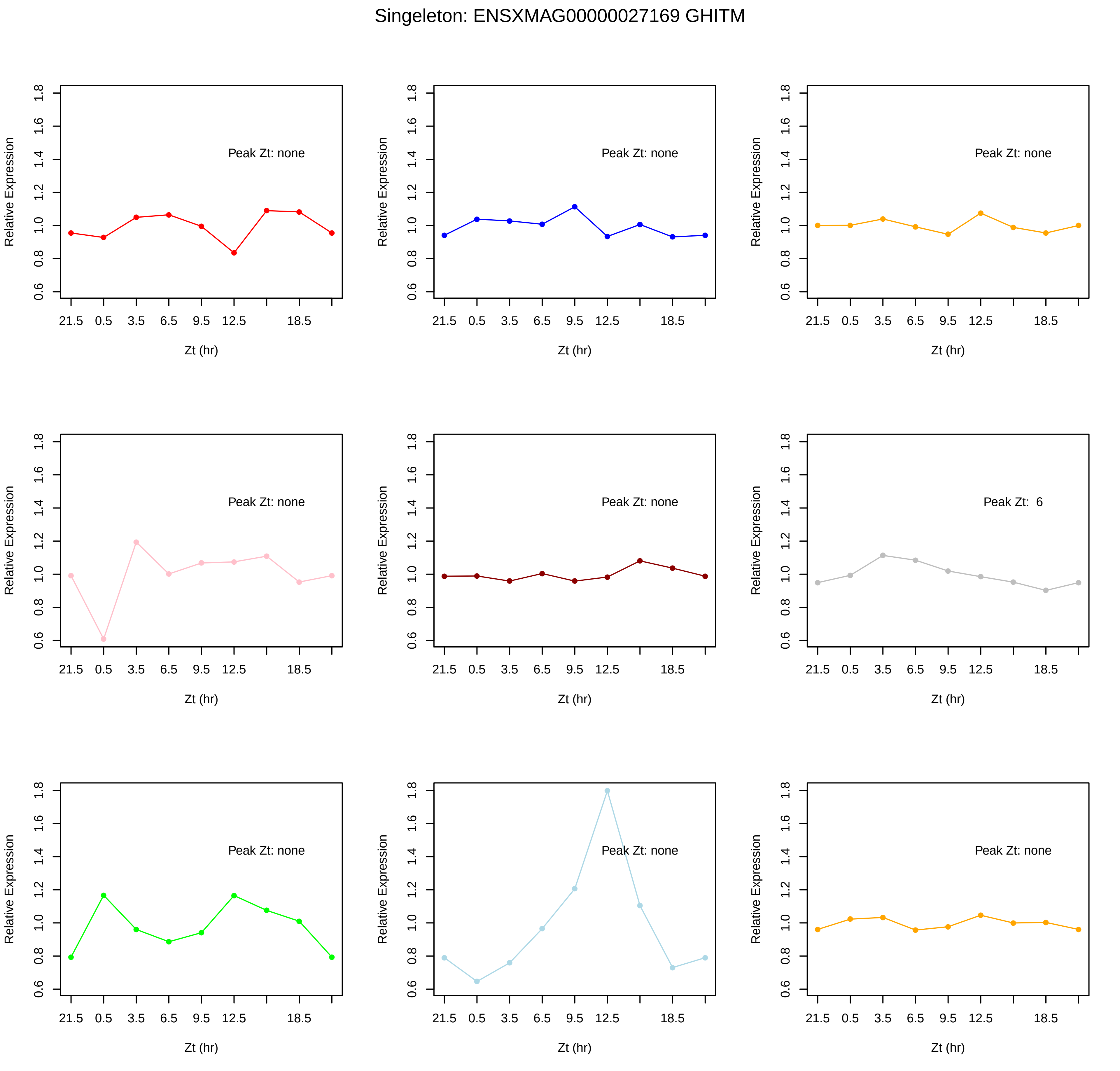

Singeleton: ENSXMAG00000027169 GHITM
Relative Expression
Zt (hr)
Peak Zt: none
Peak Zt: 6
21.5
0.5
3.5
6.5
9.5
12.5
18.5
0.6
0.8
1.0
1.2
1.4
1.6
1.8

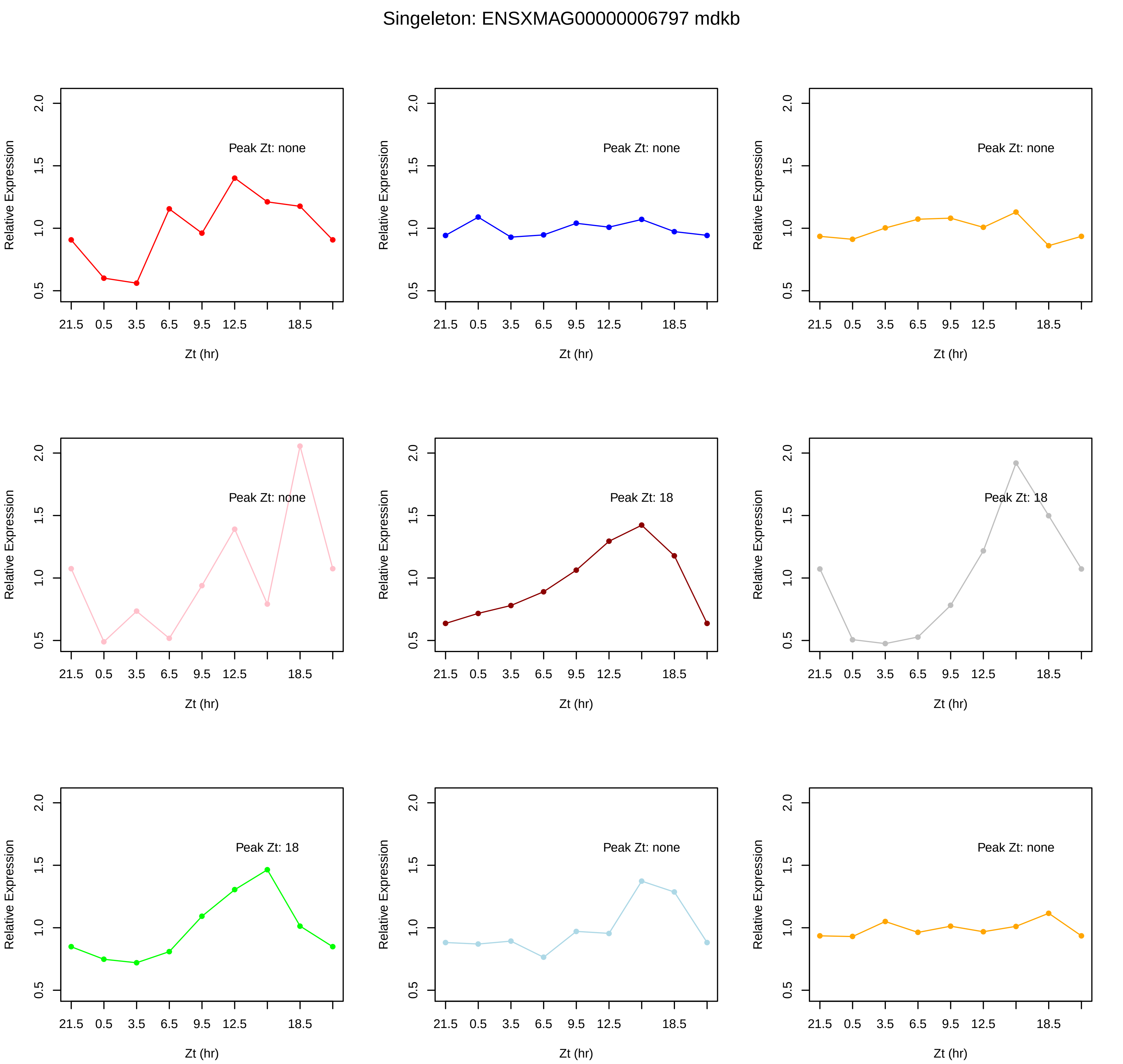
Singeleton: ENSXMAG00000006797 mdkb
Peak Zt: none
Peak Zt: none
Peak Zt: none
Peak Zt: none
Peak Zt: 18
Peak Zt: 18
Peak Zt: 18
Peak Zt: none
Peak Zt: none
Relative Expression
Zt (hr)

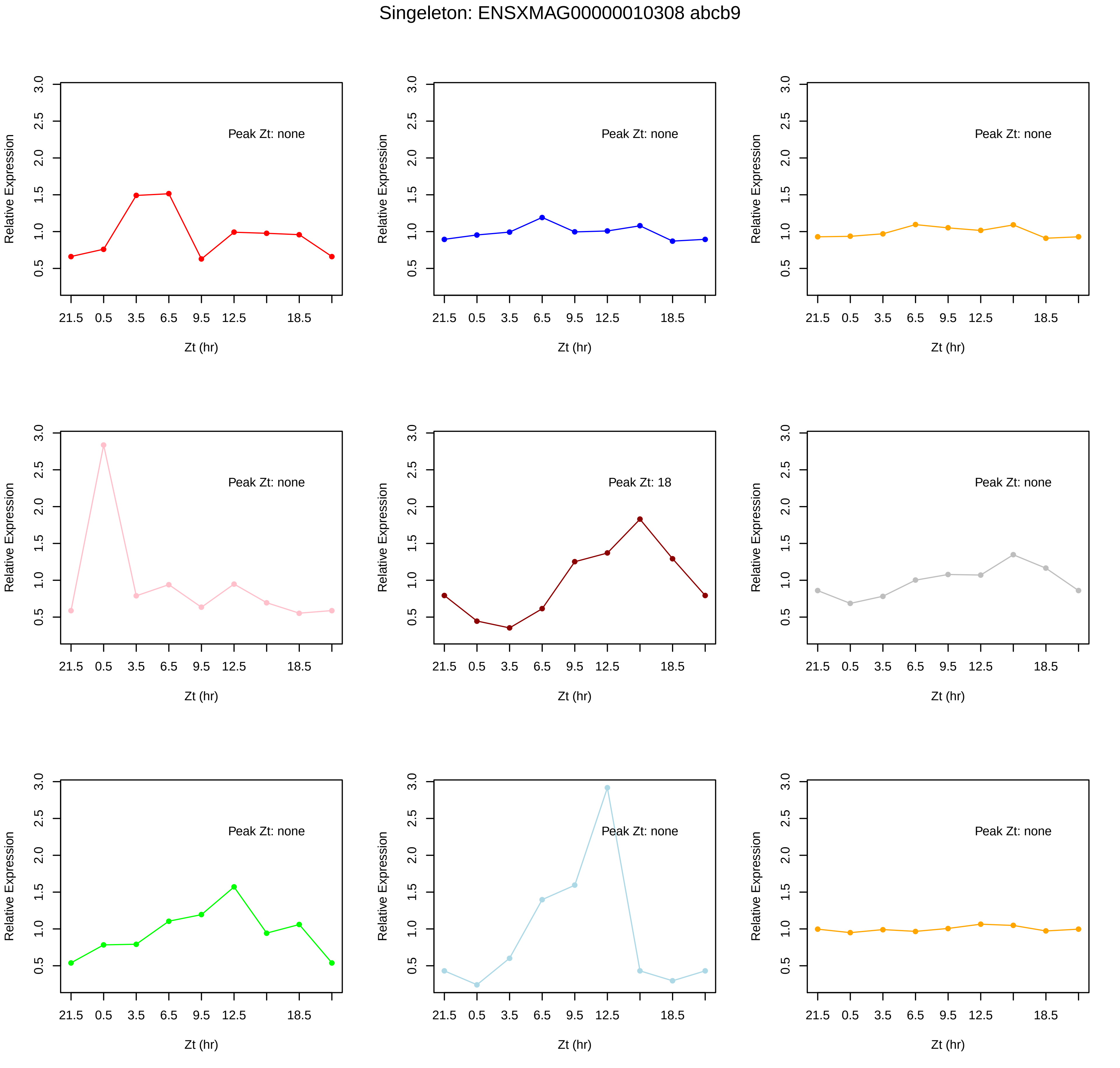

Singleton: ENSXMAG00000010308 abcb9
Peak Zt: none
Peak Zt: none
Peak Zt: none
Peak Zt: none
Peak Zt: 18
Peak Zt: none
Peak Zt: none
Peak Zt: none
Peak Zt: none
Relative Expression
Zt (hr)
21.5
0.5
3.5
6.5
9.5
12.5
18.5

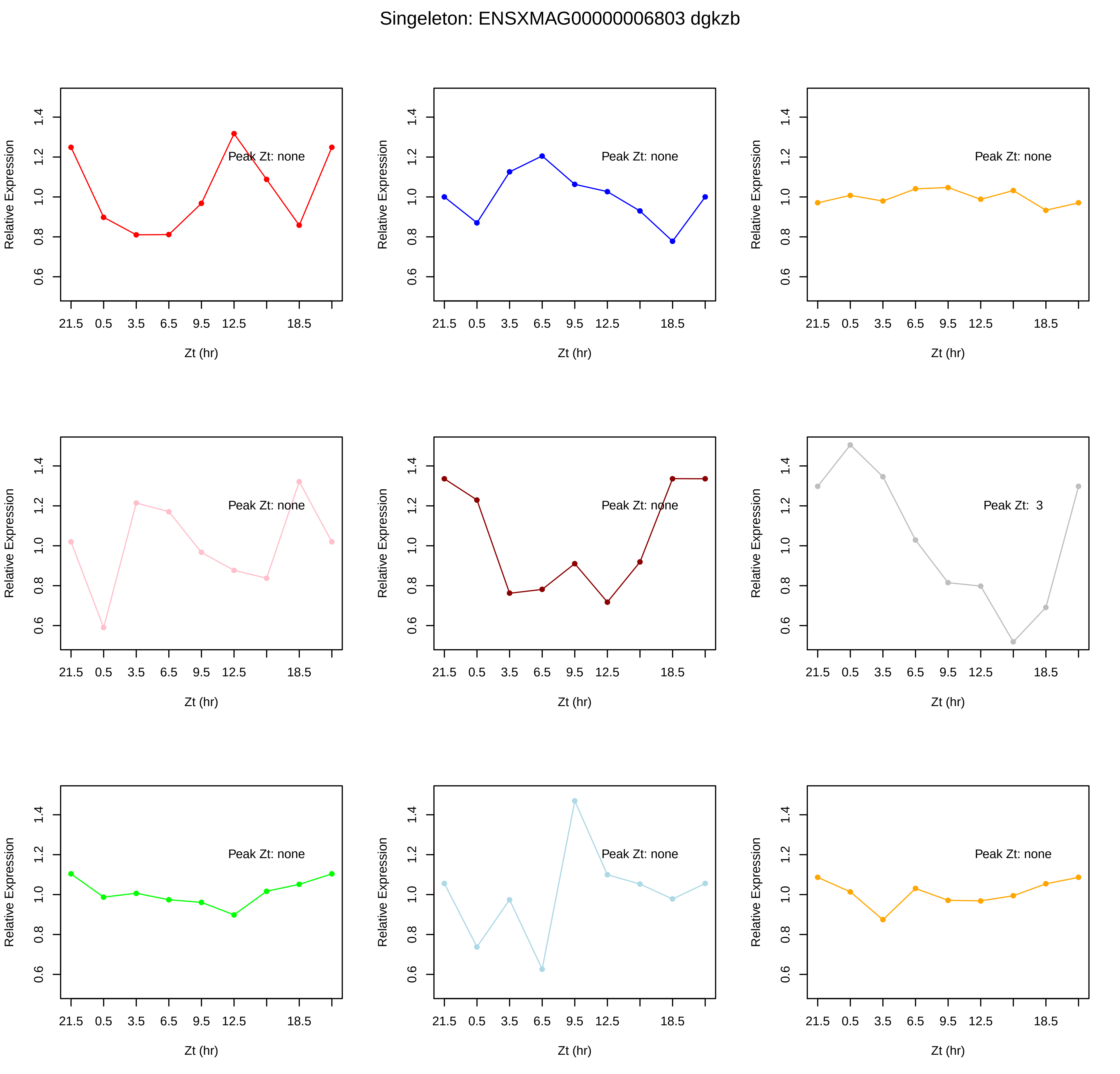

Singeleton: ENSXMAG00000006803 dgkzb
Peak Zt: none
Peak Zt: none
Peak Zt: none
Peak Zt: none
Peak Zt: none
Peak Zt: 3
Peak Zt: none
Peak Zt: none
Peak Zt: none
Relative Expression
Zt (hr)
21.5
0.5
3.5
6.5
9.5
12.5
18.5
0.6
0.8
1.0
1.2
1.4

Singleton: ENSXMAG00000009064 acss2

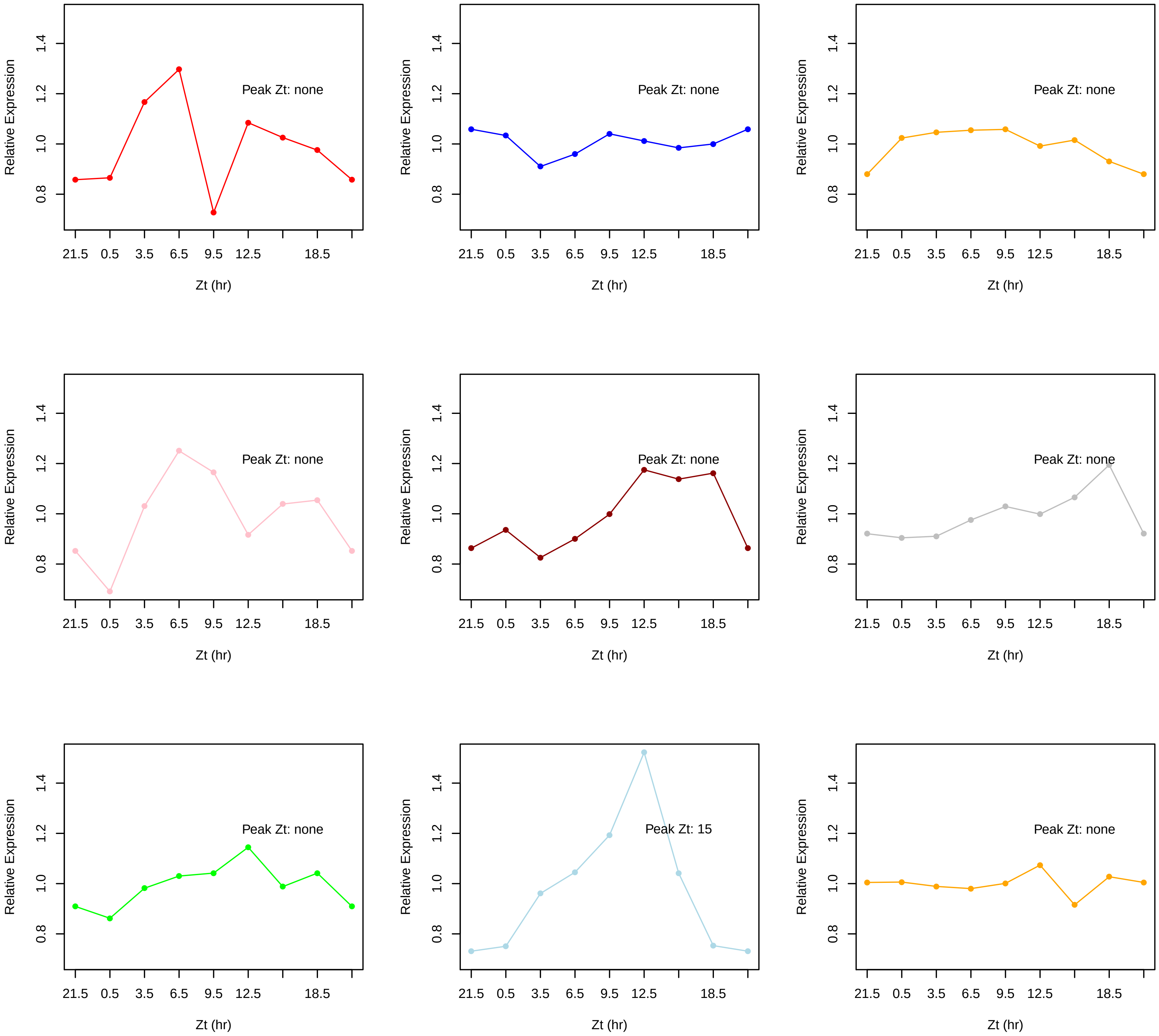

Singeleton: ENSXMAG00000000343 zgc:194678

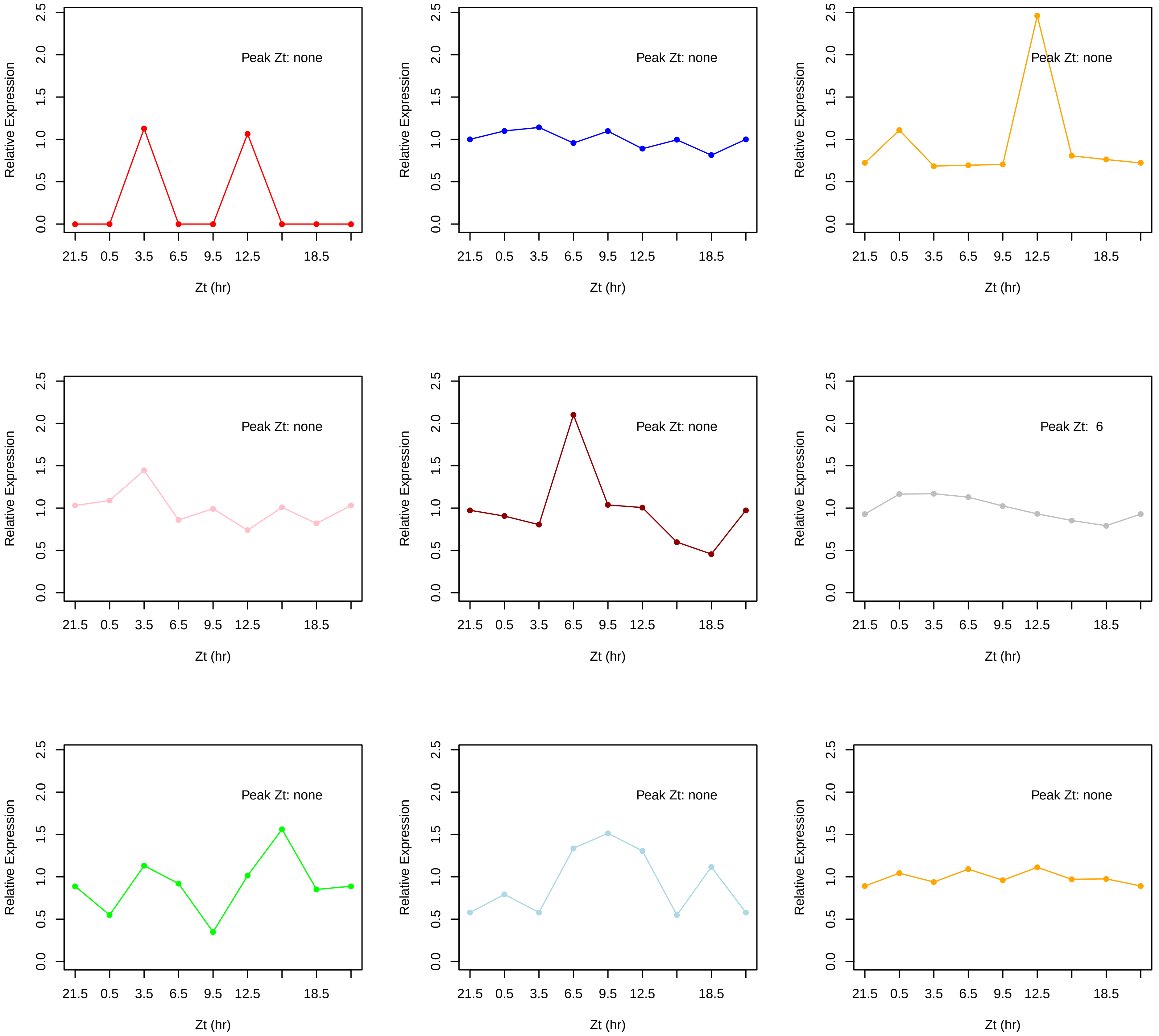

Singleton: ENSXMAG00000026619 rpp21

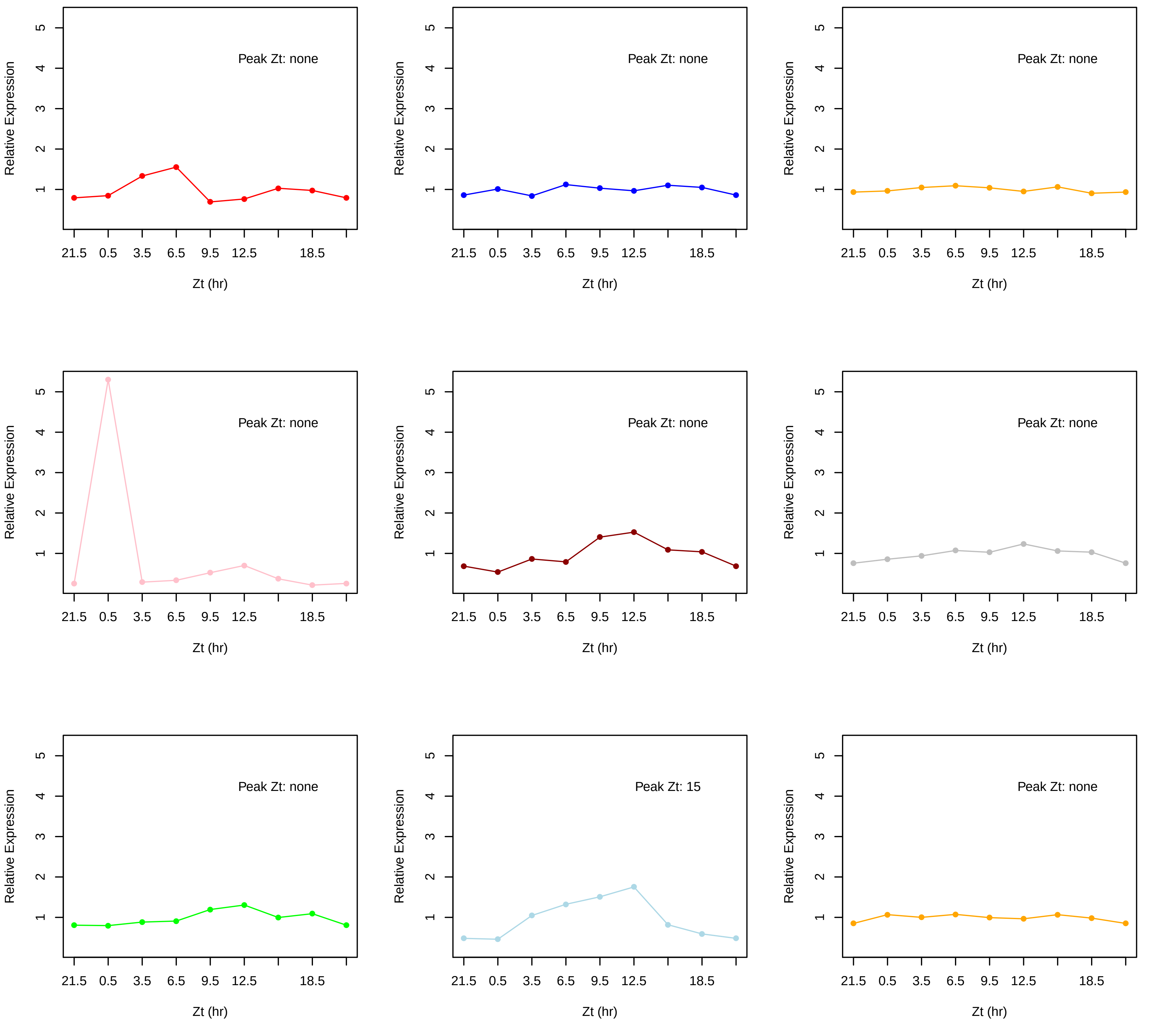

Singeleton: ENSXMAG00000007942 cpda

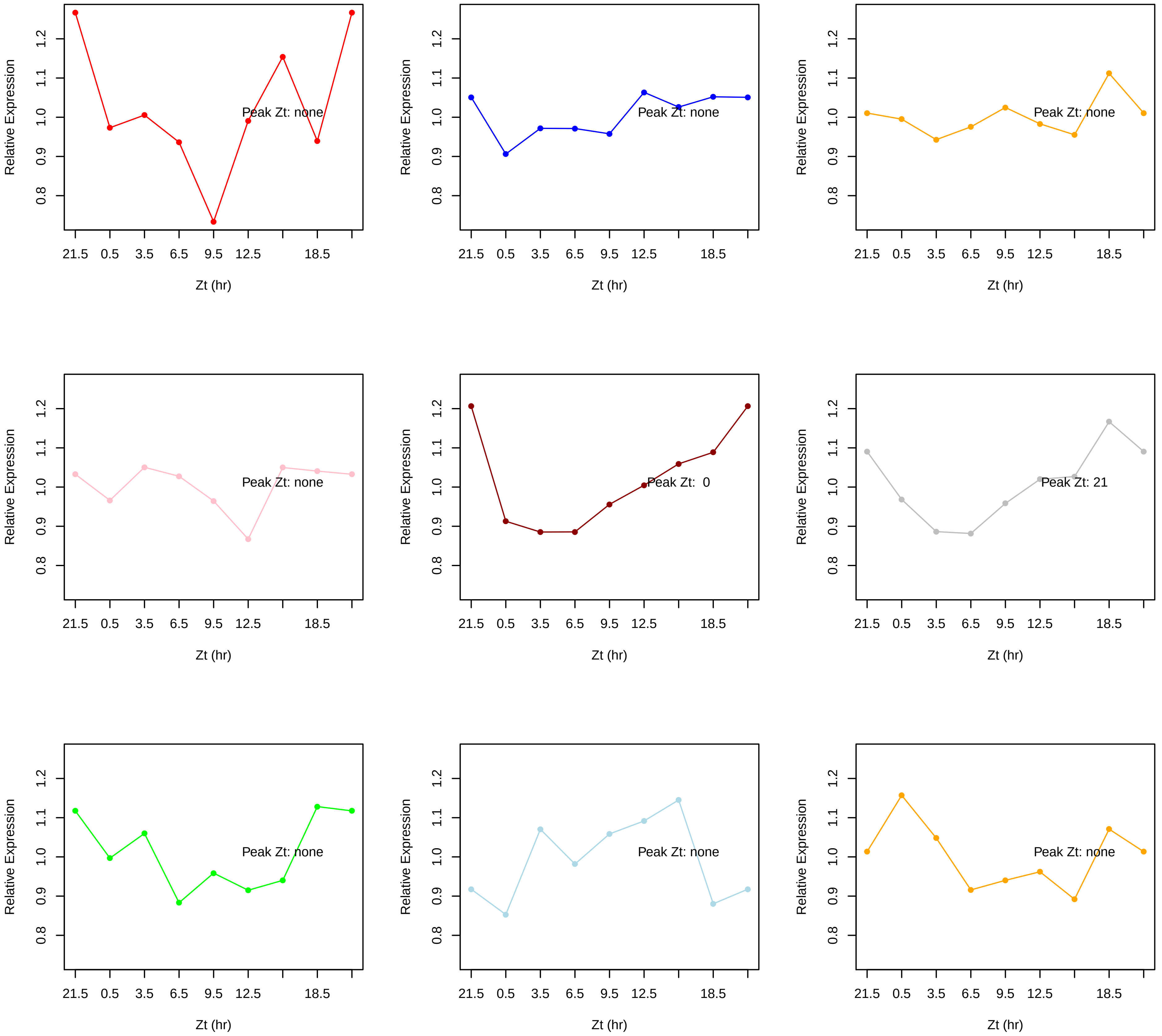

Singeleton: ENSXMAG00000003554 tsc22d1

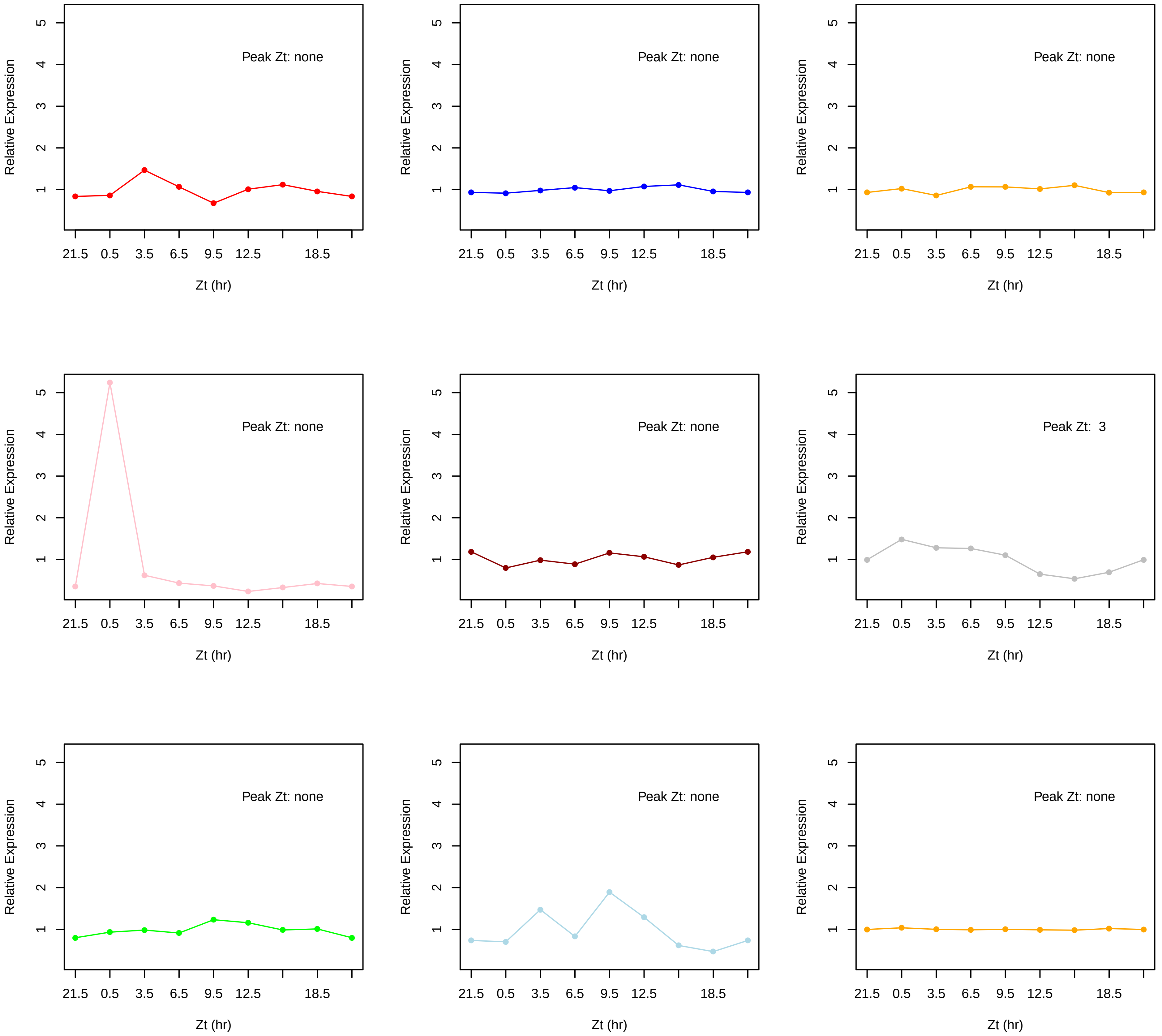

Singeleton: ENSXMAG00000013256

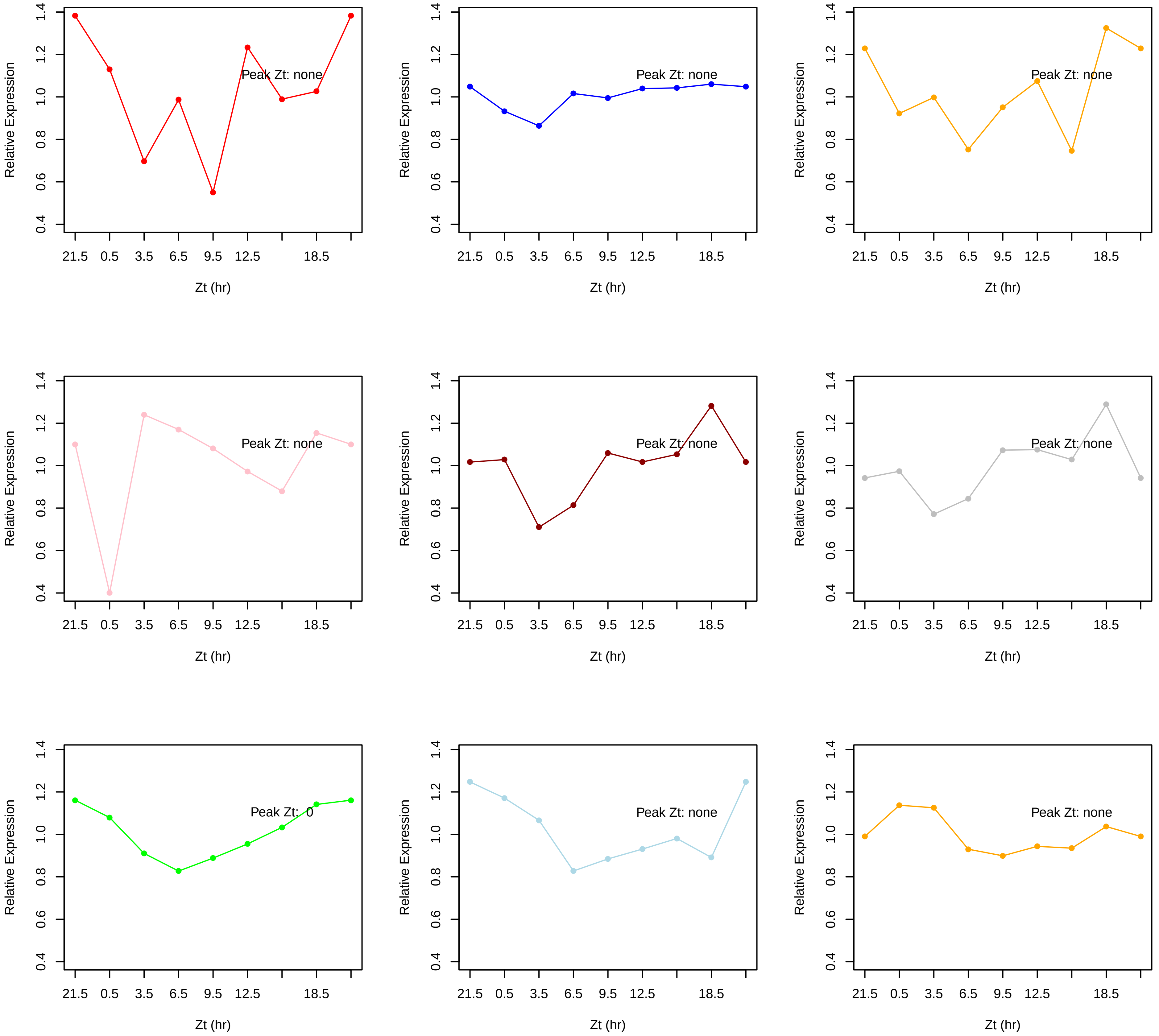

Singeleton: ENSXMAG00000022410 arl6ip4

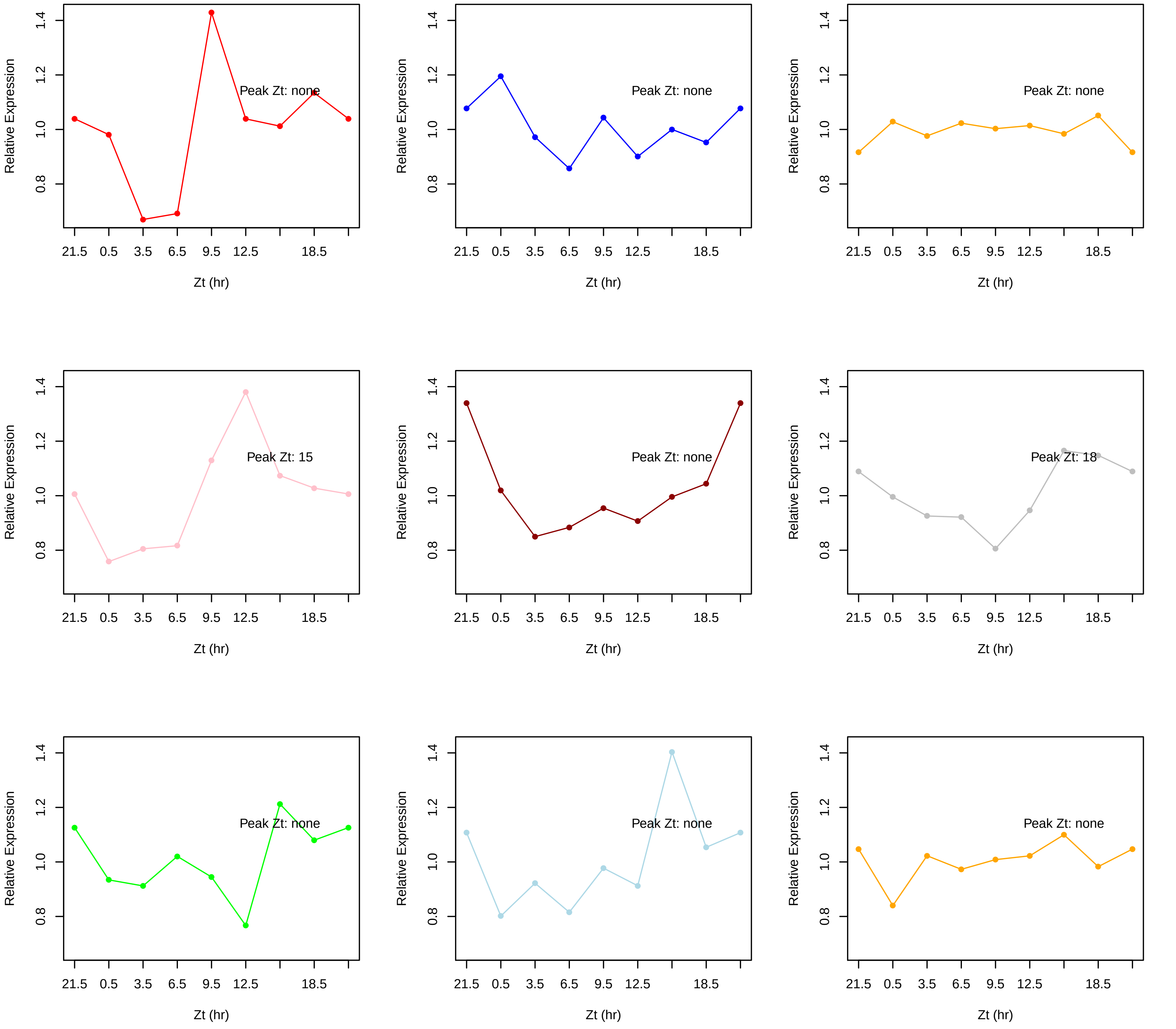

Singeleton: ENSXMAG00000010053 keap1a

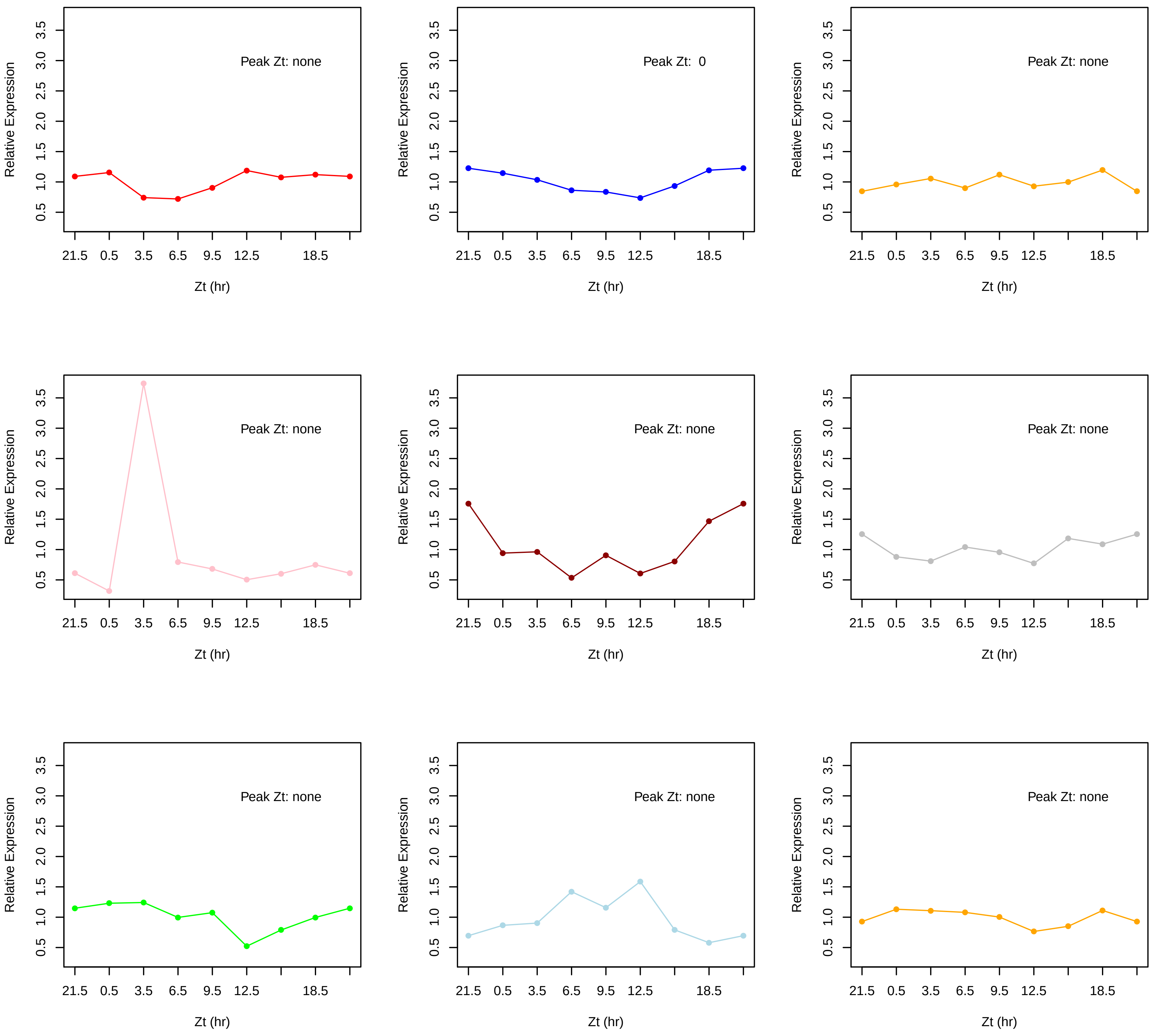

Singeleton: ENSXMAG00000025236 mapre1b

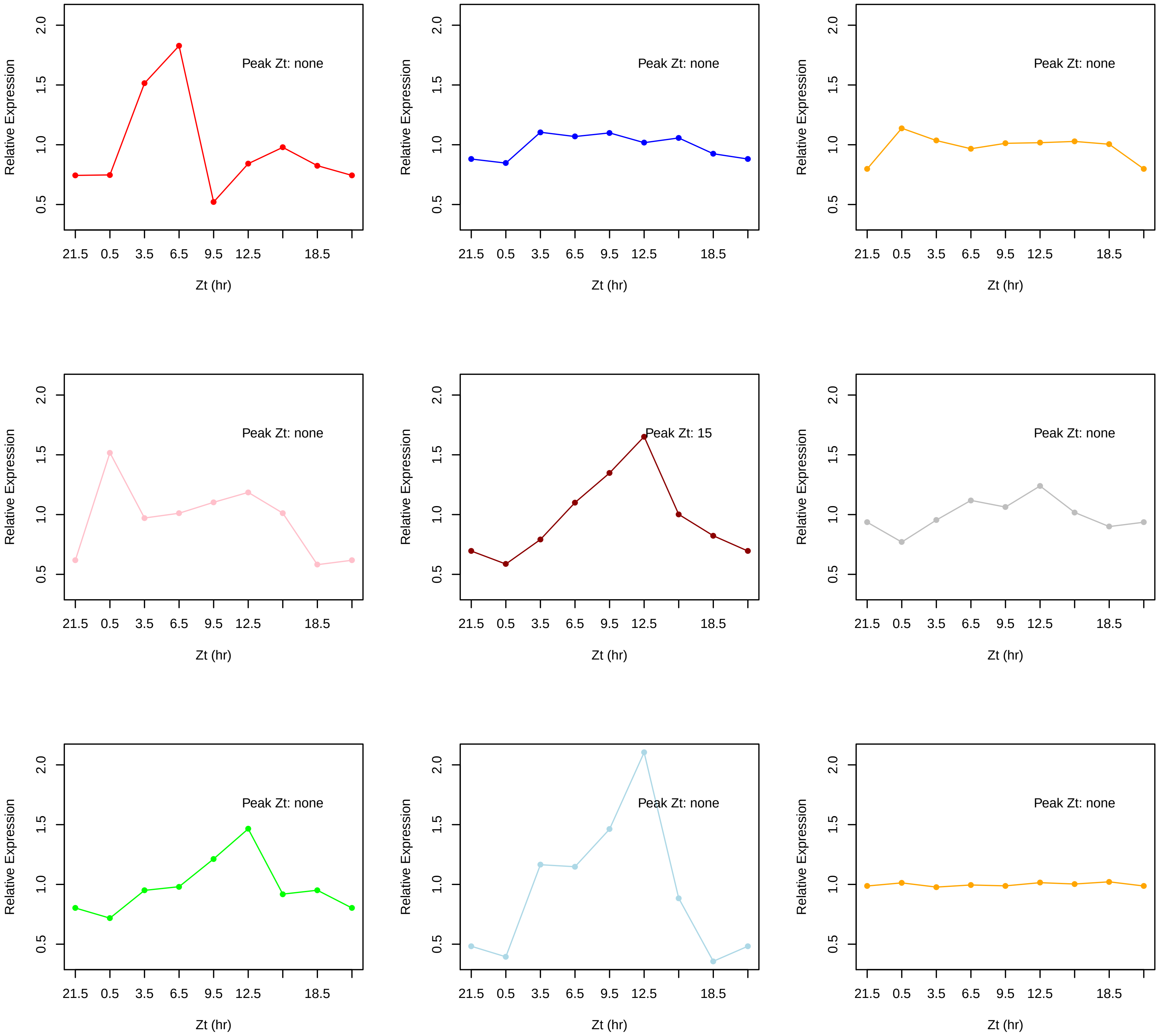

Singleton: ENSXMAG00000023550 aqp12

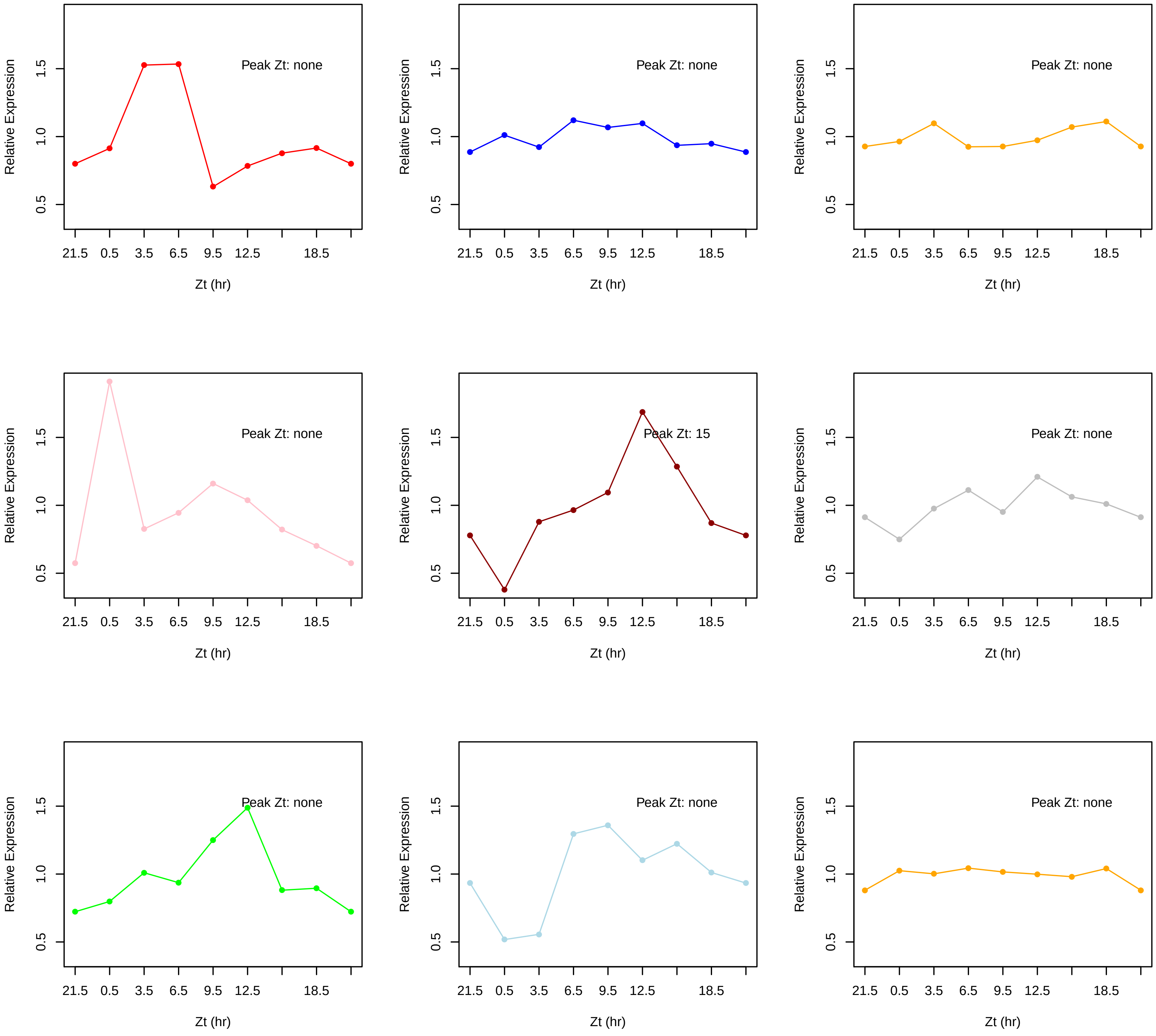

Singeleton: ENSXMAG00000015299 prpf40a

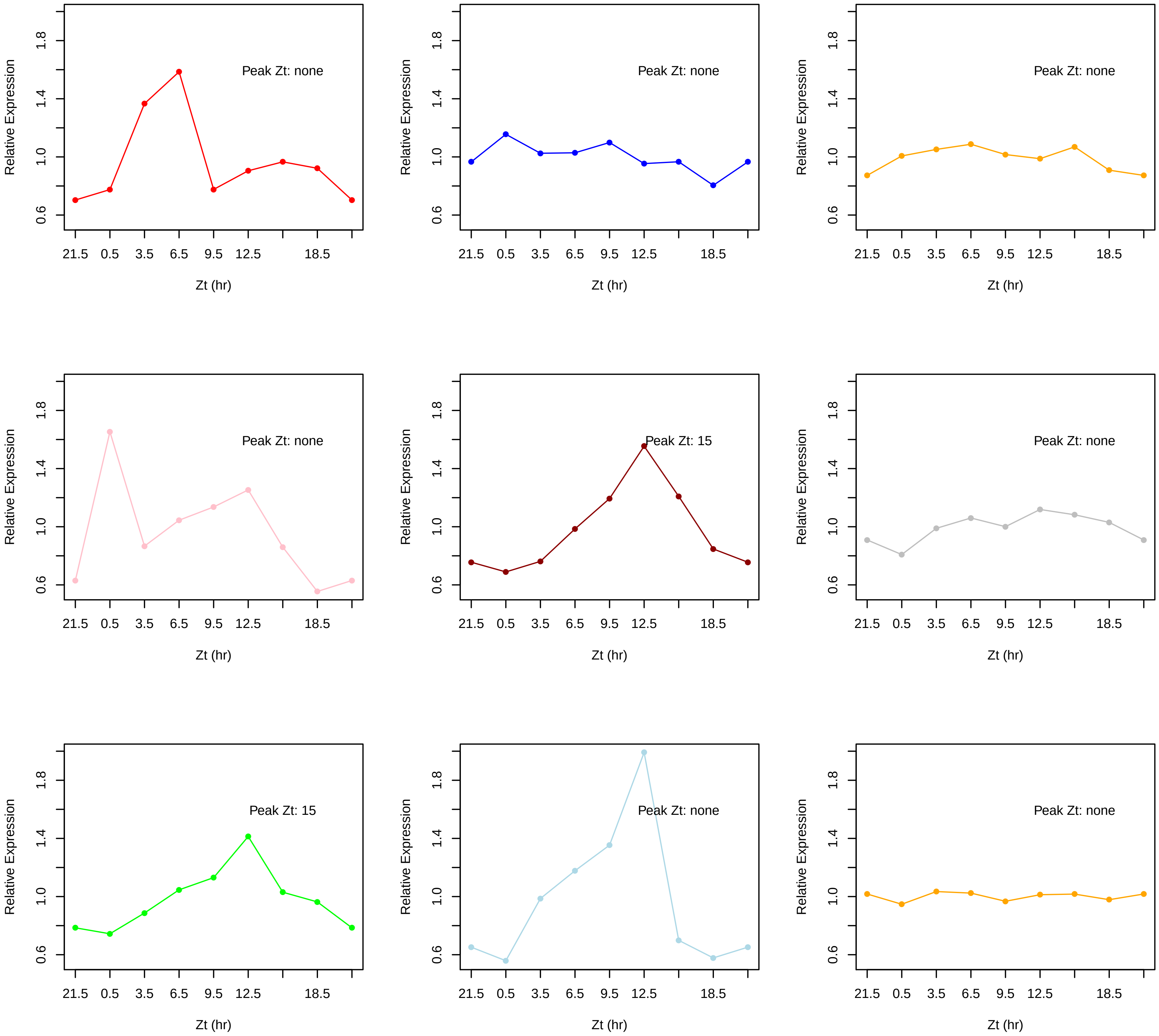

Singleton: ENSXMAG00000006678 ddx31

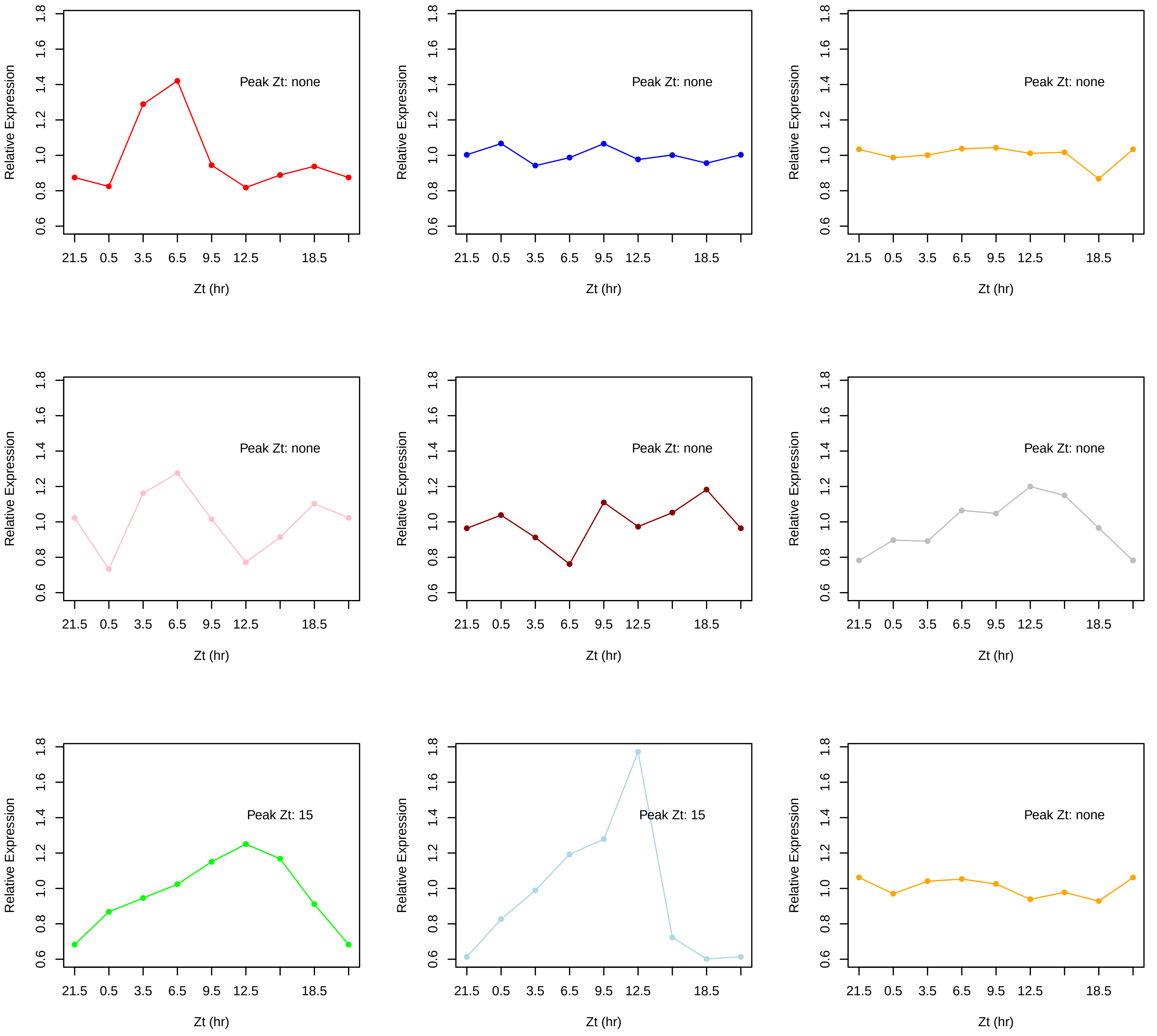

Singleton: ENSXMAG00000001678 dctn5

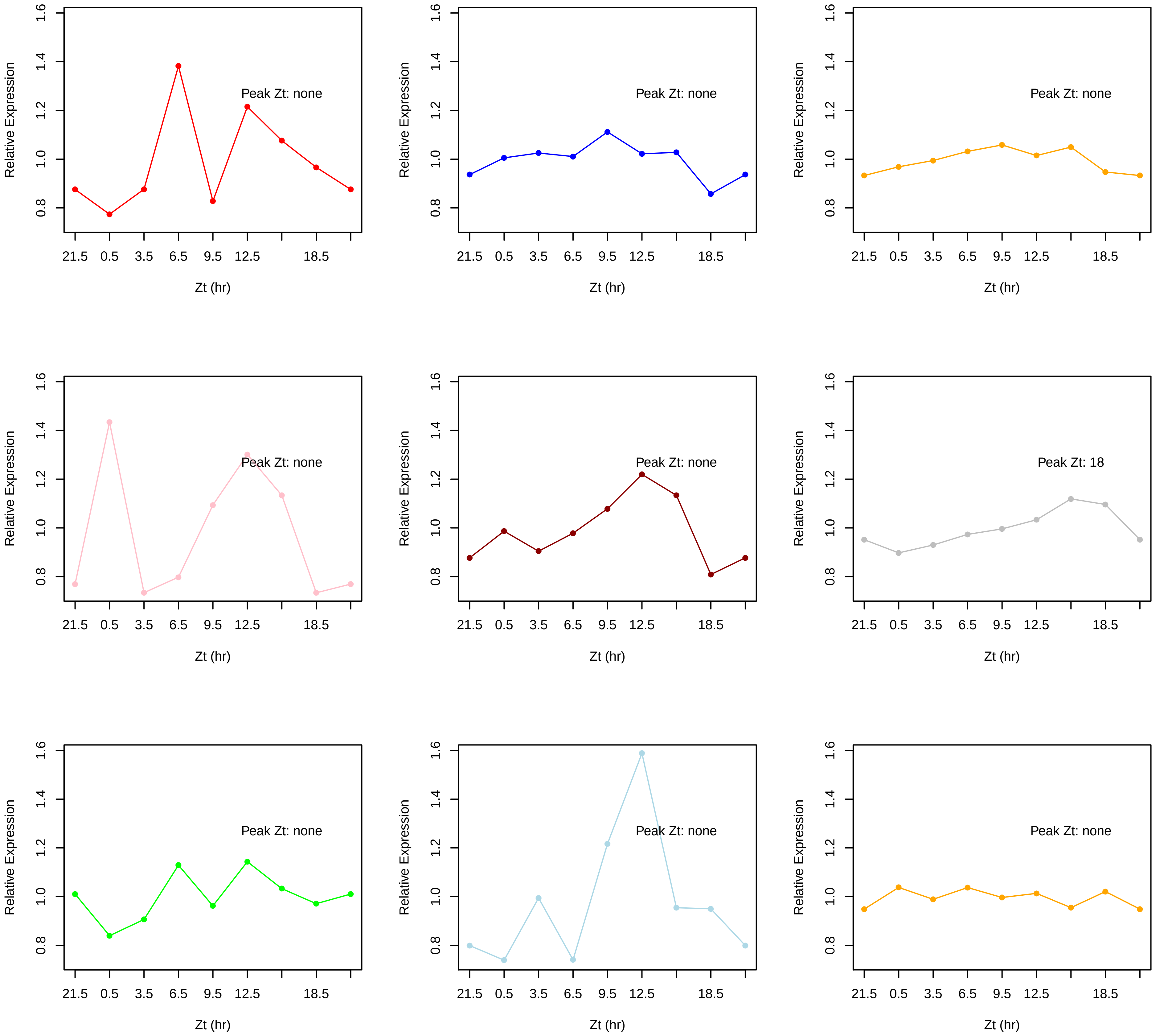

Singleton: ENSXMAG00000004872 pex10

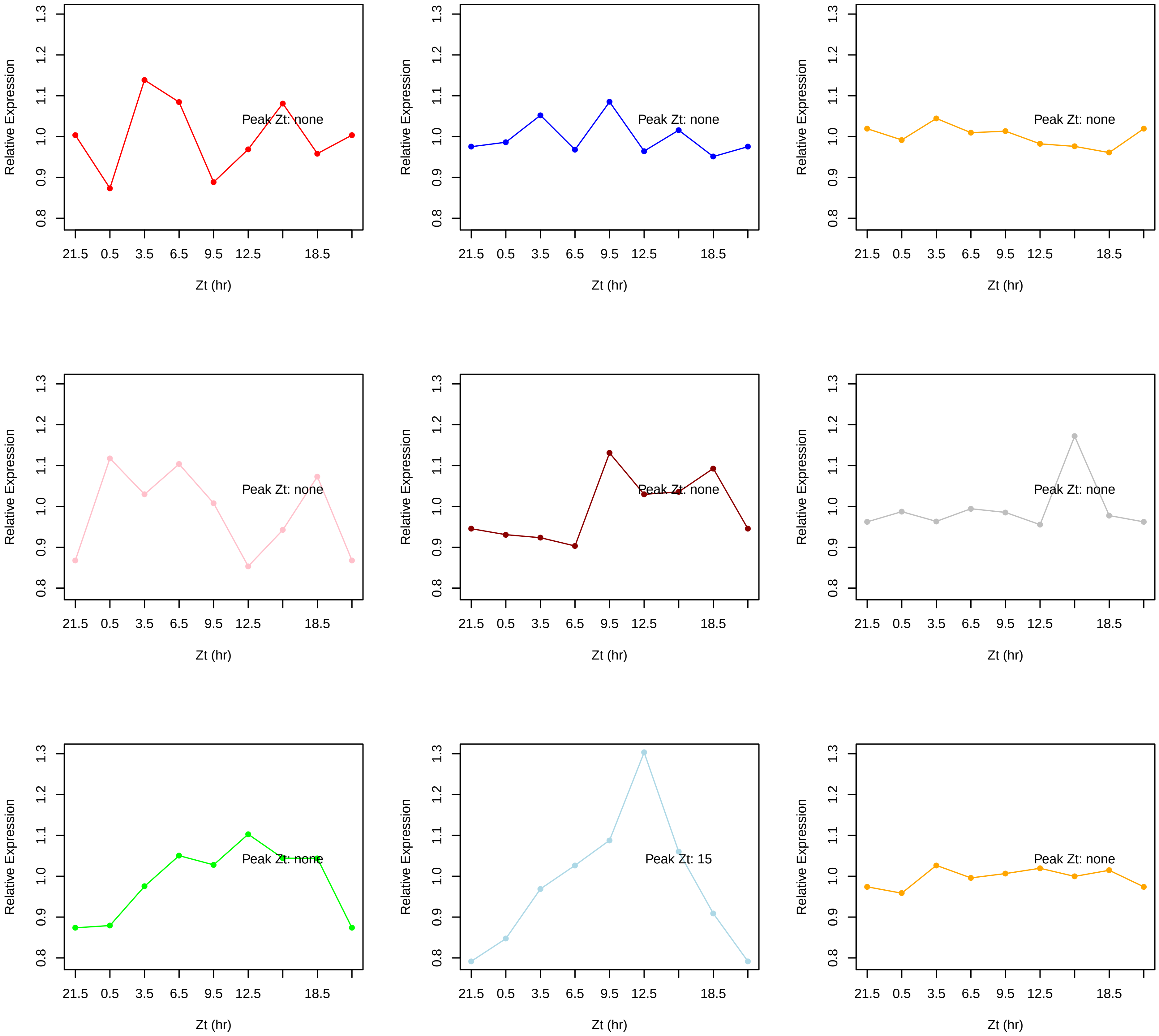

Singeleton: ENSXMAG00000006522 xrcc1

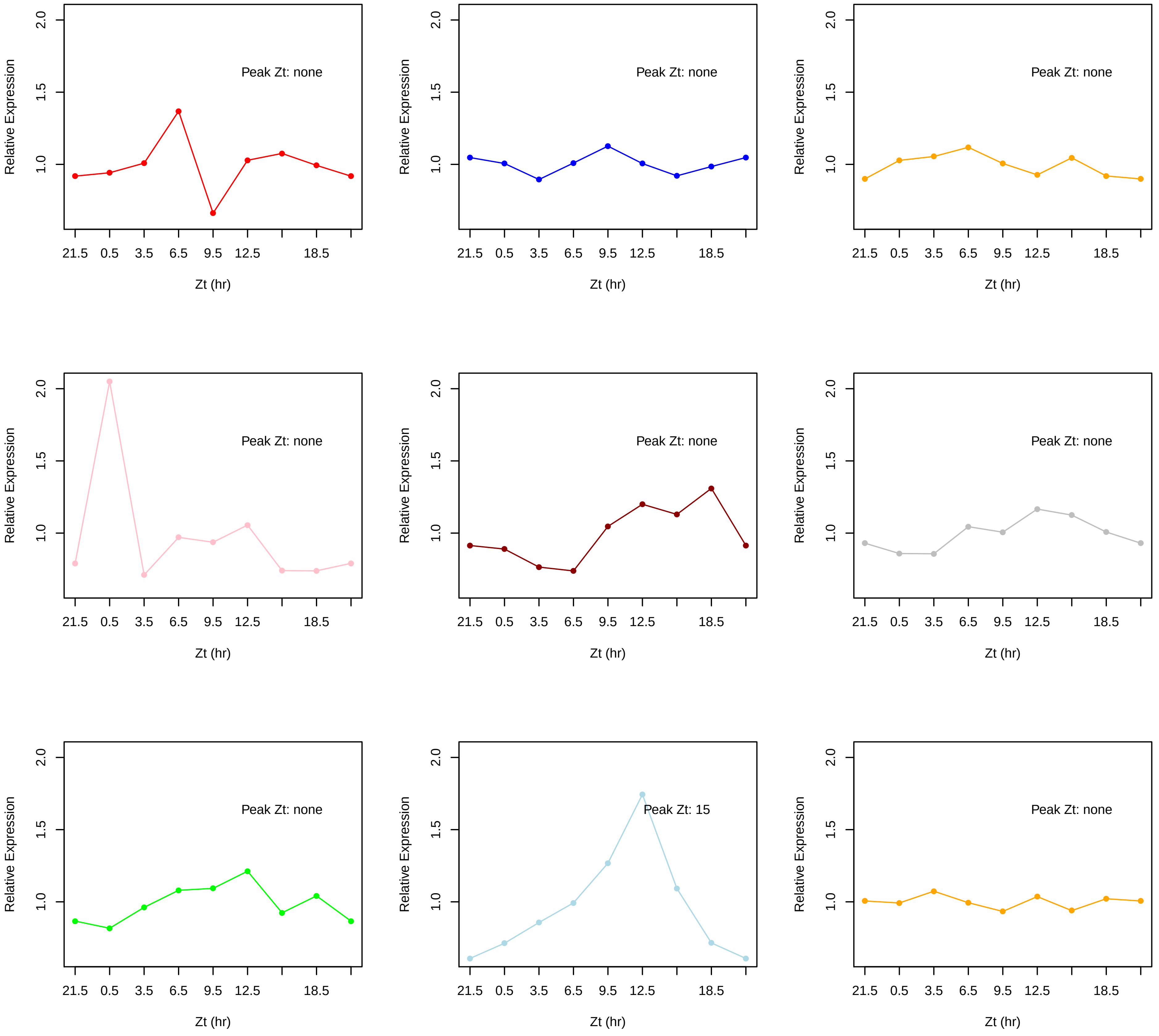

Singleton: ENSXMAG00000001626 hyou1

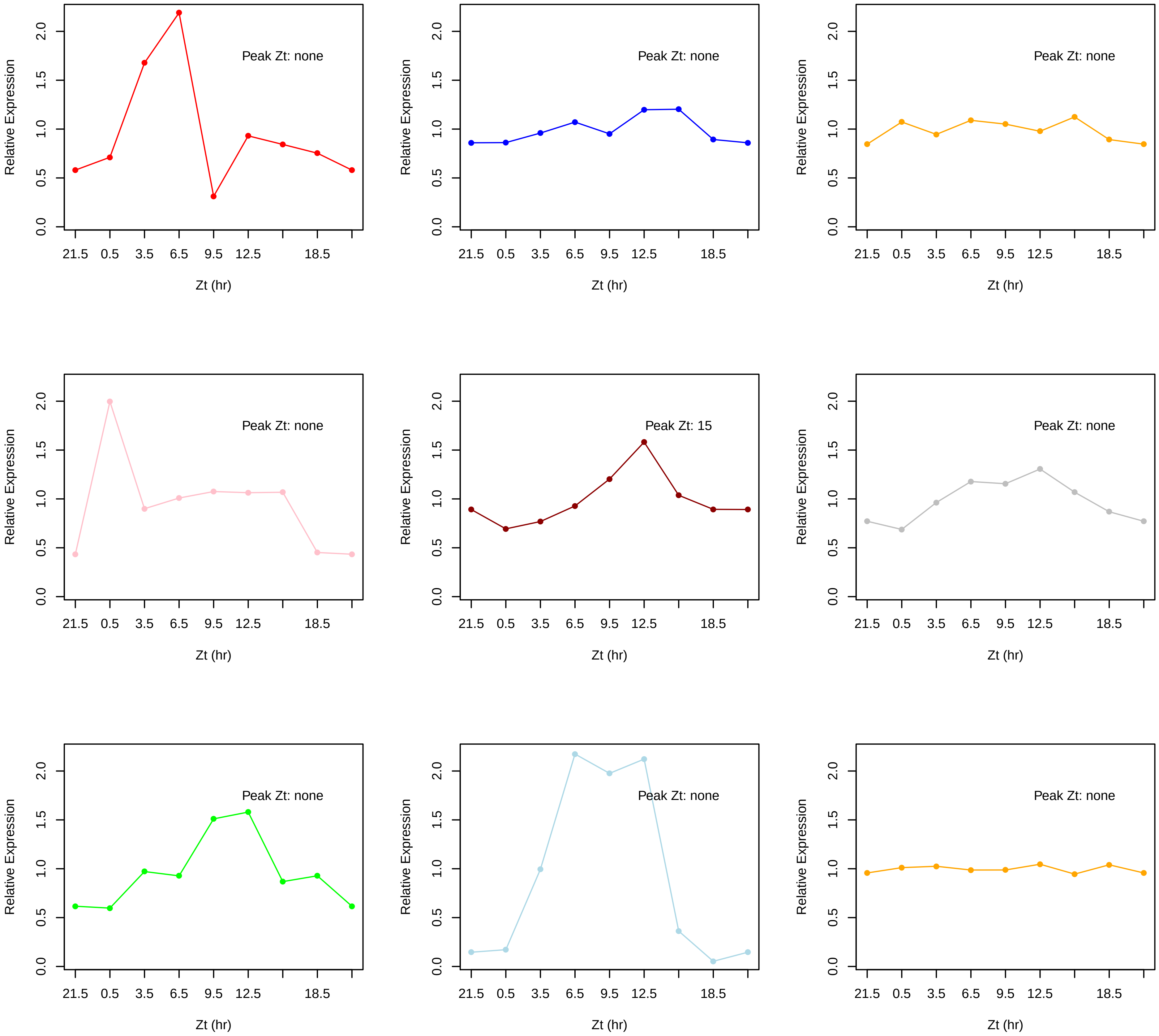

Singeleton: ENSXMAG00000003015 rgra

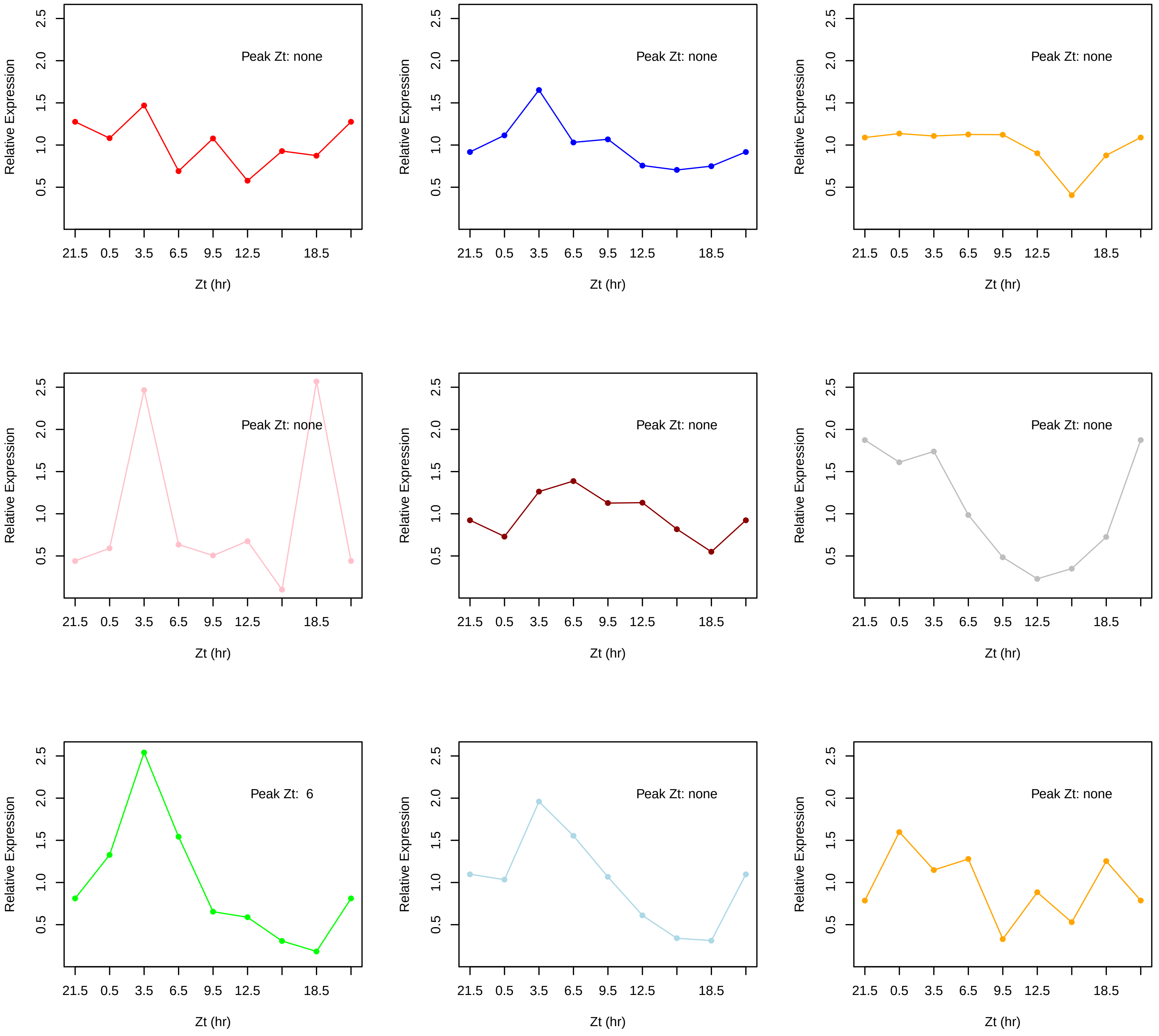

Singeleton: ENSXMAG00000002084 sox17

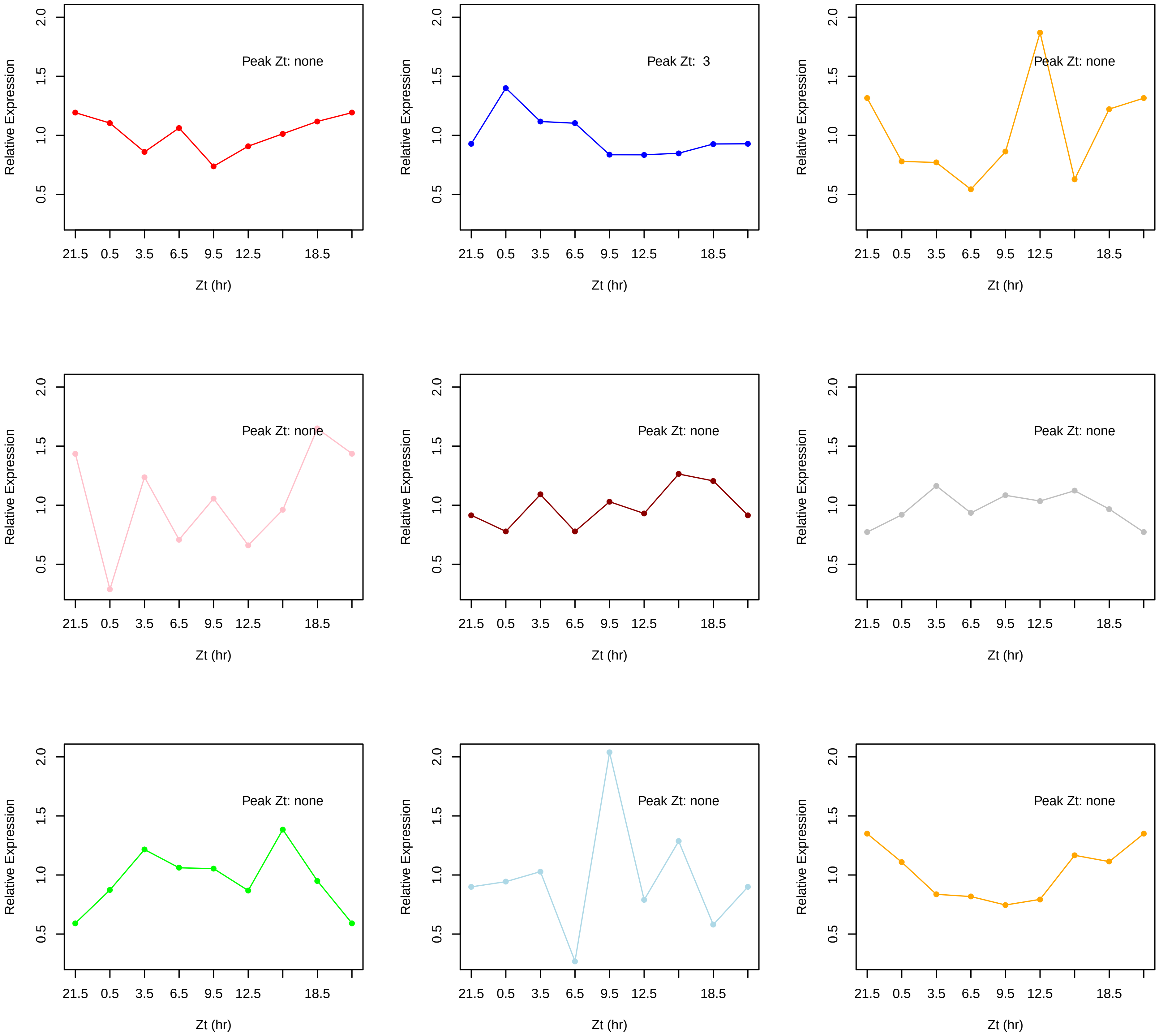

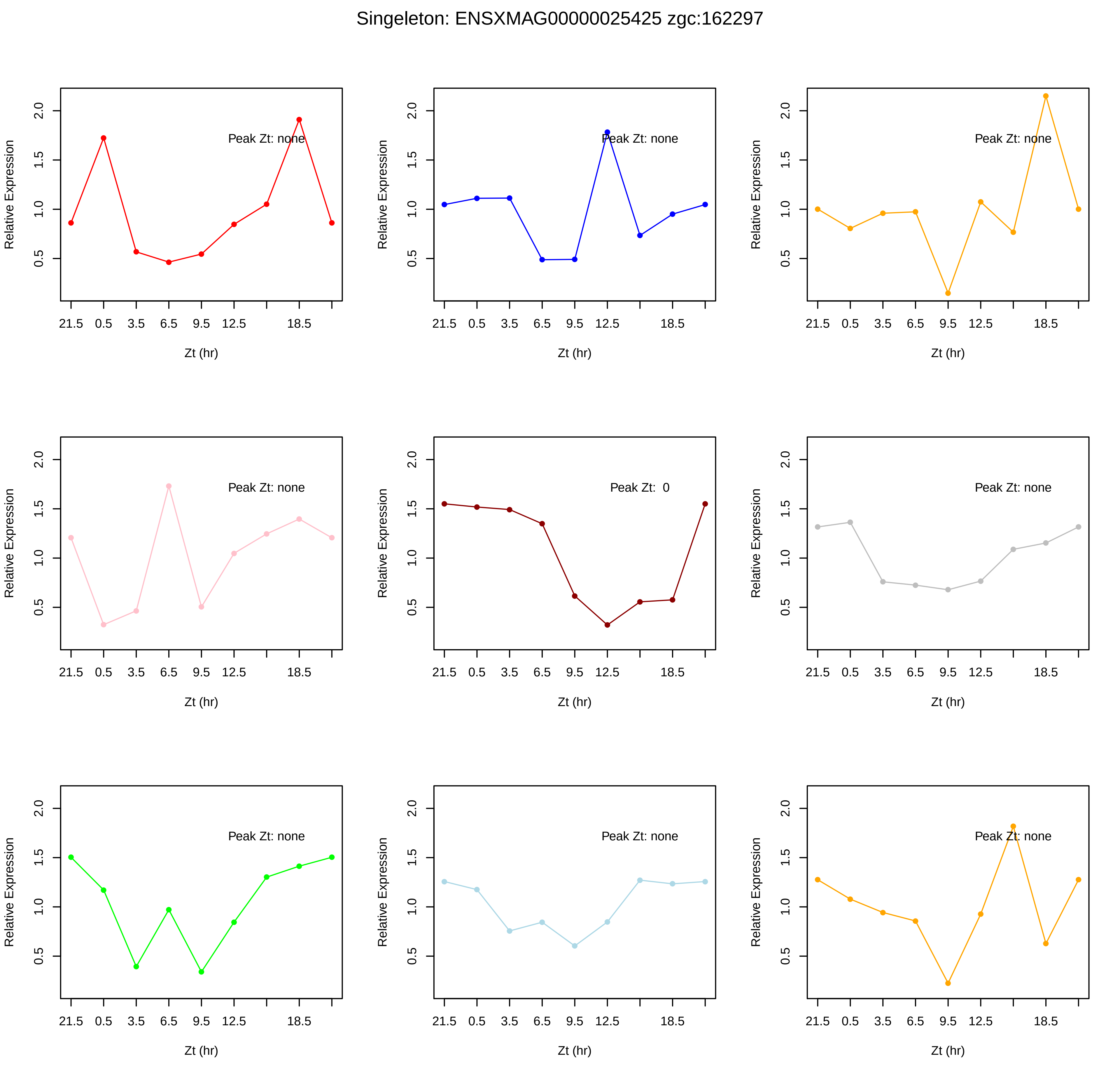

Singeleton: ENSXMAG00000025425 zgc:162297
Relative Expression
Zt (hr)
Peak Zt: none
Peak Zt: none
Peak Zt: none
Peak Zt: none
Peak Zt: 0
Peak Zt: none
Peak Zt: none
Peak Zt: none
Peak Zt: none

Singeleton: ENSXMAG00000017829 SP1

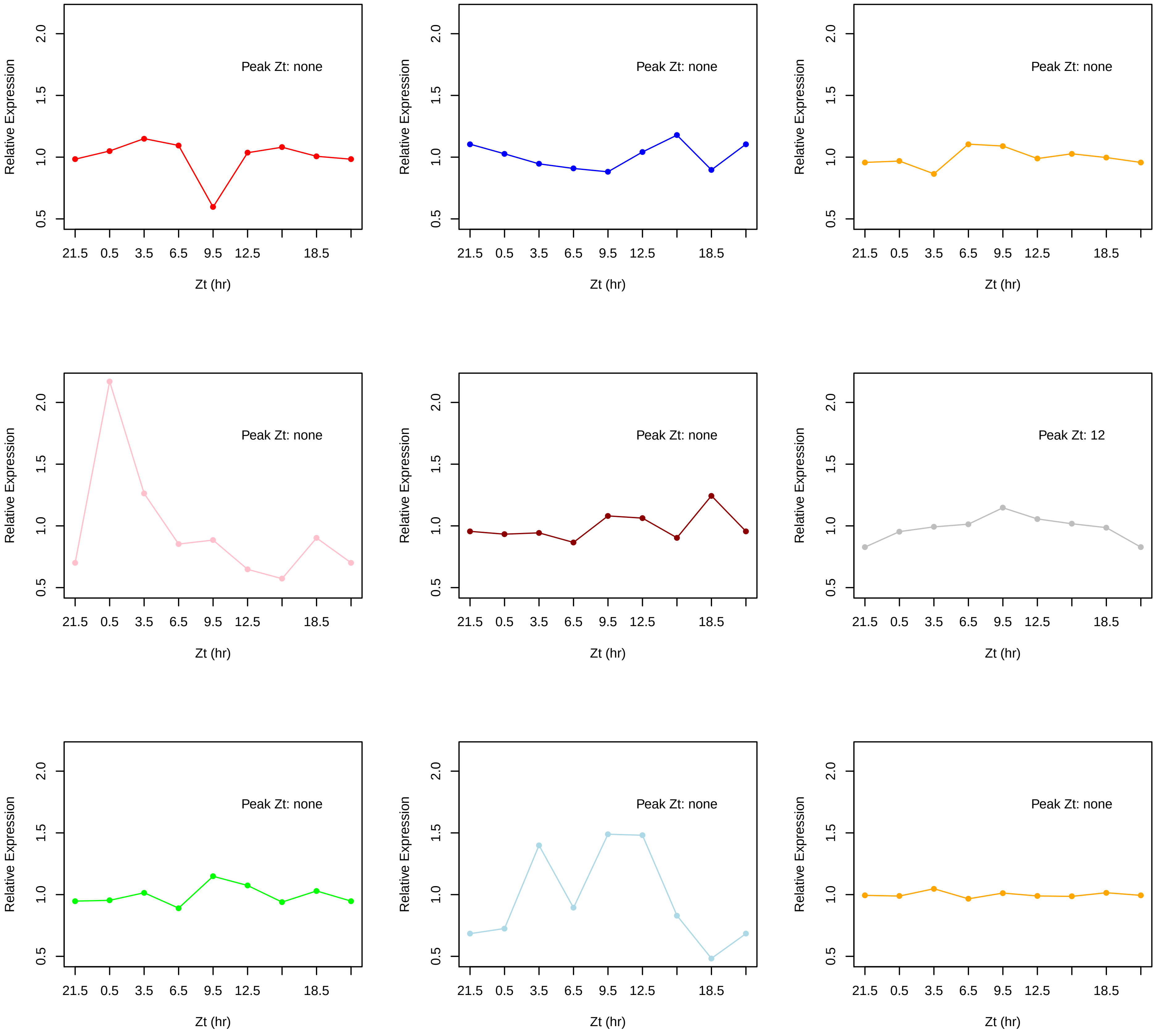

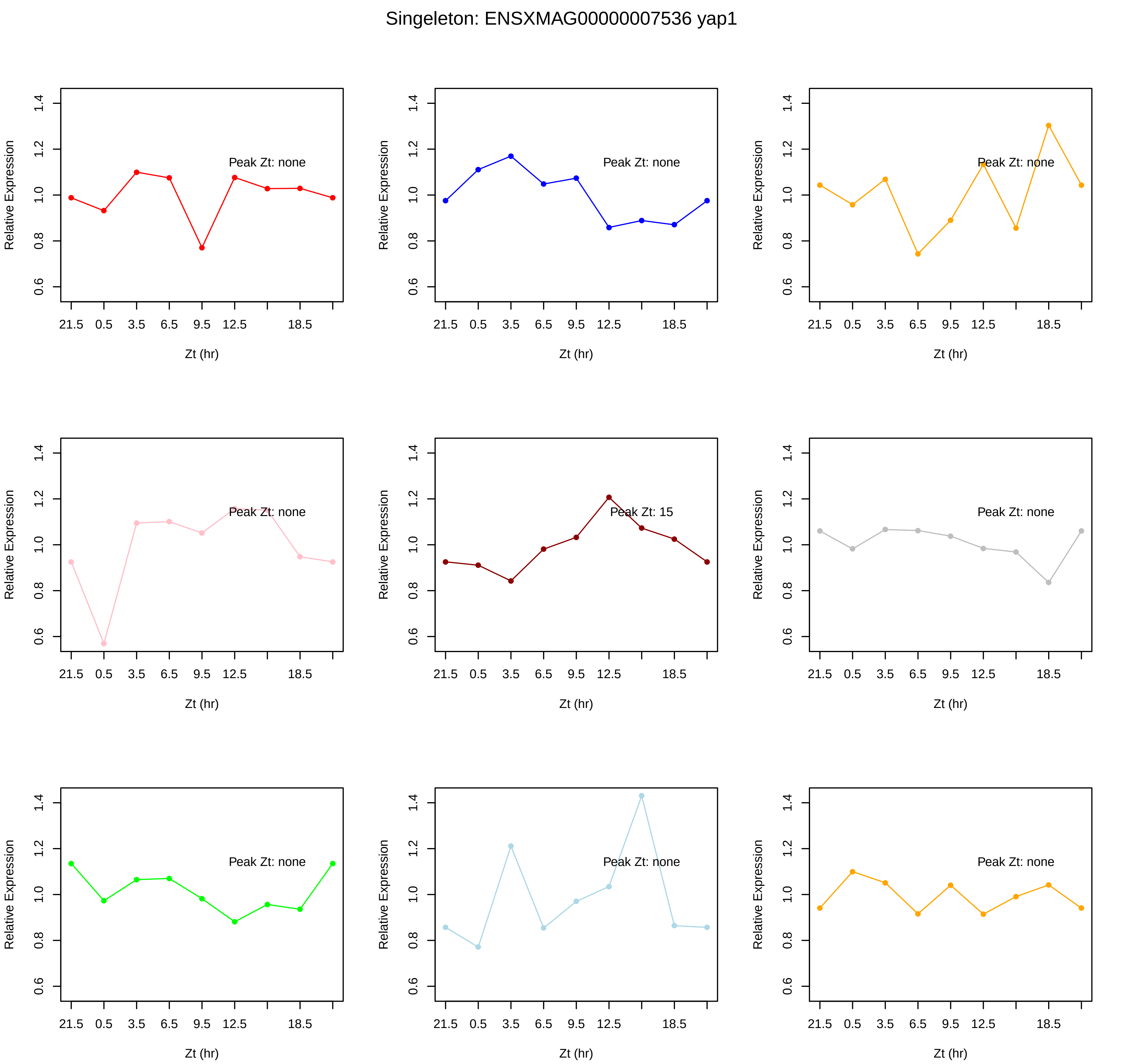
Singeleton: ENSXMAG00000007536 yap1
Relative Expression
Zt (hr)
21.5 0.5 3.5 6.5 9.5 12.5 18.5
0.6 0.8 1.0 1.2 1.4
Peak Zt: none
Peak Zt: none
Peak Zt: none
Peak Zt: none
Peak Zt: 15
Peak Zt: none
Peak Zt: none
Peak Zt: none
Peak Zt: none

Singeleton: ENSXMAG00000012171 nlk2

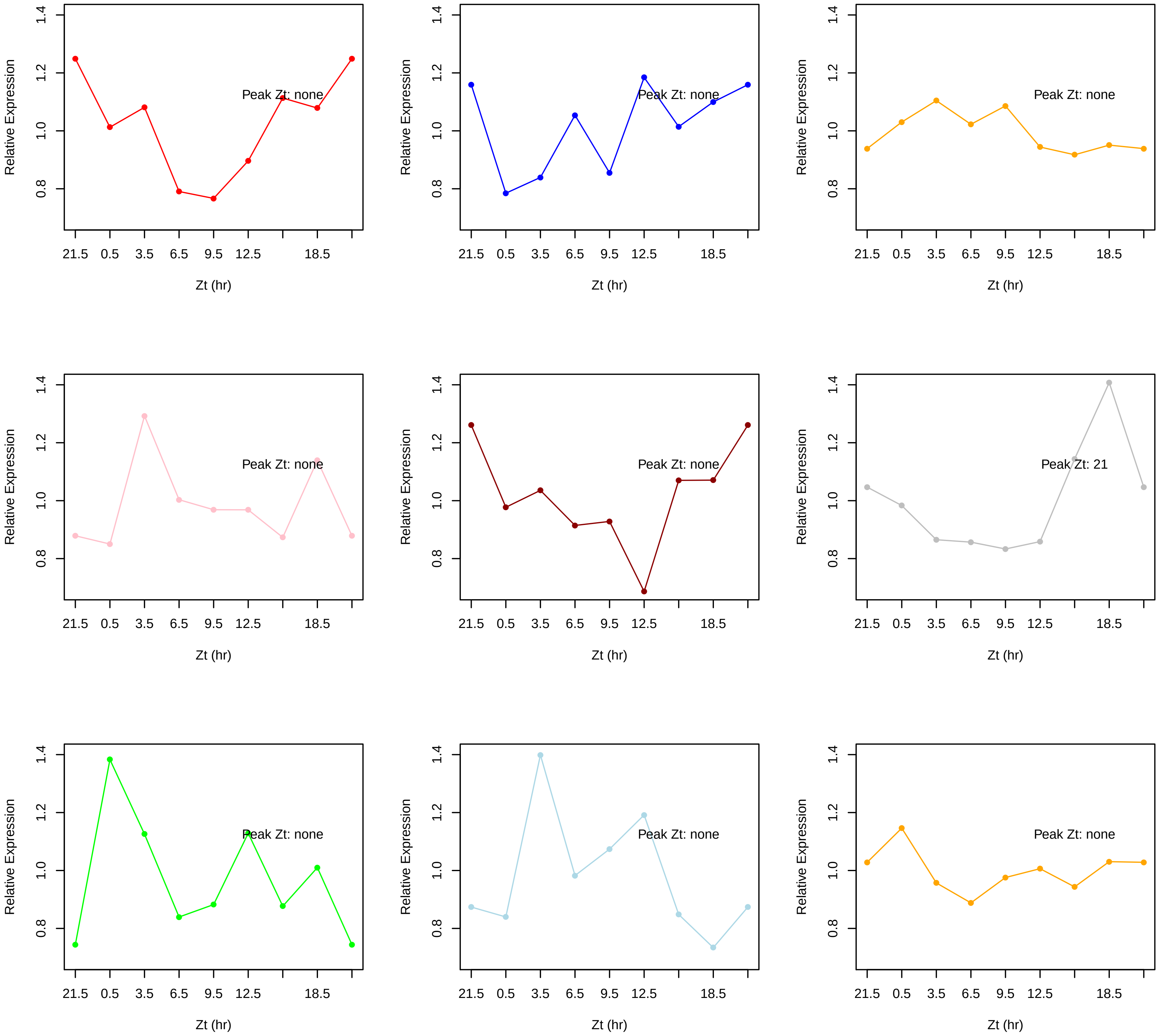

Singeleton: ENSXMAG00000002087 mrpl15

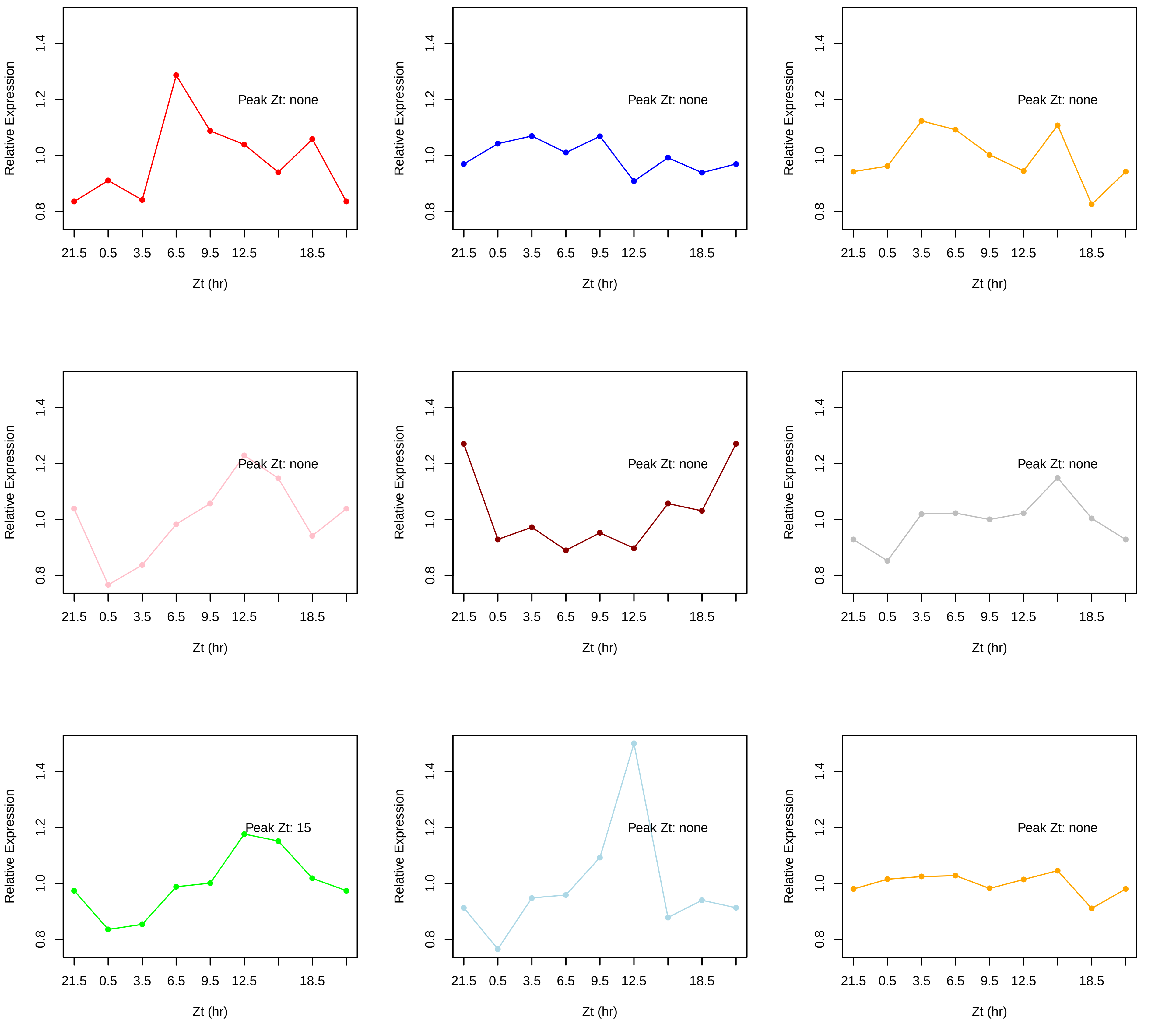

Singleton: ENSXMAG00000028105

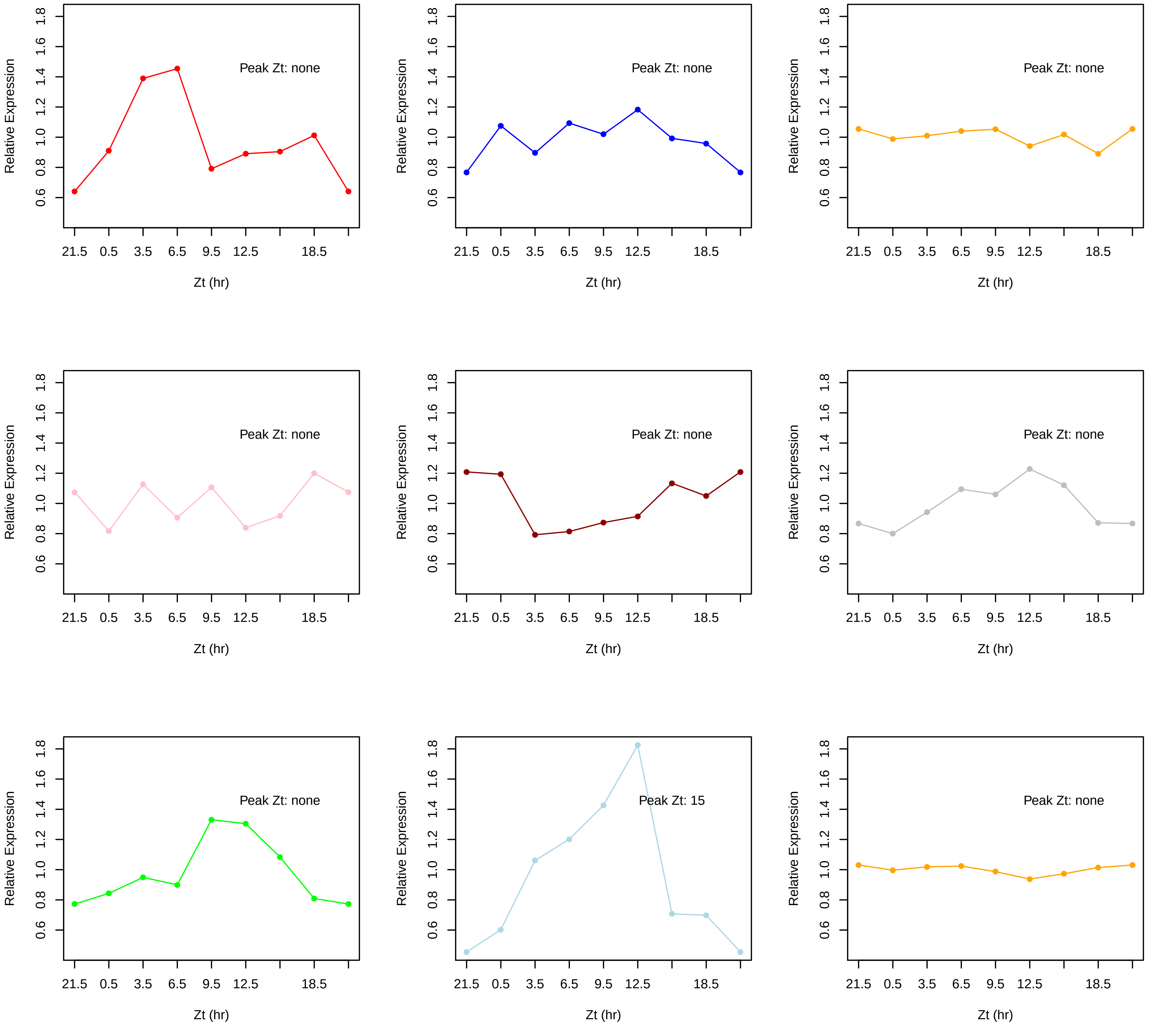

Singeleton: ENSXMAG00000006362

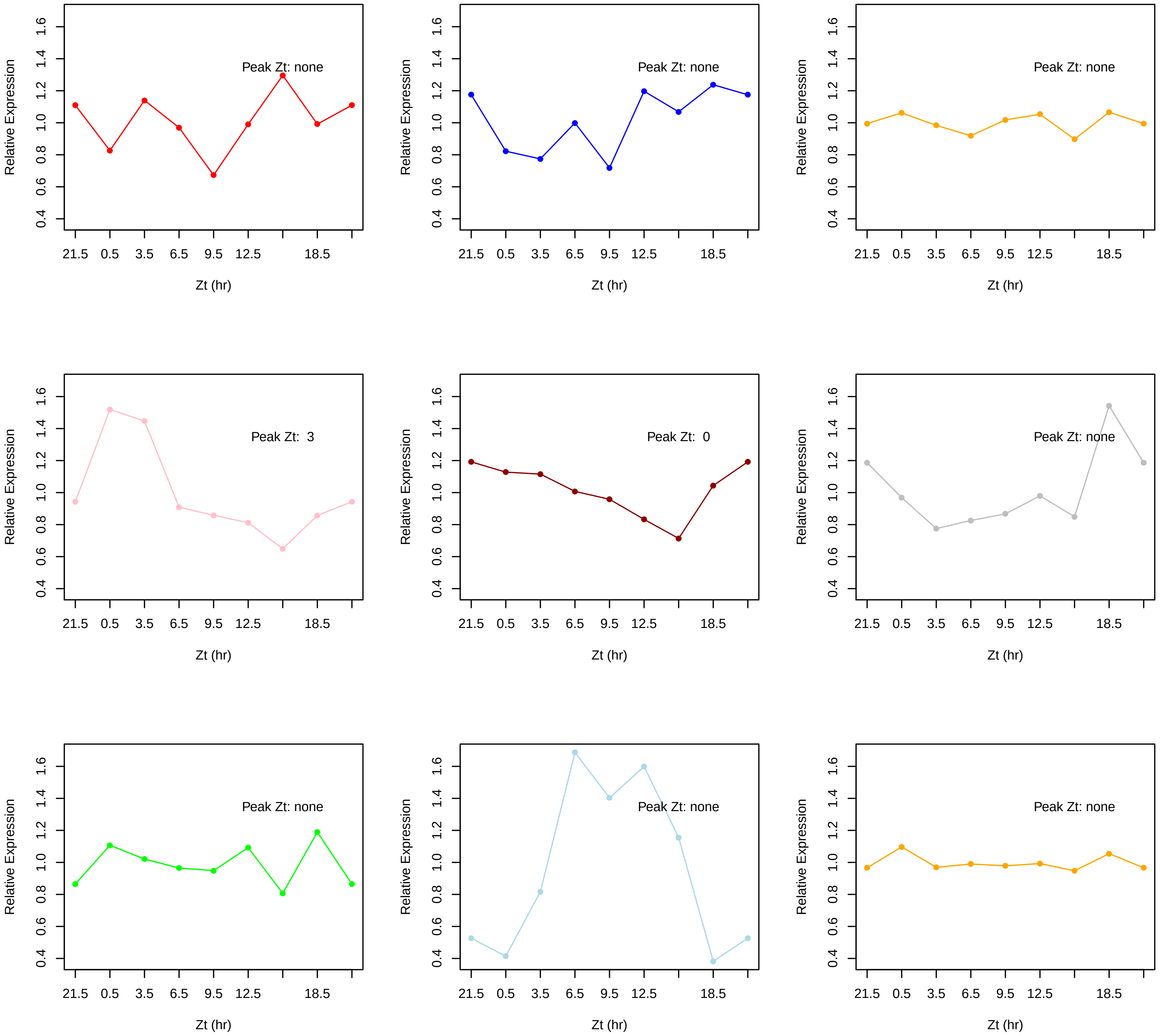

Singeleton: ENSXMAG00000014098 ccdc12

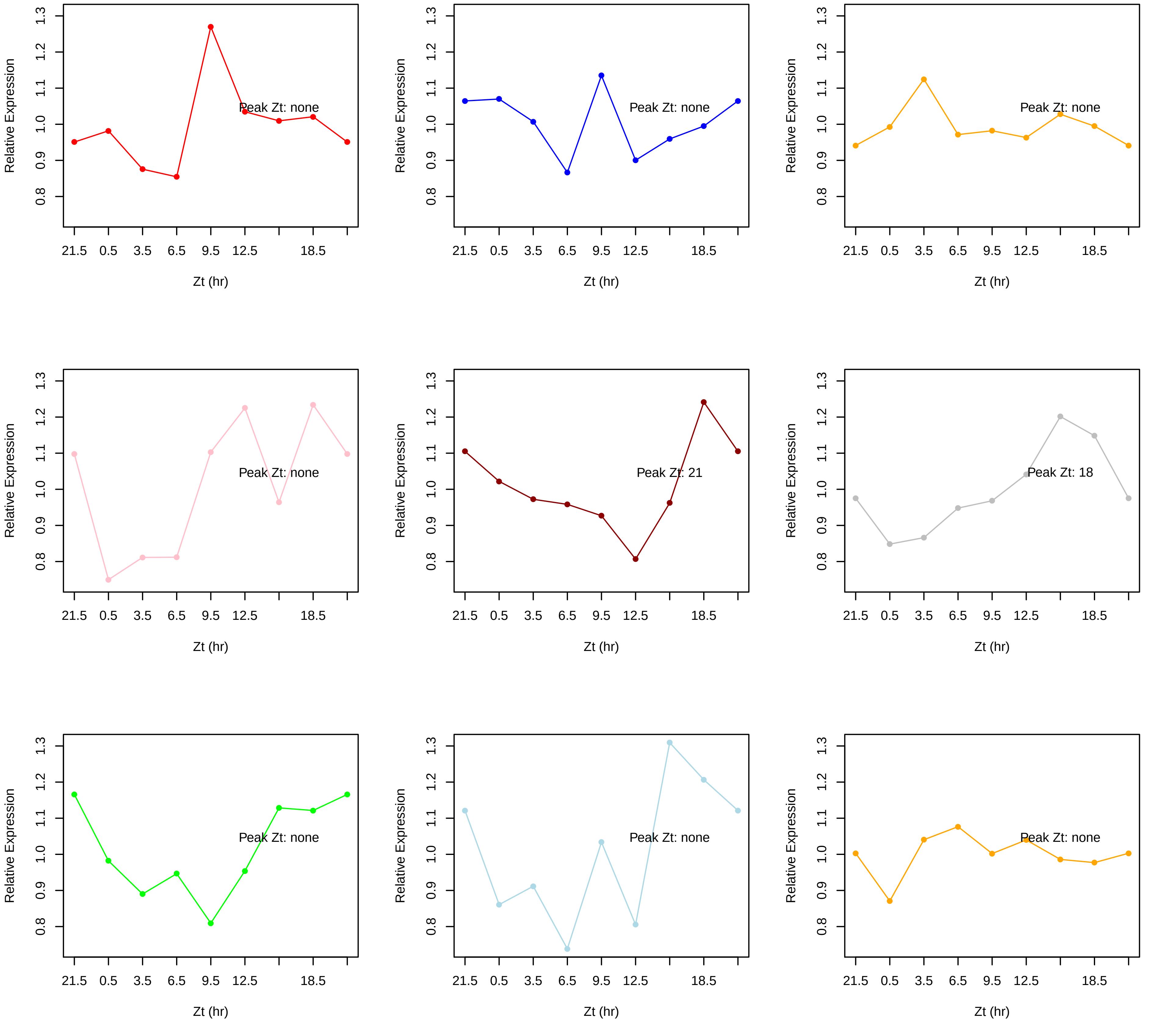

Singeleton: ENSXMAG00000025017 dnajc15

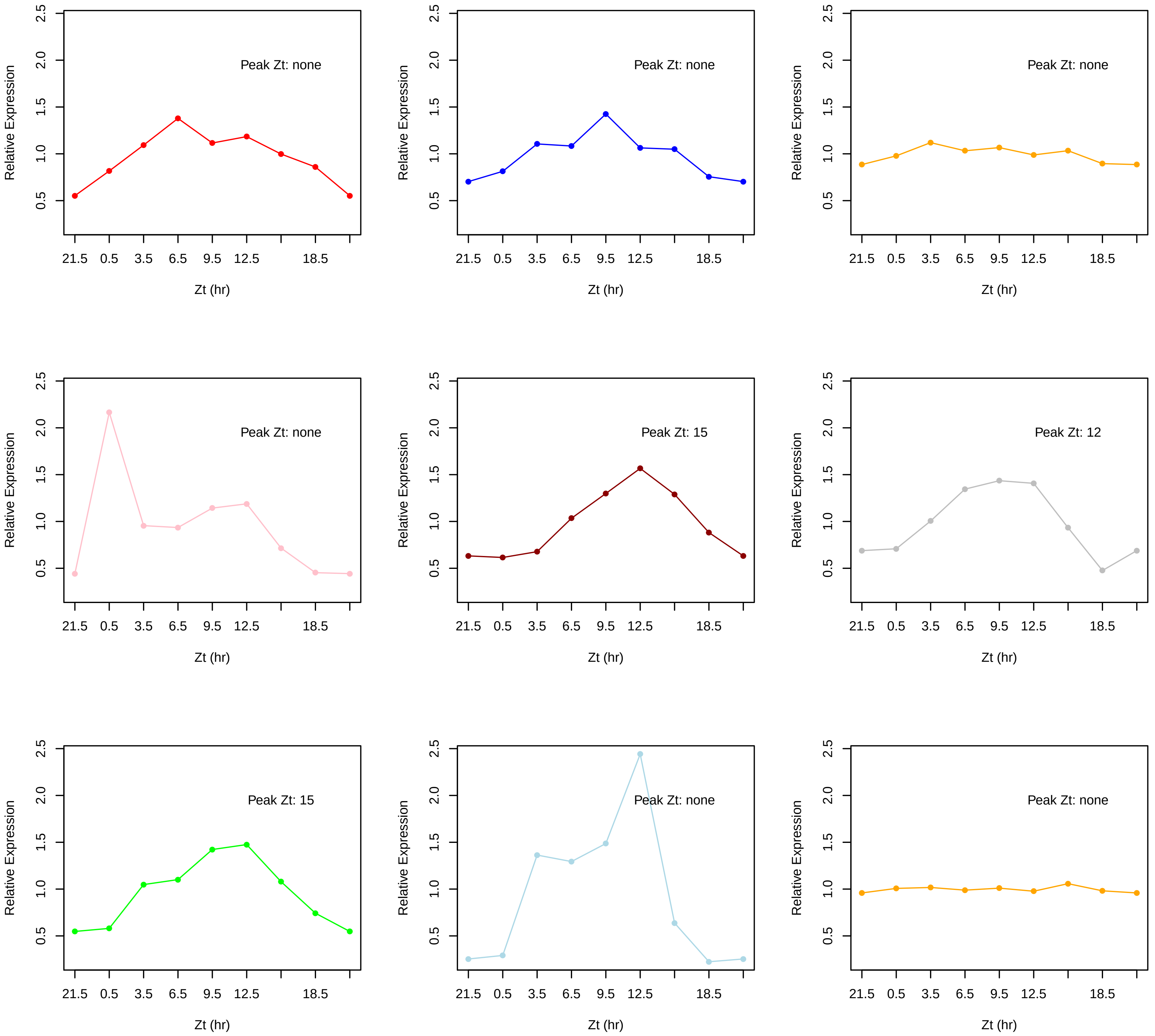

Singeleton: ENSXMAG00000007534

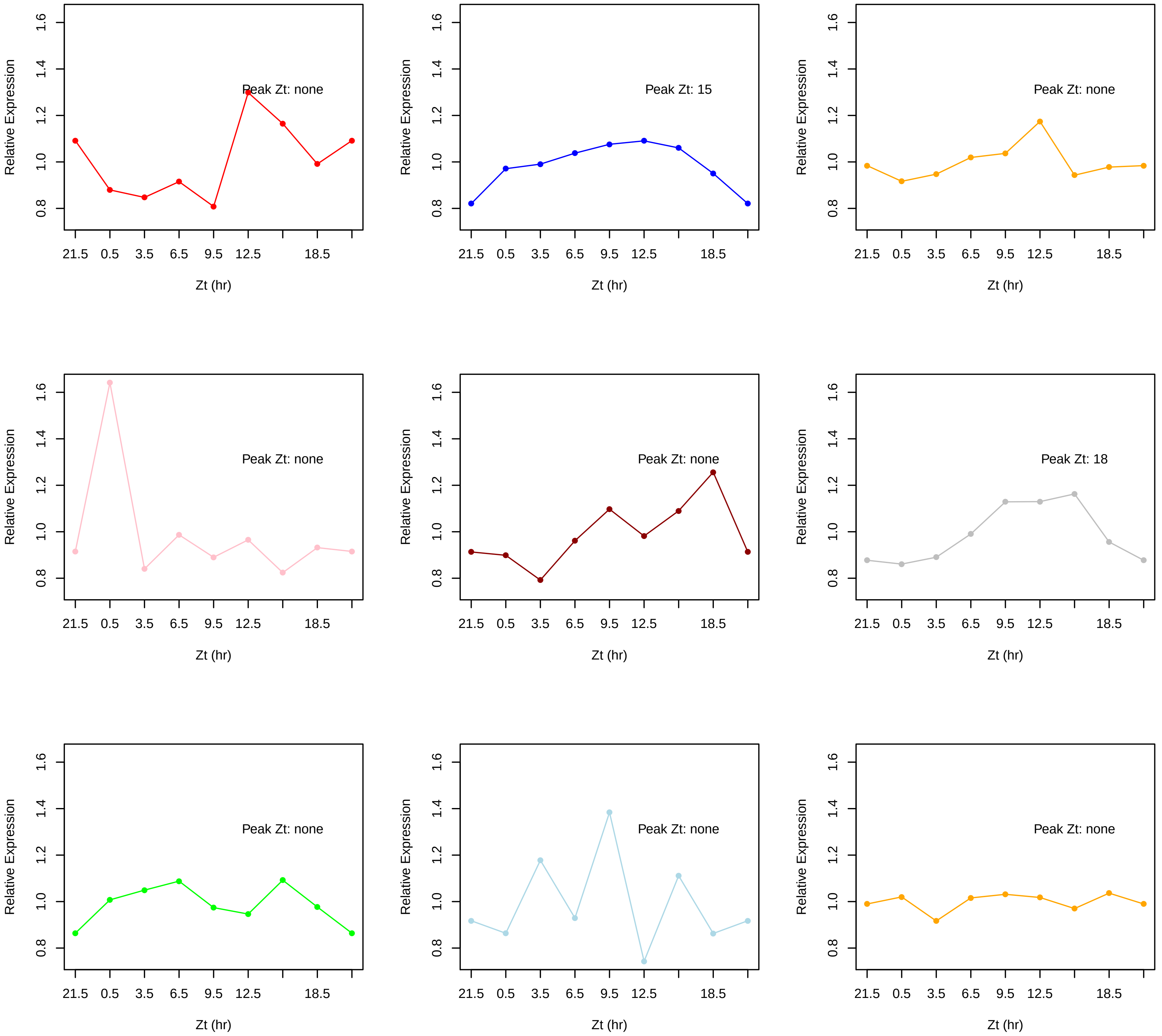

Singeleton: ENSXMAG00000013286 si:zfos−452g4.1

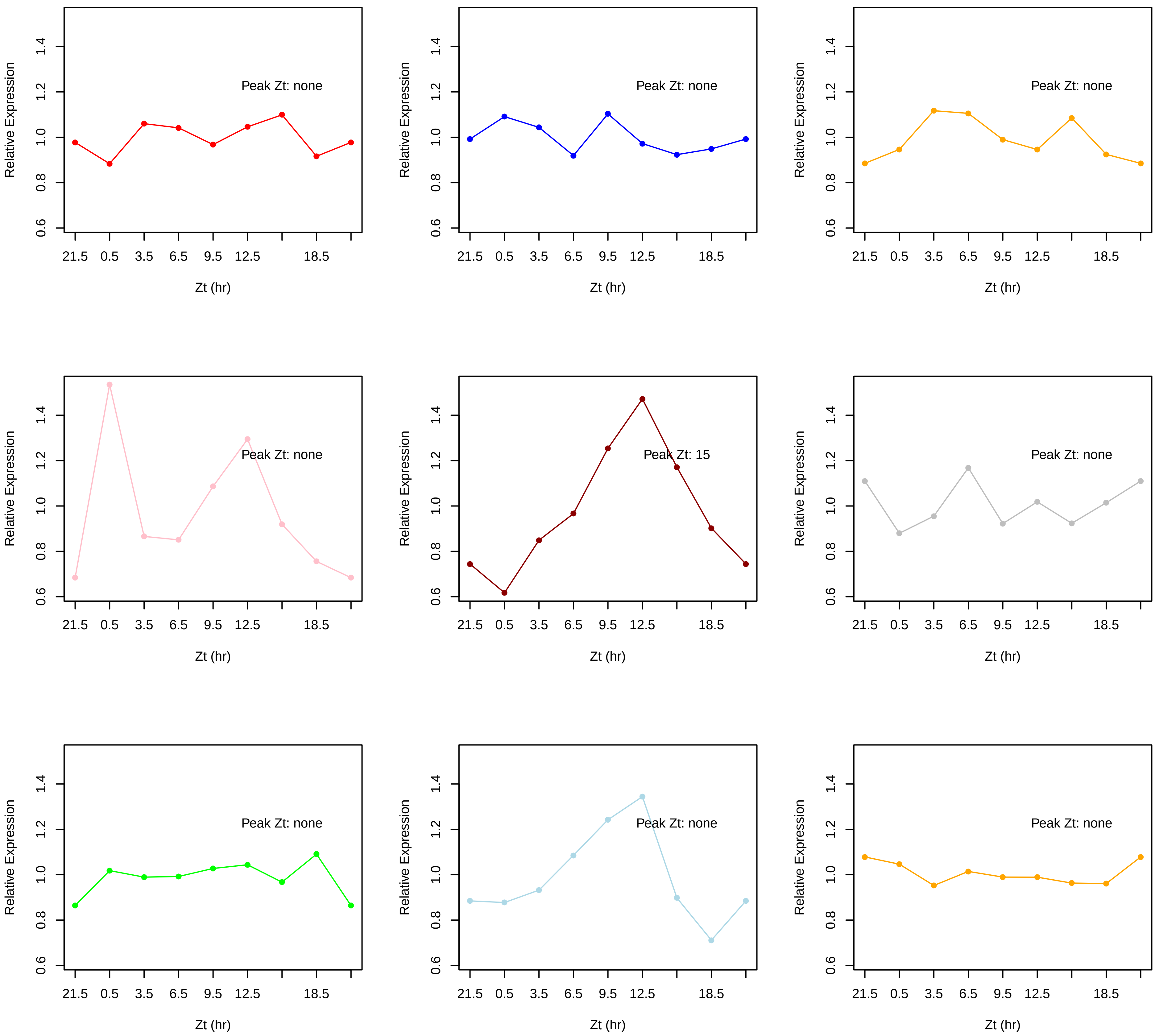

Singeleton: ENSXMAG00000017586 tcea1

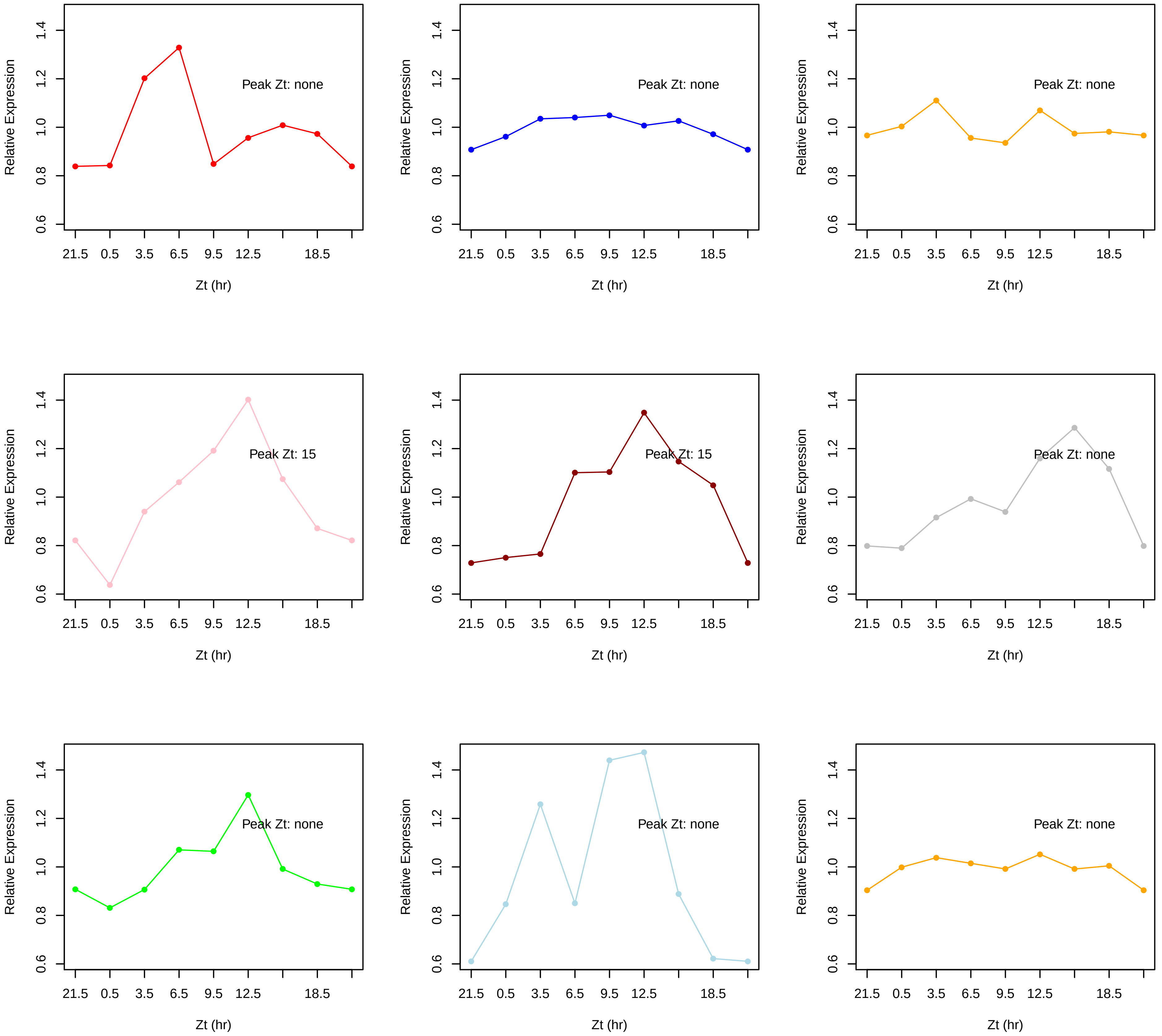

Singeleton: ENSXMAG00000019147 uri1

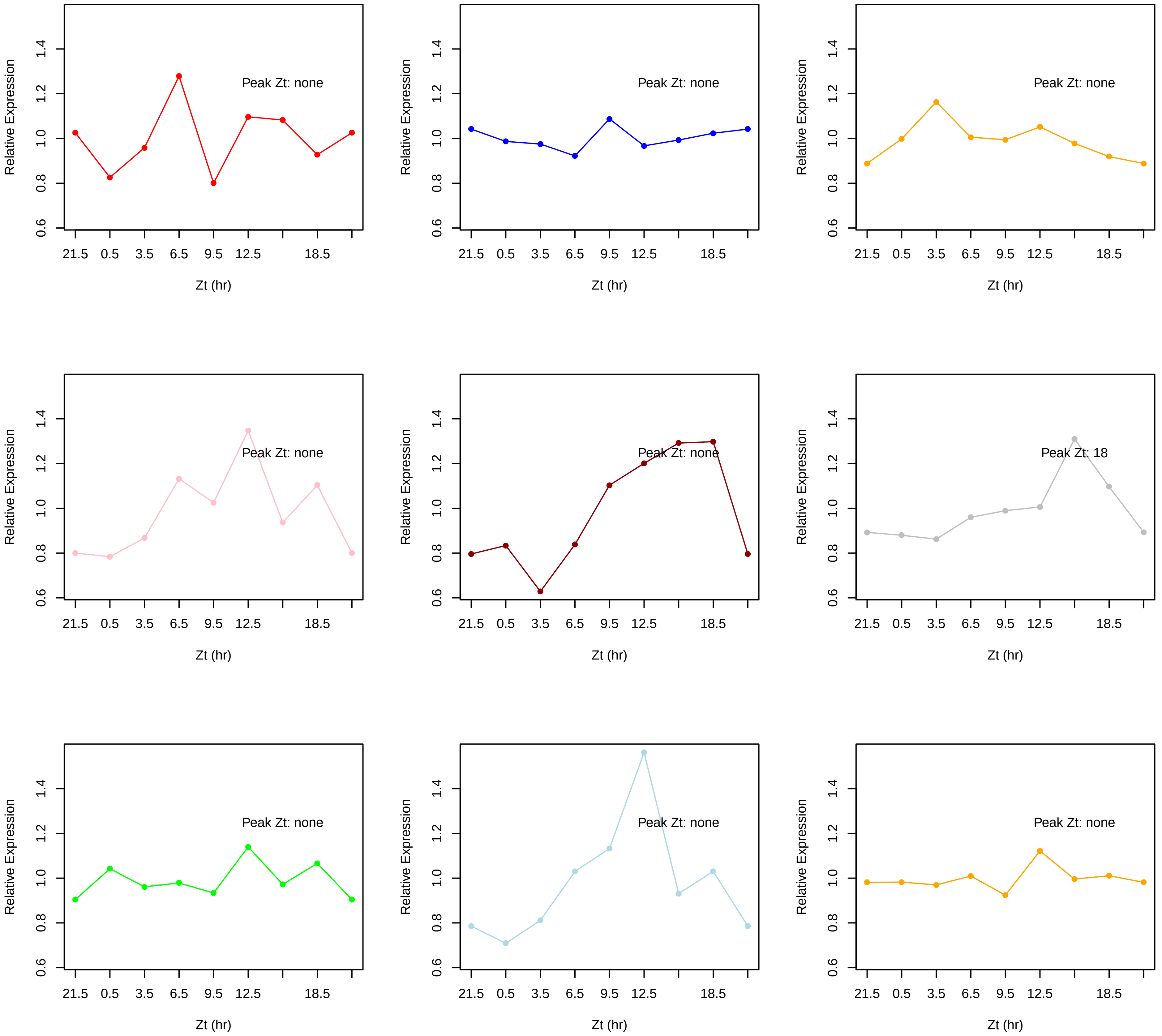

Singeleton: ENSXMAG00000009115 dnmt3bb.1

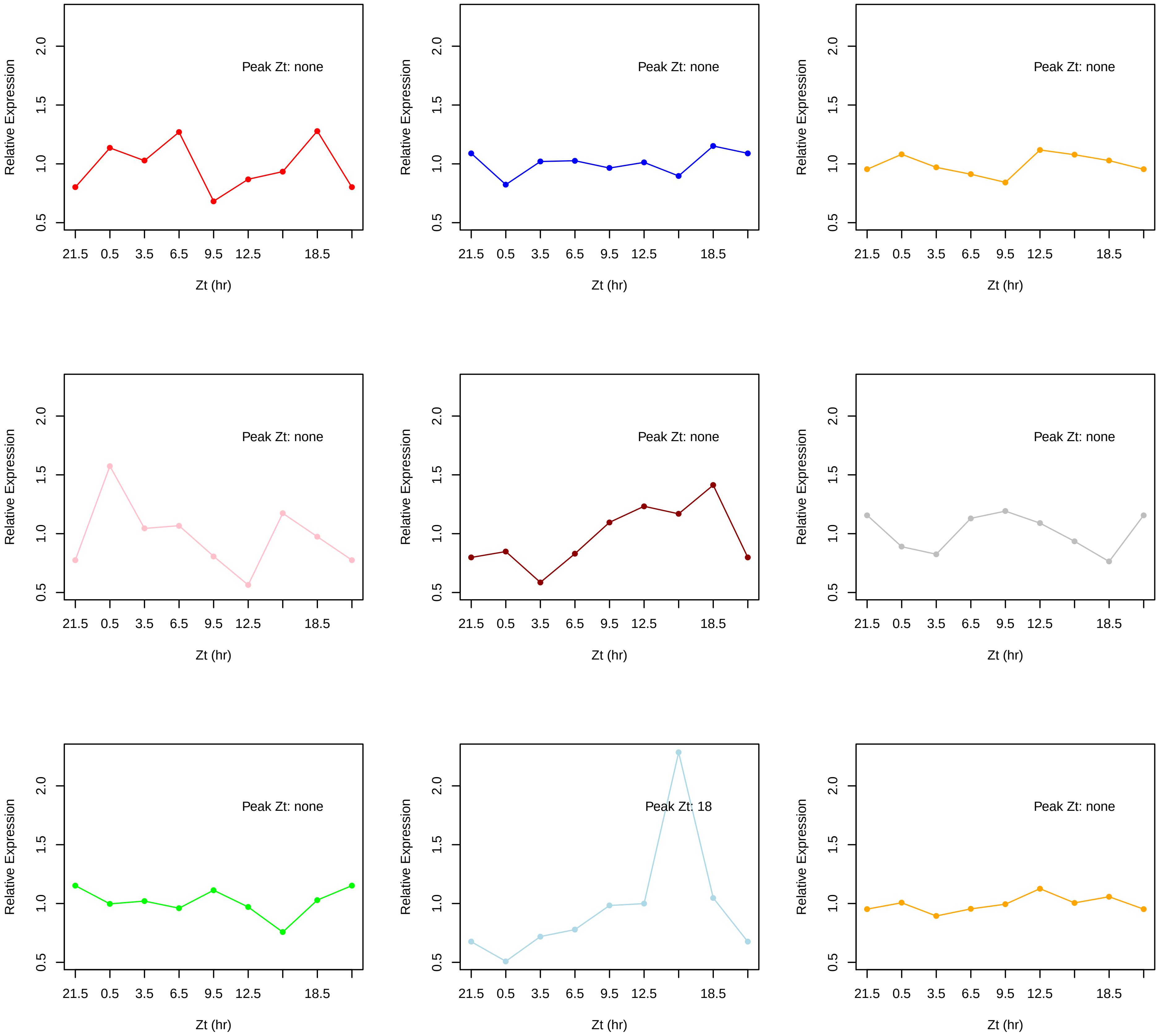

Singleton: ENSXMAG00000018842 pde8b

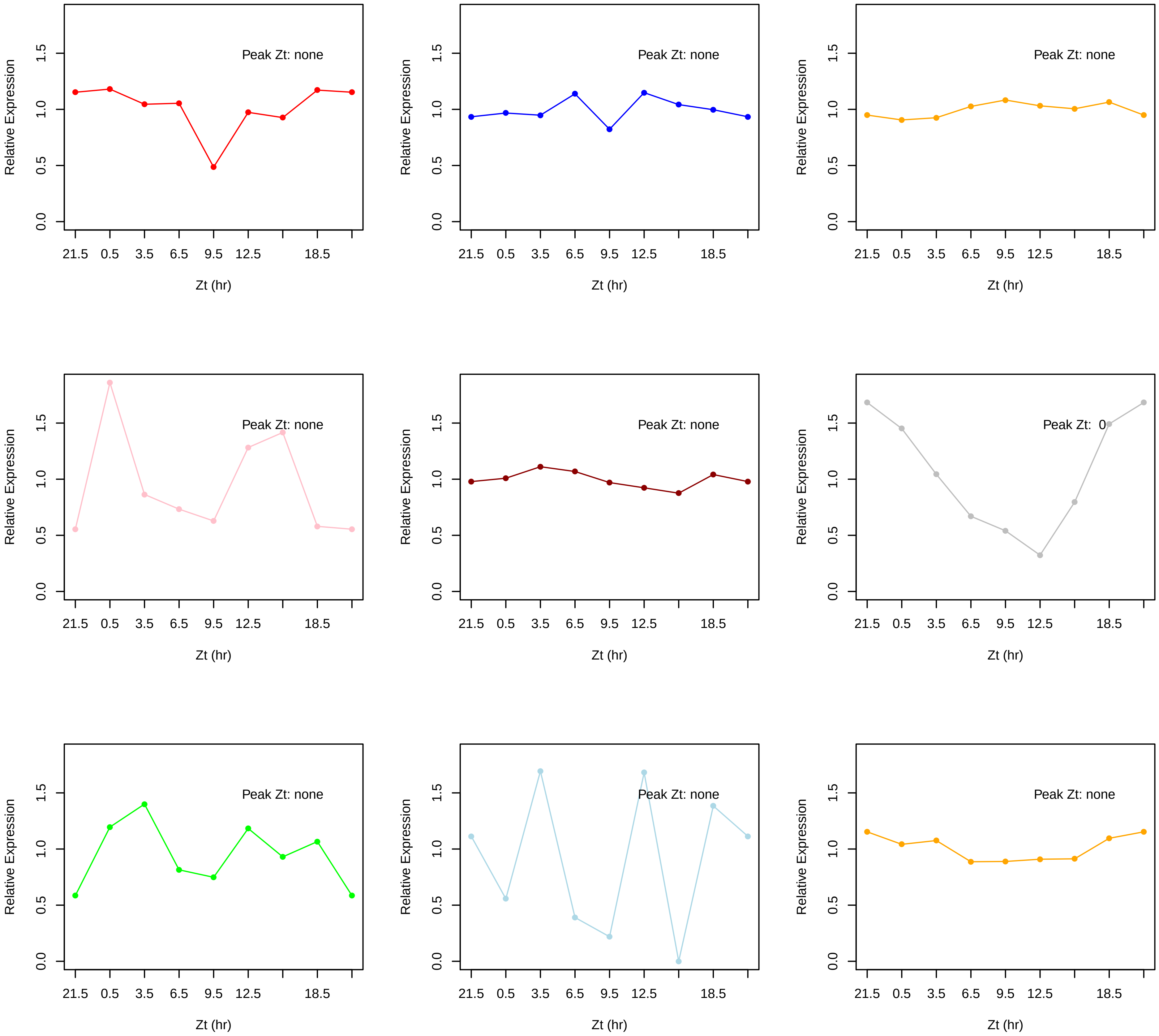

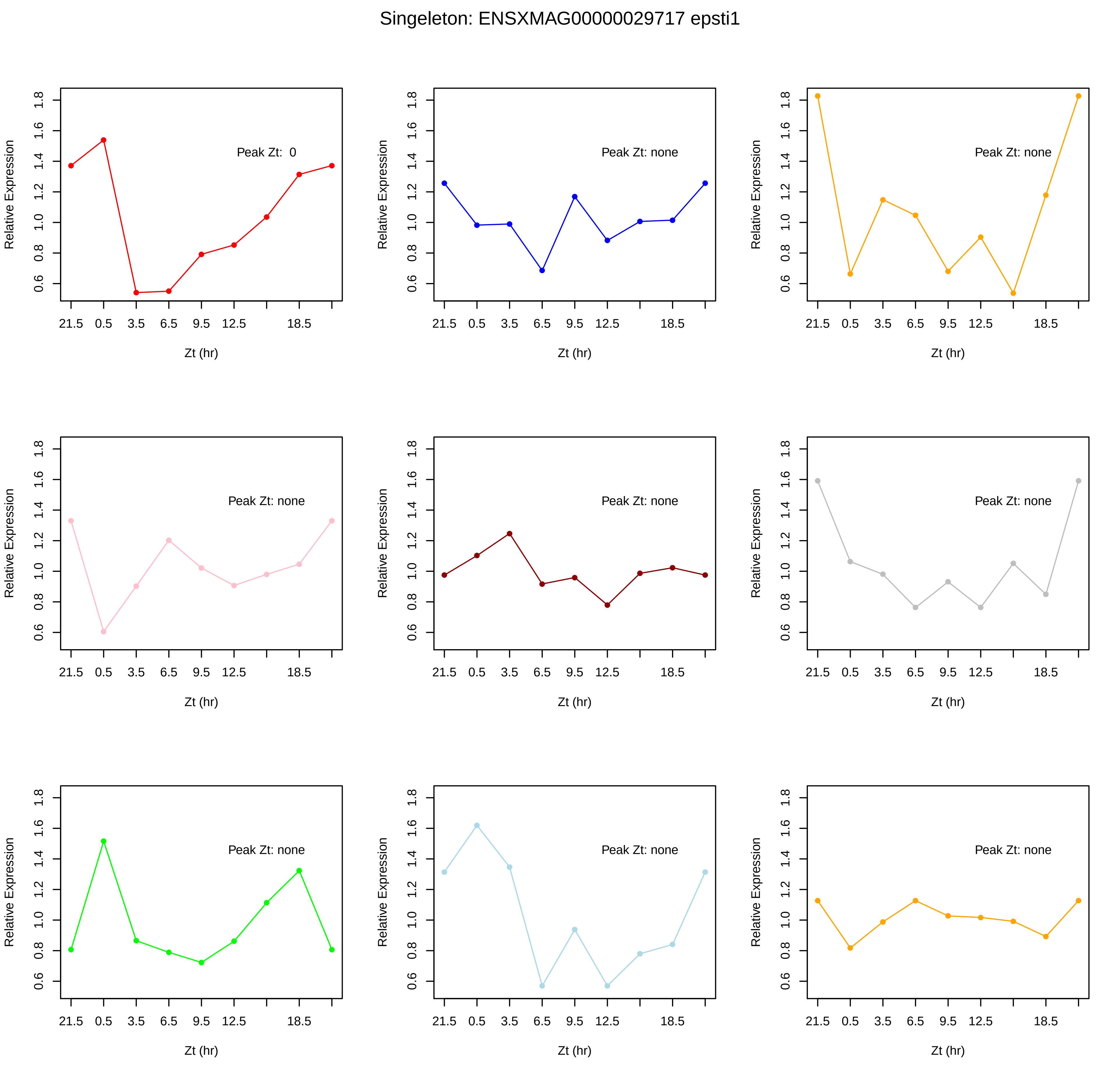
Singeleton: ENSXMAG00000029717 epsti1
Peak Zt: 0
Peak Zt: none
Relative Expression
Zt (hr)
21.5 0.5 3.5 6.5 9.5 12.5 18.5
0.6 0.8 1.0 1.2 1.4 1.6 1.8

Singeleton: ENSXMAG00000005621 pomgnt1

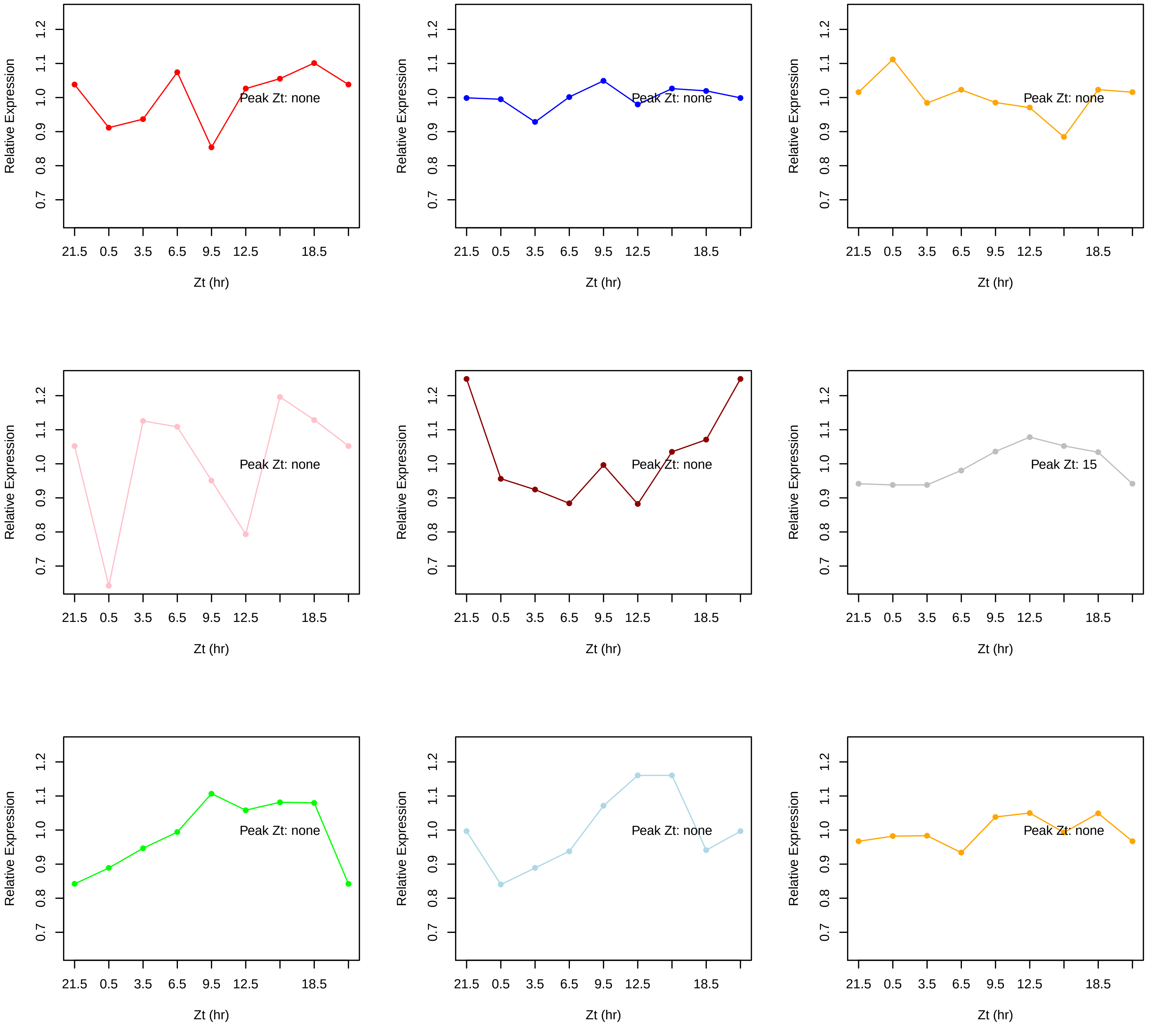

Singeleton: ENSXMAG00000013230 akirin1

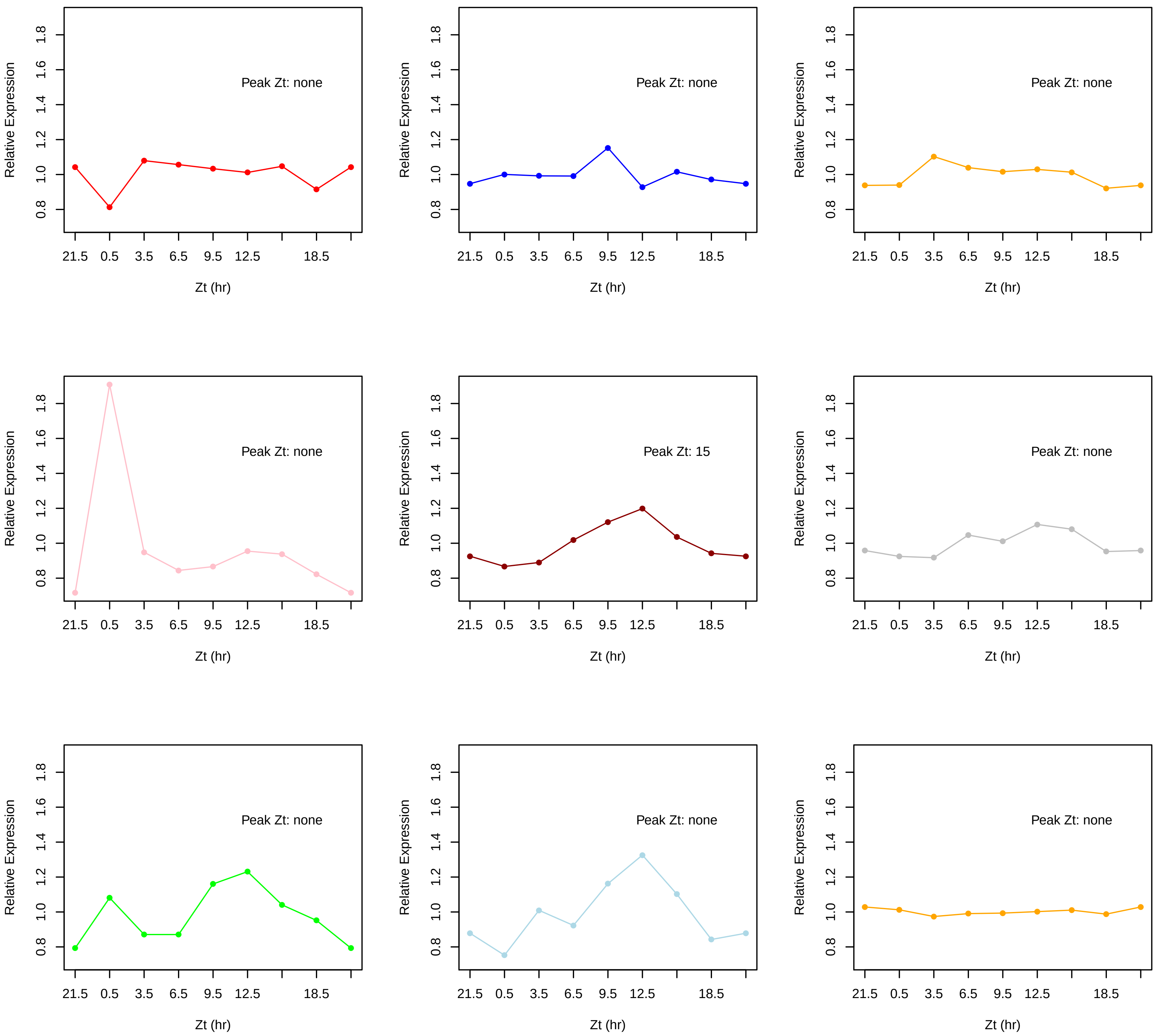

Singeleton: ENSXMAG00000017836 tarbp2

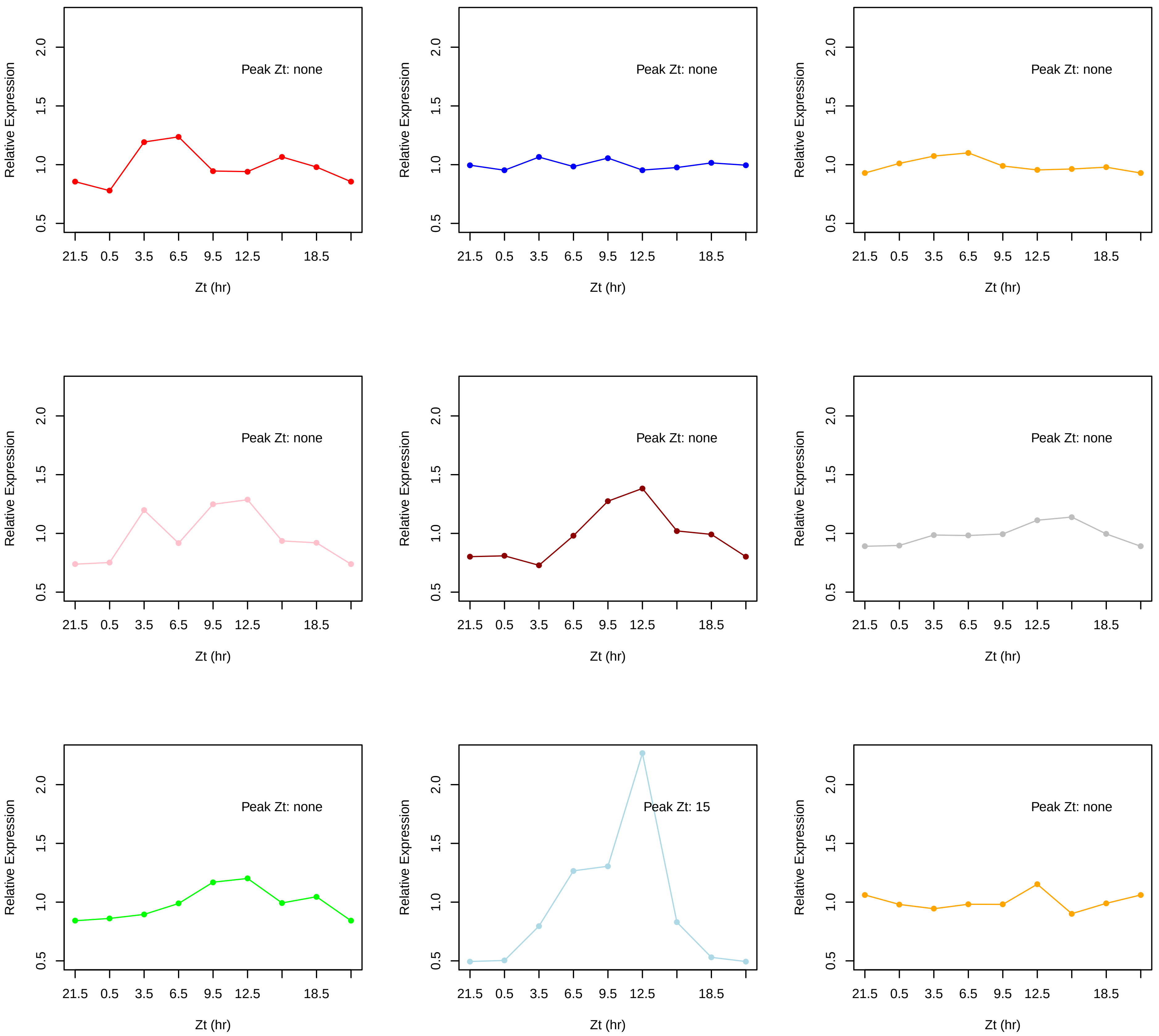

Singleton: ENSXMAG00000027320 tsc1b

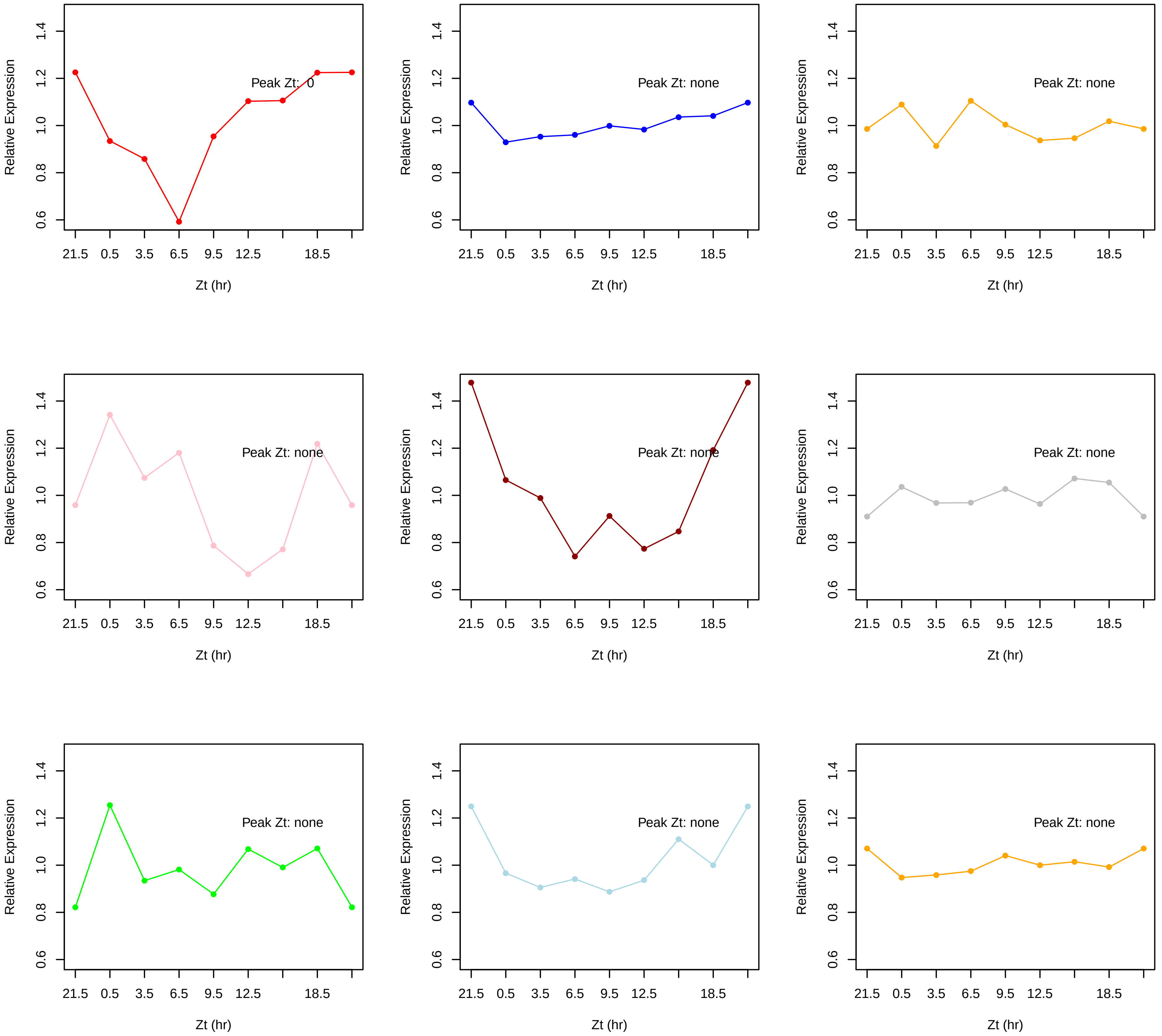

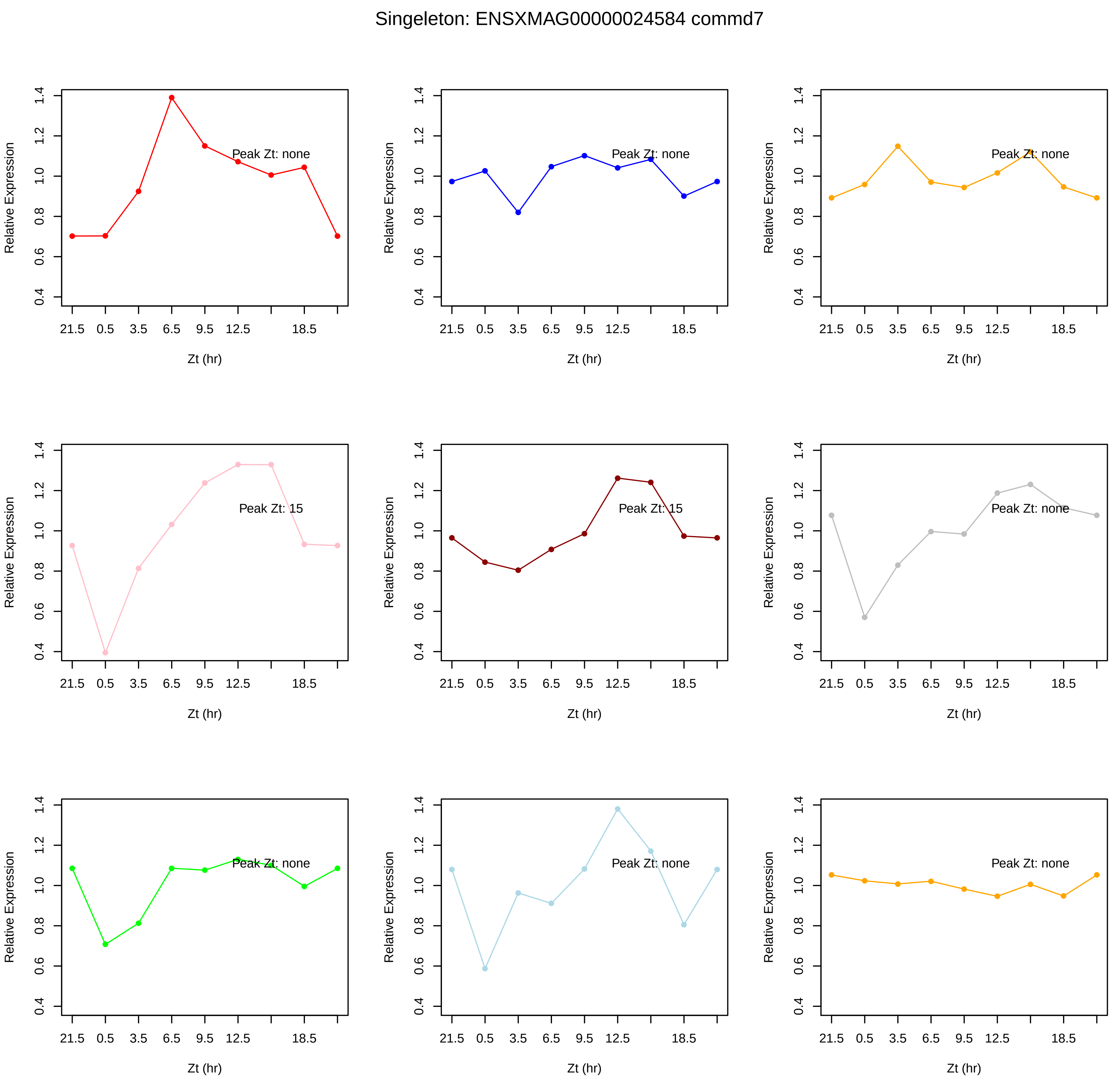
Singeleton: ENSXMAG00000024584 commd7
Relative Expression
Zt (hr)
21.5
0.5
3.5
6.5
9.5
12.5
18.5
0.4
0.6
0.8
1.0
1.2
1.4
Peak Zt: none
Peak Zt: 15

Singeleton: ENSXMAG00000014438 tbrg4

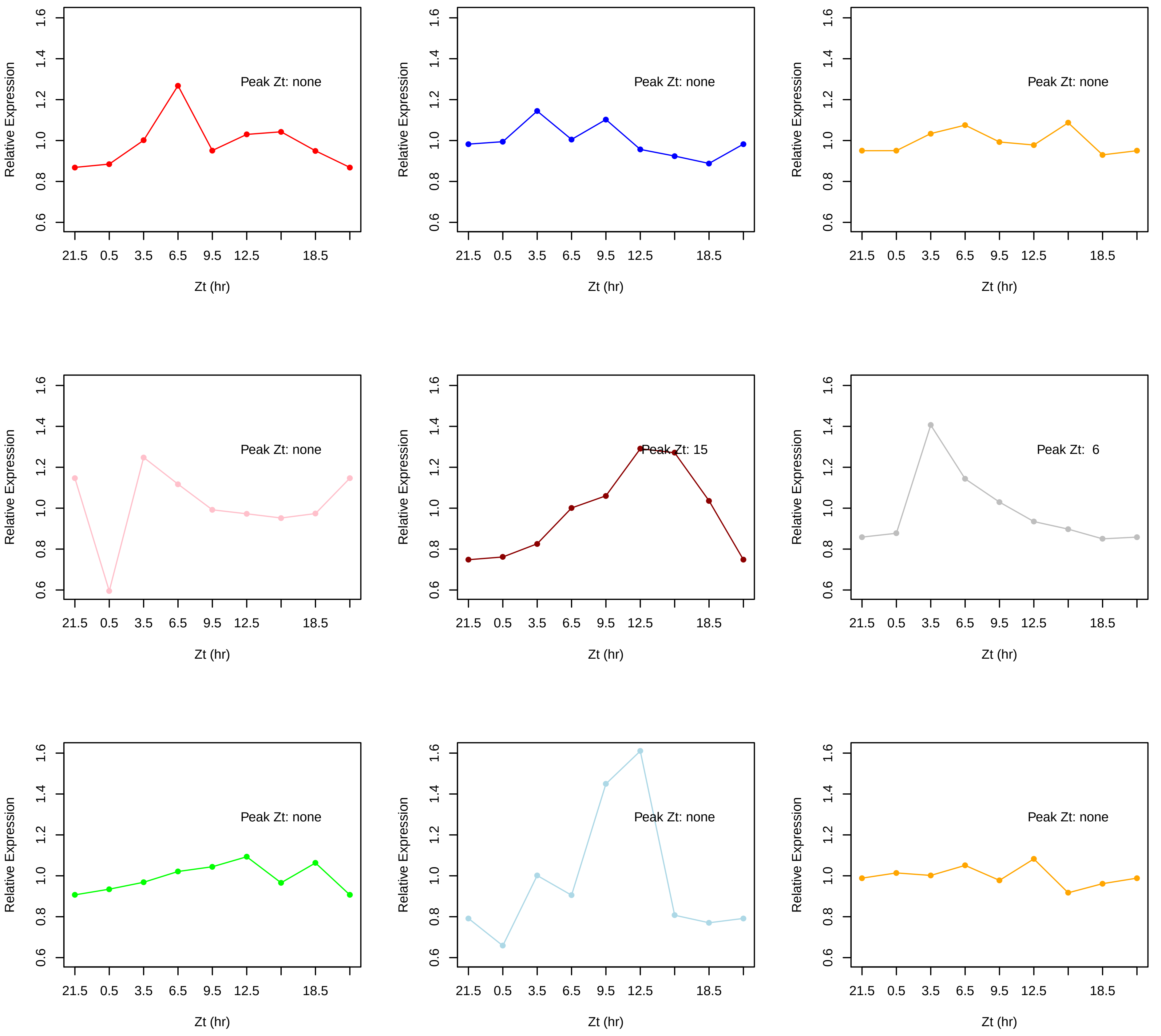

Singeleton: ENSXMAG00000014061 wscd1b

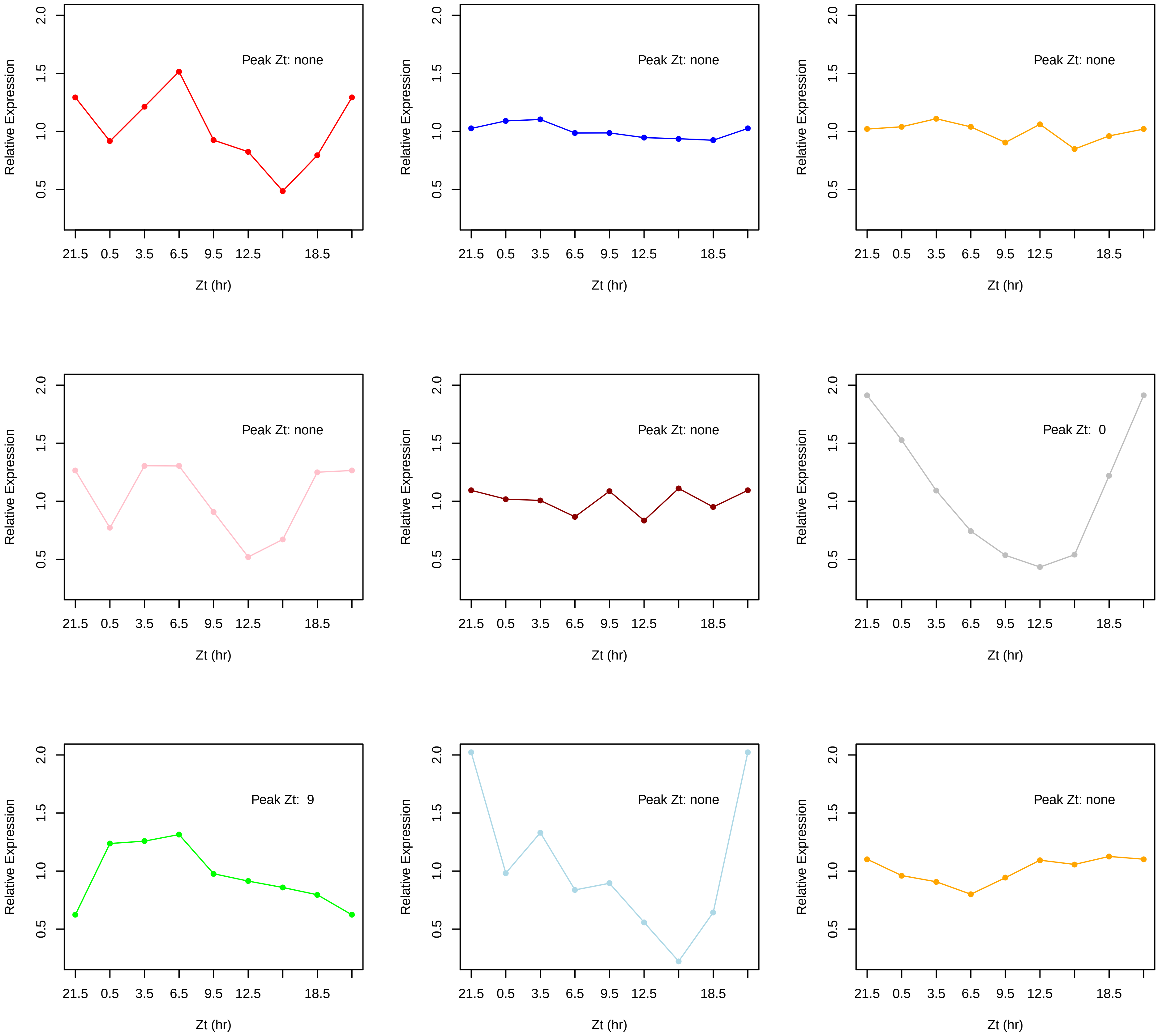

Singeleton: ENSXMAG00000002008 skia

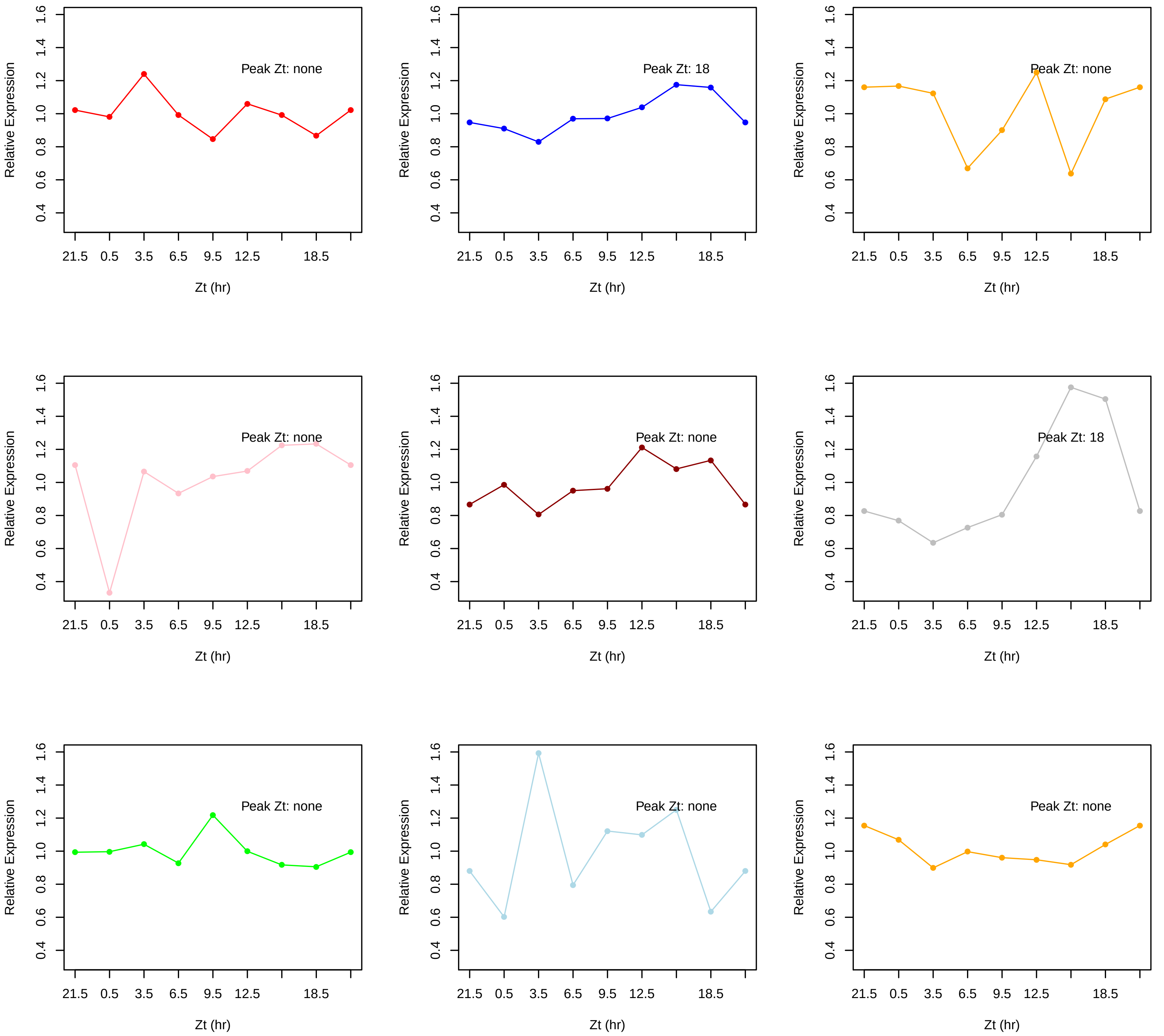

Singeleton: ENSXMAG00000014282 sdf2

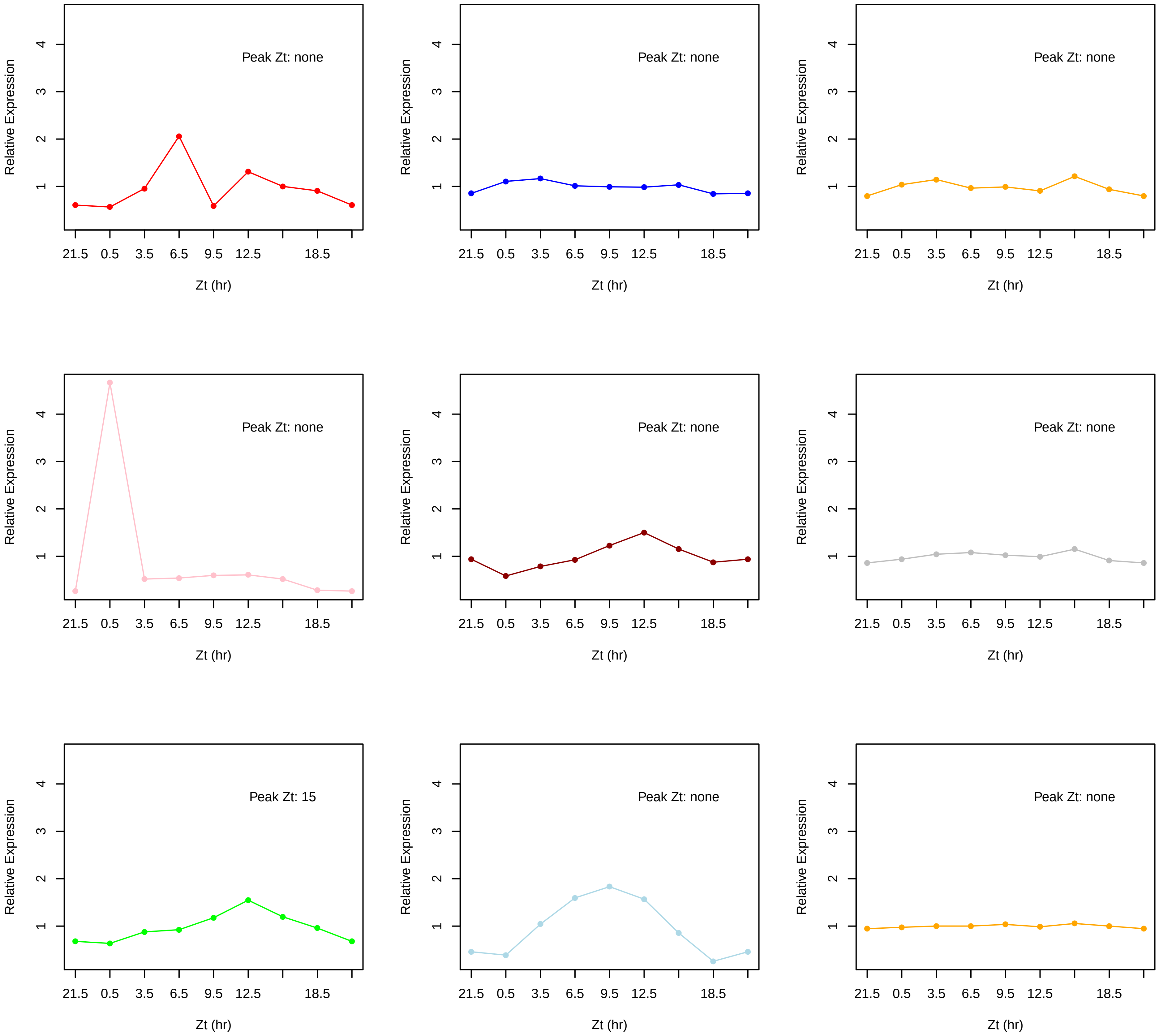

Singleton: ENSXMAG00000015288

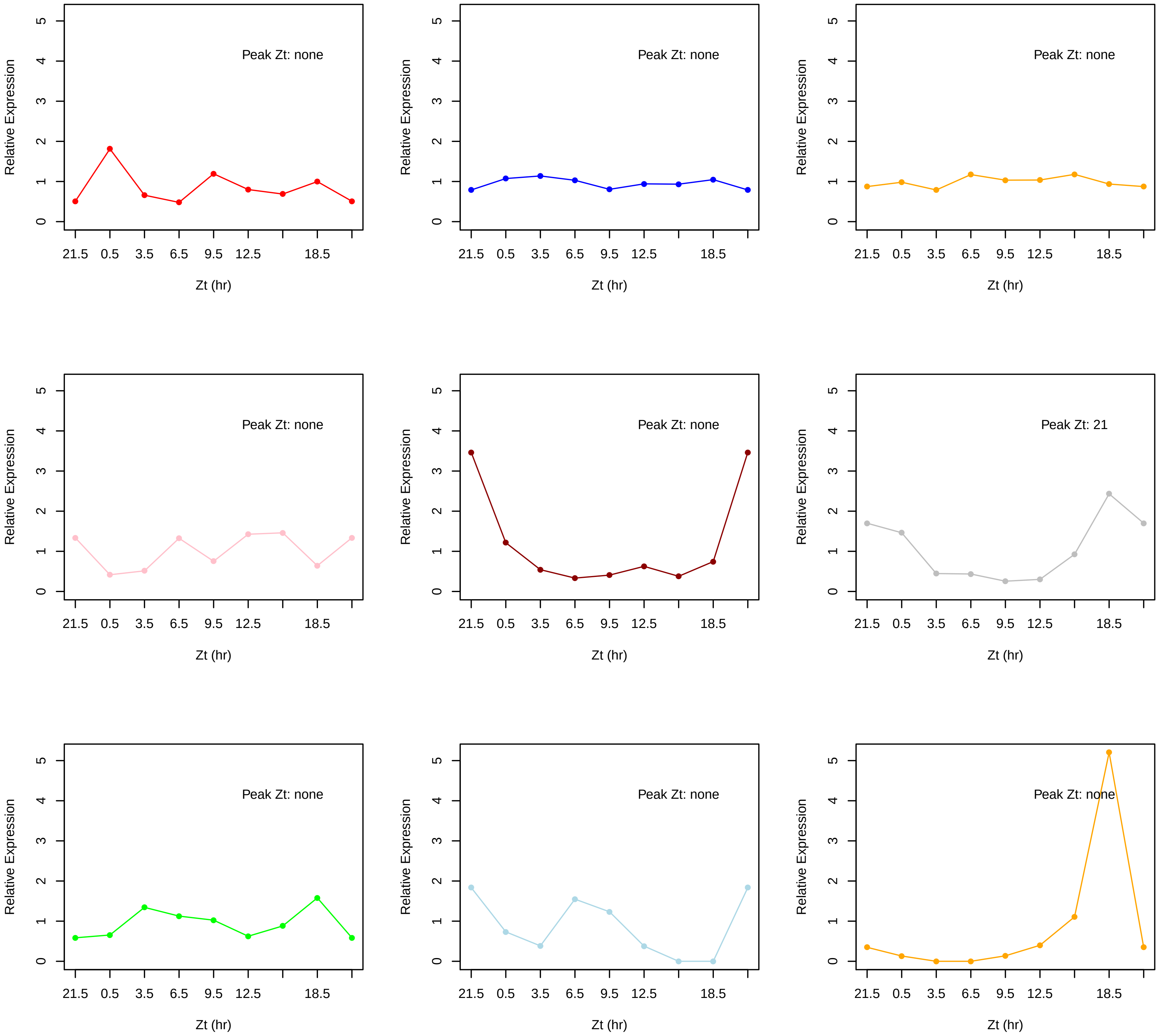

Singeleton: ENSXMAG00000002016 prkcz

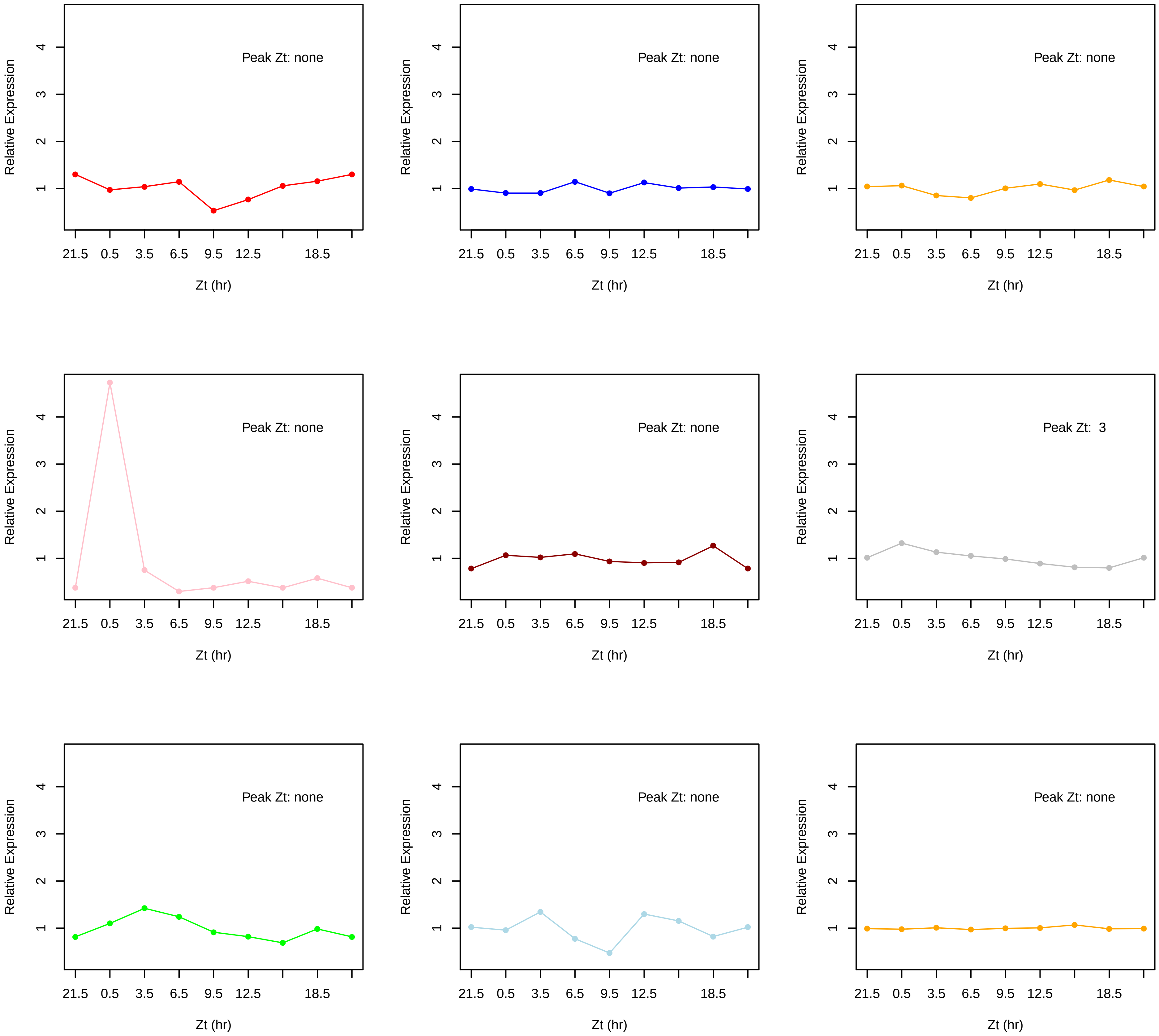

Singeleton: ENSXMAG00000016150

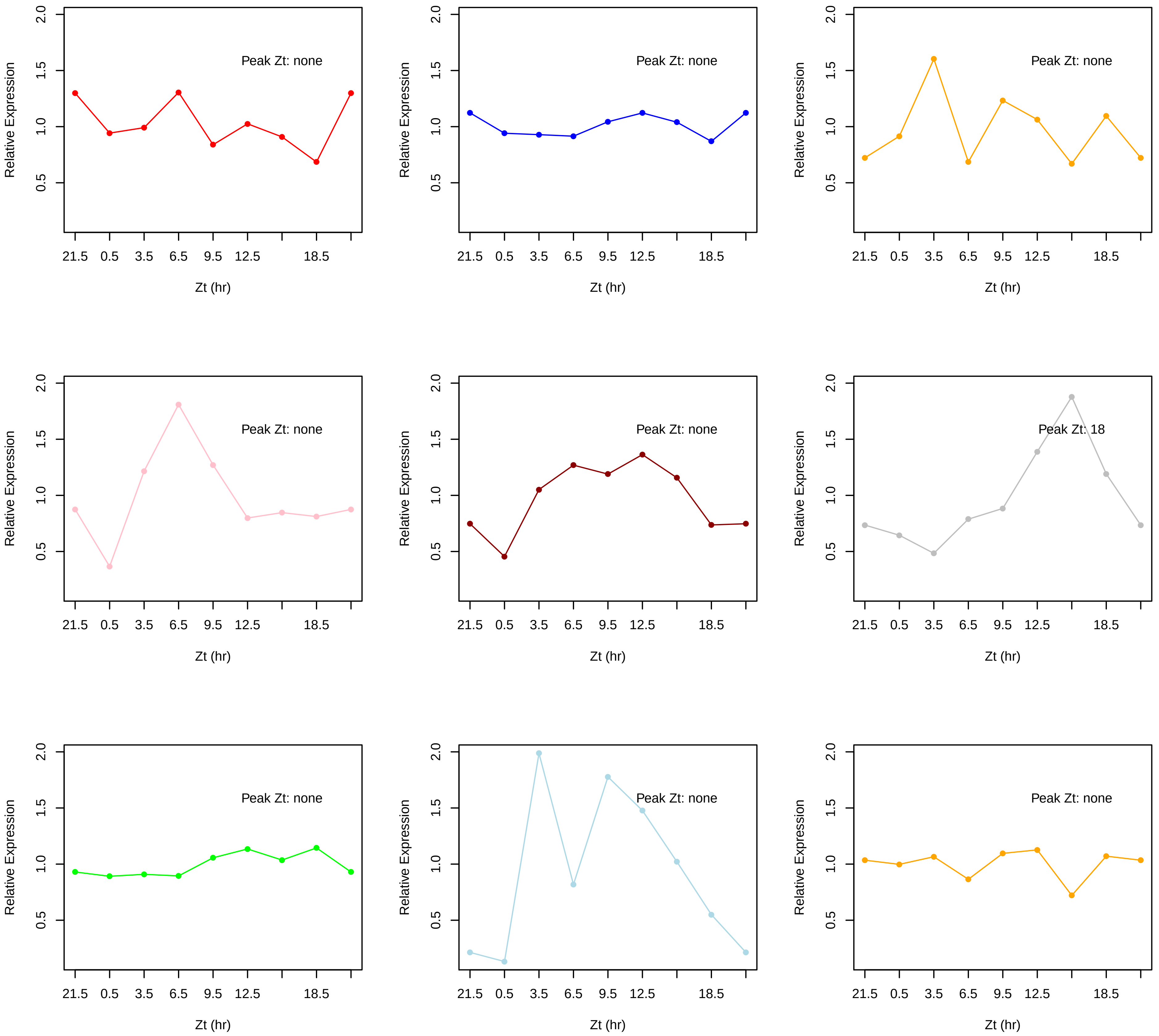

Singeleton: ENSXMAG00000017842 igsf8

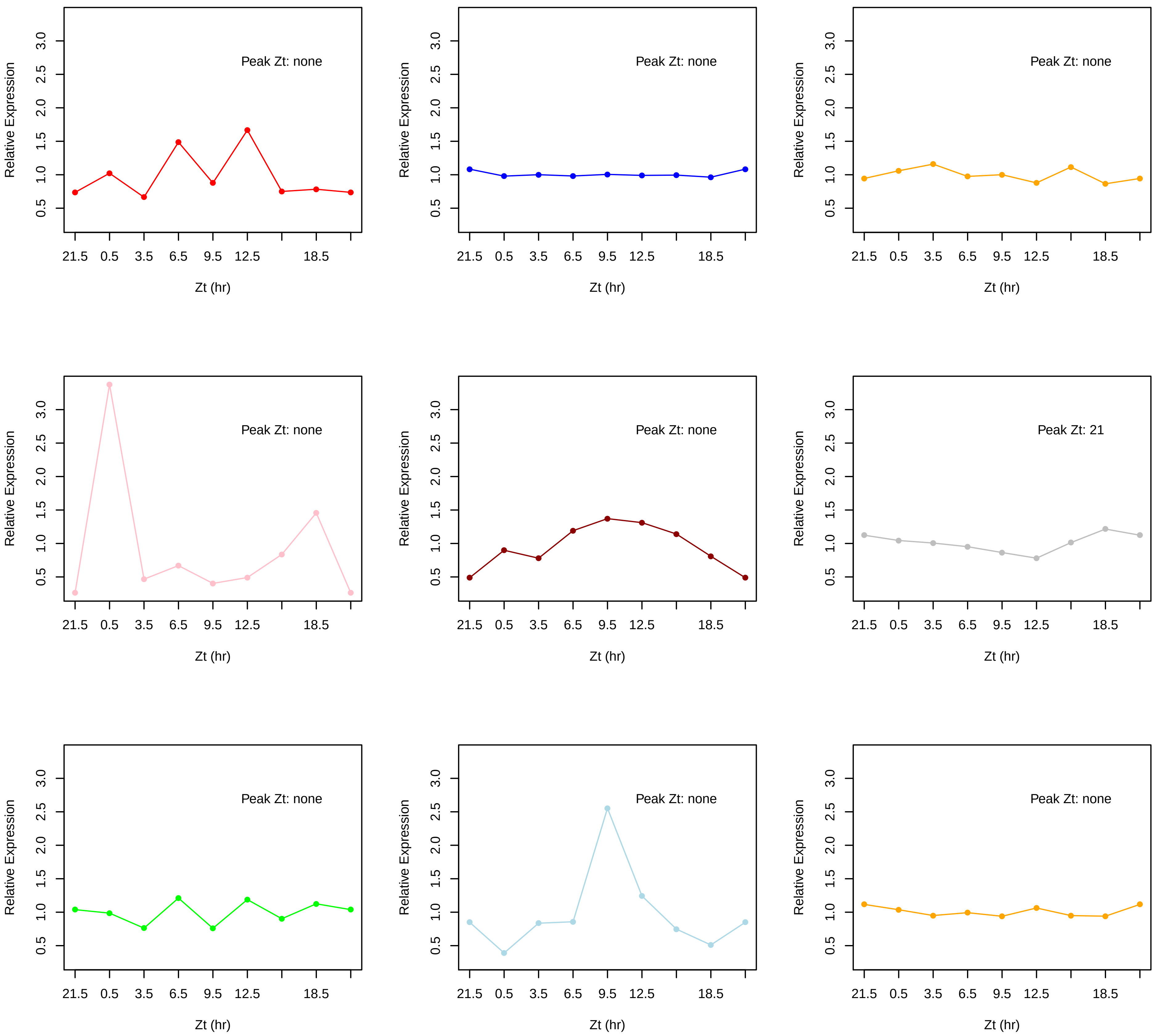

Singeleton: ENSXMAG00000027589 gfi1b

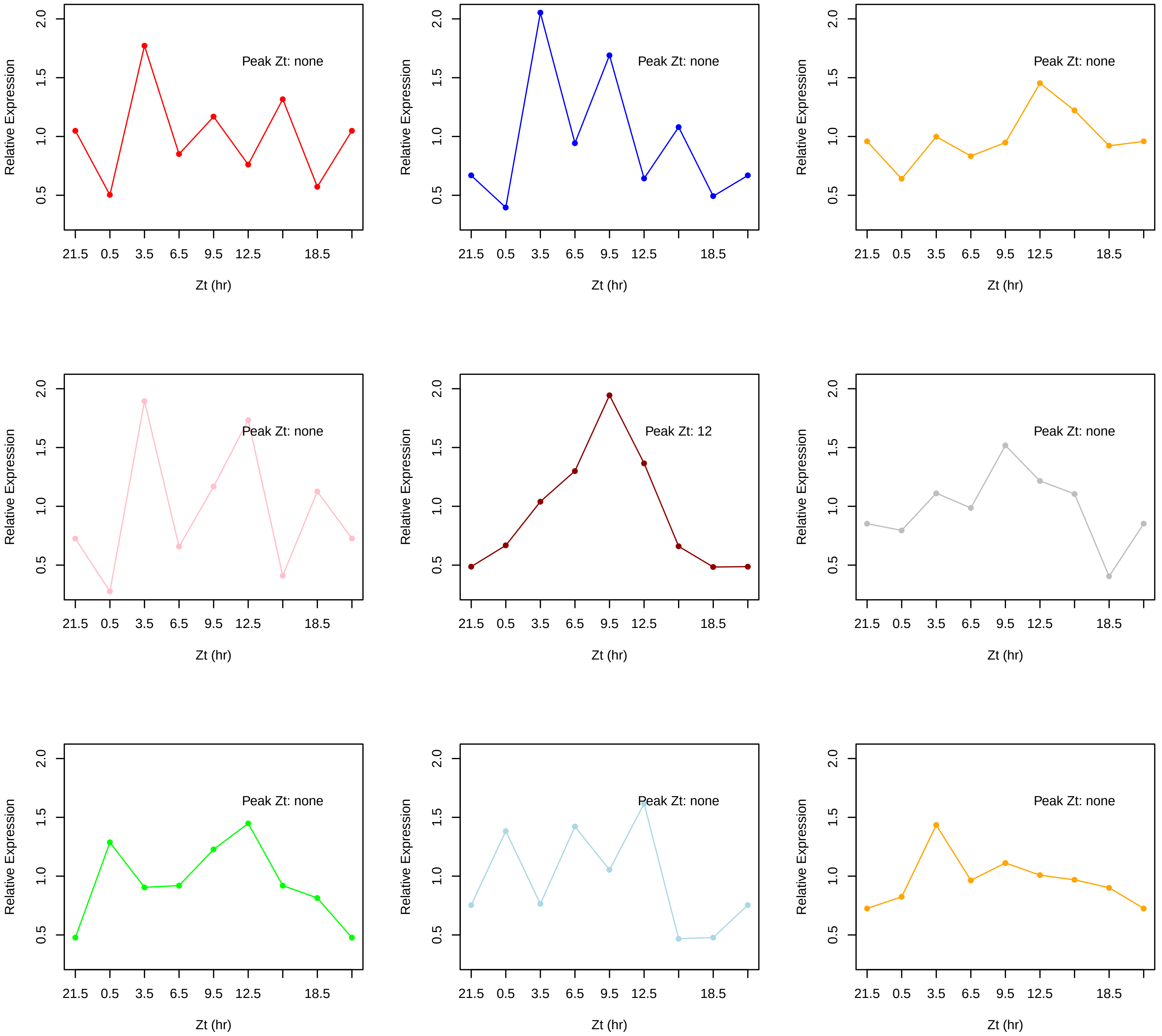

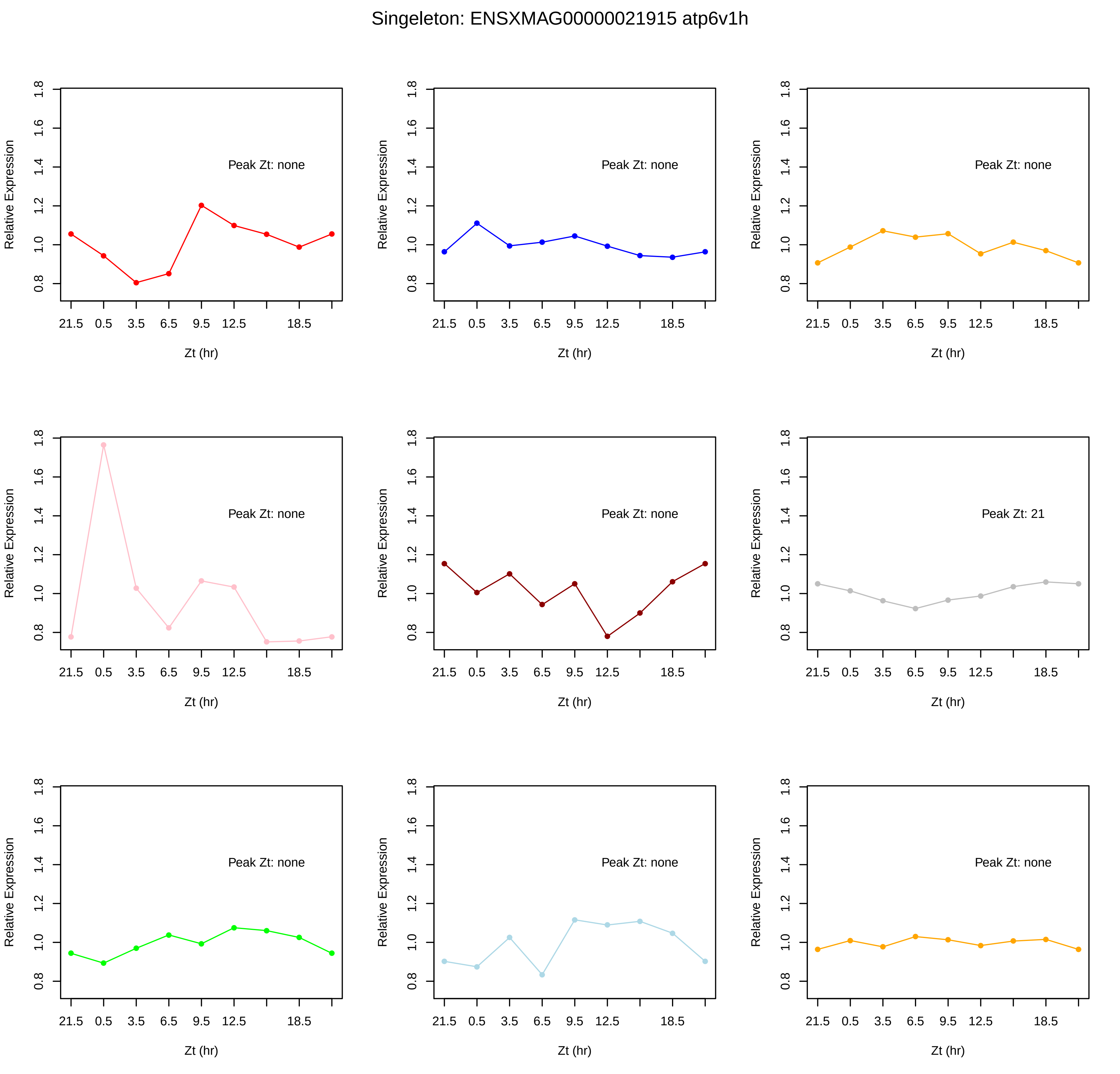

Singeleton: ENSXMAG00000021915 atp6v1h
Peak Zt: none
Peak Zt: none
Peak Zt: none
Peak Zt: none
Peak Zt: none
Peak Zt: 21
Peak Zt: none
Peak Zt: none
Peak Zt: none
Relative Expression
Zt (hr)

Singleton: ENSXMAG00000009147 nol4lb

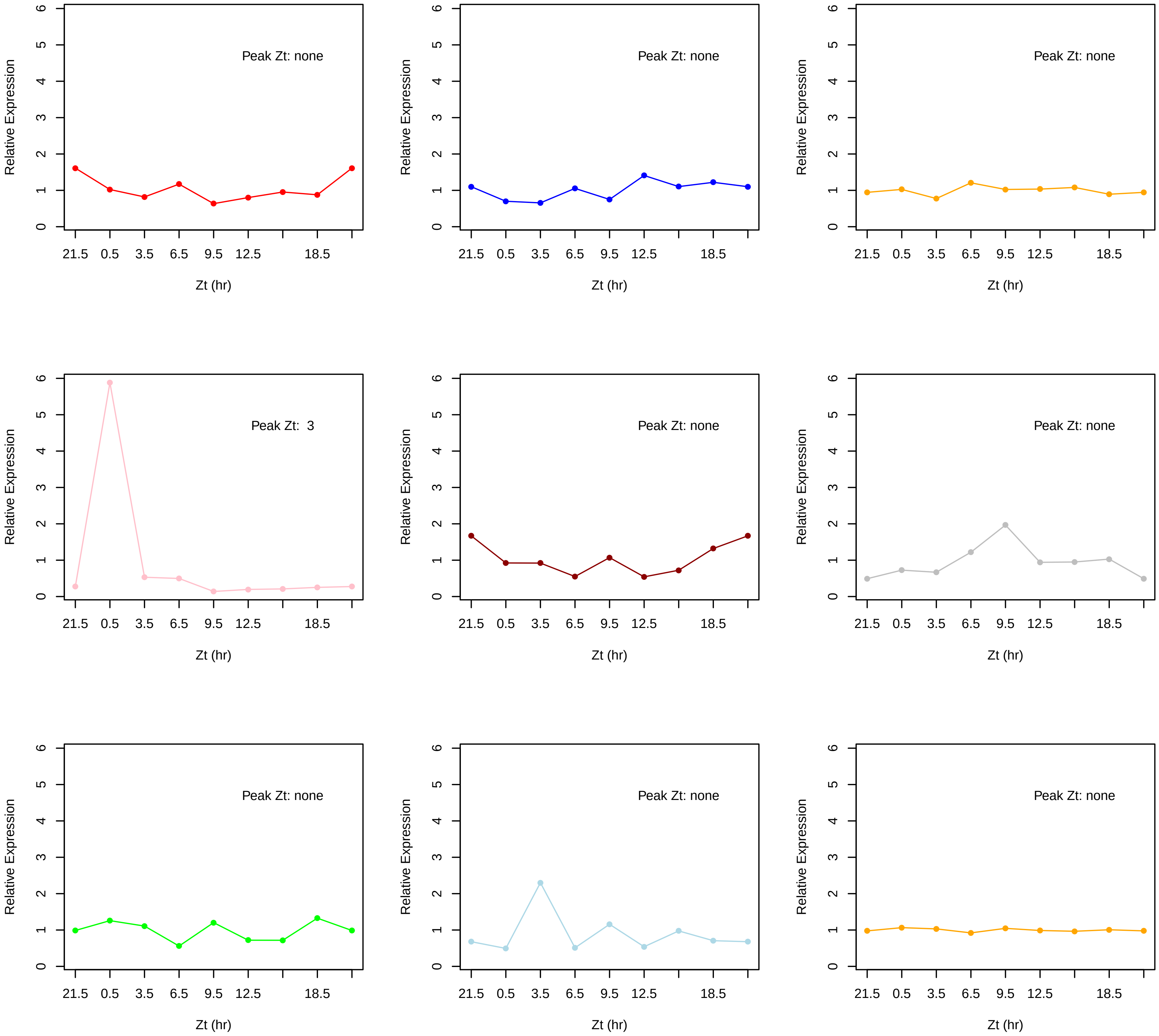

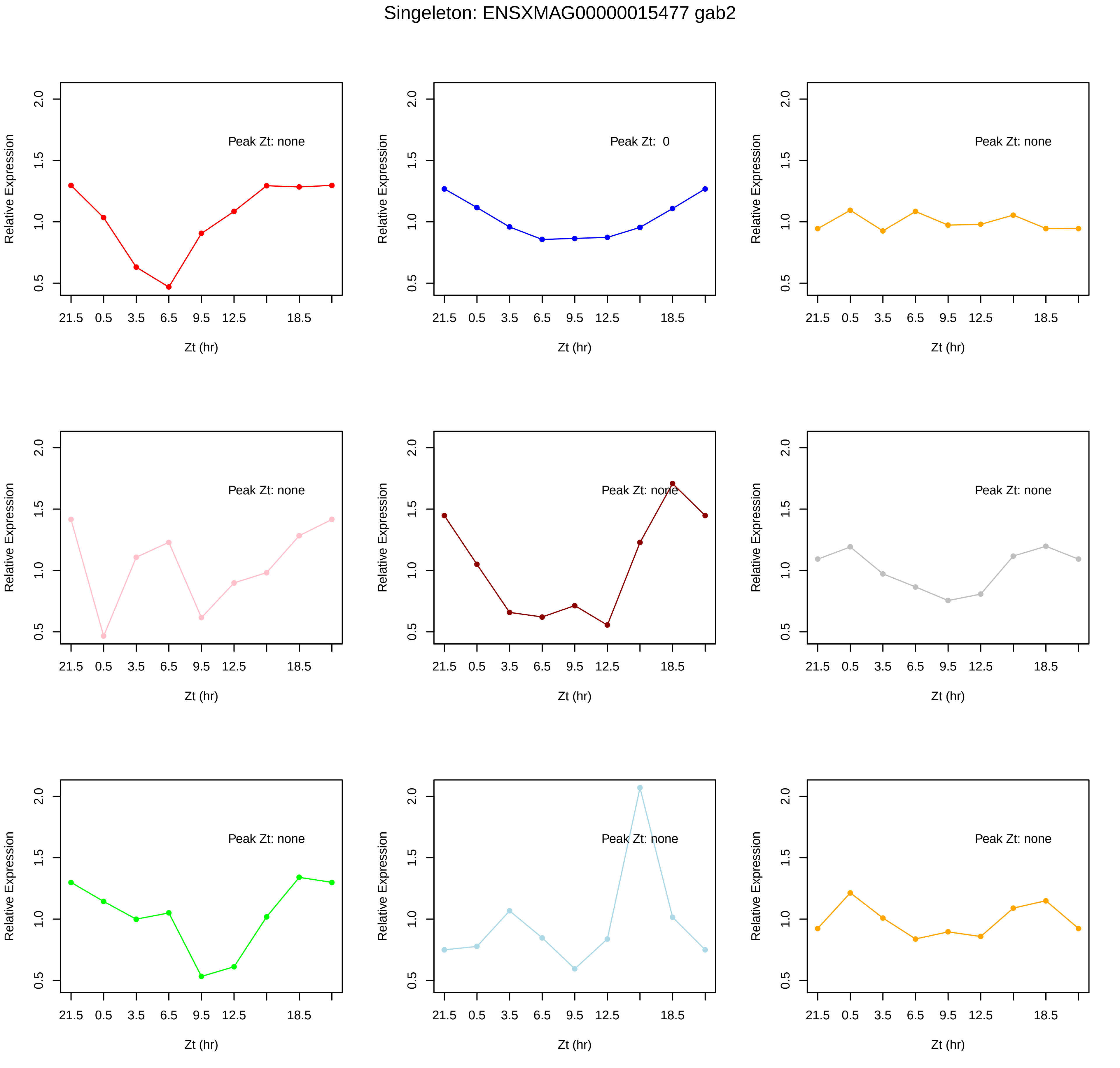
Singeleton: ENSXMAG00000015477 gab2
Peak Zt: none
Peak Zt: 0
Peak Zt: none
Peak Zt: none
Peak Zt: none
Peak Zt: none
Peak Zt: none
Peak Zt: none
Peak Zt: none
Relative Expression
Zt (hr)
21.5
0.5
3.5
6.5
9.5
12.5
18.5
0.5
1.0
1.5
2.0

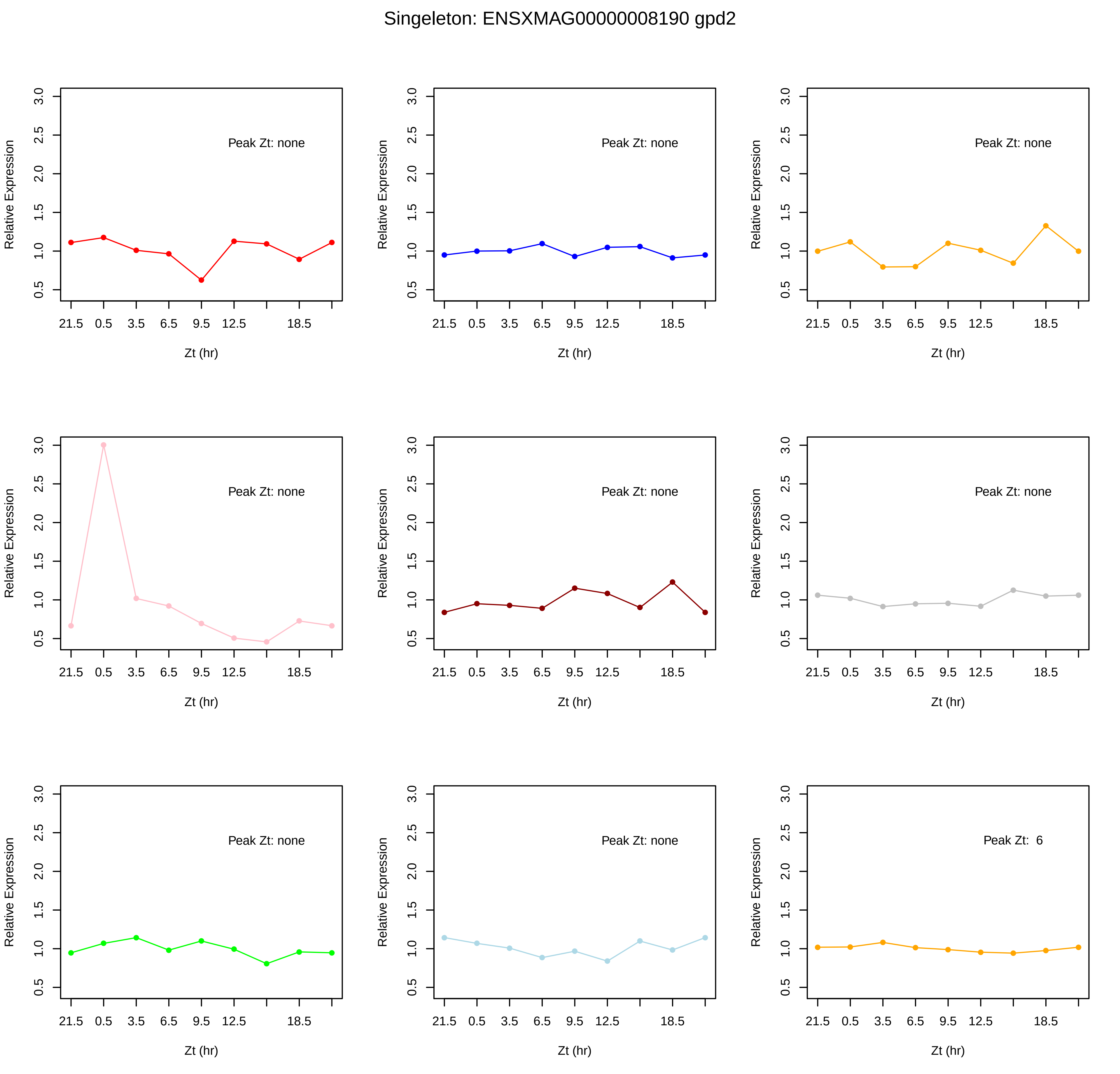
Singeleton: ENSXMAG00000008190 gpd2
Peak Zt: none
Peak Zt: none
Peak Zt: none
Peak Zt: none
Peak Zt: none
Peak Zt: none
Peak Zt: none
Peak Zt: none
Peak Zt: 6
Relative Expression
Zt (hr)
21.5
0.5
3.5
6.5
9.5
12.5
18.5
0.5
1.0
1.5
2.0
2.5
3.0

Singeleton: ENSXMAG00000002990 cyhr1

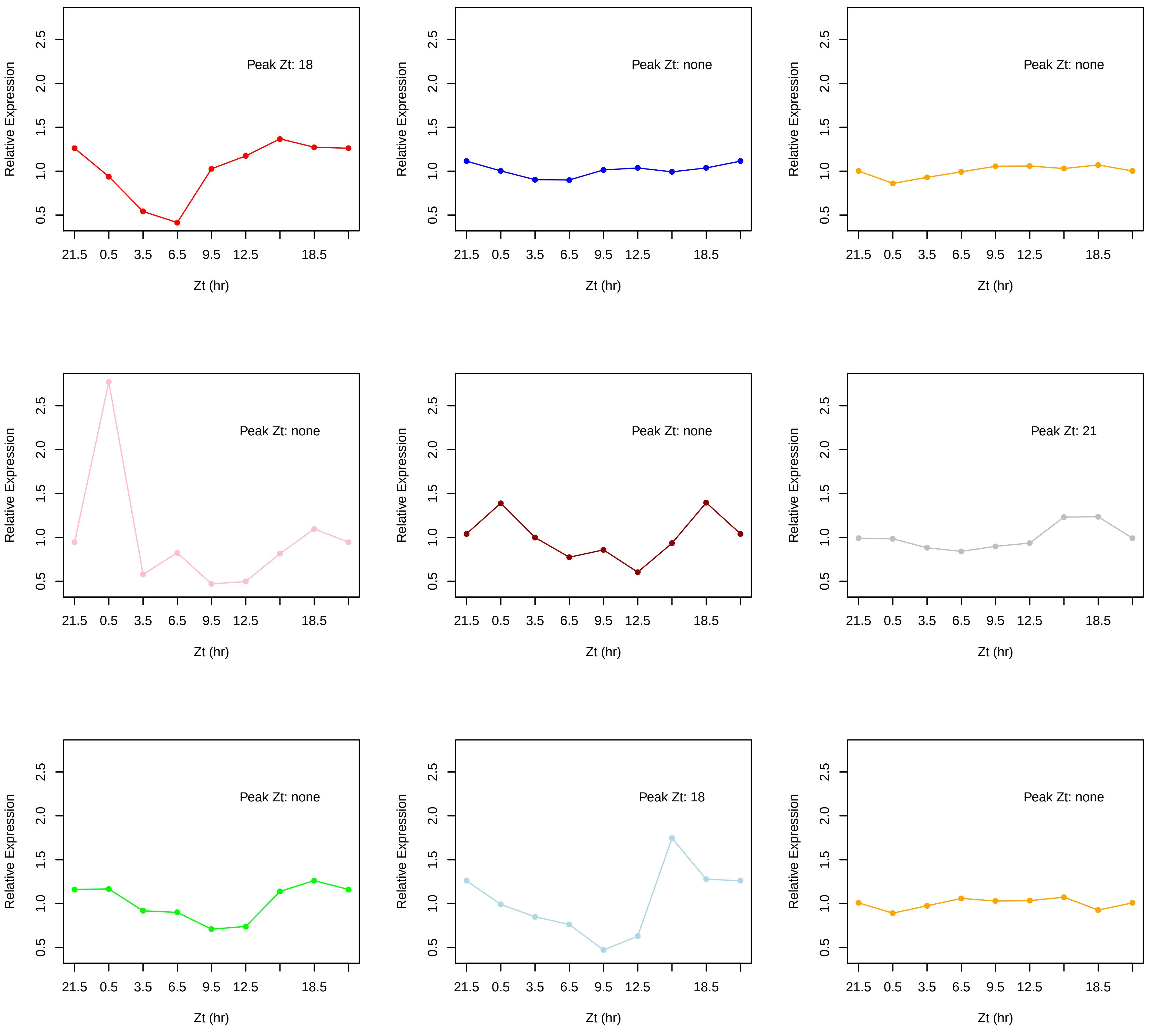

Singleton: ENSXMAG00000013967 gtf3c5

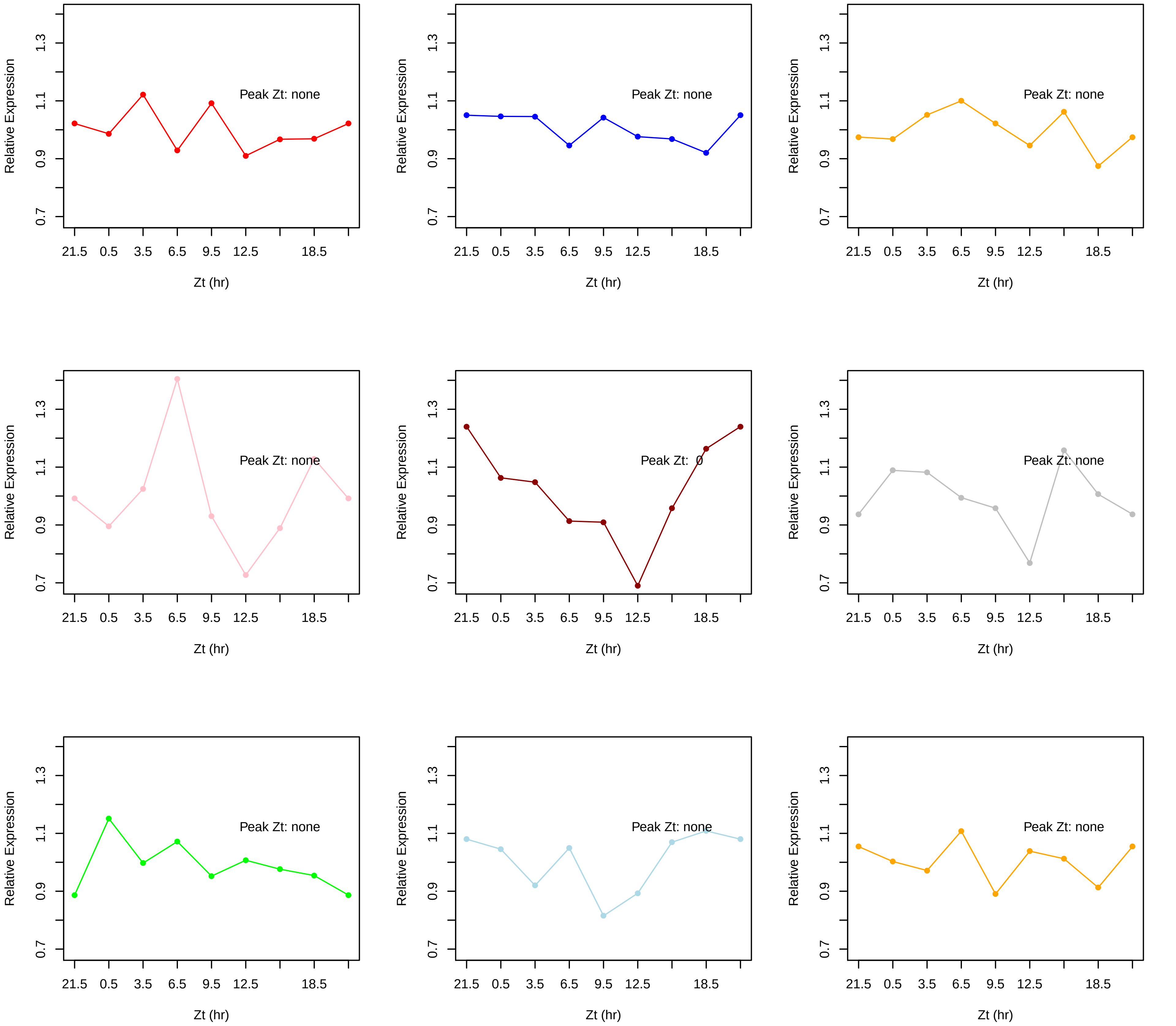

Singleton: ENSXMAG00000027331 akap11

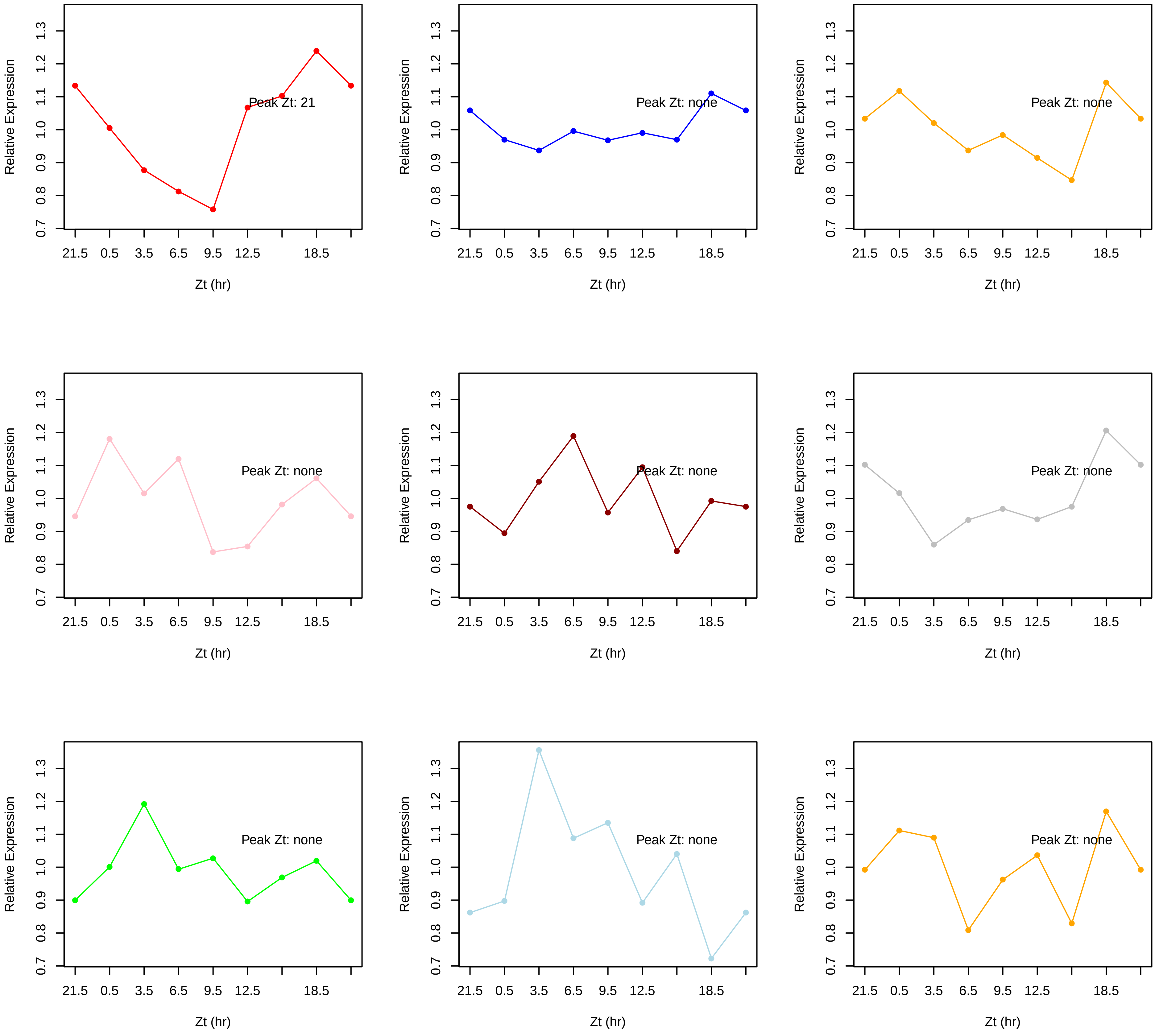

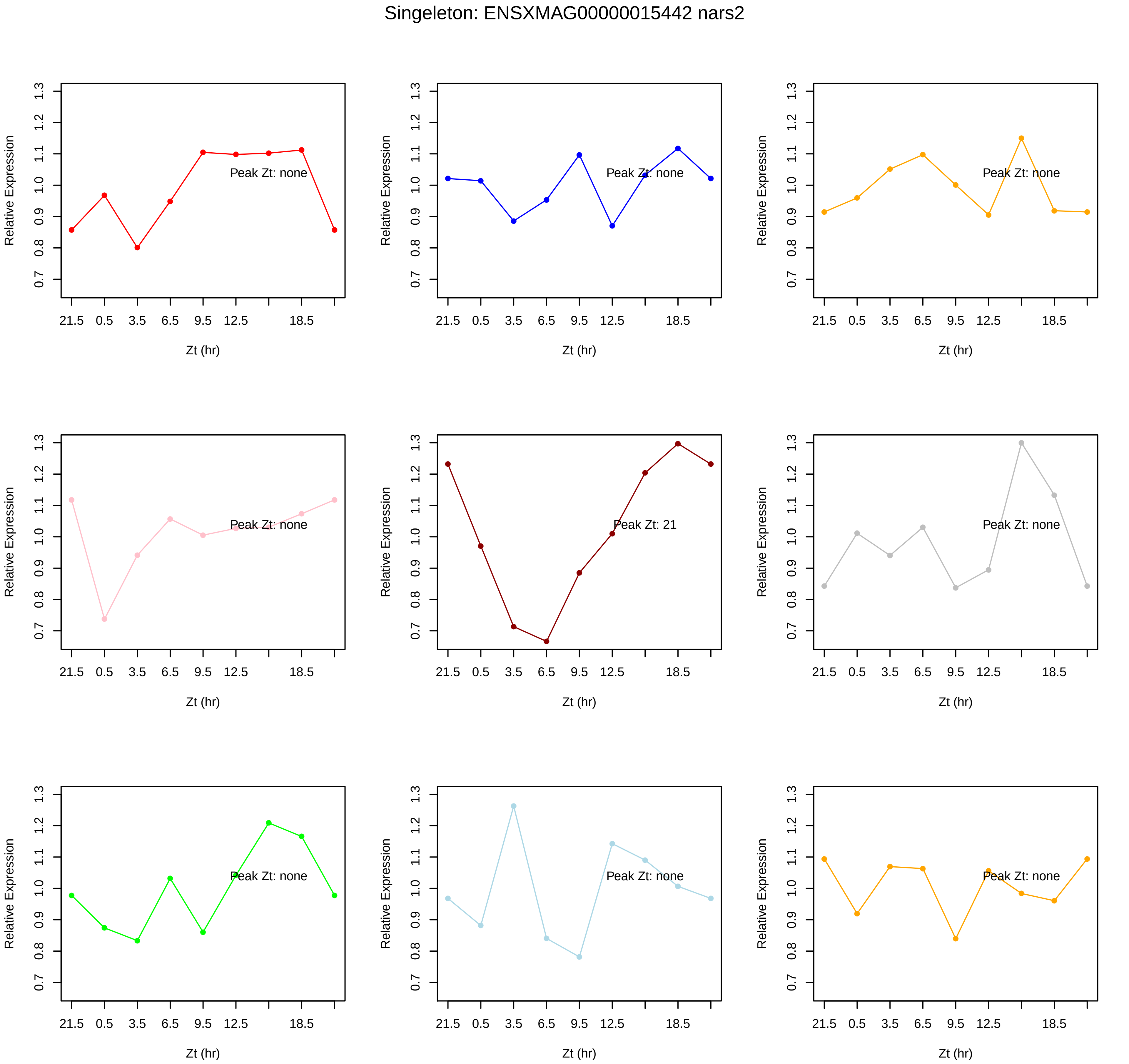
Singleton: ENSXMAG00000015442 nars2
Relative Expression
Zt (hr)
21.5
0.5
3.5
6.5
9.5
12.5
18.5
0.7
0.8
0.9
1.0
1.1
1.2
1.3
Peak Zt: none
Peak Zt: 21

Singeleton: ENSXMAG00000024381 tbca

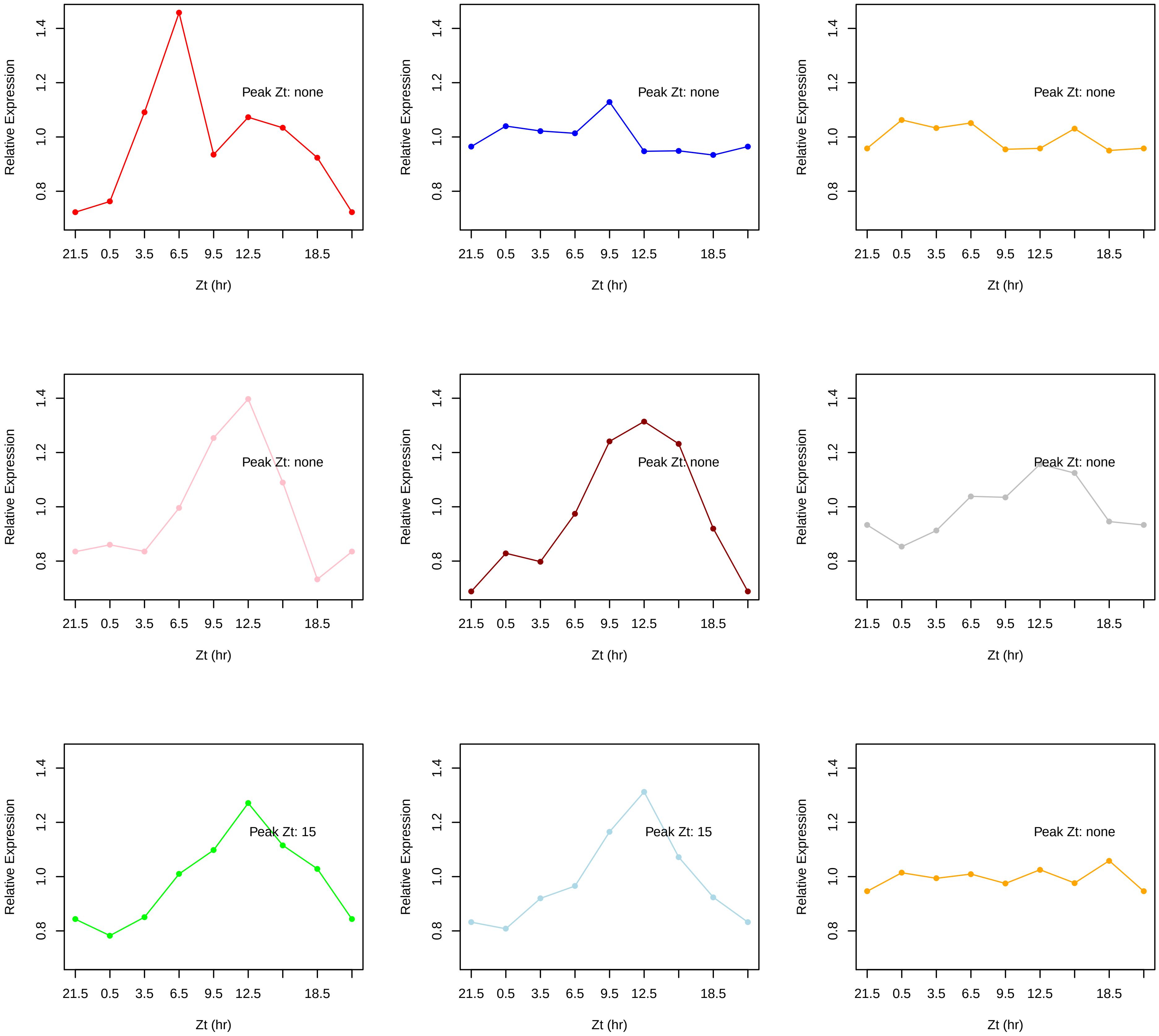

Singeleton: ENSXMAG00000018959 rb1cc1

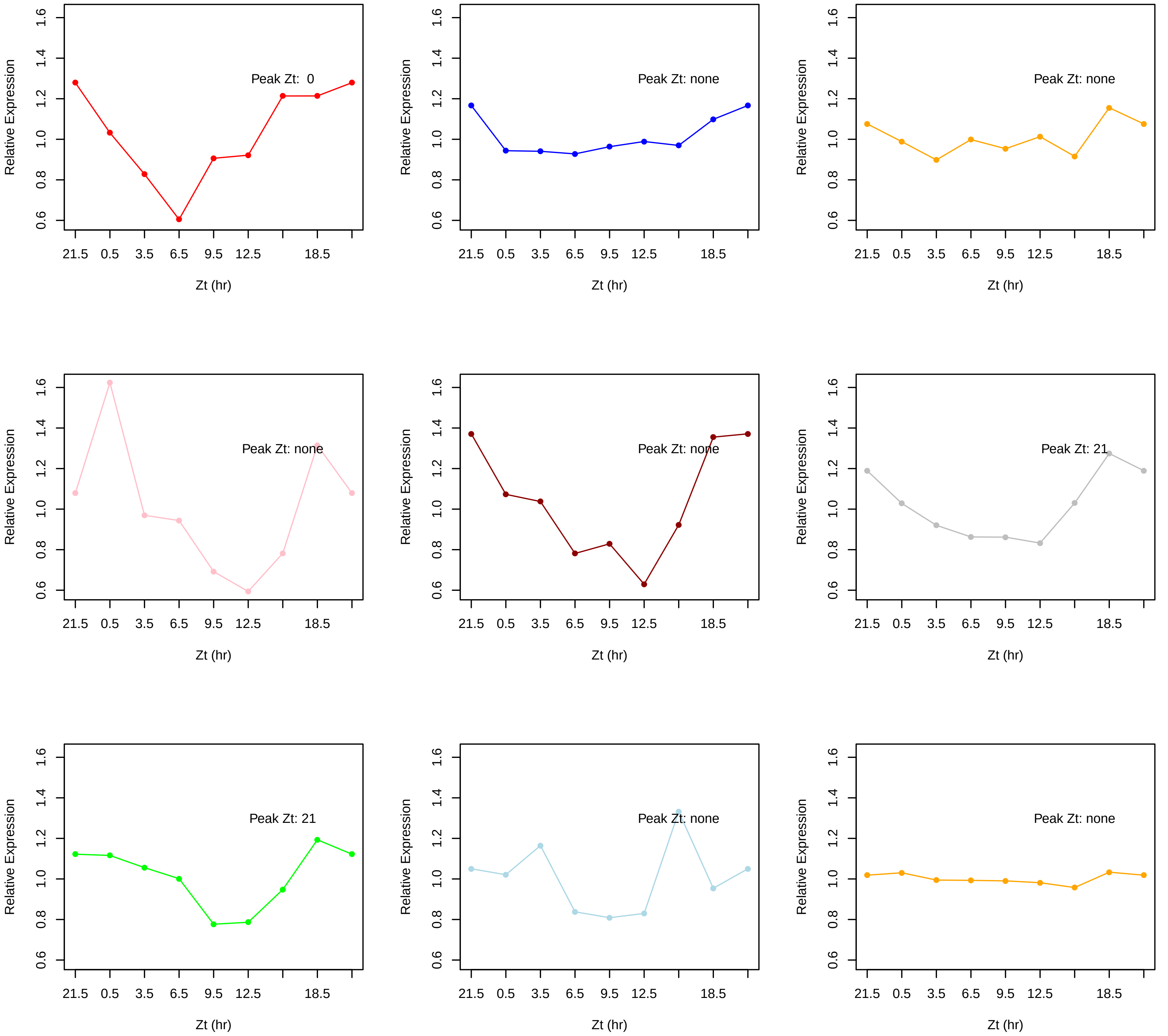

Singeleton: ENSXMAG00000028404 znf507

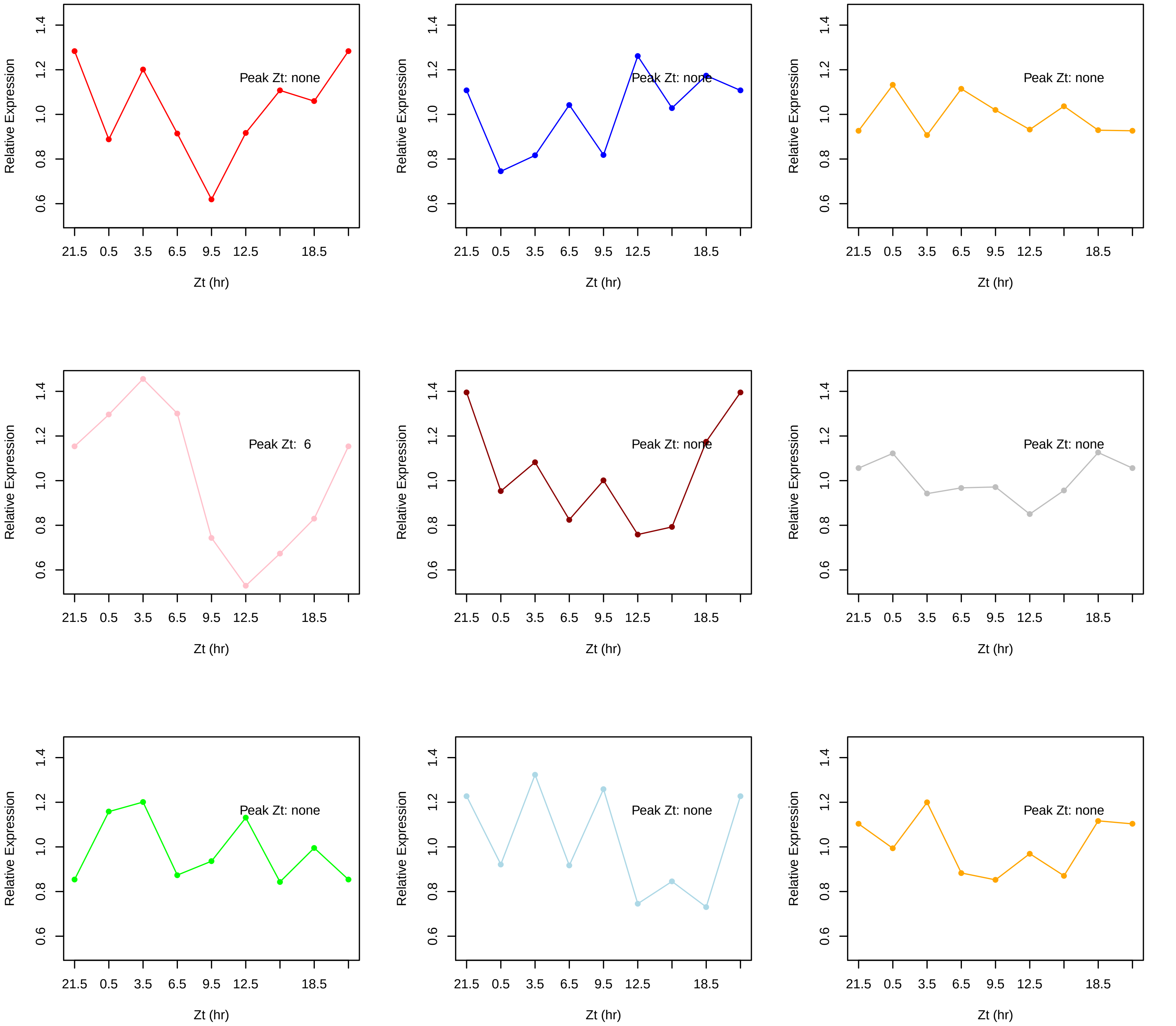

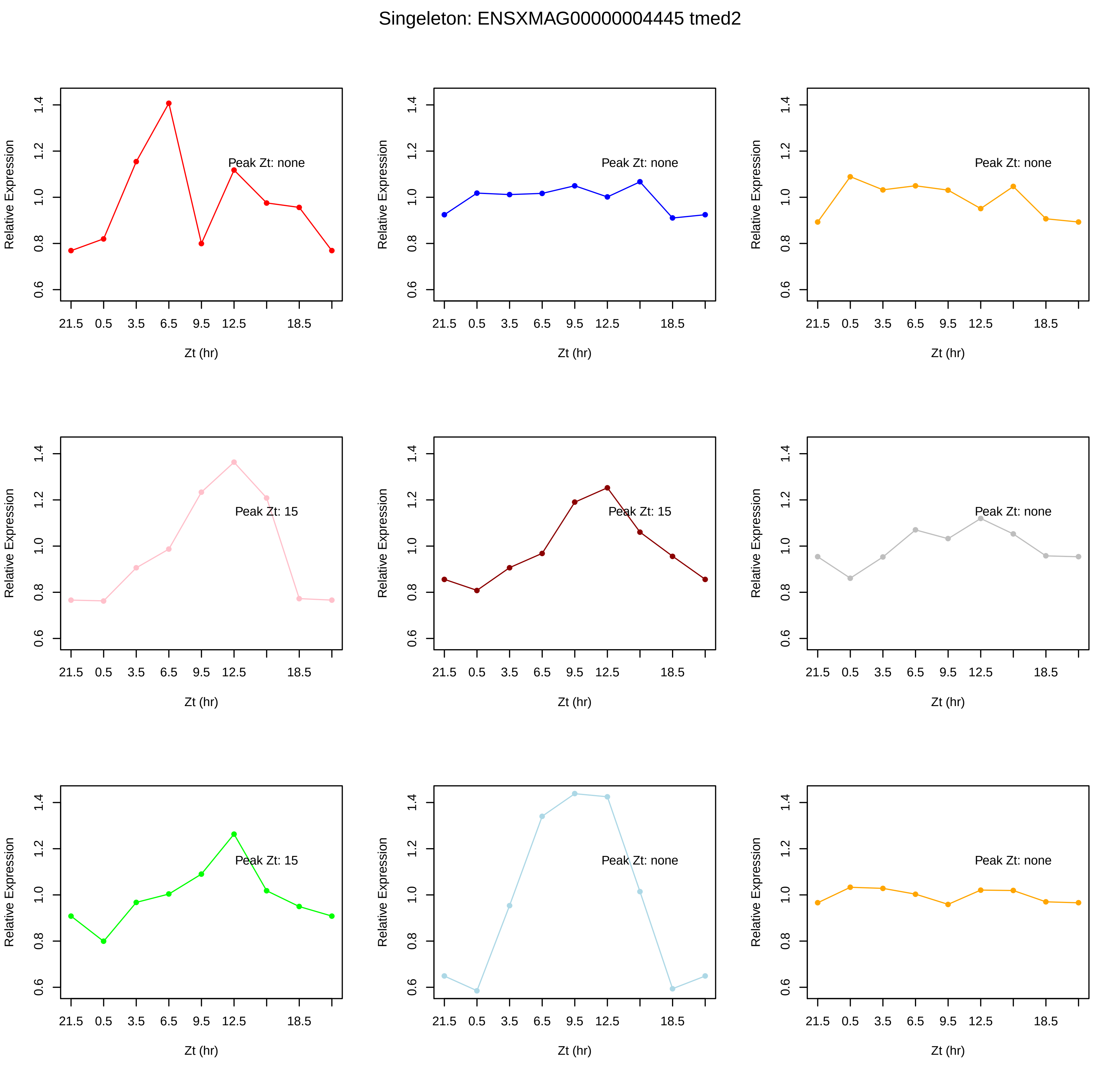
Singleton: ENSXMAG00000004445 tmed2
Peak Zt: none
Peak Zt: none
Peak Zt: none
Peak Zt: 15
Peak Zt: 15
Peak Zt: none
Peak Zt: 15
Peak Zt: none
Peak Zt: none
Relative Expression
Zt (hr)
21.5 0.5 3.5 6.5 9.5 12.5 18.5
0.6 0.8 1.0 1.2 1.4

Singleton: ENSXMAG00000013219 utp11l

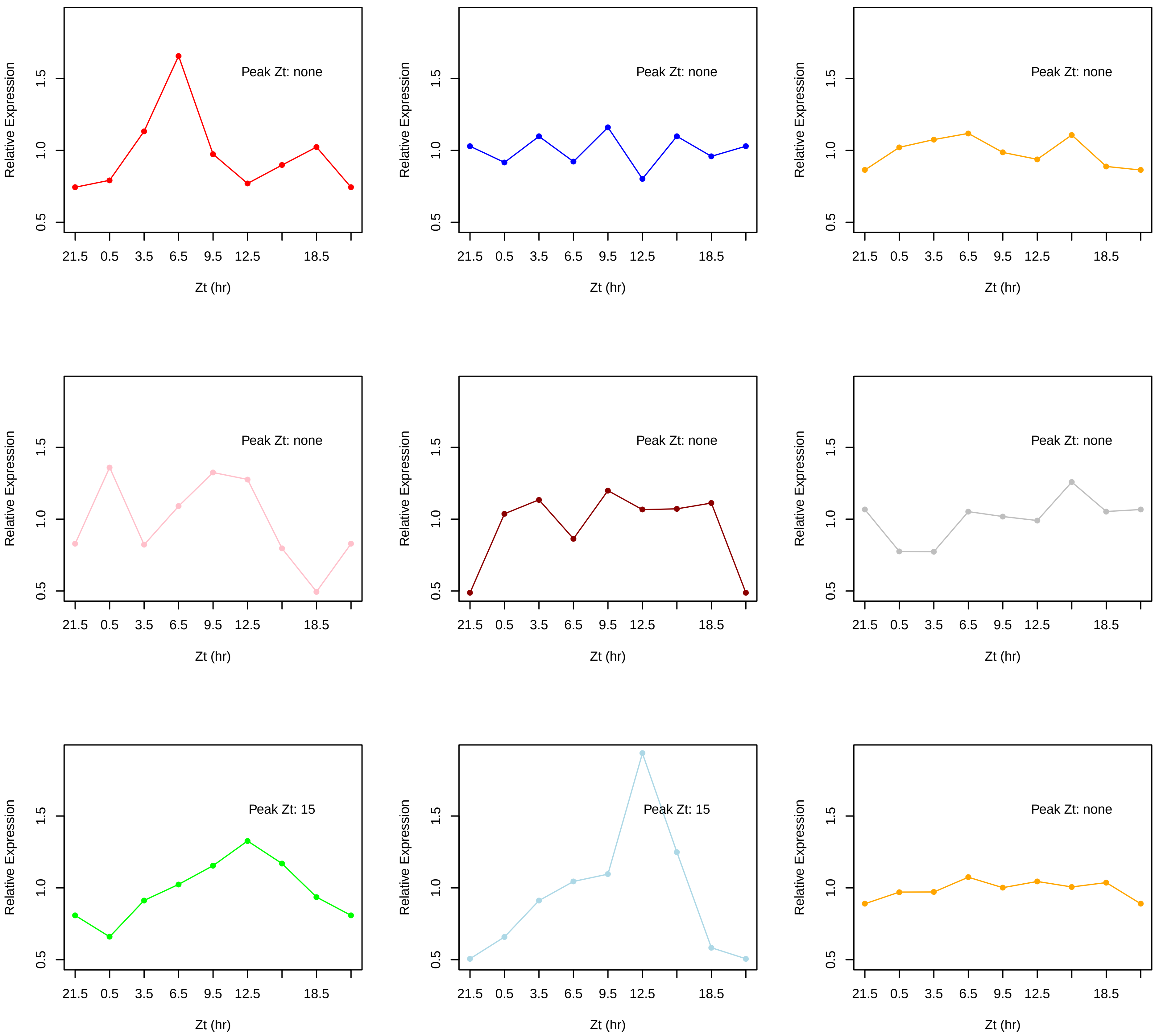

Singeleton: ENSXMAG00000007213 dgkh

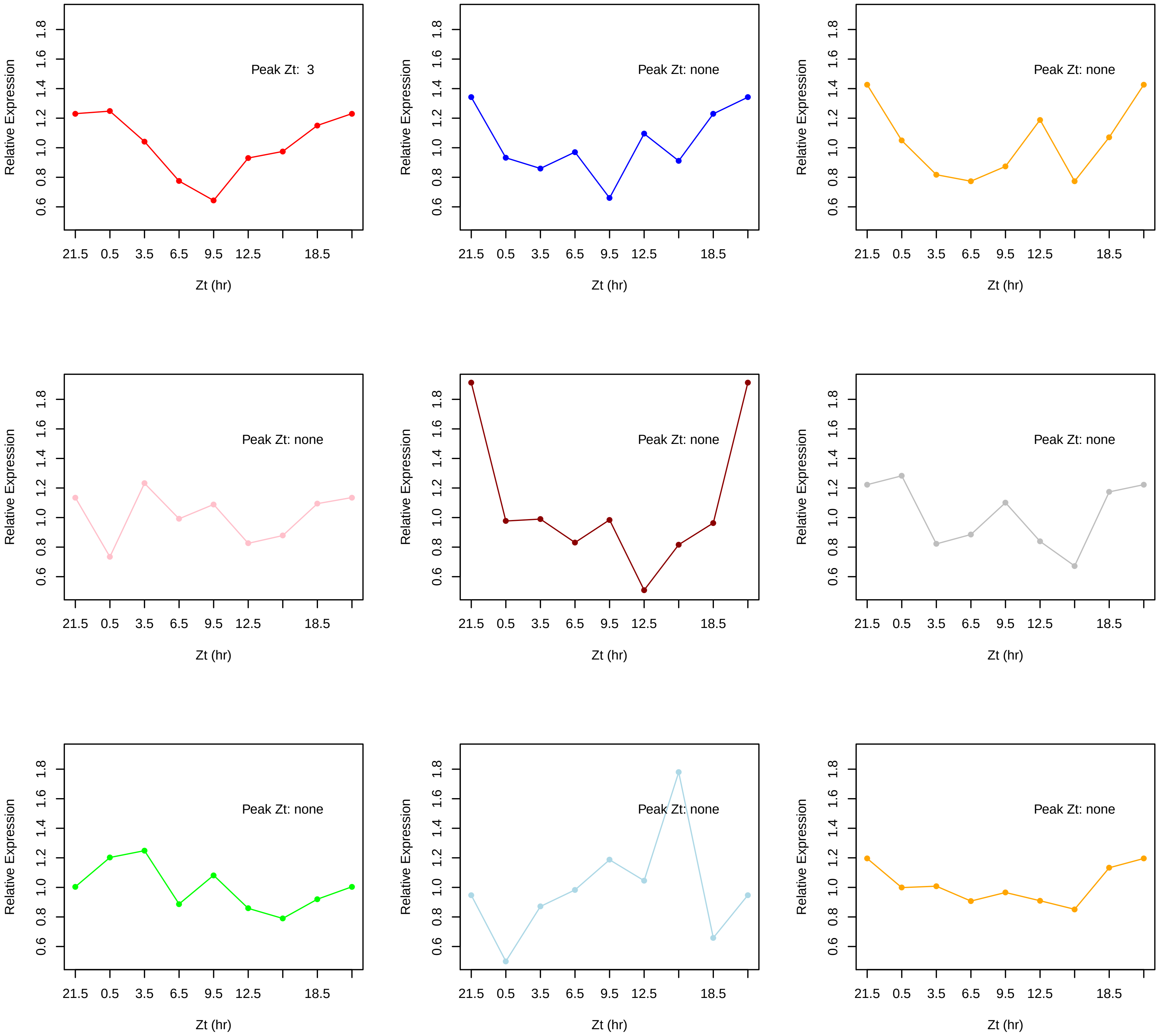

Singeleton: ENSXMAG00000008587 eif4e2rs1

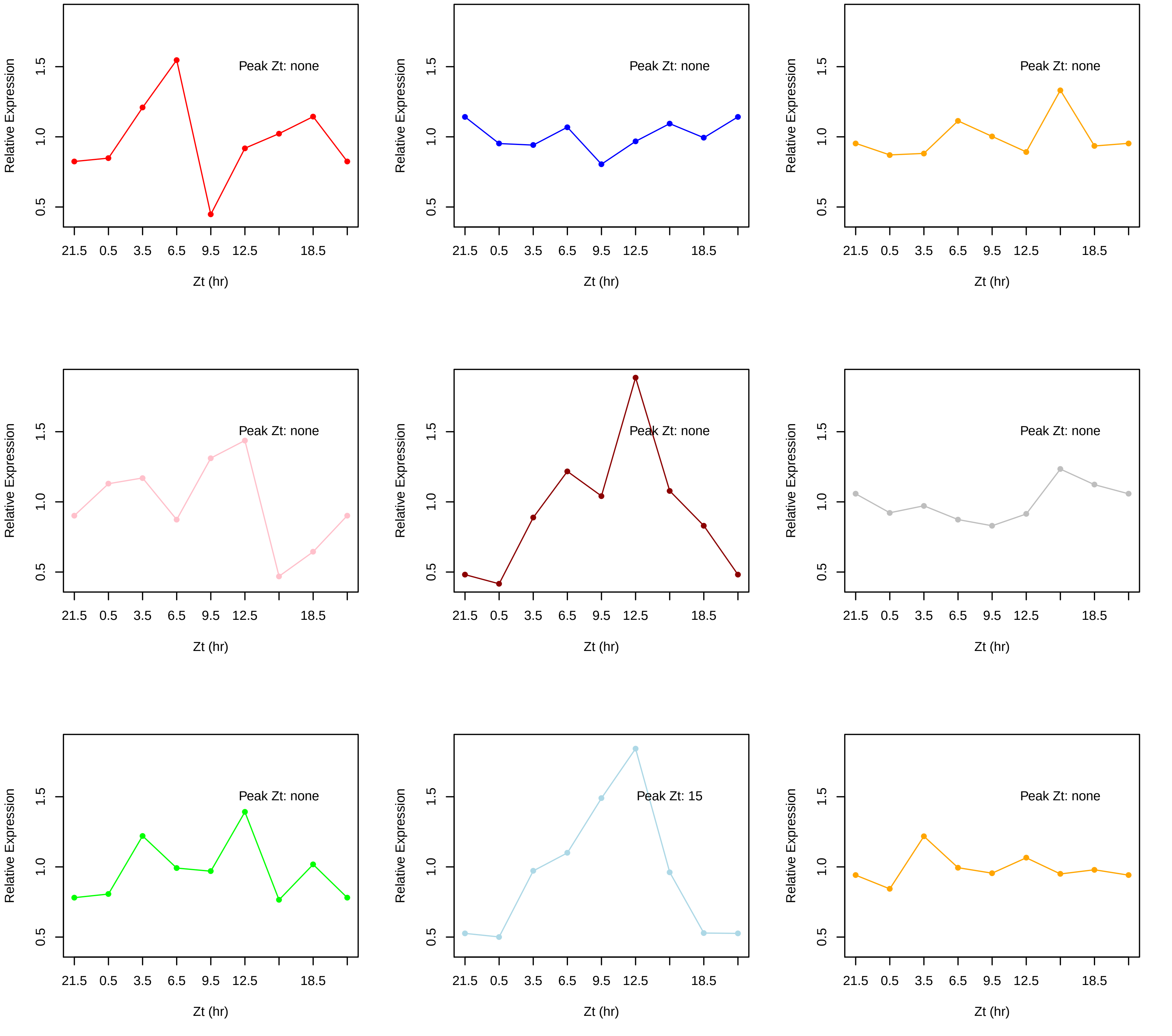

Singleton: ENSXMAG00000007490 tenm4

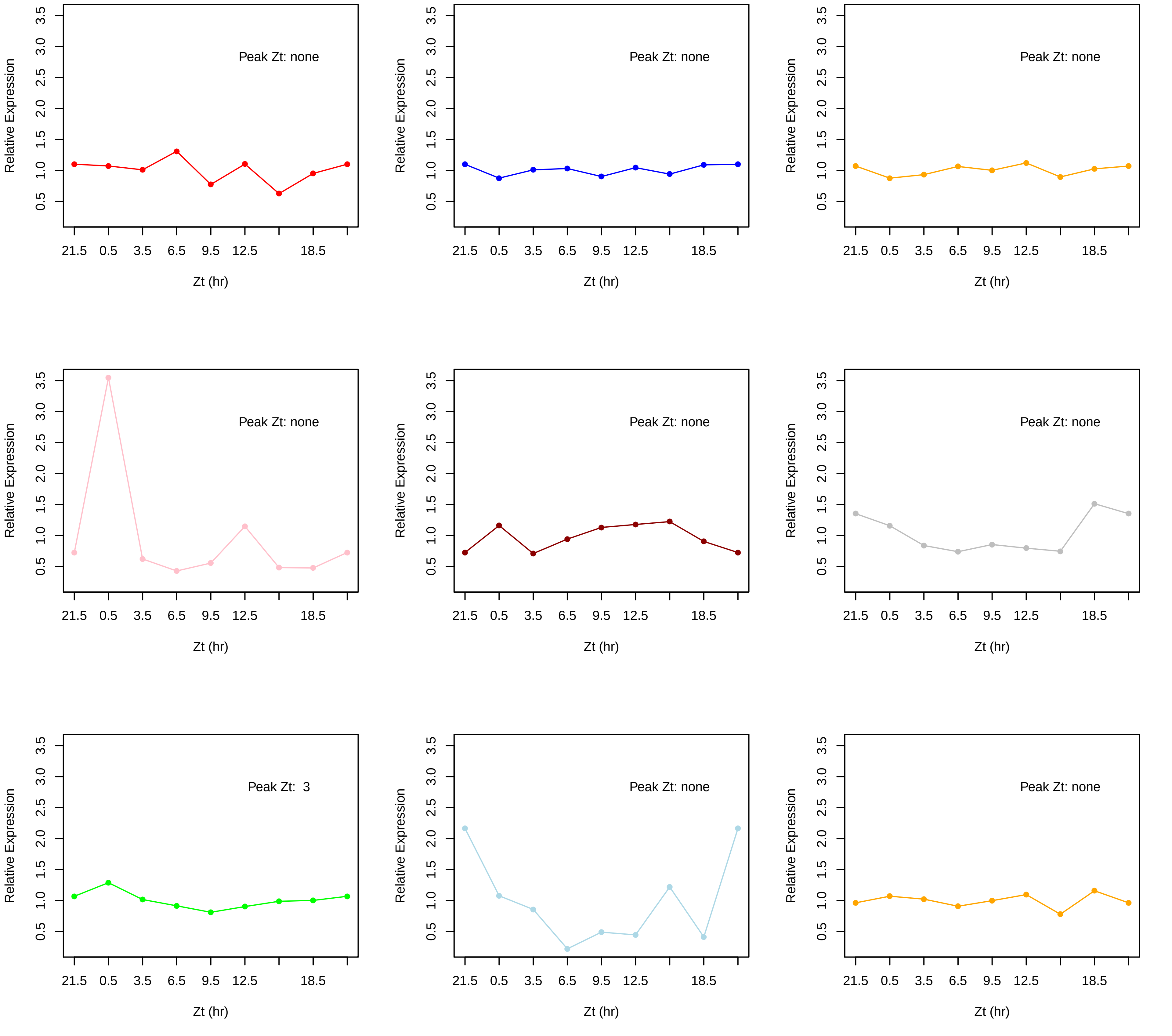

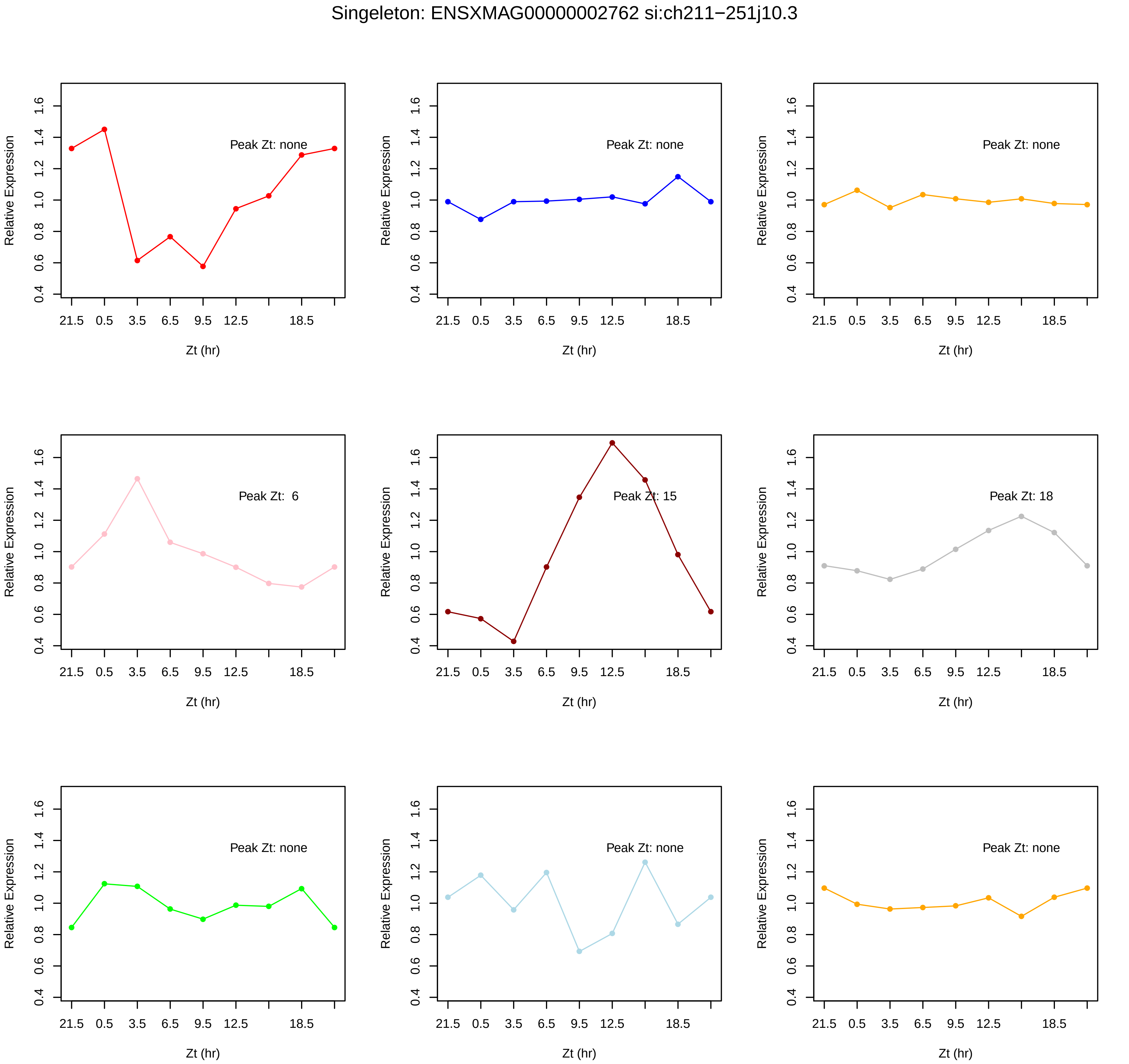
Singeleton: ENSXMAG00000002762 si:ch211−251j10.3
Peak Zt: none
Peak Zt: none
Peak Zt: none
Peak Zt: 6
Peak Zt: 15
Peak Zt: 18
Peak Zt: none
Peak Zt: none
Peak Zt: none
Relative Expression
Zt (hr)
21.5
0.5
3.5
6.5
9.5
12.5
18.5
0.4
0.6
0.8
1.0
1.2
1.4
1.6

Singeleton: ENSXMAG00000024630 dpy19l3

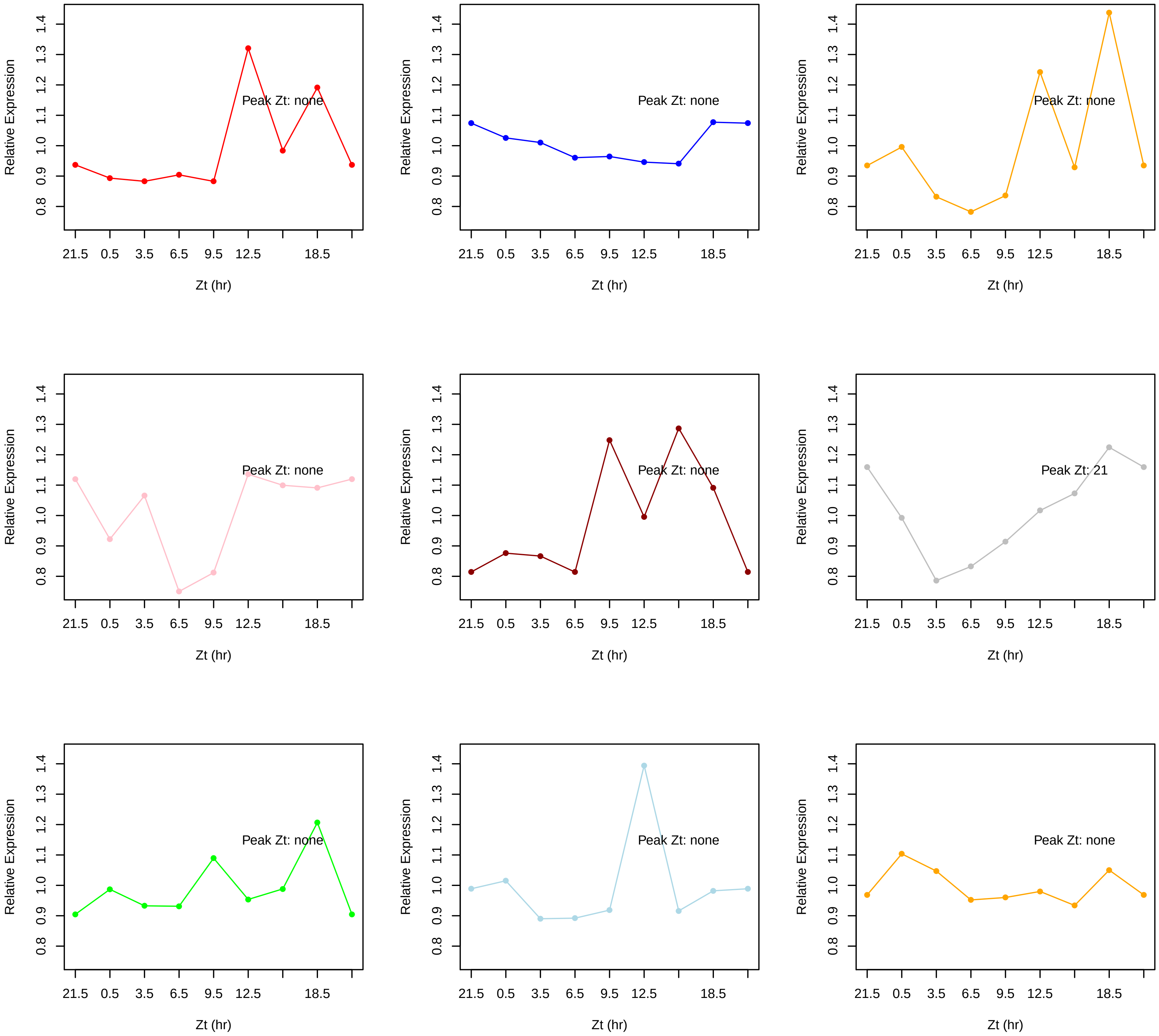

Singeleton: ENSXMAG00000012854 dot1l

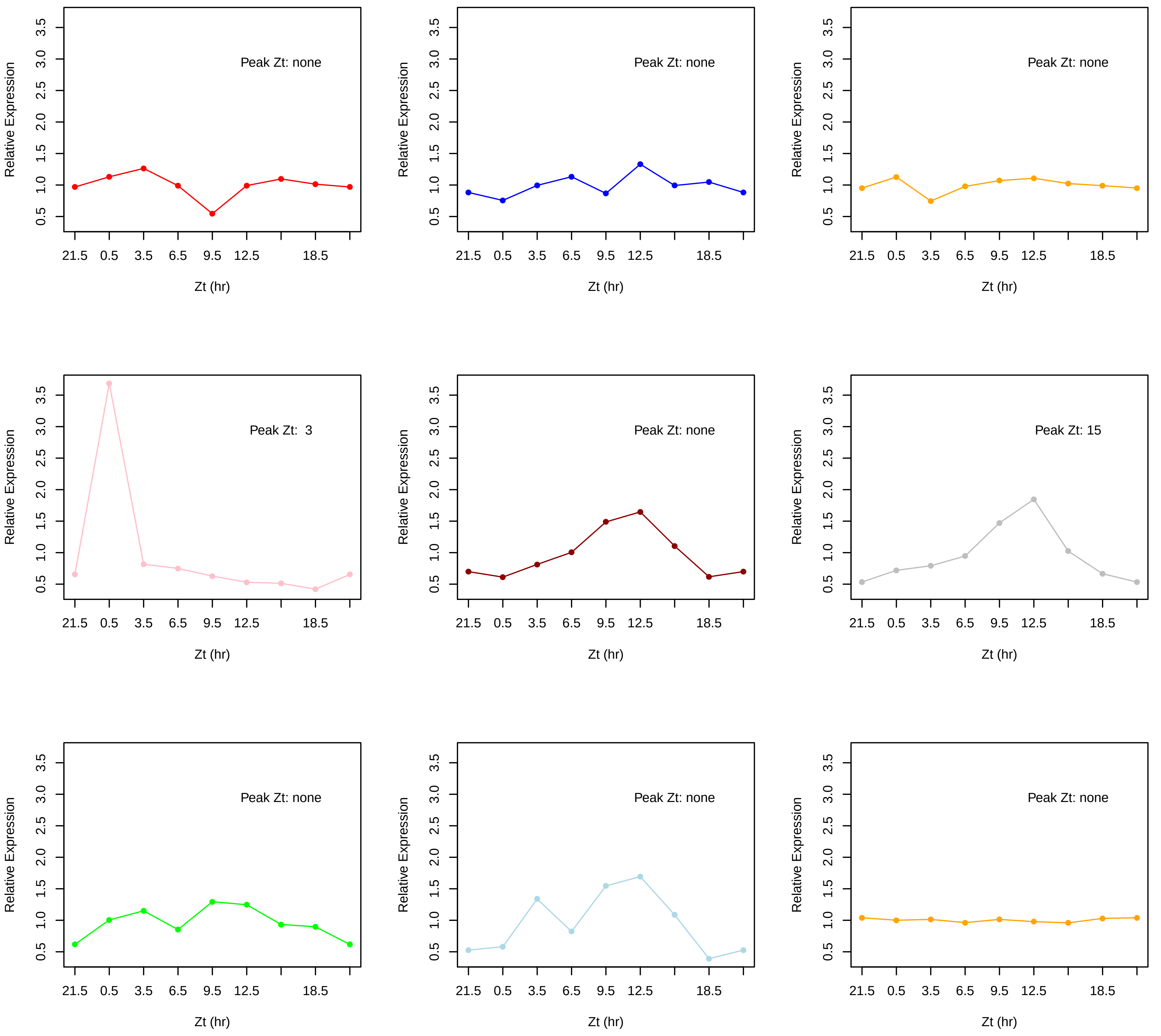

Singeleton: ENSXMAG00000021003 rbp4

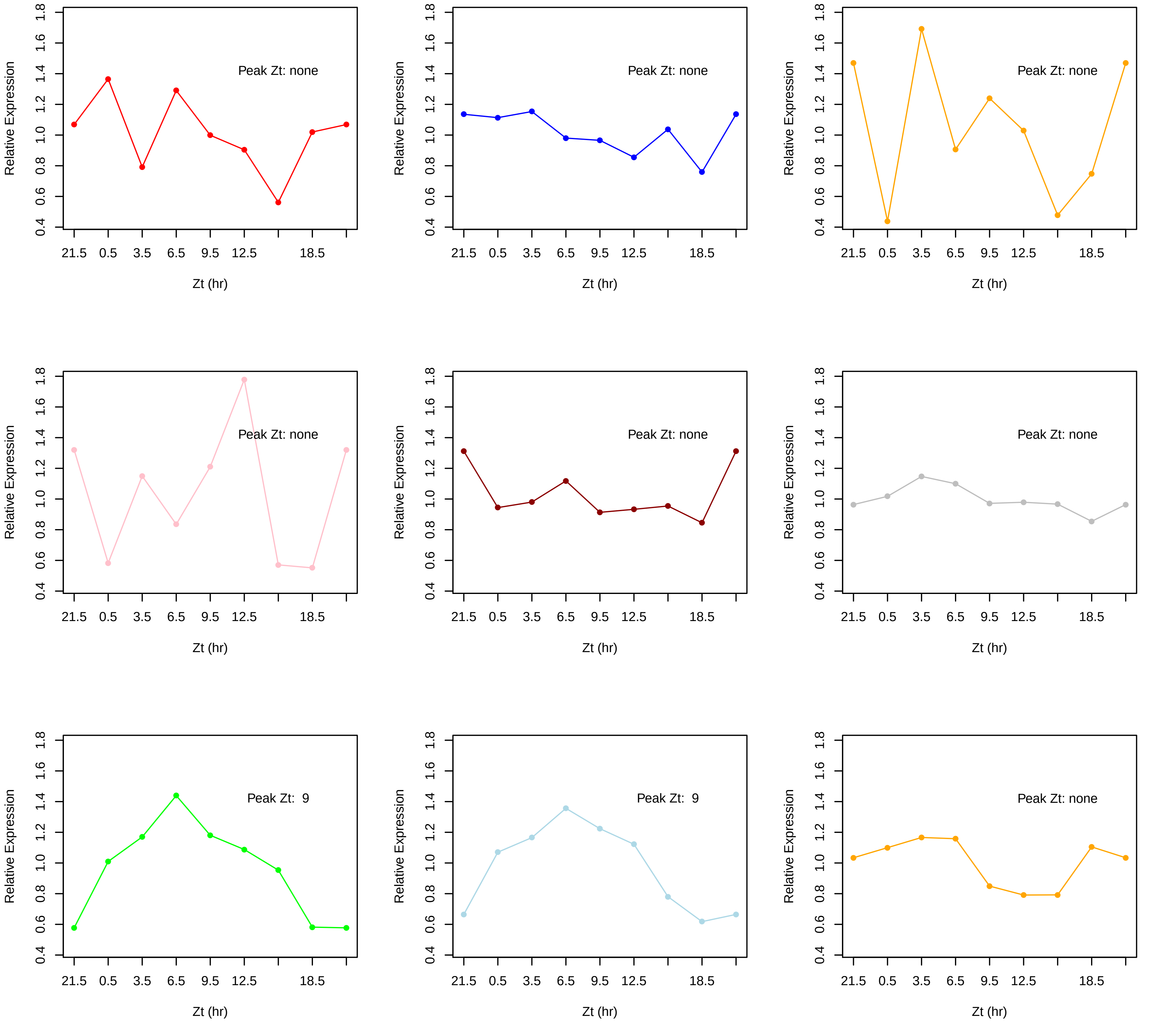

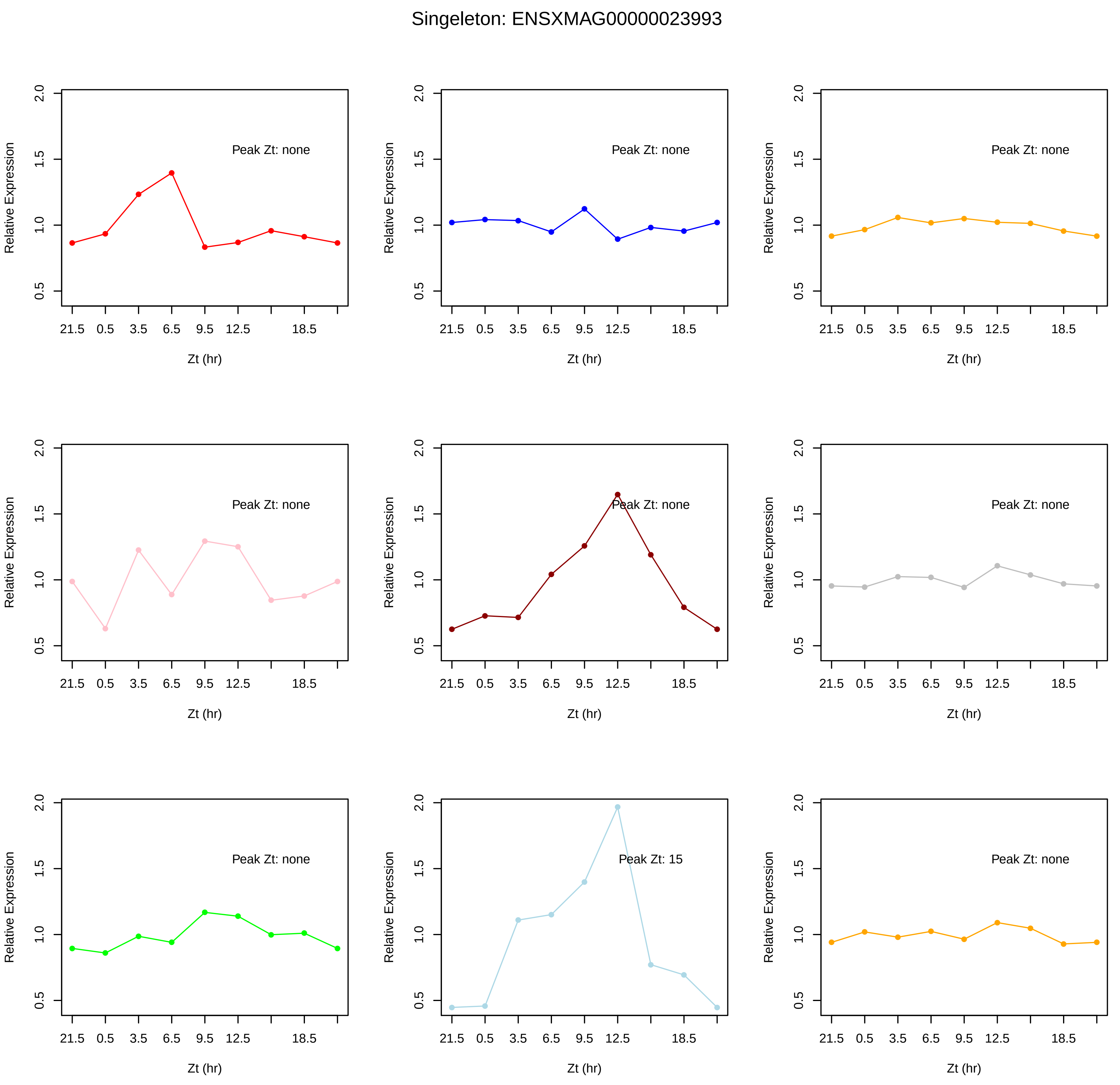
Singeleton: ENSXMAG00000023993
Peak Zt: none
Peak Zt: none
Peak Zt: none
Peak Zt: none
Peak Zt: none
Peak Zt: none
Peak Zt: none
Peak Zt: 15
Peak Zt: none
Relative Expression
Zt (hr)
21.5
0.5
3.5
6.5
9.5
12.5
18.5
0.5
1.0
1.5
2.0

Singeleton: ENSXMAG00000018753 lhfpl2a

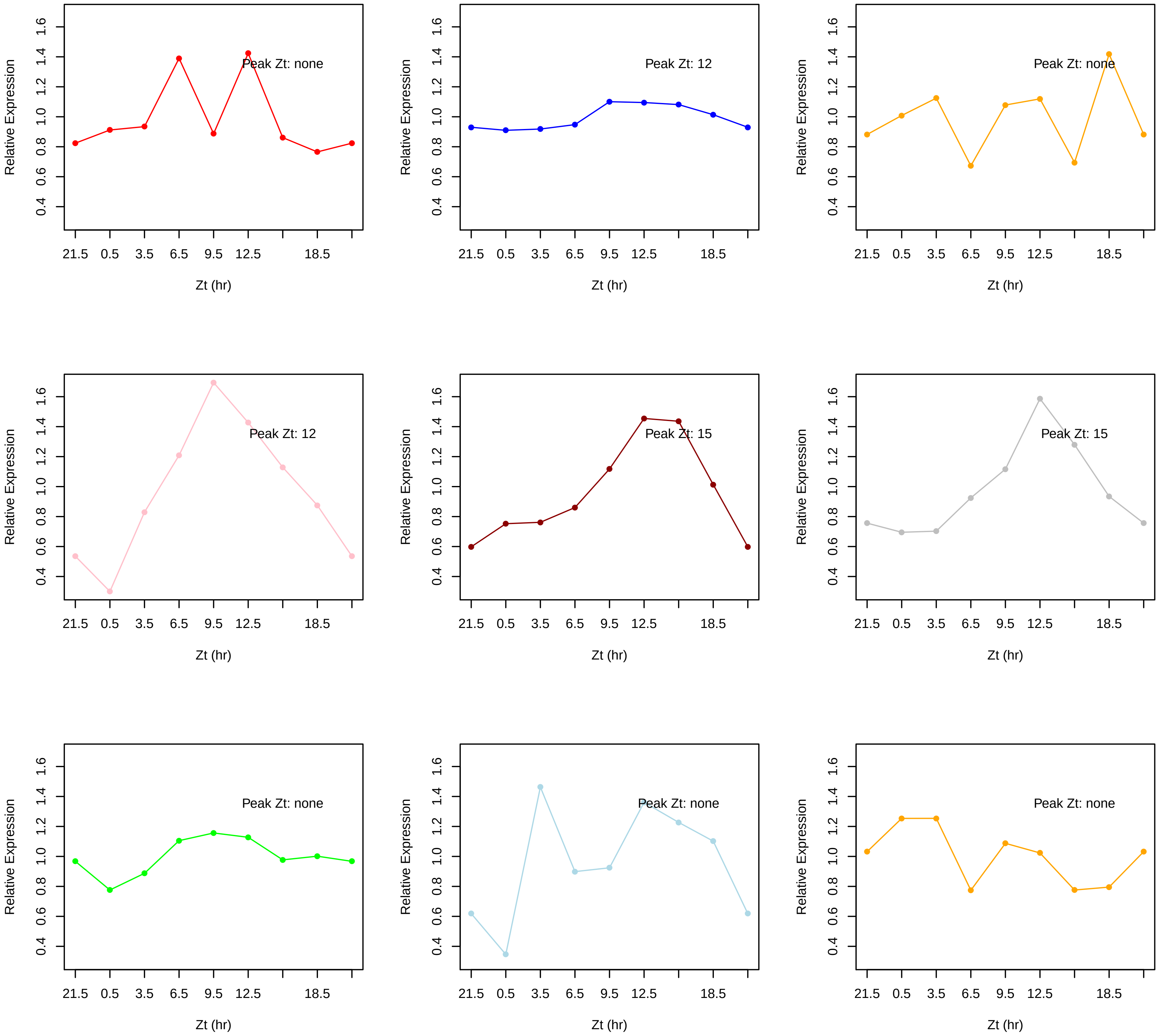

Singleton: ENSXMAG00000004262 pofut1

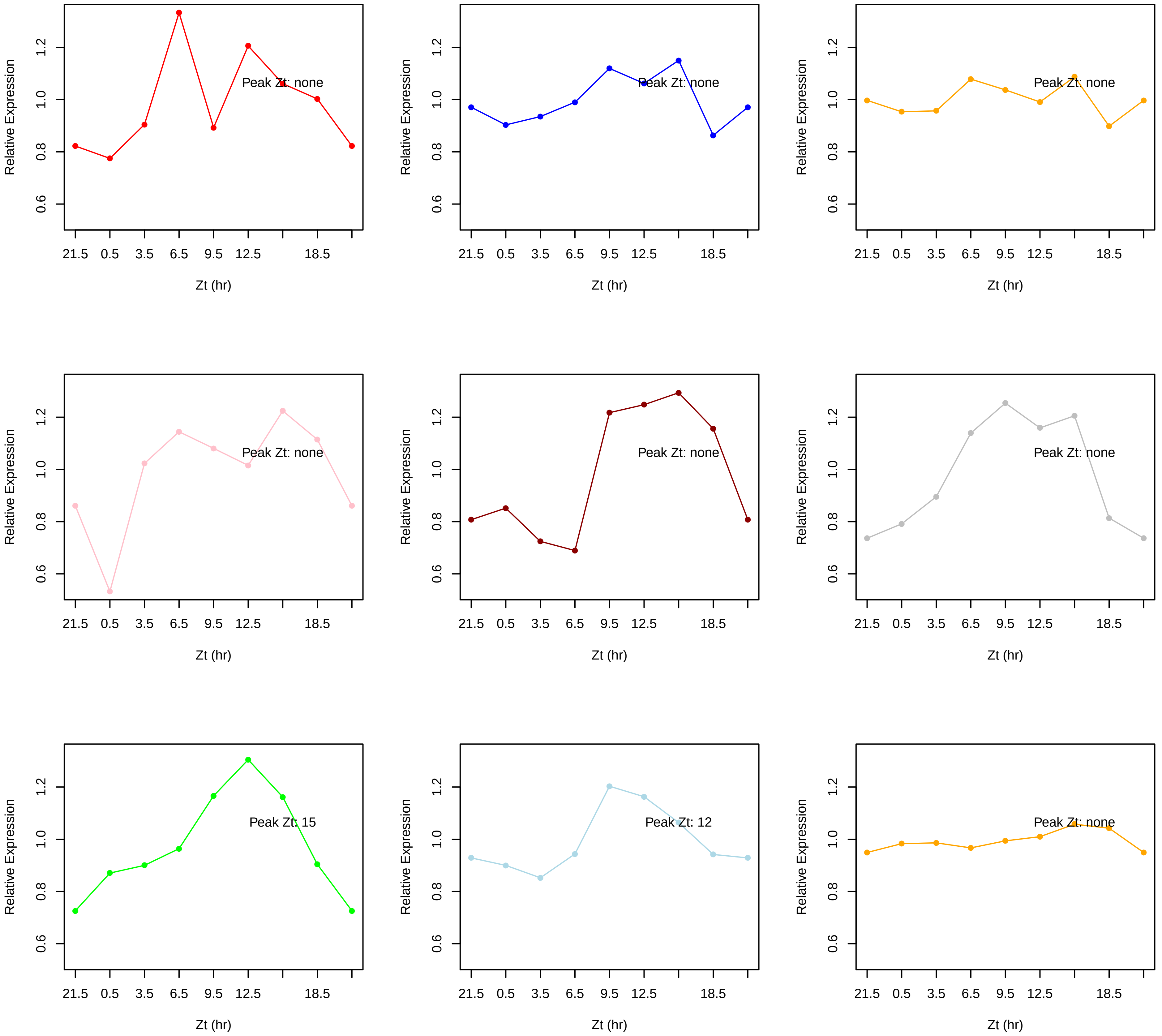

Singeleton: ENSXMAG00000003783 zer1

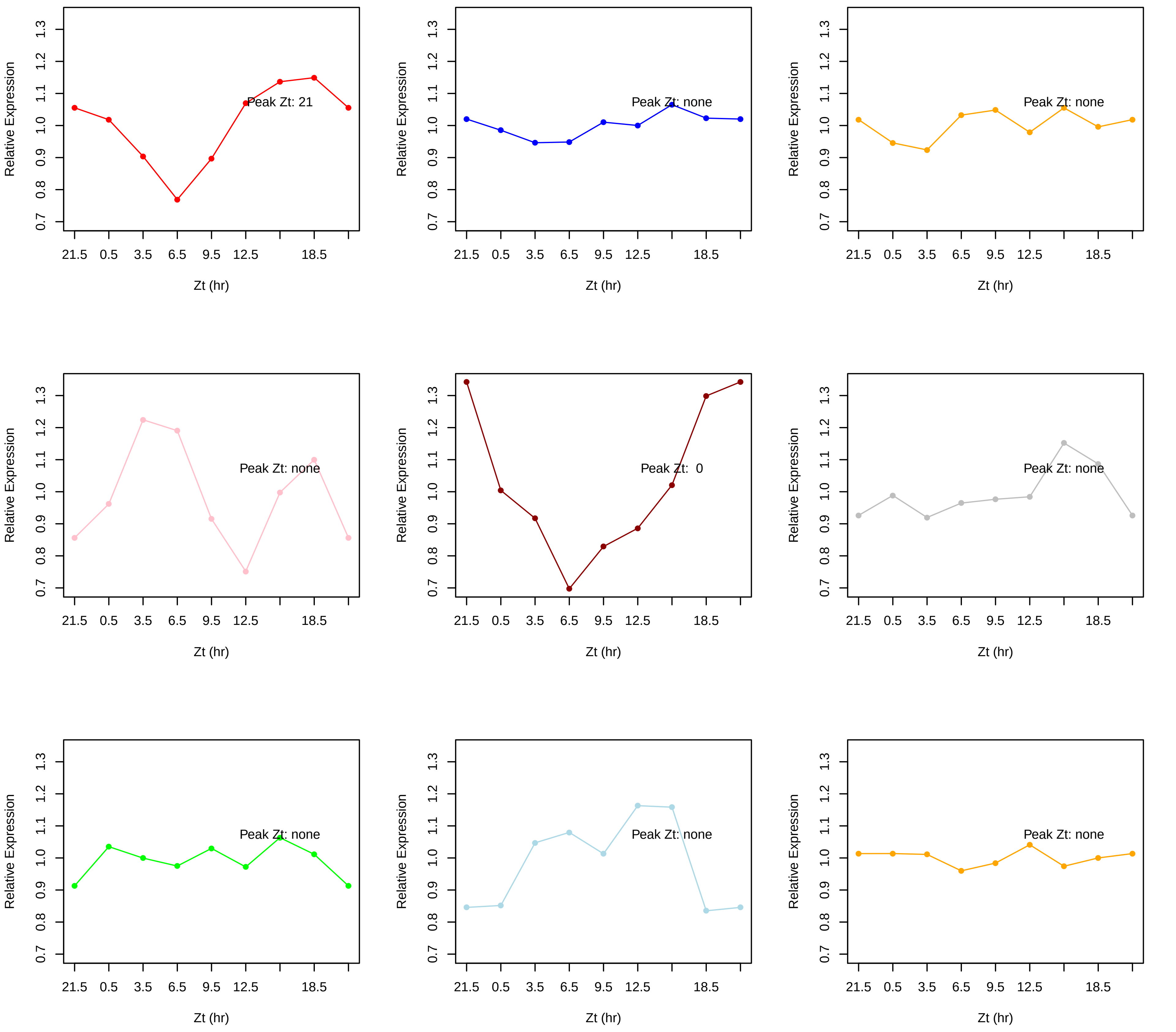

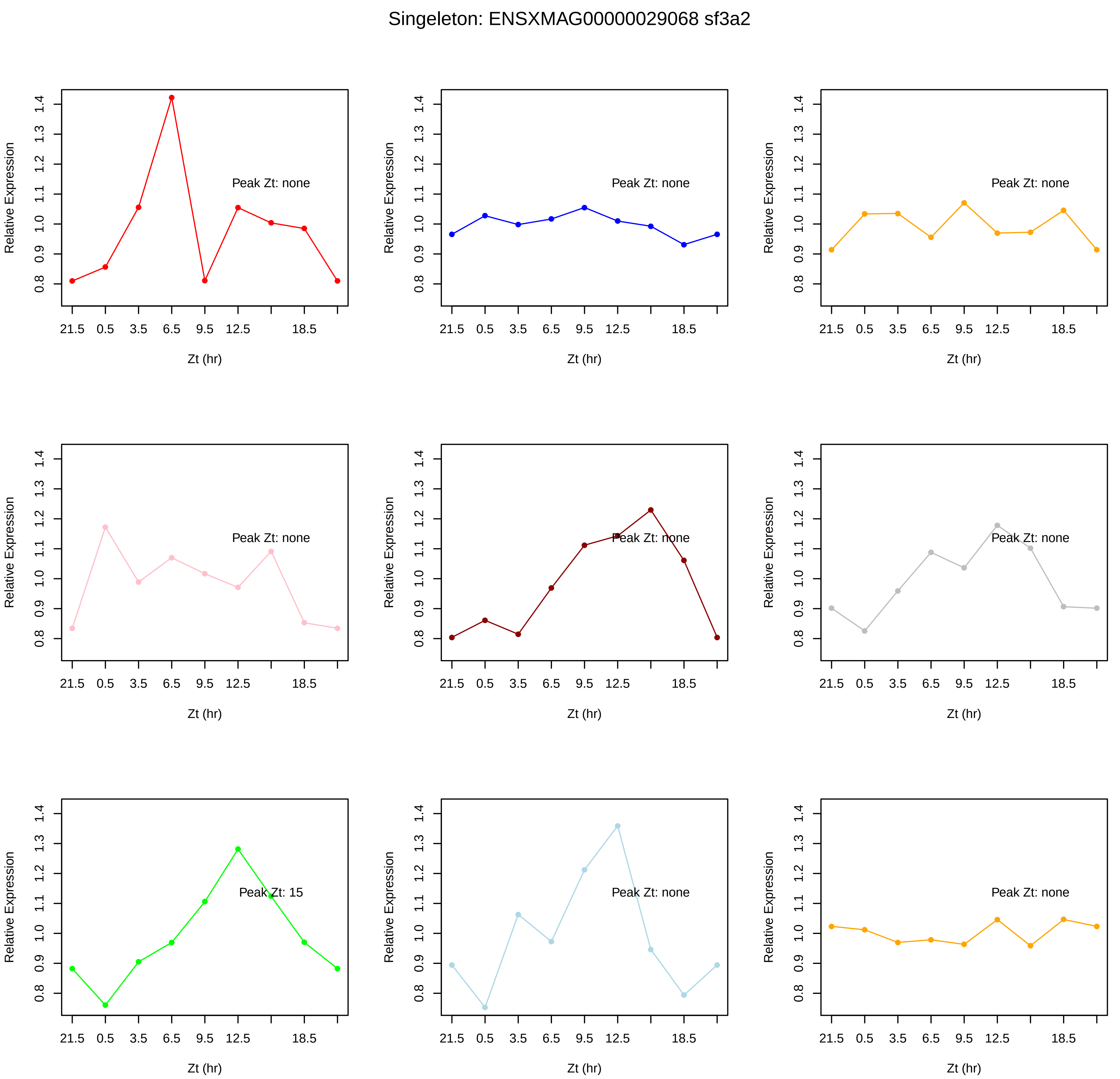
Singeleton: ENSXMAG00000029068 sf3a2
Relative Expression
Zt (hr)
0.8
0.9
1.0
1.1
1.2
1.3
1.4
21.5
0.5
3.5
6.5
9.5
12.5
18.5
Peak Zt: none
Peak Zt: 15

Singeleton: ENSXMAG00000007481 prcp

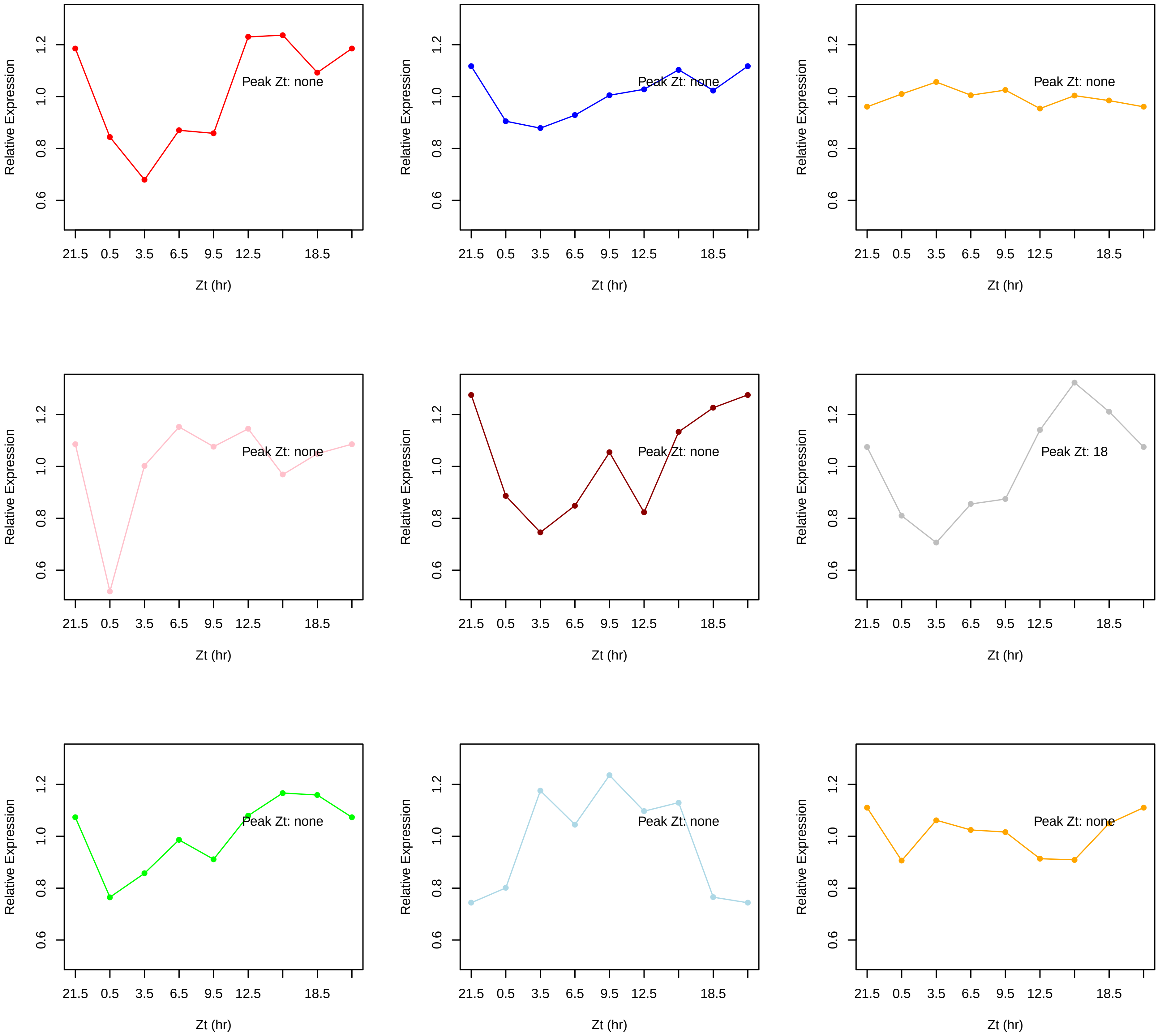

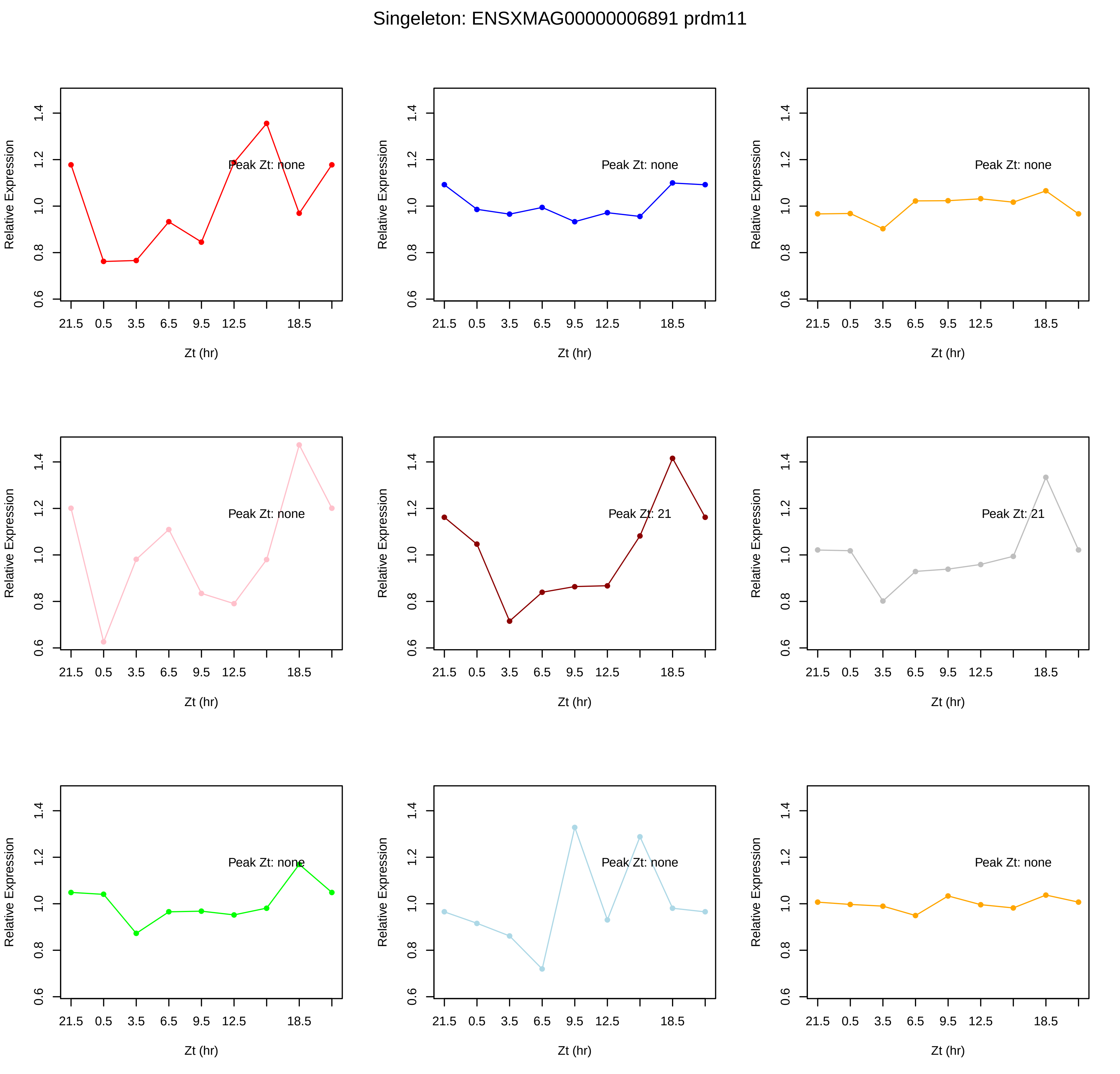
Singeleton: ENSXMAG00000006891 prdm11
Relative Expression
Zt (hr)
21.5
0.5
3.5
6.5
9.5
12.5
18.5
0.6
0.8
1.0
1.2
1.4
Peak Zt: none
Peak Zt: 21

Singleton: ENSXMAG00000027972 eif2b1

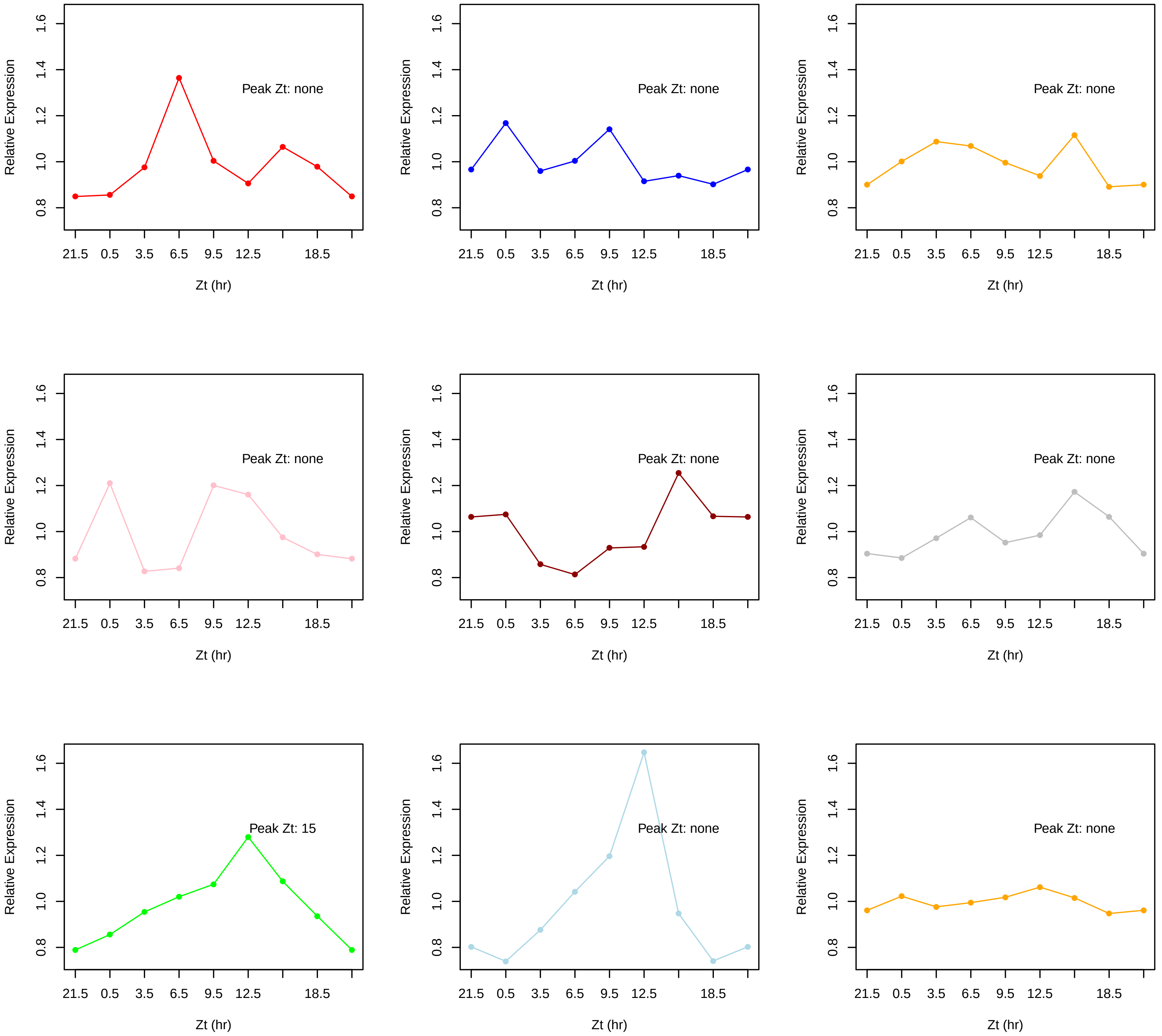

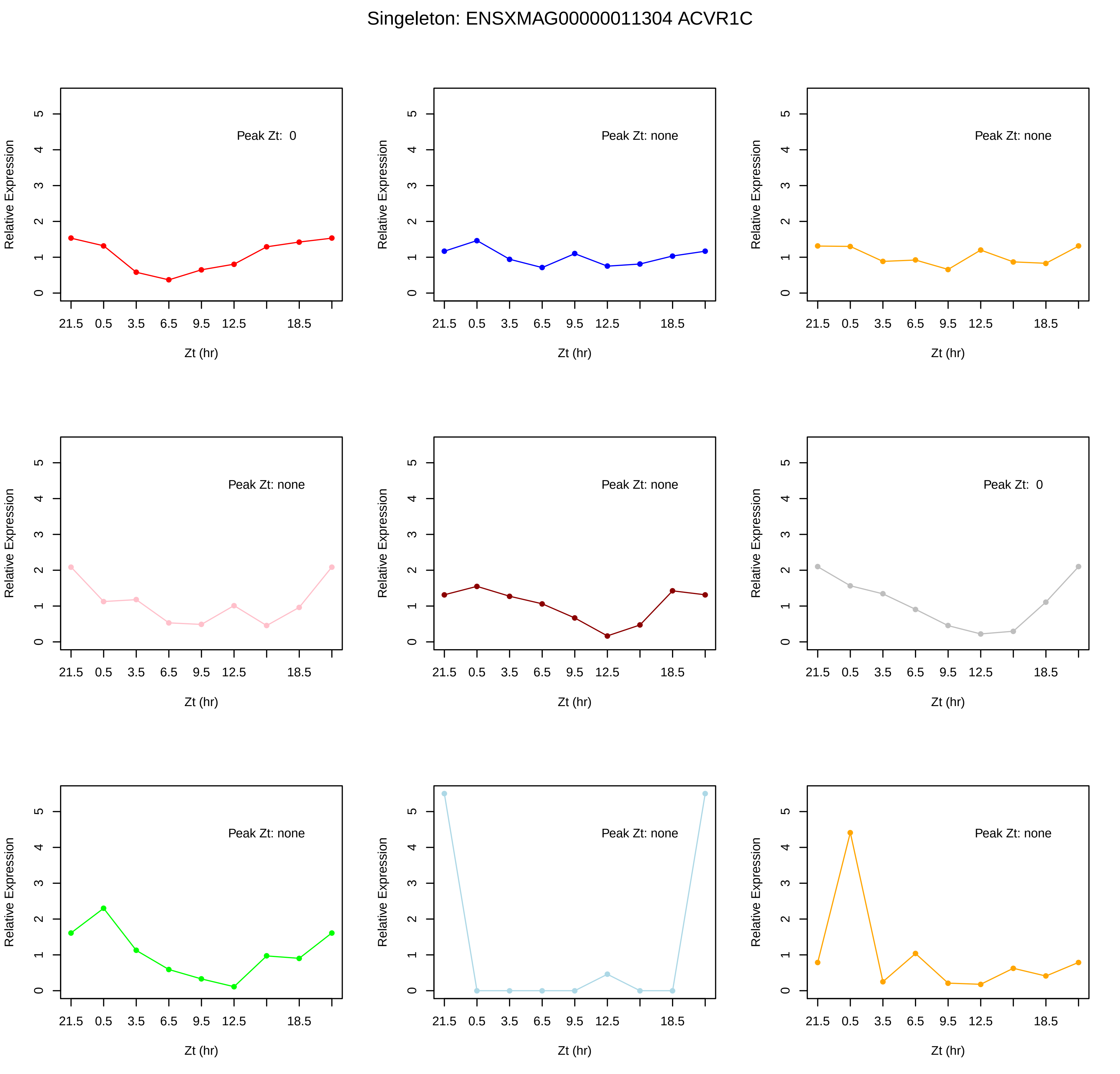
Singeleton: ENSXMAG00000011304 ACVR1C
Peak Zt: 0
Peak Zt: none
Peak Zt: none
Peak Zt: none
Peak Zt: none
Peak Zt: 0
Peak Zt: none
Peak Zt: none
Peak Zt: none
Relative Expression
Zt (hr)
21.5
0.5
3.5
6.5
9.5
12.5
18.5
0
1
2
3
4
5

Singleton: ENSXMAG00000007965 pde6c

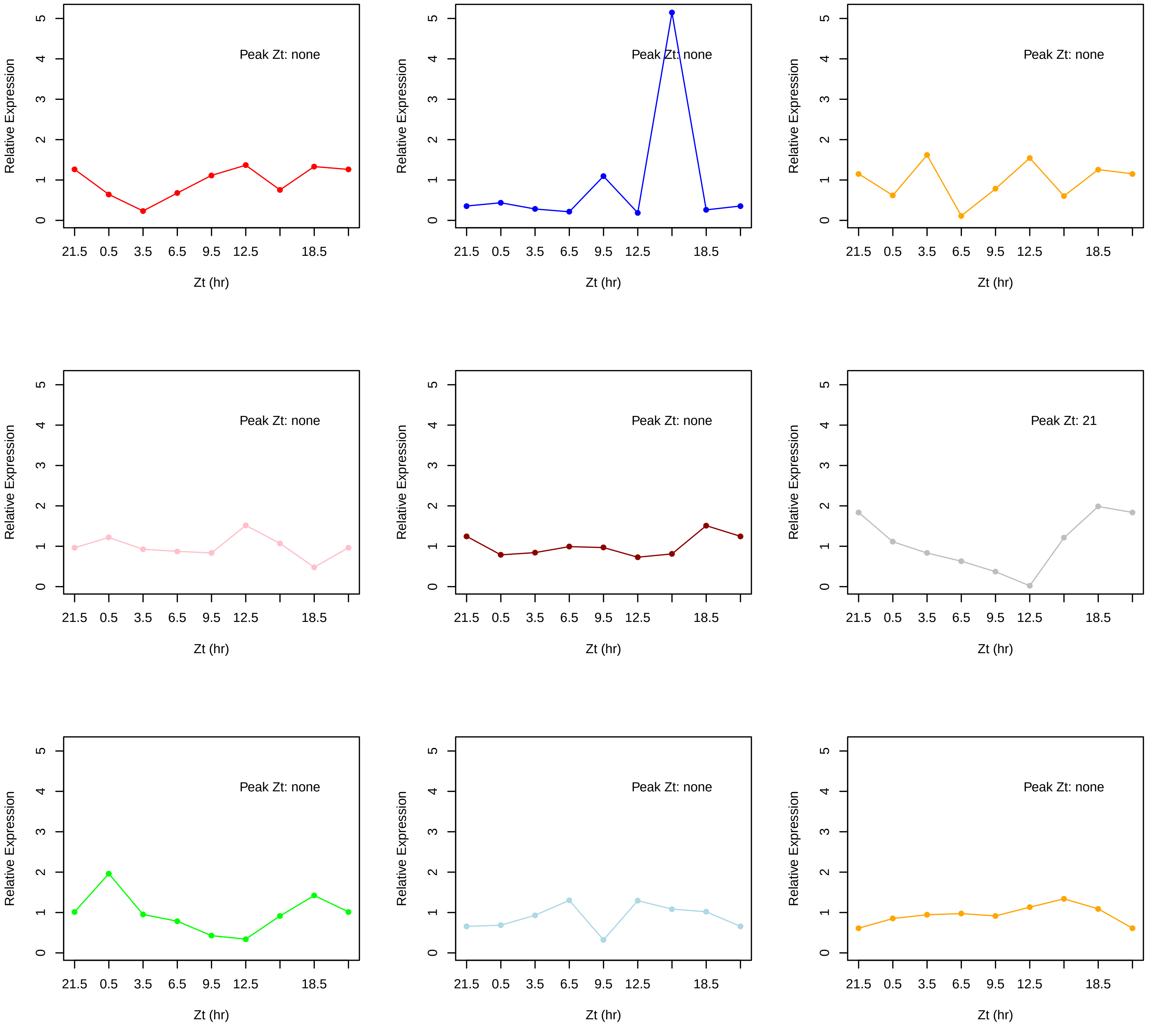

Singeleton: ENSXMAG00000012844 amh

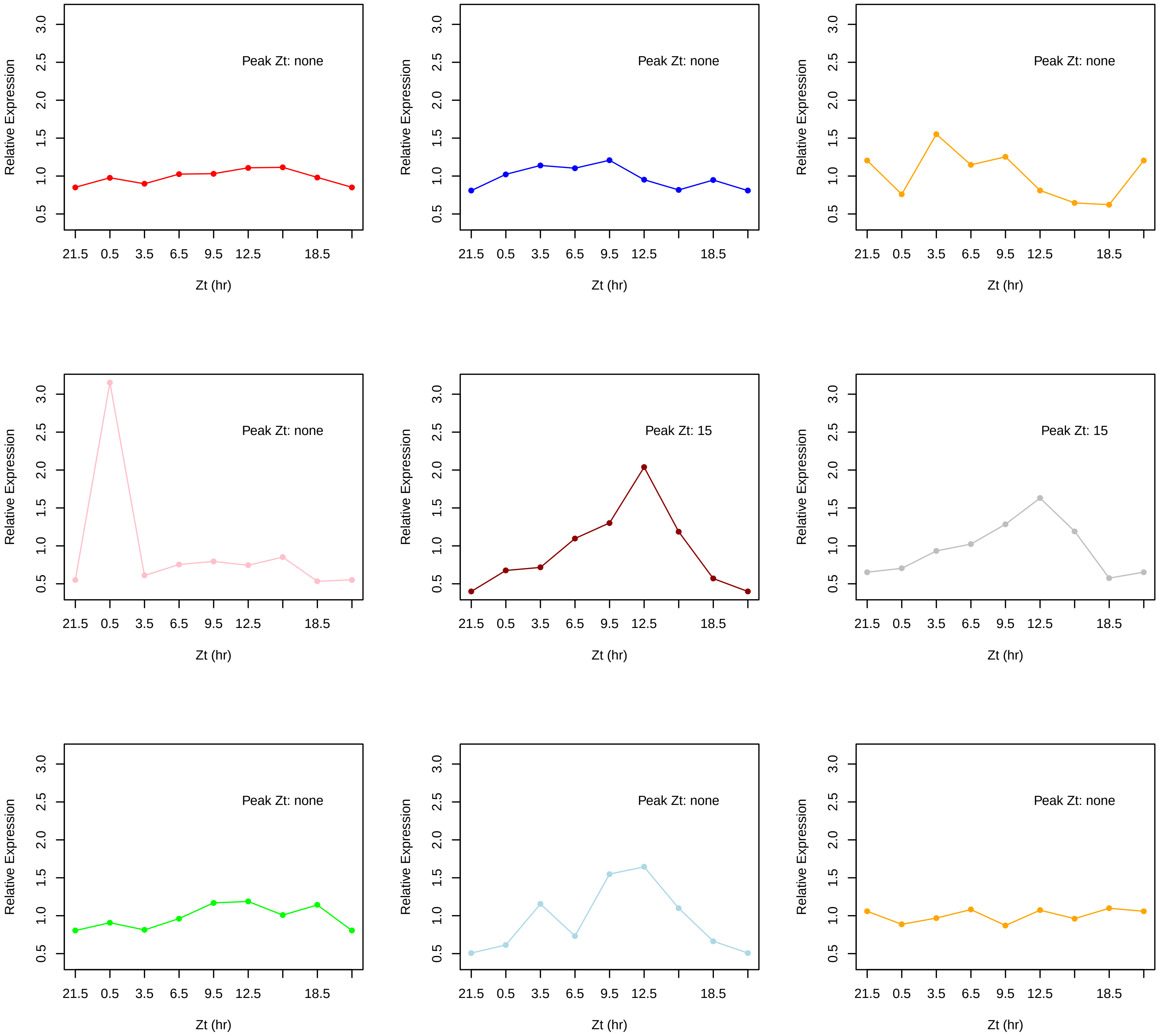

Singeleton: ENSXMAG00000028160 ptgesl

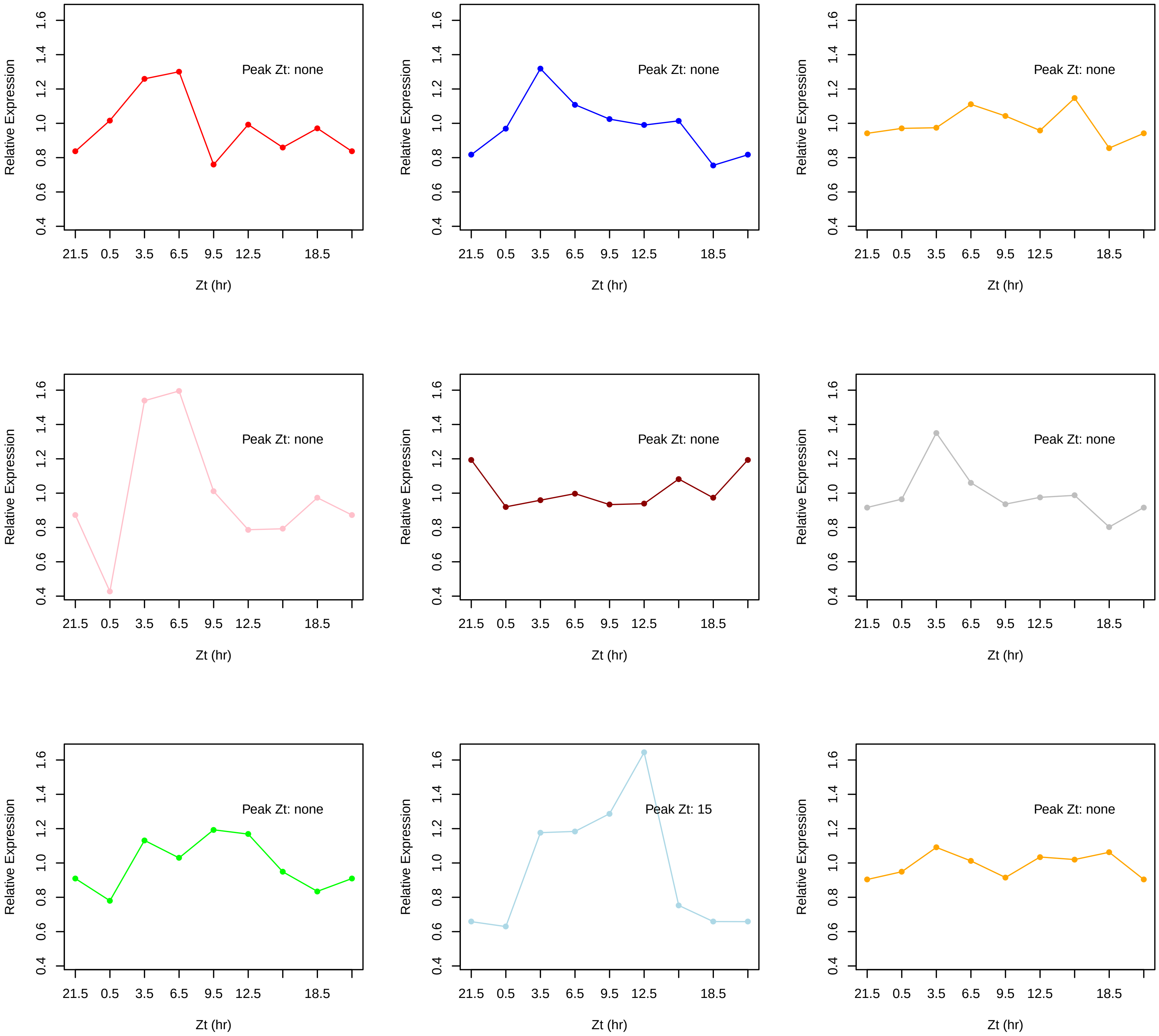

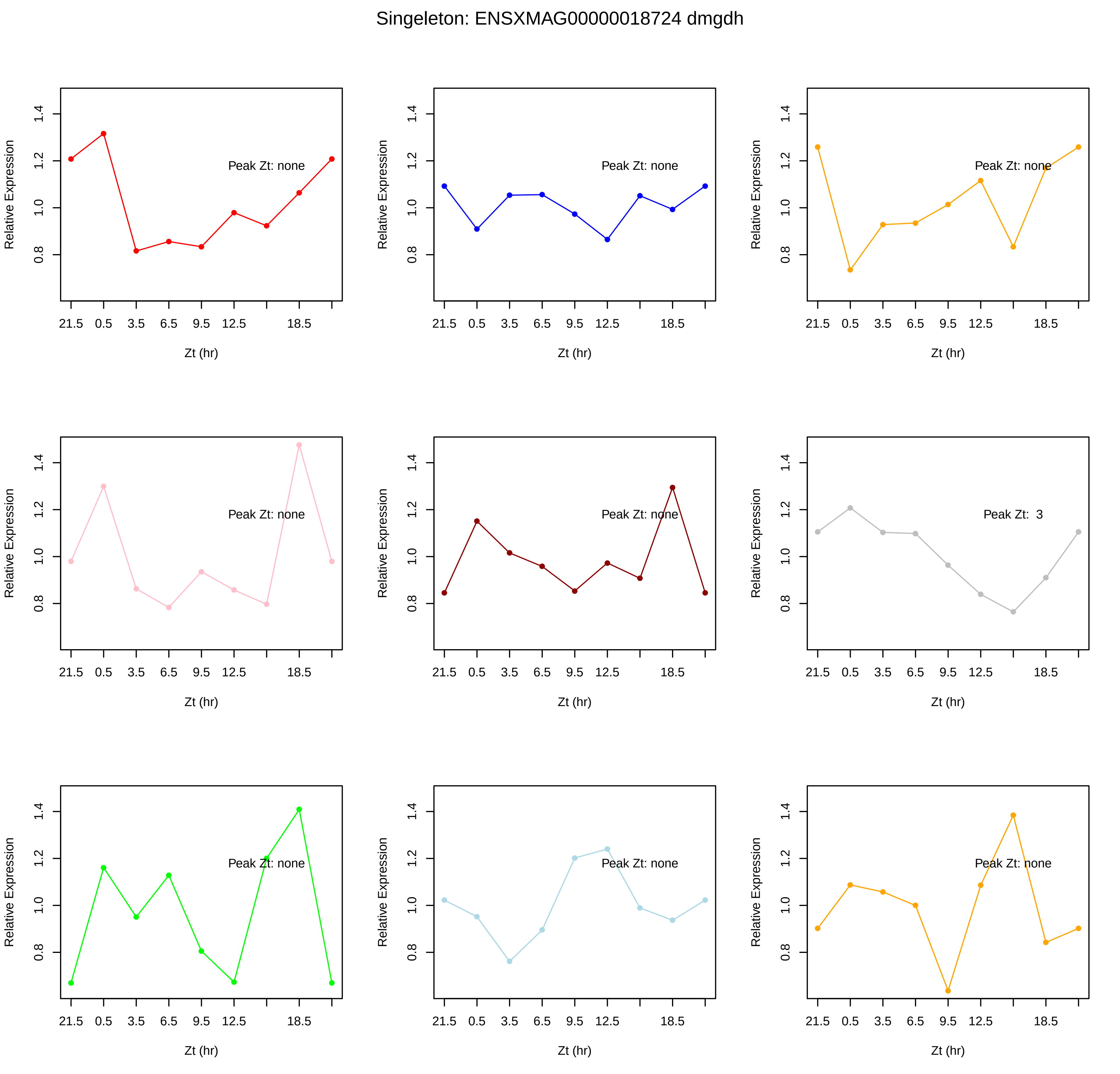
Singeleton: ENSXMAG00000018724 dmgdh
Relative Expression
Zt (hr)
Peak Zt: none
Peak Zt: 3
21.5
0.5
3.5
6.5
9.5
12.5
18.5
0.8
1.0
1.2
1.4

Singeleton: ENSXMAG00000004229 tm9sf4

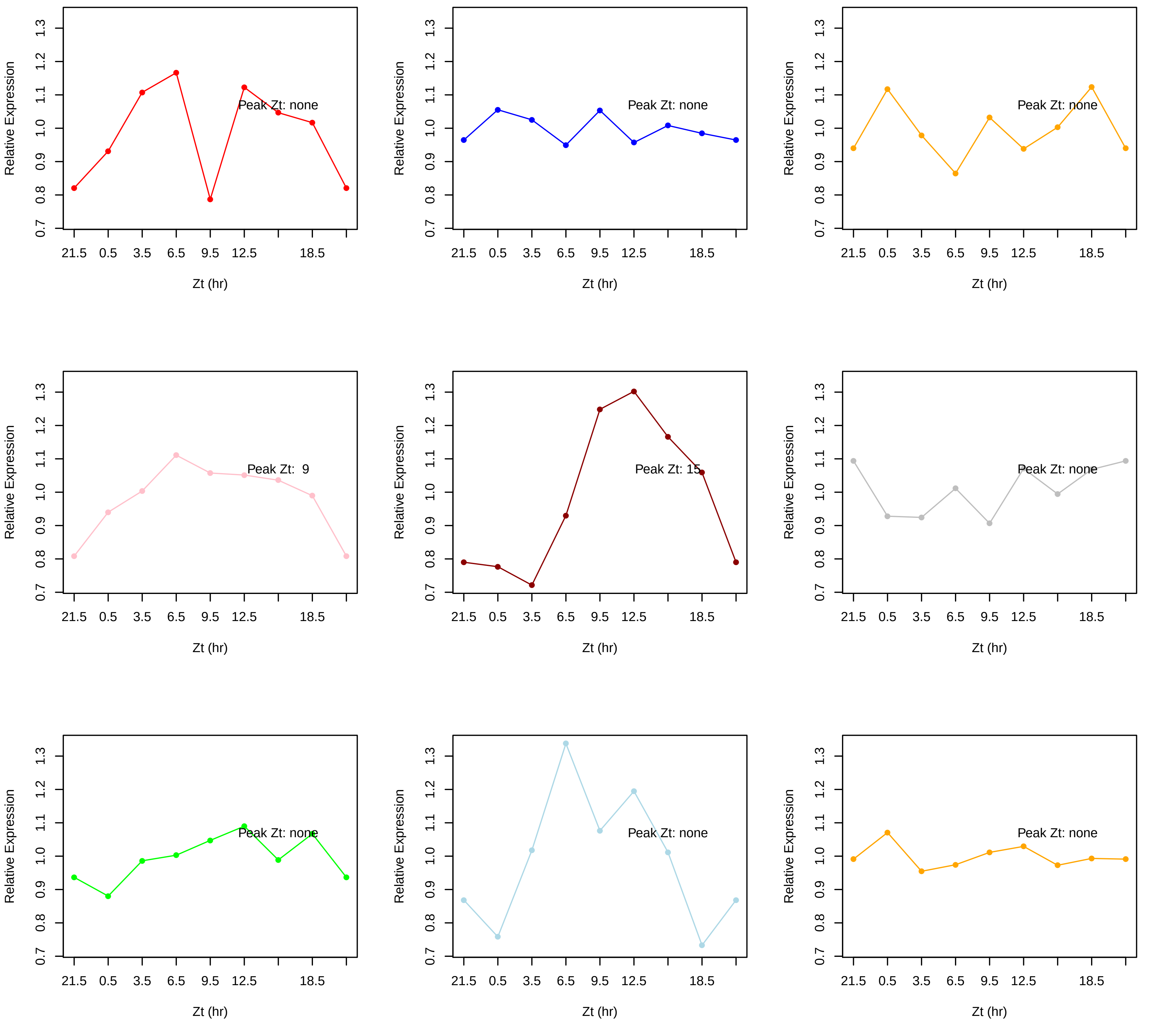

Singeleton: ENSXMAG00000010912

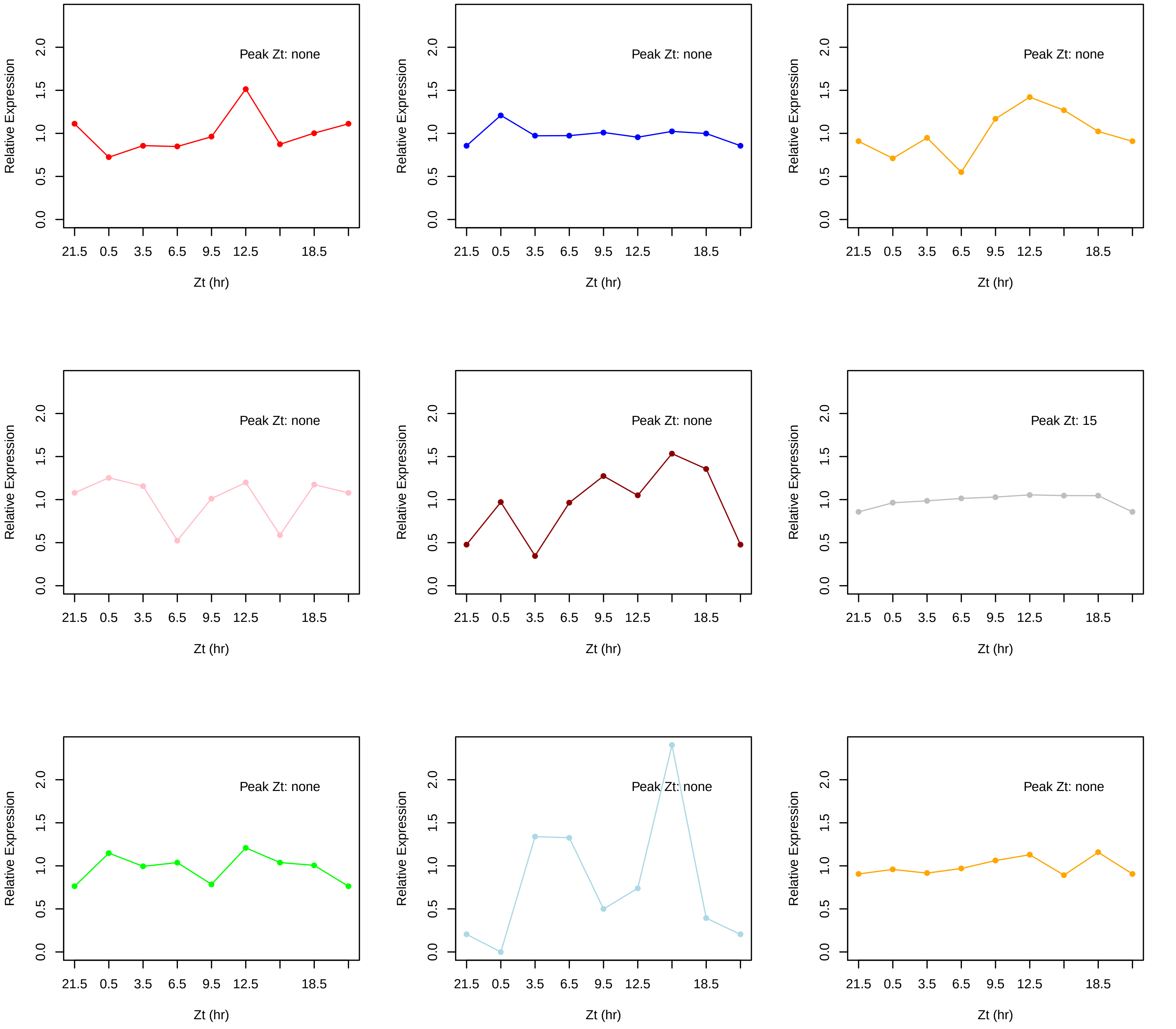

Singleton: ENSXMAG00000011868 traf4a

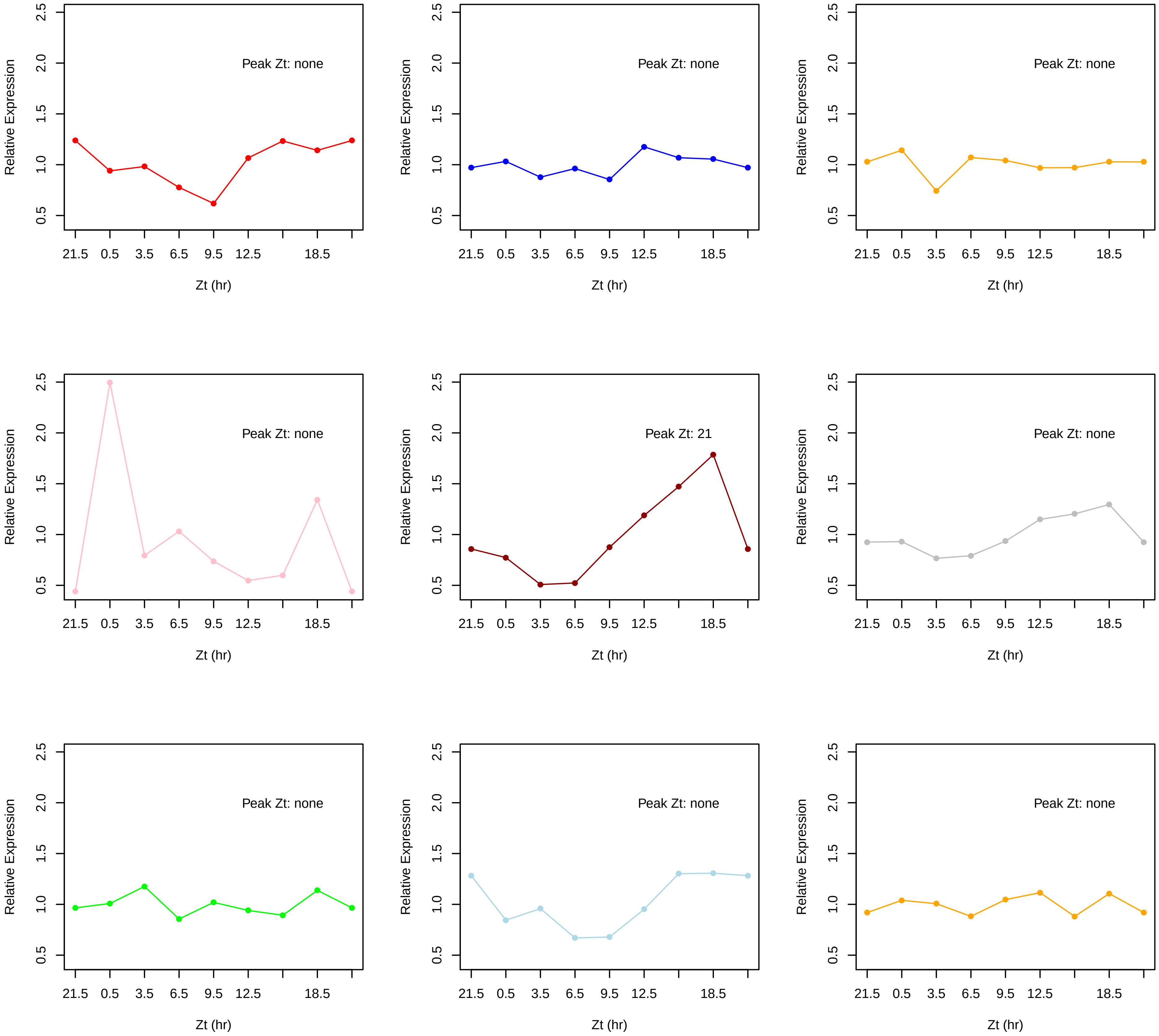

Singleton: ENSXMAG00000012841 oaz1a

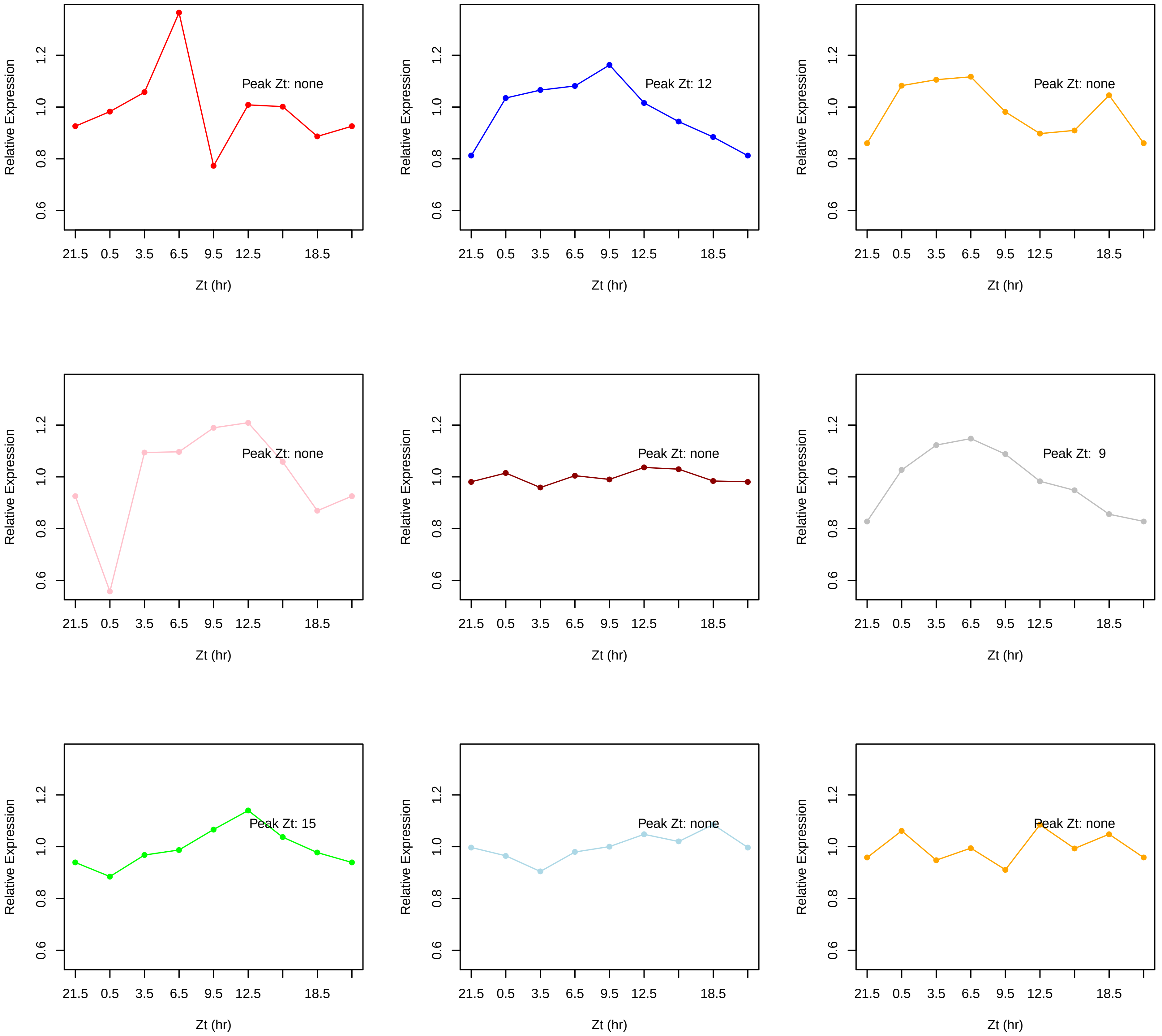

Singeleton: ENSXMAG00000029107

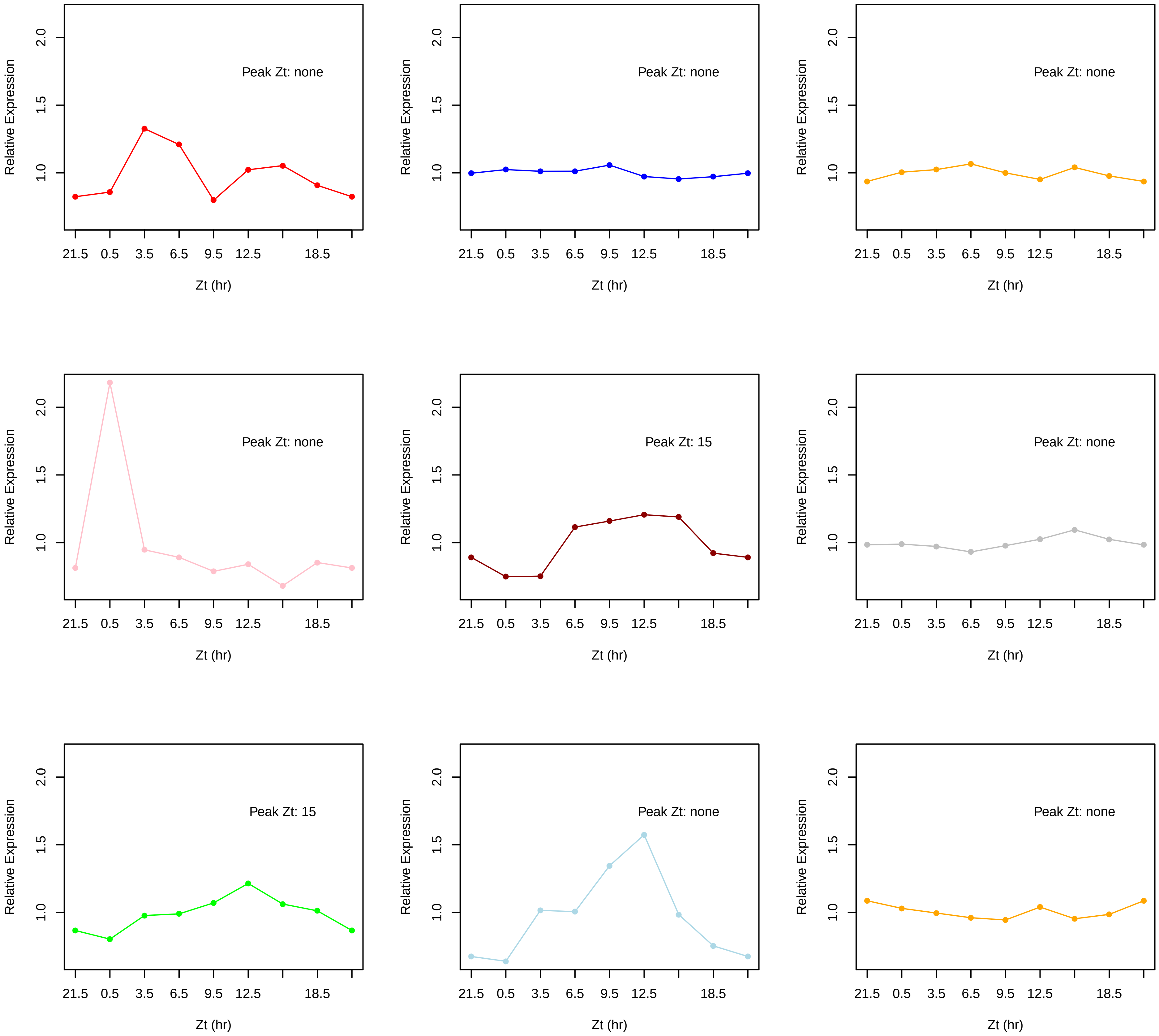

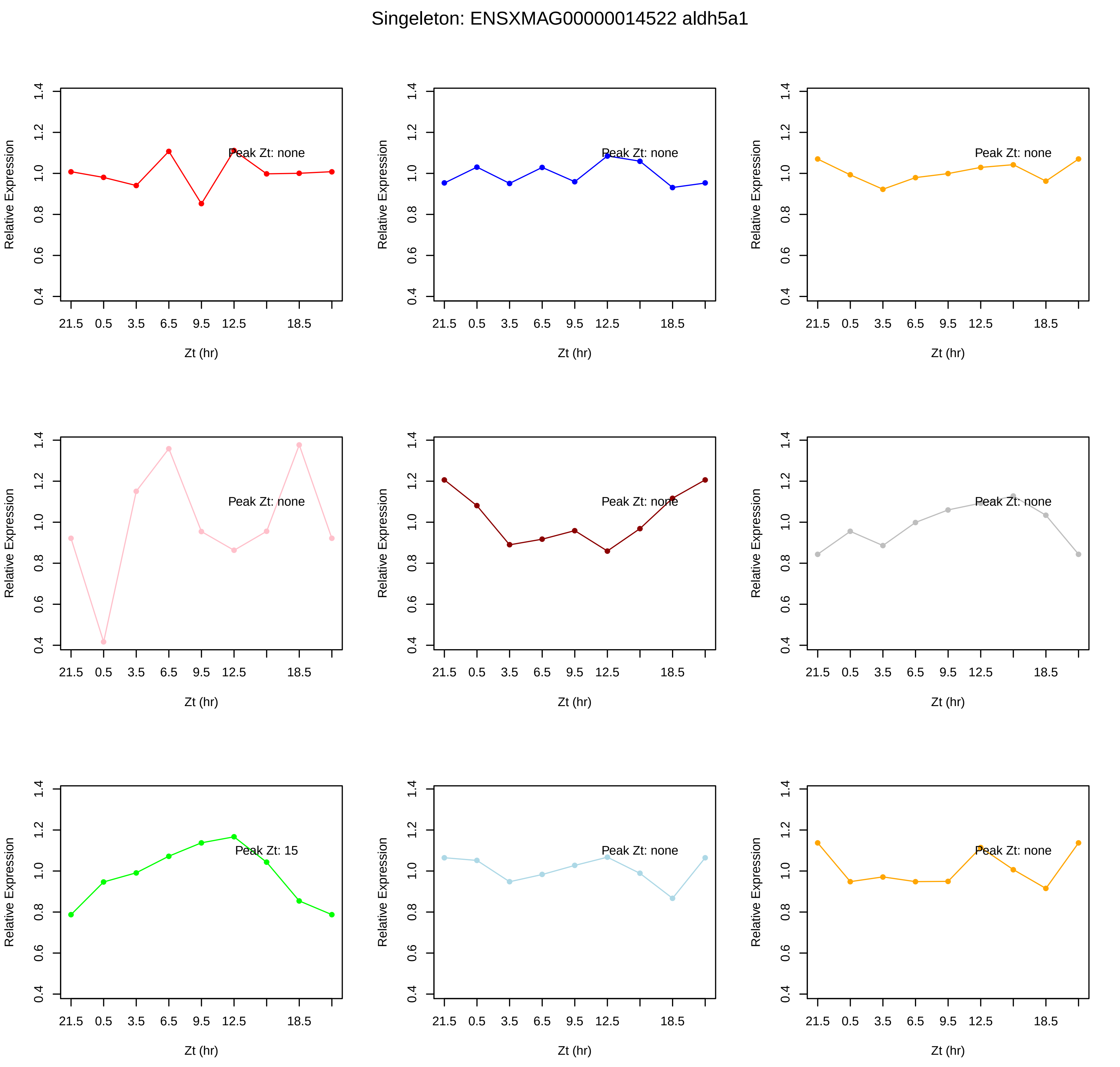
Singeleton: ENSXMAG00000014522 aldh5a1
Relative Expression
Zt (hr)
21.5
0.5
3.5
6.5
9.5
12.5
18.5
0.4
0.6
0.8
1.0
1.2
1.4
Peak Zt: none
Peak Zt: 15

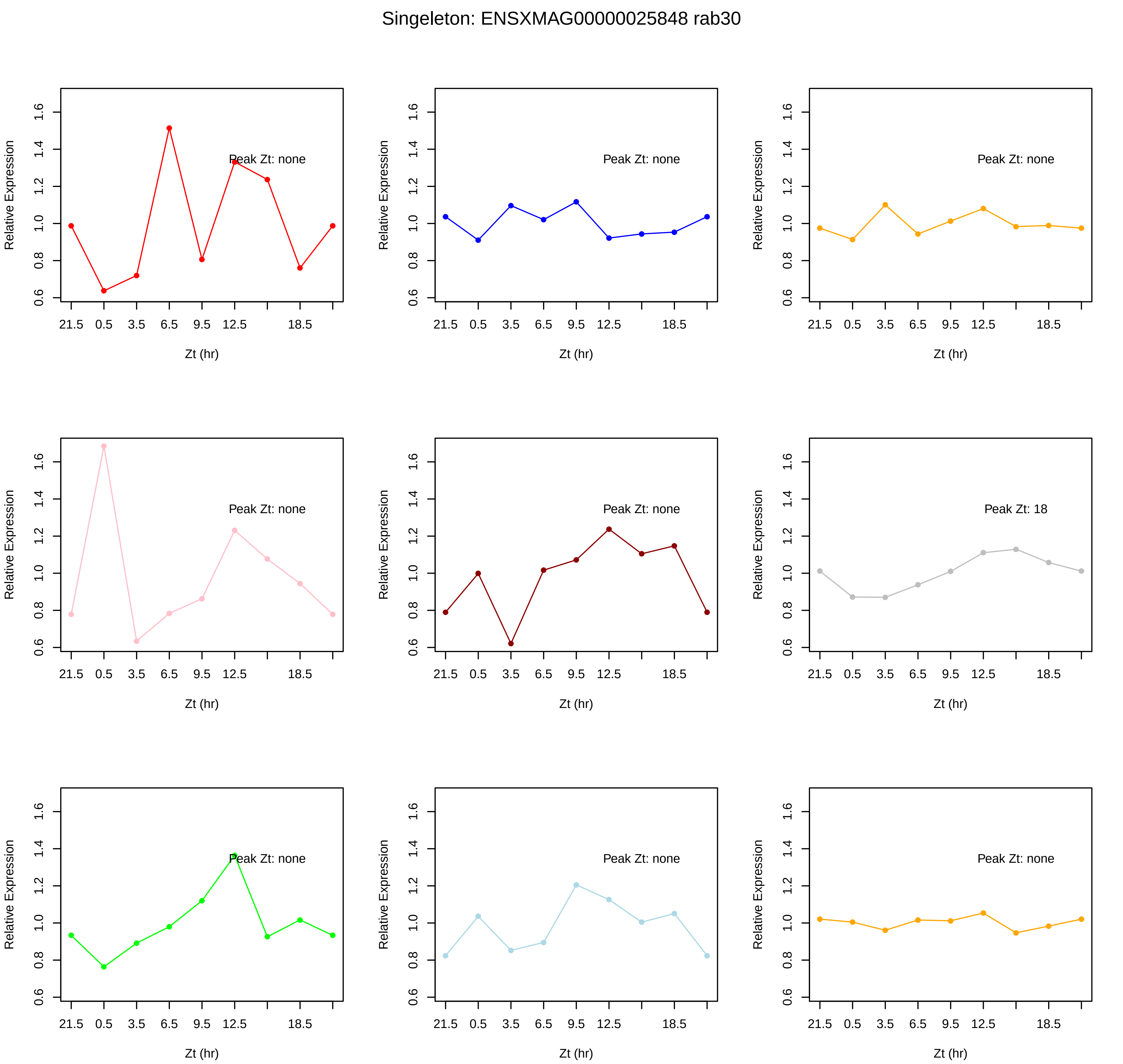

Singleton: ENSXMAG00000025848 rab30
Relative Expression
Zt (hr)
21.5
0.5
3.5
6.5
9.5
12.5
18.5
0.6
0.8
1.0
1.2
1.4
1.6
Peak Zt: none
Peak Zt: 18

Singleton: ENSXMAG00000012394 epn2

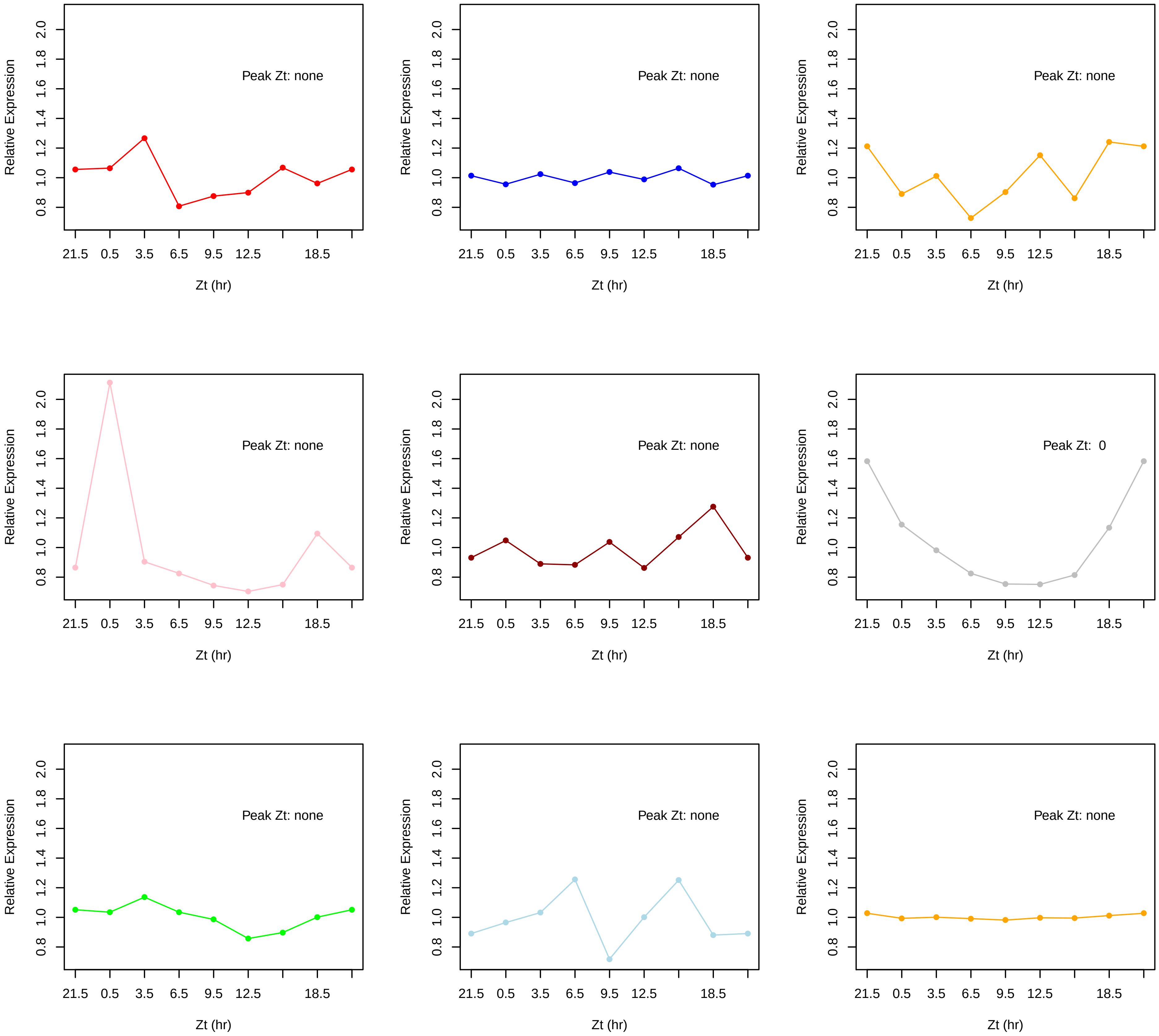

Singeleton: ENSXMAG00000007172 rgcc

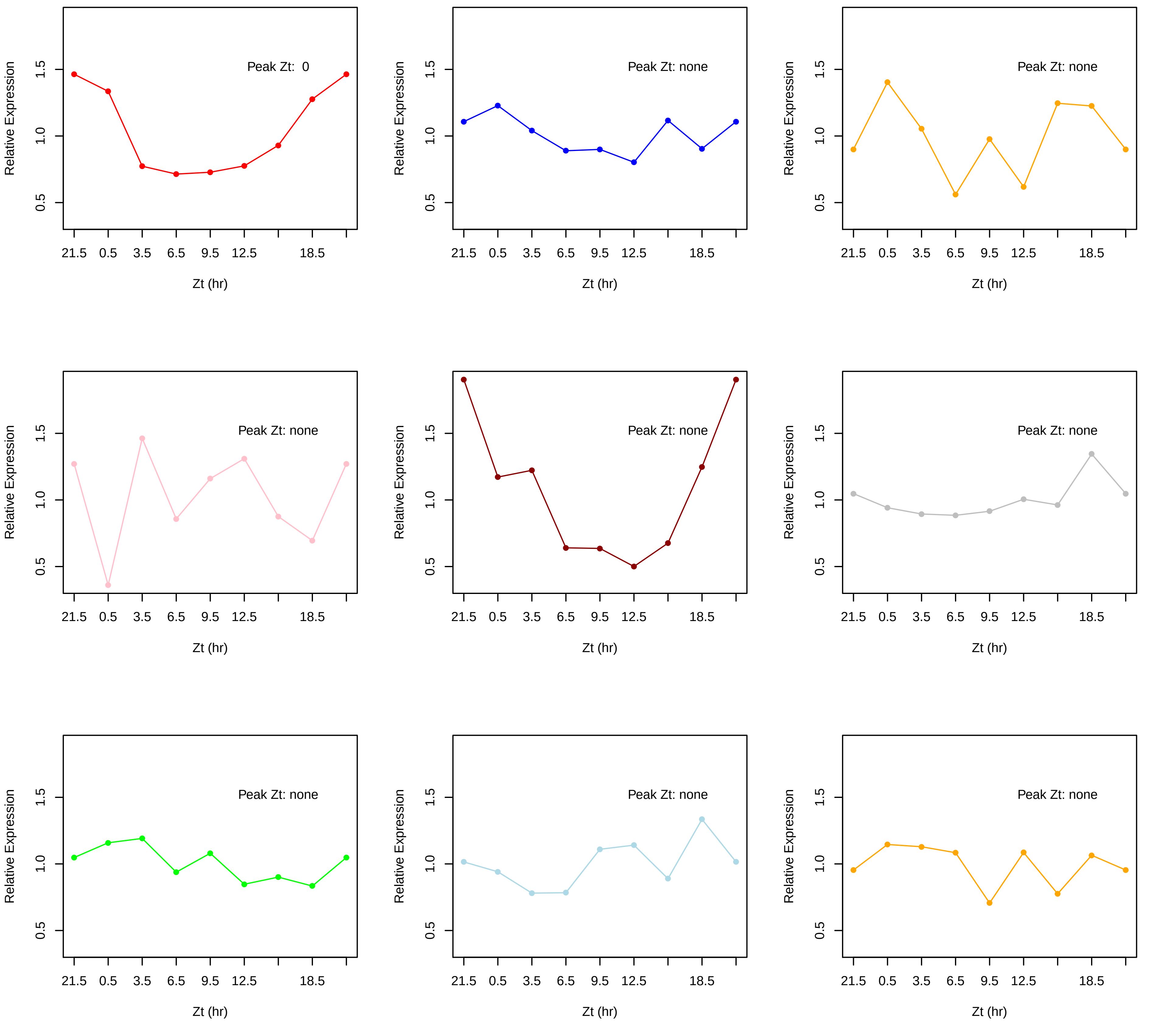

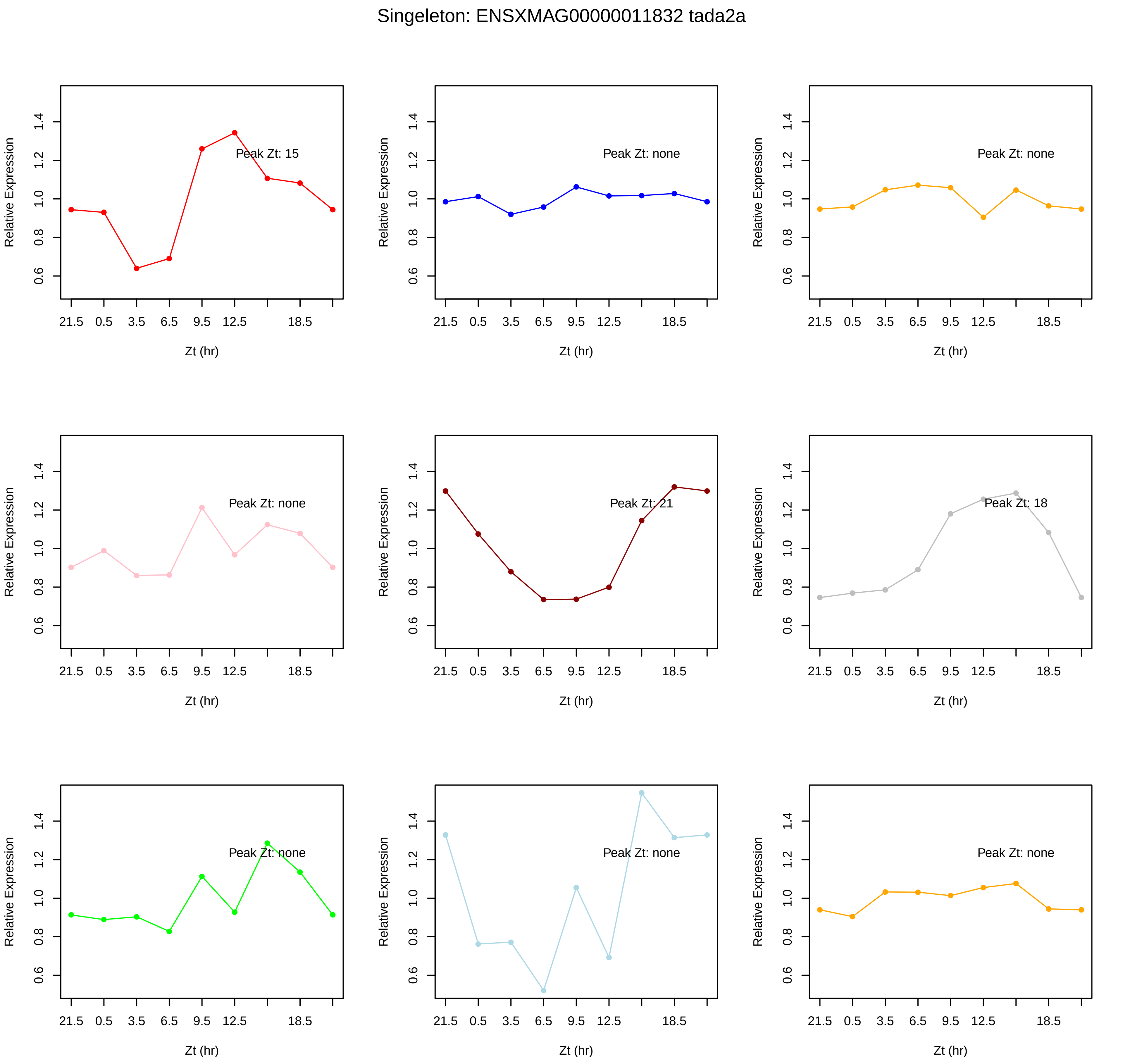
Singeleton: ENSXMAG00000011832 tada2a
Peak Zt: 15
Peak Zt: none
Peak Zt: none
Peak Zt: none
Peak Zt: 21
Peak Zt: 18
Peak Zt: none
Peak Zt: none
Peak Zt: none
Relative Expression
Zt (hr)
21.5
0.5
3.5
6.5
9.5
12.5
18.5
0.6
0.8
1.0
1.2
1.4

Singleton: ENSXMAG00000022506 SLC35G1

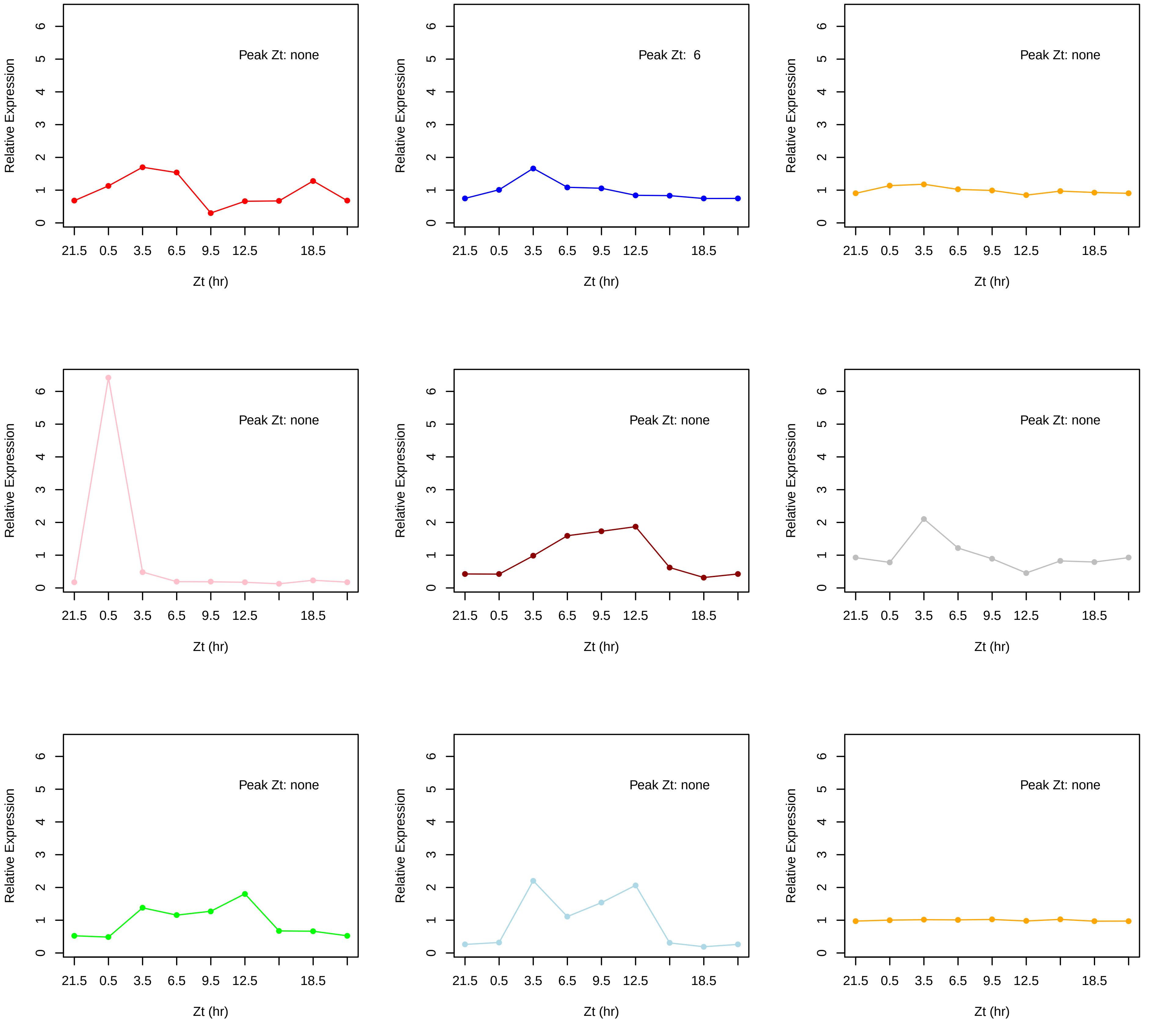

Singeleton: ENSXMAG00000018710 JMY

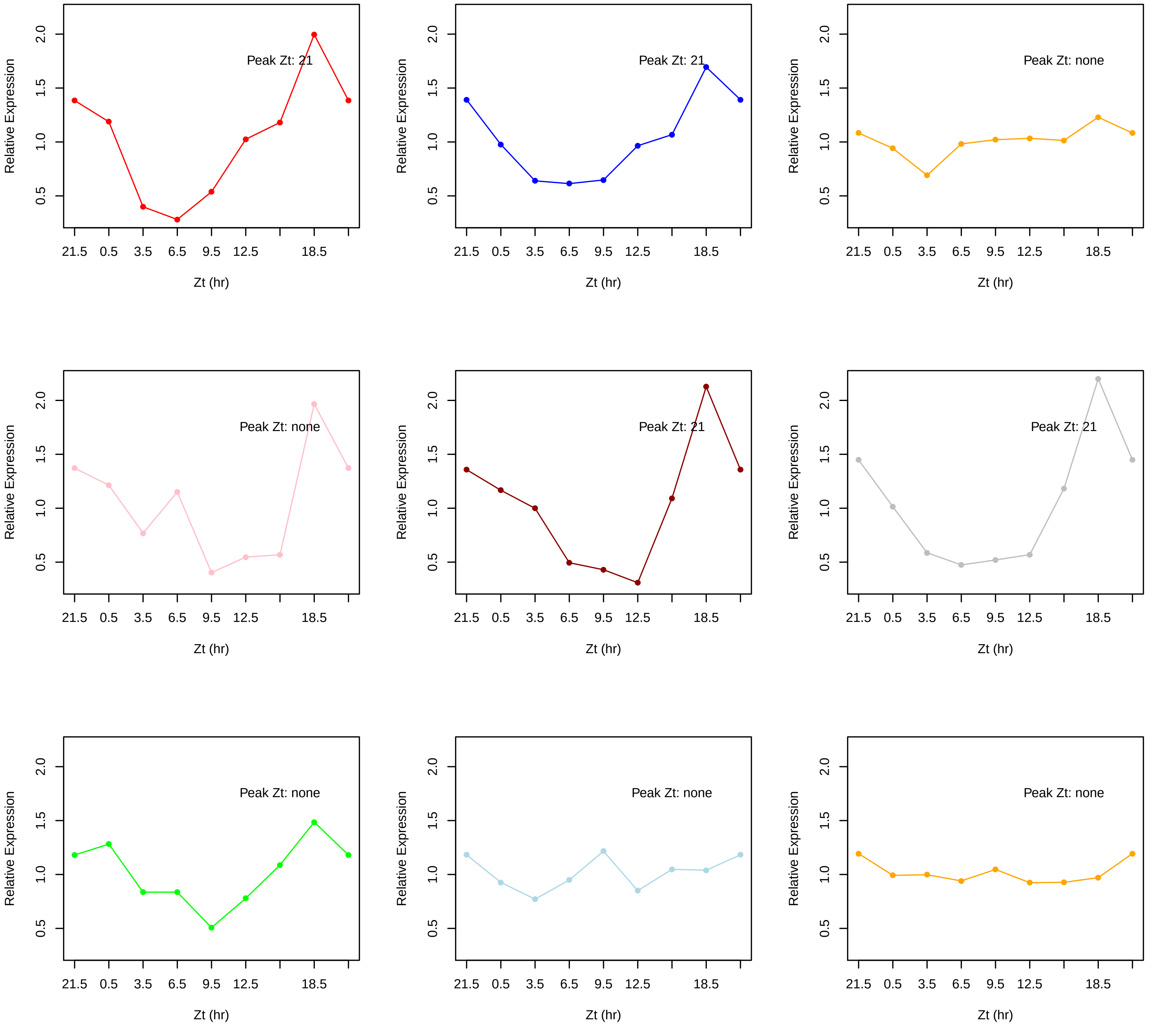

Singeleton: ENSXMAG00000018411 gpbp1l1

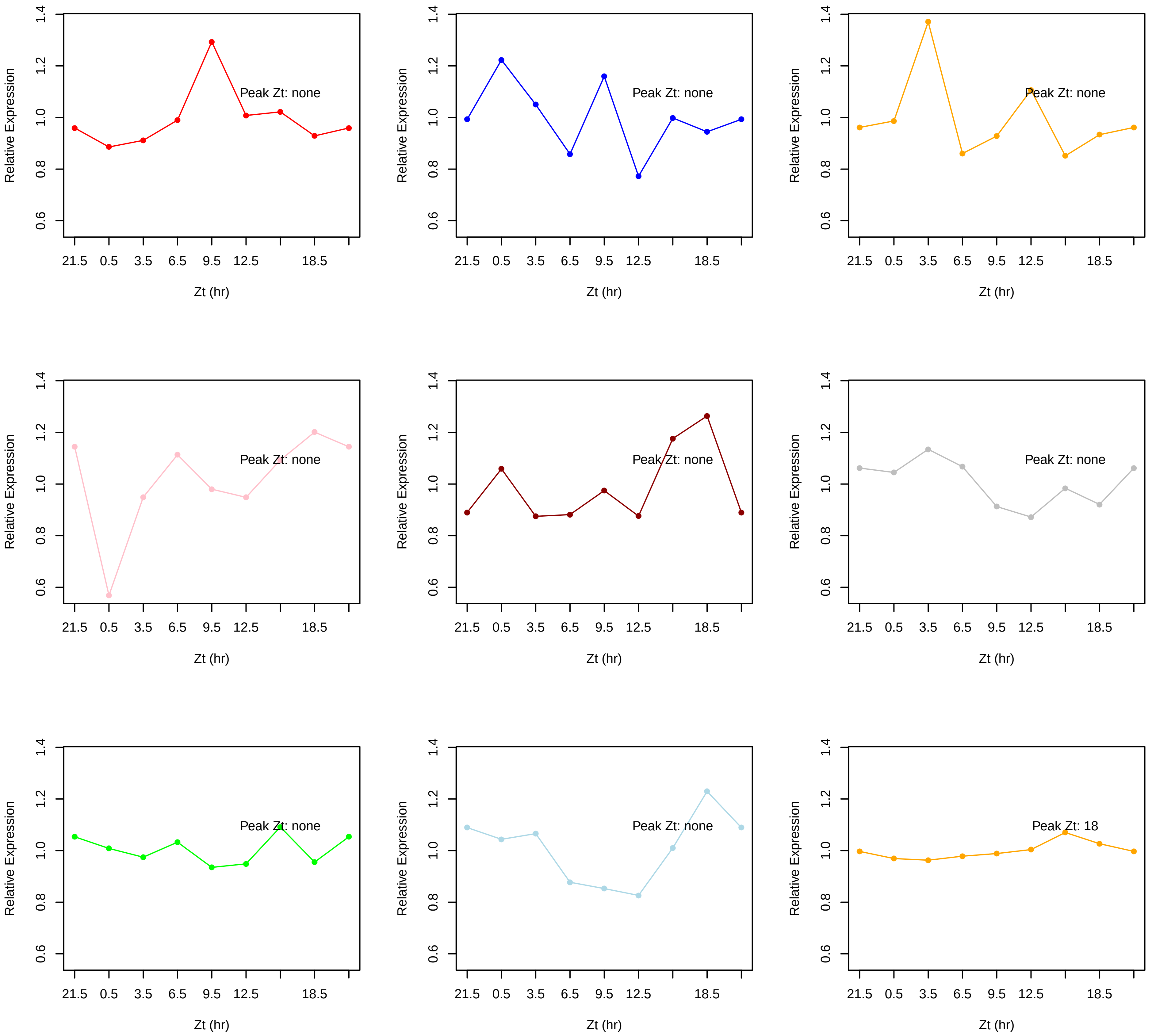

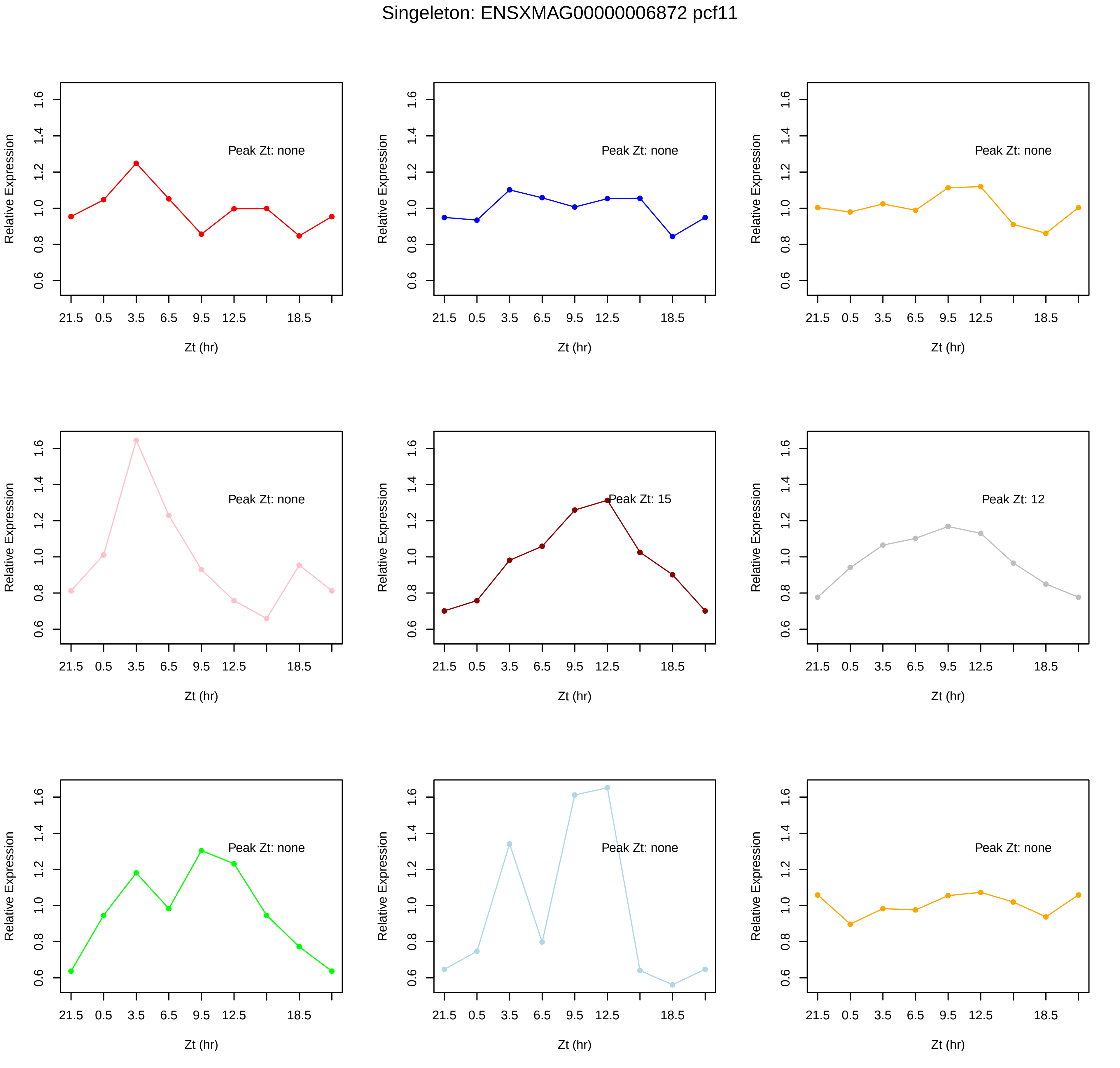

Singeleton: ENSXMAG00000006872 pcf11
Peak Zt: none
Peak Zt: none
Peak Zt: none
Peak Zt: none
Peak Zt: 15
Peak Zt: 12
Peak Zt: none
Peak Zt: none
Peak Zt: none
Relative Expression
Zt (hr)
21.5 0.5 3.5 6.5 9.5 12.5 18.5
0.6 0.8 1.0 1.2 1.4 1.6

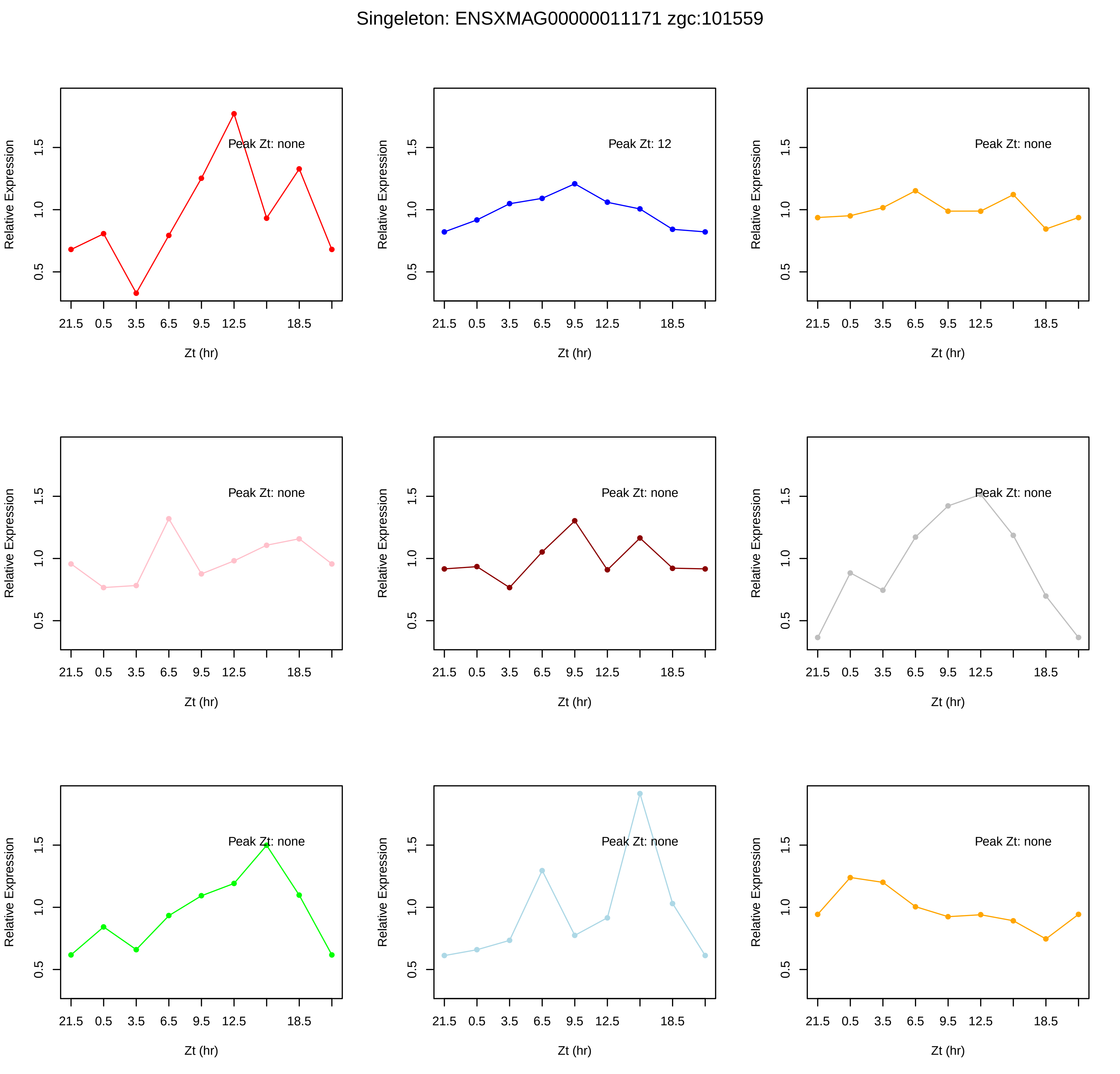
Singeleton: ENSXMAG00000011171 zgc:101559
Peak Zt: none
Peak Zt: 12
Peak Zt: none
Peak Zt: none
Peak Zt: none
Peak Zt: none
Peak Zt: none
Peak Zt: none
Peak Zt: none
Relative Expression
Zt (hr)
21.5
0.5
3.5
6.5
9.5
12.5
18.5
0.5
1.0
1.5

Singeleton: ENSXMAG00000017939 ARHGAP9

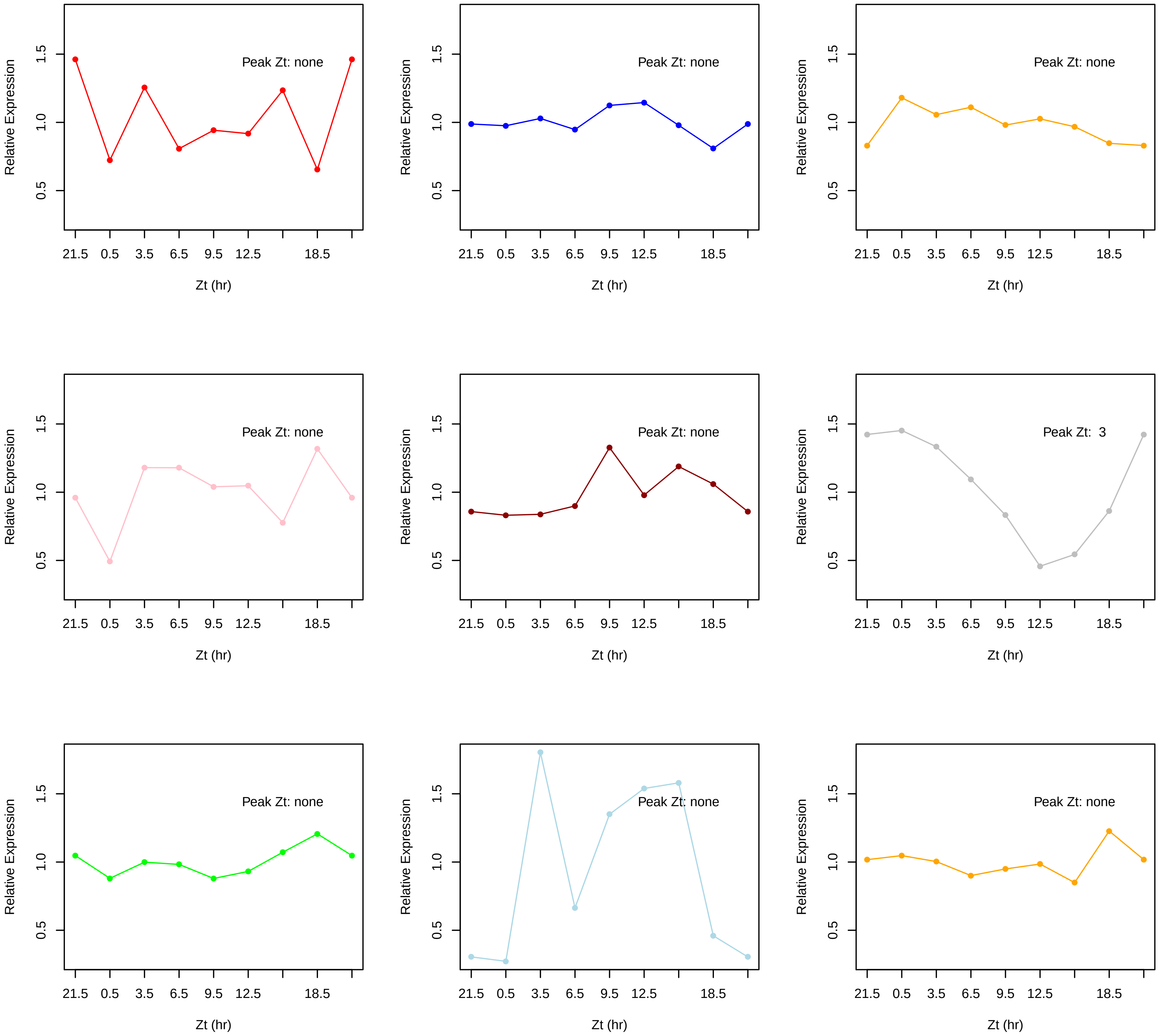

Singleton: ENSXMAG00000002812

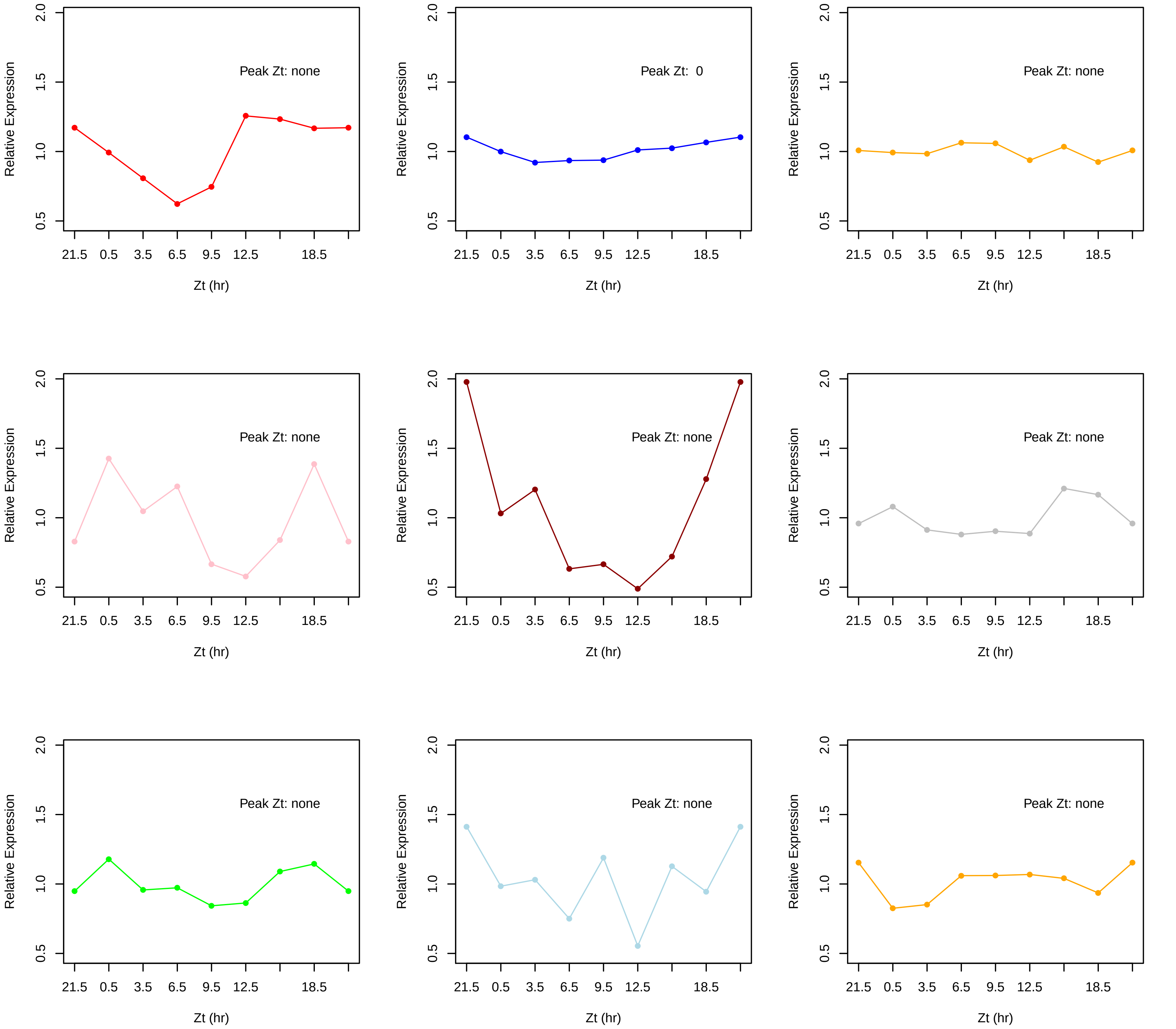

Singeleton: ENSXMAG00000029433 pcmtd1

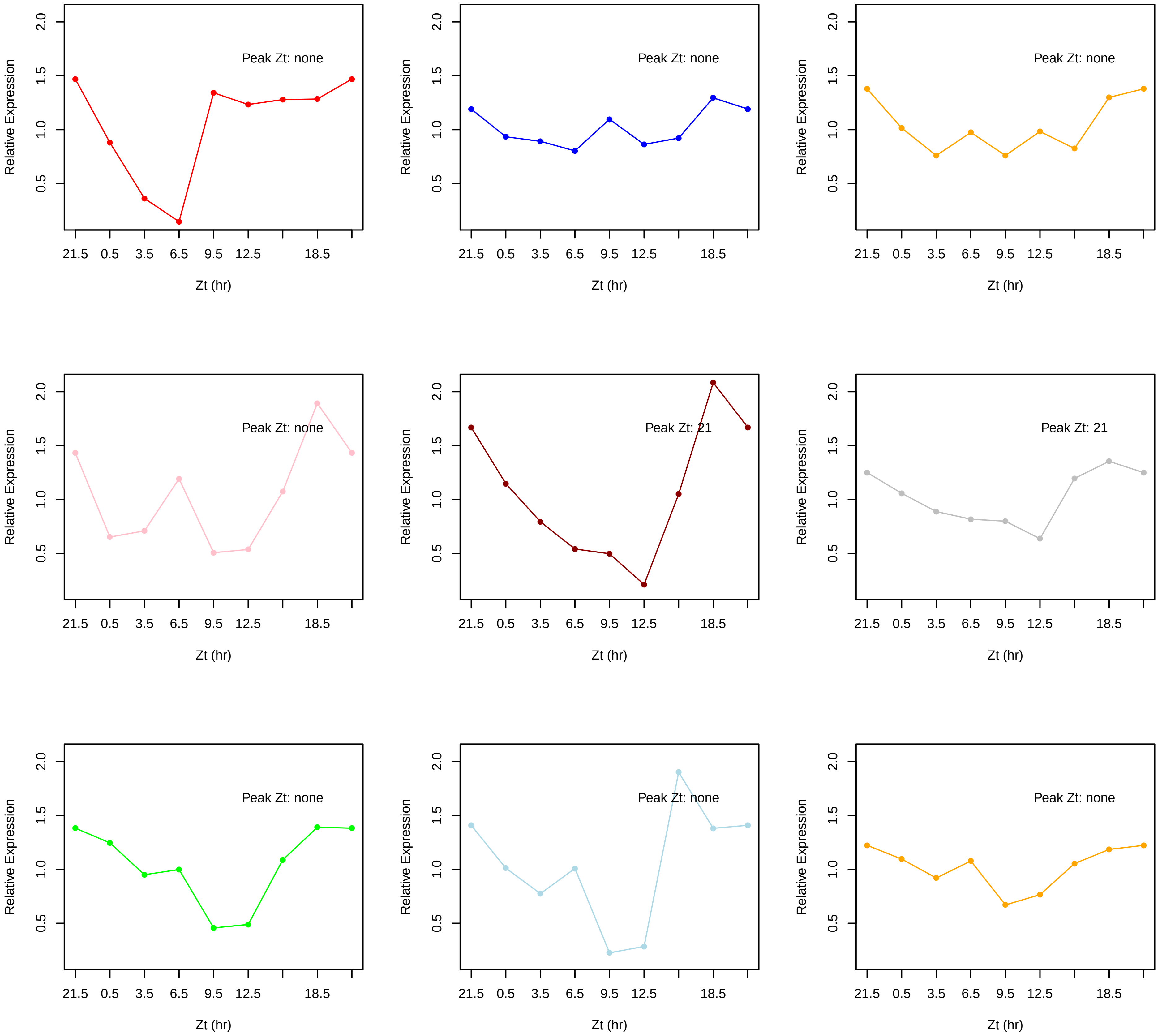

Singeleton: ENSXMAG00000010906 si:dkeyp−69c1.6

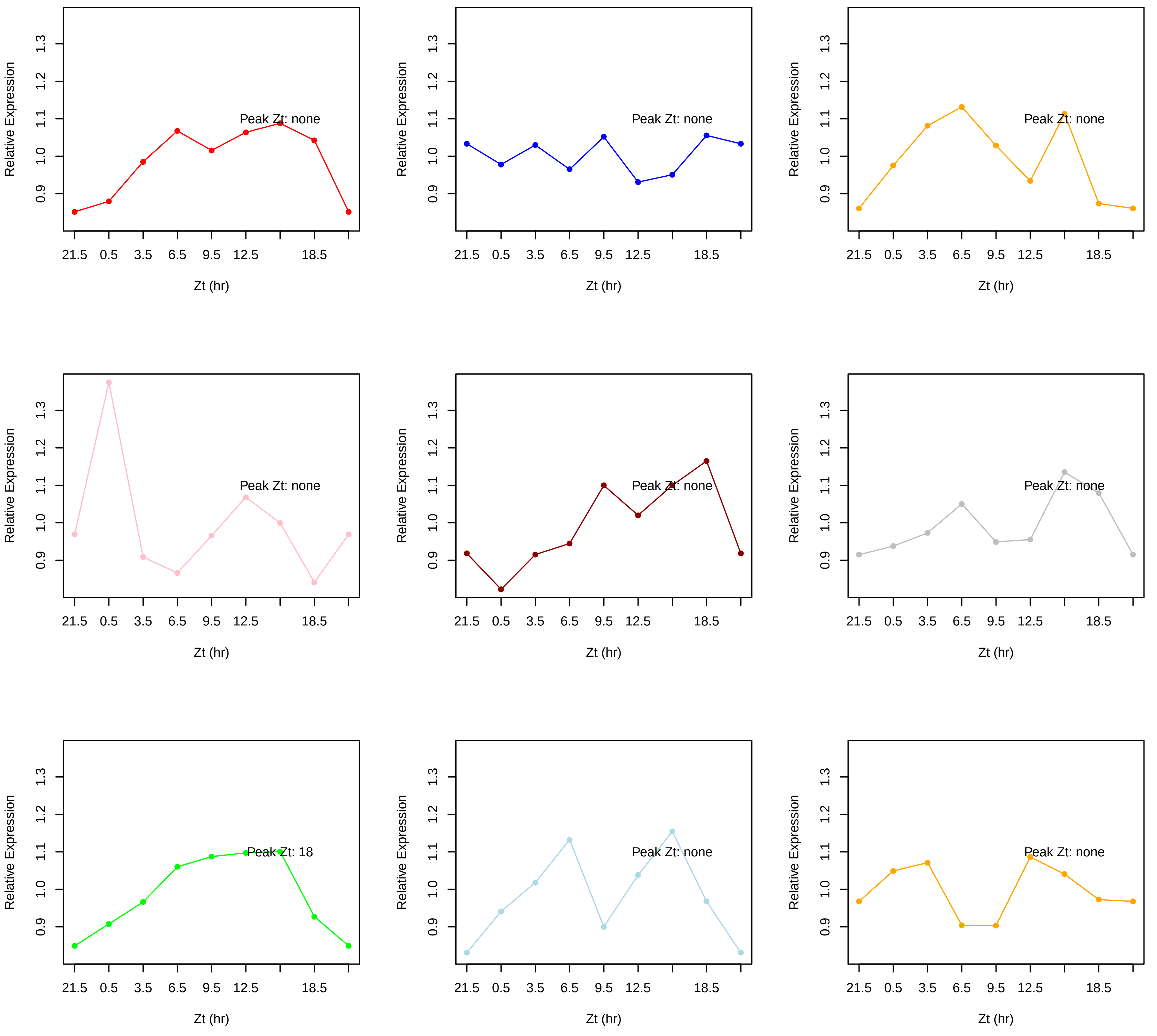

Singeleton: ENSXMAG00000012210 cep104

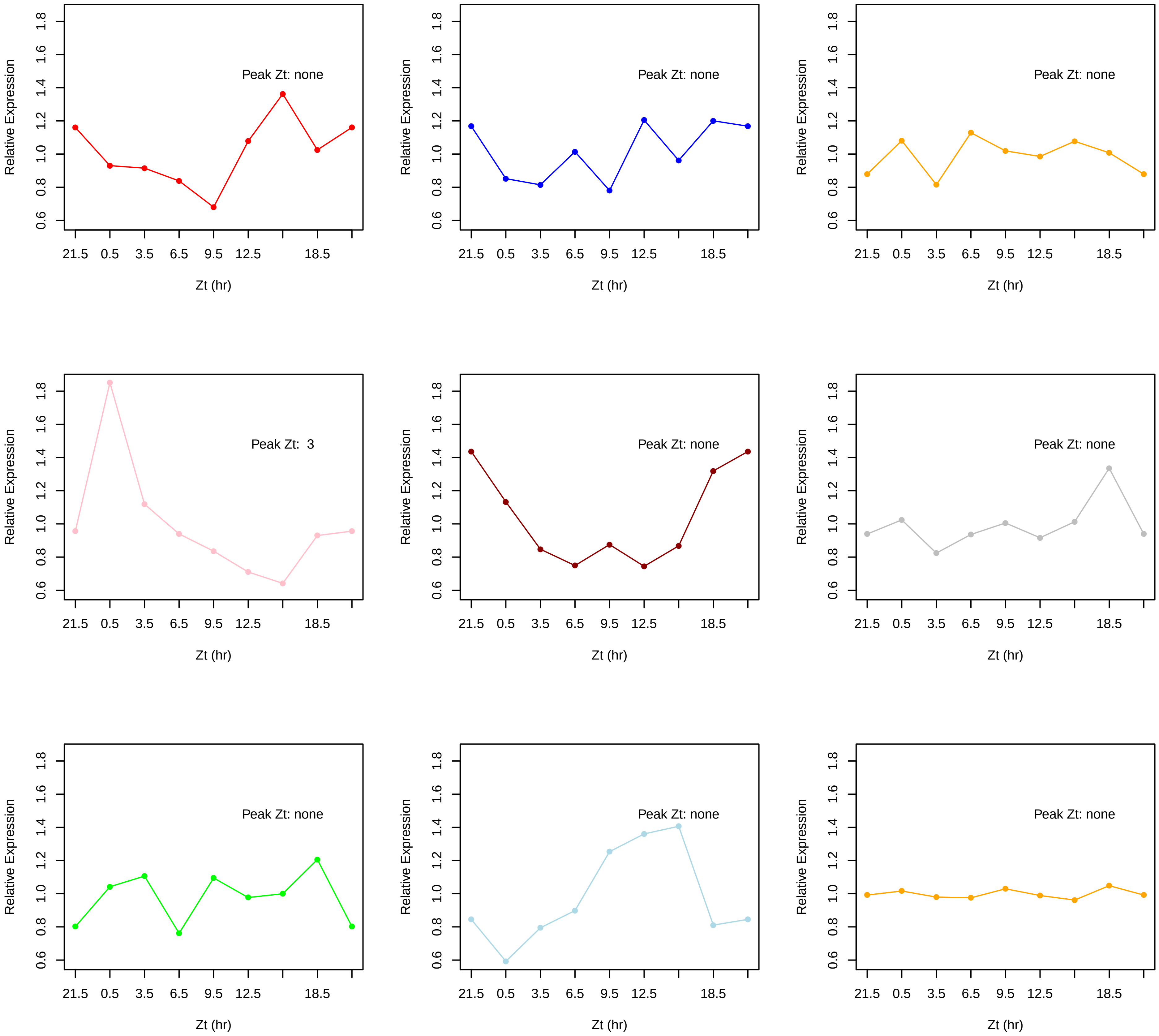

Singeleton: ENSXMAG00000015003 alg3

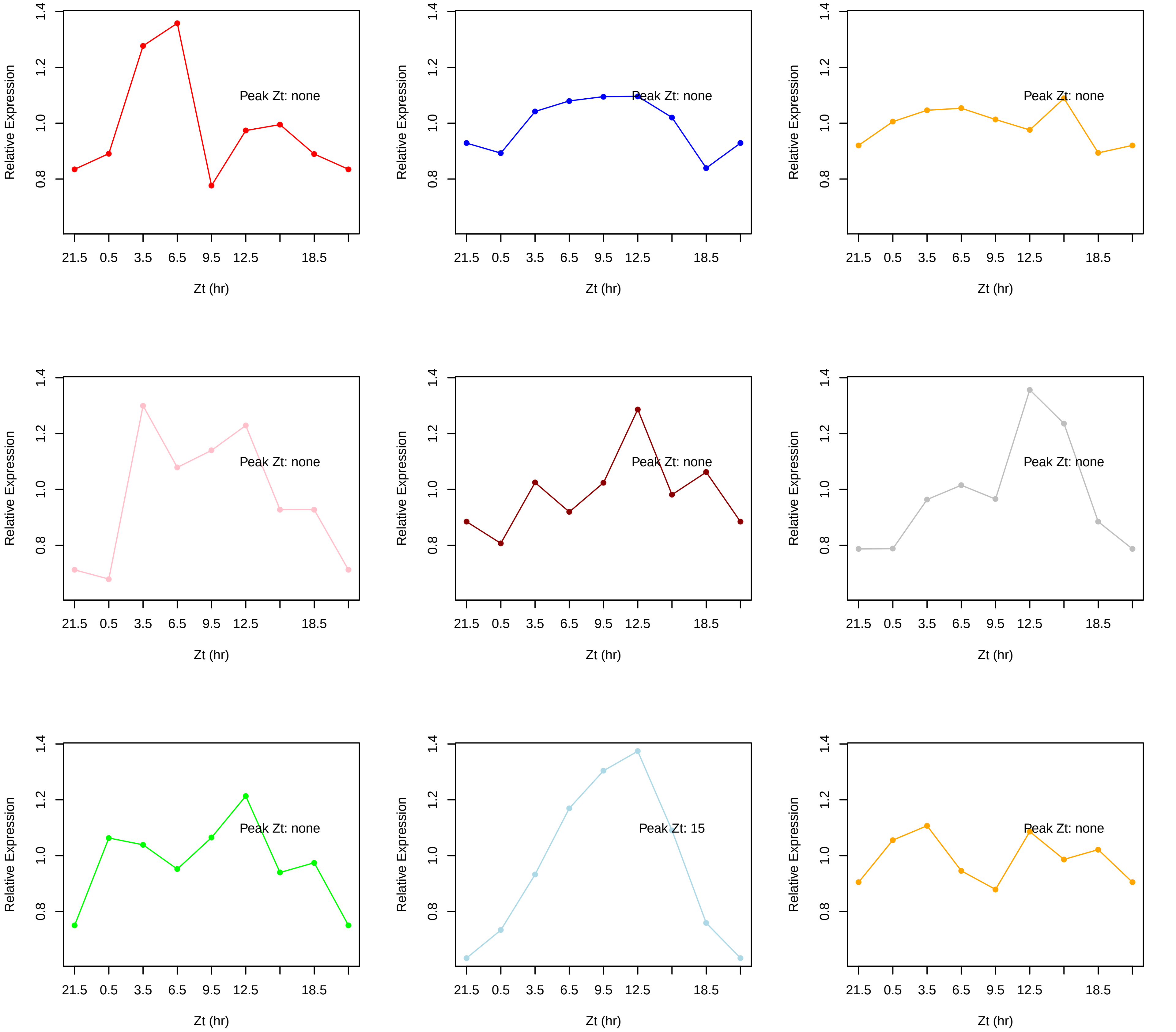

Singeleton: ENSXMAG00000001162 dek

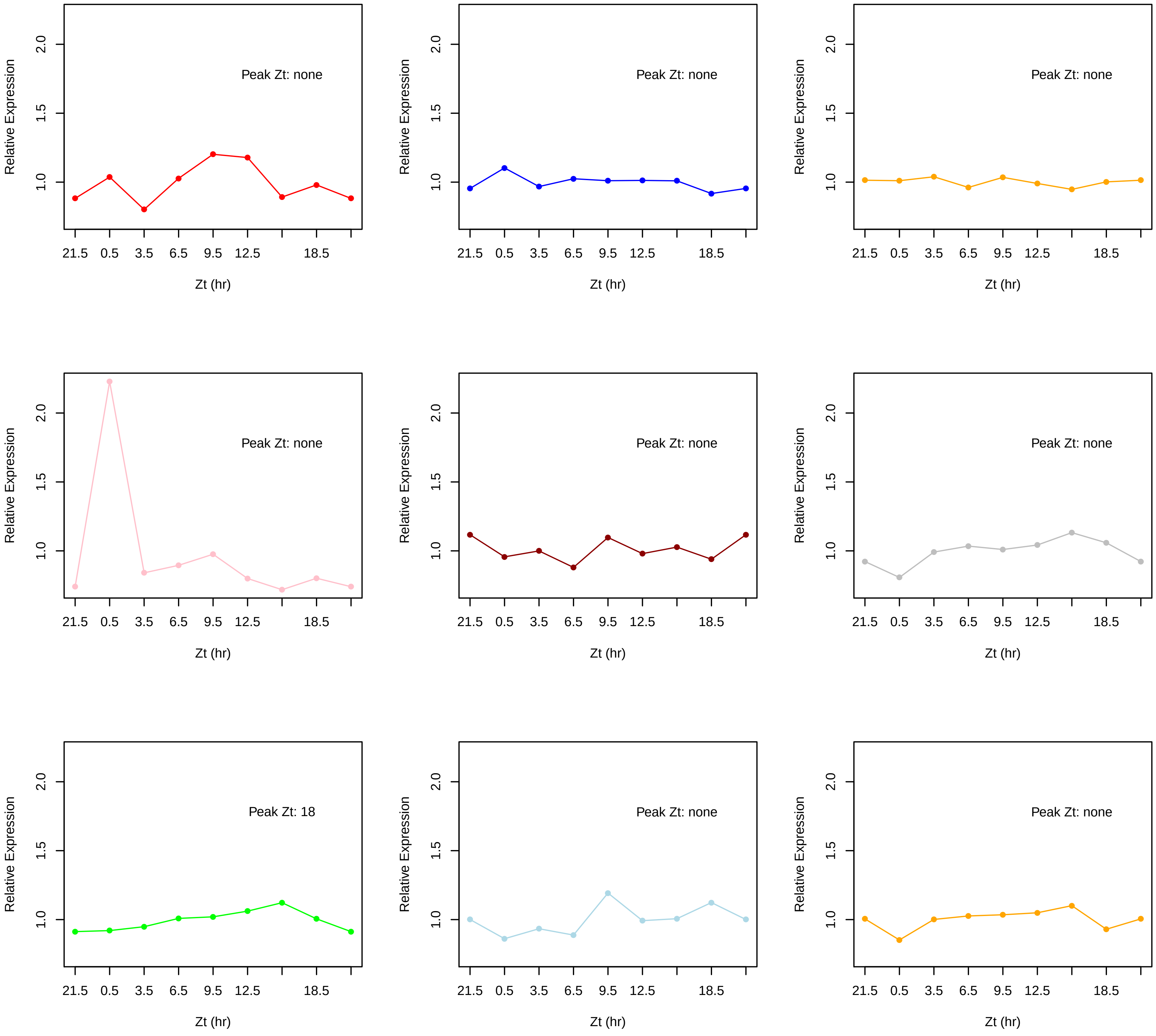

Singleton: ENSXMAG00000011363 PKP4

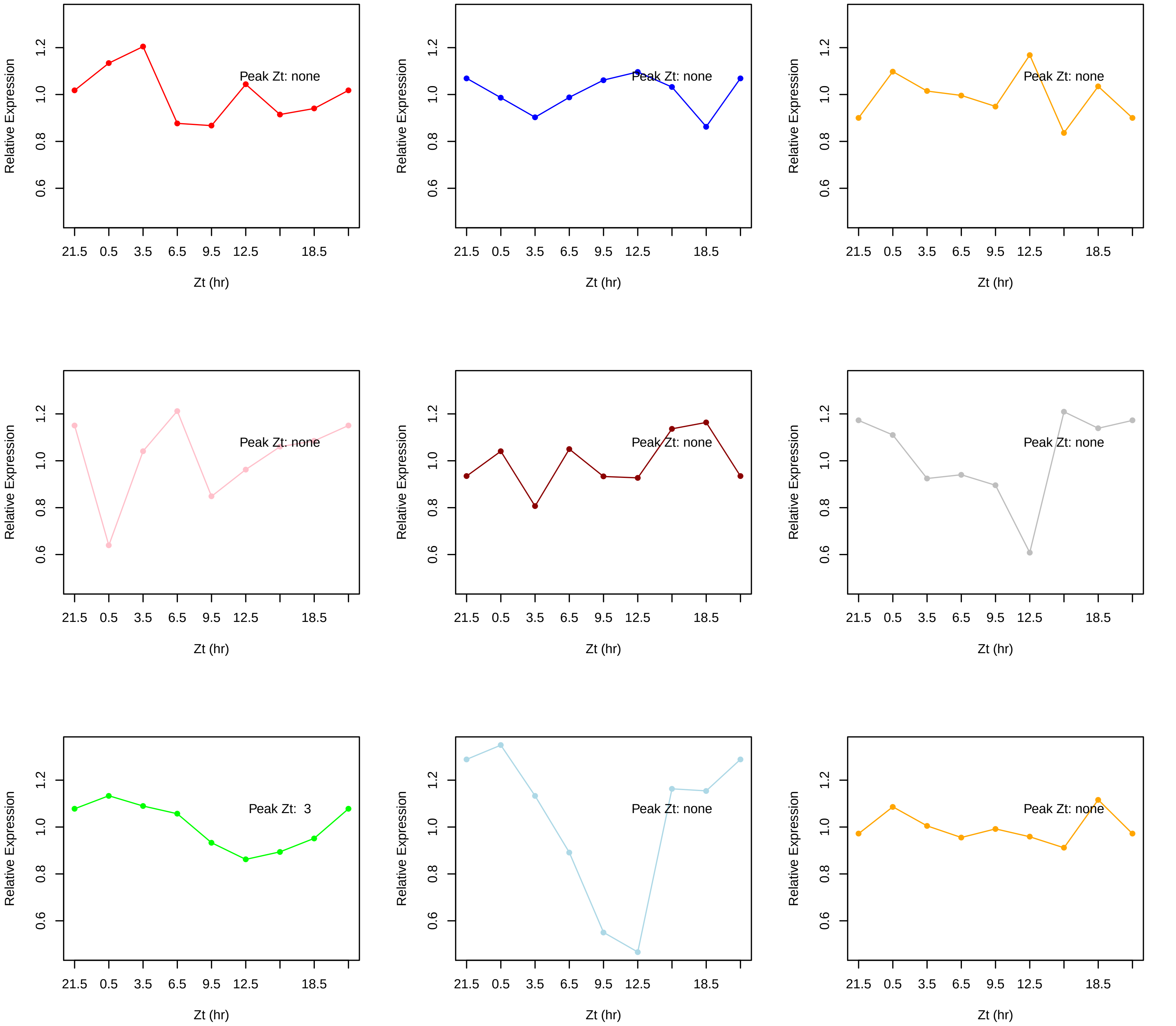

Singeleton: ENSXMAG00000018060 TCTN2

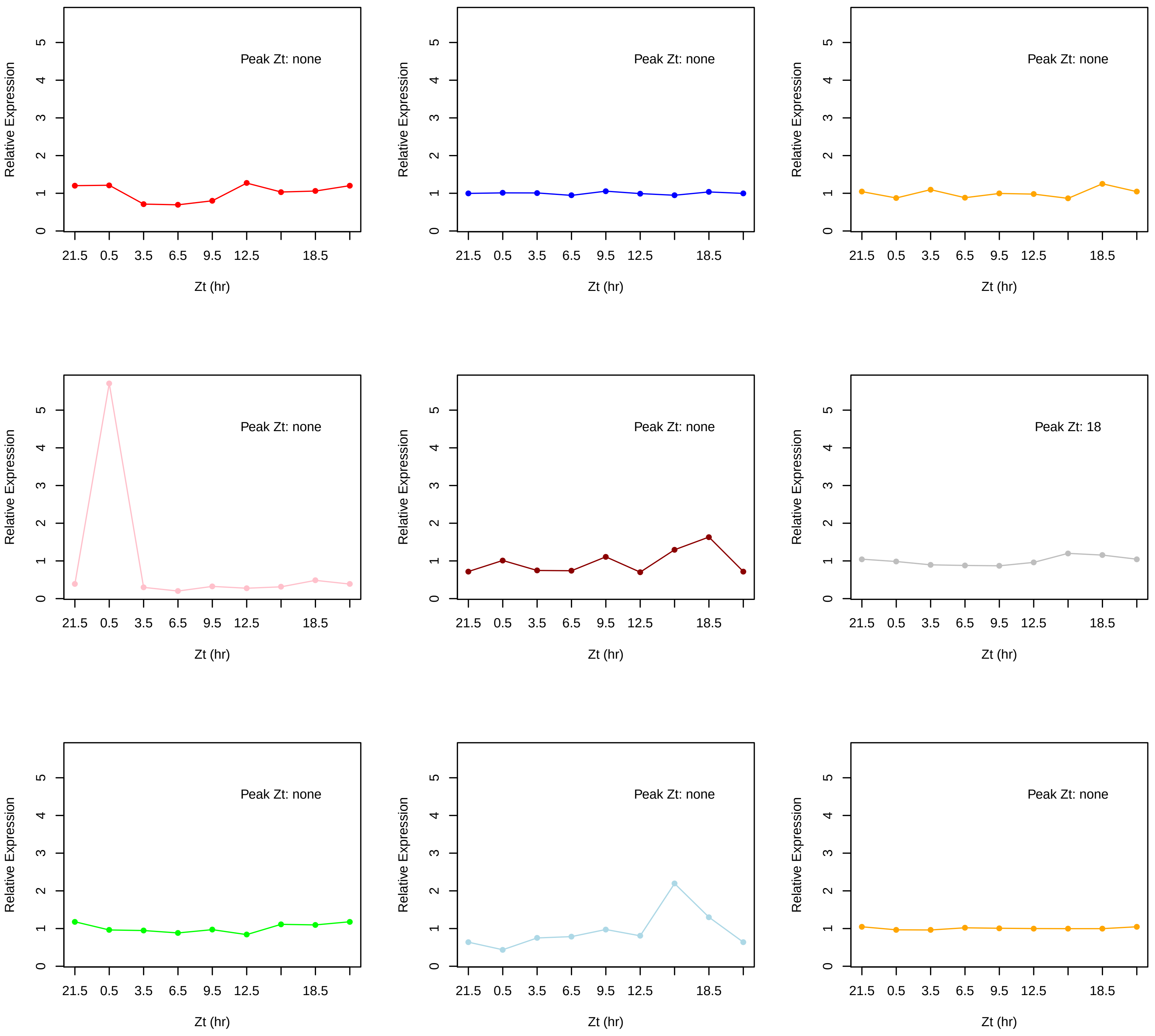

Singeleton: ENSXMAG00000022051 slco4a1

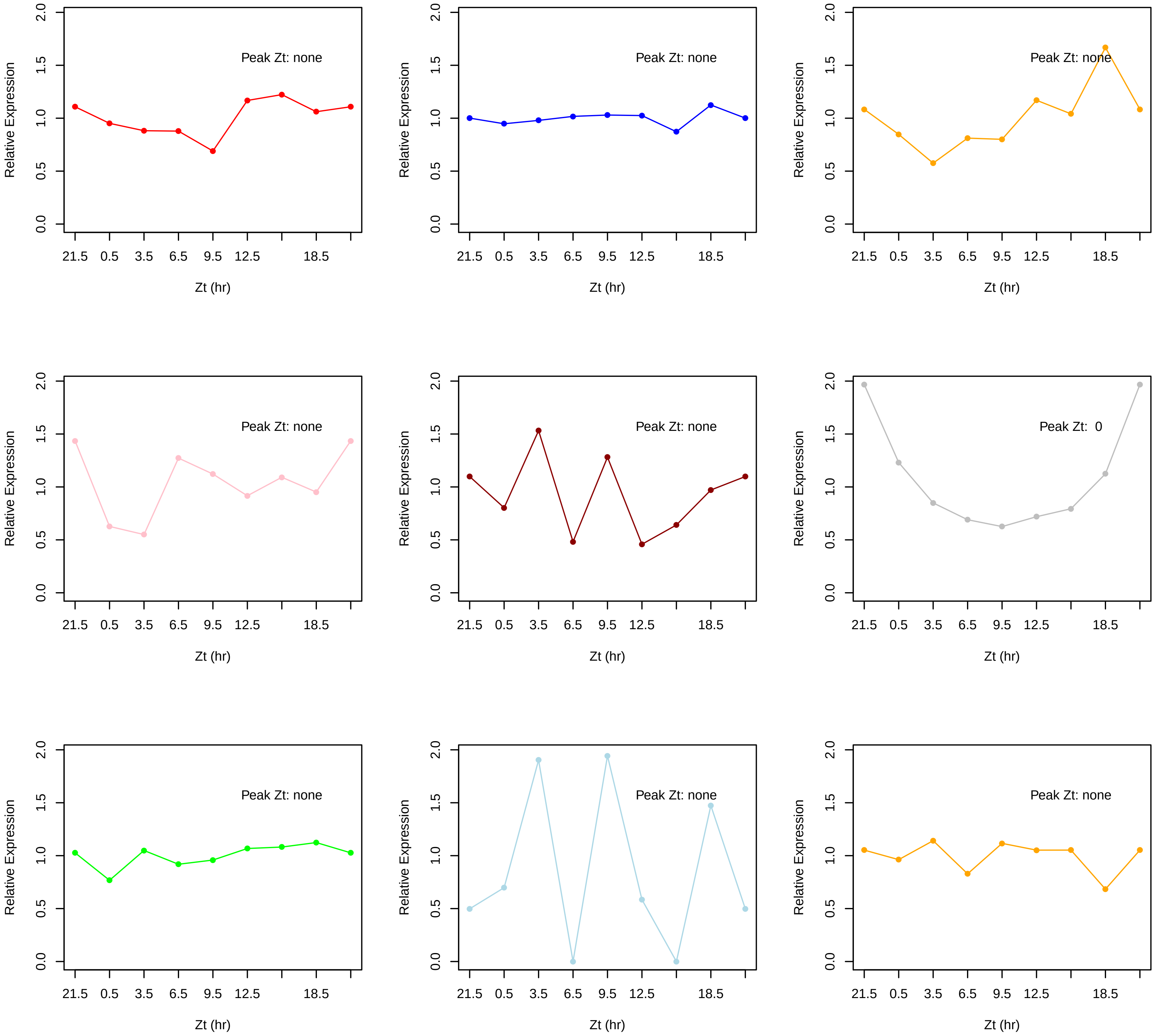

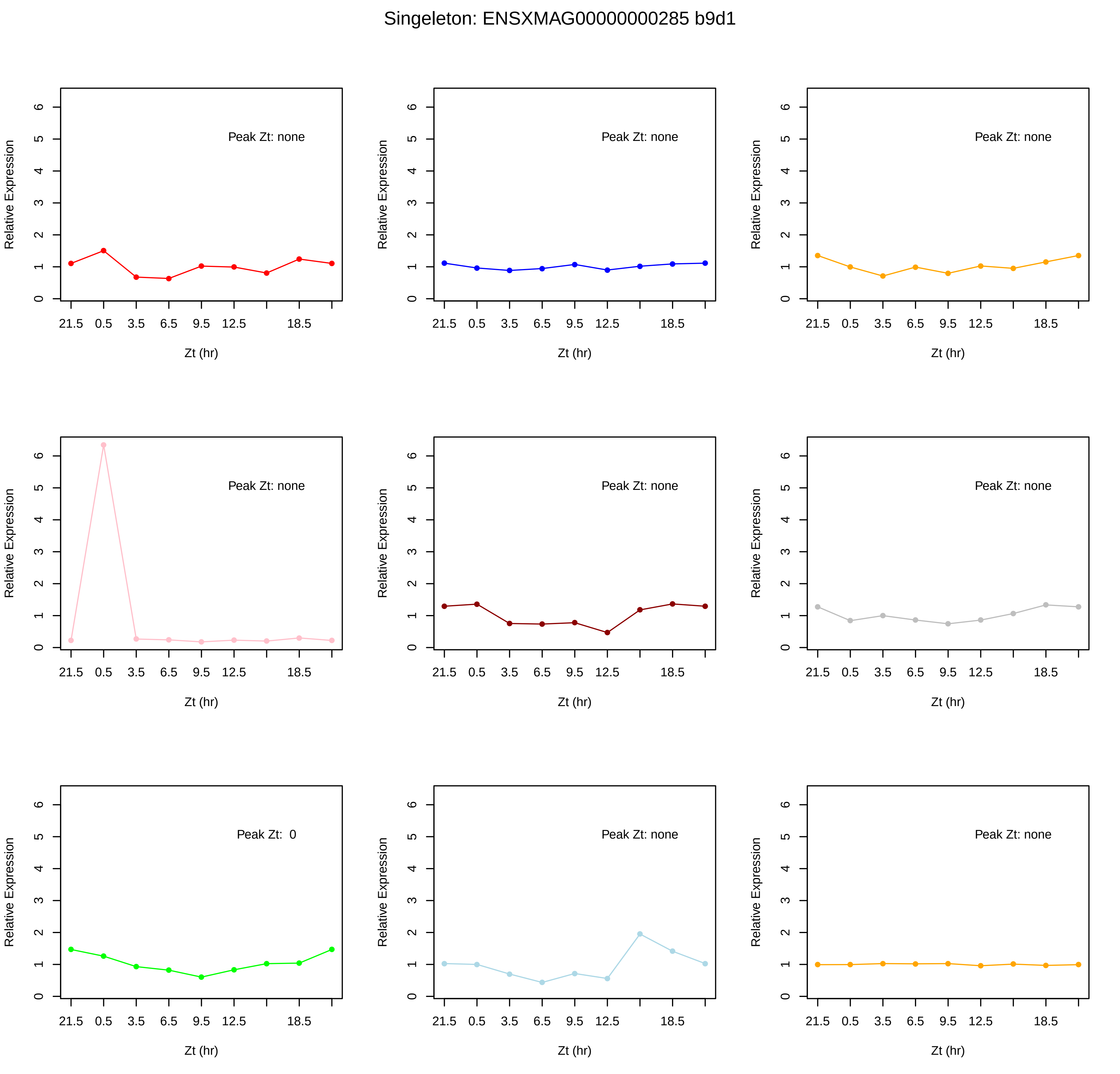
Singeleton: ENSXMAG00000000285 b9d1
Peak Zt: none
Peak Zt: 0
Relative Expression
Zt (hr)
21.5 0.5 3.5 6.5 9.5 12.5 18.5

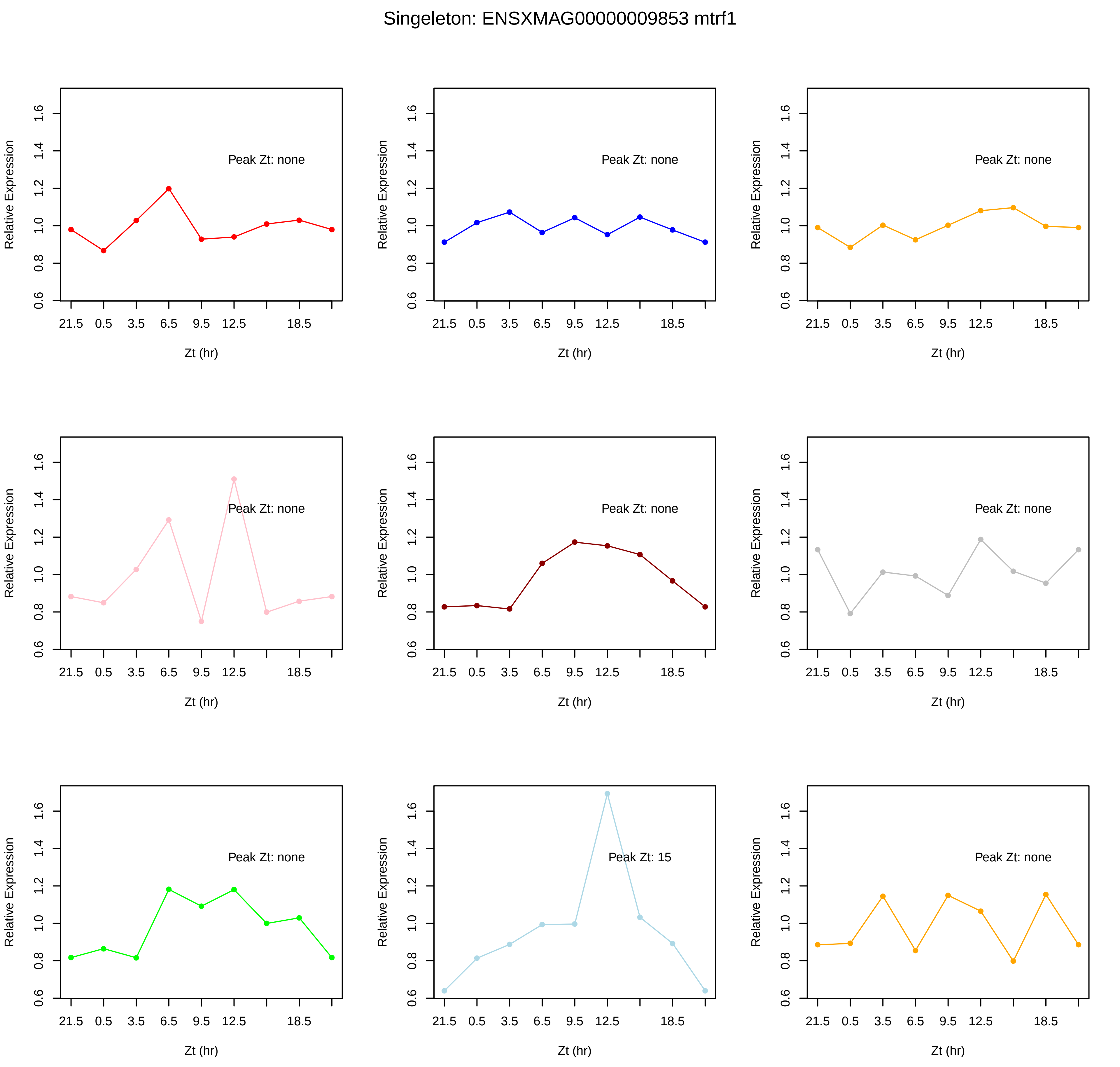

Singeleton: ENSXMAG00000009853 mtrf1
Relative Expression
Zt (hr)
0.6
0.8
1.0
1.2
1.4
1.6
21.5
0.5
3.5
6.5
9.5
12.5
18.5
Peak Zt: none
Peak Zt: 15

Singeleton: ENSXMAG00000001158 KCNV1

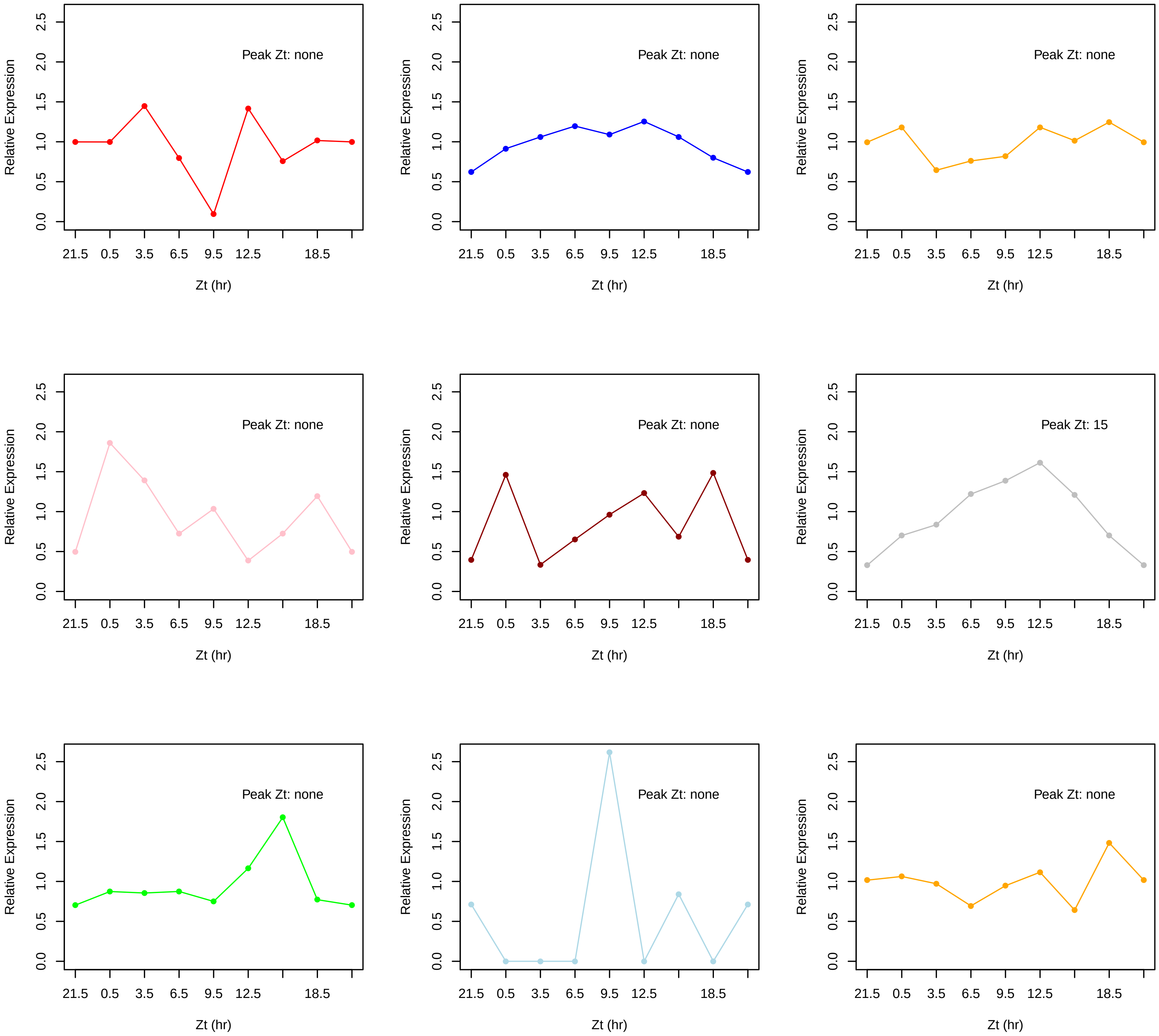

Singeleton: ENSXMAG00000019006 sntg1

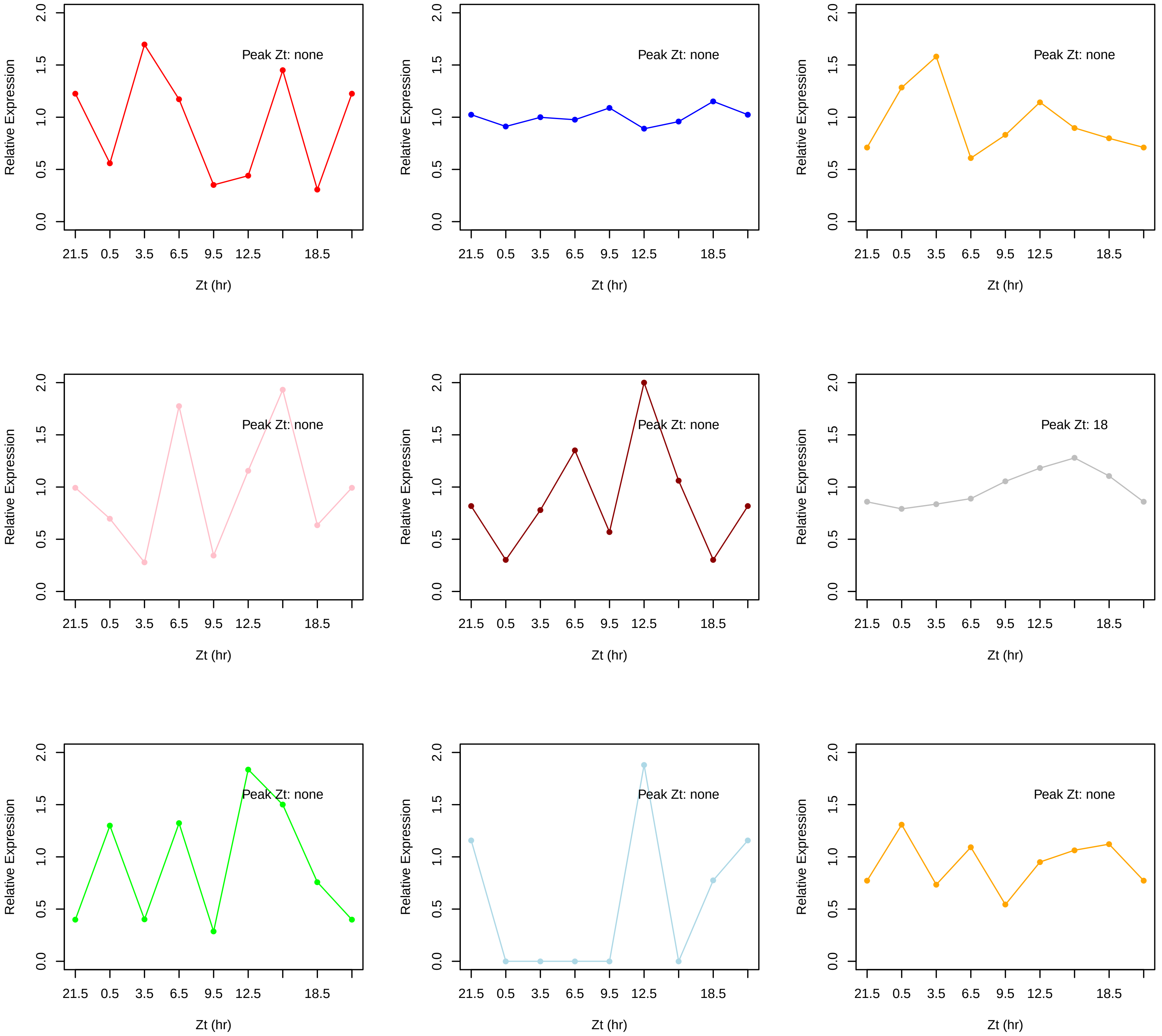

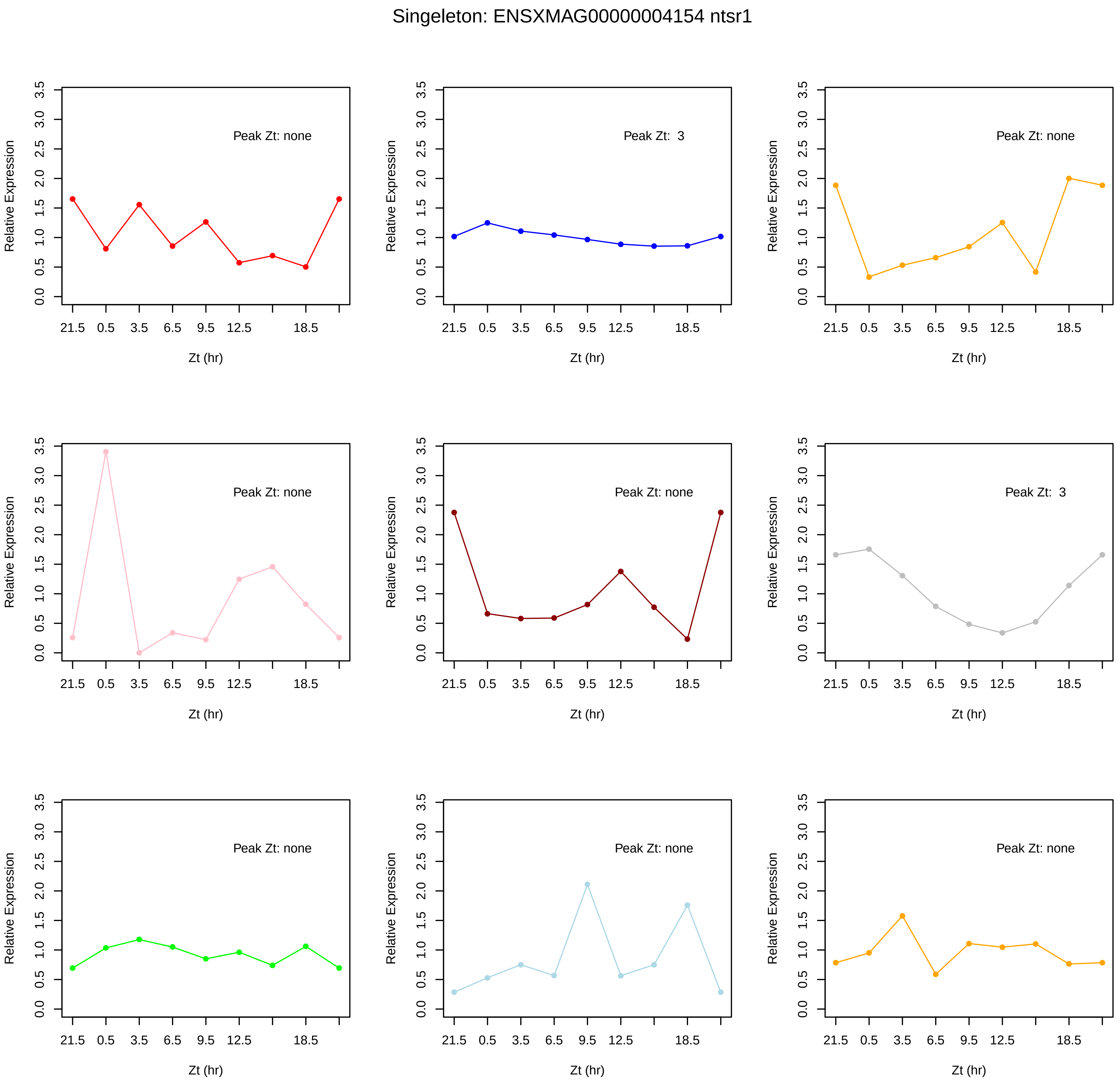
Singeleton: ENSXMAG00000004154 ntsr1
Peak Zt: none
Peak Zt: 3
Peak Zt: none
Peak Zt: none
Peak Zt: none
Peak Zt: 3
Peak Zt: none
Peak Zt: none
Peak Zt: none
Relative Expression
Zt (hr)
21.5
0.5
3.5
6.5
9.5
12.5
18.5
0.0
0.5
1.0
1.5
2.0
2.5
3.0
3.5

Singleton: ENSXMAG00000021529 foxq2

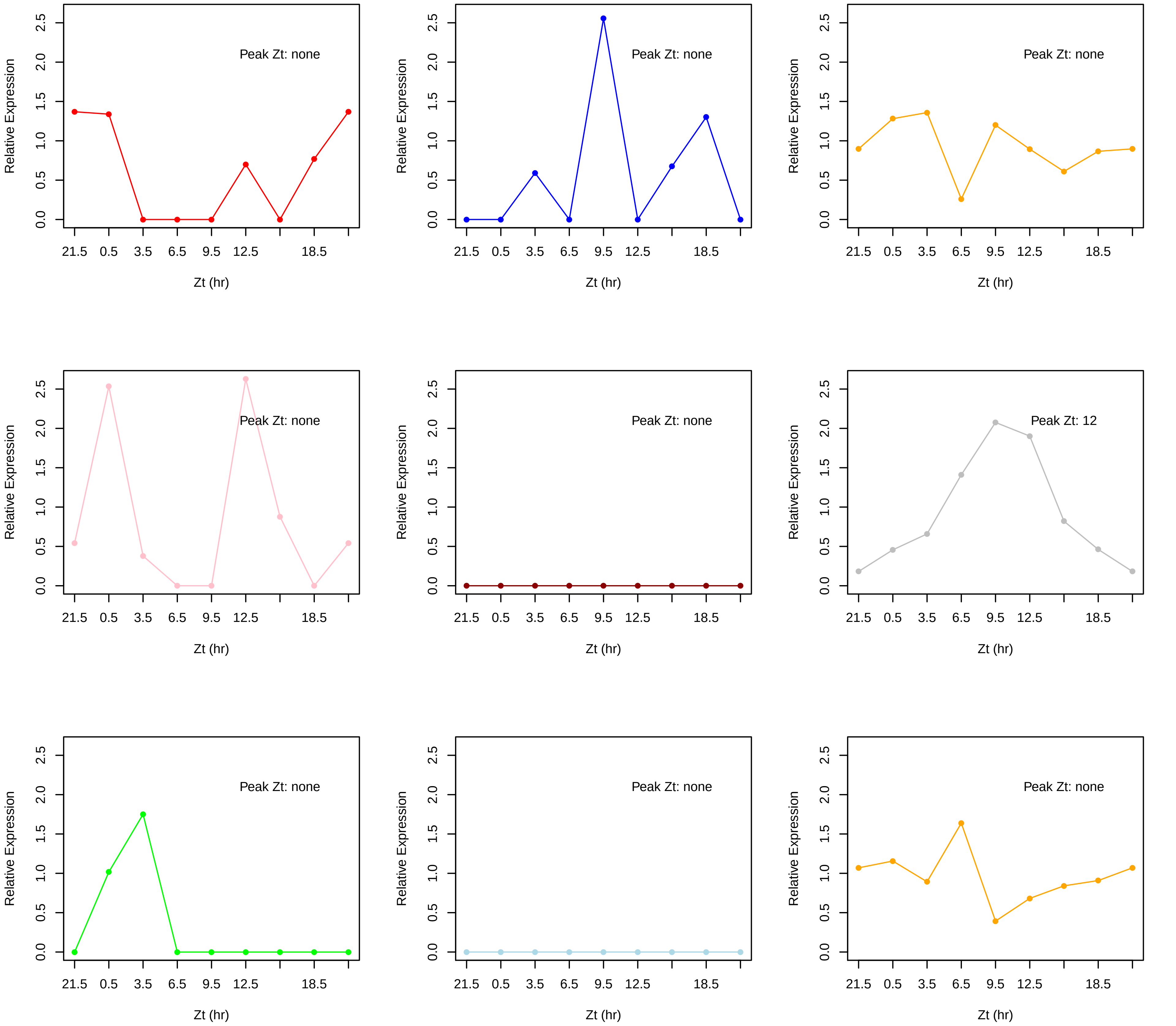

Singeleton: ENSXMAG00000015545 noc3l

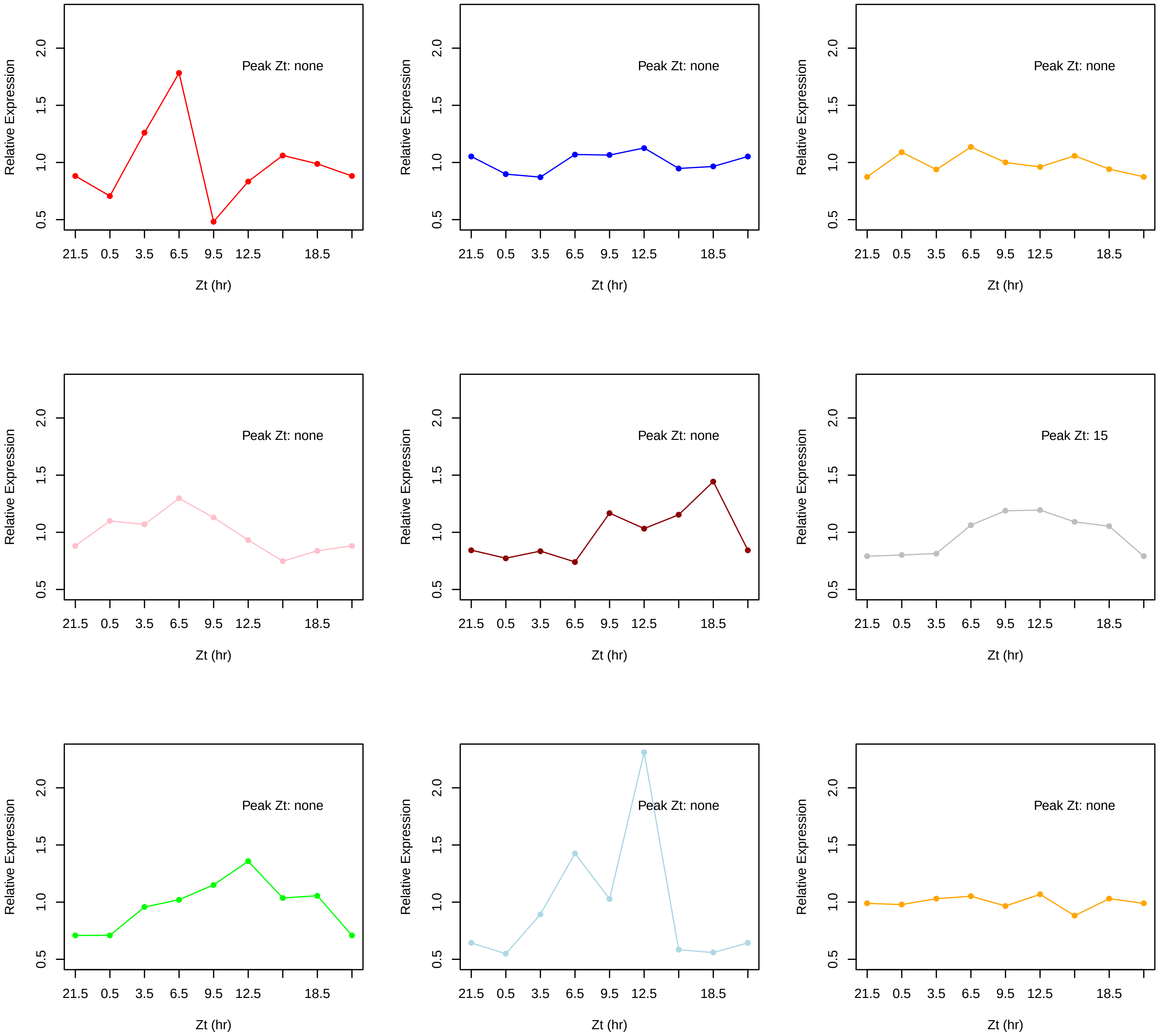

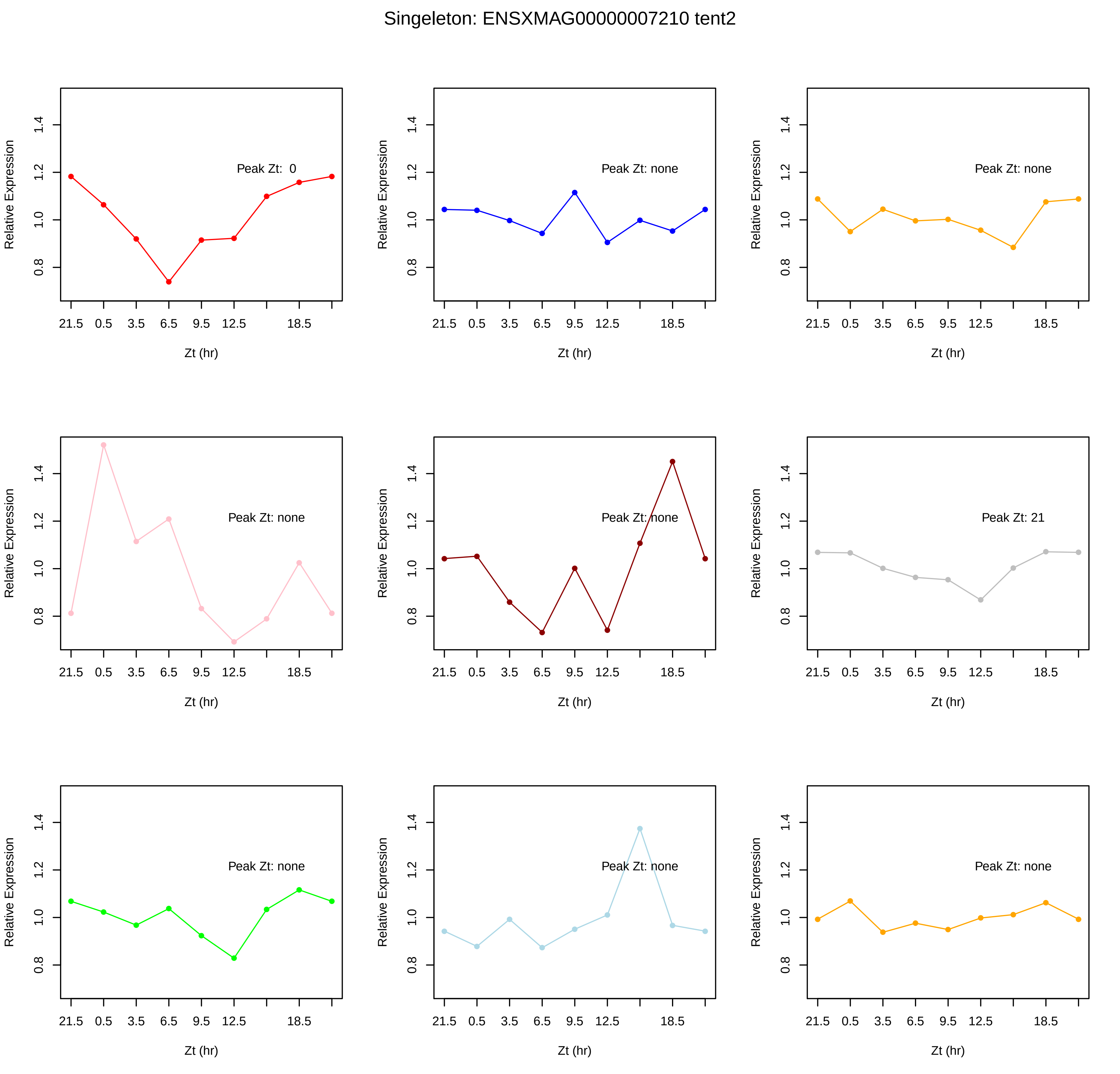
Singeleton: ENSXMAG00000007210 tent2
Peak Zt: 0
Peak Zt: none
Peak Zt: none
Peak Zt: none
Peak Zt: none
Peak Zt: 21
Peak Zt: none
Peak Zt: none
Peak Zt: none
Relative Expression
Zt (hr)
21.5
0.5
3.5
6.5
9.5
12.5
18.5
0.8
1.0
1.2
1.4

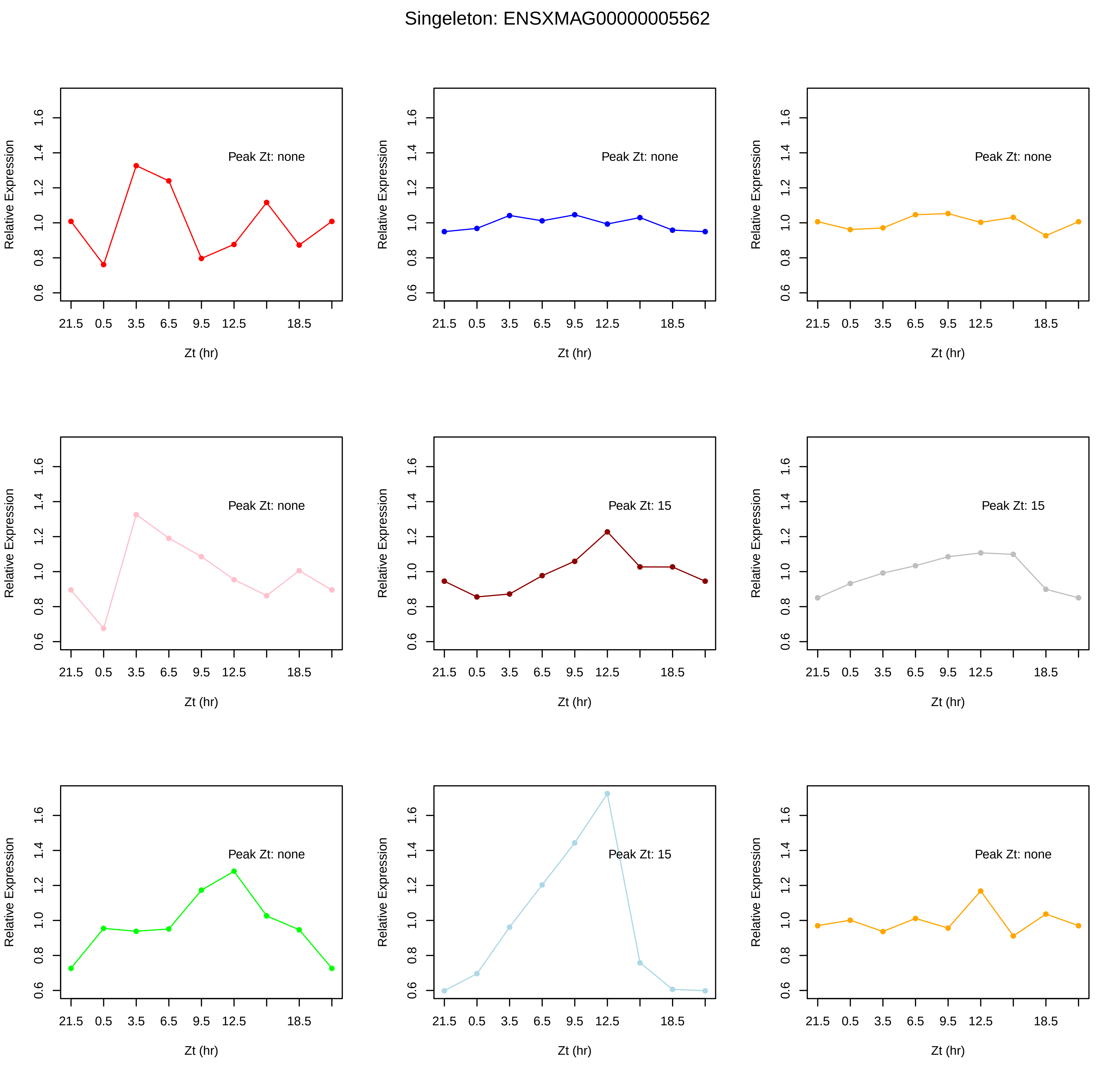
Singeleton: ENSXMAG00000005562
Peak Zt: none
Peak Zt: none
Peak Zt: none
Peak Zt: none
Peak Zt: 15
Peak Zt: 15
Peak Zt: none
Peak Zt: 15
Peak Zt: none
Relative Expression
Zt (hr)
21.5
0.5
3.5
6.5
9.5
12.5
18.5
0.6
0.8
1.0
1.2
1.4
1.6

Singleton: ENSXMAG00000010799 heatr6

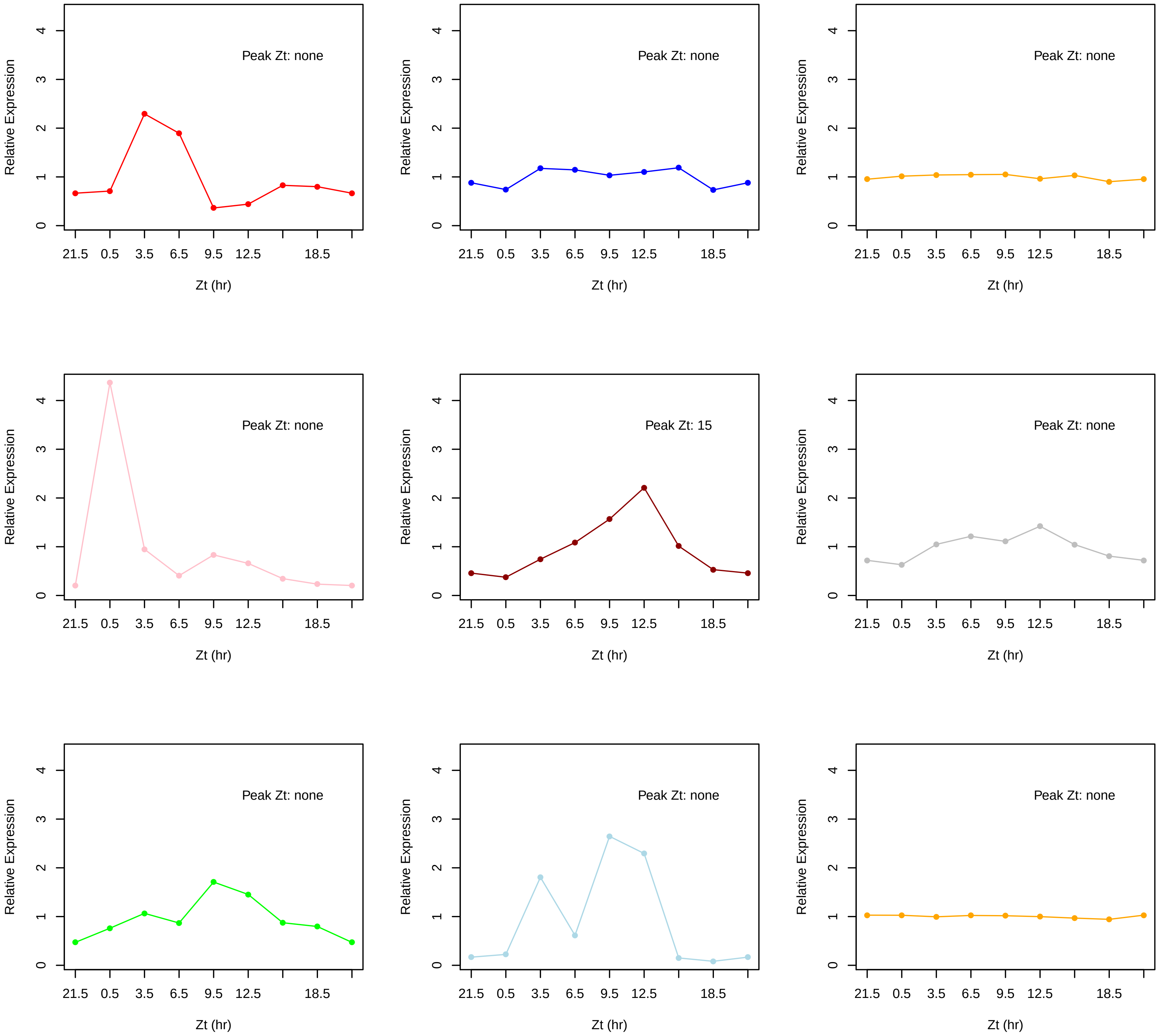

Singeleton: ENSXMAG00000025060 alg8

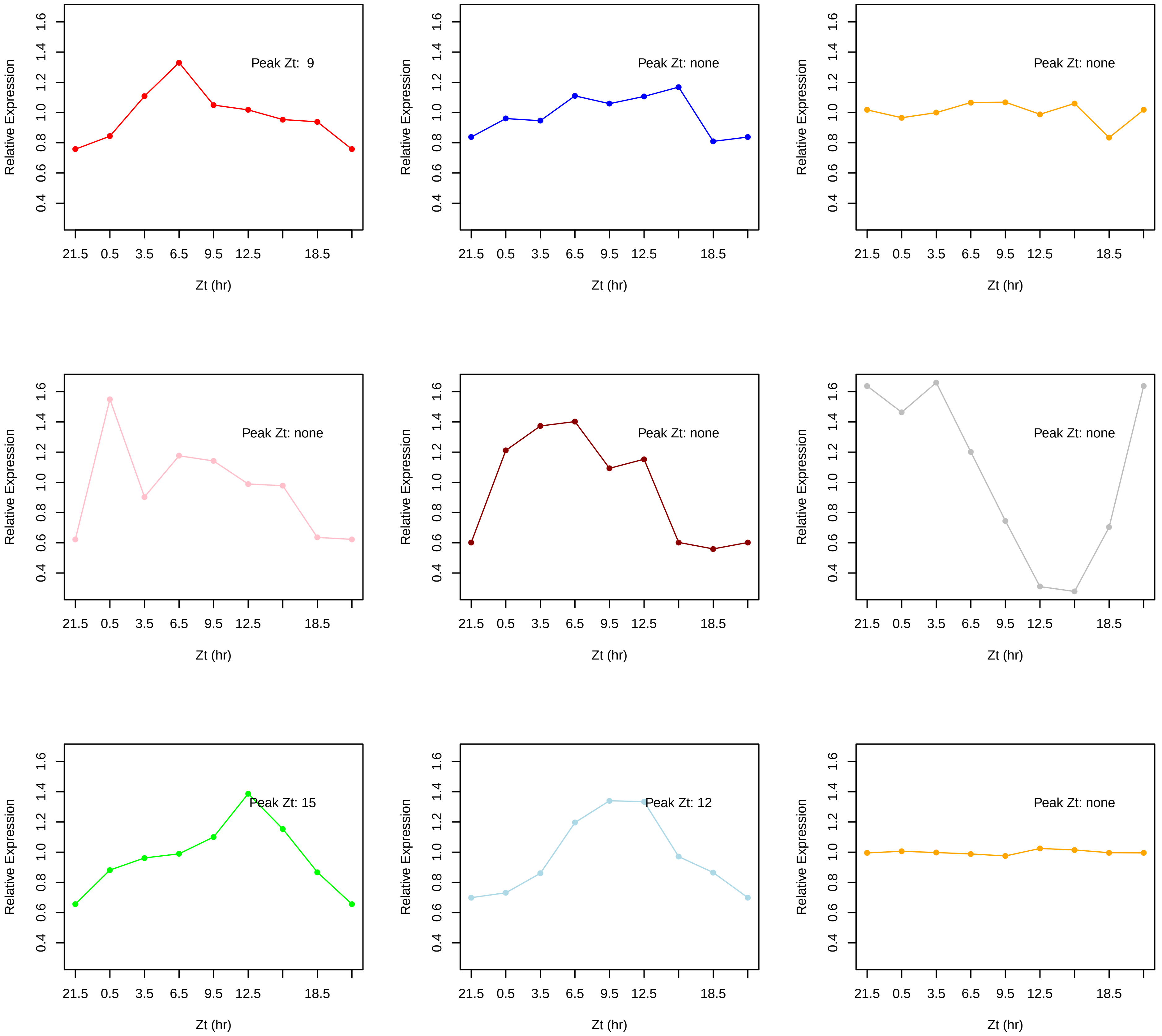

Singeleton: ENSXMAG00000012134 adgrg1

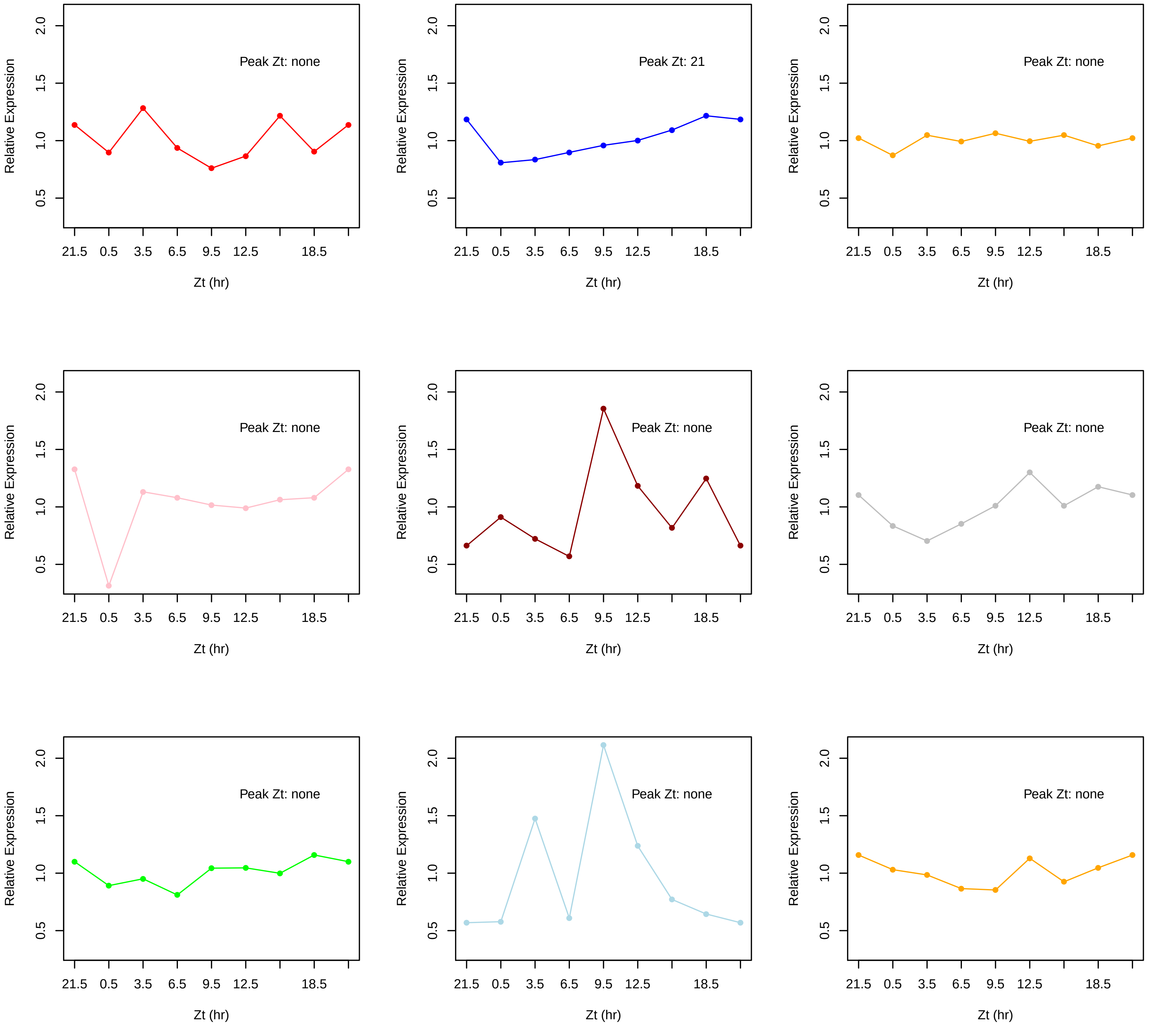

Singeleton: ENSXMAG00000028817 rspo1

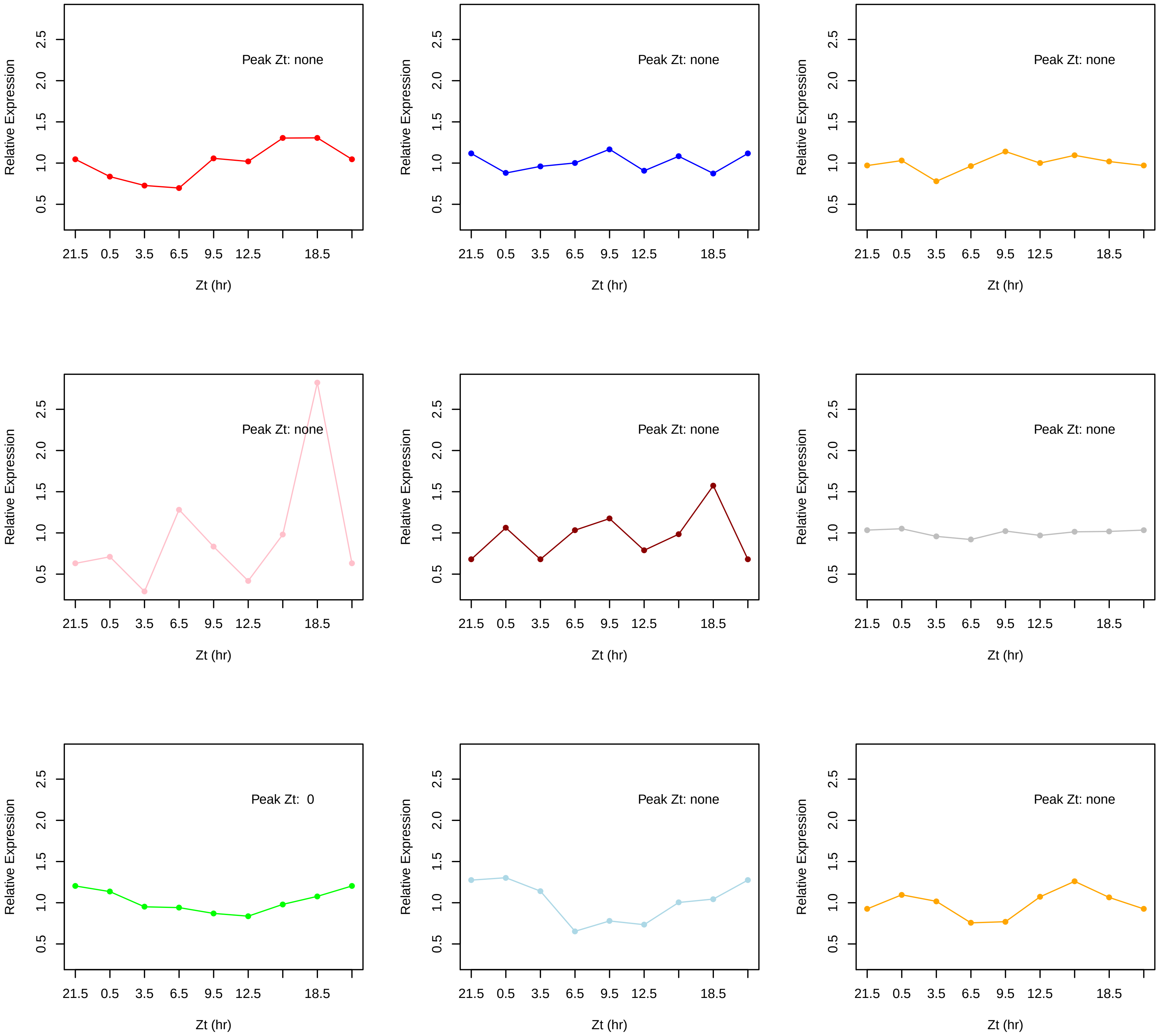

Singeleton: ENSXMAG00000021264 pias4a

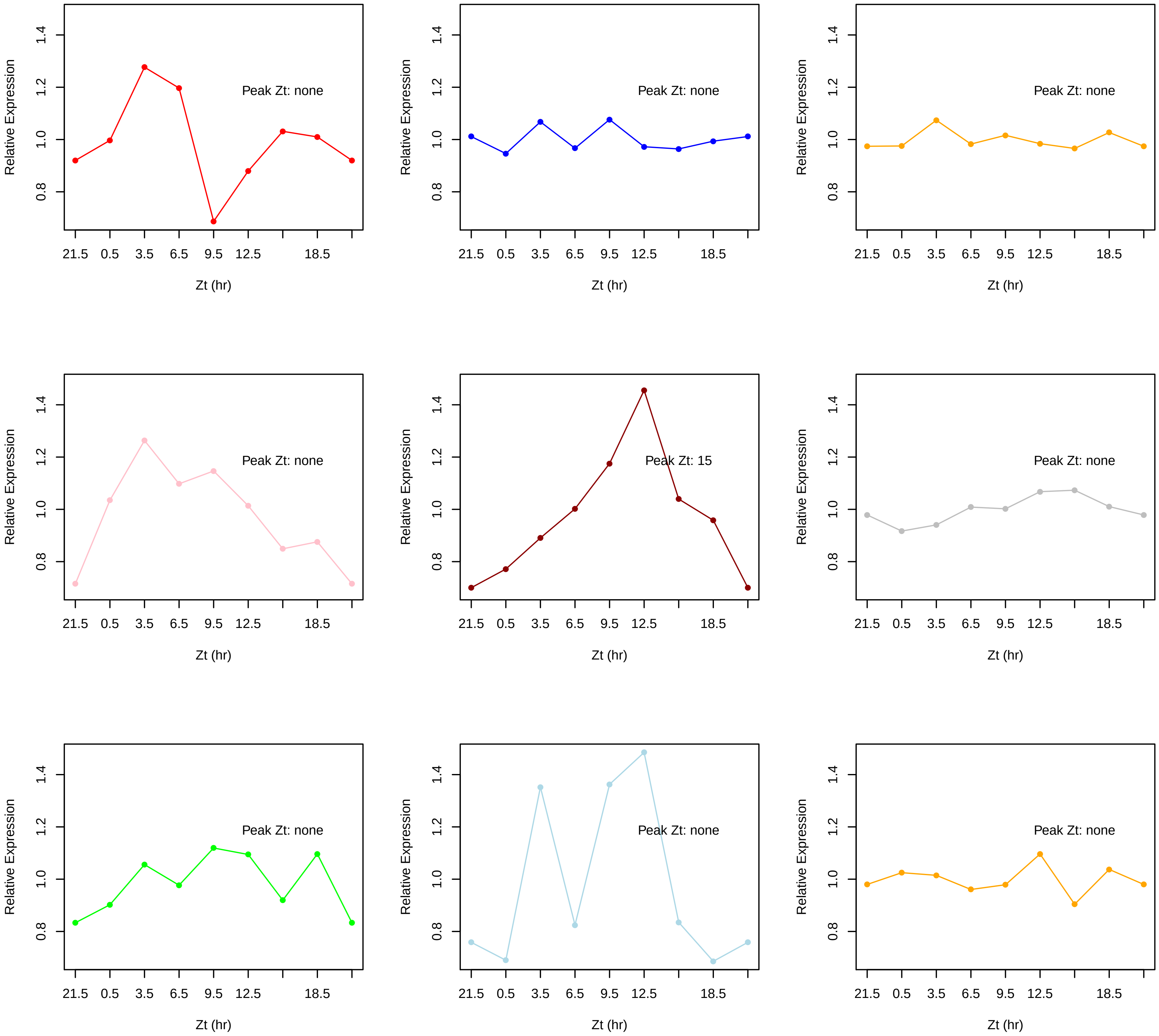

Singeleton: ENSXMAG00000011383 tanc1b

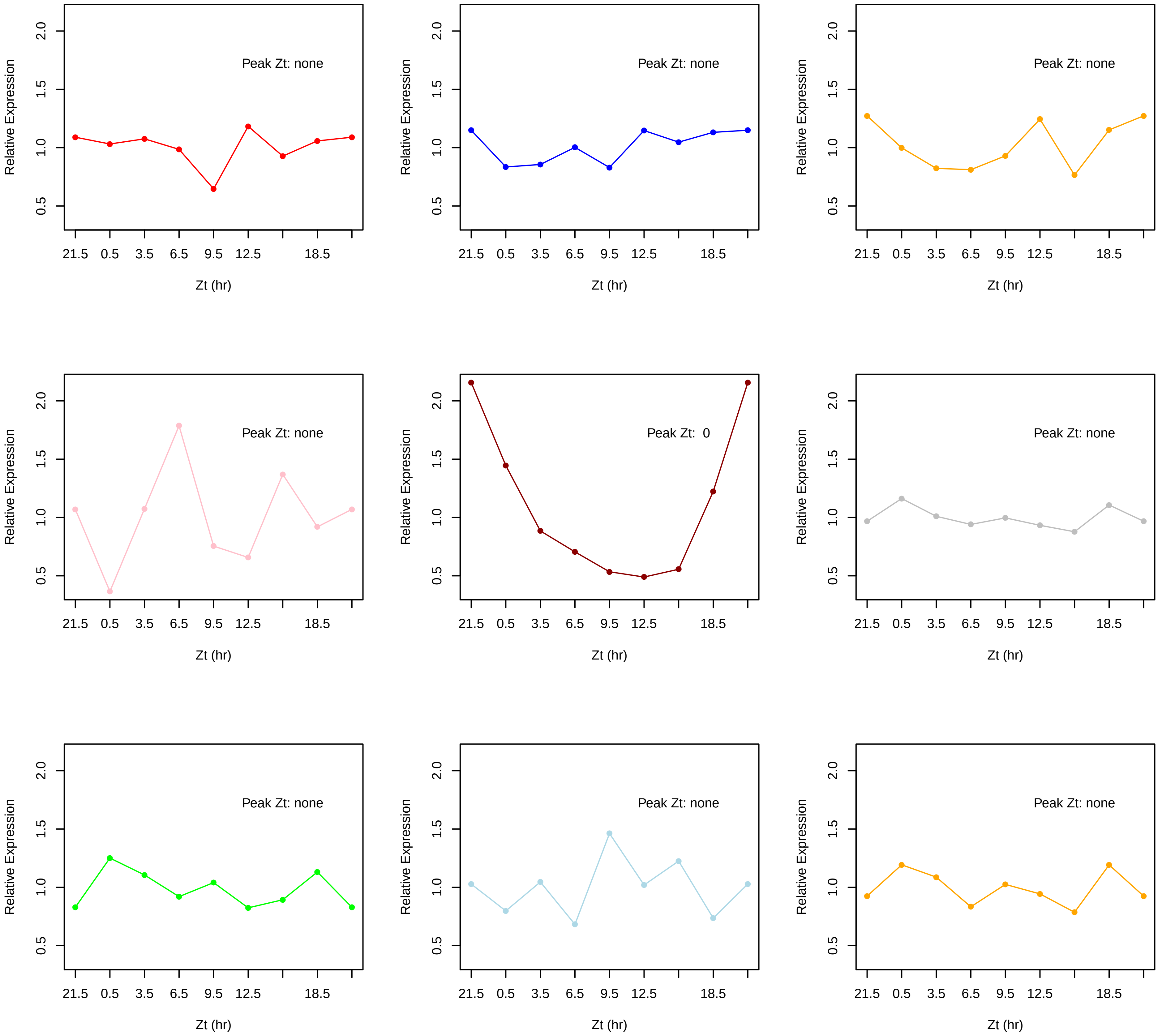

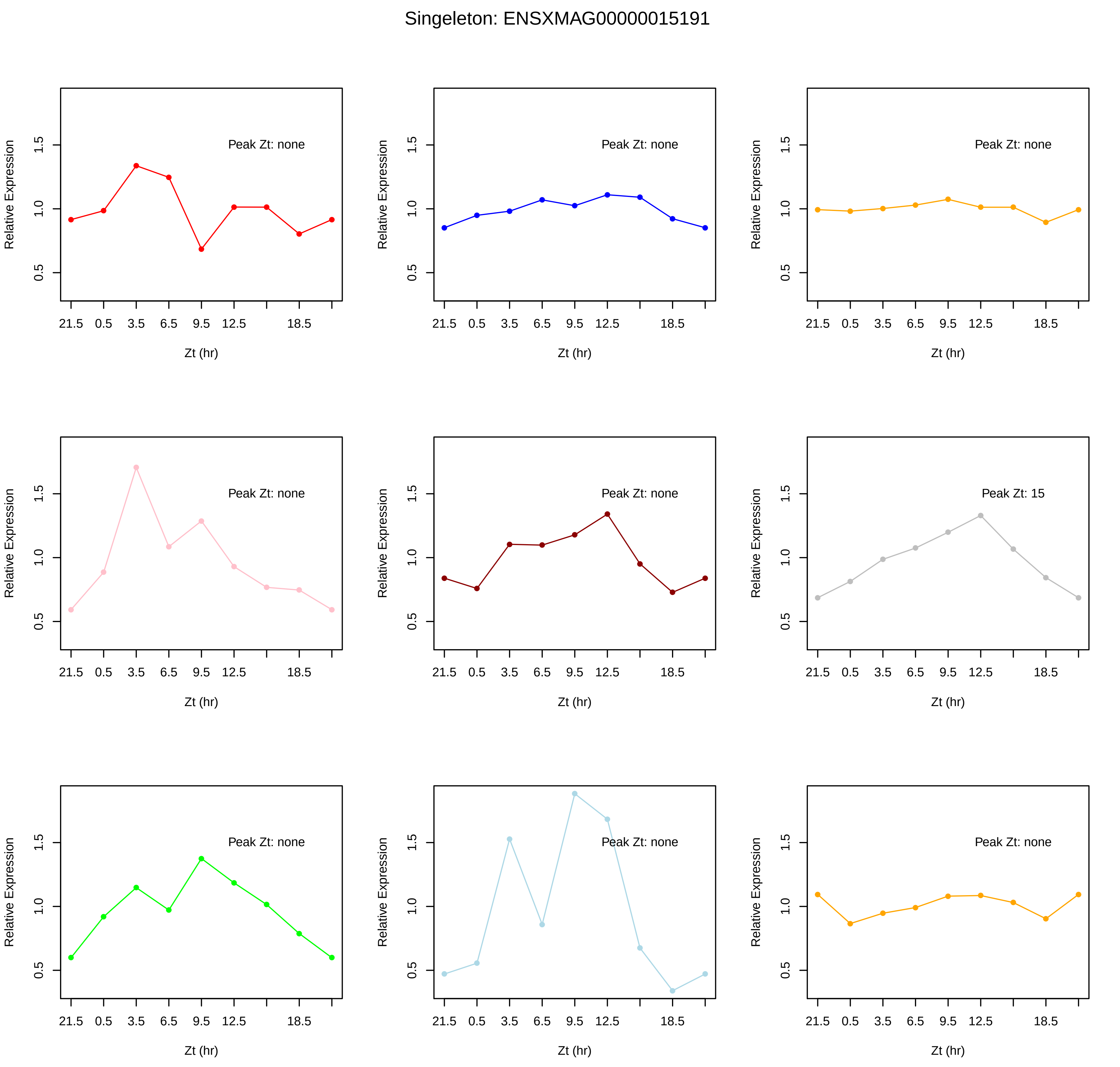
Singeleton: ENSXMAG00000015191
Peak Zt: none
Peak Zt: none
Peak Zt: none
Peak Zt: none
Peak Zt: none
Peak Zt: 15
Peak Zt: none
Peak Zt: none
Peak Zt: none
Relative Expression
Zt (hr)
21.5
0.5
3.5
6.5
9.5
12.5
18.5
0.5
1.0
1.5

Singleton: ENSXMAG00000007026 cdca8

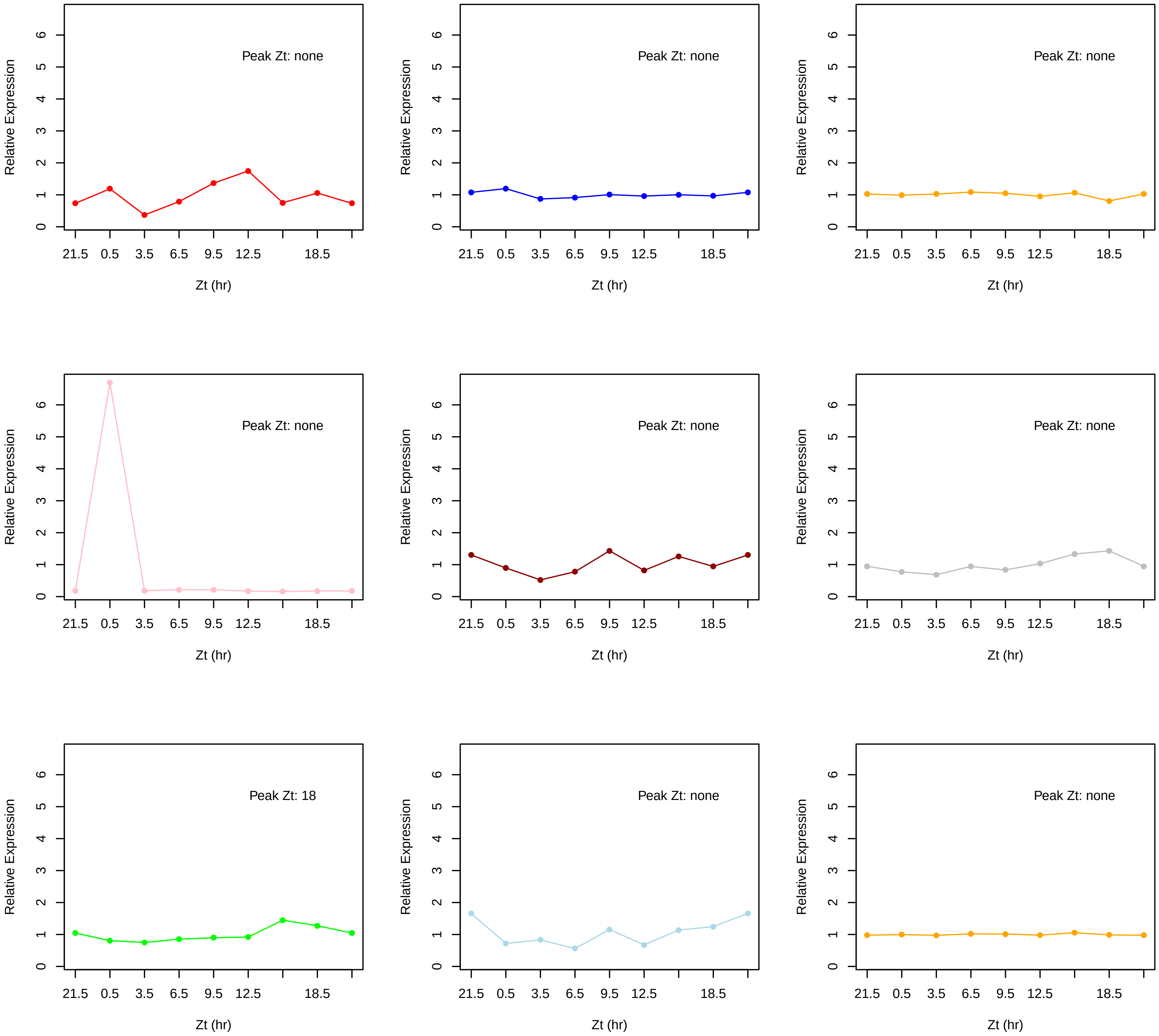

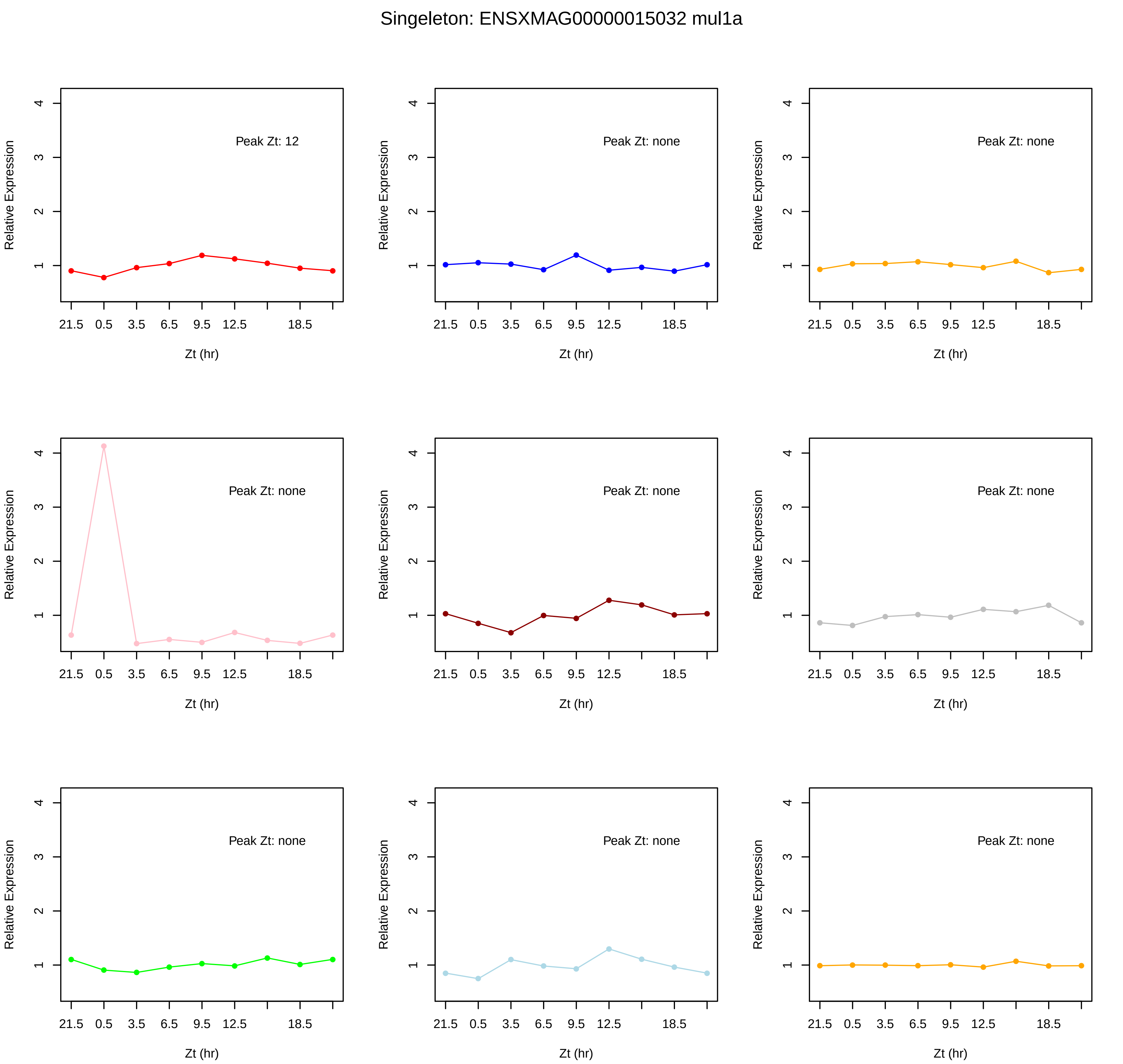
Singleton: ENSXMAG00000015032 mul1a
Peak Zt: 12
Peak Zt: none
Peak Zt: none
Peak Zt: none
Peak Zt: none
Peak Zt: none
Peak Zt: none
Peak Zt: none
Peak Zt: none
Relative Expression
Zt (hr)
21.5
0.5
3.5
6.5
9.5
12.5
18.5

Singeleton: ENSXMAG00000027295 rflna

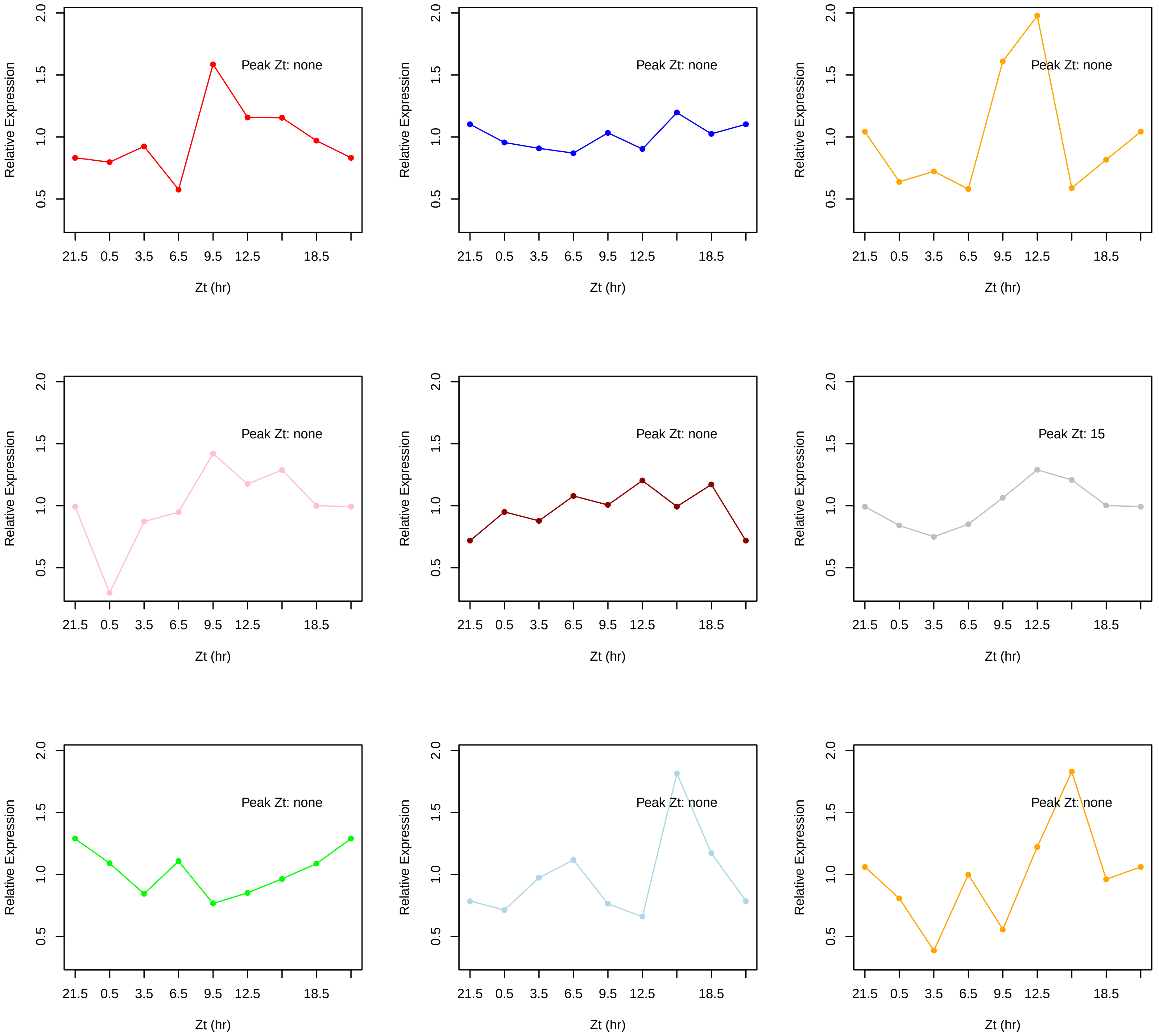

Singleton: ENSXMAG00000024196 prdx1

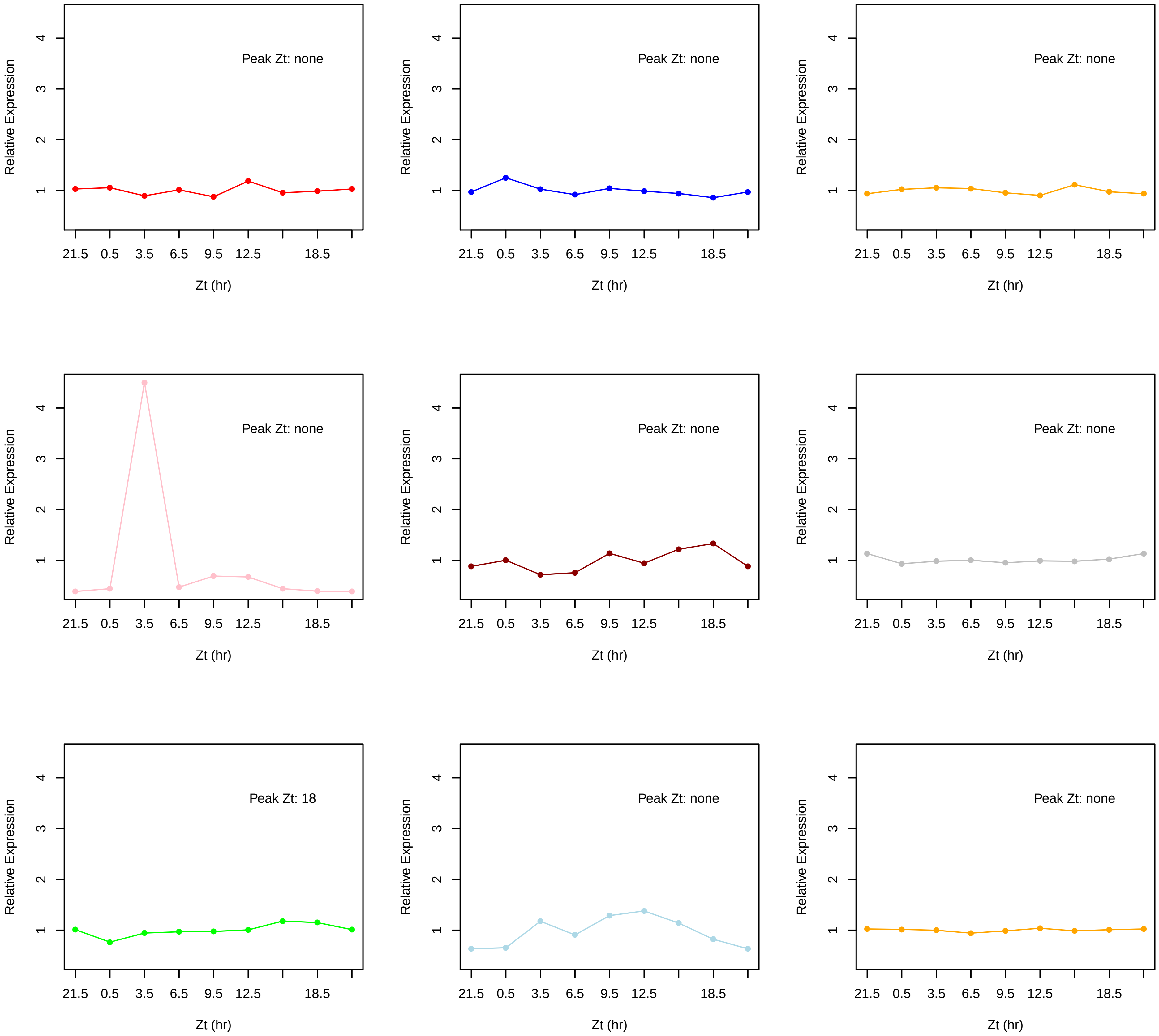

Singleton: ENSXMAG00000004886 ppp1r3ca

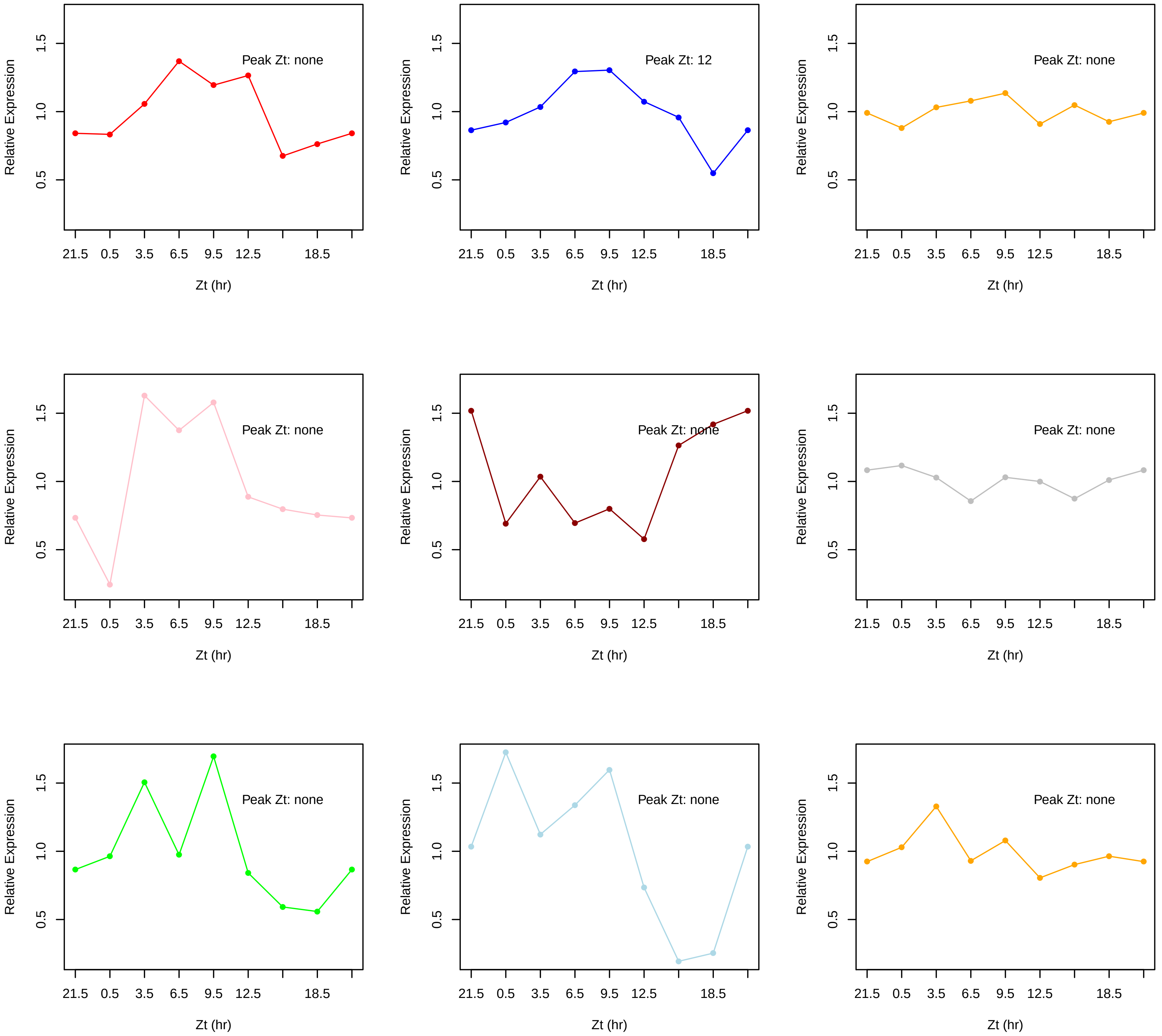

Singleton: ENSXMAG00000019038 ube2v2

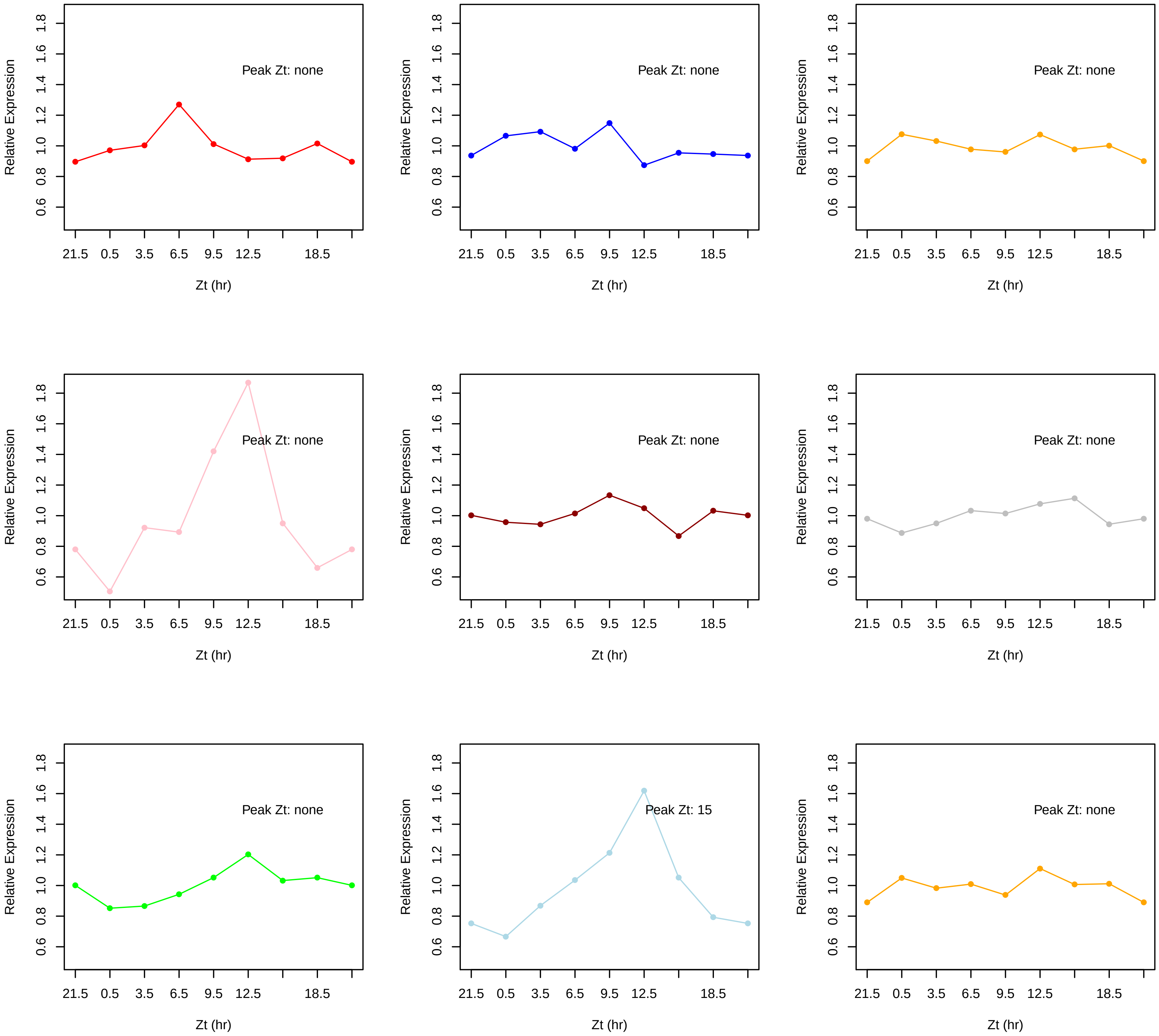

Singeleton: ENSXMAG00000010775 pigs

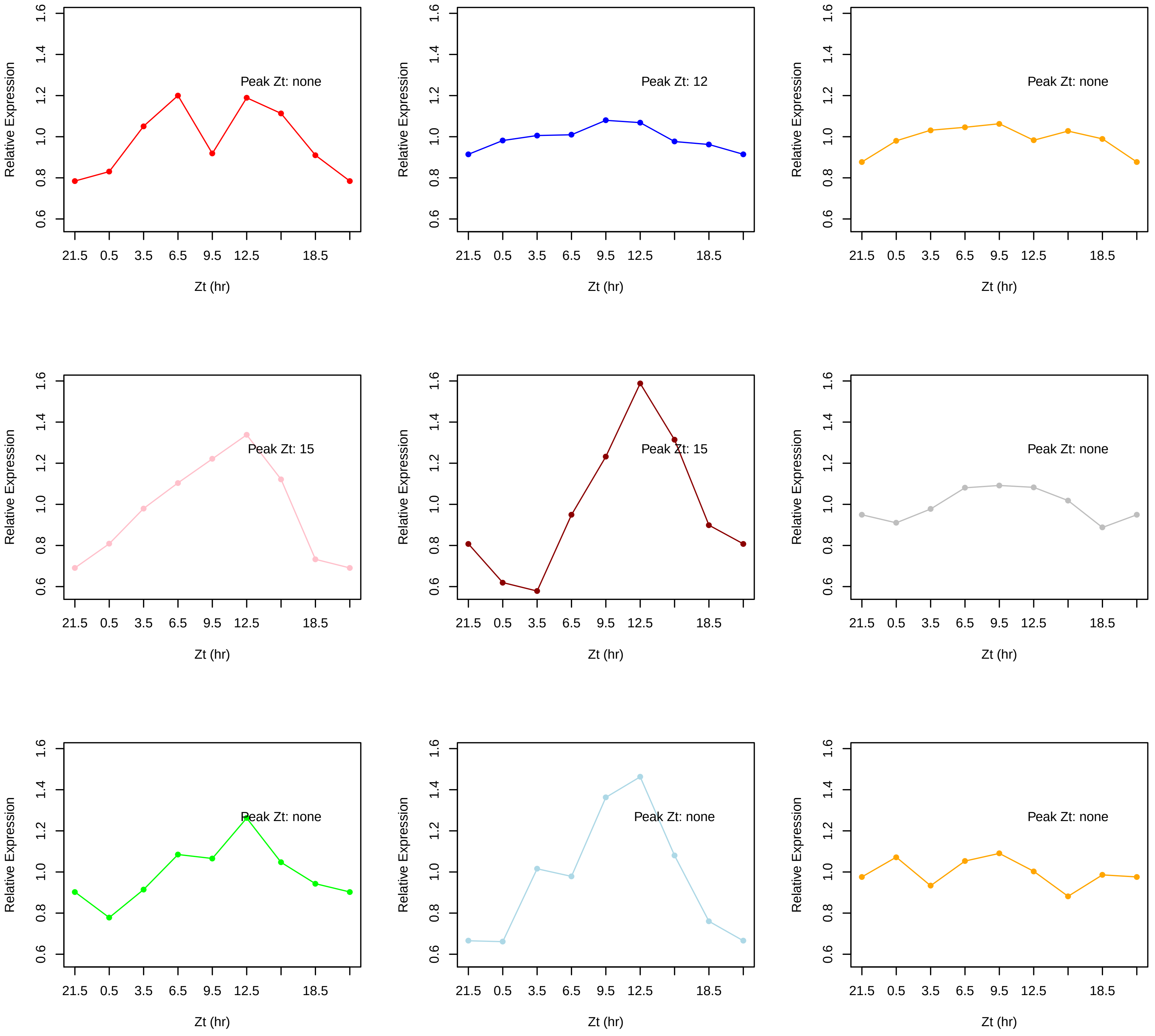

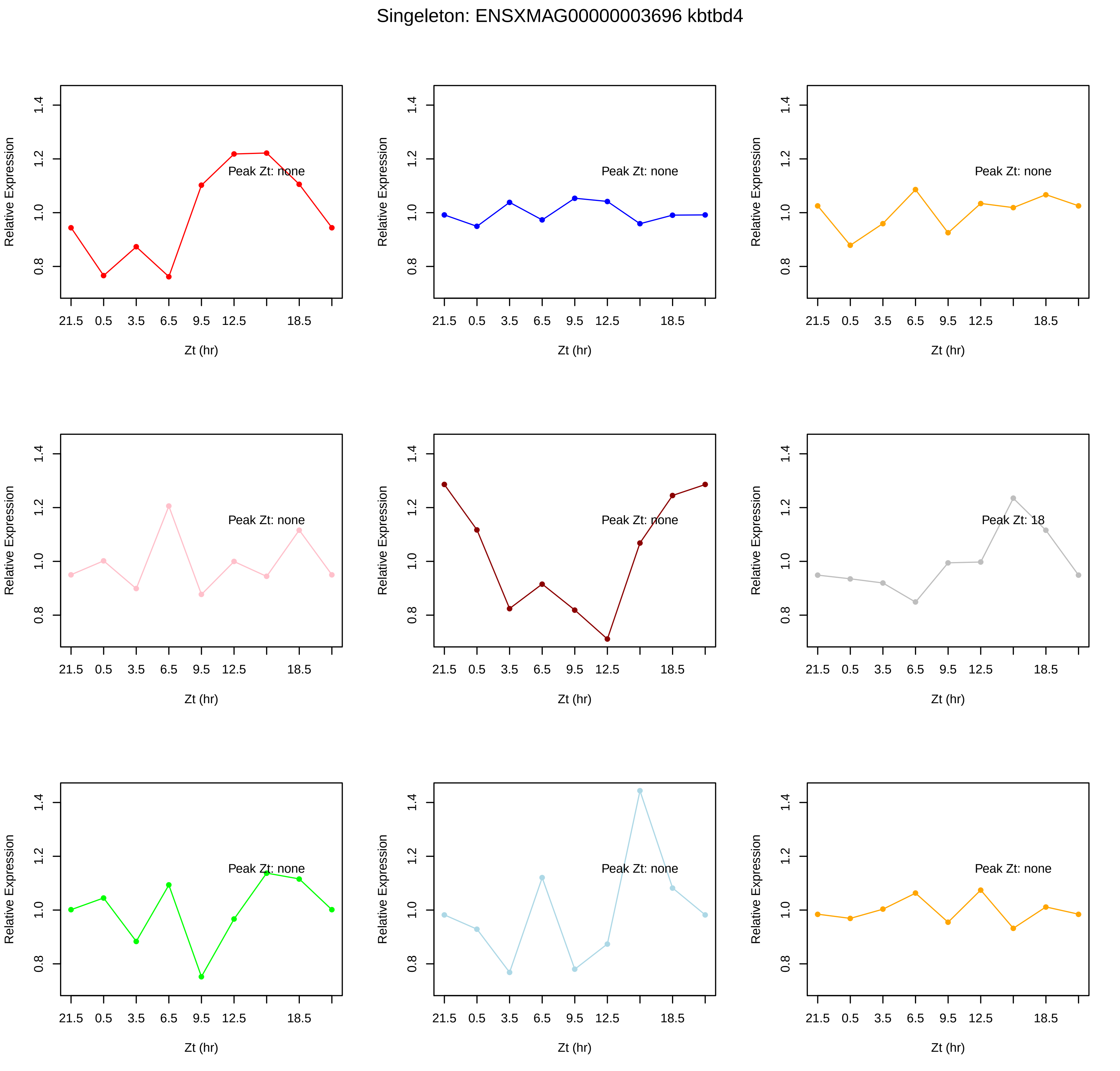

Singeleton: ENSXMAG00000003696 kbtbd4
Relative Expression
Zt (hr)
Peak Zt: none
Peak Zt: 18
21.5
0.5
3.5
6.5
9.5
12.5
18.5
0.8
1.0
1.2
1.4

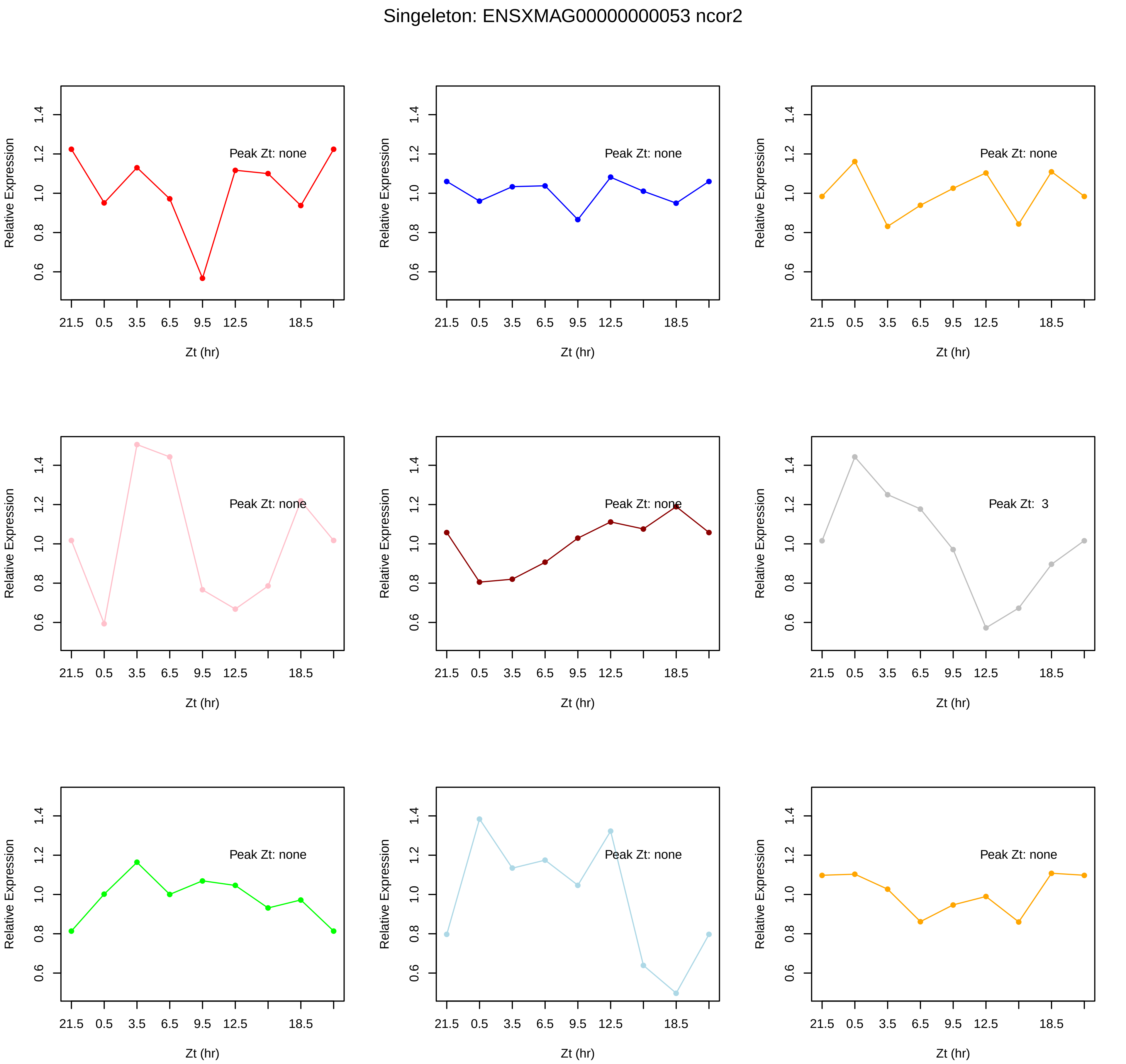
Singeleton: ENSXMAG00000000053 ncor2
Peak Zt: none
Peak Zt: none
Peak Zt: none
Peak Zt: none
Peak Zt: none
Peak Zt: 3
Peak Zt: none
Peak Zt: none
Peak Zt: none
Relative Expression
Zt (hr)
21.5
0.5
3.5
6.5
9.5
12.5
18.5
0.6
0.8
1.0
1.2
1.4

Singeleton: ENSXMAG00000001723 baz2ba

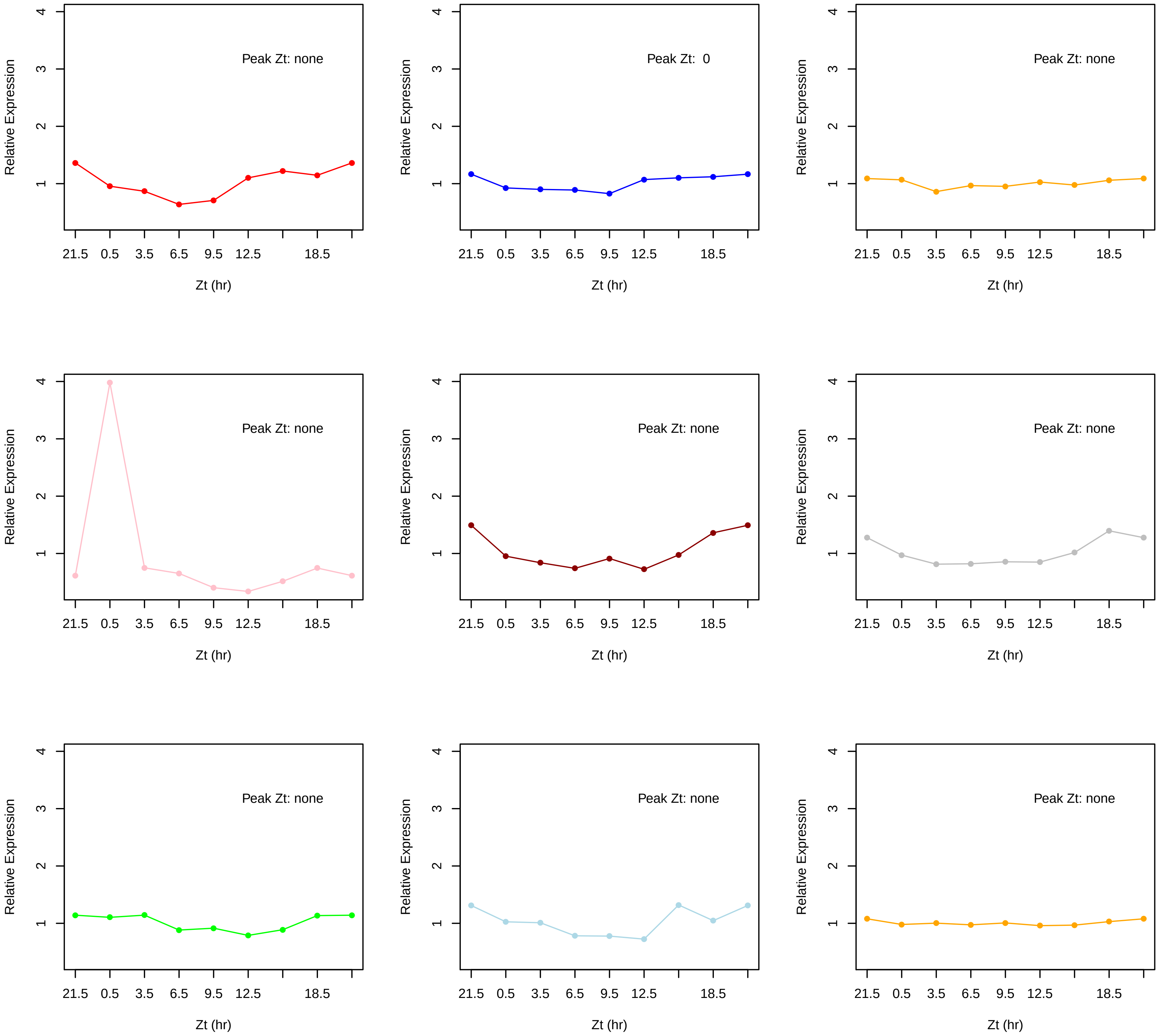

Singeleton: ENSXMAG00000002845 seta

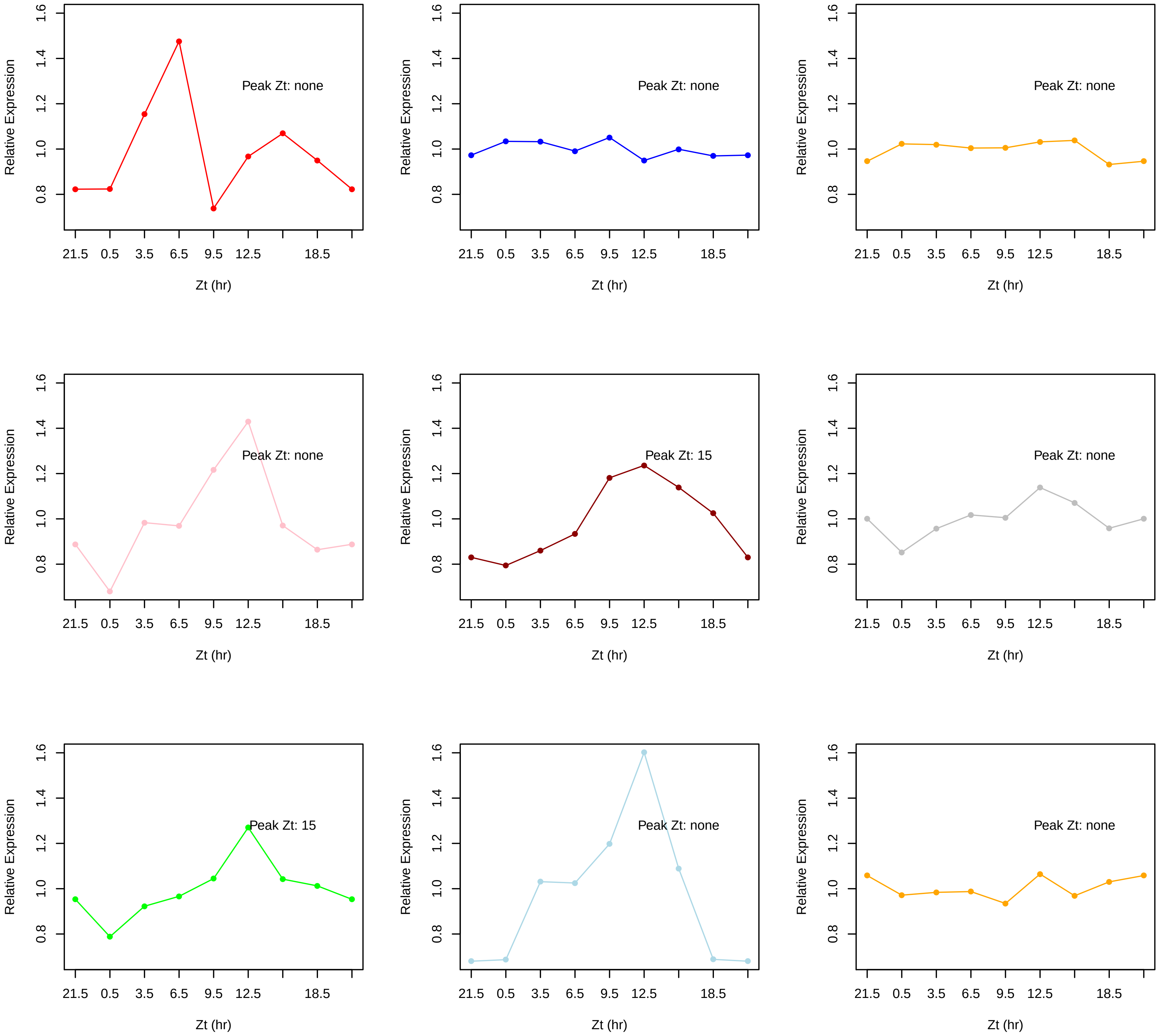

Singleton: ENSXMAG00000027001 si:dkeyp−94h10.5

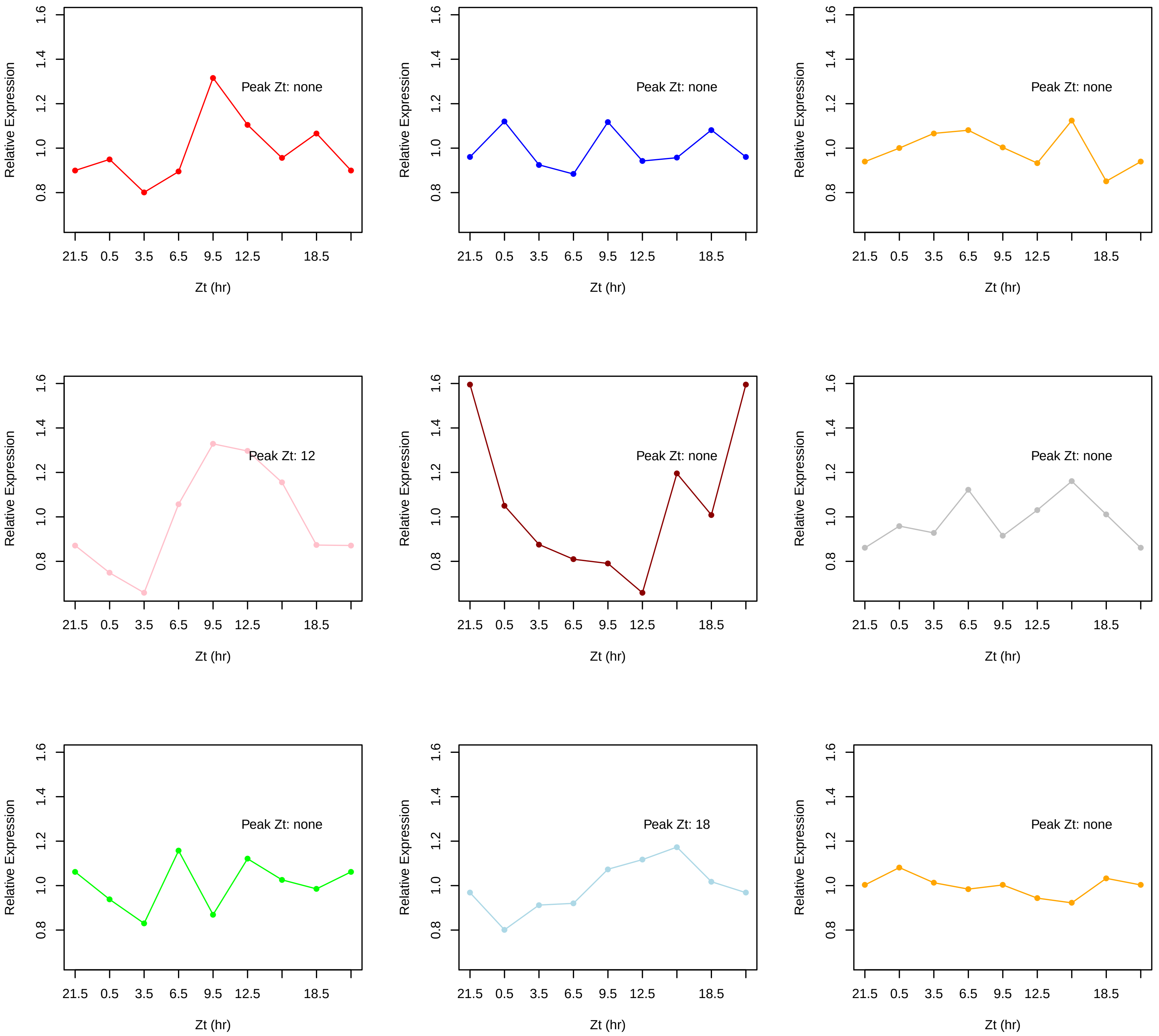

Singeleton: ENSXMAG00000011150

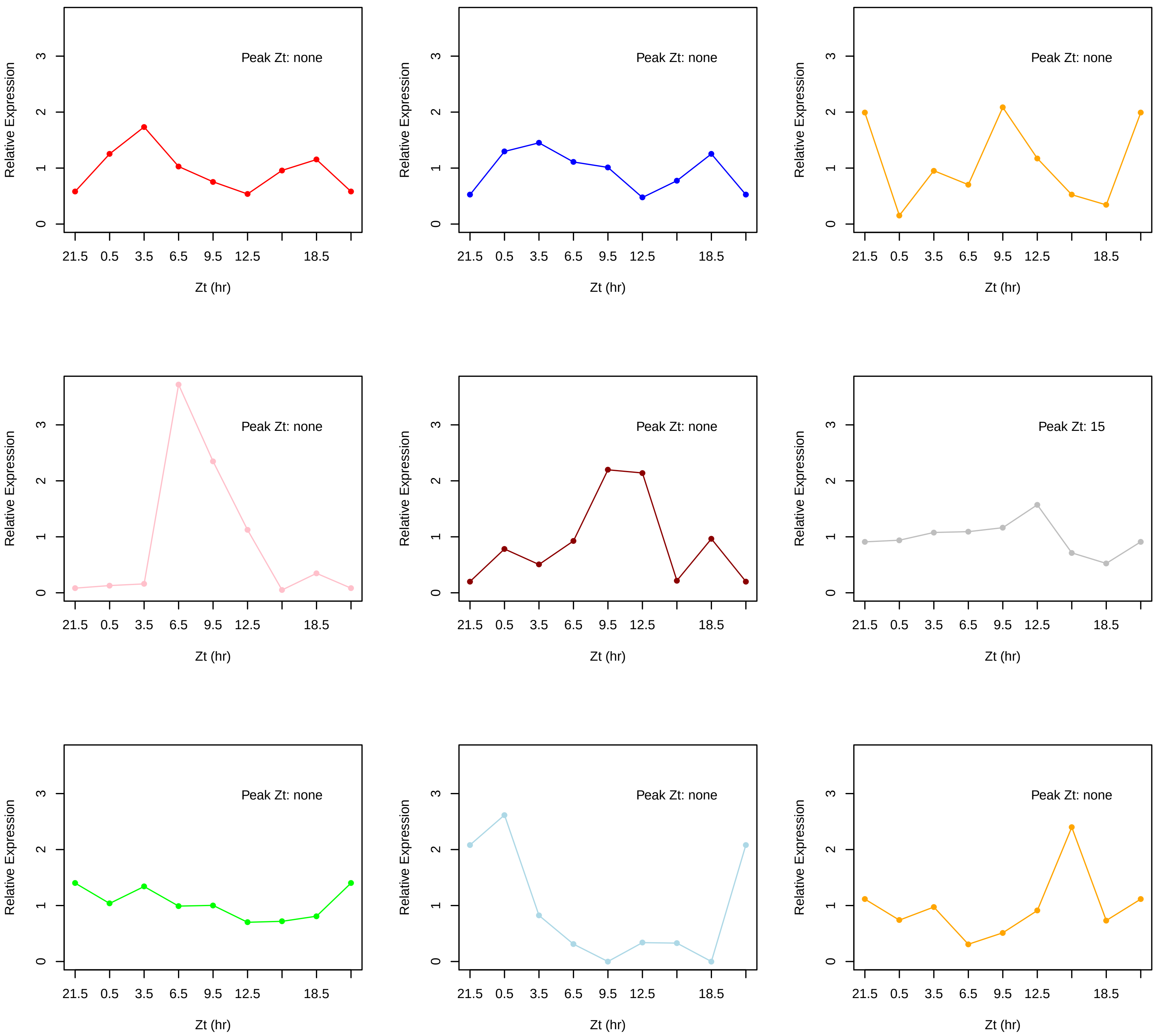

Singeleton: ENSXMAG00000010758 aldocb

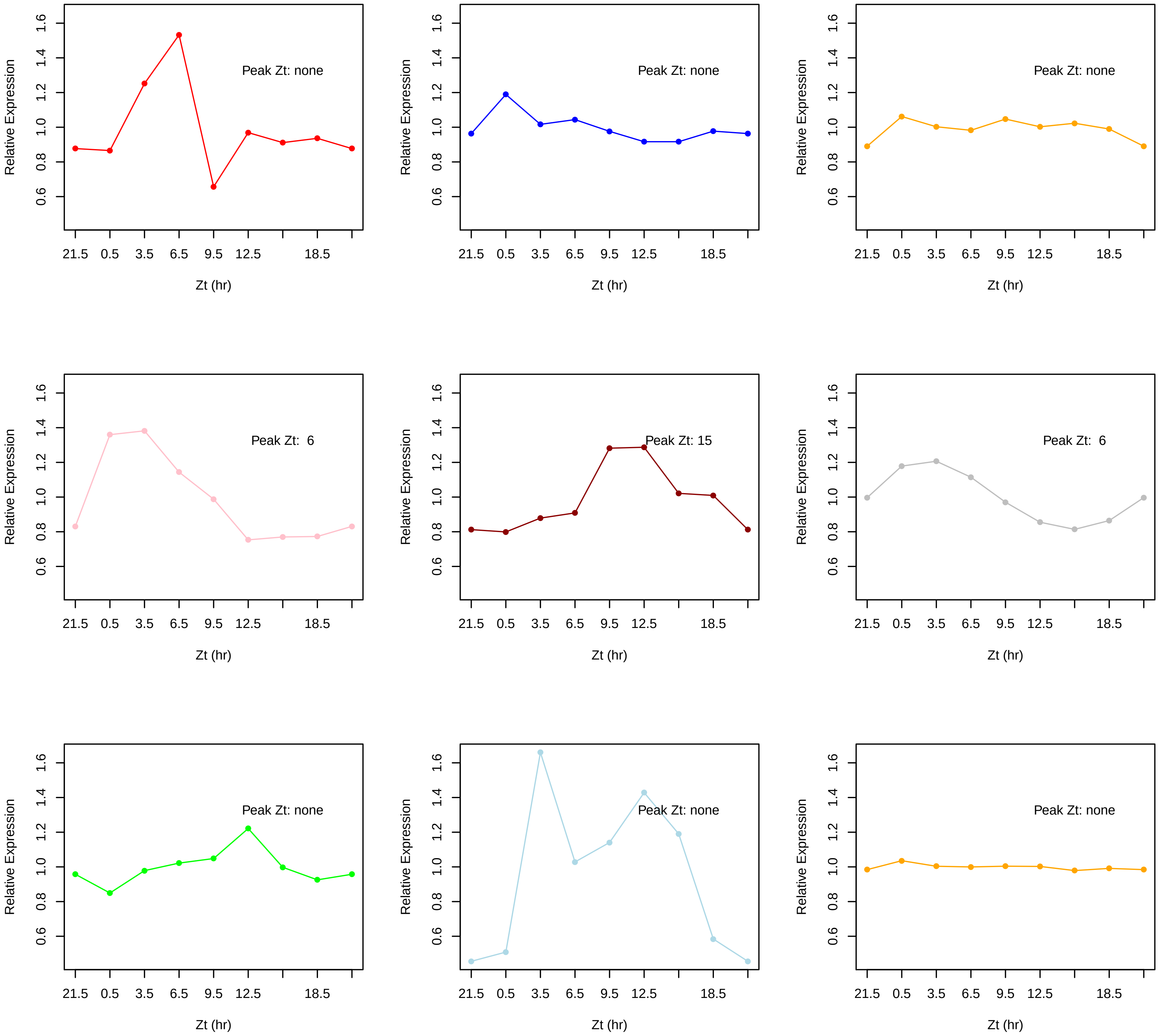

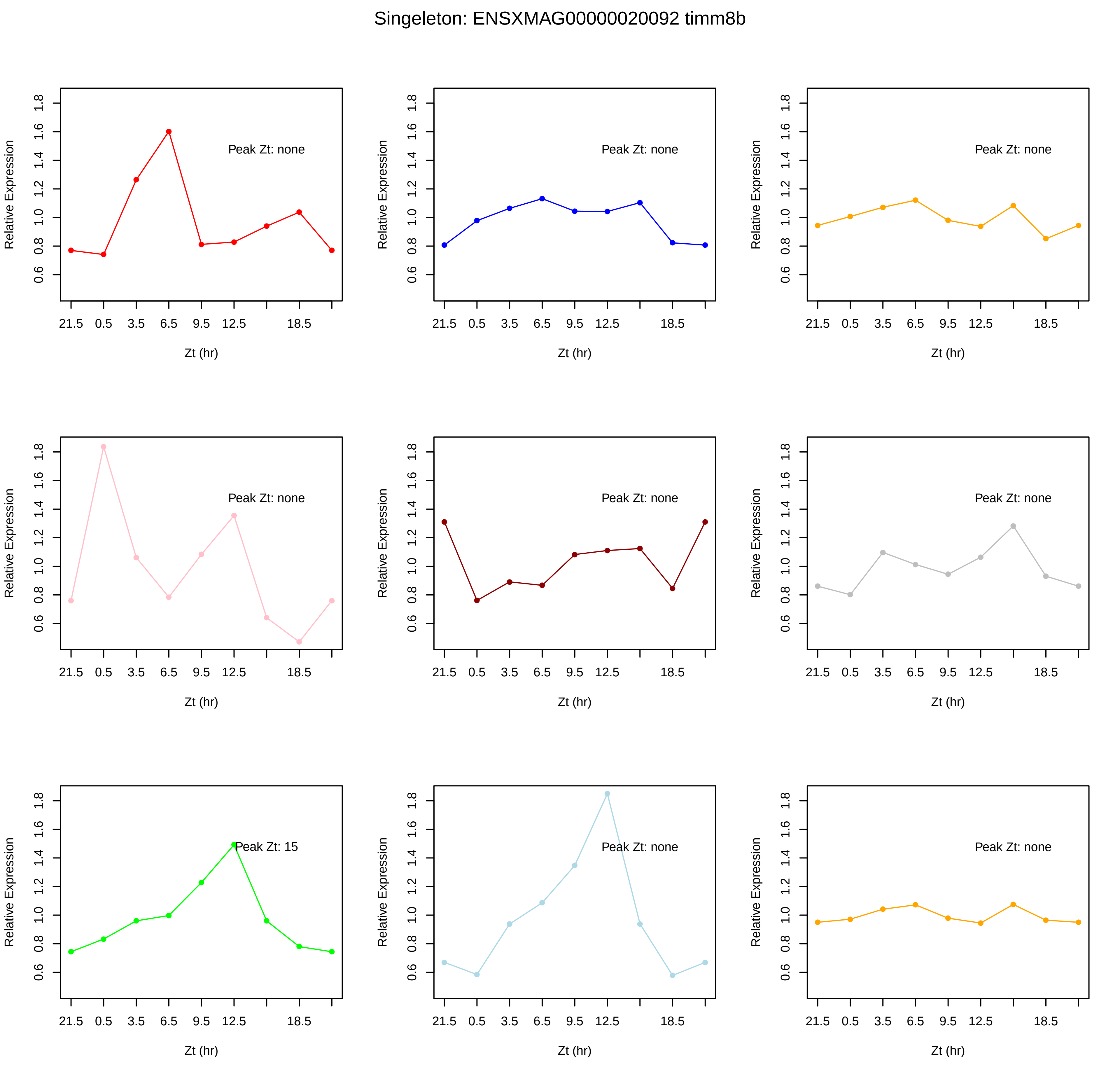
Singeleton: ENSXMAG00000020092 timm8b
Peak Zt: none
Peak Zt: none
Peak Zt: none
Peak Zt: none
Peak Zt: none
Peak Zt: none
Peak Zt: 15
Peak Zt: none
Peak Zt: none
Relative Expression
Zt (hr)
21.5
0.5
3.5
6.5
9.5
12.5
18.5
0.6
0.8
1.0
1.2
1.4
1.6
1.8

Singeleton: ENSXMAG00000002675

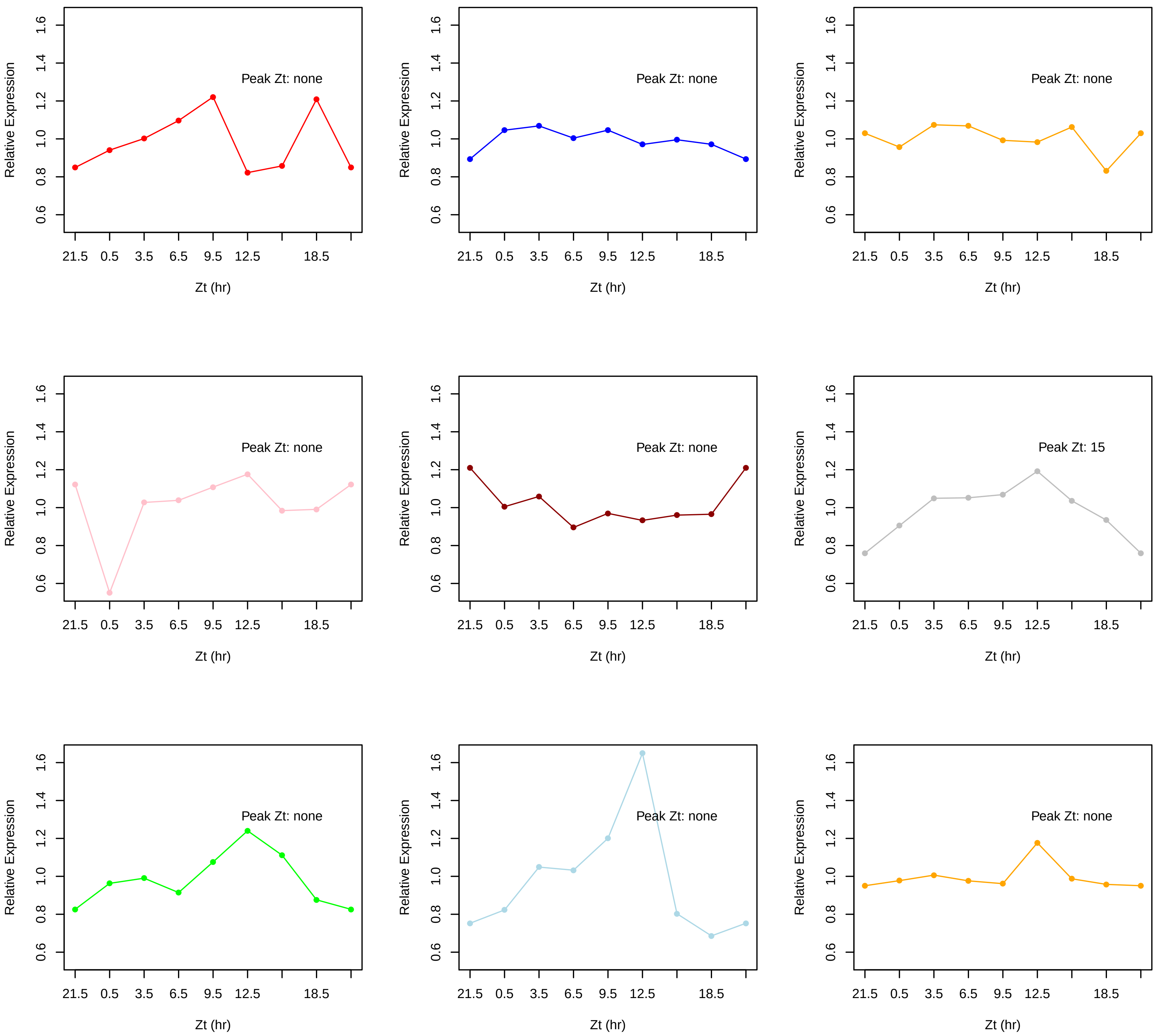

Singleton: ENSXMAG00000015080 klhl24b

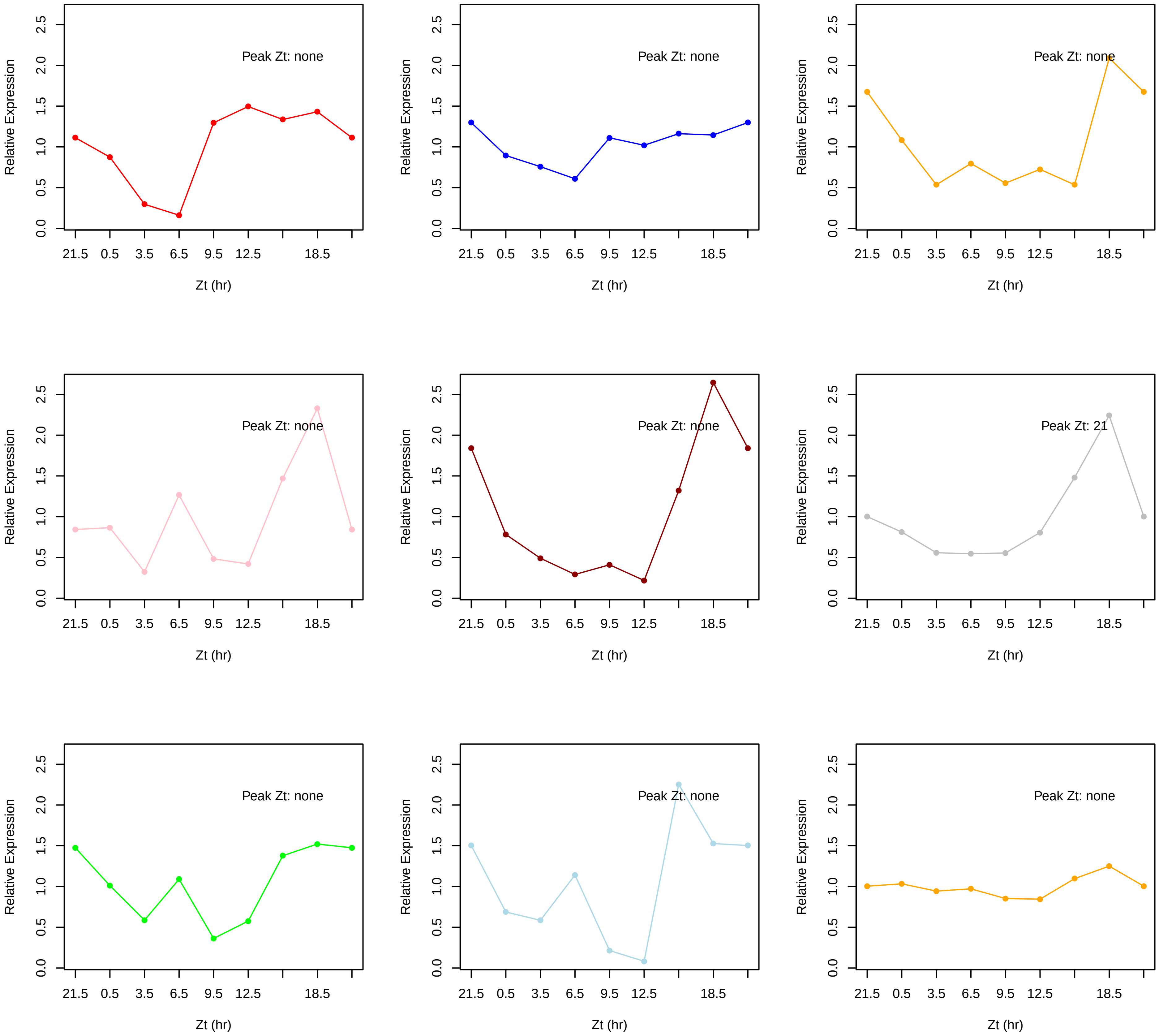

Singleton: ENSXMAG00000012036 p4ha3

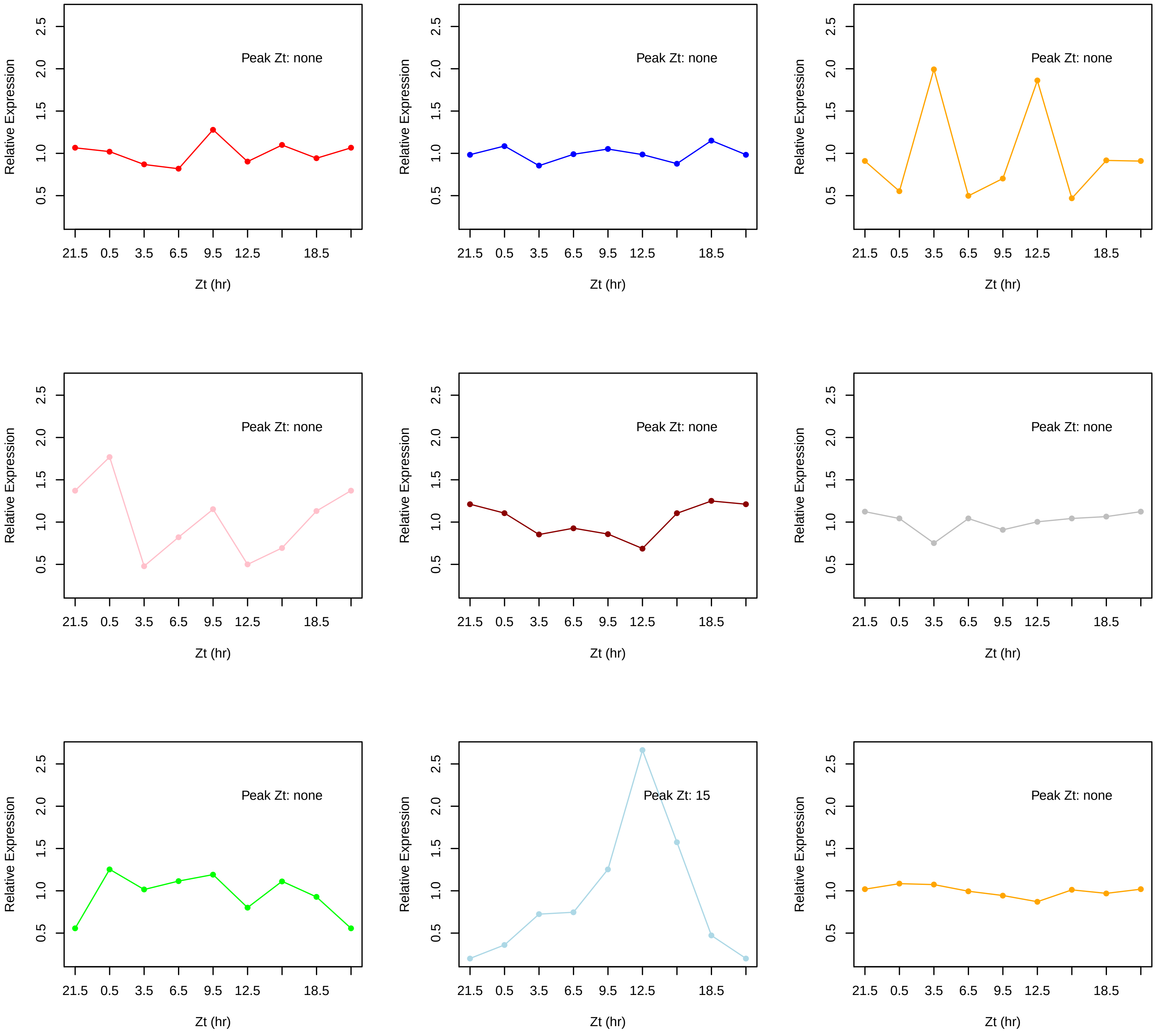

Singeleton: ENSXMAG00000001736 march7

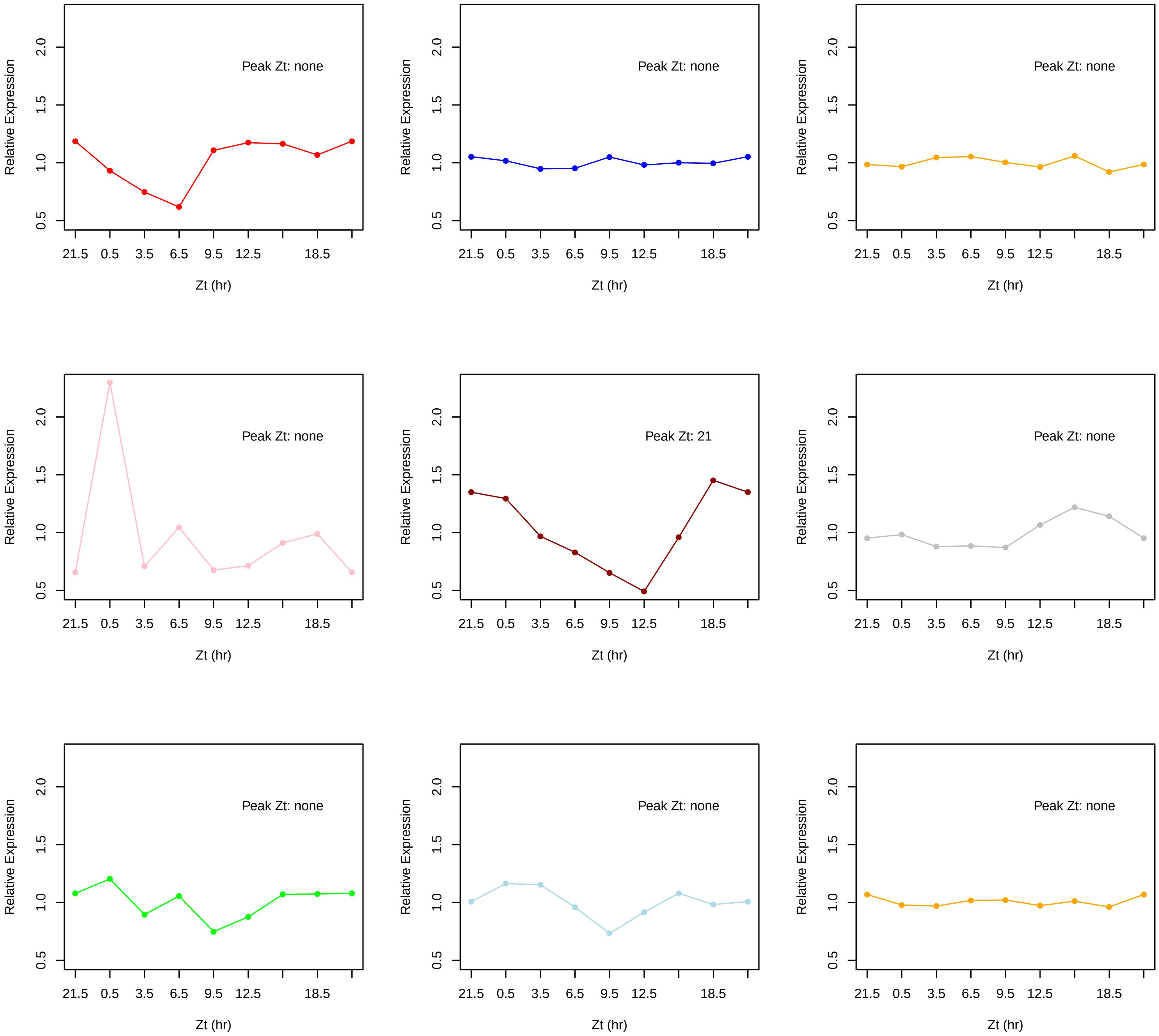

Singleton: ENSXMAG00000000964 noc4l

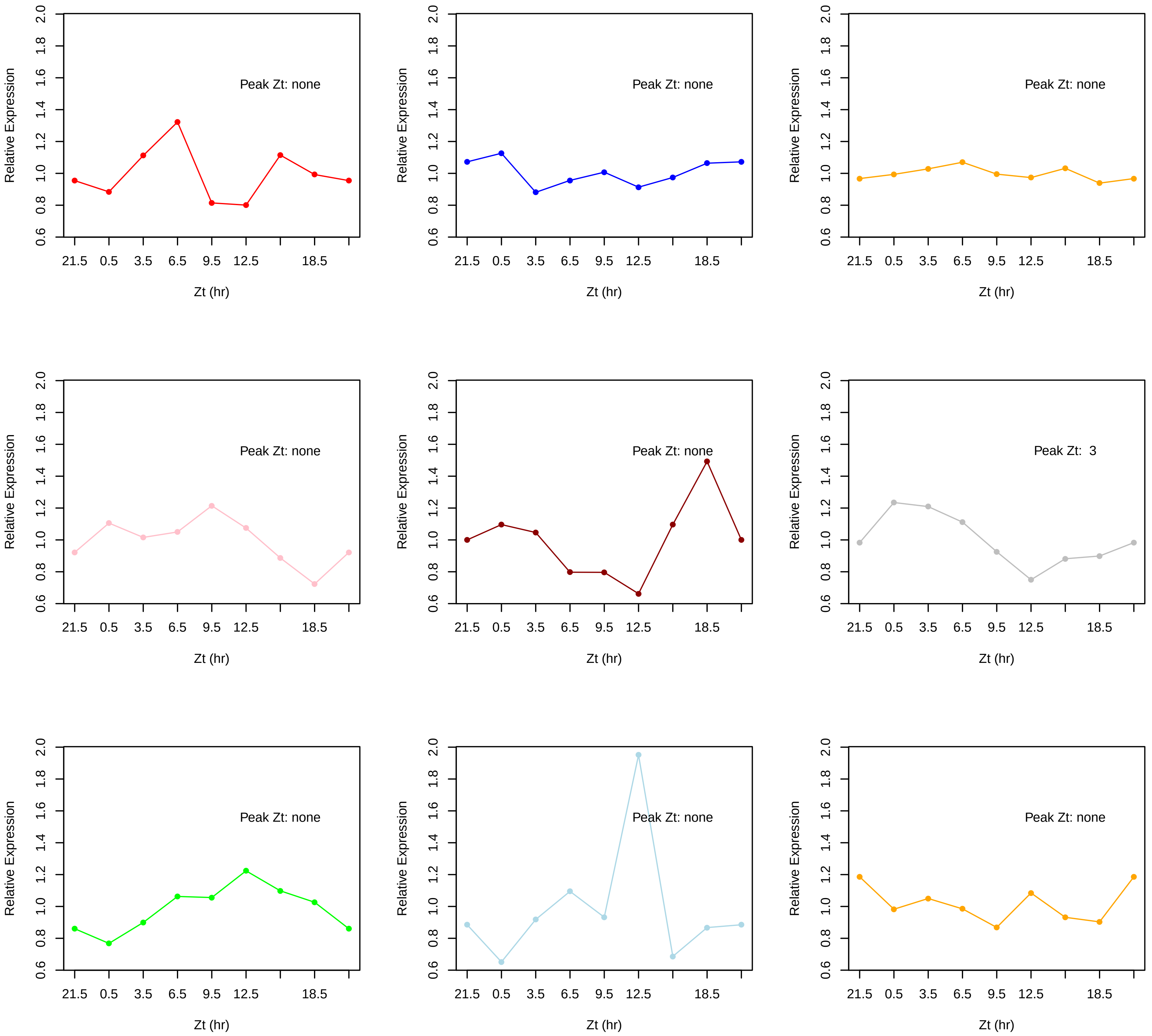

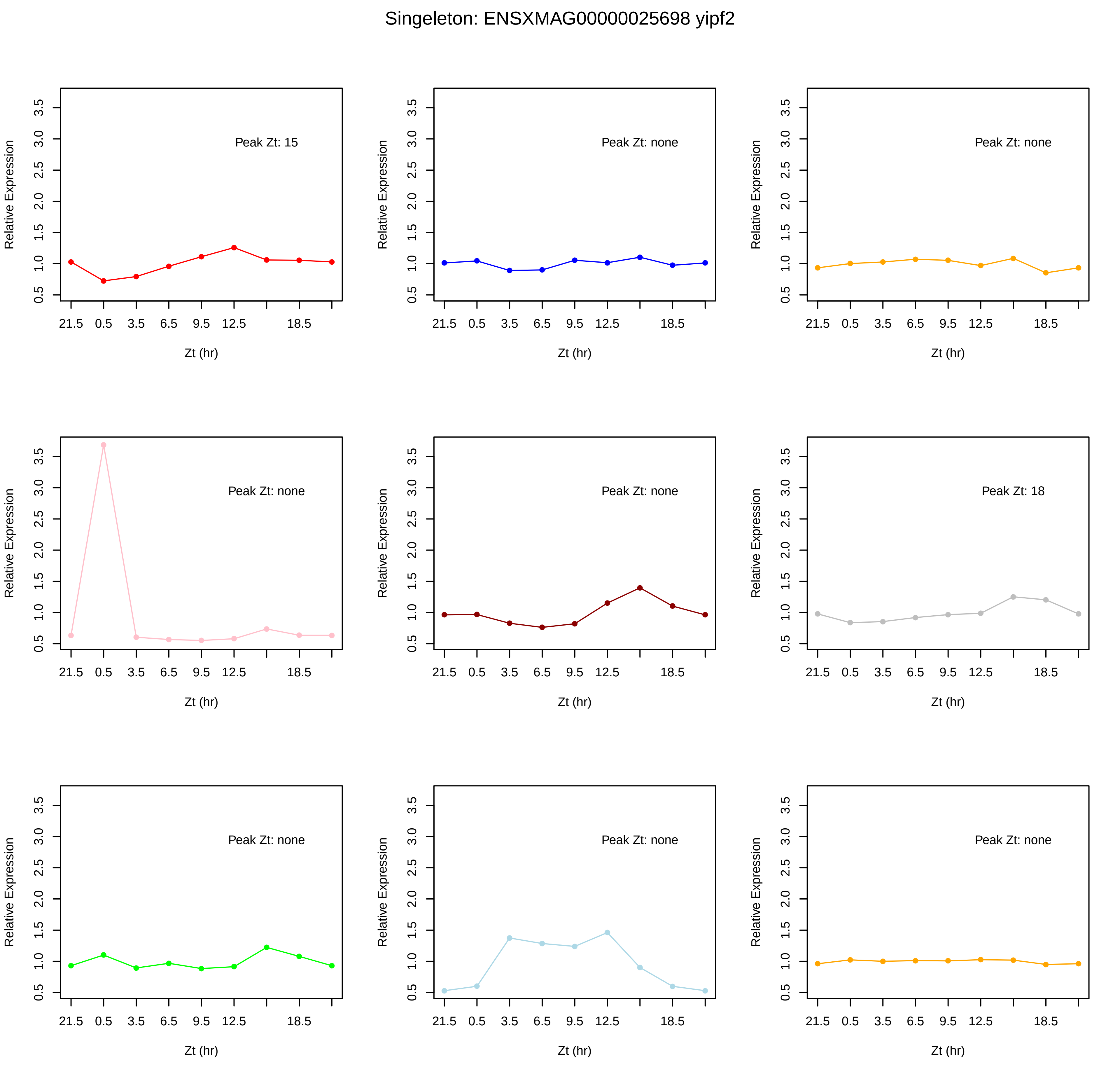
Singeleton: ENSXMAG00000025698 yipf2
Peak Zt: 15
Peak Zt: none
Peak Zt: none
Peak Zt: none
Peak Zt: none
Peak Zt: 18
Peak Zt: none
Peak Zt: none
Peak Zt: none
Relative Expression
Zt (hr)
21.5
0.5
3.5
6.5
9.5
12.5
18.5
0.5
1.0
1.5
2.0
2.5
3.0
3.5

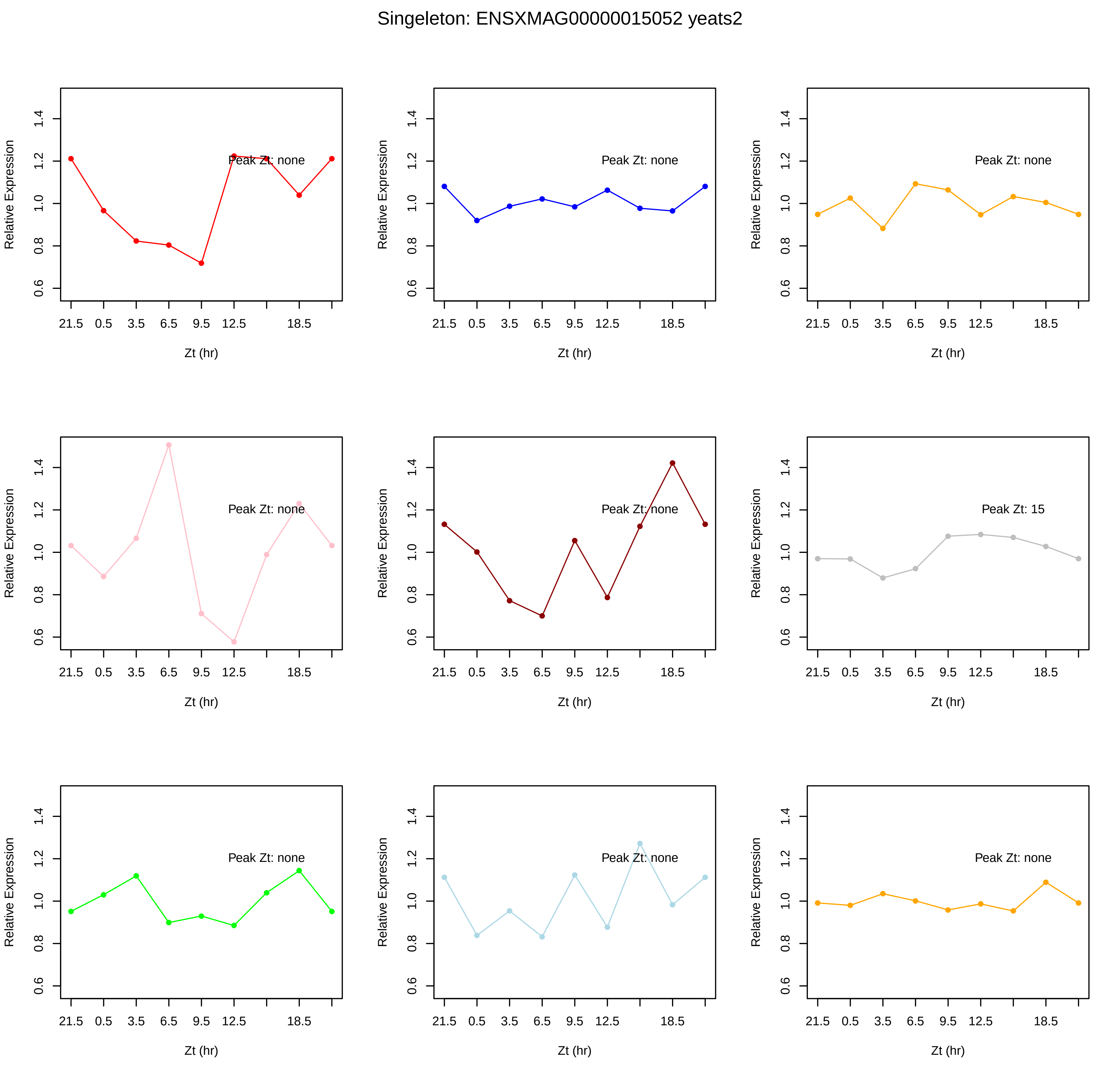
Singeleton: ENSXMAG00000015052 yeats2
Relative Expression
Zt (hr)
Peak Zt: none
Peak Zt: none
Peak Zt: none
Peak Zt: none
Peak Zt: none
Peak Zt: 15
Peak Zt: none
Peak Zt: none
Peak Zt: none

Singeleton: ENSXMAG00000001468 gfer

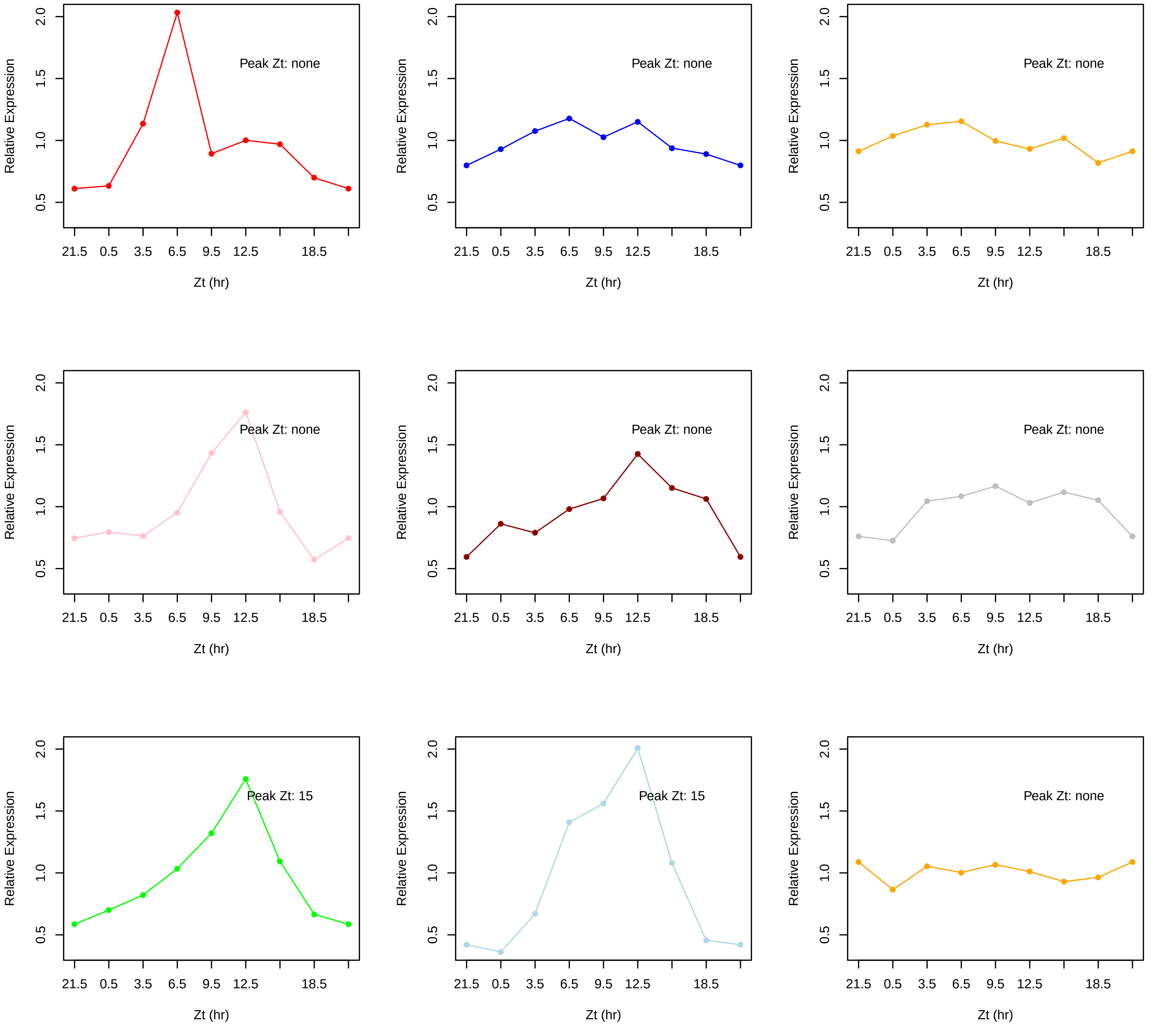

Singleton: ENSXMAG00000006311 tmco1

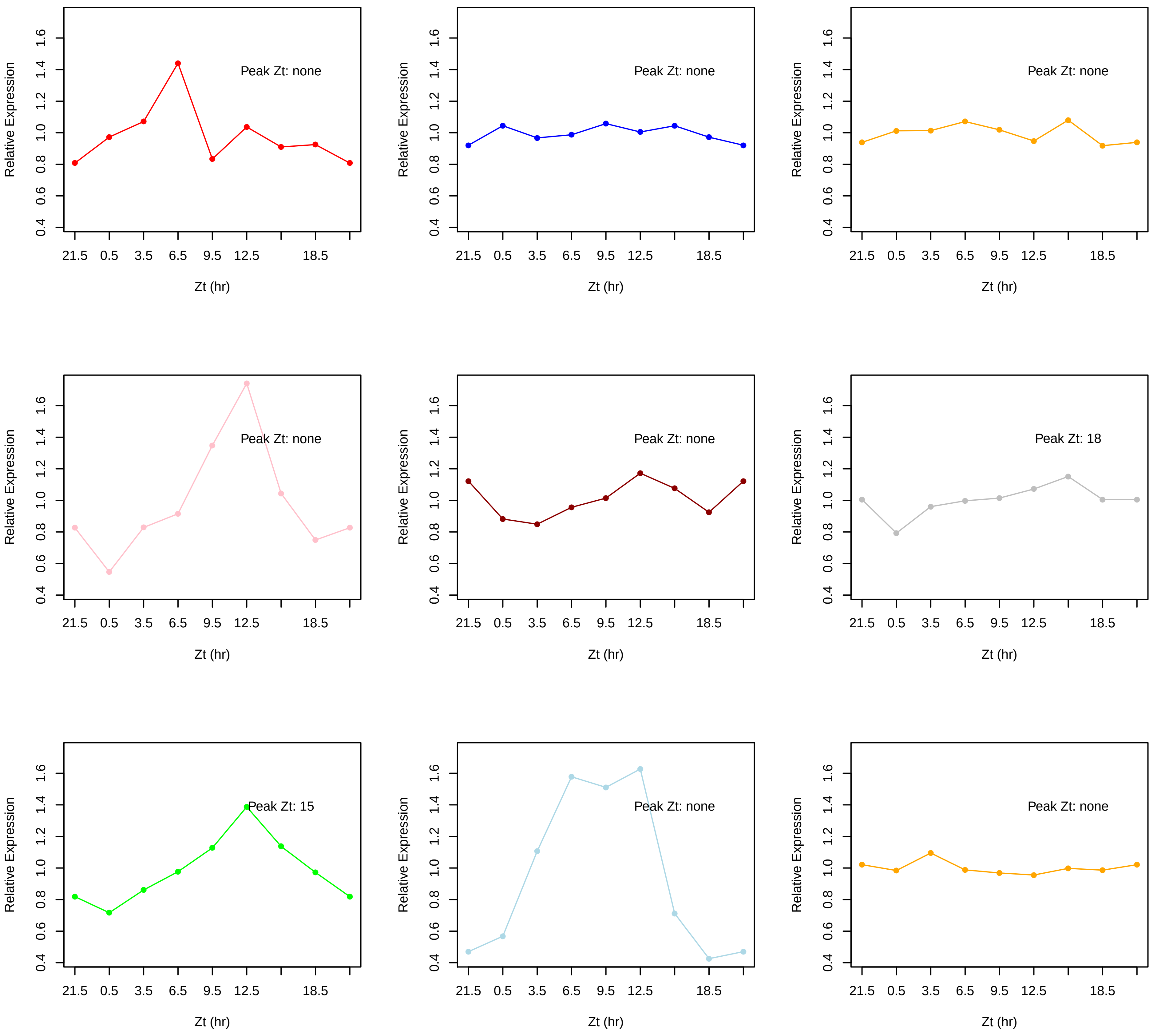

Singeleton: ENSXMAG00000011978 ppme1

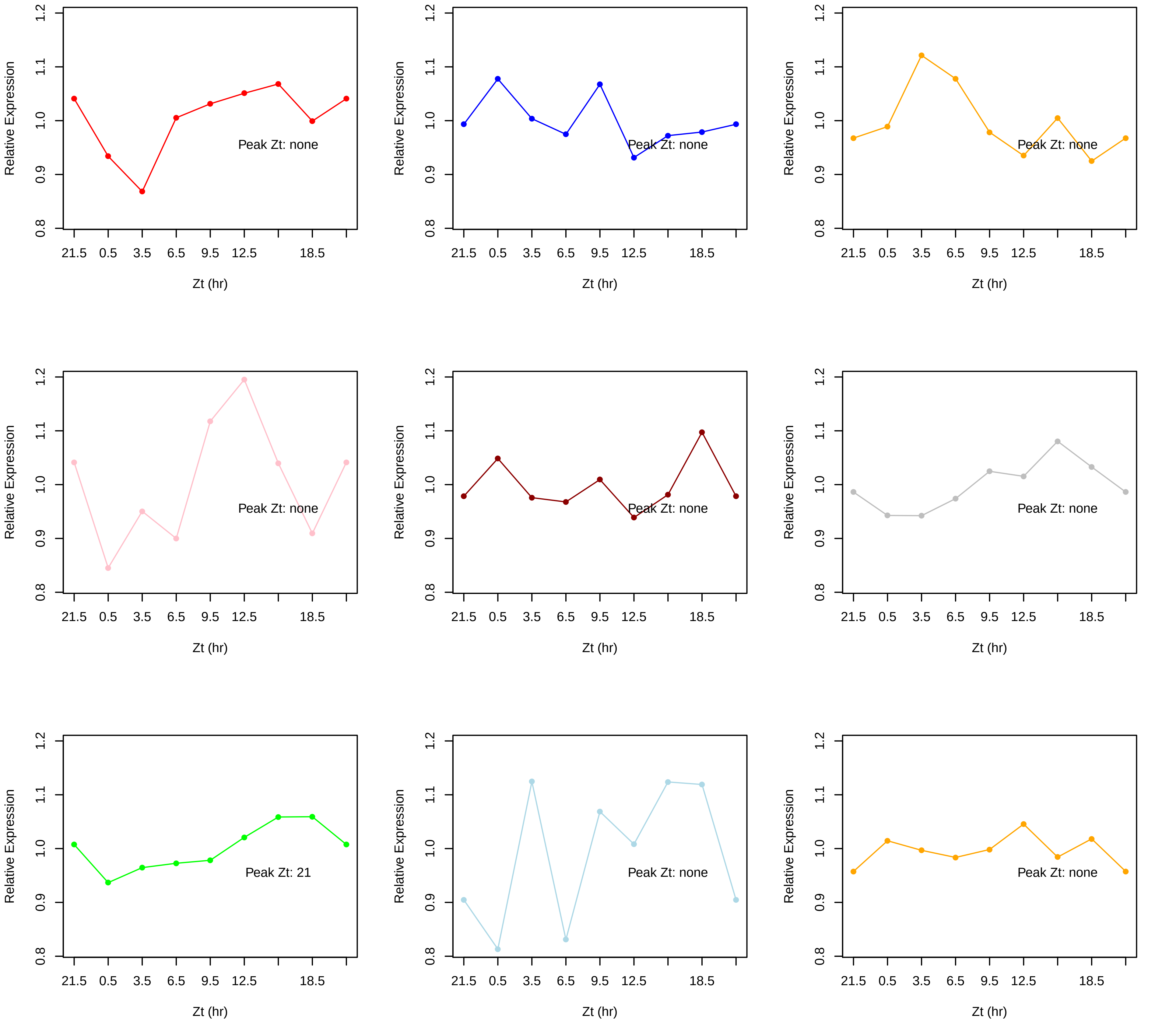

Singeleton: ENSXMAG00000019394 gabrd

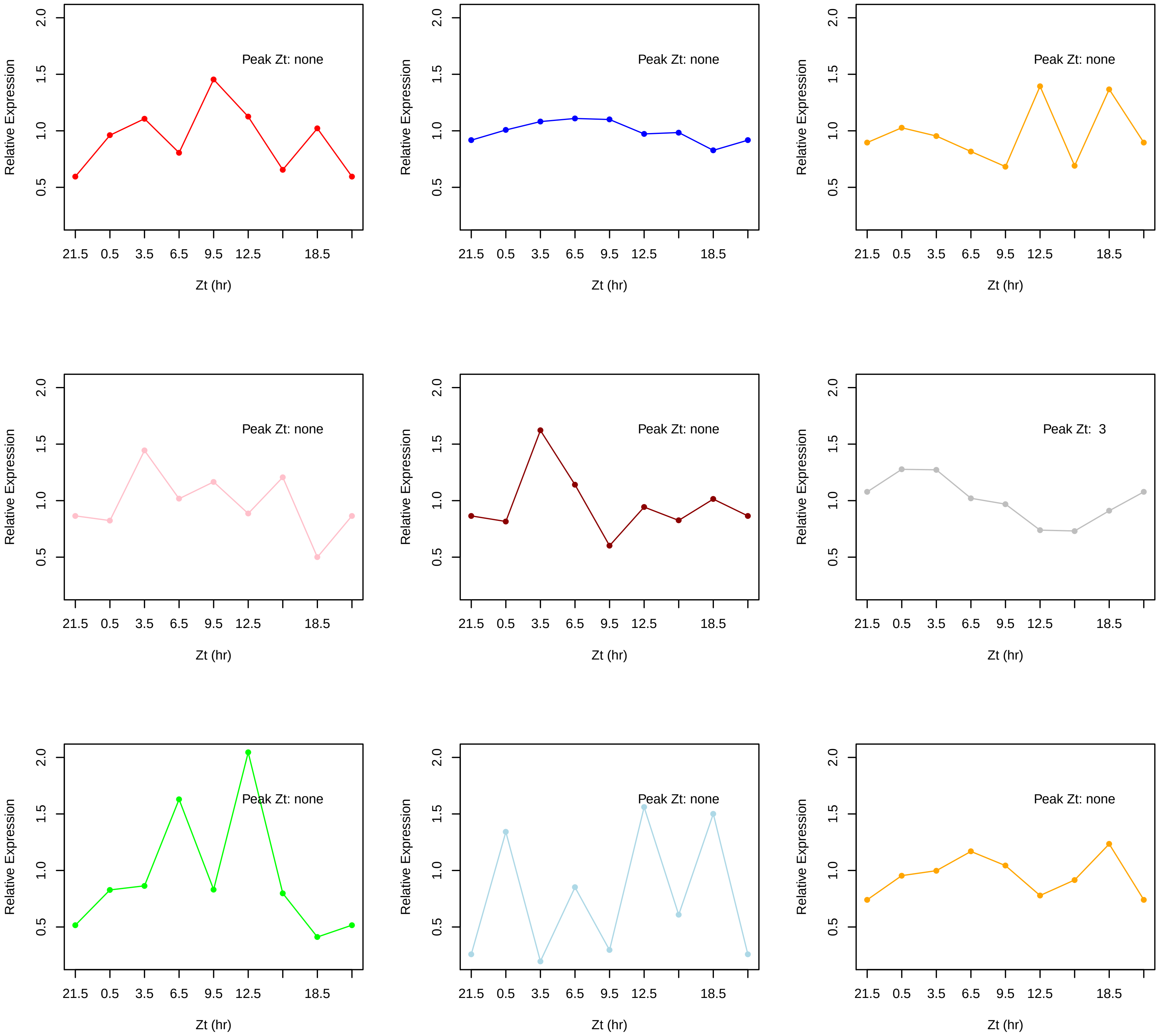

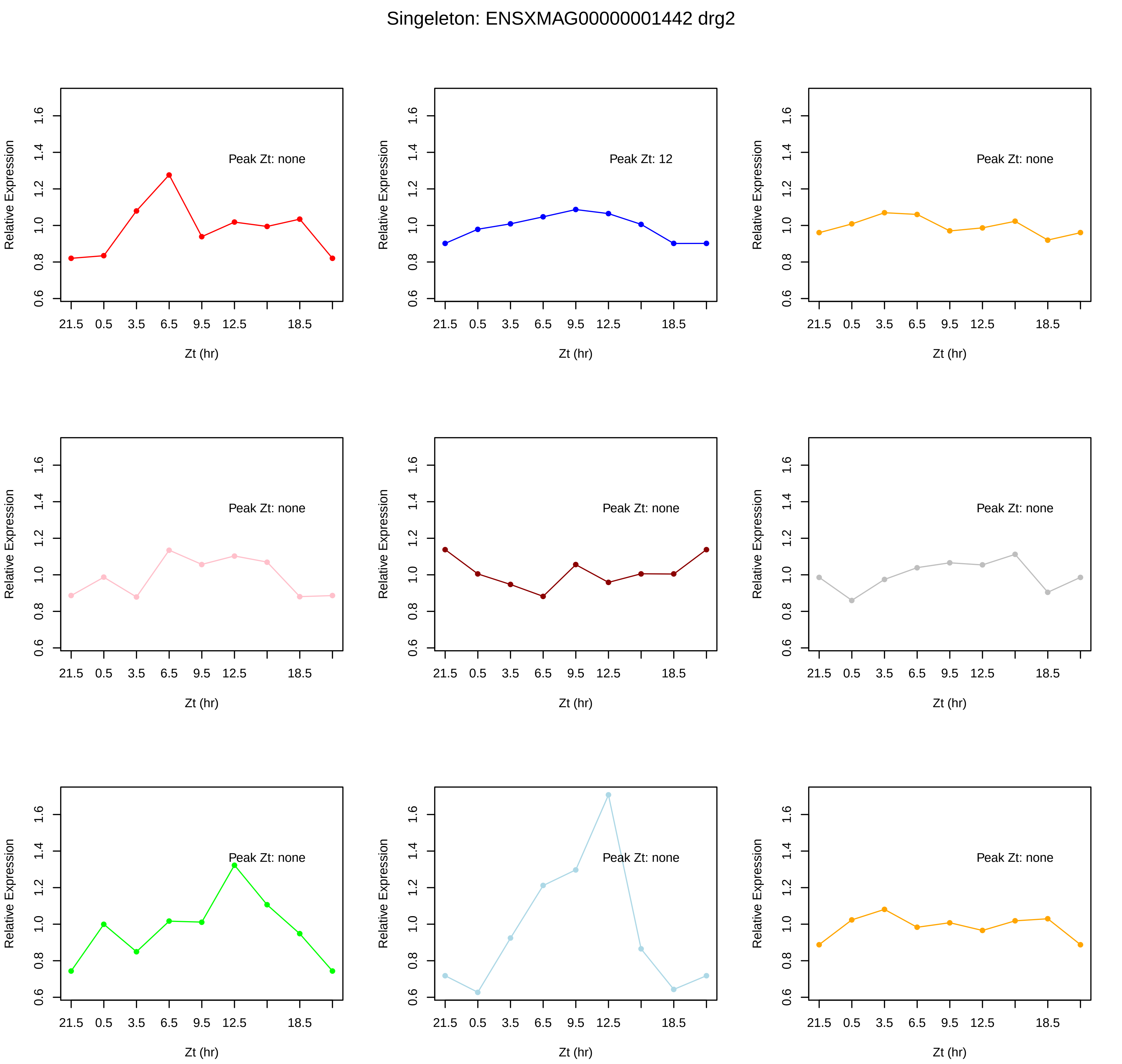

Singeleton: ENSXMAG00000001442 drg2
Peak Zt: none
Peak Zt: 12
Peak Zt: none
Peak Zt: none
Peak Zt: none
Peak Zt: none
Peak Zt: none
Peak Zt: none
Peak Zt: none
Relative Expression
Zt (hr)

Singeleton: ENSXMAG00000016749 uap1l1

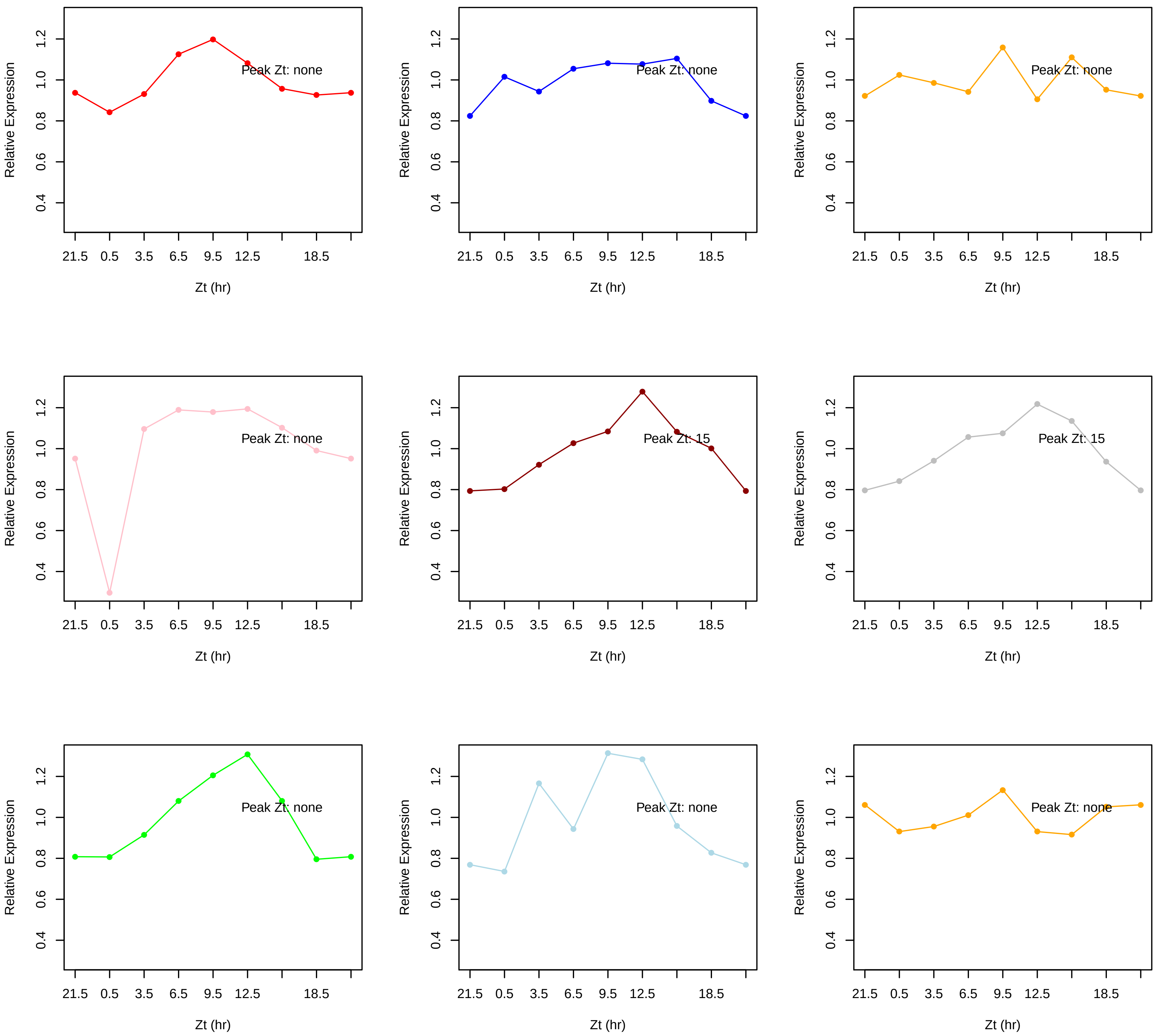

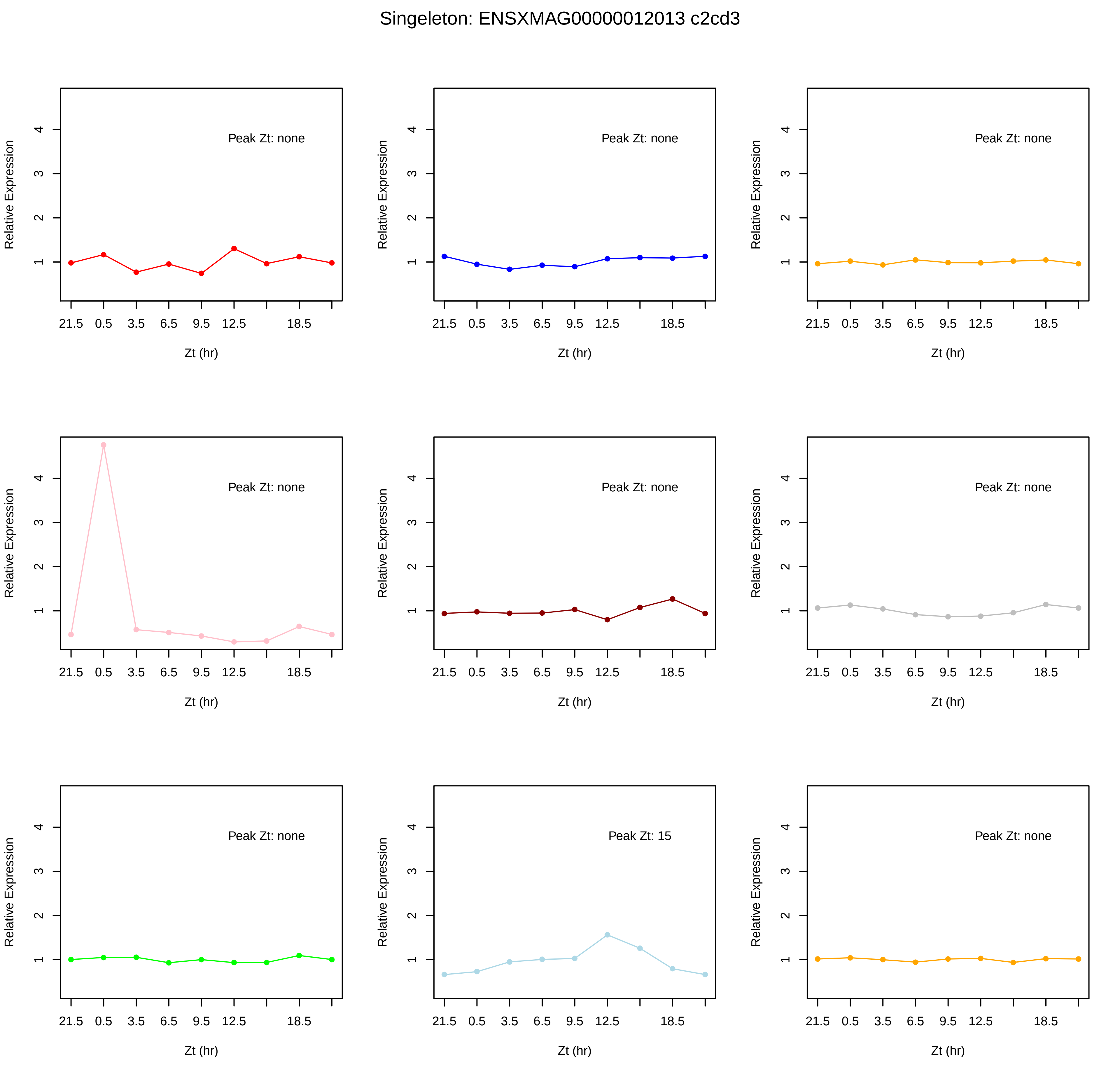
Singleton: ENSXMAG00000012013 c2cd3
Peak Zt: none
Peak Zt: none
Peak Zt: none
Peak Zt: none
Peak Zt: none
Peak Zt: none
Peak Zt: none
Peak Zt: 15
Peak Zt: none
Relative Expression
Zt (hr)
21.5
0.5
3.5
6.5
9.5
12.5
18.5
1
2
3
4

Singeleton: ENSXMAG00000016678 cox4i2

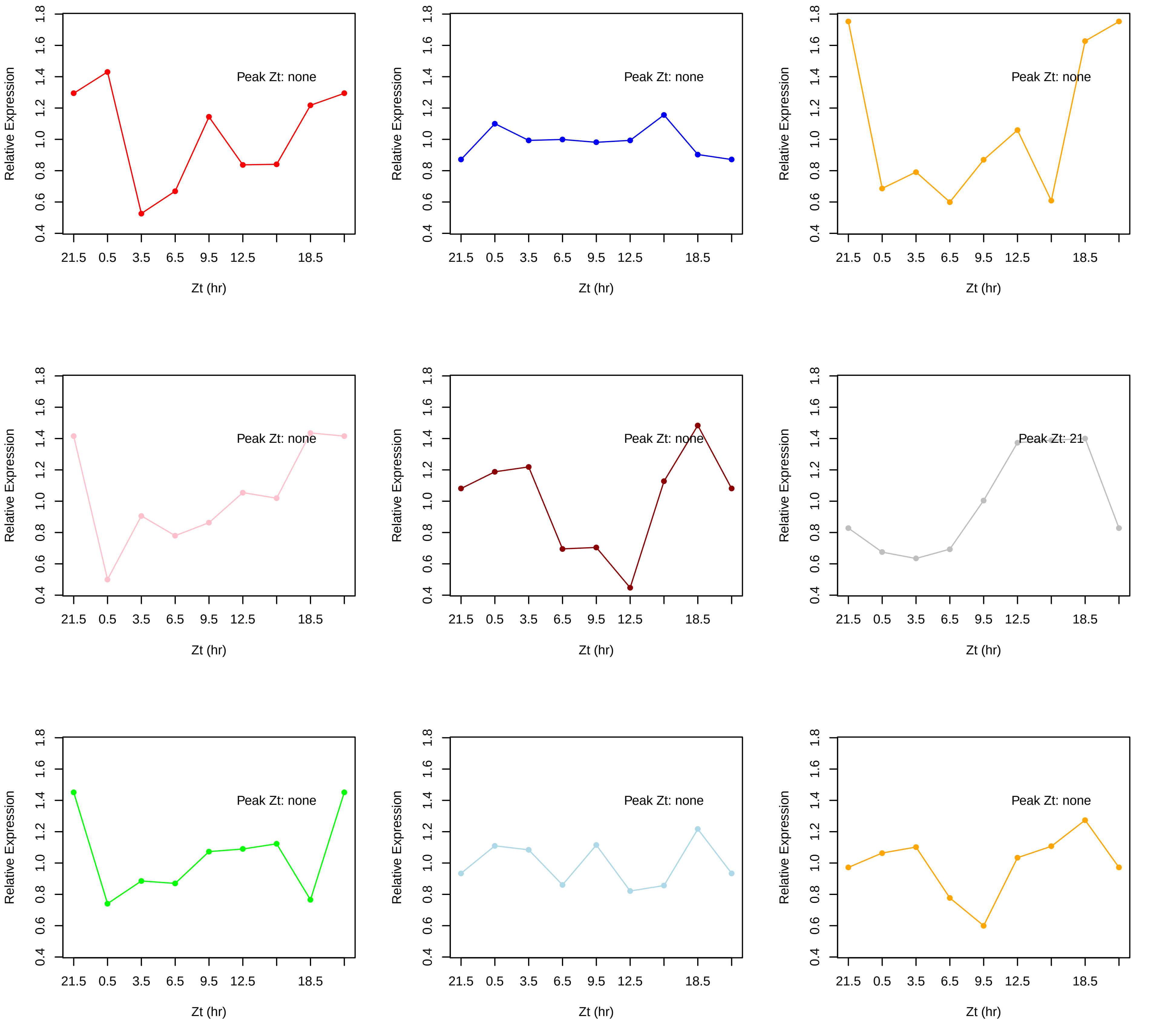

Singeleton: ENSXMAG00000020970 cacna1db

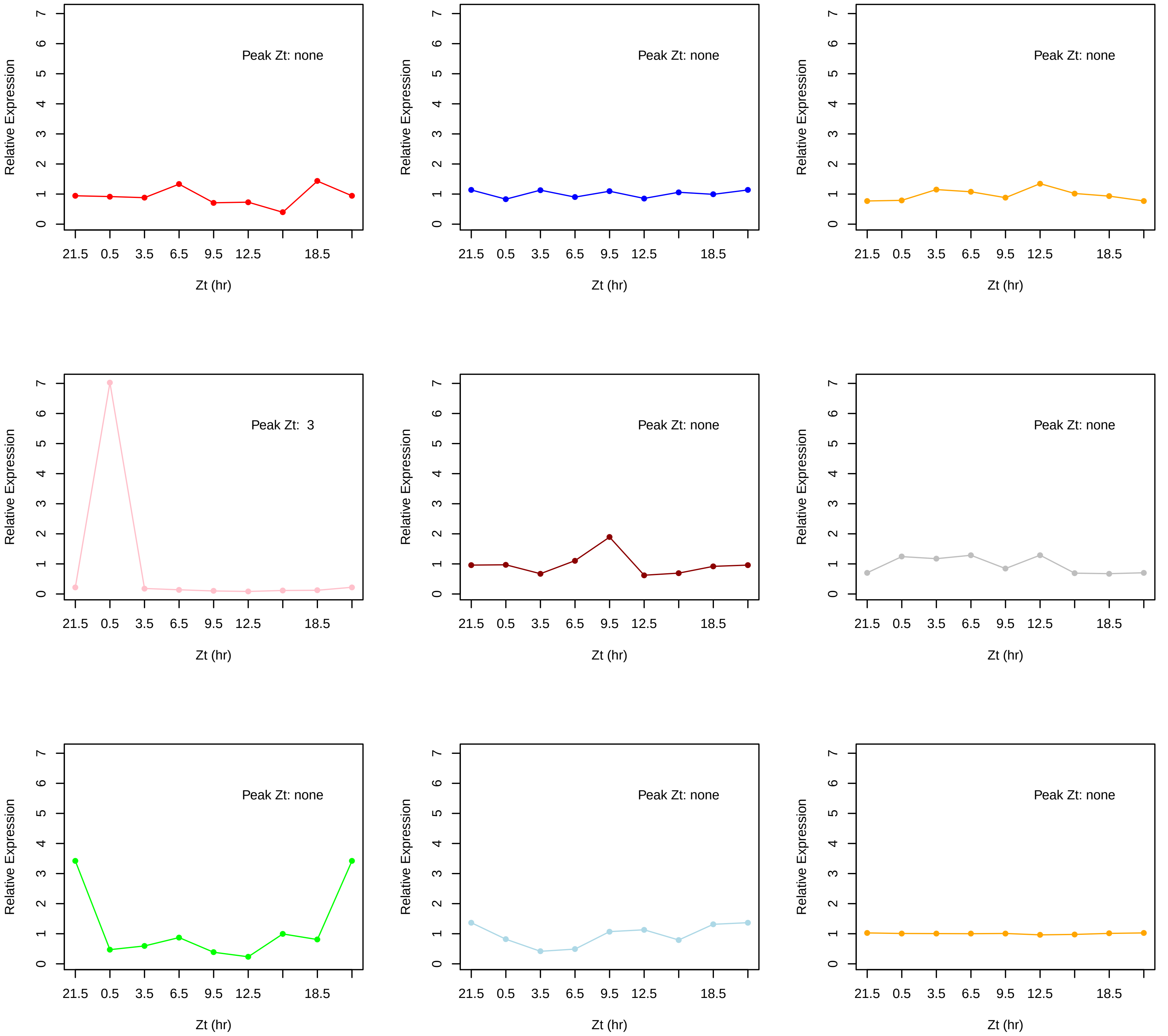

Singeleton: ENSXMAG00000006297 magoh

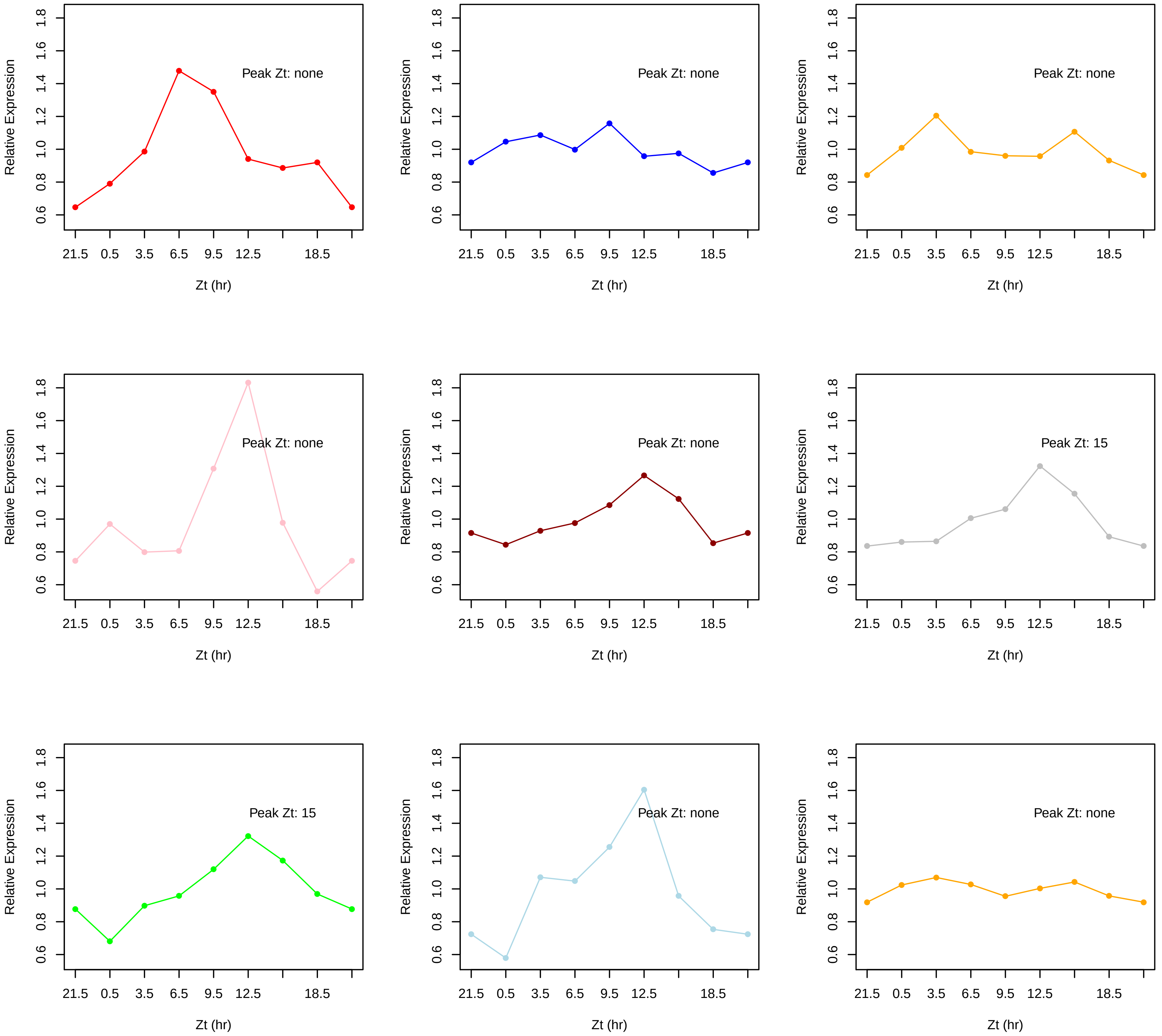

Singeleton: ENSXMAG00000015445 lipf

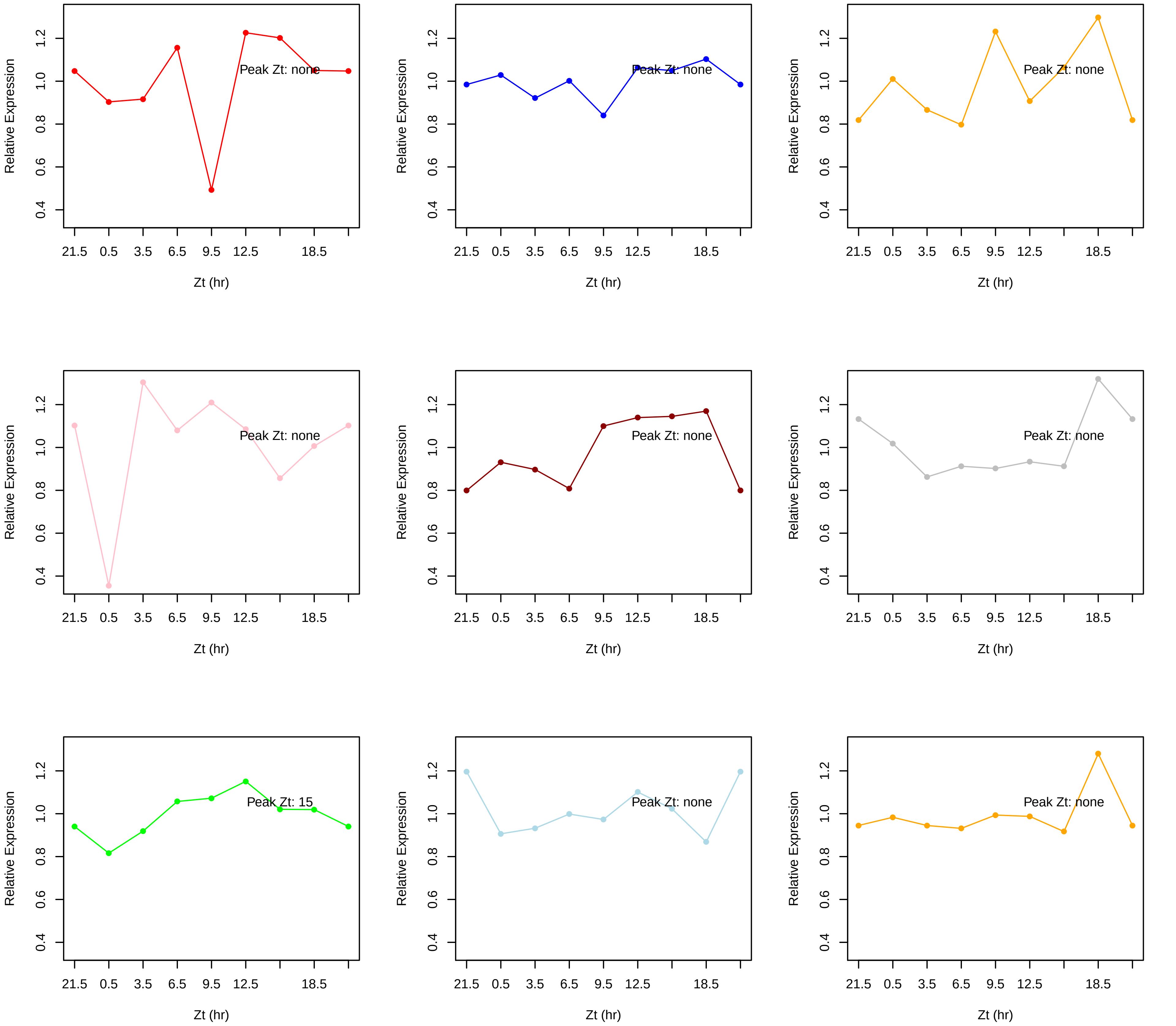

Singeleton: ENSXMAG00000002539 clpp

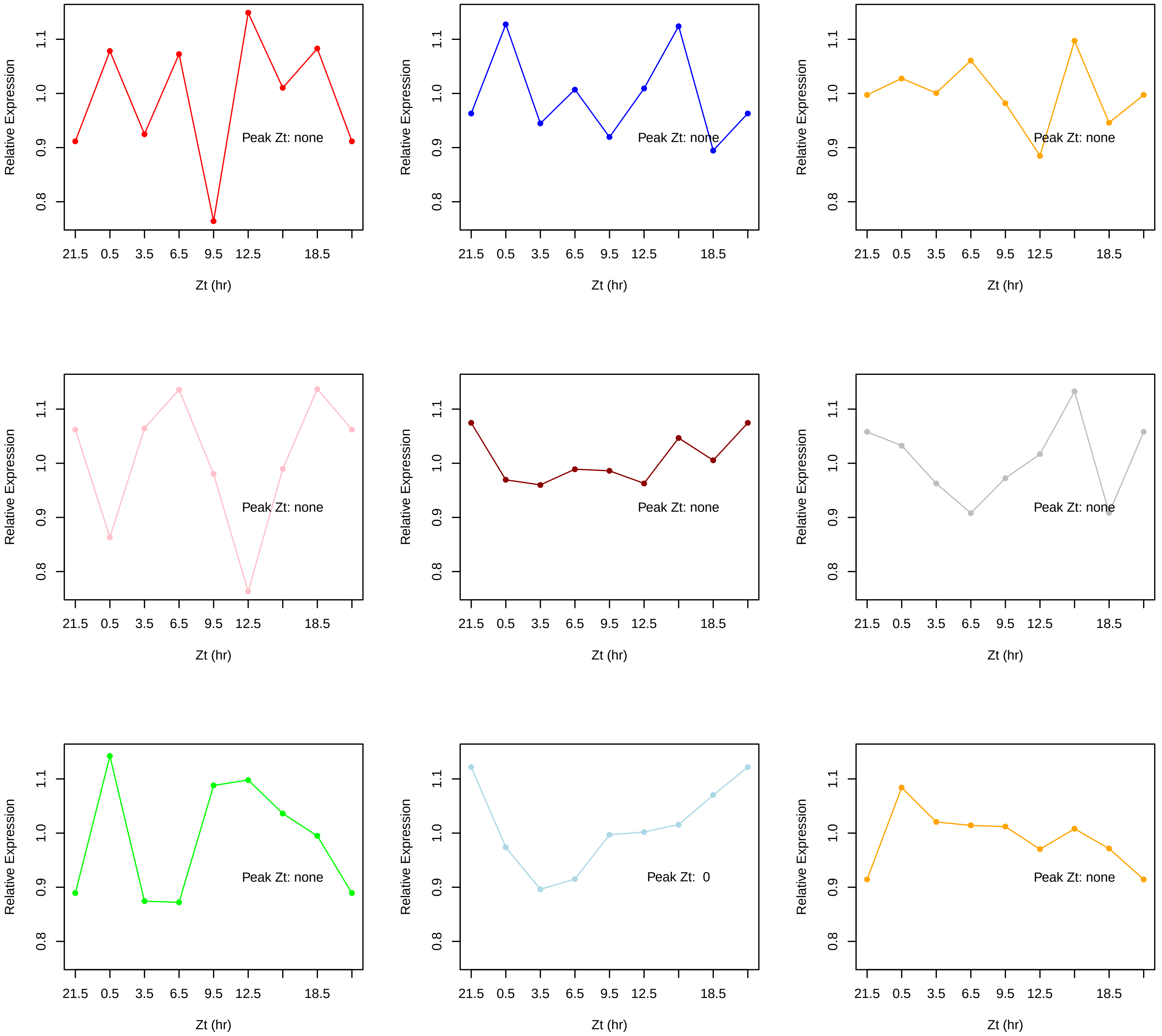

Singeleton: ENSXMAG00000007316 cdk7

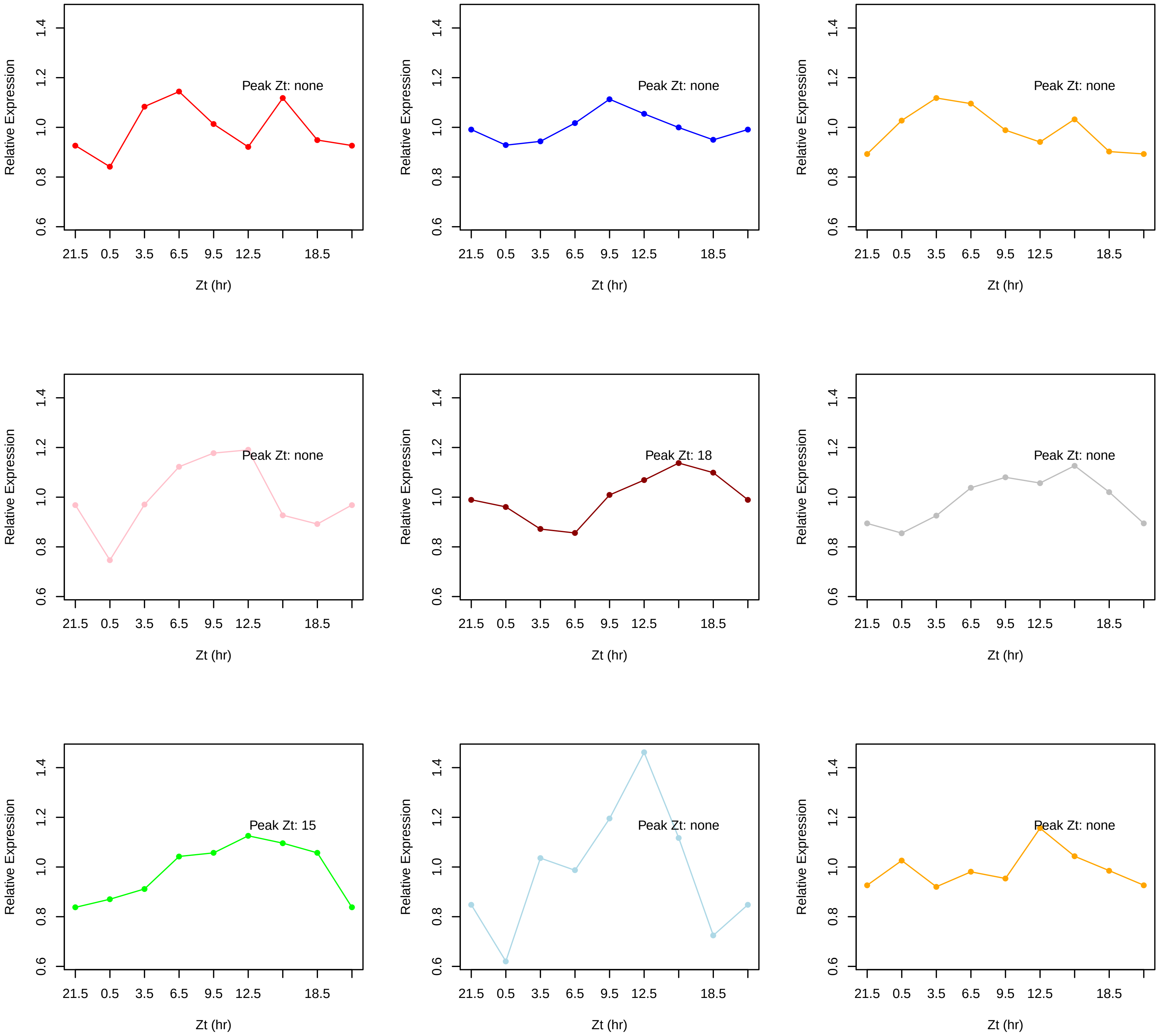

Singeleton: ENSXMAG00000014181 dhps

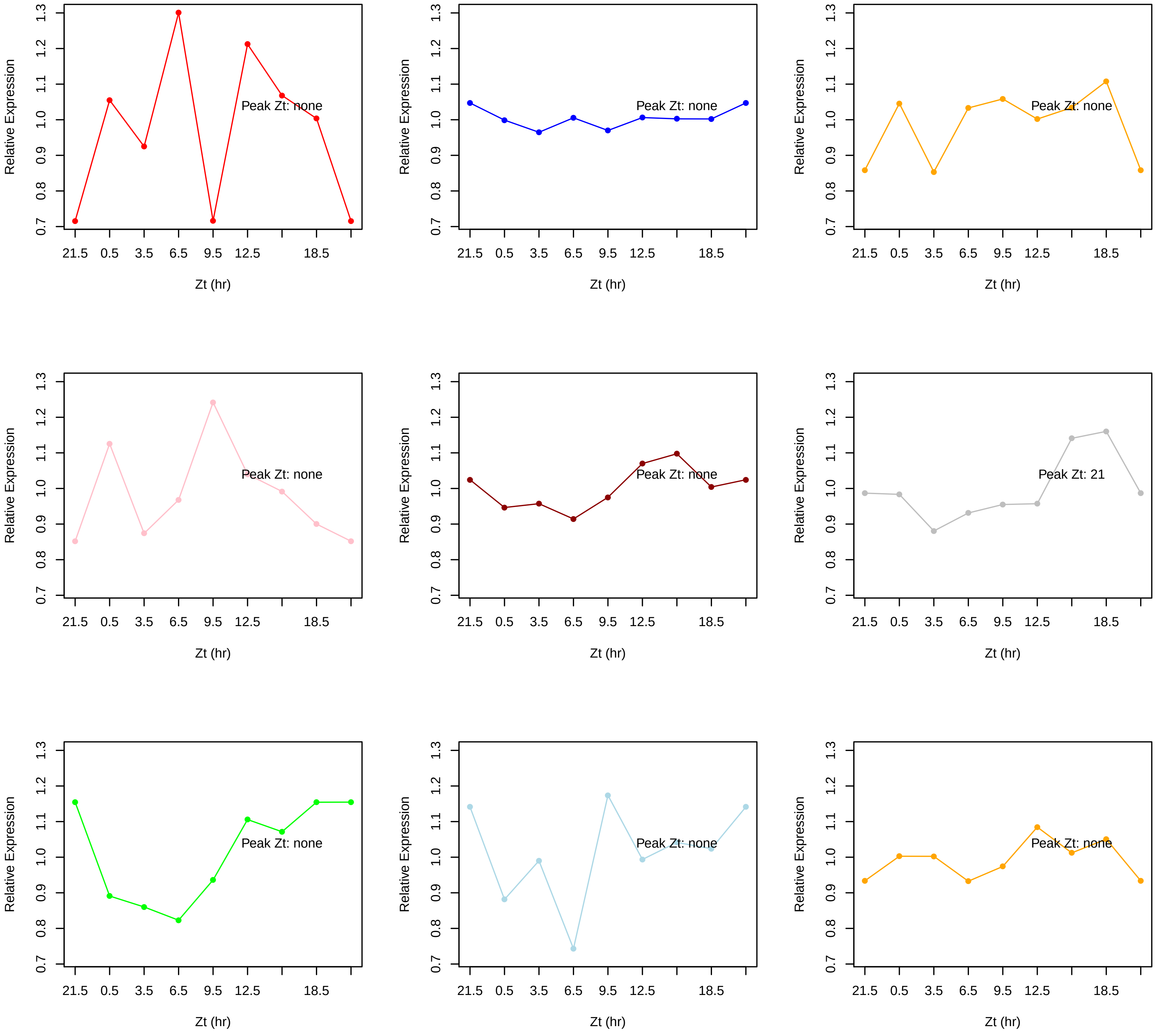

Singeleton: ENSXMAG00000015026 rnls

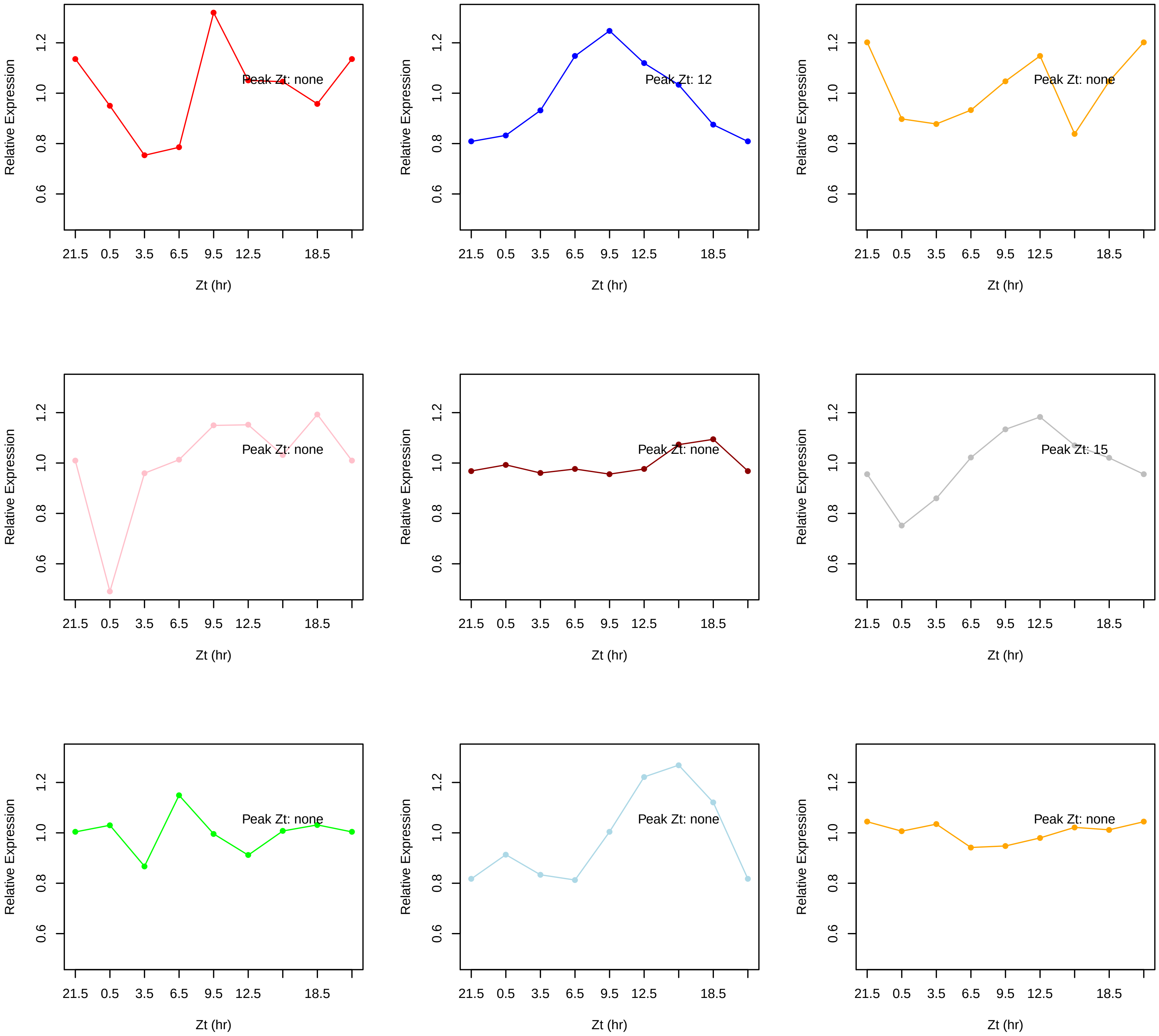

Singleton: ENSXMAG00000023272 si:ch211−284b7.3

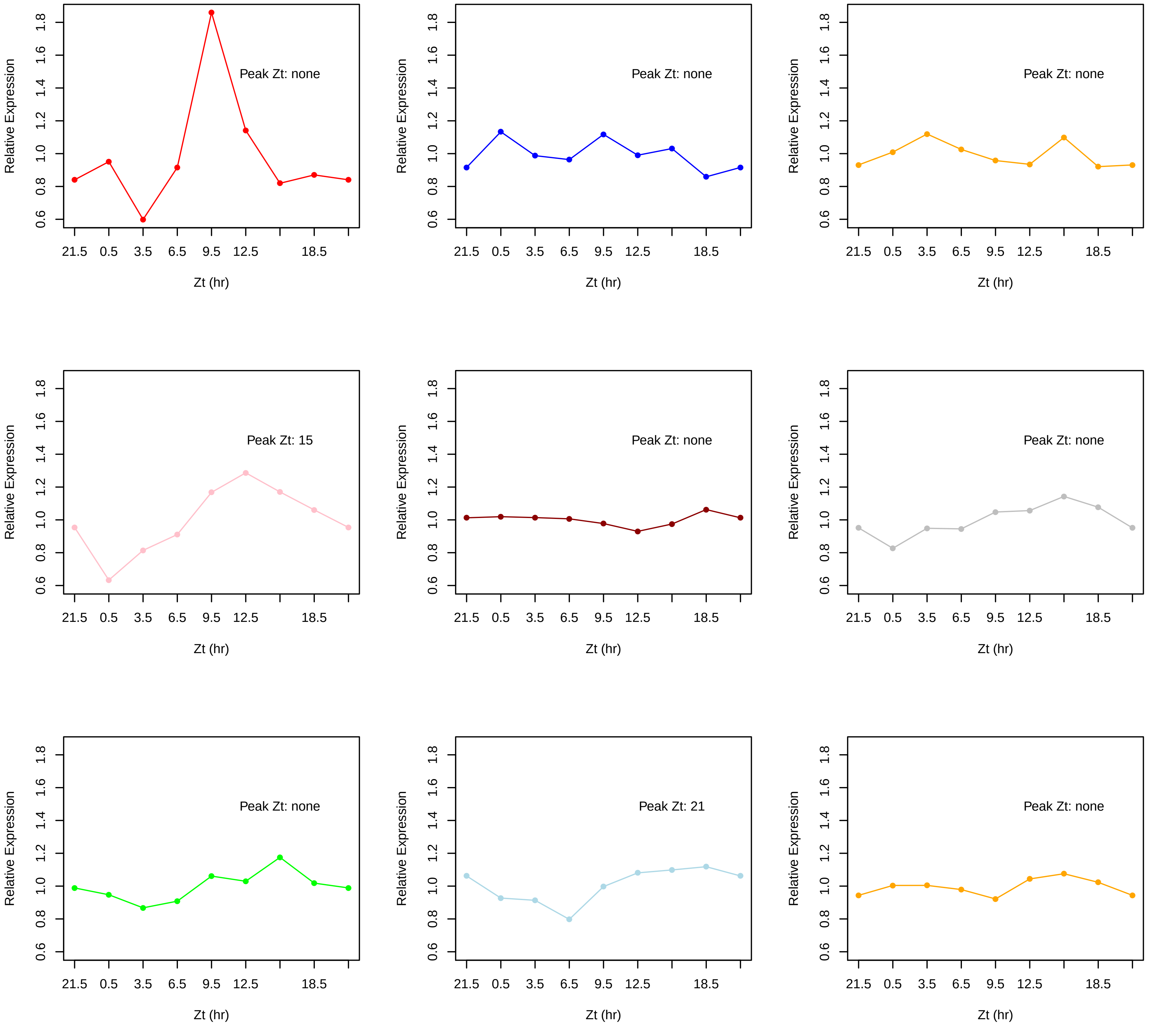

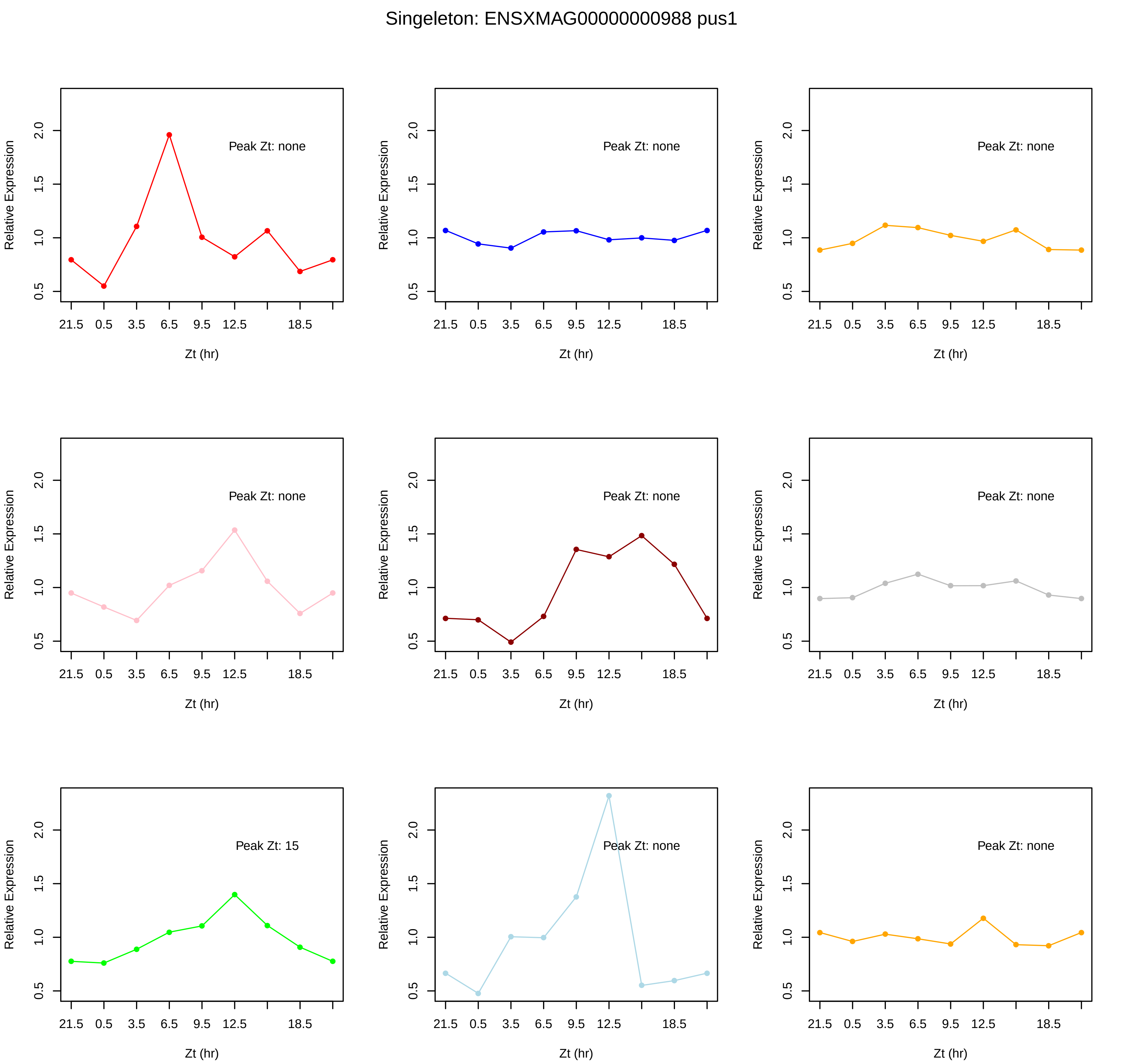

Singeleton: ENSXMAG00000000988 pus1
Peak Zt: none
Peak Zt: none
Peak Zt: none
Peak Zt: none
Peak Zt: none
Peak Zt: none
Peak Zt: 15
Peak Zt: none
Peak Zt: none
Relative Expression
Zt (hr)
21.5
0.5
3.5
6.5
9.5
12.5
18.5
0.5
1.0
1.5
2.0

Singeleton: ENSXMAG00000019068 mapre2

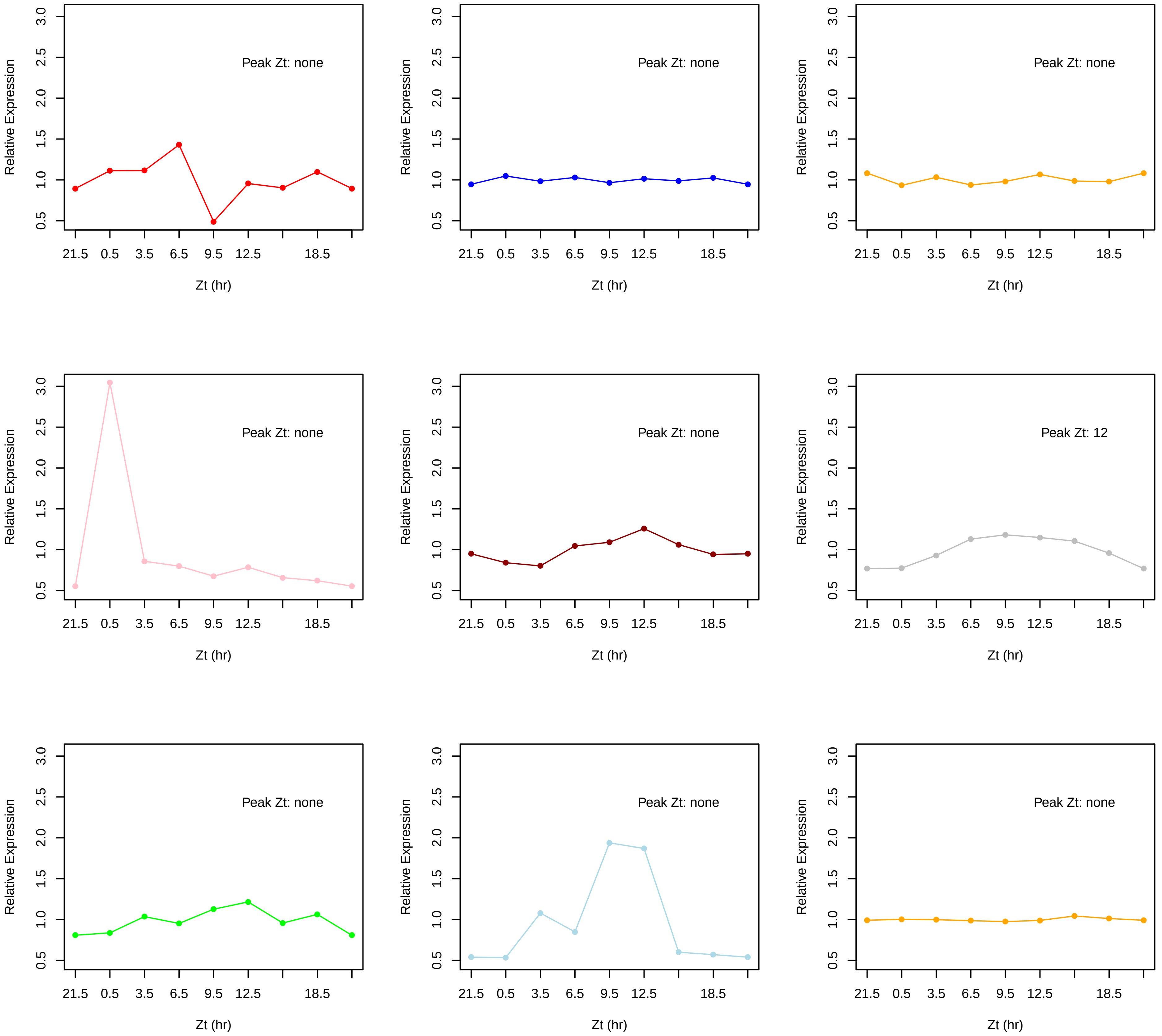

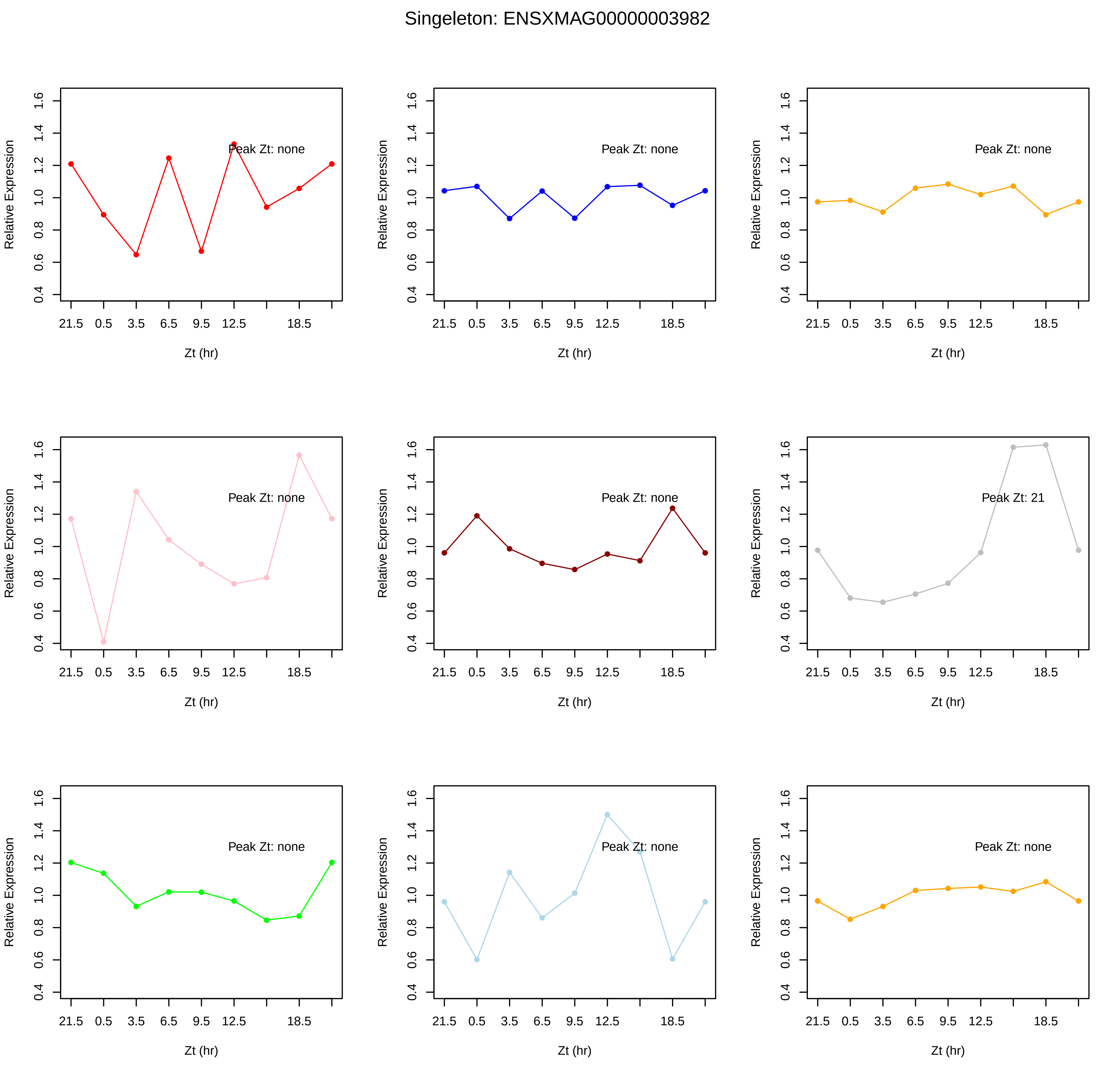

Singeleton: ENSXMAG00000003982
Peak Zt: none
Peak Zt: none
Peak Zt: none
Peak Zt: none
Peak Zt: none
Peak Zt: 21
Peak Zt: none
Peak Zt: none
Peak Zt: none
Relative Expression
Zt (hr)
21.5 0.5 3.5 6.5 9.5 12.5 18.5
0.4 0.6 0.8 1.0 1.2 1.4 1.6

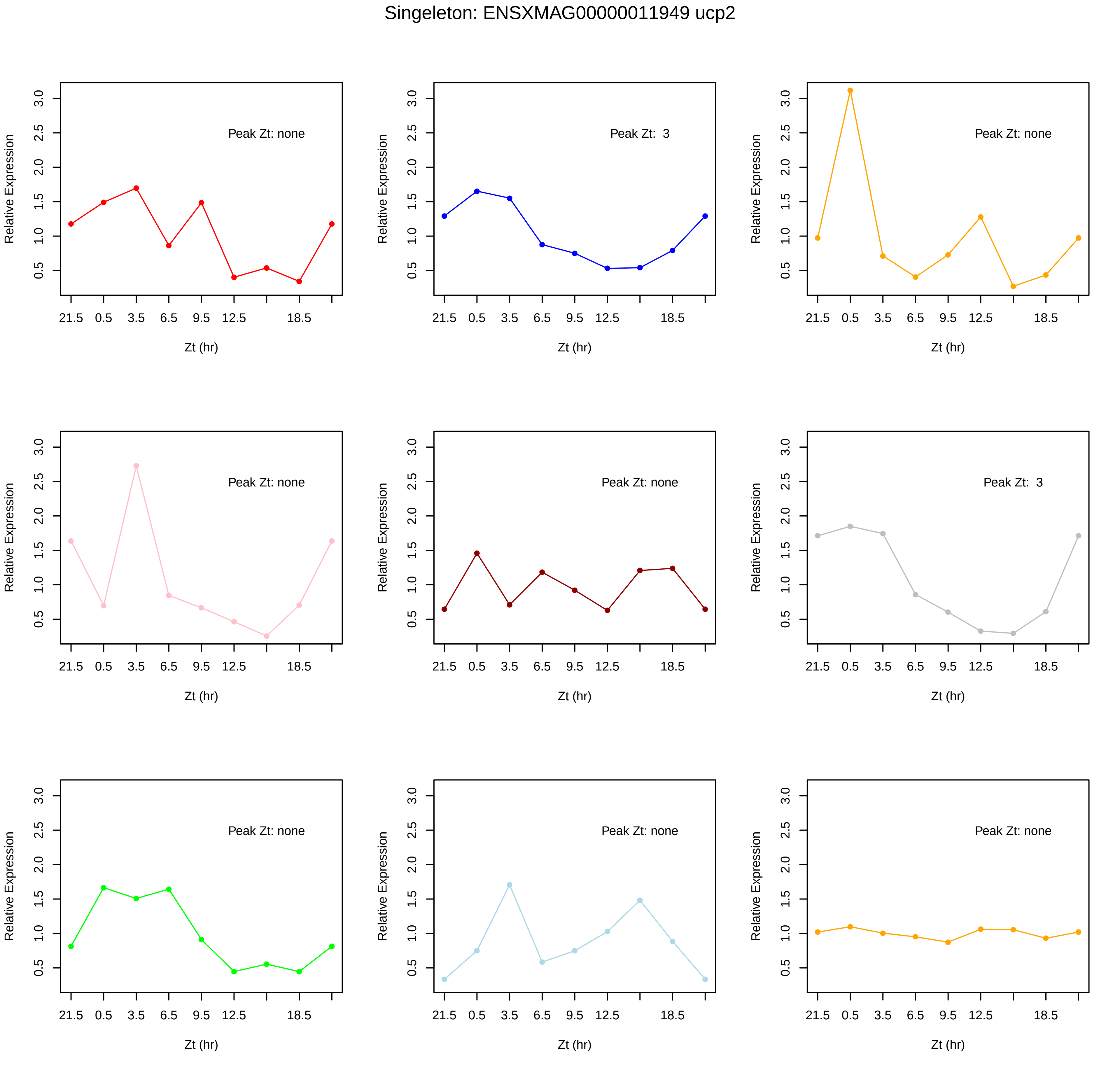

Singeleton: ENSXMAG00000011949 ucp2
Peak Zt: none
Peak Zt: 3
Peak Zt: none
Peak Zt: none
Peak Zt: none
Peak Zt: 3
Peak Zt: none
Peak Zt: none
Peak Zt: none
Relative Expression
Zt (hr)

Singeleton: ENSXMAG00000002466 ulk1b

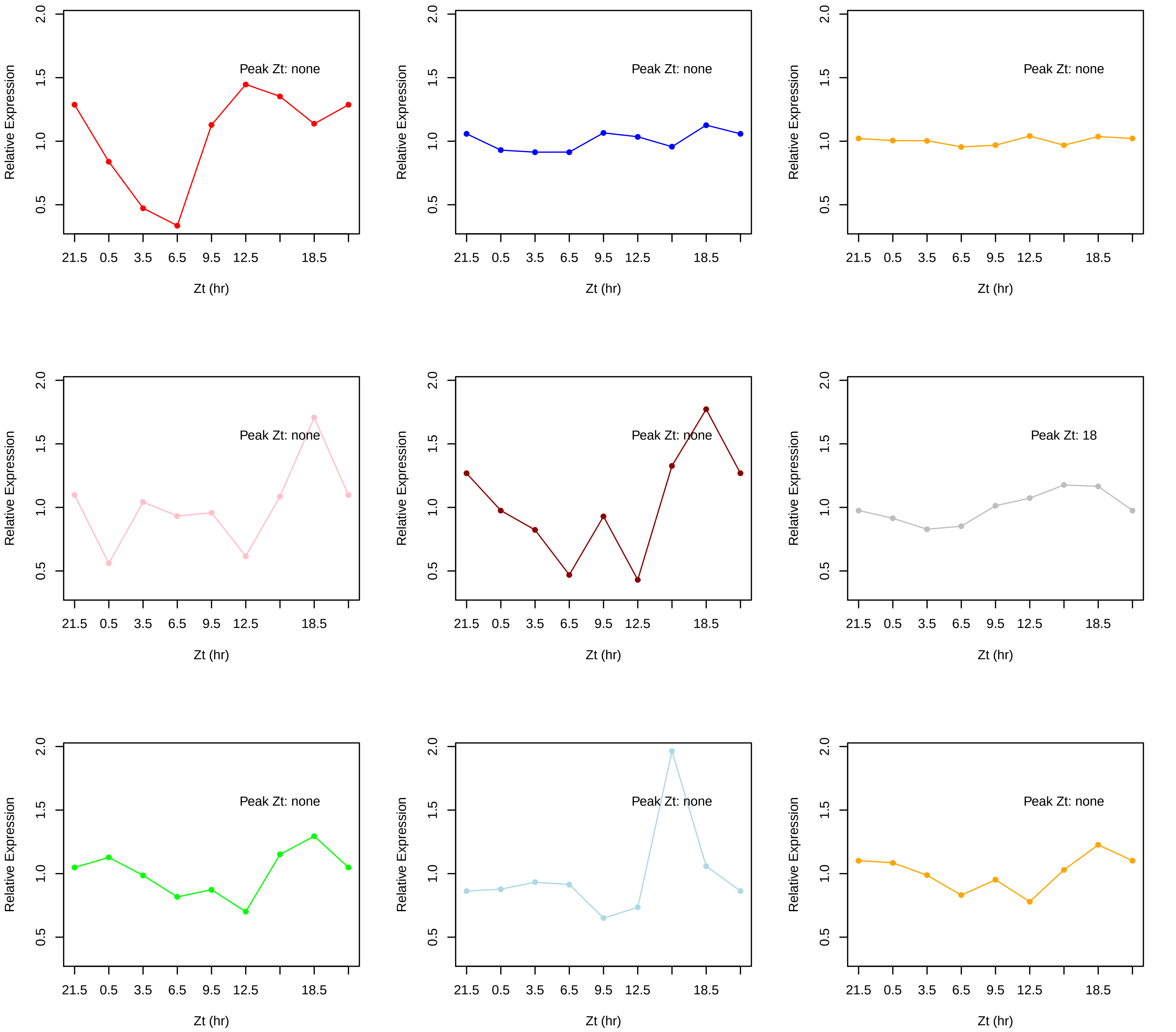

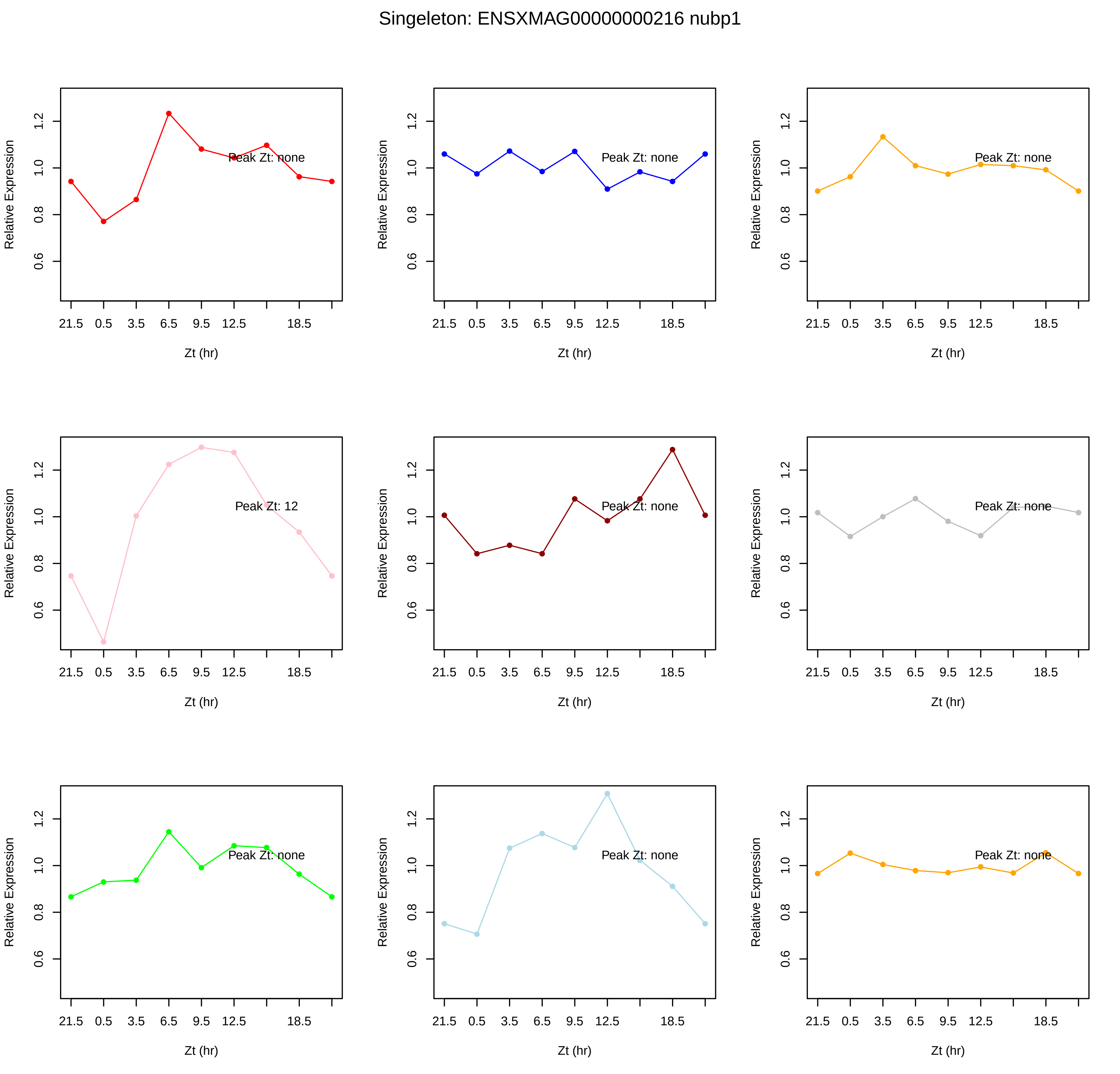
Singeleton: ENSXMAG00000000216 nubp1
Relative Expression
Zt (hr)
Peak Zt: none
Peak Zt: 12
21.5
0.5
3.5
6.5
9.5
12.5
18.5
0.6
0.8
1.0
1.2

Singeleton: ENSXMAG00000006291 cpt2

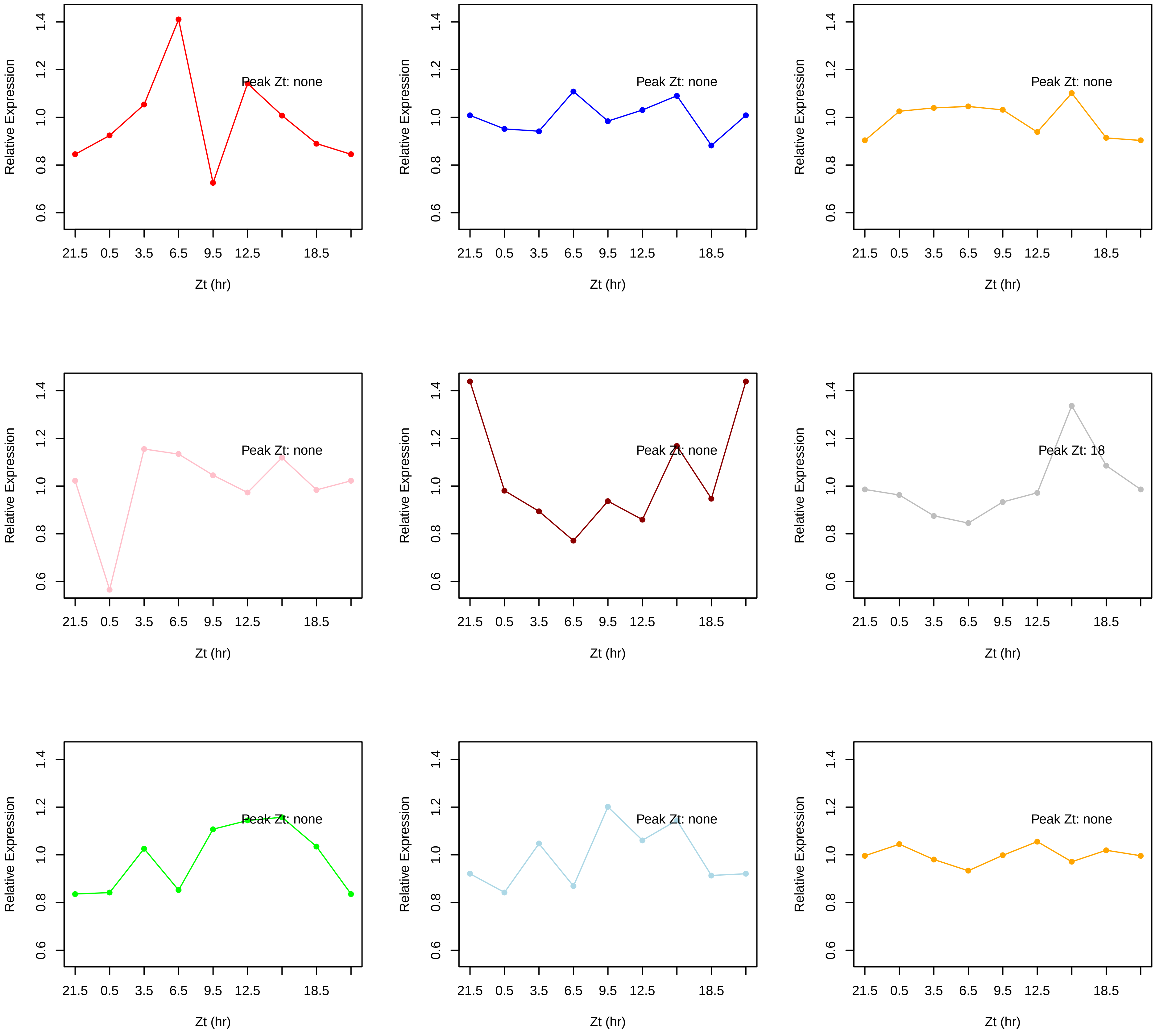

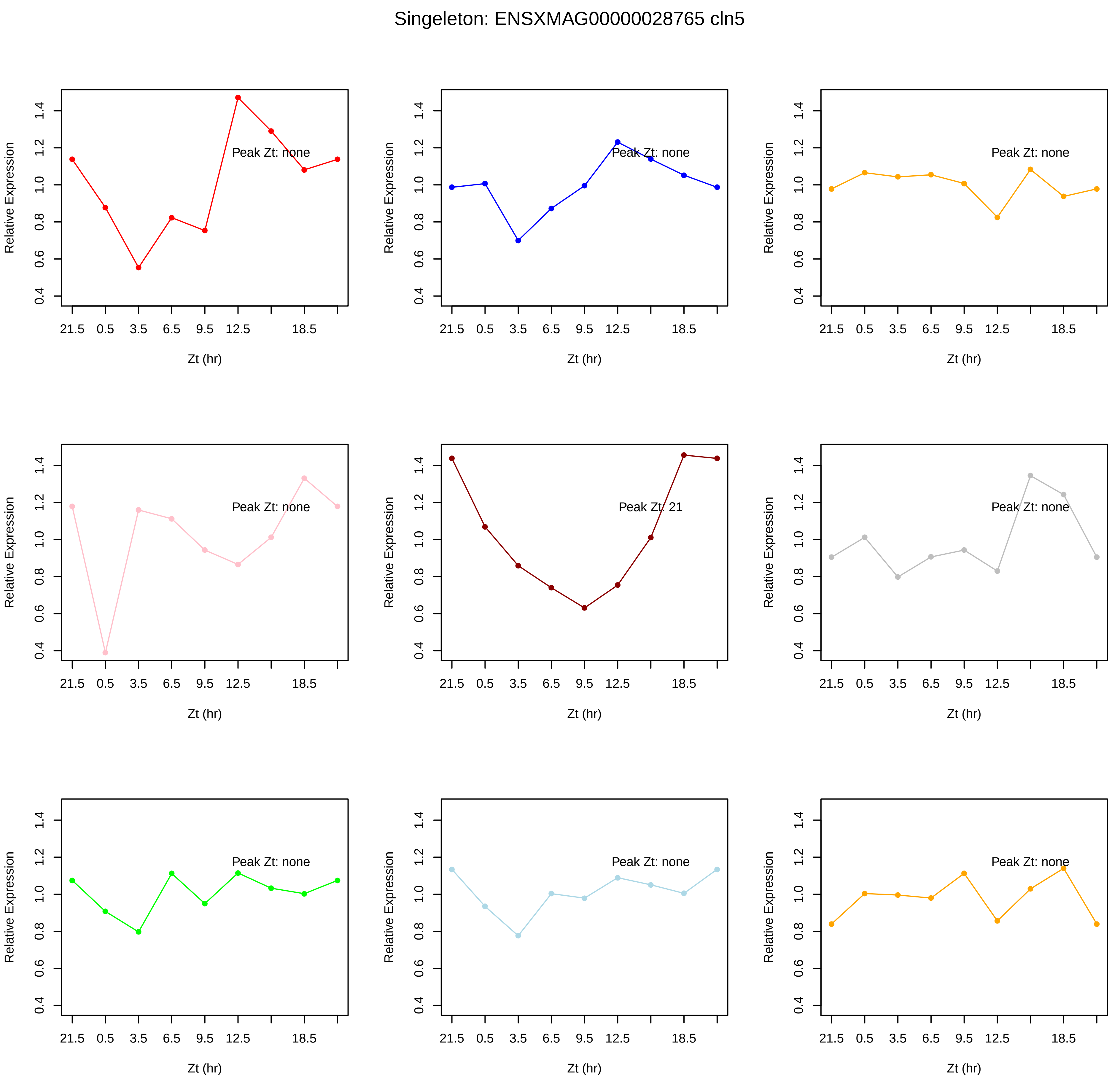

Singeleton: ENSXMAG00000028765 cln5
Peak Zt: none
Peak Zt: none
Peak Zt: none
Peak Zt: none
Peak Zt: 21
Peak Zt: none
Peak Zt: none
Peak Zt: none
Peak Zt: none
Relative Expression
Zt (hr)
0.4
0.6
0.8
1.0
1.2
1.4
21.5
0.5
3.5
6.5
9.5
12.5
18.5

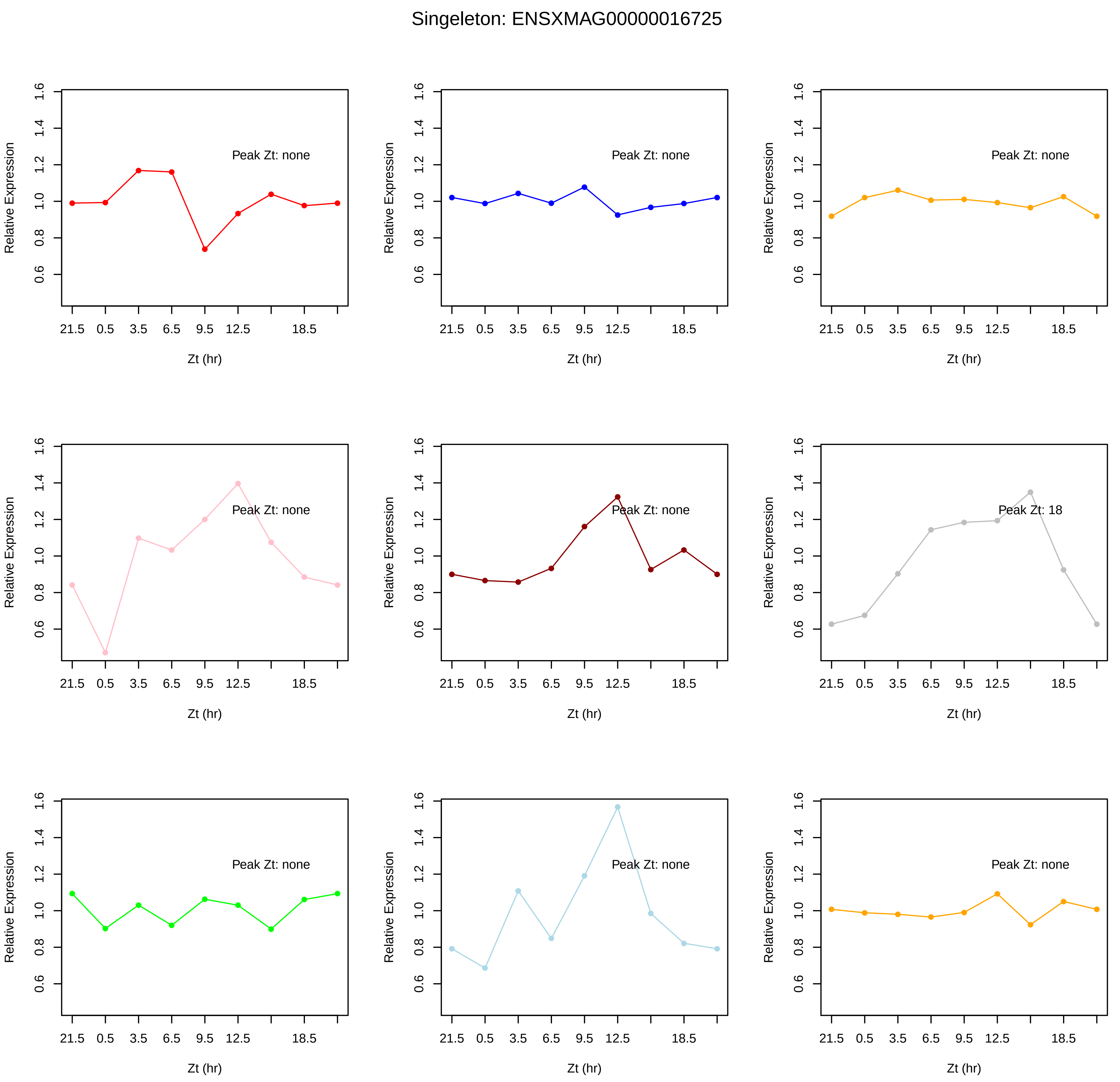
Singeleton: ENSXMAG00000016725
Relative Expression
Zt (hr)
0.6 0.8 1.0 1.2 1.4 1.6
21.5 0.5 3.5 6.5 9.5 12.5 18.5
Peak Zt: none
Peak Zt: none
Peak Zt: none
Peak Zt: none
Peak Zt: none
Peak Zt: 18
Peak Zt: none
Peak Zt: none
Peak Zt: none

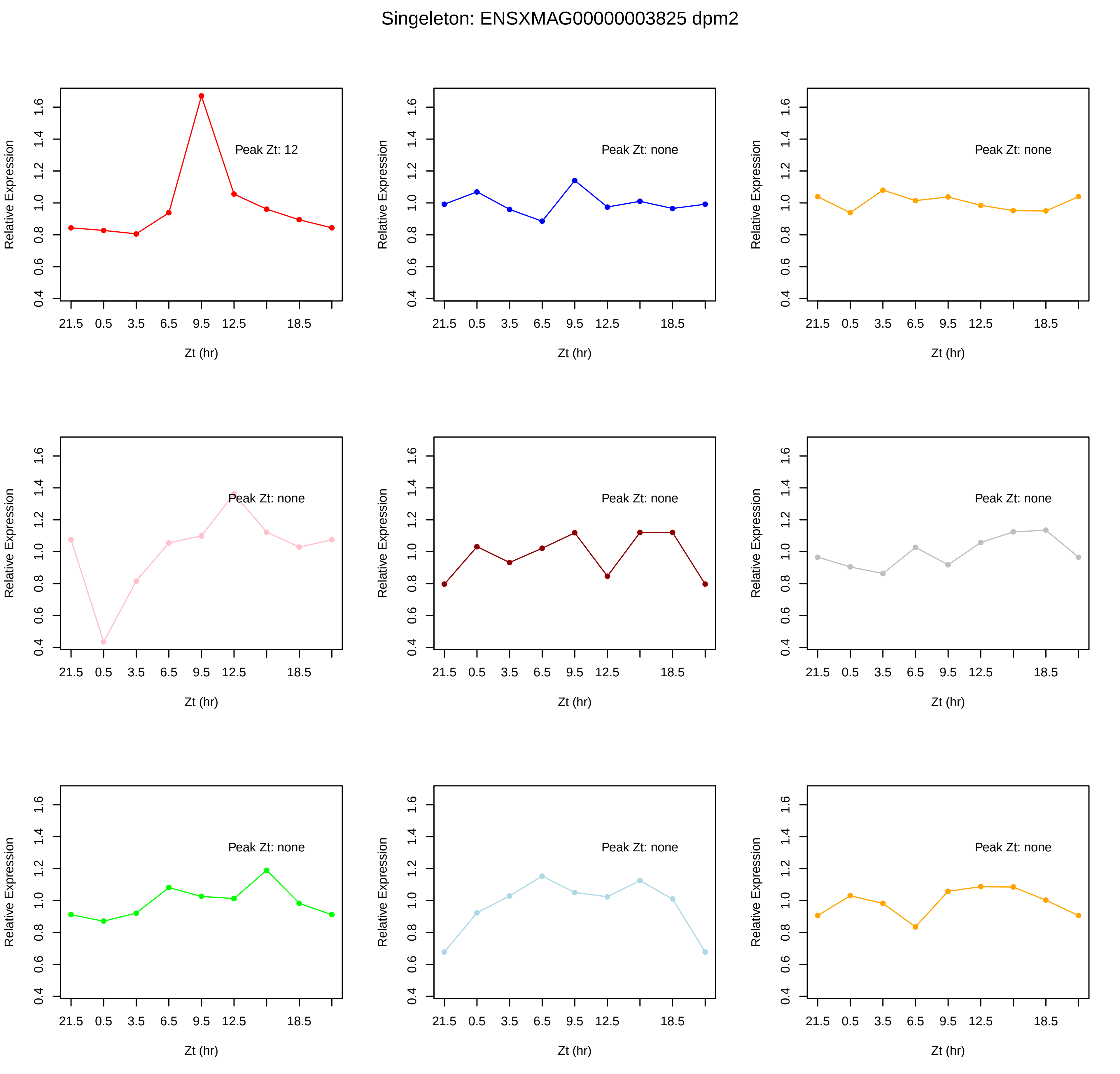

Singeleton: ENSXMAG00000003825 dpm2
Peak Zt: 12
Peak Zt: none
Peak Zt: none
Peak Zt: none
Peak Zt: none
Peak Zt: none
Peak Zt: none
Peak Zt: none
Peak Zt: none
Relative Expression
Zt (hr)
21.5
0.5
3.5
6.5
9.5
12.5
18.5
0.4
0.6
0.8
1.0
1.2
1.4
1.6

Singeleton: ENSXMAG00000000986 sb:cb649

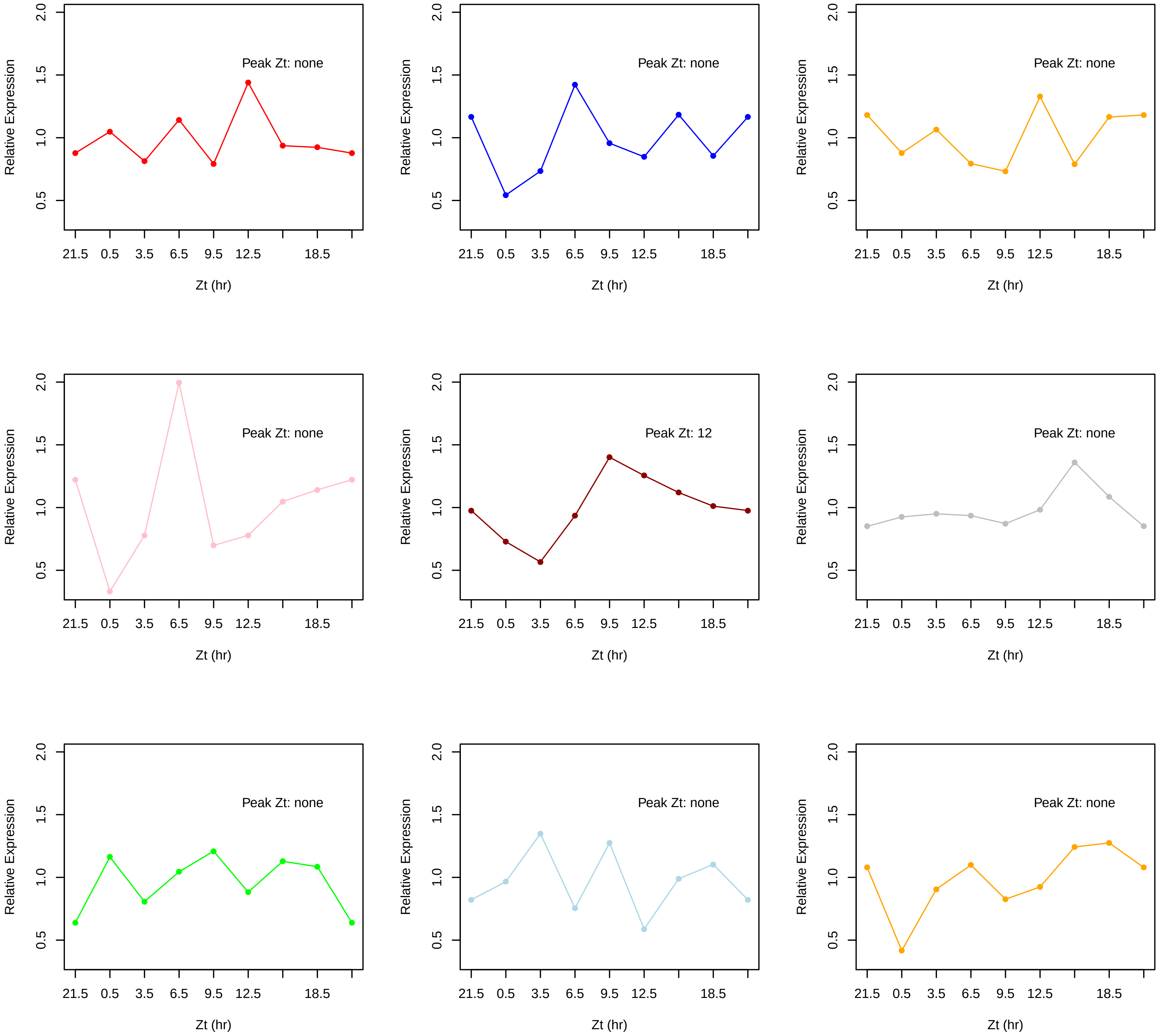

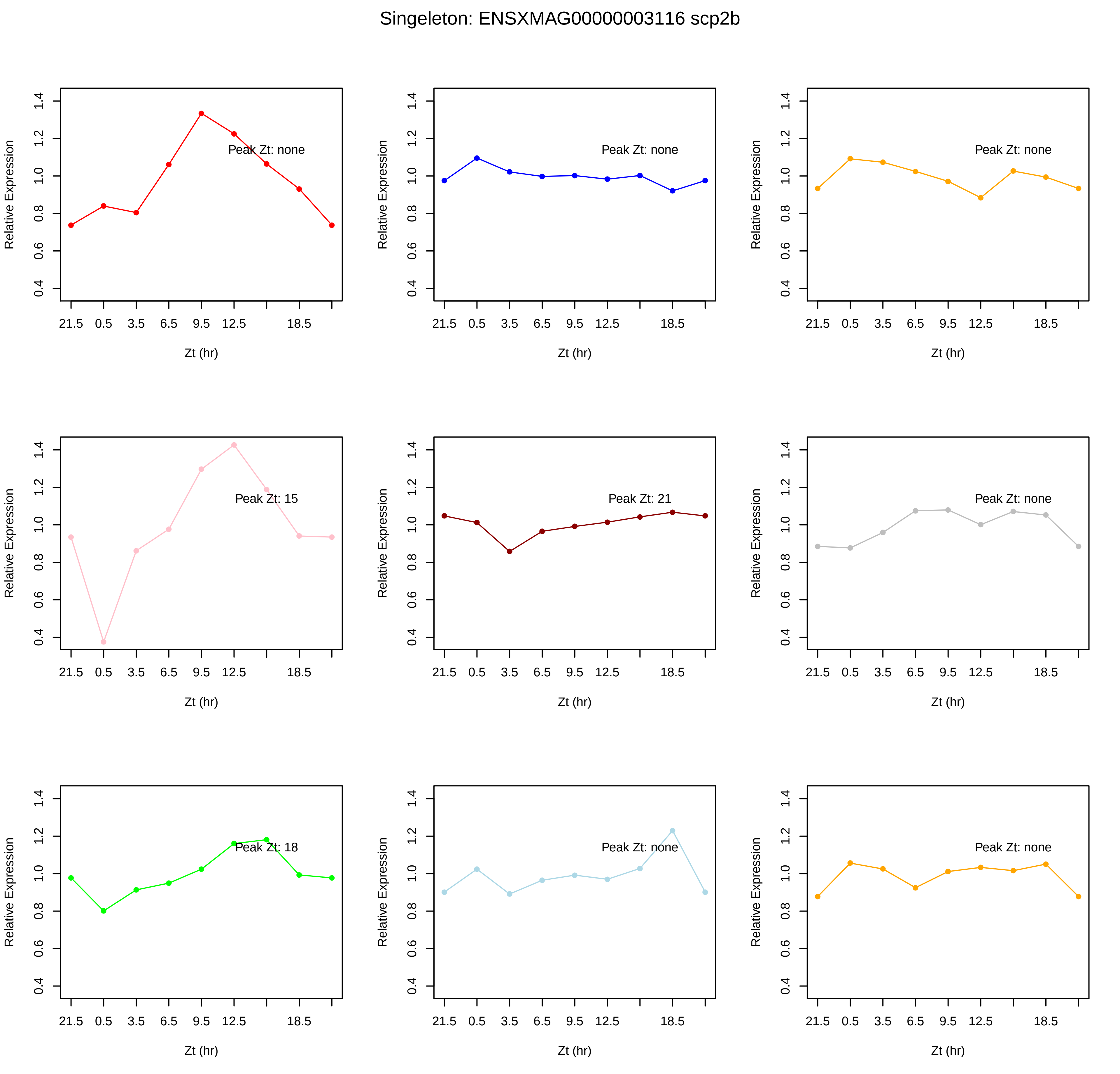

Singeleton: ENSXMAG00000003116 scp2b
Peak Zt: none
Peak Zt: none
Peak Zt: none
Peak Zt: 15
Peak Zt: 21
Peak Zt: none
Peak Zt: 18
Peak Zt: none
Peak Zt: none
Relative Expression
Zt (hr)
21.5
0.5
3.5
6.5
9.5
12.5
18.5
0.4
0.6
0.8
1.0
1.2
1.4

Singleton: ENSXMAG00000019269 agbl2

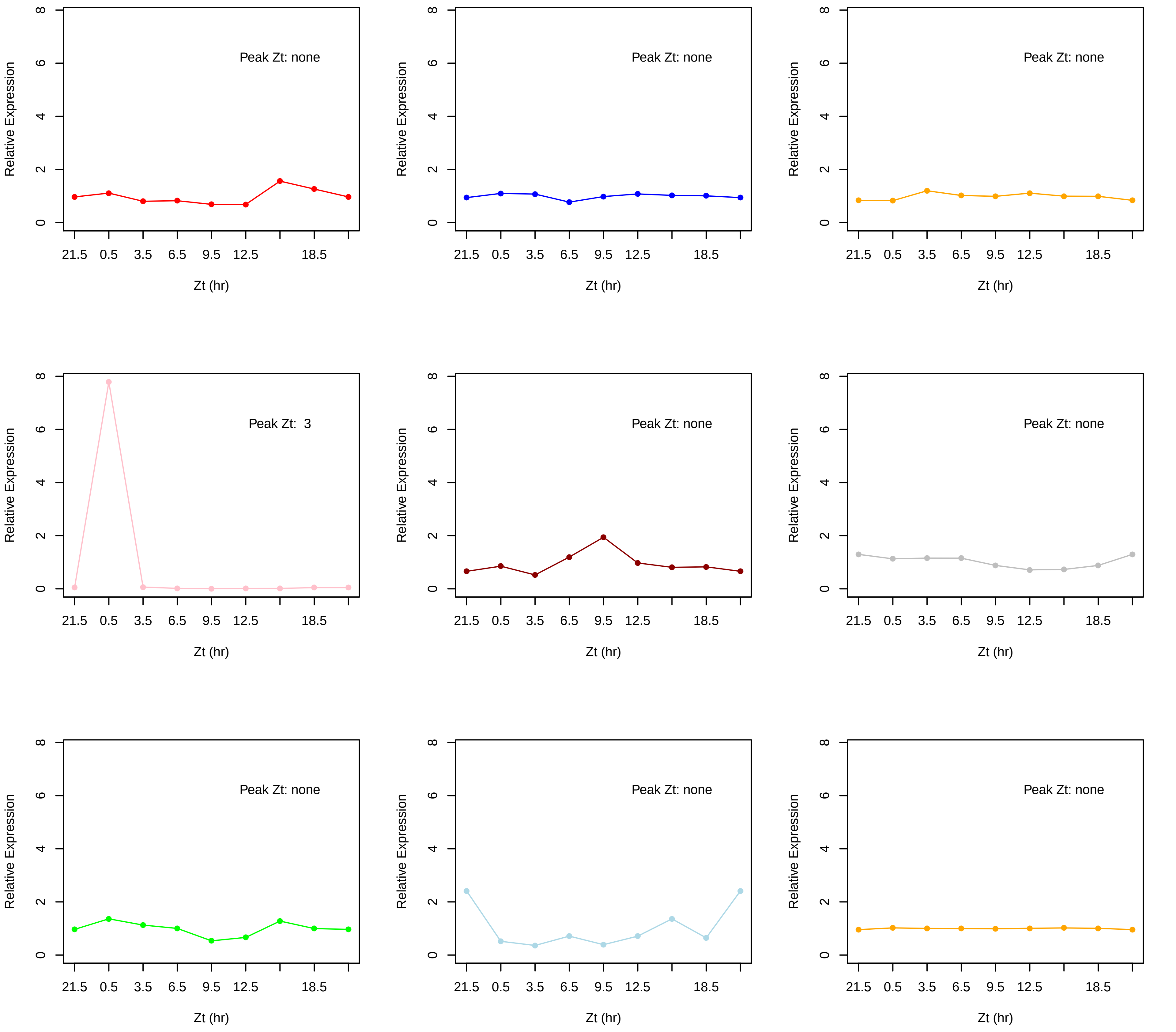

Singeleton: ENSXMAG00000005898 sorl1

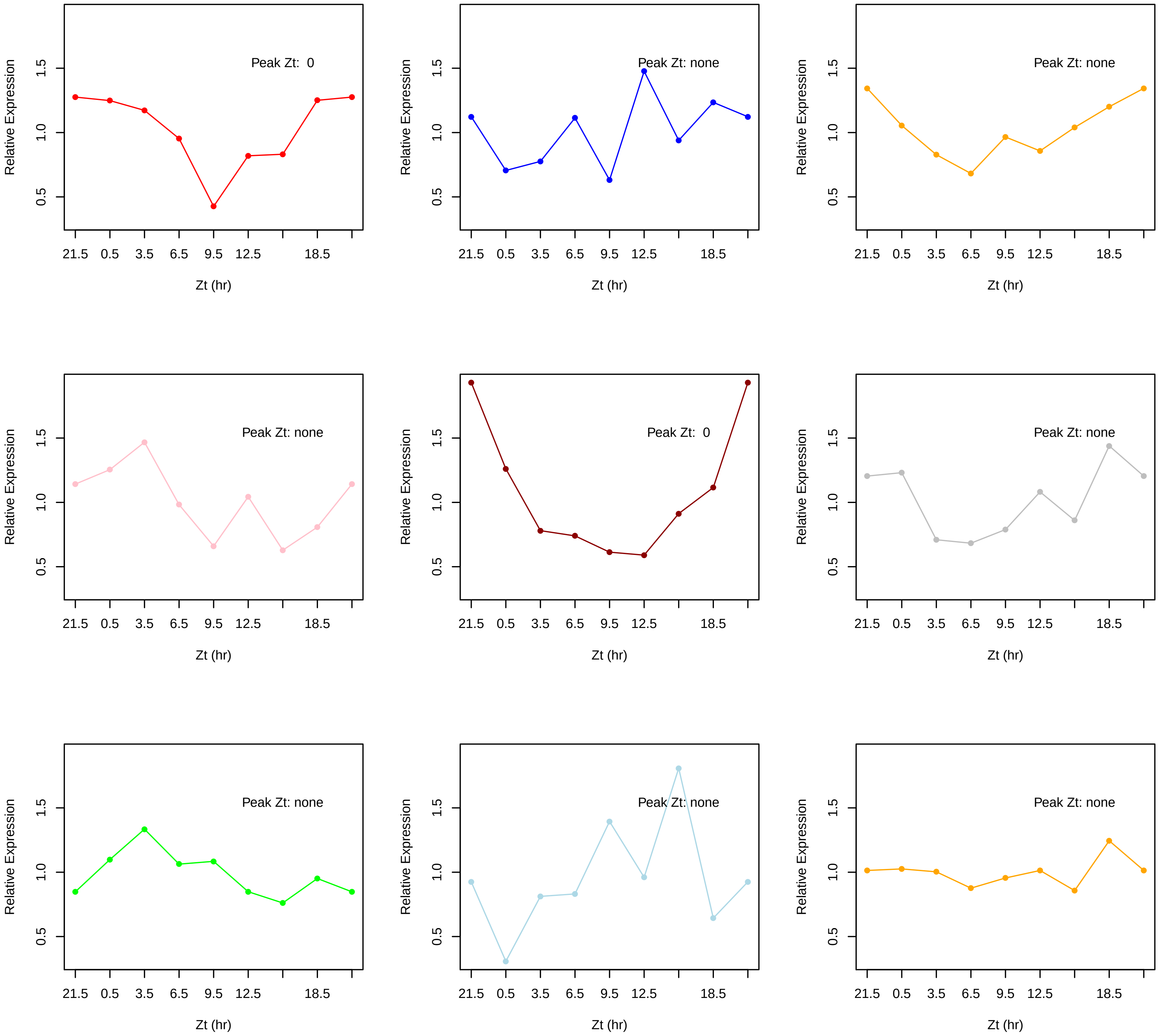

Singleton: ENSXMAG00000019309 btbd2b

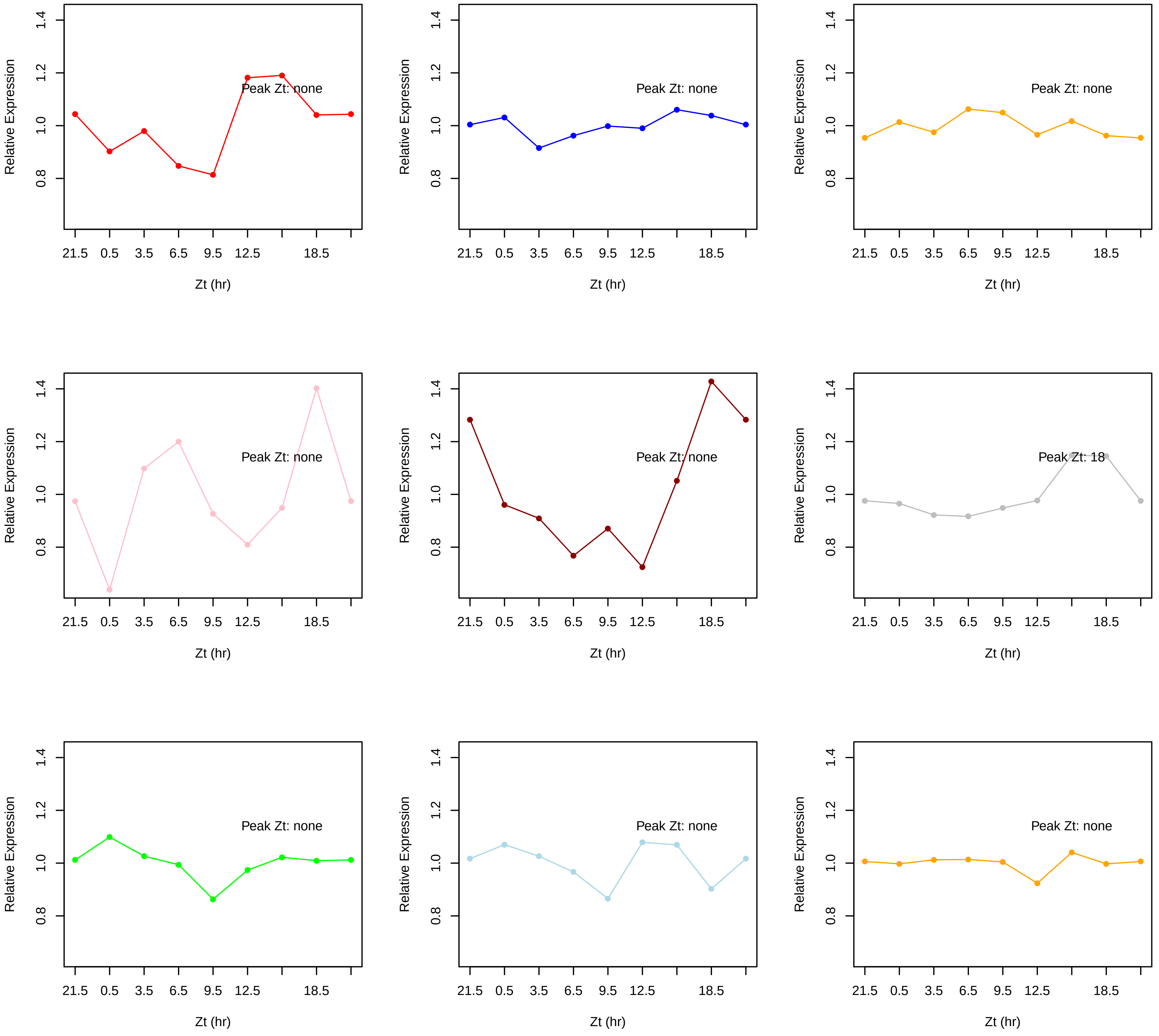

Singleton: ENSXMAG00000019390 NADK

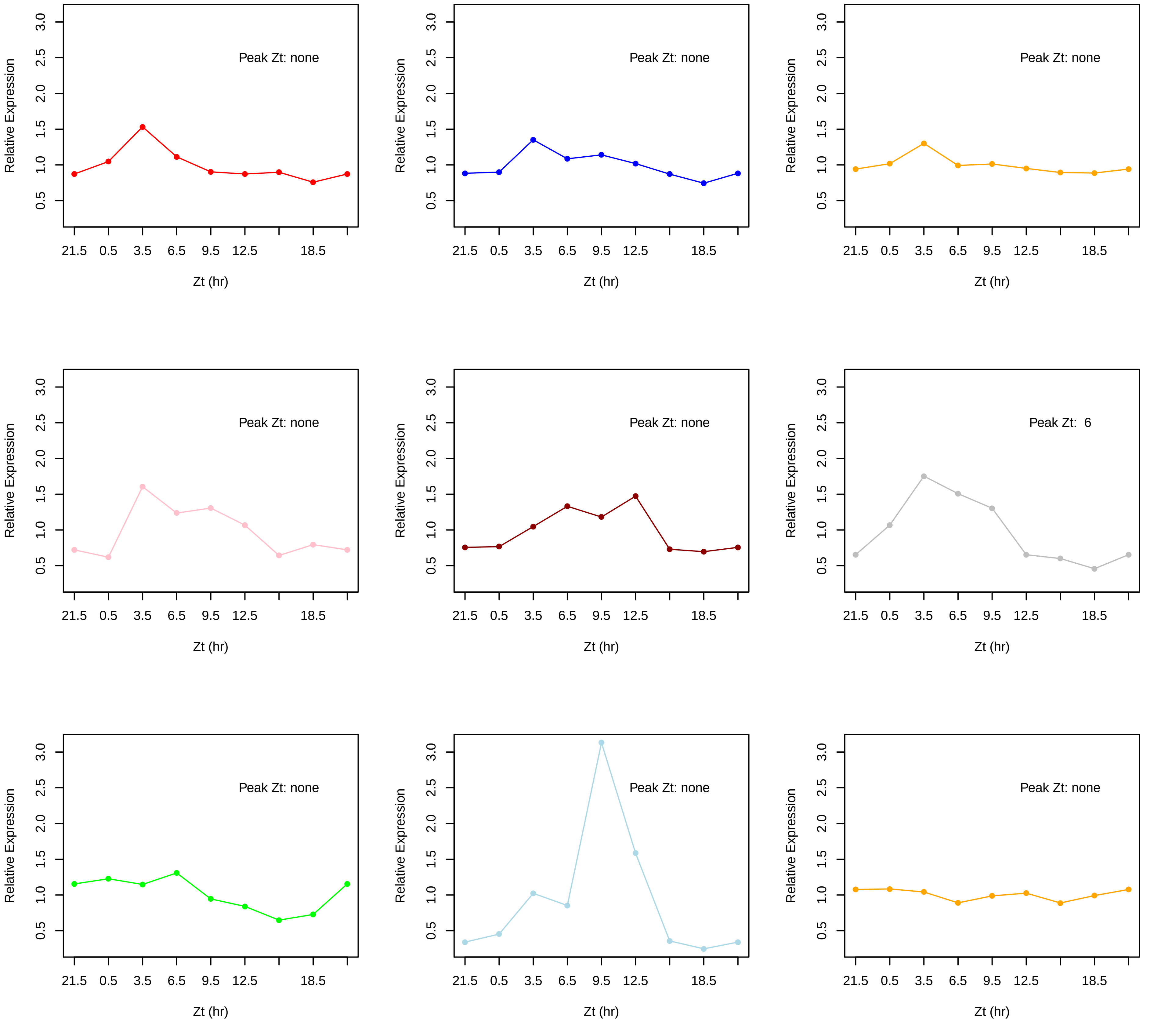

Singeleton: ENSXMAG00000029474 pip5kl1

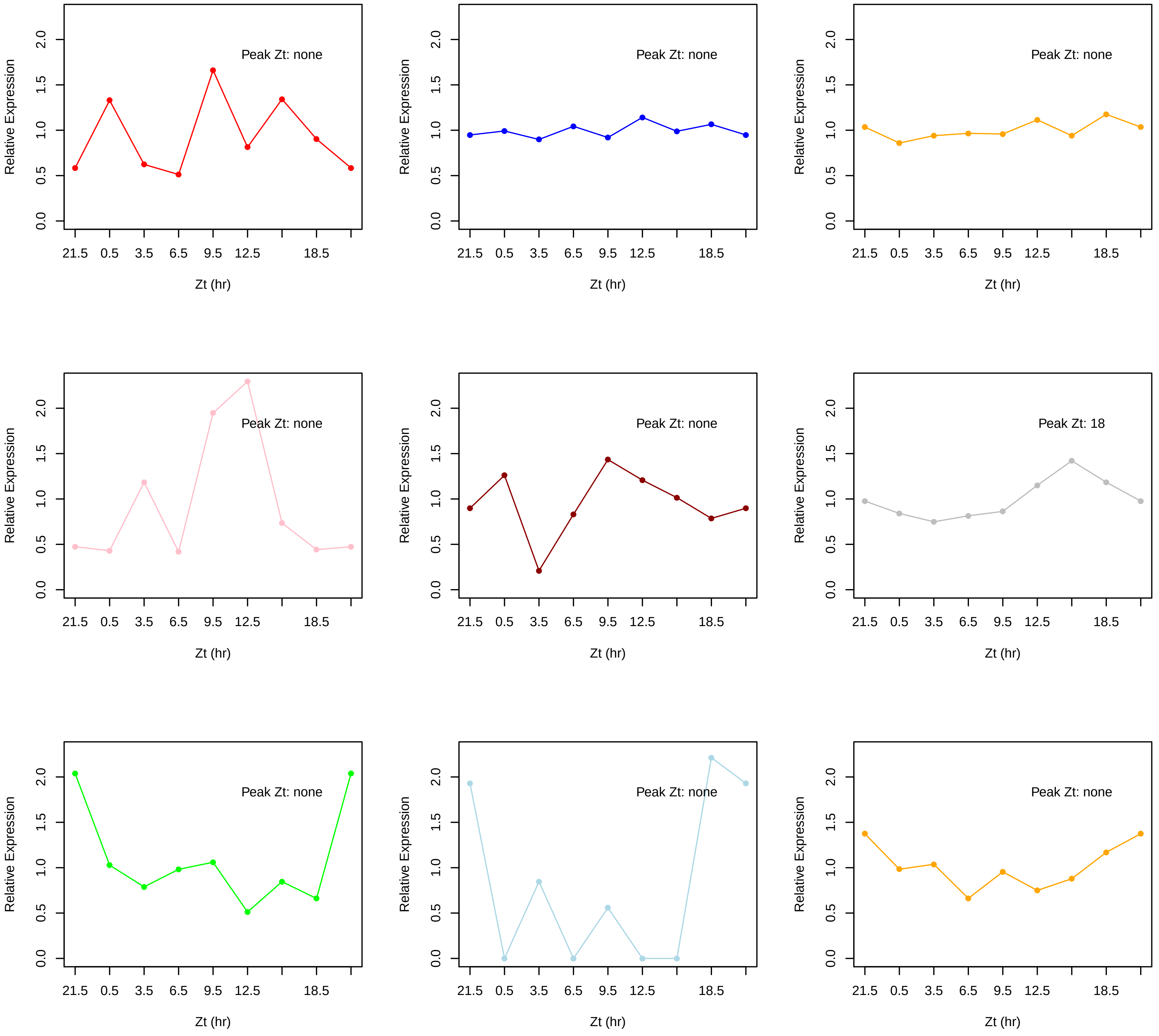

Singeleton: ENSXMAG00000029603 mtch2

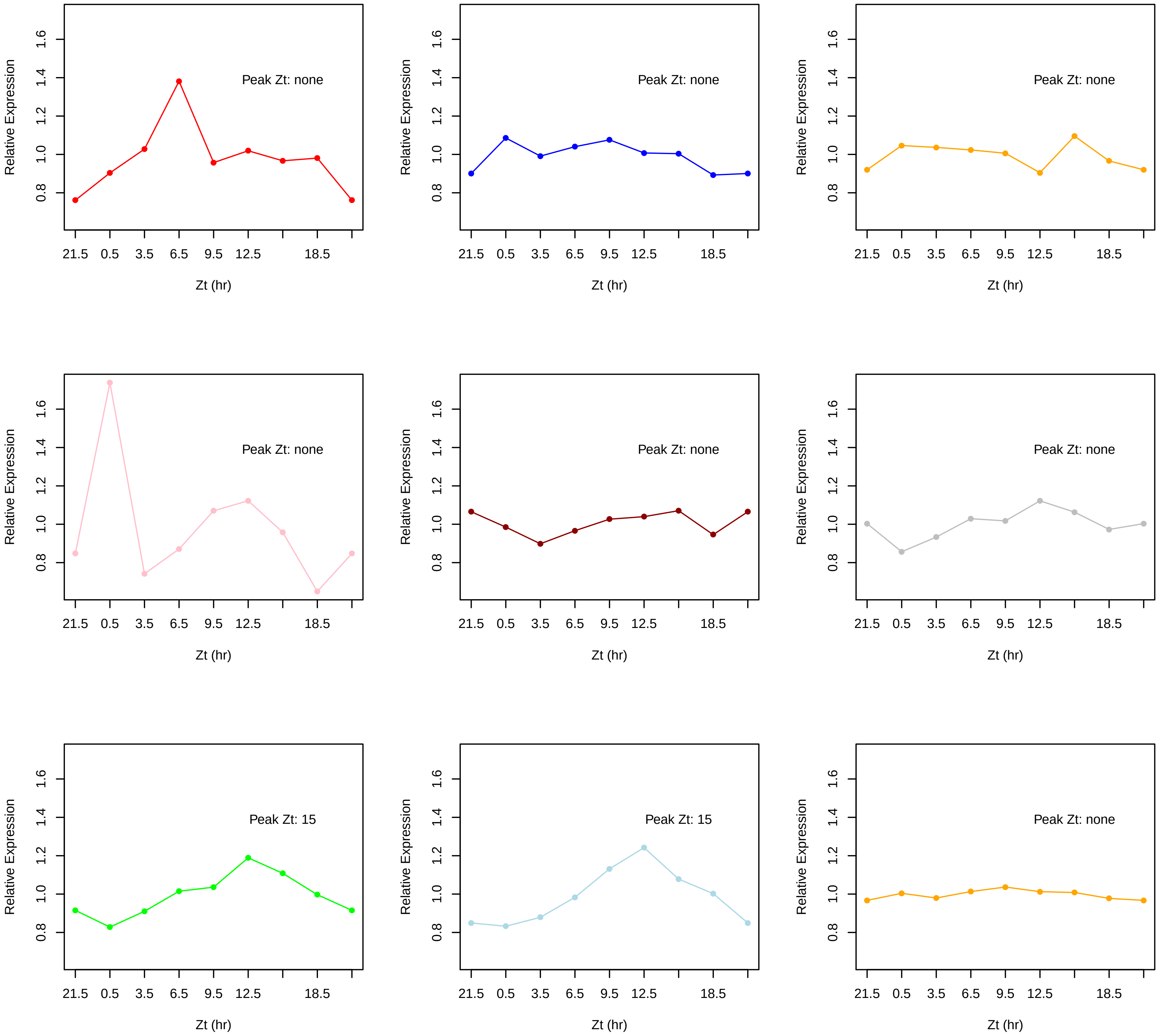

Singeleton: ENSXMAG00000003118 podn

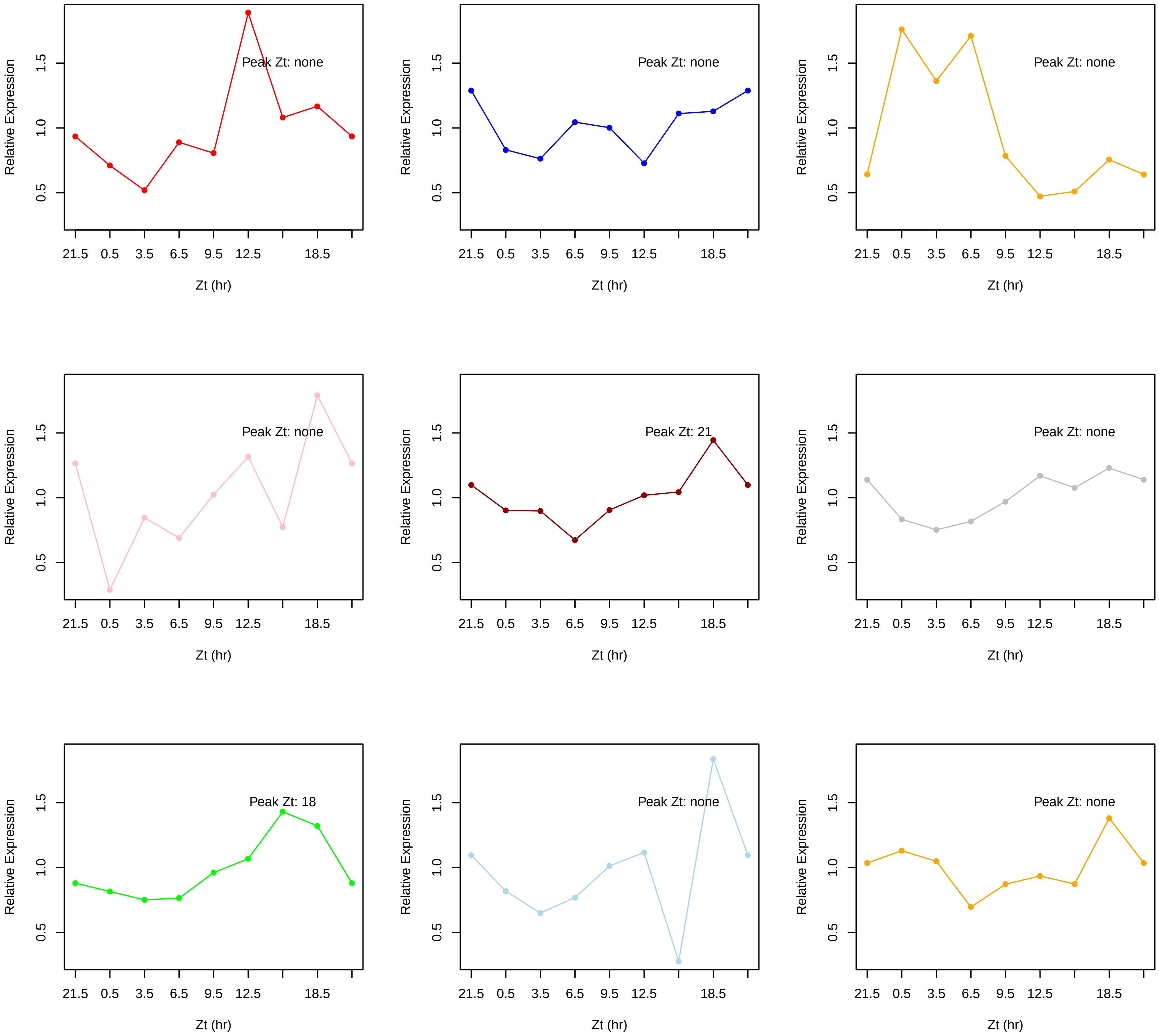

Singeleton: ENSXMAG00000000221 emp2

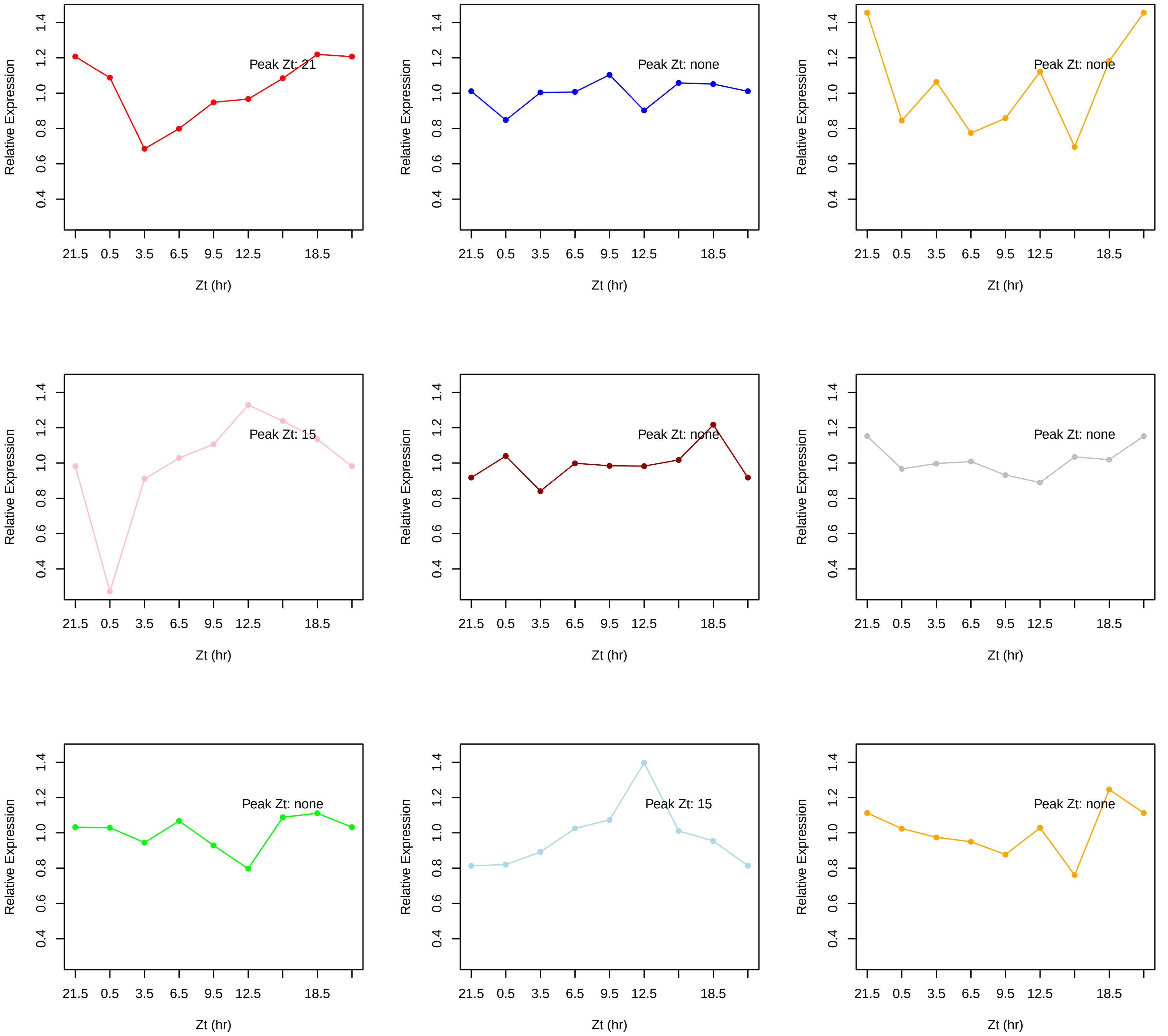

Singeleton: ENSXMAG00000014445 atad1b

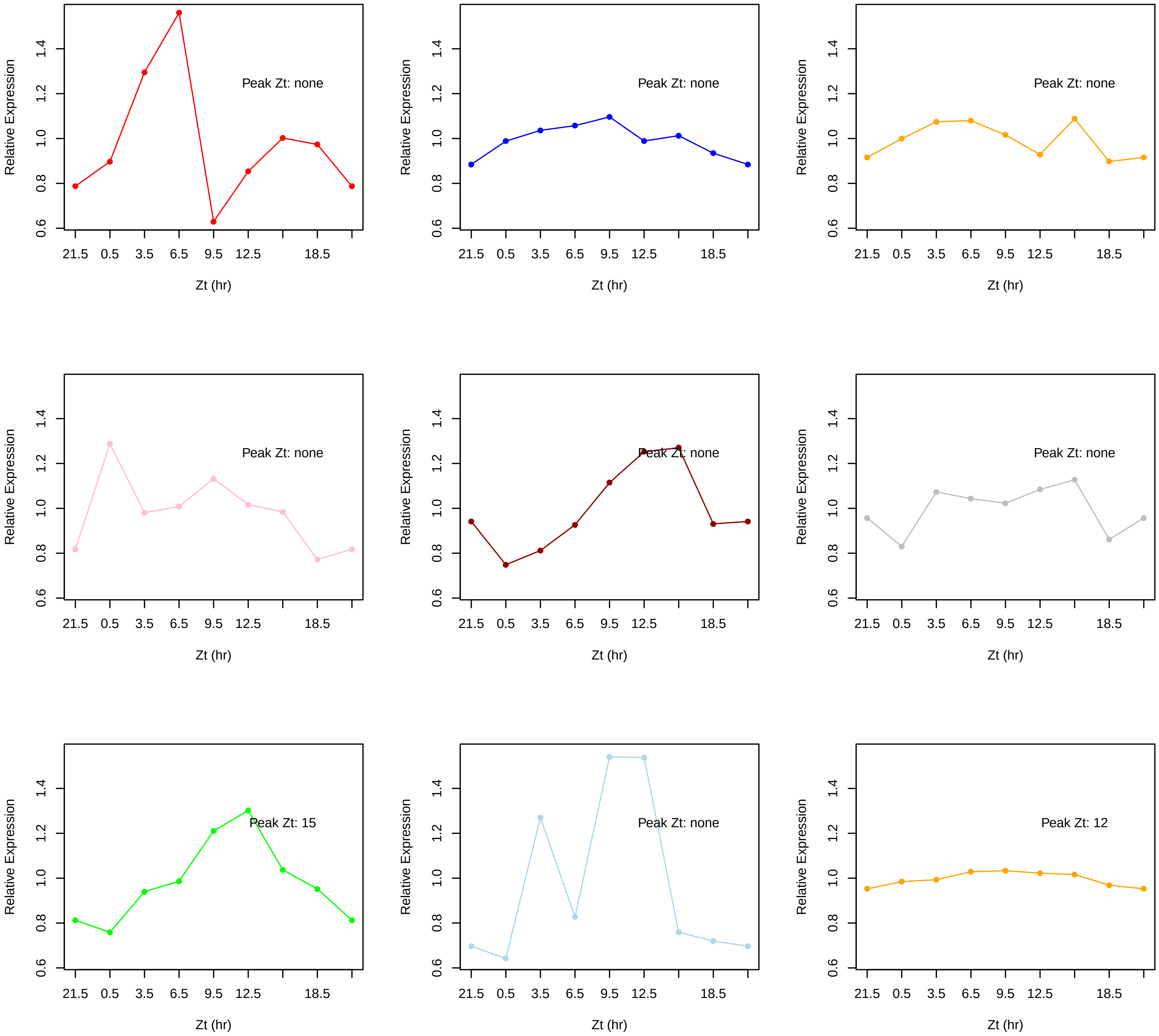

Singeleton: ENSXMAG00000014950 psmc3

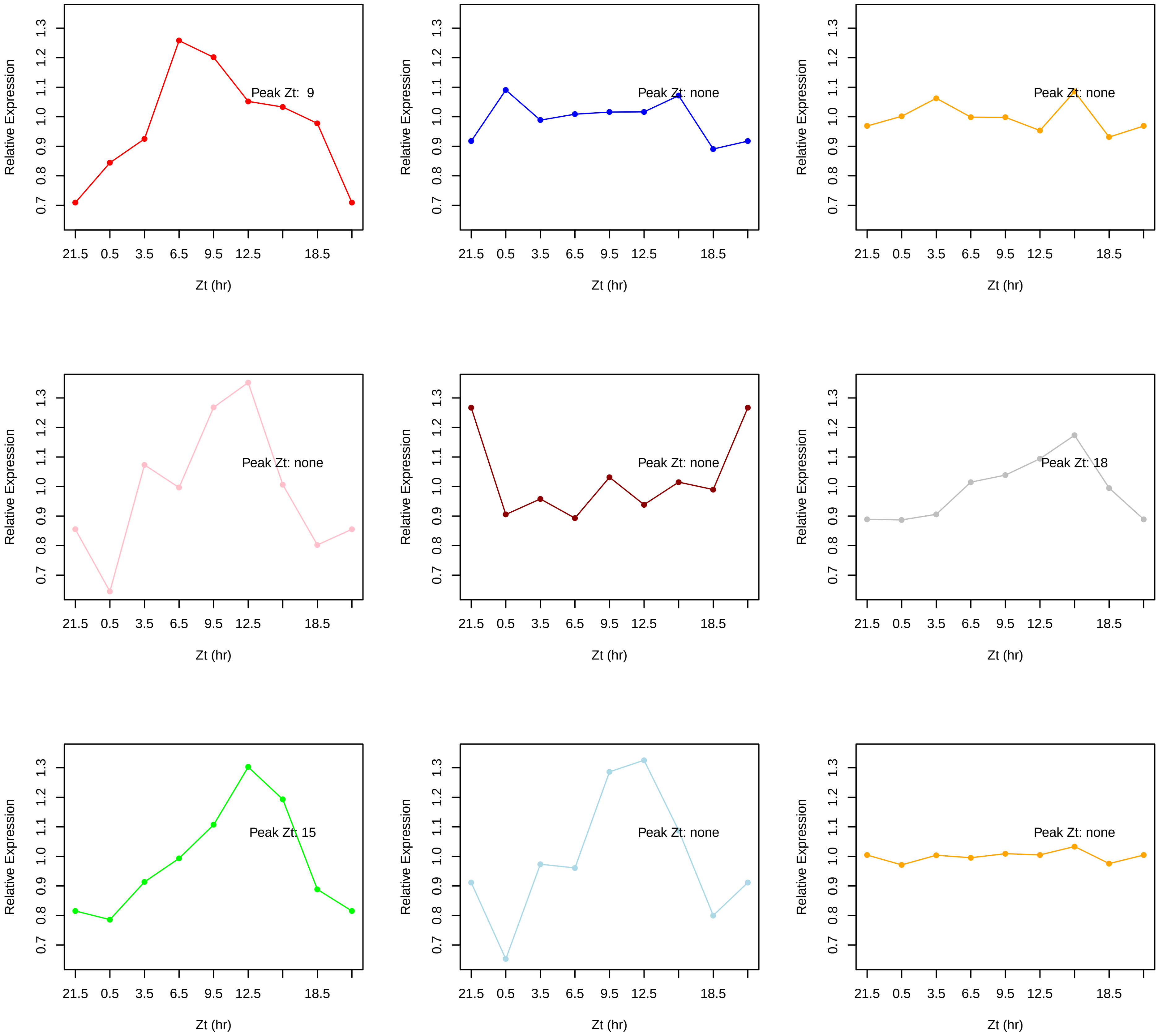

Singeleton: ENSXMAG00000009020 sfswap

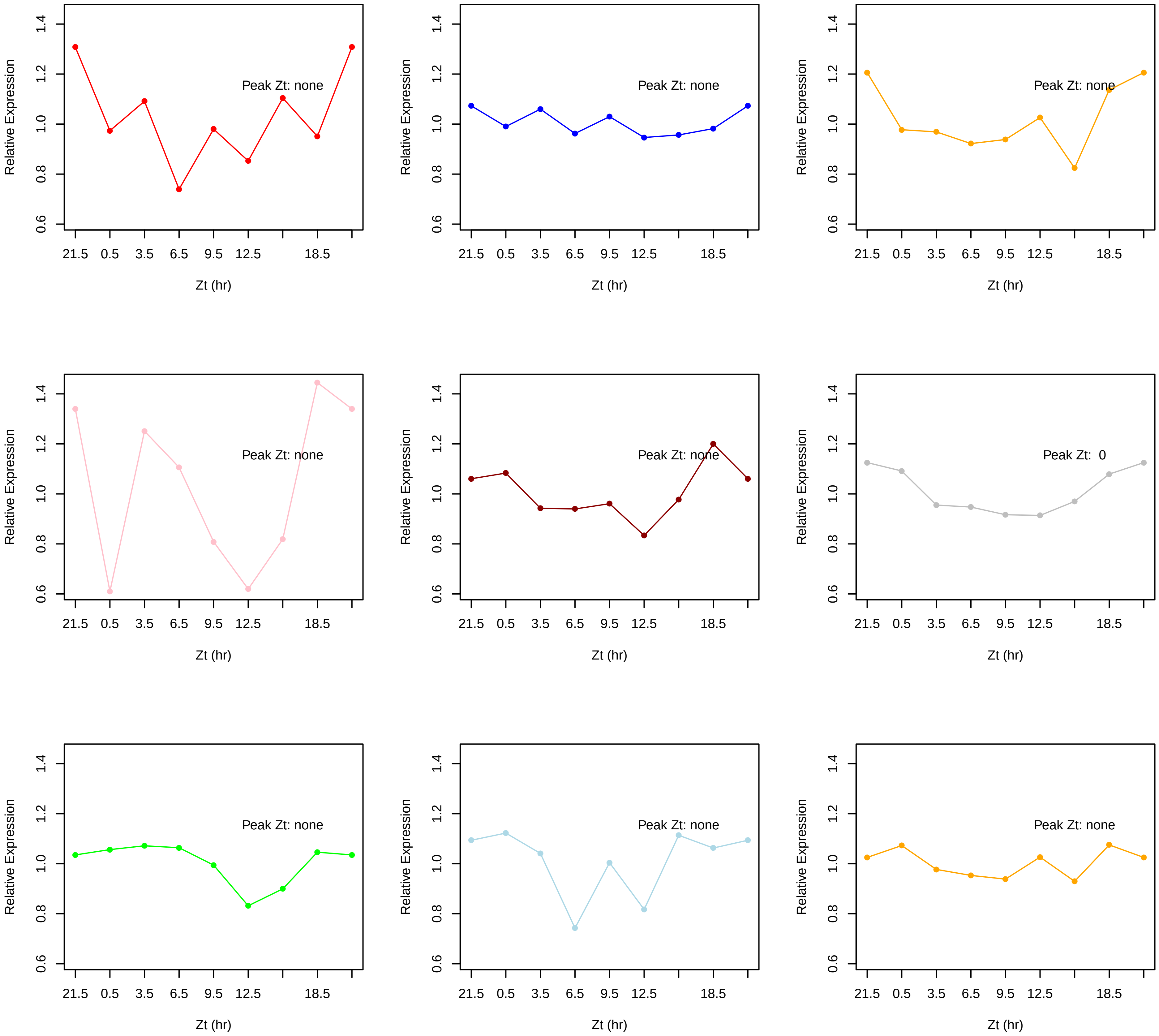

Singleton: ENSXMAG00000025720 TMEM208

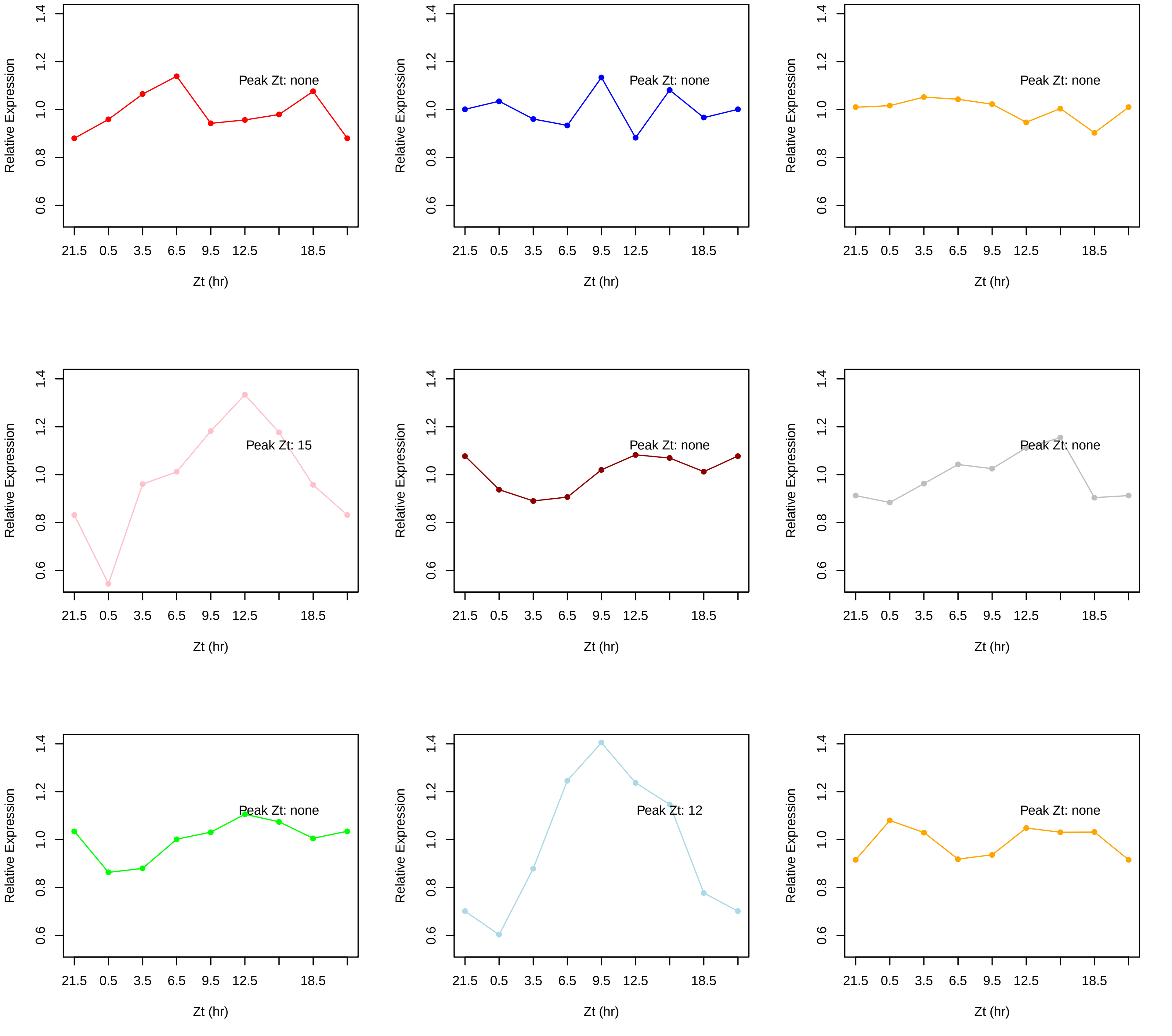

Singeleton: ENSXMAG00000027841 stx16

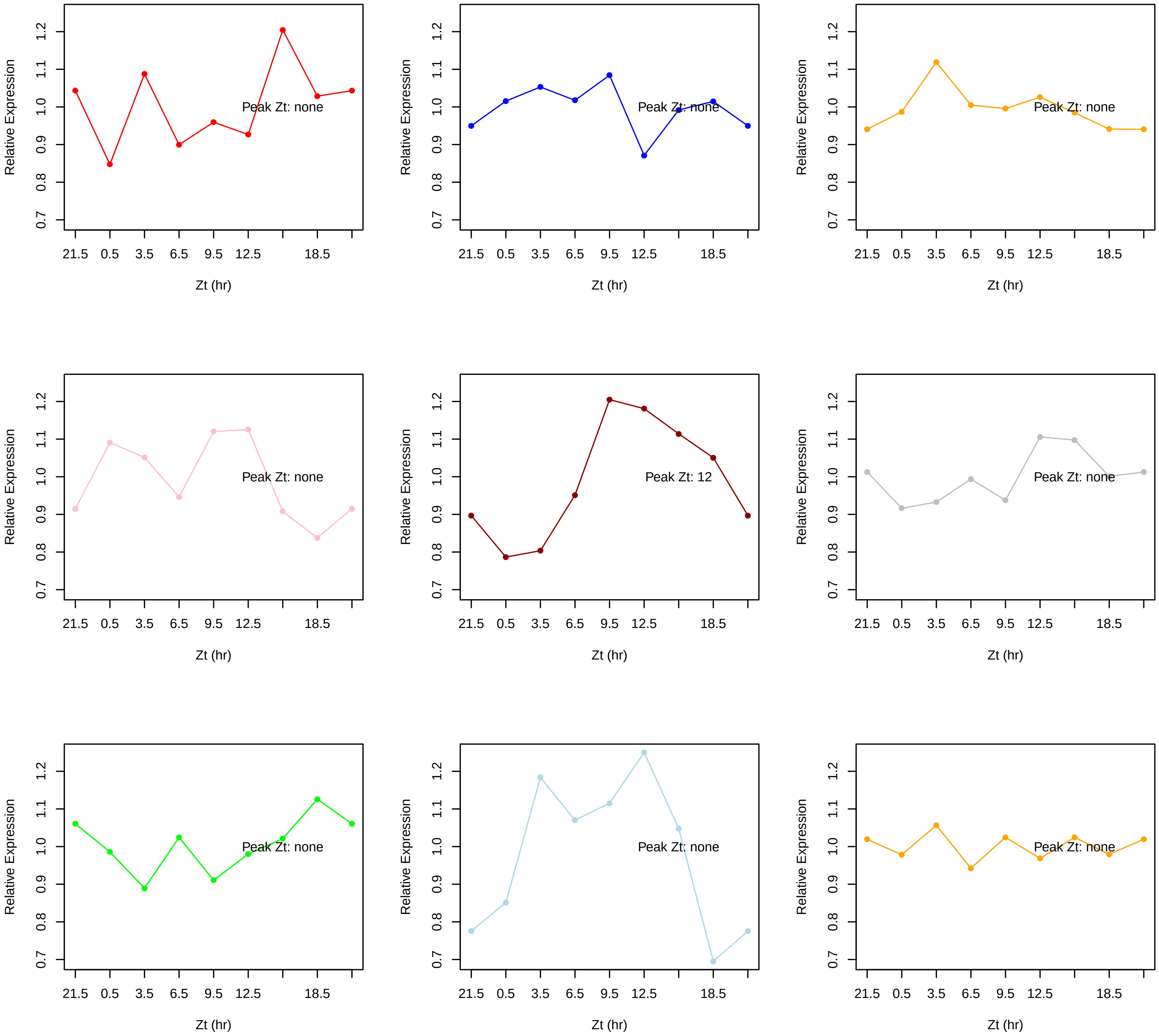

Singeleton: ENSXMAG00000019389 SLC35E2A

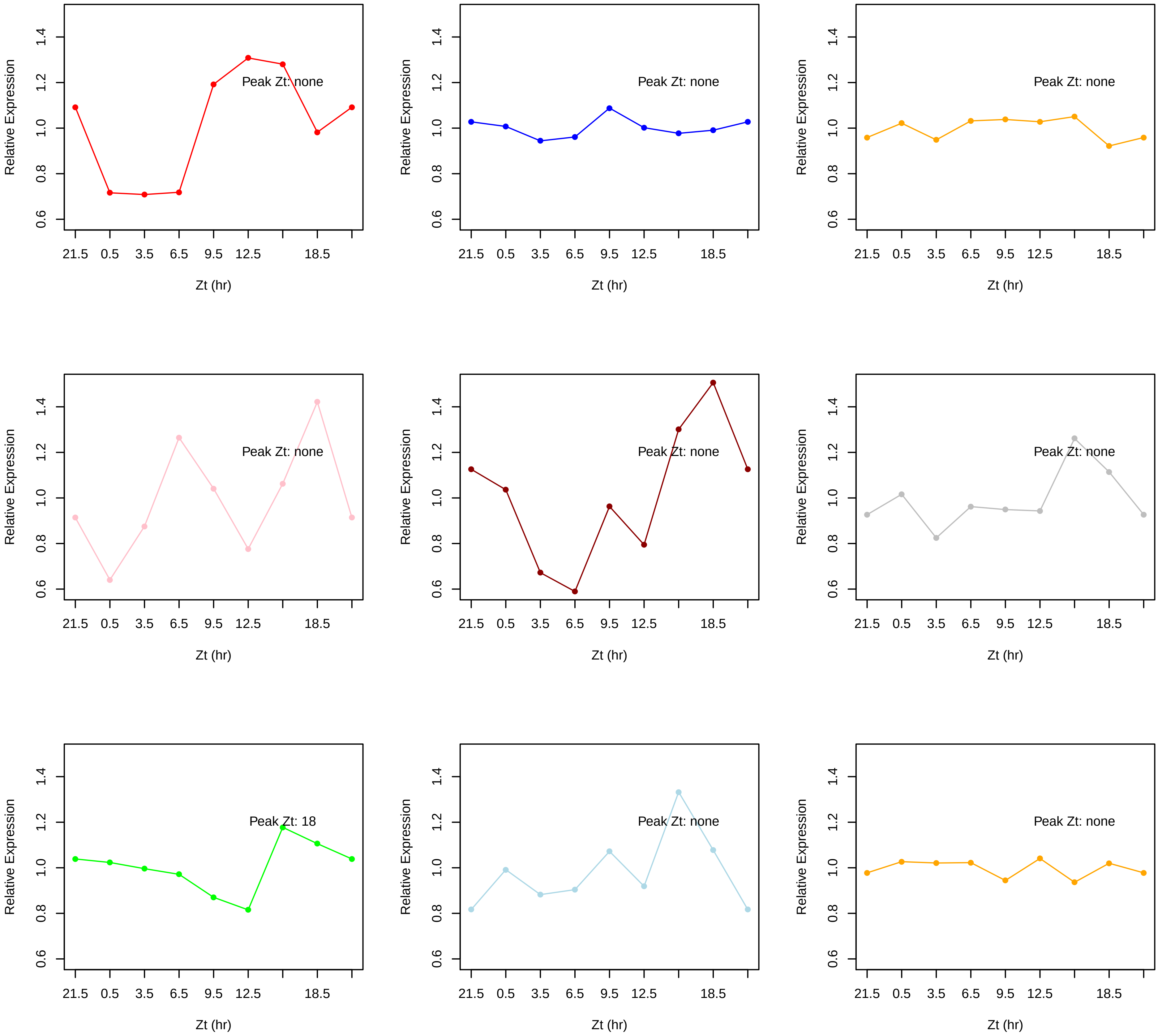

Singeleton: ENSXMAG00000013882 chn2

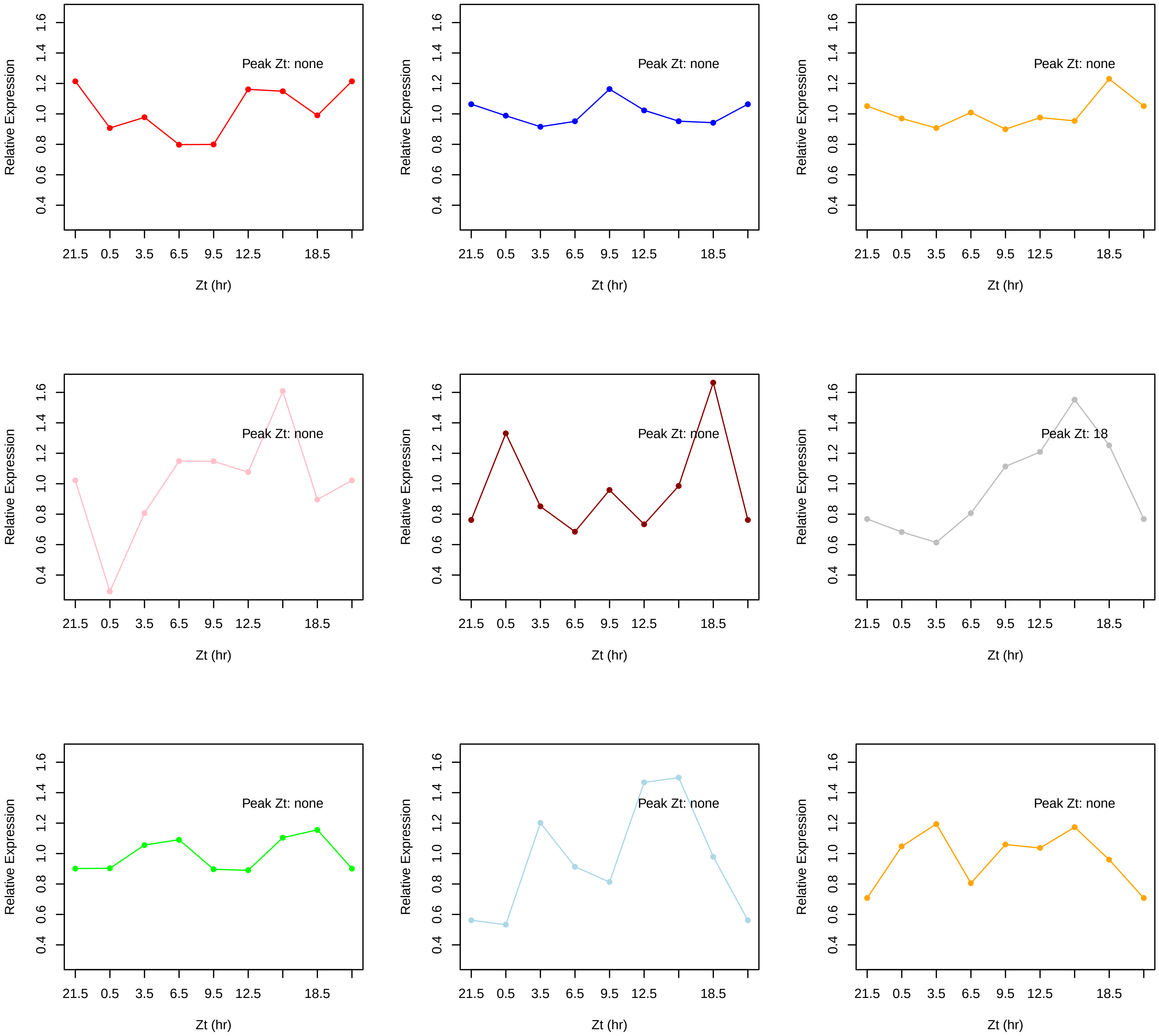

Singeleton: ENSXMAG00000005693 uck1

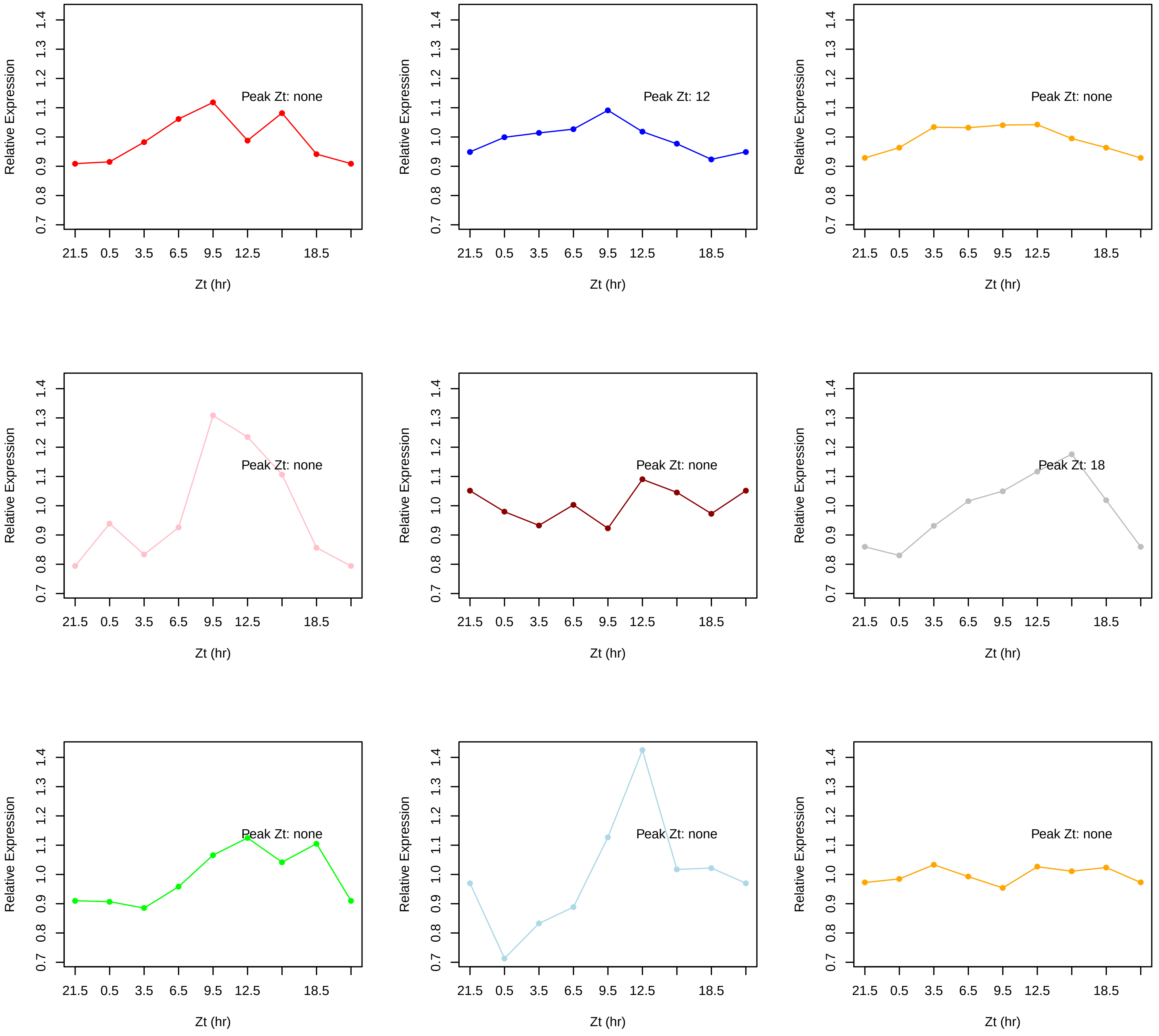

Singeleton: ENSXMAG00000012962 papss2a

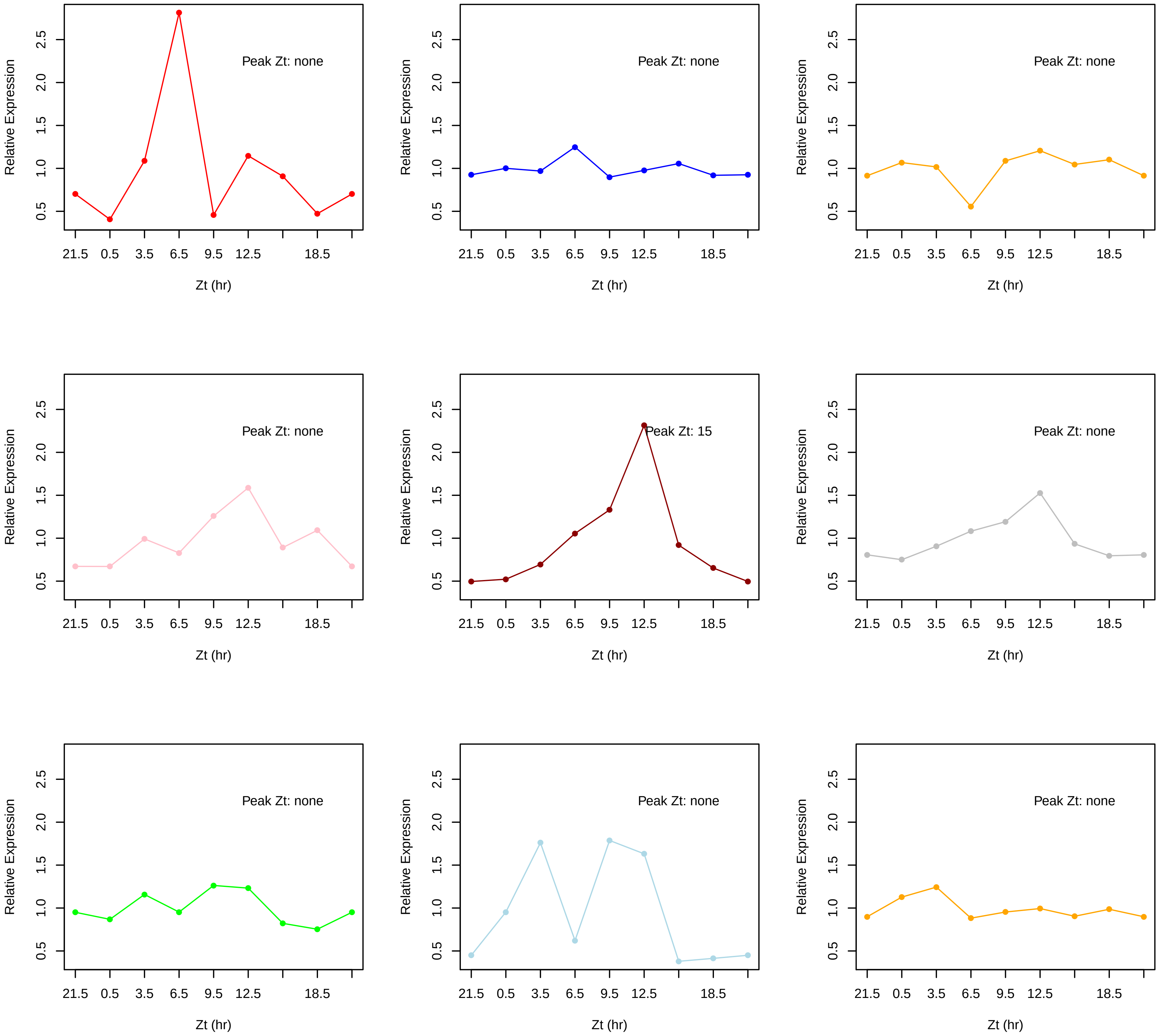

Singeleton: ENSXMAG00000000055 adgrd1

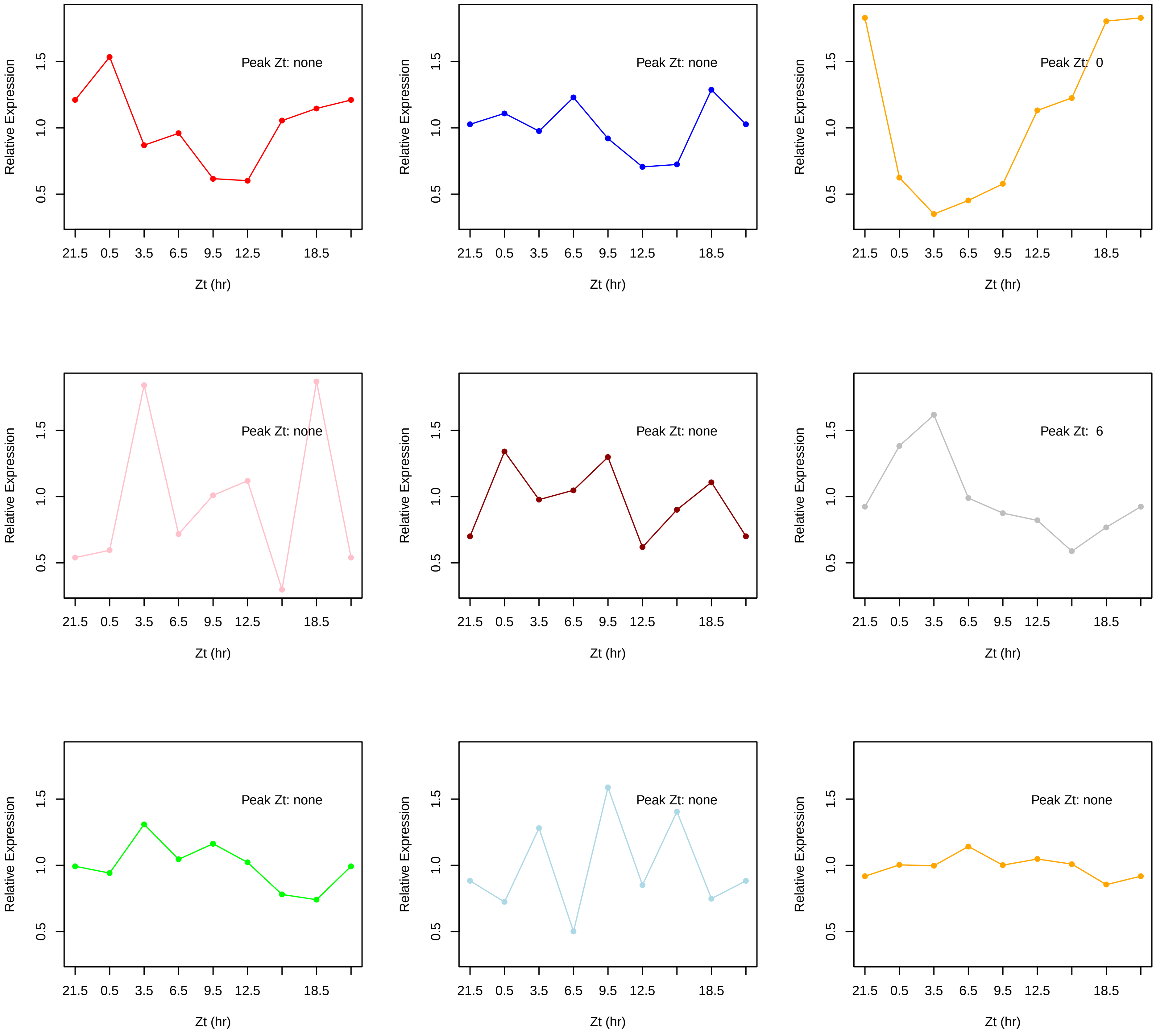

Singleton: ENSXMAG00000012387

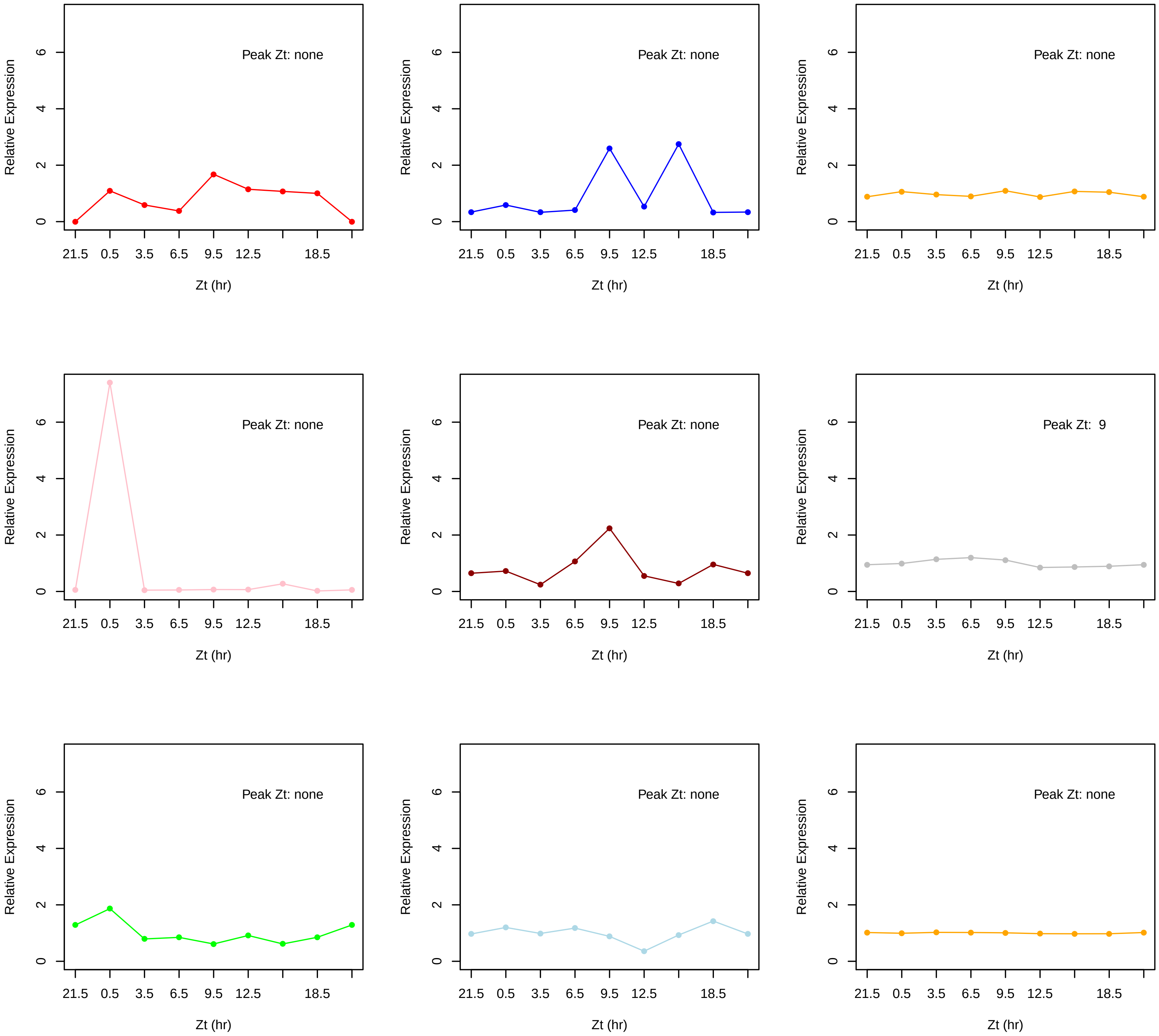

Singleton: ENSXMAG00000013030 lrrc29

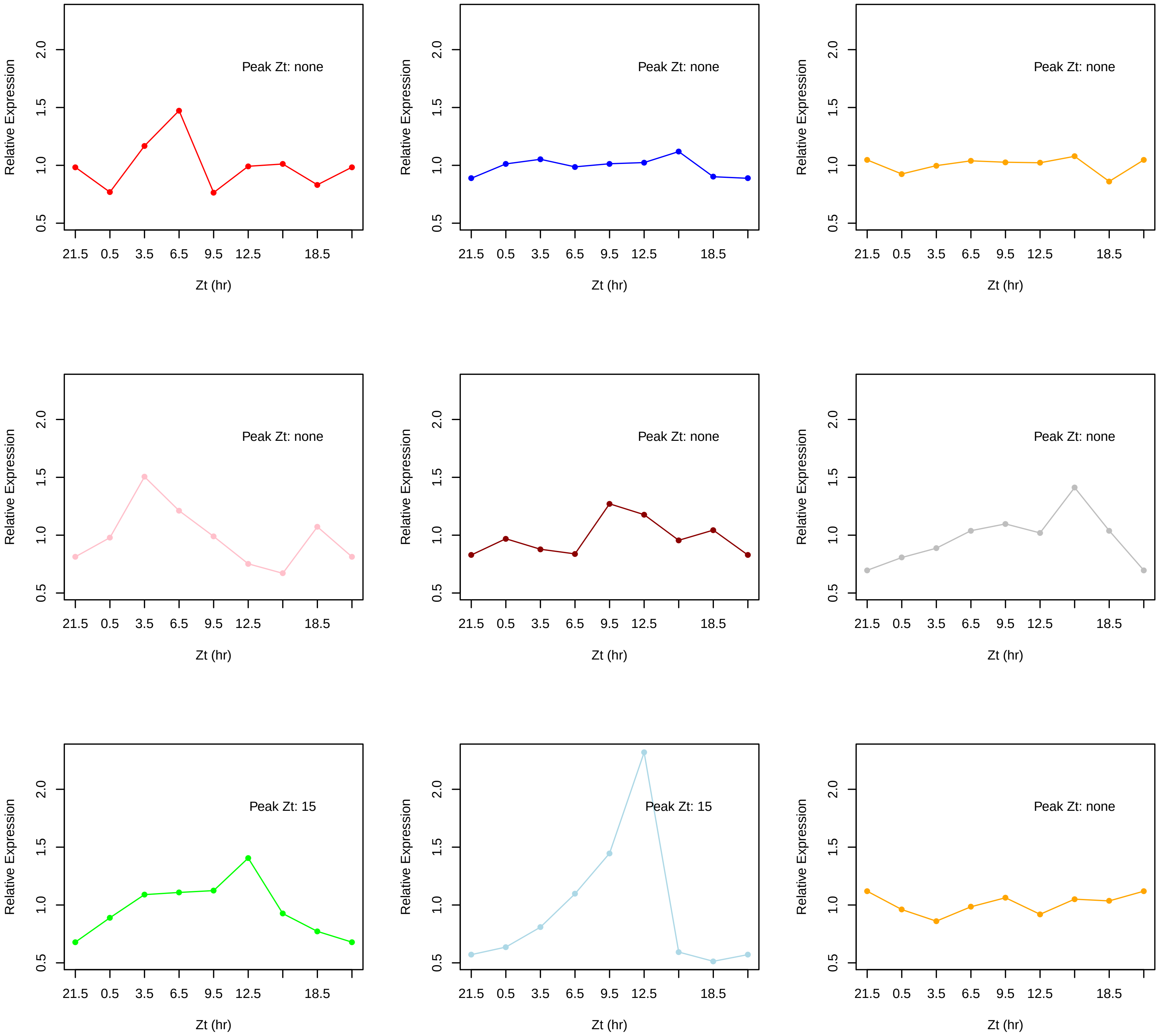

Singeleton: ENSXMAG00000003419 npepl1

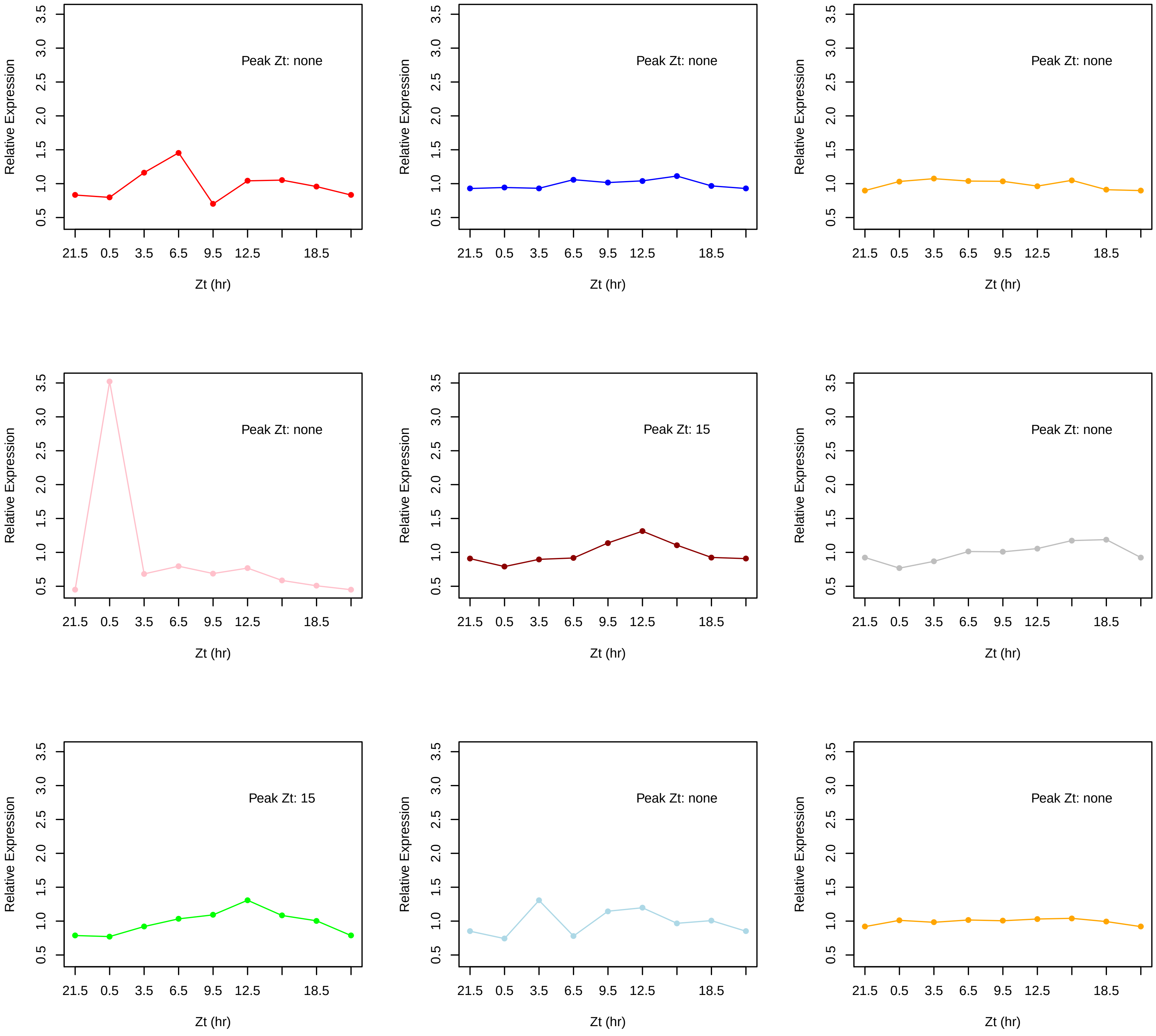

Singeleton: ENSXMAG00000012515 cdk11b

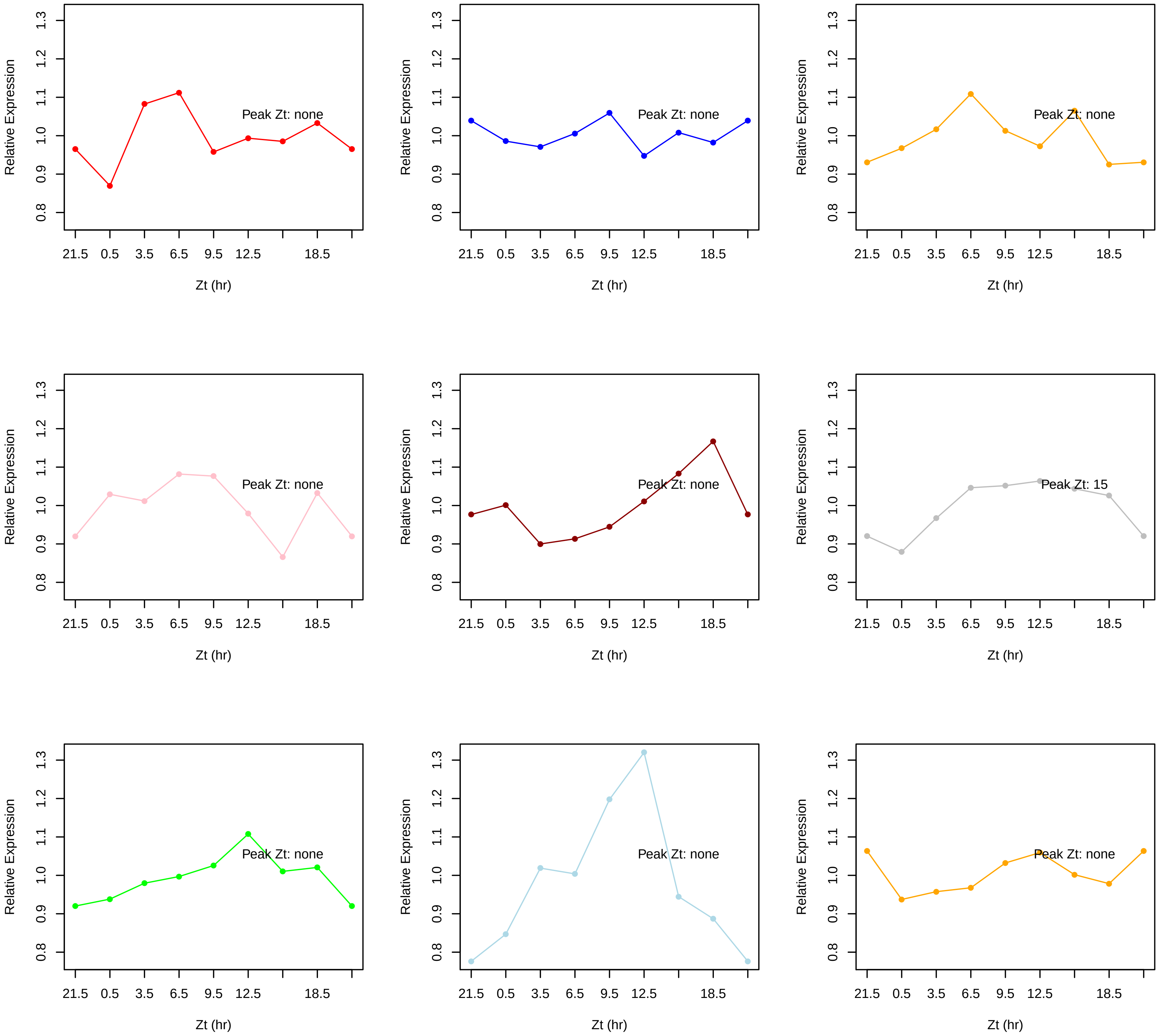

Singeleton: ENSXMAG00000012876 khsrp

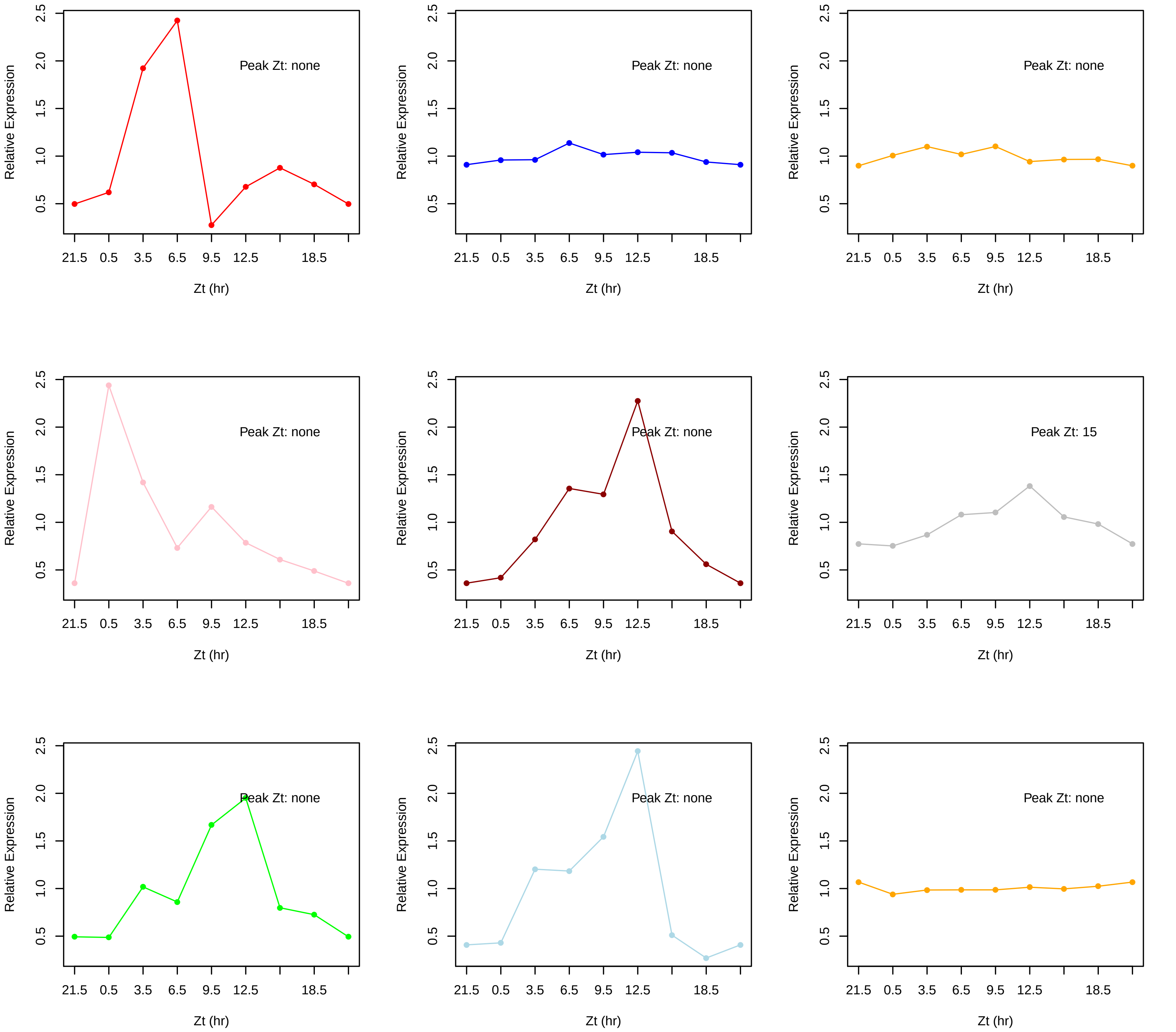

Singleton: ENSXMAG00000006704 pomt1

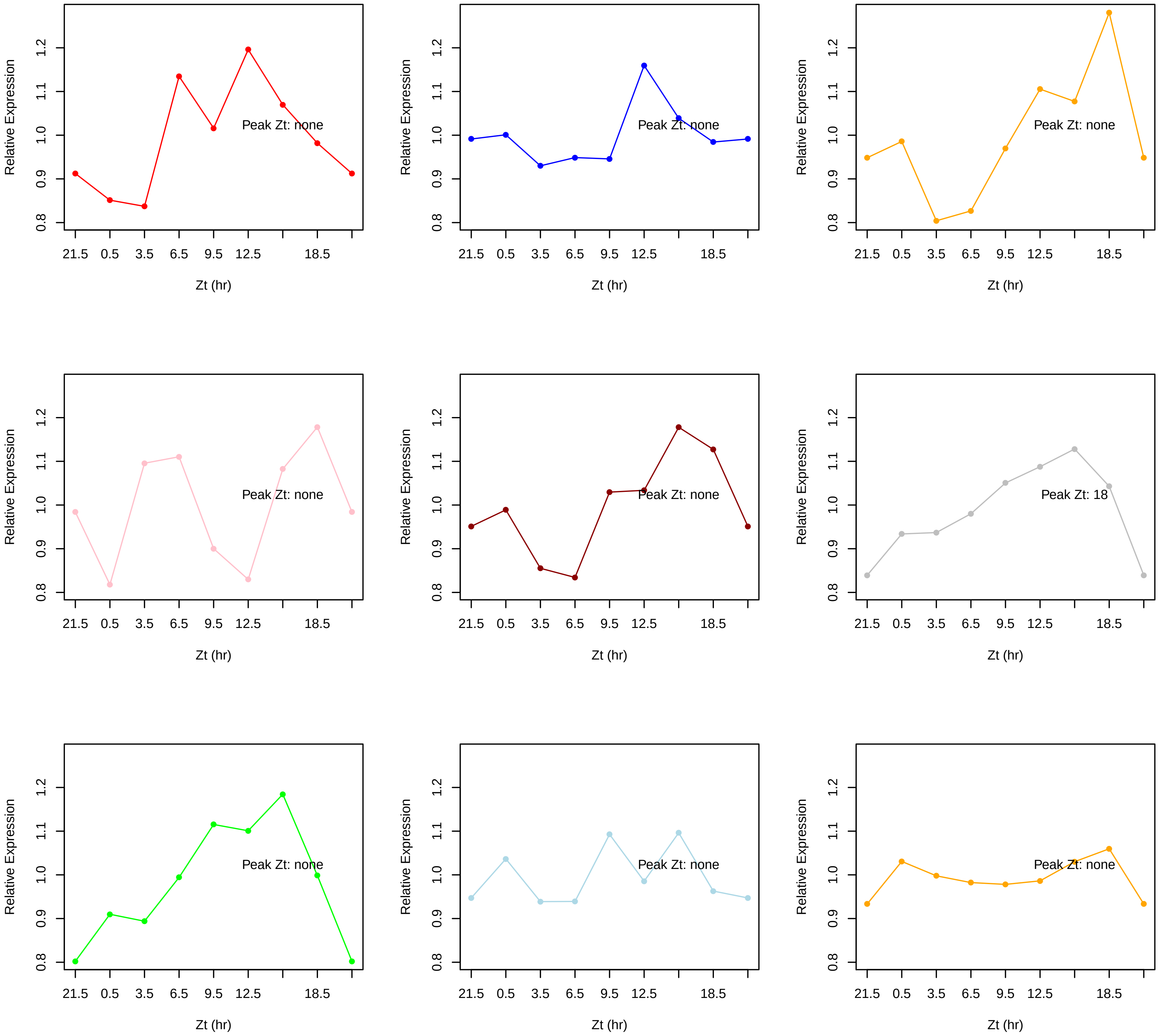

Singleton: ENSXMAG00000010735

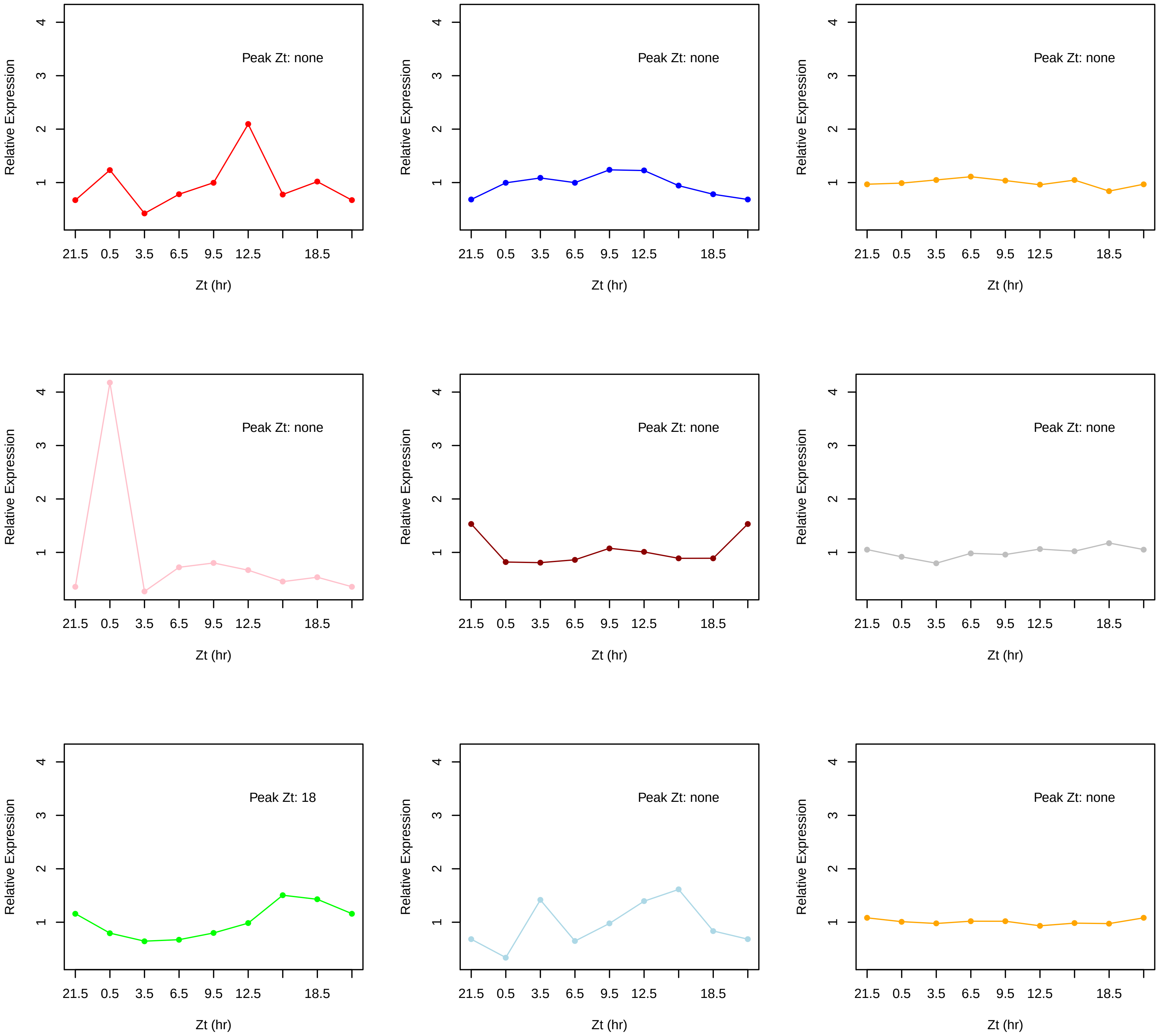

Singeleton: ENSXMAG00000028966 BACE1

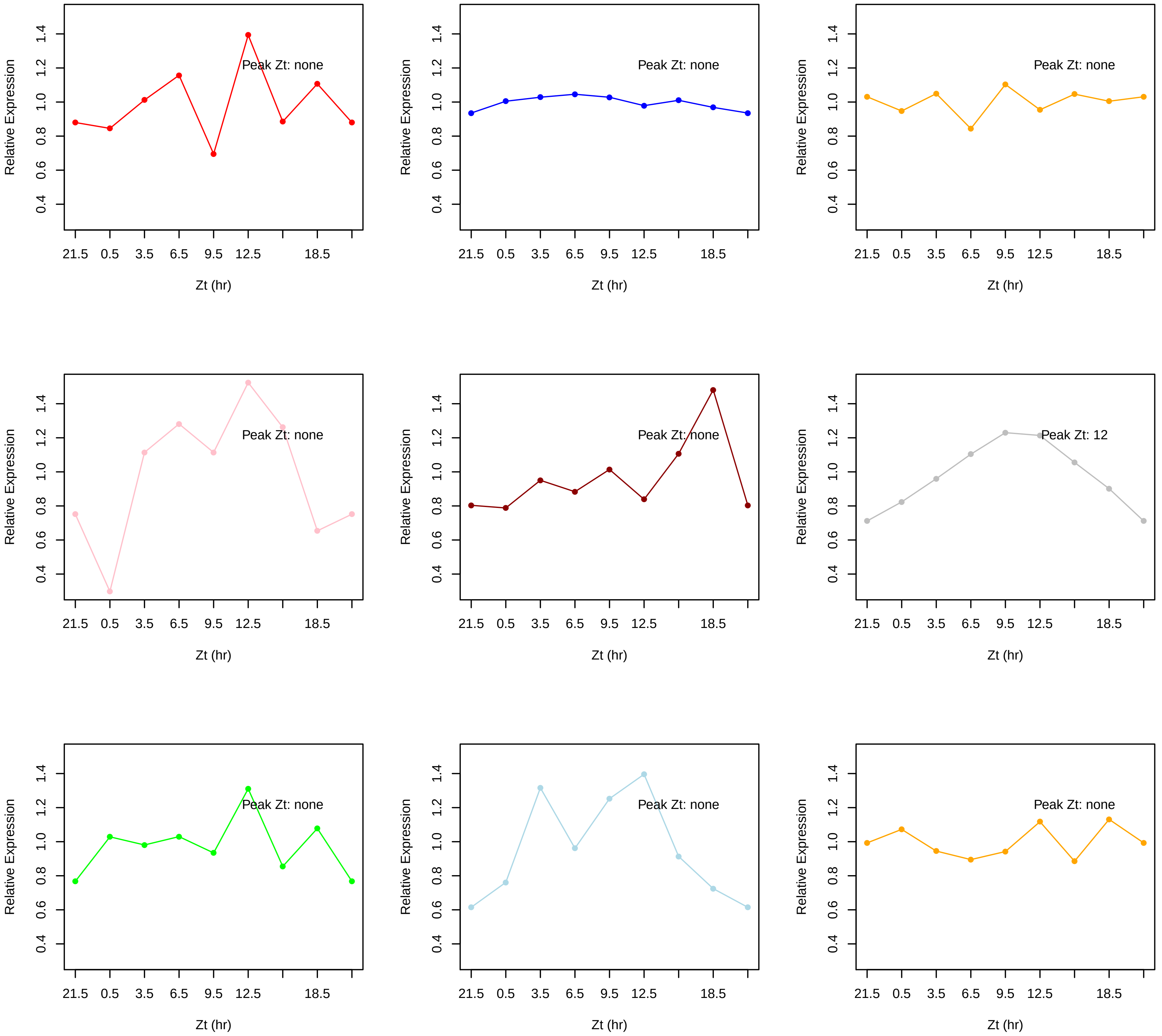

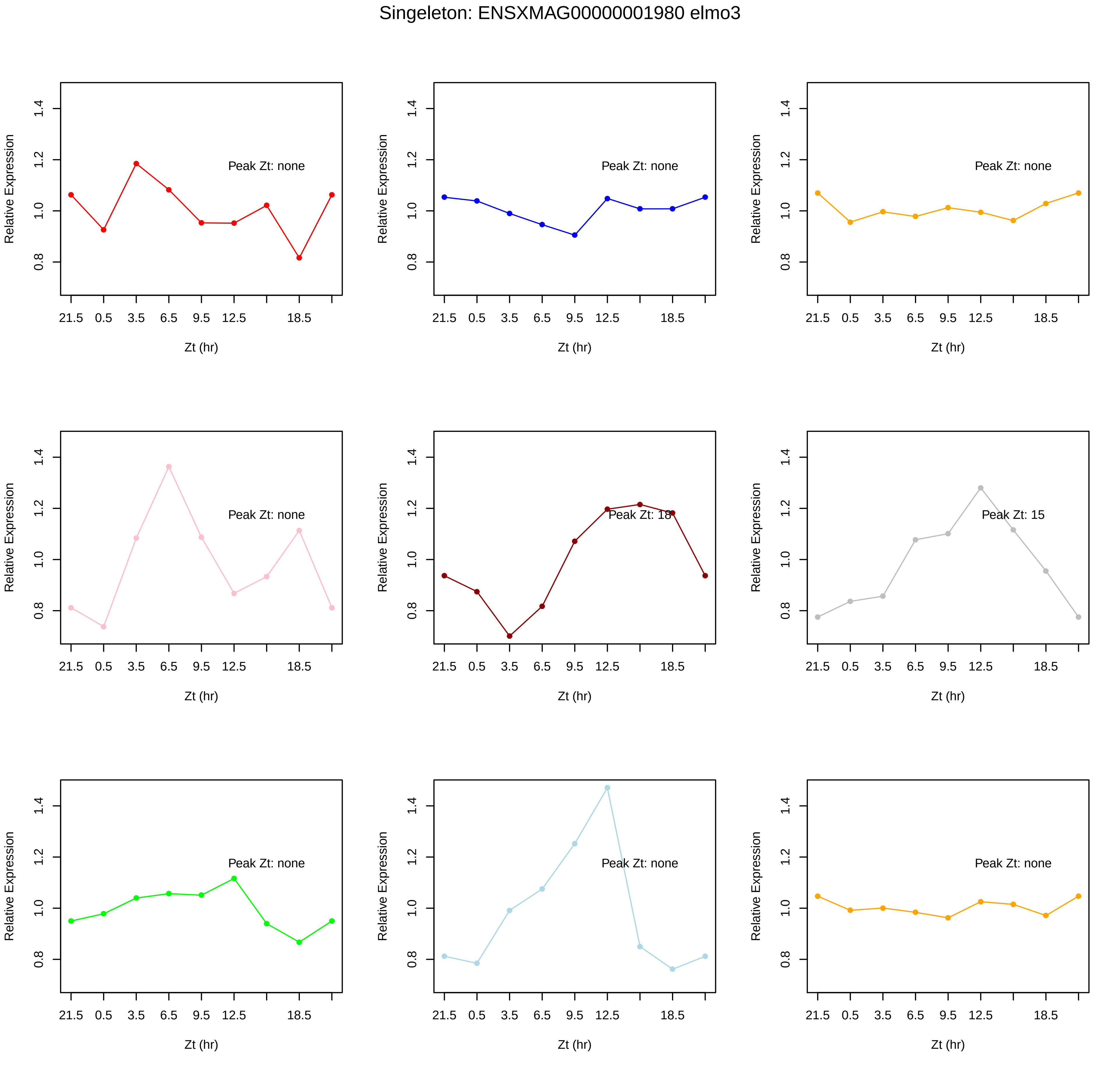
Singleton: ENSXMAG00000001980 elmo3
Relative Expression
Zt (hr)
21.5
0.5
3.5
6.5
9.5
12.5
18.5
0.8
1.0
1.2
1.4
Peak Zt: none
Peak Zt: 18
Peak Zt: 15

Singeleton: ENSXMAG00000026143 prpf38a

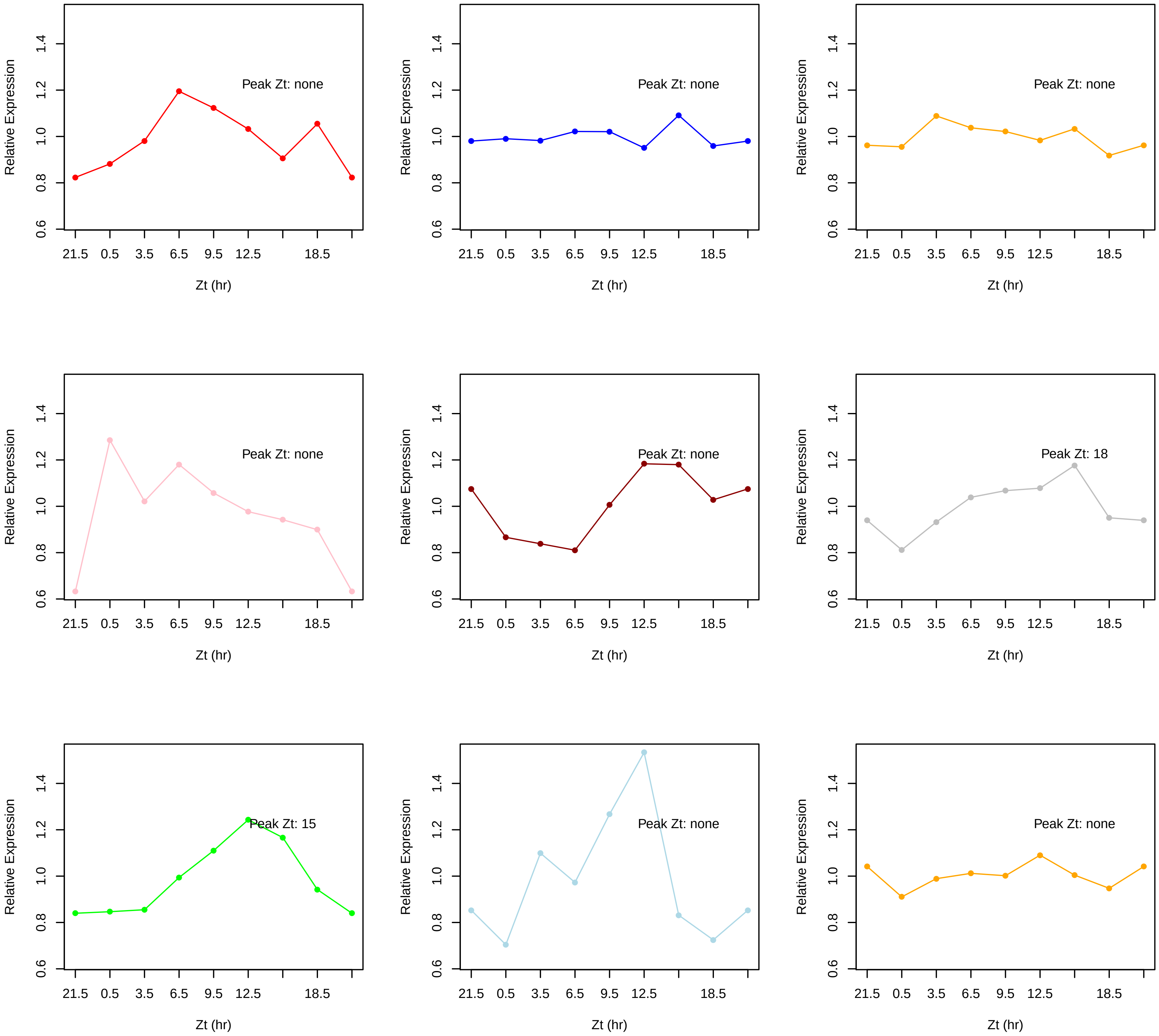

Singeleton: ENSXMAG00000003244 rabl3

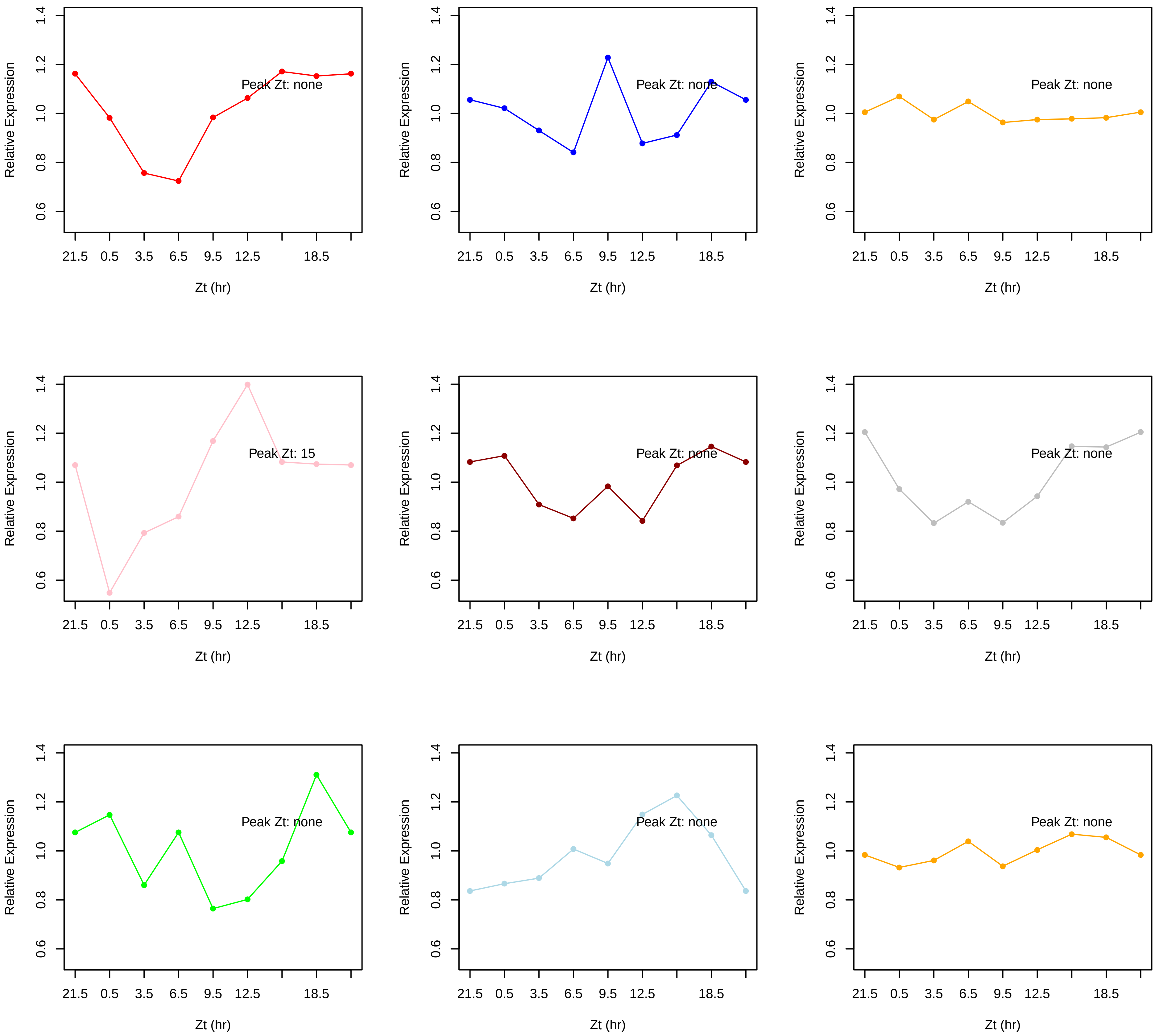

Singleton: ENSXMAG00000012904

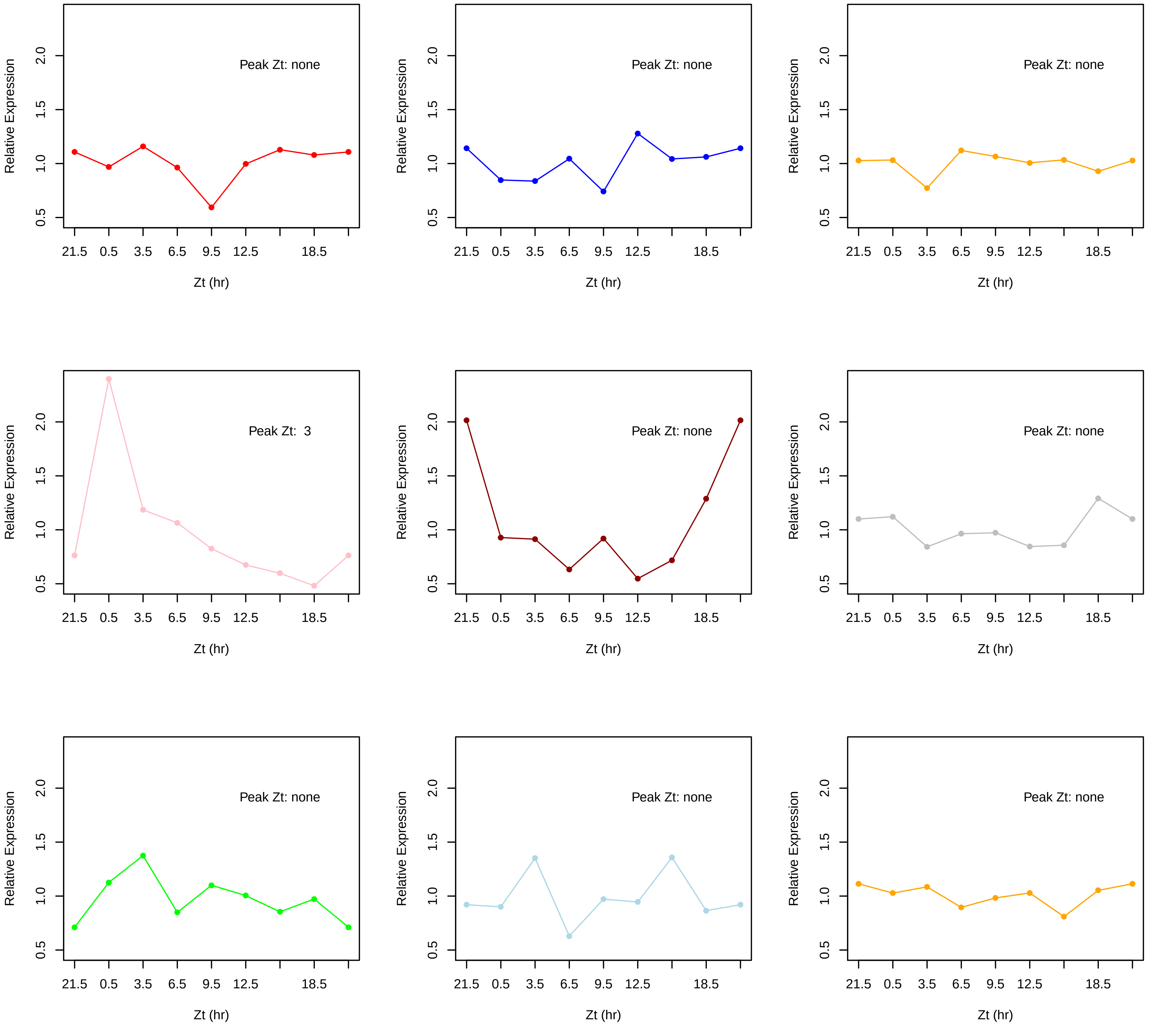

Singeleton: ENSXMAG00000026997 relt

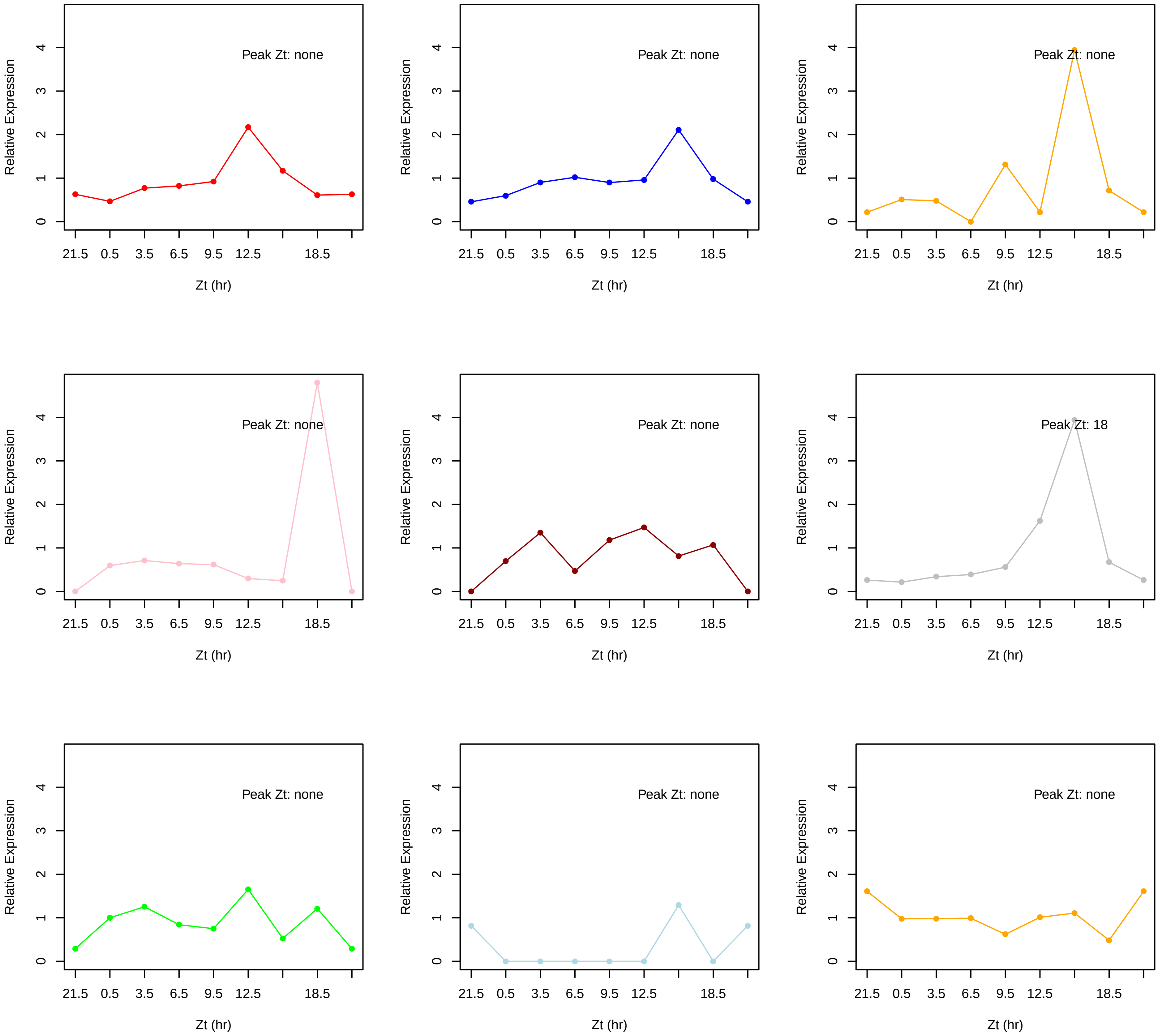

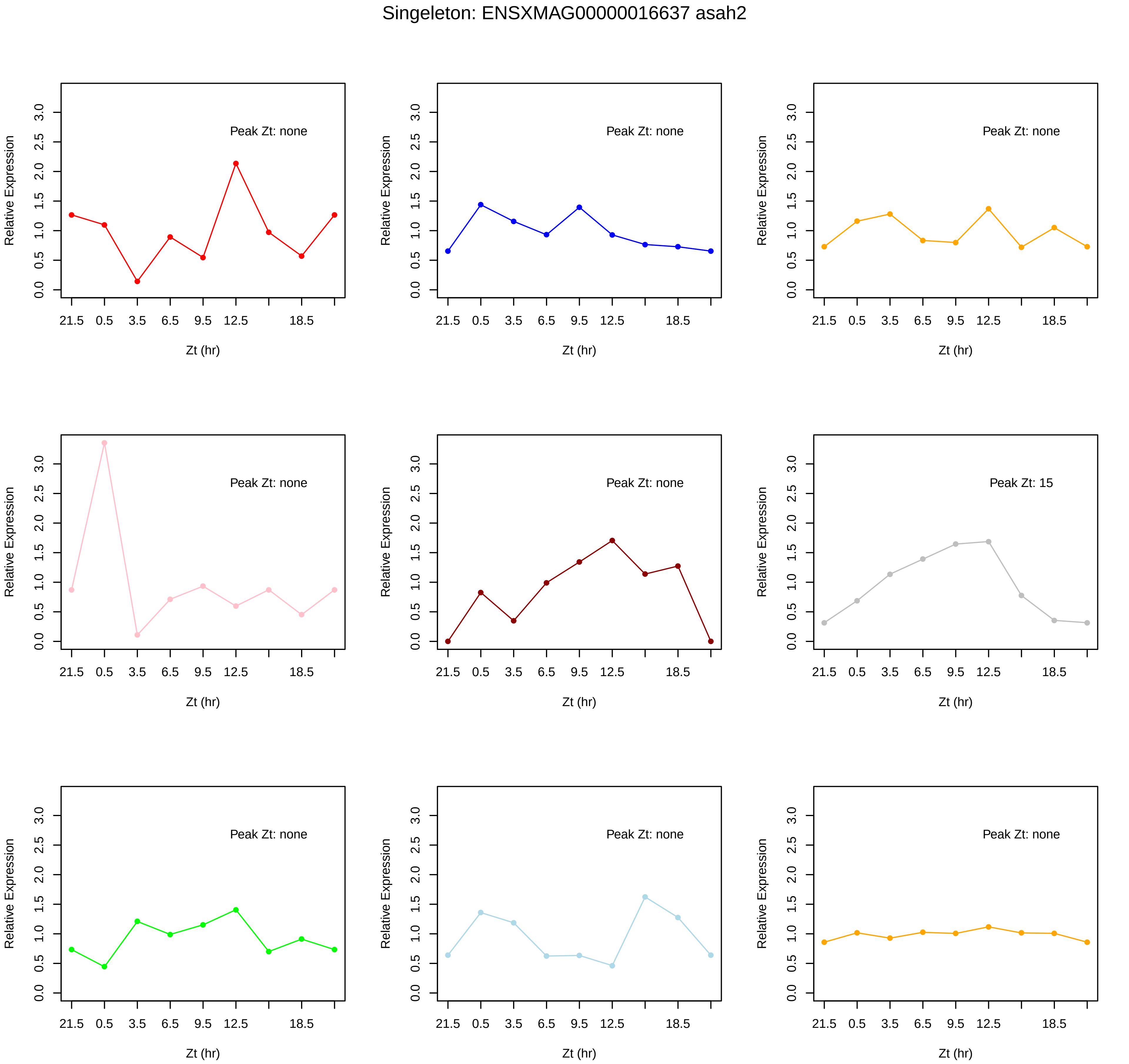
Singleton: ENSXMAG00000016637 asah2
Peak Zt: none
Peak Zt: none
Peak Zt: none
Peak Zt: none
Peak Zt: none
Peak Zt: 15
Peak Zt: none
Peak Zt: none
Peak Zt: none
Relative Expression
Zt (hr)
21.5
0.5
3.5
6.5
9.5
12.5
18.5
0.0
0.5
1.0
1.5
2.0
2.5
3.0

Singeleton: ENSXMAG00000012509 MMP23B

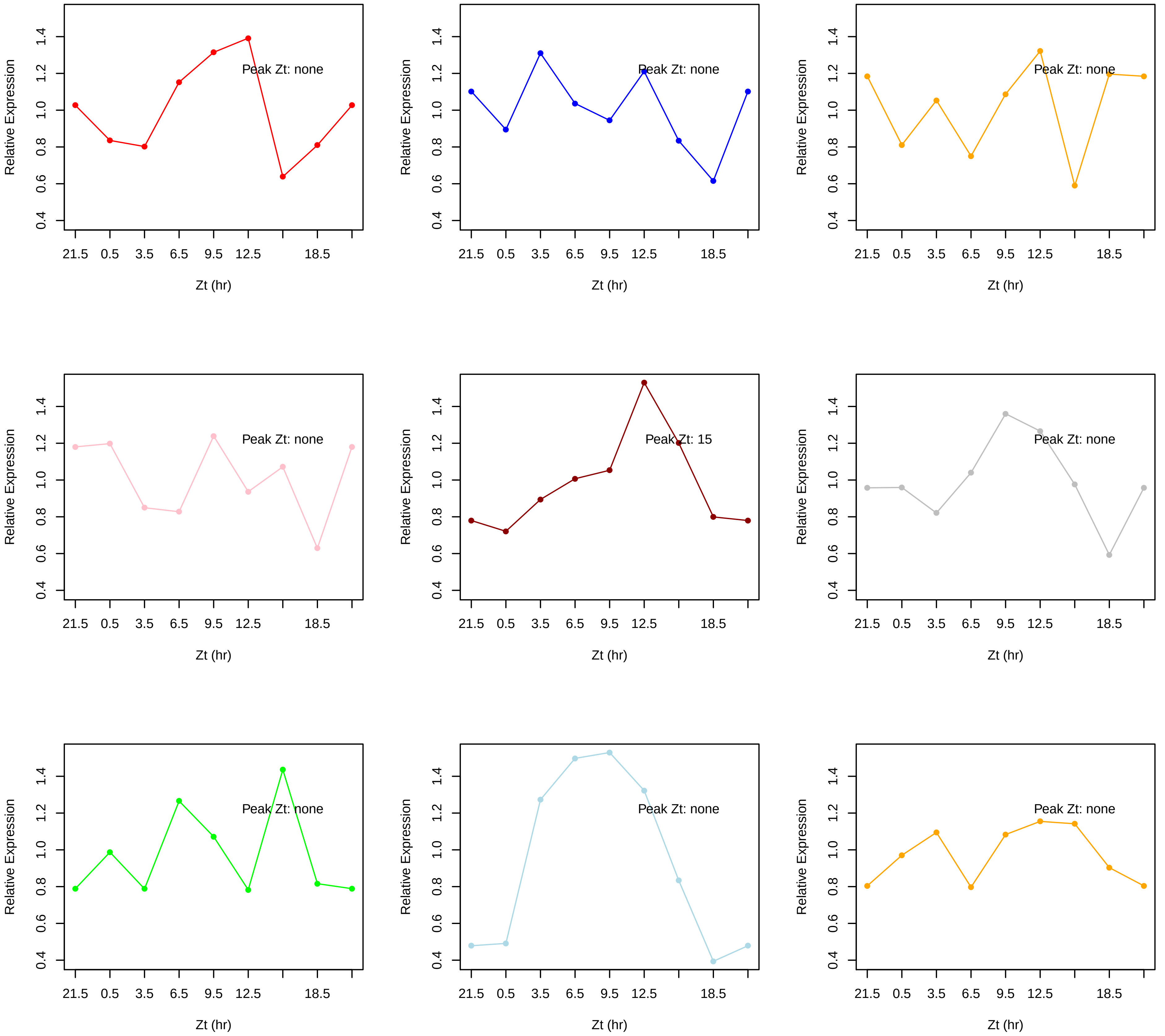

Singeleton: ENSXMAG00000005881 fkbp7

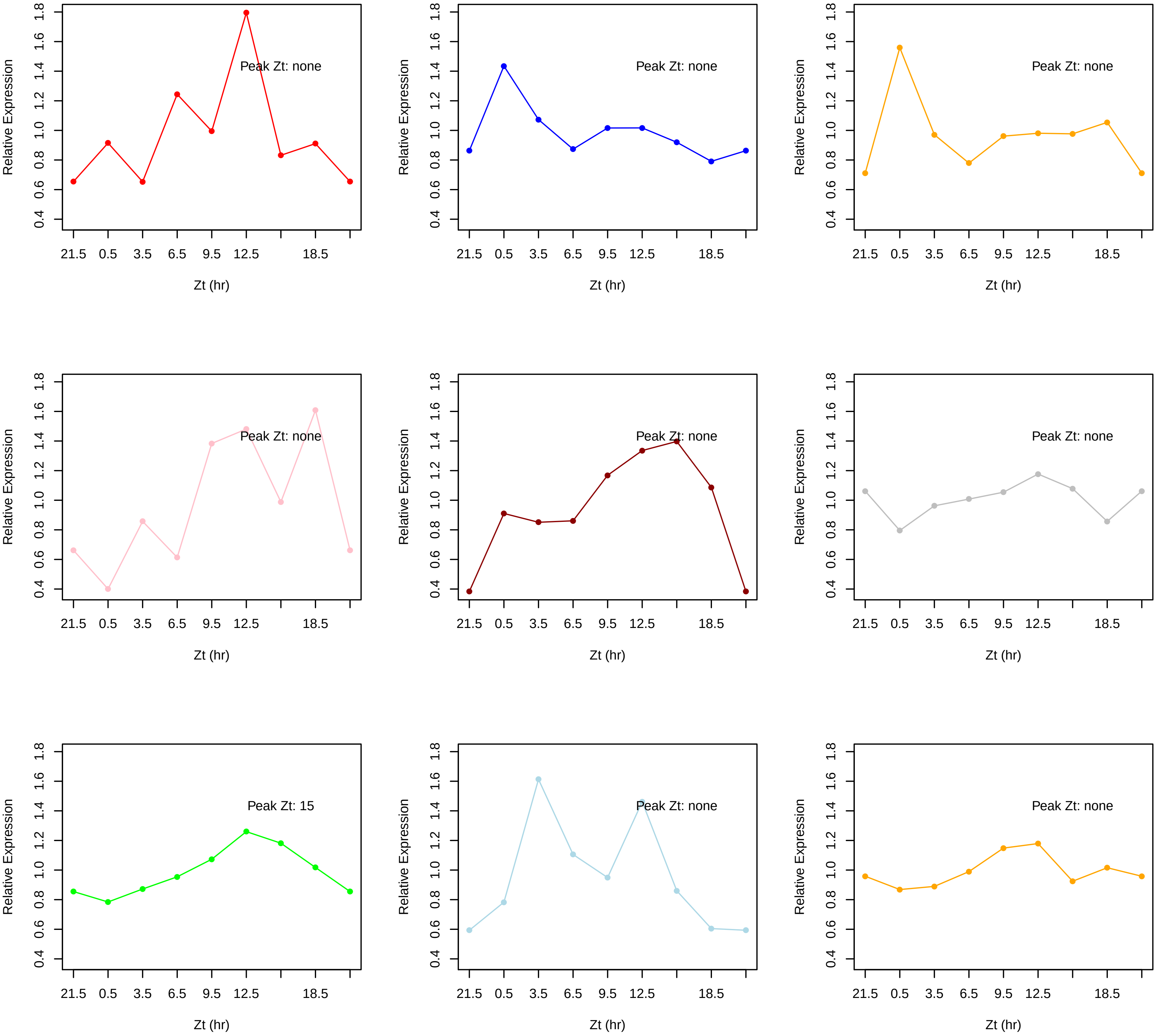

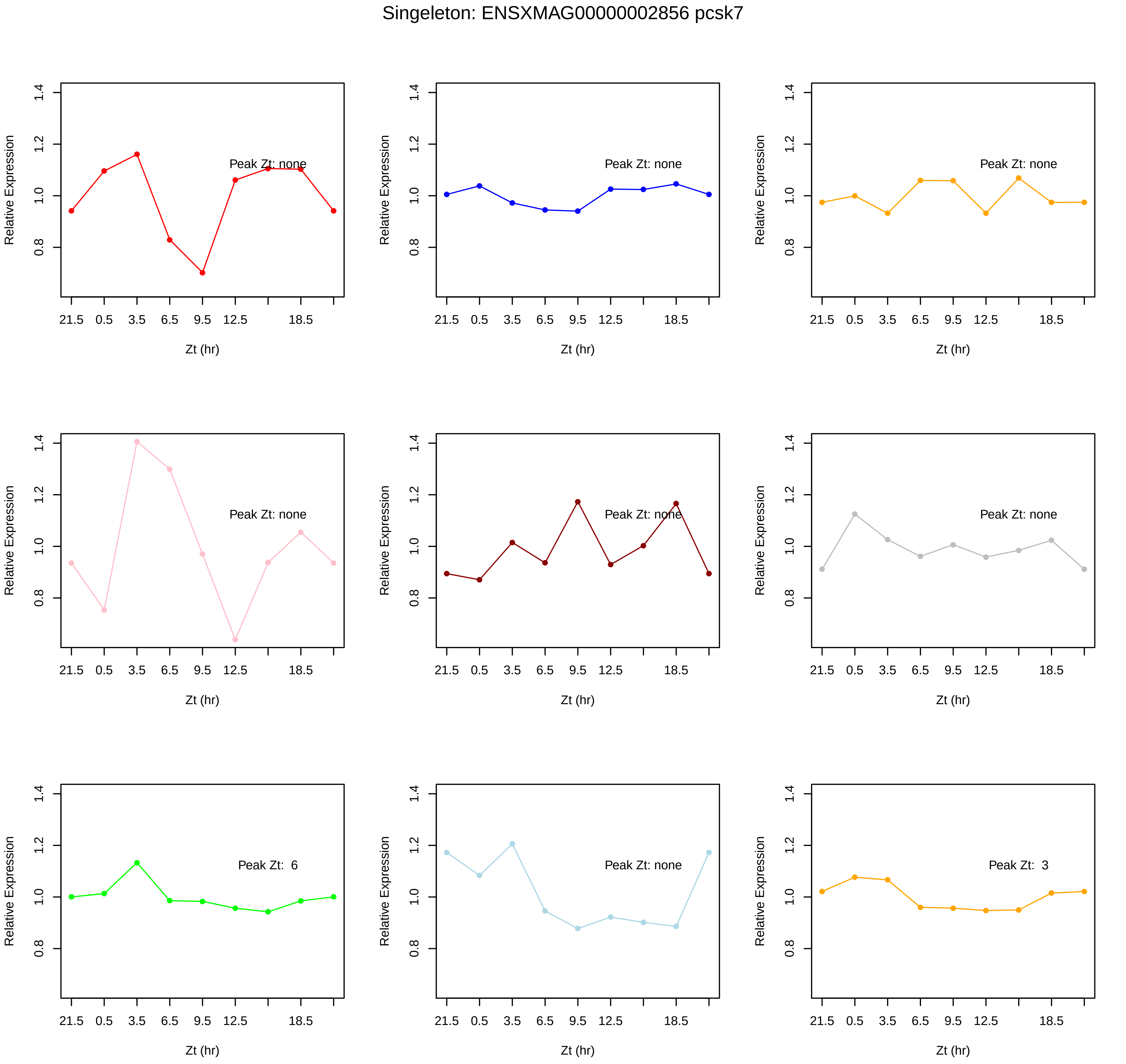
Singleton: ENSXMAG00000002856 pcsk7
Peak Zt: none
Peak Zt: none
Peak Zt: none
Peak Zt: none
Peak Zt: none
Peak Zt: none
Peak Zt: 6
Peak Zt: none
Peak Zt: 3
Relative Expression
Zt (hr)
21.5
0.5
3.5
6.5
9.5
12.5
18.5
0.8
1.0
1.2
1.4

Singeleton: ENSXMAG00000012623 KHDC4

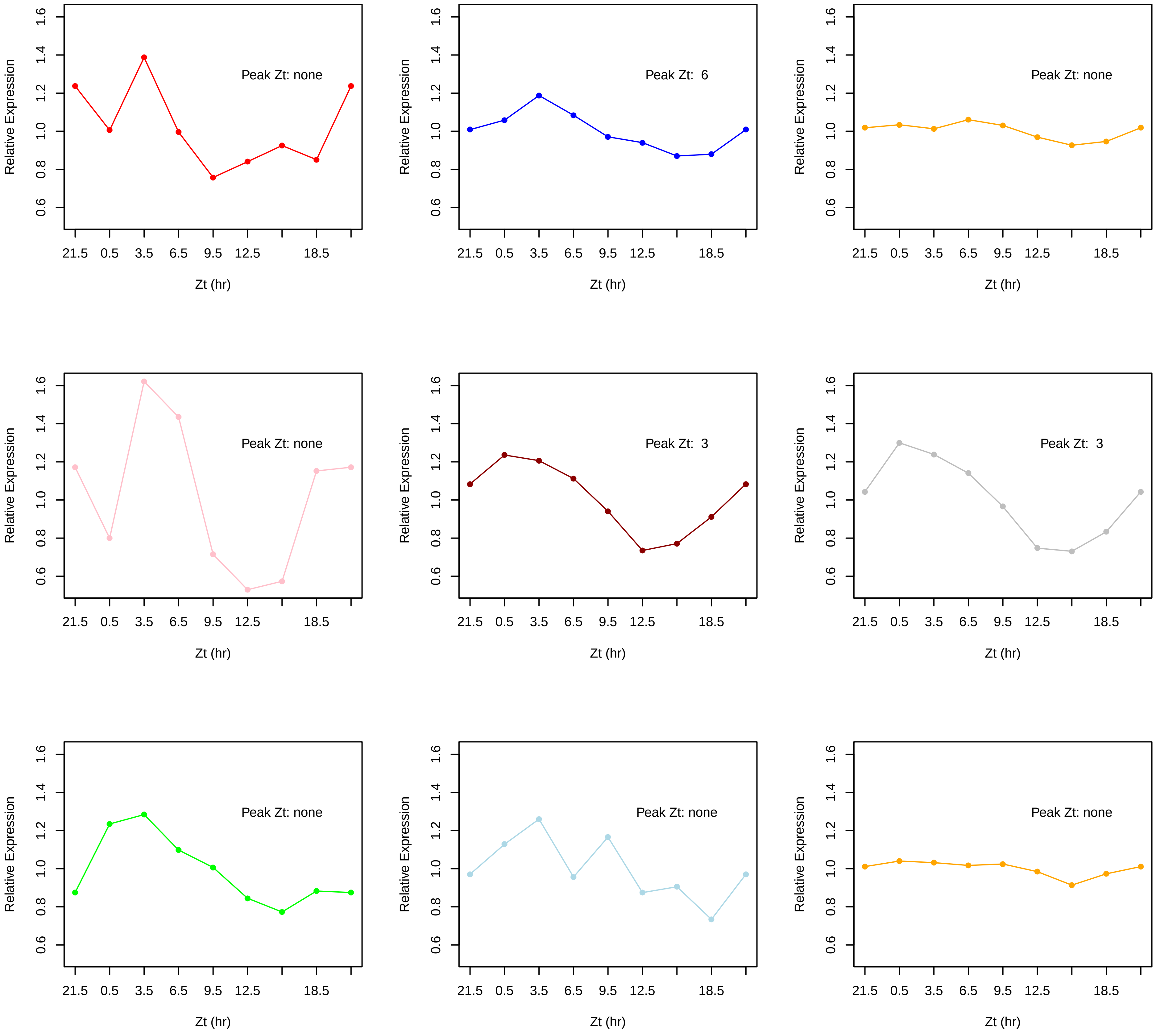

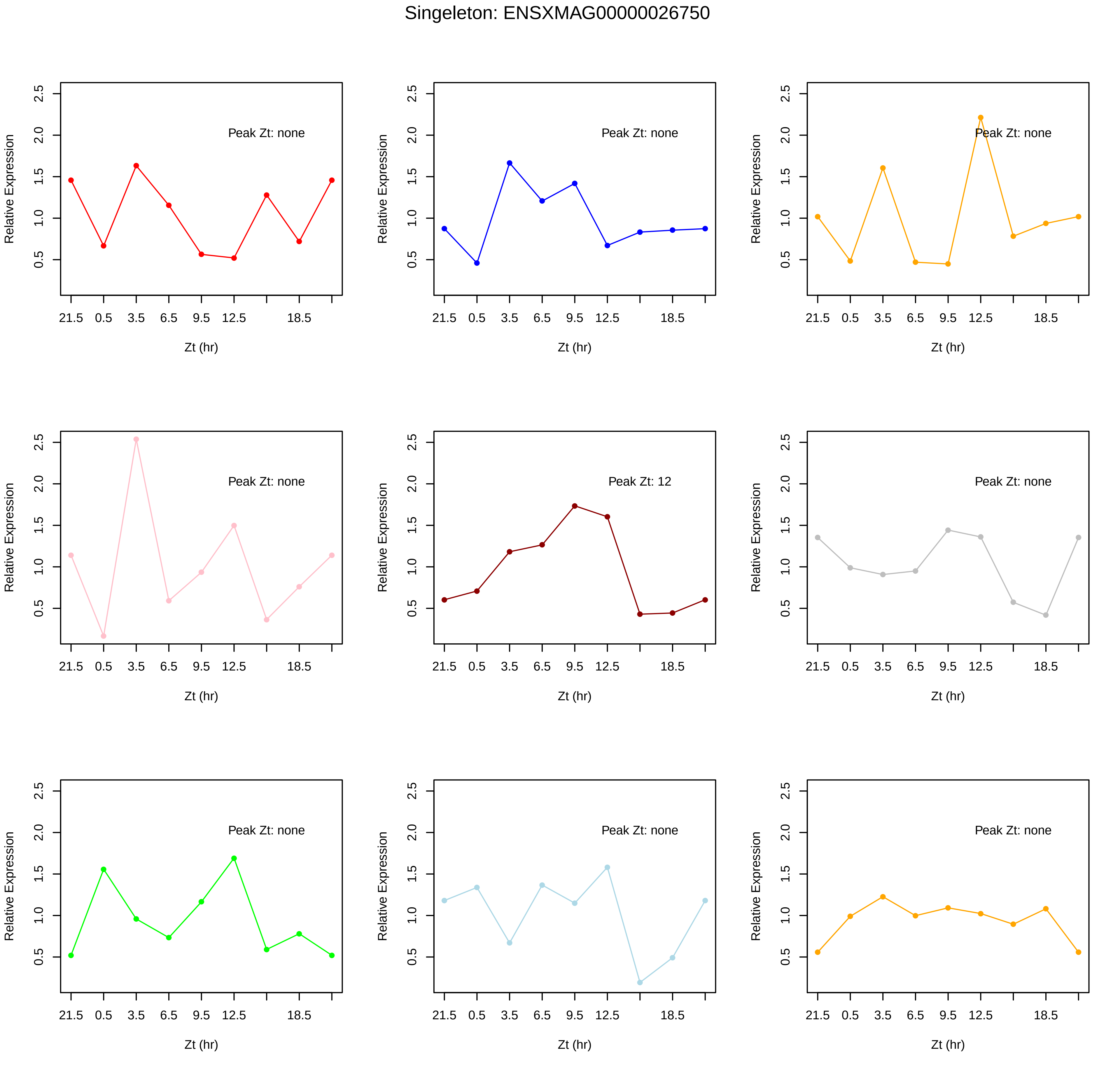
Singeleton: ENSXMAG00000026750
Peak Zt: none
Peak Zt: none
Peak Zt: none
Peak Zt: none
Peak Zt: 12
Peak Zt: none
Peak Zt: none
Peak Zt: none
Peak Zt: none
Relative Expression
Zt (hr)
21.5 0.5 3.5 6.5 9.5 12.5 18.5
0.5 1.0 1.5 2.0 2.5

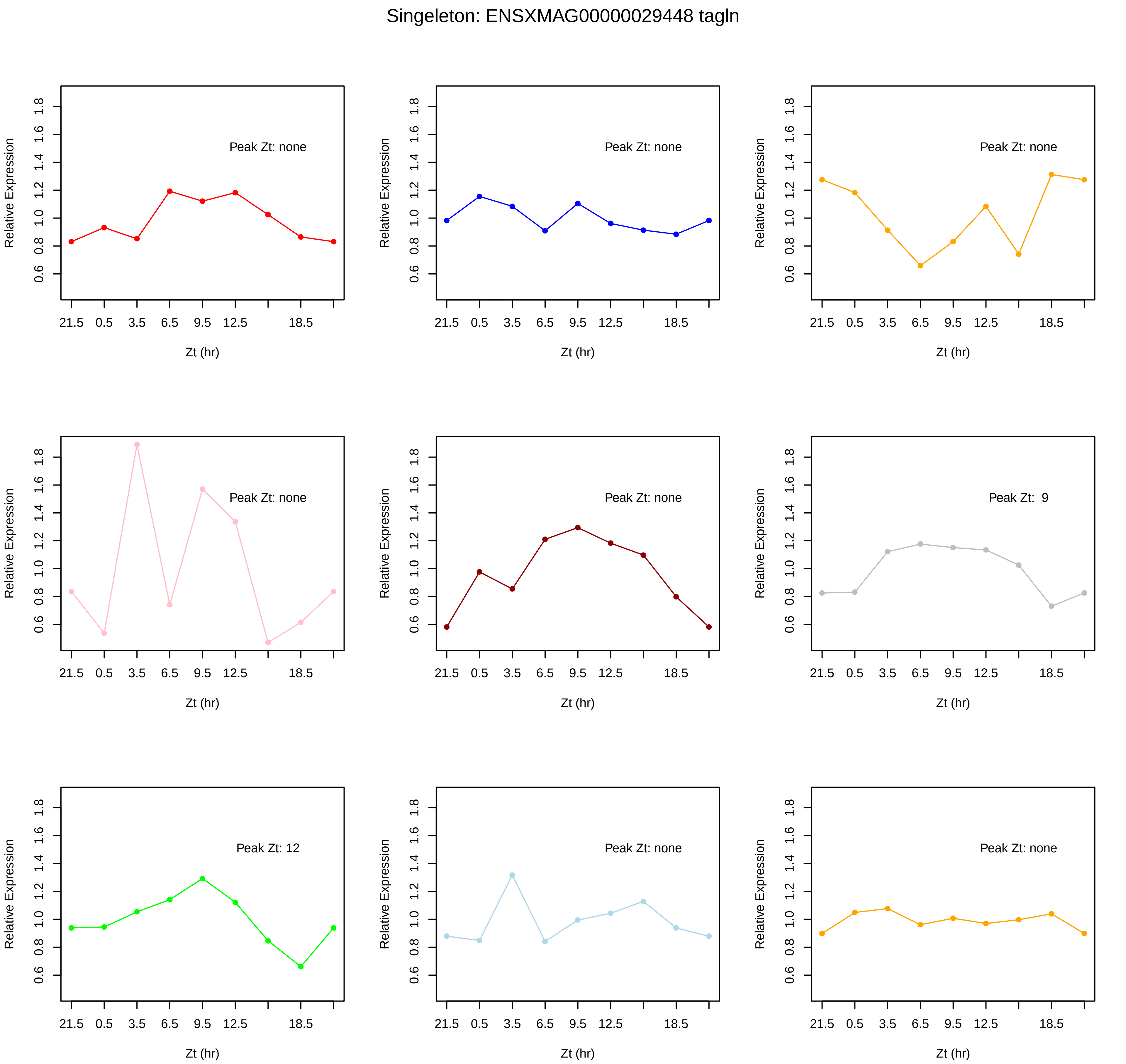
Singeleton: ENSXMAG00000029448 tagln
Peak Zt: none
Peak Zt: none
Peak Zt: none
Peak Zt: none
Peak Zt: none
Peak Zt: 9
Peak Zt: 12
Peak Zt: none
Peak Zt: none
Relative Expression
Zt (hr)
21.5
0.5
3.5
6.5
9.5
12.5
18.5
0.6
0.8
1.0
1.2
1.4
1.6
1.8

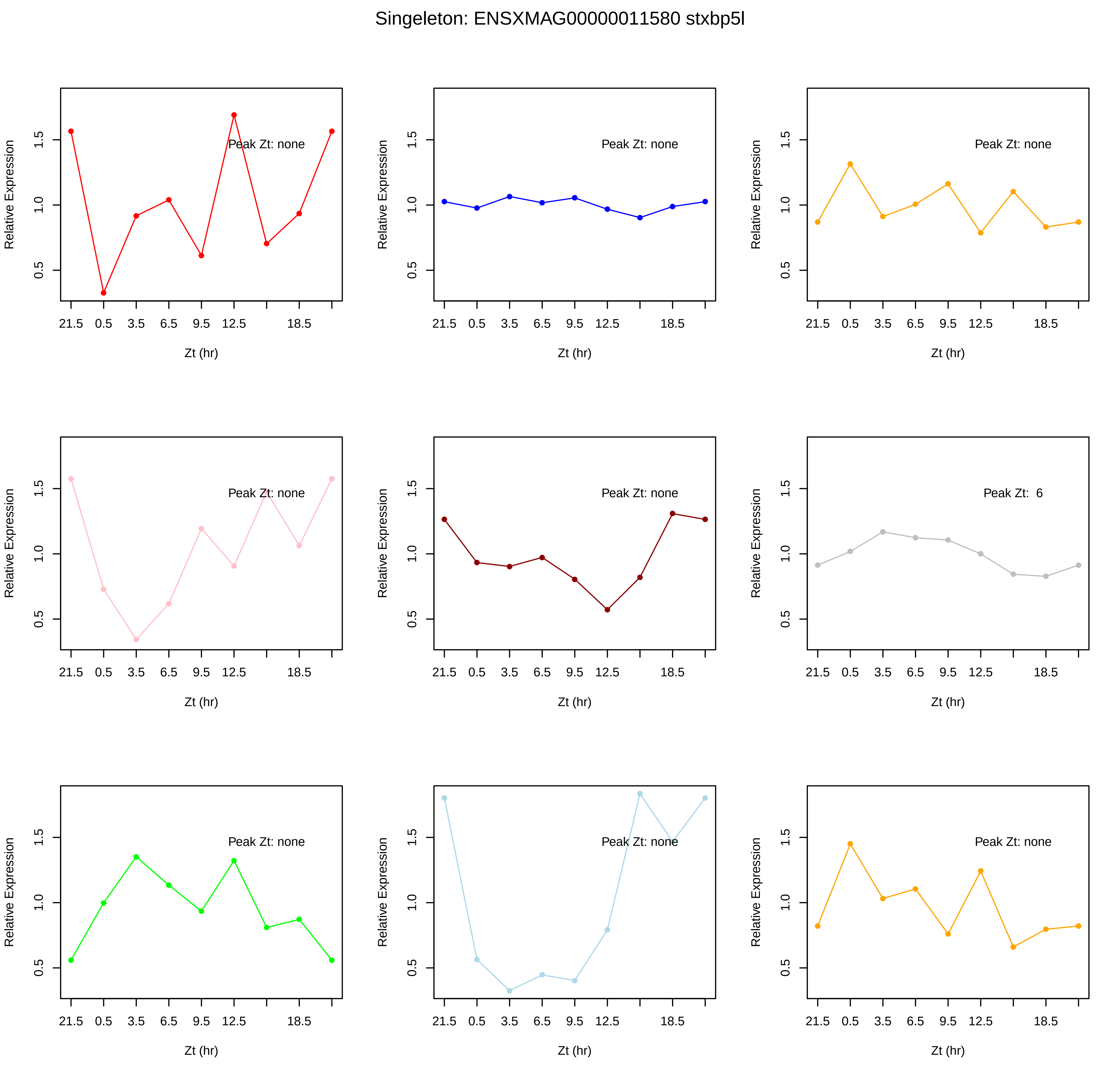
Singeleton: ENSXMAG00000011580 stxbp5l
Peak Zt: none
Peak Zt: none
Peak Zt: none
Peak Zt: none
Peak Zt: none
Peak Zt: 6
Peak Zt: none
Peak Zt: none
Peak Zt: none
Relative Expression
Zt (hr)
21.5
0.5
3.5
6.5
9.5
12.5
18.5
0.5
1.0
1.5

Singeleton: ENSXMAG00000004256 sept2

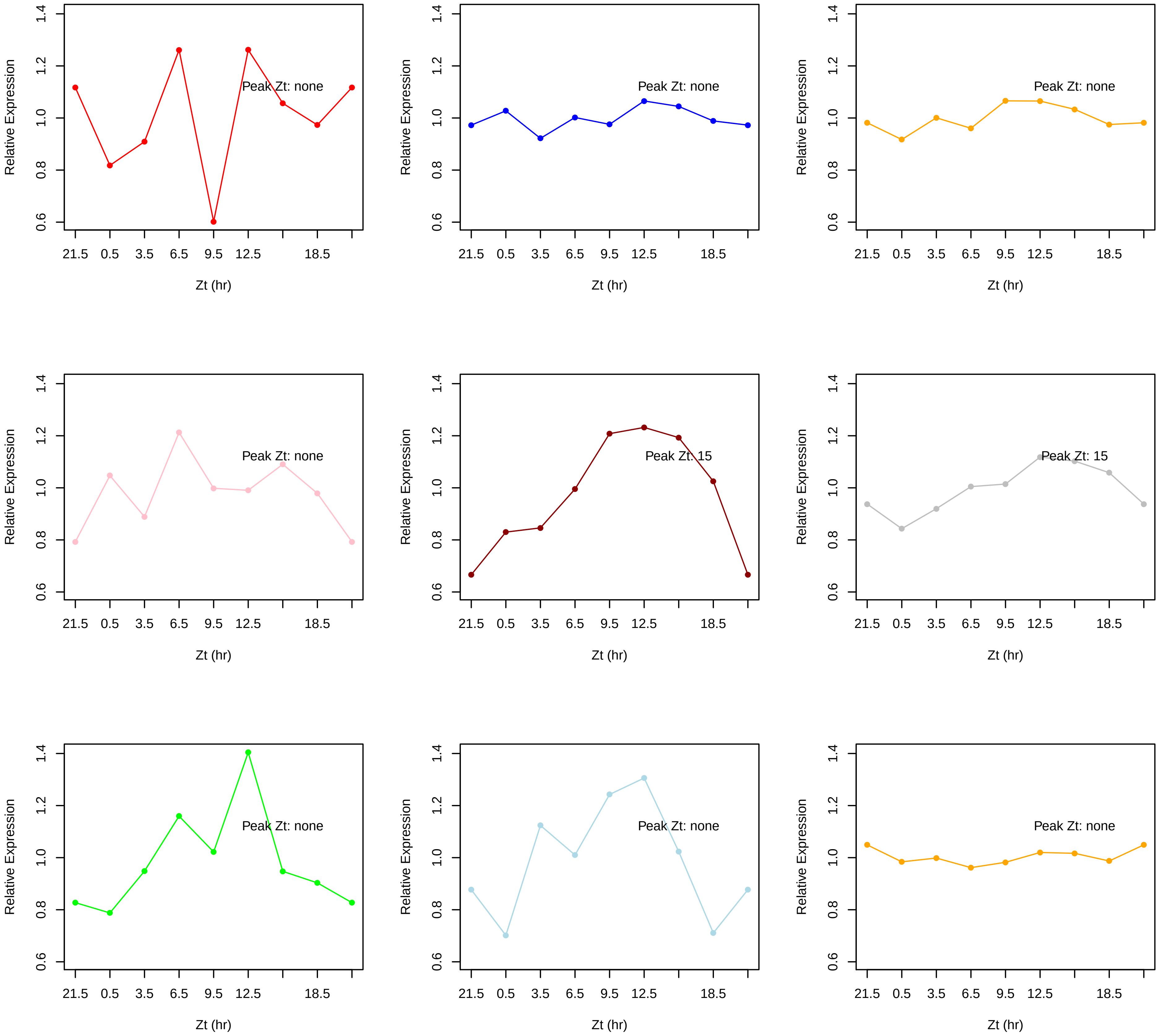

Singeleton: ENSXMAG00000006024 SETD6

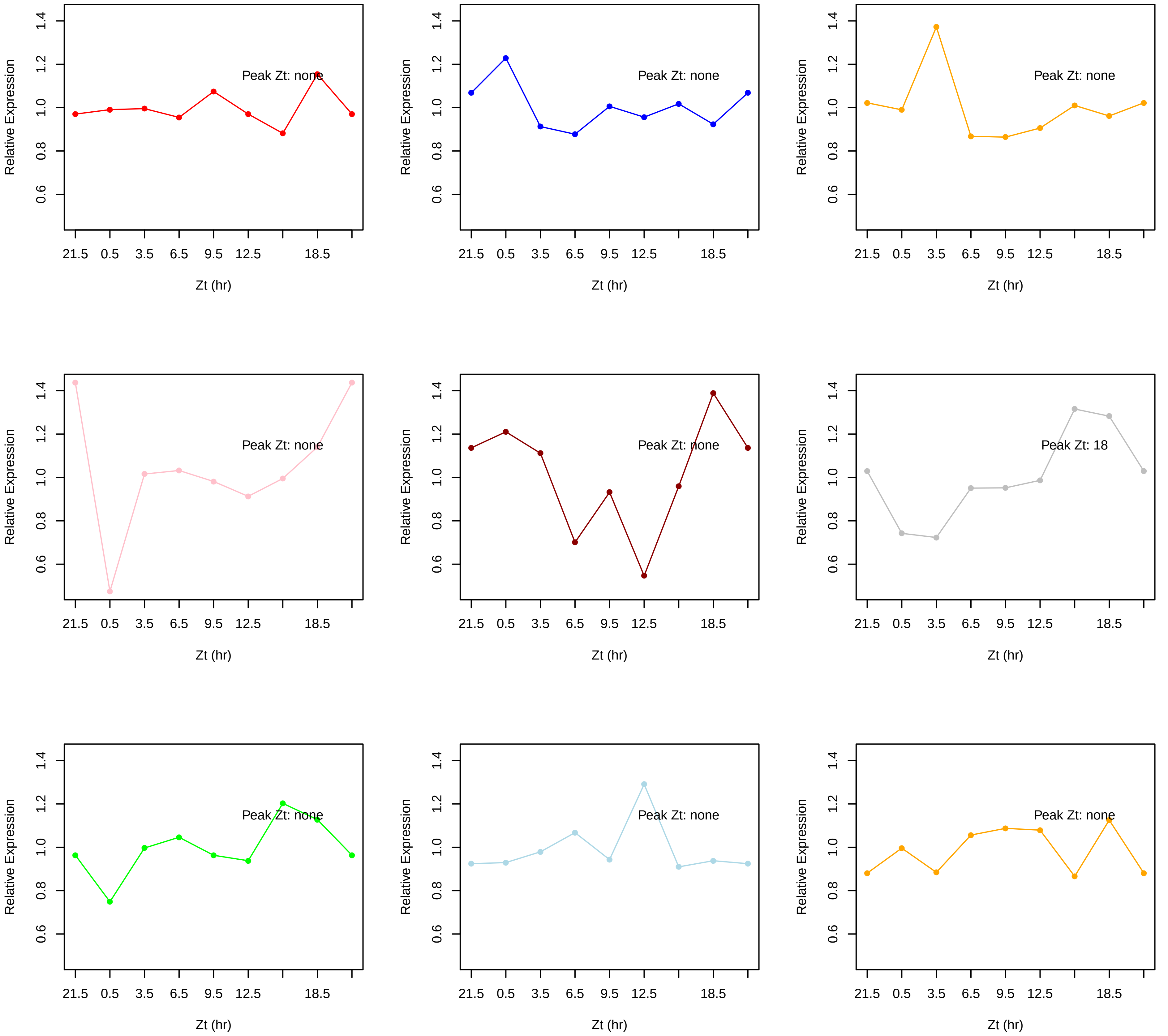

Singeleton: ENSXMAG00000025054 ARHGEF17

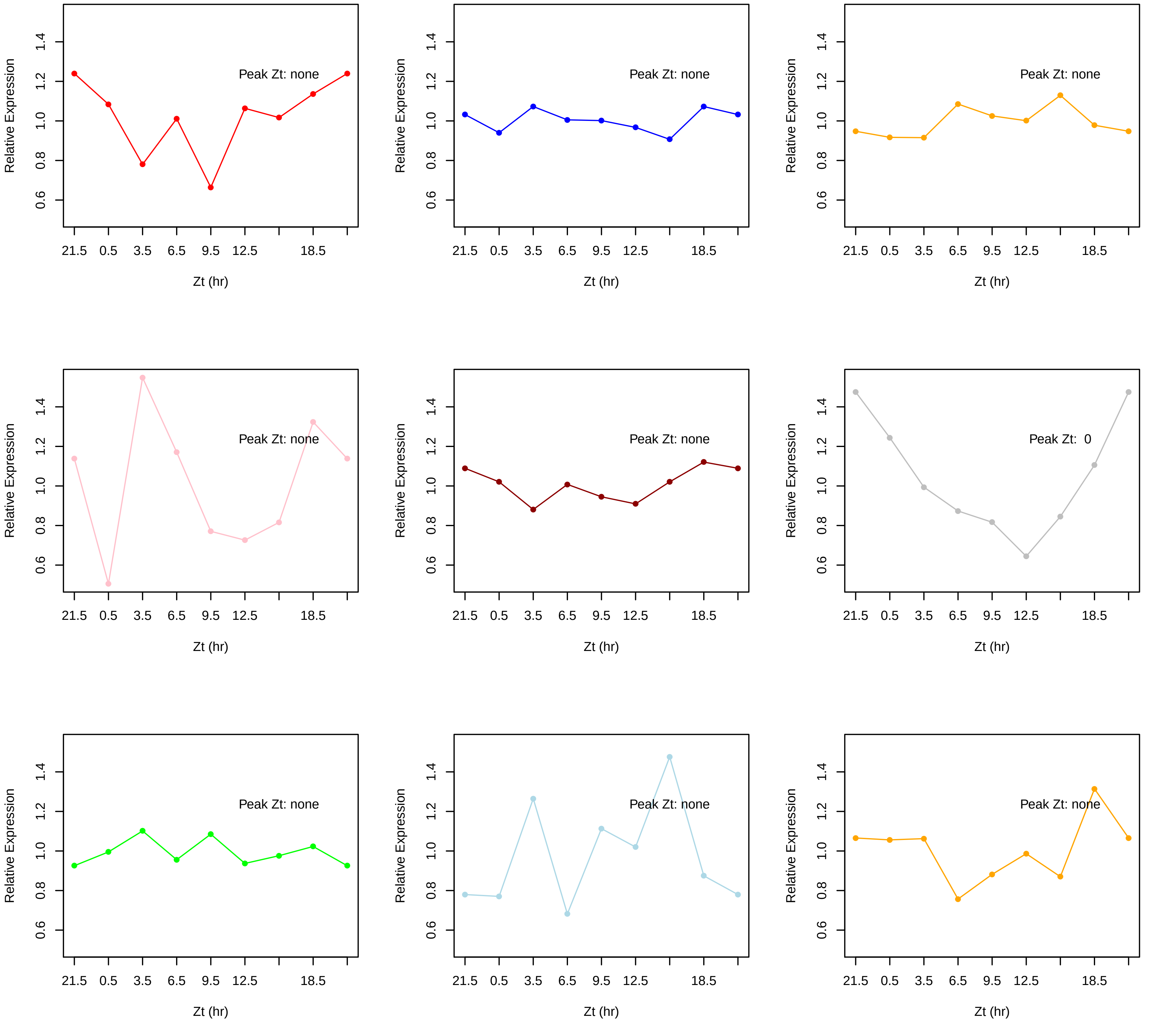

Singeleton: ENSXMAG00000029555 atp5f1e

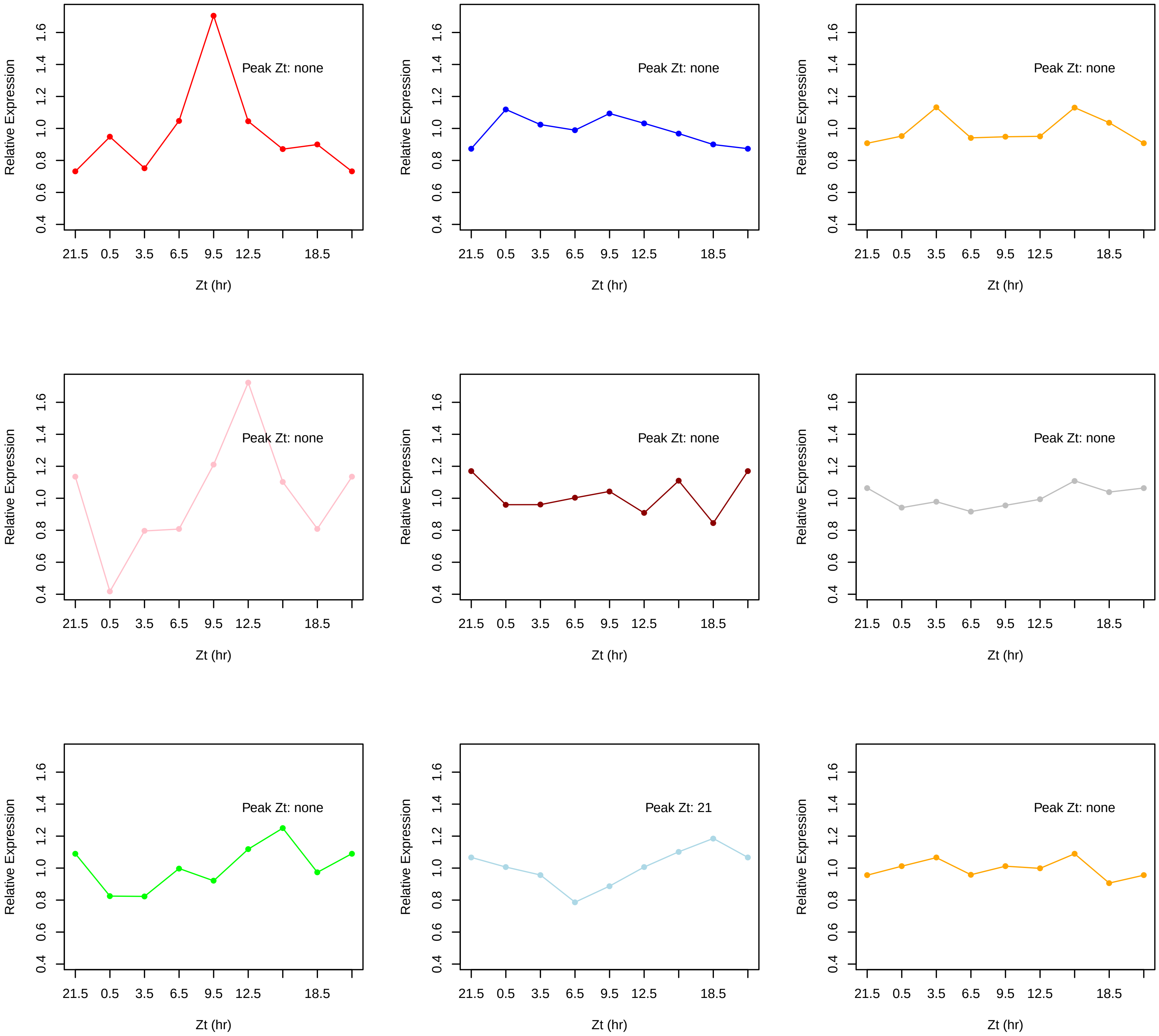

Singleton: ENSXMAG00000016658 AQP4

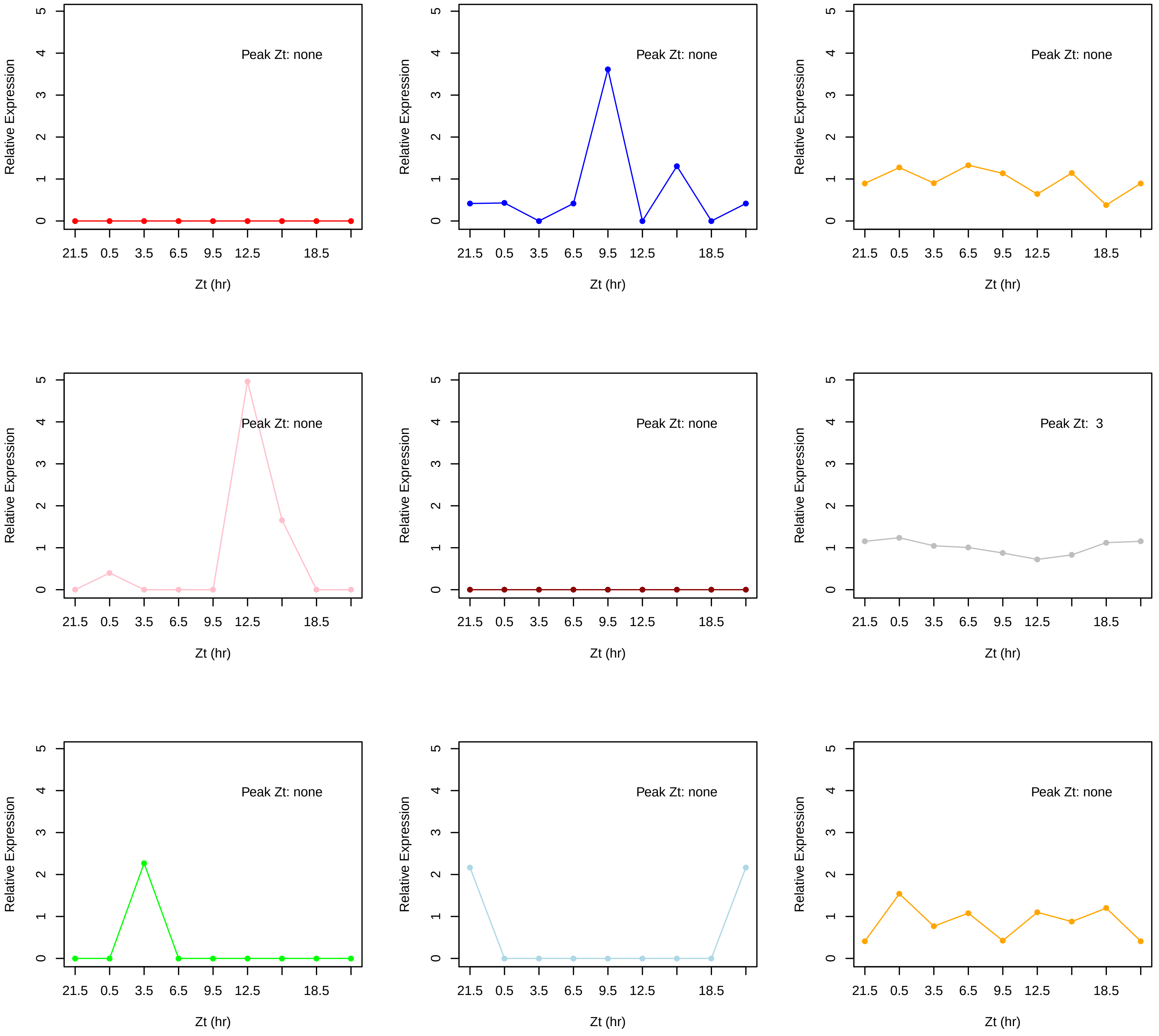

Singeleton: ENSXMAG00000025056 arhgdig

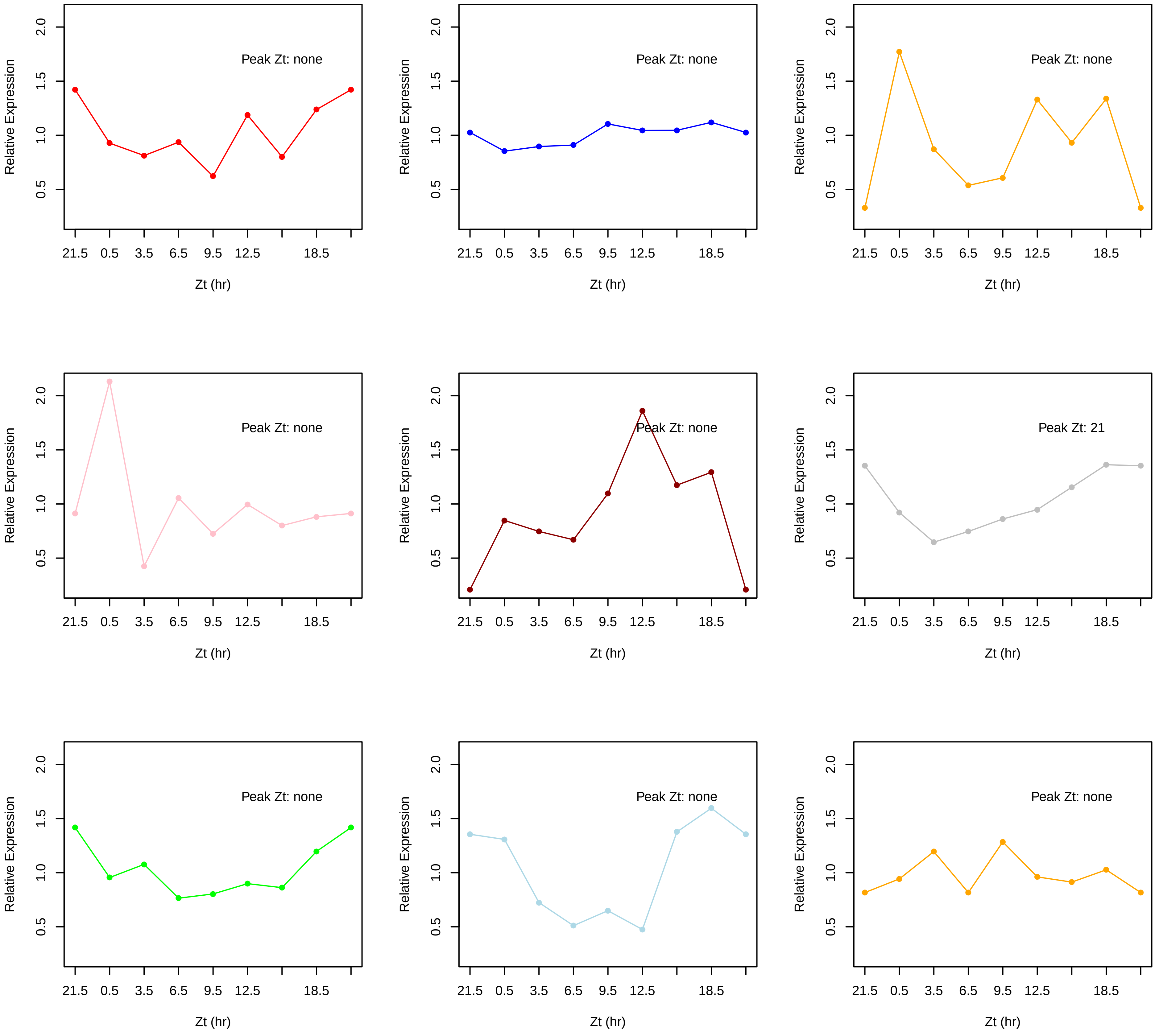

Singeleton: ENSXMAG00000001648 hoxc11a

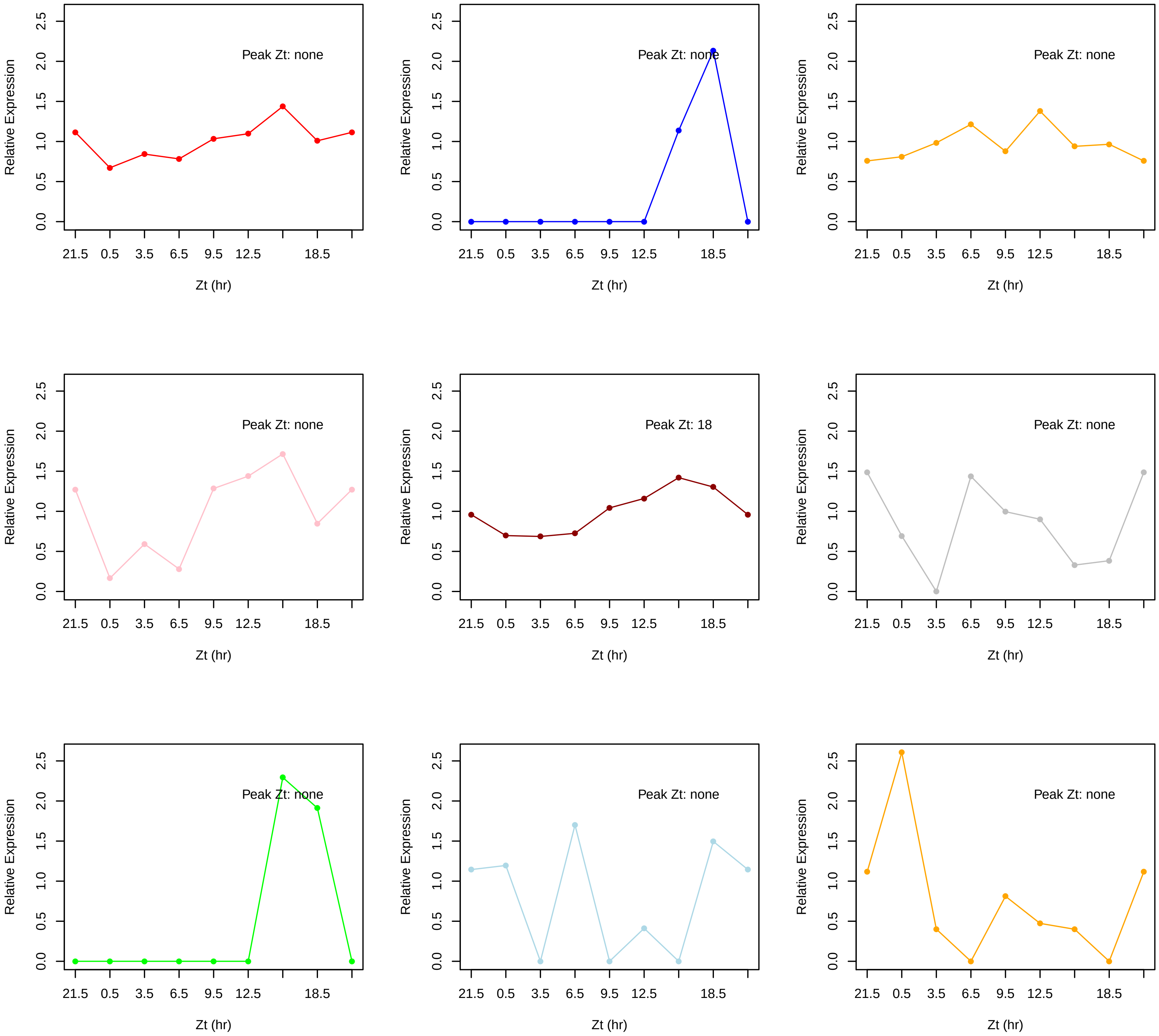

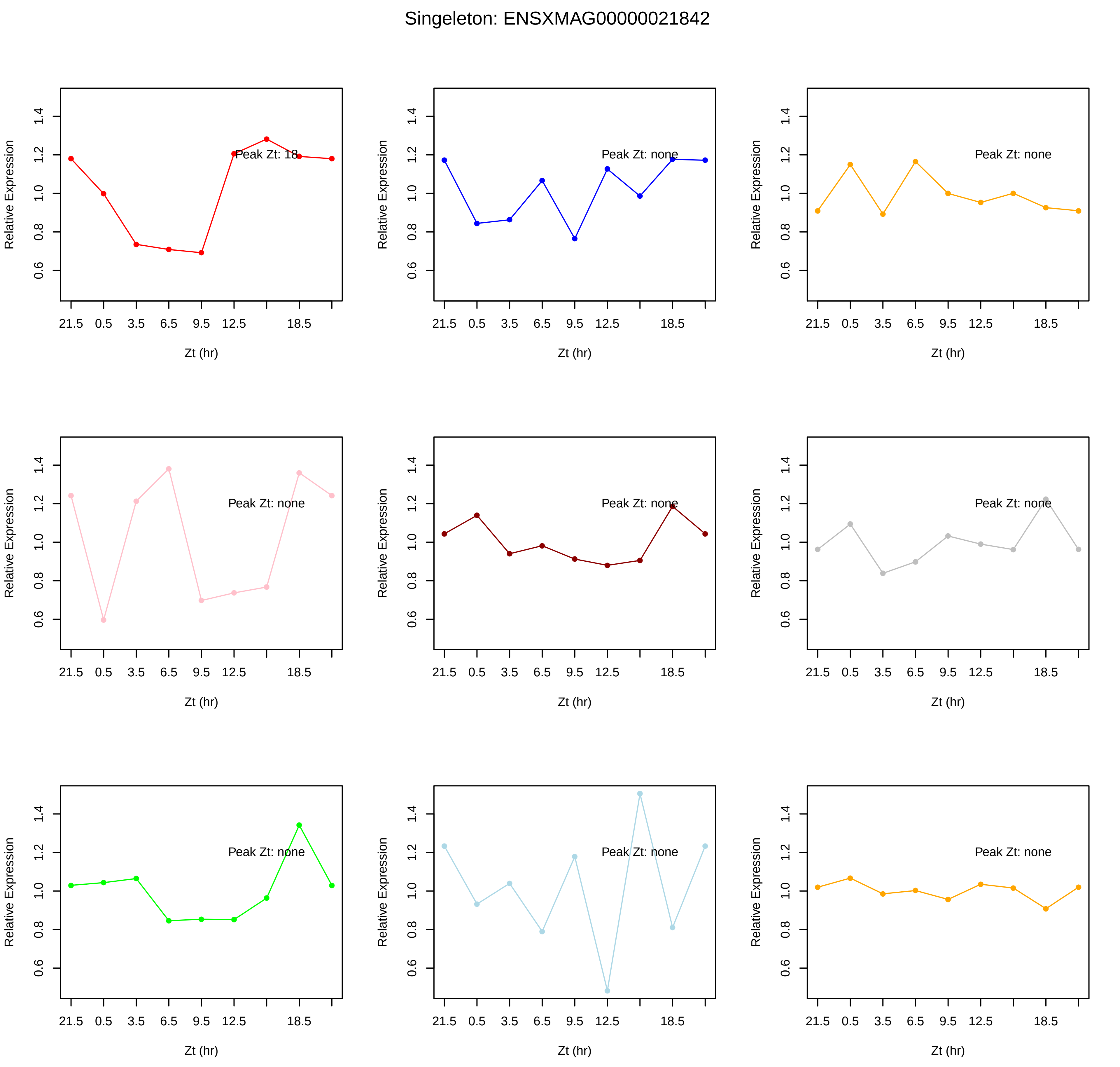

Singeleton: ENSXMAG00000021842
Peak Zt: 18
Peak Zt: none
Peak Zt: none
Peak Zt: none
Peak Zt: none
Peak Zt: none
Peak Zt: none
Peak Zt: none
Peak Zt: none
Relative Expression
Zt (hr)
21.5
0.5
3.5
6.5
9.5
12.5
18.5
0.6
0.8
1.0
1.2
1.4

Singeleton: ENSXMAG00000016653 KCTD1

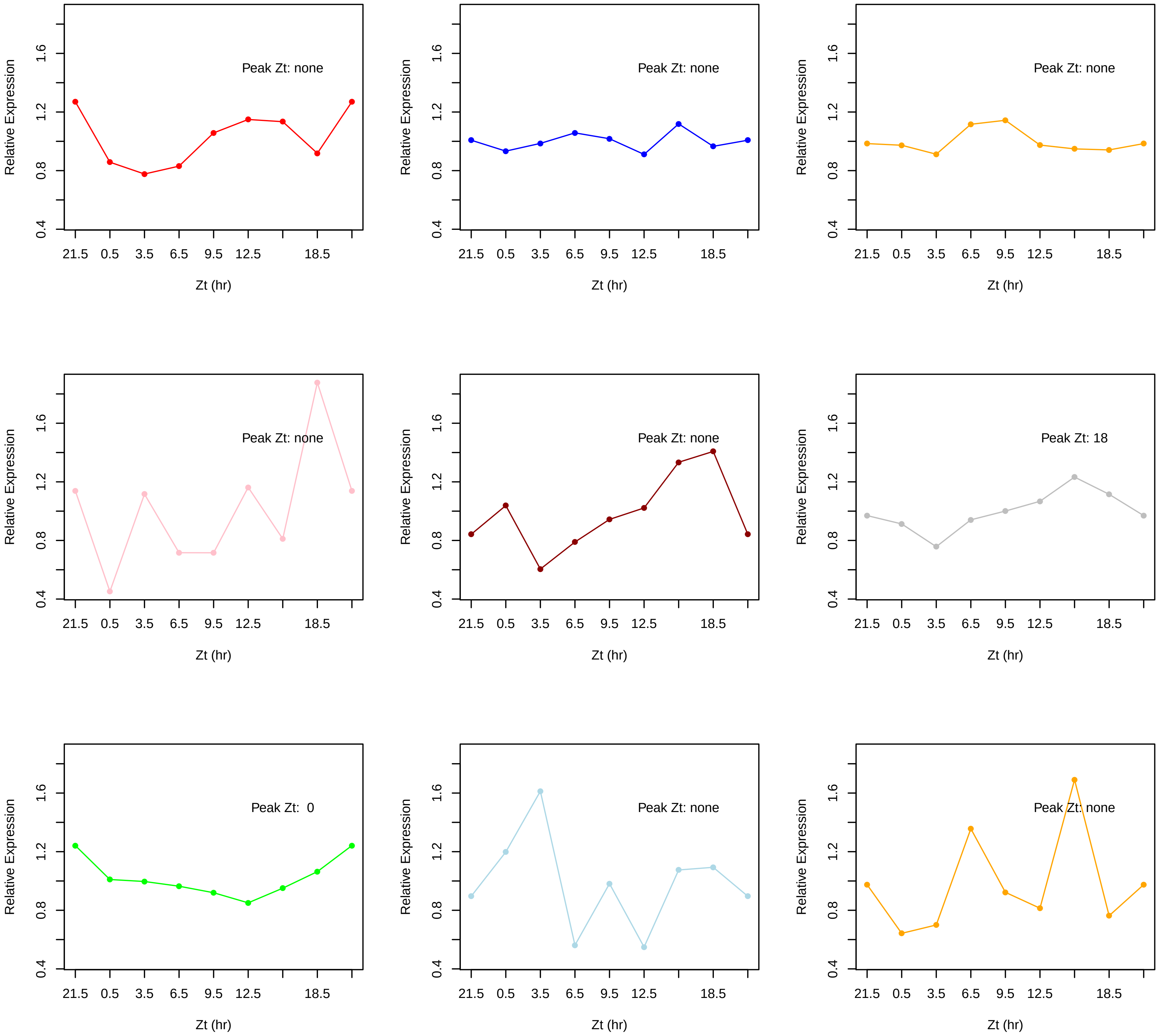

Singeleton: ENSXMAG00000019093

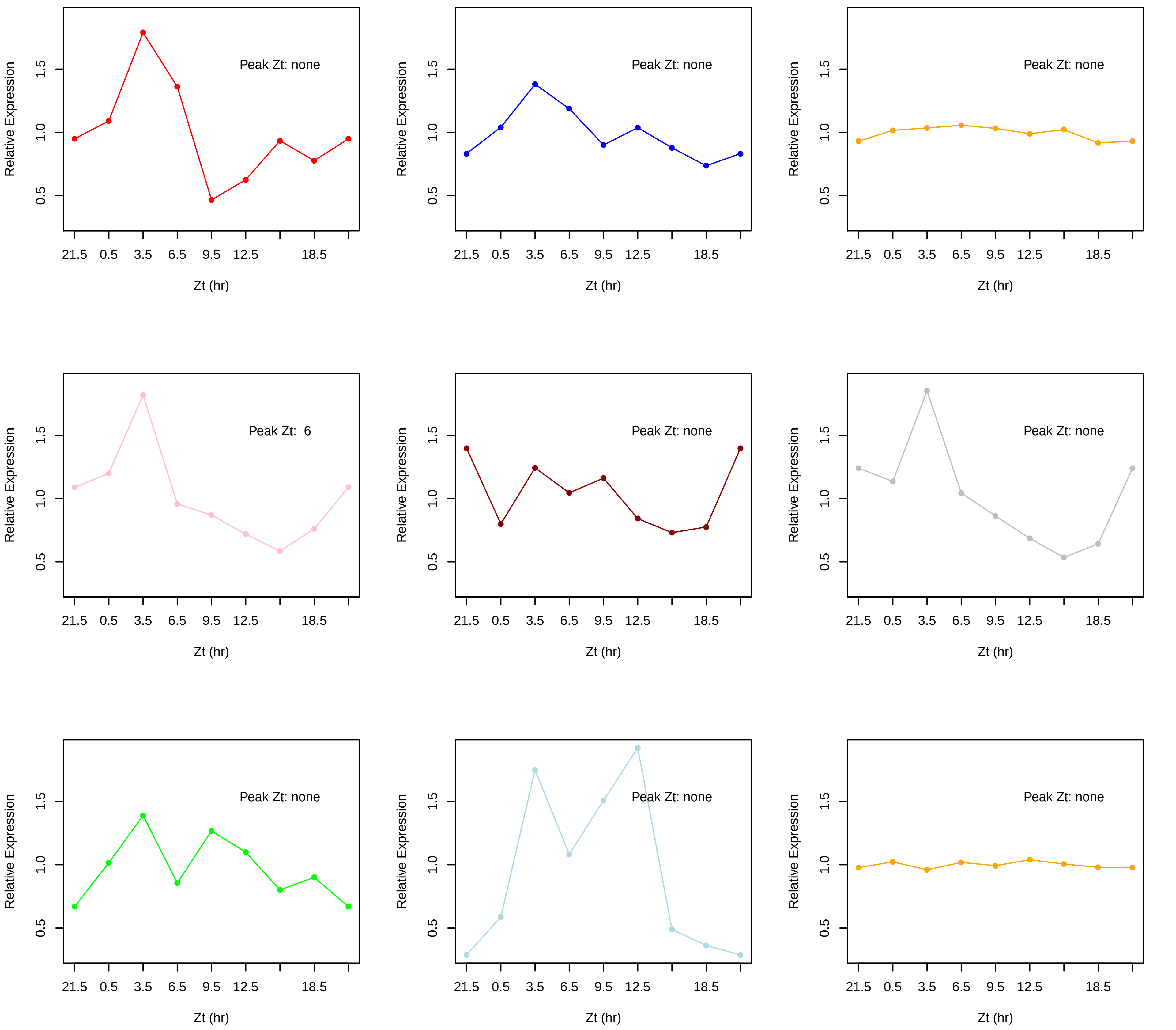

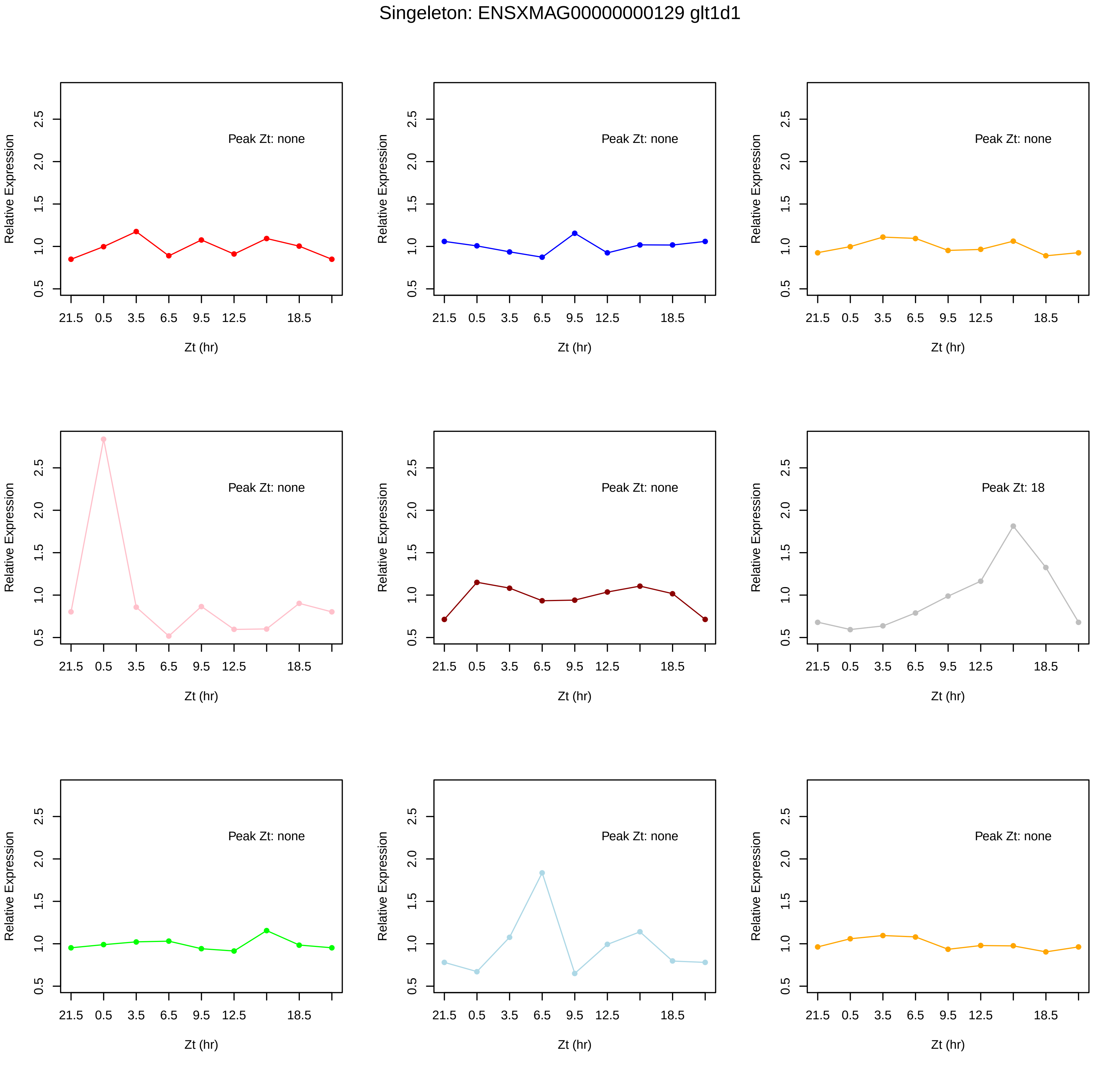
Singleton: ENSXMAG00000000129 glt1d1
Peak Zt: none
Peak Zt: none
Peak Zt: none
Peak Zt: none
Peak Zt: none
Peak Zt: 18
Peak Zt: none
Peak Zt: none
Peak Zt: none
Relative Expression
Zt (hr)
21.5
0.5
3.5
6.5
9.5
12.5
18.5
0.5
1.0
1.5
2.0
2.5

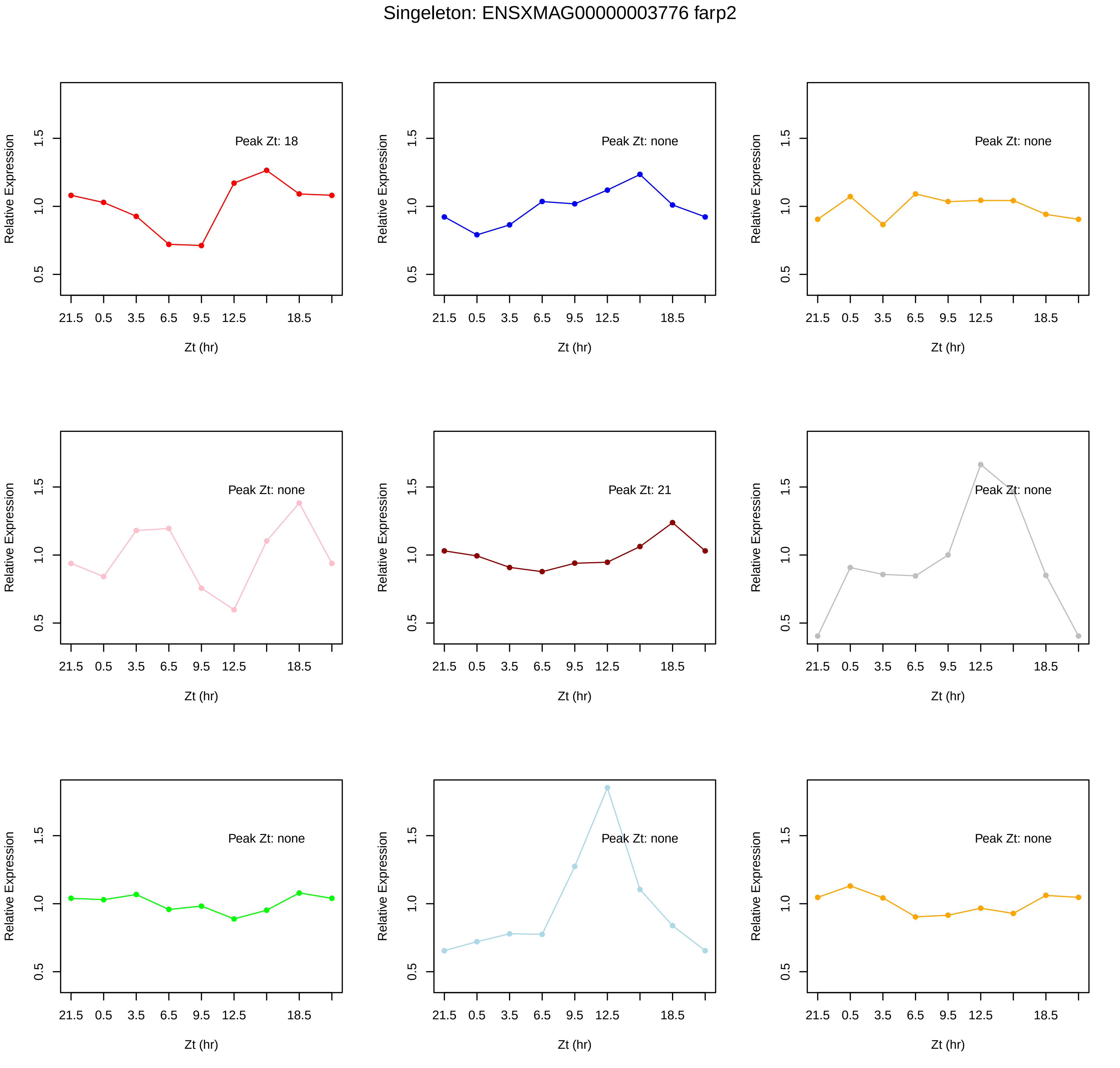
Singeleton: ENSXMAG00000003776 farp2
Peak Zt: 18
Peak Zt: none
Peak Zt: none
Peak Zt: none
Peak Zt: 21
Peak Zt: none
Peak Zt: none
Peak Zt: none
Peak Zt: none
Relative Expression
Zt (hr)
21.5
0.5
3.5
6.5
9.5
12.5
18.5
0.5
1.0
1.5

Singeleton: ENSXMAG00000000131 slc15a4

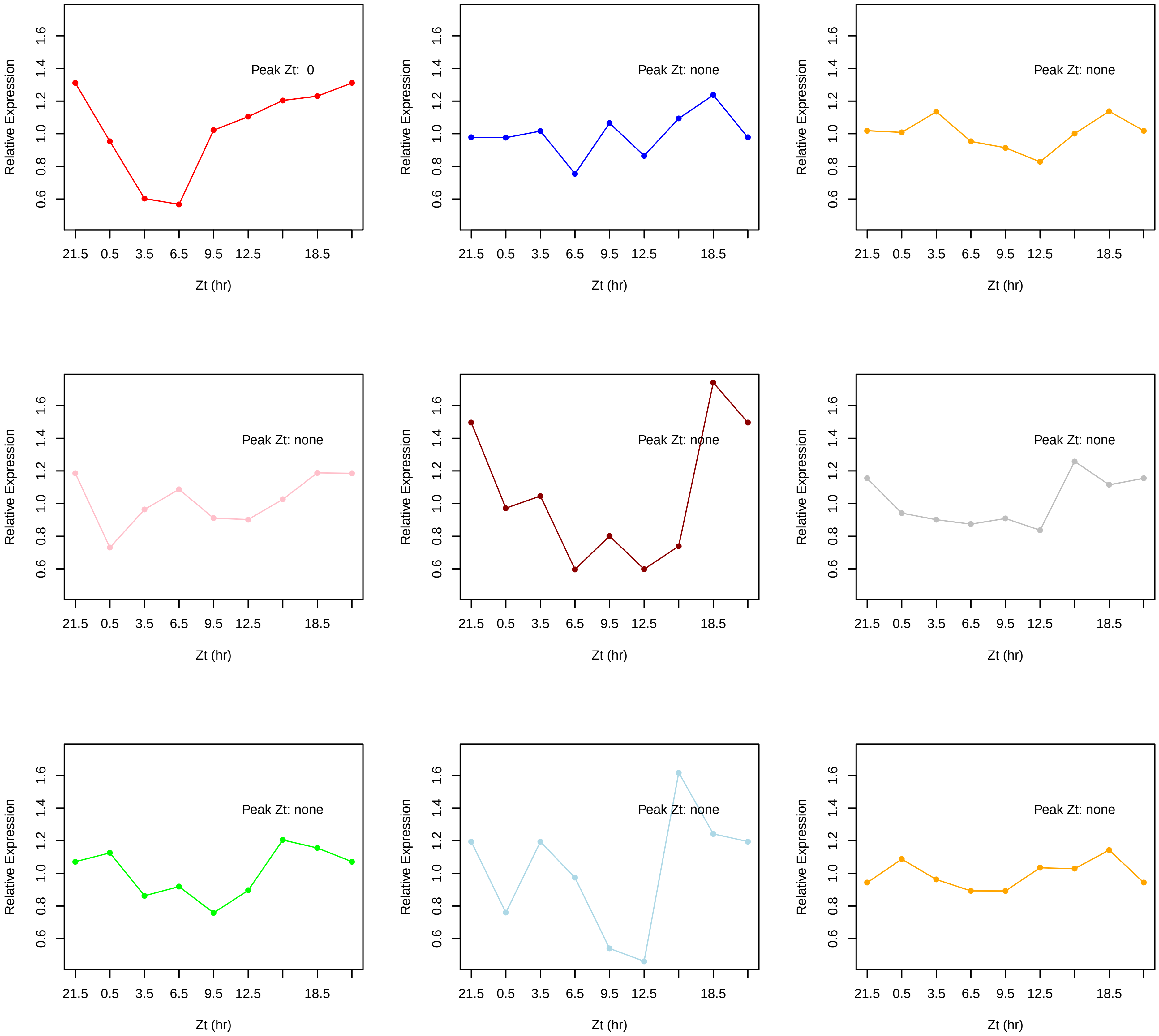

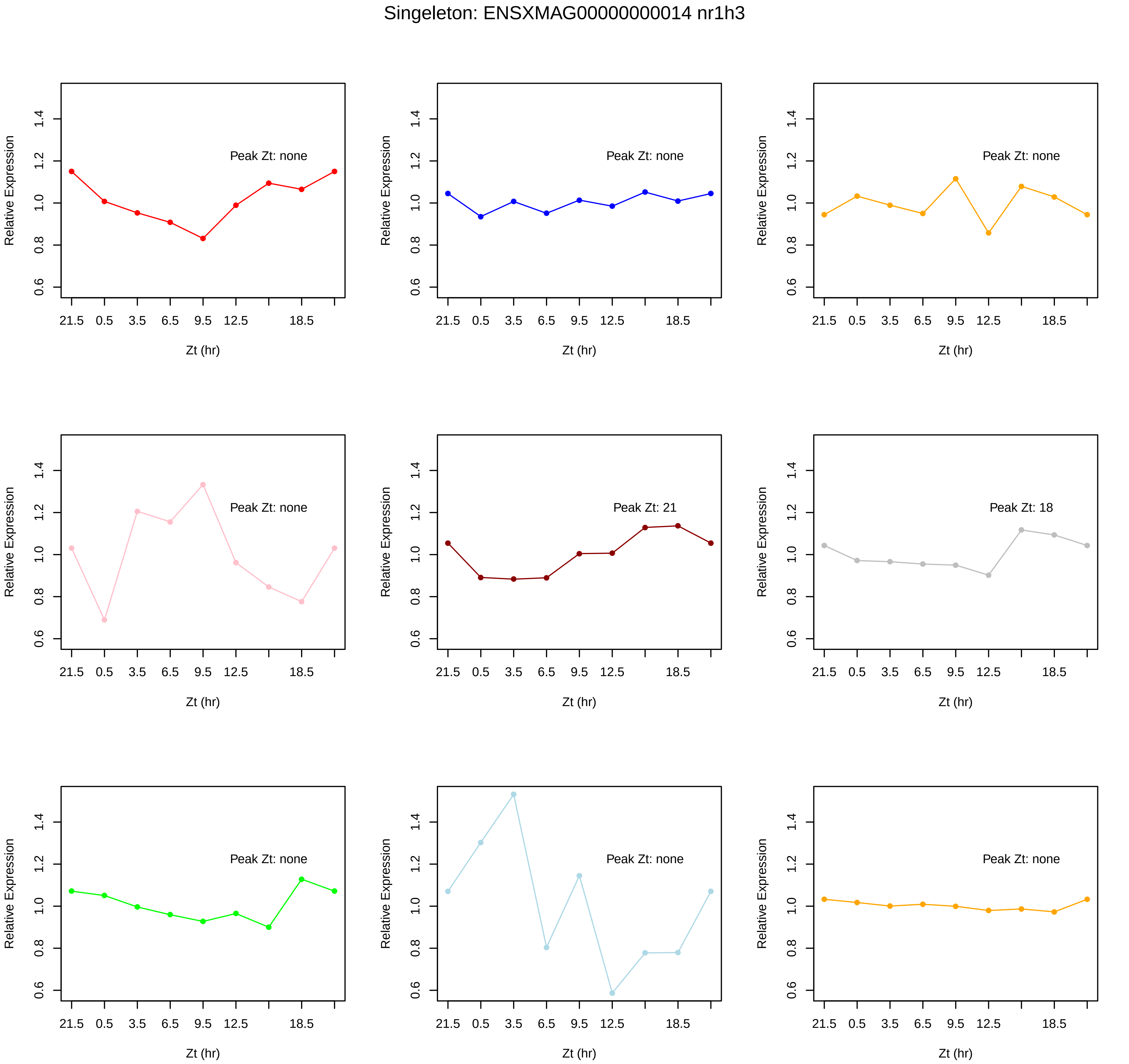

Singleton: ENSXMAG00000000014 nr1h3
Peak Zt: none
Peak Zt: none
Peak Zt: none
Peak Zt: none
Peak Zt: 21
Peak Zt: 18
Peak Zt: none
Peak Zt: none
Peak Zt: none
Relative Expression
Zt (hr)
0.6
0.8
1.0
1.2
1.4
21.5
0.5
3.5
6.5
9.5
12.5
18.5

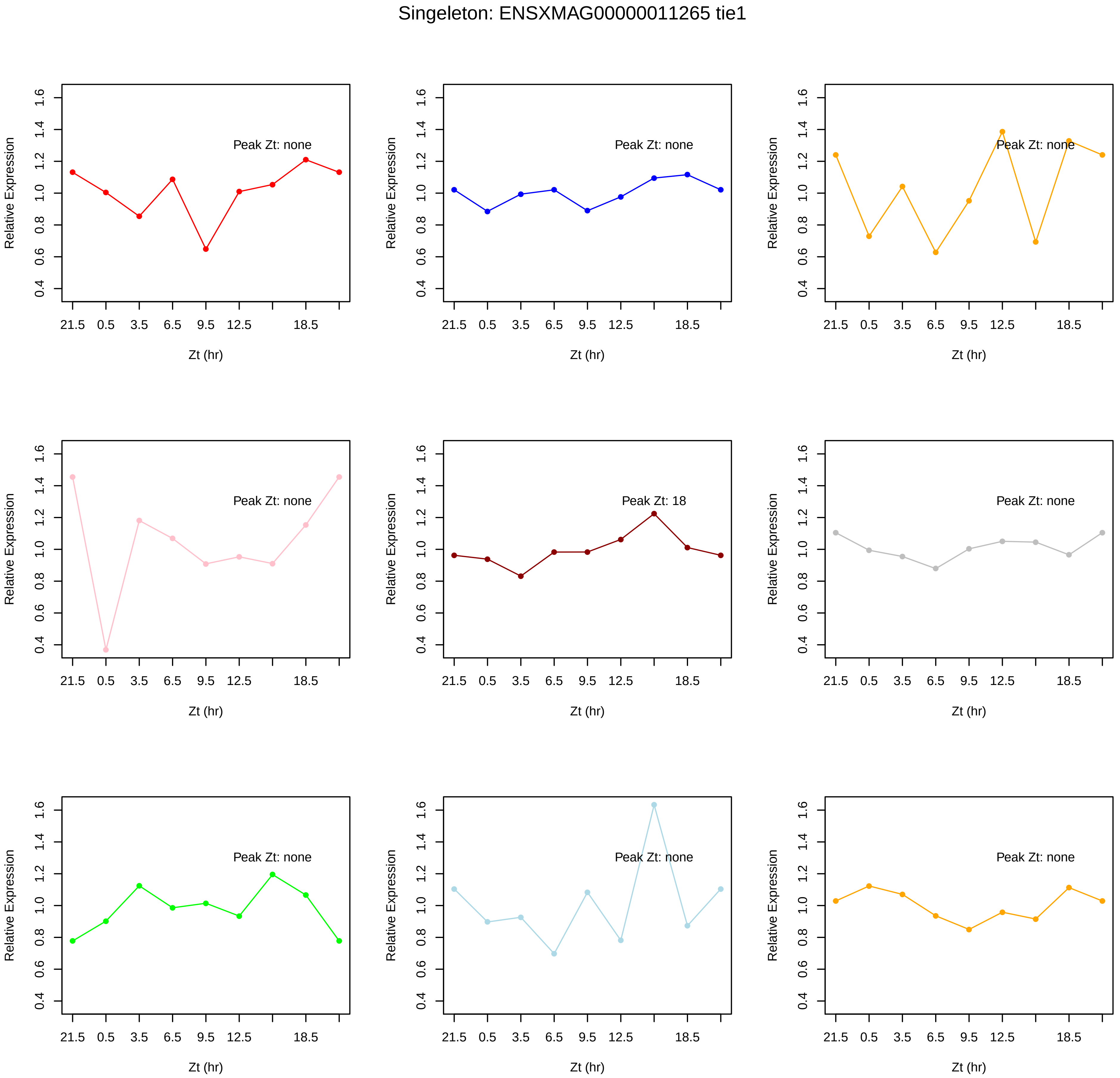
Singeleton: ENSXMAG00000011265 tie1
Relative Expression
Zt (hr)
21.5 0.5 3.5 6.5 9.5 12.5 18.5
0.4 0.6 0.8 1.0 1.2 1.4 1.6
Peak Zt: none
Peak Zt: none
Peak Zt: none
Peak Zt: none
Peak Zt: 18
Peak Zt: none
Peak Zt: none
Peak Zt: none
Peak Zt: none

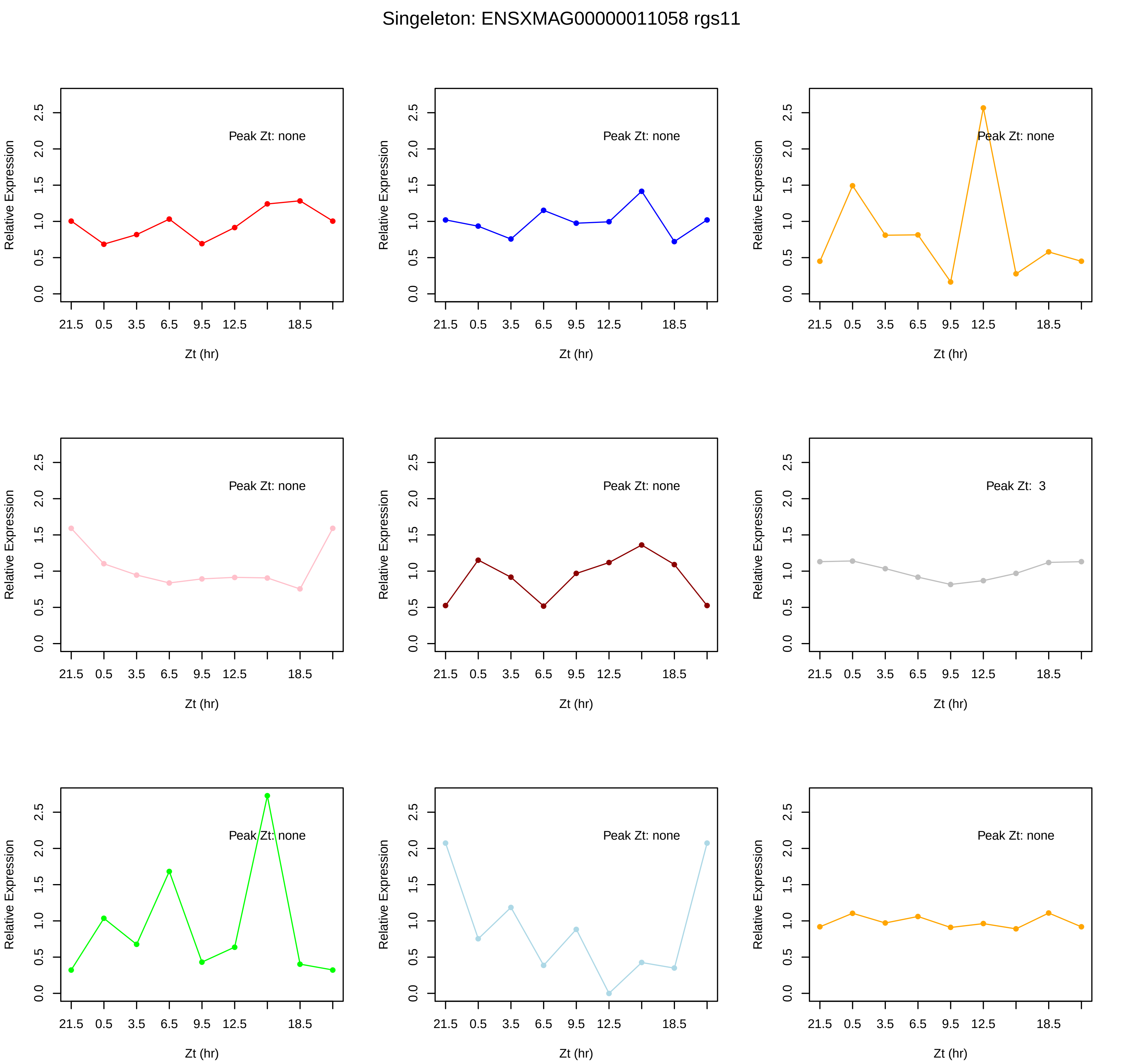
Singleton: ENSXMAG00000011058 rgs11
Peak Zt: none
Peak Zt: none
Peak Zt: none
Peak Zt: none
Peak Zt: none
Peak Zt: 3
Peak Zt: none
Peak Zt: none
Peak Zt: none
Relative Expression
Zt (hr)
21.5
0.5
3.5
6.5
9.5
12.5
18.5
0.0
0.5
1.0
1.5
2.0
2.5

Singleton: ENSXMAG00000016260 itga10

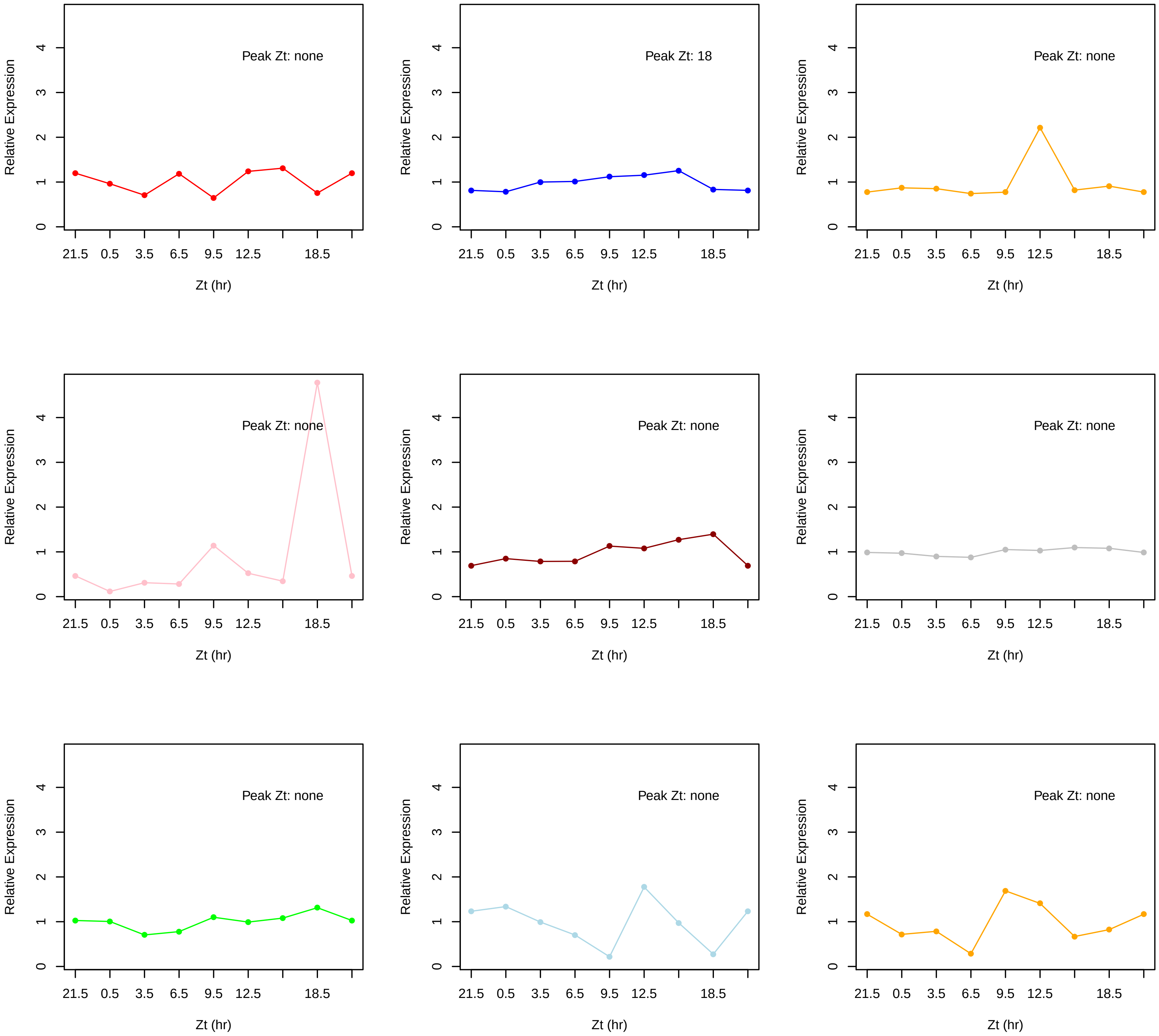

Singeleton: ENSXMAG00000016624 psma8

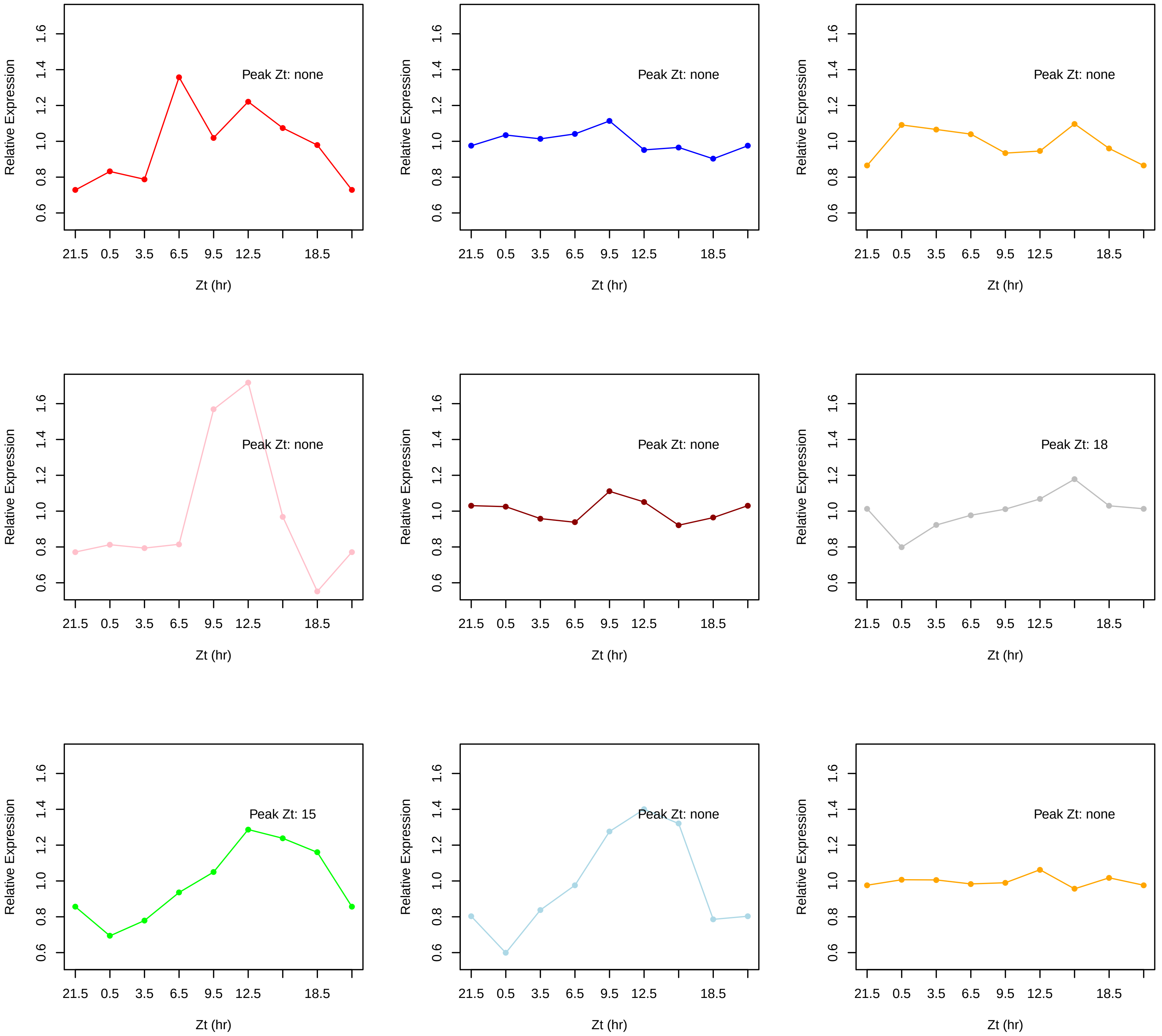

Singeleton: ENSXMAG00000027011 tp53inp1

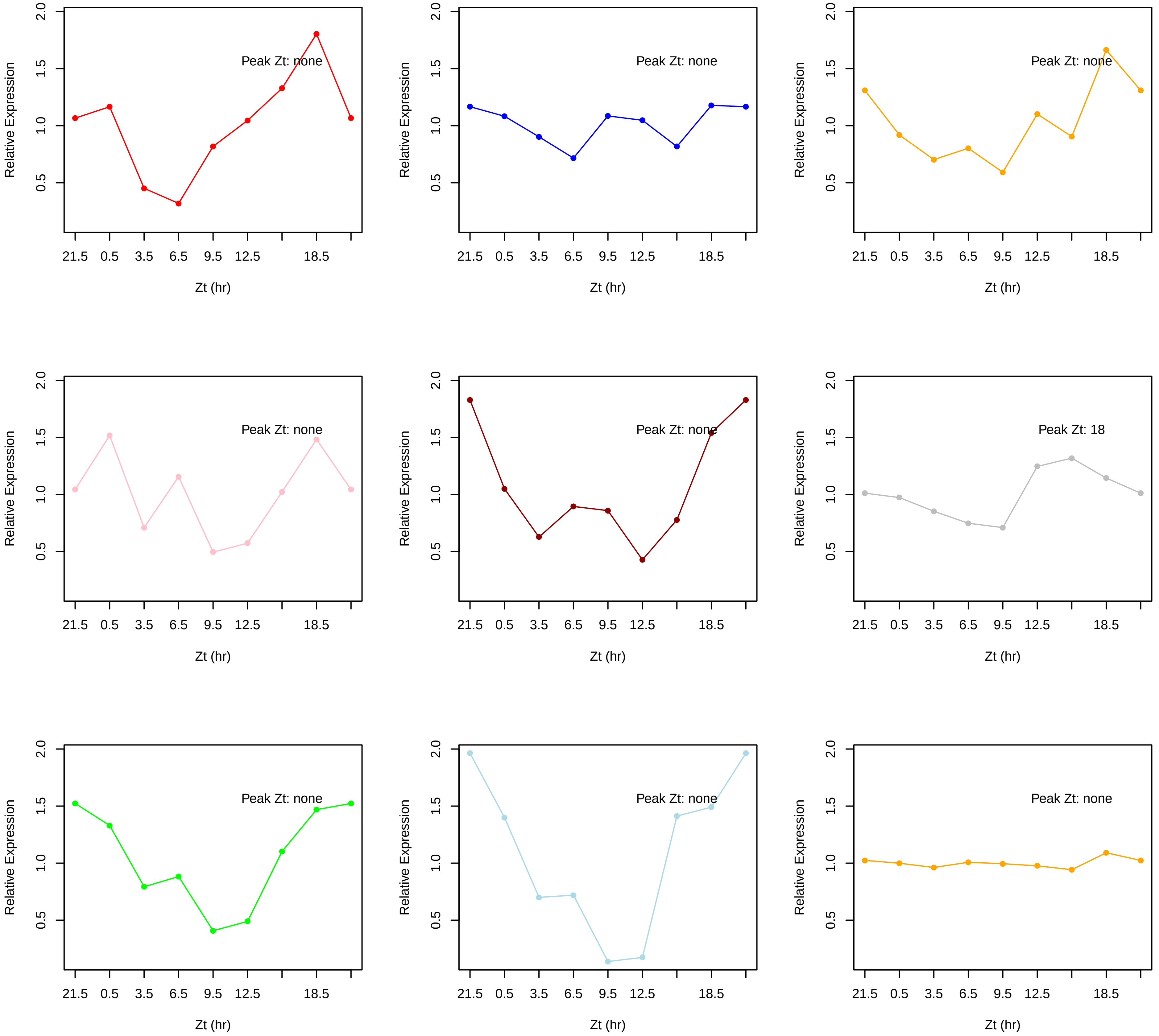

Singeleton: ENSXMAG00000013381 tle2a

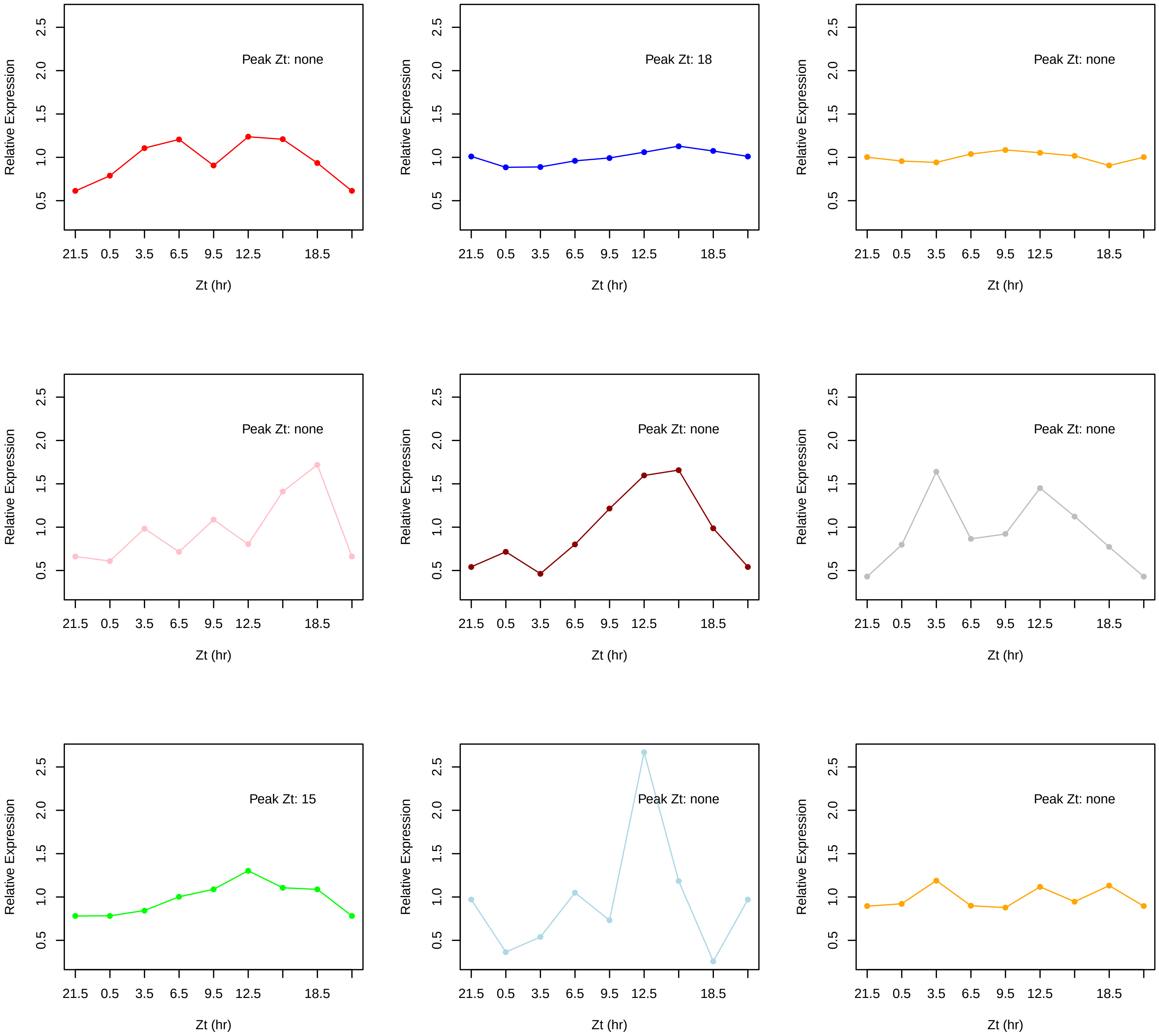

Singleton: ENSXMAG00000007362 erap2

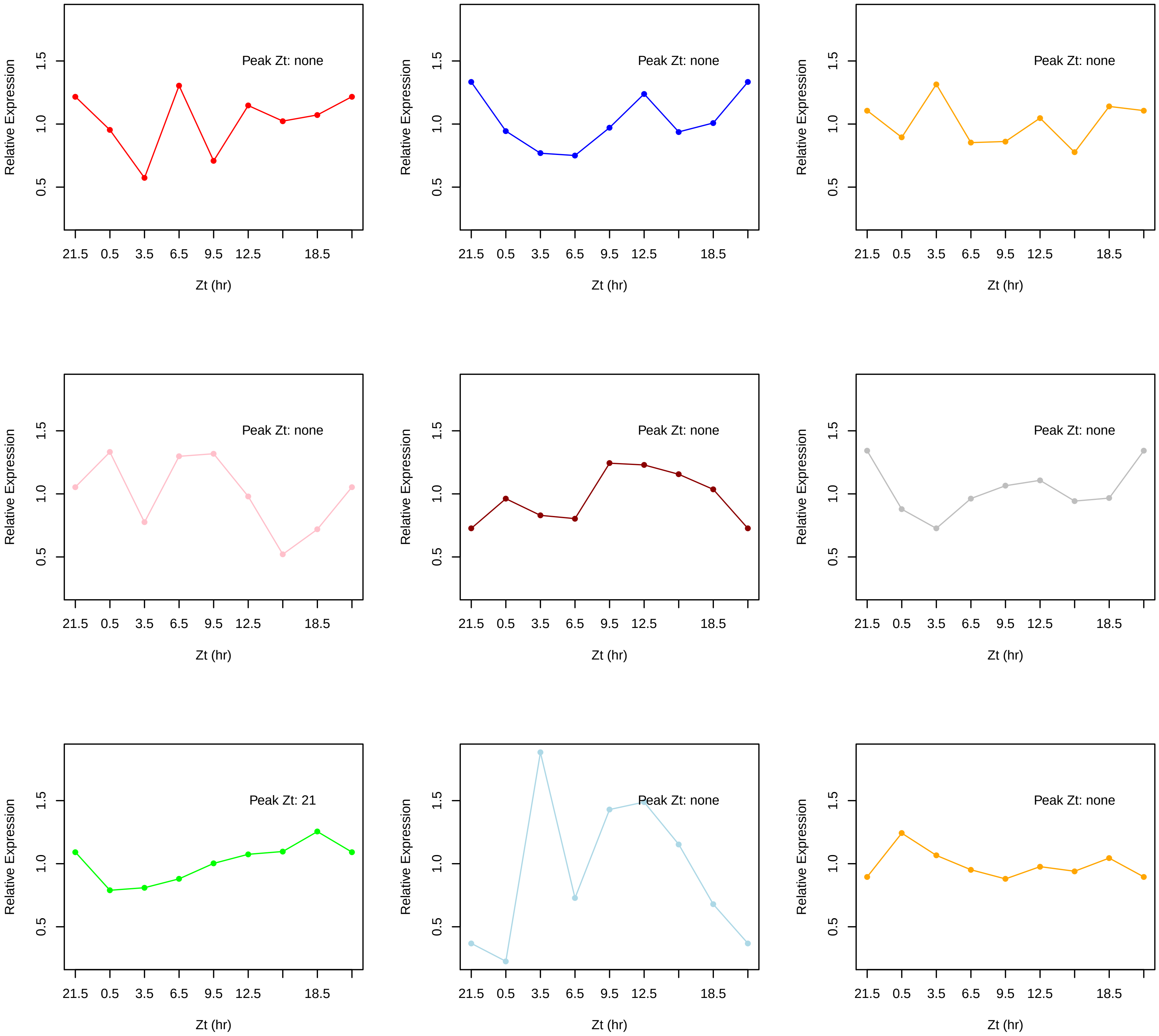

Singleton: ENSXMAG00000005965 lamc3

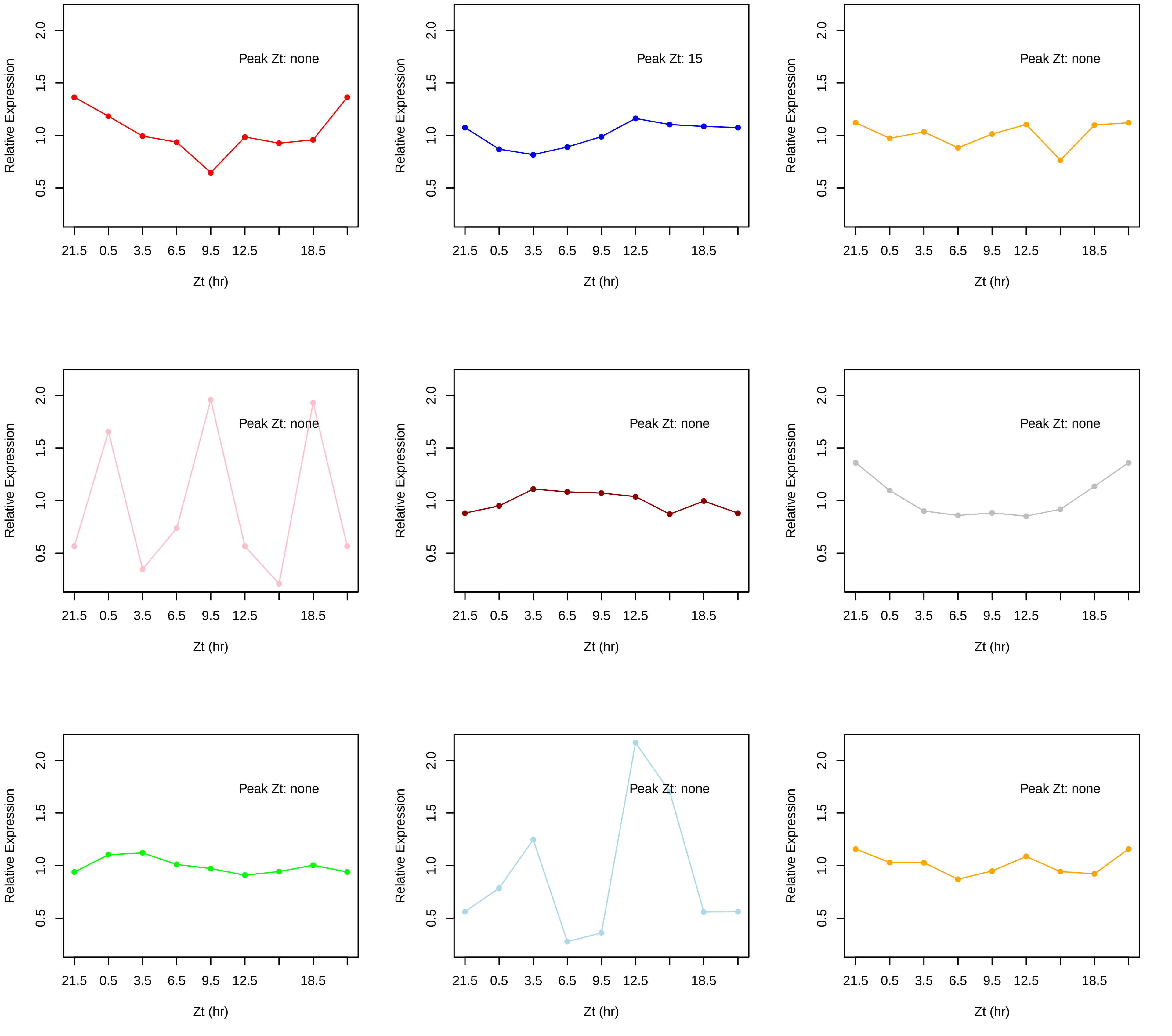

Singeleton: ENSXMAG00000025009

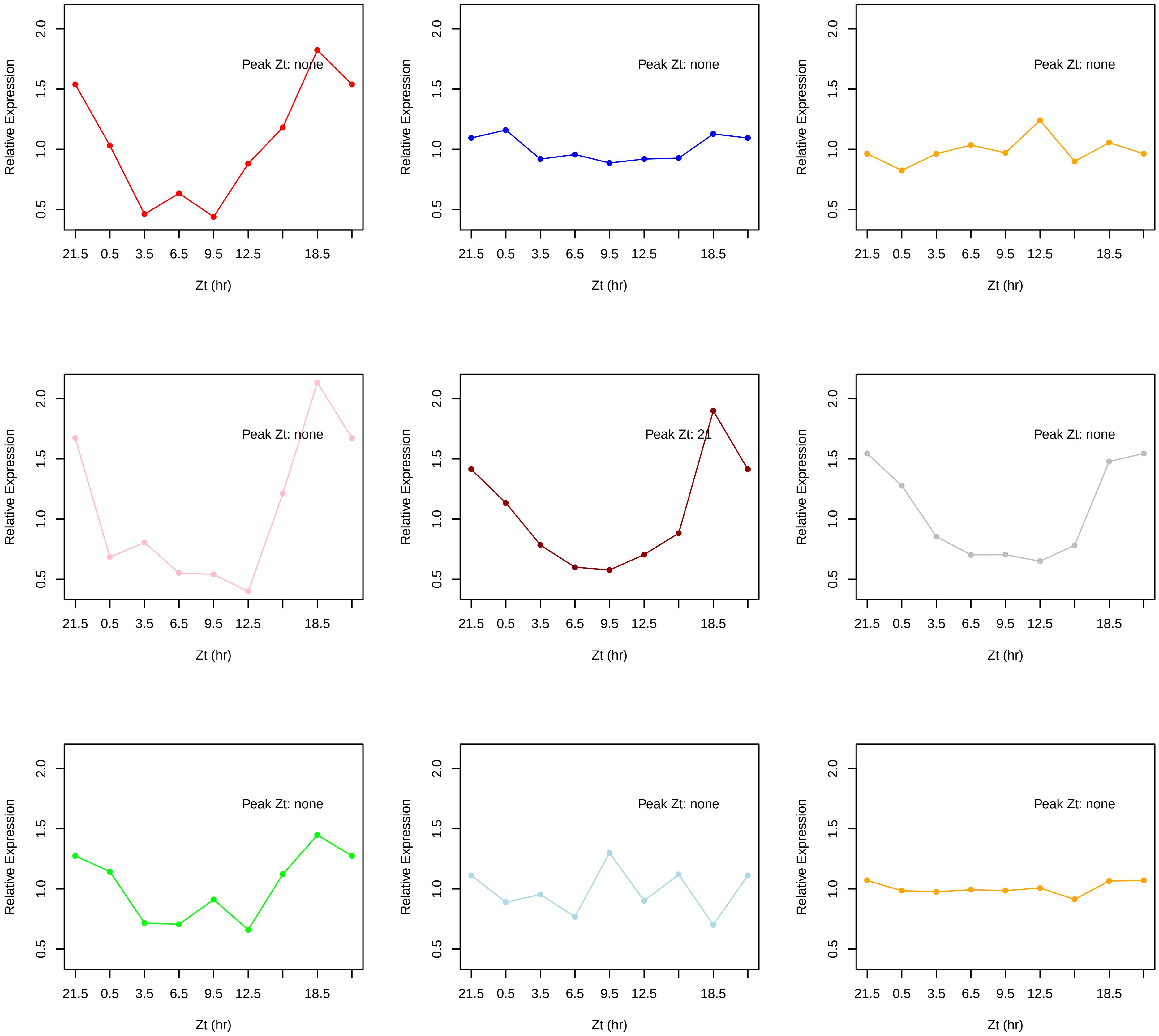

Singeleton: ENSXMAG00000004304 stk25b

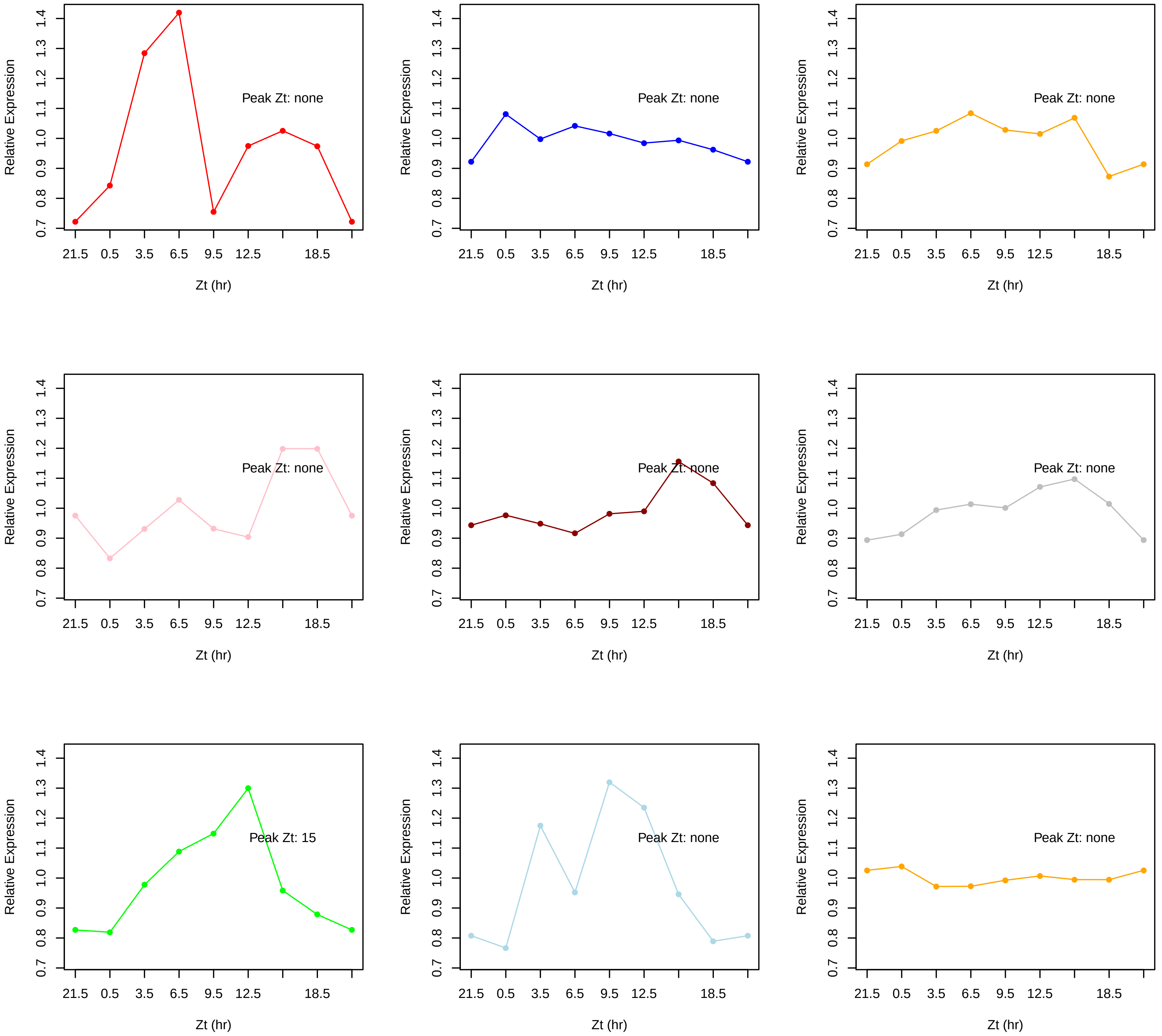

Singeleton: ENSXMAG00000003779 KIF18A

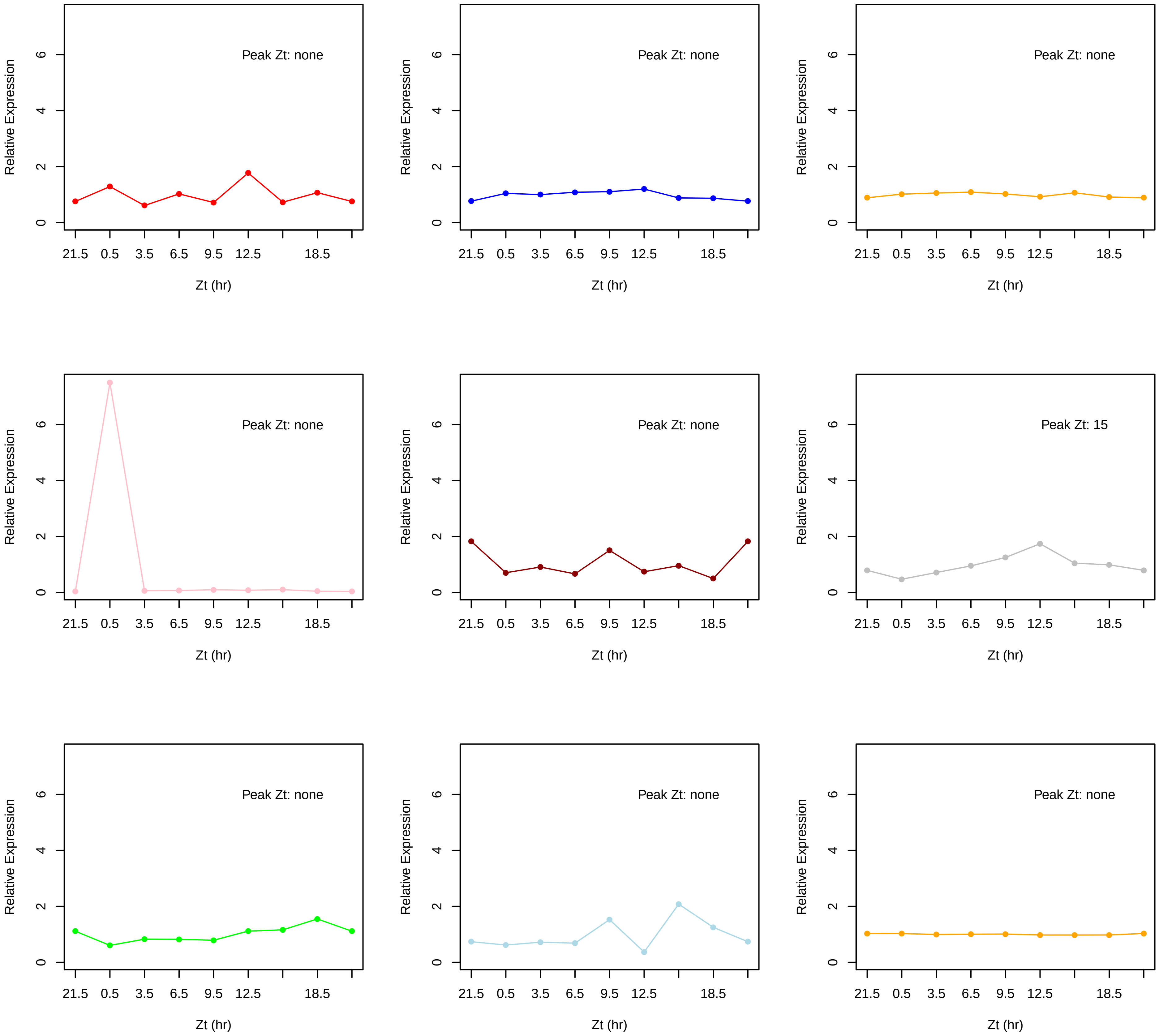

Singeleton: ENSXMAG00000011310 ap1m3

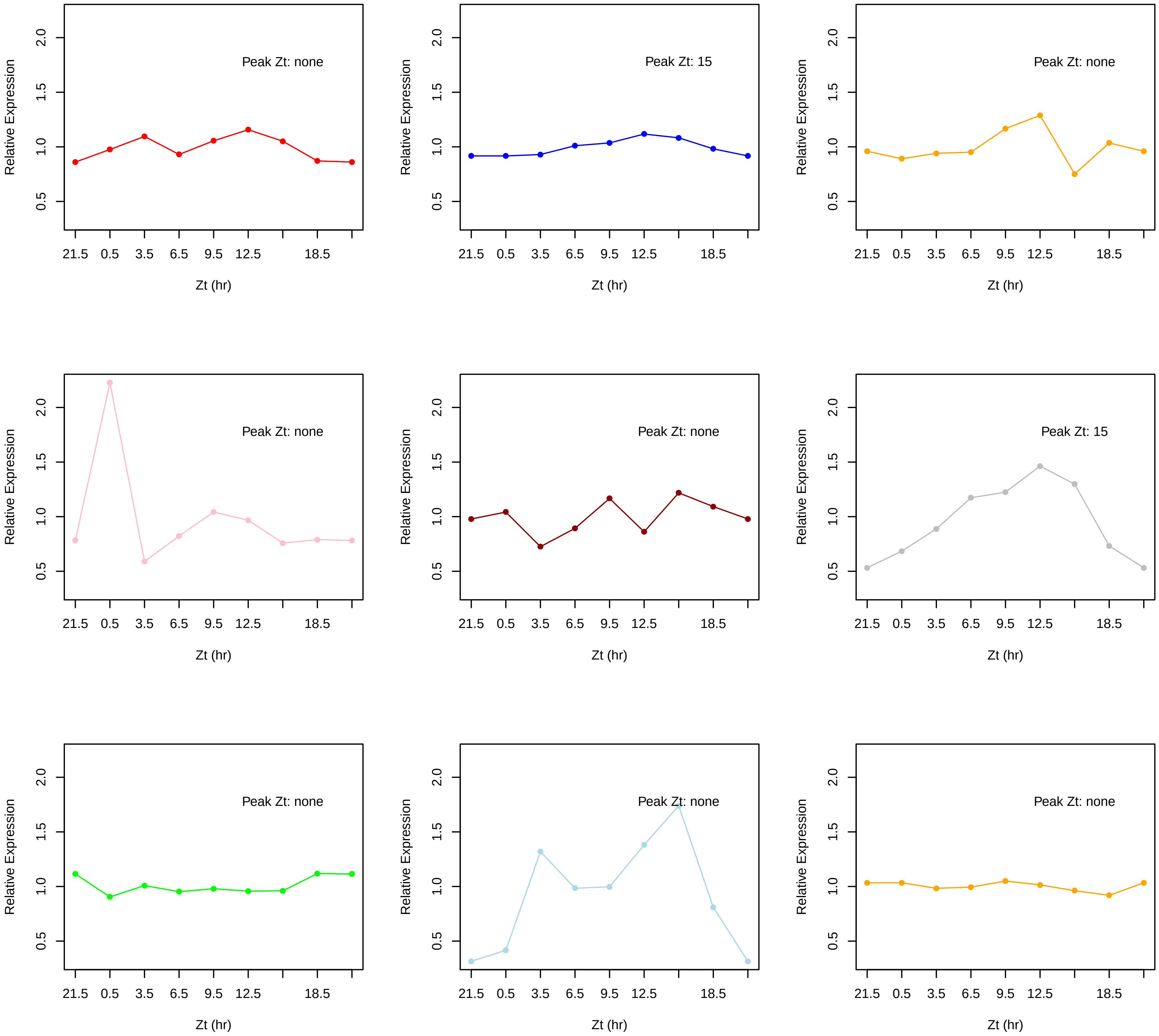

Singeleton: ENSXMAG00000029699 gna11a

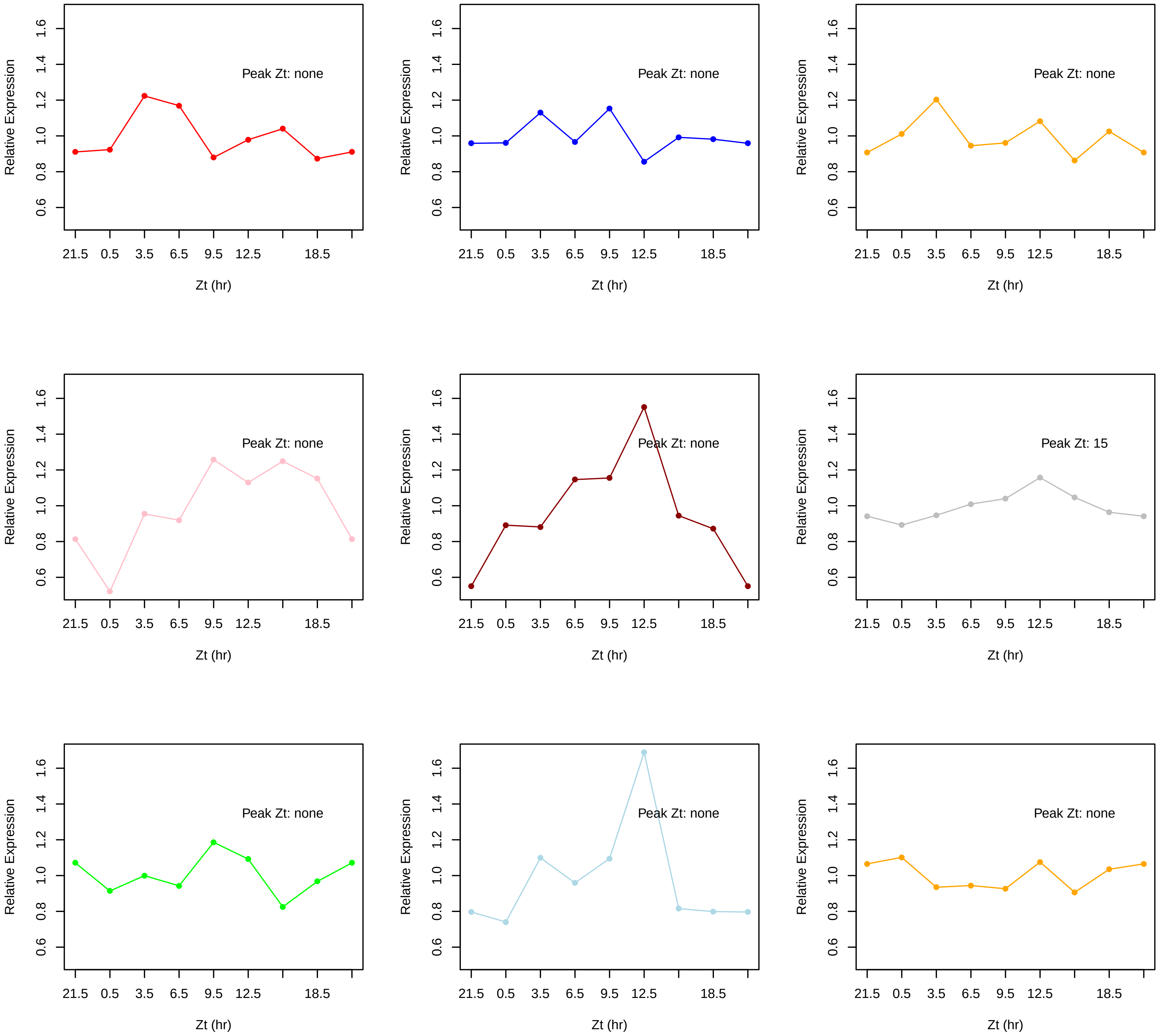

Singeleton: ENSXMAG00000027608 rgmb

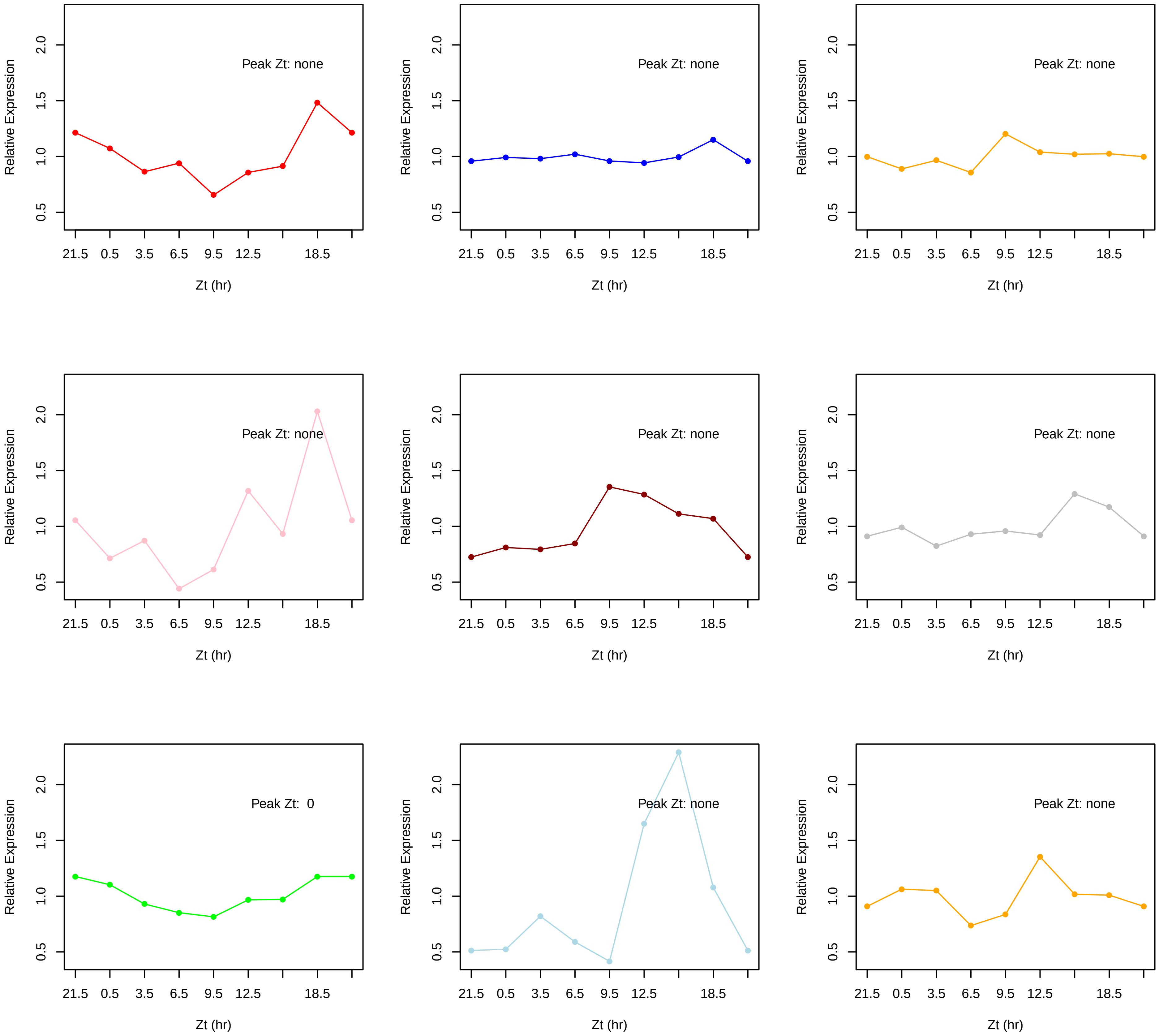

Singeleton: ENSXMAG00000016577 ZNF521

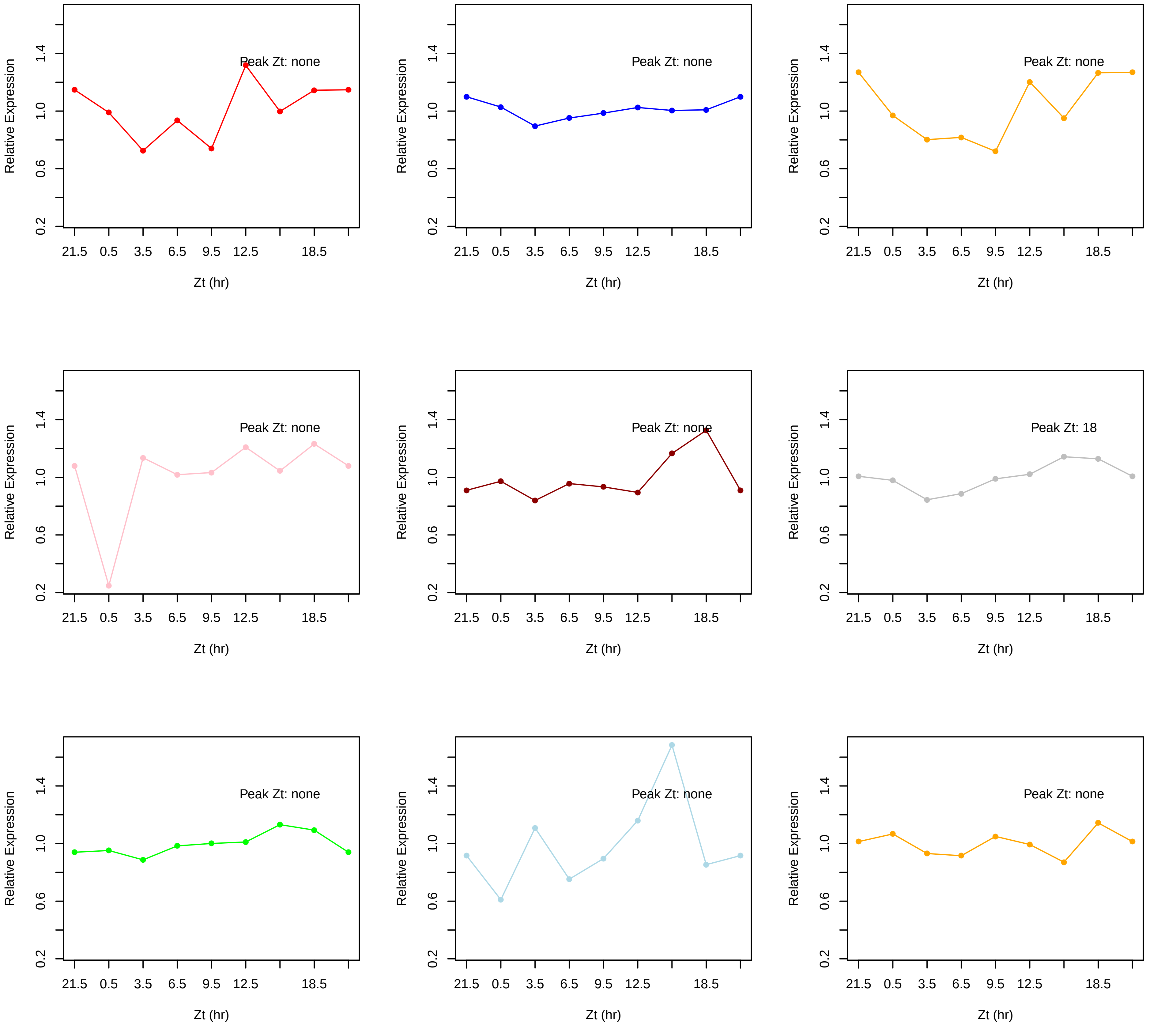

Singeleton: ENSXMAG00000001387 sestd1

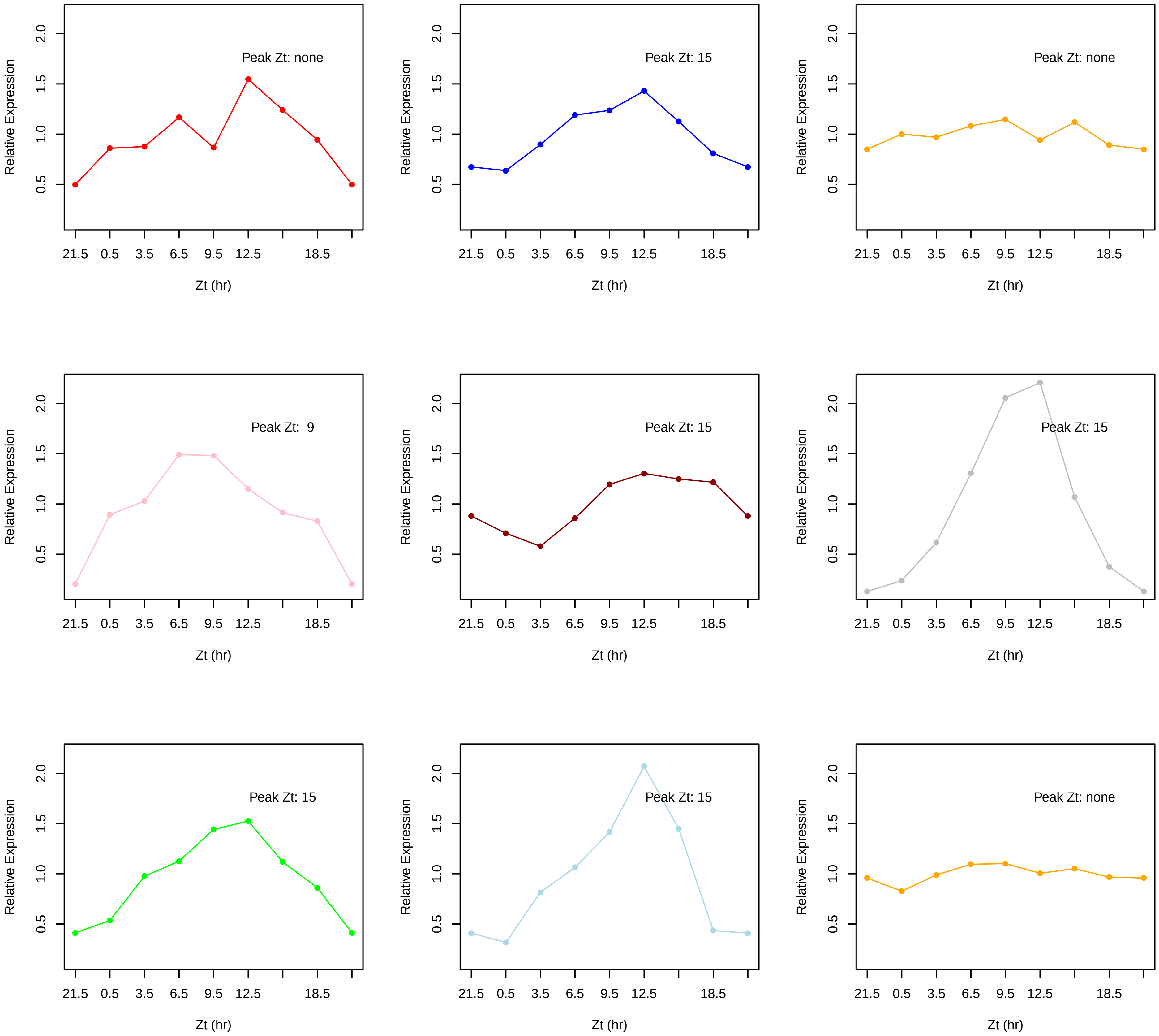

Singleton: ENSXMAG00000013765 wdr83

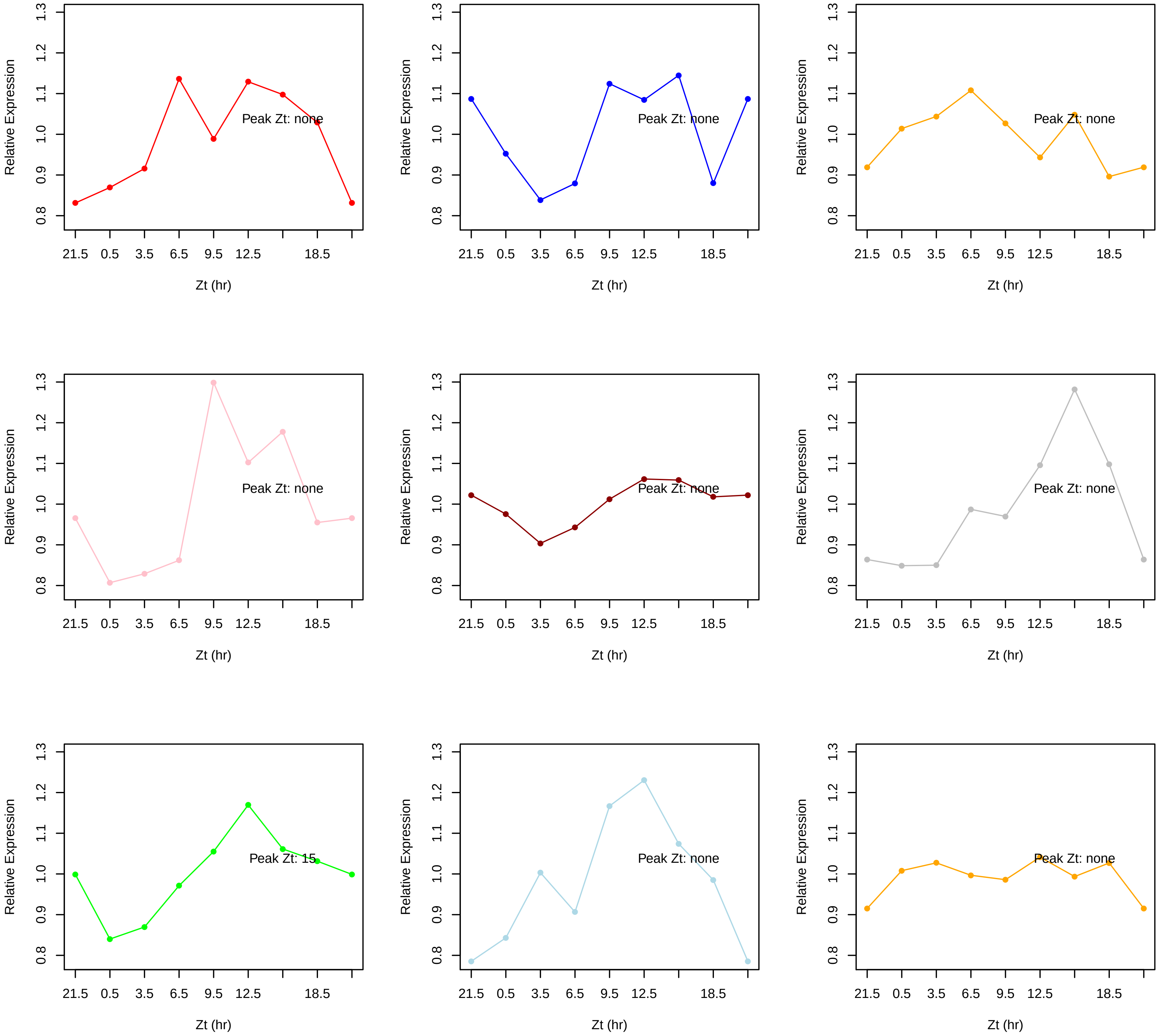

Singleton: ENSXMAG00000016215 polr3c

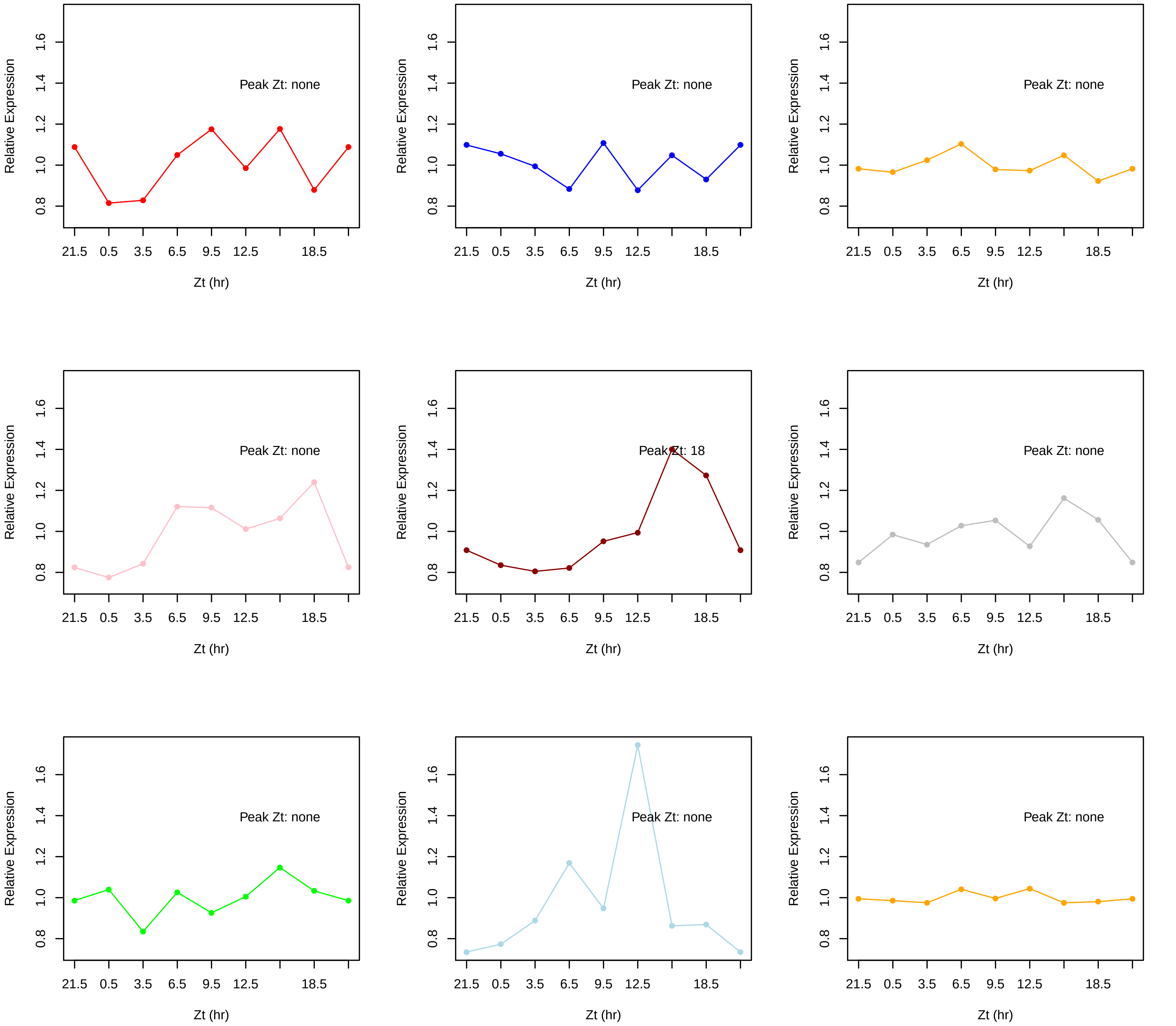

Singeleton: ENSXMAG00000003742 lgr4

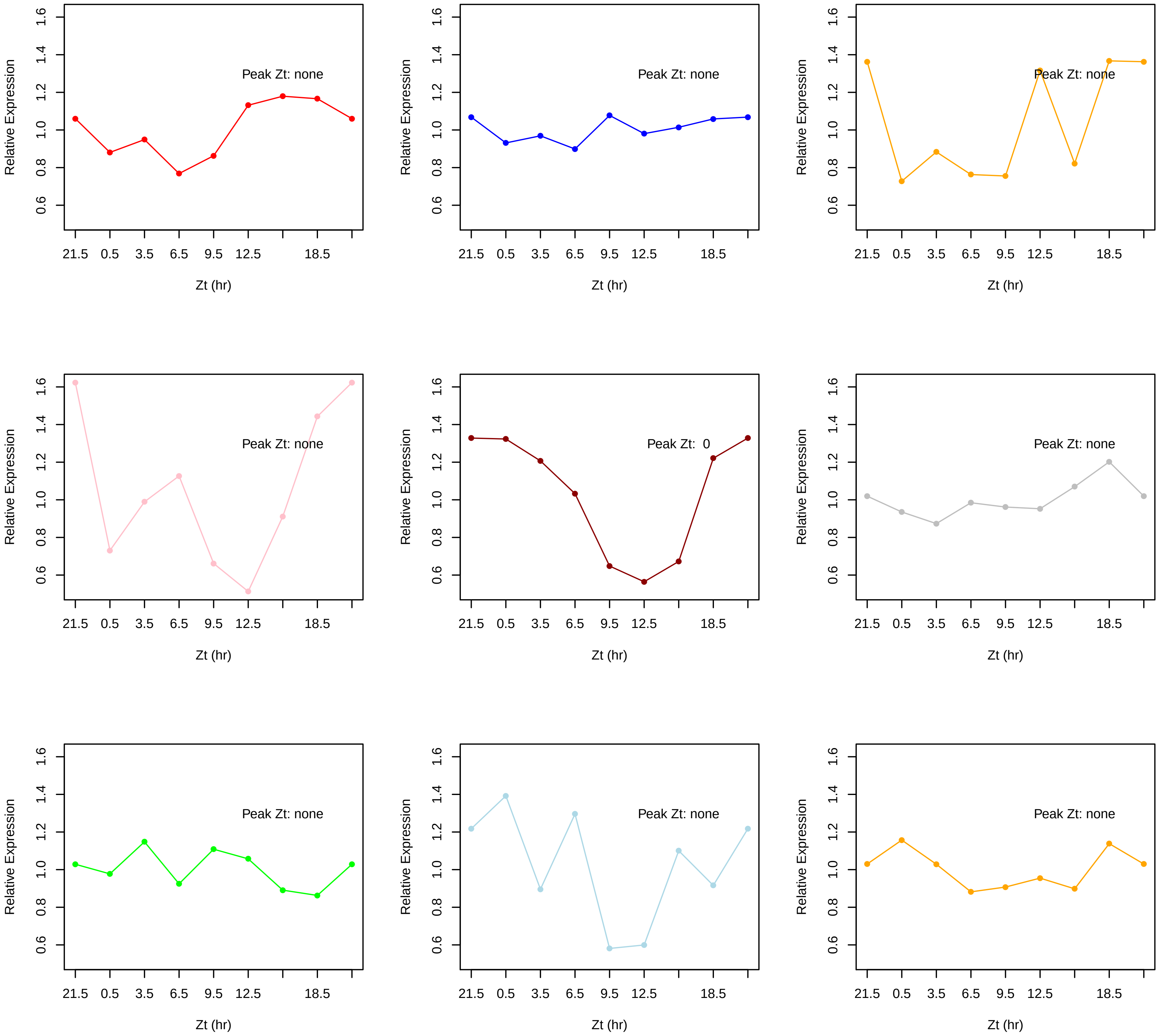

Singleton: ENSXMAG00000019055 actr5

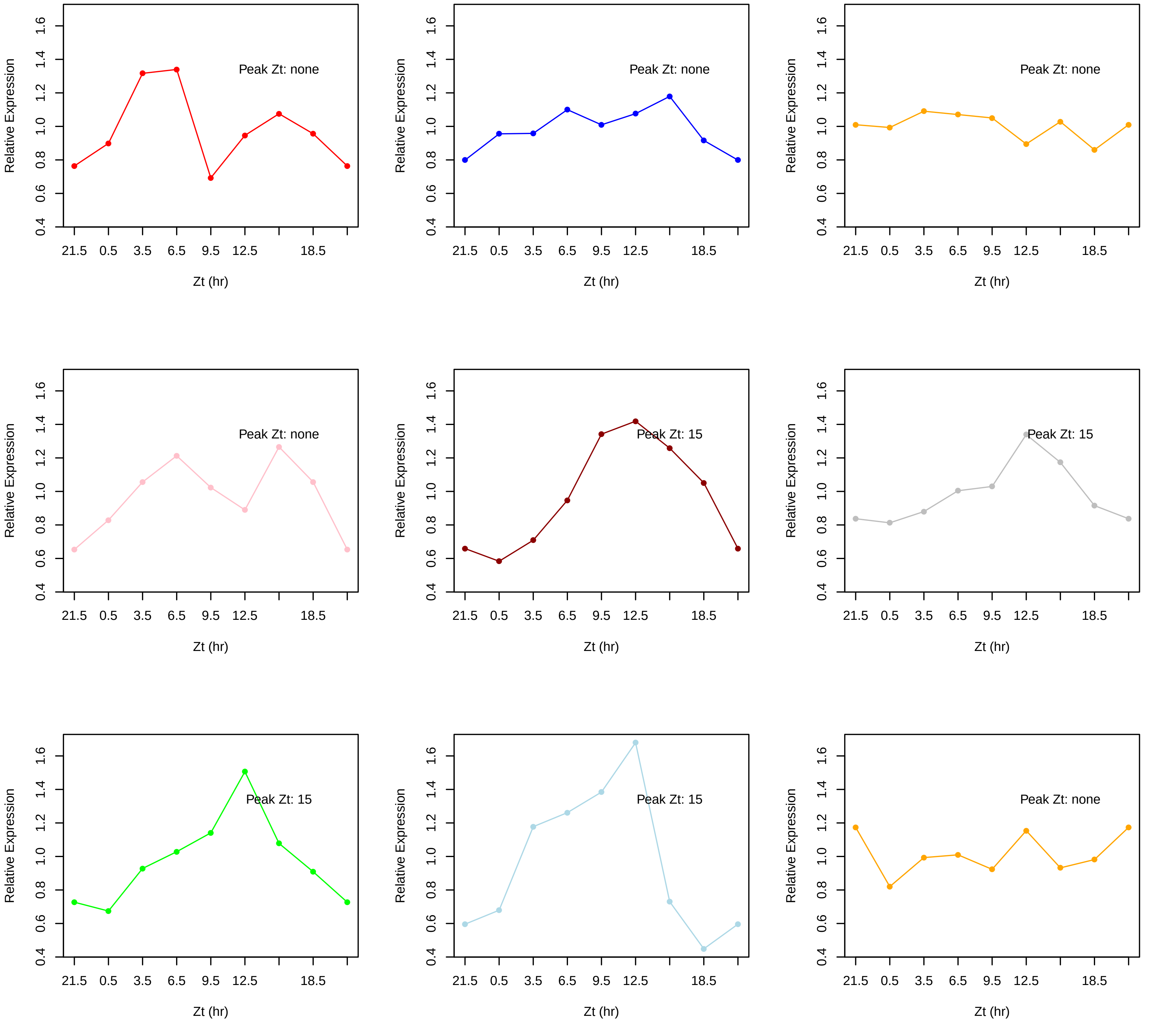

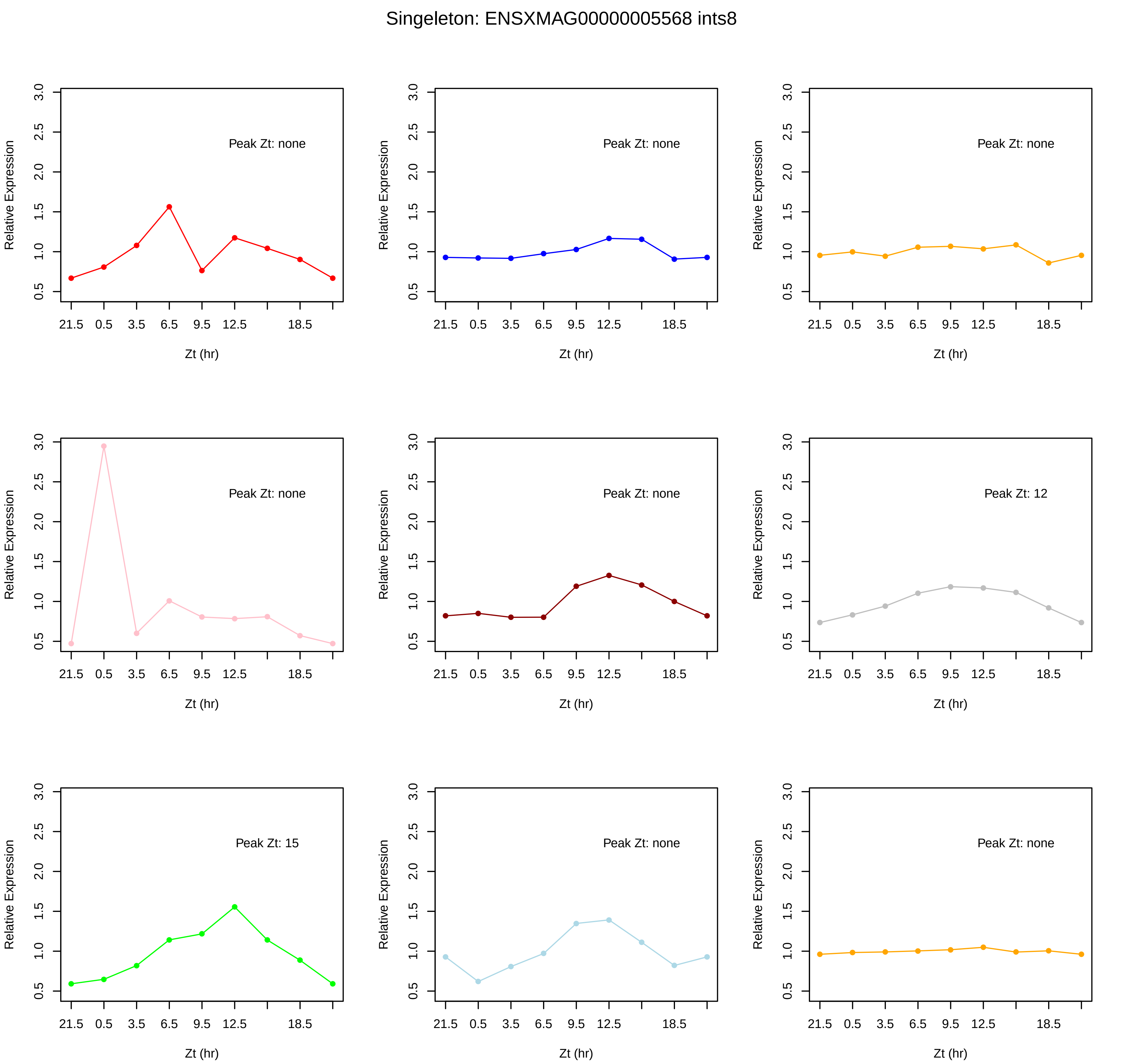

Singeleton: ENSXMAG00000005568 ints8
Peak Zt: none
Peak Zt: none
Peak Zt: none
Peak Zt: none
Peak Zt: none
Peak Zt: 12
Peak Zt: 15
Peak Zt: none
Peak Zt: none
Relative Expression
Zt (hr)

Singeleton: ENSXMAG00000010524 slc38a7

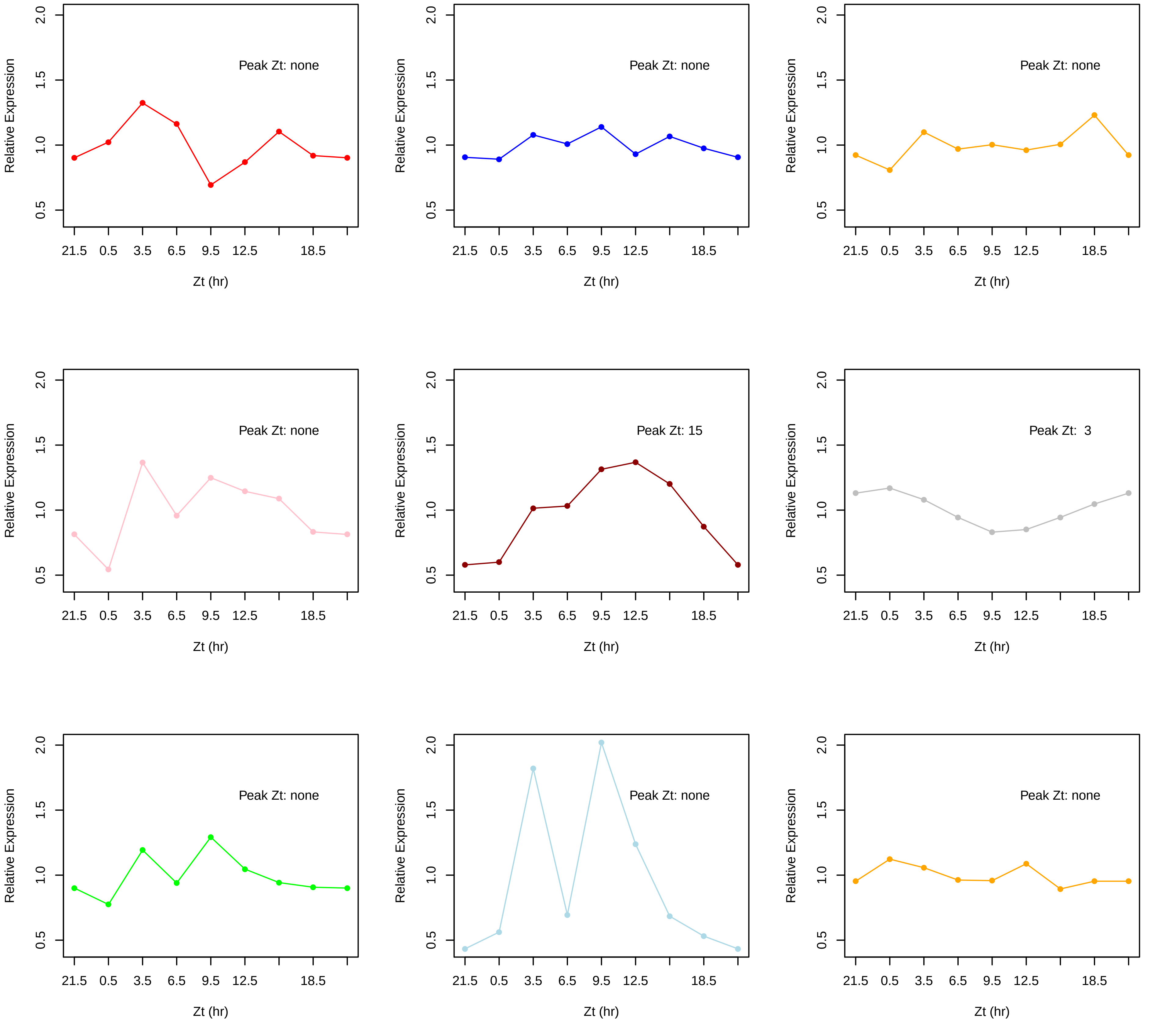

Singeleton: ENSXMAG00000015857 smpd4

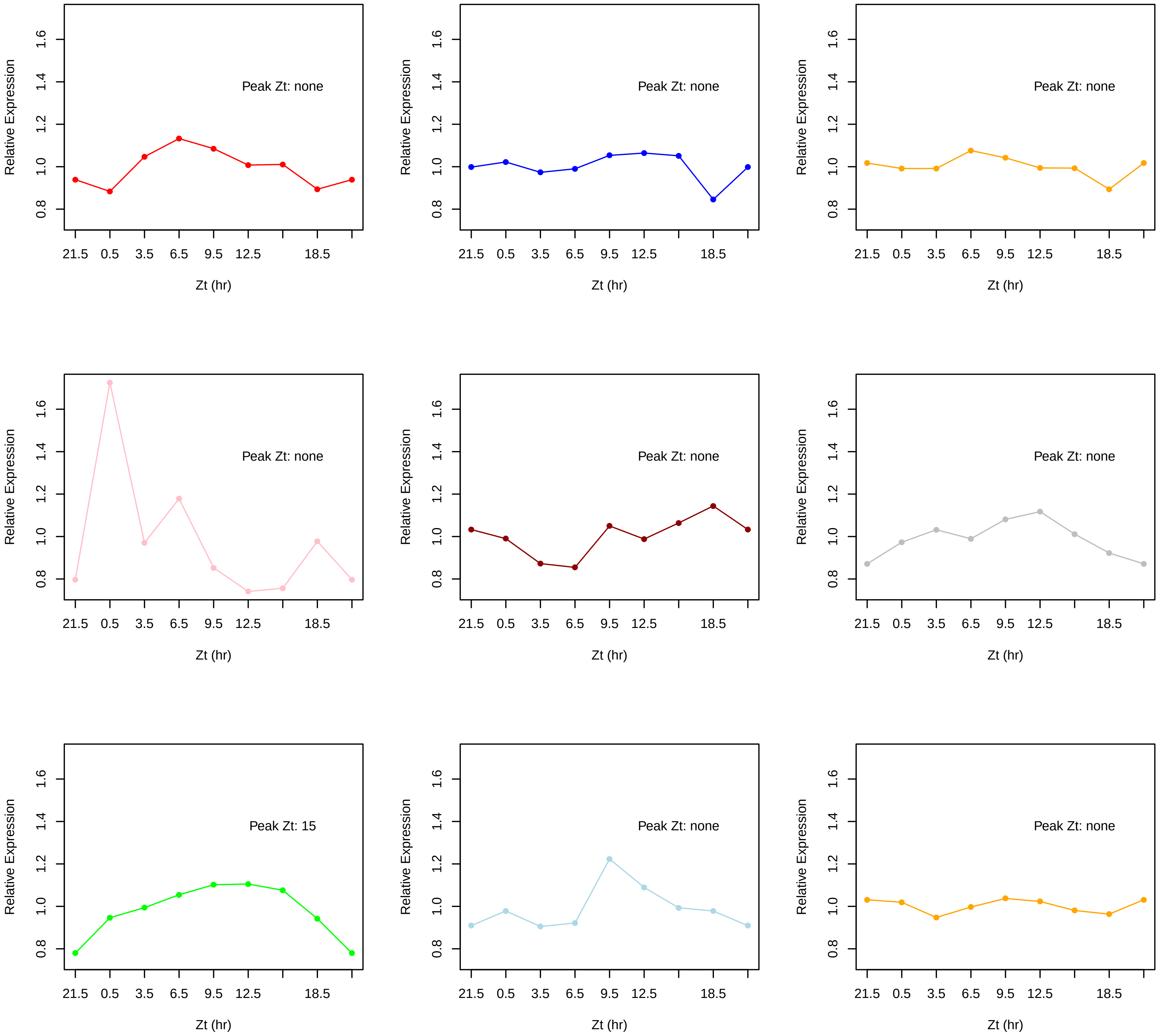

Singeleton: ENSXMAG00000000980 oxnad1

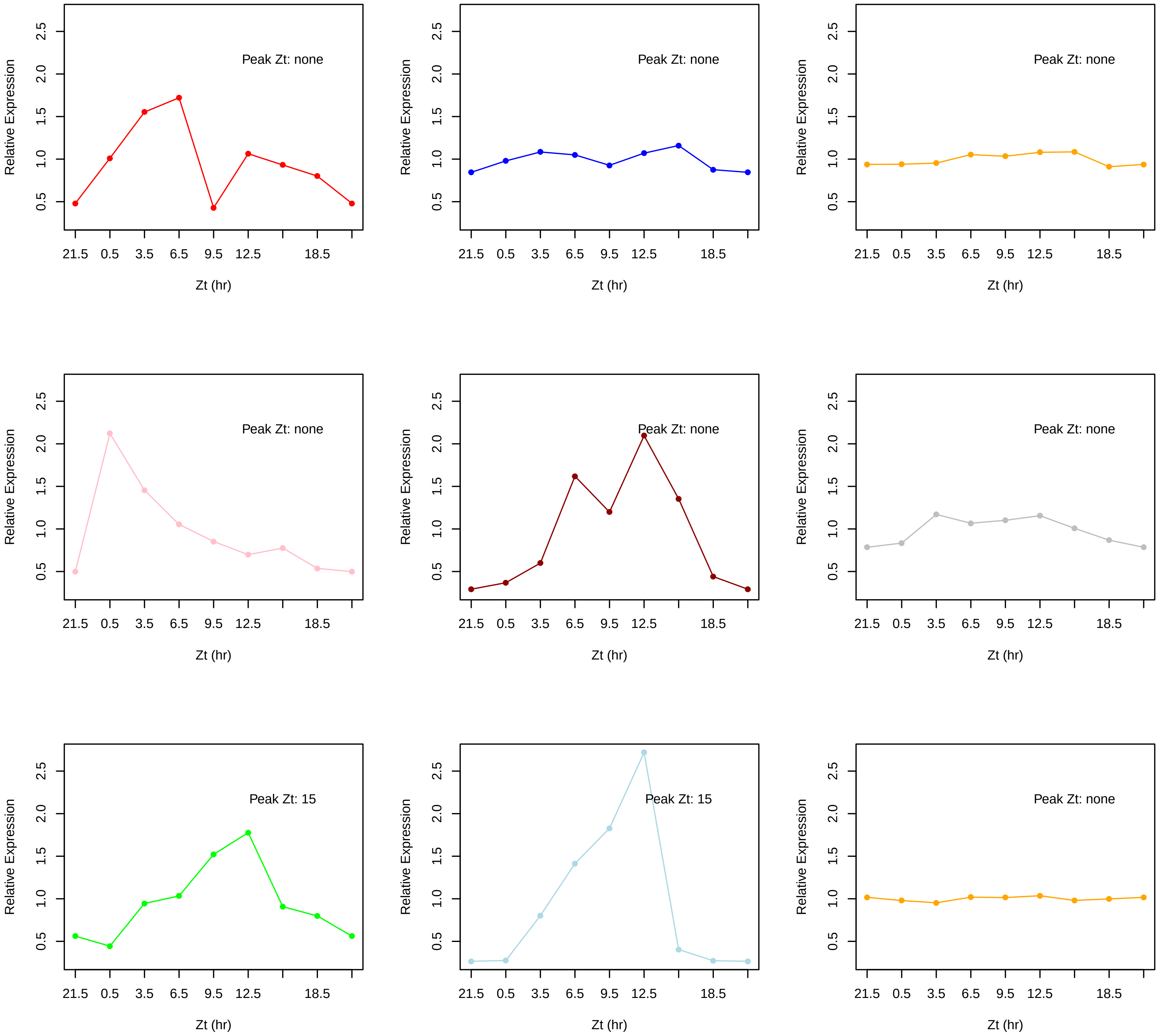

Singleton: ENSXMAG00000008080 phf19

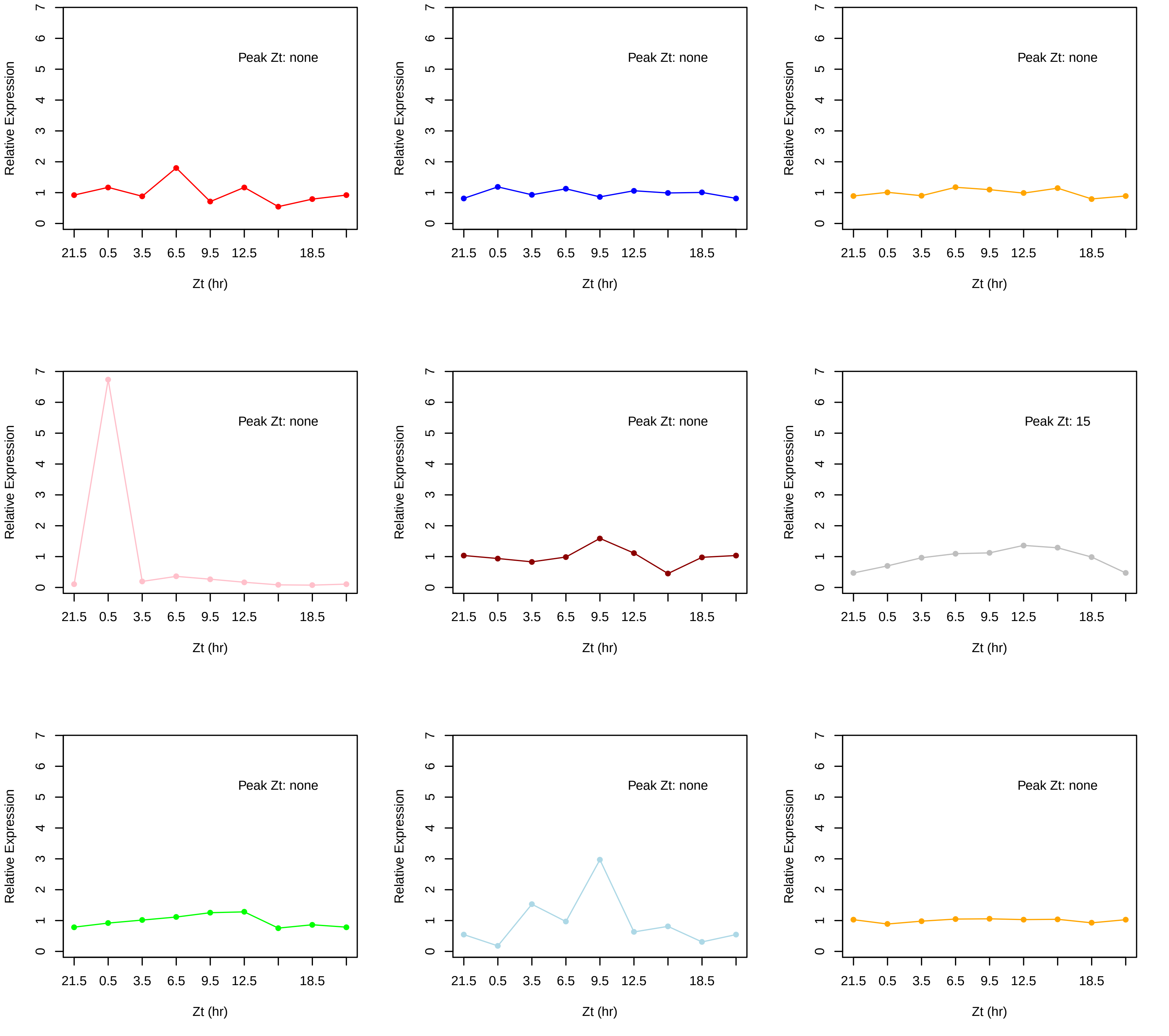

Singeleton: ENSXMAG00000021818 smug1

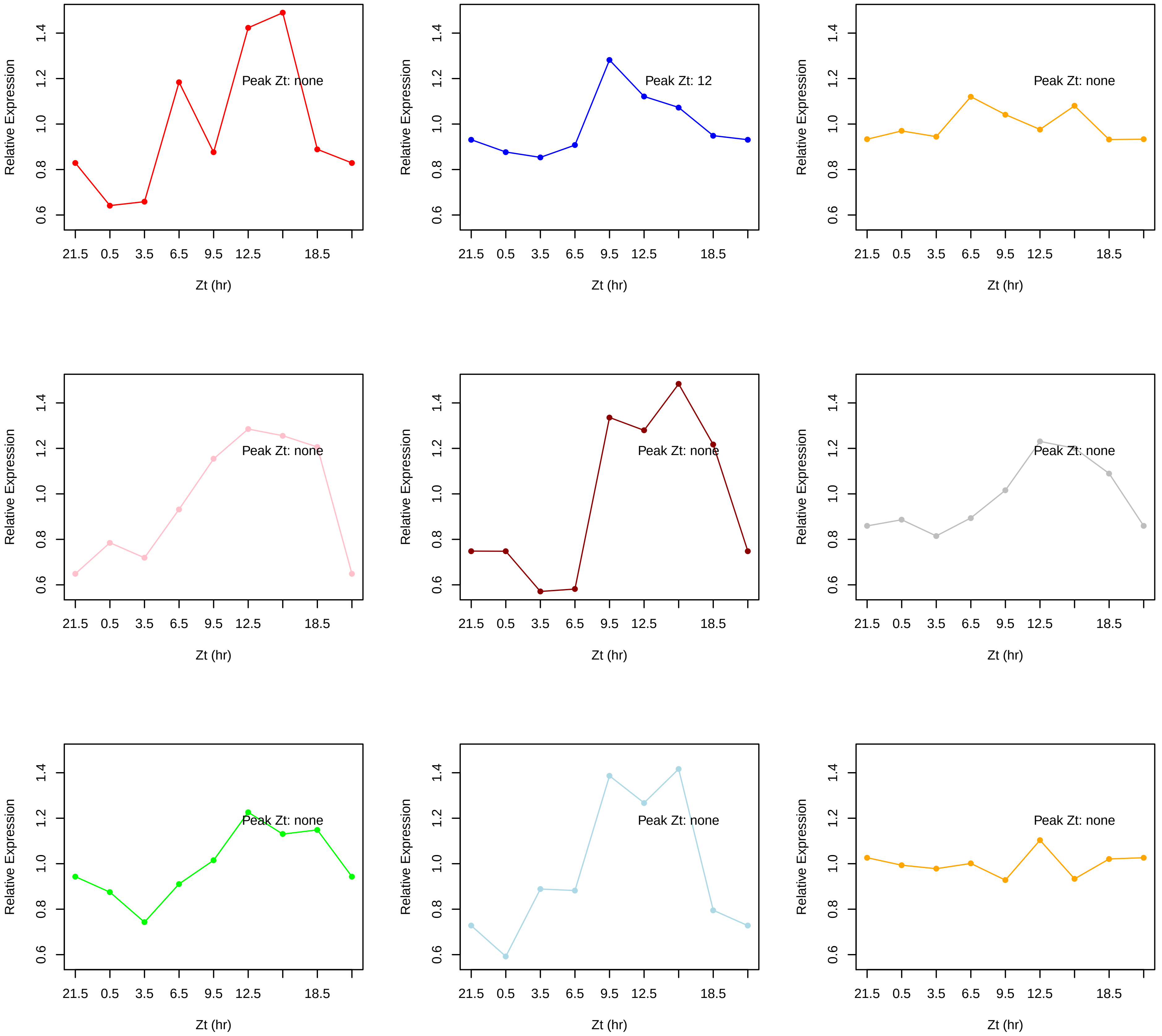

Singeleton: ENSXMAG00000010321 smg6

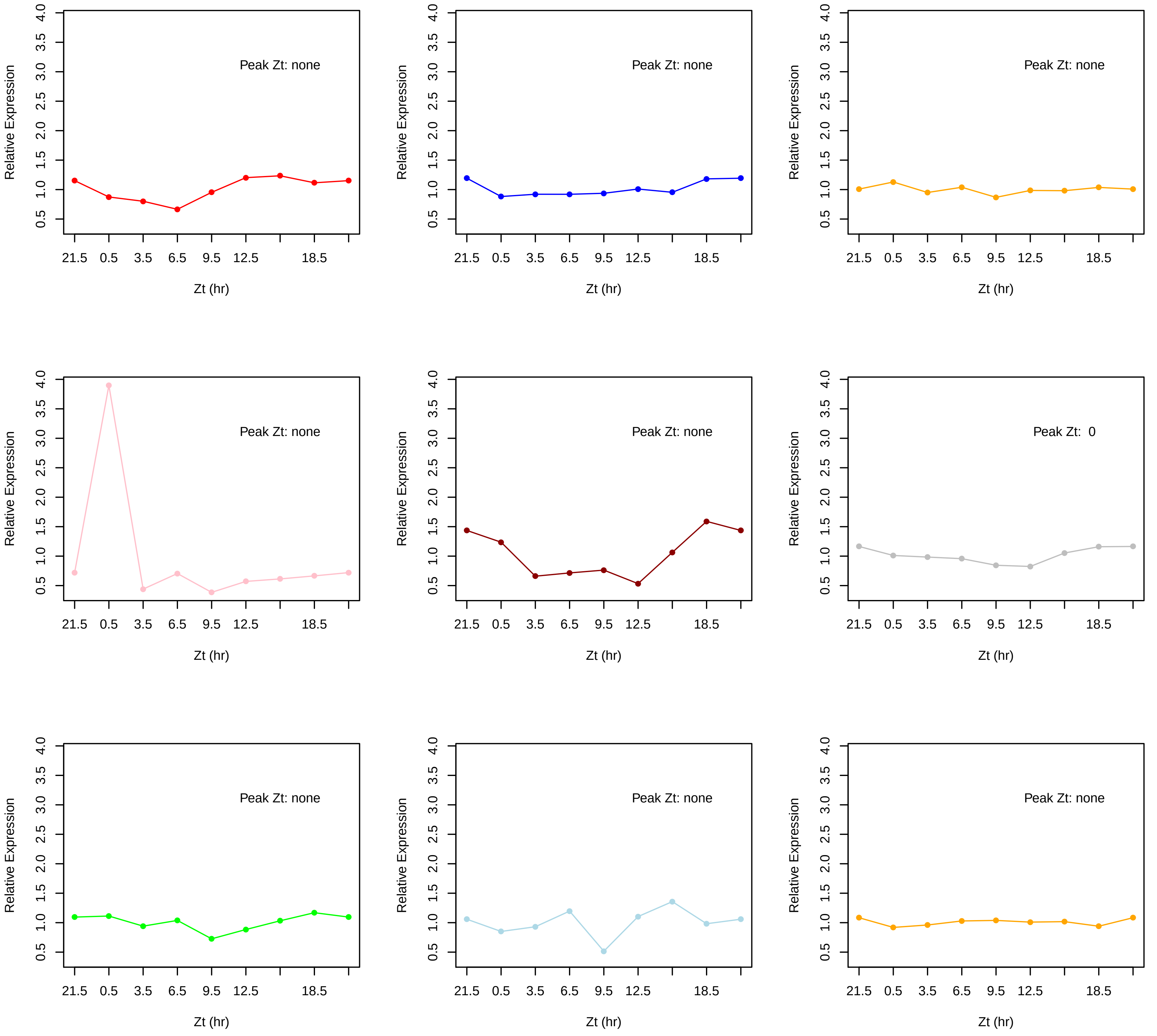

Singeleton: ENSXMAG00000003514 ap3d1

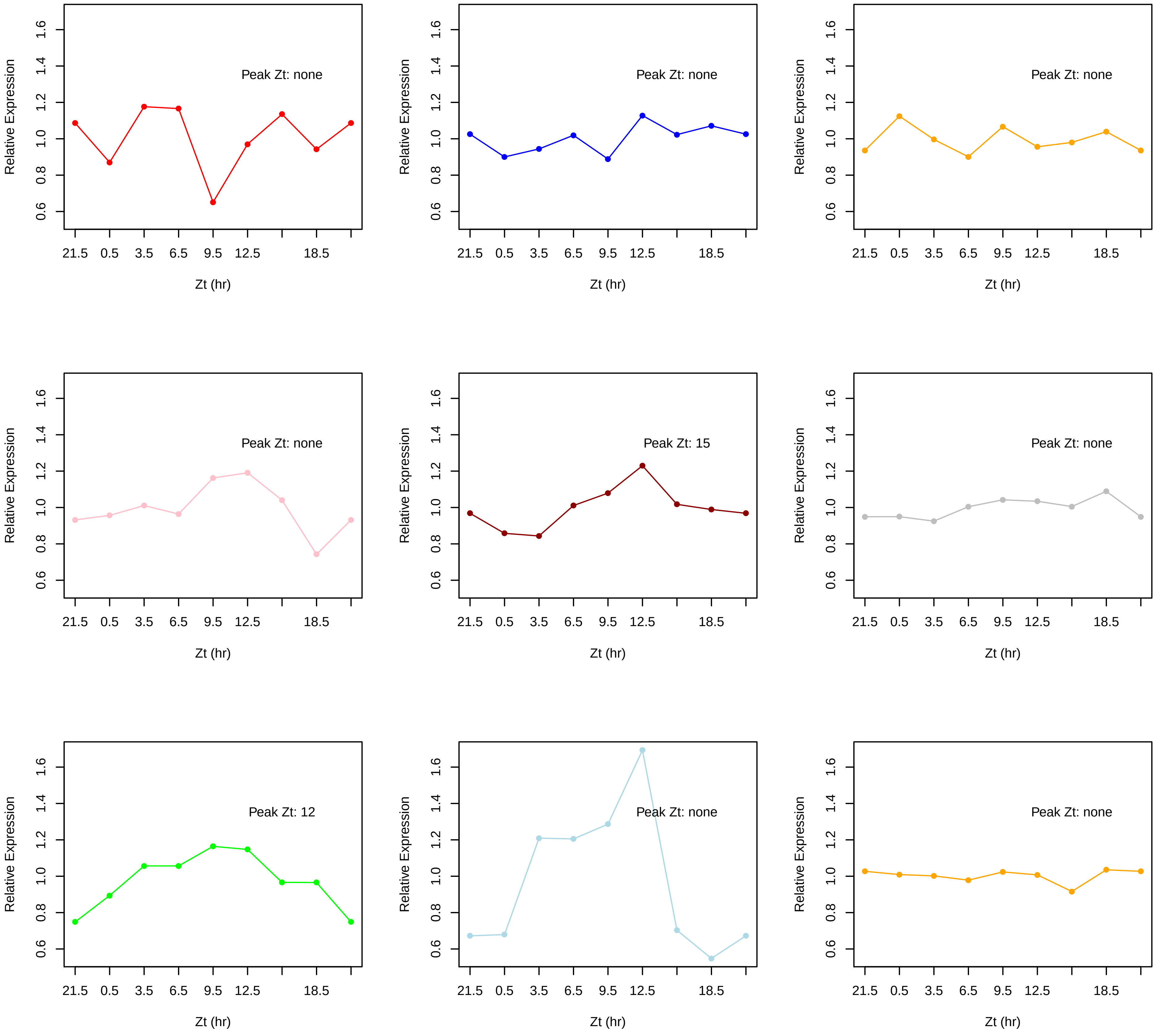

Singeleton: ENSXMAG00000023857 tfam

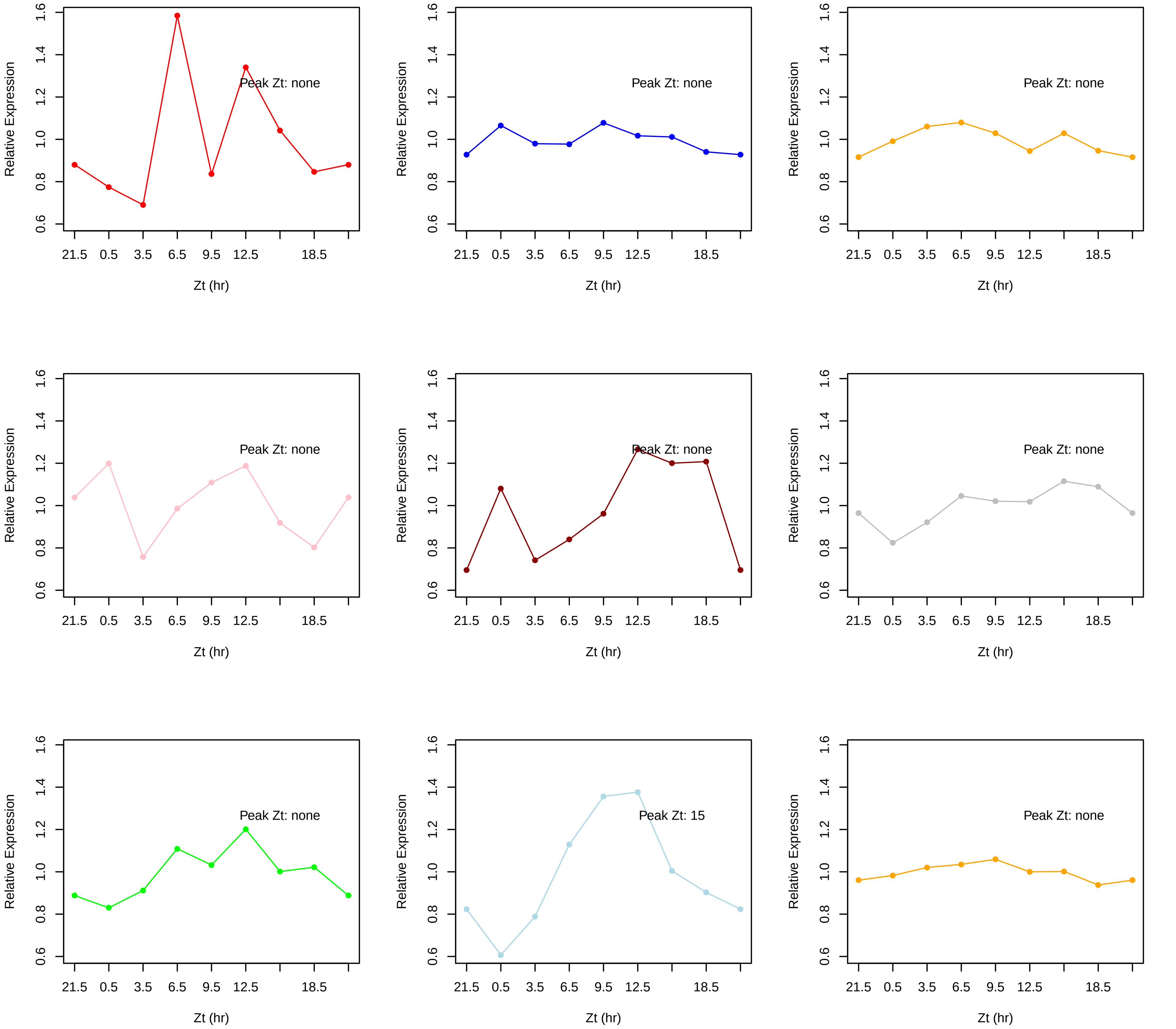

Singeleton: ENSXMAG00000013201 cwc22

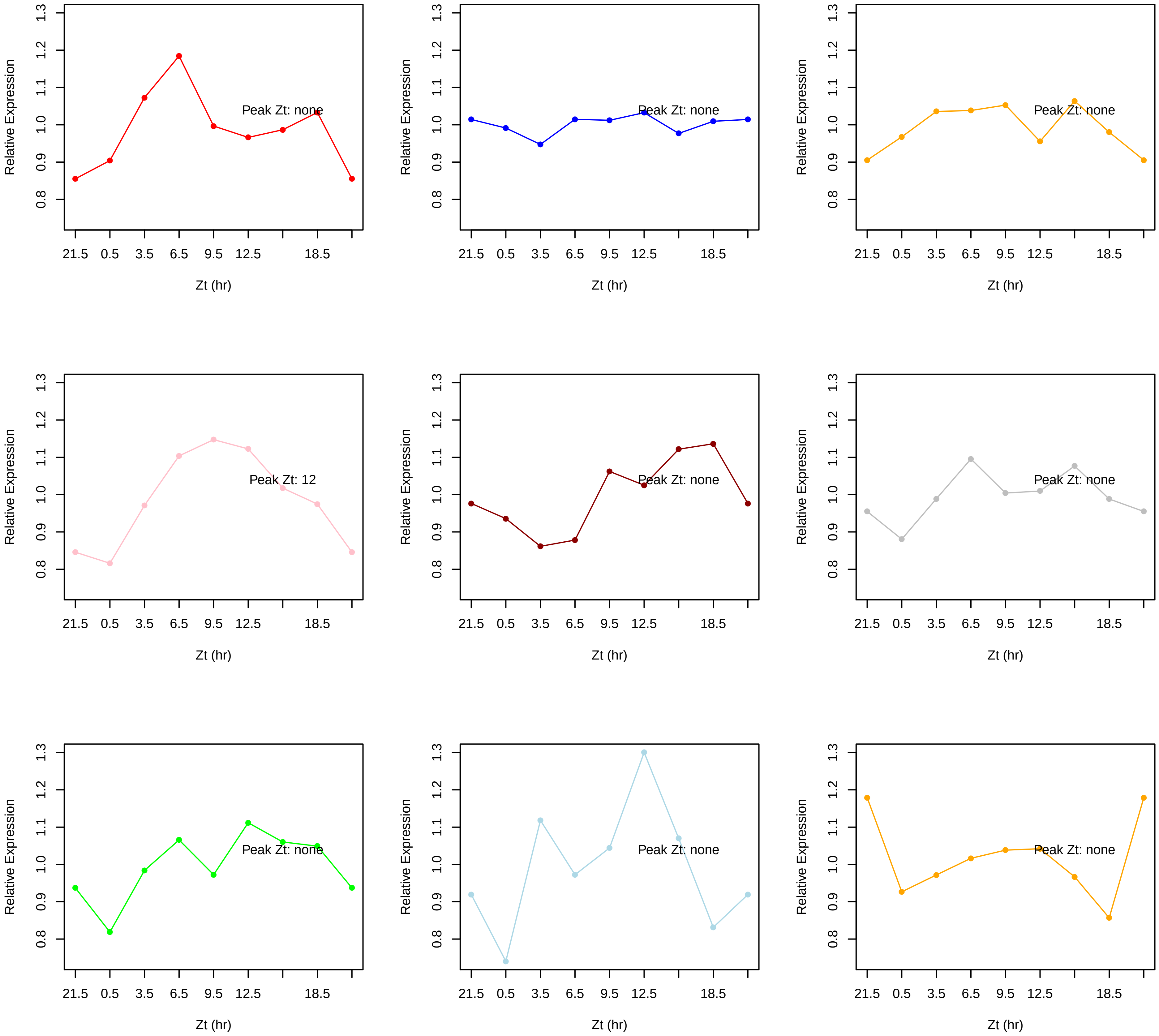

Singeleton: ENSXMAG00000023539 cbx5

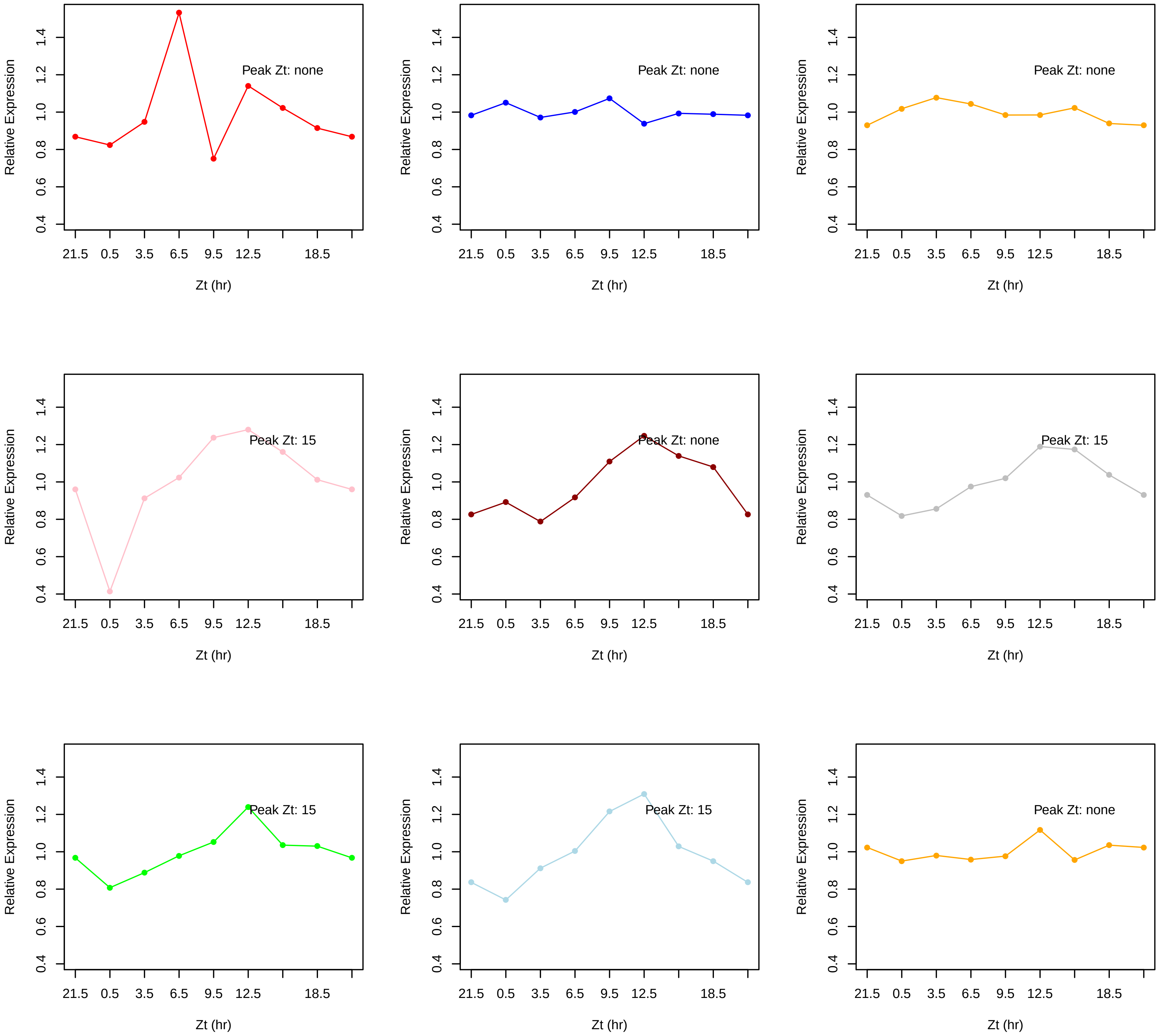

Singleton: ENSXMAG00000000010 bbox1

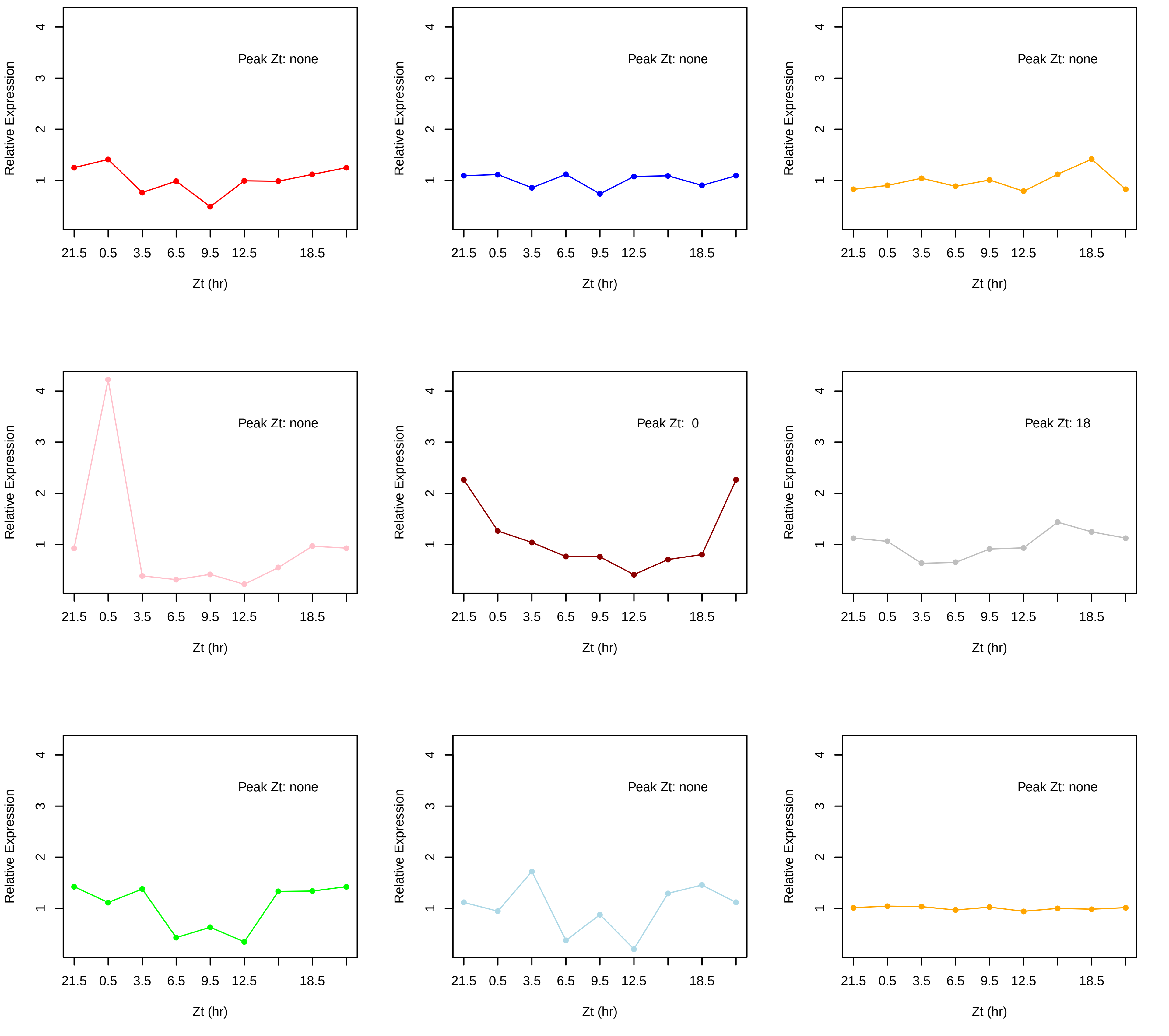

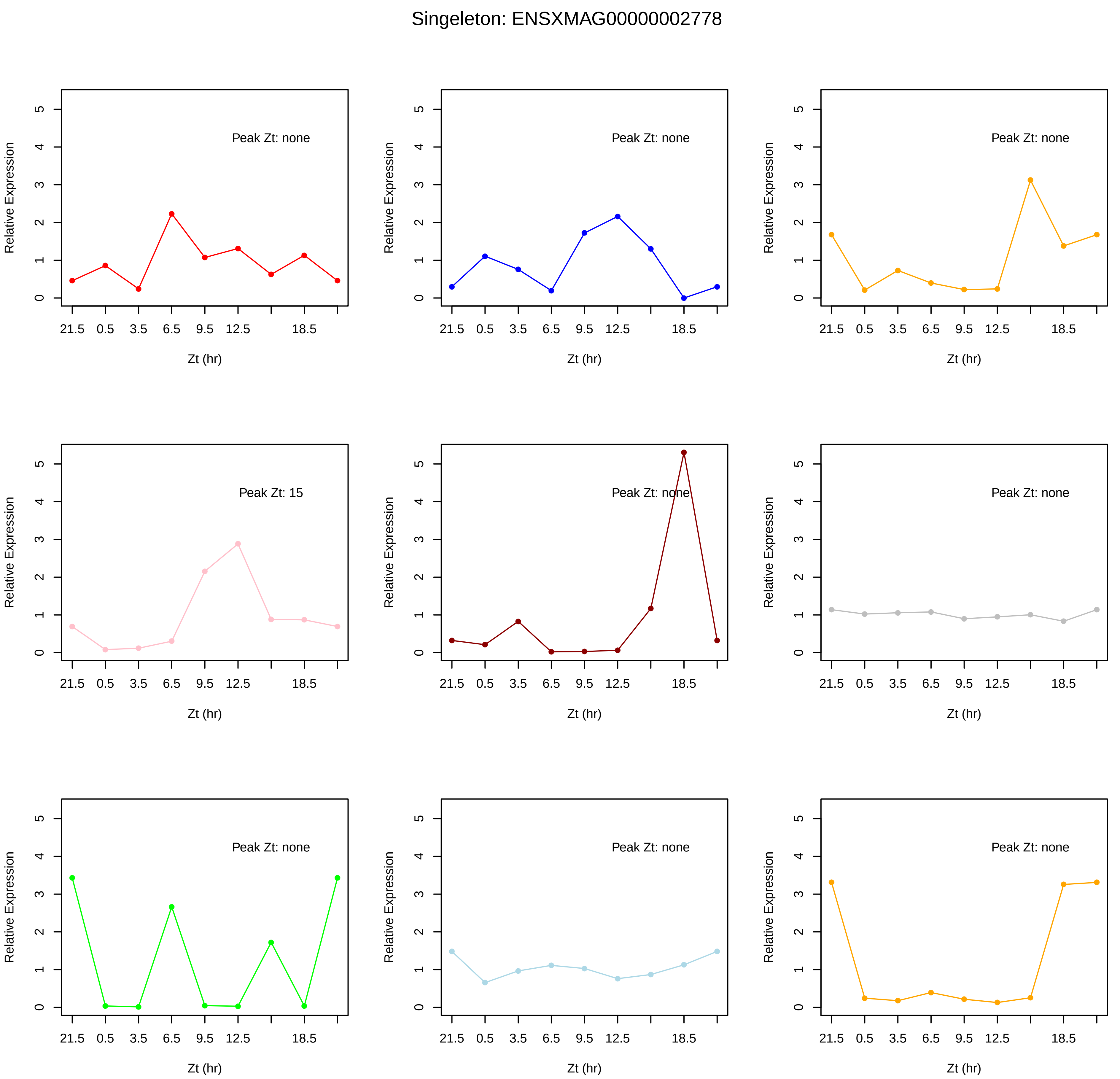

Singeleton: ENSXMAG00000002778
Peak Zt: none
Peak Zt: none
Peak Zt: none
Peak Zt: 15
Peak Zt: none
Peak Zt: none
Peak Zt: none
Peak Zt: none
Peak Zt: none
Relative Expression
Zt (hr)
21.5 0.5 3.5 6.5 9.5 12.5 18.5
0 1 2 3 4 5

Singeleton: ENSXMAG00000018961 med15

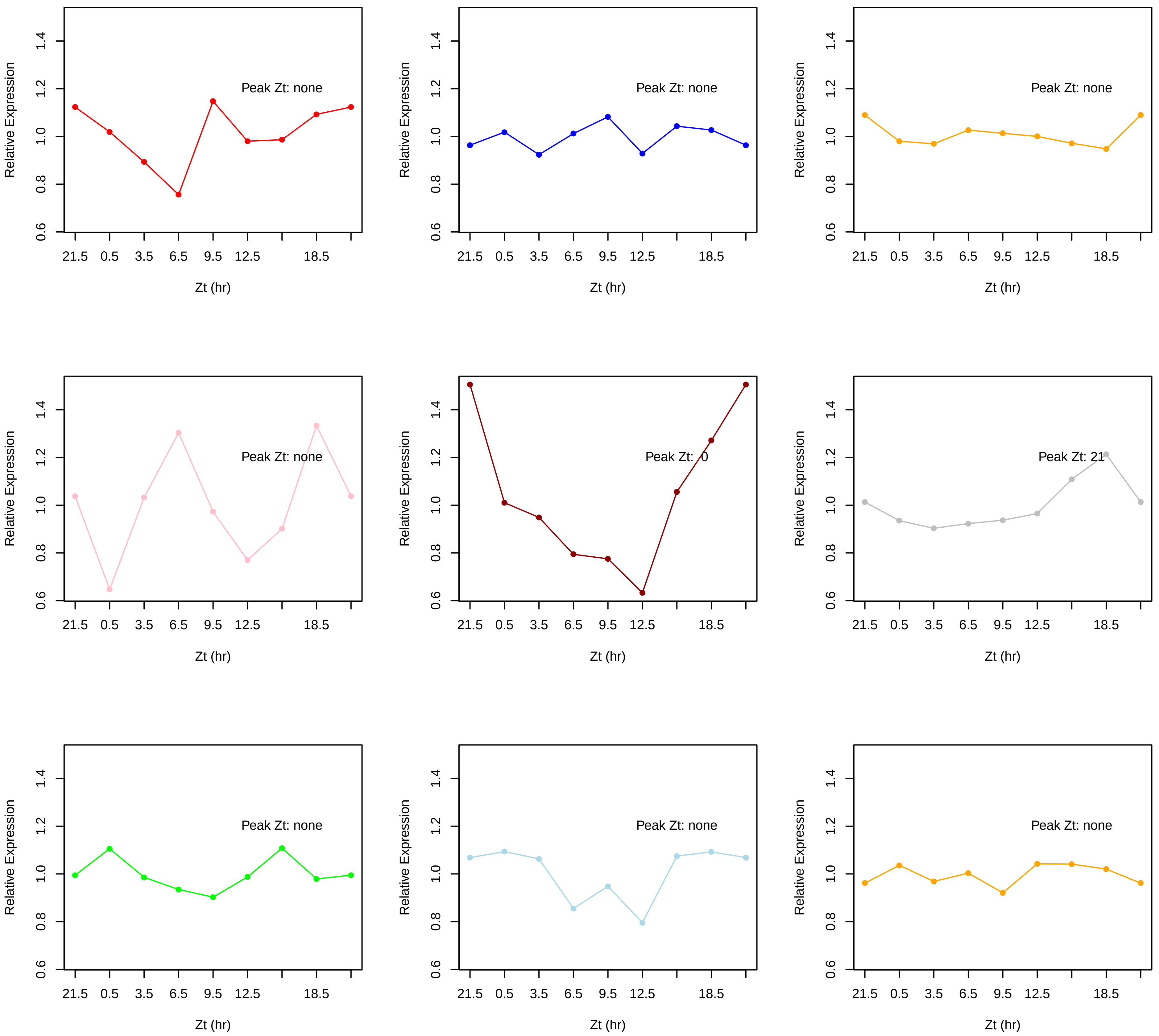

Singeleton: ENSXMAG00000003762 atg4b

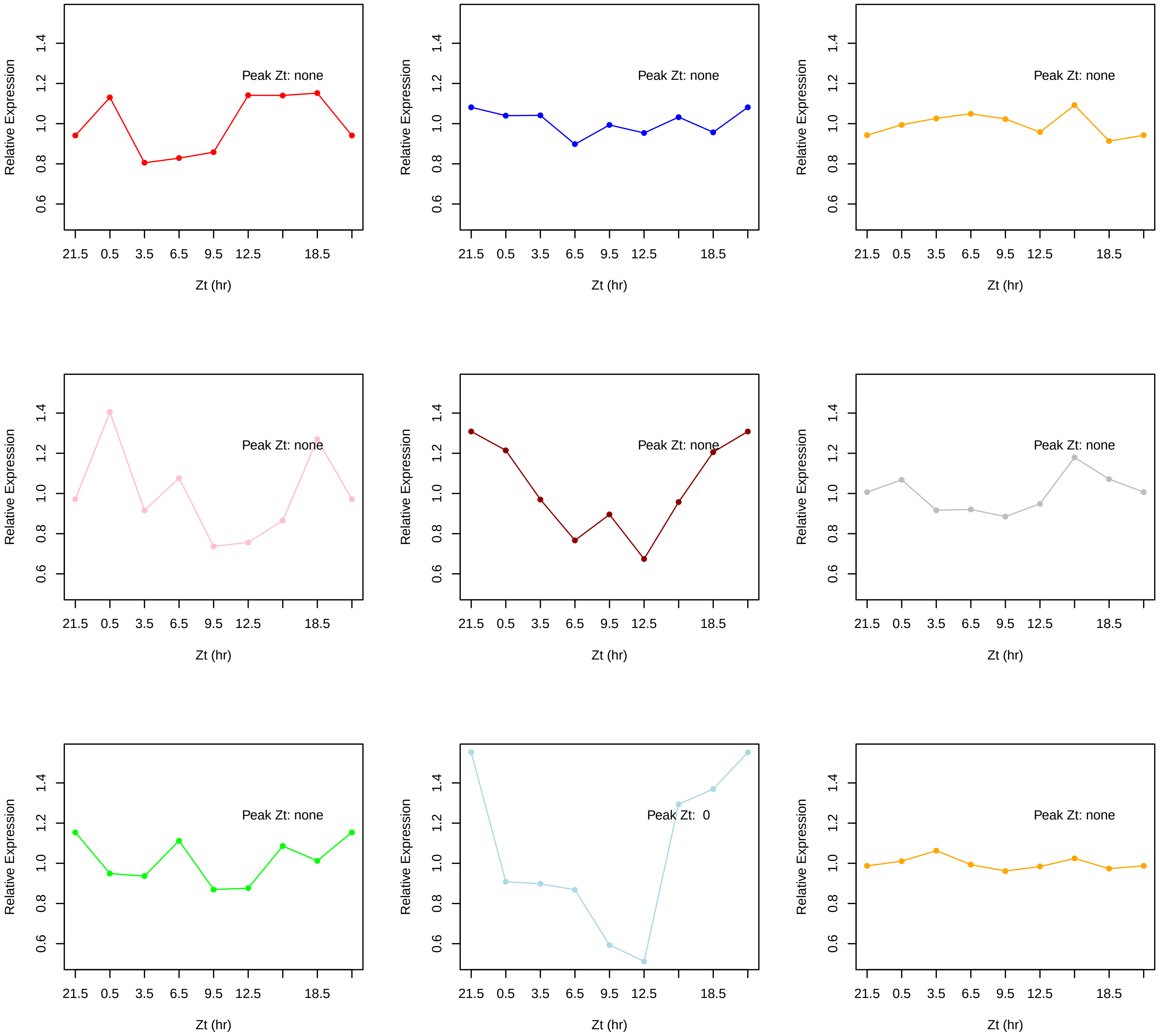

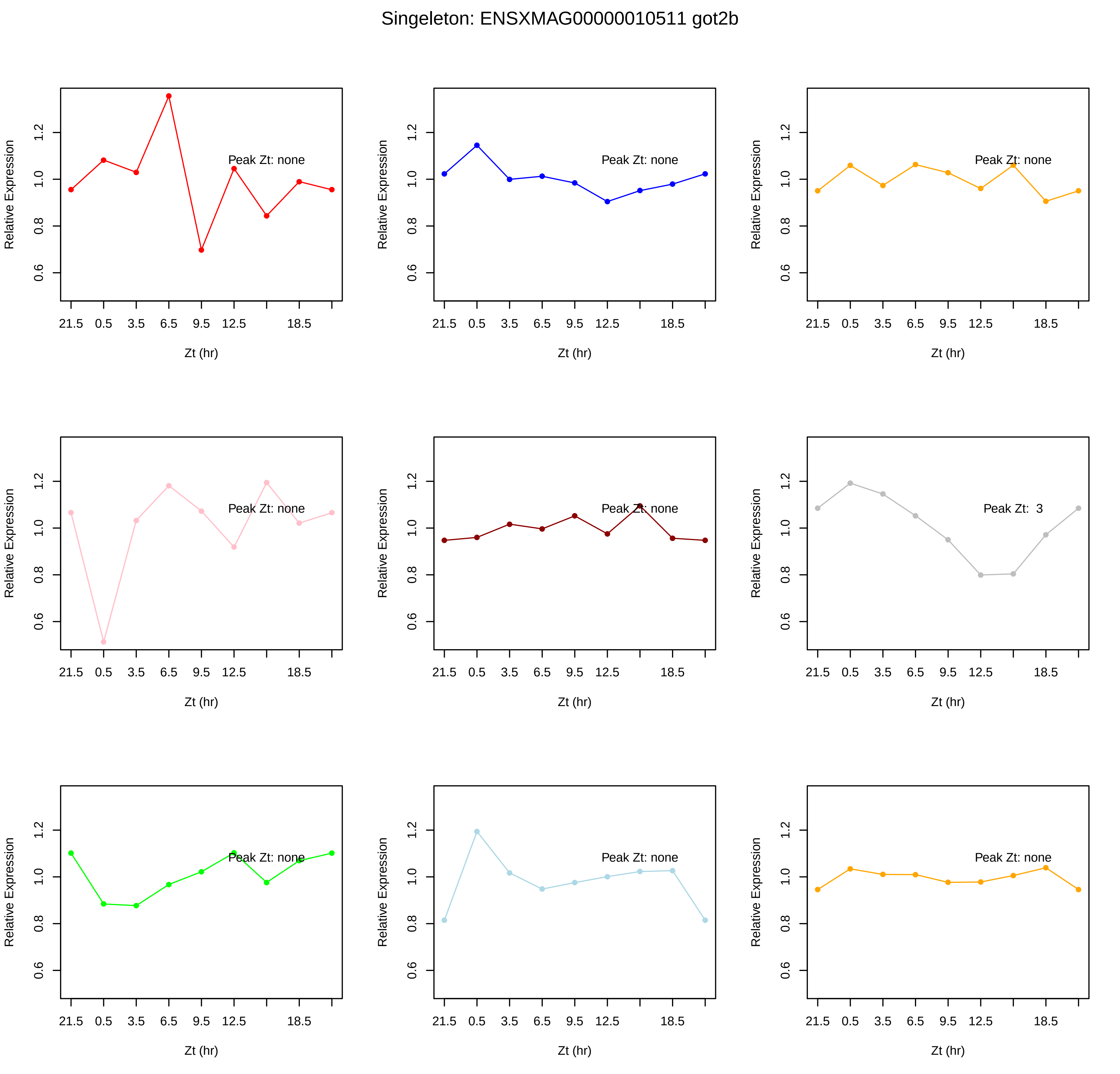
Singleton: ENSXMAG00000010511 got2b
Relative Expression
Zt (hr)
21.5
0.5
3.5
6.5
9.5
12.5
18.5
0.6
0.8
1.0
1.2
Peak Zt: none
Peak Zt: 3

Singeleton: ENSXMAG00000018656 plcl2

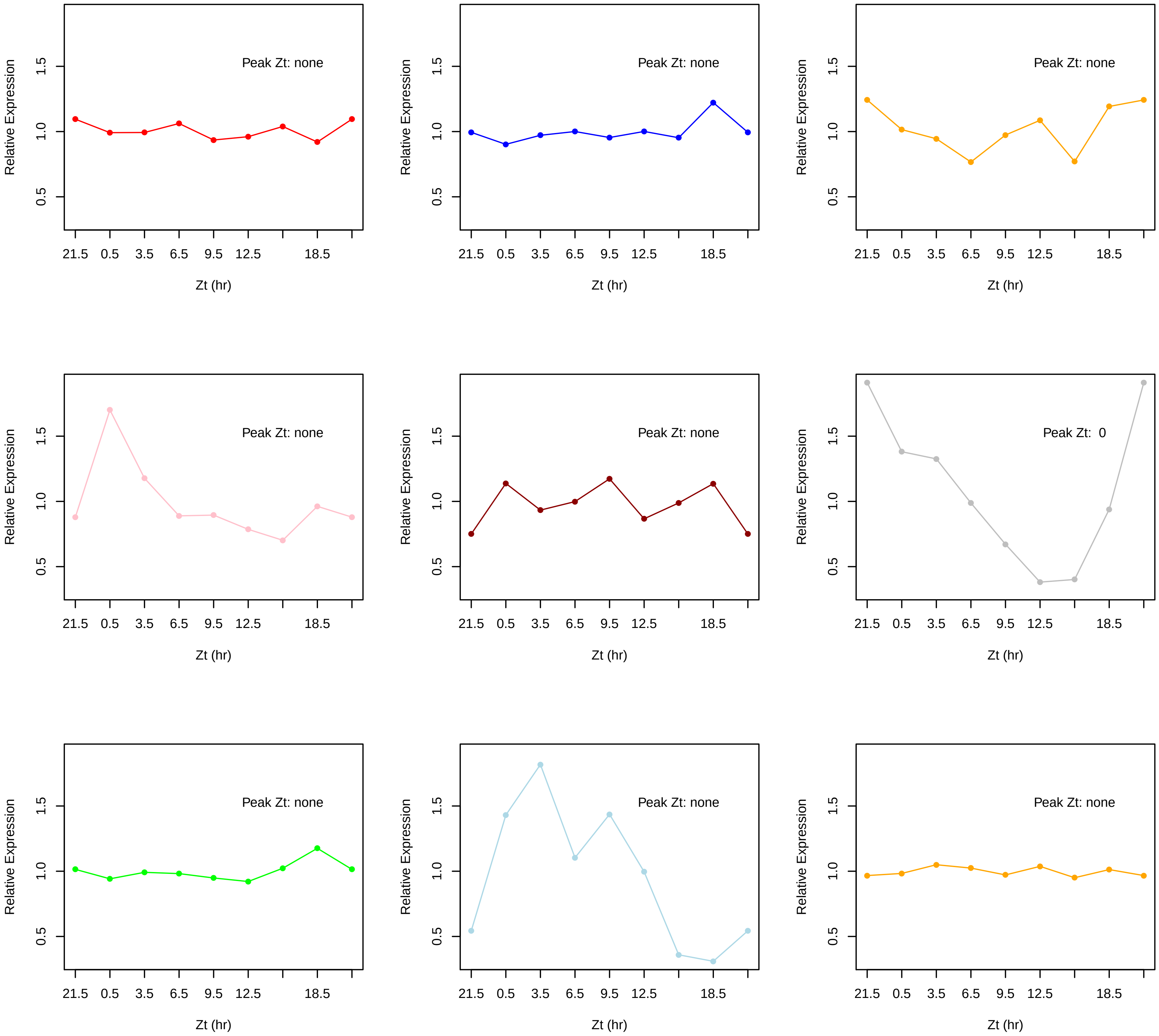

Singeleton: ENSXMAG00000002831

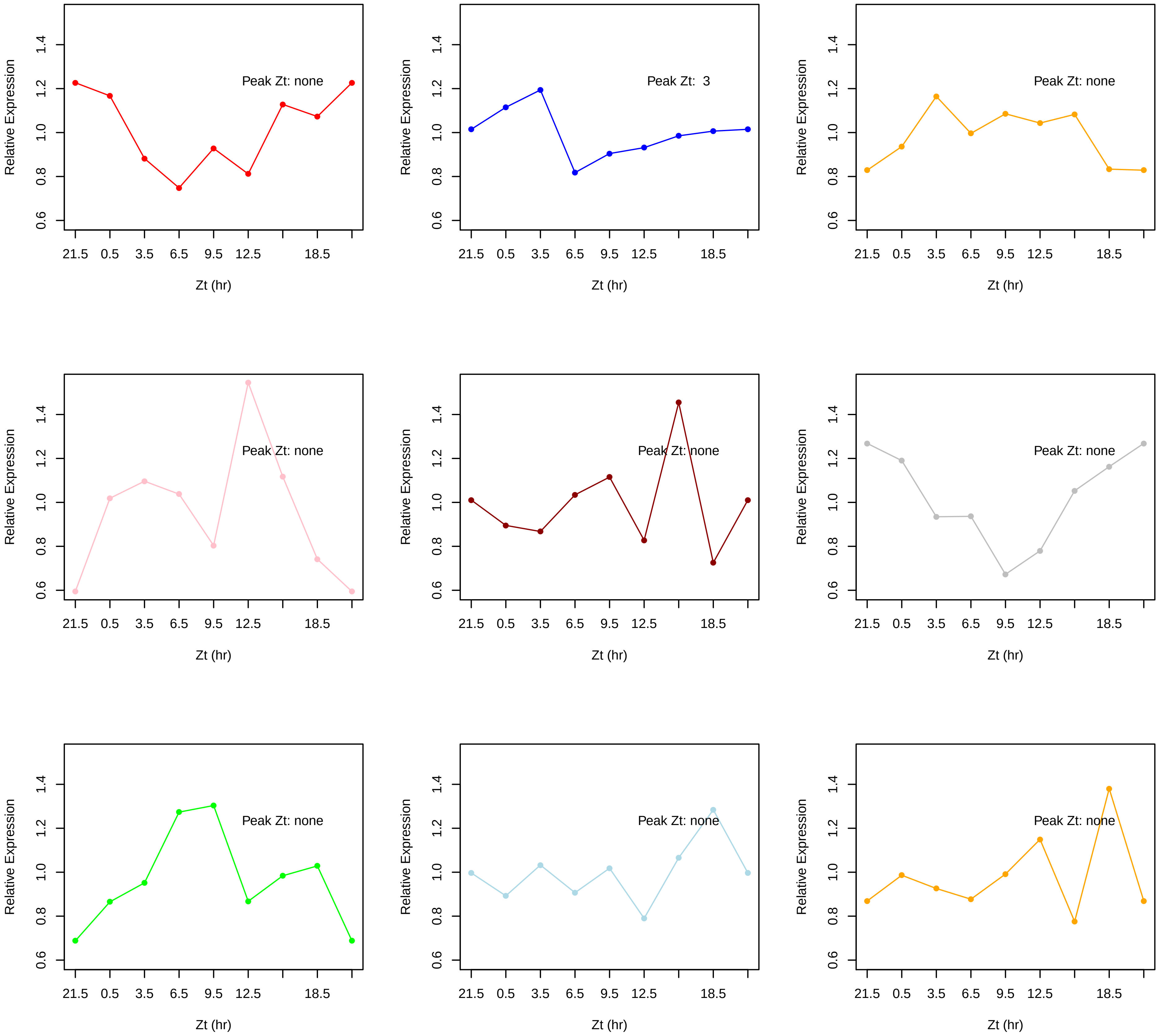

Singeleton: ENSXMAG00000013823 trmt1

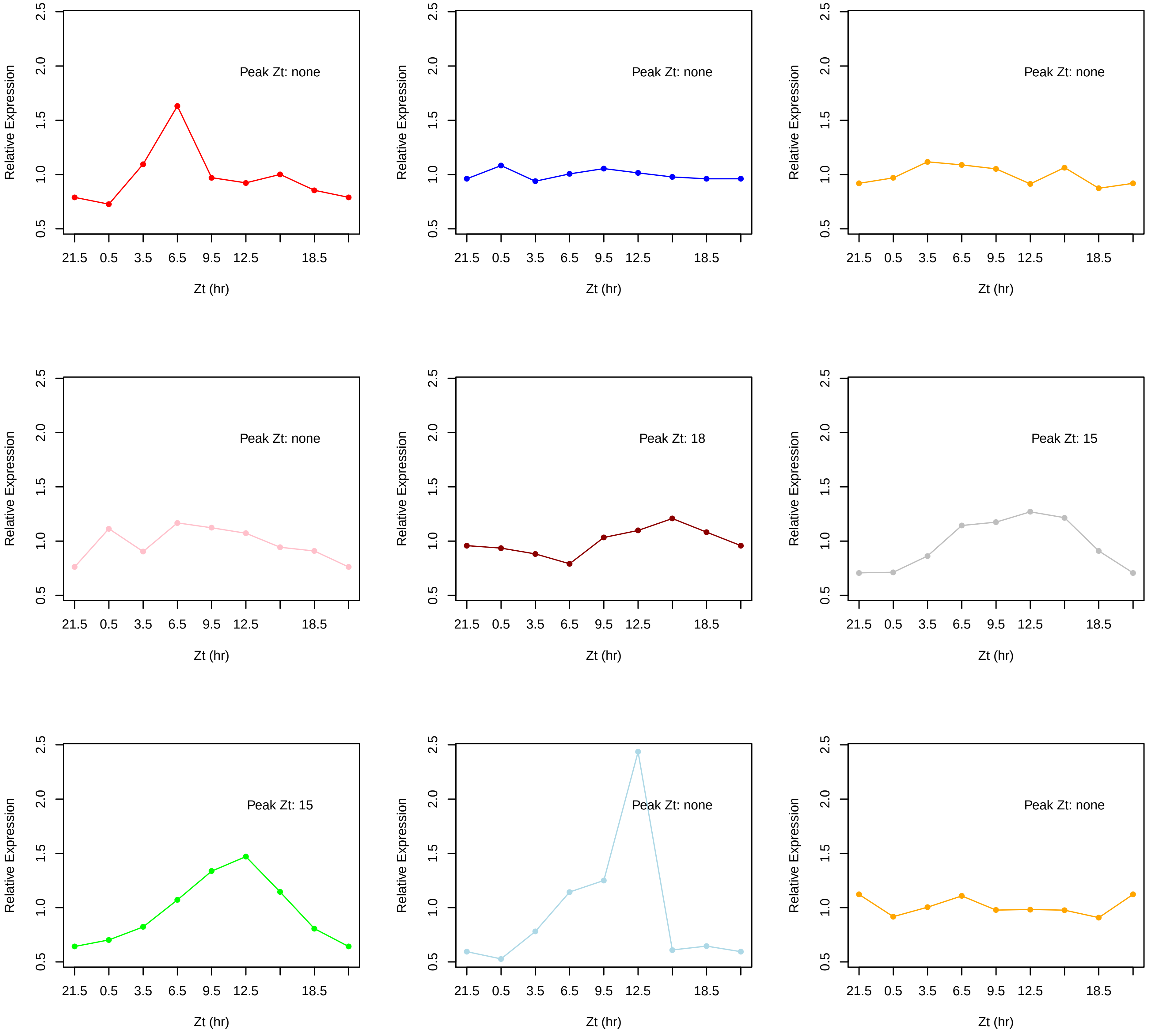

Singeleton: ENSXMAG00000011275 srm

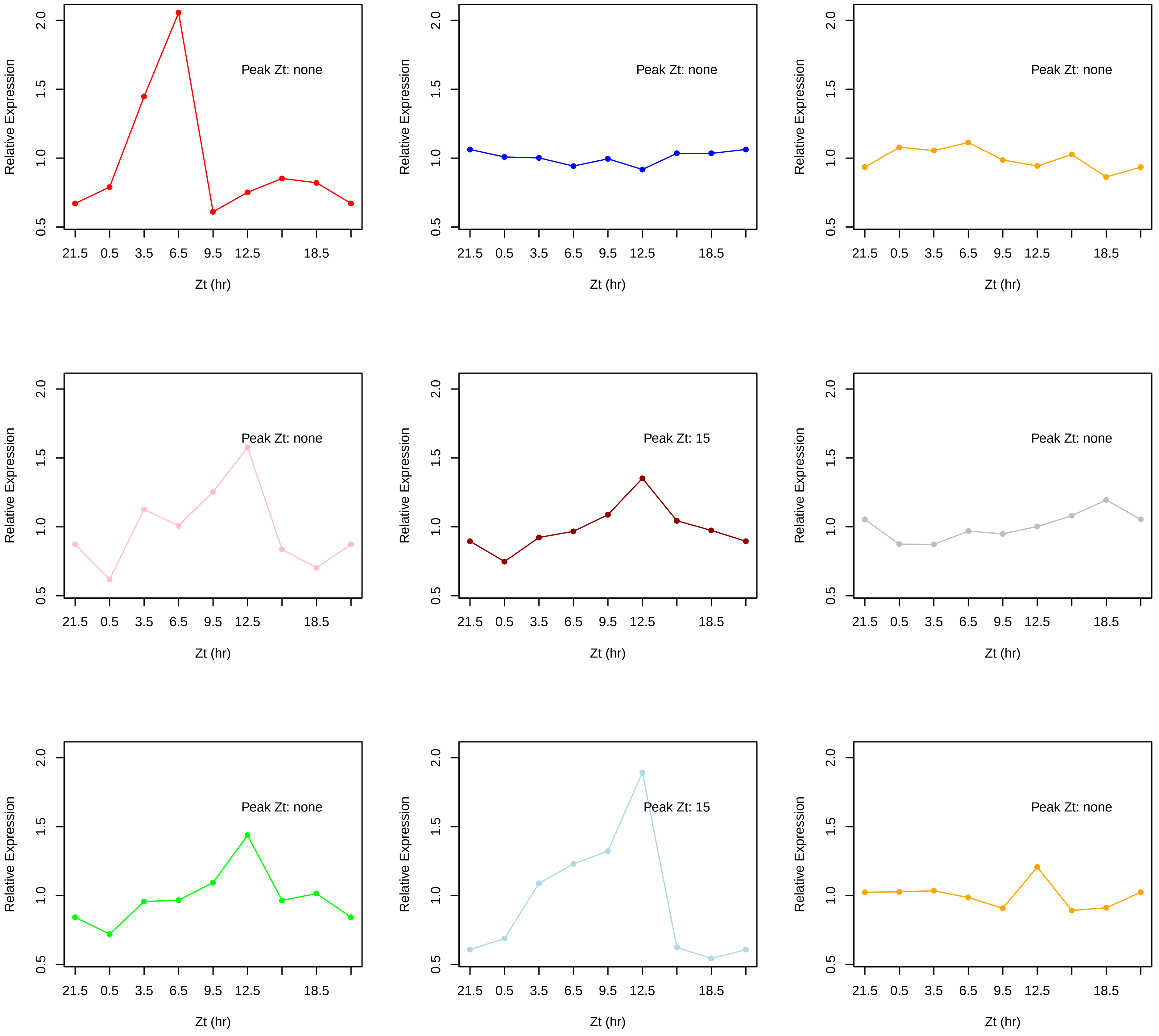

Singeleton: ENSXMAG00000022643 ankrd50l

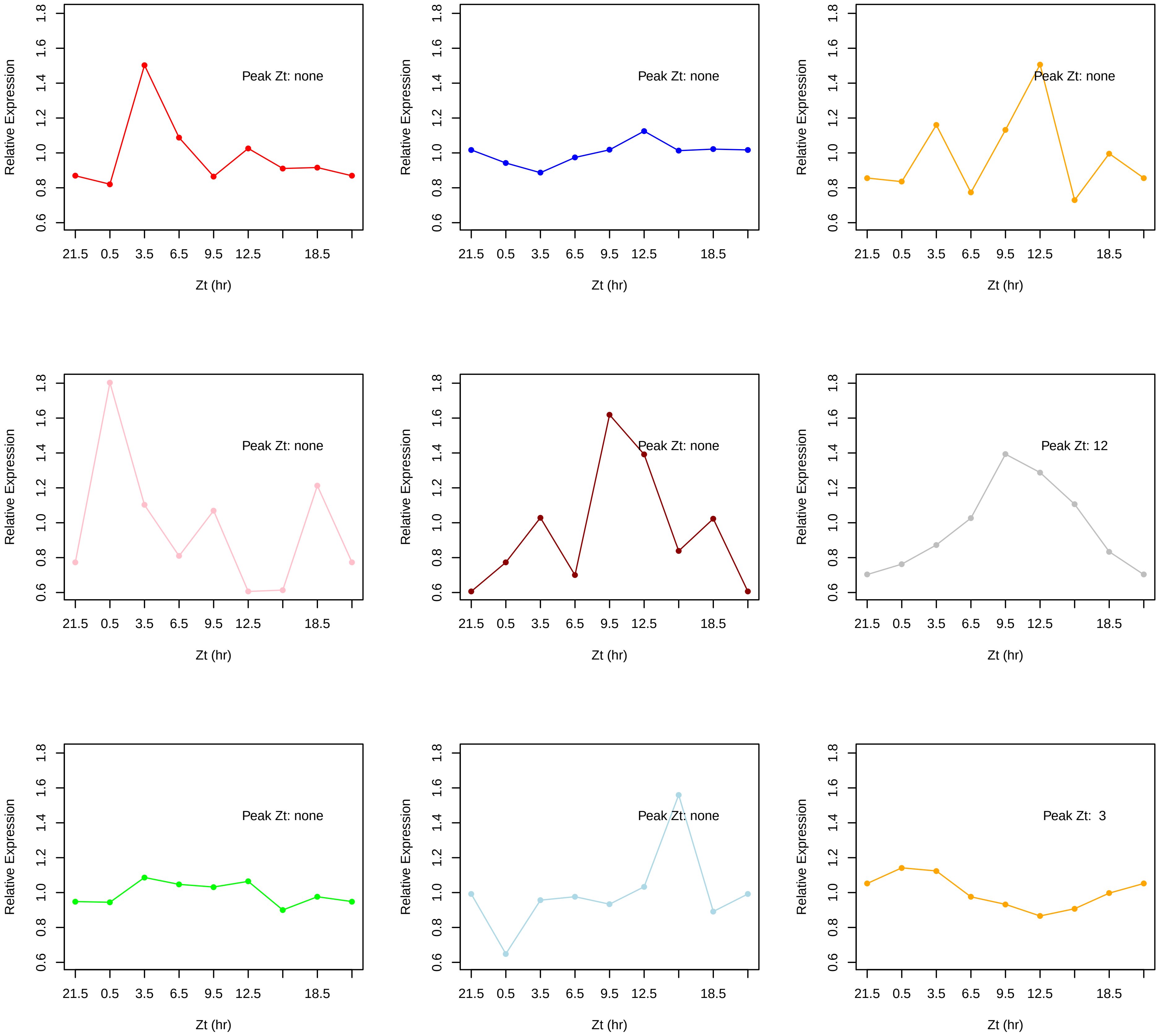

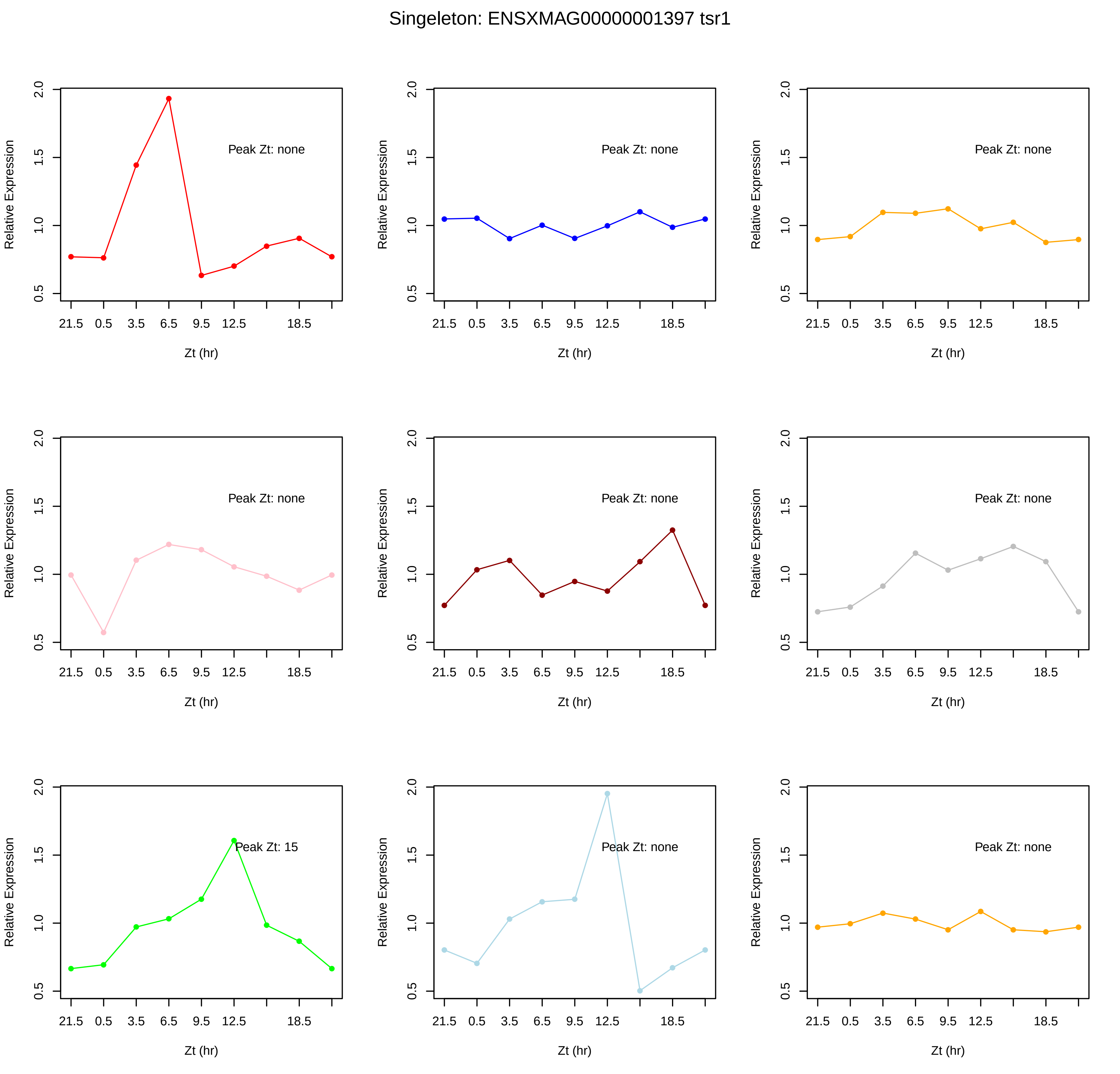
Singeleton: ENSXMAG00000001397 tsr1
Relative Expression
Zt (hr)
Peak Zt: none
Peak Zt: 15
21.5
0.5
3.5
6.5
9.5
12.5
18.5
0.5
1.0
1.5
2.0

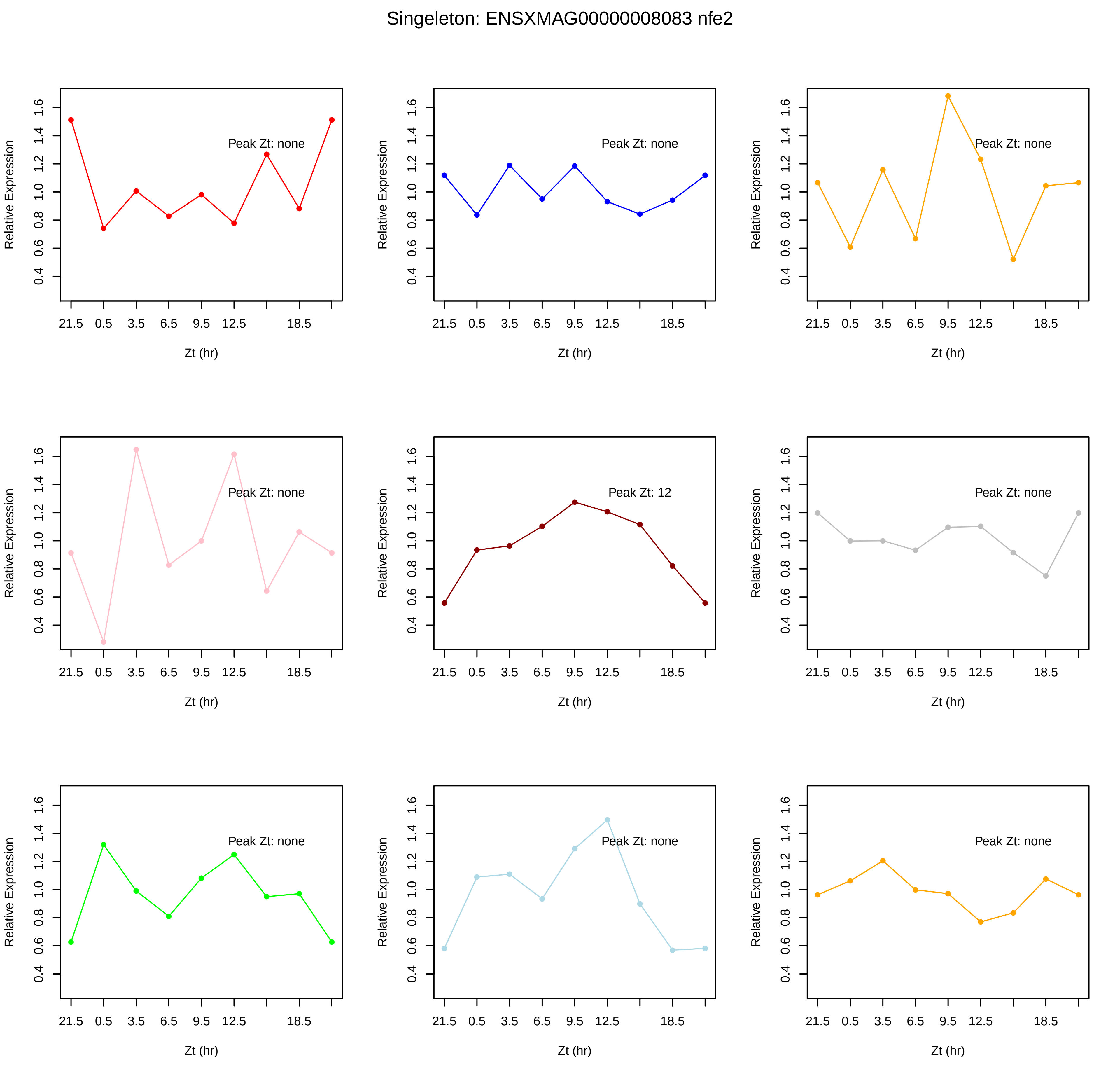

Singeleton: ENSXMAG00000008083 nfe2
Relative Expression
Zt (hr)
Peak Zt: none
Peak Zt: none
Peak Zt: none
Peak Zt: none
Peak Zt: 12
Peak Zt: none
Peak Zt: none
Peak Zt: none
Peak Zt: none

Singeleton: ENSXMAG00000007368 bicc1a

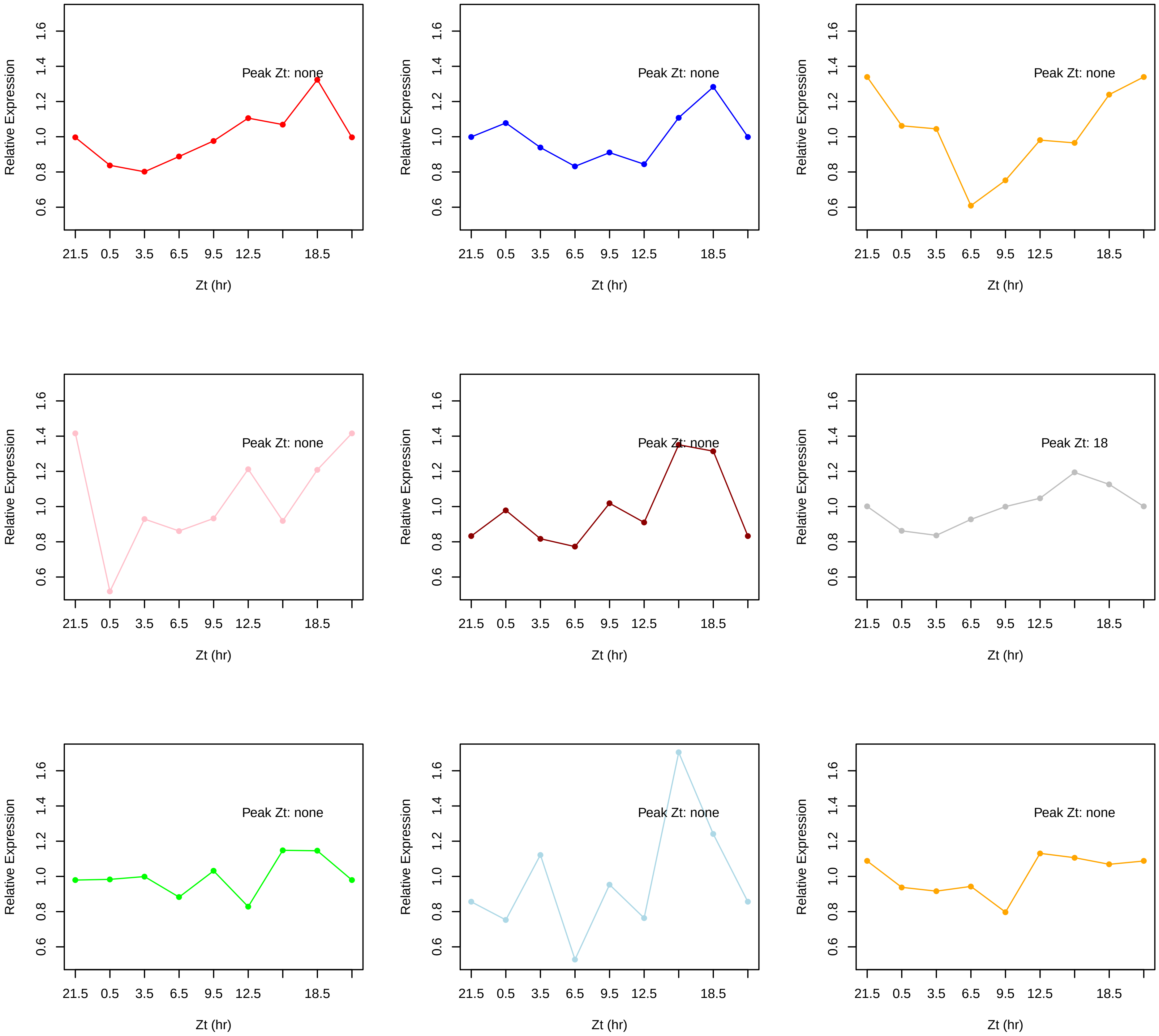

Singeleton: ENSXMAG00000010288 taok1b

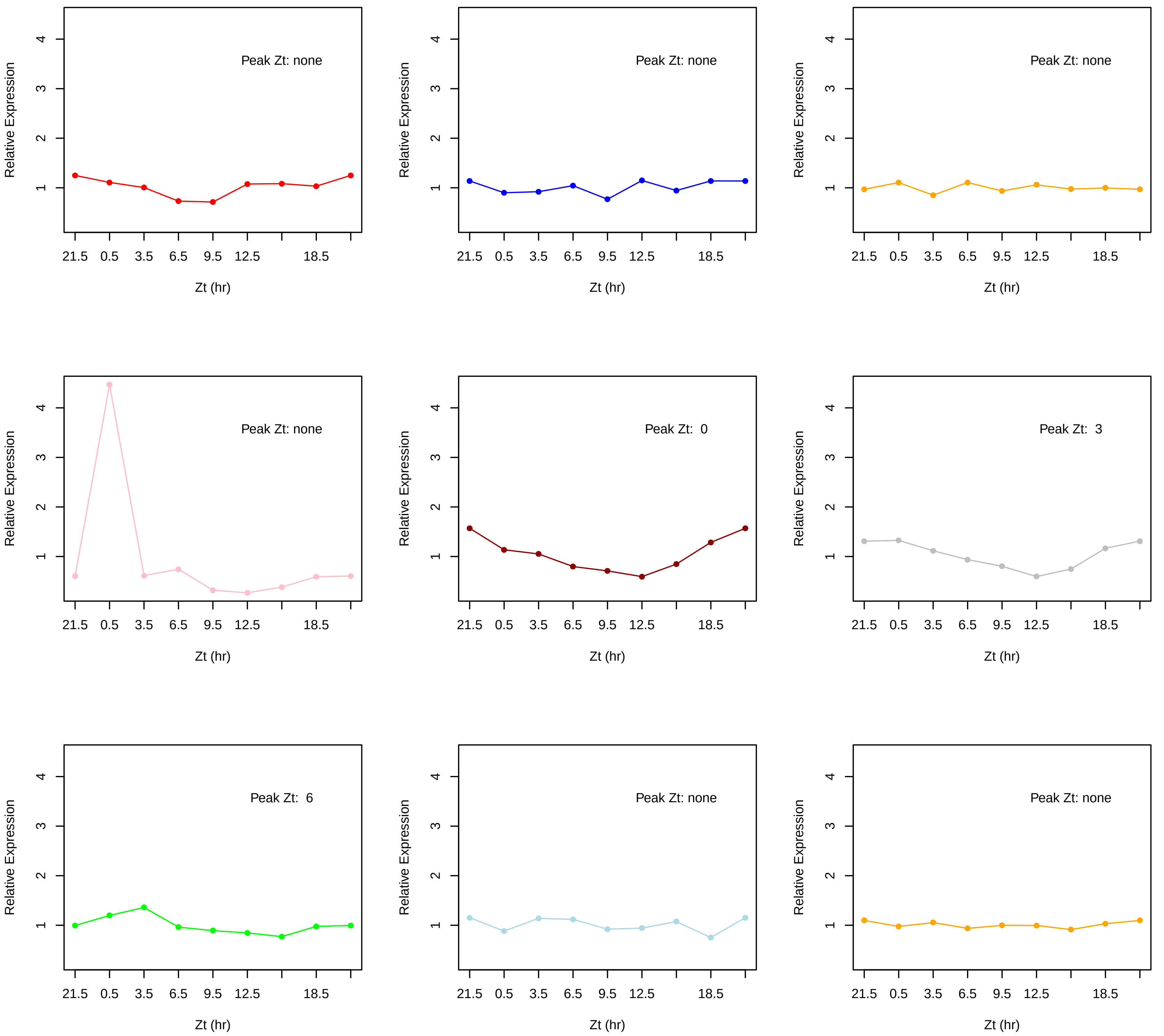

Singeleton: ENSXMAG00000006916 ano3

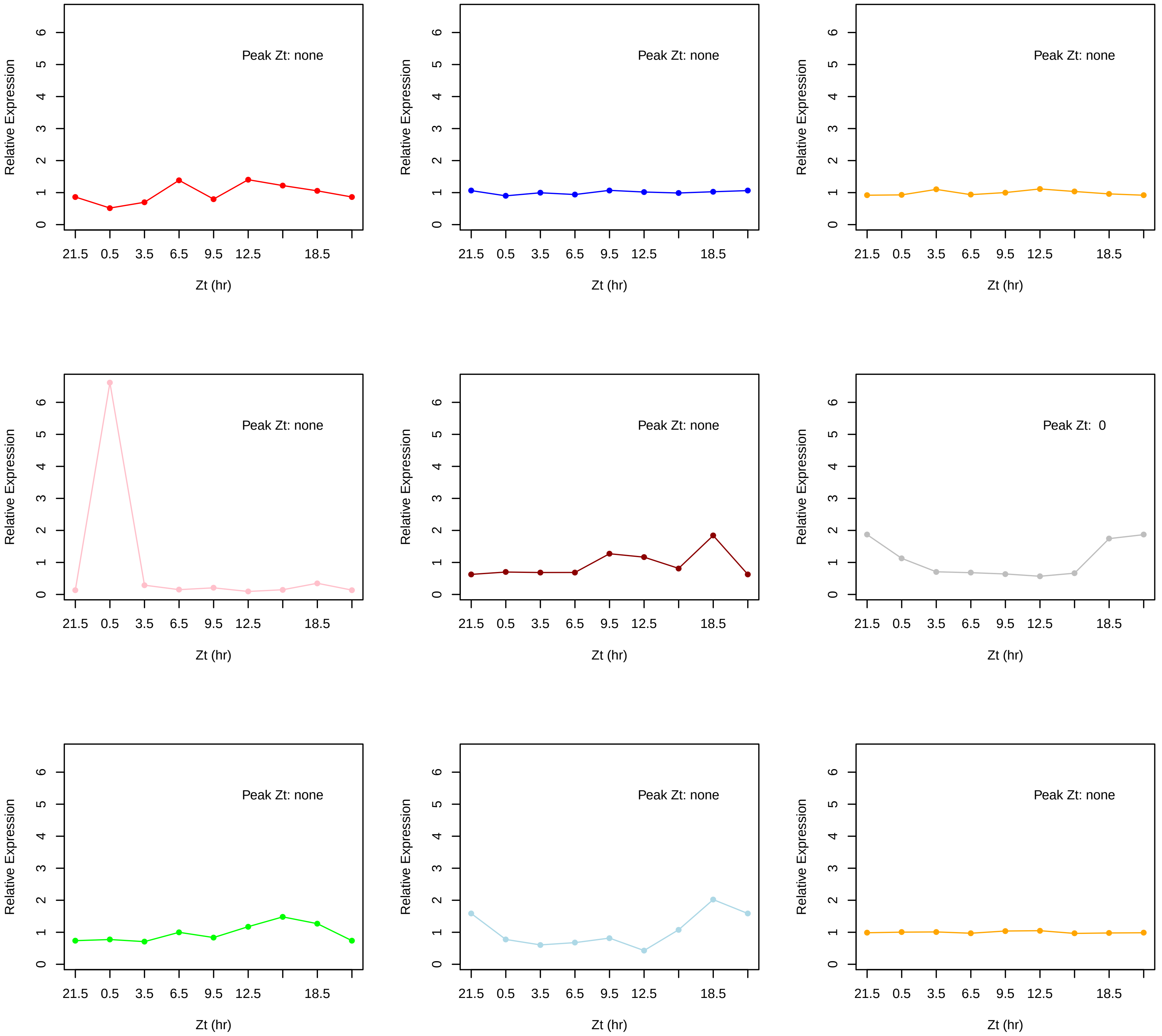

Singeleton: ENSXMAG00000008084 copz1

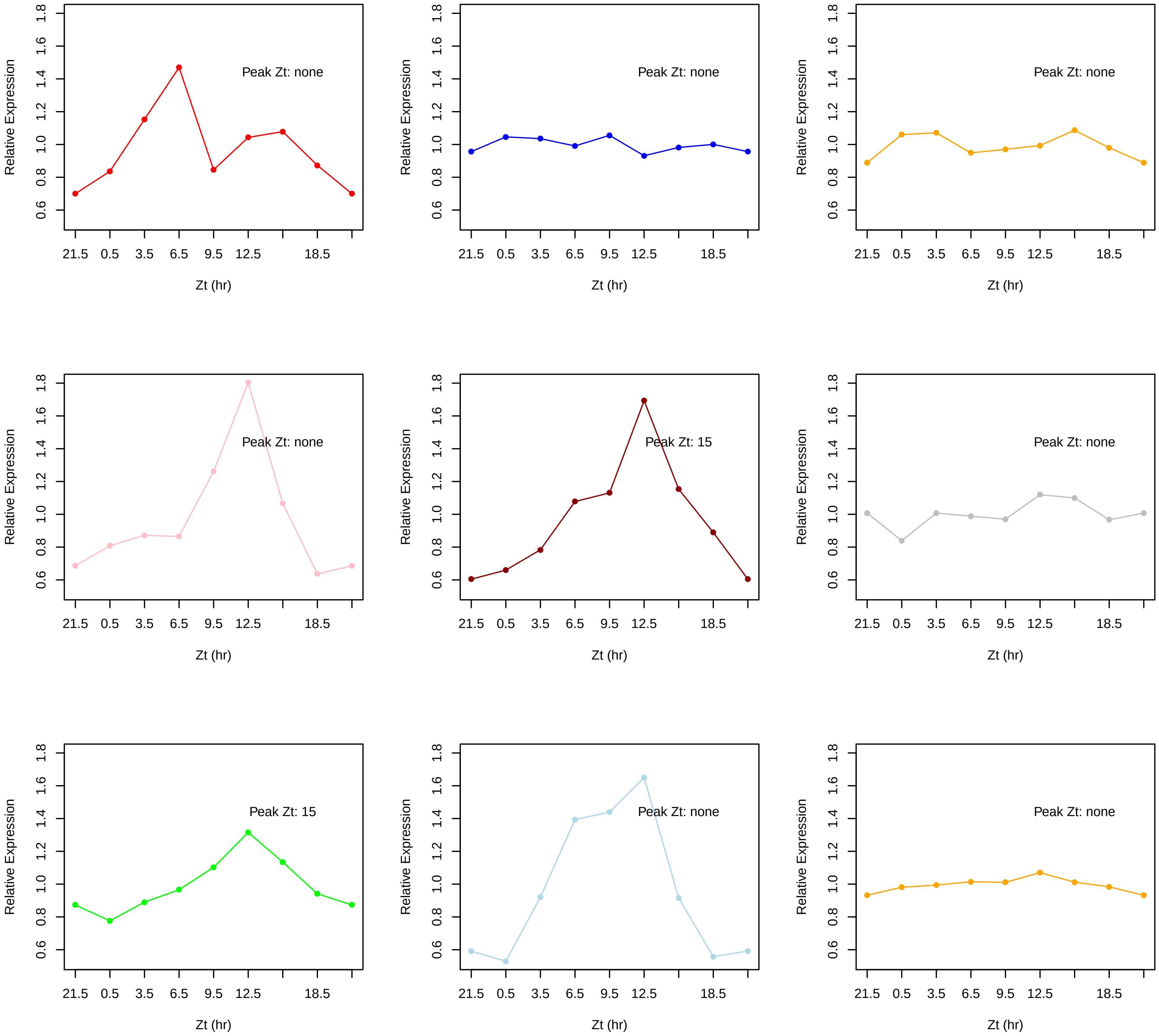

Singleton: ENSXMAG00000004537 clns1a

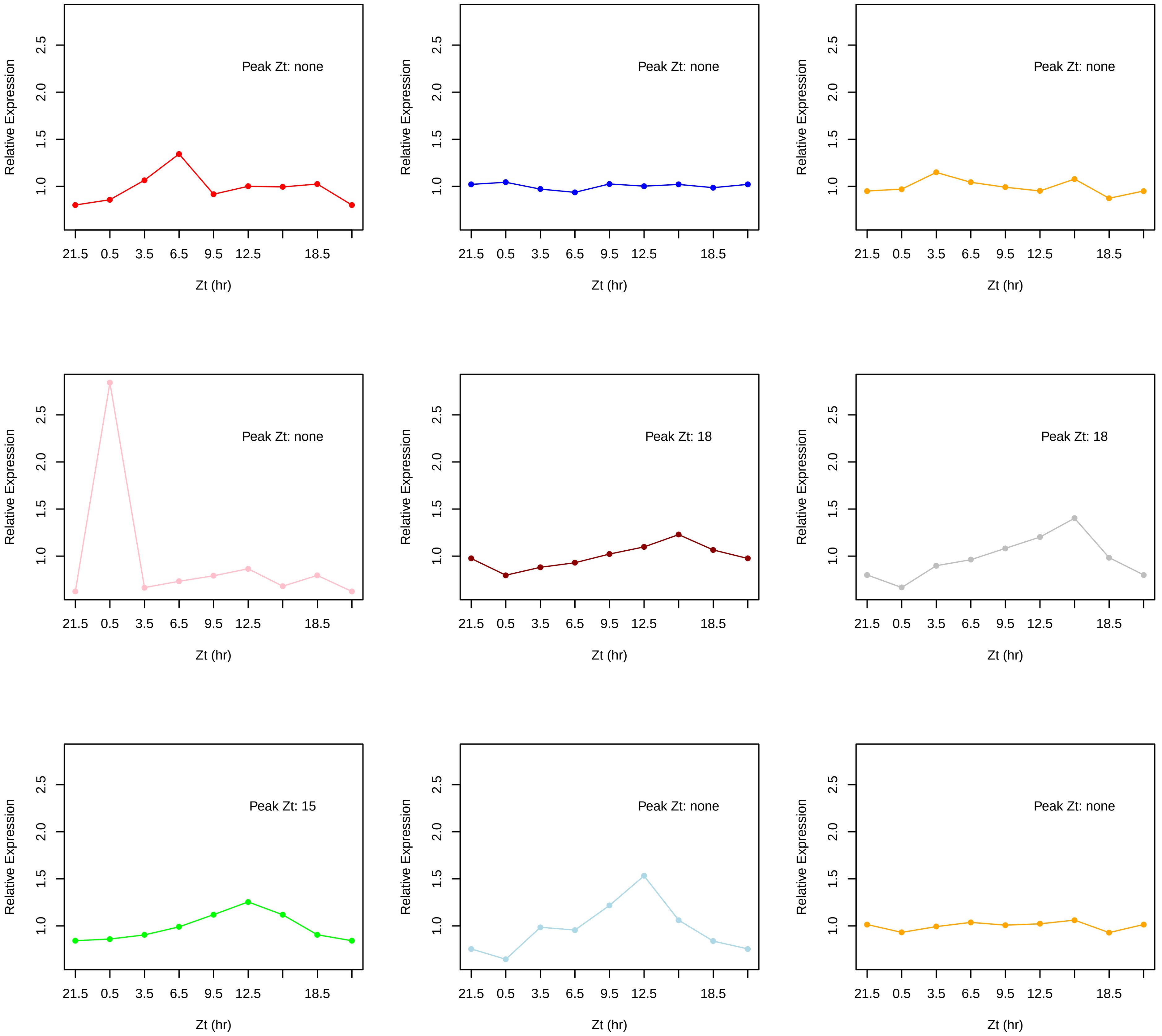

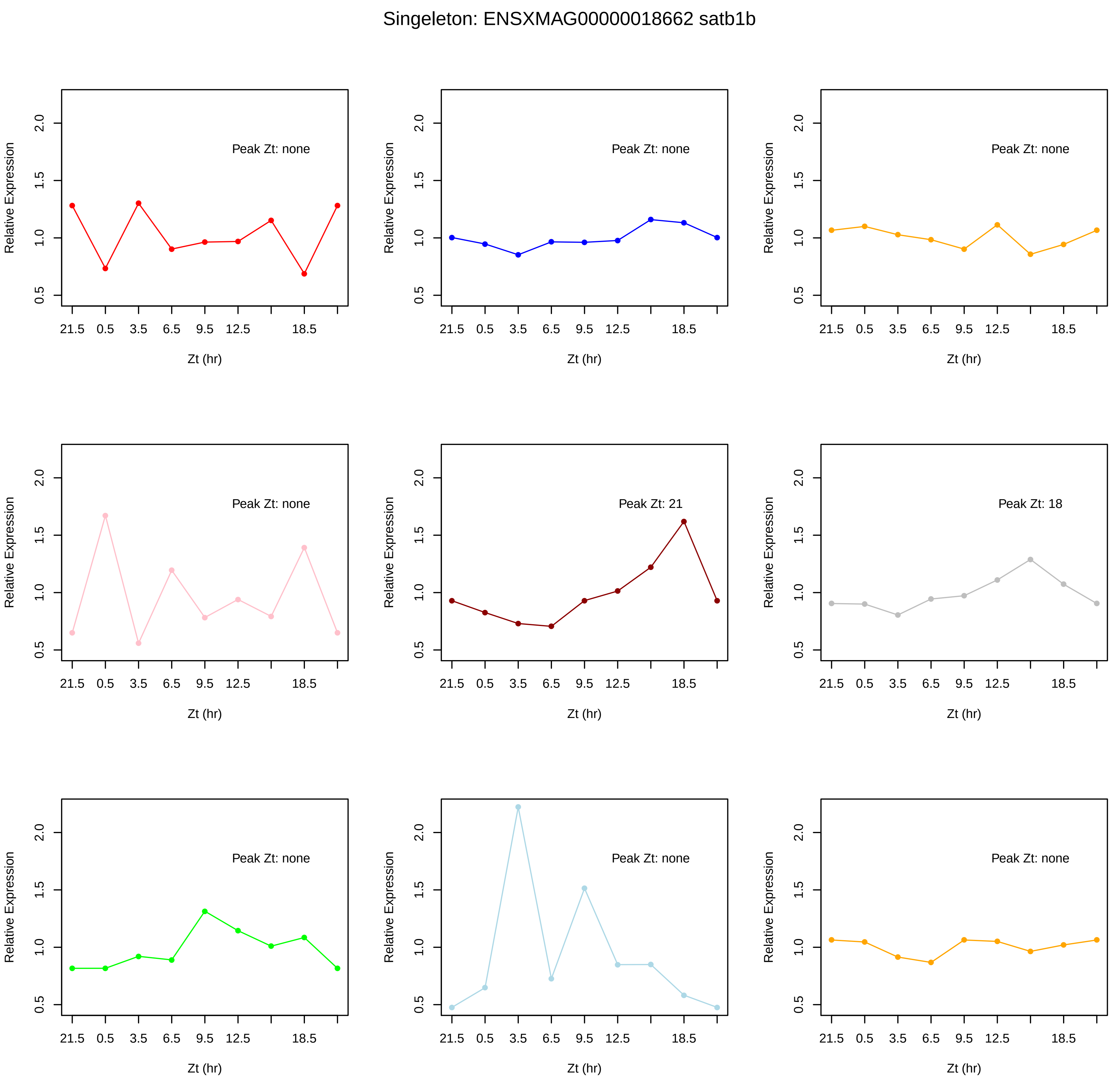
Singeleton: ENSXMAG00000018662 satb1b
Peak Zt: none
Peak Zt: none
Peak Zt: none
Peak Zt: none
Peak Zt: 21
Peak Zt: 18
Peak Zt: none
Peak Zt: none
Peak Zt: none
Relative Expression
Zt (hr)
21.5 0.5 3.5 6.5 9.5 12.5 18.5
0.5 1.0 1.5 2.0

Singeleton: ENSXMAG00000011870 scamp4

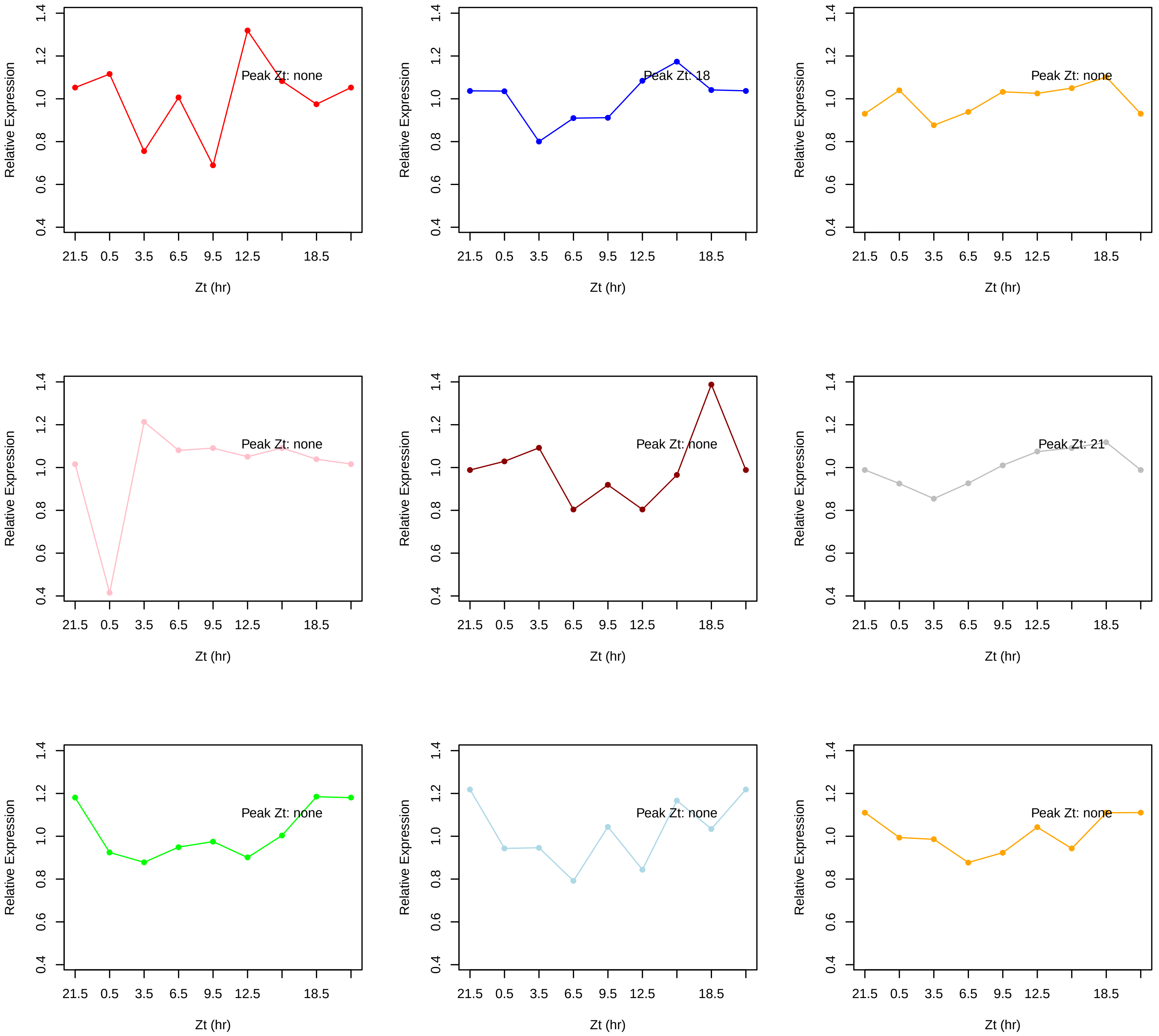

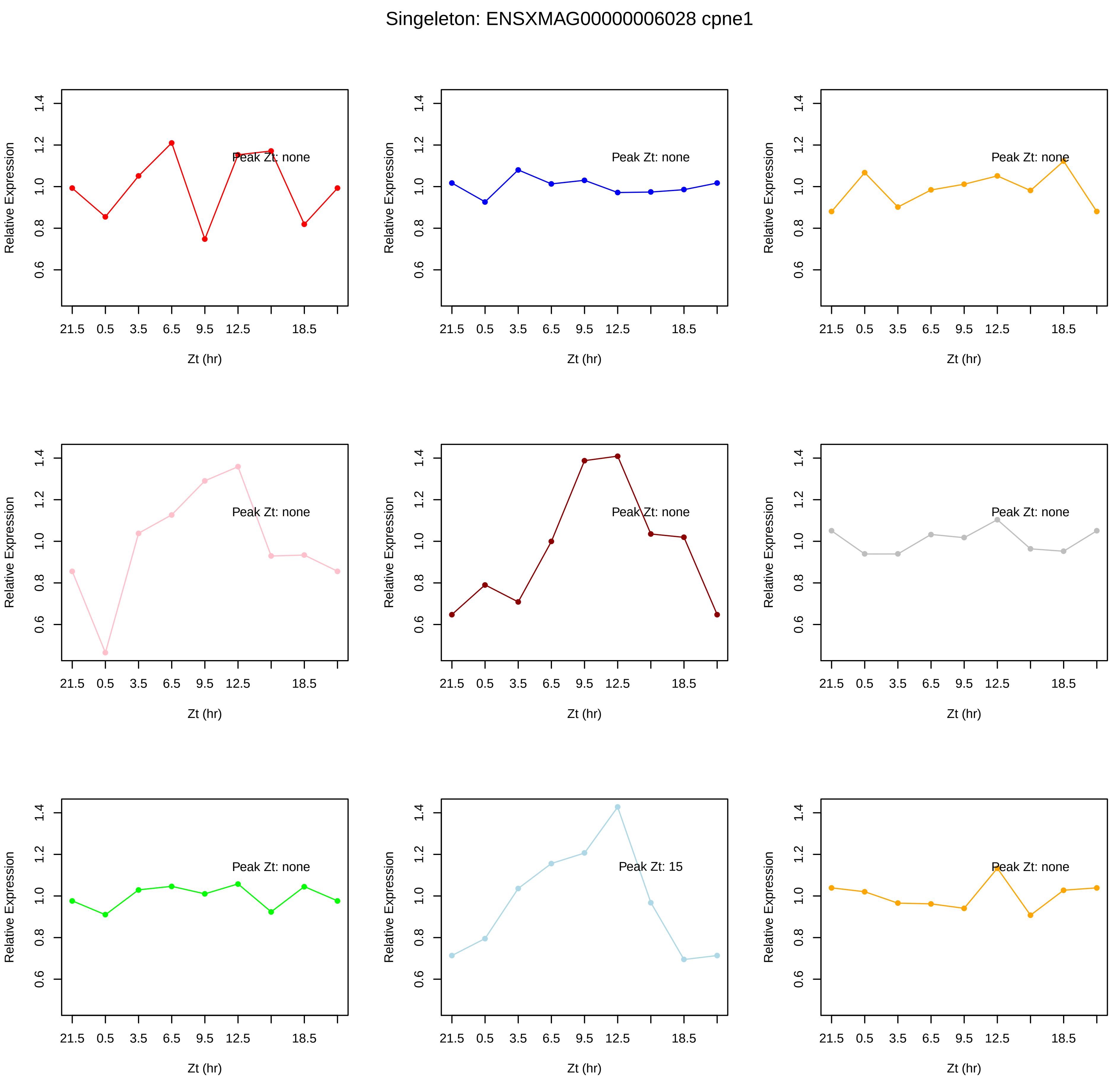

Singleton: ENSXMAG00000006028 cpne1
Peak Zt: none
Peak Zt: none
Peak Zt: none
Peak Zt: none
Peak Zt: none
Peak Zt: none
Peak Zt: none
Peak Zt: 15
Peak Zt: none
Relative Expression
Zt (hr)

Singeleton: ENSXMAG00000001520 cerkl

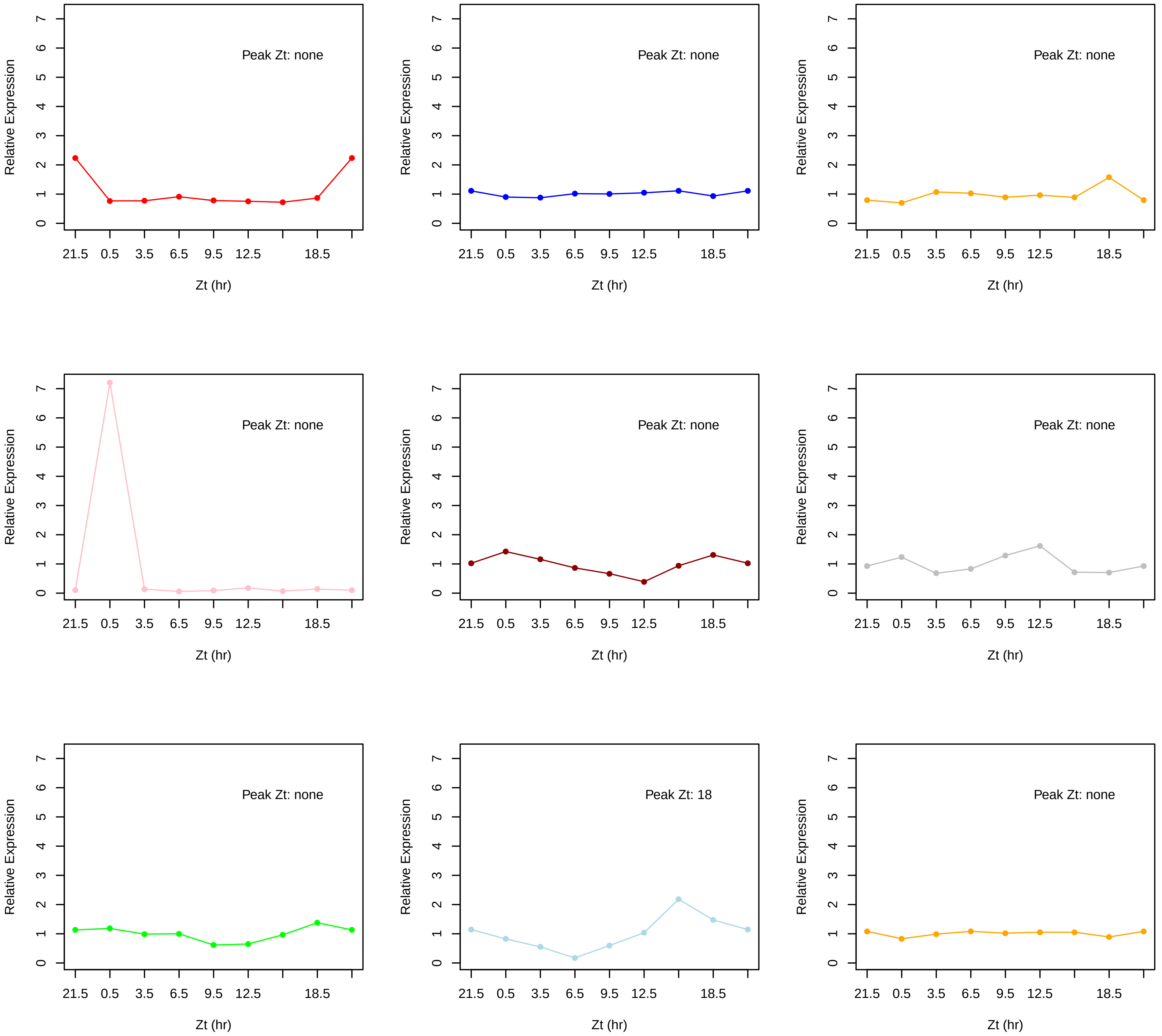

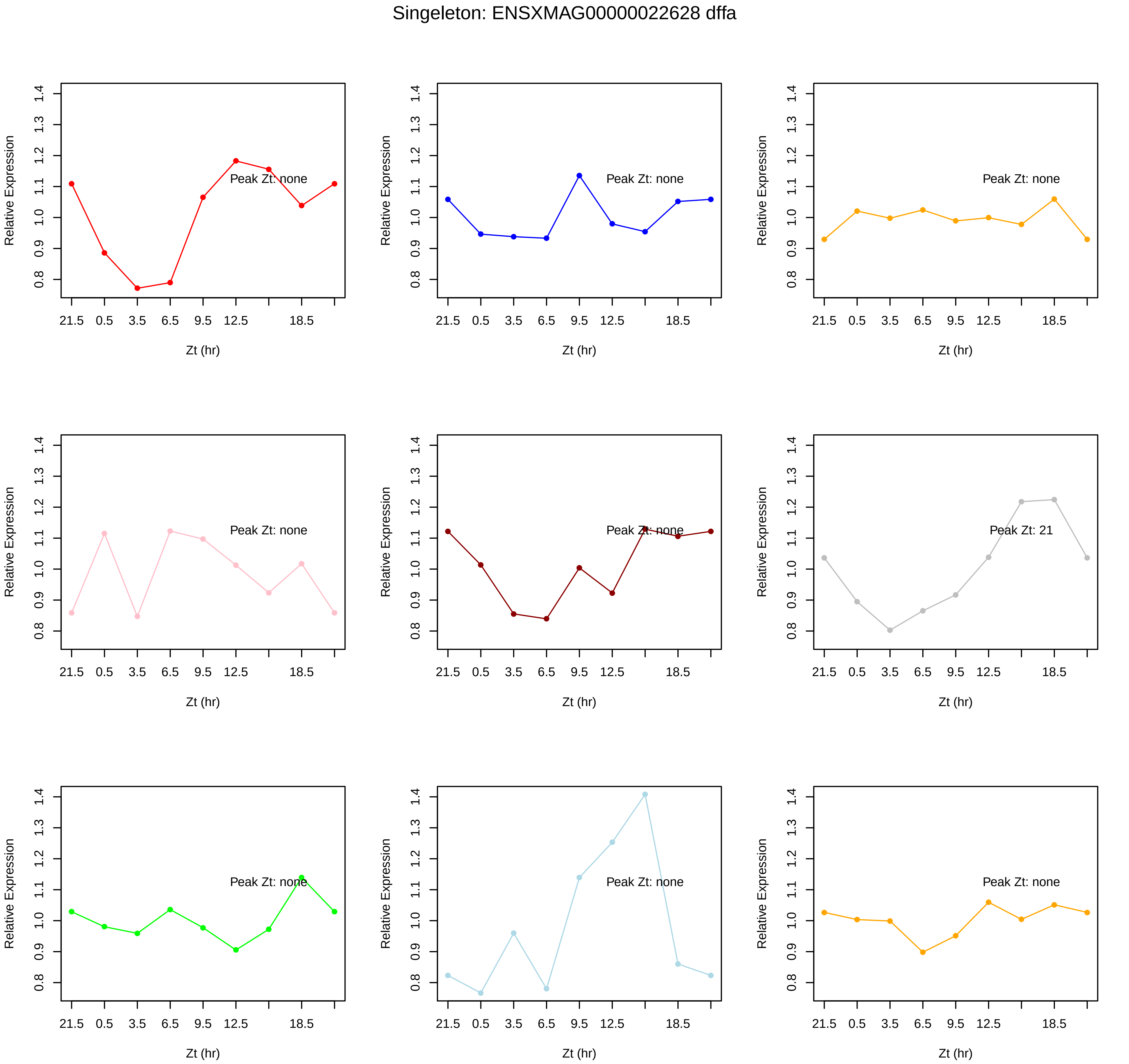
Singeleton: ENSXMAG00000022628 dffa
Relative Expression
Zt (hr)
21.5 0.5 3.5 6.5 9.5 12.5 18.5
0.8 0.9 1.0 1.1 1.2 1.3 1.4
Peak Zt: none
Peak Zt: none
Peak Zt: none
Peak Zt: none
Peak Zt: none
Peak Zt: 21
Peak Zt: none
Peak Zt: none
Peak Zt: none

Singeleton: ENSXMAG00000026069 crkl

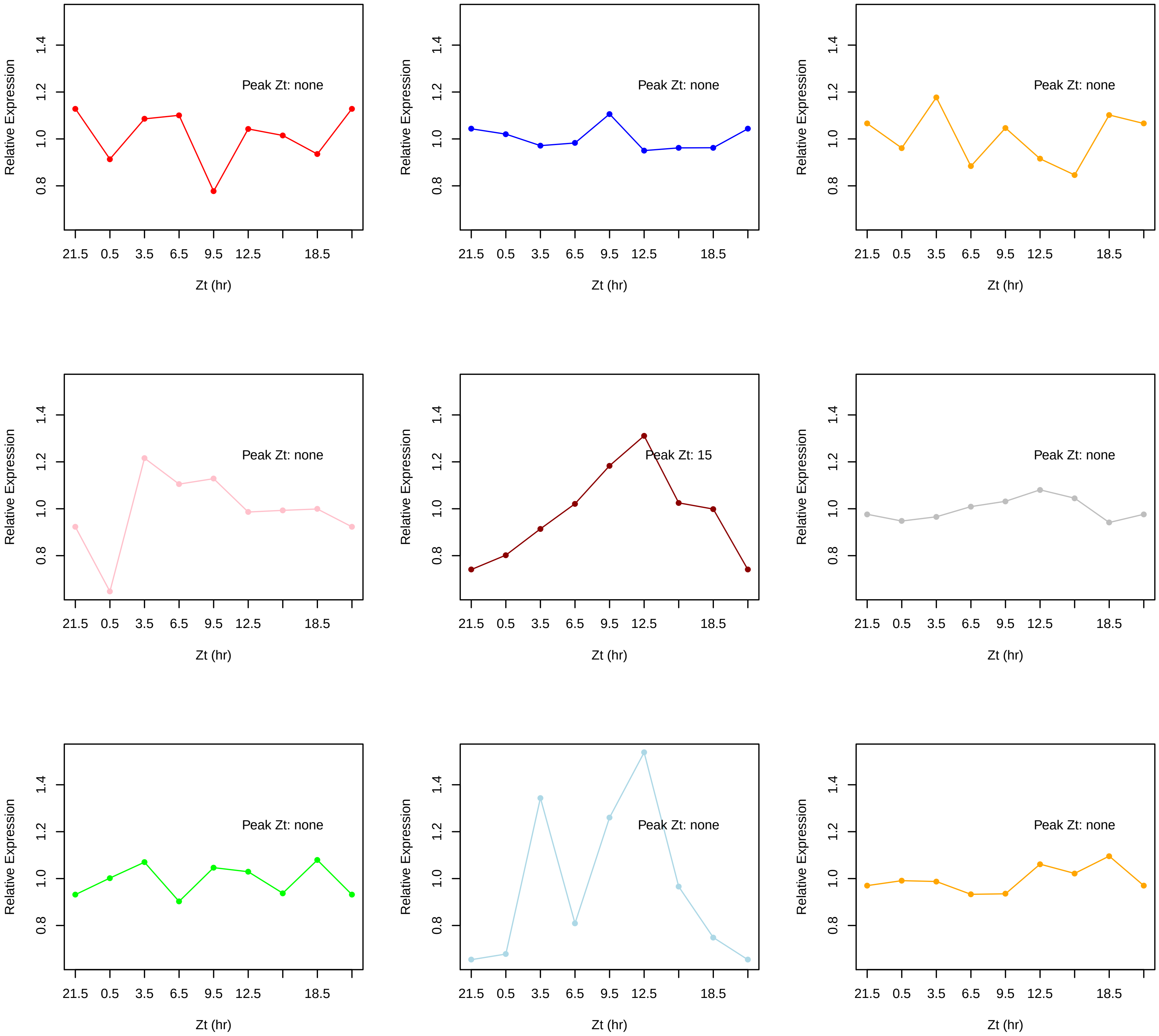

Singleton: ENSXMAG00000003740 zgc:103559

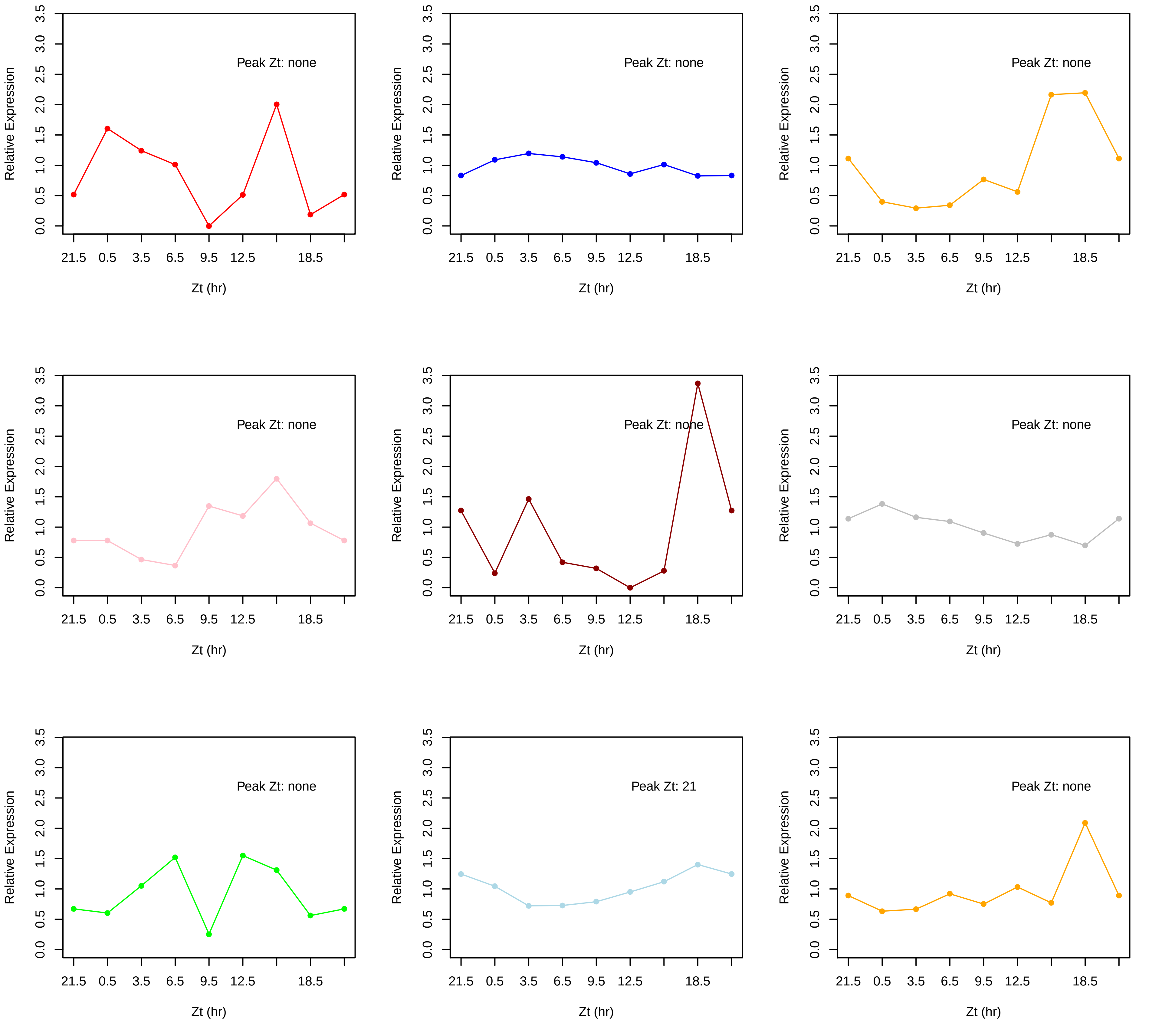

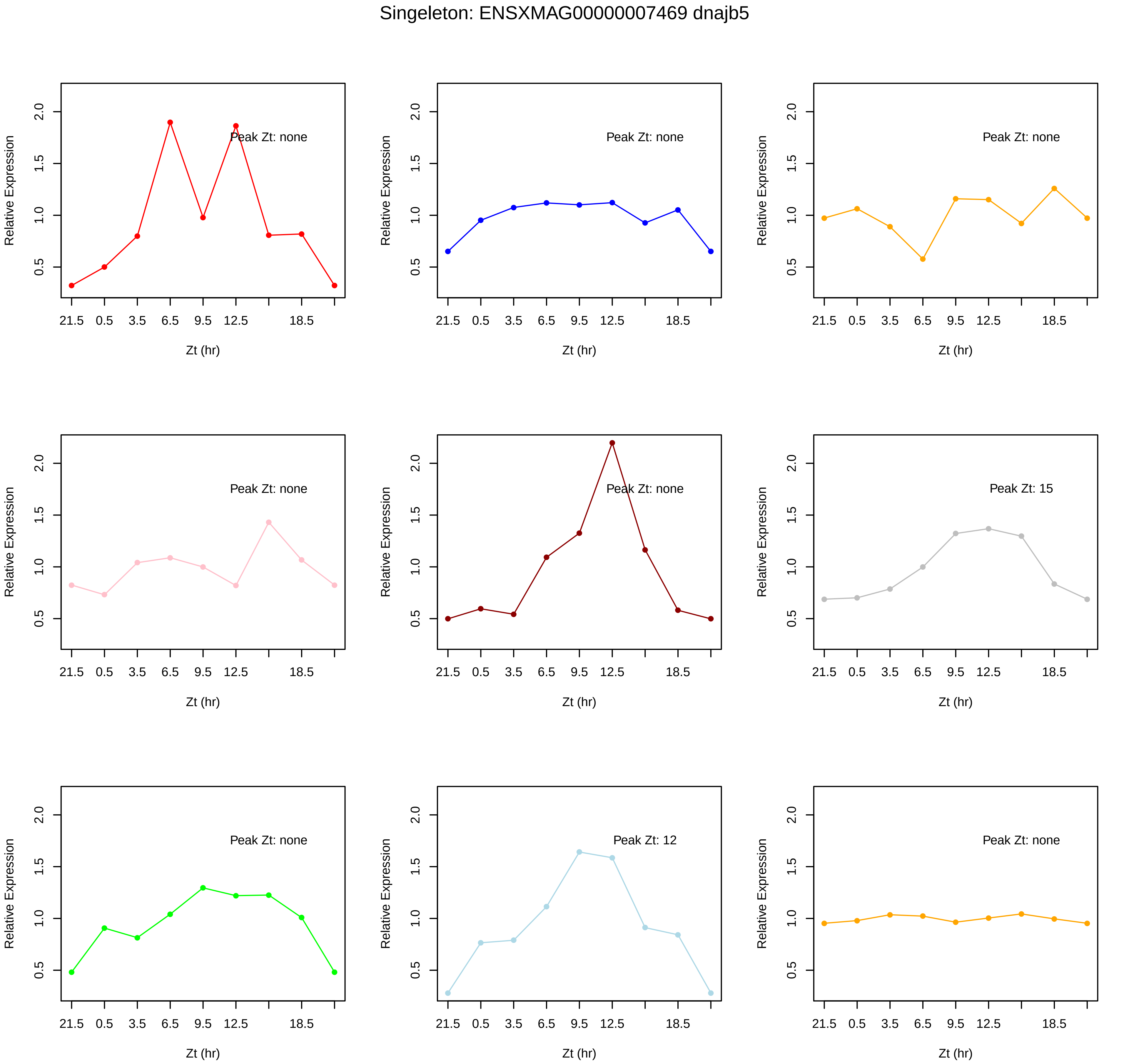
Singeleton: ENSXMAG00000007469 dnajb5
Relative Expression
Zt (hr)
21.5
0.5
3.5
6.5
9.5
12.5
18.5
0.5
1.0
1.5
2.0
Peak Zt: none
Peak Zt: none
Peak Zt: none
Peak Zt: none
Peak Zt: none
Peak Zt: 15
Peak Zt: none
Peak Zt: 12
Peak Zt: none

Singeleton: ENSXMAG00000012029 git1

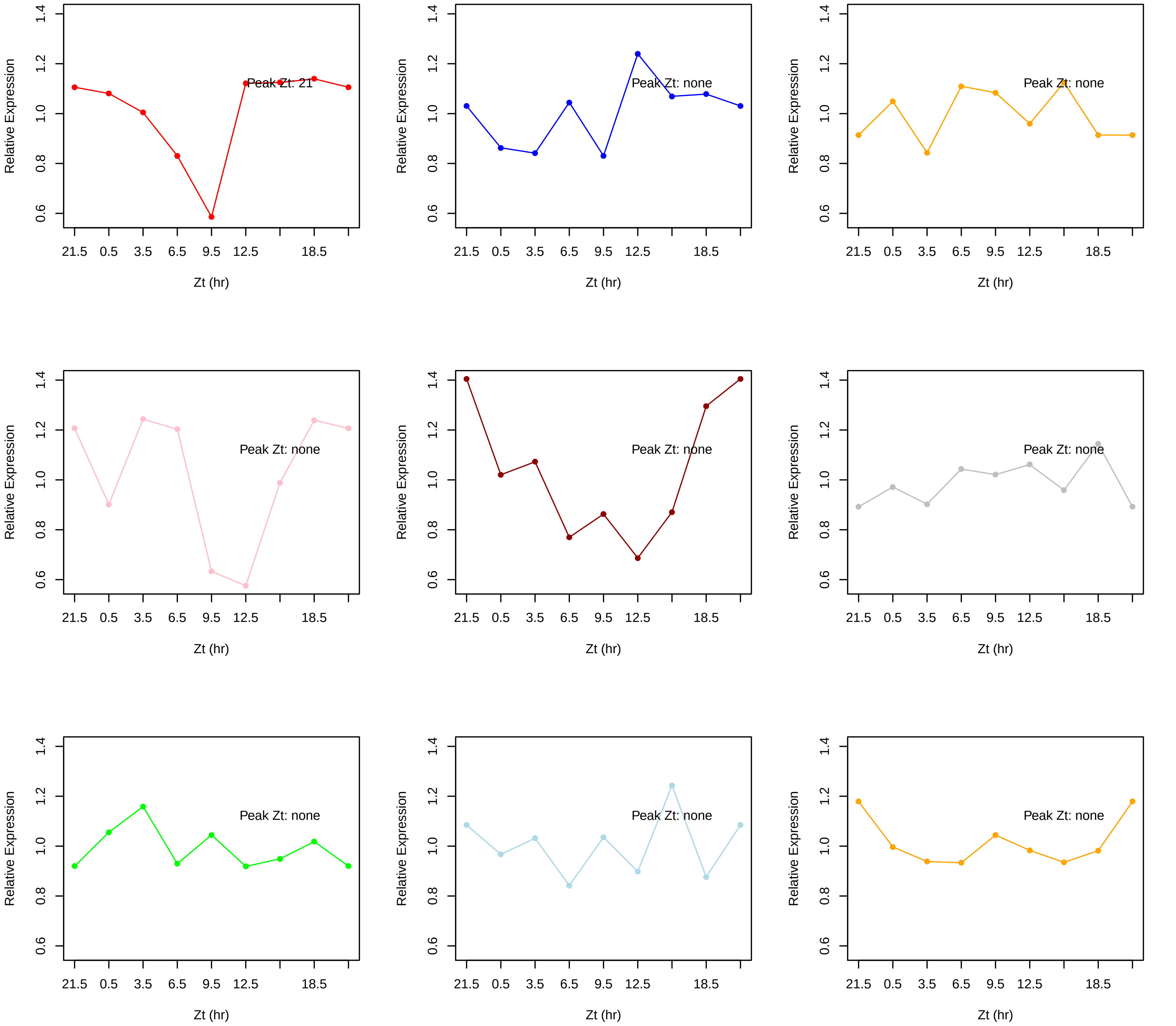

Singleton: ENSXMAG00000011294 pex14

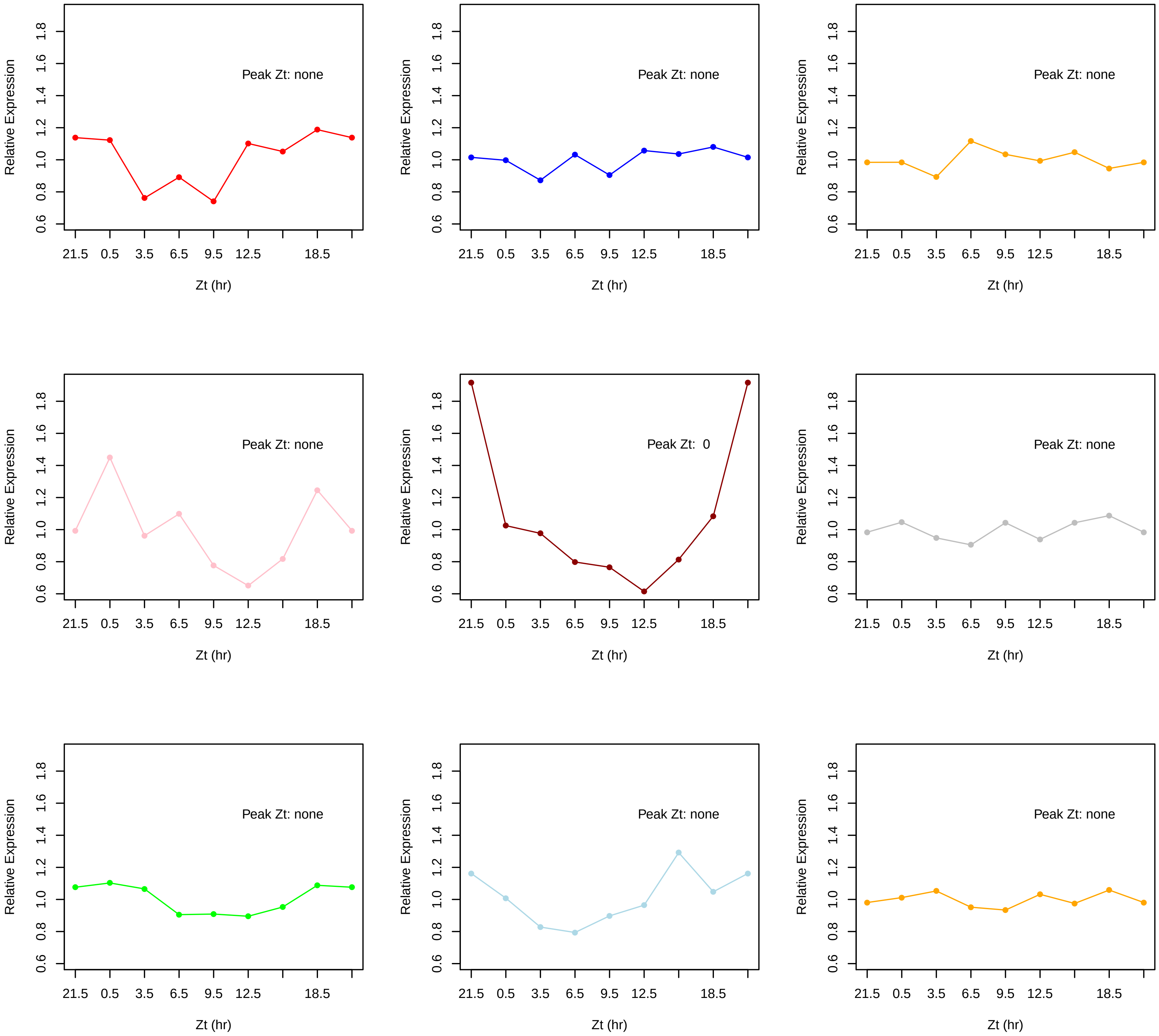

Singeleton: ENSXMAG00000004548 pak1

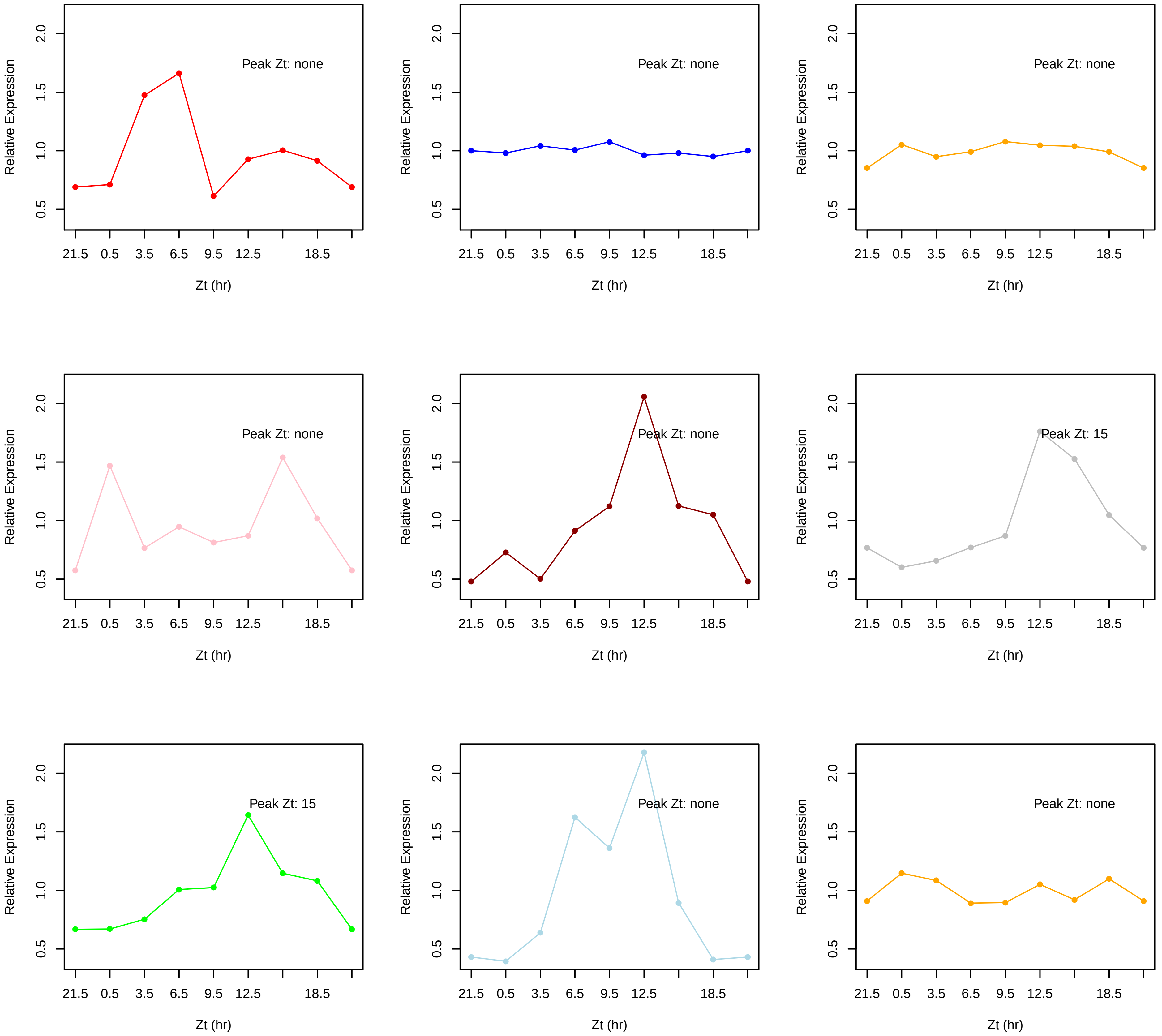

Singeleton: ENSXMAG00000007474 akr1a1a

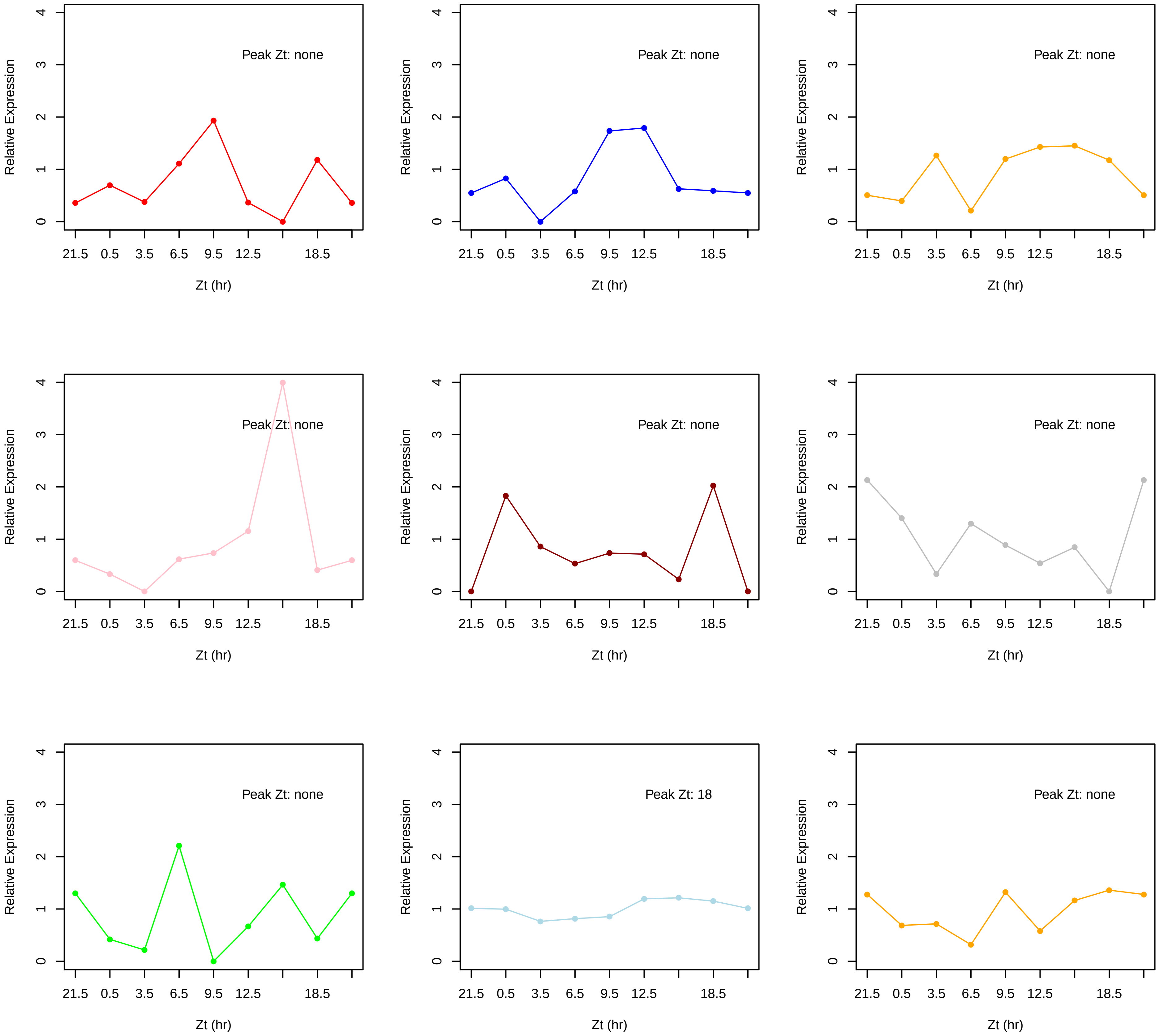

Singeleton: ENSXMAG00000026779 znf740b

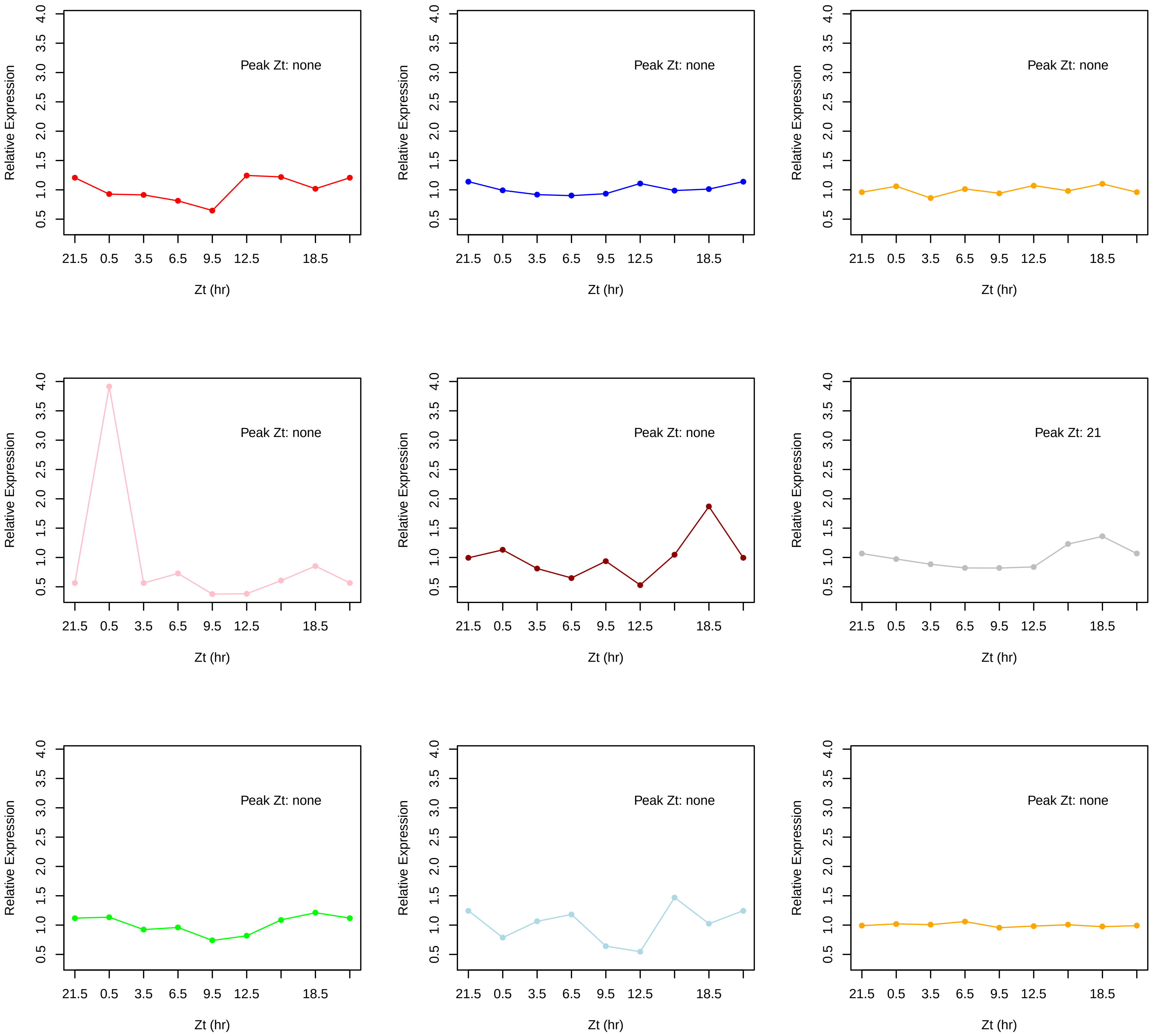

Singeleton: ENSXMAG00000016600 setdb1b

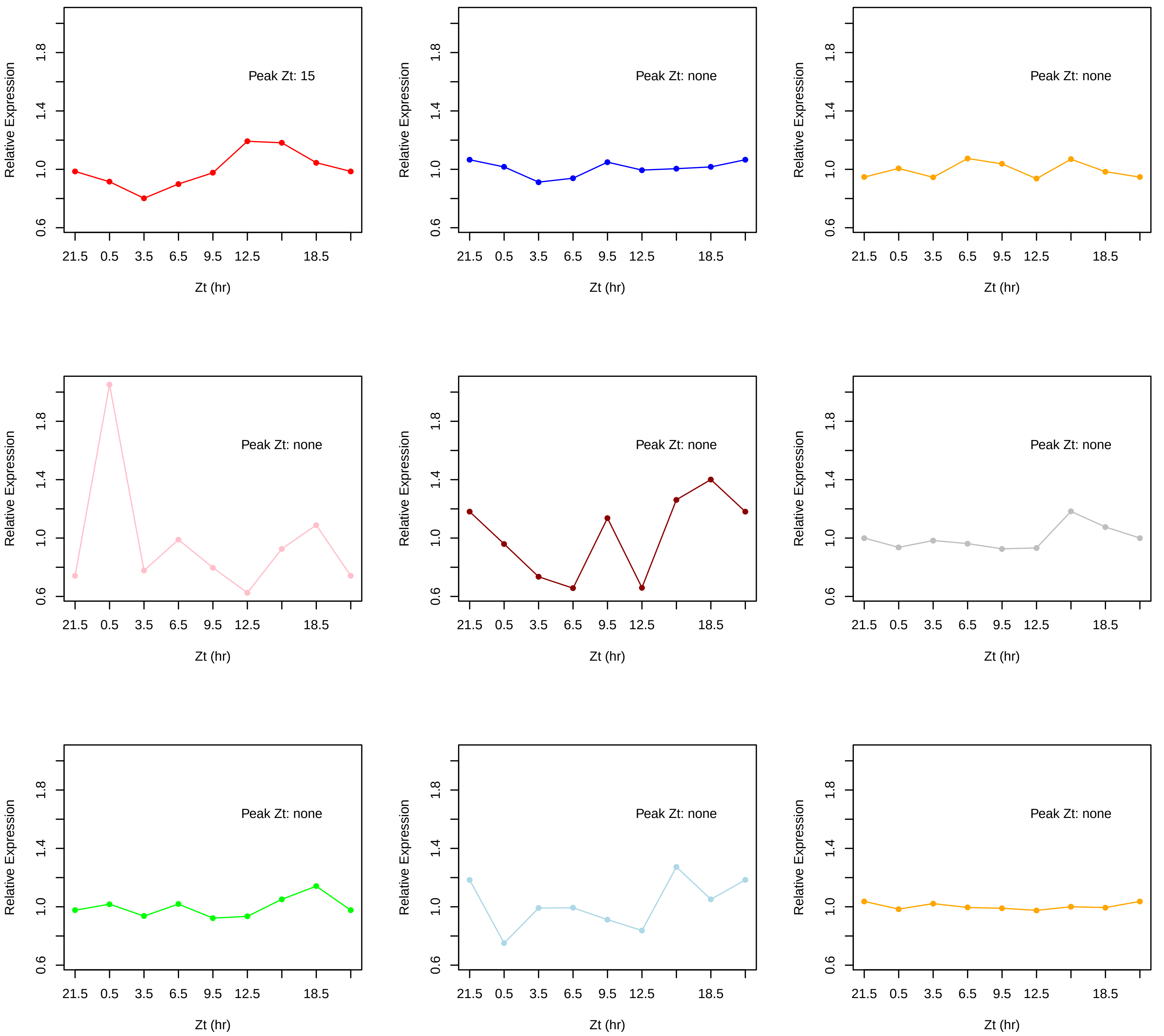

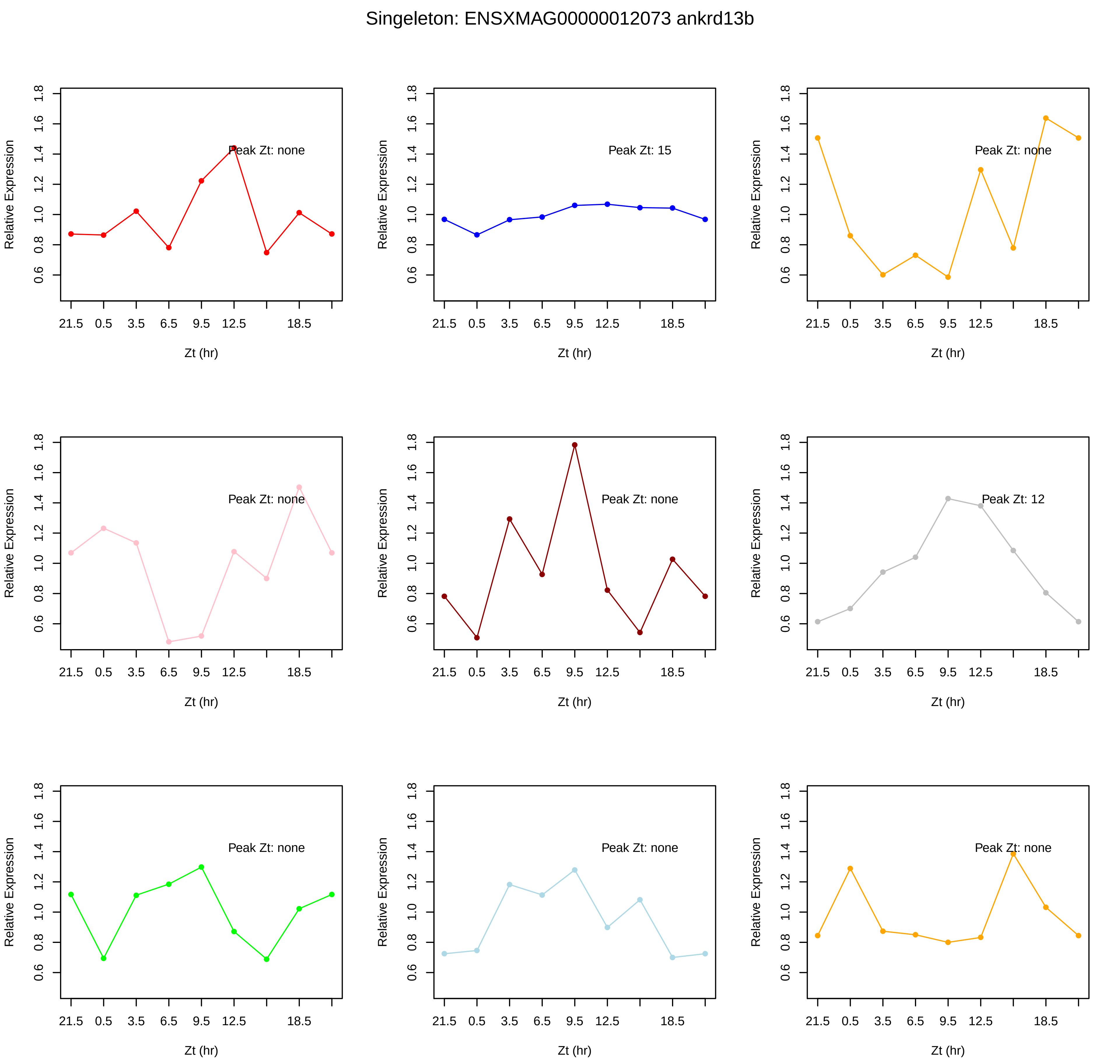

Singeleton: ENSXMAG00000012073 ankrd13b
Peak Zt: none
Peak Zt: 15
Peak Zt: none
Peak Zt: none
Peak Zt: none
Peak Zt: 12
Peak Zt: none
Peak Zt: none
Peak Zt: none
Relative Expression
Zt (hr)
21.5
0.5
3.5
6.5
9.5
12.5
18.5
0.6
0.8
1.0
1.2
1.4
1.6
1.8

Singeleton: ENSXMAG00000012252 PMM2

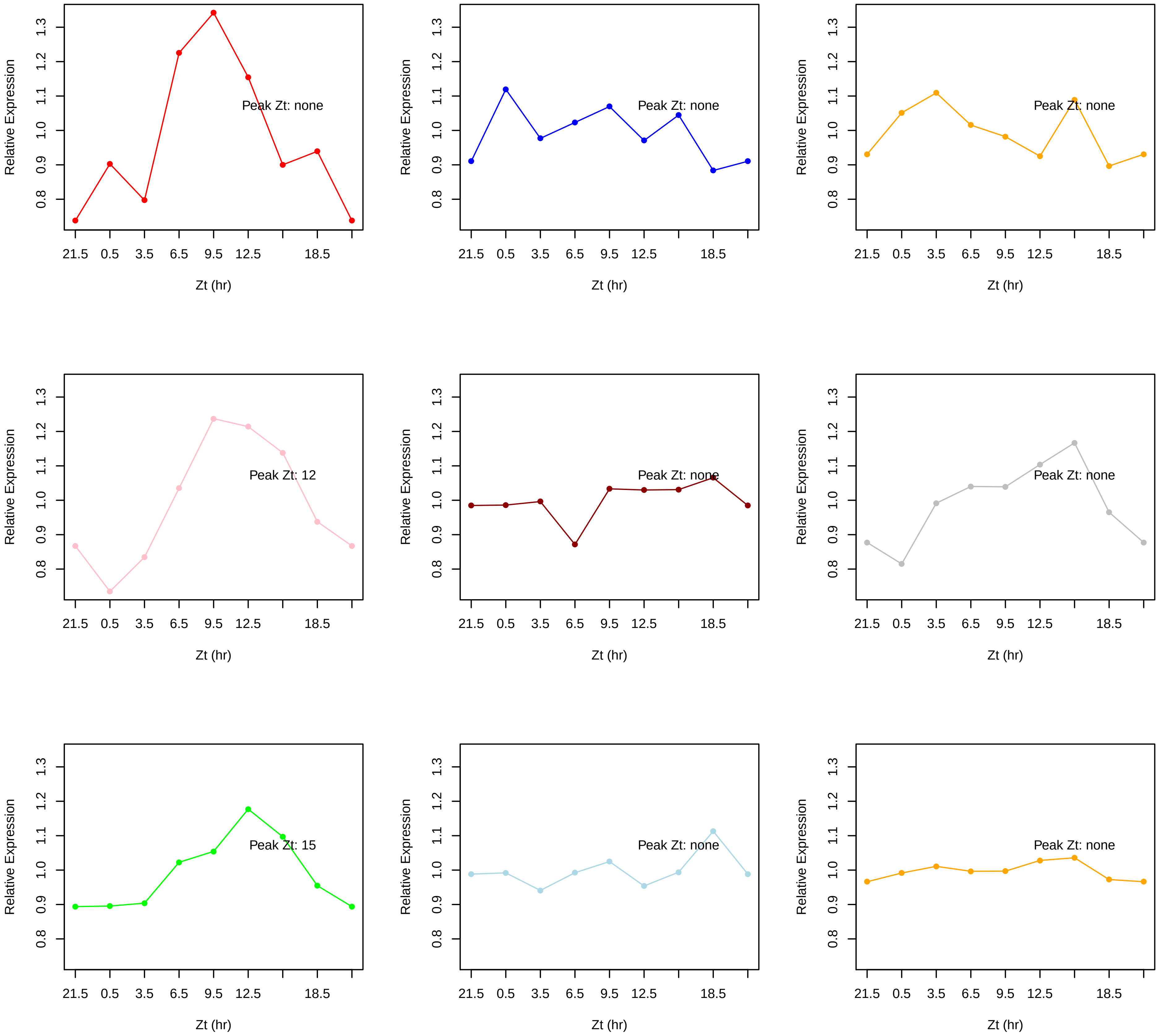

Singeleton: ENSXMAG00000003666 cabin1

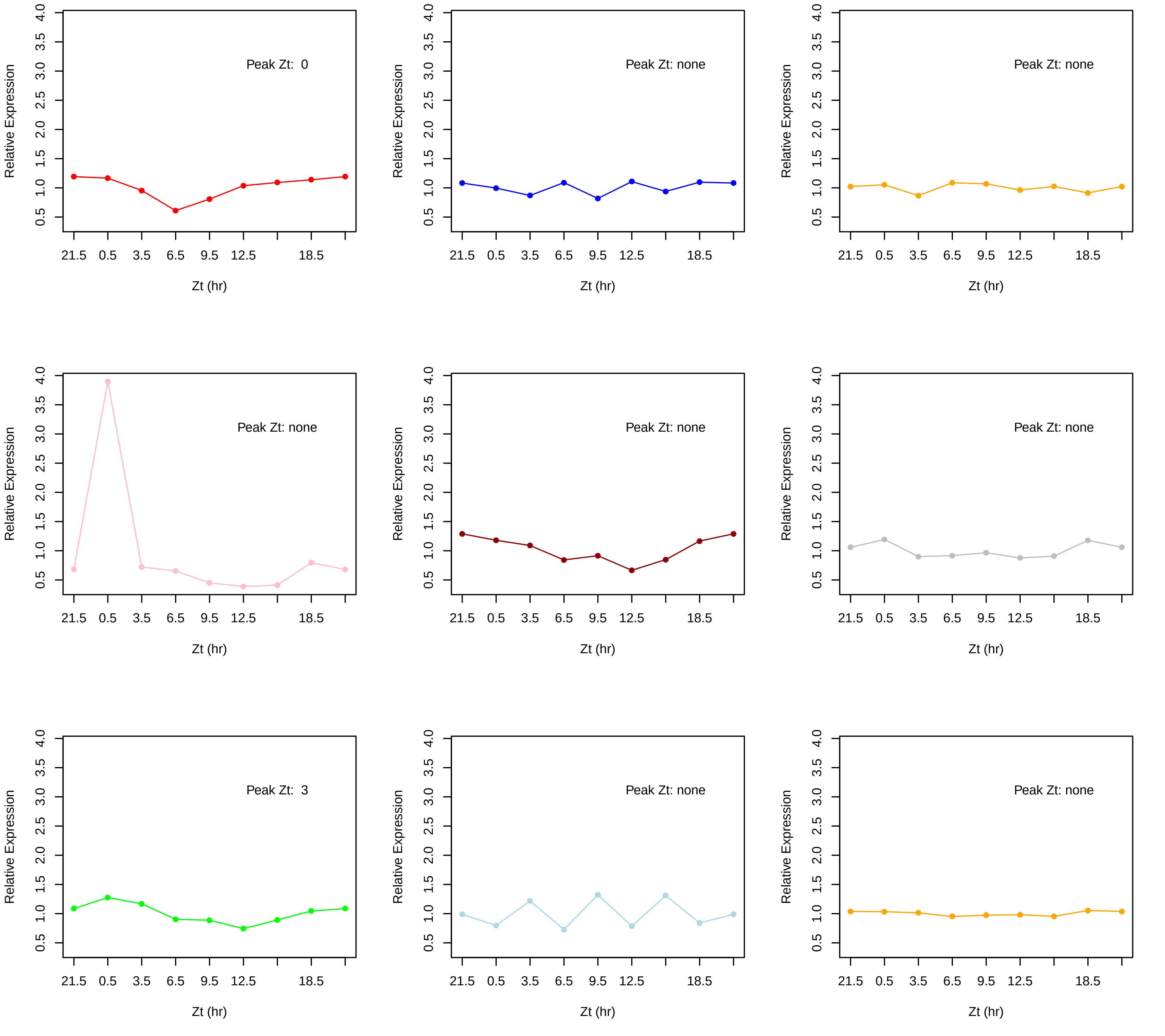

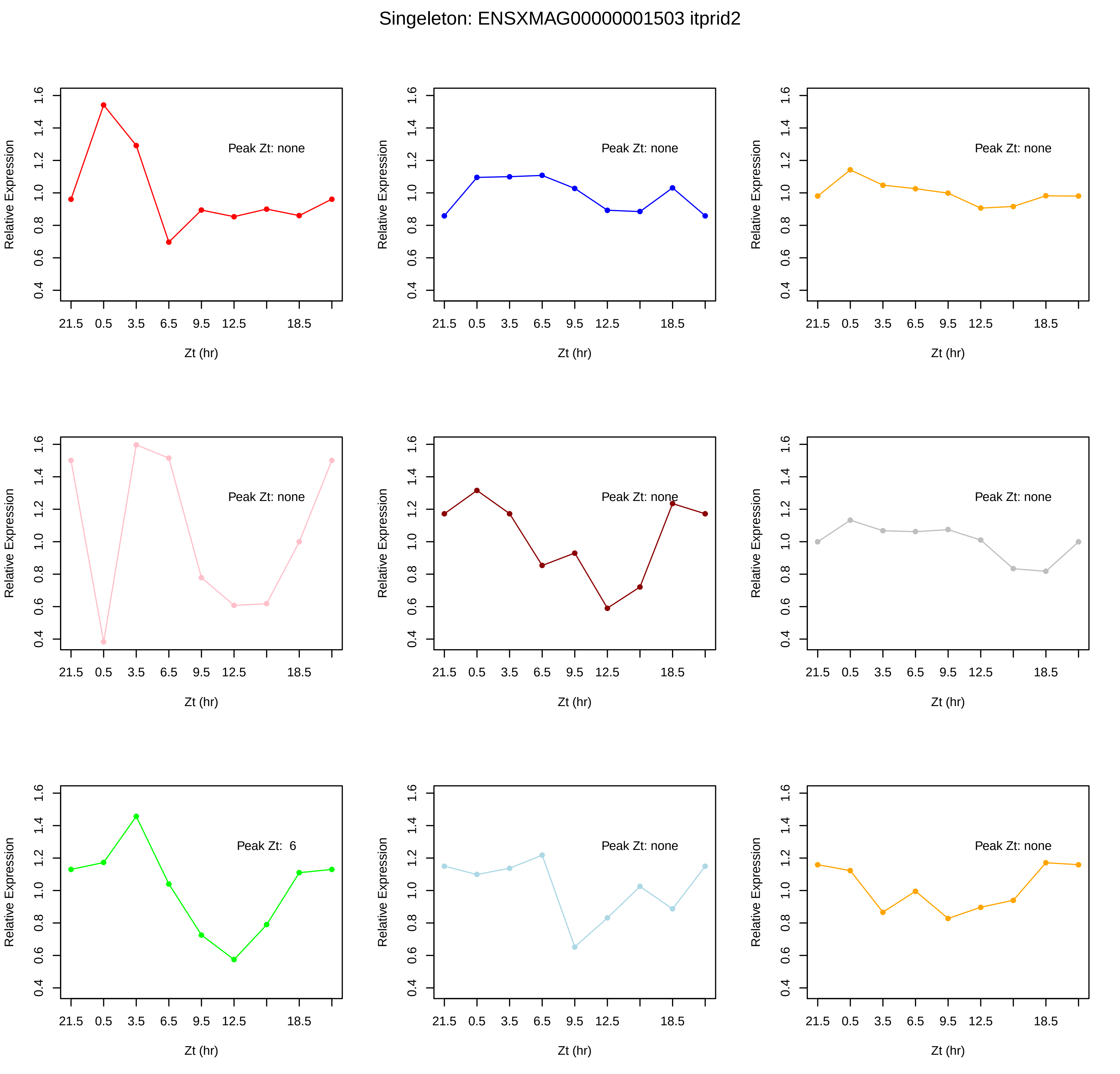

Singleton: ENSXMAG00000001503 itprid2
Peak Zt: none
Peak Zt: none
Peak Zt: none
Peak Zt: none
Peak Zt: none
Peak Zt: none
Peak Zt: 6
Peak Zt: none
Peak Zt: none
Relative Expression
Zt (hr)

Singeleton: ENSXMAG00000007485 vcp

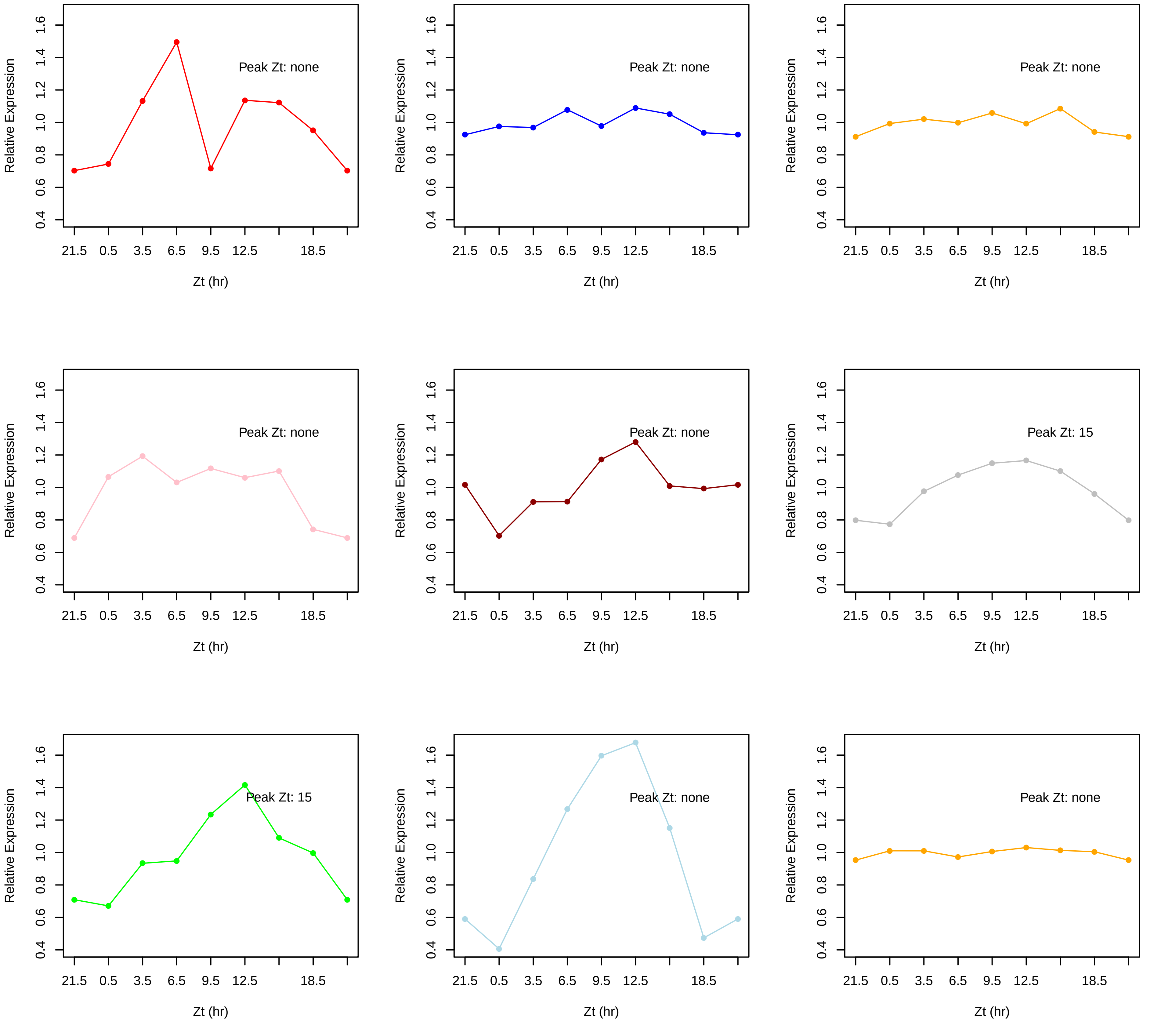

Singeleton: ENSXMAG00000027144 zgc:174906

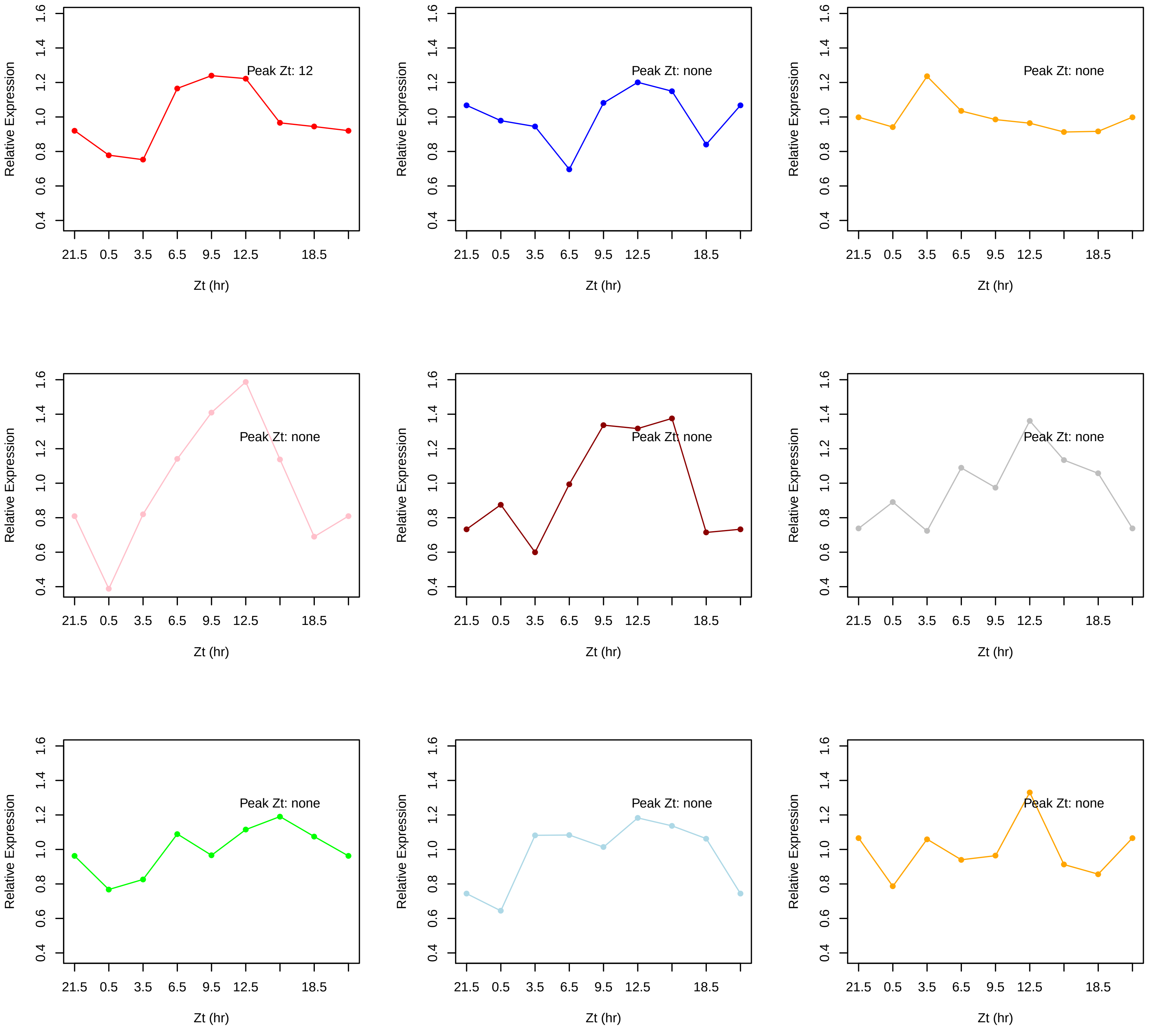

Singeleton: ENSXMAG00000002810 poglut1

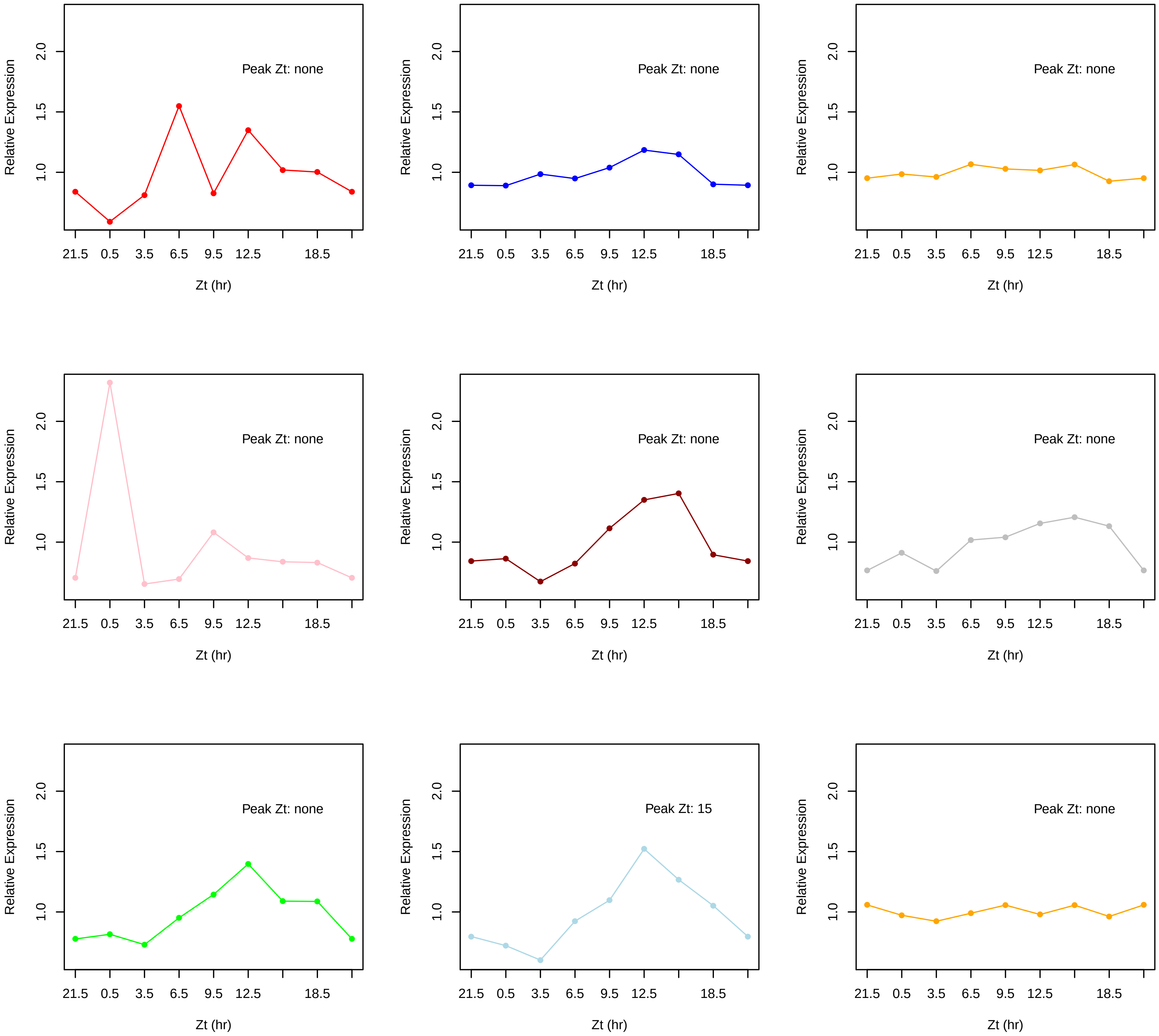

Singeleton: ENSXMAG00000002791 pnkp

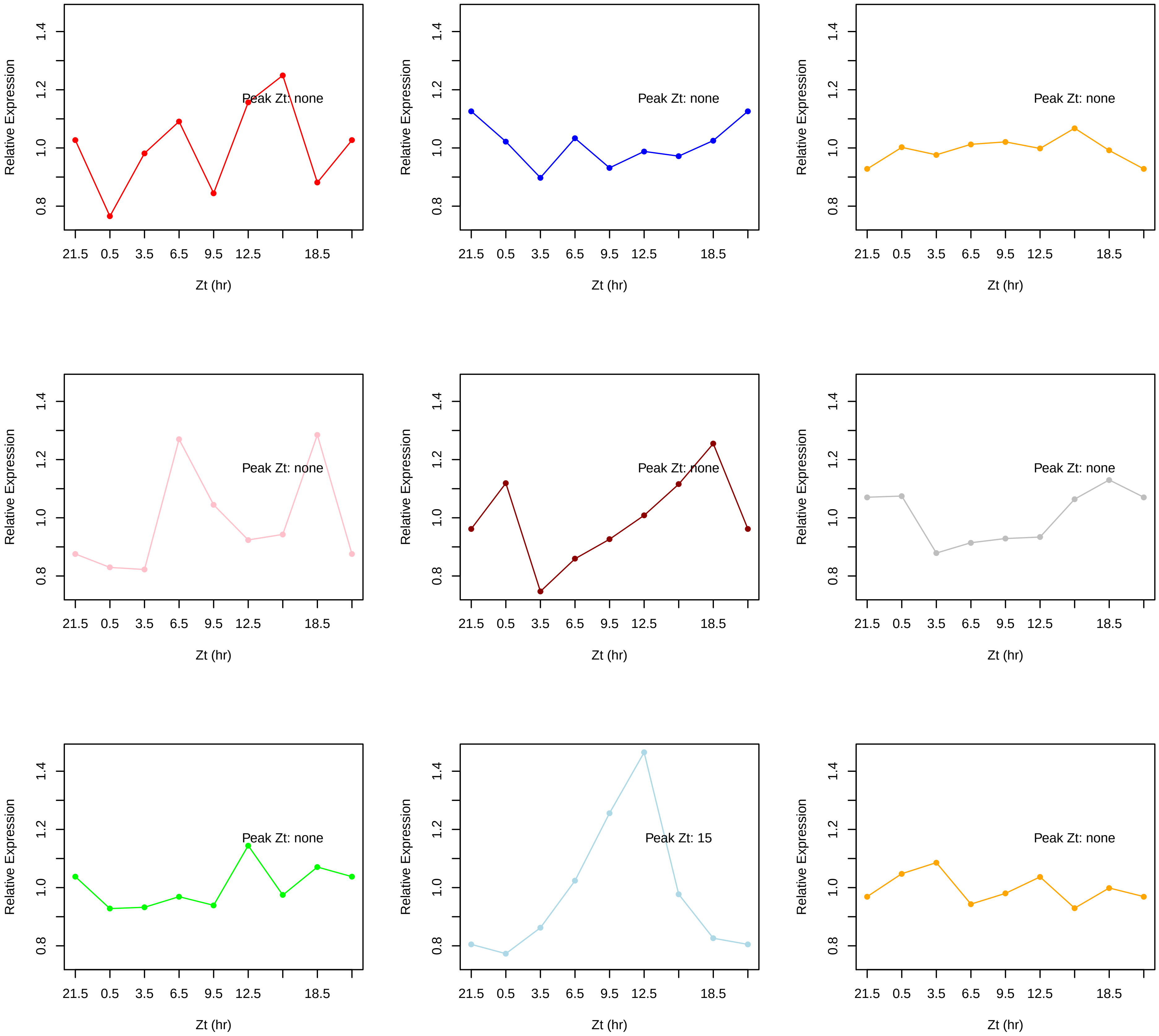

Singeleton: ENSXMAG00000002559 abat

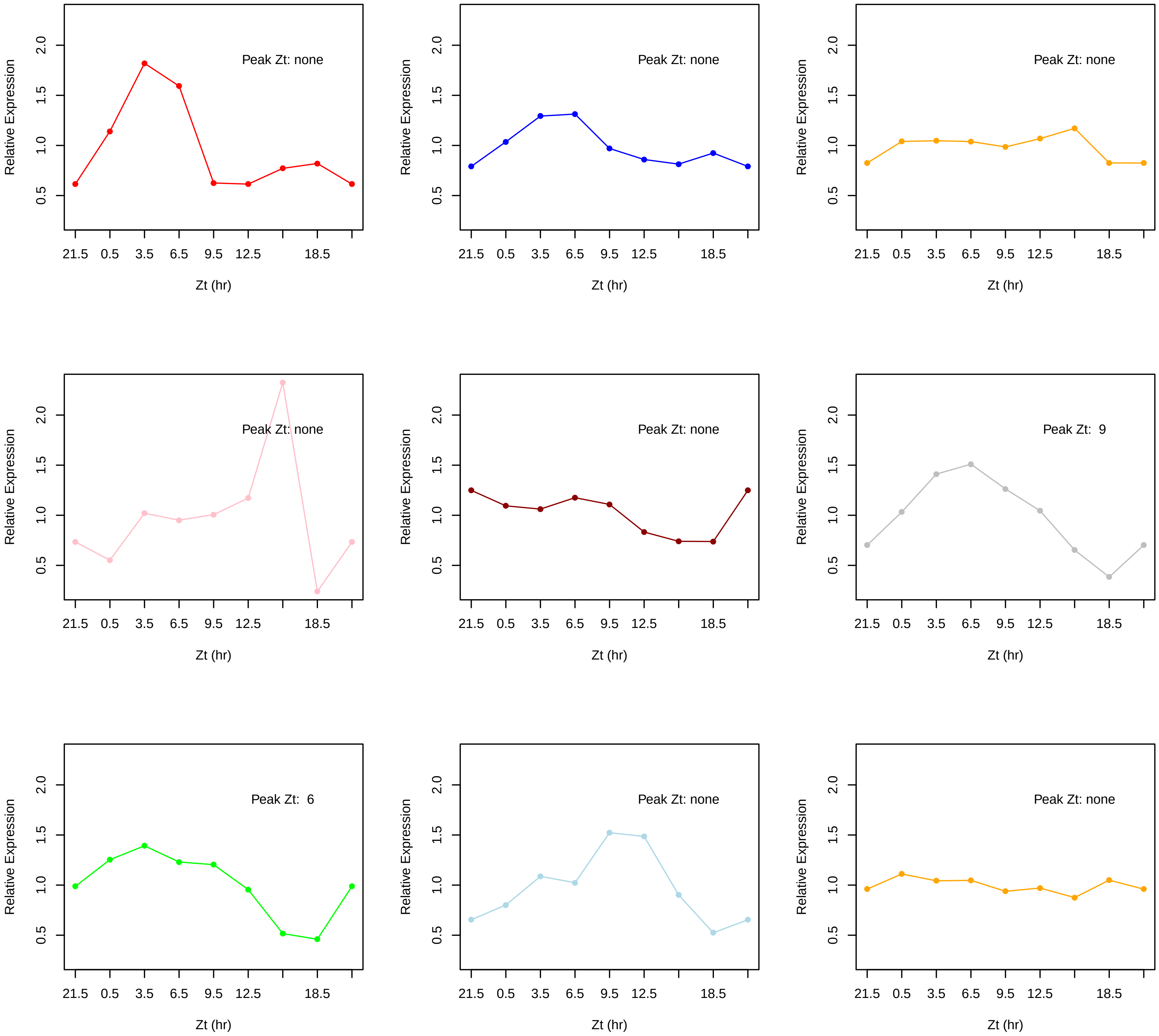

Singeleton: ENSXMAG00000018468 tsfm

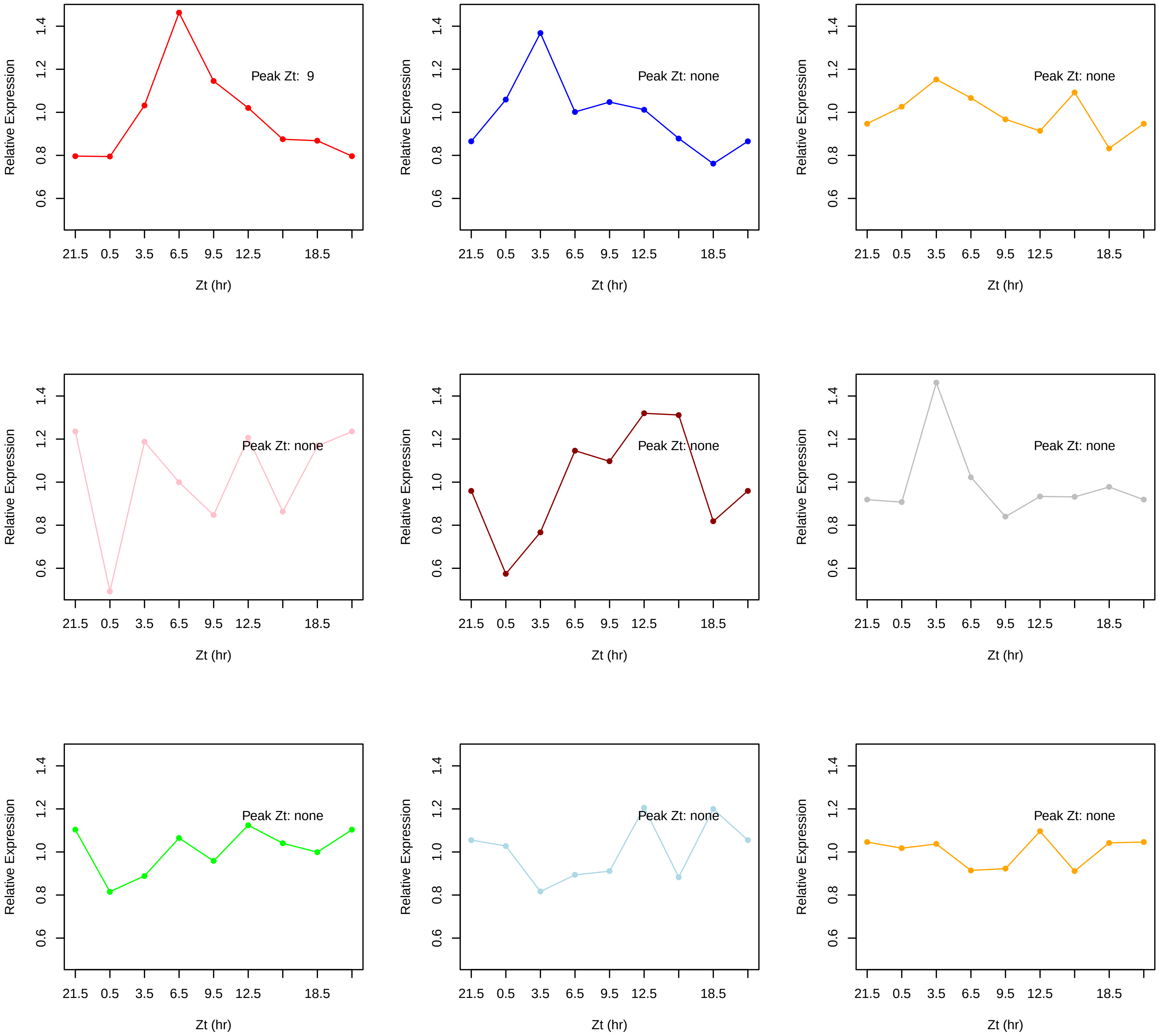

Singleton: ENSXMAG00000003253 kat2b

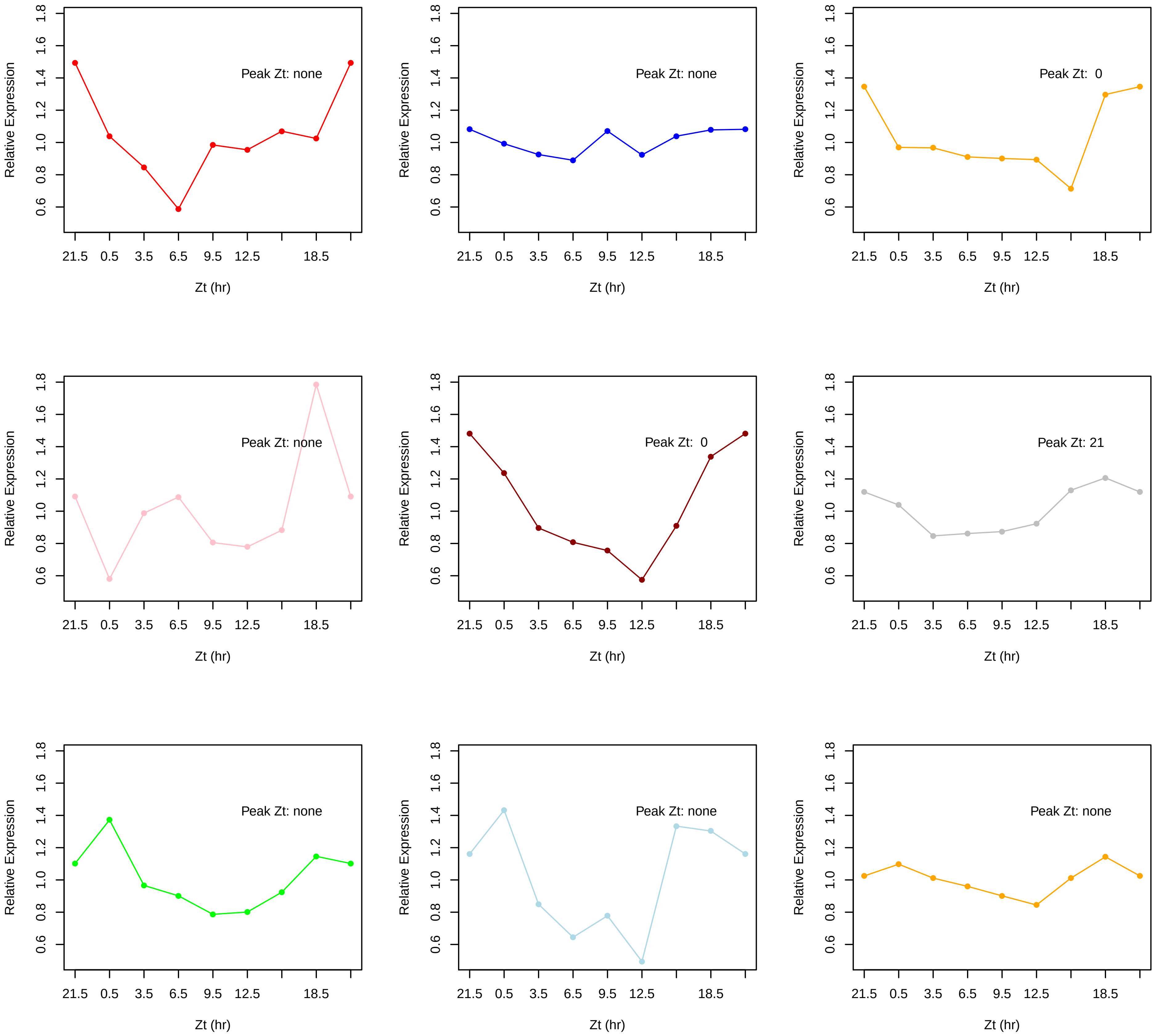

Singeleton: ENSXMAG00000026091

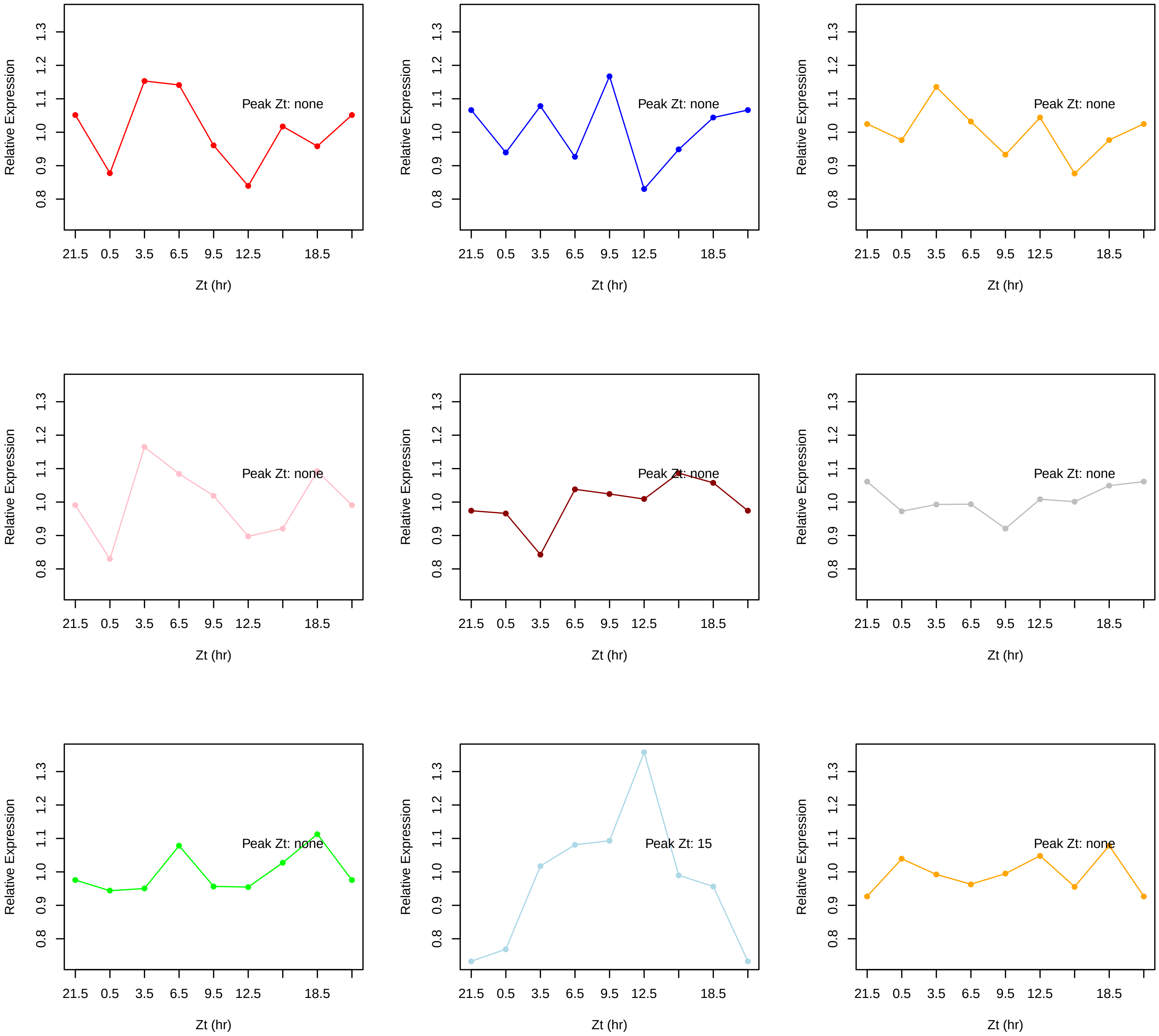

Singeleton: ENSXMAG00000003822 mettl22

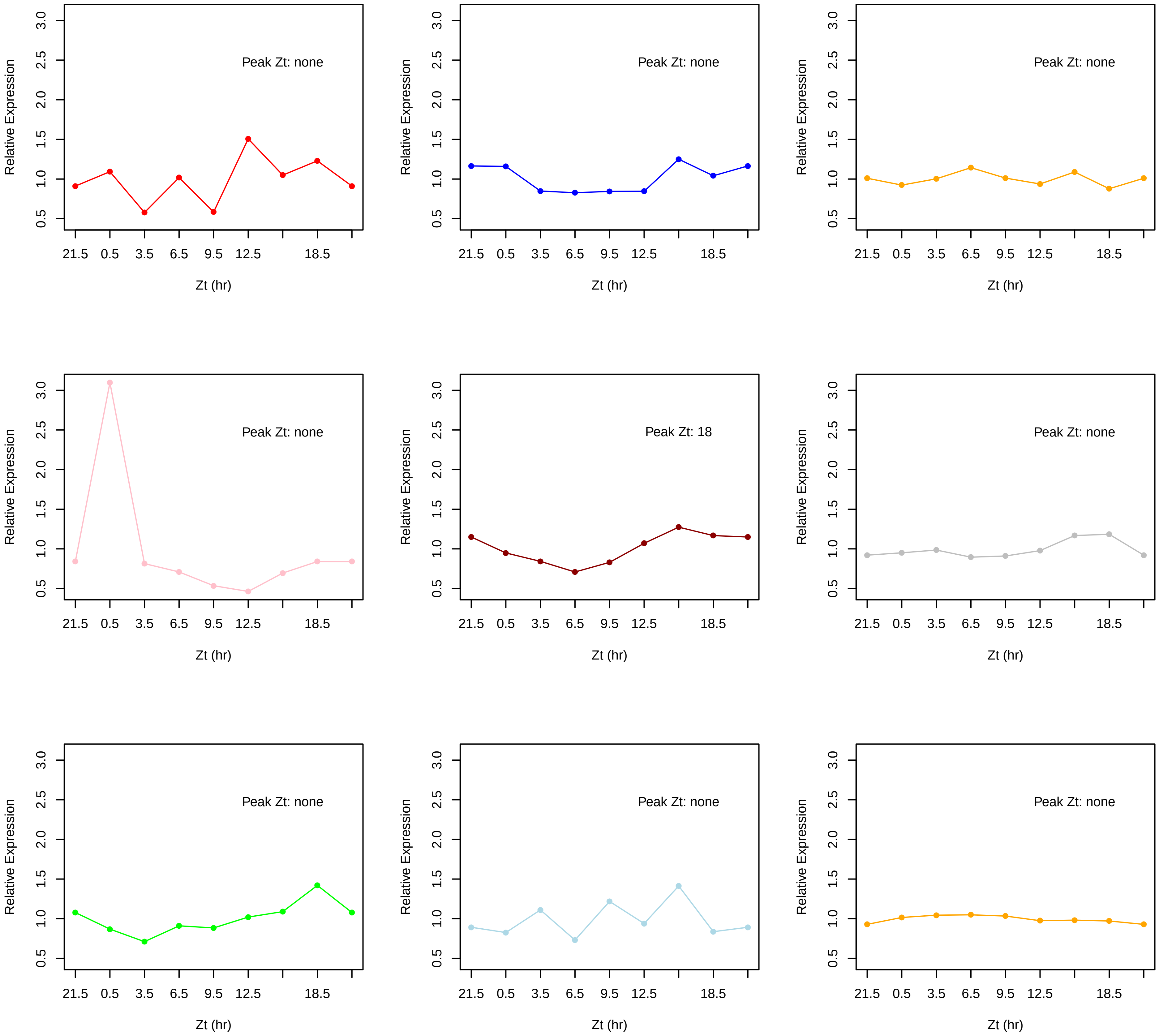

Singleton: ENSXMAG00000006473 si:ch211−214c7.5

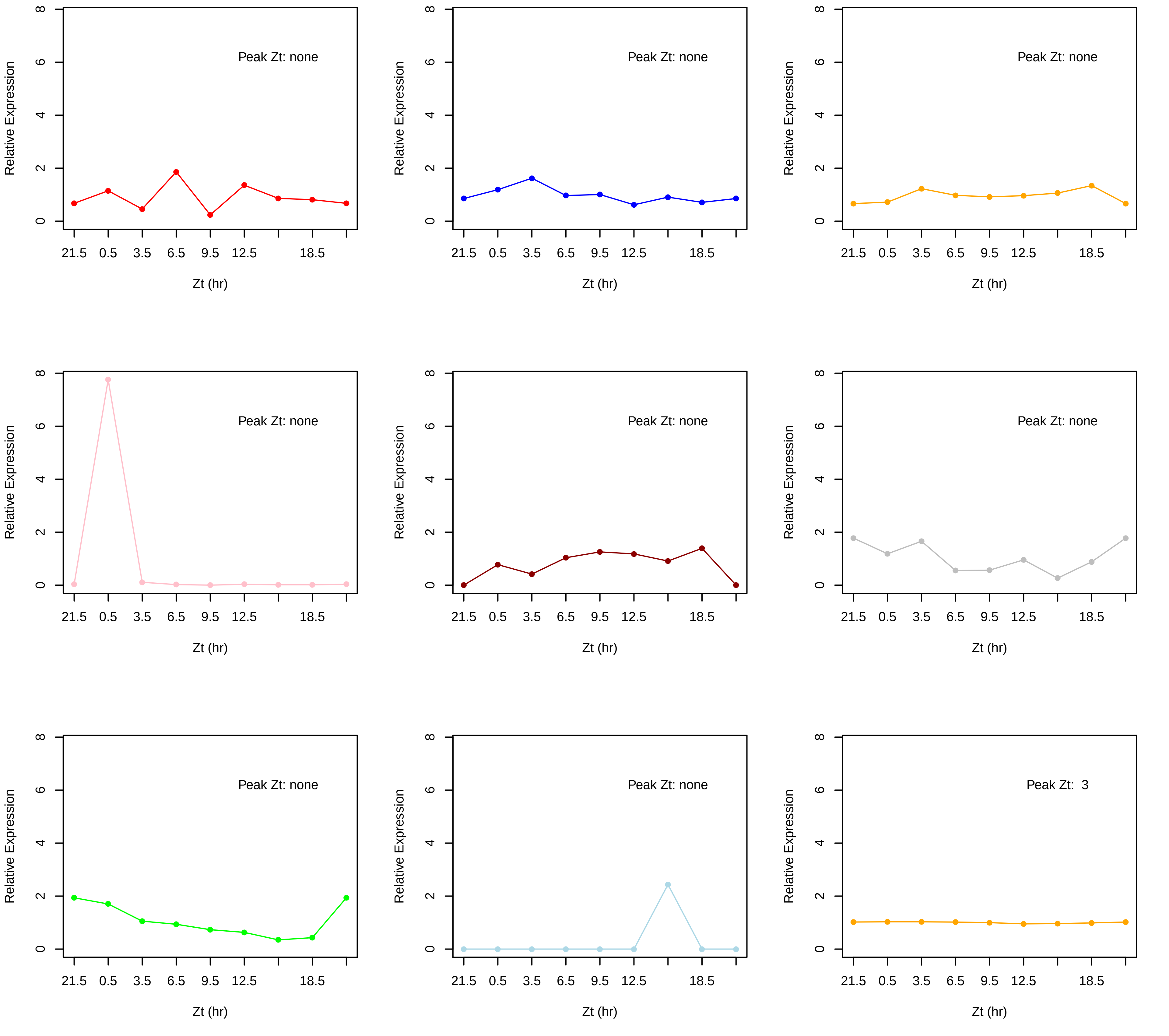

Singleton: ENSXMAG00000002074 MYL6

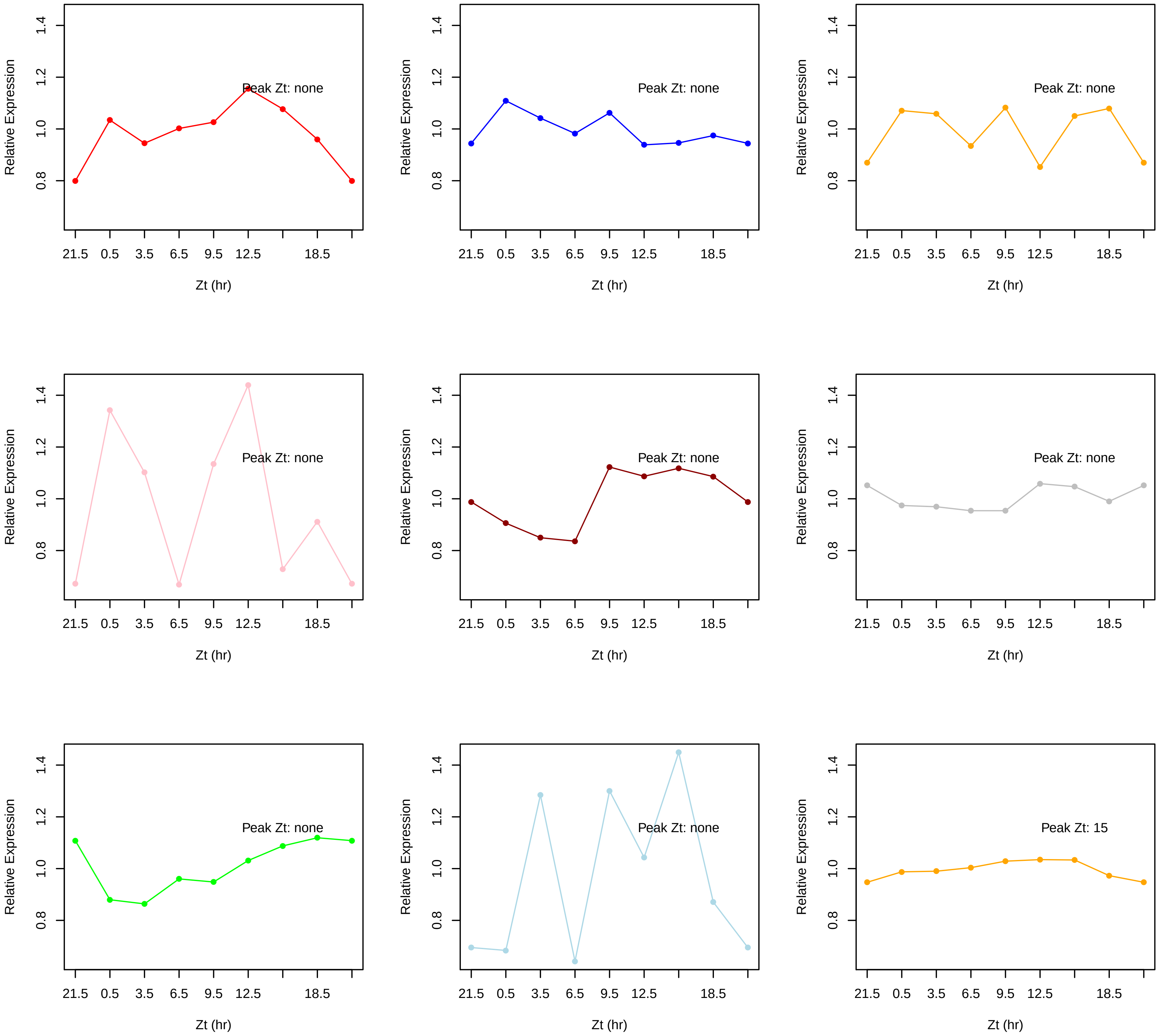

Singeleton: ENSXMAG00000011389 atg4c

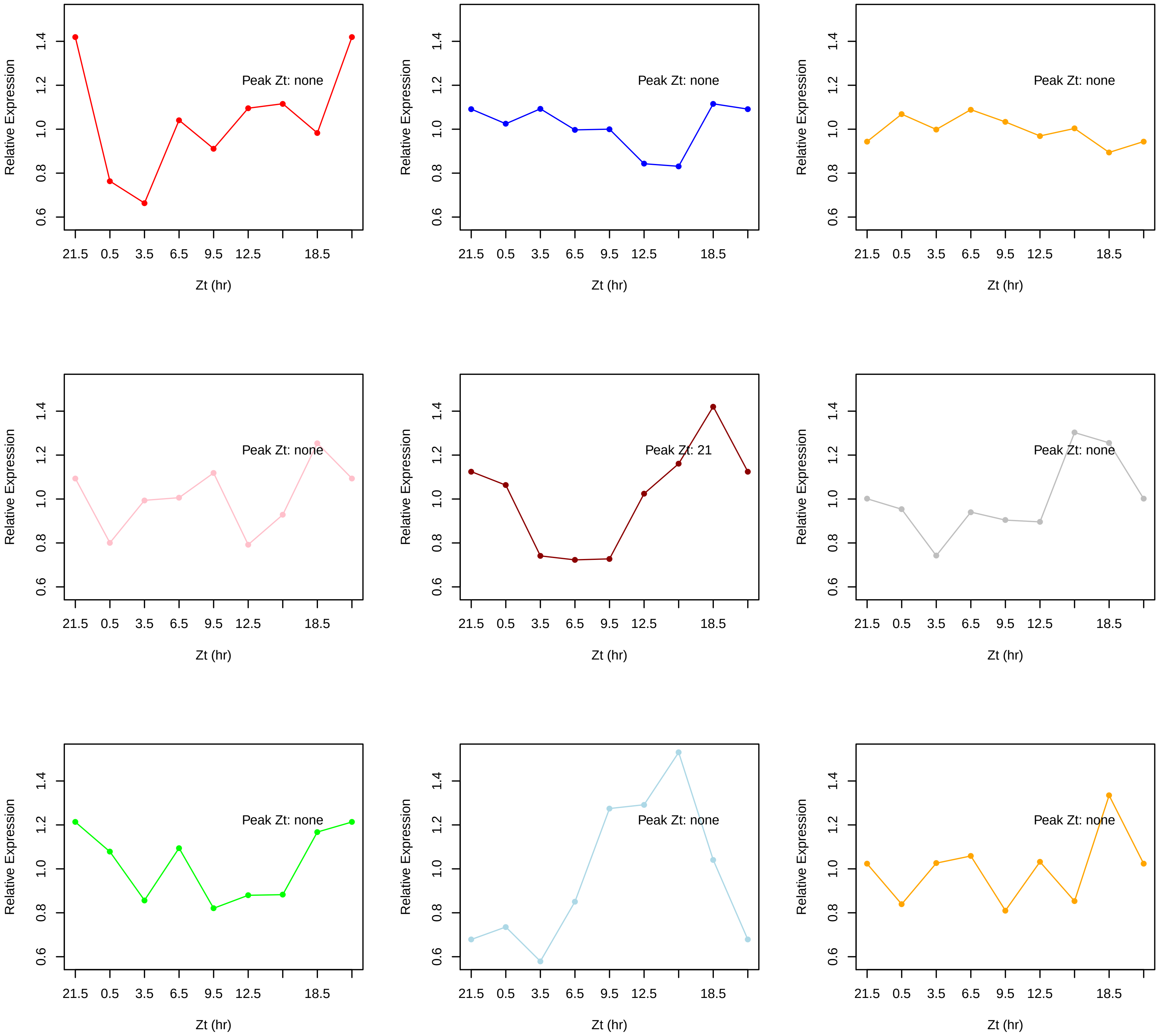

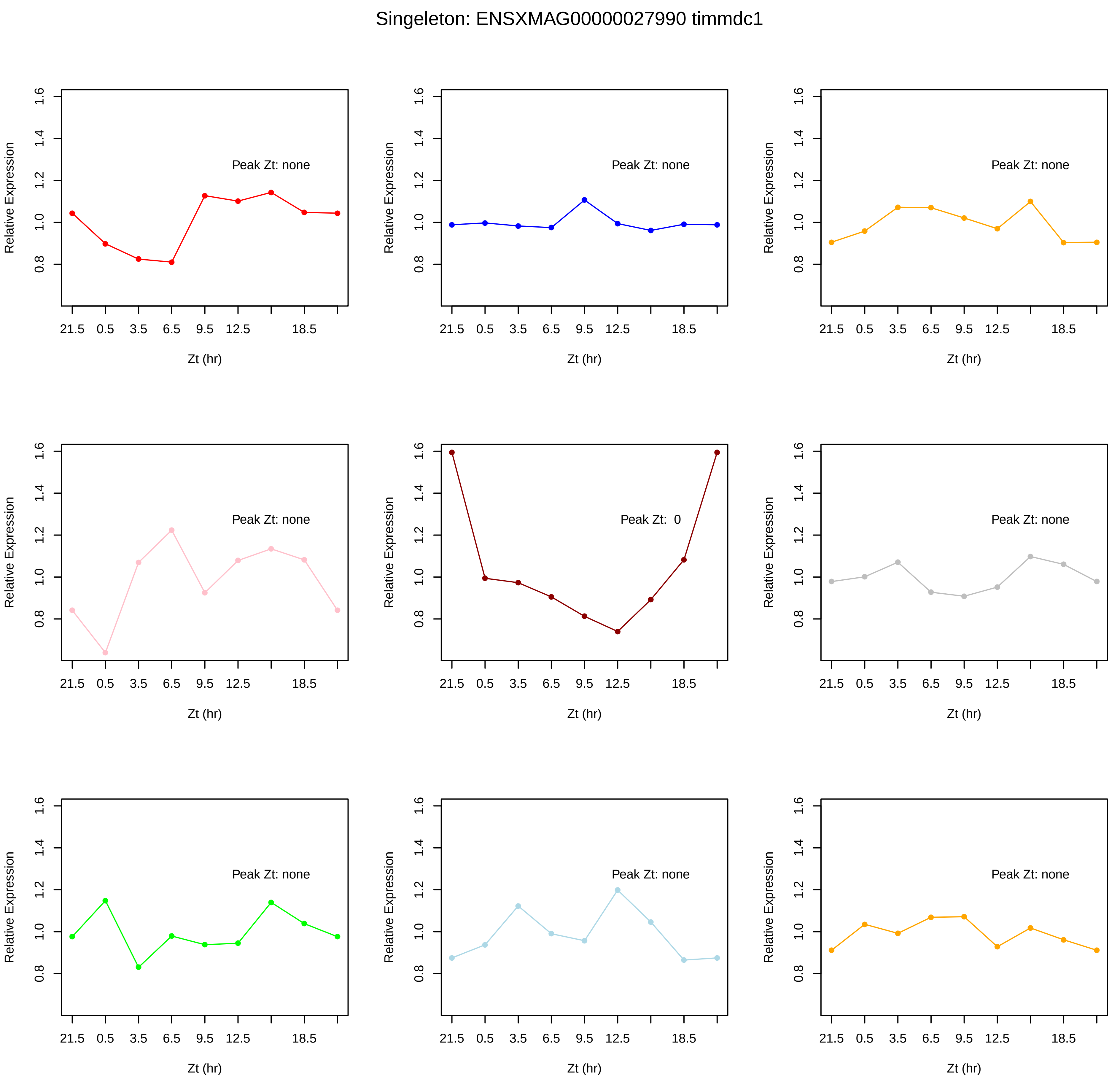
Singeleton: ENSXMAG00000027990 timmdc1
Relative Expression
Zt (hr)
Peak Zt: none
Peak Zt: 0
21.5
0.5
3.5
6.5
9.5
12.5
18.5
0.8
1.0
1.2
1.4
1.6

Singeleton: ENSXMAG00000003795 ccdc6a

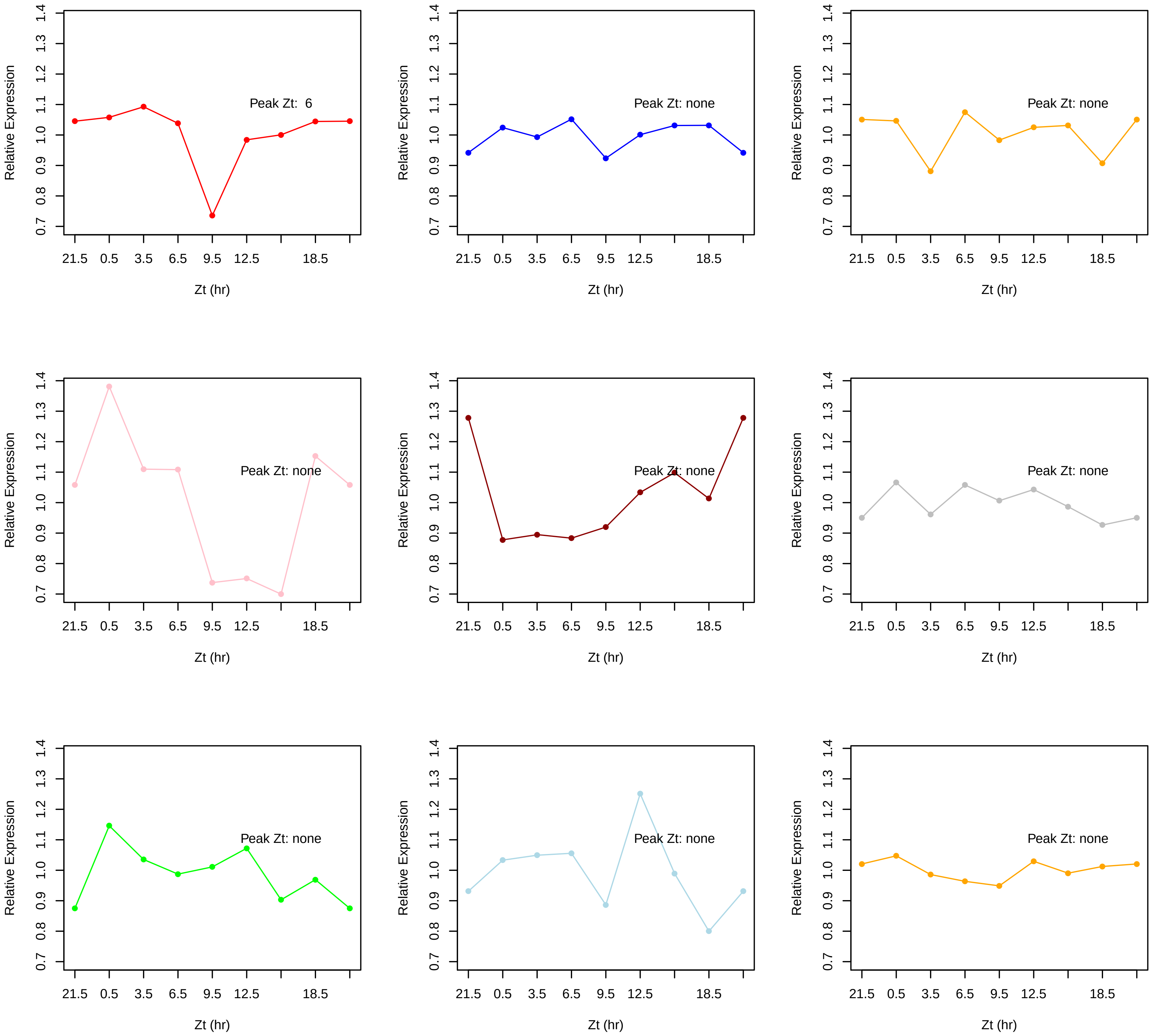

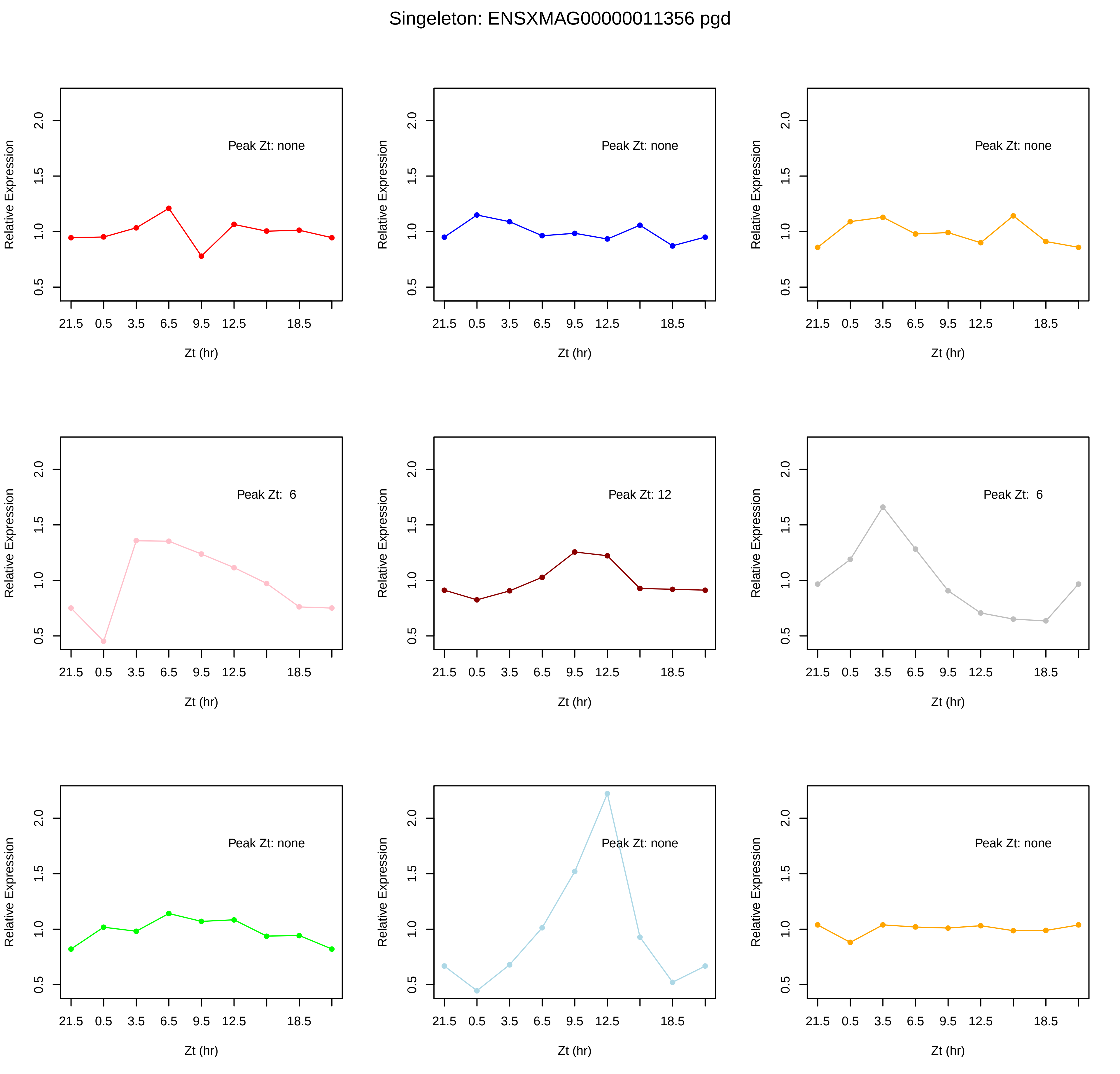
Singeleton: ENSXMAG00000011356 pgd
Peak Zt: none
Peak Zt: none
Peak Zt: none
Peak Zt: 6
Peak Zt: 12
Peak Zt: 6
Peak Zt: none
Peak Zt: none
Peak Zt: none
Relative Expression
Zt (hr)
21.5
0.5
3.5
6.5
9.5
12.5
18.5
0.5
1.0
1.5
2.0

Singeleton: ENSXMAG00000002171 alg6

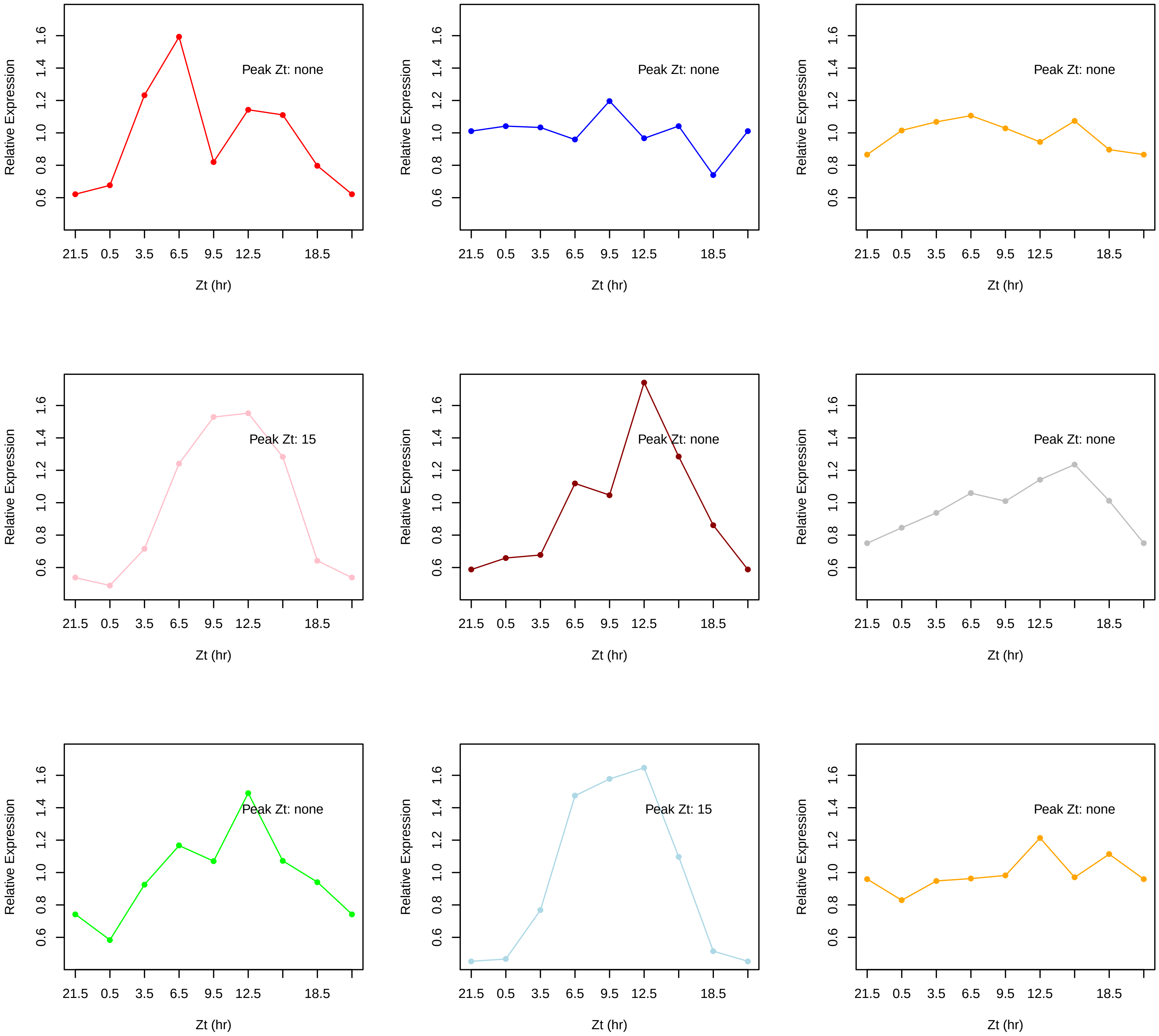

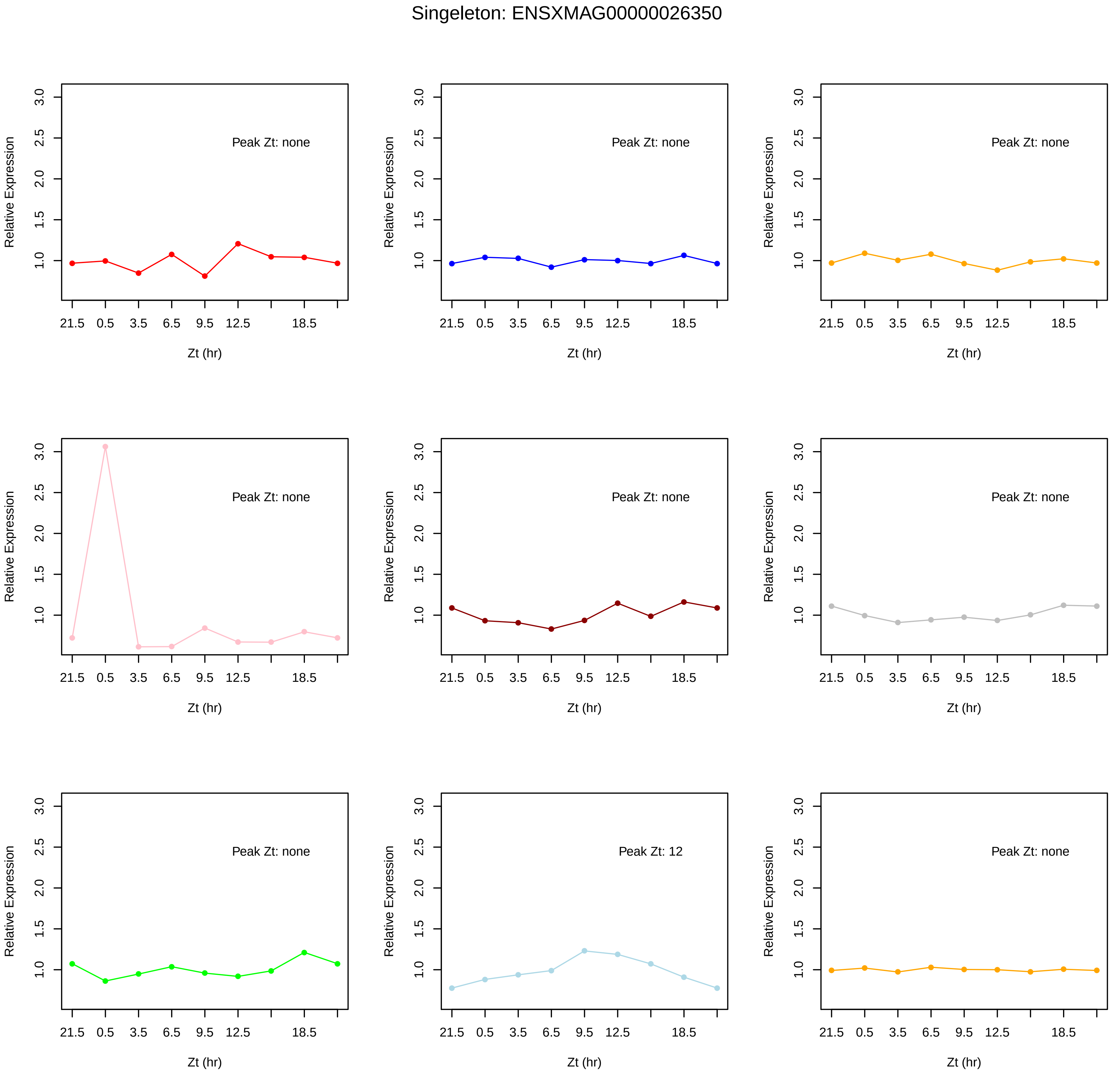

Singeleton: ENSXMAG00000026350
Peak Zt: none
Peak Zt: none
Peak Zt: none
Peak Zt: none
Peak Zt: none
Peak Zt: none
Peak Zt: none
Peak Zt: 12
Peak Zt: none
Relative Expression
Zt (hr)
21.5 0.5 3.5 6.5 9.5 12.5 18.5
1.0 1.5 2.0 2.5 3.0

Singleton: ENSXMAG00000003247

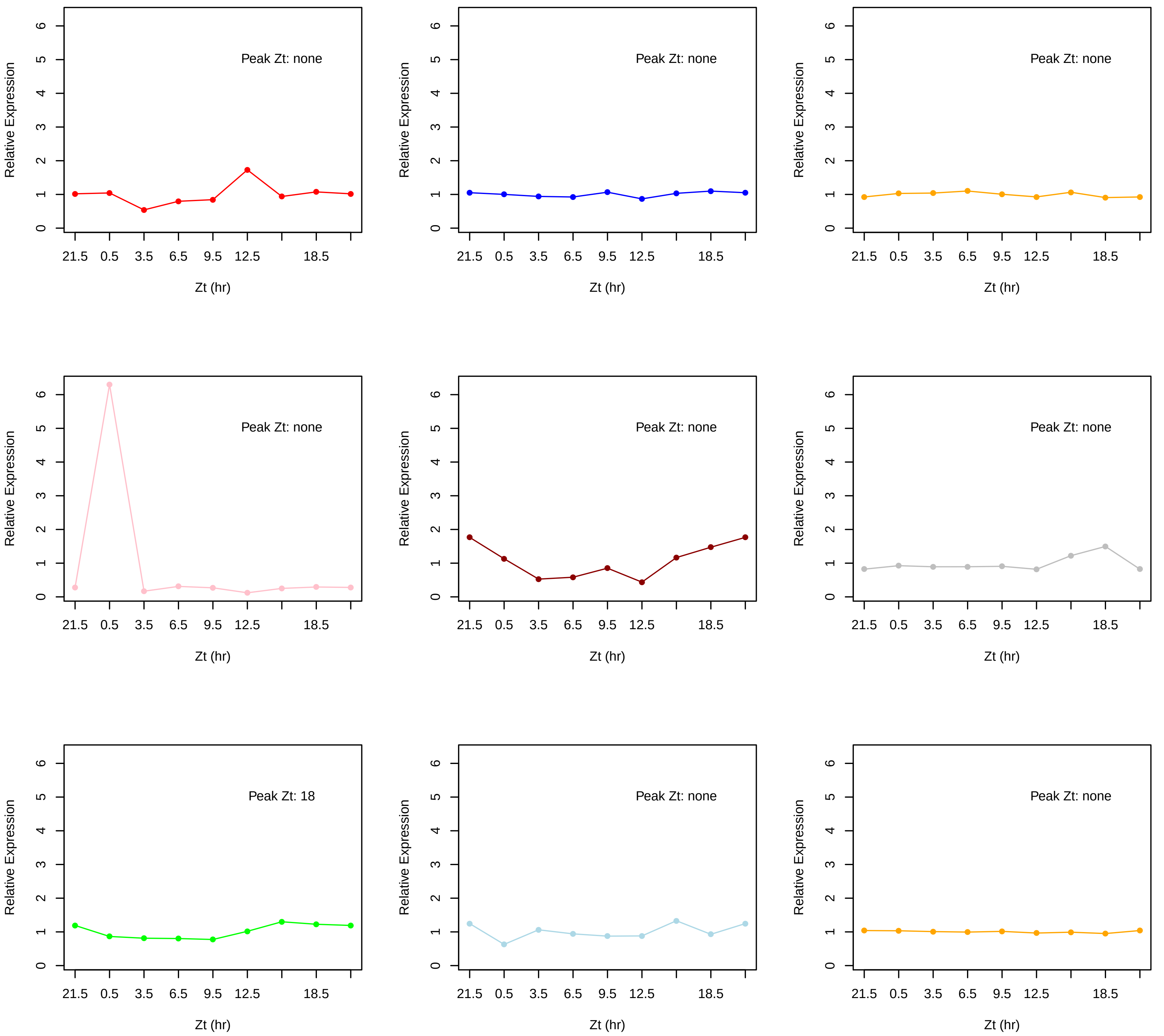

Singleton: ENSXMAG00000019083 PGM1

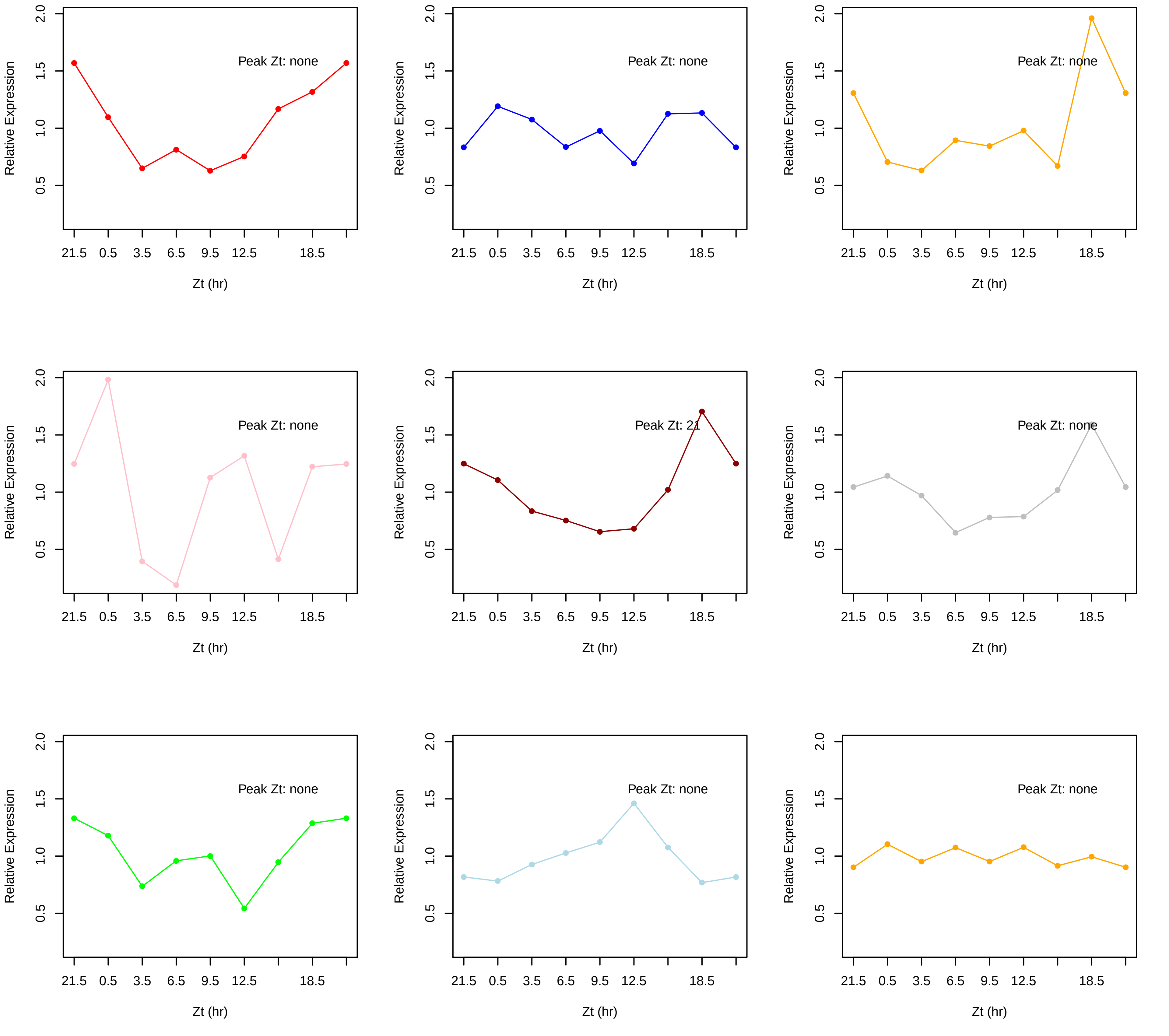

Singleton: ENSXMAG00000011842 ubxn6

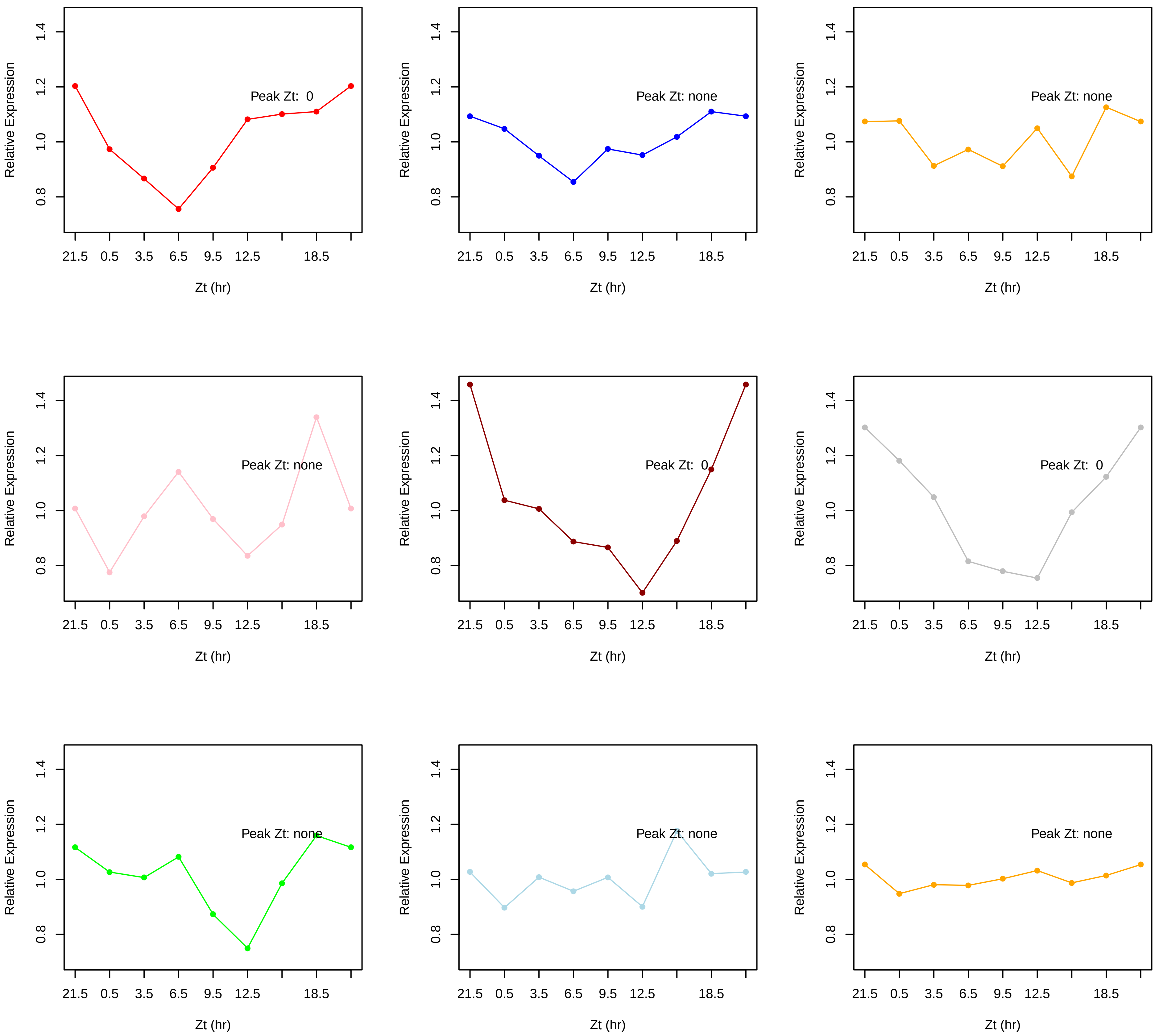

Singeleton: ENSXMAG00000012109 slc6a4a

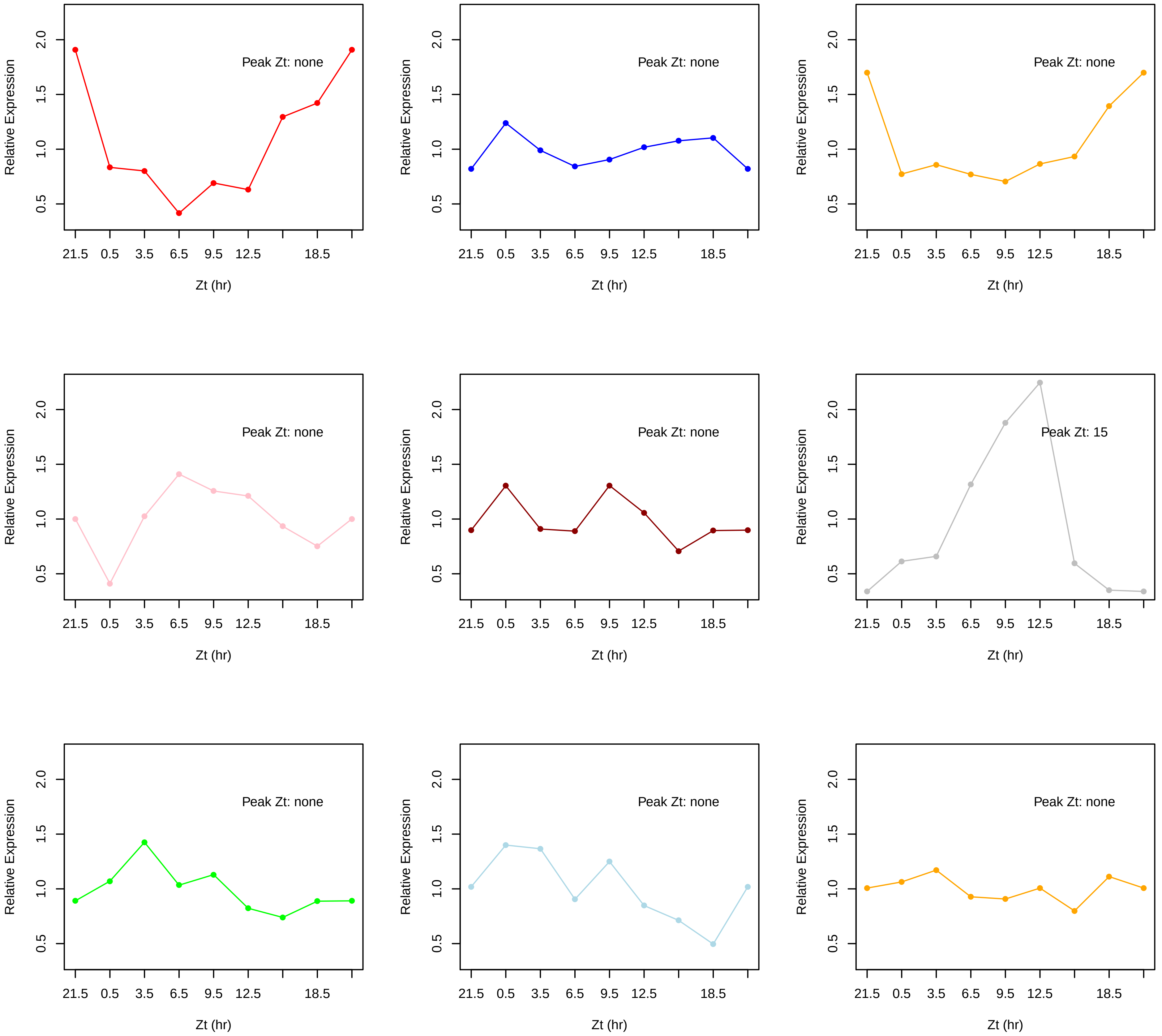

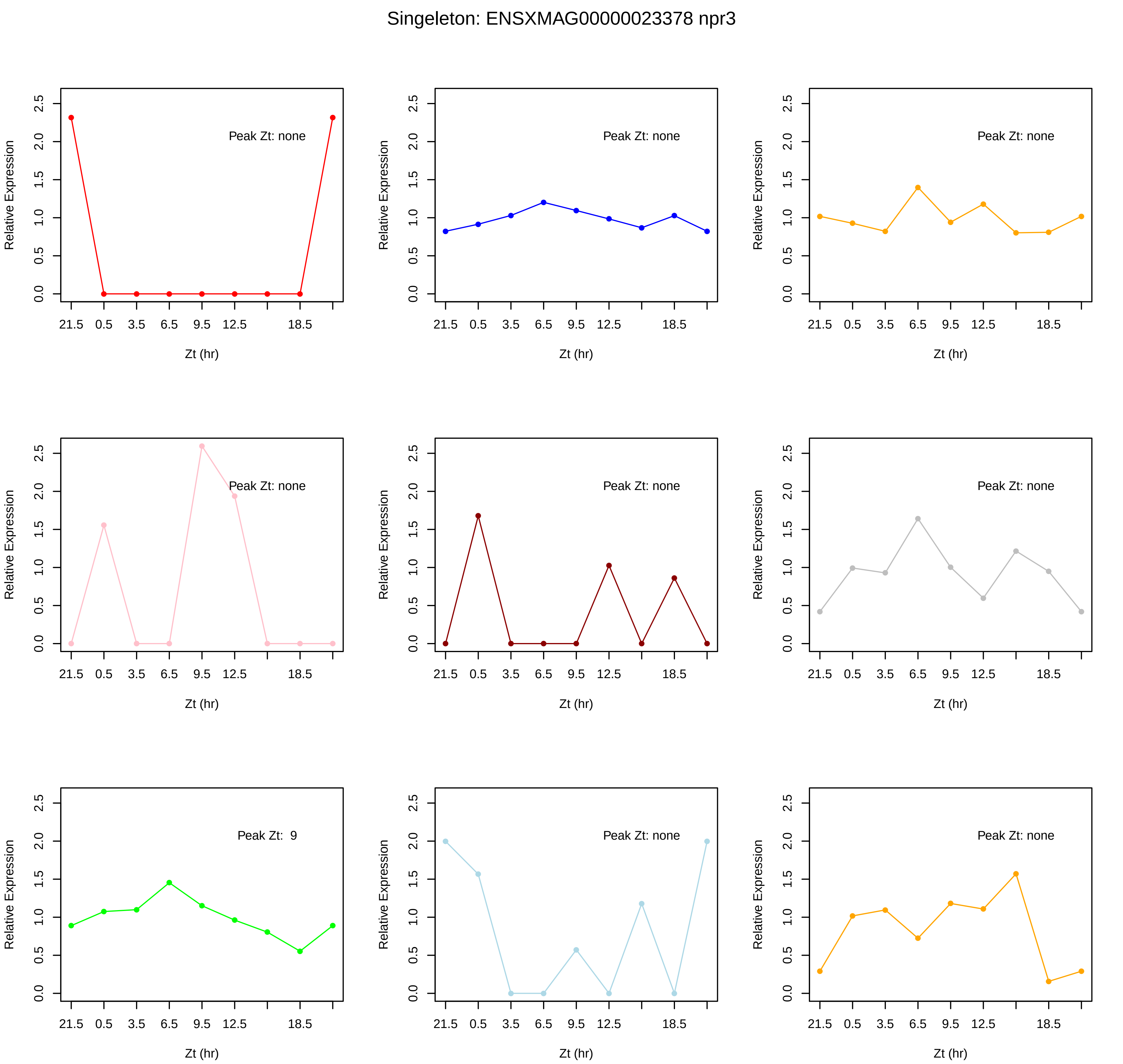

Singeleton: ENSXMAG00000023378 npr3
Peak Zt: none
Peak Zt: none
Peak Zt: none
Peak Zt: none
Peak Zt: none
Peak Zt: none
Peak Zt: 9
Peak Zt: none
Peak Zt: none
Relative Expression
Zt (hr)
21.5
0.5
3.5
6.5
9.5
12.5
18.5
0.0
0.5
1.0
1.5
2.0
2.5

Singeleton: ENSXMAG00000003505 hdgfl2

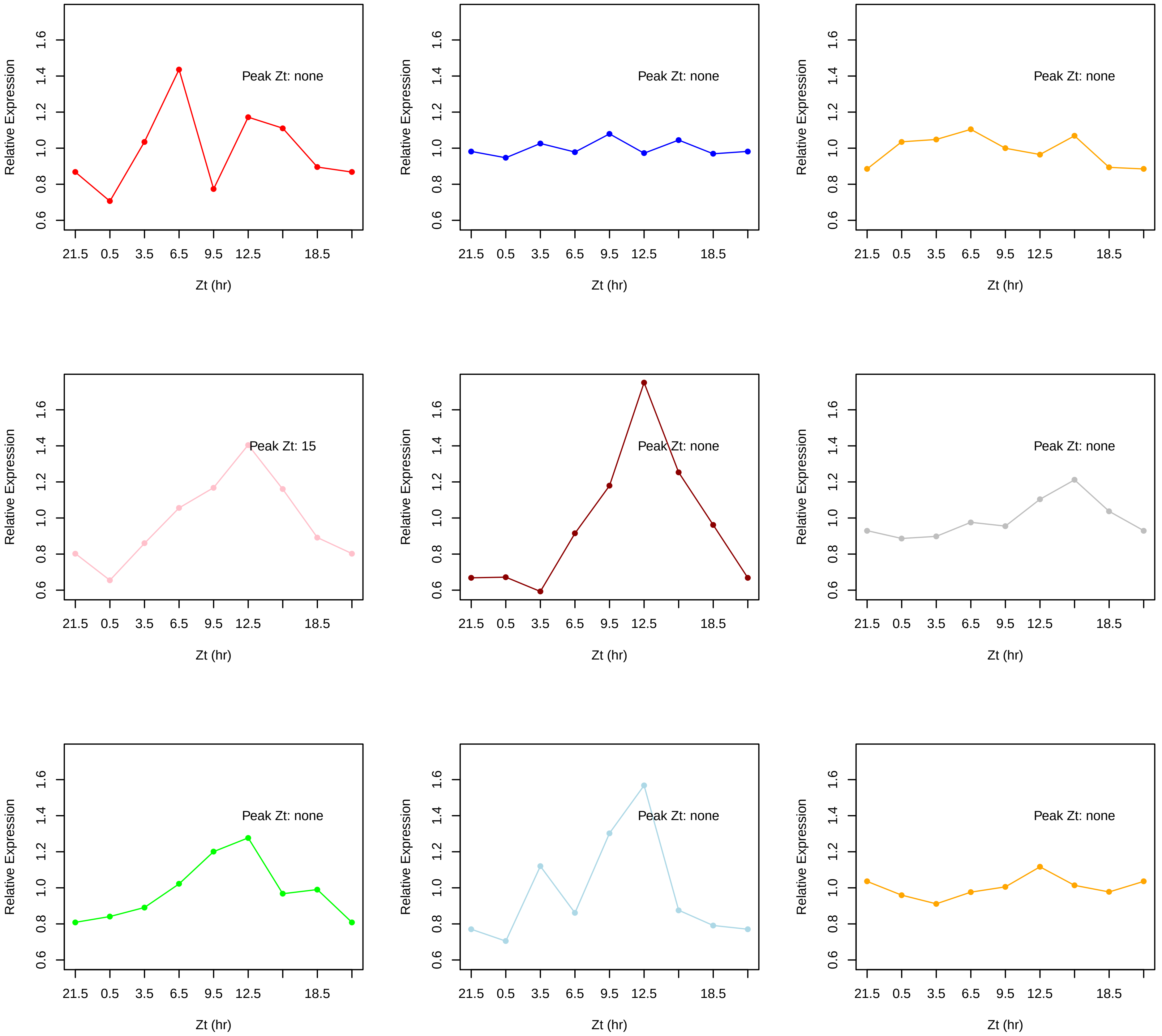

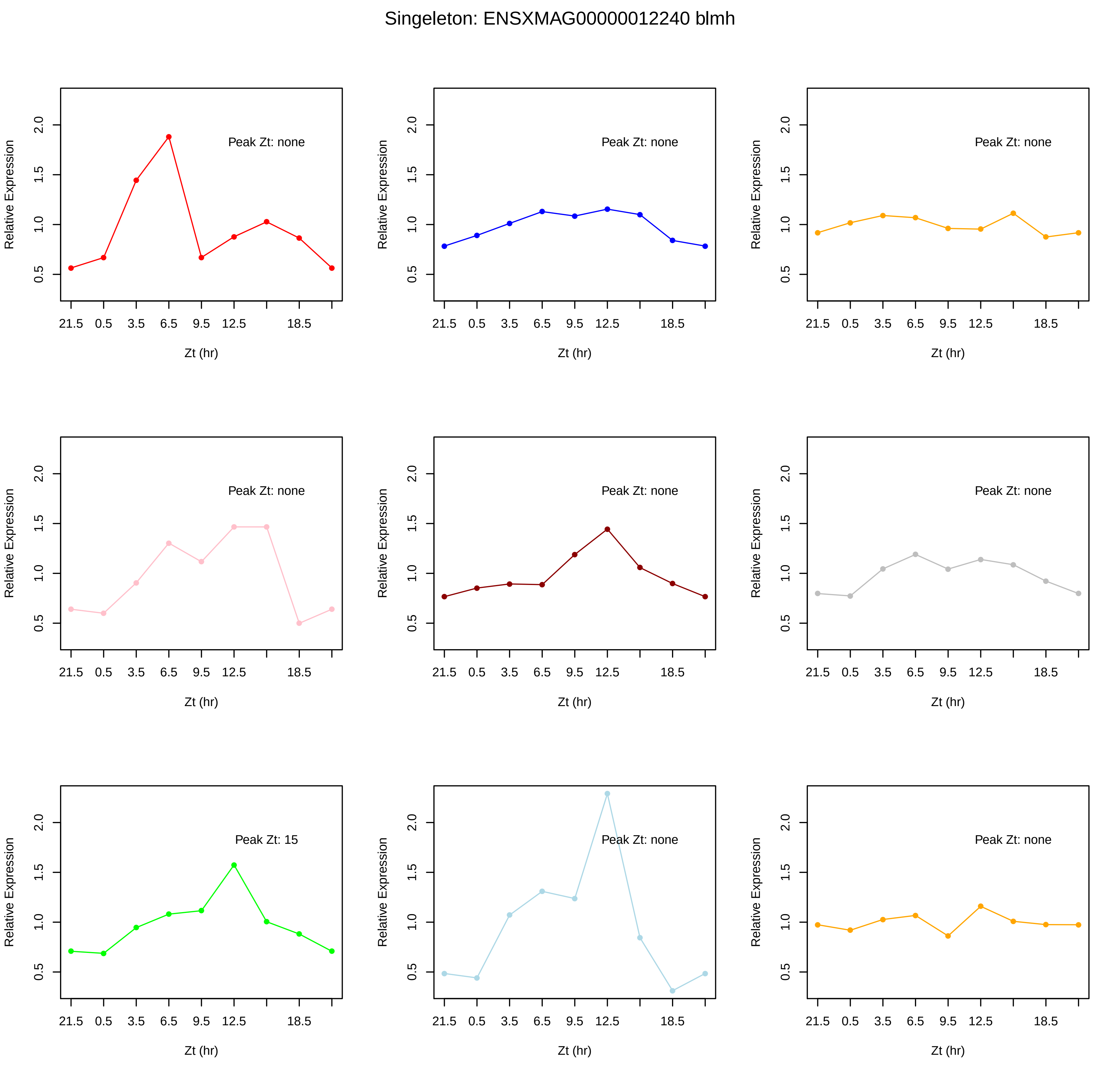
Singeleton: ENSXMAG00000012240 blmh
Relative Expression
Zt (hr)
21.5
0.5
3.5
6.5
9.5
12.5
18.5
0.5
1.0
1.5
2.0
Peak Zt: none
Peak Zt: 15

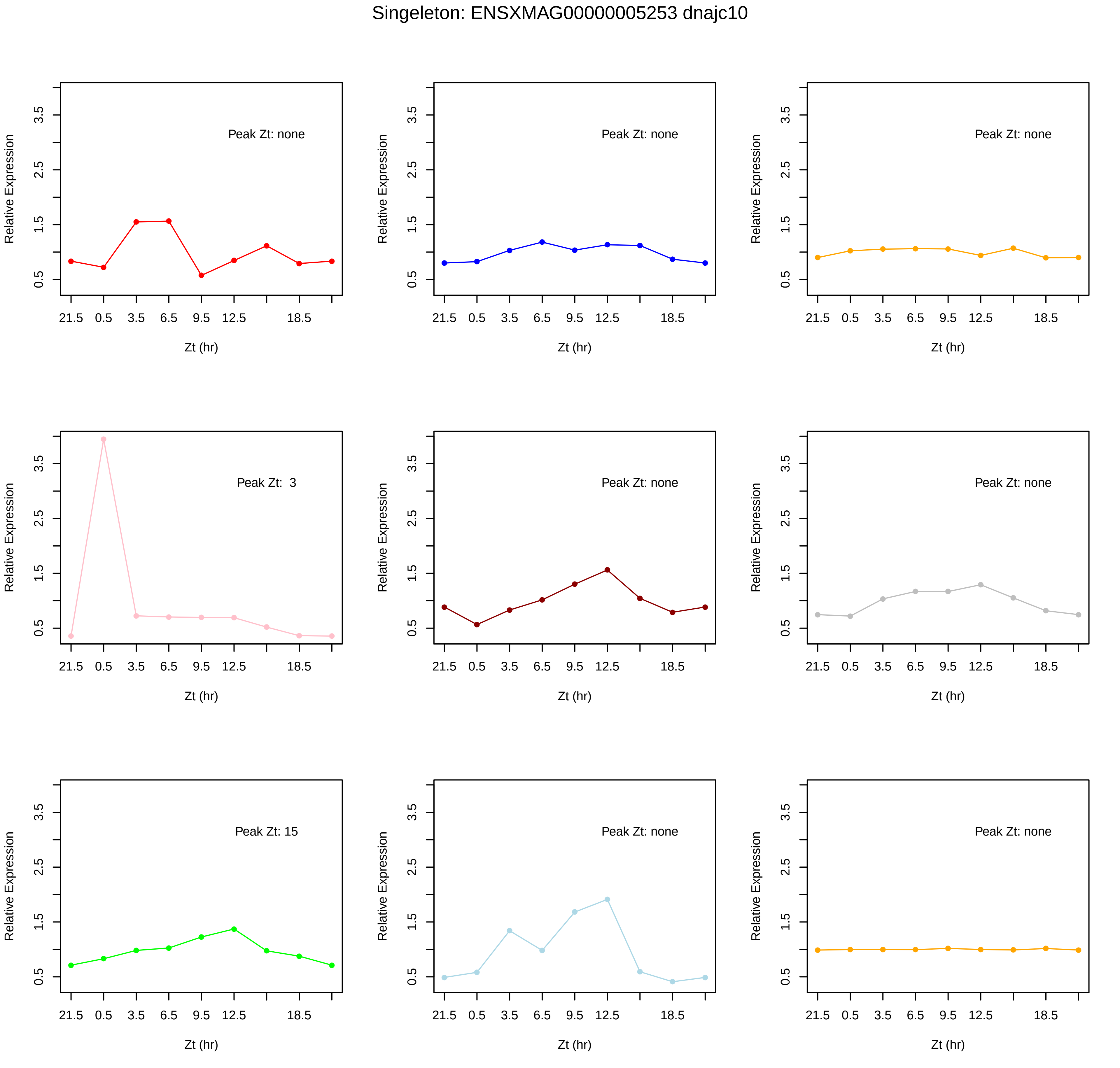
Singeleton: ENSXMAG00000005253 dnajc10
Peak Zt: none
Peak Zt: none
Peak Zt: none
Peak Zt: 3
Peak Zt: none
Peak Zt: none
Peak Zt: 15
Peak Zt: none
Peak Zt: none
Relative Expression
Zt (hr)
21.5
0.5
3.5
6.5
9.5
12.5
18.5
0.5
1.5
2.5
3.5

Singleton: ENSXMAG00000024794 acvr1l

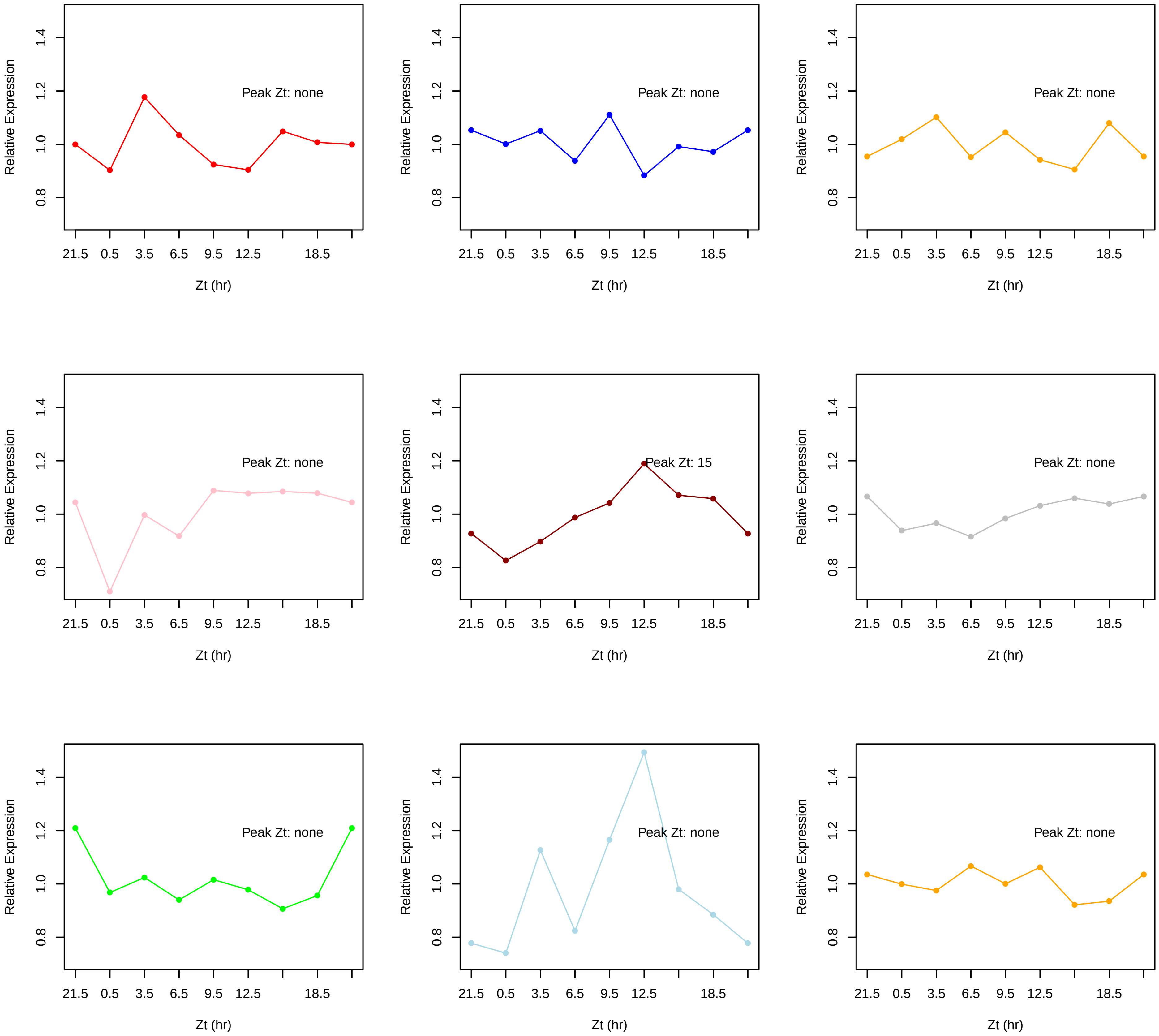

Singeleton: ENSXMAG00000005735 edc4

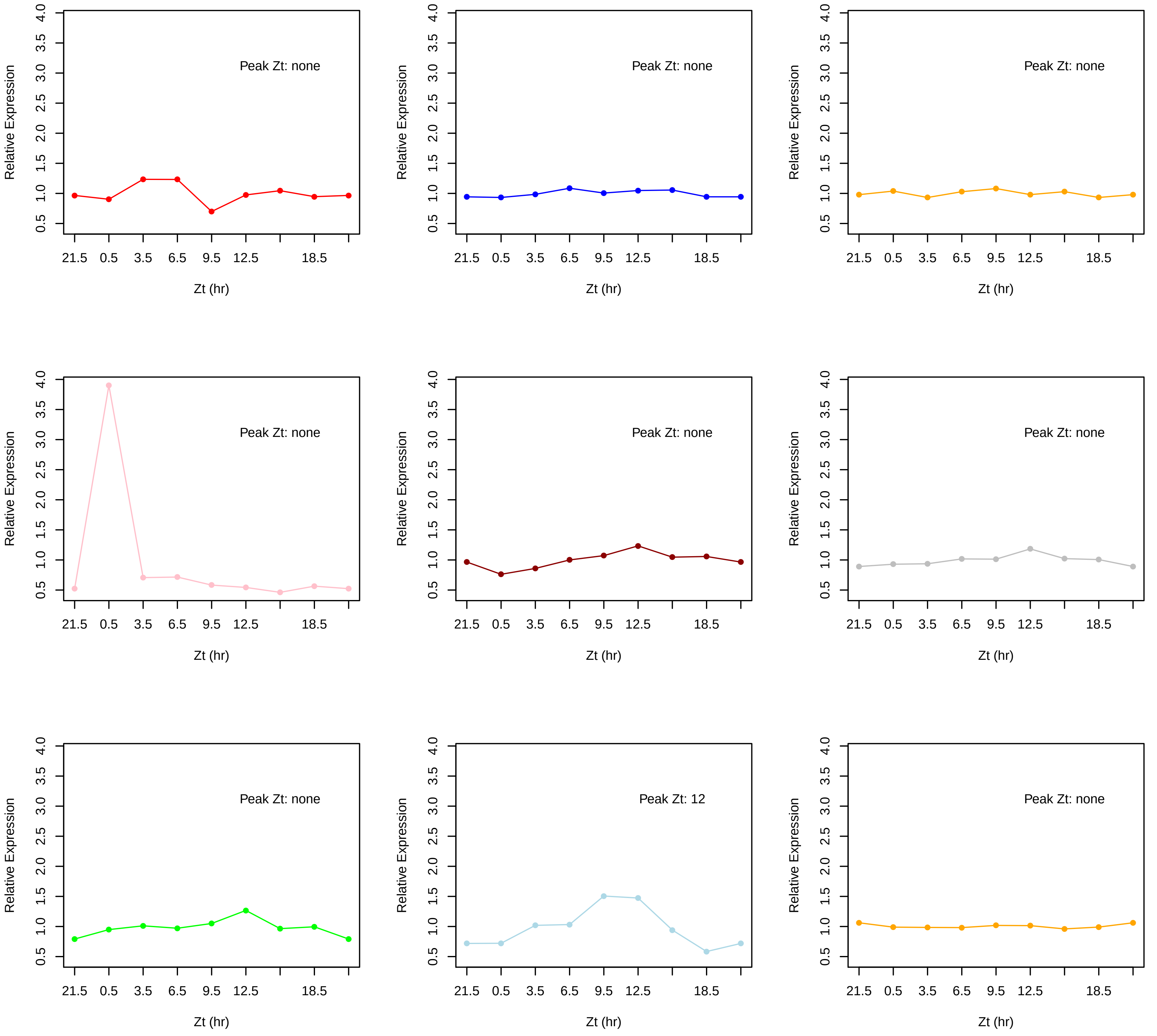

Singeleton: ENSXMAG00000015500 slc2a11a

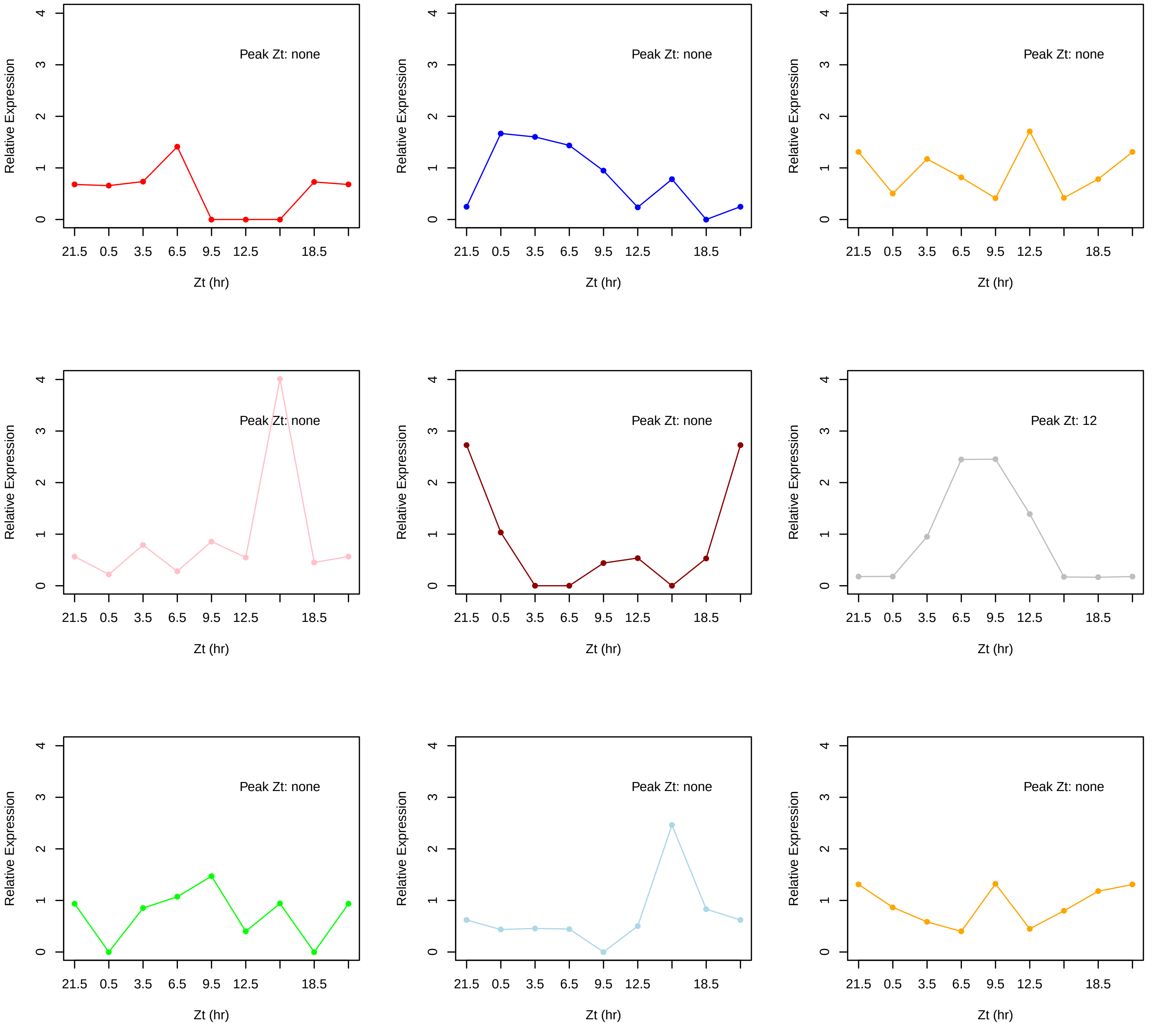

Singeleton: ENSXMAG00000004471 dcun1d5

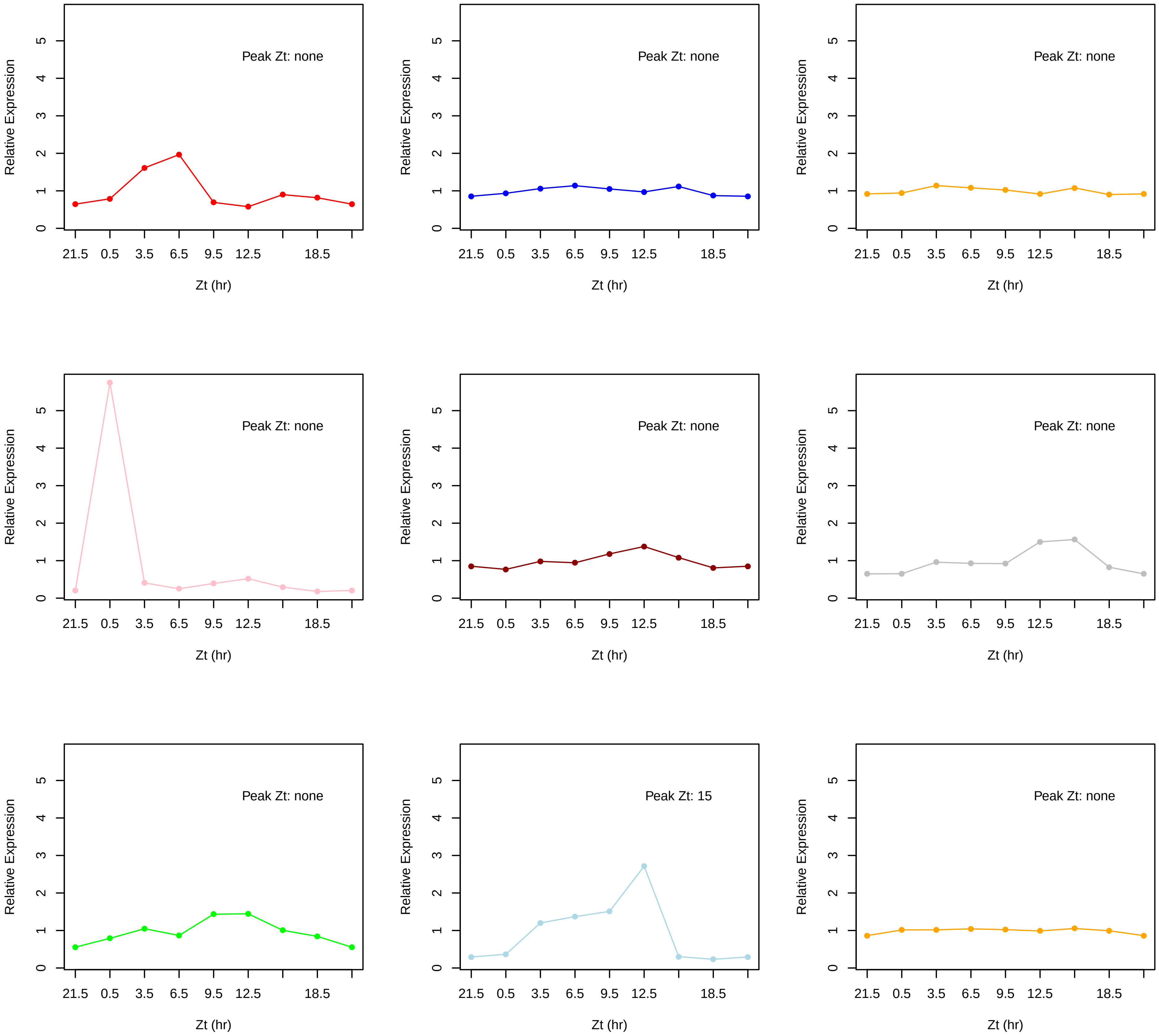

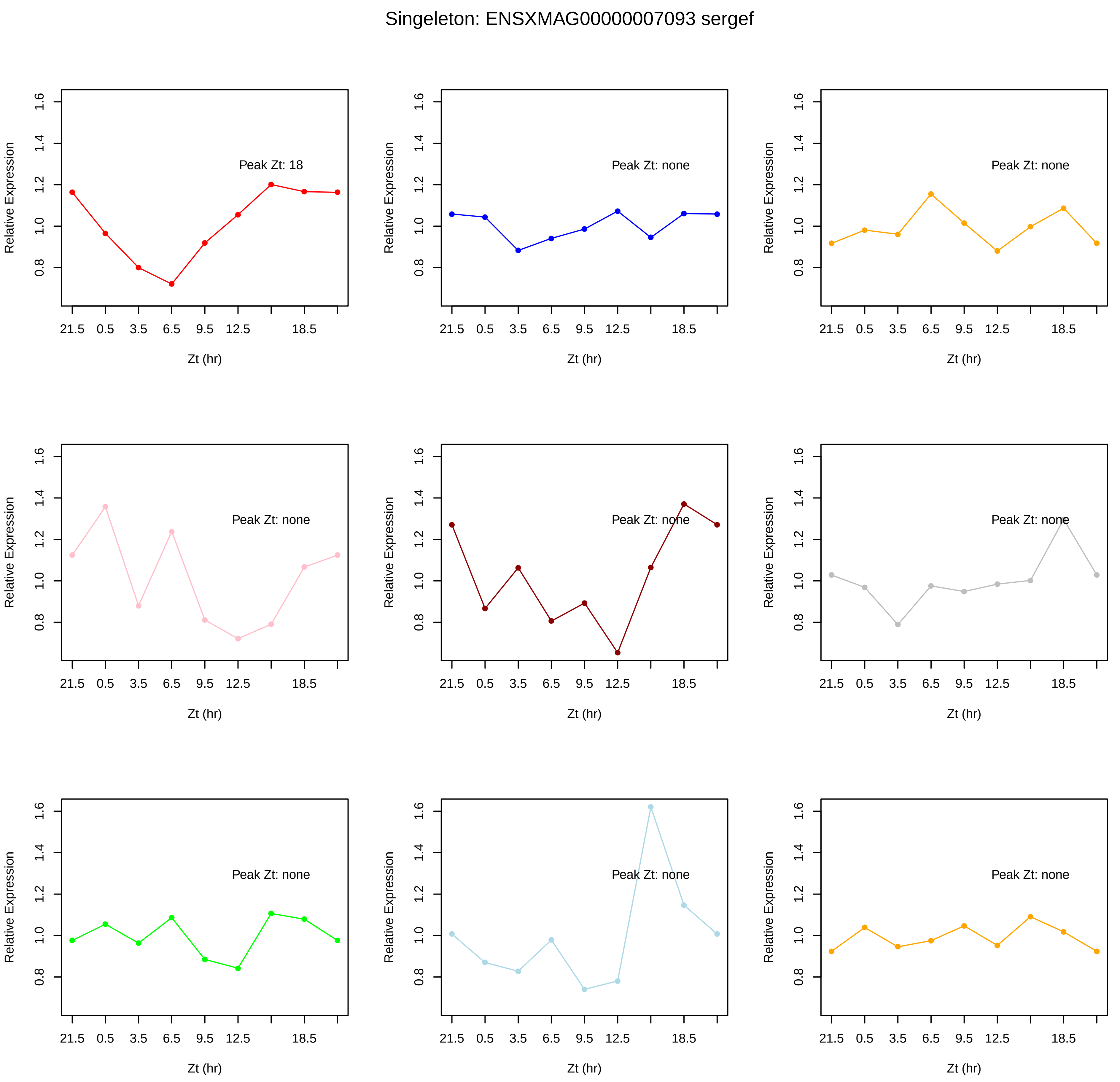
Singeleton: ENSXMAG00000007093 sergef
Peak Zt: 18
Peak Zt: none
Peak Zt: none
Peak Zt: none
Peak Zt: none
Peak Zt: none
Peak Zt: none
Peak Zt: none
Peak Zt: none
Relative Expression
Zt (hr)
21.5 0.5 3.5 6.5 9.5 12.5 18.5
0.8 1.0 1.2 1.4 1.6

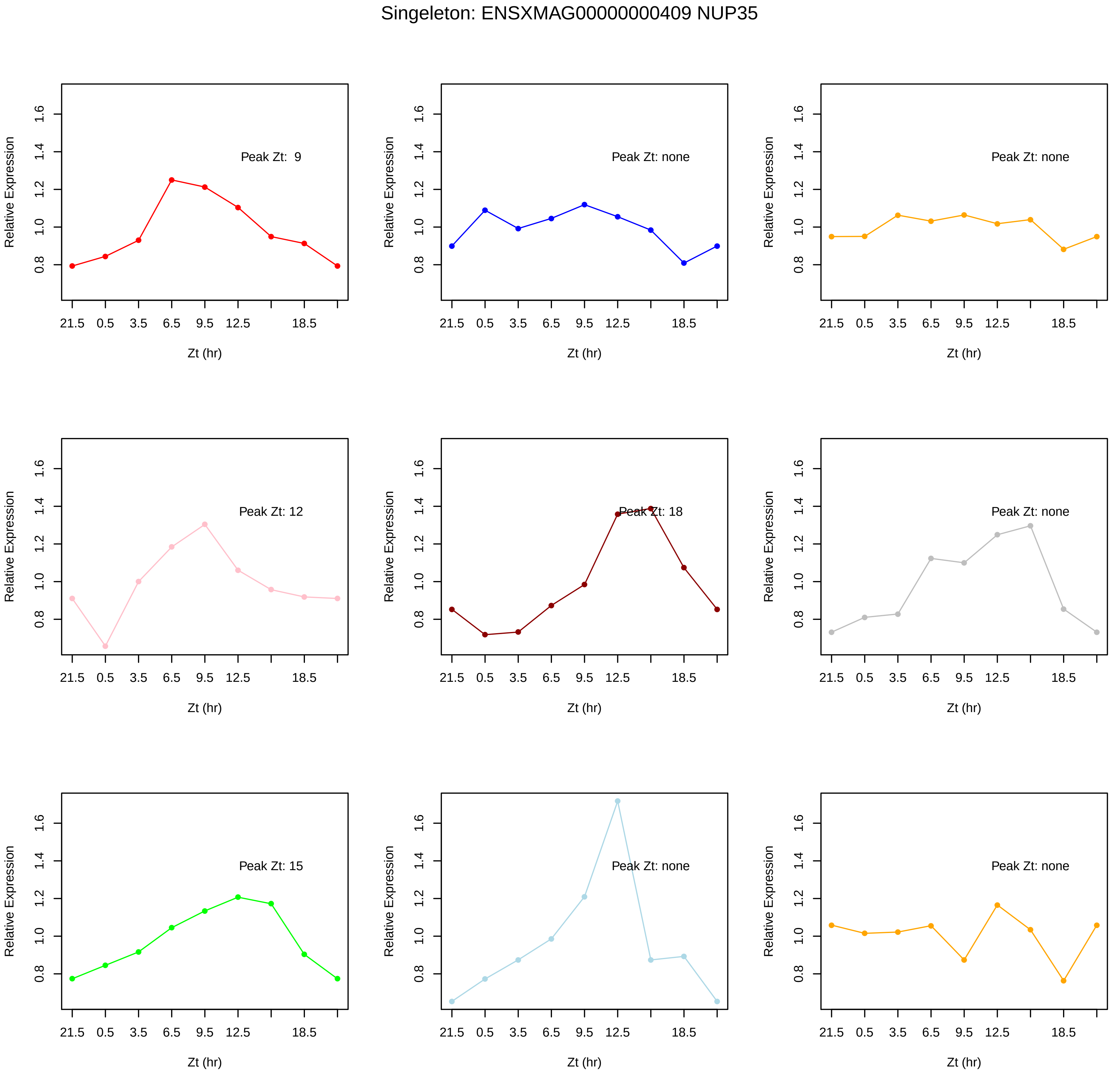
Singeleton: ENSXMAG00000000409 NUP35
Peak Zt: 9
Peak Zt: none
Peak Zt: none
Peak Zt: 12
Peak Zt: 18
Peak Zt: none
Peak Zt: 15
Peak Zt: none
Peak Zt: none
Relative Expression
Zt (hr)
21.5
0.5
3.5
6.5
9.5
12.5
18.5
0.8
1.0
1.2
1.4
1.6

Singeleton: ENSXMAG00000013356 zfr

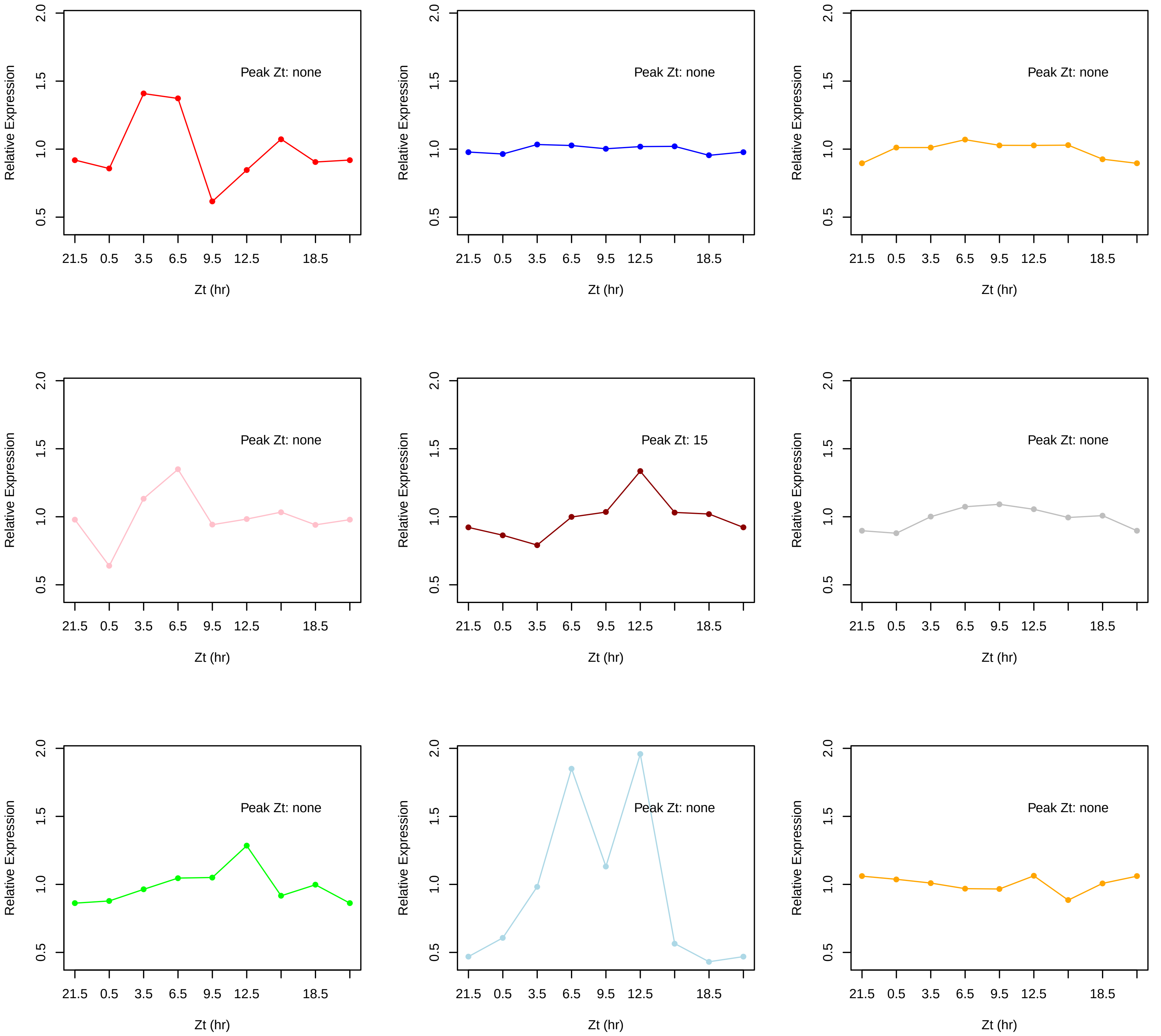

Singleton: ENSXMAG00000027307 cryzl1

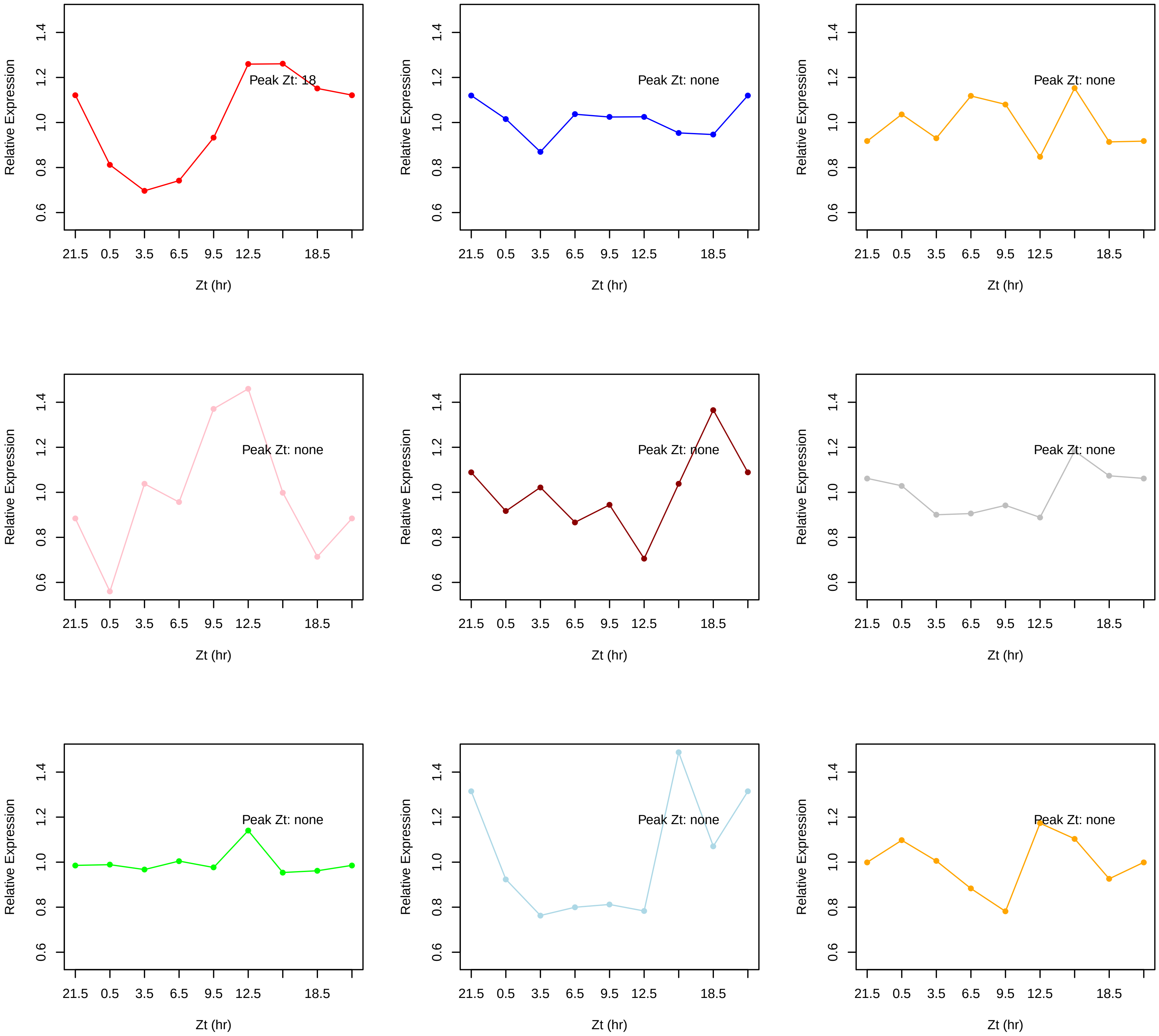

Singeleton: ENSXMAG00000029874 tmem26a

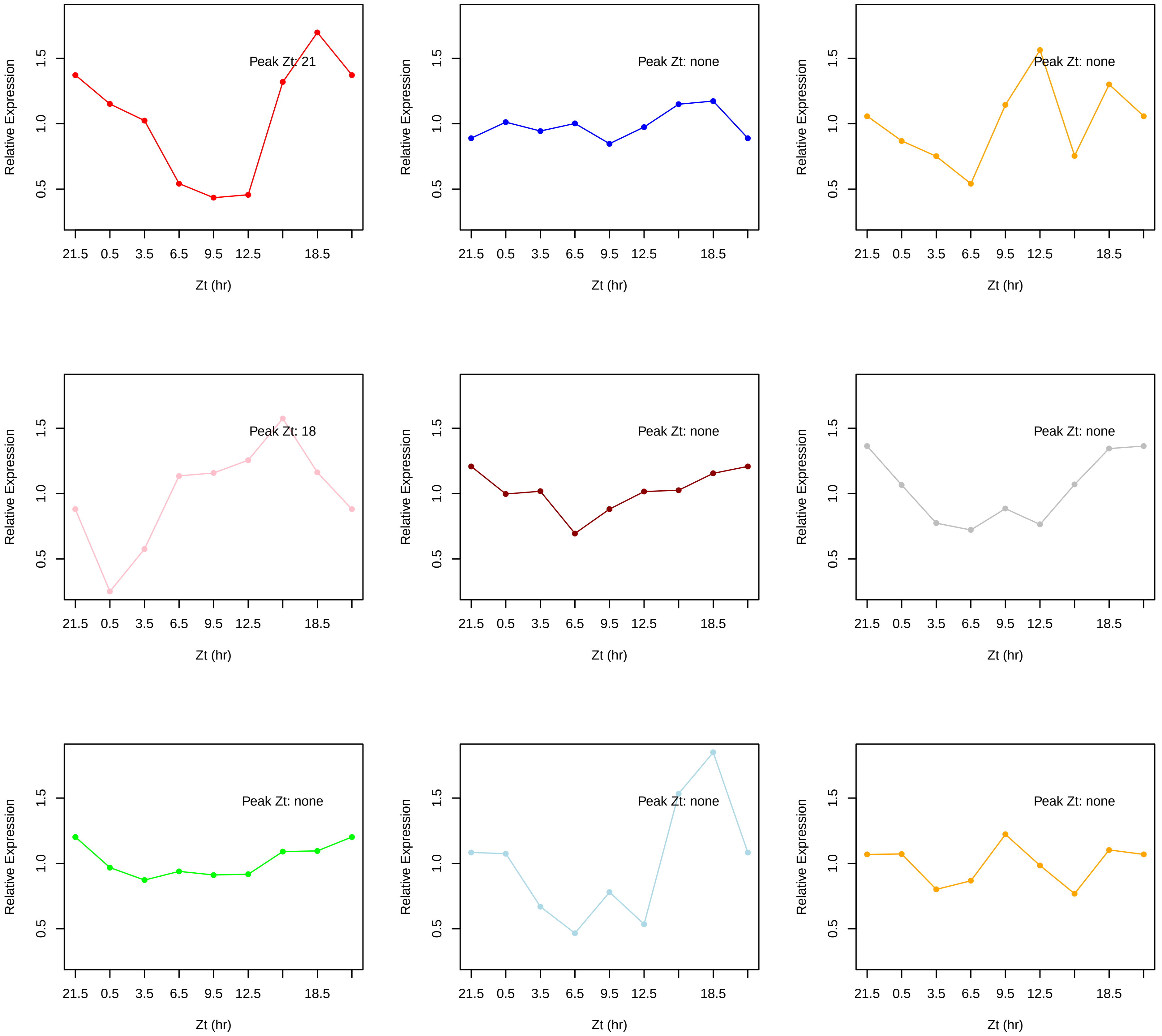

Singeleton: ENSXMAG00000005770 nutf2

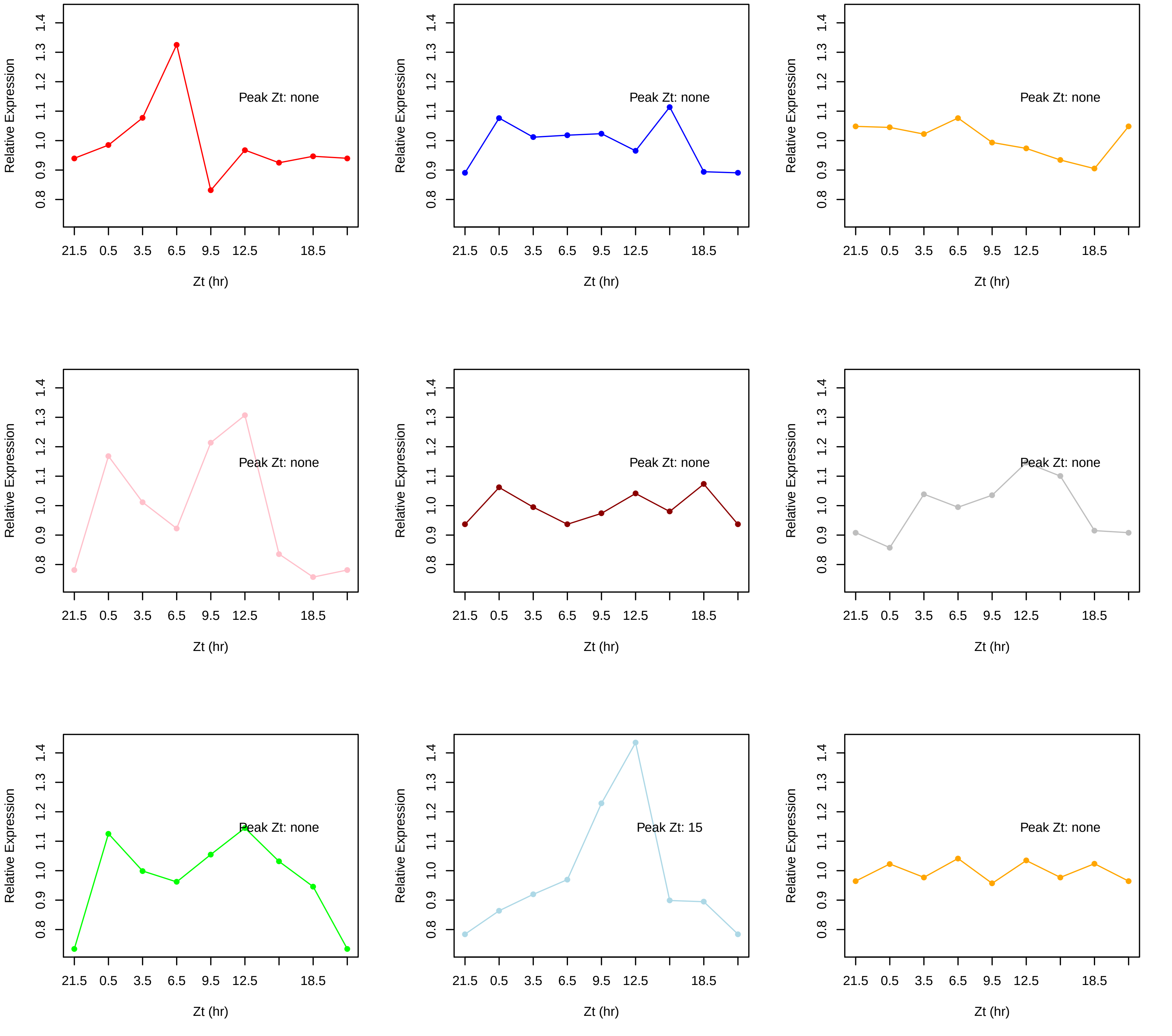

Singeleton: ENSXMAG00000025409 slc25a1a

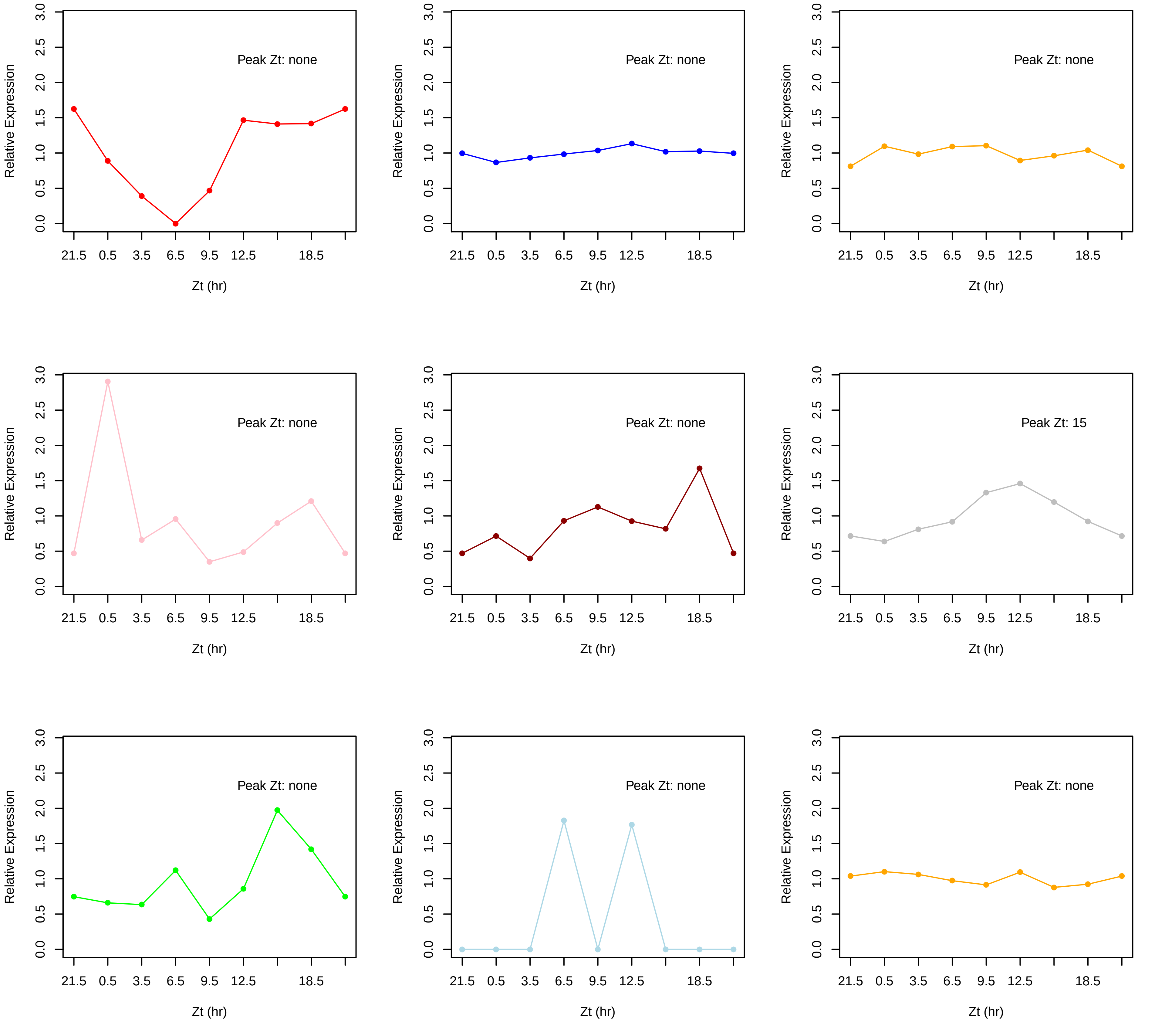

Singeleton: ENSXMAG00000025517

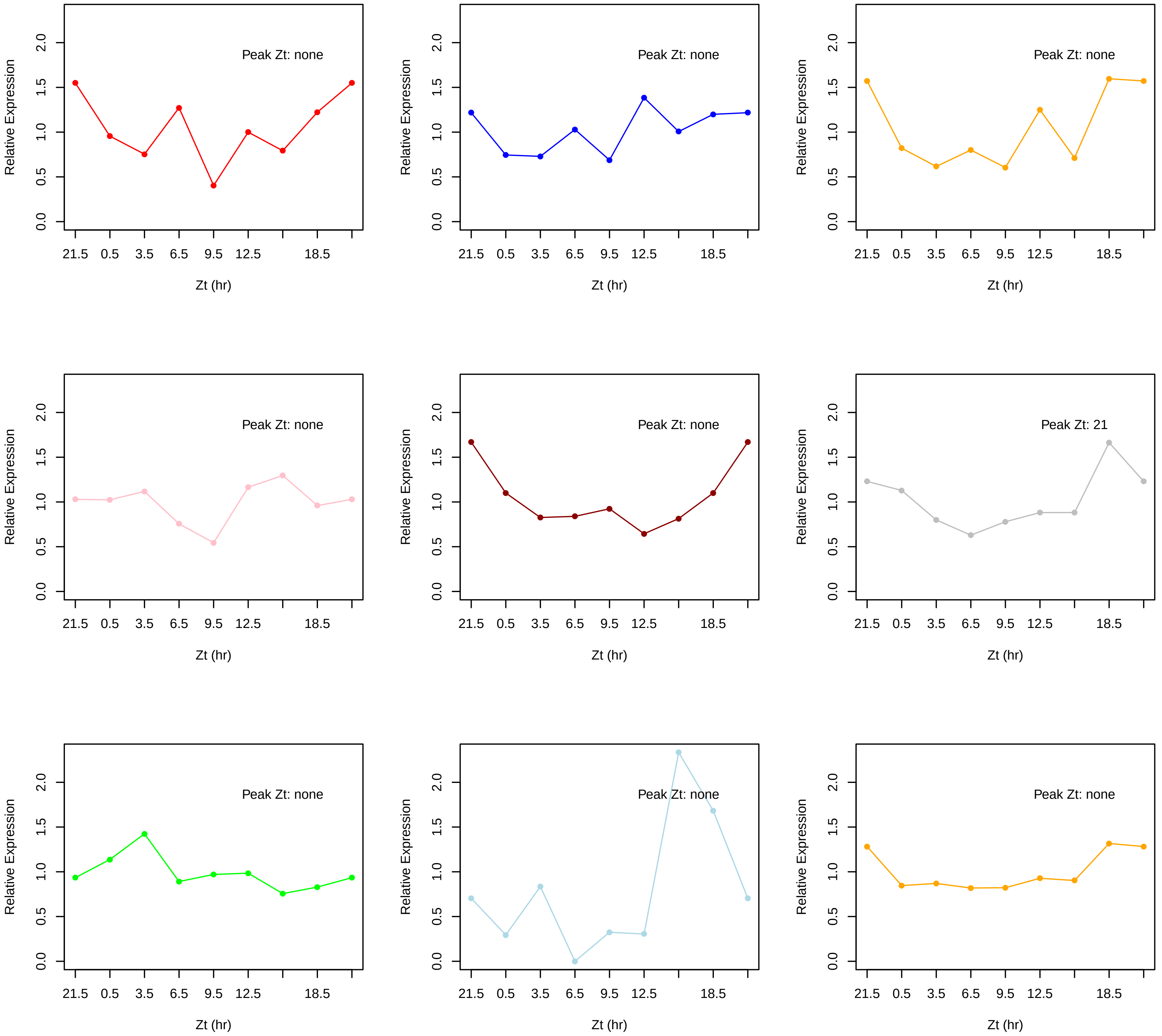

Singeleton: ENSXMAG00000002196 cachd1

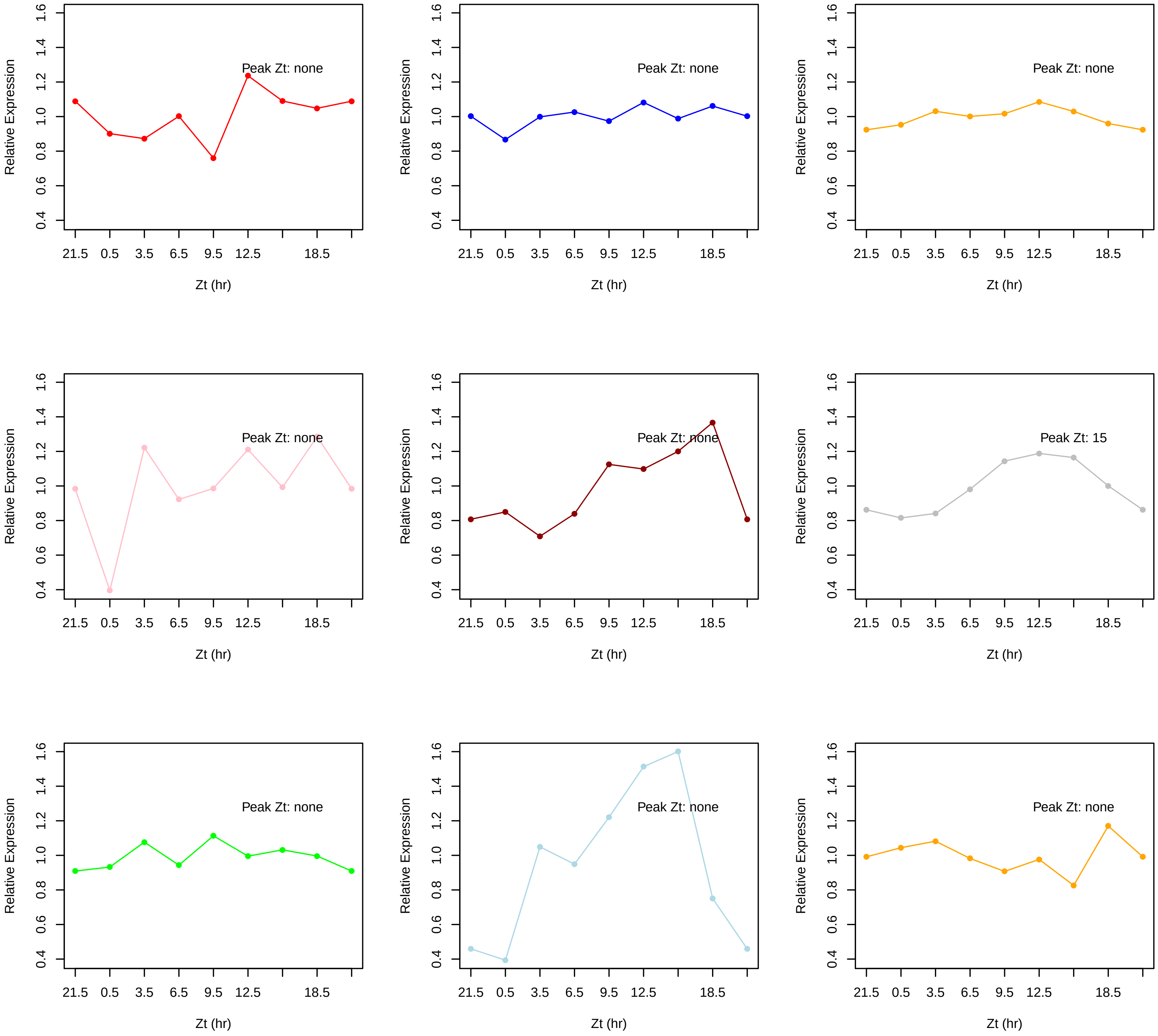

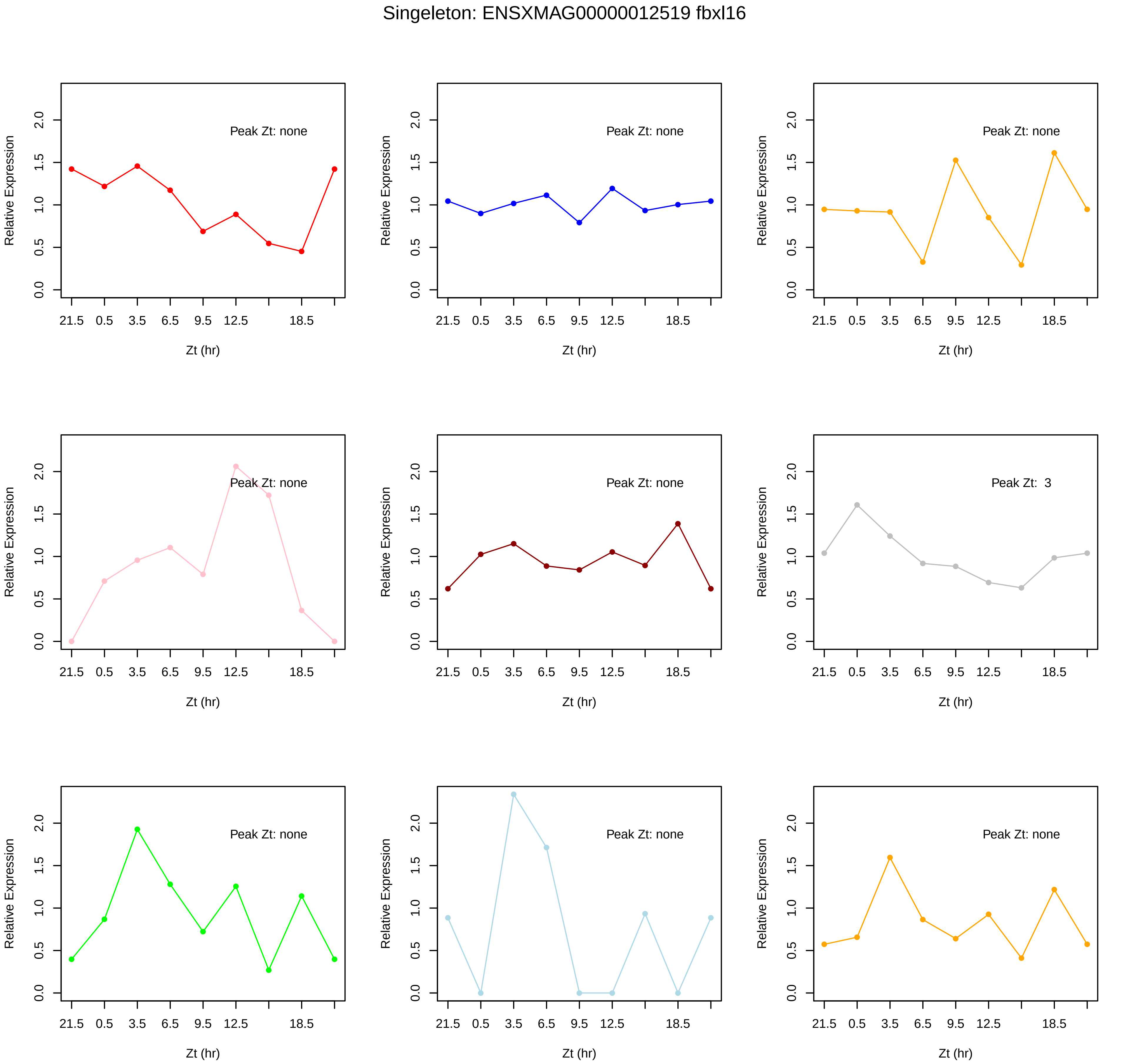

Singleton: ENSXMAG00000012519 fbxl16
Peak Zt: none
Peak Zt: none
Peak Zt: none
Peak Zt: none
Peak Zt: none
Peak Zt: 3
Peak Zt: none
Peak Zt: none
Peak Zt: none
Relative Expression
Zt (hr)
21.5
0.5
3.5
6.5
9.5
12.5
18.5
0.0
0.5
1.0
1.5
2.0

Singeleton: ENSXMAG00000009880 uggt1

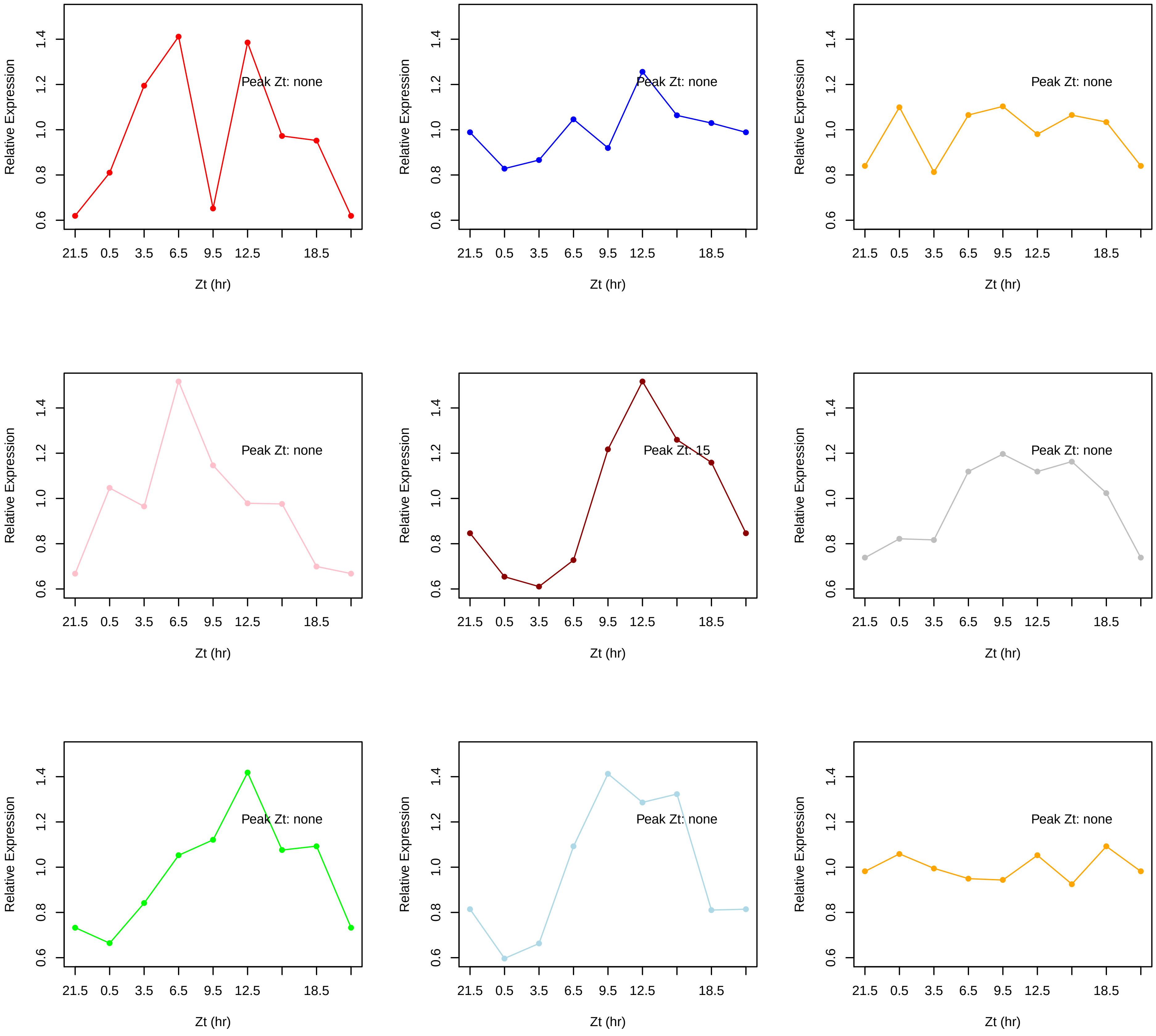

Singeleton: ENSXMAG00000012224 pltp

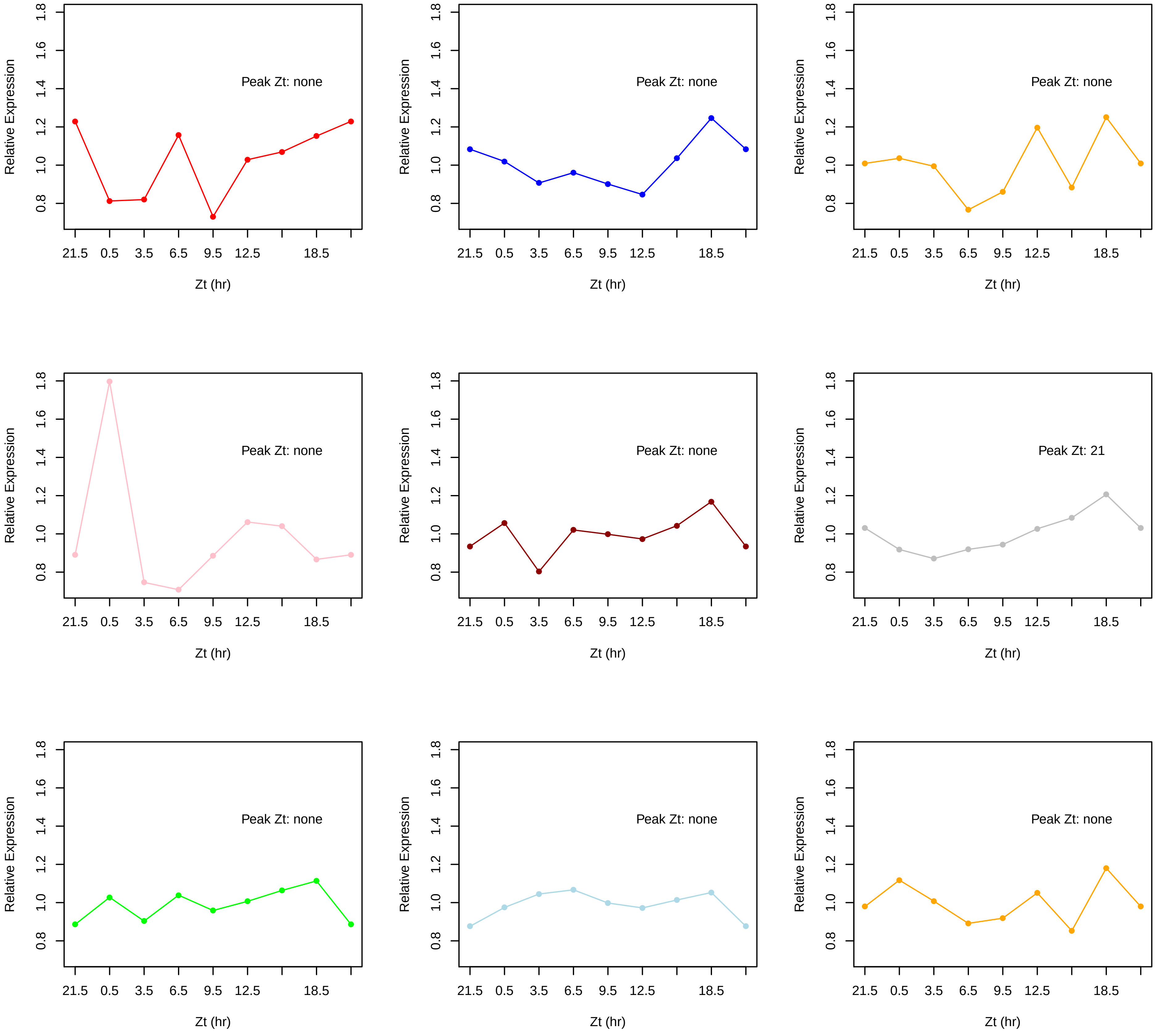

Singleton: ENSXMAG00000003973 pdzd2

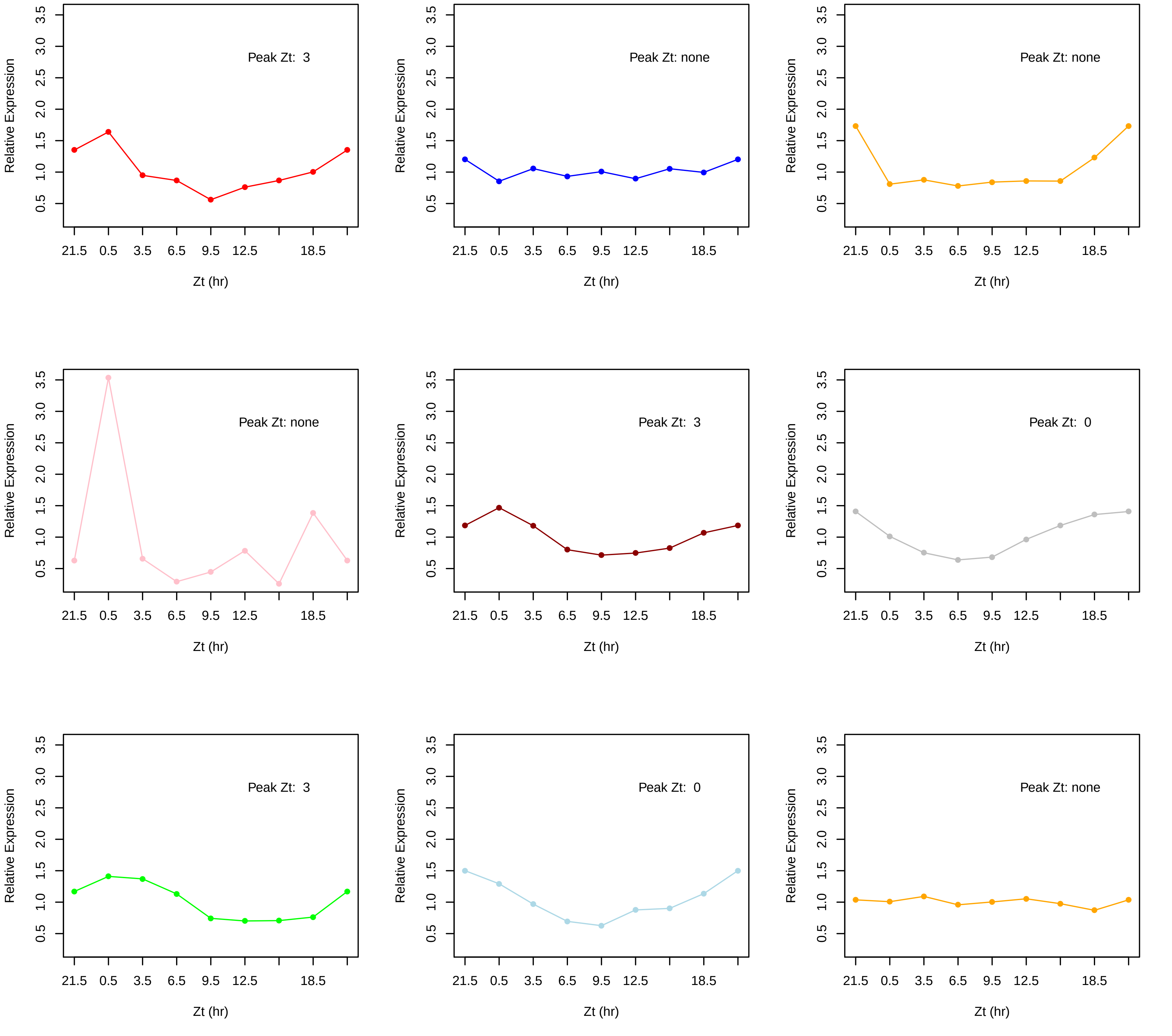

Singeleton: ENSXMAG00000012302 elmod1

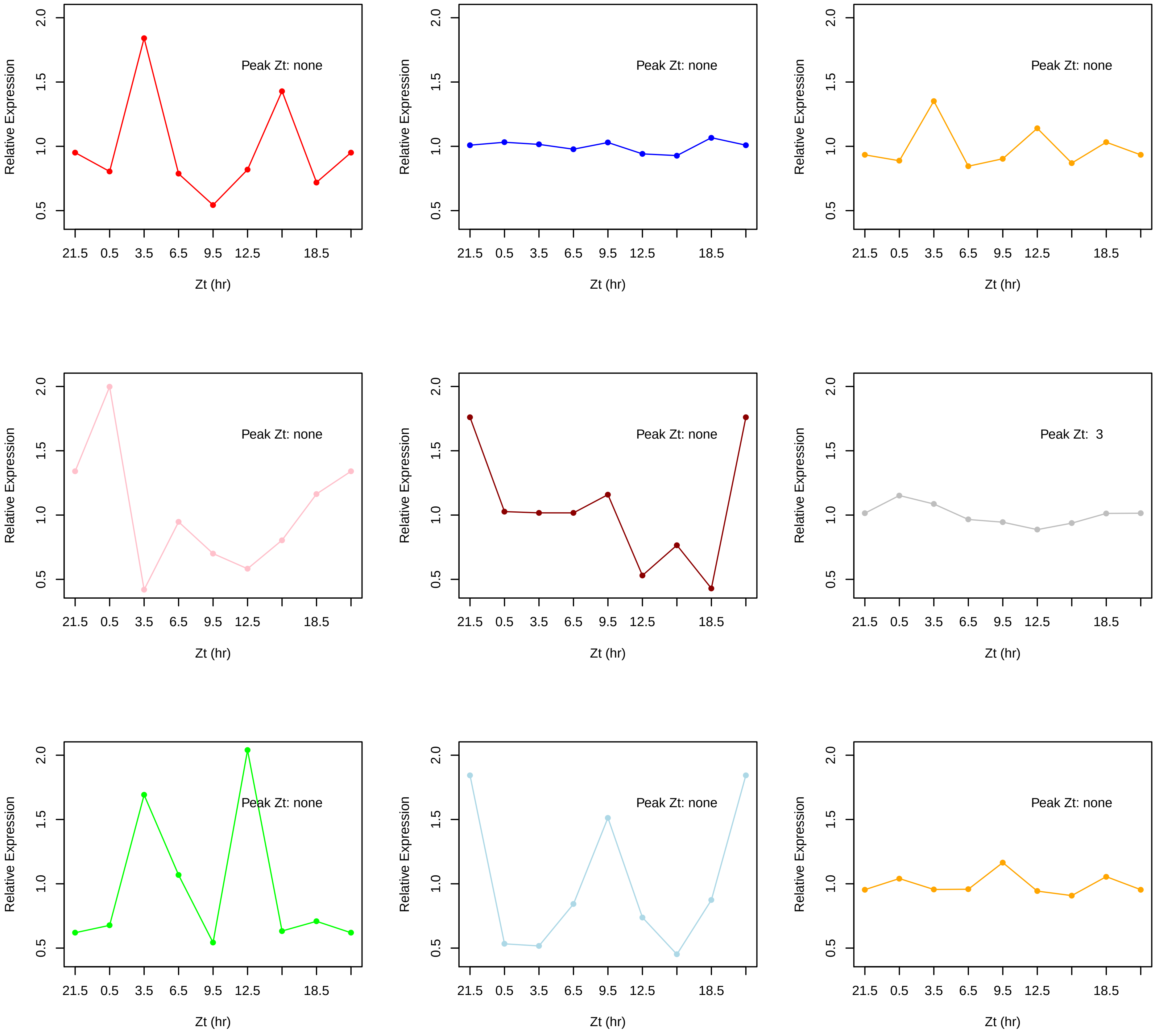

Singeleton: ENSXMAG00000012347 ctsa

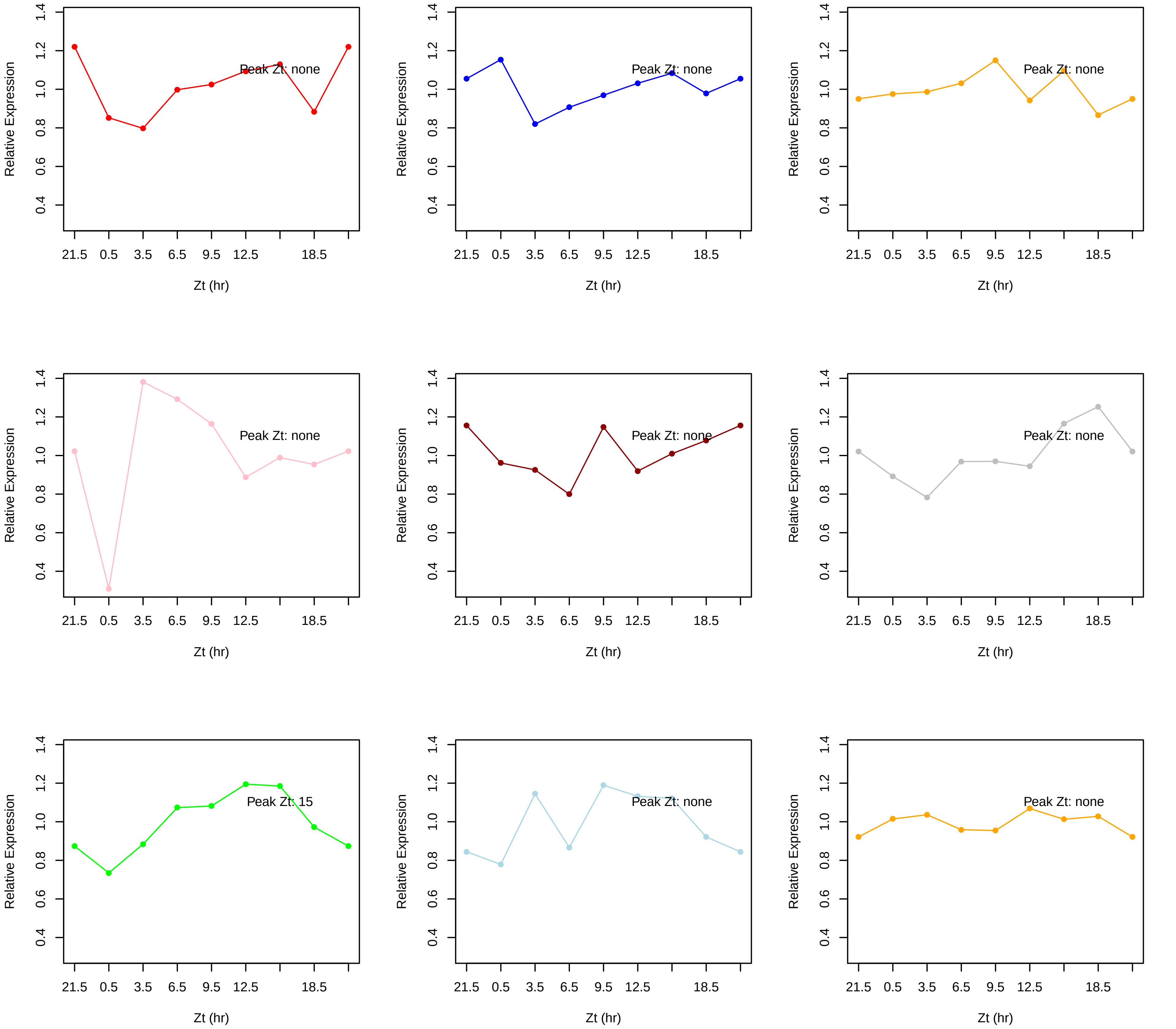

Singleton: ENSXMAG00000004151 dop1b

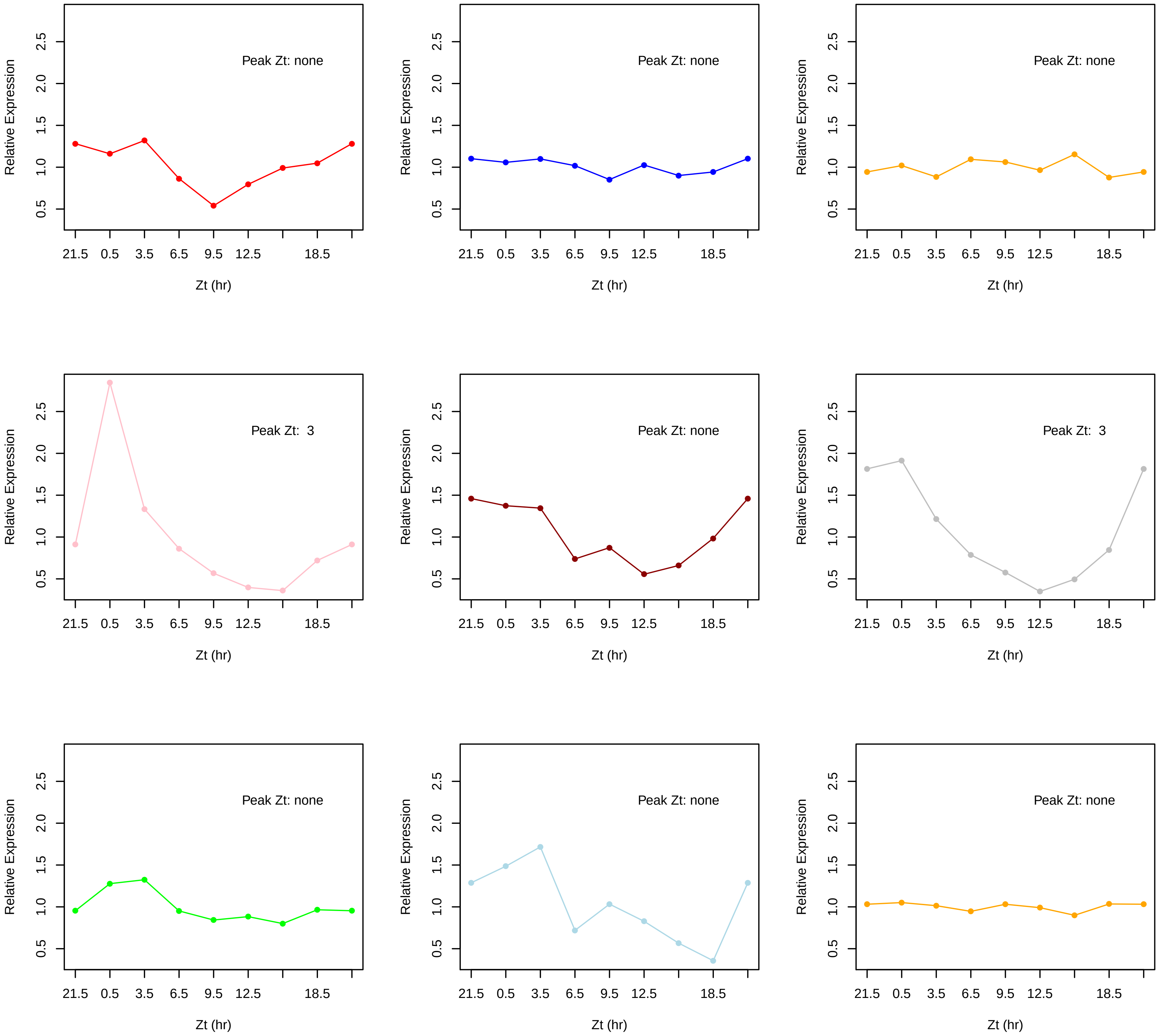

Singeleton: ENSXMAG00000024562 mif

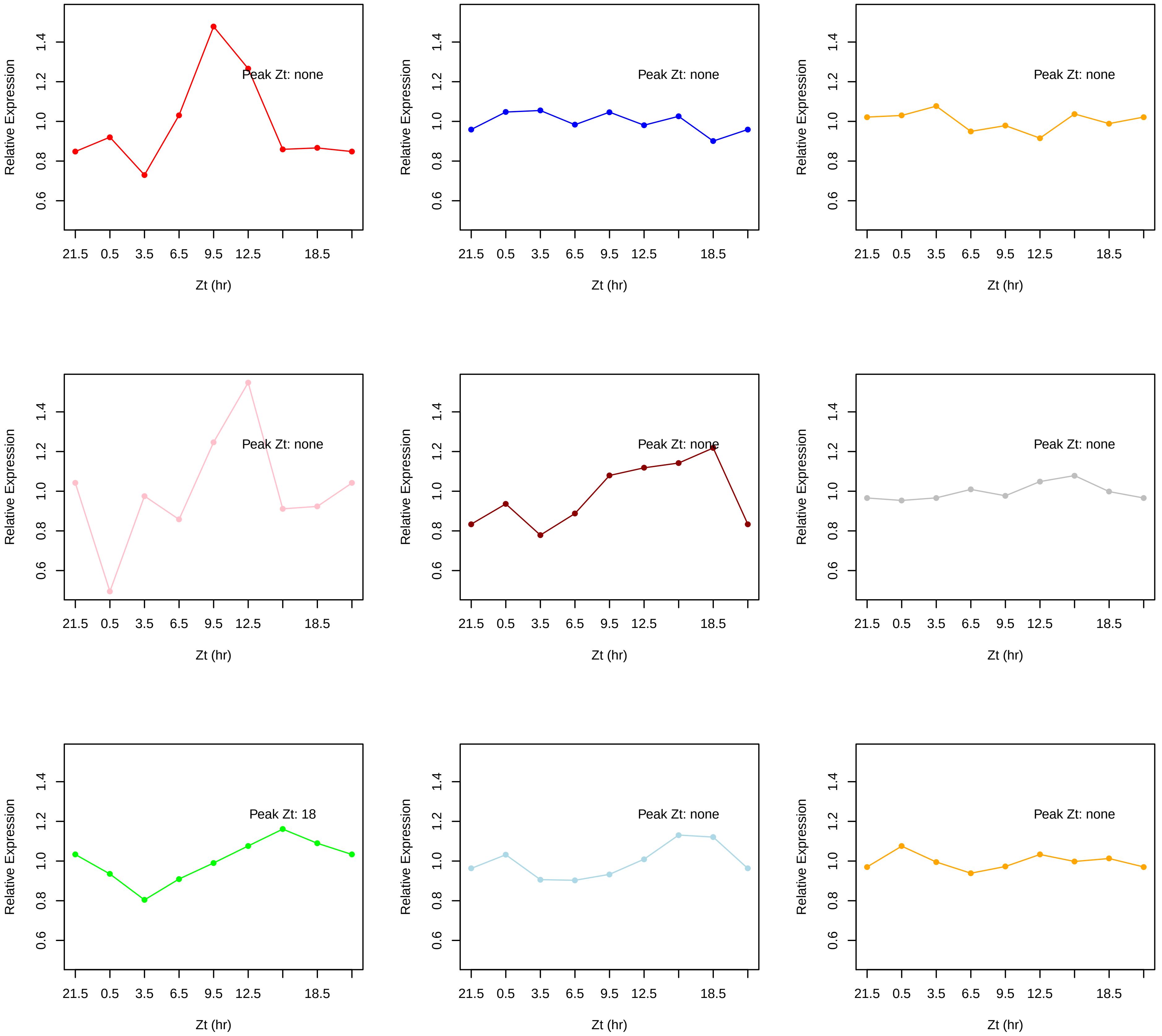

Singleton: ENSXMAG00000022609 si:dkey−3h3.3

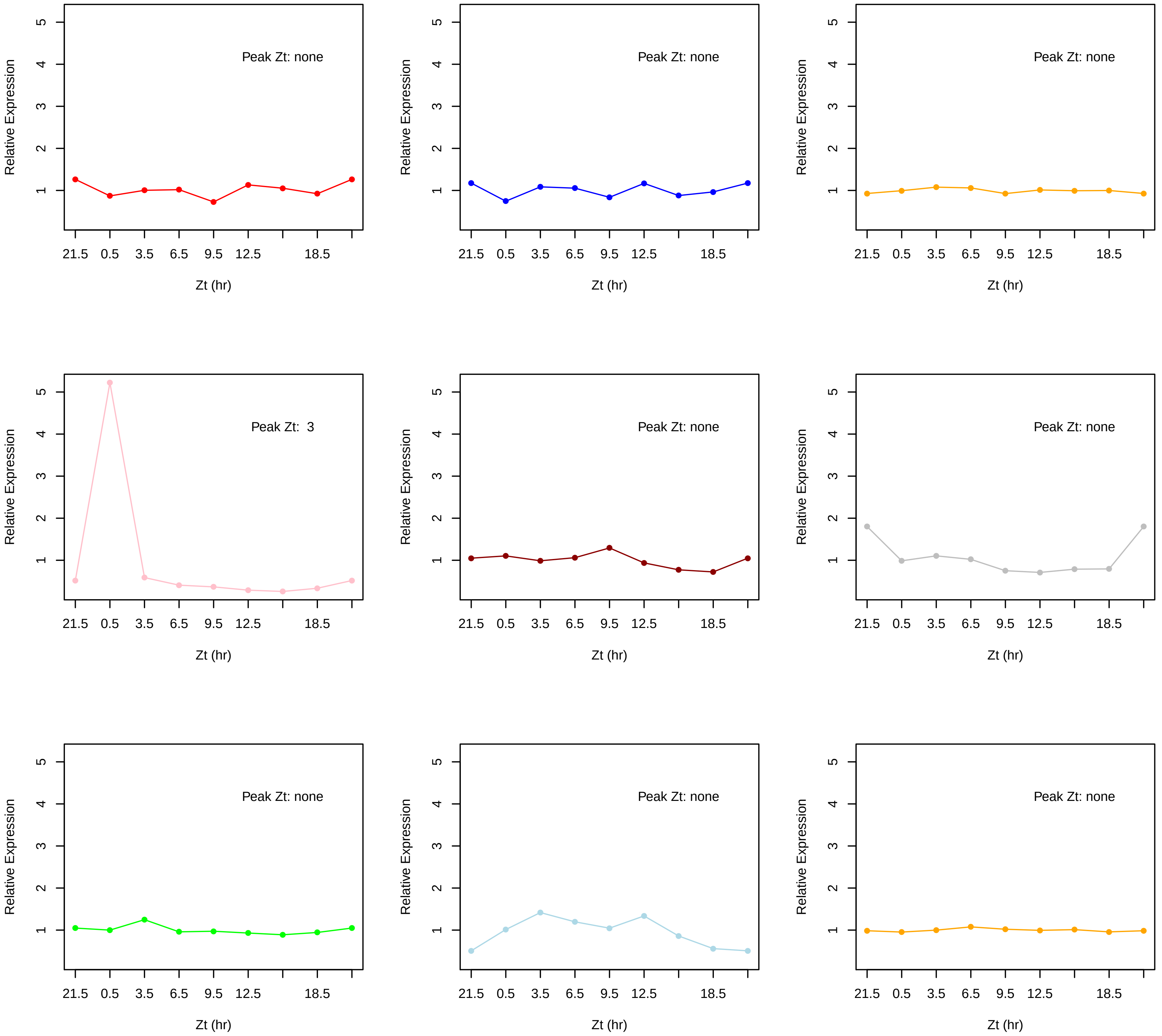

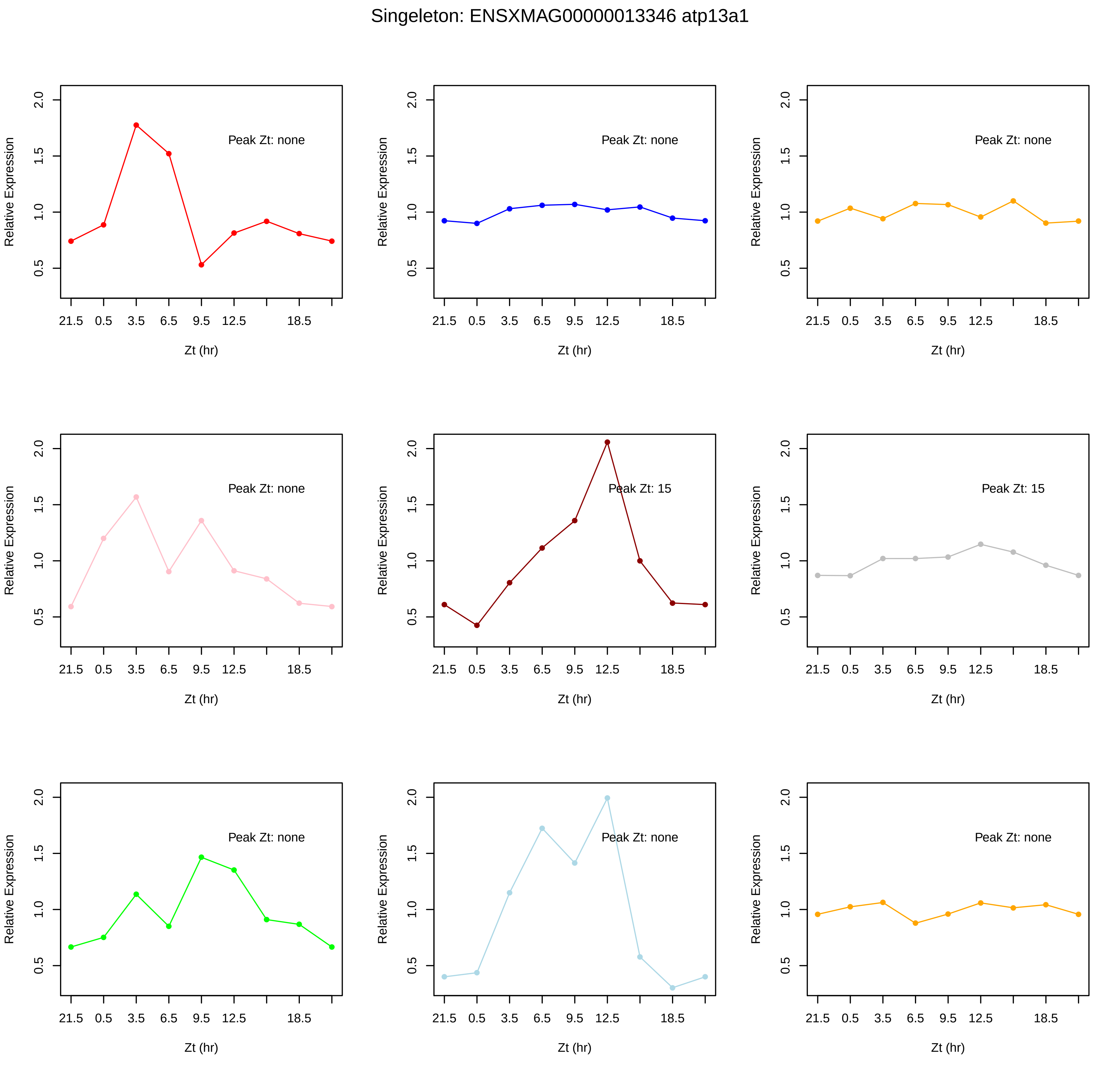

Singeleton: ENSXMAG00000013346 atp13a1
Peak Zt: none
Peak Zt: none
Peak Zt: none
Peak Zt: none
Peak Zt: 15
Peak Zt: 15
Peak Zt: none
Peak Zt: none
Peak Zt: none
Relative Expression
Zt (hr)
21.5
0.5
3.5
6.5
9.5
12.5
18.5
0.5
1.0
1.5
2.0

Singeleton: ENSXMAG00000003966 hps5

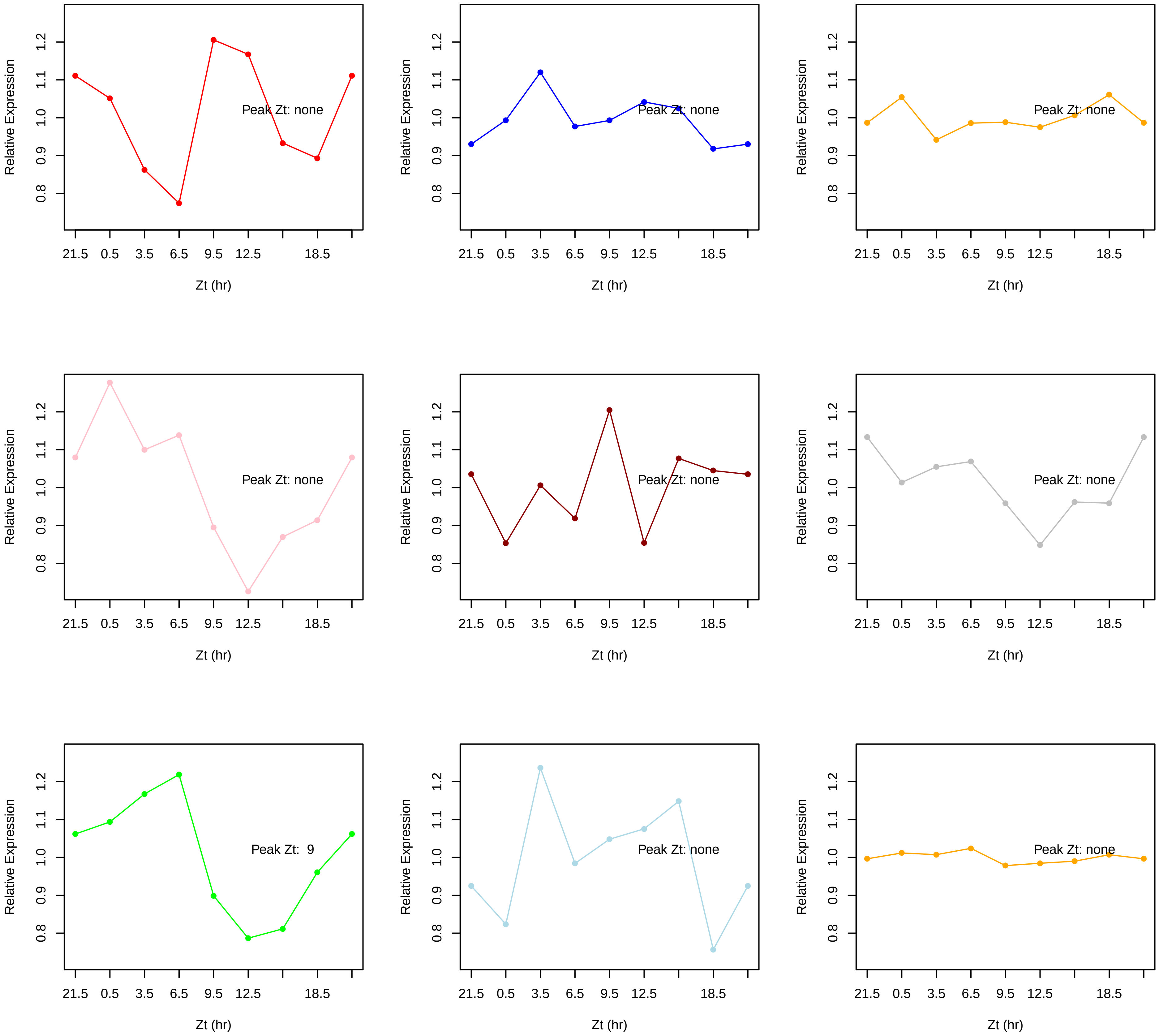

Singeleton: ENSXMAG00000002212 jak1

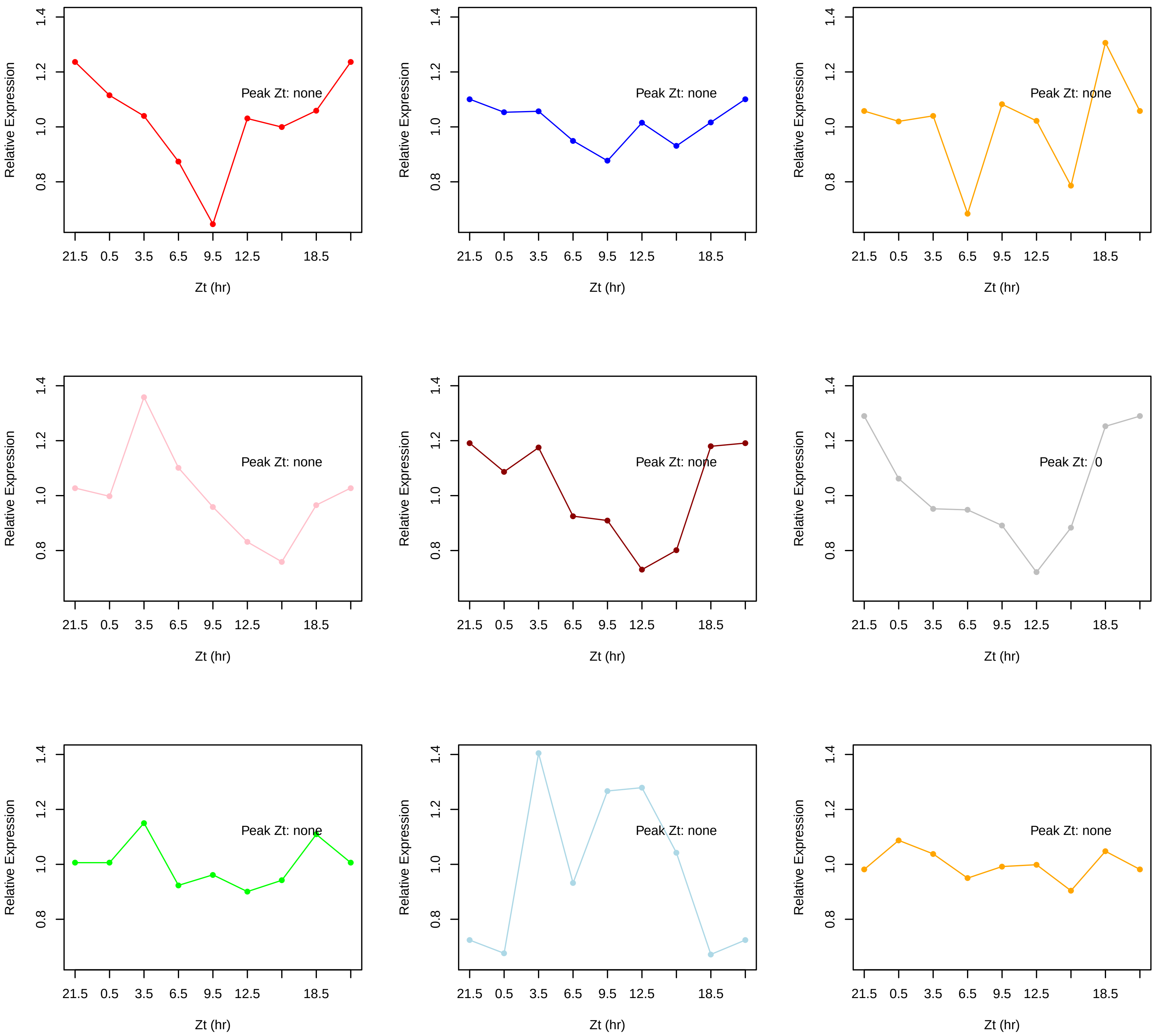

Singleton: ENSXMAG00000027181 arid5b

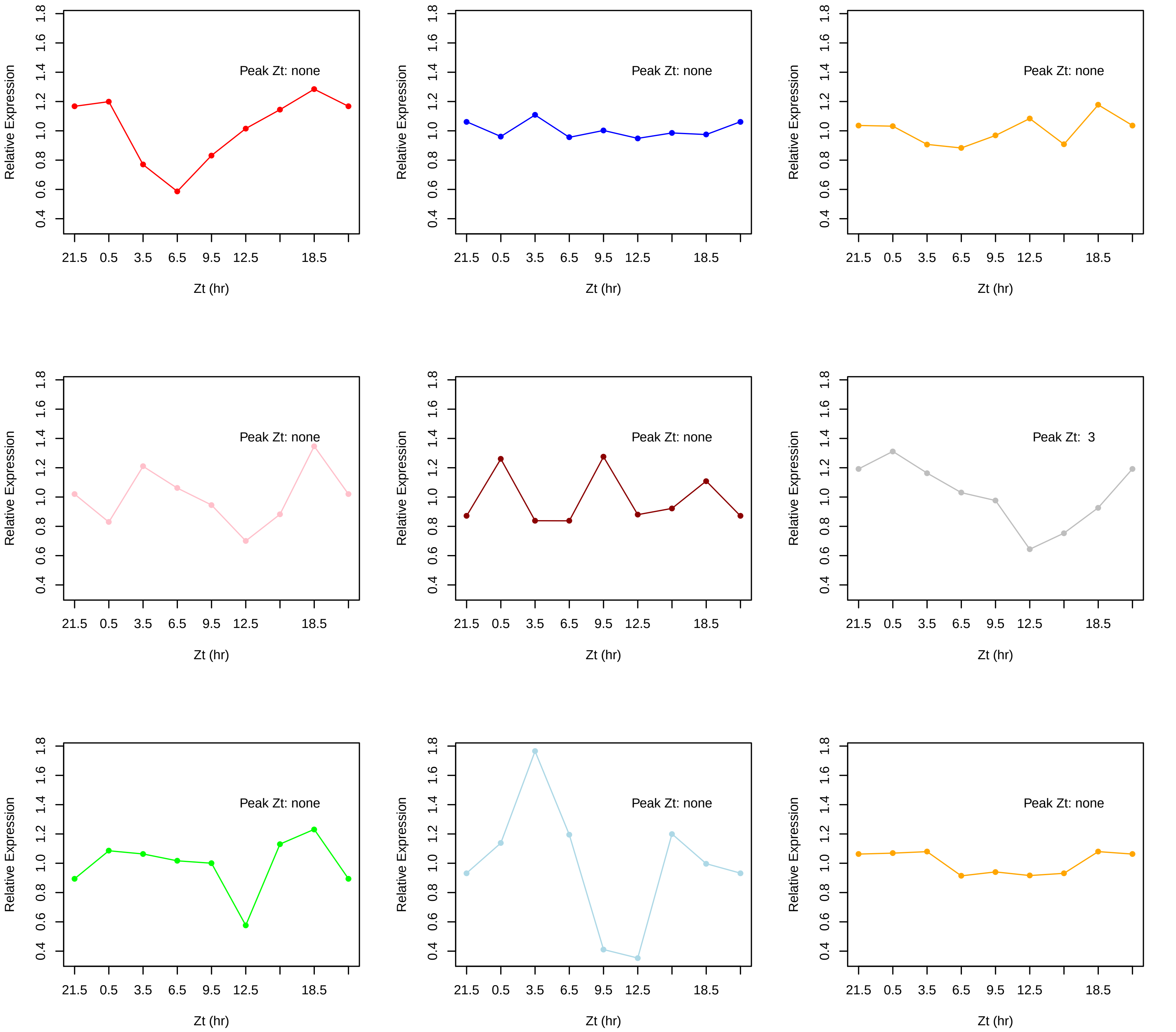

Singleton: ENSXMAG00000014704

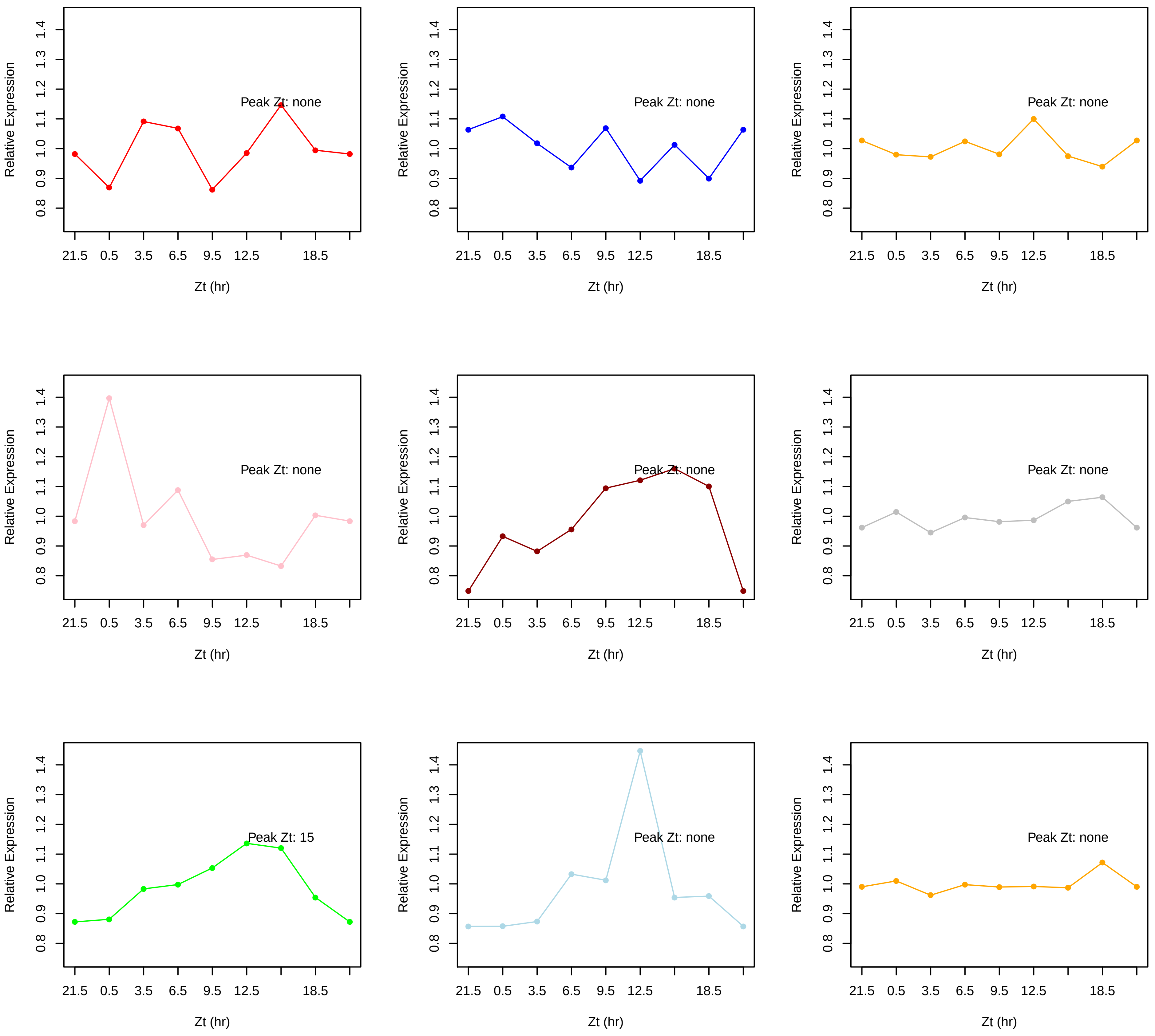

Singleton: ENSXMAG00000019223

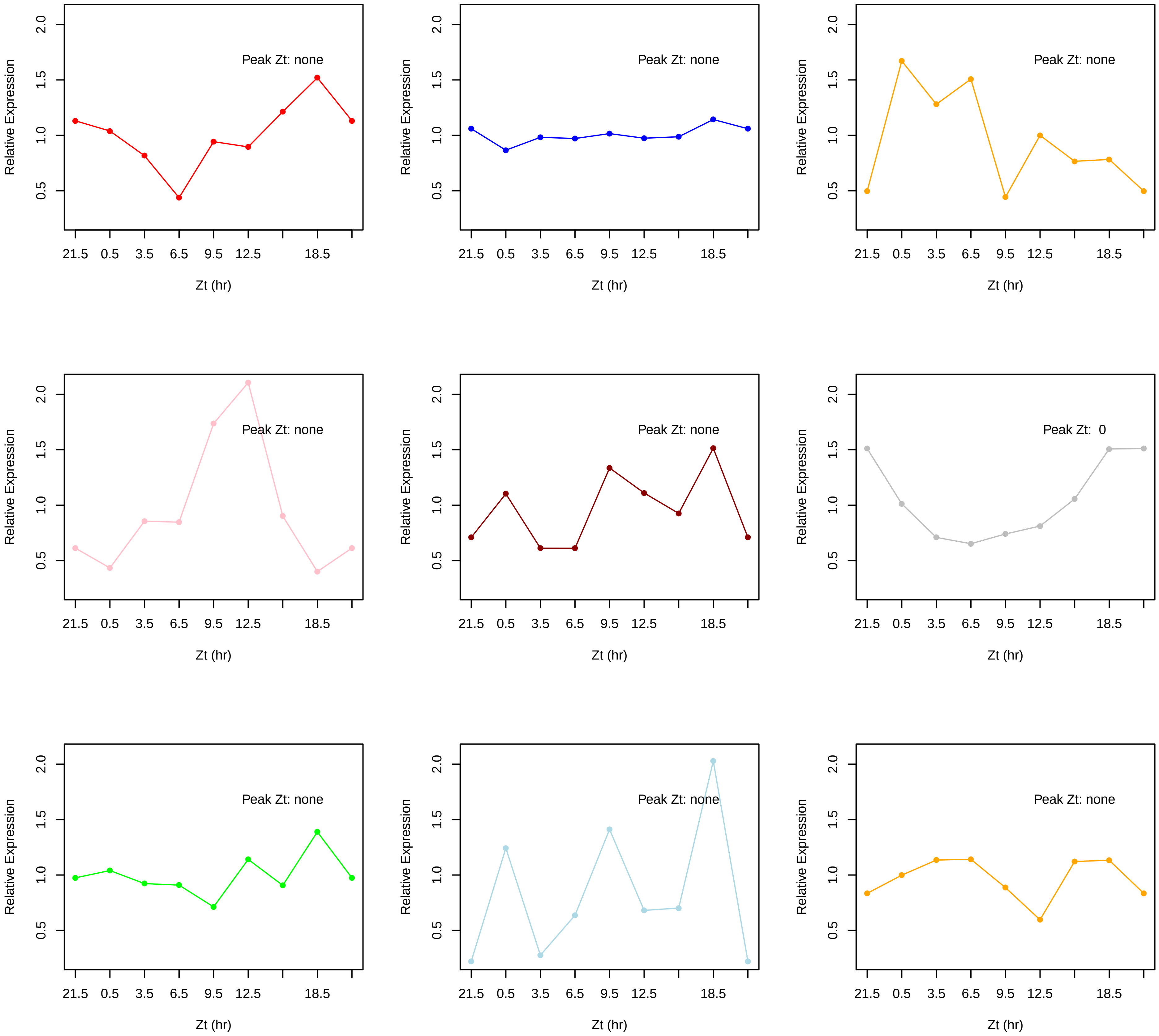

Singleton: ENSXMAG00000003986 gtf2h1

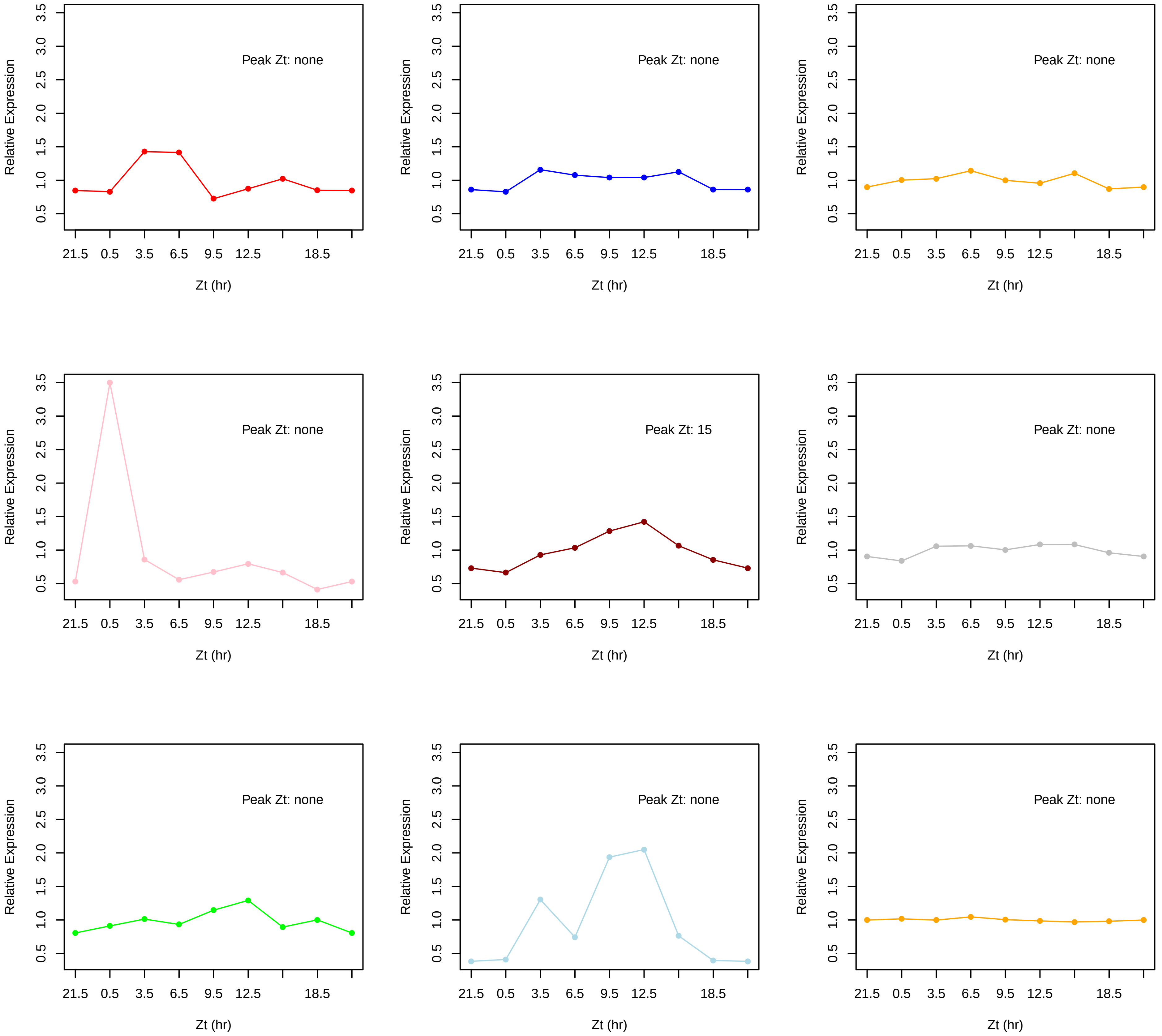

Singleton: ENSXMAG00000018196 rtkn2a

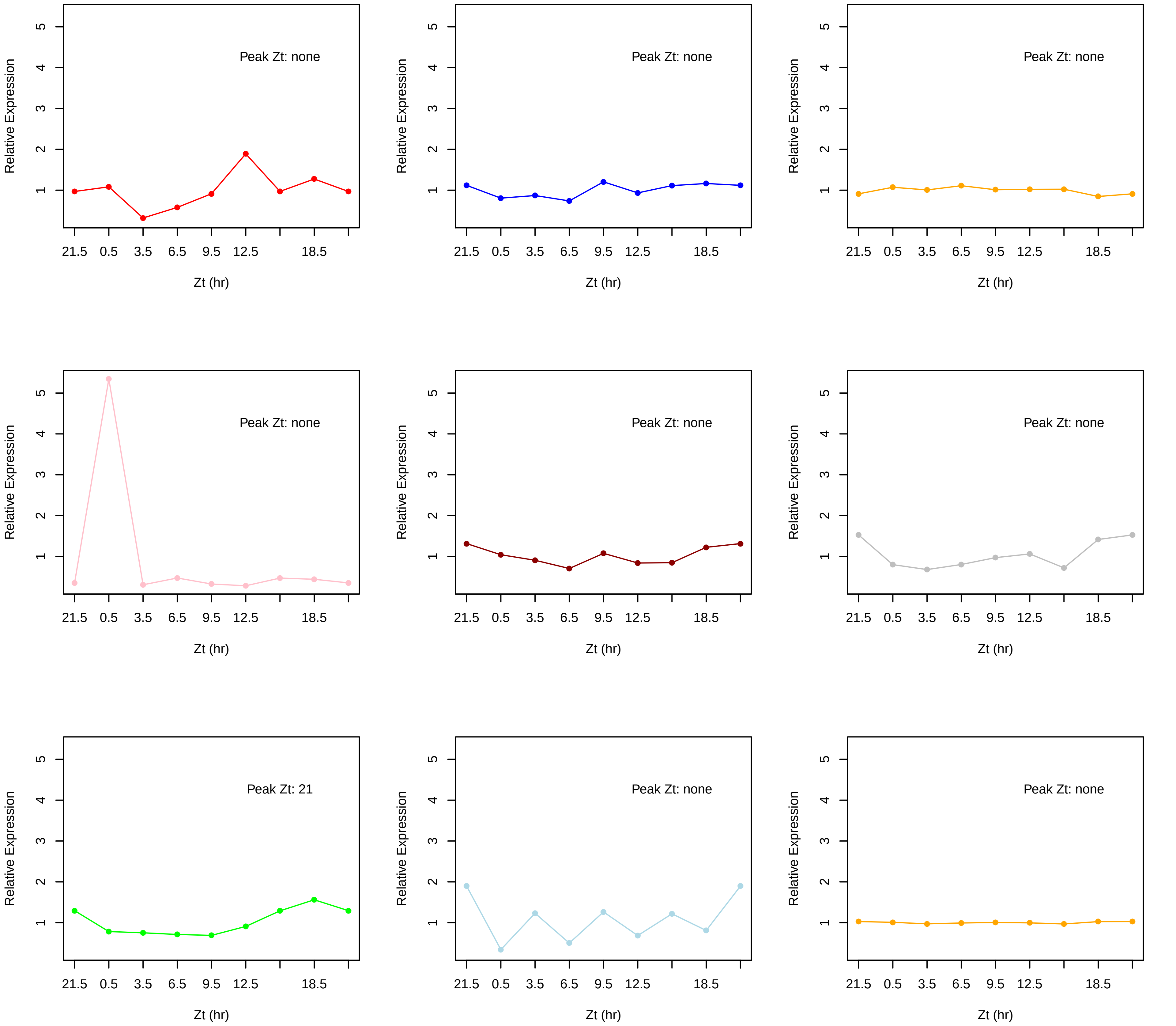

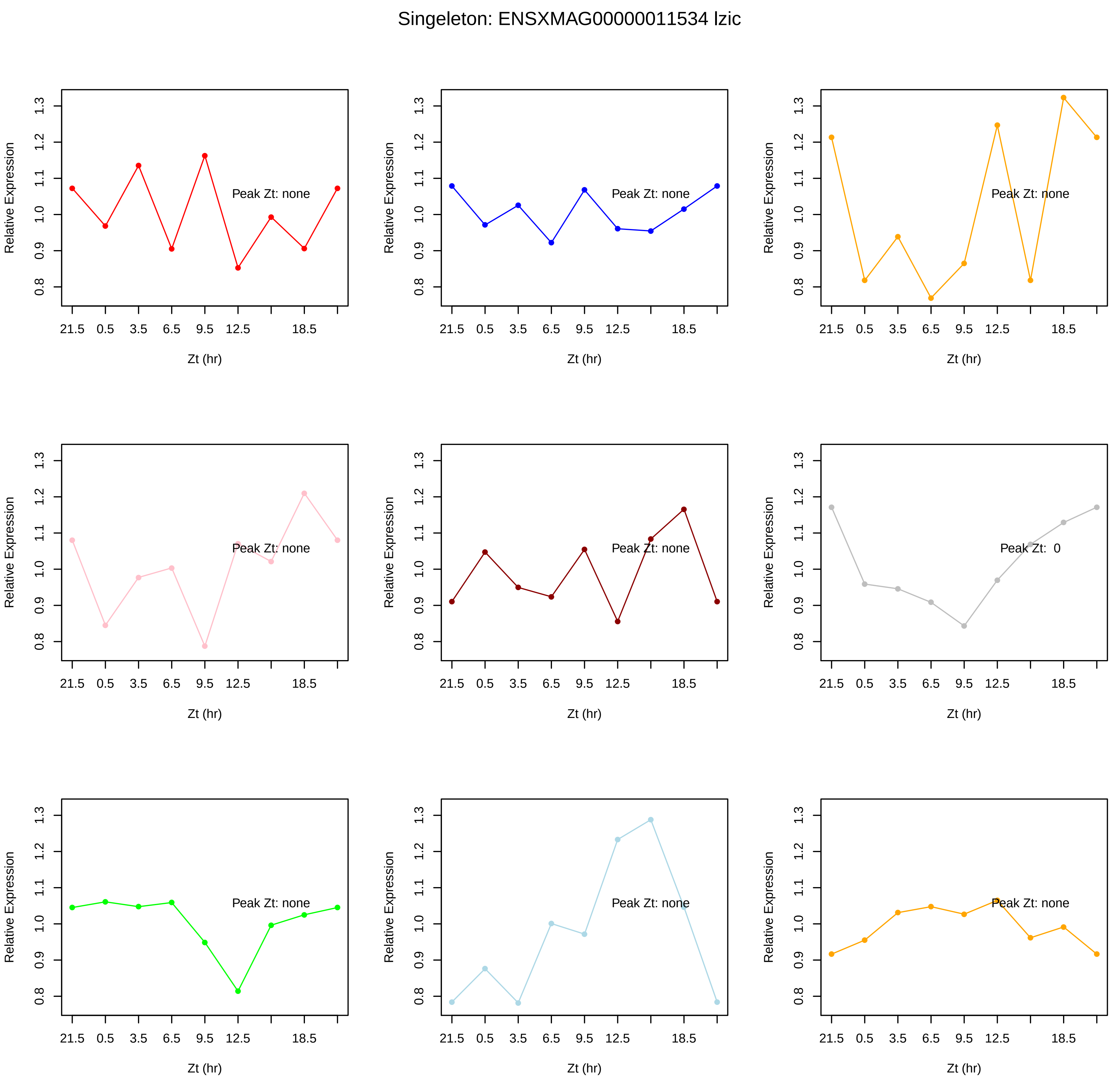

Singeleton: ENSXMAG00000011534 lzic
Peak Zt: none
Peak Zt: none
Peak Zt: none
Peak Zt: none
Peak Zt: none
Peak Zt: 0
Peak Zt: none
Peak Zt: none
Peak Zt: none
Relative Expression
Zt (hr)
21.5
0.5
3.5
6.5
9.5
12.5
18.5
0.8
0.9
1.0
1.1
1.2
1.3

Singleton: ENSXMAG00000028313 krtcap2

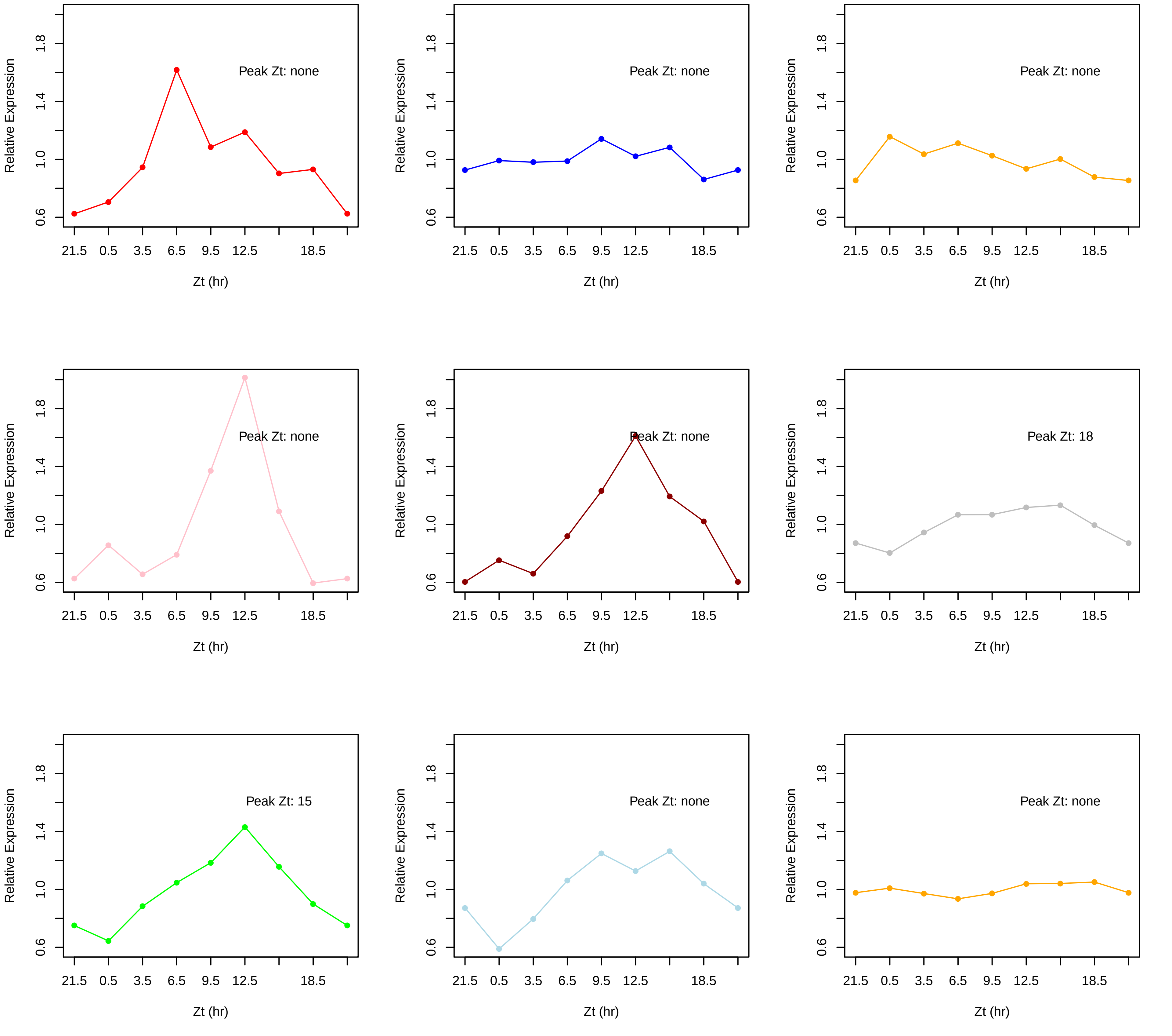

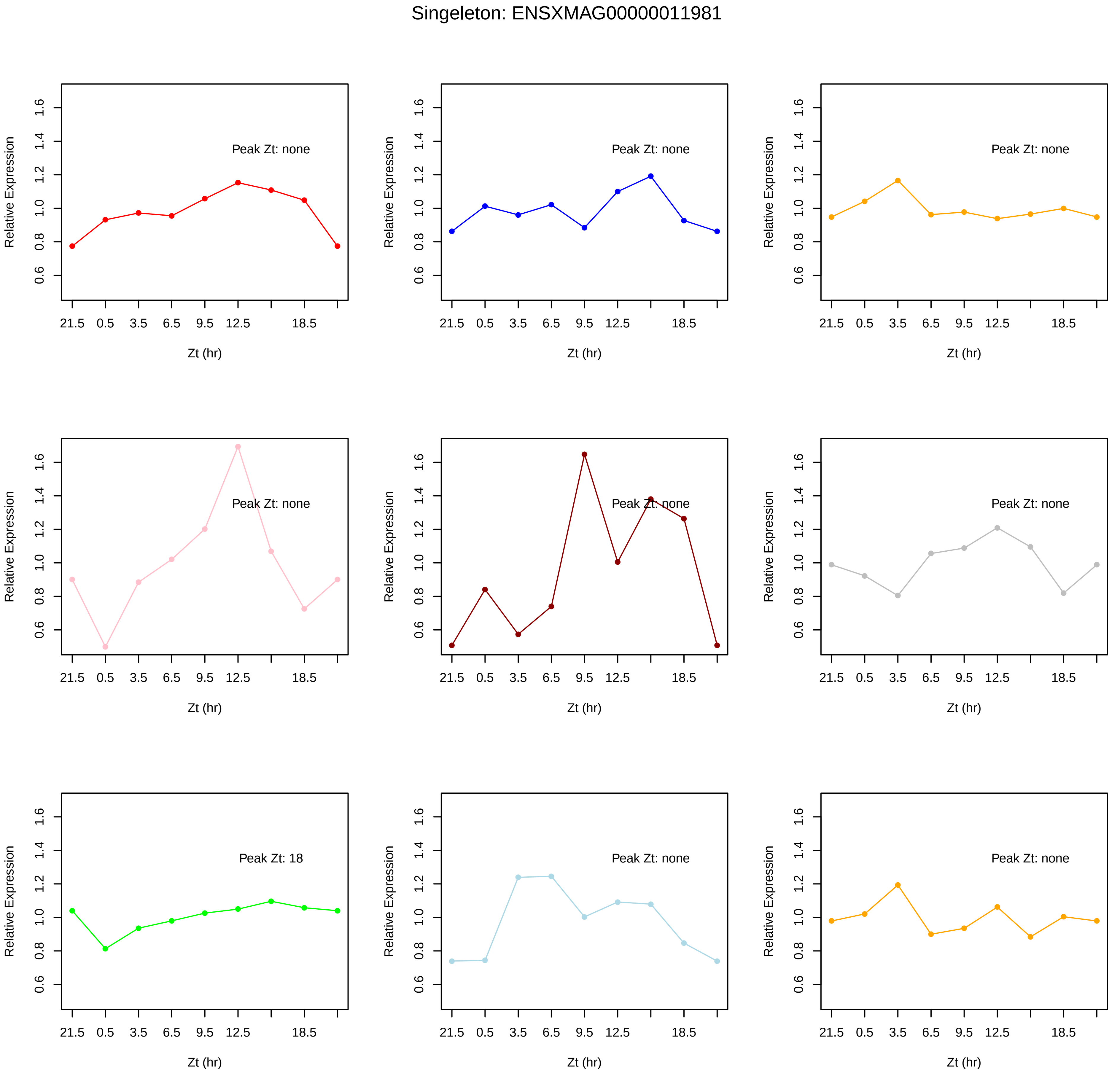

Singeleton: ENSXMAG00000011981
Peak Zt: none
Peak Zt: none
Peak Zt: none
Peak Zt: none
Peak Zt: none
Peak Zt: none
Peak Zt: 18
Peak Zt: none
Peak Zt: none
Relative Expression
Zt (hr)

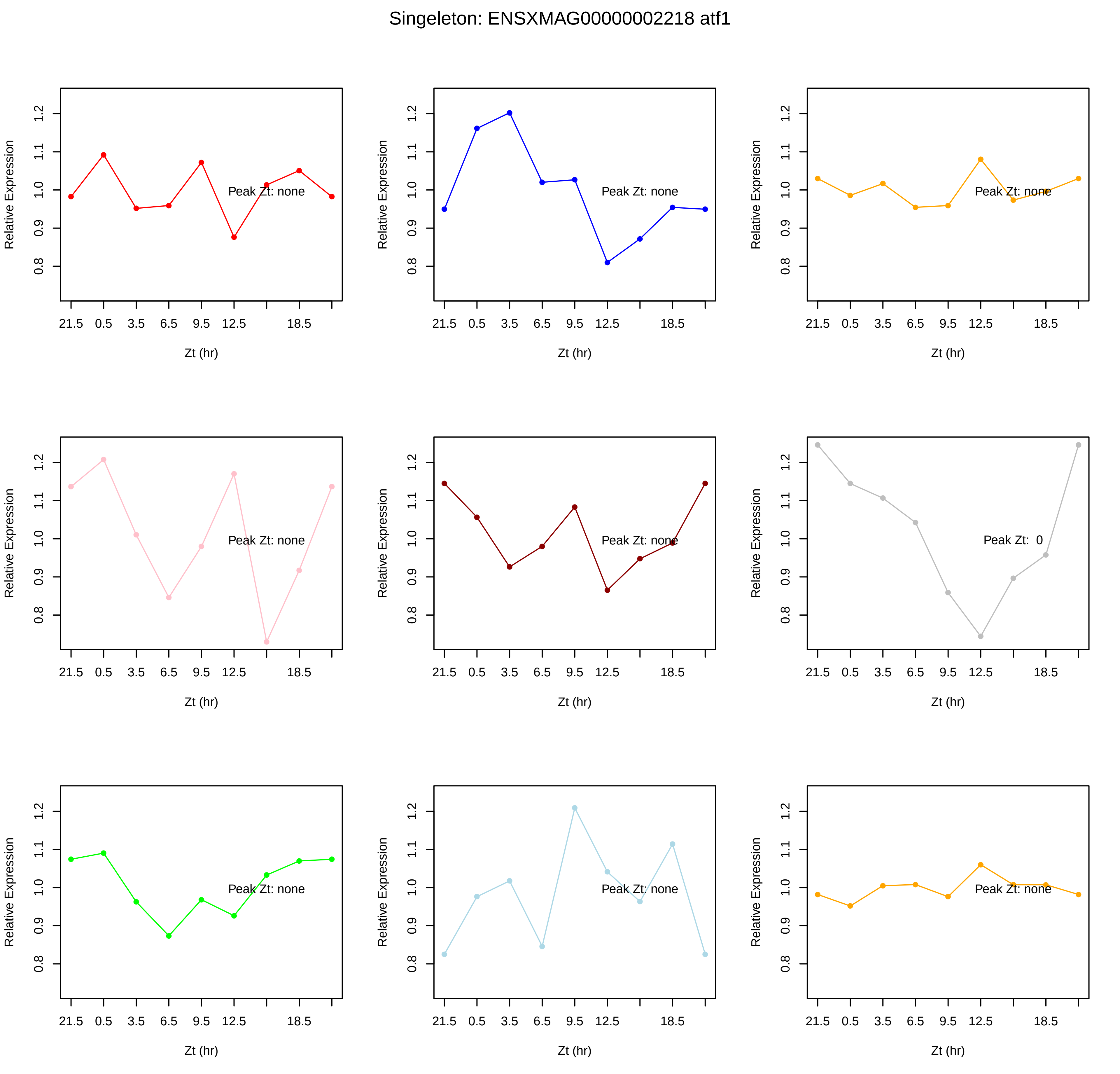
Singeleton: ENSXMAG00000002218 atf1
Relative Expression
Zt (hr)
21.5
0.5
3.5
6.5
9.5
12.5
18.5
0.8
0.9
1.0
1.1
1.2
Peak Zt: none
Peak Zt: 0

Singeleton: ENSXMAG00000022447

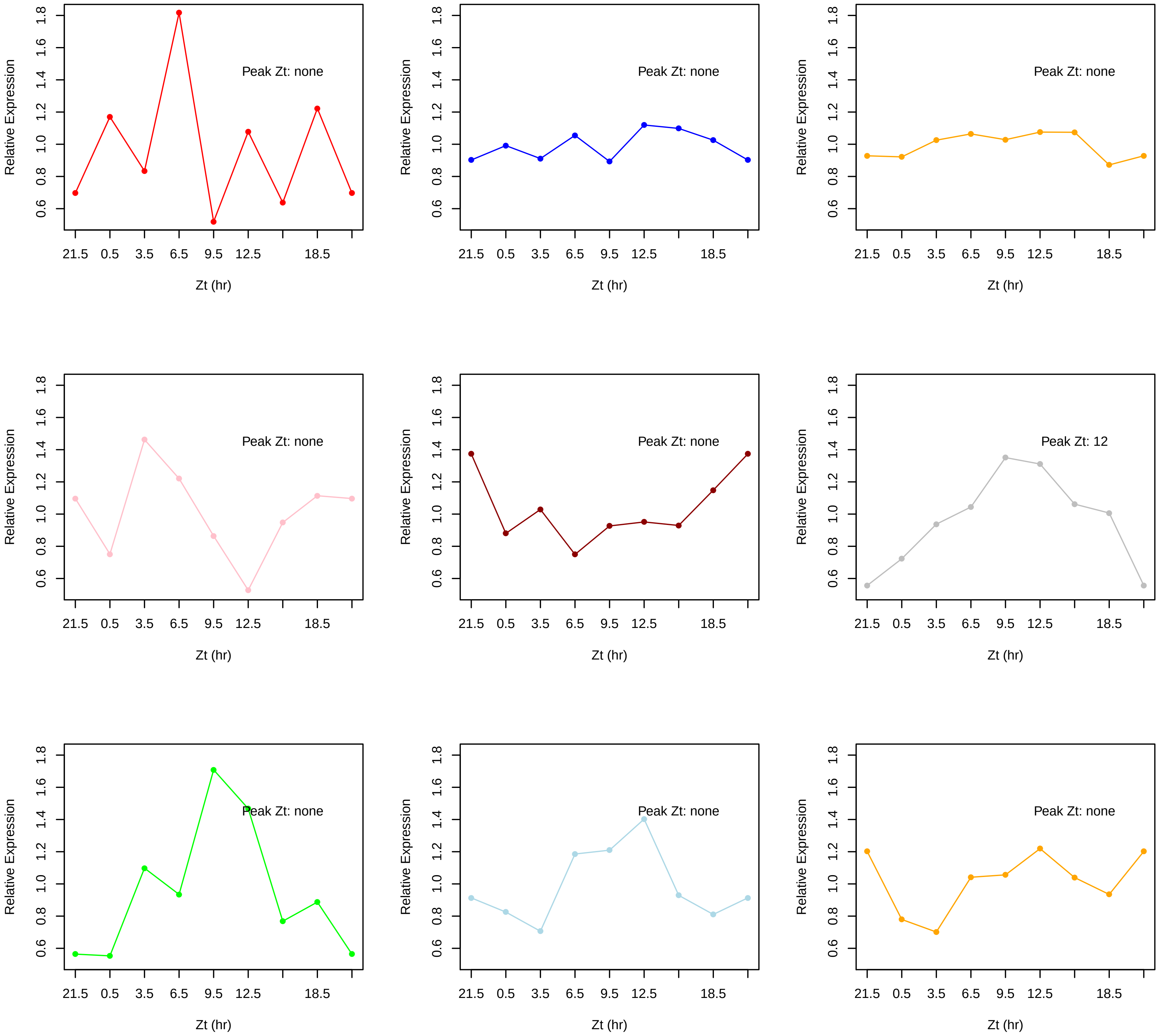

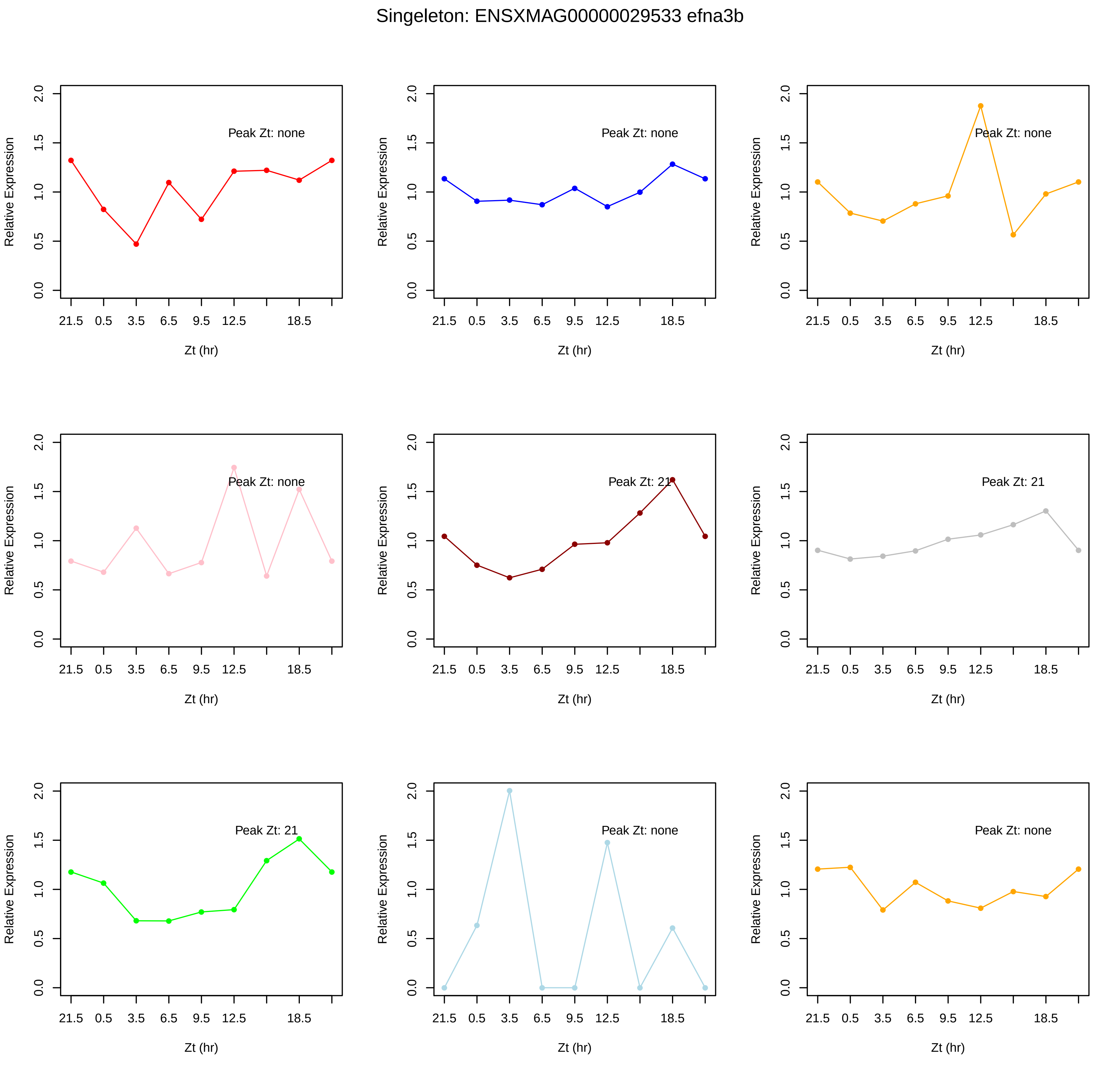
Singeleton: ENSXMAG00000029533 efna3b
Peak Zt: none
Peak Zt: none
Peak Zt: none
Peak Zt: none
Peak Zt: 21
Peak Zt: 21
Peak Zt: 21
Peak Zt: none
Peak Zt: none
Relative Expression
Zt (hr)
0.0
0.5
1.0
1.5
2.0
21.5
0.5
3.5
6.5
9.5
12.5
18.5

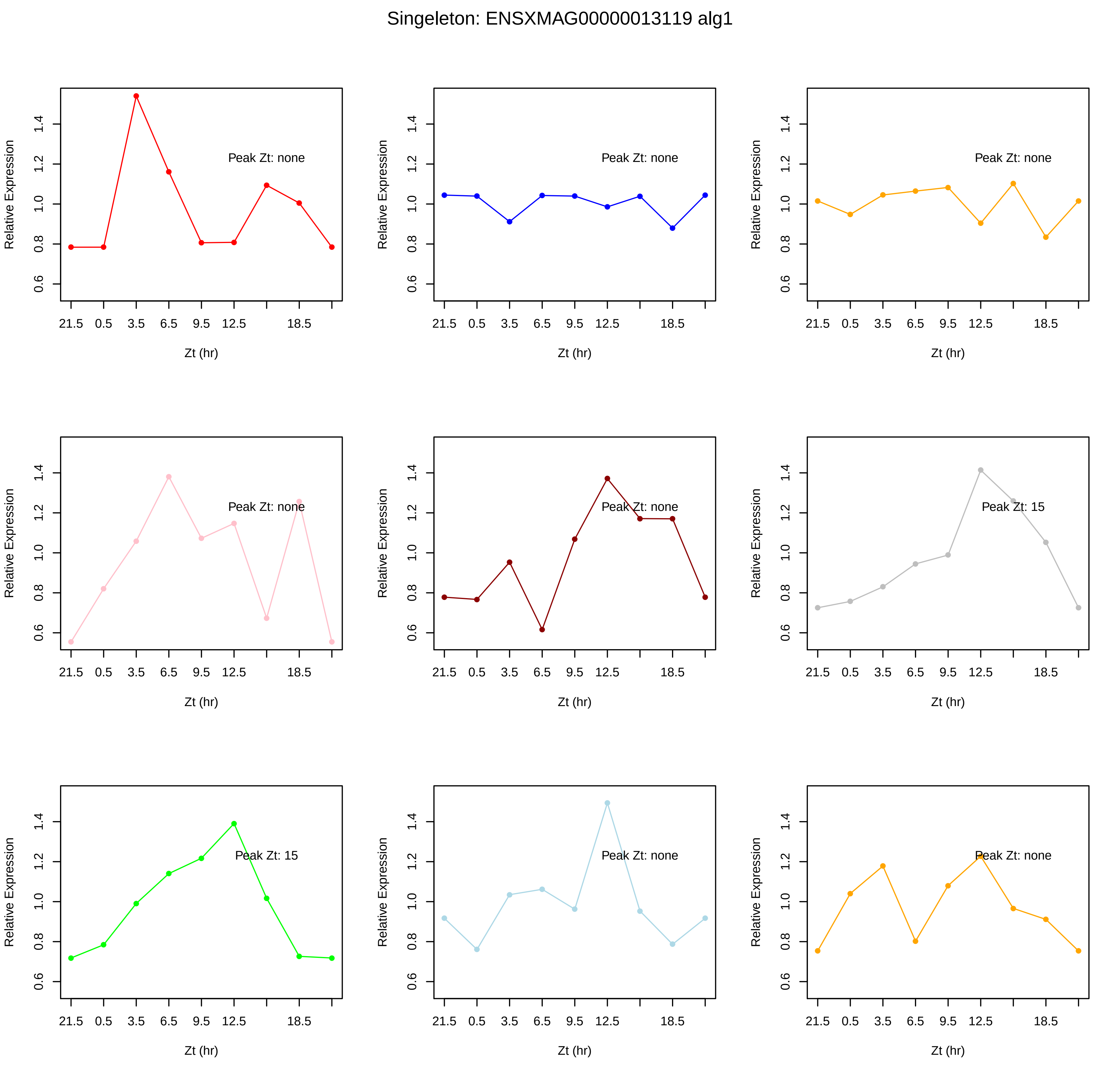
Singeleton: ENSXMAG00000013119 alg1
Peak Zt: none
Peak Zt: none
Peak Zt: none
Peak Zt: none
Peak Zt: none
Peak Zt: 15
Peak Zt: 15
Peak Zt: none
Peak Zt: none
Relative Expression
Zt (hr)
21.5 0.5 3.5 6.5 9.5 12.5 18.5
0.6 0.8 1.0 1.2 1.4

Singeleton: ENSXMAG00000017066 thrb

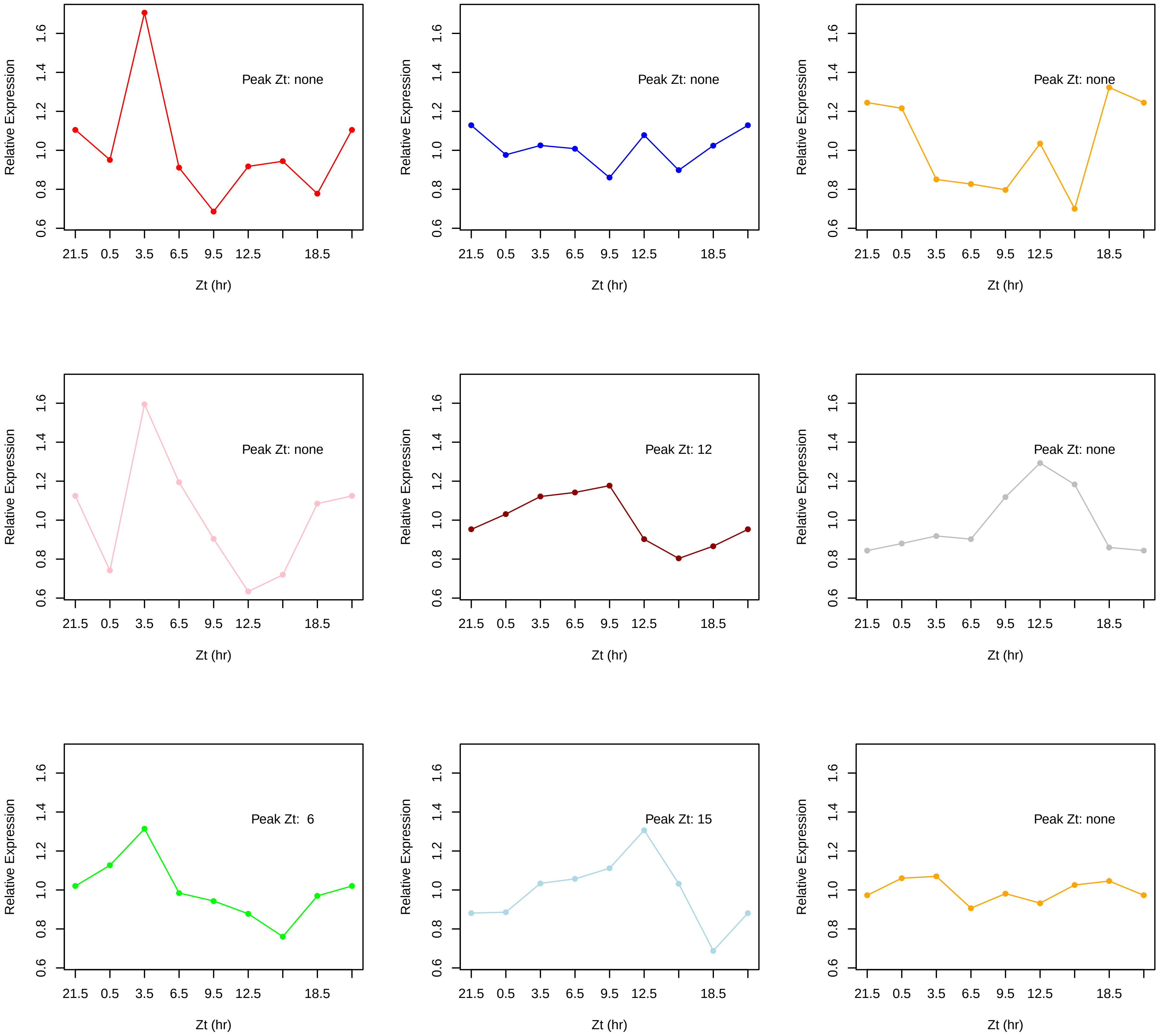

Singeleton: ENSXMAG00000029666 ctnnbip1

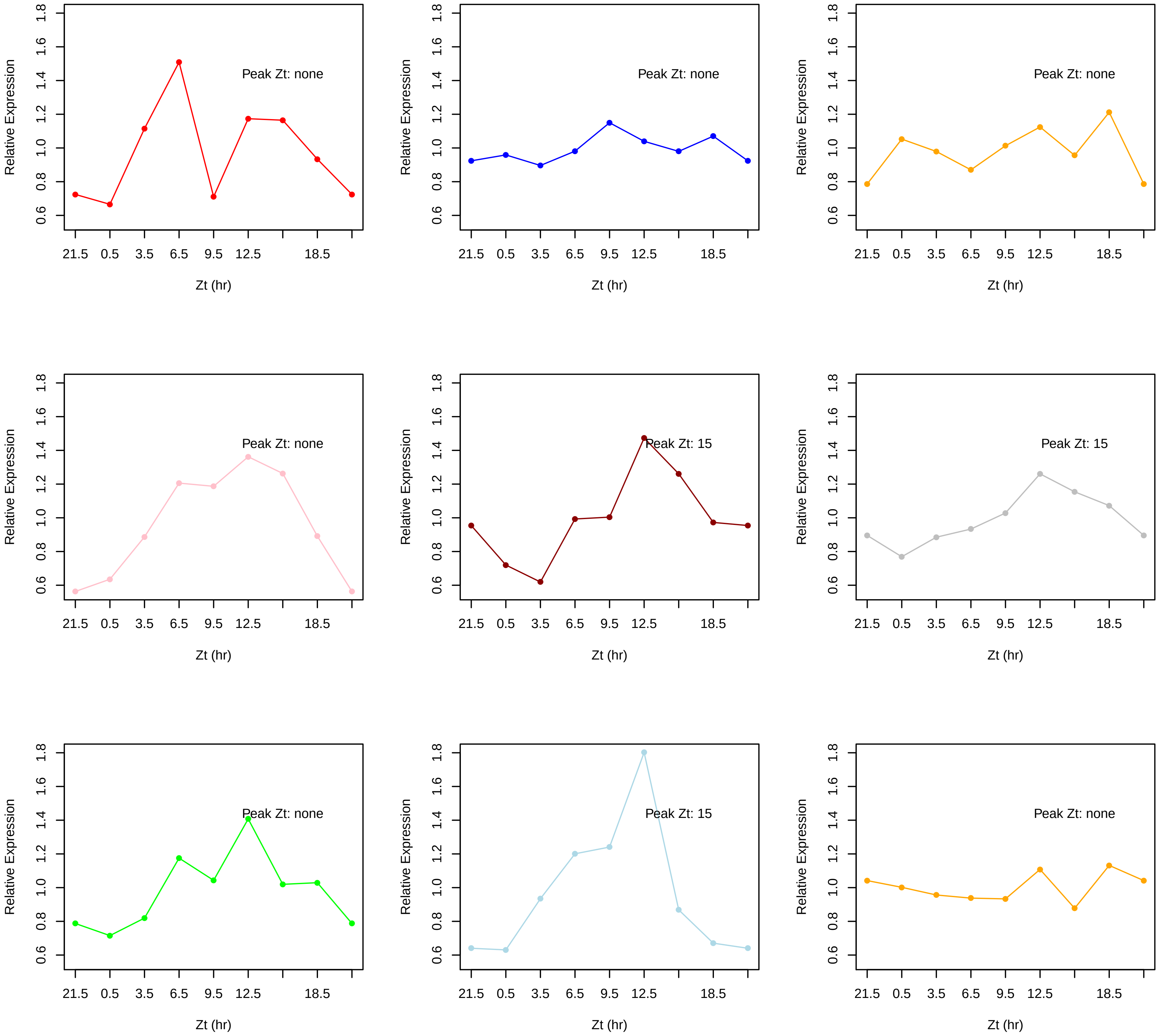

Singeleton: ENSXMAG00000003573 fam57a

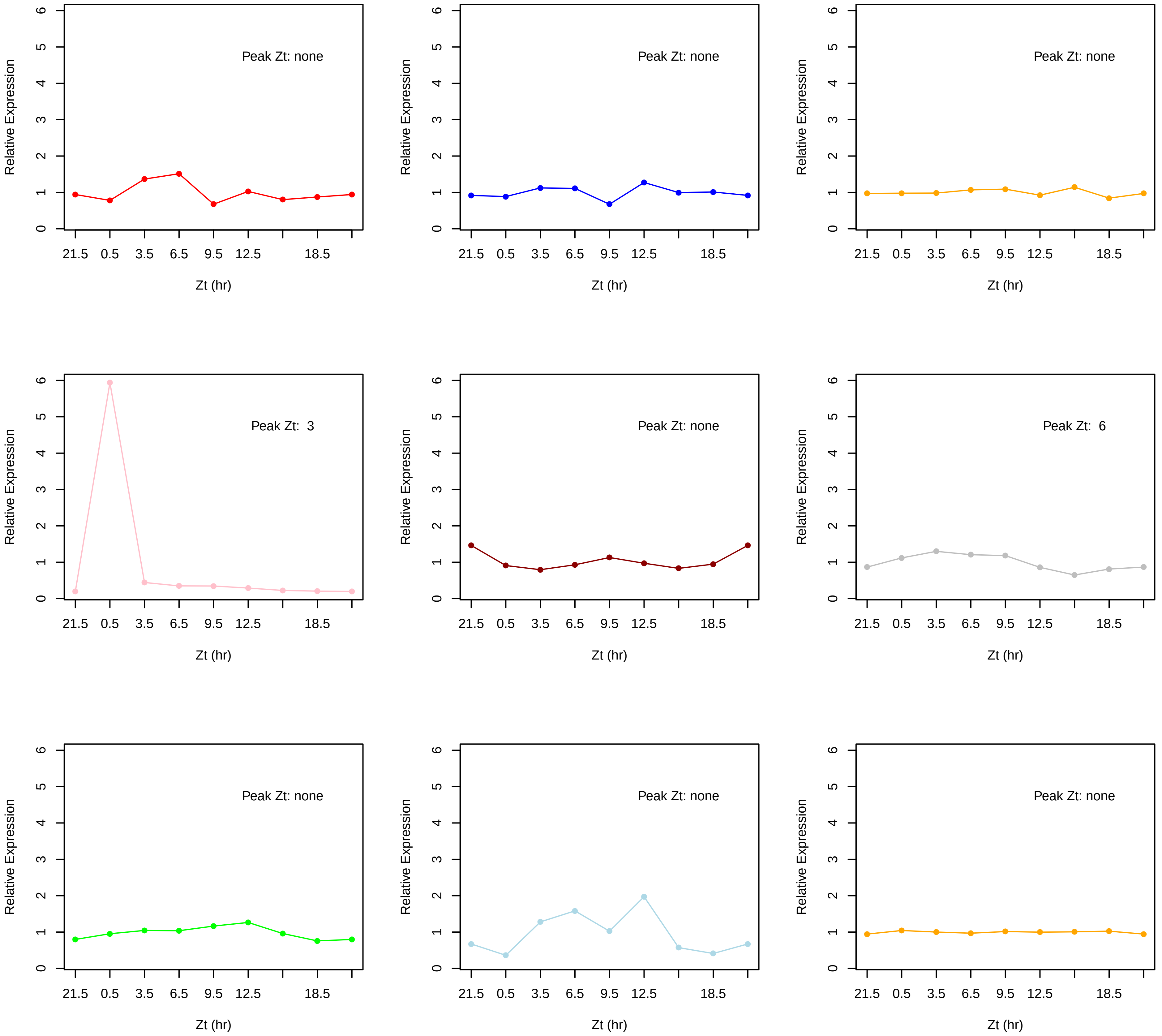

Singeleton: ENSXMAG00000002240 dnajc6

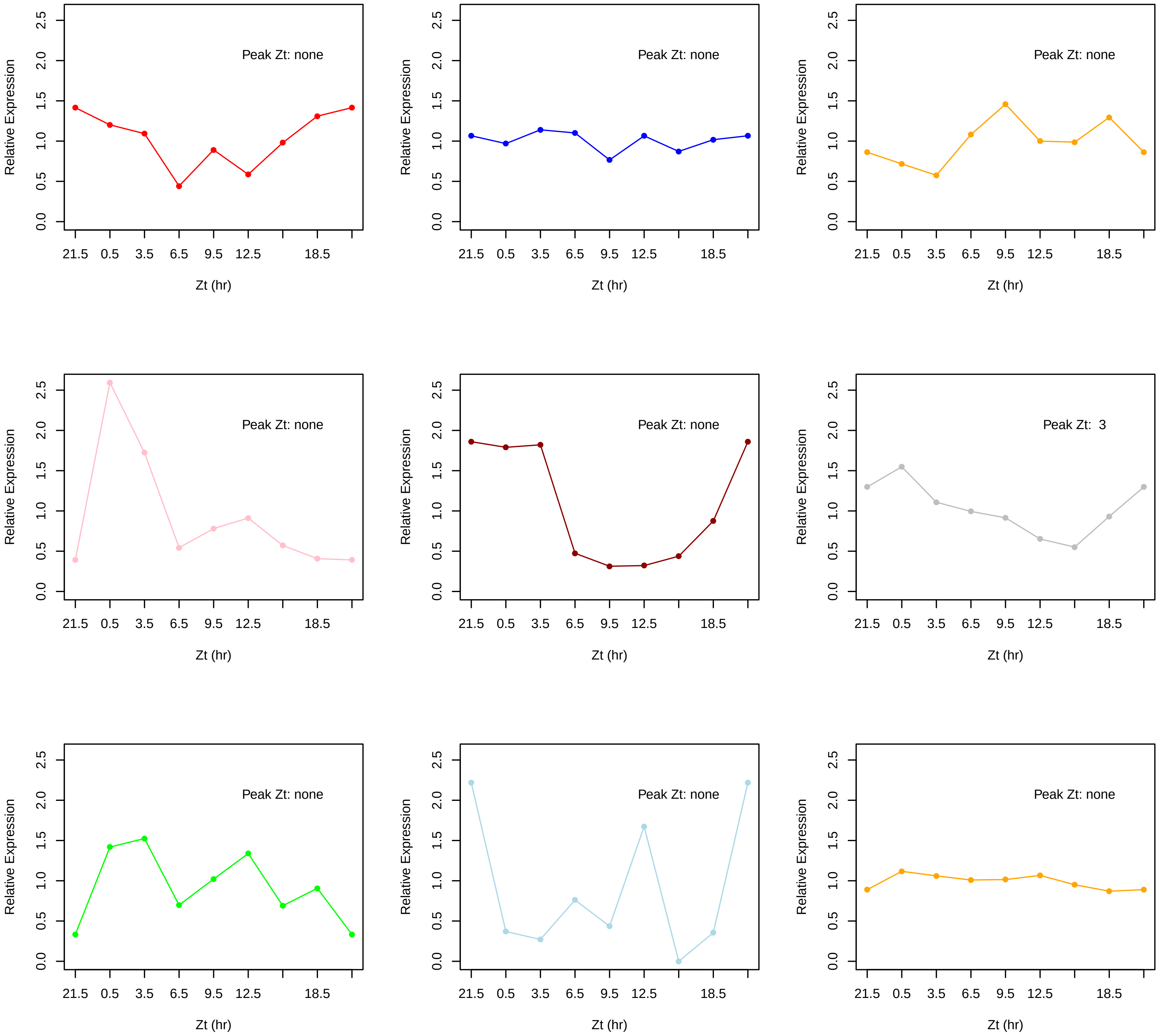

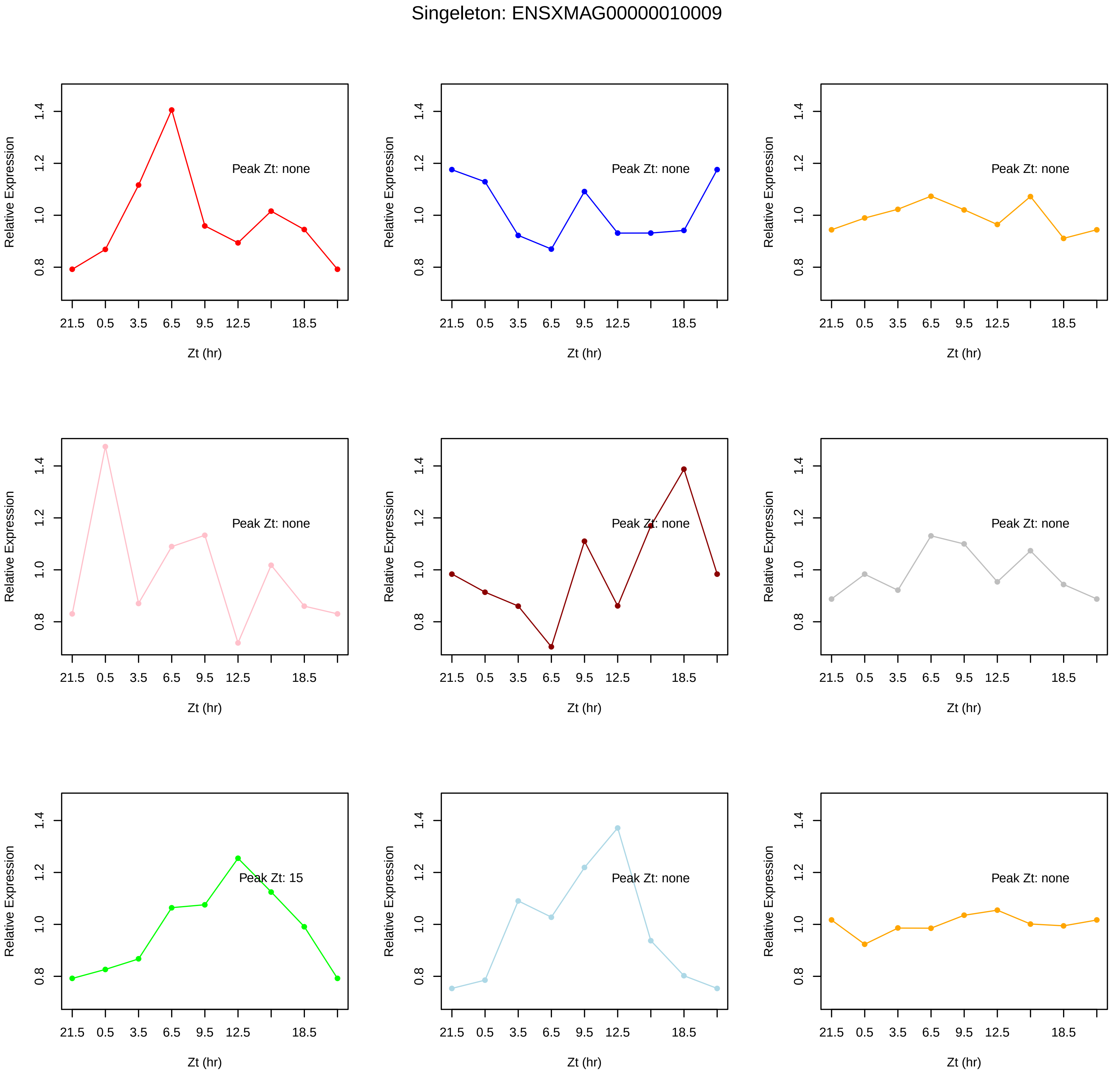
Singeleton: ENSXMAG00000010009
Peak Zt: none
Peak Zt: none
Peak Zt: none
Peak Zt: none
Peak Zt: none
Peak Zt: none
Peak Zt: 15
Peak Zt: none
Peak Zt: none
Relative Expression
Zt (hr)
0.8
1.0
1.2
1.4
21.5
0.5
3.5
6.5
9.5
12.5
18.5

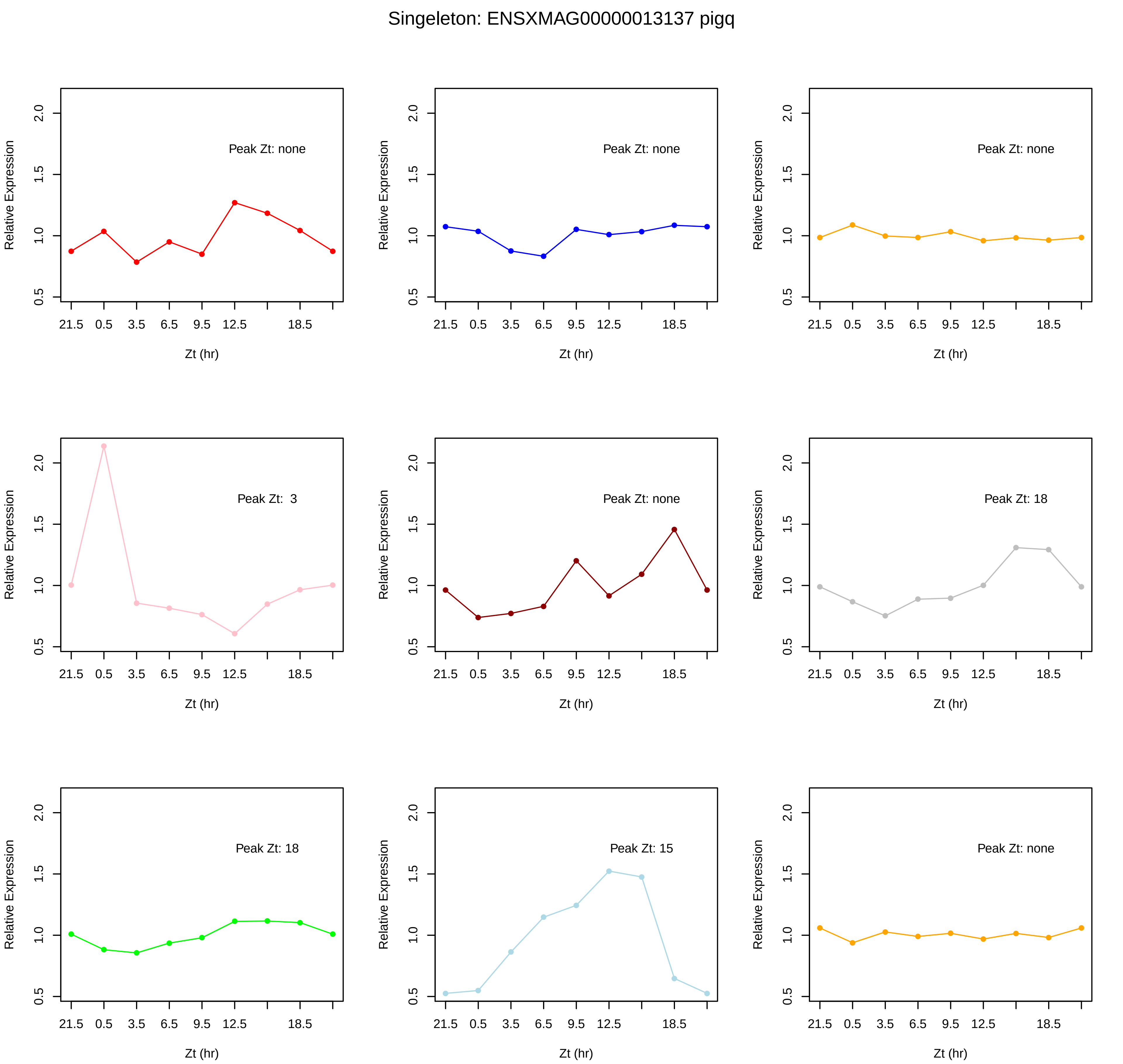
Singeleton: ENSXMAG00000013137 pigq
Peak Zt: none
Peak Zt: none
Peak Zt: none
Peak Zt: 3
Peak Zt: none
Peak Zt: 18
Peak Zt: 18
Peak Zt: 15
Peak Zt: none
Relative Expression
Zt (hr)
21.5
0.5
3.5
6.5
9.5
12.5
18.5
0.5
1.0
1.5
2.0

Singeleton: ENSXMAG00000024956 tnfrsf1a

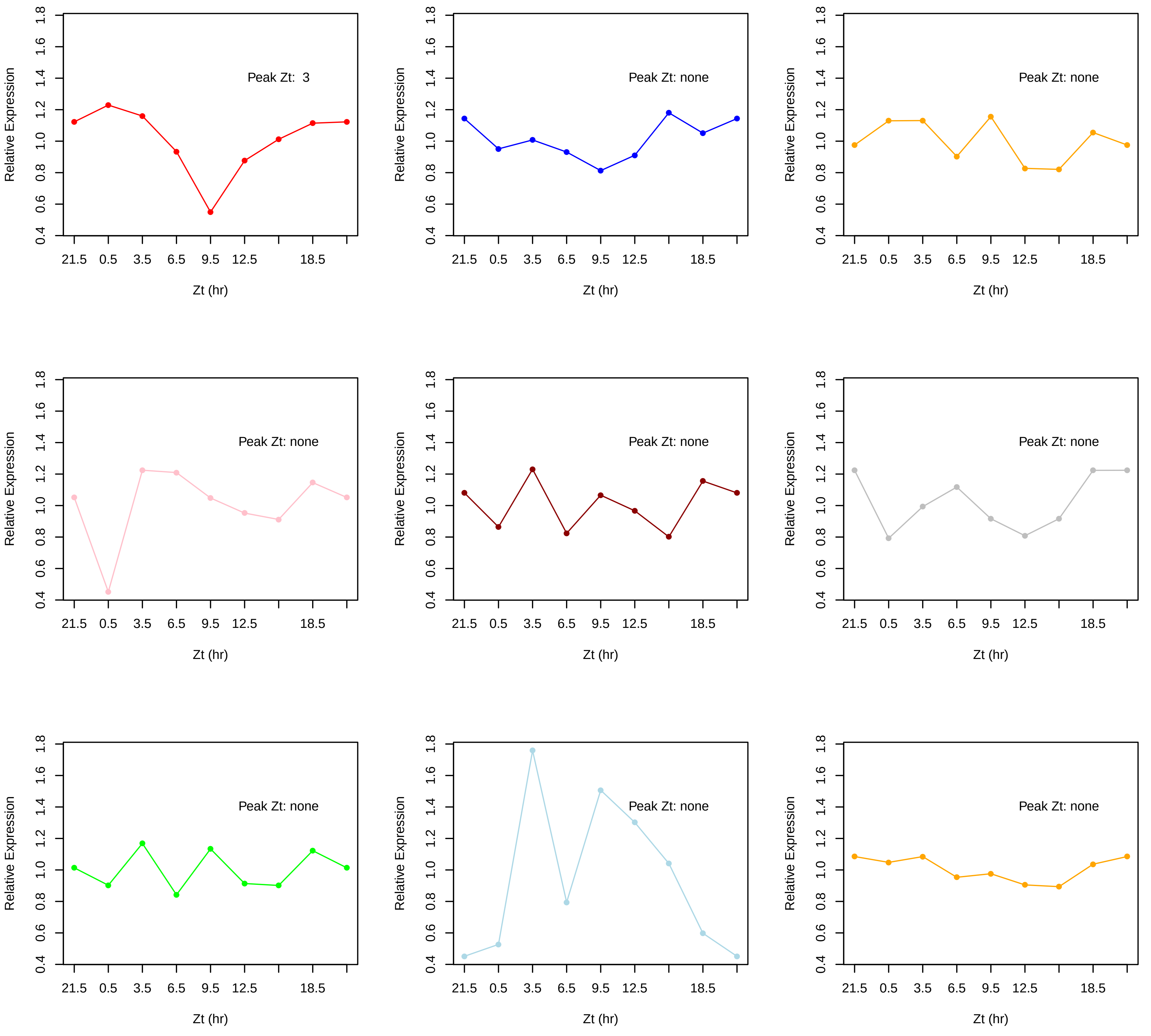

Singeleton: ENSXMAG00000014154 jmjd1cb

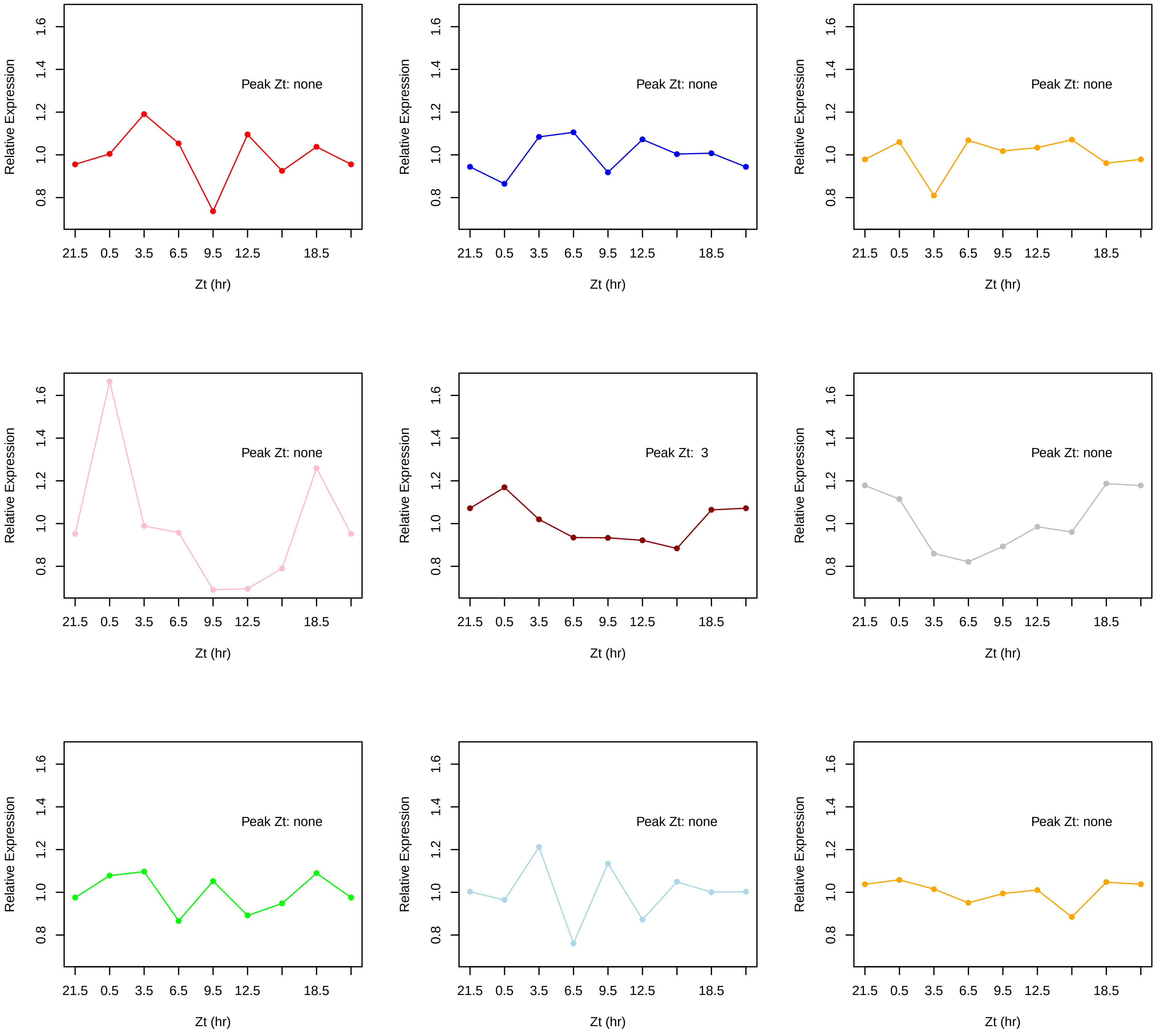

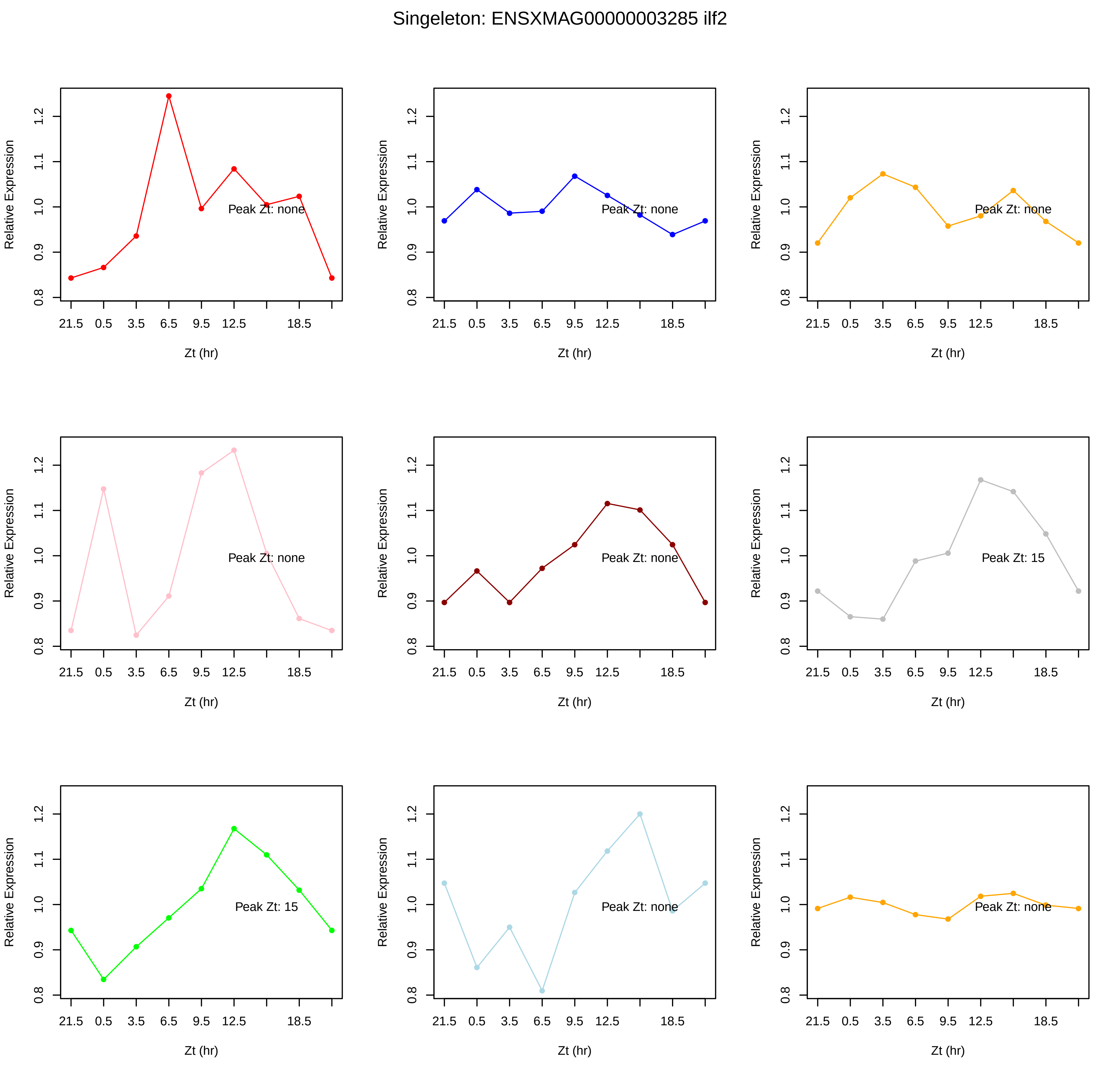
Singeleton: ENSXMAG00000003285 ilf2
Relative Expression
Zt (hr)
Peak Zt: none
Peak Zt: 15
21.5
0.5
3.5
6.5
9.5
12.5
18.5
0.8
0.9
1.0
1.1
1.2

Singeleton: ENSXMAG00000023048 LEPROT

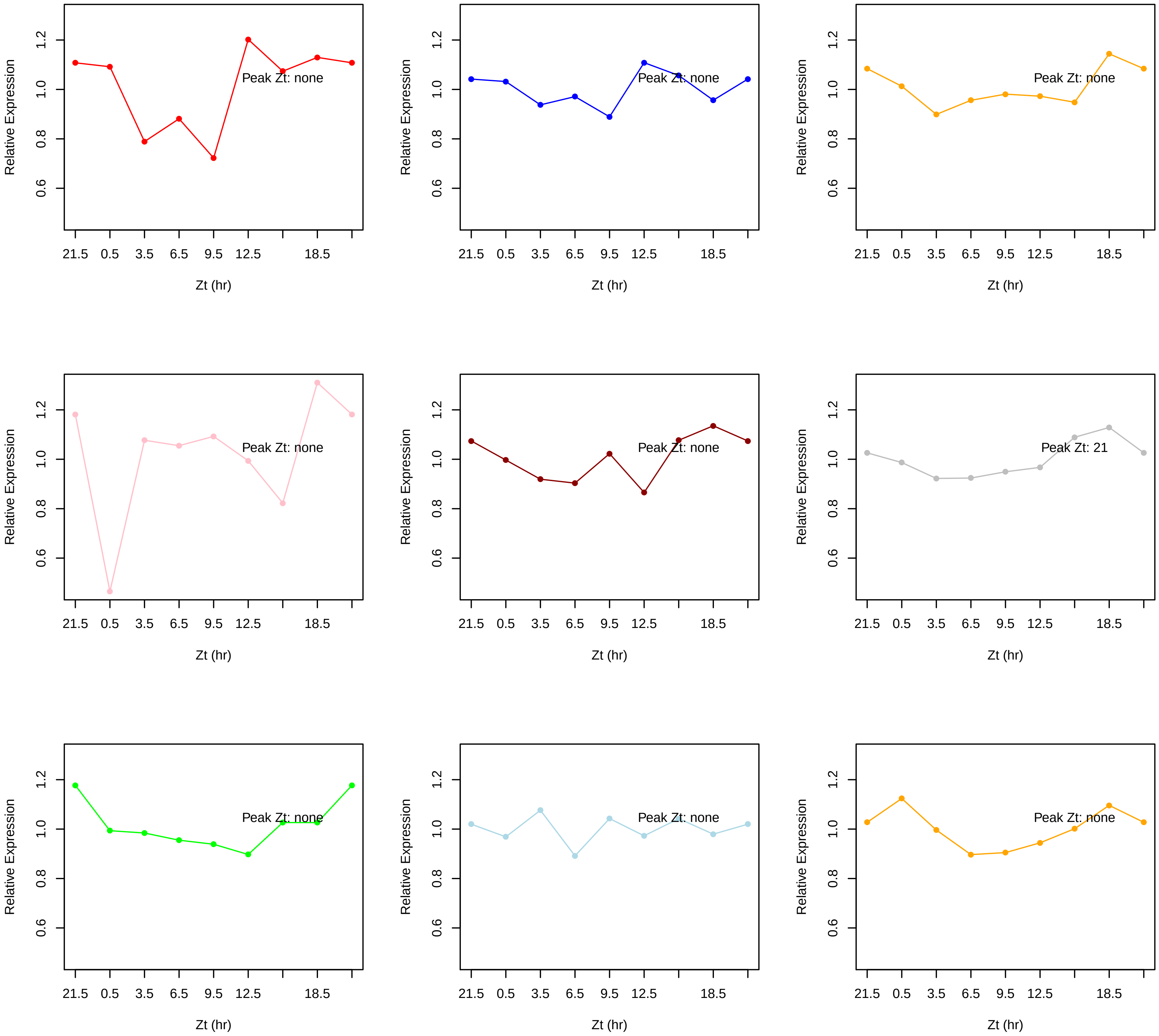

Singeleton: ENSXMAG00000018718

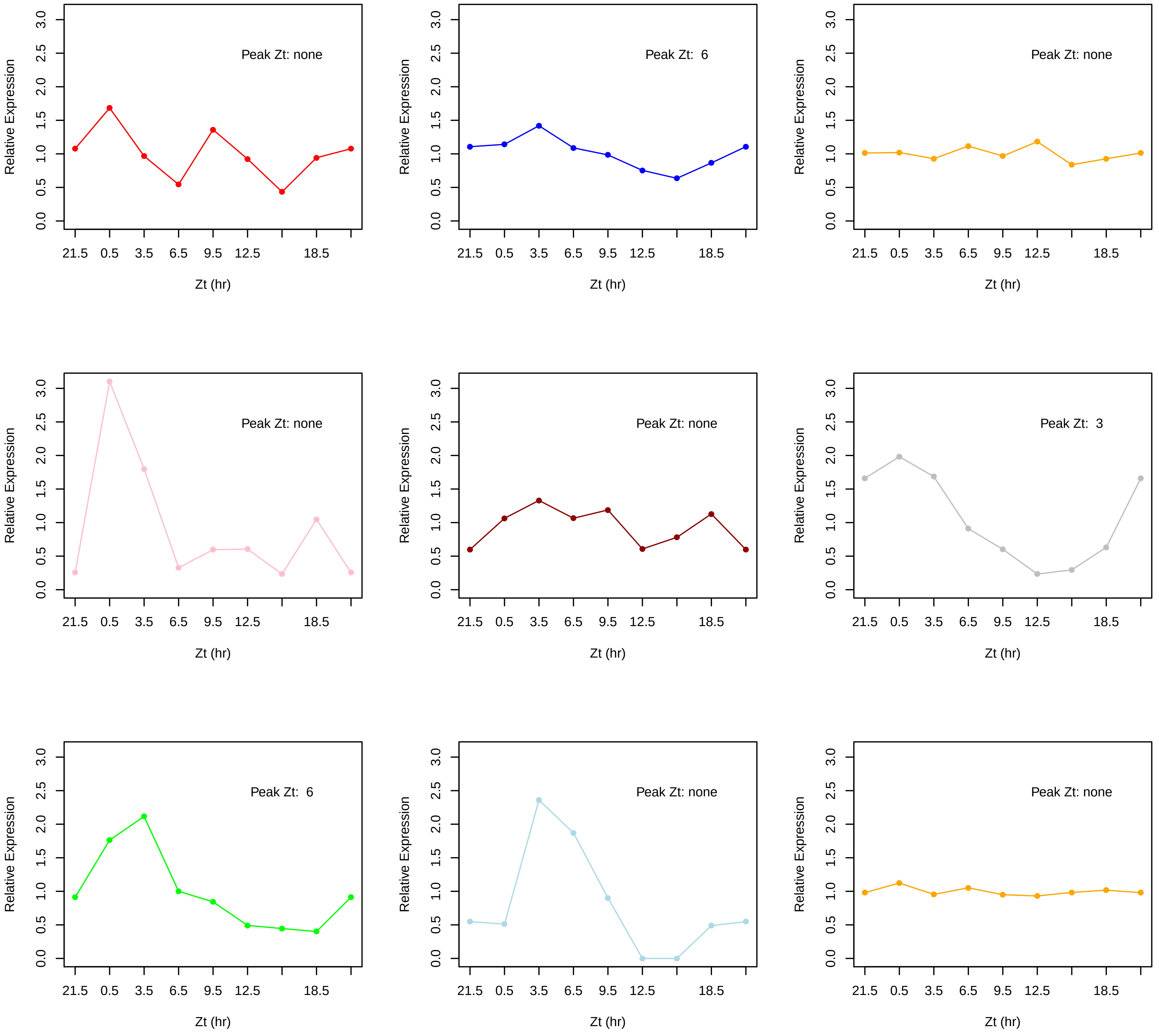

Singleton: ENSXMAG00000016826 gstt1a

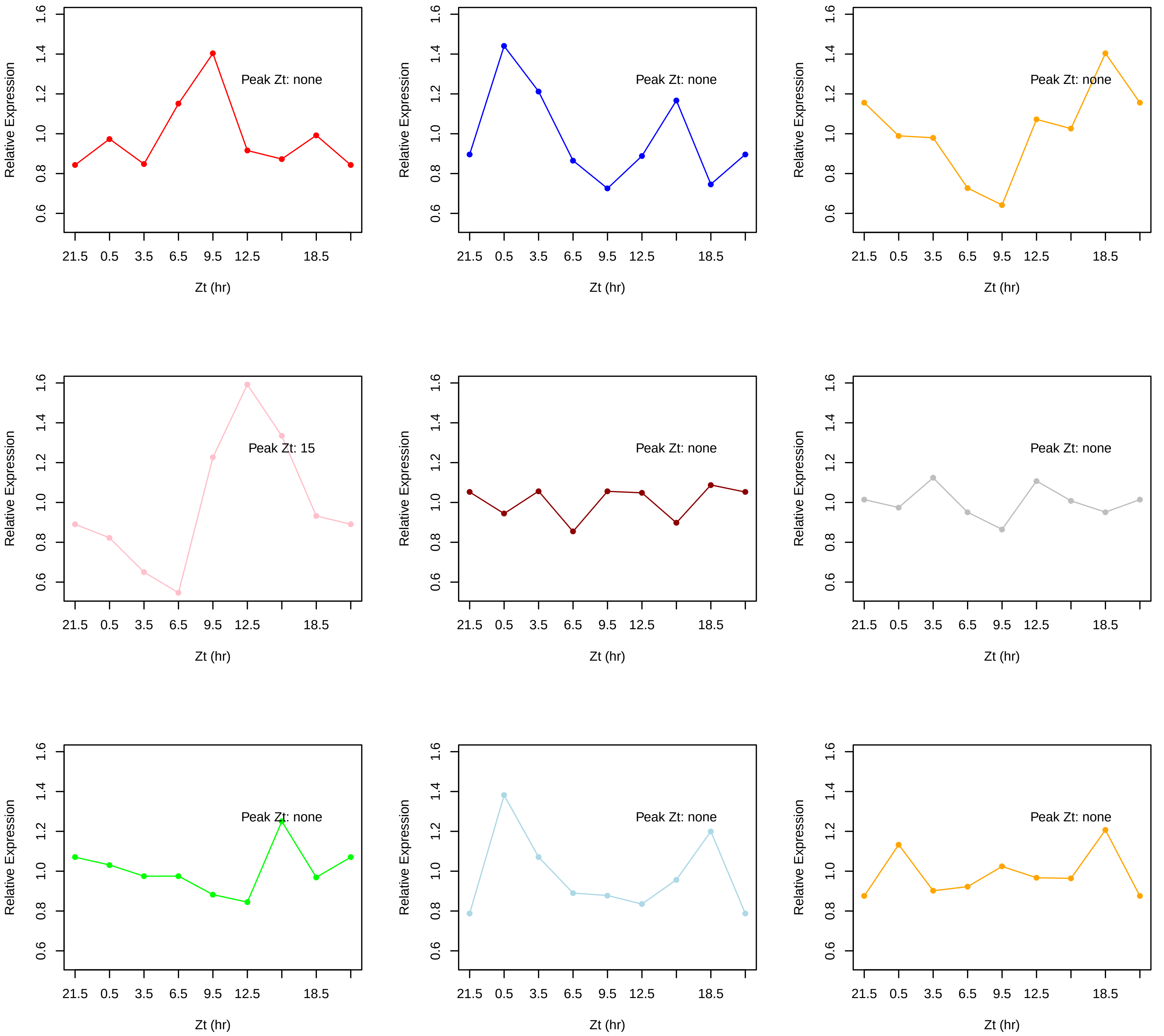

Singeleton: ENSXMAG00000002246 lepr

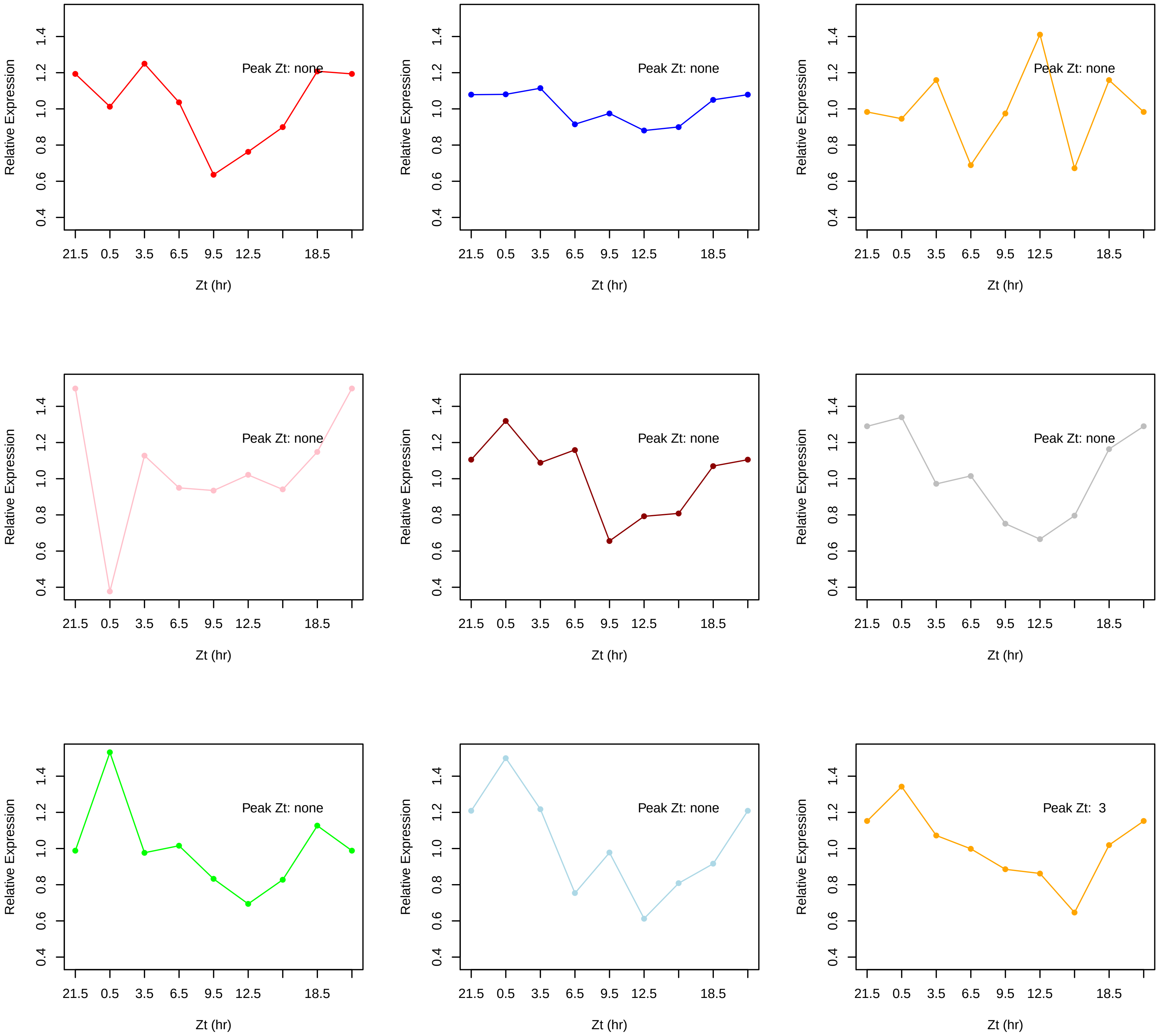

Singeleton: ENSXMAG00000012658

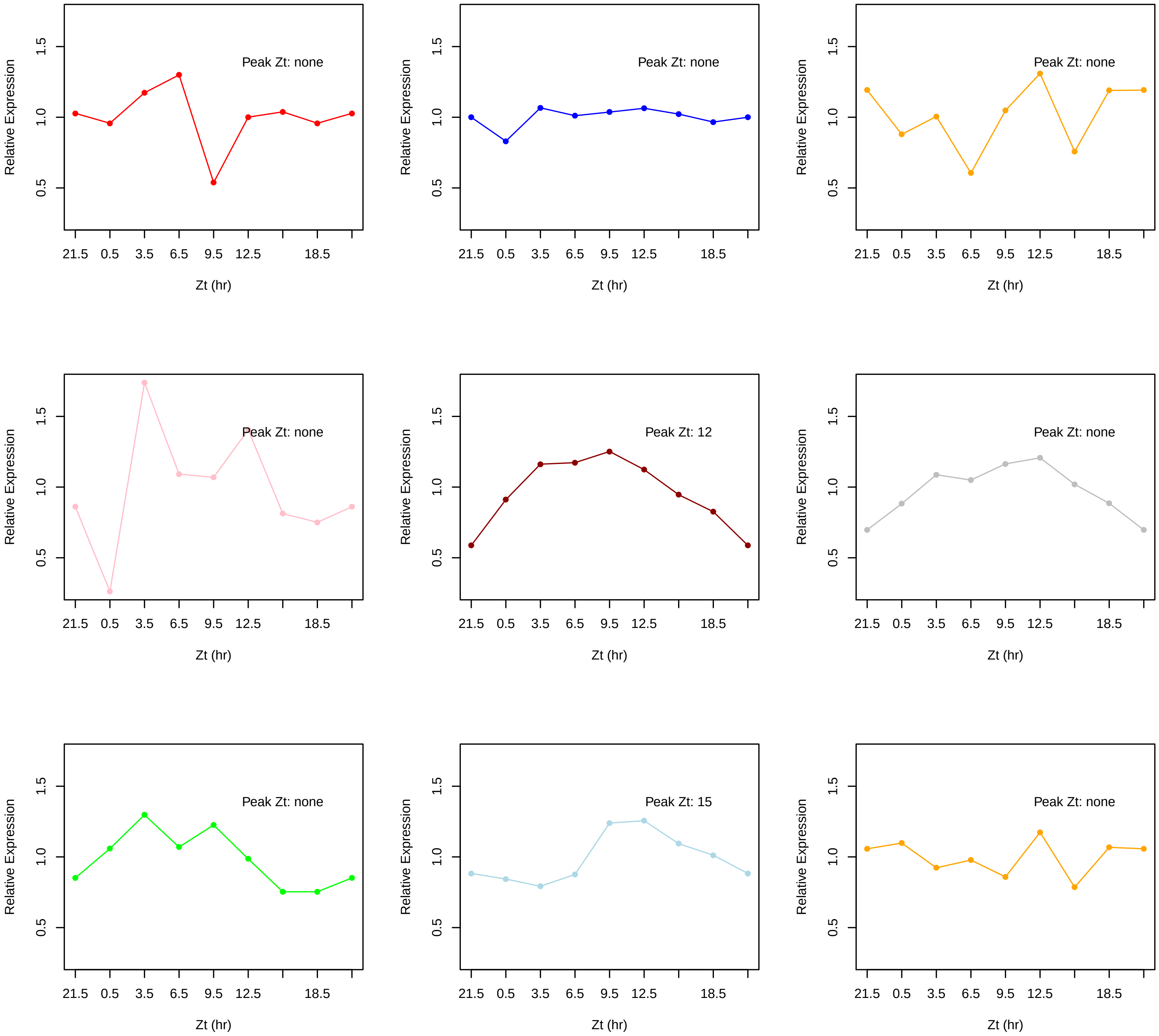

Singleton: ENSXMAG00000016936 top2b

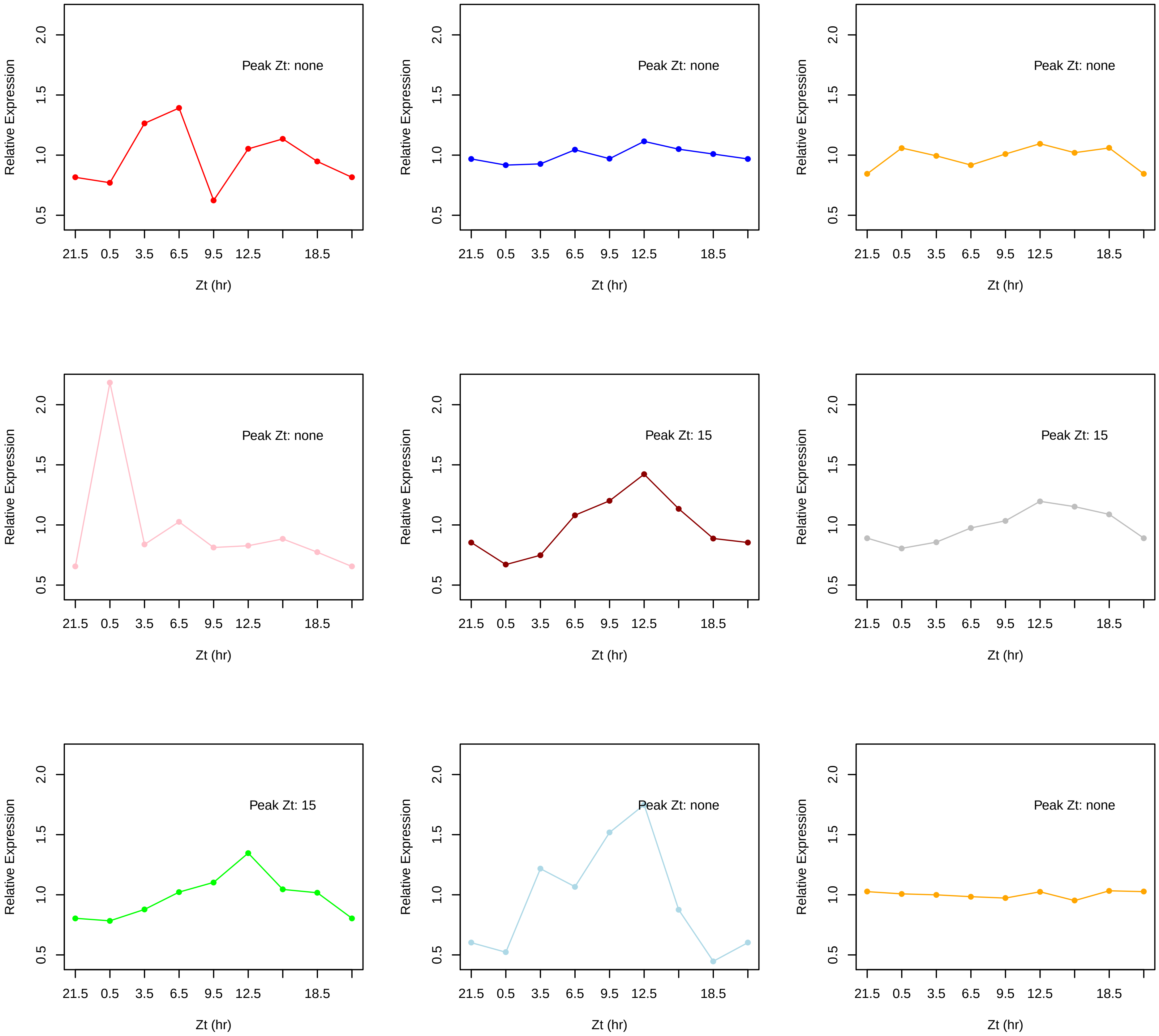

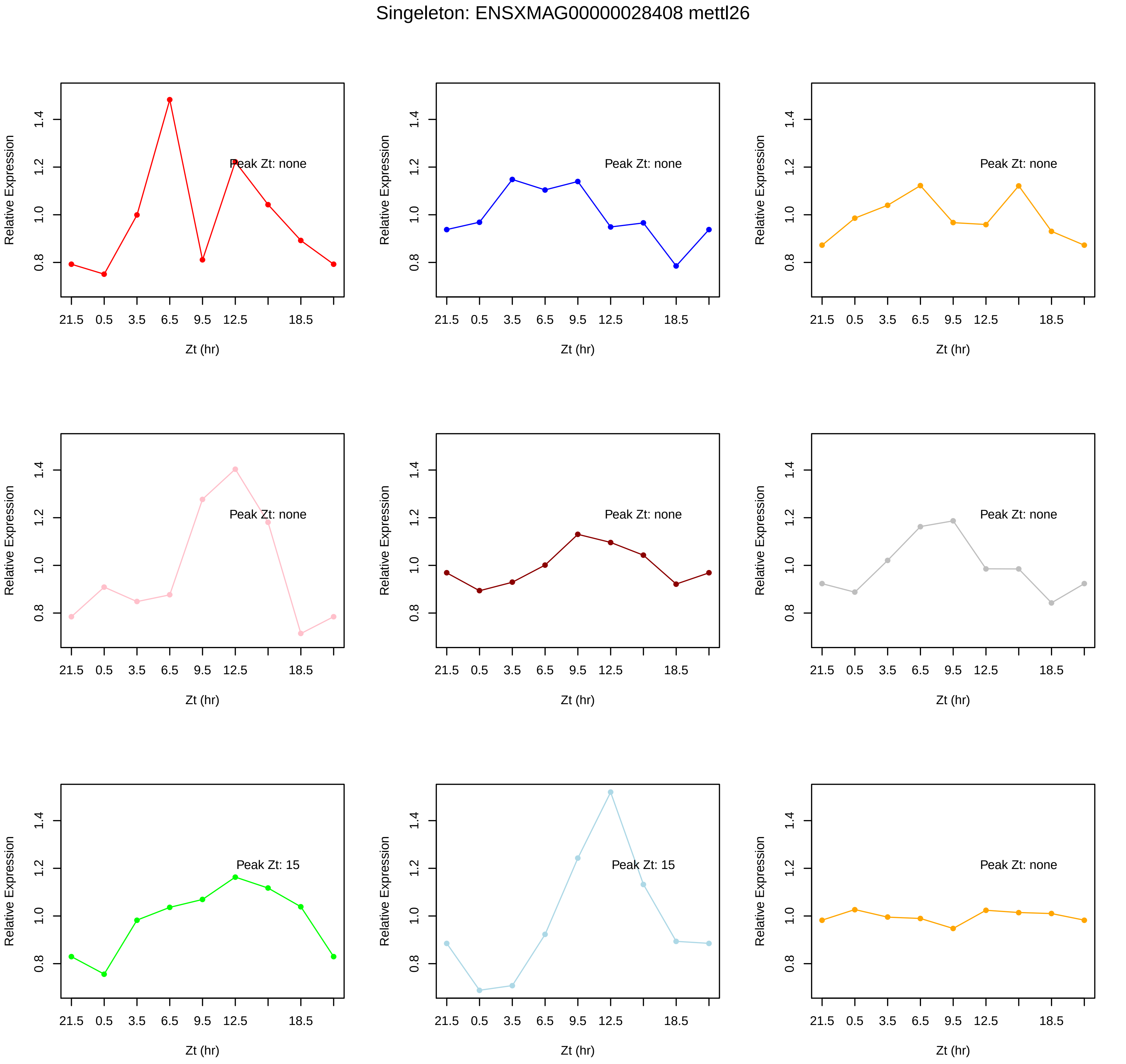
Singeleton: ENSXMAG00000028408 mettl26
Relative Expression
Zt (hr)
21.5 0.5 3.5 6.5 9.5 12.5 18.5
0.8 1.0 1.2 1.4
Peak Zt: none
Peak Zt: none
Peak Zt: none
Peak Zt: none
Peak Zt: none
Peak Zt: none
Peak Zt: 15
Peak Zt: 15
Peak Zt: none

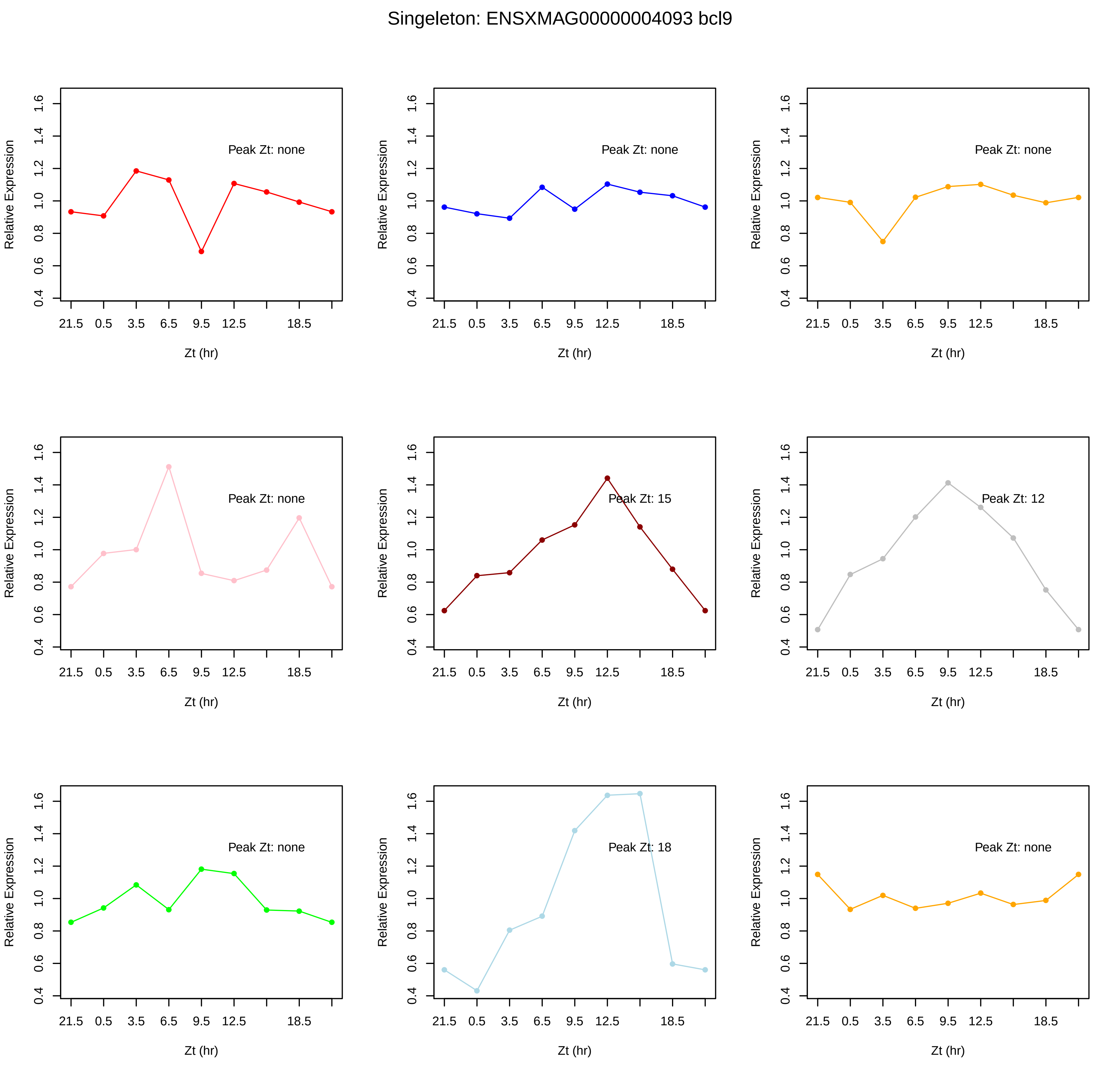

Singeleton: ENSXMAG00000004093 bcl9
Peak Zt: none
Peak Zt: none
Peak Zt: none
Peak Zt: none
Peak Zt: 15
Peak Zt: 12
Peak Zt: none
Peak Zt: 18
Peak Zt: none
Relative Expression
Zt (hr)
21.5
0.5
3.5
6.5
9.5
12.5
18.5

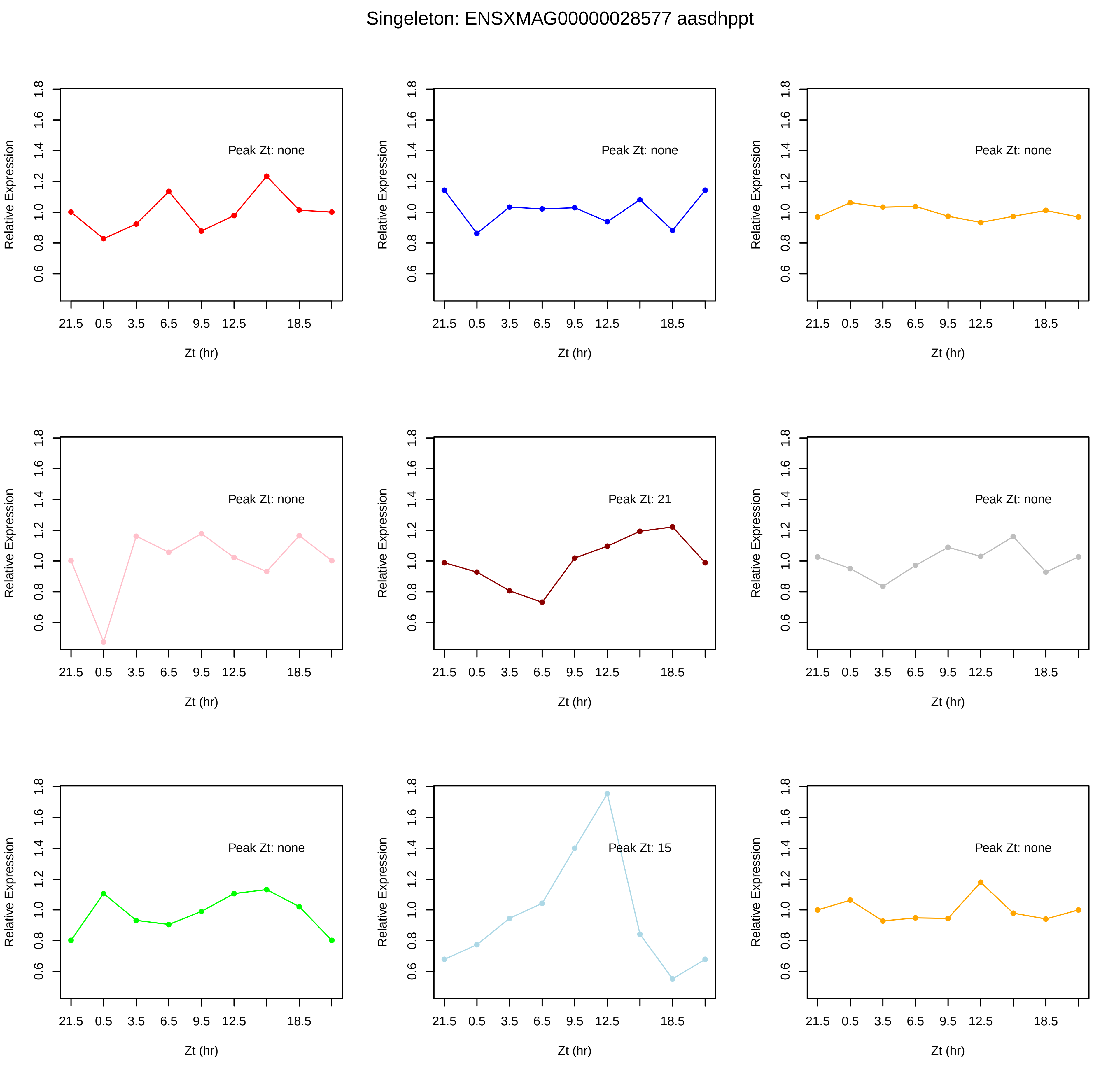

Singeleton: ENSXMAG00000028577 aasdhppt
Peak Zt: none
Peak Zt: none
Peak Zt: none
Peak Zt: none
Peak Zt: 21
Peak Zt: none
Peak Zt: none
Peak Zt: 15
Peak Zt: none
Relative Expression
Zt (hr)

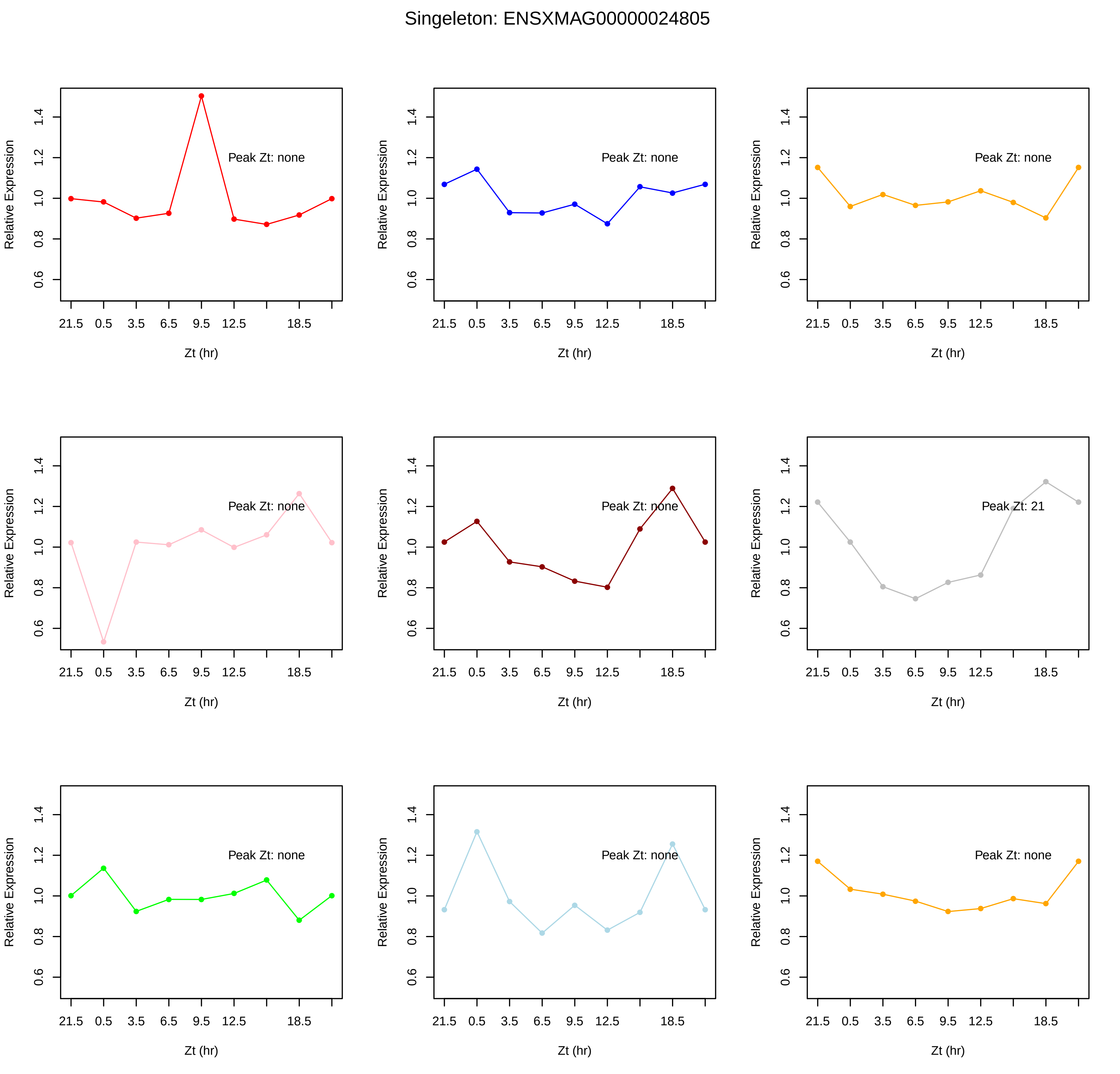

Singeleton: ENSXMAG00000024805
Relative Expression
Zt (hr)
Peak Zt: none
Peak Zt: none
Peak Zt: none
Peak Zt: none
Peak Zt: none
Peak Zt: 21
Peak Zt: none
Peak Zt: none
Peak Zt: none

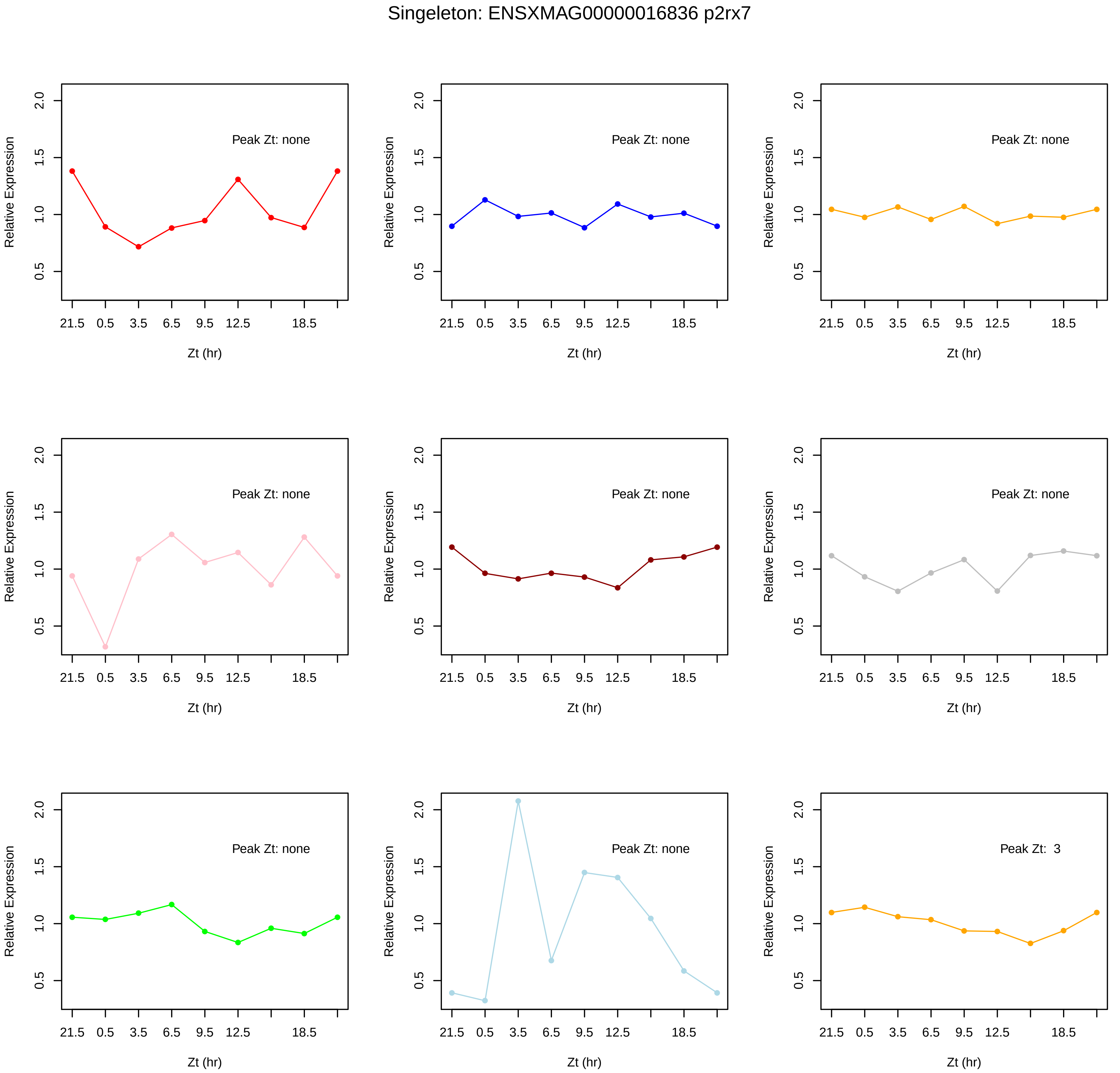

Singleton: ENSXMAG00000016836 p2rx7
Peak Zt: none
Peak Zt: none
Peak Zt: none
Peak Zt: none
Peak Zt: none
Peak Zt: none
Peak Zt: none
Peak Zt: none
Peak Zt: 3
Relative Expression
Zt (hr)
21.5 0.5 3.5 6.5 9.5 12.5 18.5
0.5 1.0 1.5 2.0

Singeleton: ENSXMAG00000017462 ints3

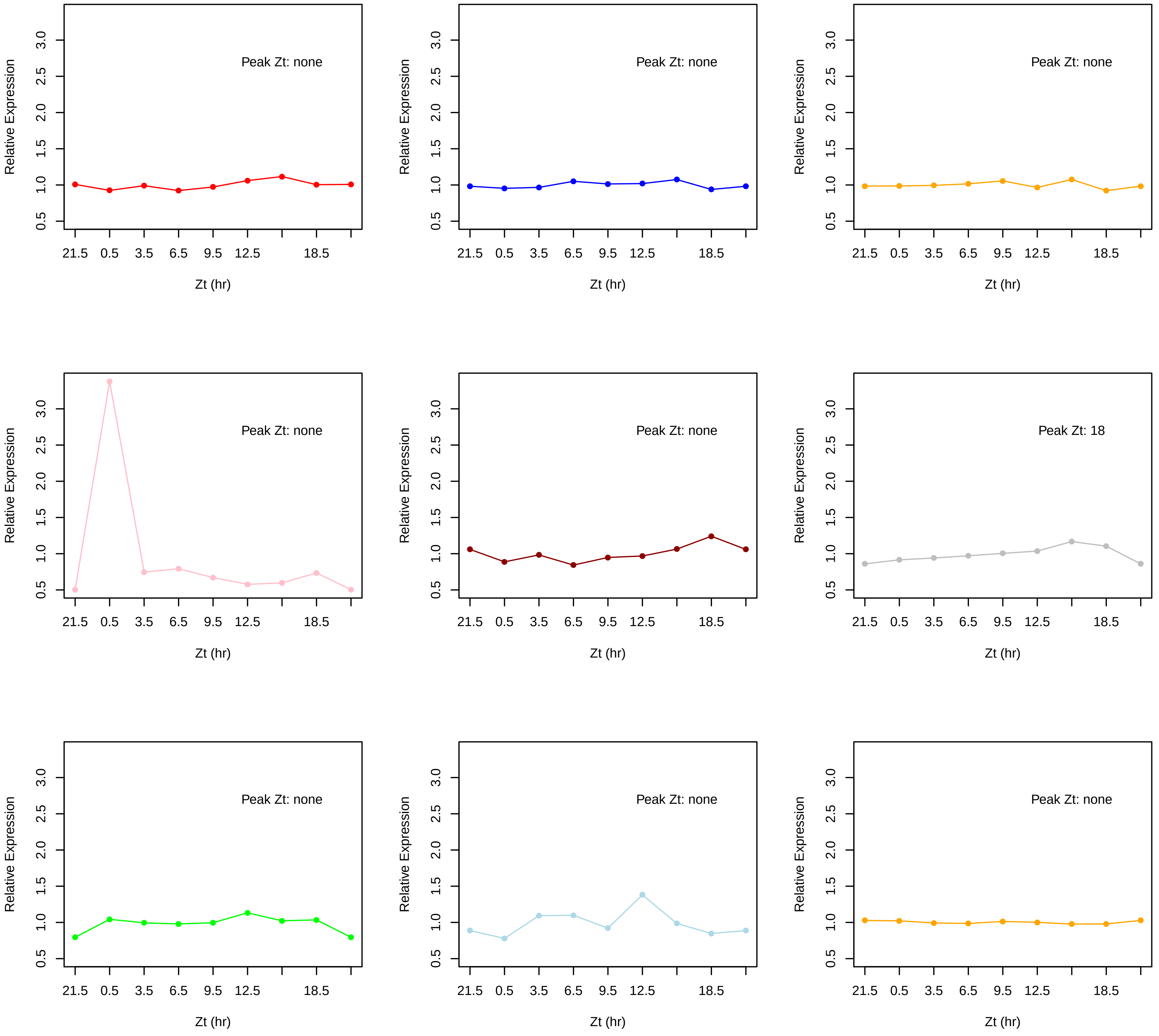

Singeleton: ENSXMAG00000014232

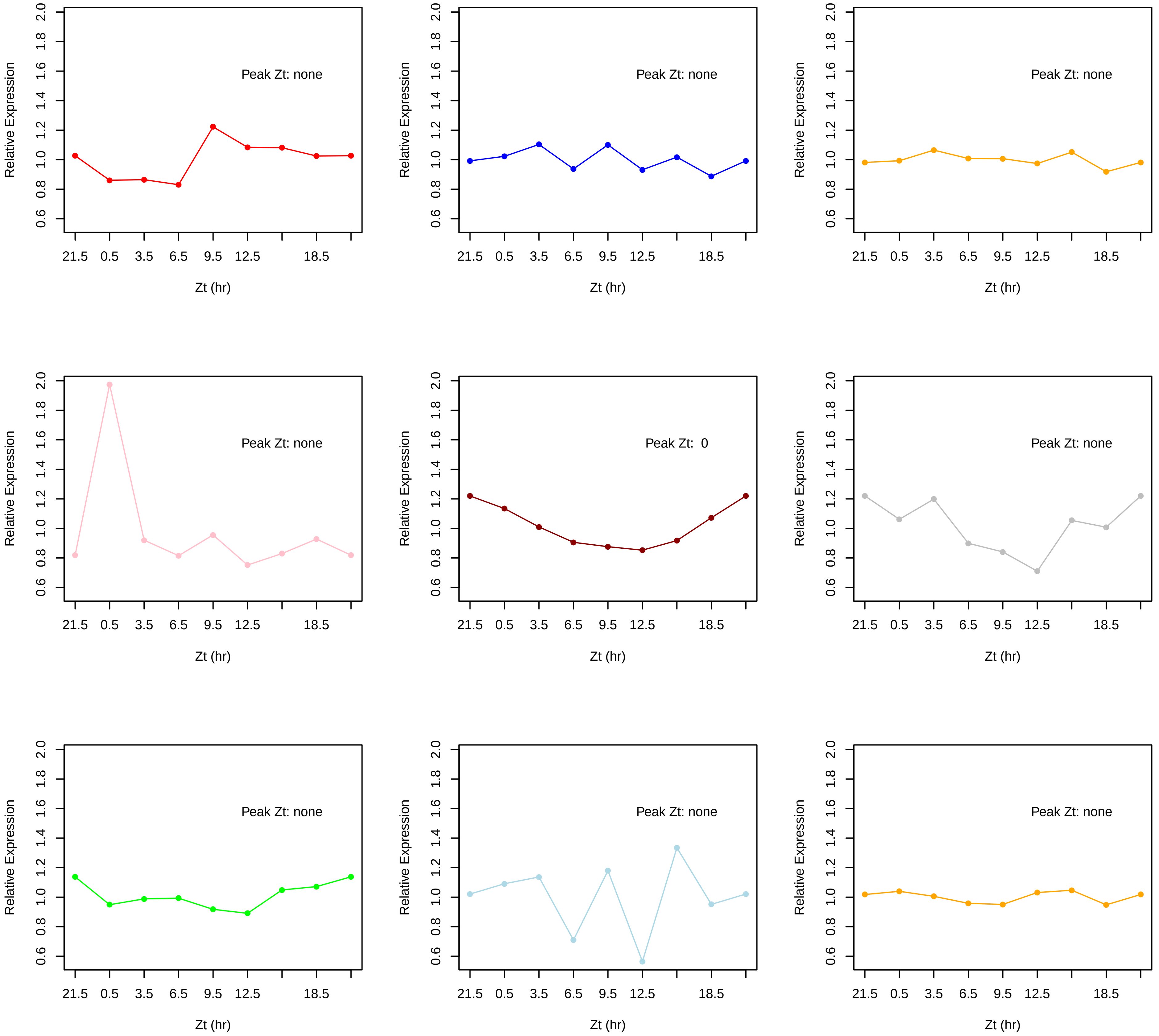

Singeleton: ENSXMAG00000023912 acp6

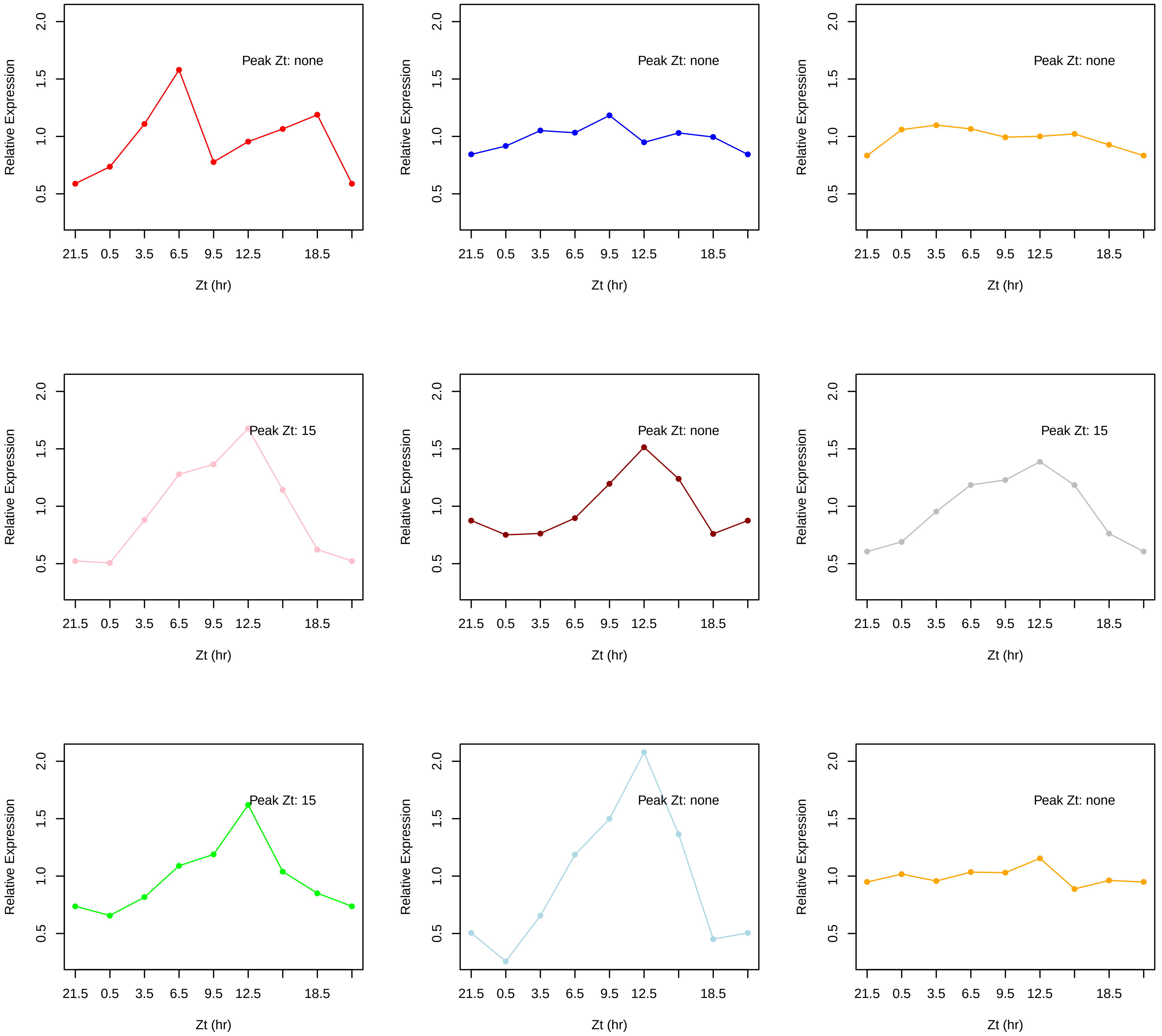

Singeleton: ENSXMAG00000016915 ngly1

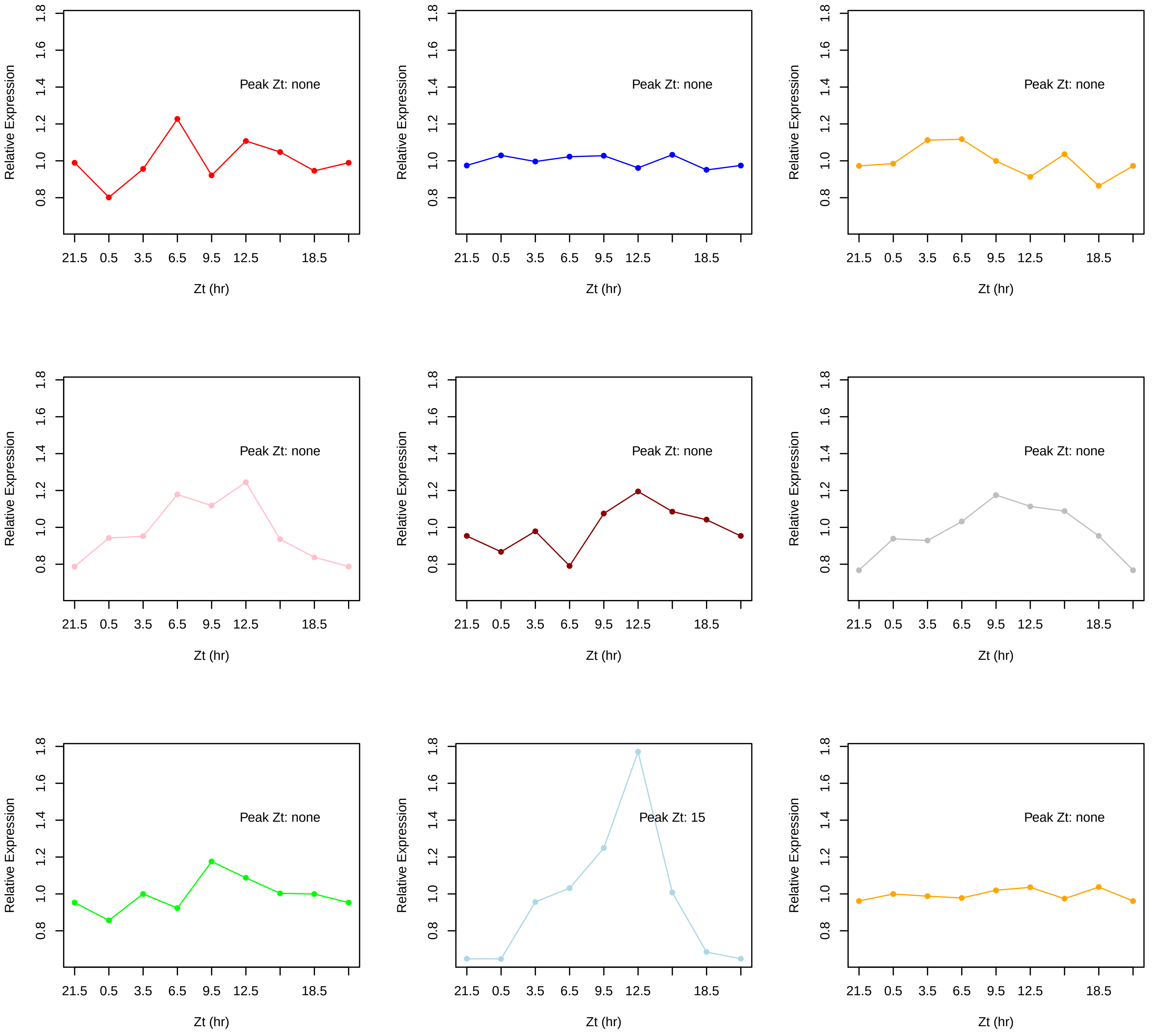

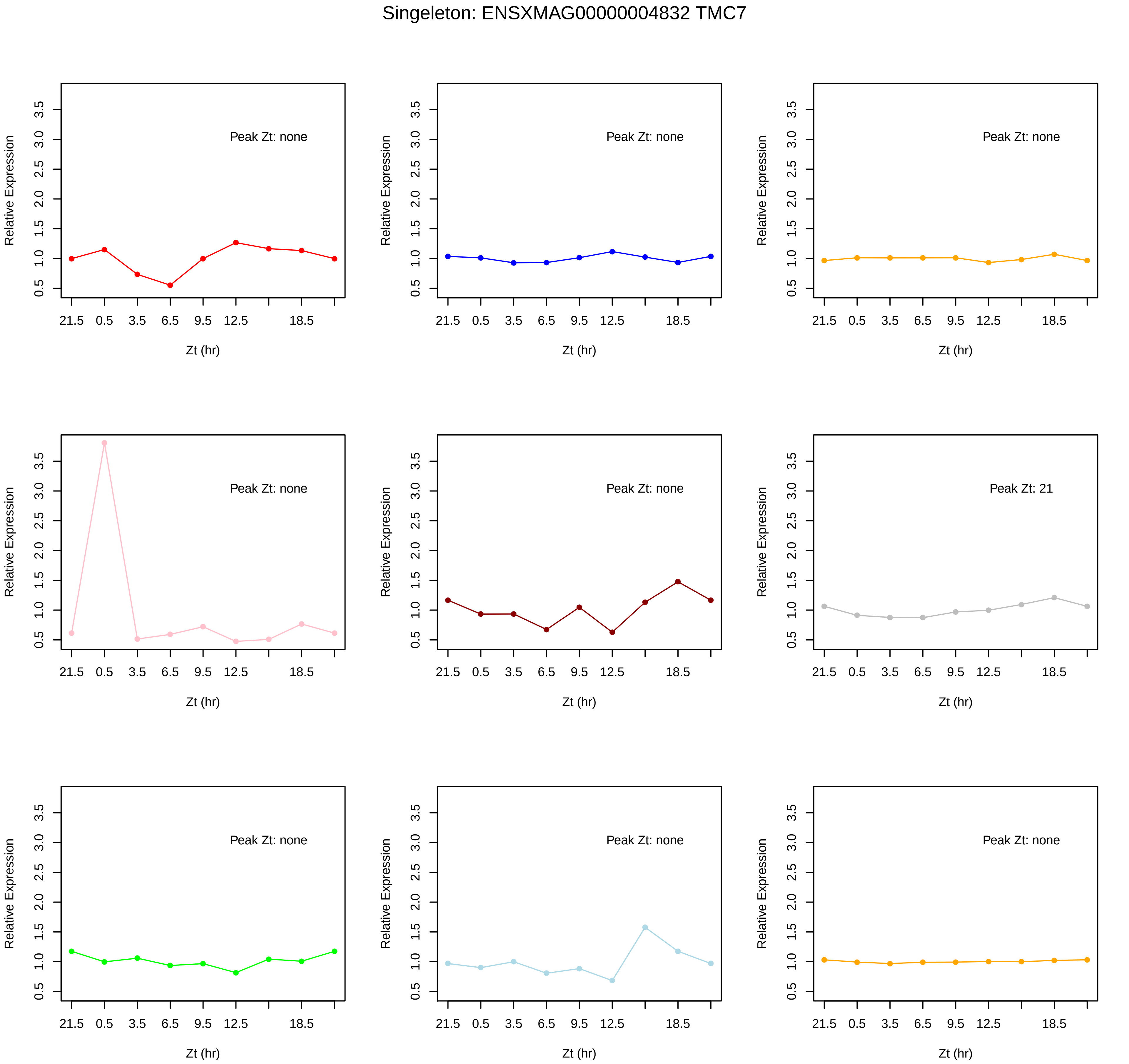

Singleton: ENSXMAG00000004832 TMC7
Peak Zt: none
Peak Zt: none
Peak Zt: none
Peak Zt: none
Peak Zt: none
Peak Zt: 21
Peak Zt: none
Peak Zt: none
Peak Zt: none
Relative Expression
Zt (hr)
21.5
0.5
3.5
6.5
9.5
12.5
18.5

Singeleton: ENSXMAG00000010188 fsta

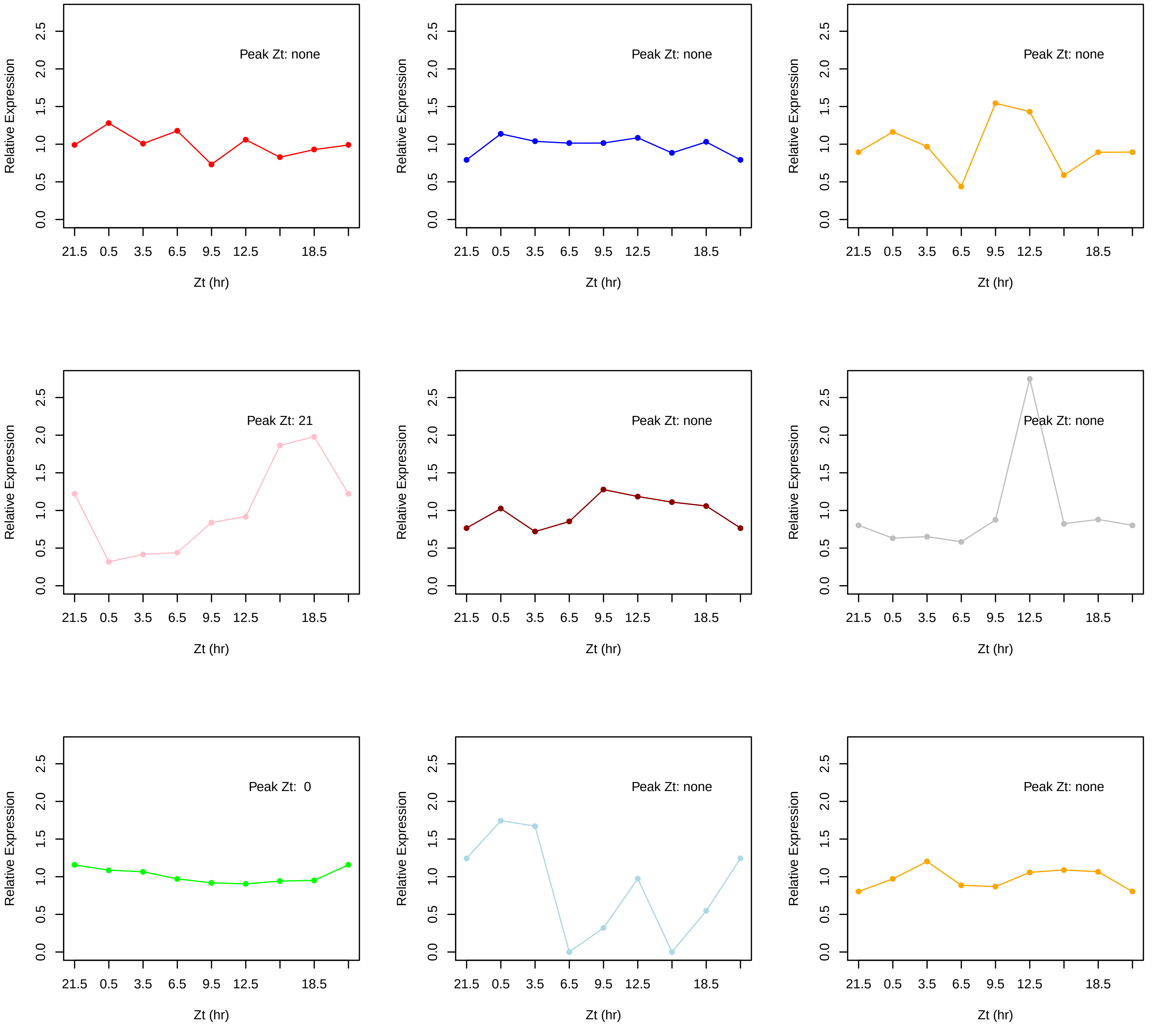

Singleton: ENSXMAG00000005099 syt11b

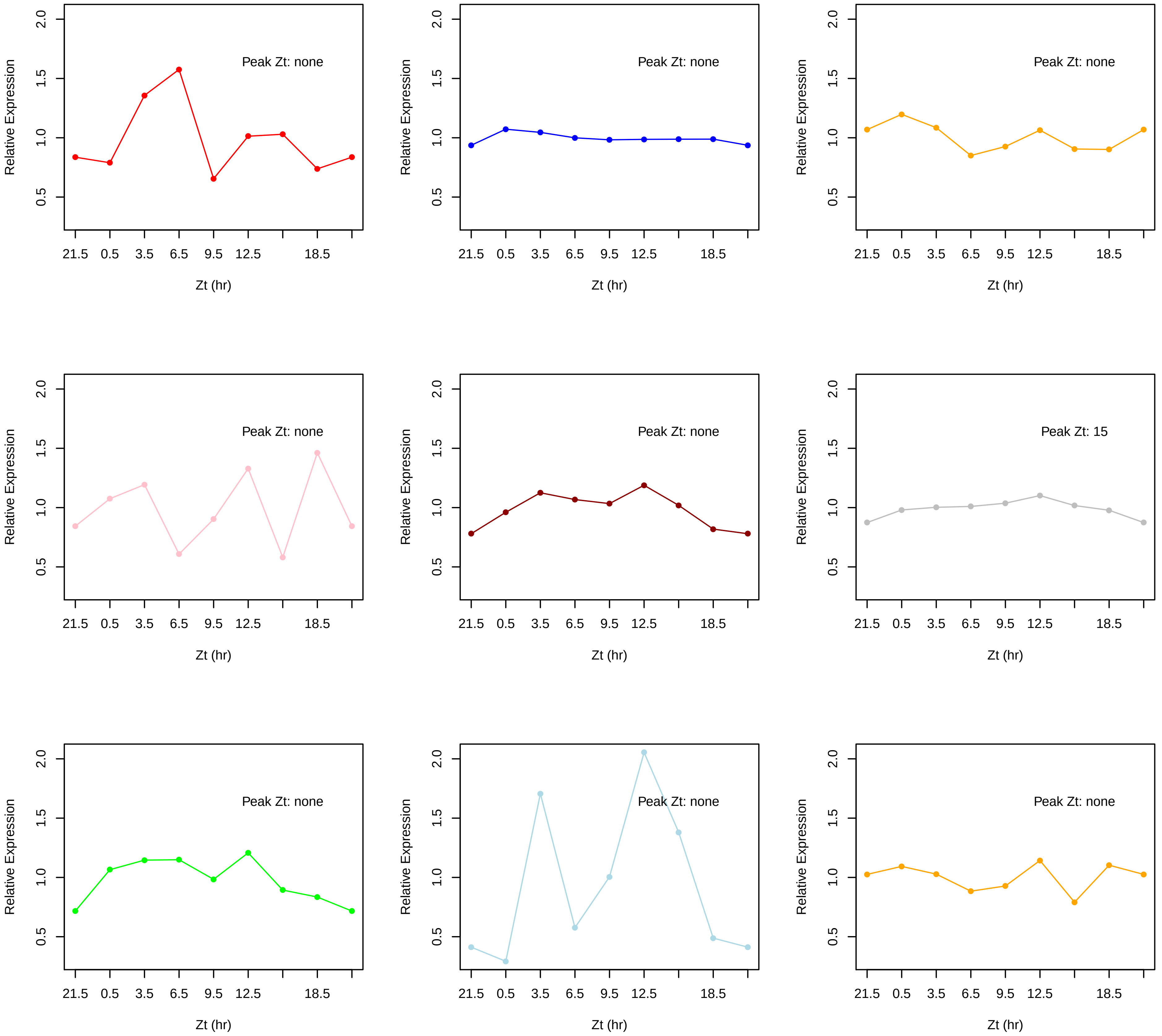

Singleton: ENSXMAG00000017400 amfra

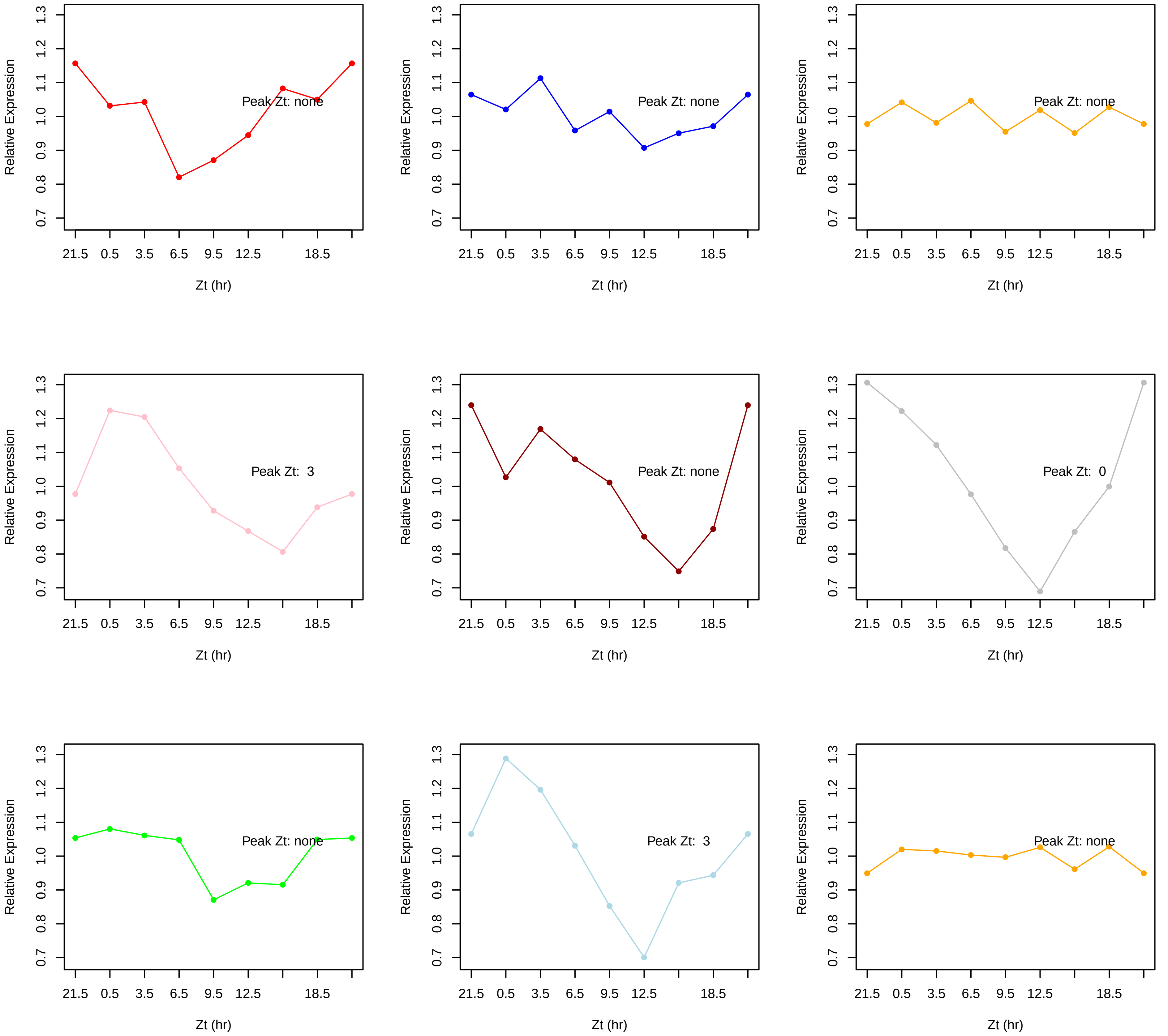

Singeleton: ENSXMAG00000011619 tmem201

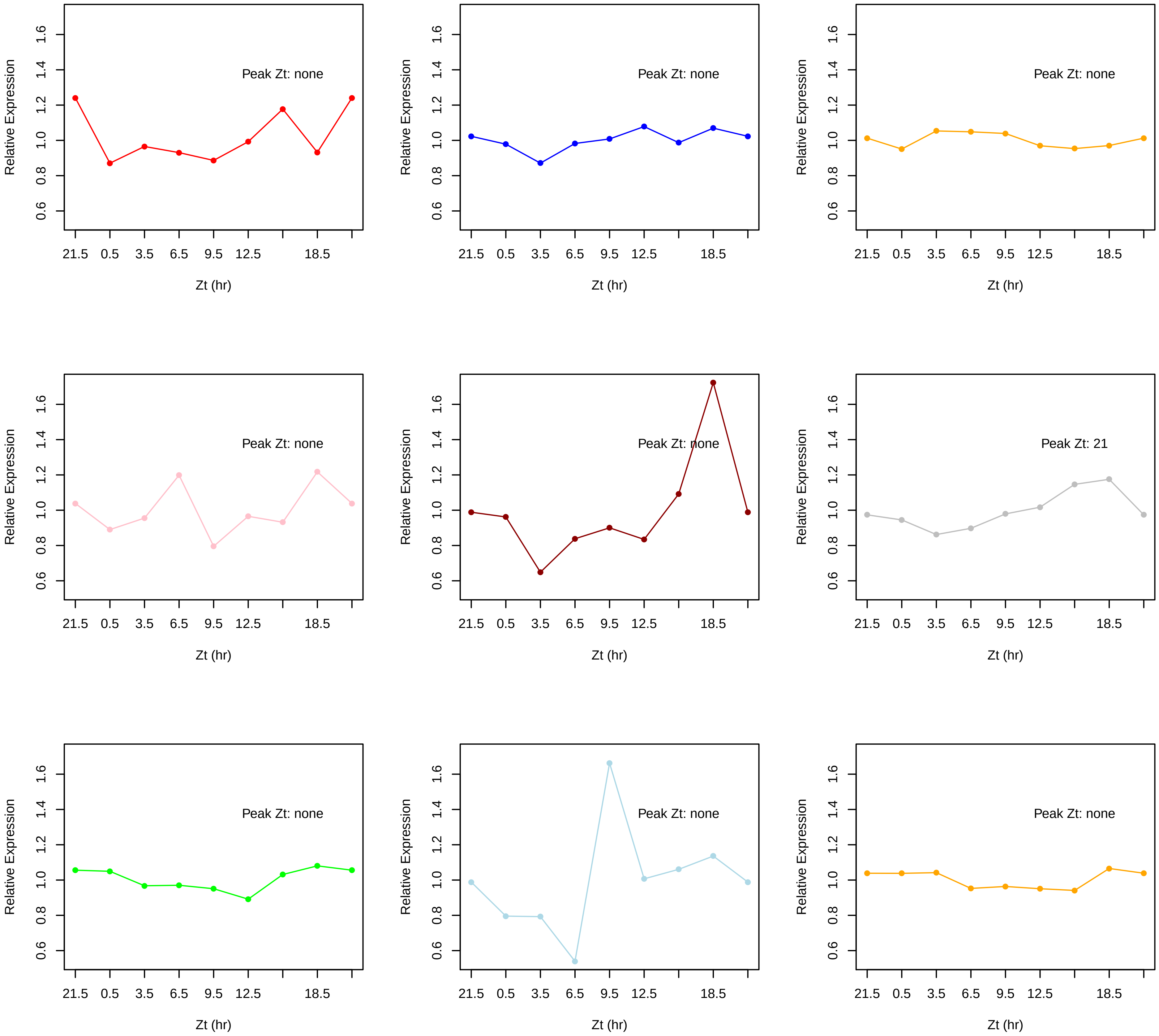

Singleton: ENSXMAG00000015165

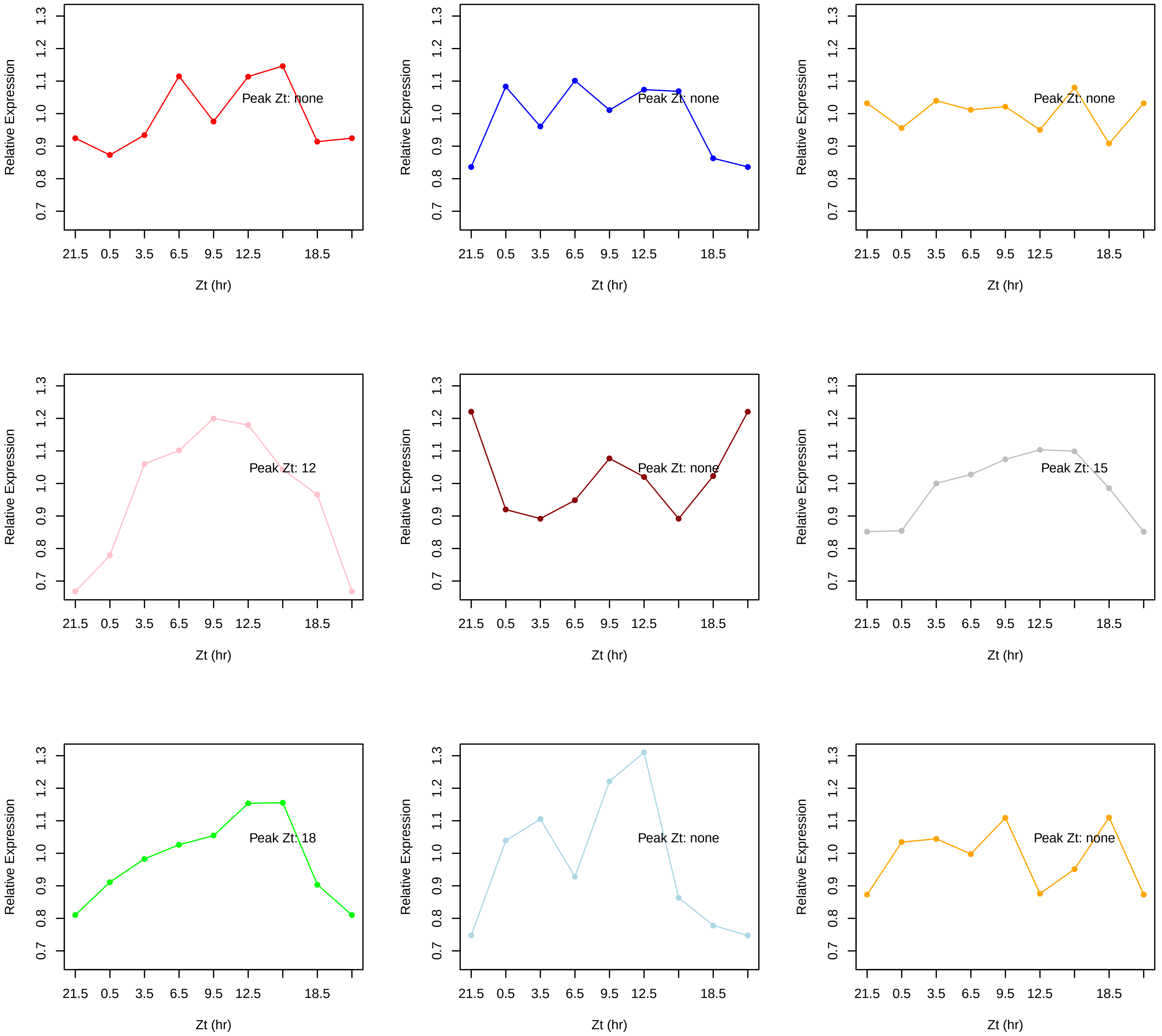

Singleton: ENSXMAG00000009950 doc2d

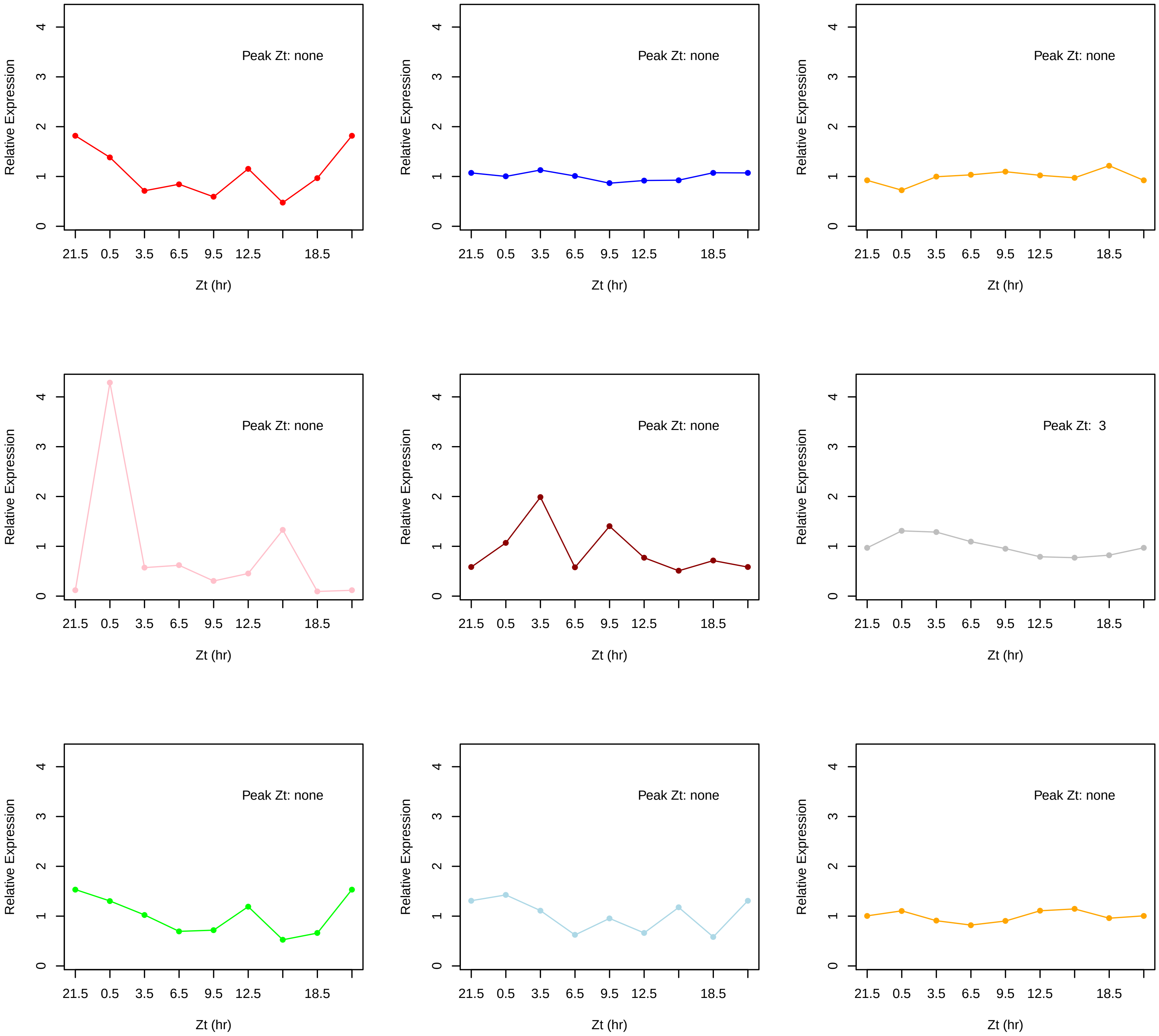

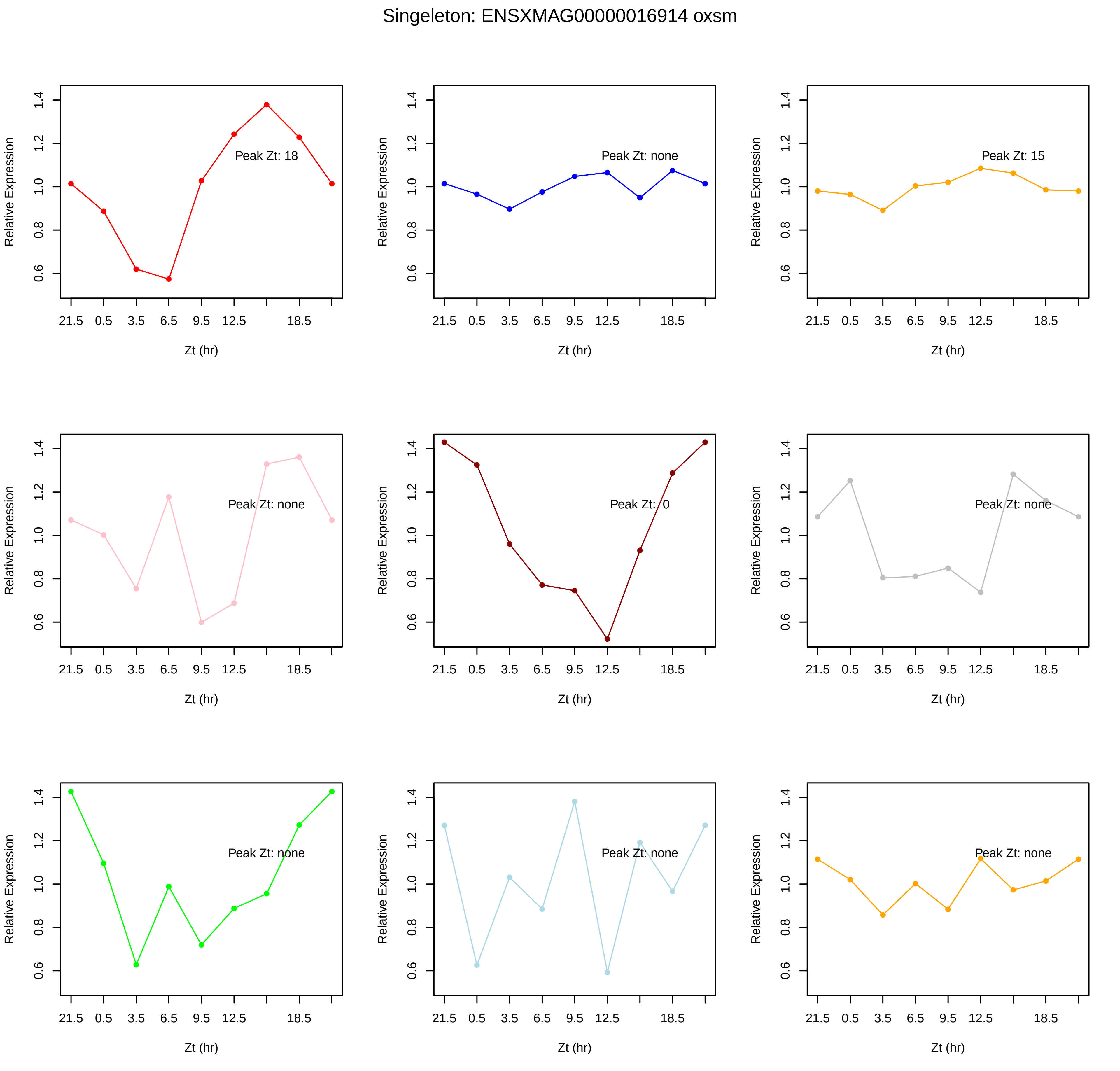
Singeleton: ENSXMAG00000016914 oxsm
Relative Expression
Zt (hr)
21.5 0.5 3.5 6.5 9.5 12.5 18.5
0.6 0.8 1.0 1.2 1.4
Peak Zt: 18
Peak Zt: none
Peak Zt: 15
Peak Zt: none
Peak Zt: 0
Peak Zt: none
Peak Zt: none
Peak Zt: none
Peak Zt: none

Singeleton: ENSXMAG00000010194 ndufs4

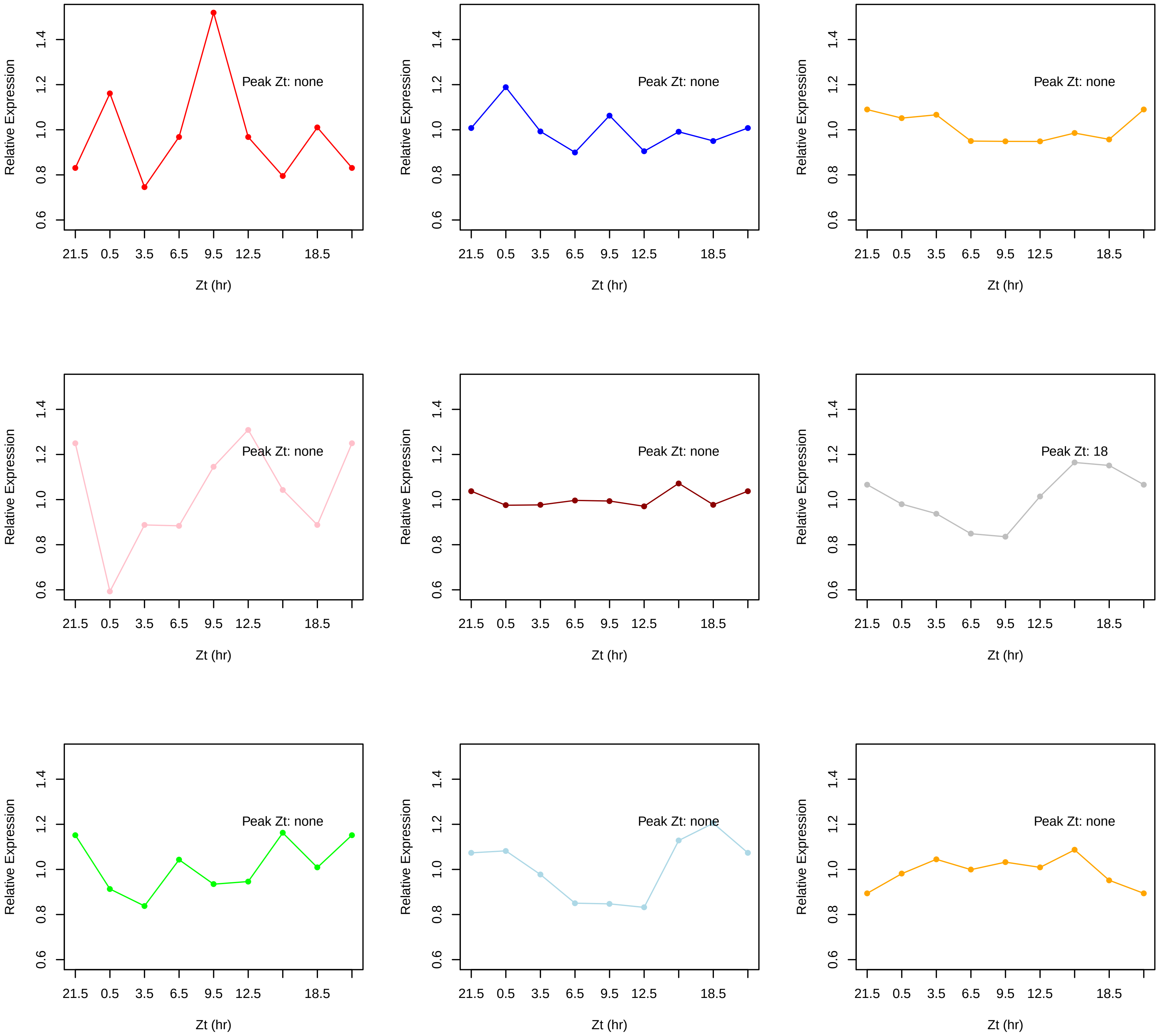

Singleton: ENSXMAG00000004835 nomo

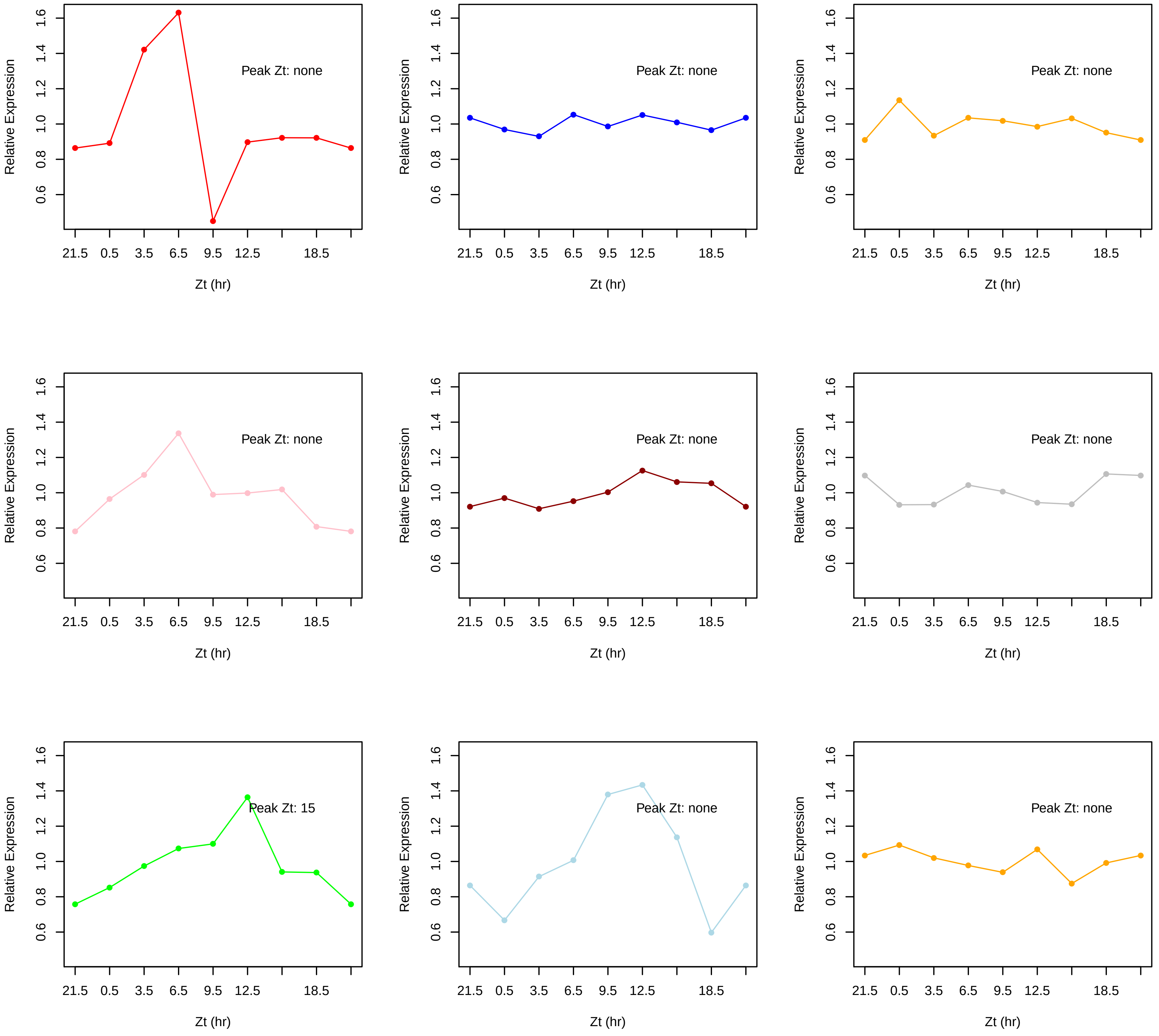

Singeleton: ENSXMAG00000013544 si:dkey−21a6.5

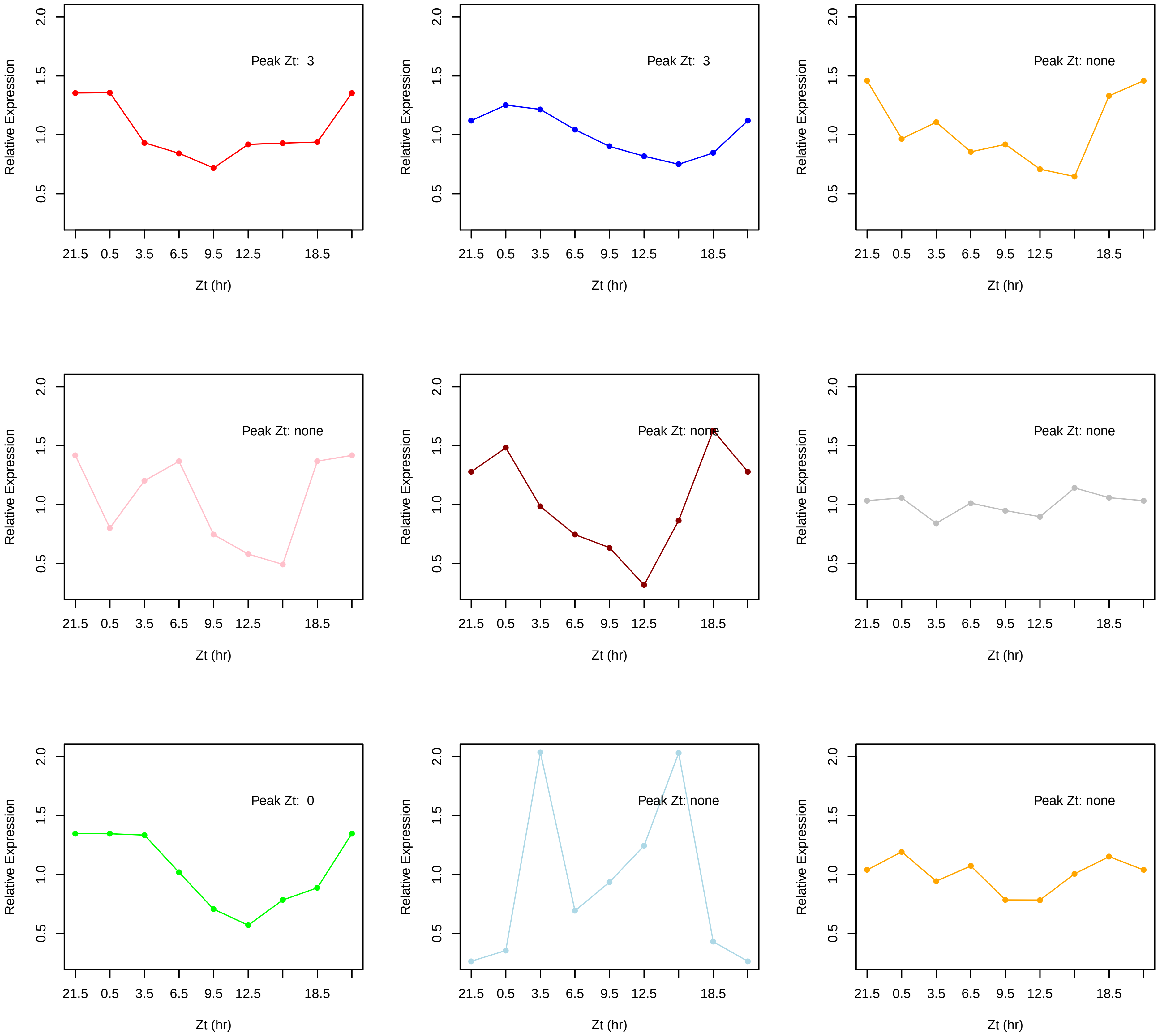

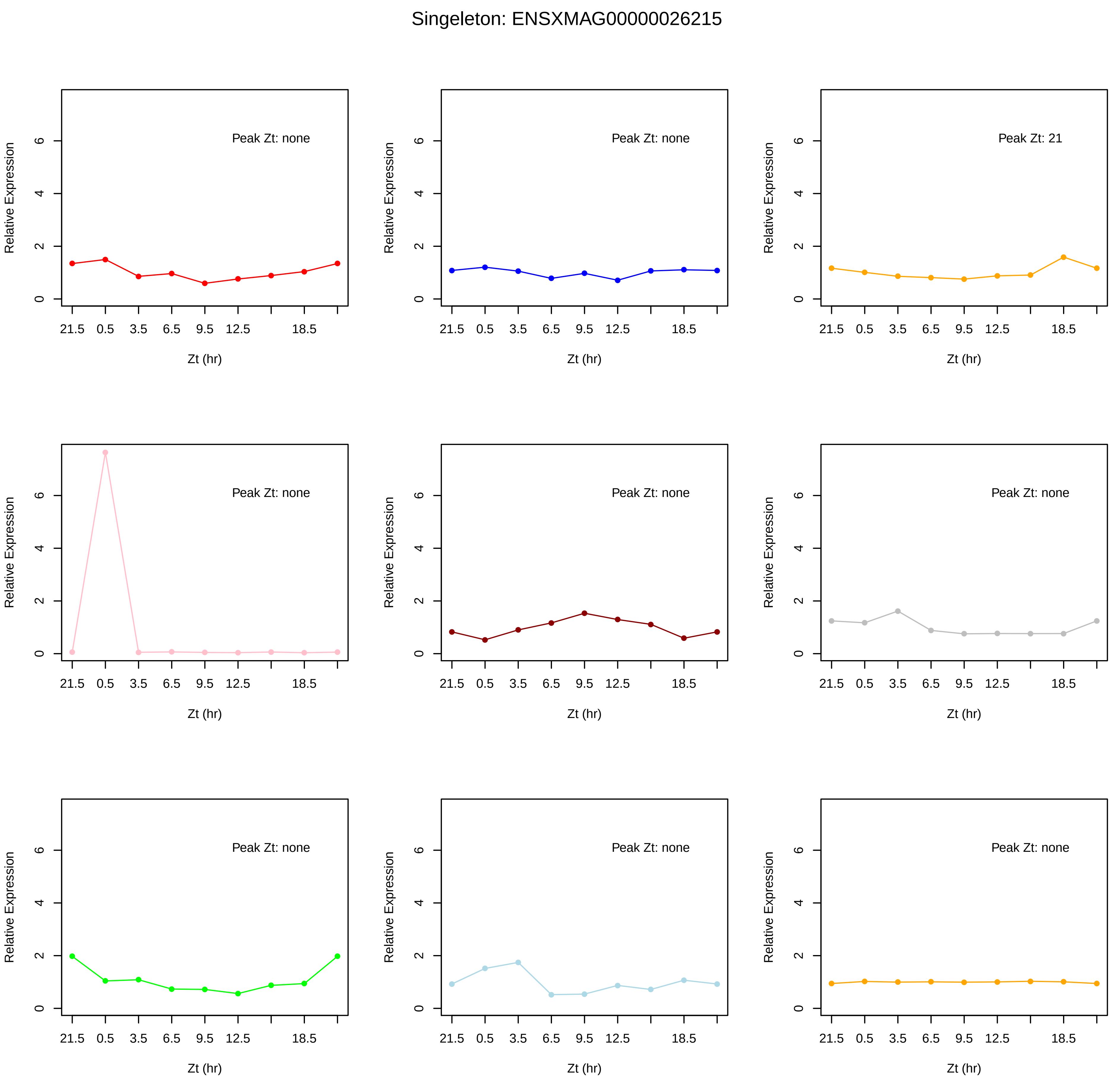
Singeleton: ENSXMAG00000026215
Peak Zt: none
Peak Zt: none
Peak Zt: 21
Peak Zt: none
Peak Zt: none
Peak Zt: none
Peak Zt: none
Peak Zt: none
Peak Zt: none
Relative Expression
Zt (hr)
21.5
0.5
3.5
6.5
9.5
12.5
18.5
0
2
4
6

Singeleton: ENSXMAG00000007889 dpep2

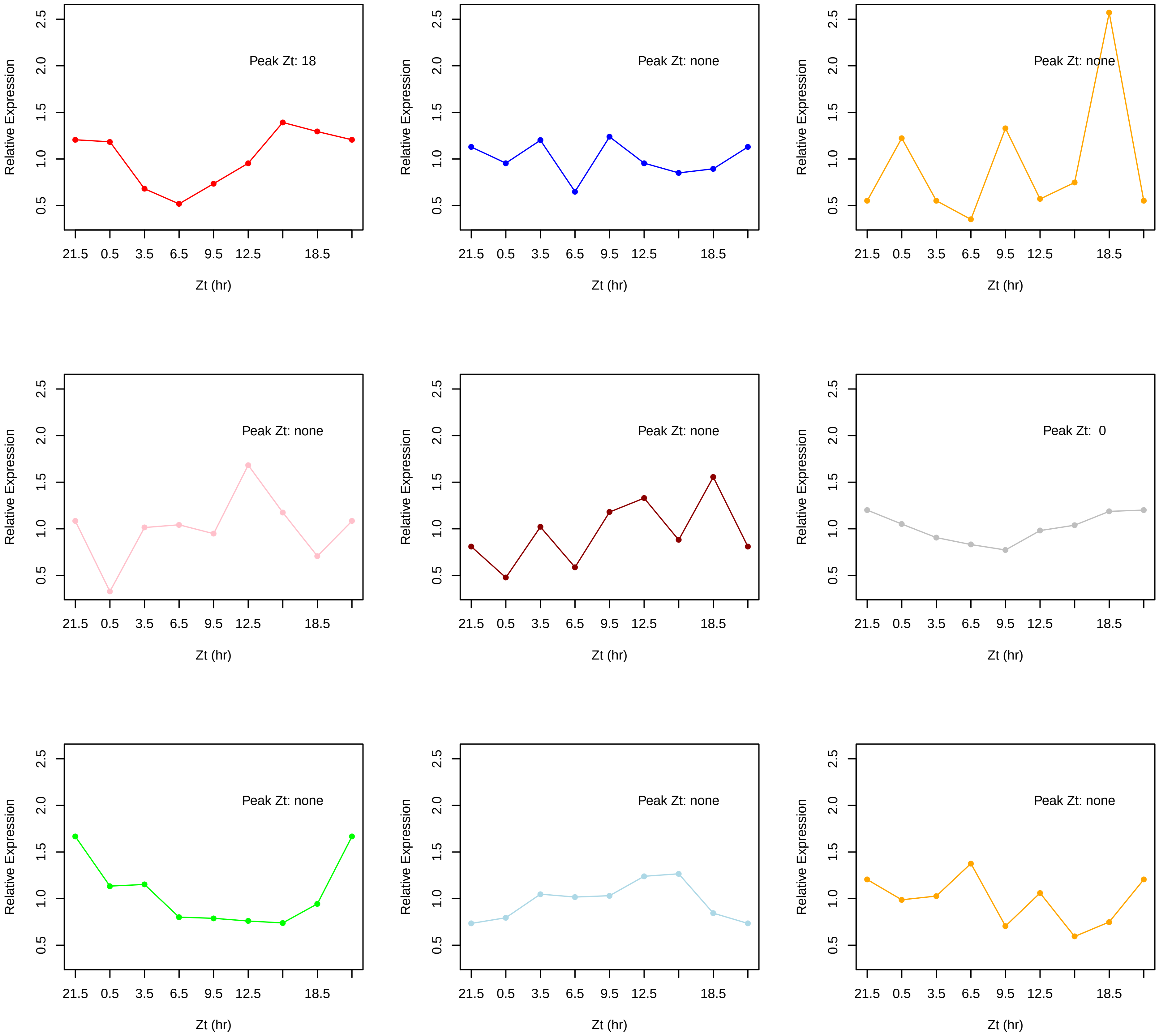

Singeleton: ENSXMAG00000029796 rtf2

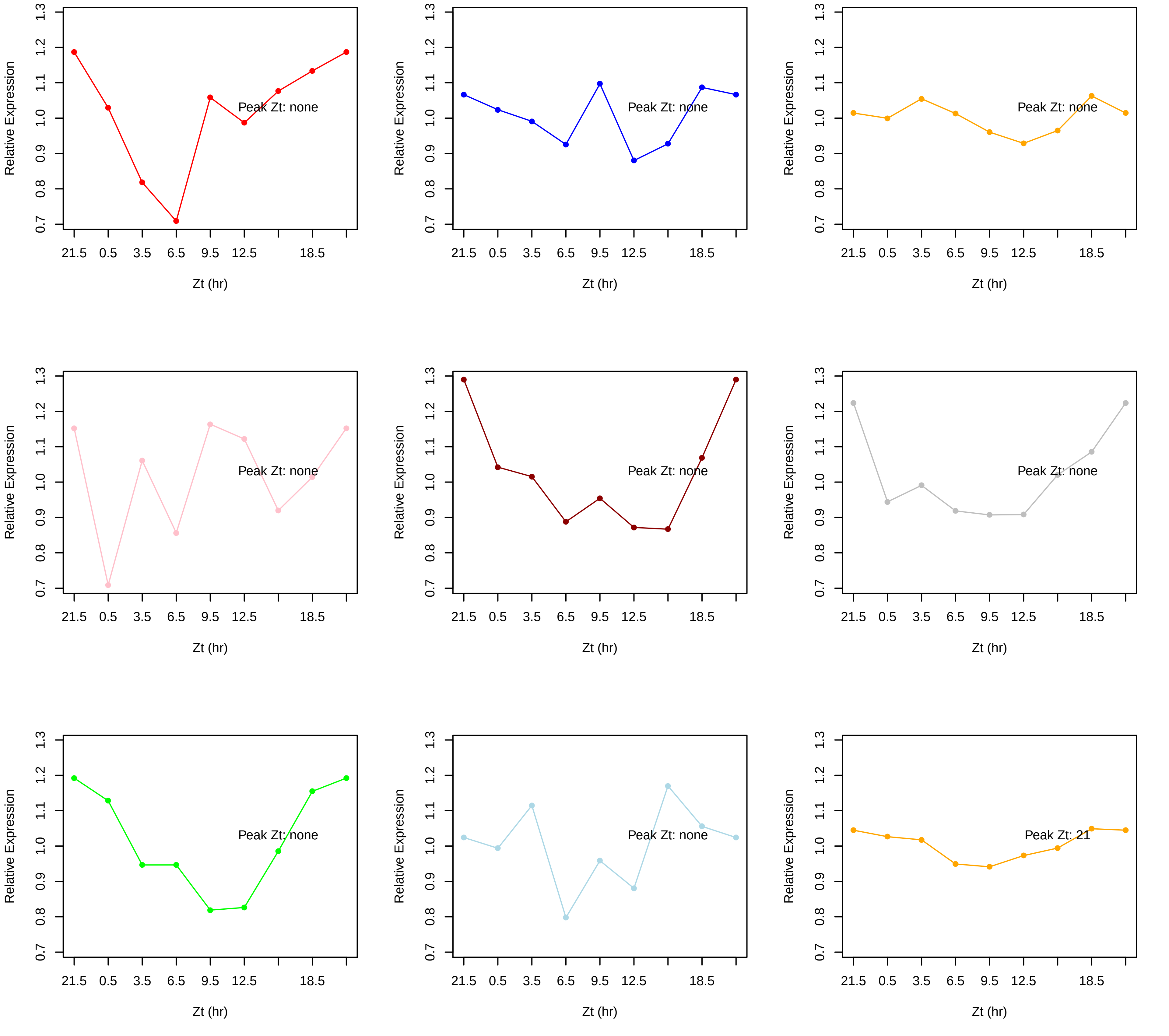

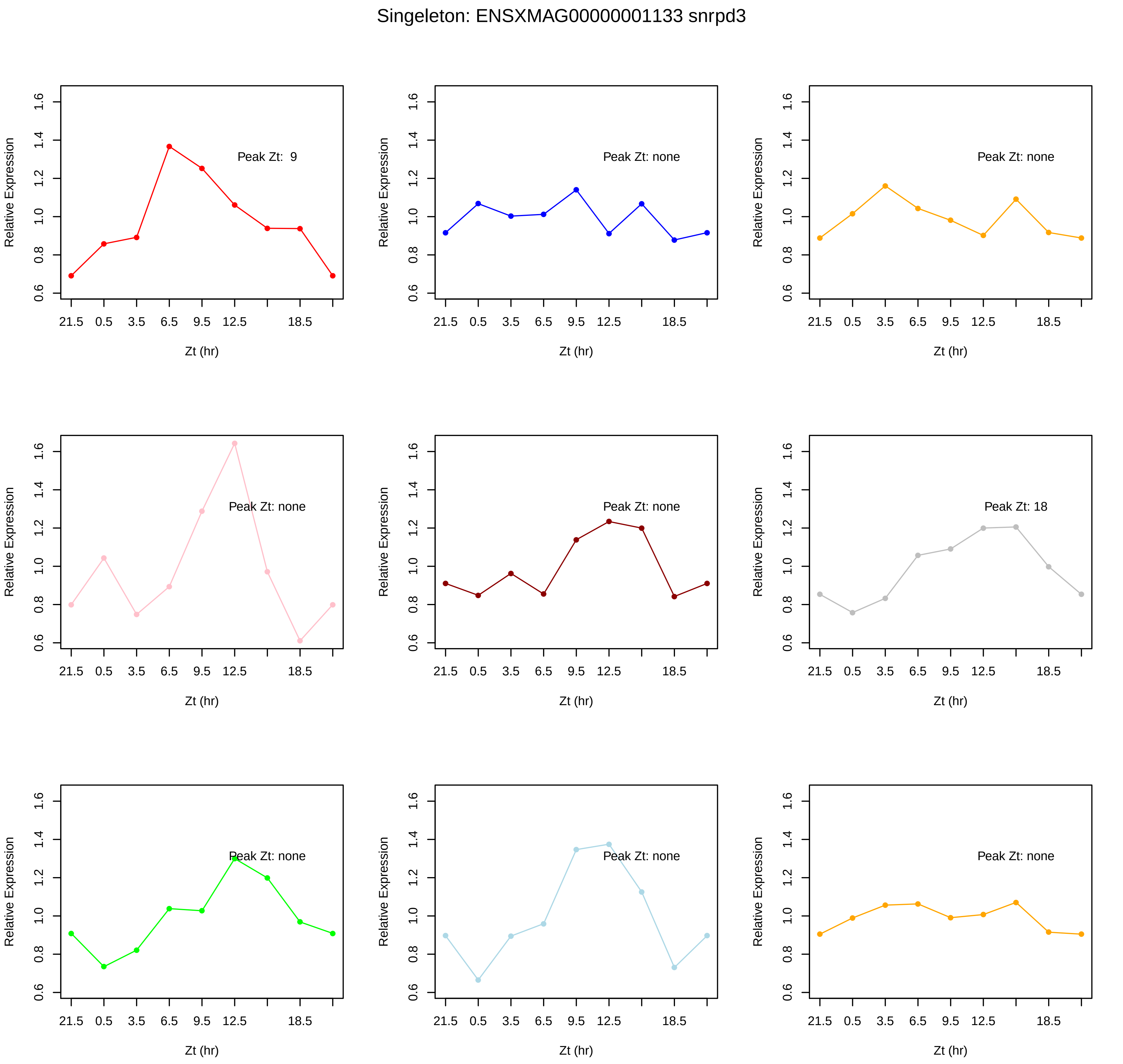
Singeleton: ENSXMAG00000001133 snrpd3
Peak Zt: 9
Peak Zt: none
Peak Zt: none
Peak Zt: none
Peak Zt: none
Peak Zt: 18
Peak Zt: none
Peak Zt: none
Peak Zt: none
Relative Expression
Zt (hr)
21.5
0.5
3.5
6.5
9.5
12.5
18.5
0.6
0.8
1.0
1.2
1.4
1.6

Singeleton: ENSXMAG00000017201 tnk2b

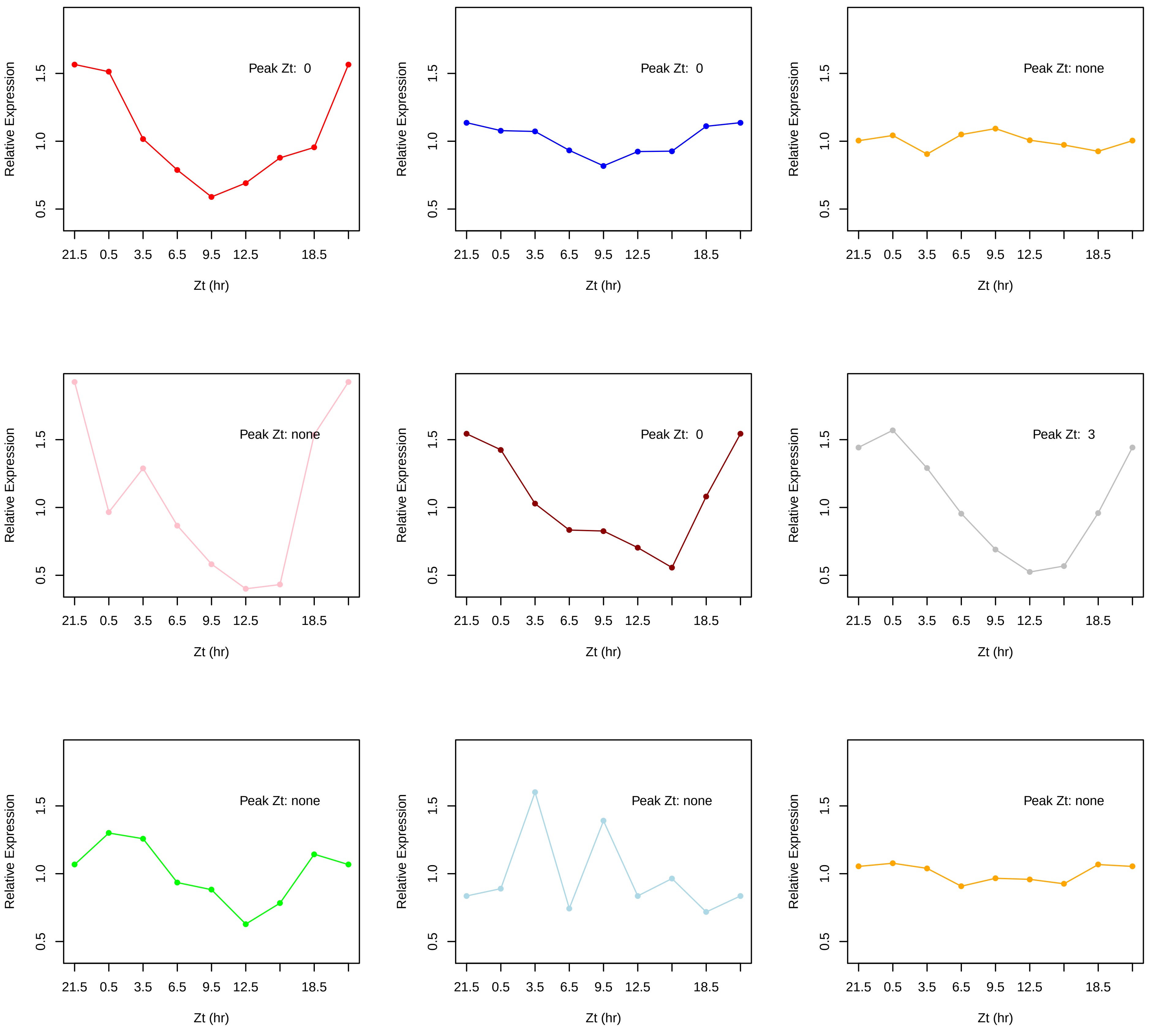

Singeleton: ENSXMAG00000028723 slc25a33

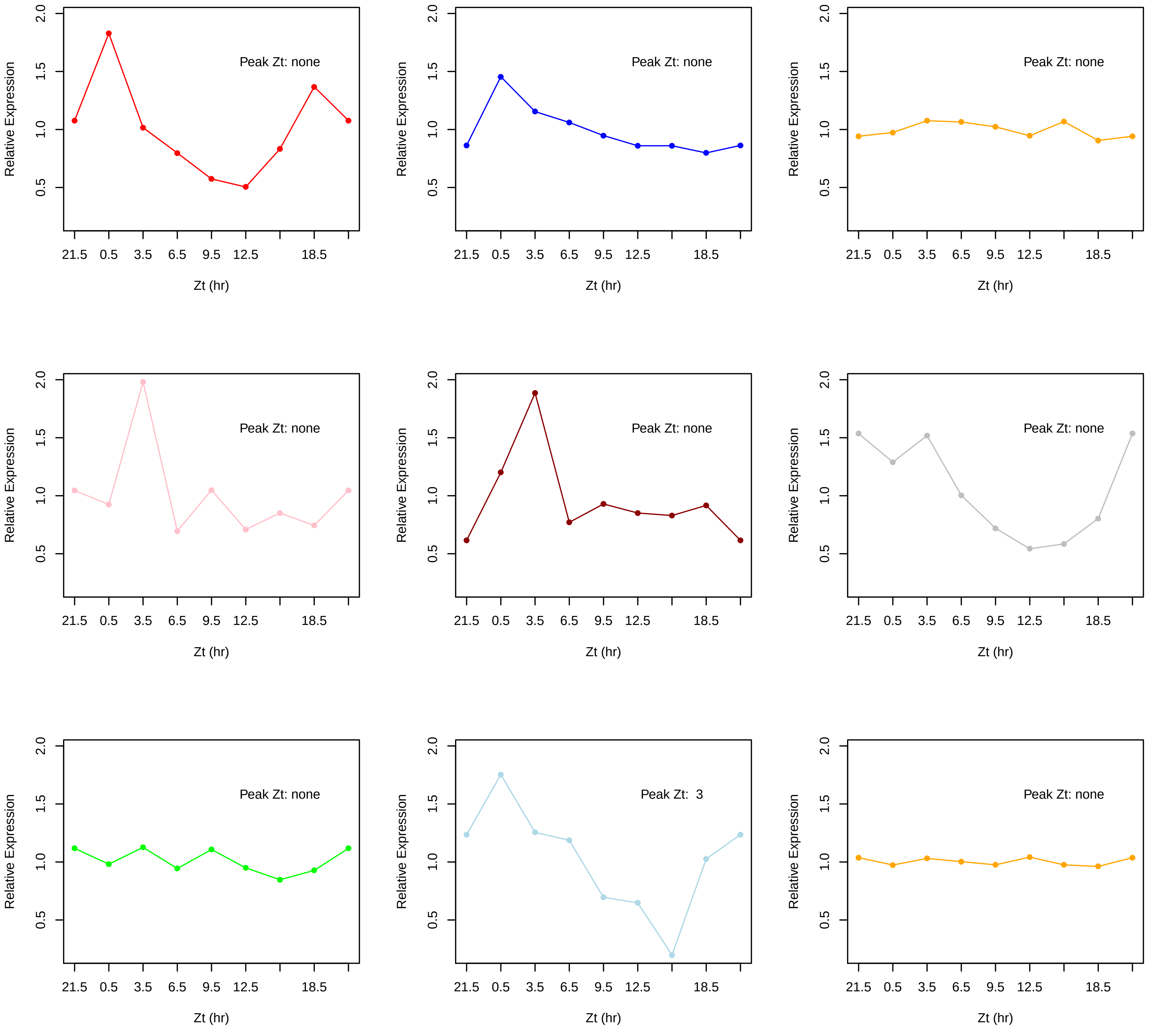

Singeleton: ENSXMAG00000004889 cyp26c1

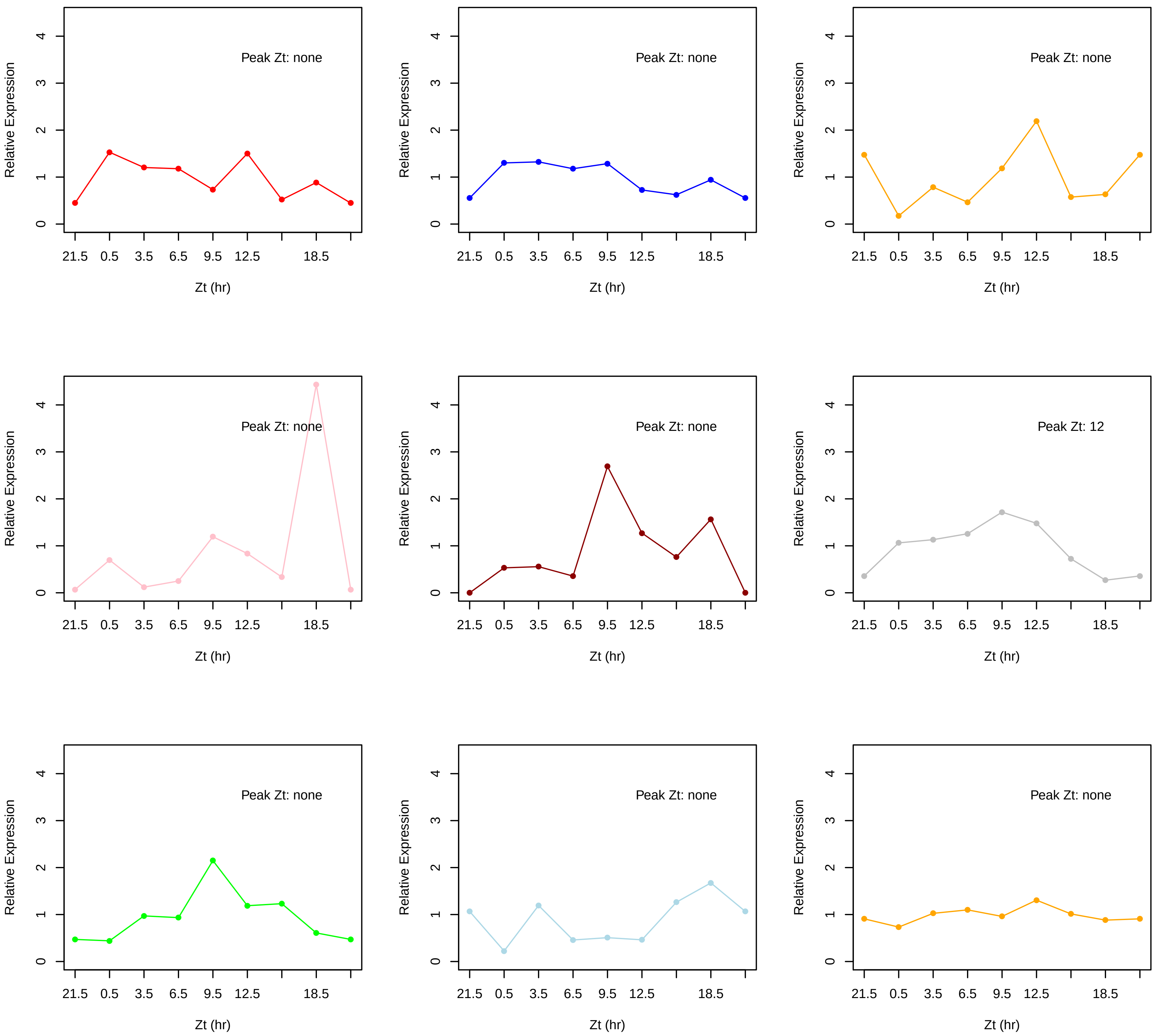

Singeleton: ENSXMAG00000011455 ncapd2

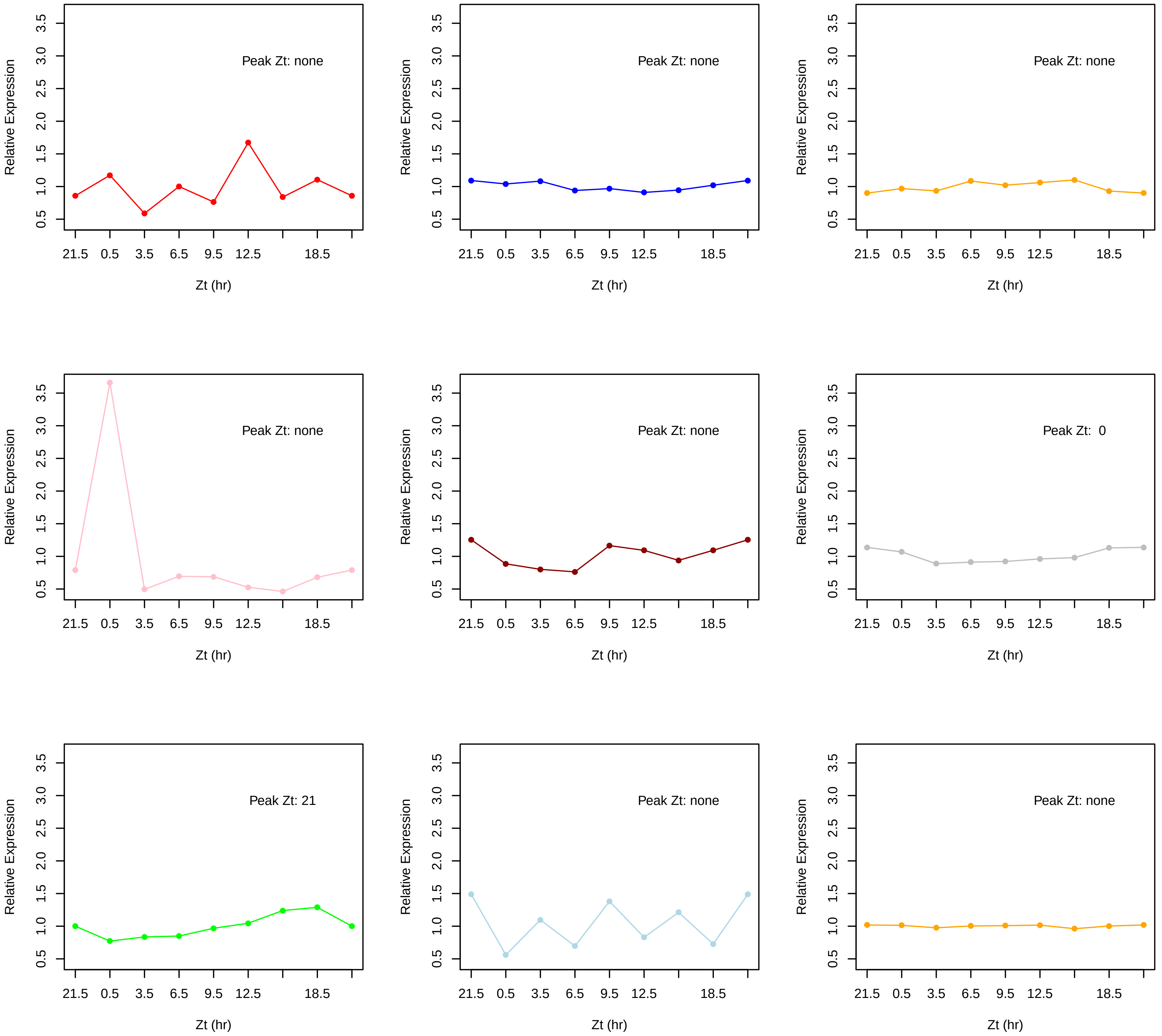

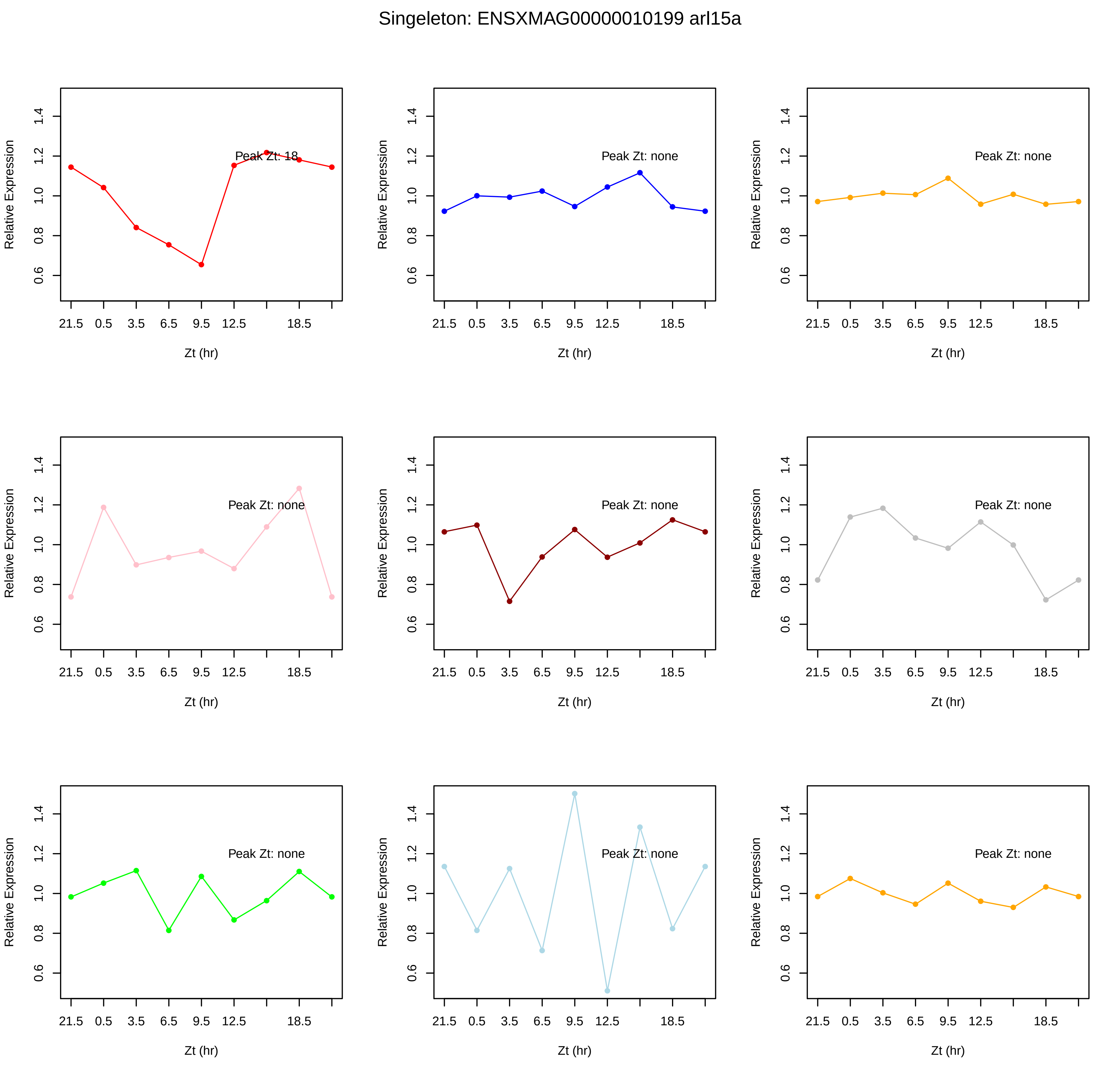
Singleton: ENSXMAG00000010199 arl15a
Relative Expression
Zt (hr)
21.5
0.5
3.5
6.5
9.5
12.5
18.5
0.6
0.8
1.0
1.2
1.4
Peak Zt: 18
Peak Zt: none

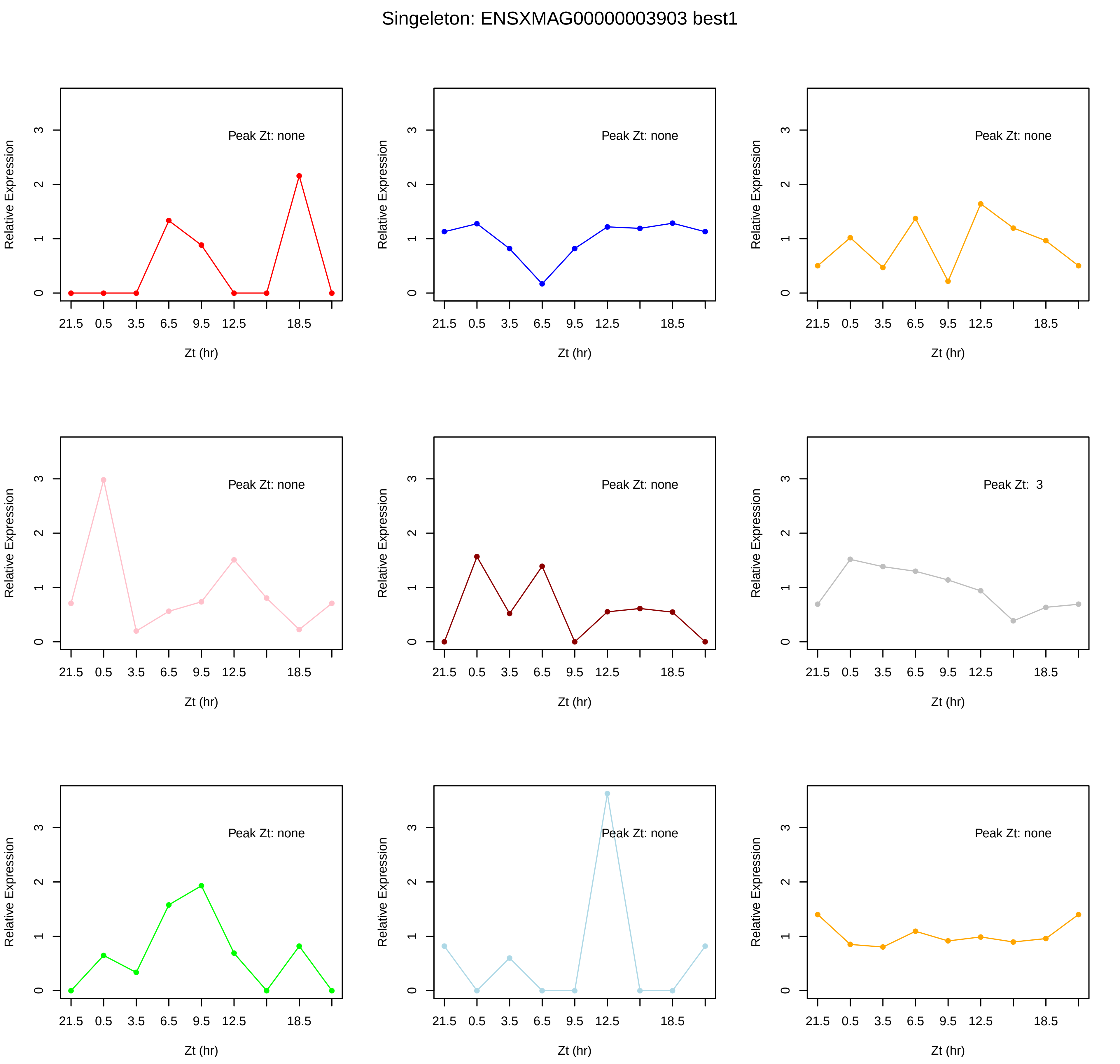
Singleton: ENSXMAG00000003903 best1
Peak Zt: none
Peak Zt: none
Peak Zt: none
Peak Zt: none
Peak Zt: none
Peak Zt: 3
Peak Zt: none
Peak Zt: none
Peak Zt: none
Relative Expression
Zt (hr)
21.5
0.5
3.5
6.5
9.5
12.5
18.5

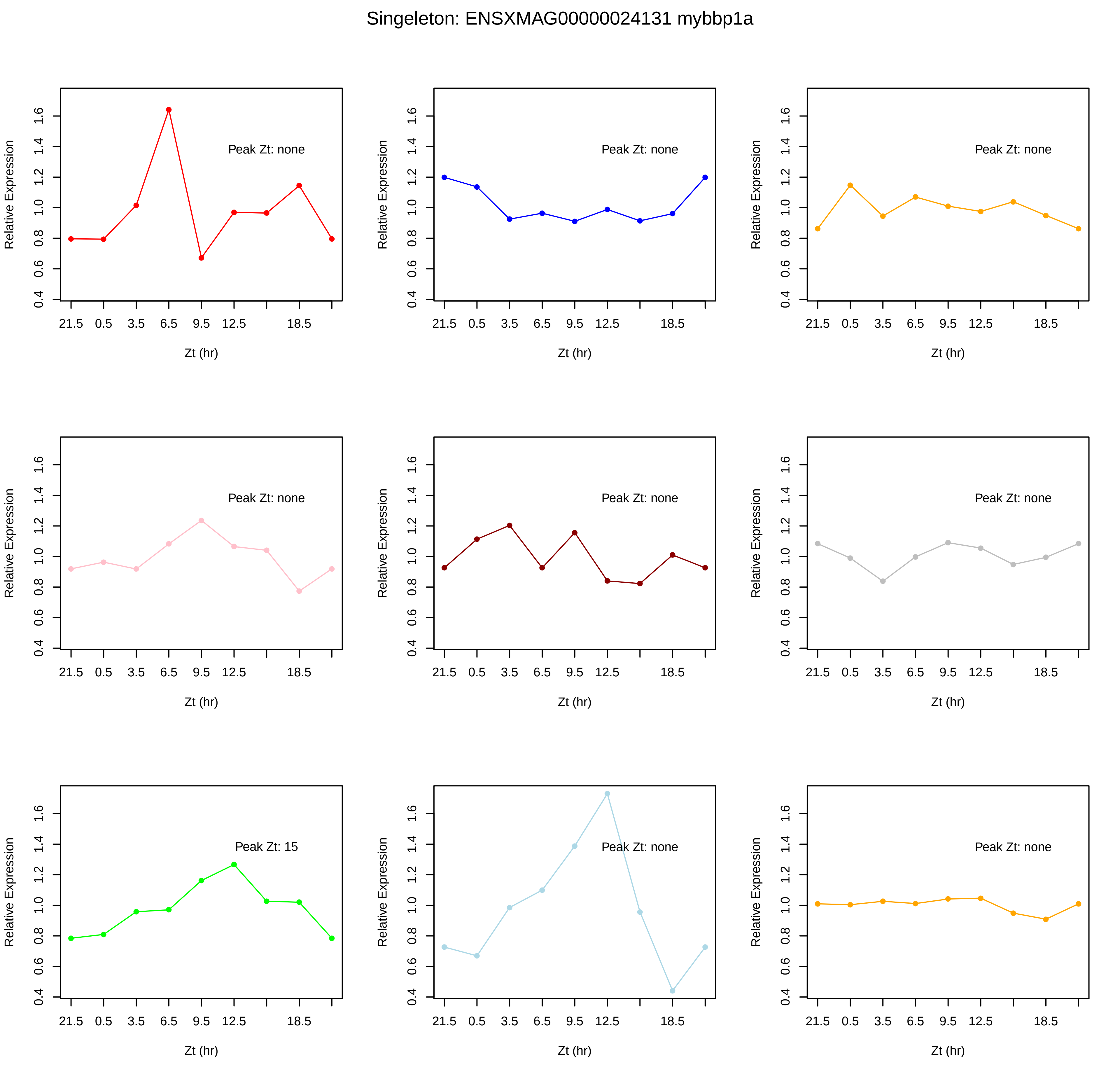
Singeleton: ENSXMAG00000024131 mybbp1a
Peak Zt: none
Peak Zt: none
Peak Zt: none
Peak Zt: none
Peak Zt: none
Peak Zt: none
Peak Zt: 15
Peak Zt: none
Peak Zt: none
Relative Expression
Zt (hr)
21.5 0.5 3.5 6.5 9.5 12.5 18.5
0.4 0.6 0.8 1.0 1.2 1.4 1.6

Singeleton: ENSXMAG00000018737 sept7b

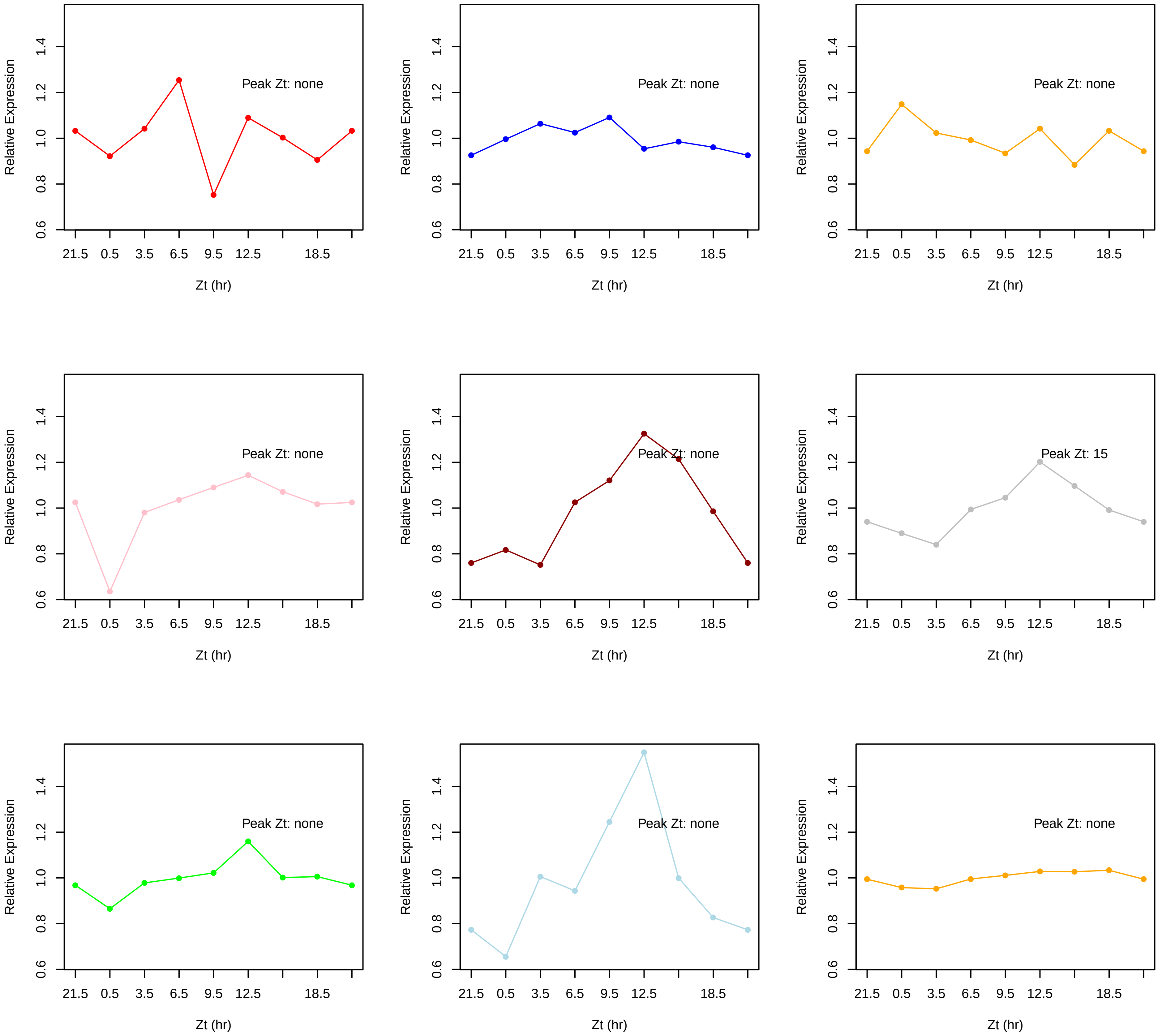

Singeleton: ENSXMAG00000028576 mtx2

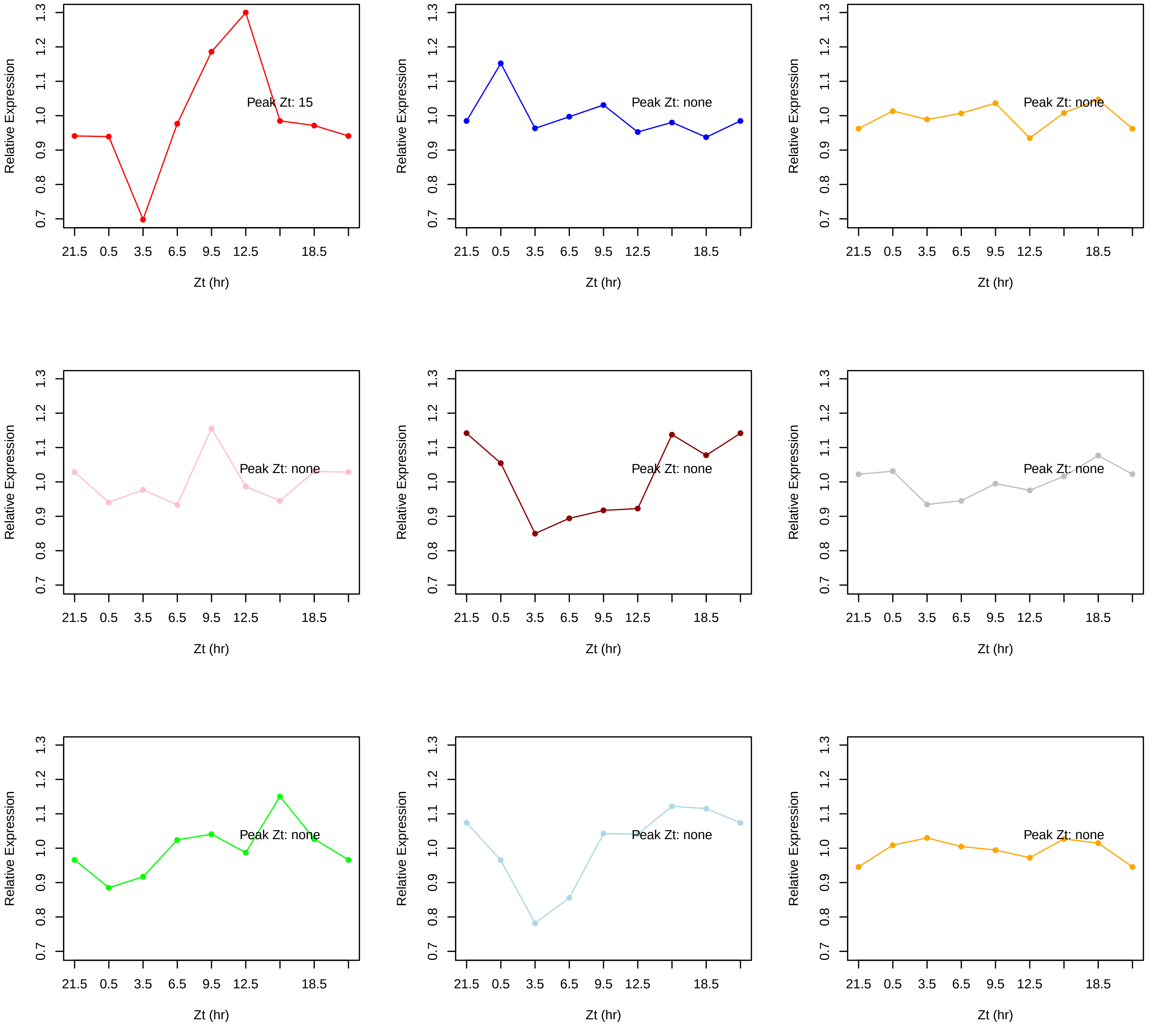

Singleton: ENSXMAG00000030053 spsb1

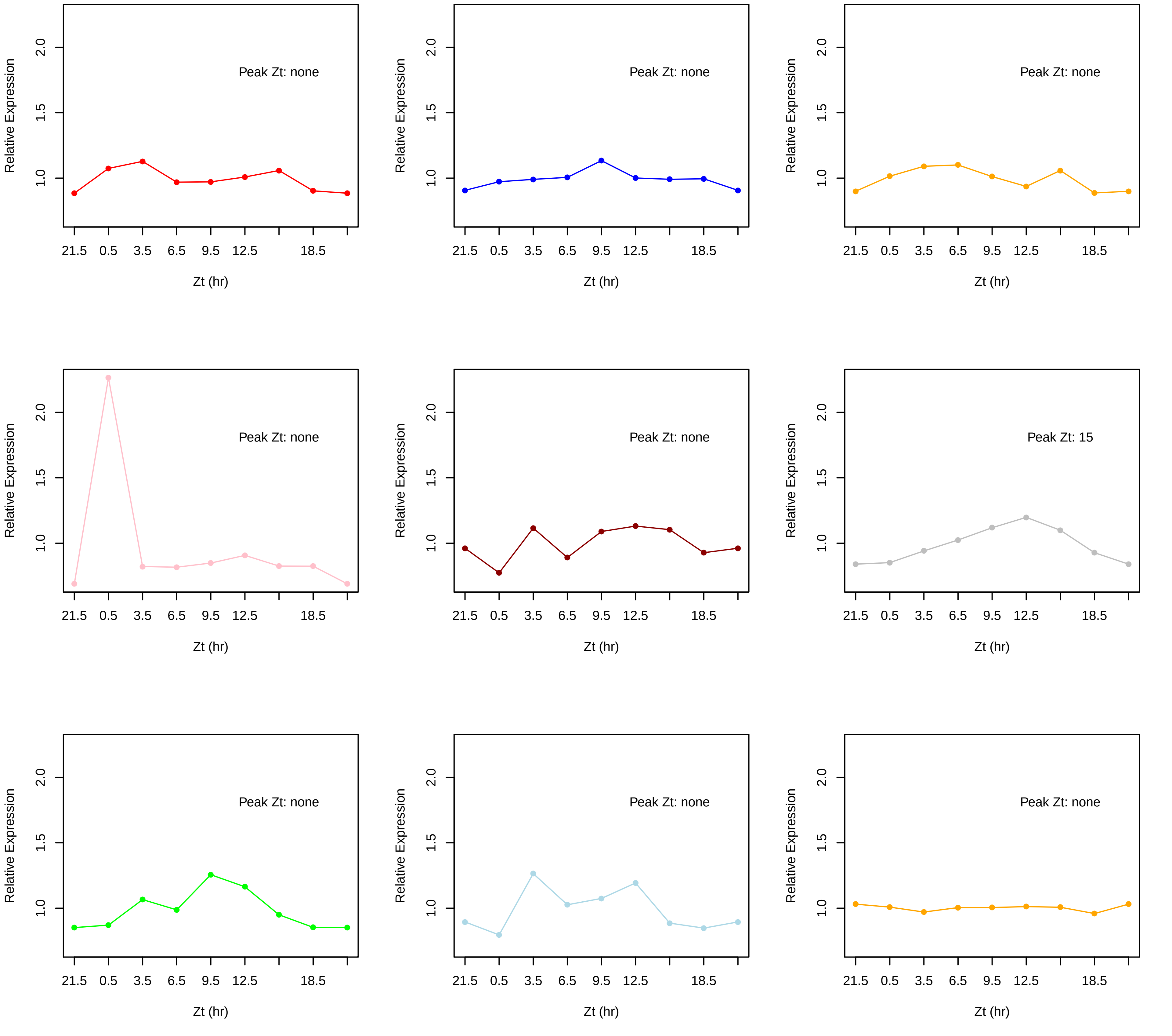

Singeleton: ENSXMAG00000029281 lrrc58b

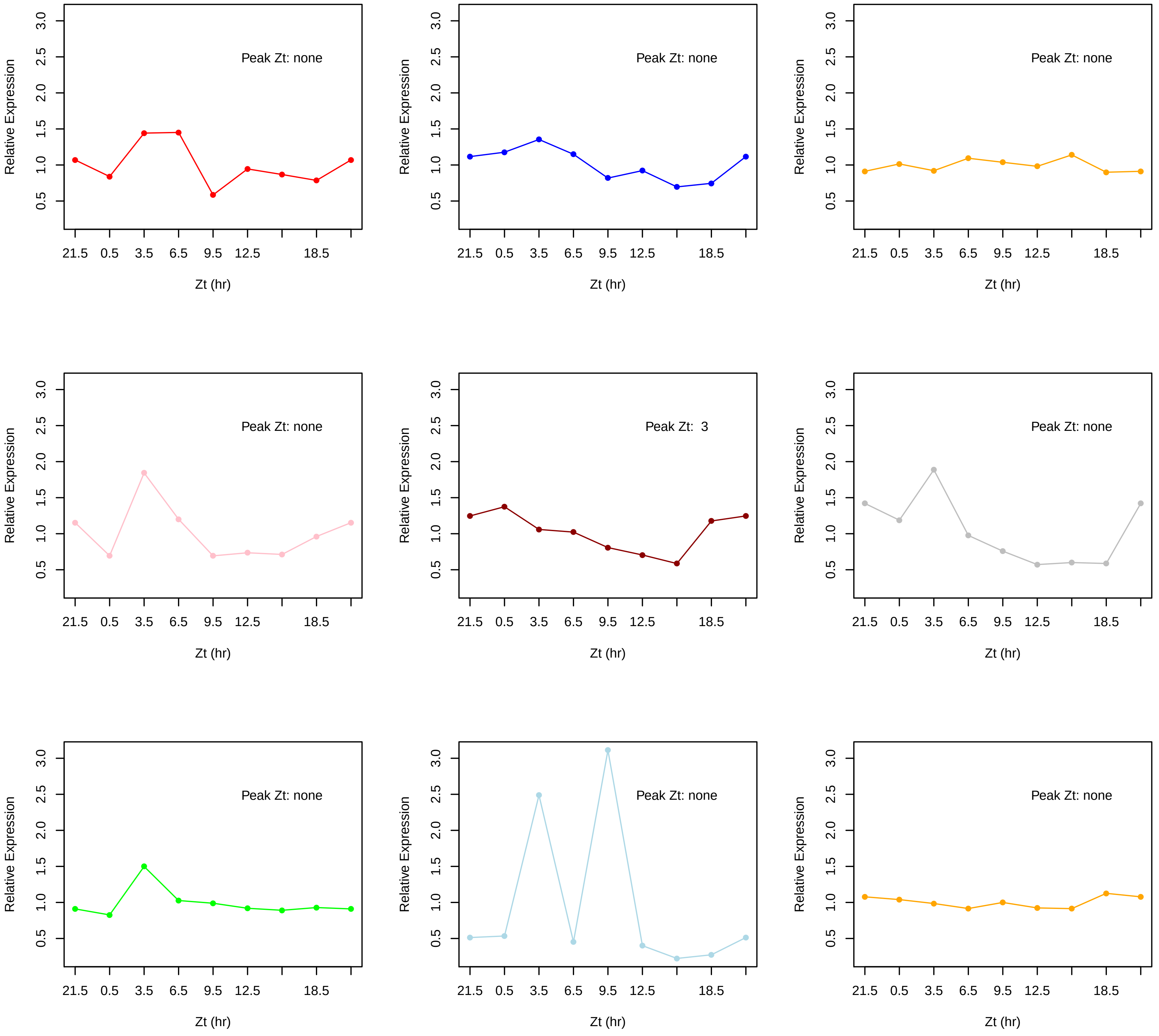

Singeleton: ENSXMAG00000026202 rab3il1

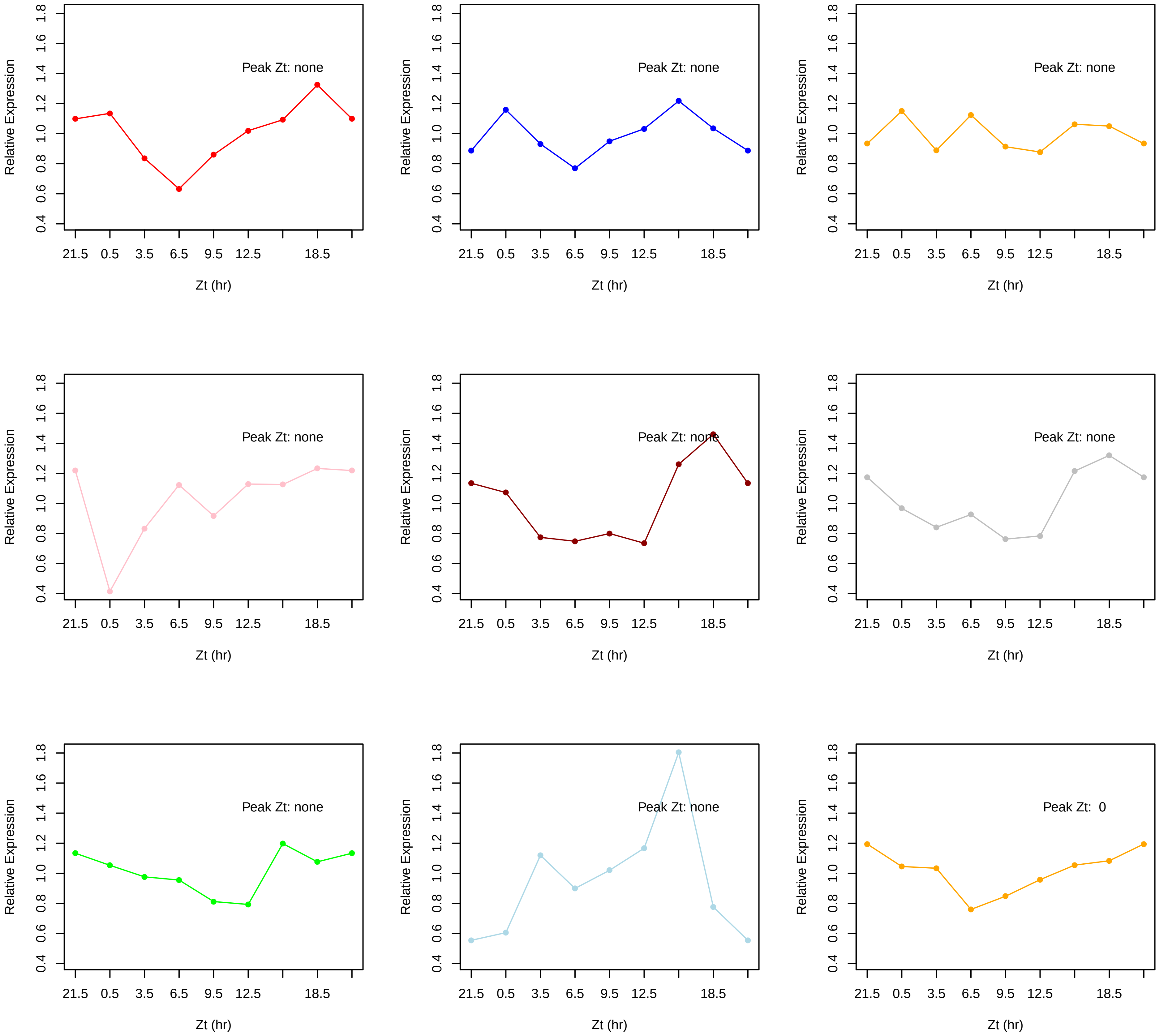

Singeleton: ENSXMAG00000027691 zgc:171704

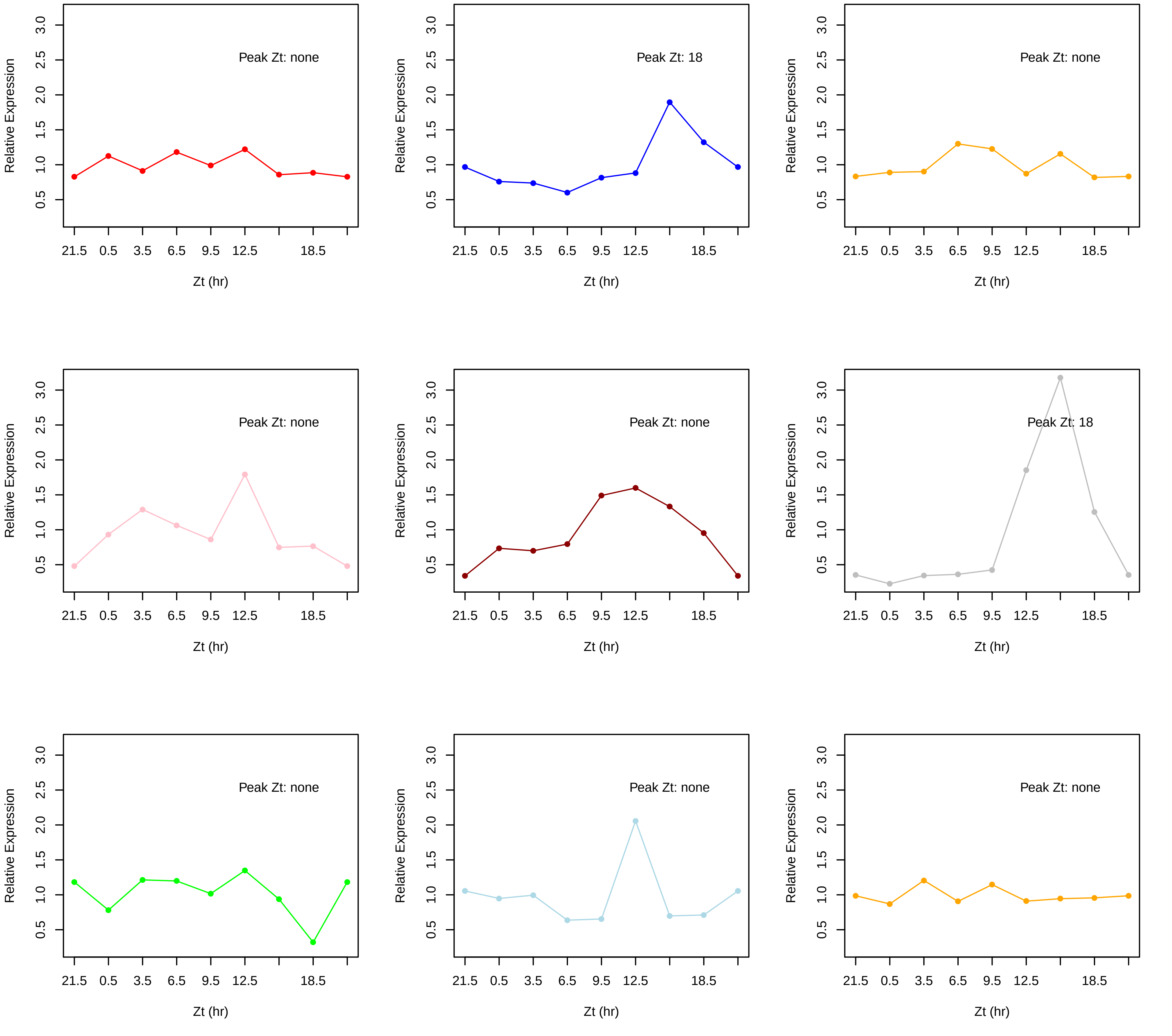

Singeleton: ENSXMAG00000010641 pitpnab

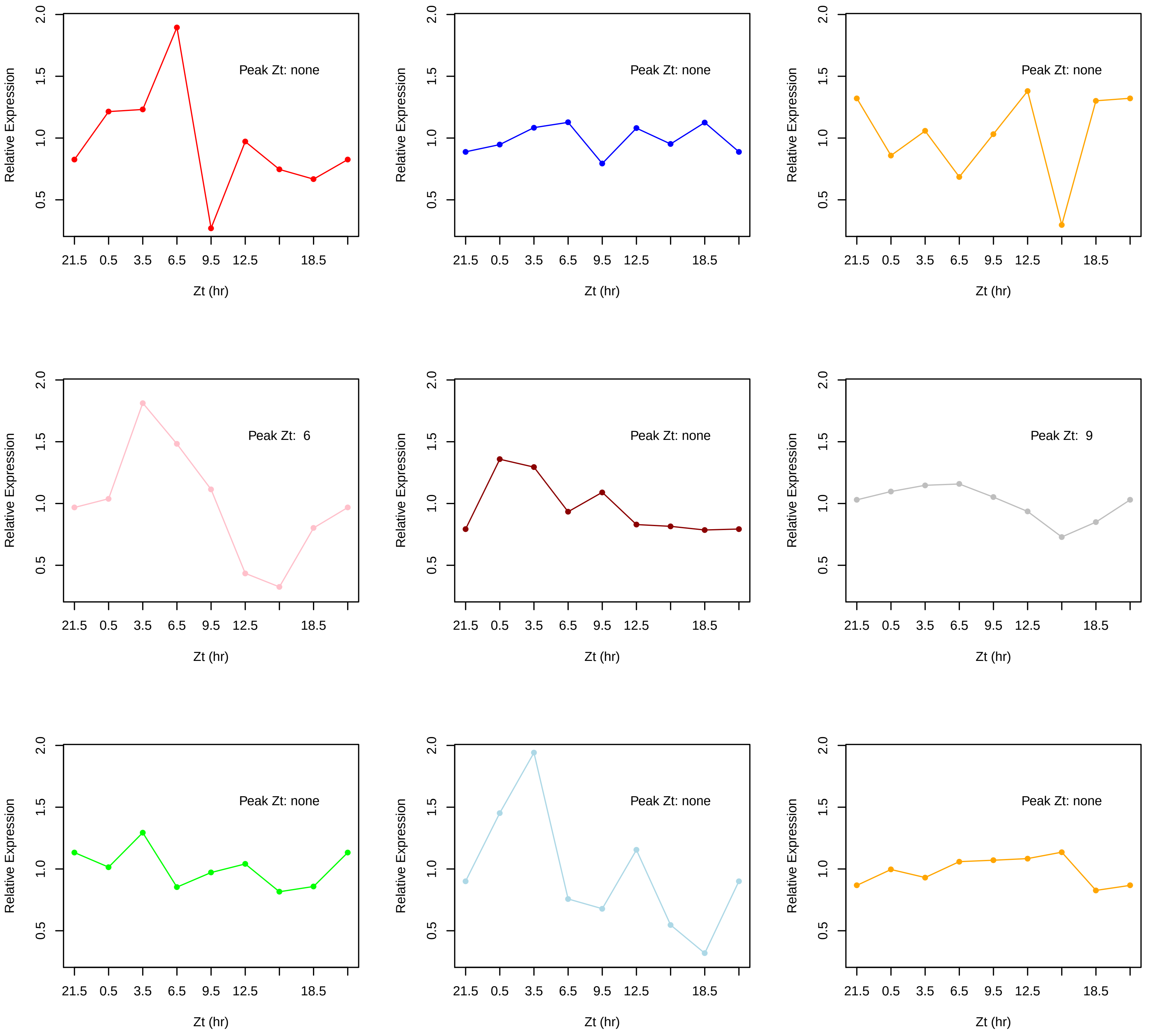

Singeleton: ENSXMAG00000021494 pdgfd

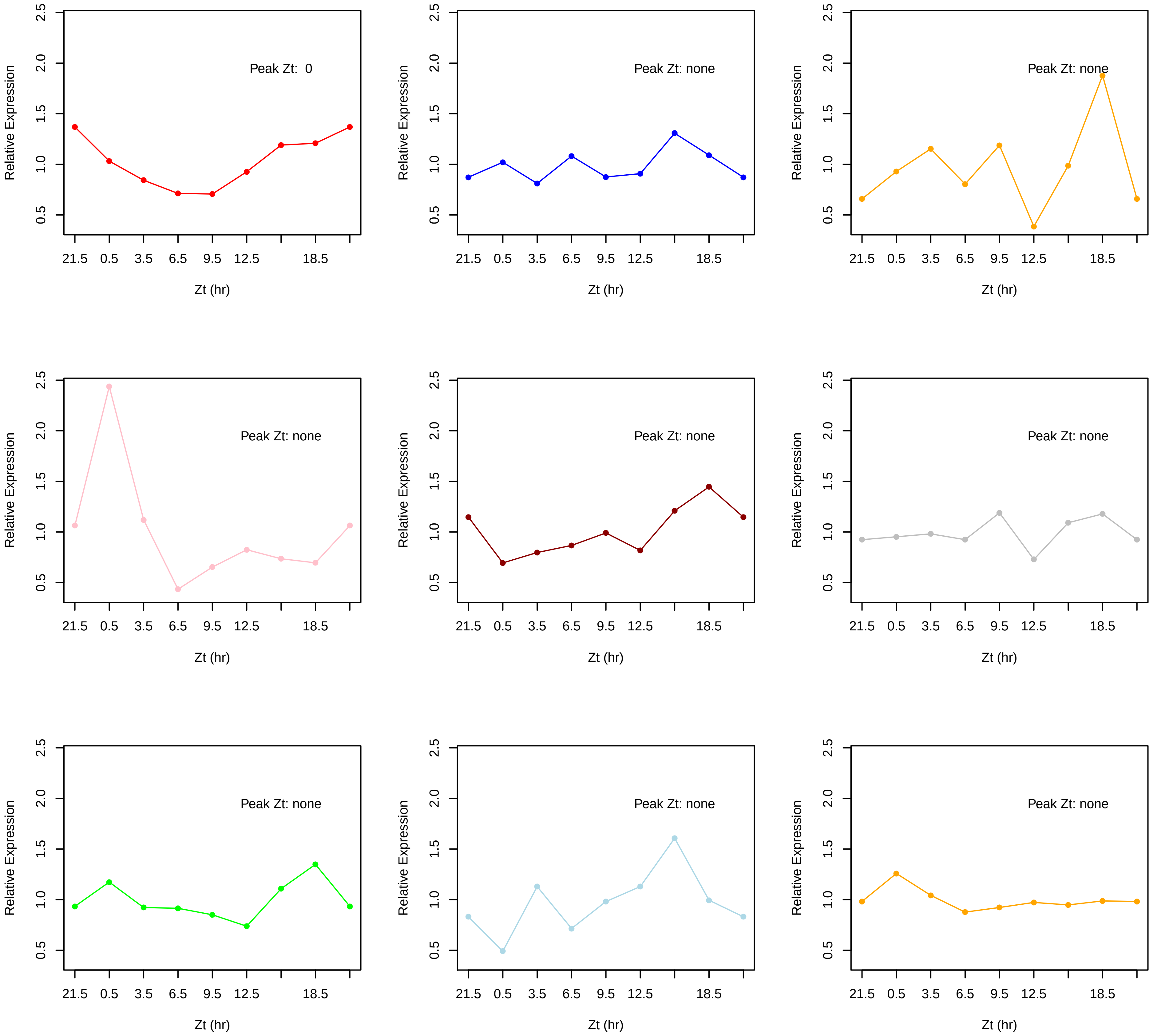

Singeleton: ENSXMAG00000004411 upb1

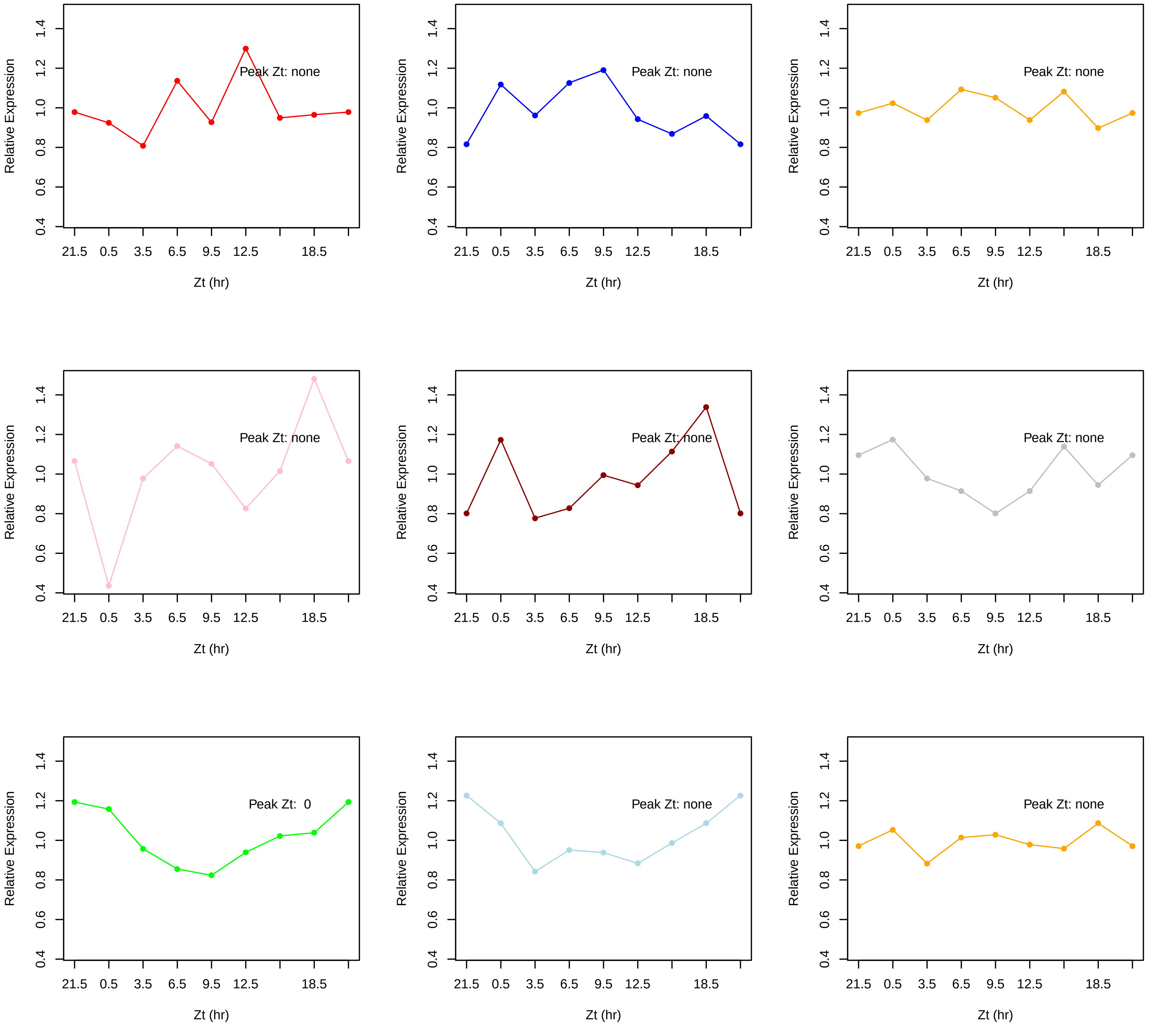

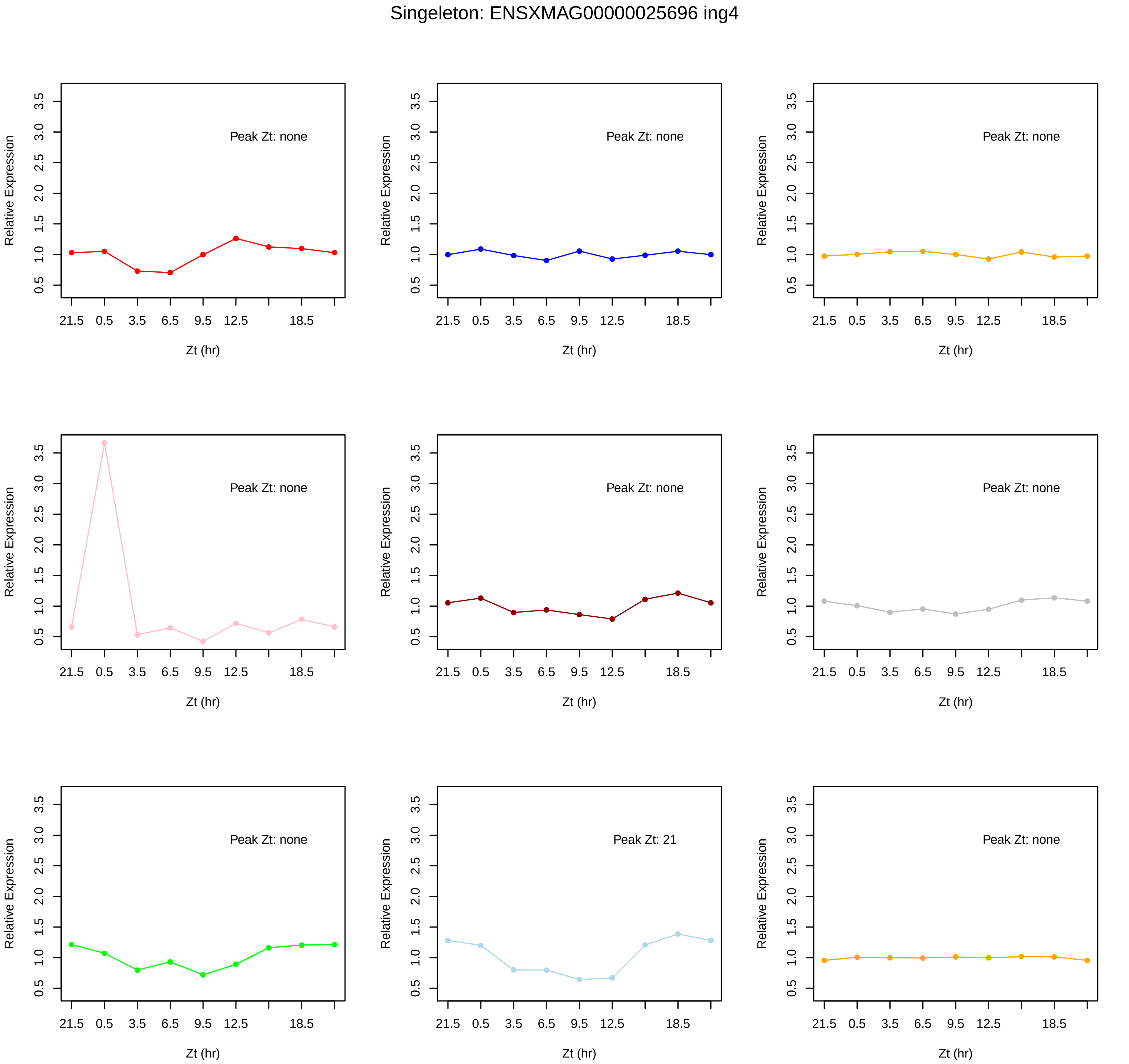
Singeleton: ENSXMAG00000025696 ing4
Peak Zt: none
Peak Zt: none
Peak Zt: none
Peak Zt: none
Peak Zt: none
Peak Zt: none
Peak Zt: none
Peak Zt: 21
Peak Zt: none
Relative Expression
Zt (hr)
21.5
0.5
3.5
6.5
9.5
12.5
18.5

Singeleton: ENSXMAG00000007834 slc6a2

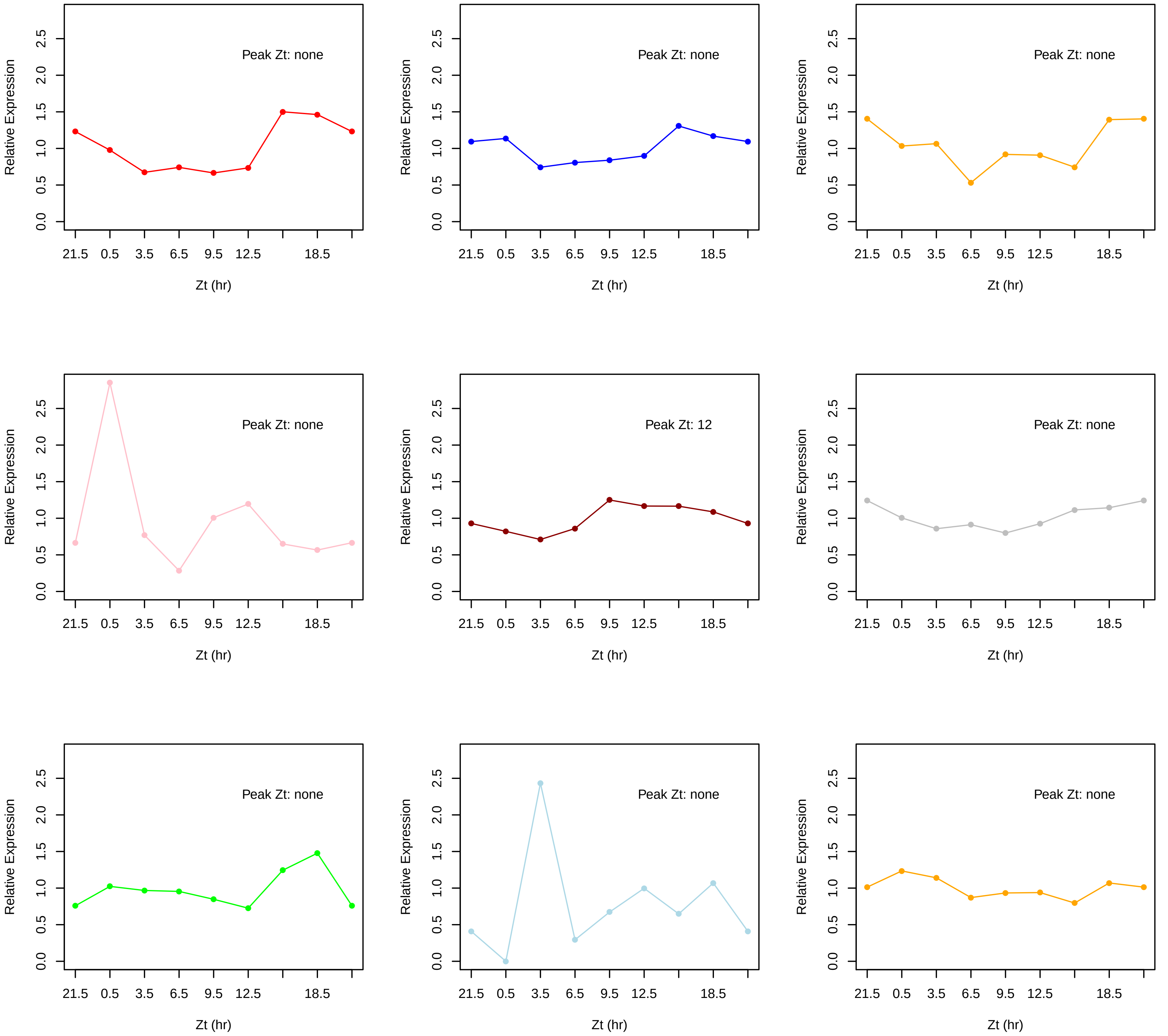

Singleton: ENSXMAG00000010593 KIAA0895

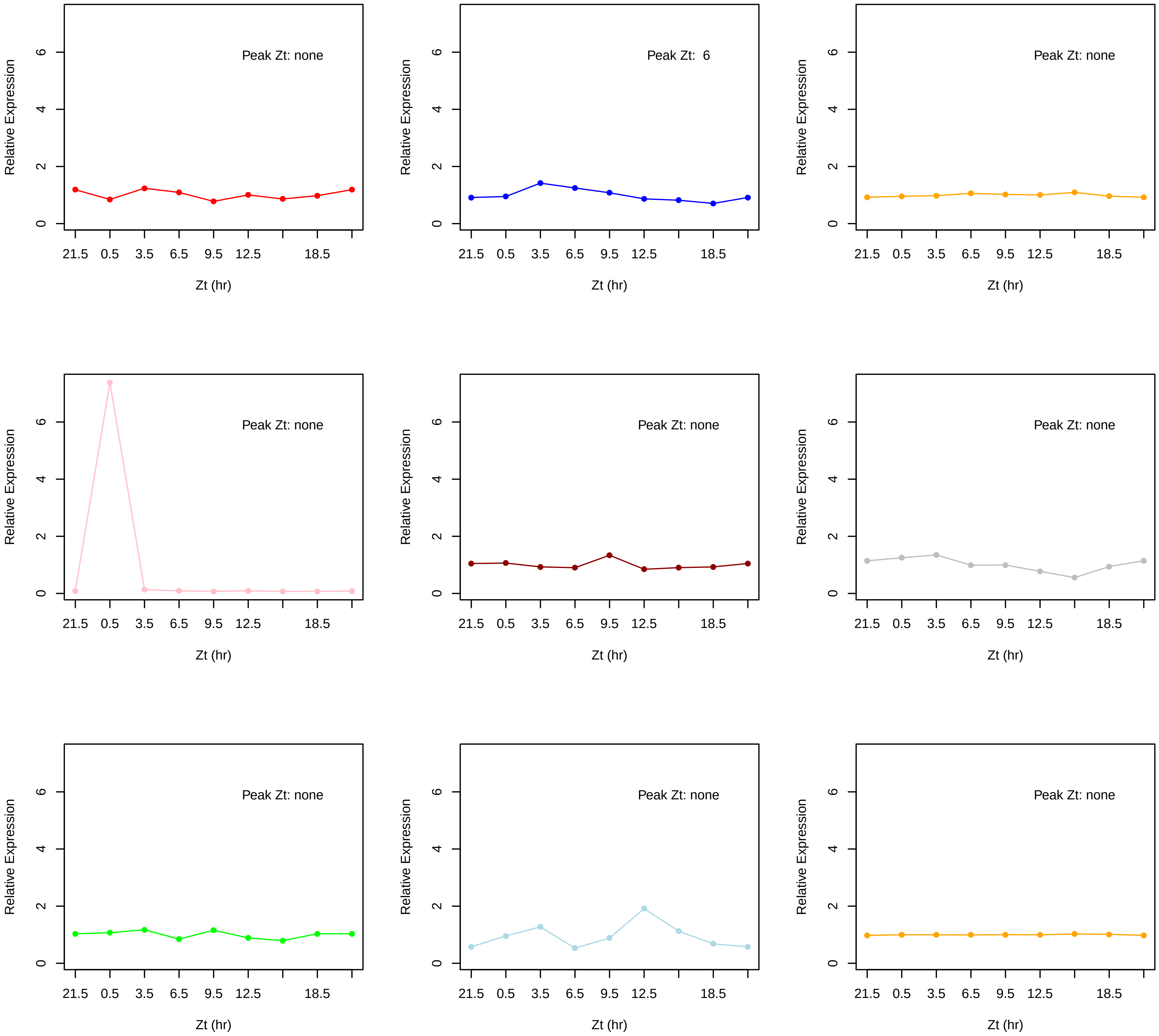

Singleton: ENSXMAG00000024650 pygo2

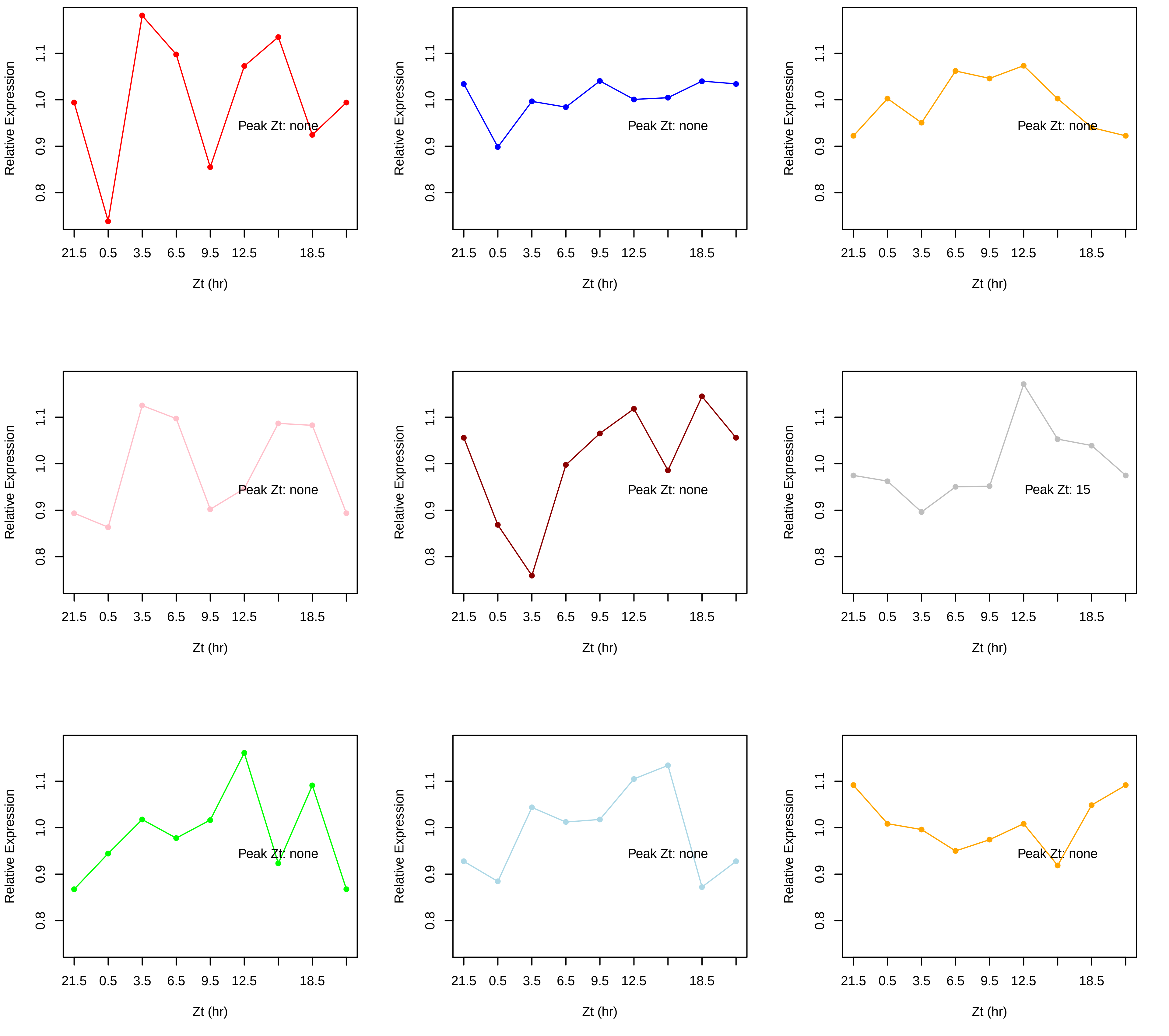

Singleton: ENSXMAG00000011483 nop2

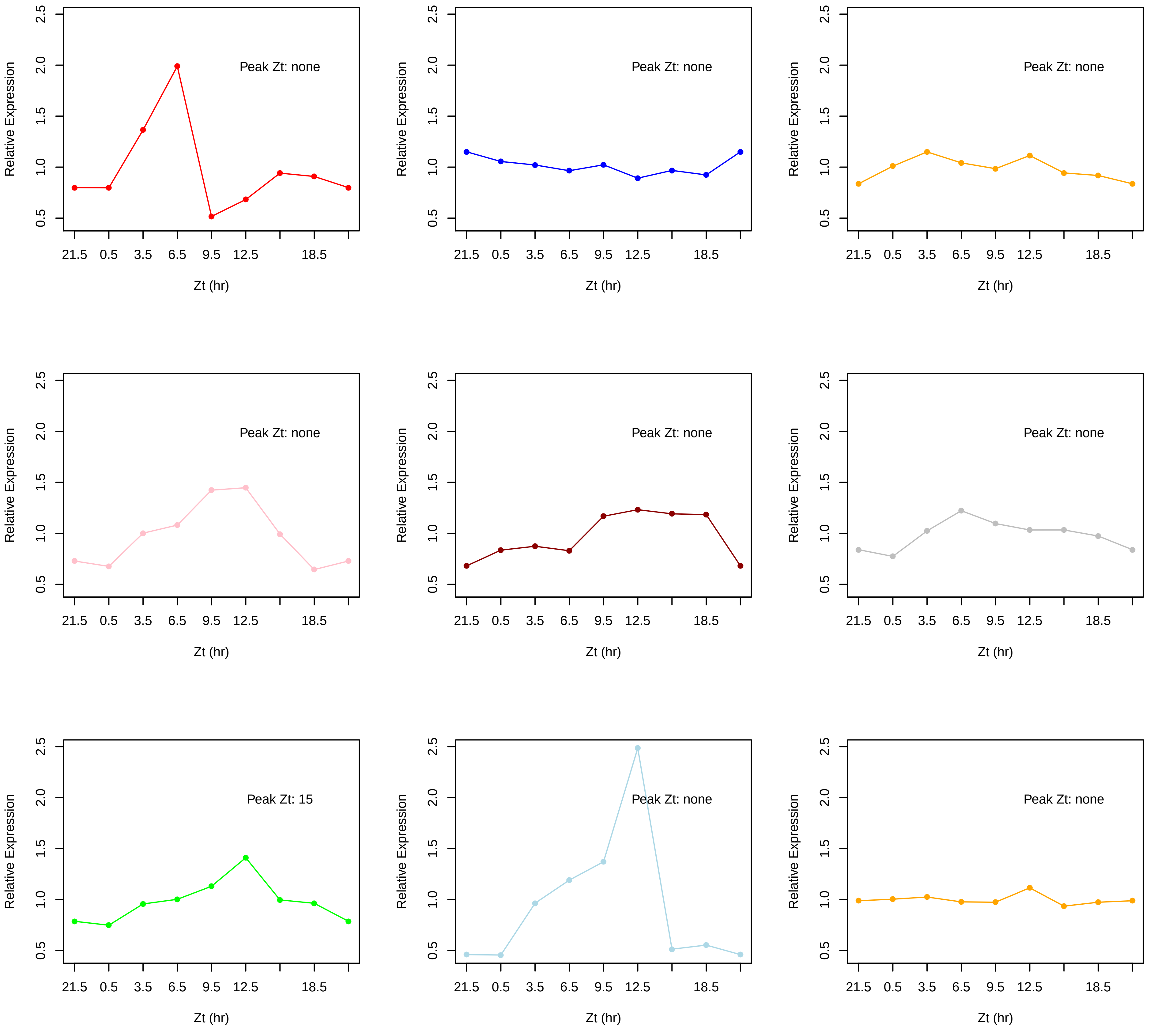

Singleton: ENSXMAG00000024168

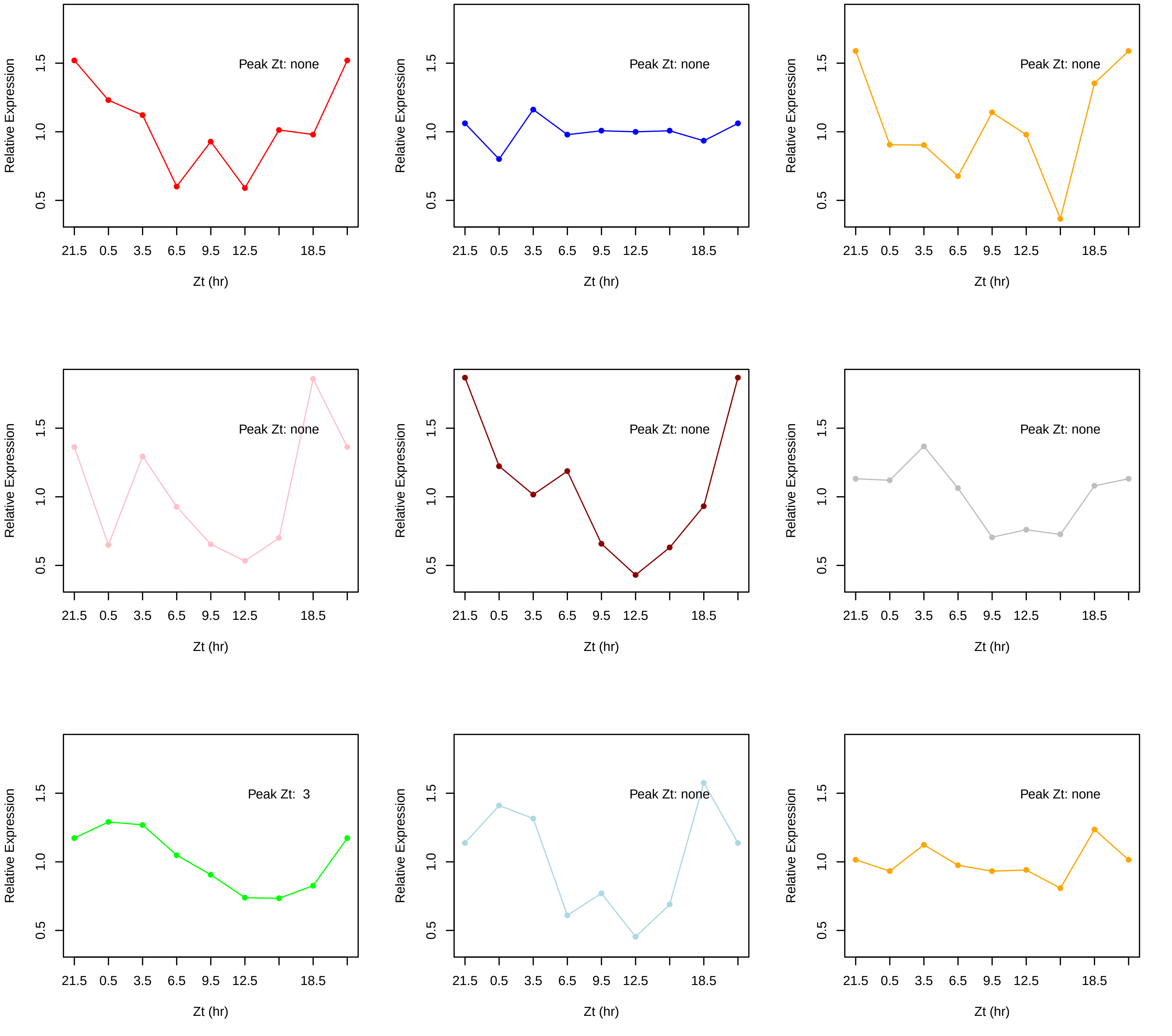

Singleton: ENSXMAG00000010057 wdfy3

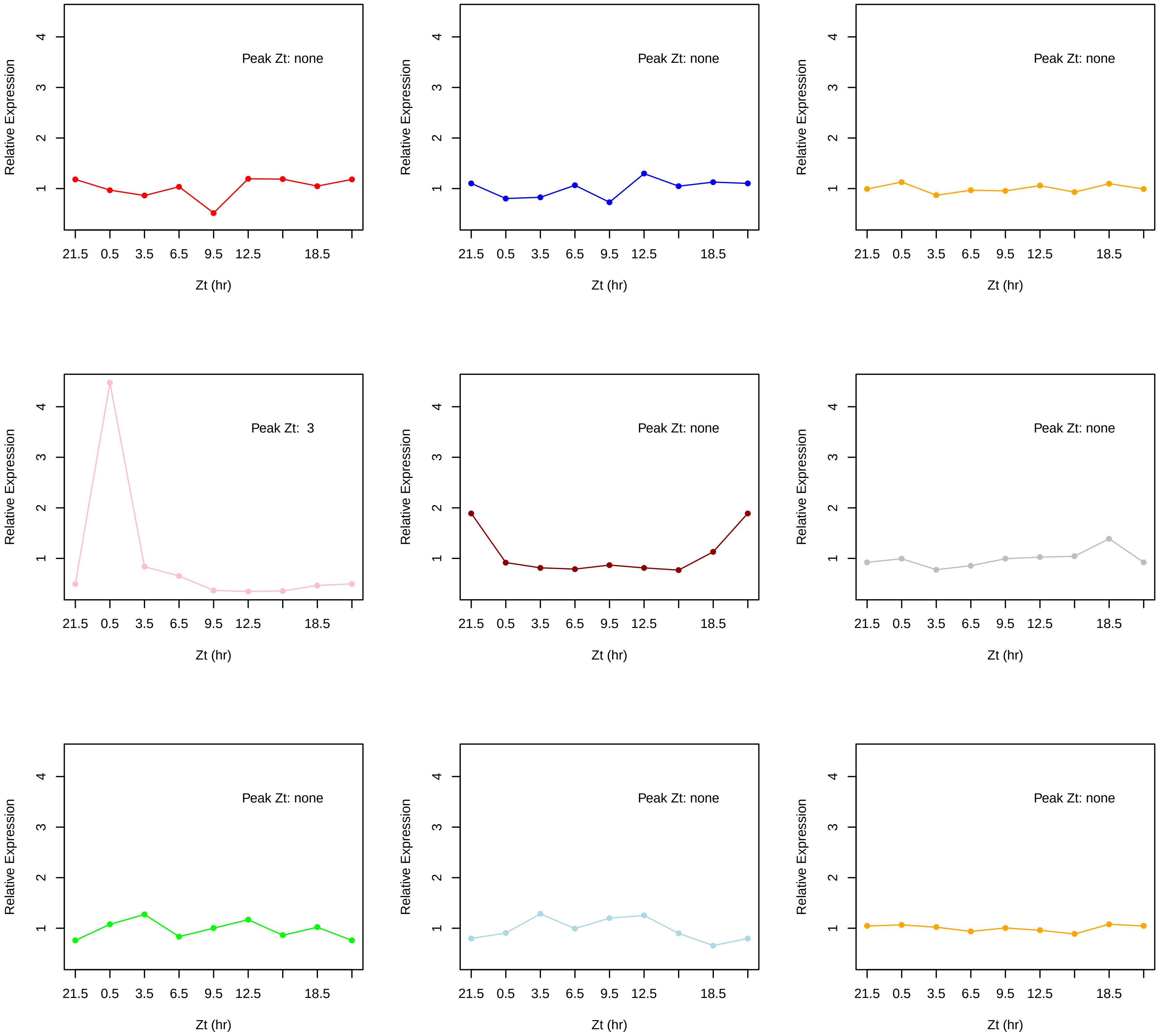

Singleton: ENSXMAG00000007415 ANLN

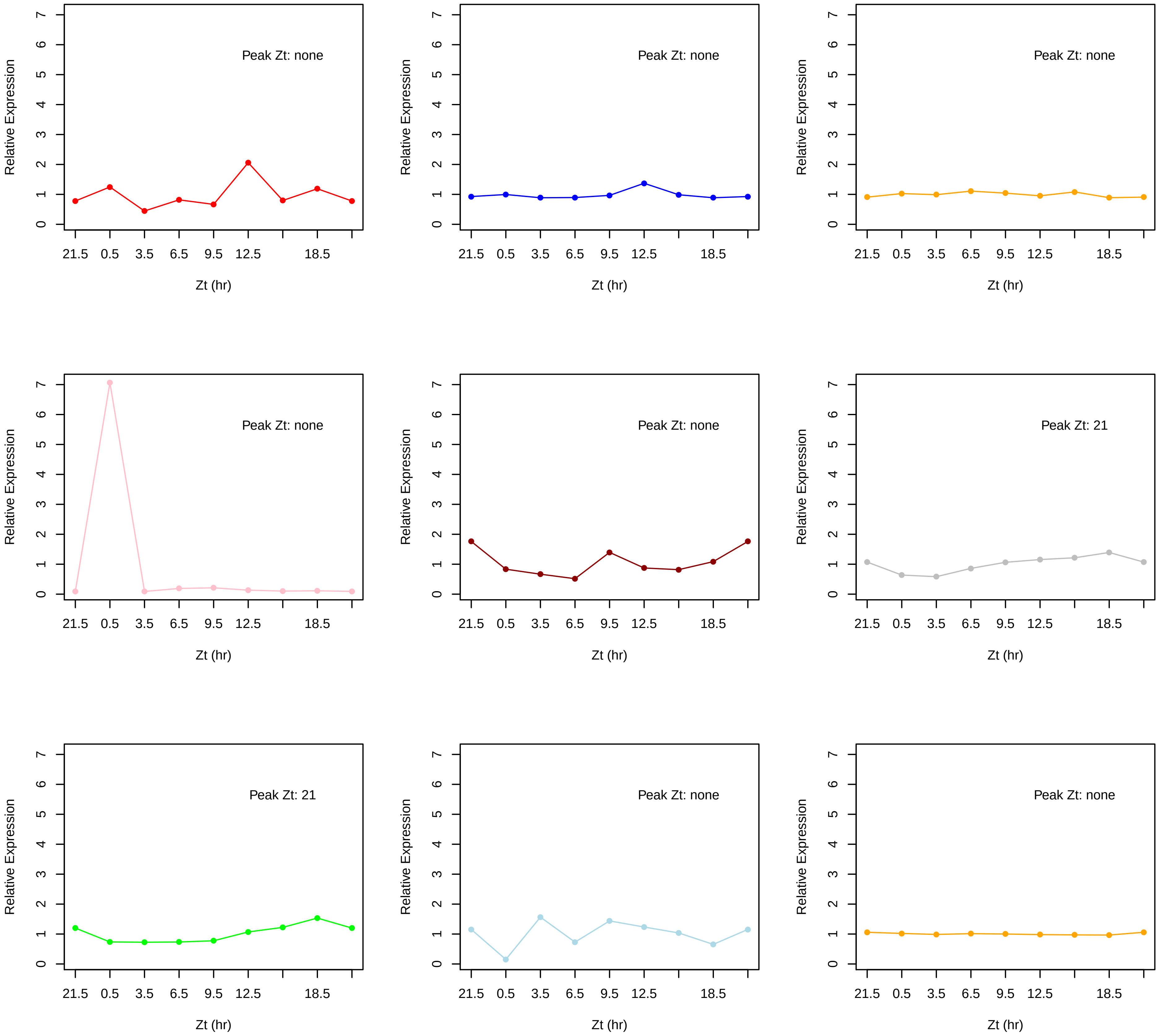

Singleton: ENSXMAG00000007819 lpcat2

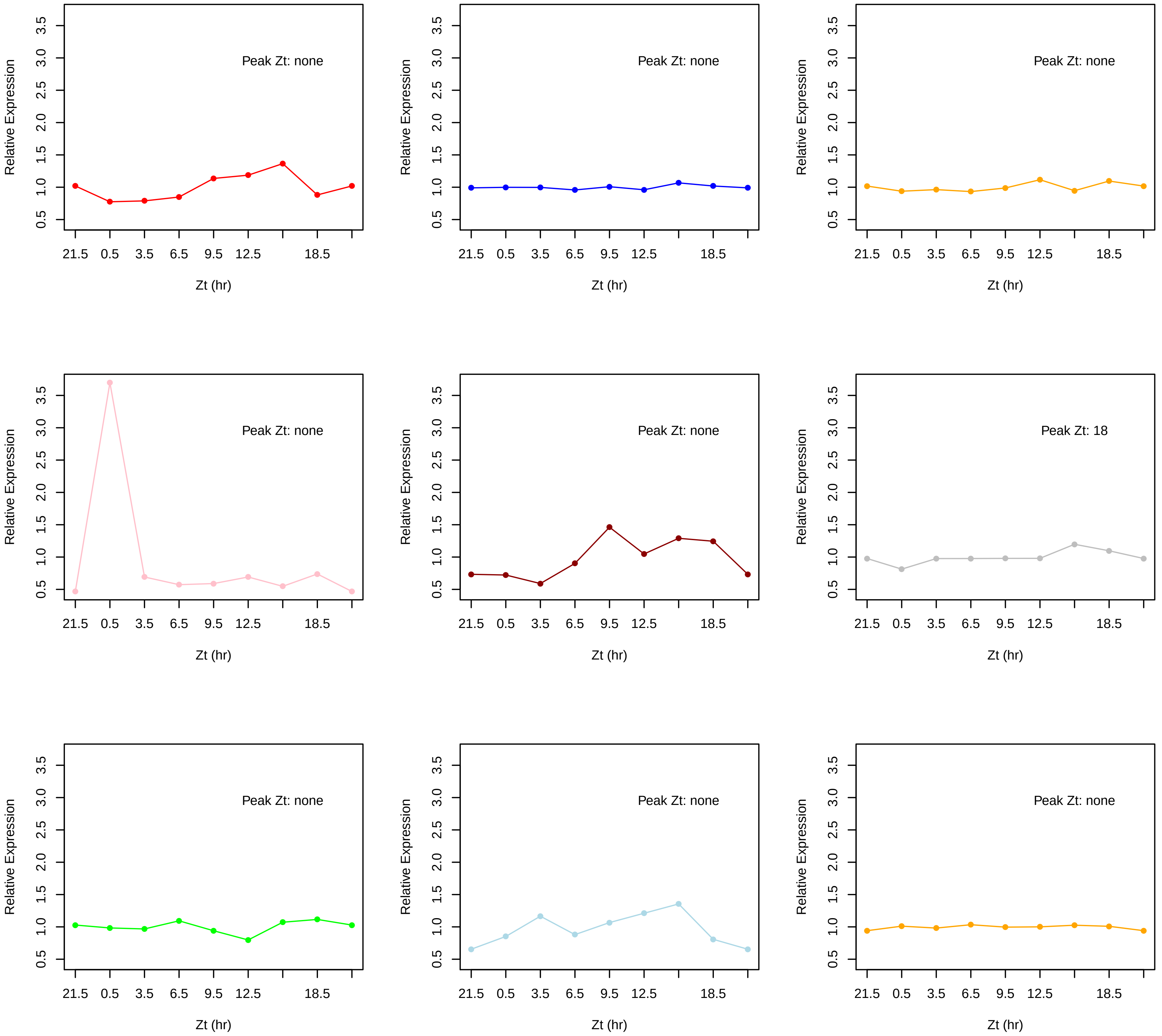

Singeleton: ENSXMAG00000002379

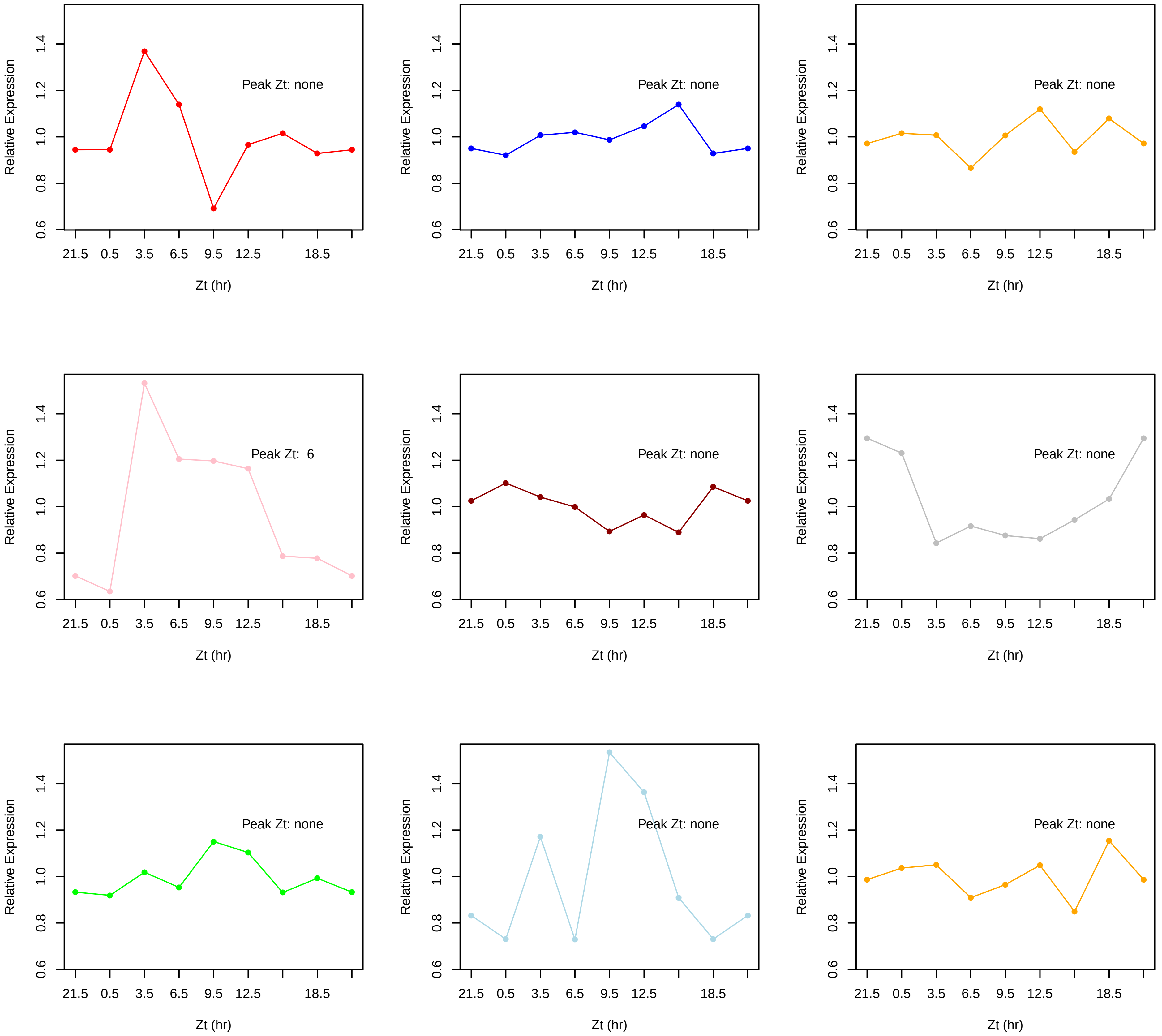

Singeleton: ENSXMAG00000004383 specc1la

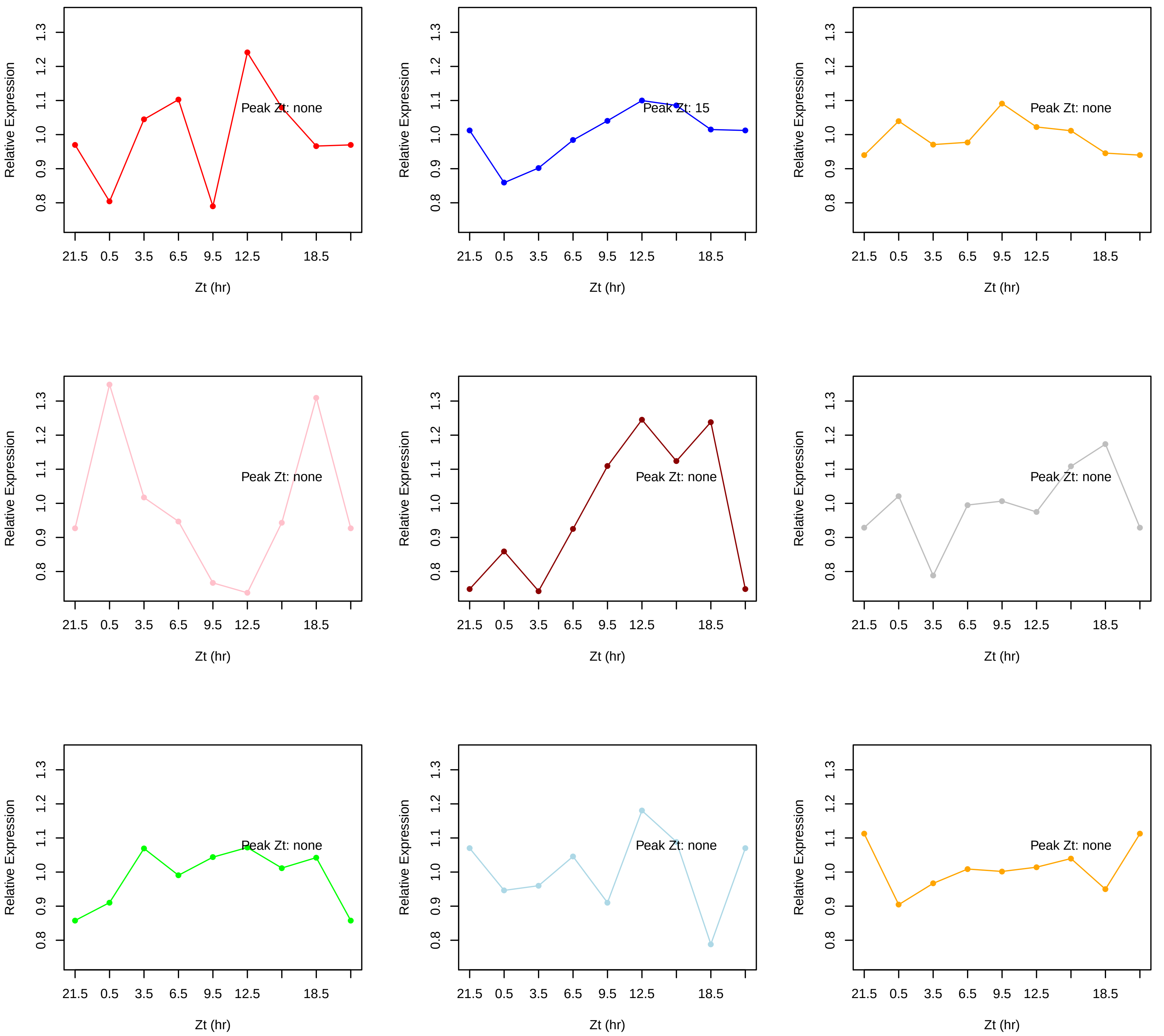

Singleton: ENSXMAG00000015892 coro1b

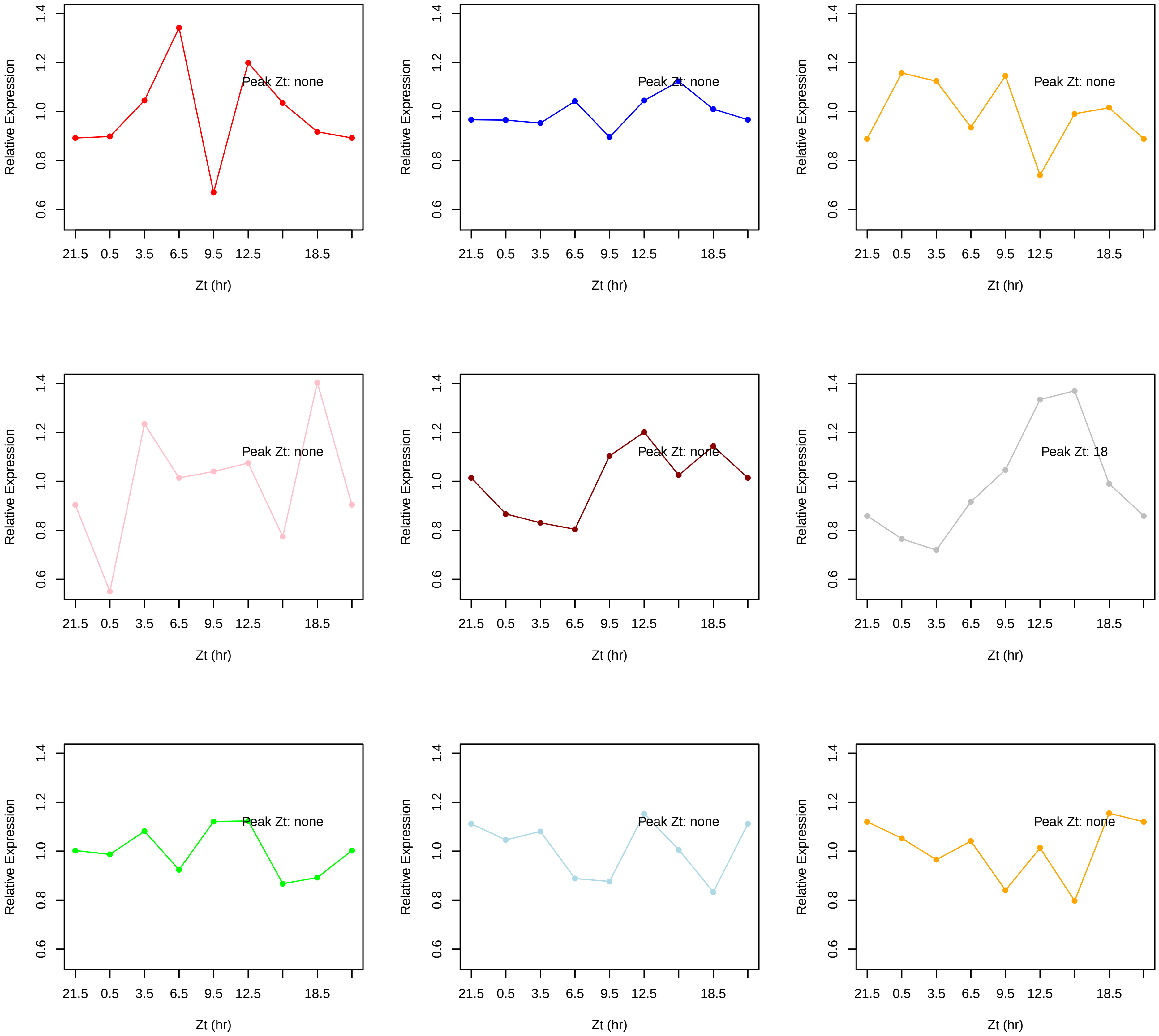

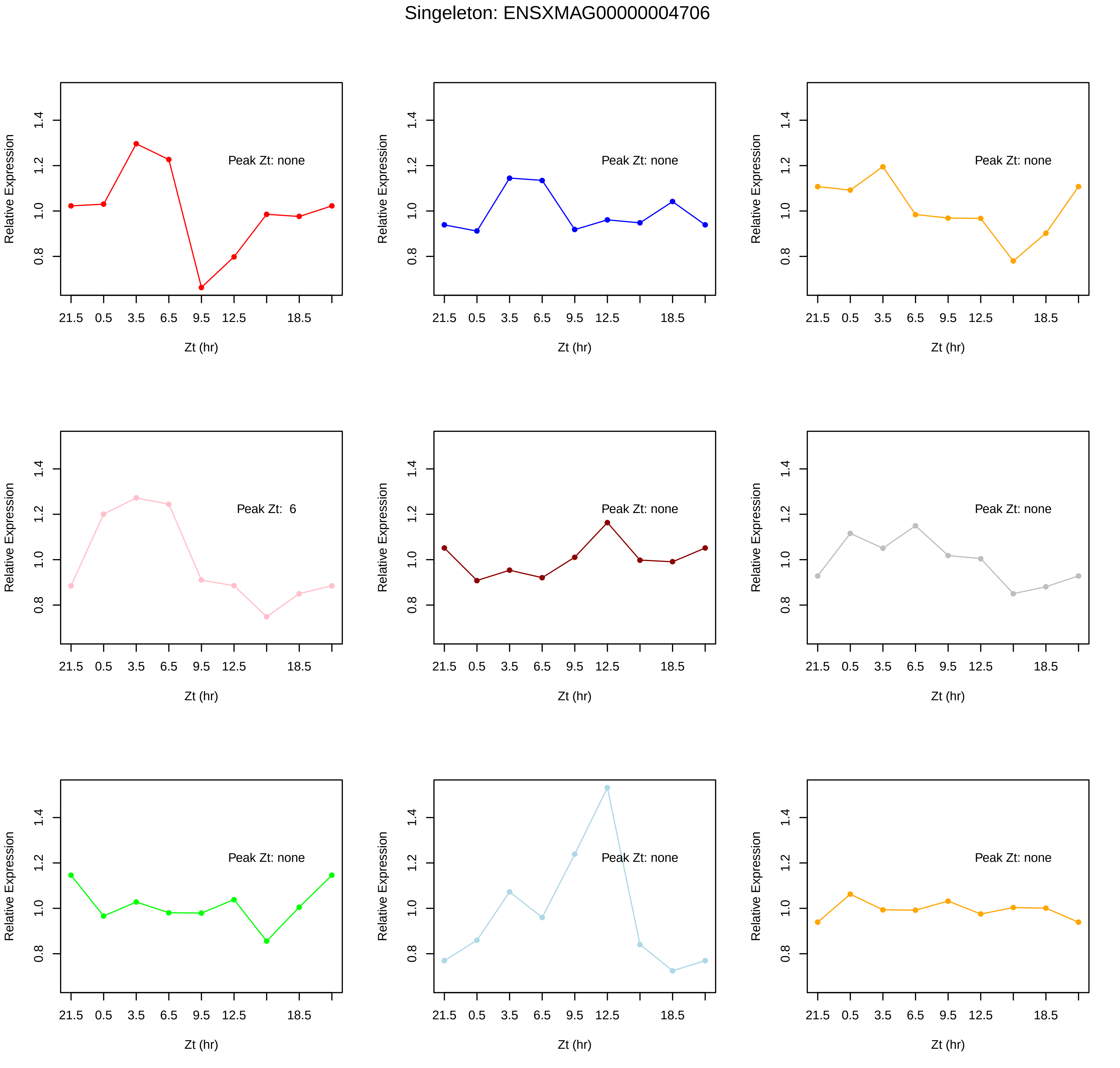
Singeleton: ENSXMAG00000004706
Peak Zt: none
Peak Zt: none
Peak Zt: none
Peak Zt: 6
Peak Zt: none
Peak Zt: none
Peak Zt: none
Peak Zt: none
Peak Zt: none
Relative Expression
Zt (hr)
21.5
0.5
3.5
6.5
9.5
12.5
18.5
0.8
1.0
1.2
1.4

Singeleton: ENSXMAG00000009818 gpr157

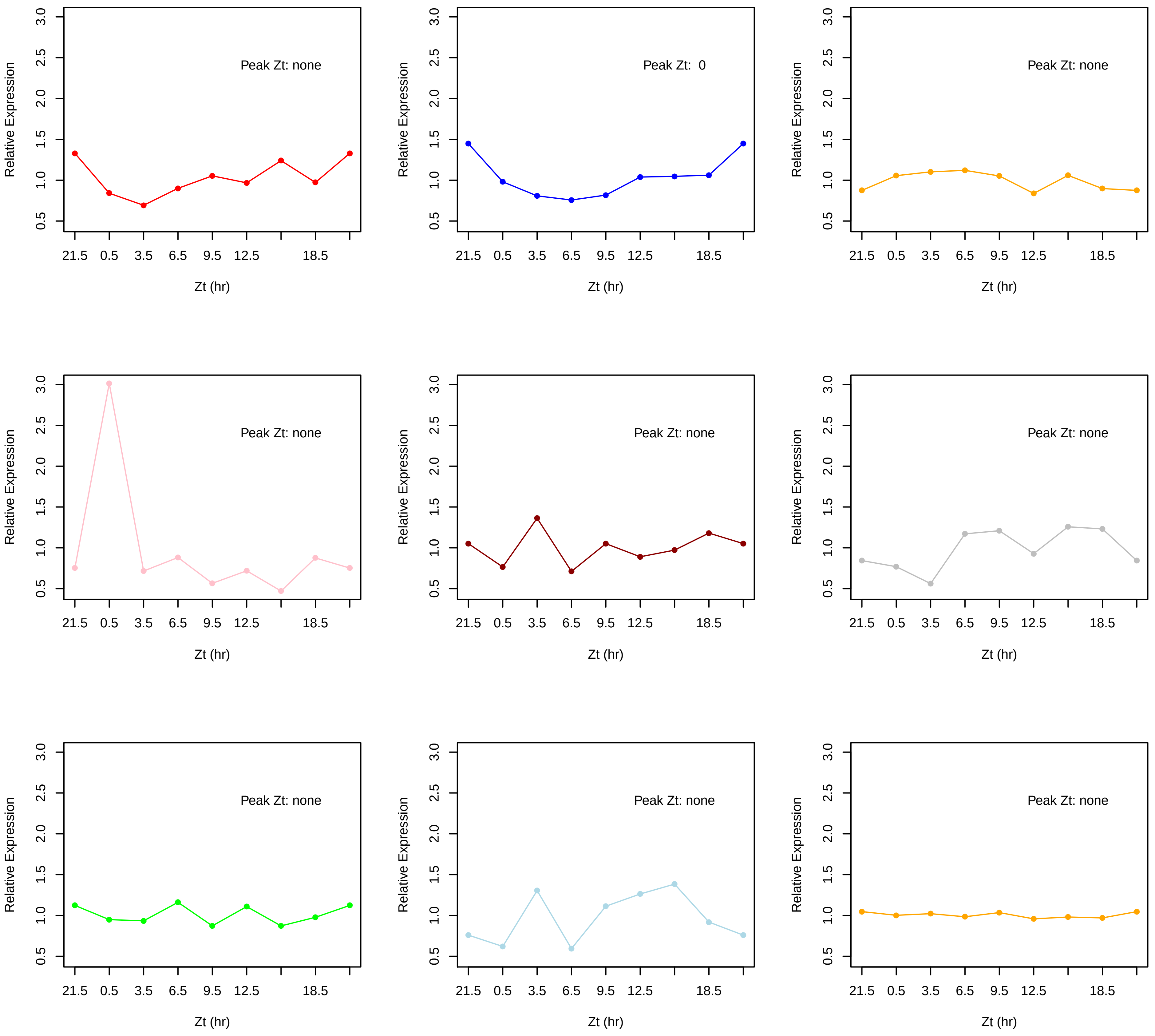

Singleton: ENSXMAG00000002410 zmp:0000001048

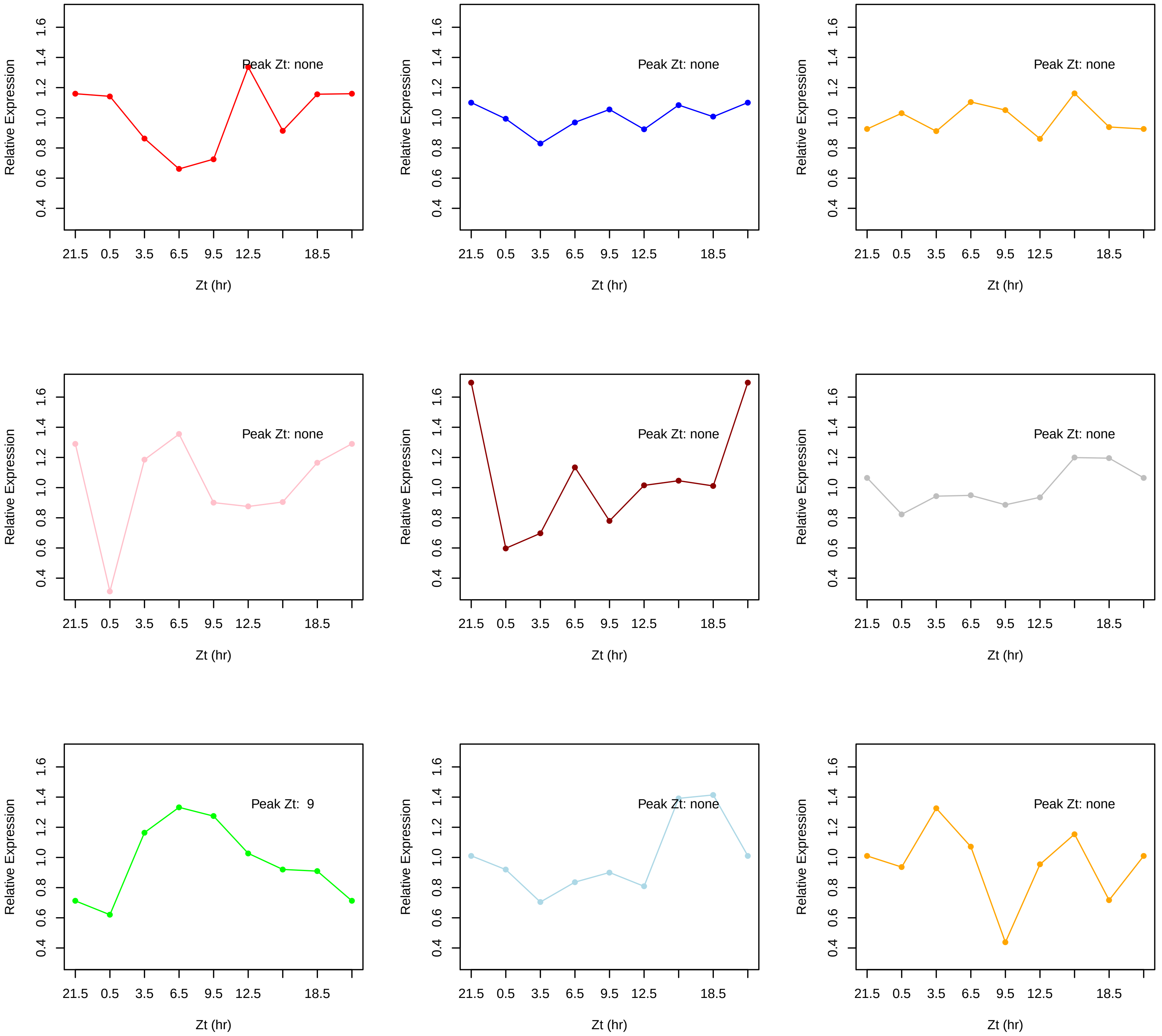

Singeleton: ENSXMAG00000010458 ccndbp1

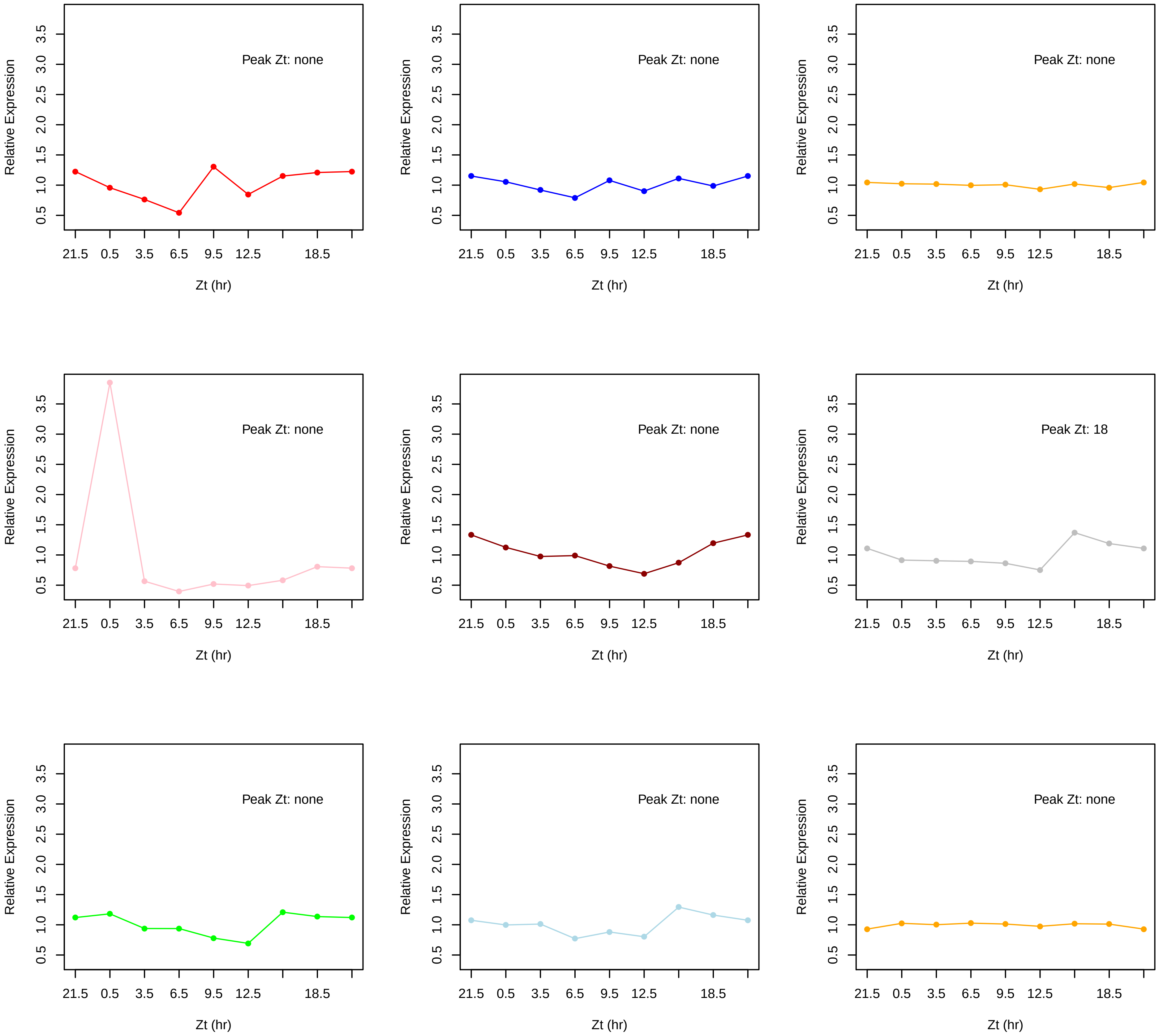

Singeleton: ENSXMAG00000003923 slc2a5

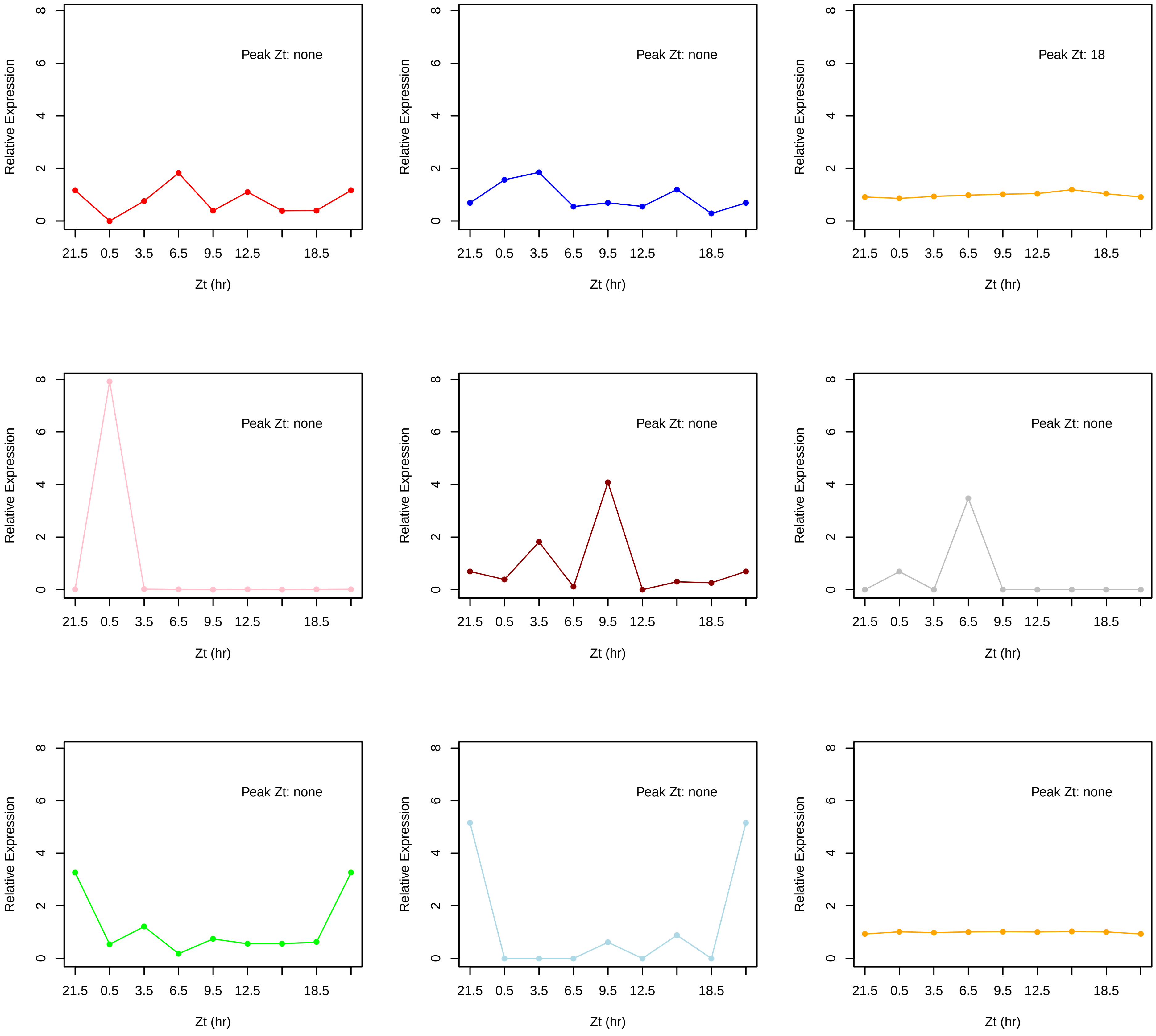

Singeleton: ENSXMAG00000010519 larp4aa

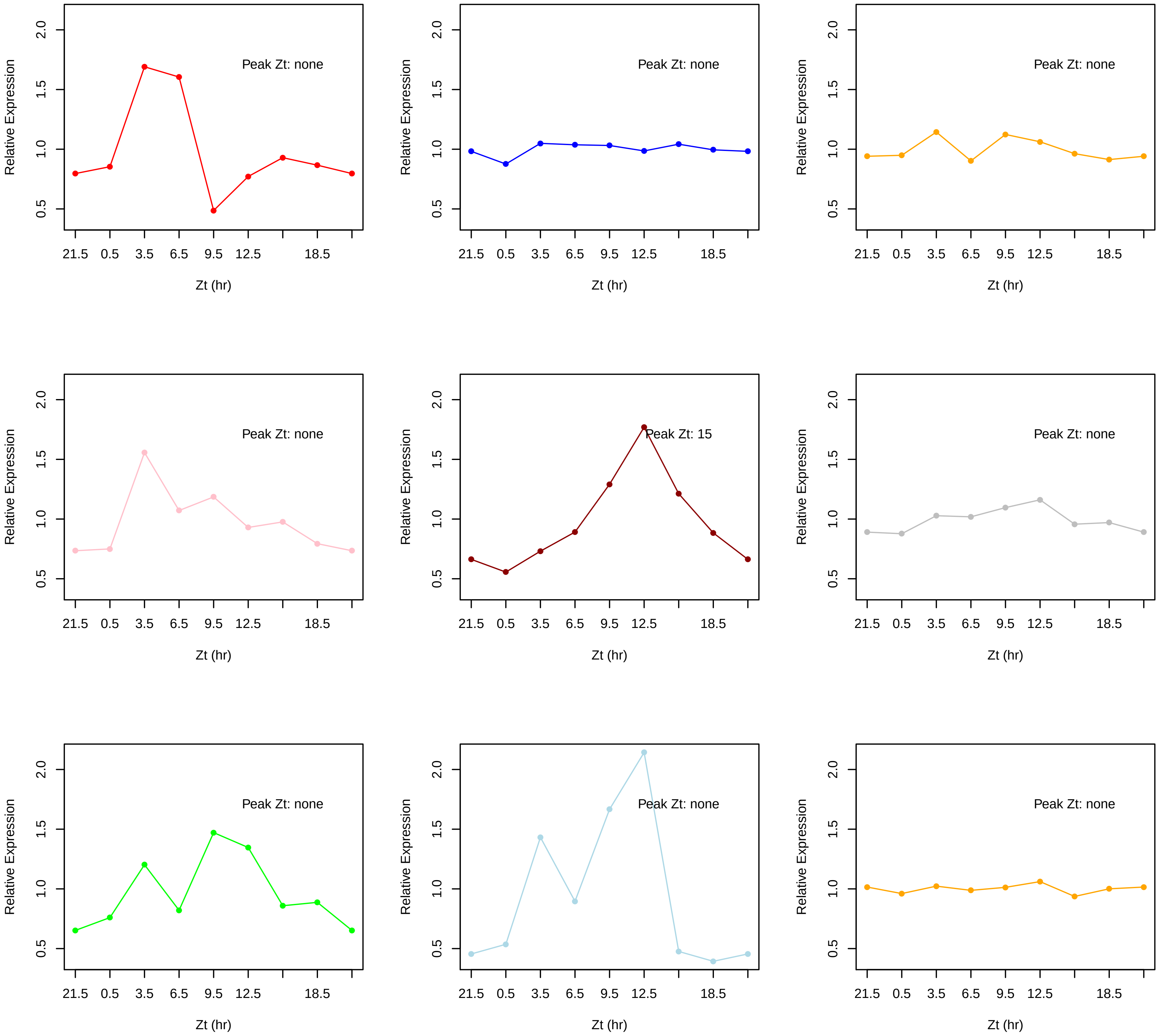

Singeleton: ENSXMAG00000028963 tmem135

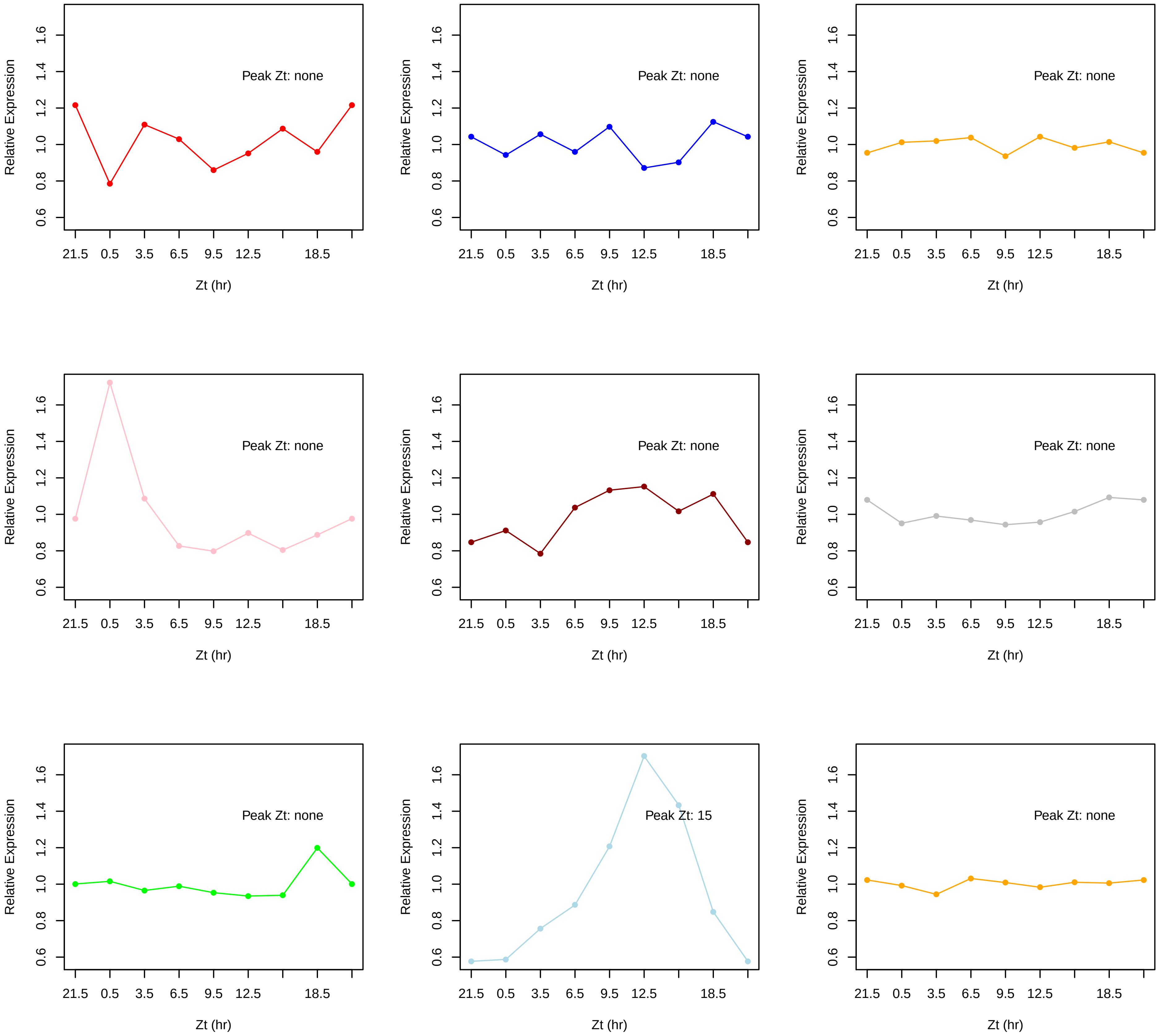

Singeleton: ENSXMAG00000023881 rp9

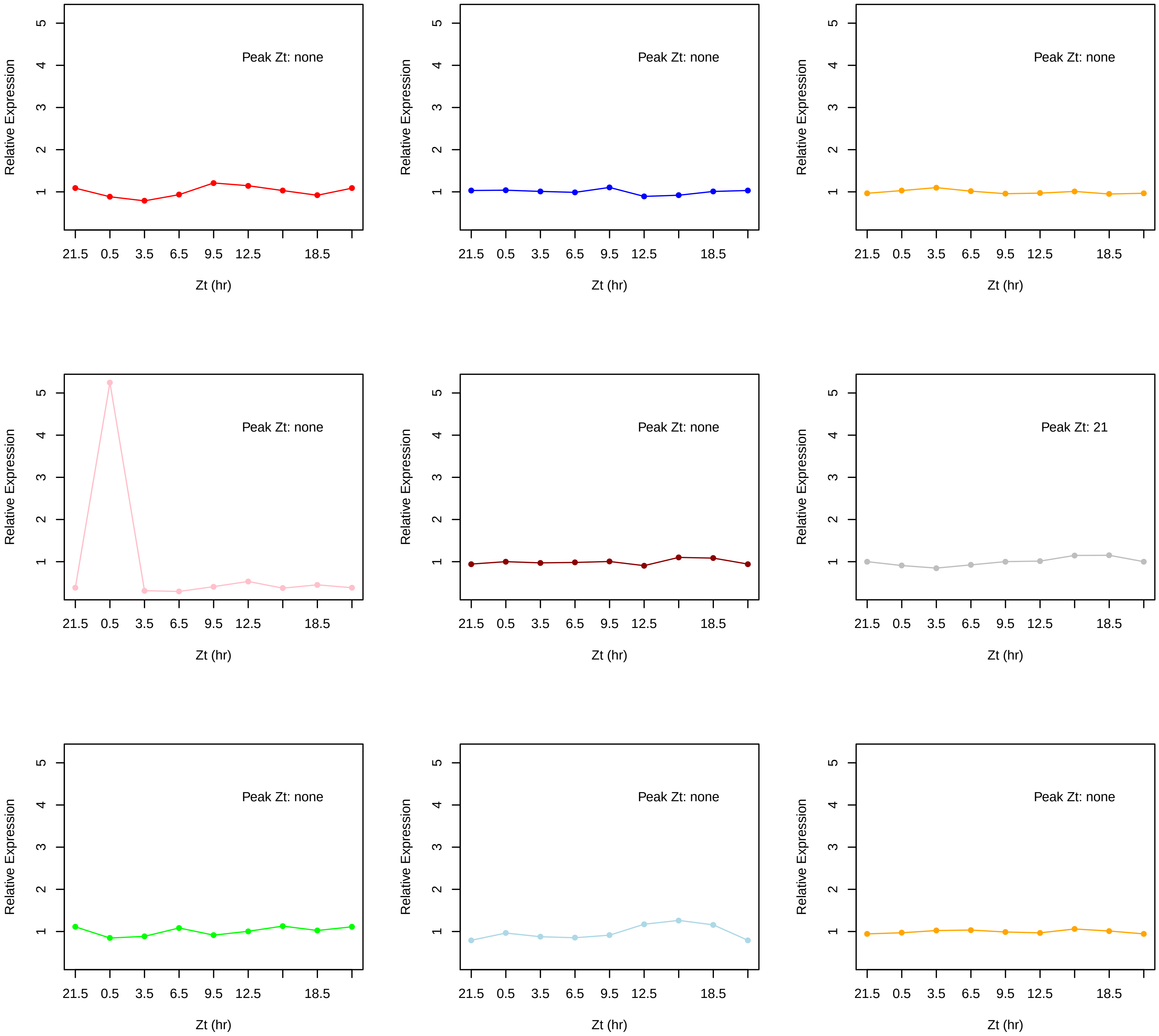

Singeleton: ENSXMAG00000002155 pfdn5

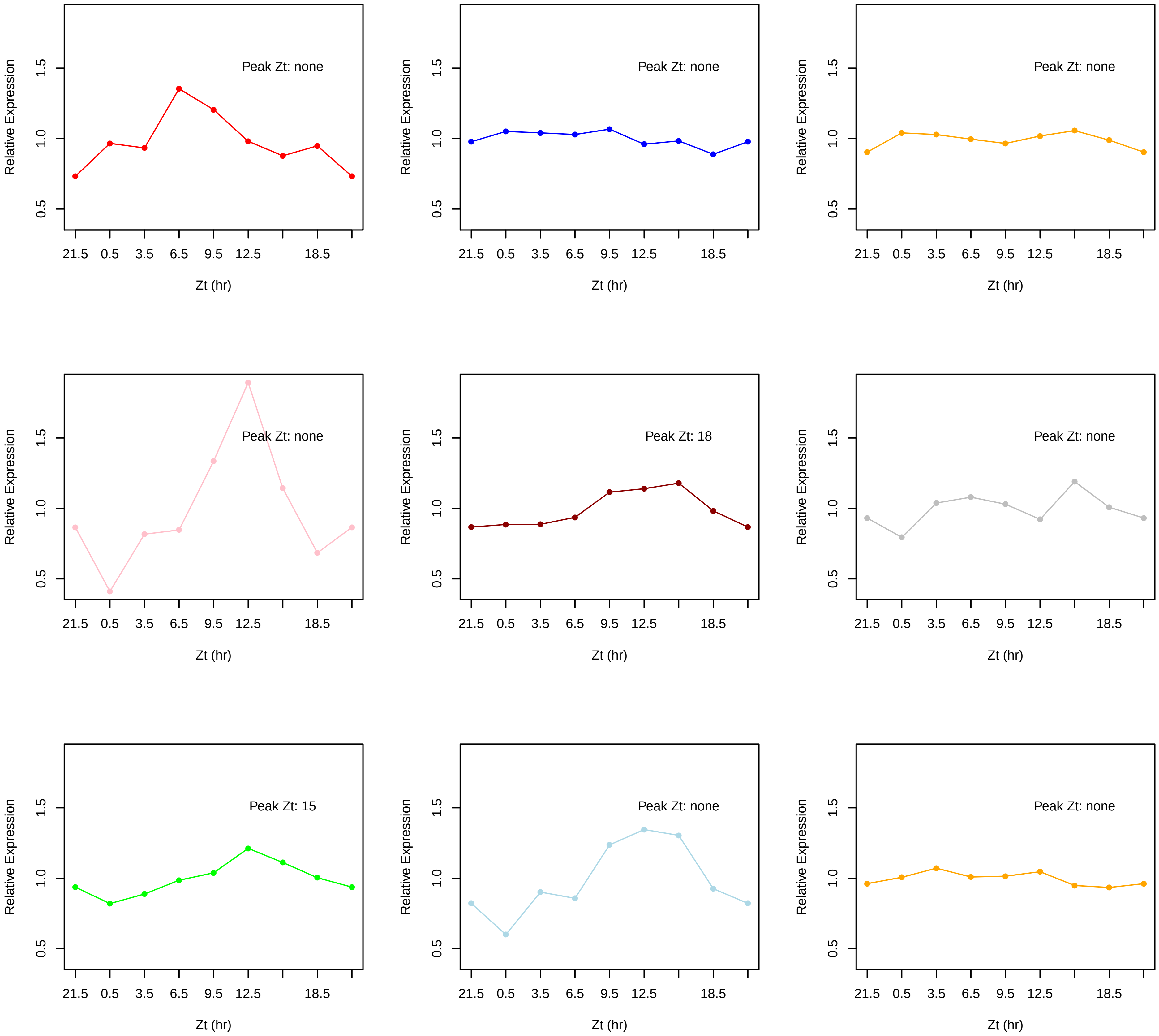

Singleton: ENSXMAG00000002797 si:dkey−184p18.2

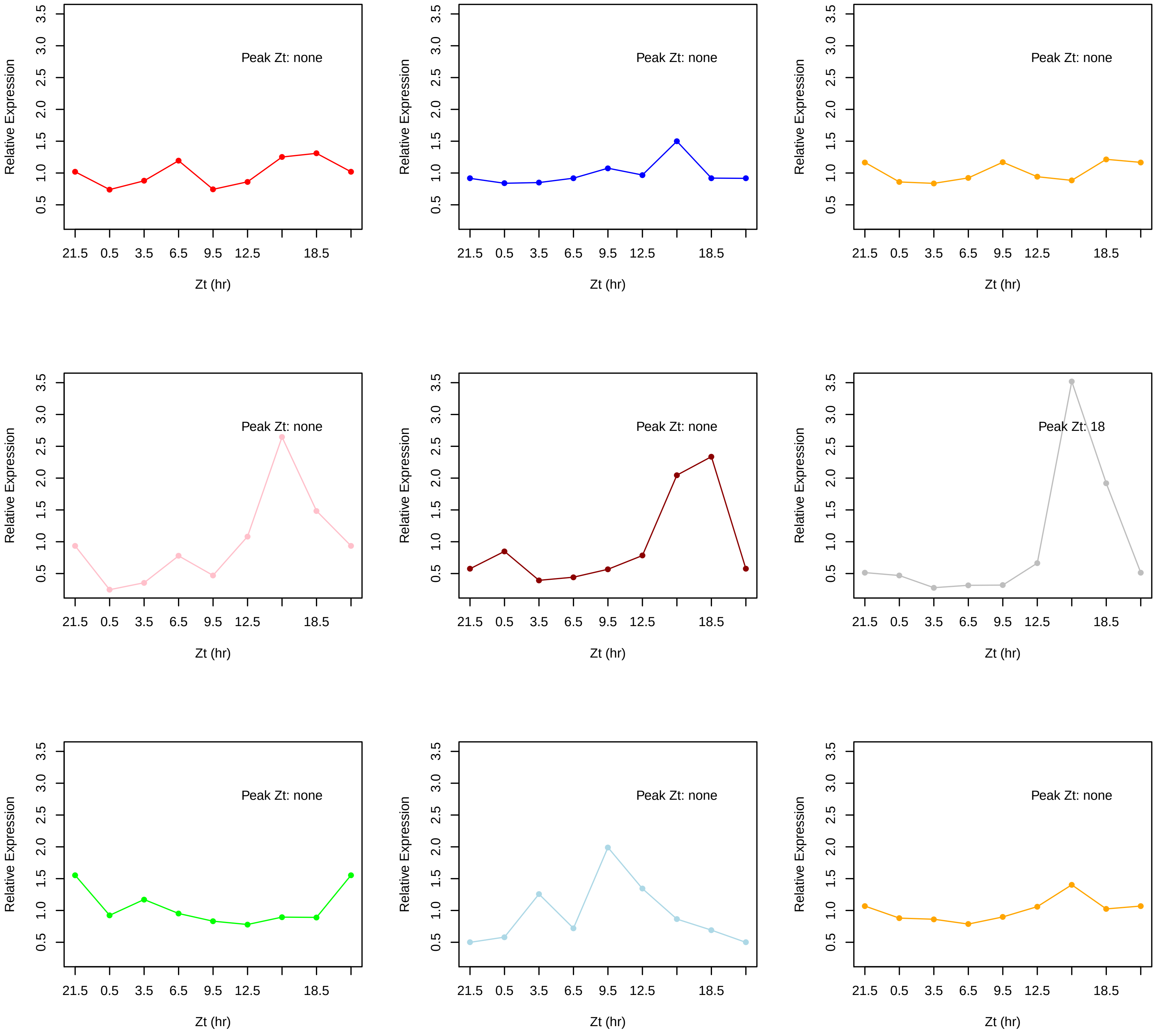

Singleton: ENSXMAG00000010000 prkg2

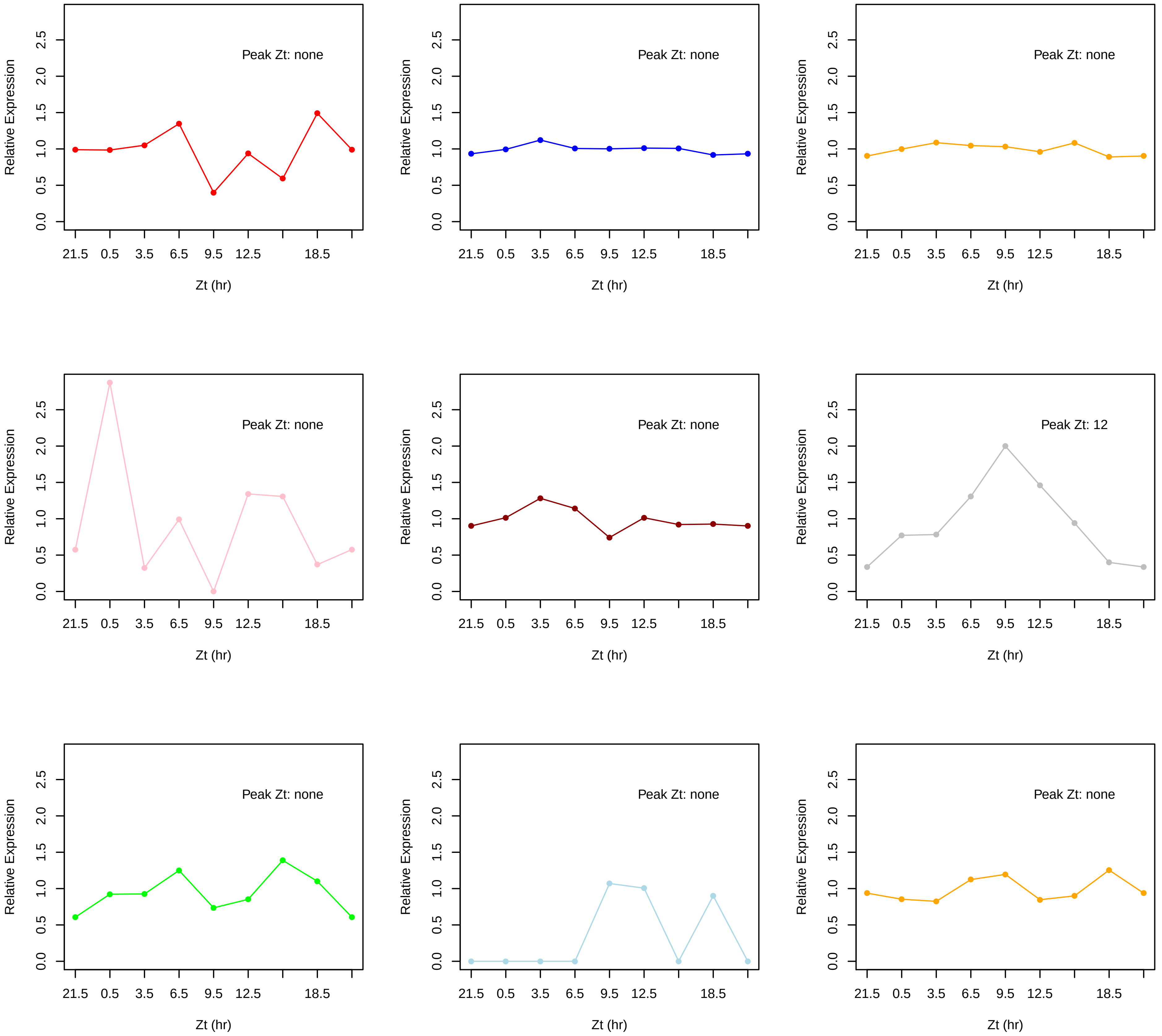

Singeleton: ENSXMAG00000028932

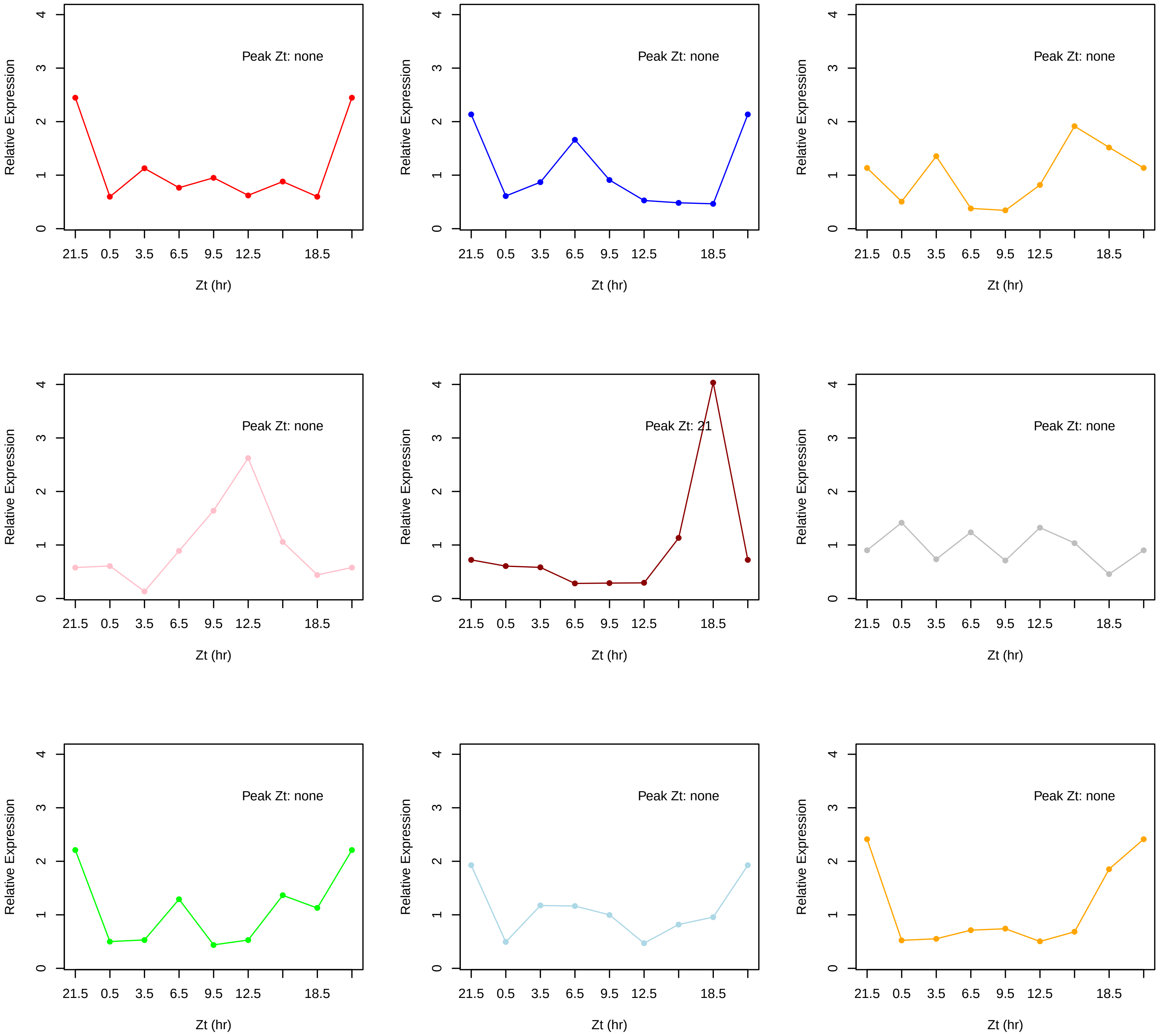

Singeleton: ENSXMAG00000006728 rpgrip1l

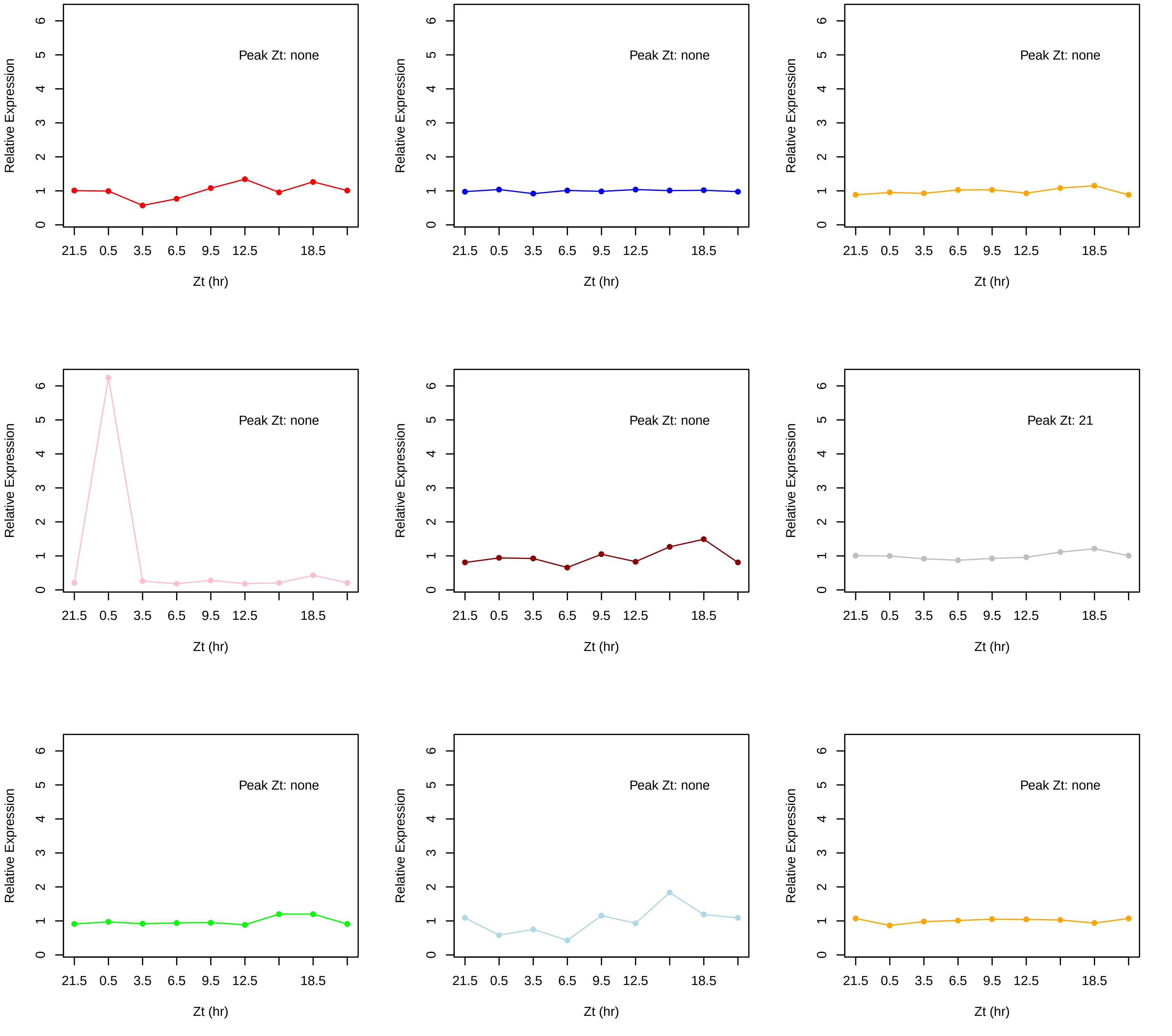

Singeleton: ENSXMAG00000002452 LRRC75A

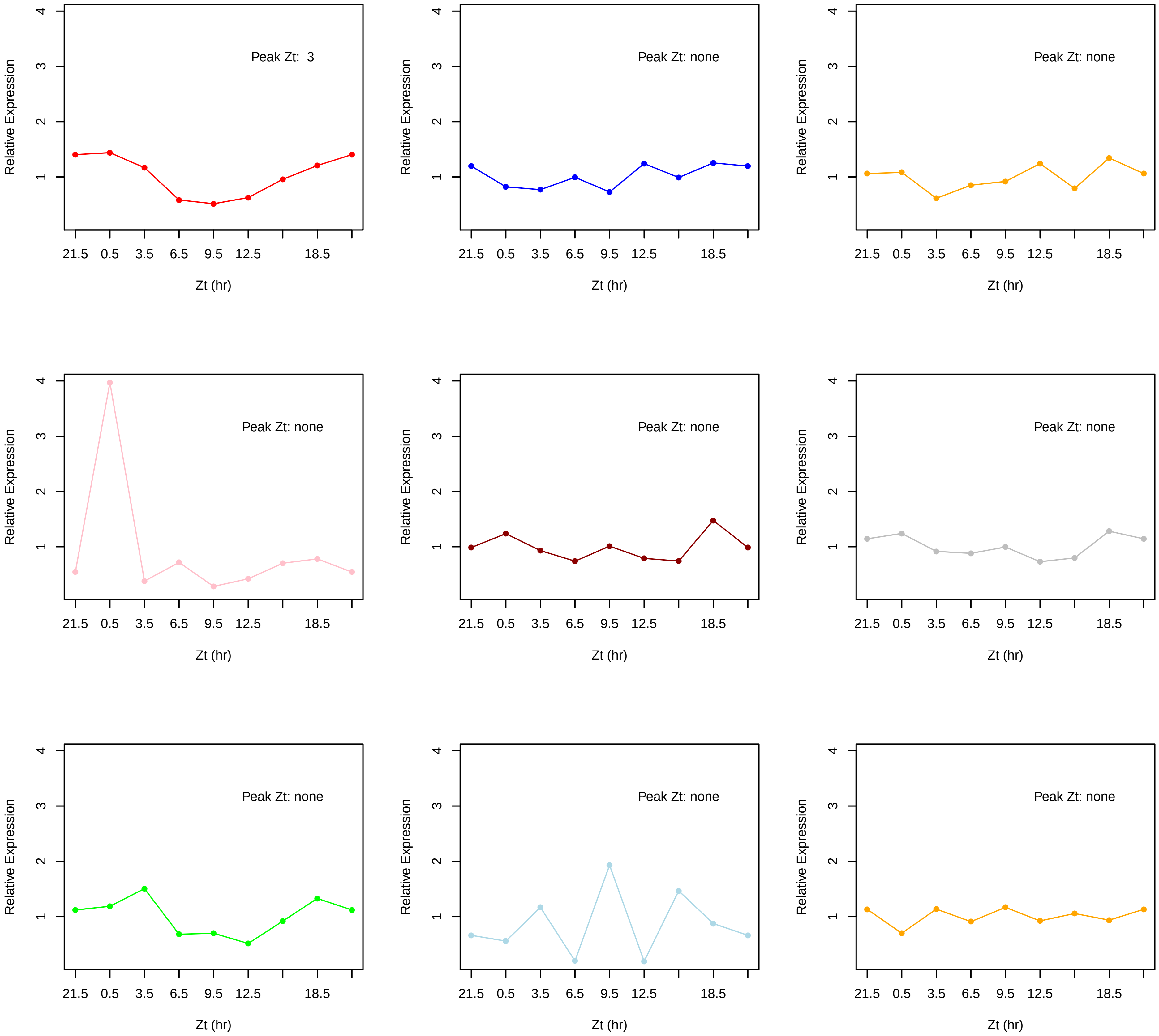

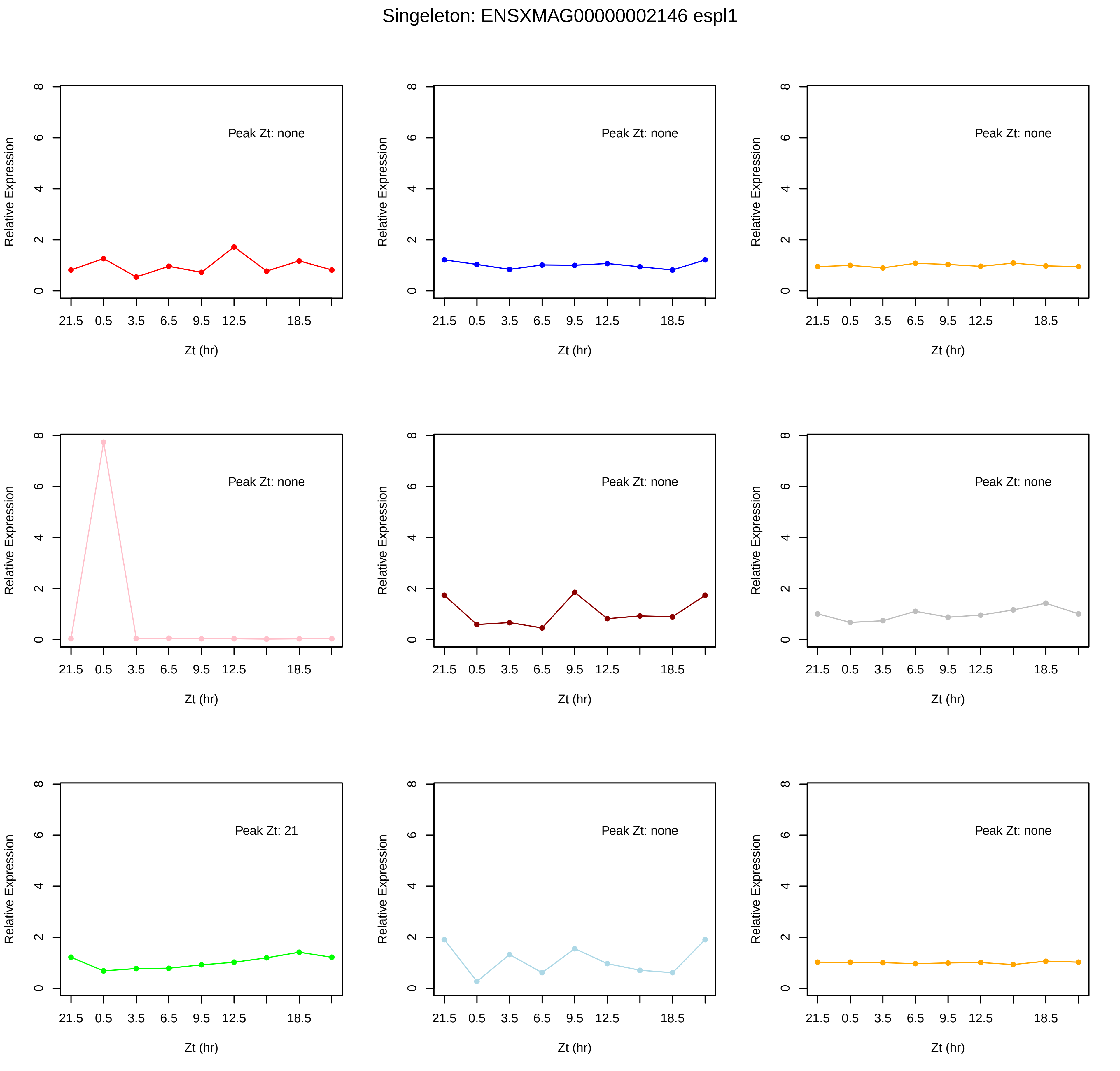
Singeleton: ENSXMAG00000002146 espl1
Peak Zt: none
Peak Zt: none
Peak Zt: none
Peak Zt: none
Peak Zt: none
Peak Zt: none
Peak Zt: 21
Peak Zt: none
Peak Zt: none
Relative Expression
Zt (hr)
21.5
0.5
3.5
6.5
9.5
12.5
18.5

Singeleton: ENSXMAG00000019165 chmp4bb

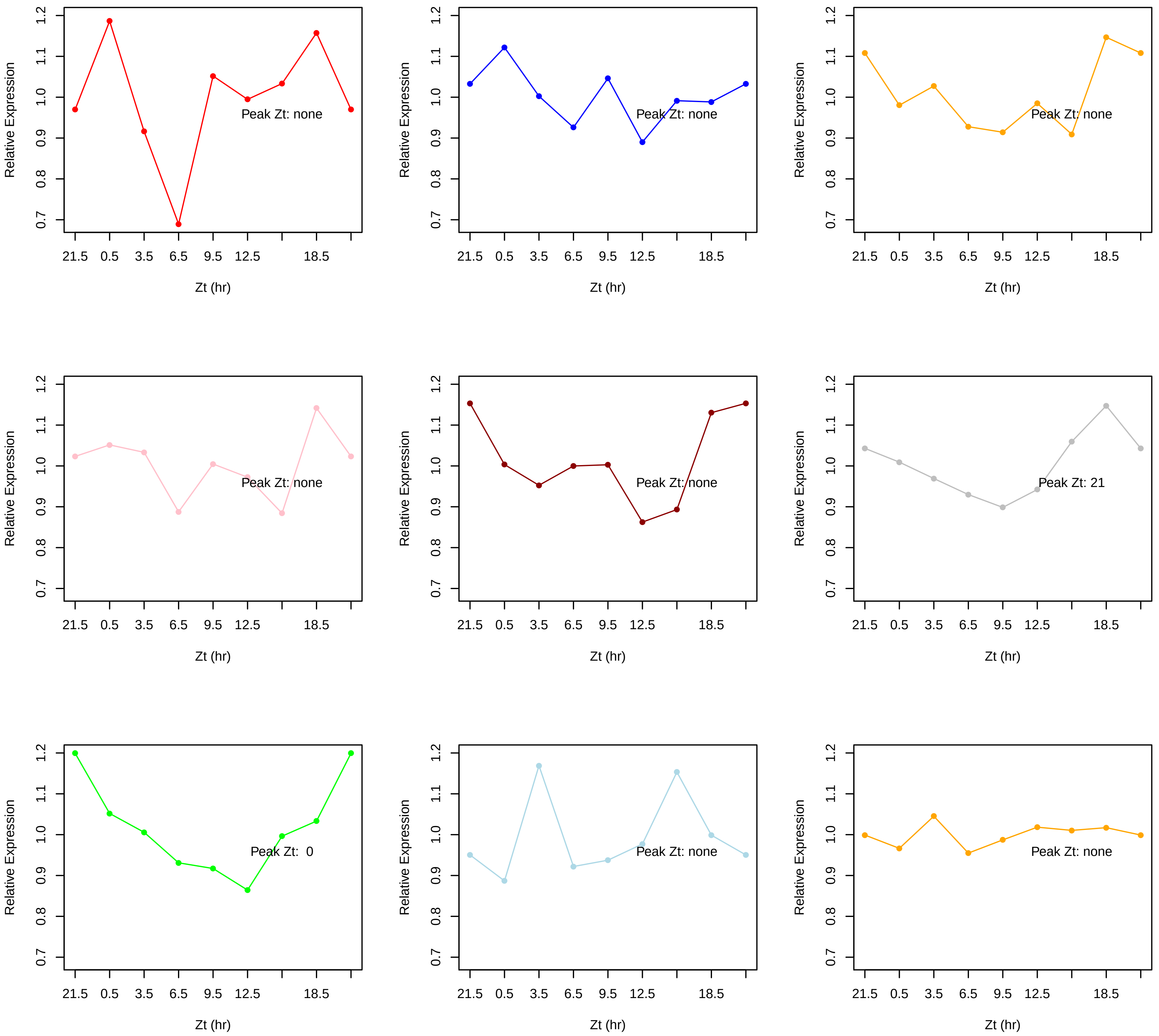

Singleton: ENSXMAG00000011735 uap1

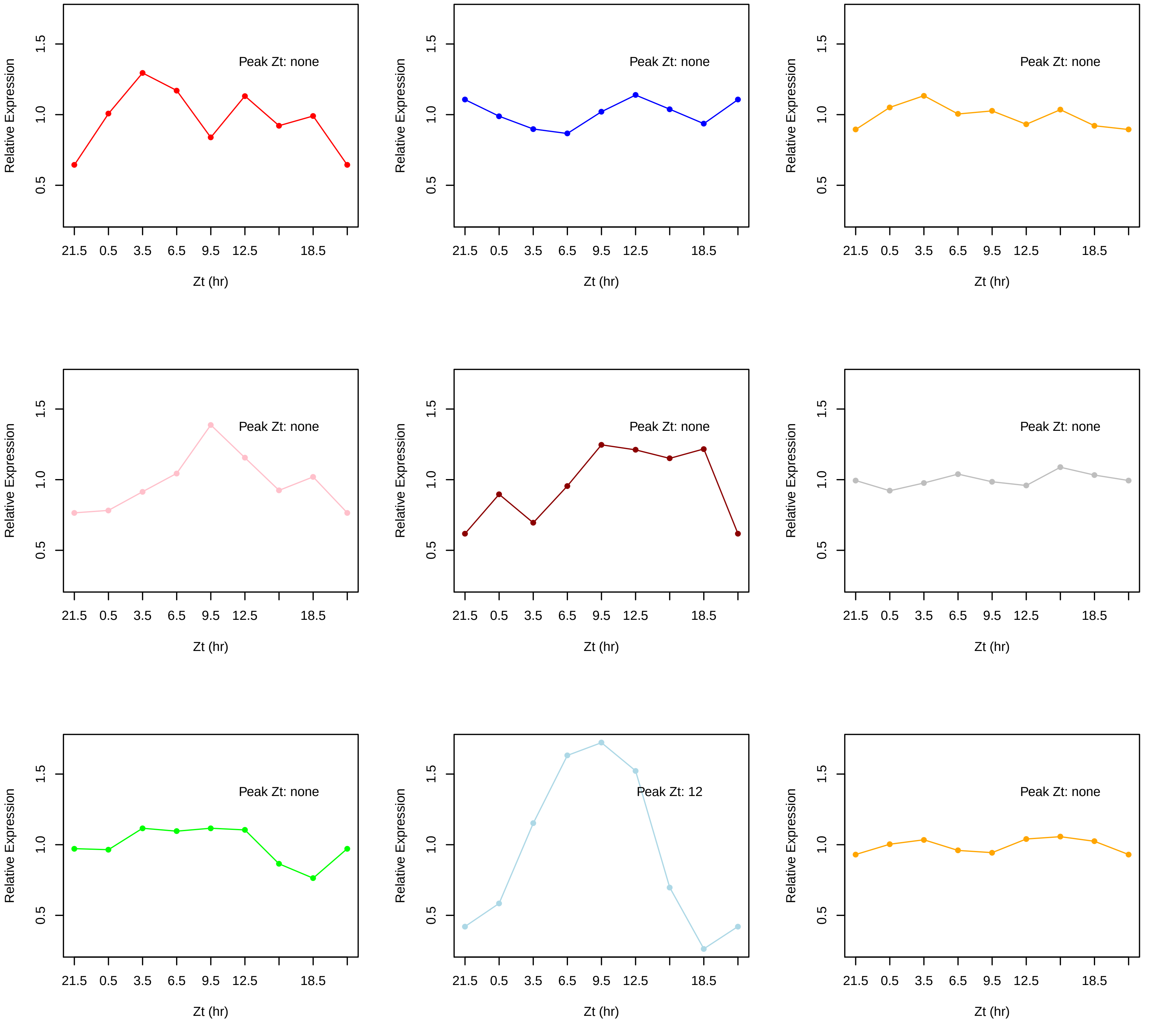

Singeleton: ENSXMAG00000025829 evx2

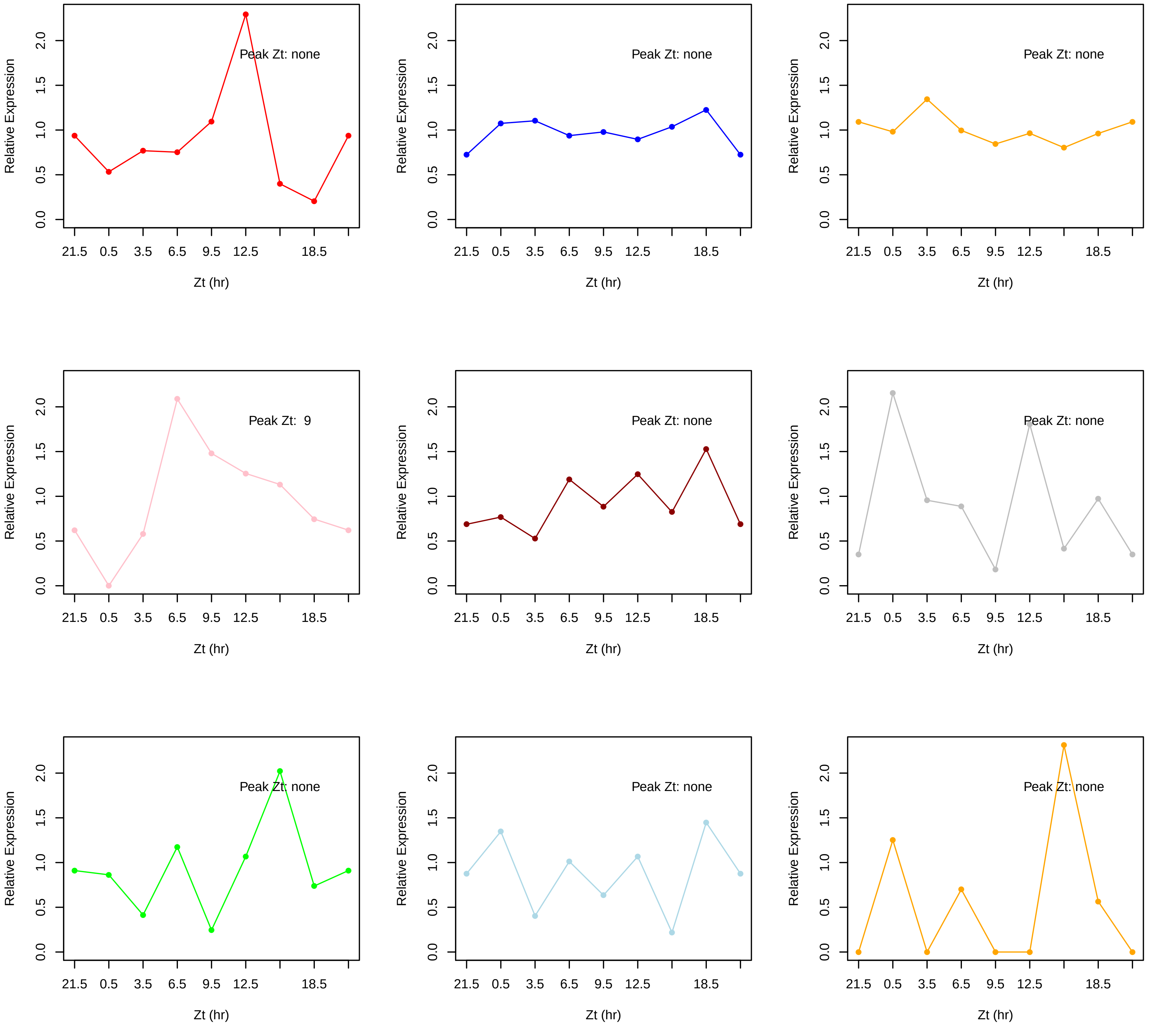

Singeleton: ENSXMAG00000016056 uhmk1

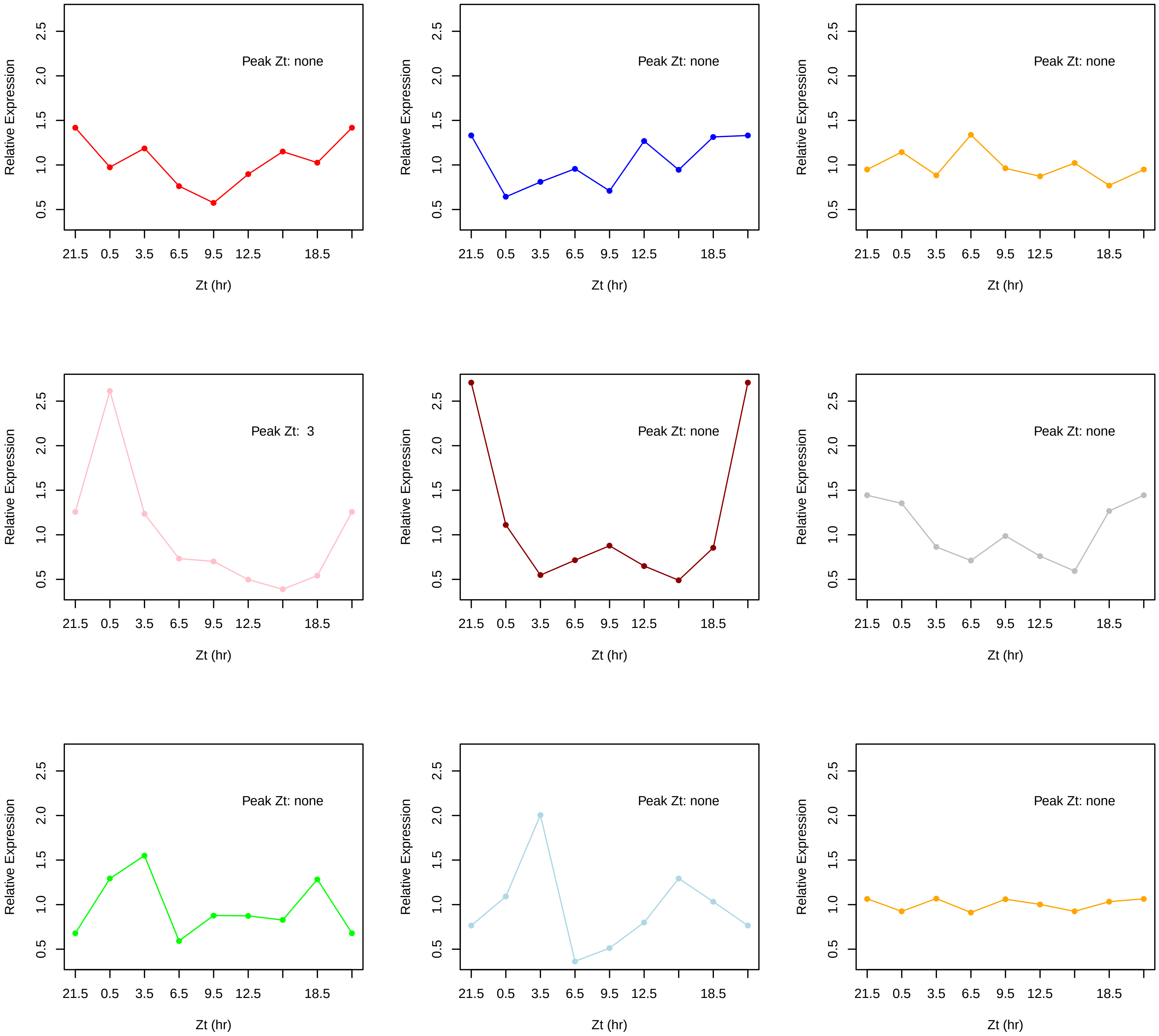

Singleton: ENSXMAG00000020271 gpr84

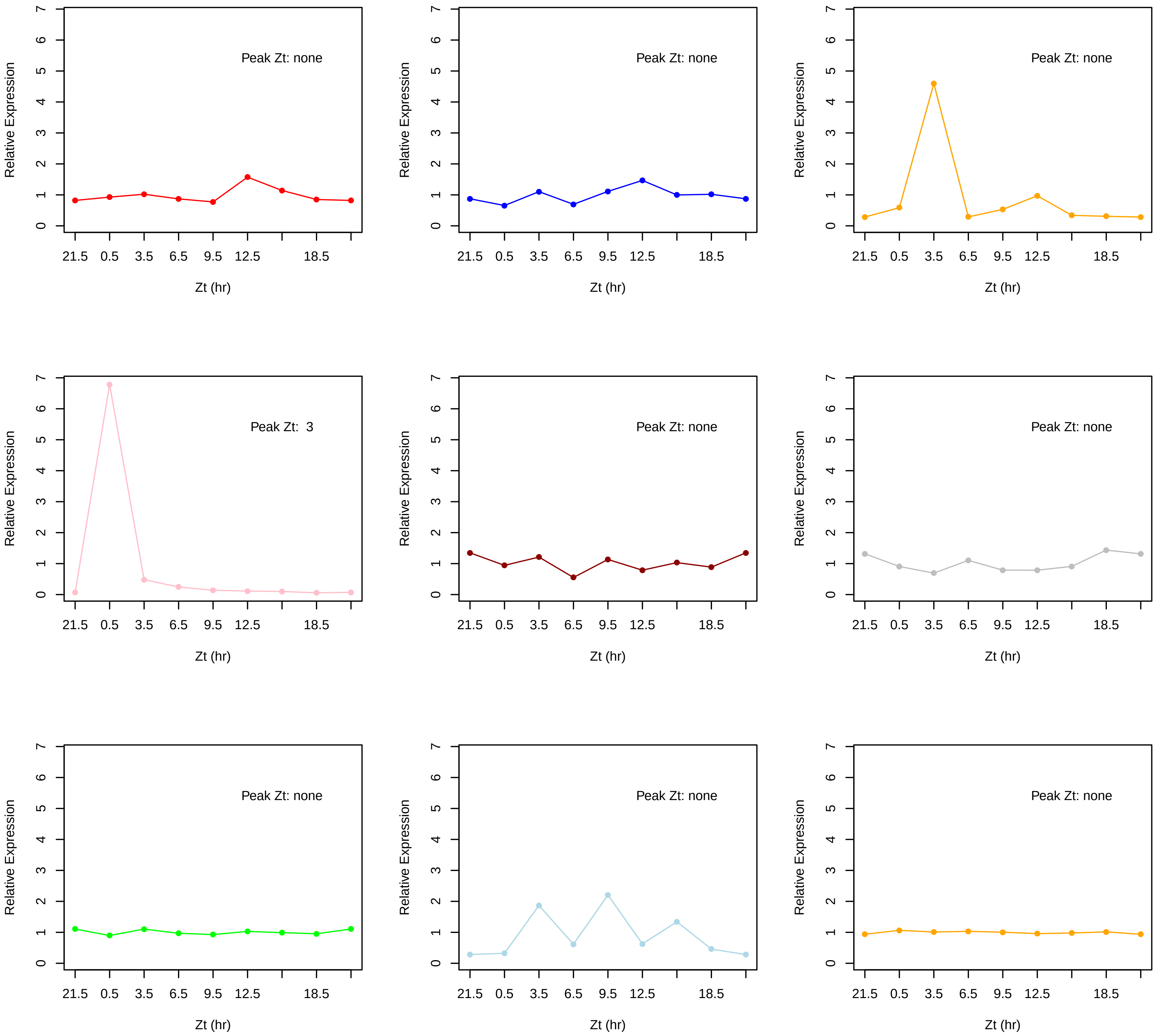

Singeleton: ENSXMAG00000005841 nos1apa

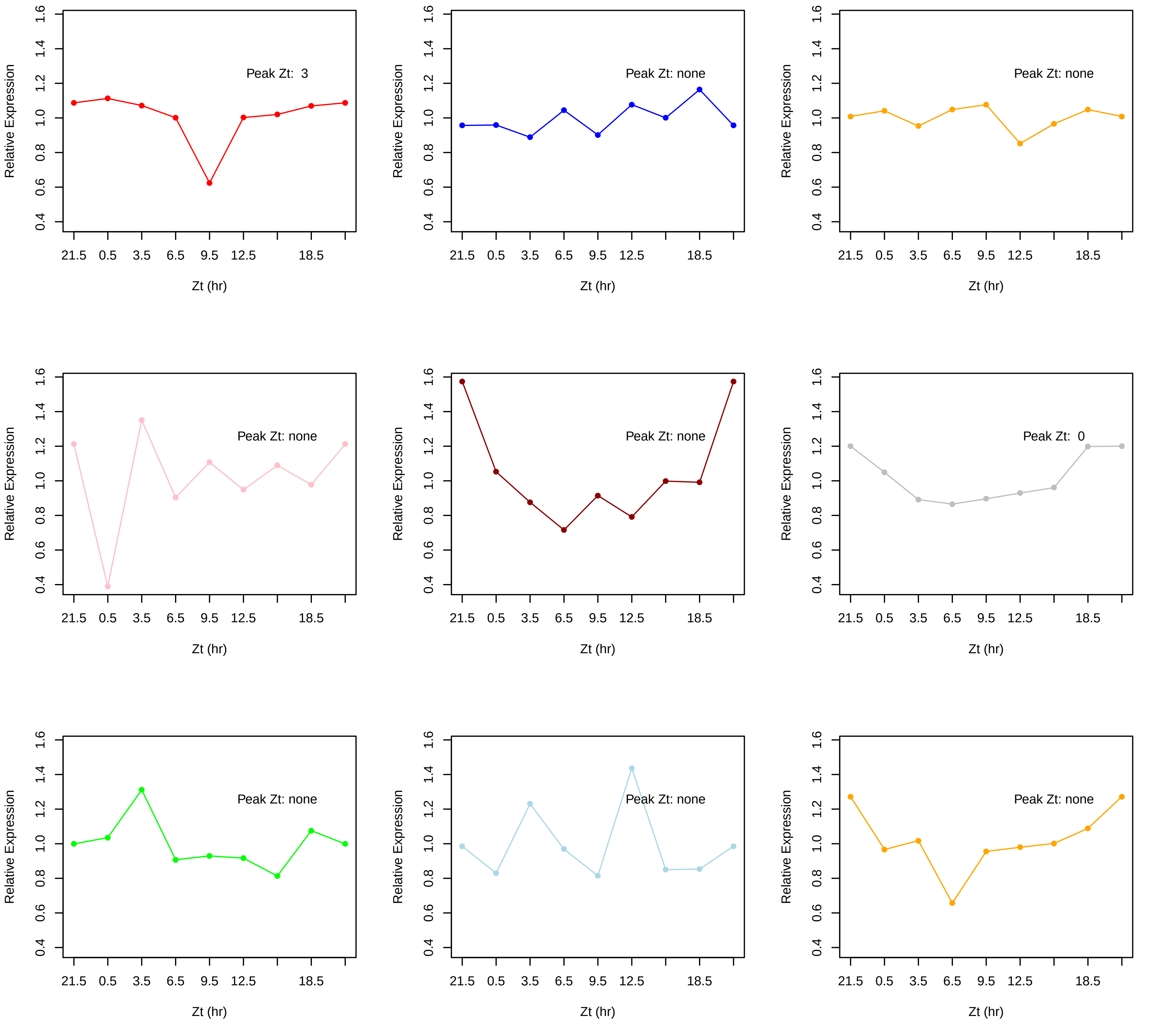

Singleton: ENSXMAG00000024897 sec62

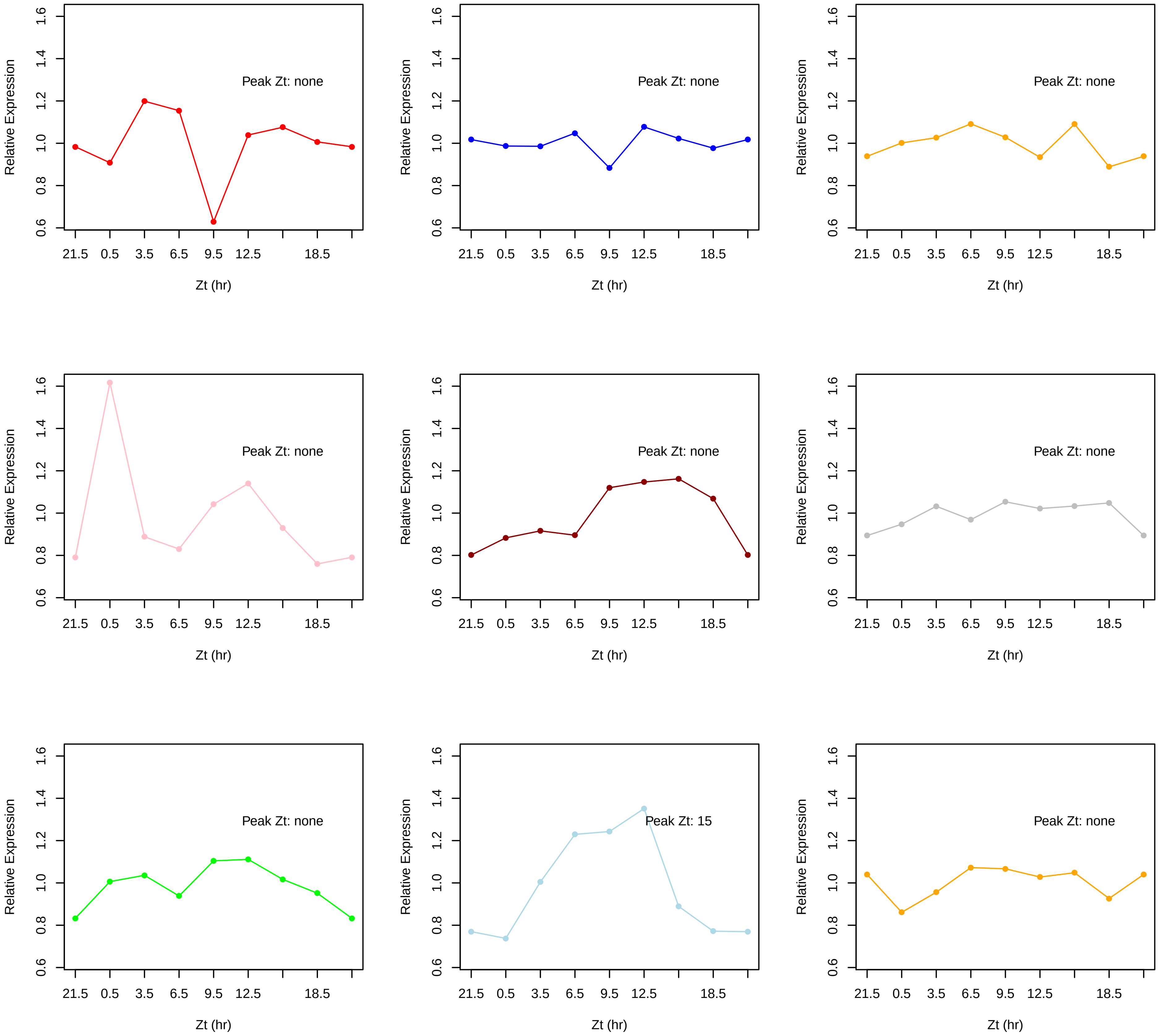

Singleton: ENSXMAG00000019172 eif2s2

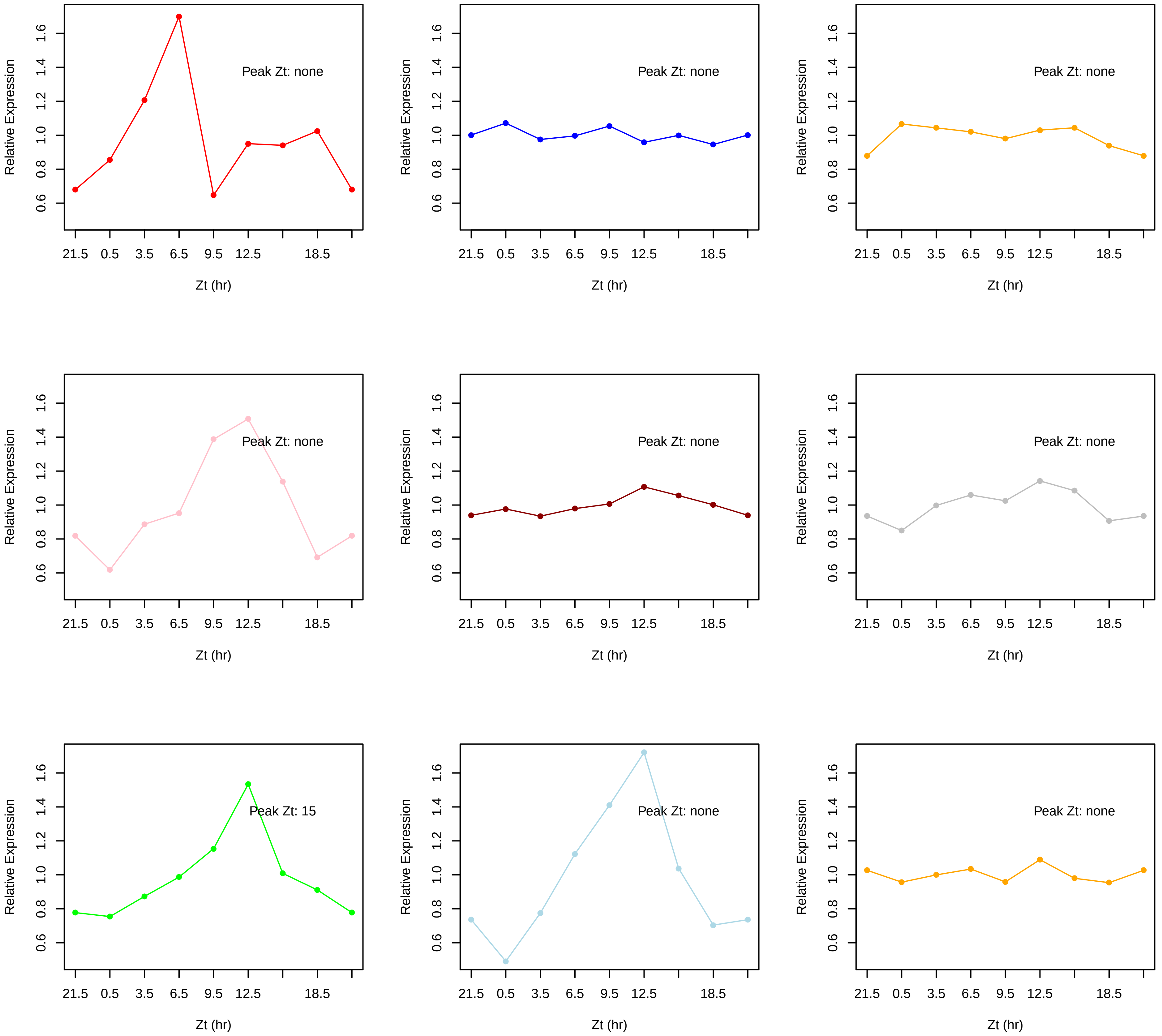

Singeleton: ENSXMAG00000005694 rgs4

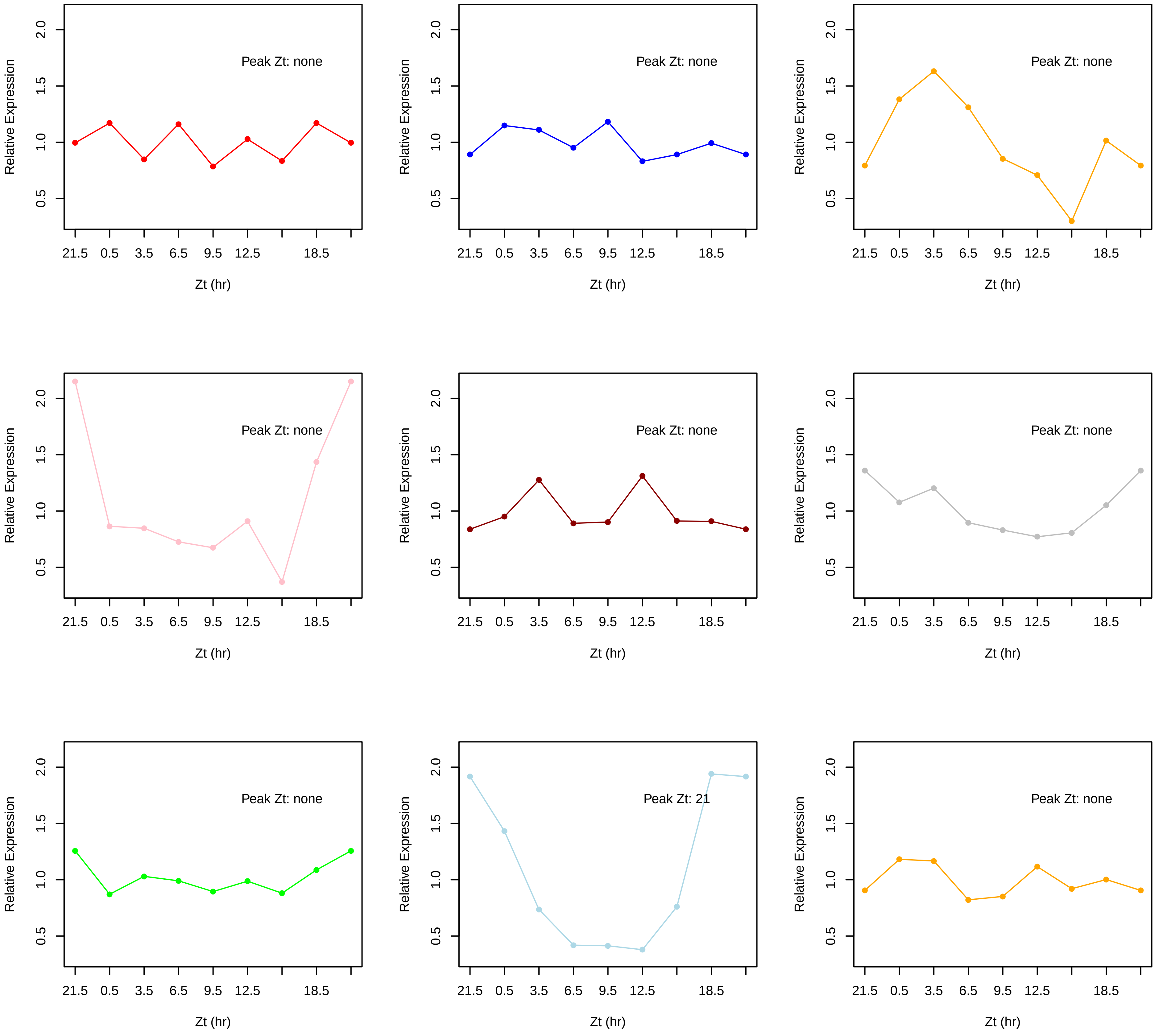

Singeleton: ENSXMAG00000003860 samd11

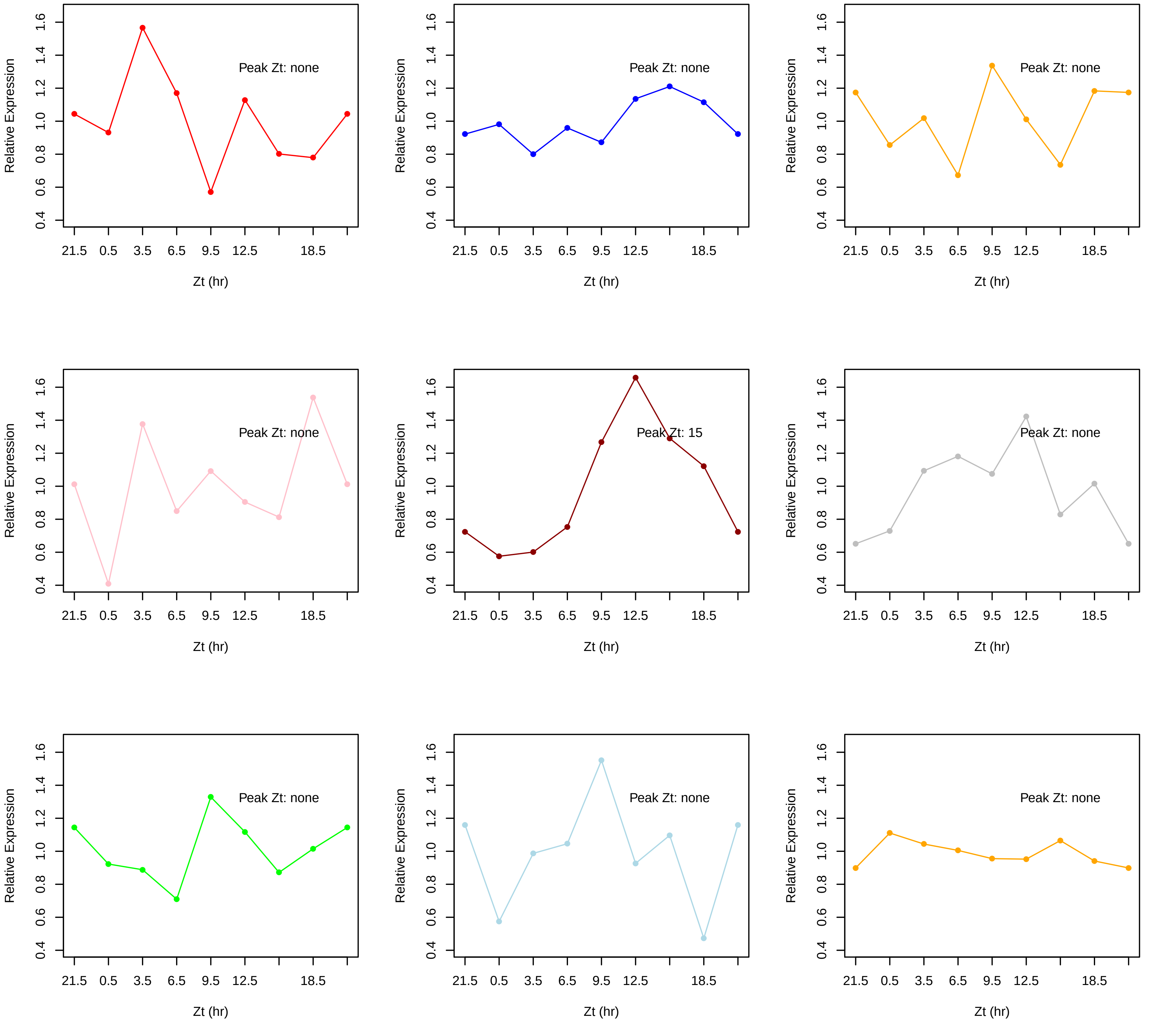

Singeleton: ENSXMAG00000017601

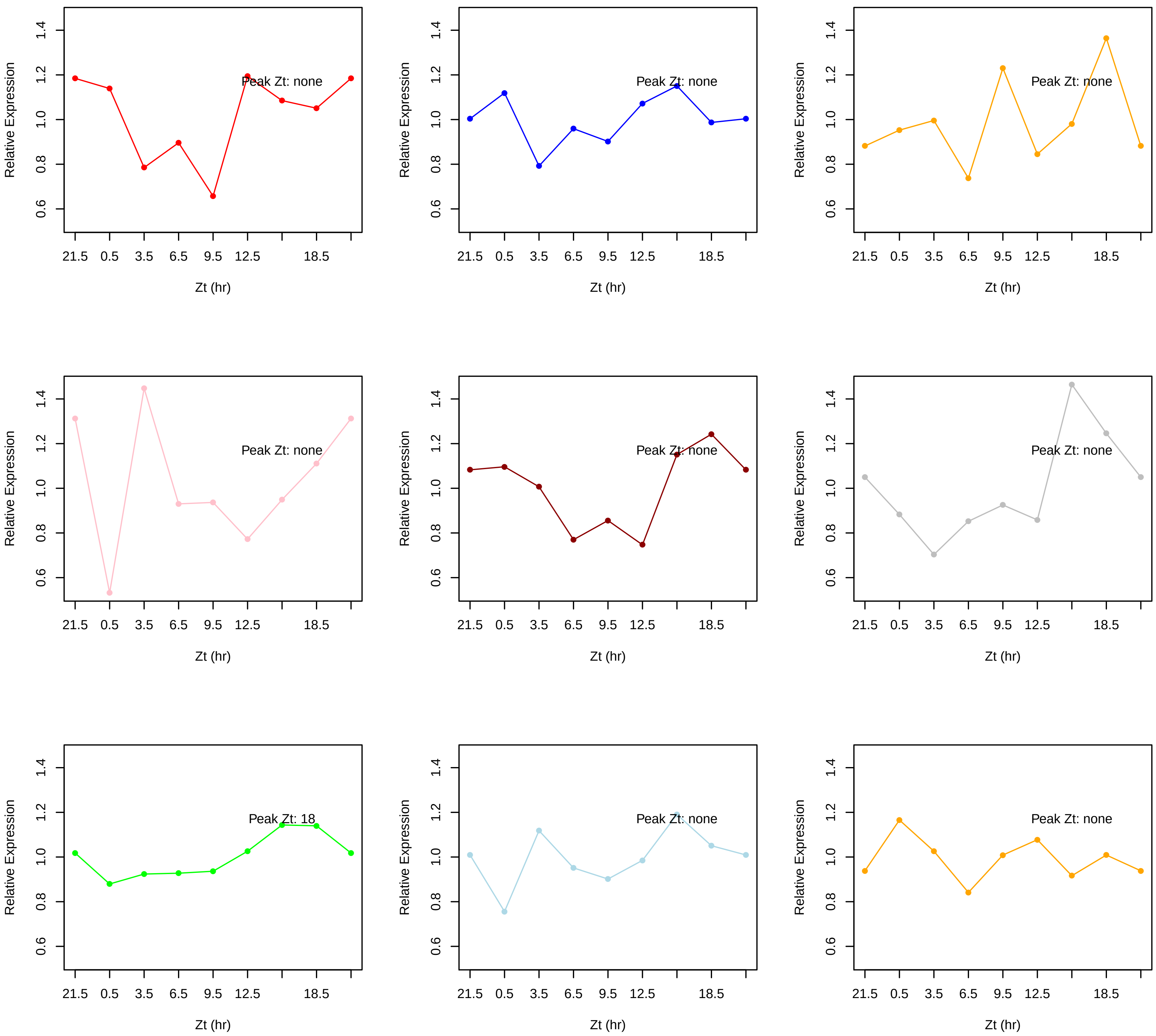

Singeleton: ENSXMAG00000005651 rgs5a

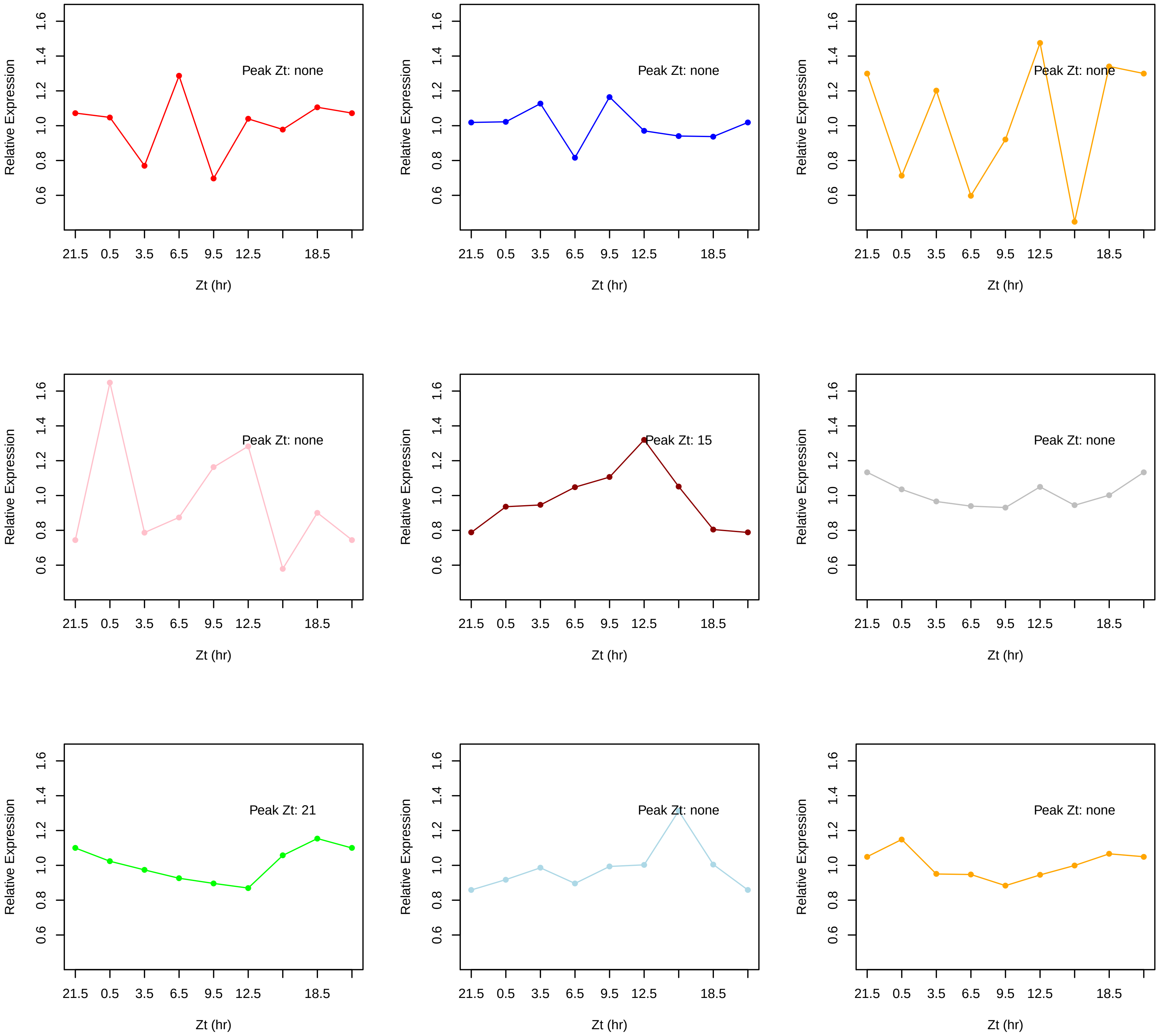

Singeleton: ENSXMAG00000000397 atf2

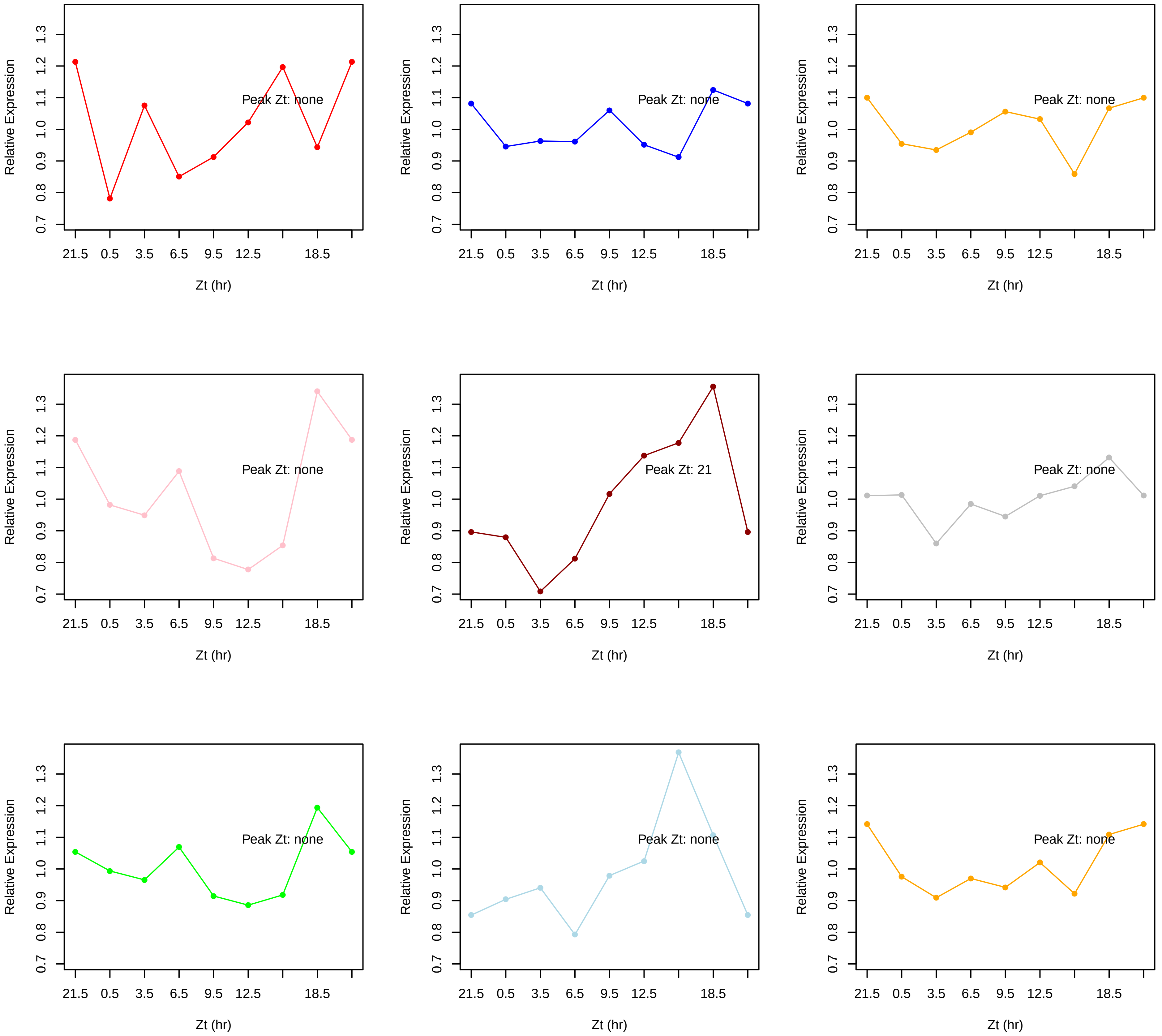

Singeleton: ENSXMAG00000016192 chd9

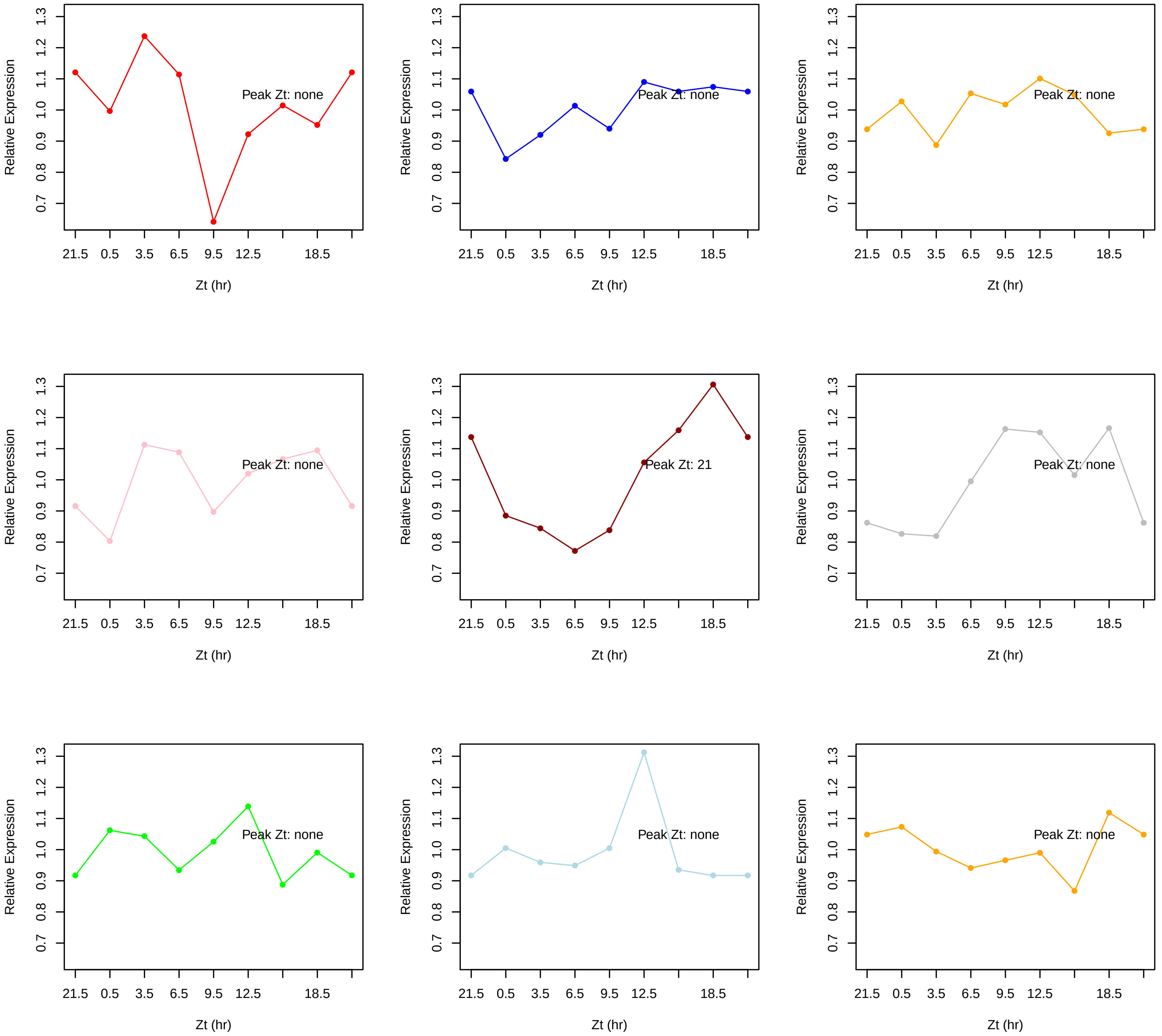

Singleton: ENSXMAG00000003835 noc2l

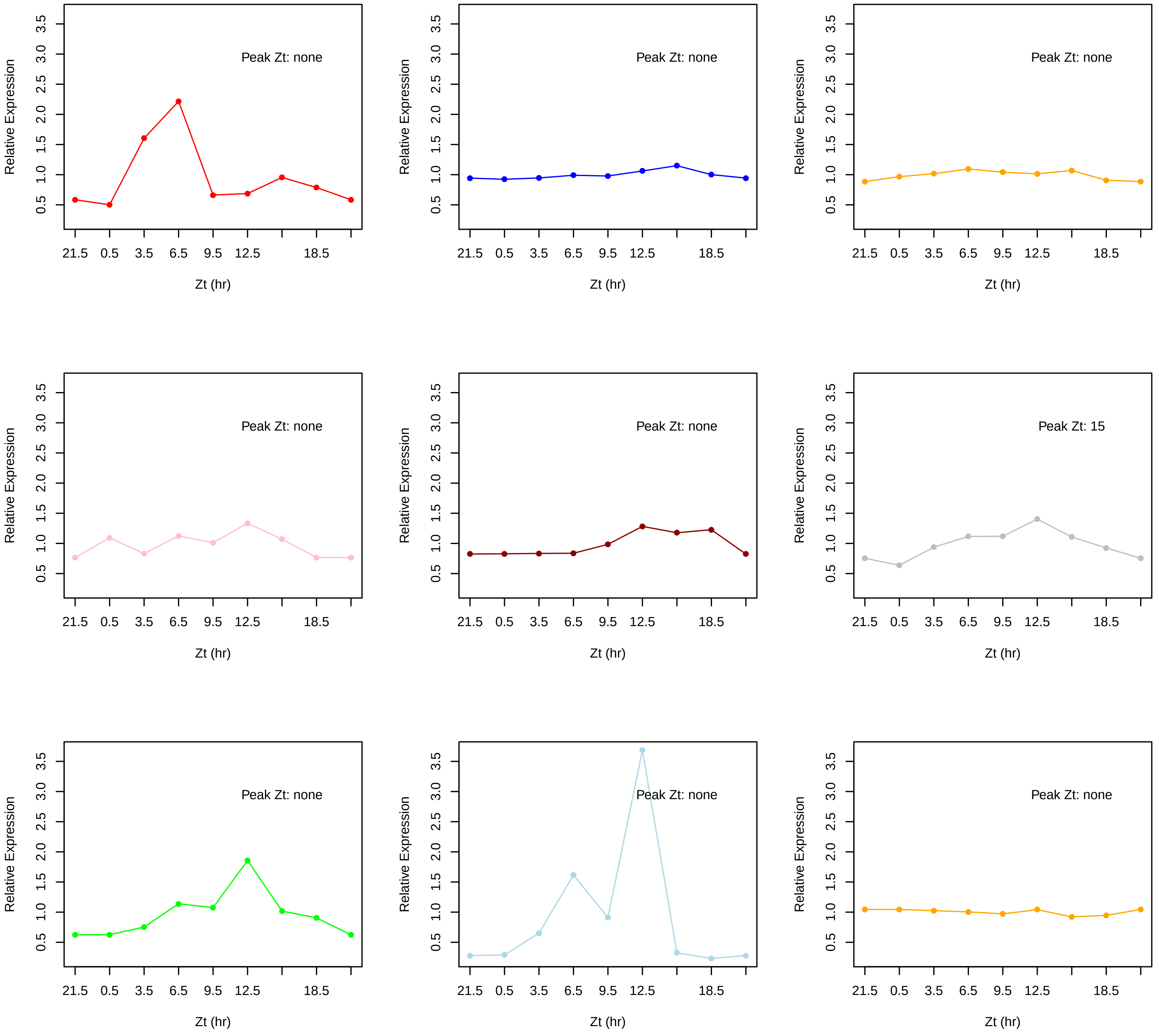

Singeleton: ENSXMAG00000014544 cyc1

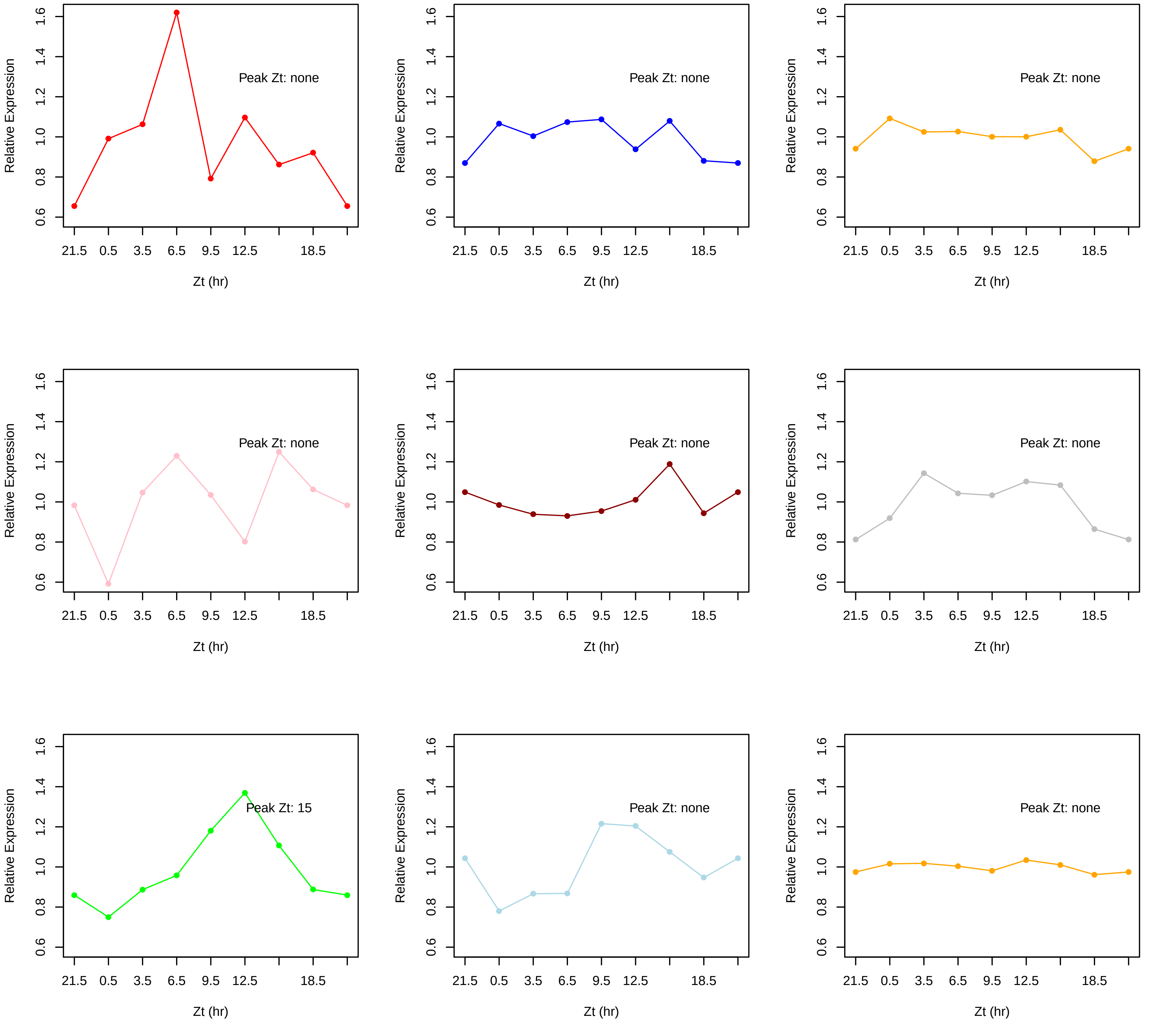

Singeleton: ENSXMAG00000000481 prkci

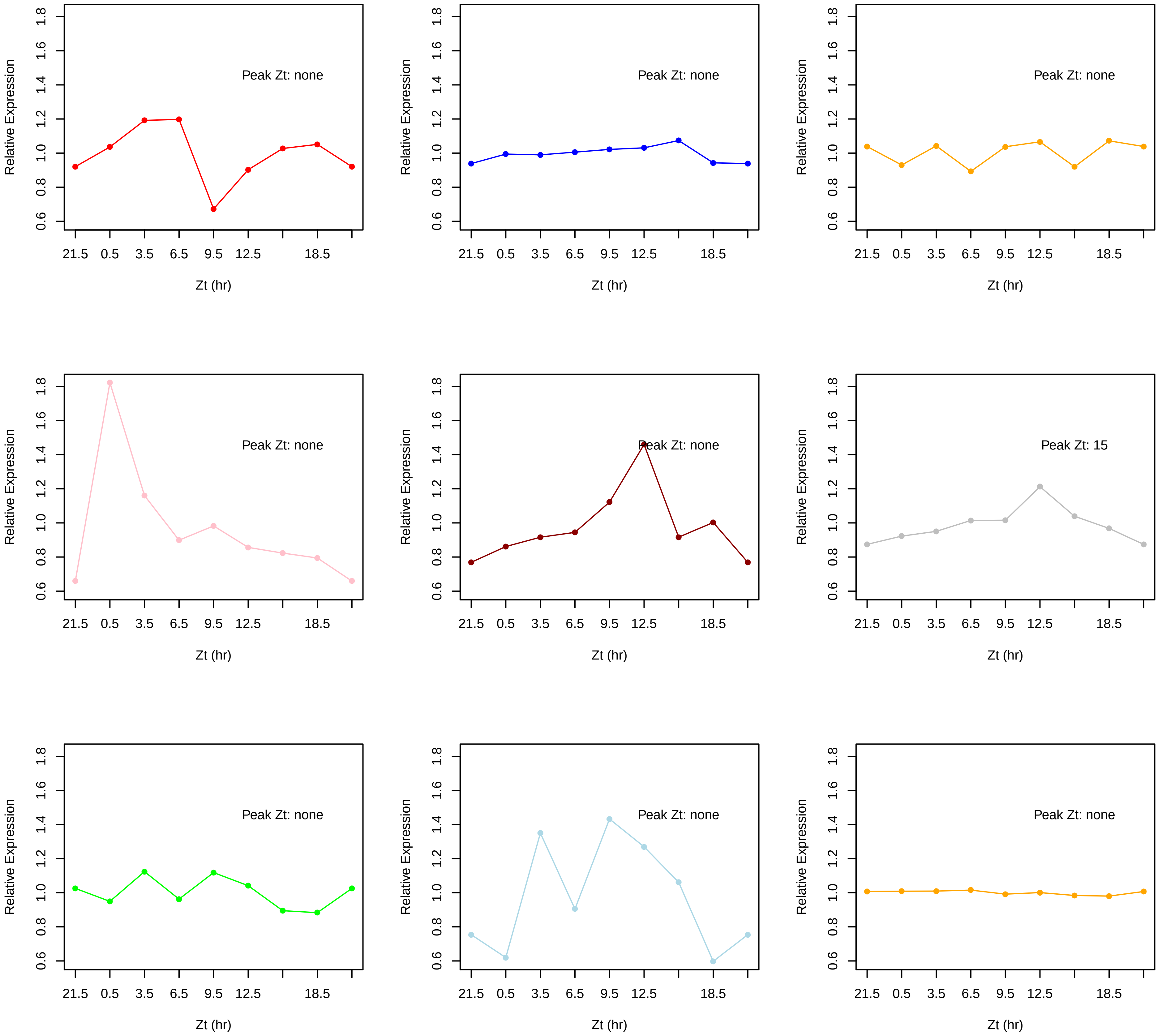

Singeleton: ENSXMAG00000015557 PIN1

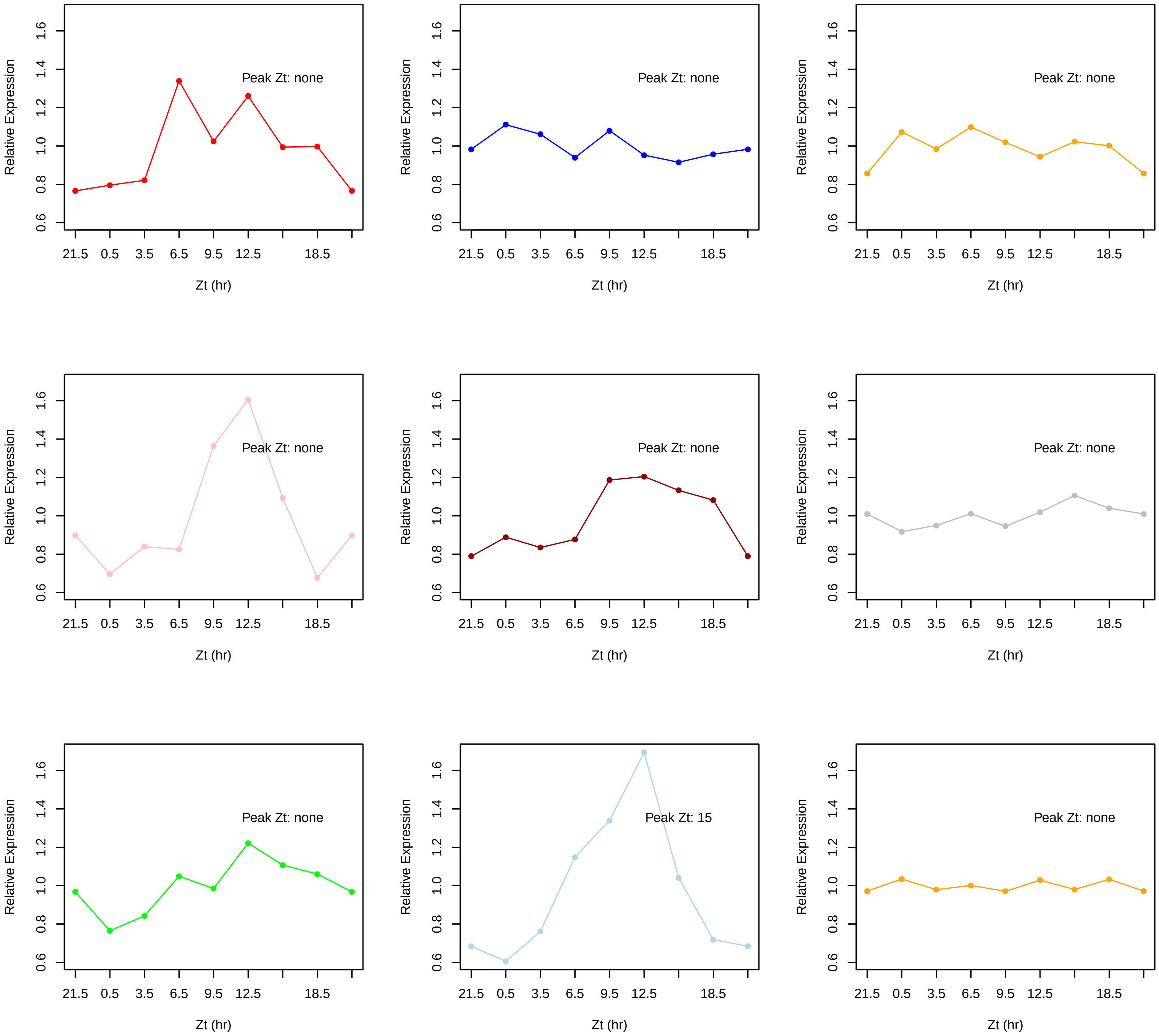

Singeleton: ENSXMAG00000017707 chordc1b

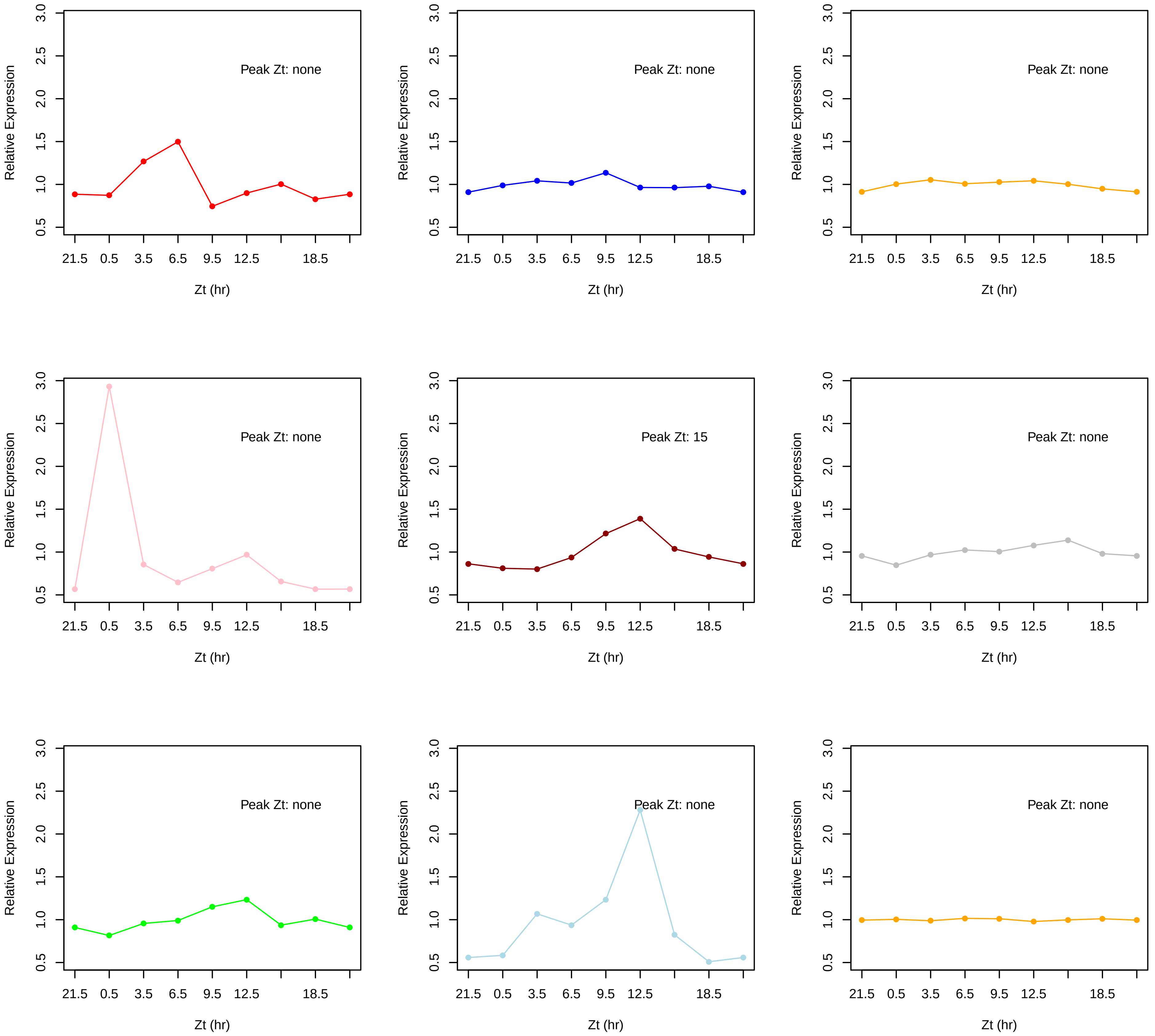

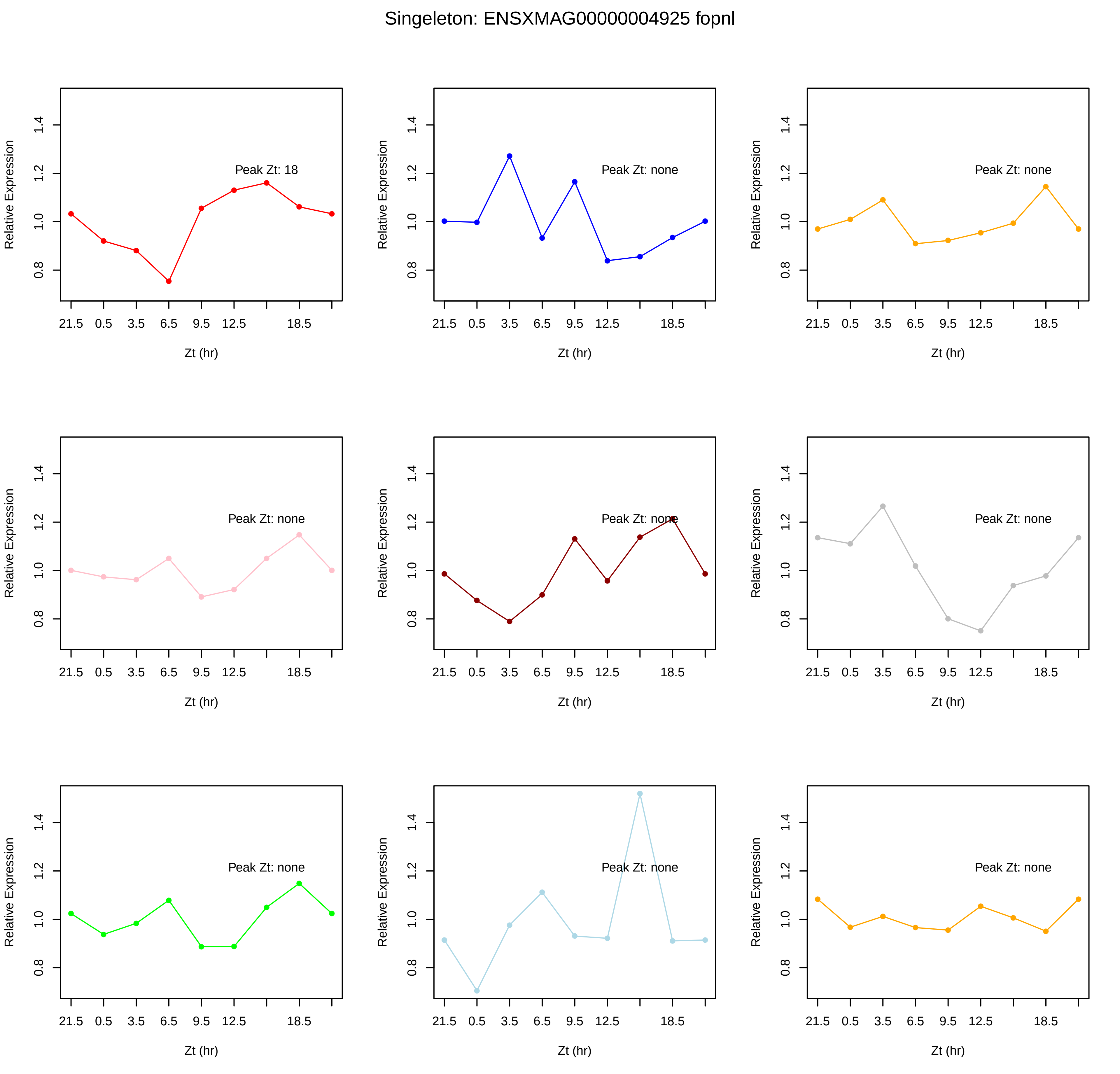

Singeleton: ENSXMAG00000004925 fopnl
Peak Zt: 18
Peak Zt: none
Relative Expression
Zt (hr)
21.5
0.5
3.5
6.5
9.5
12.5
18.5
0.8
1.0
1.2
1.4

Singleton: ENSXMAG00000003804 klhl17

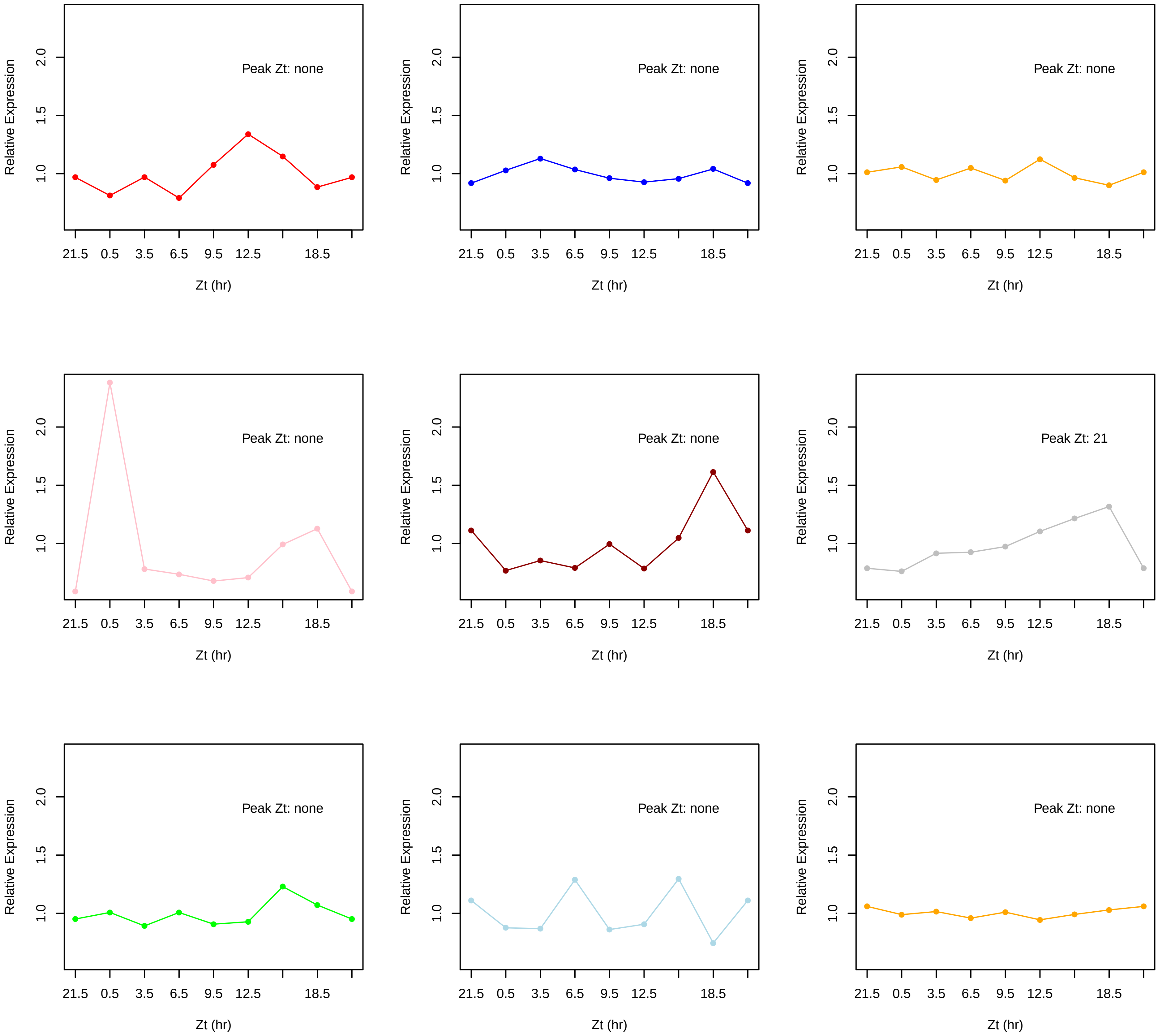

Singleton: ENSXMAG00000015563 VAV1

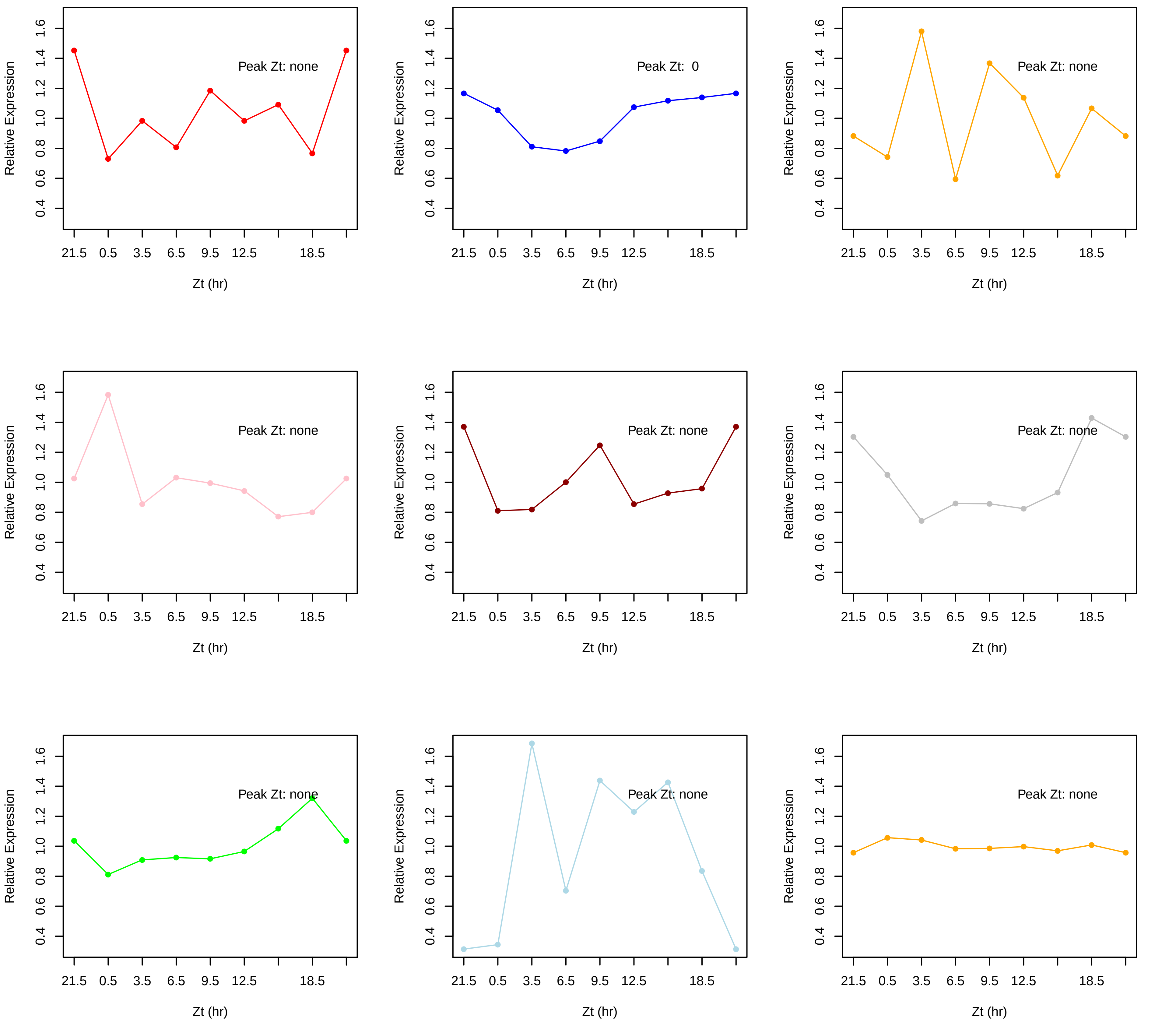

Singeleton: ENSXMAG00000017493 si:dkey−27c15.3

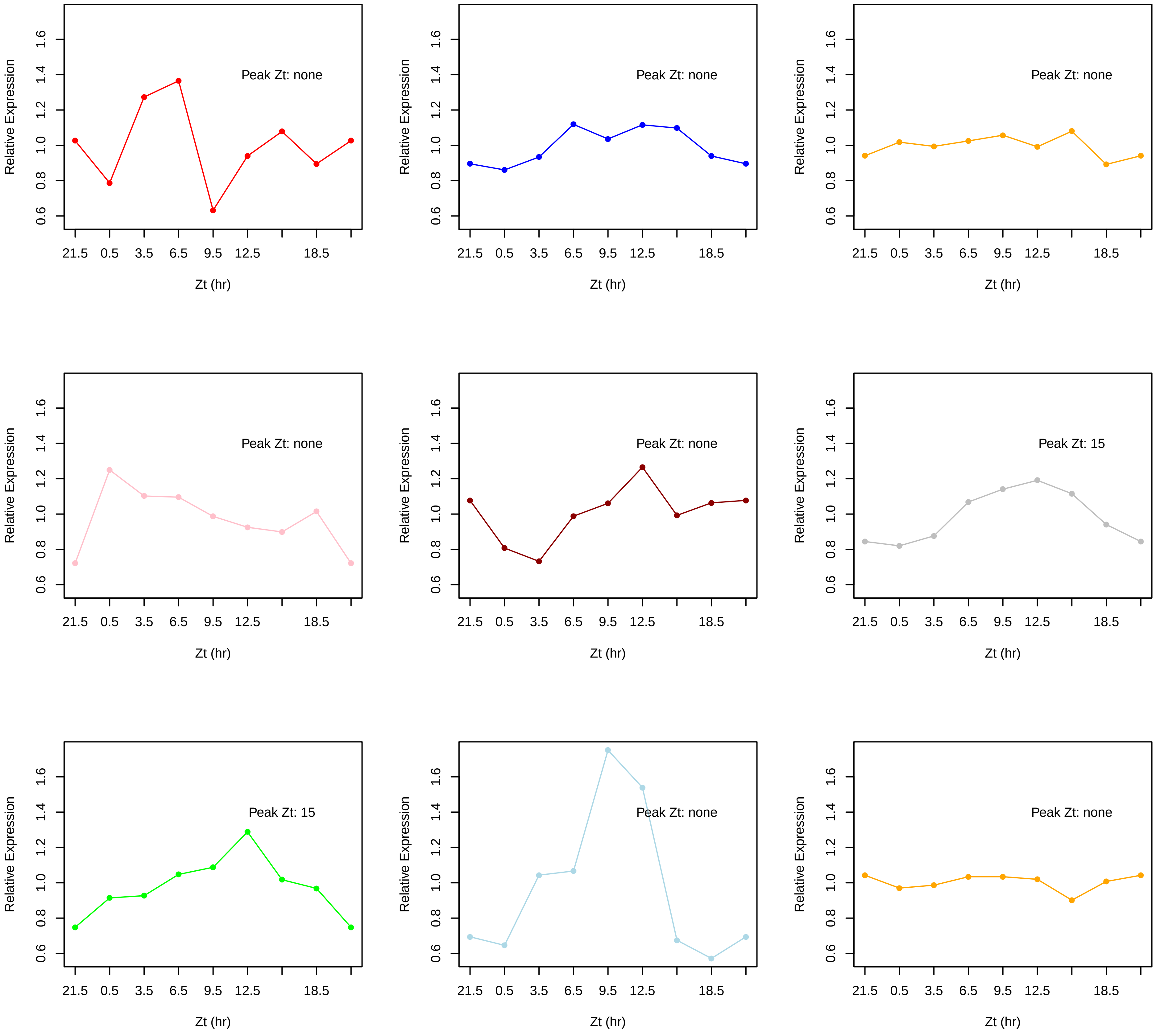

Singeleton: ENSXMAG00000024591

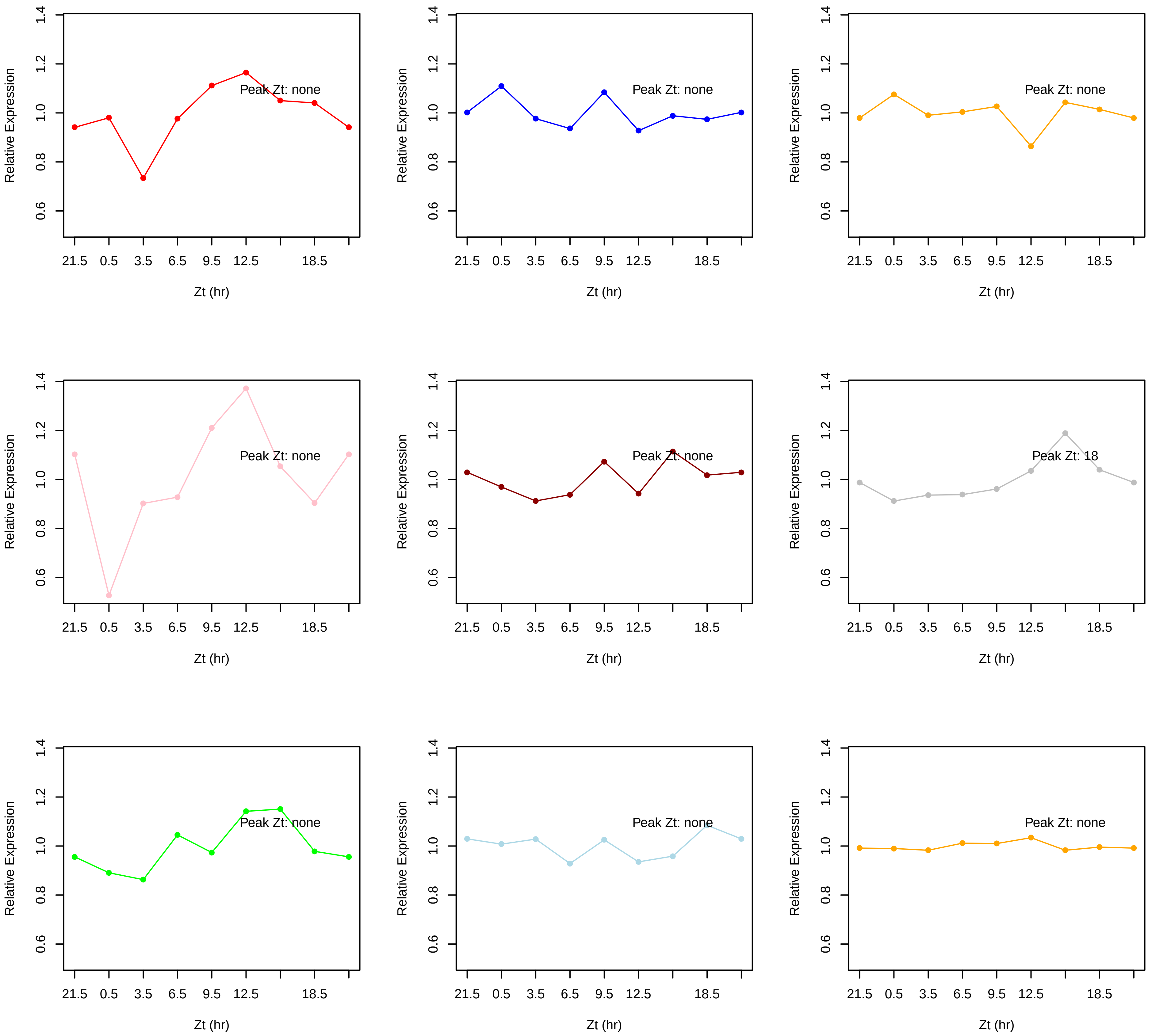

Singeleton: ENSXMAG00000007597 vox

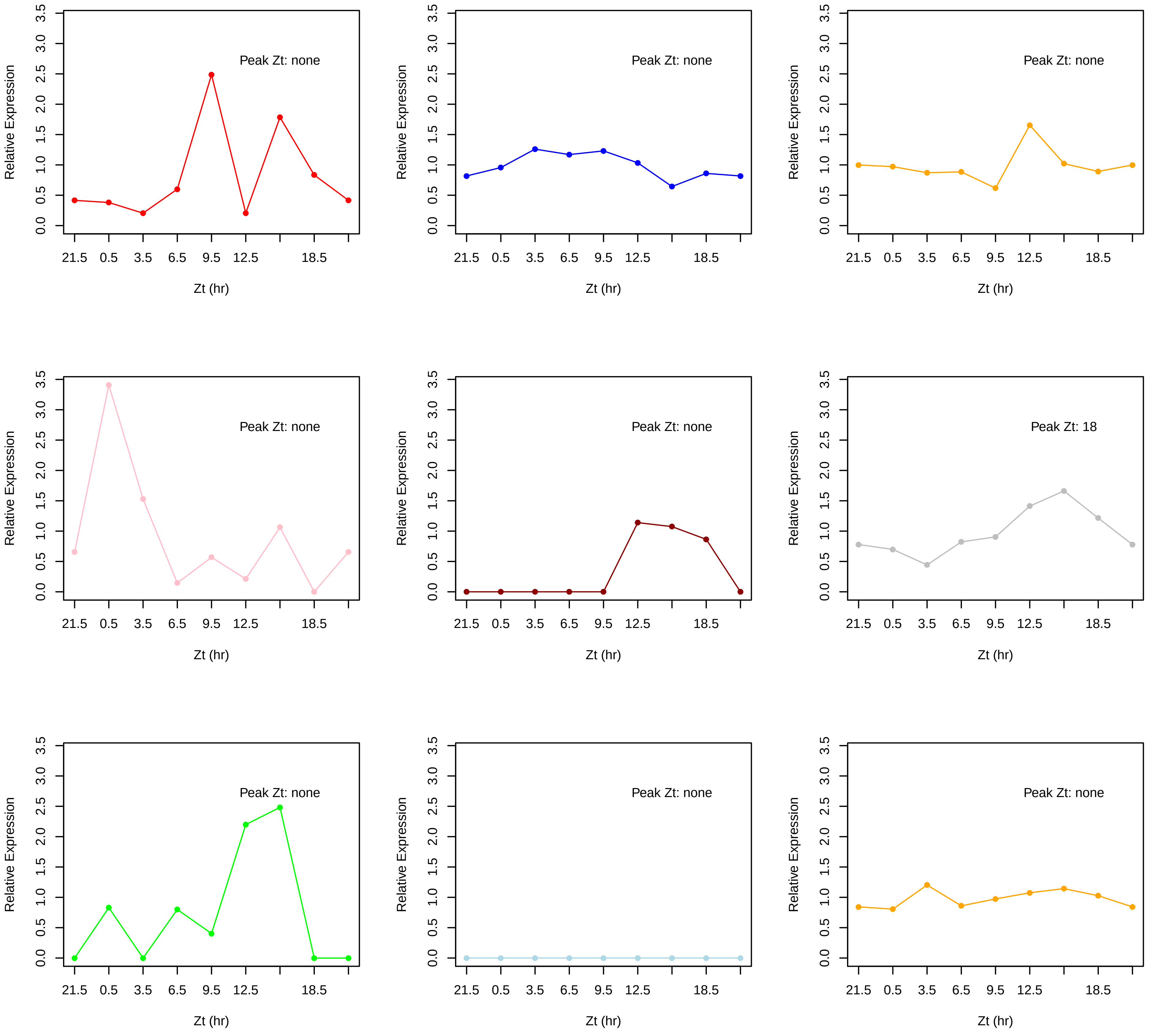

Singeleton: ENSXMAG00000003803 plekhn1

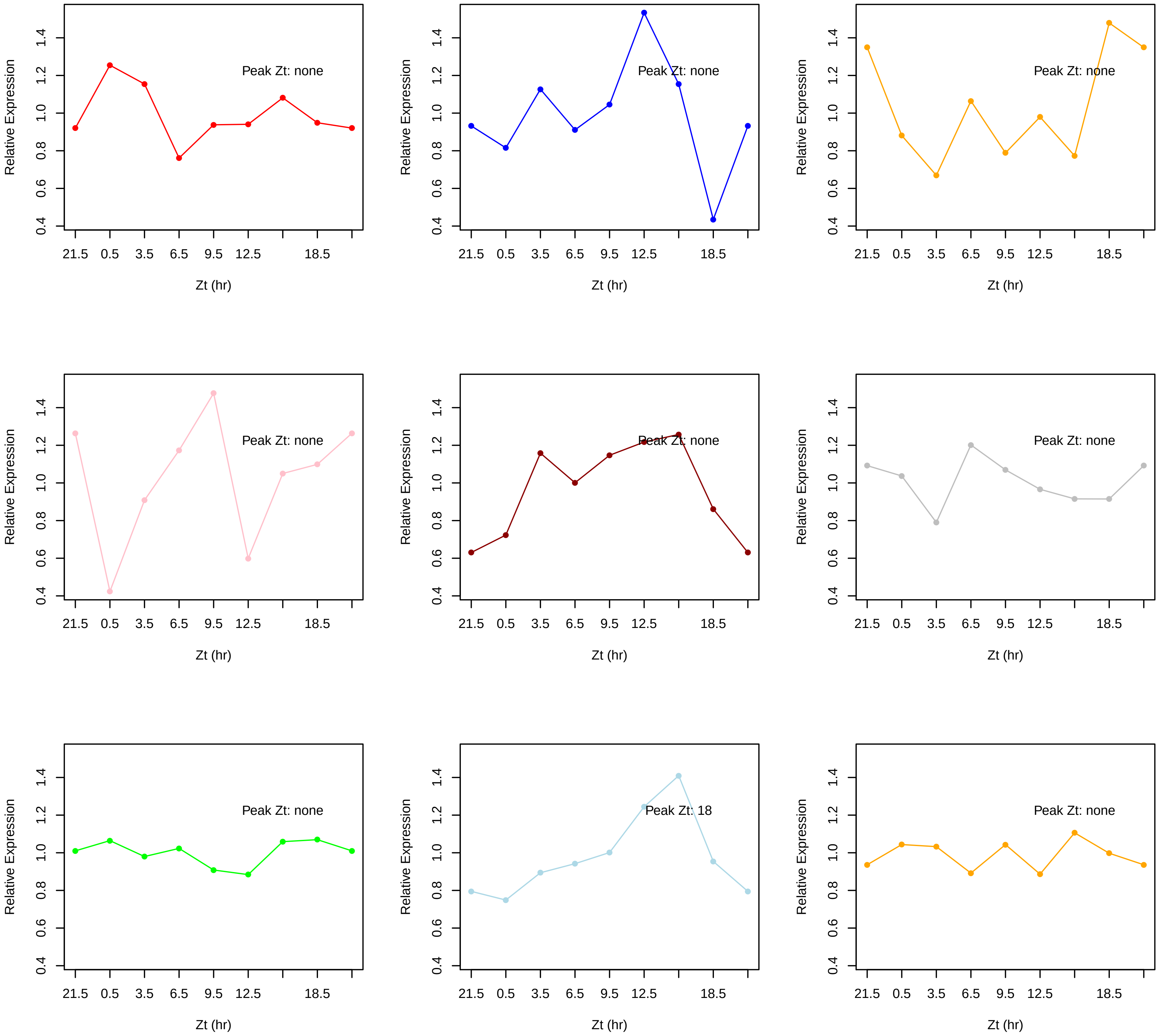

Singeleton: ENSXMAG00000030098 ssr2

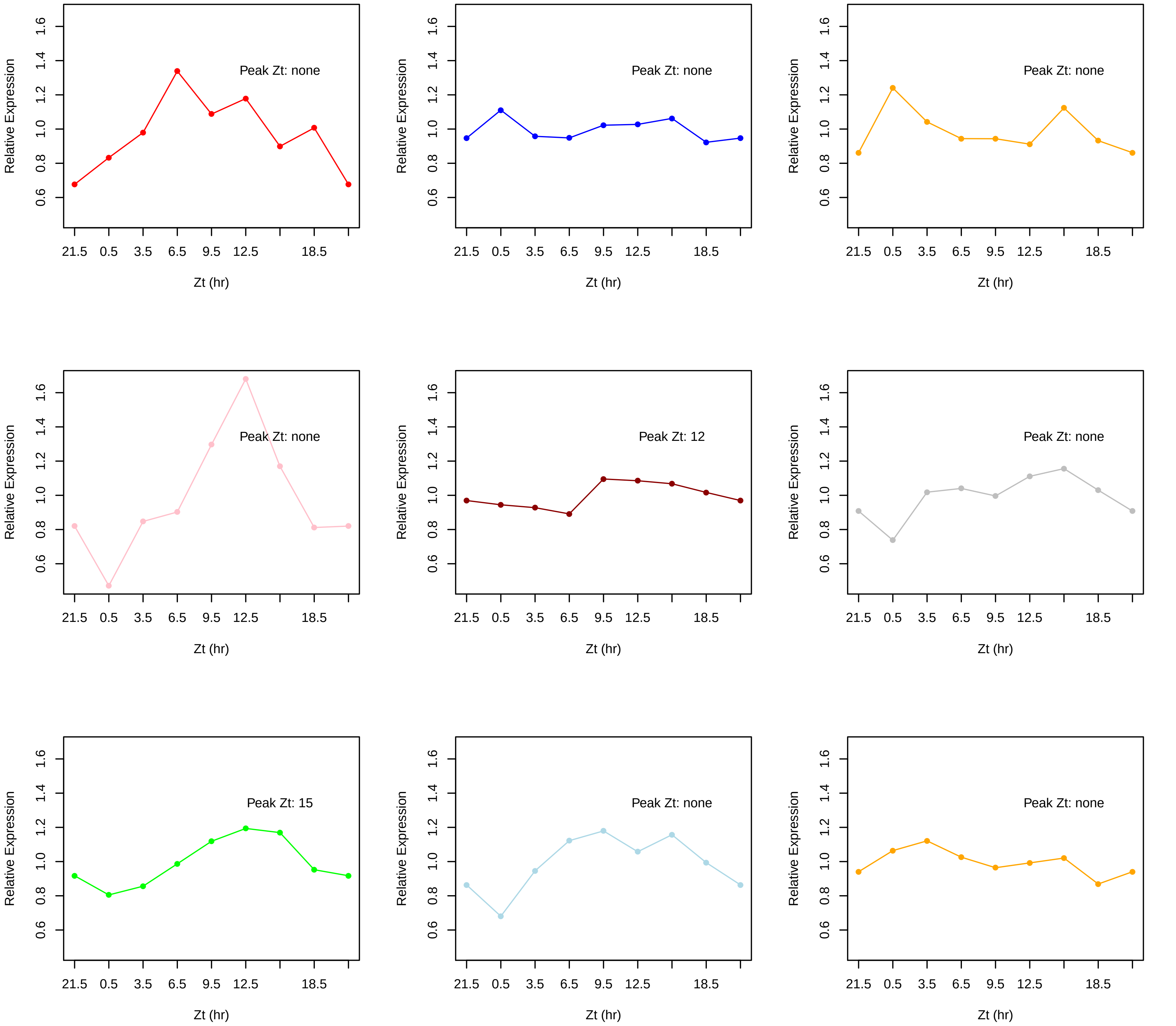

Singleton: ENSXMAG00000013336 osgn1

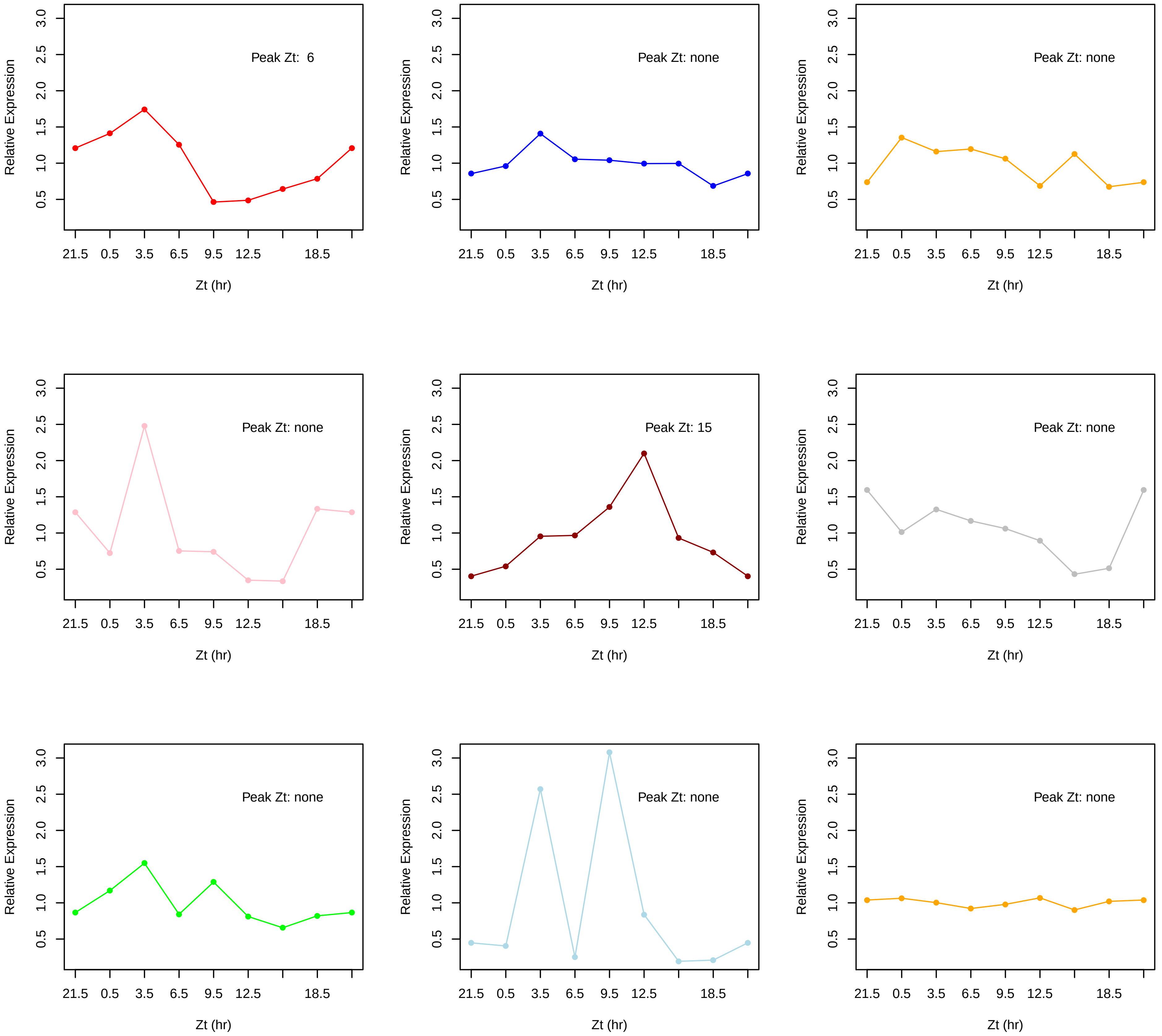

Singeleton: ENSXMAG00000028637 her9

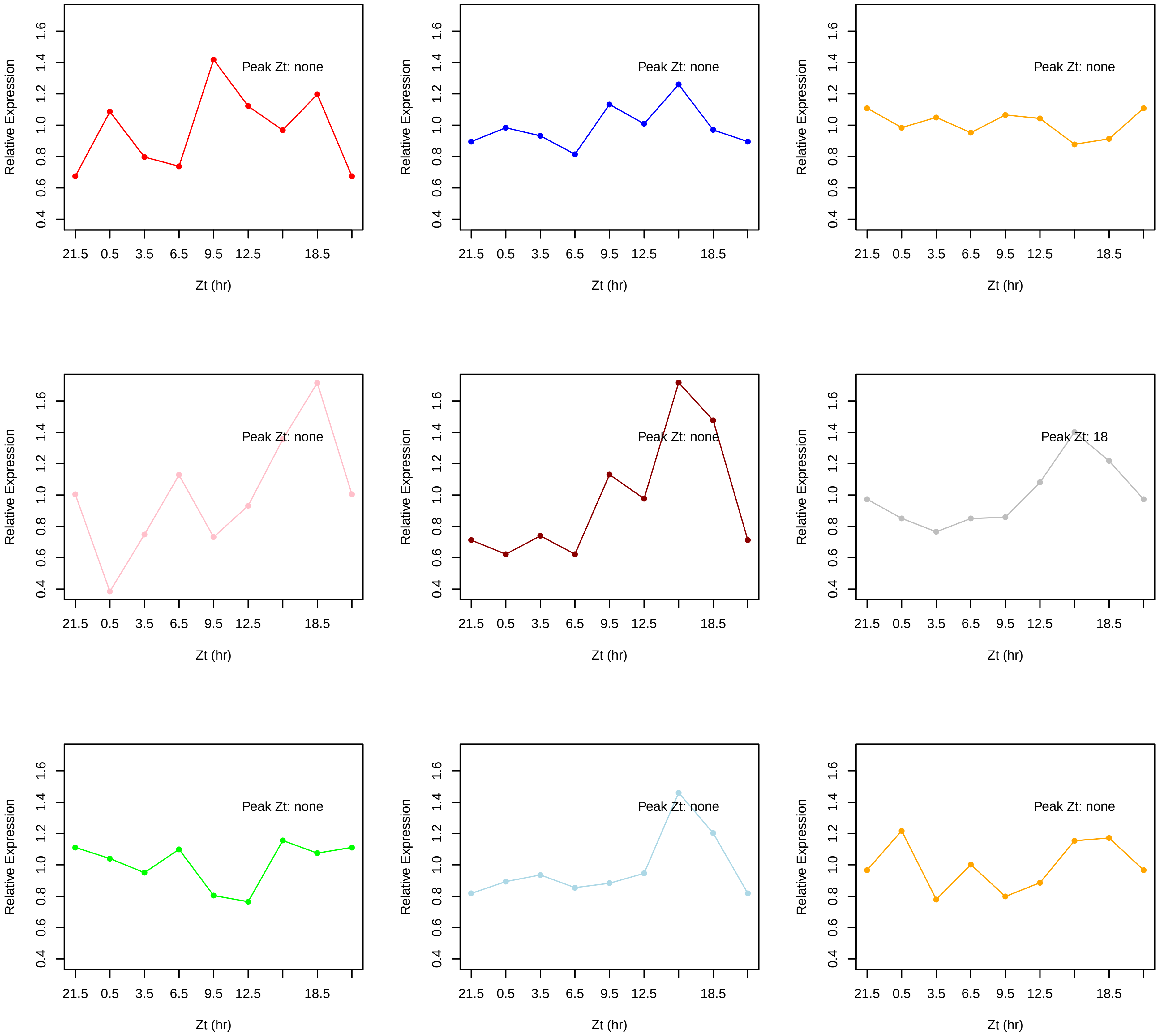

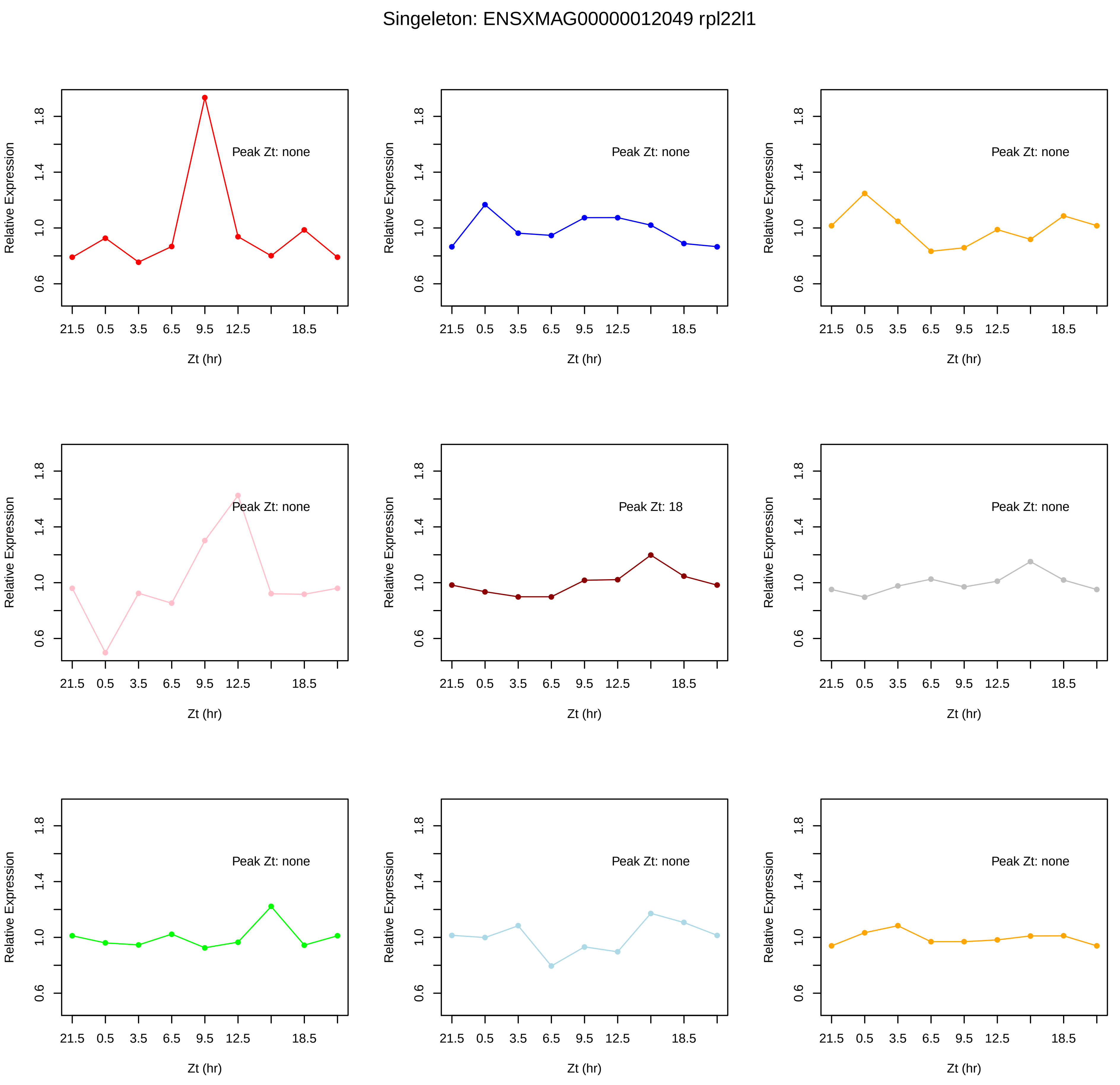

Singeleton: ENSXMAG00000012049 rpl22l1
Peak Zt: none
Peak Zt: none
Peak Zt: none
Peak Zt: none
Peak Zt: 18
Peak Zt: none
Peak Zt: none
Peak Zt: none
Peak Zt: none
Relative Expression
Zt (hr)
21.5 0.5 3.5 6.5 9.5 12.5 18.5
0.6 1.0 1.4 1.8

Singeleton: ENSXMAG00000000799 clcn4

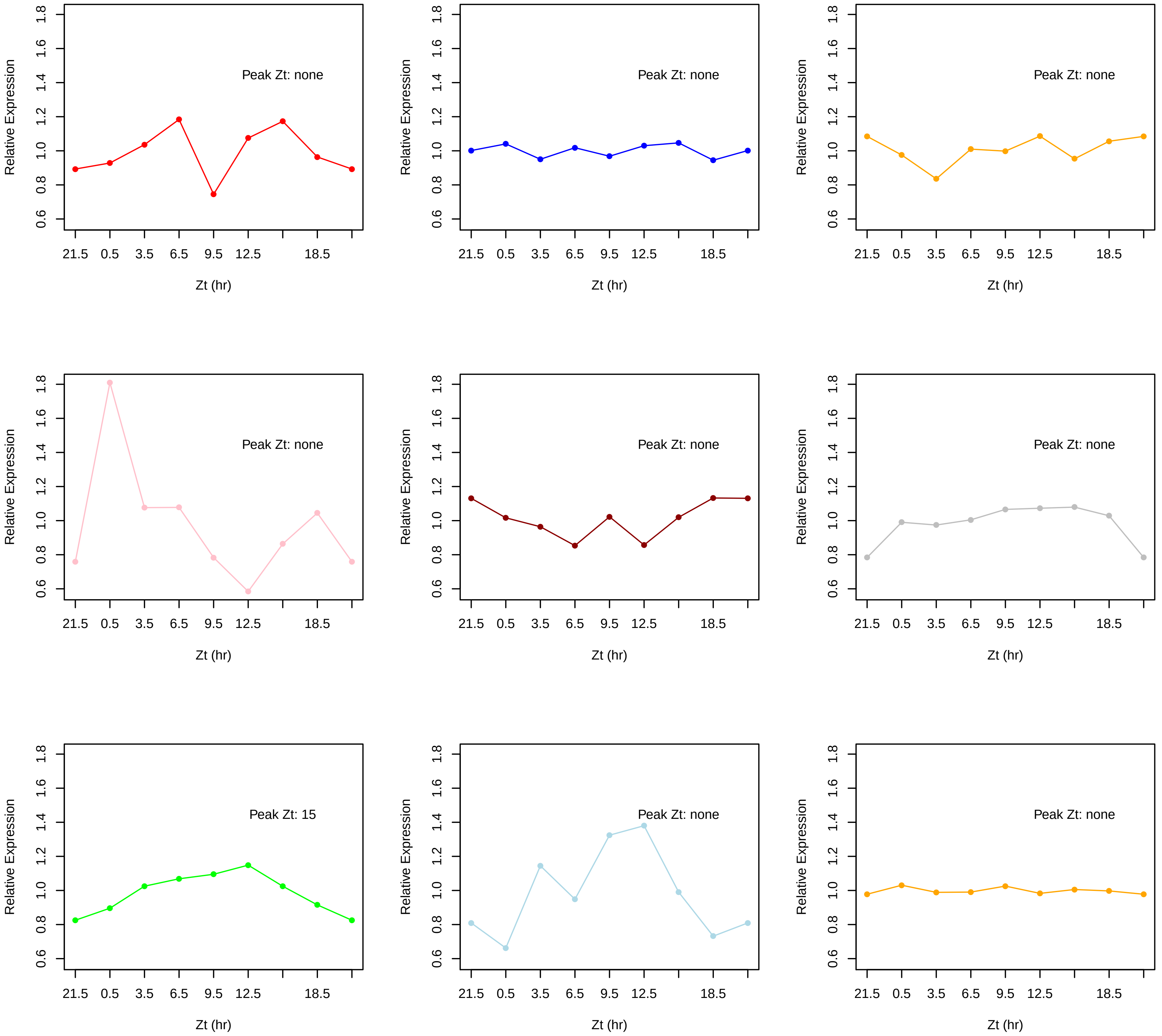

Singeleton: ENSXMAG00000017758 mtnr1bb

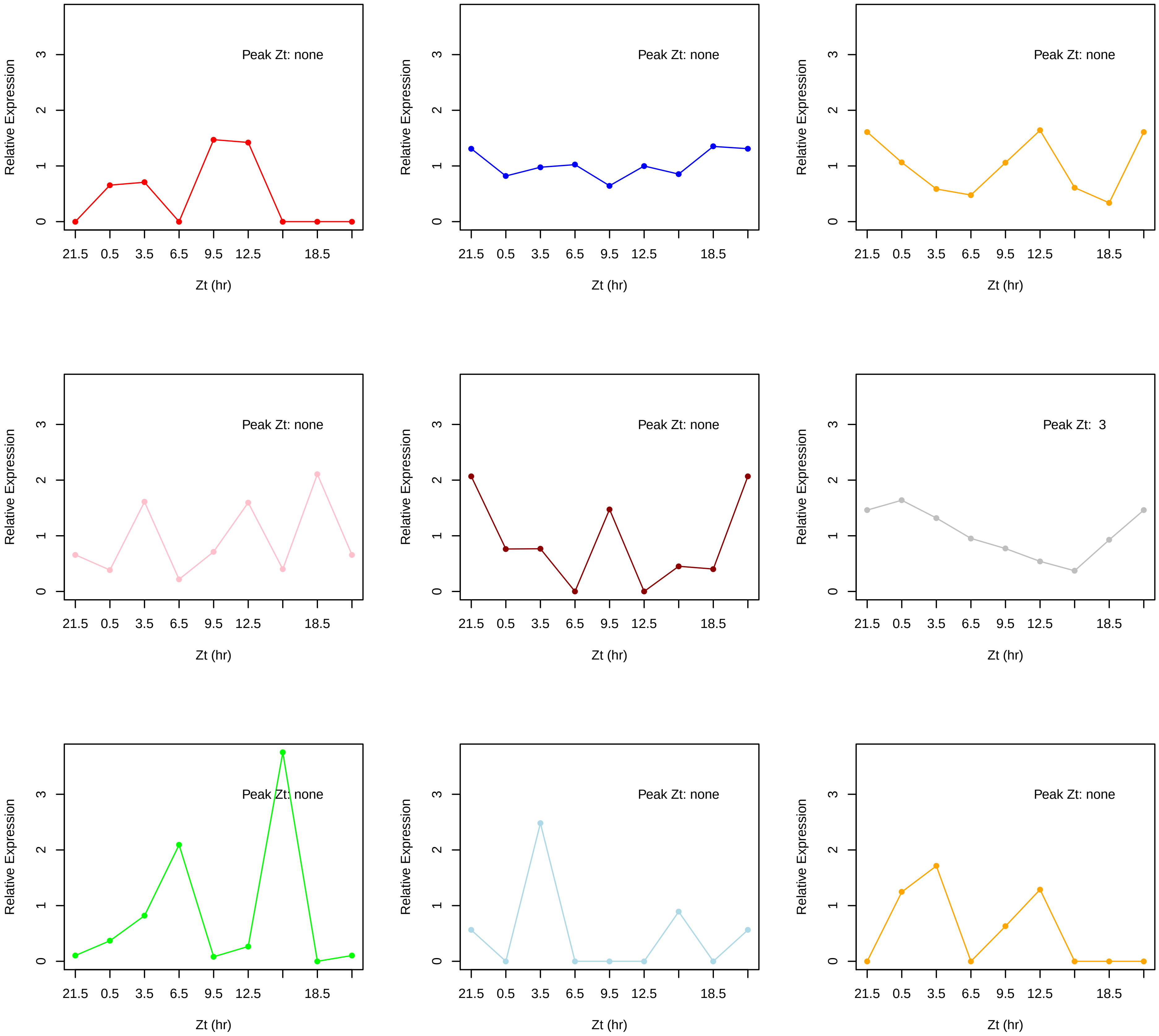

Singeleton: ENSXMAG00000005057 C16orf45

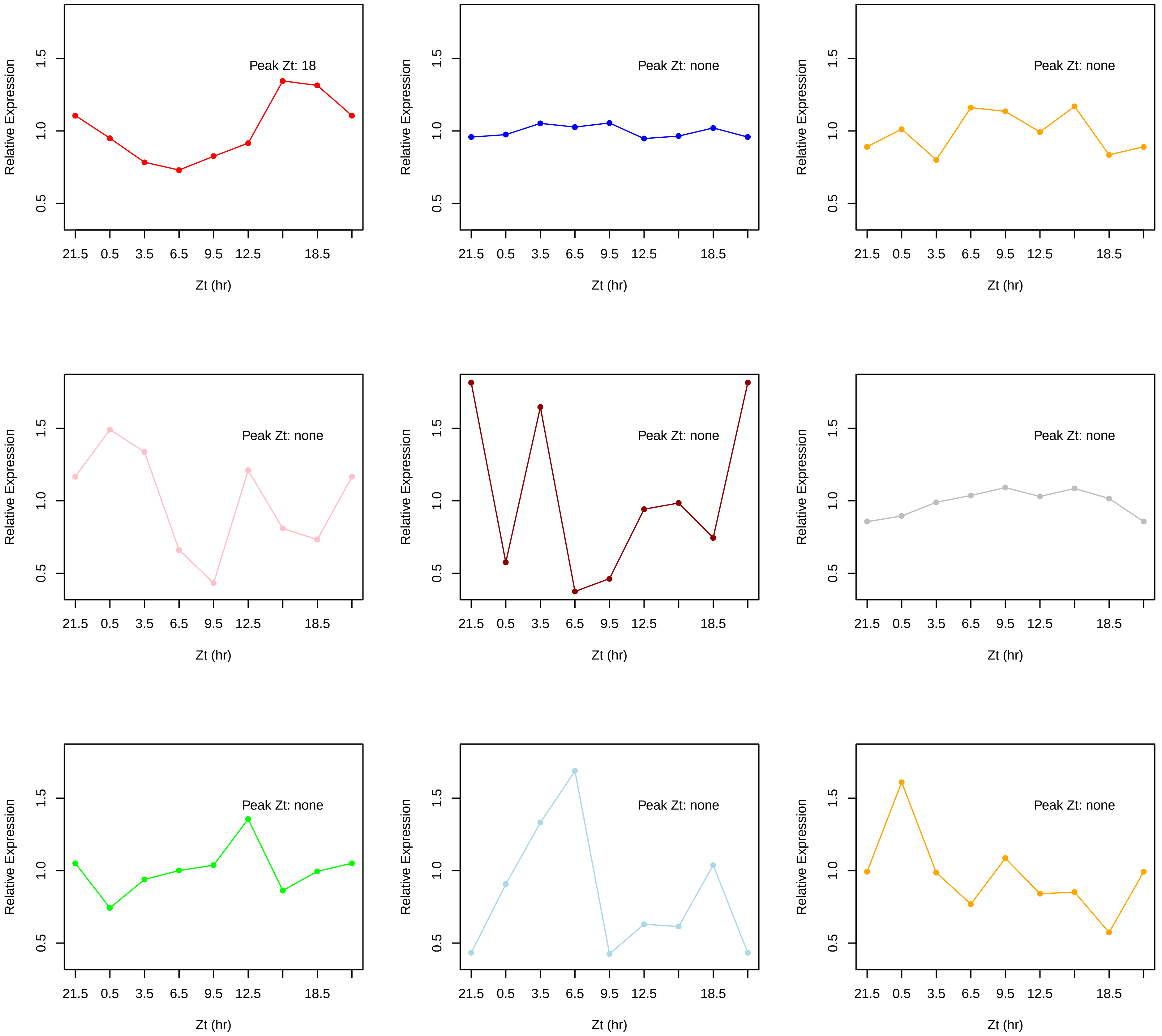

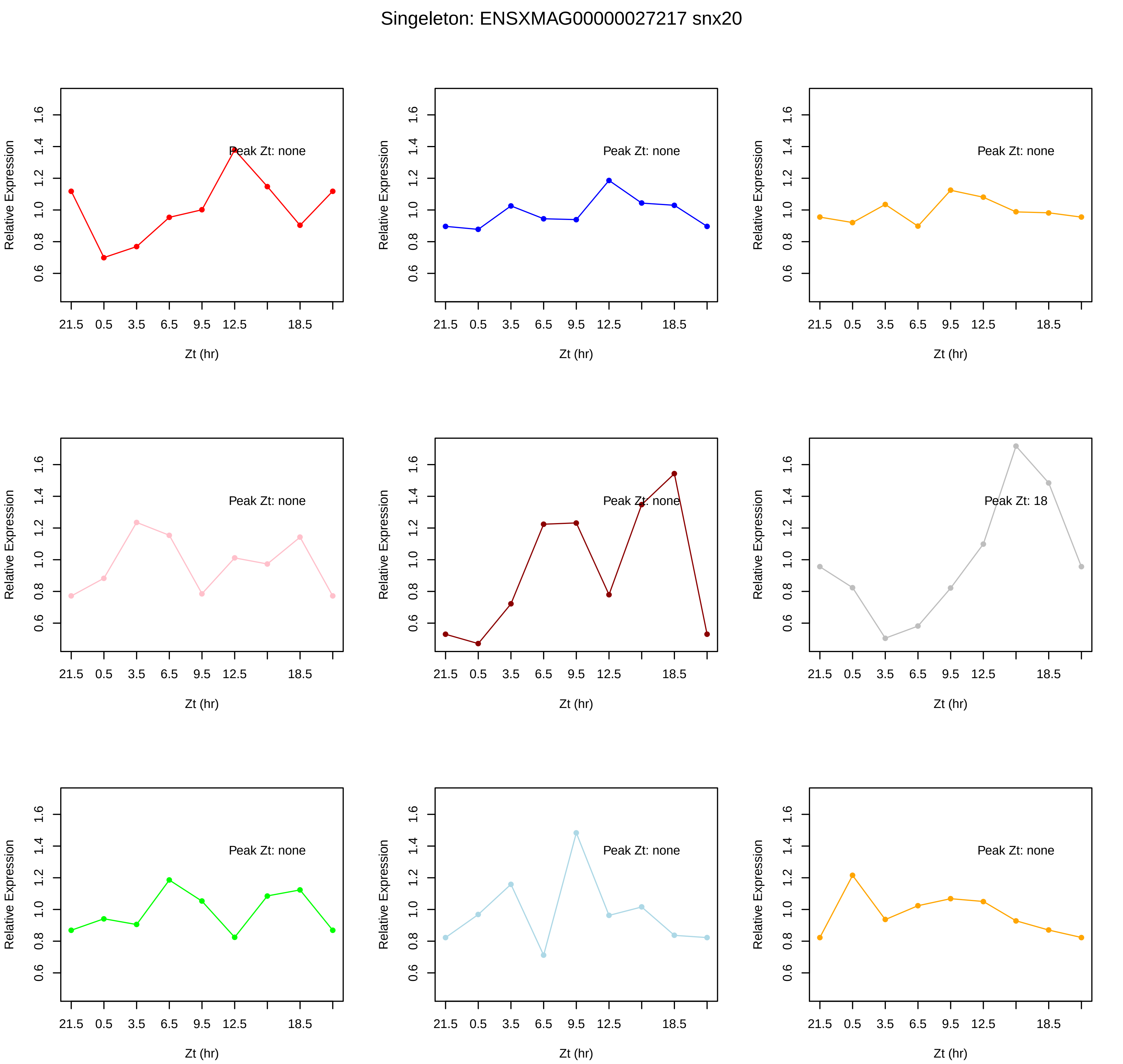

Singleton: ENSXMAG00000027217 snx20
Peak Zt: none
Peak Zt: none
Peak Zt: none
Peak Zt: none
Peak Zt: none
Peak Zt: 18
Peak Zt: none
Peak Zt: none
Peak Zt: none
Relative Expression
Zt (hr)
21.5
0.5
3.5
6.5
9.5
12.5
18.5
0.6
0.8
1.0
1.2
1.4
1.6

Singeleton: ENSXMAG00000000264 ptprt

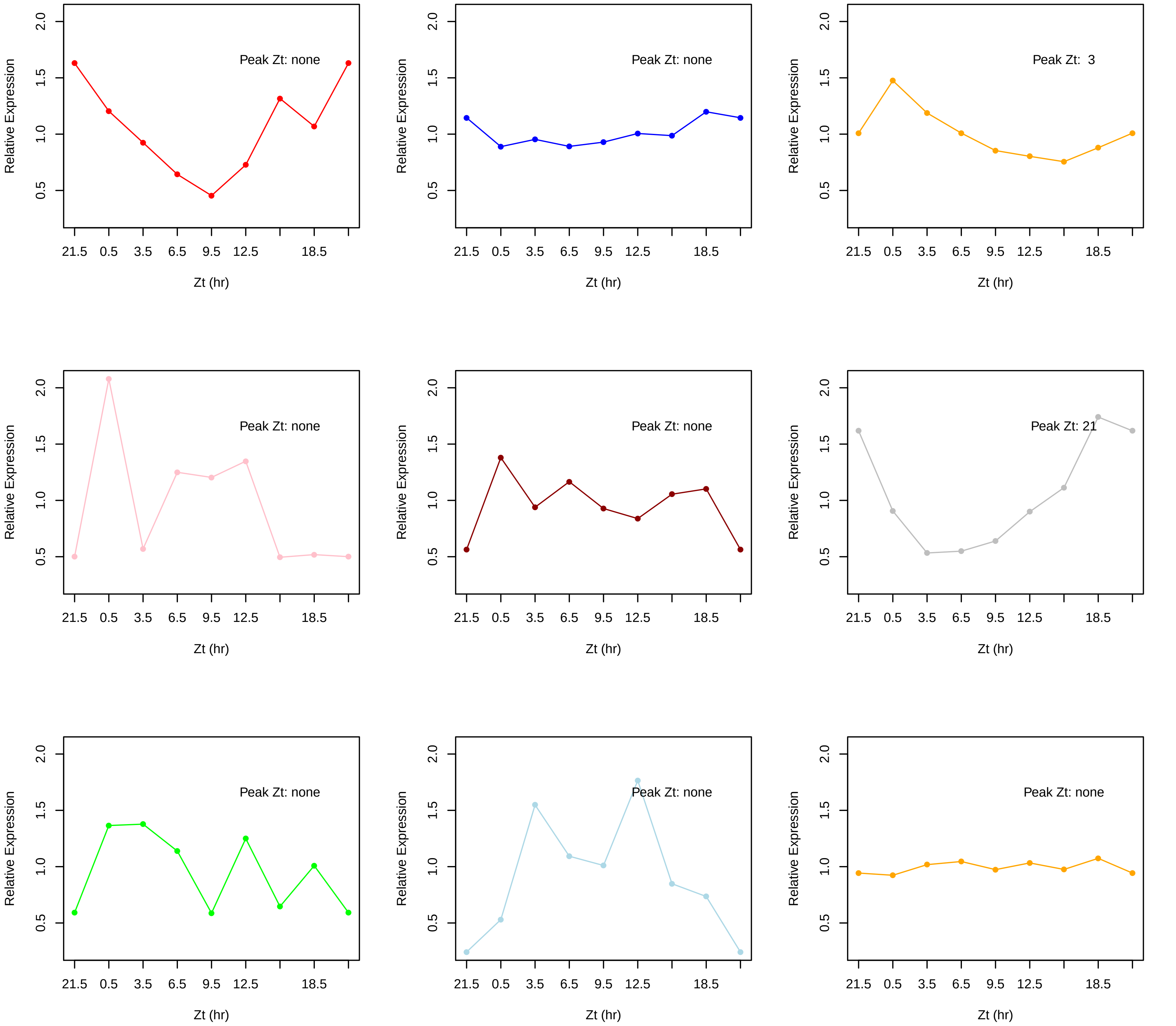

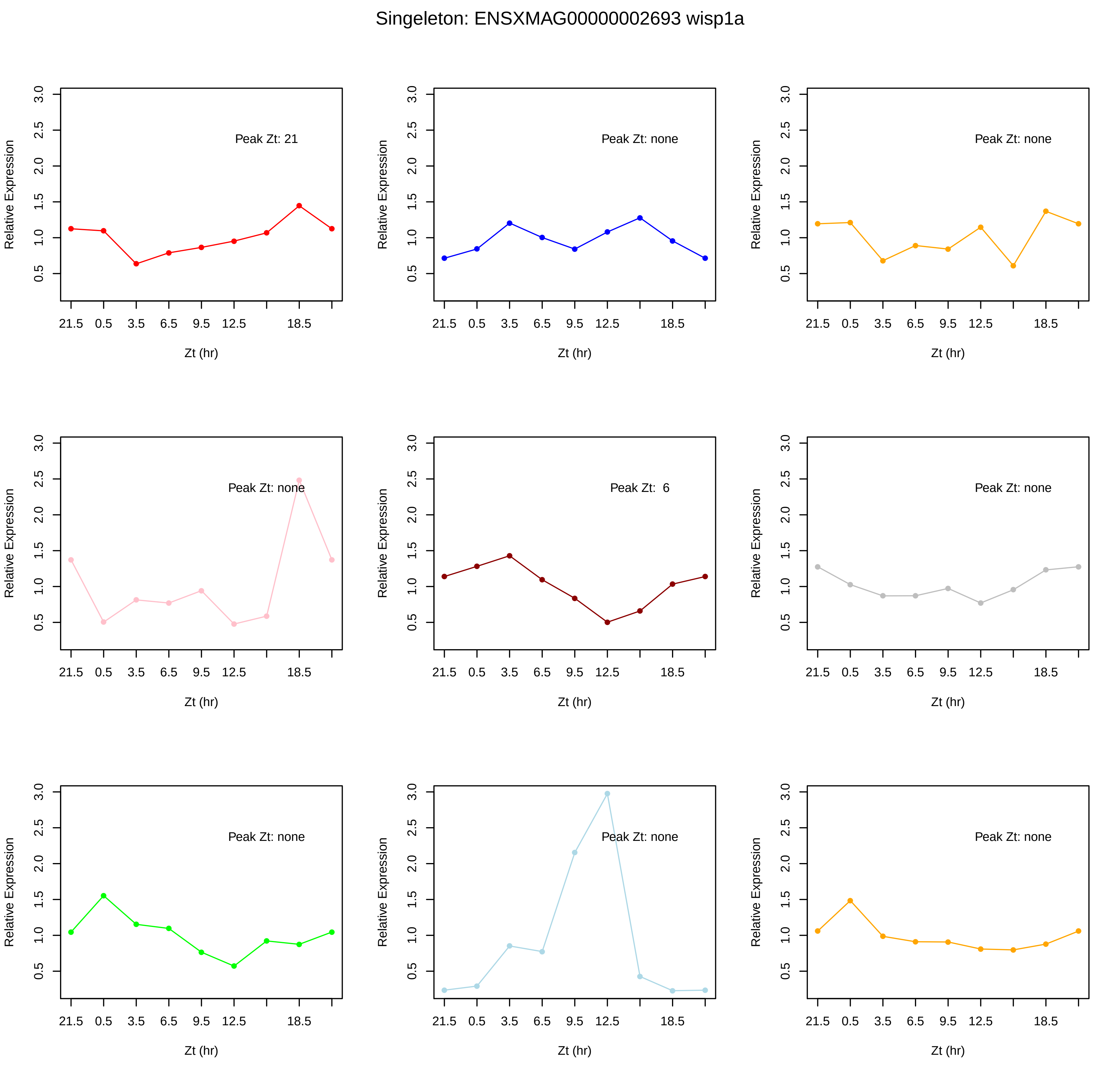

Singeleton: ENSXMAG00000002693 wisp1a
Peak Zt: 21
Peak Zt: none
Peak Zt: none
Peak Zt: none
Peak Zt: 6
Peak Zt: none
Peak Zt: none
Peak Zt: none
Peak Zt: none
Relative Expression
Zt (hr)
21.5
0.5
3.5
6.5
9.5
12.5
18.5
0.5
1.0
1.5
2.0
2.5
3.0

Singeleton: ENSXMAG00000009913 bmp2k

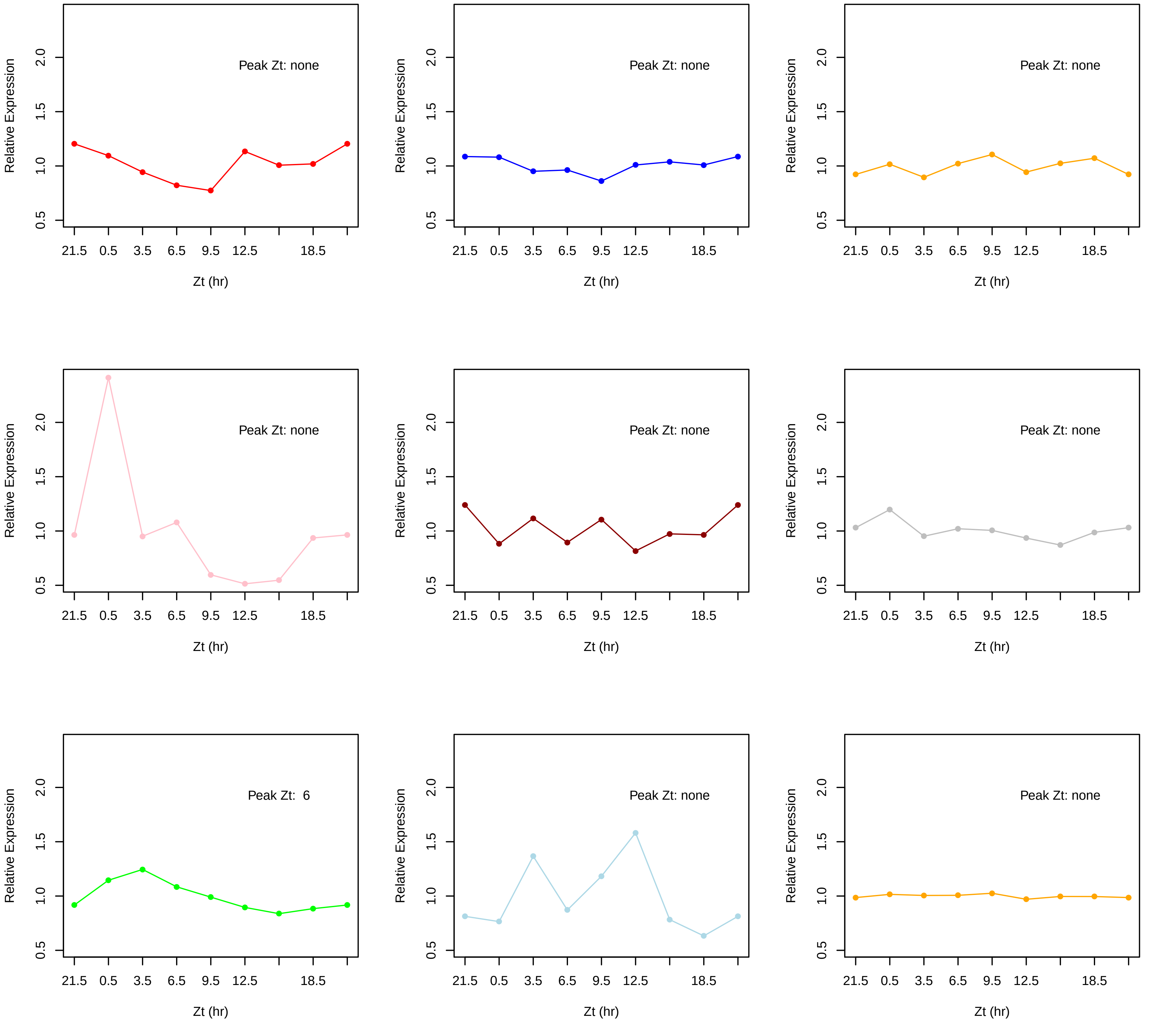

Singeleton: ENSXMAG00000012317 ptpn11b

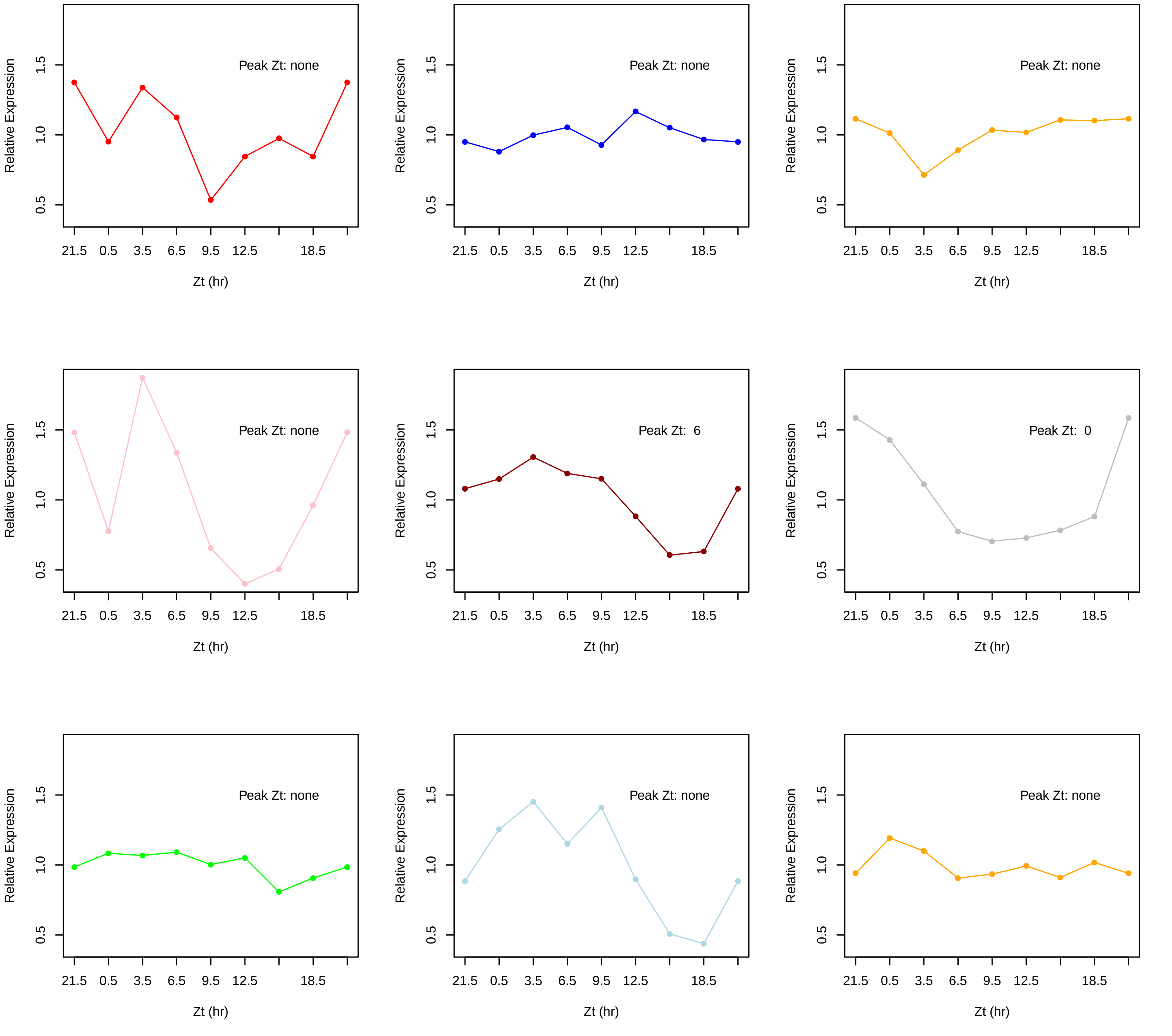

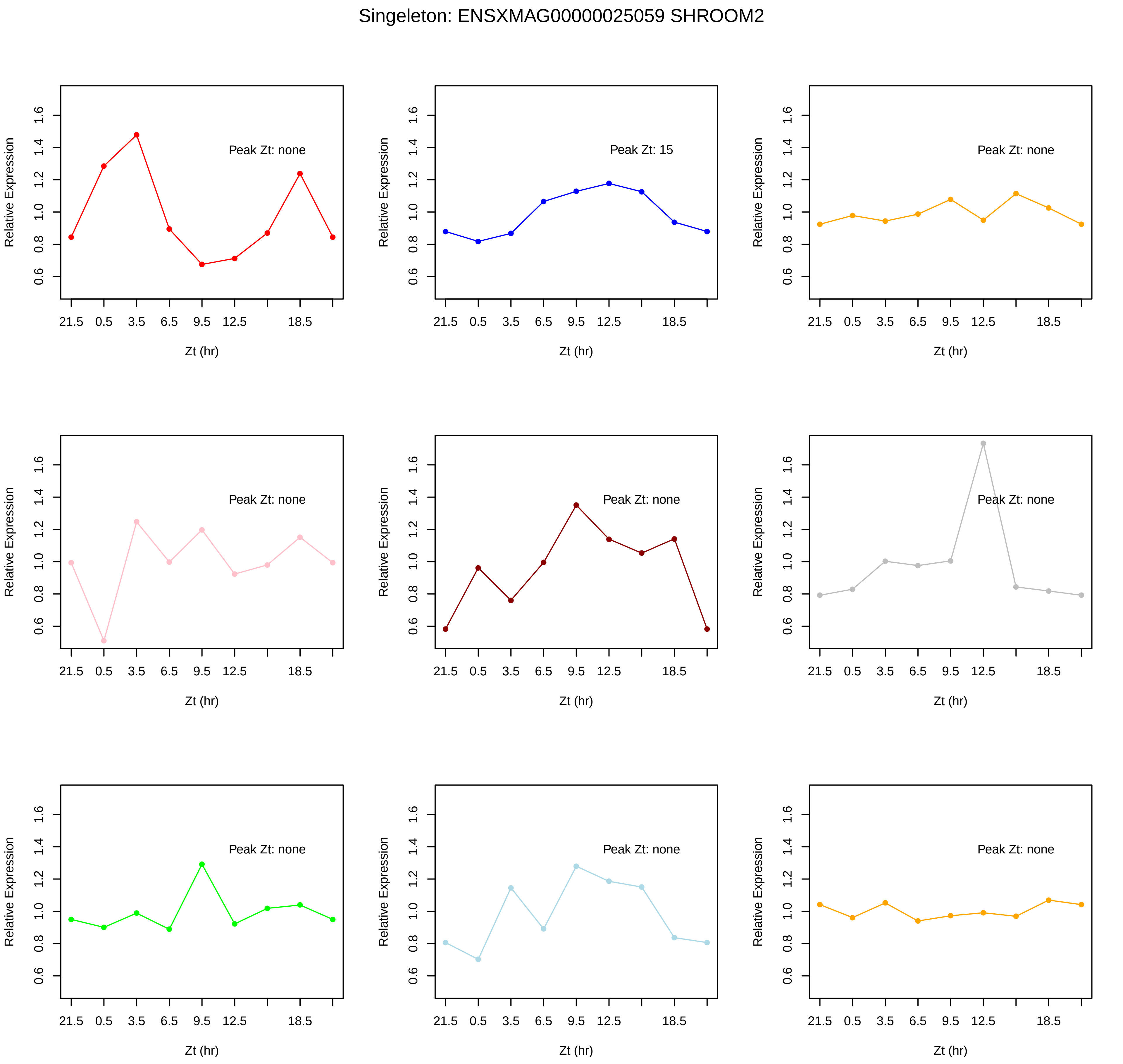
Singeleton: ENSXMAG00000025059 SHROOM2
Peak Zt: none
Peak Zt: 15
Peak Zt: none
Peak Zt: none
Peak Zt: none
Peak Zt: none
Peak Zt: none
Peak Zt: none
Peak Zt: none
Relative Expression
Zt (hr)
21.5
0.5
3.5
6.5
9.5
12.5
18.5
0.6
0.8
1.0
1.2
1.4
1.6

Singleton: ENSXMAG00000016741 si:dkey−240h12.4

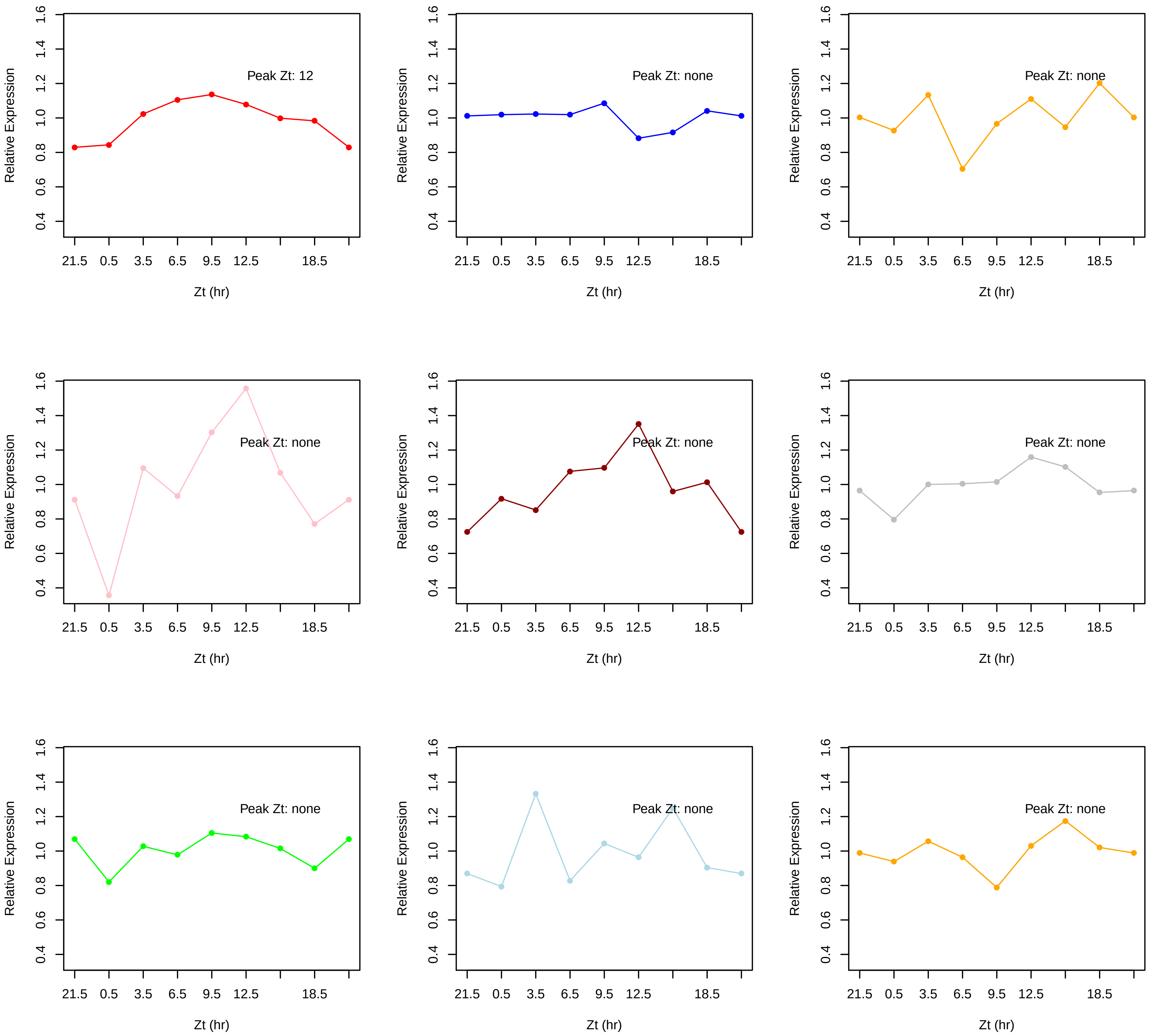

Singeleton: ENSXMAG00000010629 zgc:66440

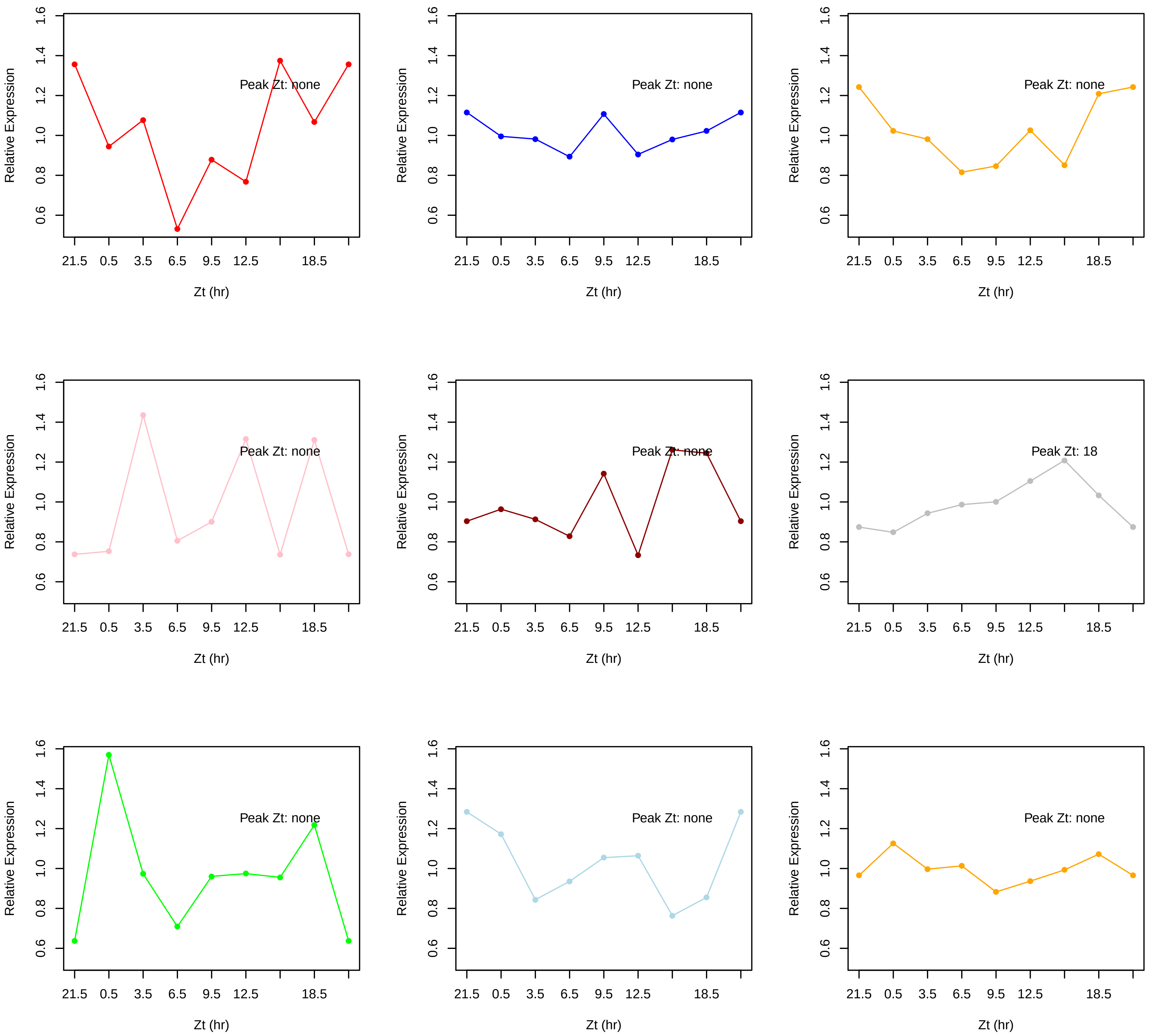

Singeleton: ENSXMAG00000016346 brd7

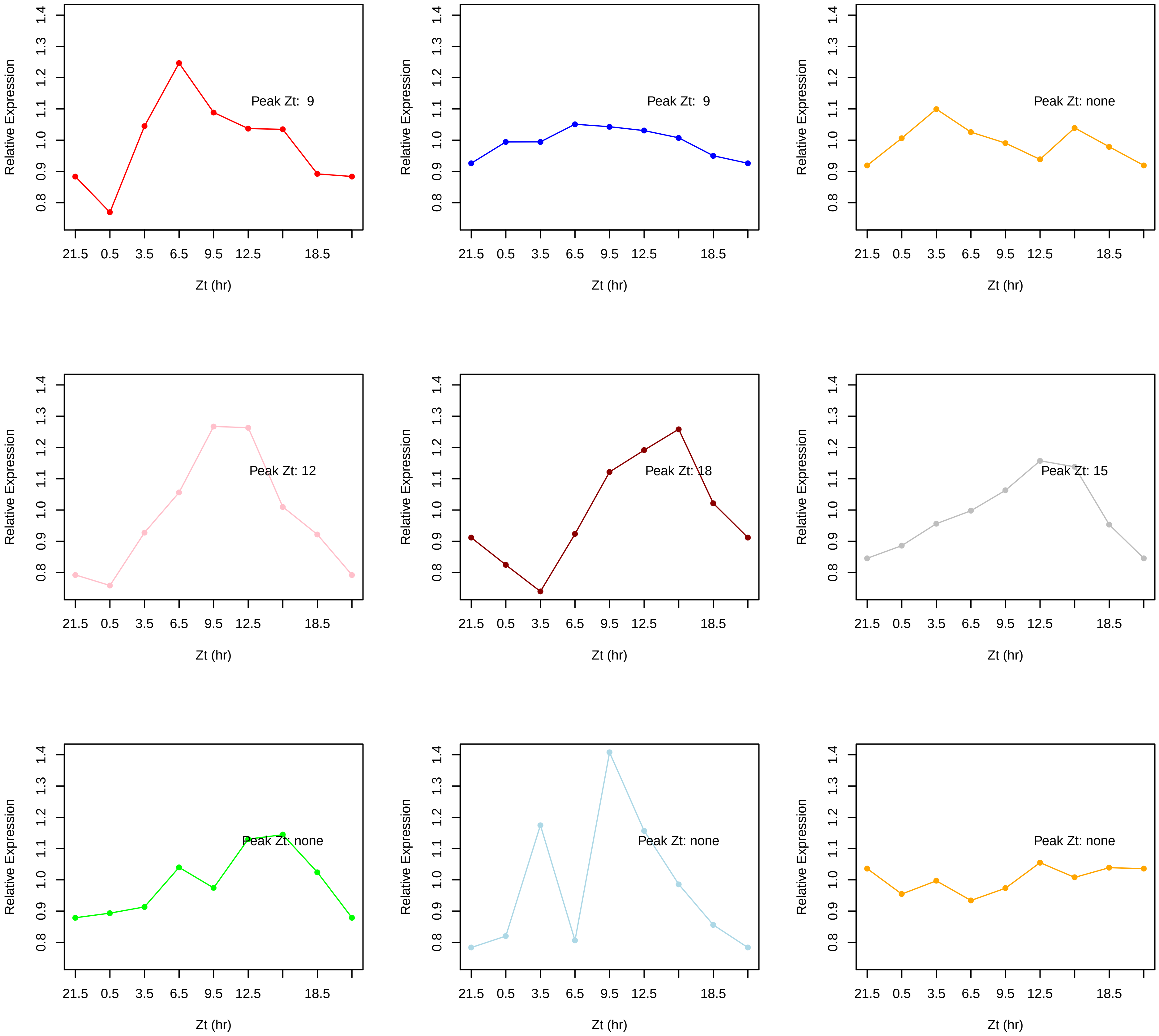

Singeleton: ENSXMAG00000012503 mtpap

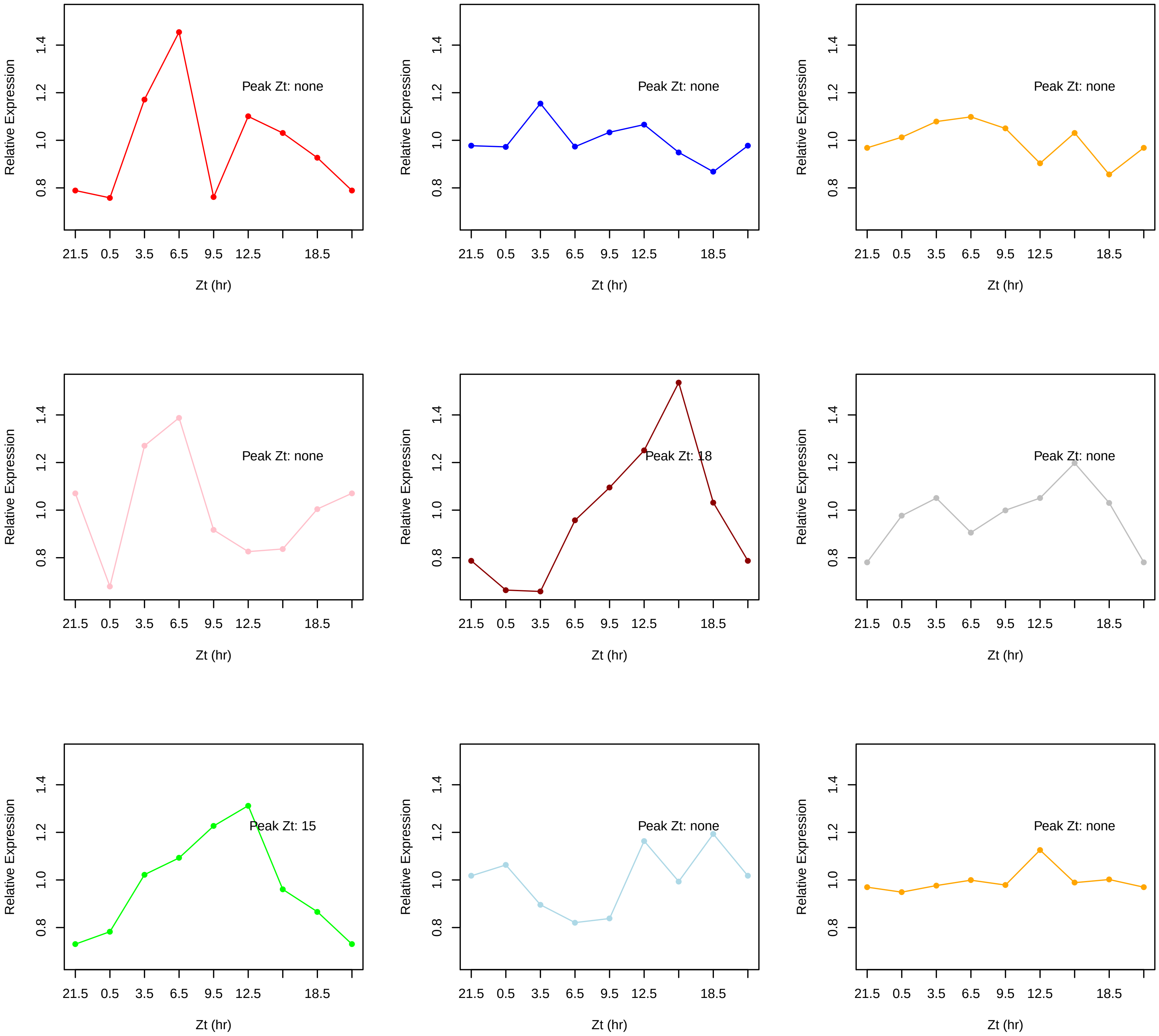

Singeleton: ENSXMAG00000021699 PPP1CC

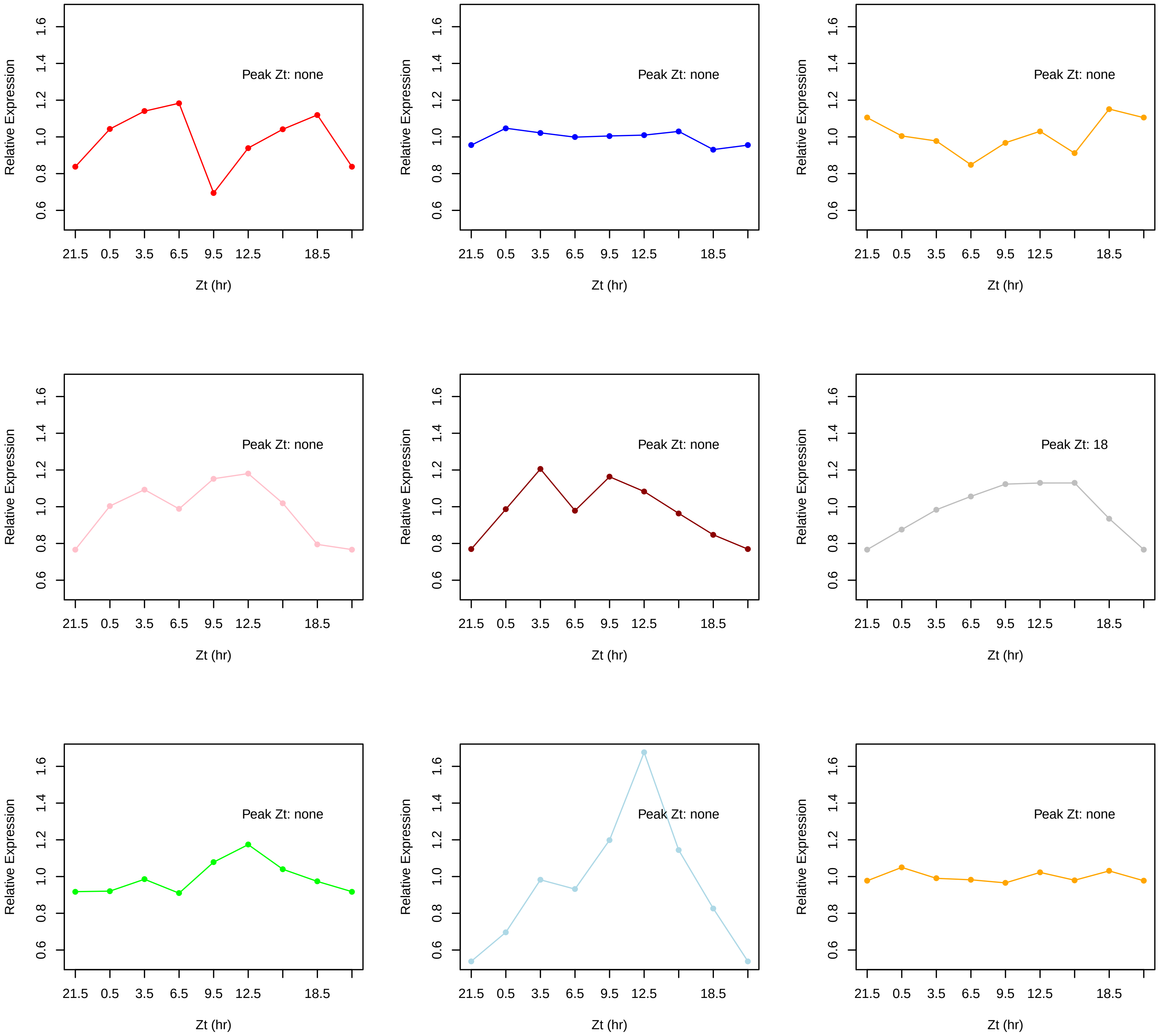

Singleton: ENSXMAG00000002929 fras1

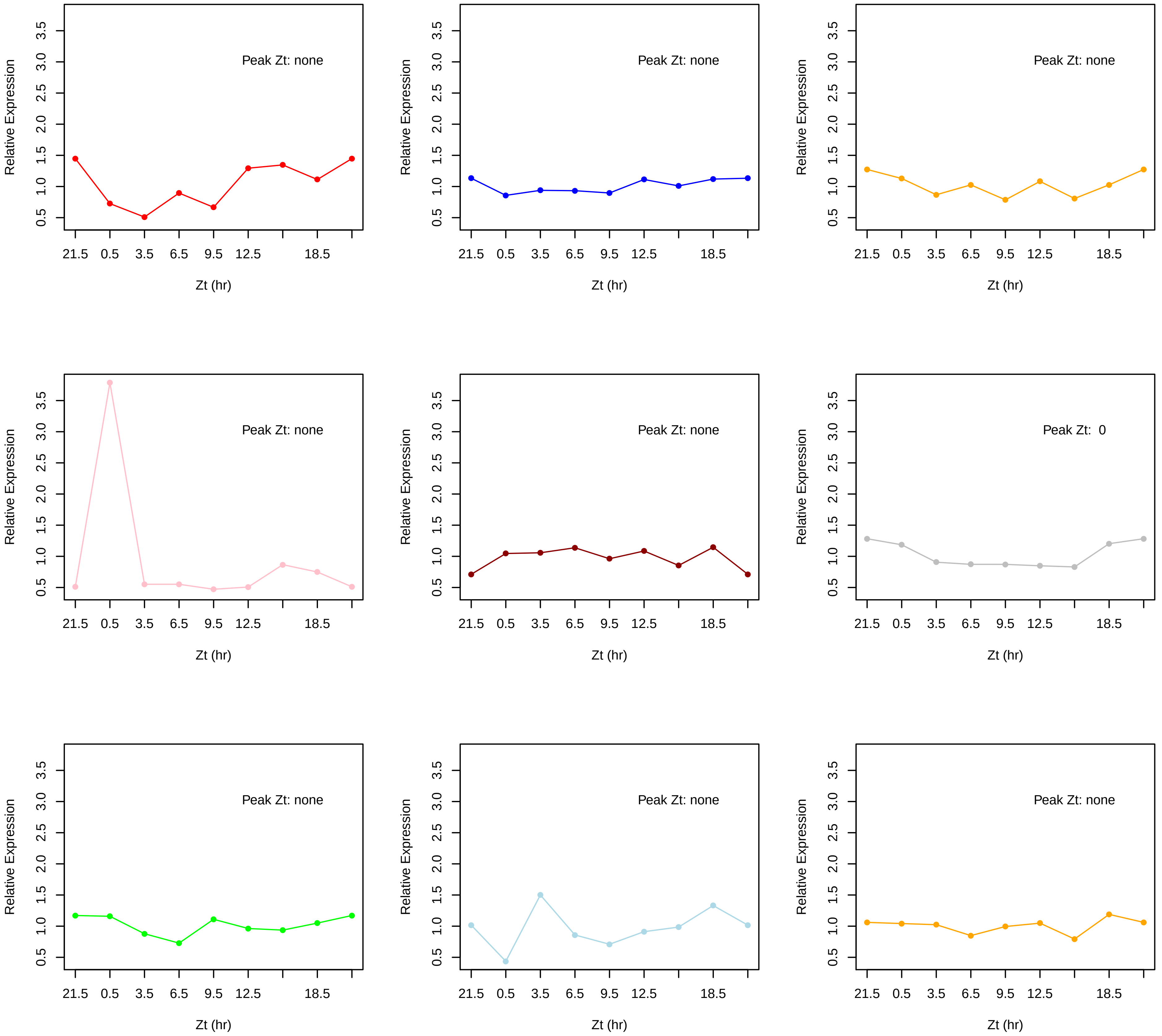

Singeleton: ENSXMAG00000022332 map3k8

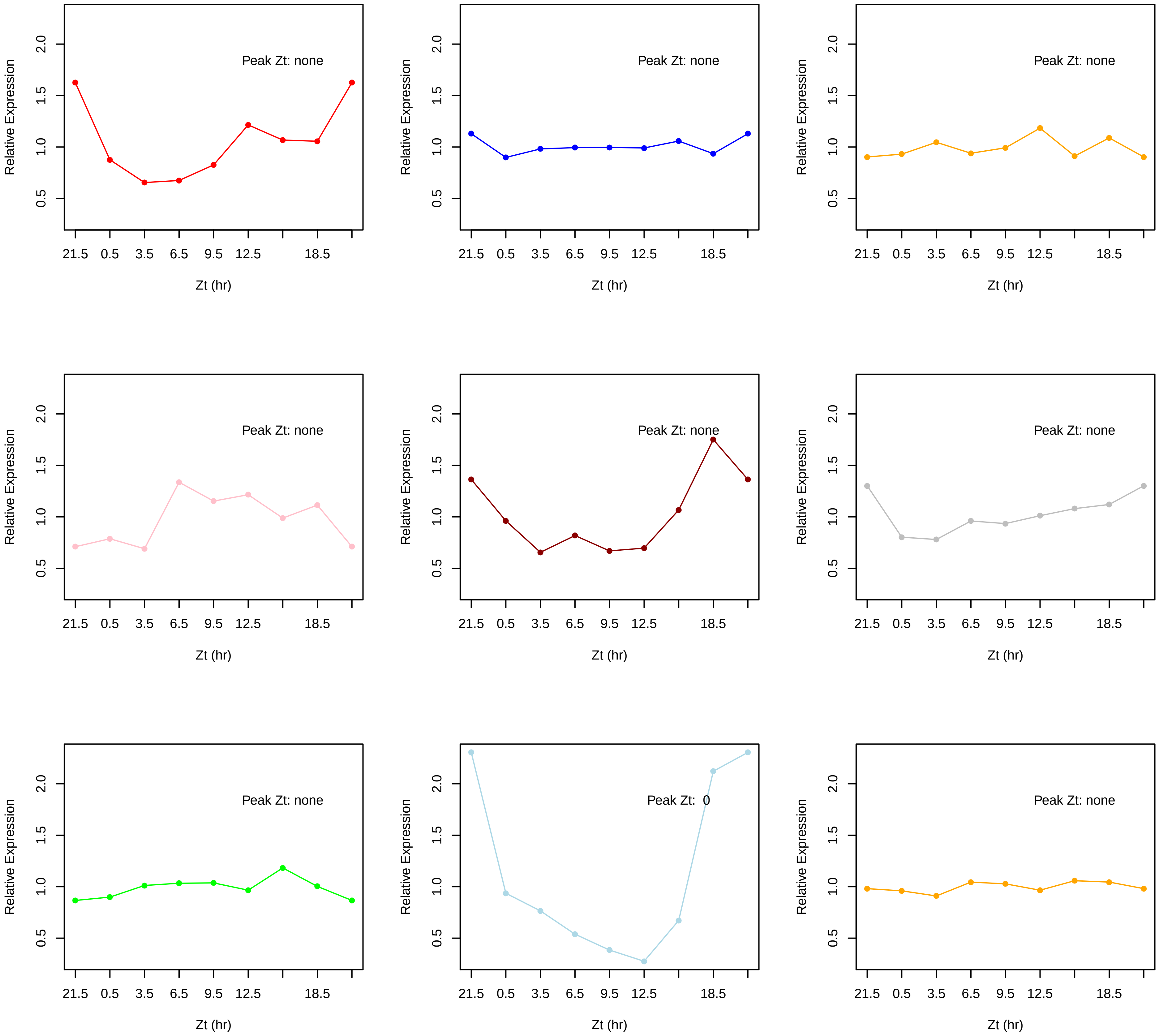

Singeleton: ENSXMAG00000017818

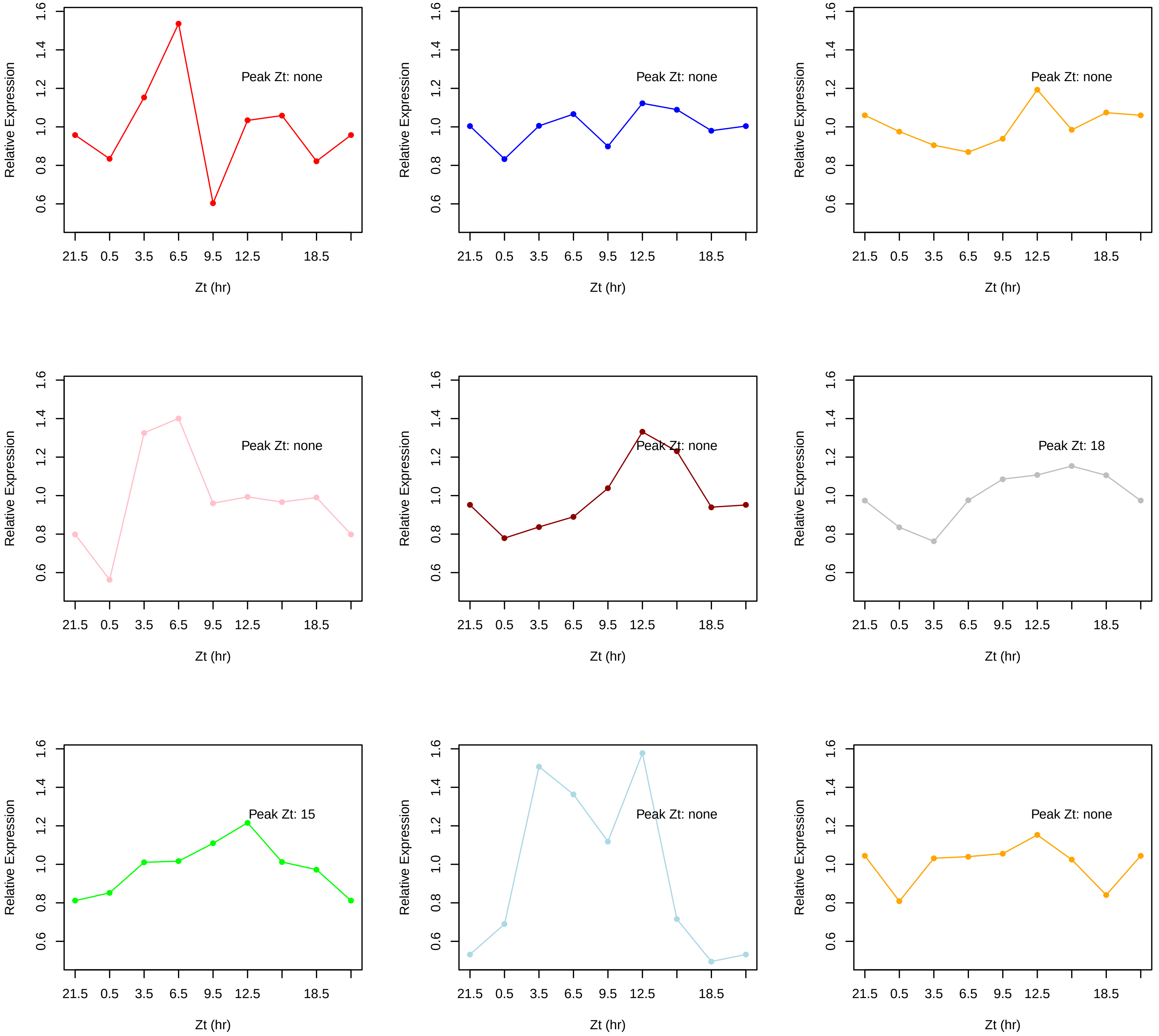

Singeleton: ENSXMAG00000018281

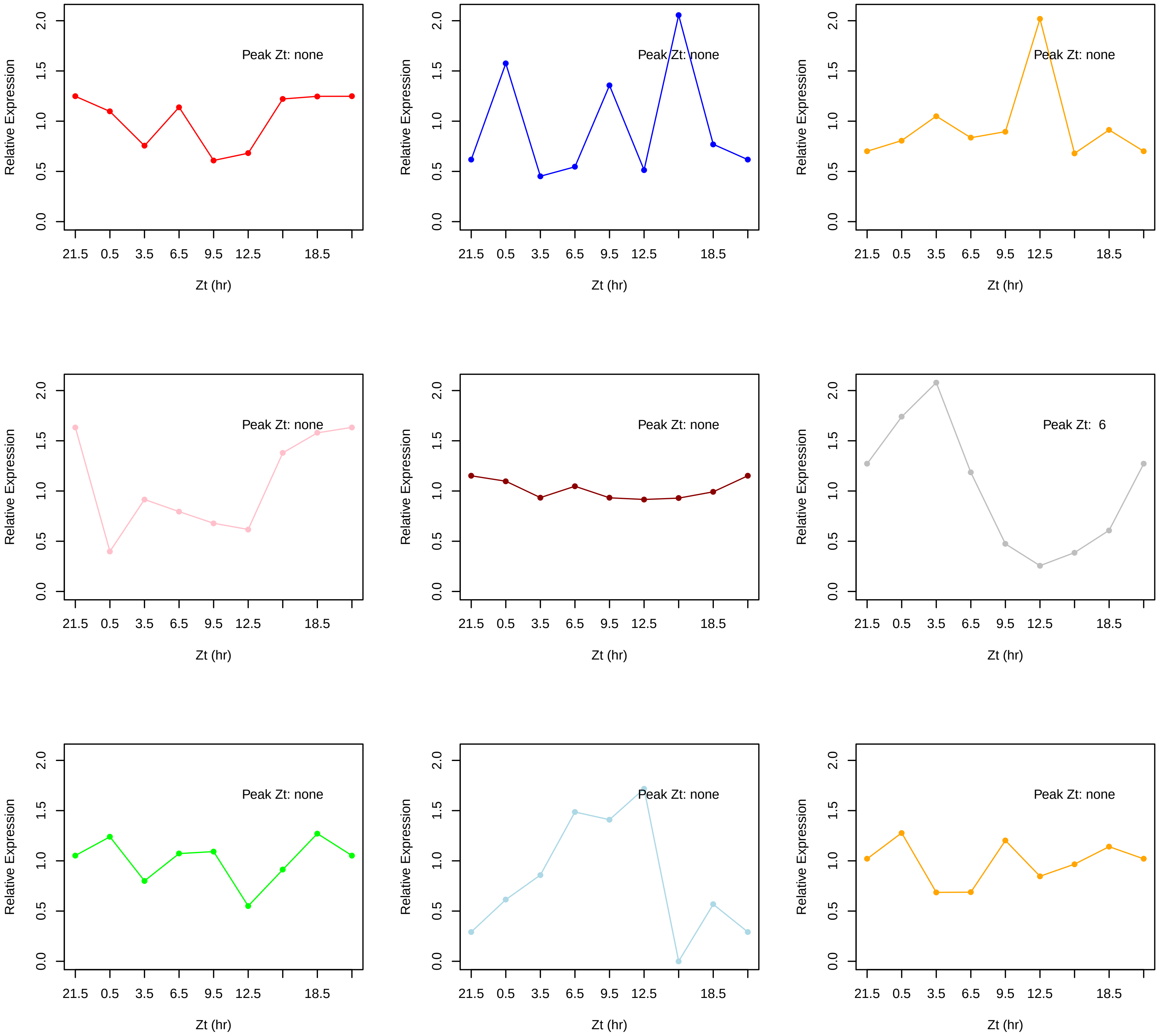

Singeleton: ENSXMAG00000014582 rbl1

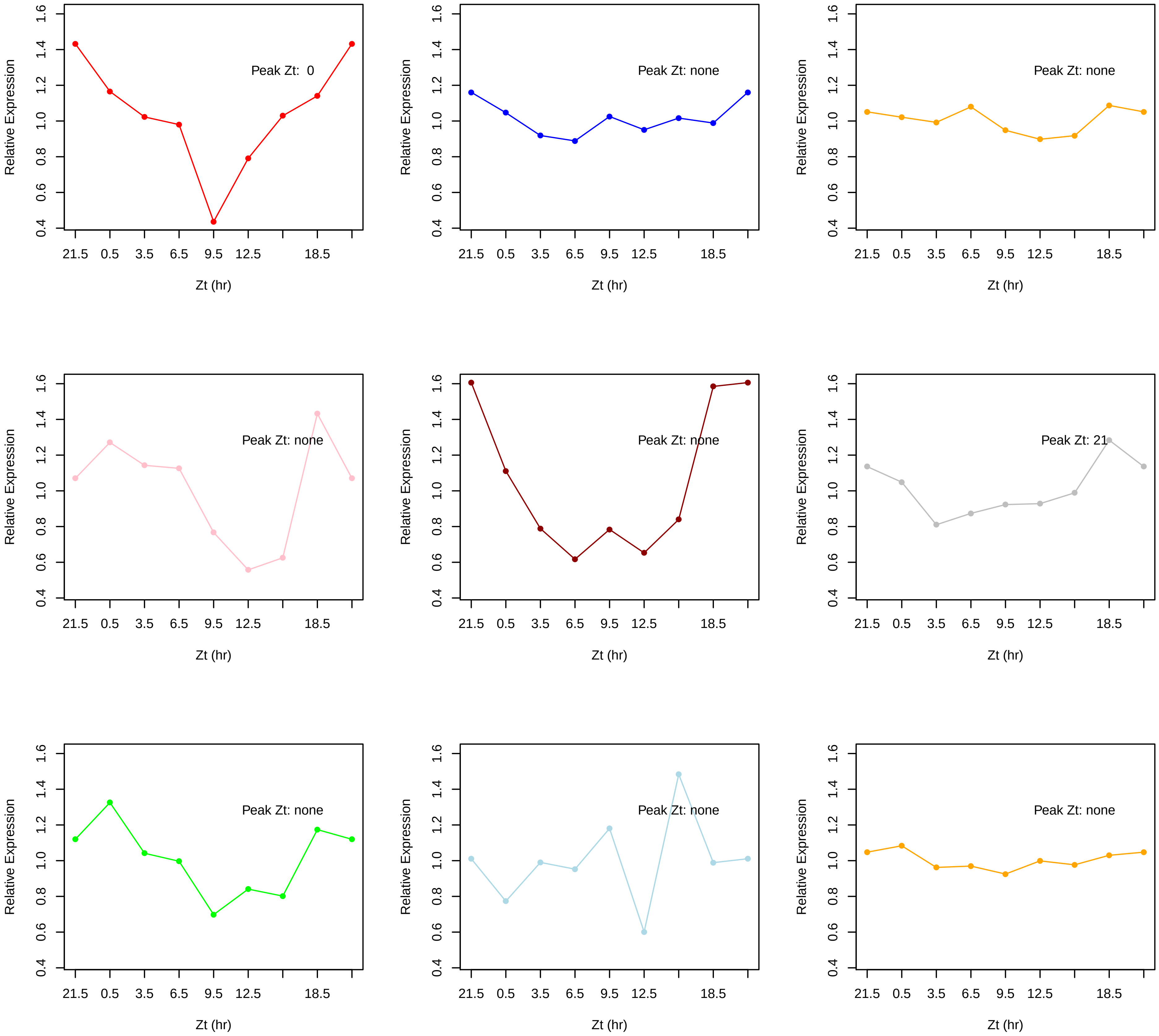

Singeleton: ENSXMAG00000002691 uchl5

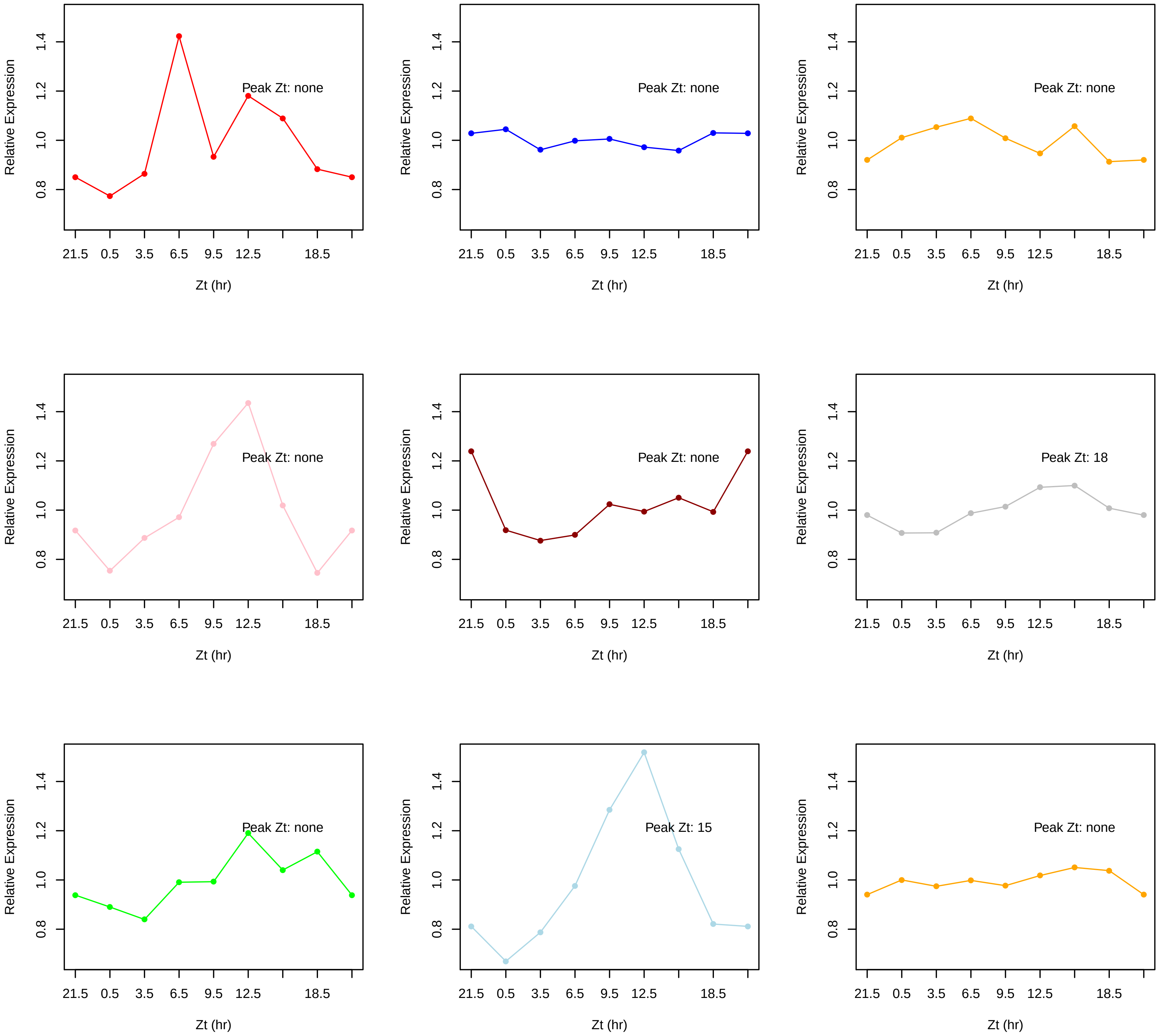

Singeleton: ENSXMAG00000022646

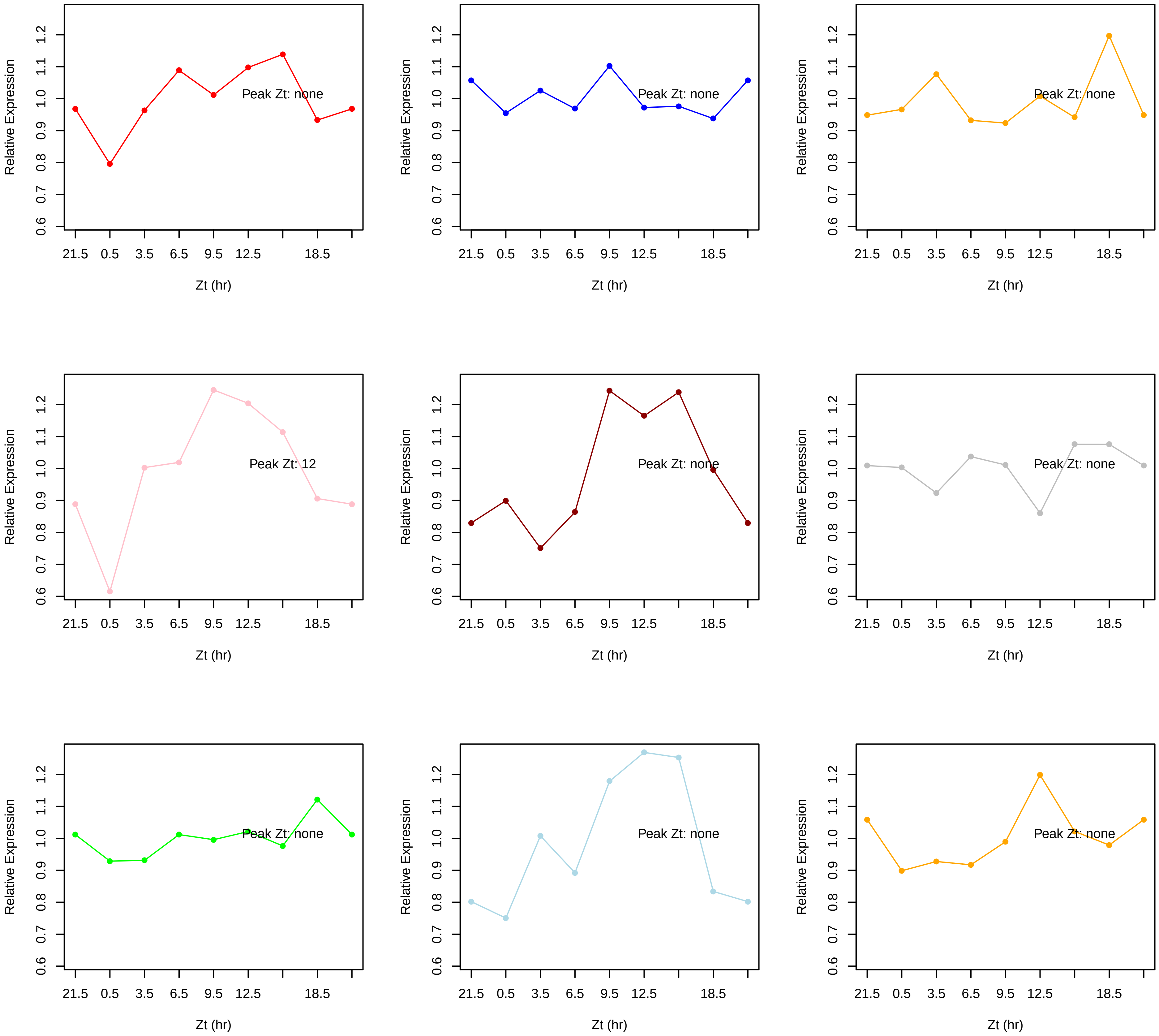

Singeleton: ENSXMAG00000028708 ppan

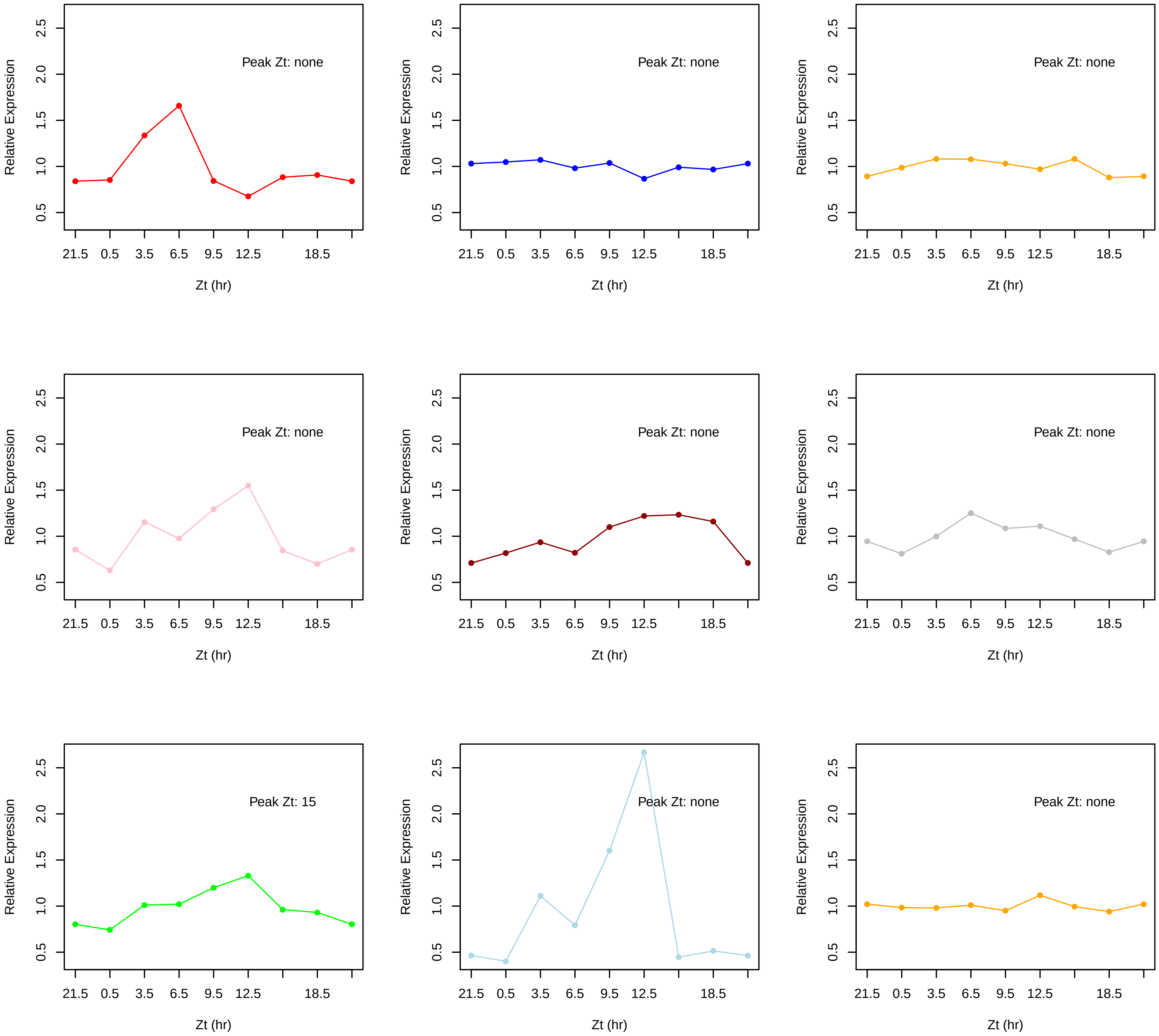

Singeleton: ENSXMAG00000026424

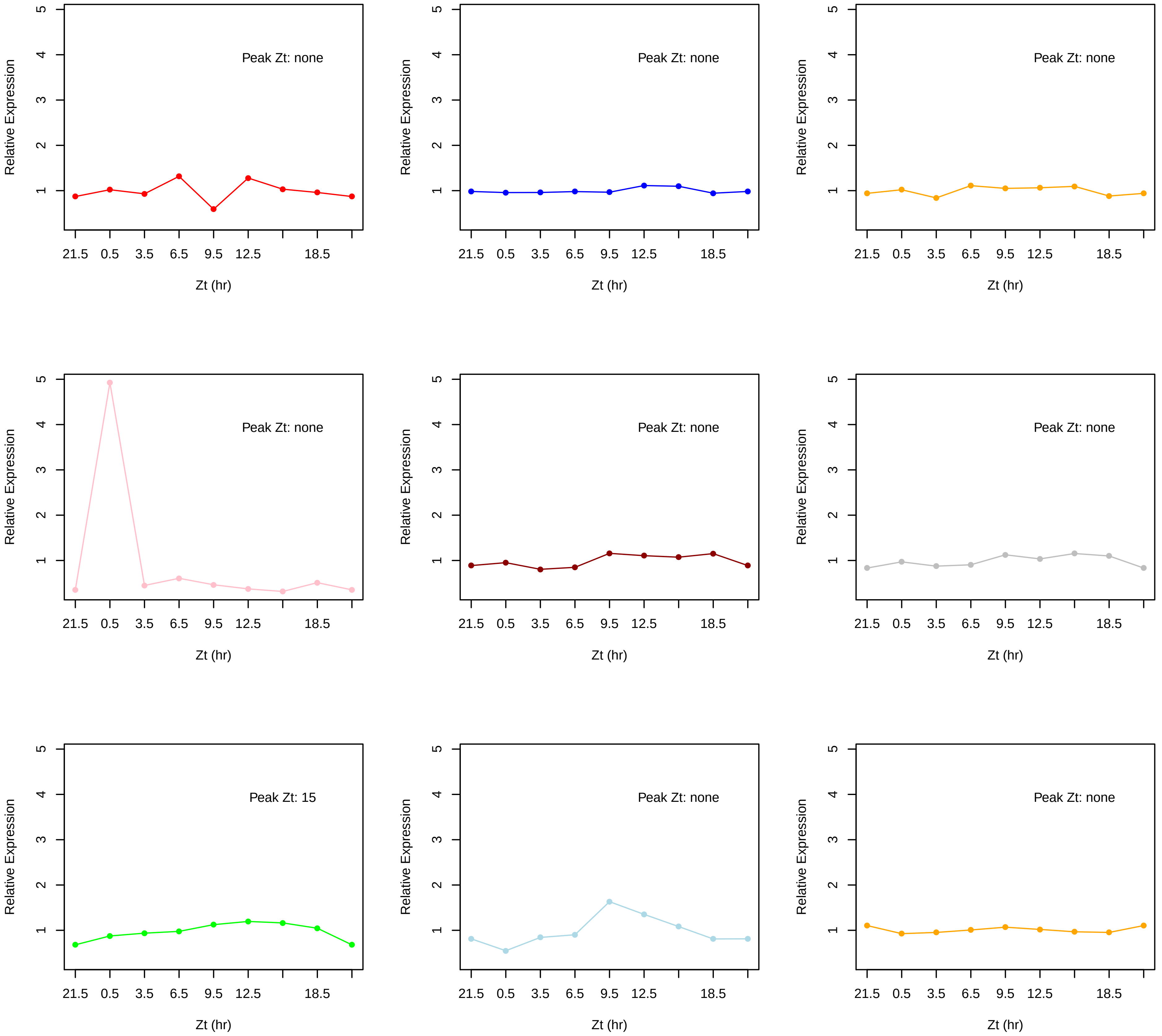

Singleton: ENSXMAG00000004825 trove2

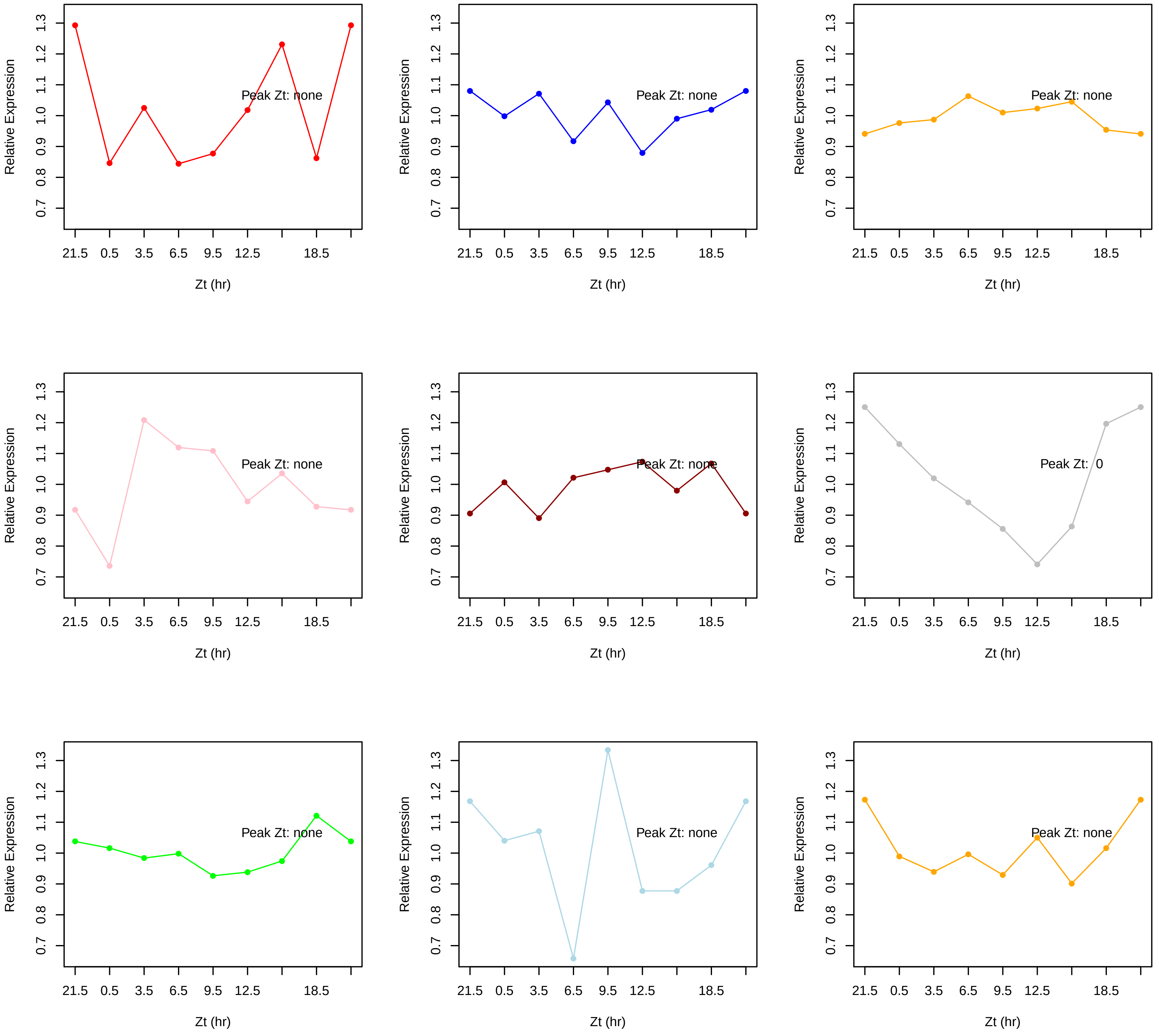

Singeleton: ENSXMAG00000010106 rrn3

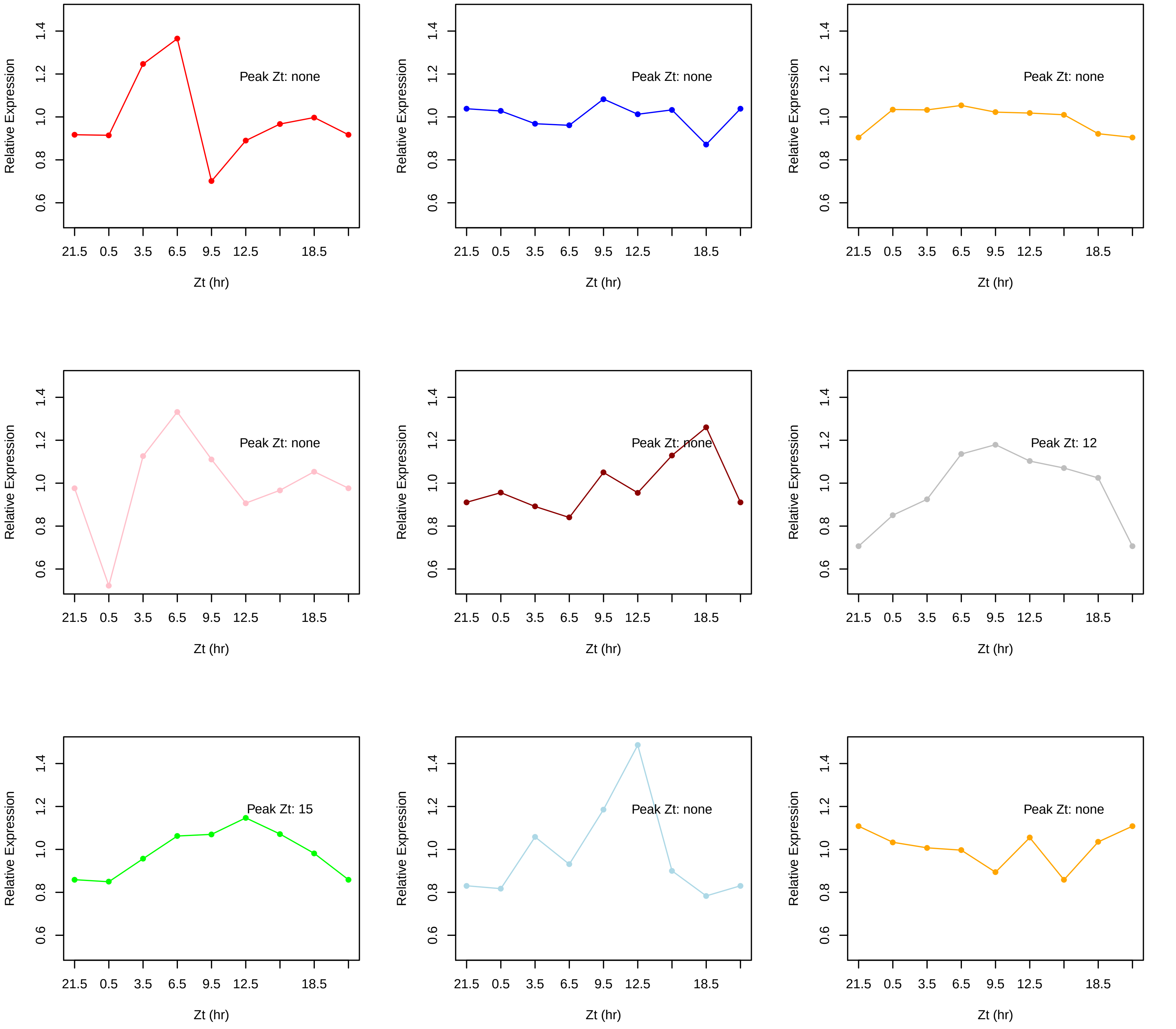

Singeleton: ENSXMAG00000011227 mlf2

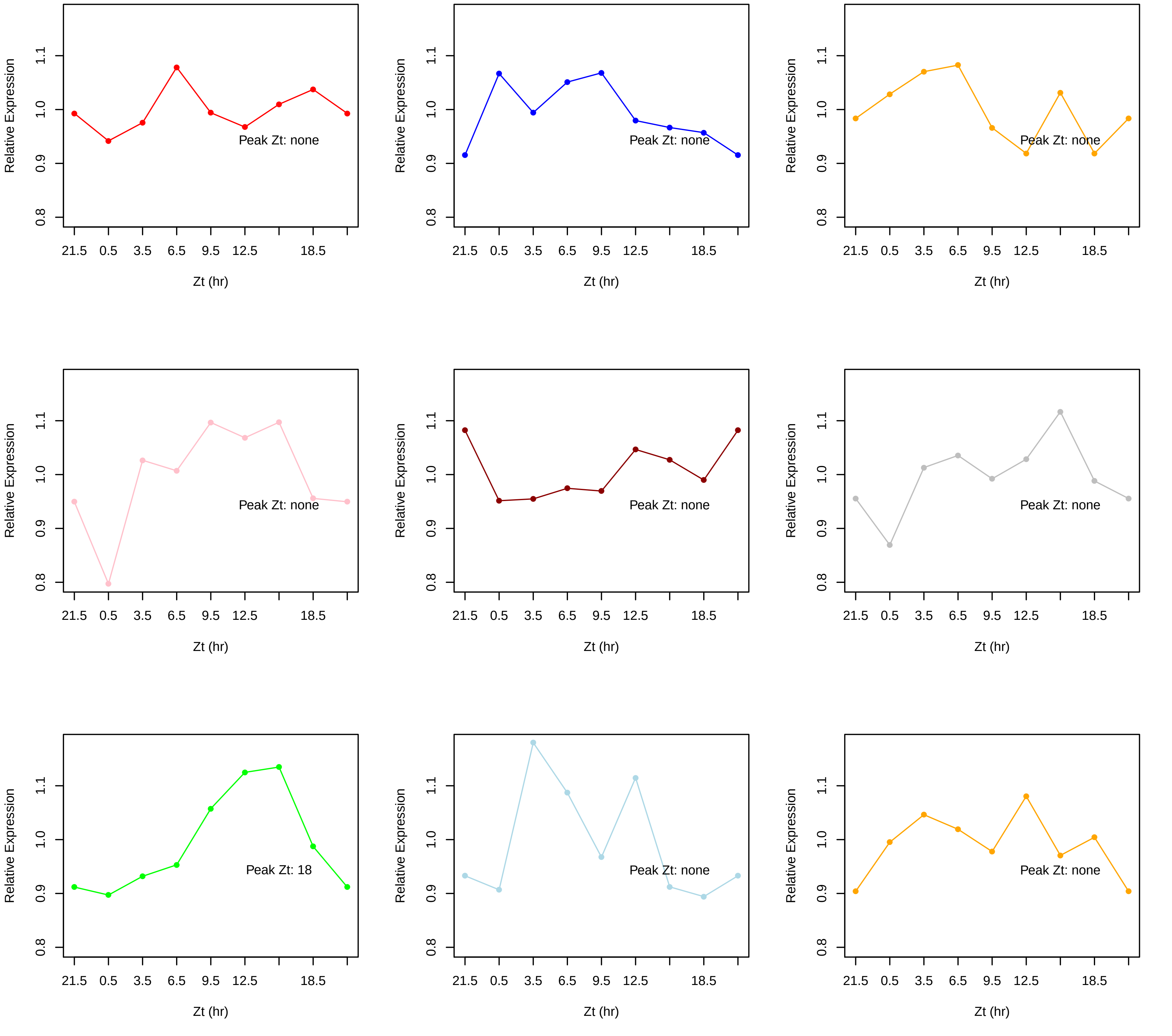

Singeleton: ENSXMAG00000002687 glrx2

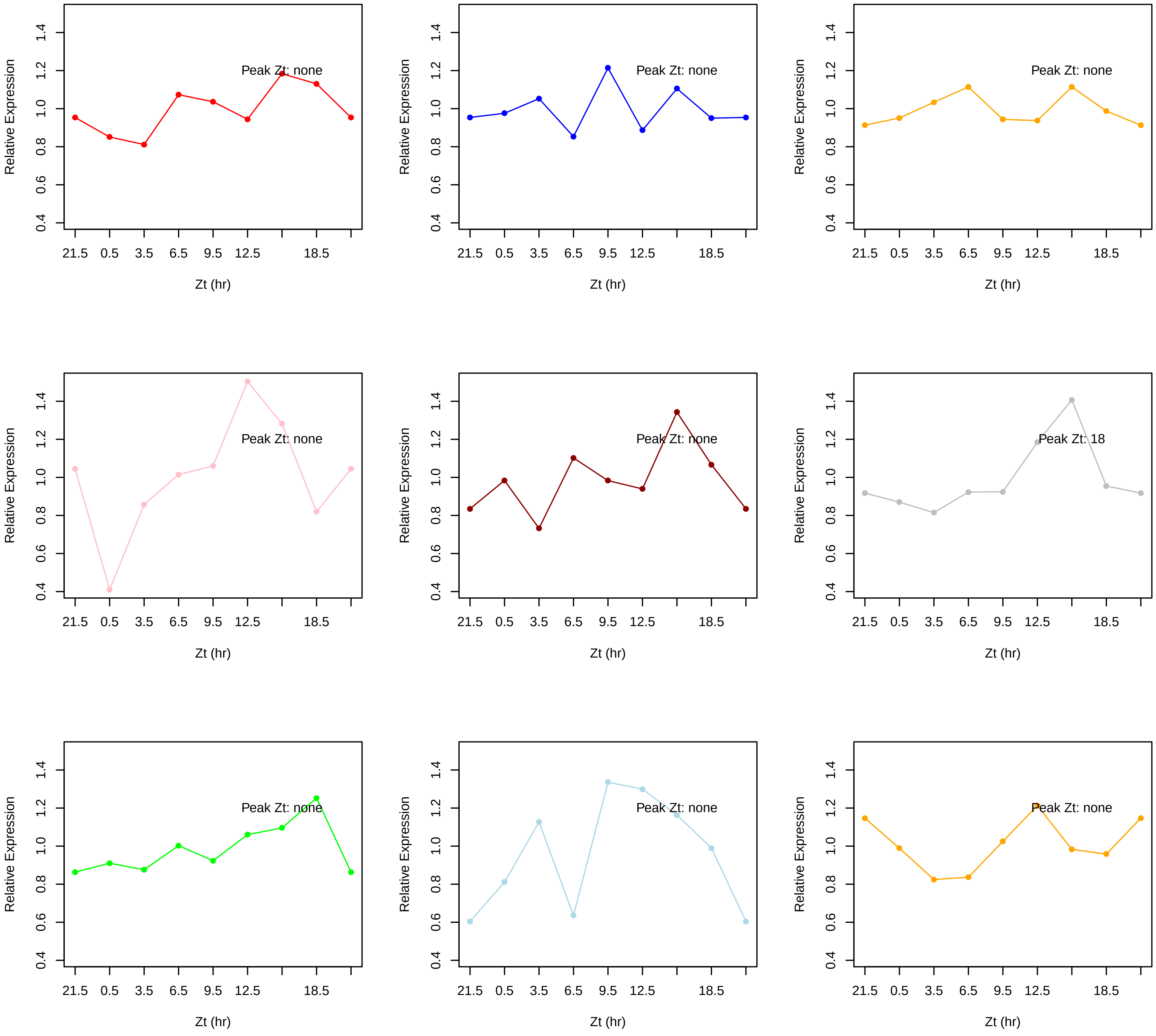

Singleton: ENSXMAG00000012157 ripor3

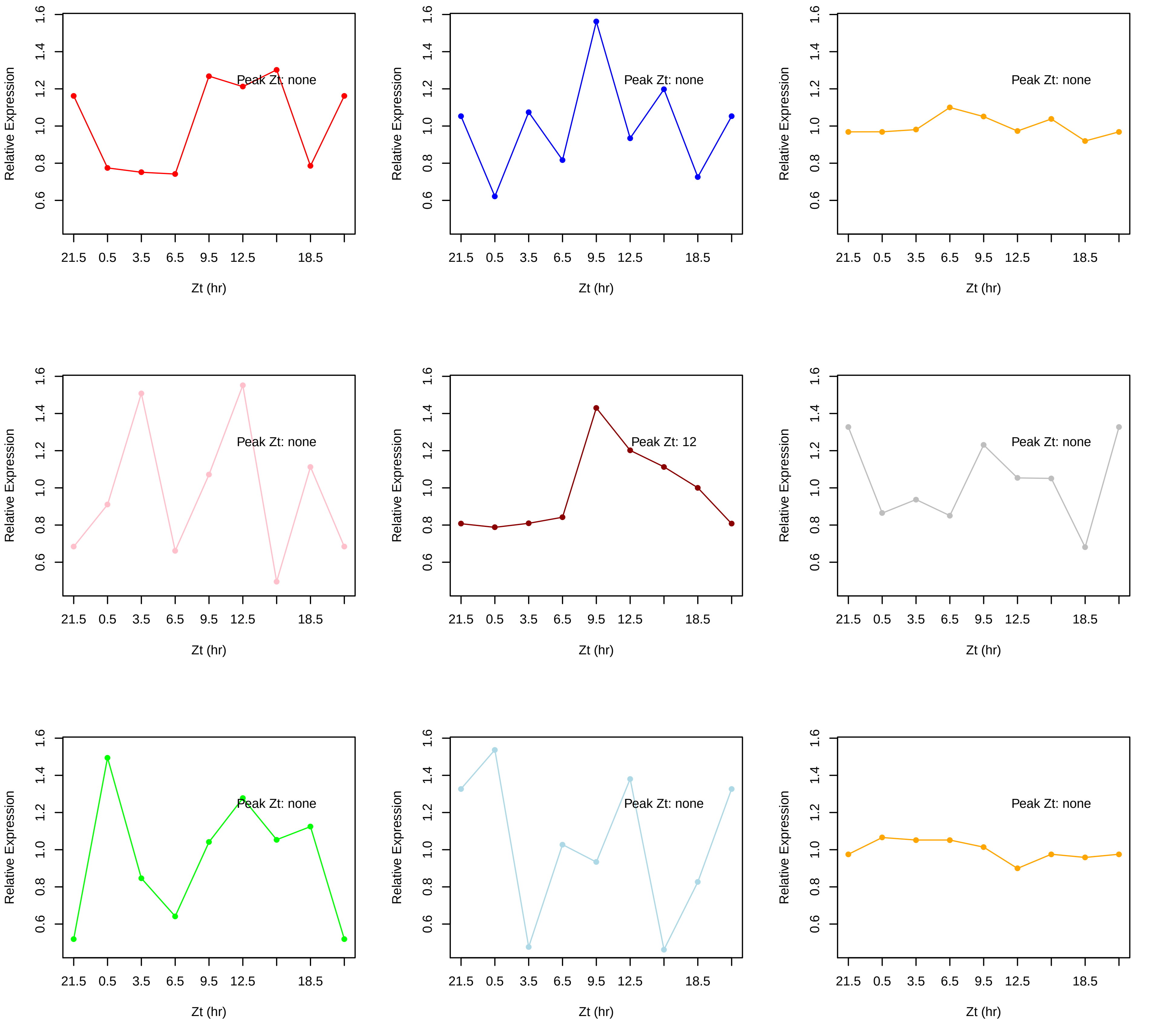

Singleton: ENSXMAG00000004807 cdc73

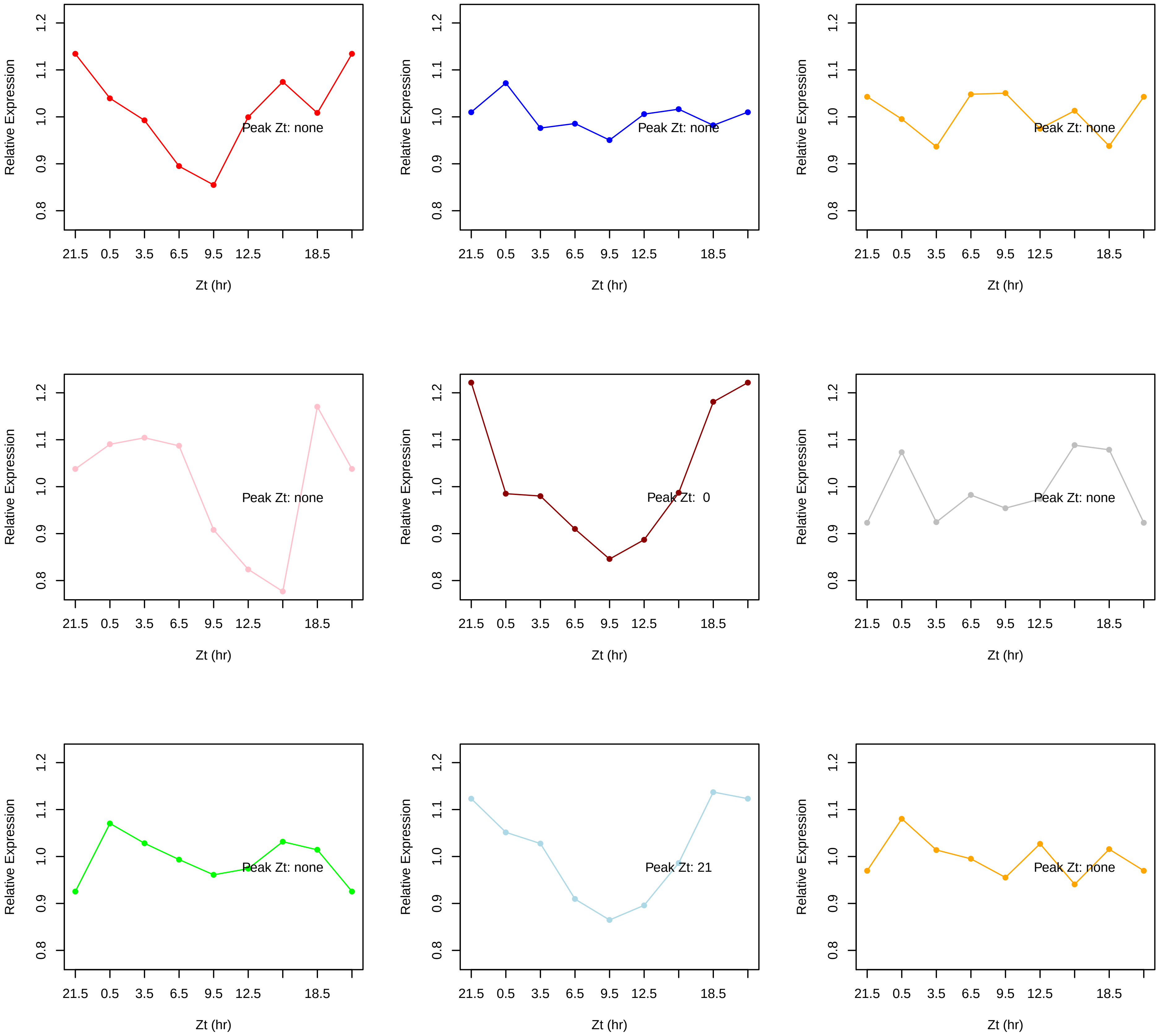

Singleton: ENSXMAG00000007752 med17

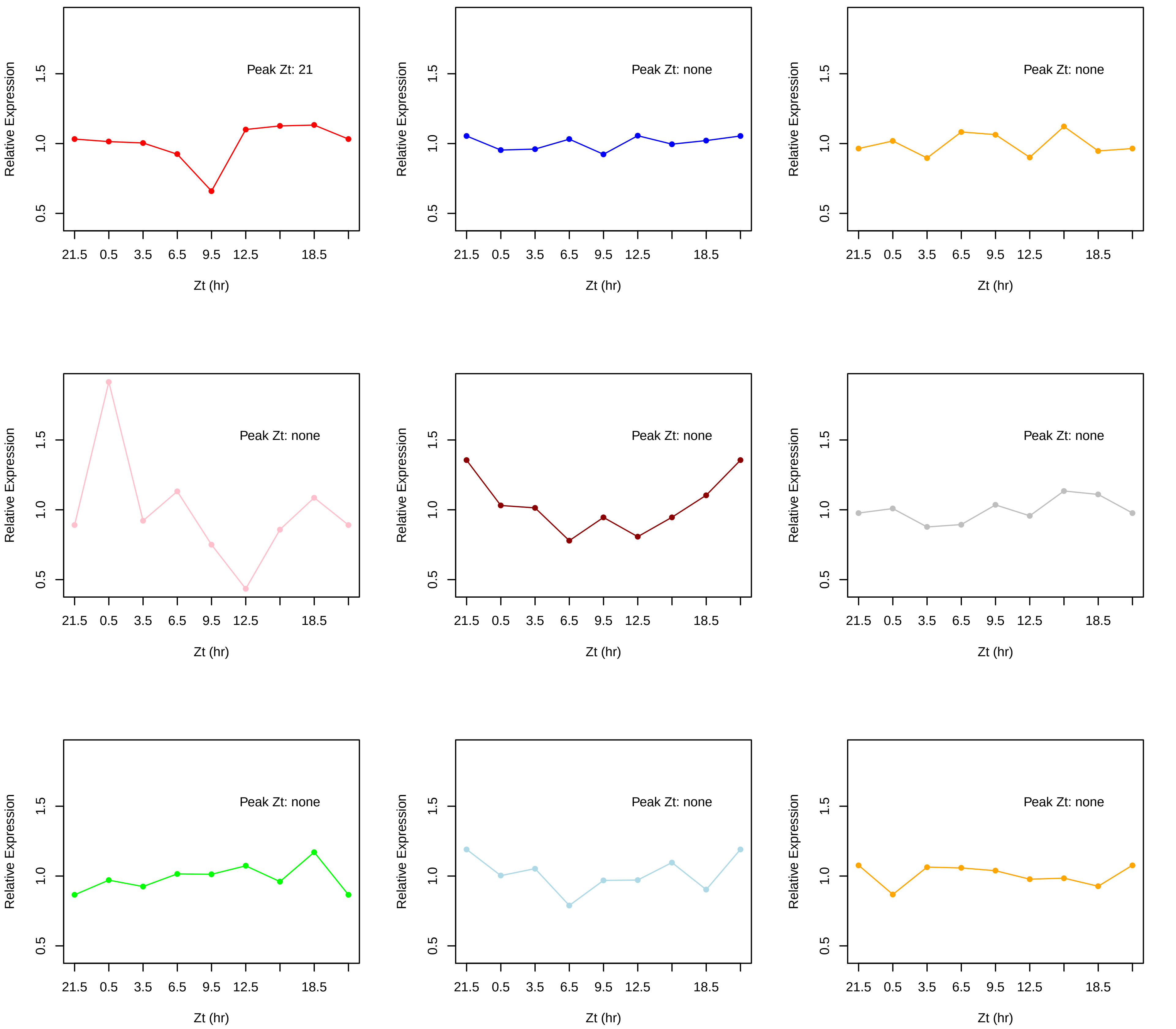

Singleton: ENSXMAG00000002920 mrpl1

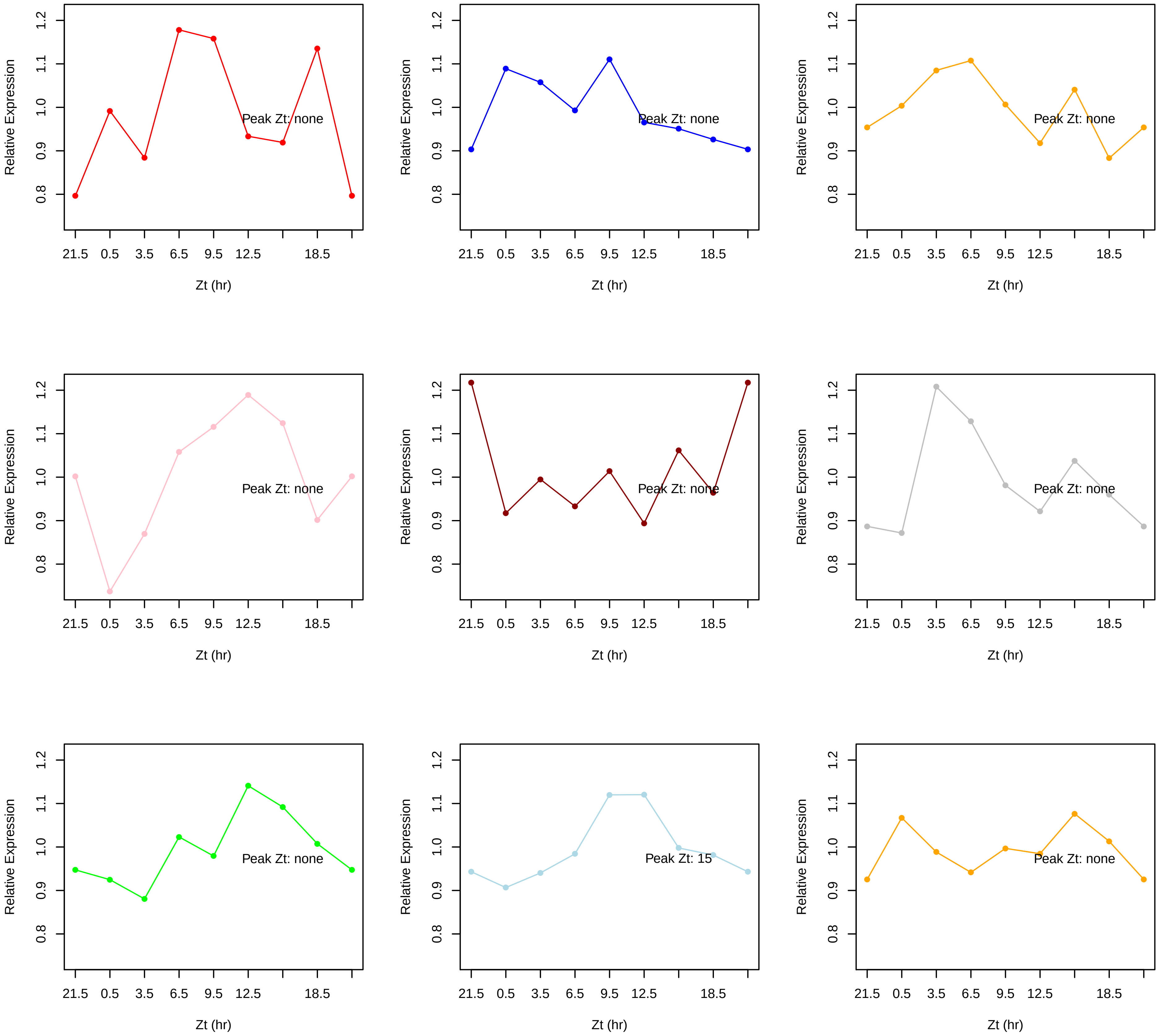

Singleton: ENSXMAG00000023205 tent4b

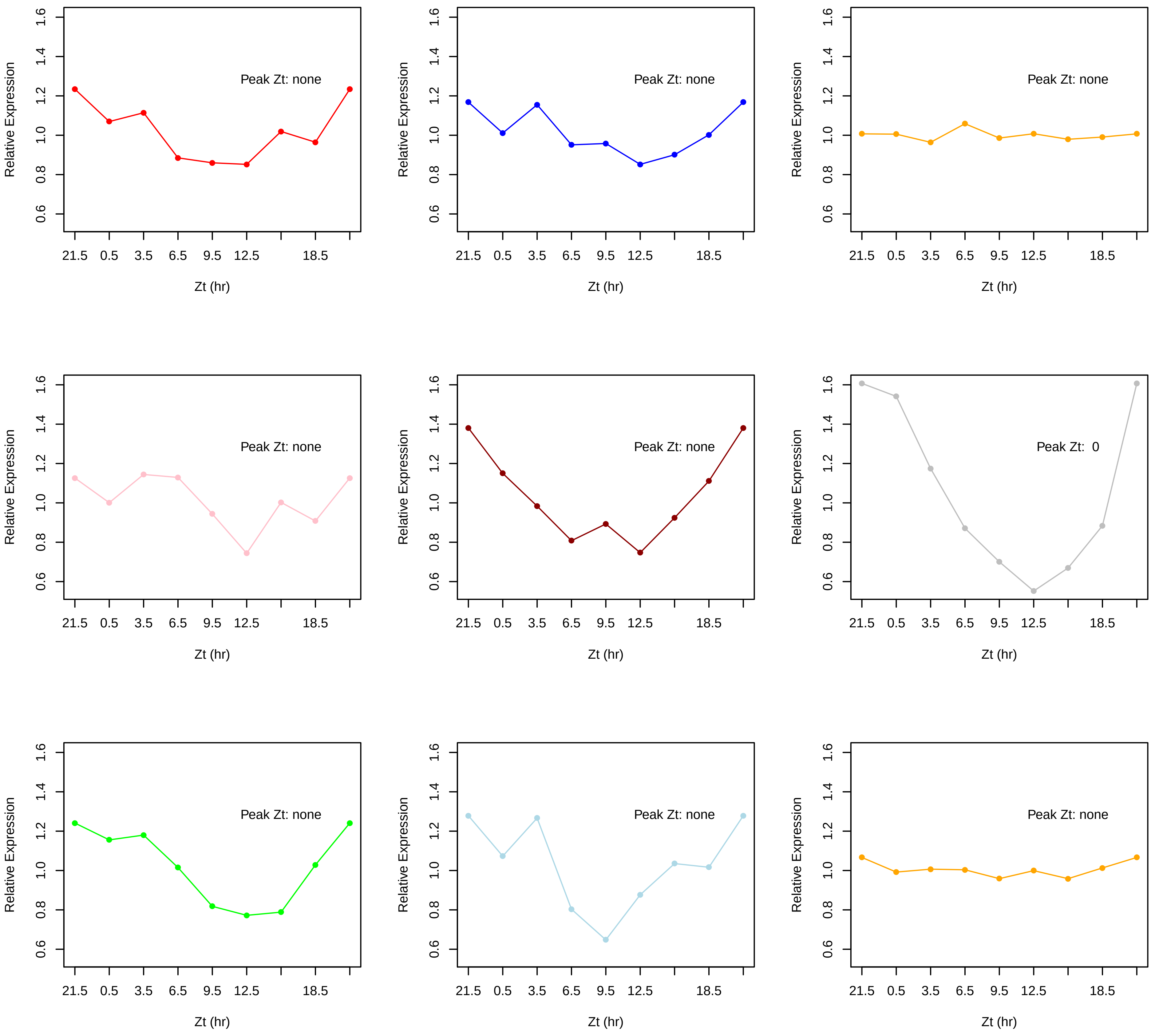

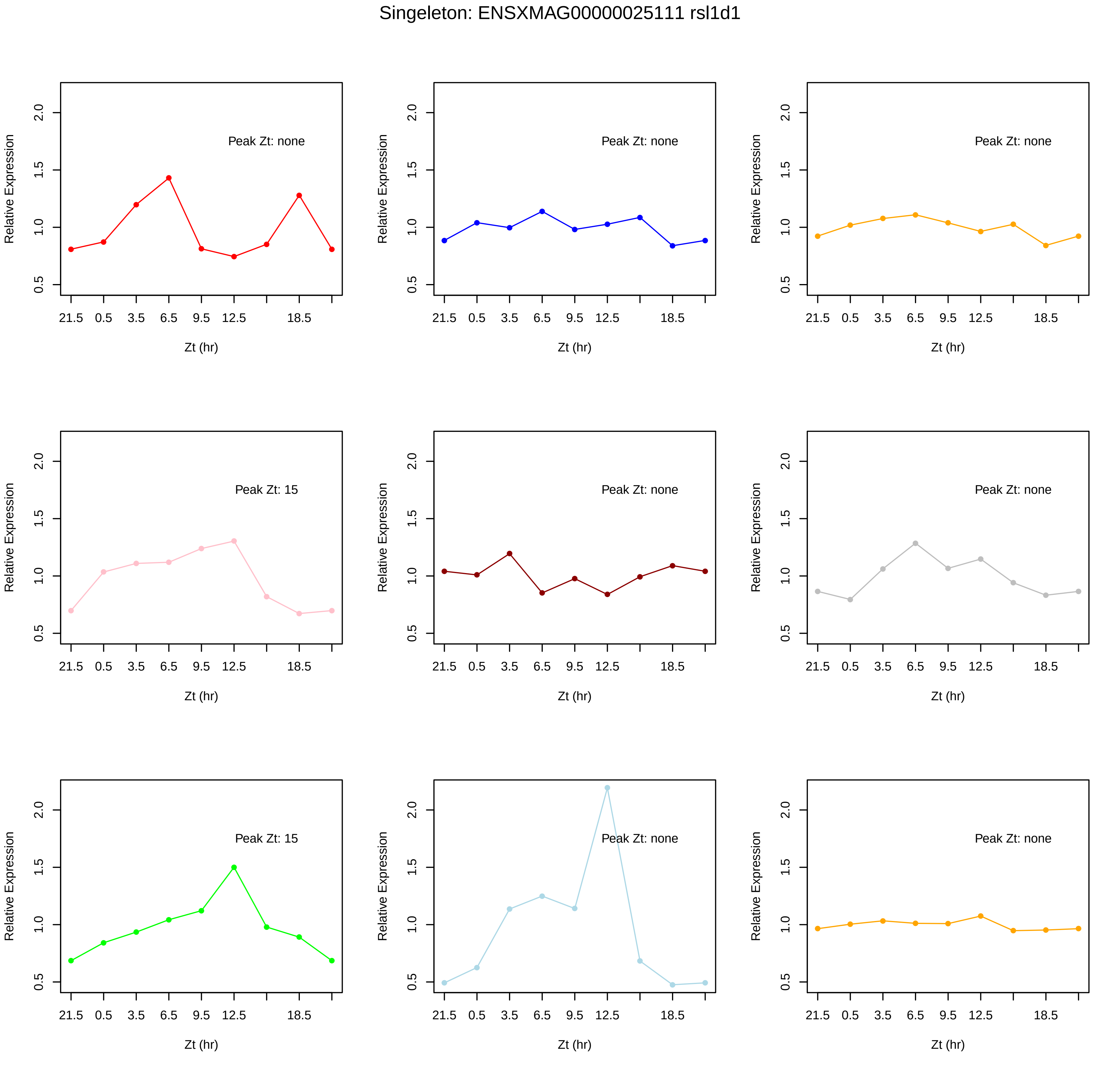
Singleton: ENSXMAG00000025111 rsl1d1
Peak Zt: none
Peak Zt: none
Peak Zt: none
Peak Zt: 15
Peak Zt: none
Peak Zt: none
Peak Zt: 15
Peak Zt: none
Peak Zt: none
Relative Expression
Zt (hr)
21.5
0.5
3.5
6.5
9.5
12.5
18.5
0.5
1.0
1.5
2.0

Singleton: ENSXMAG00000002900 cnot6l

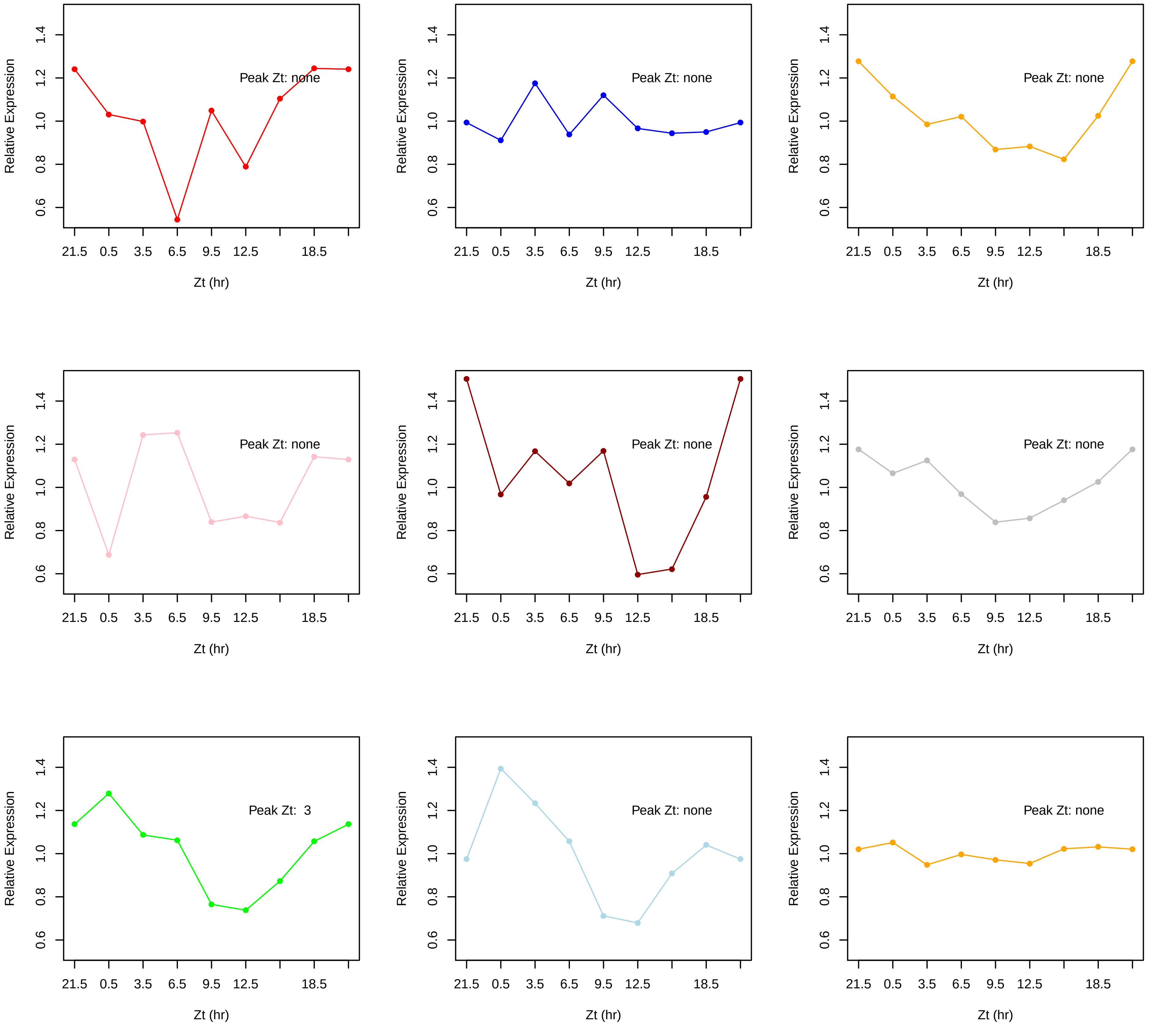

Singeleton: ENSXMAG00000029130 TMEM74B

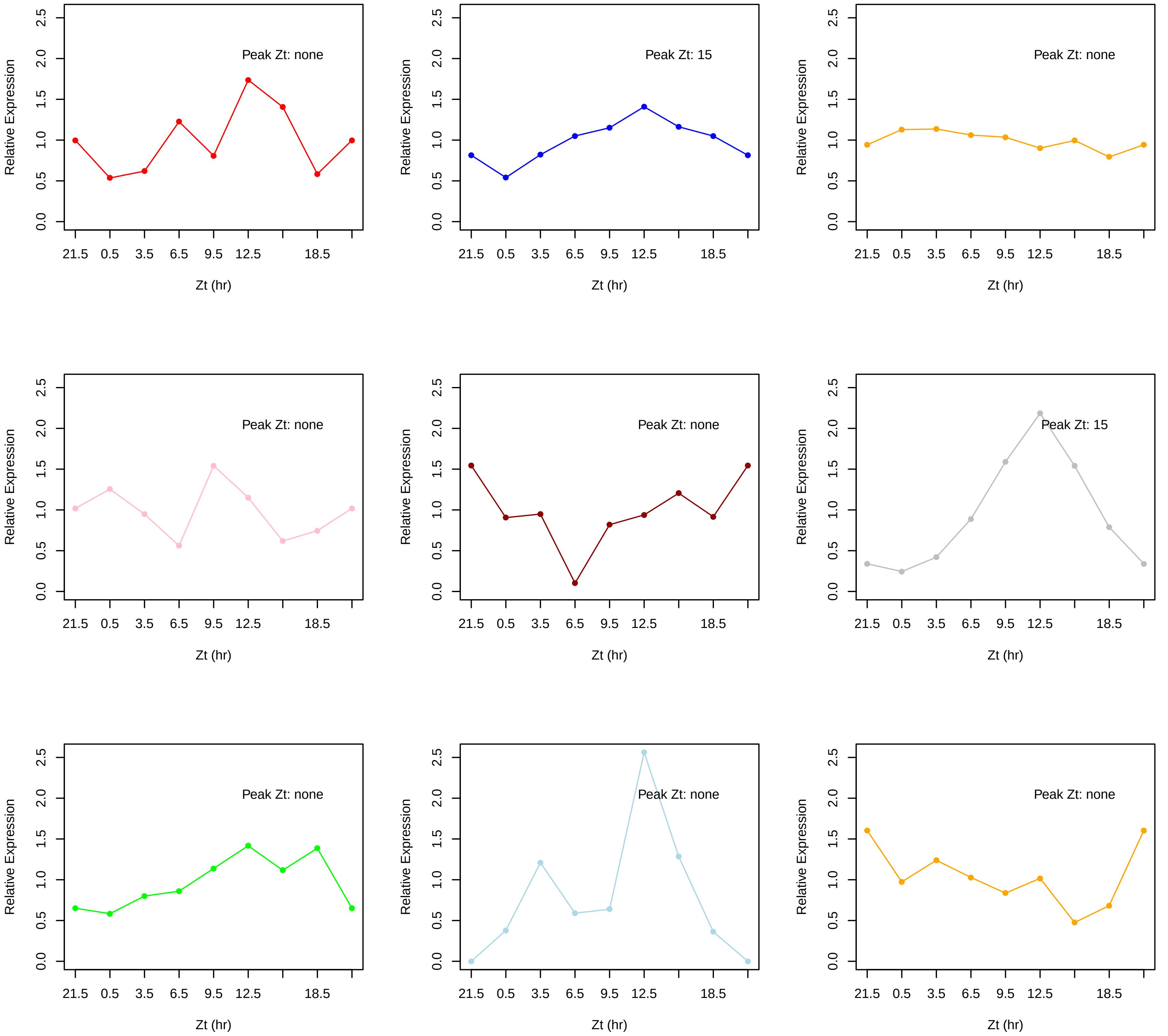

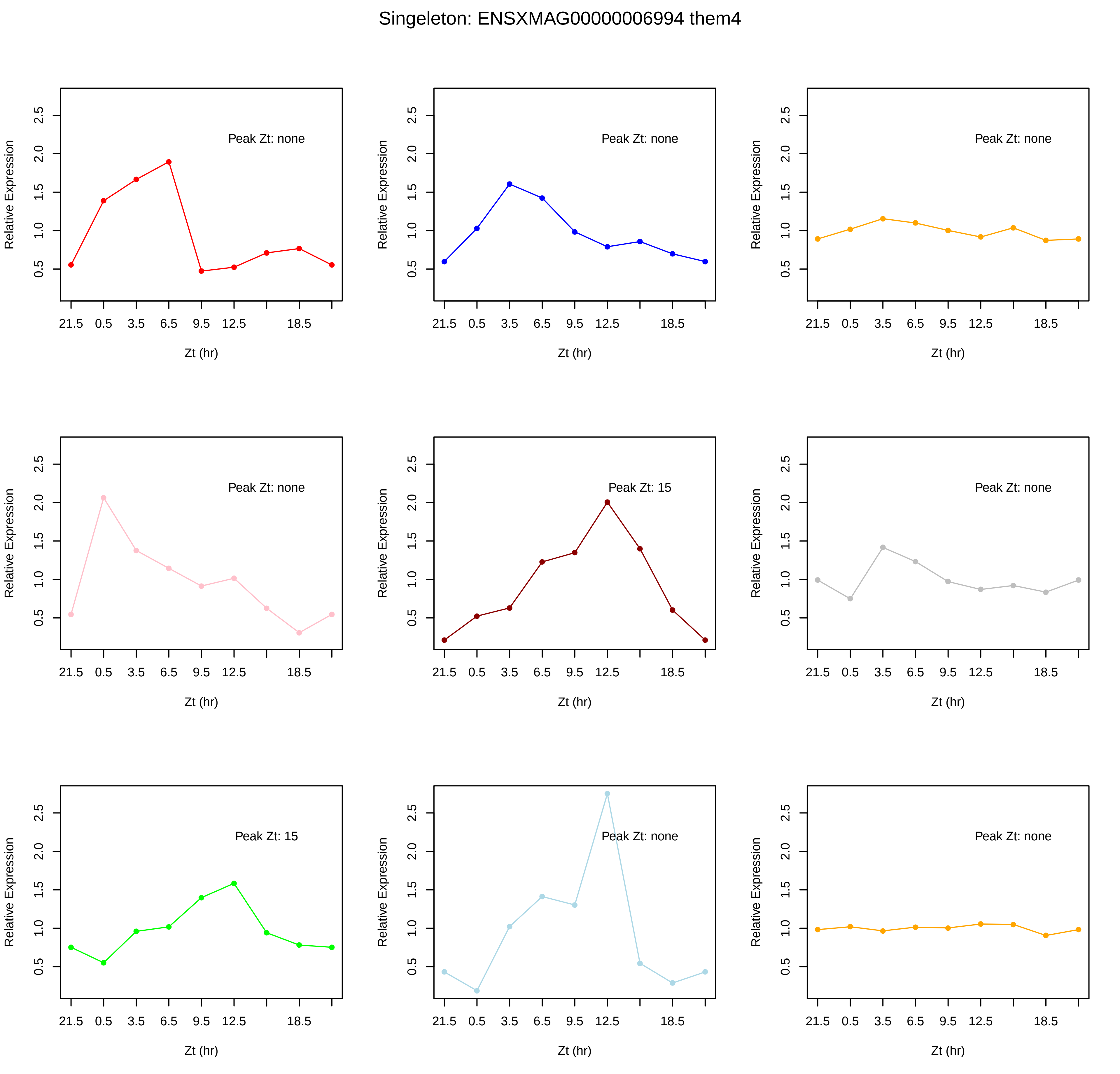
Singeleton: ENSXMAG00000006994 them4
Peak Zt: none
Peak Zt: none
Peak Zt: none
Peak Zt: none
Peak Zt: 15
Peak Zt: none
Peak Zt: 15
Peak Zt: none
Peak Zt: none
Relative Expression
Zt (hr)
21.5 0.5 3.5 6.5 9.5 12.5 18.5
0.5 1.0 1.5 2.0 2.5

Singleton: ENSXMAG00000002032 phka2

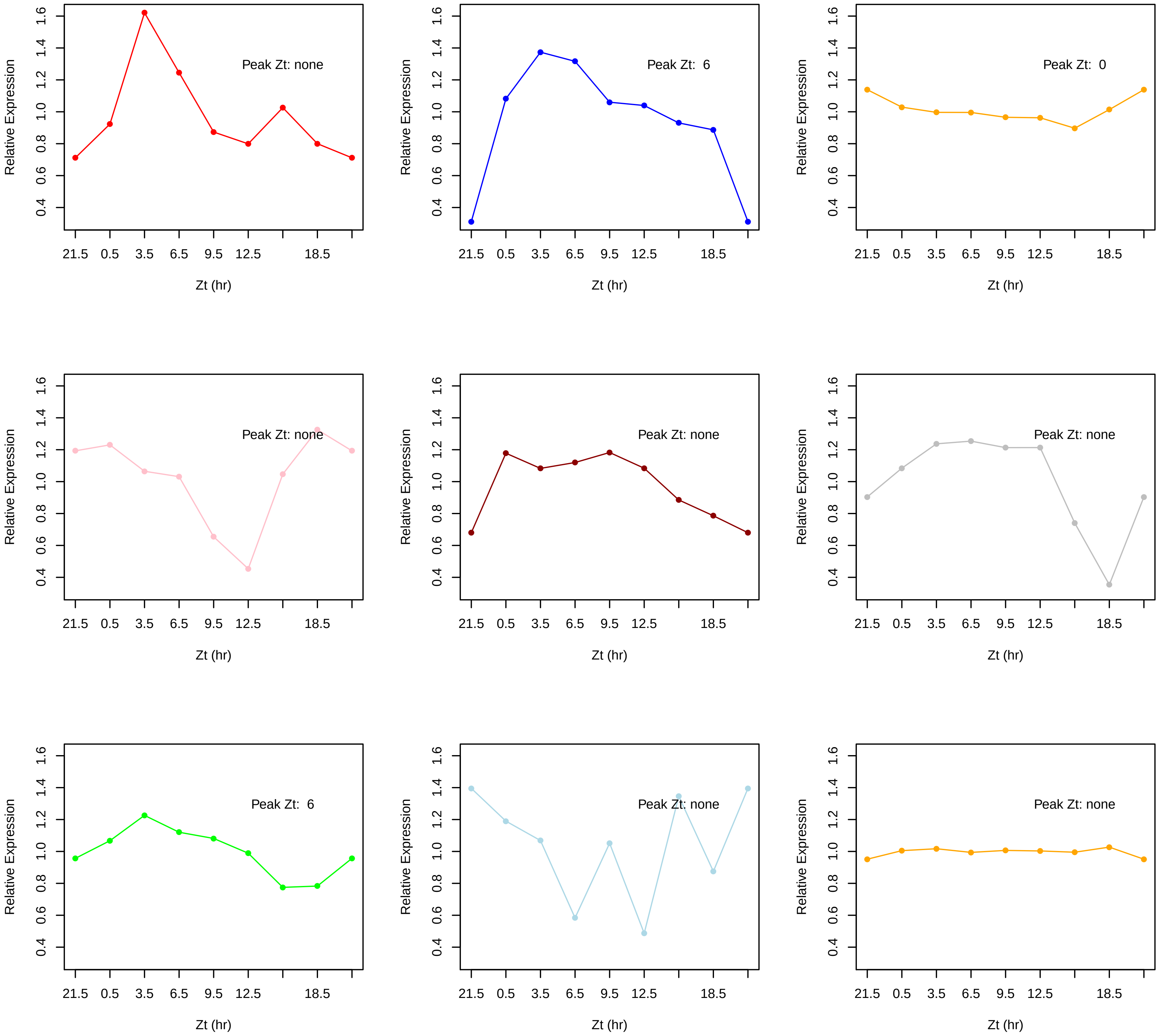

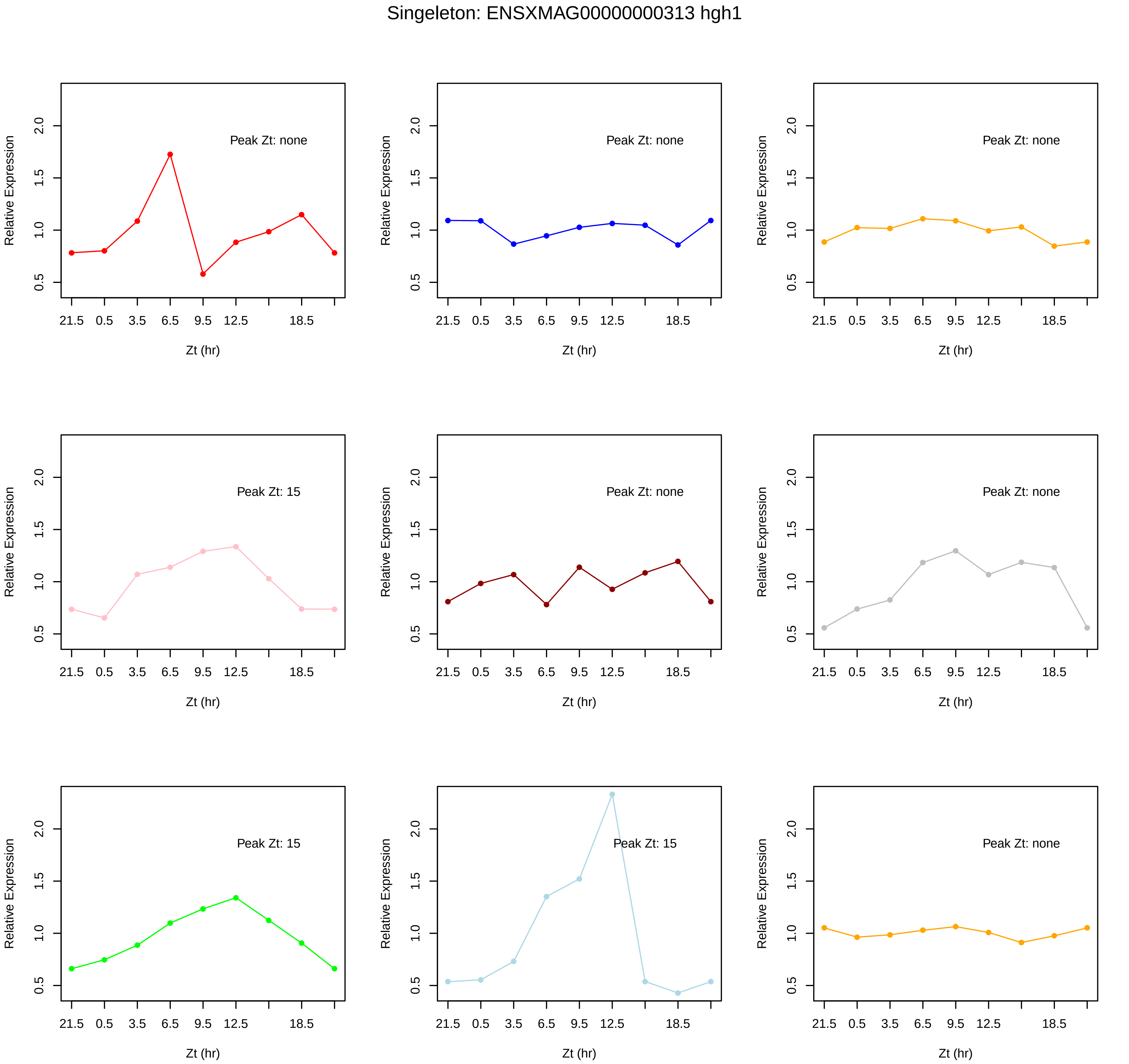

Singeleton: ENSXMAG00000000313 hgh1
Peak Zt: none
Peak Zt: none
Peak Zt: none
Peak Zt: 15
Peak Zt: none
Peak Zt: none
Peak Zt: 15
Peak Zt: 15
Peak Zt: none
Relative Expression
Zt (hr)
21.5
0.5
3.5
6.5
9.5
12.5
18.5
0.5
1.0
1.5
2.0

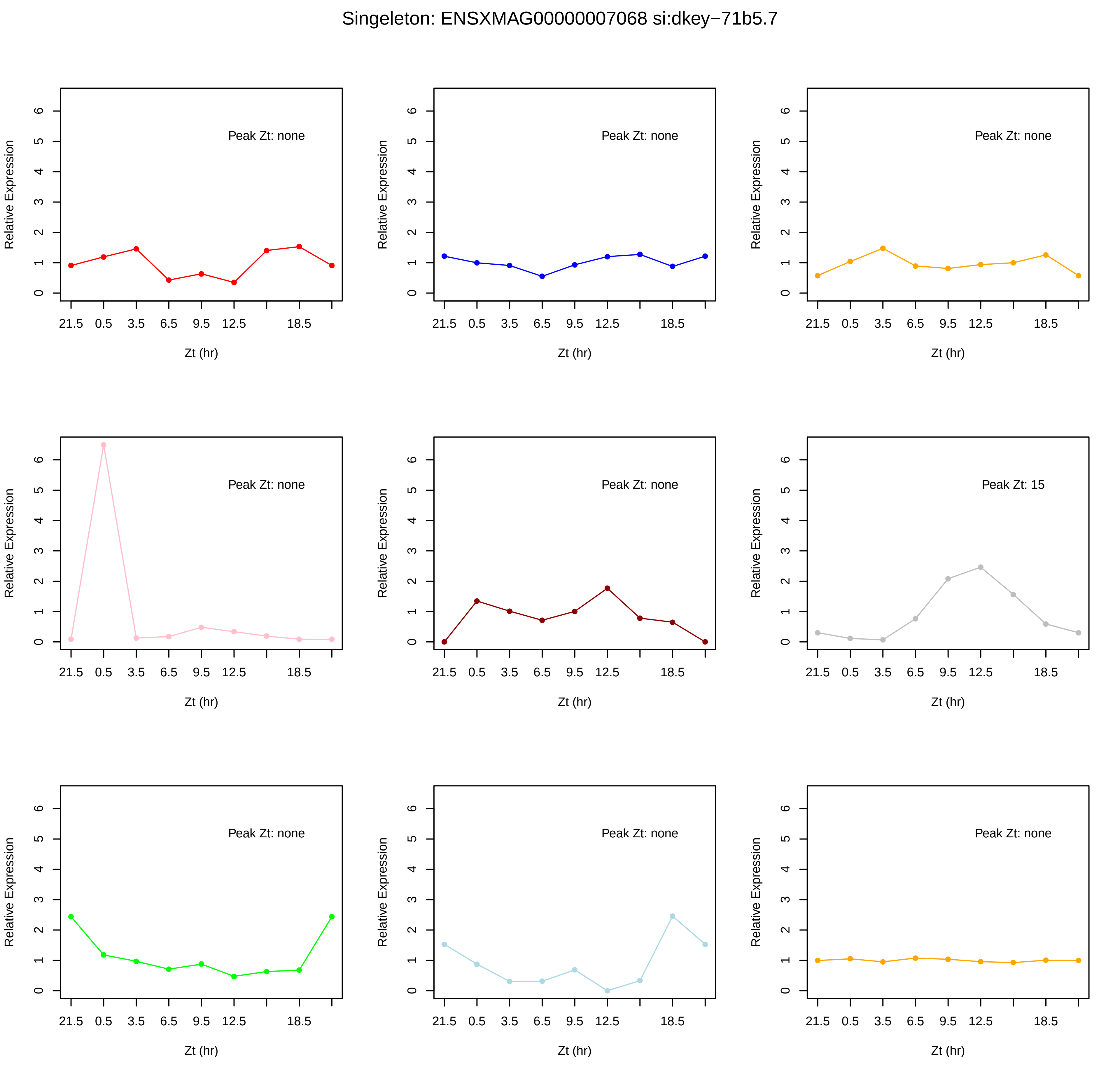
Singeleton: ENSXMAG00000007068 si:dkey−71b5.7
Peak Zt: none
Peak Zt: none
Peak Zt: none
Peak Zt: none
Peak Zt: none
Peak Zt: 15
Peak Zt: none
Peak Zt: none
Peak Zt: none
Relative Expression
Zt (hr)
21.5 0.5 3.5 6.5 9.5 12.5 18.5

Singeleton: ENSXMAG00000010603 hephl1b

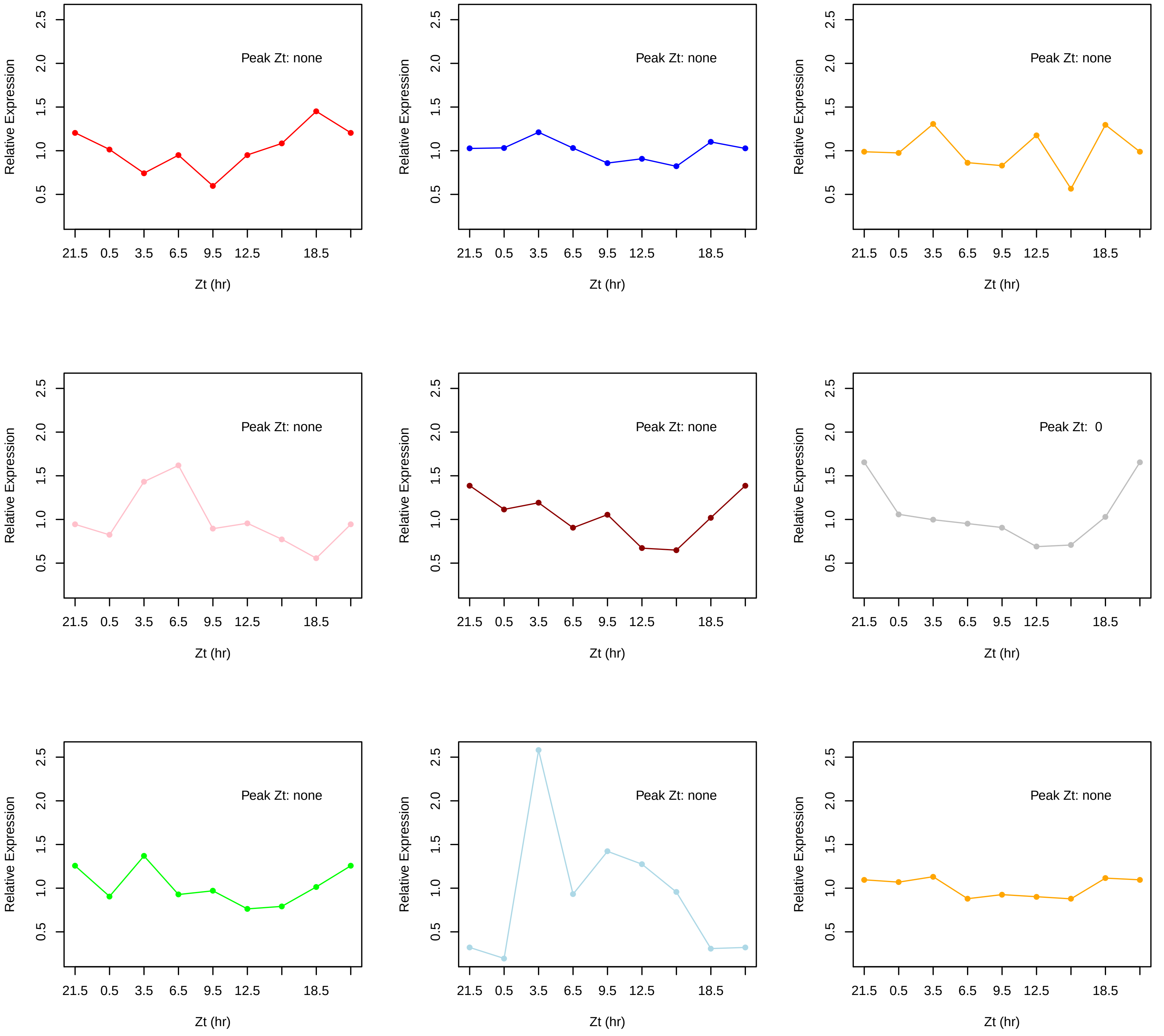

Singeleton: ENSXMAG00000018075 ascc2

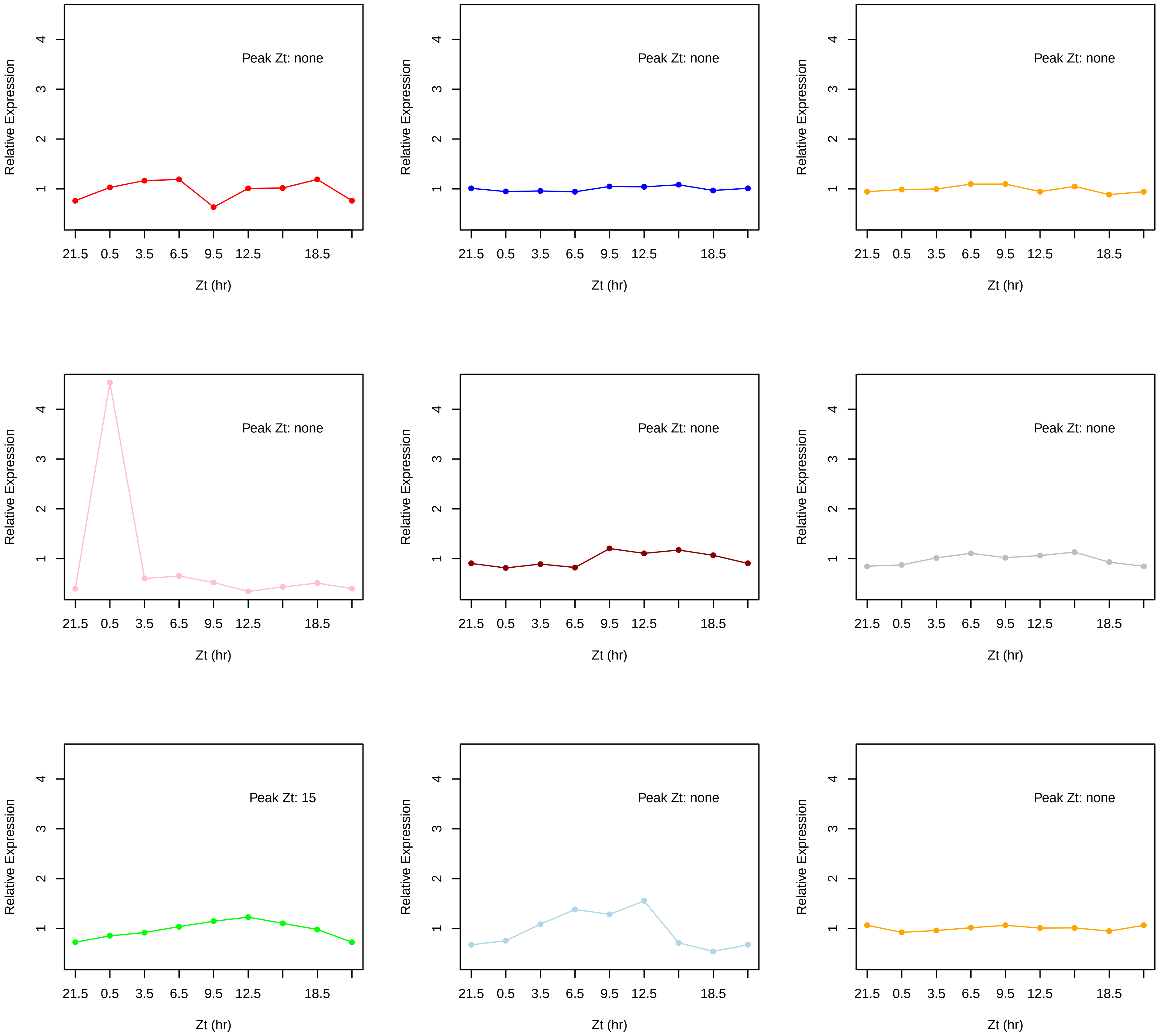

Singleton: ENSXMAG00000027907 si:ch211−212o1.2

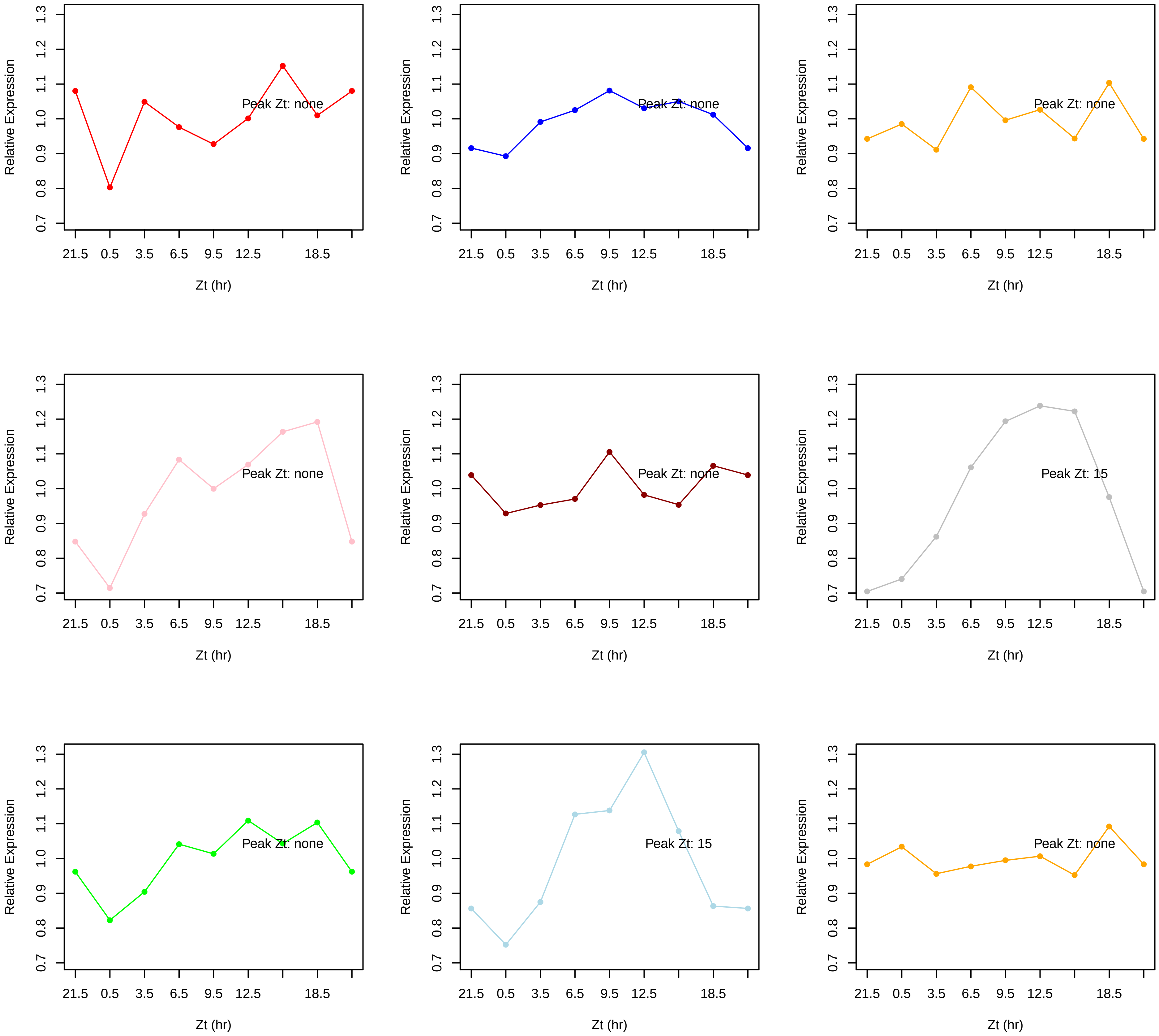

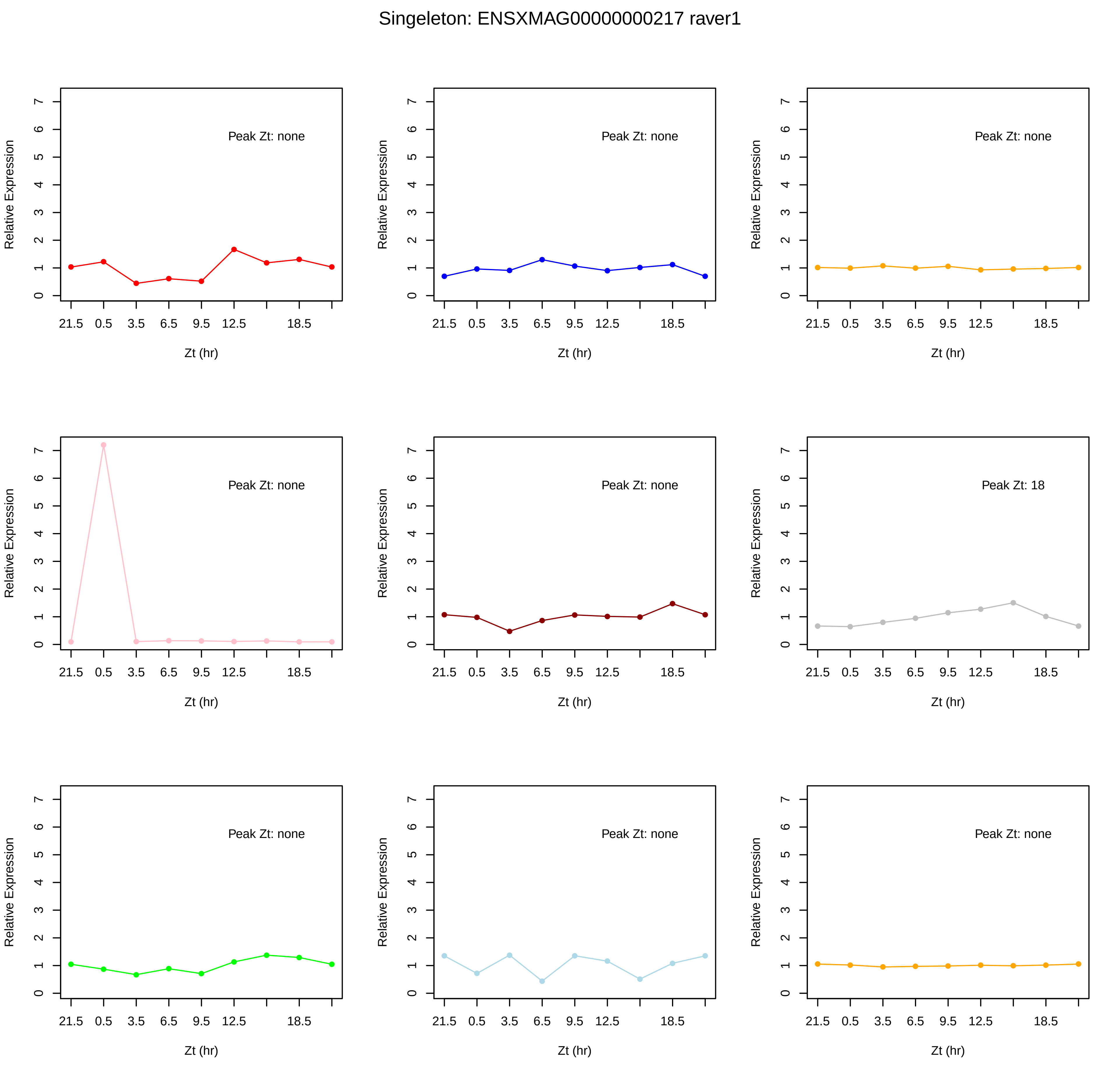
Singleton: ENSXMAG00000000217 raver1
Relative Expression
Zt (hr)
21.5
0.5
3.5
6.5
9.5
12.5
18.5
Peak Zt: none
Peak Zt: 18

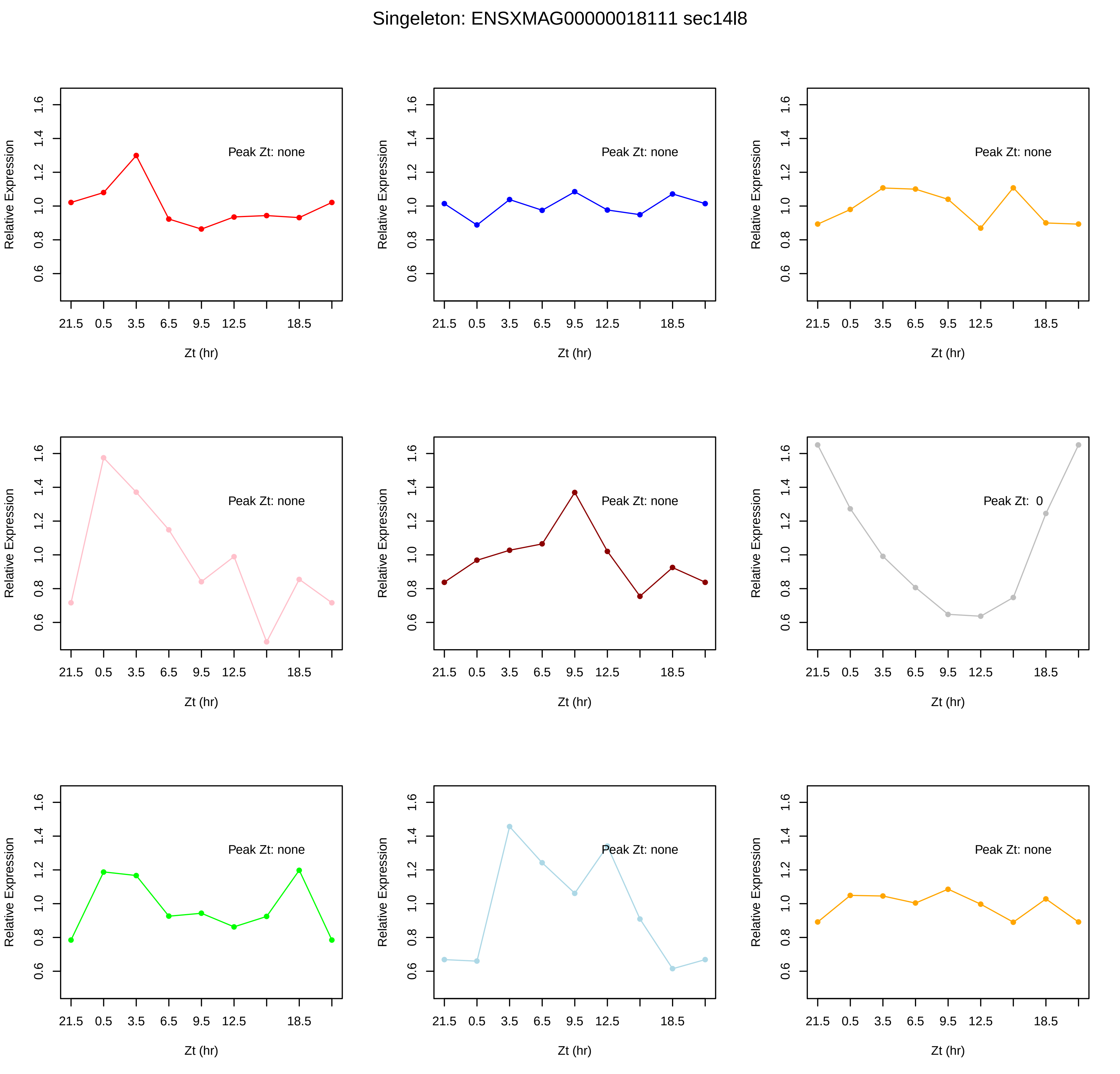

Singeleton: ENSXMAG00000018111 sec14l8
Peak Zt: none
Peak Zt: none
Peak Zt: none
Peak Zt: none
Peak Zt: none
Peak Zt: 0
Peak Zt: none
Peak Zt: none
Peak Zt: none
Relative Expression
Zt (hr)
21.5
0.5
3.5
6.5
9.5
12.5
18.5
0.6
0.8
1.0
1.2
1.4
1.6

Singleton: ENSXMAG00000002022 ppef1

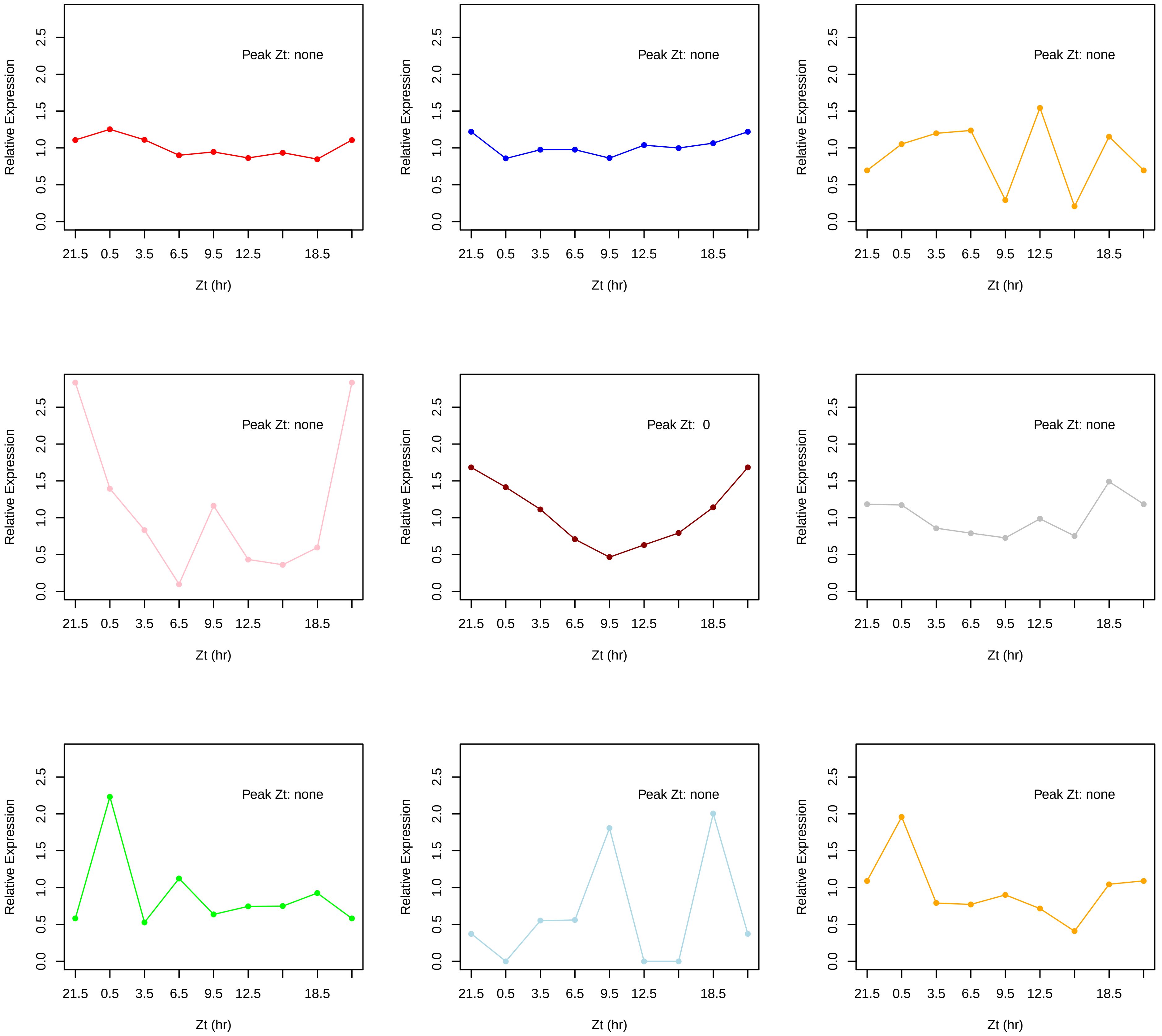

Singeleton: ENSXMAG00000007167

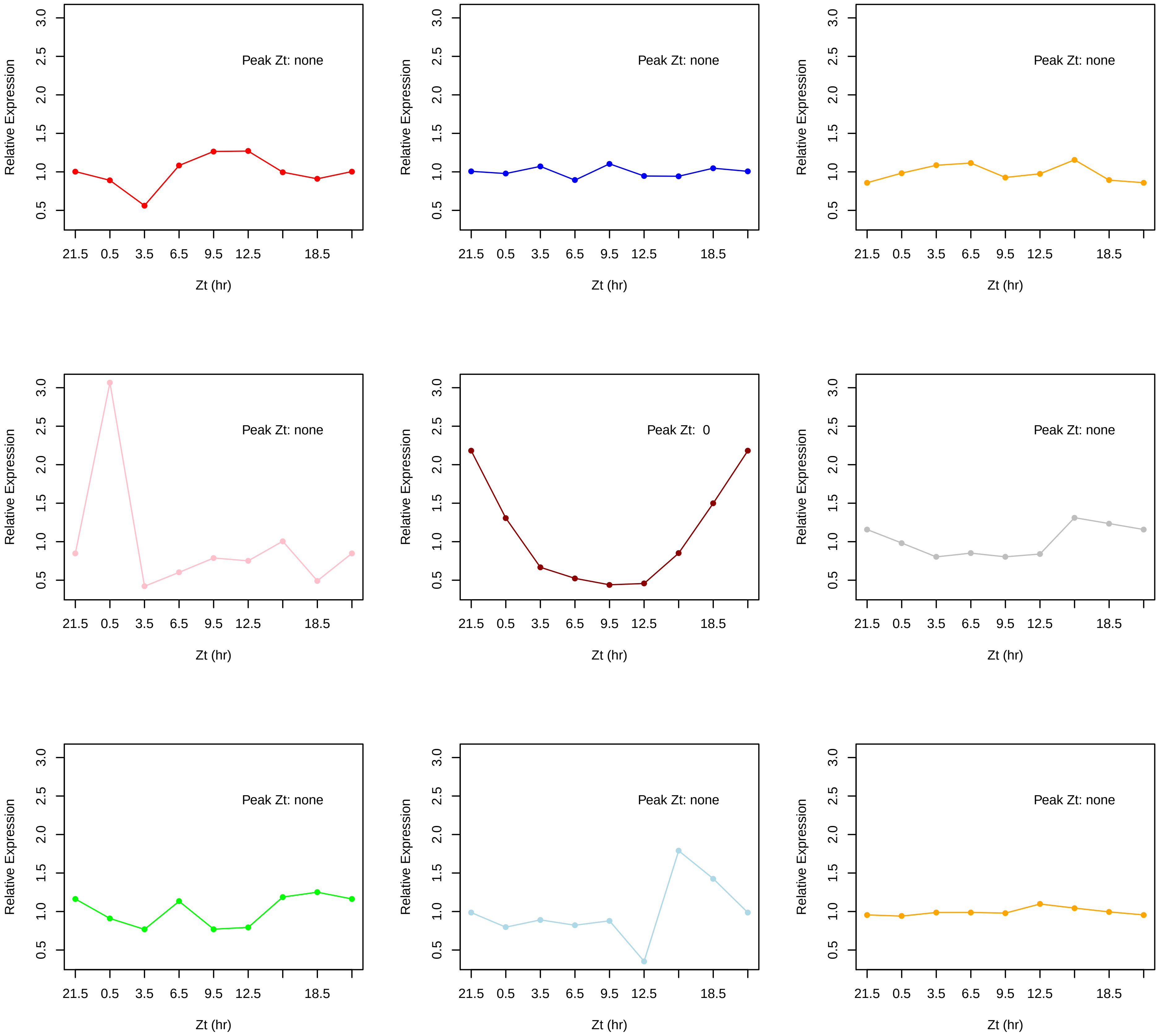

Singeleton: ENSXMAG00000022295 cbln1

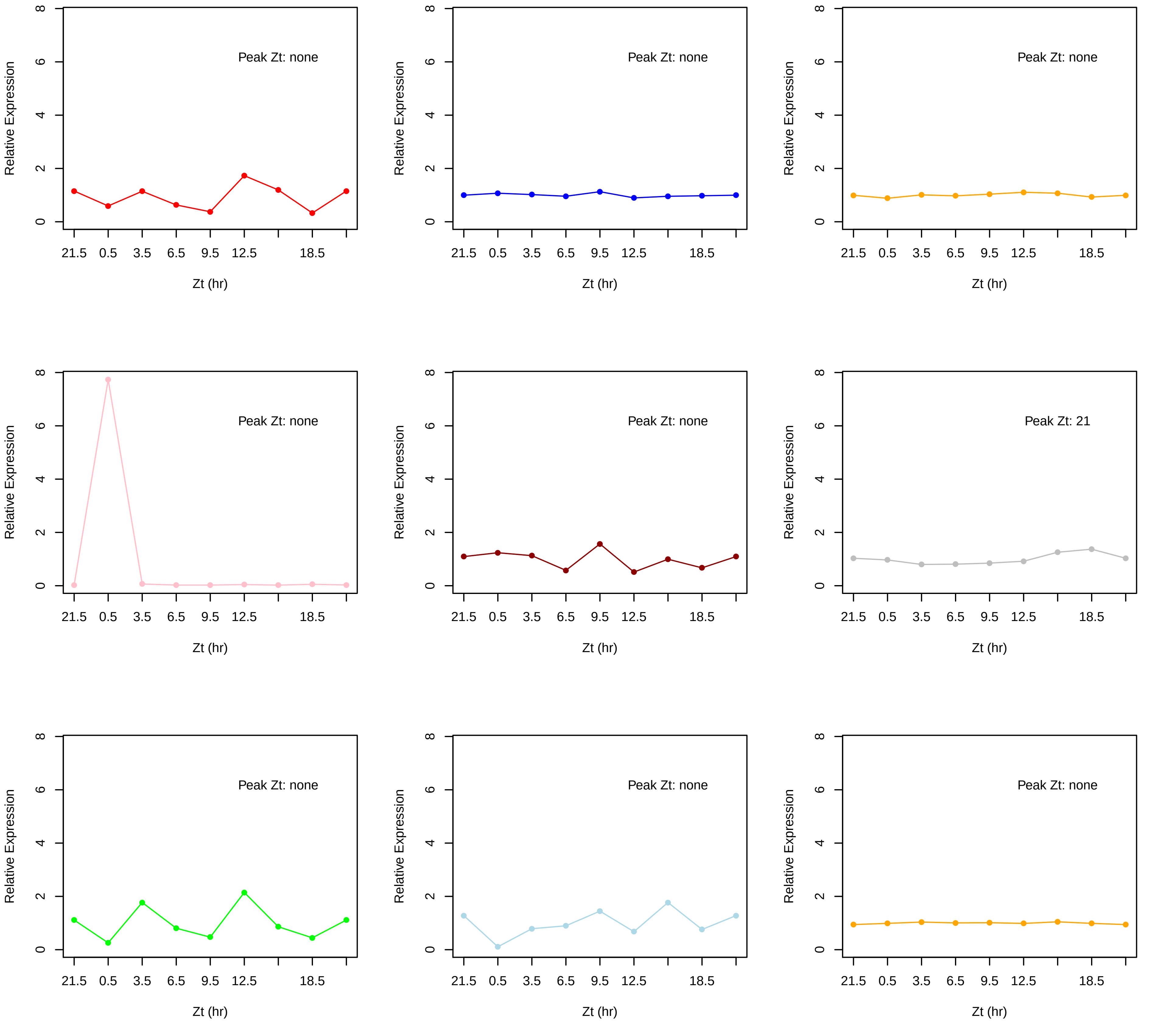

Singeleton: ENSXMAG00000004003 mre11a

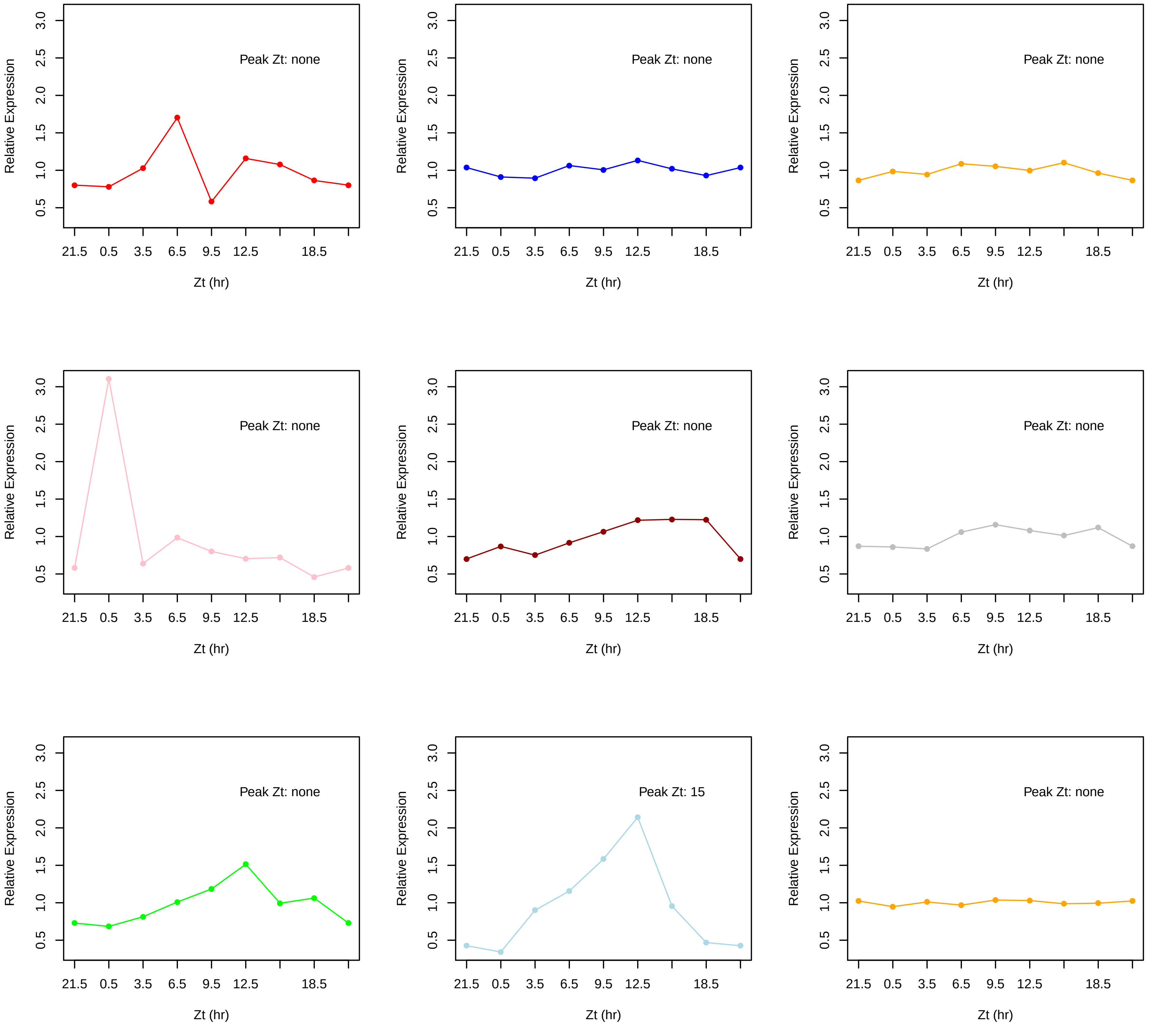

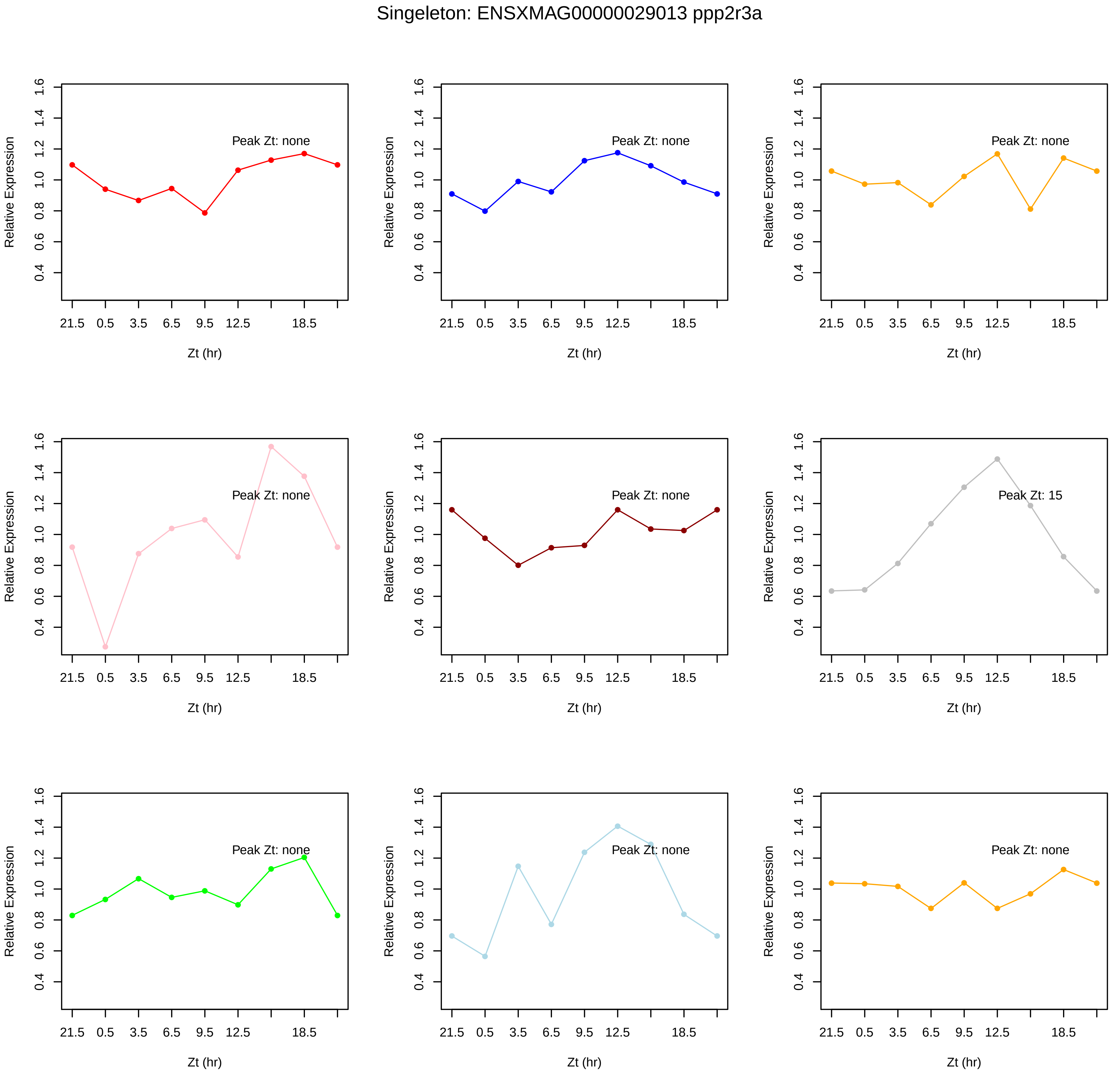
Singeleton: ENSXMAG00000029013 ppp2r3a
Peak Zt: none
Peak Zt: none
Peak Zt: none
Peak Zt: none
Peak Zt: none
Peak Zt: 15
Peak Zt: none
Peak Zt: none
Peak Zt: none
Relative Expression
Zt (hr)
21.5 0.5 3.5 6.5 9.5 12.5 18.5
0.4 0.6 0.8 1.0 1.2 1.4 1.6

Singeleton: ENSXMAG00000027324 c1galt1a

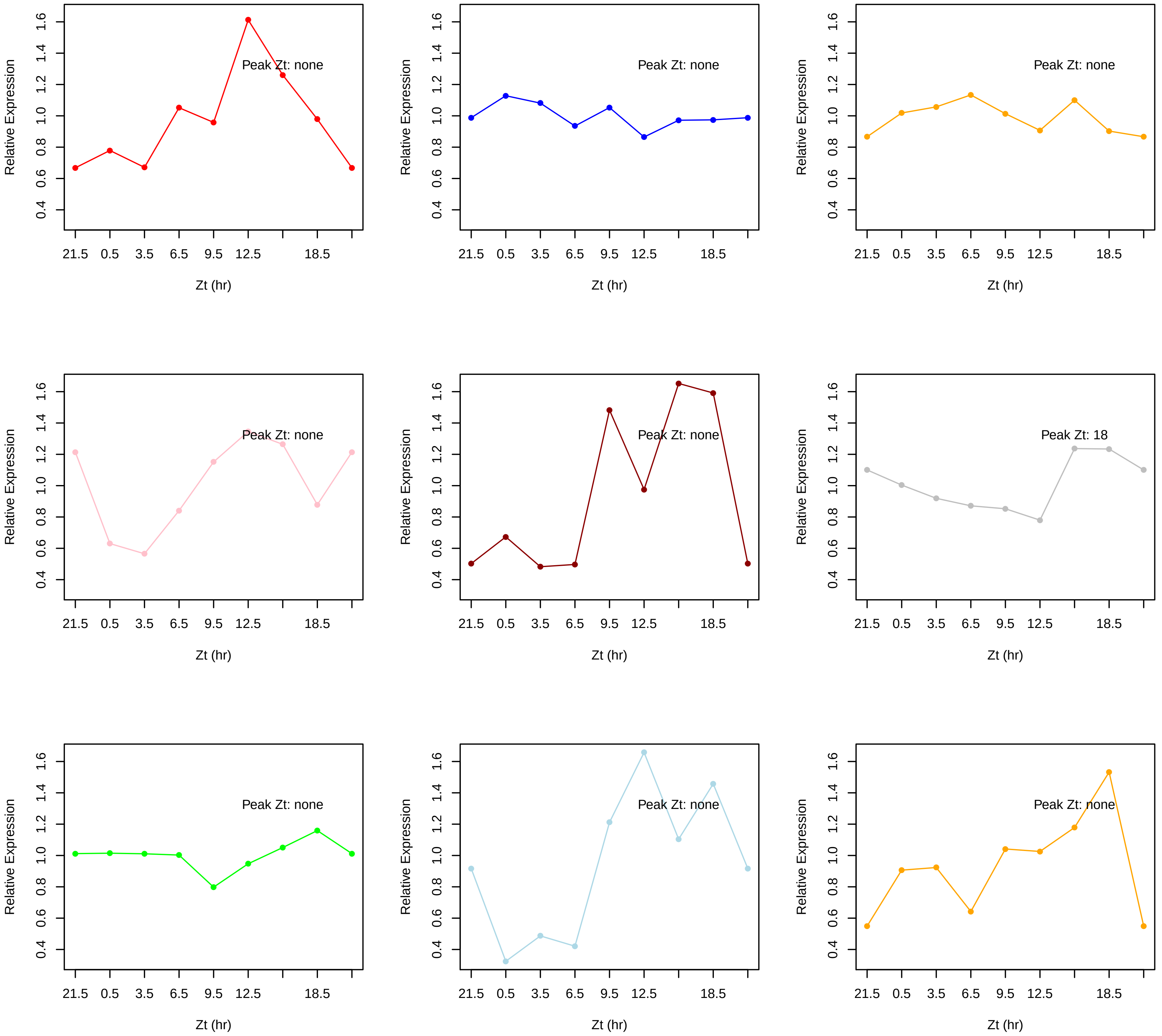

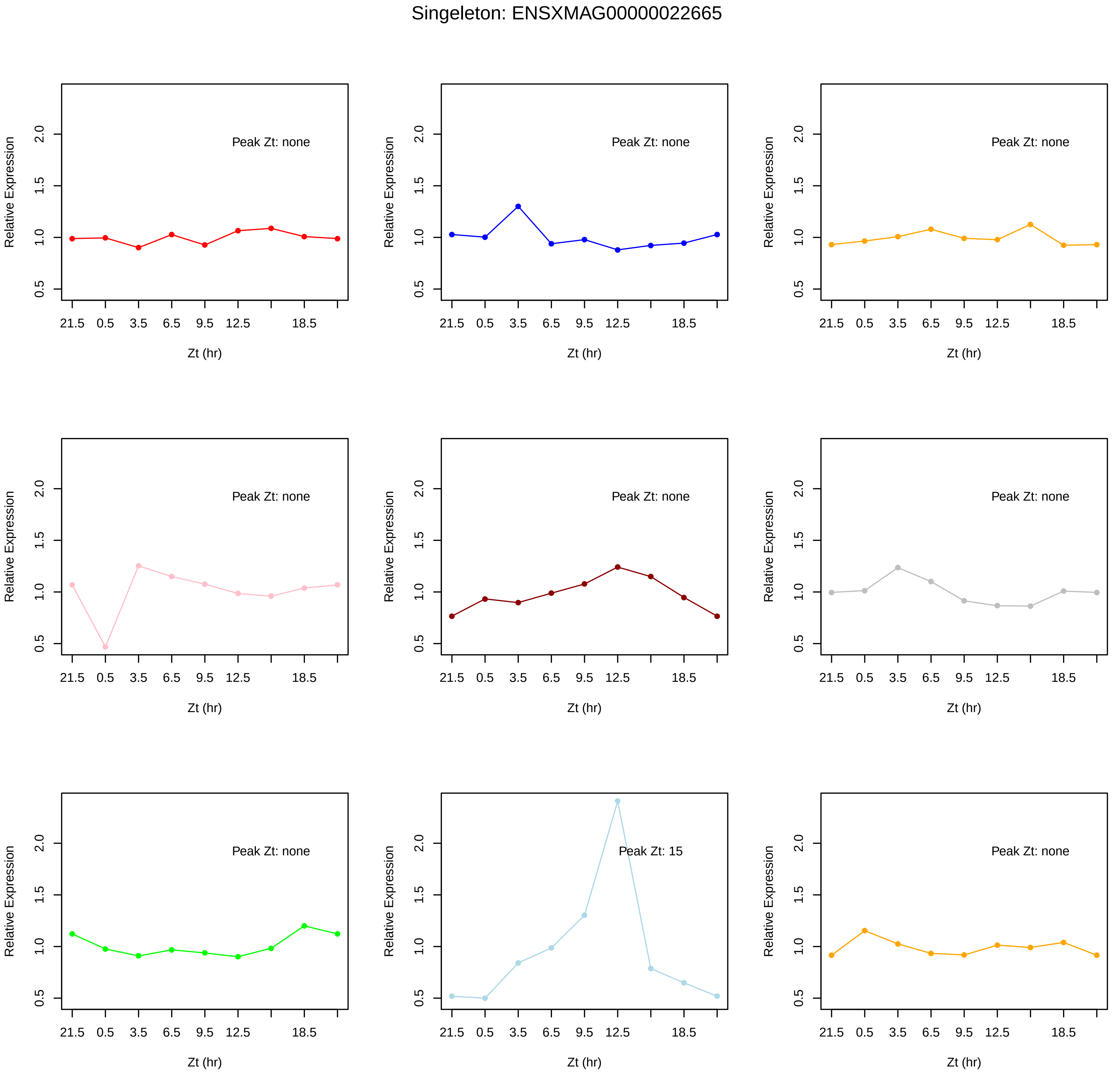
Singeleton: ENSXMAG00000022665
Peak Zt: none
Peak Zt: none
Peak Zt: none
Peak Zt: none
Peak Zt: none
Peak Zt: none
Peak Zt: none
Peak Zt: 15
Peak Zt: none
Relative Expression
Zt (hr)
21.5 0.5 3.5 6.5 9.5 12.5 18.5
0.5 1.0 1.5 2.0

Singeleton: ENSXMAG00000007074 atp8b2

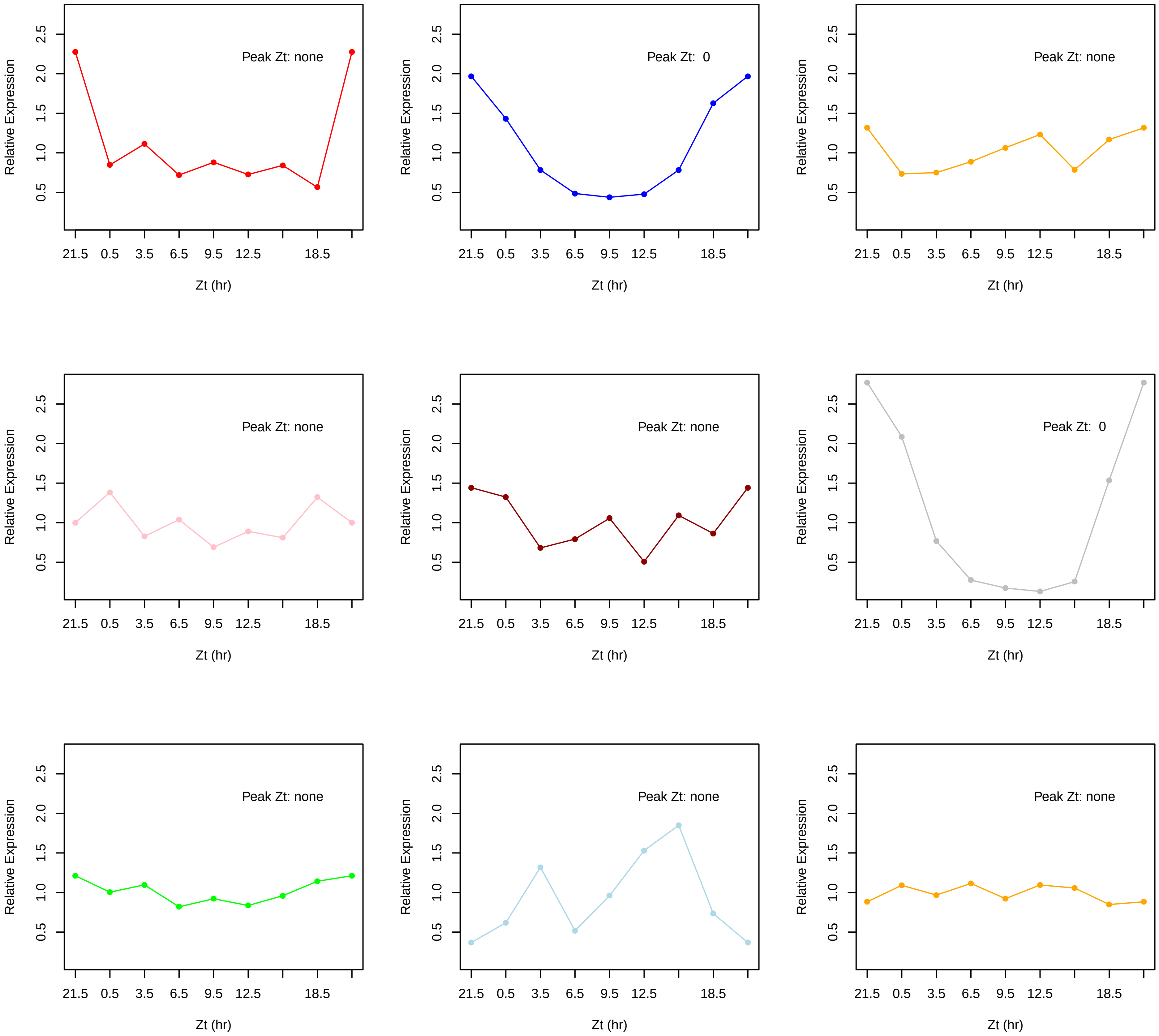

Singeleton: ENSXMAG00000005213 ppp1r9ala

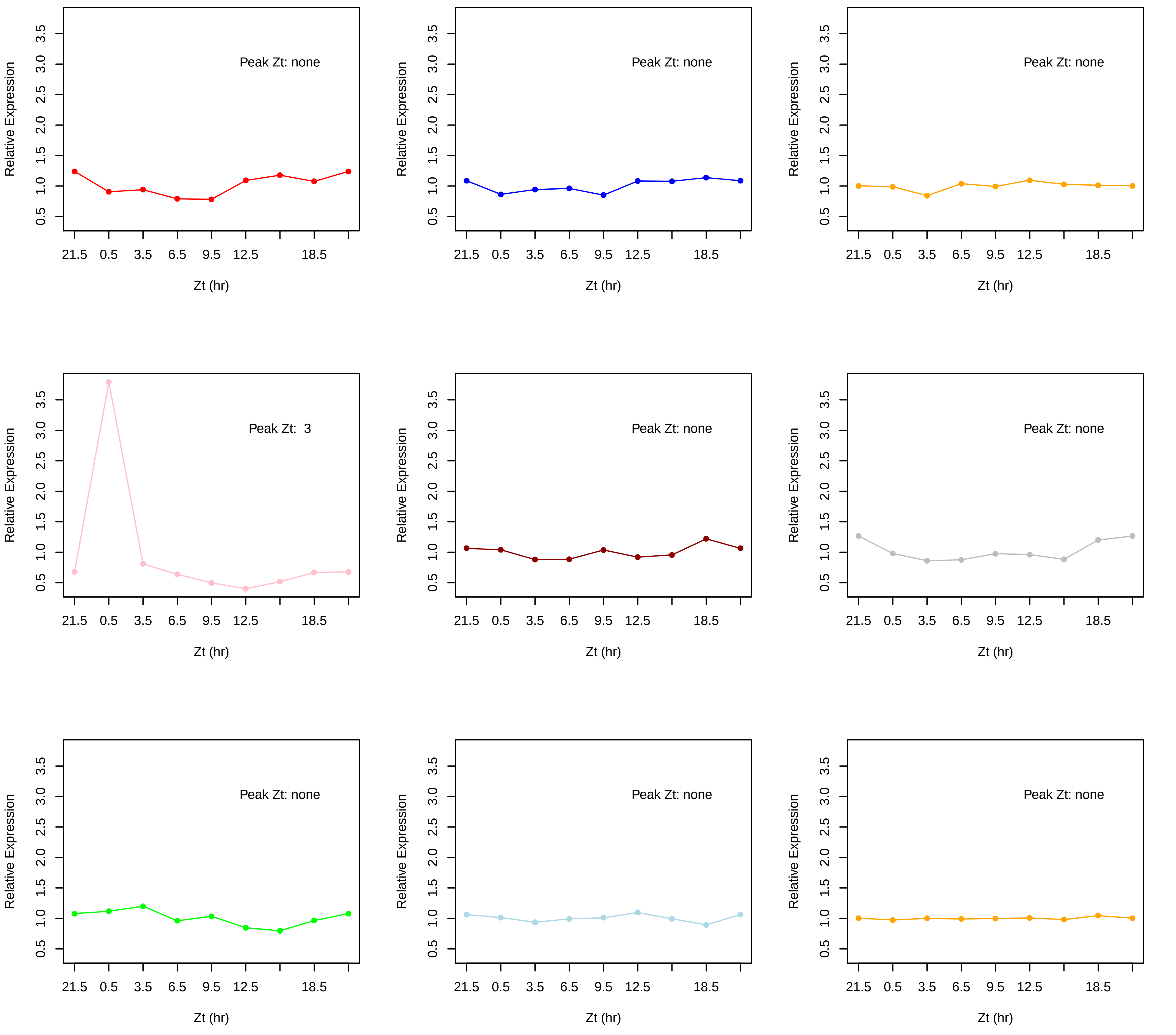

Singeleton: ENSXMAG00000000220 tyk2

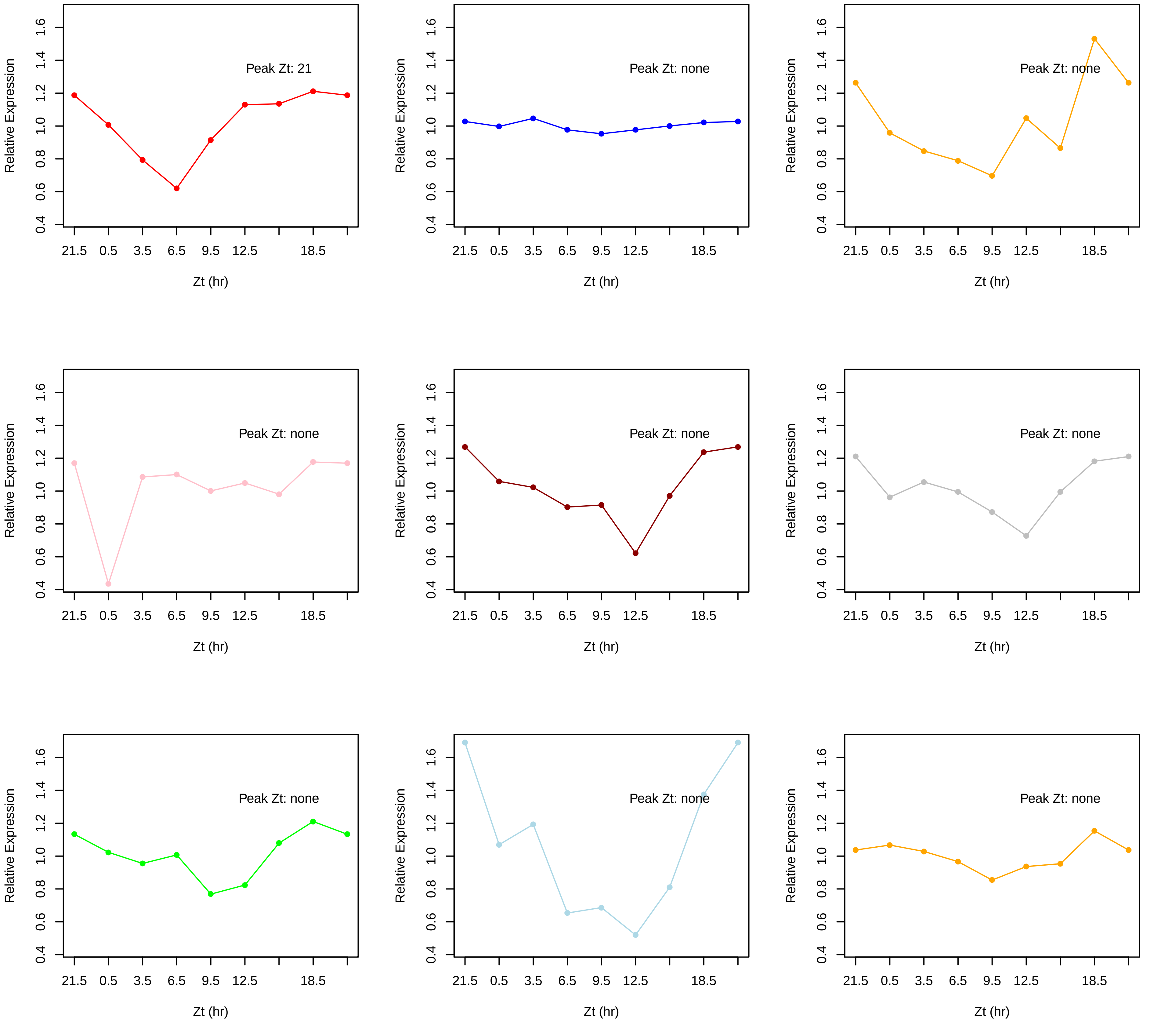

Singeleton: ENSXMAG00000002036 os9

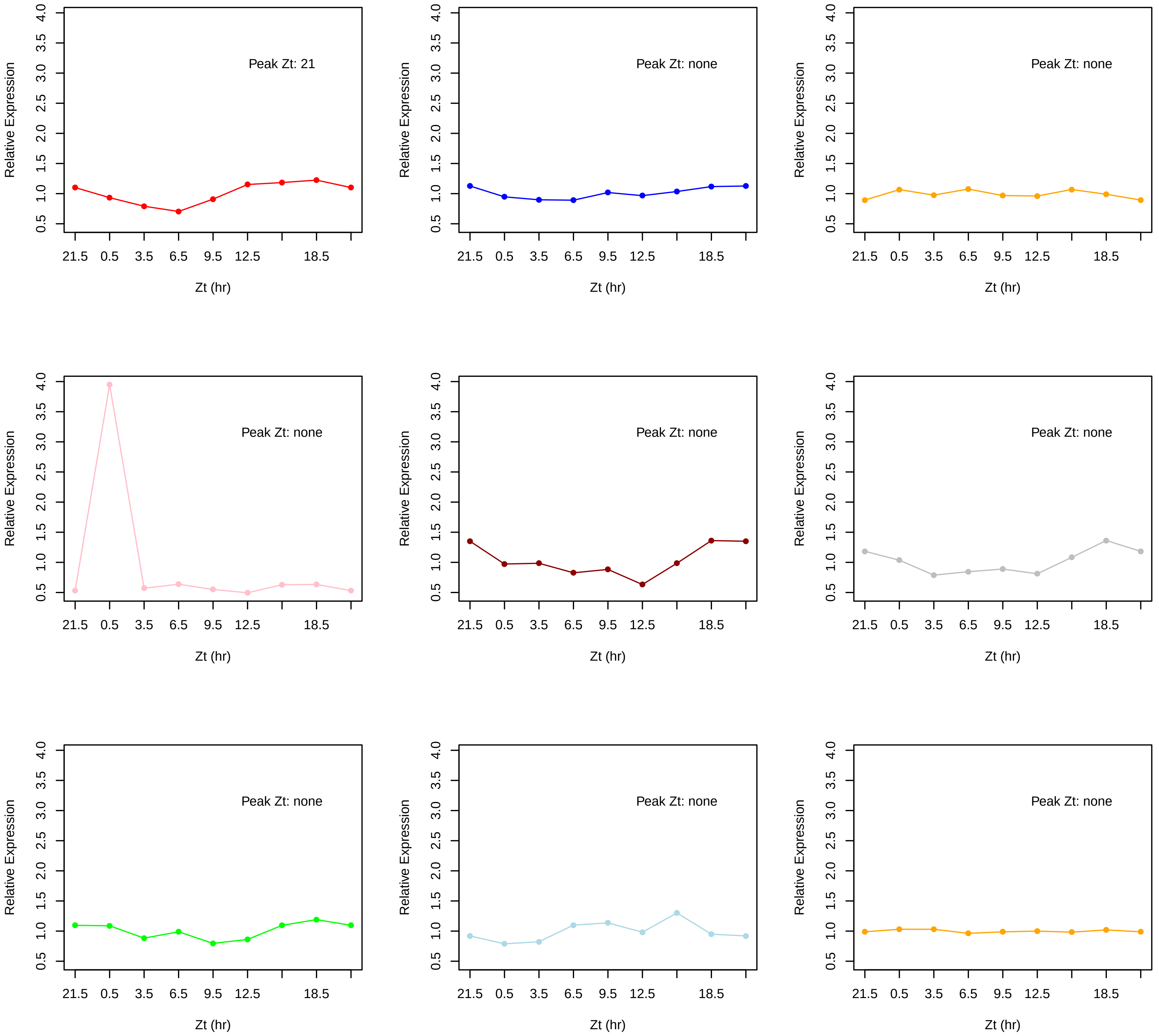

Singeleton: ENSXMAG00000009888 ankrd49

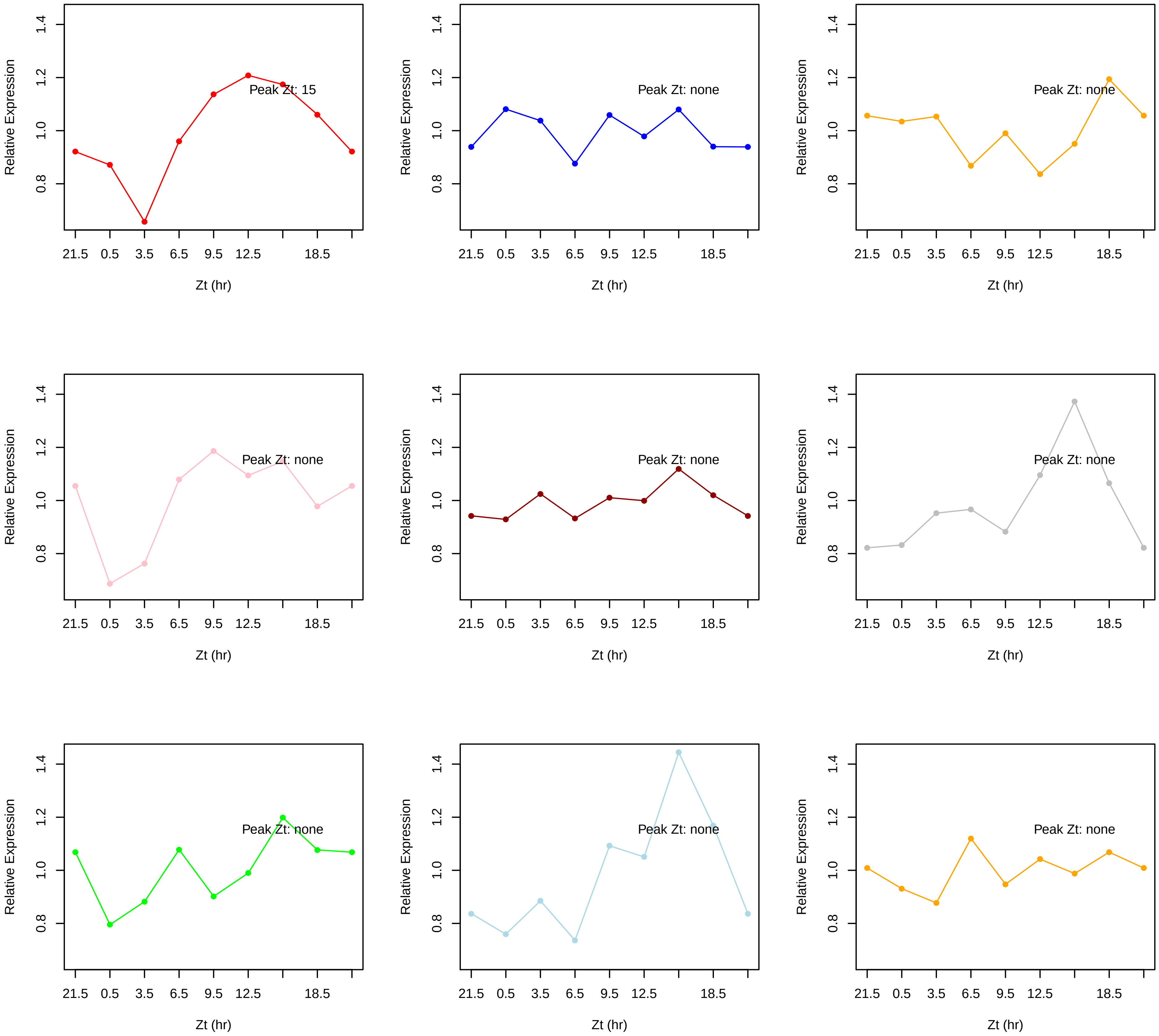

Singeleton: ENSXMAG00000009136 tomm34

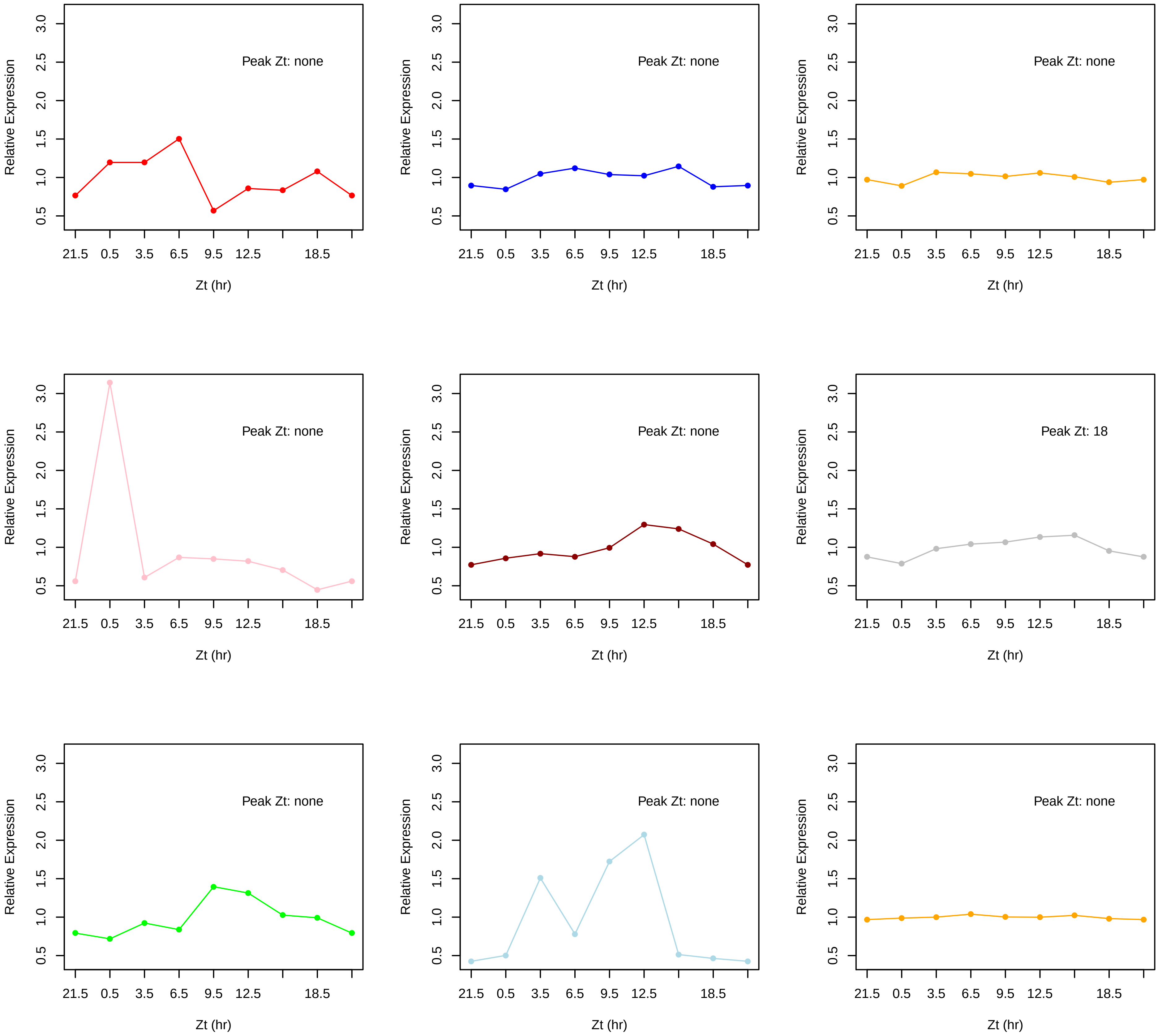

Singleton: ENSXMAG00000005122 itga6a

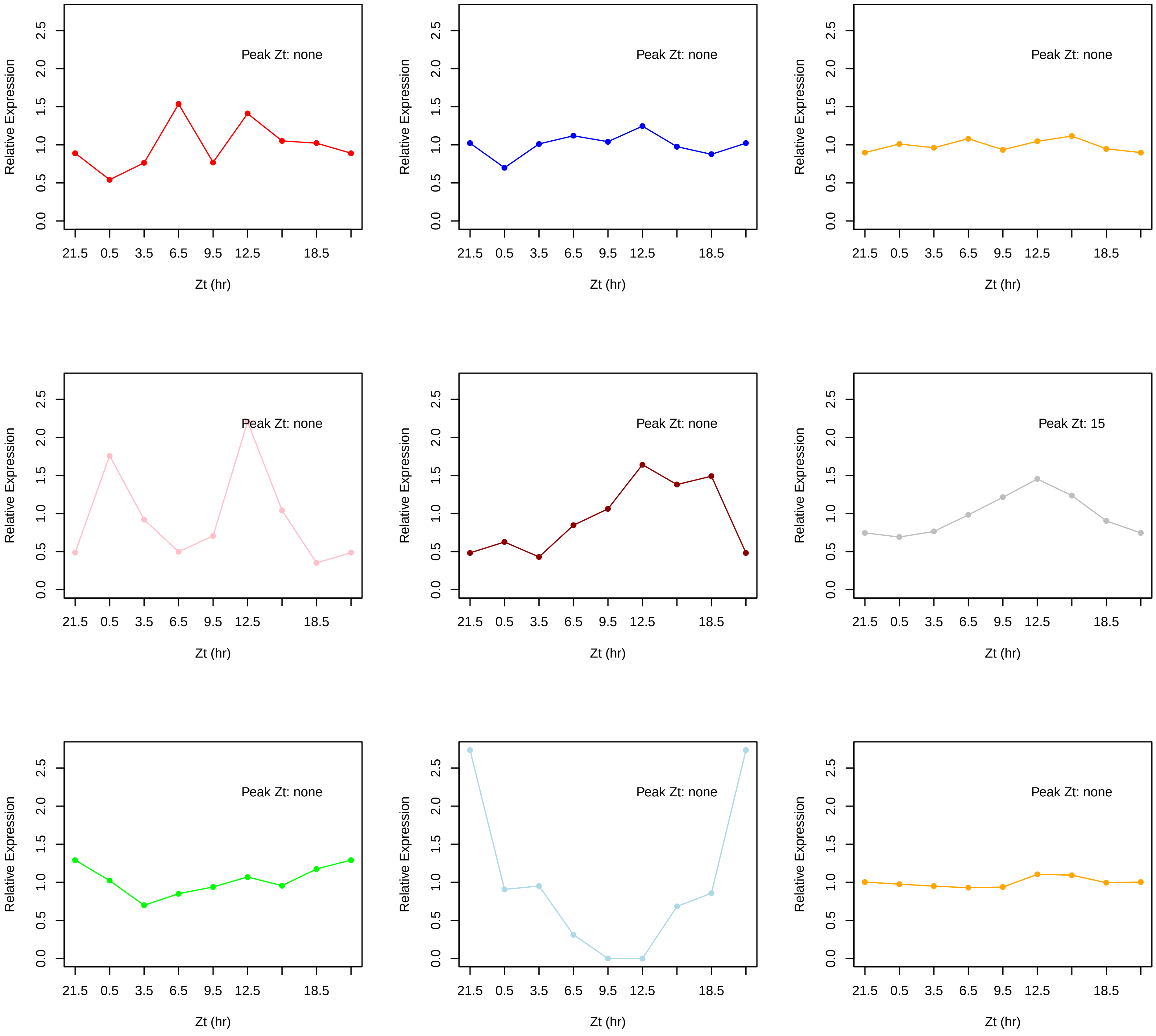

Singeleton: ENSXMAG00000002440 dennd1b

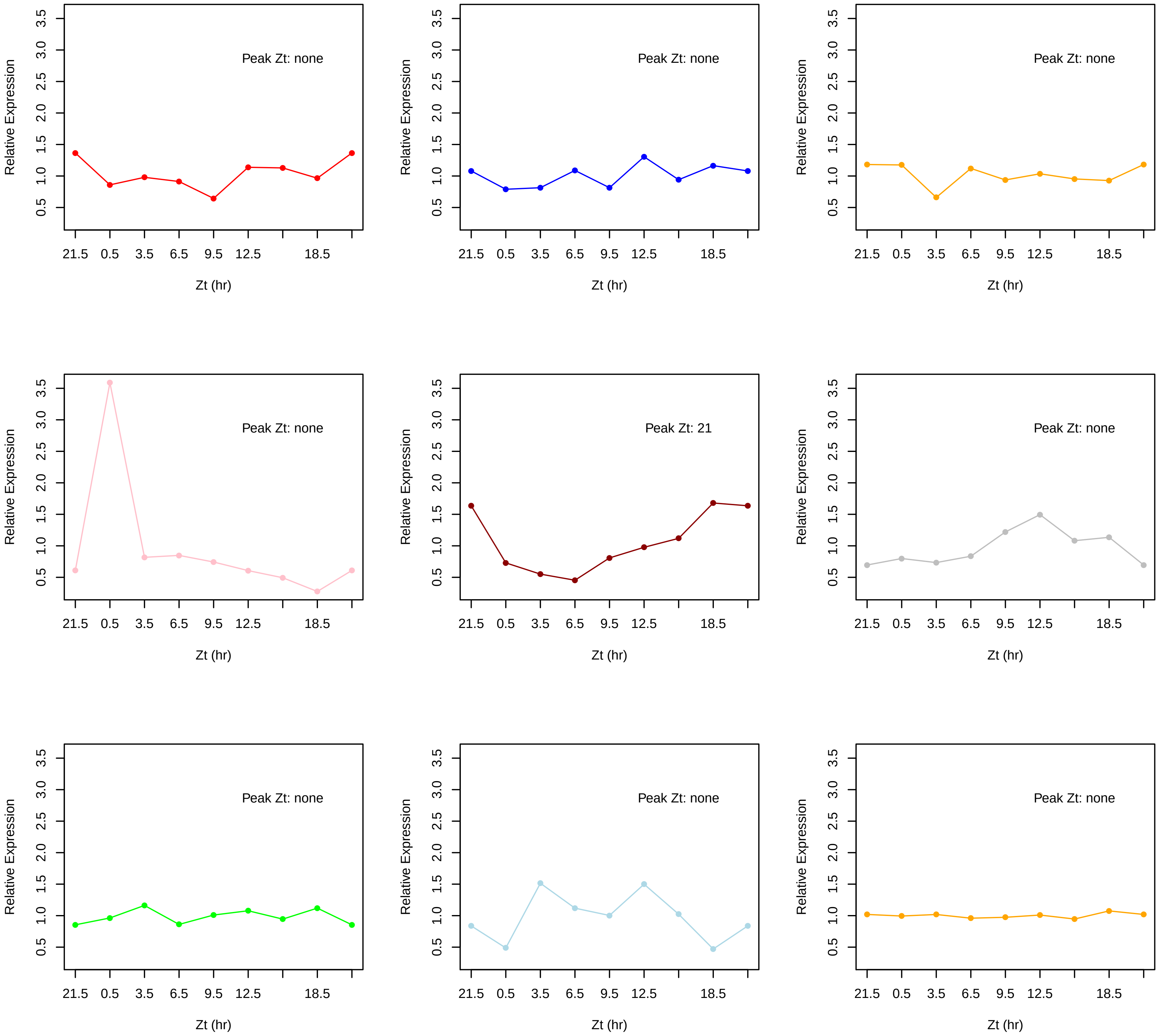

Singeleton: ENSXMAG00000009959 slc26a10

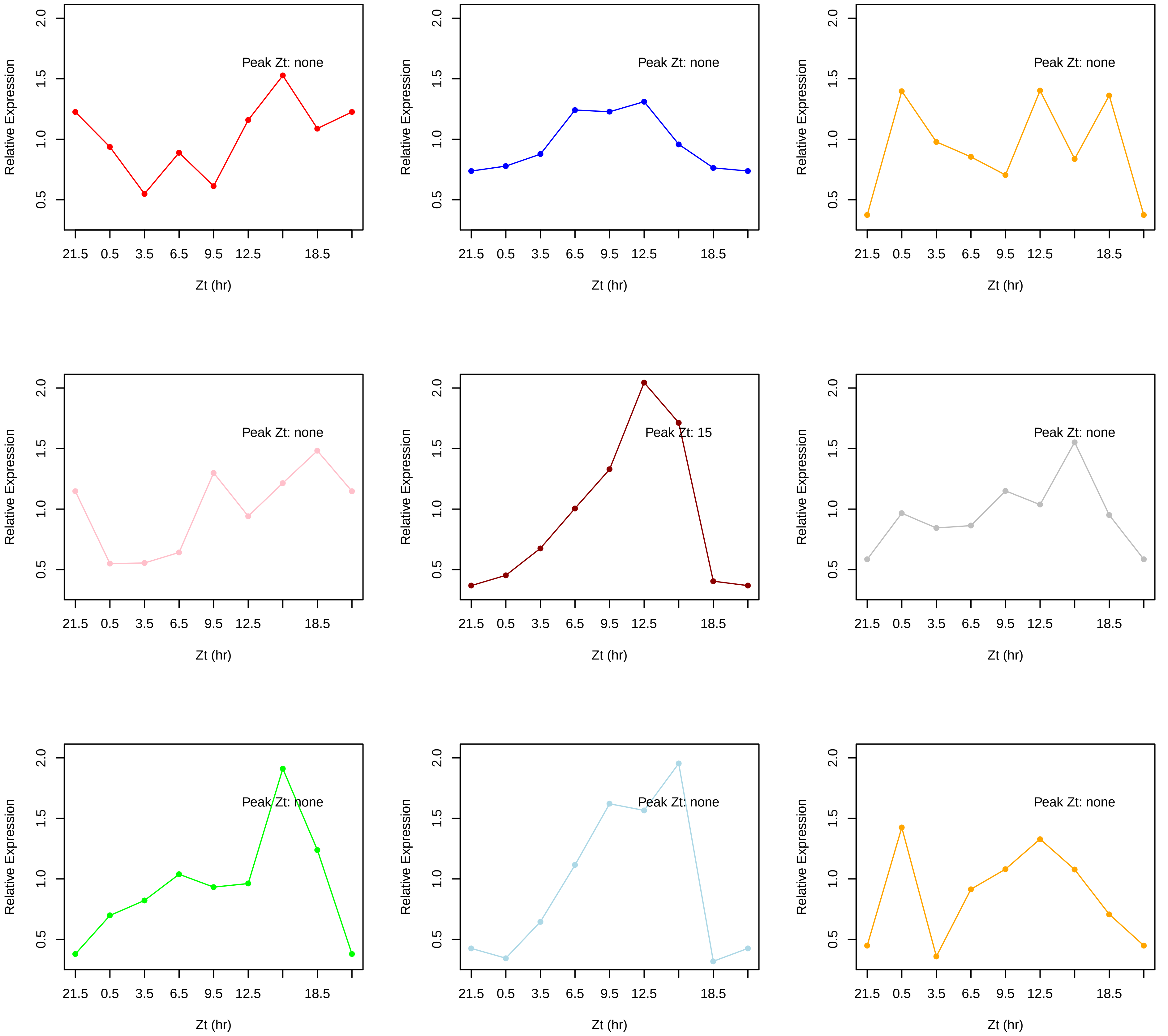

Singleton: ENSXMAG00000000225 capzb

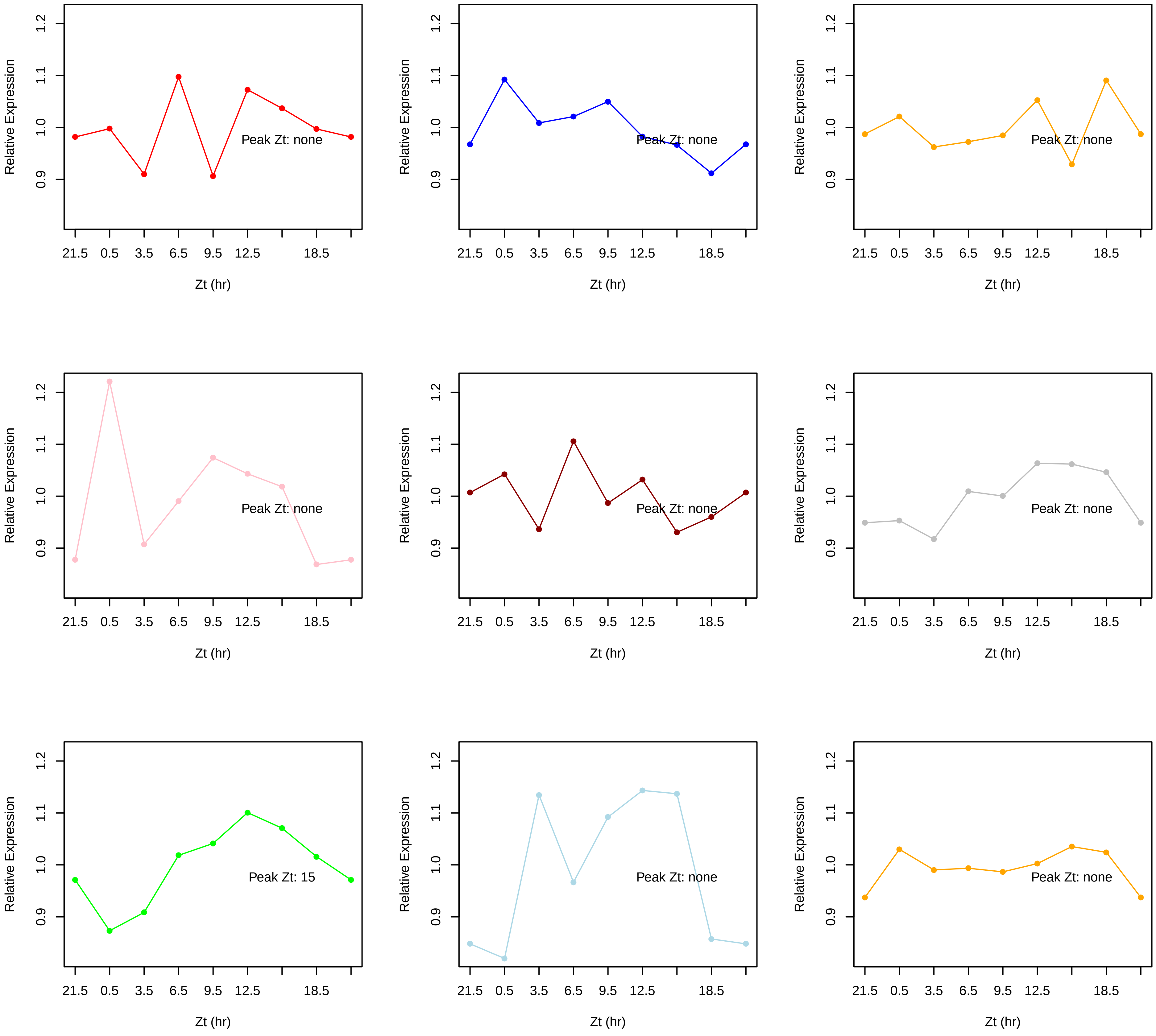

Singeleton: ENSXMAG00000009241 stag1b

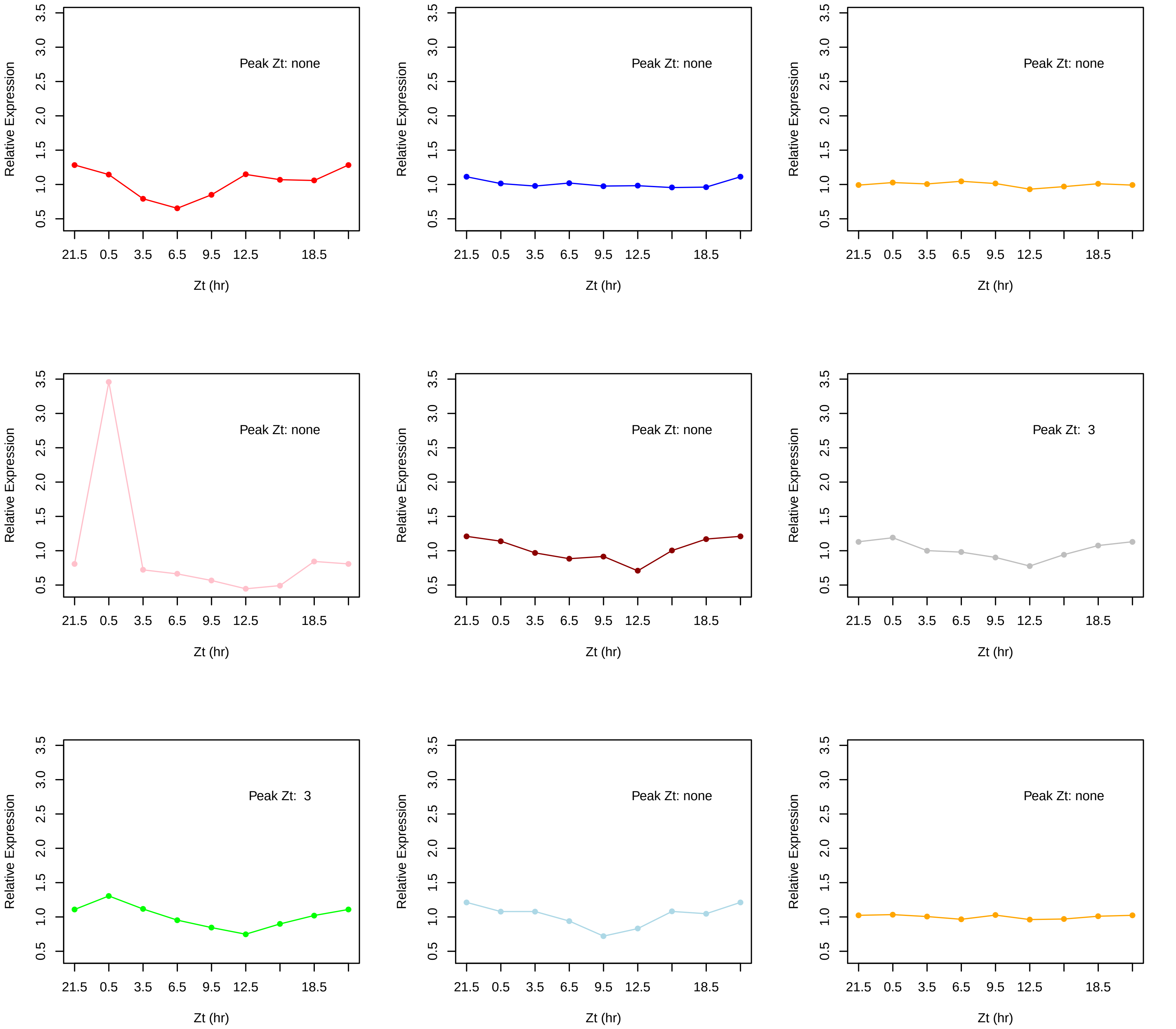

Singleton: ENSXMAG00000011472 cwc15

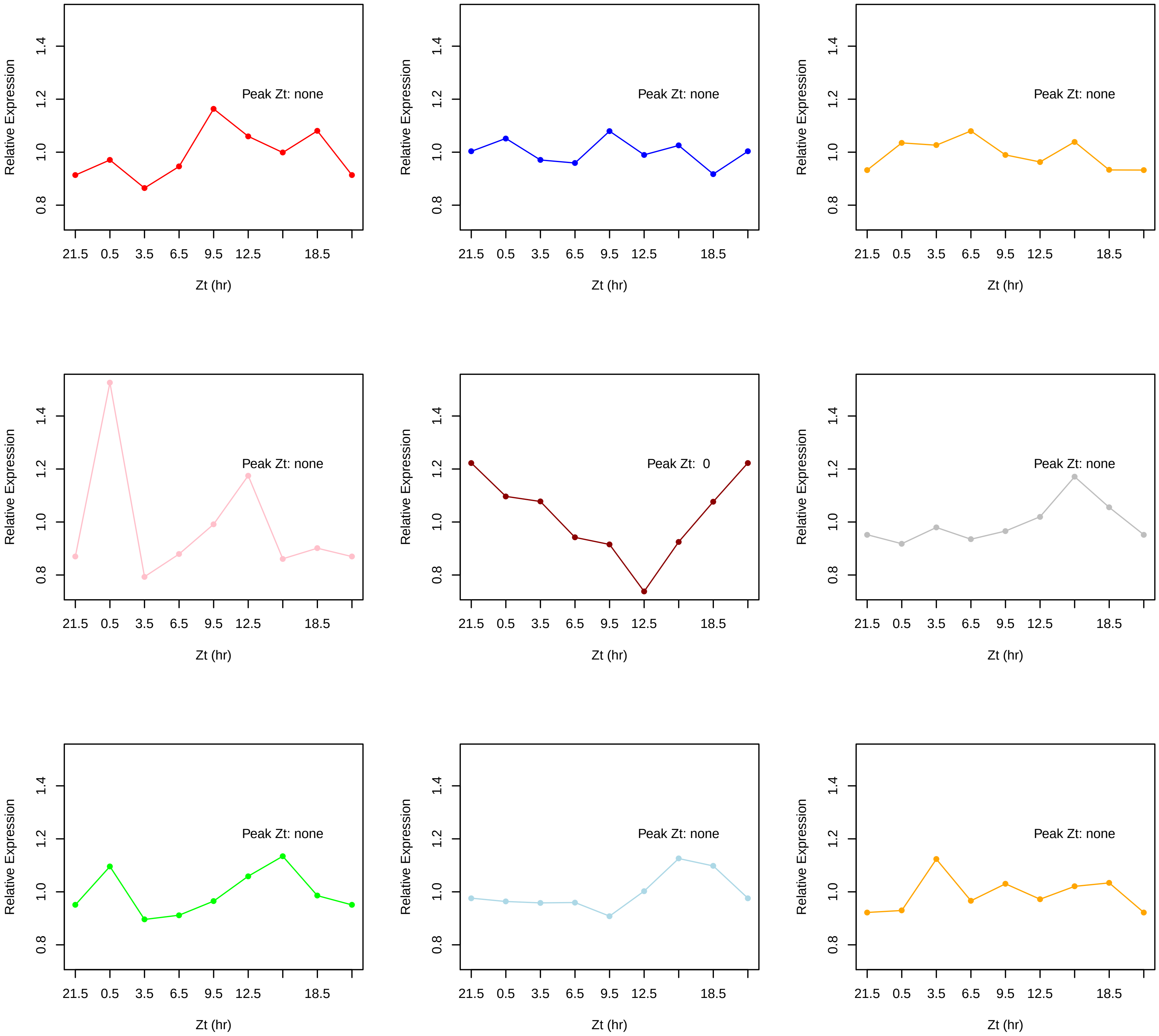

Singleton: ENSXMAG00000005396

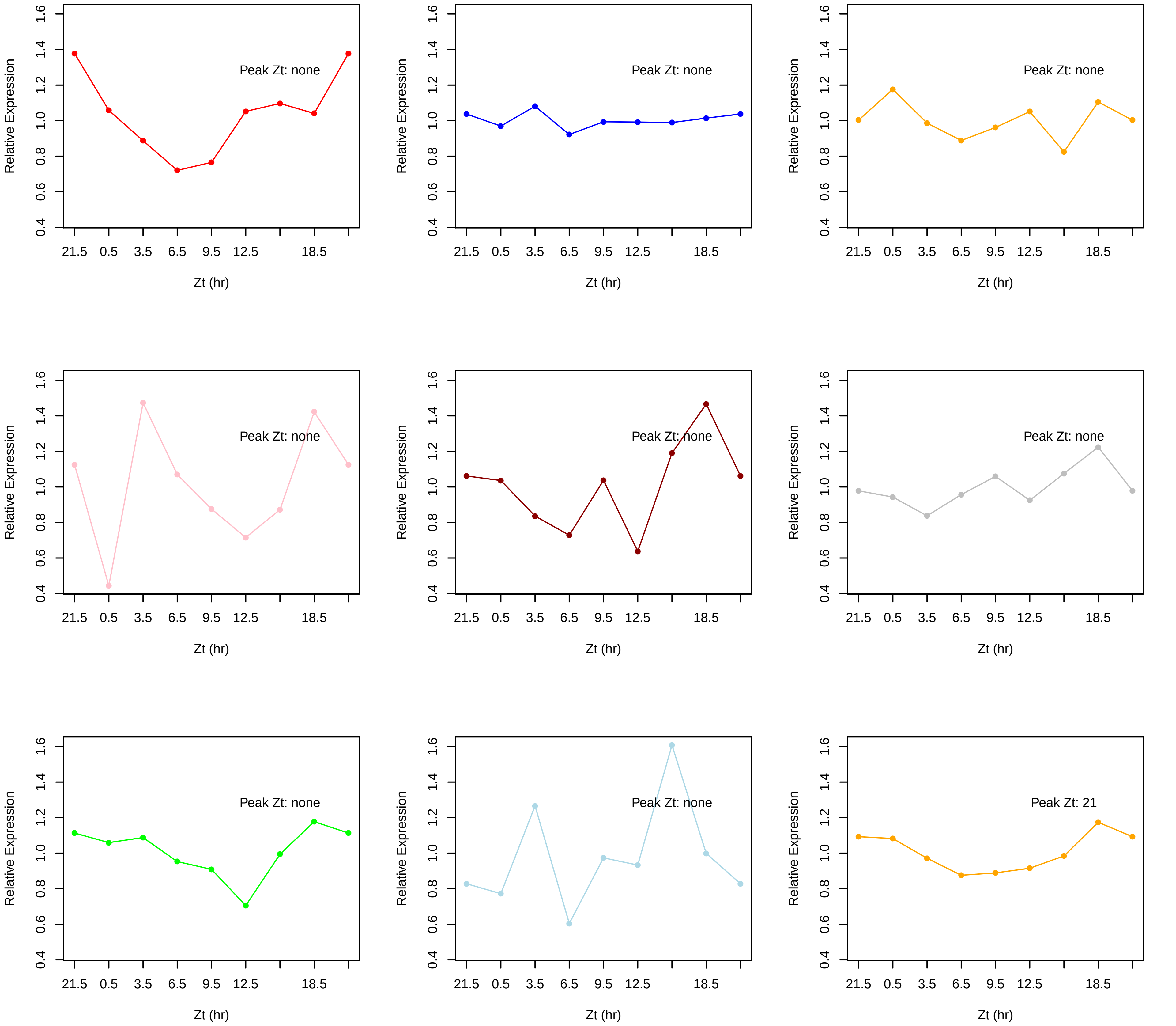

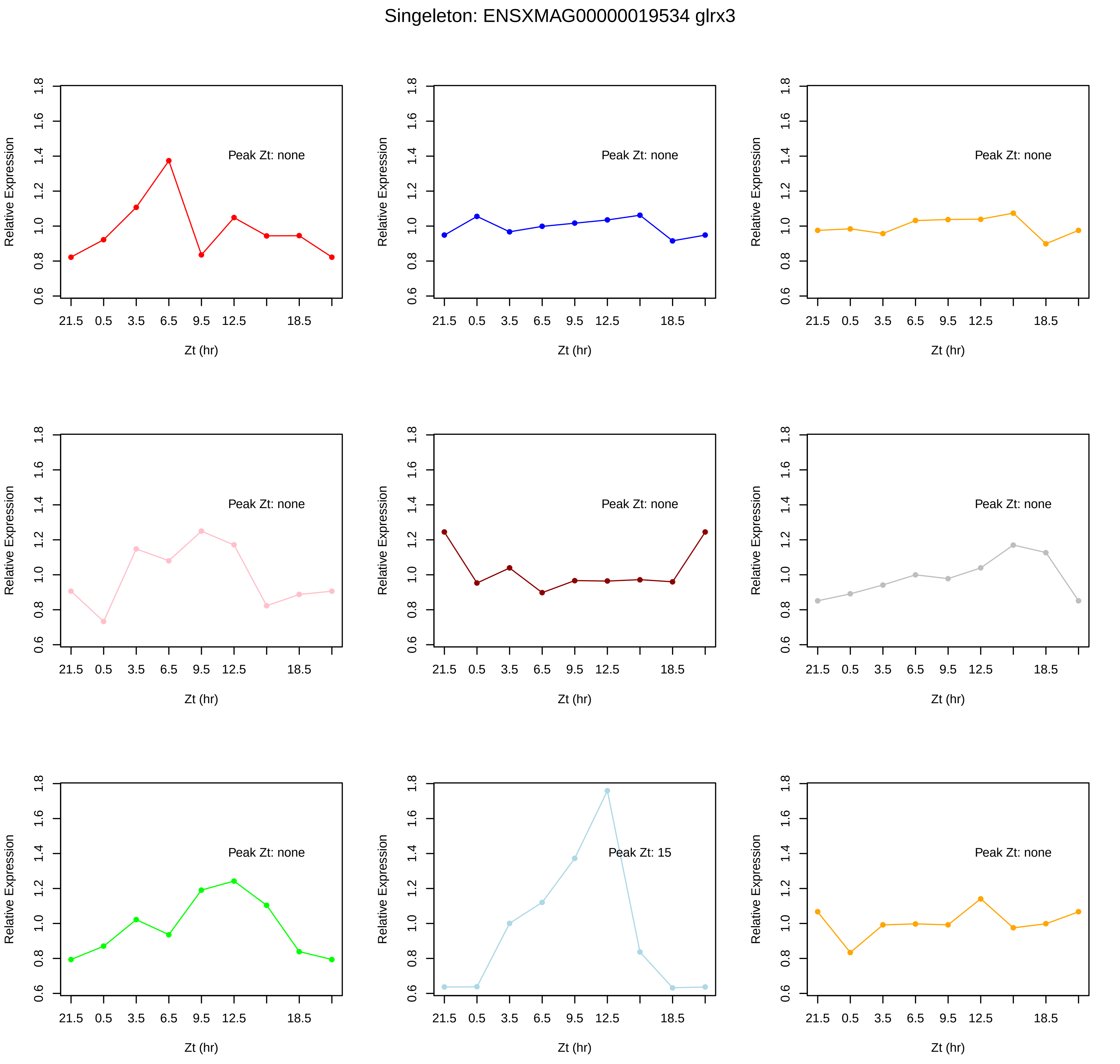
Singeleton: ENSXMAG00000019534 glrx3
Relative Expression
Zt (hr)
Peak Zt: none
Peak Zt: 15
0.6
0.8
1.0
1.2
1.4
1.6
1.8
21.5
0.5
3.5
6.5
9.5
12.5
18.5

Singeleton: ENSXMAG00000014085 cog4

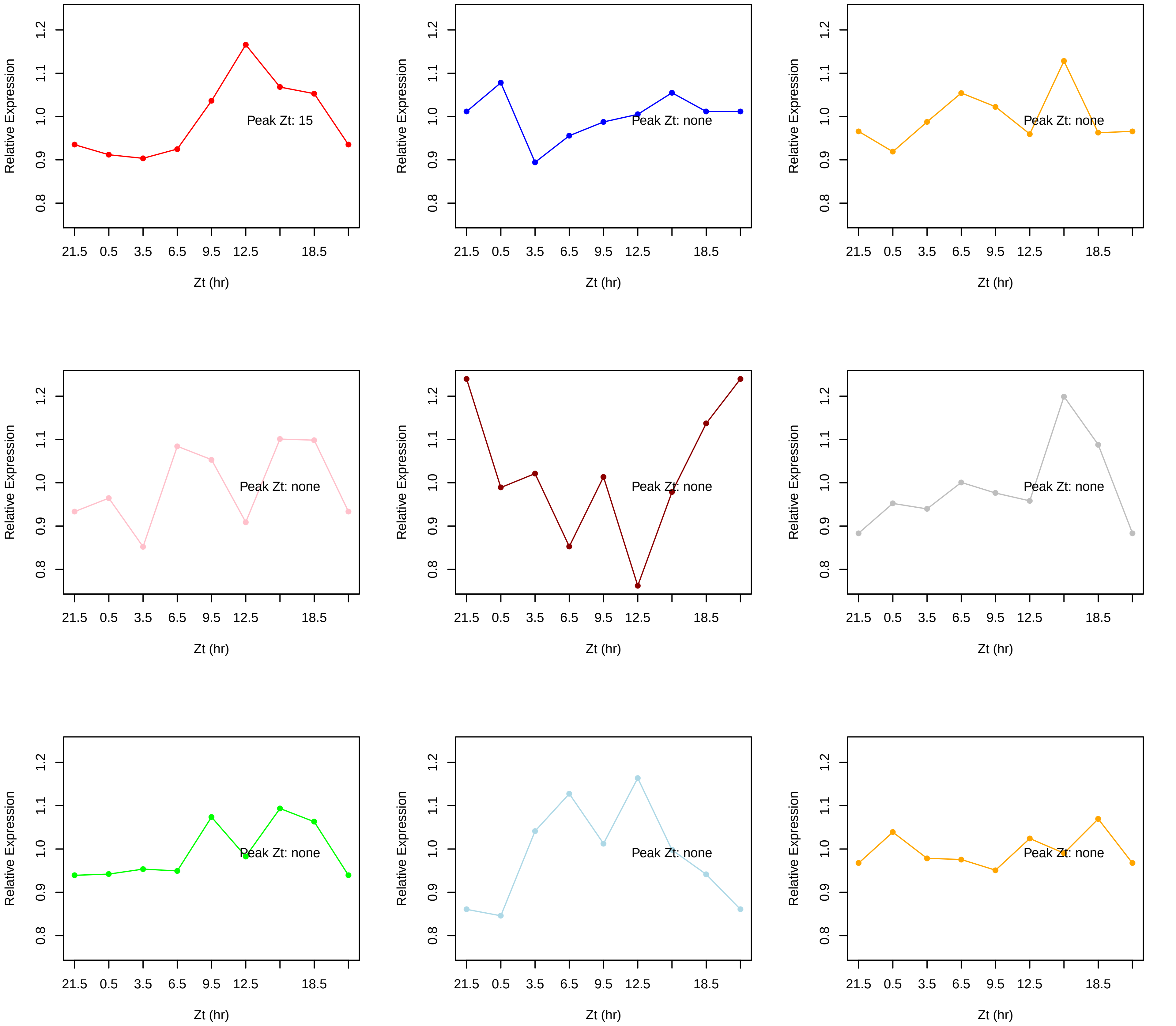

Singeleton: ENSXMAG00000019347 si:dkey−43k4.5

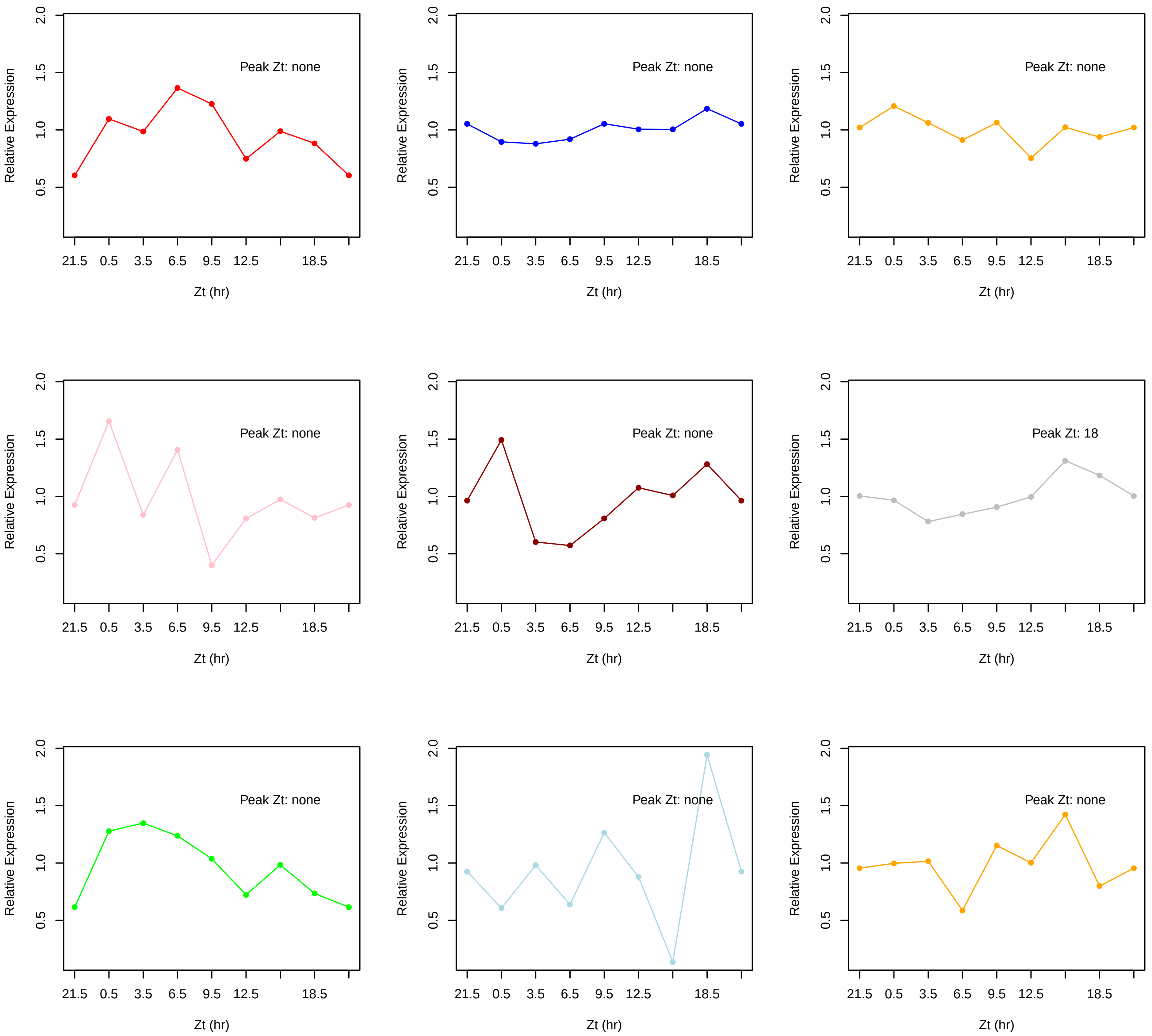

Singleton: ENSXMAG00000025654 polr3k

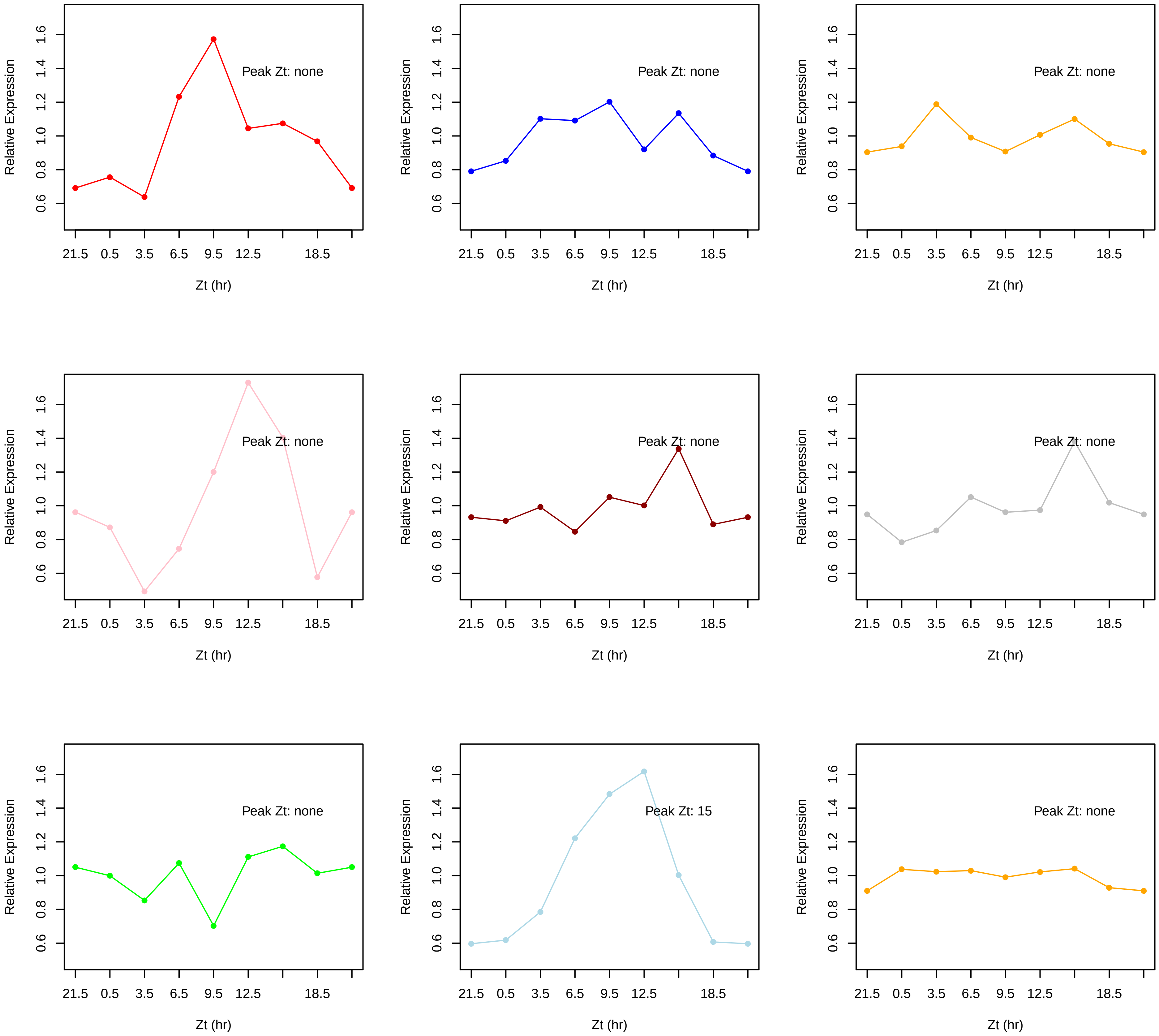

Singeleton: ENSXMAG00000015355 zgc:63863

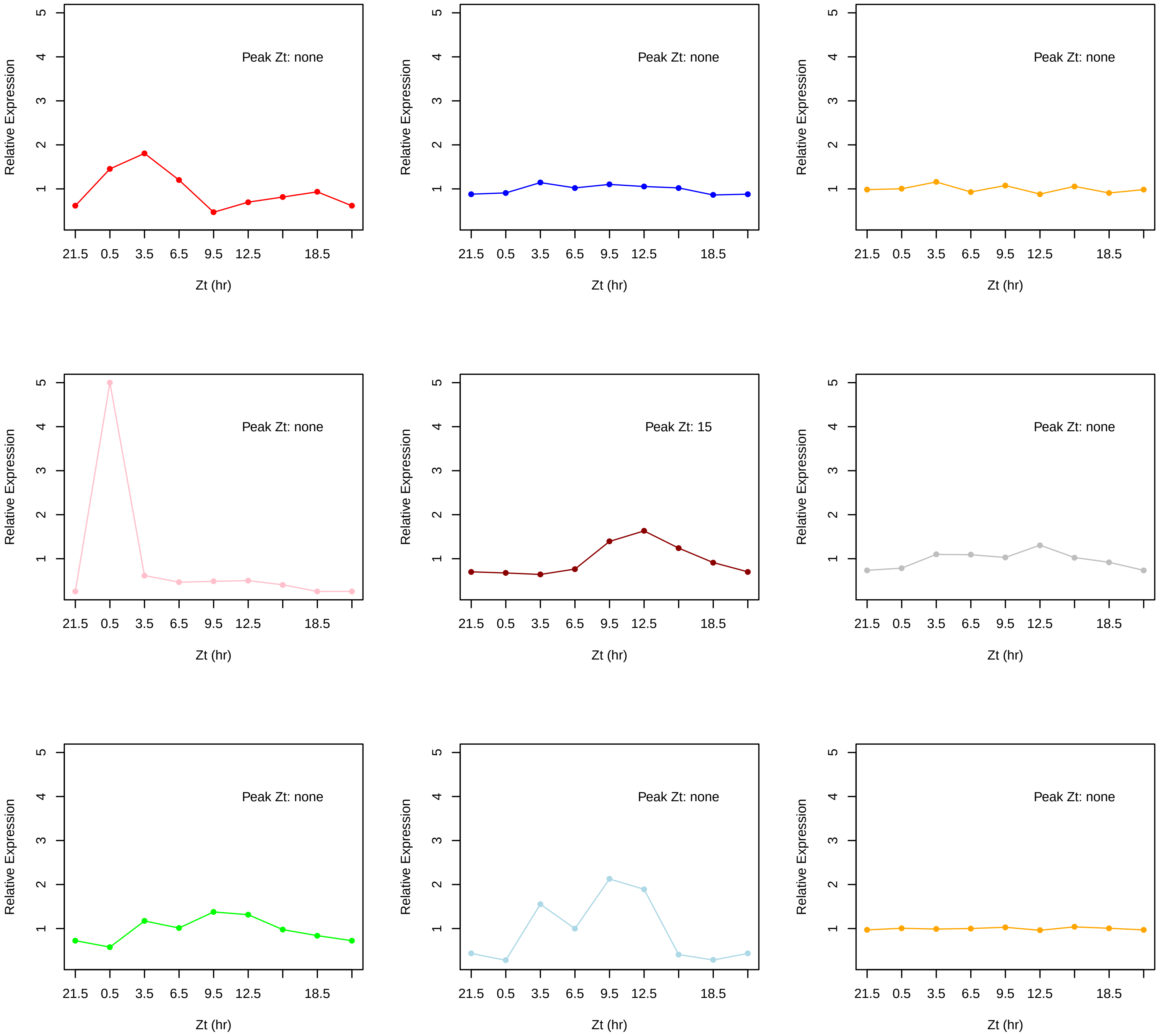

Singeleton: ENSXMAG00000010164 ARHGEF25

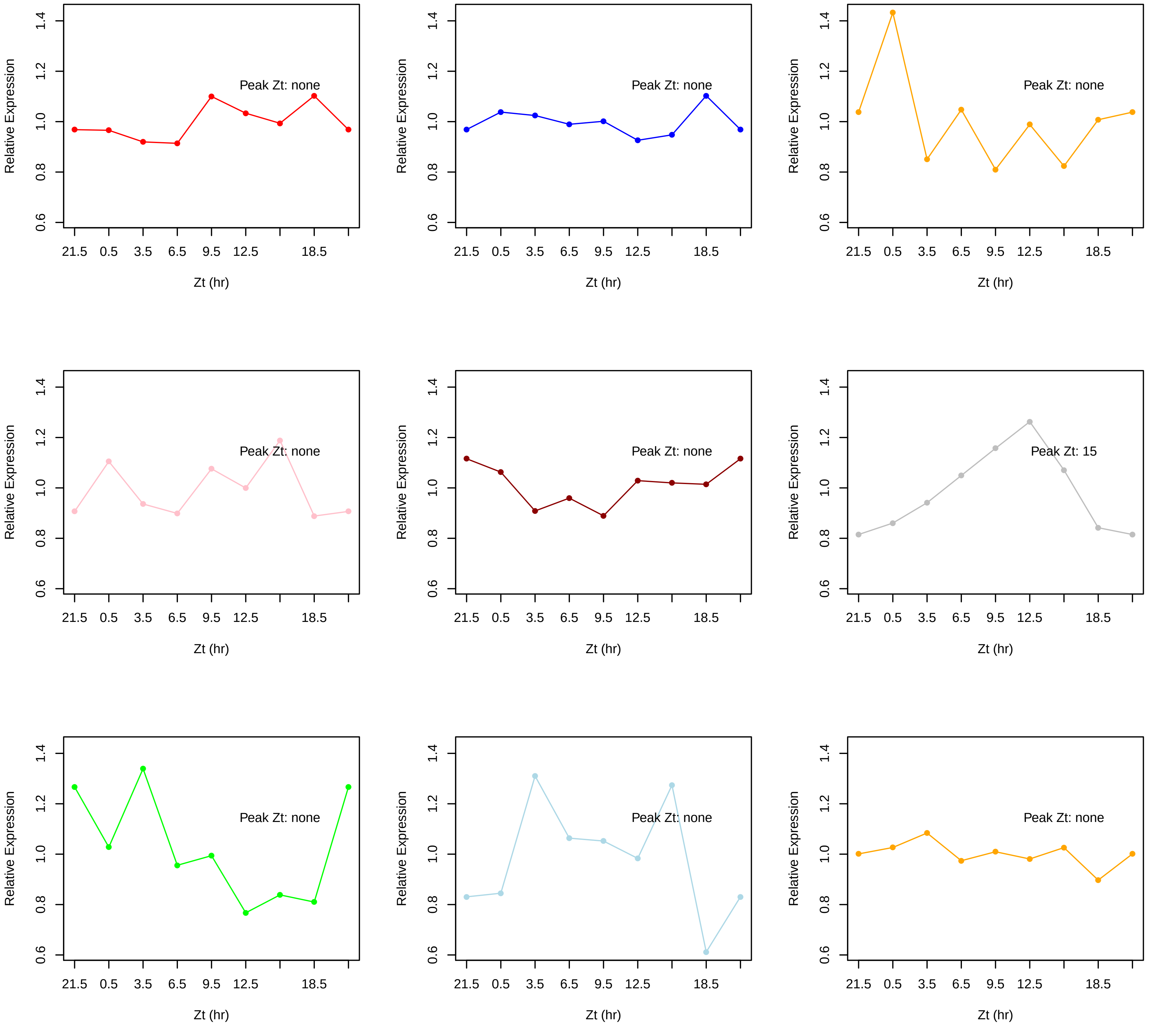

Singeleton: ENSXMAG00000014867 ubap2l

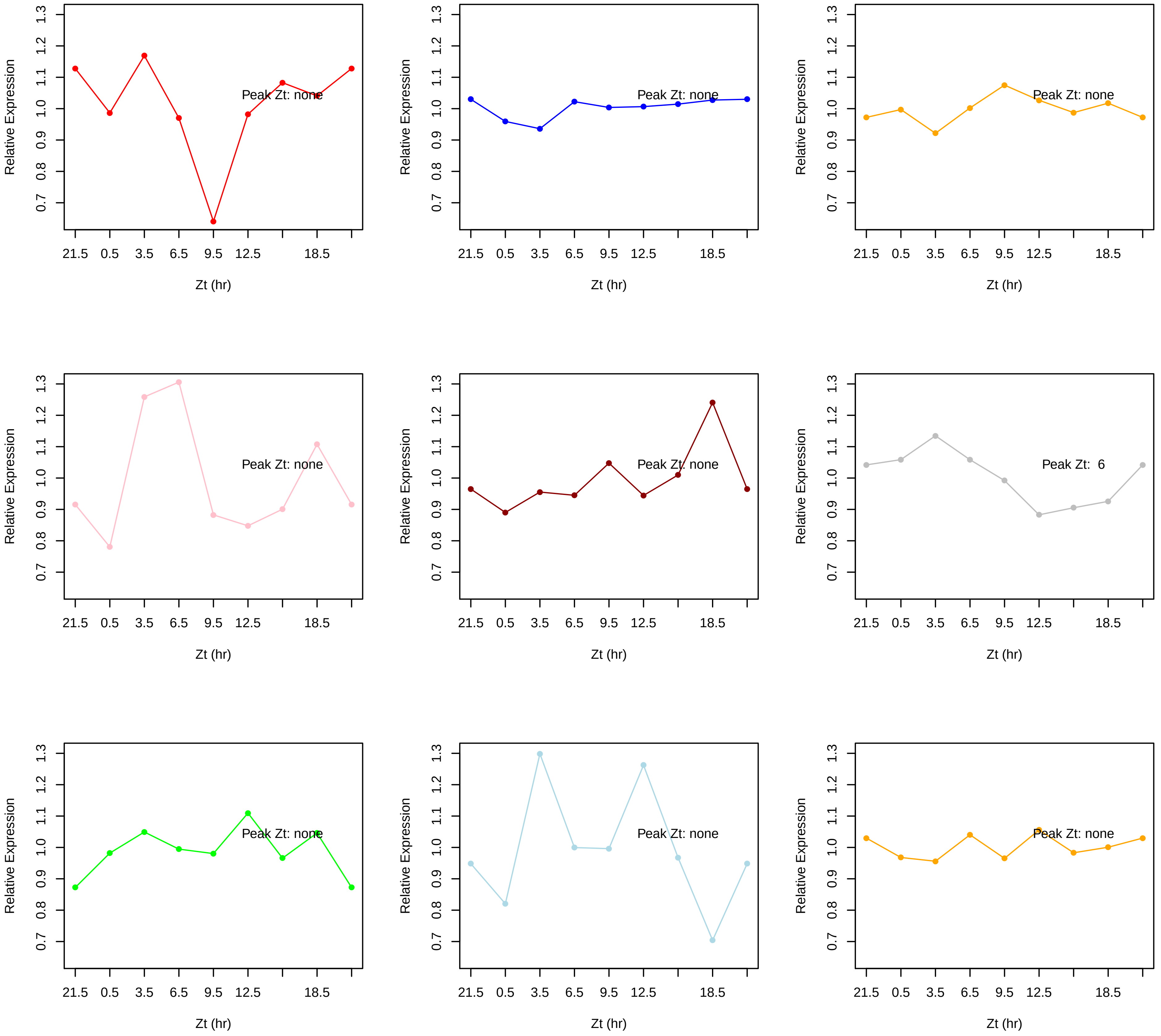

Singeleton: ENSXMAG00000015508 plk2b

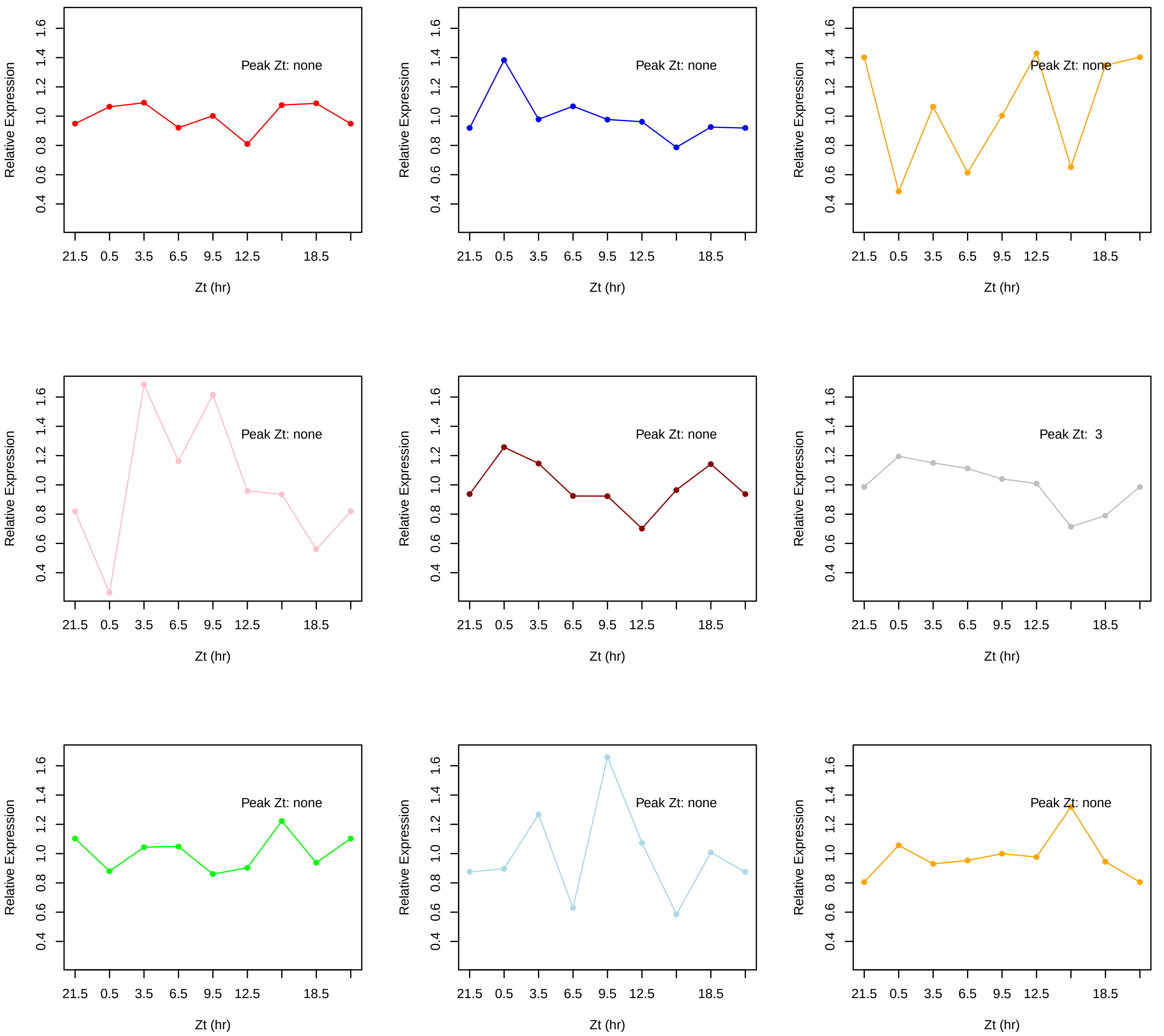

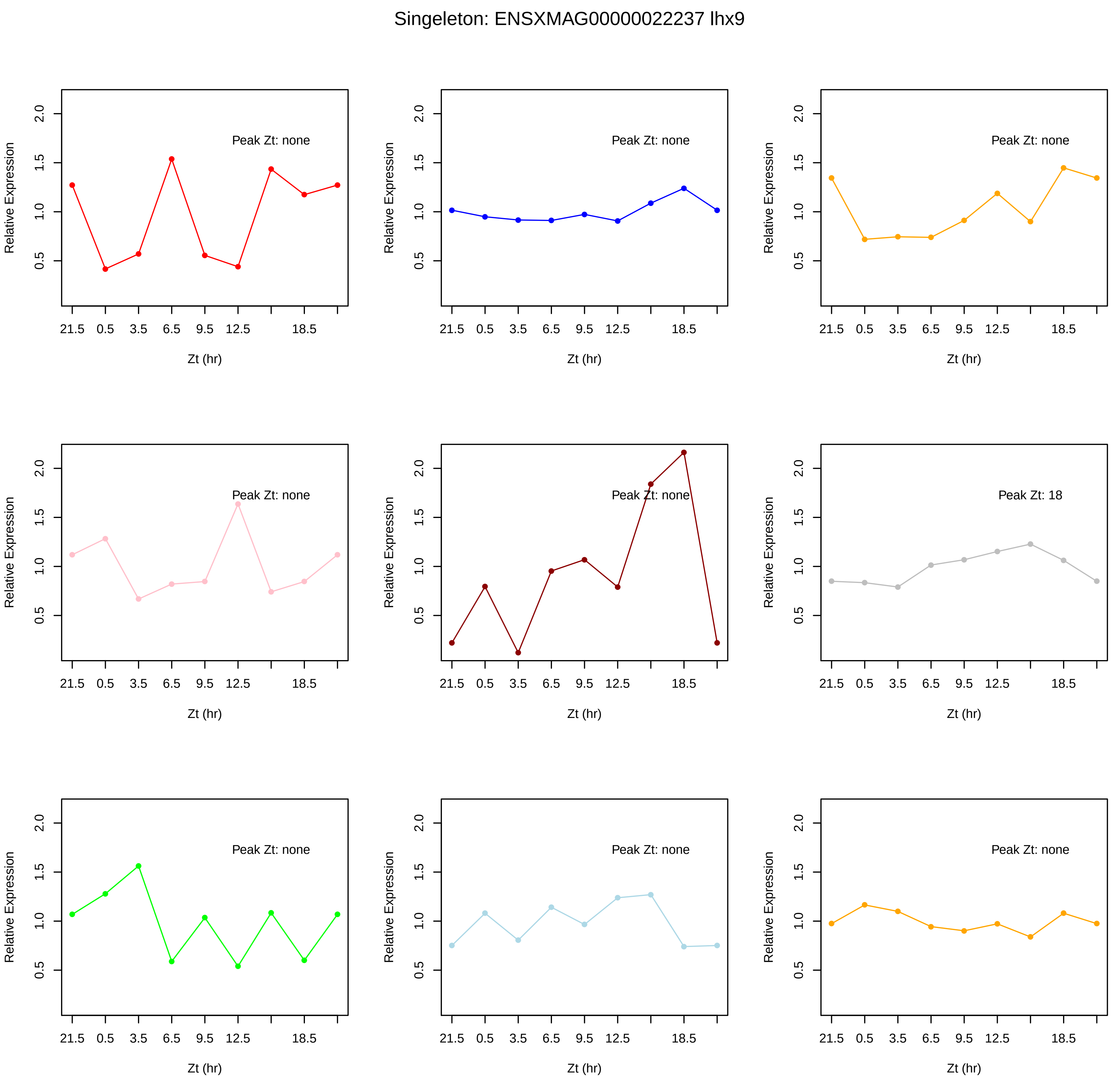
Singeleton: ENSXMAG00000022237 lhx9
Peak Zt: none
Peak Zt: none
Peak Zt: none
Peak Zt: none
Peak Zt: none
Peak Zt: 18
Peak Zt: none
Peak Zt: none
Peak Zt: none
Relative Expression
Zt (hr)
21.5 0.5 3.5 6.5 9.5 12.5 18.5
0.5 1.0 1.5 2.0

Singleton: ENSXMAG00000000113 cdip1

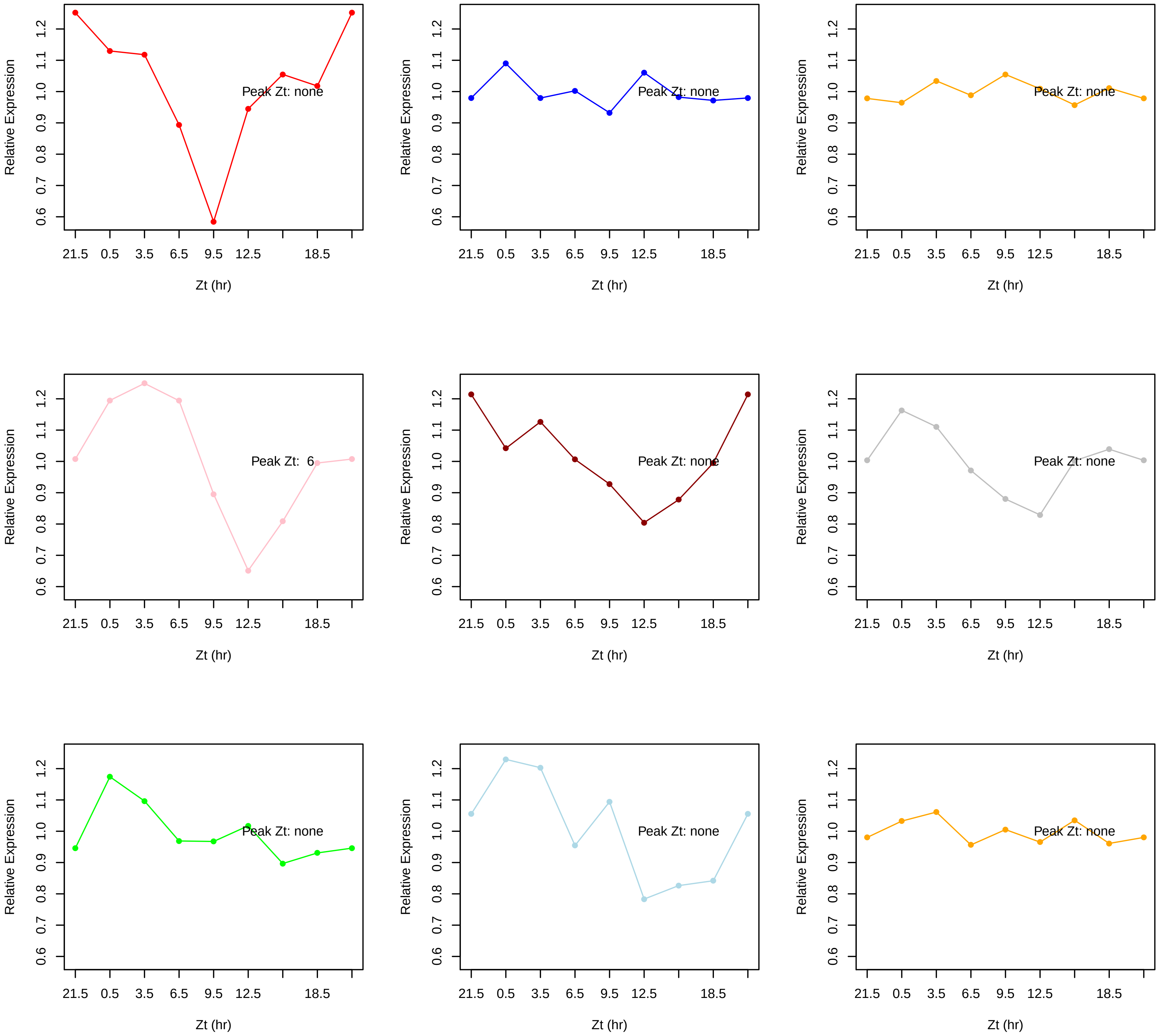

Singeleton: ENSXMAG00000005372 tbx3a

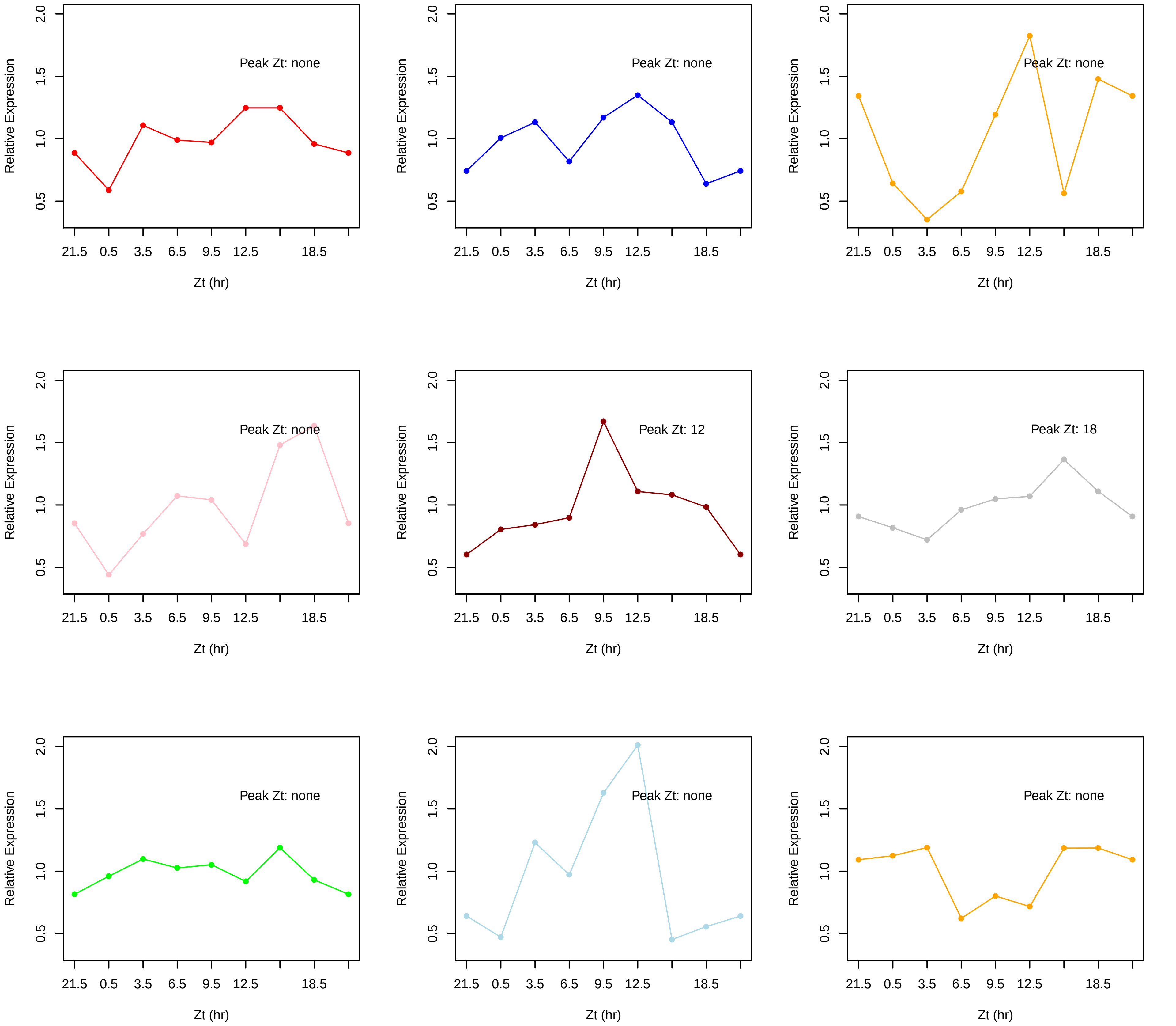

Singeleton: ENSXMAG00000006699 fuk

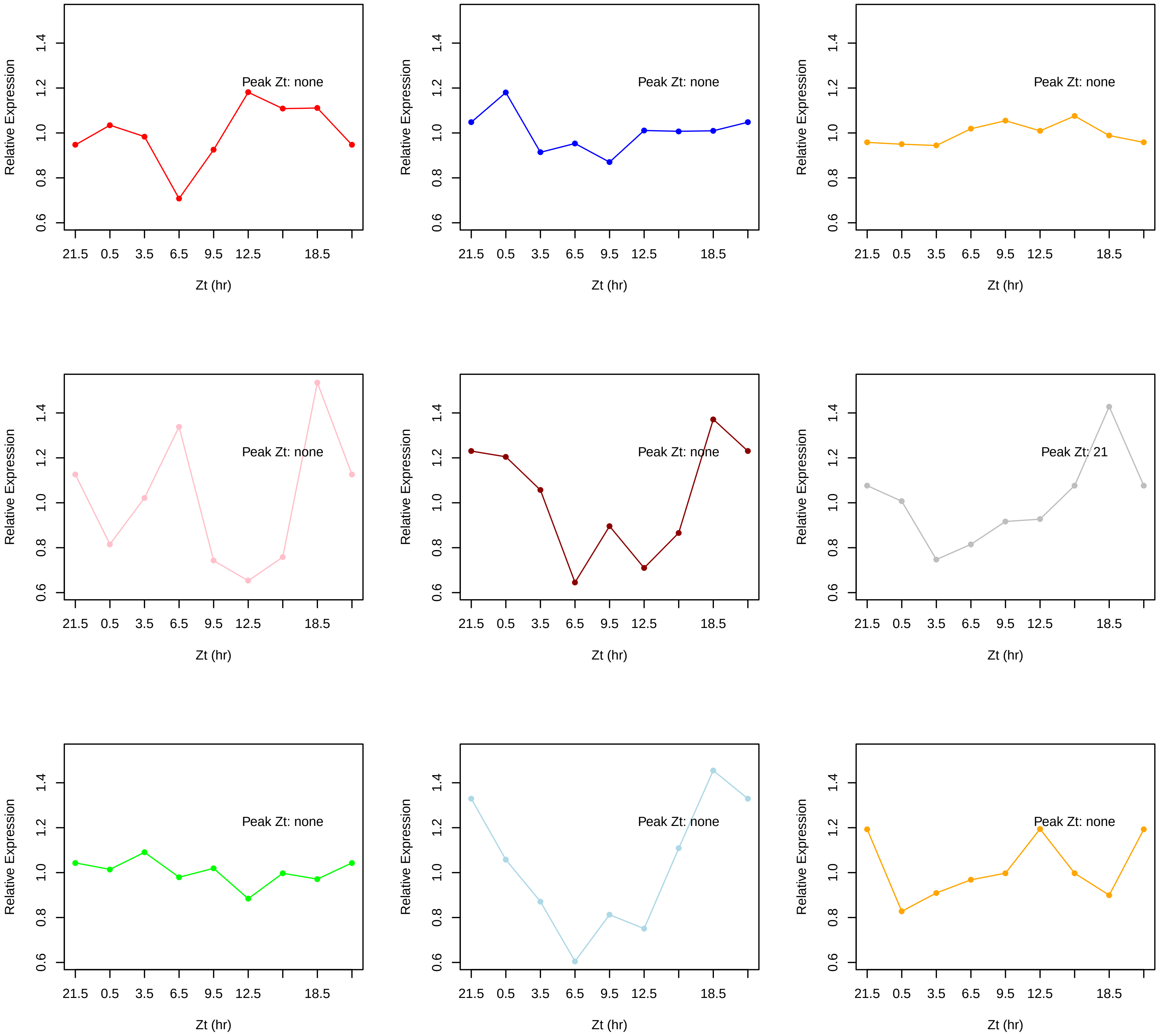

Singleton: ENSXMAG00000029896 nck1b

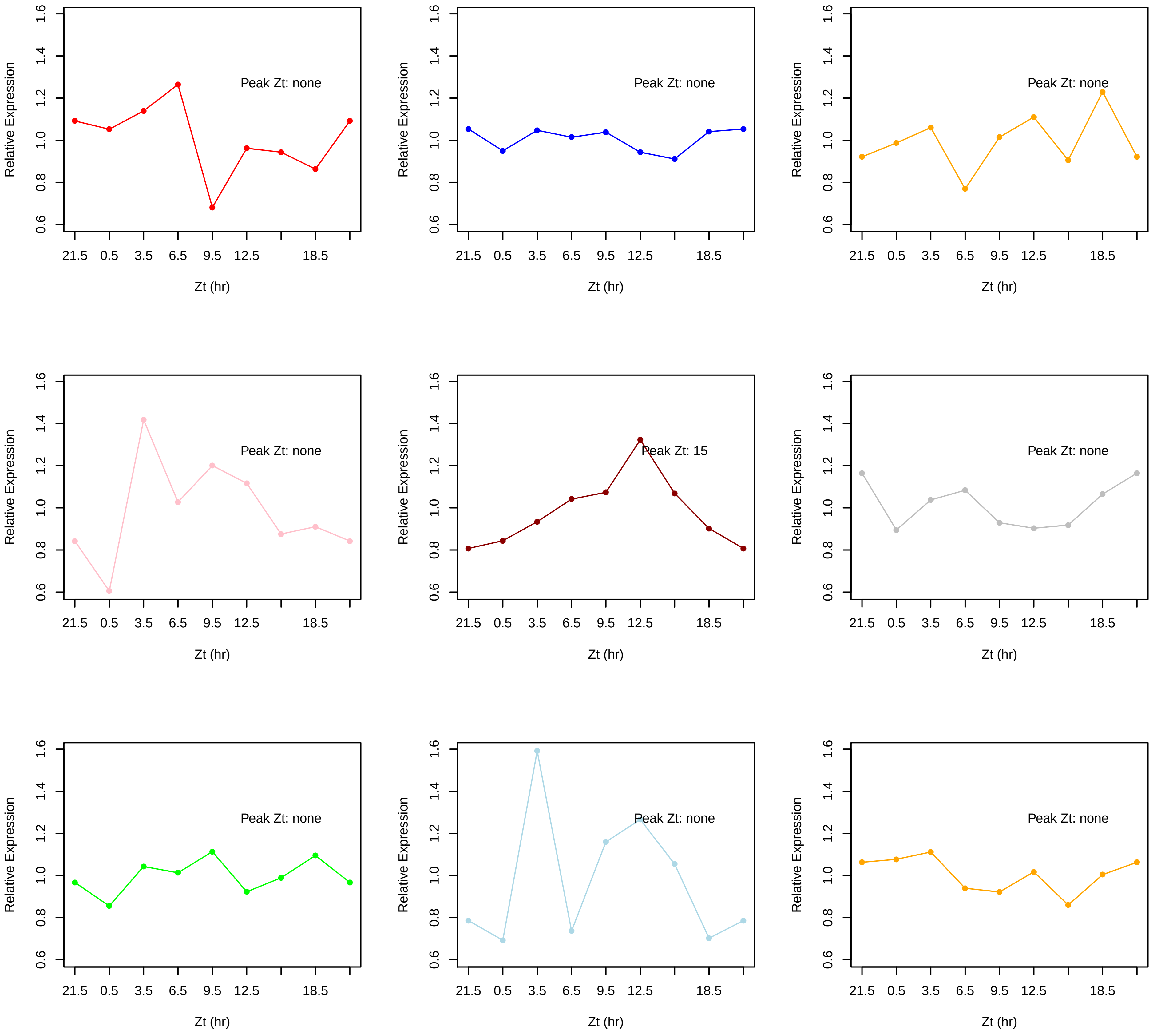

Singeleton: ENSXMAG00000015399 cfap20

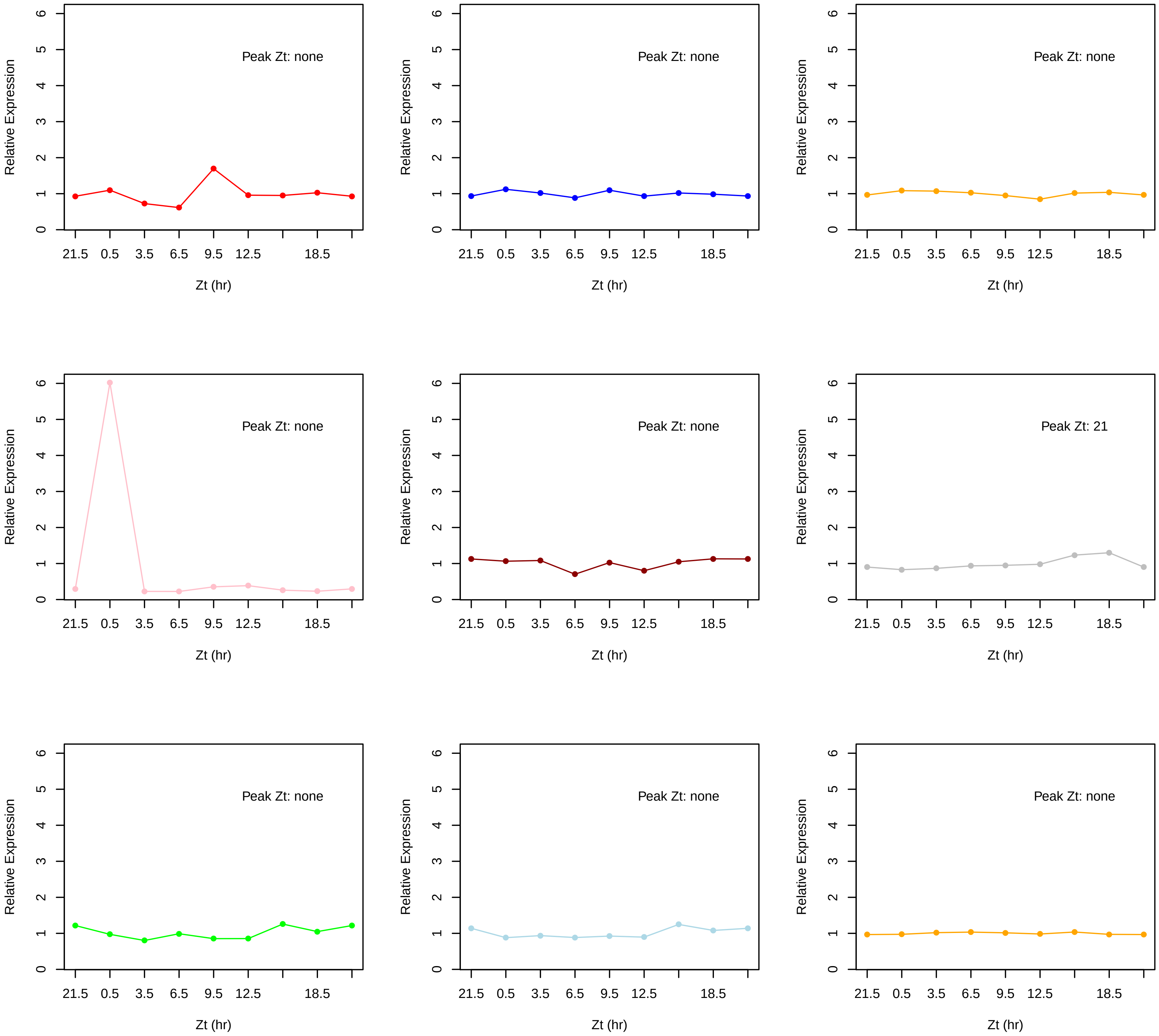

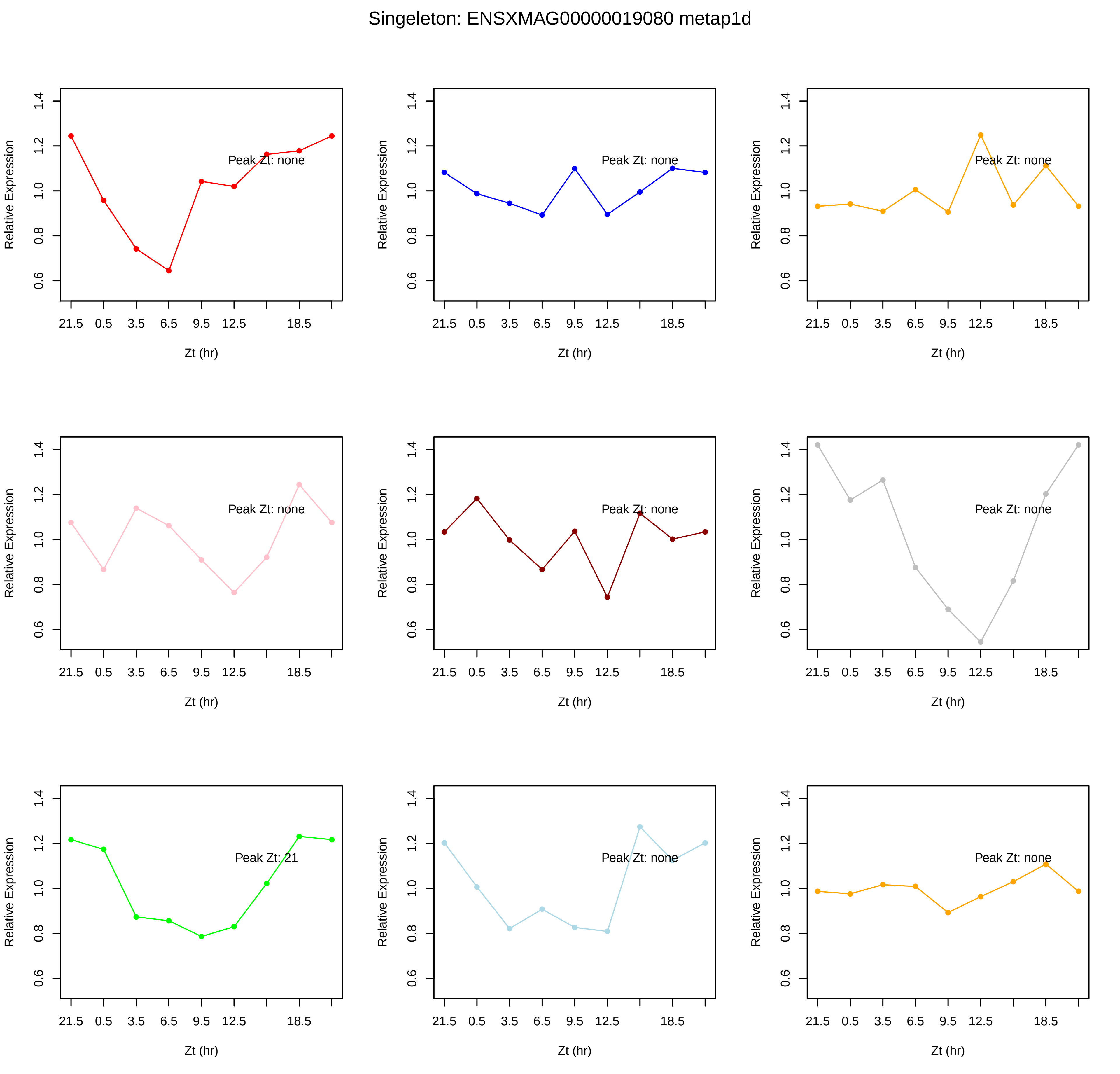

Singeleton: ENSXMAG00000019080 metap1d
Relative Expression
Zt (hr)
21.5
0.5
3.5
6.5
9.5
12.5
18.5
0.6
0.8
1.0
1.2
1.4
Peak Zt: none
Peak Zt: 21

Singeleton: ENSXMAG00000015537 si:dkey−190g11.3

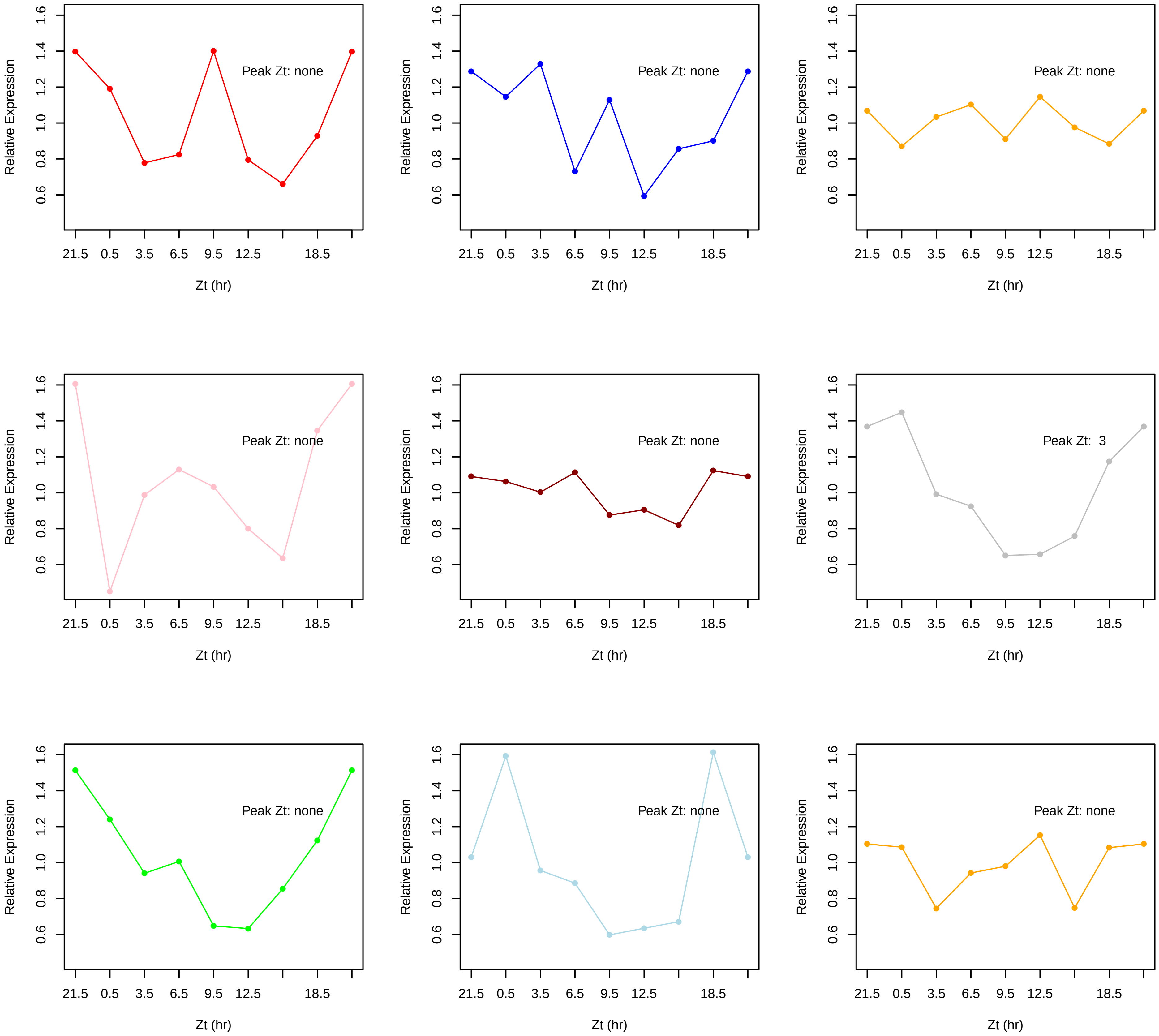

Singeleton: ENSXMAG00000025287 nek7

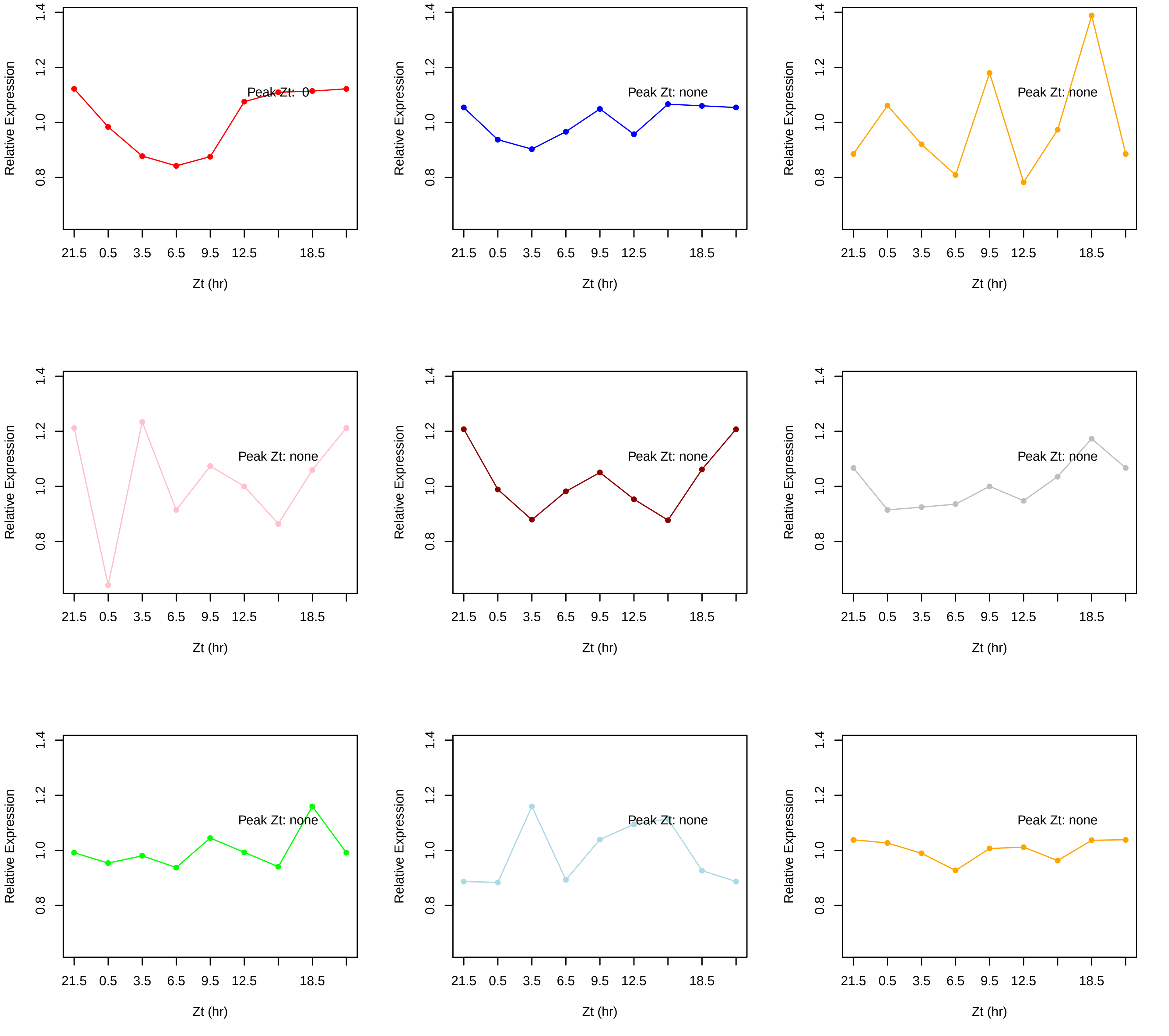

Singeleton: ENSXMAG00000000117 mgrn1b

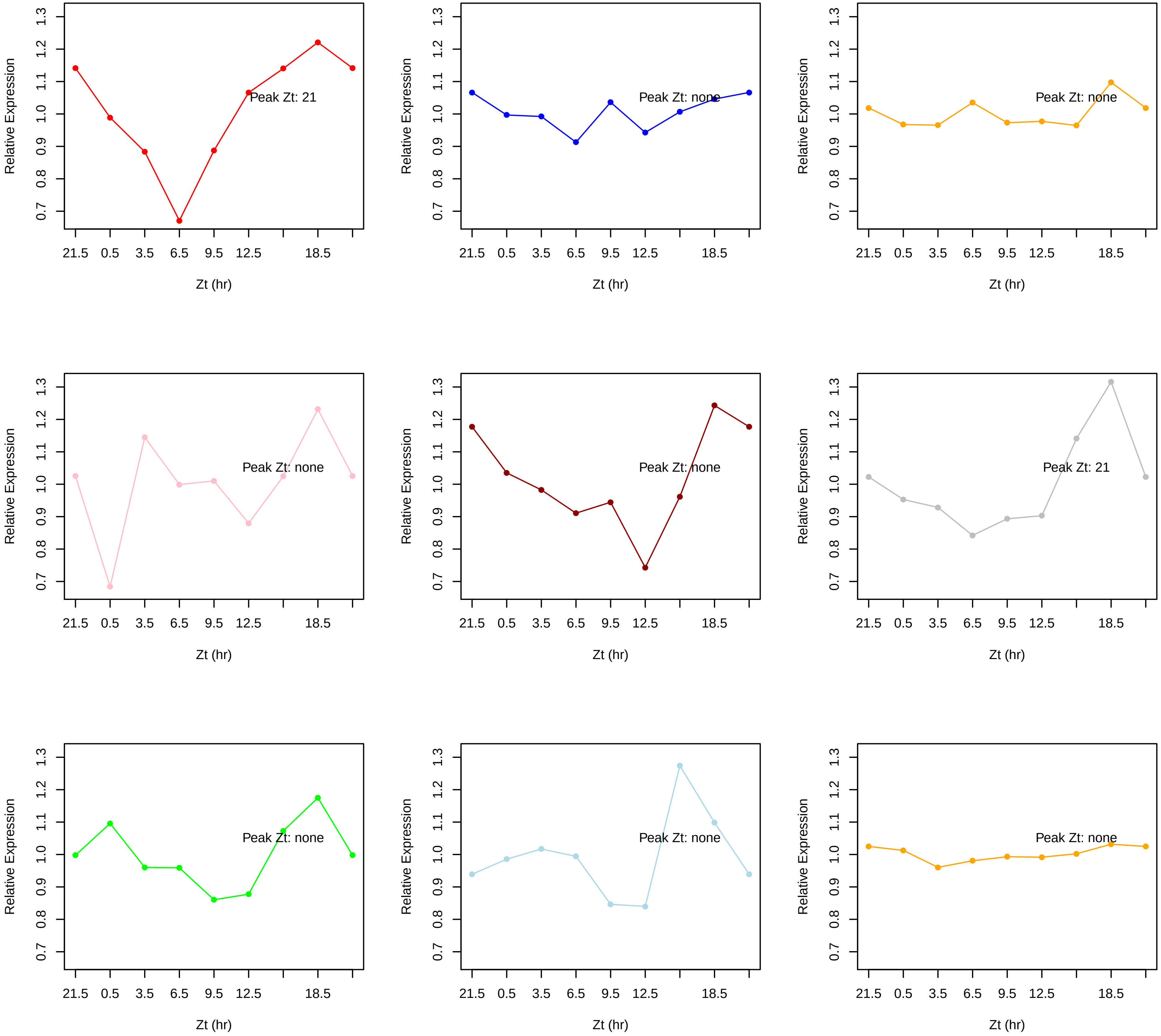

Singeleton: ENSXMAG00000016601 ATAD3A

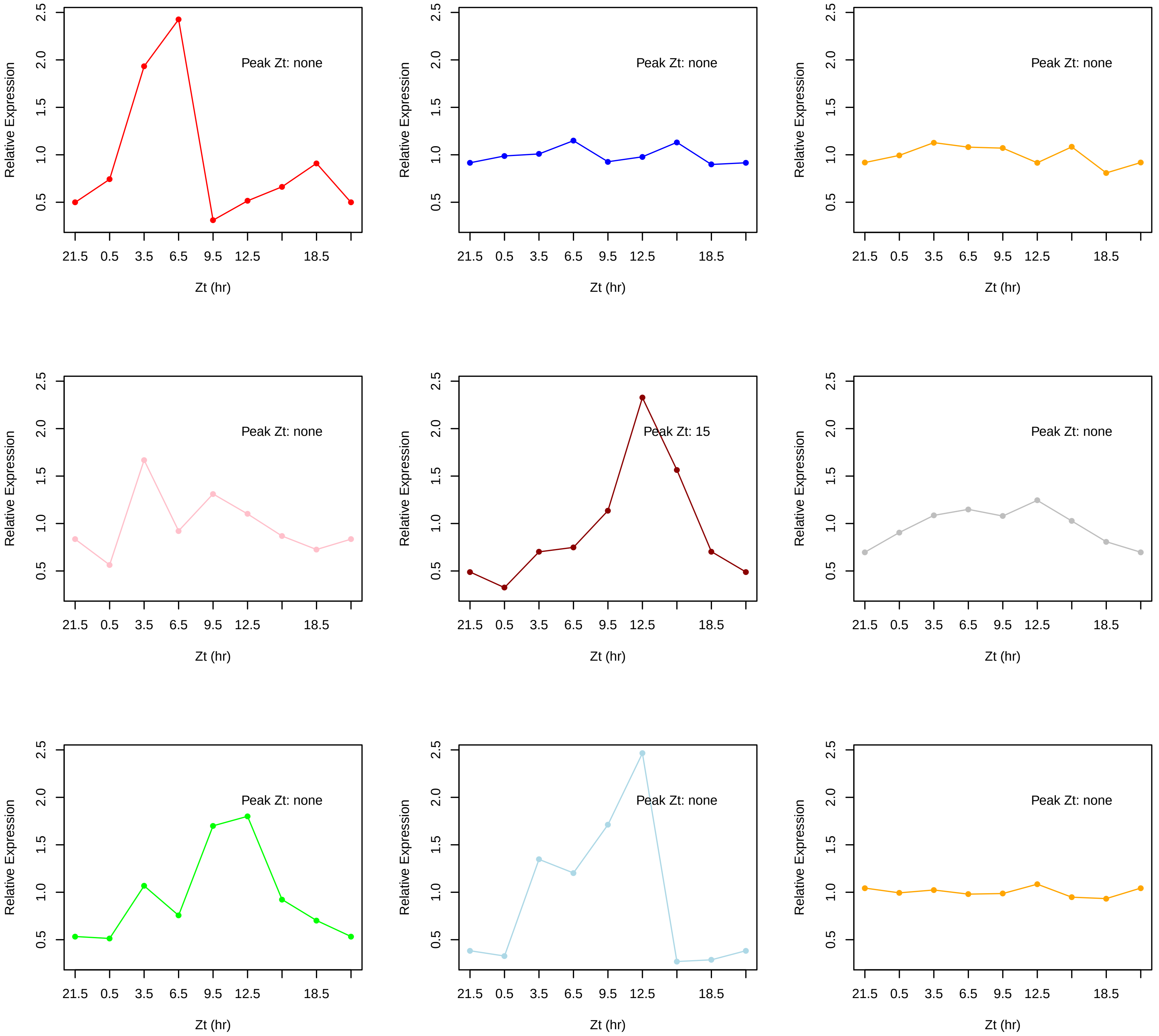

Singleton: ENSXMAG00000008320 pdss1

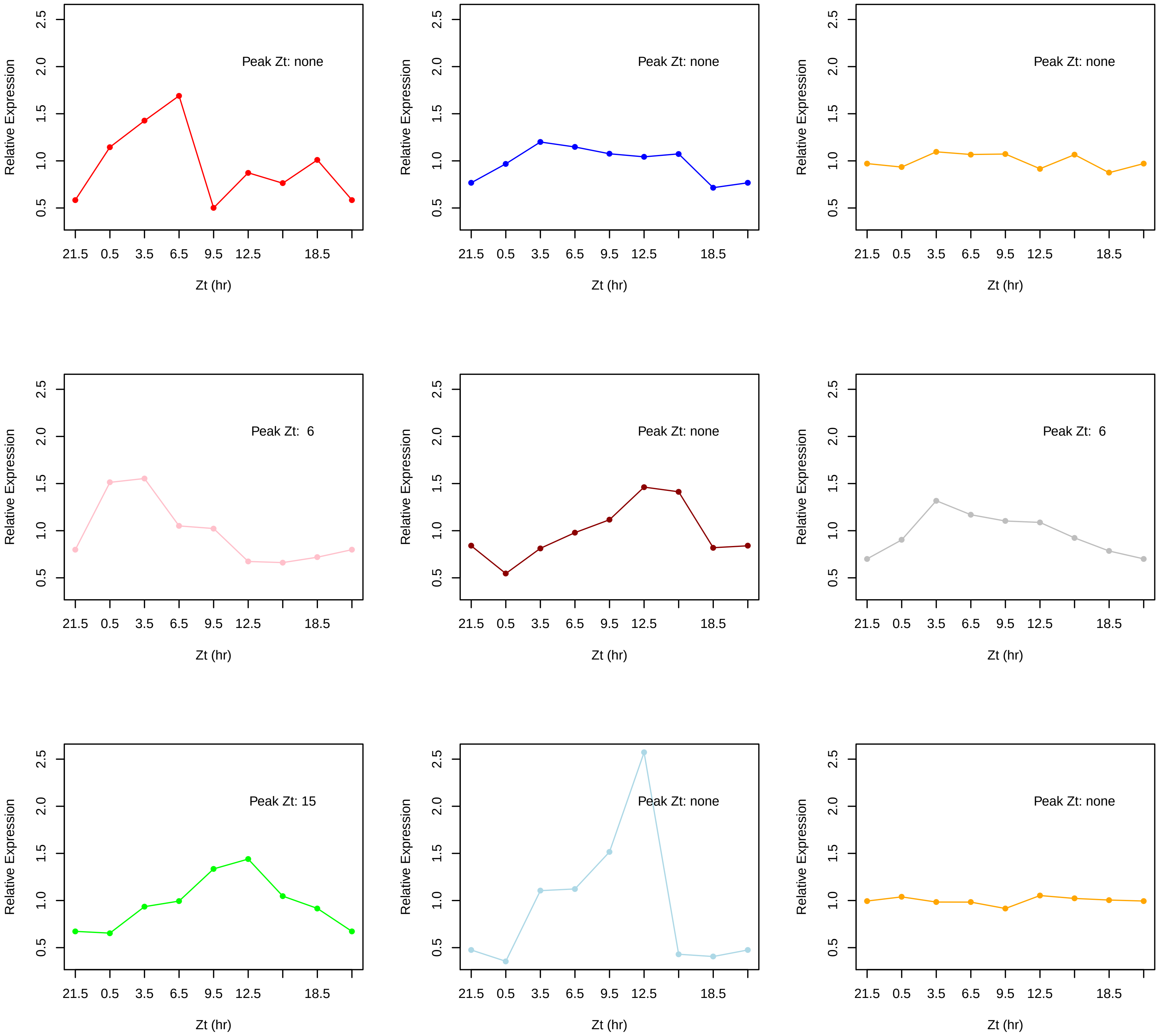

Singeleton: ENSXMAG00000009324 hltf

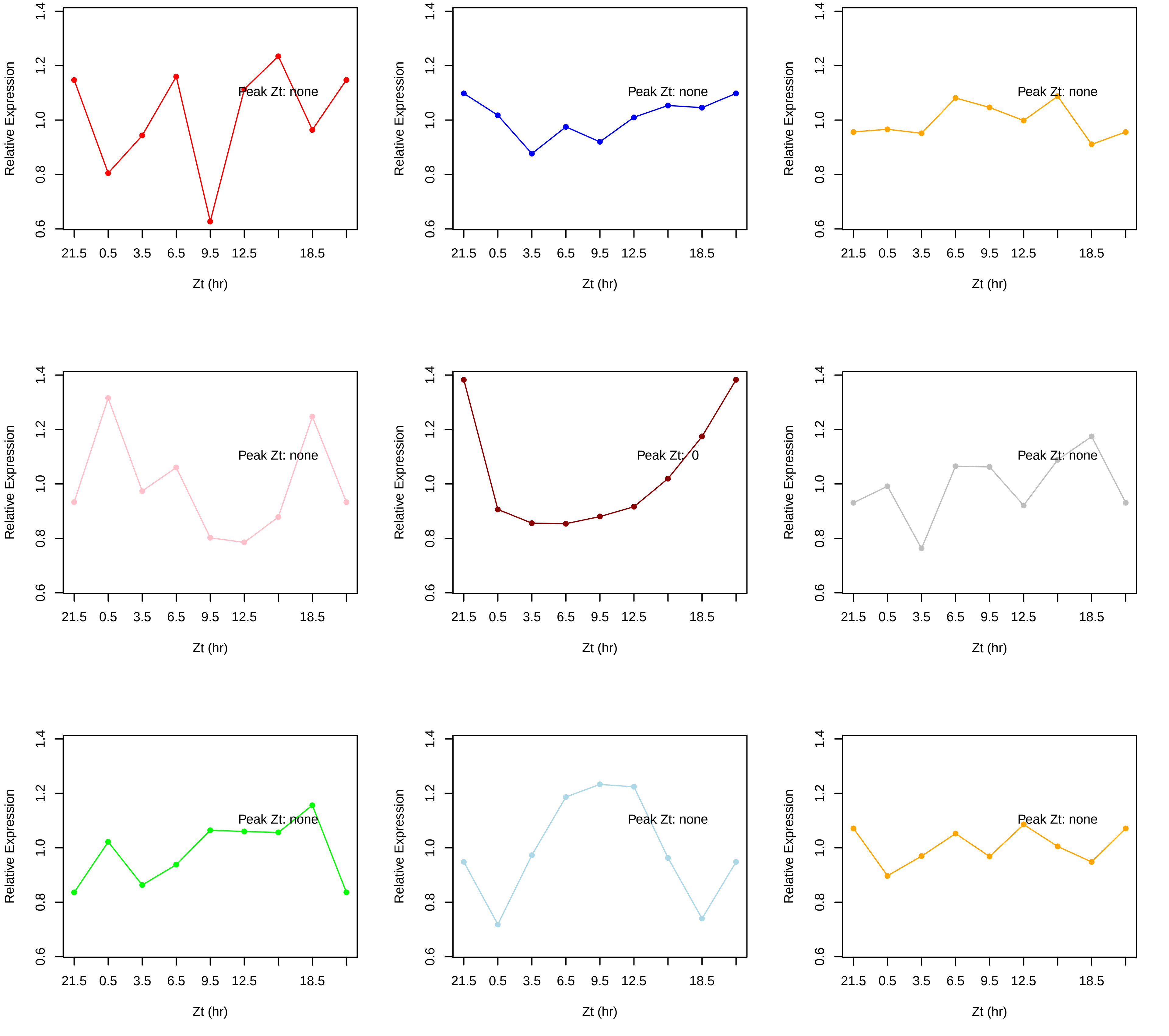

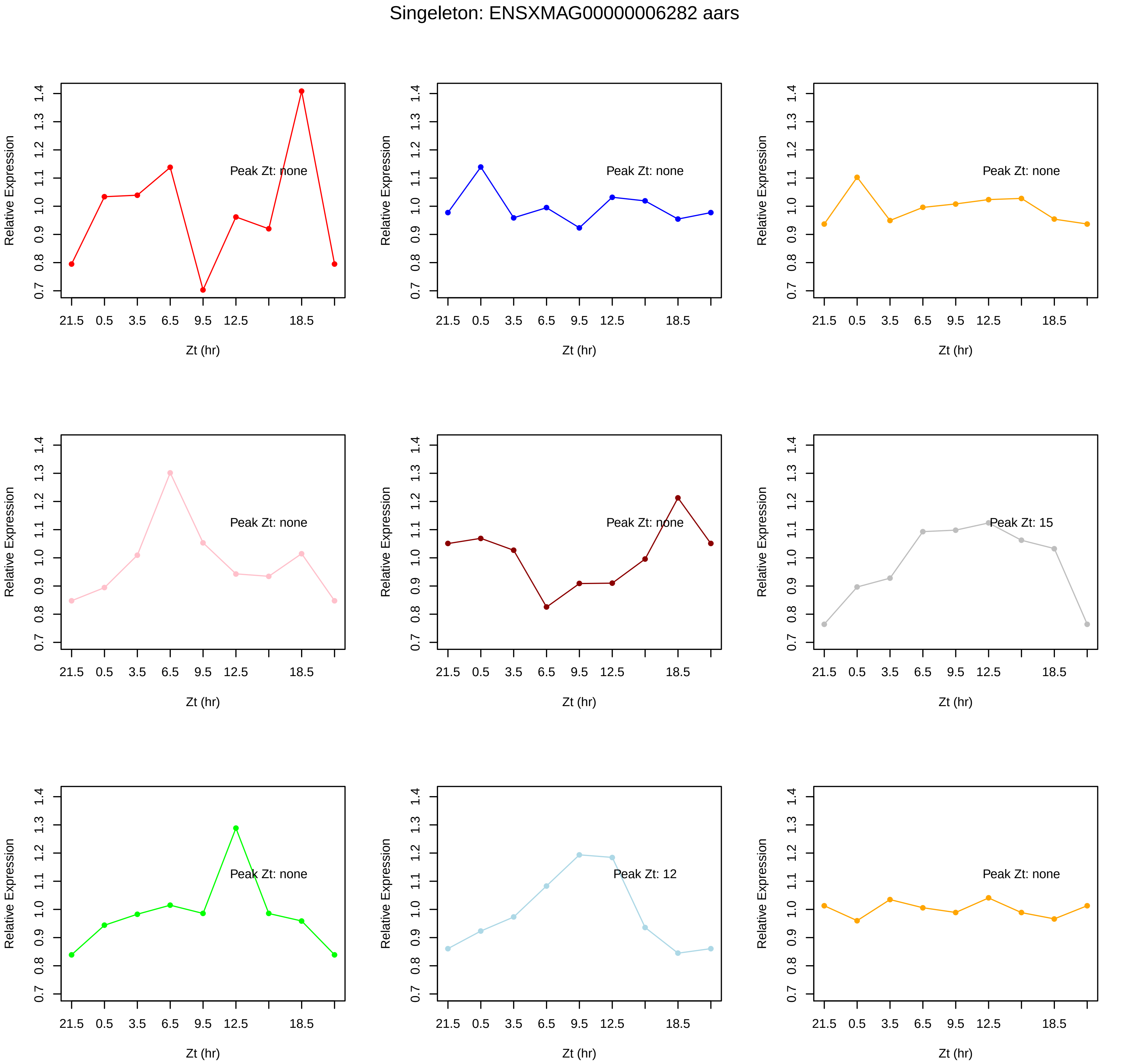
Singeleton: ENSXMAG00000006282 aars
Relative Expression
Zt (hr)
21.5 0.5 3.5 6.5 9.5 12.5 18.5
0.7 0.8 0.9 1.0 1.1 1.2 1.3 1.4
Peak Zt: none
Peak Zt: none
Peak Zt: none
Peak Zt: none
Peak Zt: none
Peak Zt: 15
Peak Zt: none
Peak Zt: 12
Peak Zt: none

Singleton: ENSXMAG00000014627

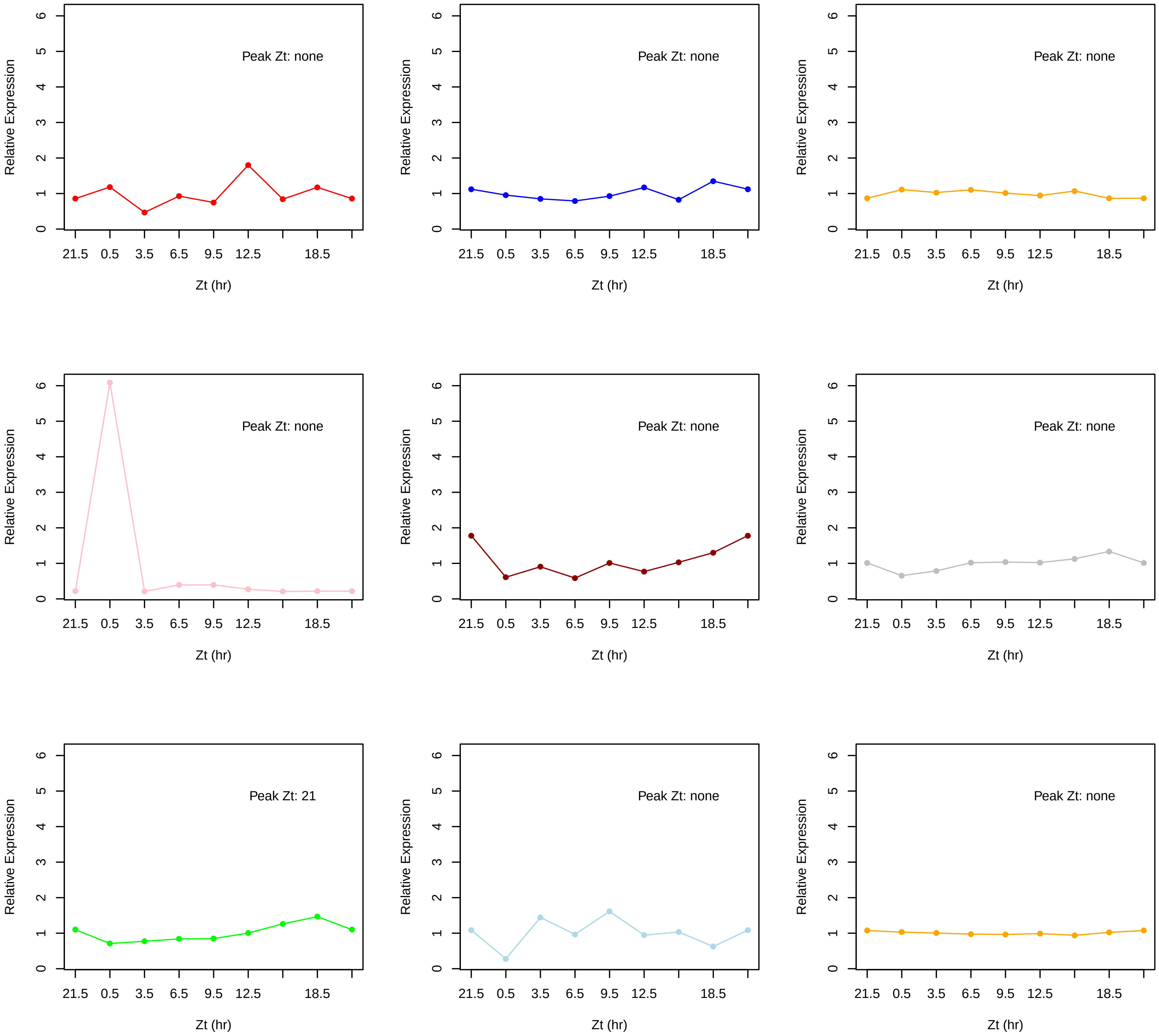

Singeleton: ENSXMAG00000027995 shc1

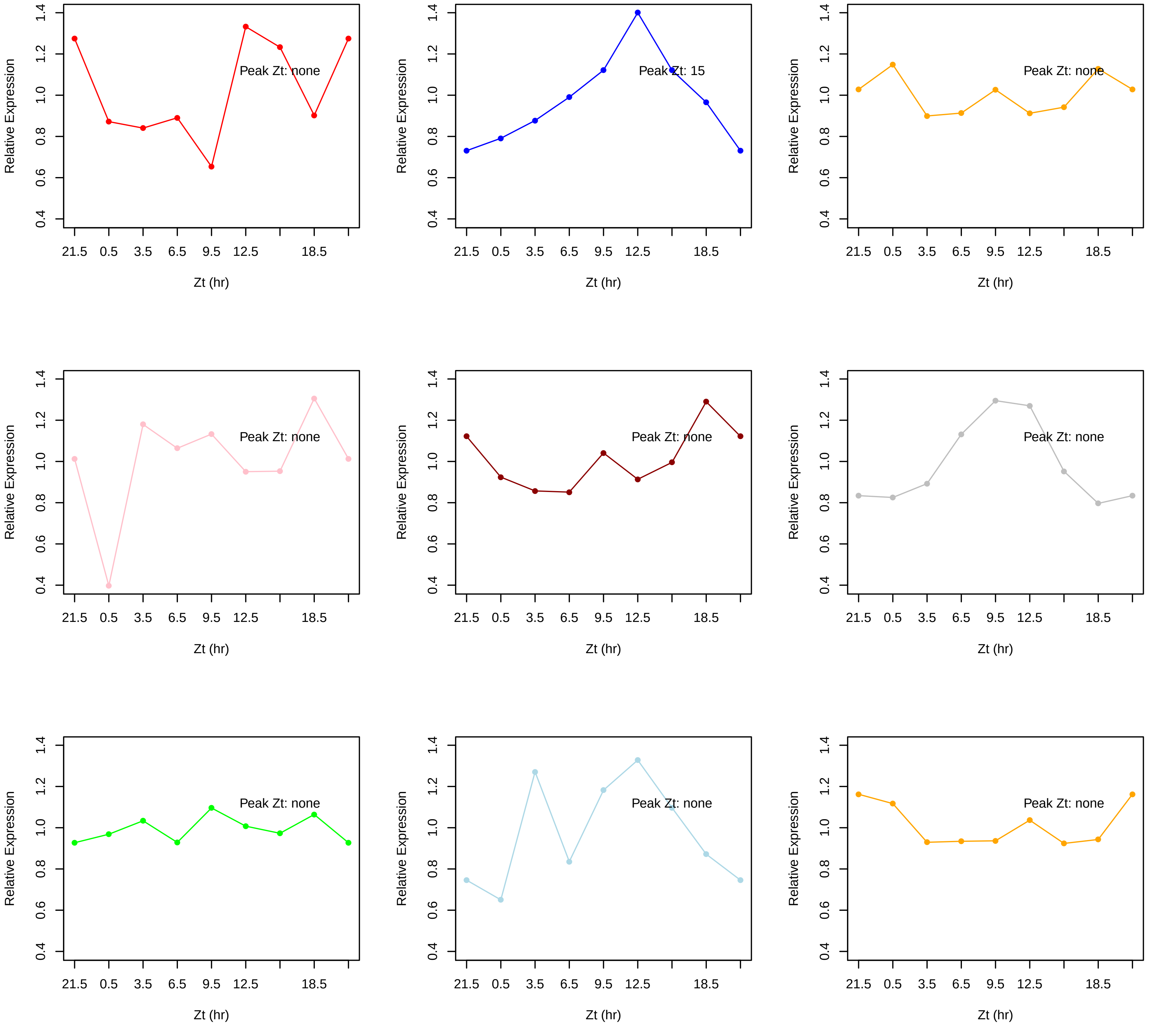

Singeleton: ENSXMAG00000002012

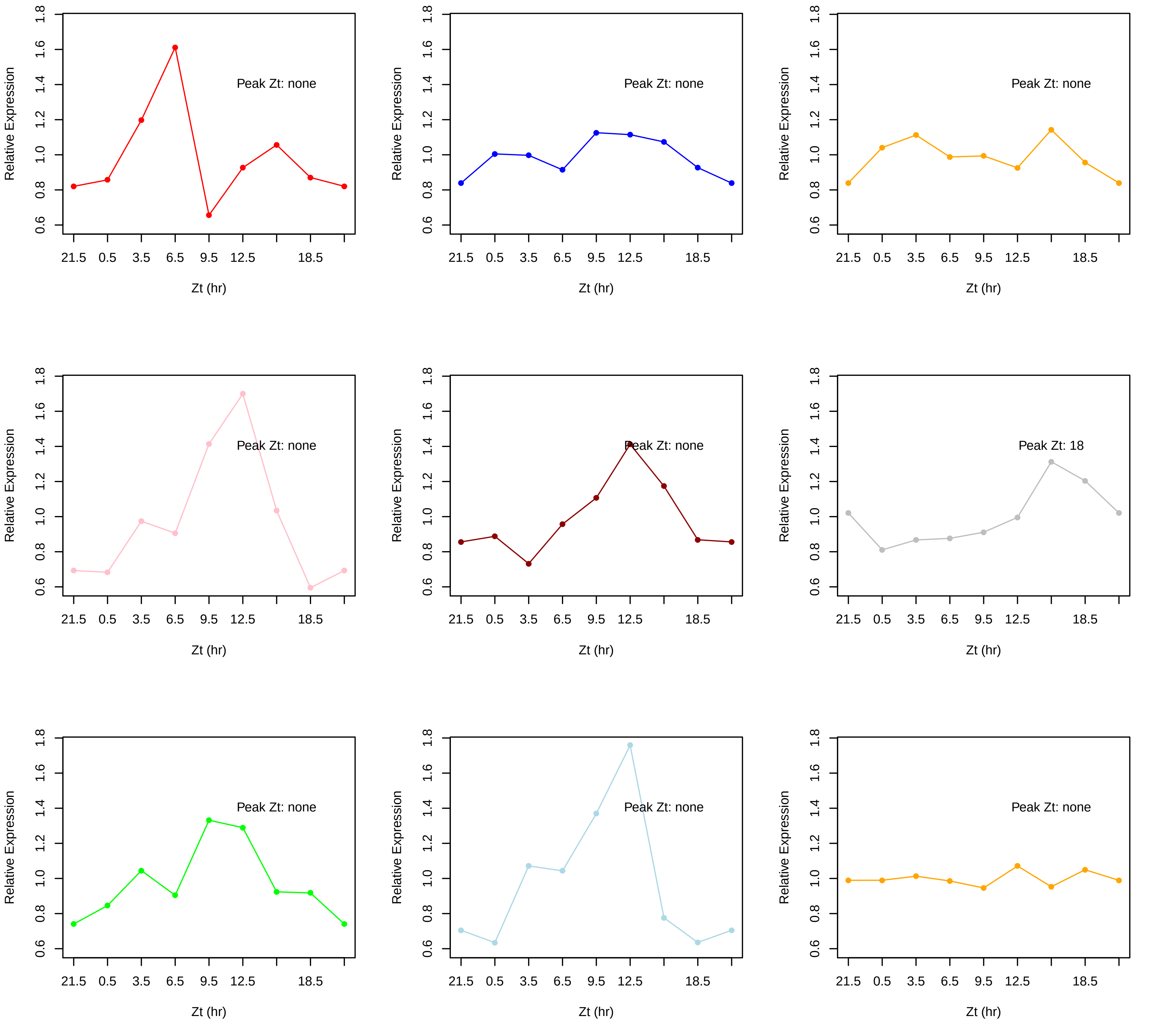

Singeleton: ENSXMAG00000005906

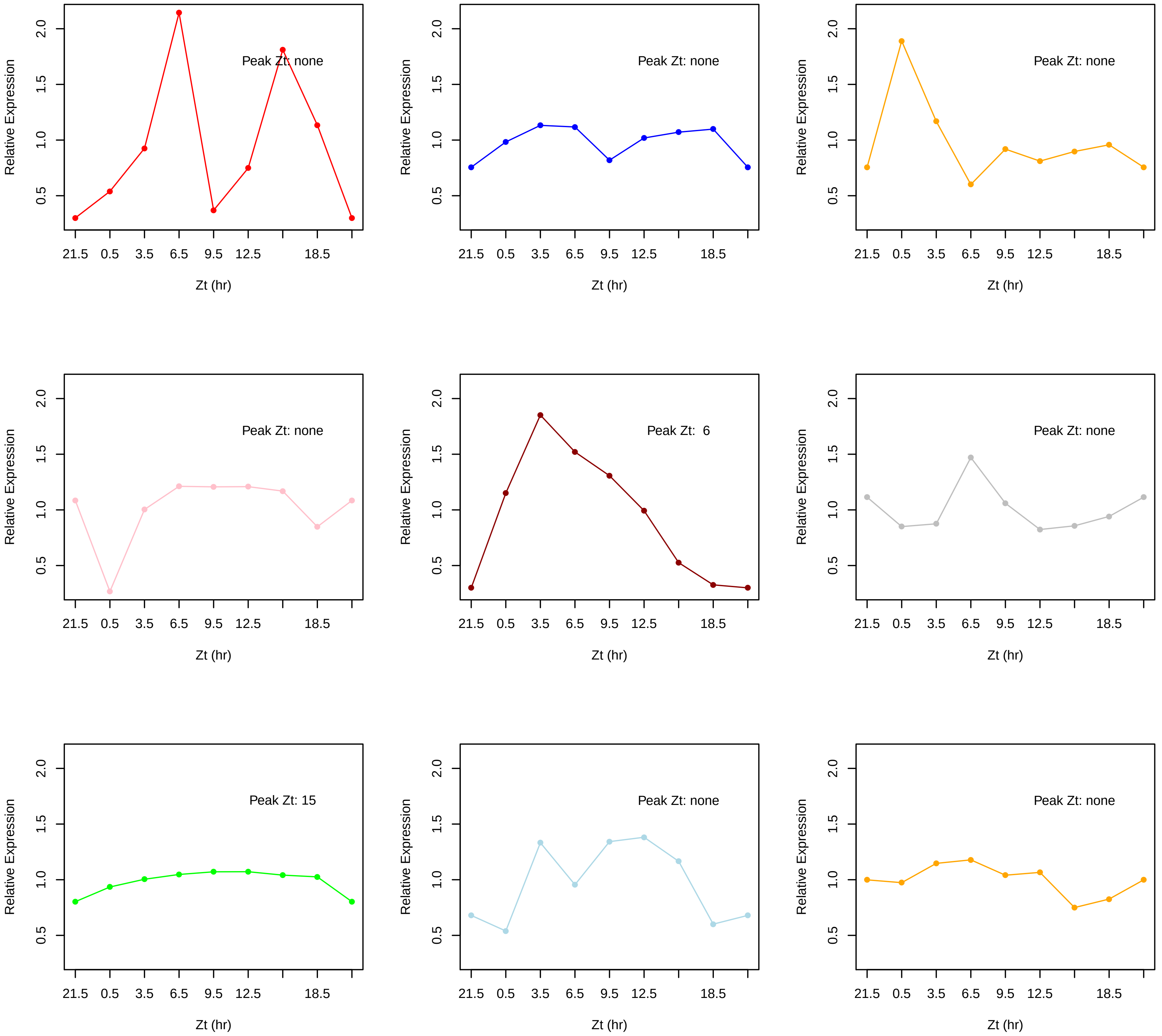

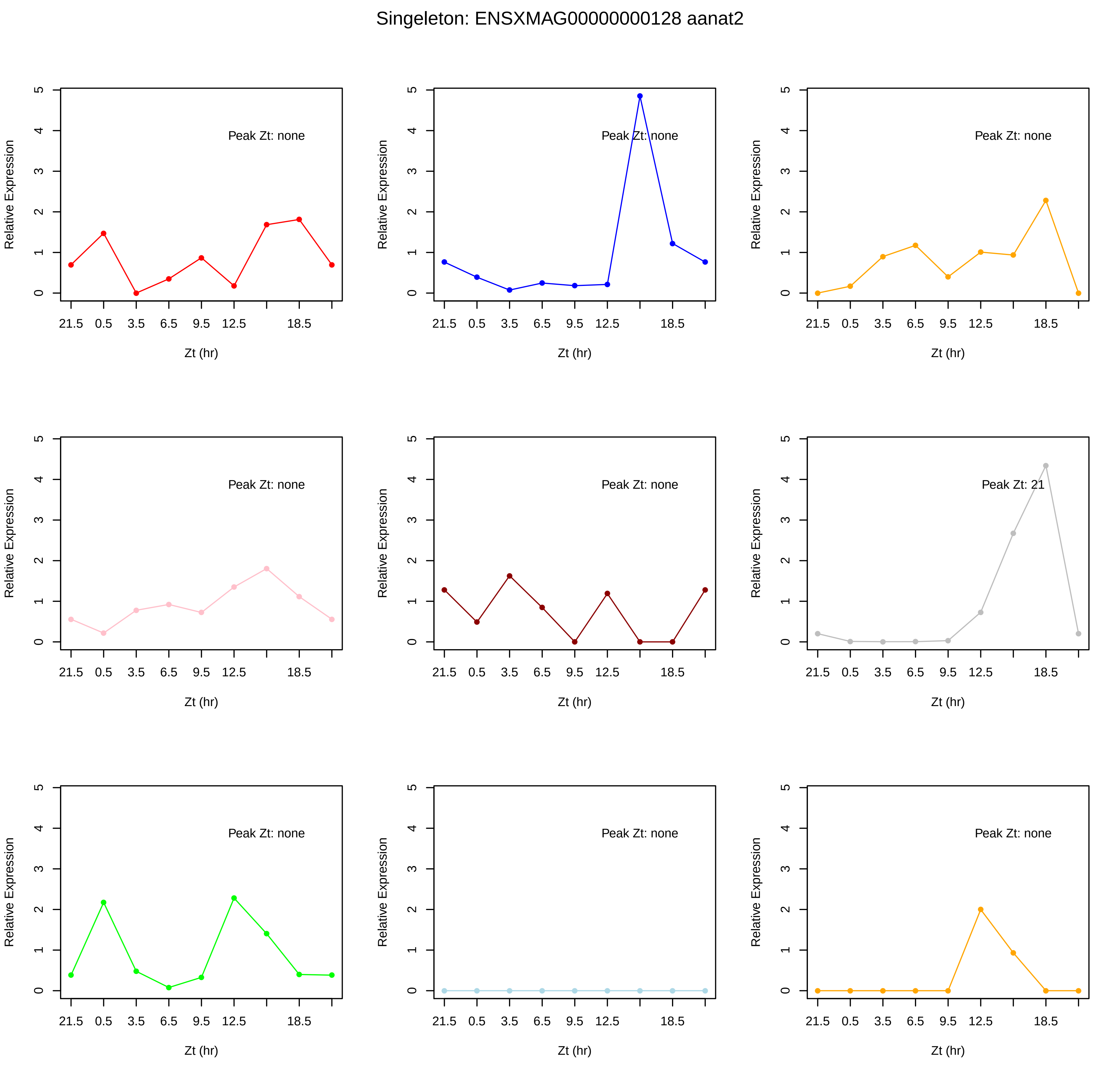

Singeleton: ENSXMAG00000000128 aanat2
Peak Zt: none
Peak Zt: none
Peak Zt: none
Peak Zt: none
Peak Zt: none
Peak Zt: 21
Peak Zt: none
Peak Zt: none
Peak Zt: none
Relative Expression
Zt (hr)
21.5
0.5
3.5
6.5
9.5
12.5
18.5

Singeleton: ENSXMAG00000023365 ptpn11a

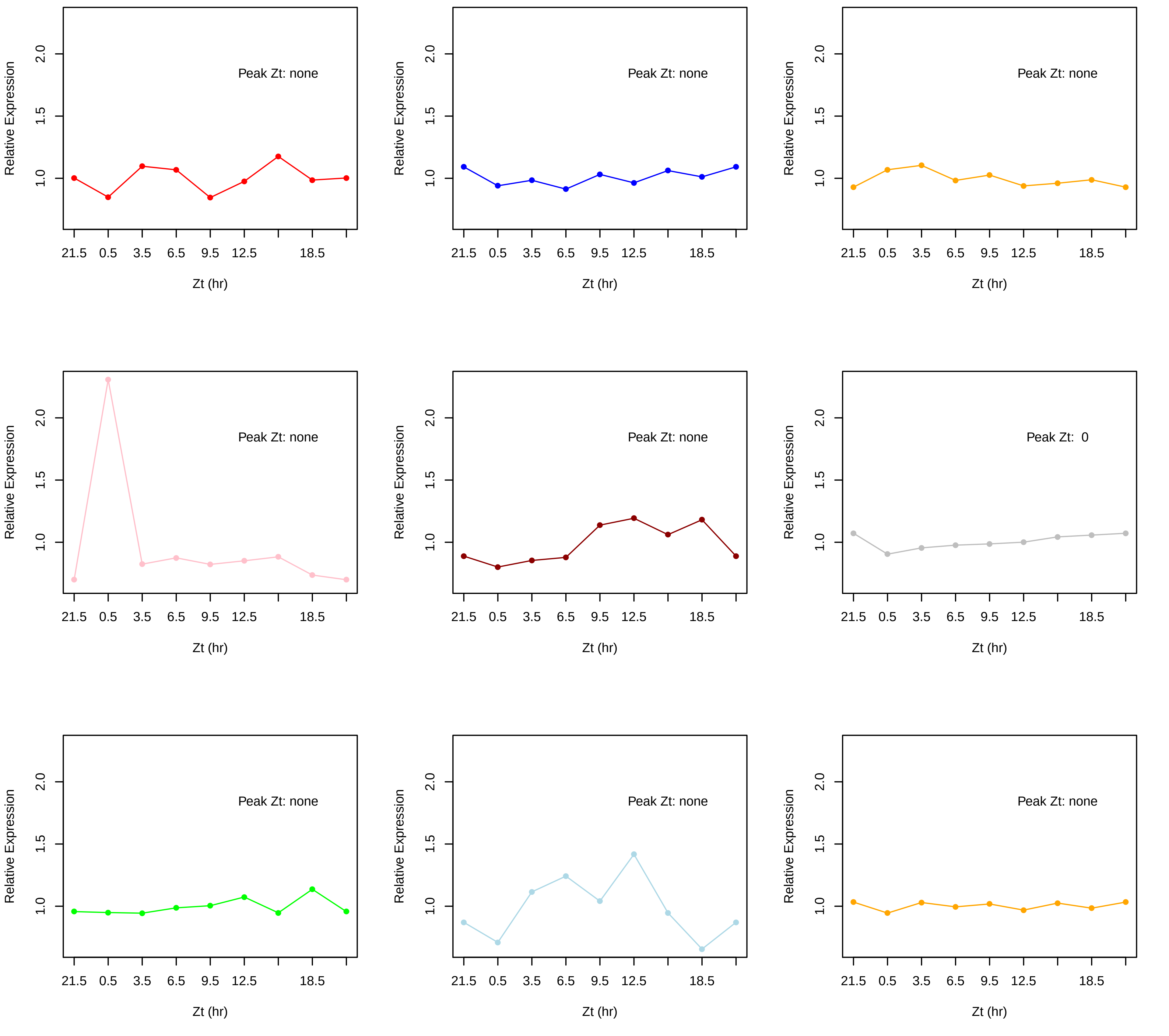

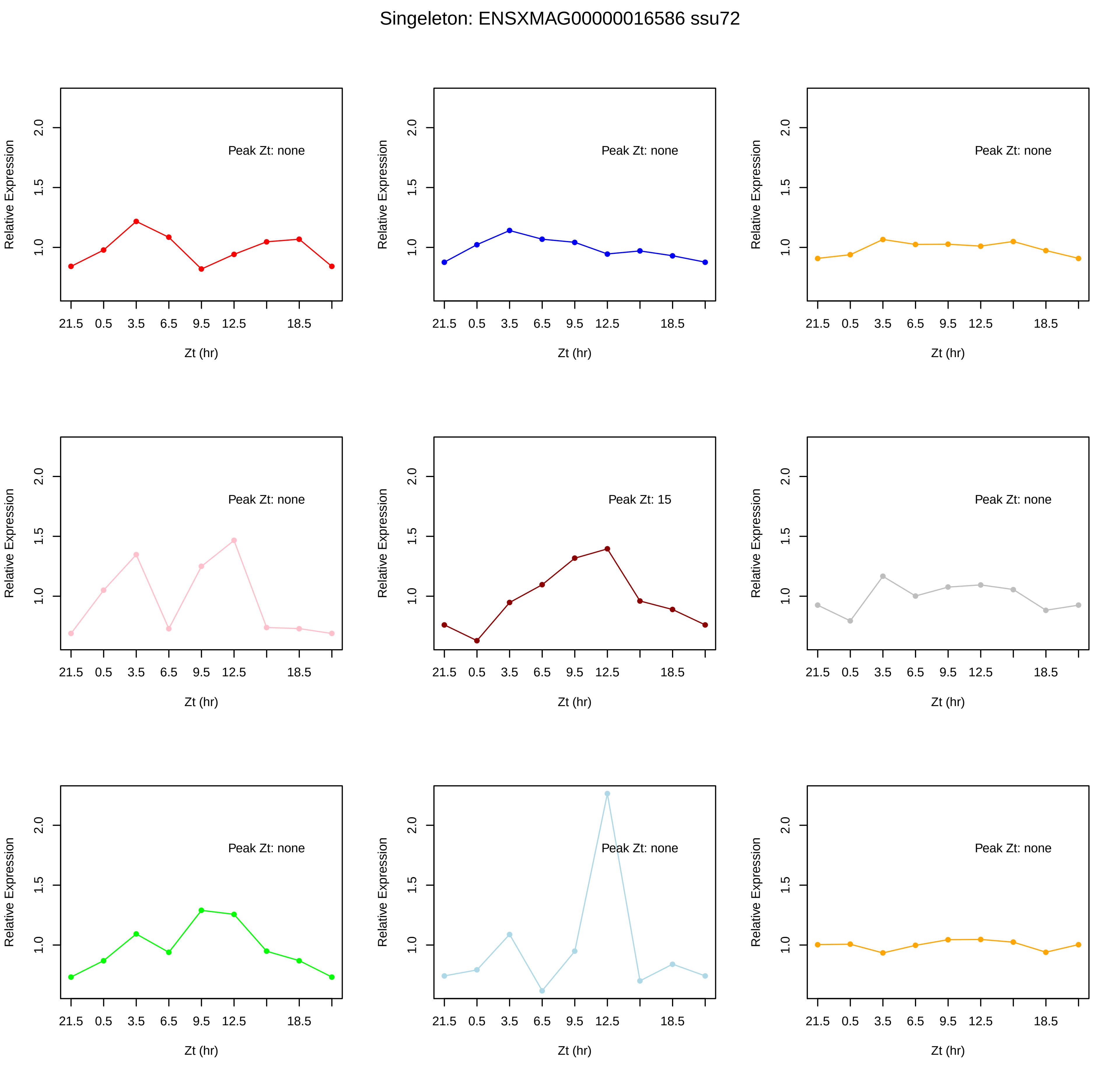

Singleton: ENSXMAG00000016586 ssu72
Peak Zt: none
Peak Zt: none
Peak Zt: none
Peak Zt: none
Peak Zt: 15
Peak Zt: none
Peak Zt: none
Peak Zt: none
Peak Zt: none
Relative Expression
Zt (hr)
21.5
0.5
3.5
6.5
9.5
12.5
18.5
1.0
1.5
2.0

Singleton: ENSXMAG00000024549 prune

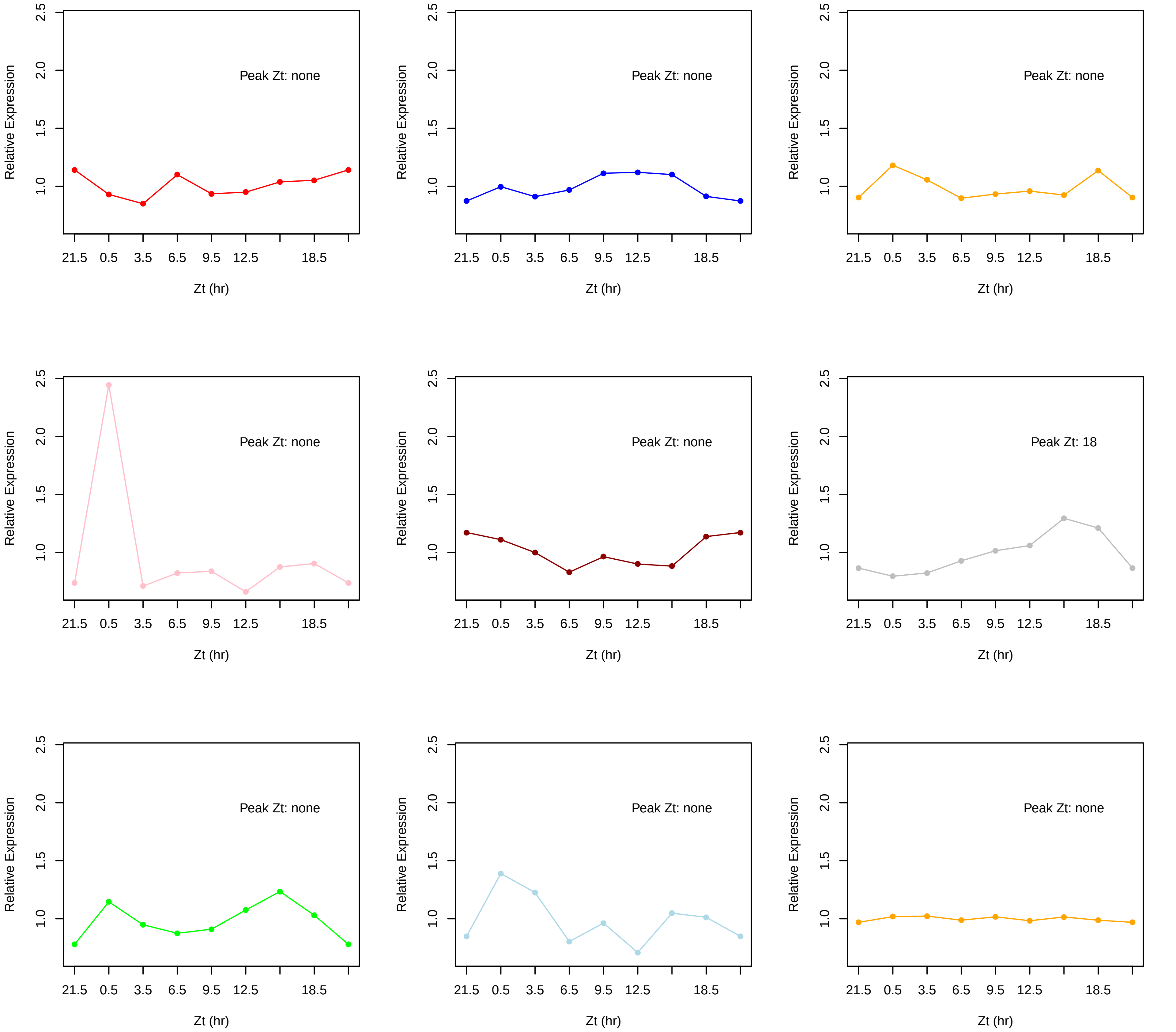

Singeleton: ENSXMAG00000007931 ecsit

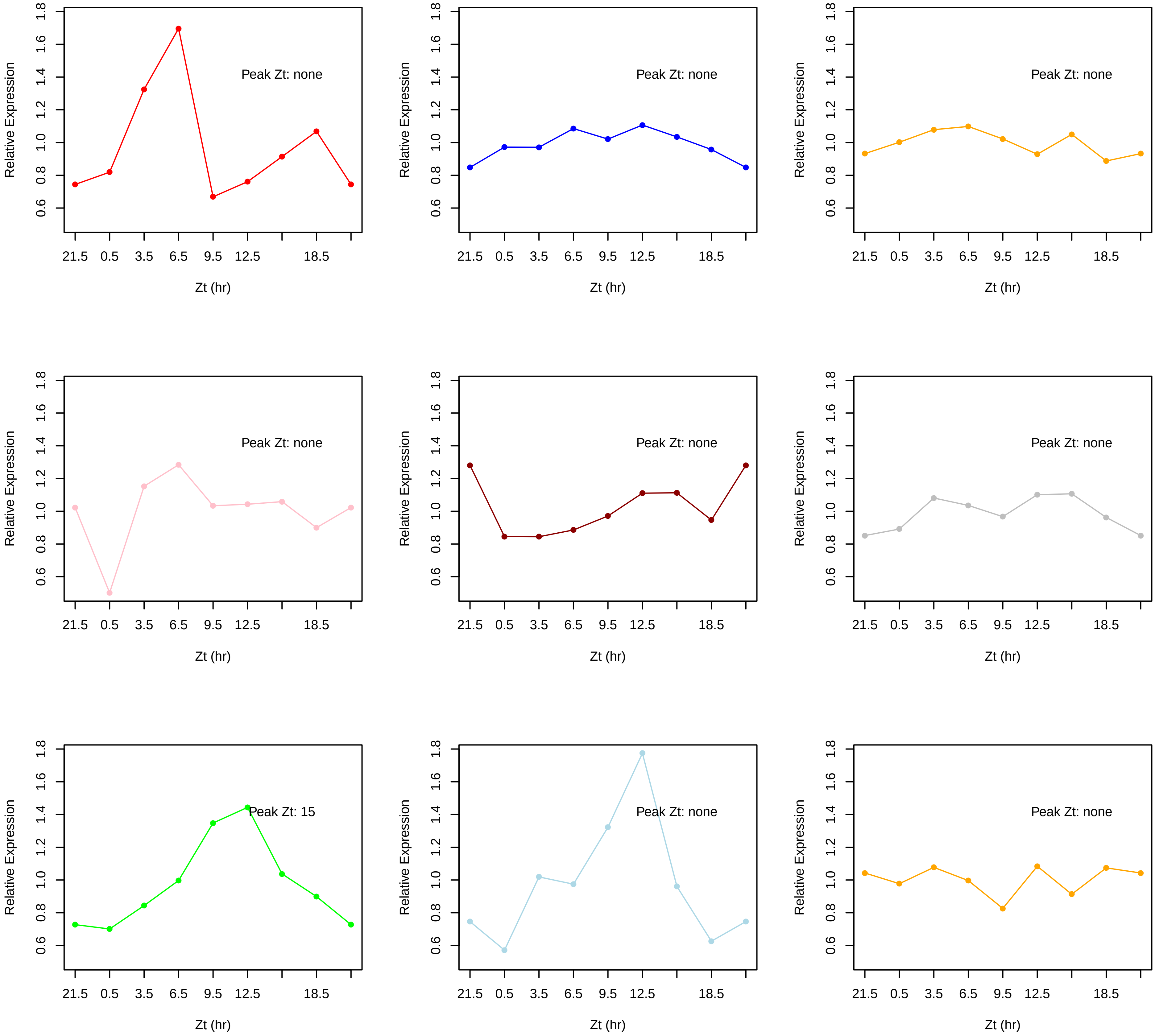

Singleton: ENSXMAG00000011186 lpcat3

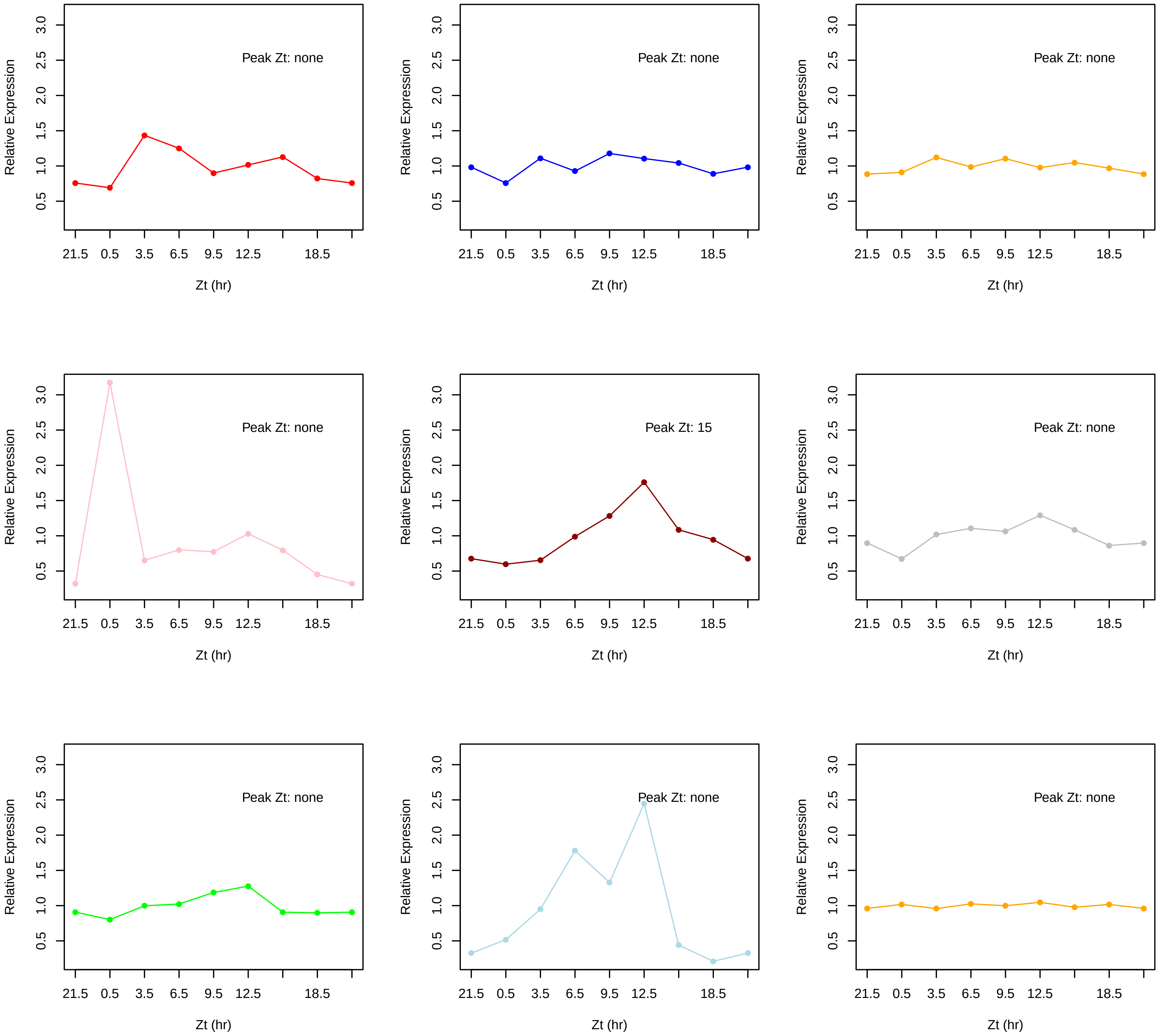

Singeleton: ENSXMAG00000021268 pllp

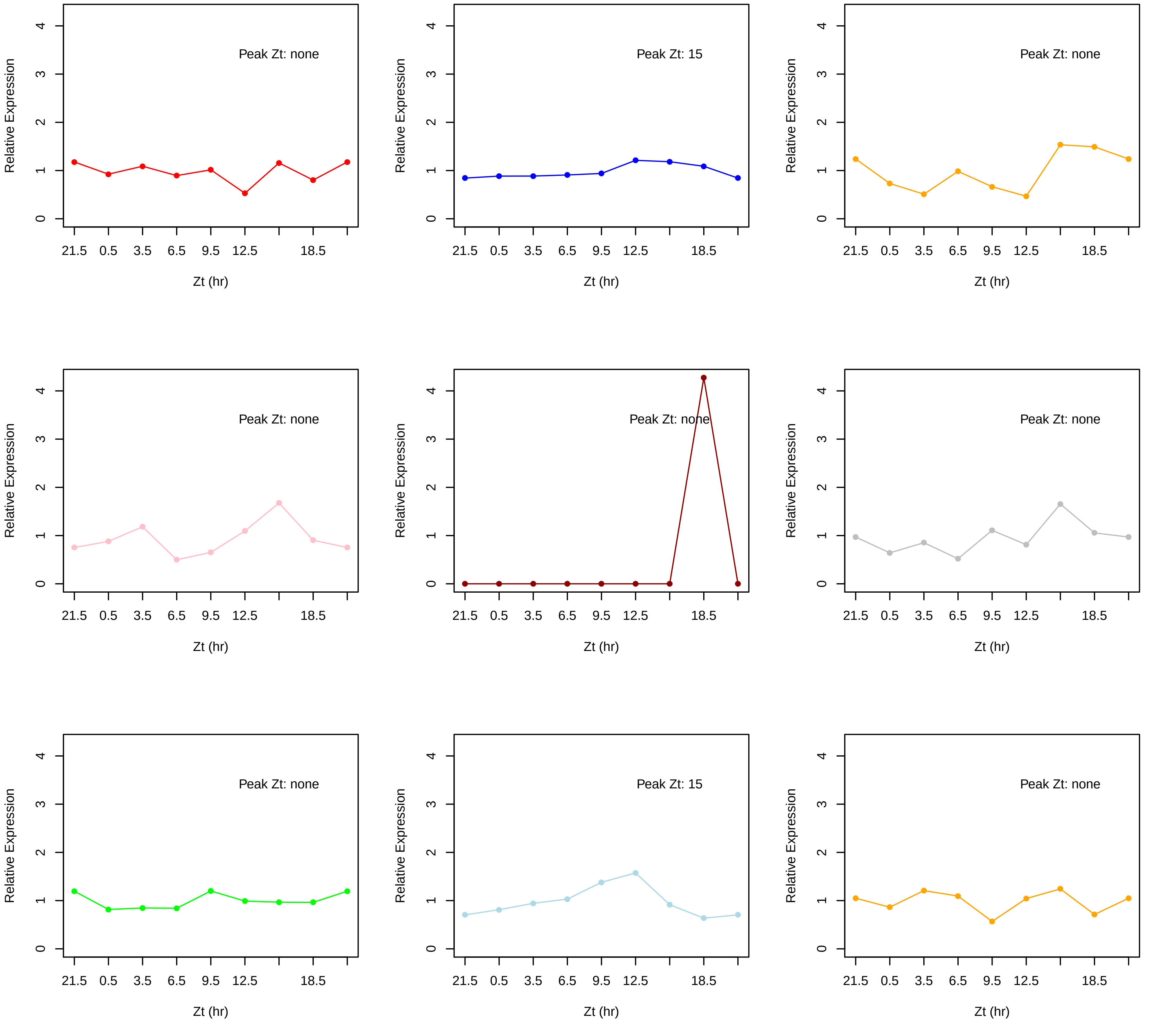

Singleton: ENSXMAG00000011155 clstn3

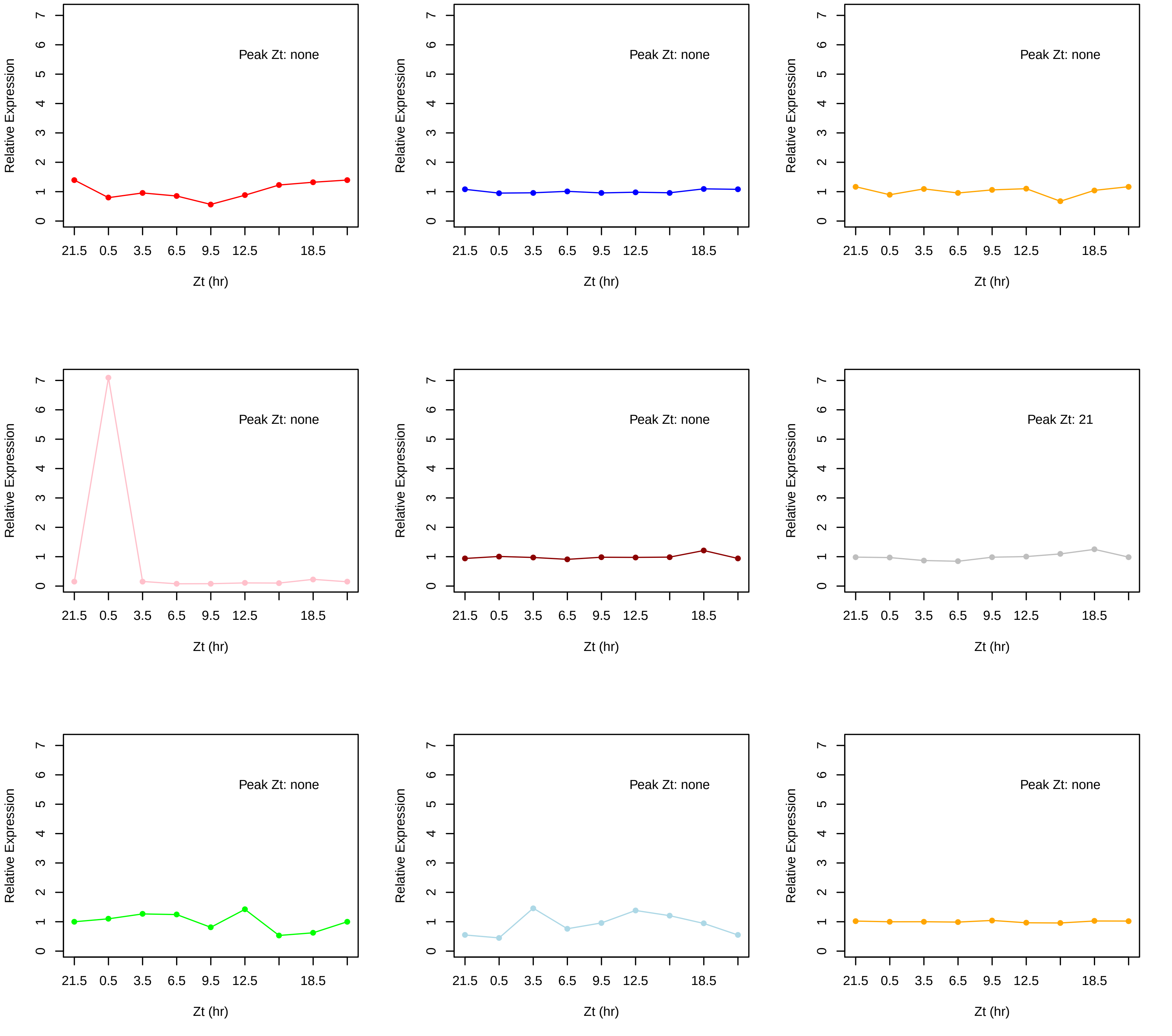

Singeleton: ENSXMAG00000011069 stard10

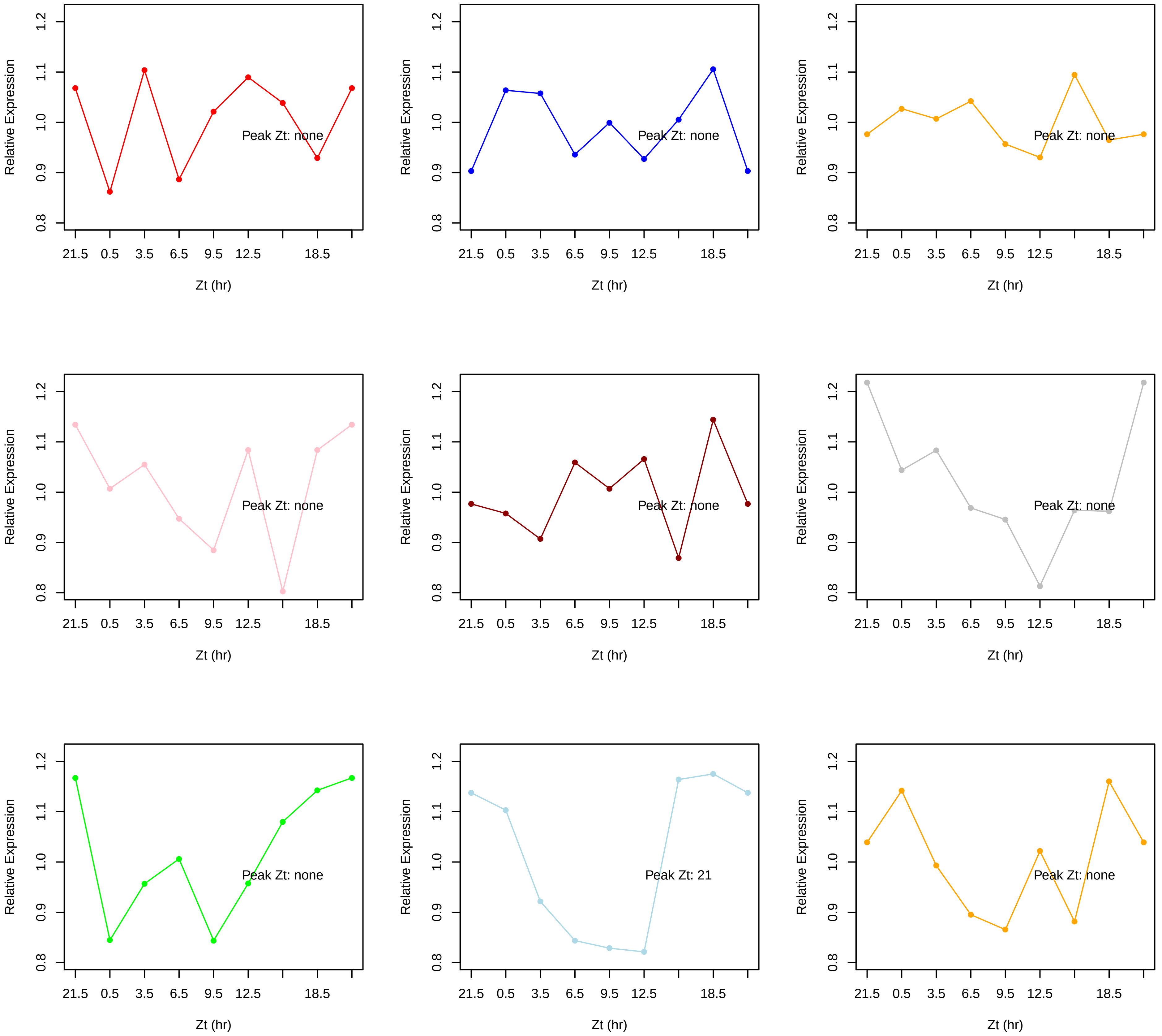

Singeleton: ENSXMAG00000013018 ARL2BP

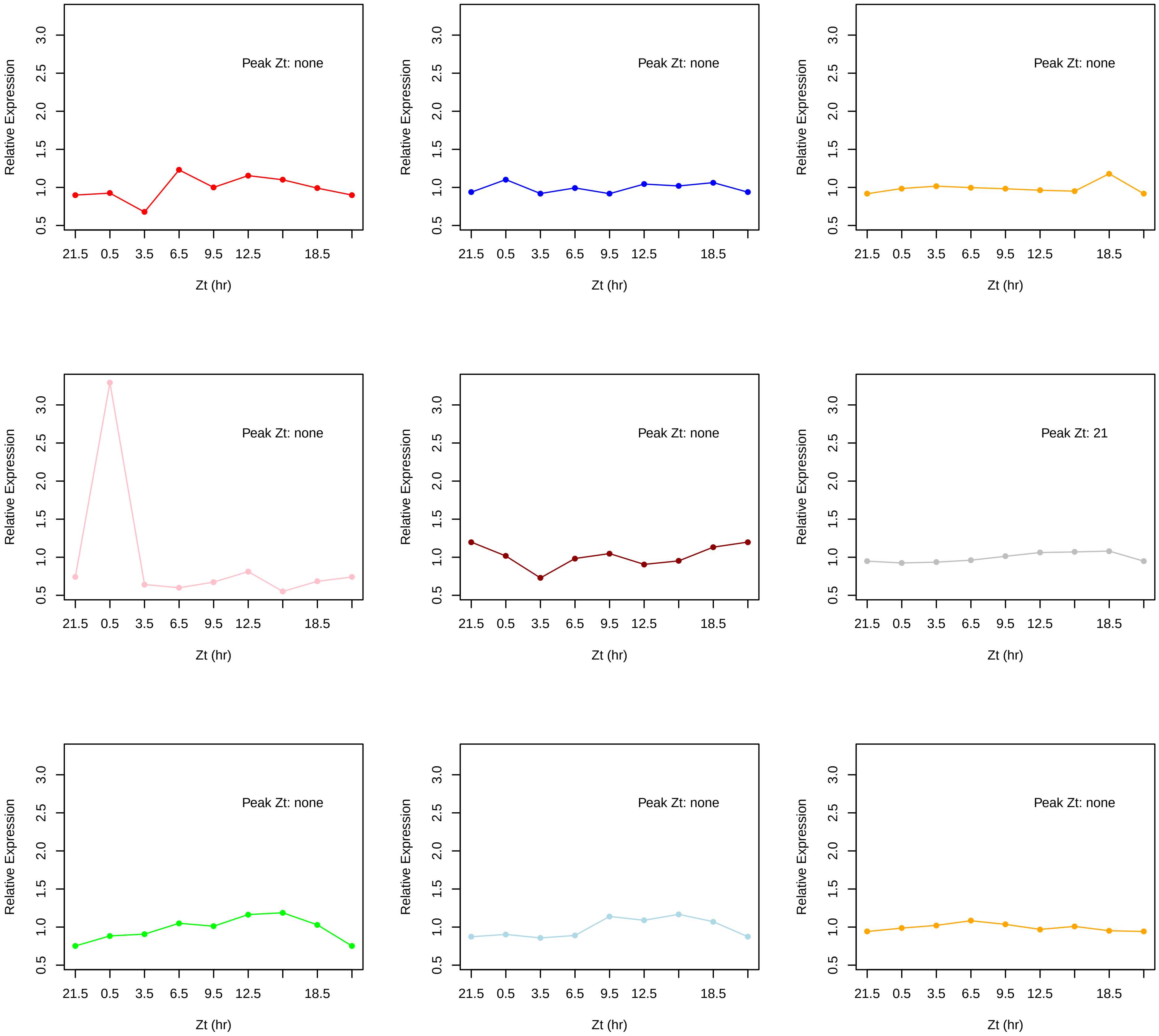

Singleton: ENSXMAG00000009067

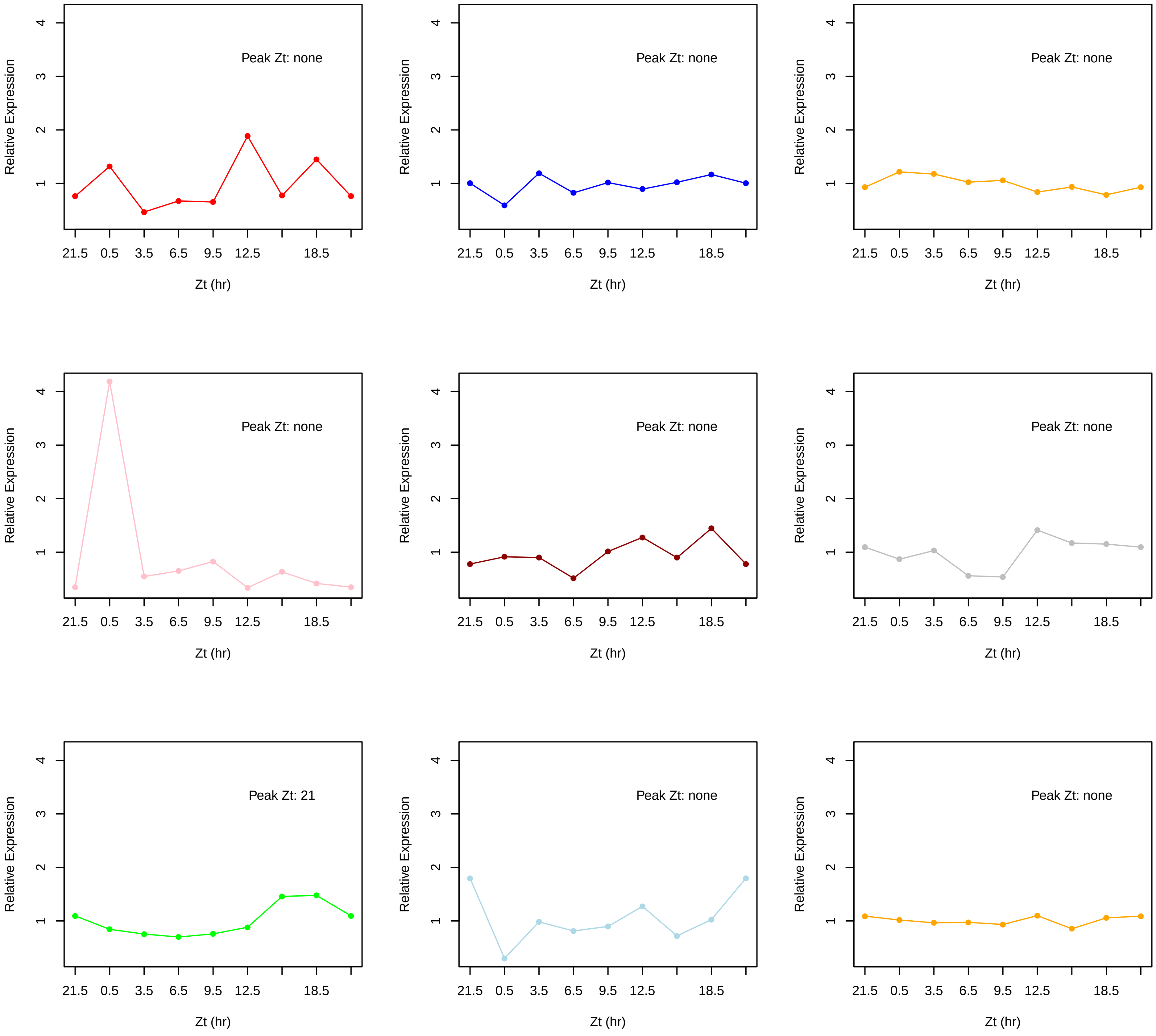

Singeleton: ENSXMAG00000013026 RSPRY1

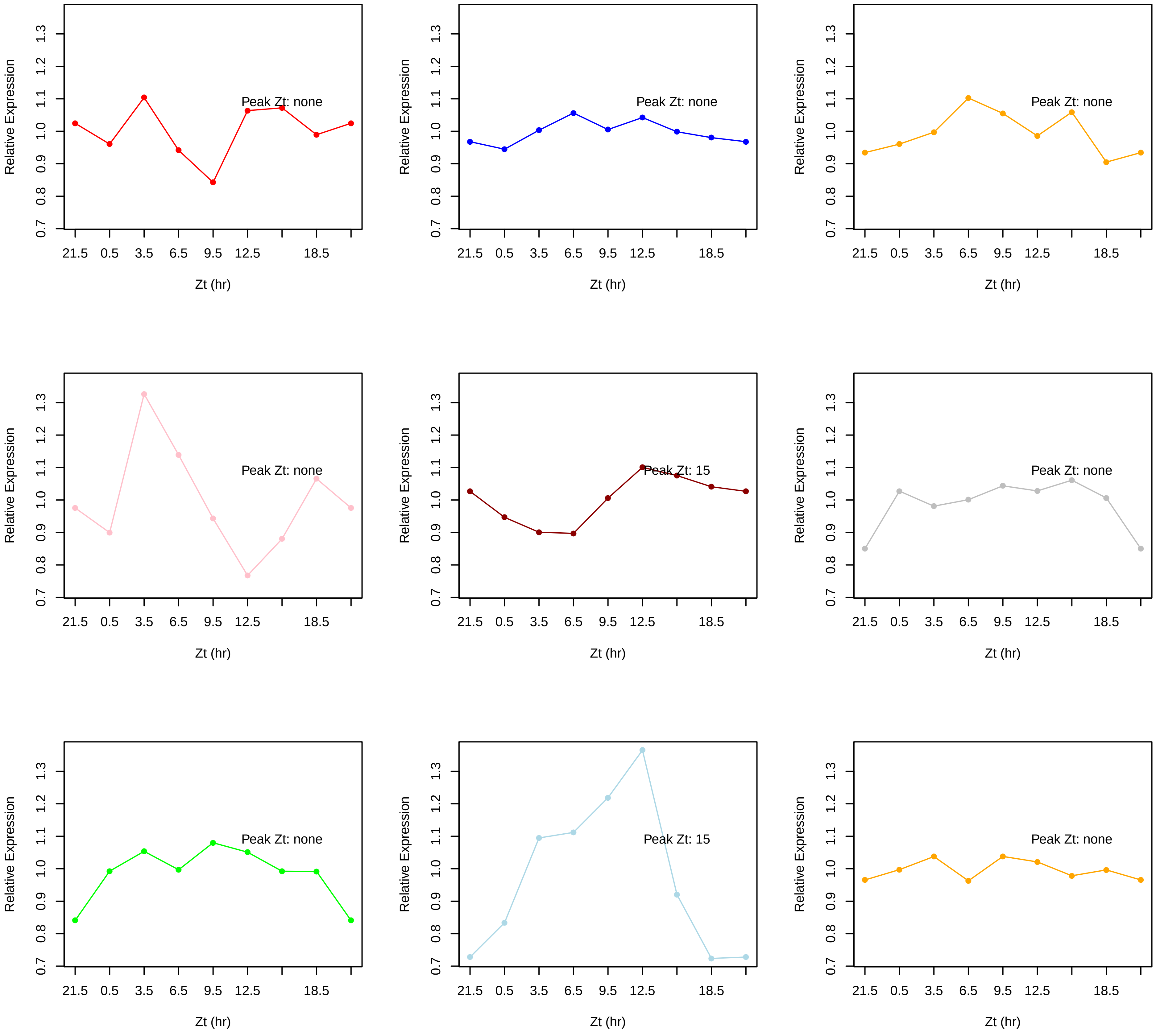

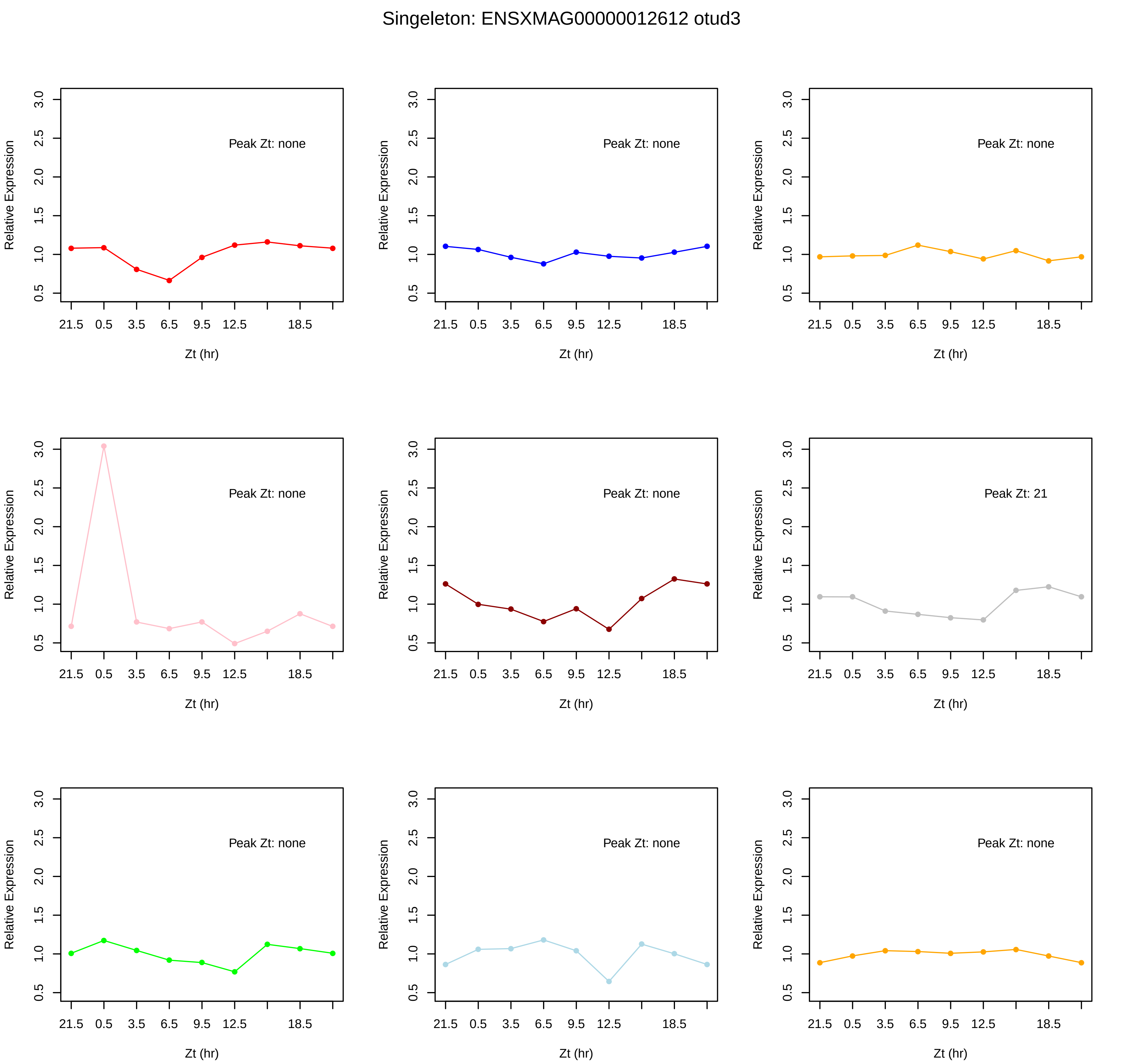
Singleton: ENSXMAG00000012612 otud3
Peak Zt: none
Peak Zt: none
Peak Zt: none
Peak Zt: none
Peak Zt: none
Peak Zt: 21
Peak Zt: none
Peak Zt: none
Peak Zt: none
Relative Expression
Zt (hr)
21.5
0.5
3.5
6.5
9.5
12.5
18.5
0.5
1.0
1.5
2.0
2.5
3.0

Singeleton: ENSXMAG00000008275 myo3a

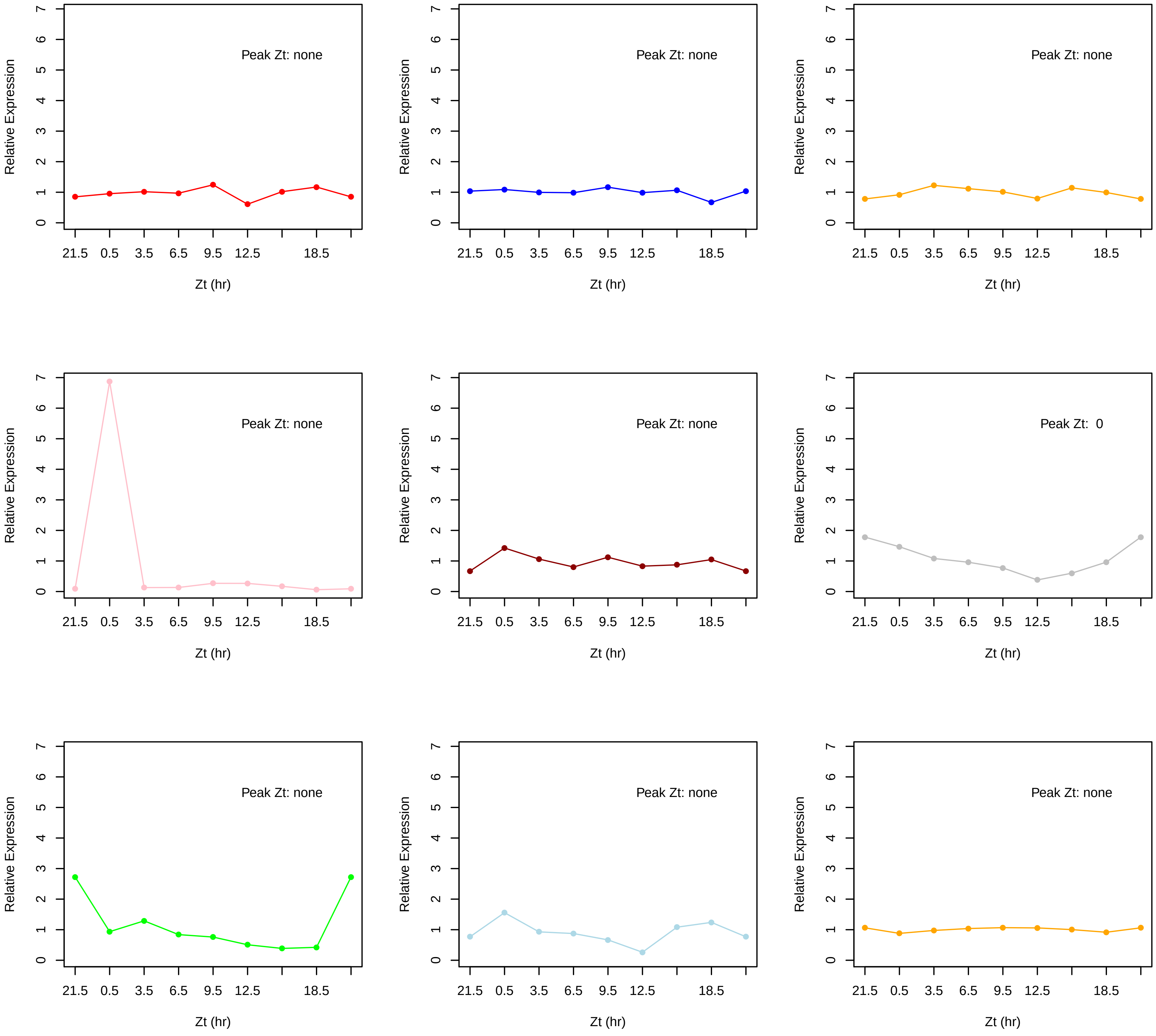

Singeleton: ENSXMAG00000021448 keap1b

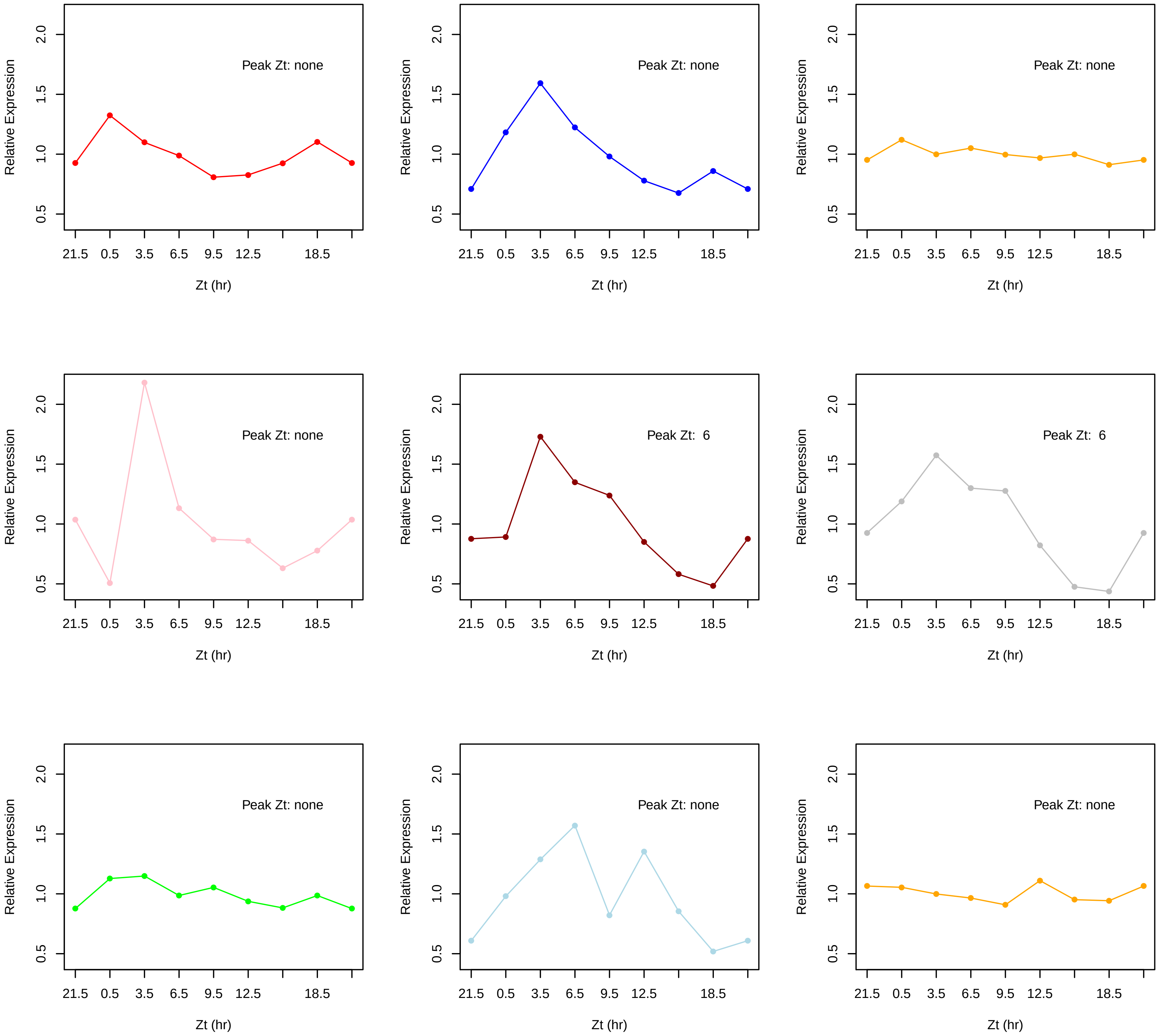

Singeleton: ENSXMAG00000023187 dnajc22

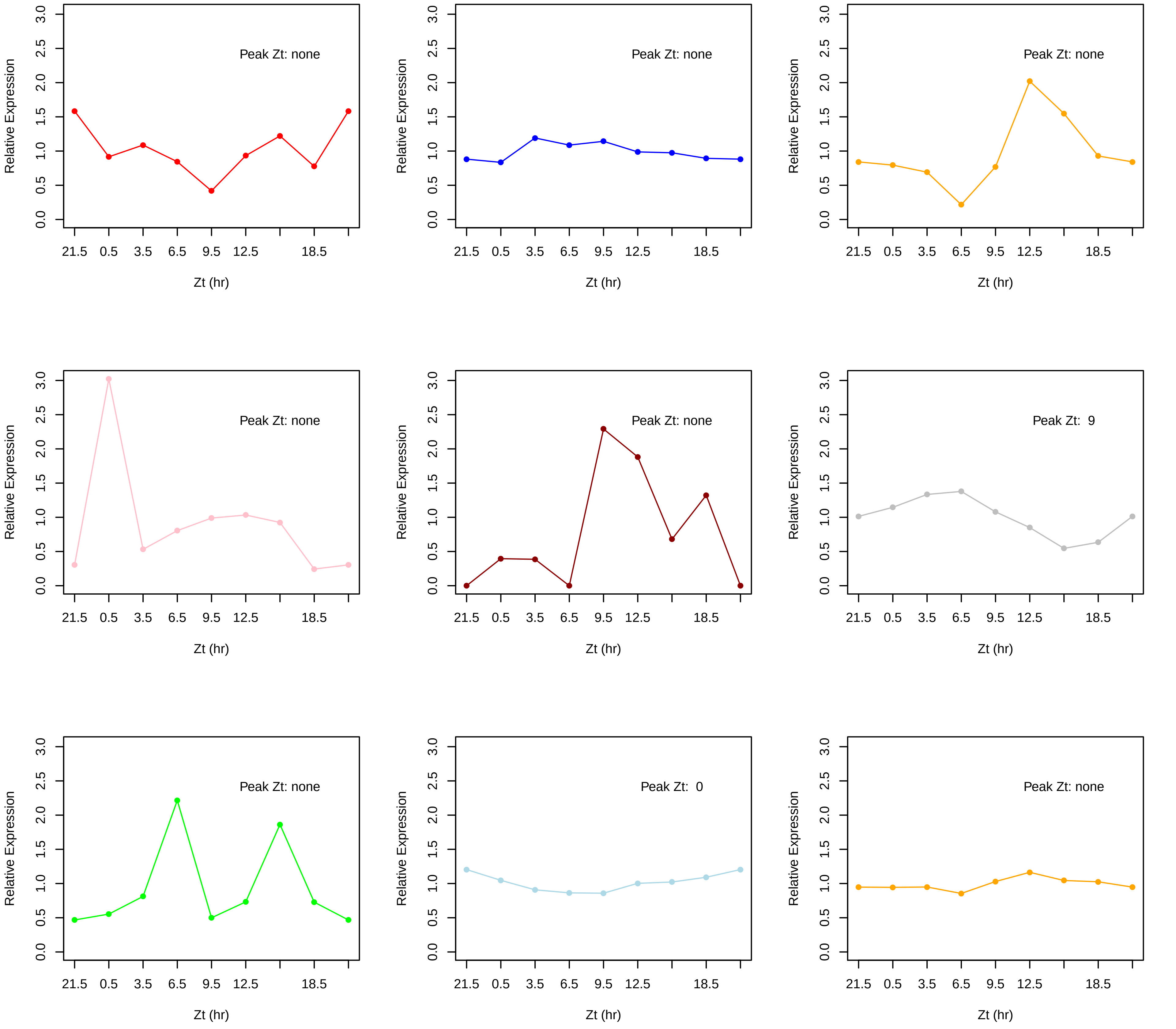

Singeleton: ENSXMAG00000023922 fam83c

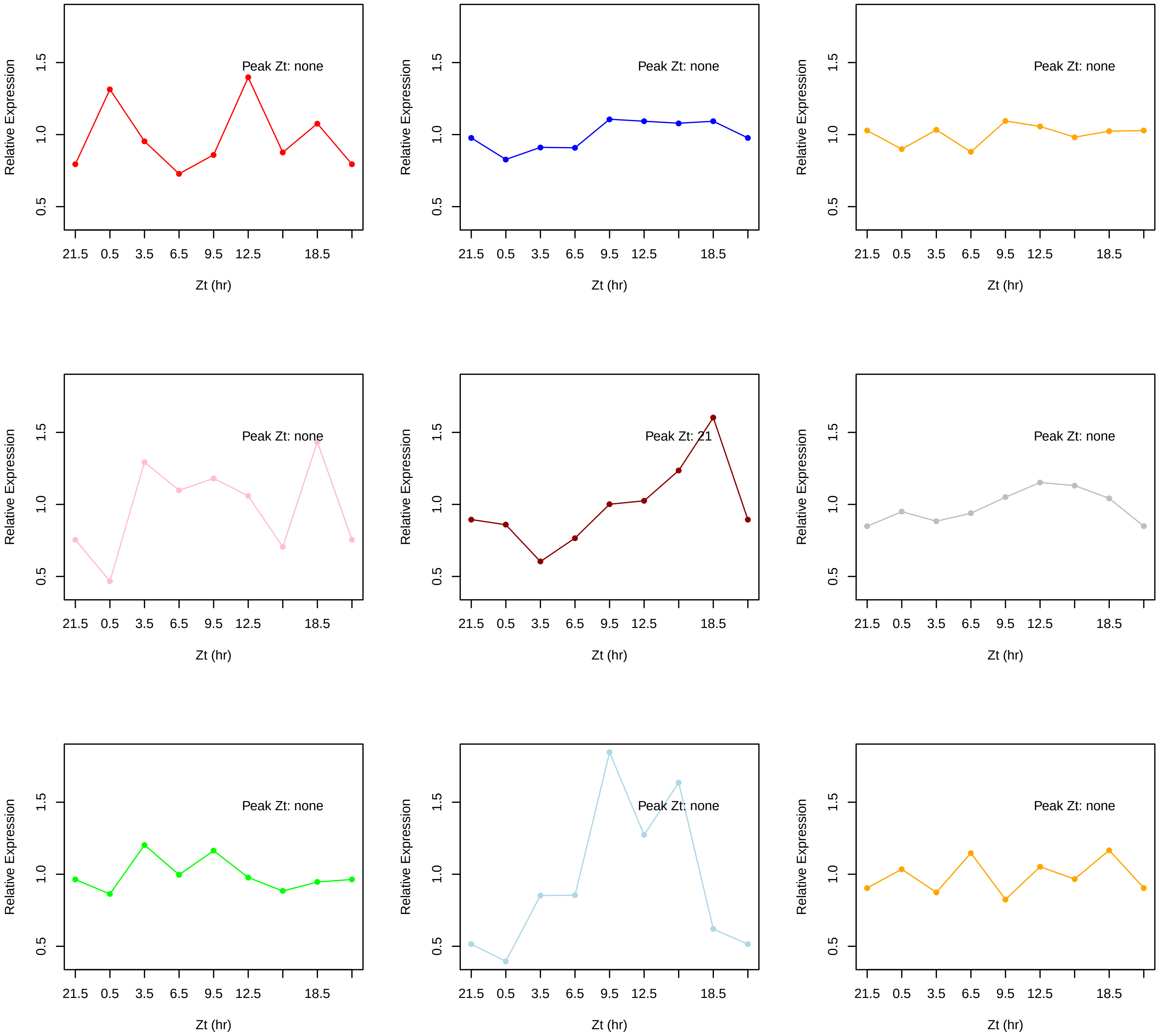

Singeleton: ENSXMAG00000008609 camsap2b

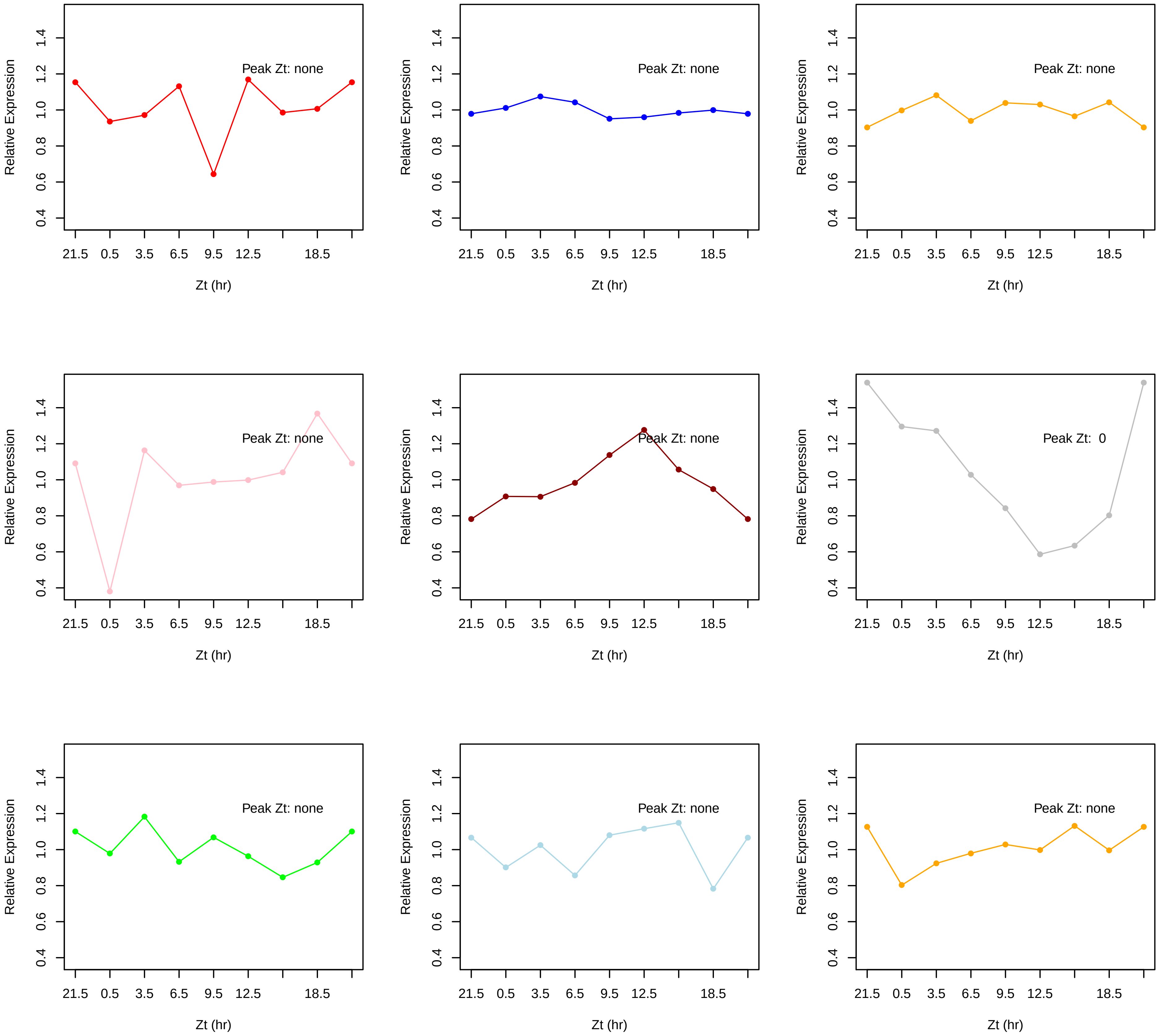

Singeleton: ENSXMAG00000015573 elovl7a

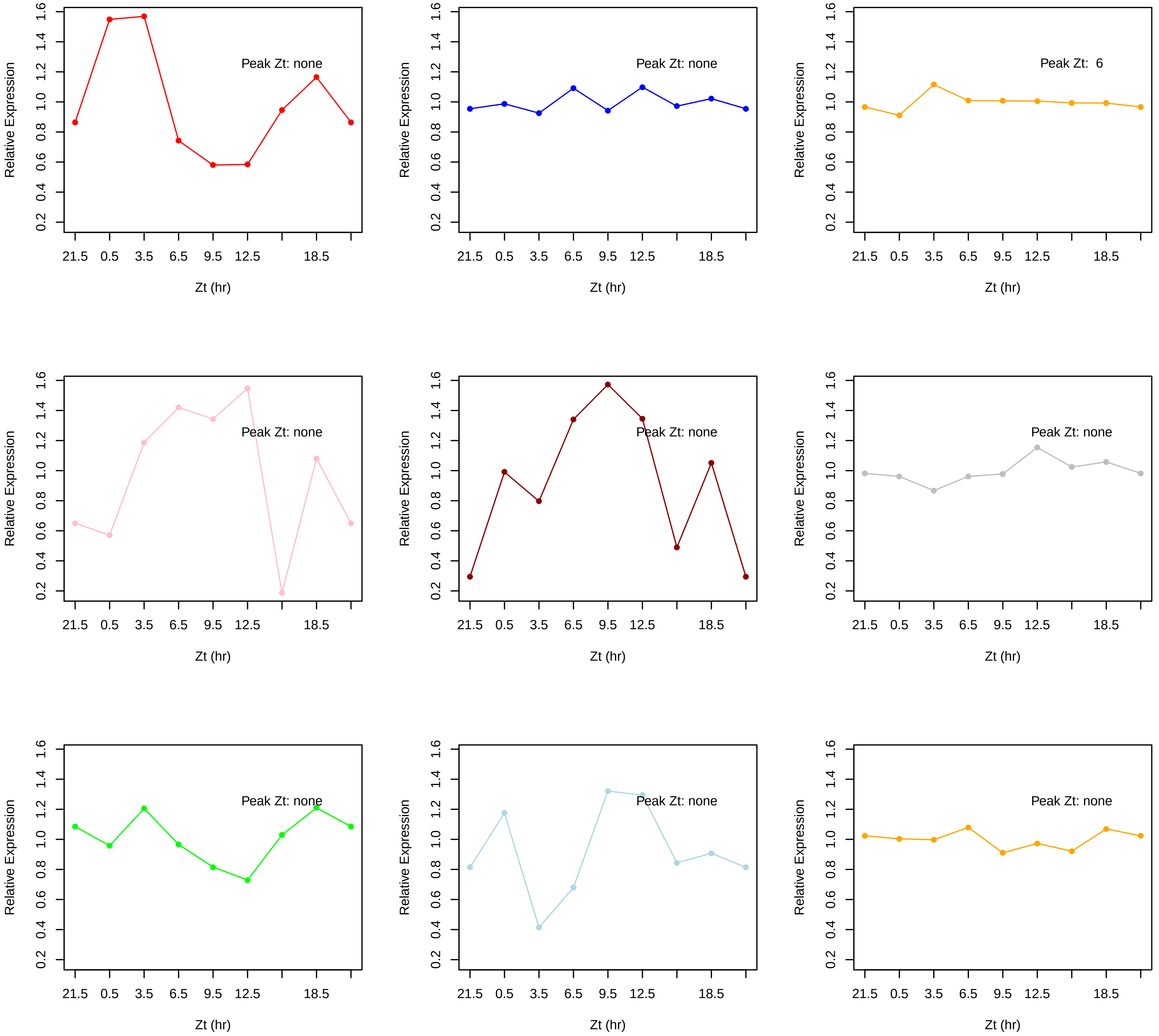

Singeleton: ENSXMAG00000011133 pex5

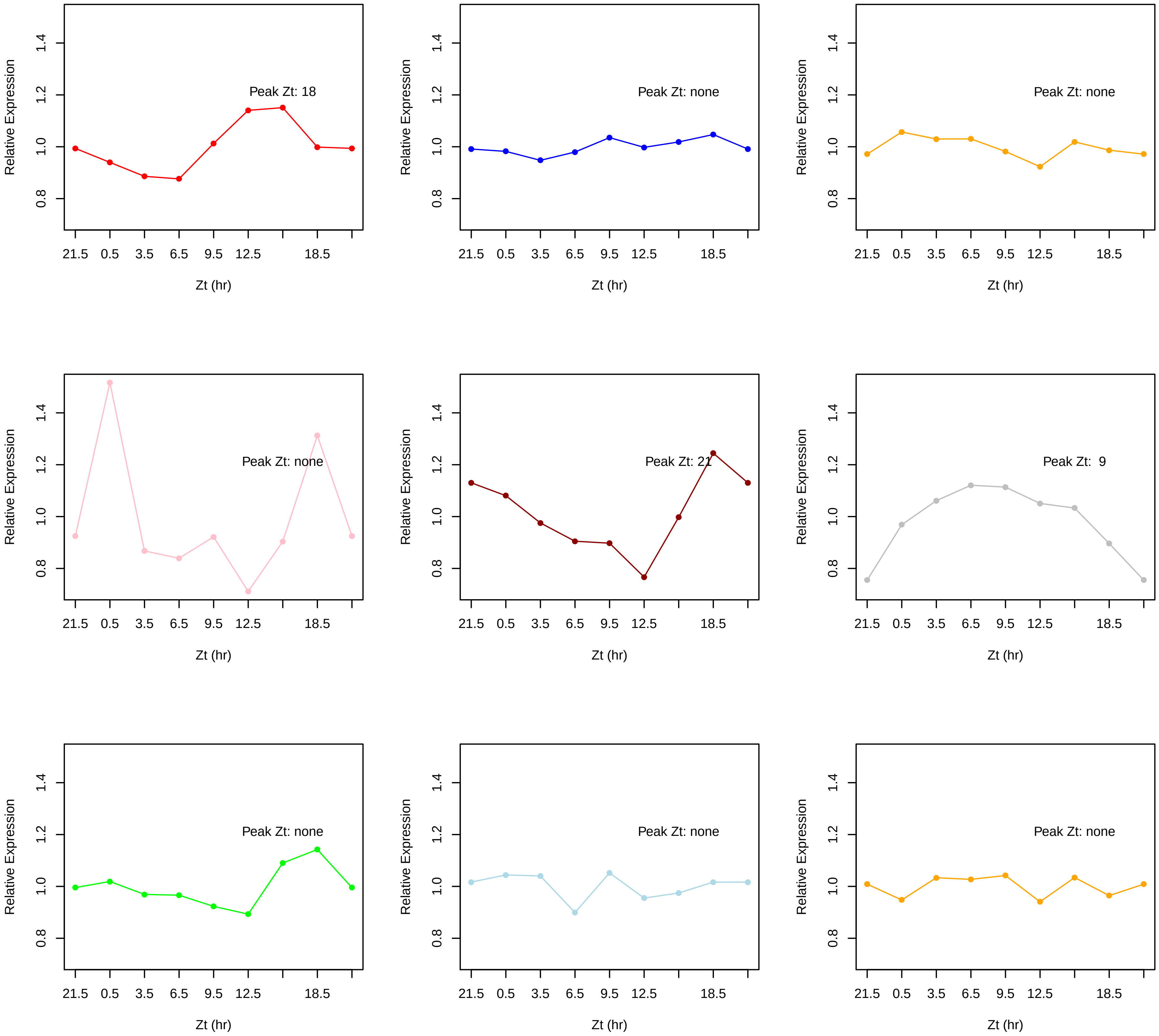

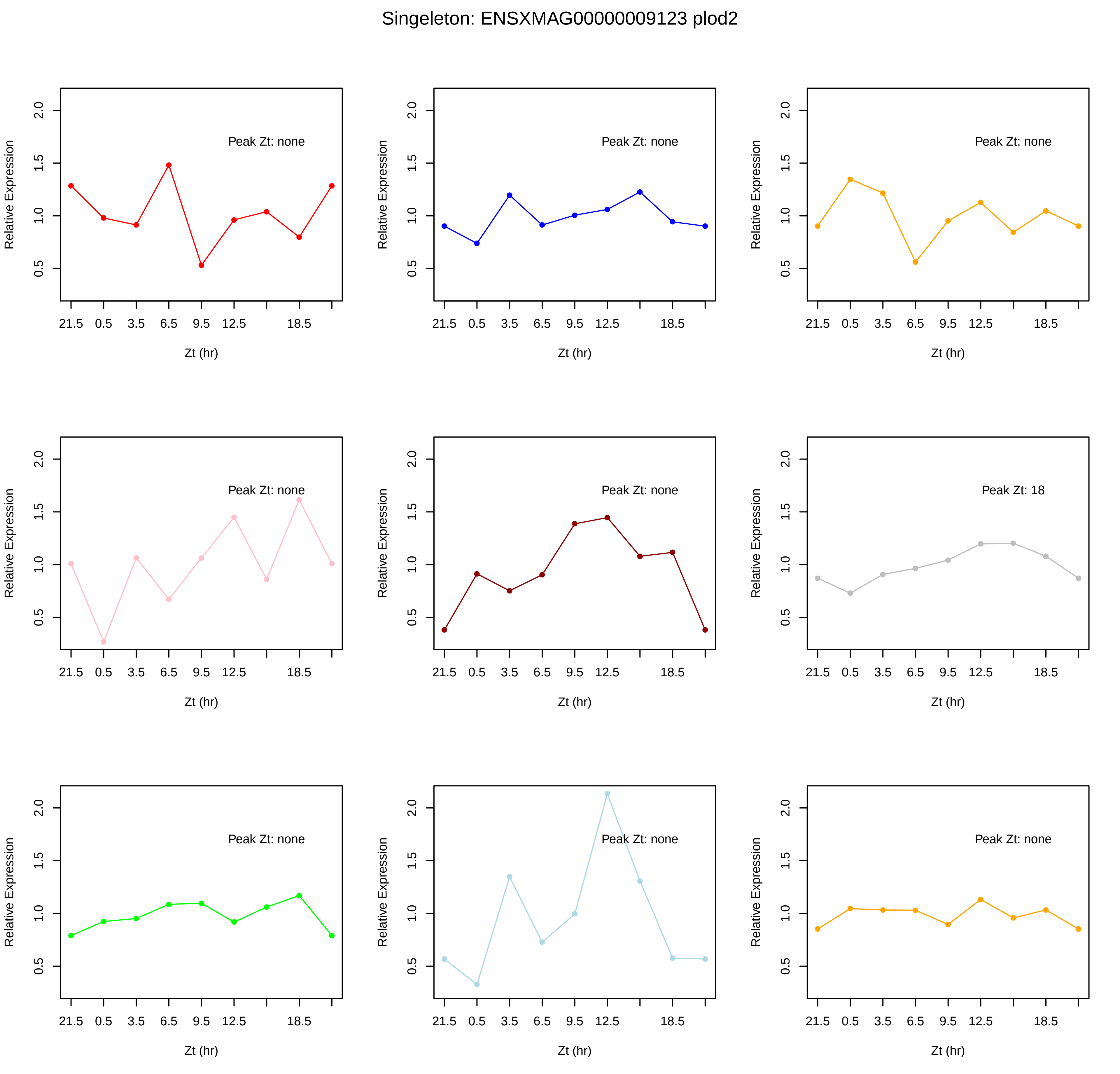
Singeleton: ENSXMAG00000009123 plod2
Peak Zt: none
Peak Zt: none
Peak Zt: none
Peak Zt: none
Peak Zt: none
Peak Zt: 18
Peak Zt: none
Peak Zt: none
Peak Zt: none
Relative Expression
Zt (hr)
21.5
0.5
3.5
6.5
9.5
12.5
18.5
0.5
1.0
1.5
2.0

Singeleton: ENSXMAG00000015583 ercc8

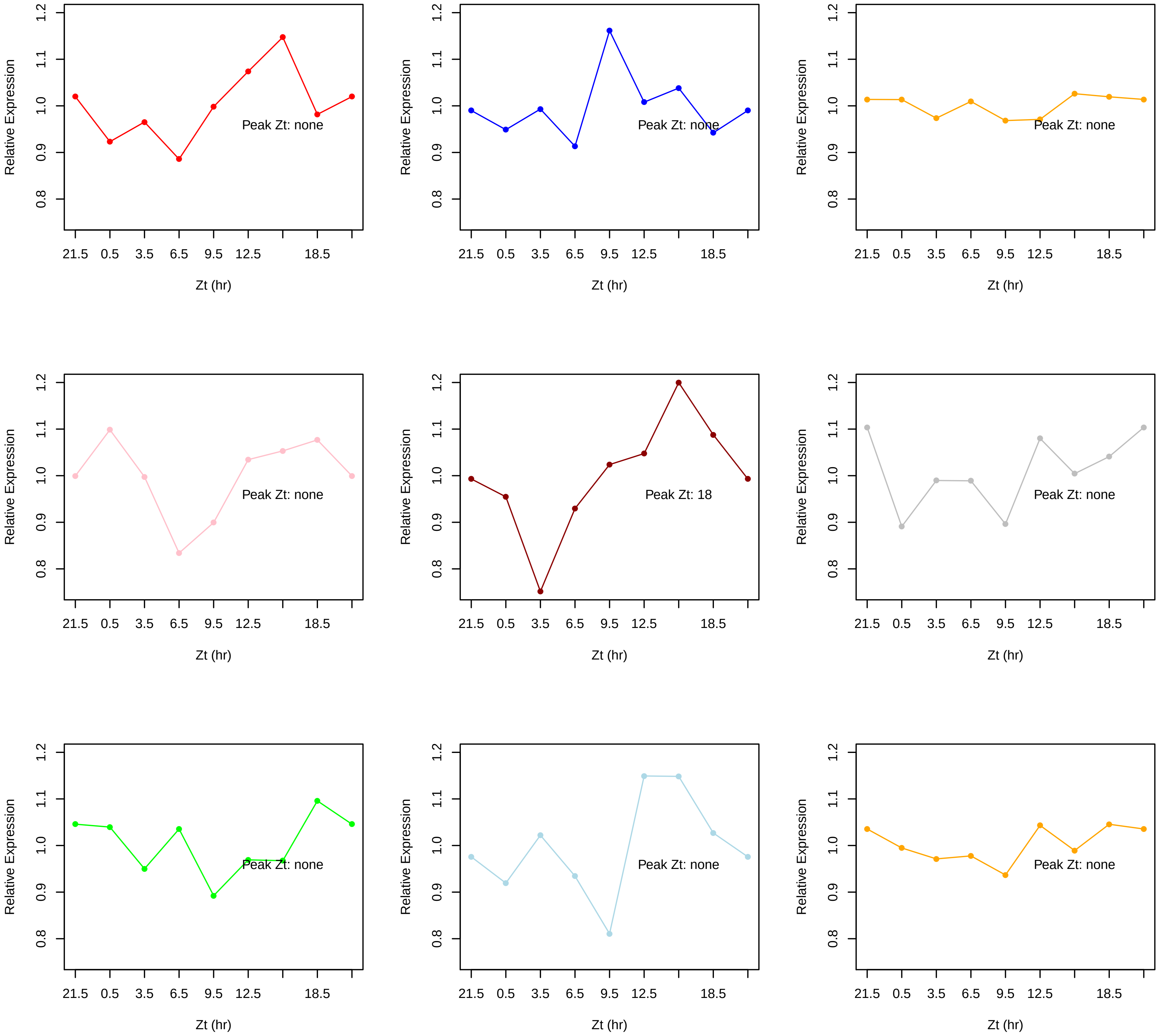

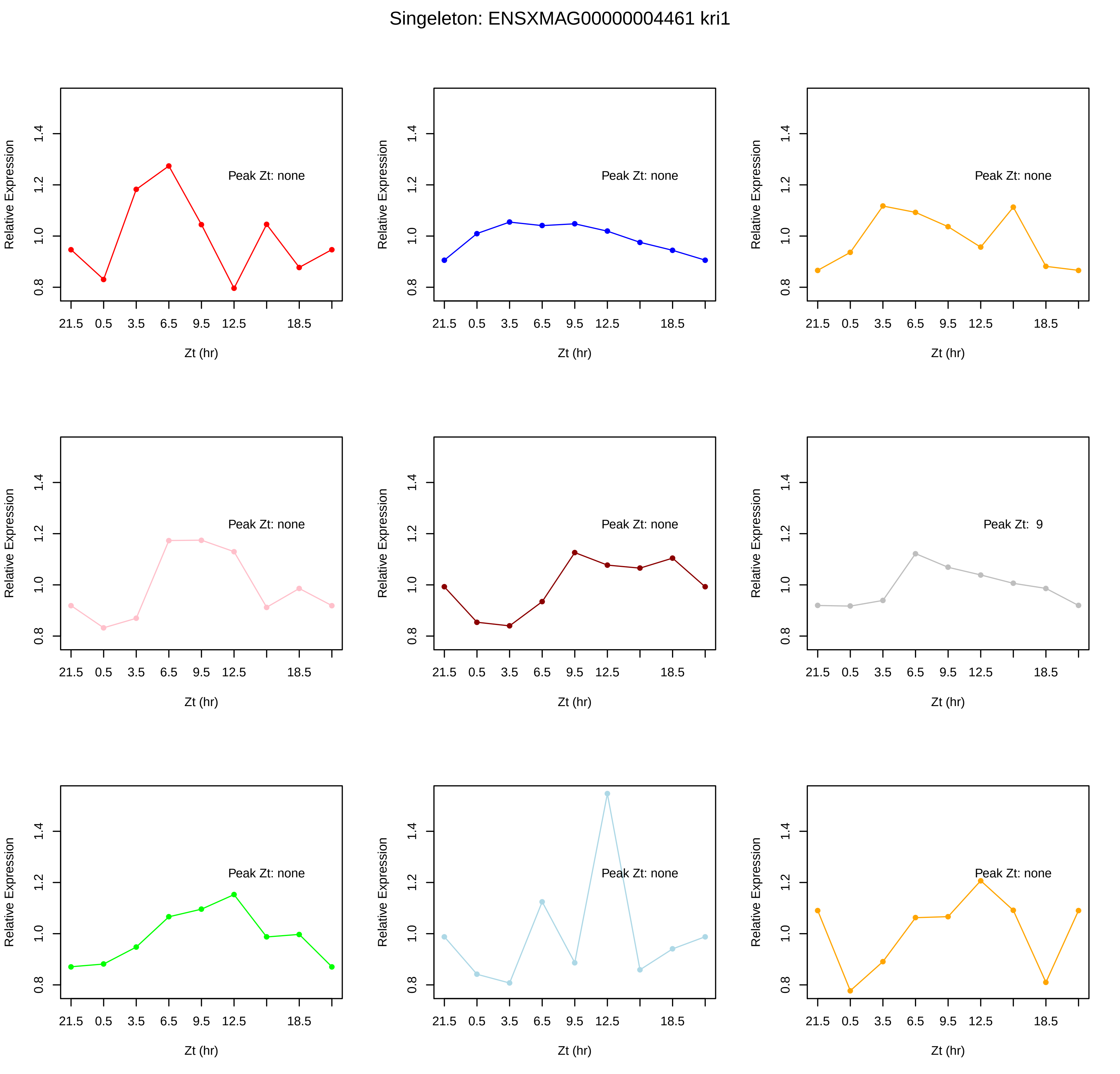
Singeleton: ENSXMAG00000004461 kri1
Peak Zt: none
Peak Zt: none
Peak Zt: none
Peak Zt: none
Peak Zt: none
Peak Zt: 9
Peak Zt: none
Peak Zt: none
Peak Zt: none
Relative Expression
Zt (hr)
21.5 0.5 3.5 6.5 9.5 12.5 18.5
0.8 1.0 1.2 1.4

Singeleton: ENSXMAG00000001928 pir

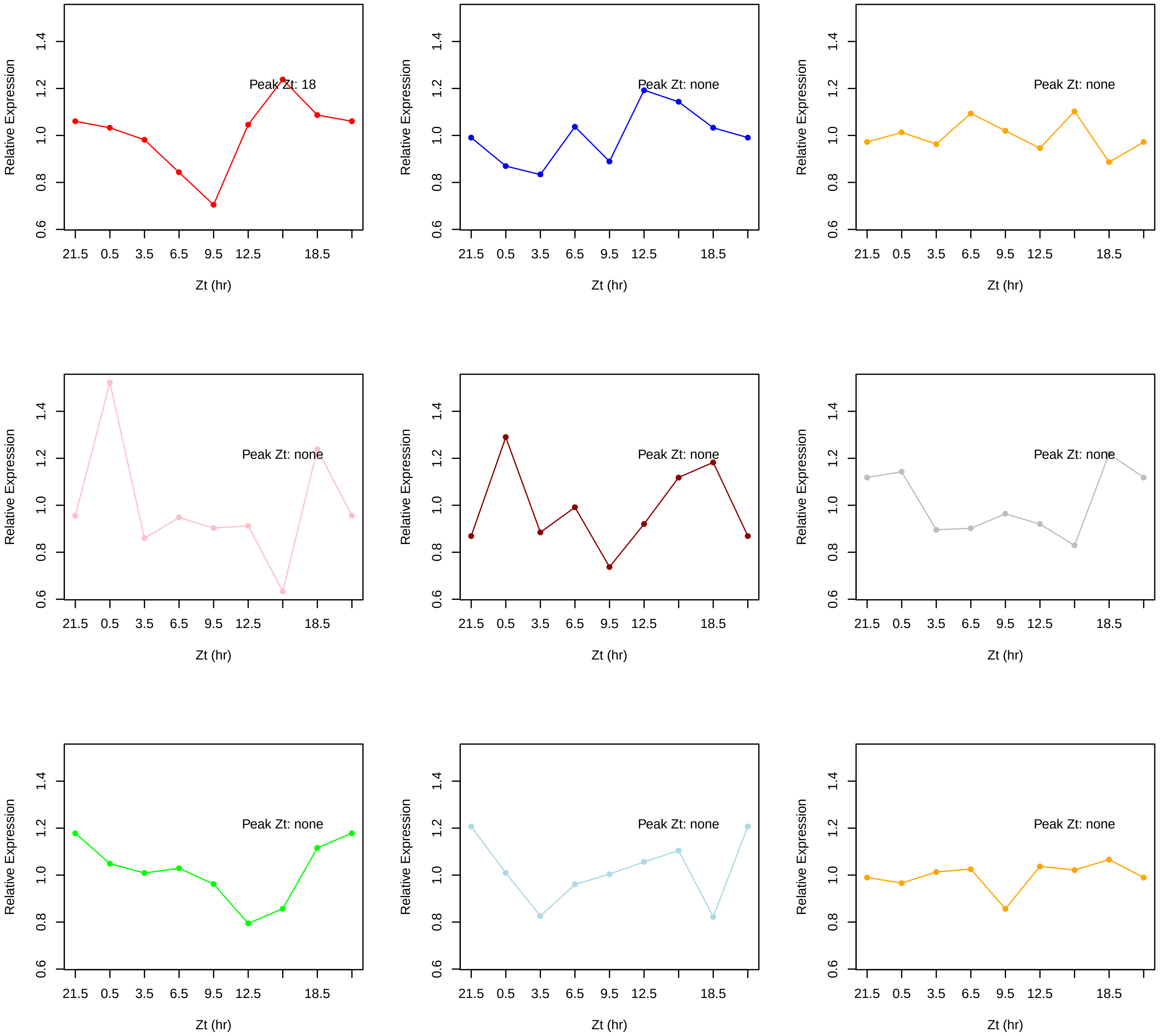

Singeleton: ENSXMAG00000001985 fkbp11

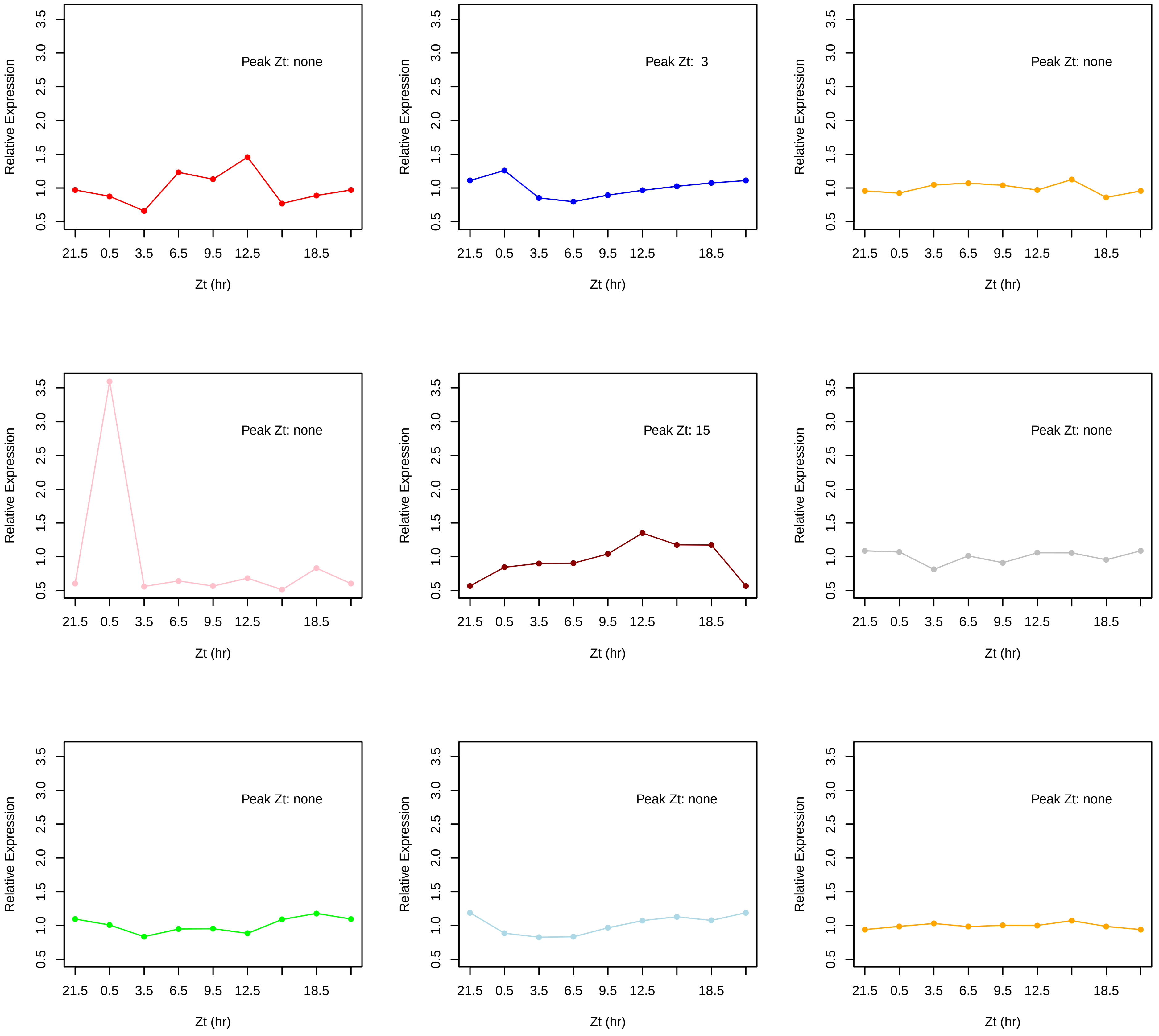

Singeleton: ENSXMAG00000029512 dia1b

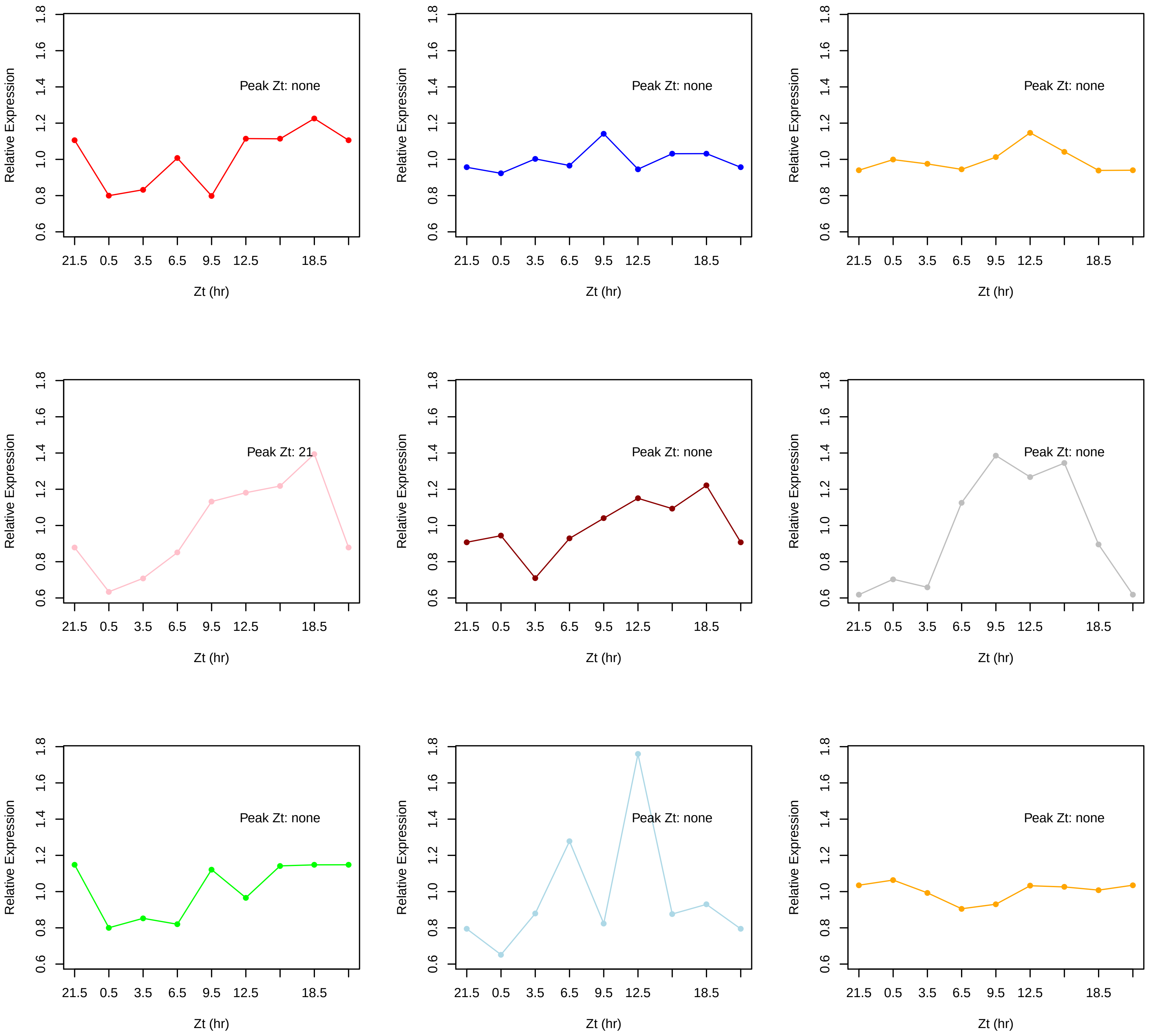

Singeleton: ENSXMAG00000027789 anapc15

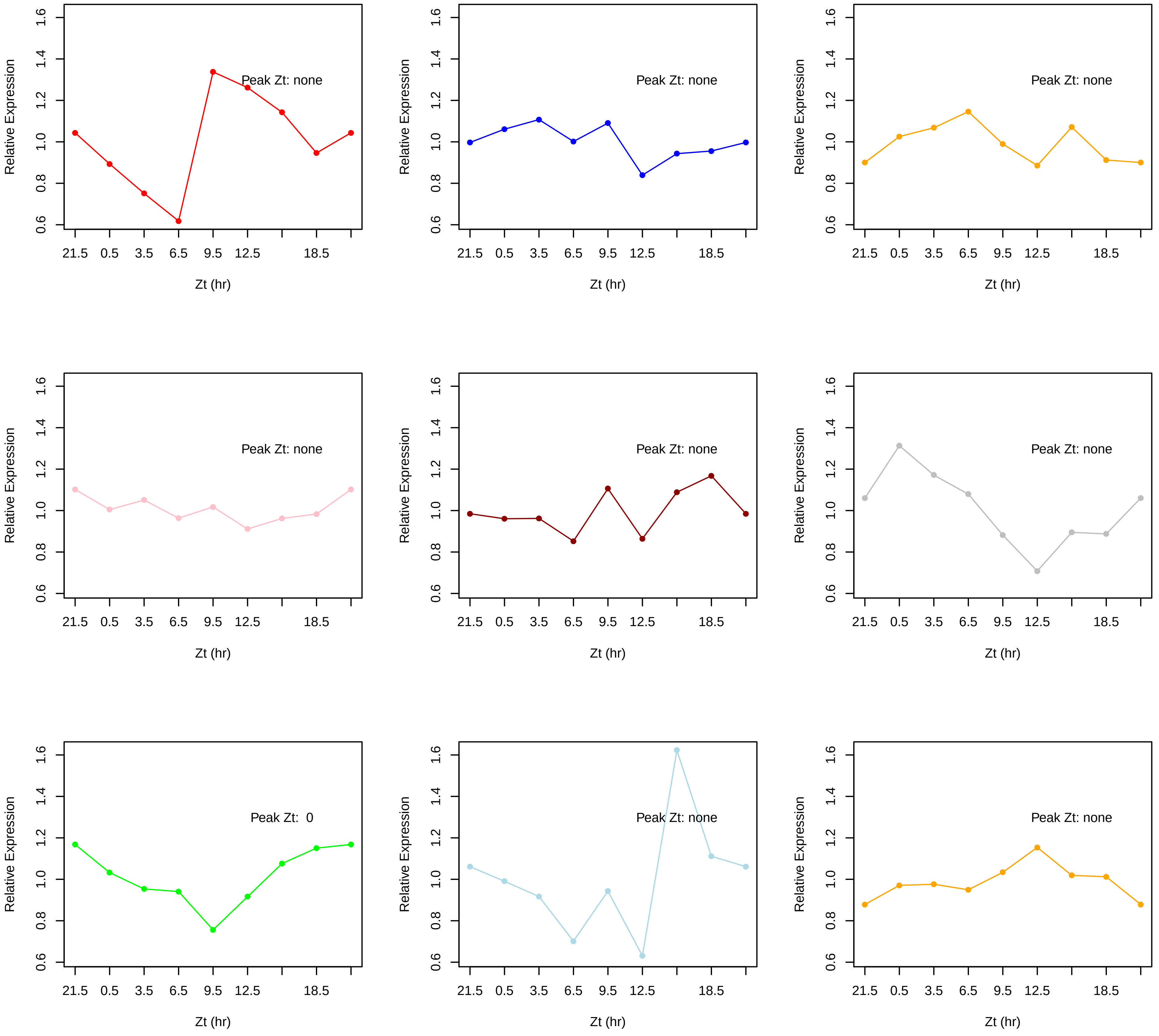

Singeleton: ENSXMAG00000011089 p3h3

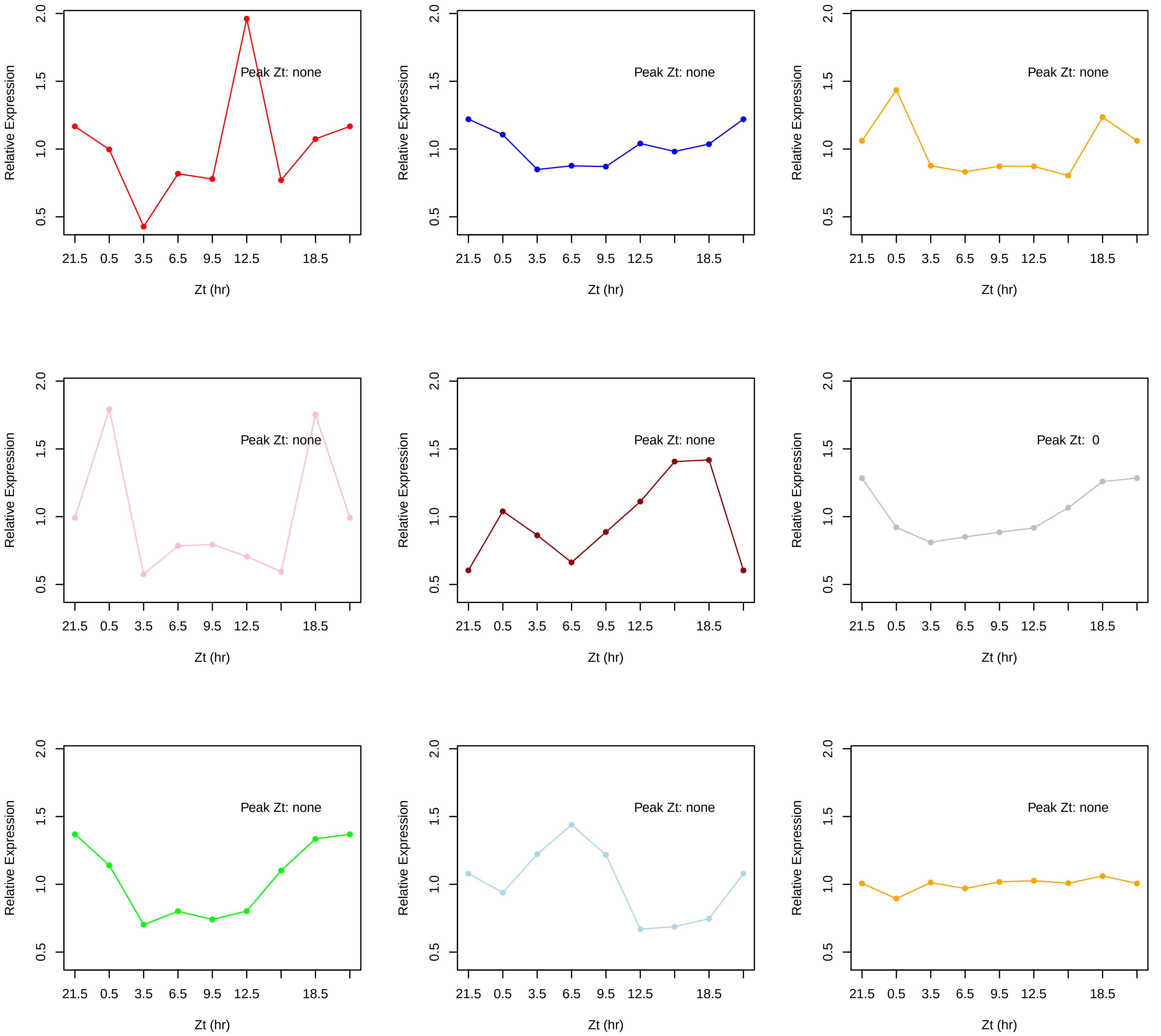

Singeleton: ENSXMAG00000022548 wnt1

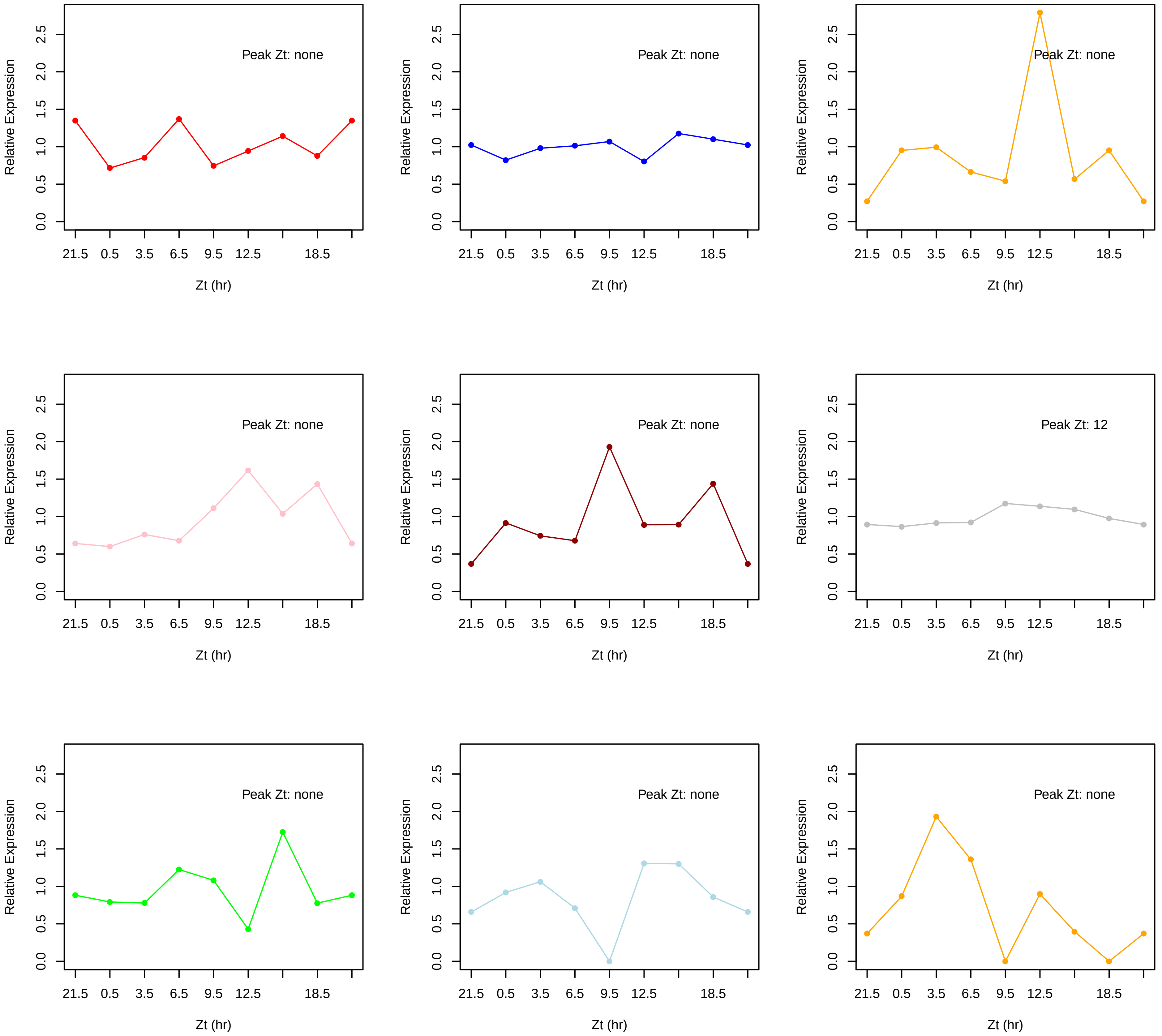

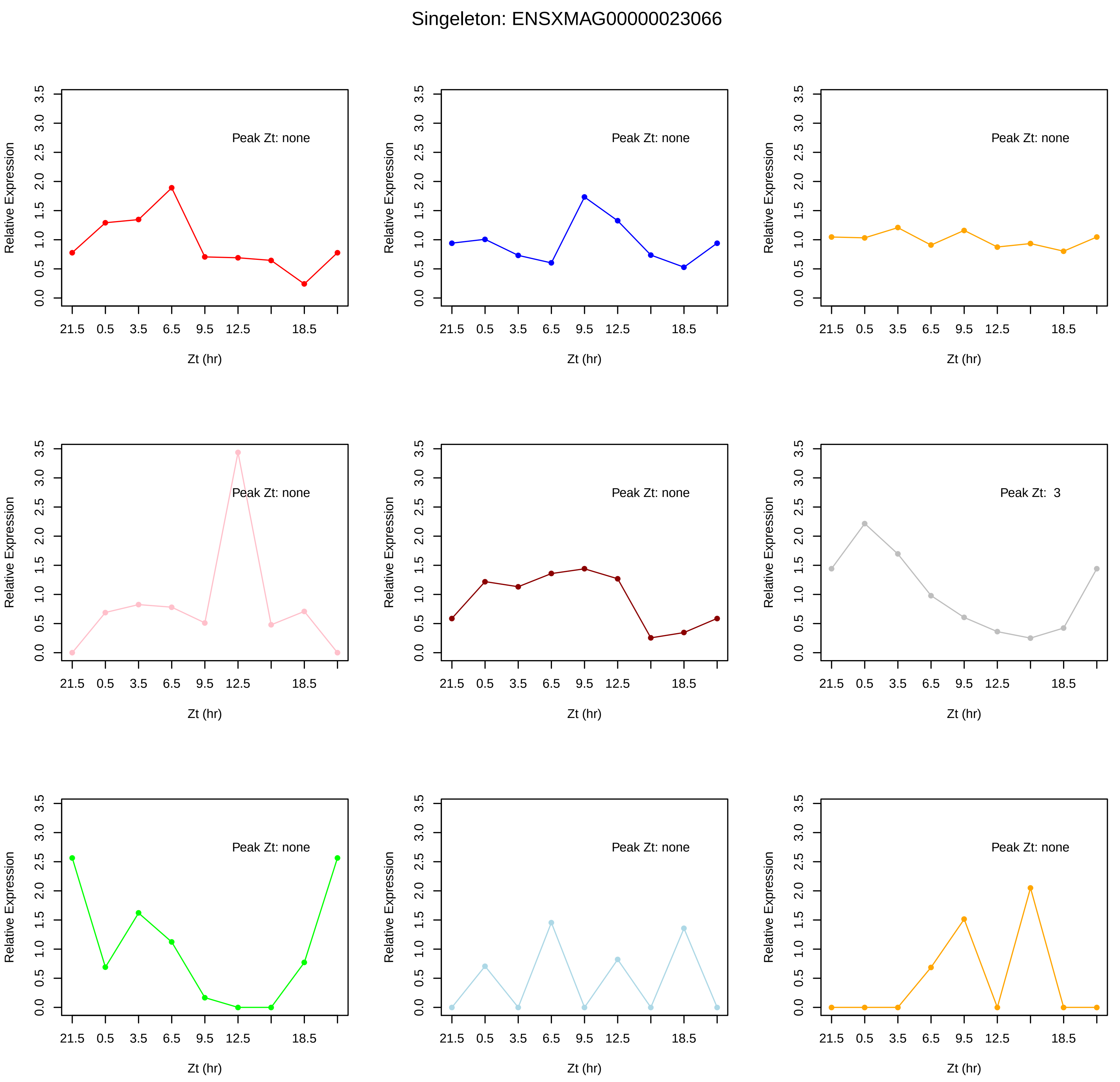
Singeleton: ENSXMAG00000023066
Peak Zt: none
Peak Zt: none
Peak Zt: none
Peak Zt: none
Peak Zt: none
Peak Zt: 3
Peak Zt: none
Peak Zt: none
Peak Zt: none
Relative Expression
Zt (hr)
21.5
0.5
3.5
6.5
9.5
12.5
18.5
0.0
0.5
1.0
1.5
2.0
2.5
3.0
3.5

Singeleton: ENSXMAG00000028482 lysmd1

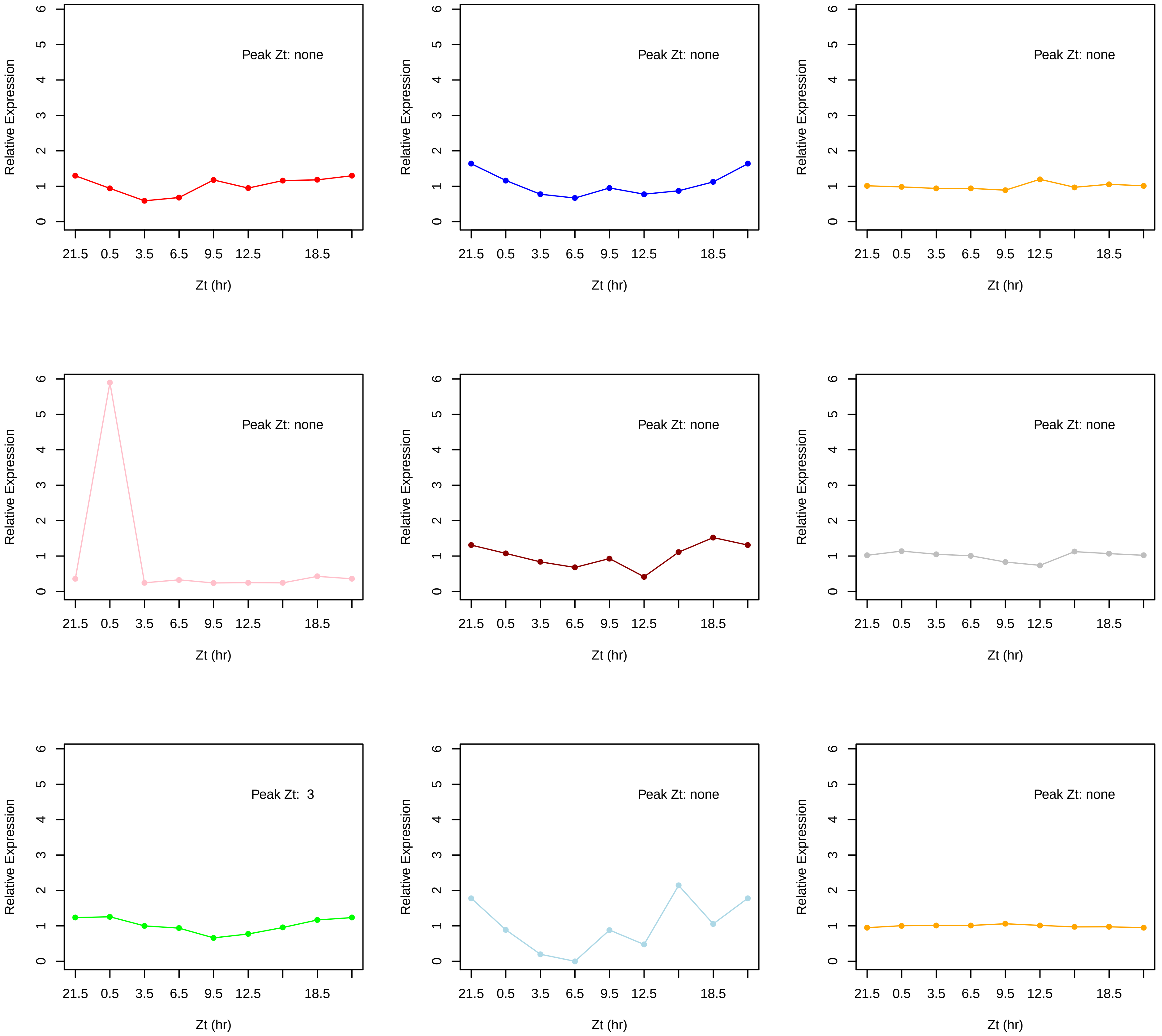

Singeleton: ENSXMAG00000001924 TTC28

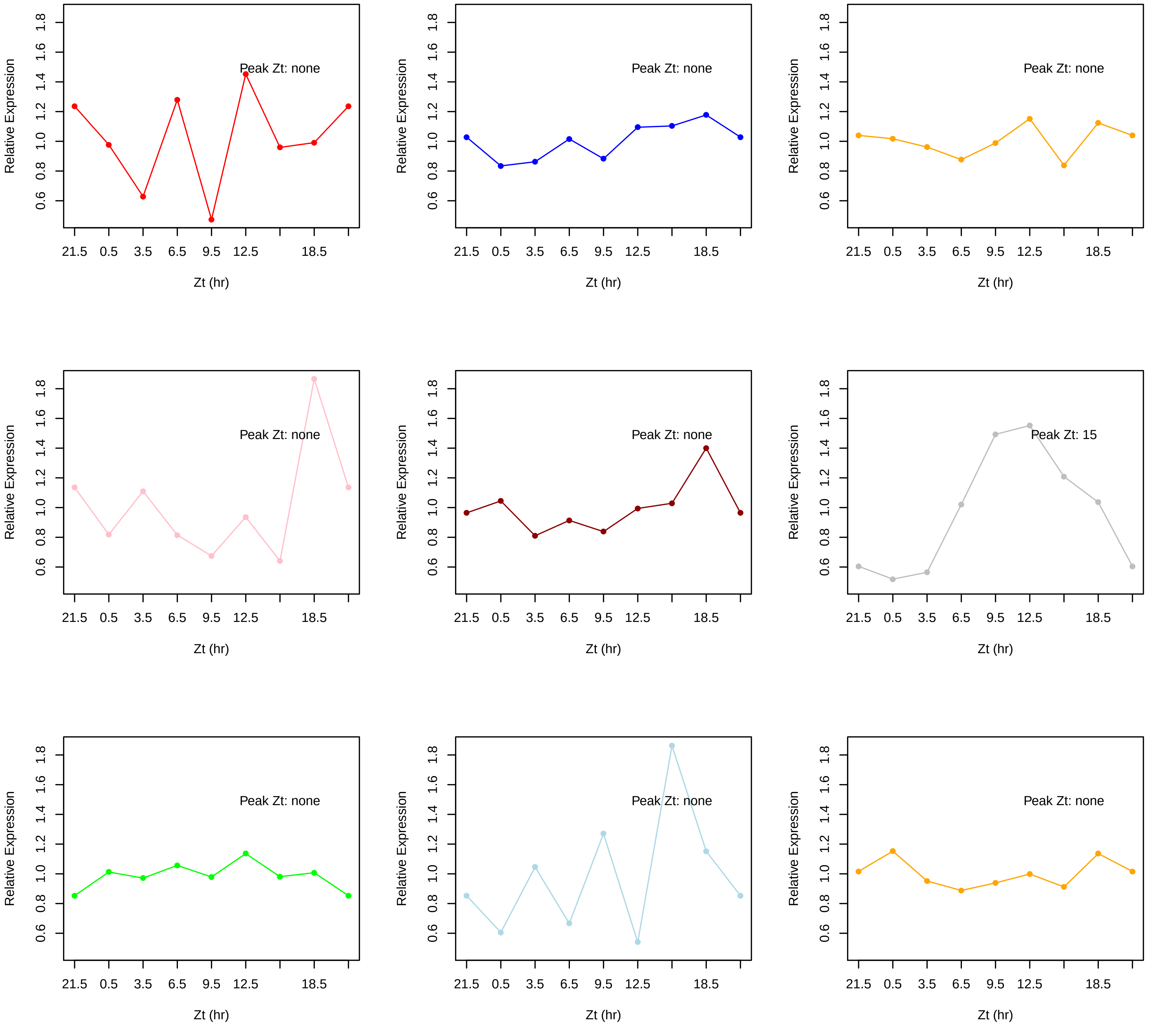

Singleton: ENSXMAG00000021046 sfrp2l

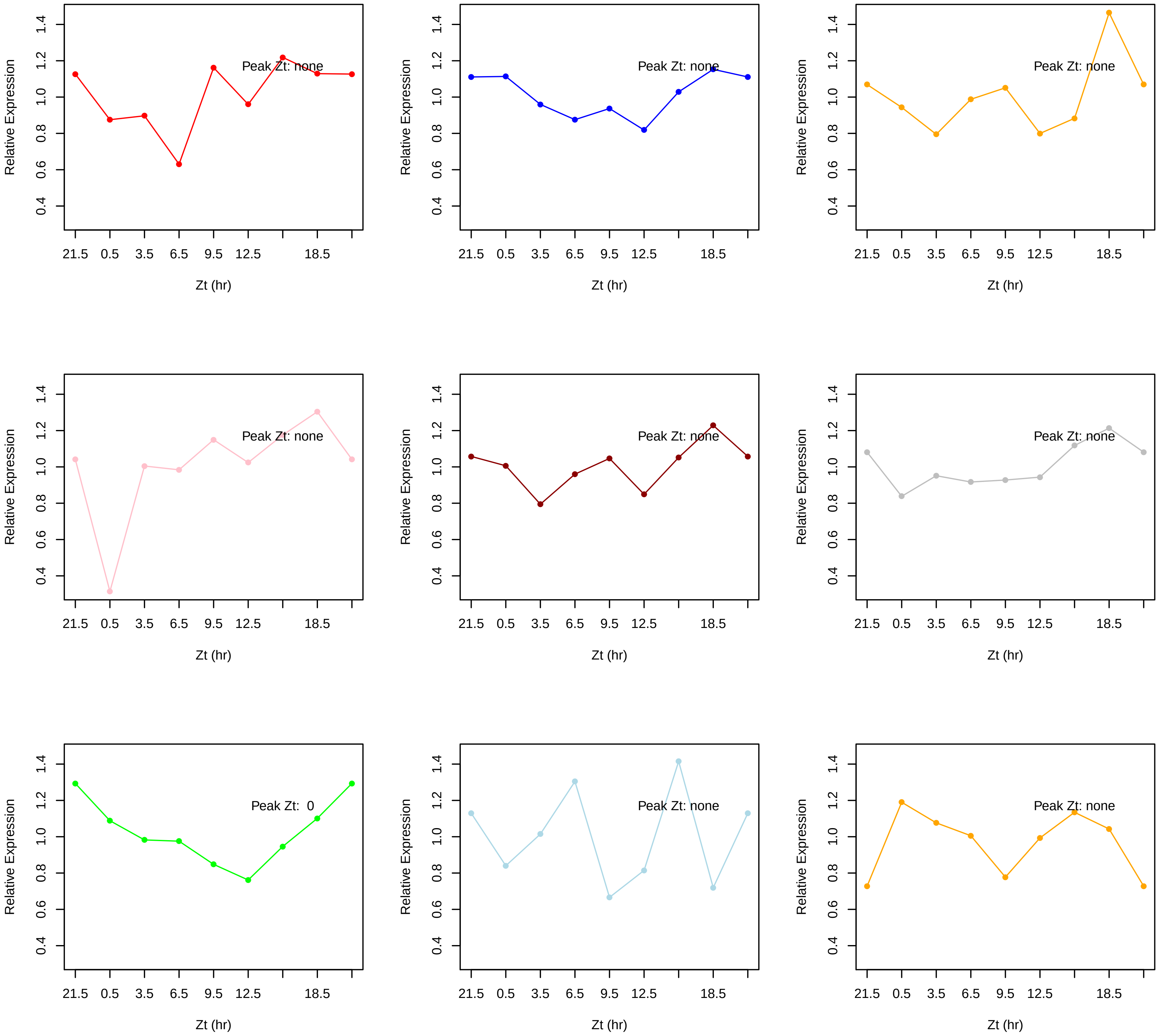

Singeleton: ENSXMAG00000026503 lamtor1

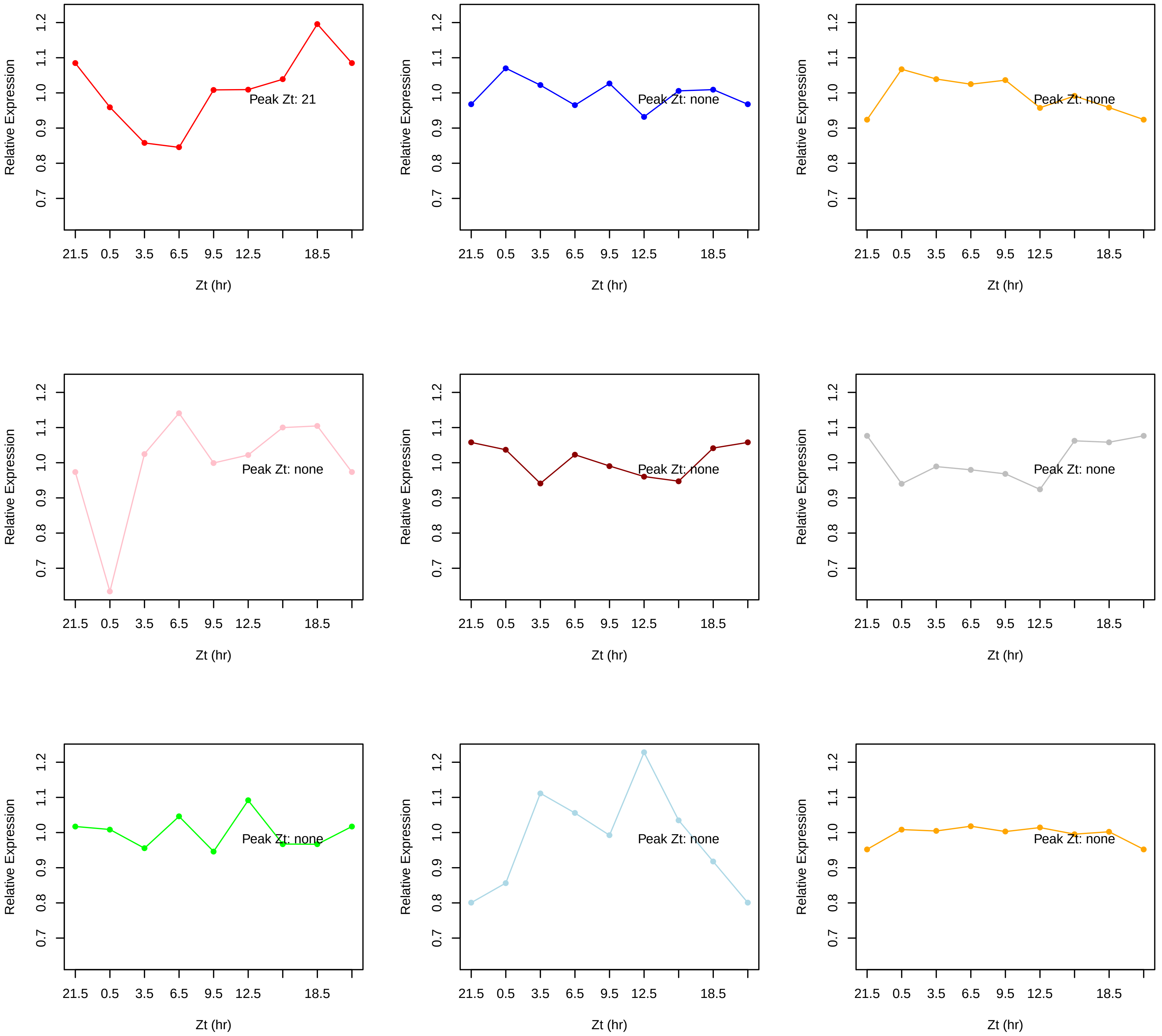

Singeleton: ENSXMAG00000002608 u2surp

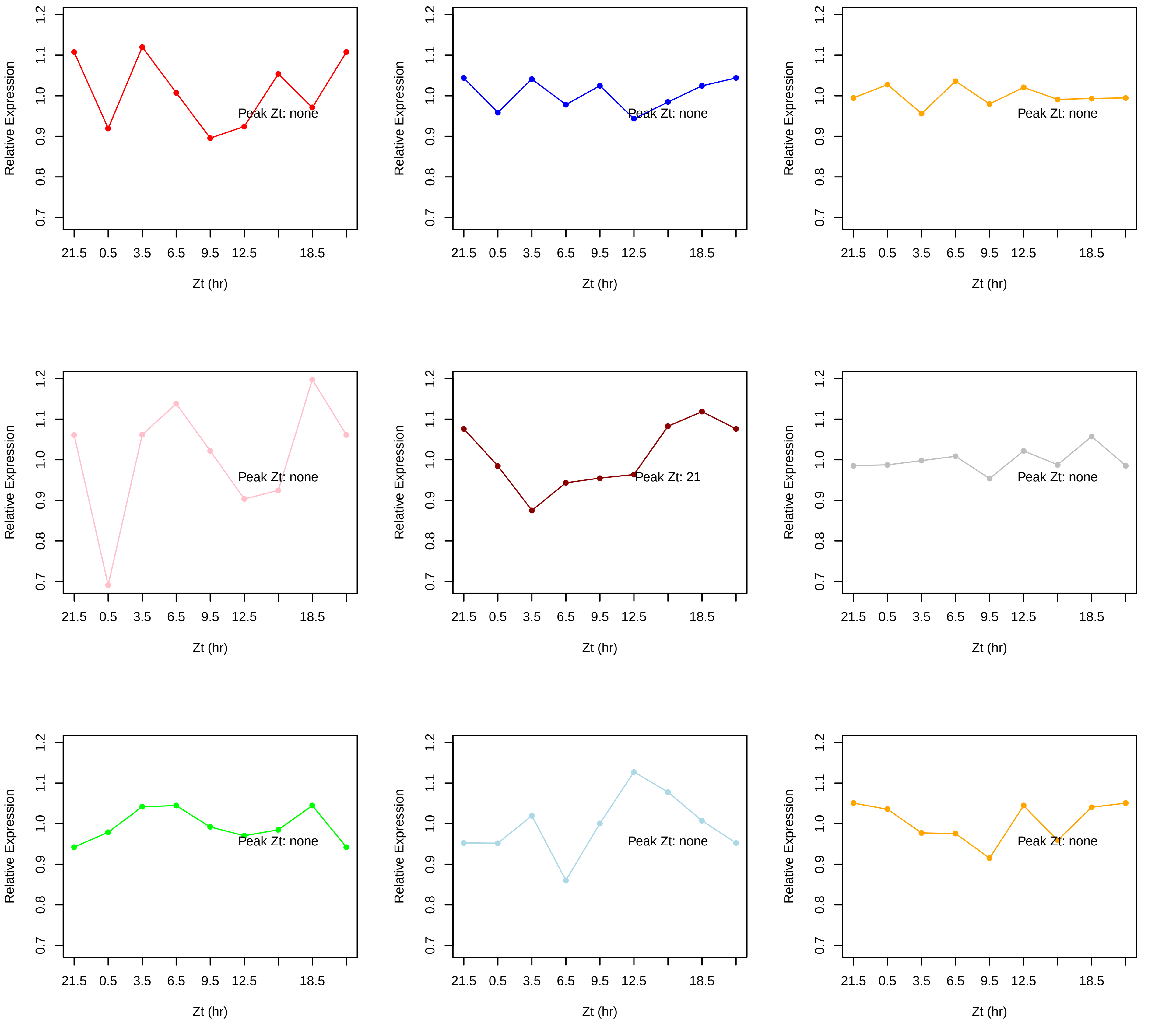

Singeleton: ENSXMAG00000015722 herpud1

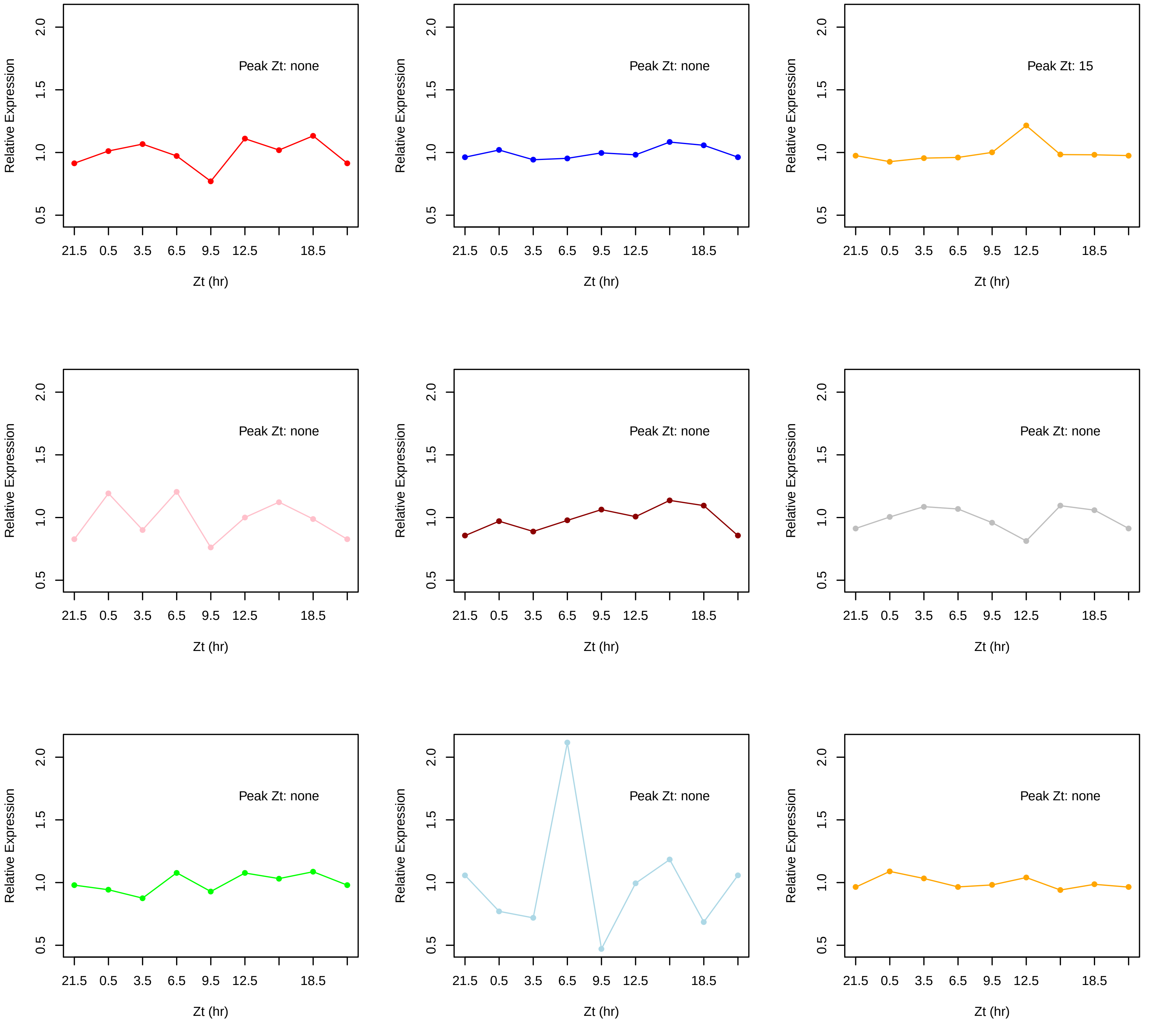

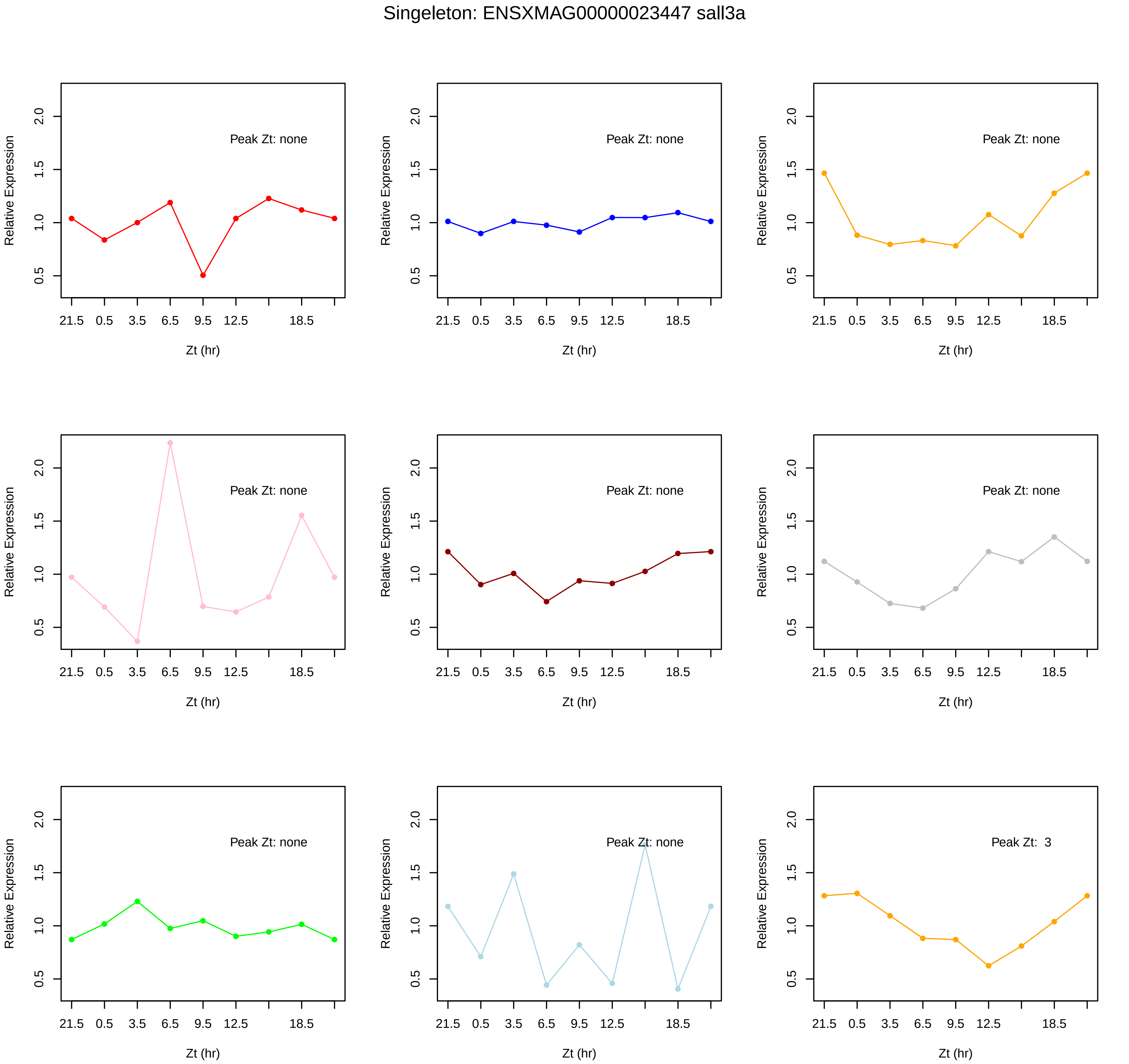
Singleton: ENSXMAG00000023447 sall3a
Peak Zt: none
Peak Zt: none
Peak Zt: none
Peak Zt: none
Peak Zt: none
Peak Zt: none
Peak Zt: none
Peak Zt: none
Peak Zt: 3
Relative Expression
Zt (hr)
21.5 0.5 3.5 6.5 9.5 12.5 18.5
0.5 1.0 1.5 2.0

Singeleton: ENSXMAG00000003802 vps72a

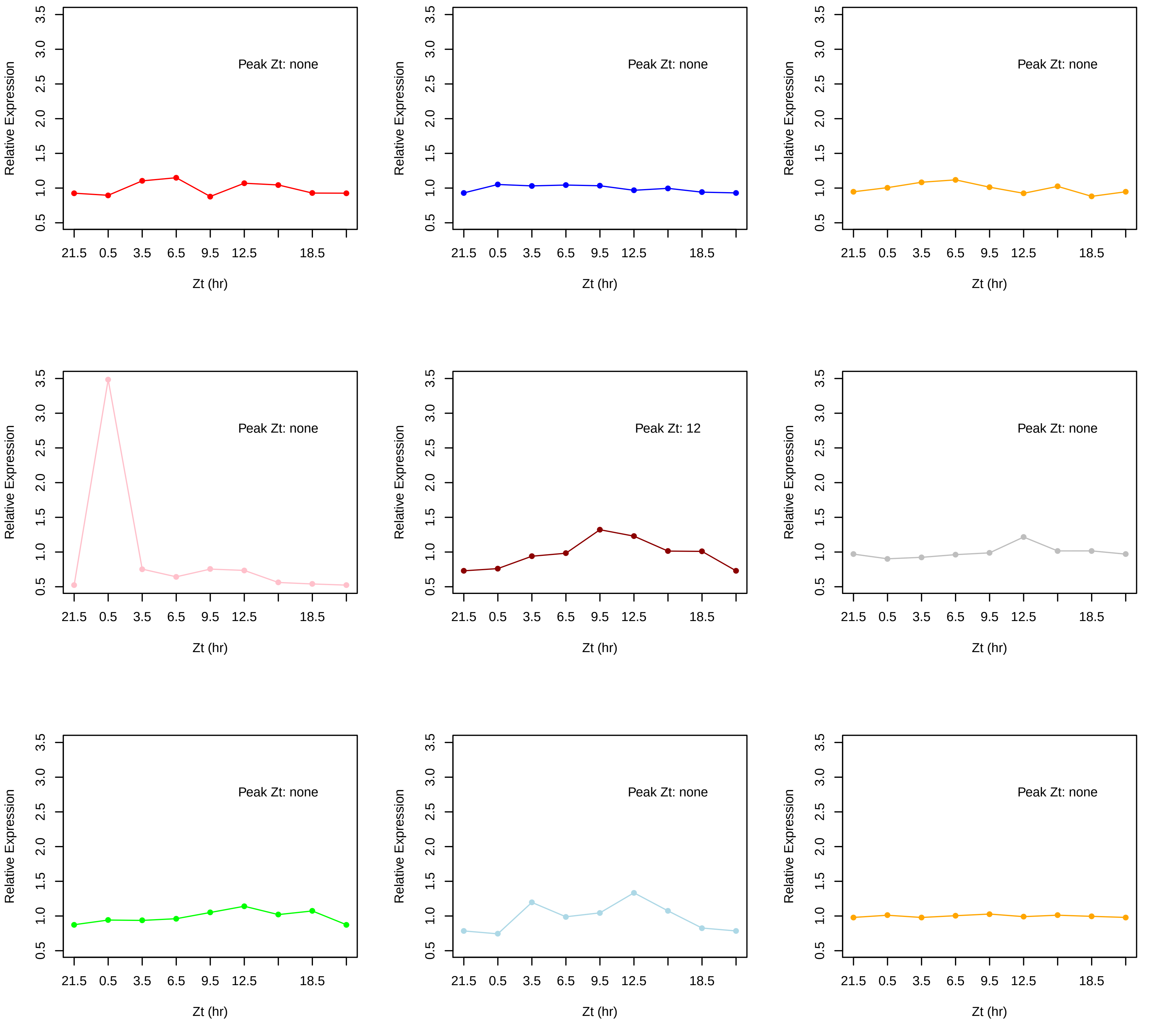

Singleton: ENSXMAG00000001915 mn1b

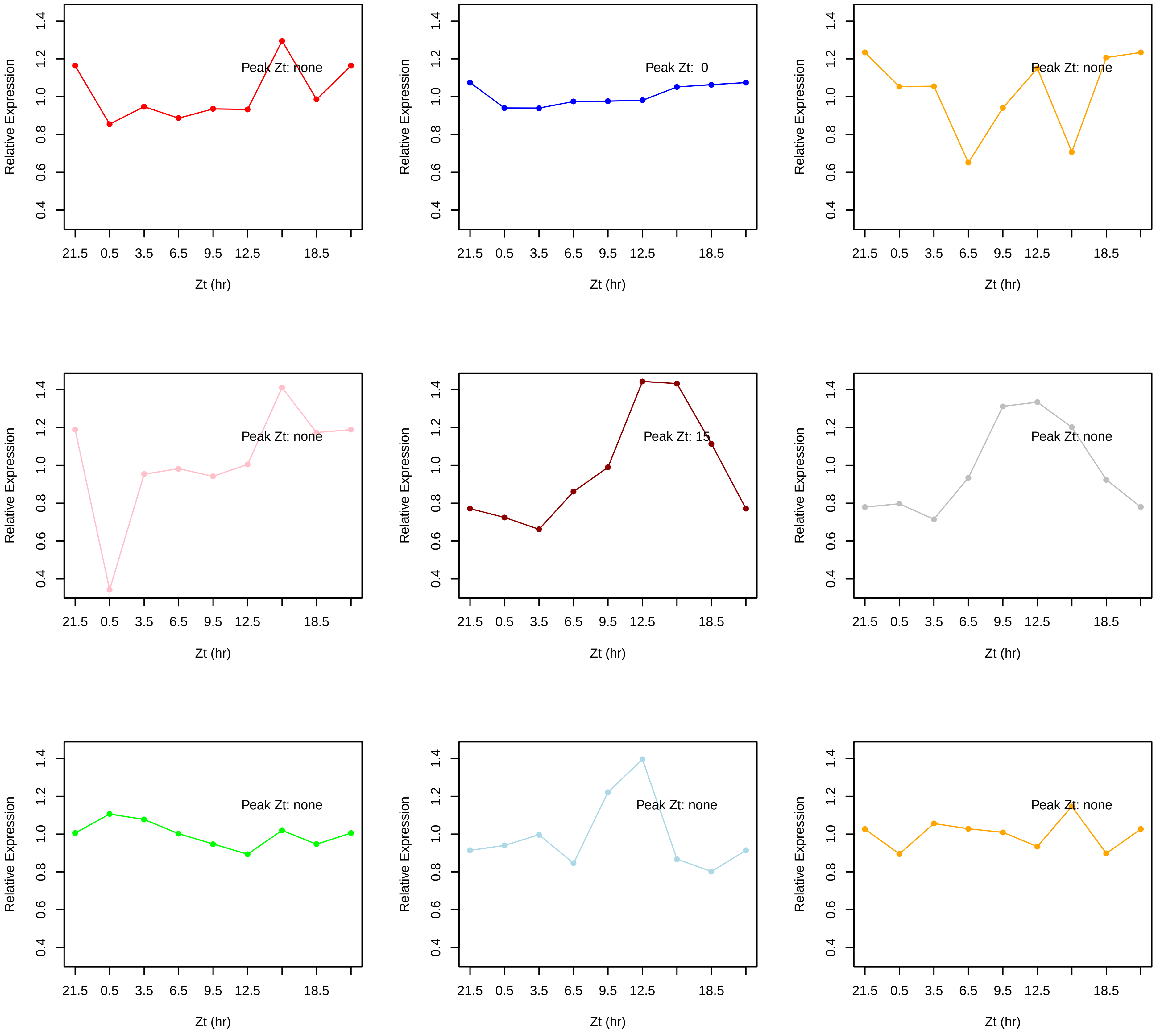

Singleton: ENSXMAG00000023269 necap2

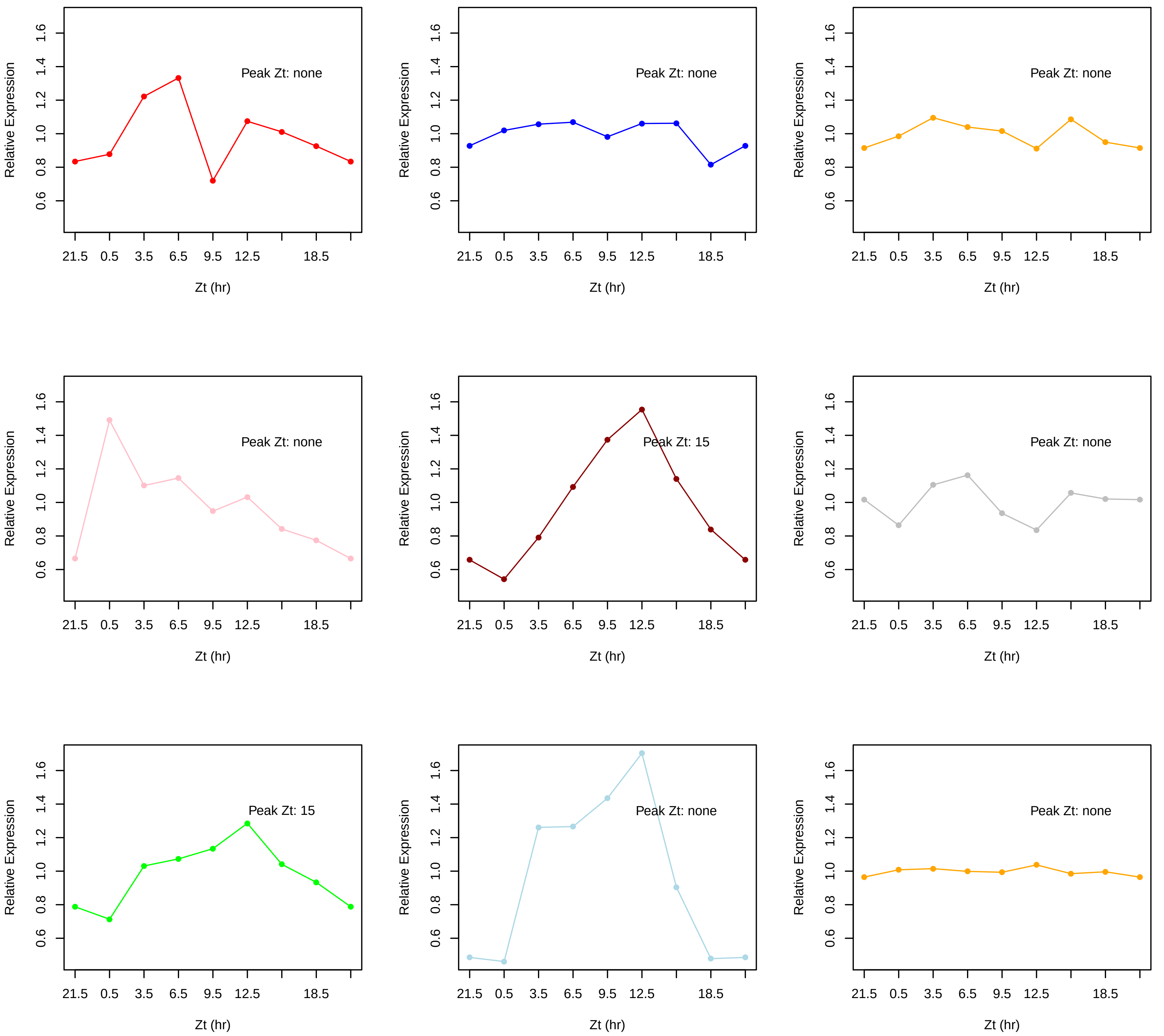

Singeleton: ENSXMAG00000029932 xk

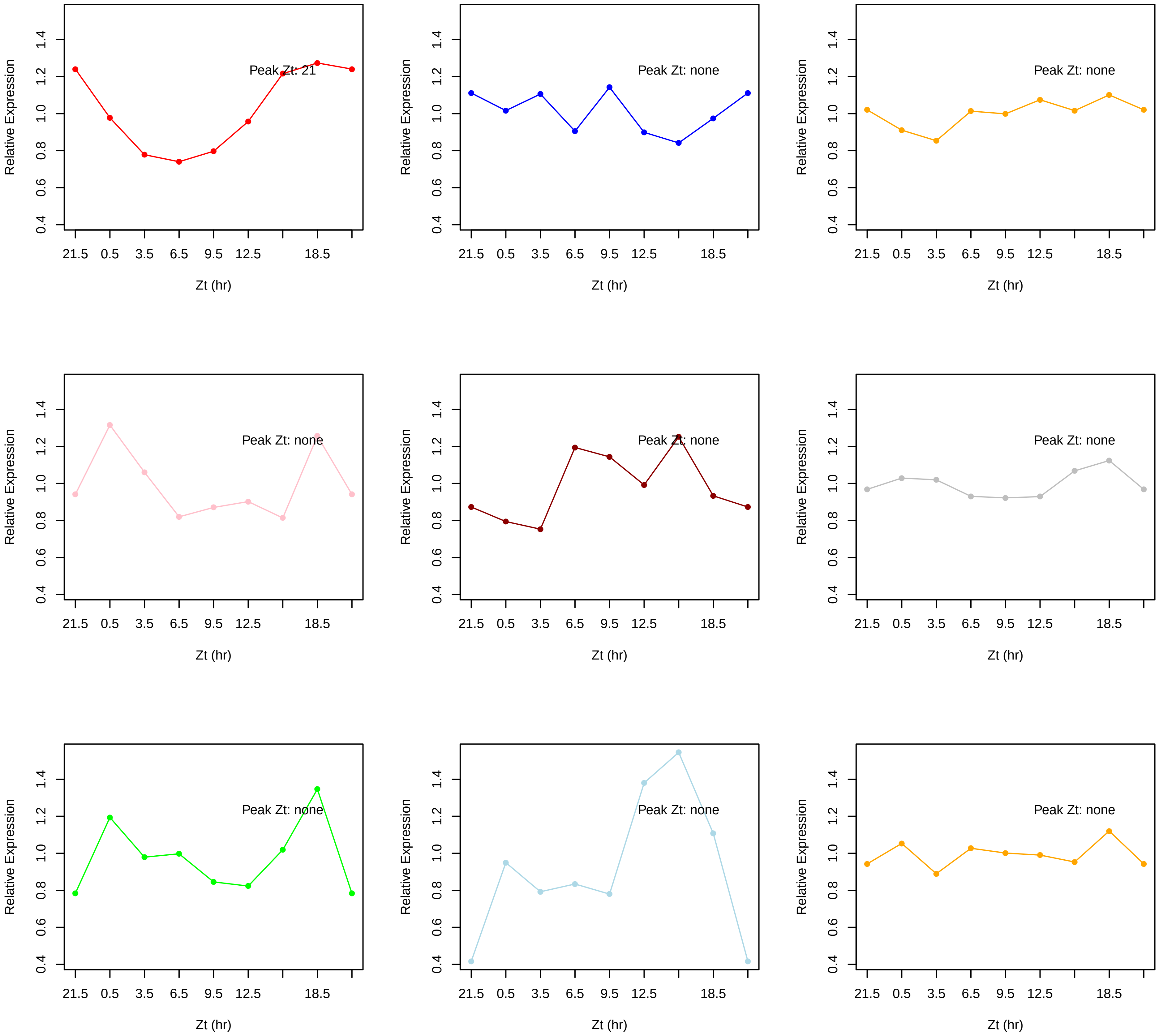

Singeleton: ENSXMAG00000007571 adam15

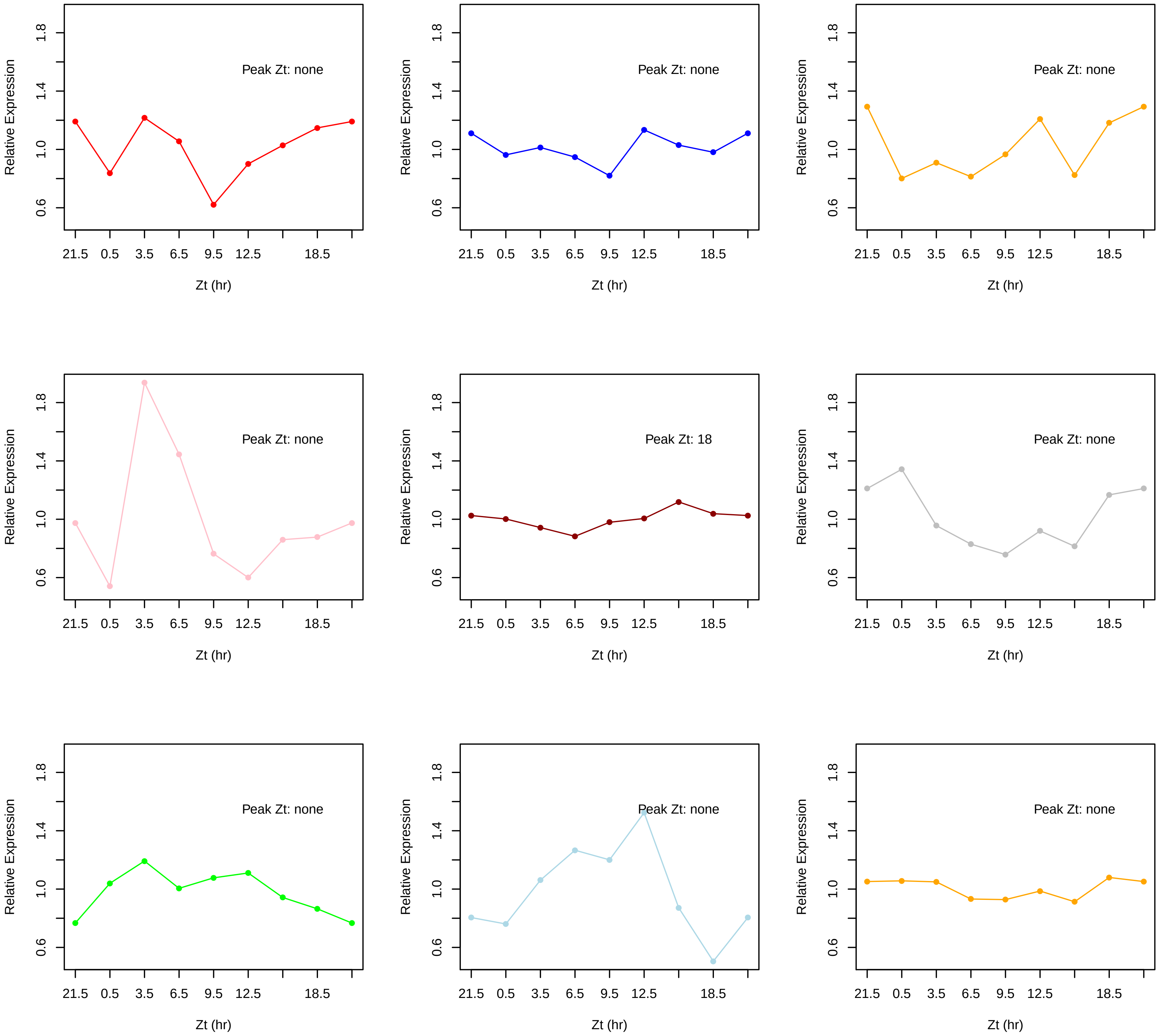

Singleton: ENSXMAG00000020947 dcst1

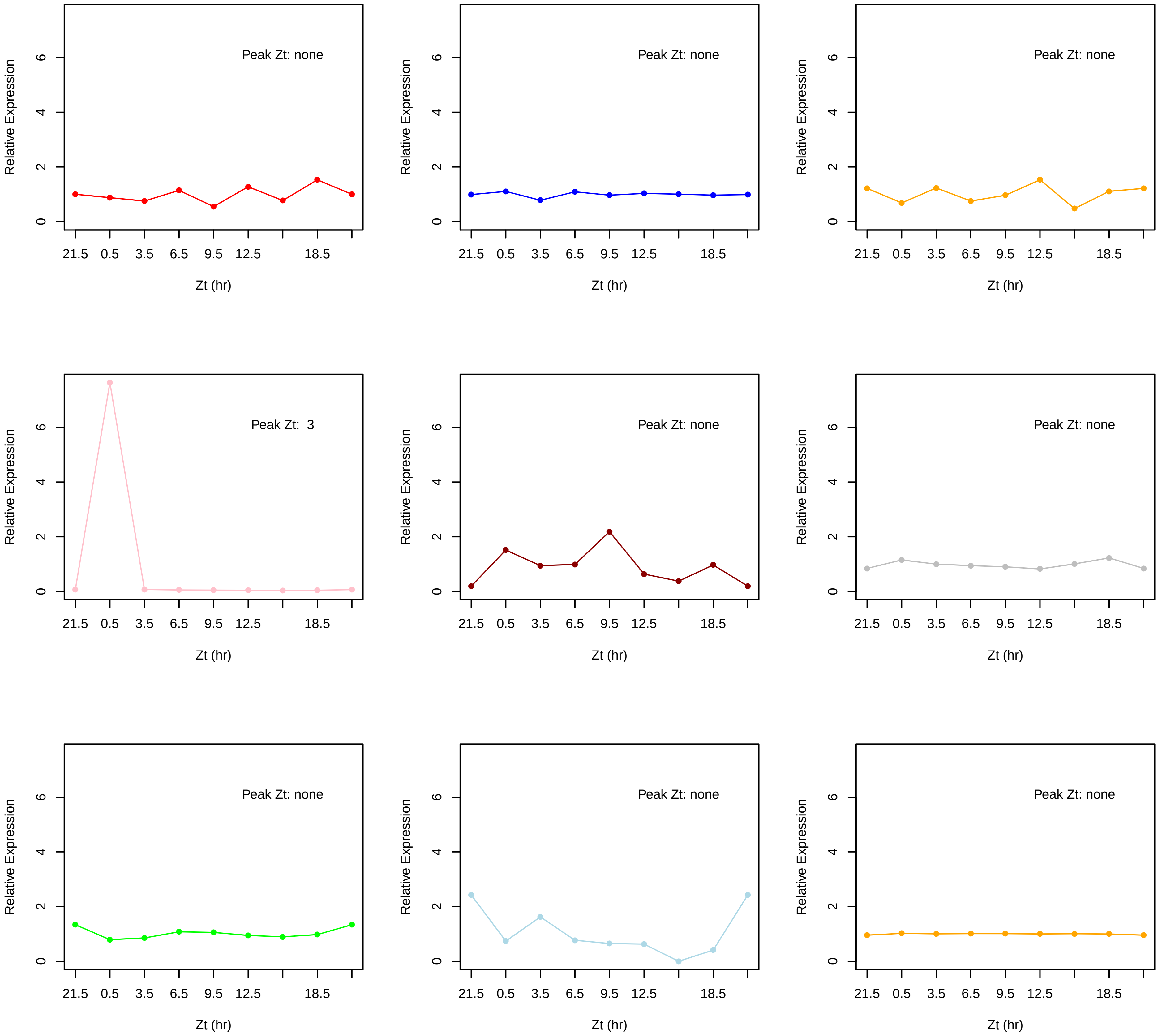

Singleton: ENSXMAG00000027193 fbxo42

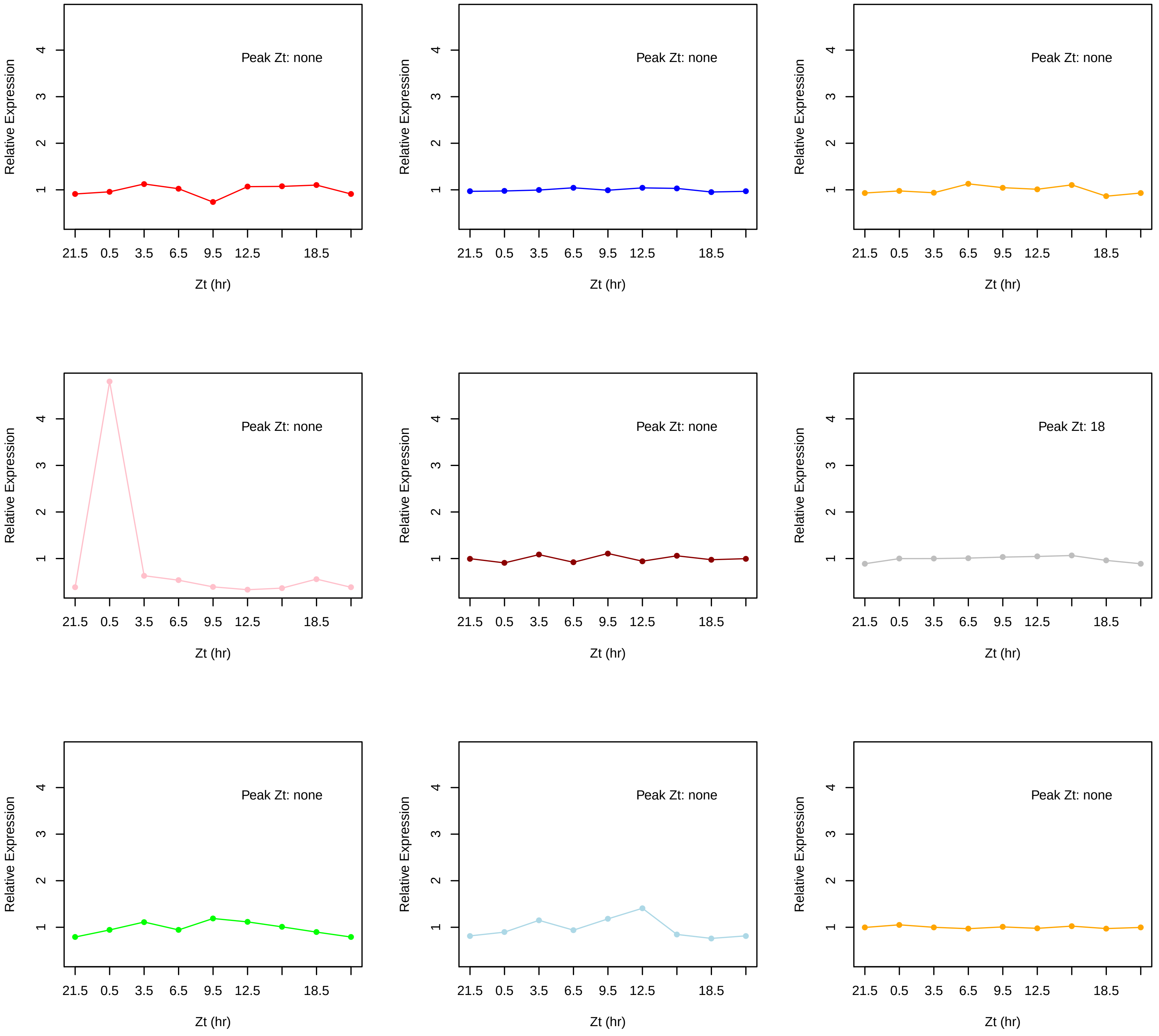

Singleton: ENSXMAG00000013927 nup93

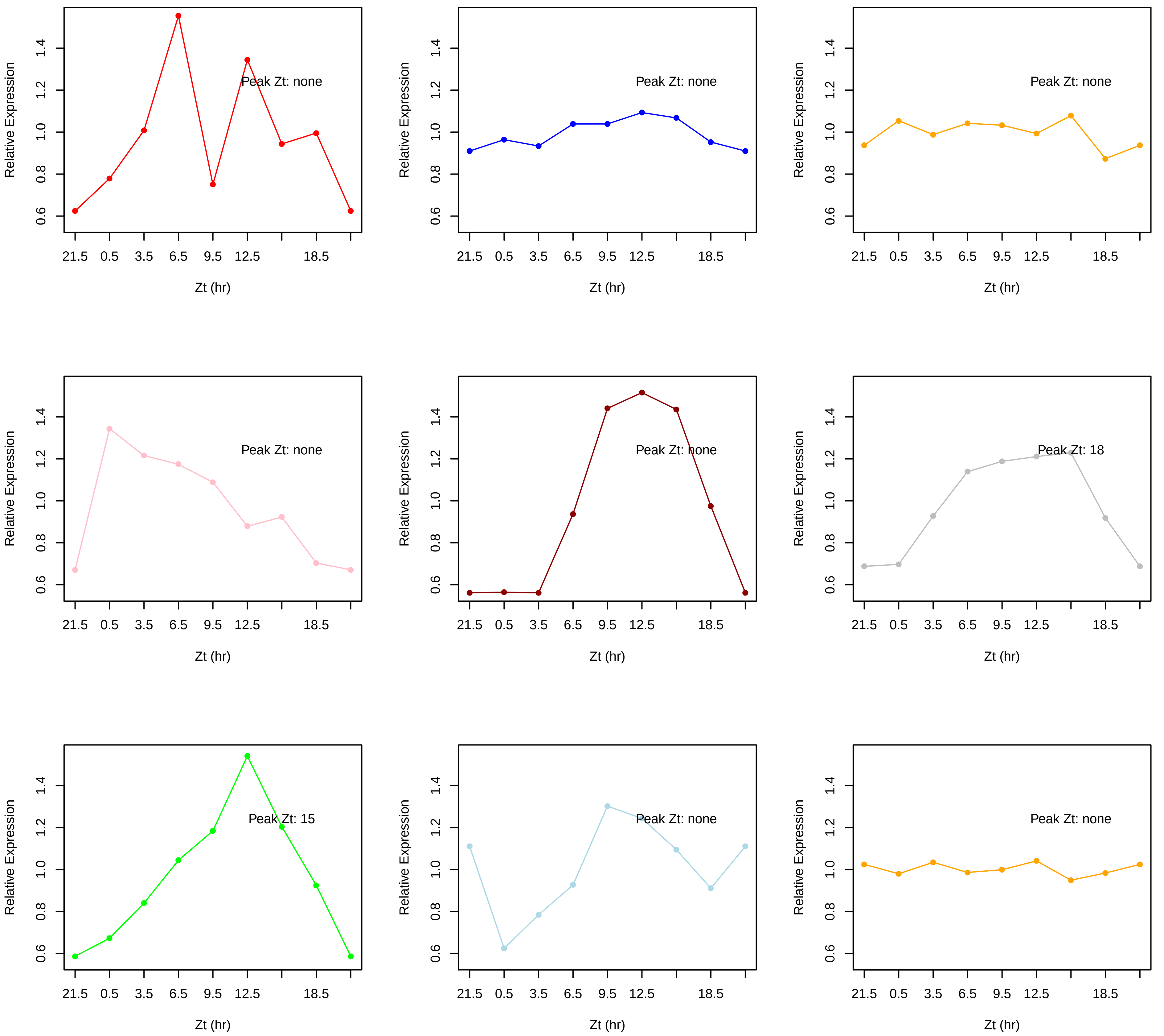

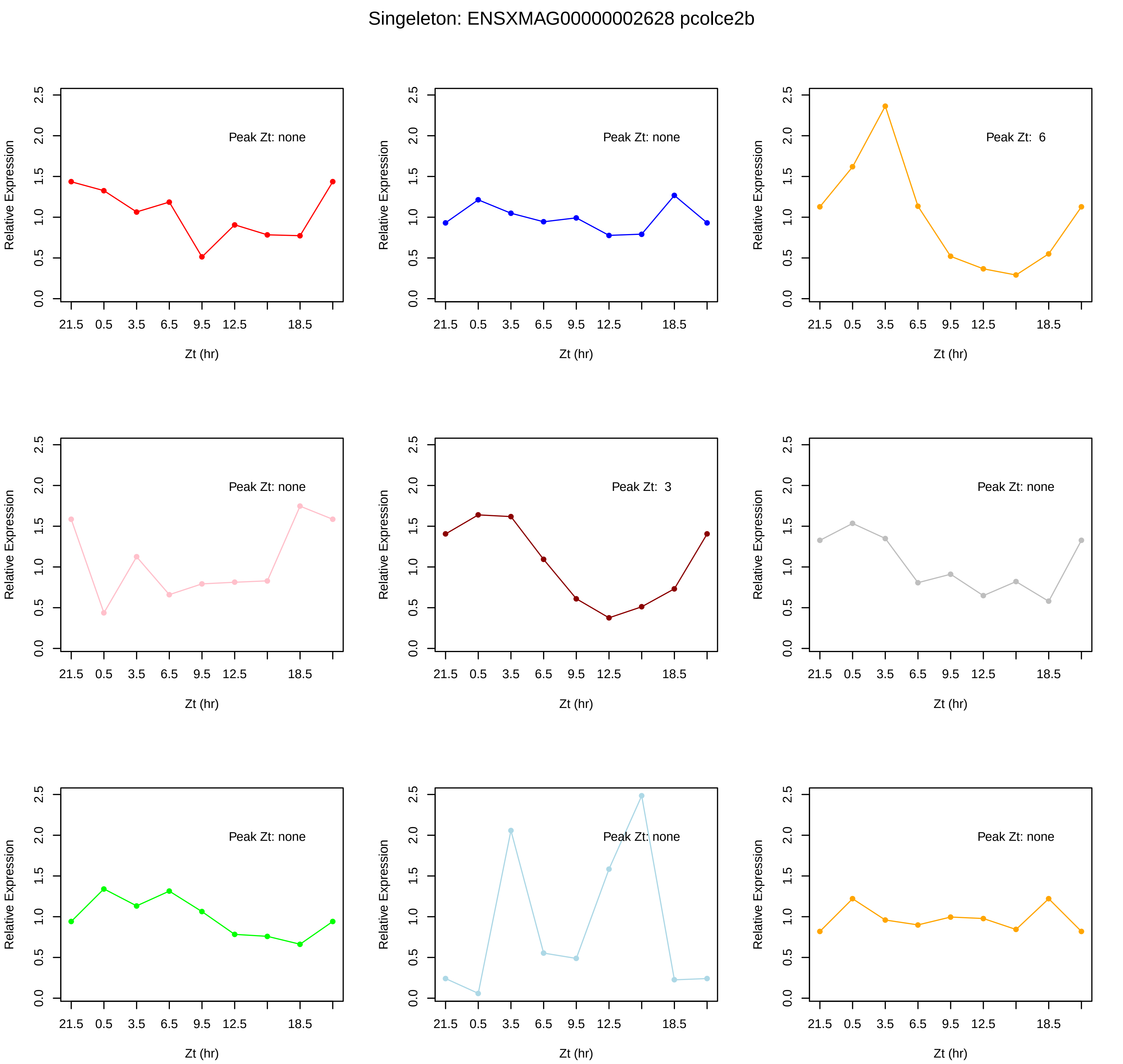

Singeleton: ENSXMAG00000002628 pcolce2b
Peak Zt: none
Peak Zt: none
Peak Zt: 6
Peak Zt: none
Peak Zt: 3
Peak Zt: none
Peak Zt: none
Peak Zt: none
Peak Zt: none
Relative Expression
Zt (hr)
0.0
0.5
1.0
1.5
2.0
2.5
21.5
0.5
3.5
6.5
9.5
12.5
18.5

Singeleton: ENSXMAG00000007857 cpe

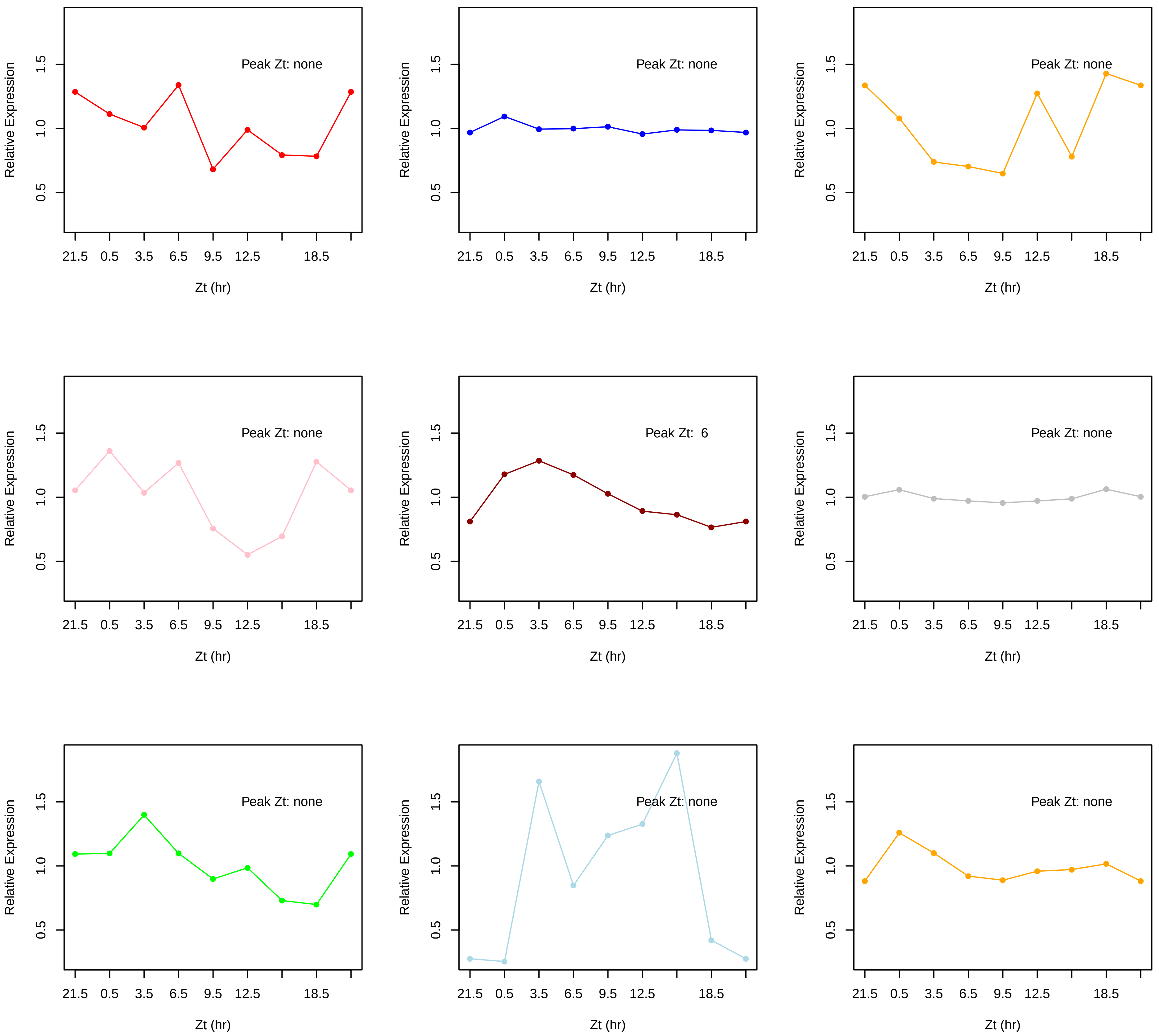

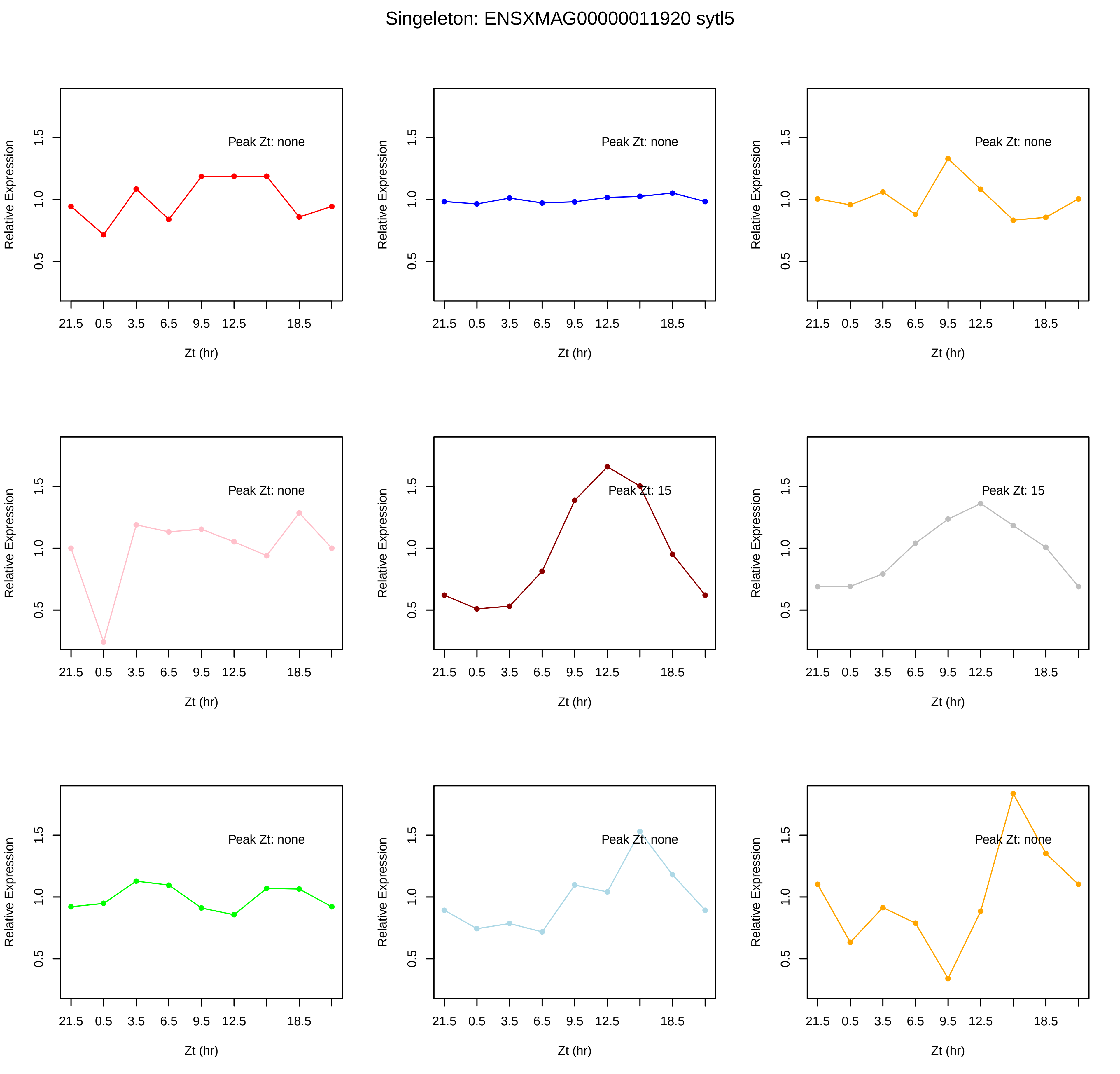
Singeleton: ENSXMAG00000011920 sytl5
Peak Zt: none
Peak Zt: none
Peak Zt: none
Peak Zt: none
Peak Zt: 15
Peak Zt: 15
Peak Zt: none
Peak Zt: none
Peak Zt: none
Relative Expression
Zt (hr)
0.5
1.0
1.5
21.5
0.5
3.5
6.5
9.5
12.5
18.5

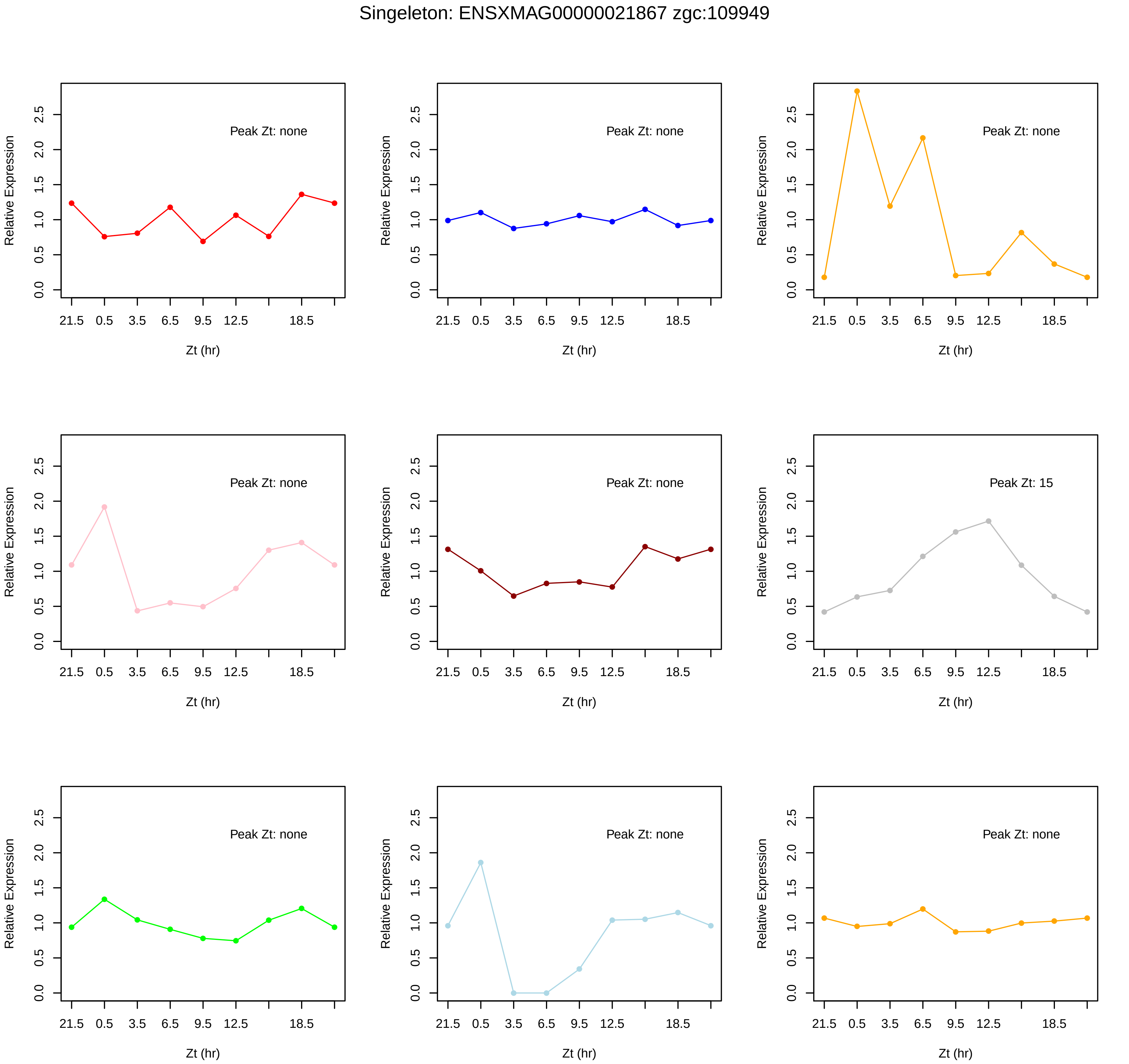

Singeleton: ENSXMAG00000021867 zgc:109949
Peak Zt: none
Peak Zt: none
Peak Zt: none
Peak Zt: none
Peak Zt: none
Peak Zt: 15
Peak Zt: none
Peak Zt: none
Peak Zt: none
Relative Expression
Zt (hr)
21.5 0.5 3.5 6.5 9.5 12.5 18.5
0.0 0.5 1.0 1.5 2.0 2.5

Singeleton: ENSXMAG00000016520 uros

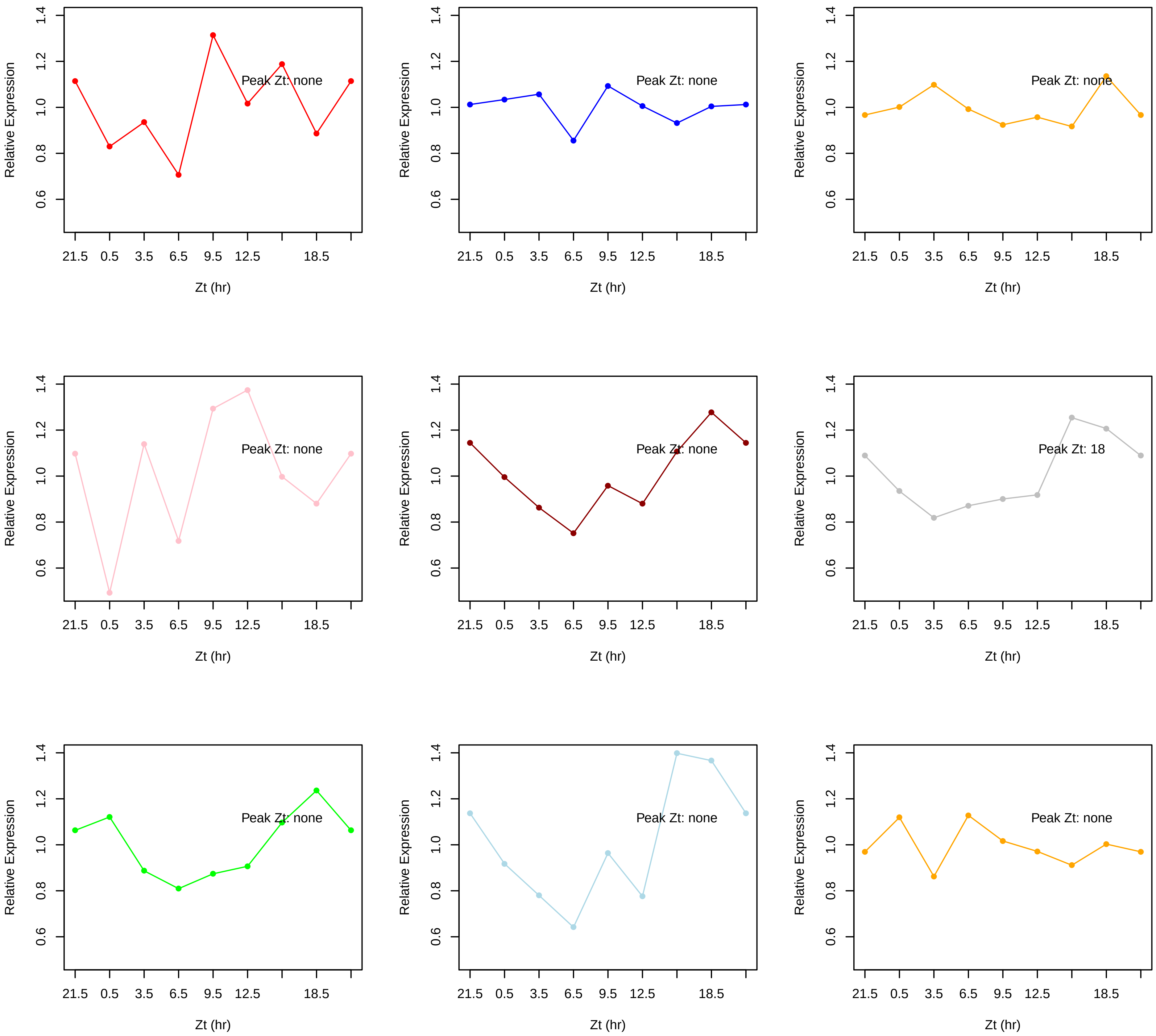

Singeleton: ENSXMAG00000018643 fam234a

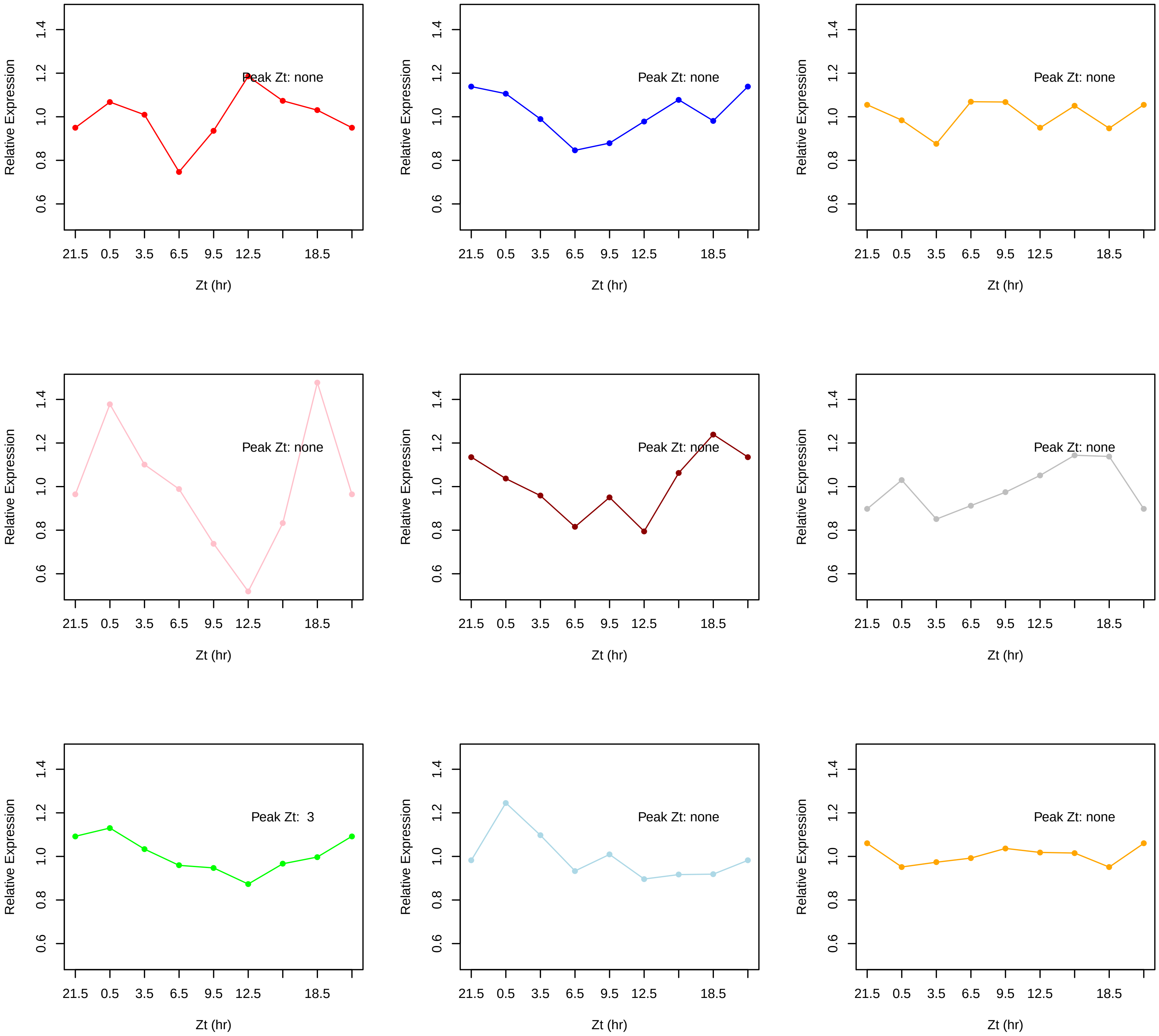

Singeleton: ENSXMAG00000002645 trpc1

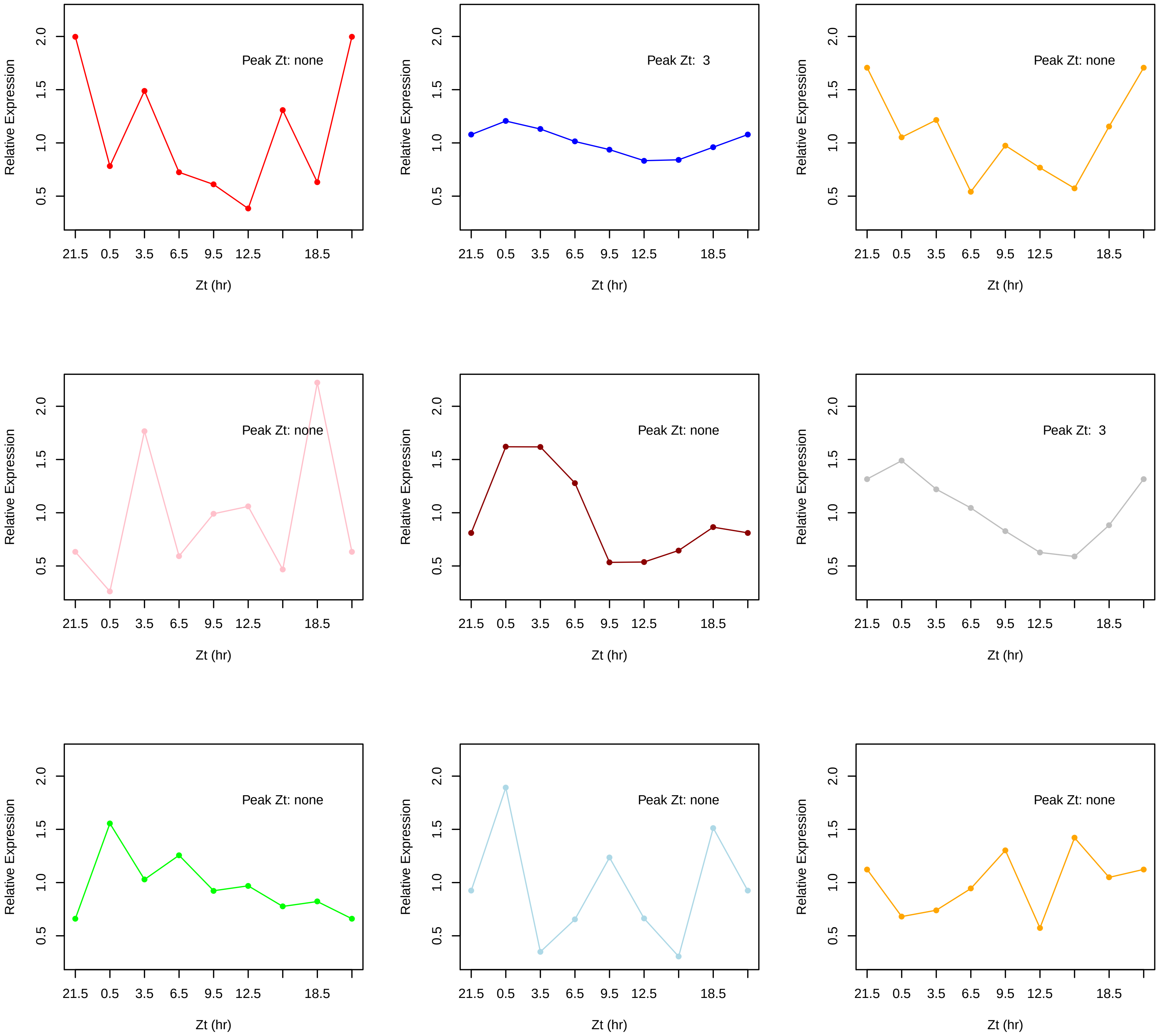

Singeleton: ENSXMAG00000010407 rnf121

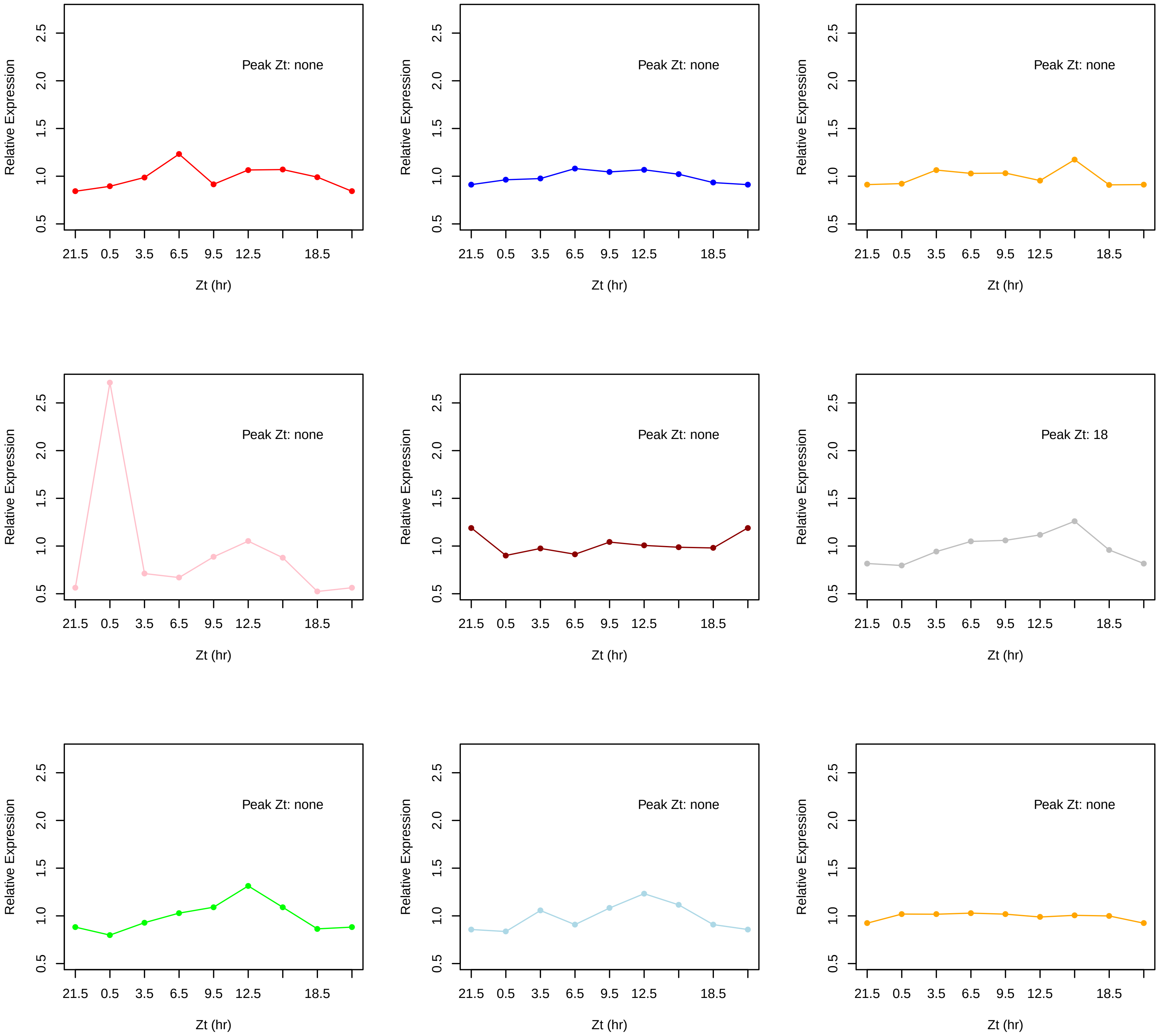

Singeleton: ENSXMAG00000019893 smim13

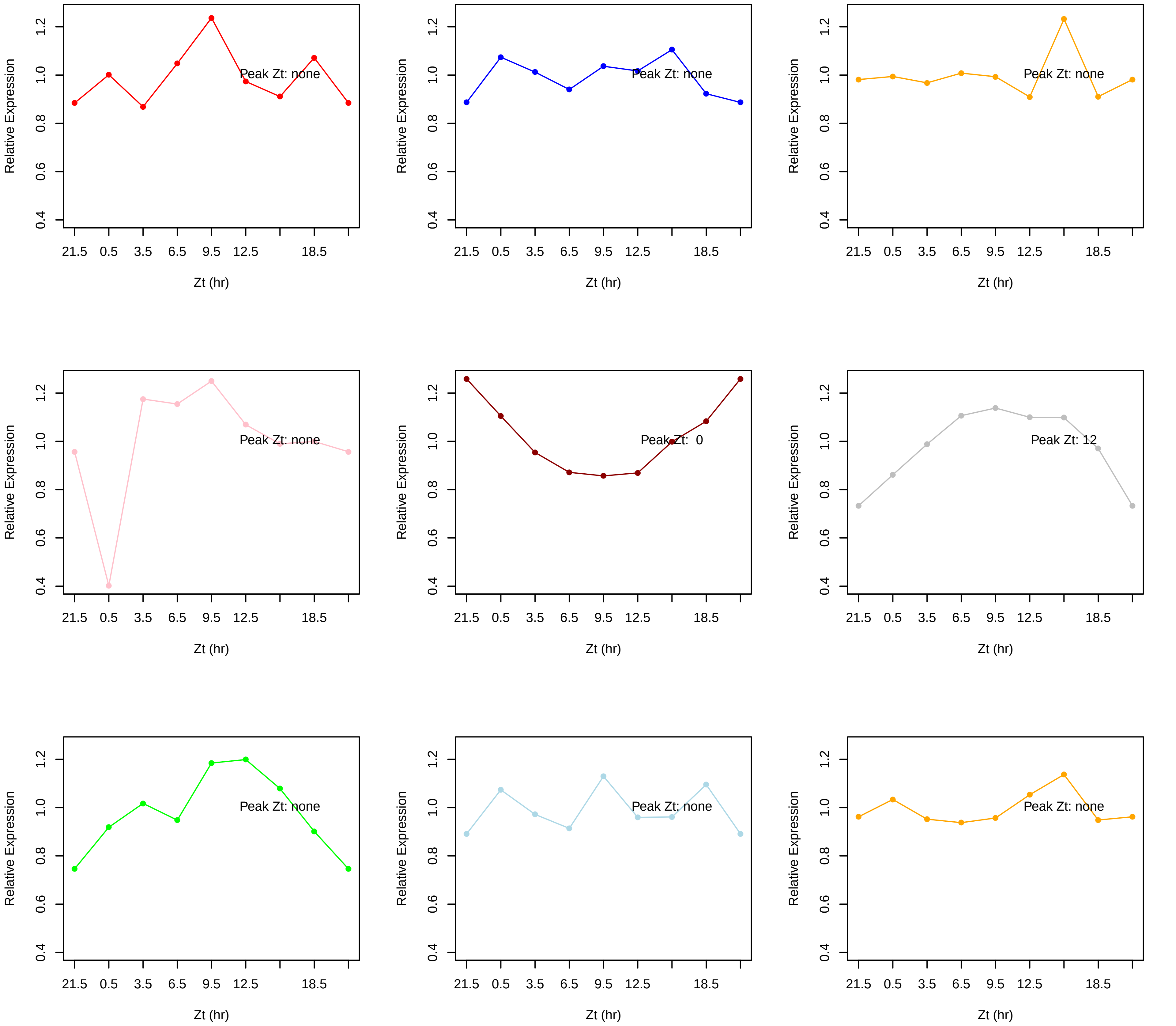

Singleton: ENSXMAG00000010372 trpc2b

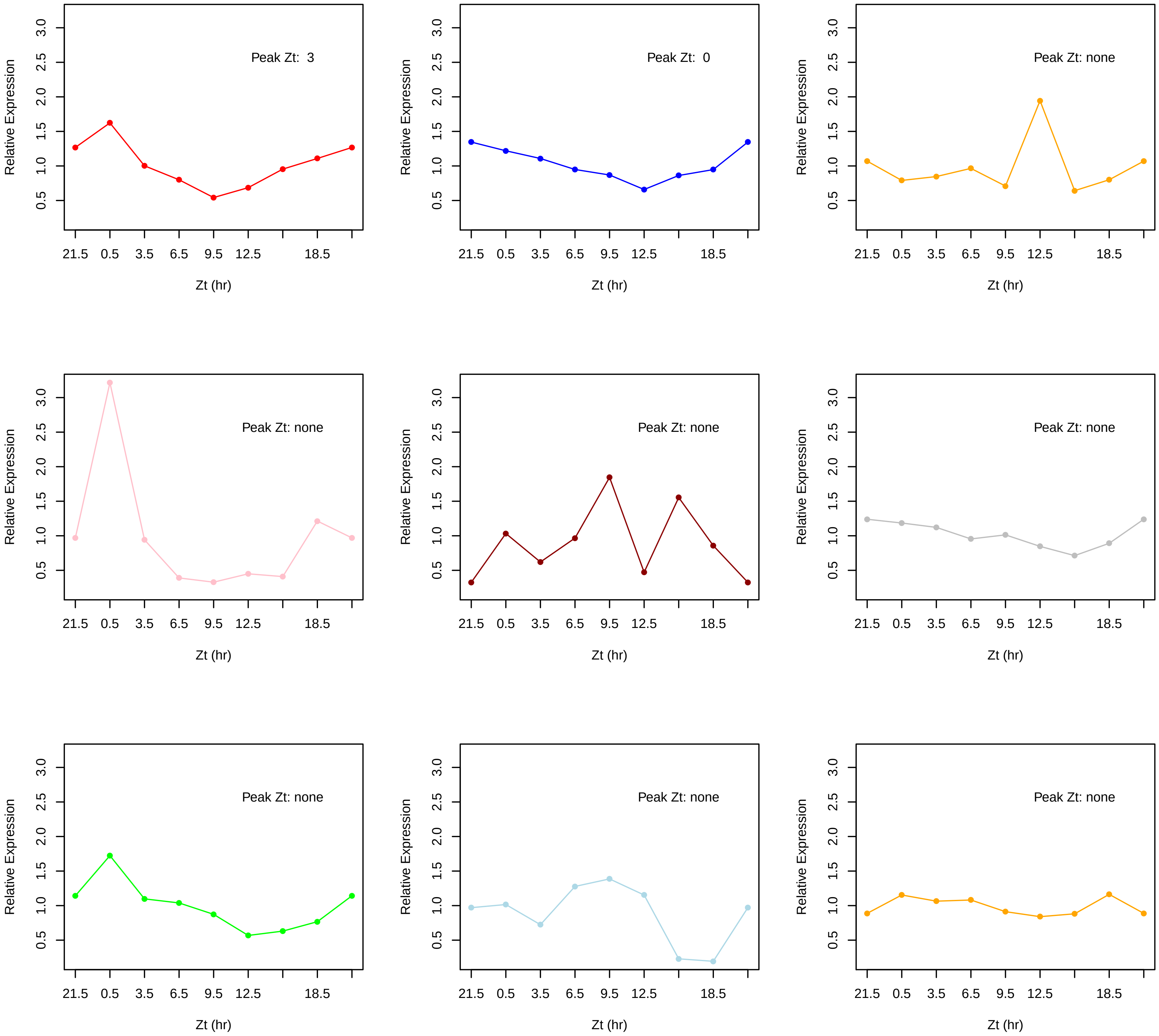

Singeleton: ENSXMAG00000005915

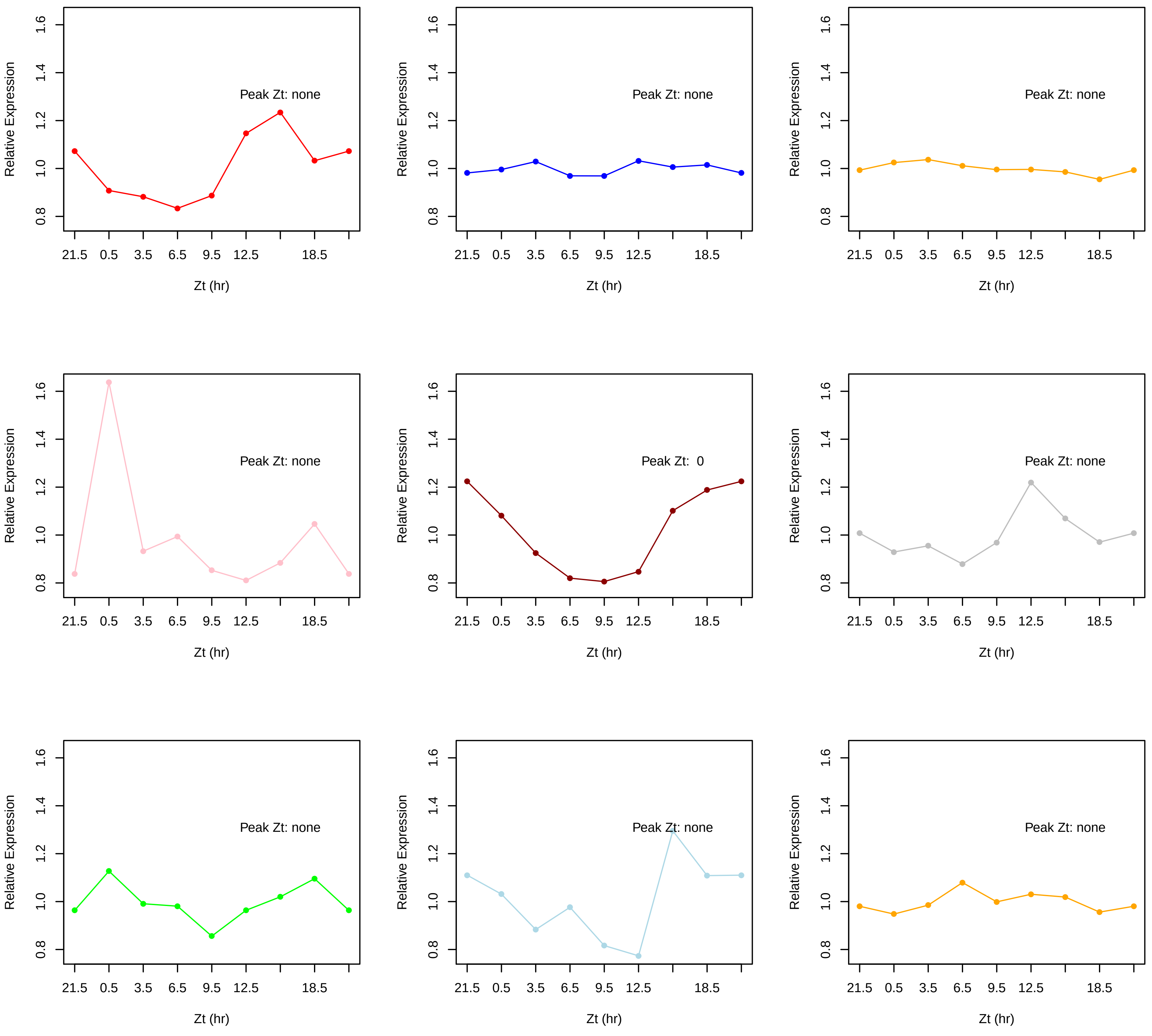

Singeleton: ENSXMAG00000017736 timm10

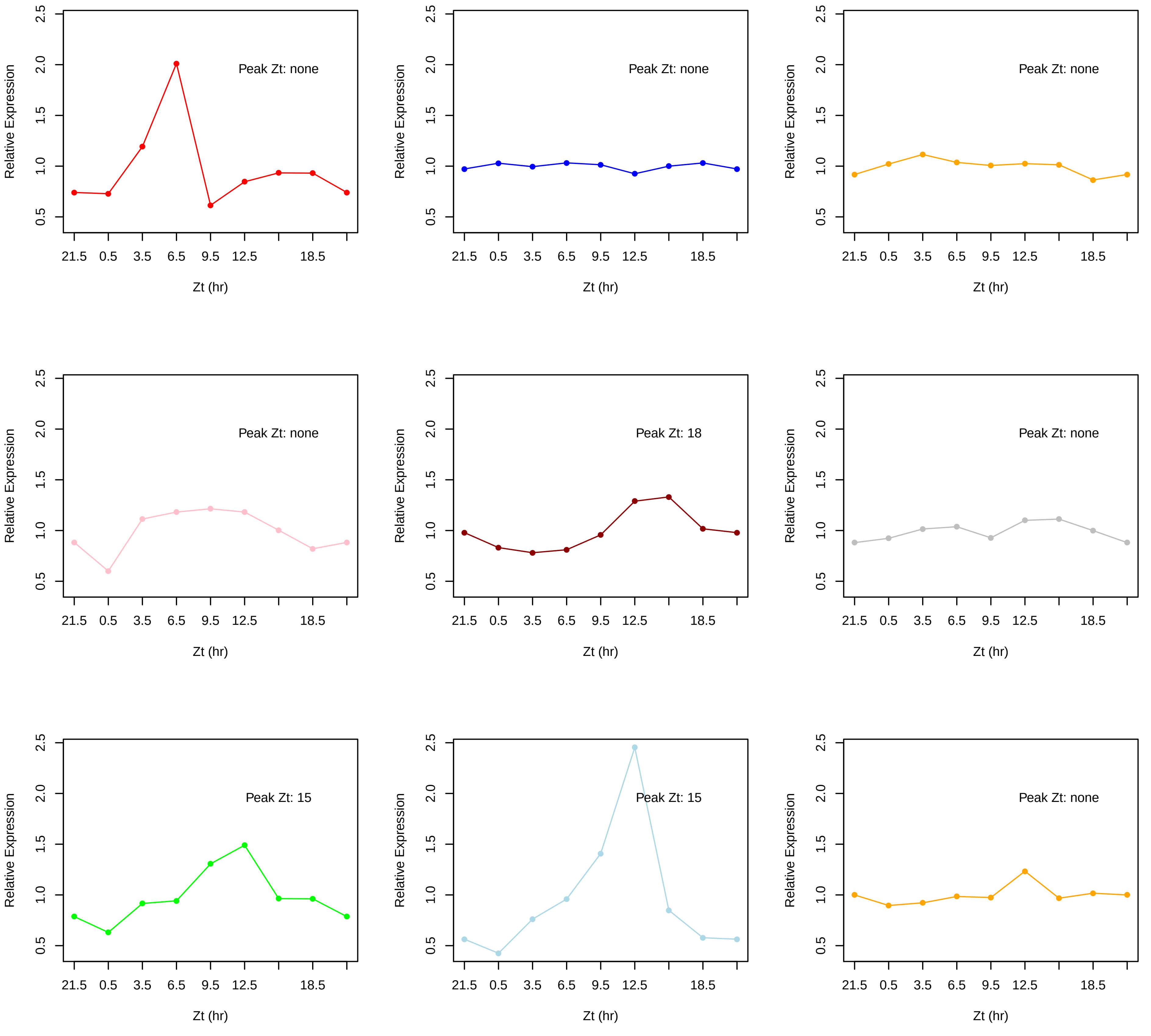

Singeleton: ENSXMAG00000014106 gnao1b

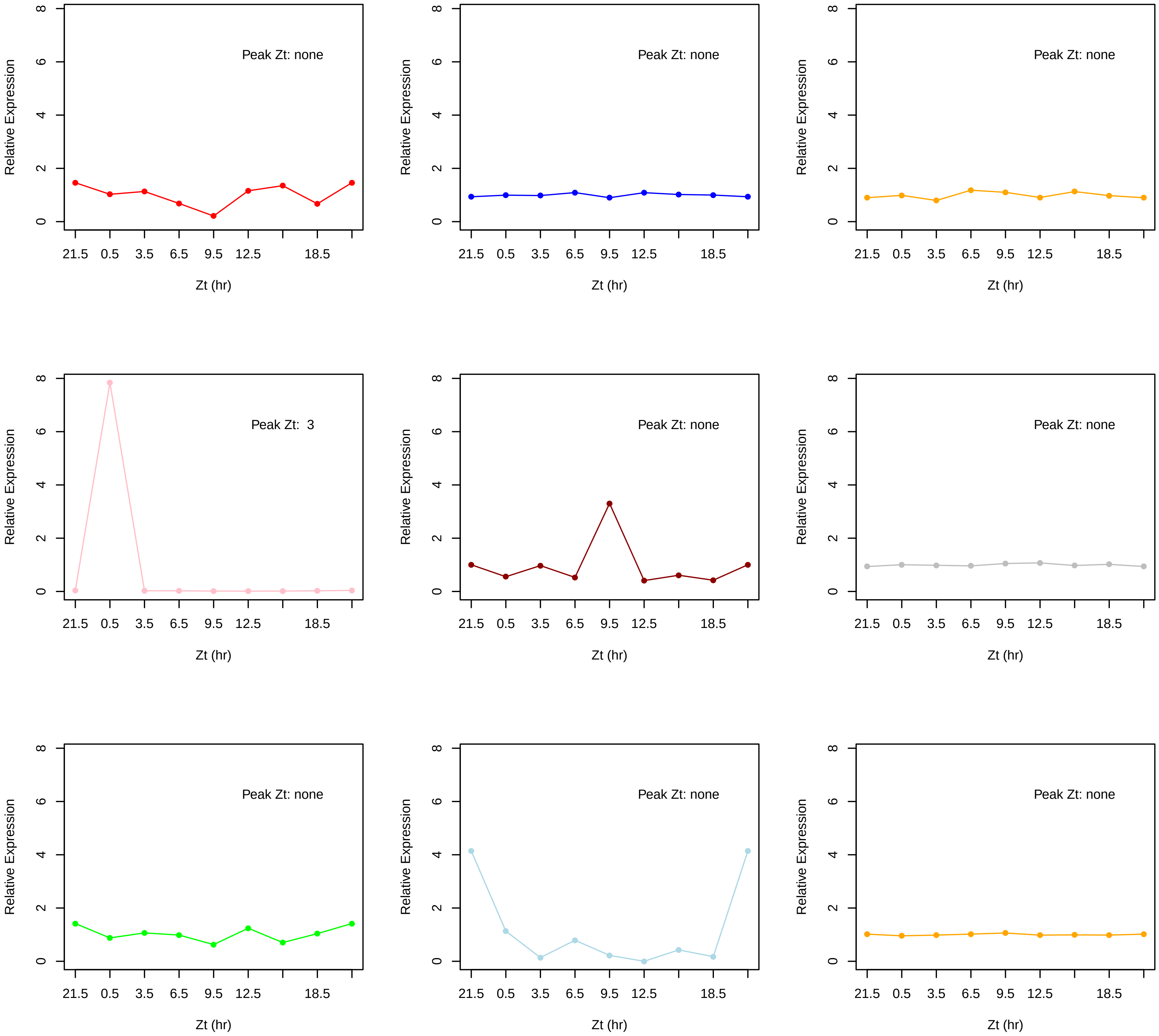

Singeleton: ENSXMAG00000004888 ssb

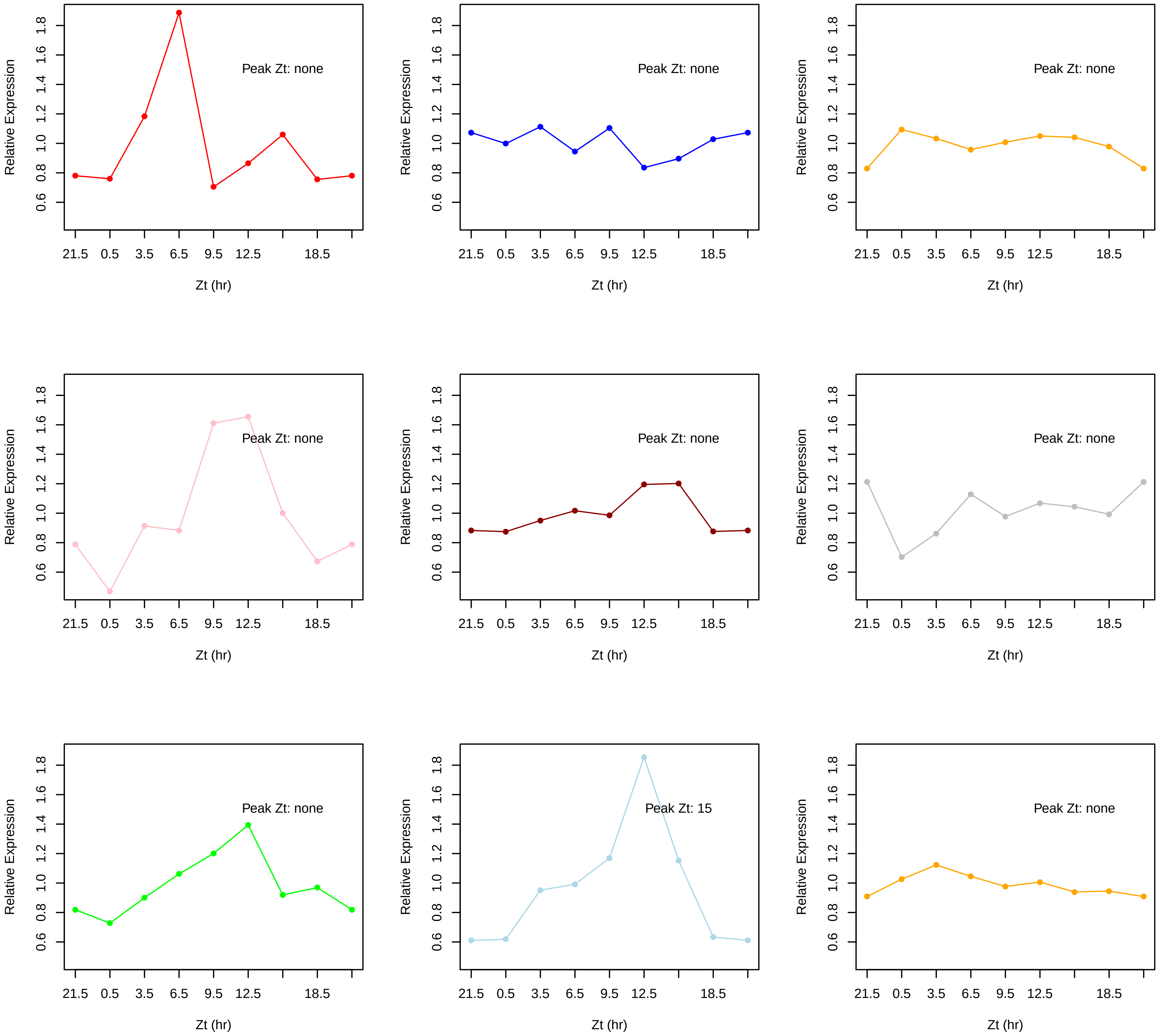

Singleton: ENSXMAG00000004883 klhl23

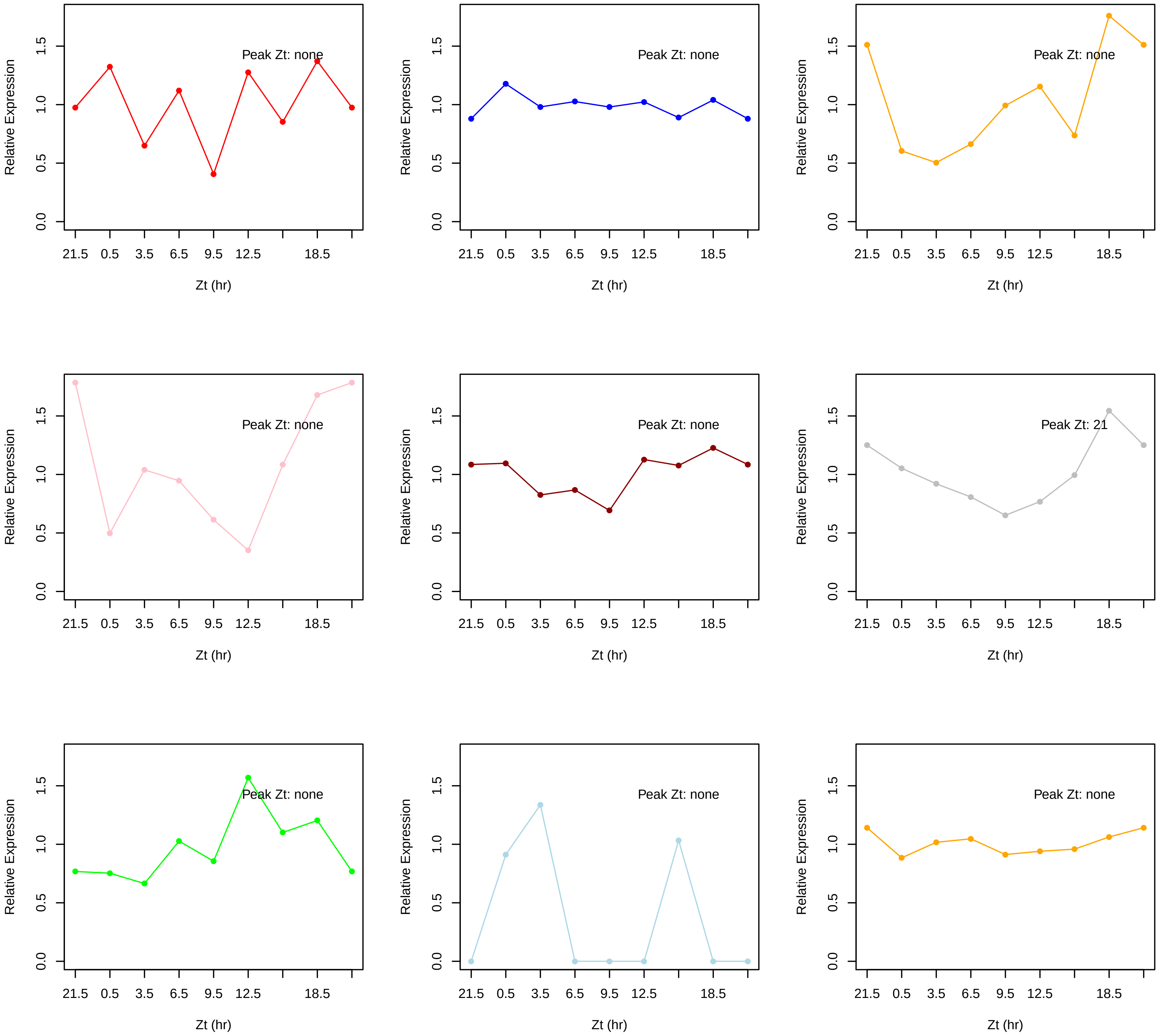

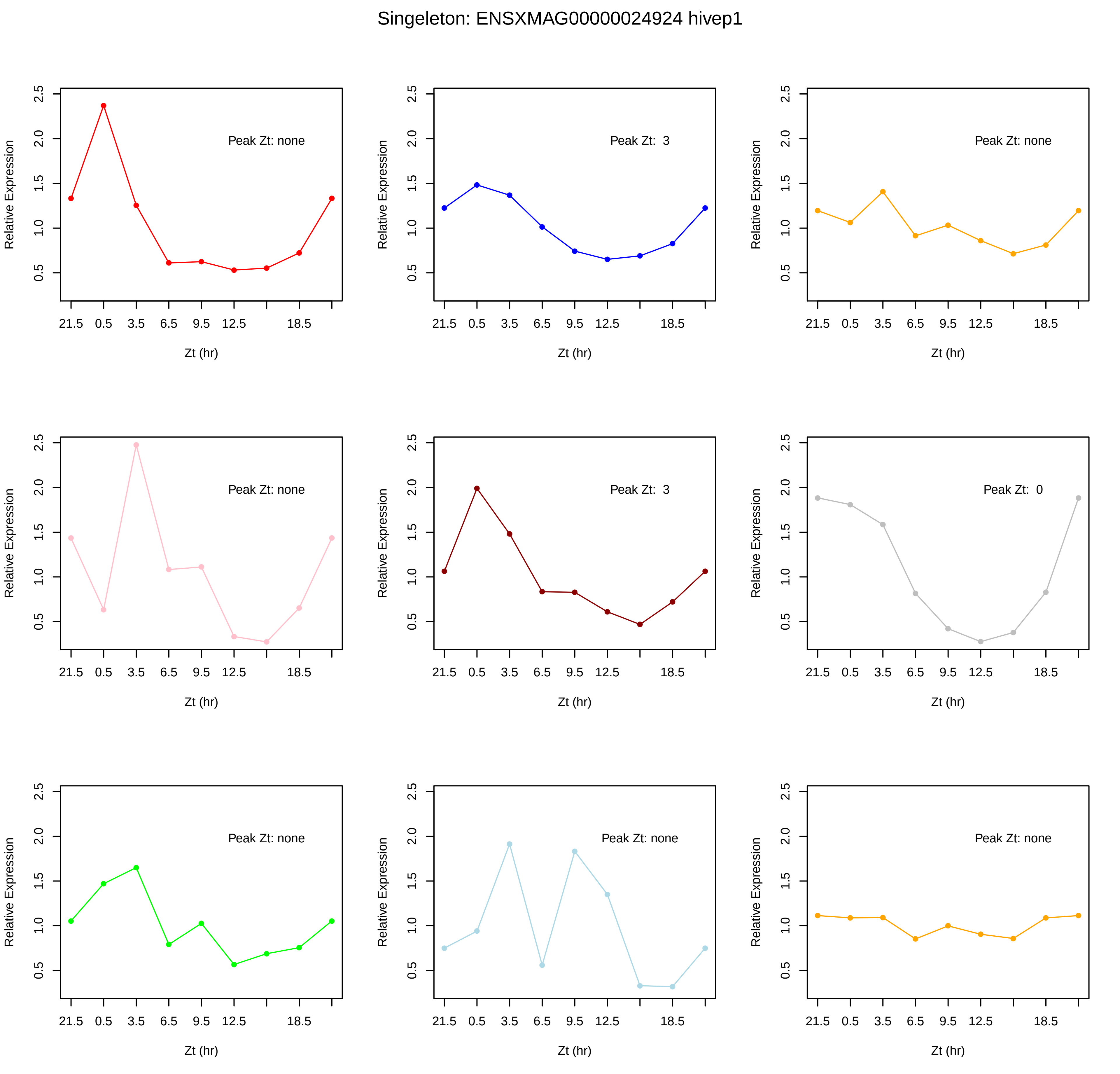
Singleton: ENSXMAG00000024924 hivep1
Peak Zt: none
Peak Zt: 3
Peak Zt: none
Peak Zt: none
Peak Zt: 3
Peak Zt: 0
Peak Zt: none
Peak Zt: none
Peak Zt: none
Relative Expression
Zt (hr)
21.5
0.5
3.5
6.5
9.5
12.5
18.5
0.5
1.0
1.5
2.0
2.5

Singeleton: ENSXMAG00000008976 ctsk

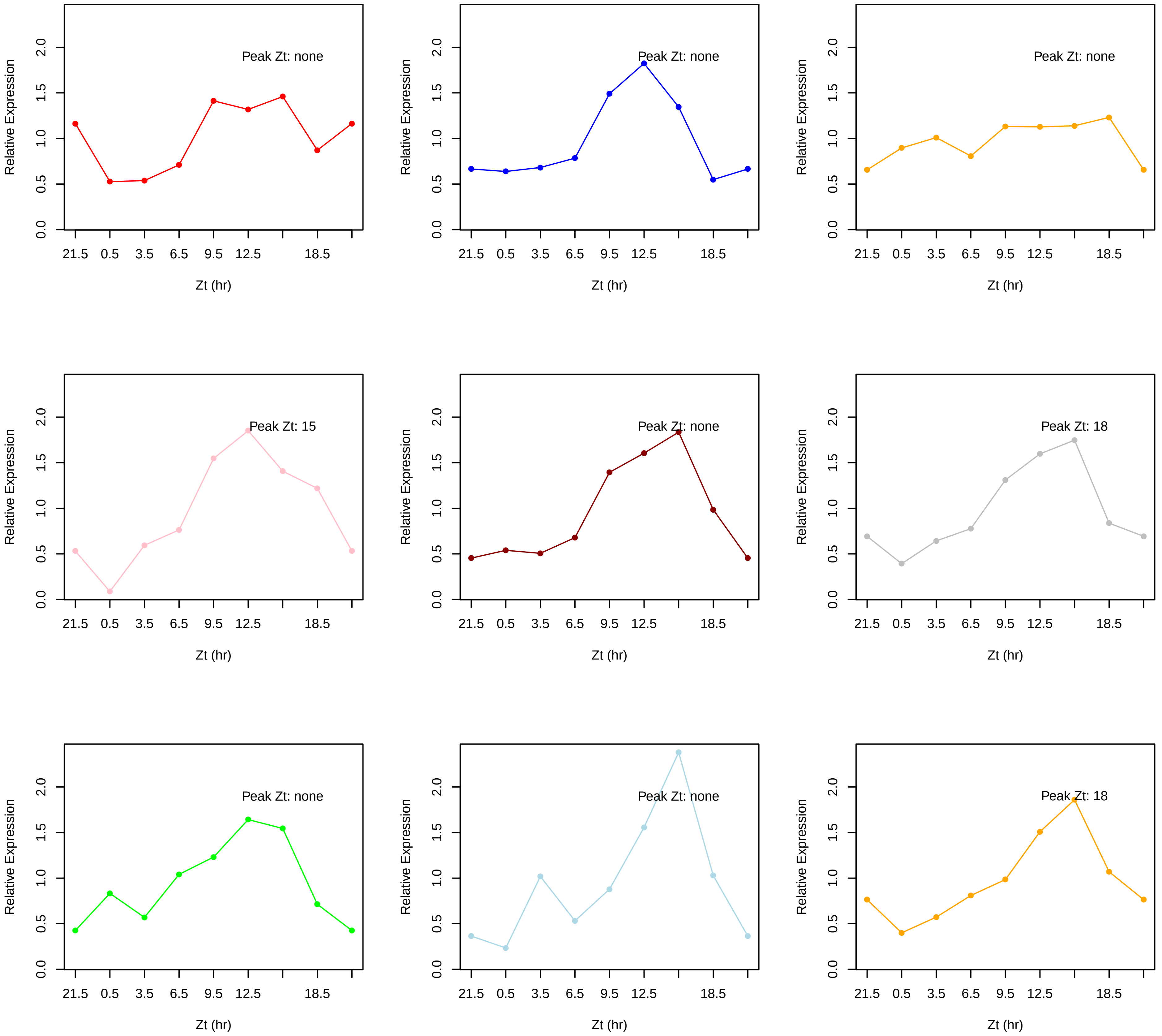

Singeleton: ENSXMAG00000018816 lyrm1

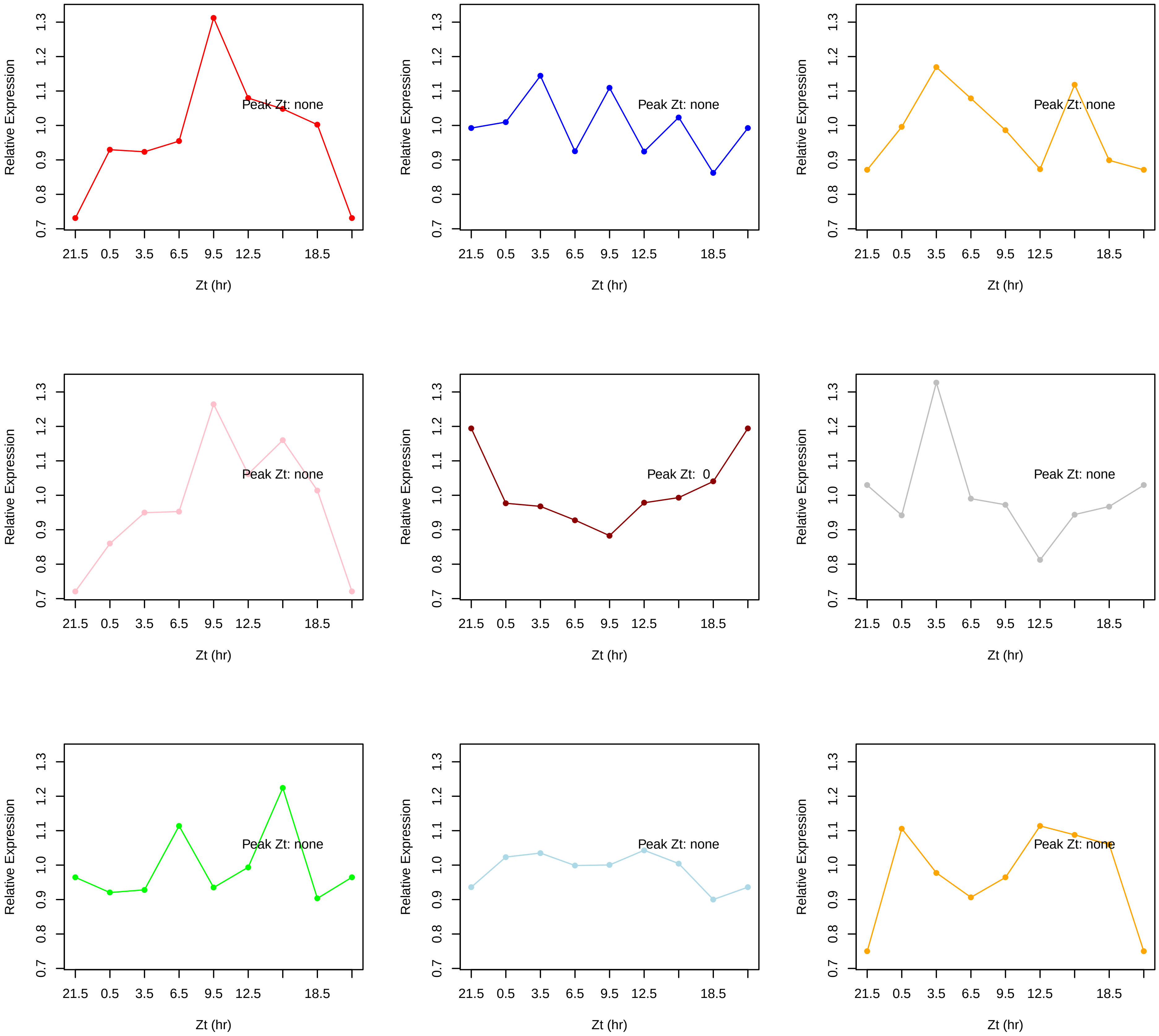

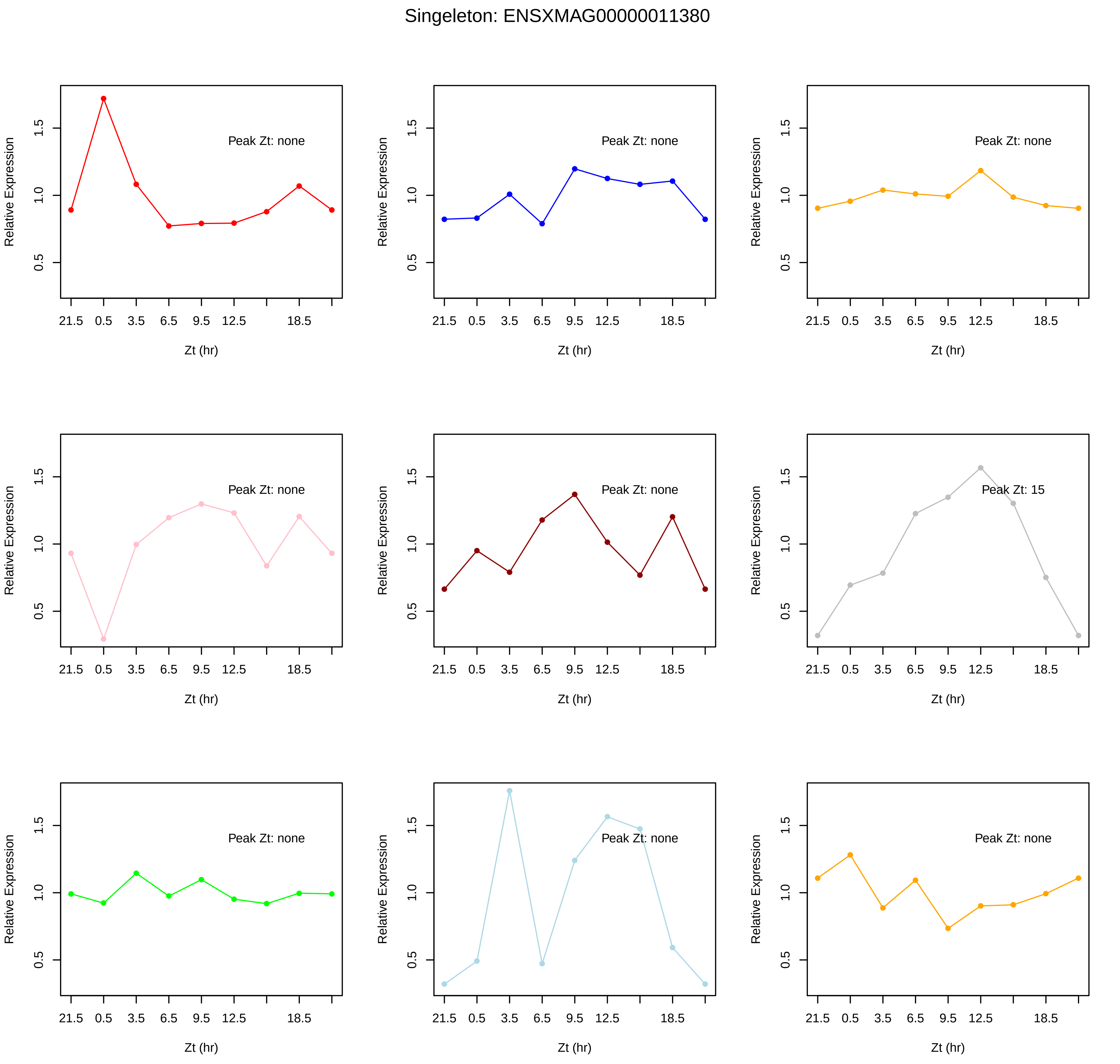
Singeleton: ENSXMAG00000011380
Peak Zt: none
Peak Zt: none
Peak Zt: none
Peak Zt: none
Peak Zt: none
Peak Zt: 15
Peak Zt: none
Peak Zt: none
Peak Zt: none
Relative Expression
Zt (hr)
21.5 0.5 3.5 6.5 9.5 12.5 18.5
0.5 1.0 1.5

Singeleton: ENSXMAG00000012938

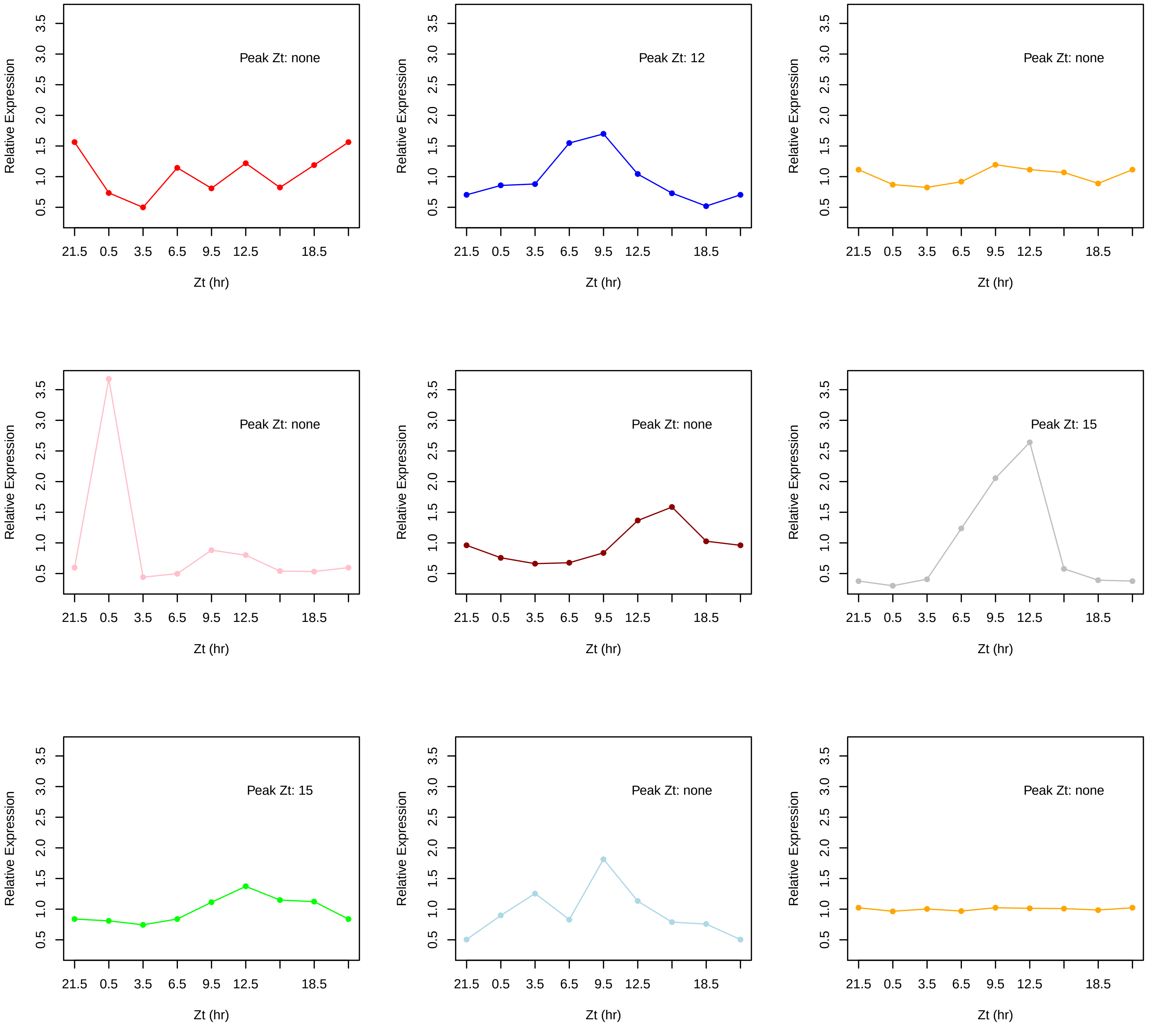

Singleton: ENSXMAG00000014366 phgdh

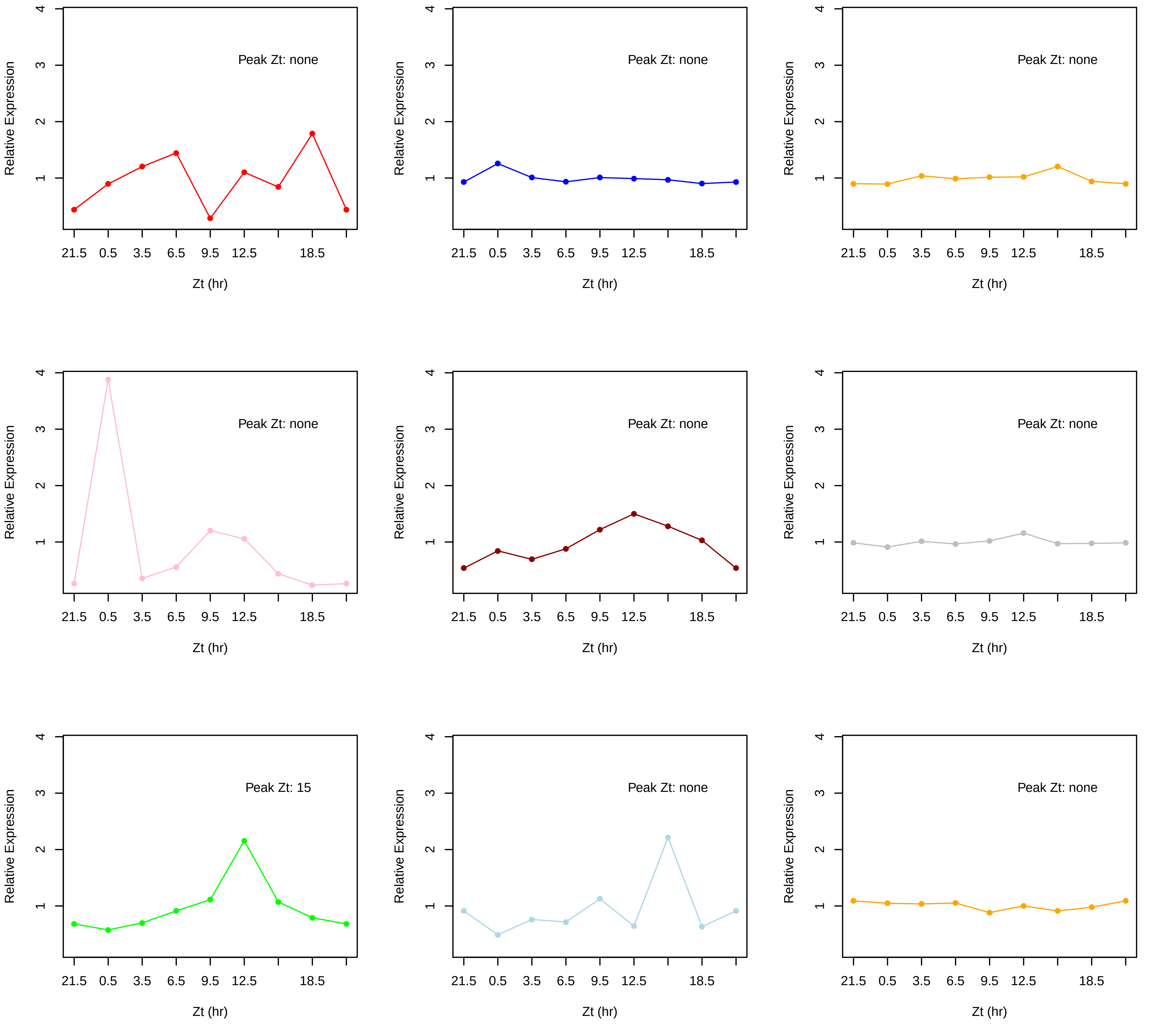

Singleton: ENSXMAG00000013372 si:ch211−113g11.6

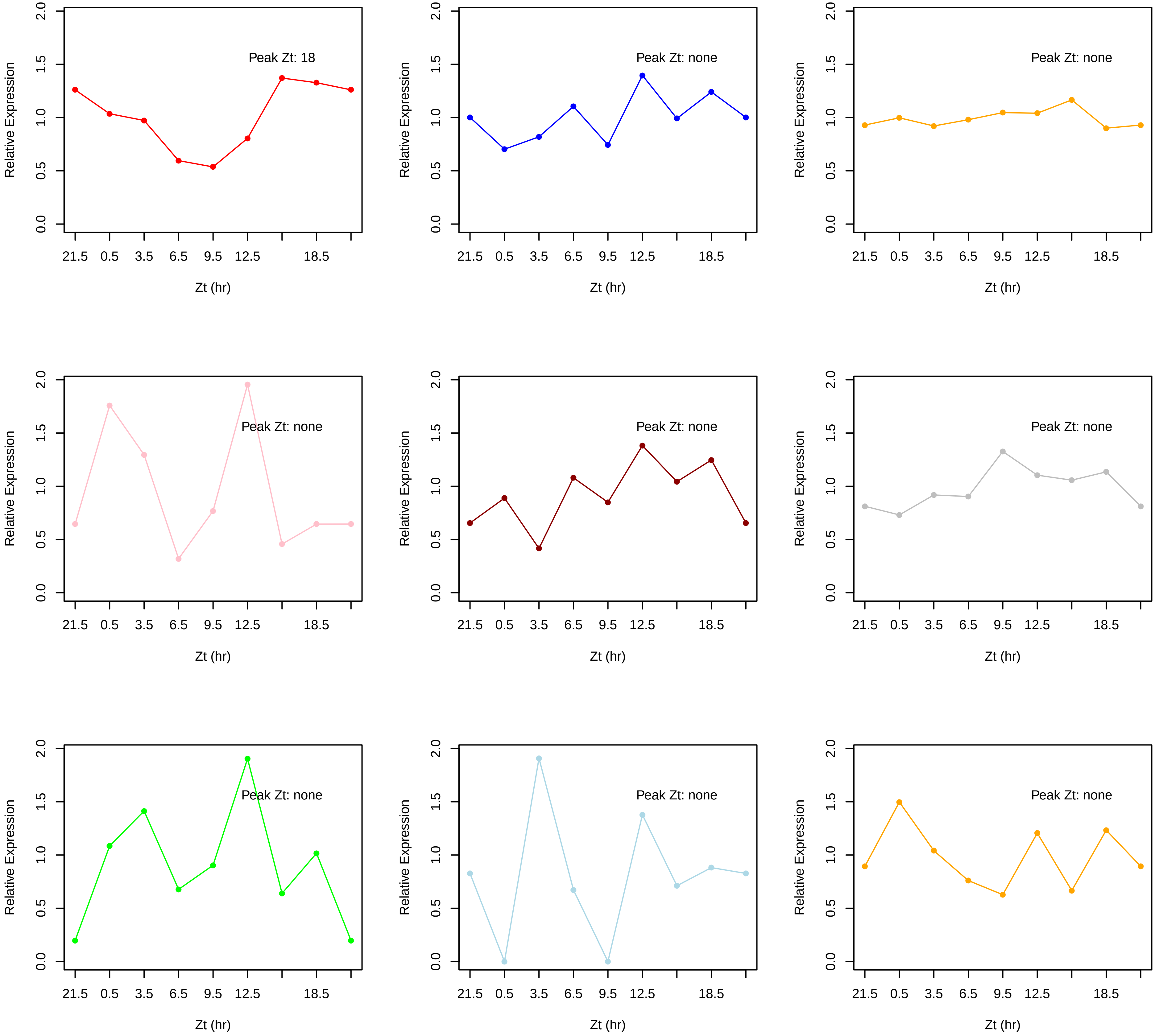

Singeleton: ENSXMAG00000008741 rad23aa

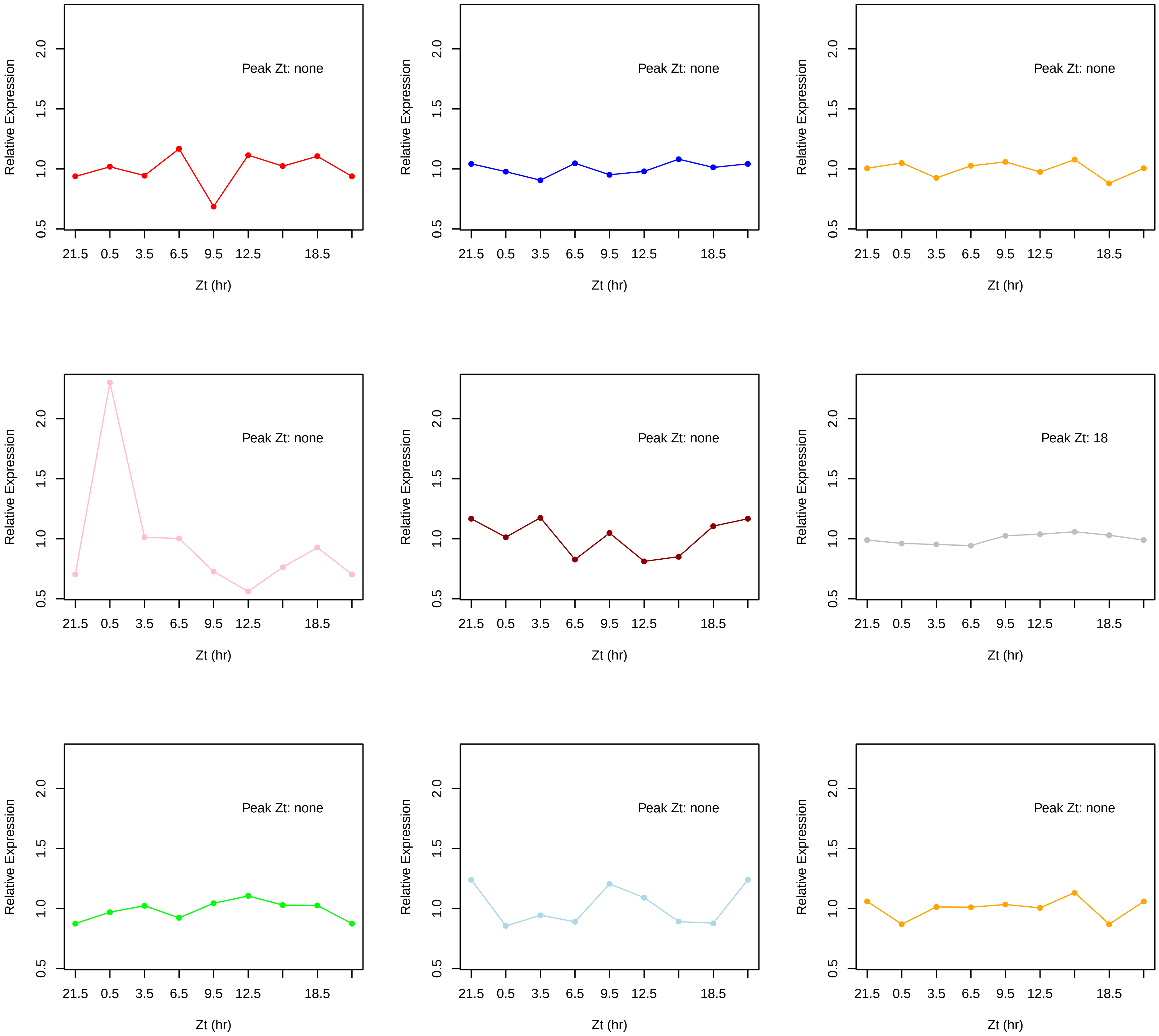

Singleton: ENSXMAG00000005930 naa25

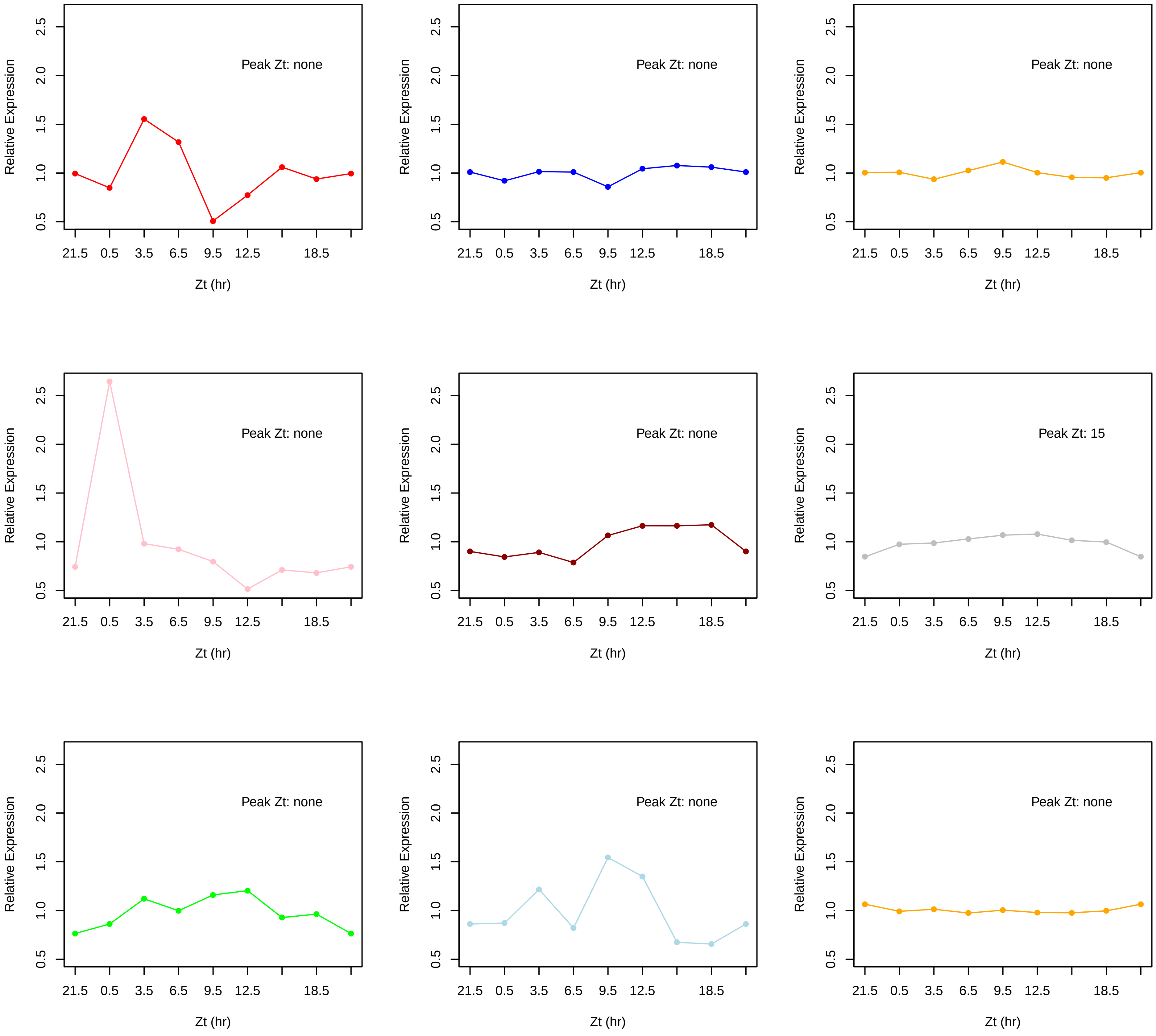

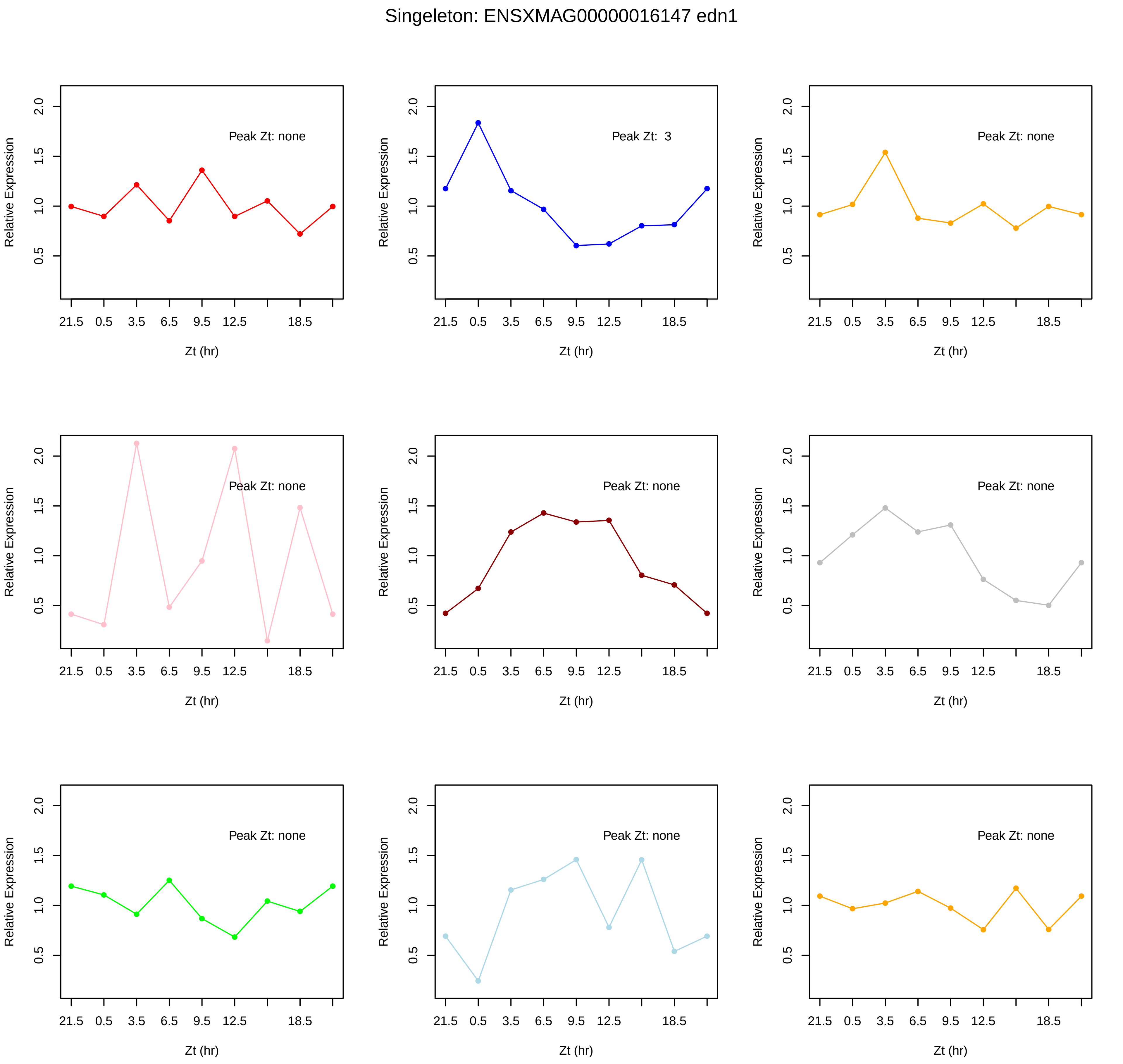
Singeleton: ENSXMAG00000016147 edn1
Peak Zt: none
Peak Zt: 3
Peak Zt: none
Peak Zt: none
Peak Zt: none
Peak Zt: none
Peak Zt: none
Peak Zt: none
Peak Zt: none
Relative Expression
Zt (hr)
21.5 0.5 3.5 6.5 9.5 12.5 18.5
0.5 1.0 1.5 2.0

Singeleton: ENSXMAG00000026346 mboat7

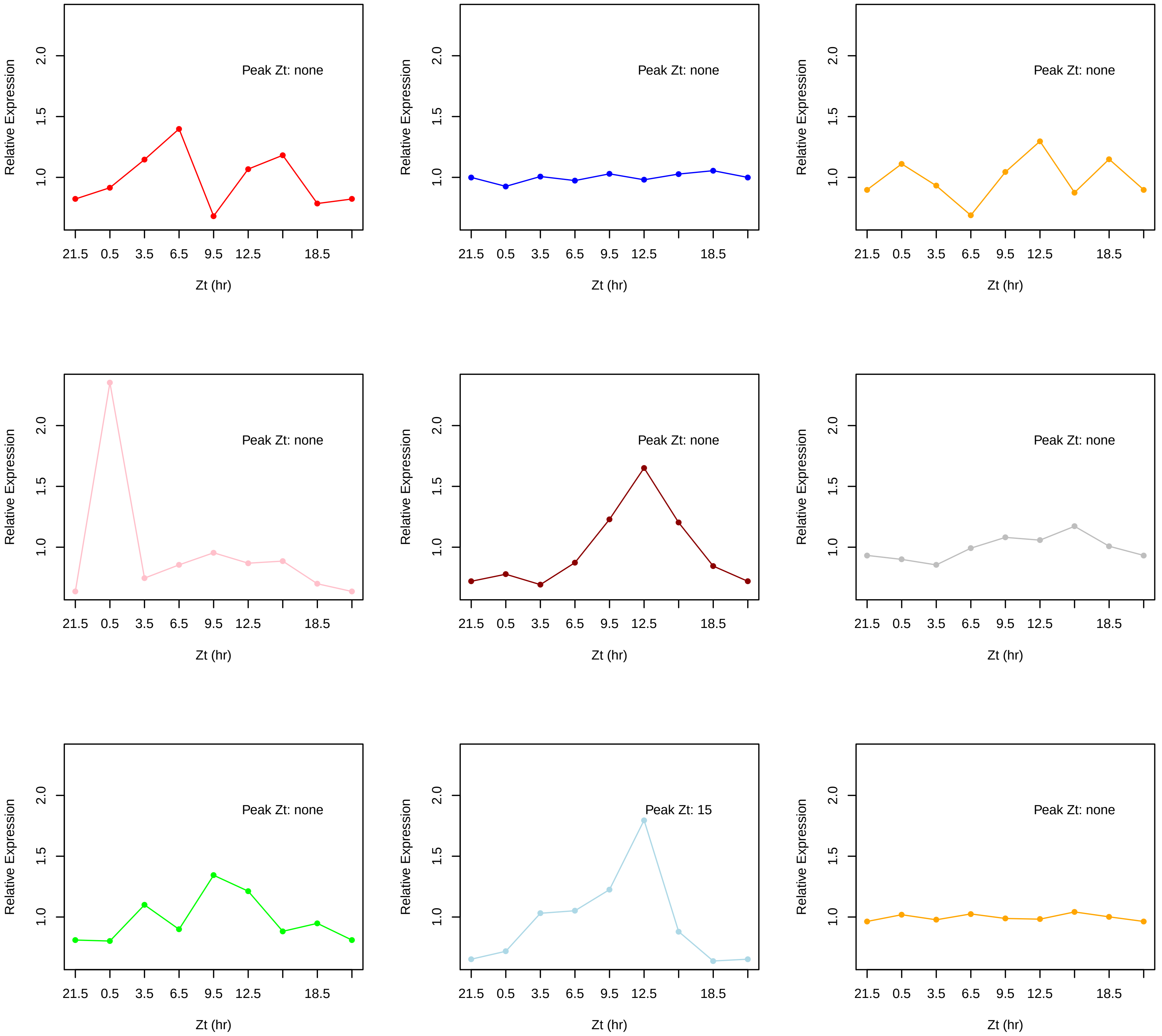

Singeleton: ENSXMAG00000015880 plcxd3

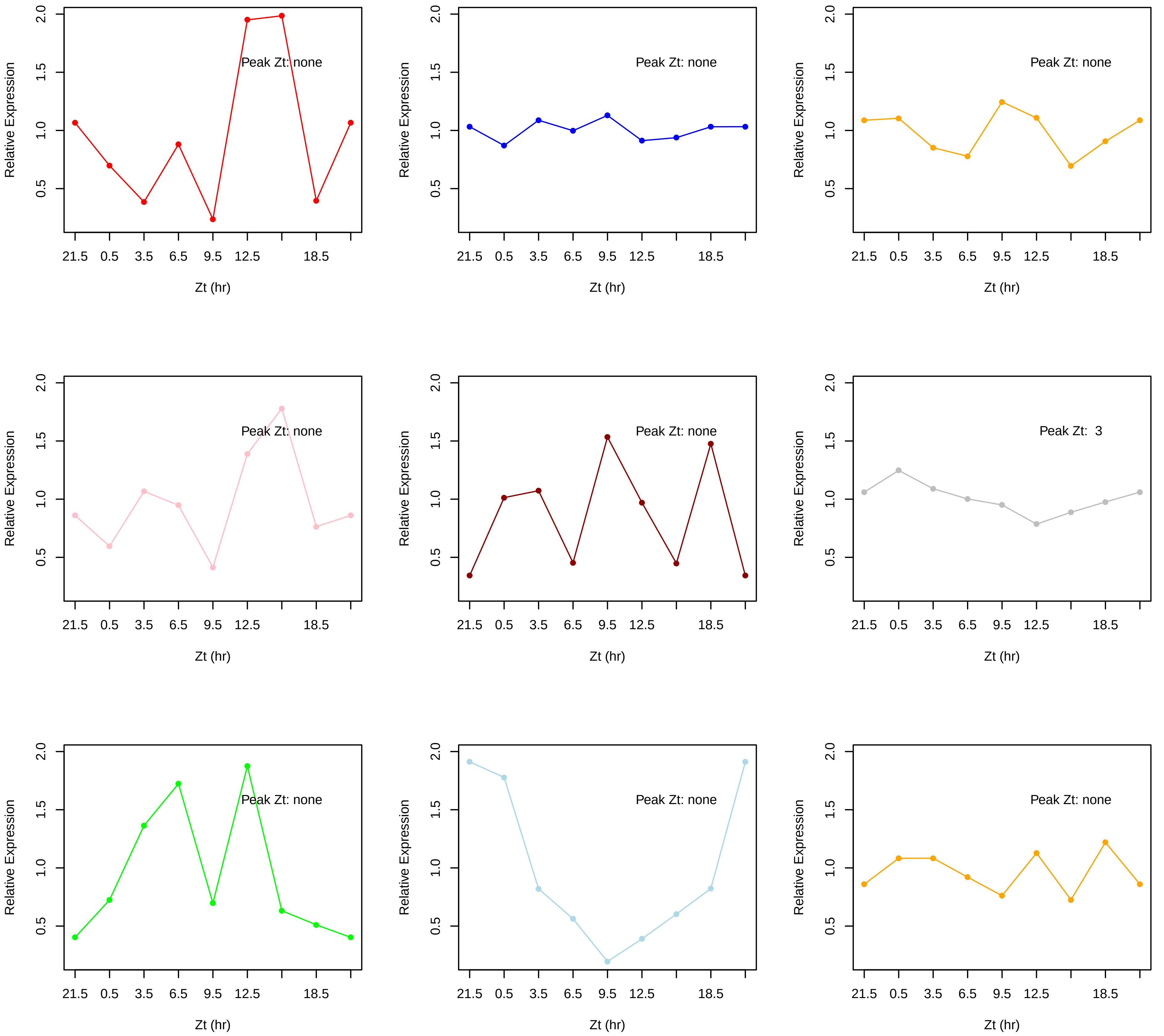

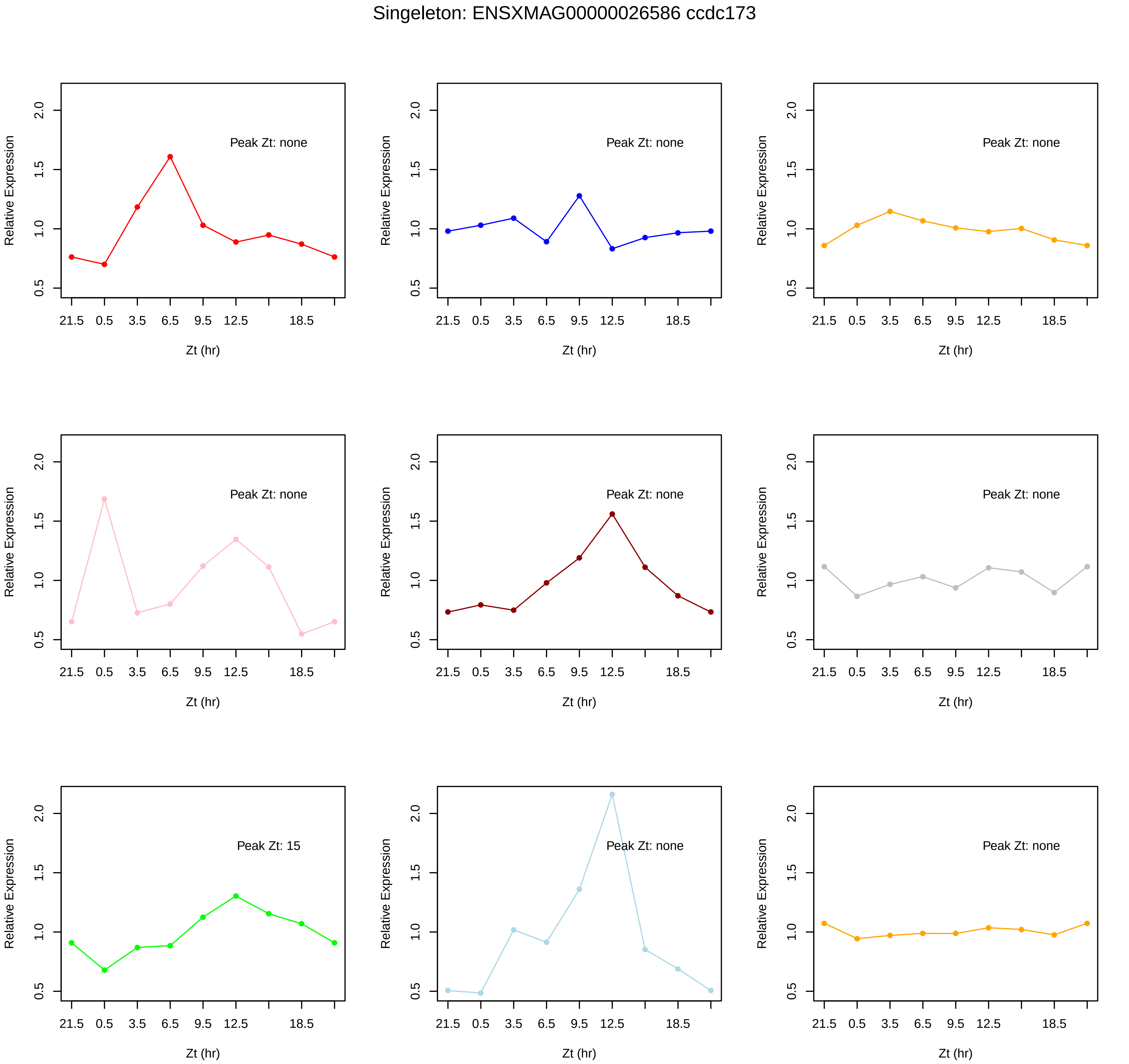
Singeleton: ENSXMAG00000026586 ccdc173
Peak Zt: none
Peak Zt: none
Peak Zt: none
Peak Zt: none
Peak Zt: none
Peak Zt: none
Peak Zt: 15
Peak Zt: none
Peak Zt: none
Relative Expression
Zt (hr)
21.5
0.5
3.5
6.5
9.5
12.5
18.5

Singeleton: ENSXMAG00000023877 stim1a

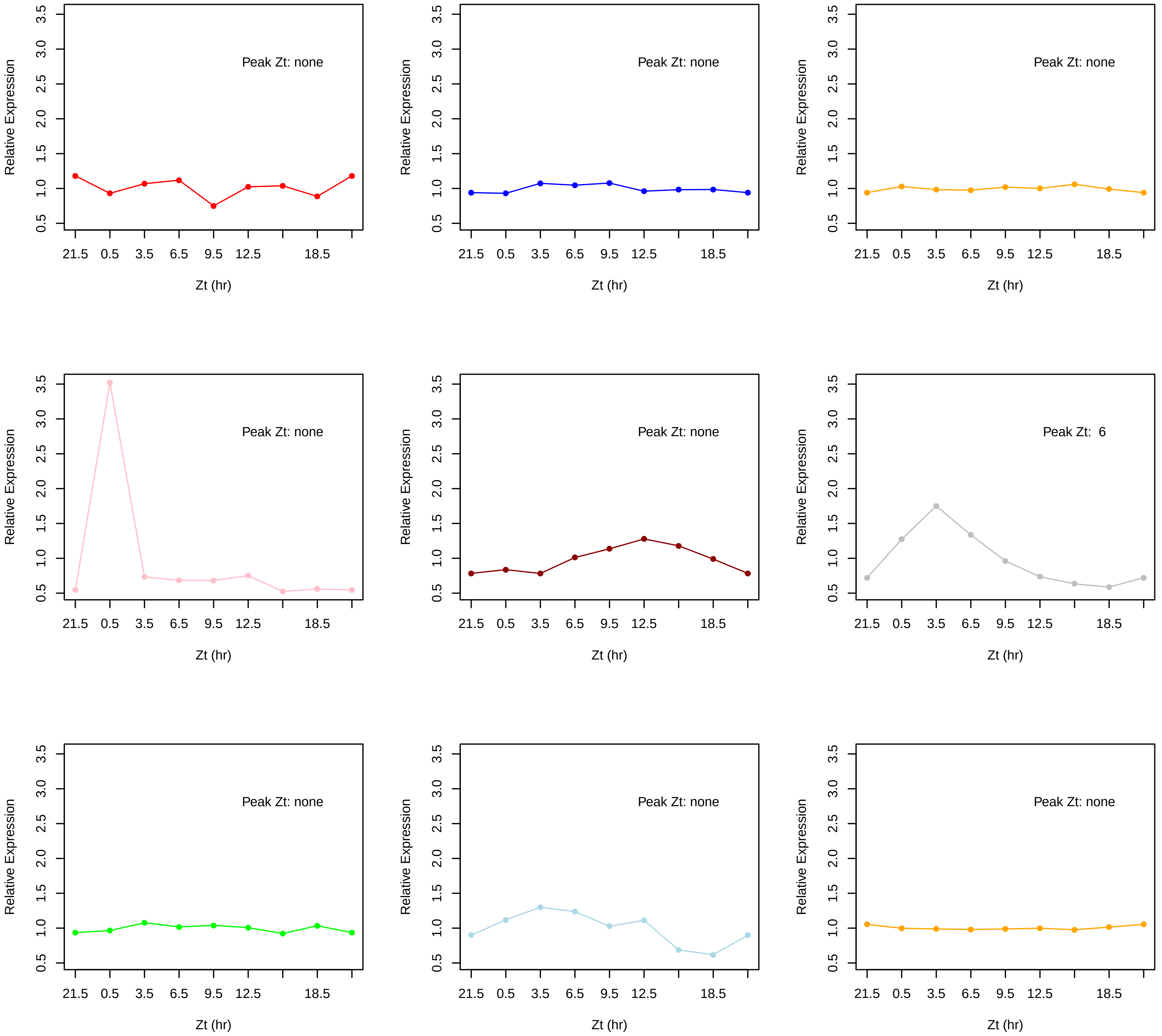

Singleton: ENSXMAG00000011494

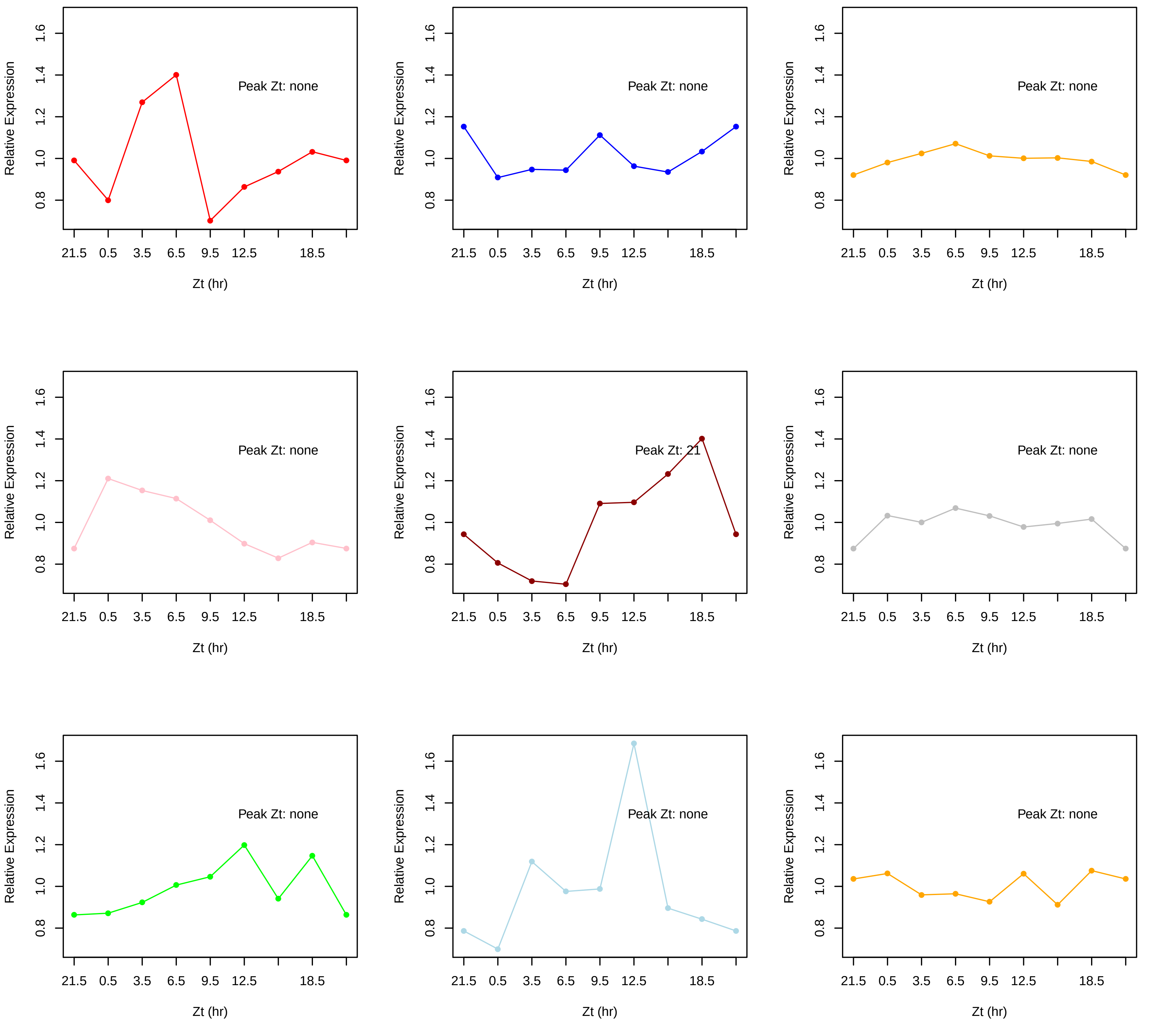

Singeleton: ENSXMAG00000012557 zranb1b

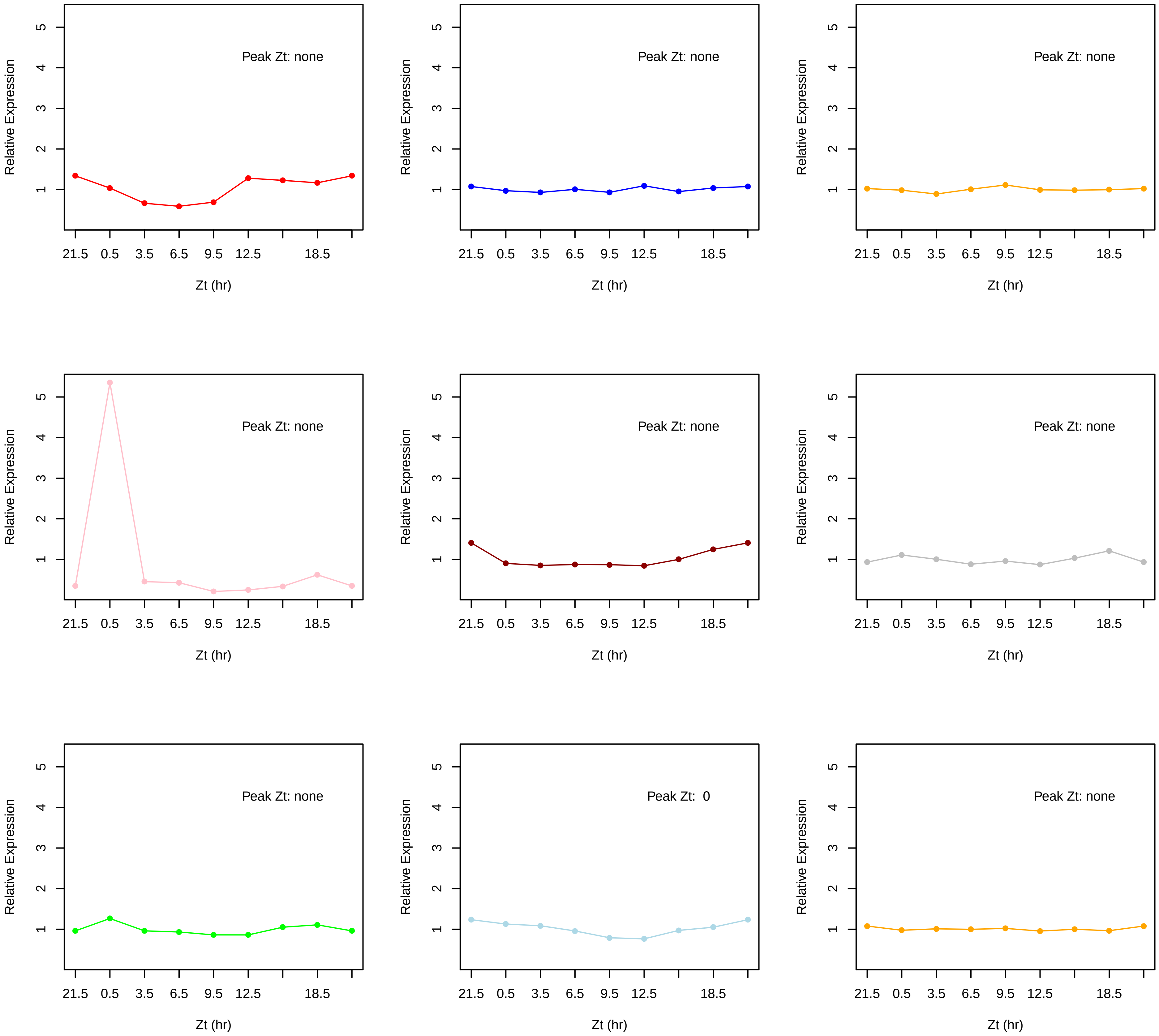

Singeleton: ENSXMAG00000014150 ogfod1

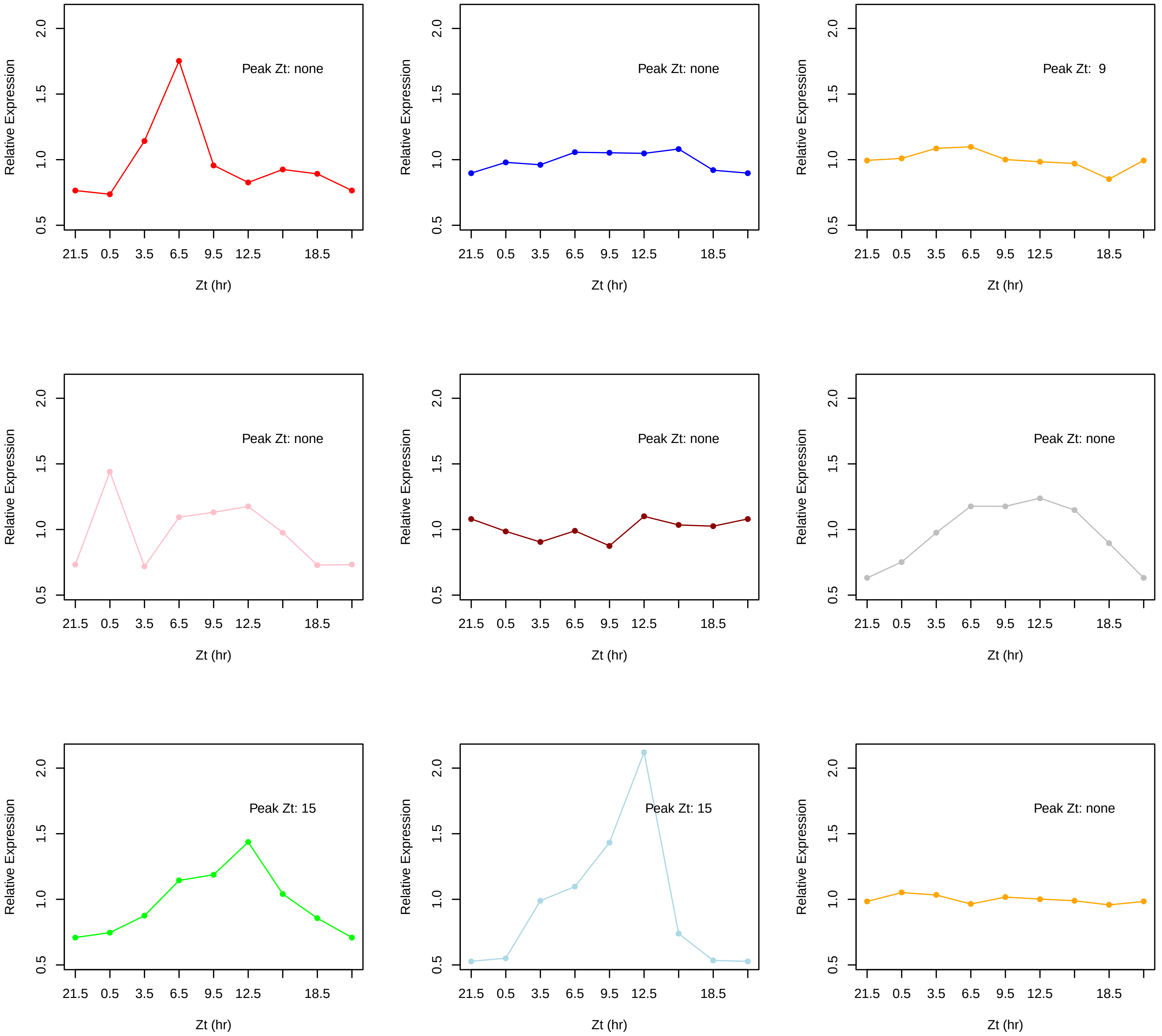

Singeleton: ENSXMAG00000013367 tlr18

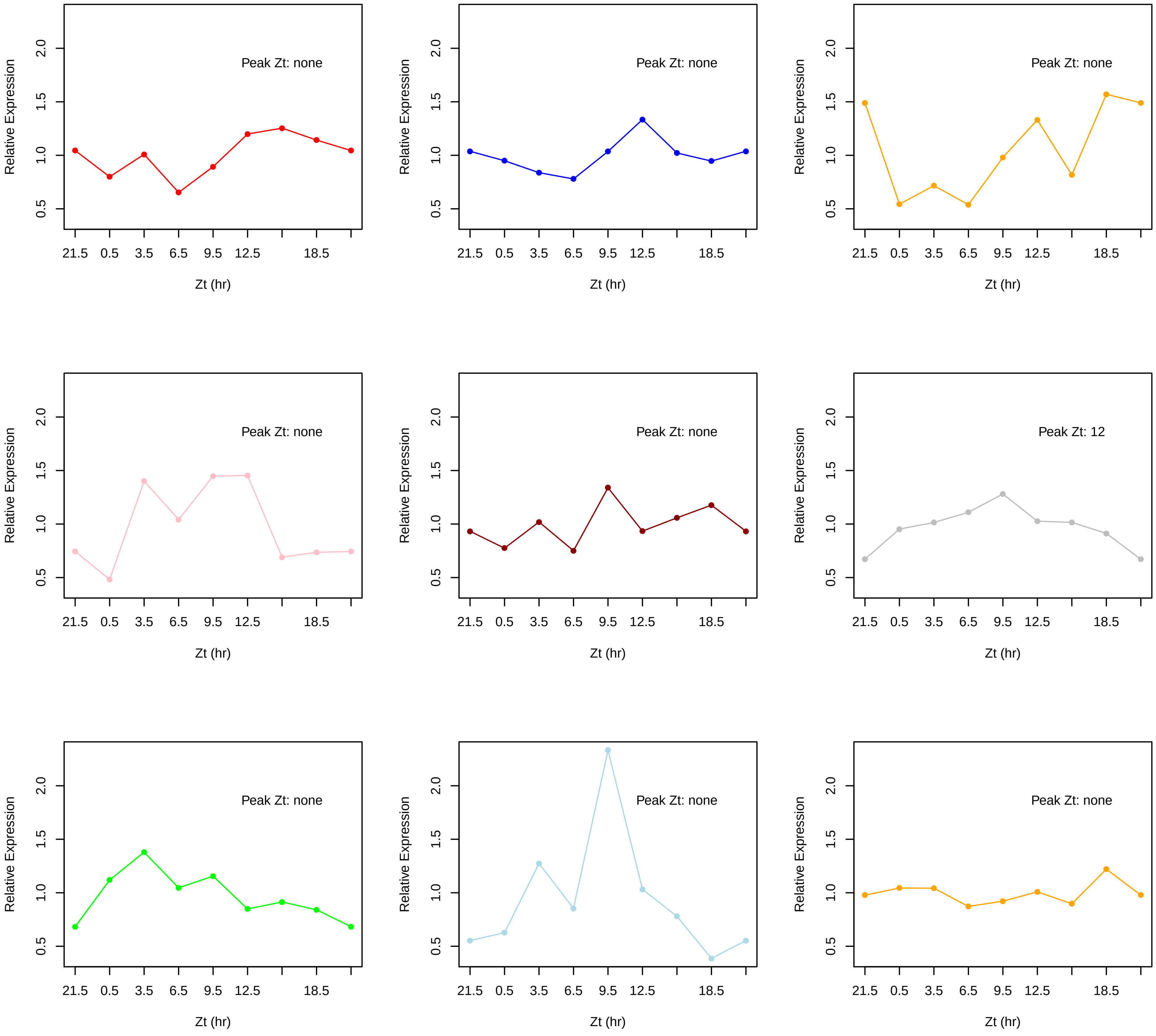

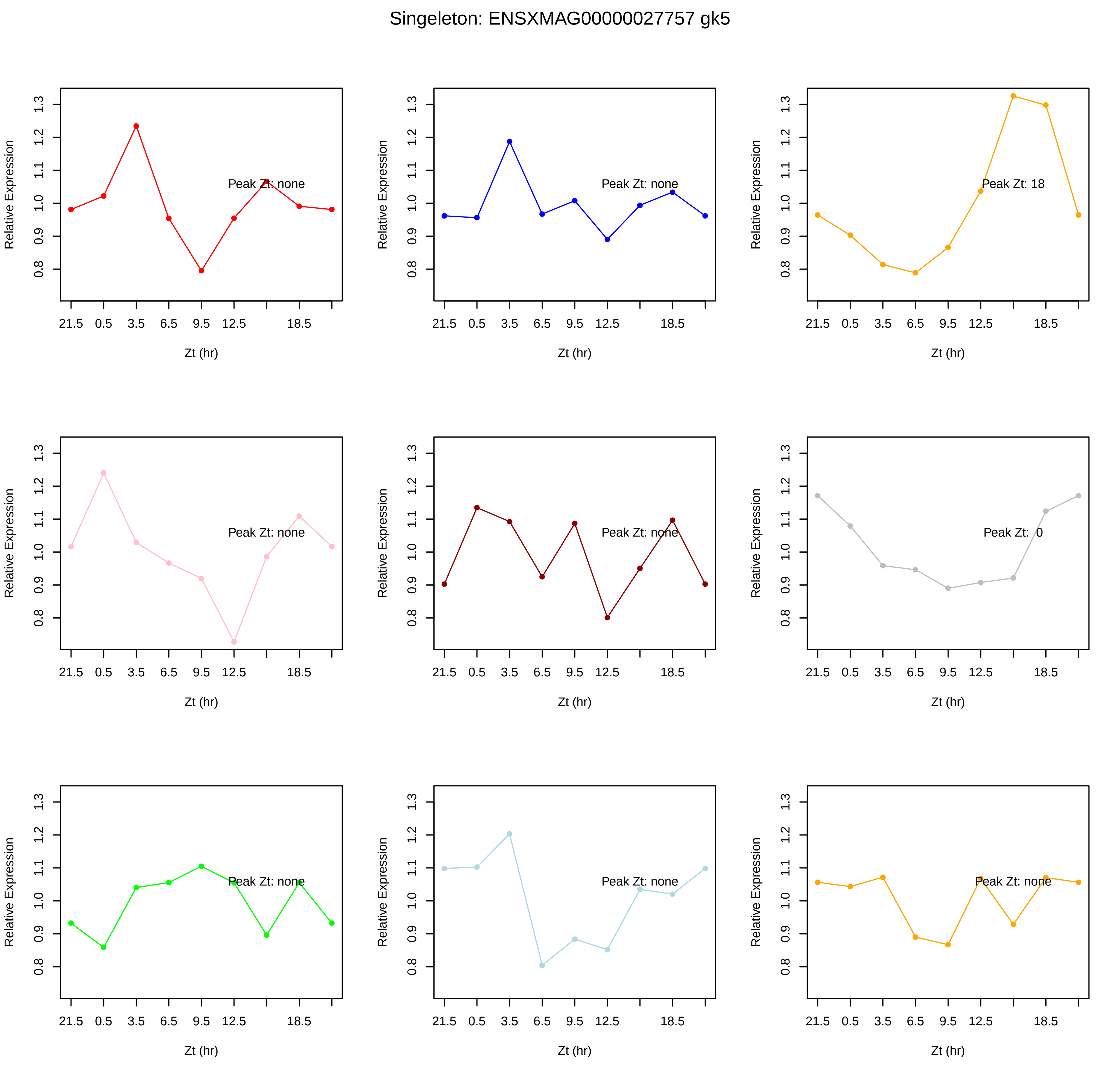
Singeleton: ENSXMAG00000027757 gk5
Relative Expression
Zt (hr)
21.5 0.5 3.5 6.5 9.5 12.5 18.5
0.8 0.9 1.0 1.1 1.2 1.3
Peak Zt: none
Peak Zt: none
Peak Zt: 18
Peak Zt: none
Peak Zt: none
Peak Zt: 0
Peak Zt: none
Peak Zt: none
Peak Zt: none

Singeleton: ENSXMAG00000000449 hpdl

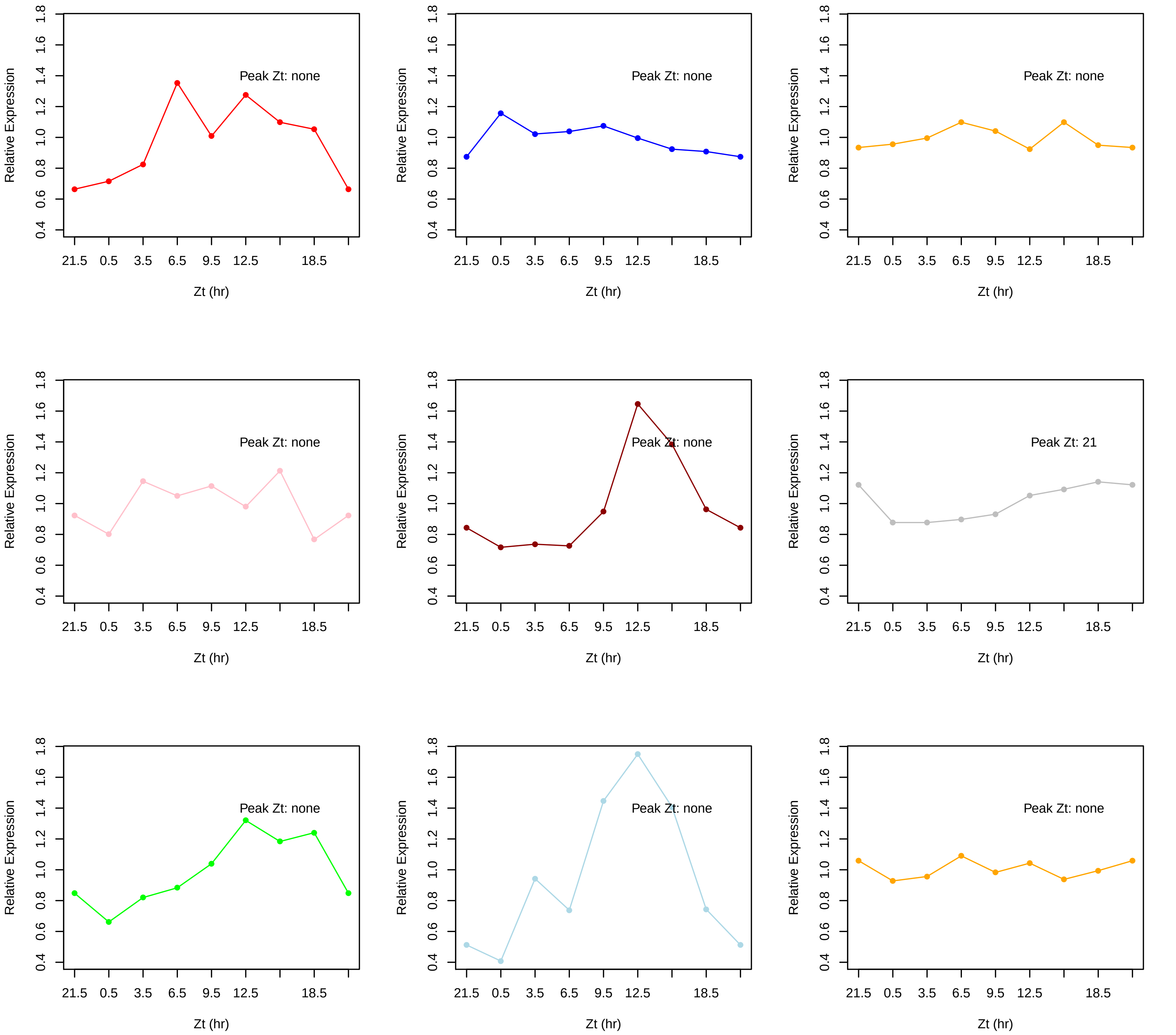

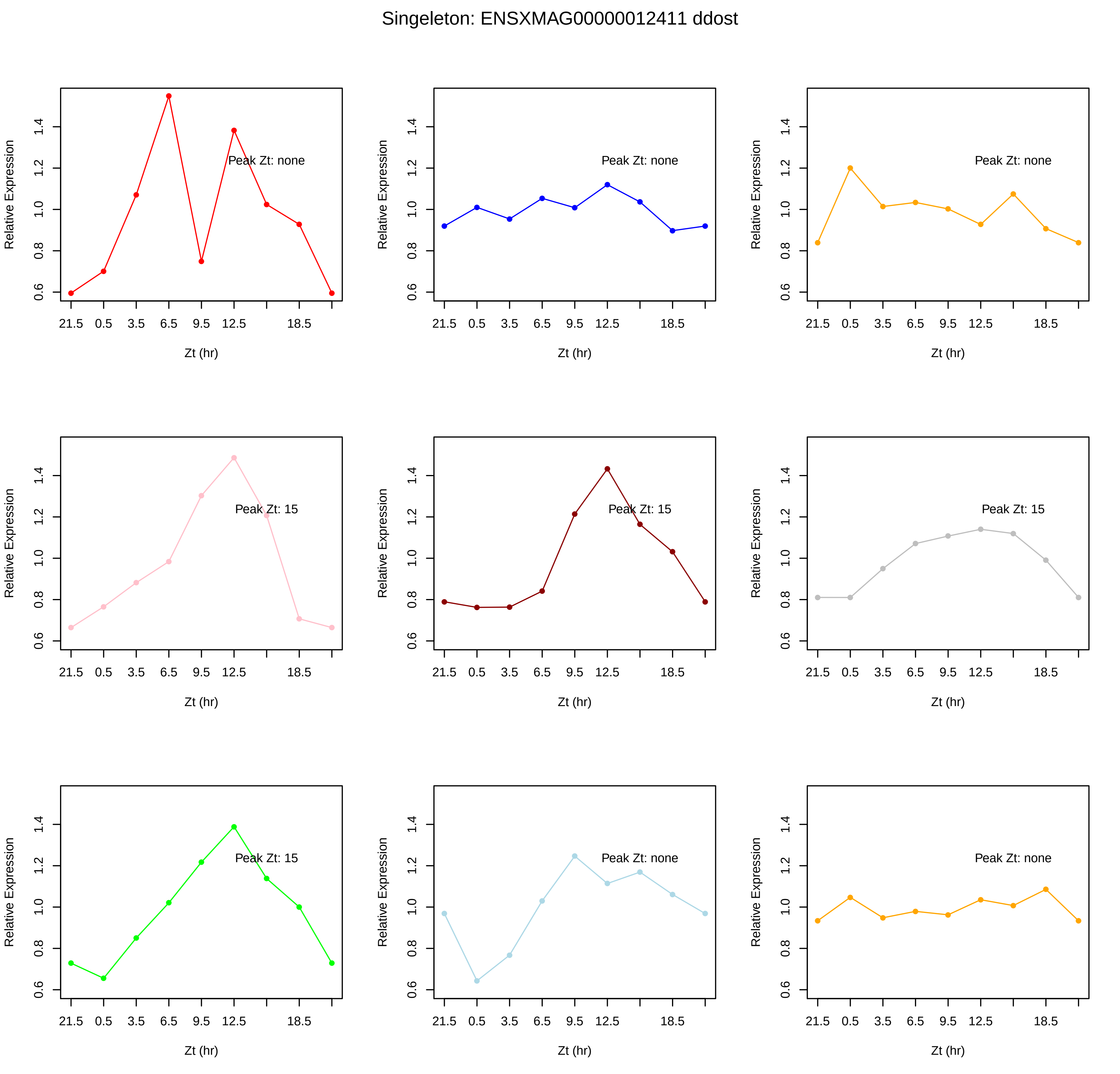

Singeleton: ENSXMAG00000012411 ddost
Peak Zt: none
Peak Zt: none
Peak Zt: none
Peak Zt: 15
Peak Zt: 15
Peak Zt: 15
Peak Zt: 15
Peak Zt: none
Peak Zt: none
Relative Expression
Zt (hr)
21.5
0.5
3.5
6.5
9.5
12.5
18.5

Singeleton: ENSXMAG00000014172 nudt21

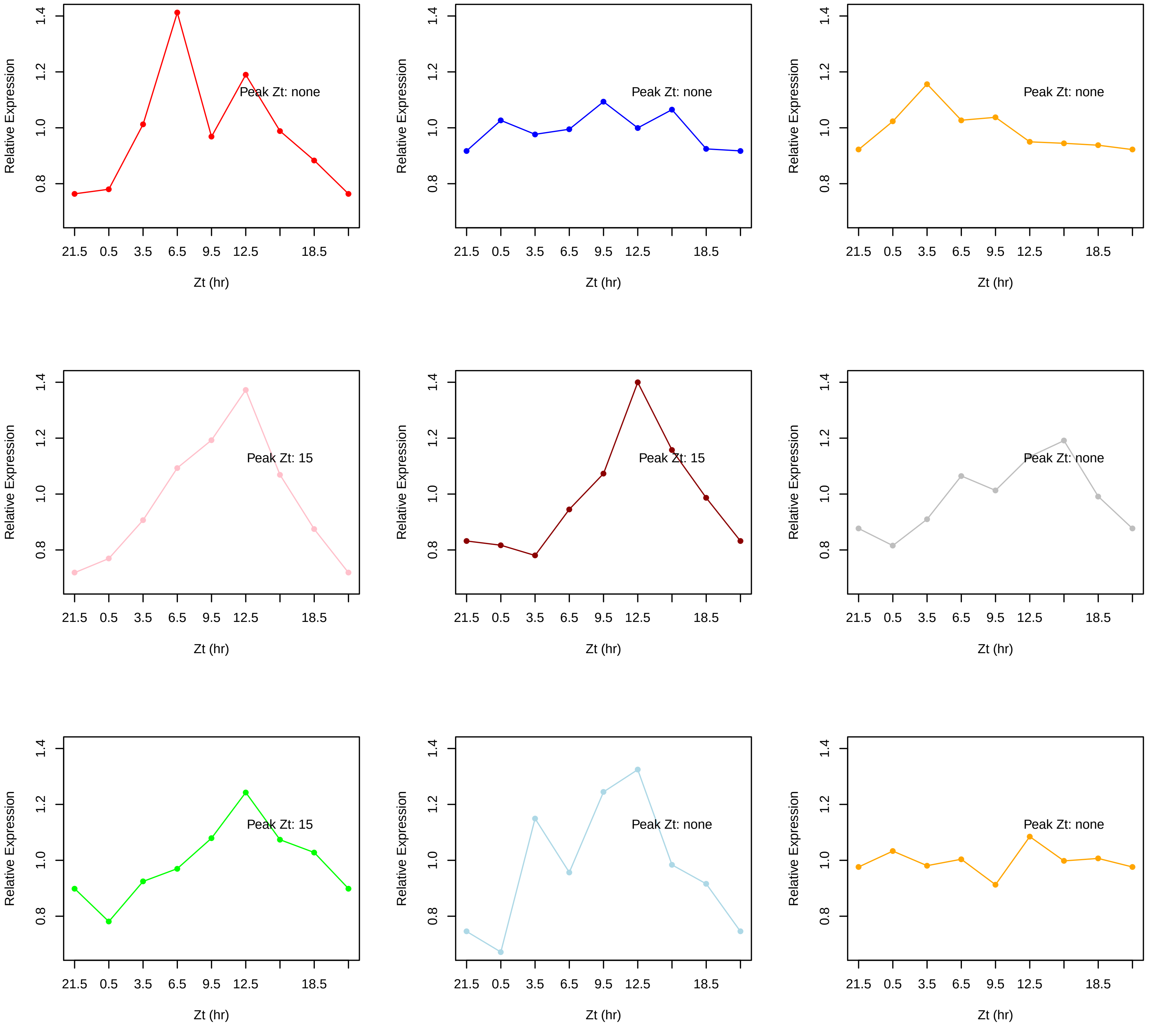

Singeleton: ENSXMAG00000004132 slc35e4

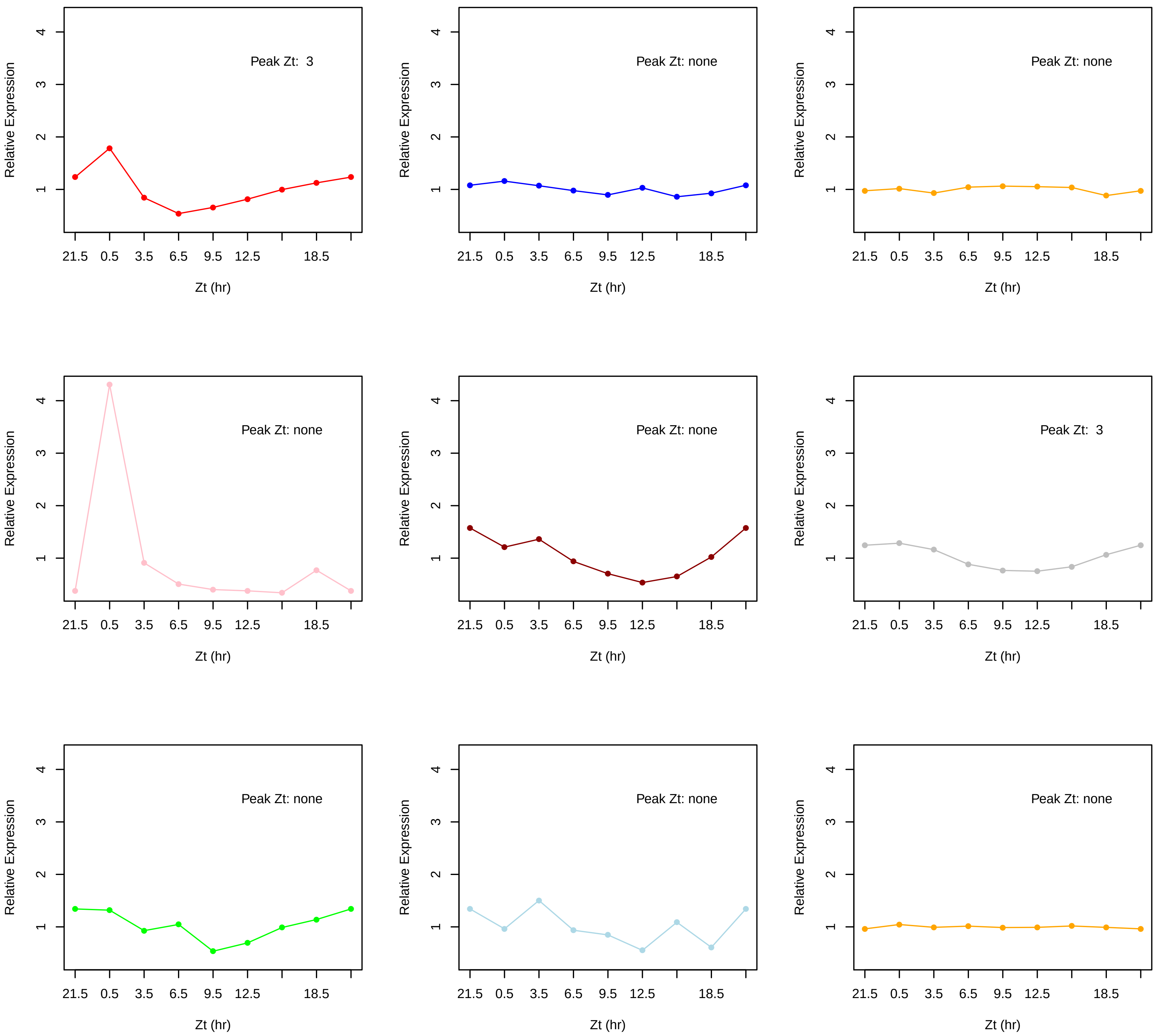

Singleton: ENSXMAG00000014336 fastkd1

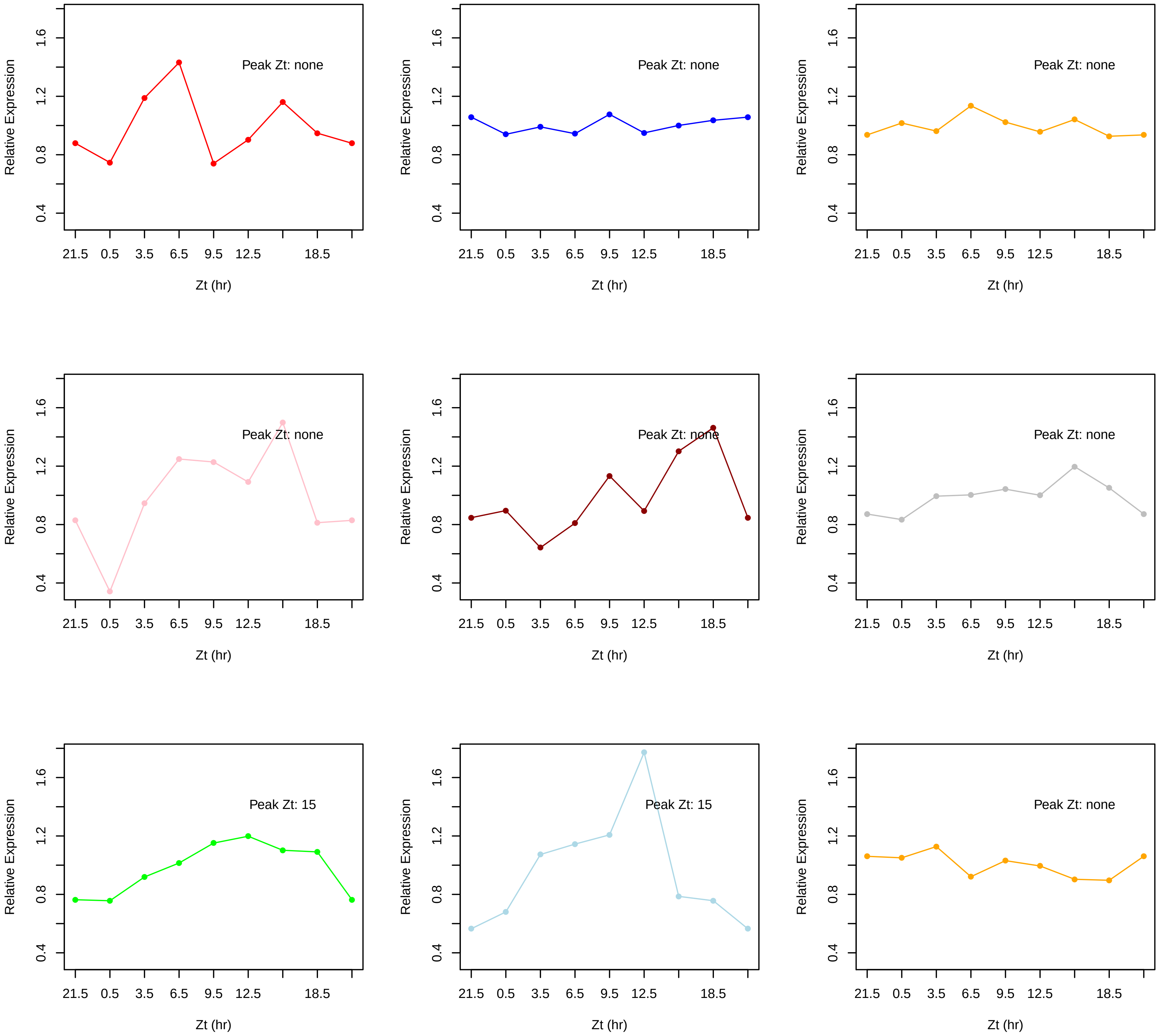

Singeleton: ENSXMAG00000002141 rapgef2

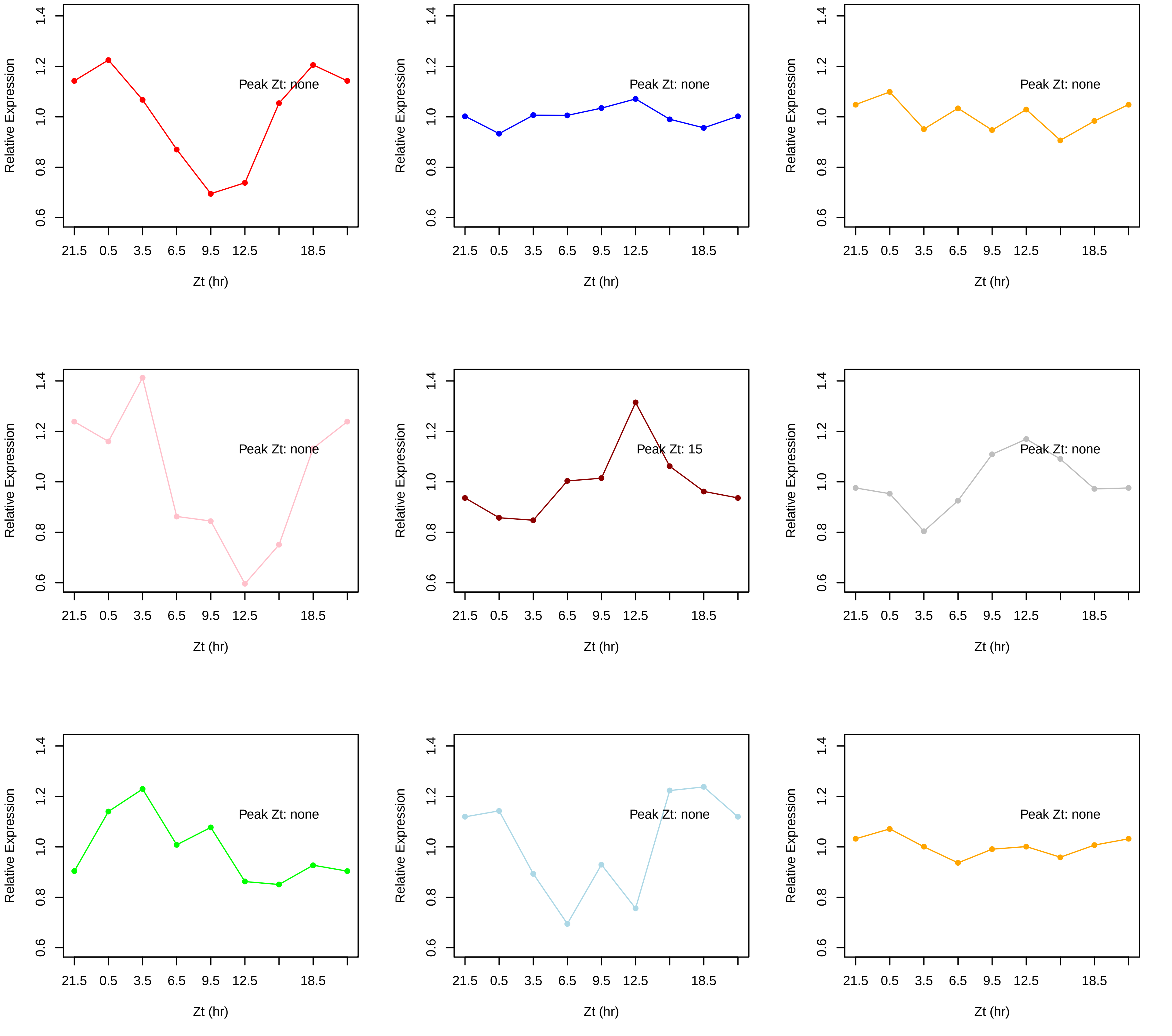

Singeleton: ENSXMAG00000015108 prcc

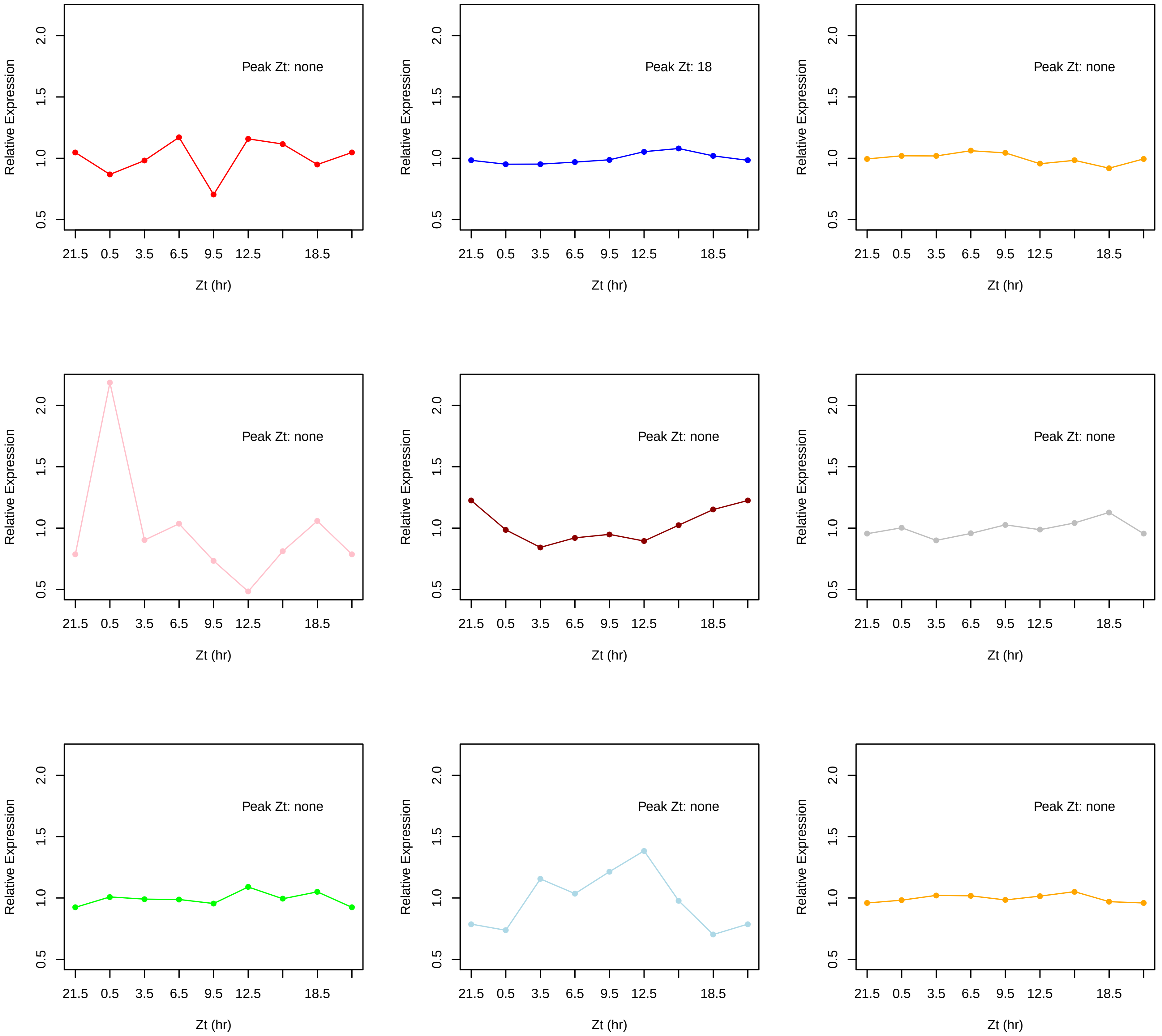

Singleton: ENSXMAG00000020876 bcar1

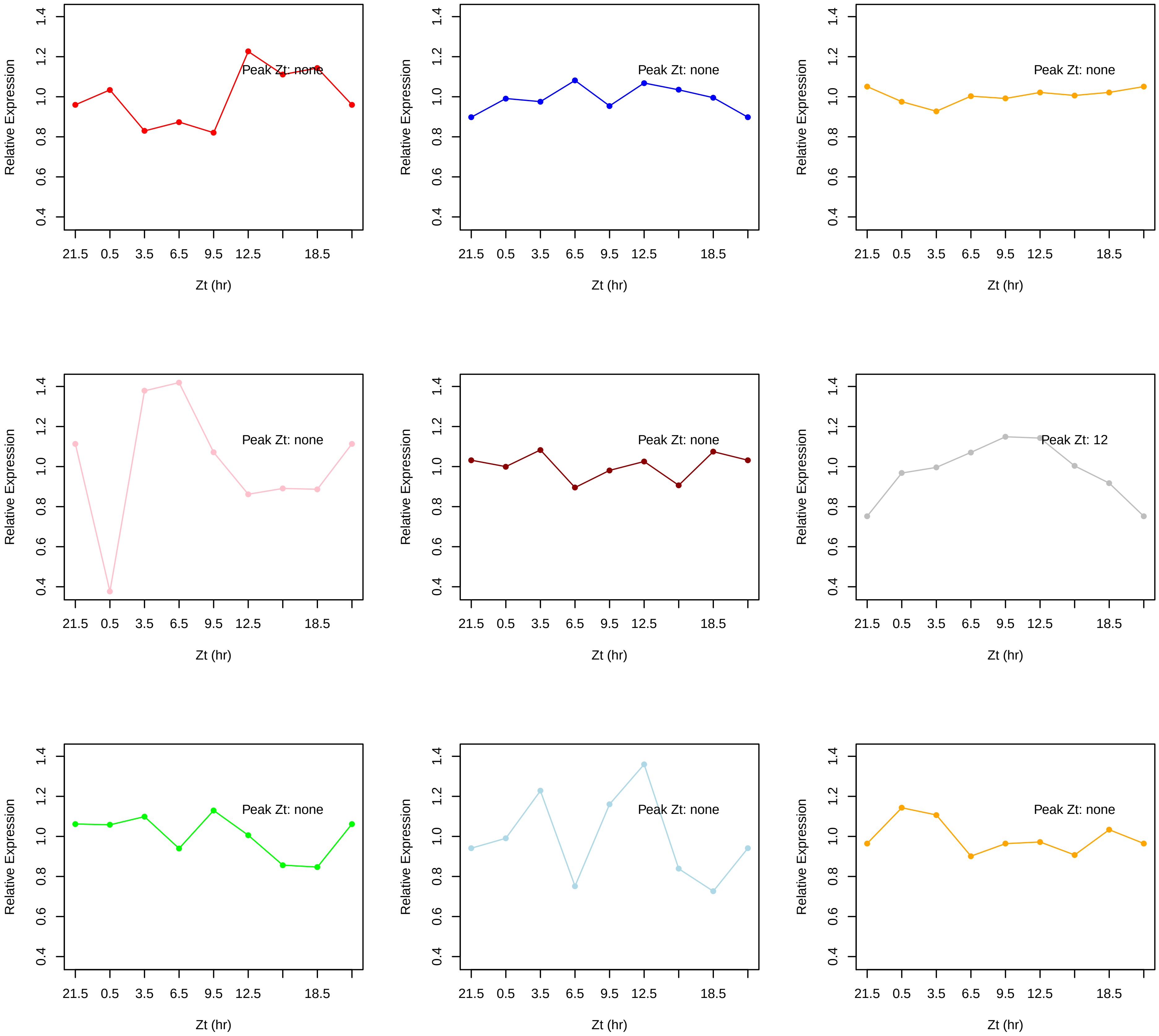

Singeleton: ENSXMAG00000017900

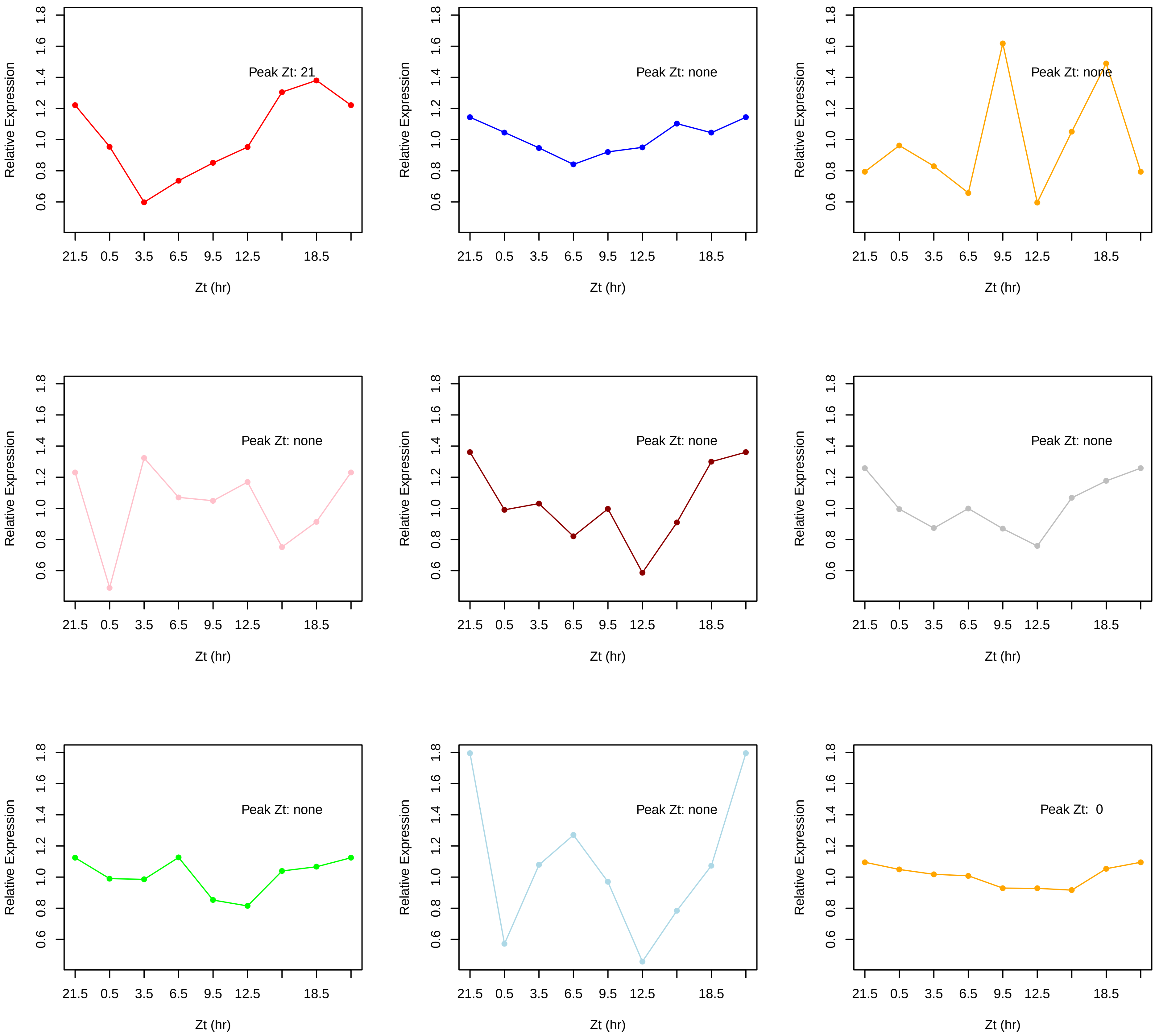

Singeleton: ENSXMAG00000016673 ddi2

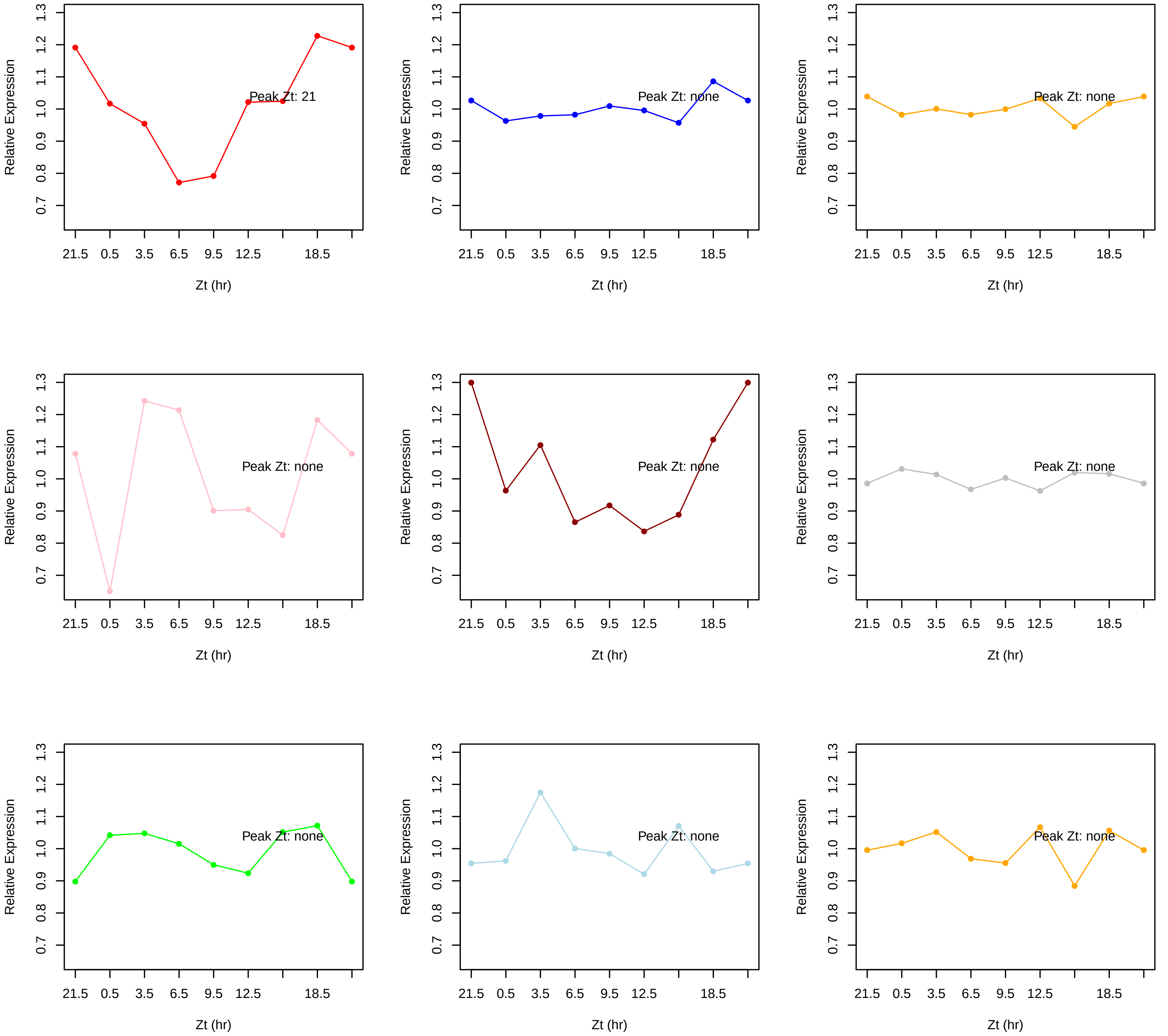

Singeleton: ENSXMAG00000012537 EEF1AKMT2

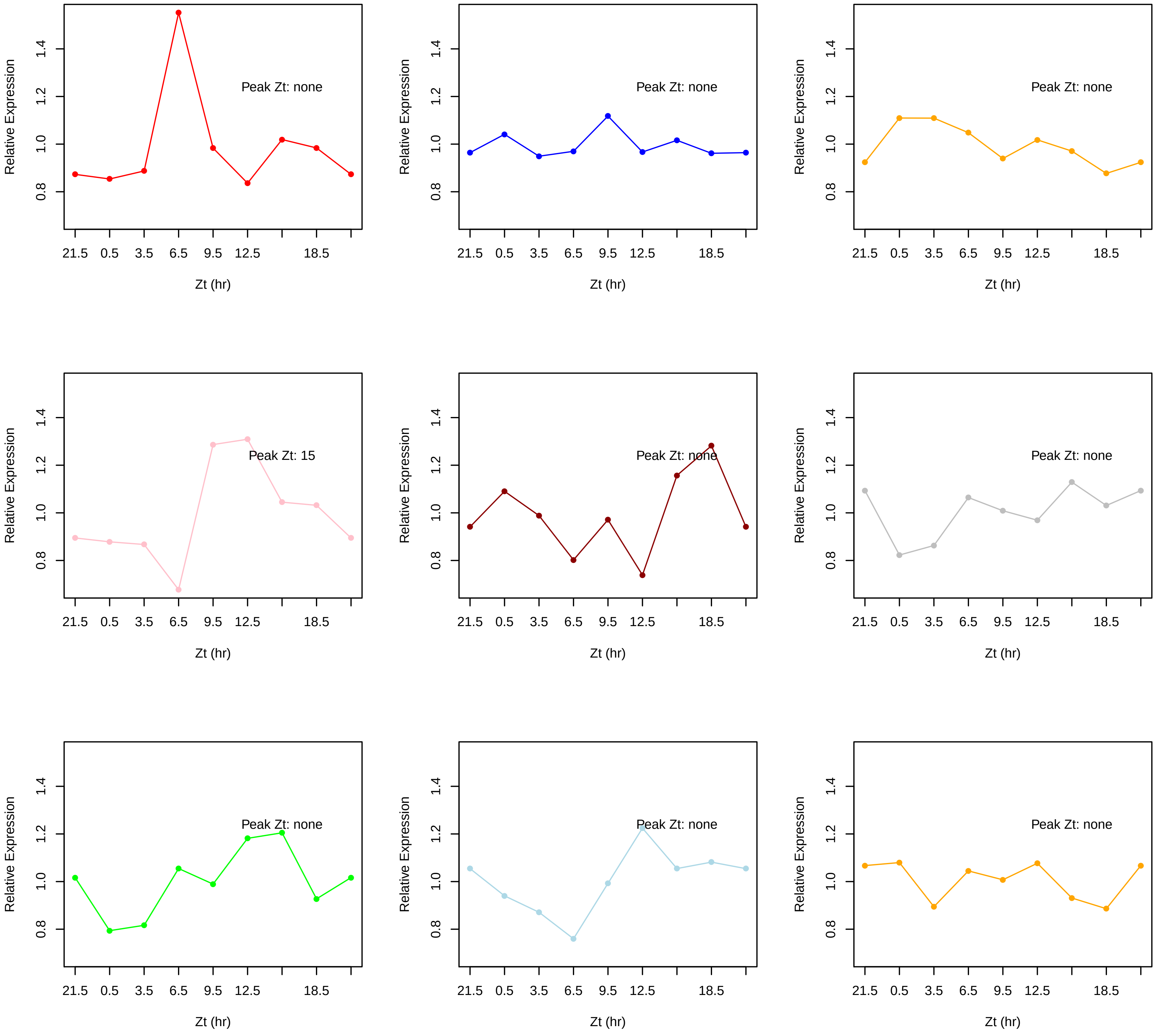

Singleton: ENSXMAG00000017910 si:ch211−102c2.7

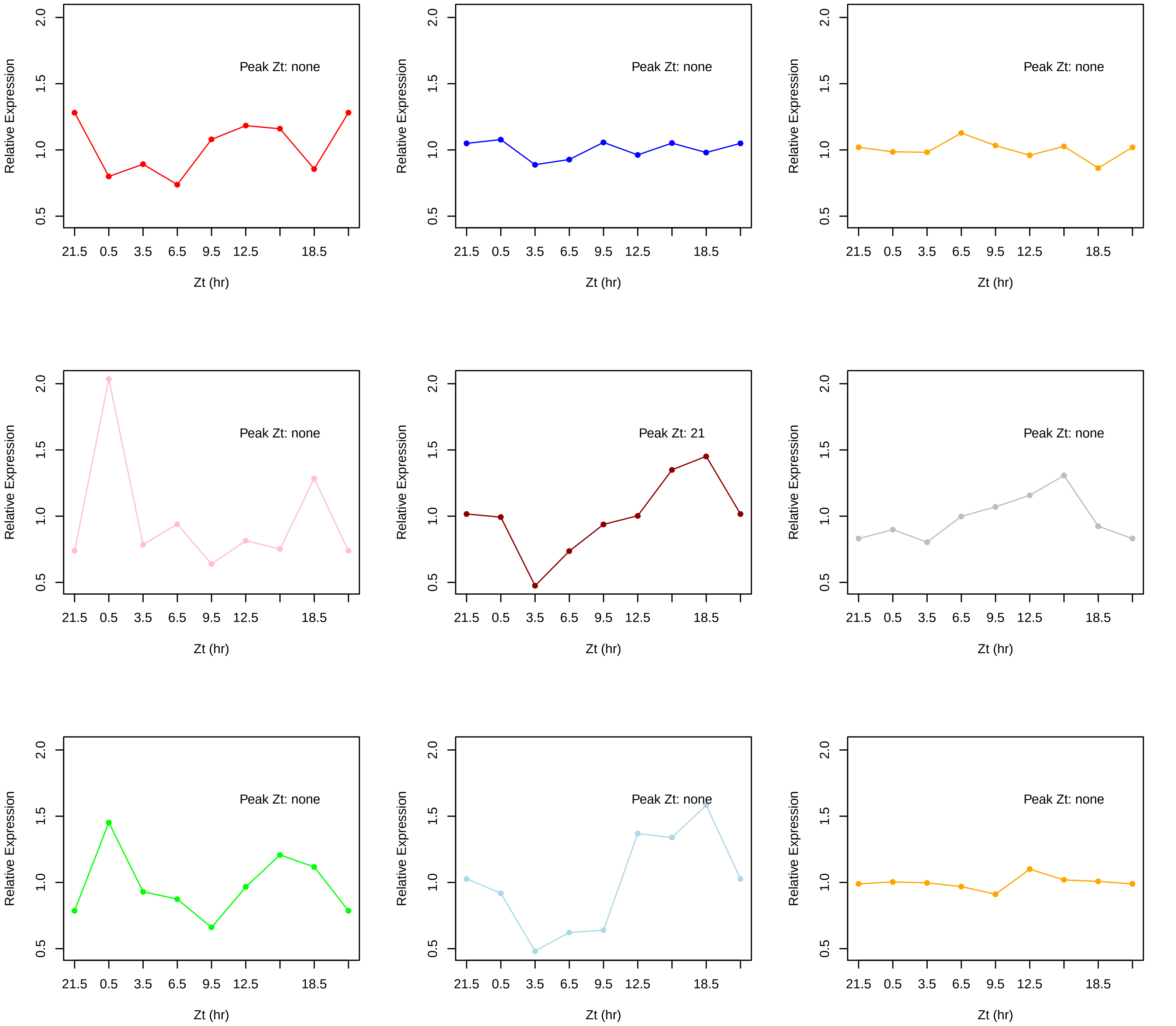

Singeleton: ENSXMAG00000014110 zgc:158403

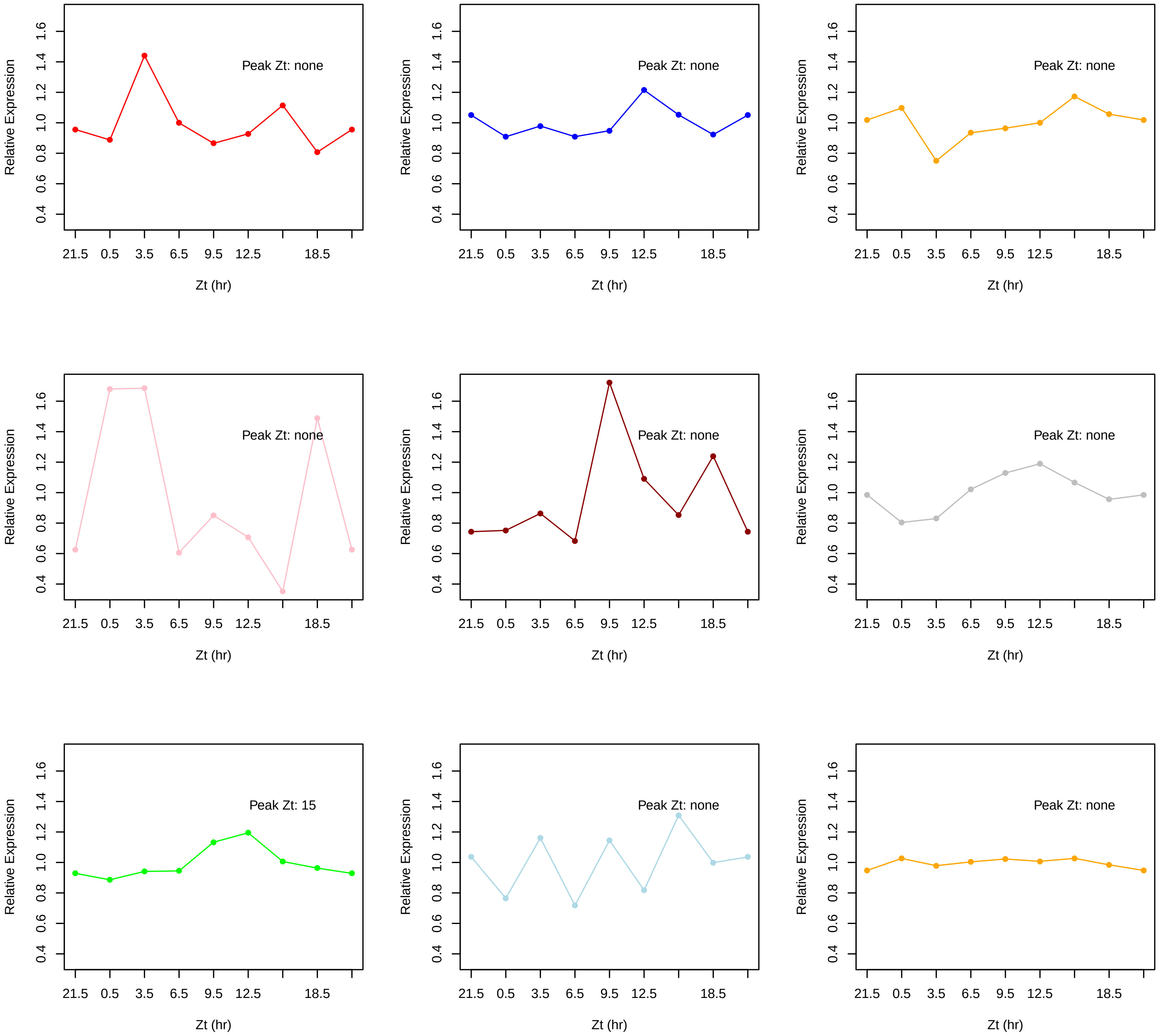

Singleton: ENSXMAG00000025761 cep72

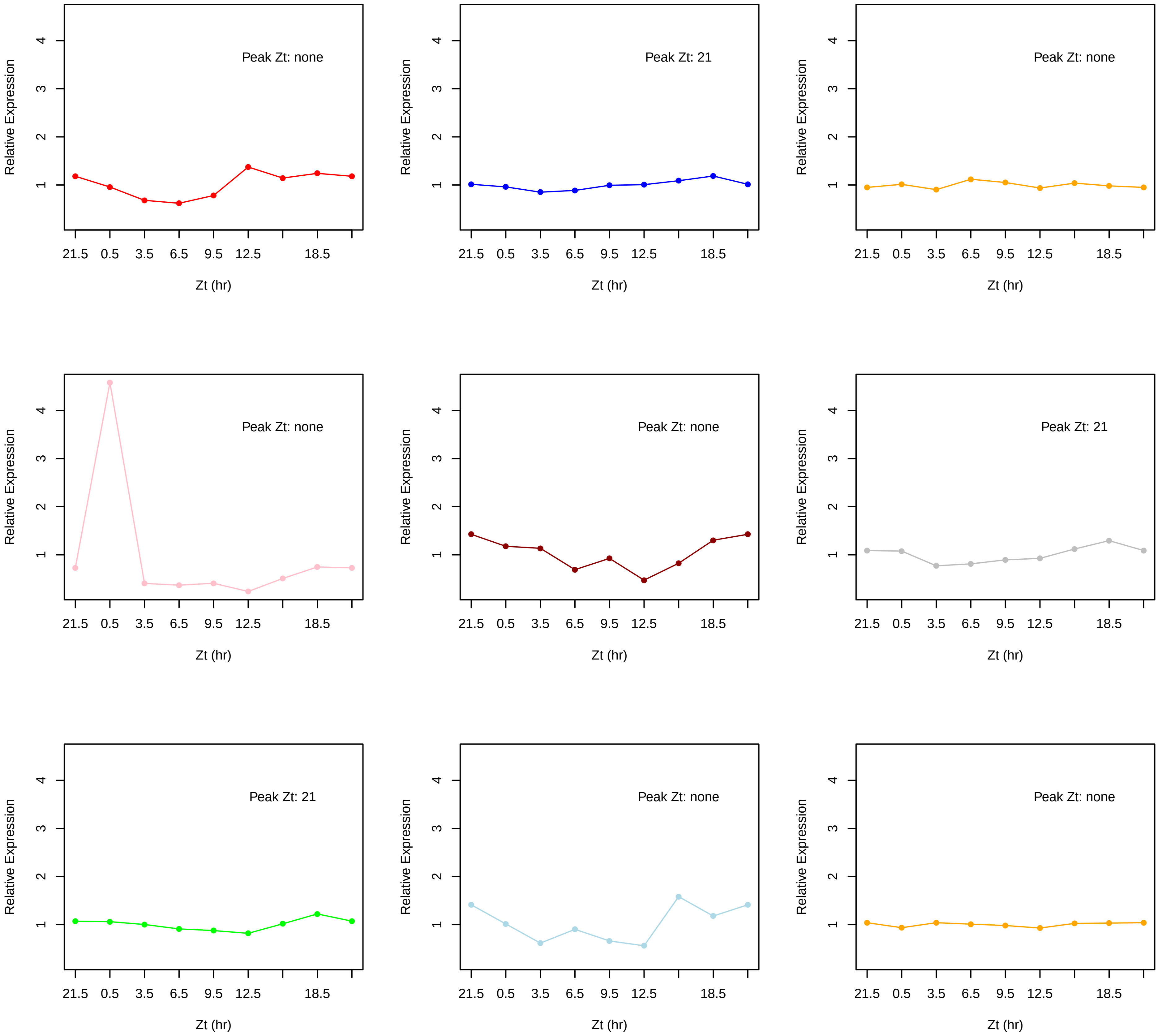

Singeleton: ENSXMAG00000011979 u2af2b

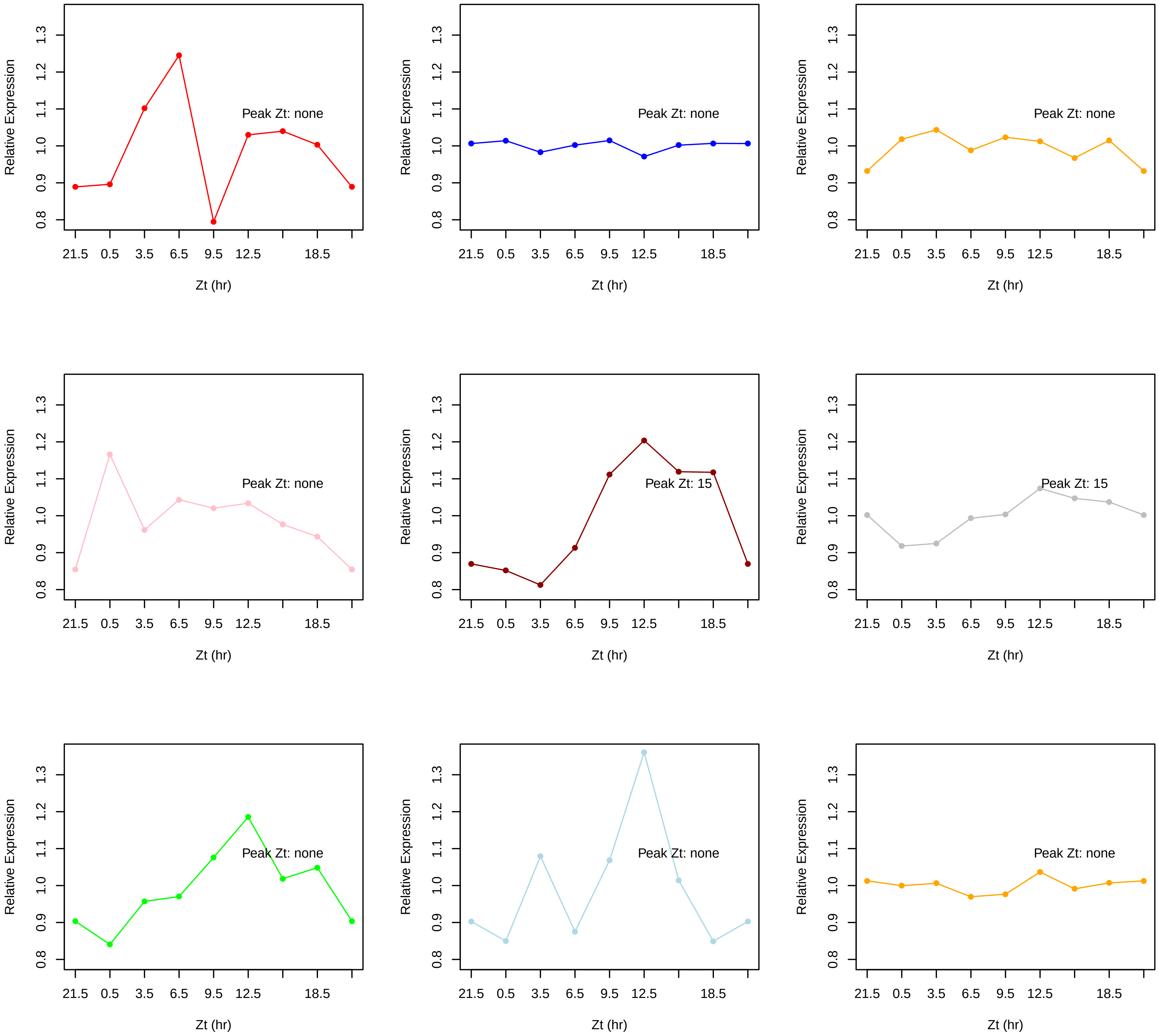

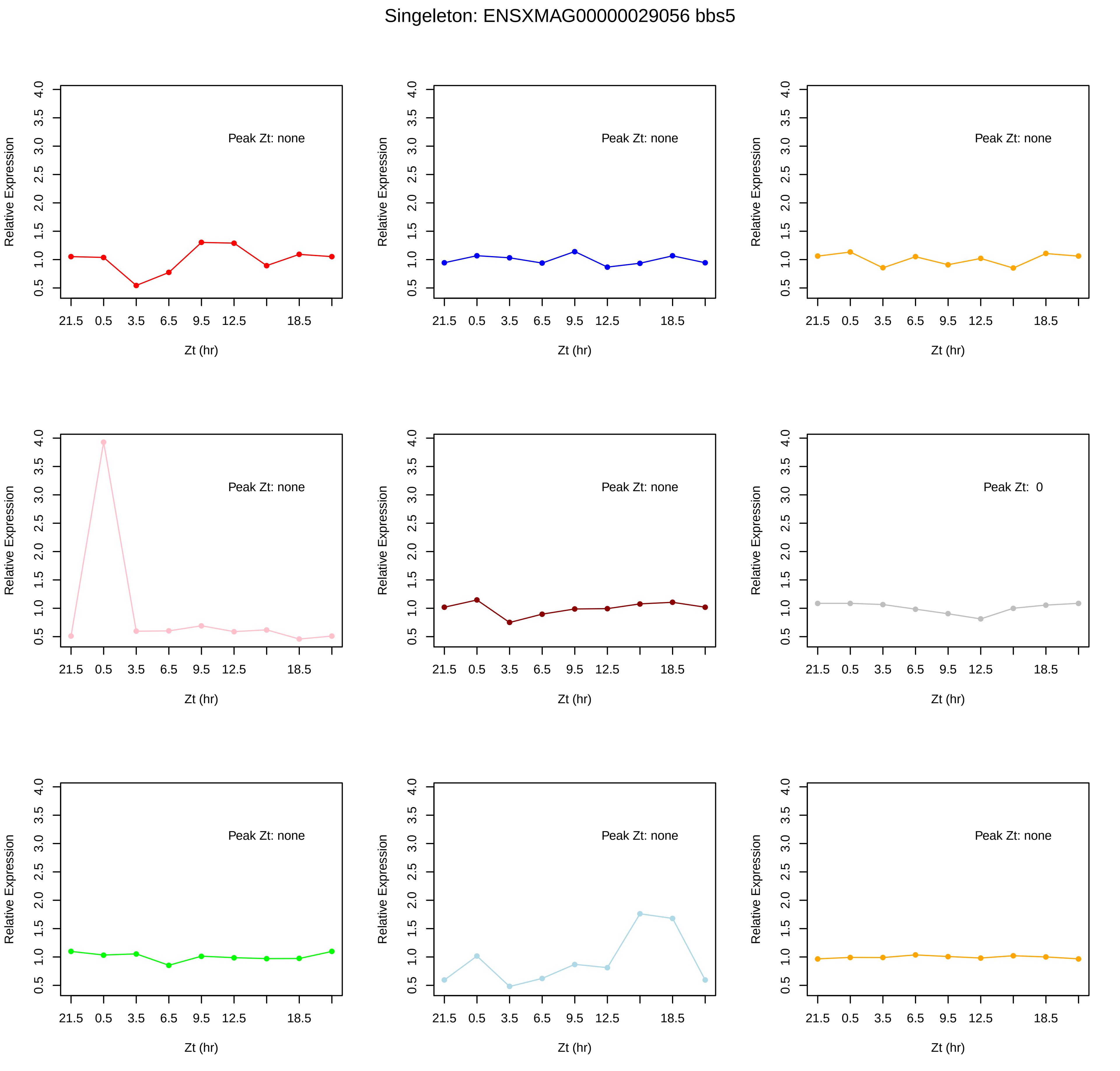
Singeleton: ENSXMAG00000029056 bbs5
Peak Zt: none
Peak Zt: none
Peak Zt: none
Peak Zt: none
Peak Zt: none
Peak Zt: 0
Peak Zt: none
Peak Zt: none
Peak Zt: none
Relative Expression
Zt (hr)
21.5
0.5
3.5
6.5
9.5
12.5
18.5

Singleton: ENSXMAG00000014084 TPPP

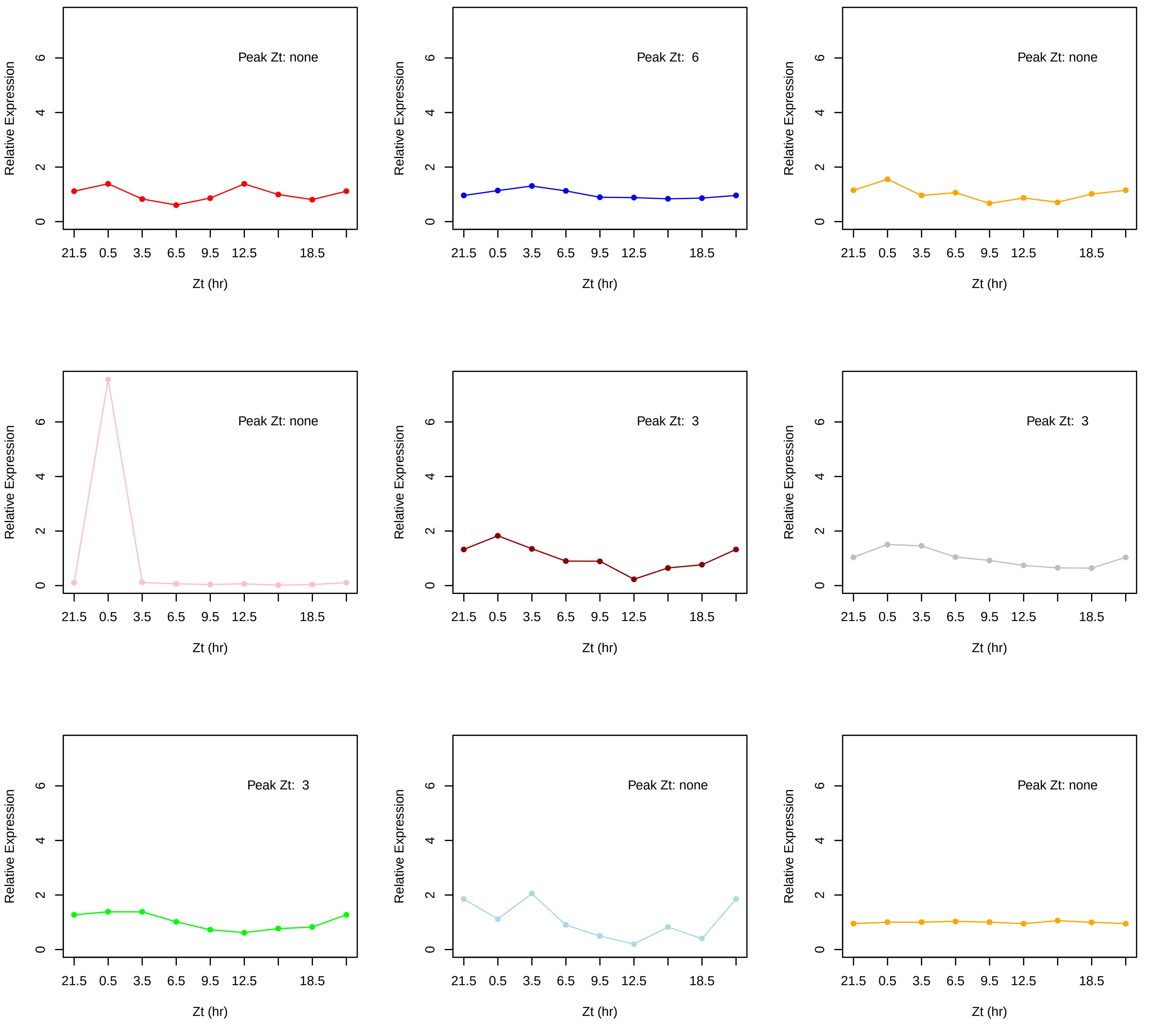

Singleton: ENSXMAG00000017366 esrp2

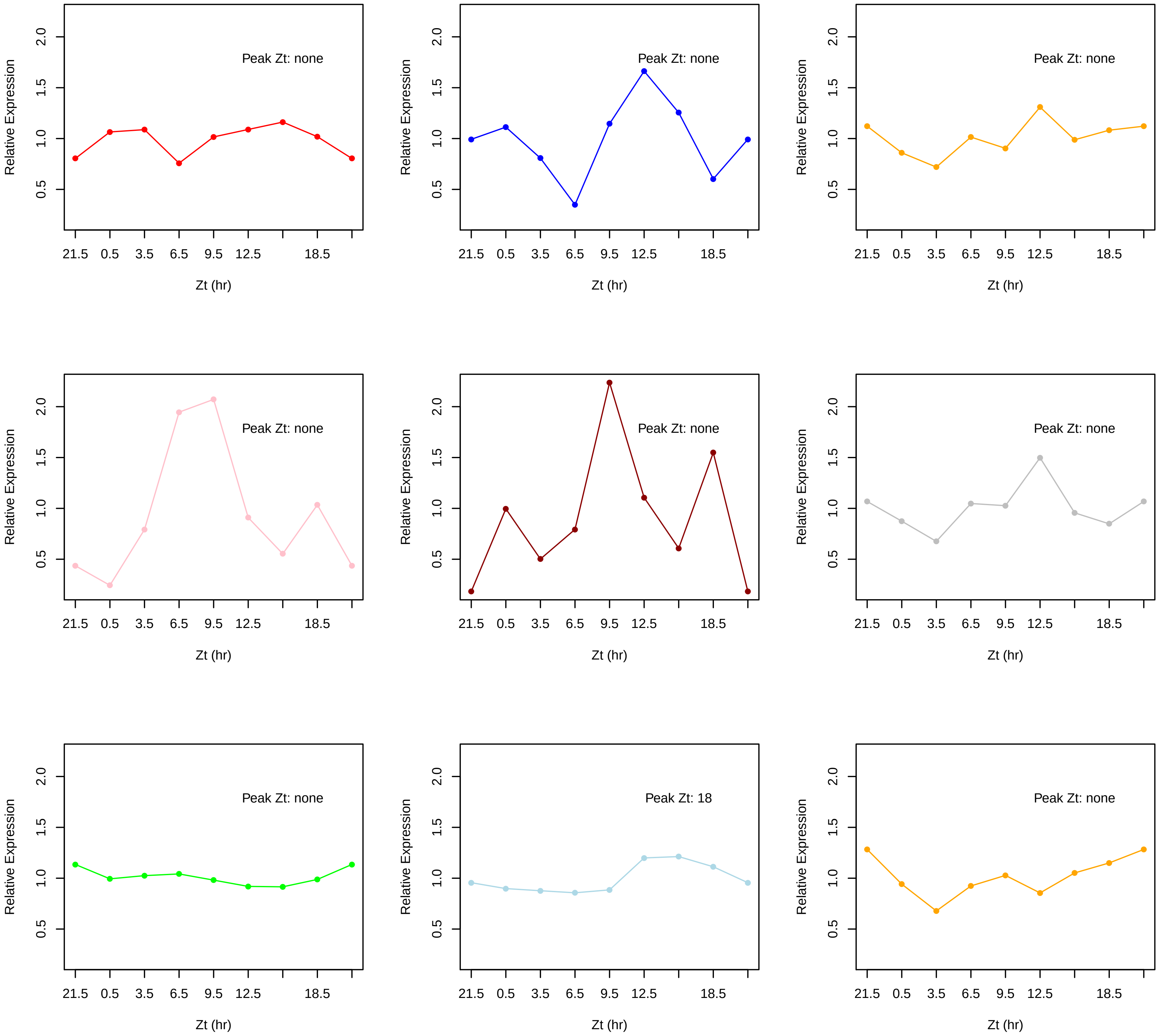

Singeleton: ENSXMAG00000026480 rnf7

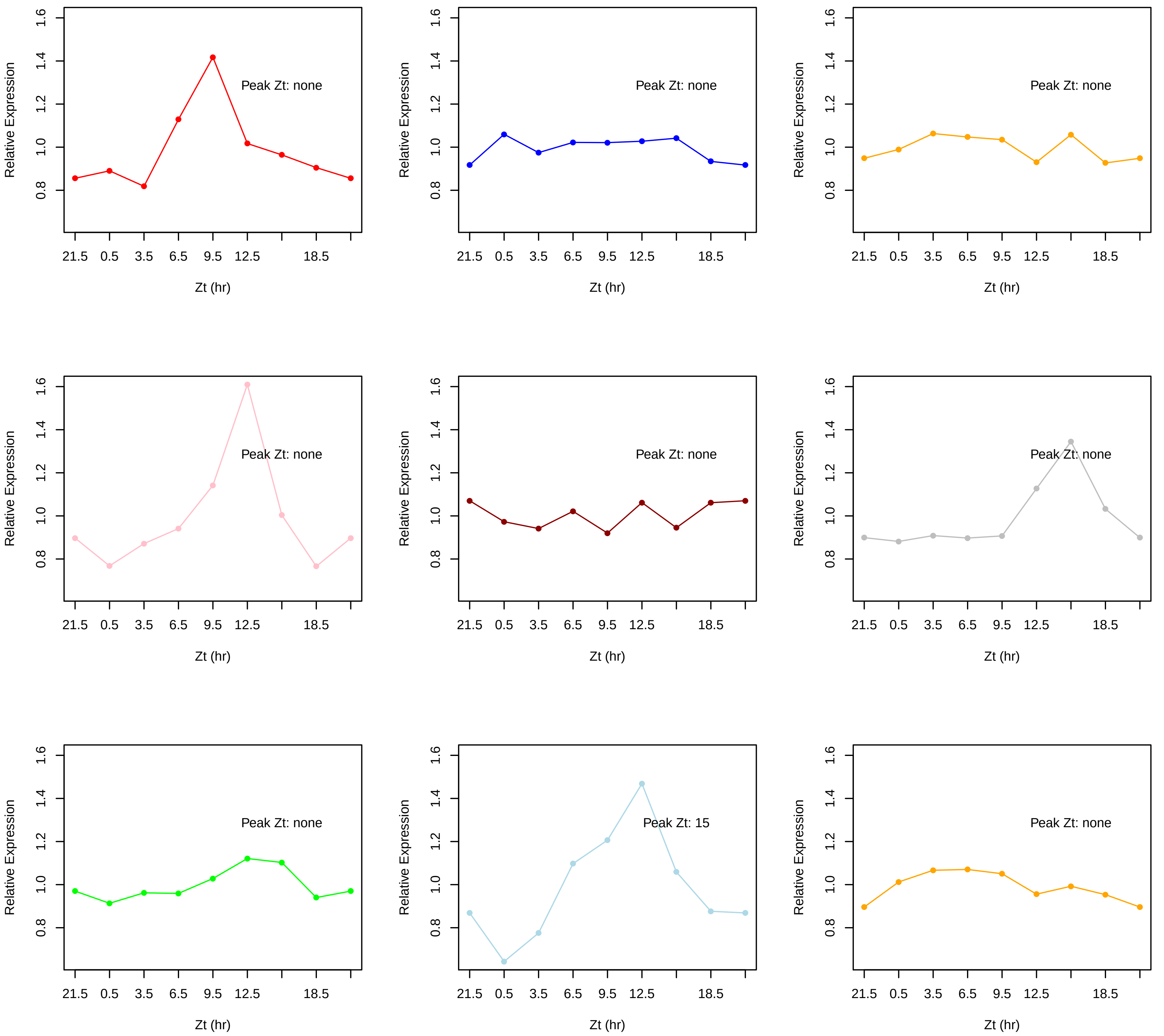

Singeleton: ENSXMAG00000016538 lhpp

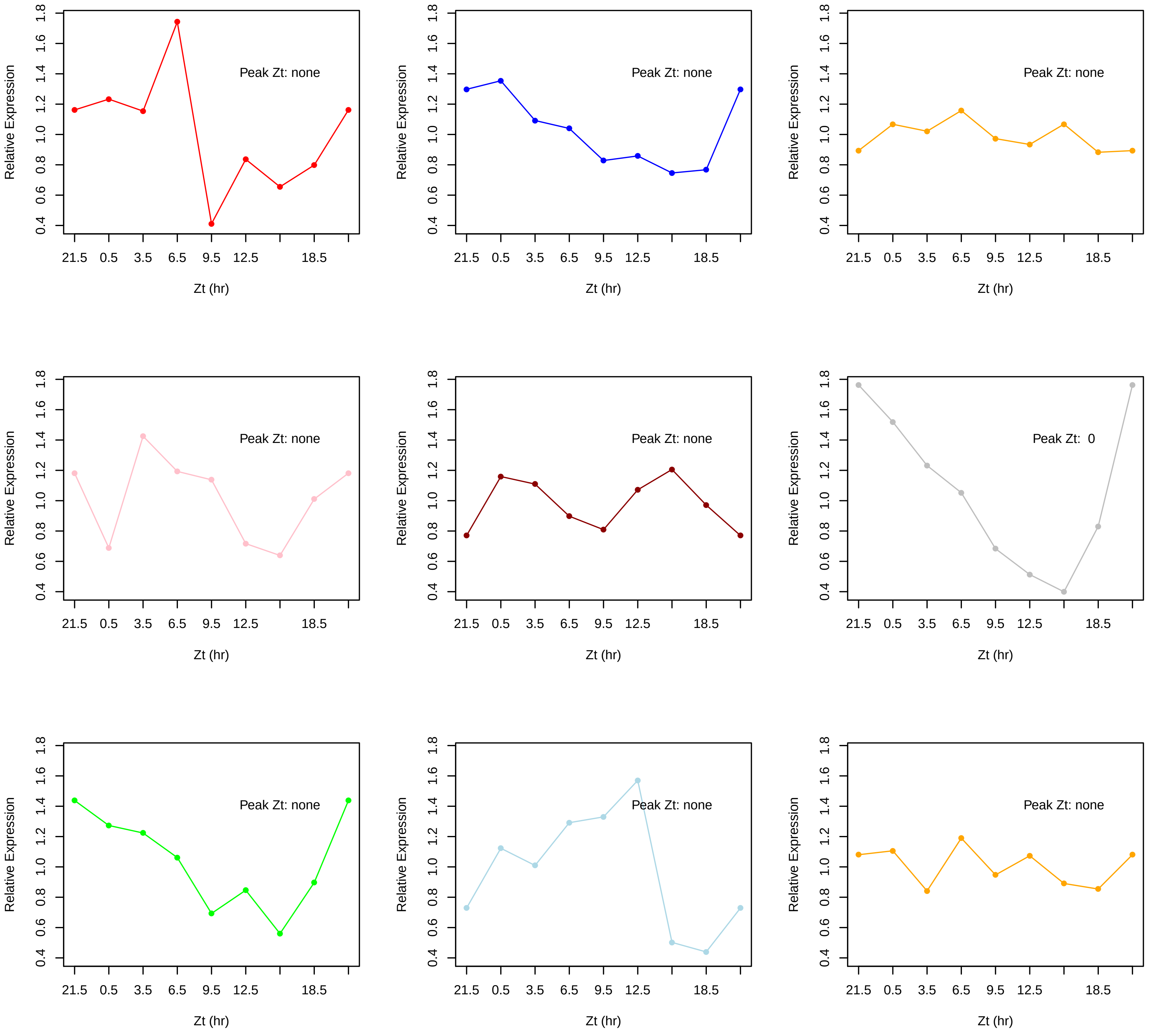

Singeleton: ENSXMAG00000011967 si:dkey−17m8.1

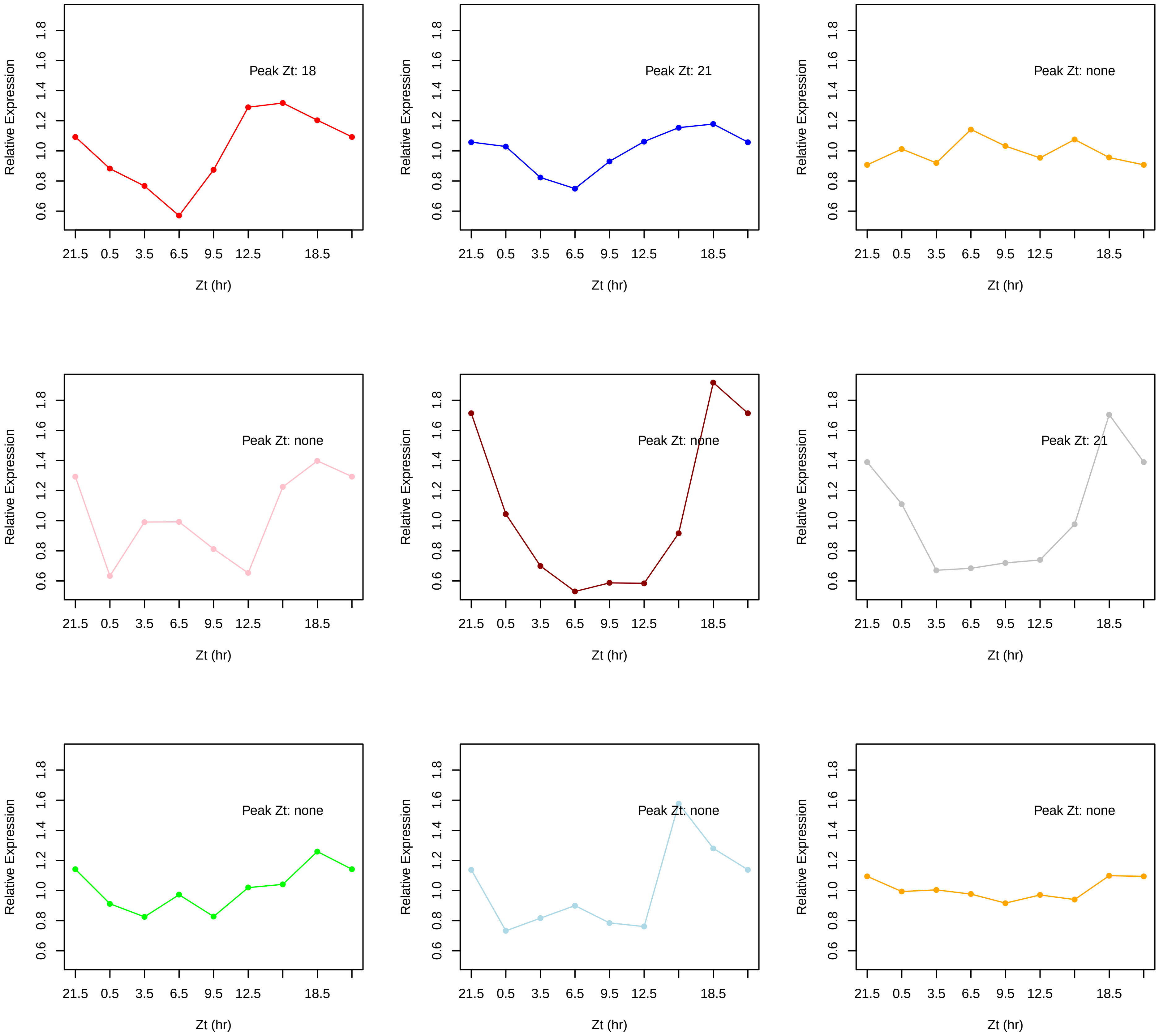

Singeleton: ENSXMAG00000001102 STK19

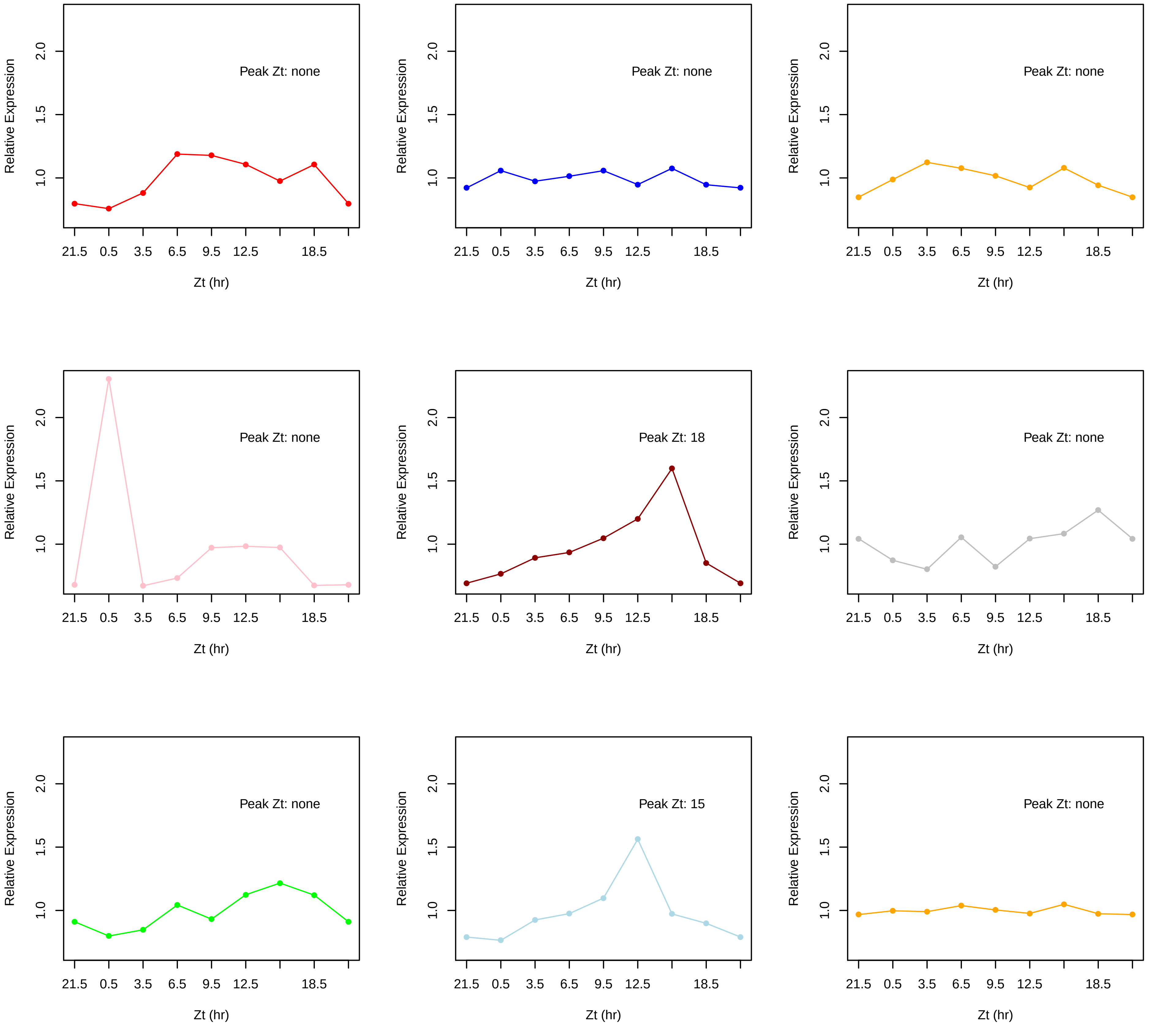

Singeleton: ENSXMAG00000009783 clp1

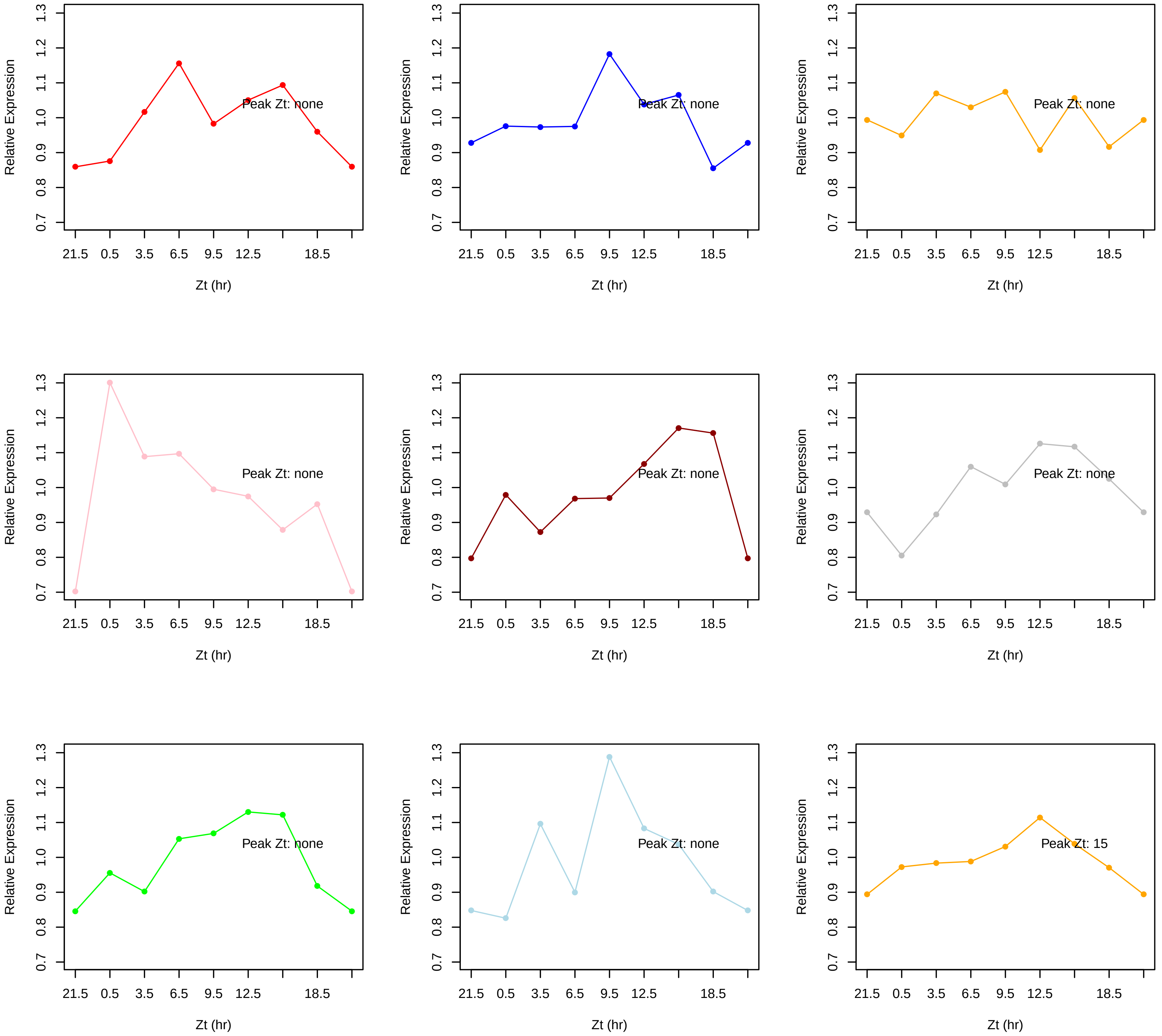

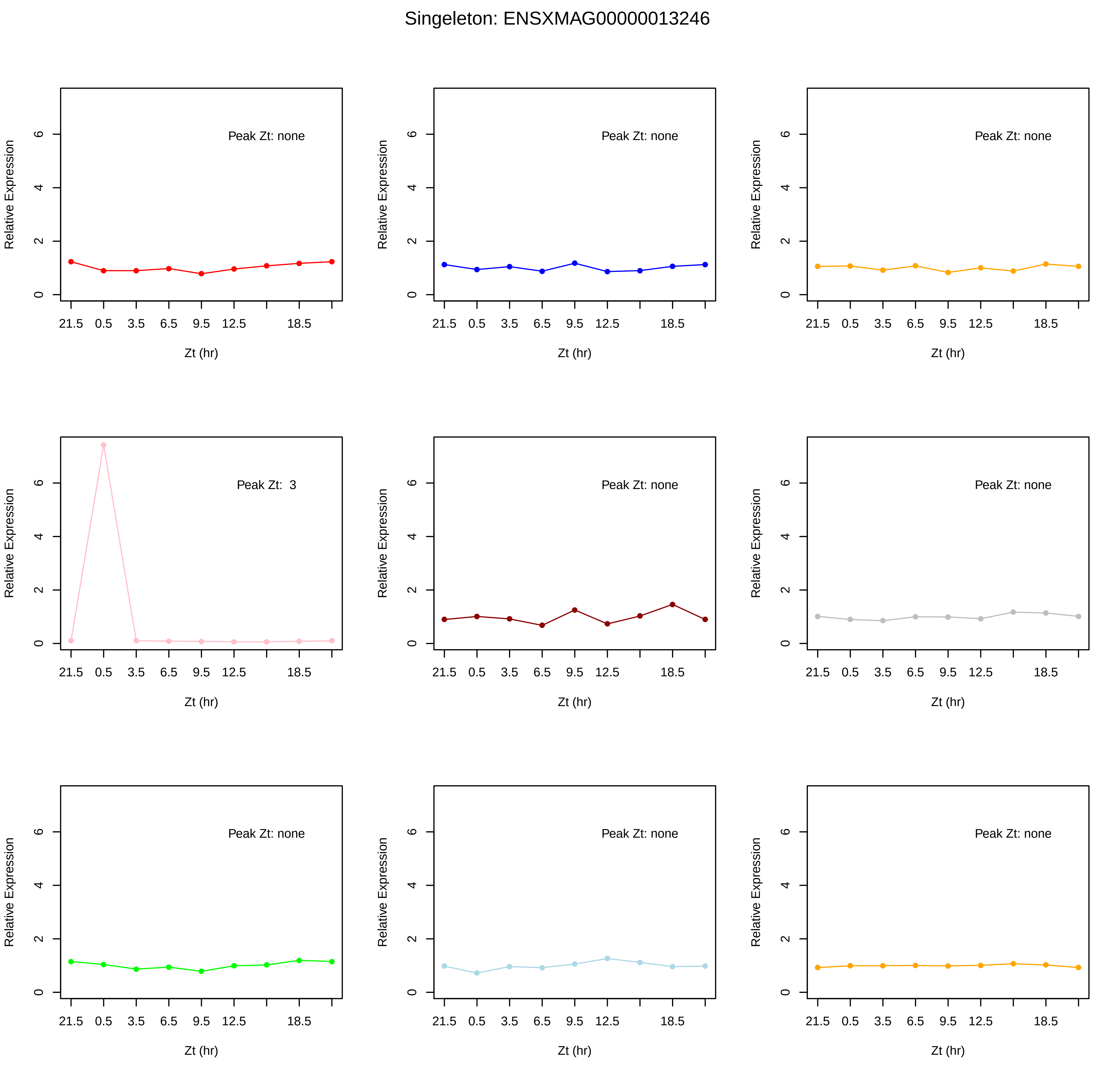

Singleton: ENSXMAG00000013246
Peak Zt: none
Peak Zt: none
Peak Zt: none
Peak Zt: 3
Peak Zt: none
Peak Zt: none
Peak Zt: none
Peak Zt: none
Peak Zt: none
Relative Expression
Zt (hr)
21.5
0.5
3.5
6.5
9.5
12.5
18.5
0
2
4
6

Singeleton: ENSXMAG00000002165 ppid

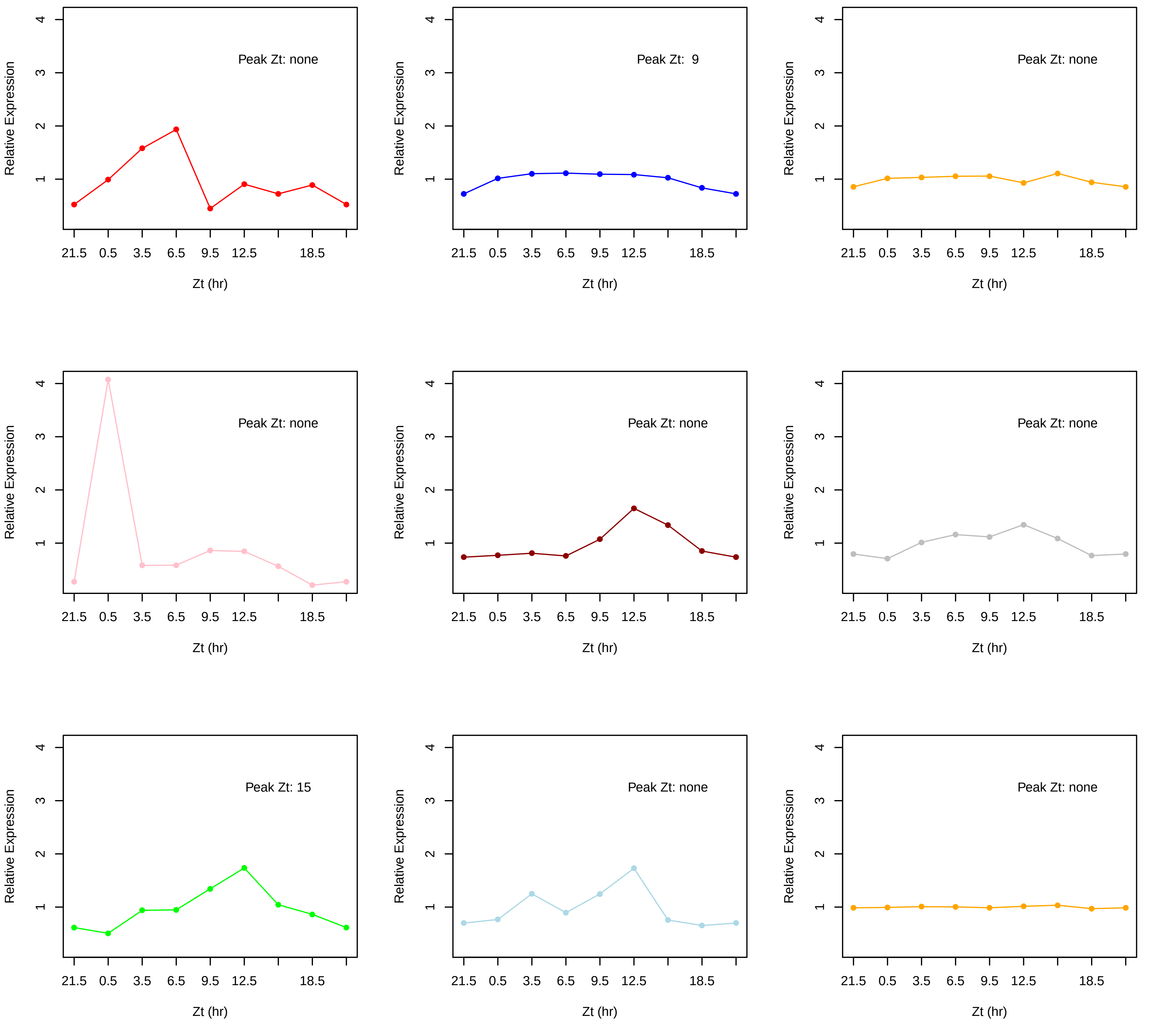

Singeleton: ENSXMAG00000012291 nfatc3b

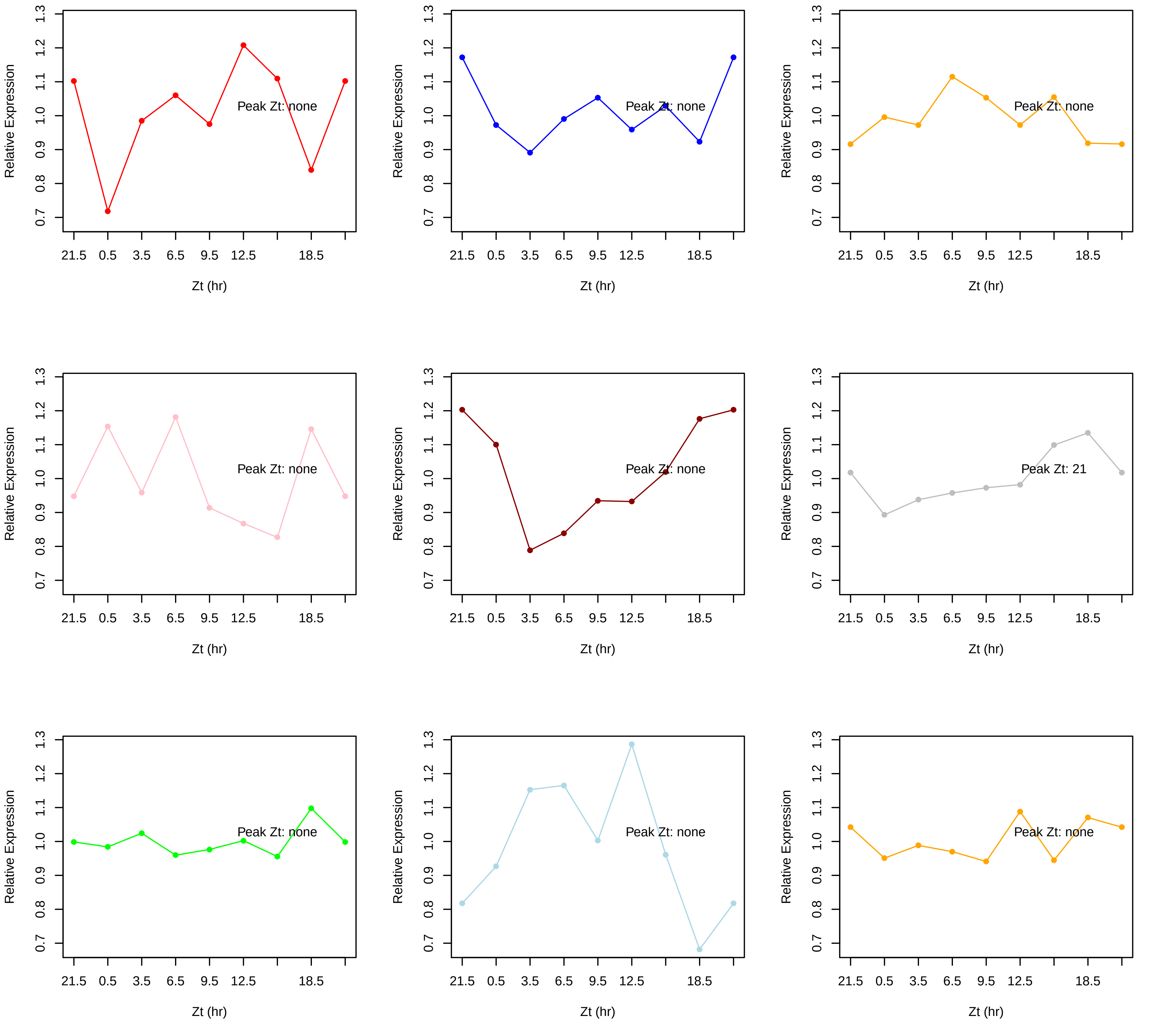

Singleton: ENSXMAG00000015937

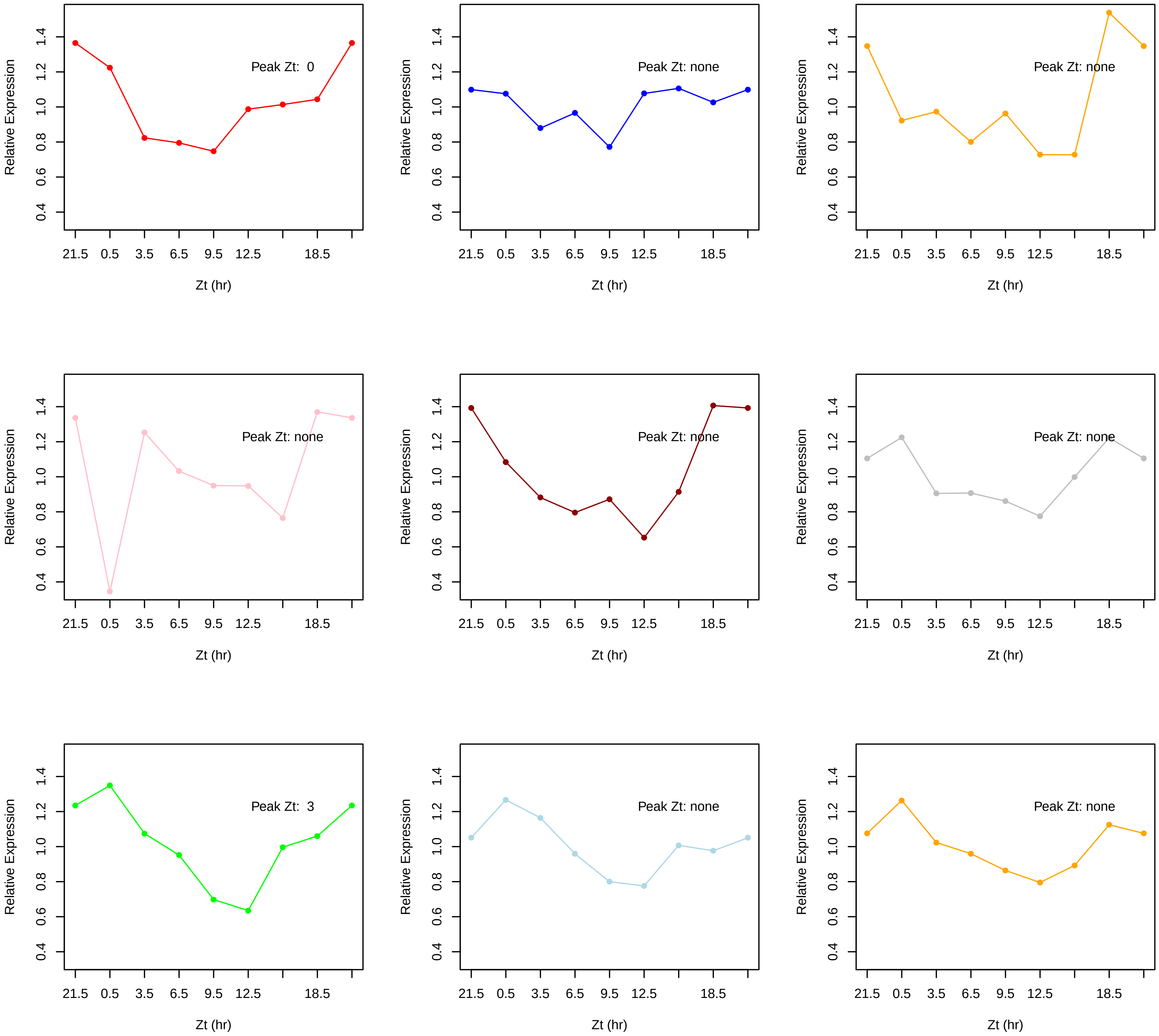

Singeleton: ENSXMAG00000014068 brd9

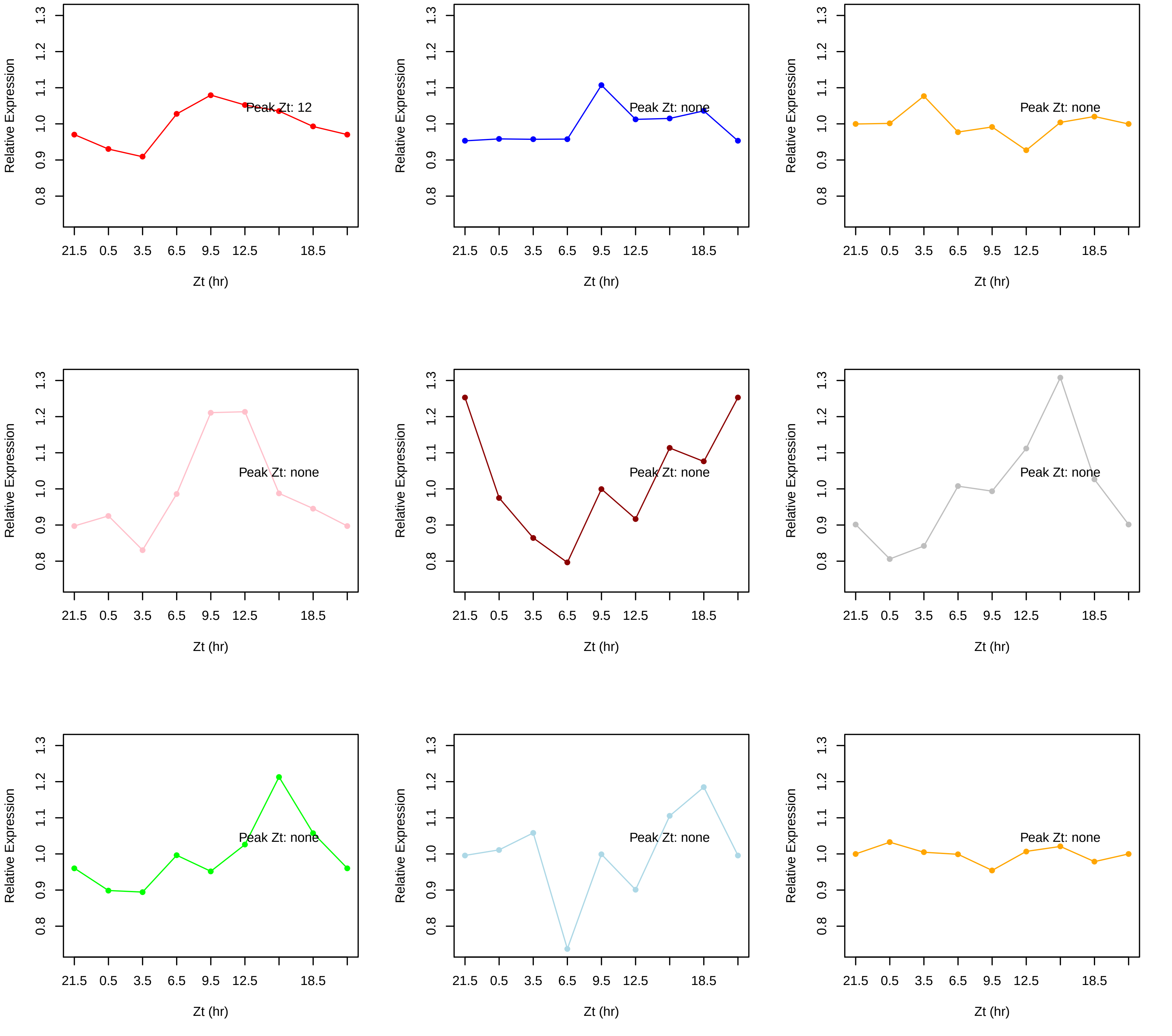

Singeleton: ENSXMAG00000012268 dus2

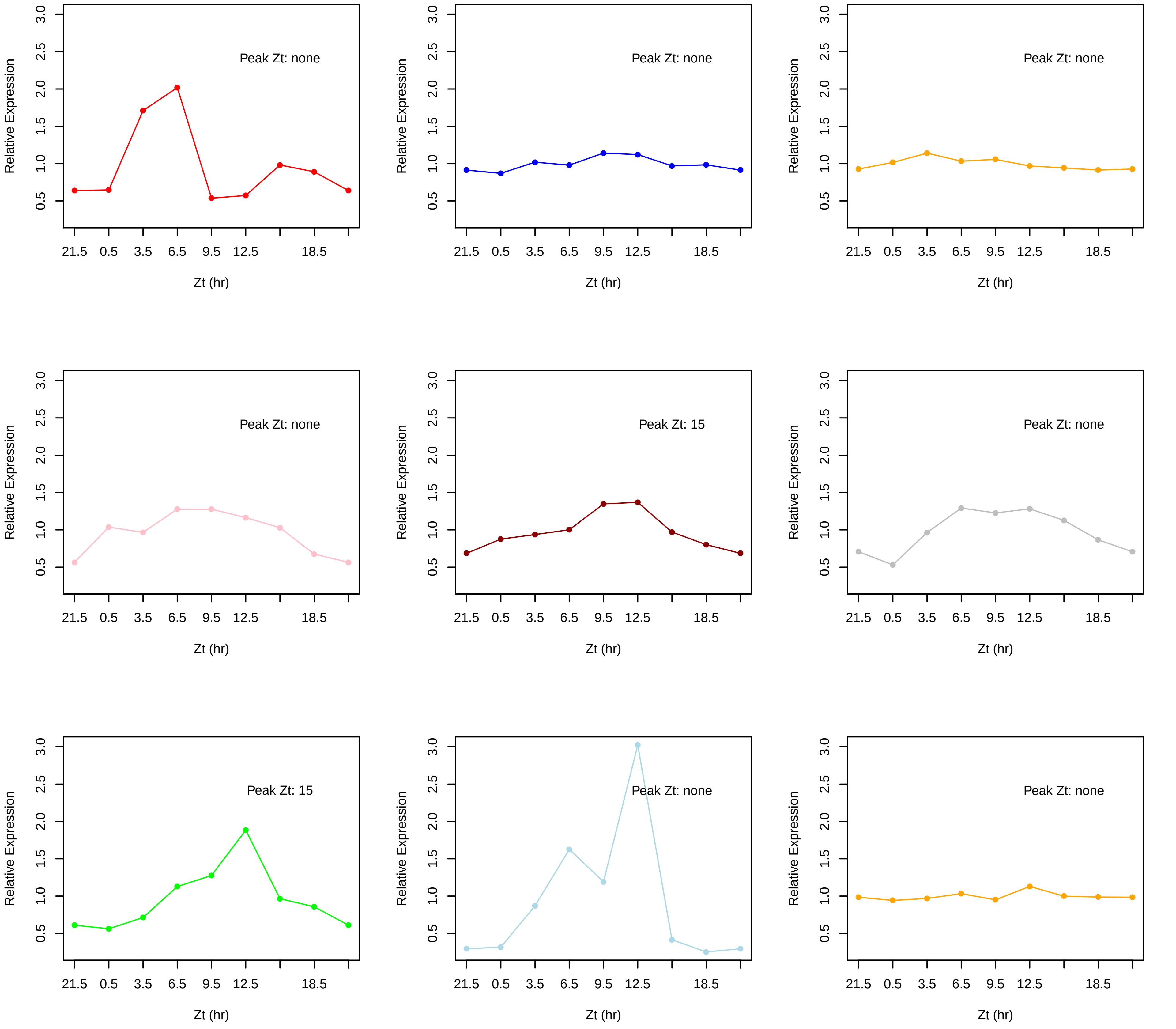

Singeleton: ENSXMAG00000016554 oat

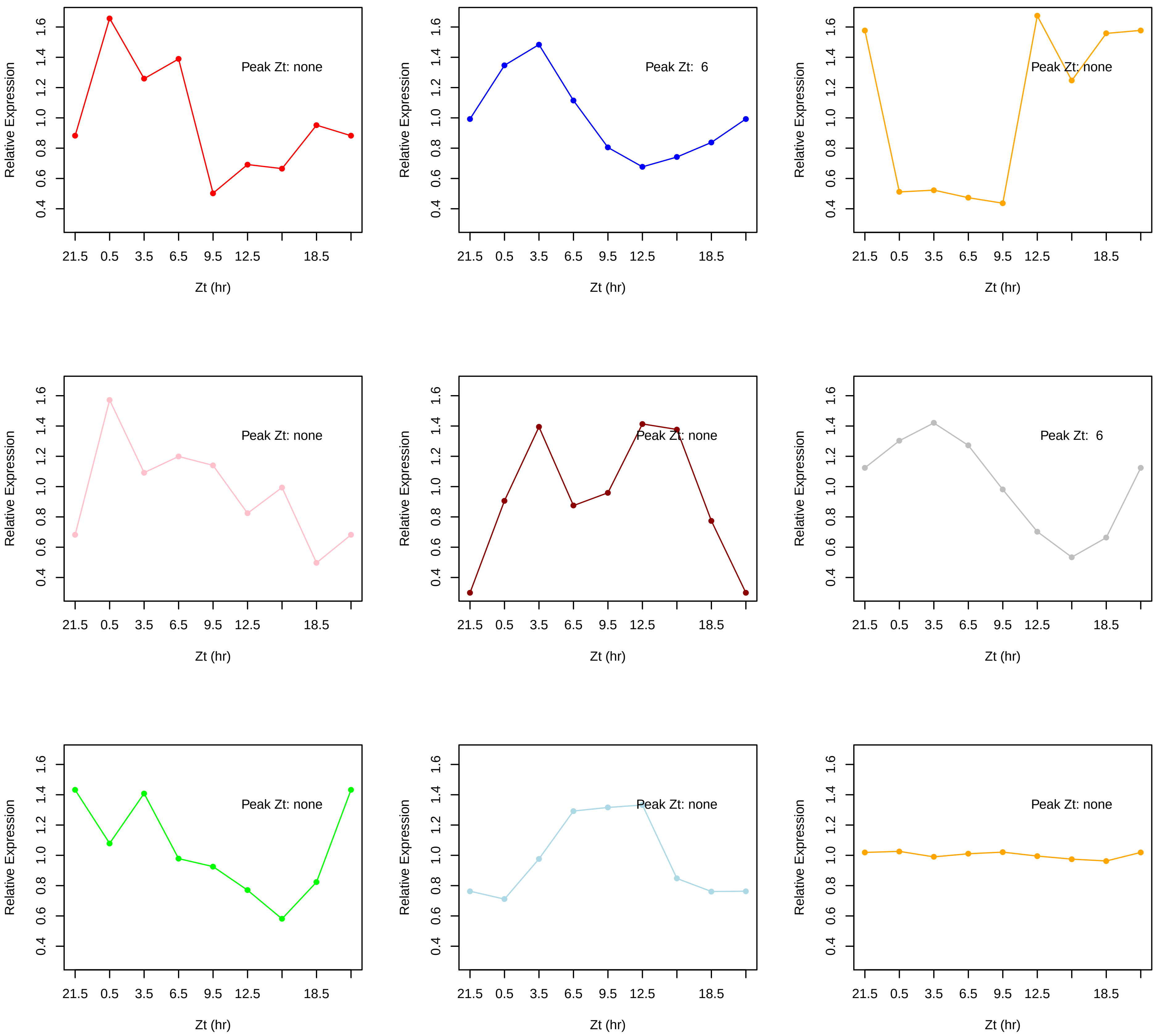

Singleton: ENSXMAG00000001067

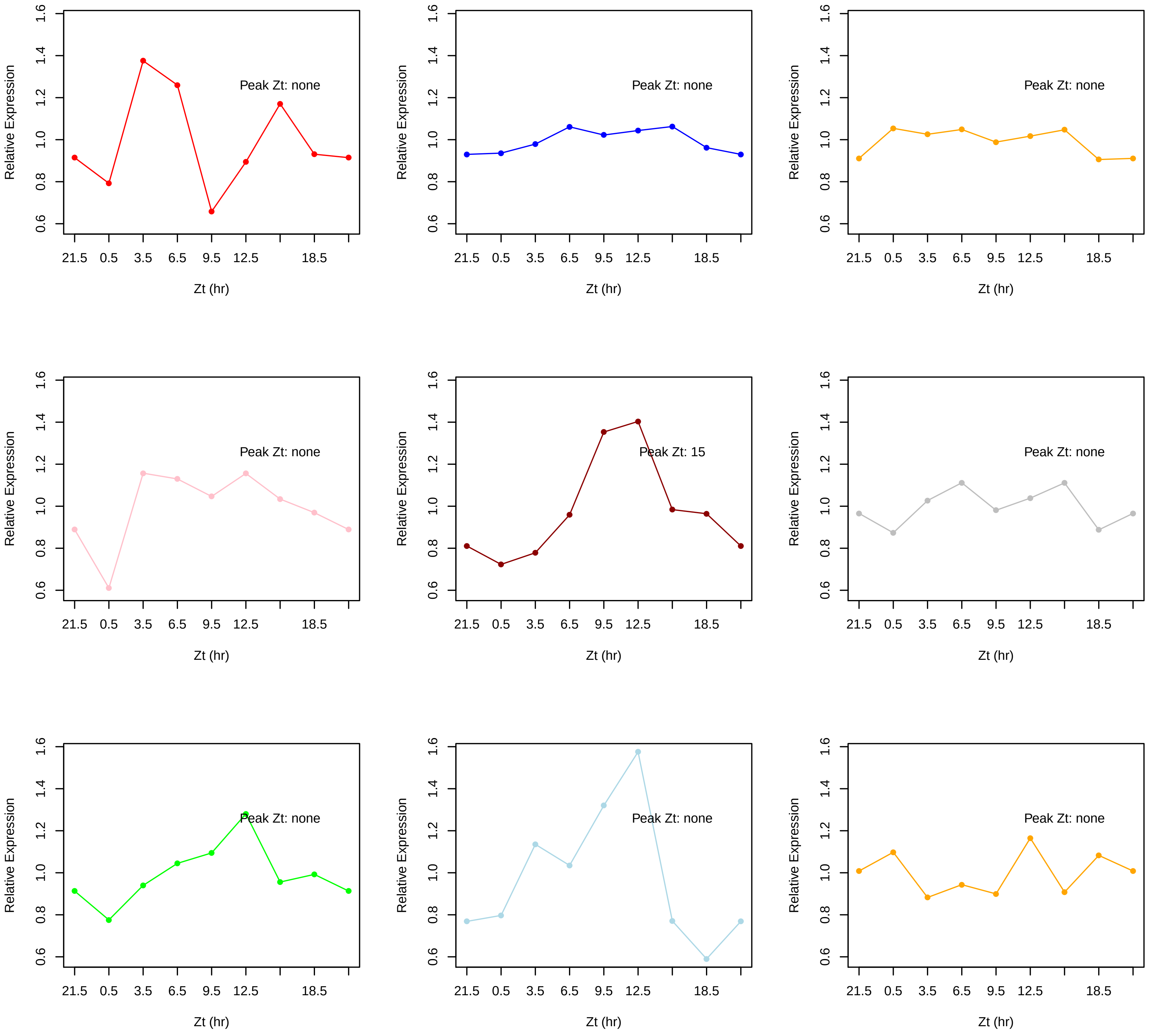

Singeleton: ENSXMAG00000015995 rp2

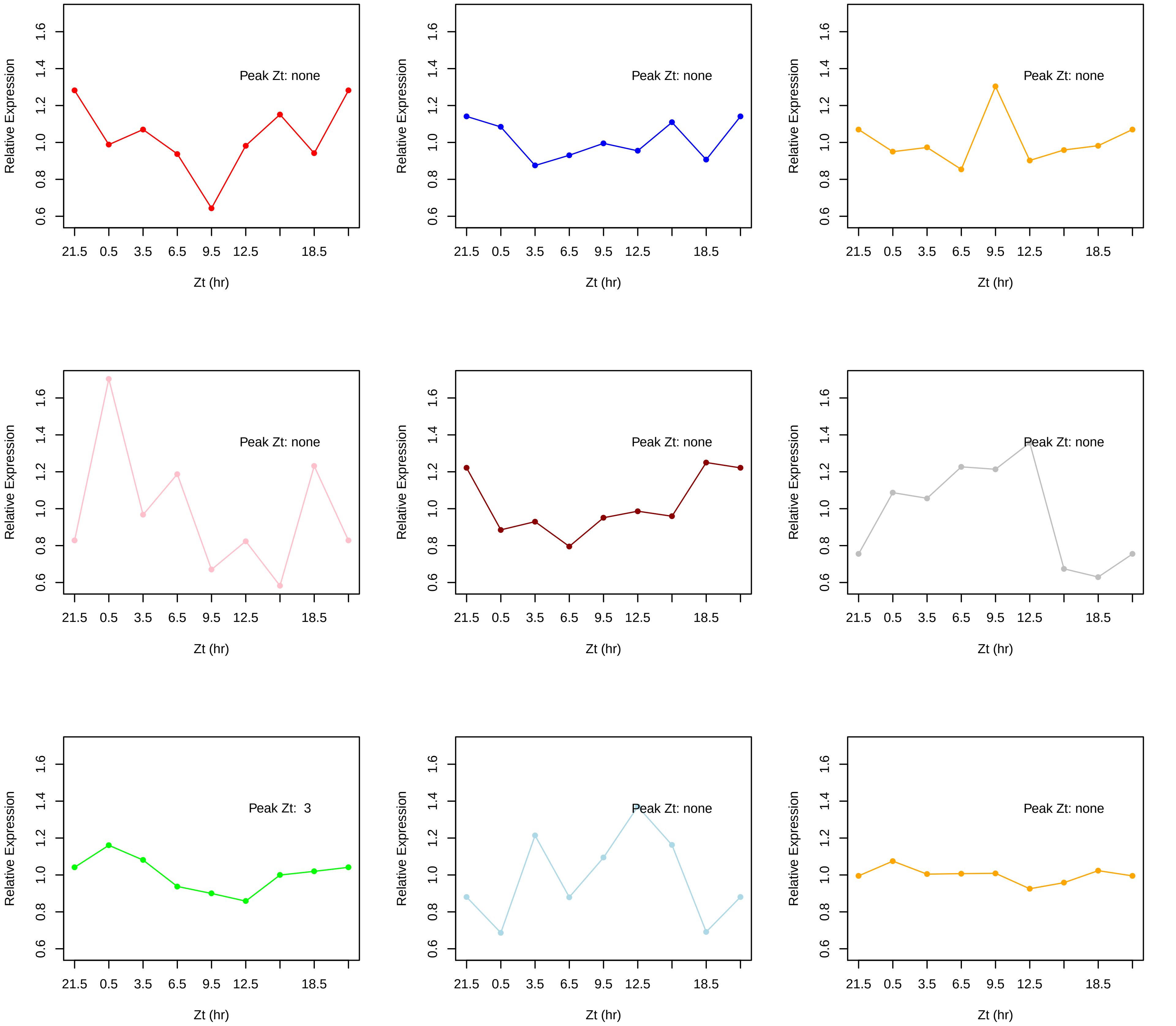

Singeleton: ENSXMAG00000017933 ewsr1b

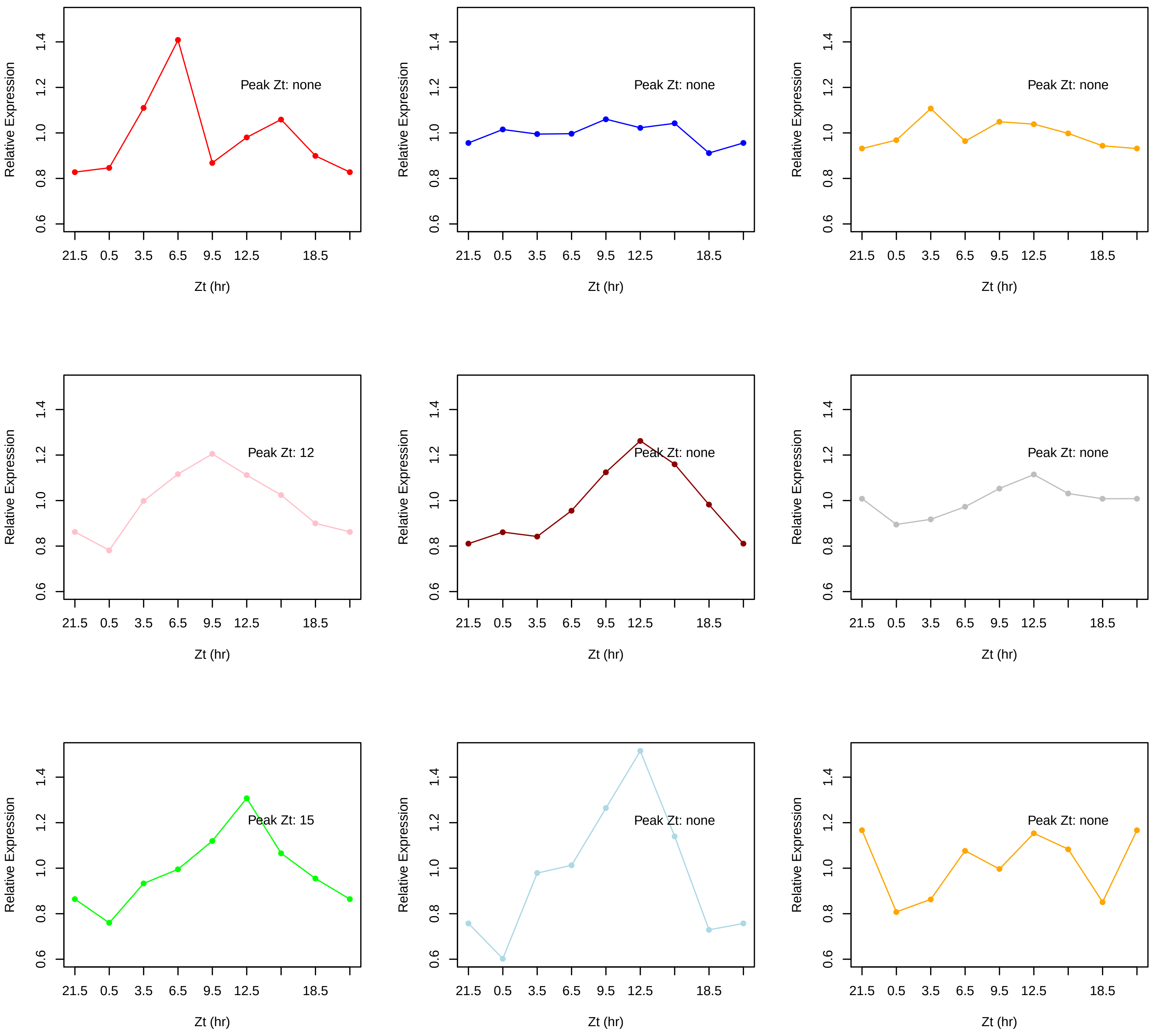

Singeleton: ENSXMAG00000010855 RASA2

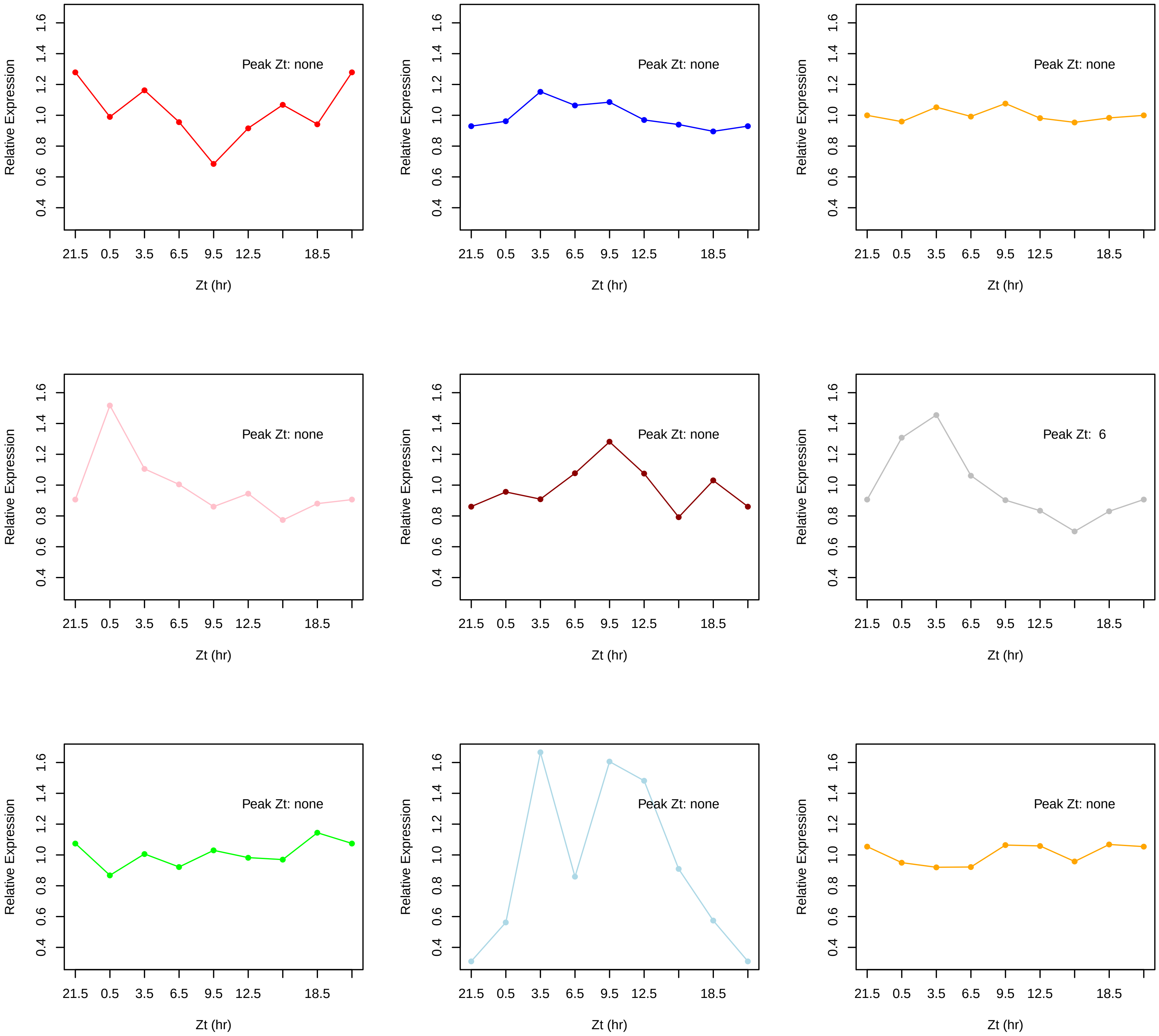

Singeleton: ENSXMAG00000008485 prpf19

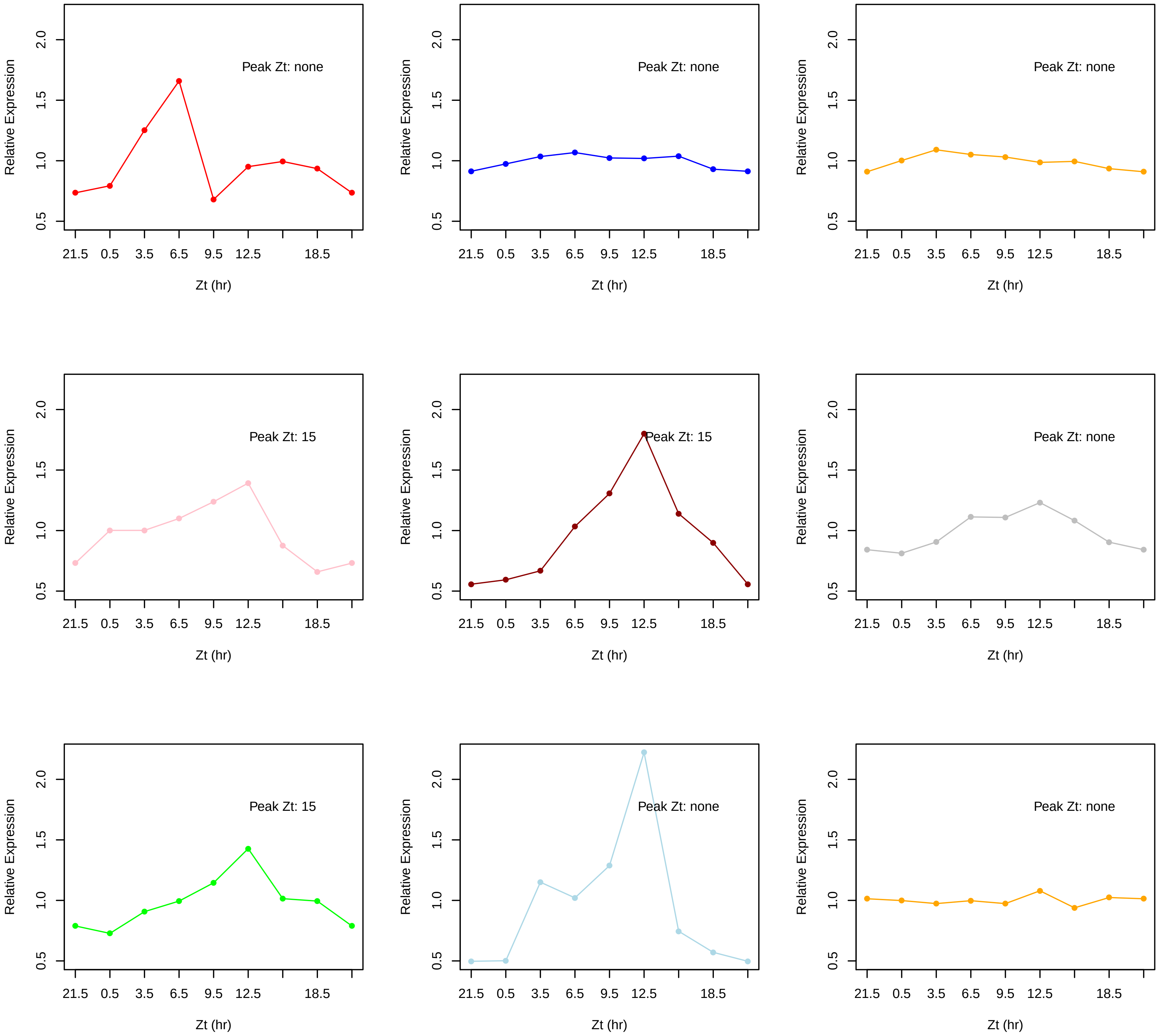

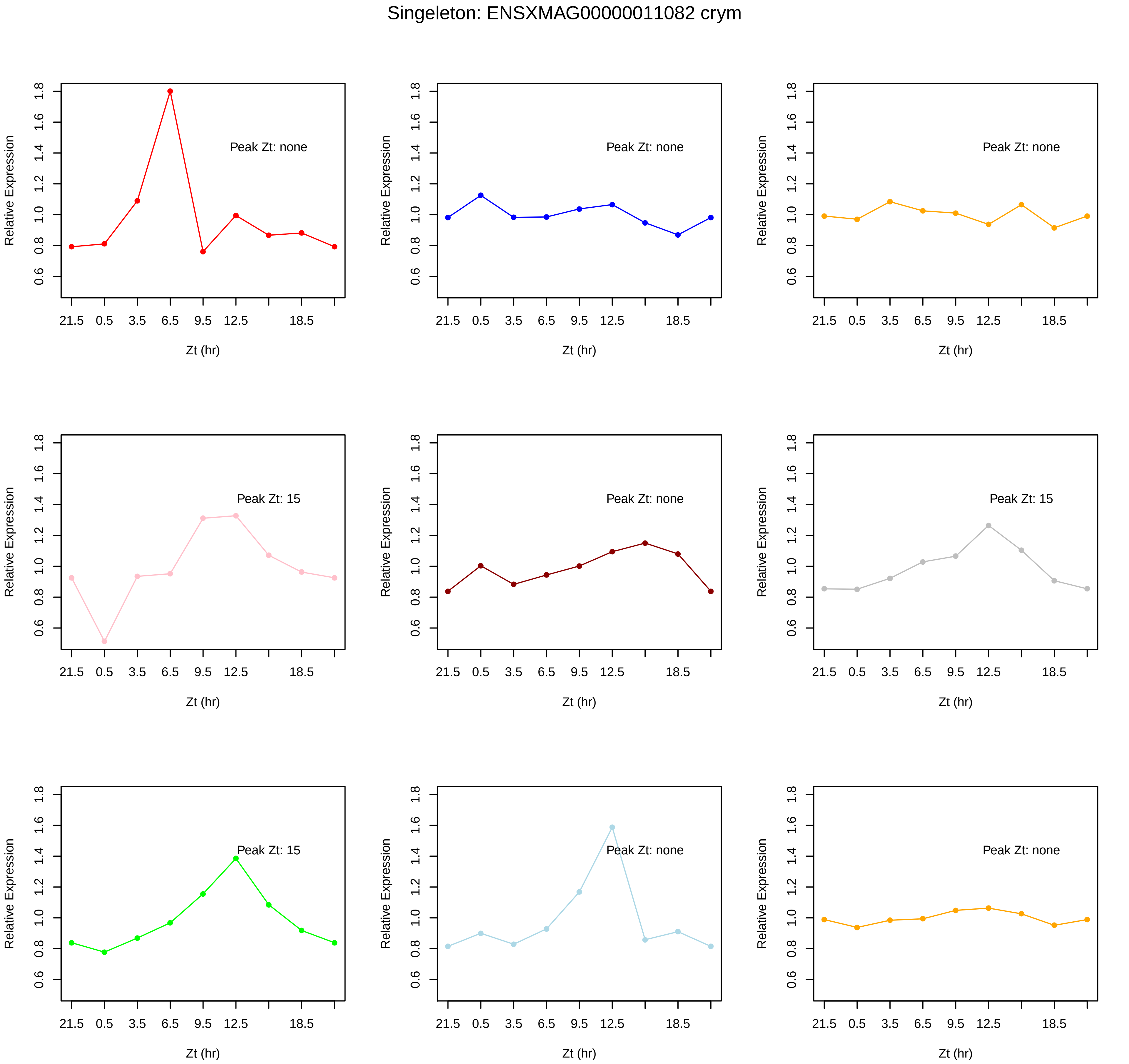
Singeleton: ENSXMAG00000011082 crym
Peak Zt: none
Peak Zt: none
Peak Zt: none
Peak Zt: 15
Peak Zt: none
Peak Zt: 15
Peak Zt: 15
Peak Zt: none
Peak Zt: none
Relative Expression
Zt (hr)
21.5
0.5
3.5
6.5
9.5
12.5
18.5
0.6
0.8
1.0
1.2
1.4
1.6
1.8

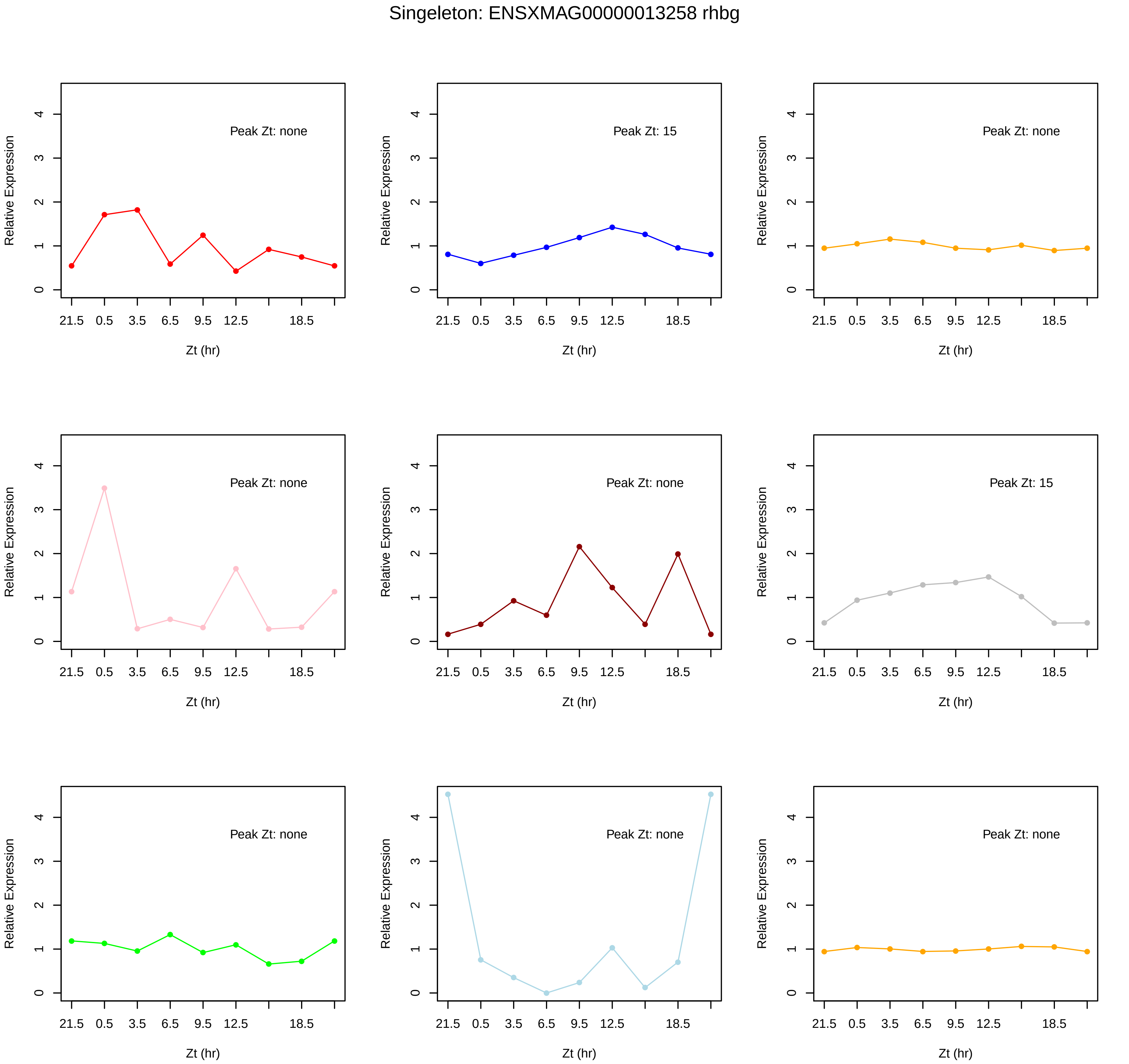
Singeleton: ENSXMAG00000013258 rhbg
Peak Zt: none
Peak Zt: 15
Peak Zt: none
Peak Zt: none
Peak Zt: none
Peak Zt: 15
Peak Zt: none
Peak Zt: none
Peak Zt: none
Relative Expression
Zt (hr)
21.5 0.5 3.5 6.5 9.5 12.5 18.5
0 1 2 3 4

Singleton: ENSXMAG00000001021

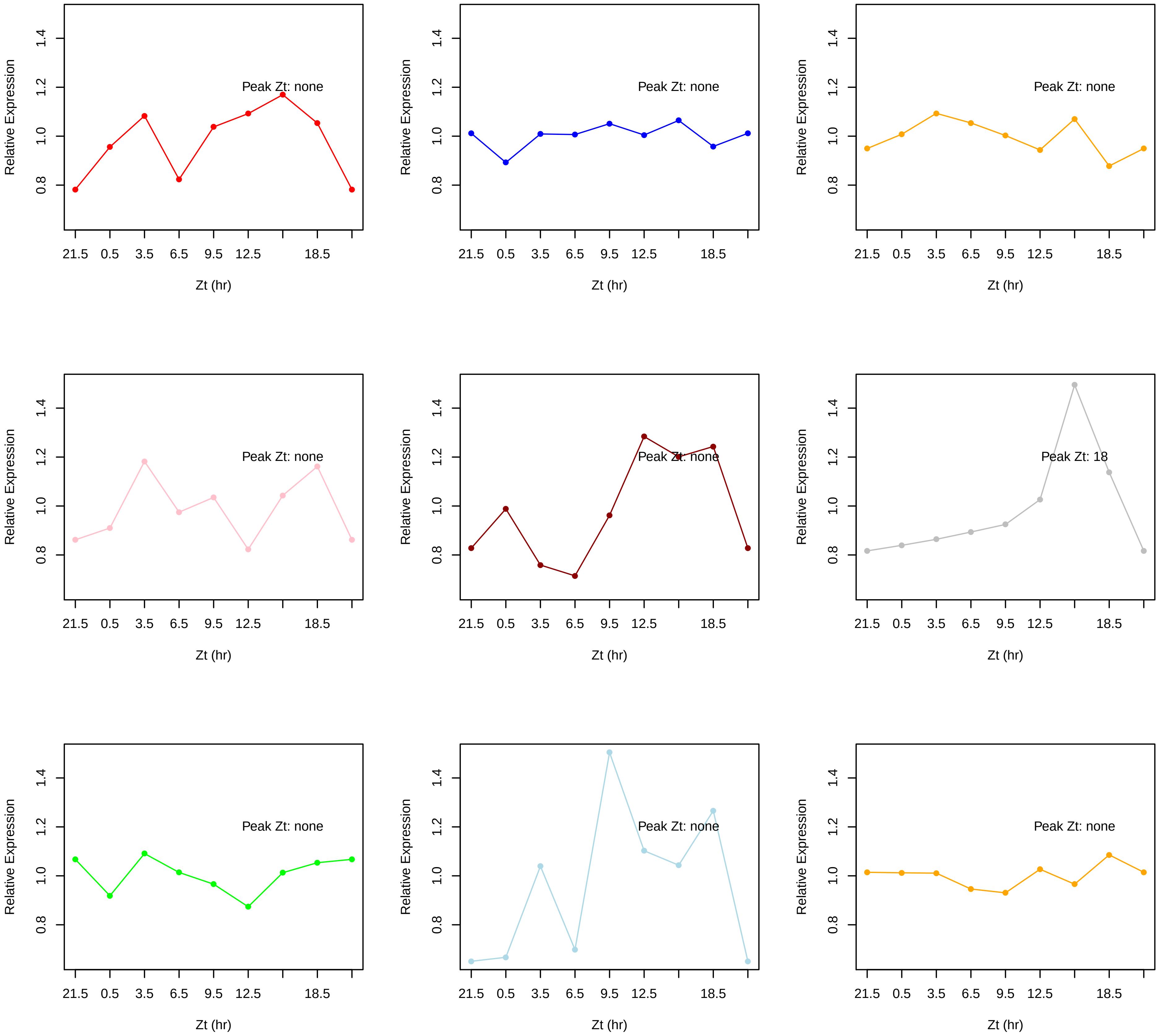

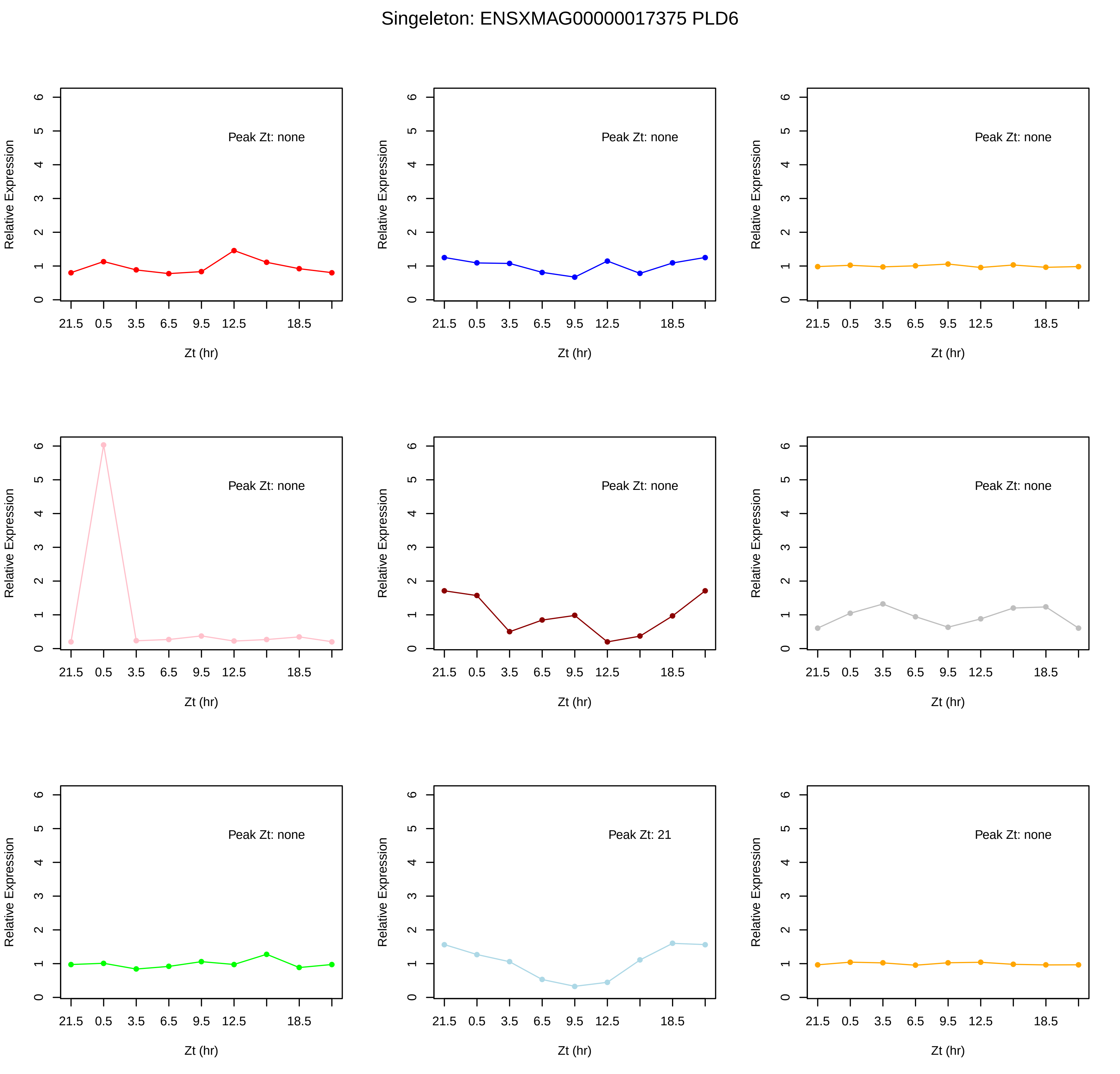

Singleton: ENSXMAG00000017375 PLD6
Peak Zt: none
Peak Zt: none
Peak Zt: none
Peak Zt: none
Peak Zt: none
Peak Zt: none
Peak Zt: none
Peak Zt: 21
Peak Zt: none
Relative Expression
Zt (hr)
21.5 0.5 3.5 6.5 9.5 12.5 18.5

Singeleton: ENSXMAG00000016574 CPXM2

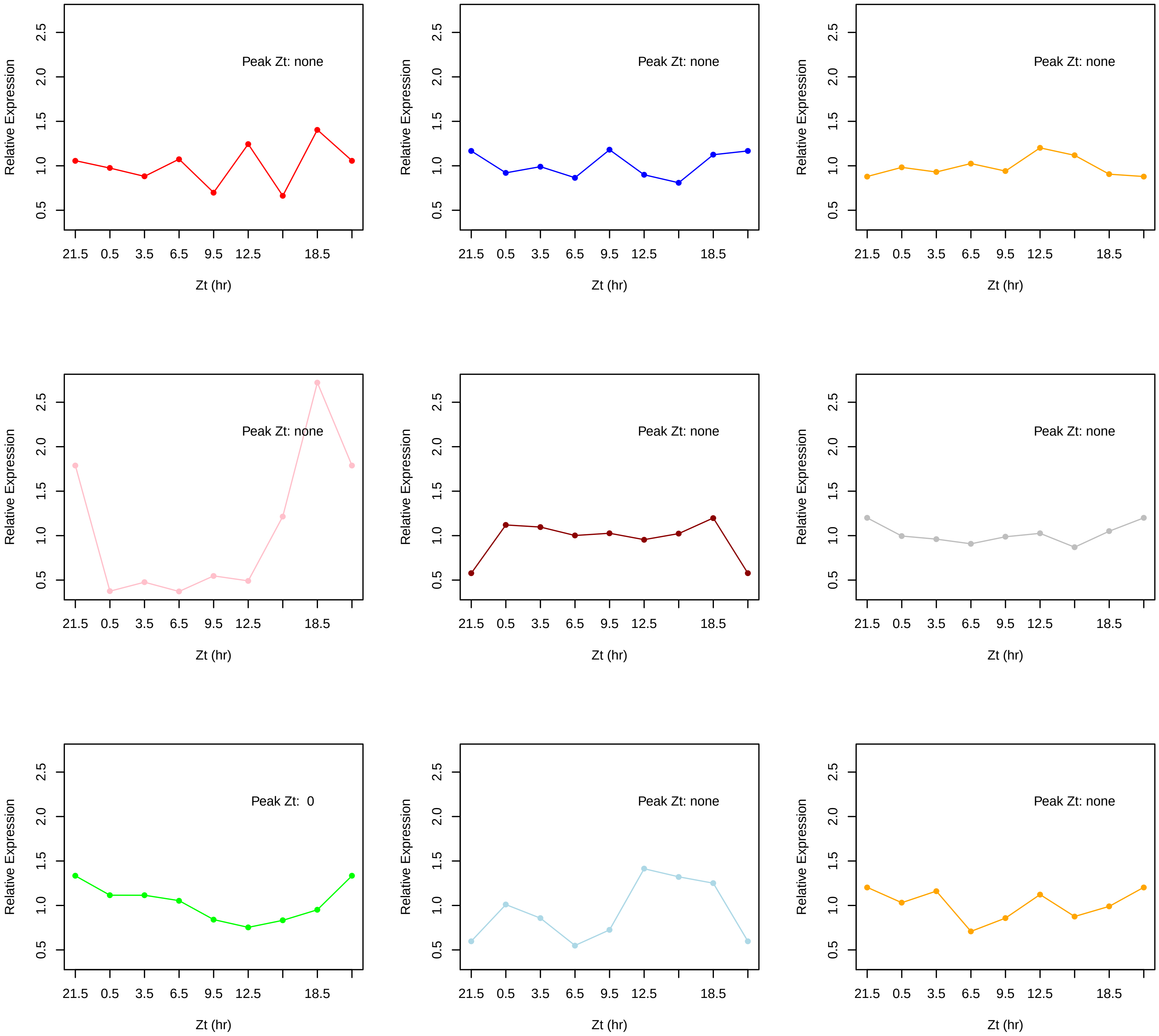

Singeleton: ENSXMAG00000015950 creb3l3l

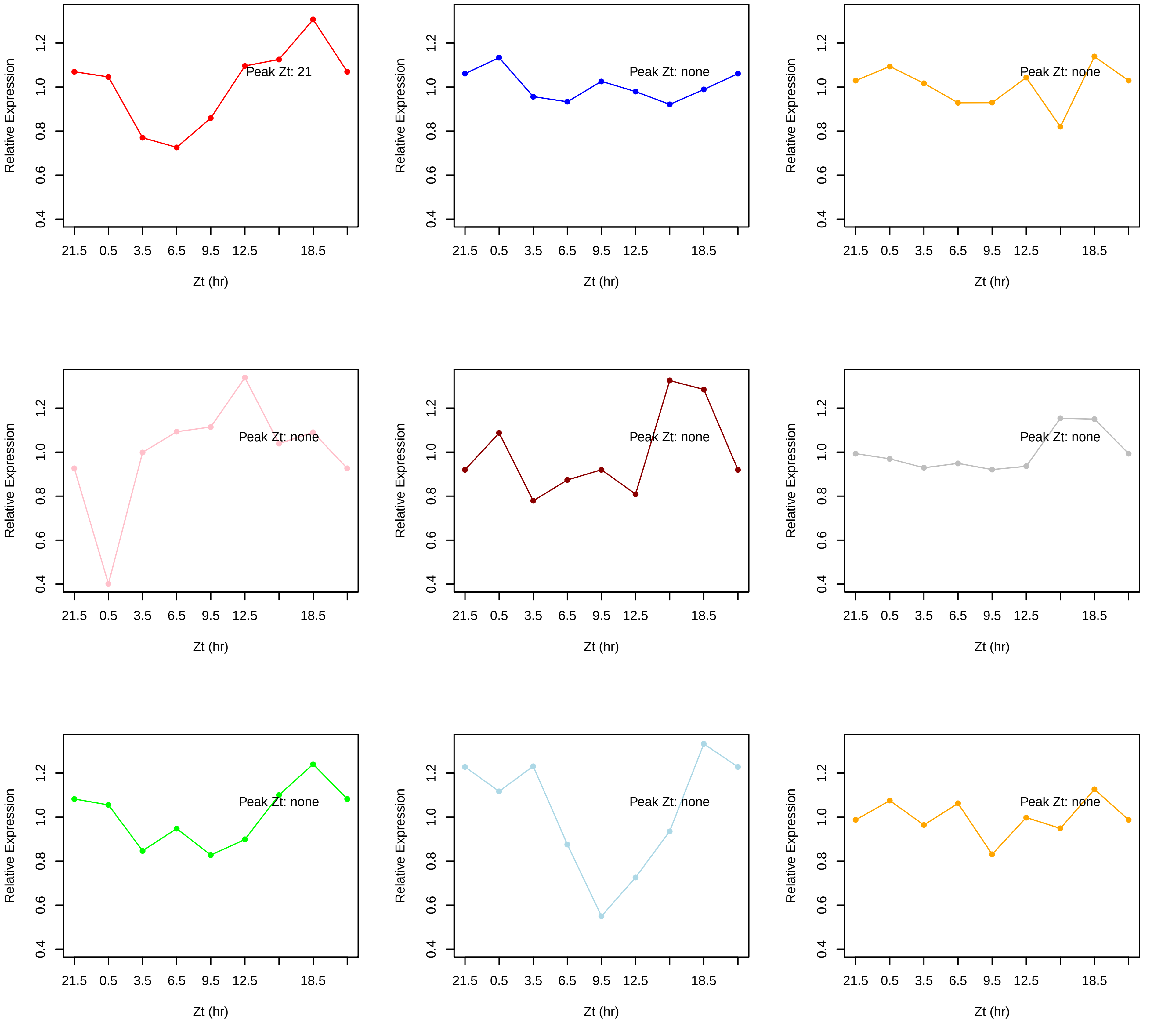

Singleton: ENSXMAG00000014229 necap1

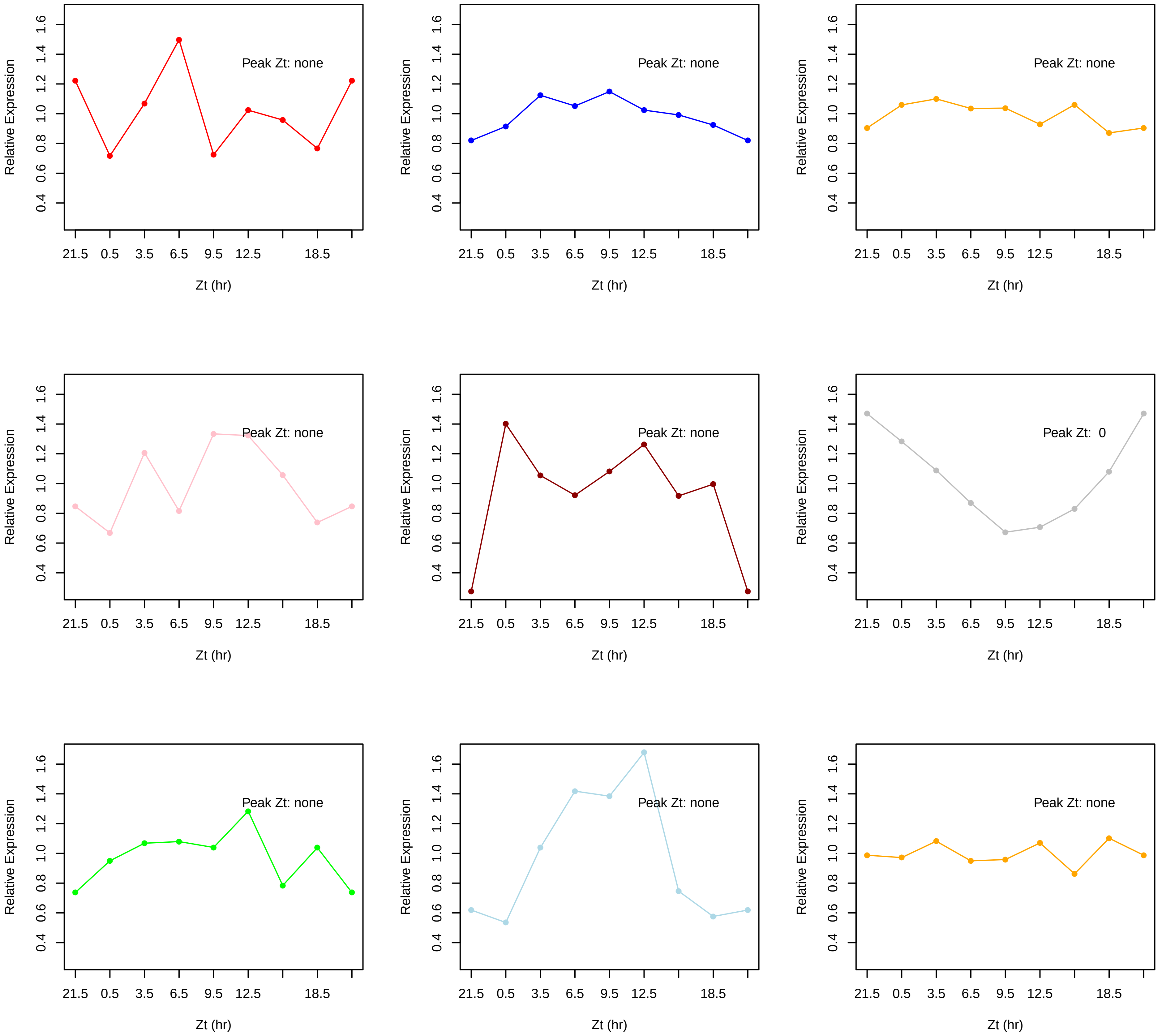

Singeleton: ENSXMAG00000002199 fam198b

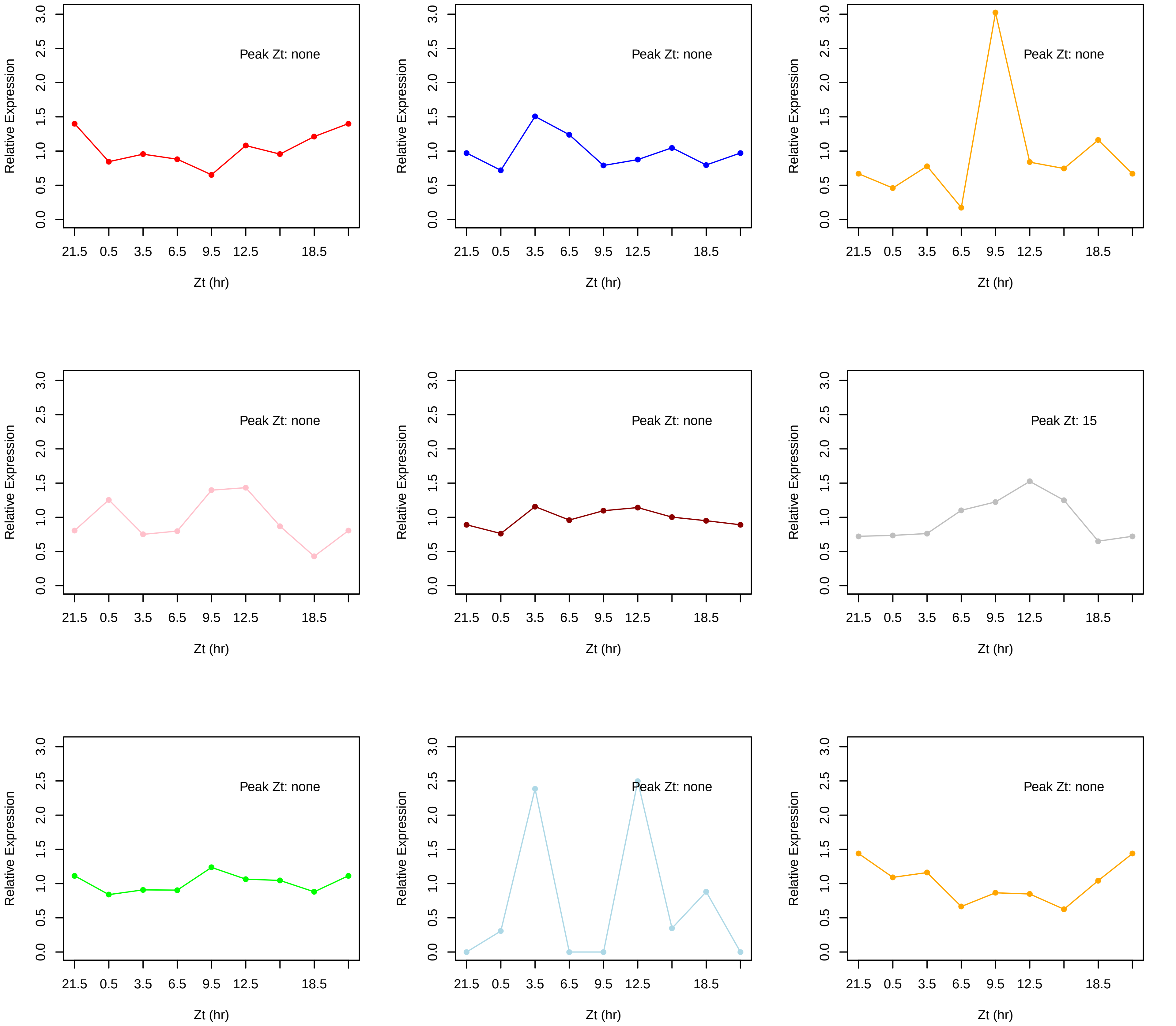

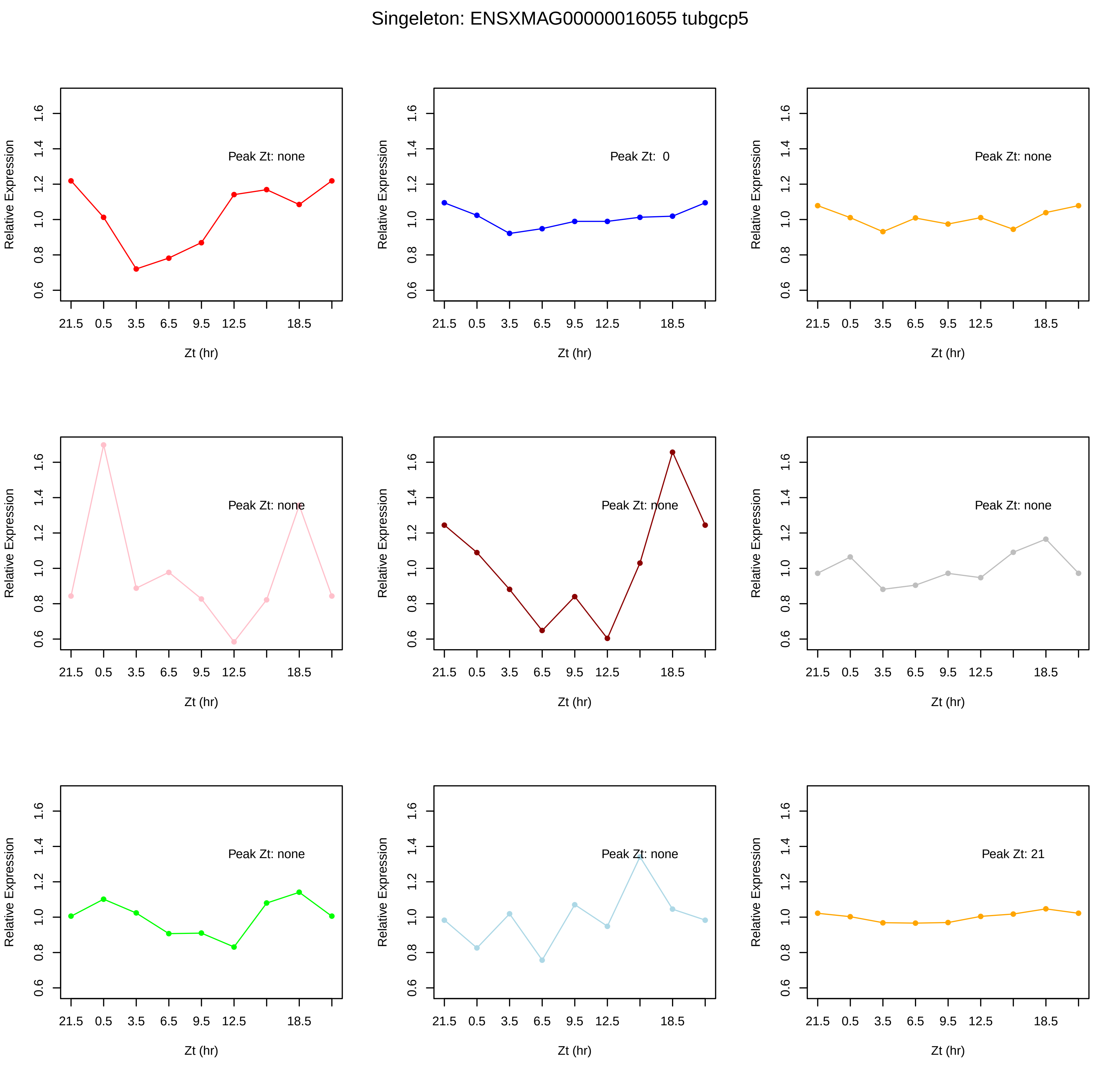
Singeleton: ENSXMAG00000016055 tubgcp5
Relative Expression
Zt (hr)
21.5 0.5 3.5 6.5 9.5 12.5 18.5
0.6 0.8 1.0 1.2 1.4 1.6
Peak Zt: none
Peak Zt: 0
Peak Zt: none
Peak Zt: none
Peak Zt: none
Peak Zt: none
Peak Zt: none
Peak Zt: none
Peak Zt: 21

Singeleton: ENSXMAG00000021572 lrrc14b

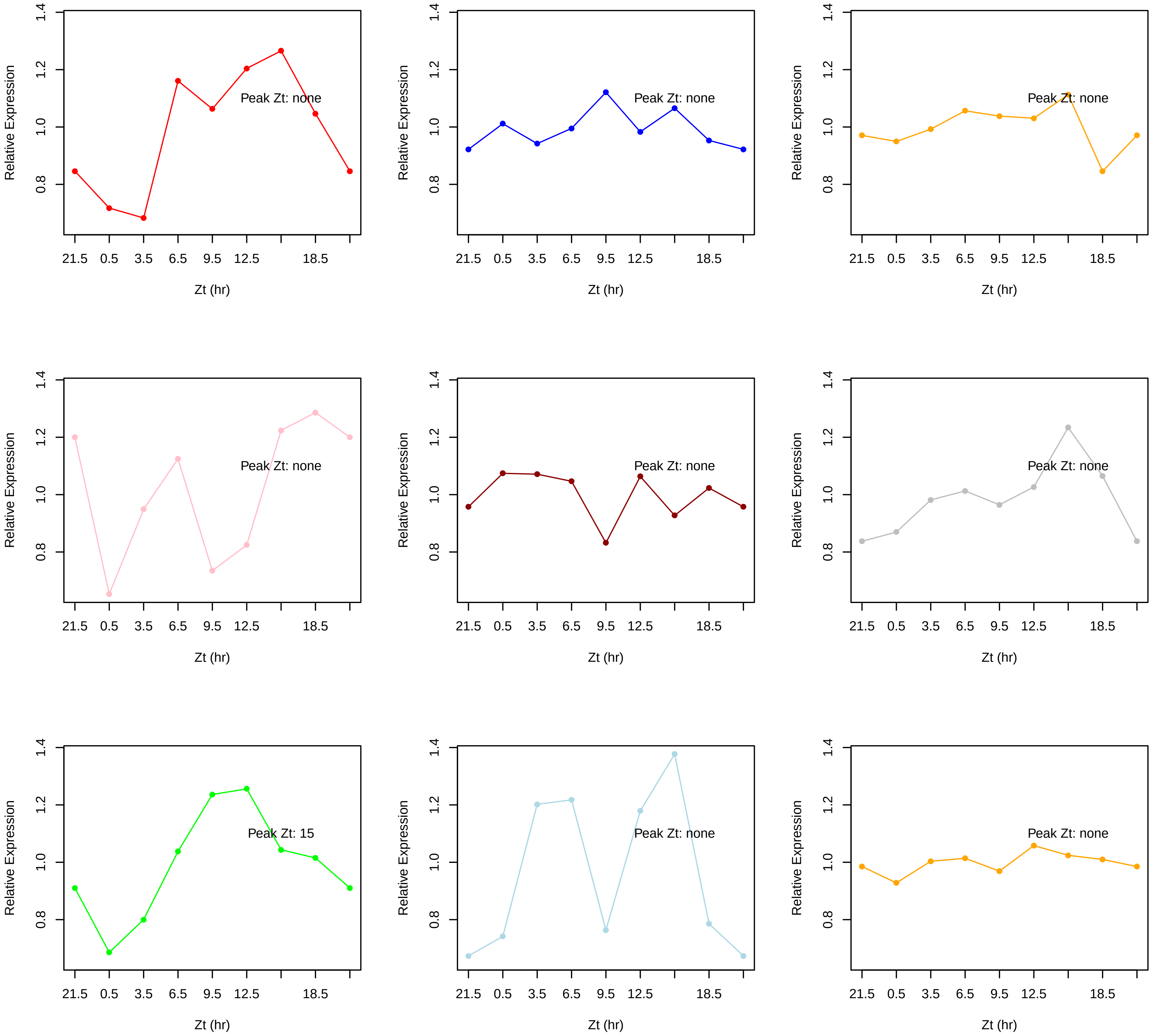

Singeleton: ENSXMAG00000011915 foxj2

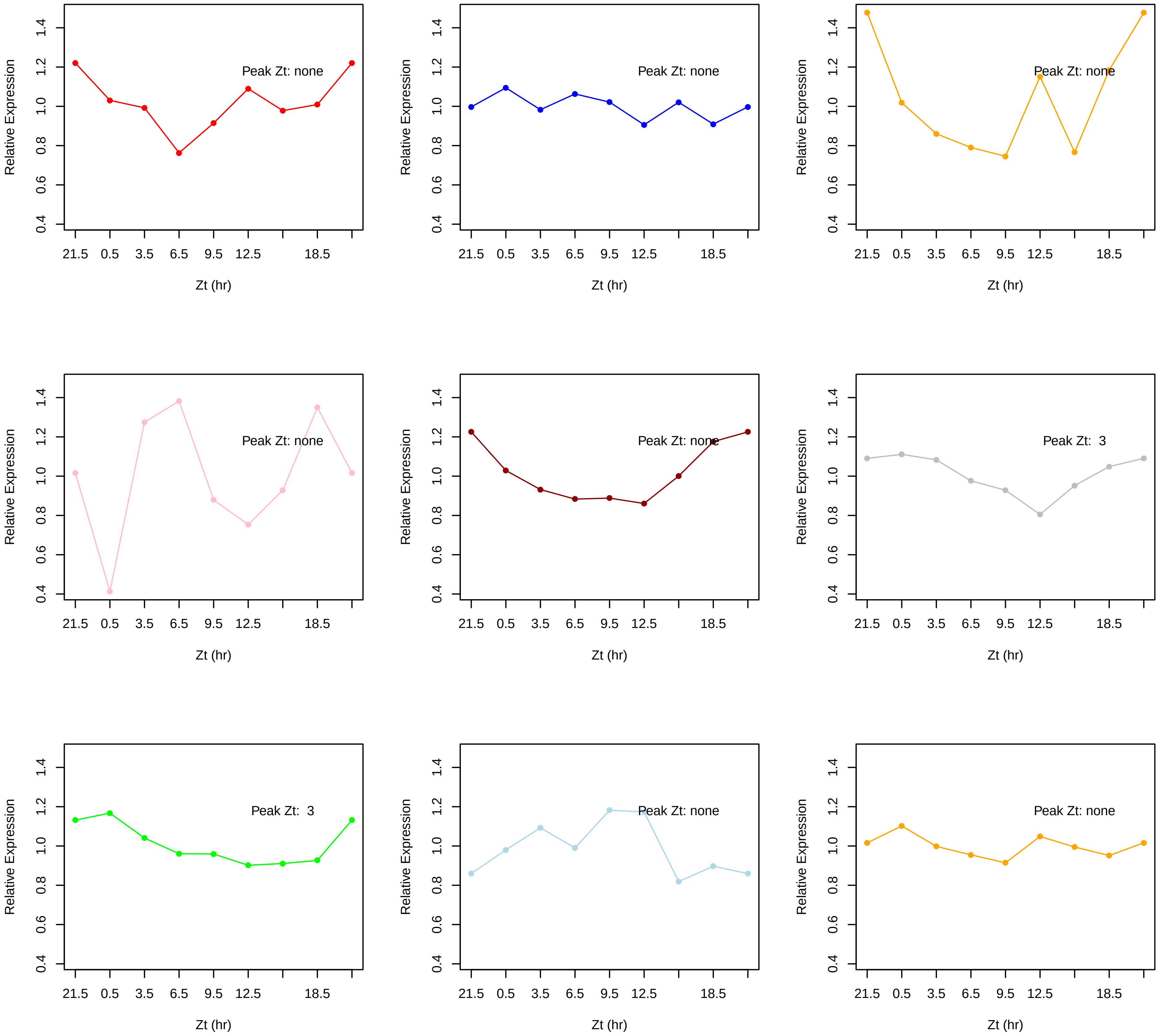

Singleton: ENSXMAG00000015952 gadd45ga

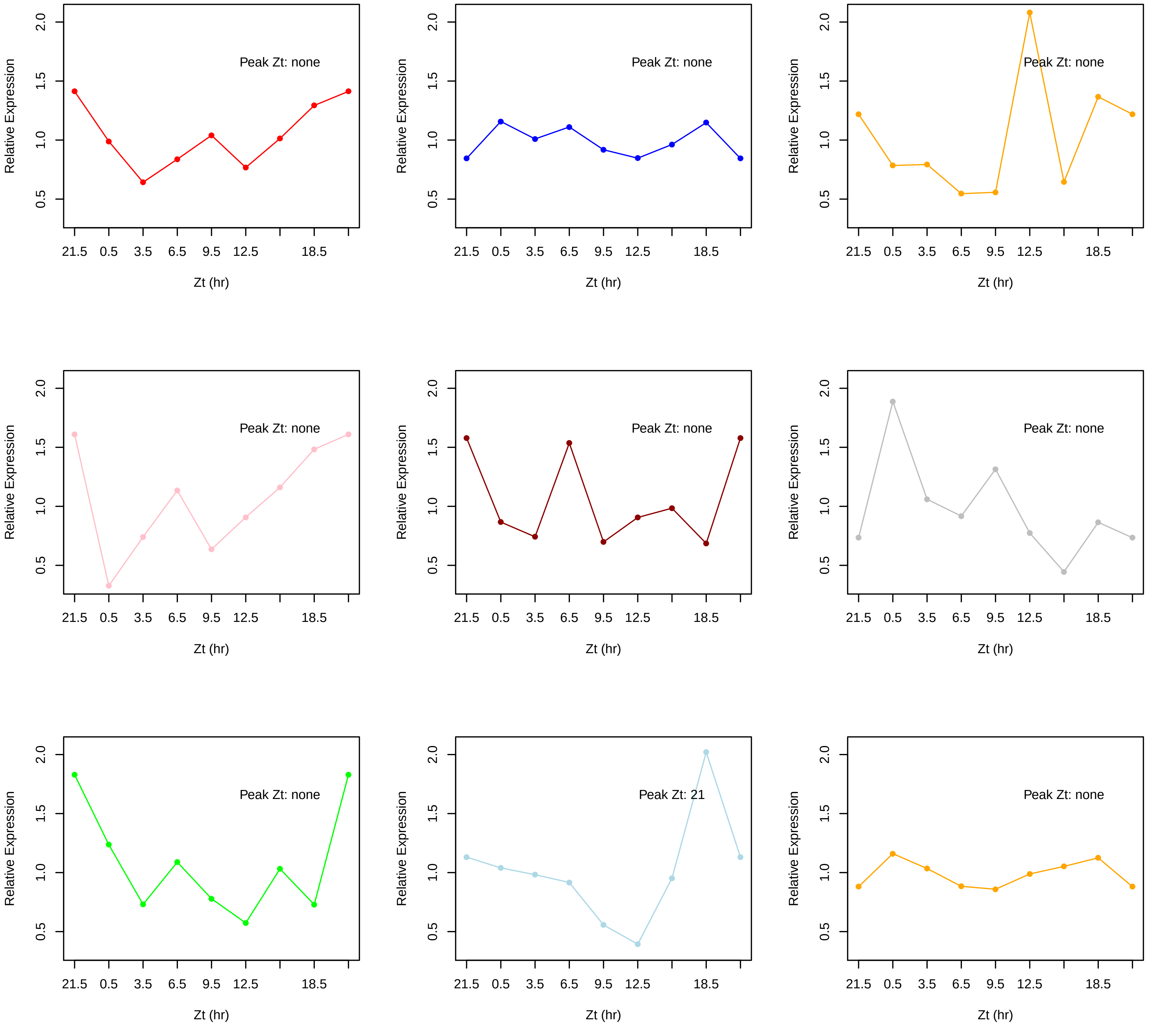

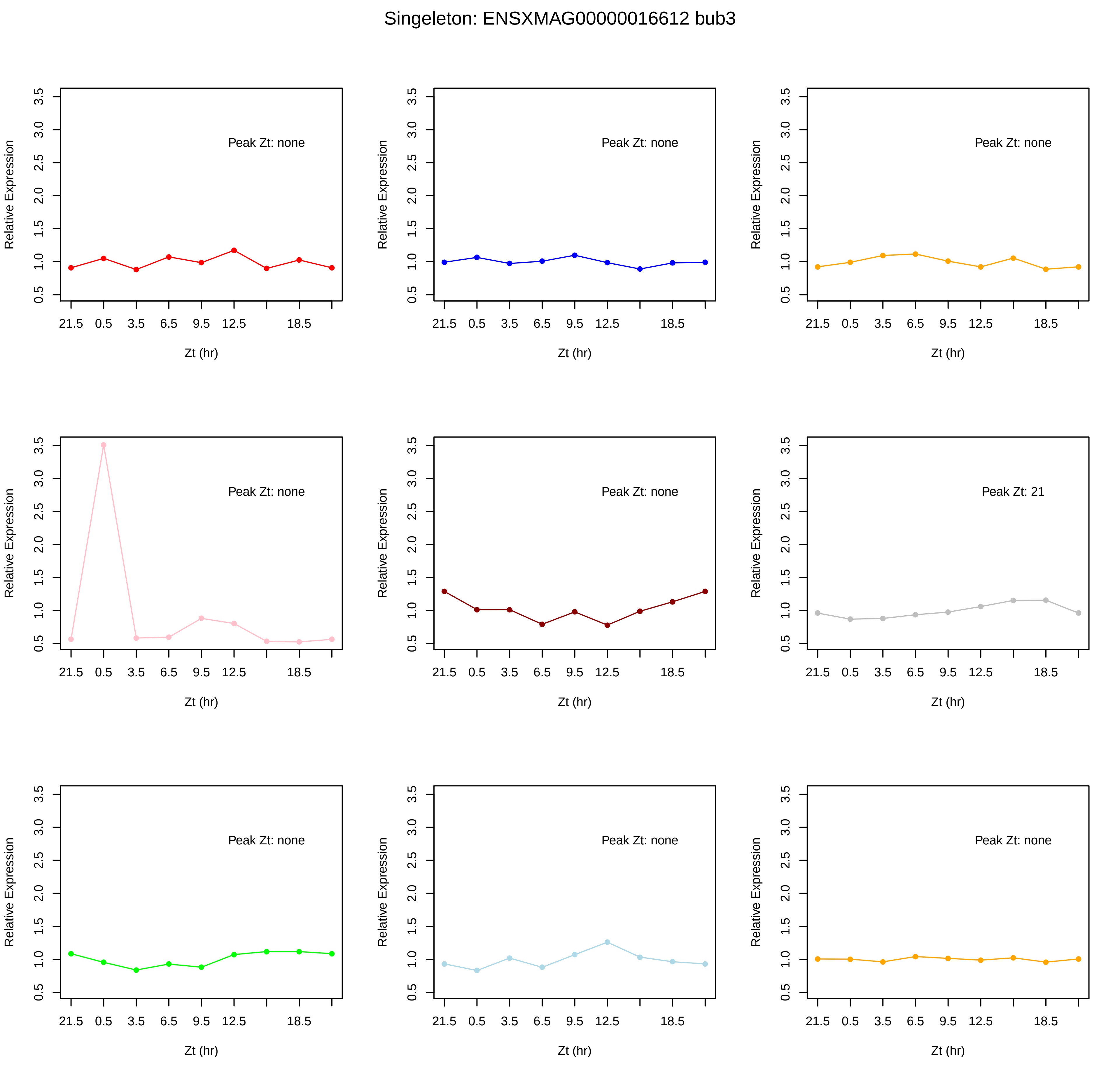

Singeleton: ENSXMAG00000016612 bub3
Peak Zt: none
Peak Zt: none
Peak Zt: none
Peak Zt: none
Peak Zt: none
Peak Zt: 21
Peak Zt: none
Peak Zt: none
Peak Zt: none
Relative Expression
Zt (hr)

Singeleton: ENSXMAG00000011810 slc2a3b

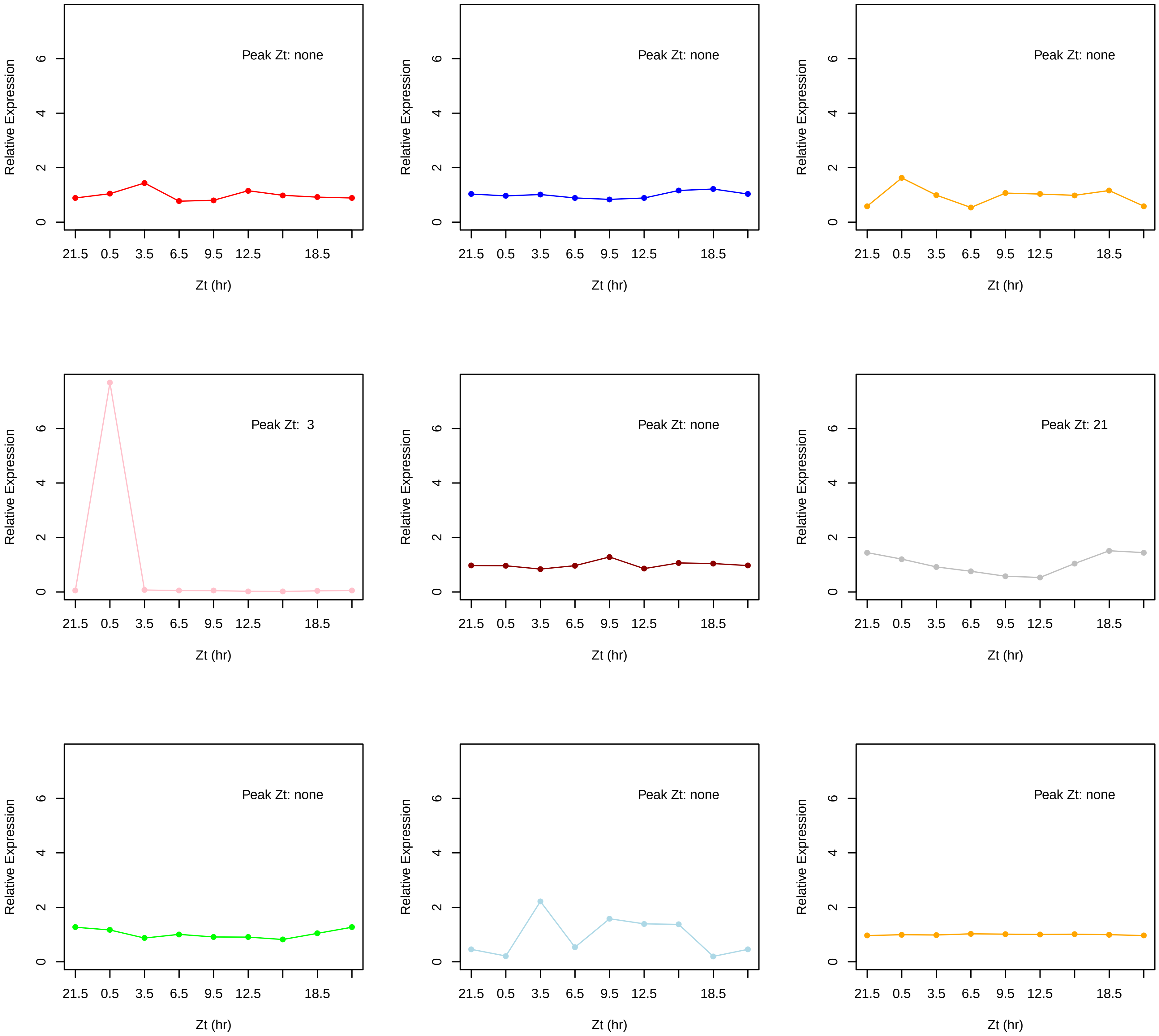

Singeleton: ENSXMAG00000025218 tmem56b

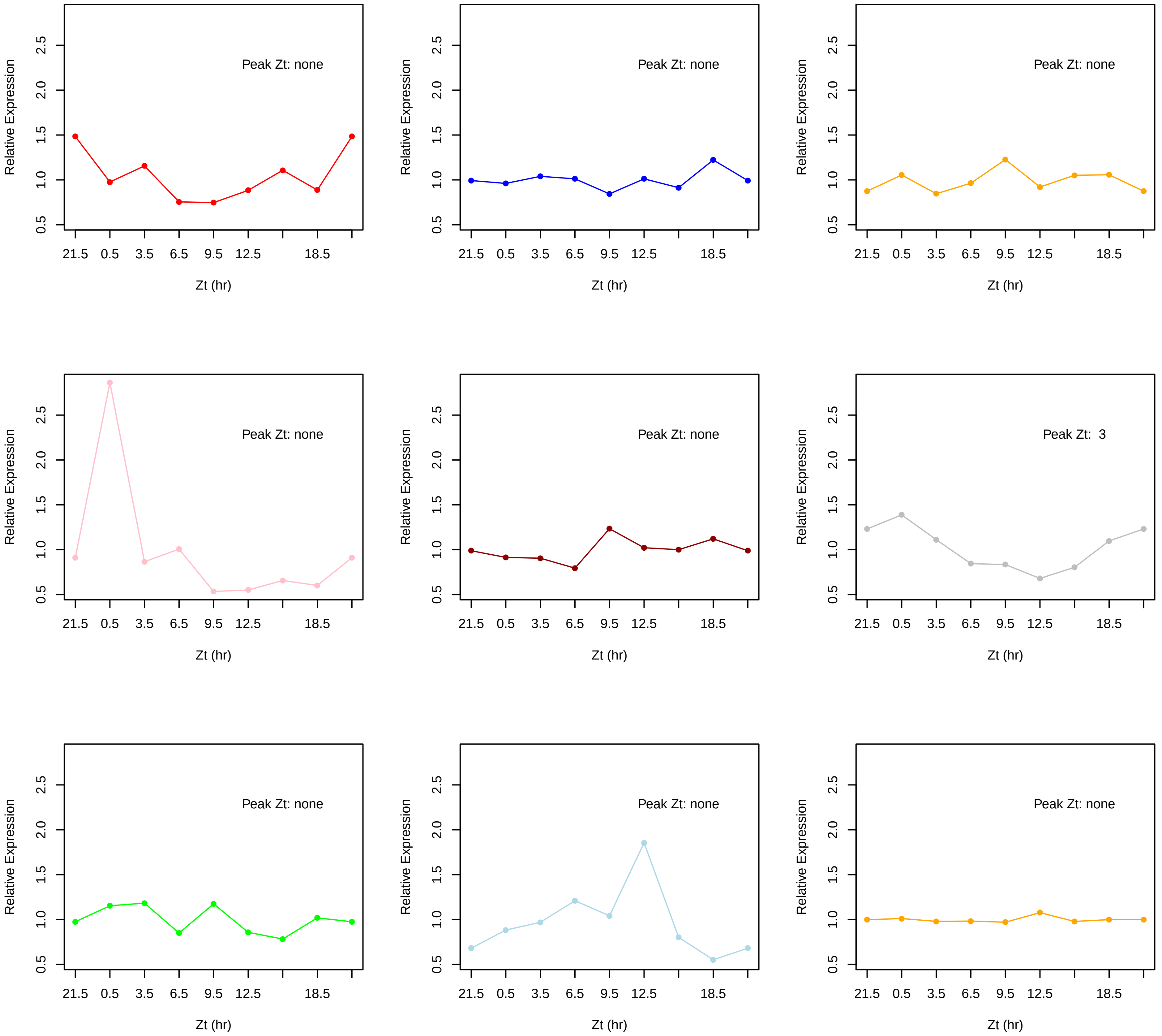

Singeleton: ENSXMAG00000016005 tmem176

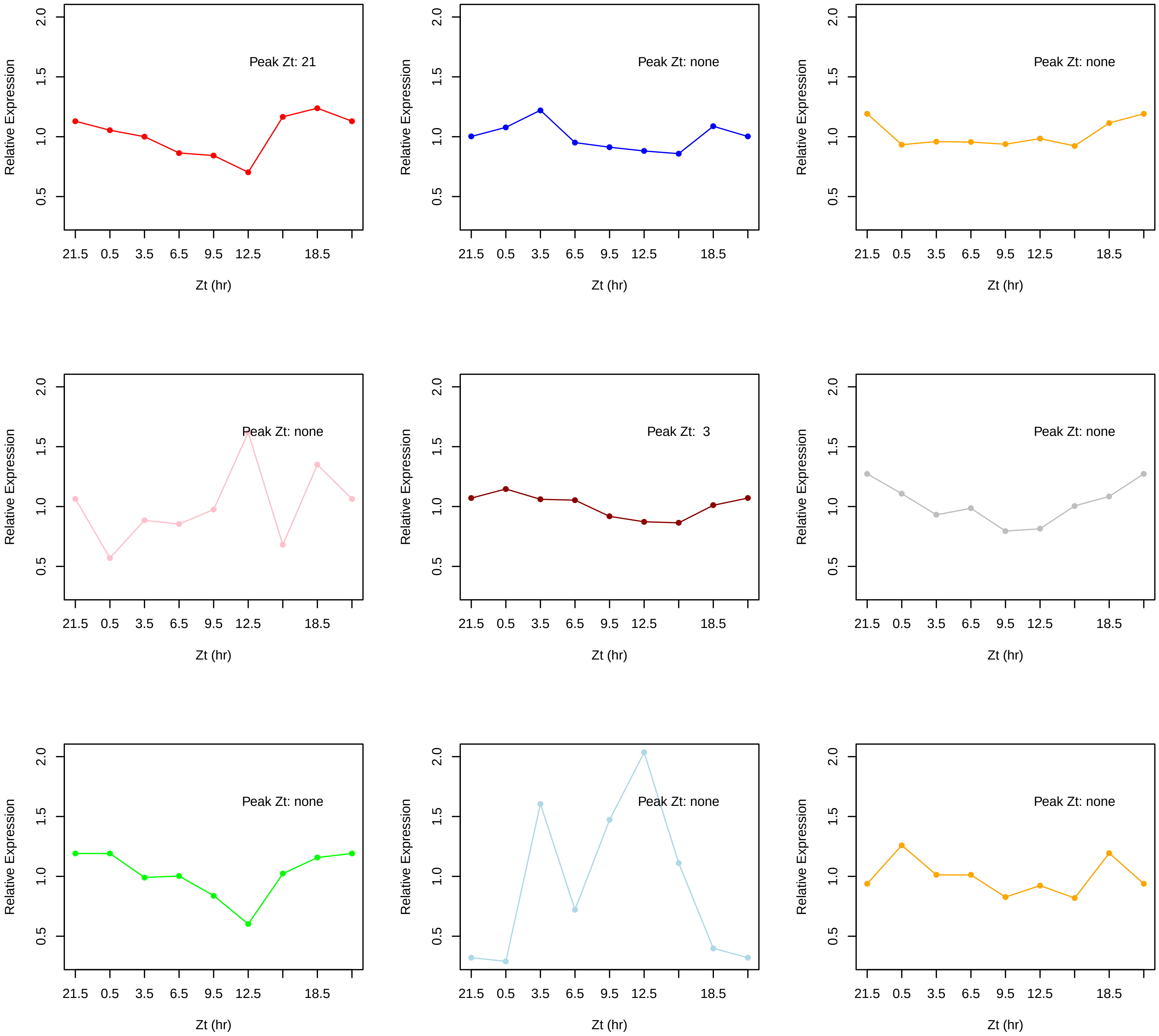

Singleton: ENSXMAG00000016009 vps37c

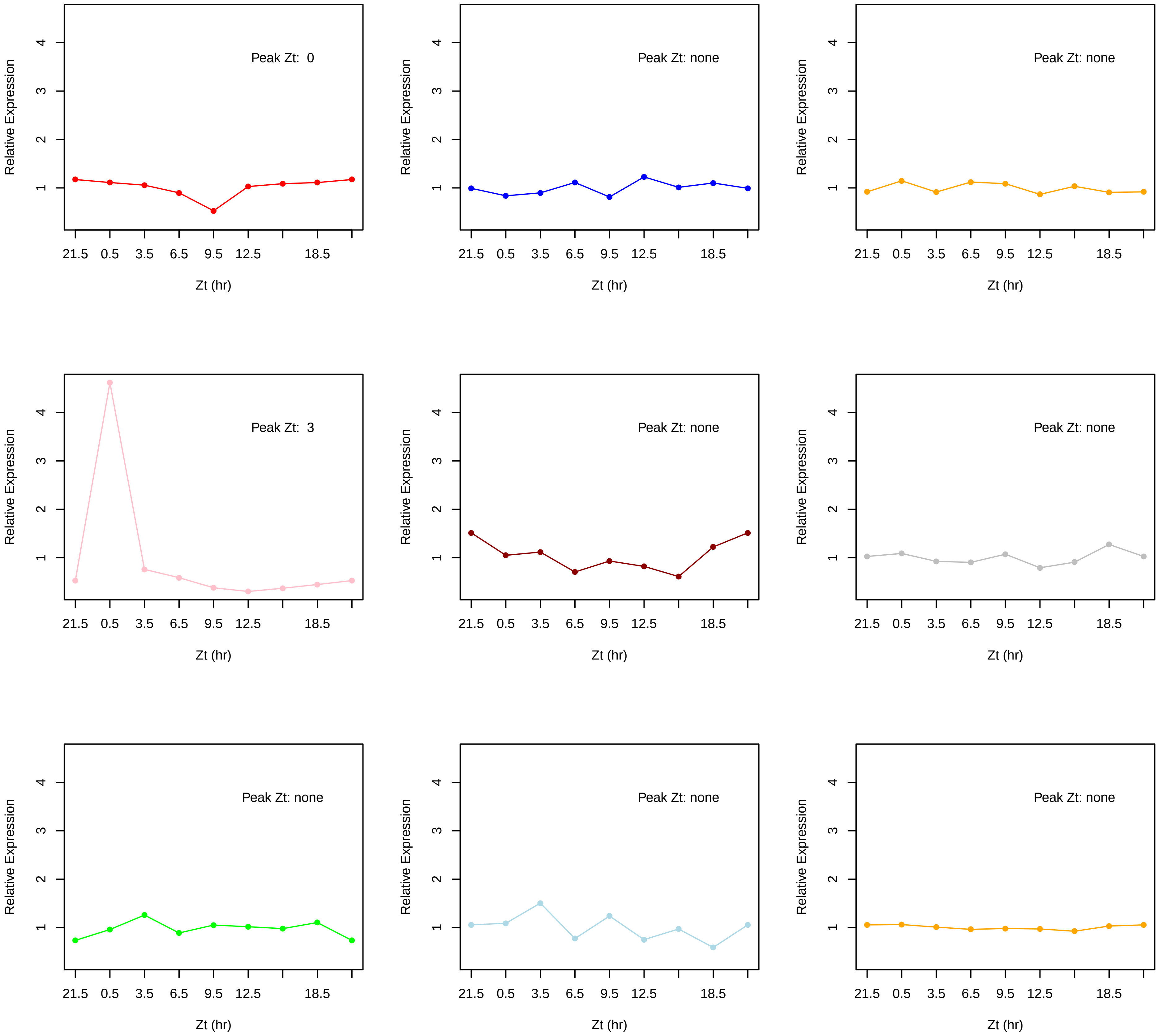

Singeleton: ENSXMAG00000000067 hccsb

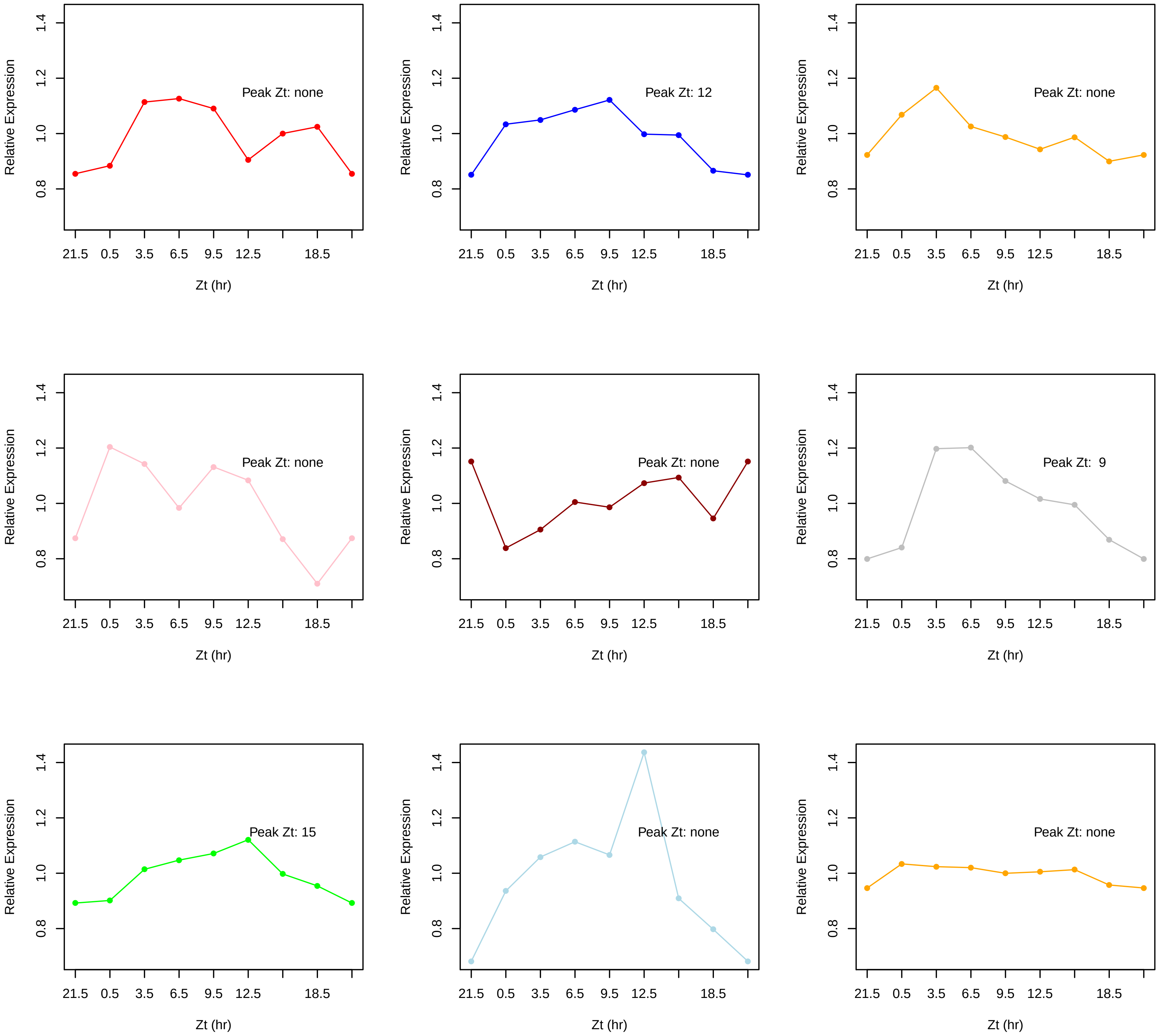

Singleton: ENSXMAG00000016124 nipa2

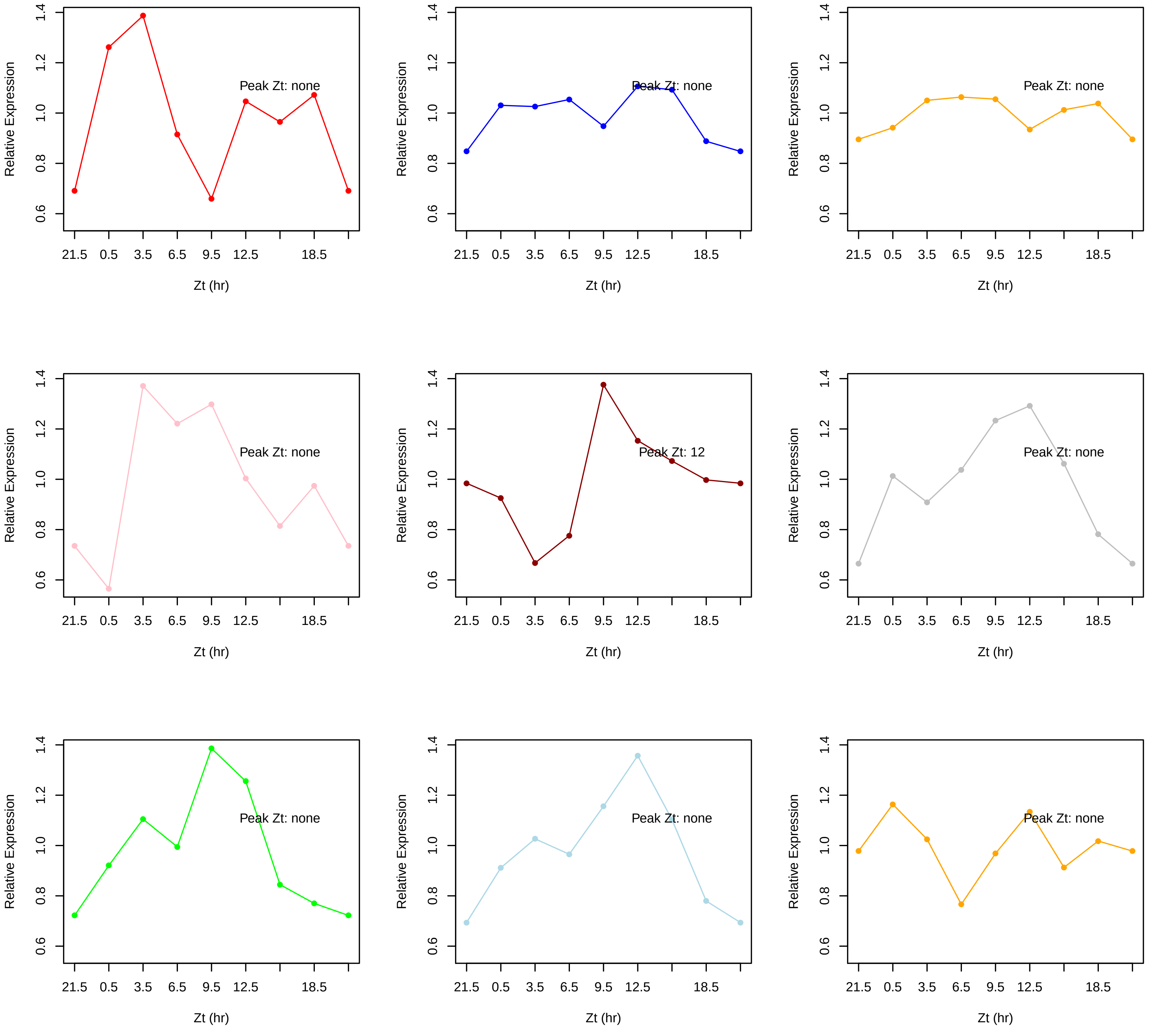

Singeleton: ENSXMAG00000012009 tppp3

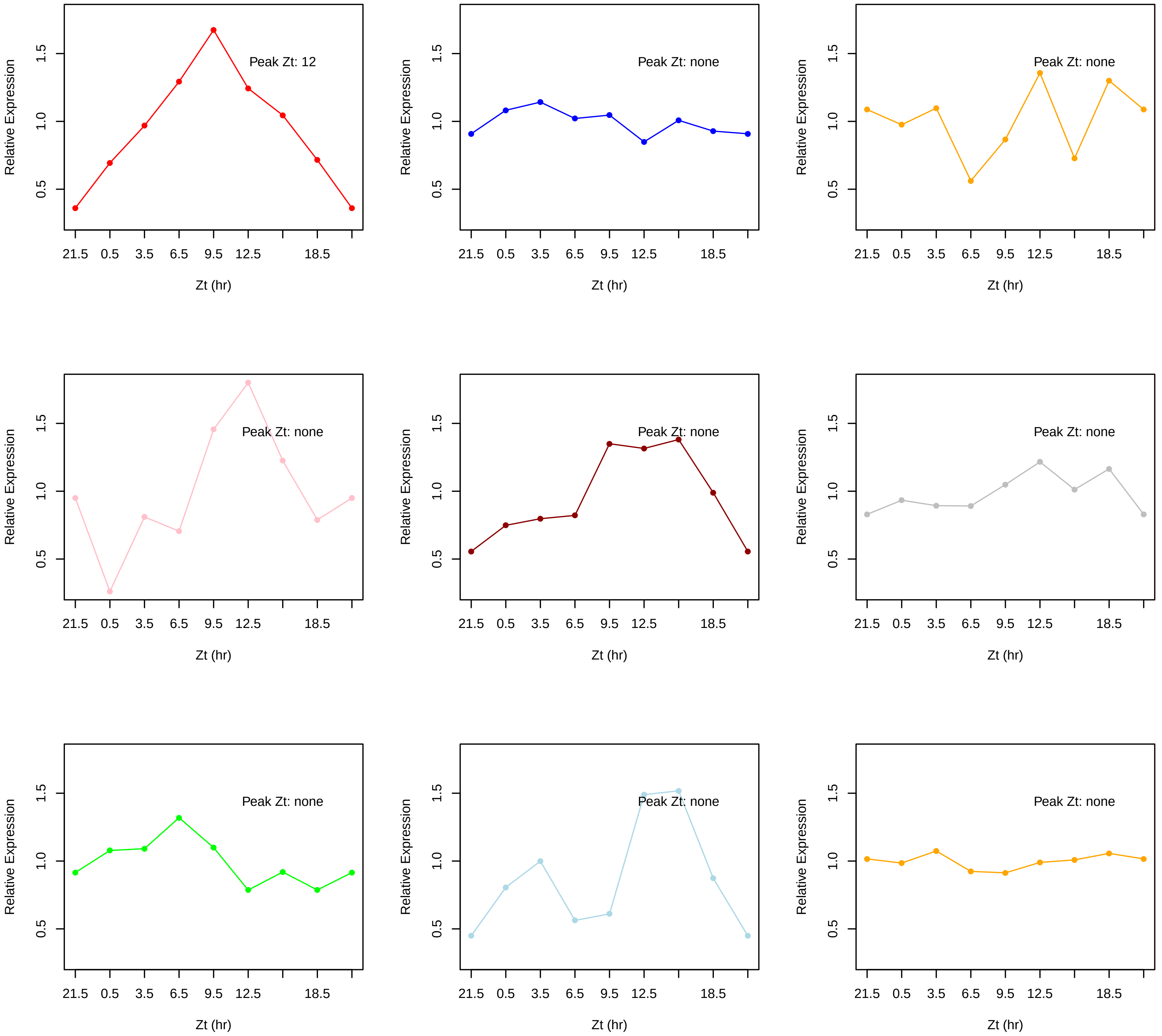

Singeleton: ENSXMAG00000019943 nfil3

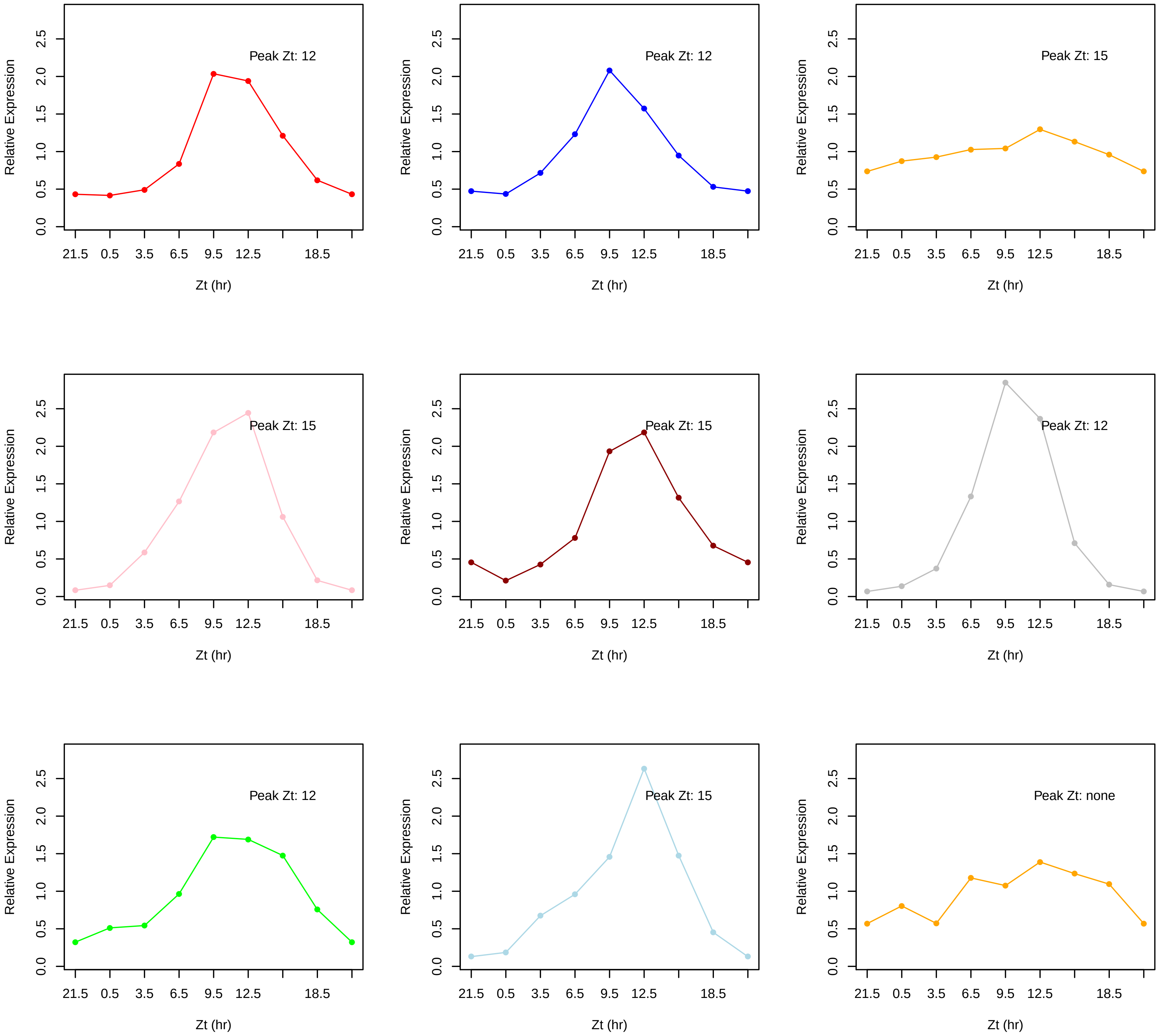

Singeleton: ENSXMAG00000000124 TMEM132D

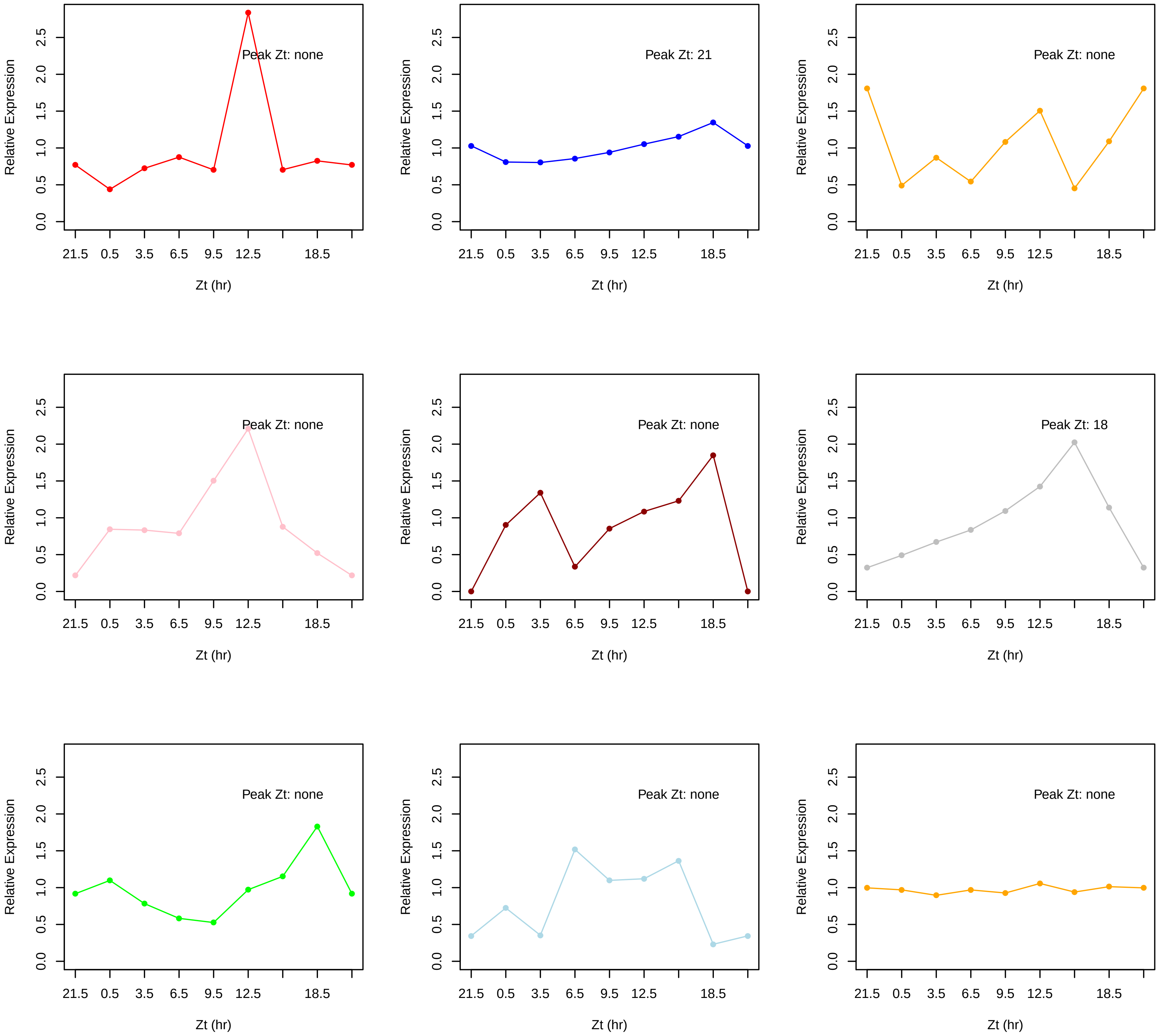

Singeleton: ENSXMAG00000015267 slc7a4

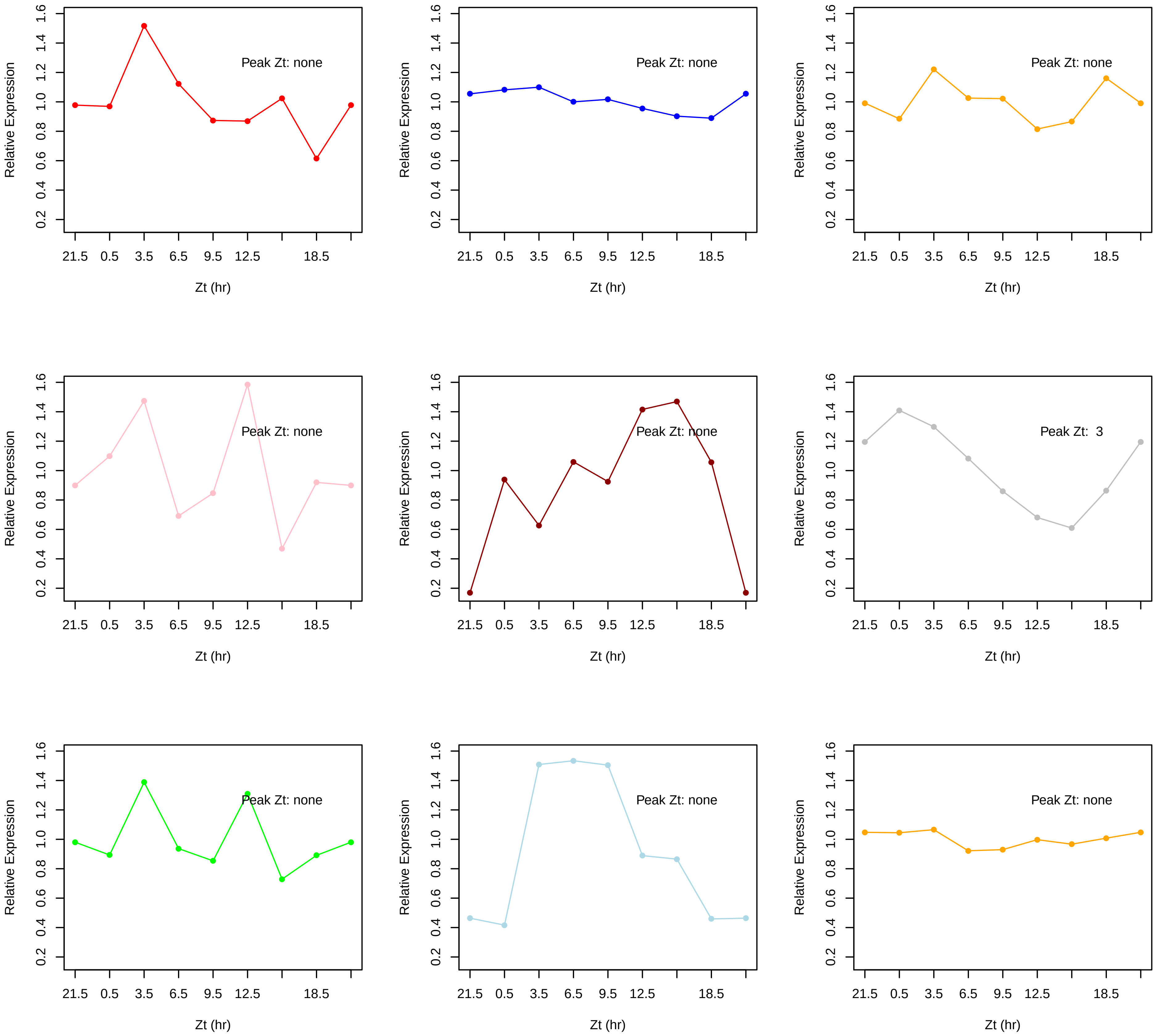

Singeleton: ENSXMAG00000015615

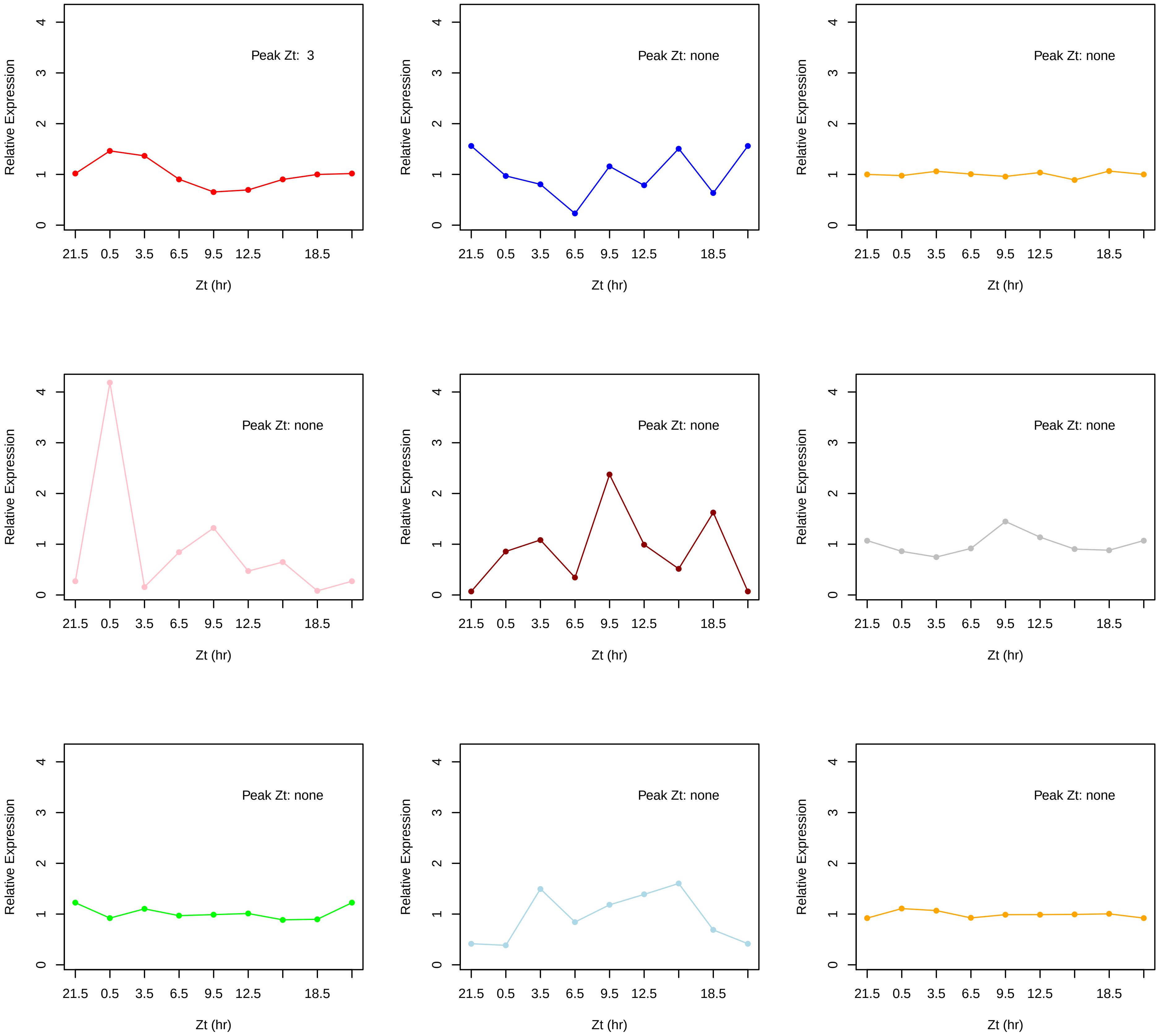

Singleton: ENSXMAG00000009669 n4bp2

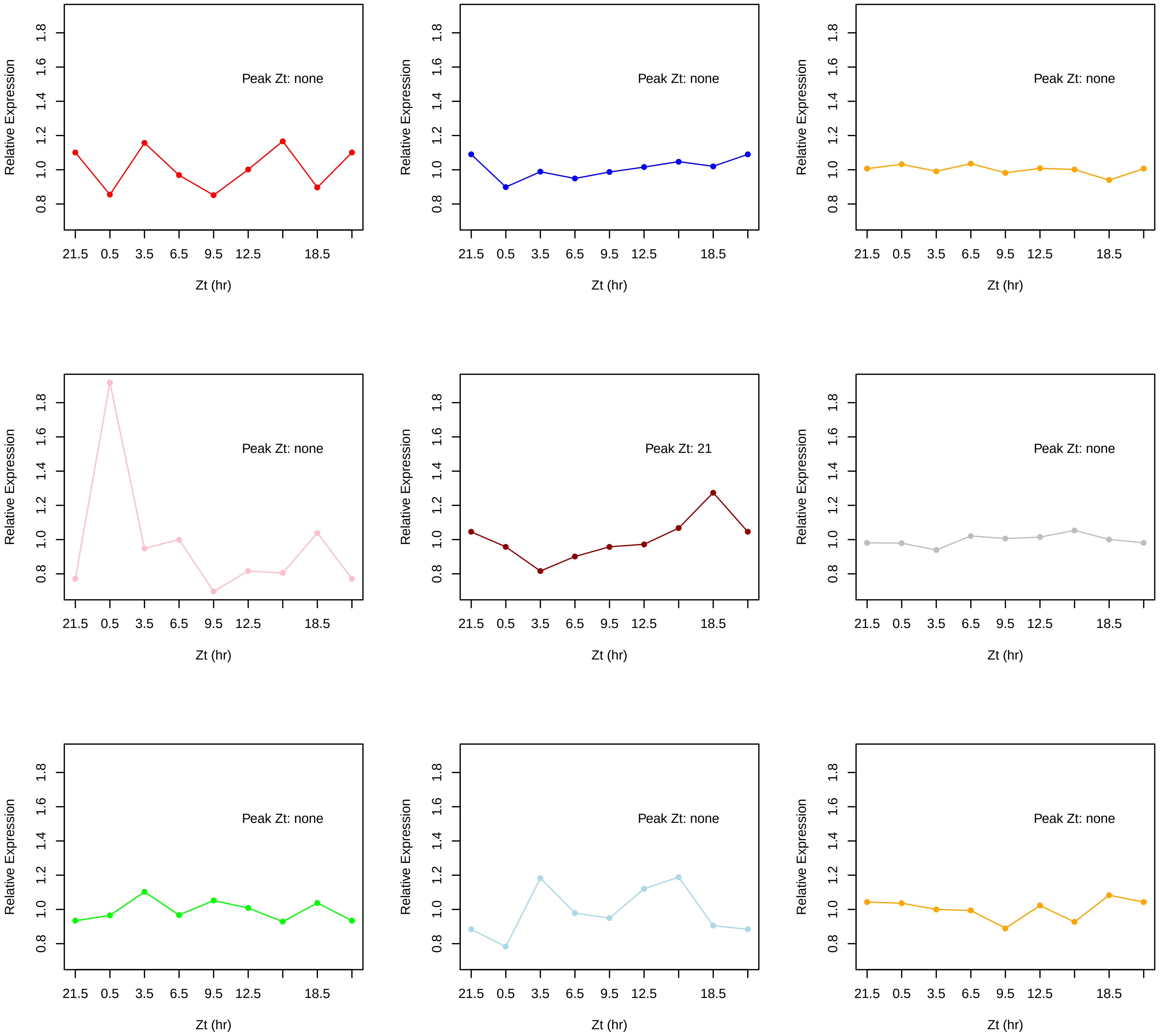

Singeleton: ENSXMAG00000021164 pid1

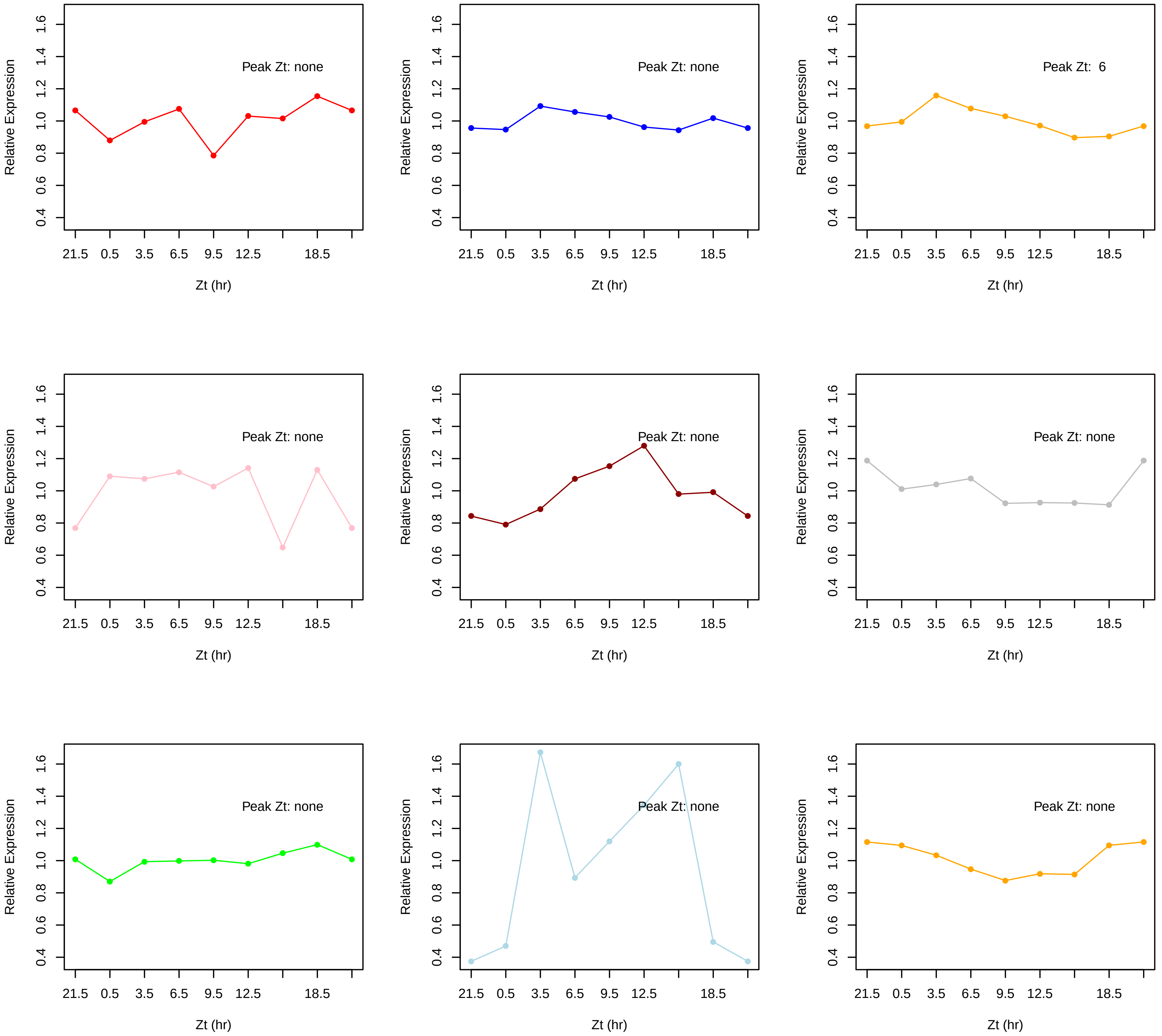

Singleton: ENSXMAG00000016133 herc2

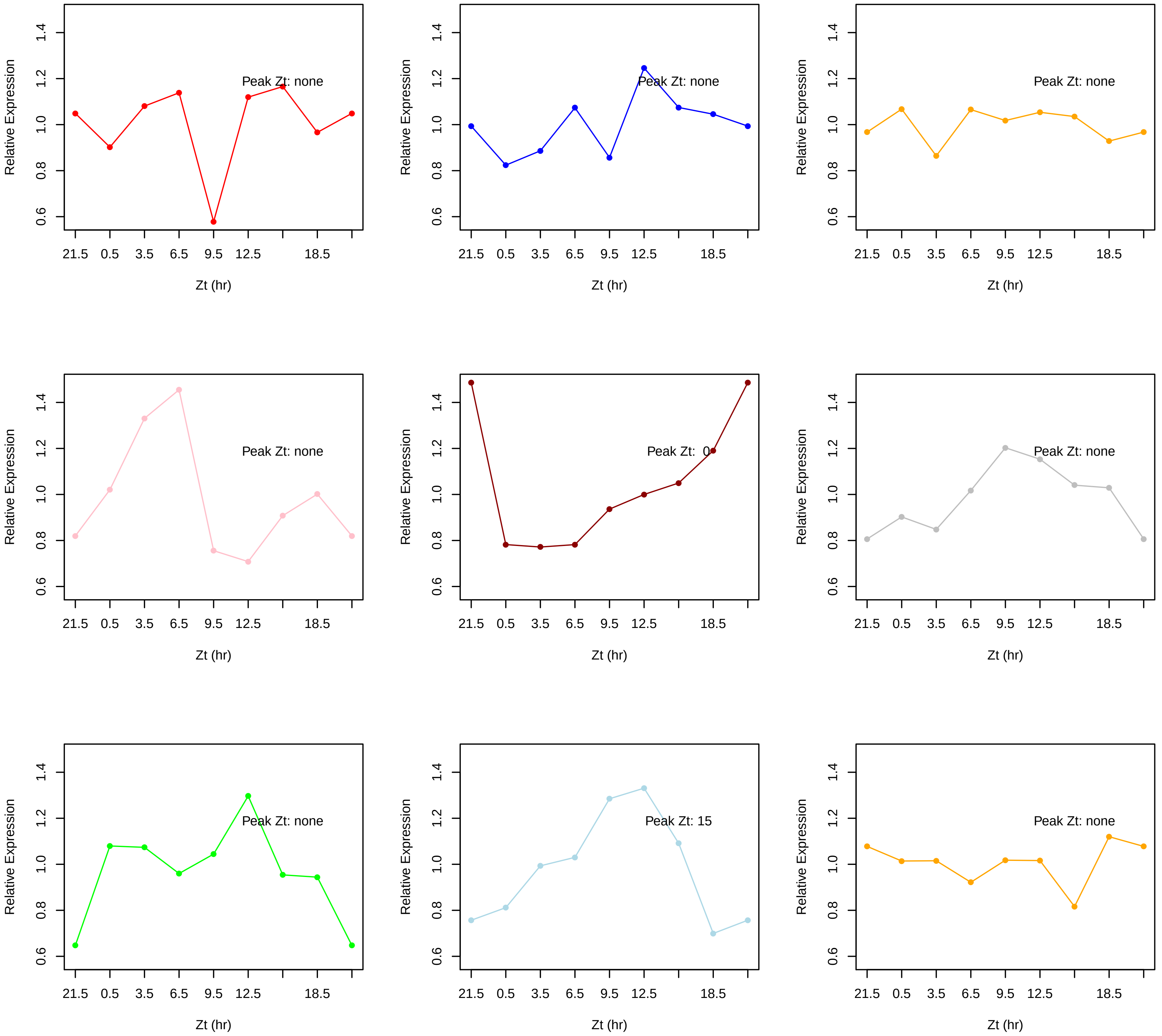

Singleton: ENSXMAG00000016125 sptlc1

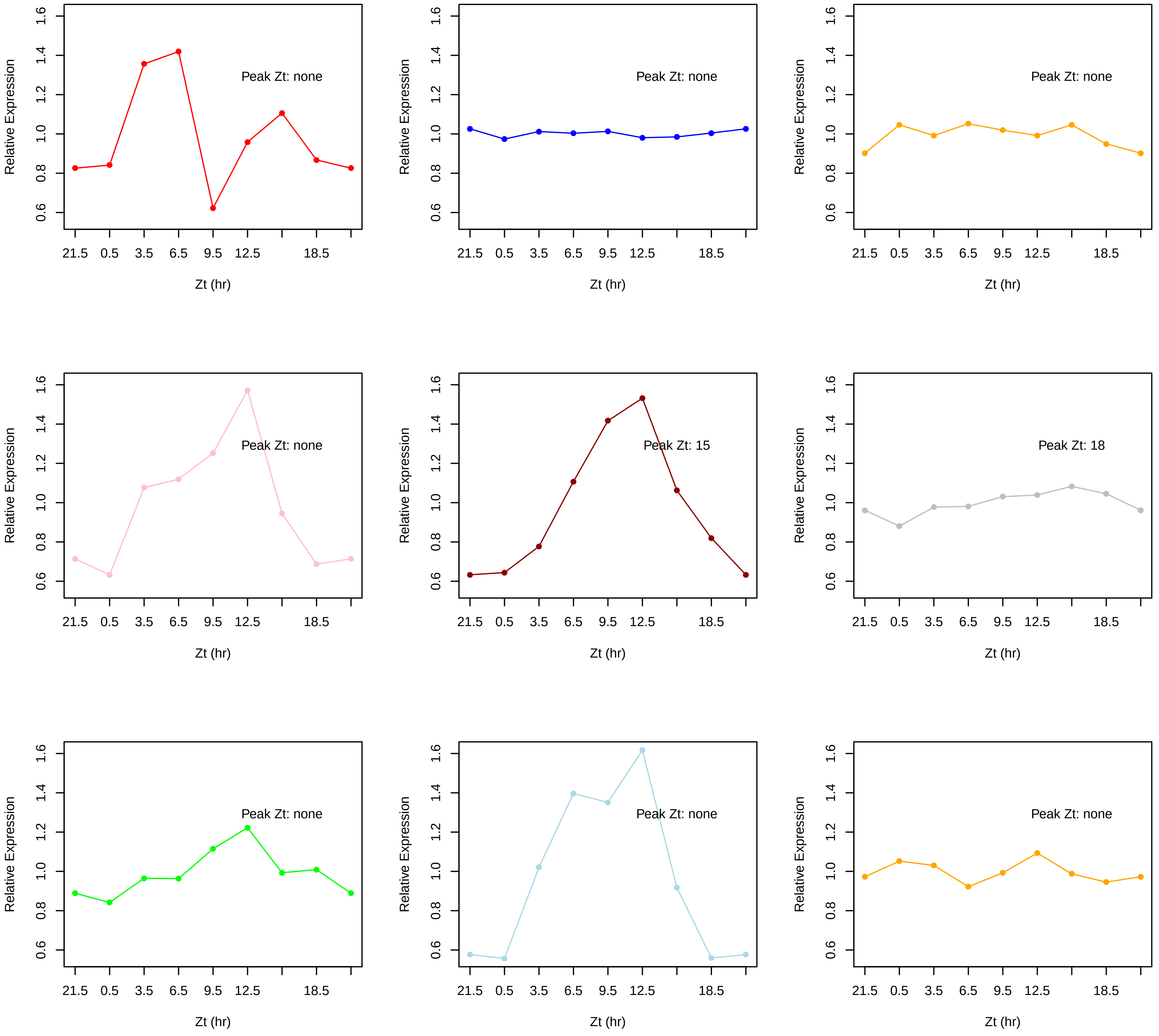

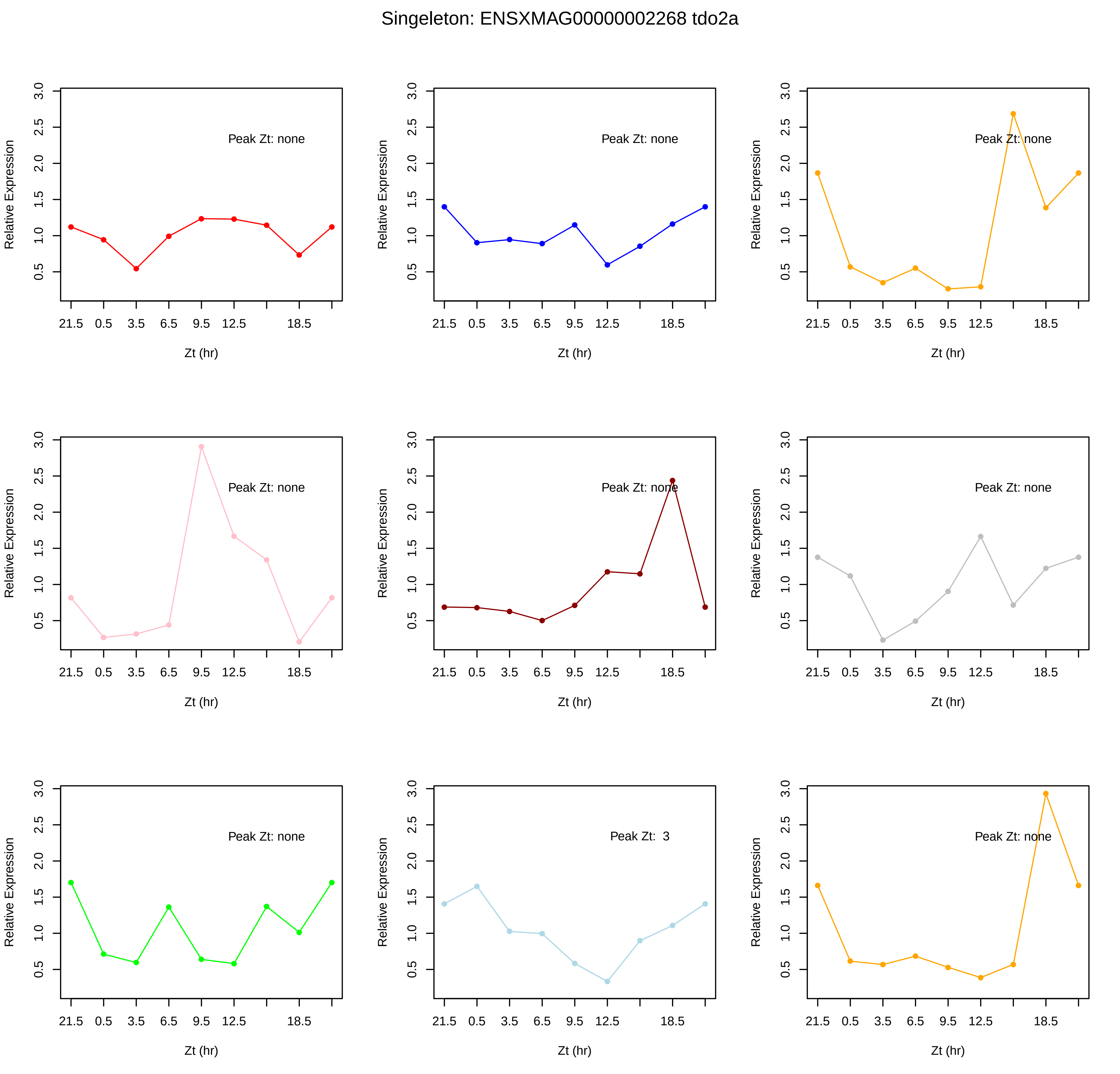
Singleton: ENSXMAG00000002268 tdo2a
Peak Zt: none
Peak Zt: none
Peak Zt: none
Peak Zt: none
Peak Zt: none
Peak Zt: none
Peak Zt: none
Peak Zt: 3
Peak Zt: none
Relative Expression
Zt (hr)
21.5
0.5
3.5
6.5
9.5
12.5
18.5
0.5
1.0
1.5
2.0
2.5
3.0

Singleton: ENSXMAG00000002753 sf3a1

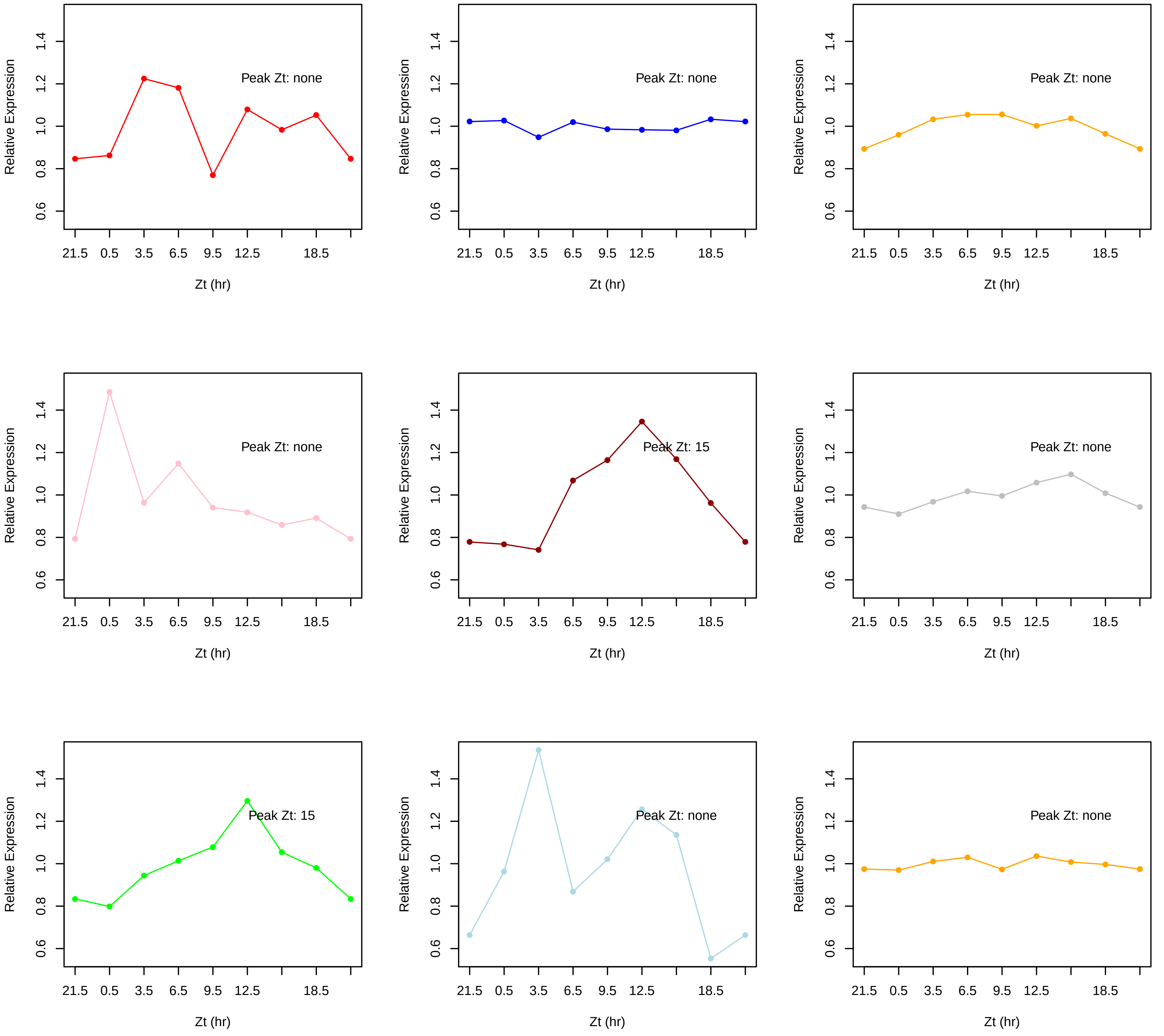

Singleton: ENSXMAG00000018645 dpydb

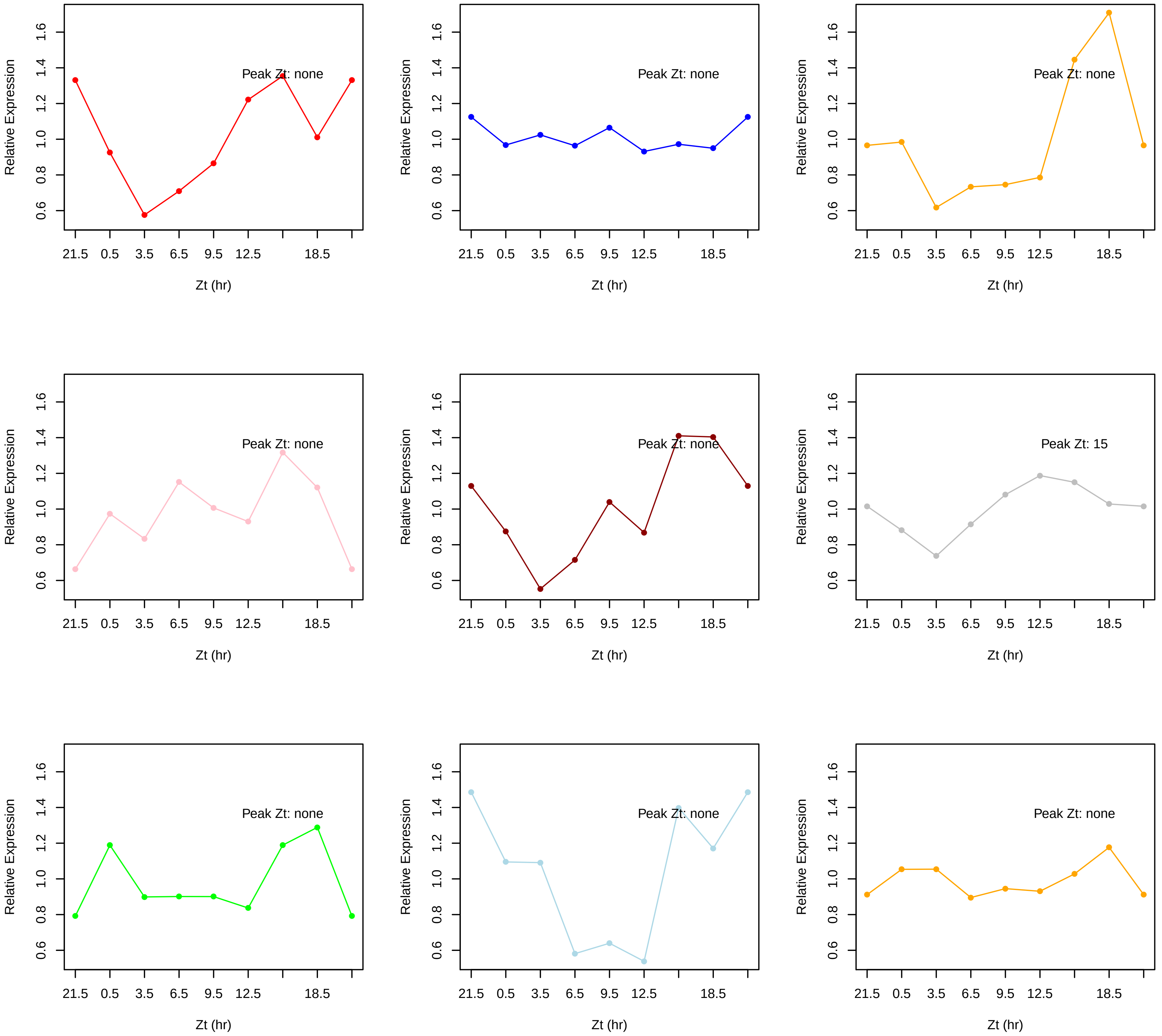

Singeleton: ENSXMAG00000005656 gucy1a1

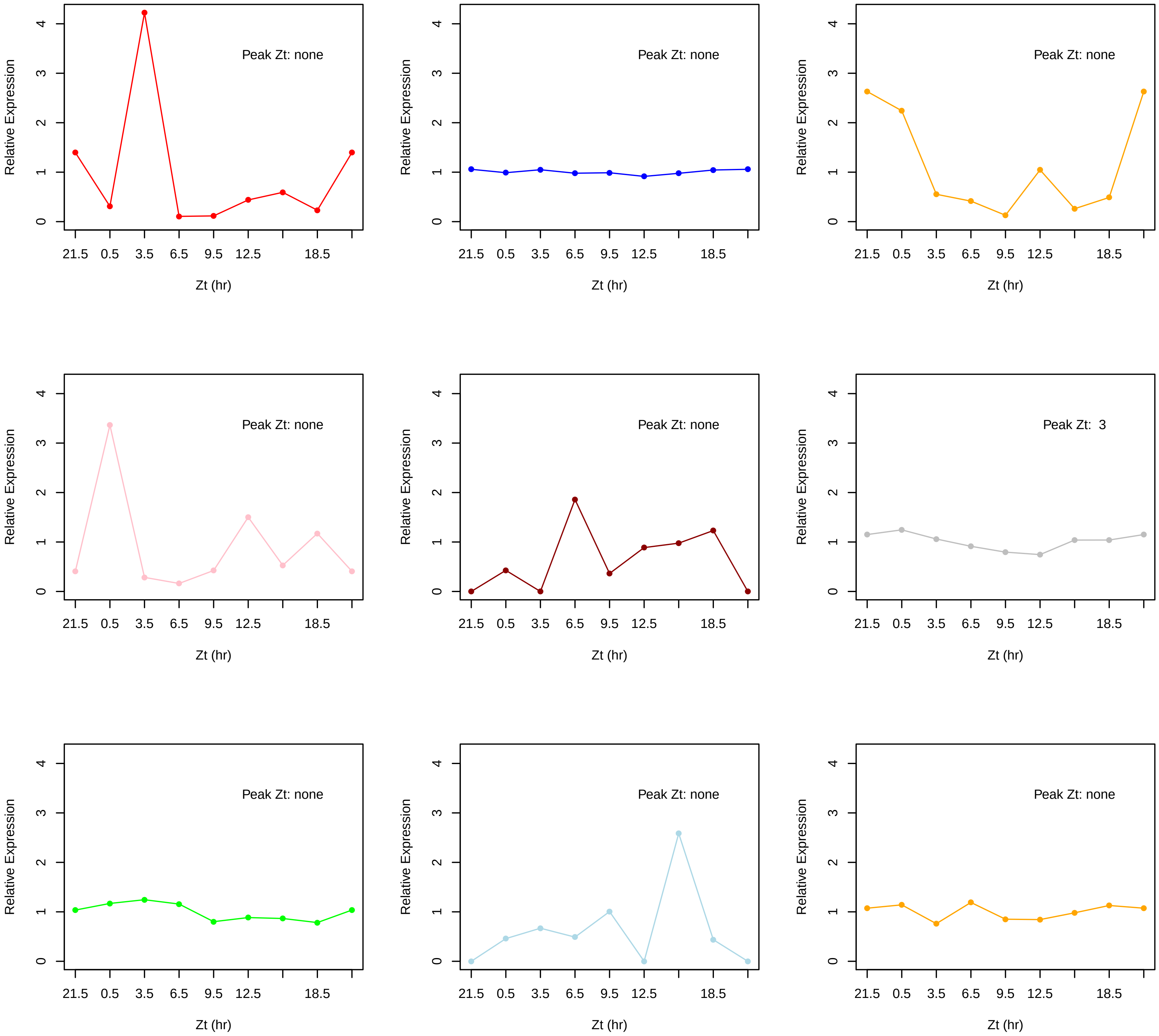

Singleton: ENSXMAG00000011685 ephb6

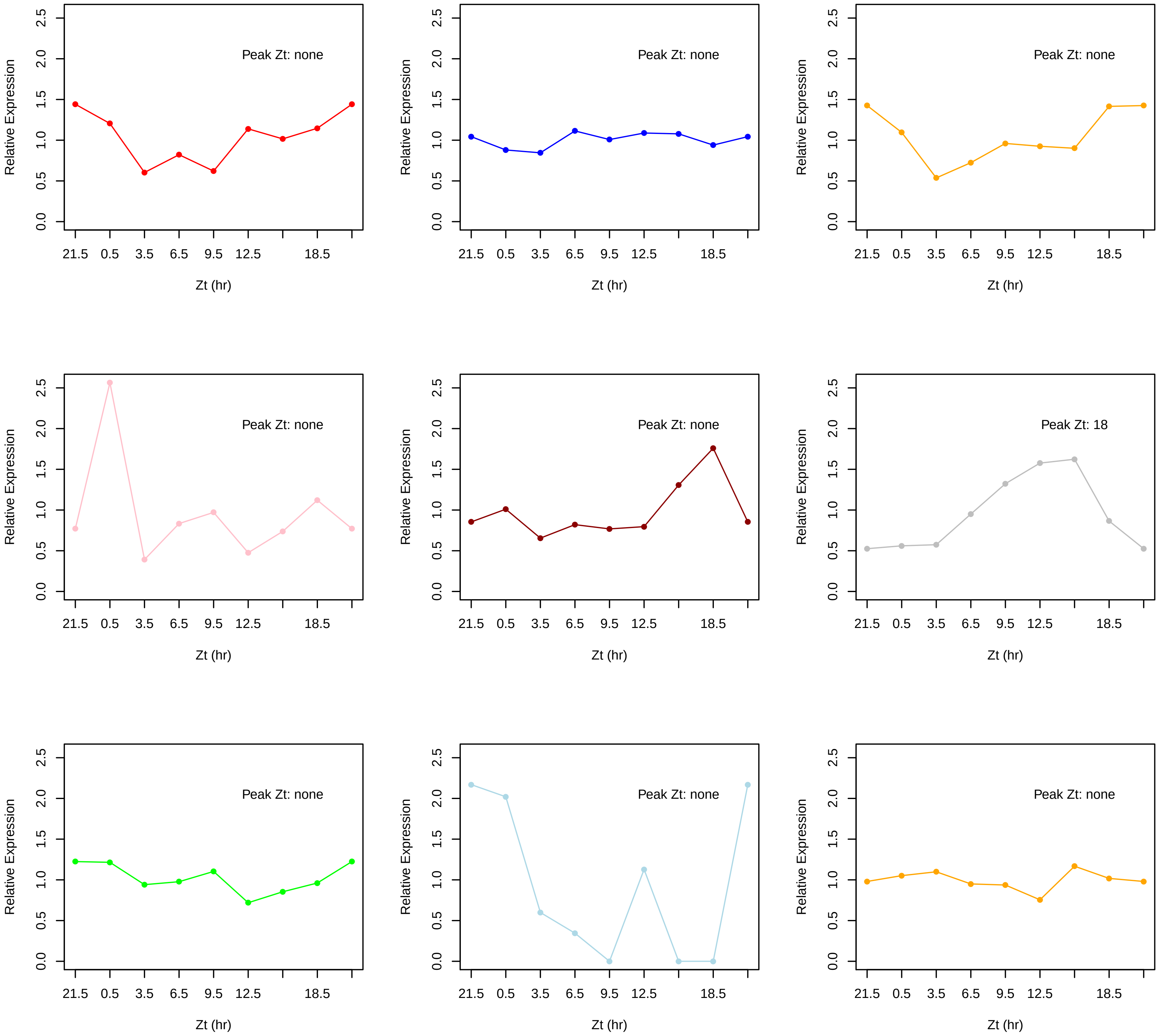

Singeleton: ENSXMAG00000009988 ankrd13d

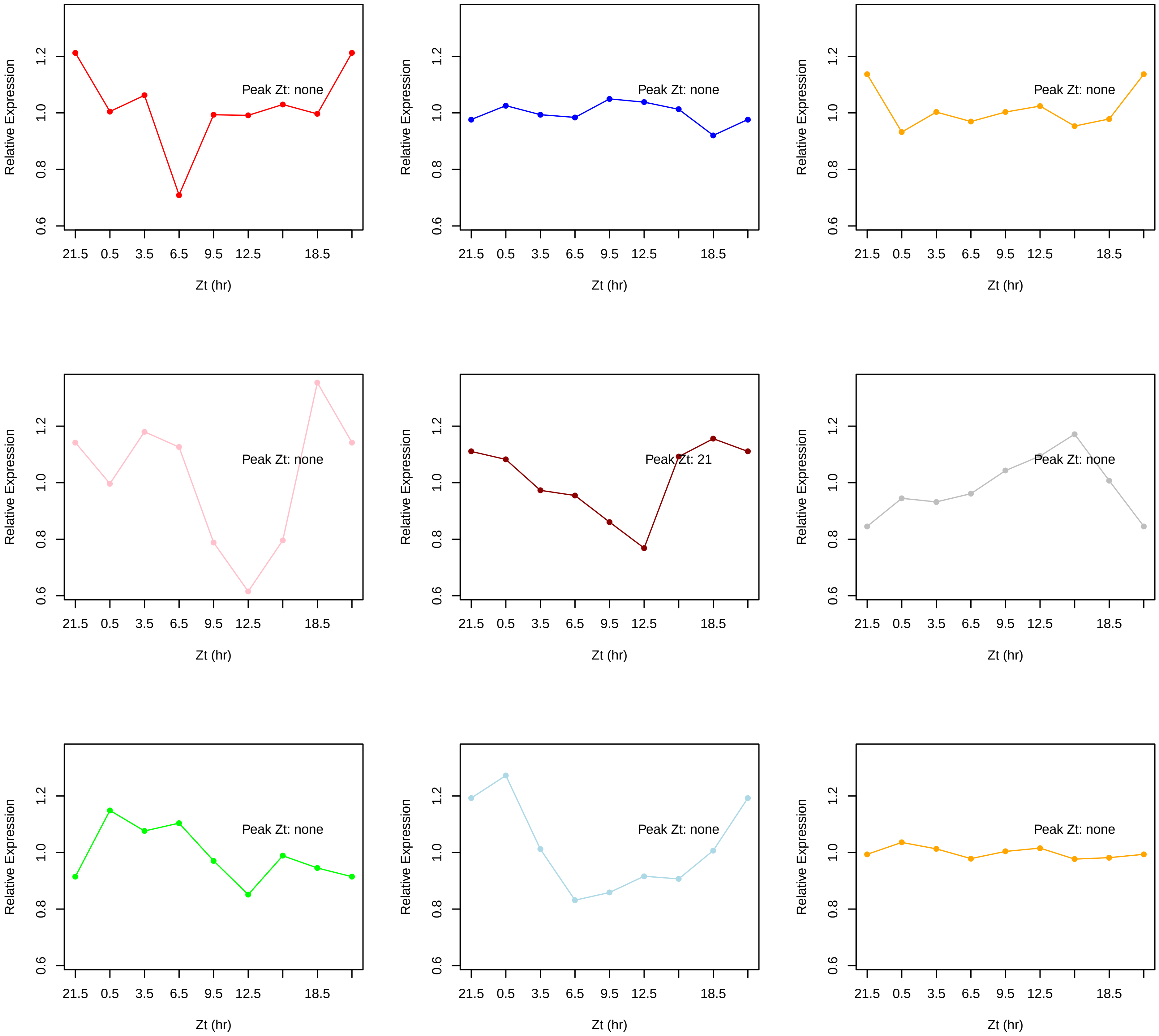

Singeleton: ENSXMAG00000010815 sphkap

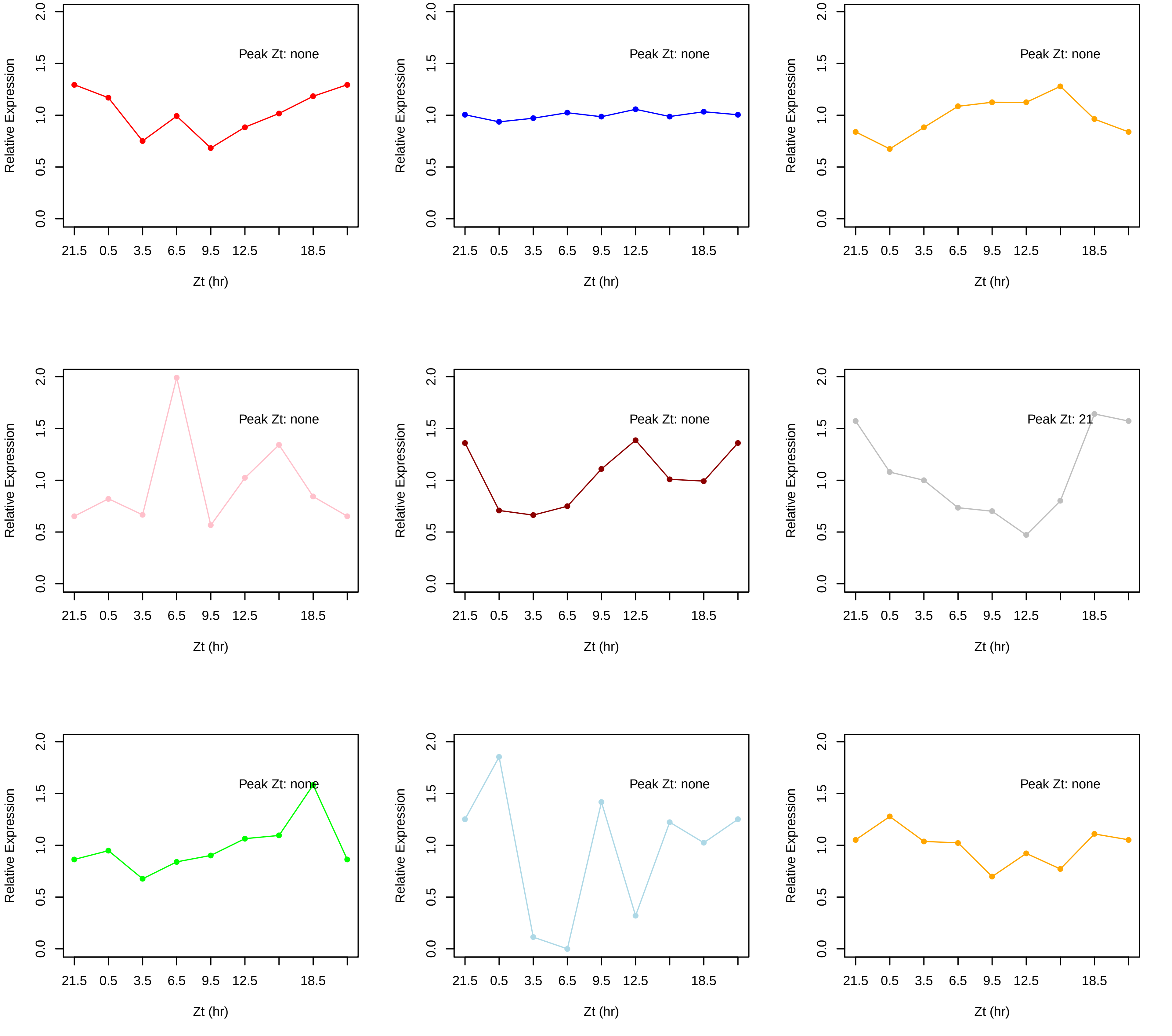

Singeleton: ENSXMAG00000015752 eef2k

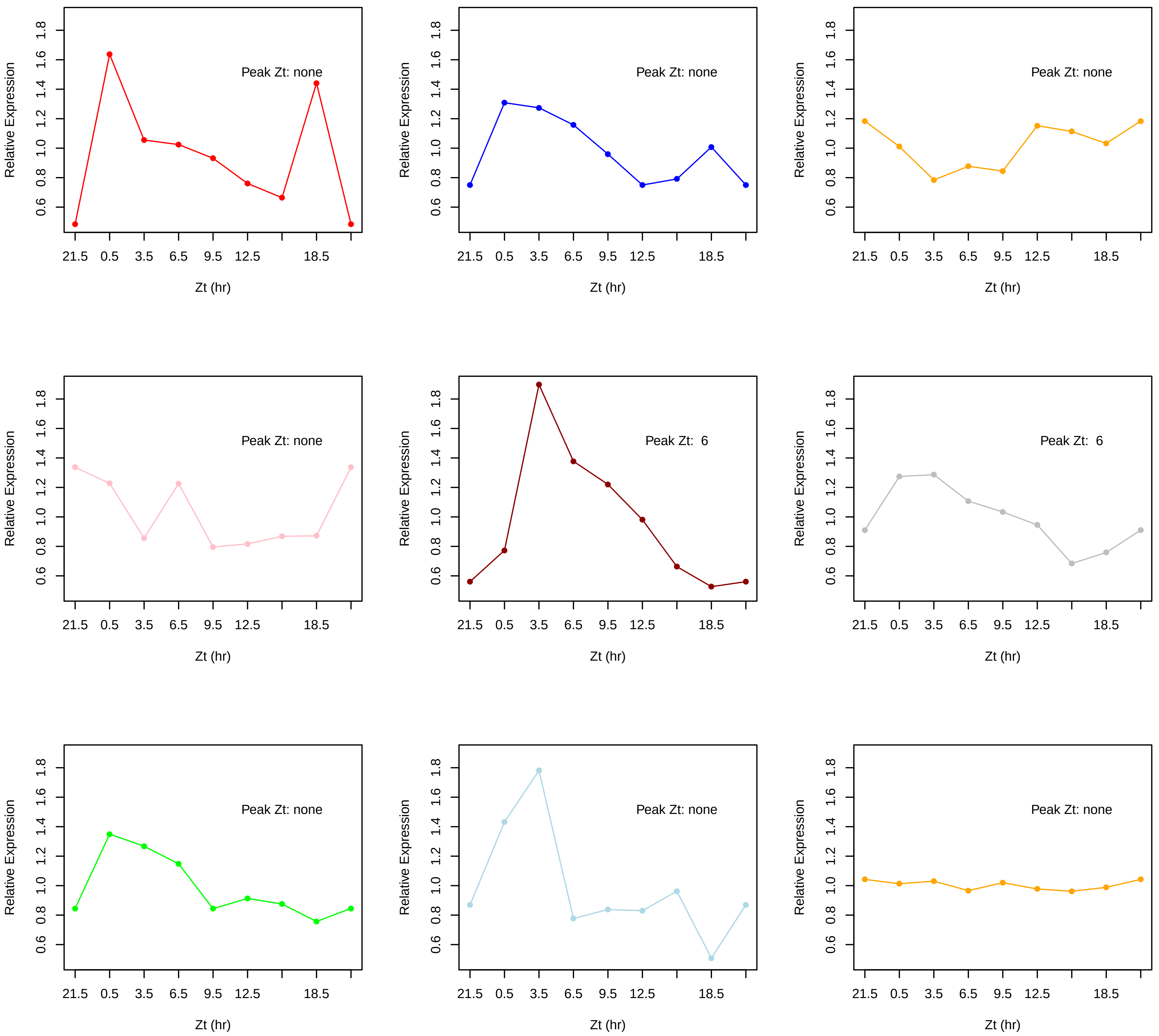

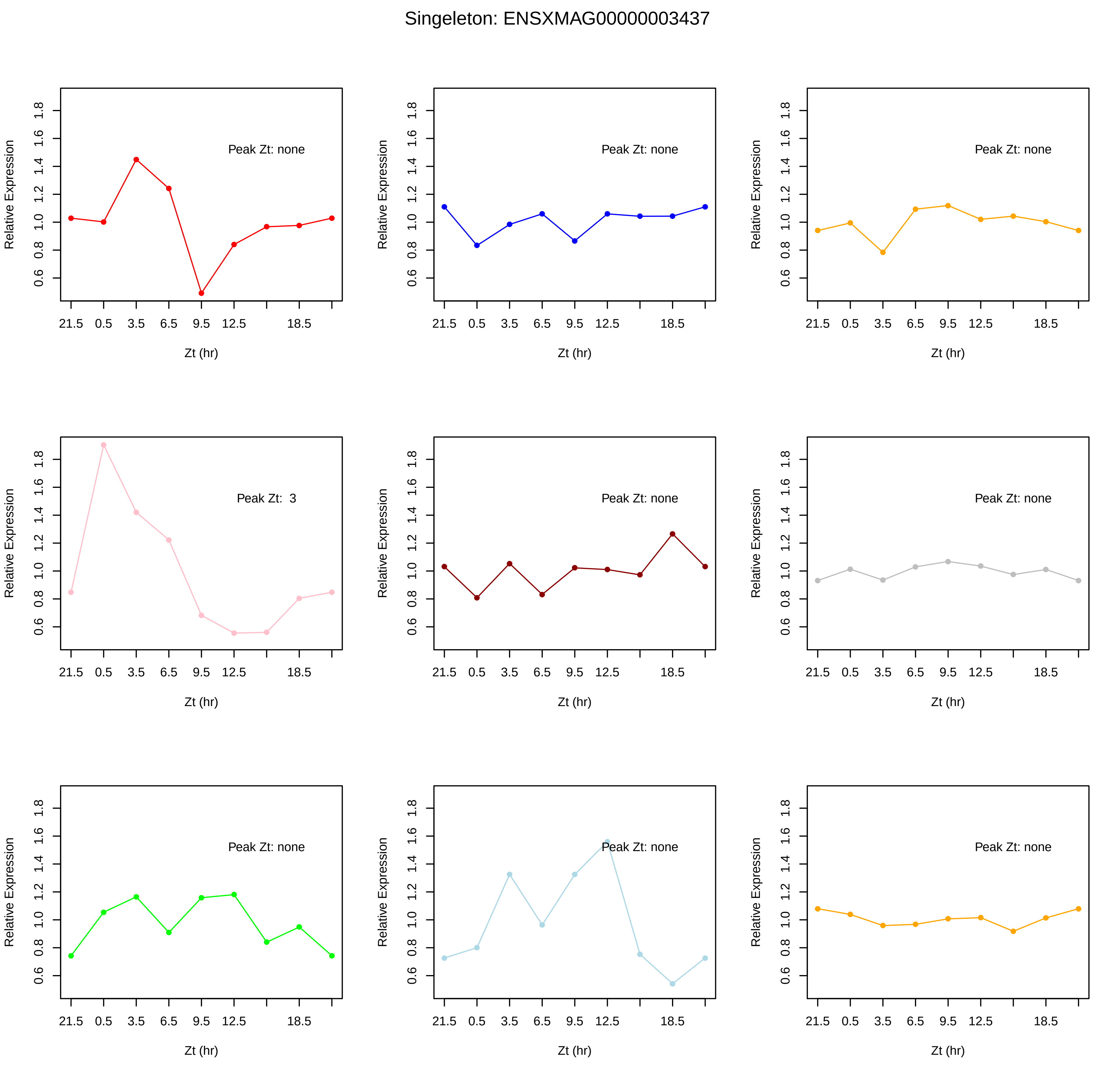
Singeleton: ENSXMAG00000003437
Peak Zt: none
Peak Zt: none
Peak Zt: none
Peak Zt: 3
Peak Zt: none
Peak Zt: none
Peak Zt: none
Peak Zt: none
Peak Zt: none
Relative Expression
Zt (hr)
21.5
0.5
3.5
6.5
9.5
12.5
18.5
0.6
0.8
1.0
1.2
1.4
1.6
1.8

Singleton: ENSXMAG00000018169 lyrm7

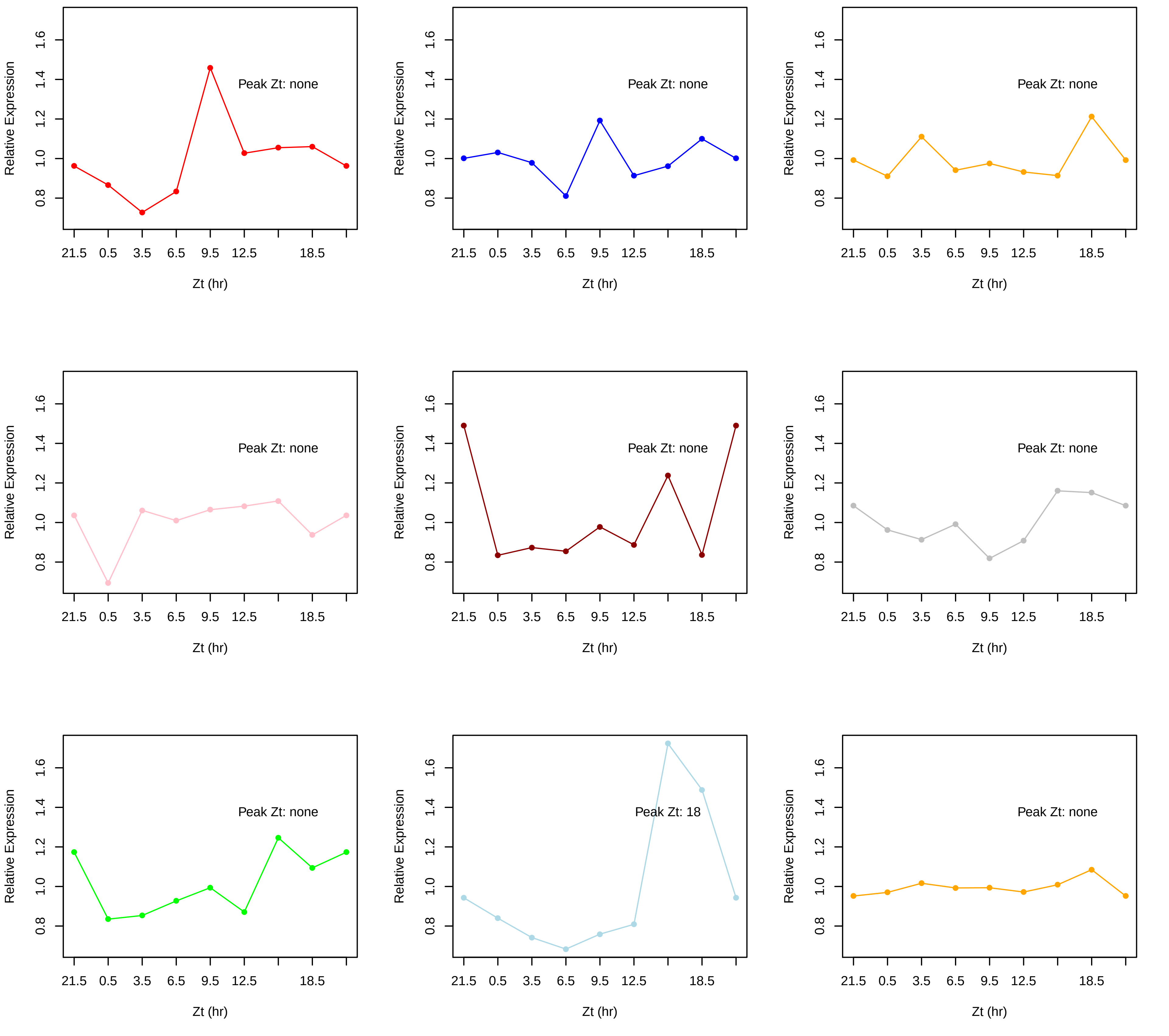

Singeleton: ENSXMAG00000011241 snx7

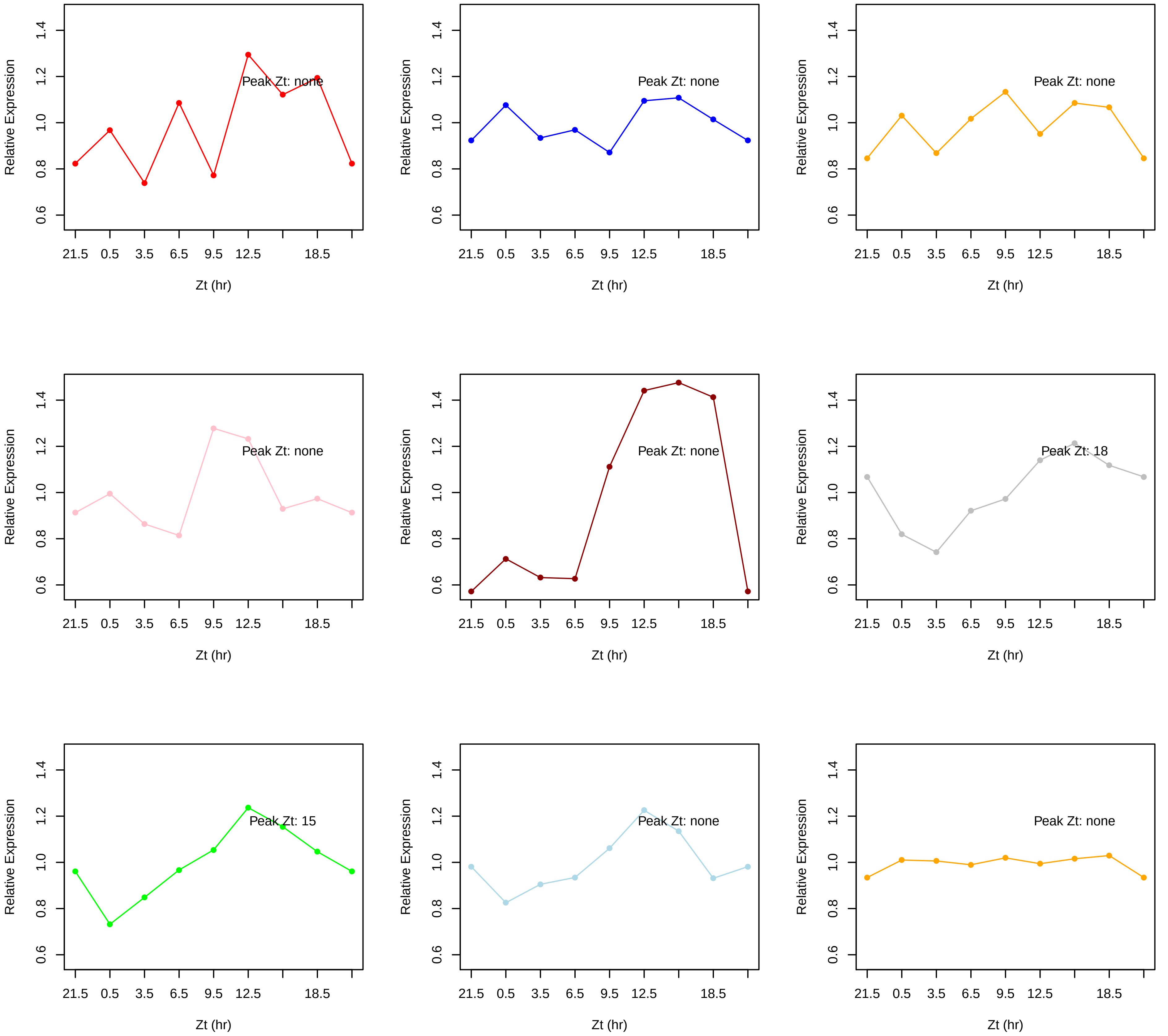

Singeleton: ENSXMAG00000013575 ndufaf6

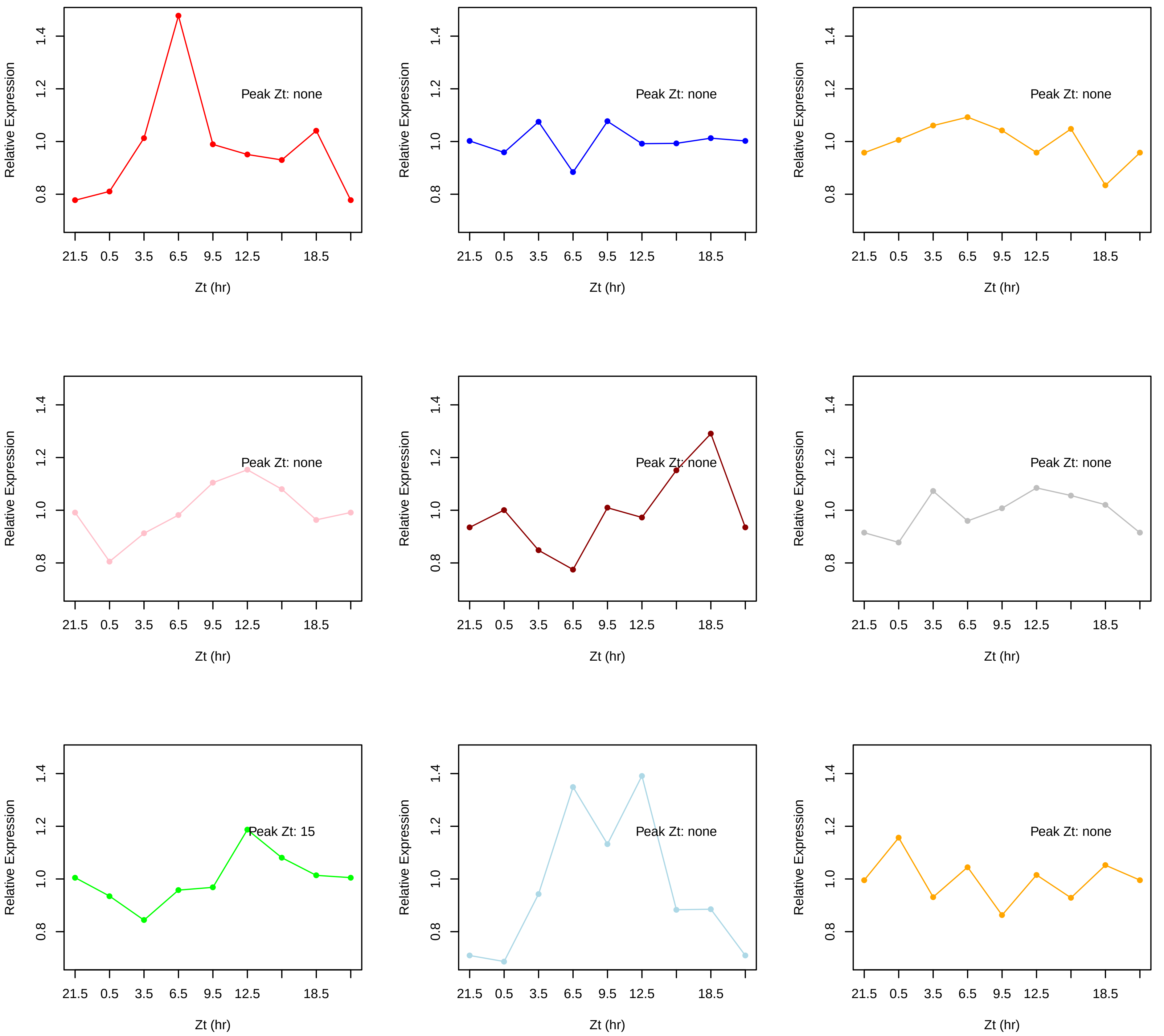

Singeleton: ENSXMAG00000016171 oca2

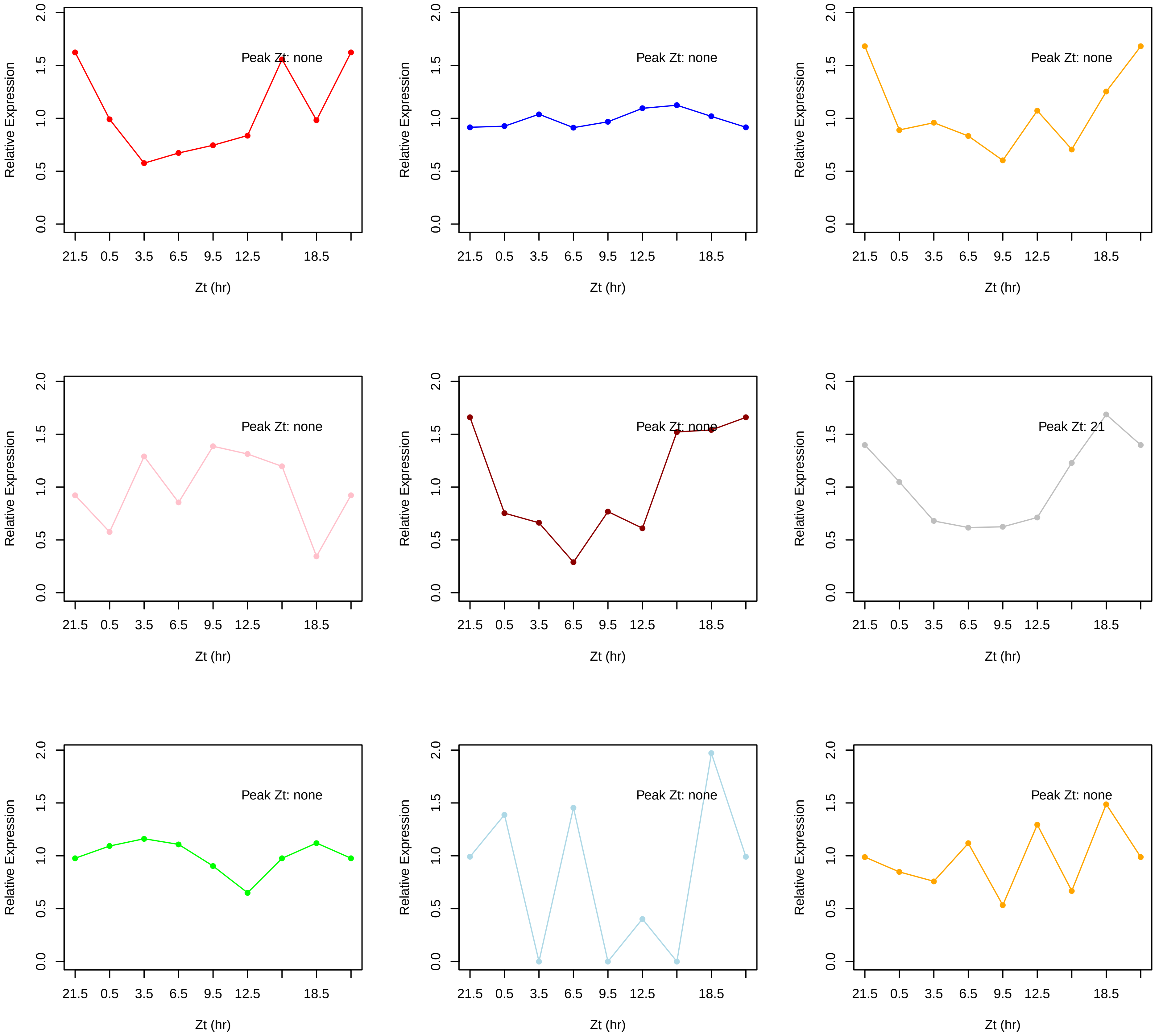

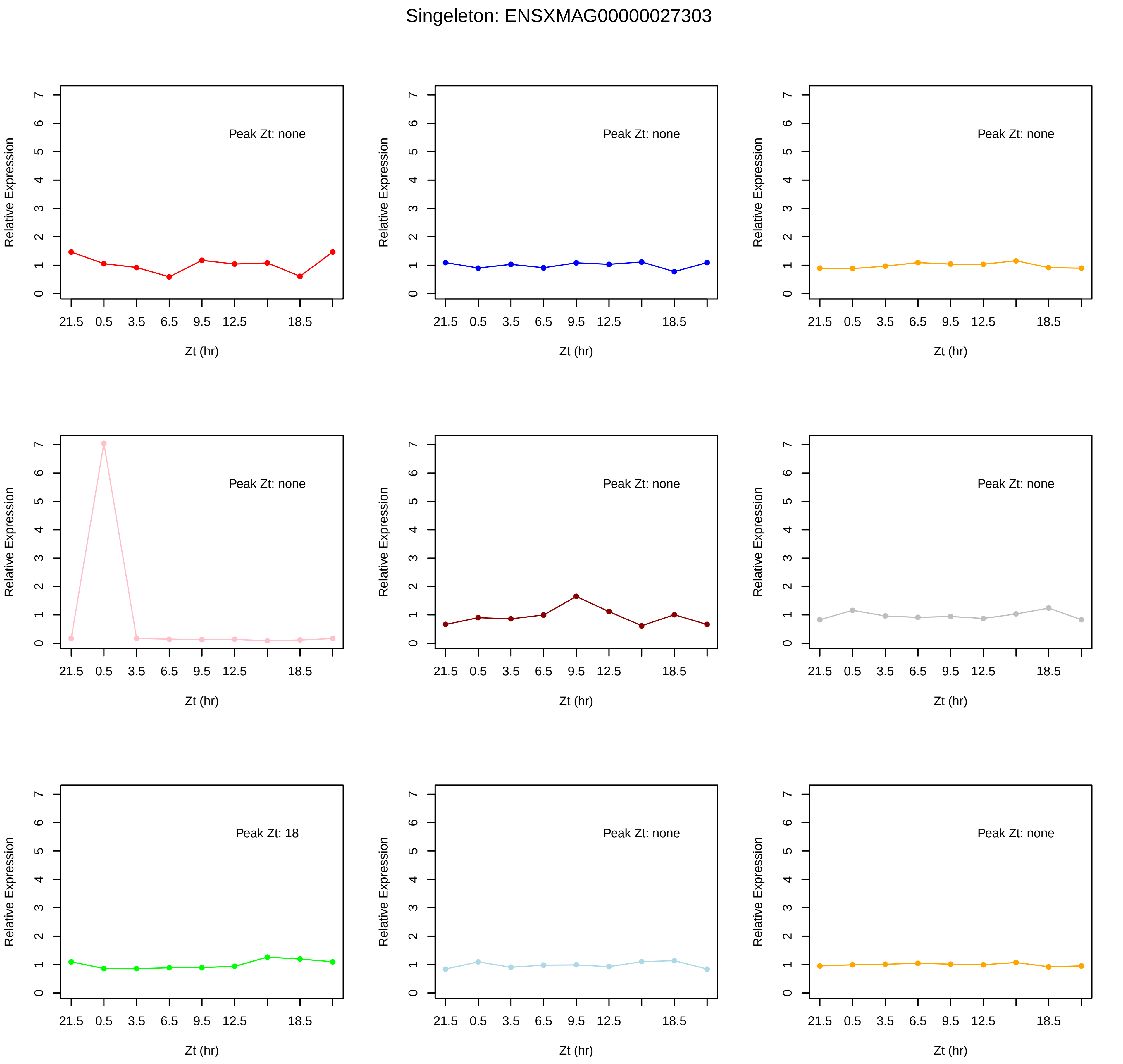
Singleton: ENSXMAG00000027303
Peak Zt: none
Peak Zt: none
Peak Zt: none
Peak Zt: none
Peak Zt: none
Peak Zt: none
Peak Zt: 18
Peak Zt: none
Peak Zt: none
Relative Expression
Zt (hr)
21.5
0.5
3.5
6.5
9.5
12.5
18.5
0
1
2
3
4
5
6
7

Singeleton: ENSXMAG00000028582 CSRNP3

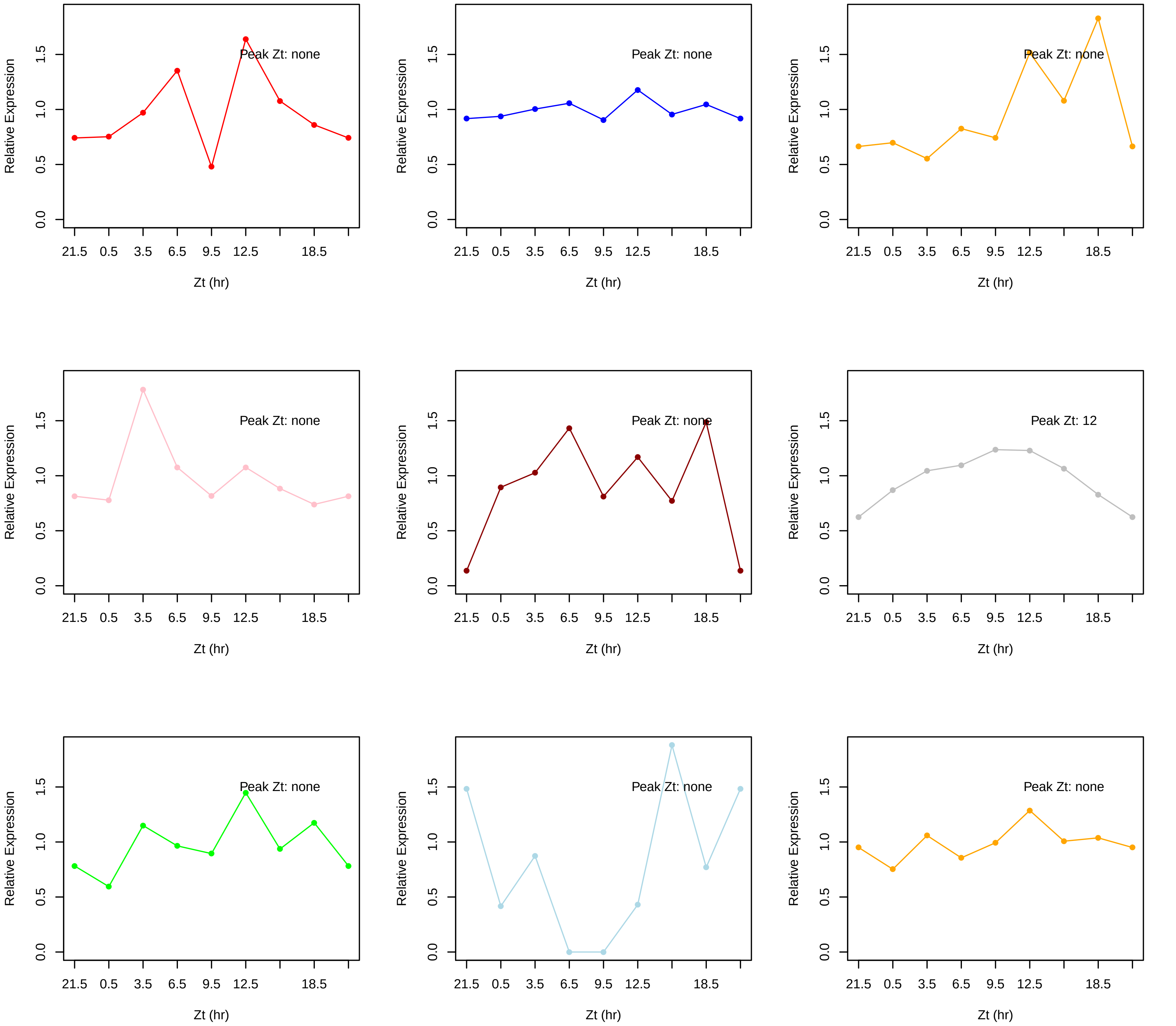

Singeleton: ENSXMAG00000020843

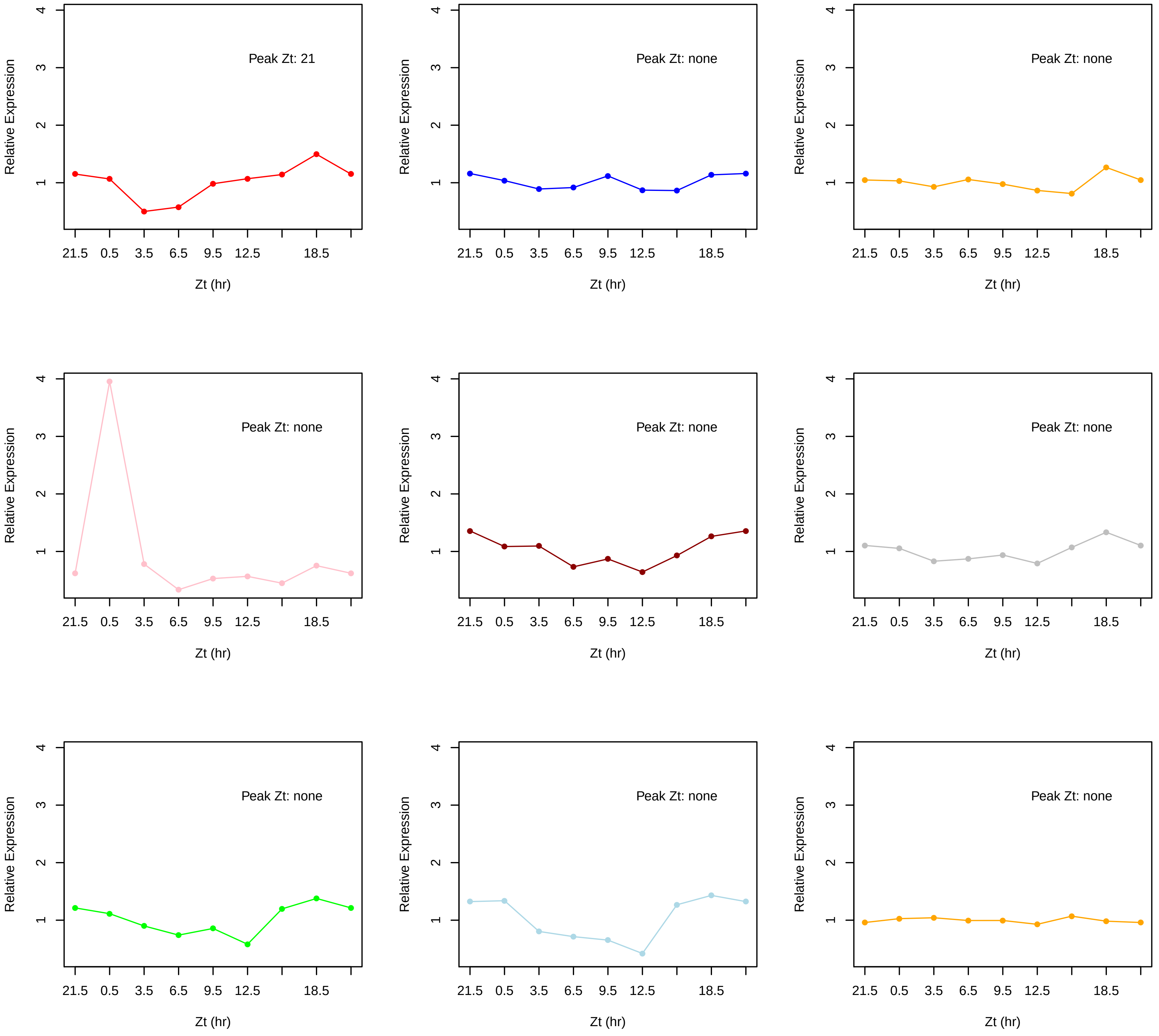

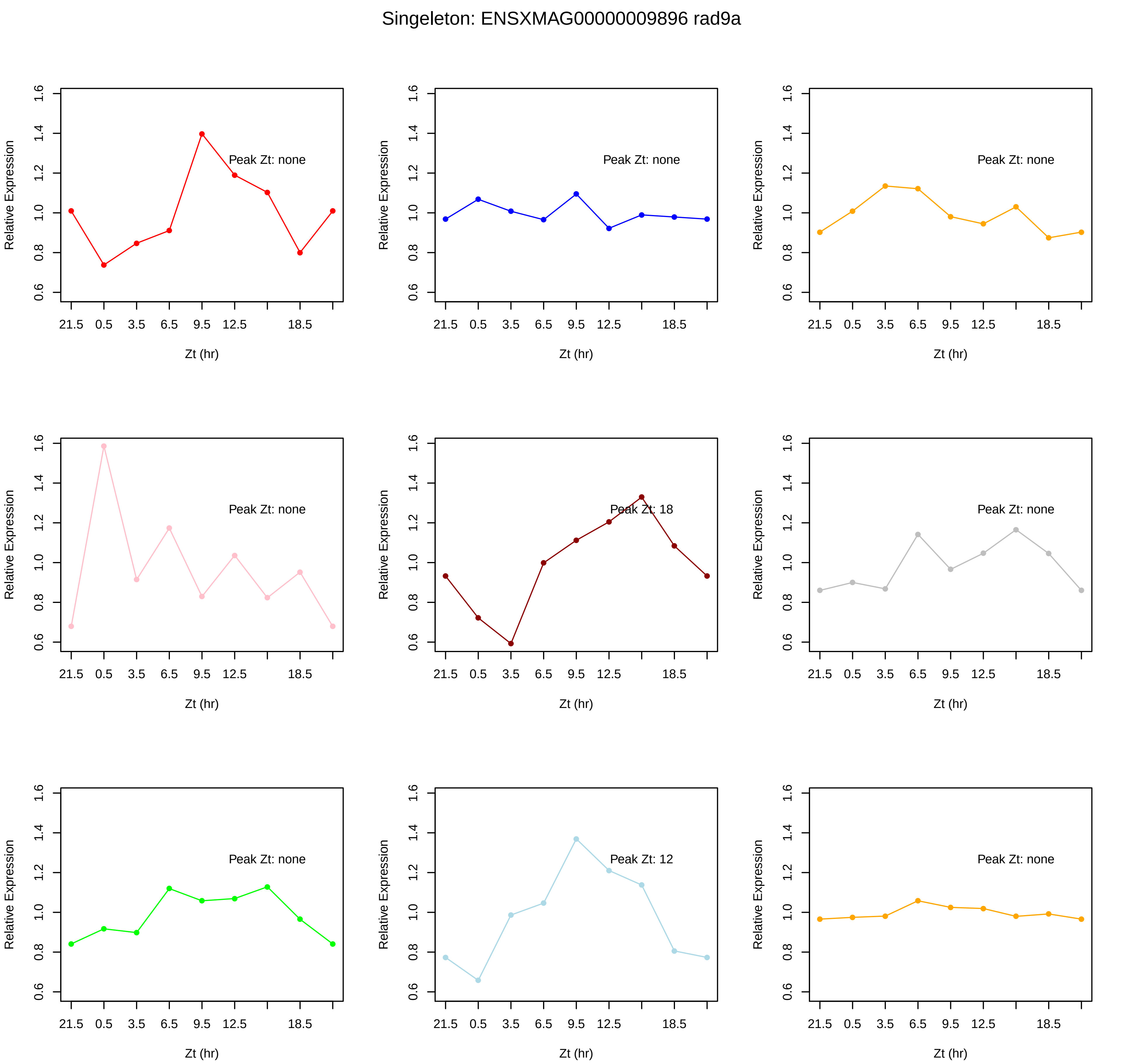

Singleton: ENSXMAG00000009896 rad9a
Peak Zt: none
Peak Zt: none
Peak Zt: none
Peak Zt: none
Peak Zt: 18
Peak Zt: none
Peak Zt: none
Peak Zt: 12
Peak Zt: none
Relative Expression
Zt (hr)
21.5
0.5
3.5
6.5
9.5
12.5
18.5
0.6
0.8
1.0
1.2
1.4
1.6

Singeleton: ENSXMAG00000011705 GALNT3

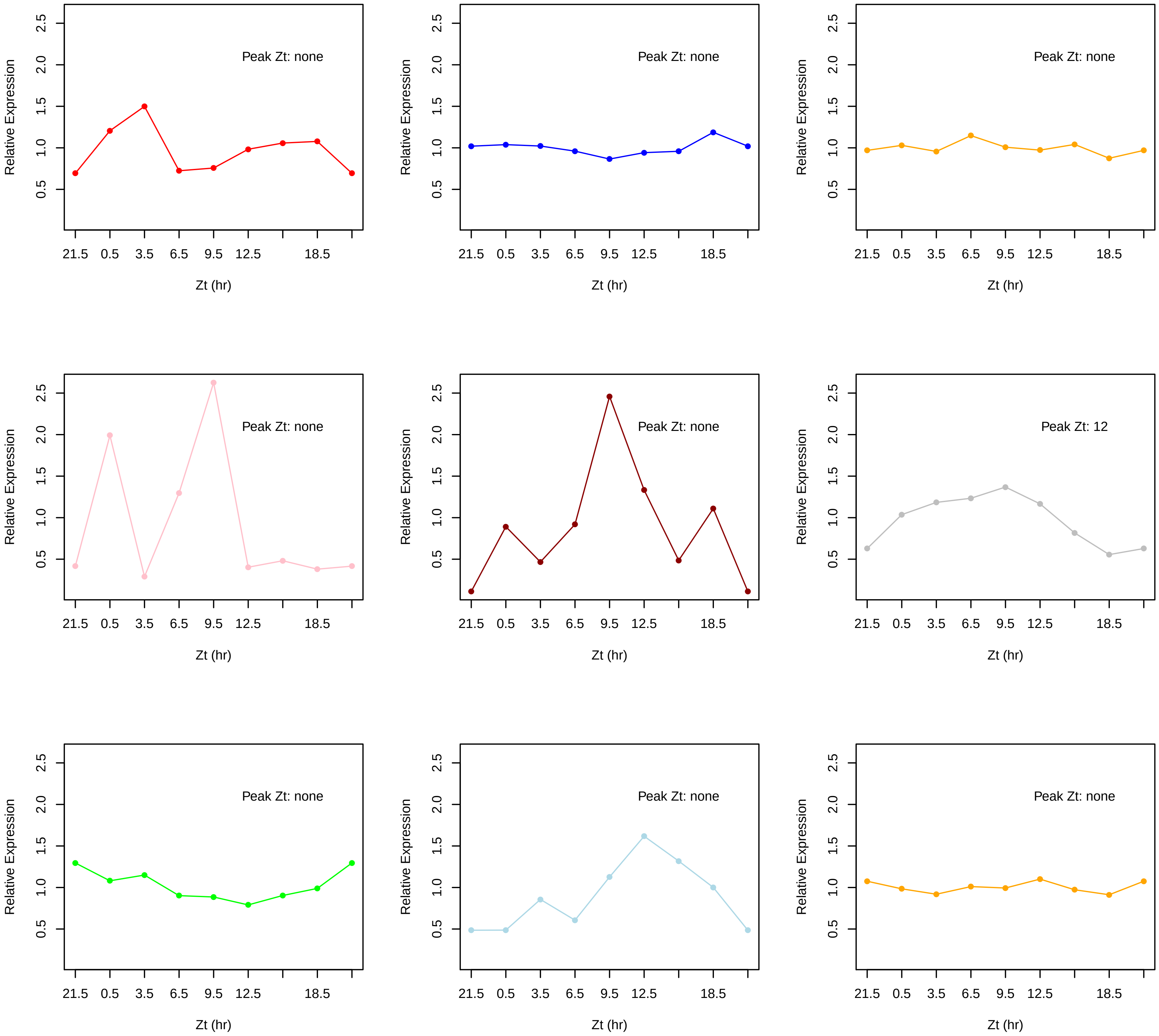

Singeleton: ENSXMAG00000003628 mylipa

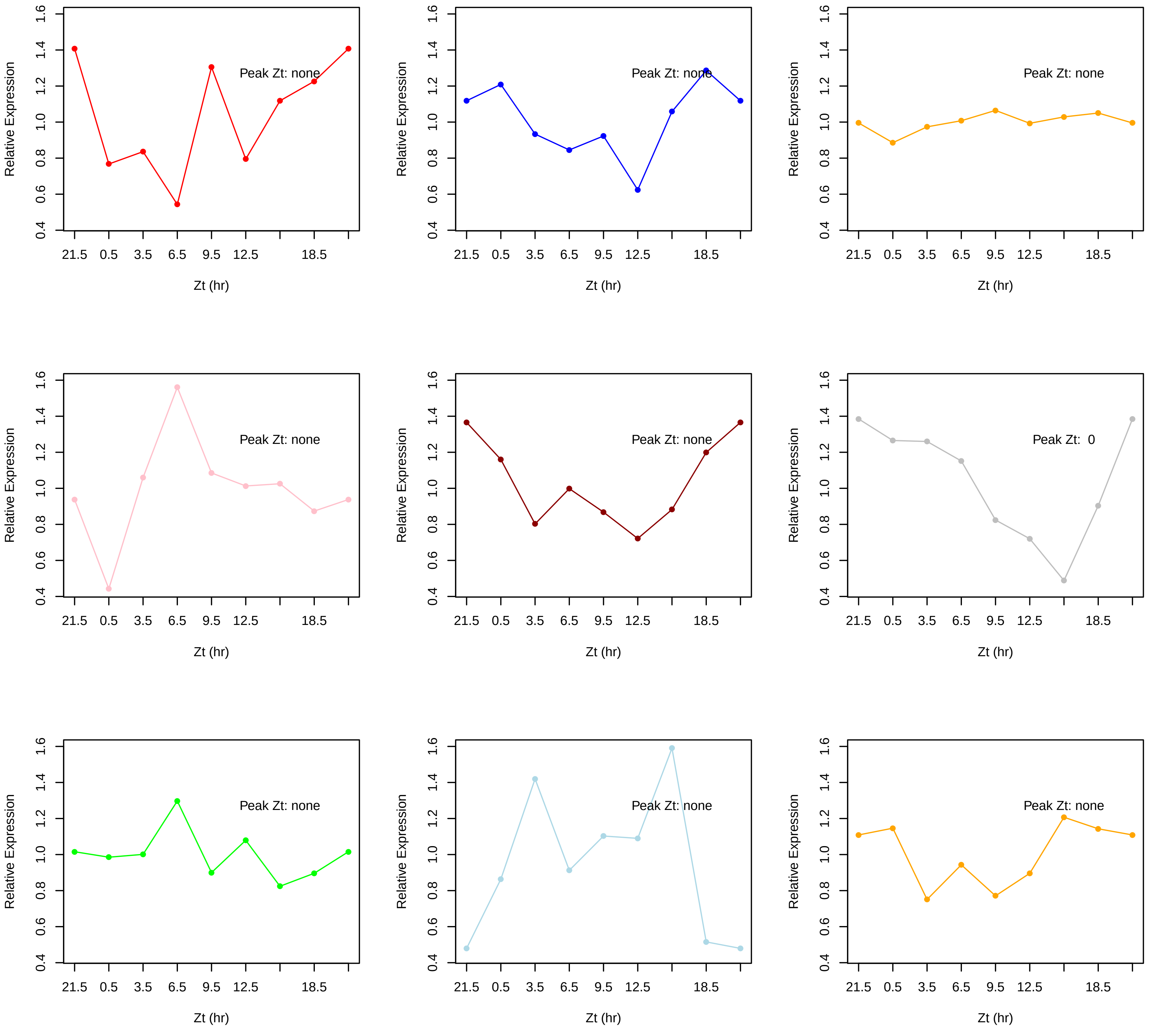

Singeleton: ENSXMAG00000029275 zgc:171489

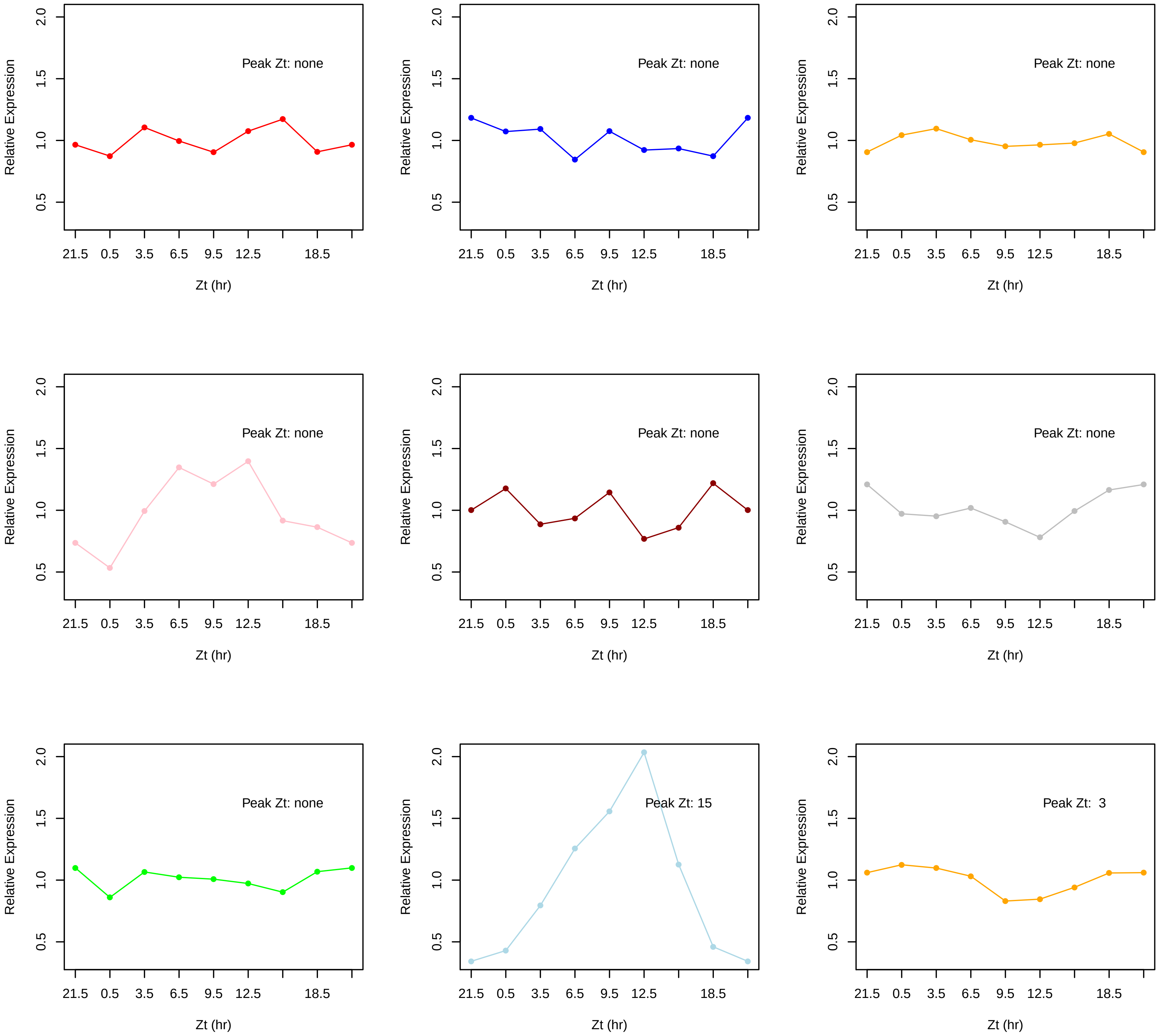

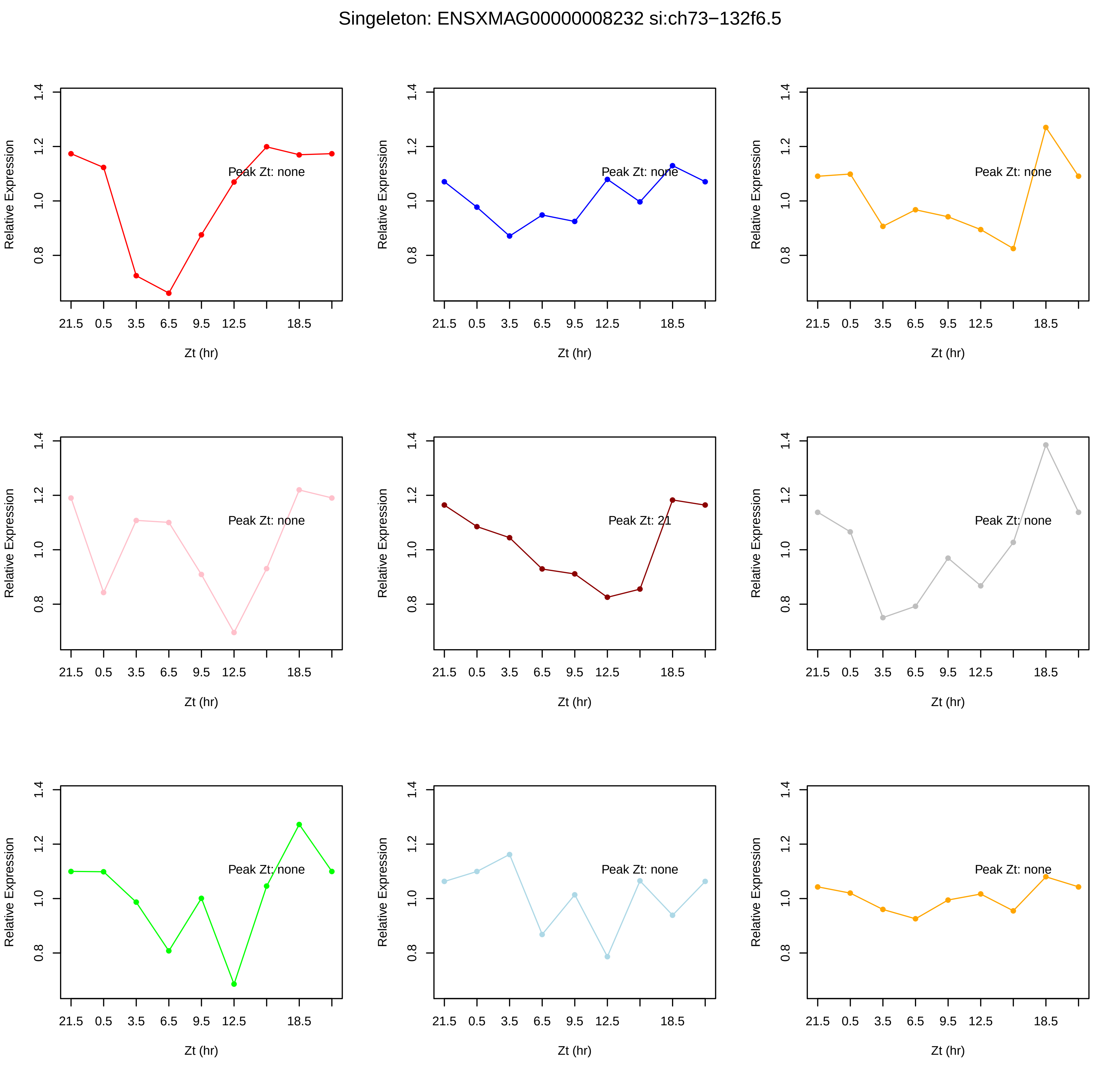

Singeleton: ENSXMAG00000008232 si:ch73−132f6.5
Peak Zt: none
Peak Zt: none
Peak Zt: none
Peak Zt: none
Peak Zt: 21
Peak Zt: none
Peak Zt: none
Peak Zt: none
Peak Zt: none
Relative Expression
Zt (hr)
21.5 0.5 3.5 6.5 9.5 12.5 18.5
0.8 1.0 1.2 1.4

Singleton: ENSXMAG00000006981 trmt9b

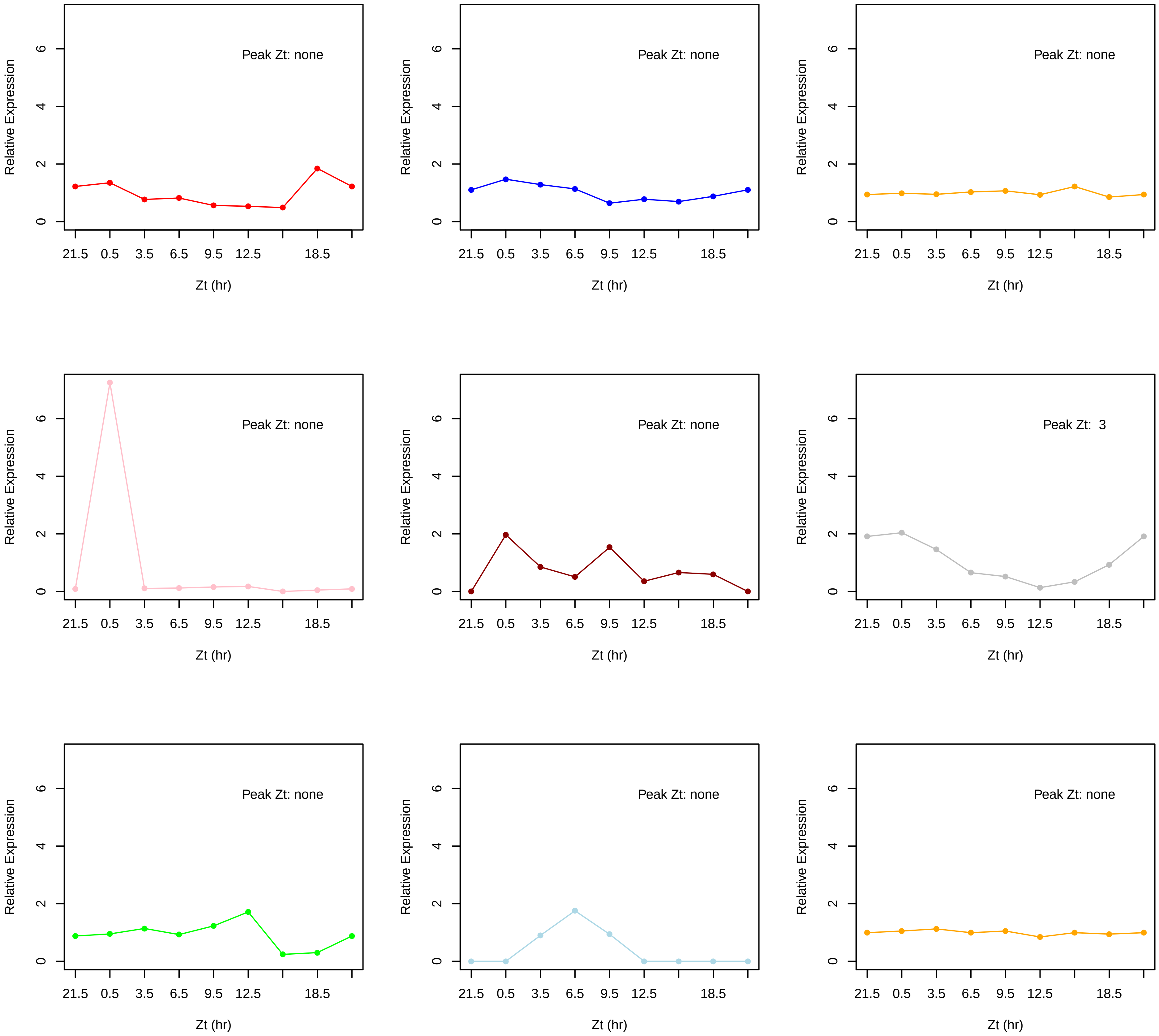

Singeleton: ENSXMAG00000021286 jarid2b

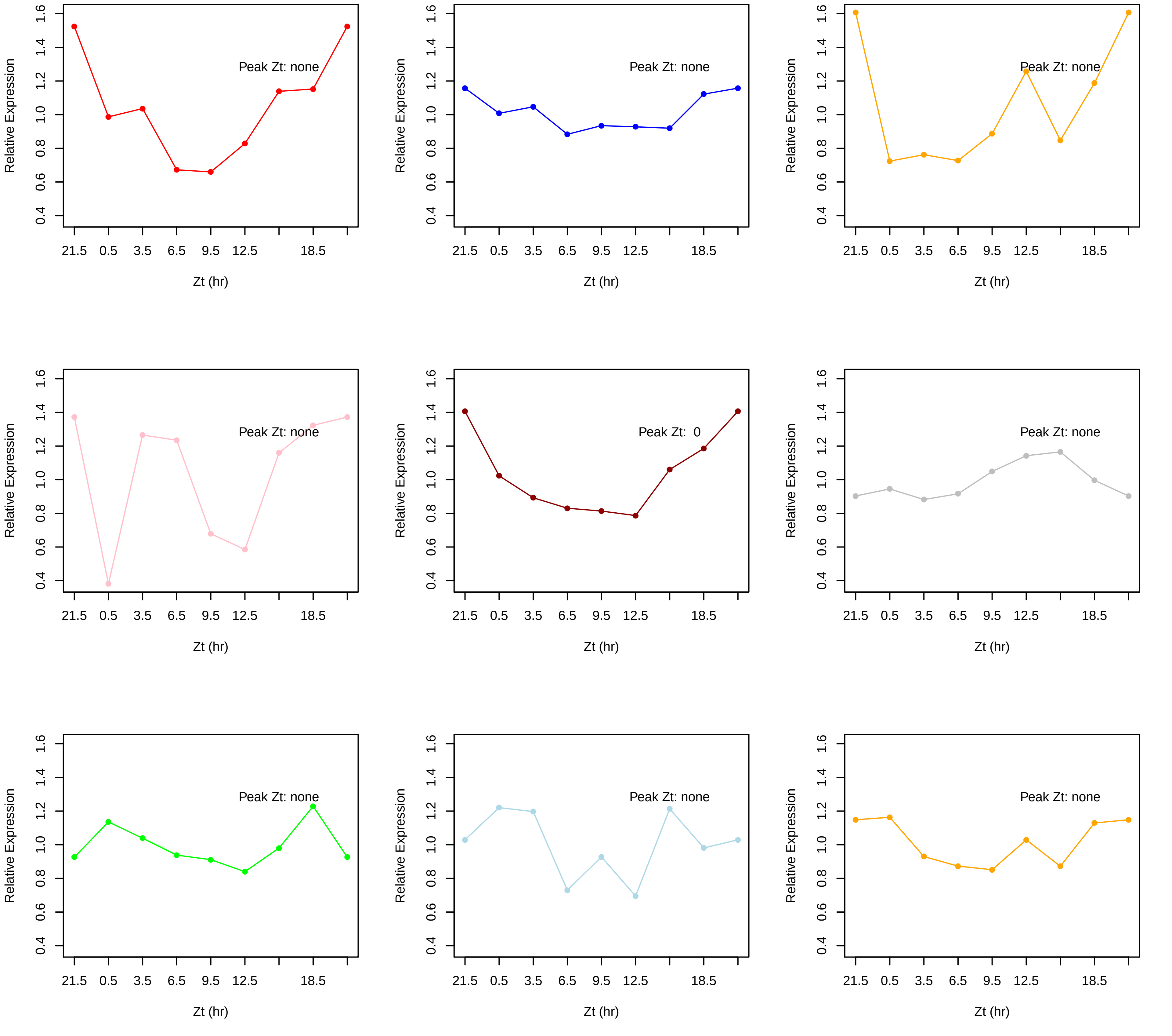

Singeleton: ENSXMAG00000026514 mettl9

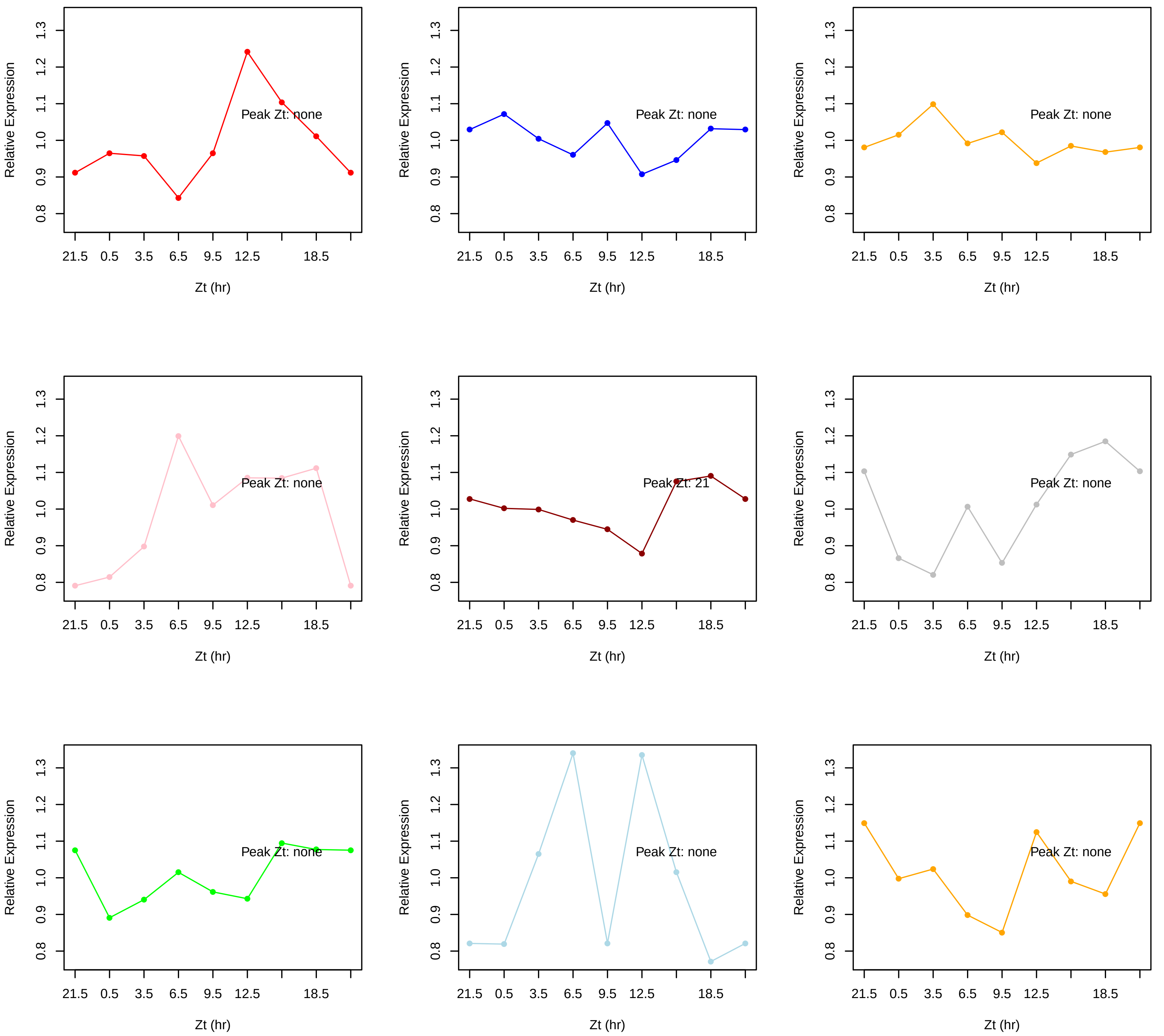

Singleton: ENSXMAG00000011652 zgc:92606

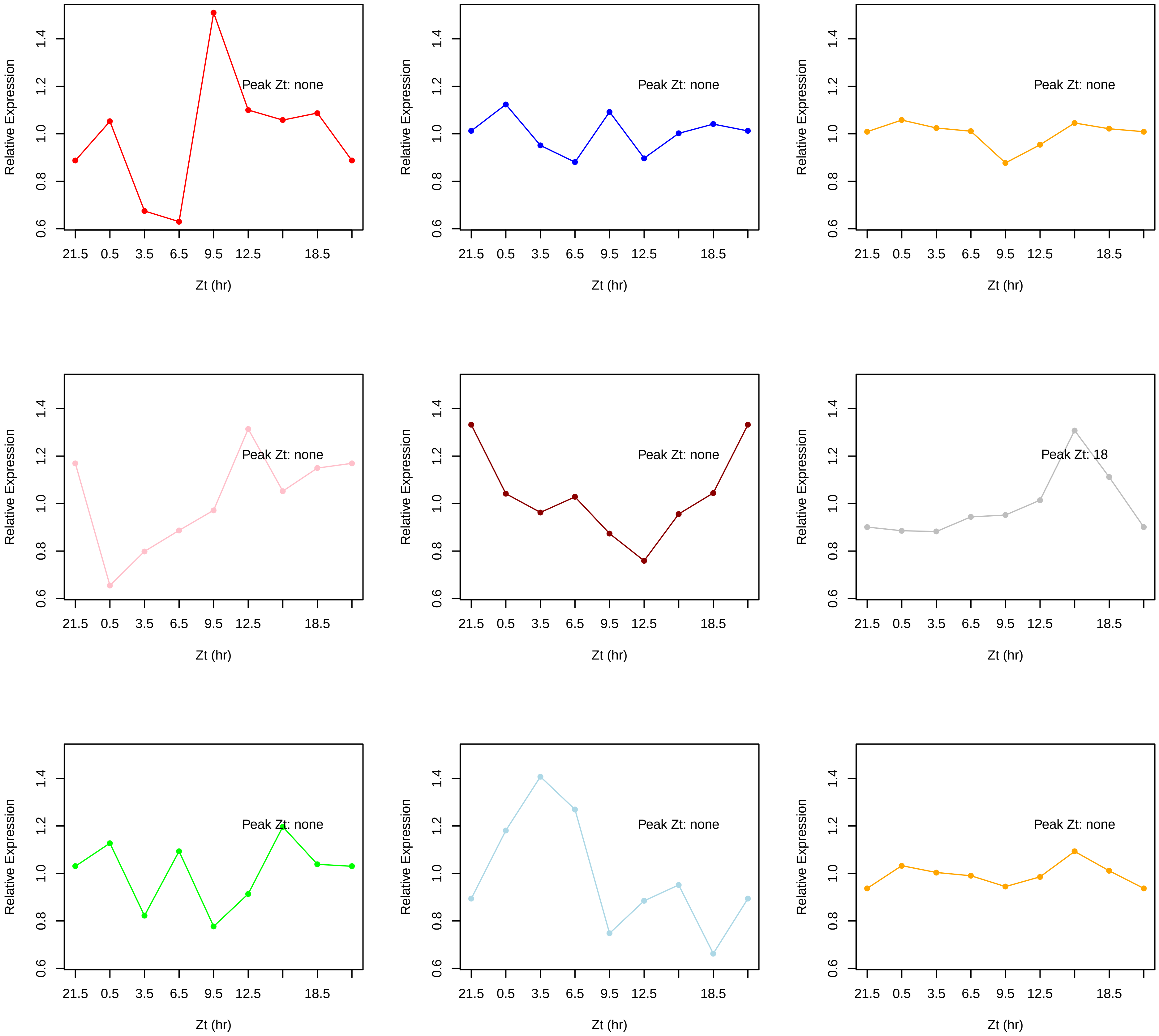

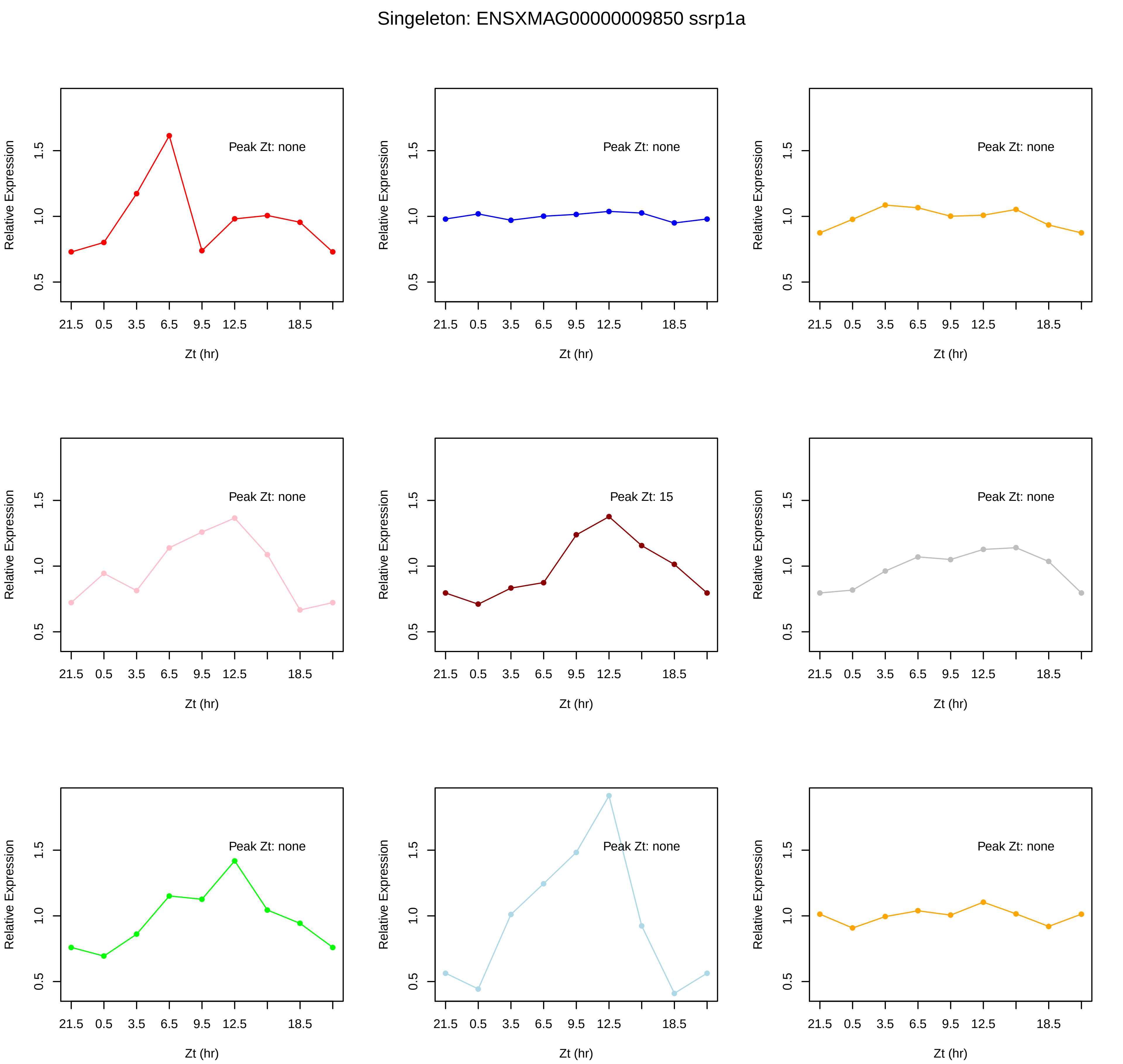
Singeleton: ENSXMAG00000009850 ssrp1a
Peak Zt: none
Peak Zt: none
Peak Zt: none
Peak Zt: none
Peak Zt: 15
Peak Zt: none
Peak Zt: none
Peak Zt: none
Peak Zt: none
Relative Expression
Zt (hr)

Singeleton: ENSXMAG00000013864 scn1lab

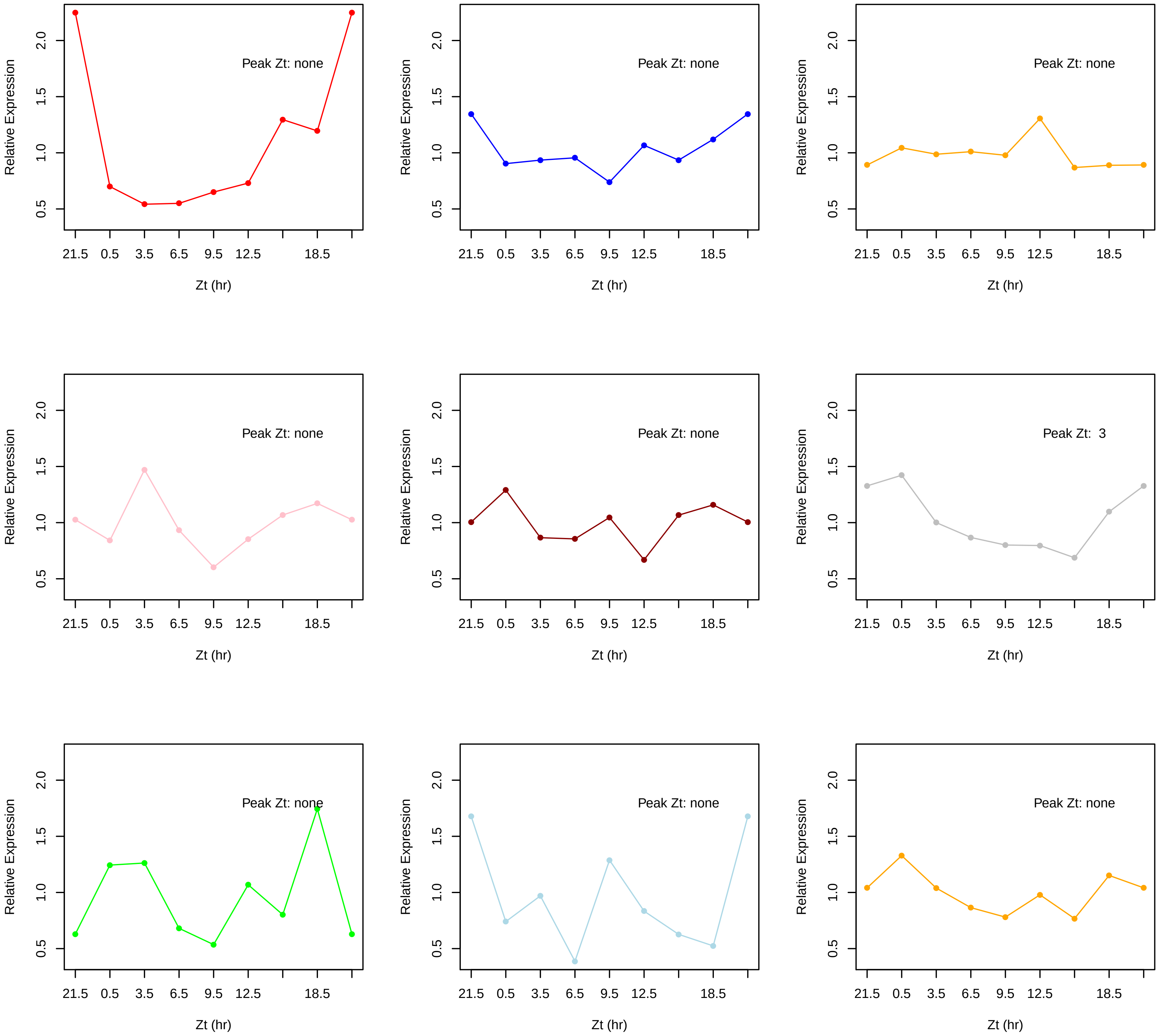

Singleton: ENSXMAG00000008234 lratb.1

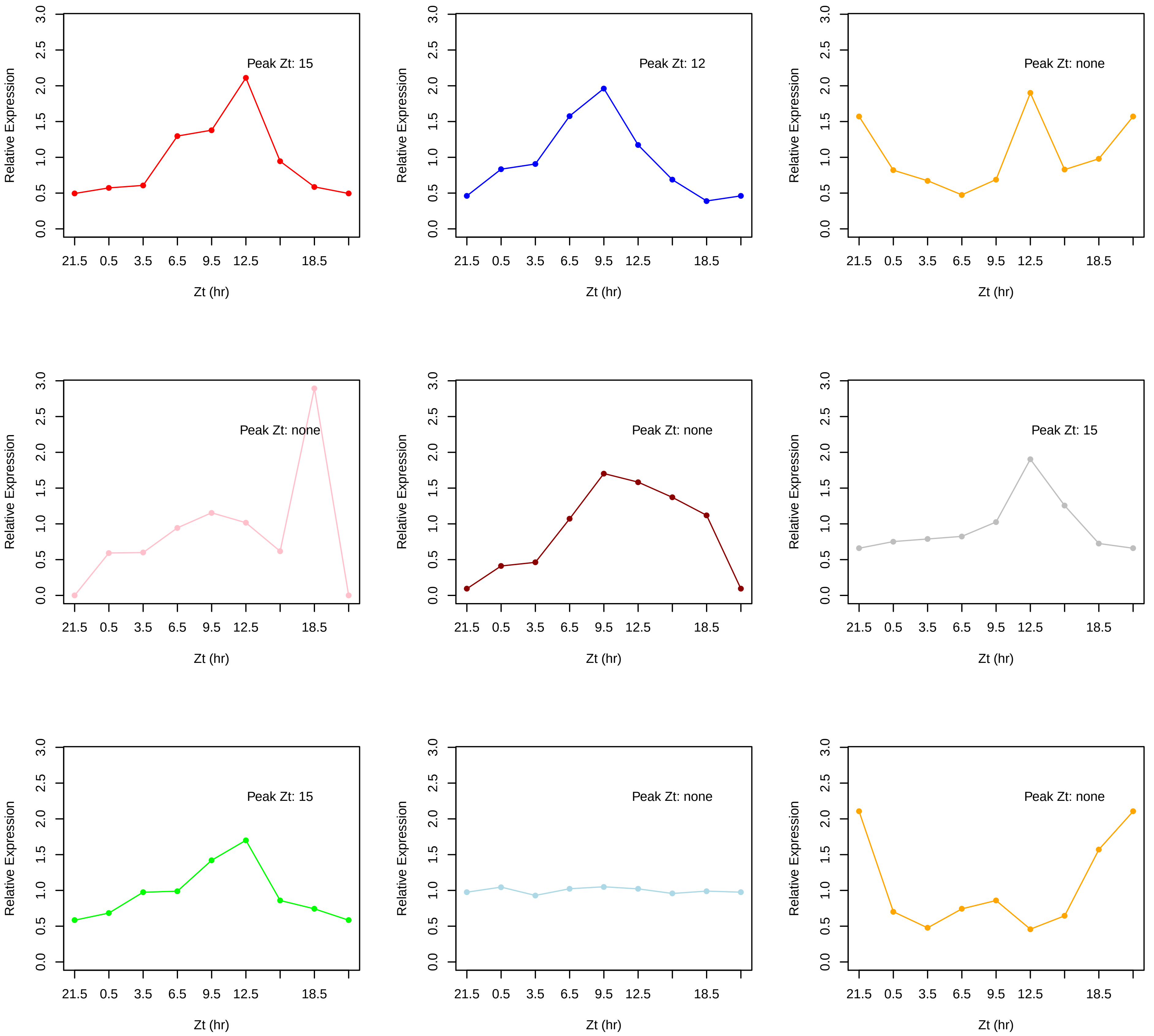

Singeleton: ENSXMAG00000011601 GCDH

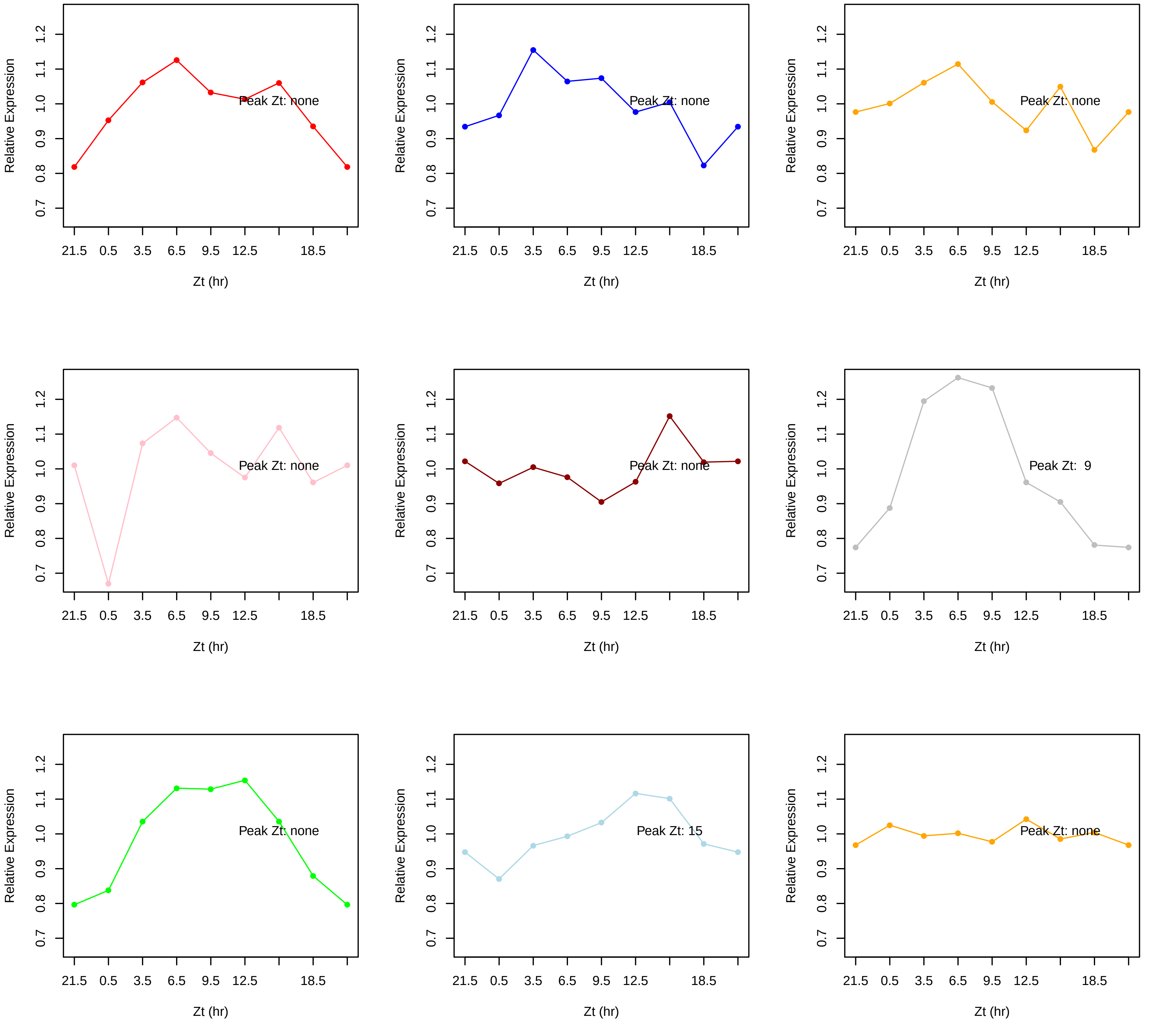

Singleton: ENSXMAG00000014027 mmp23bb

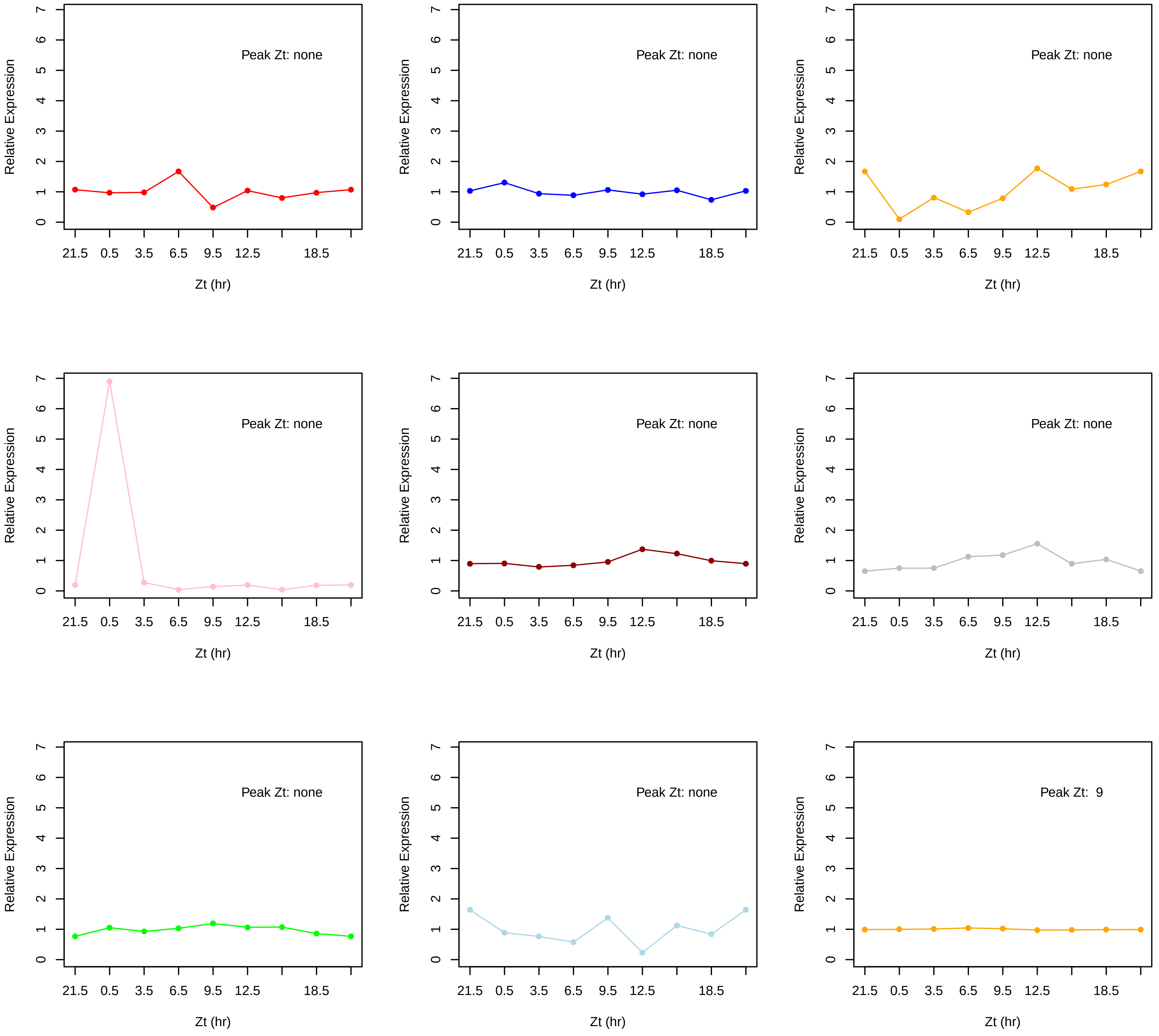

Singeleton: ENSXMAG00000016224 atp10a

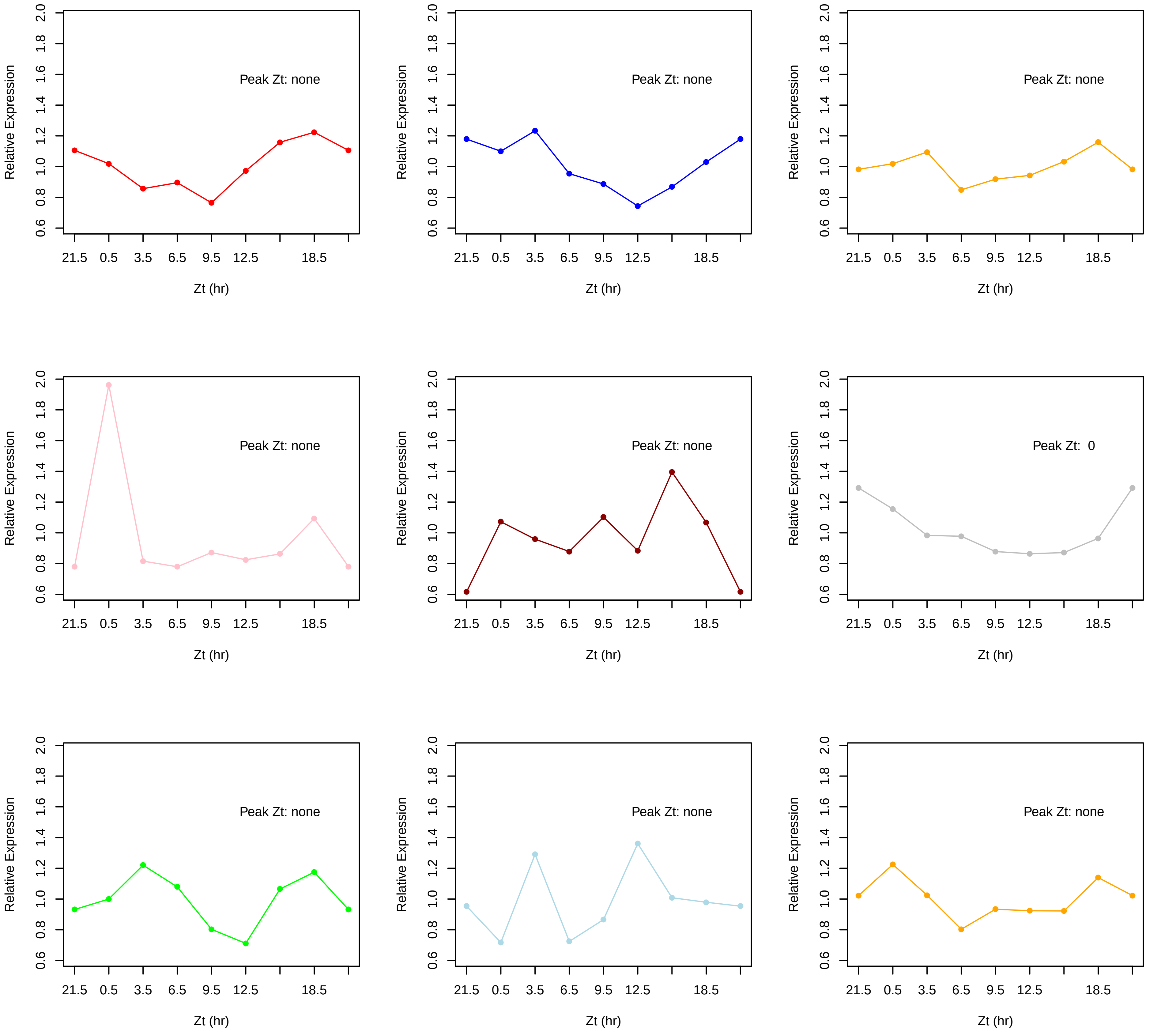

Singleton: ENSXMAG00000013484 paqr6

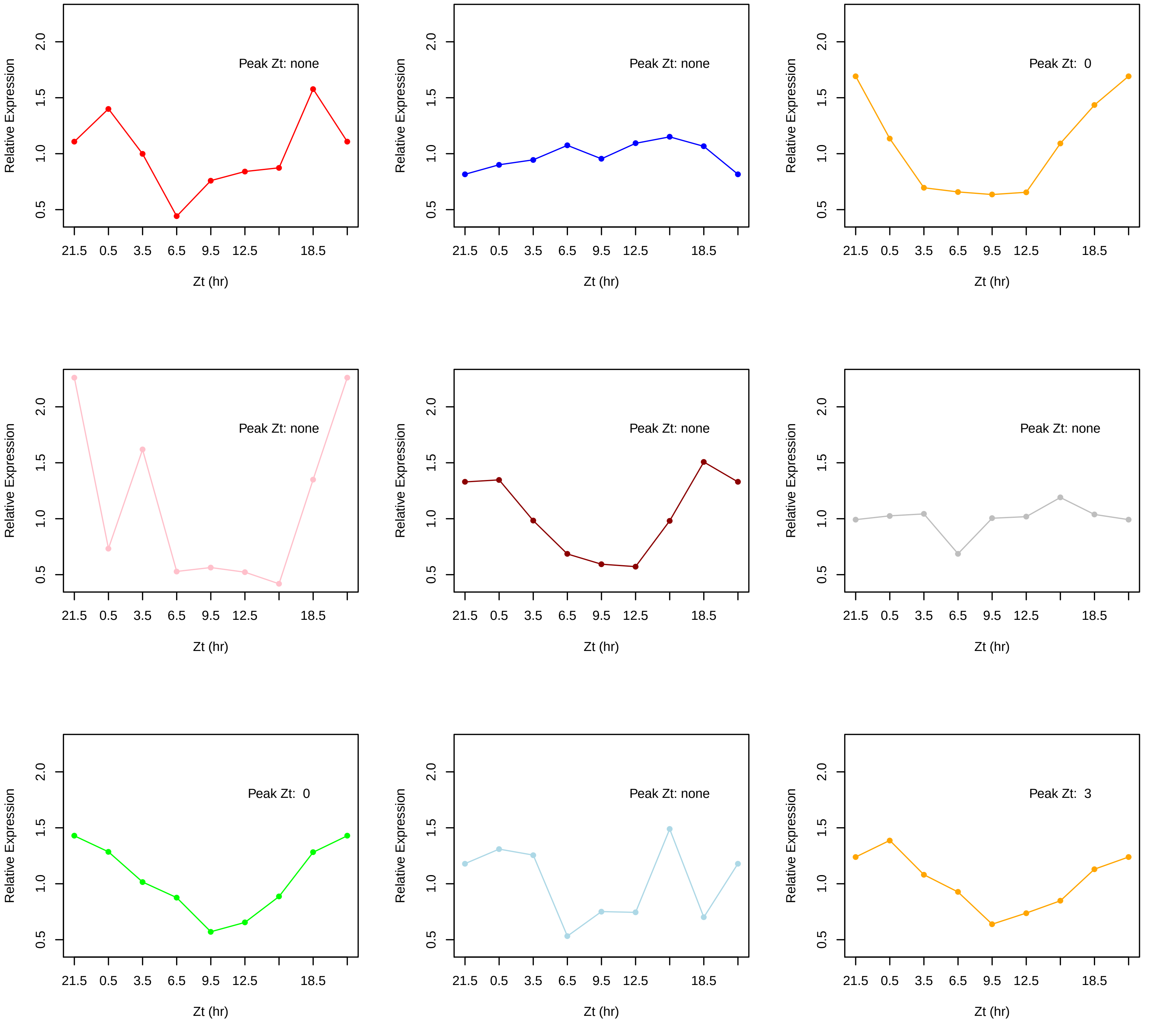

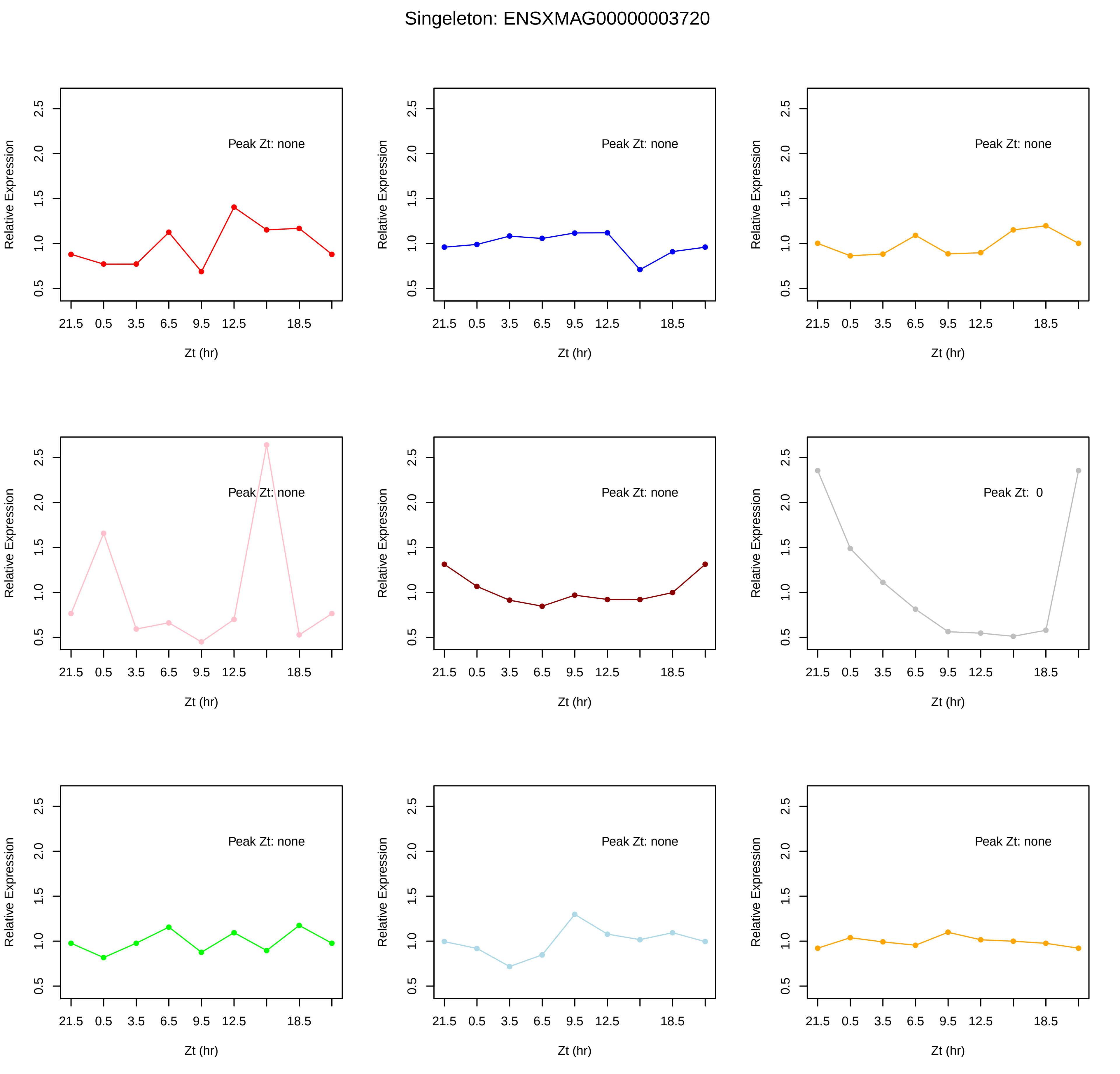
Singleton: ENSXMAG00000003720
Peak Zt: none
Peak Zt: none
Peak Zt: none
Peak Zt: none
Peak Zt: none
Peak Zt: 0
Peak Zt: none
Peak Zt: none
Peak Zt: none
Relative Expression
Zt (hr)
21.5 0.5 3.5 6.5 9.5 12.5 18.5
0.5 1.0 1.5 2.0 2.5

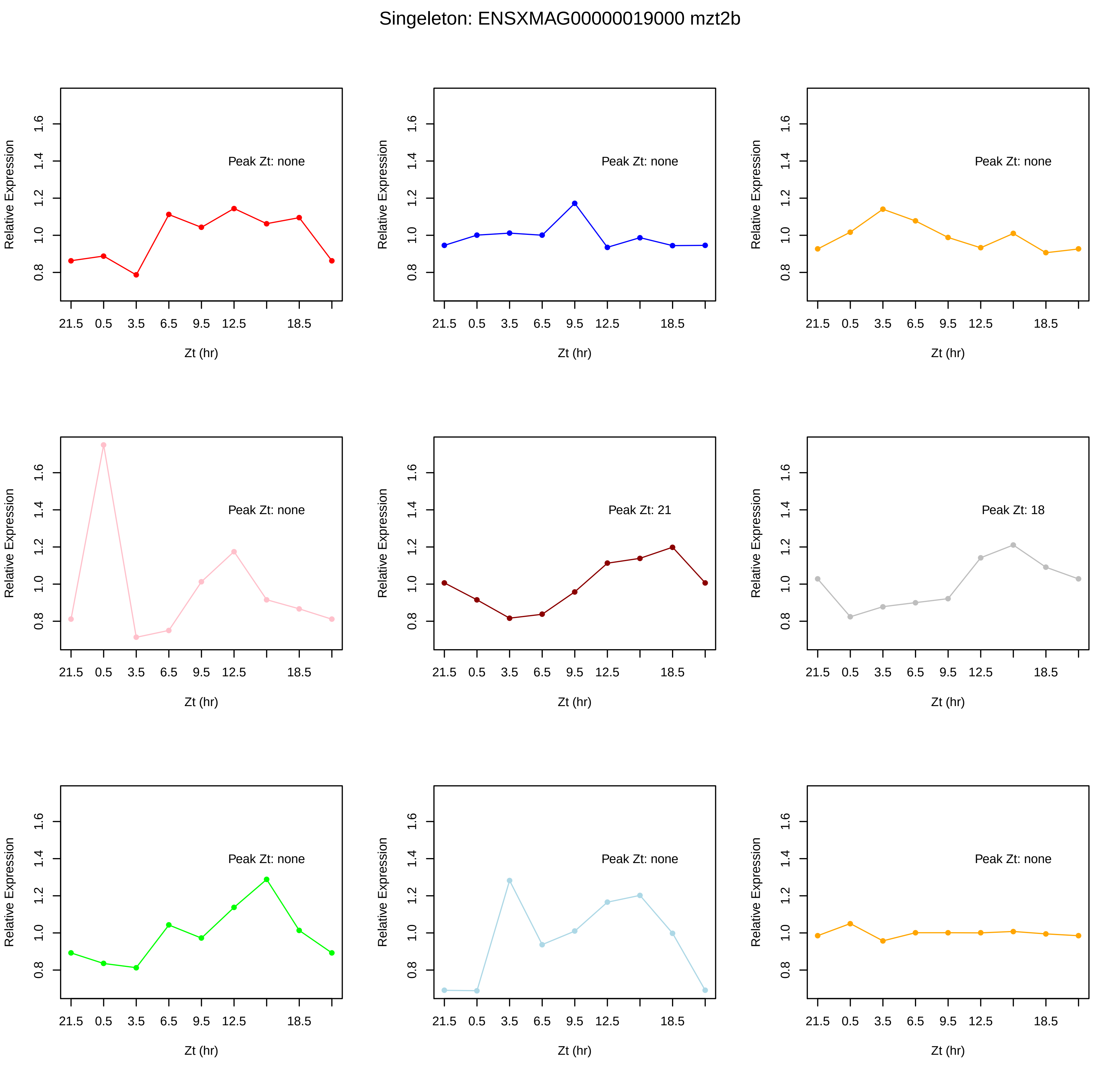

Singleton: ENSXMAG00000019000 mzt2b
Peak Zt: none
Peak Zt: none
Peak Zt: none
Peak Zt: none
Peak Zt: 21
Peak Zt: 18
Peak Zt: none
Peak Zt: none
Peak Zt: none
Relative Expression
Zt (hr)
21.5
0.5
3.5
6.5
9.5
12.5
18.5
0.8
1.0
1.2
1.4
1.6

Singeleton: ENSXMAG00000019001 ap2m1a

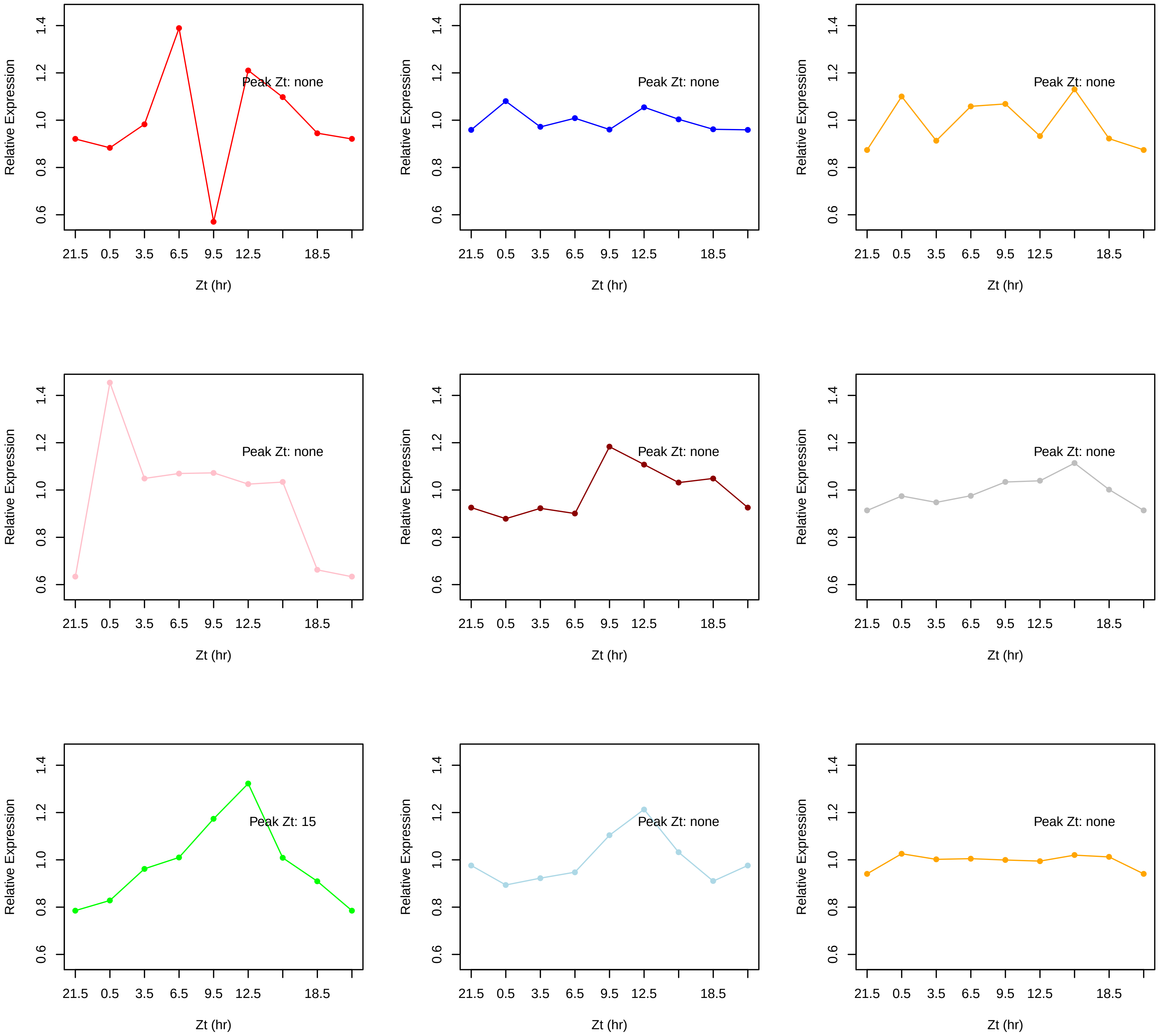

Singeleton: ENSXMAG00000017932

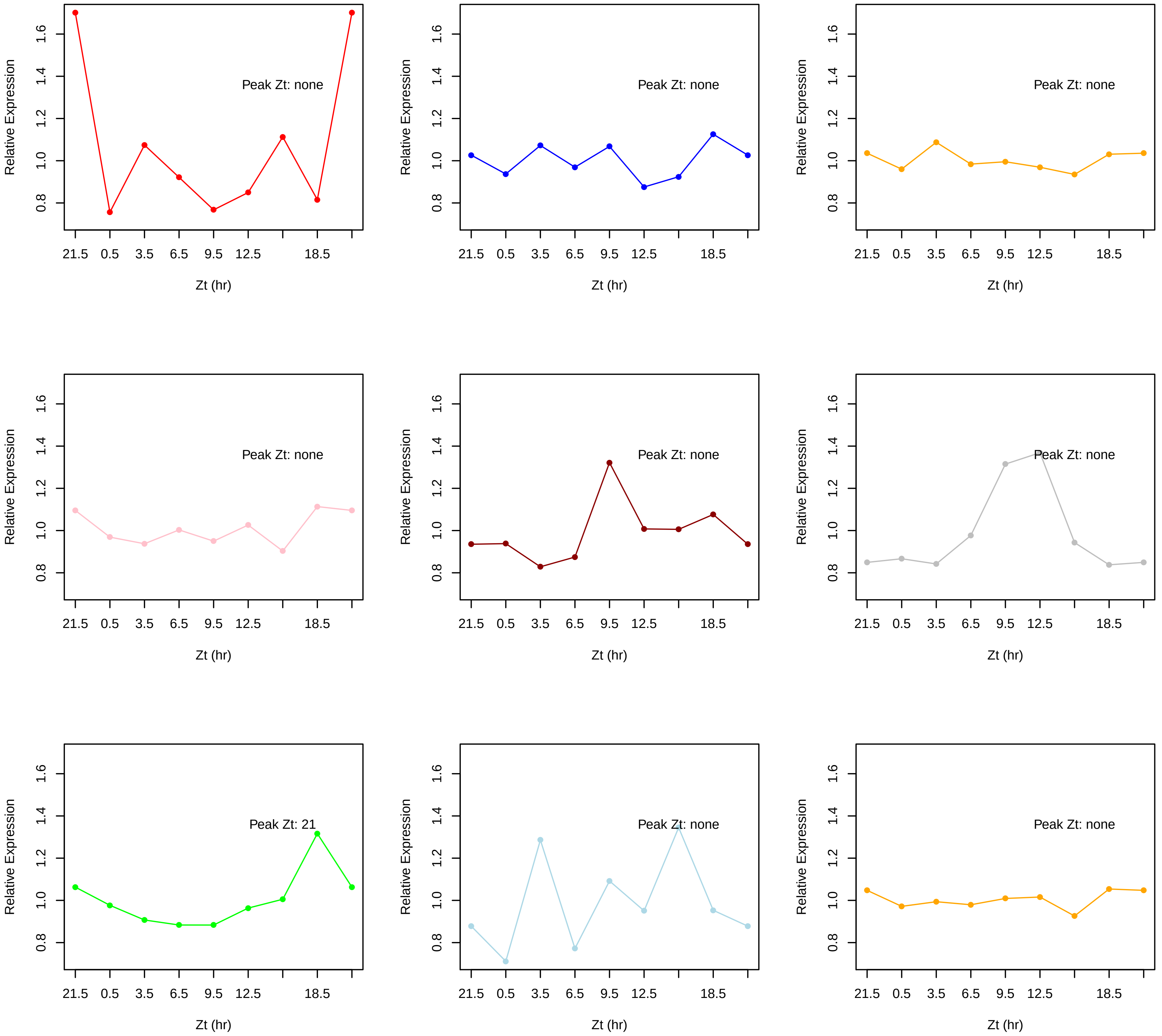

Singeleton: ENSXMAG00000003652 pard6gb

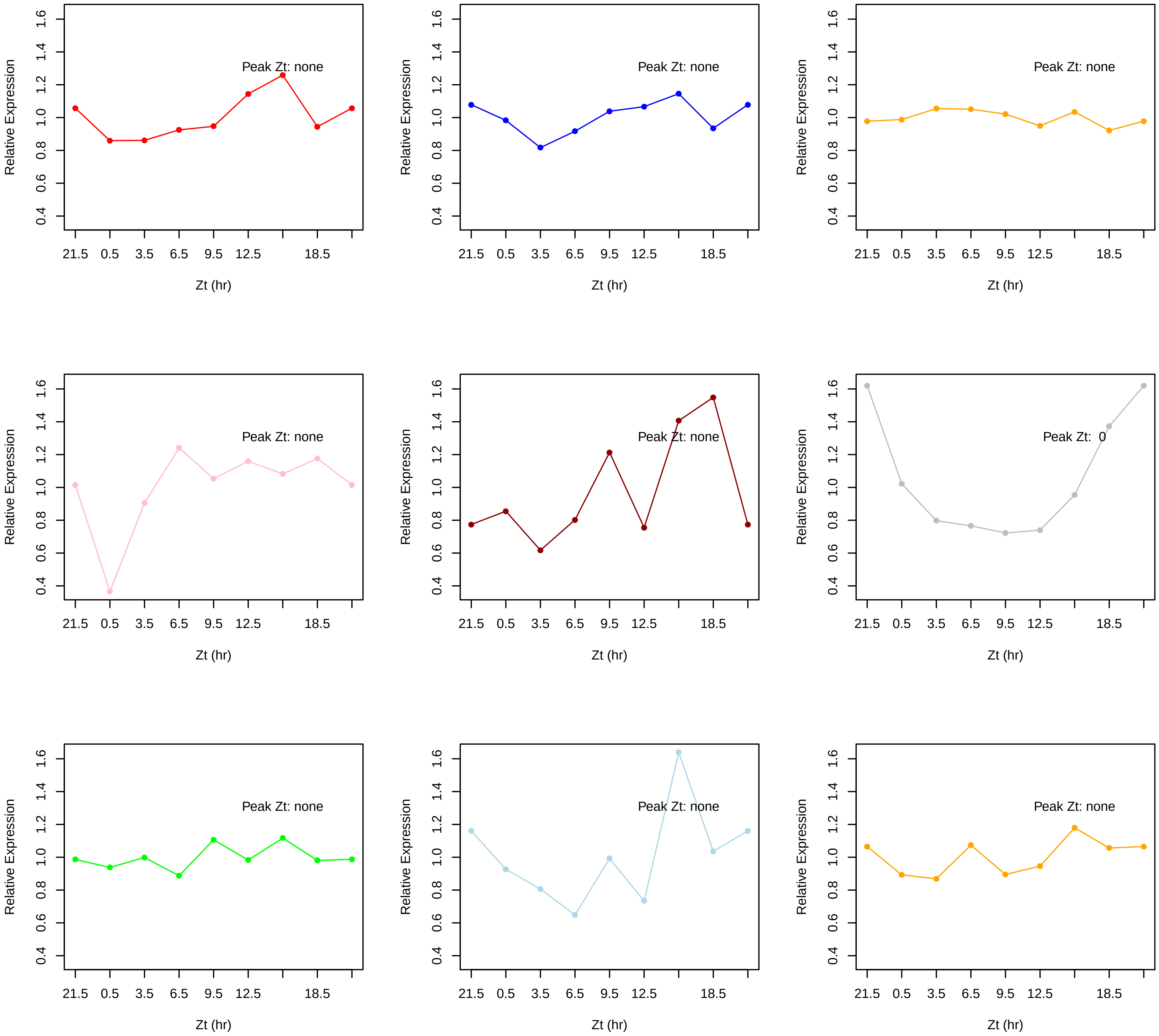

Singeleton: ENSXMAG00000003653 bloc1s4

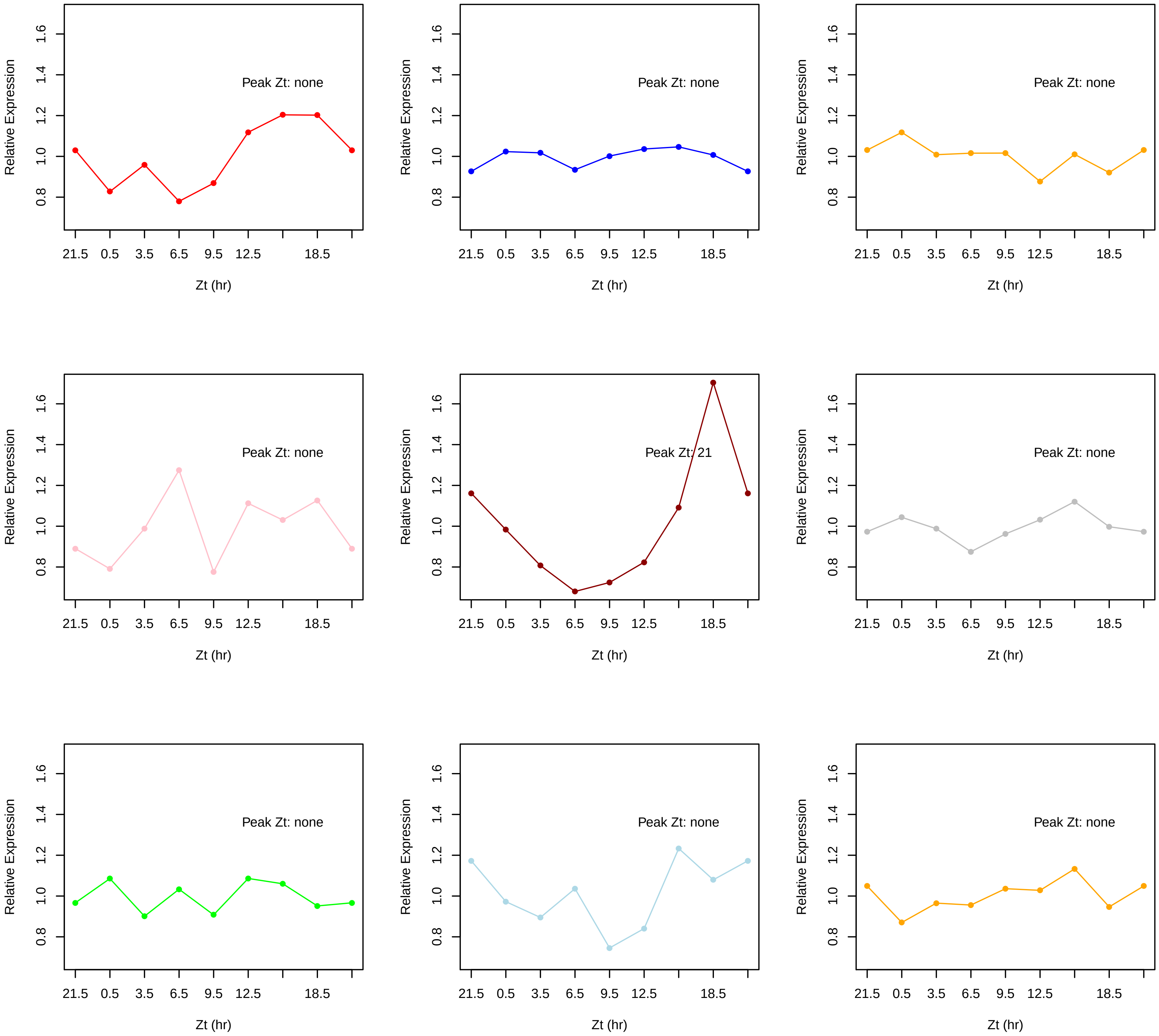

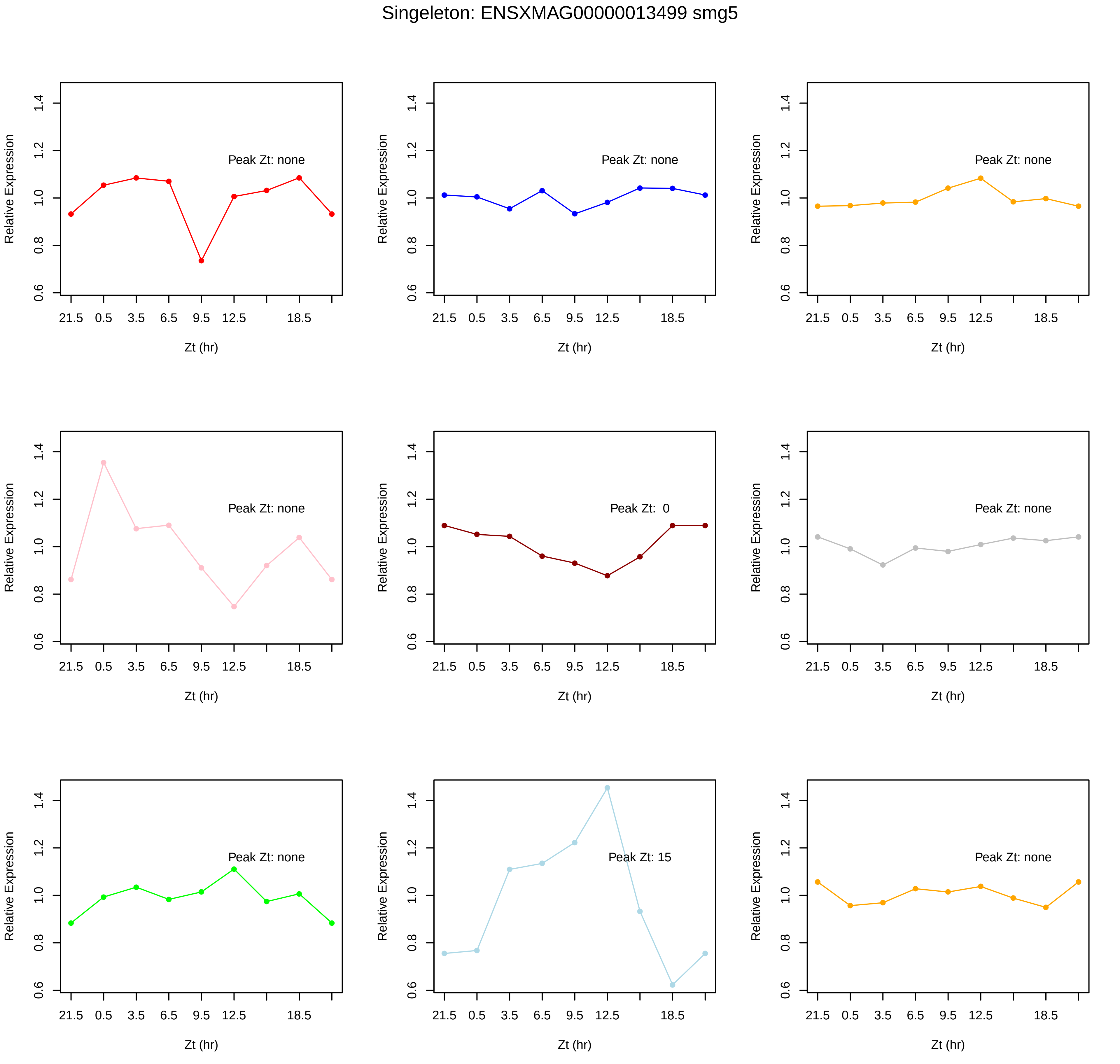
Singeleton: ENSXMAG00000013499 smg5
Relative Expression
Zt (hr)
21.5 0.5 3.5 6.5 9.5 12.5 18.5
0.6 0.8 1.0 1.2 1.4
Peak Zt: none
Peak Zt: none
Peak Zt: none
Peak Zt: none
Peak Zt: 0
Peak Zt: none
Peak Zt: none
Peak Zt: 15
Peak Zt: none

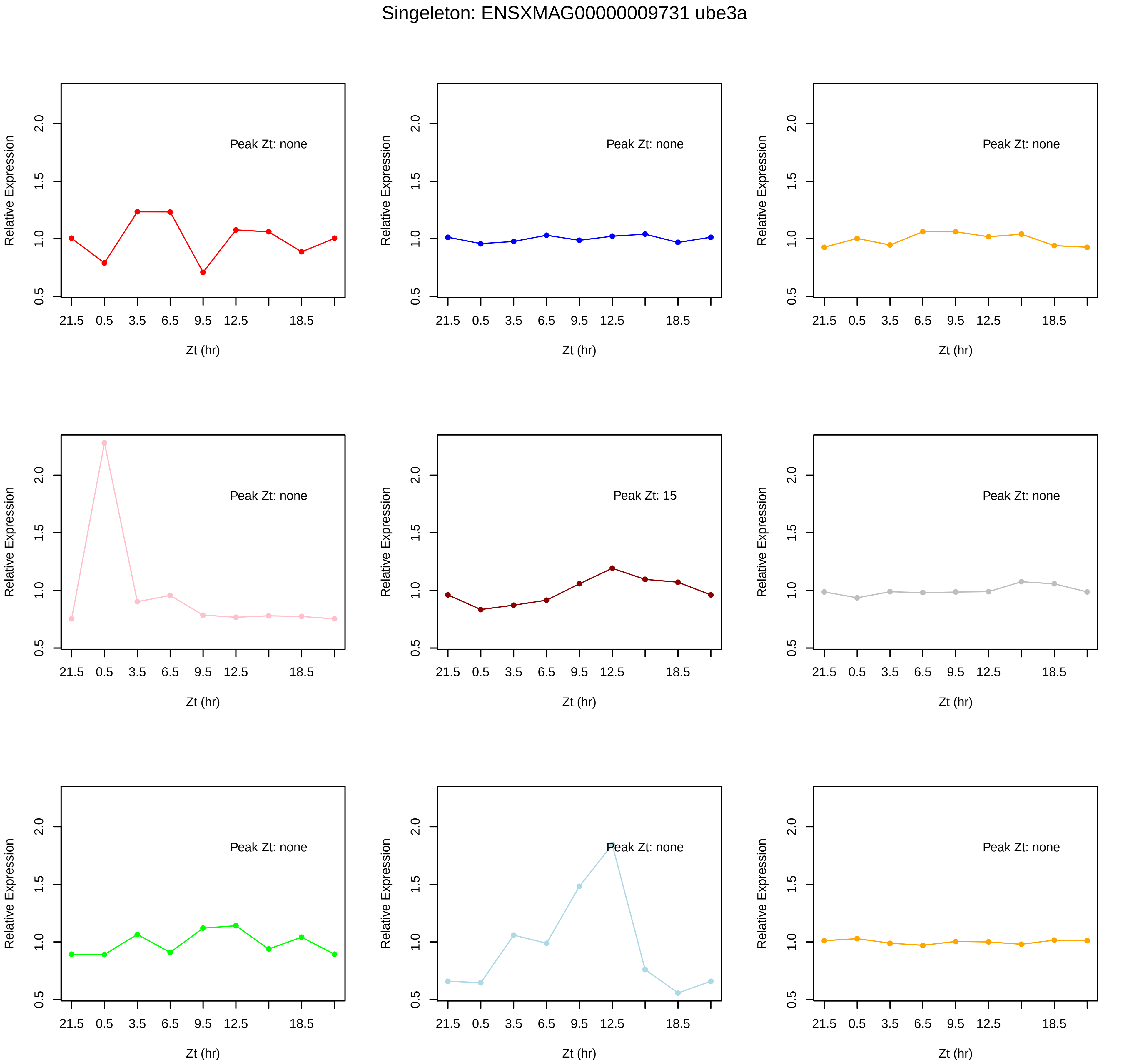

Singeleton: ENSXMAG00000009731 ube3a
Peak Zt: none
Peak Zt: none
Peak Zt: none
Peak Zt: none
Peak Zt: 15
Peak Zt: none
Peak Zt: none
Peak Zt: none
Peak Zt: none
Relative Expression
Zt (hr)
0.5
1.0
1.5
2.0
21.5
0.5
3.5
6.5
9.5
12.5
18.5

Singeleton: ENSXMAG00000029882 adnp2b

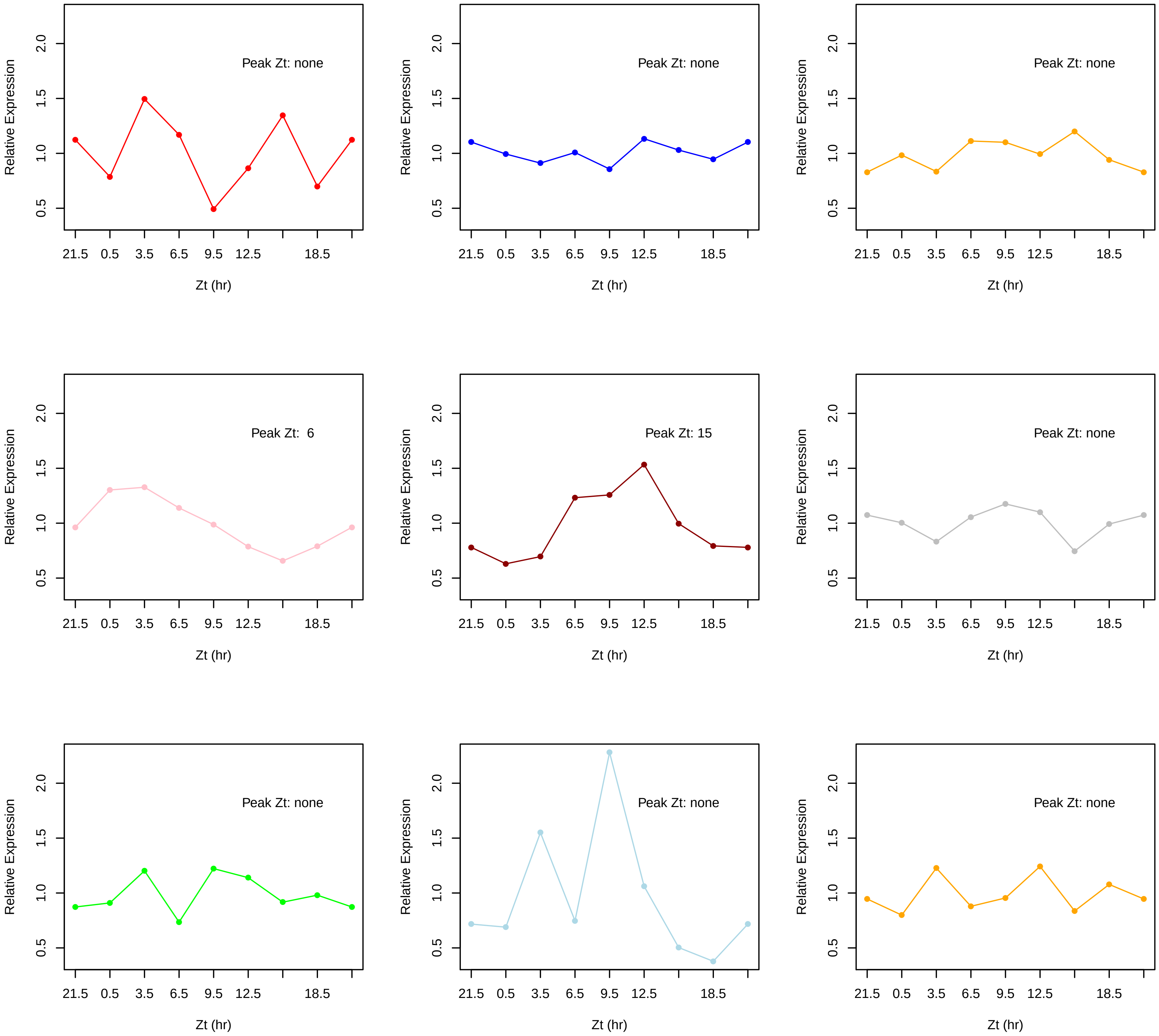

Singeleton: ENSXMAG00000007503 thoc5

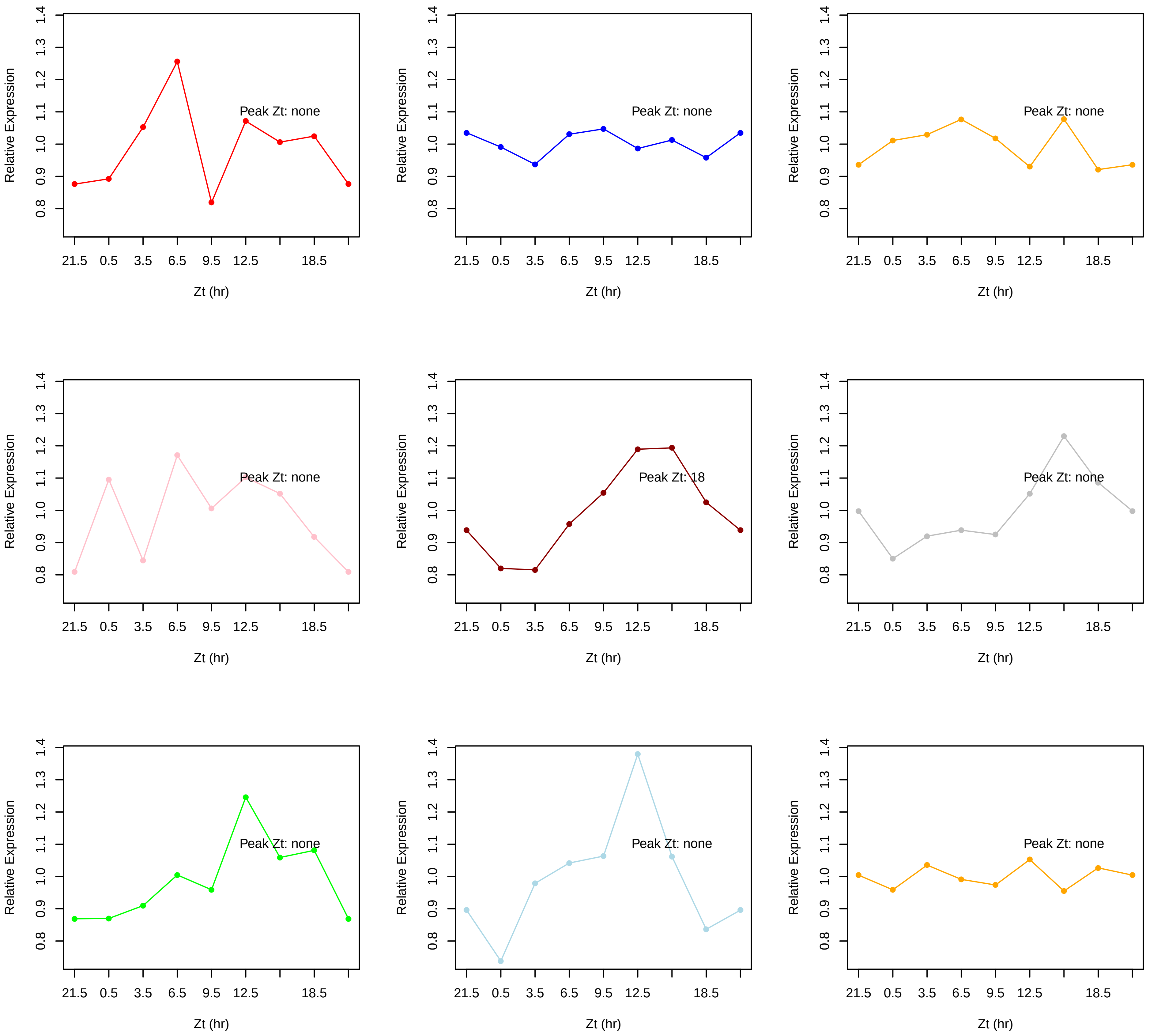

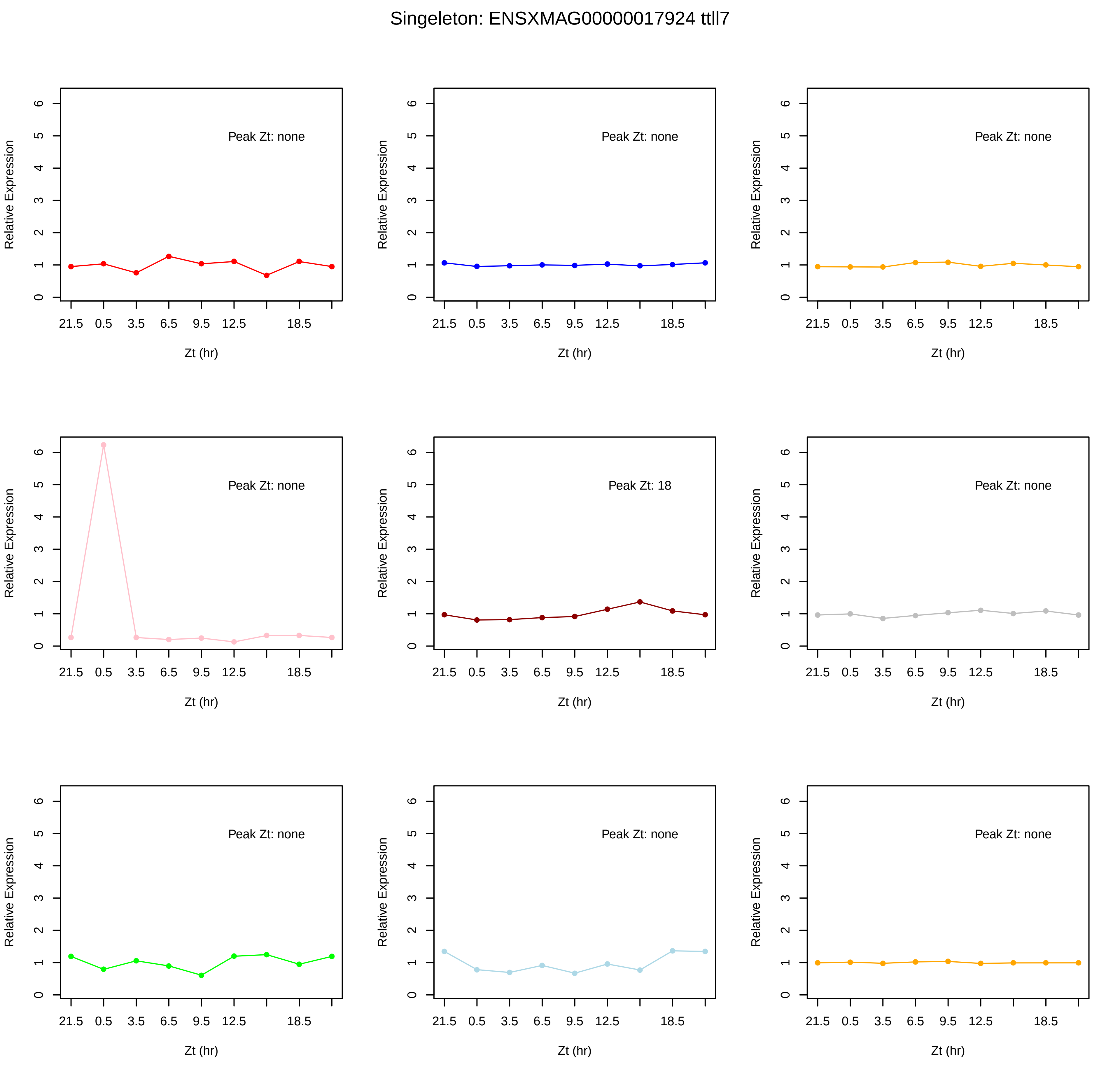
Singeleton: ENSXMAG00000017924 ttll7
Peak Zt: none
Peak Zt: none
Peak Zt: none
Peak Zt: none
Peak Zt: 18
Peak Zt: none
Peak Zt: none
Peak Zt: none
Peak Zt: none
Relative Expression
Zt (hr)
21.5 0.5 3.5 6.5 9.5 12.5 18.5
0 1 2 3 4 5 6

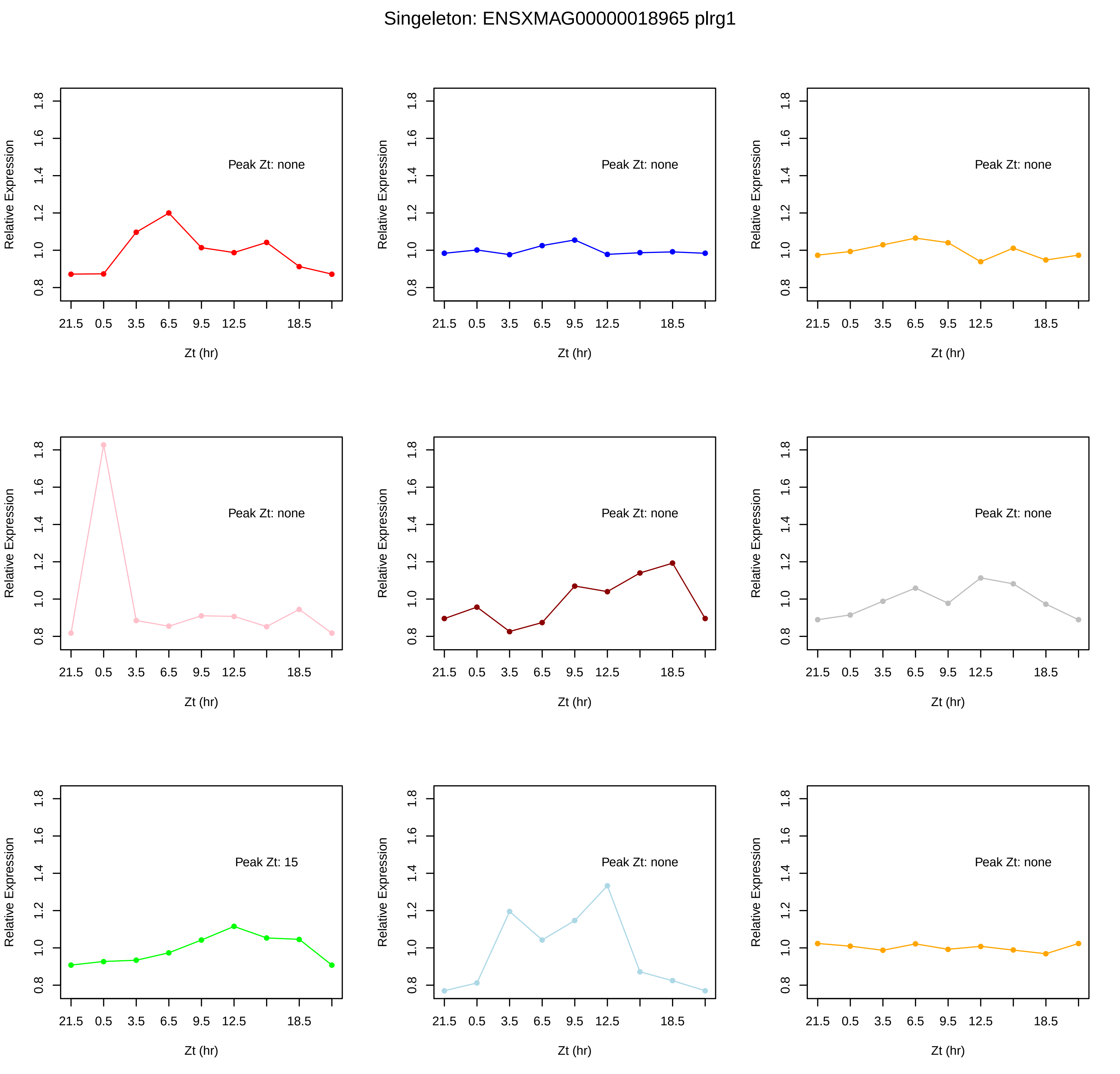
Singeleton: ENSXMAG00000018965 plrg1
Relative Expression
Zt (hr)
21.5
0.5
3.5
6.5
9.5
12.5
18.5
0.8
1.0
1.2
1.4
1.6
1.8
Peak Zt: none
Peak Zt: 15

Singeleton: ENSXMAG00000011574 GRB14

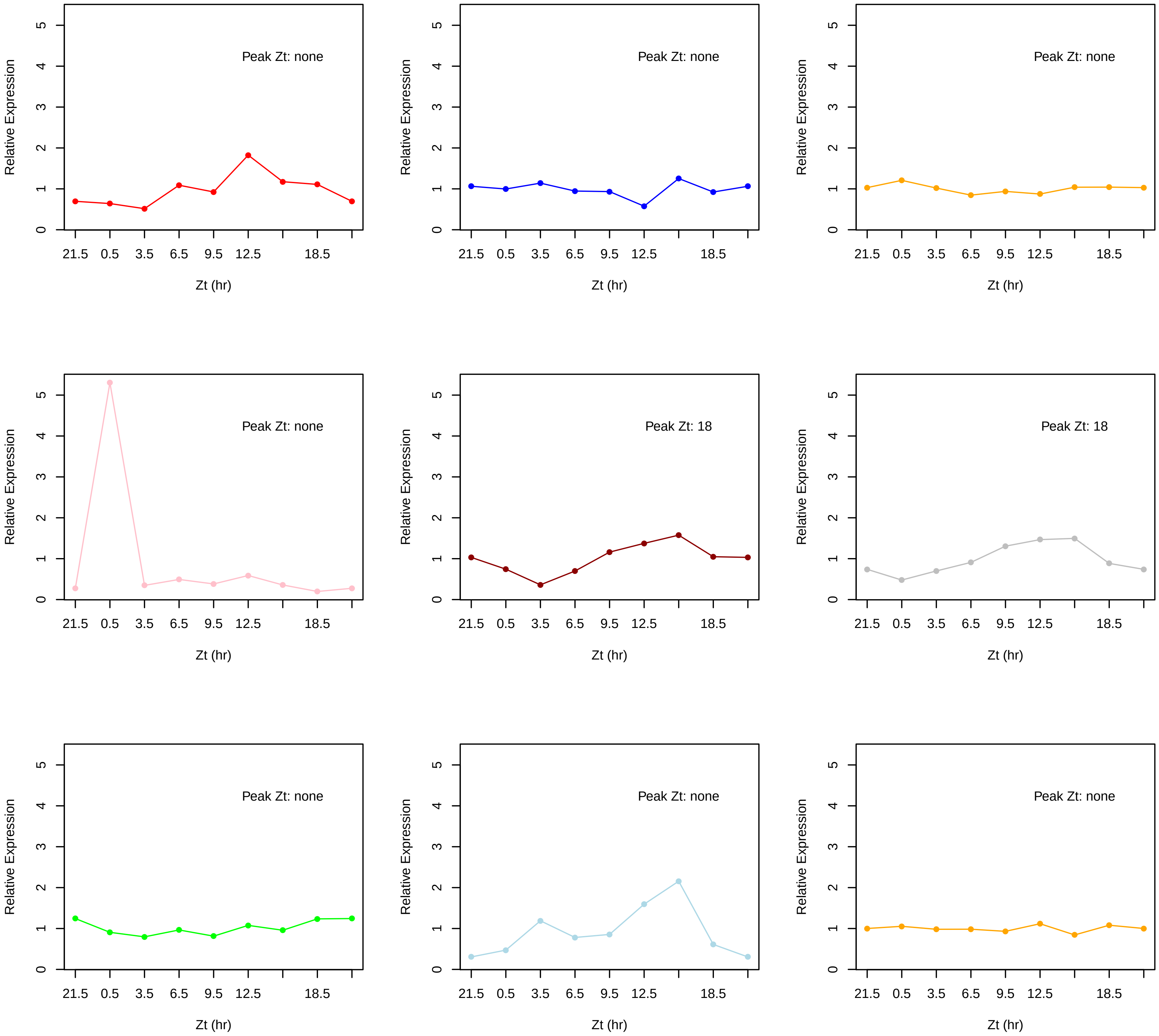

Singeleton: ENSXMAG00000011566 KCNH7

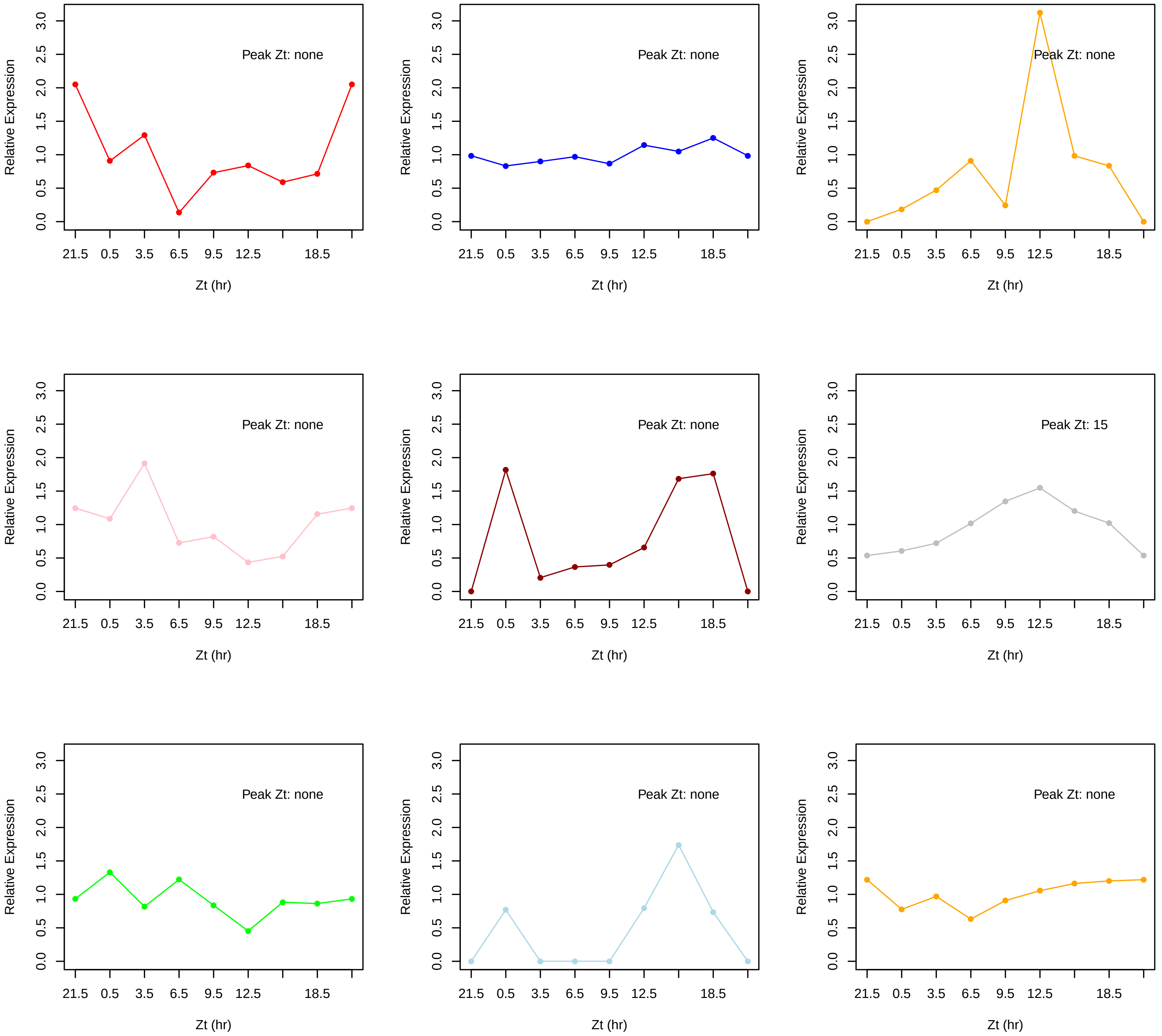

Singleton: ENSXMAG00000024011 txnl4a

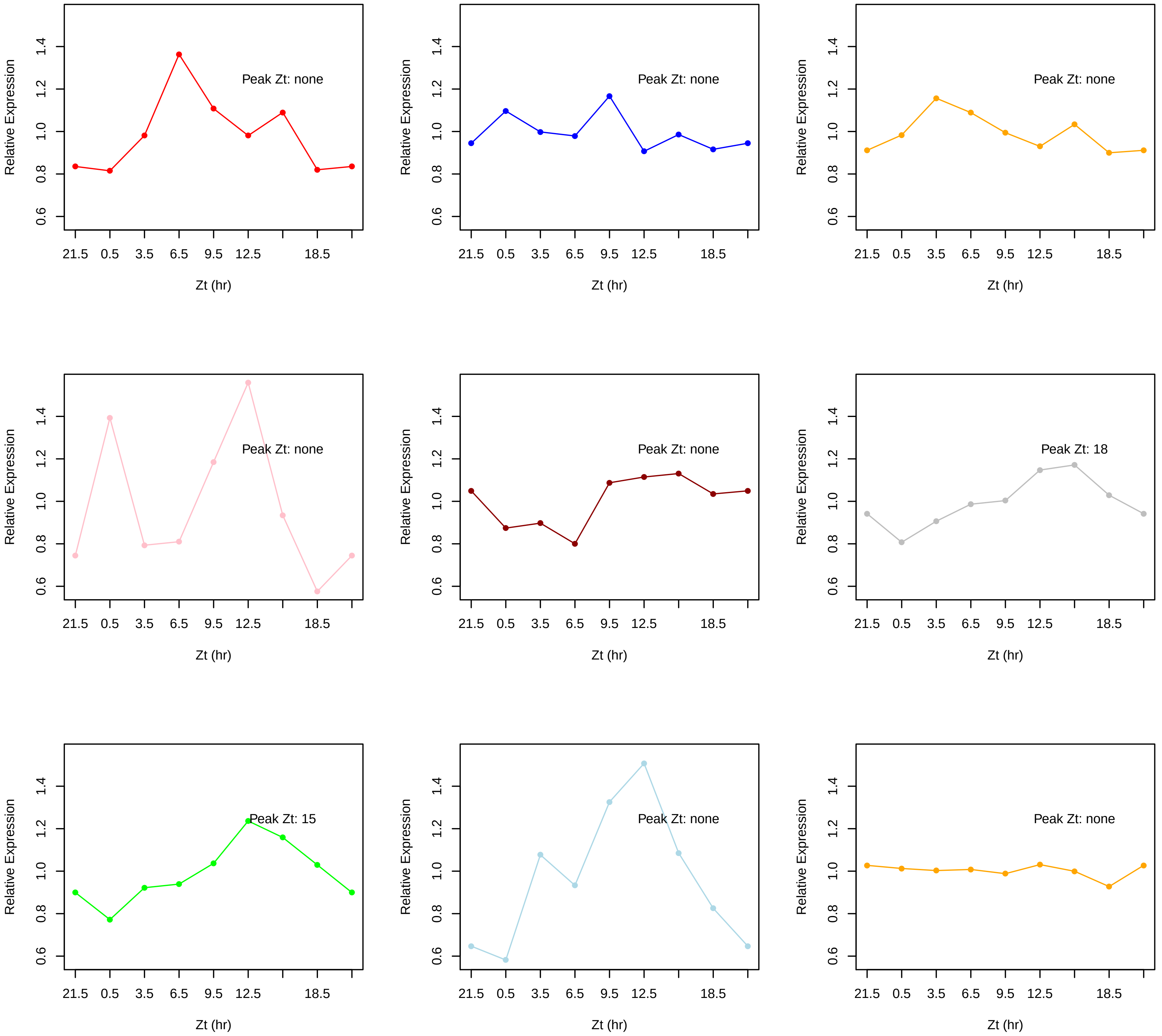

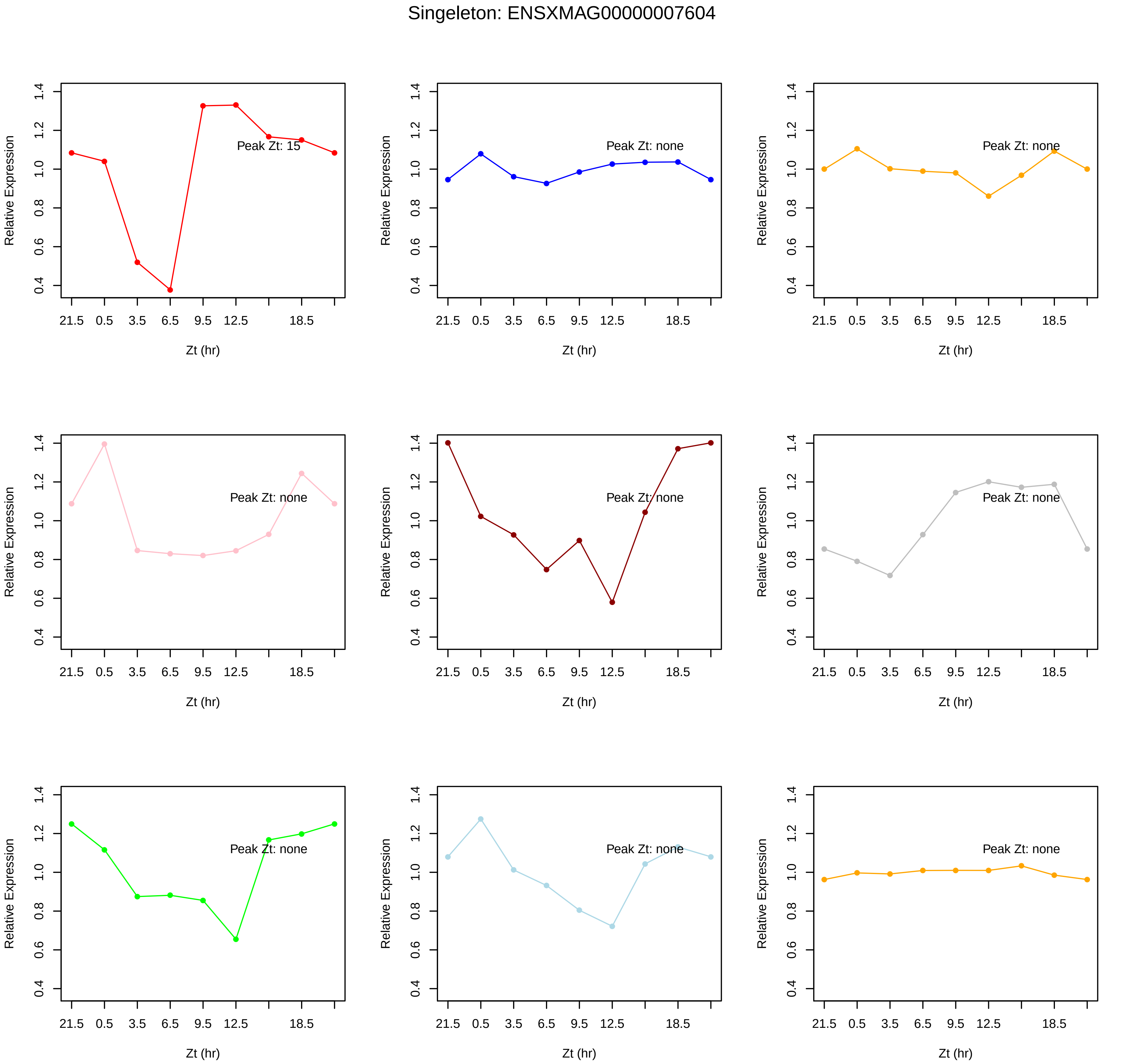
Singeleton: ENSXMAG00000007604
Peak Zt: 15
Peak Zt: none
Peak Zt: none
Peak Zt: none
Peak Zt: none
Peak Zt: none
Peak Zt: none
Peak Zt: none
Peak Zt: none
Relative Expression
Zt (hr)
21.5 0.5 3.5 6.5 9.5 12.5 18.5
0.4 0.6 0.8 1.0 1.2 1.4

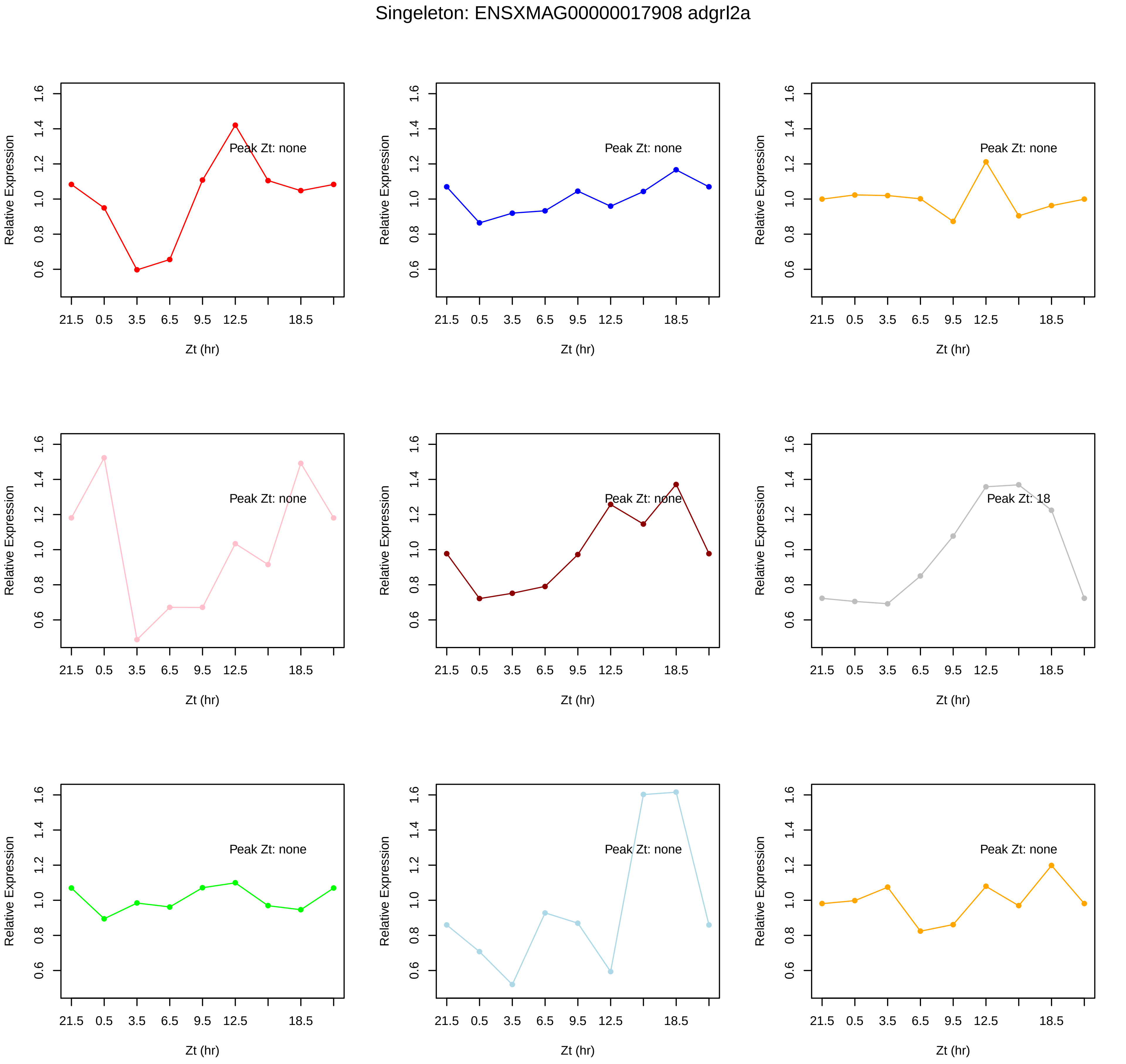

Singleton: ENSXMAG00000017908 adgrl2a
Peak Zt: none
Peak Zt: none
Peak Zt: none
Peak Zt: none
Peak Zt: none
Peak Zt: 18
Peak Zt: none
Peak Zt: none
Peak Zt: none
Relative Expression
Zt (hr)
21.5
0.5
3.5
6.5
9.5
12.5
18.5

Singeleton: ENSXMAG00000004840 myo1eb

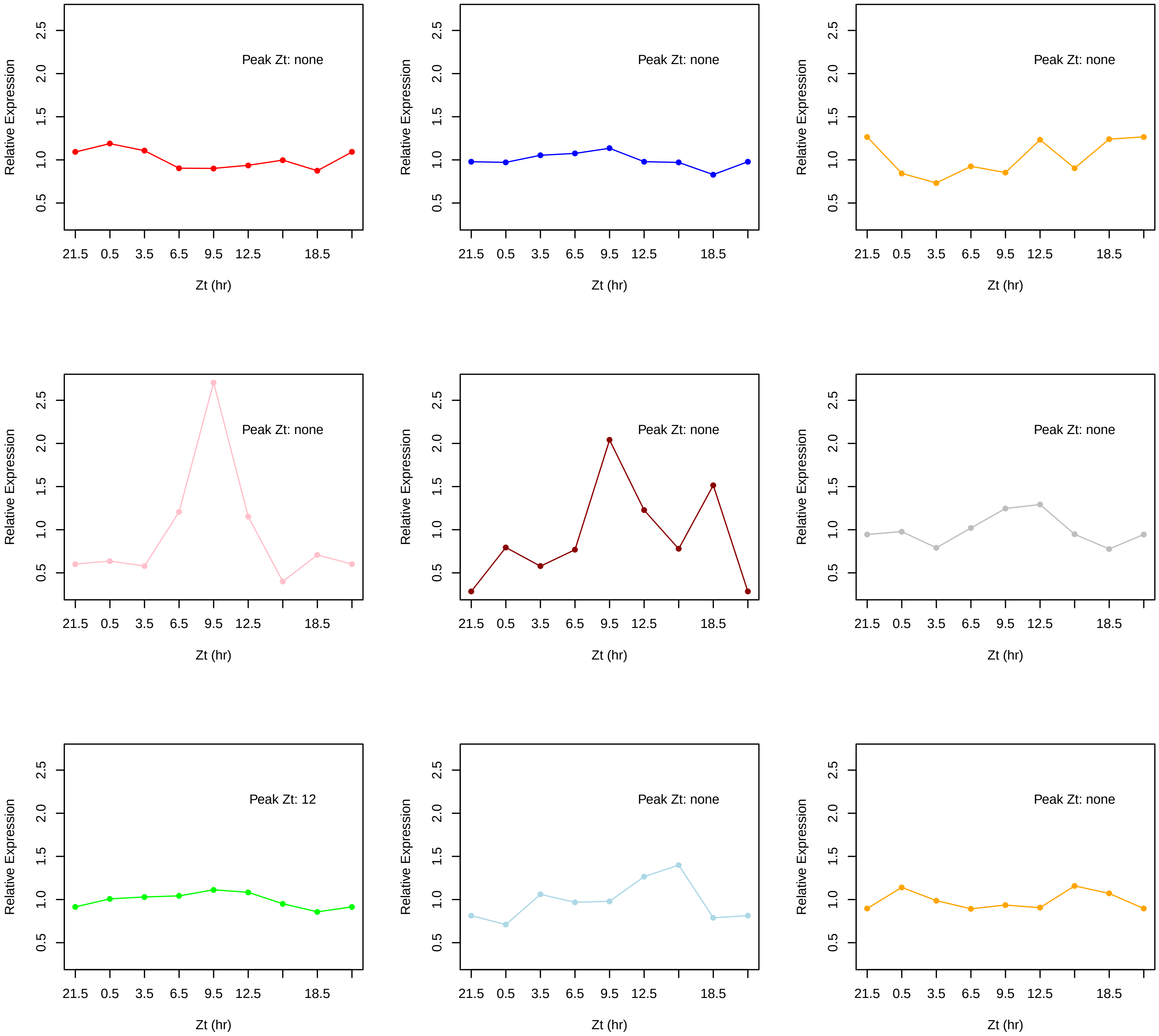

Singeleton: ENSXMAG00000014077 abcc5

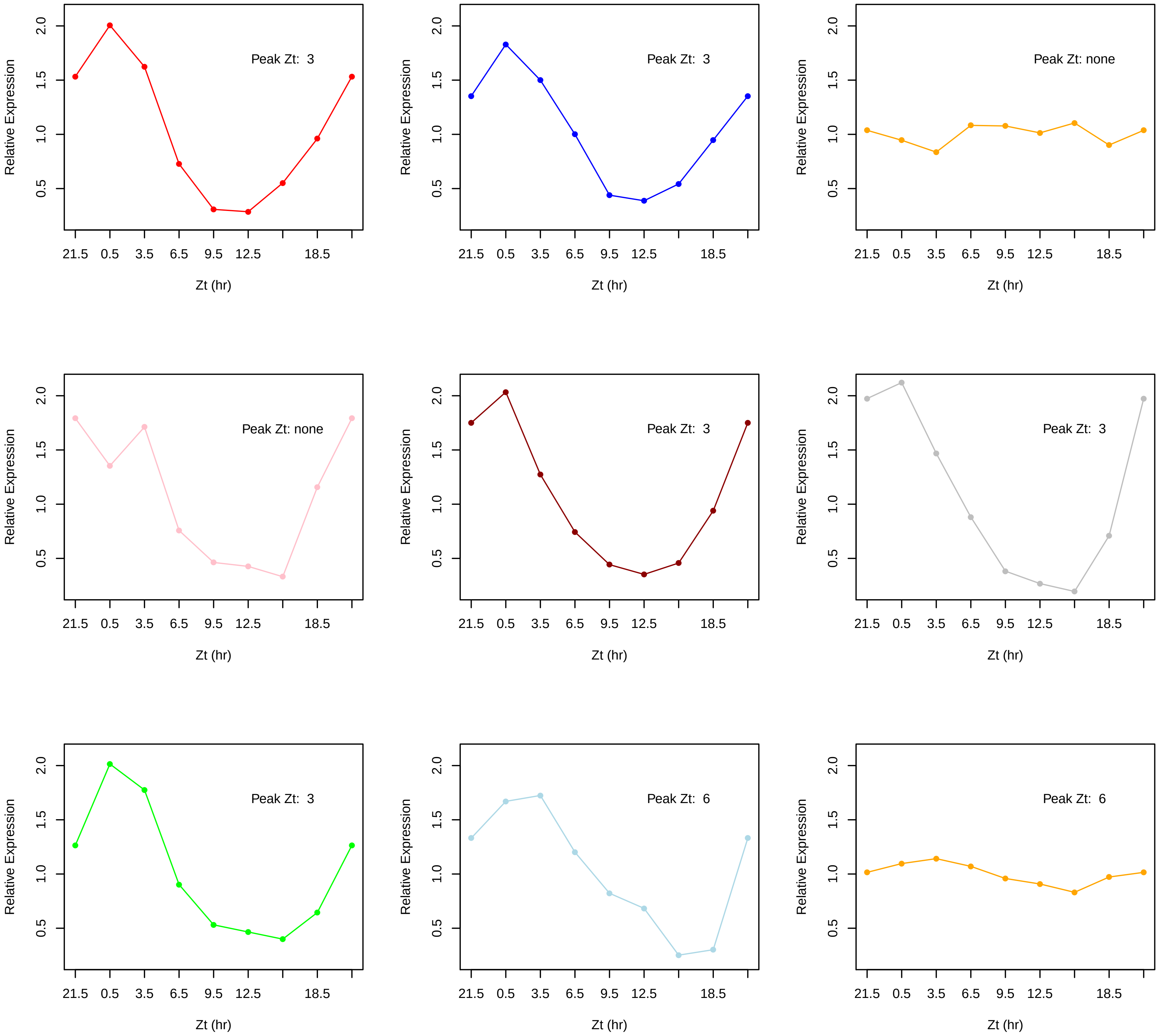

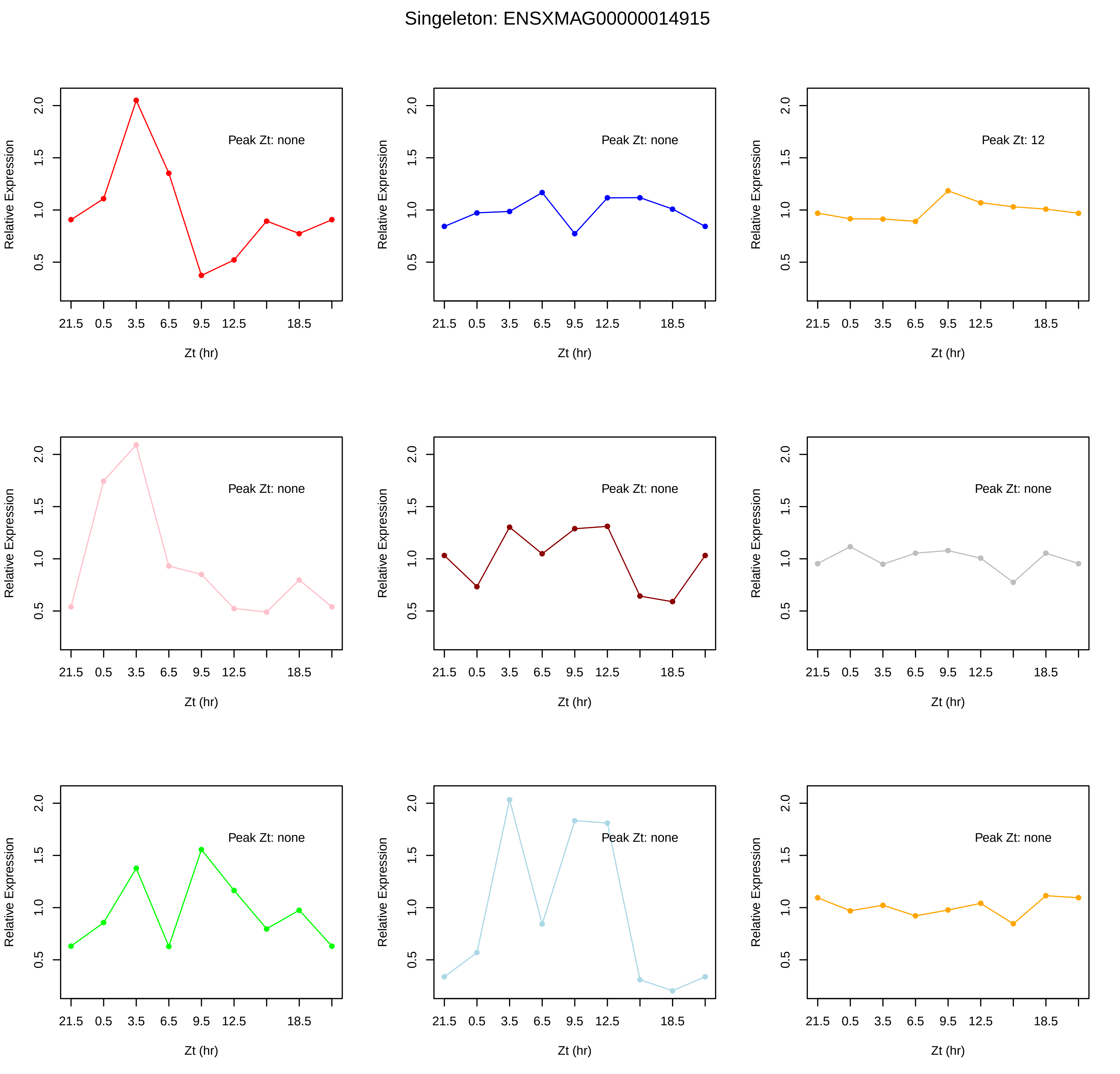
Singeleton: ENSXMAG00000014915
Peak Zt: none
Peak Zt: none
Peak Zt: 12
Peak Zt: none
Peak Zt: none
Peak Zt: none
Peak Zt: none
Peak Zt: none
Peak Zt: none
Relative Expression
Zt (hr)
21.5
0.5
3.5
6.5
9.5
12.5
18.5
0.5
1.0
1.5
2.0

Singeleton: ENSXMAG00000007594 SLC8B1

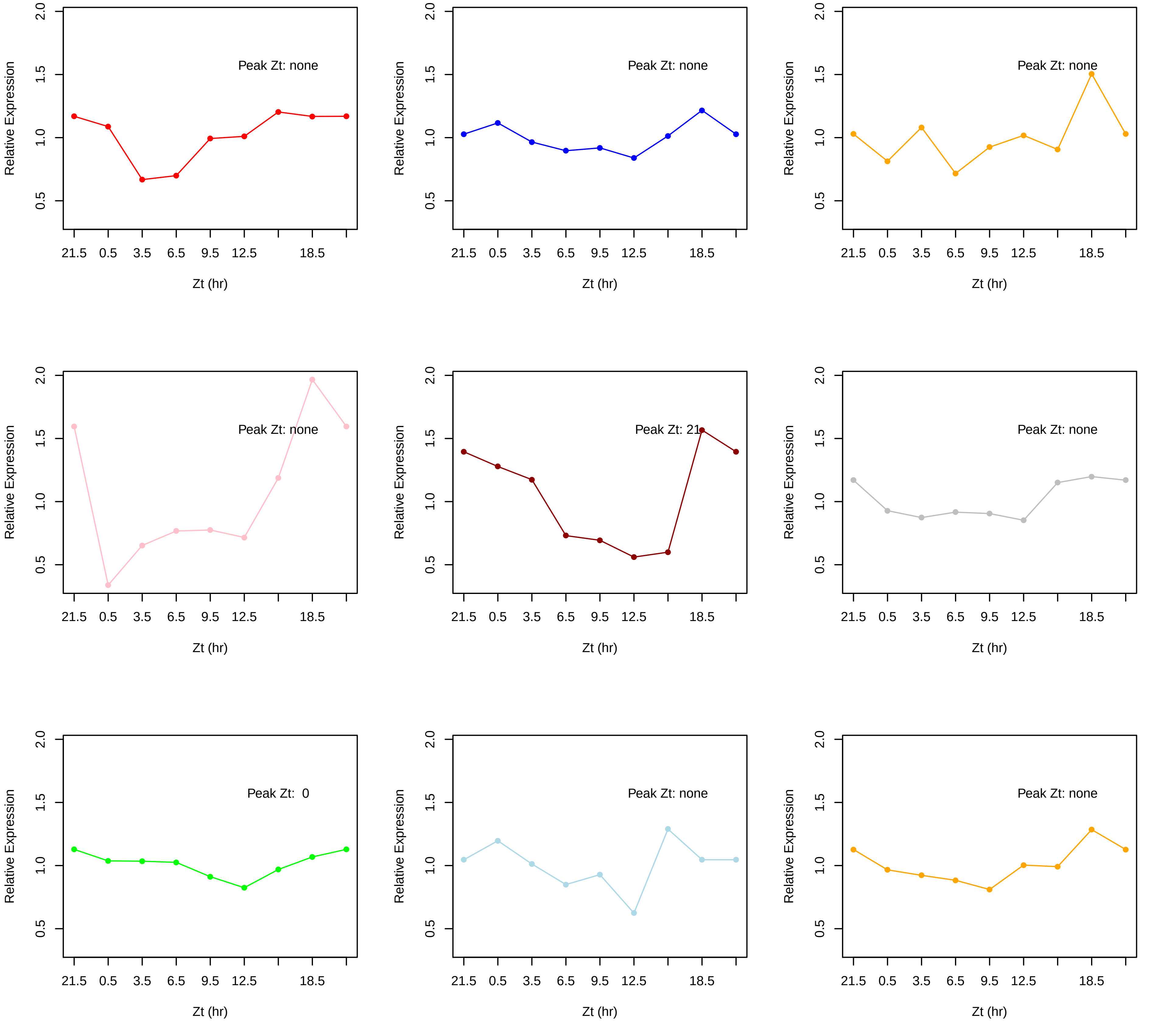

Singeleton: ENSXMAG00000013845 GCA

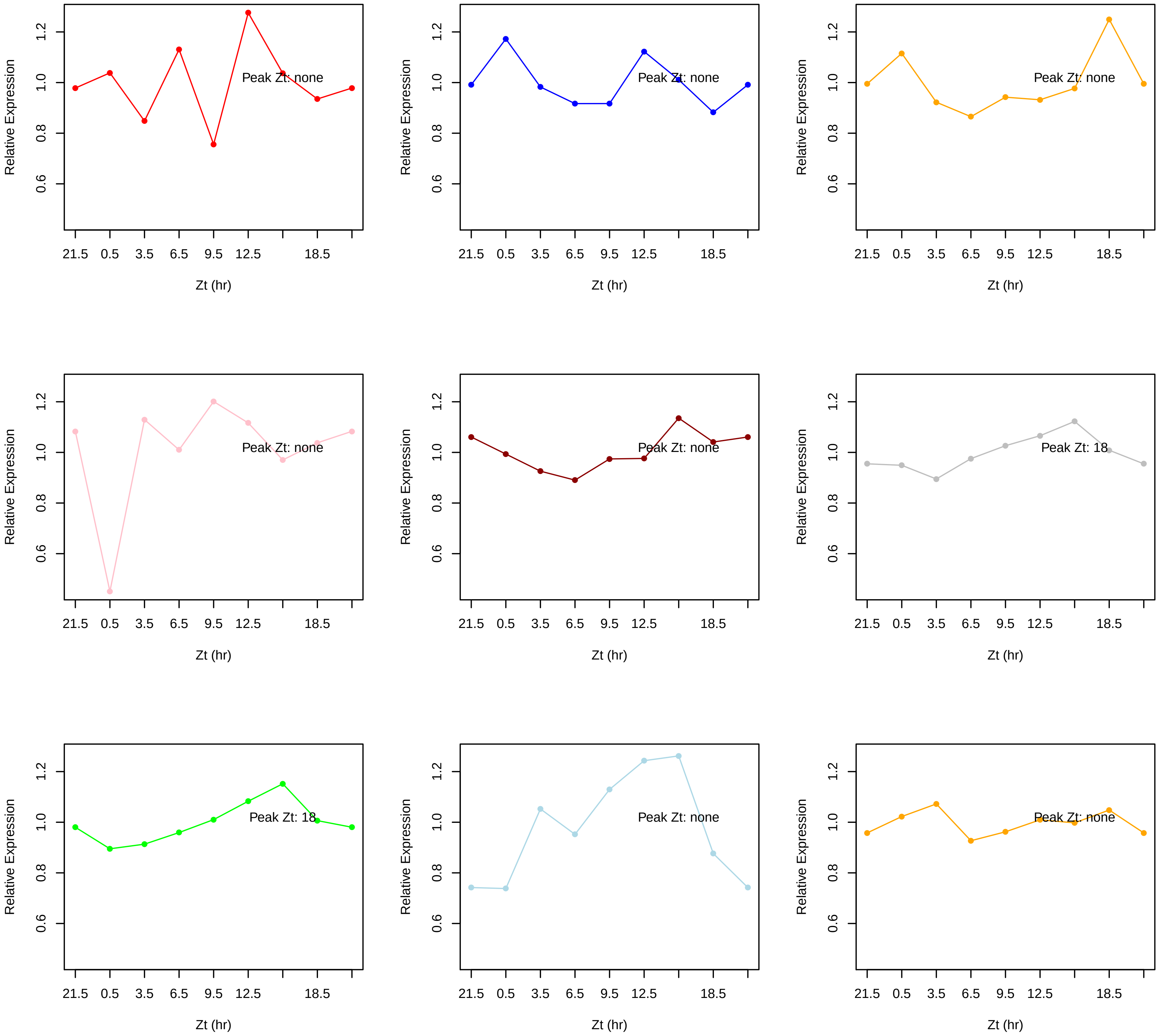

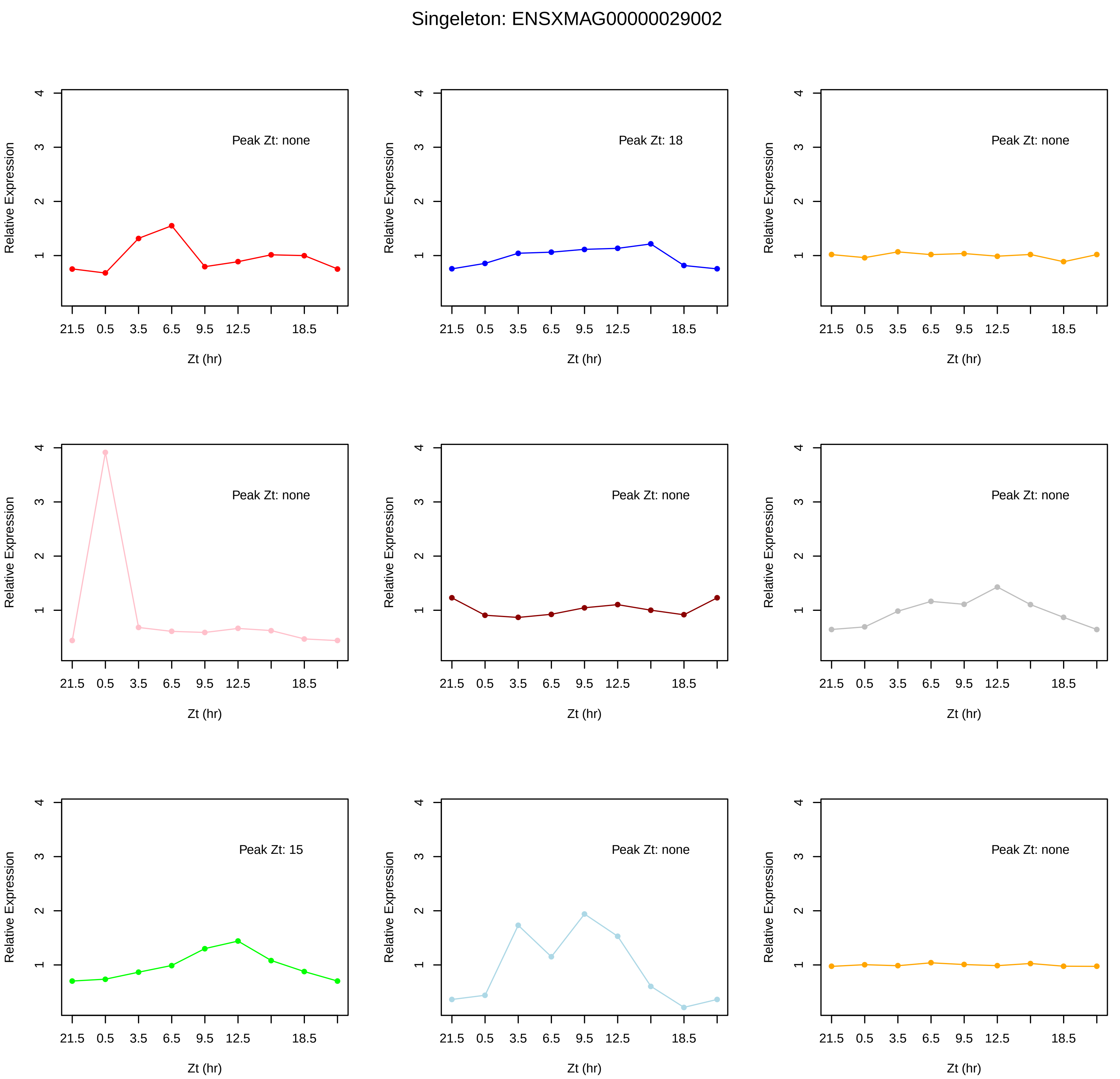
Singeleton: ENSXMAG00000029002
Peak Zt: none
Peak Zt: 18
Peak Zt: none
Peak Zt: none
Peak Zt: none
Peak Zt: none
Peak Zt: 15
Peak Zt: none
Peak Zt: none
Relative Expression
Zt (hr)
21.5
0.5
3.5
6.5
9.5
12.5
18.5
1
2
3
4

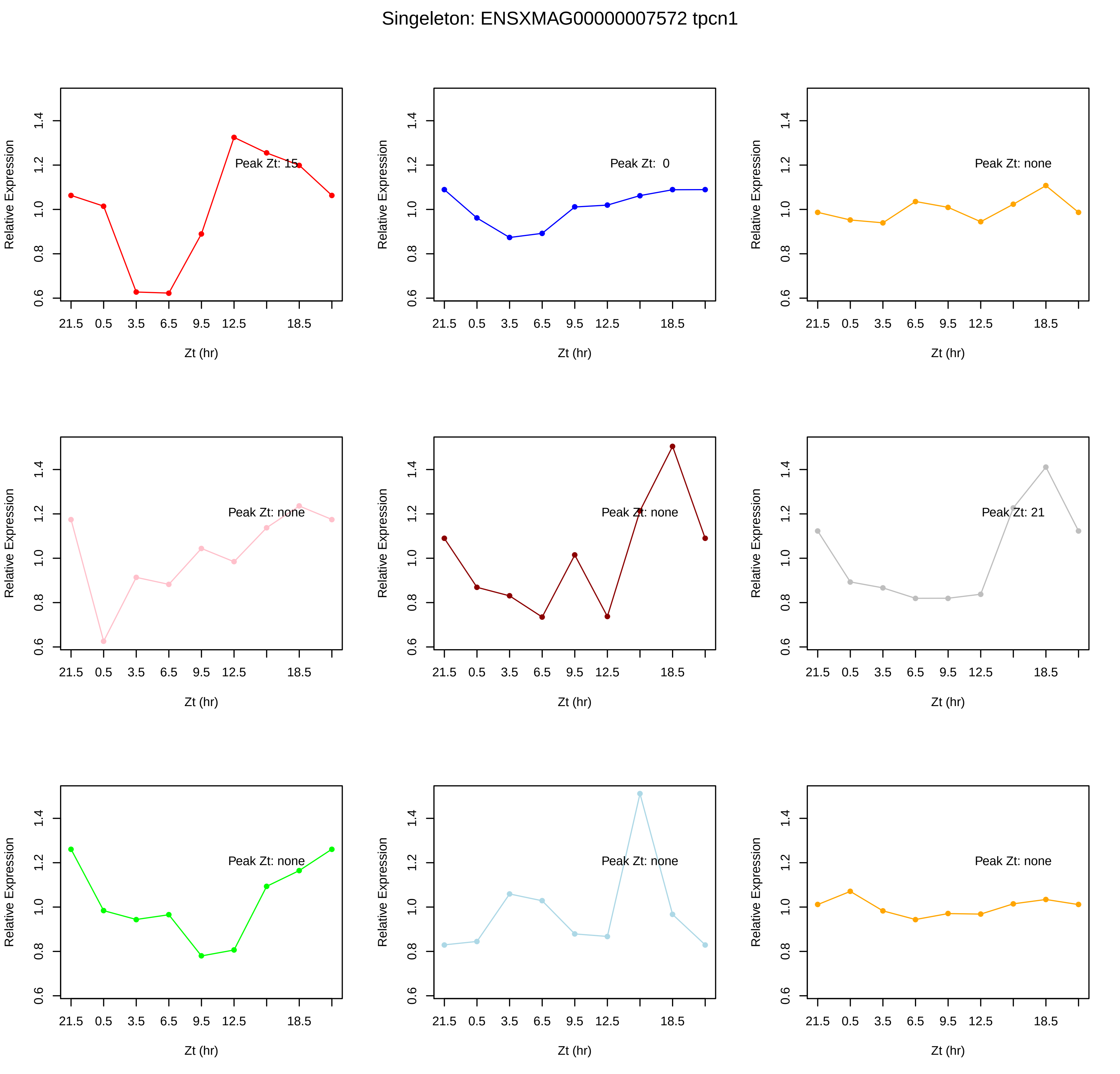
Singleton: ENSXMAG00000007572 tpcn1
Relative Expression
Zt (hr)
21.5
0.5
3.5
6.5
9.5
12.5
18.5
0.6
0.8
1.0
1.2
1.4
Peak Zt: 15
Peak Zt: 0
Peak Zt: none
Peak Zt: none
Peak Zt: none
Peak Zt: 21
Peak Zt: none
Peak Zt: none
Peak Zt: none

Singeleton: ENSXMAG00000024586 C8orf82

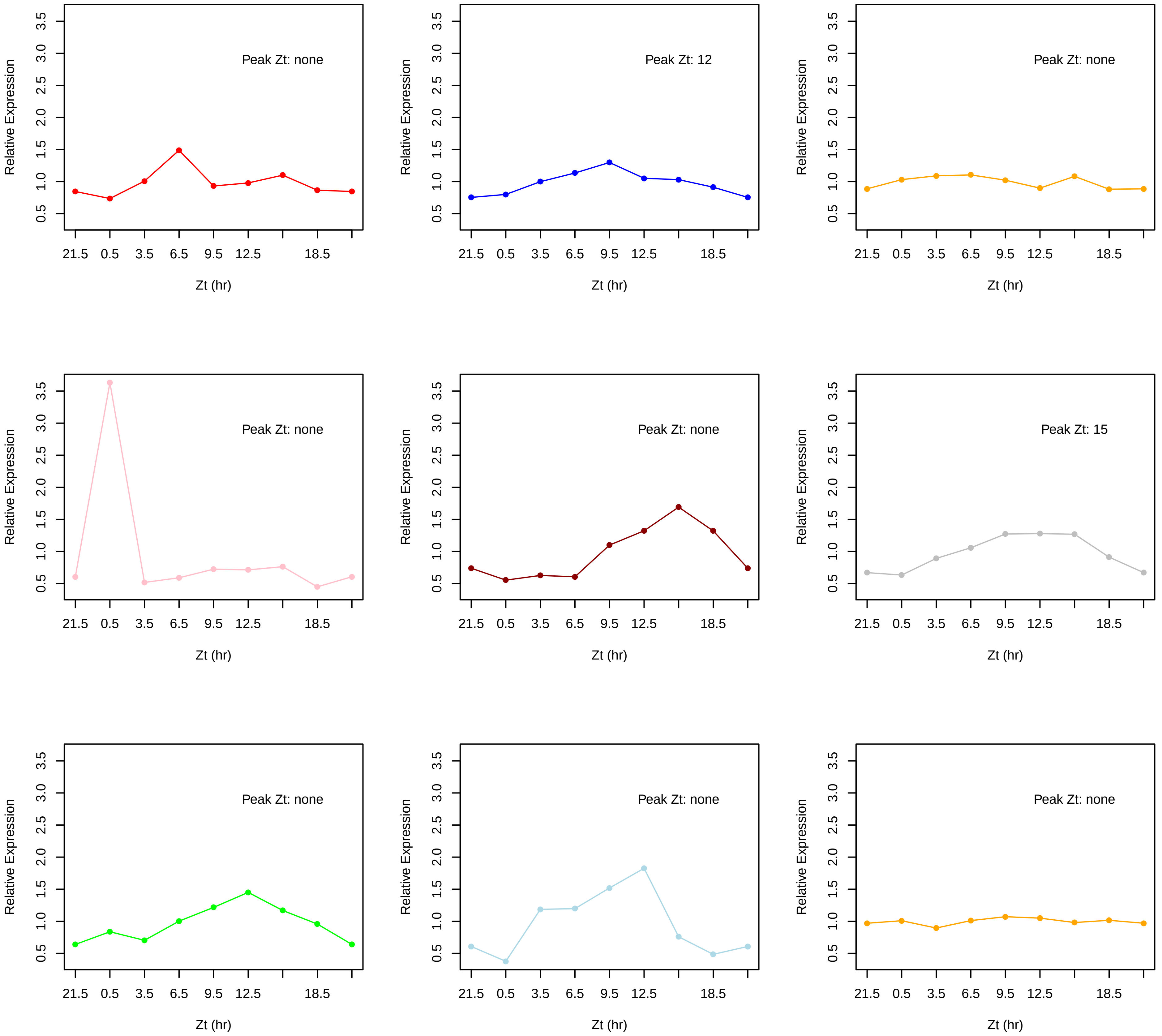

Singeleton: ENSXMAG00000029802

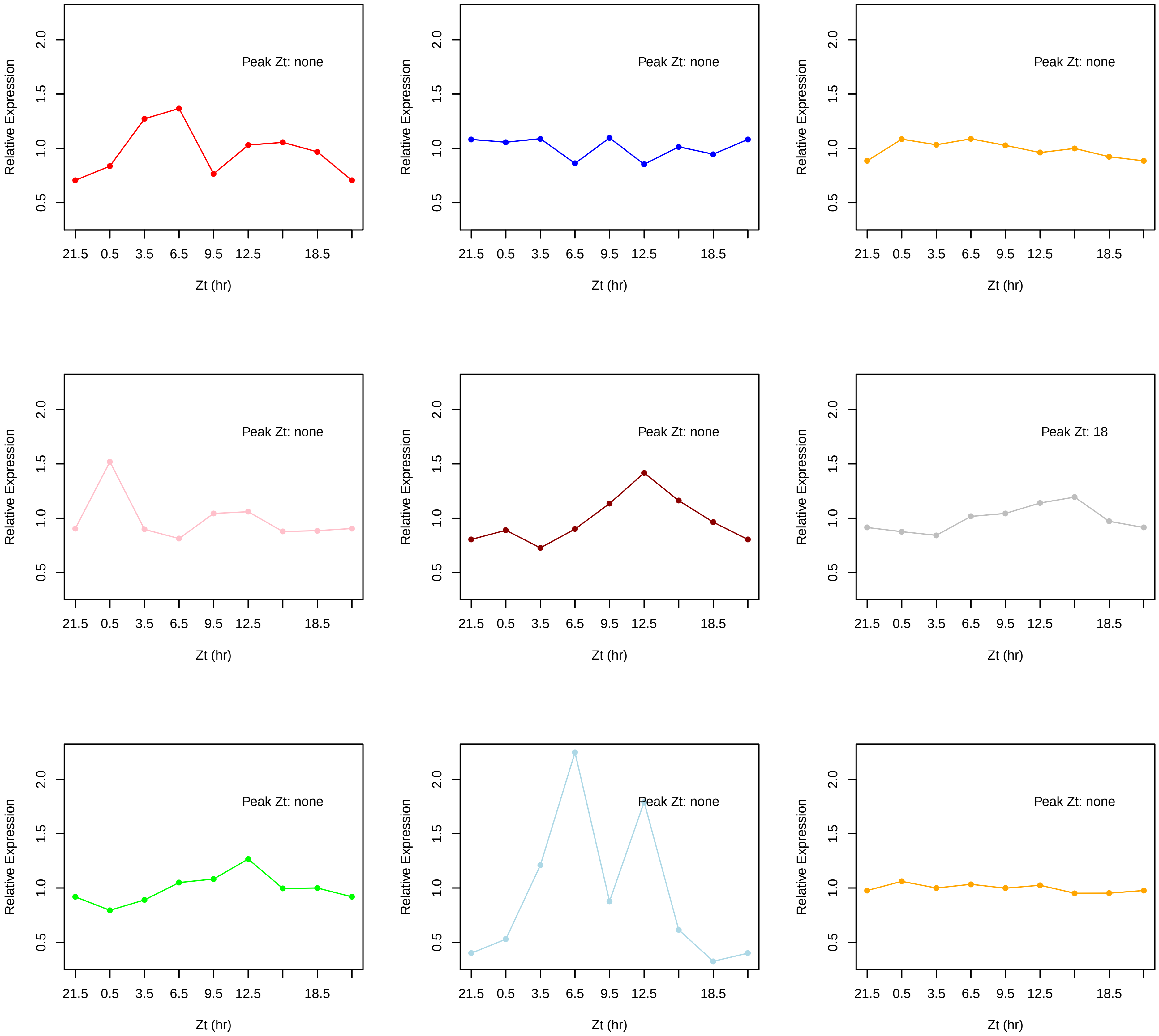

Singeleton: ENSXMAG00000005765 ctdp1

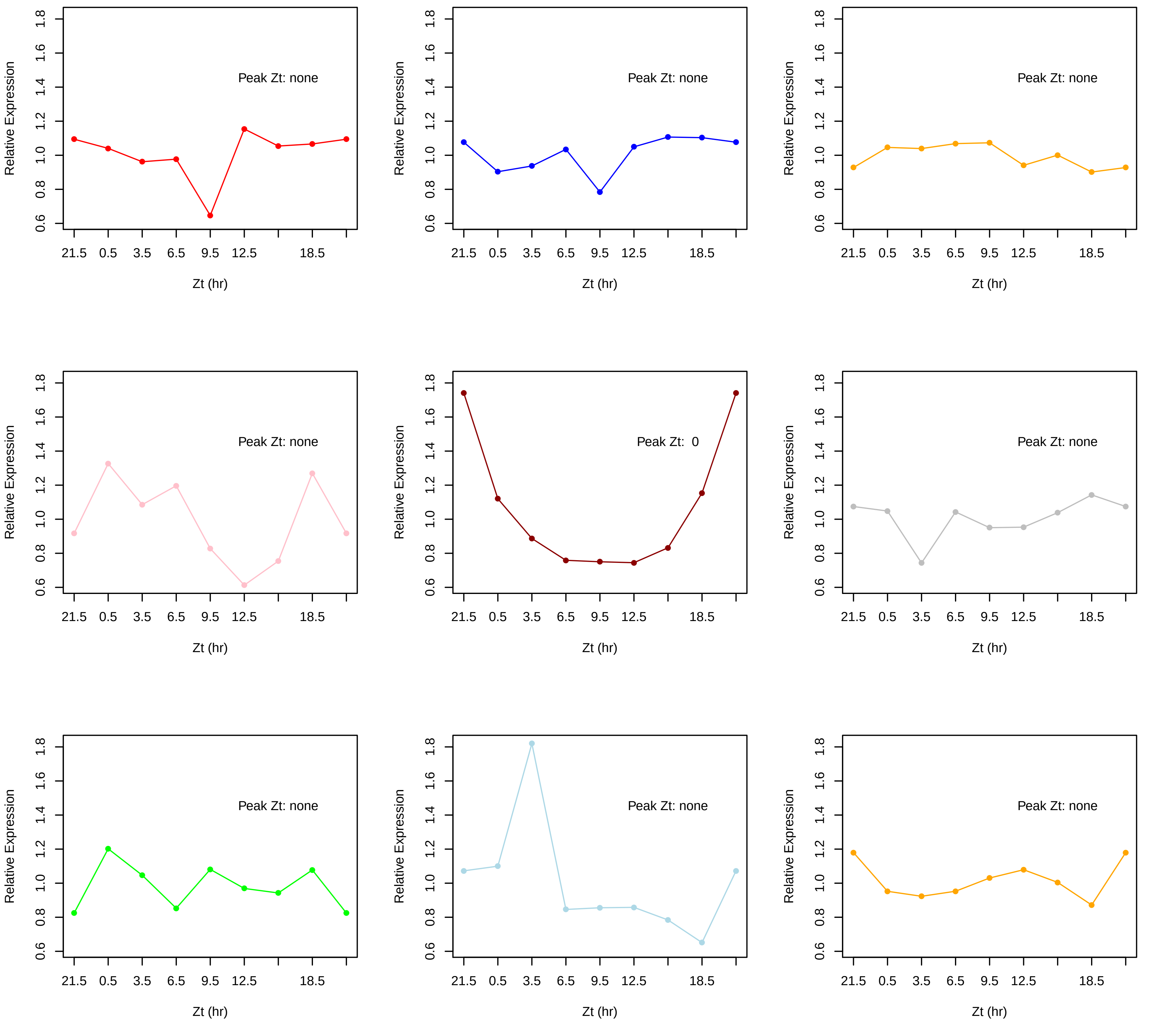

Singeleton: ENSXMAG00000014906 eif2ak1

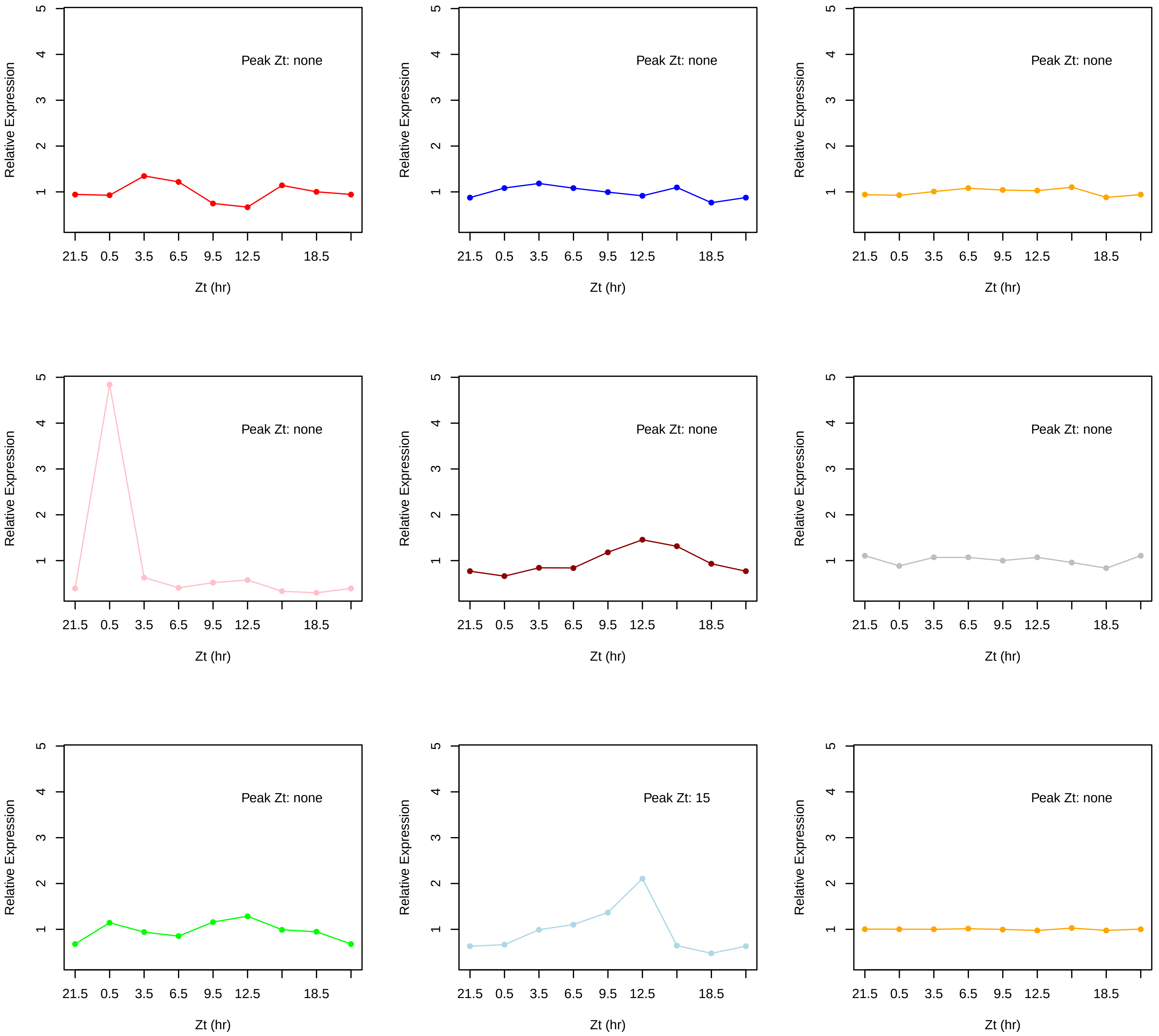

Singeleton: ENSXMAG00000011225 arhgap39

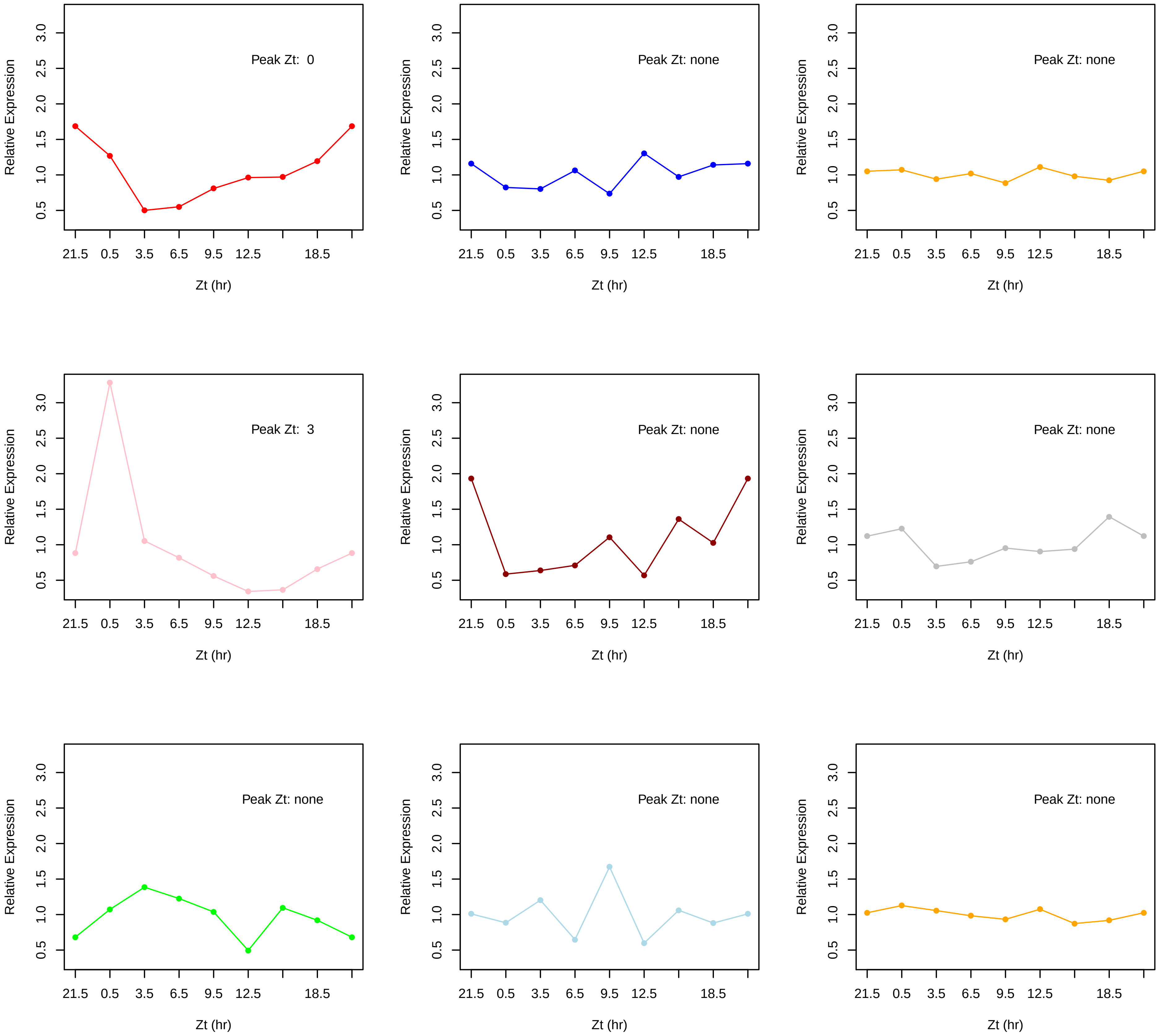

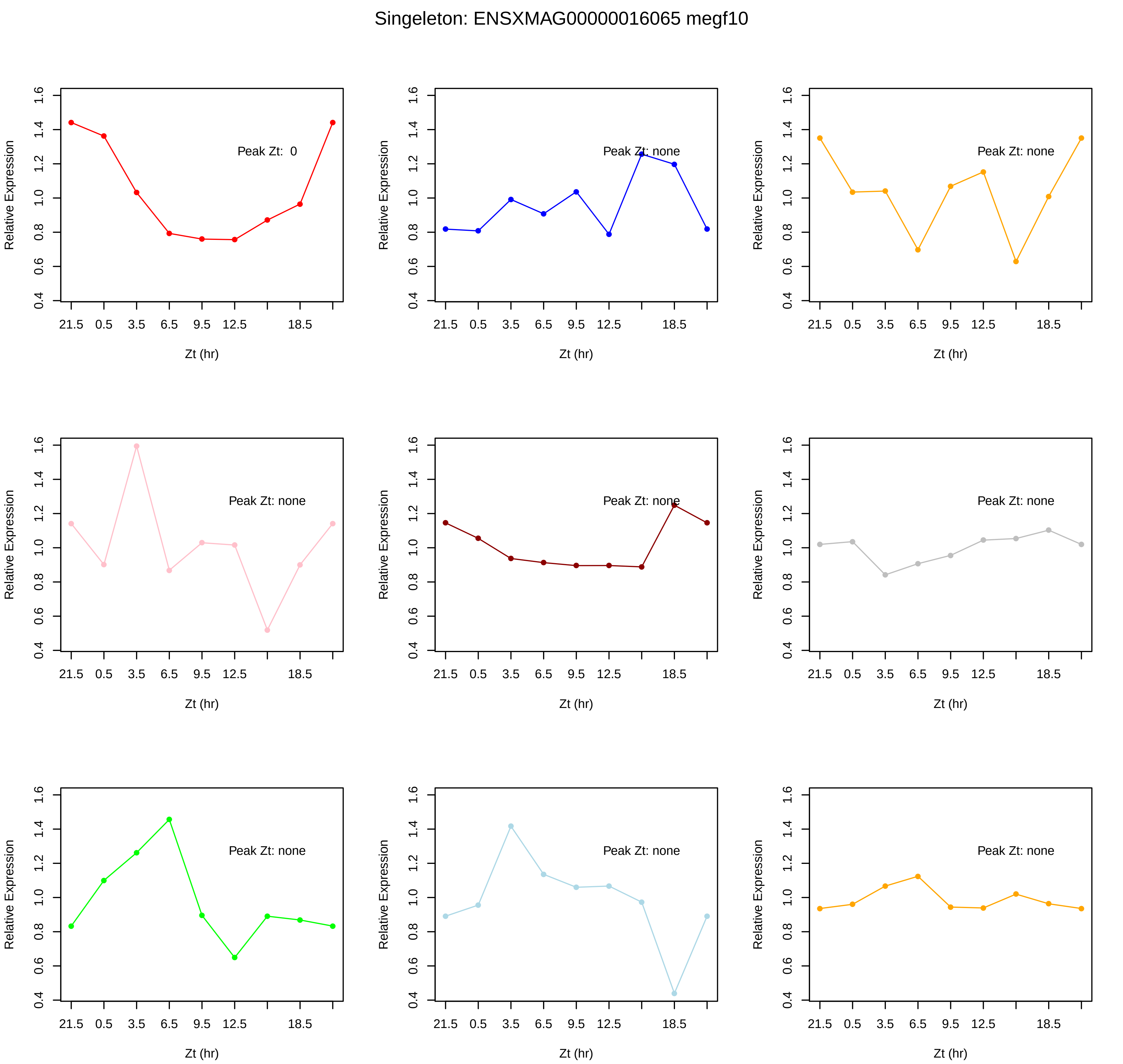
Singeleton: ENSXMAG00000016065 megf10
Peak Zt: 0
Peak Zt: none
Peak Zt: none
Peak Zt: none
Peak Zt: none
Peak Zt: none
Peak Zt: none
Peak Zt: none
Peak Zt: none
Relative Expression
Zt (hr)
21.5 0.5 3.5 6.5 9.5 12.5 18.5
0.4 0.6 0.8 1.0 1.2 1.4 1.6

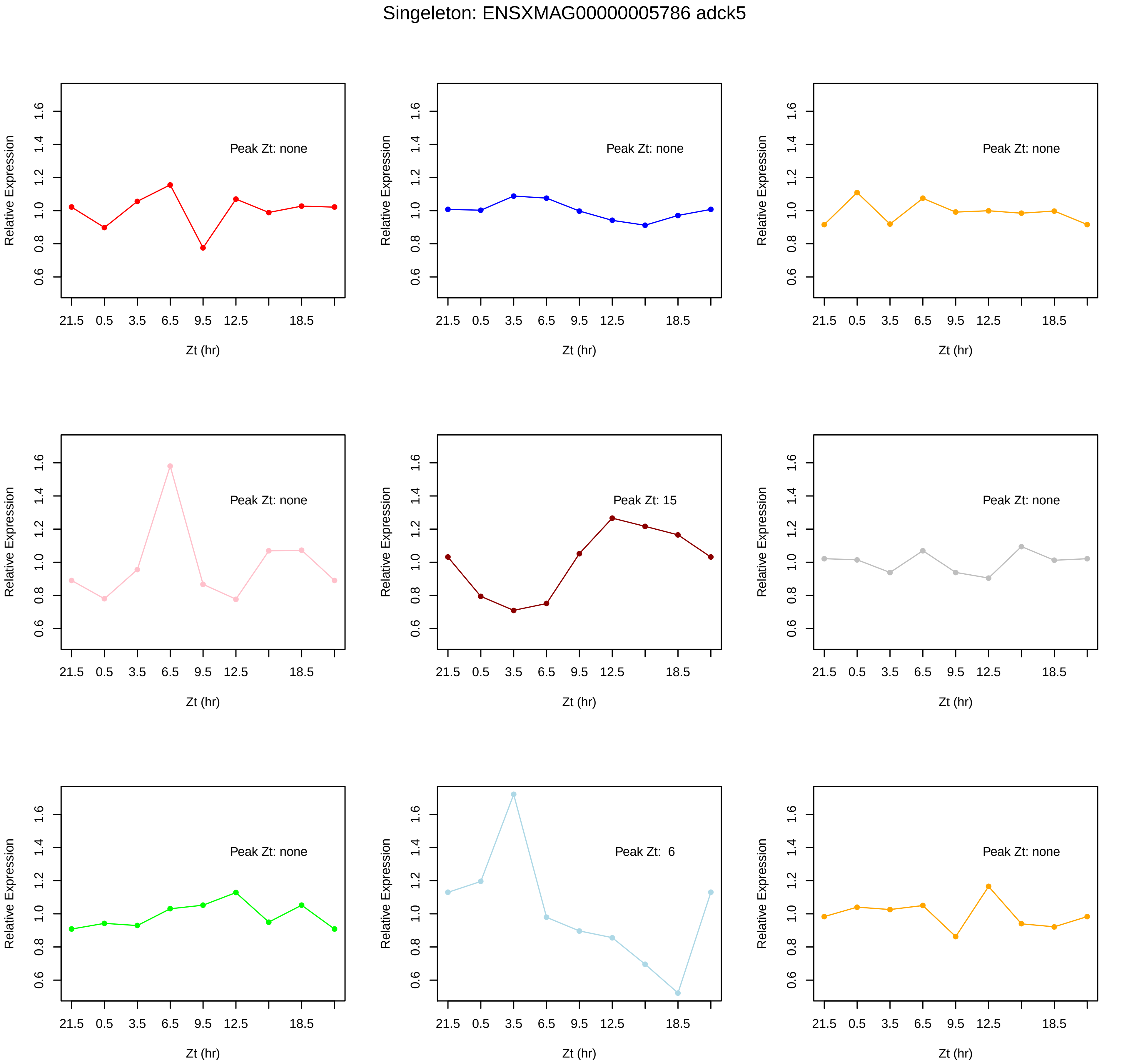
Singleton: ENSXMAG00000005786 adck5
Peak Zt: none
Peak Zt: none
Peak Zt: none
Peak Zt: none
Peak Zt: 15
Peak Zt: none
Peak Zt: none
Peak Zt: 6
Peak Zt: none
Relative Expression
Zt (hr)

Singeleton: ENSXMAG00000014055 lrch3

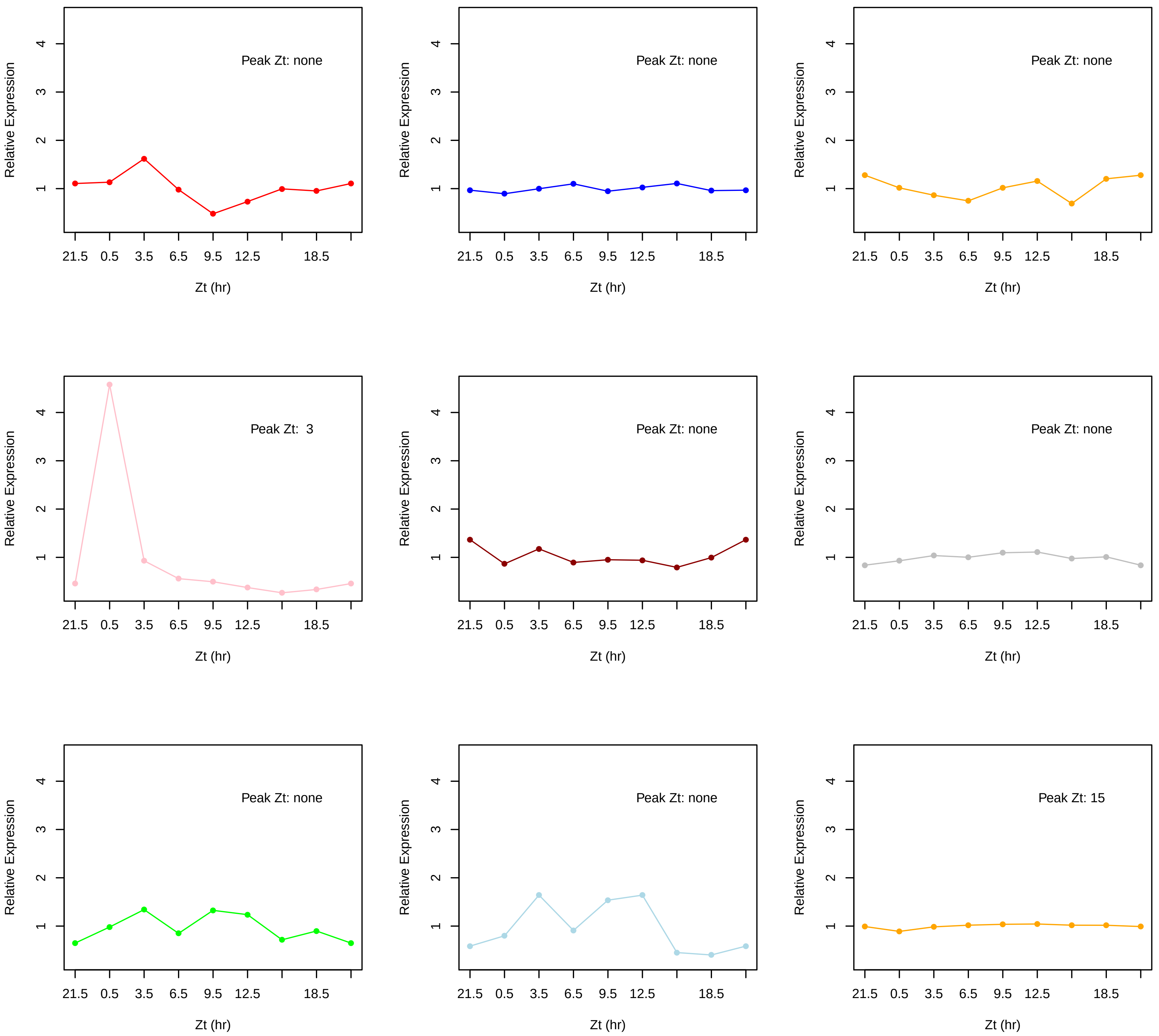

Singeleton: ENSXMAG00000008595

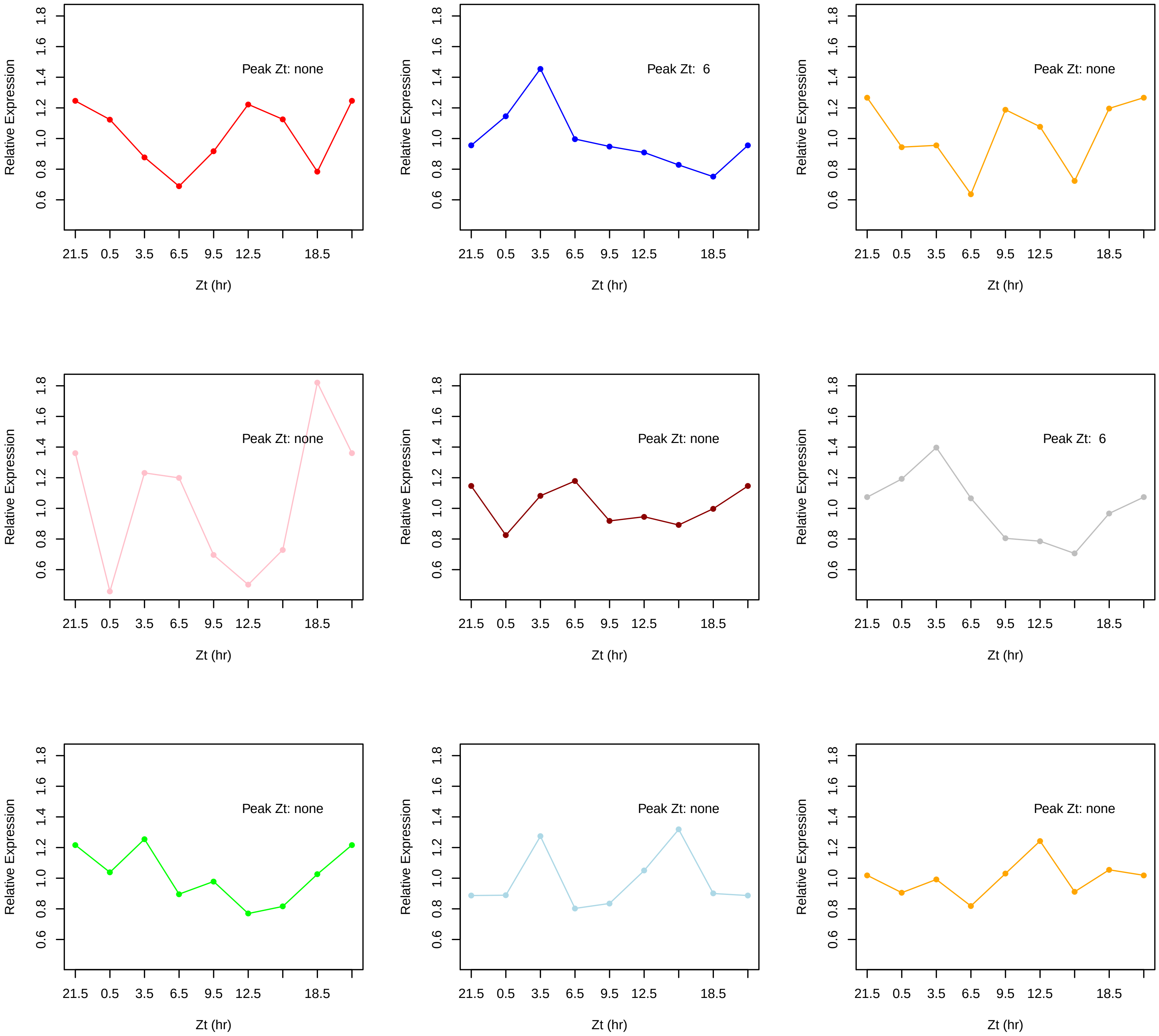

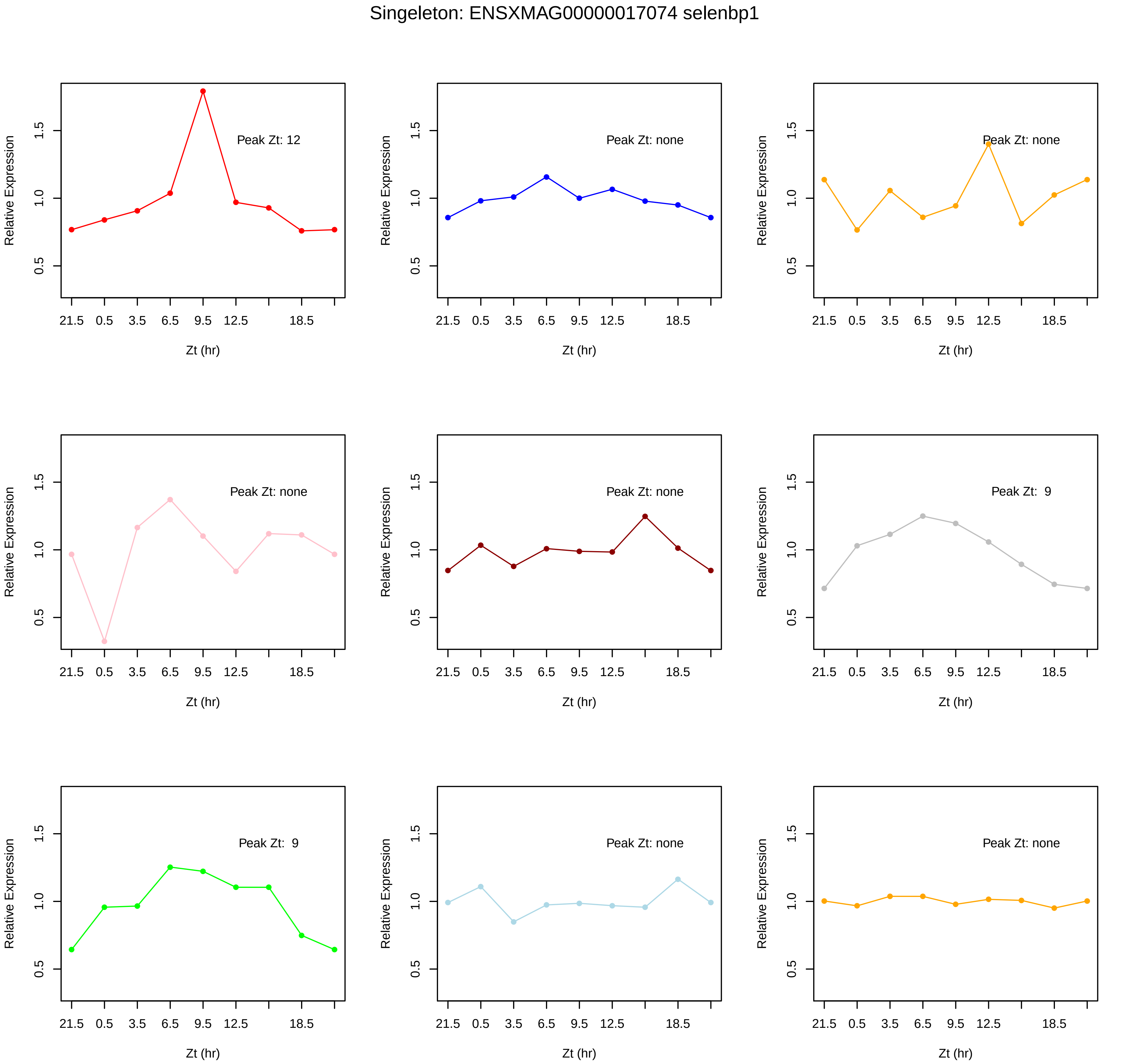
Singeleton: ENSXMAG00000017074 selenbp1
Peak Zt: 12
Peak Zt: none
Peak Zt: none
Peak Zt: none
Peak Zt: none
Peak Zt: 9
Peak Zt: 9
Peak Zt: none
Peak Zt: none
Relative Expression
Zt (hr)
21.5
0.5
3.5
6.5
9.5
12.5
18.5
0.5
1.0
1.5

Singeleton: ENSXMAG00000012211 kcnk10b

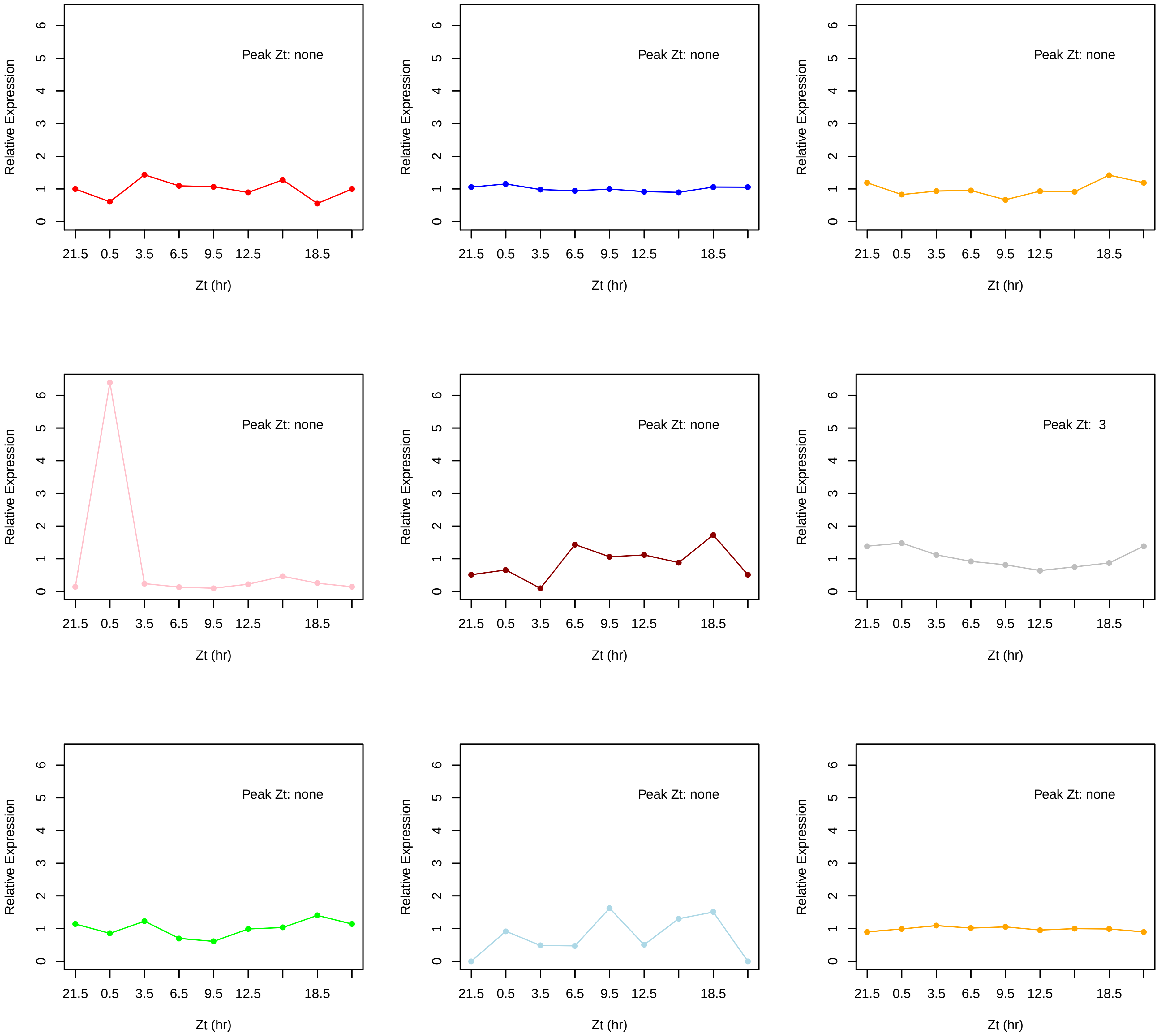

Singeleton: ENSXMAG00000005798 cpsf1

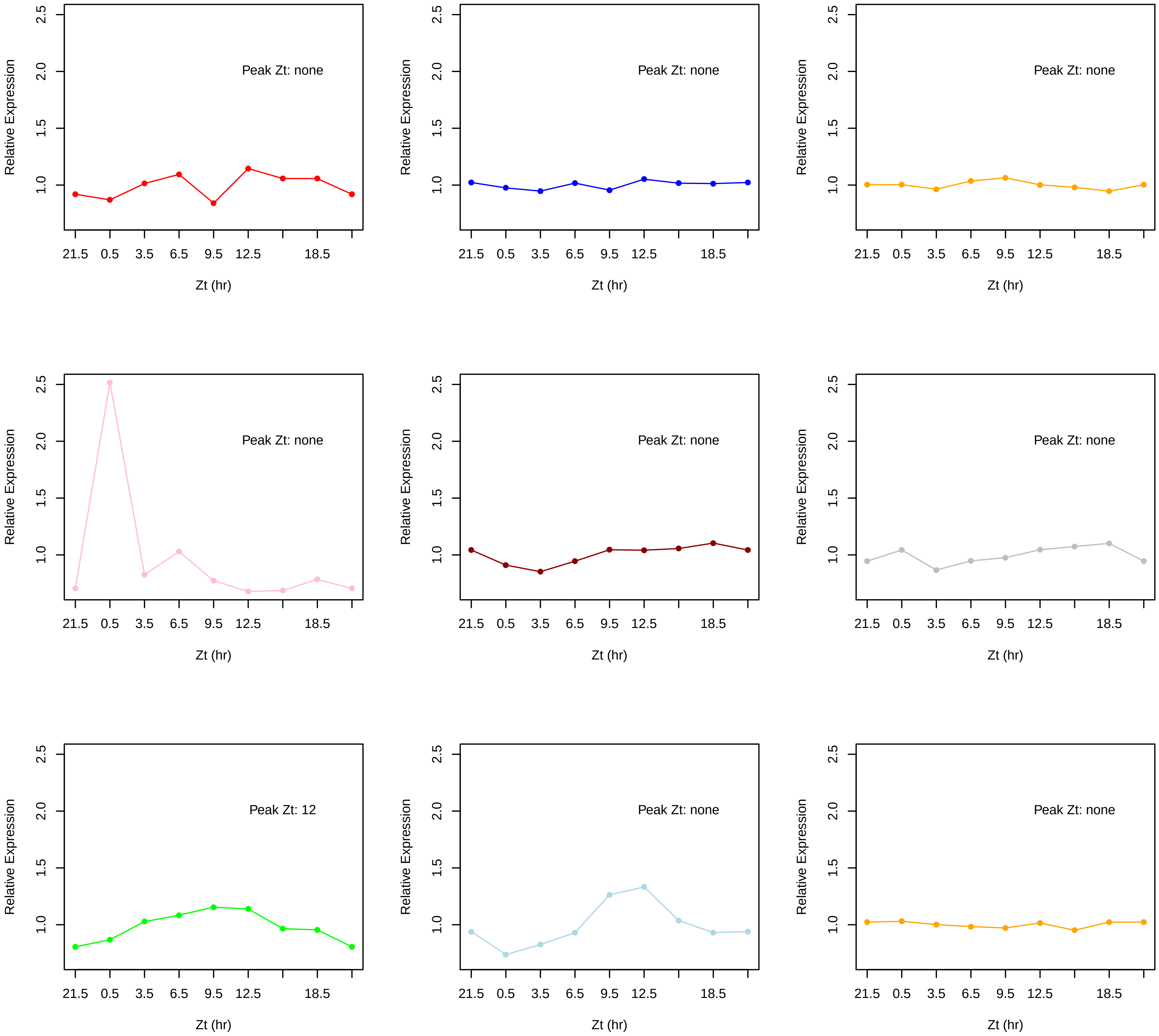

Singleton: ENSXMAG00000005312 ddx54

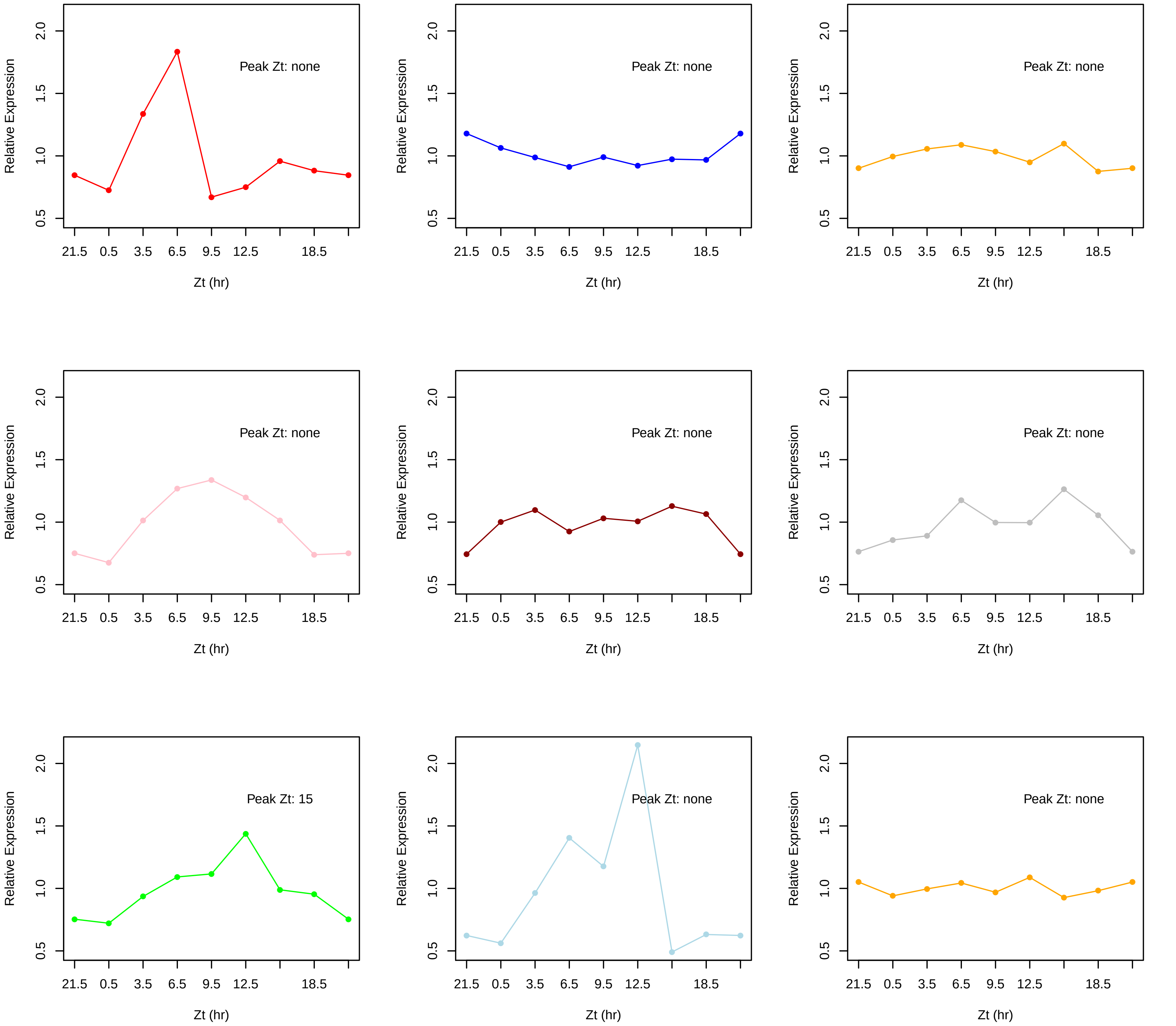

Singeleton: ENSXMAG00000008607 alg5

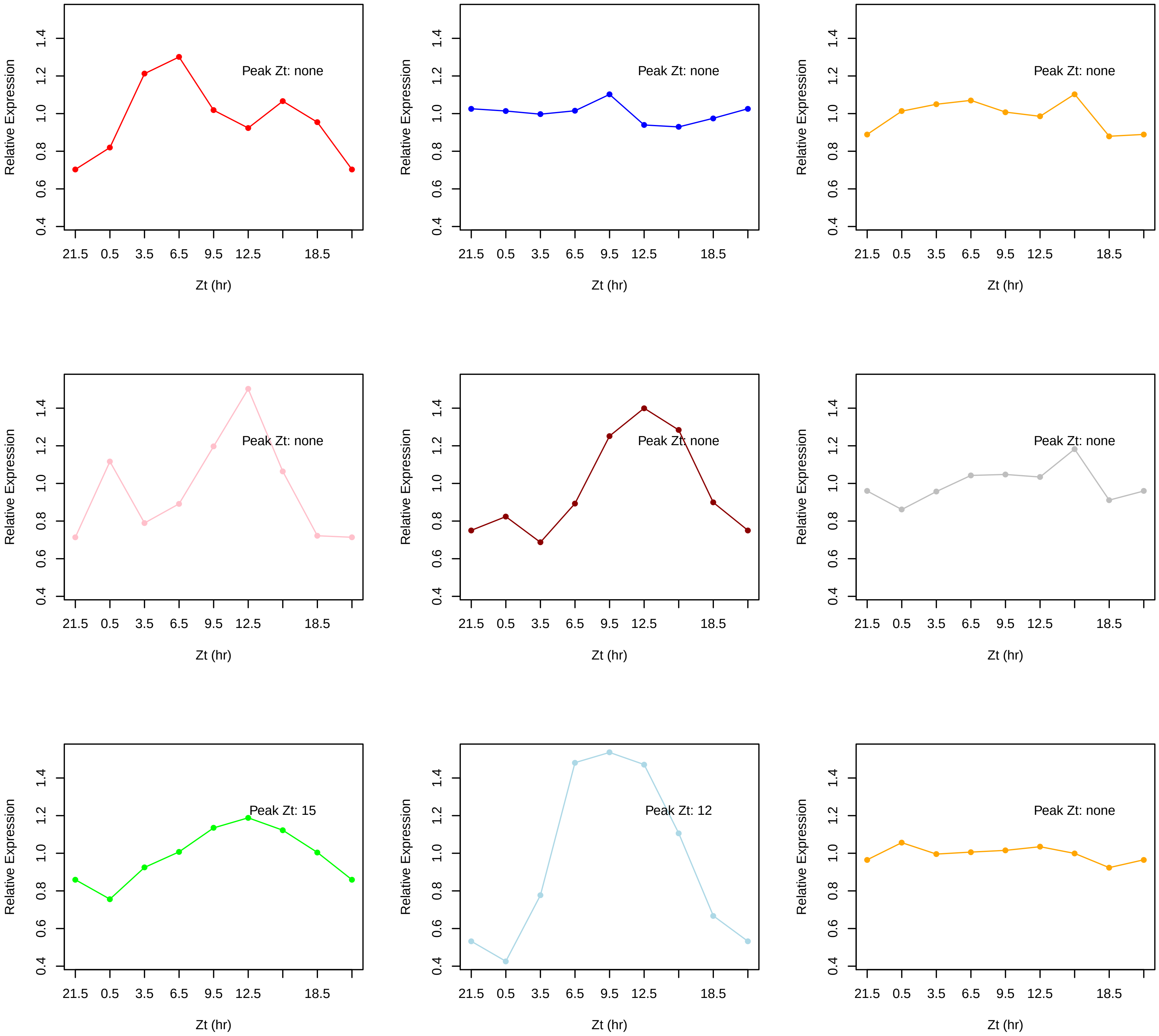

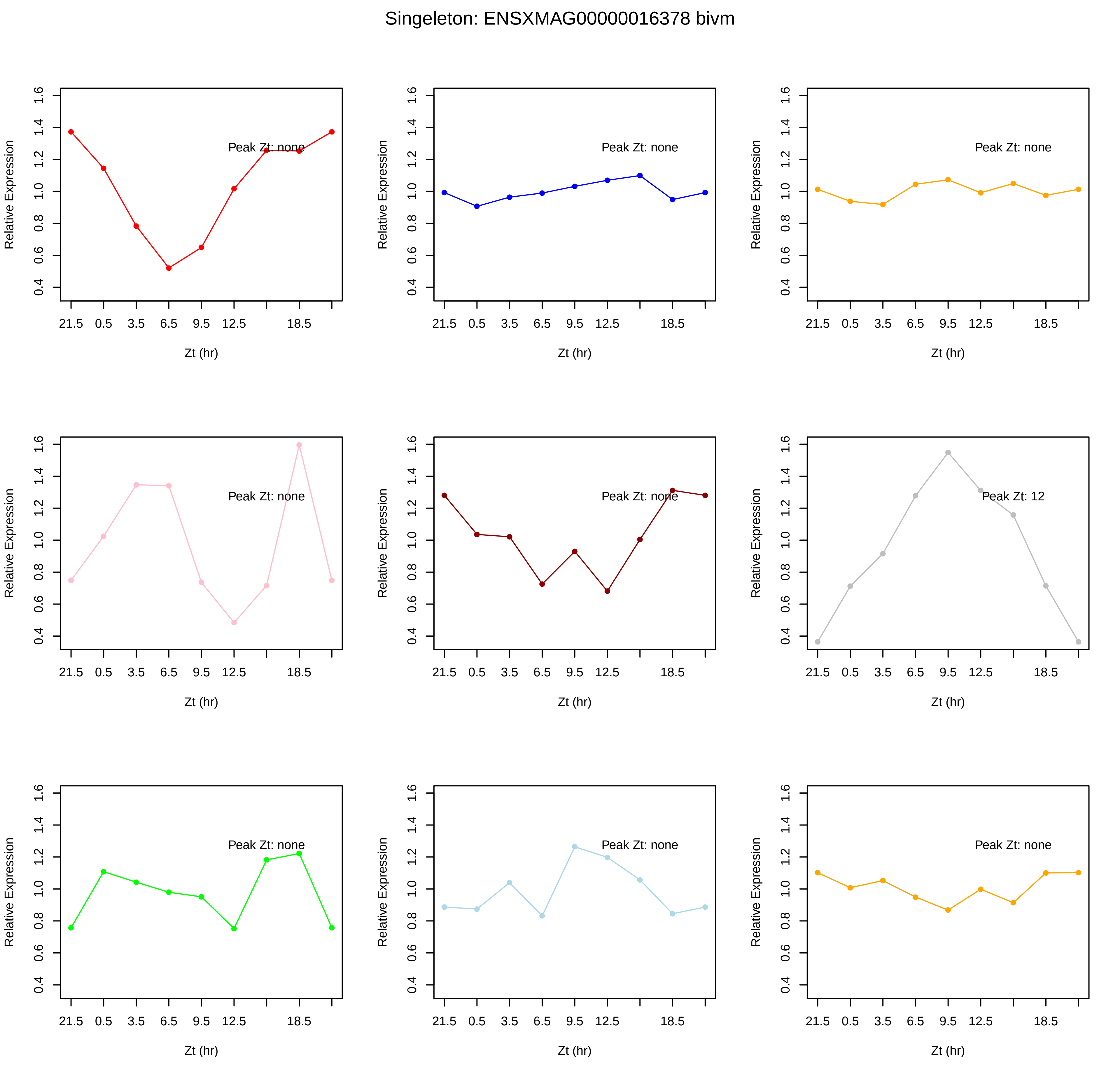
Singeleton: ENSXMAG00000016378 bivm
Peak Zt: none
Peak Zt: none
Peak Zt: none
Peak Zt: none
Peak Zt: none
Peak Zt: 12
Peak Zt: none
Peak Zt: none
Peak Zt: none
Relative Expression
Zt (hr)
21.5
0.5
3.5
6.5
9.5
12.5
18.5
0.4
0.6
0.8
1.0
1.2
1.4
1.6

Singeleton: ENSXMAG00000018569 ecd

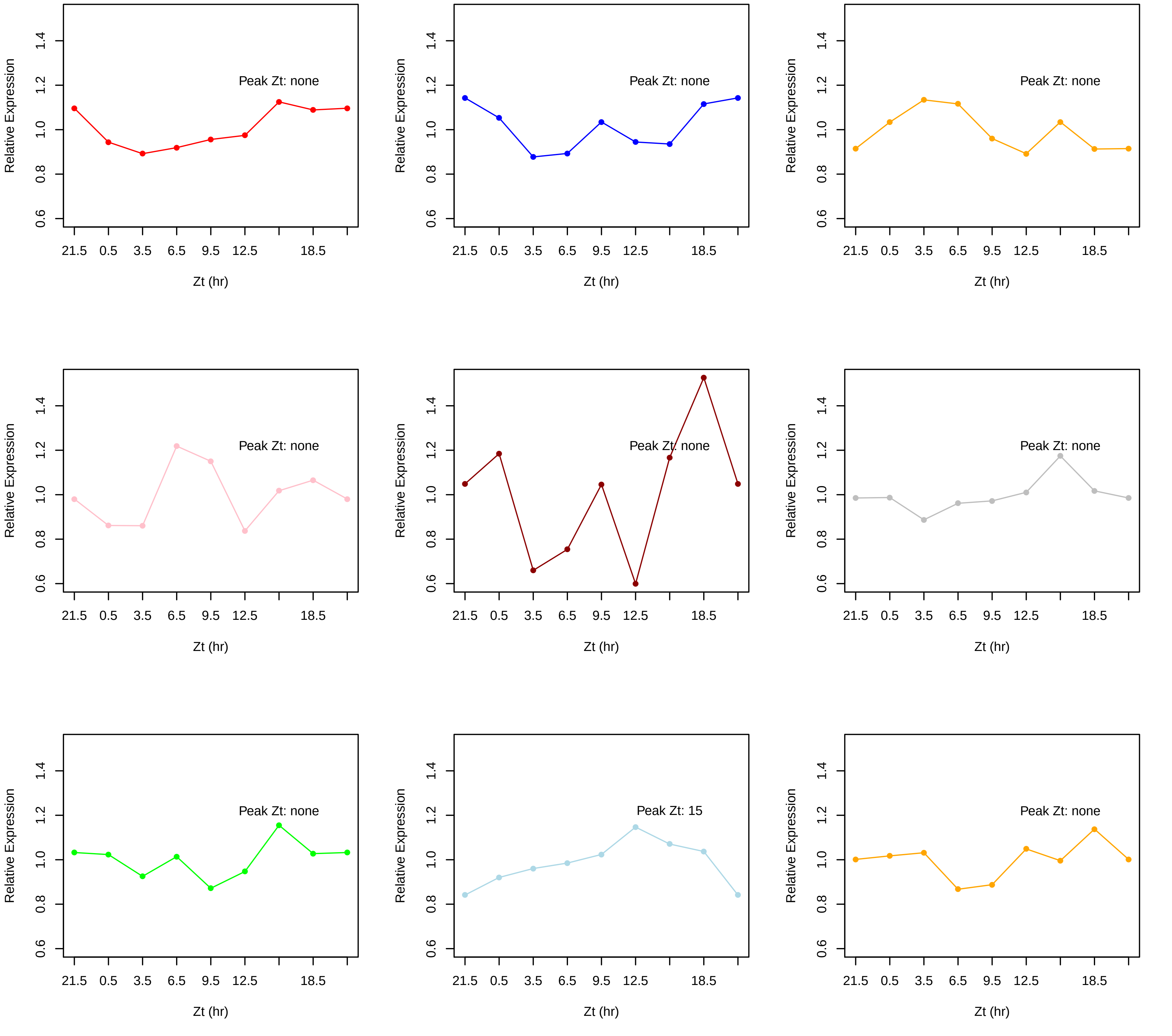

Singleton: ENSXMAG00000005783 rubcn

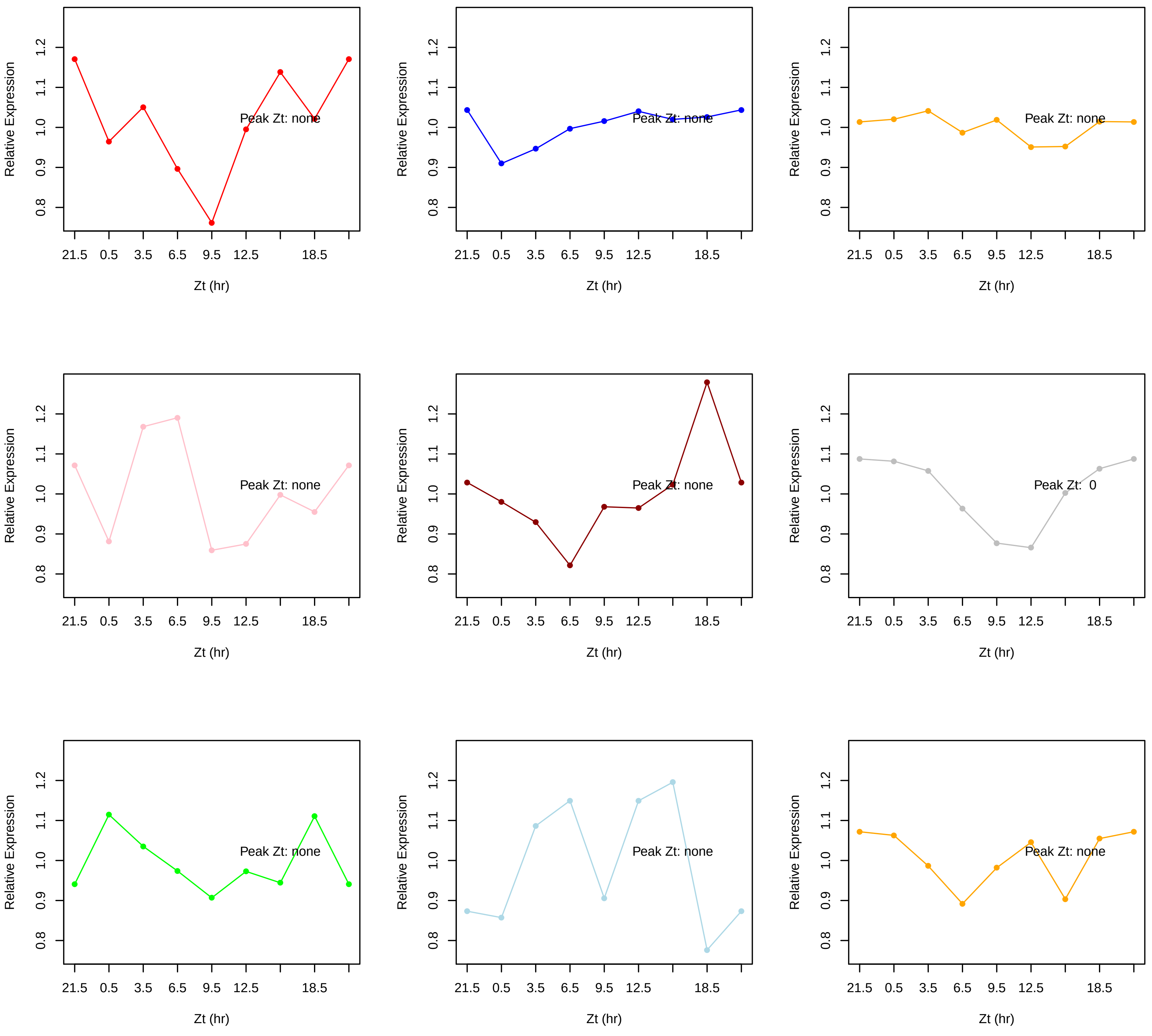

Singeleton: ENSXMAG00000025645 glrx5

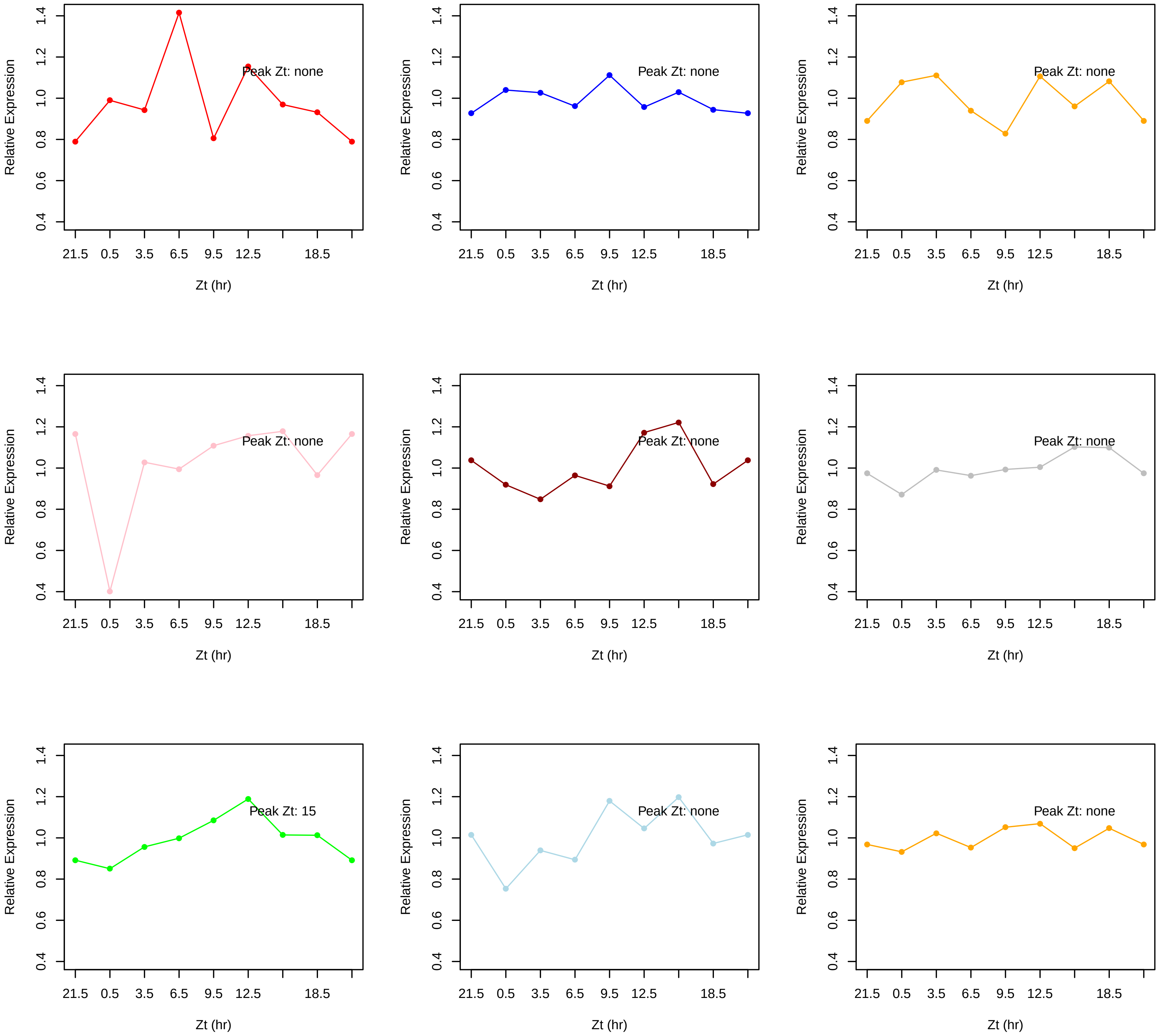

Singeleton: ENSXMAG00000005975 pycr3

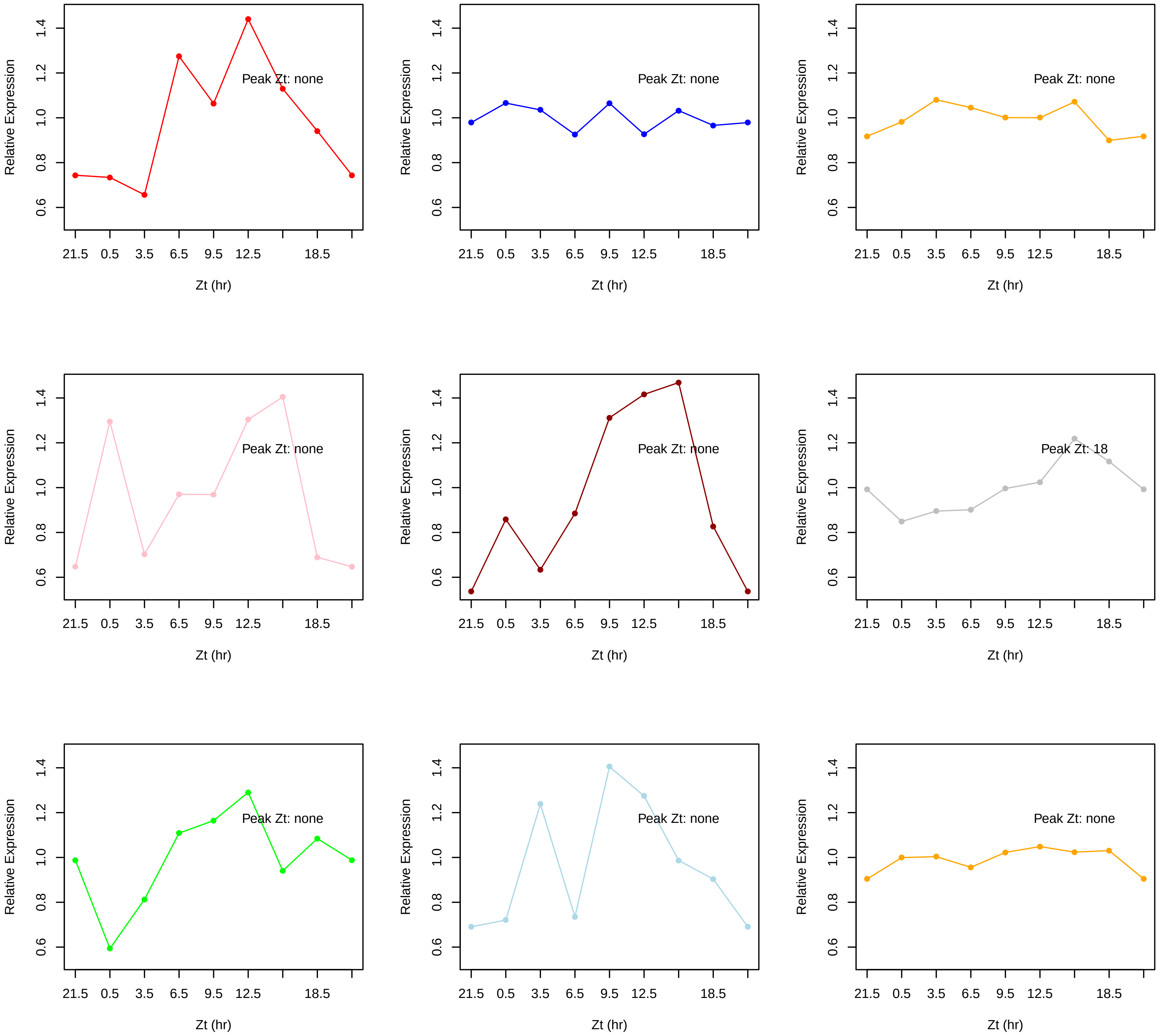

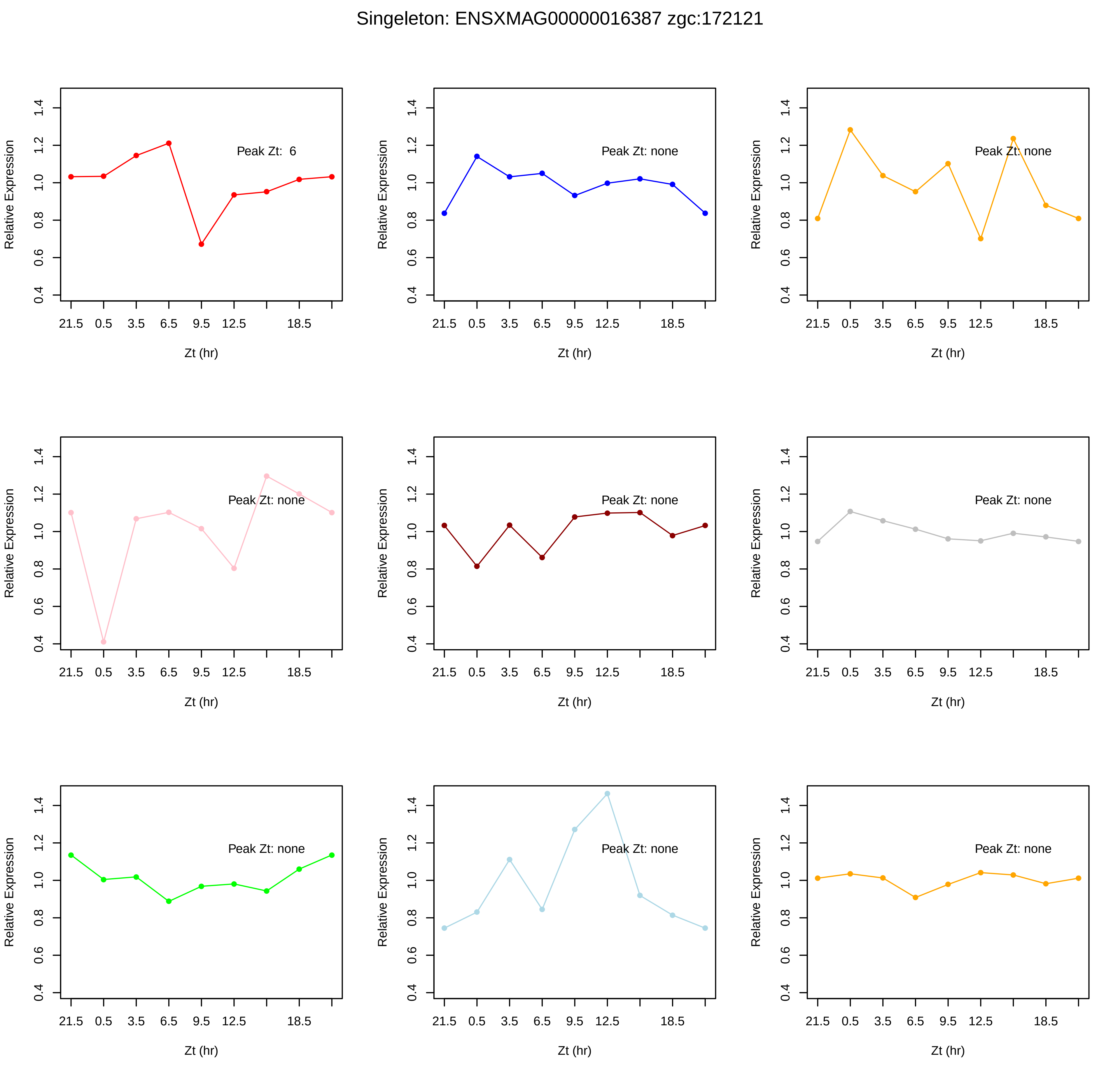

Singeleton: ENSXMAG00000016387 zgc:172121
Peak Zt: 6
Peak Zt: none
Relative Expression
Zt (hr)
21.5
0.5
3.5
6.5
9.5
12.5
18.5
0.4
0.6
0.8
1.0
1.2
1.4

Singleton: ENSXMAG00000024025 syne3

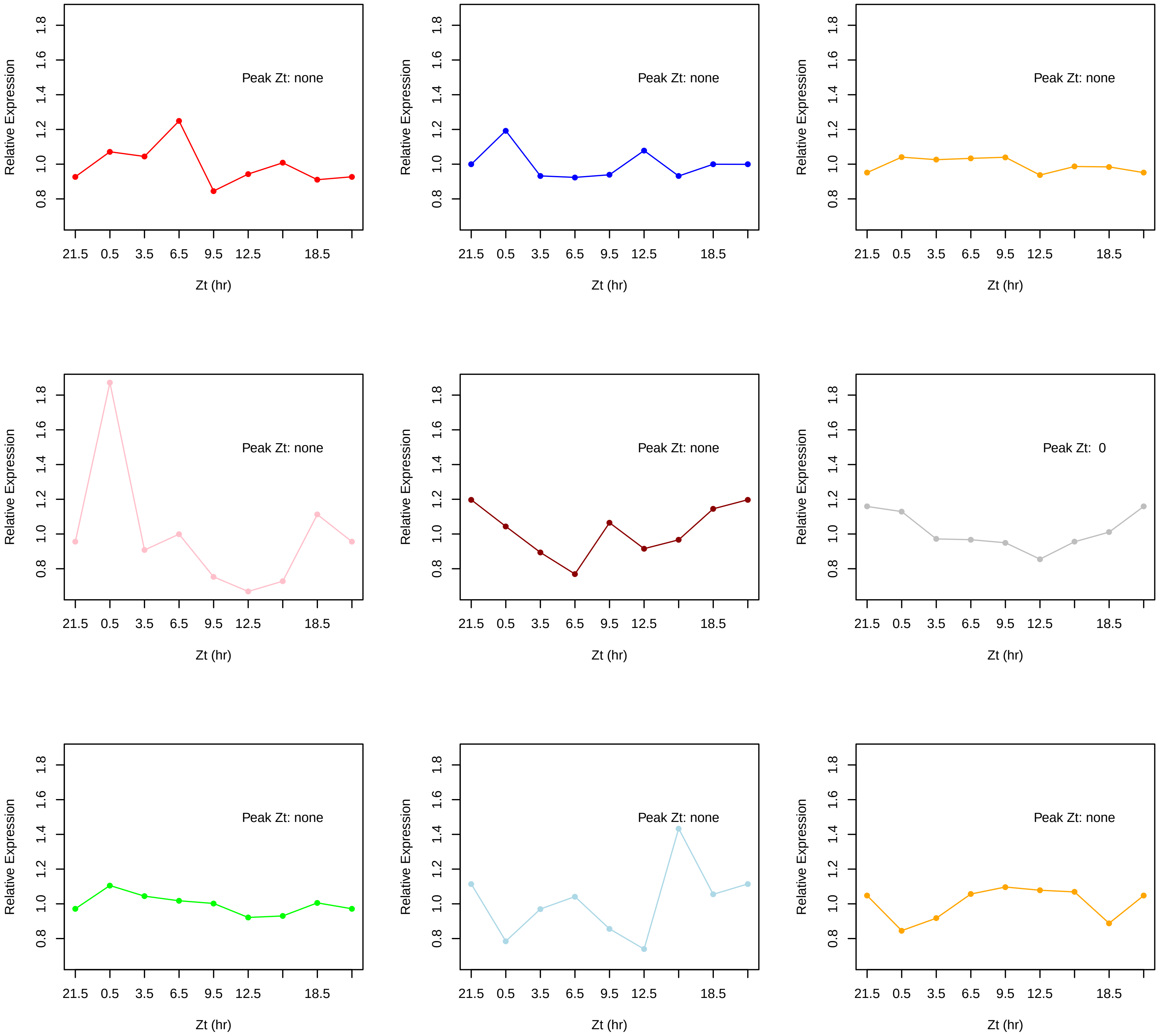

Singeleton: ENSXMAG00000005913

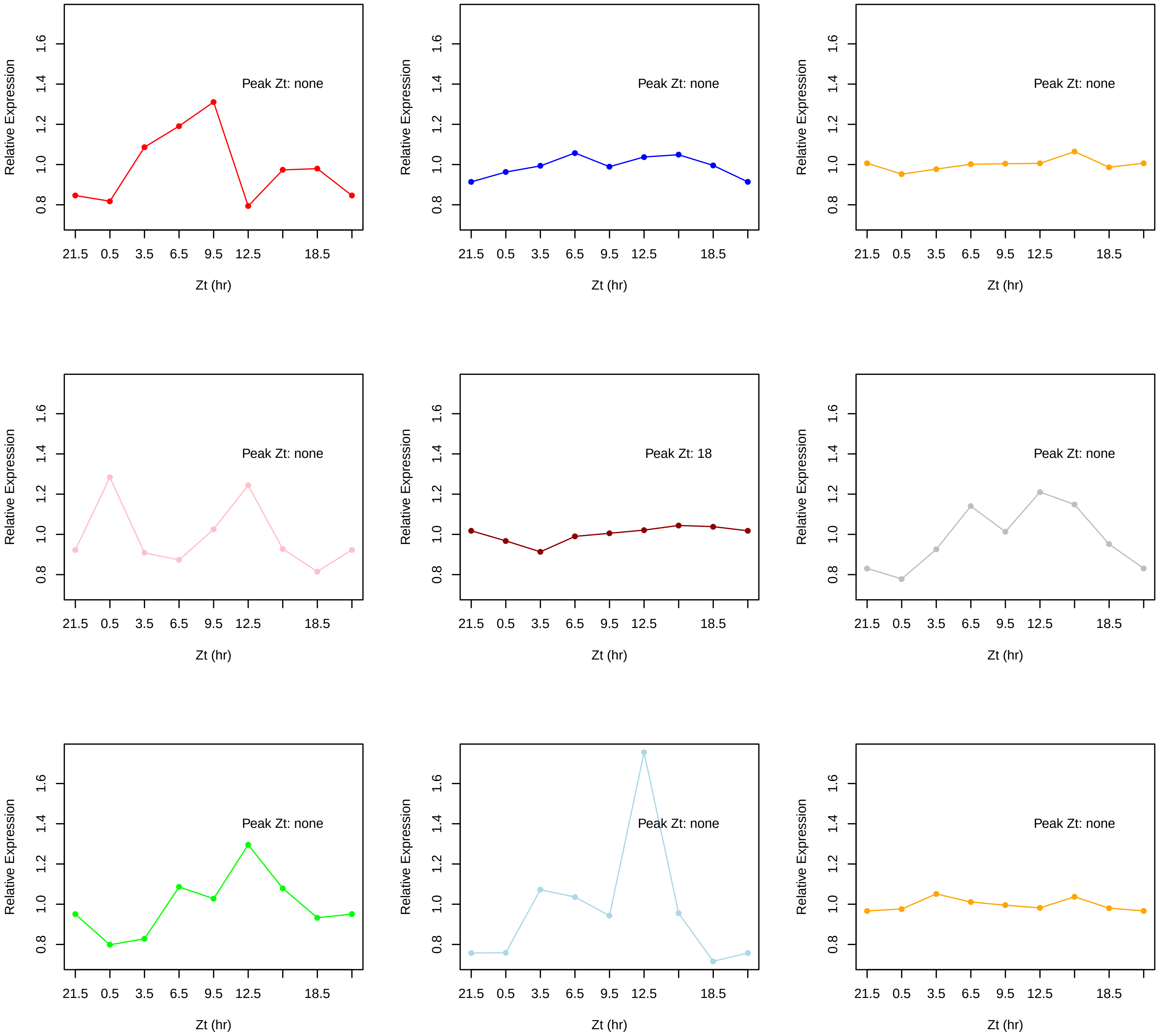

Singeleton: ENSXMAG00000014852 ccz1

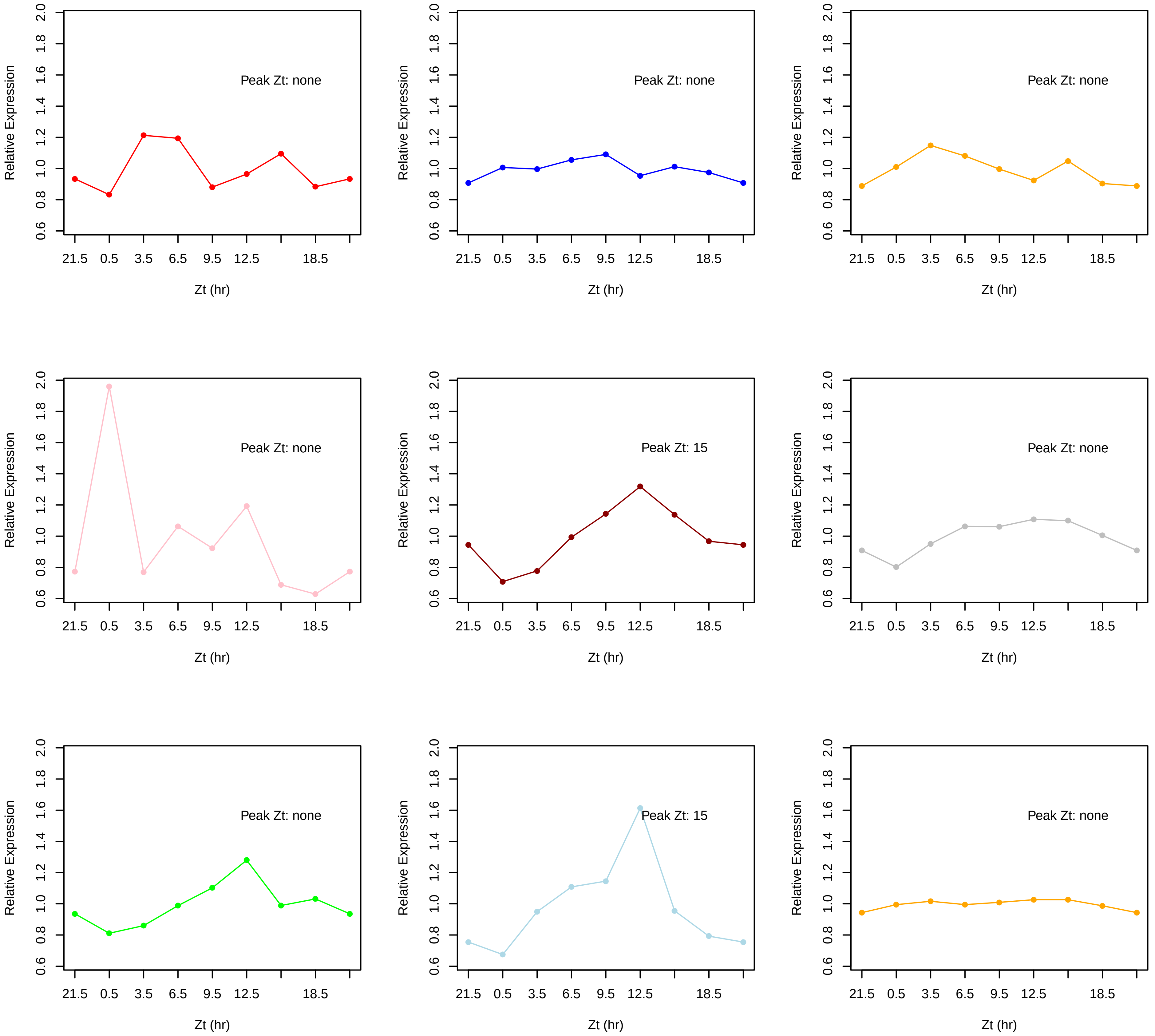

Singeleton: ENSXMAG00000001816

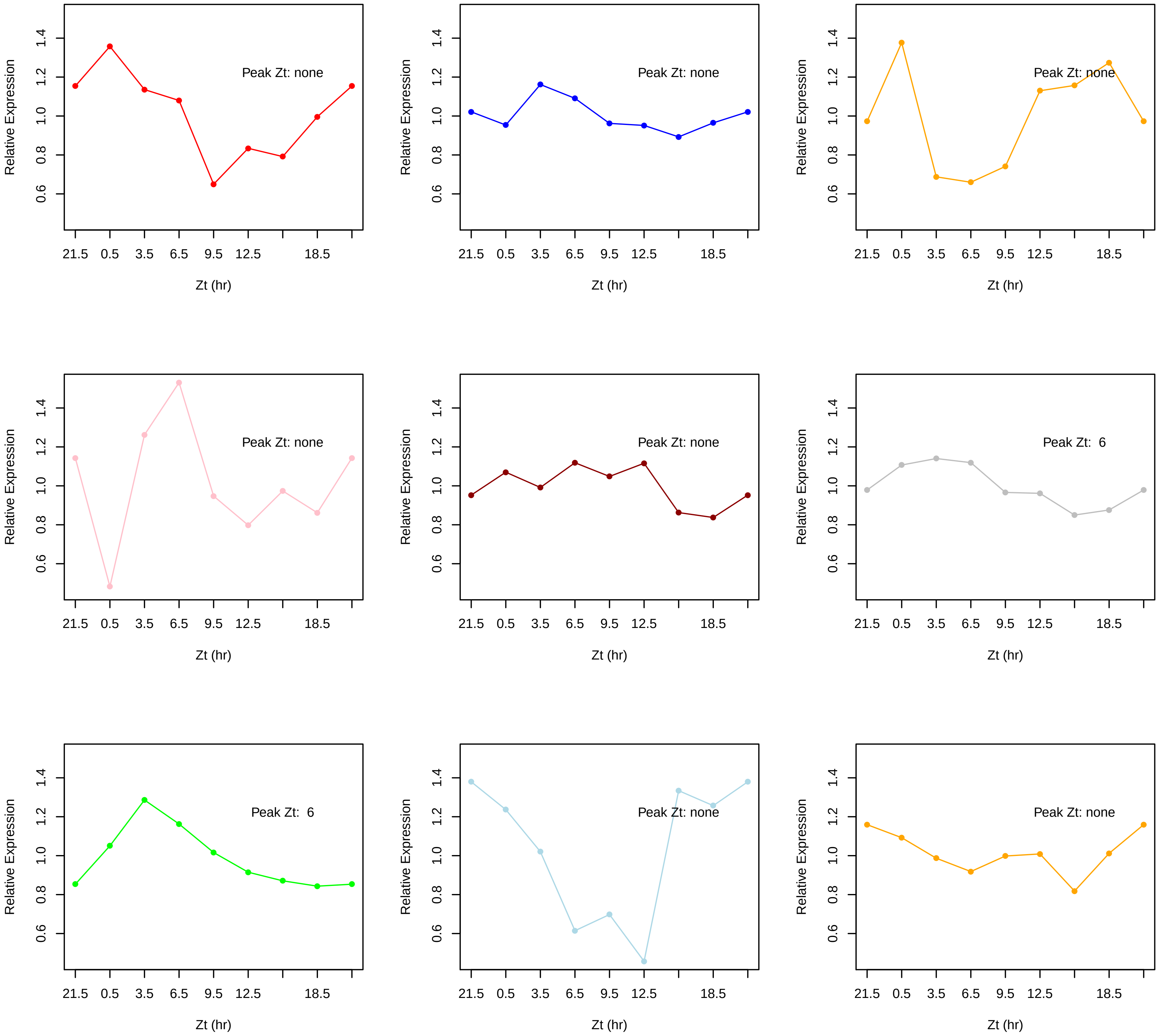

Singleton: ENSXMAG00000015721 dicer1

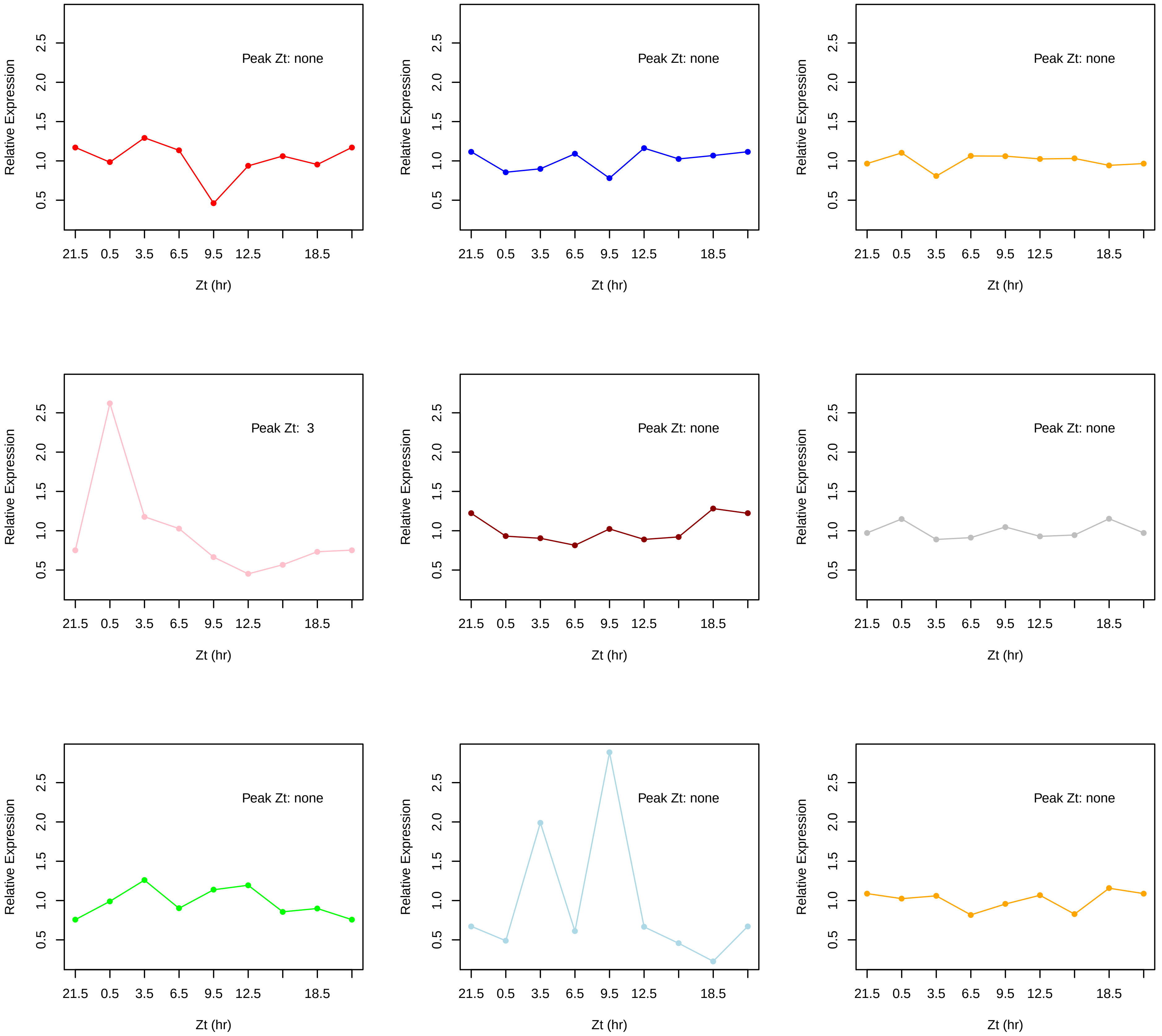

Singeleton: ENSXMAG00000004751 zgc:66475

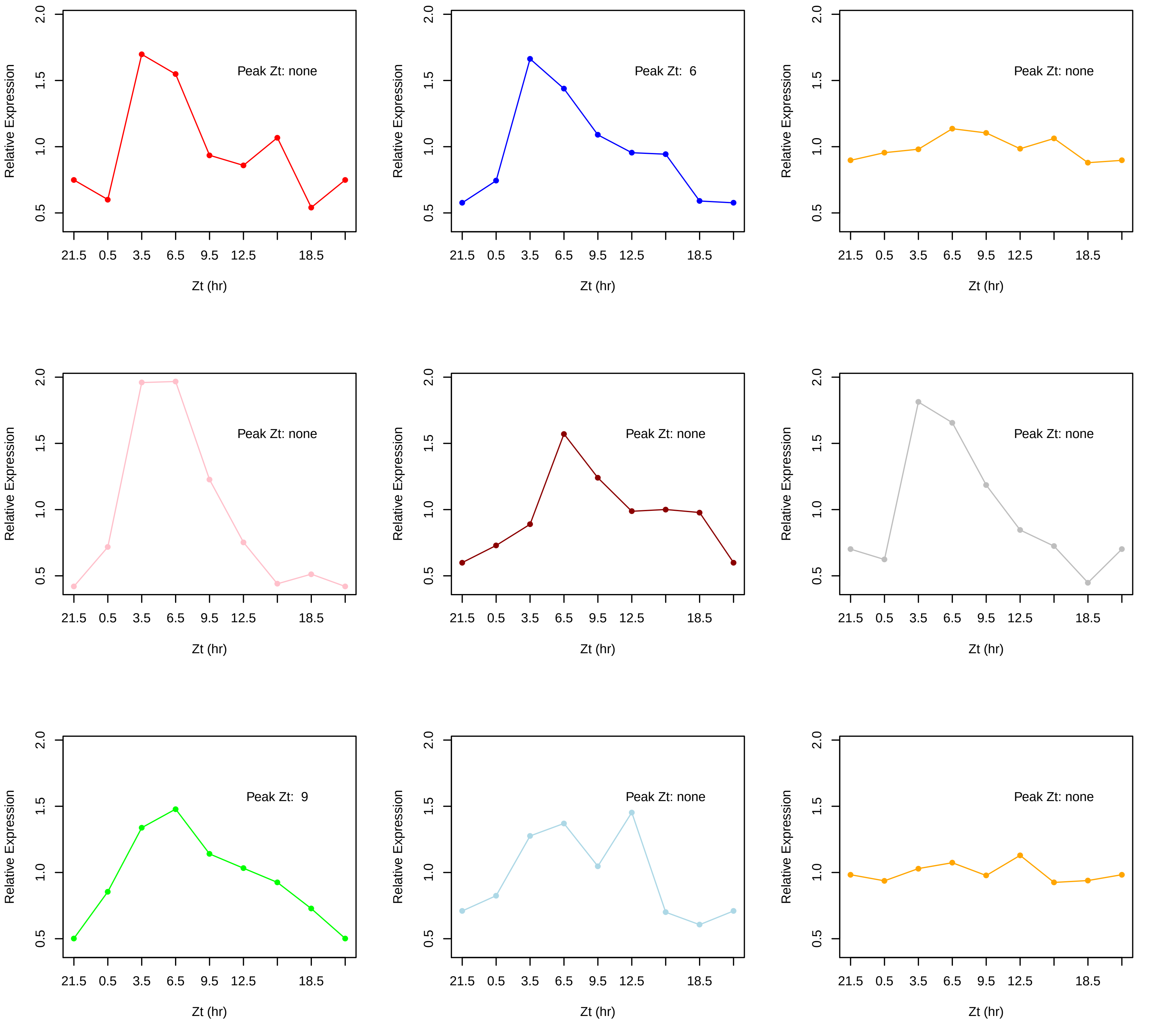

Singeleton: ENSXMAG00000017770 selenom

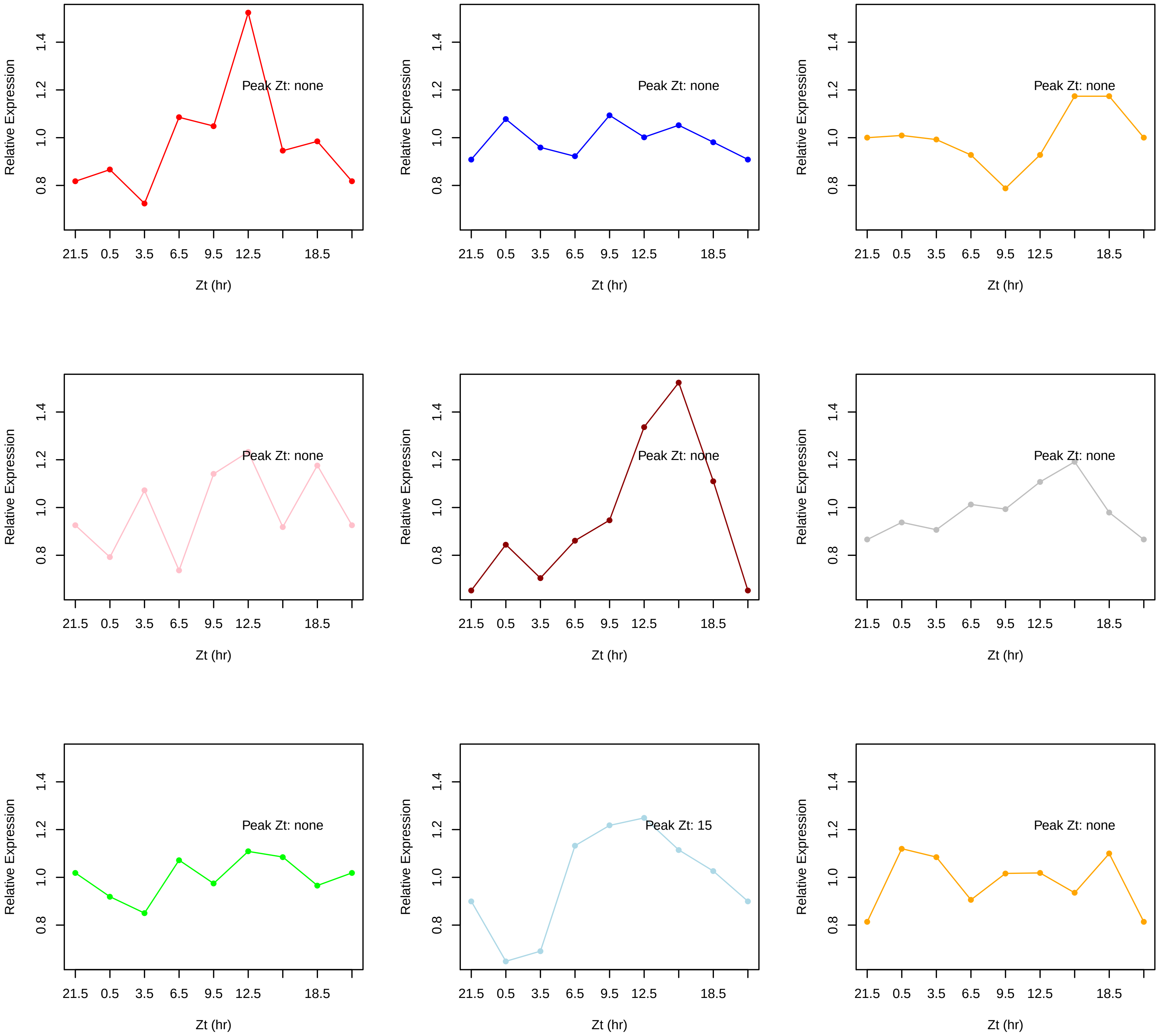

Singeleton: ENSXMAG00000008071 gatb

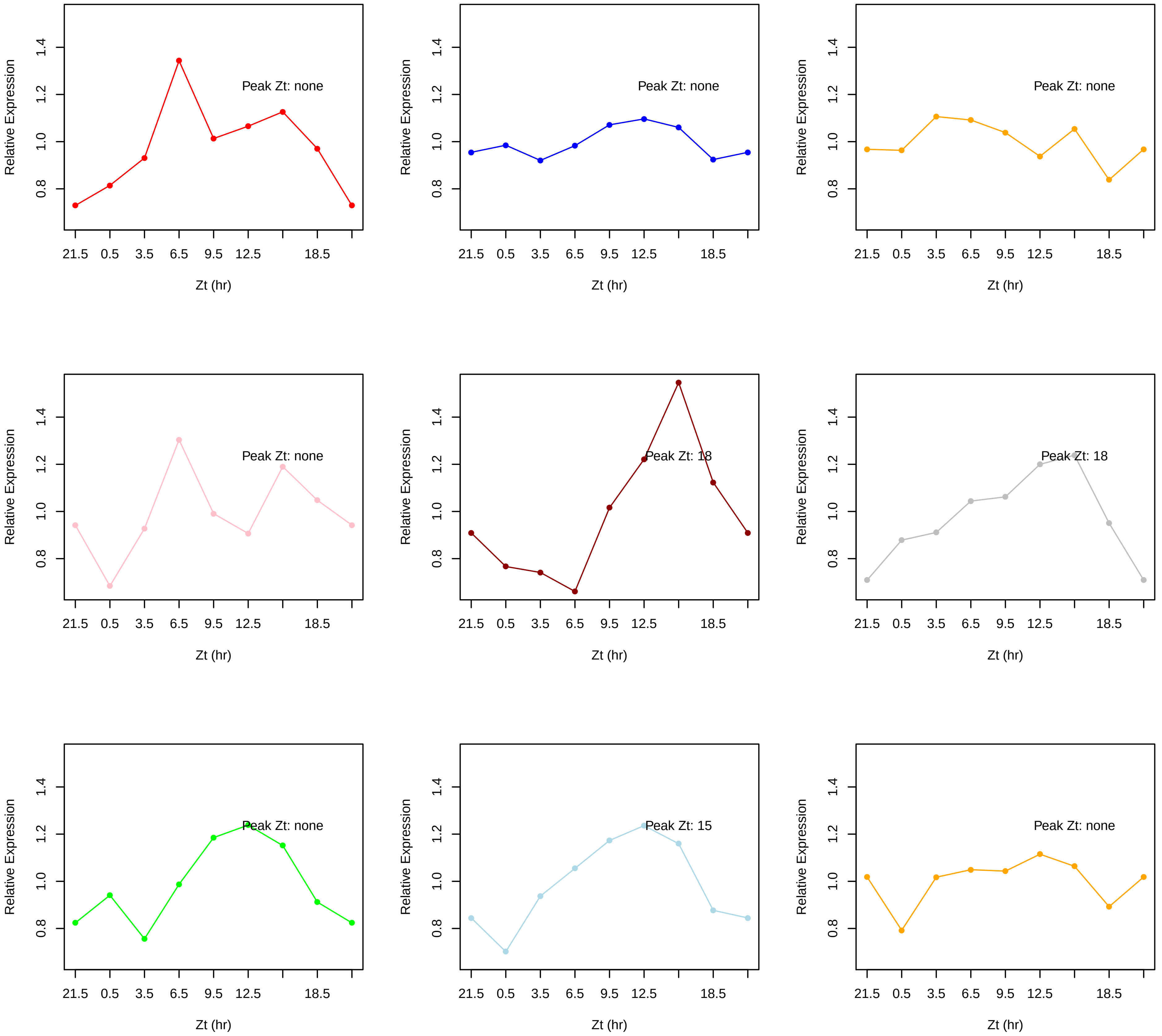

Singeleton: ENSXMAG00000028770

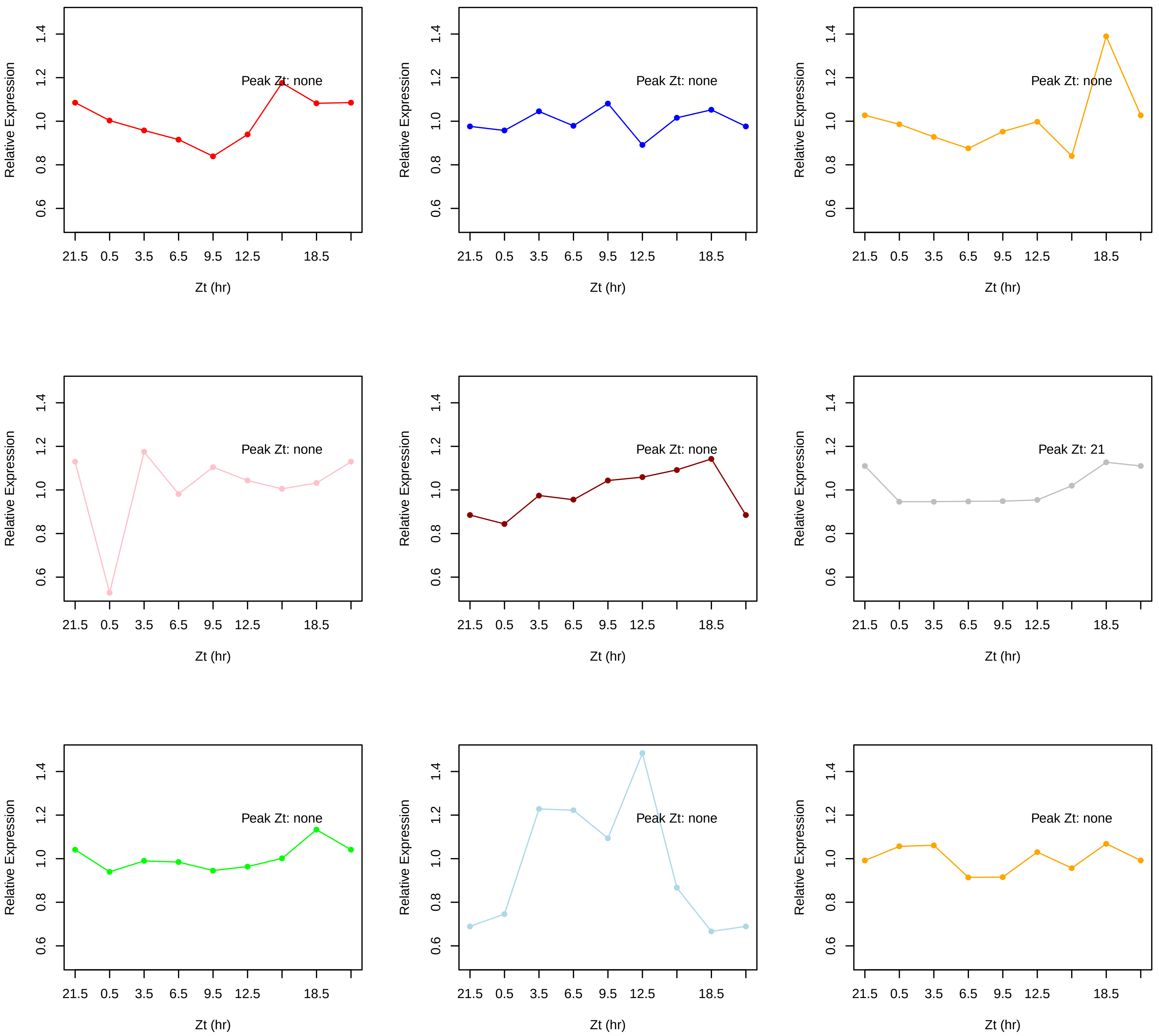

Singeleton: ENSXMAG00000019958 aldh7a1

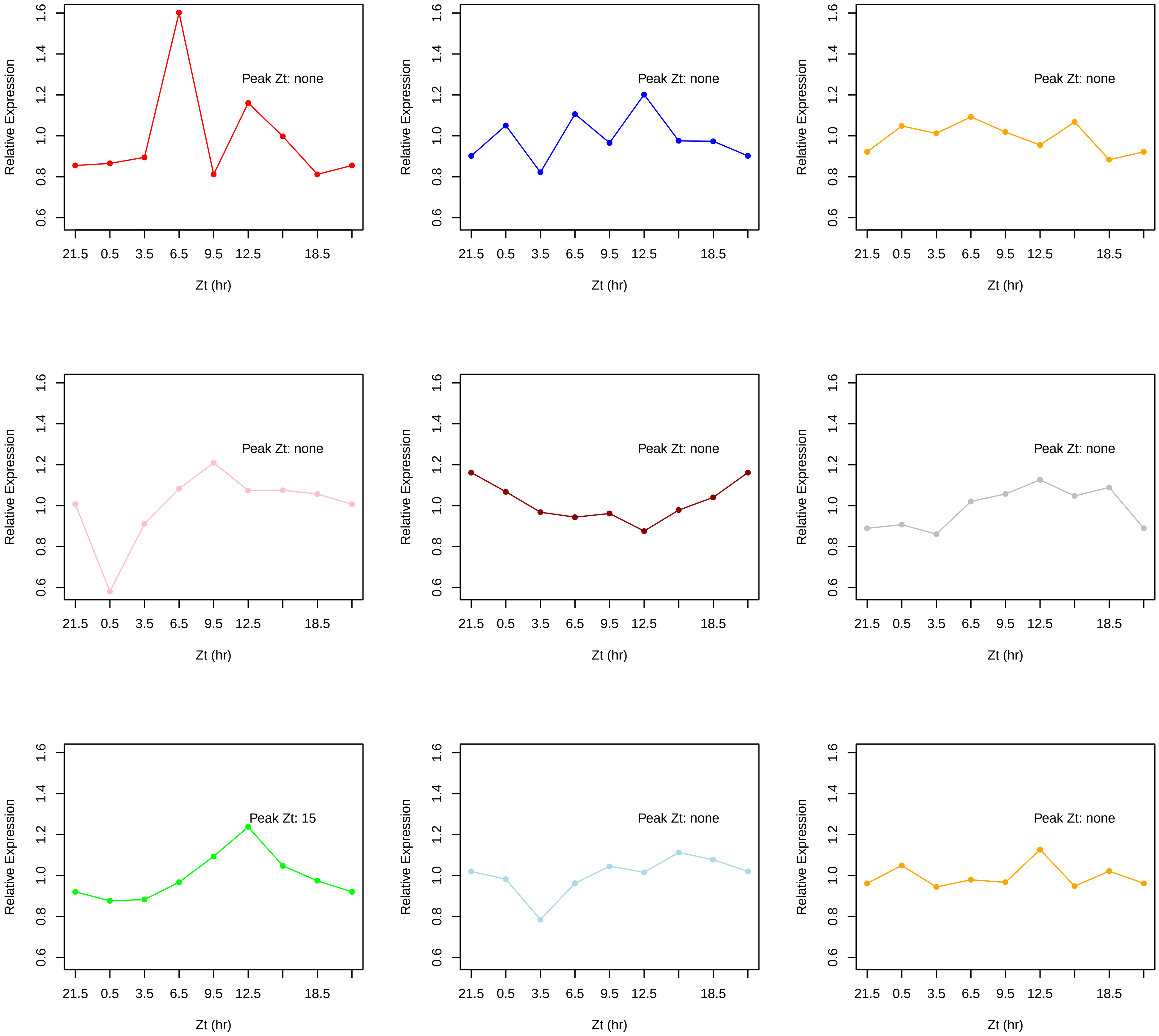

Singeleton: ENSXMAG00000016415 dpt

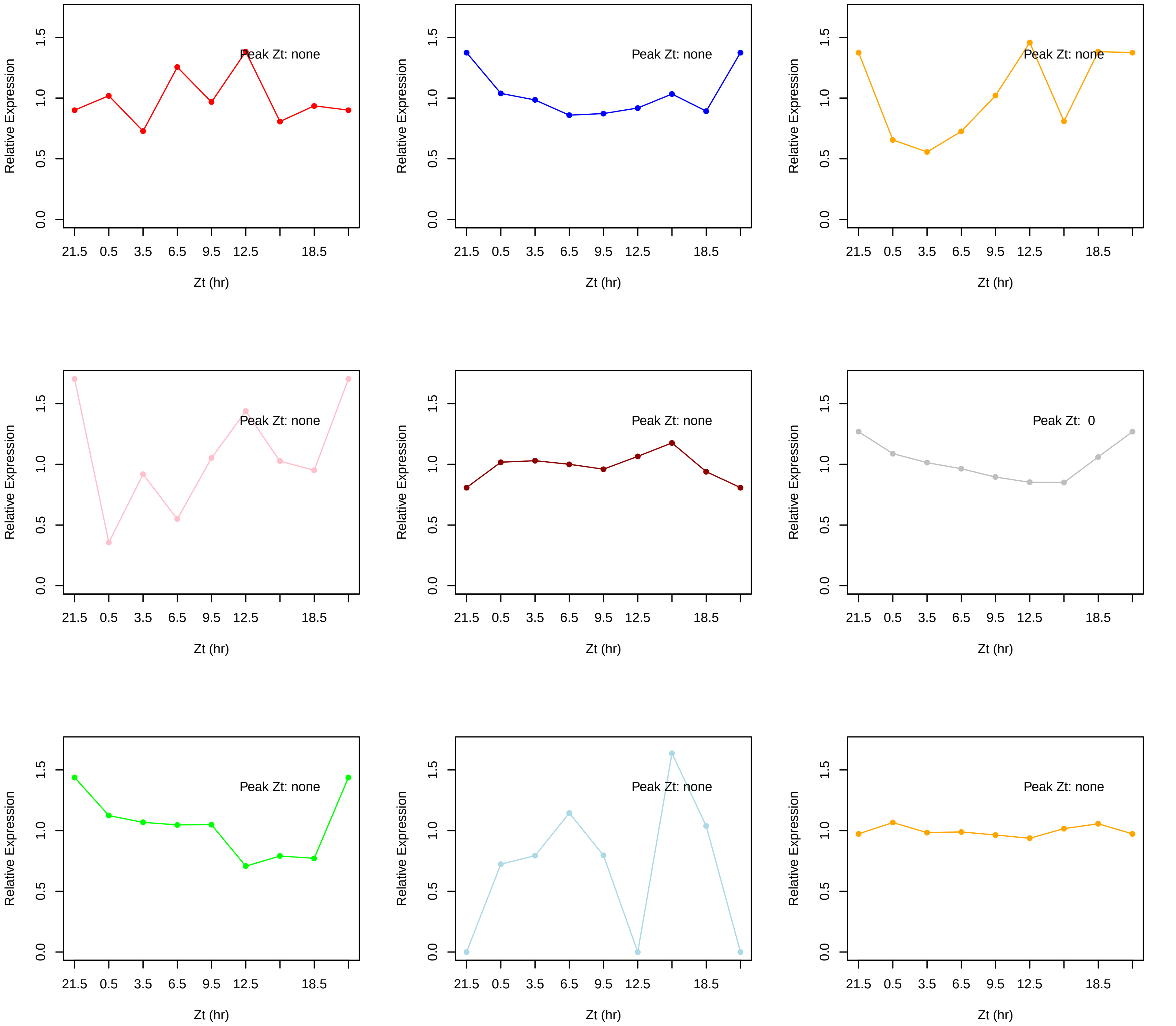

Singeleton: ENSXMAG00000013279 pgls

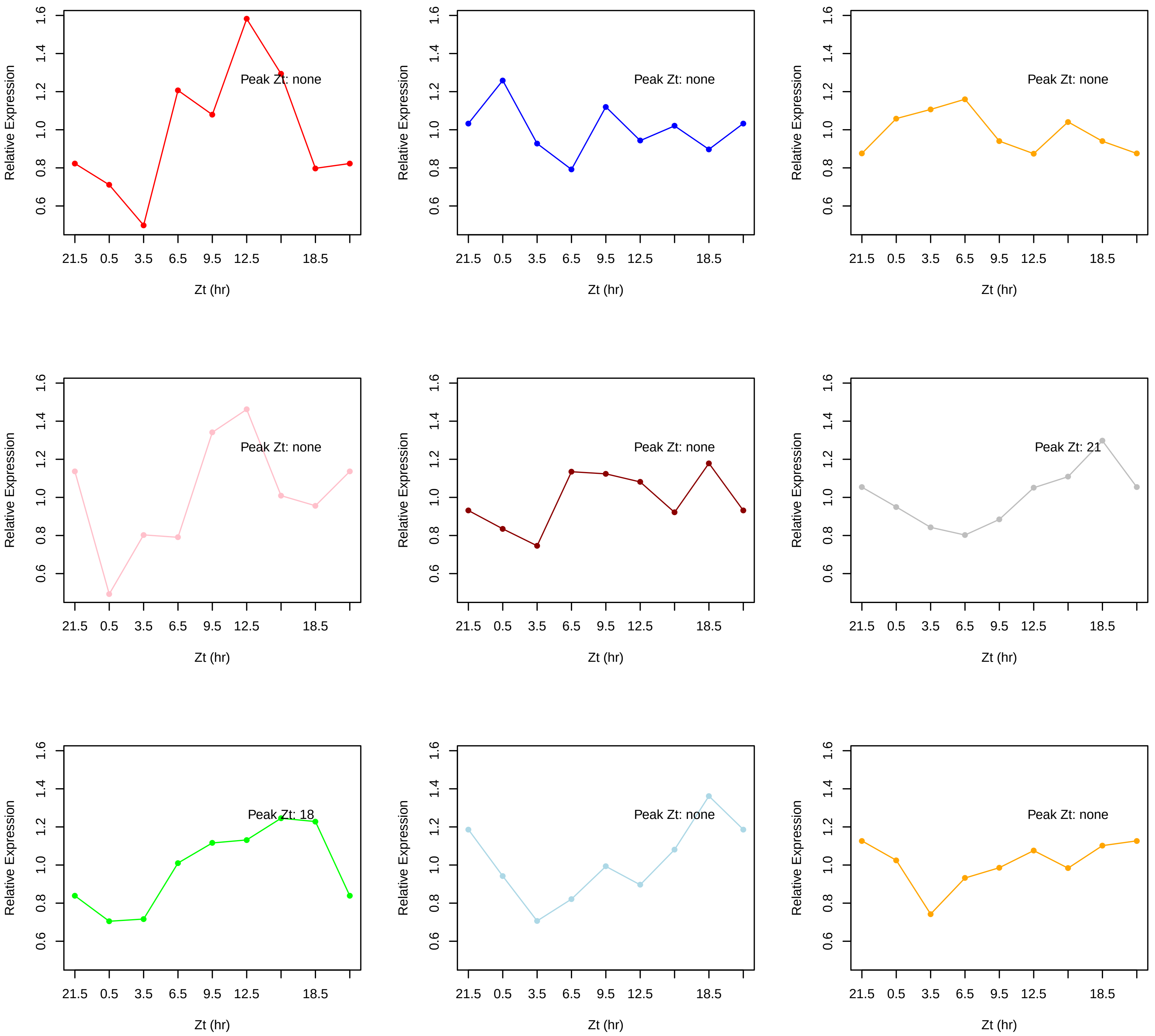

Singeleton: ENSXMAG00000022027 tctex1d2

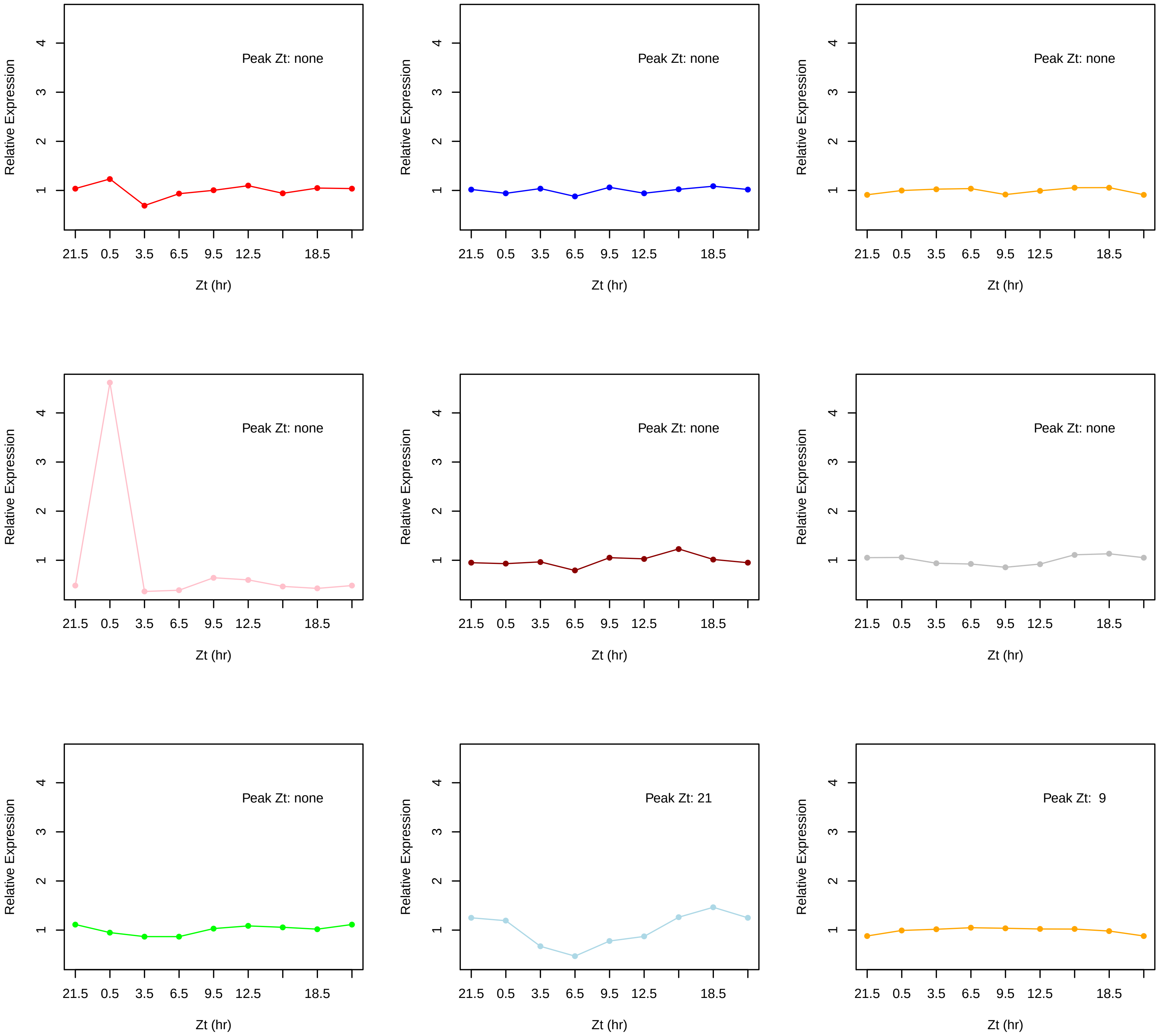

Singeleton: ENSXMAG00000015701 galcb

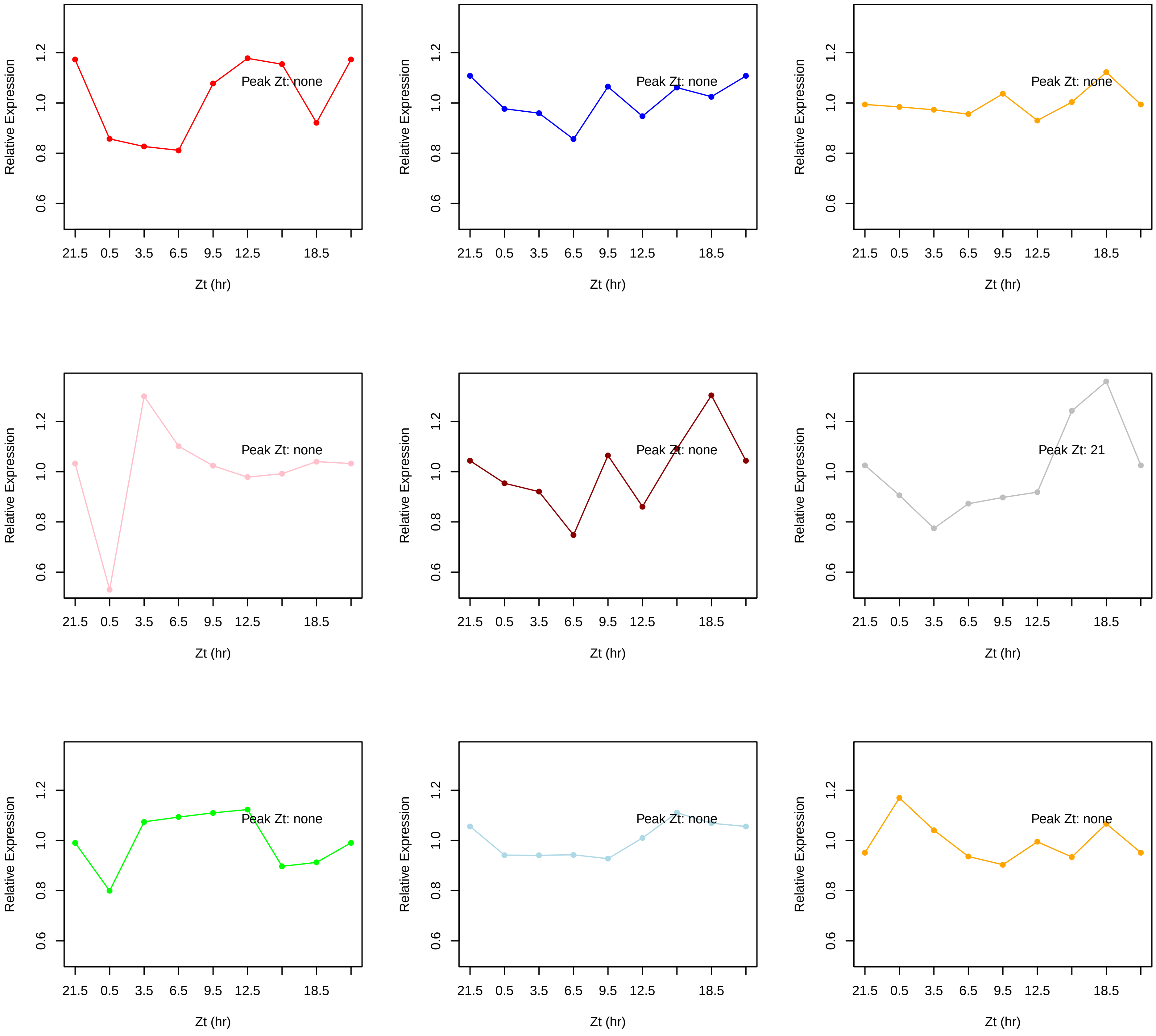

Singleton: ENSXMAG00000003385

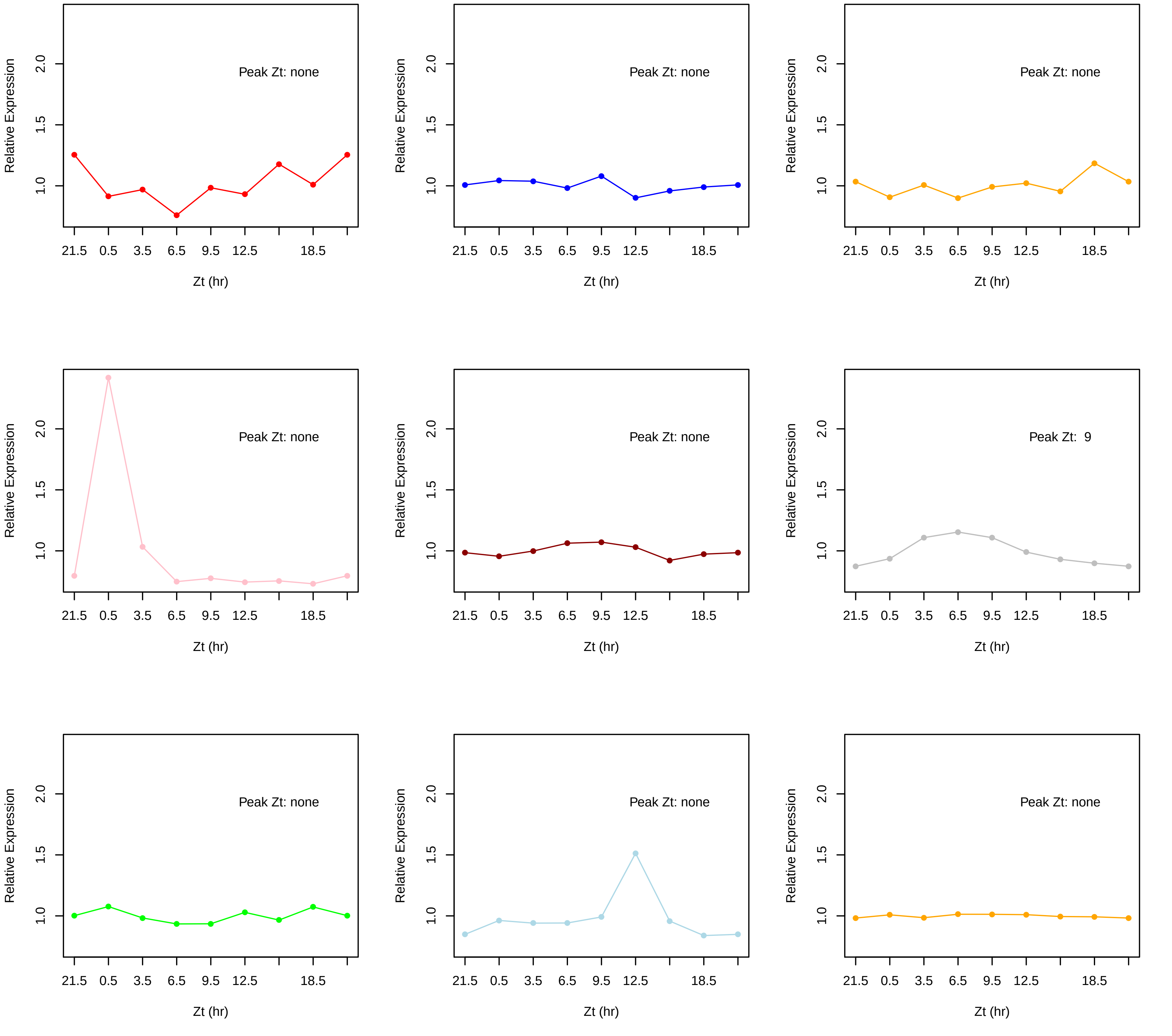

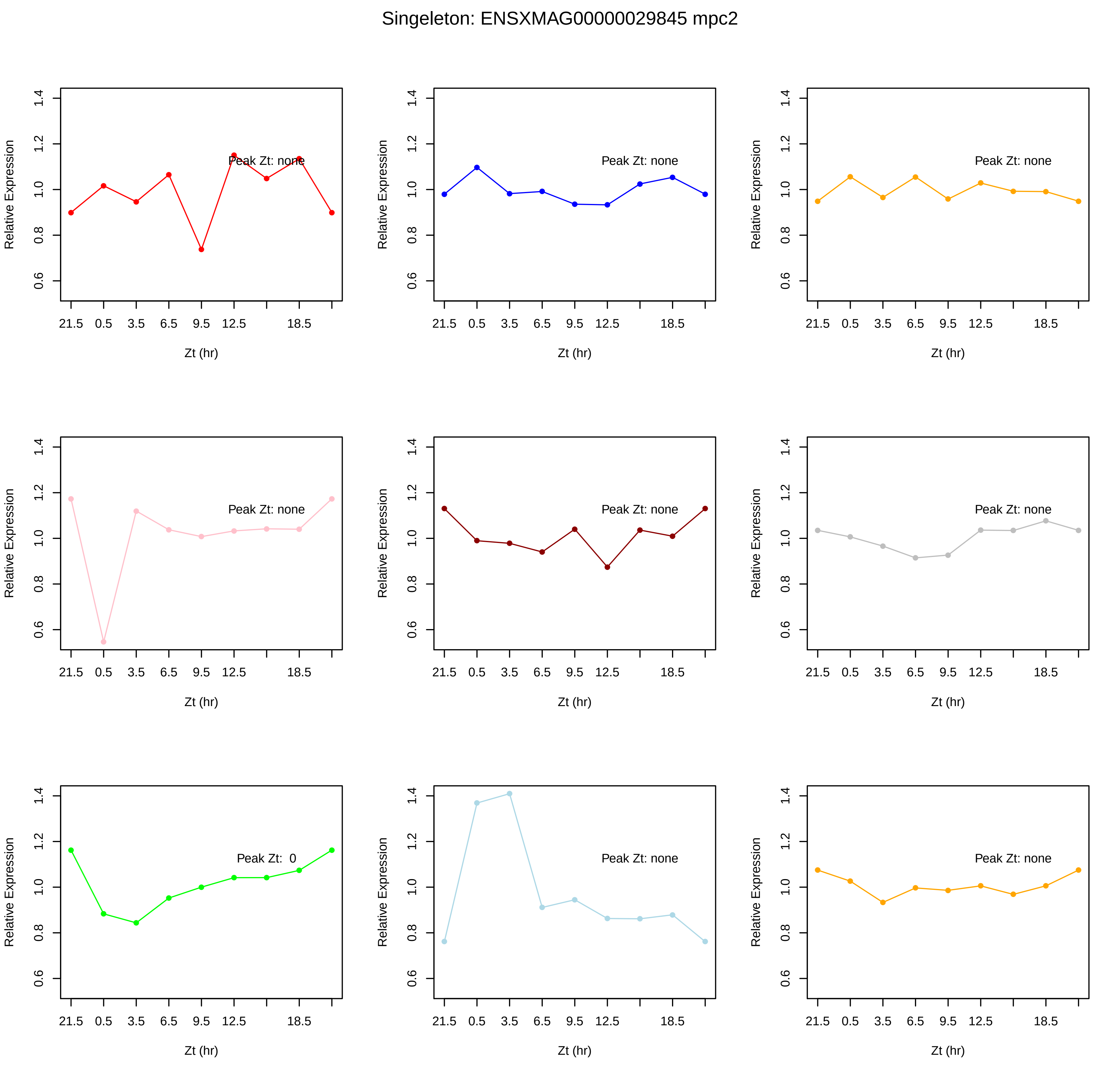
Singeleton: ENSXMAG00000029845 mpc2
Peak Zt: none
Peak Zt: none
Peak Zt: none
Peak Zt: none
Peak Zt: none
Peak Zt: none
Peak Zt: 0
Peak Zt: none
Peak Zt: none
Relative Expression
Zt (hr)
21.5 0.5 3.5 6.5 9.5 12.5 18.5
0.6 0.8 1.0 1.2 1.4

Singeleton: ENSXMAG00000006437 RHPN1

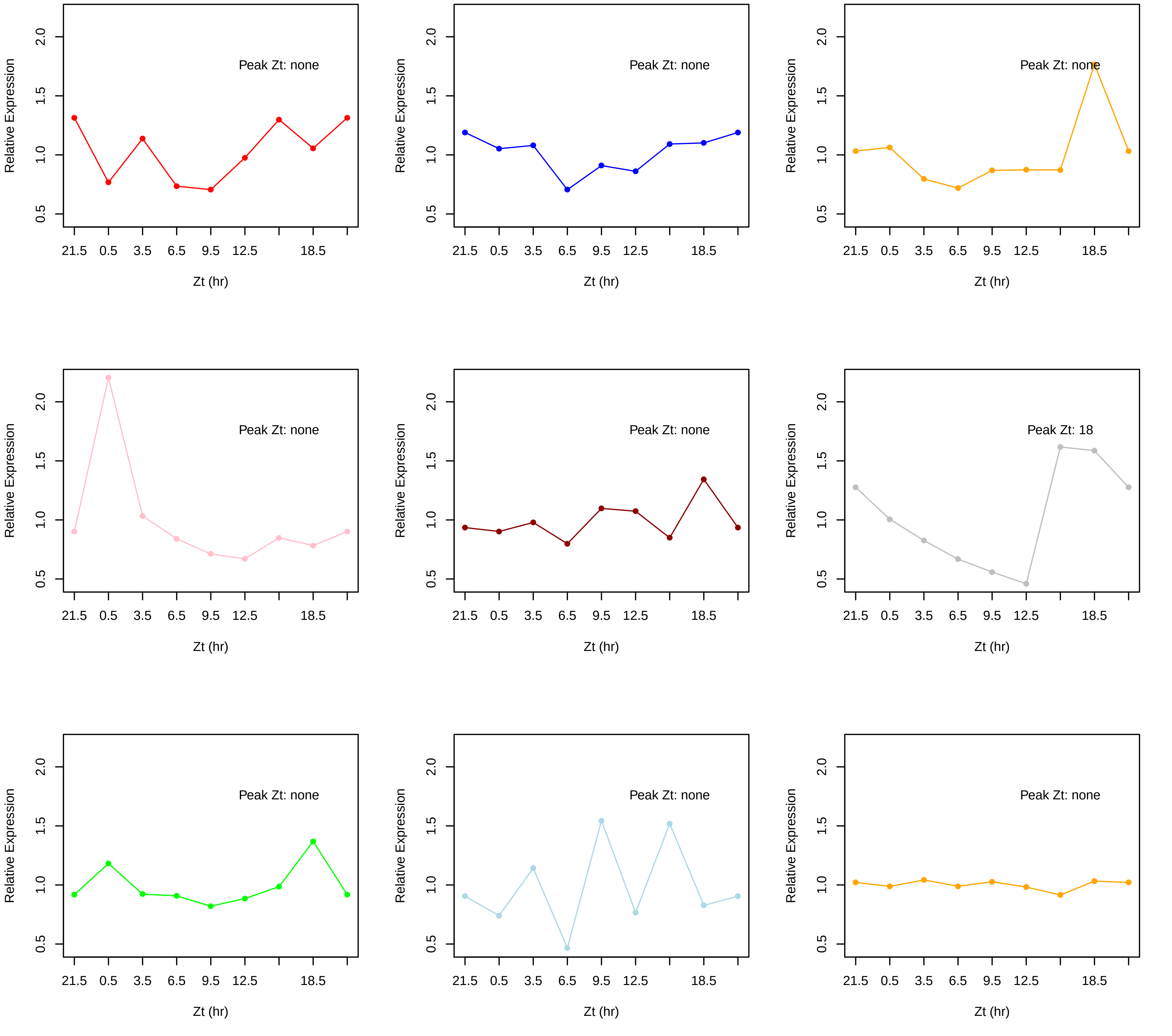

Singeleton: ENSXMAG00000029900 MCU

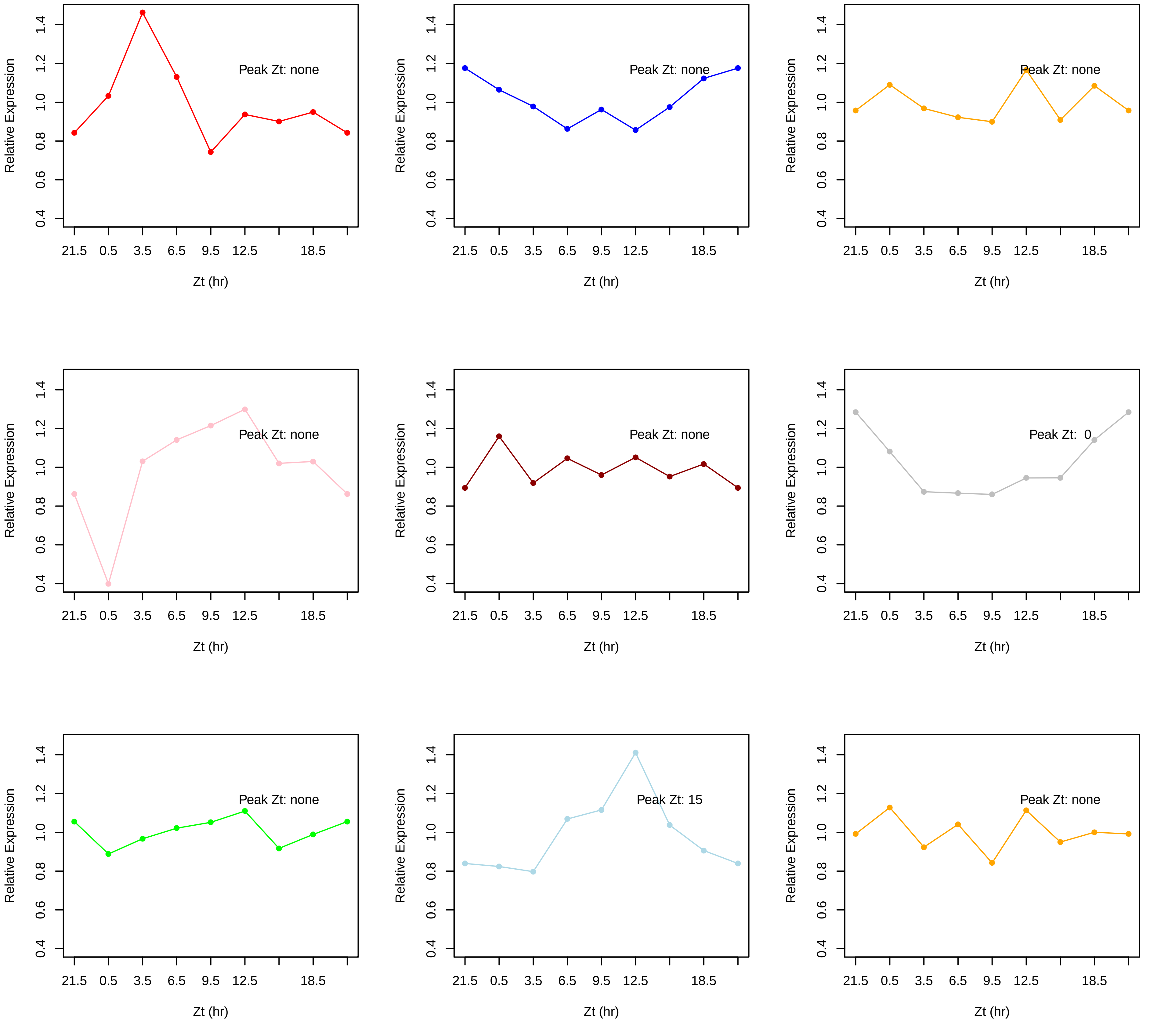

Singeleton: ENSXMAG00000001819 psmd14

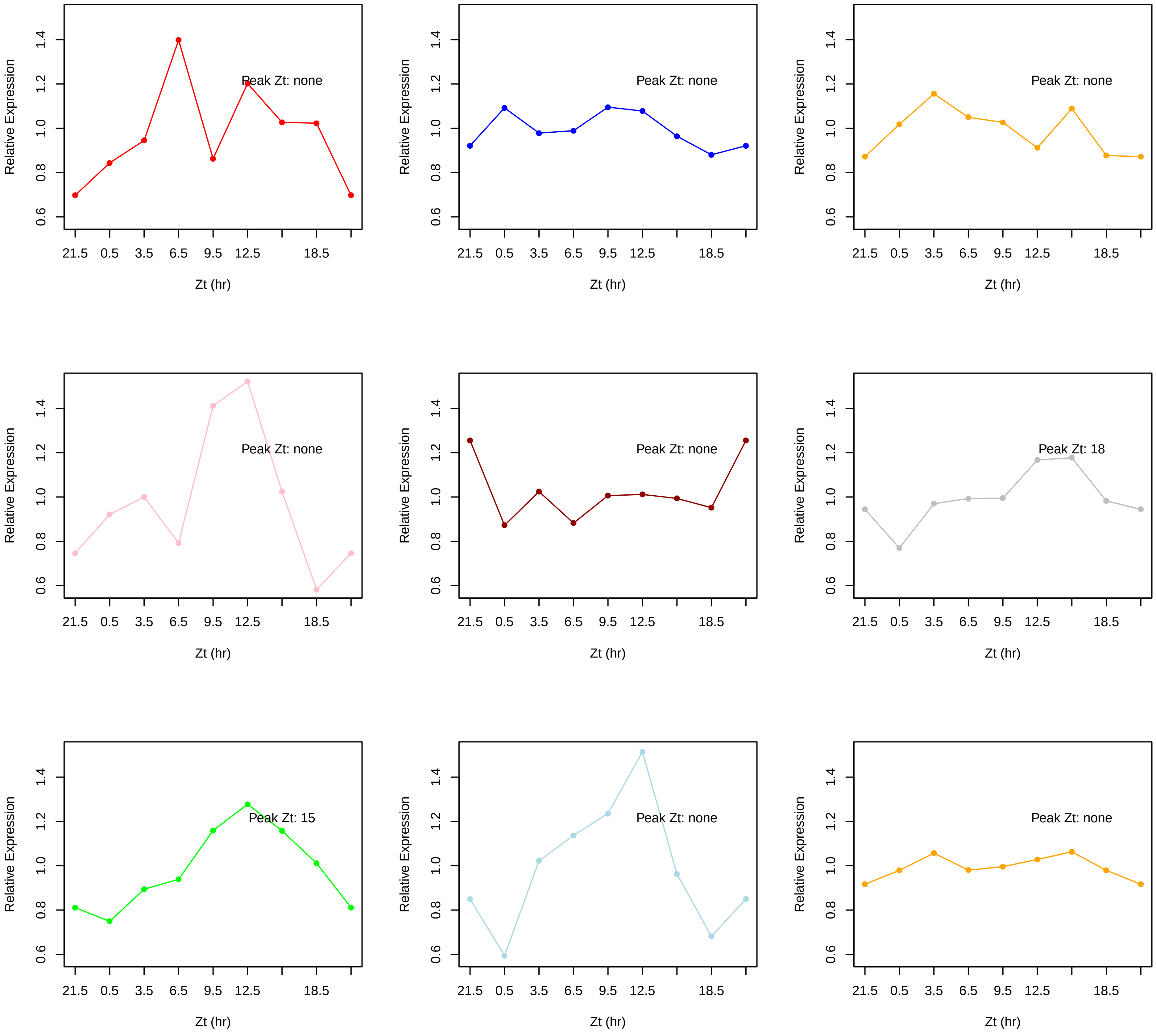

Singleton: ENSXMAG00000008941 negr1

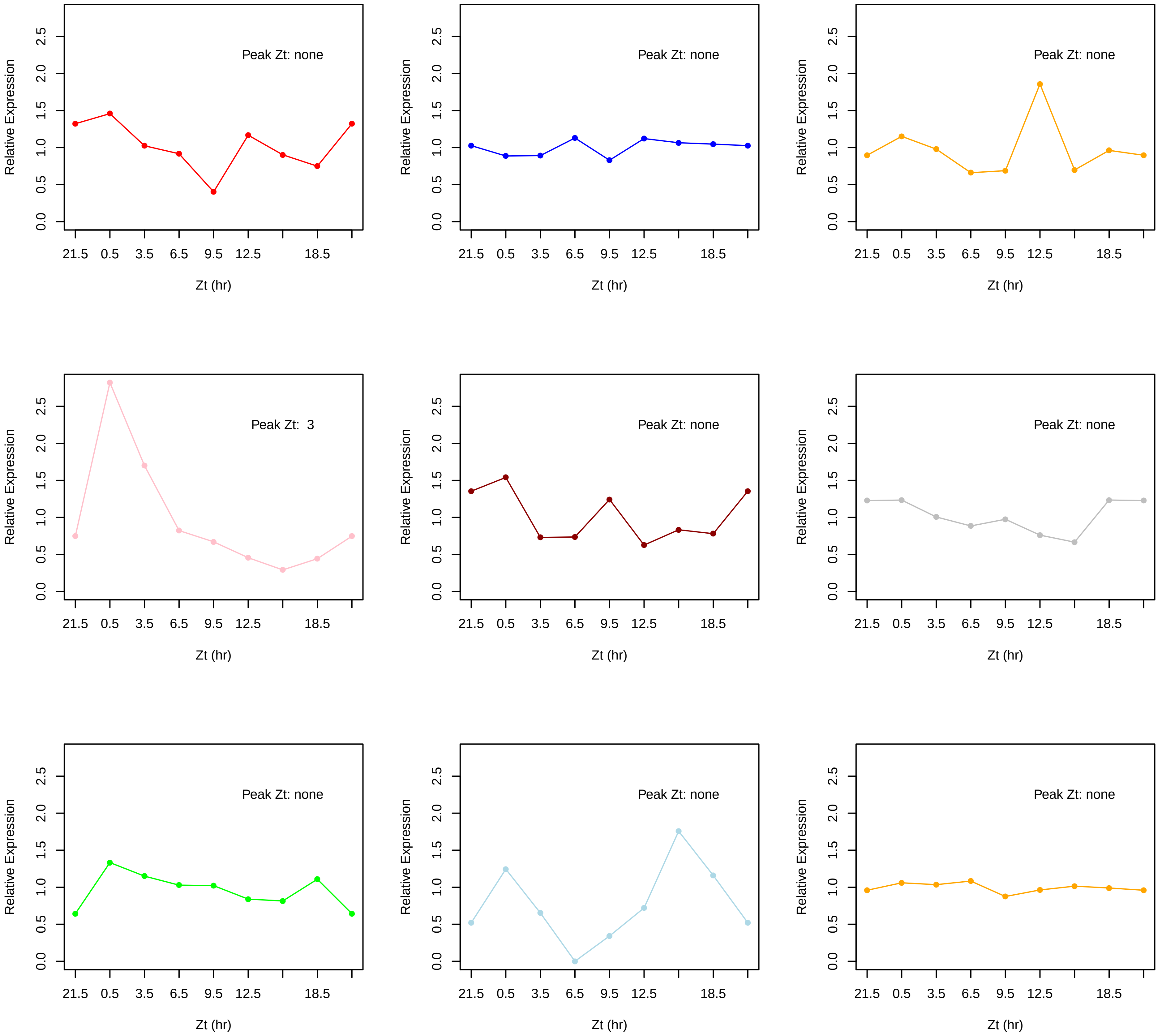

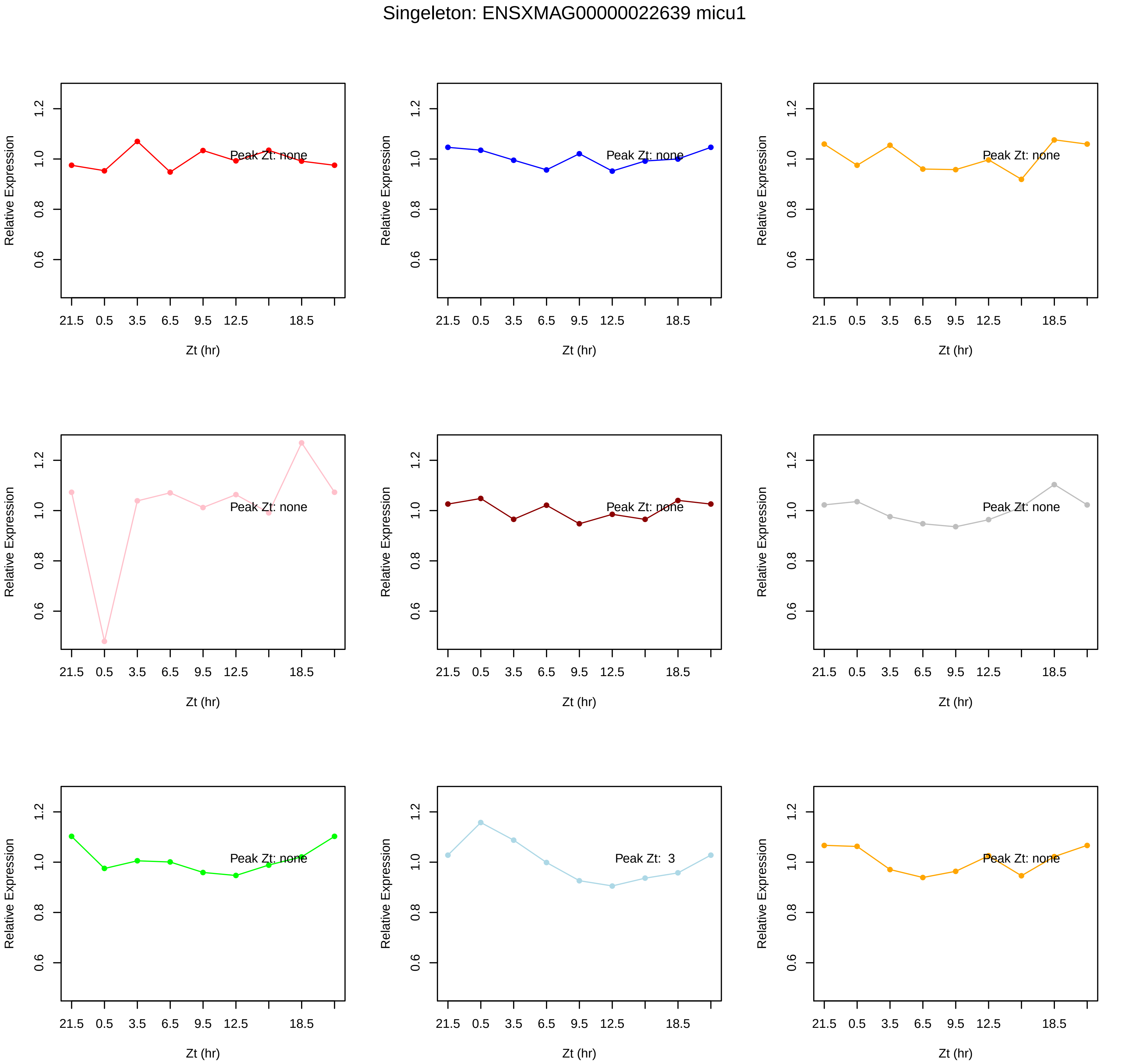
Singeleton: ENSXMAG00000022639 micu1
Relative Expression
Zt (hr)
21.5
0.5
3.5
6.5
9.5
12.5
18.5
0.6
0.8
1.0
1.2
Peak Zt: none
Peak Zt: 3

Singleton: ENSXMAG00000015748 vps28

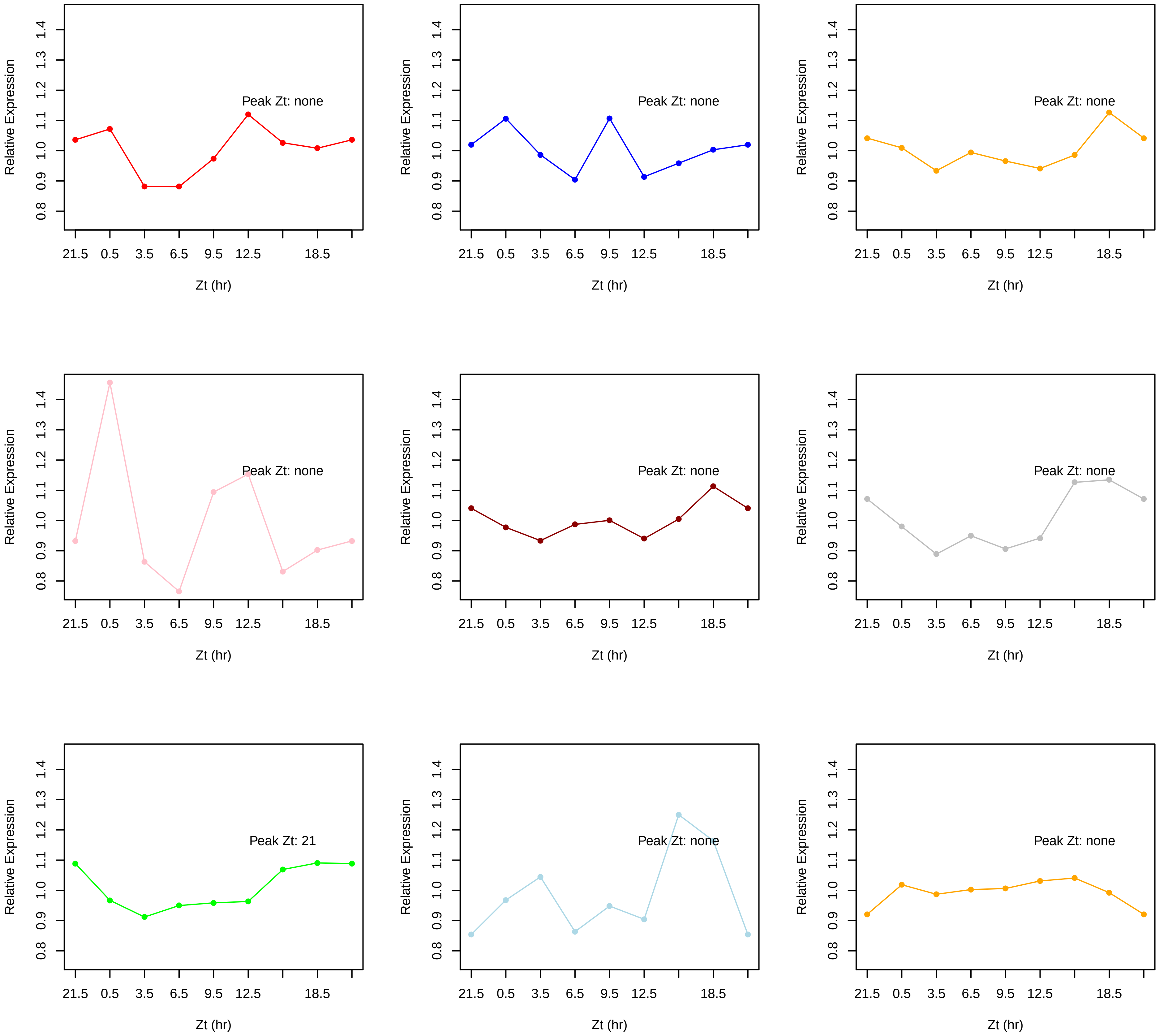

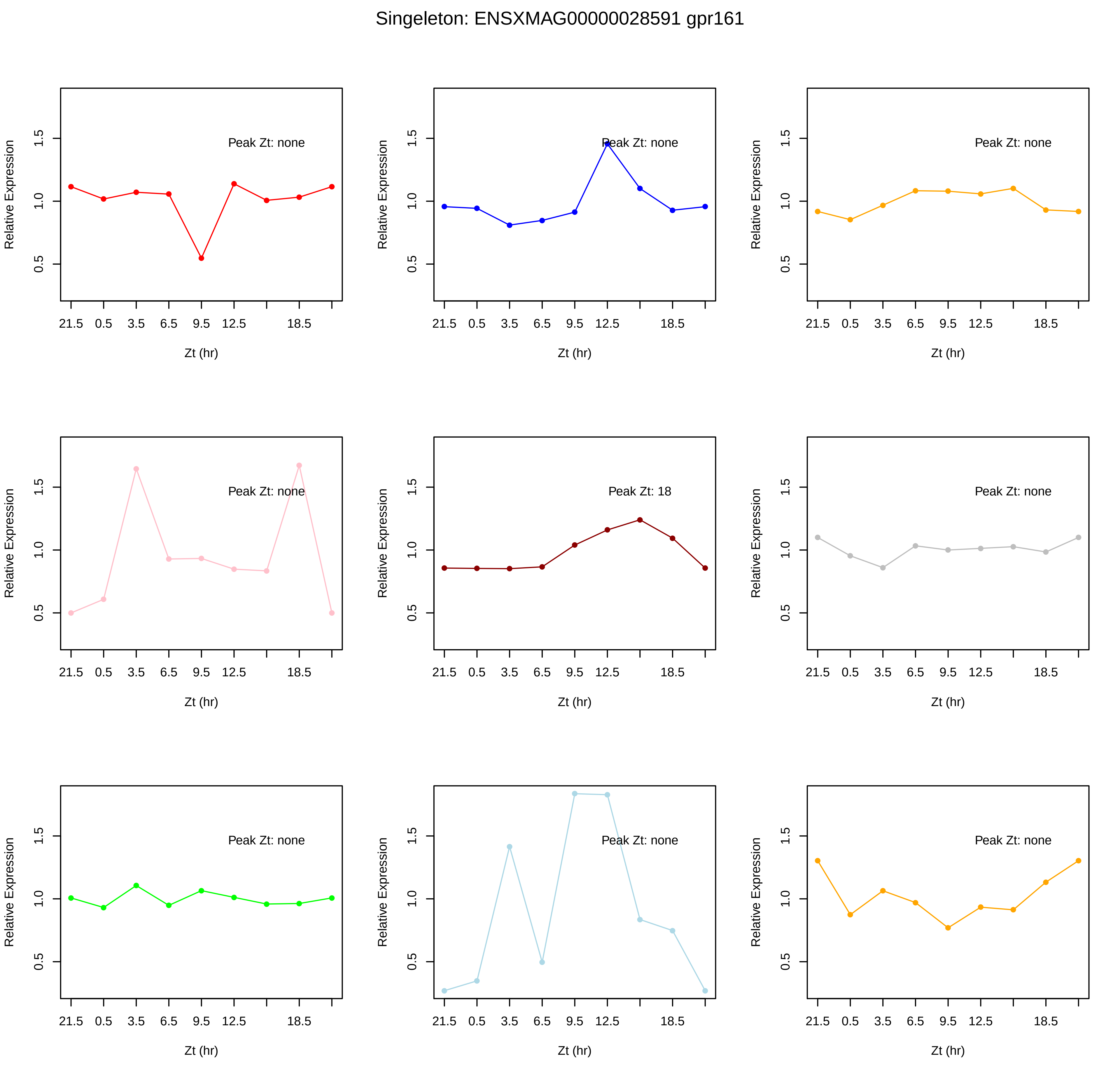
Singeleton: ENSXMAG00000028591 gpr161
Peak Zt: none
Peak Zt: none
Peak Zt: none
Peak Zt: none
Peak Zt: 18
Peak Zt: none
Peak Zt: none
Peak Zt: none
Peak Zt: none
Relative Expression
Zt (hr)
21.5
0.5
3.5
6.5
9.5
12.5
18.5
0.5
1.0
1.5

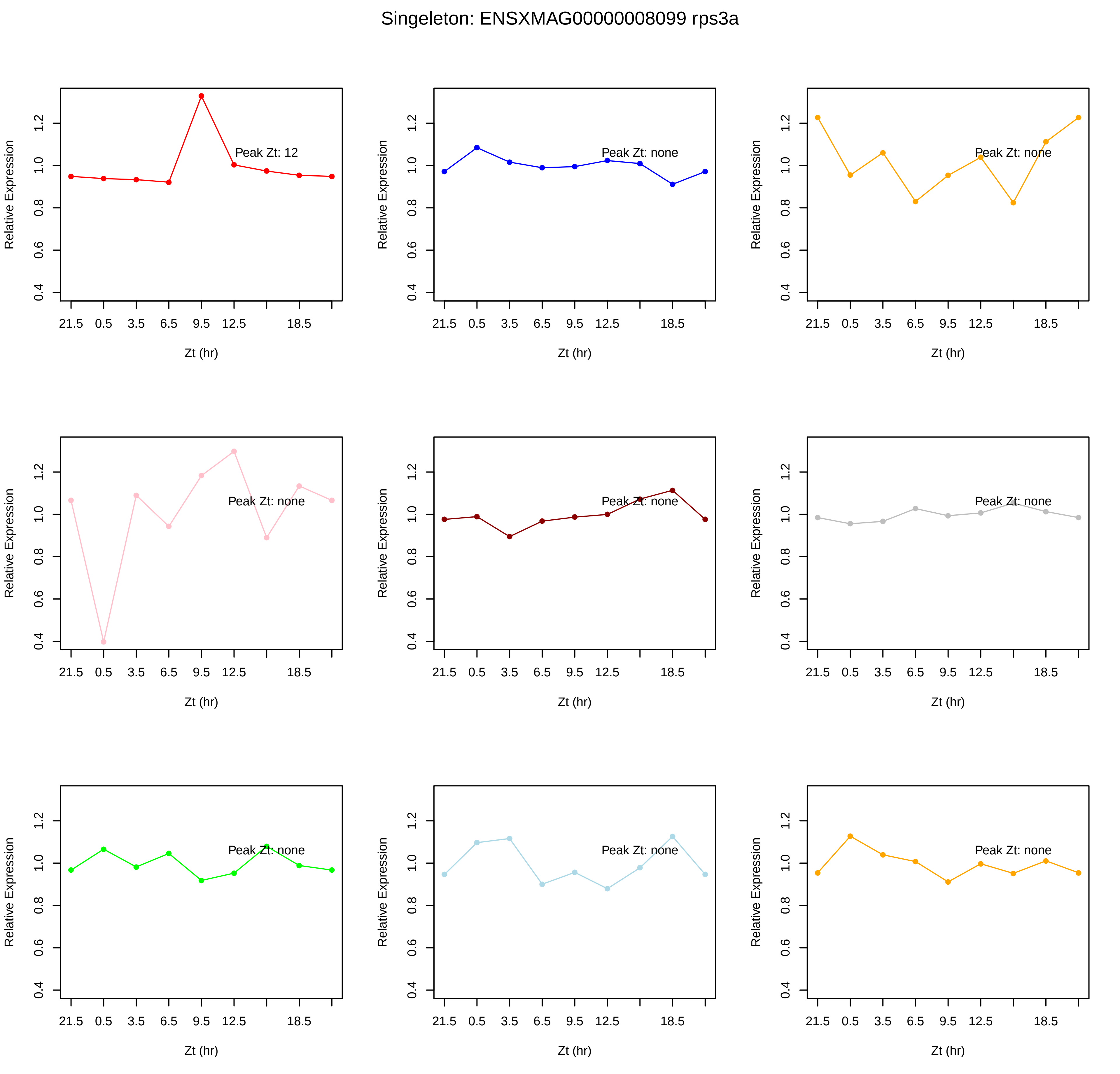

Singleton: ENSXMAG00000008099 rps3a
Relative Expression
Zt (hr)
21.5
0.5
3.5
6.5
9.5
12.5
18.5
0.4
0.6
0.8
1.0
1.2
Peak Zt: 12
Peak Zt: none

Singeleton: ENSXMAG00000005835 hykk.2

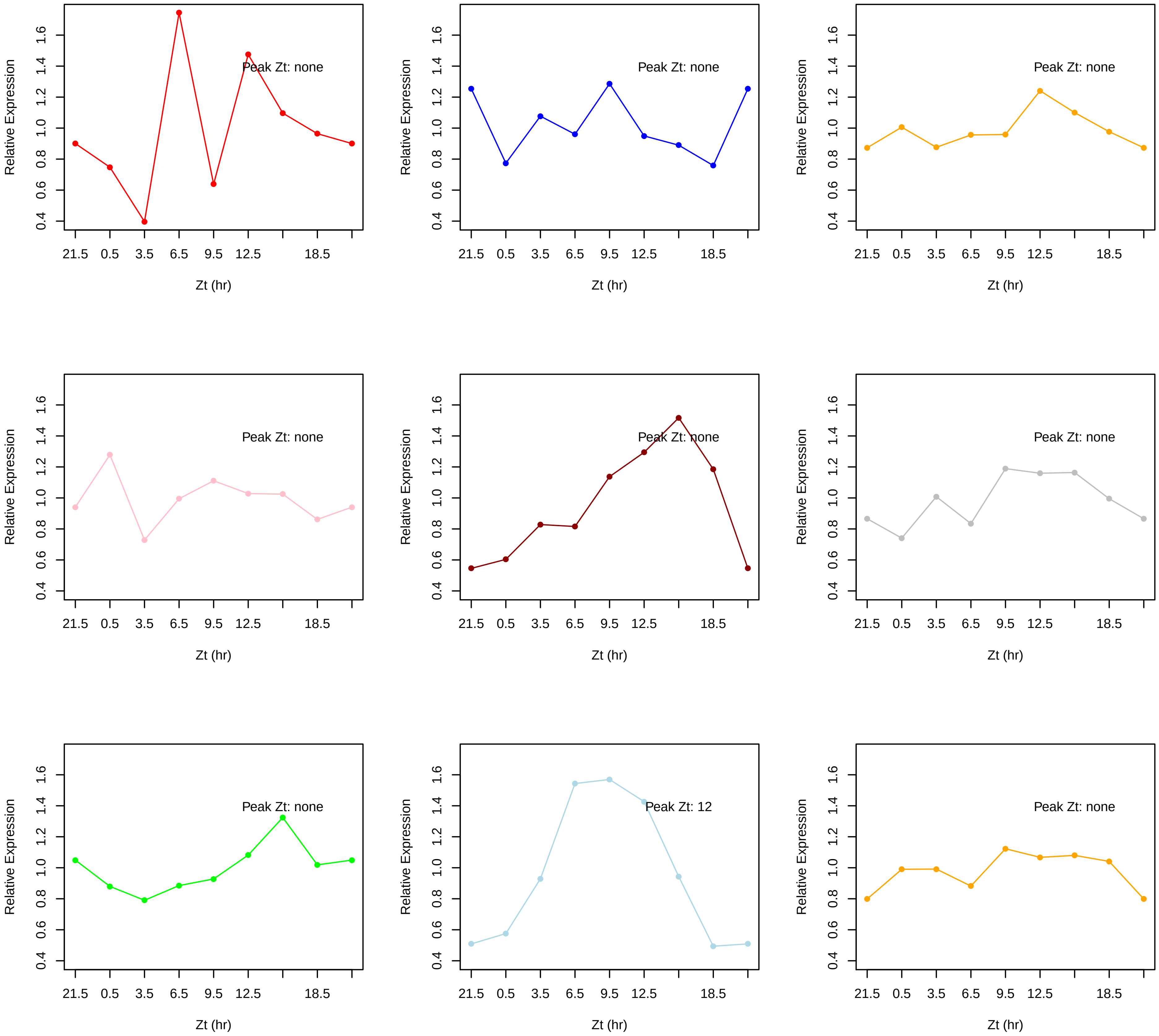

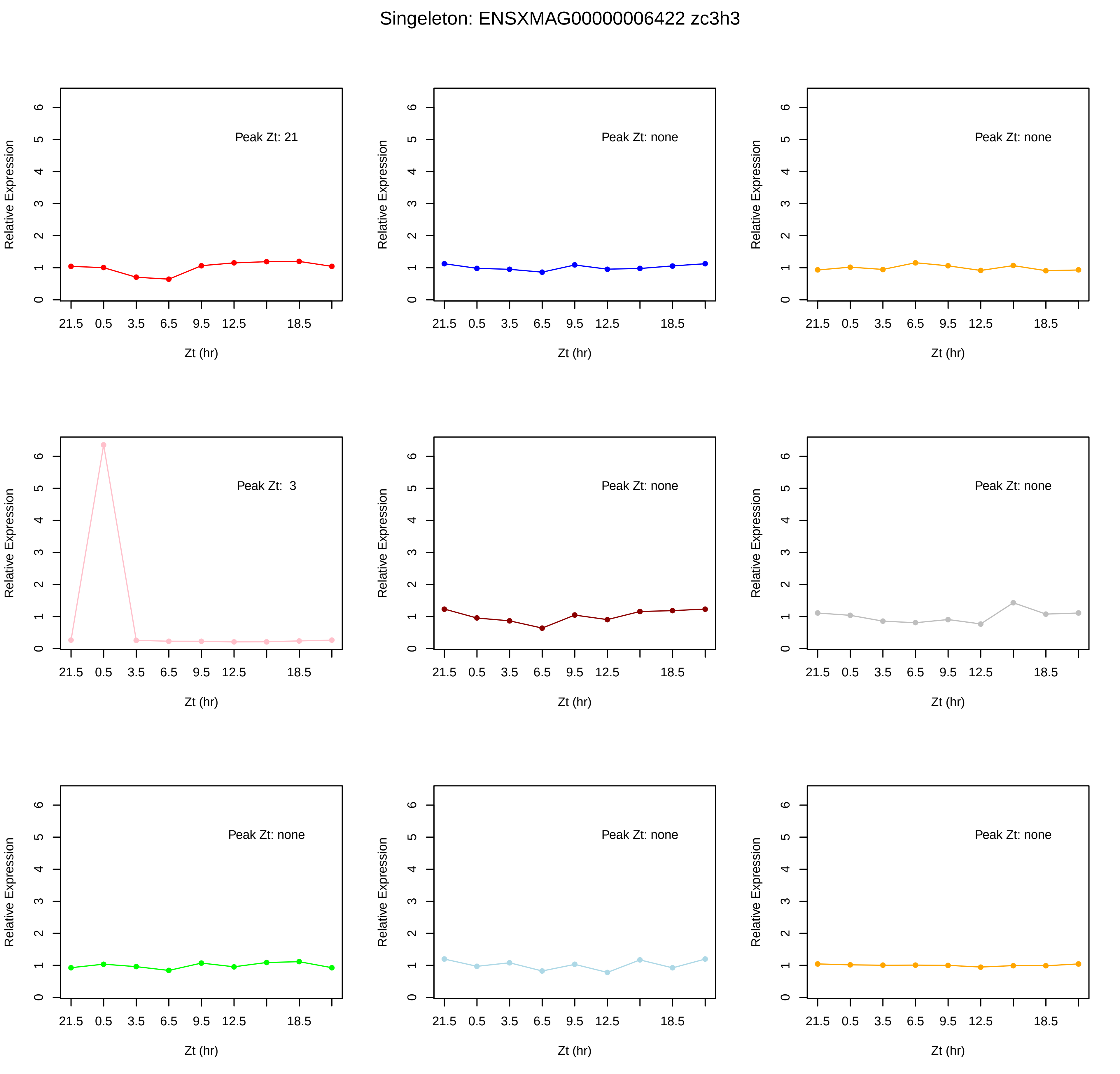
Singeleton: ENSXMAG00000006422 zc3h3
Peak Zt: 21
Peak Zt: none
Peak Zt: none
Peak Zt: 3
Peak Zt: none
Peak Zt: none
Peak Zt: none
Peak Zt: none
Peak Zt: none
Relative Expression
Zt (hr)
21.5
0.5
3.5
6.5
9.5
12.5
18.5

Singleton: ENSXMAG00000014034 lrba

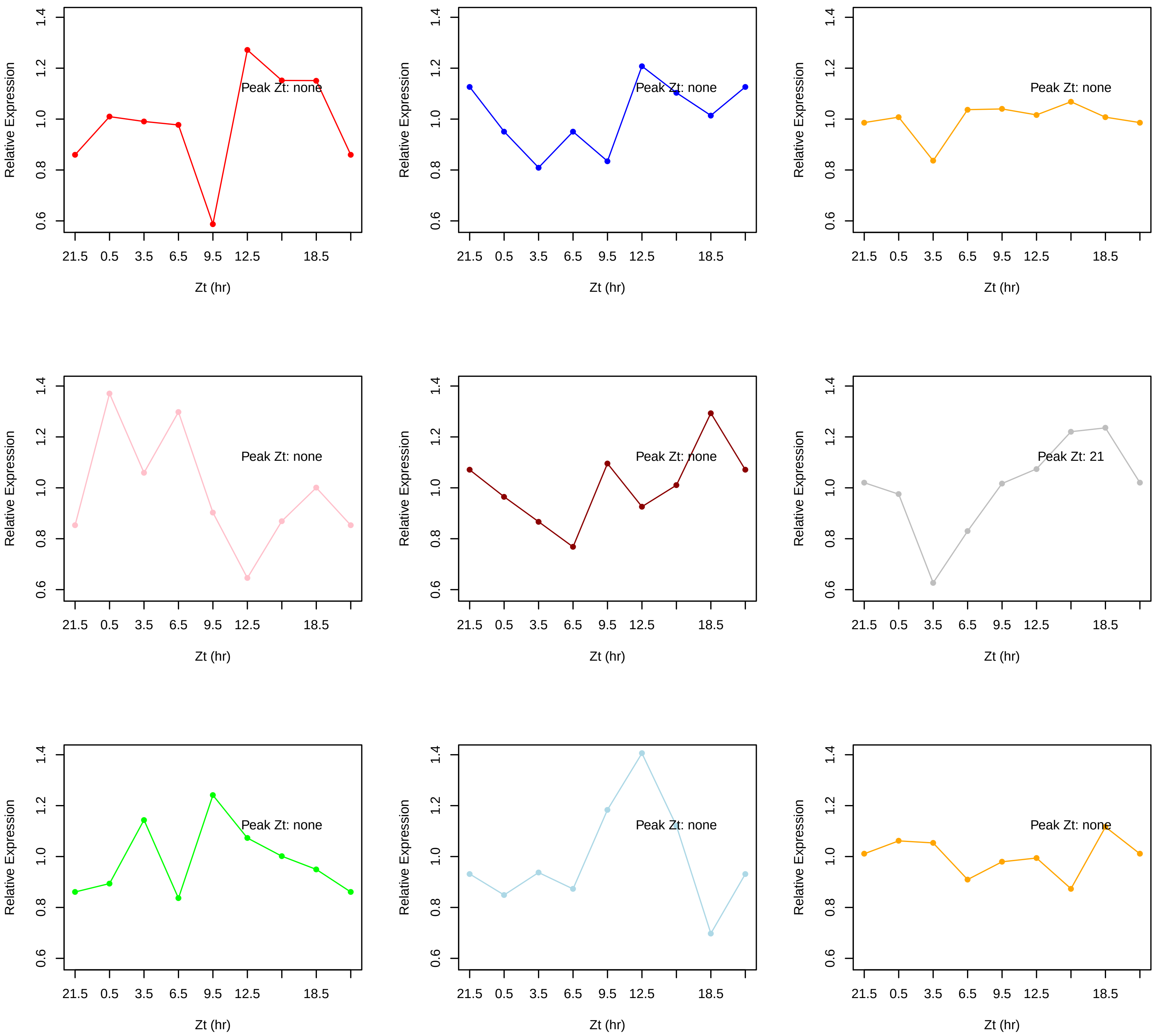

Singeleton: ENSXMAG00000004673 ephb3a

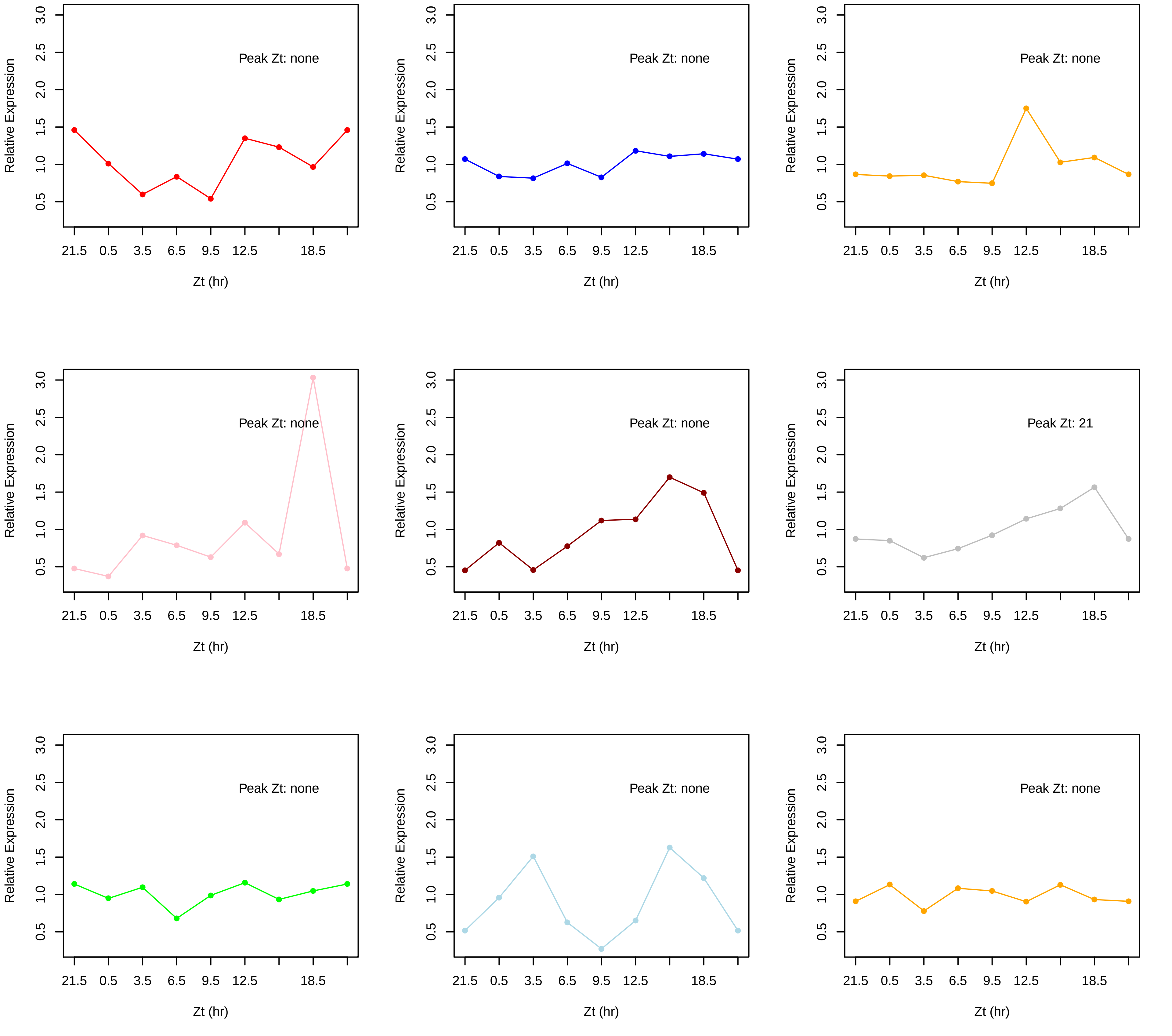

Singeleton: ENSXMAG00000015326 cep120

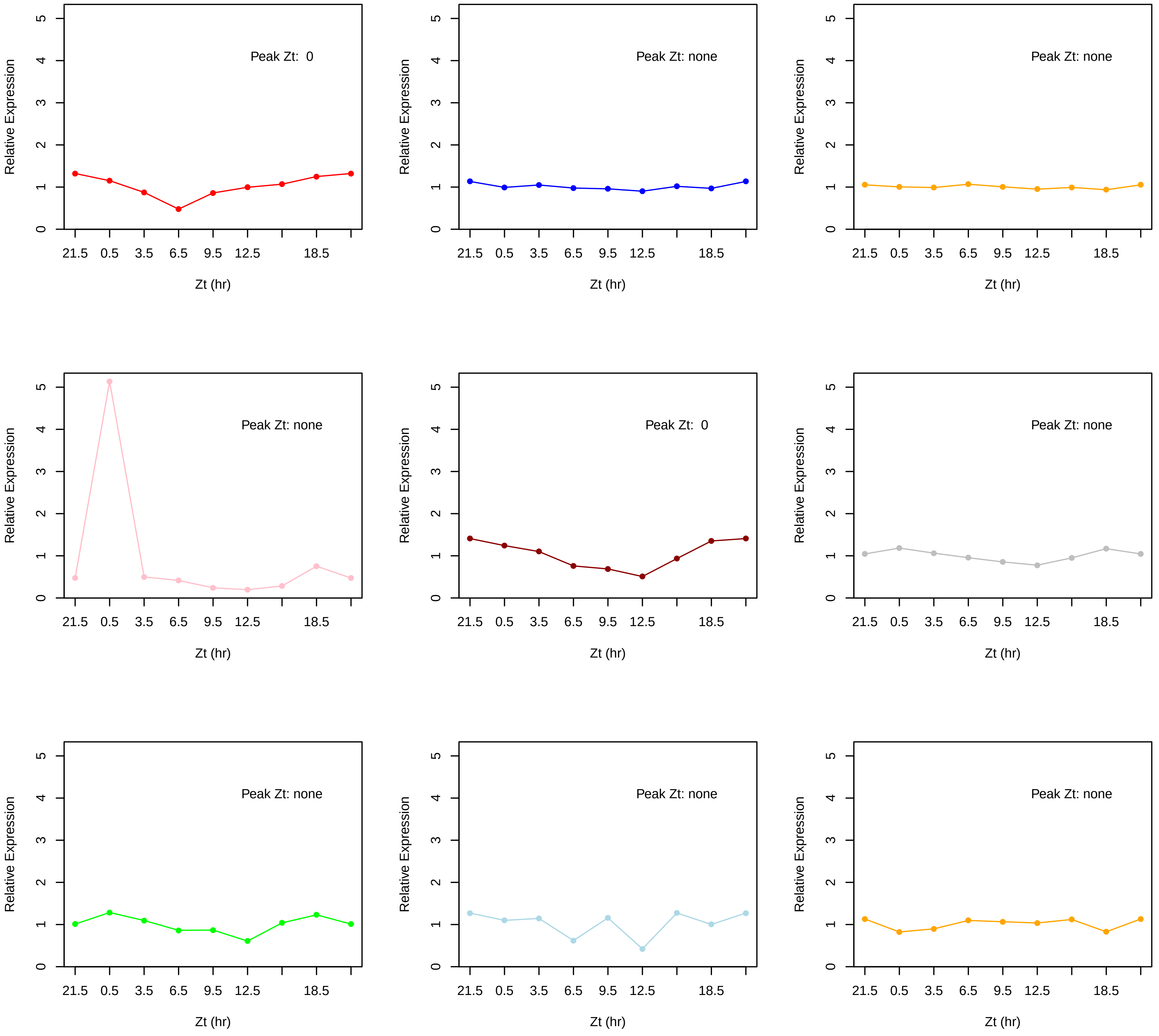

Singeleton: ENSXMAG00000004437

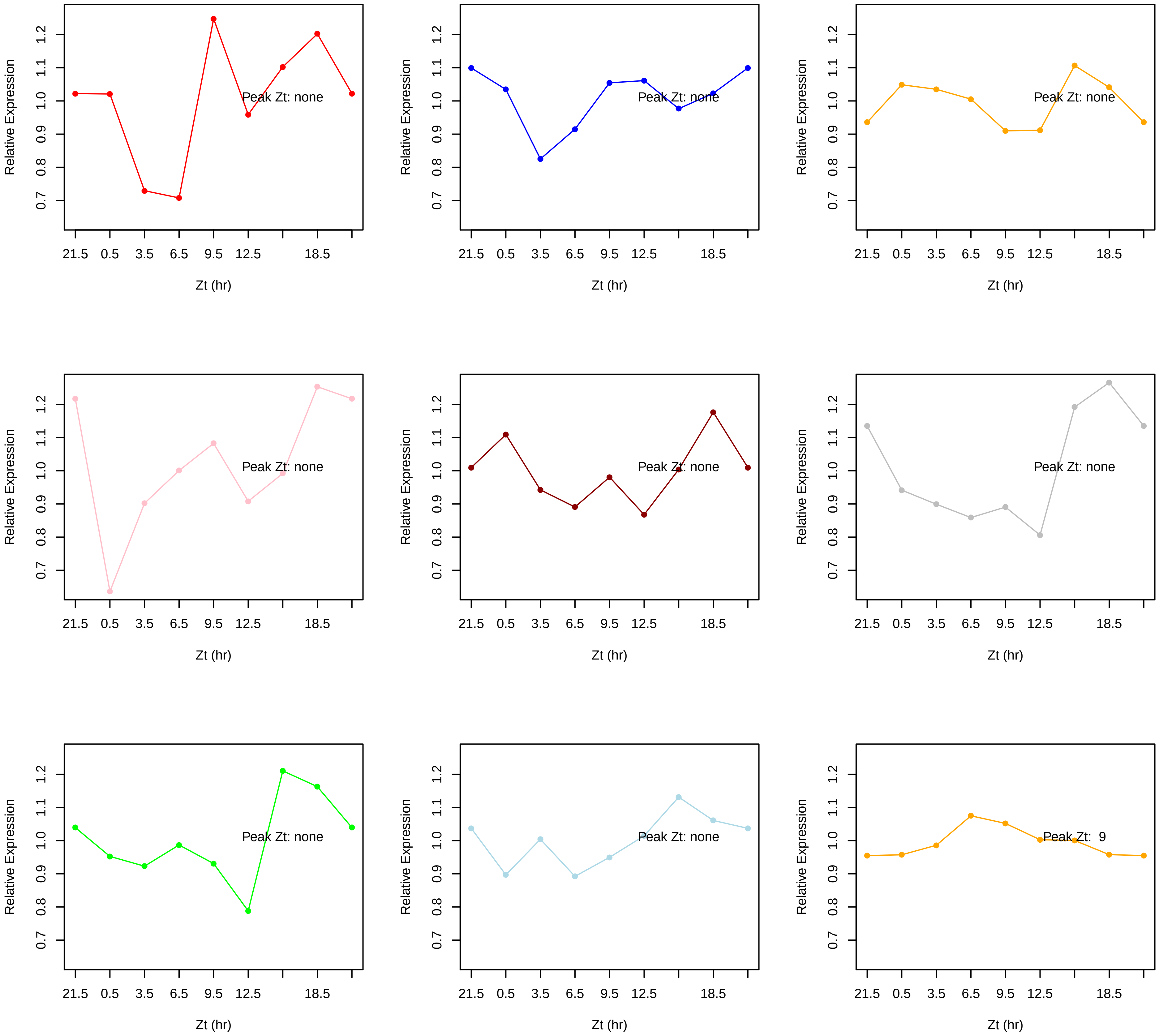

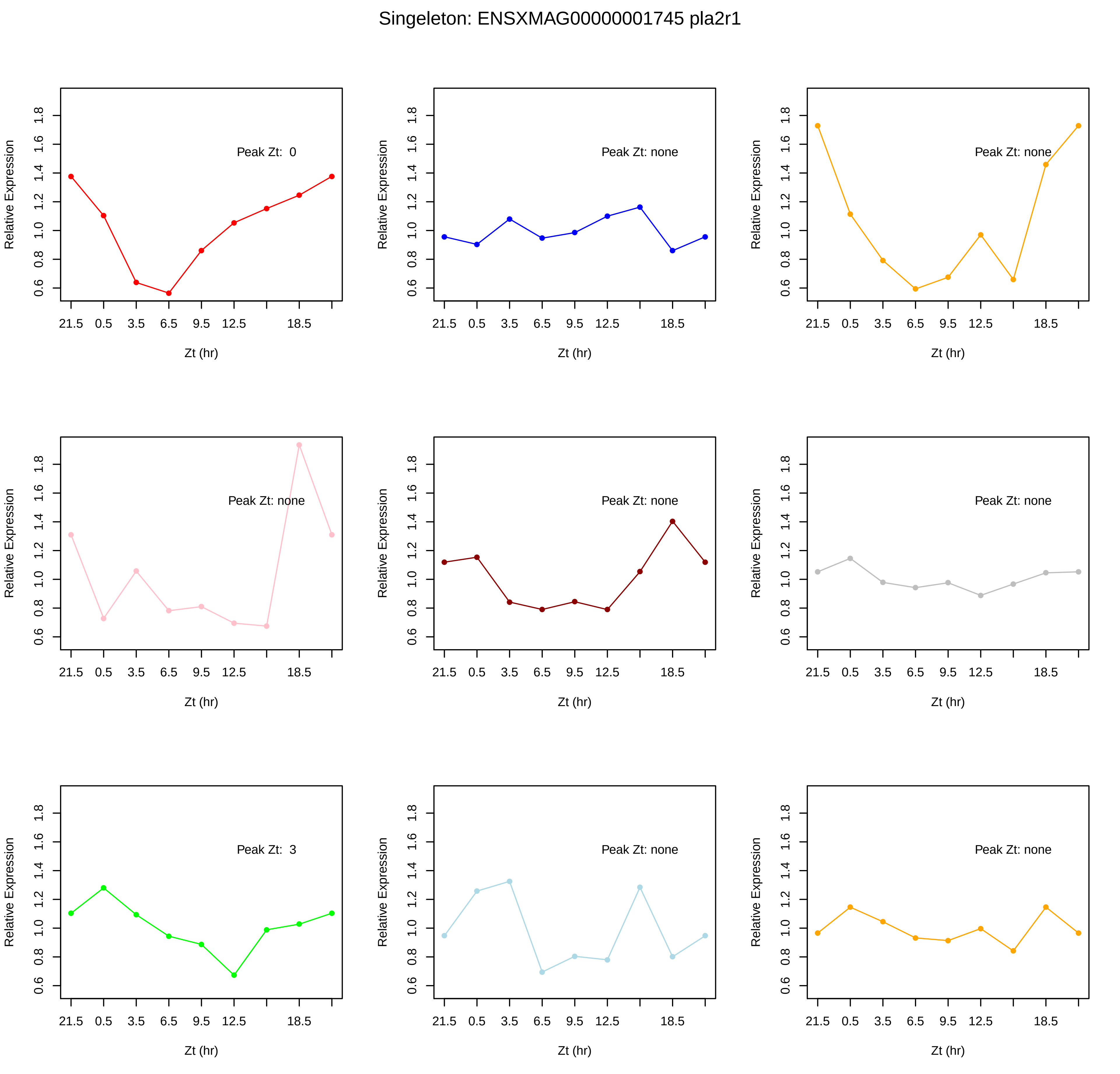
Singleton: ENSXMAG00000001745 pla2r1
Peak Zt: 0
Peak Zt: none
Peak Zt: none
Peak Zt: none
Peak Zt: none
Peak Zt: none
Peak Zt: 3
Peak Zt: none
Peak Zt: none
Relative Expression
Zt (hr)
21.5
0.5
3.5
6.5
9.5
12.5
18.5
0.6
0.8
1.0
1.2
1.4
1.6
1.8

Singeleton: ENSXMAG00000025890 slc10a2

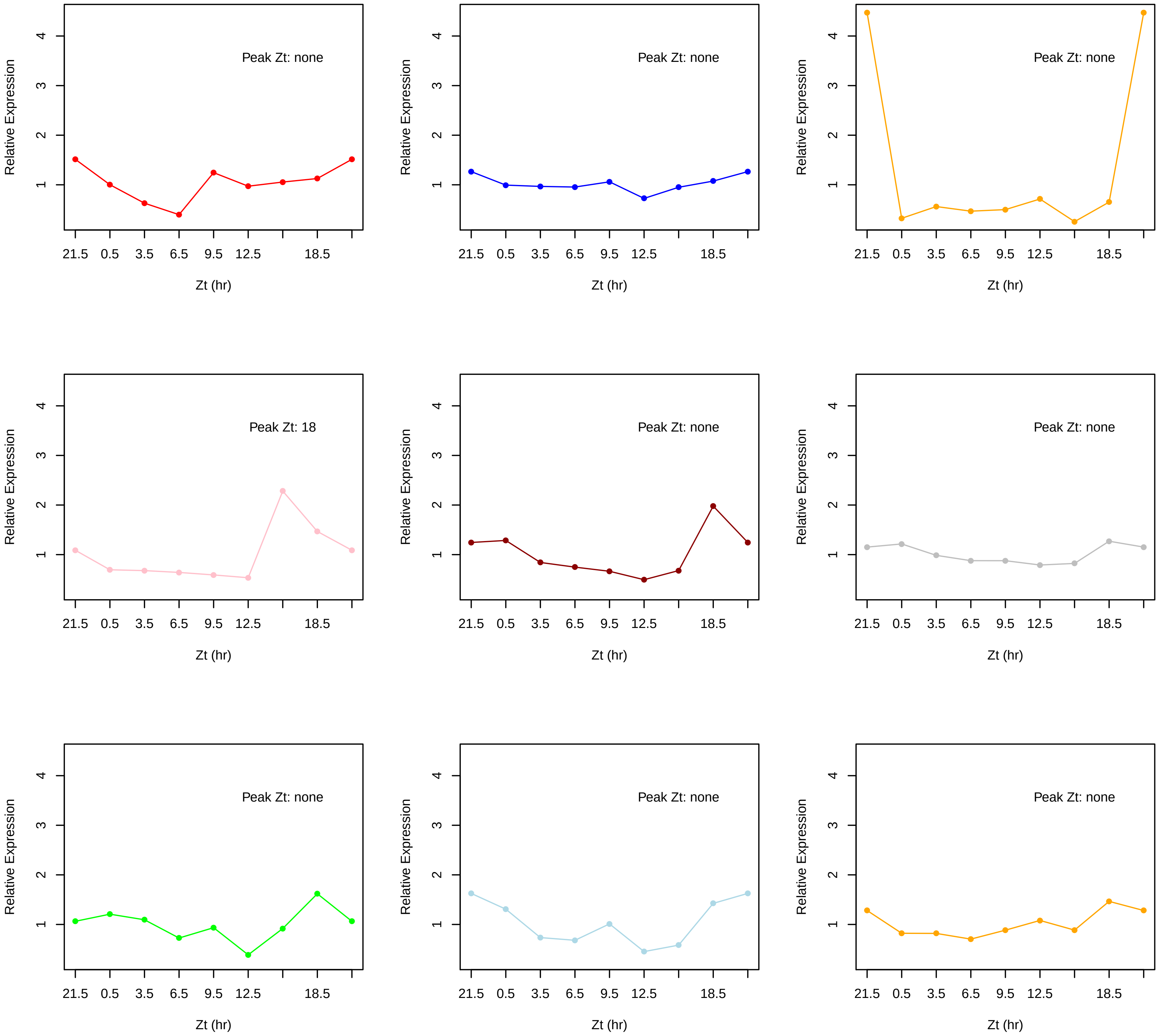

Singleton: ENSXMAG00000025776 si:ch211−121a2.4

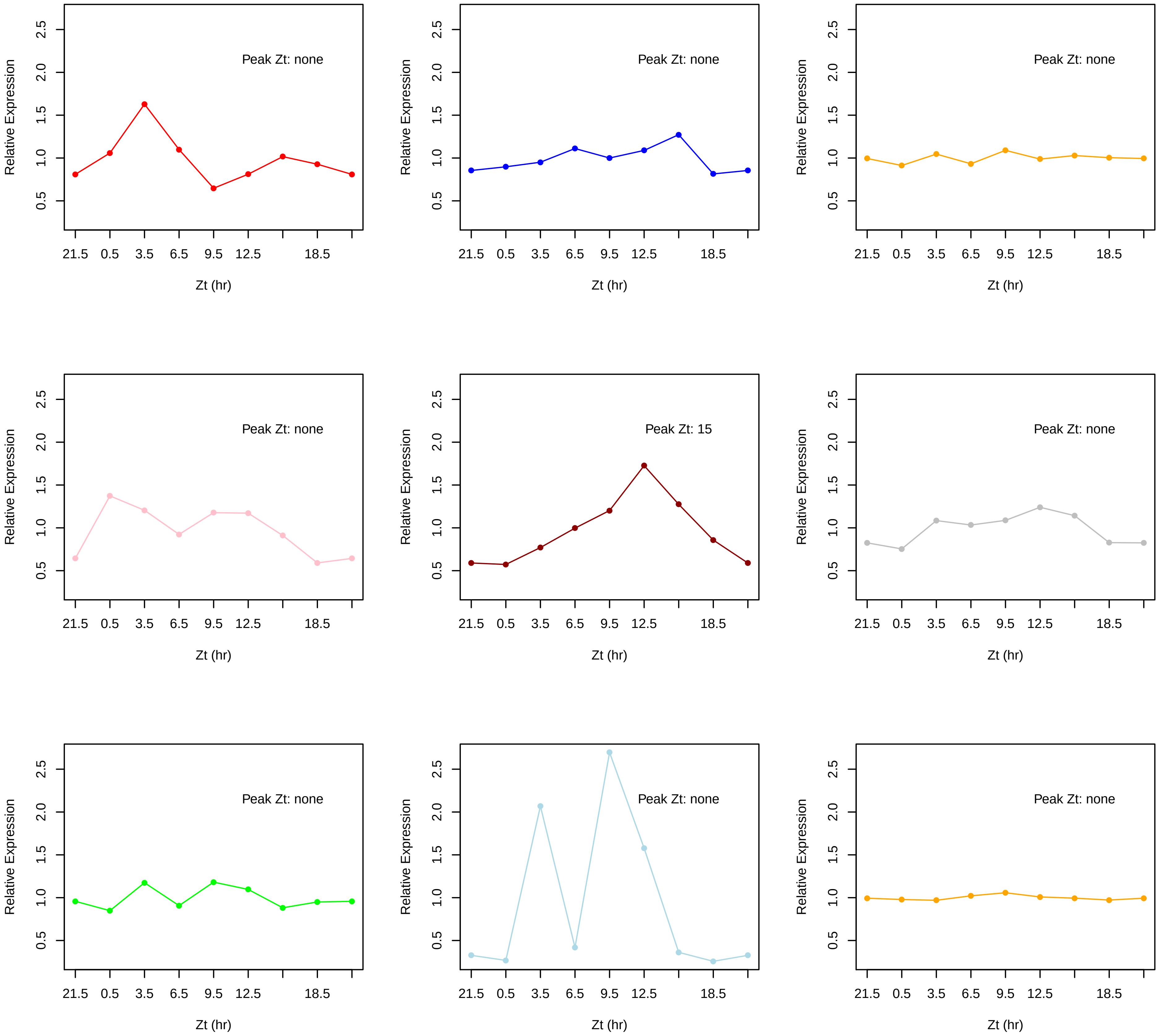

Singeleton: ENSXMAG00000007532

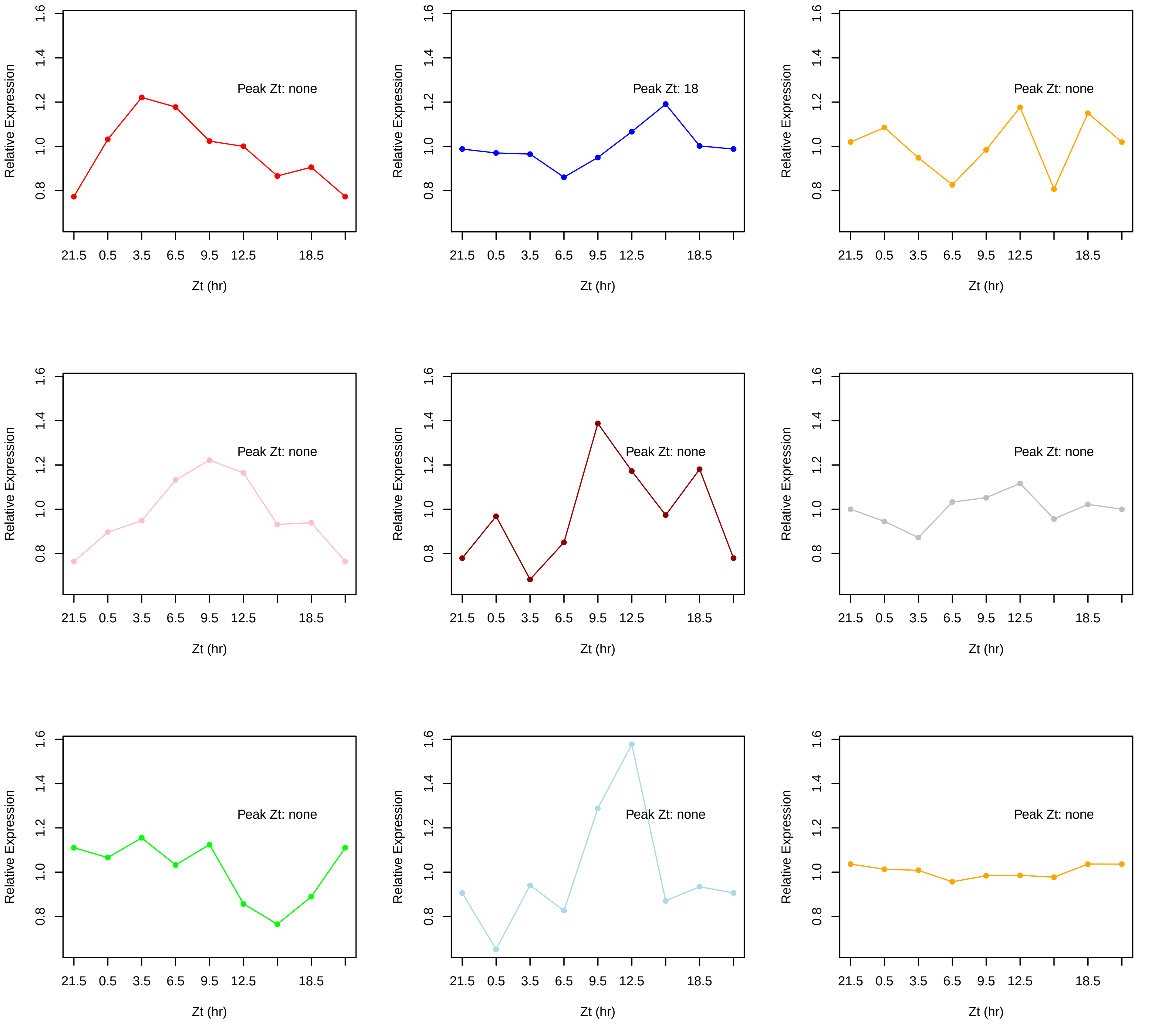

Singeleton: ENSXMAG00000022385 ttc4

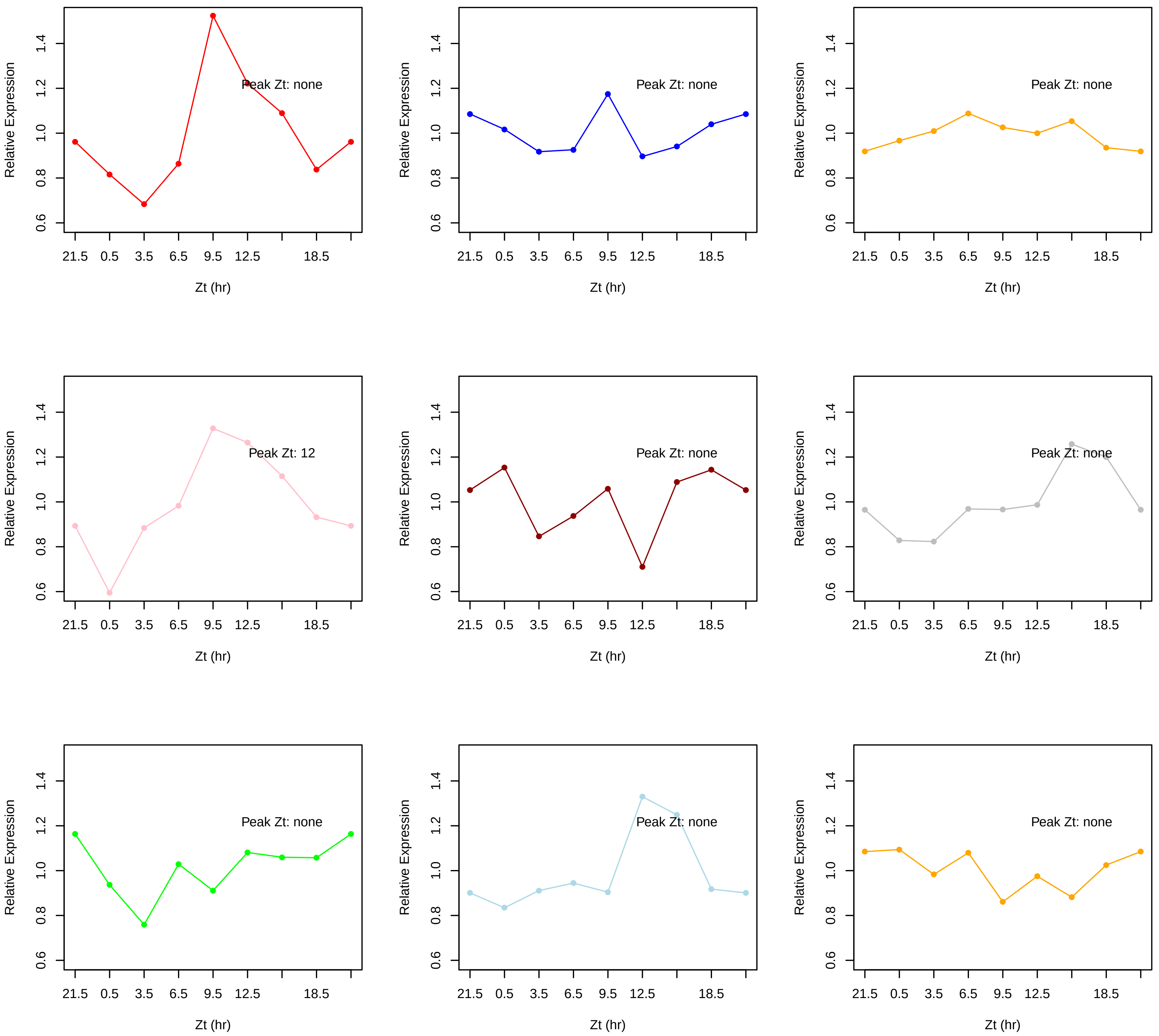

Singleton: ENSXMAG00000005316 scospondin

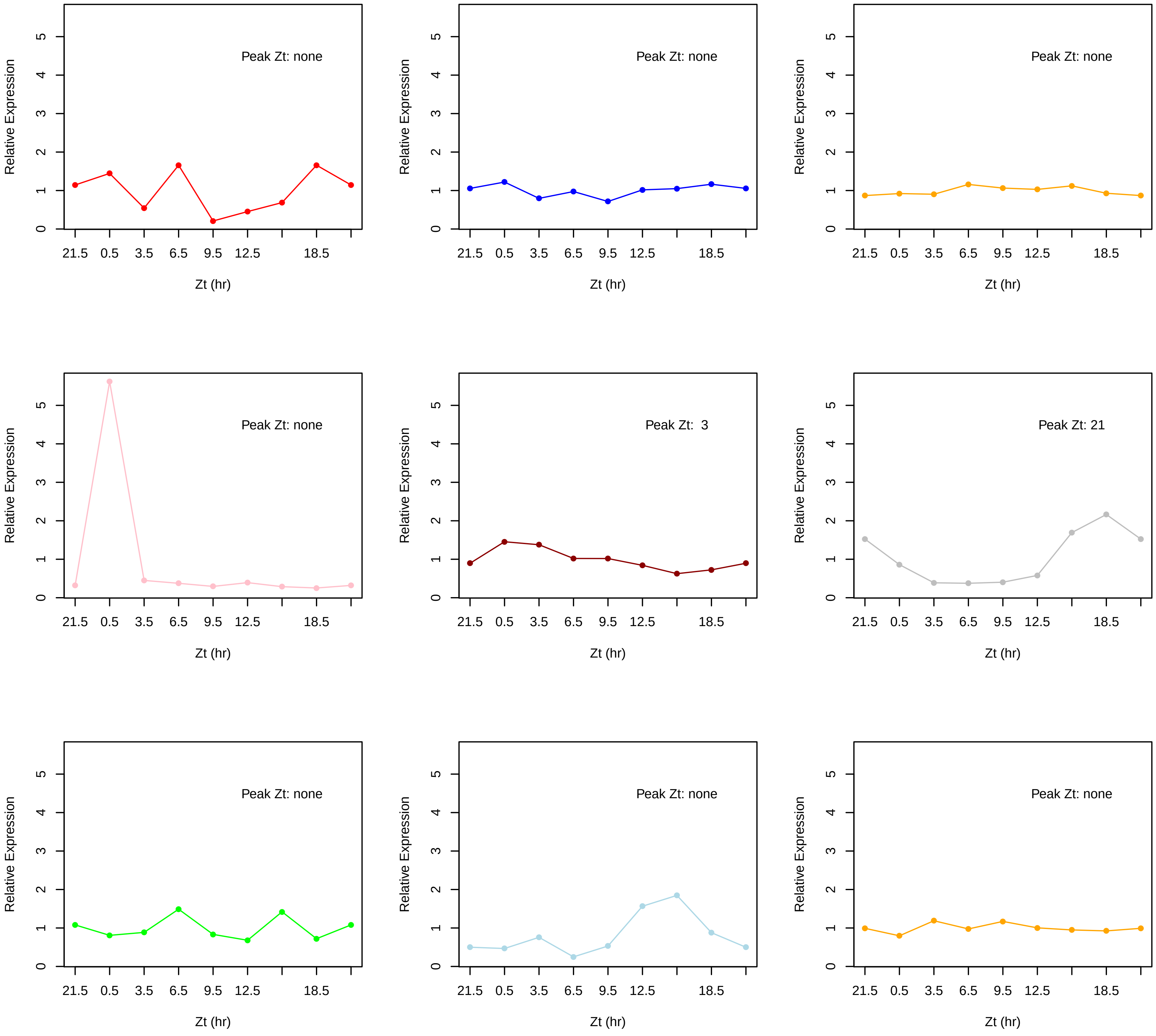

Singeleton: ENSXMAG00000001737 ly75

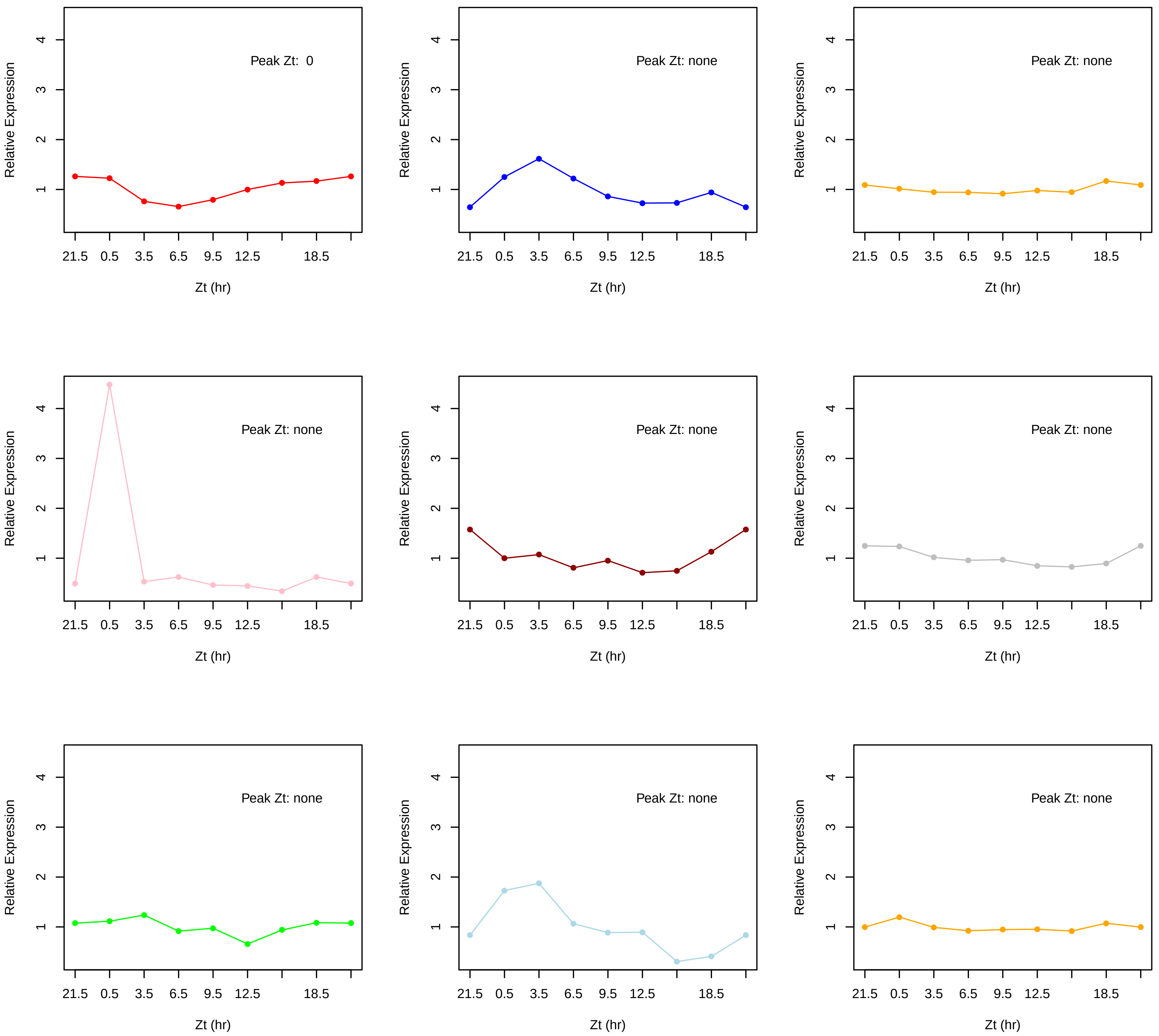

Singleton: ENSXMAG00000026862 mfsd9

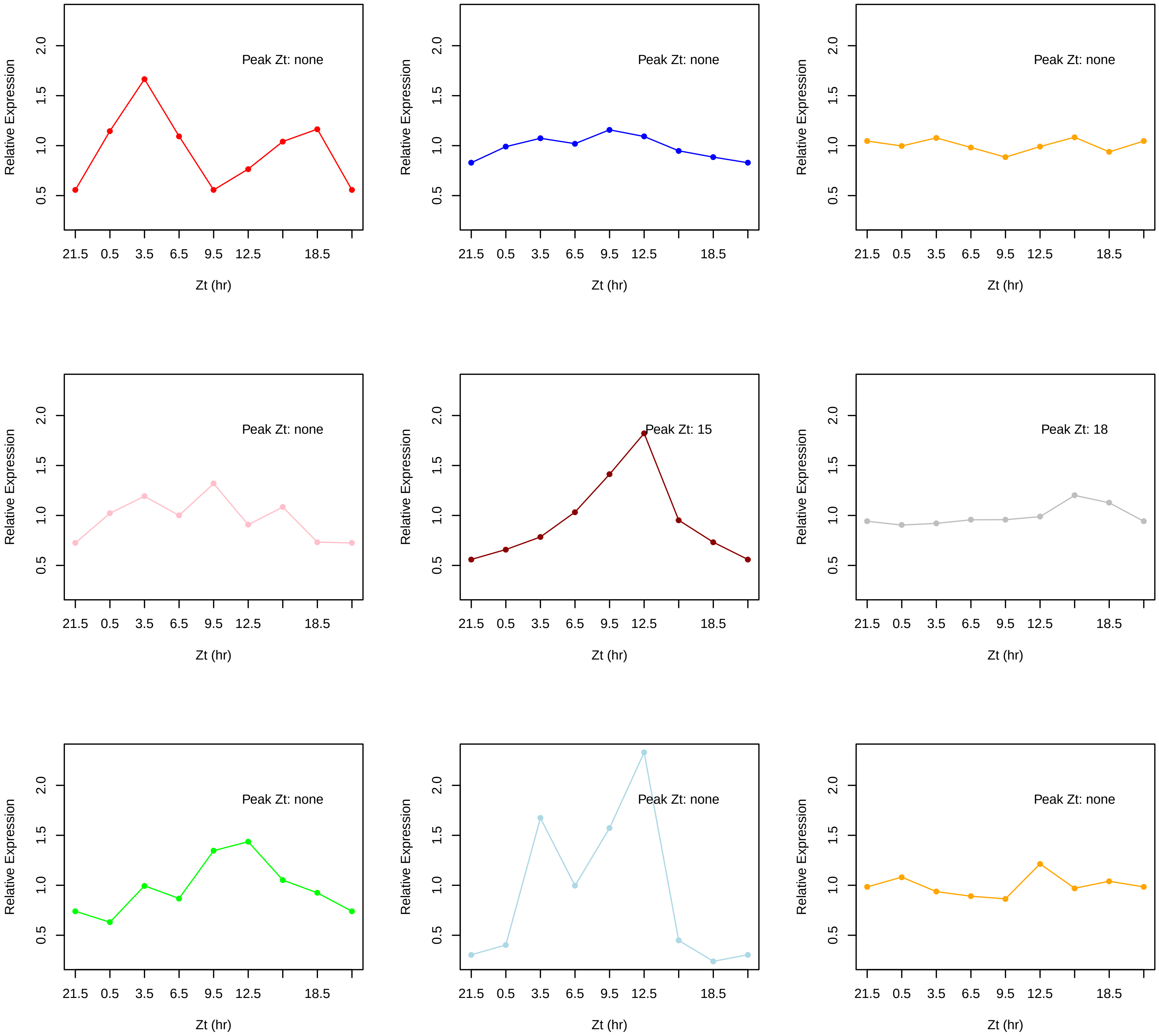

Singeleton: ENSXMAG00000024679 PPIC

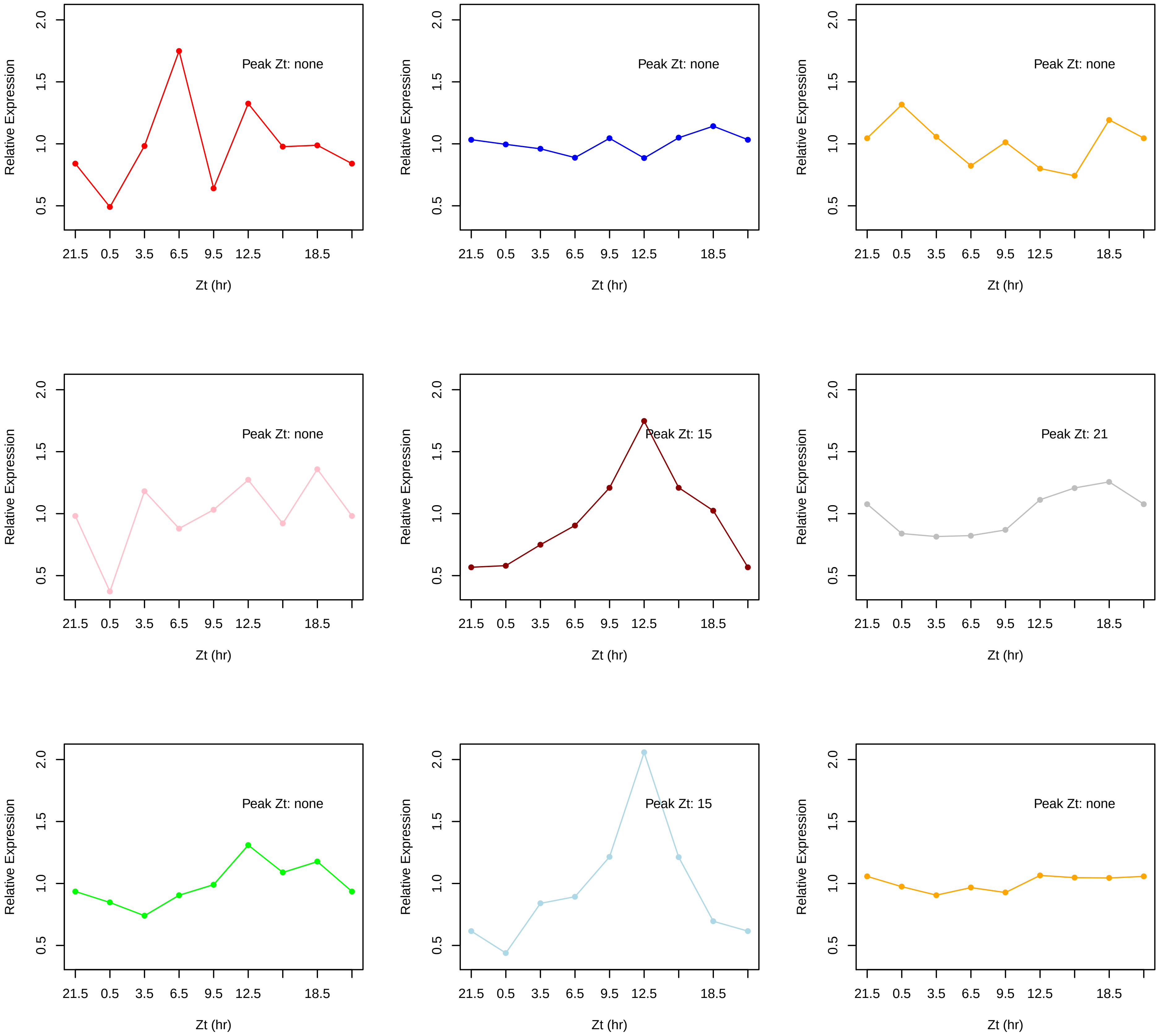

Singleton: ENSXMAG00000002269 pars2

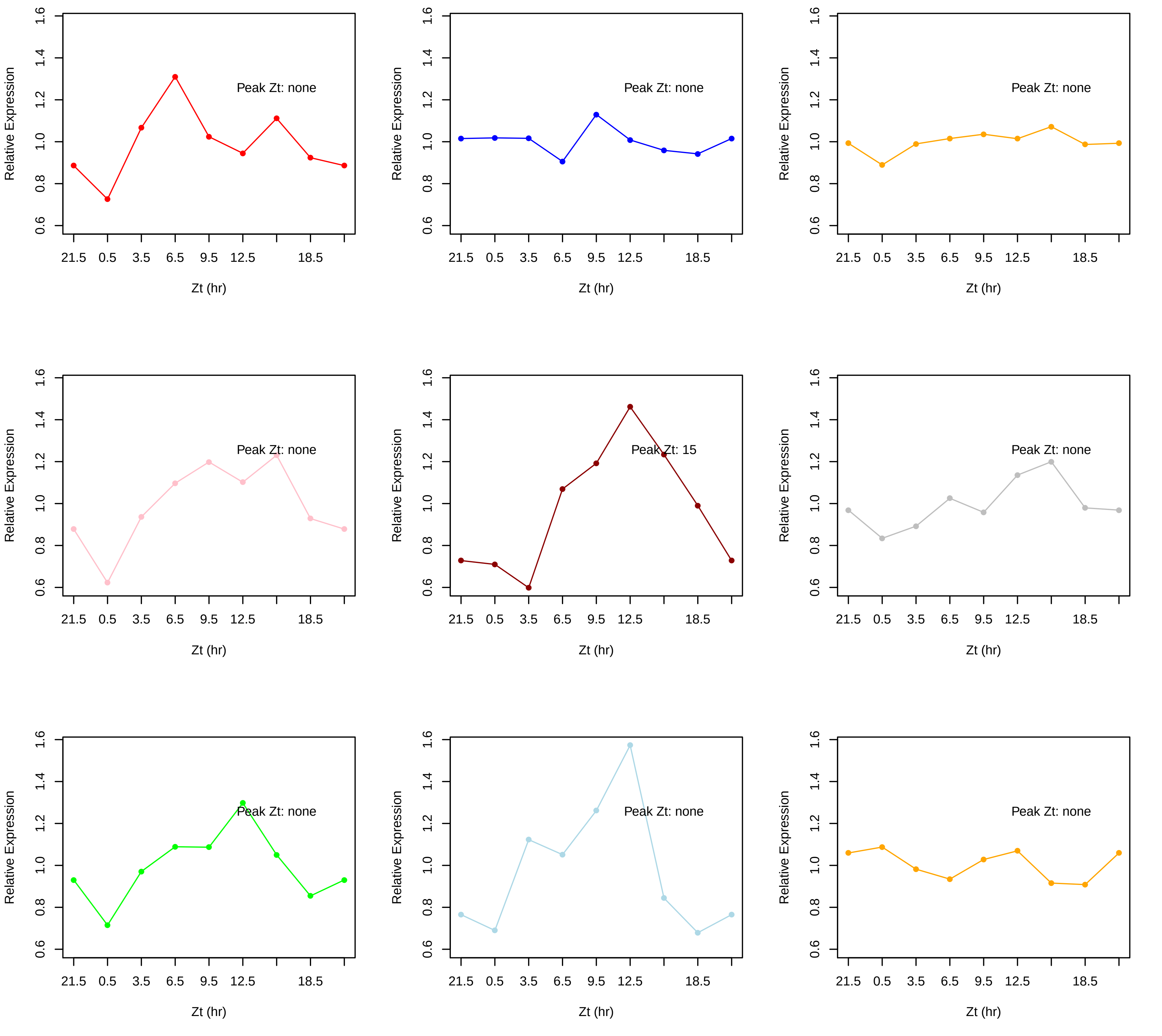

Singleton: ENSXMAG00000014399 ece2b

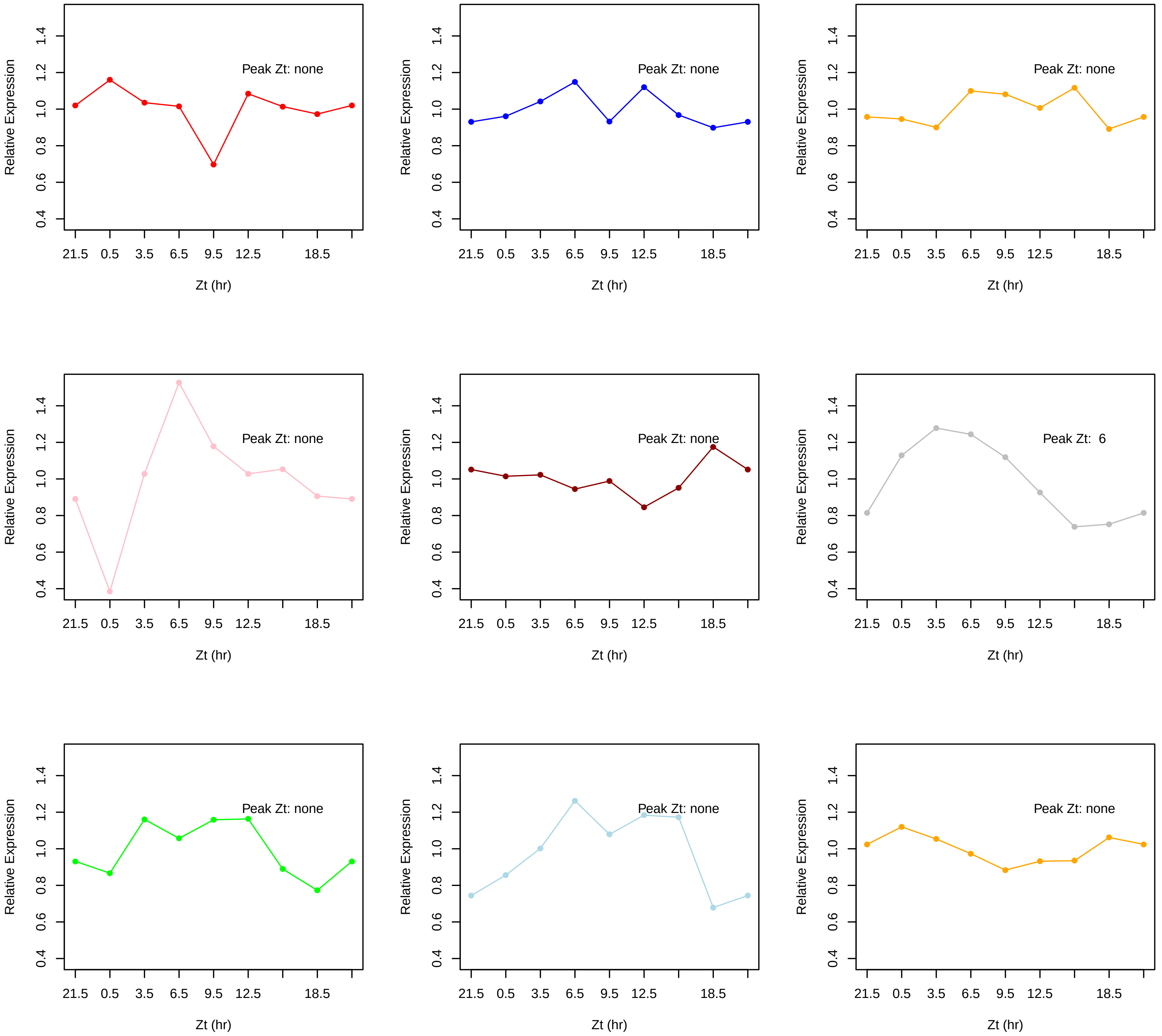

Singeleton: ENSXMAG00000006810 PNPLA6

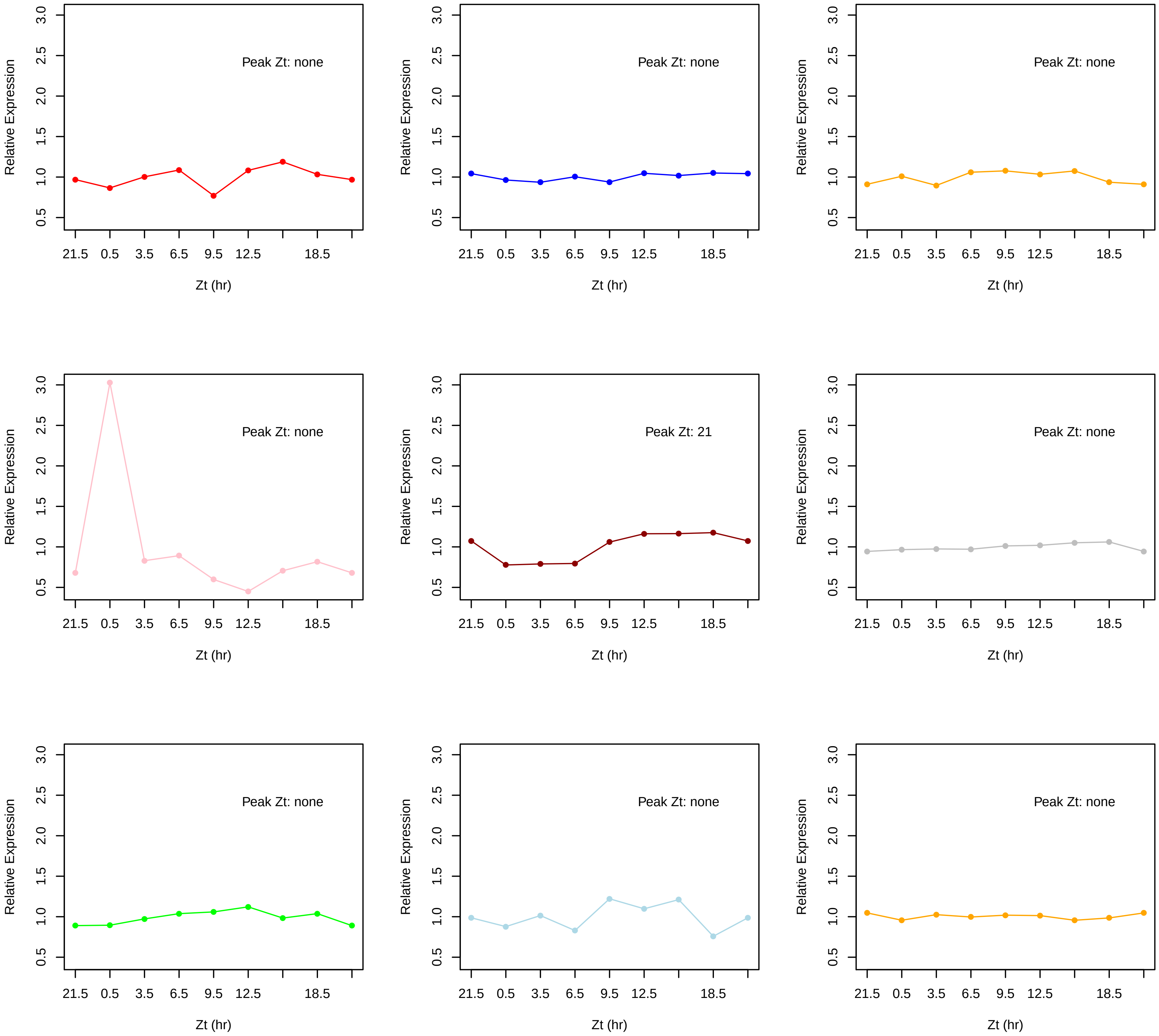

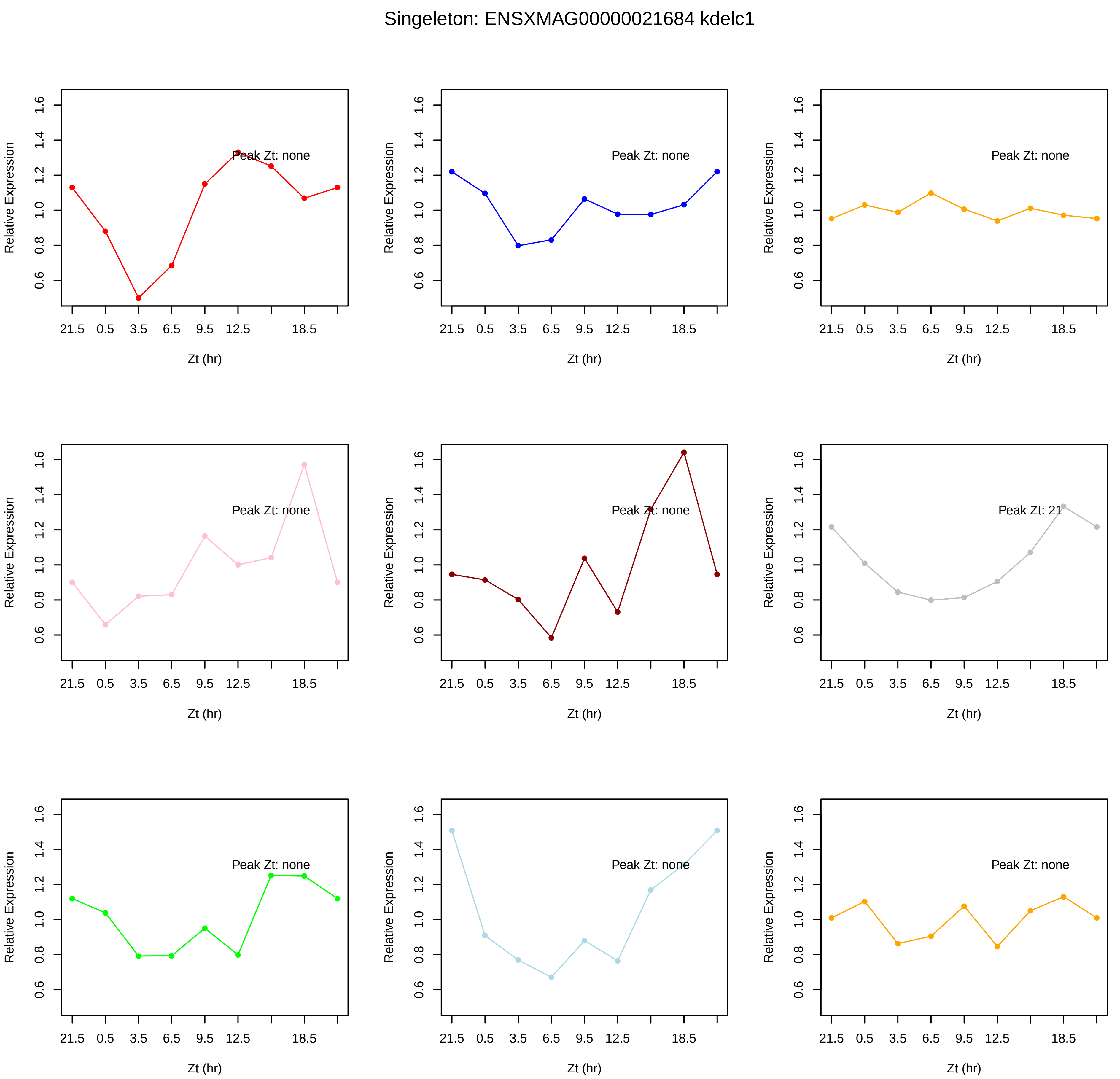

Singeleton: ENSXMAG00000021684 kdelc1
Relative Expression
Zt (hr)
21.5
0.5
3.5
6.5
9.5
12.5
18.5
0.6
0.8
1.0
1.2
1.4
1.6
Peak Zt: none
Peak Zt: 21

Singleton: ENSXMAG00000004220 snx24

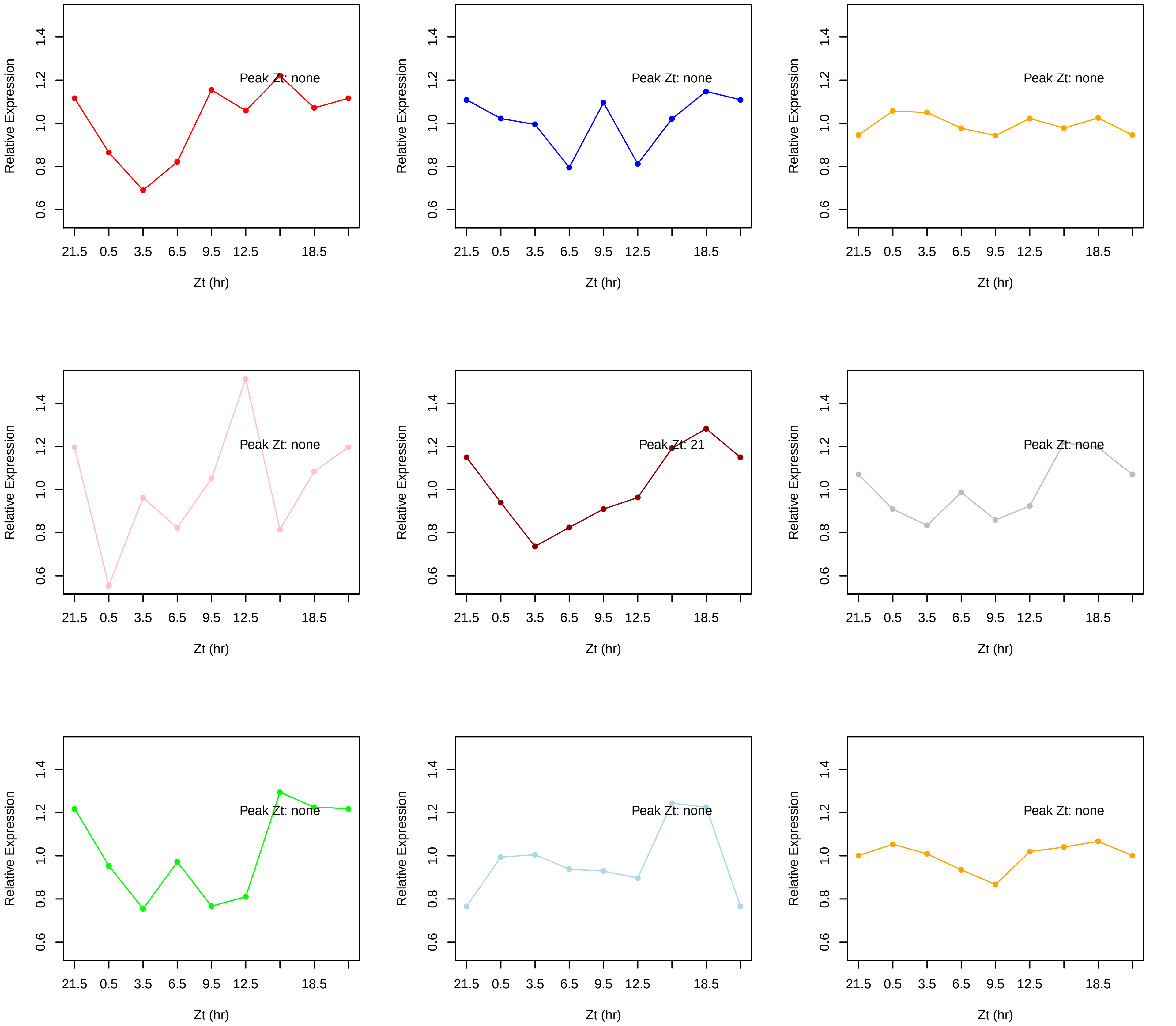

Singeleton: ENSXMAG00000028597 setd3

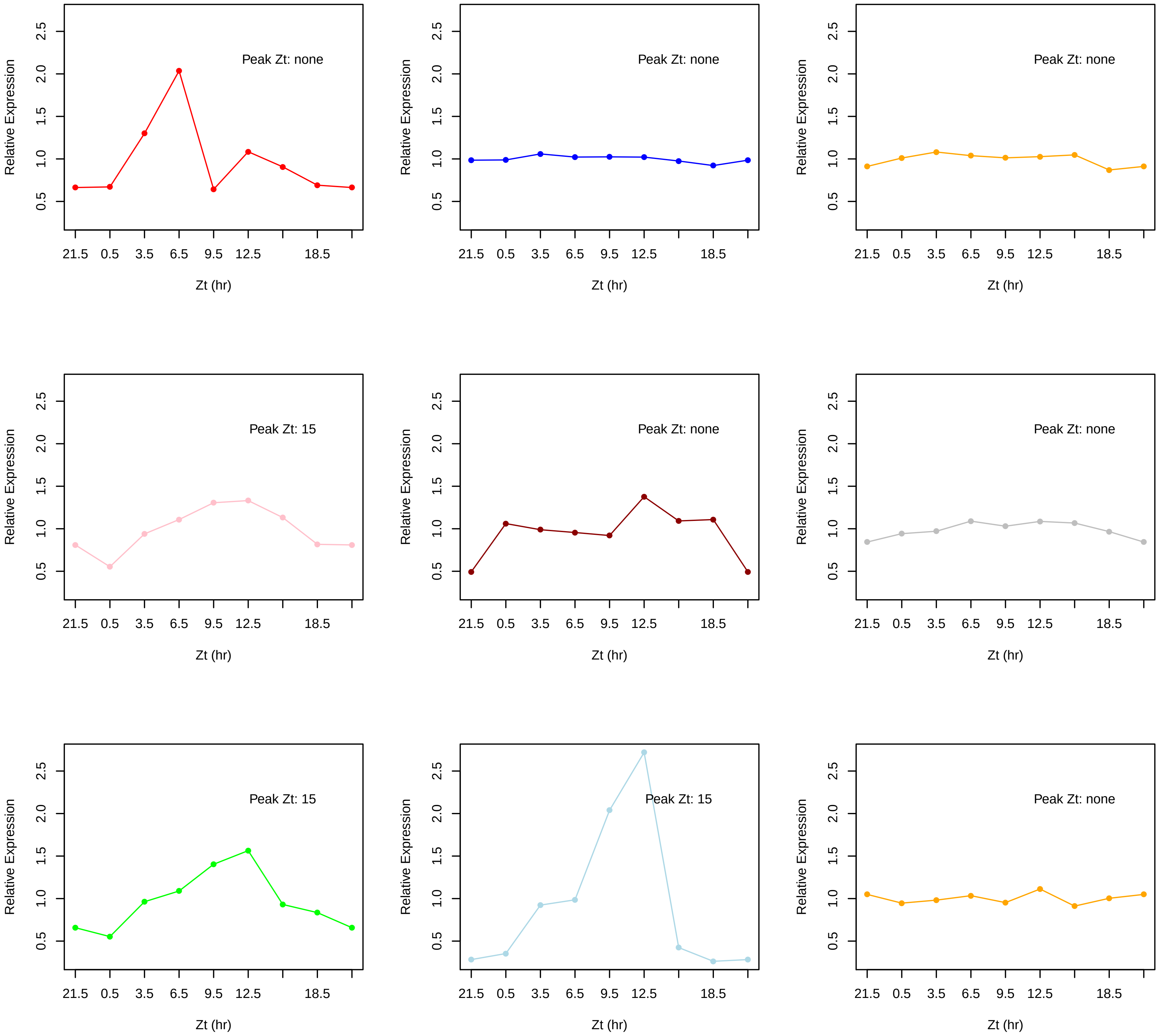

Singeleton: ENSXMAG00000029794 slc52a2

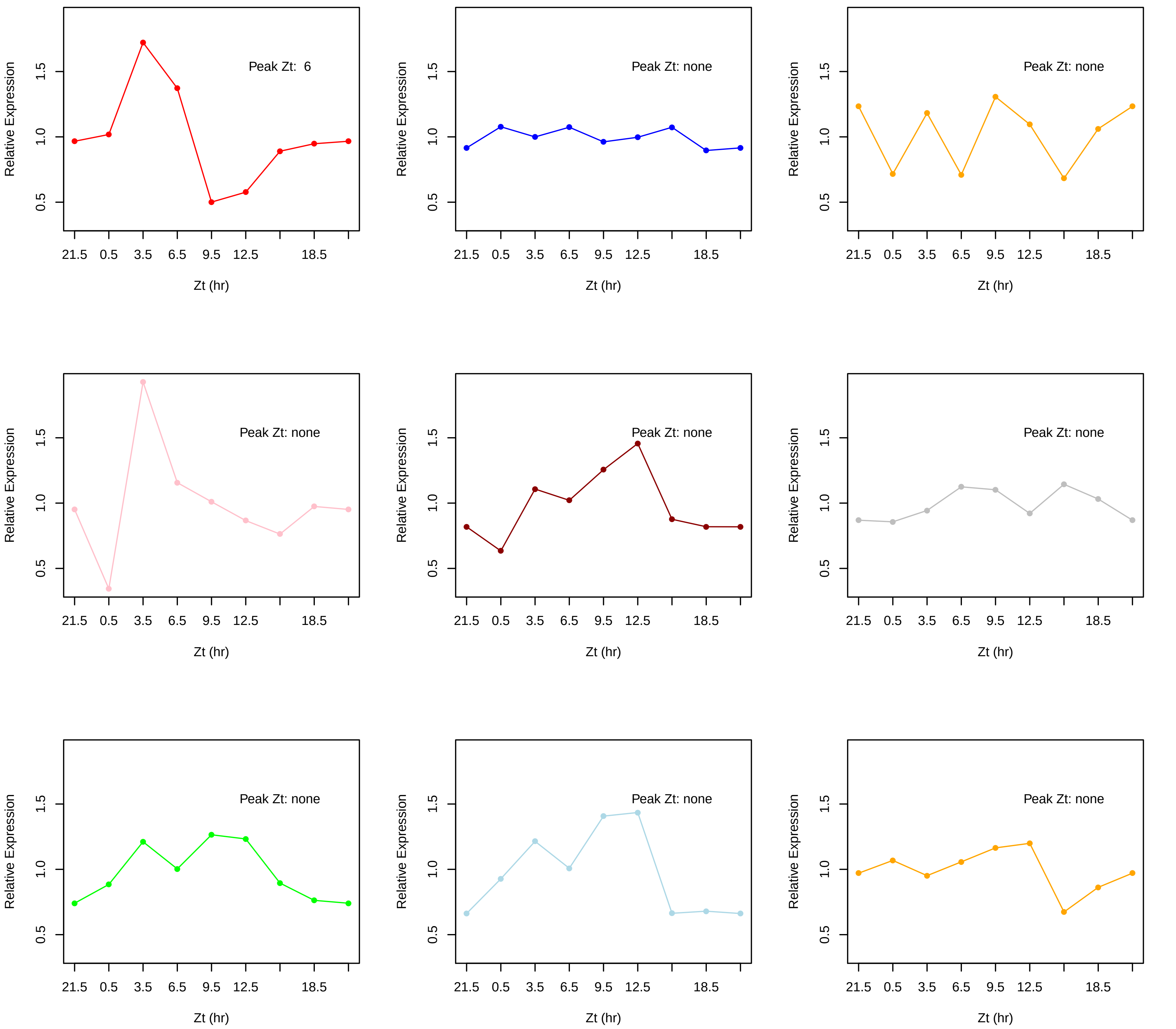

Singeleton: ENSXMAG00000017861 proser1

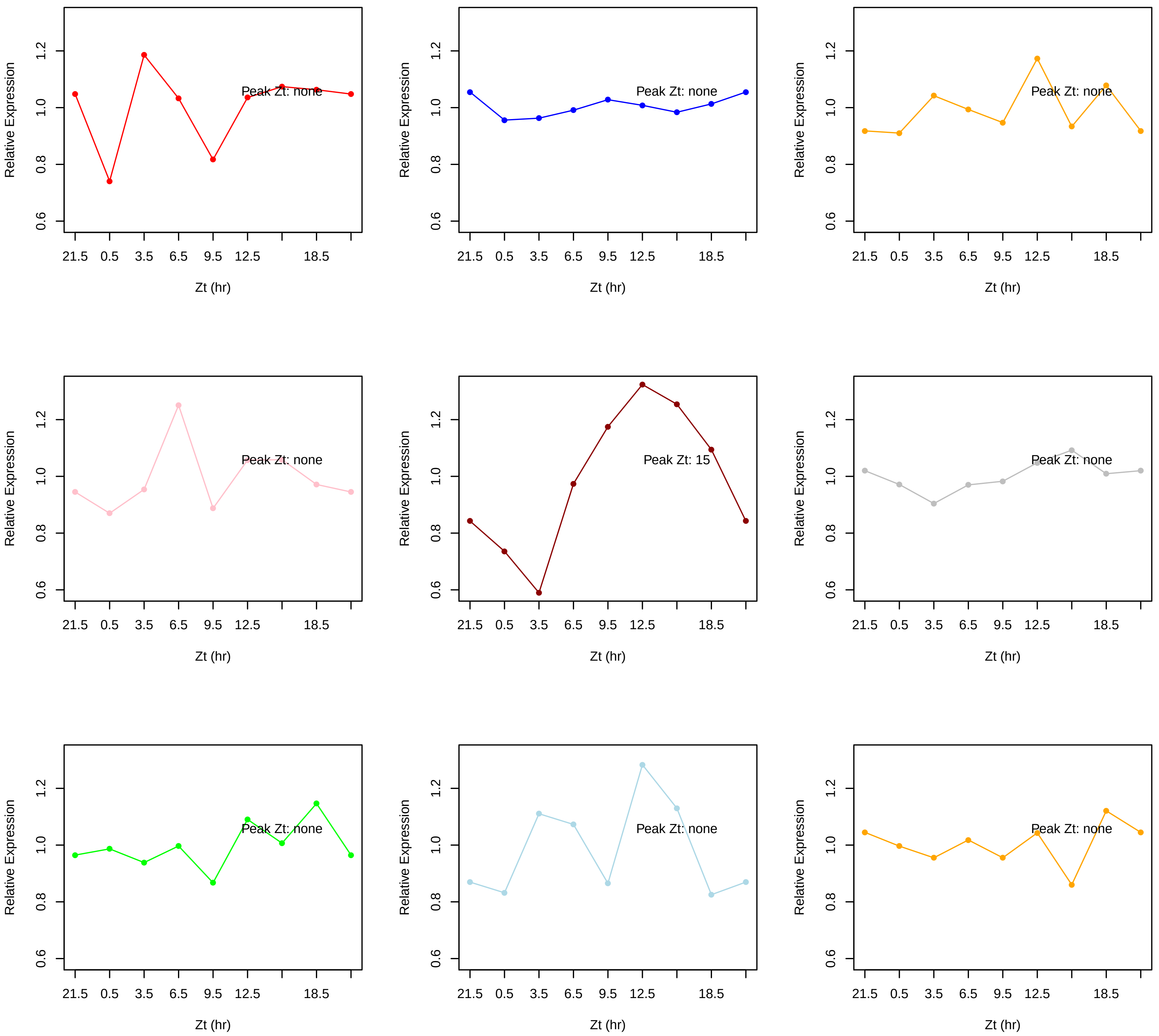

Singeleton: ENSXMAG00000004819 vsir

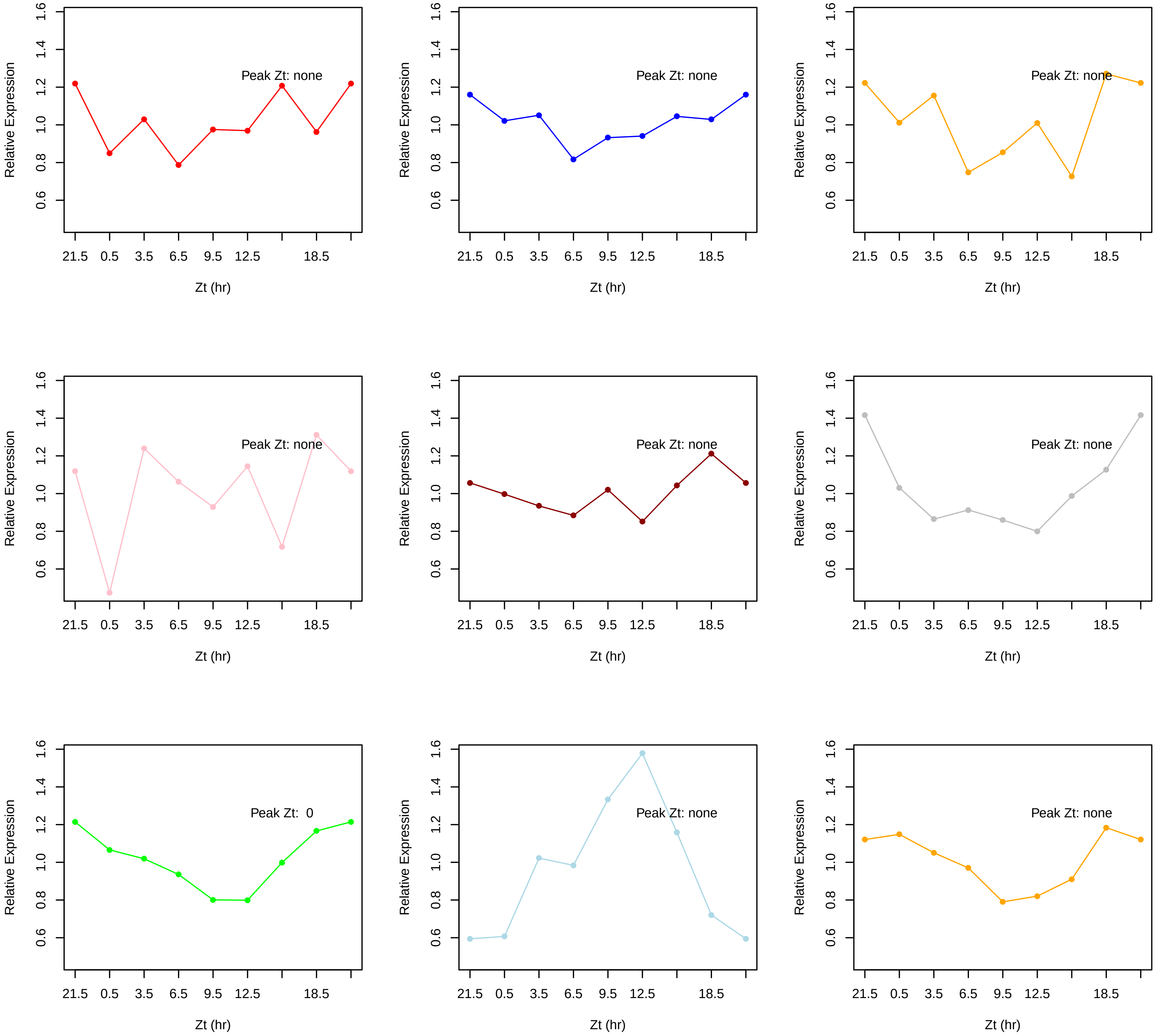

Singeleton: ENSXMAG00000015264 snx2

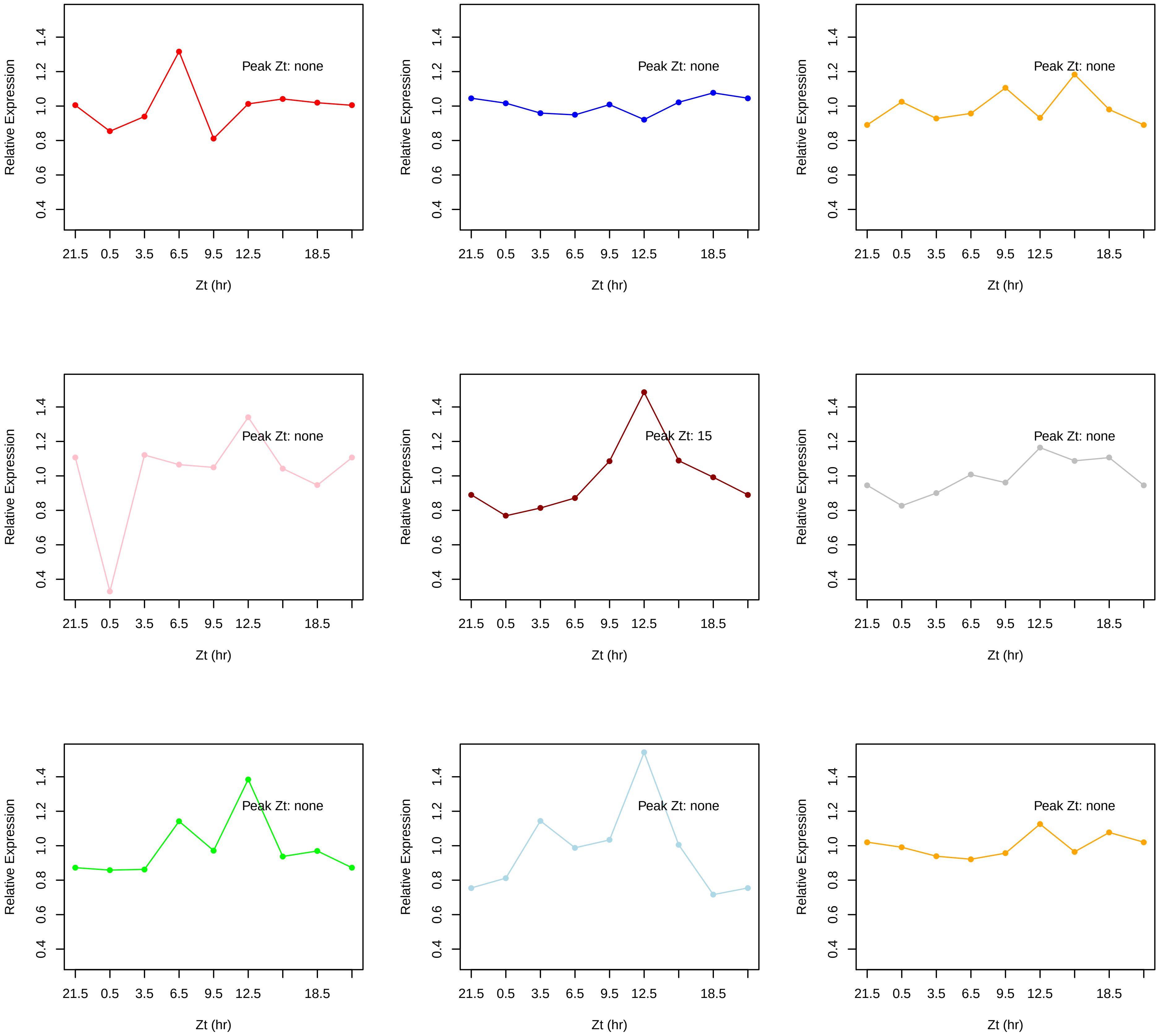

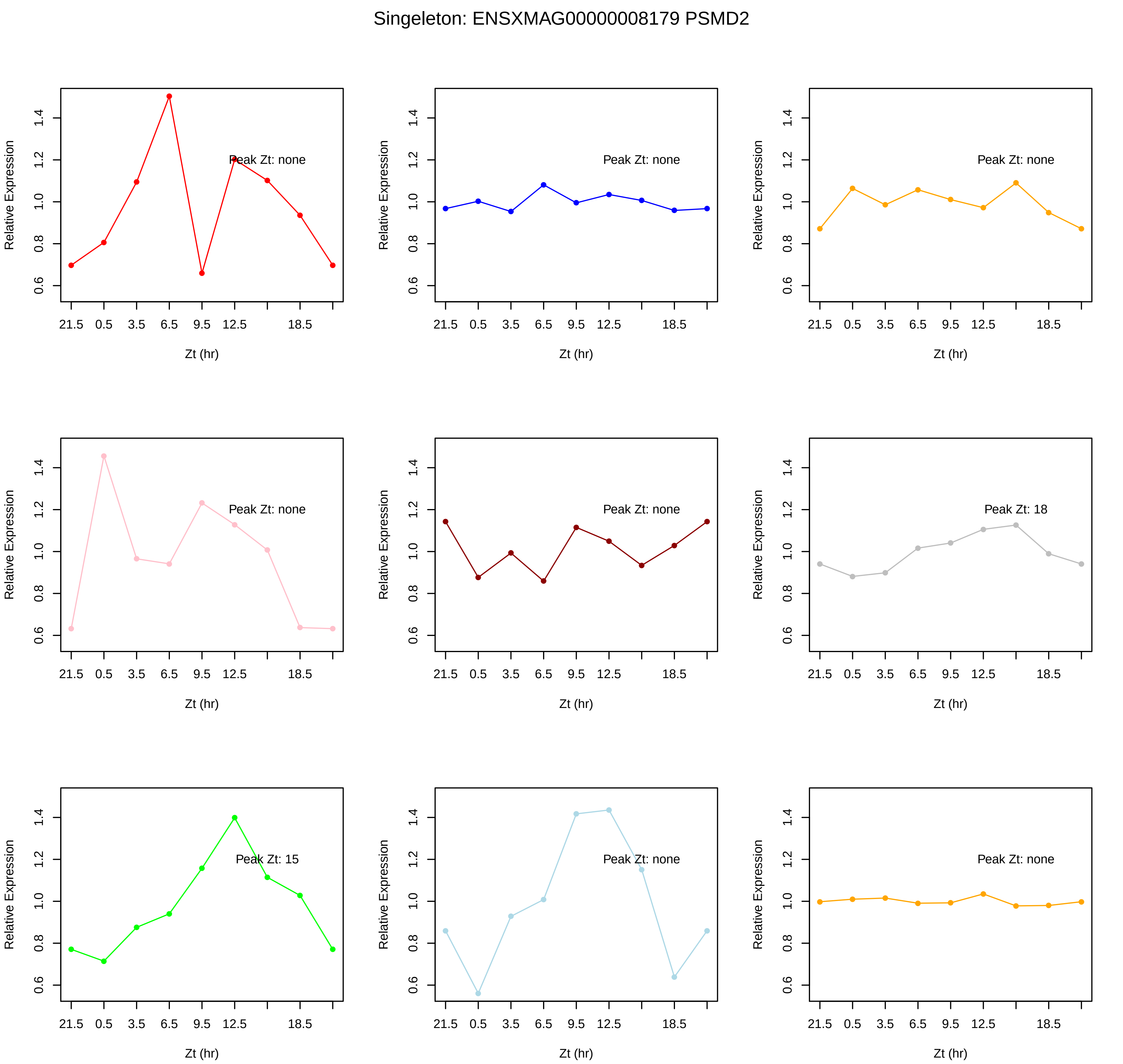
Singeleton: ENSXMAG00000008179 PSMD2
Peak Zt: none
Peak Zt: none
Peak Zt: none
Peak Zt: none
Peak Zt: none
Peak Zt: 18
Peak Zt: 15
Peak Zt: none
Peak Zt: none
Relative Expression
Zt (hr)

Singeleton: ENSXMAG00000025834 ccnk

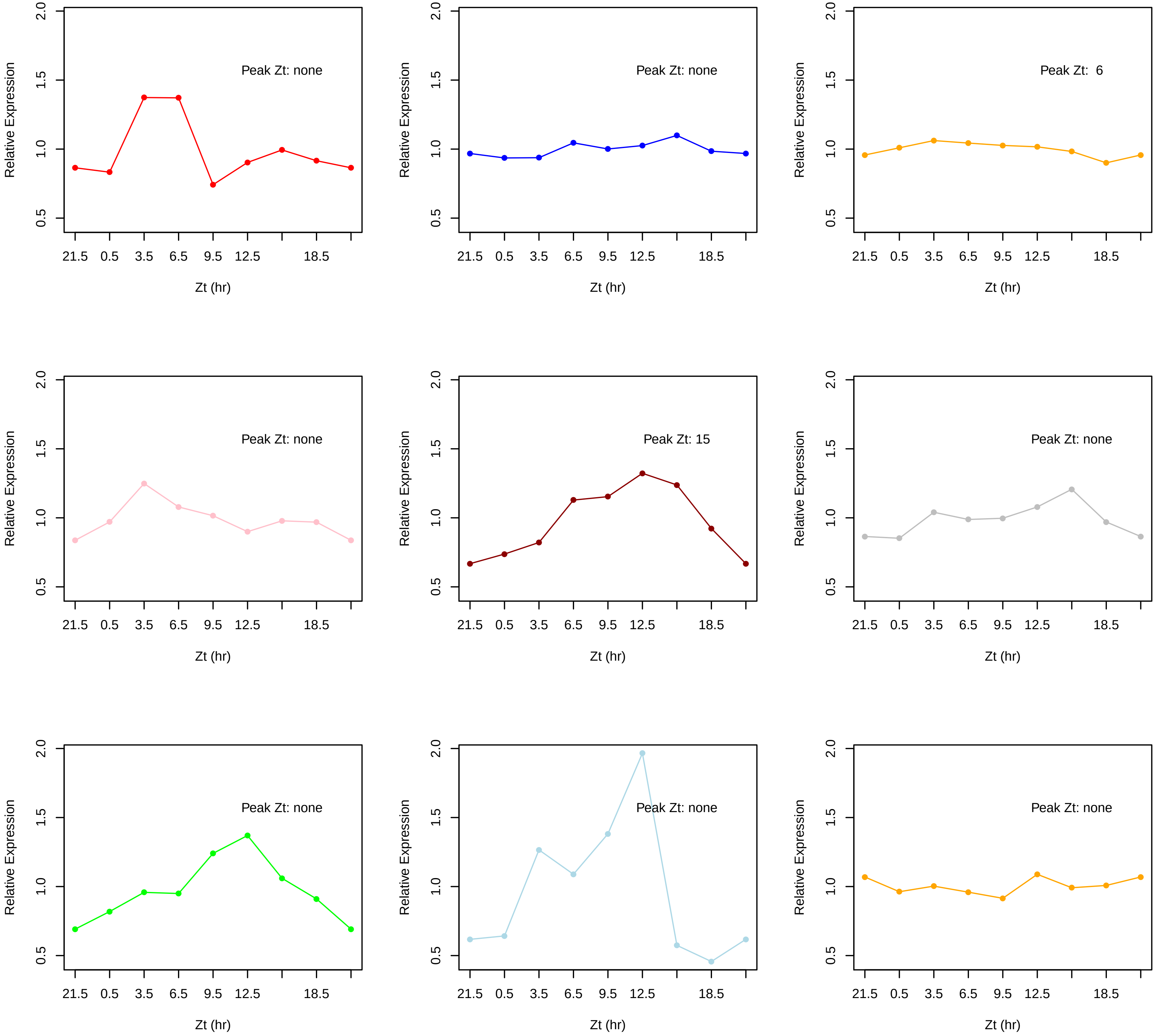

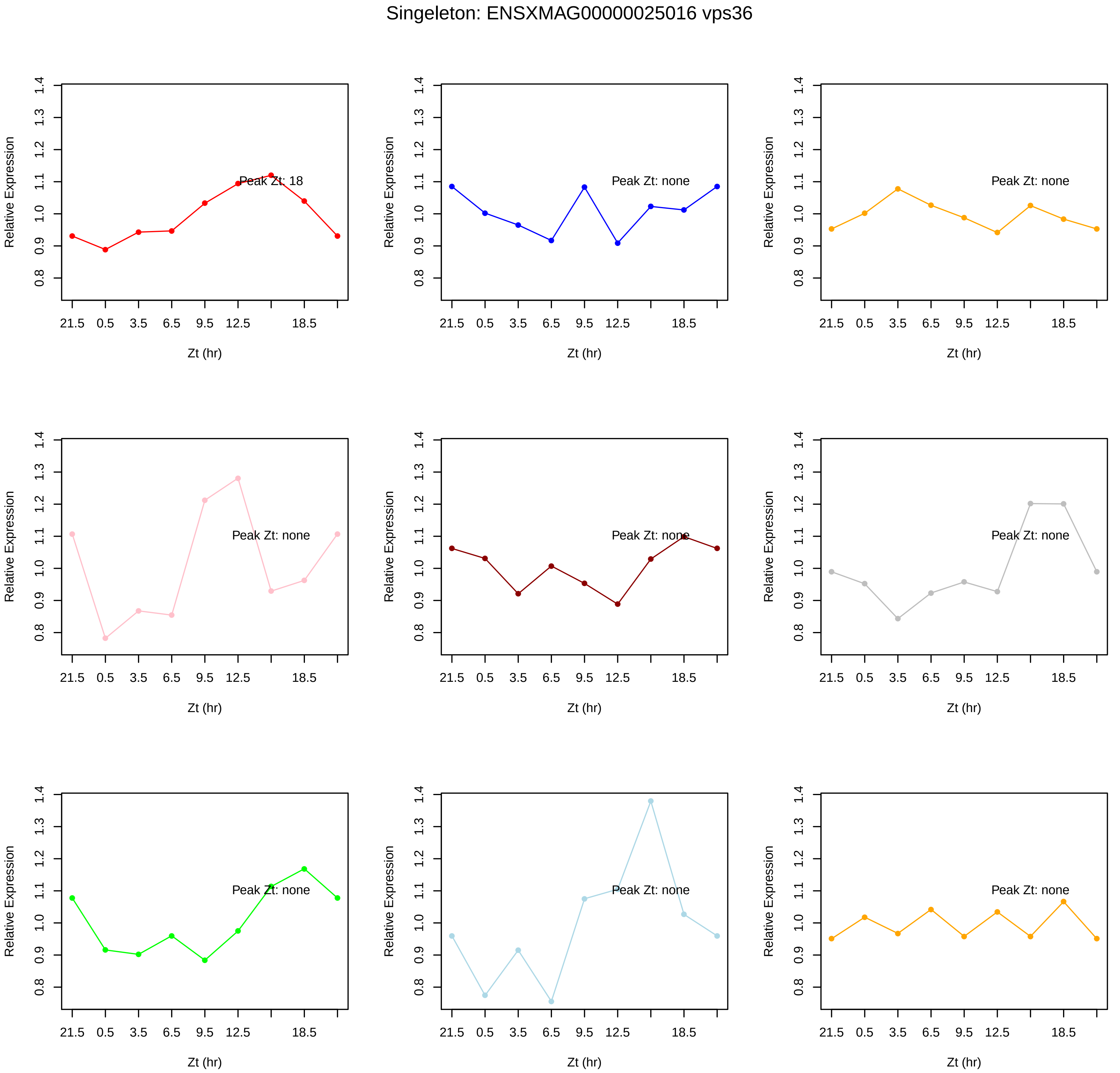

Singleton: ENSXMAG00000025016 vps36
Relative Expression
Zt (hr)
21.5 0.5 3.5 6.5 9.5 12.5 18.5
0.8 0.9 1.0 1.1 1.2 1.3 1.4
Peak Zt: 18
Peak Zt: none
Peak Zt: none
Peak Zt: none
Peak Zt: none
Peak Zt: none
Peak Zt: none
Peak Zt: none
Peak Zt: none

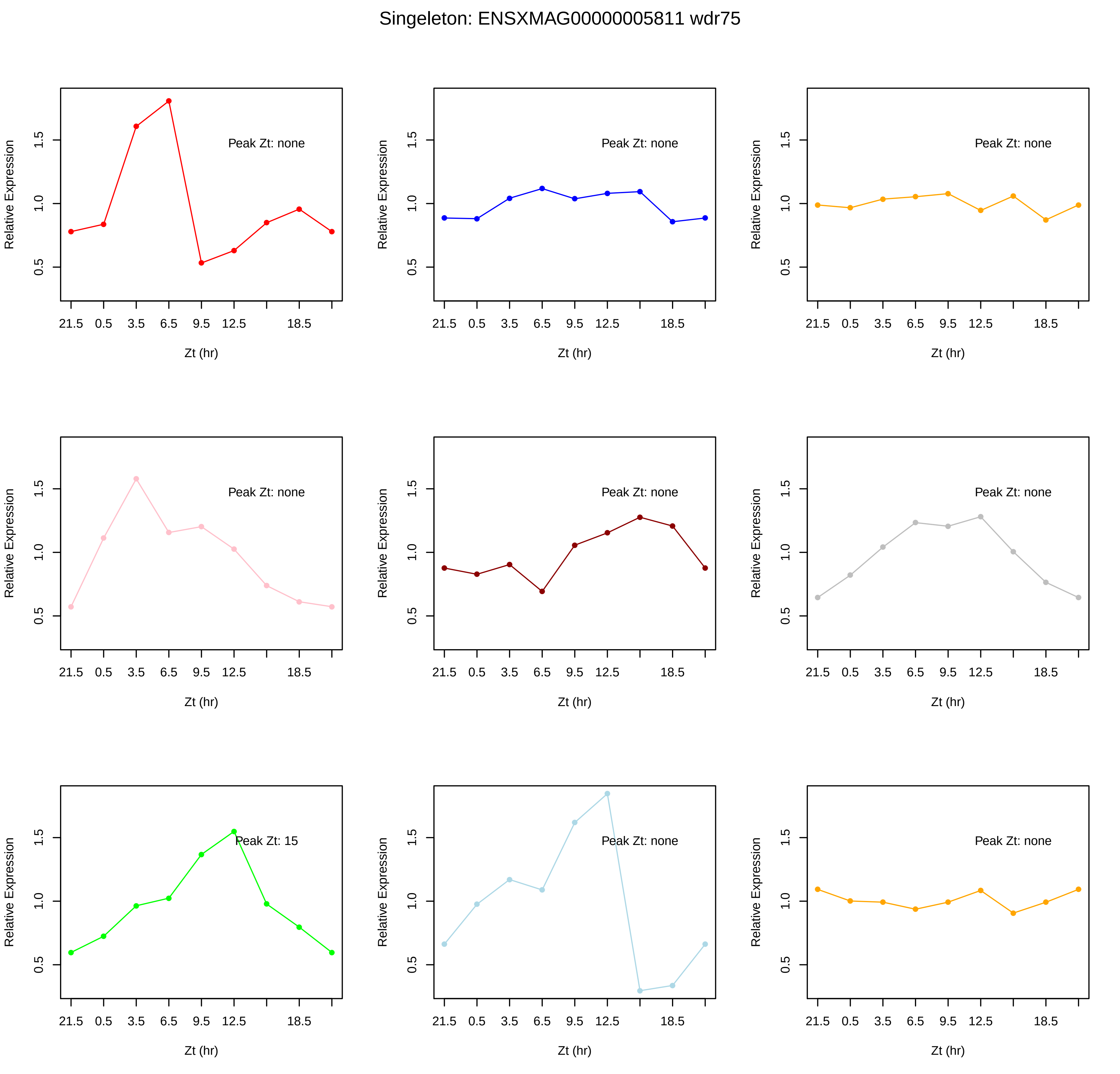
Singleton: ENSXMAG00000005811 wdr75
Peak Zt: none
Peak Zt: none
Peak Zt: none
Peak Zt: none
Peak Zt: none
Peak Zt: none
Peak Zt: 15
Peak Zt: none
Peak Zt: none
Relative Expression
Zt (hr)

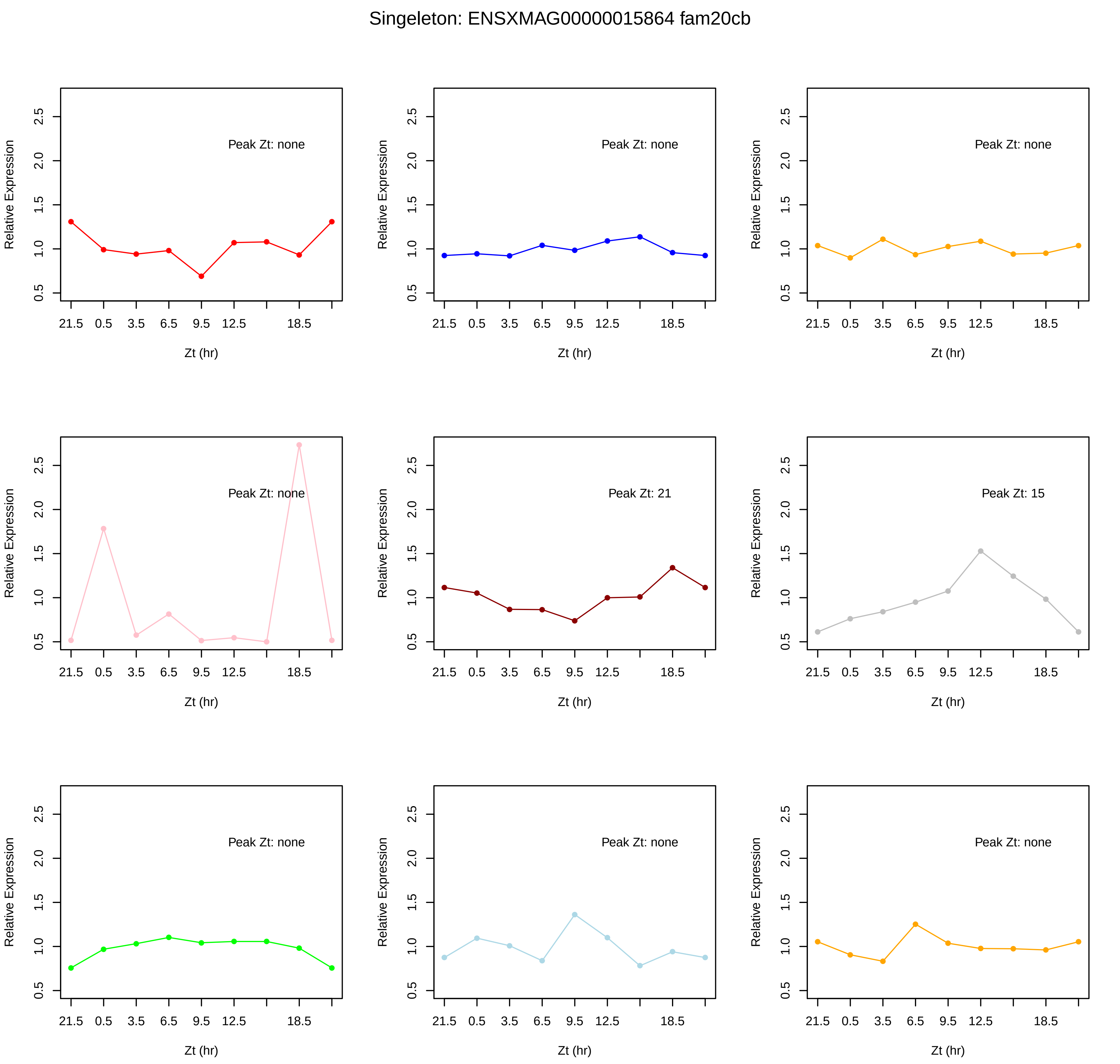
Singeleton: ENSXMAG00000015864 fam20cb
Peak Zt: none
Peak Zt: none
Peak Zt: none
Peak Zt: none
Peak Zt: 21
Peak Zt: 15
Peak Zt: none
Peak Zt: none
Peak Zt: none
Relative Expression
Zt (hr)
21.5 0.5 3.5 6.5 9.5 12.5 18.5
0.5 1.0 1.5 2.0 2.5

Singeleton: ENSXMAG00000017831 thsd1

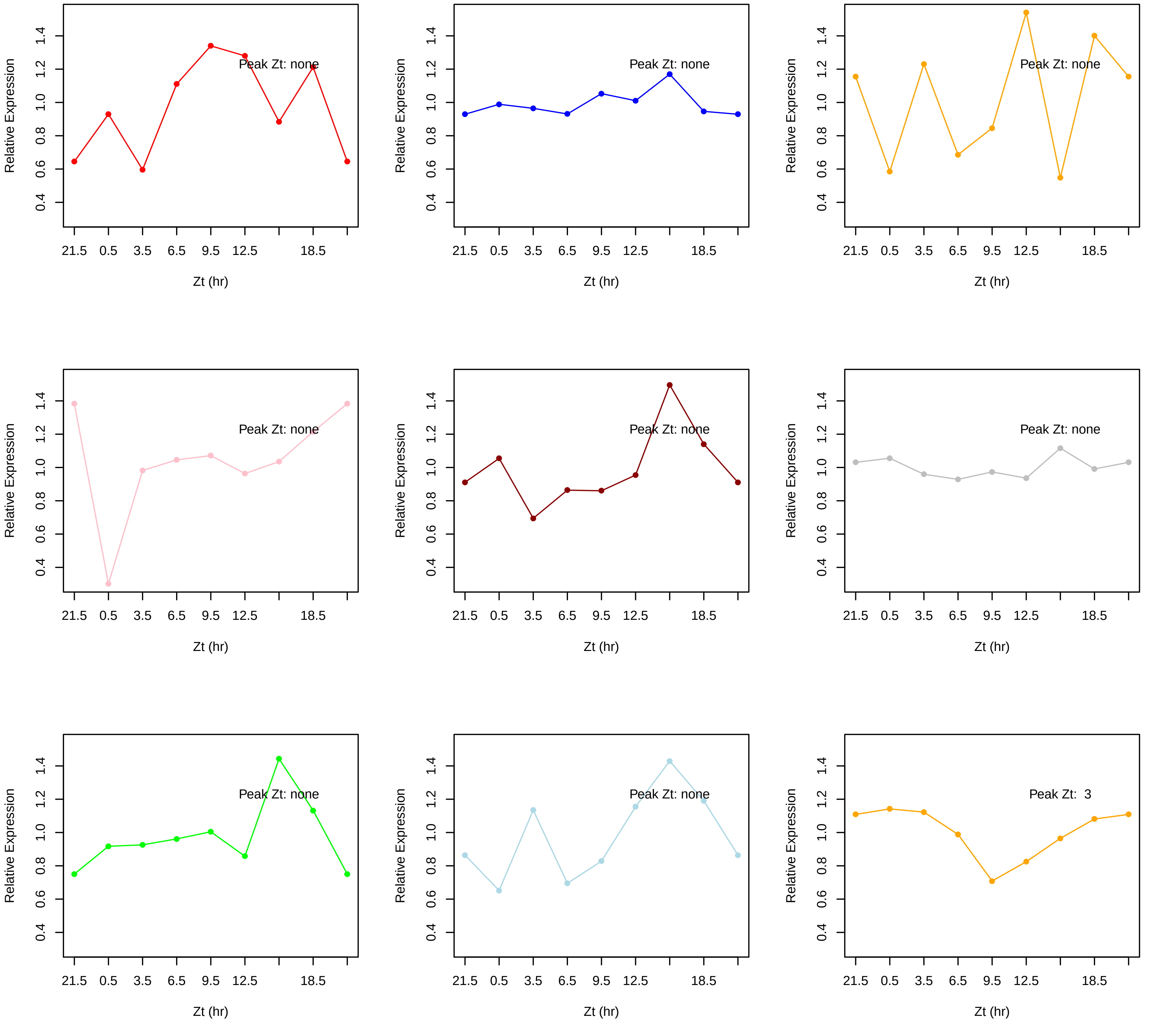

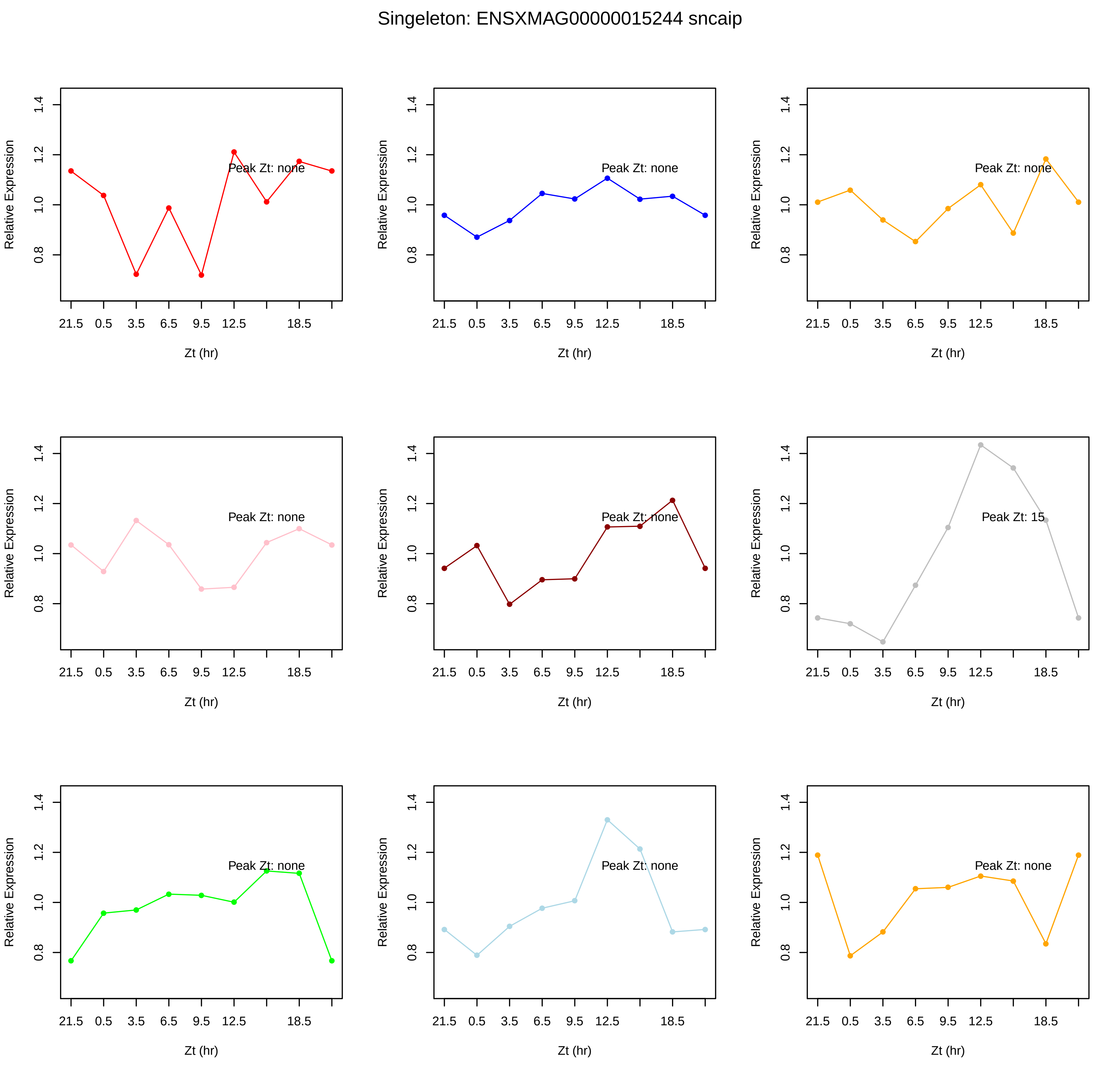

Singeleton: ENSXMAG00000015244 sncaip
Relative Expression
Zt (hr)
21.5
0.5
3.5
6.5
9.5
12.5
18.5
0.8
1.0
1.2
1.4
Peak Zt: none
Peak Zt: 15

Singeleton: ENSXMAG00000029620 slc40a1

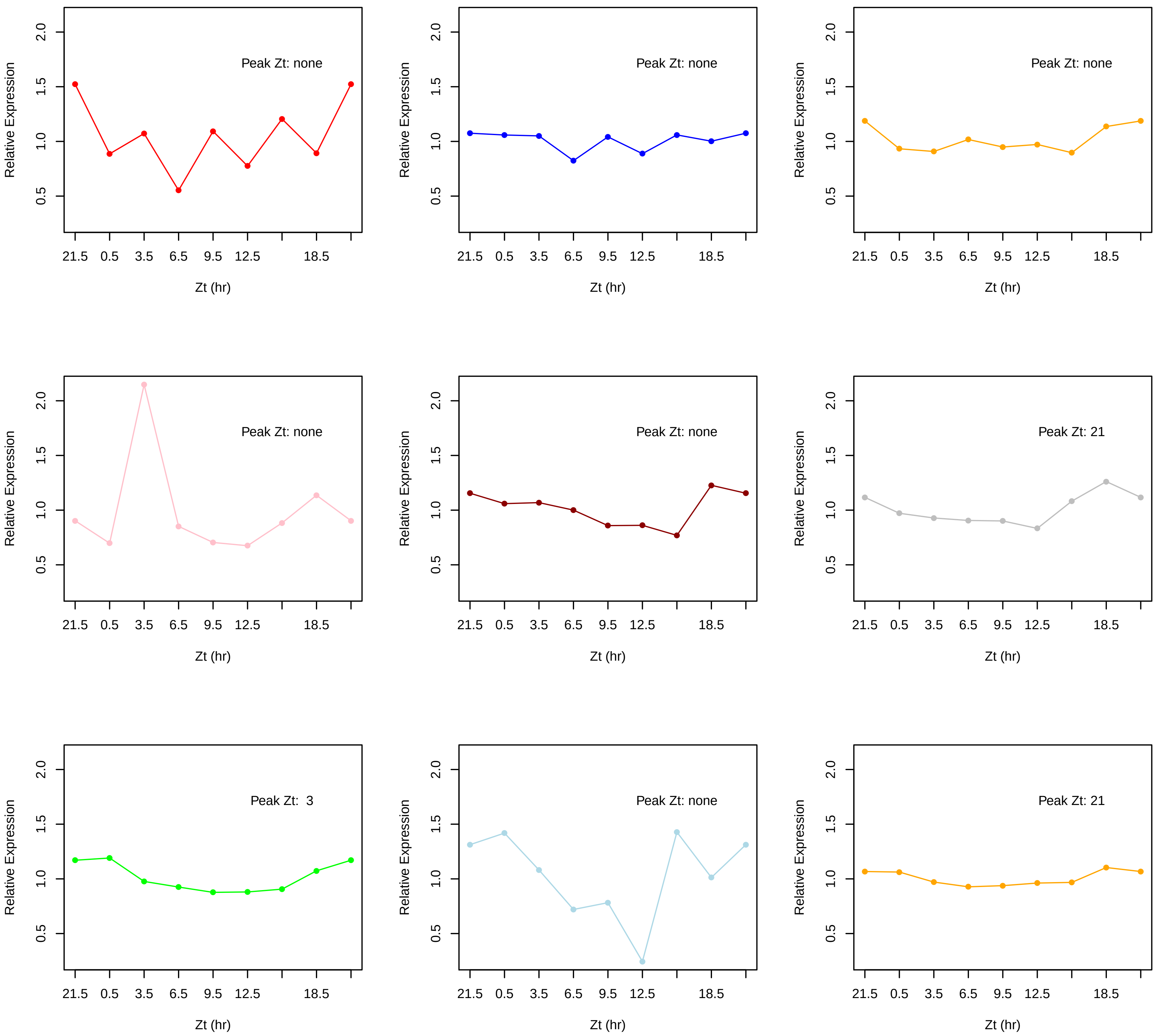

Singeleton: ENSXMAG00000028882 srd5a1

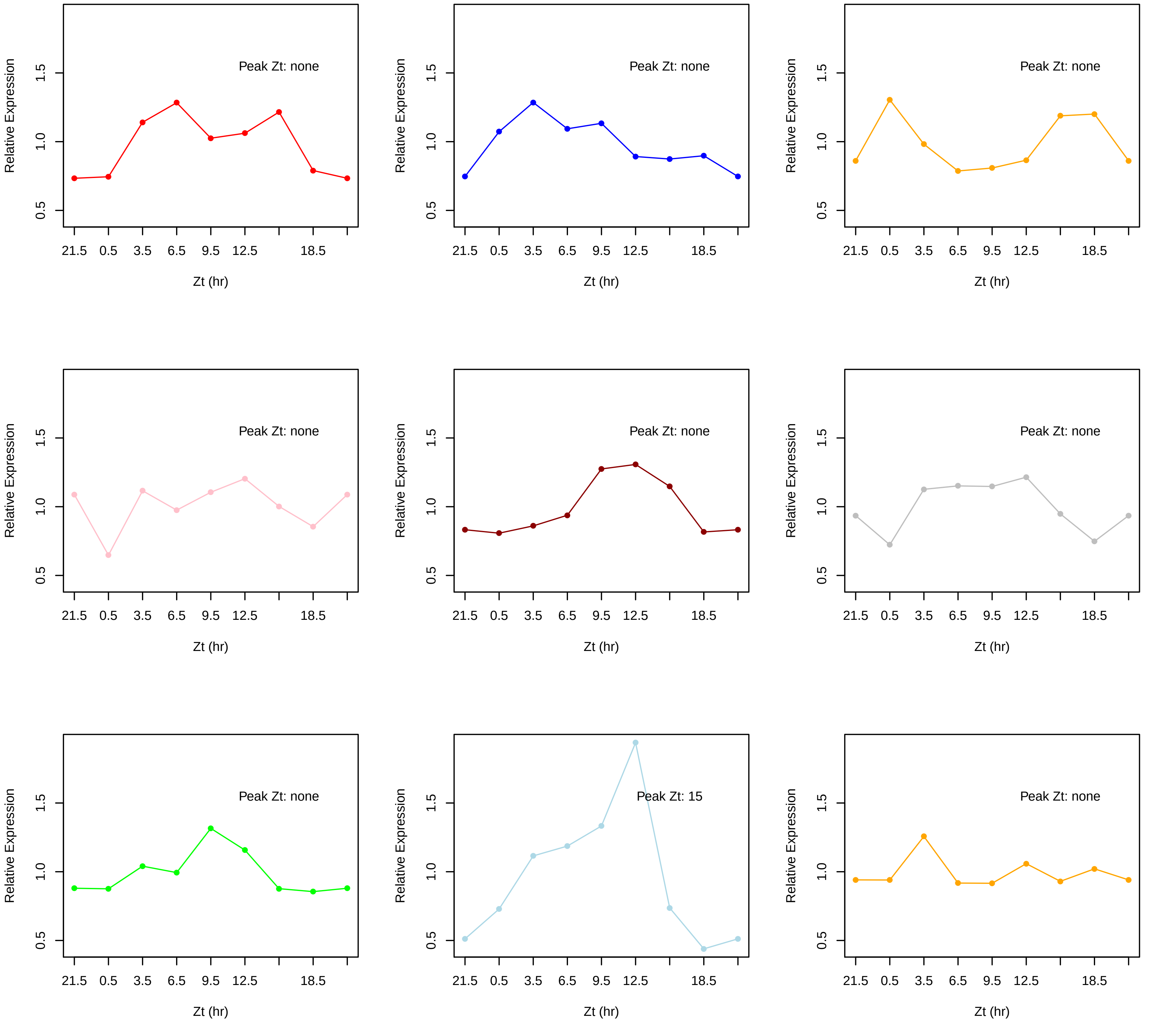

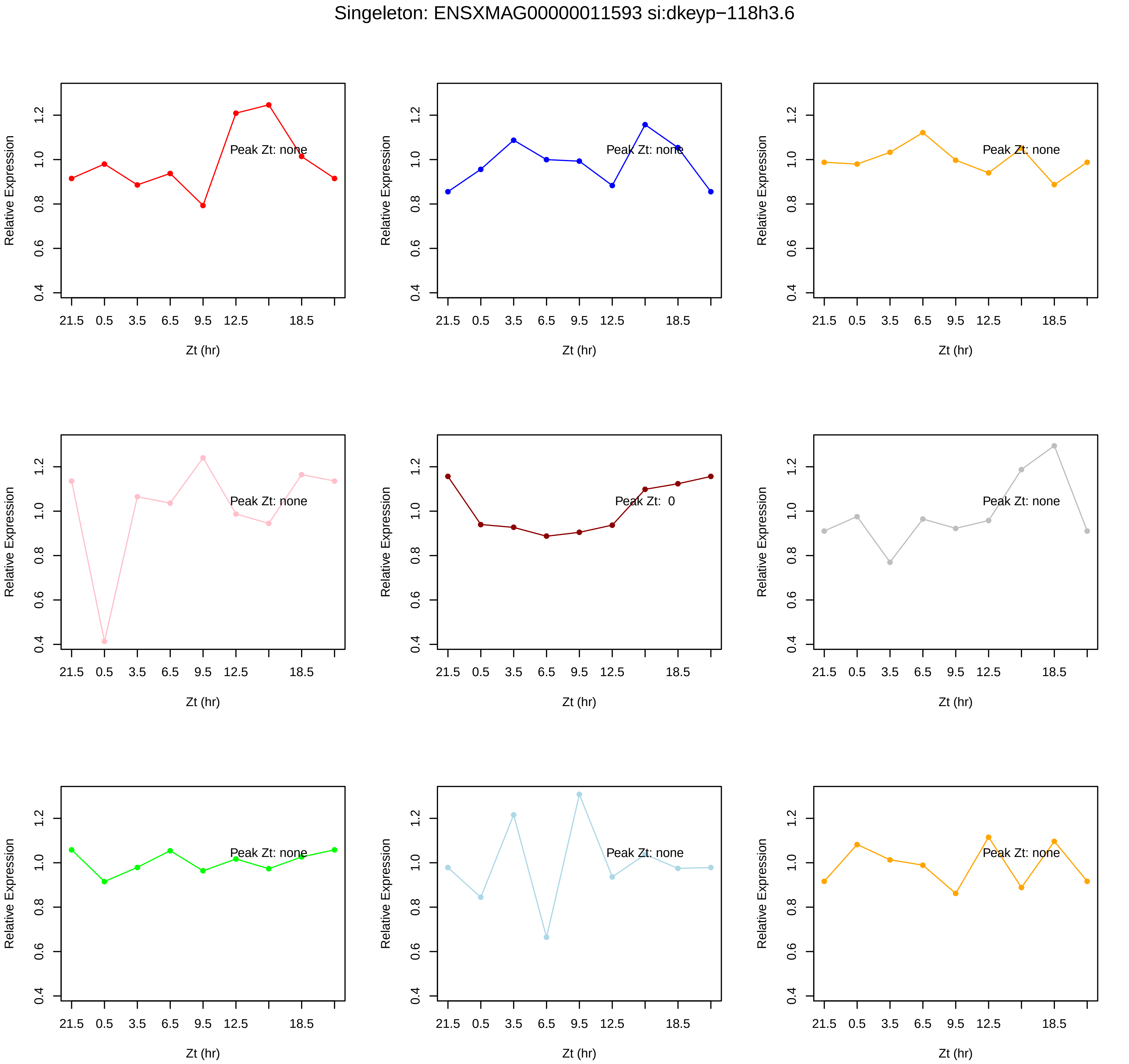
Singeleton: ENSXMAG00000011593 si:dkeyp−118h3.6
Relative Expression
Zt (hr)
21.5 0.5 3.5 6.5 9.5 12.5 18.5
0.4 0.6 0.8 1.0 1.2
Peak Zt: none
Peak Zt: none
Peak Zt: none
Peak Zt: none
Peak Zt: 0
Peak Zt: none
Peak Zt: none
Peak Zt: none
Peak Zt: none

Singeleton: ENSXMAG00000014773 rai1

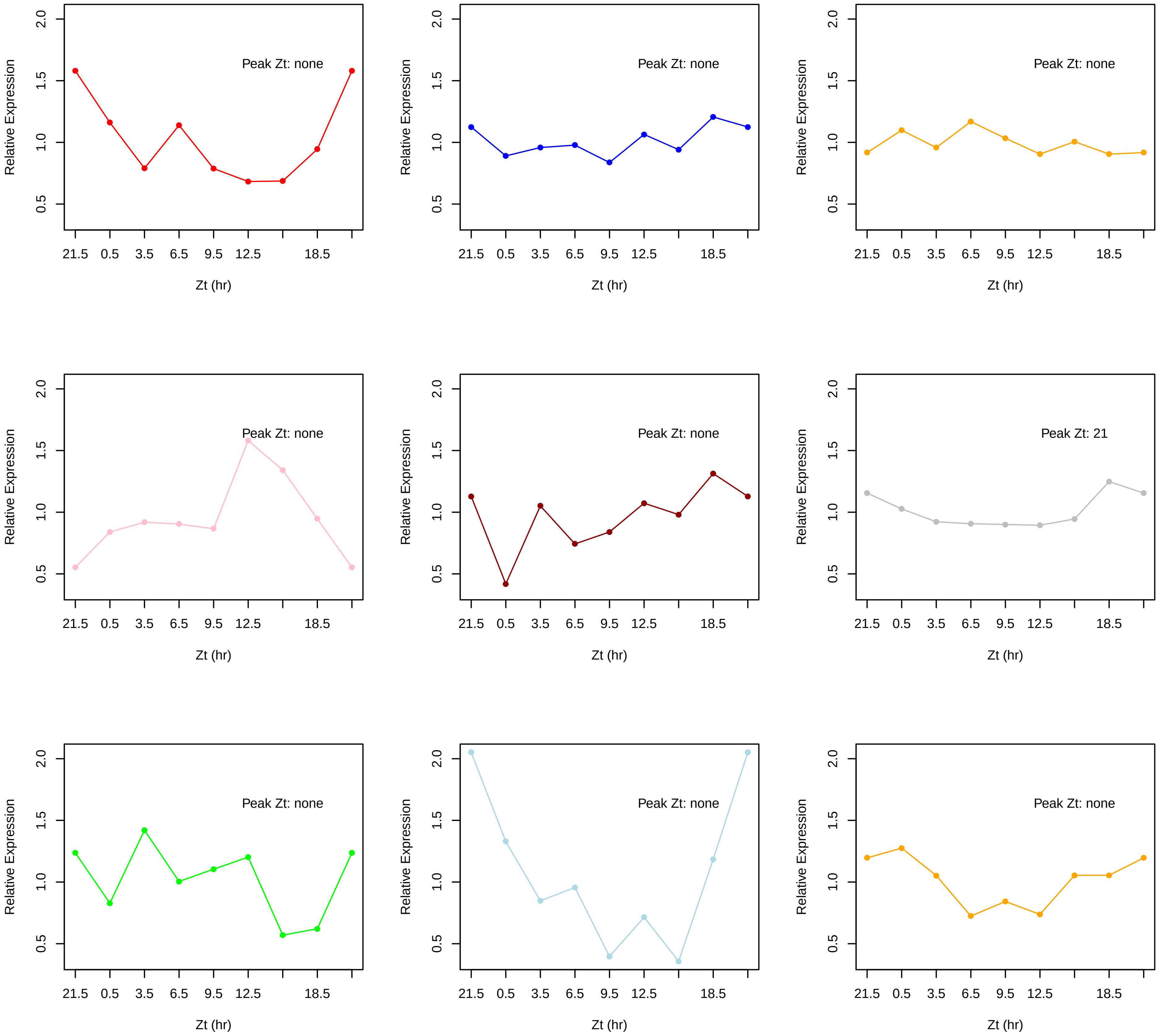

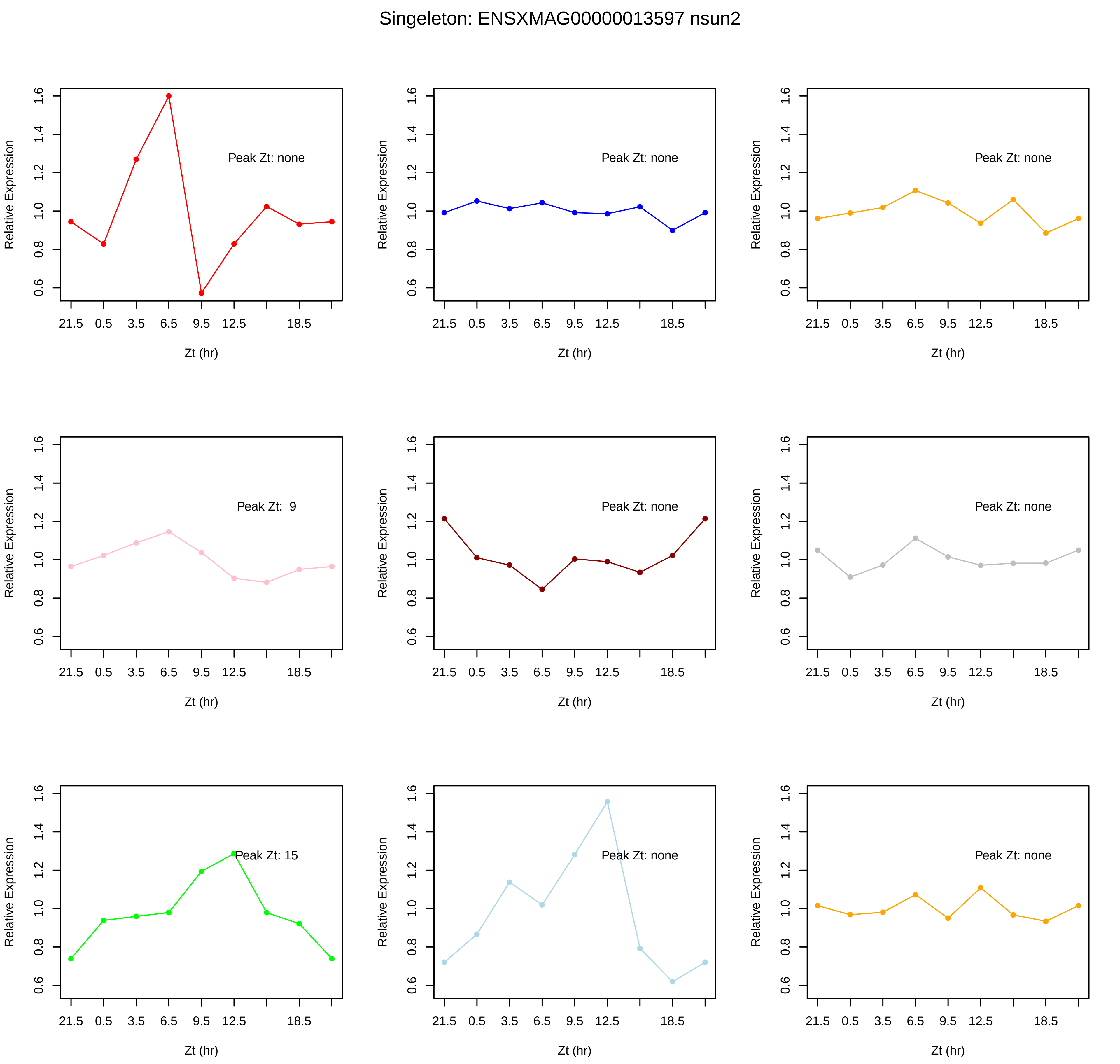

Singeleton: ENSXMAG00000013597 nsun2
Relative Expression
Zt (hr)
21.5
0.5
3.5
6.5
9.5
12.5
18.5
0.6
0.8
1.0
1.2
1.4
1.6
Peak Zt: none
Peak Zt: 9
Peak Zt: 15

Singeleton: ENSXMAG00000010894 cyp46a1.4

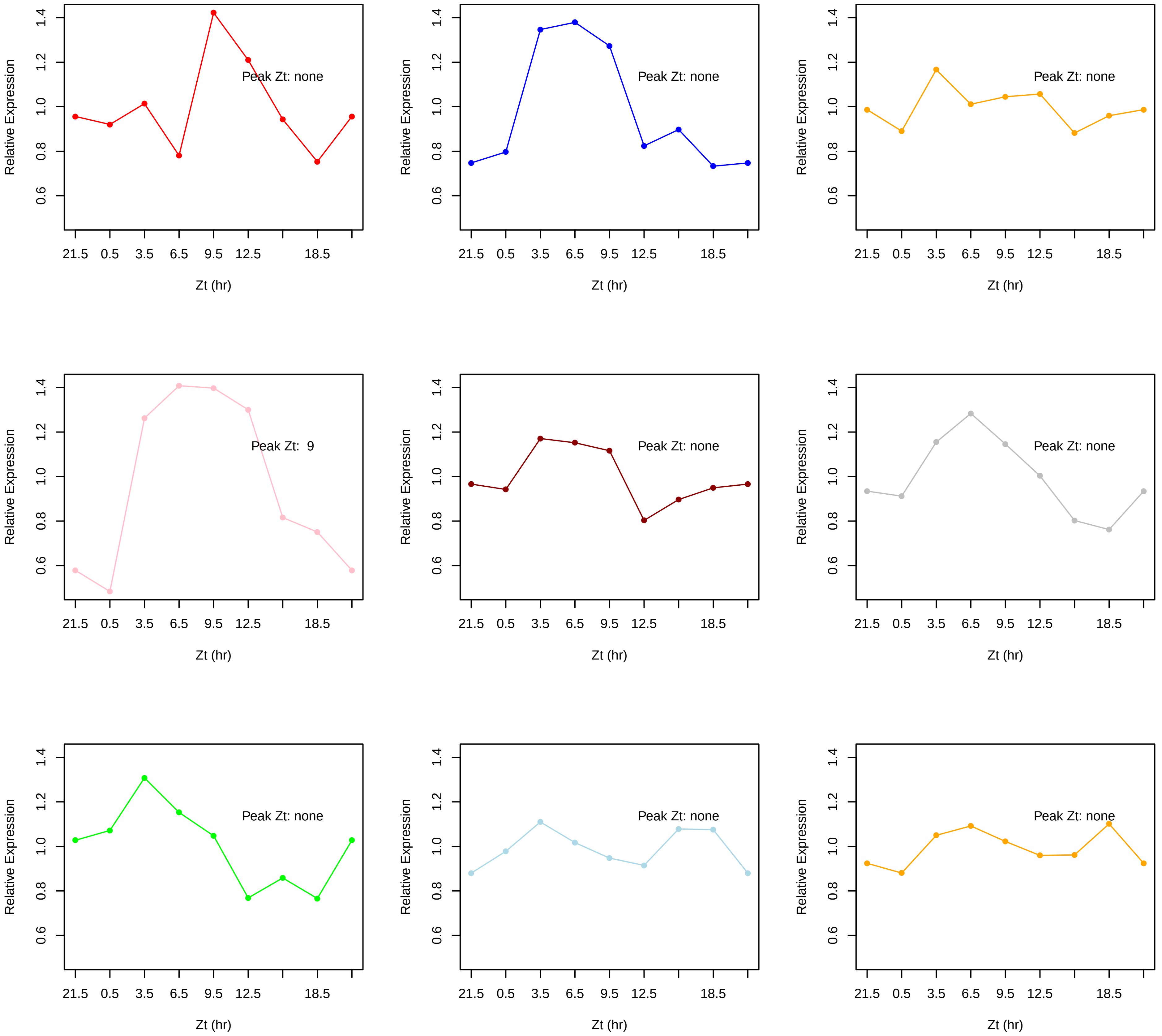

Singleton: ENSXMAG00000017138 prmt9

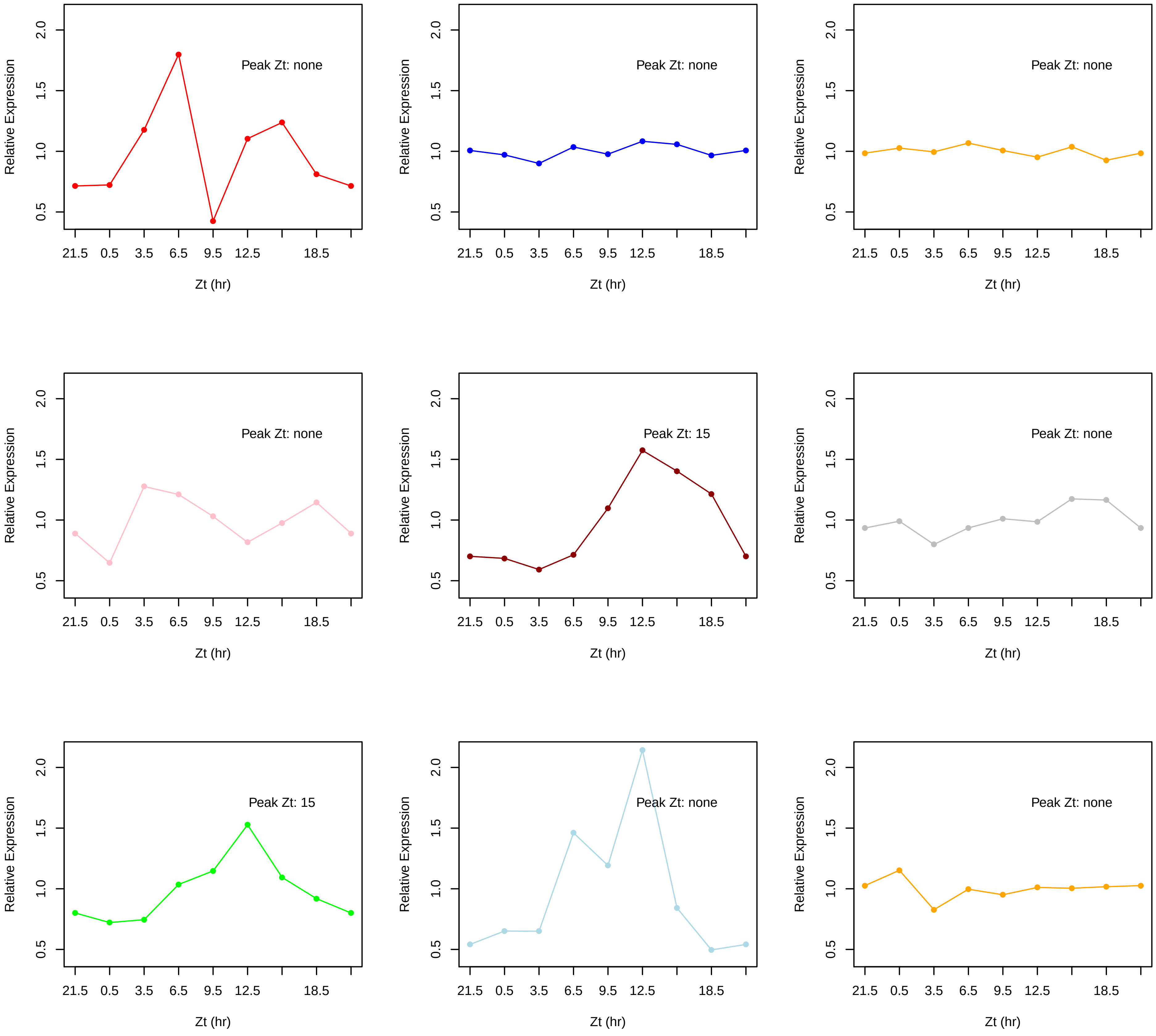

Singleton: ENSXMAG00000014774 srebf1

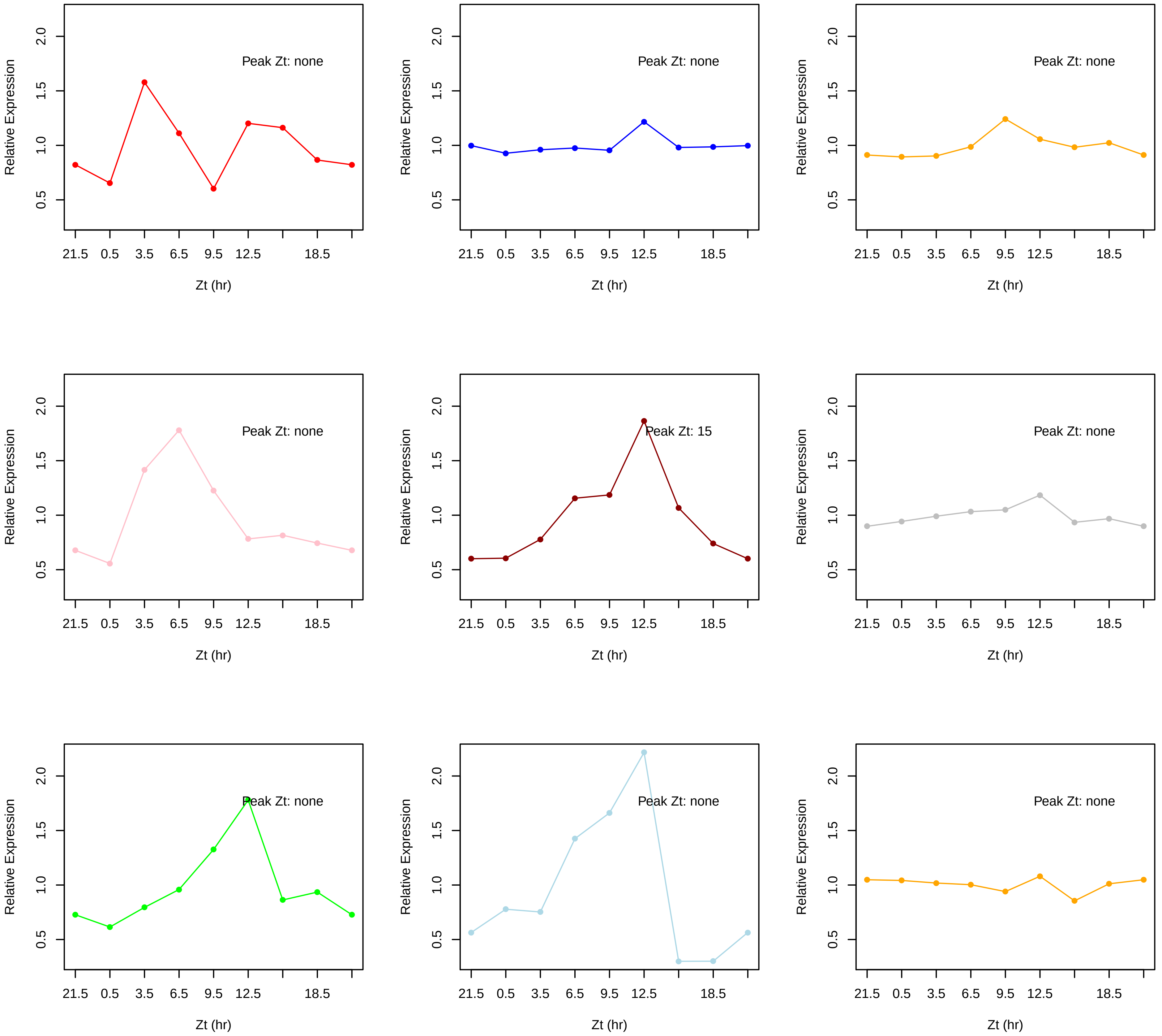

Singeleton: ENSXMAG00000013614 ube2ql1

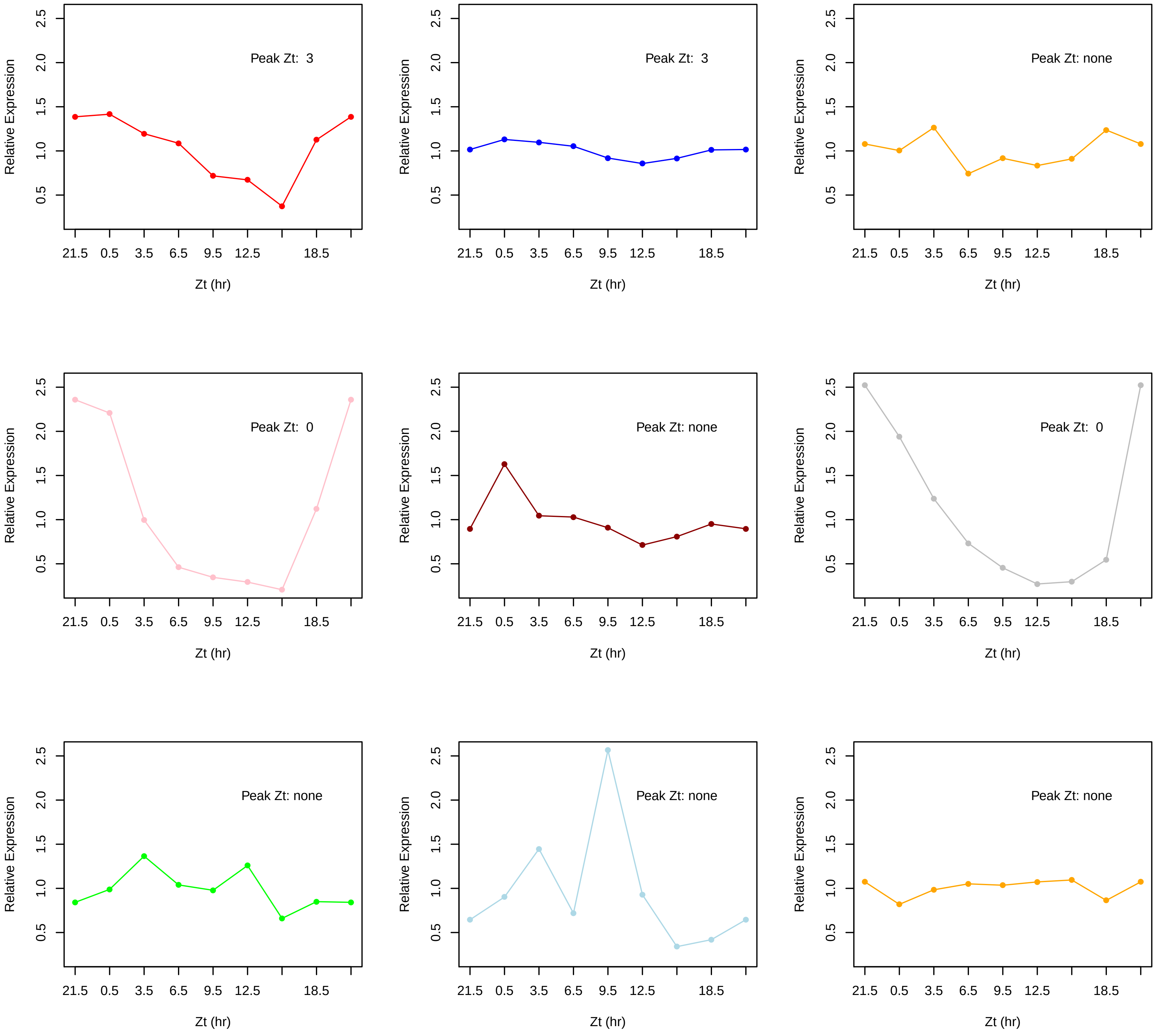

Singeleton: ENSXMAG00000011609 ecrg4b

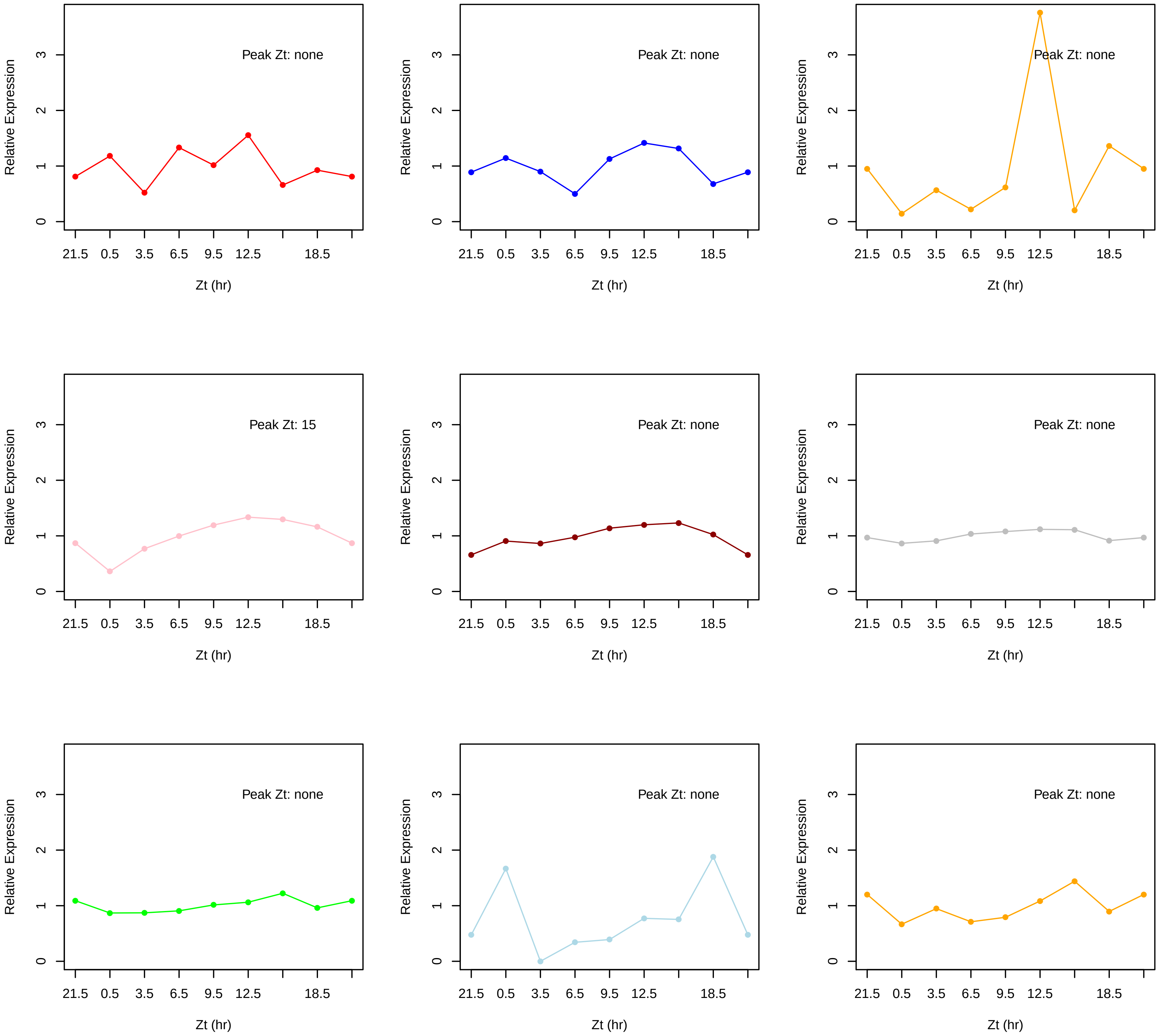

Singeleton: ENSXMAG00000028211 TOM1L2

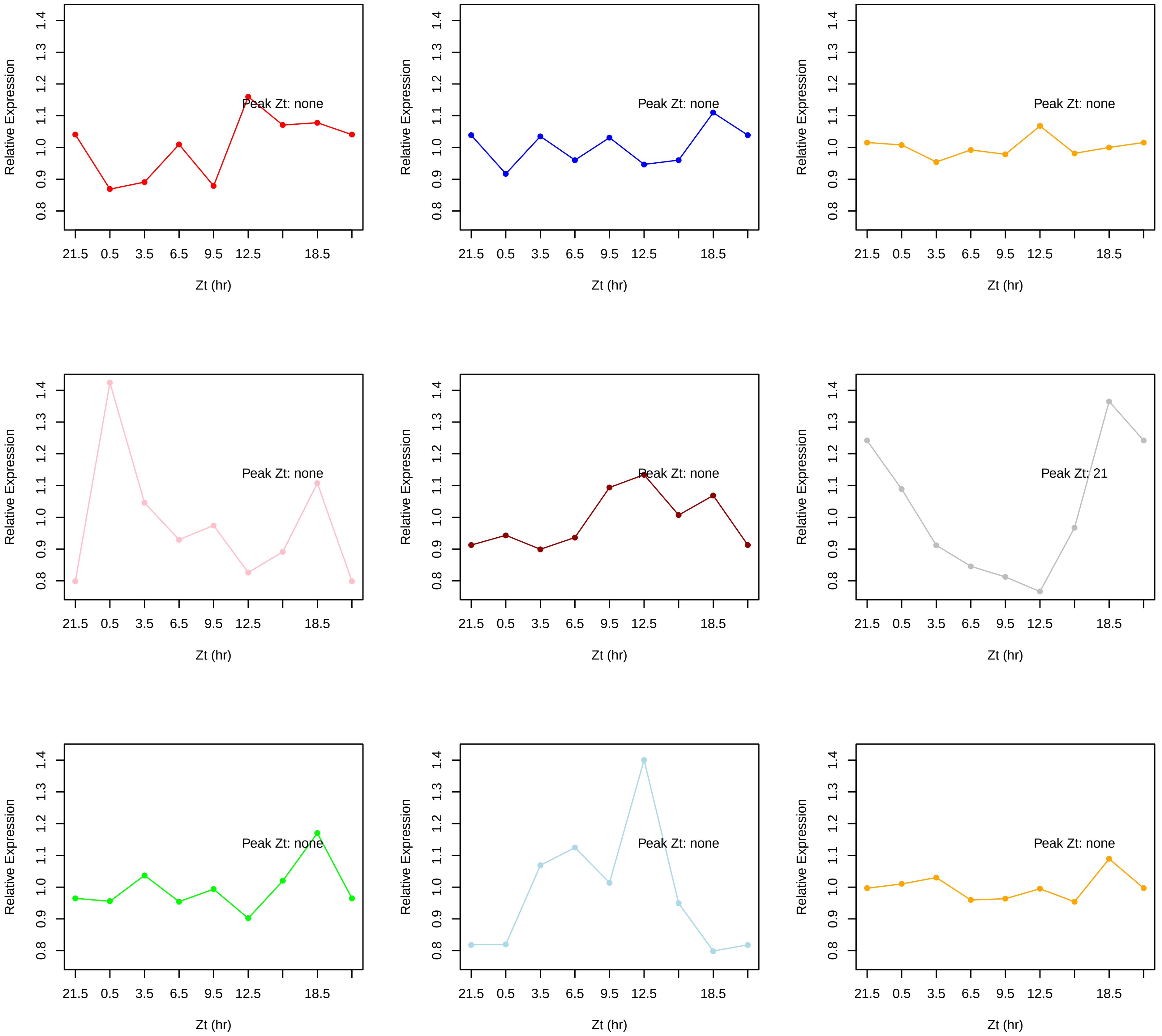

Singeleton: ENSXMAG00000022156 prr16

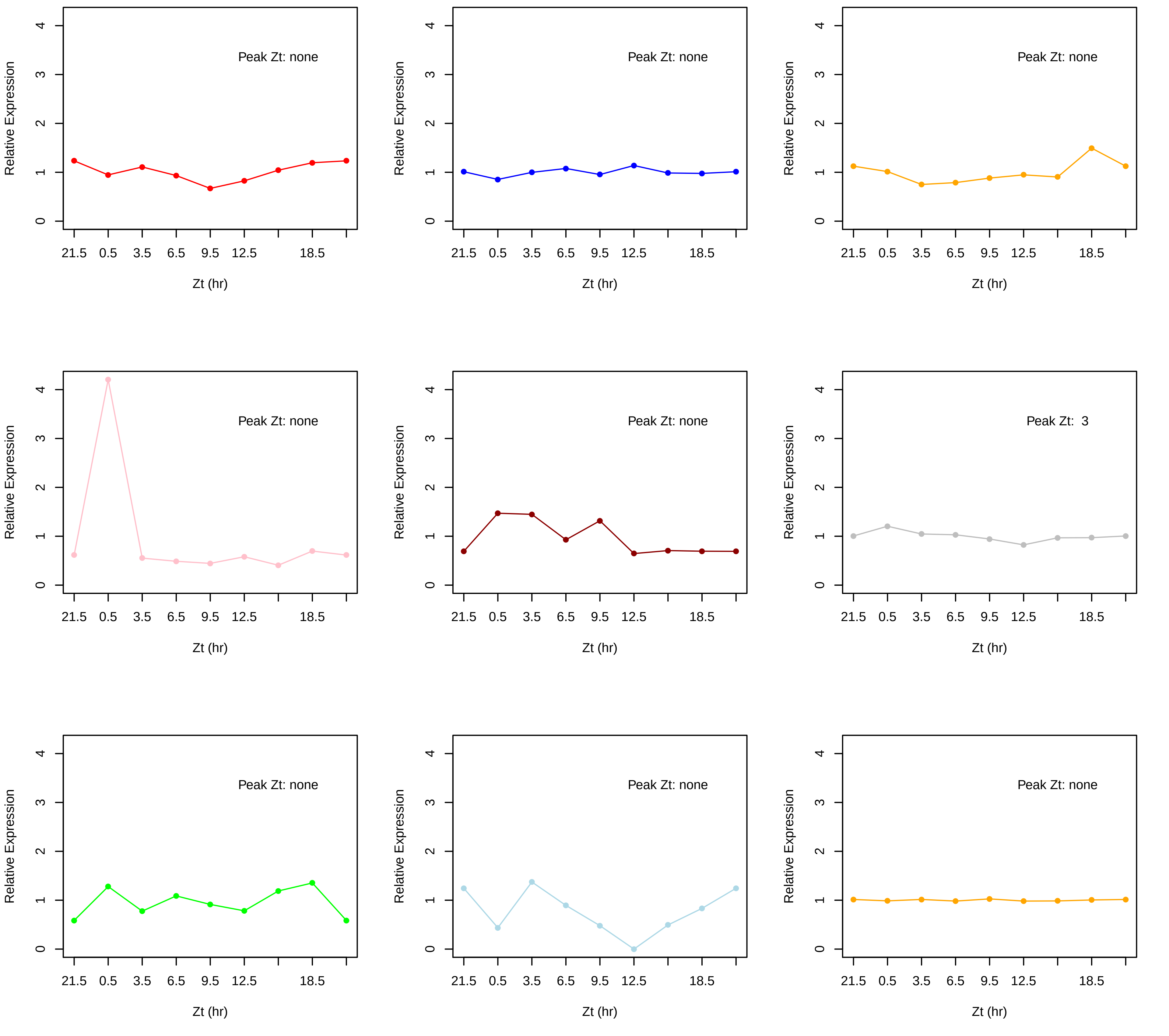

Singeleton: ENSXMAG00000011173 prkaa2

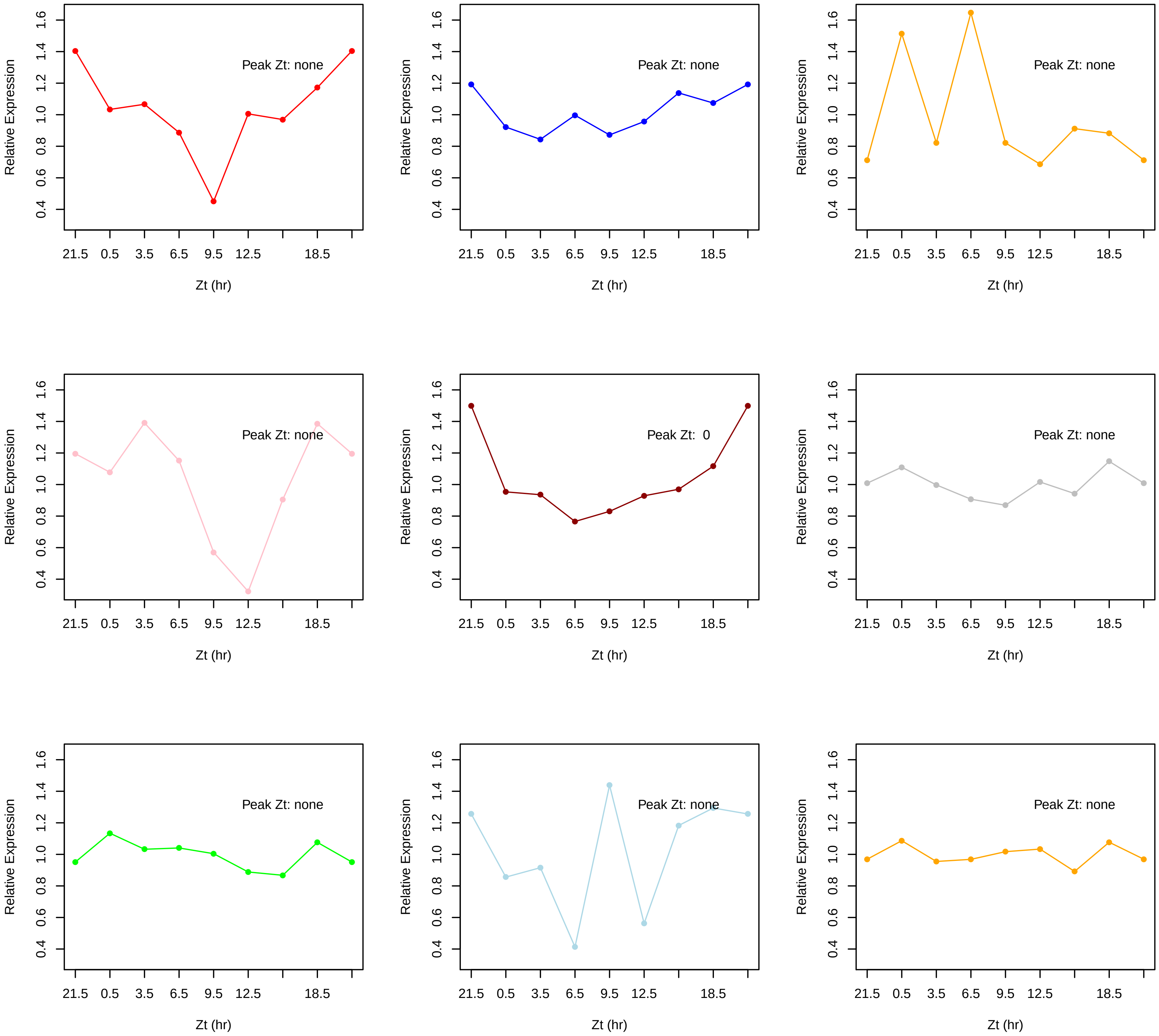

Singeleton: ENSXMAG00000028988

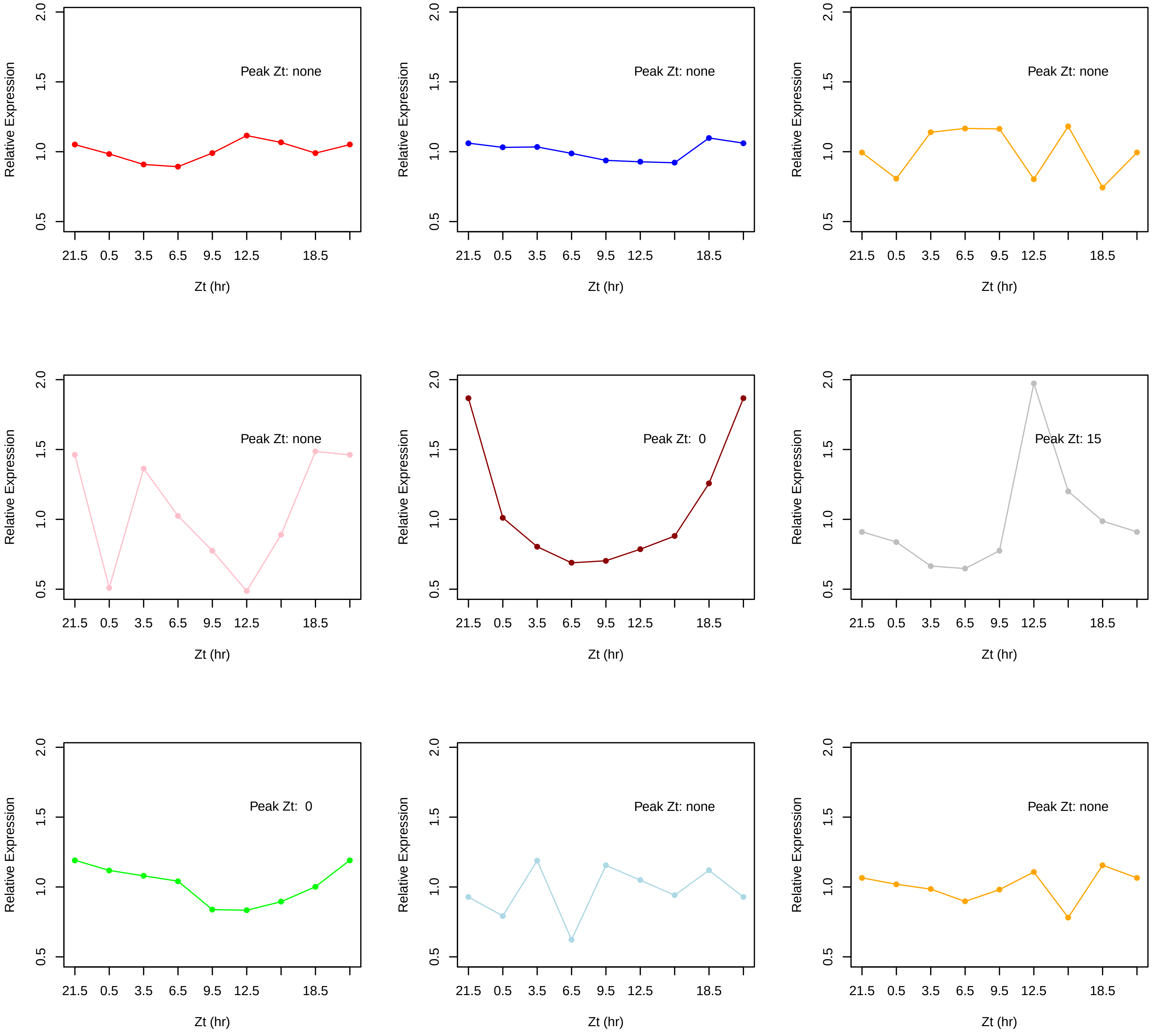

Singeleton: ENSXMAG00000008057 SYDE1

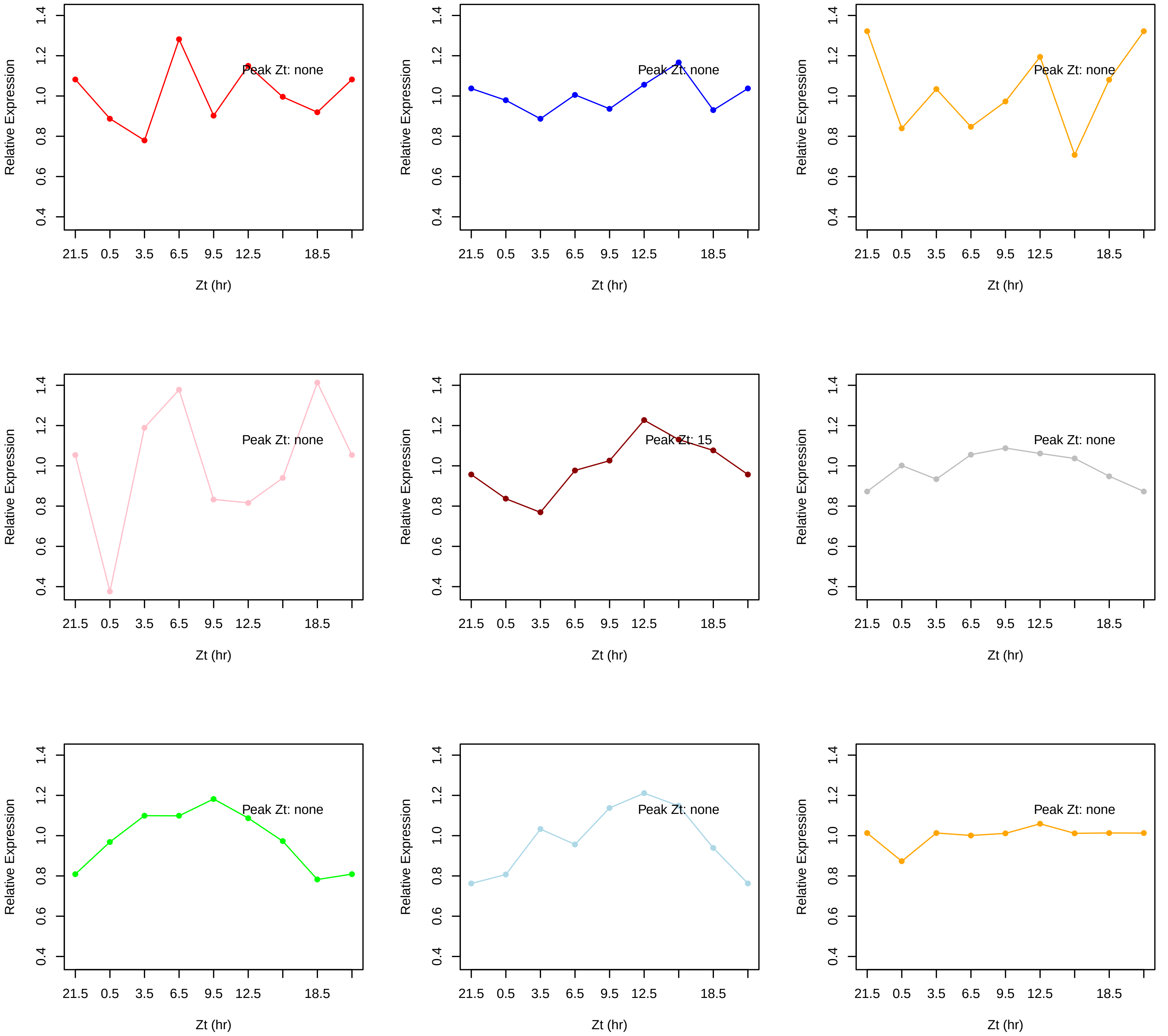

Singeleton: ENSXMAG00000010928 snw1

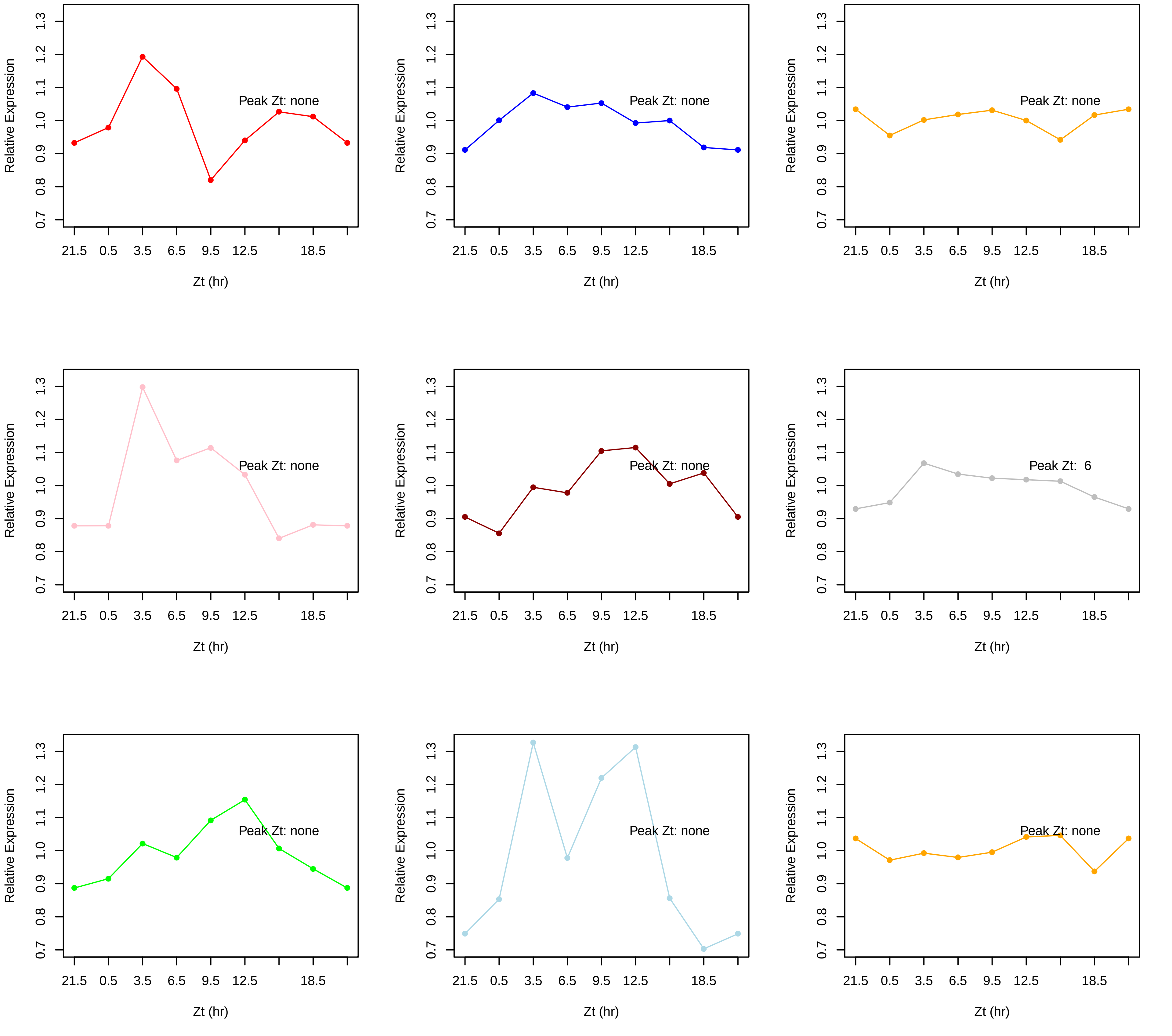

Singleton: ENSXMAG00000002340 si:ch73−174h16.4

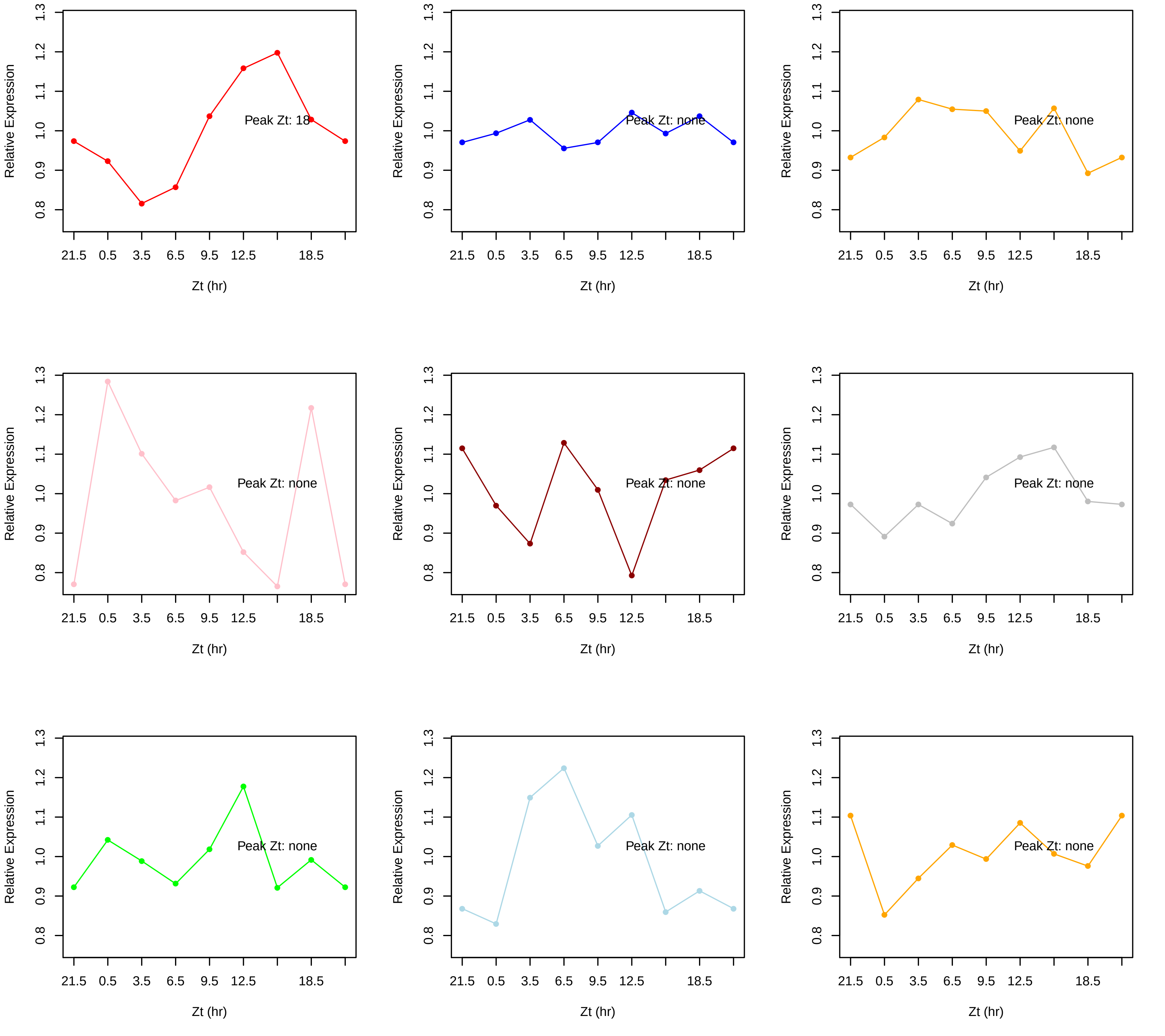

Singeleton: ENSXMAG00000000163 atpaf2

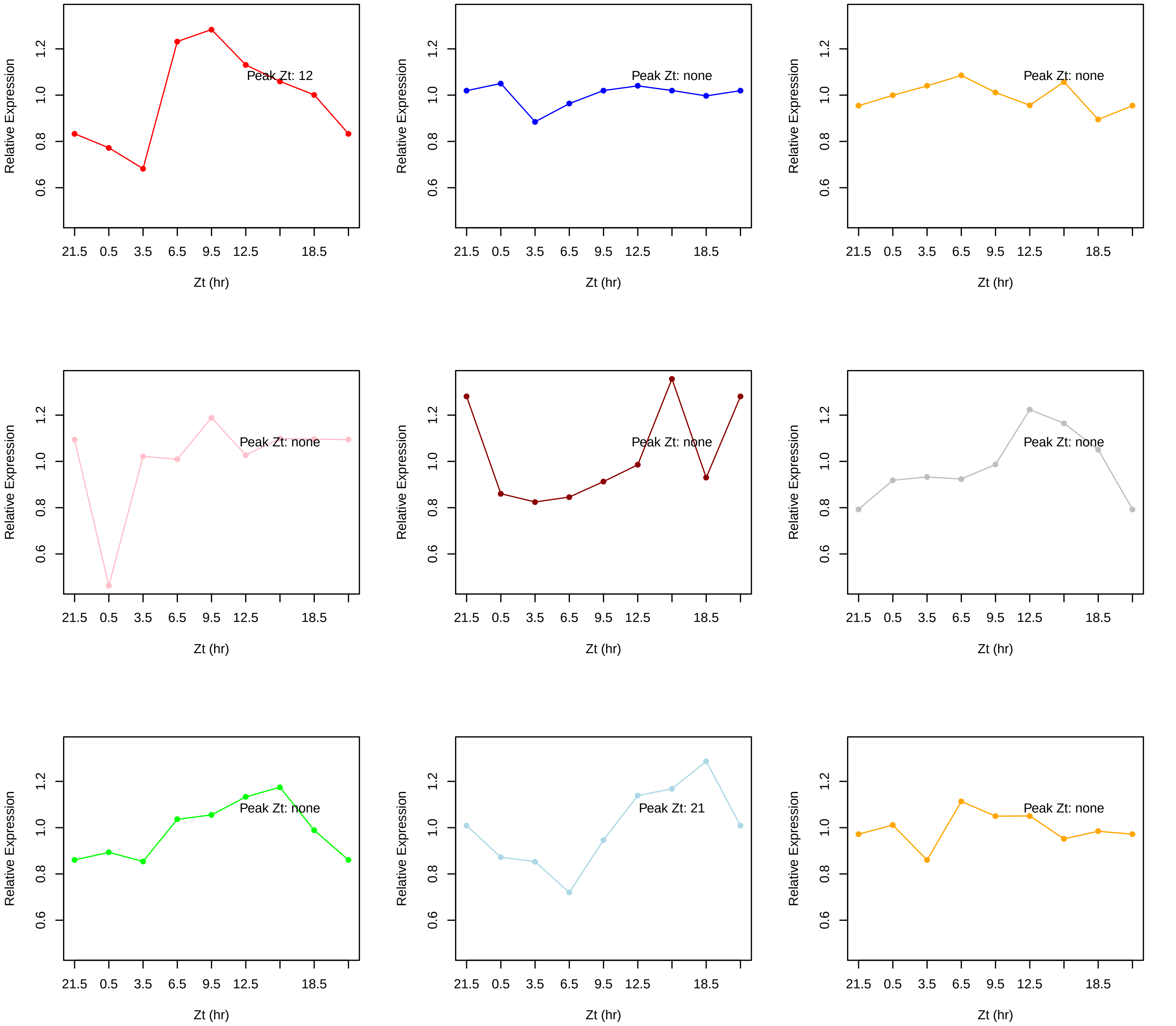

Singleton: ENSXMAG00000015645 adck1

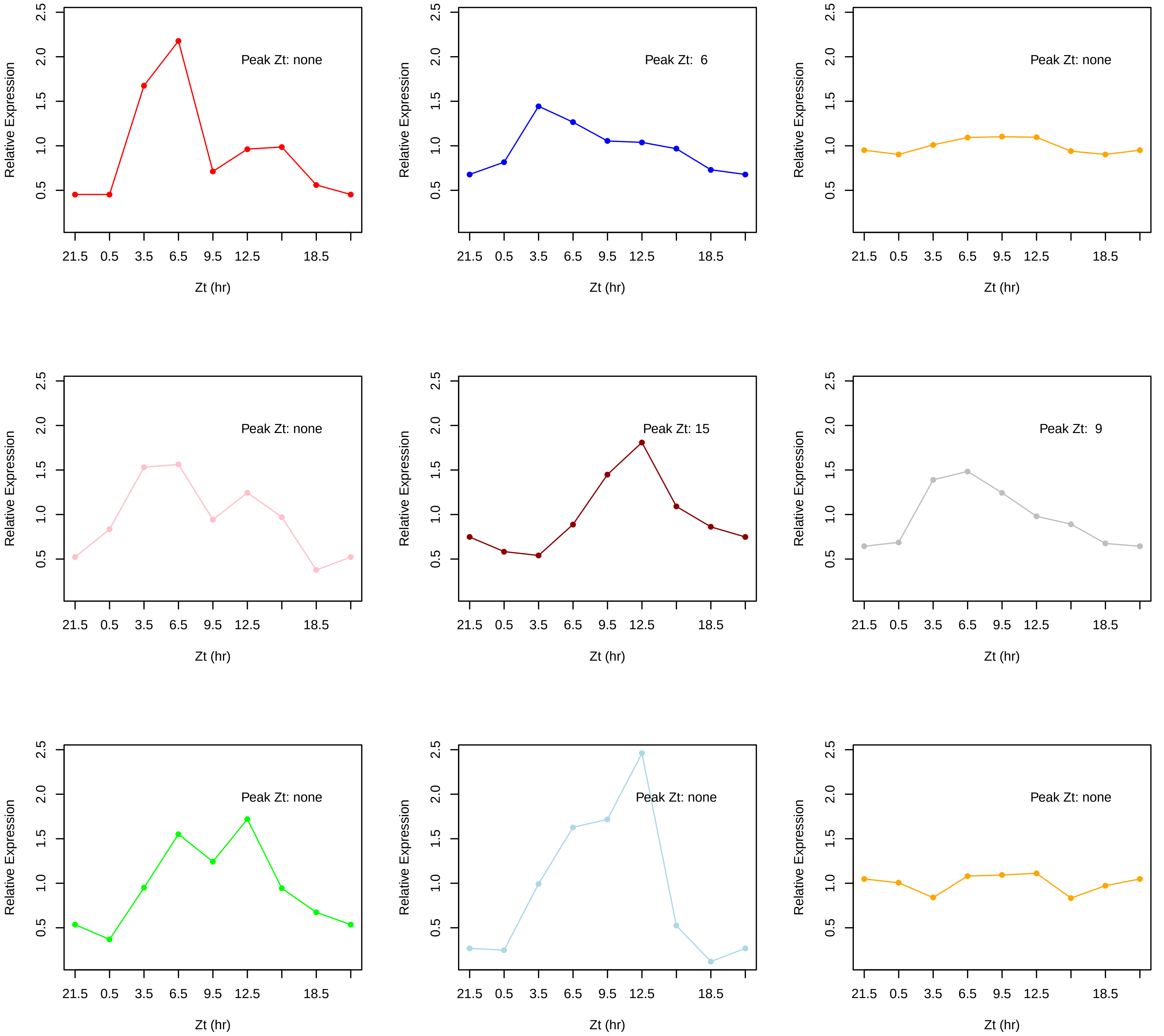

Singeleton: ENSXMAG00000007388 cog6

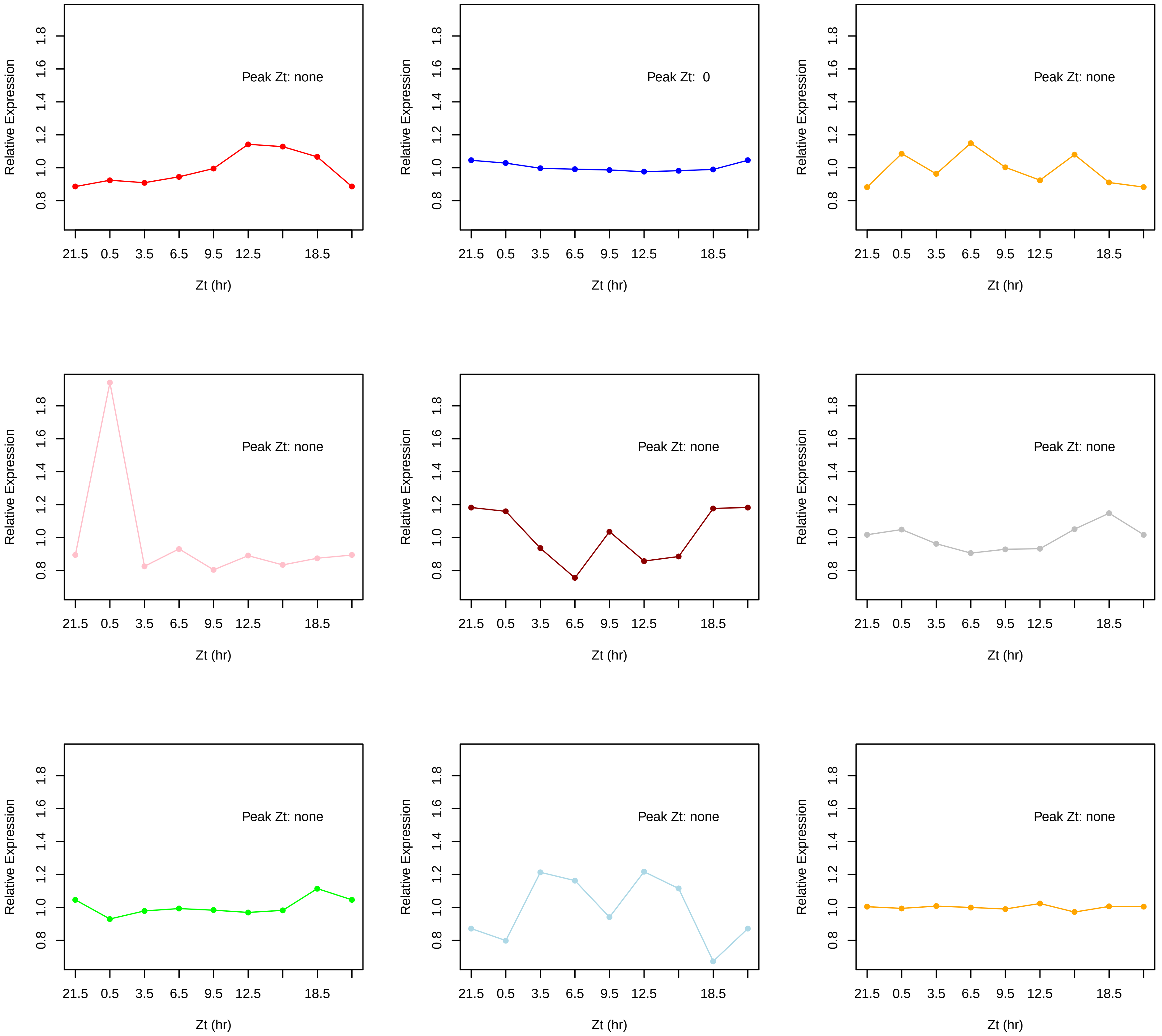

Singeleton: ENSXMAG00000018789 pou4f2

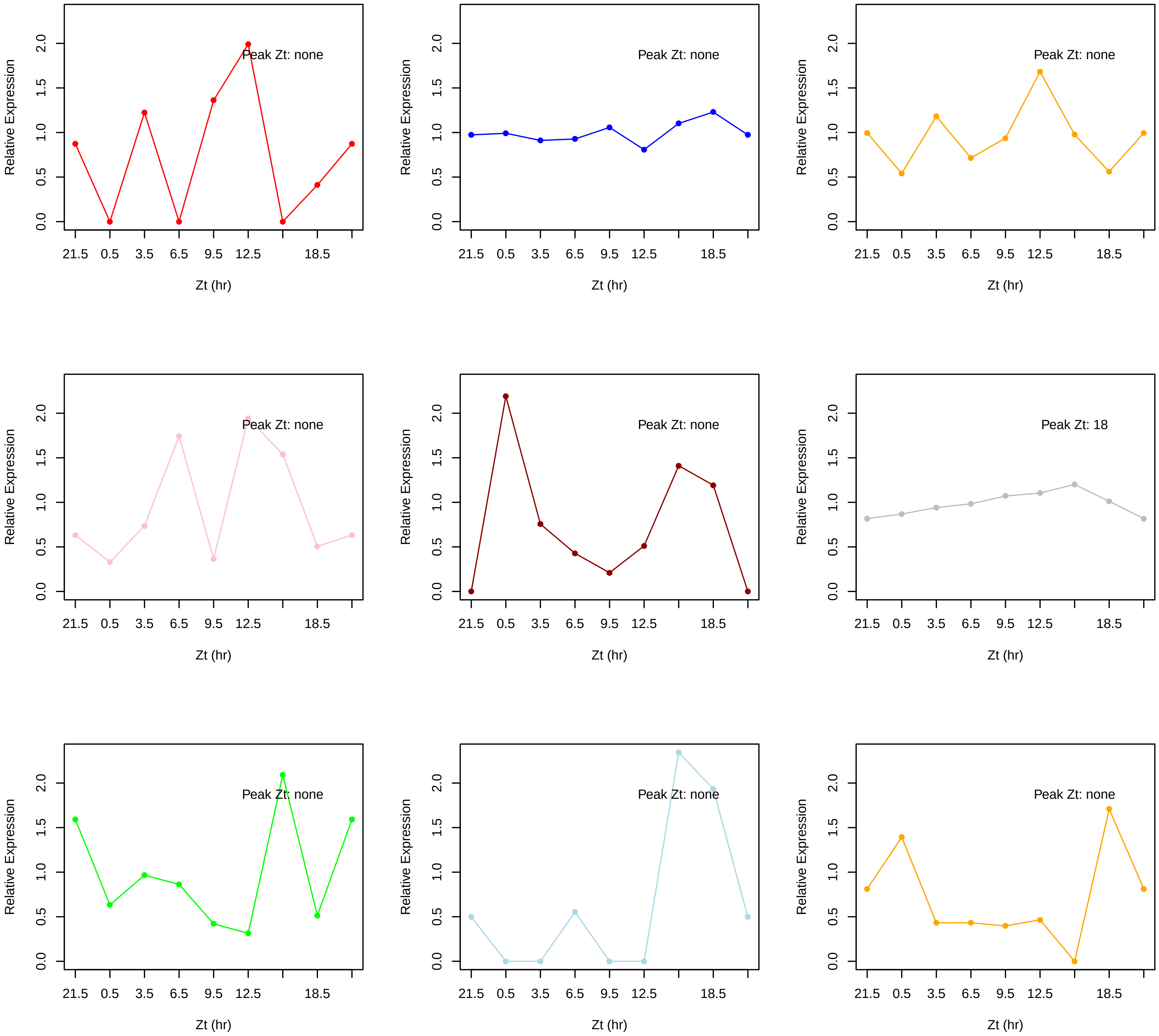

Singeleton: ENSXMAG00000002299 zgc:112356

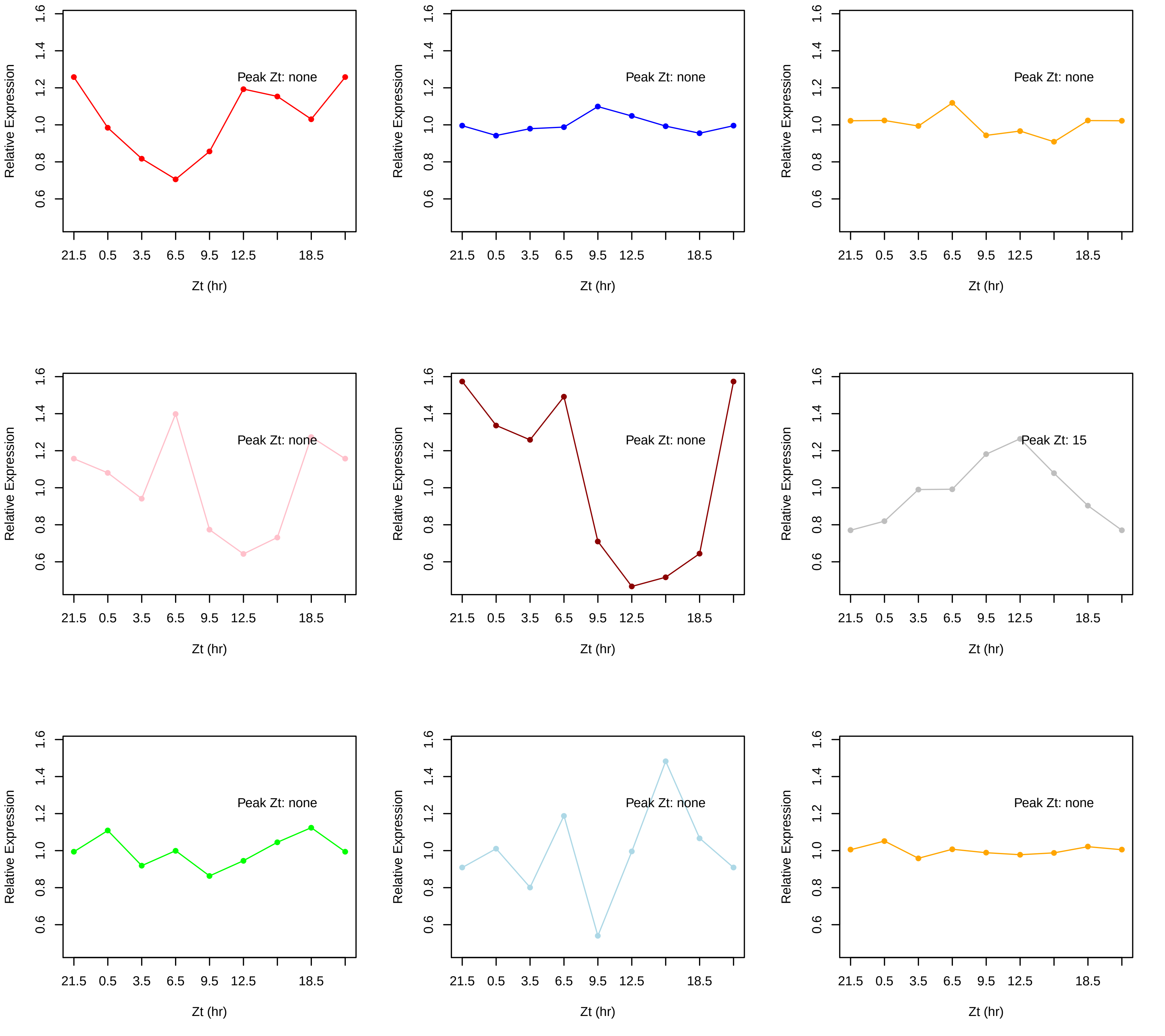

Singeleton: ENSXMAG00000011639 tmtops2b

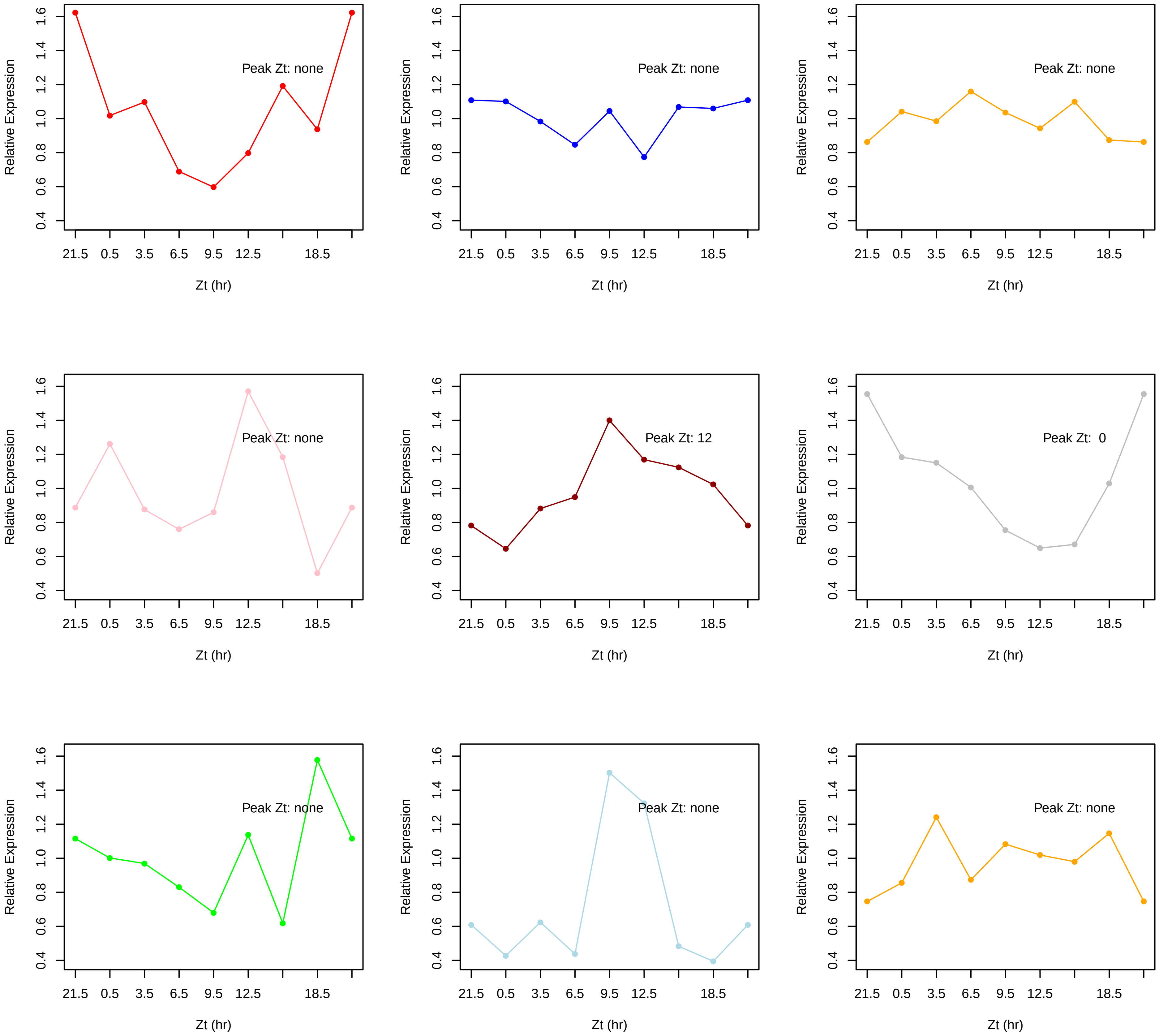

Singeleton: ENSXMAG00000001794 slc10a7

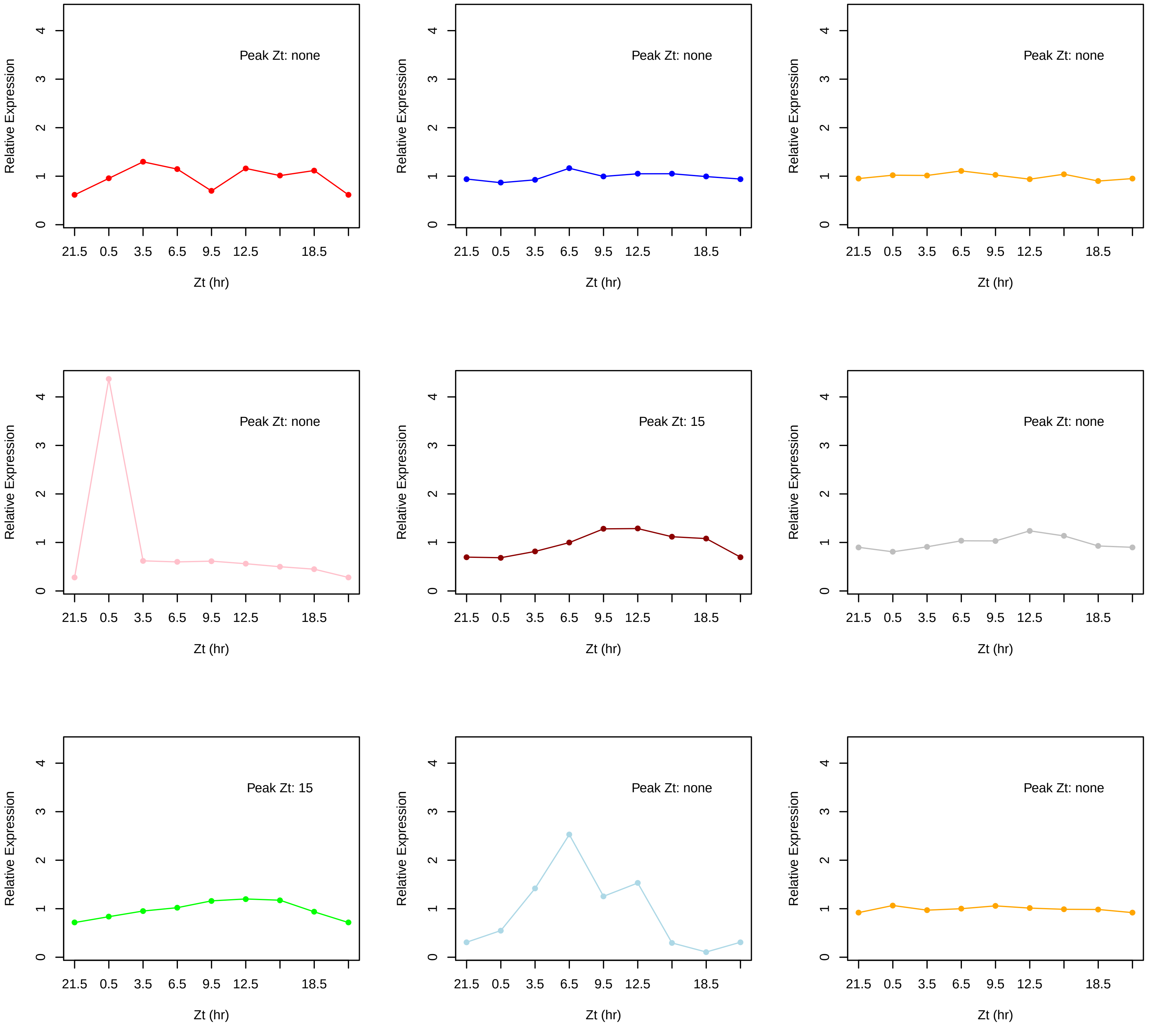

Singeleton: ENSXMAG00000006425 hibch

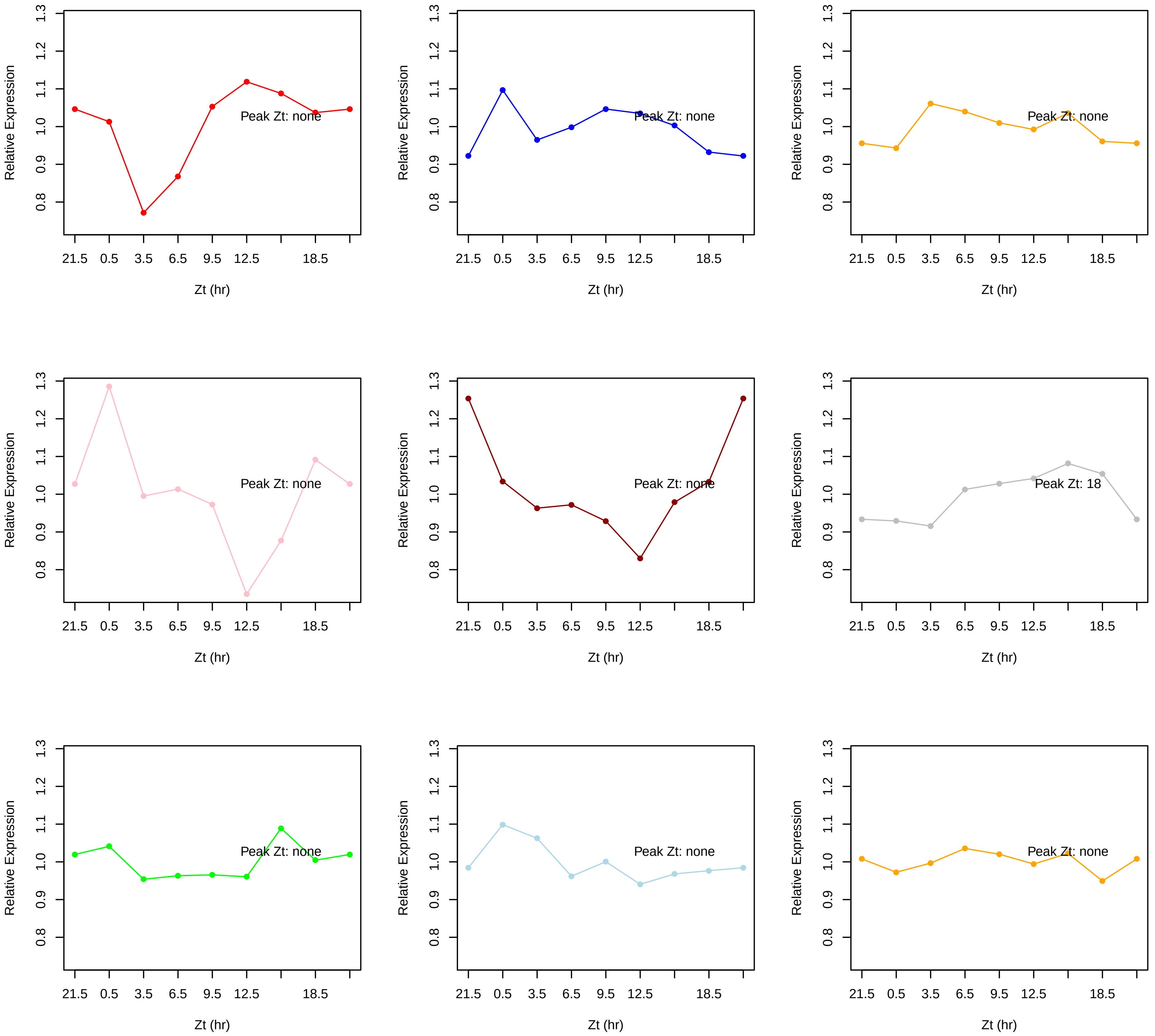

Singeleton: ENSXMAG00000015610 nrxn3a

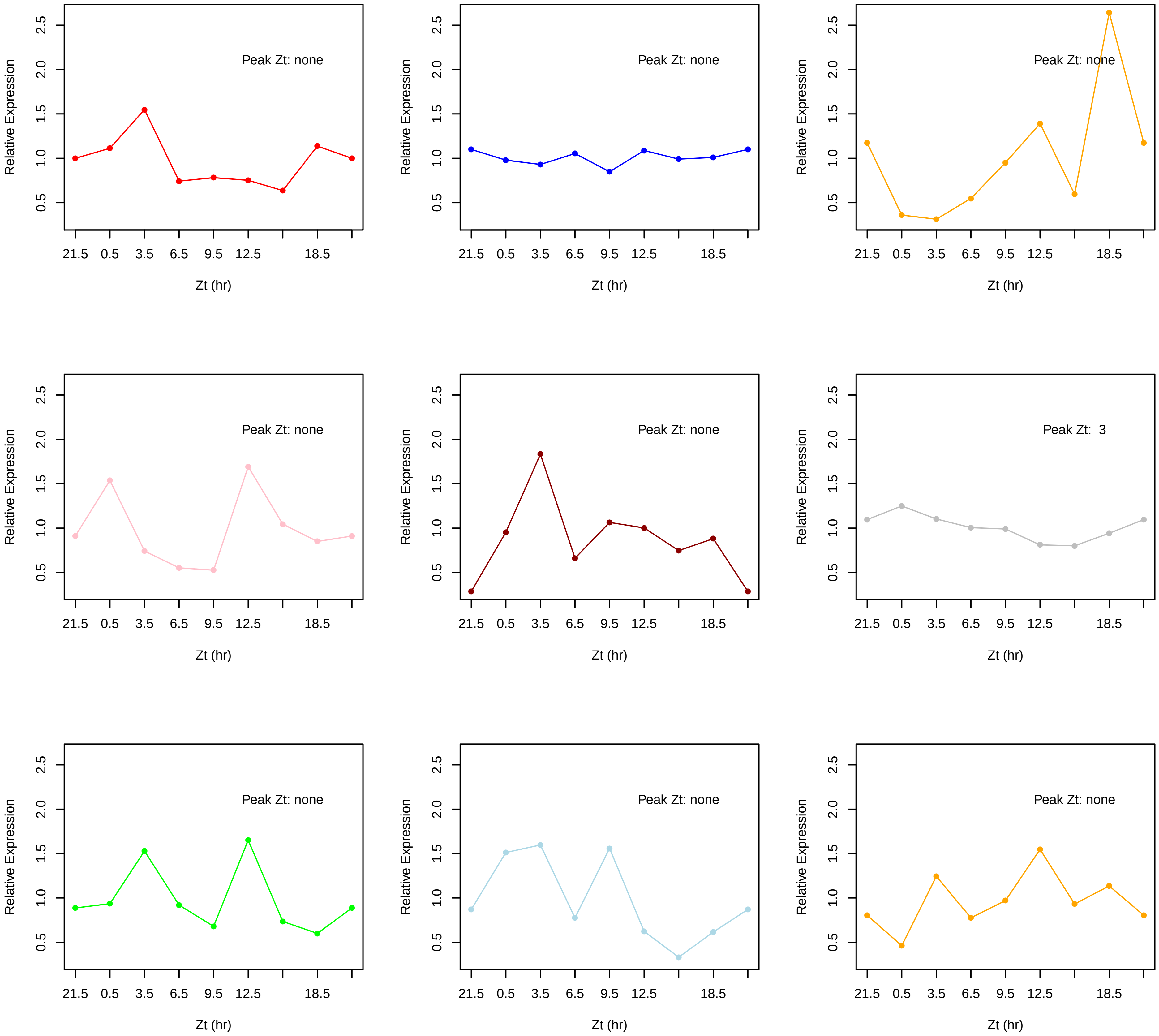

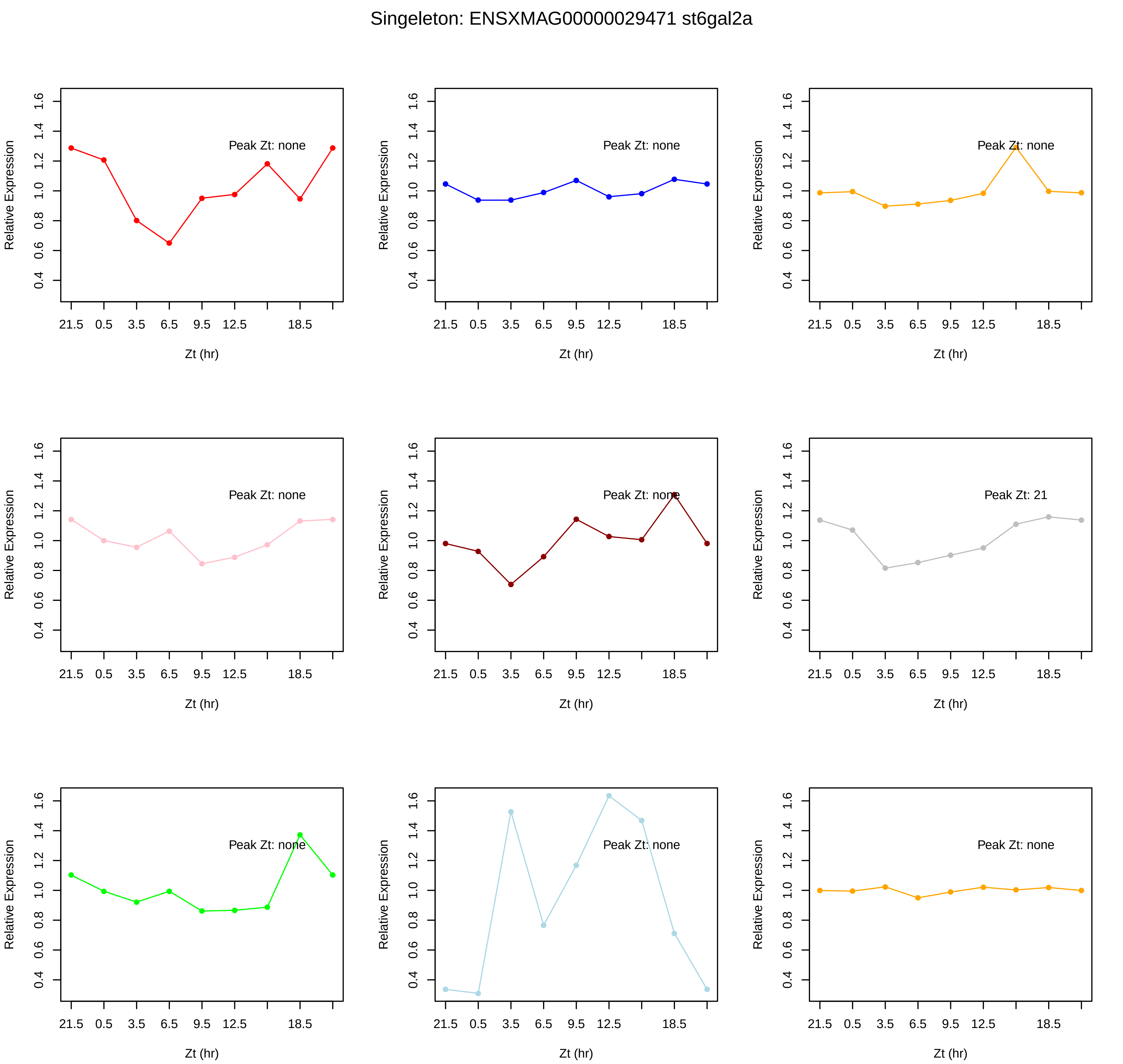
Singeleton: ENSXMAG00000029471 st6gal2a
Peak Zt: none
Peak Zt: none
Peak Zt: none
Peak Zt: none
Peak Zt: none
Peak Zt: 21
Peak Zt: none
Peak Zt: none
Peak Zt: none
Relative Expression
Zt (hr)
21.5
0.5
3.5
6.5
9.5
12.5
18.5
0.4
0.6
0.8
1.0
1.2
1.4
1.6

Singleton: ENSXMAG00000014231

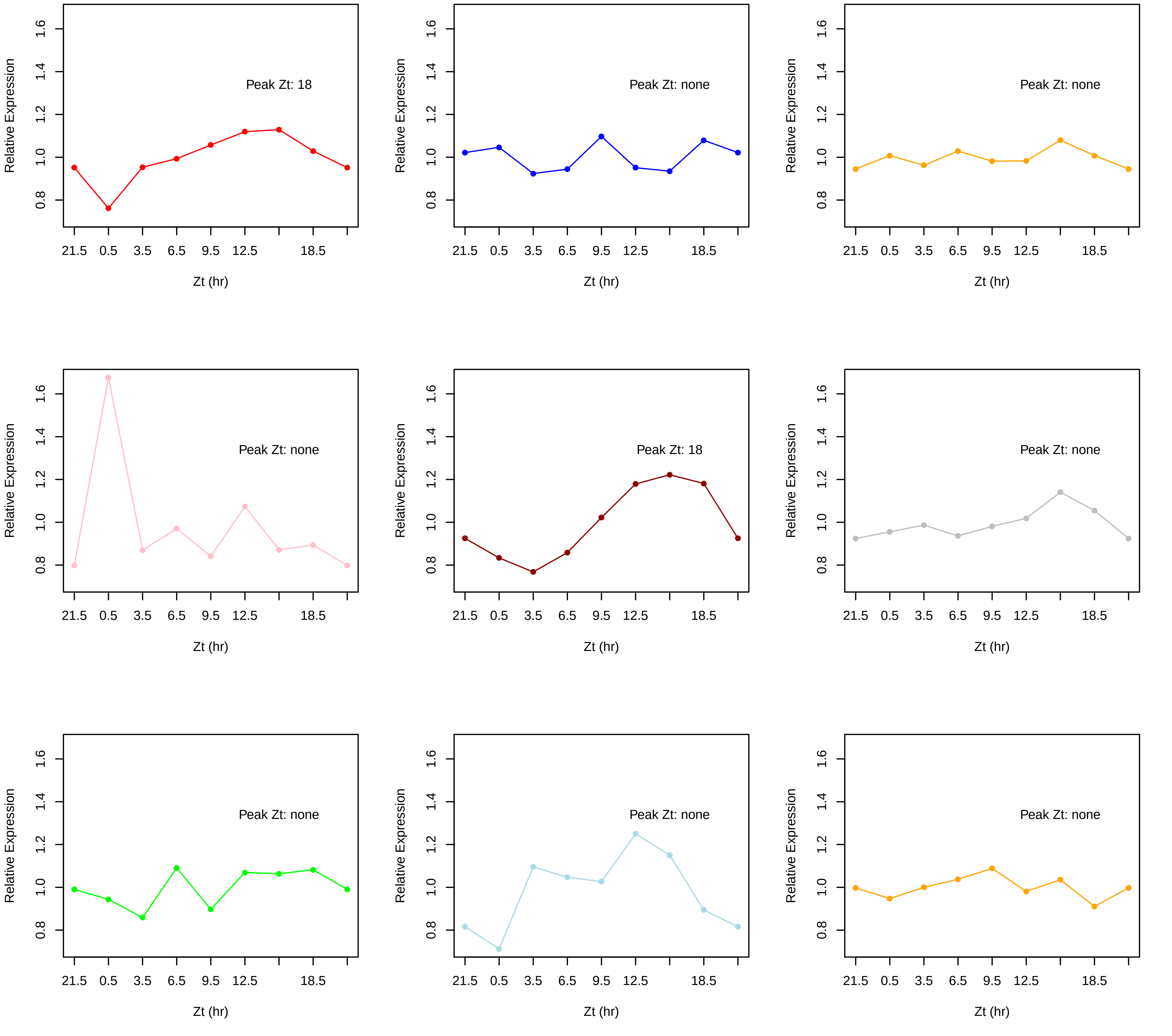

Singeleton: ENSXMAG00000018915 oplah

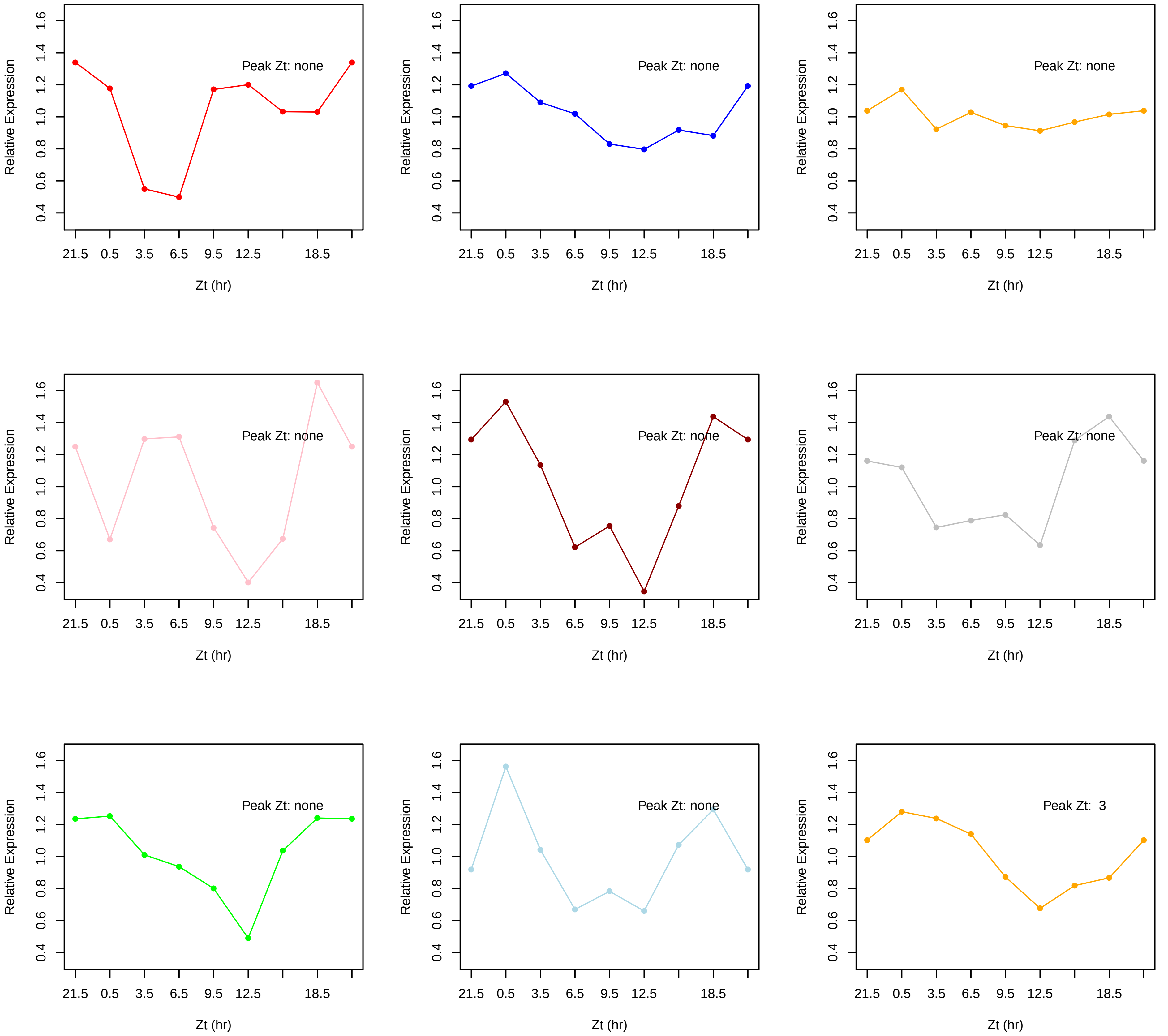

Singeleton: ENSXMAG00000003958 GPR89B

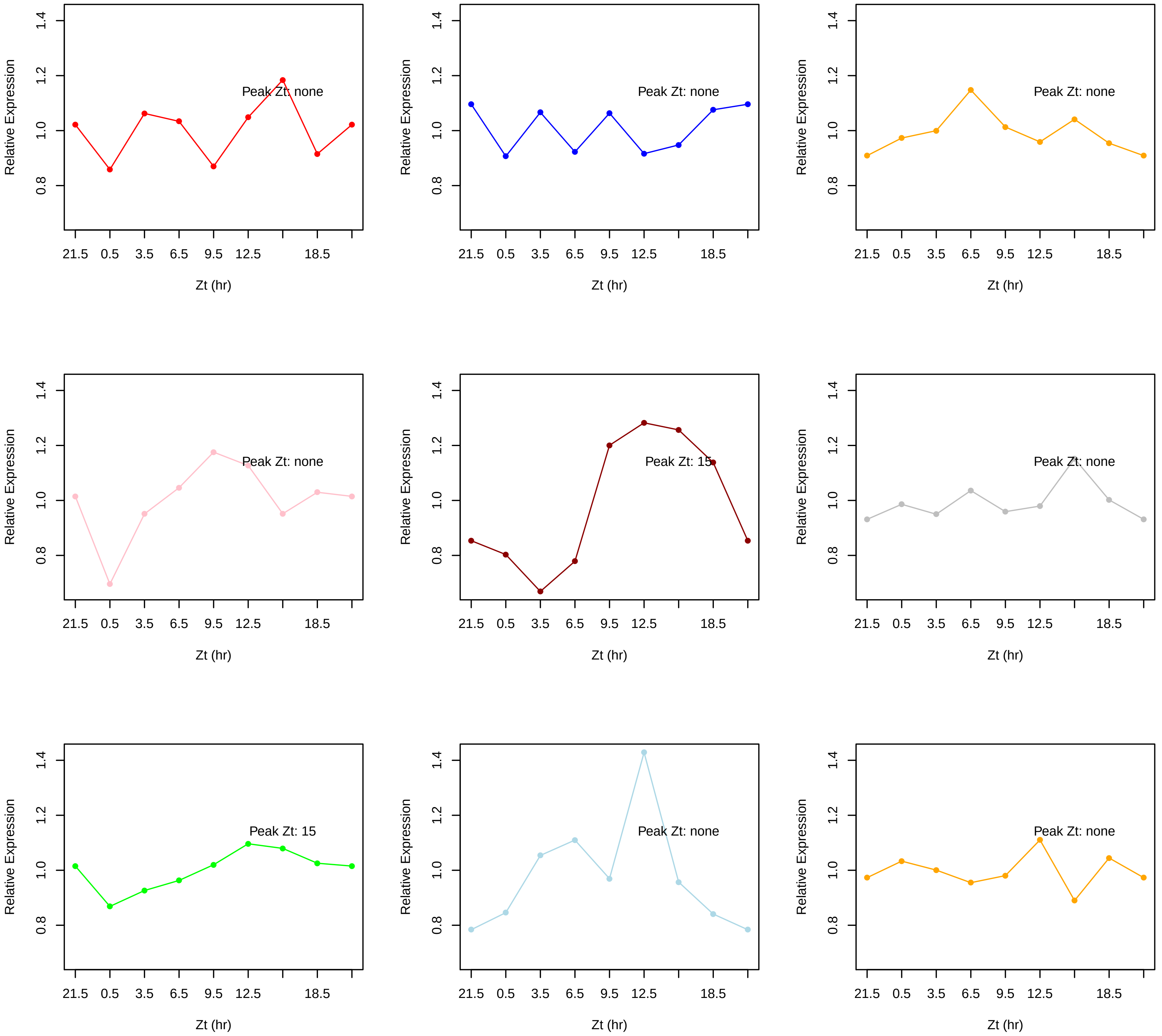

Singeleton: ENSXMAG00000016332 DMXL1

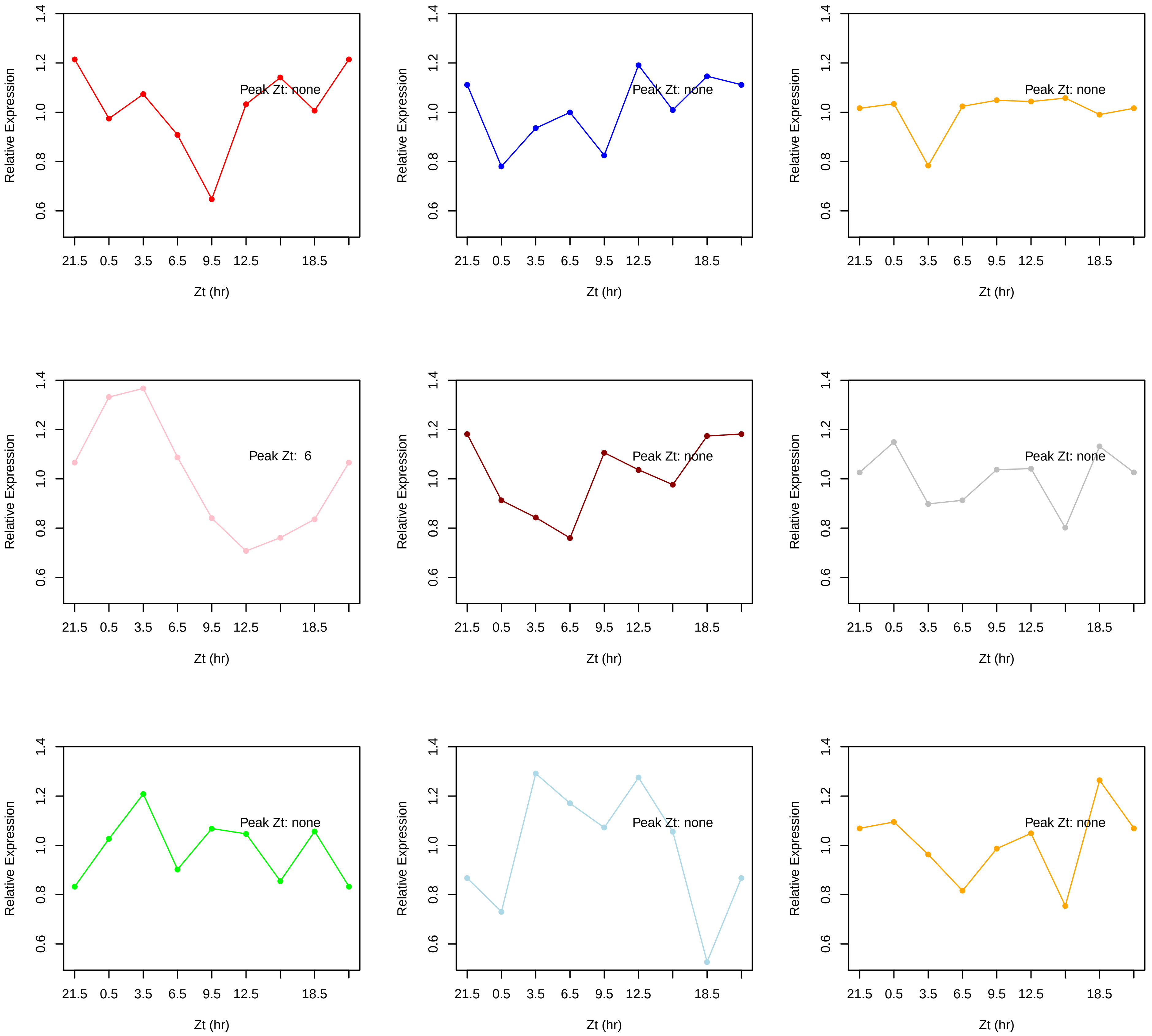

Singeleton: ENSXMAG00000014866 tbl3

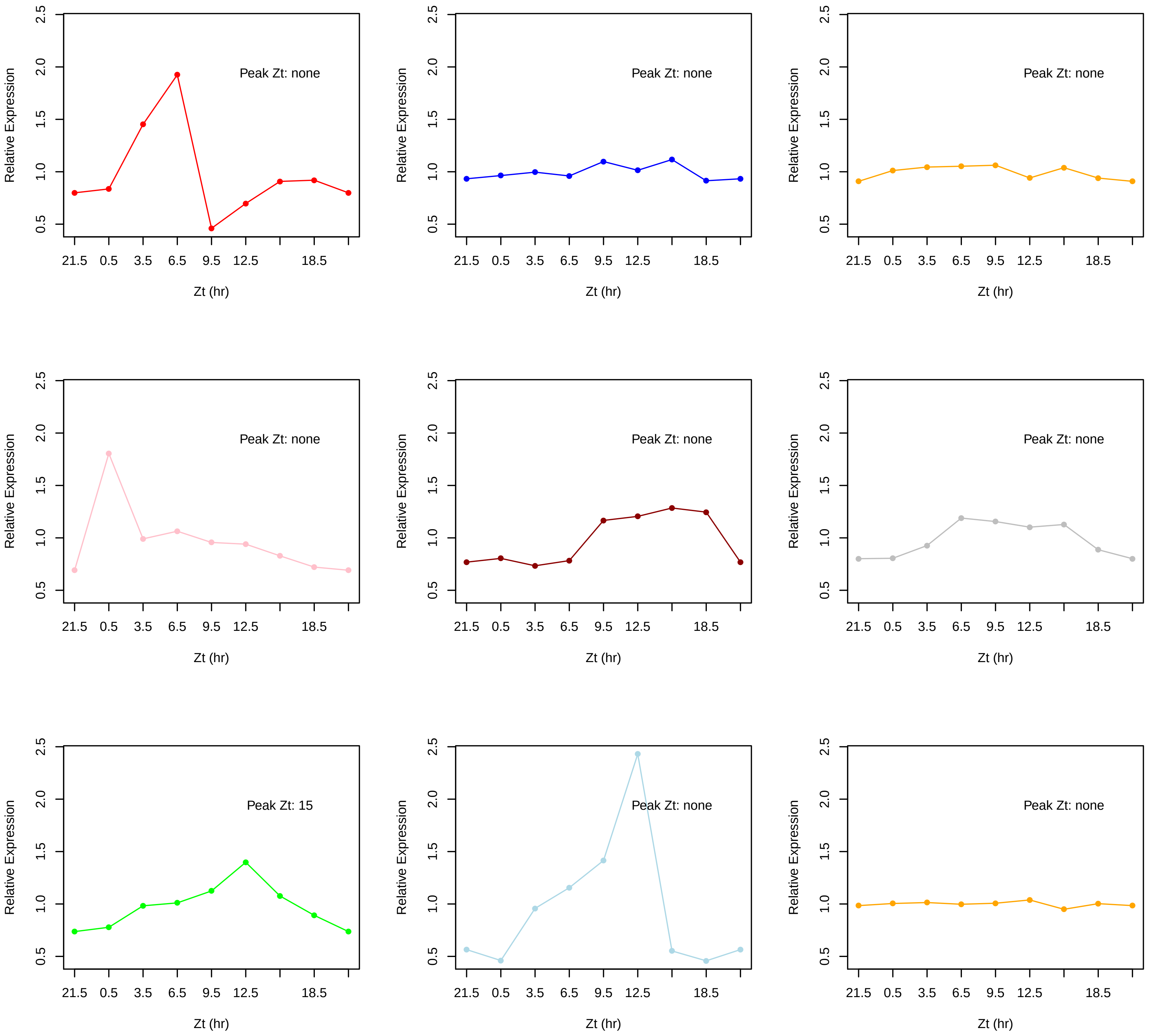

Singeleton: ENSXMAG00000018696

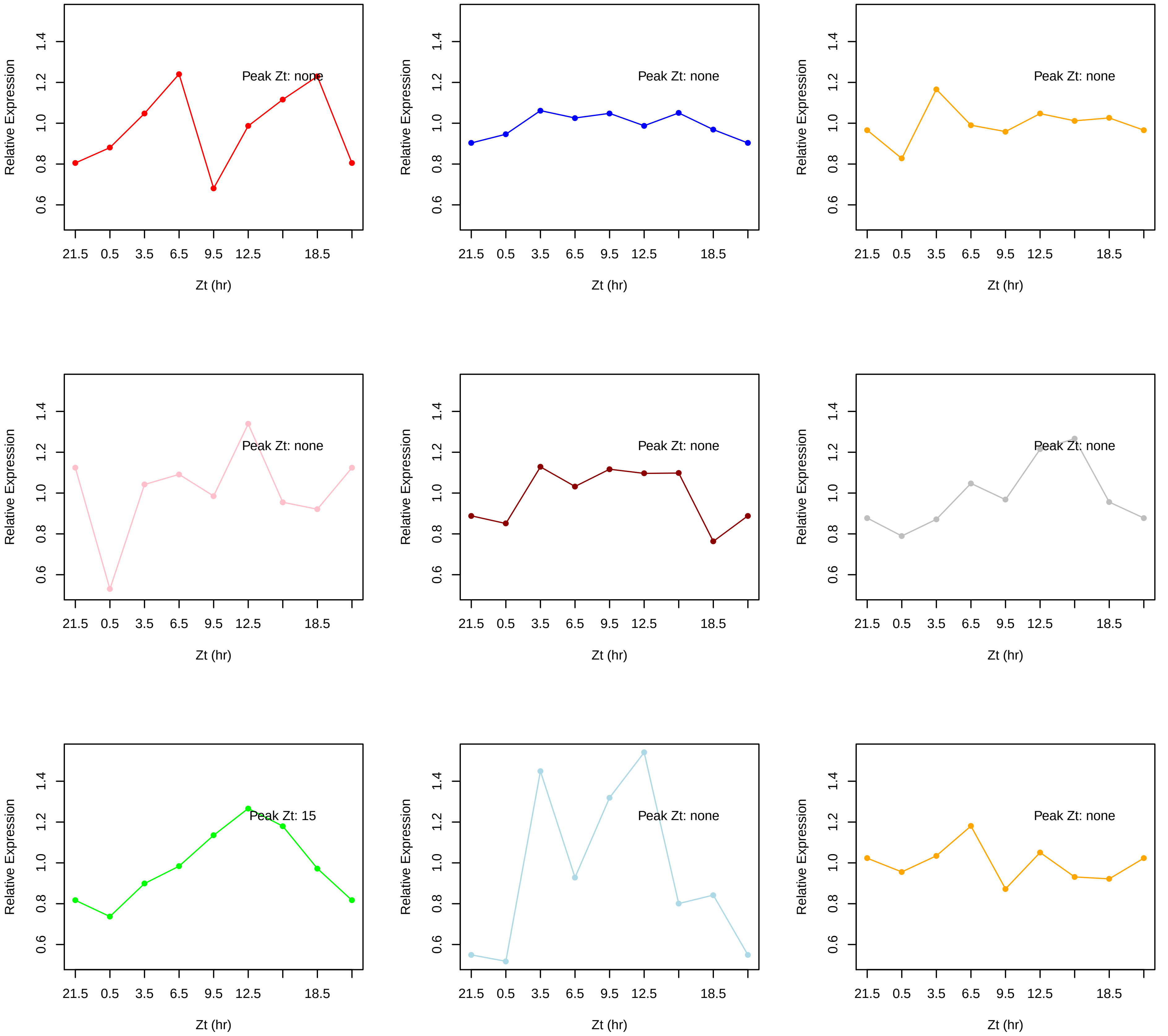

Singeleton: ENSXMAG00000007407

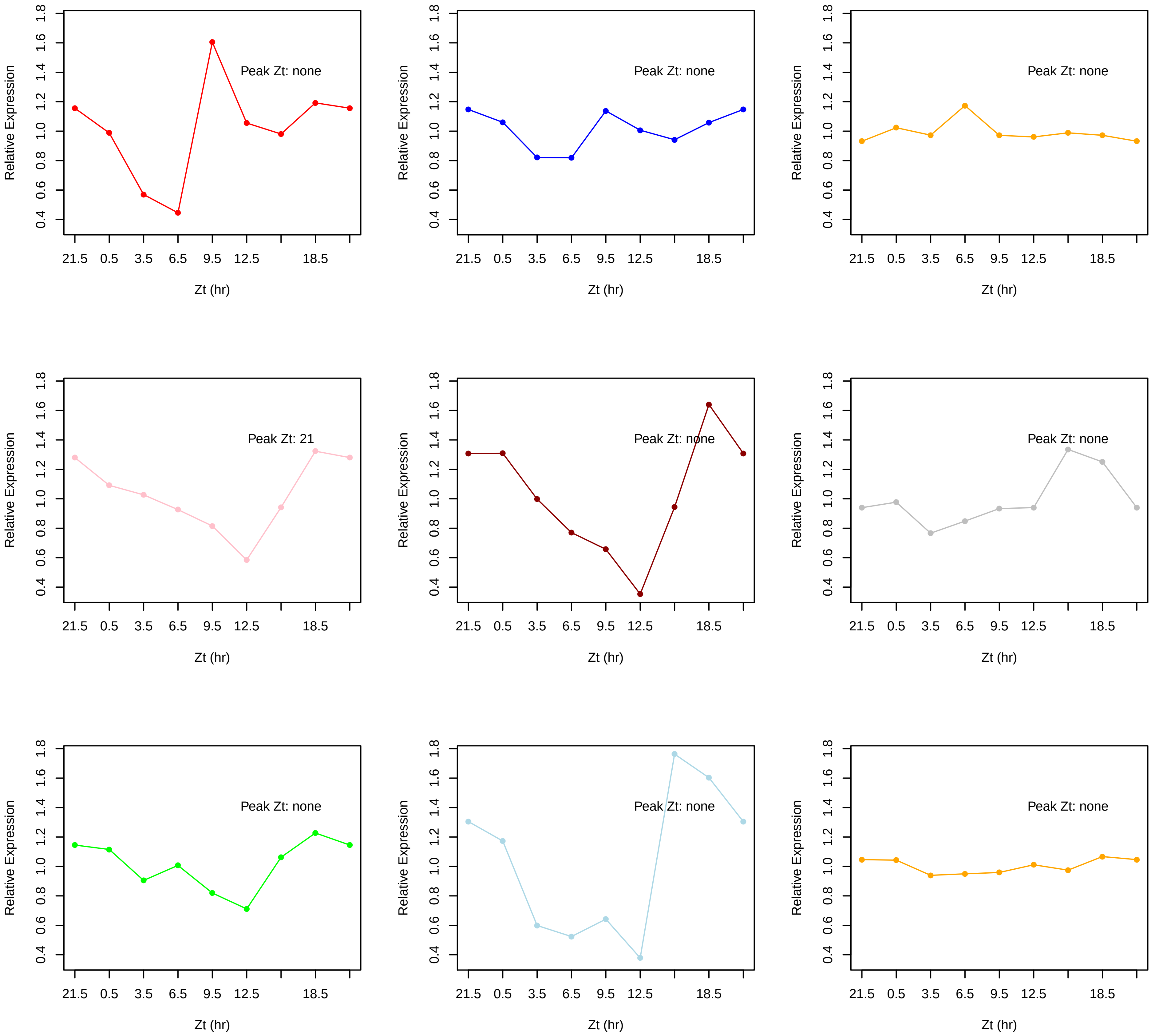

Singleton: ENSXMAG00000009615 mmaa

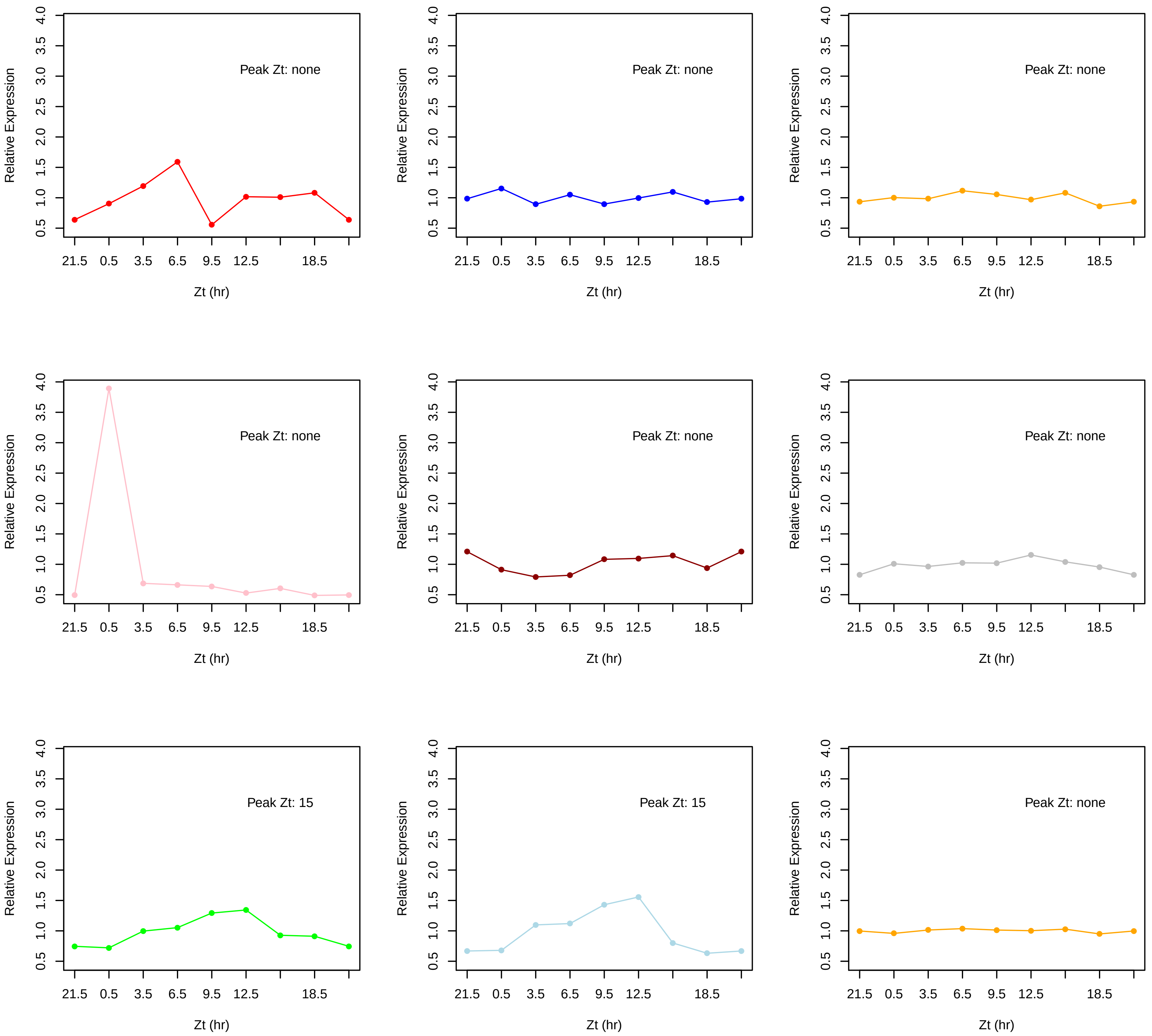

Singleton: ENSXMAG00000011200 oma1

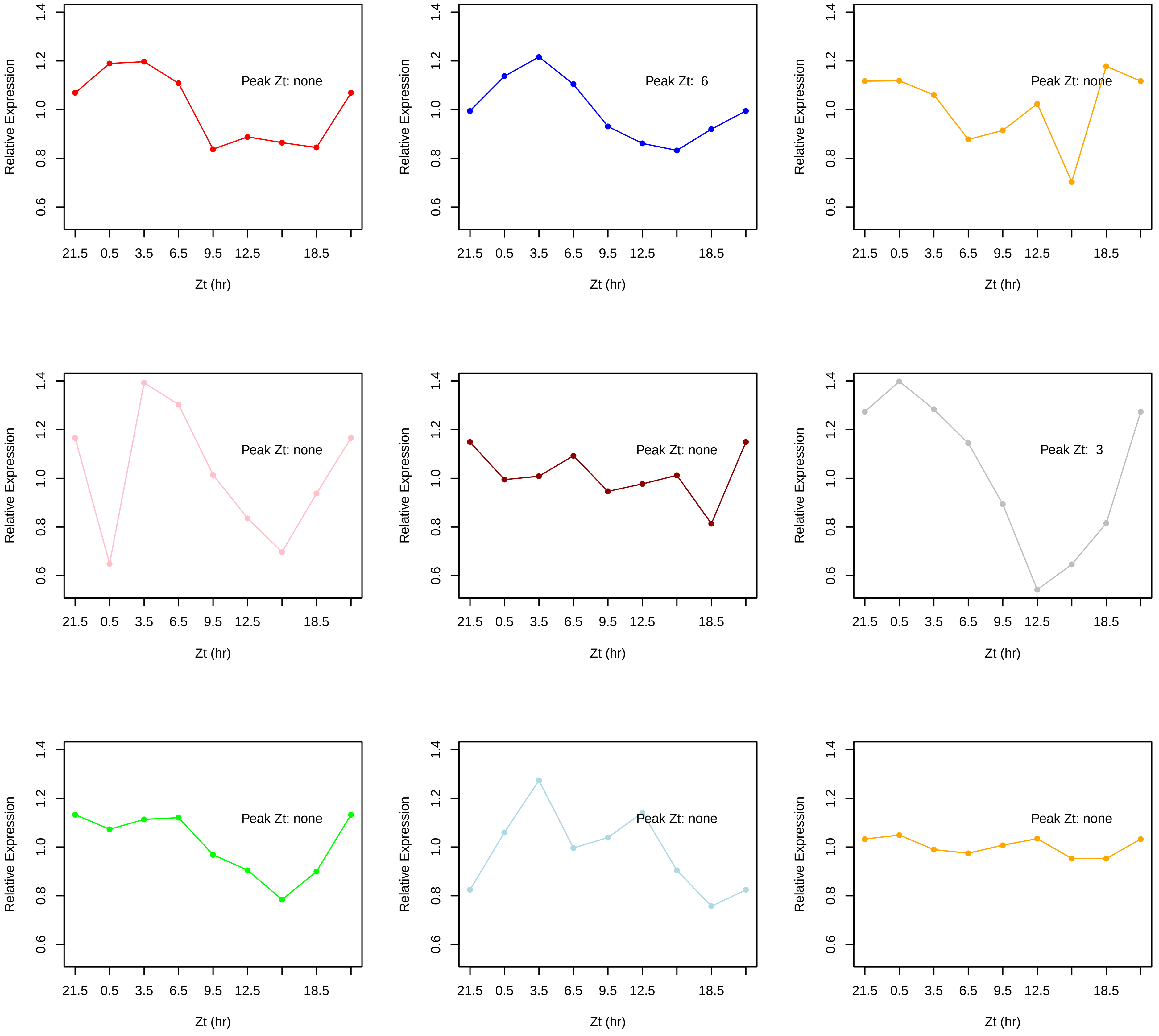

Singeleton: ENSXMAG00000006445 lss

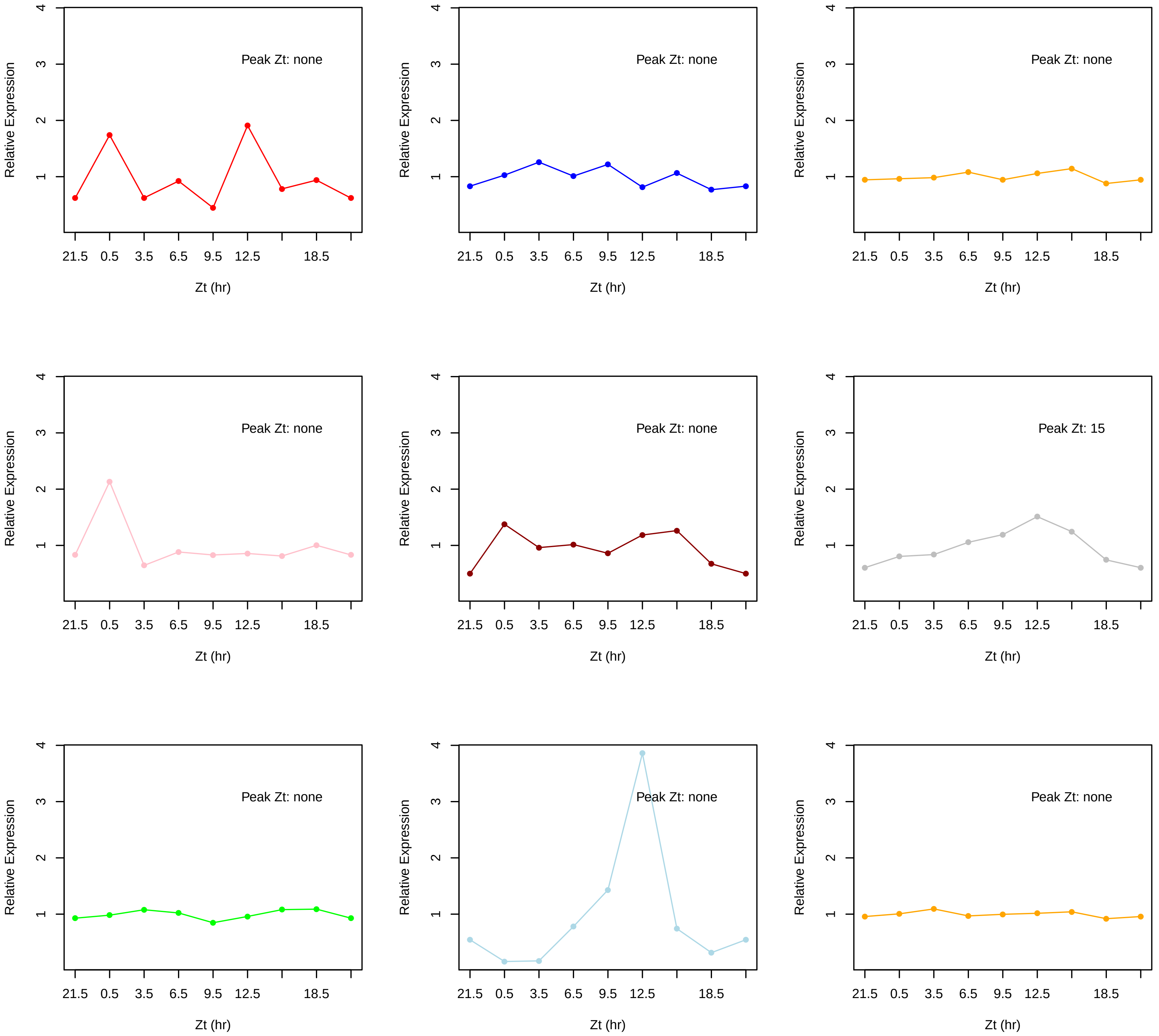

Singeleton: ENSXMAG00000024682 gpaa1

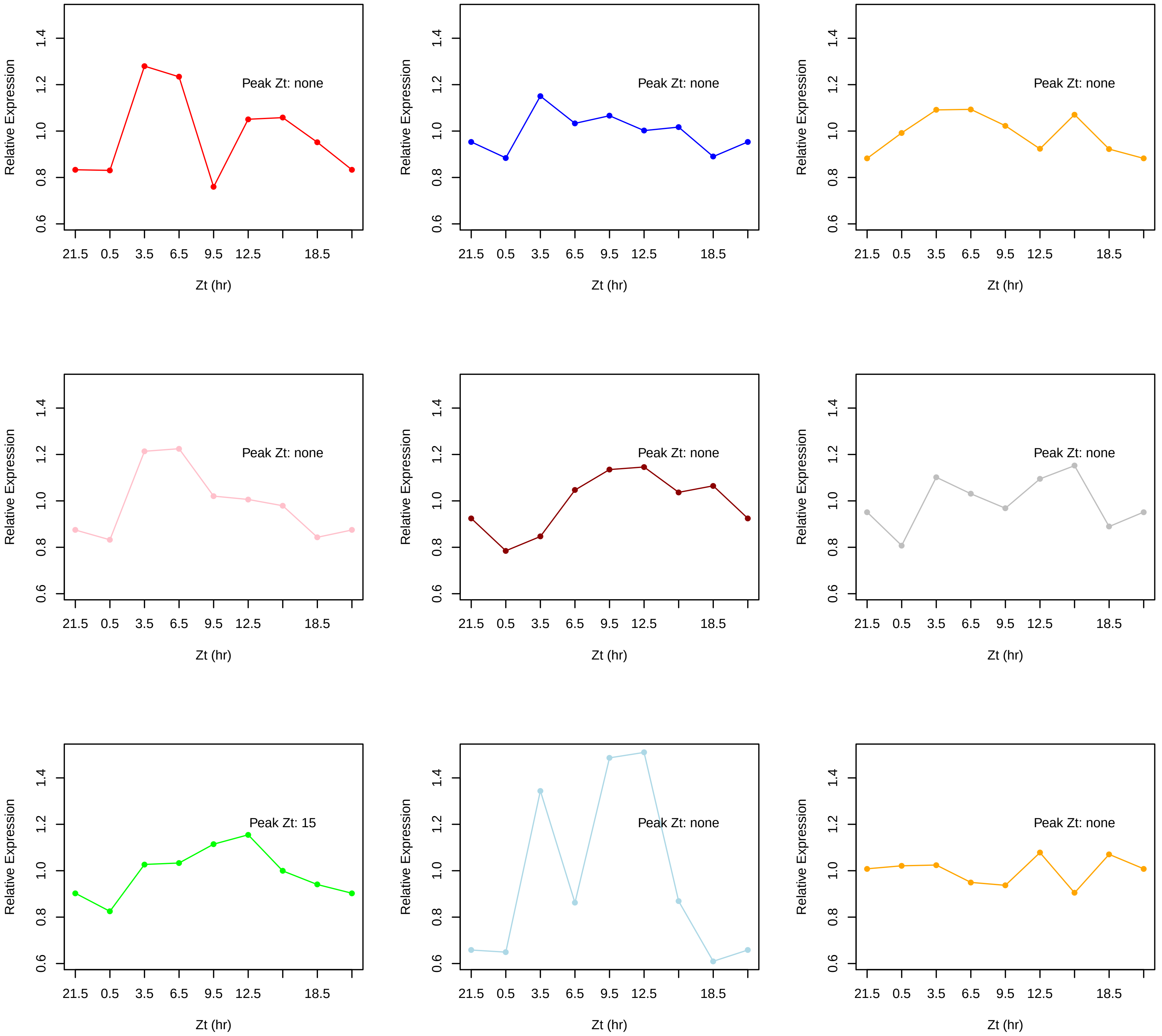

Singeleton: ENSXMAG00000022758 slc6a3

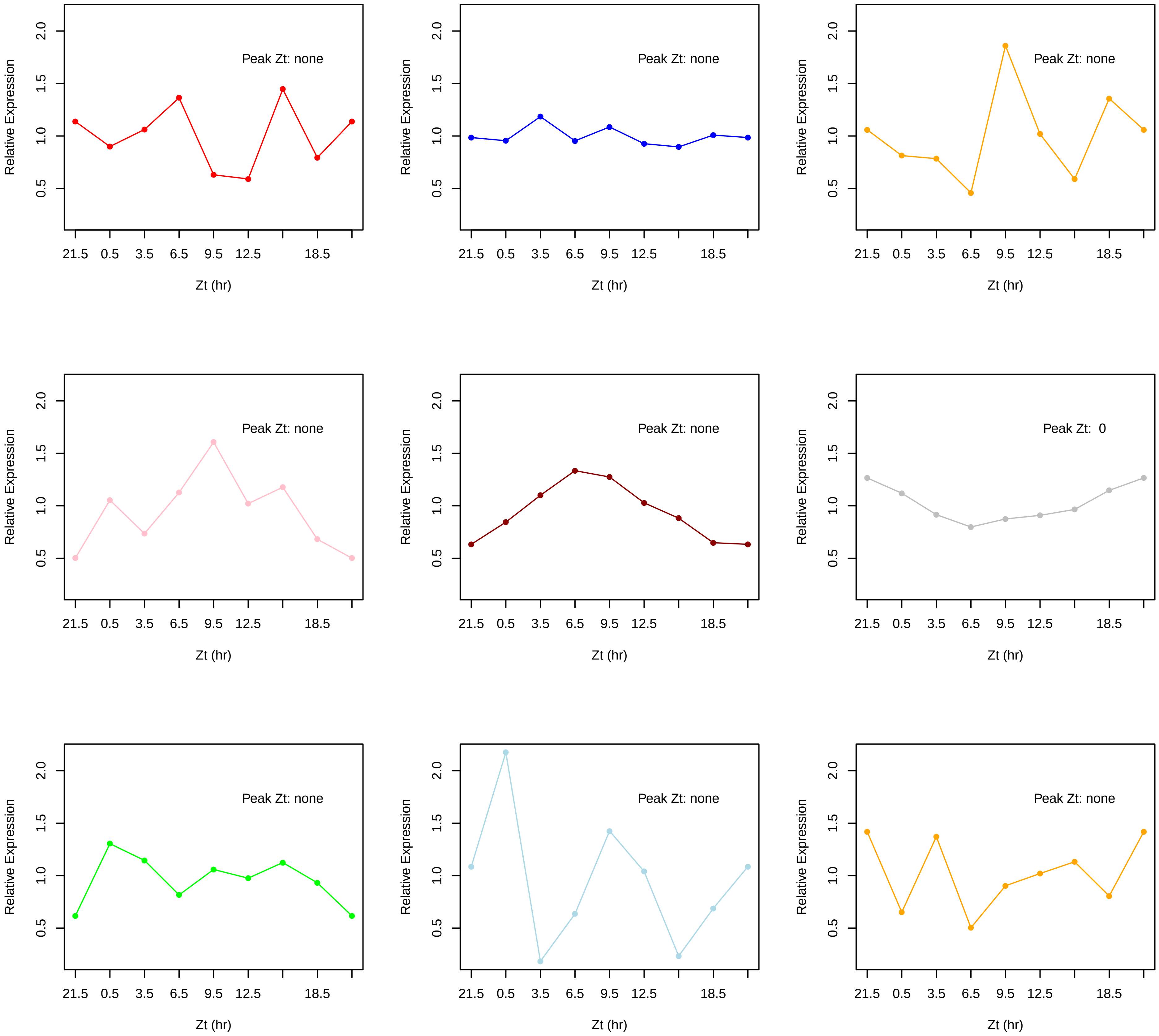

Singeleton: ENSXMAG00000011403 si:dkey−86e18.1

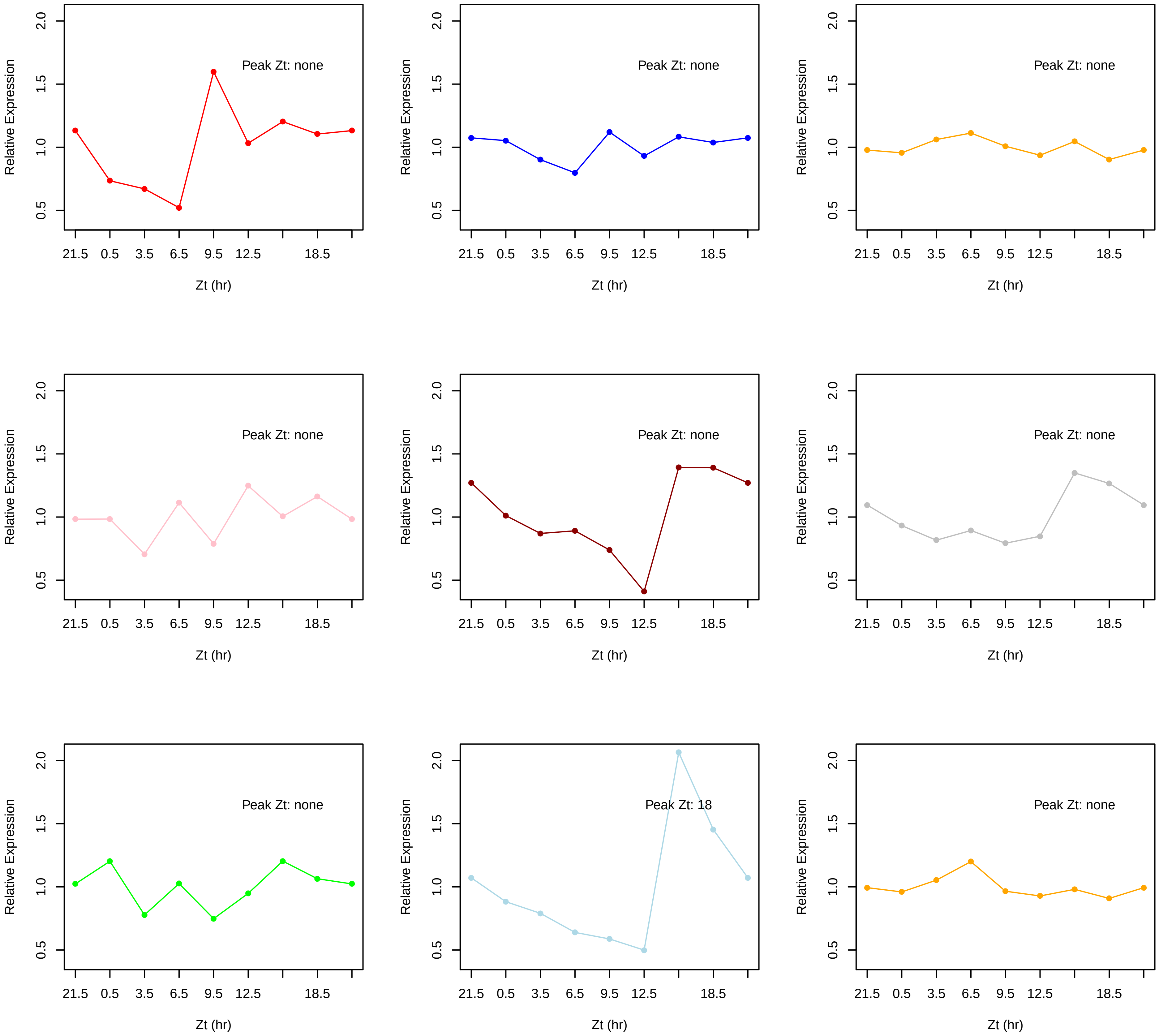

Singeleton: ENSXMAG00000015012 ybey

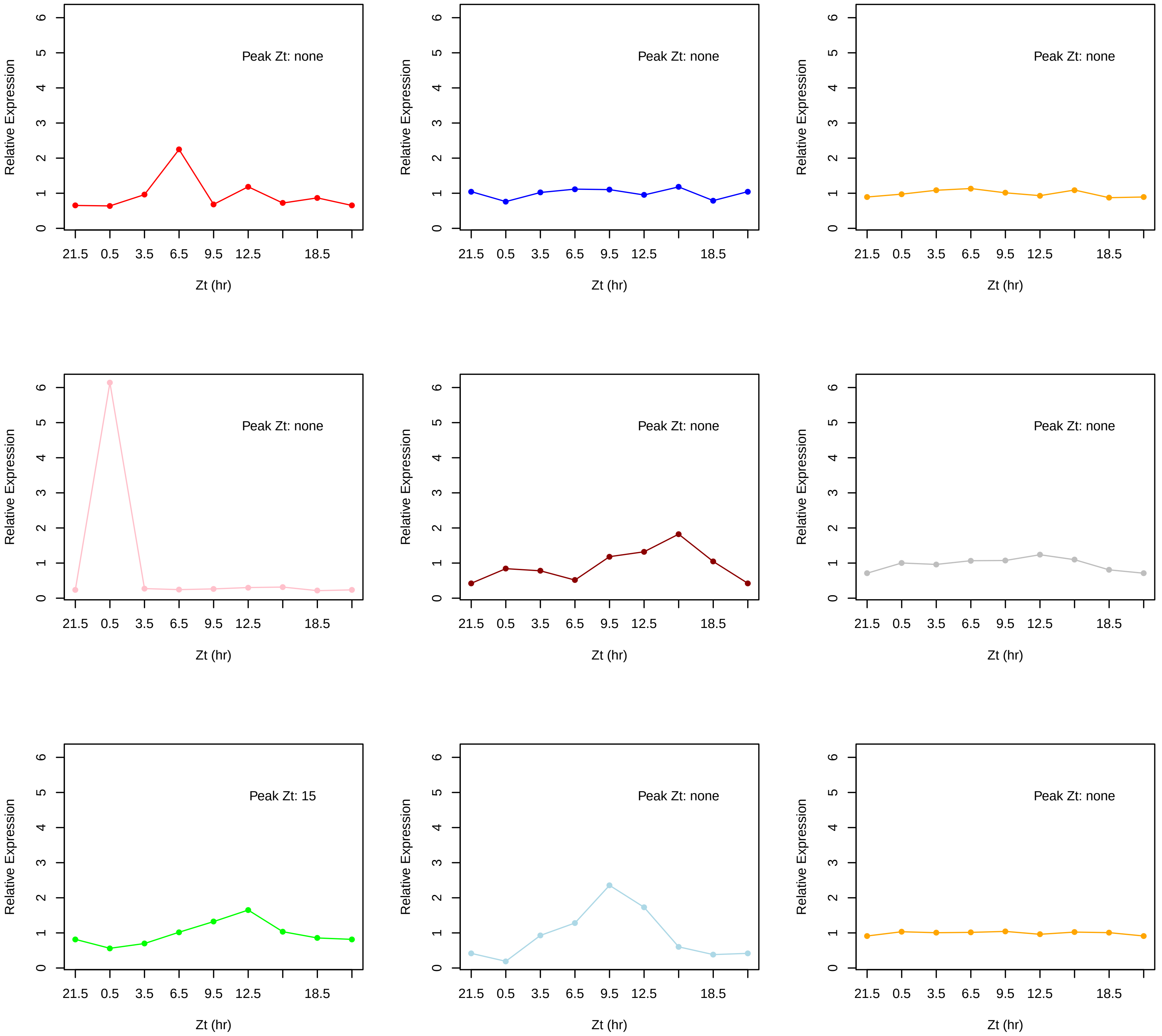

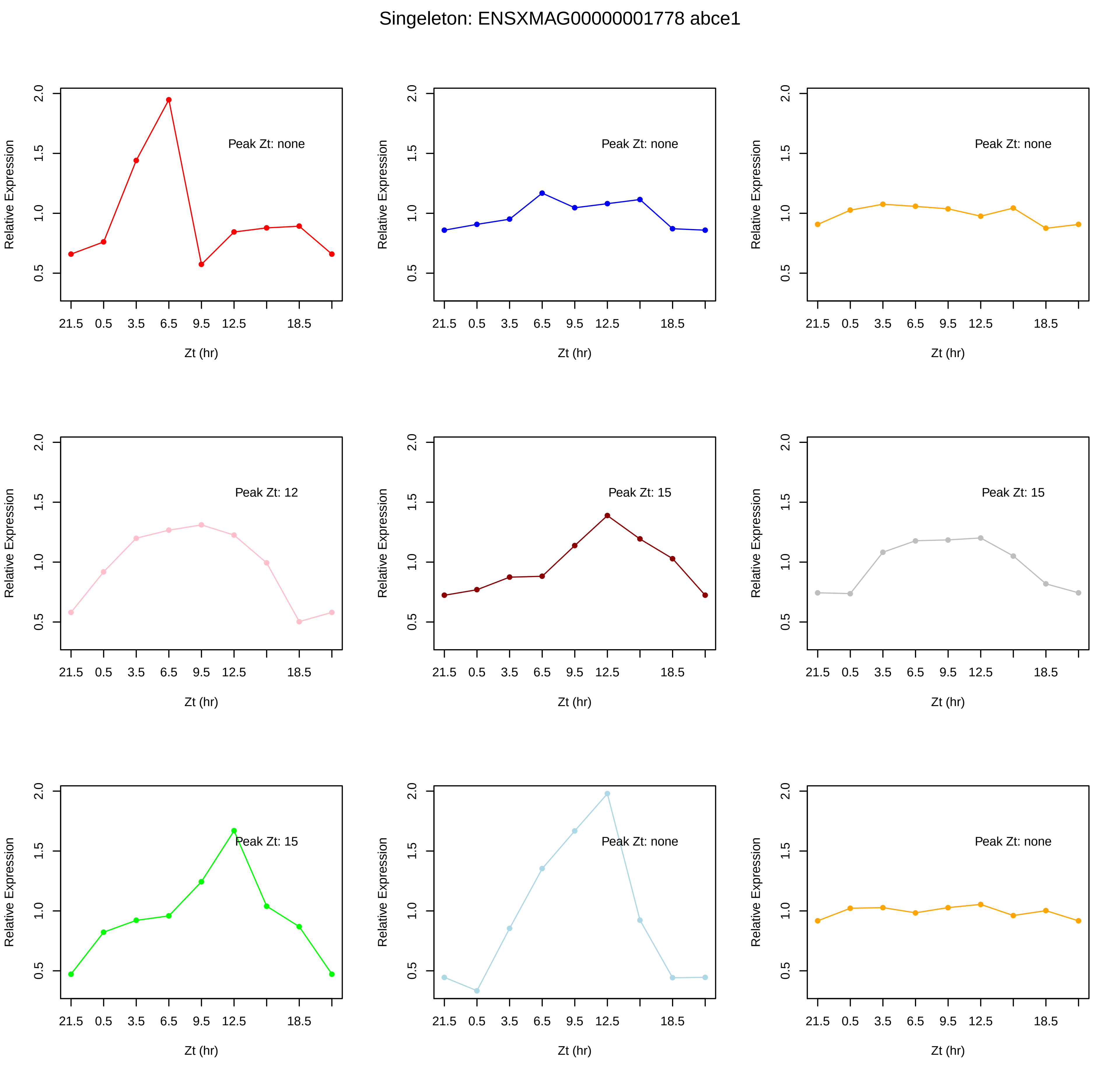

Singleton: ENSXMAG00000001778 abce1
Peak Zt: none
Peak Zt: none
Peak Zt: none
Peak Zt: 12
Peak Zt: 15
Peak Zt: 15
Peak Zt: 15
Peak Zt: none
Peak Zt: none
Relative Expression
Zt (hr)

Singeleton: ENSXMAG00000018286 fggy

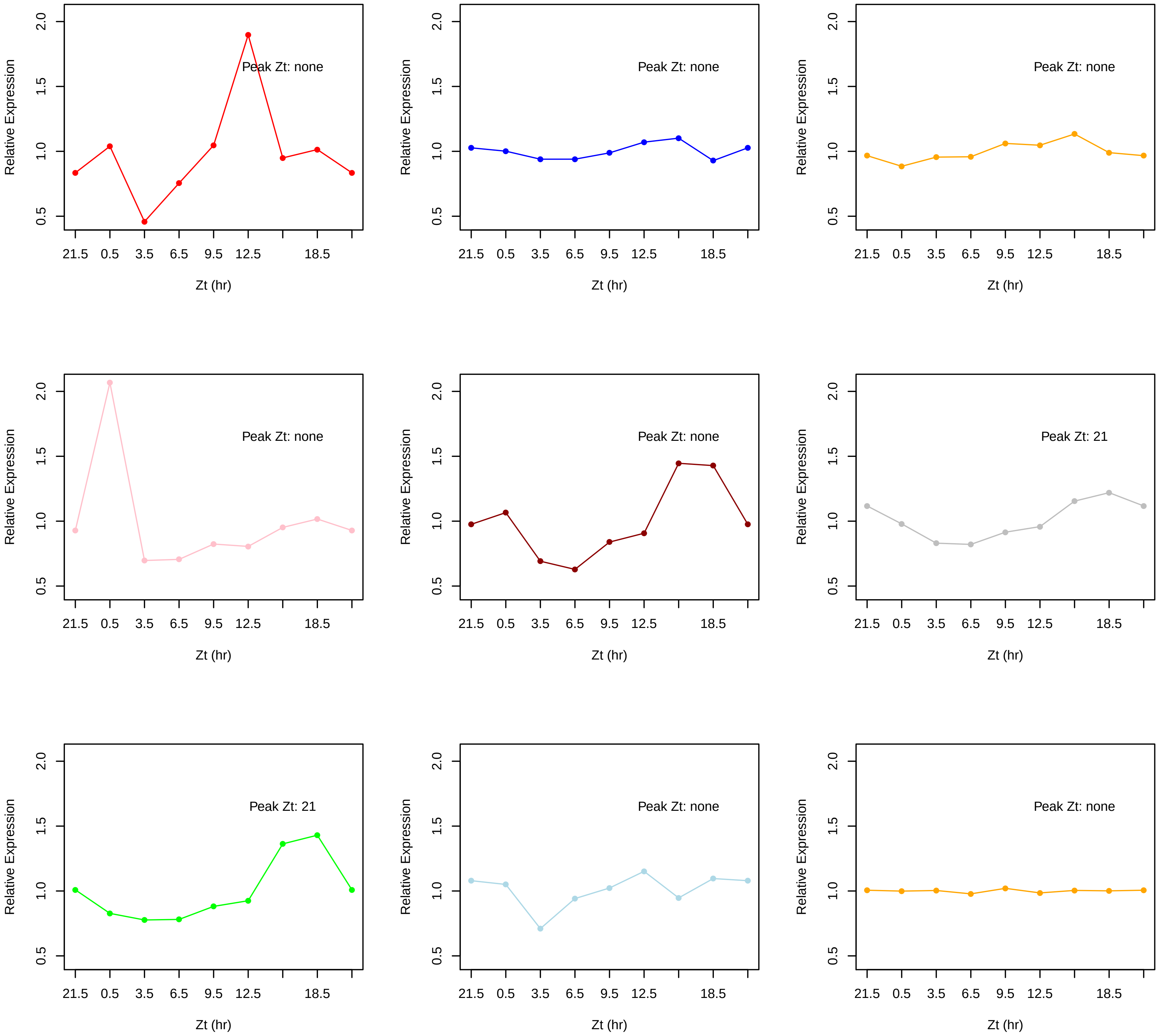

Singleton: ENSXMAG00000014971

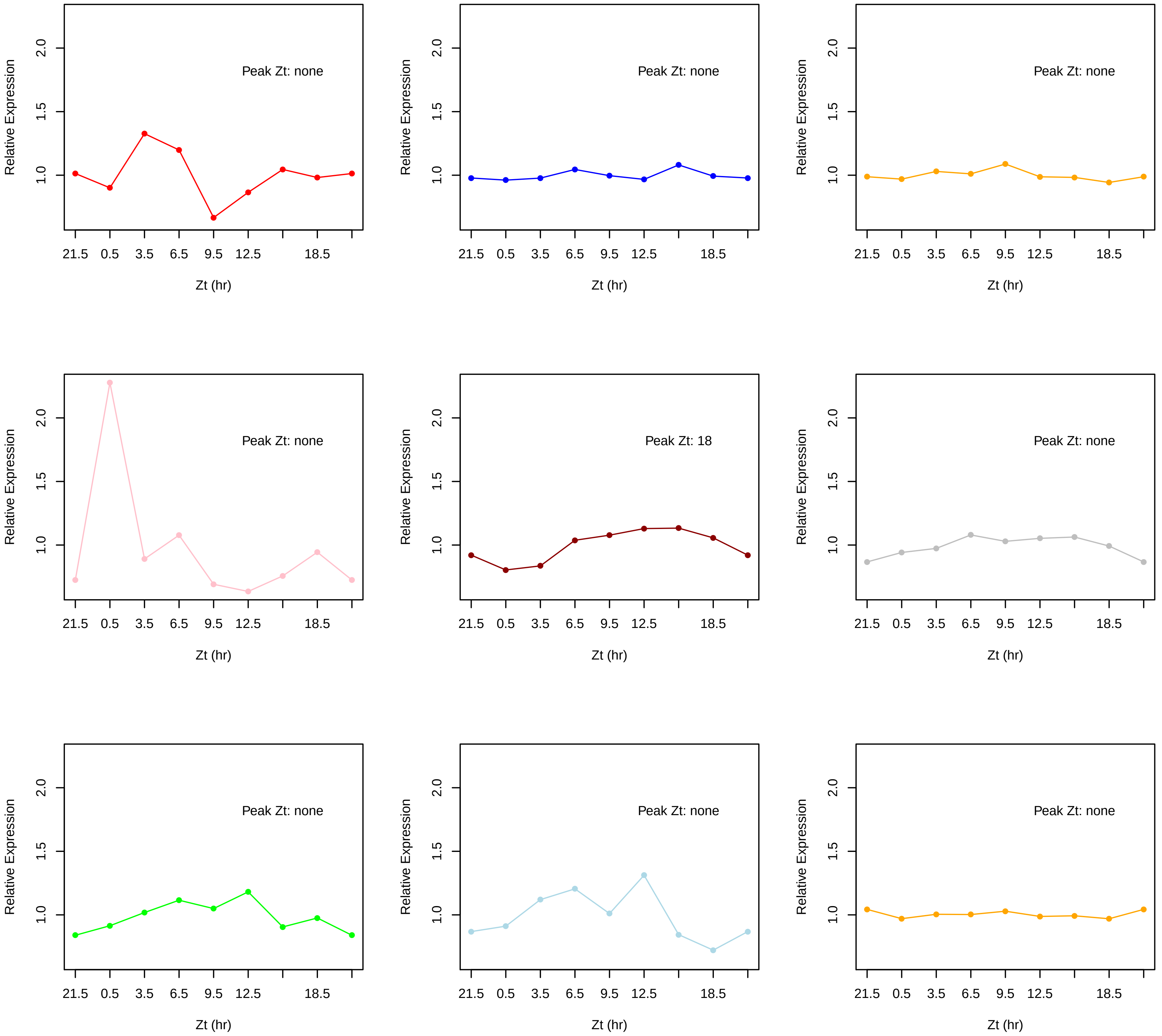

Singeleton: ENSXMAG00000001772 anapc10

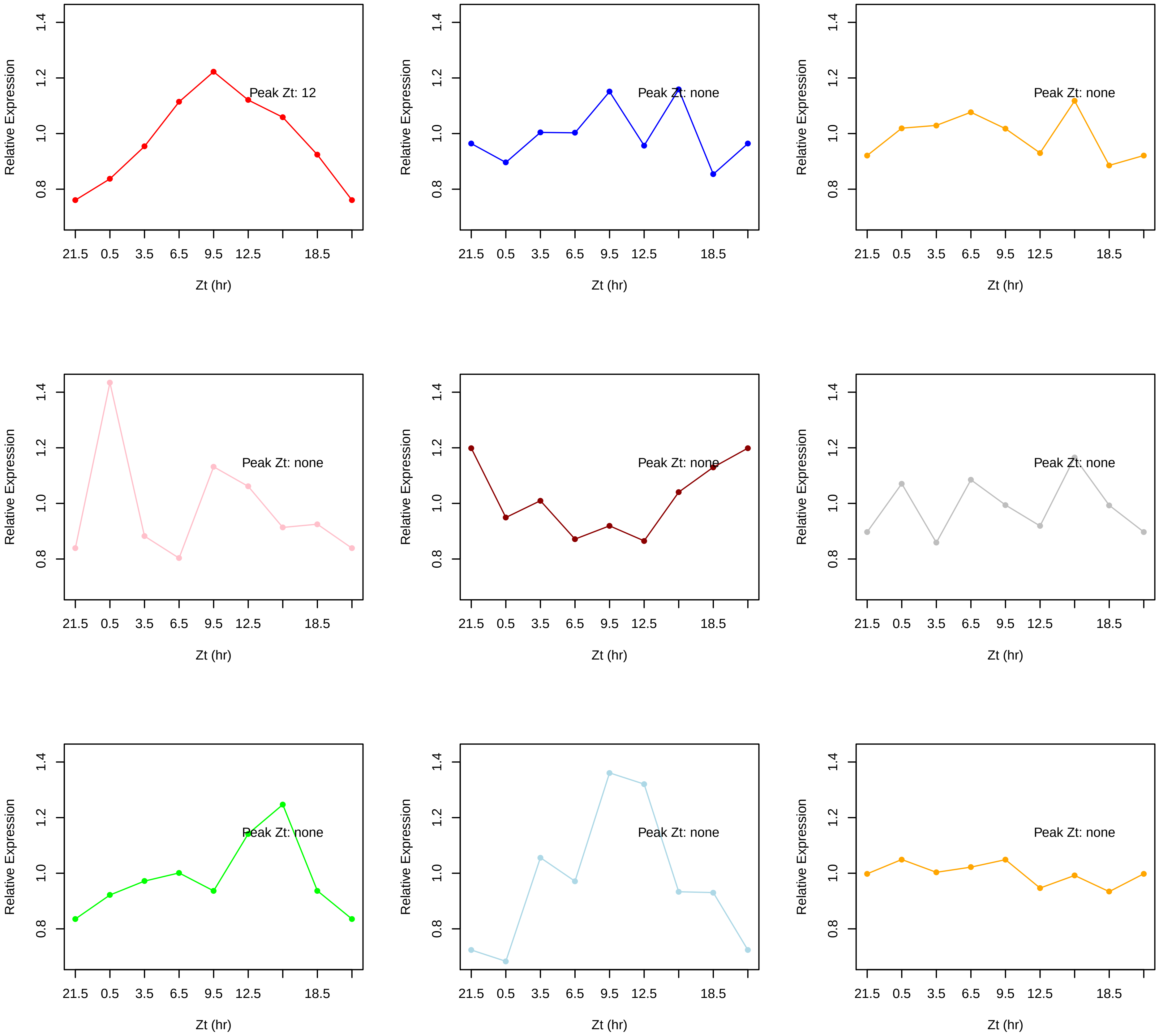

Singeleton: ENSXMAG00000029910 cdo1

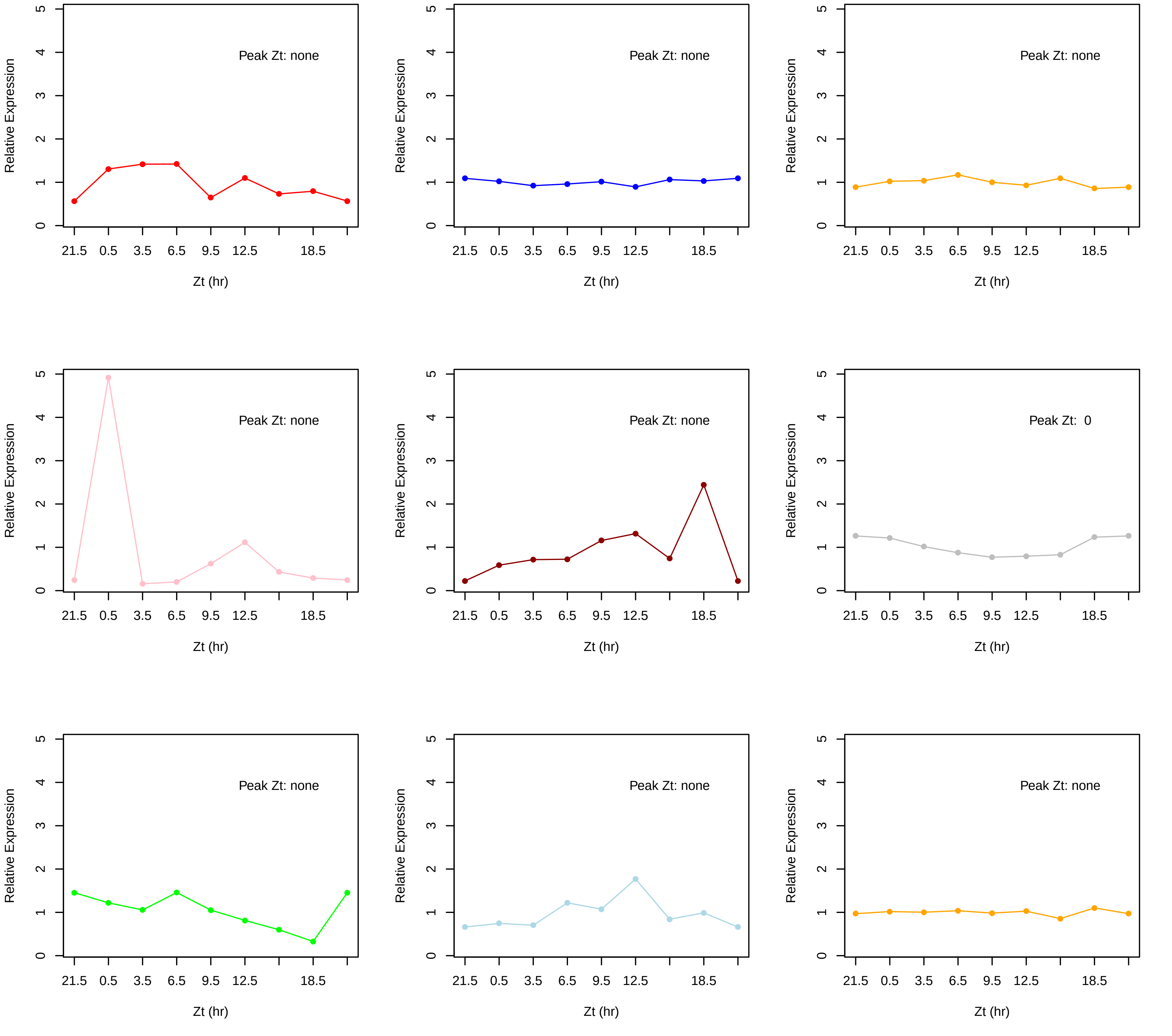

Singeleton: ENSXMAG00000012554 myo16

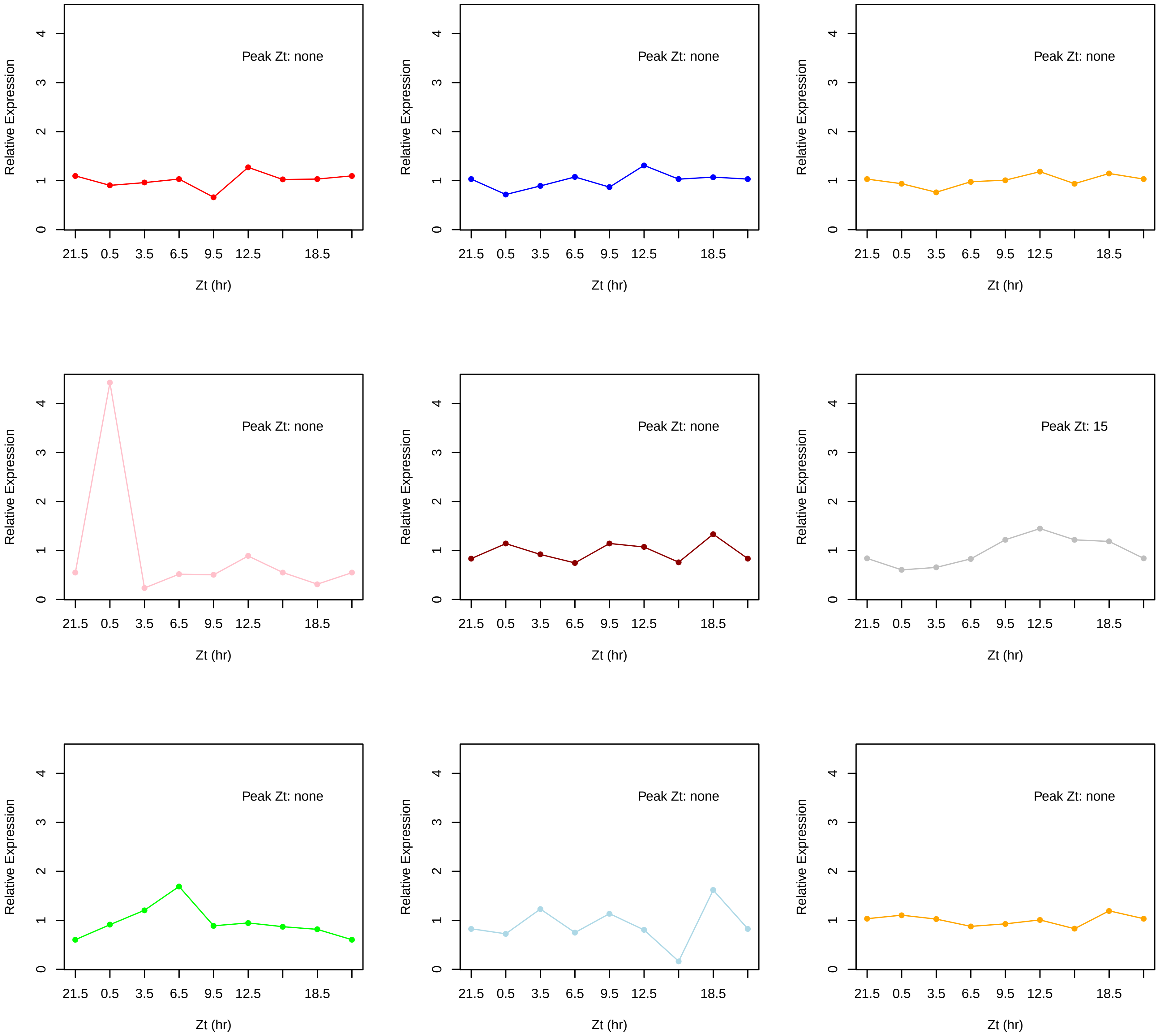

Singleton: ENSXMAG00000006474 cnot9

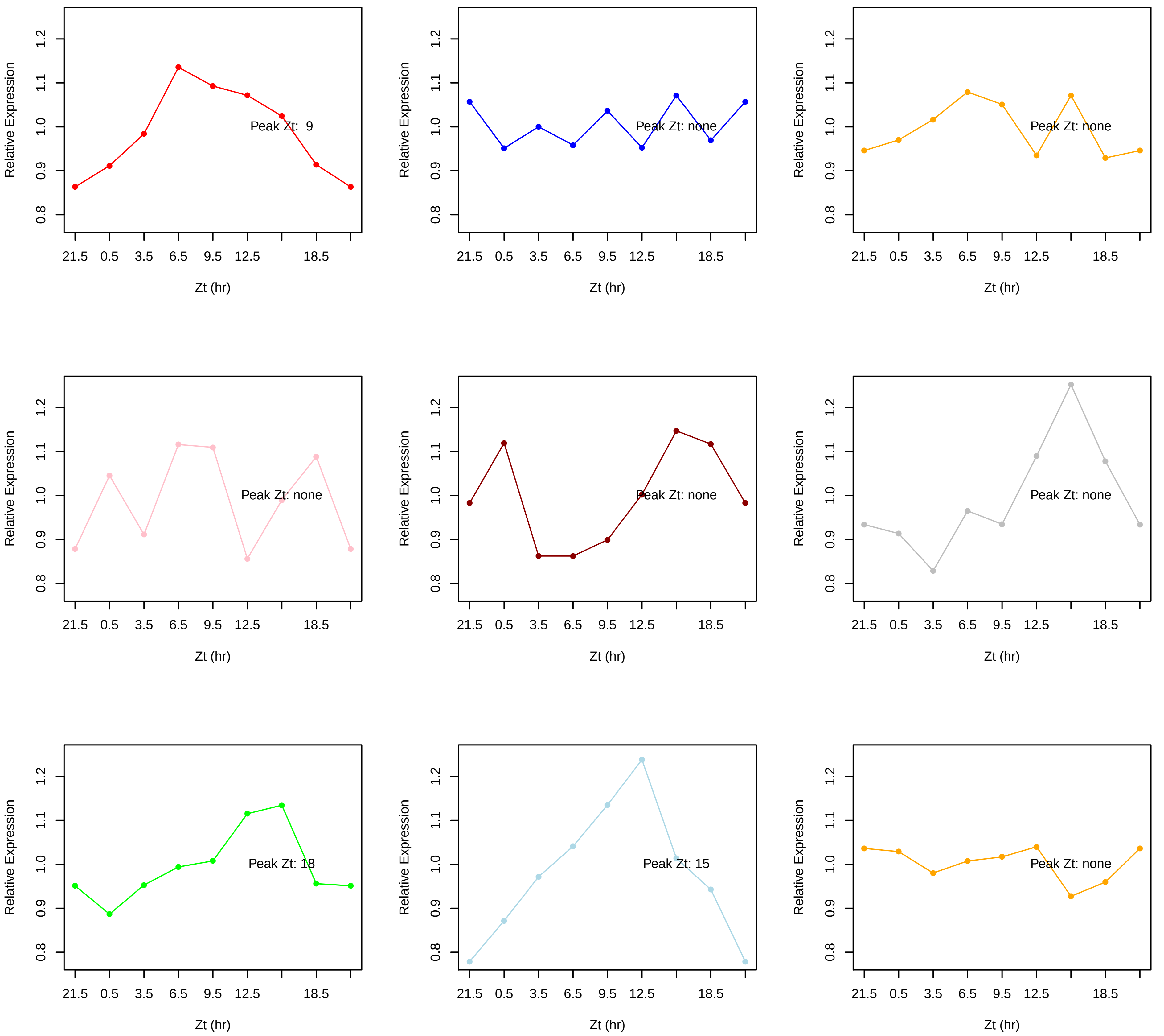

Singeleton: ENSXMAG00000009627 hhip

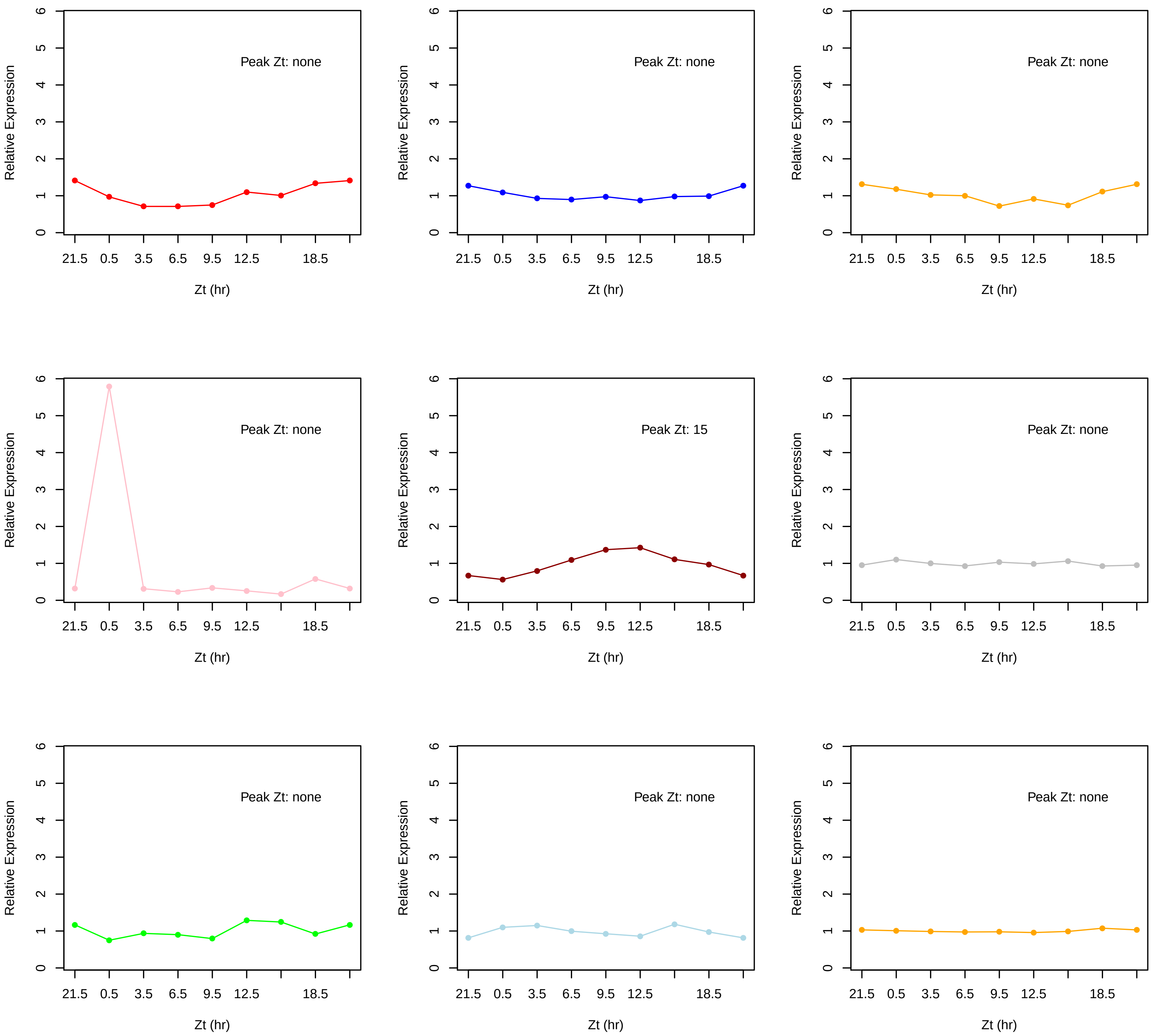

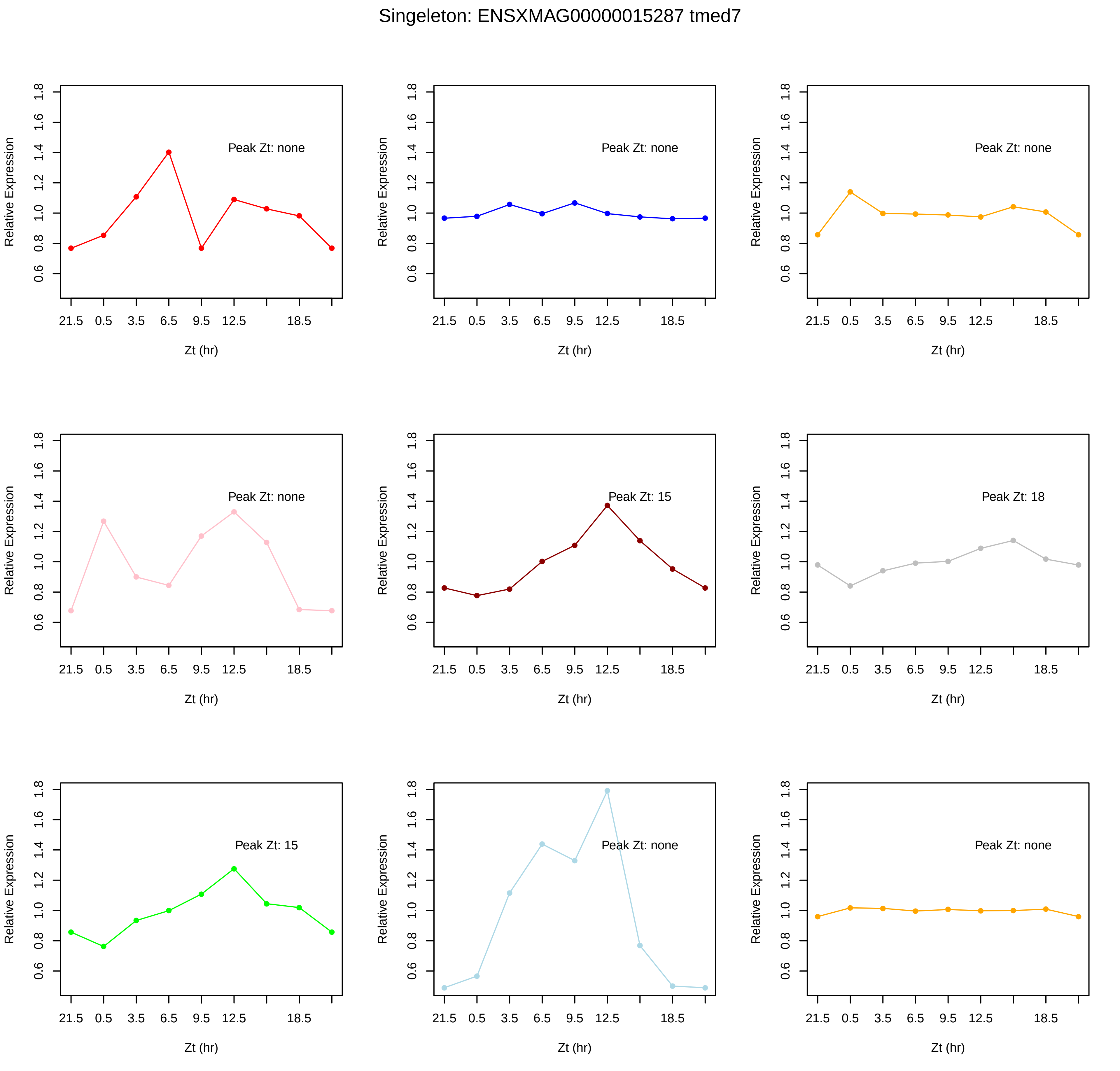
Singeleton: ENSXMAG00000015287 tmed7
Peak Zt: none
Peak Zt: none
Peak Zt: none
Peak Zt: none
Peak Zt: 15
Peak Zt: 18
Peak Zt: 15
Peak Zt: none
Peak Zt: none
Relative Expression
Zt (hr)
21.5
0.5
3.5
6.5
9.5
12.5
18.5
0.6
0.8
1.0
1.2
1.4
1.6
1.8

Singeleton: ENSXMAG00000021029 e2f3

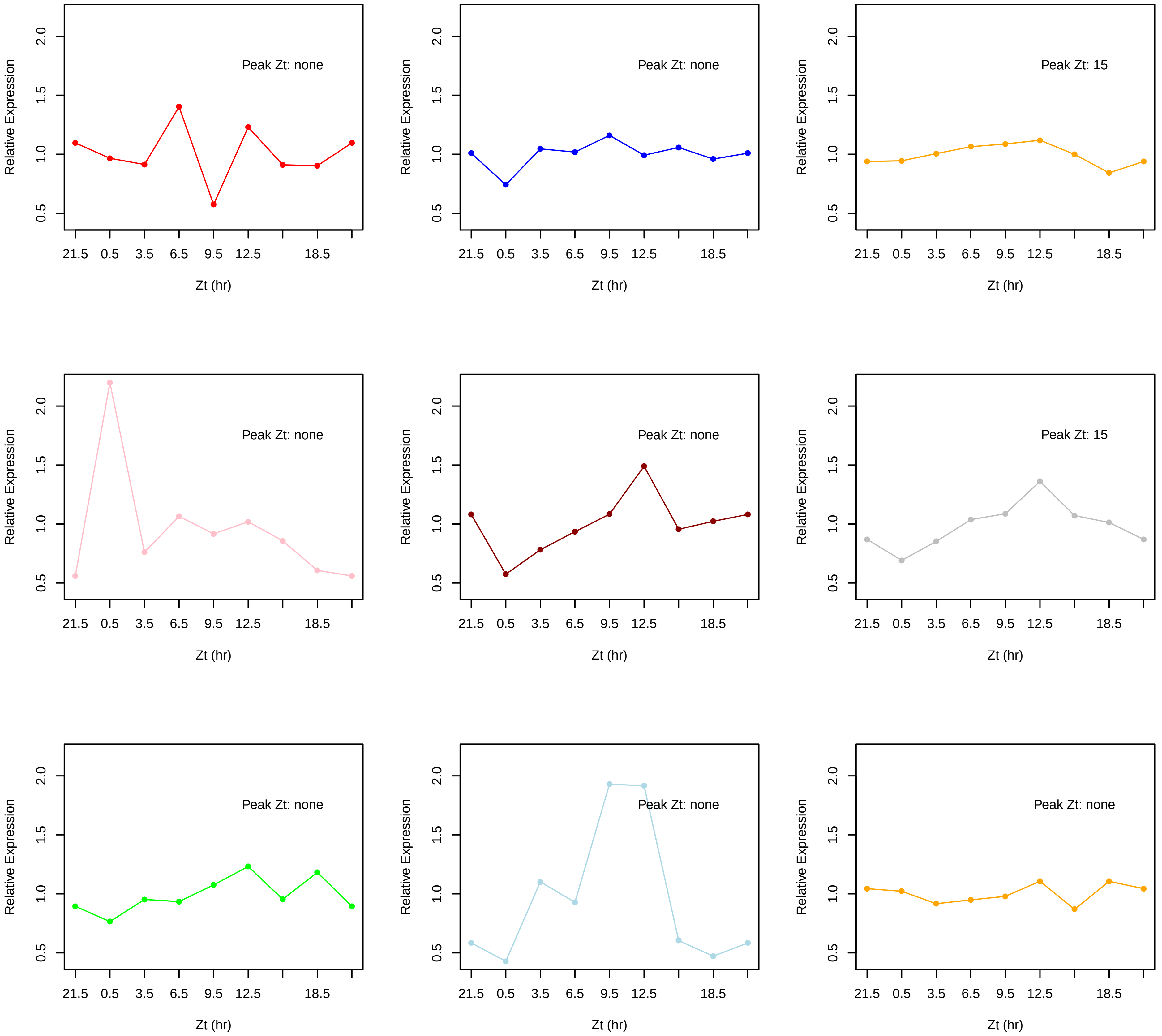

Singleton: ENSXMAG00000015297 fem1c

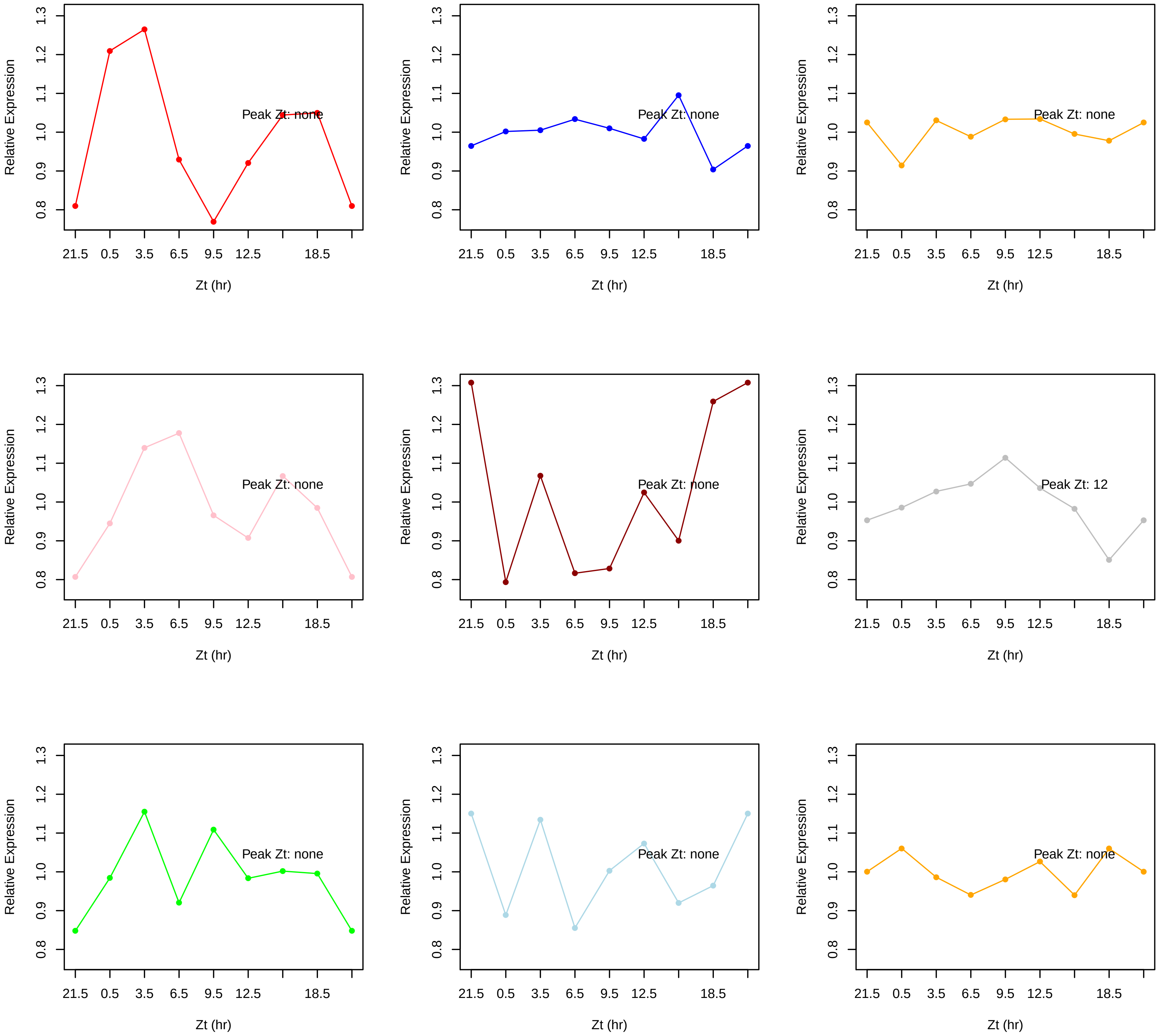

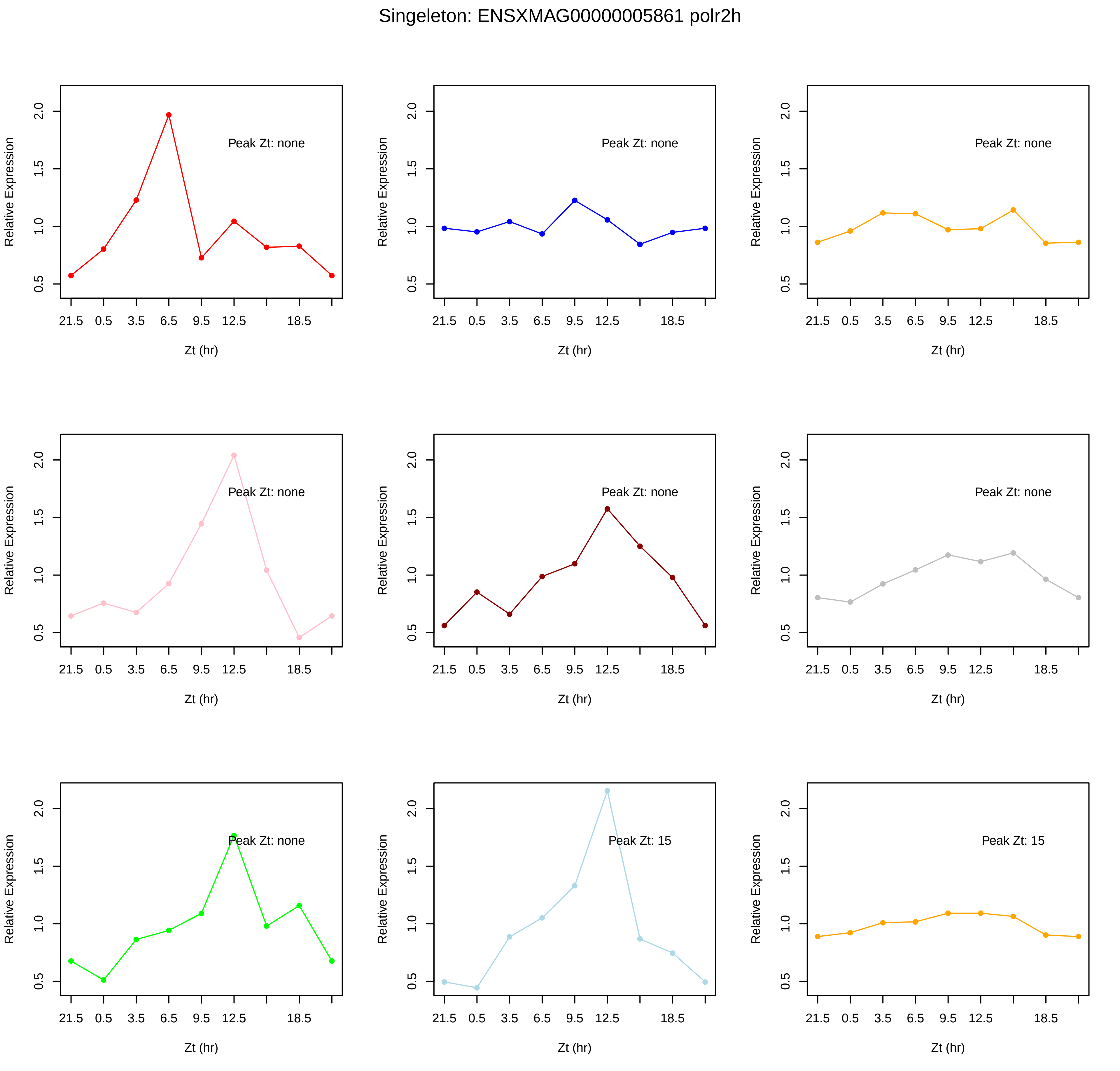
Singeleton: ENSXMAG00000005861 polr2h
Relative Expression
Zt (hr)
Peak Zt: none
Peak Zt: 15
21.5
0.5
3.5
6.5
9.5
12.5
18.5
0.5
1.0
1.5
2.0

Singleton: ENSXMAG00000009662 frem3

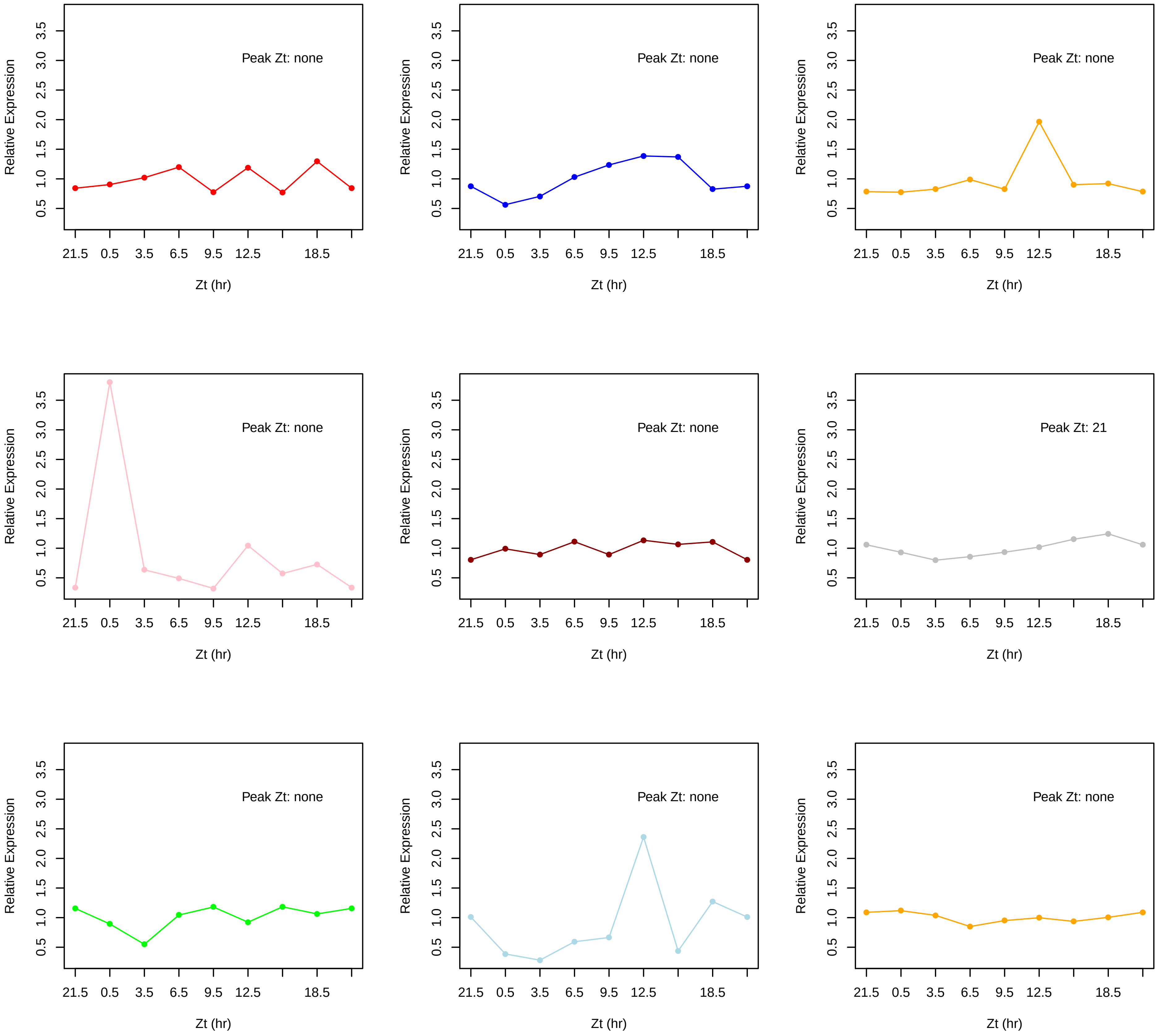

Singeleton: ENSXMAG00000025867 qtrt1

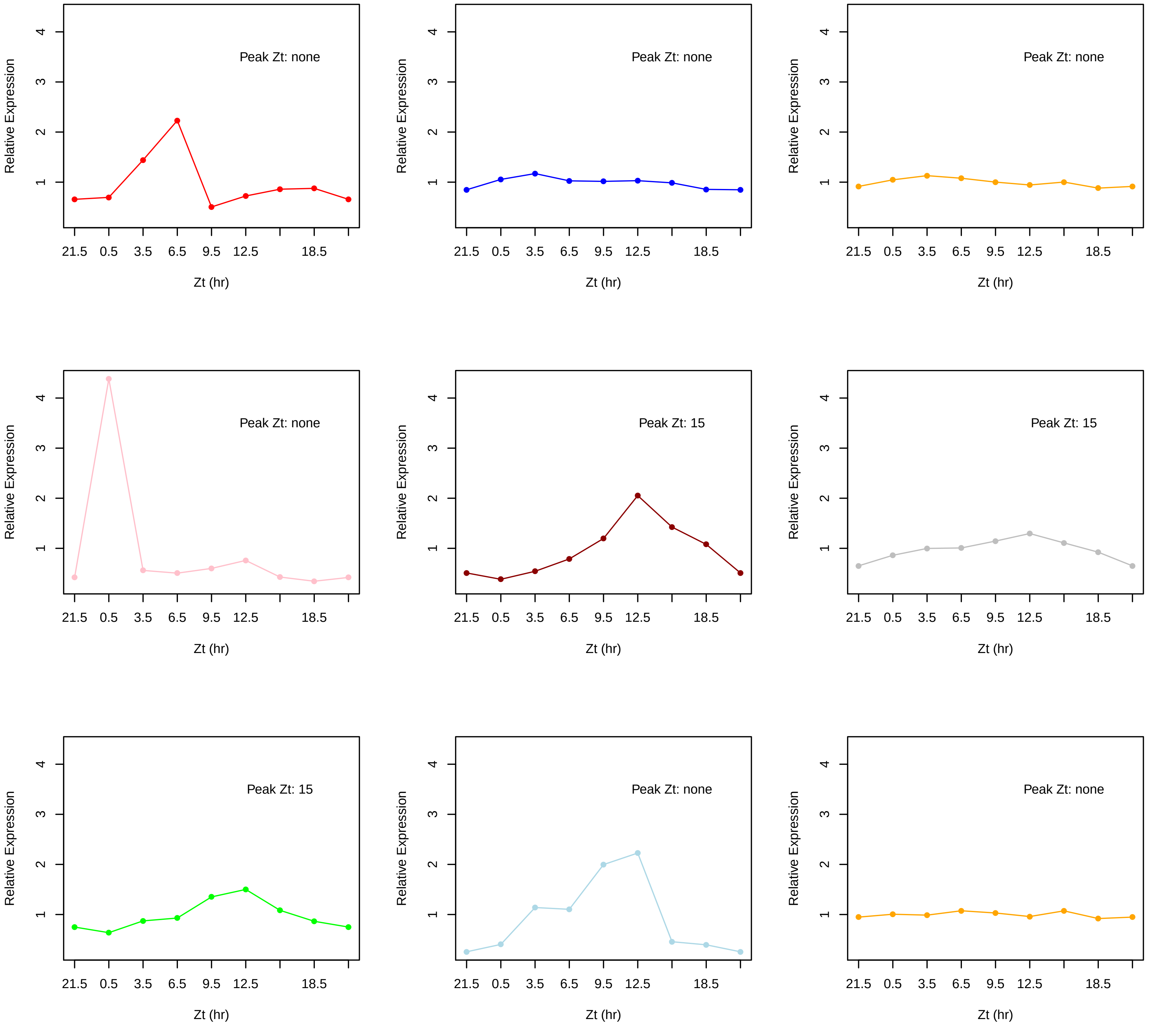

Singeleton: ENSXMAG00000000127 tbc1d24

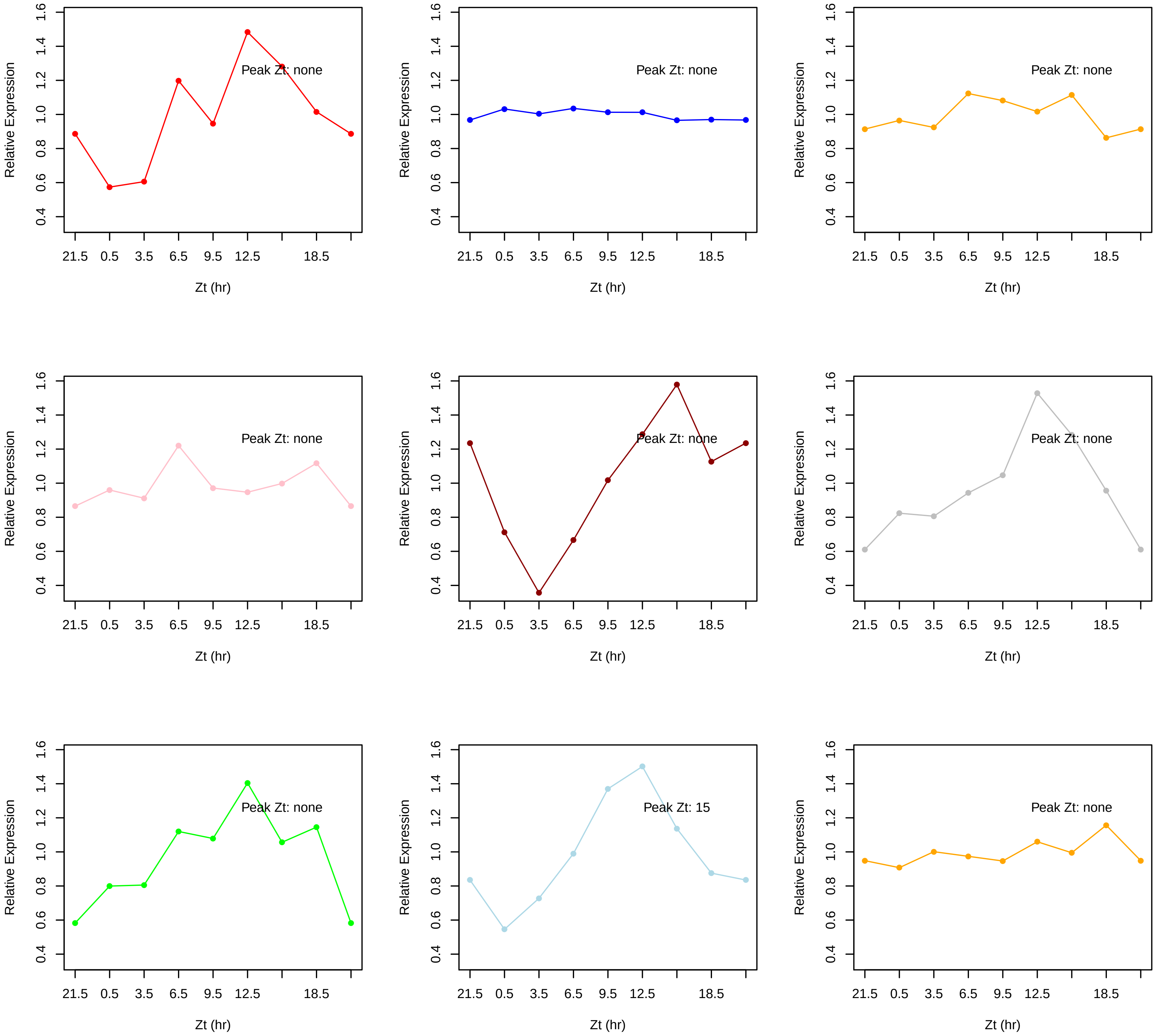

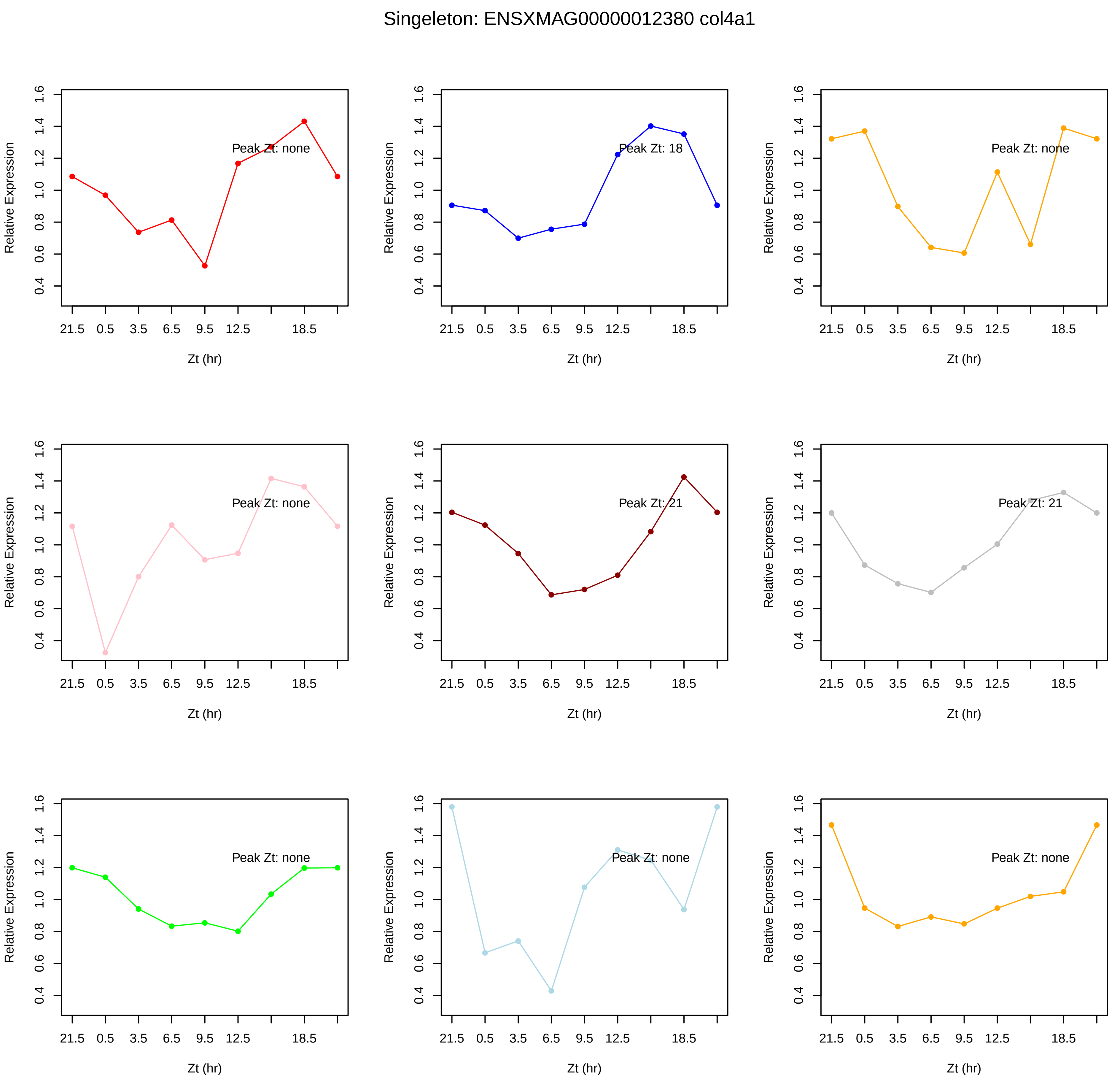
Singeleton: ENSXMAG00000012380 col4a1
Peak Zt: none
Peak Zt: 18
Peak Zt: none
Peak Zt: none
Peak Zt: 21
Peak Zt: 21
Peak Zt: none
Peak Zt: none
Peak Zt: none
Relative Expression
Zt (hr)
21.5
0.5
3.5
6.5
9.5
12.5
18.5
0.4
0.6
0.8
1.0
1.2
1.4
1.6

Singeleton: ENSXMAG00000018905 ppp1r16a

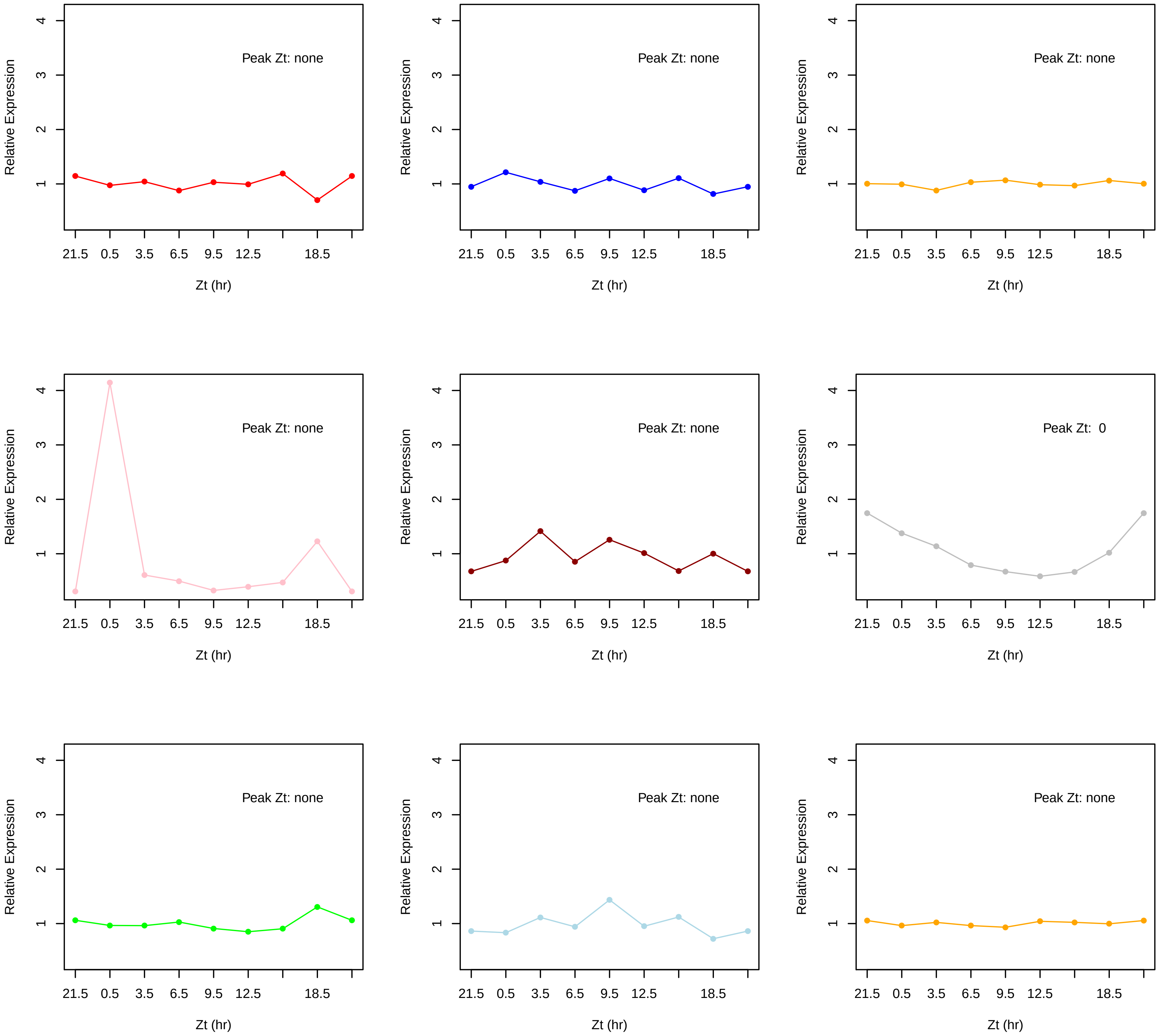

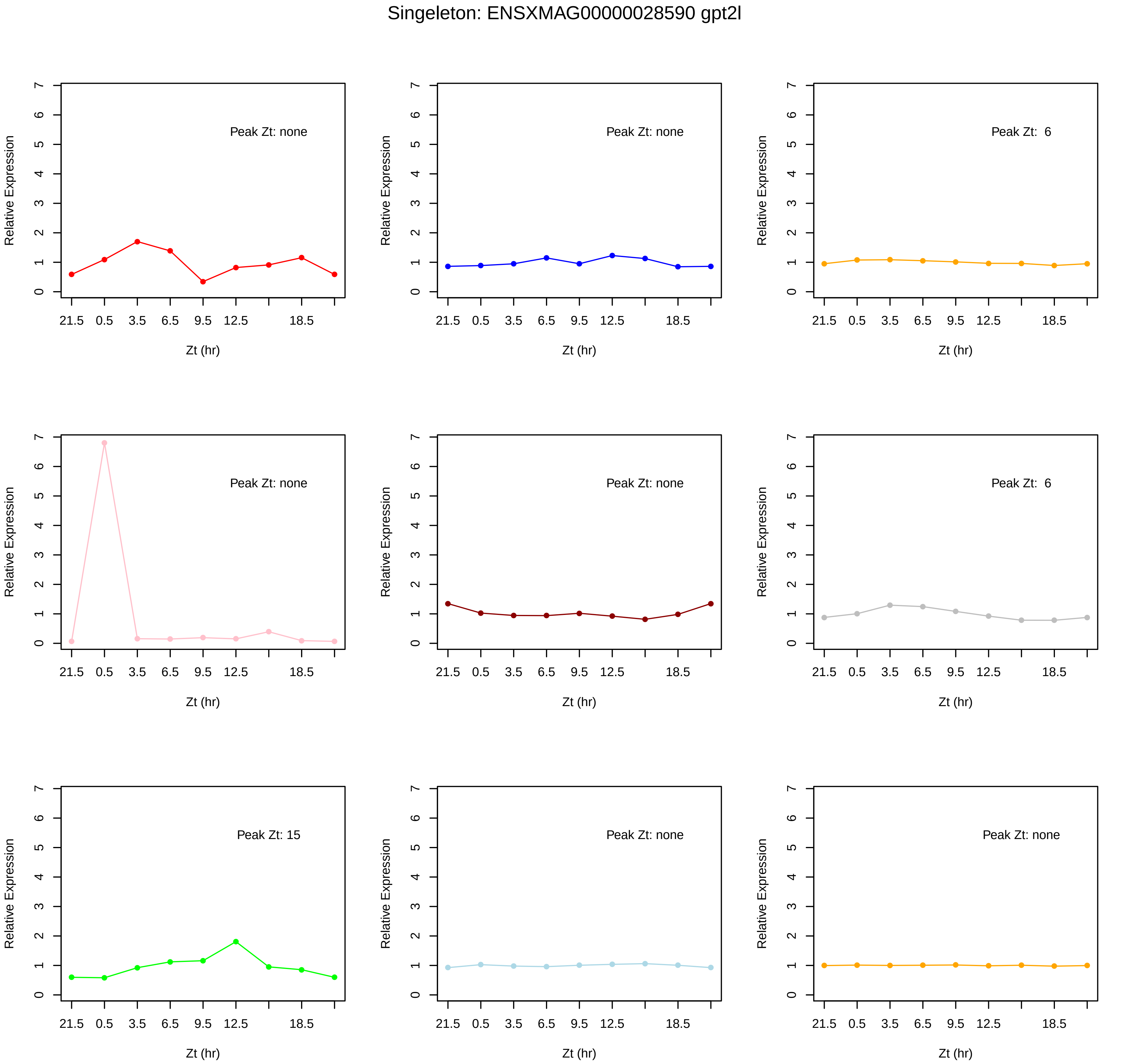

Singeleton: ENSXMAG00000028590 gpt2l
Peak Zt: none
Peak Zt: none
Peak Zt: 6
Peak Zt: none
Peak Zt: none
Peak Zt: 6
Peak Zt: 15
Peak Zt: none
Peak Zt: none
Relative Expression
Zt (hr)
21.5
0.5
3.5
6.5
9.5
12.5
18.5

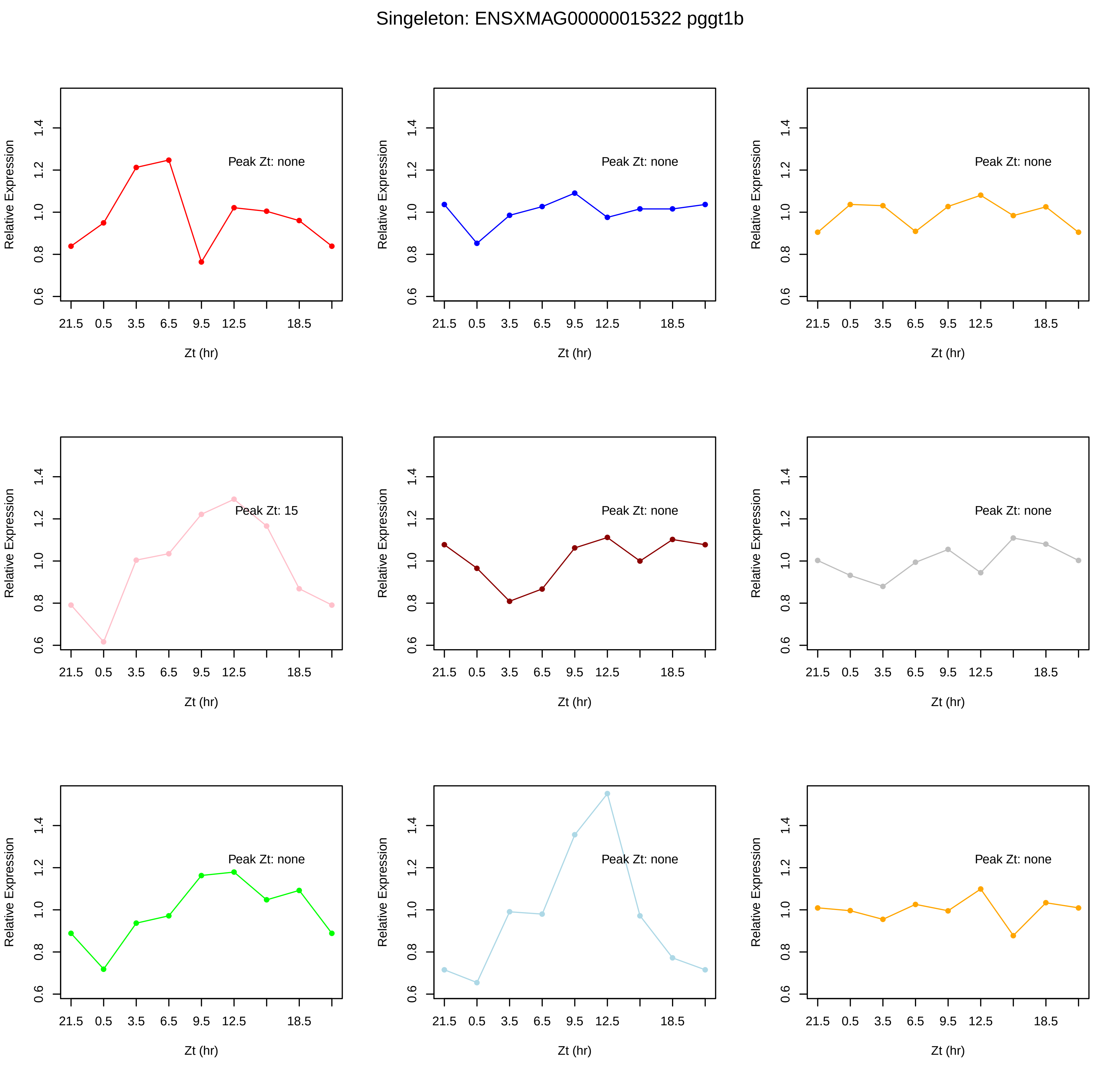
Singeleton: ENSXMAG00000015322 pggt1b
Peak Zt: none
Peak Zt: none
Peak Zt: none
Peak Zt: 15
Peak Zt: none
Peak Zt: none
Peak Zt: none
Peak Zt: none
Peak Zt: none
Relative Expression
Zt (hr)
0.6
0.8
1.0
1.2
1.4
21.5
0.5
3.5
6.5
9.5
12.5
18.5

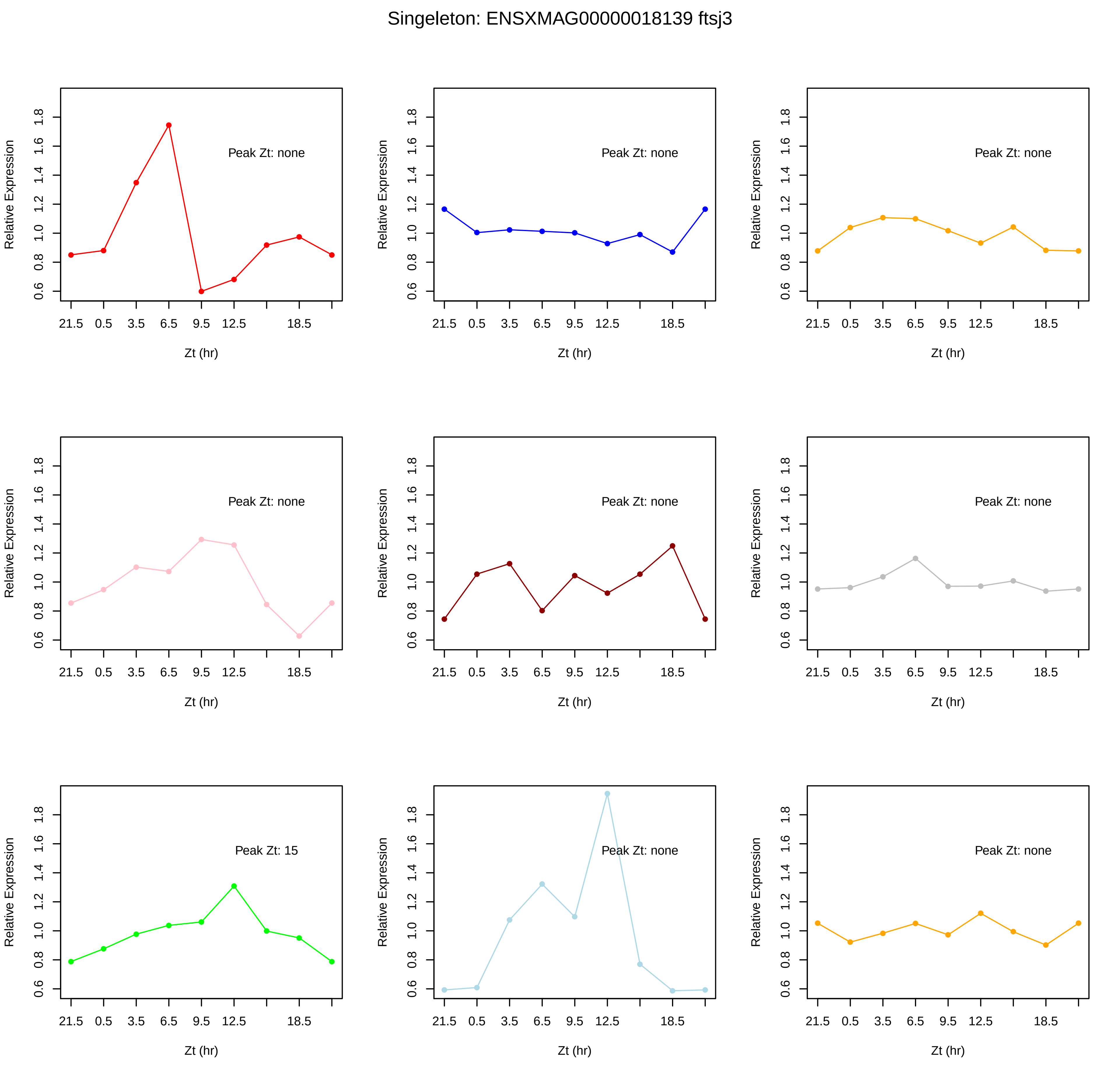

Singeleton: ENSXMAG00000018139 ftsj3
Peak Zt: none
Peak Zt: none
Peak Zt: none
Peak Zt: none
Peak Zt: none
Peak Zt: none
Peak Zt: 15
Peak Zt: none
Peak Zt: none
Relative Expression
Zt (hr)
21.5
0.5
3.5
6.5
9.5
12.5
18.5
0.6
0.8
1.0
1.2
1.4
1.6
1.8

Singleton: ENSXMAG00000015327 trim36

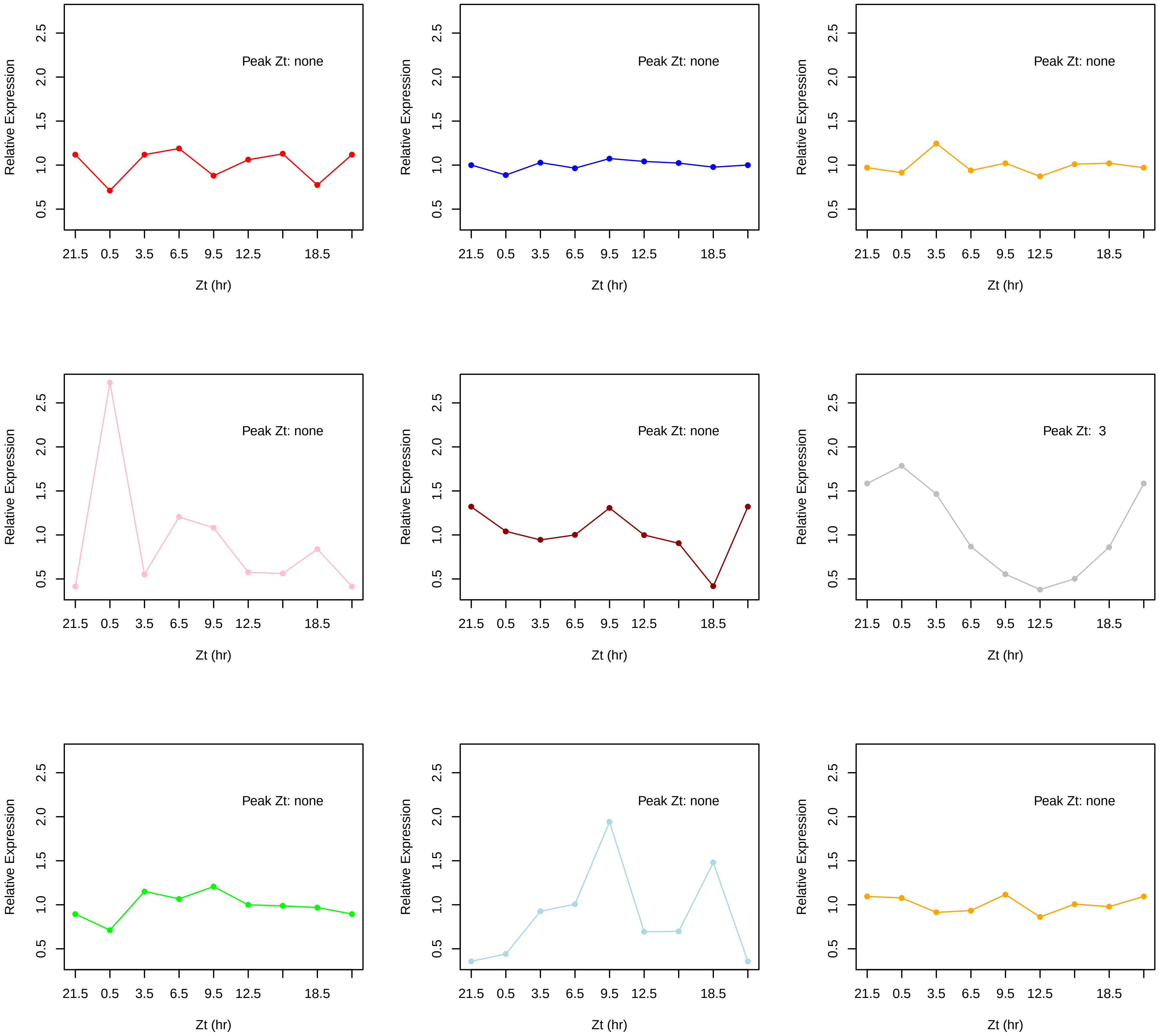

Singeleton: ENSXMAG00000021763

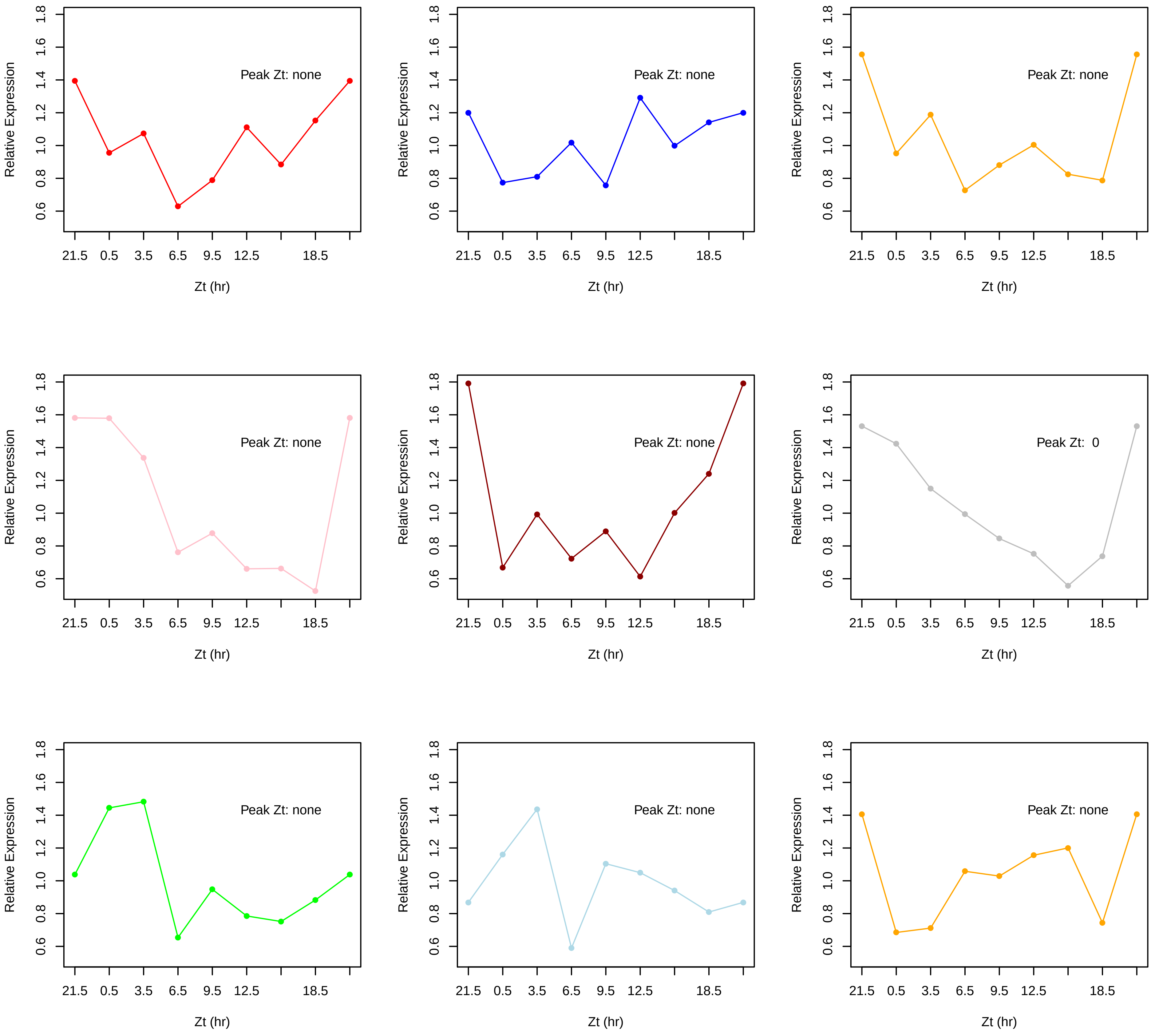

Singeleton: ENSXMAG00000009734 gab1

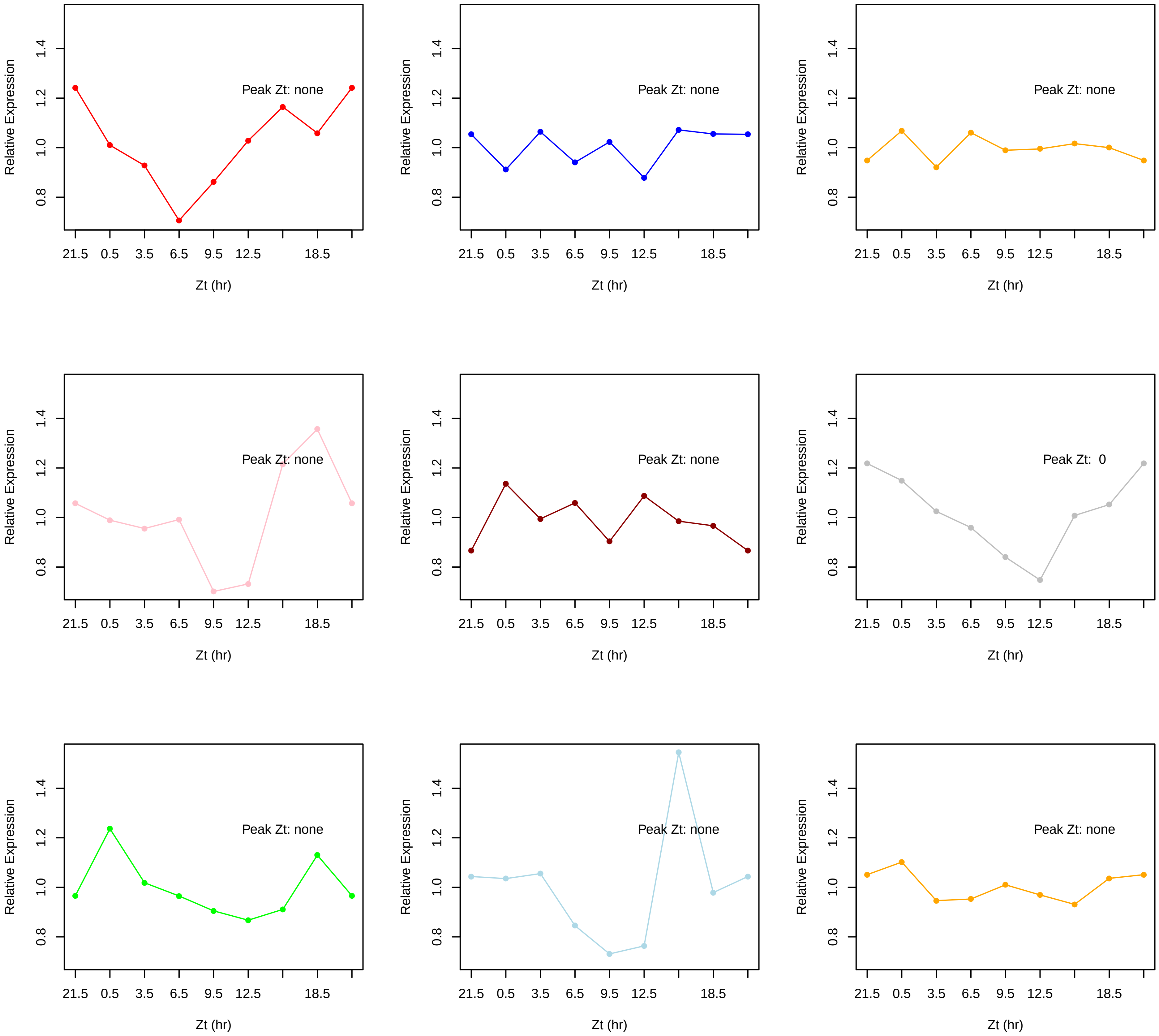

Singeleton: ENSXMAG00000010574 LTN1

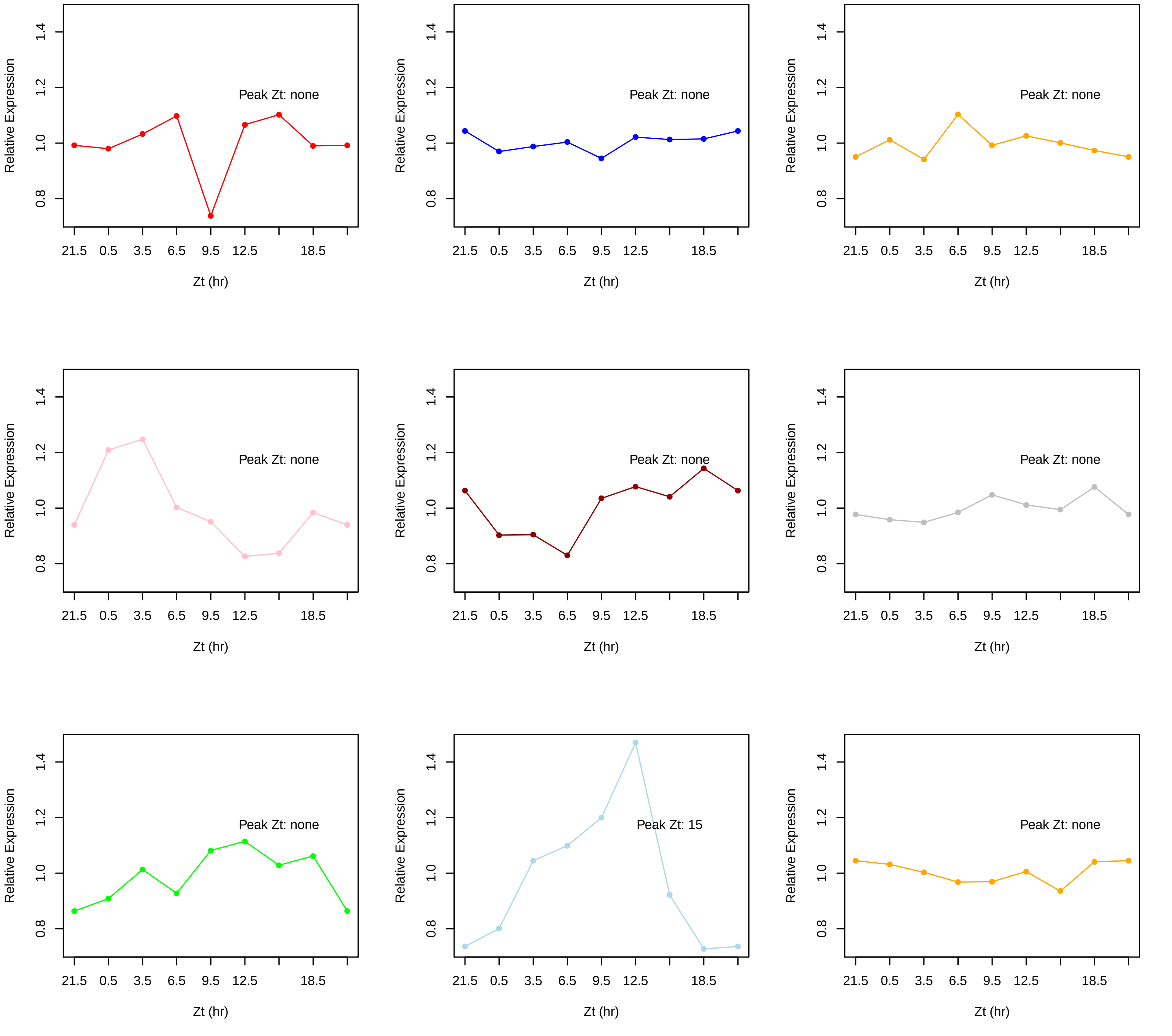

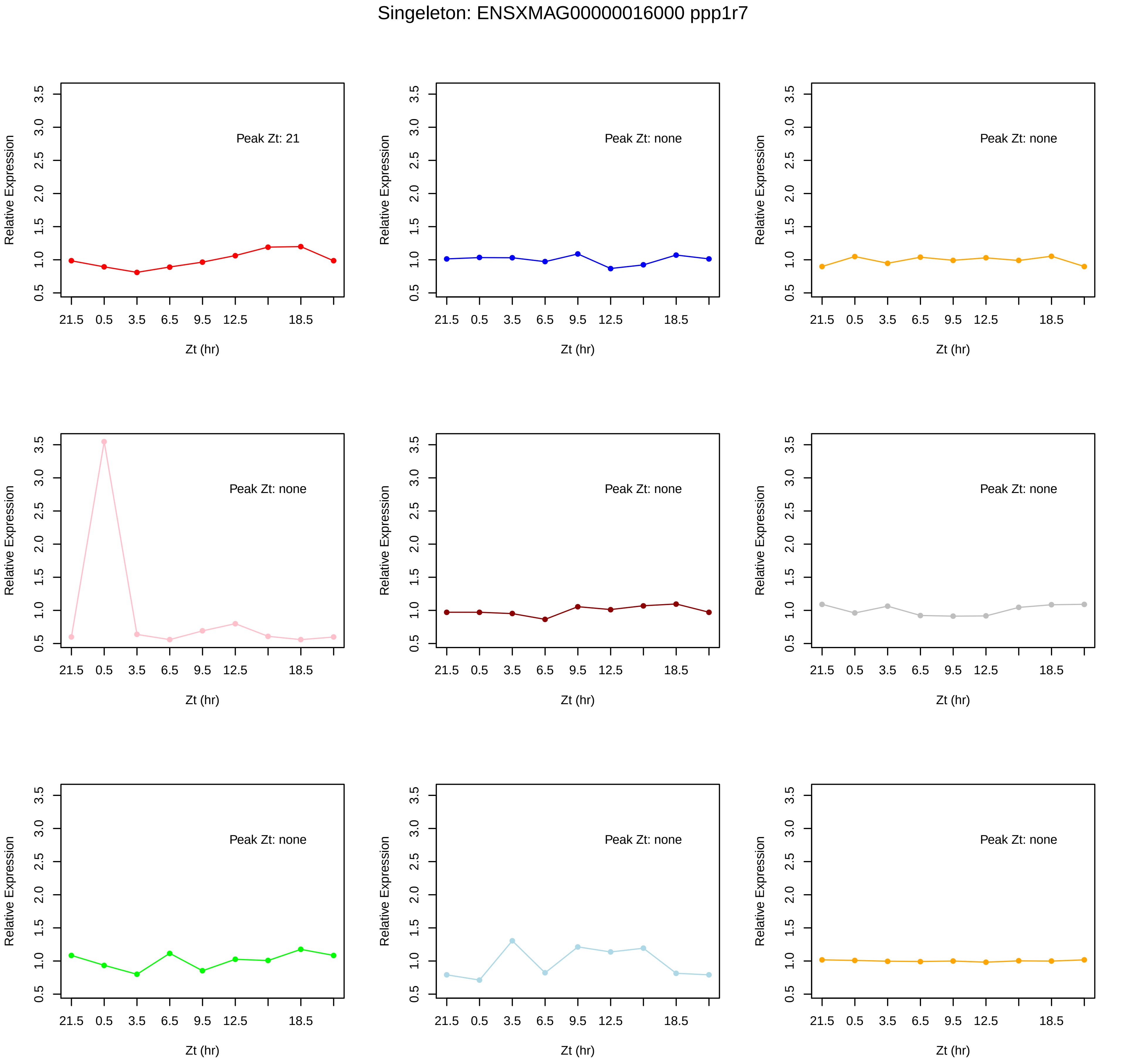
Singeleton: ENSXMAG00000016000 ppp1r7
Peak Zt: 21
Peak Zt: none
Peak Zt: none
Peak Zt: none
Peak Zt: none
Peak Zt: none
Peak Zt: none
Peak Zt: none
Peak Zt: none
Relative Expression
Zt (hr)
21.5
0.5
3.5
6.5
9.5
12.5
18.5
0.5
1.0
1.5
2.0
2.5
3.0
3.5

Singeleton: ENSXMAG00000019044 capn15

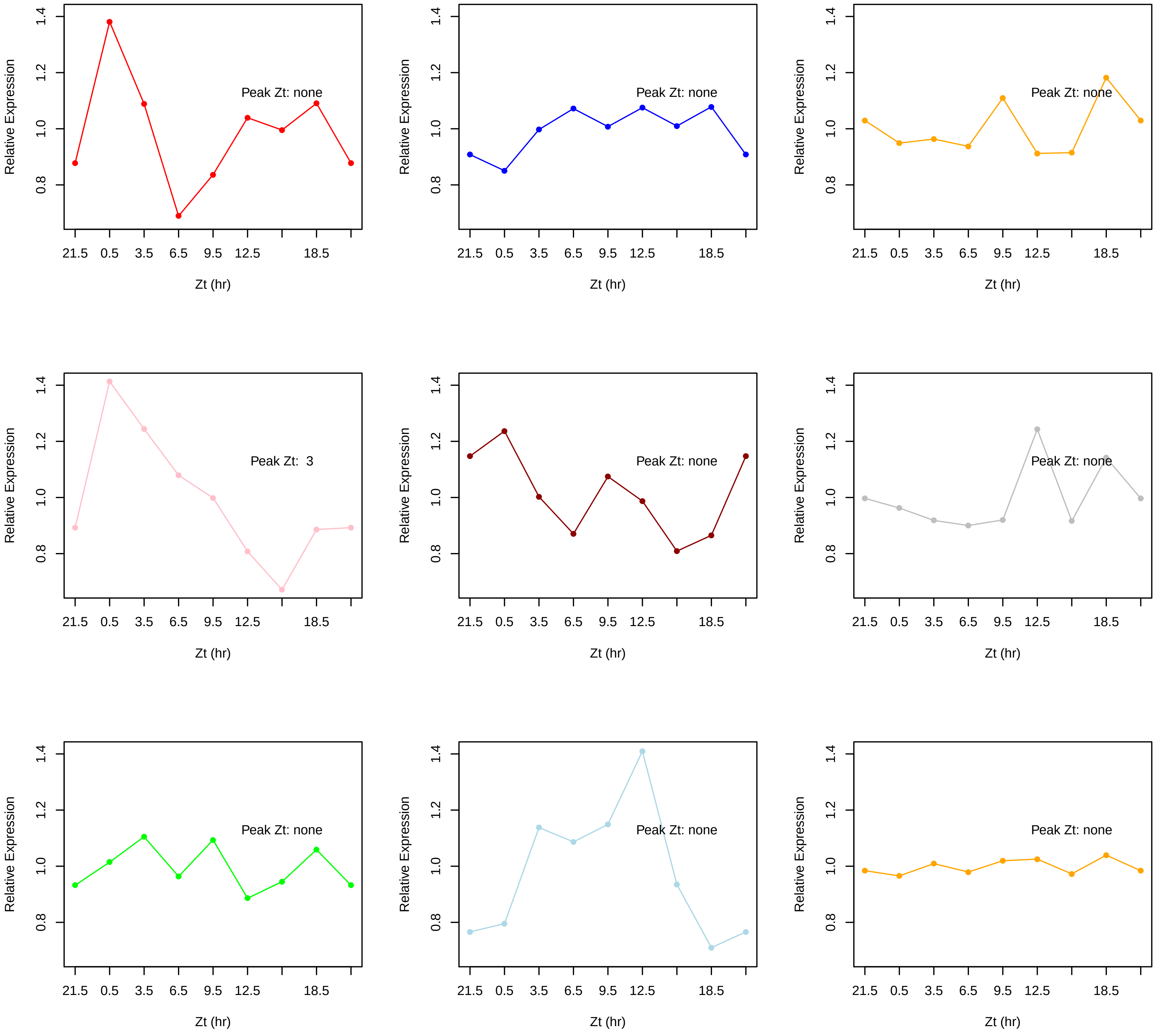

Singeleton: ENSXMAG00000013436 nr2f5

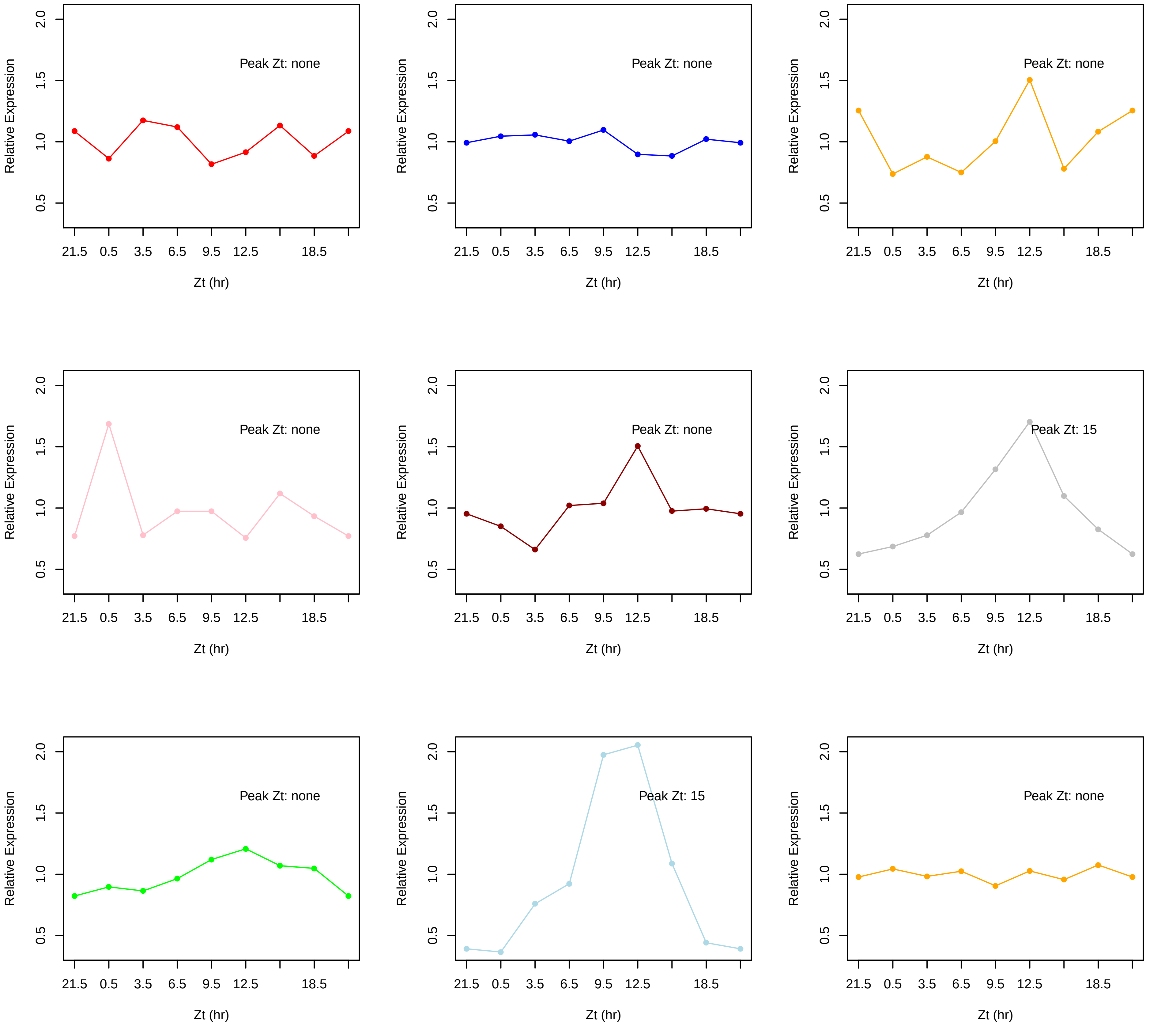

Singeleton: ENSXMAG00000013221

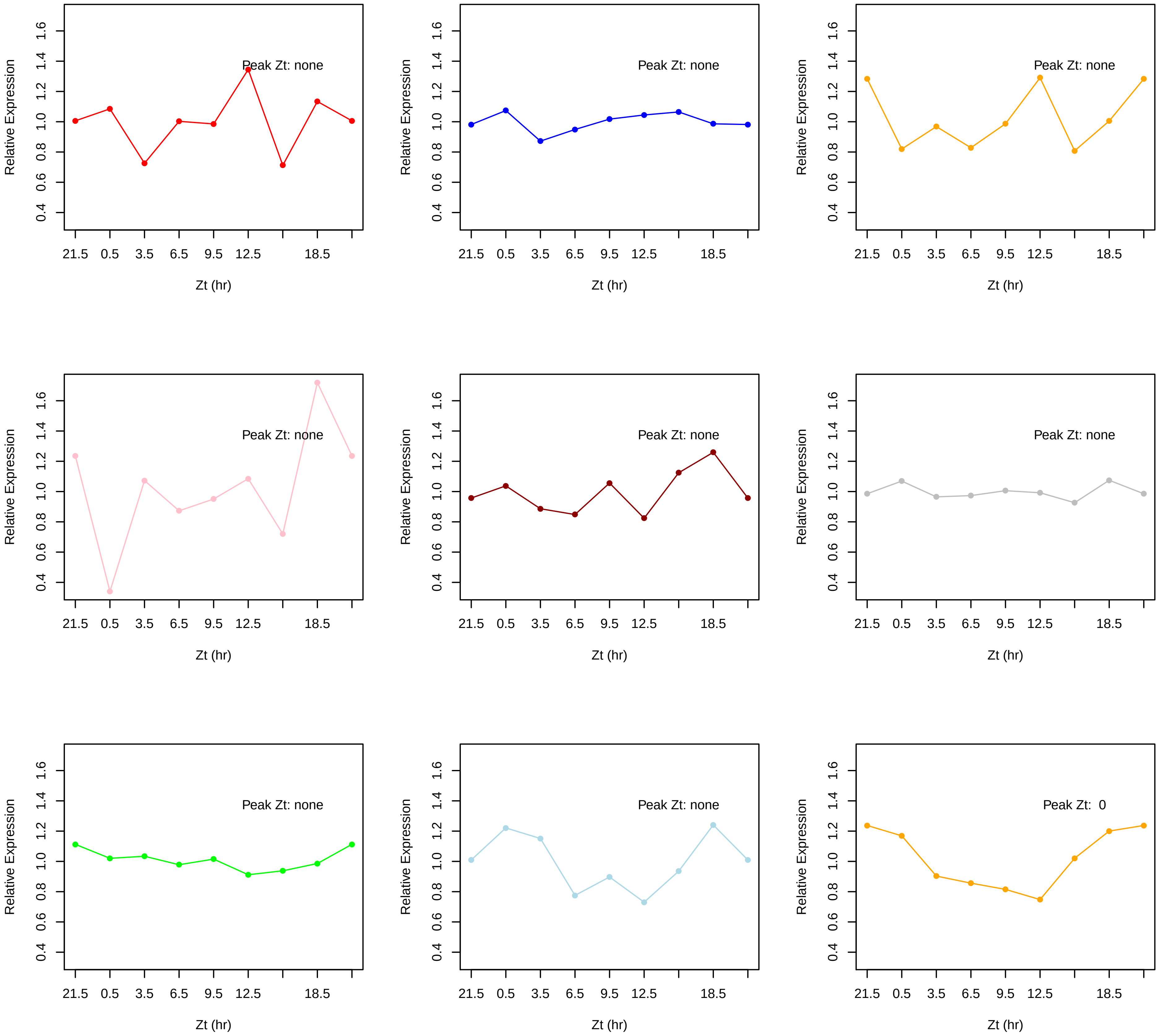

Singeleton: ENSXMAG00000001677 psmc1a

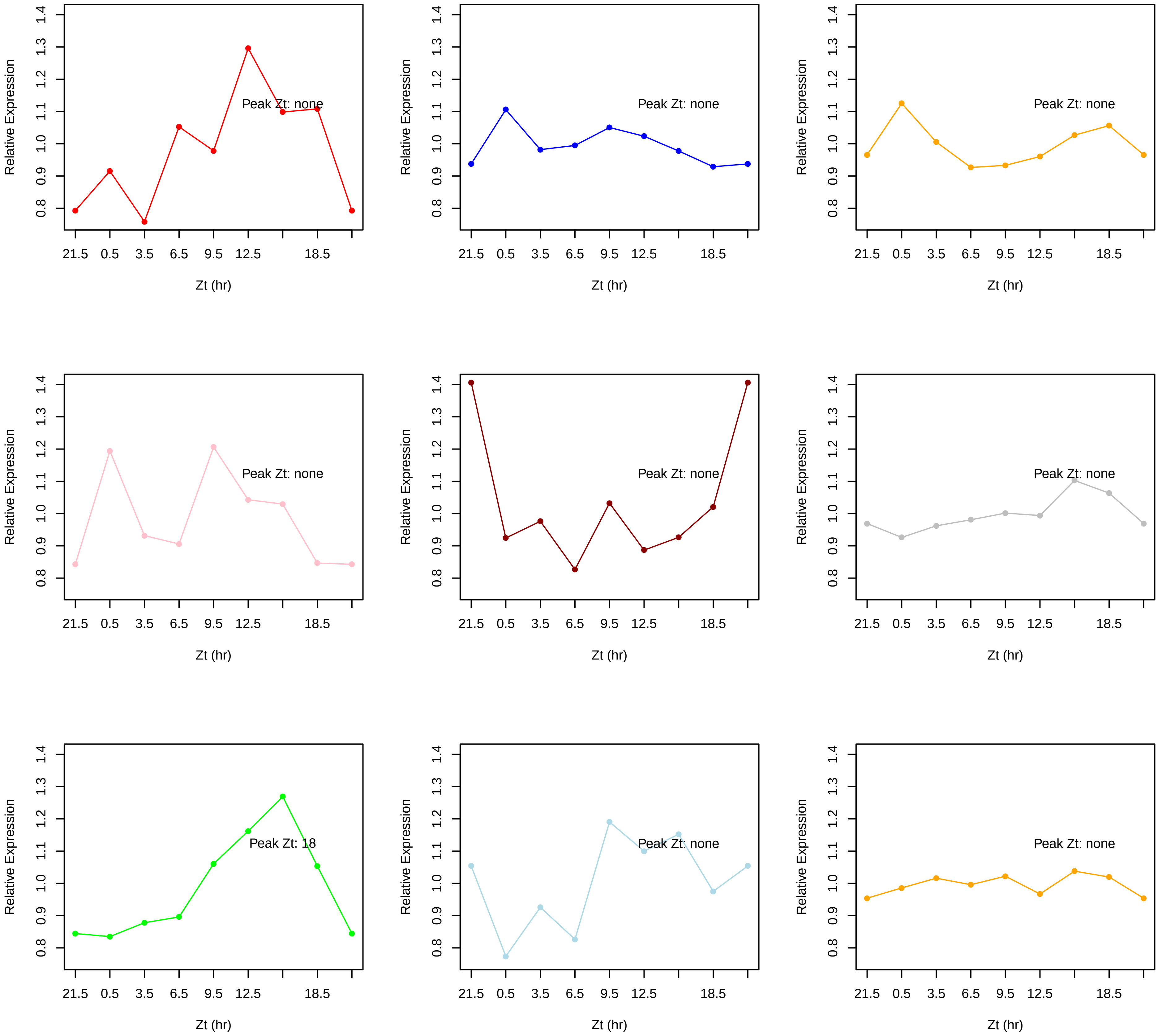

Singeleton: ENSXMAG00000009721 USP38

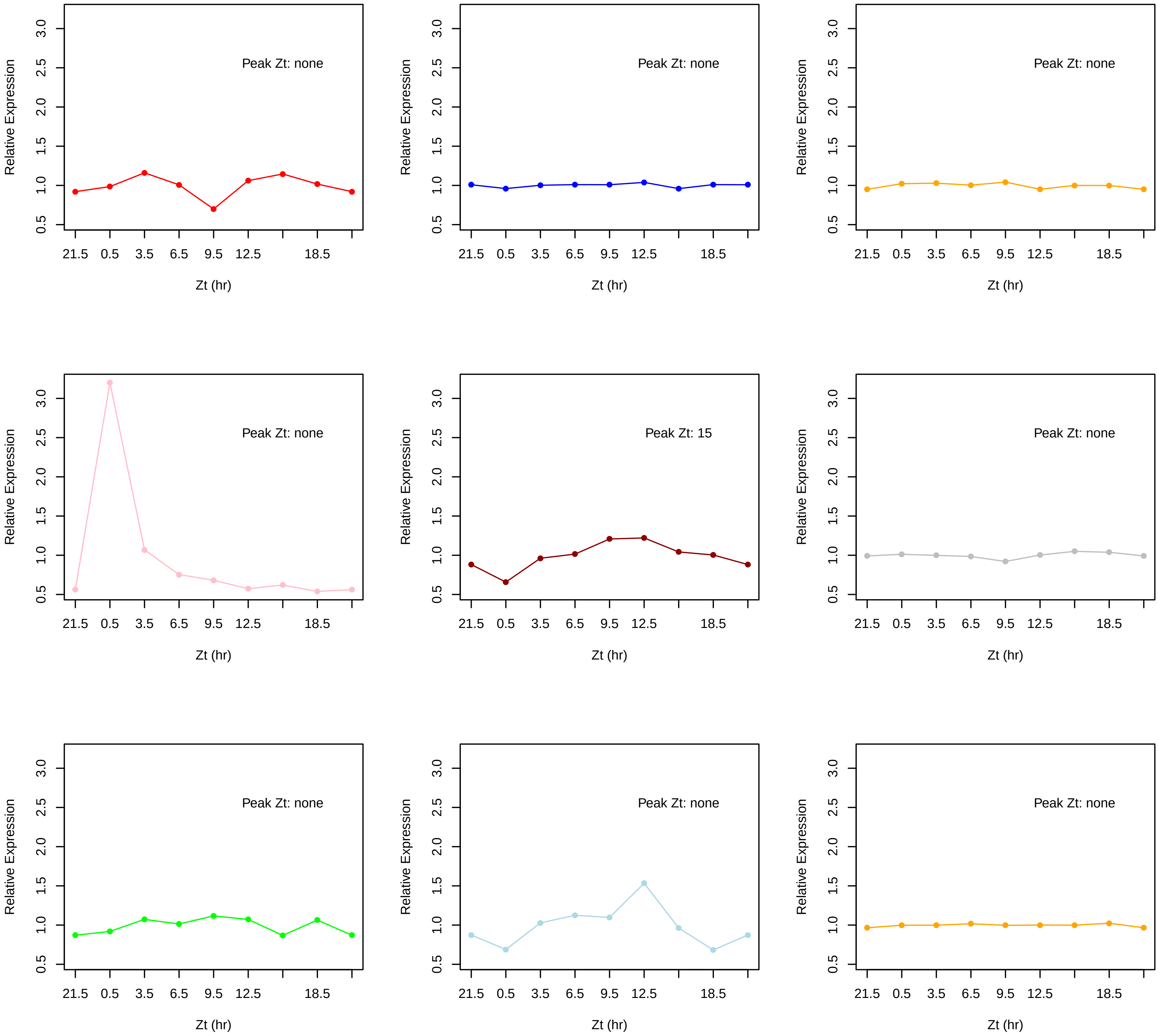

Singeleton: ENSXMAG00000007320 rwdd2b

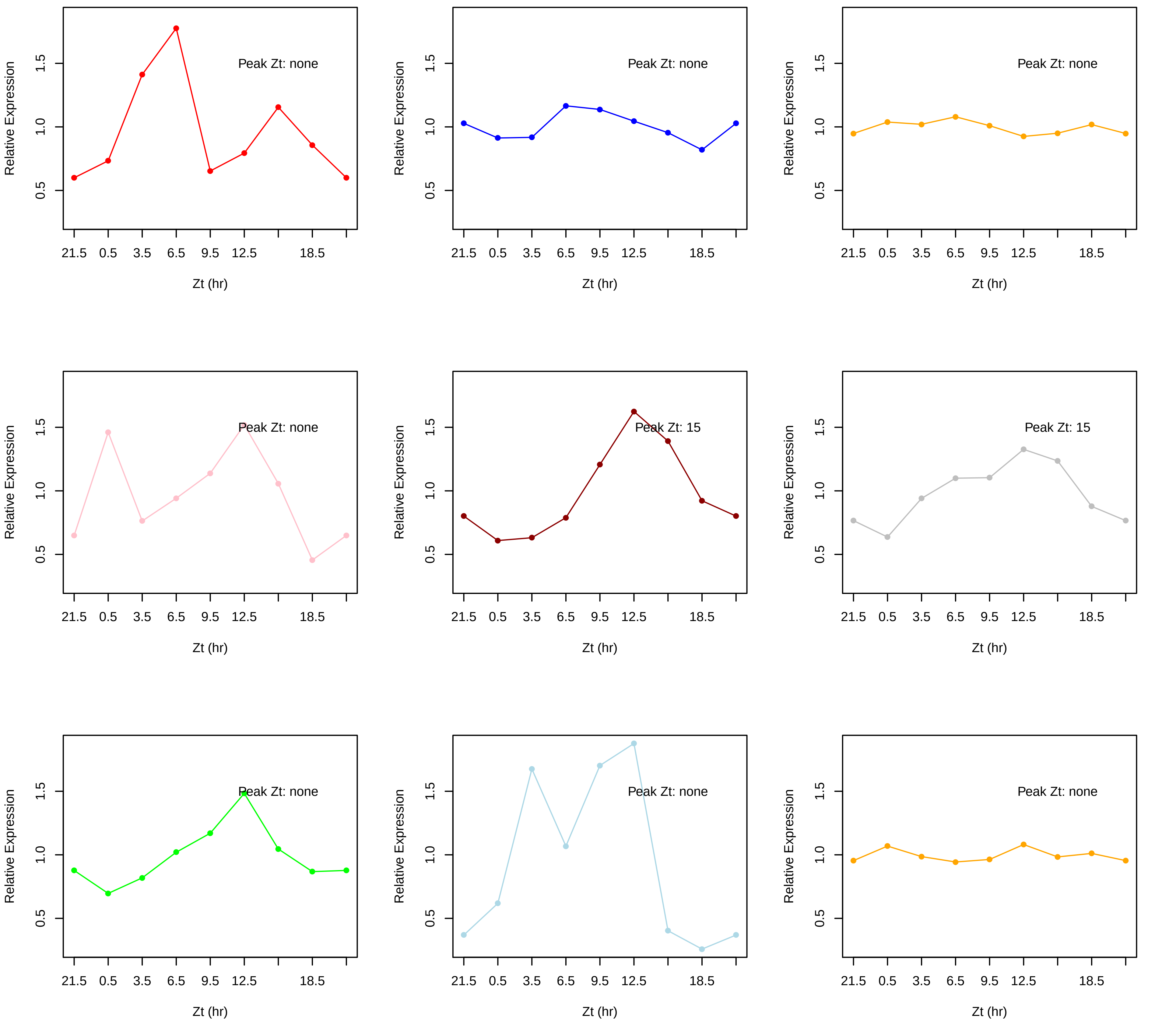

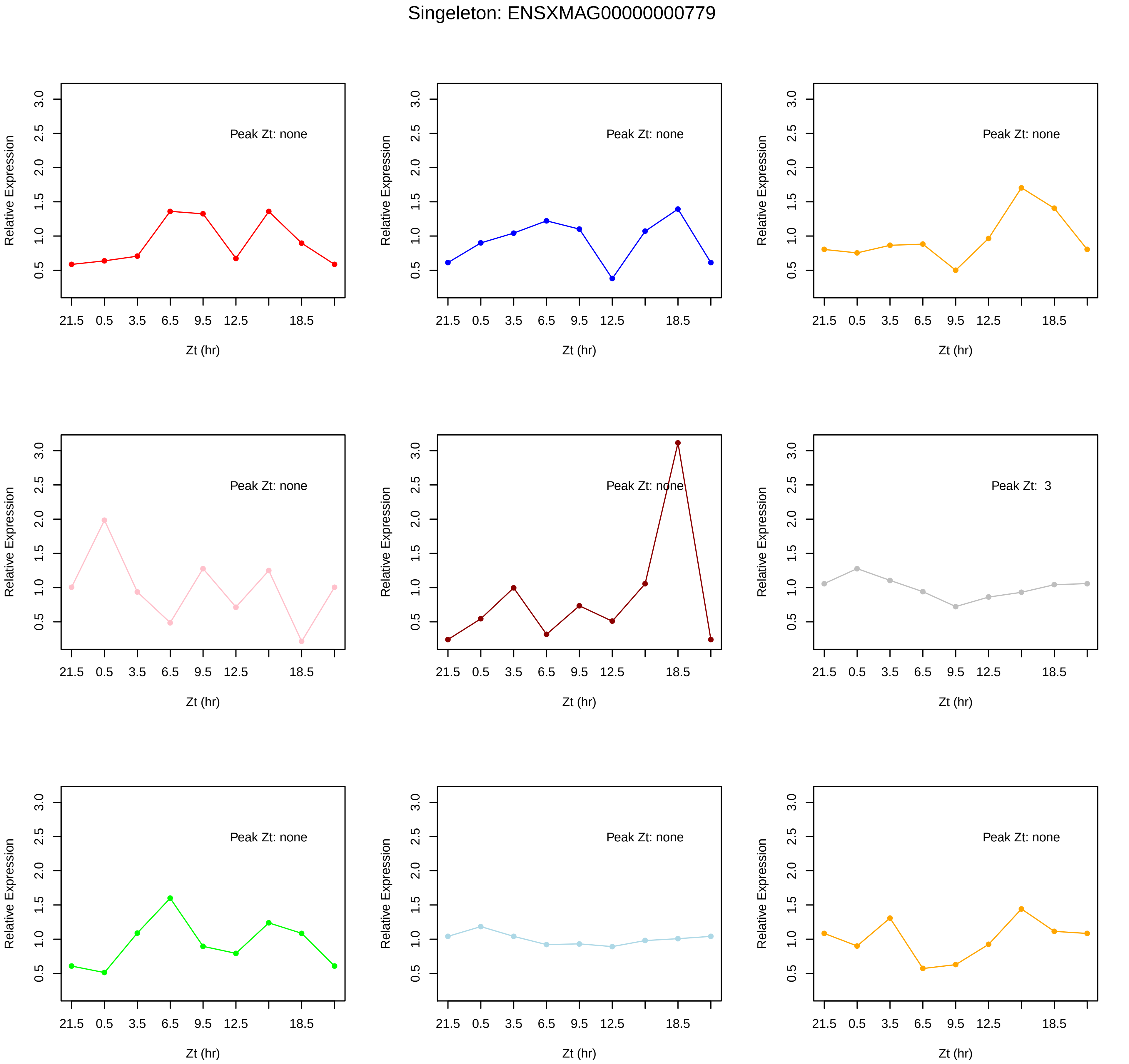
Singeleton: ENSXMAG00000000779
Peak Zt: none
Peak Zt: none
Peak Zt: none
Peak Zt: none
Peak Zt: none
Peak Zt: 3
Peak Zt: none
Peak Zt: none
Peak Zt: none
Relative Expression
Zt (hr)
21.5
0.5
3.5
6.5
9.5
12.5
18.5
0.5
1.0
1.5
2.0
2.5
3.0

Singleton: ENSXMAG00000021181 rab20

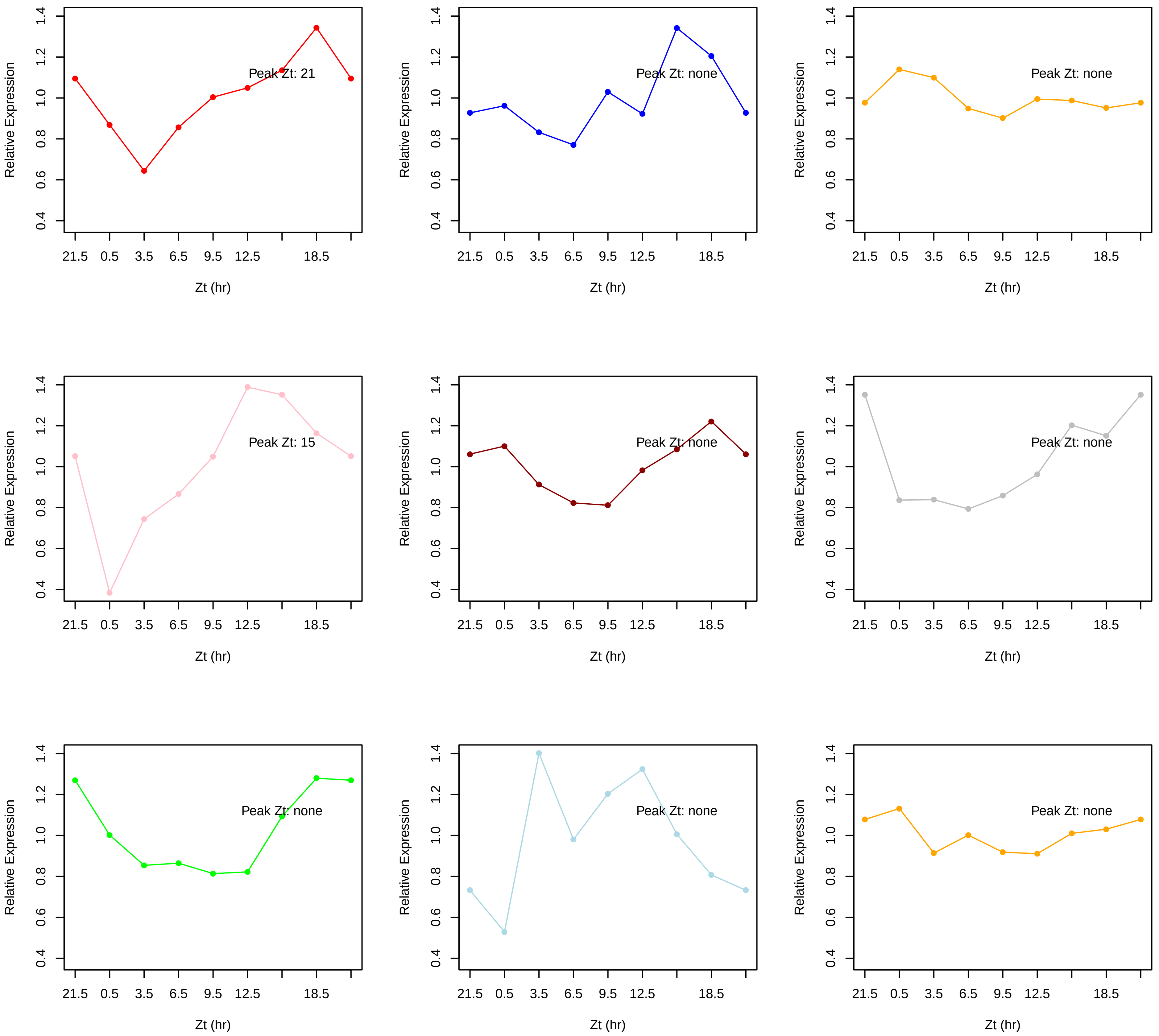

Singleton: ENSXMAG00000007314 usp16

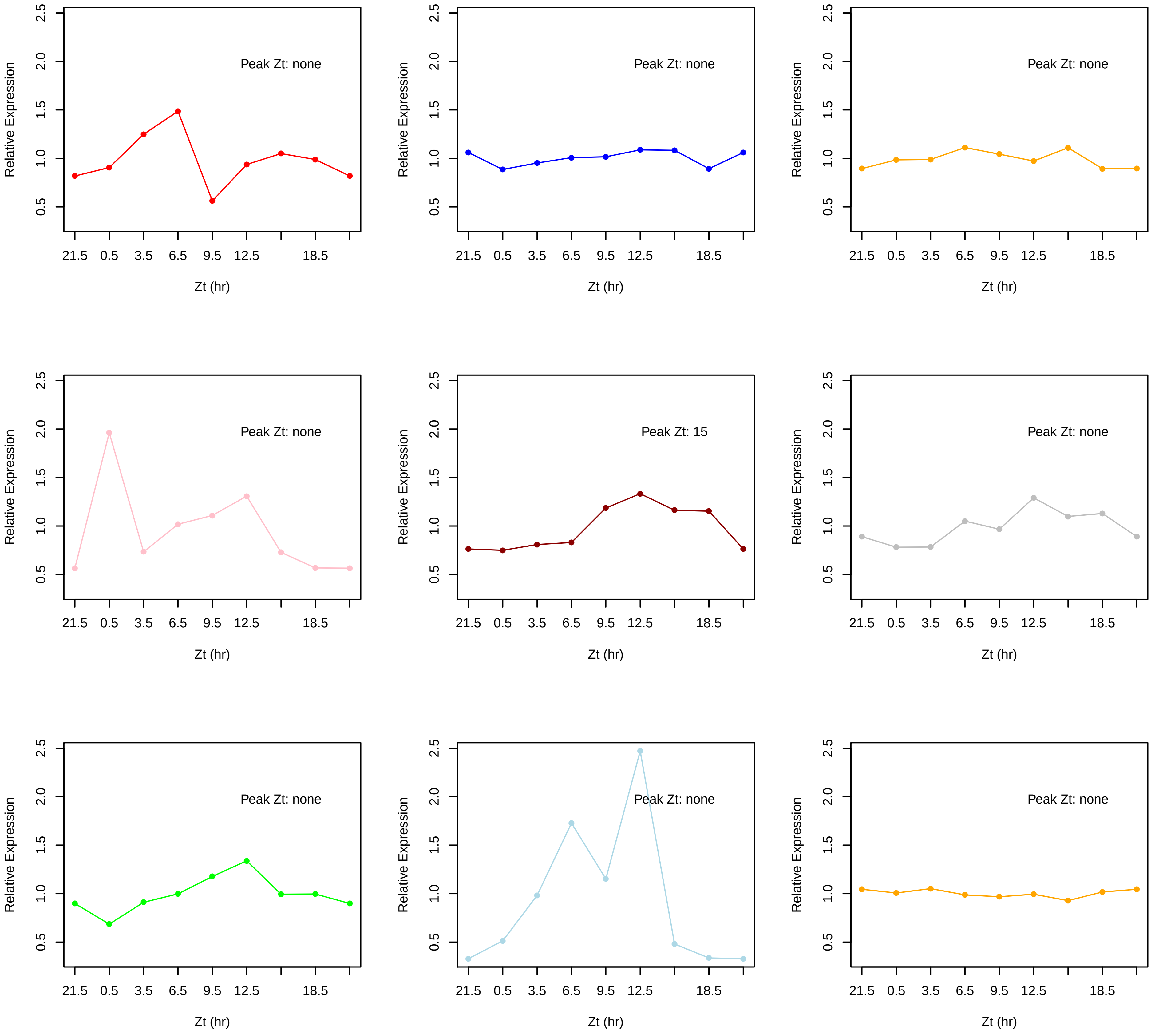

Singeleton: ENSXMAG00000012149 naxd

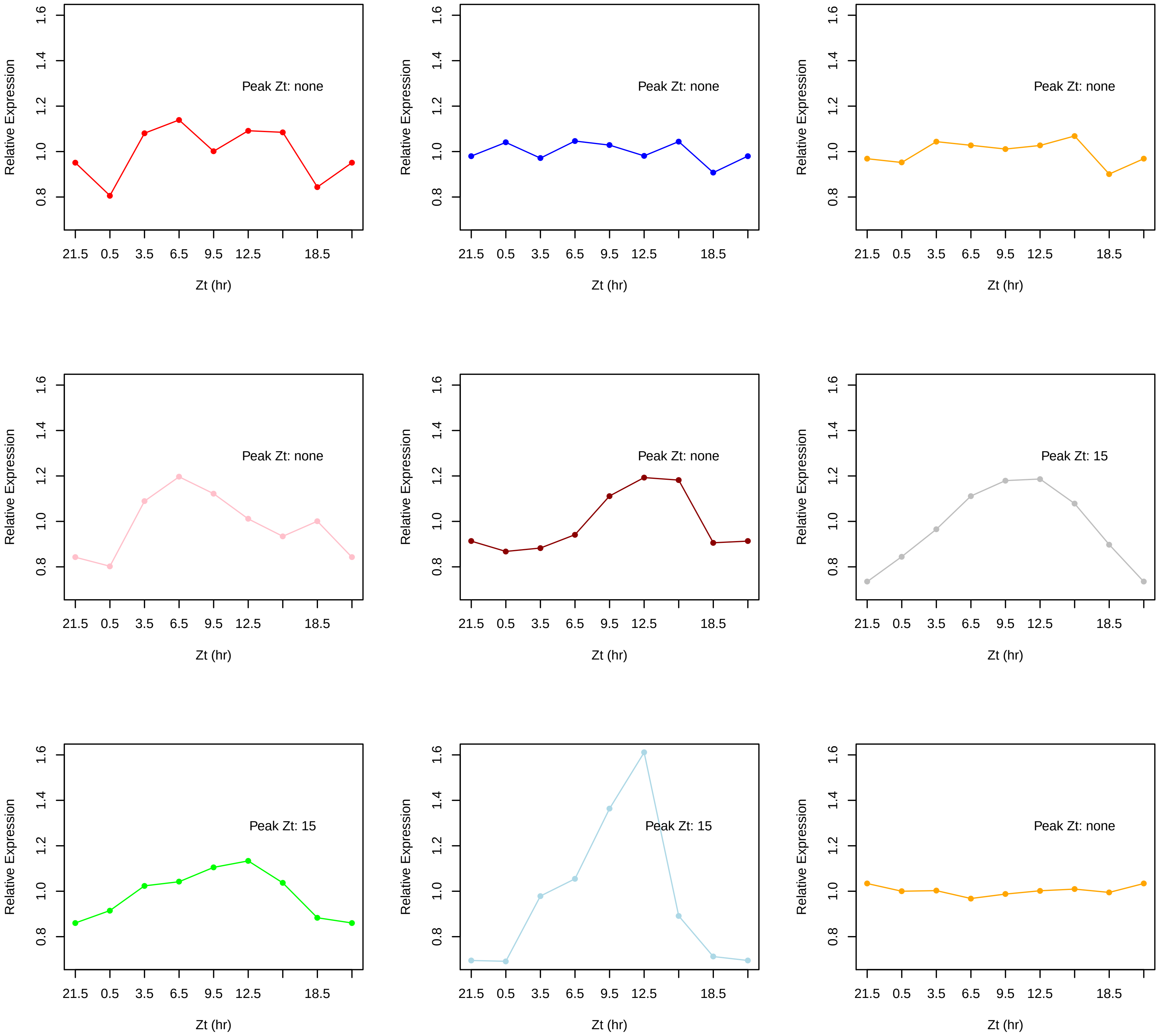

Singeleton: ENSXMAG00000023964 fam207a

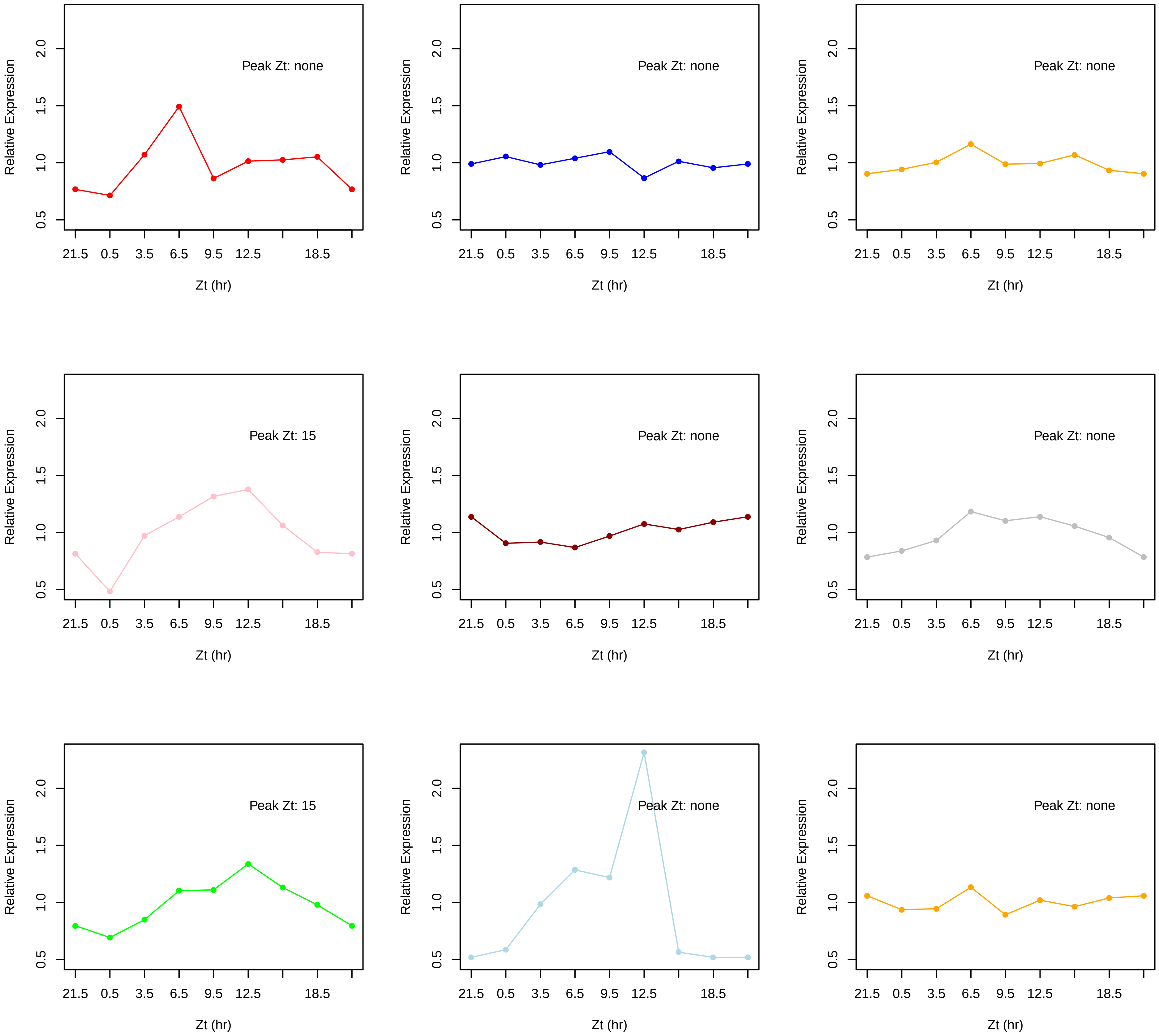

Singeleton: ENSXMAG00000001927 abcf2a

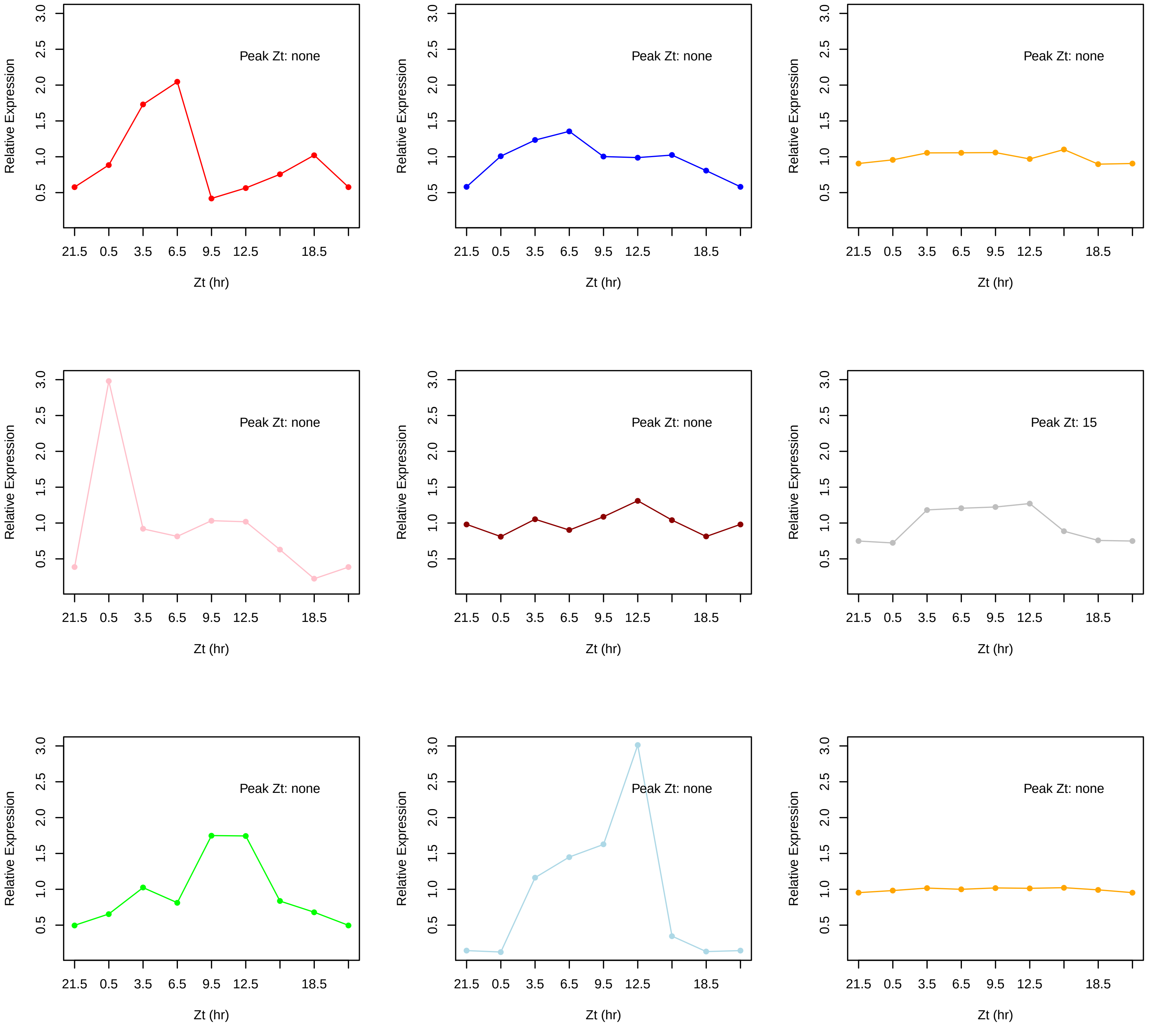

Singeleton: ENSXMAG00000029207

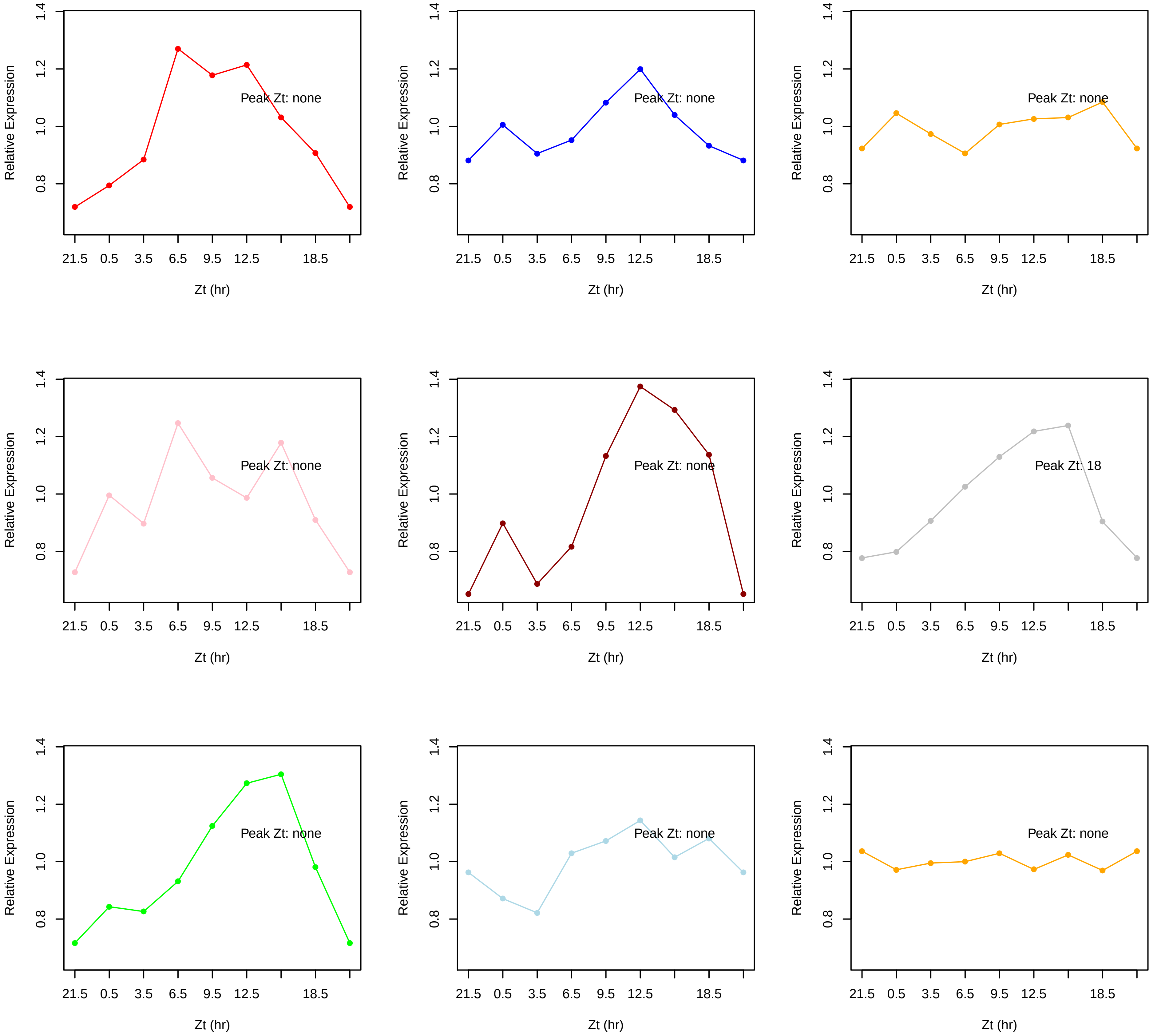

Singeleton: ENSXMAG00000012476 si:dkey−76b14.2

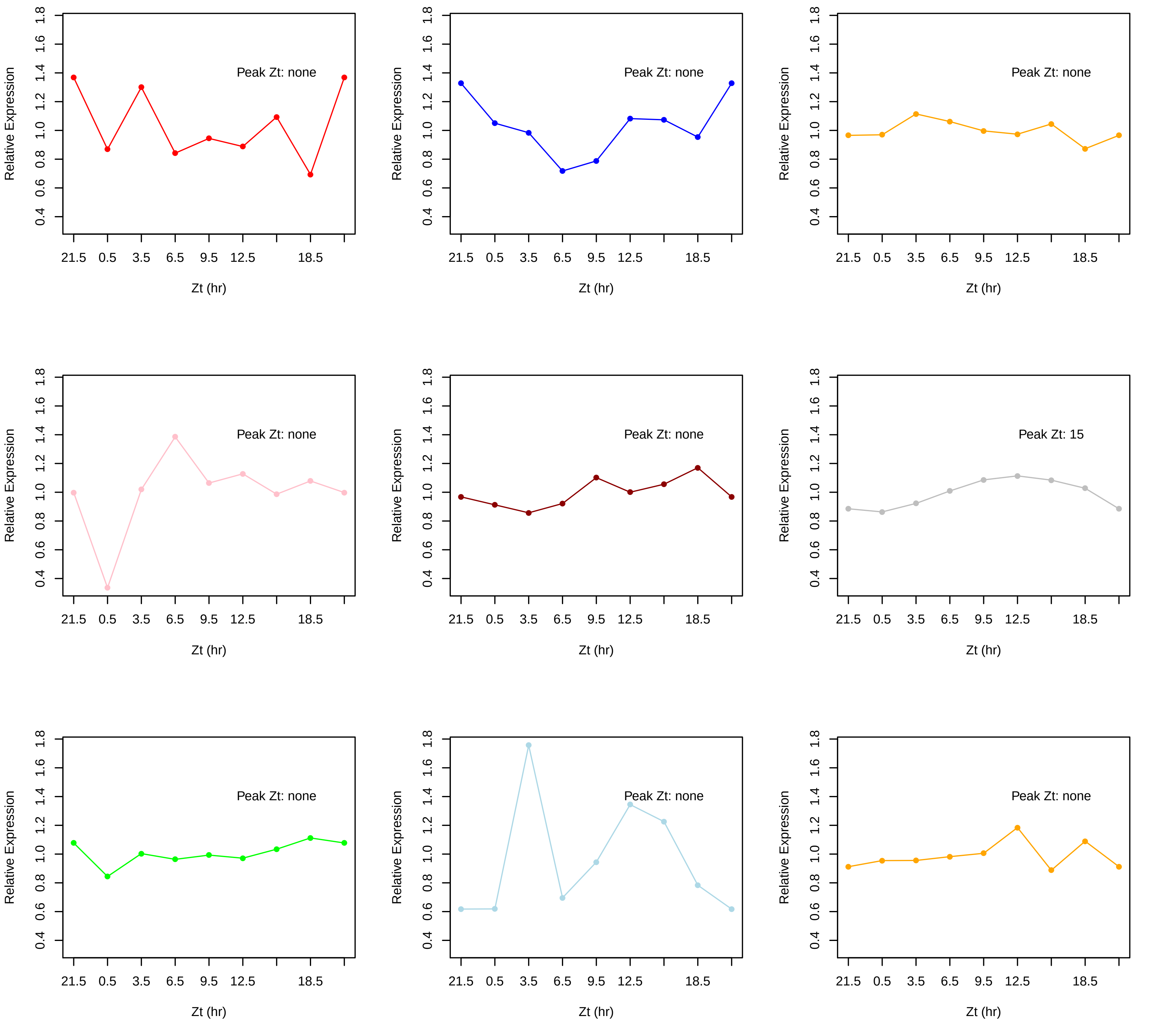

Singeleton: ENSXMAG00000017981

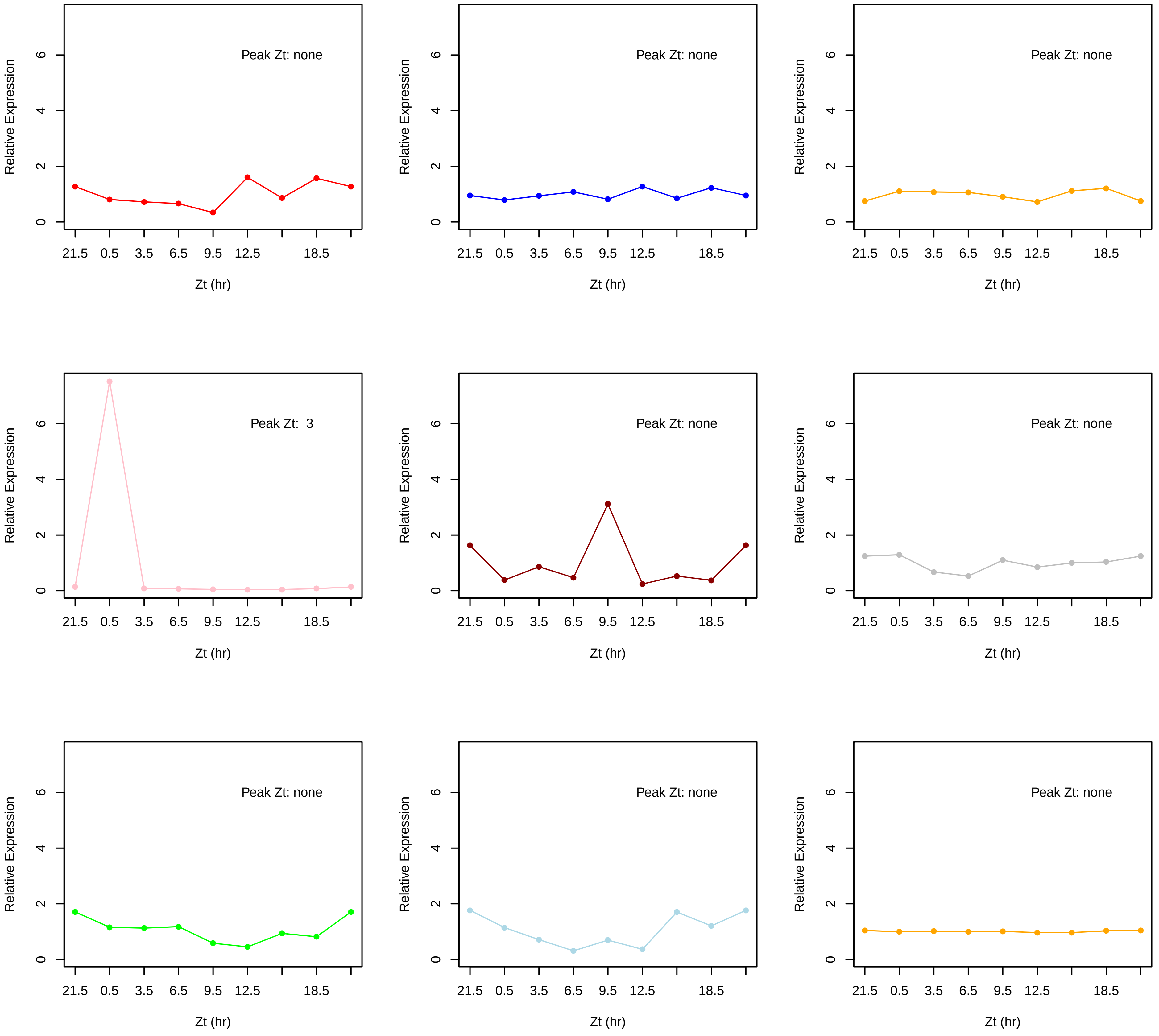

Singeleton: ENSXMAG00000003772 sf3b4

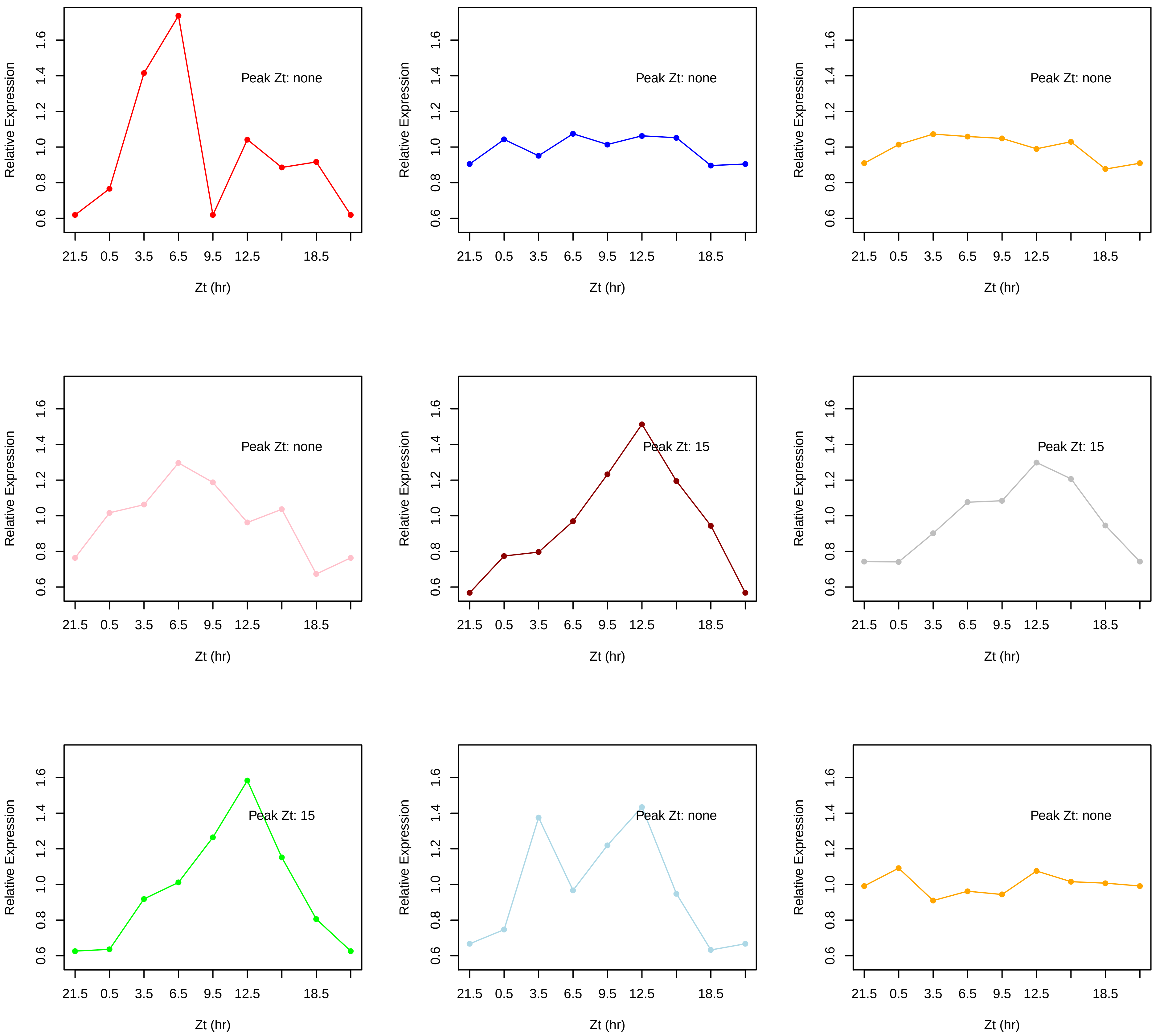

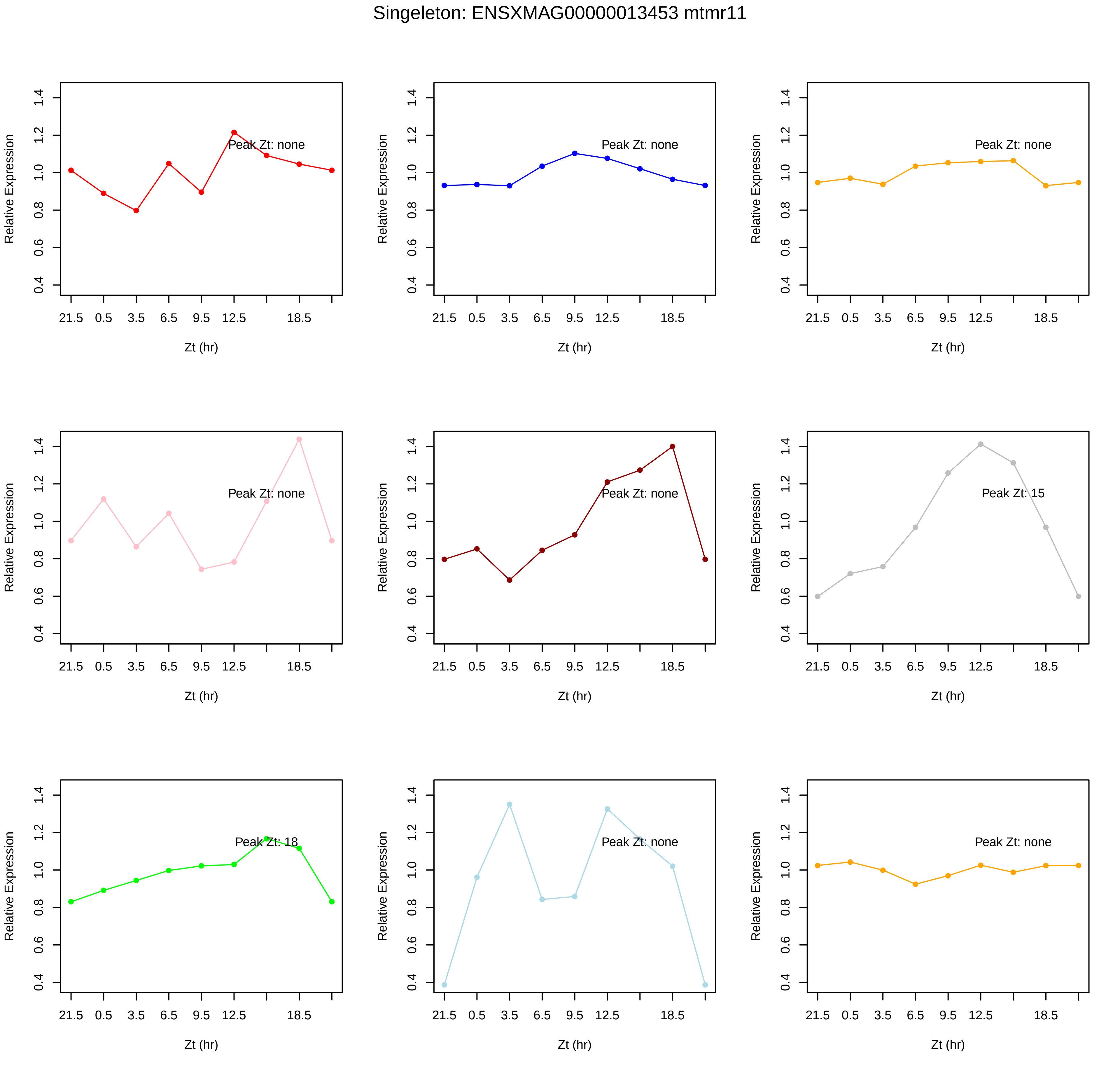
Singeleton: ENSXMAG00000013453 mtmr11
Relative Expression
Zt (hr)
21.5
0.5
3.5
6.5
9.5
12.5
18.5
0.4
0.6
0.8
1.0
1.2
1.4
Peak Zt: none
Peak Zt: 15
Peak Zt: 18

Singleton: ENSXMAG00000022630 smarcd3b

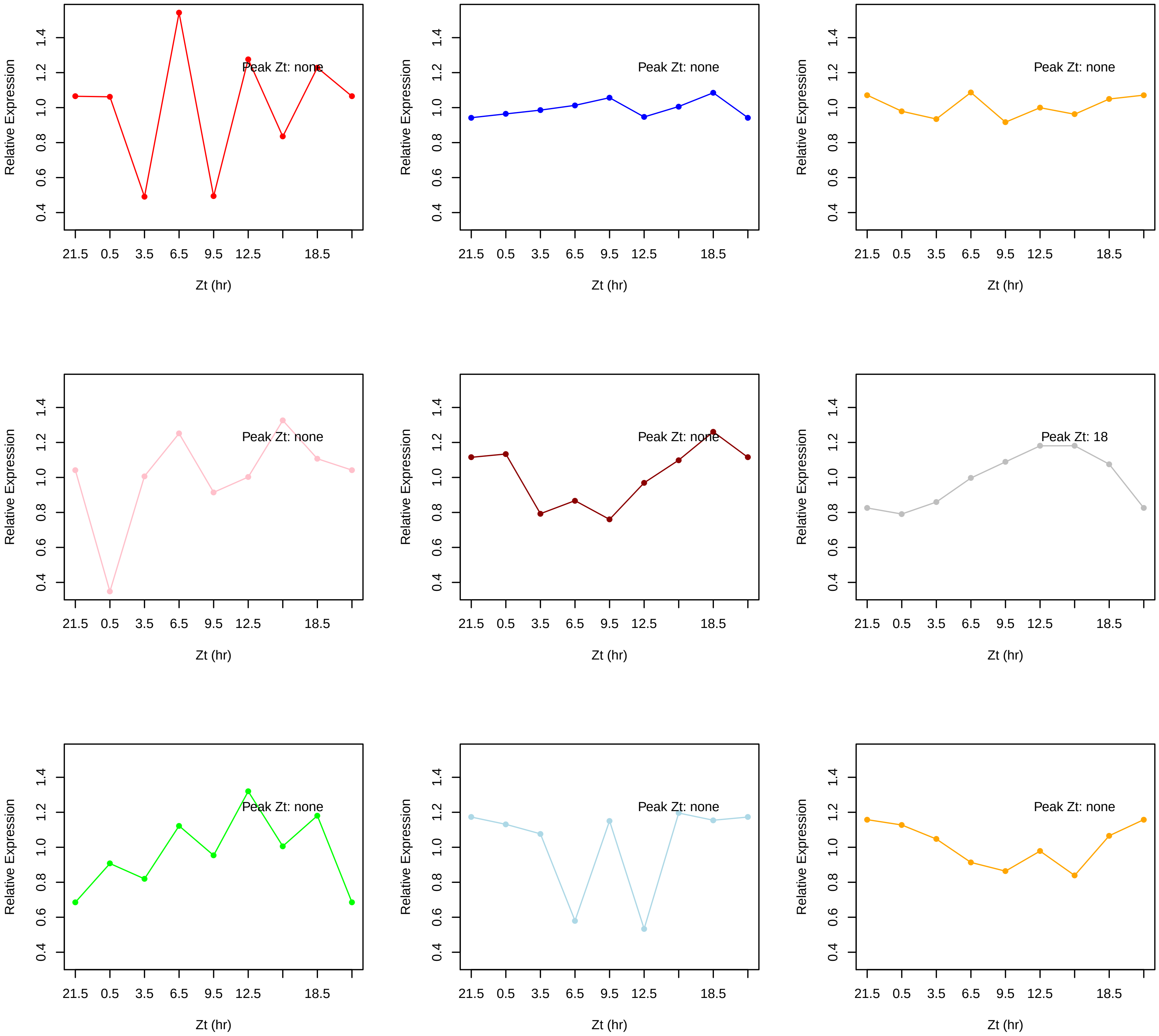

Singeleton: ENSXMAG00000002534 hps1

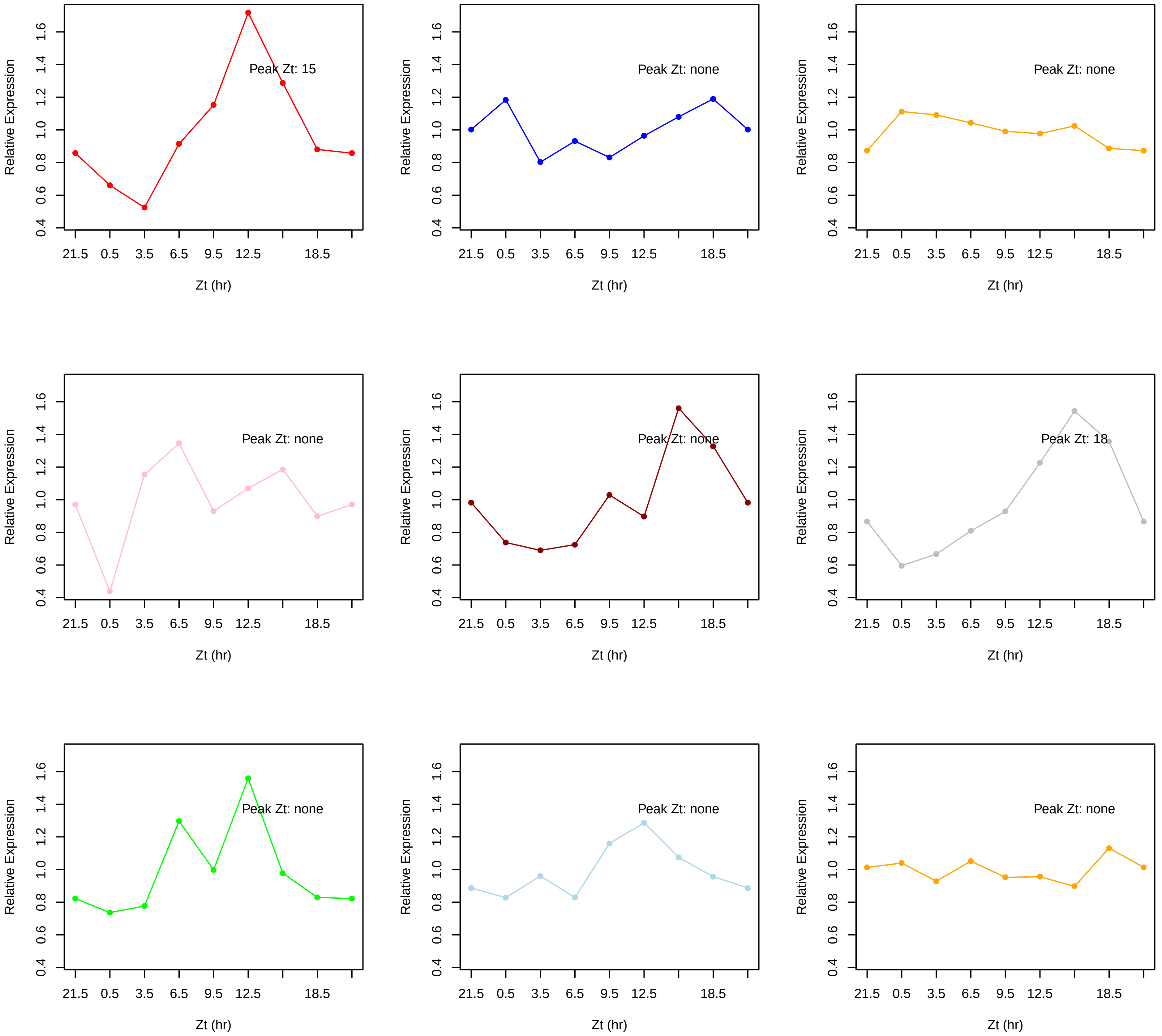

Singeleton: ENSXMAG00000002068 asb1

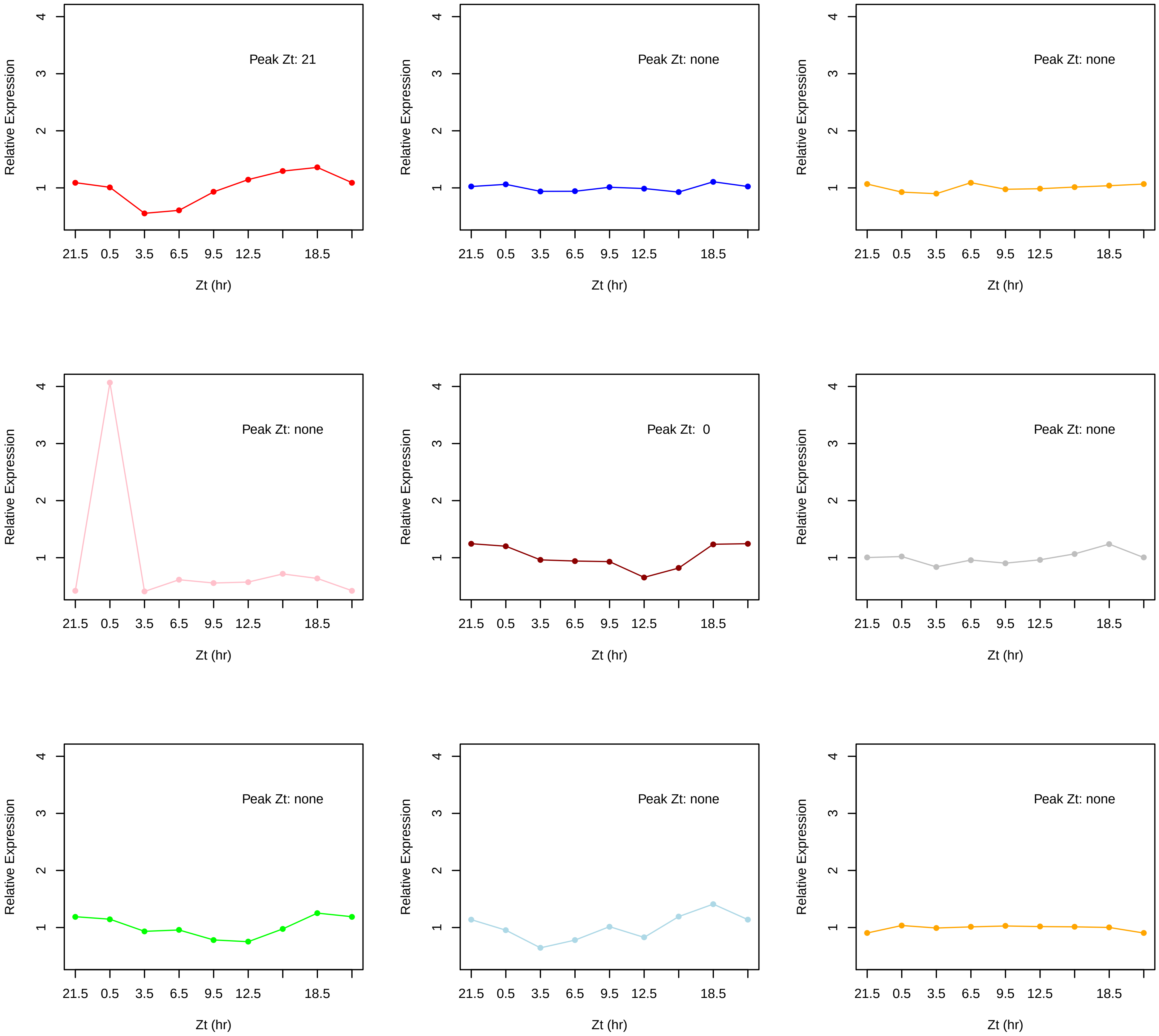

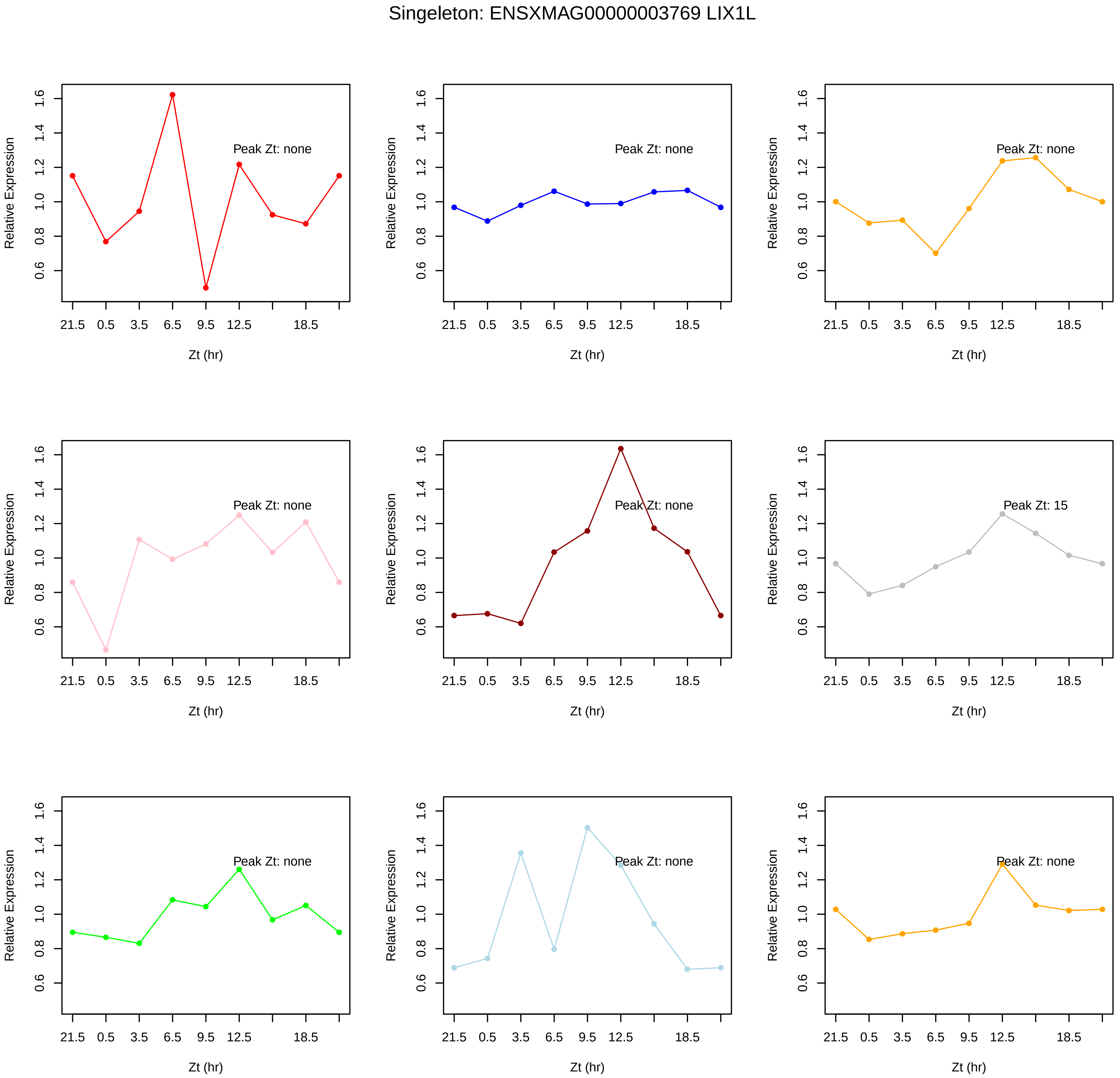
Singleton: ENSXMAG00000003769 LIX1L
Relative Expression
Zt (hr)
21.5 0.5 3.5 6.5 9.5 12.5 18.5
0.6 0.8 1.0 1.2 1.4 1.6
Peak Zt: none
Peak Zt: none
Peak Zt: none
Peak Zt: none
Peak Zt: none
Peak Zt: 15
Peak Zt: none
Peak Zt: none
Peak Zt: none

Singeleton: ENSXMAG00000009805 tbc1d9

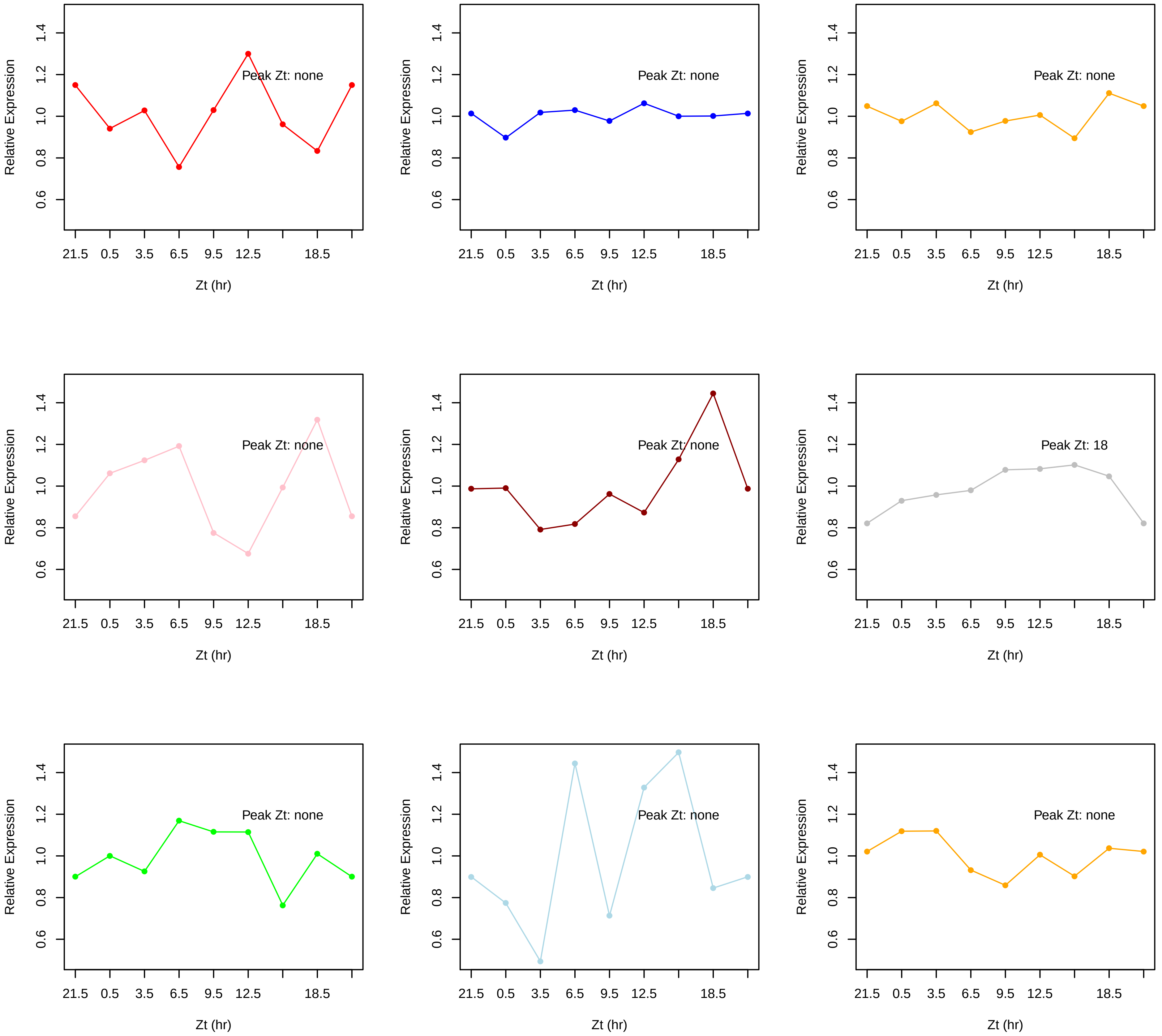

Singleton: ENSXMAG00000021827

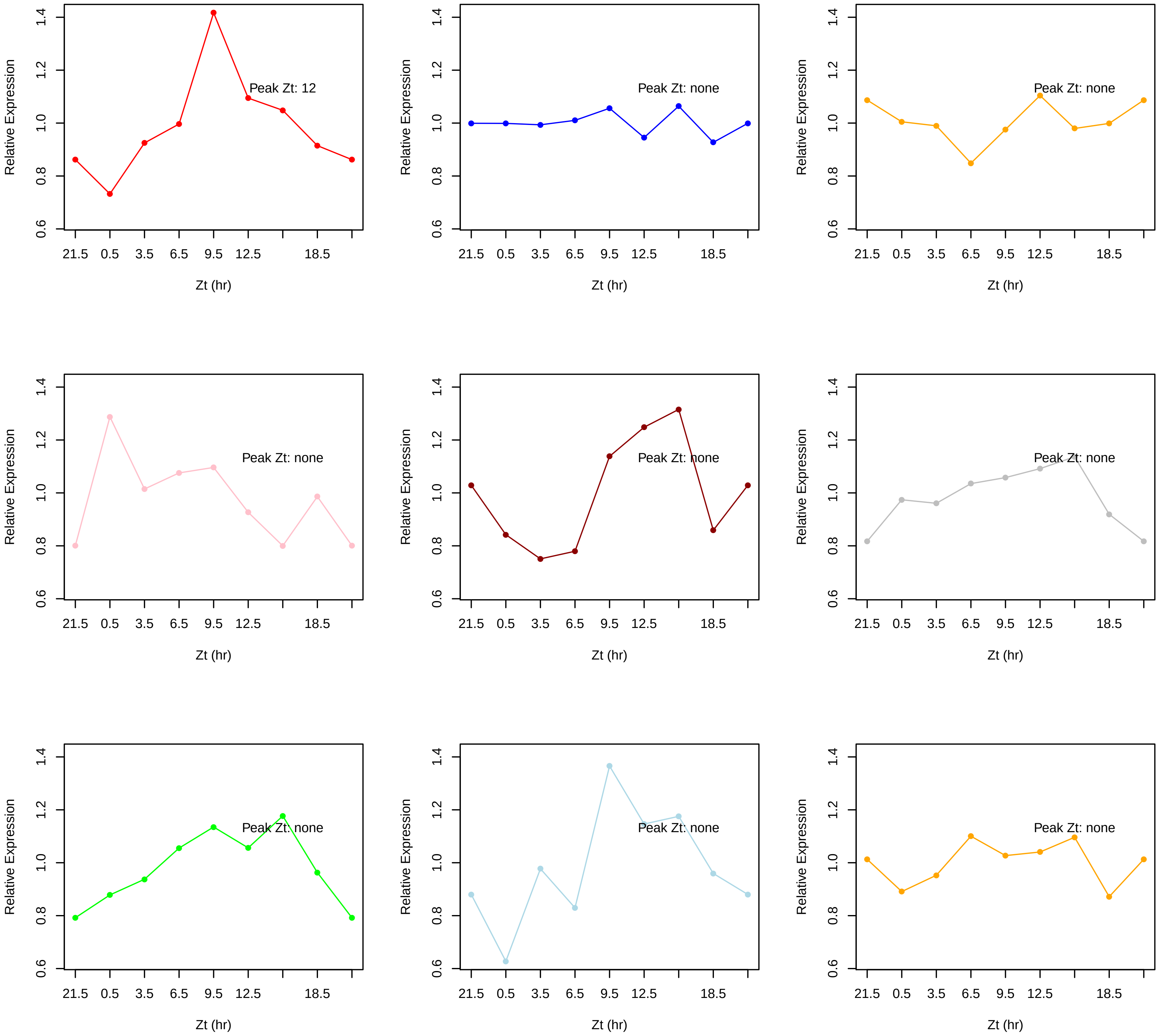

Singleton: ENSXMAG00000014037

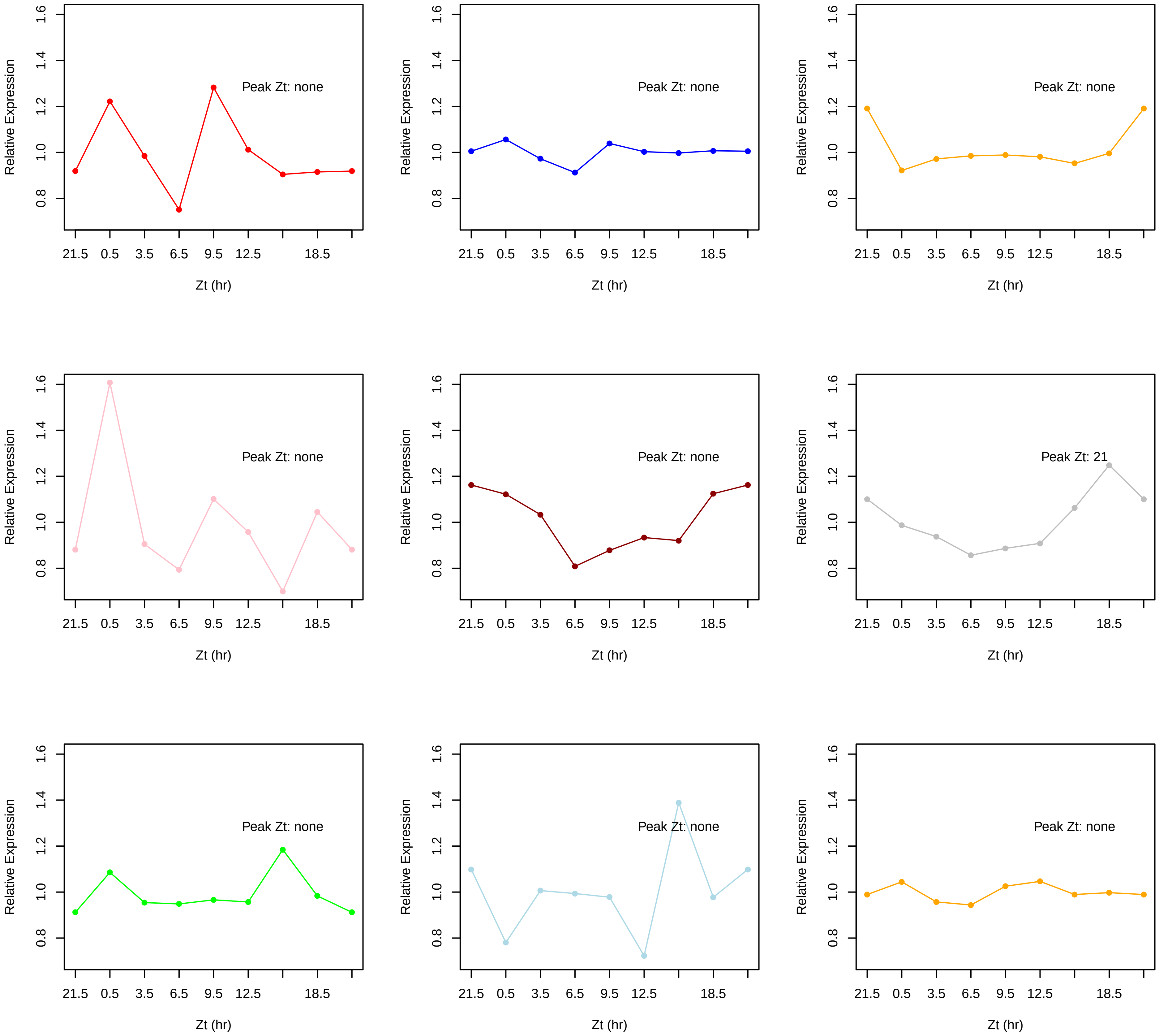

Singeleton: ENSXMAG00000006424 atpaf1

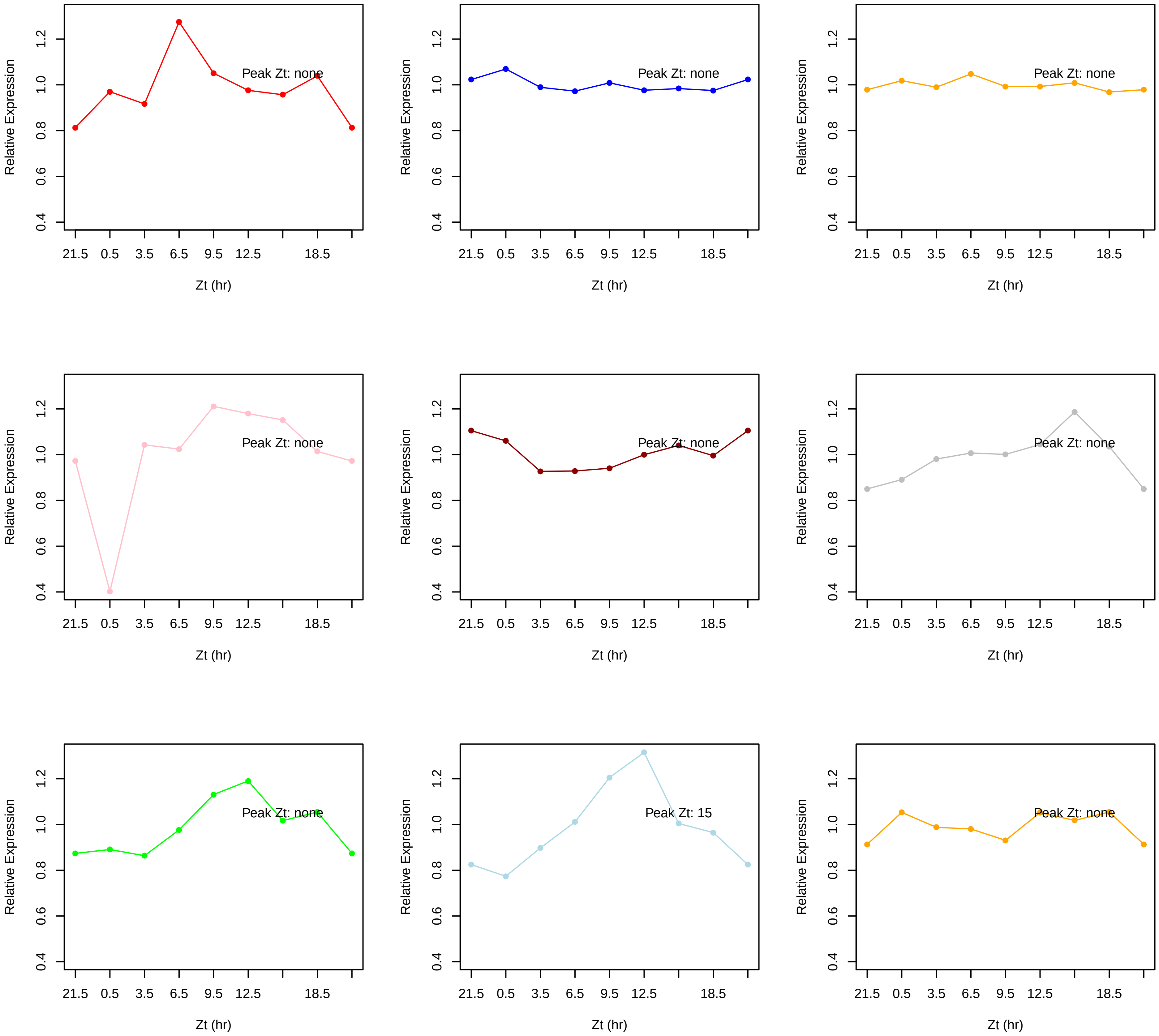

Singeleton: ENSXMAG00000008199 DDX21

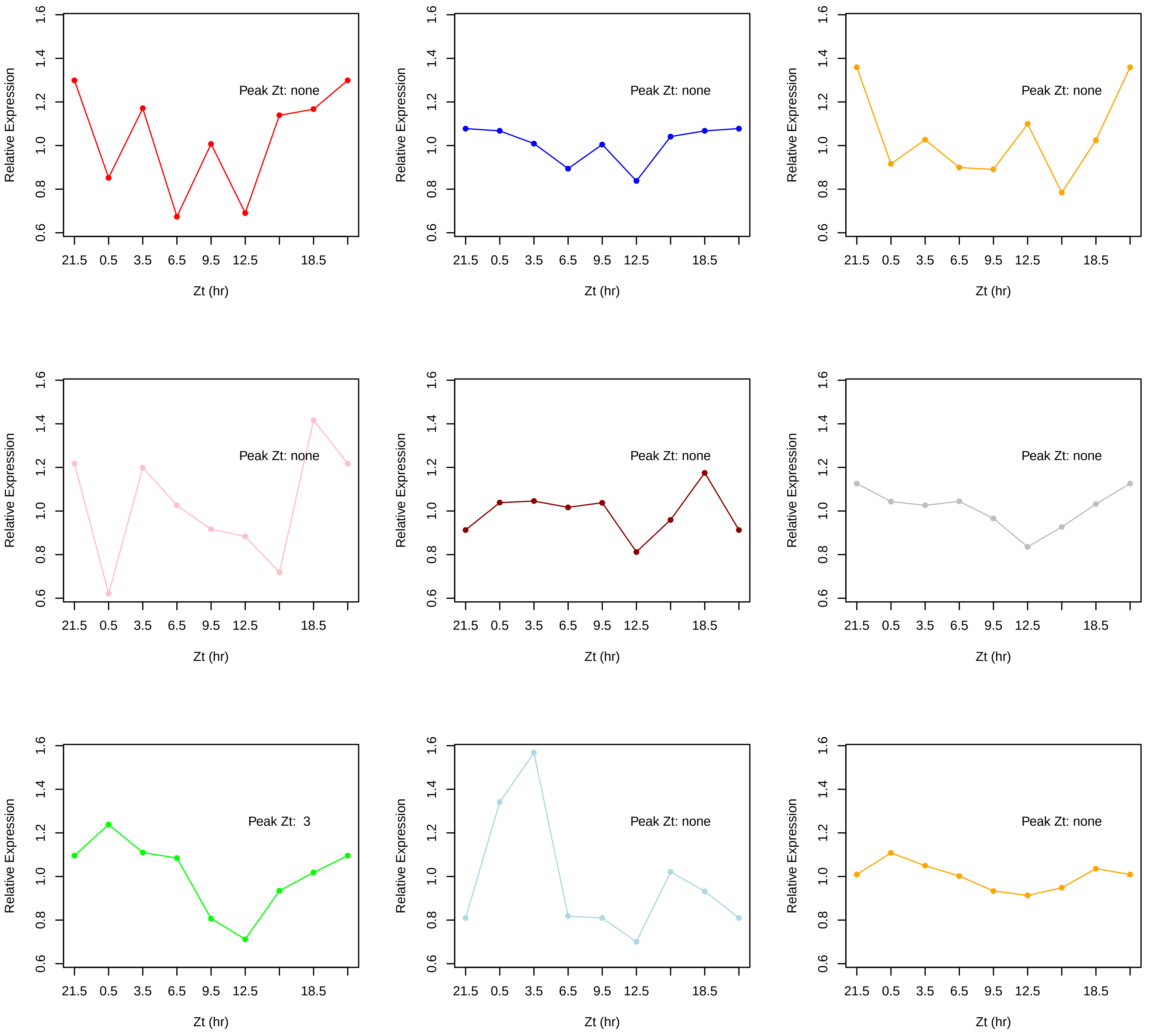

Singeleton: ENSXMAG00000008025 aamp

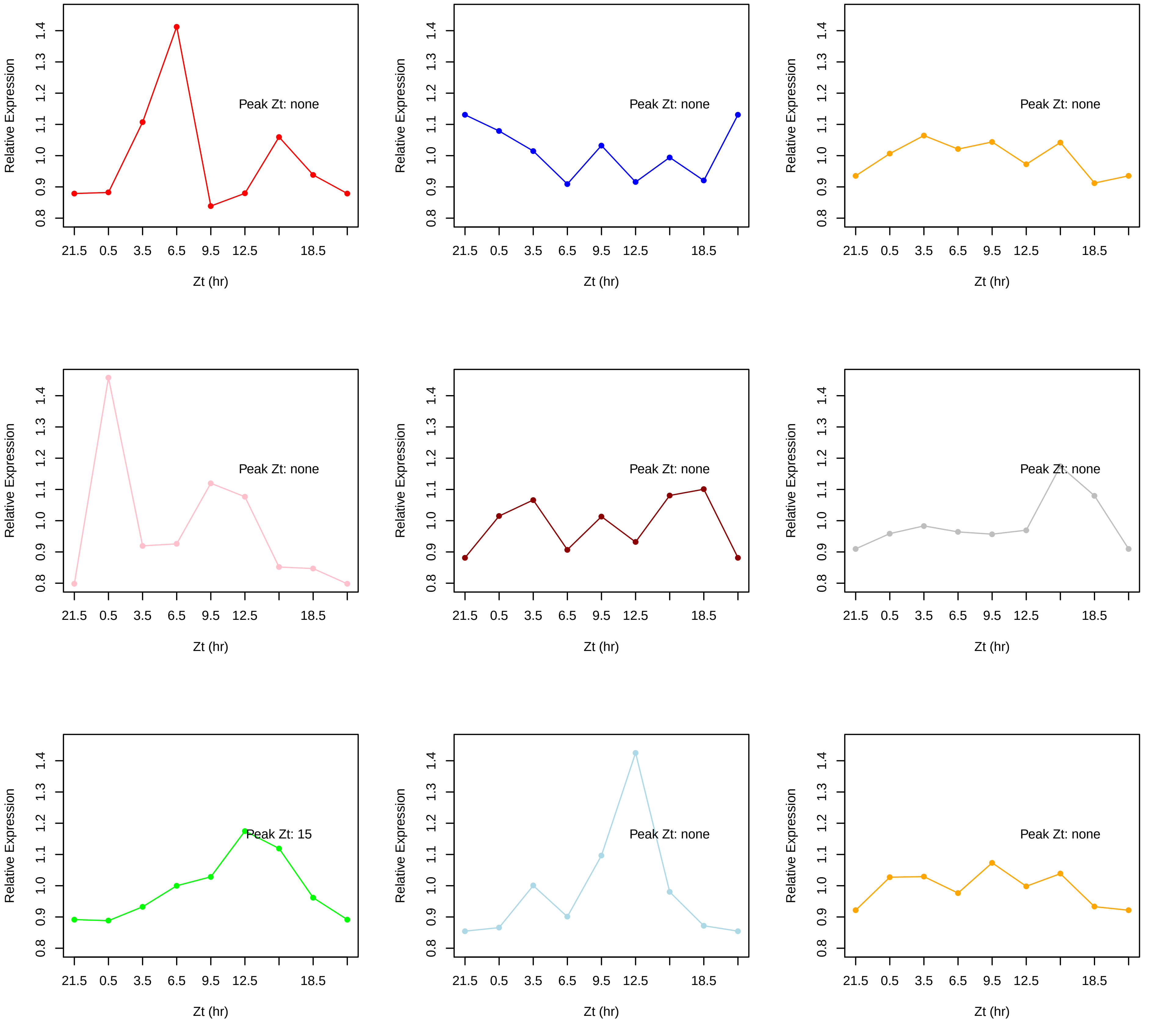

Singeleton: ENSXMAG00000009842 elmod2

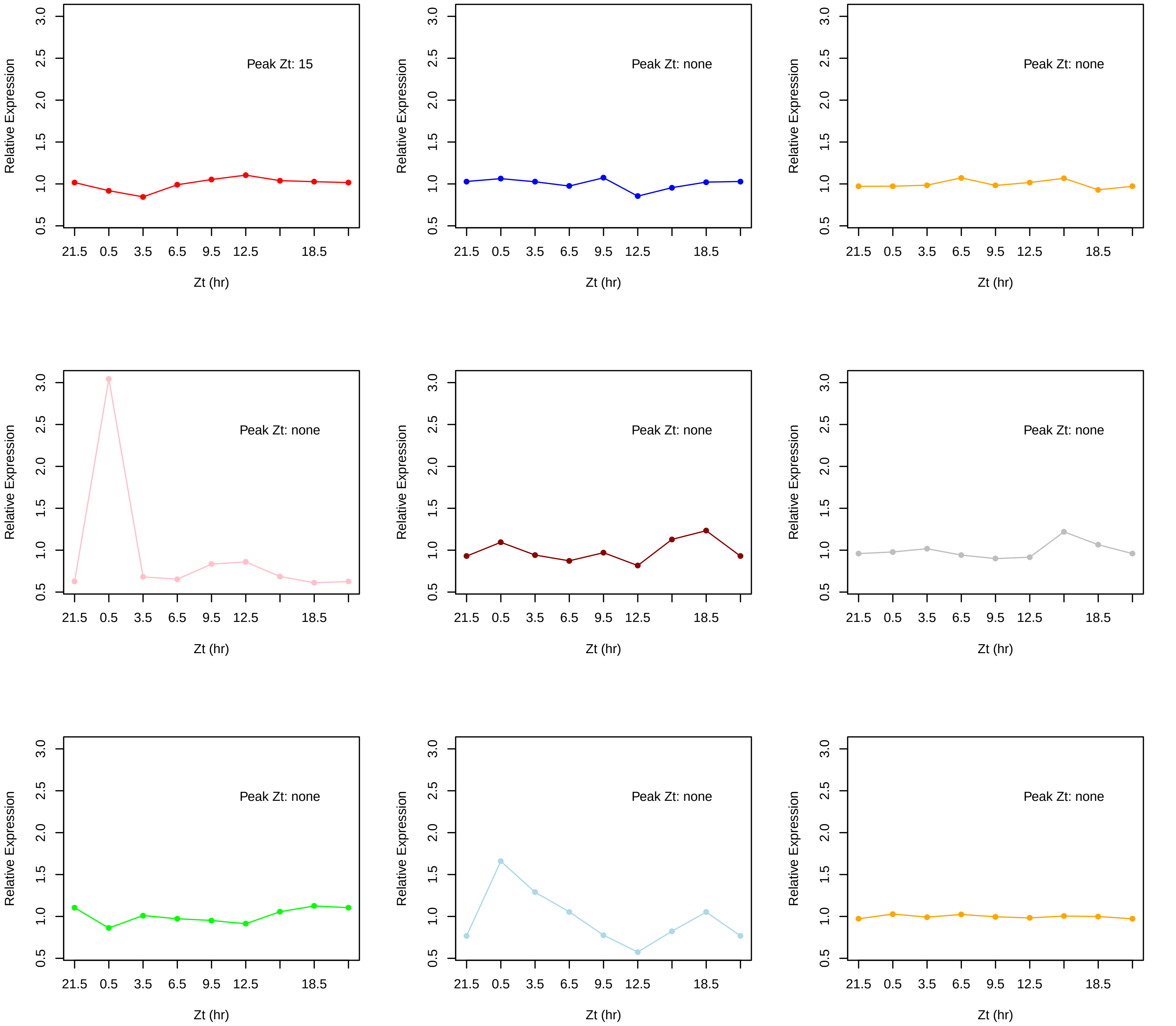

Singleton: ENSXMAG00000014516 kif1bp

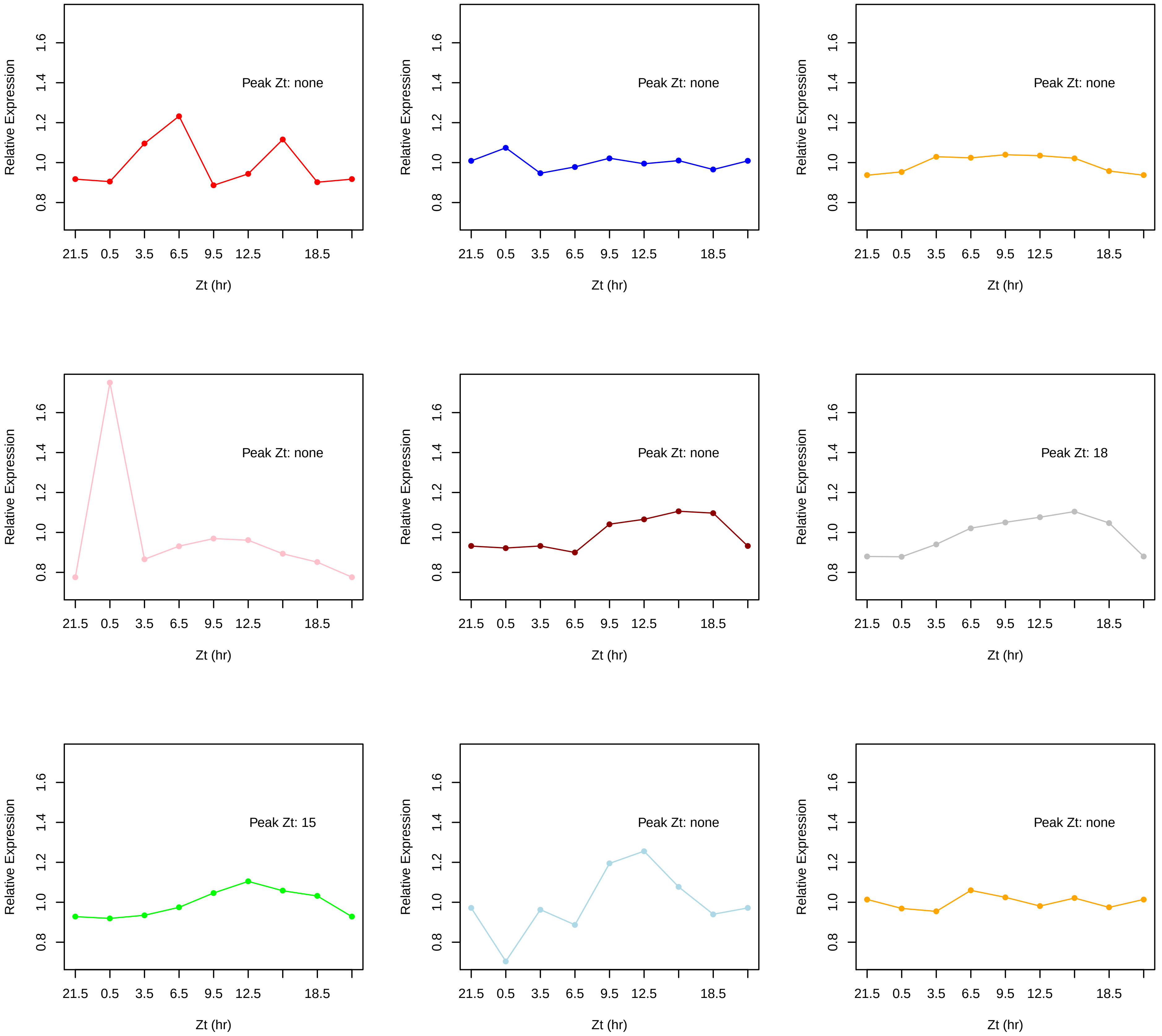

Singeleton: ENSXMAG00000020181

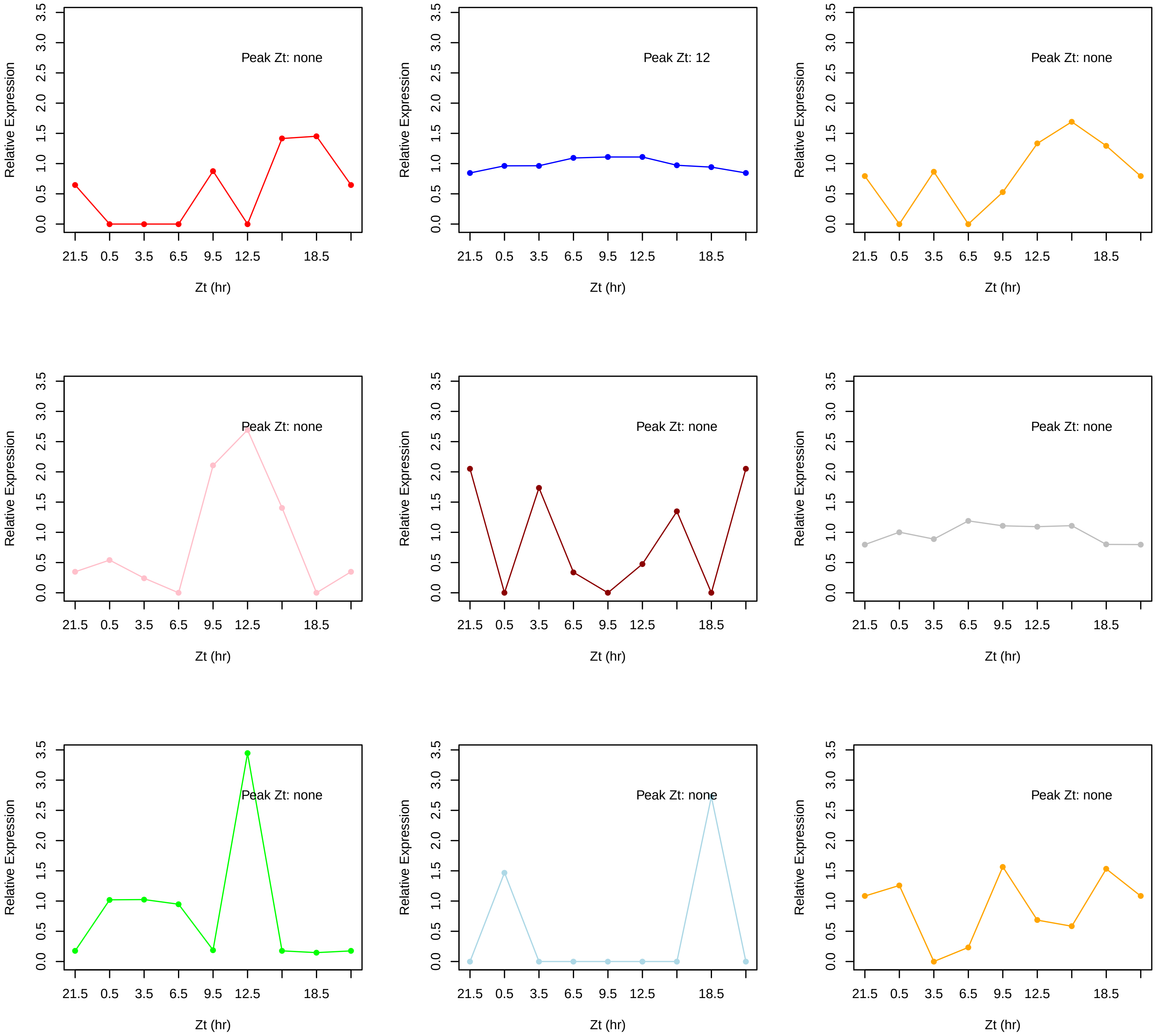

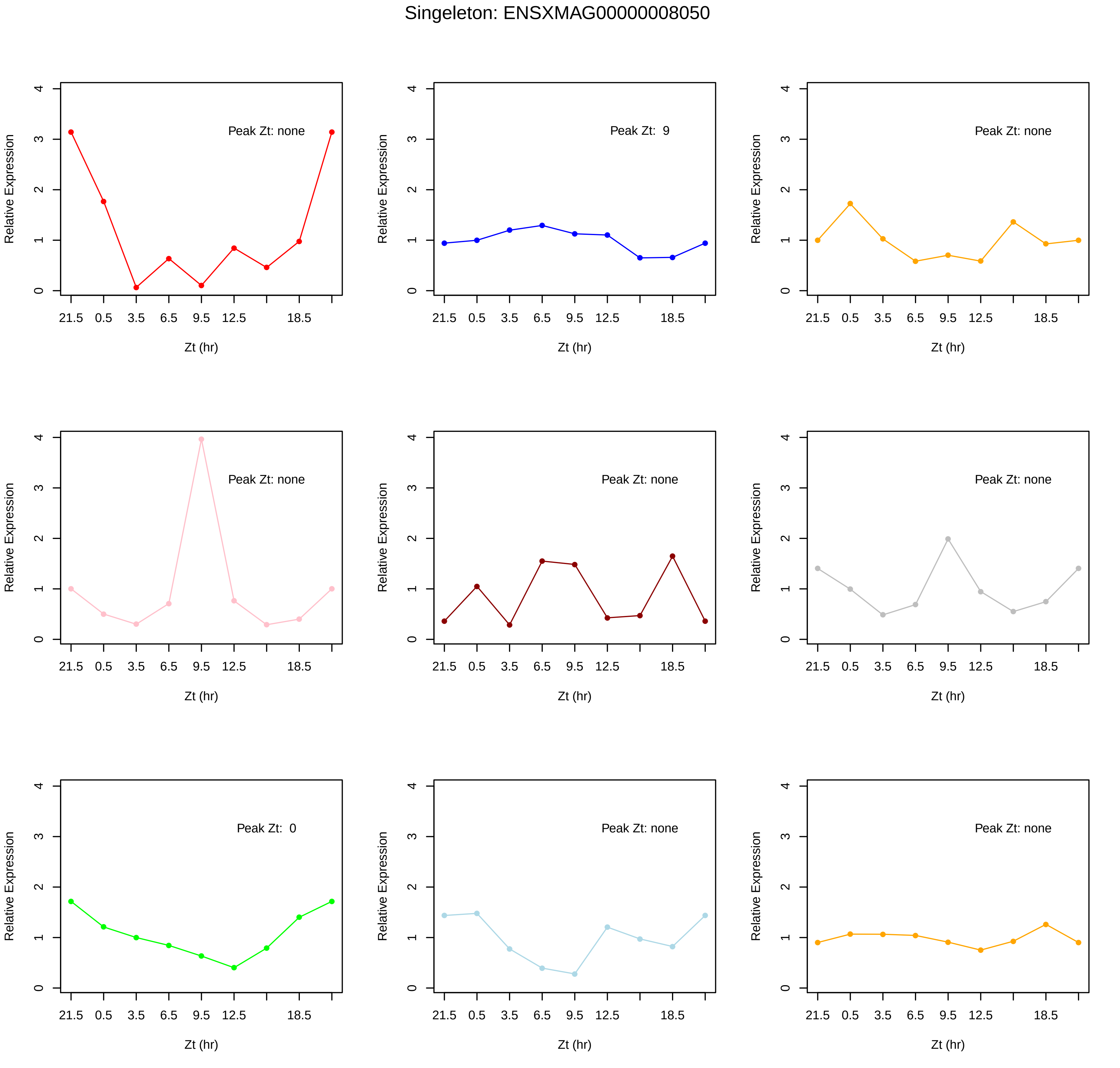

Singeleton: ENSXMAG00000008050
Peak Zt: none
Peak Zt: 9
Peak Zt: none
Peak Zt: none
Peak Zt: none
Peak Zt: none
Peak Zt: 0
Peak Zt: none
Peak Zt: none
Relative Expression
Zt (hr)
21.5
0.5
3.5
6.5
9.5
12.5
18.5

Singleton: ENSXMAG00000003297 rasal2

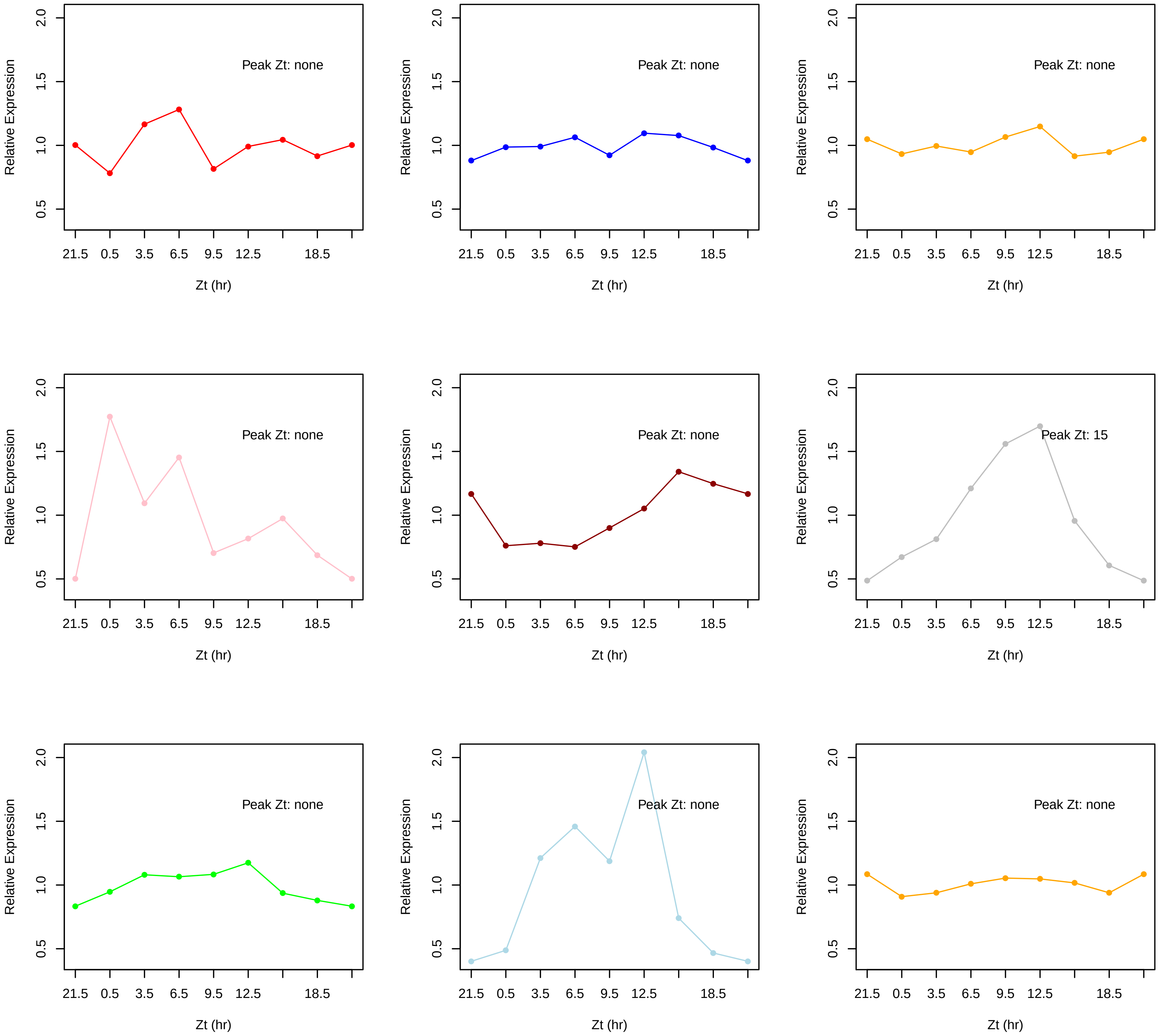

Singeleton: ENSXMAG00000017597 sod1

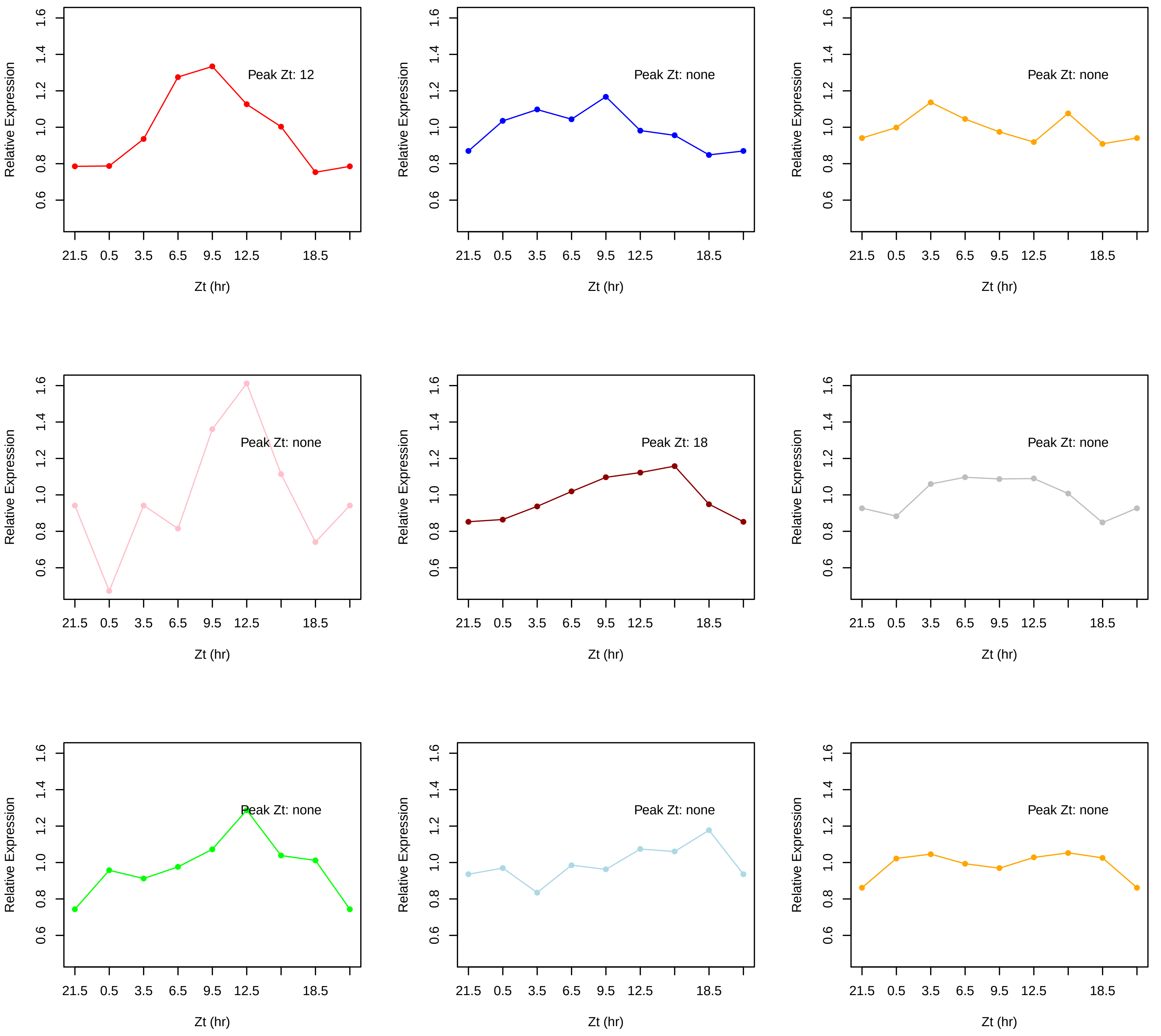

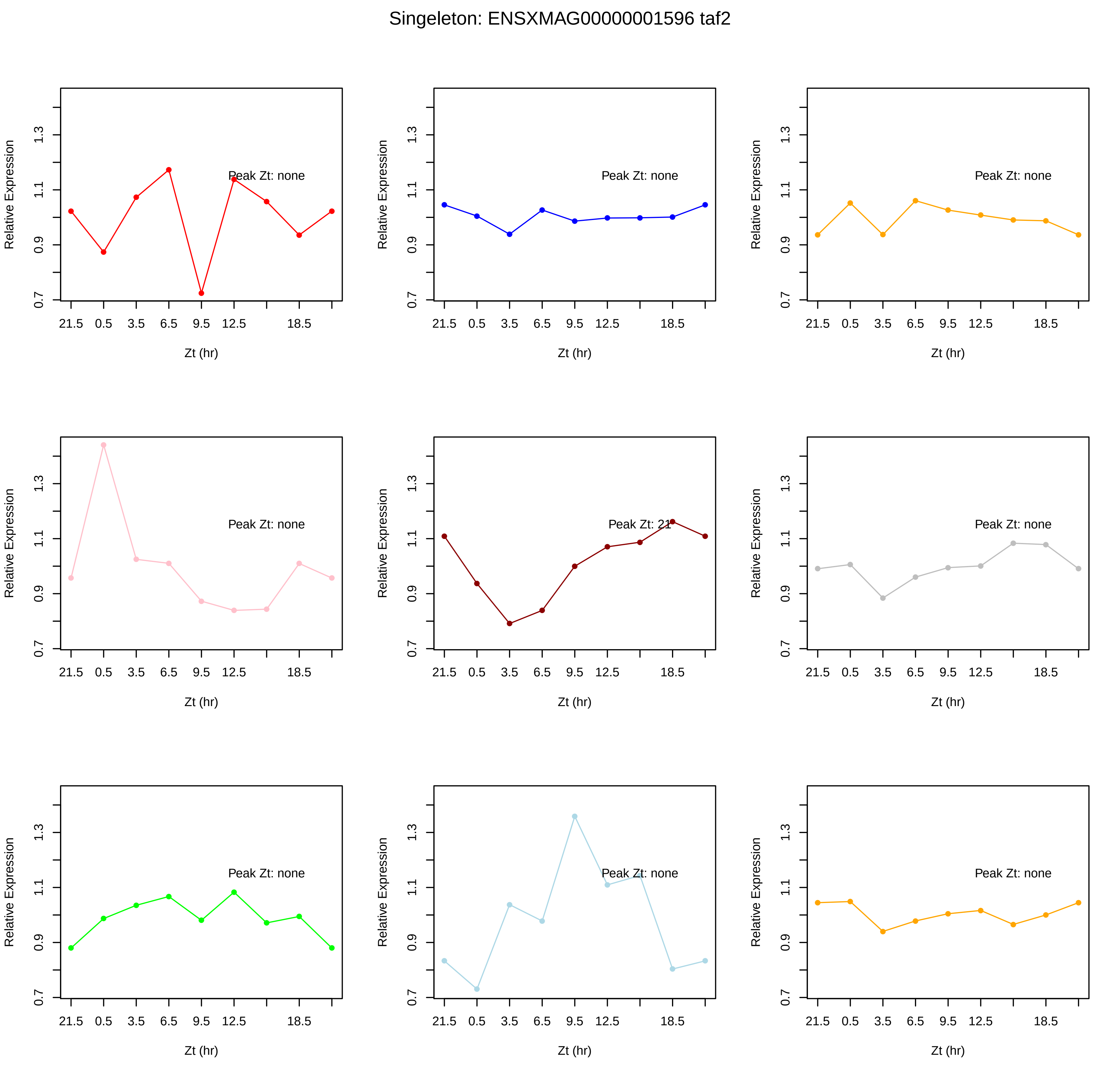
Singeleton: ENSXMAG00000001596 taf2
Relative Expression
Zt (hr)
0.7
0.9
1.1
1.3
21.5
0.5
3.5
6.5
9.5
12.5
18.5
Peak Zt: none
Peak Zt: 21

Singeleton: ENSXMAG00000009862 fam13a

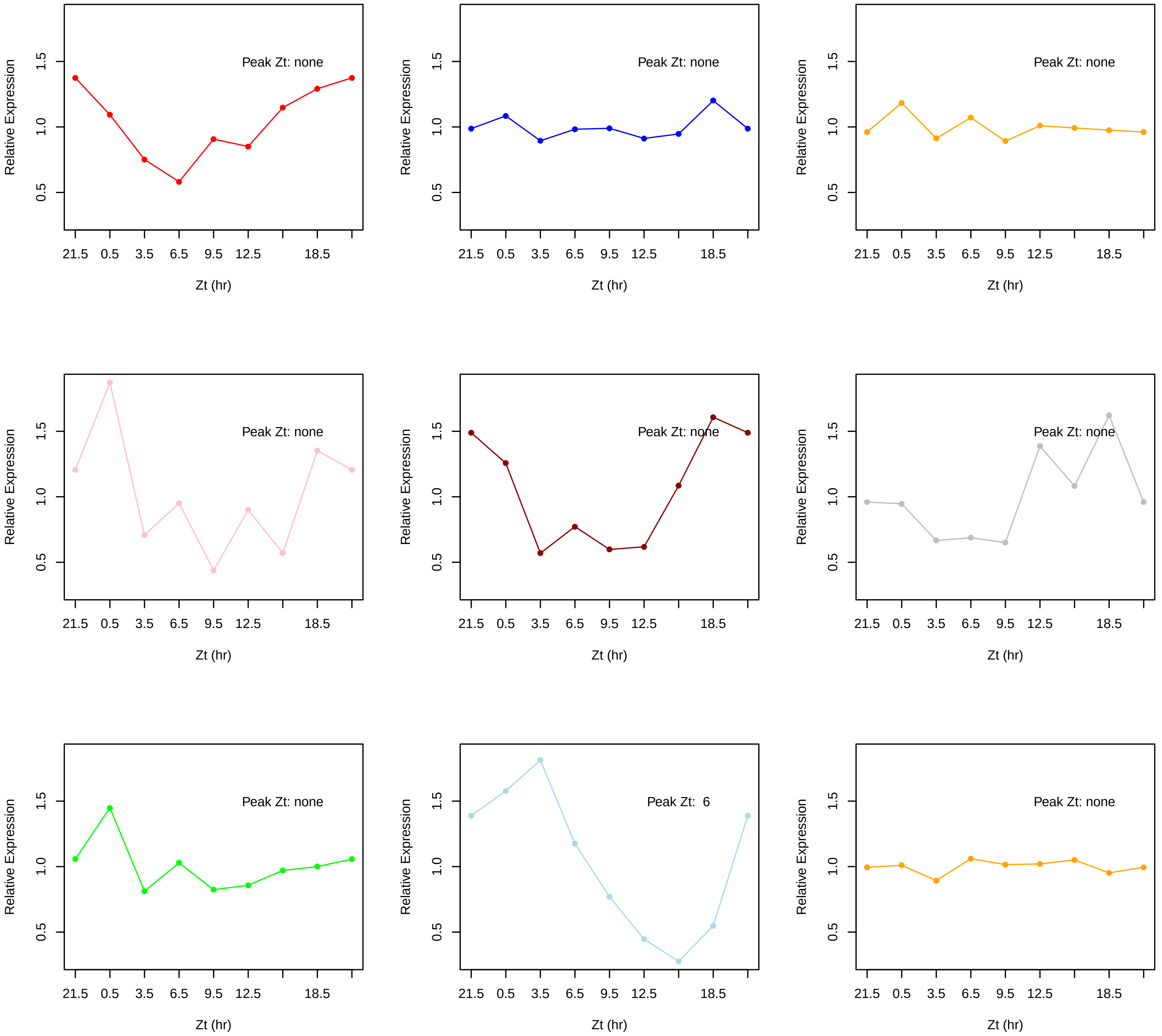

Singleton: ENSXMAG00000008074 arpc2

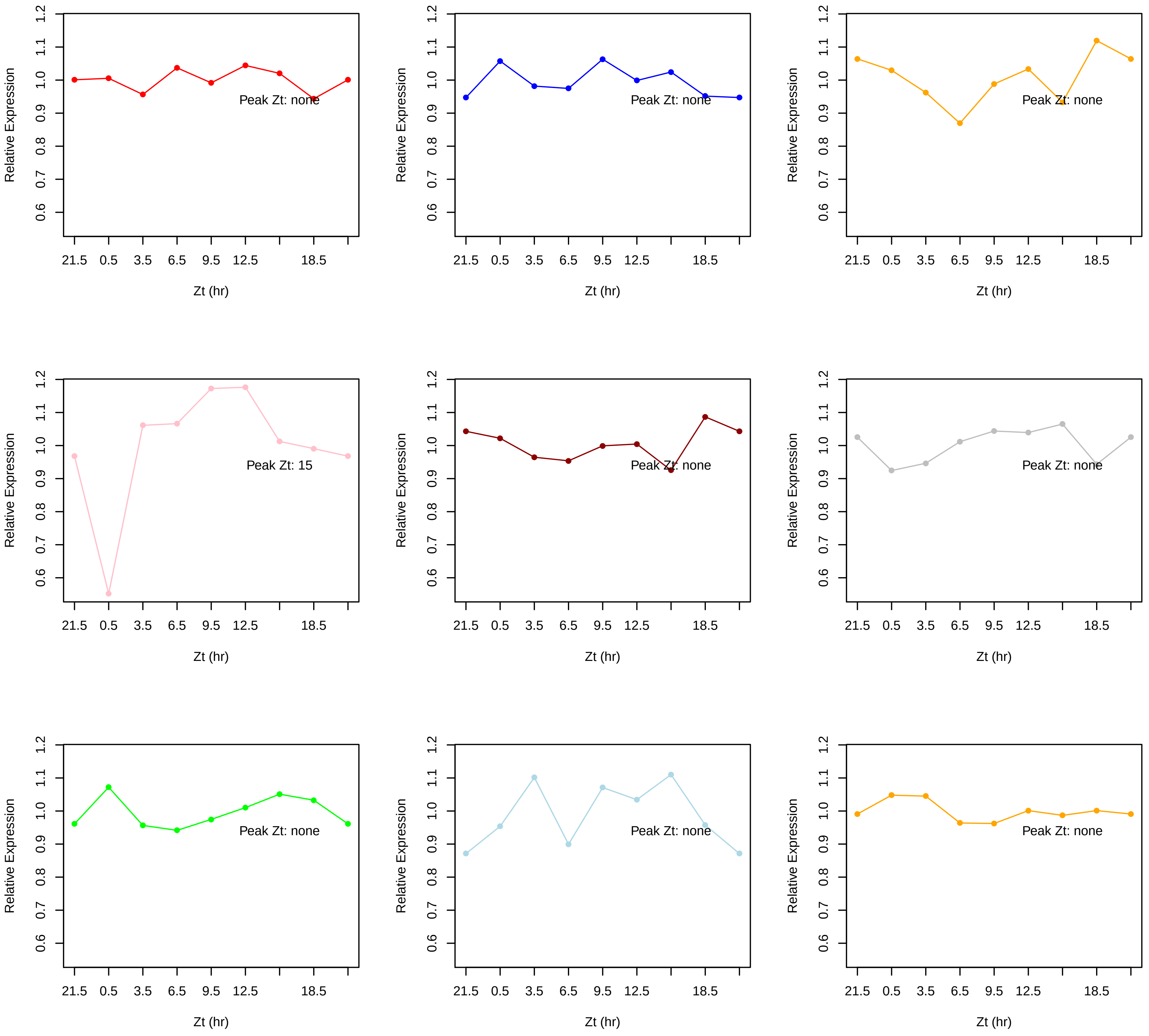

Singeleton: ENSXMAG00000009383 si:dkeyp−33b5.4

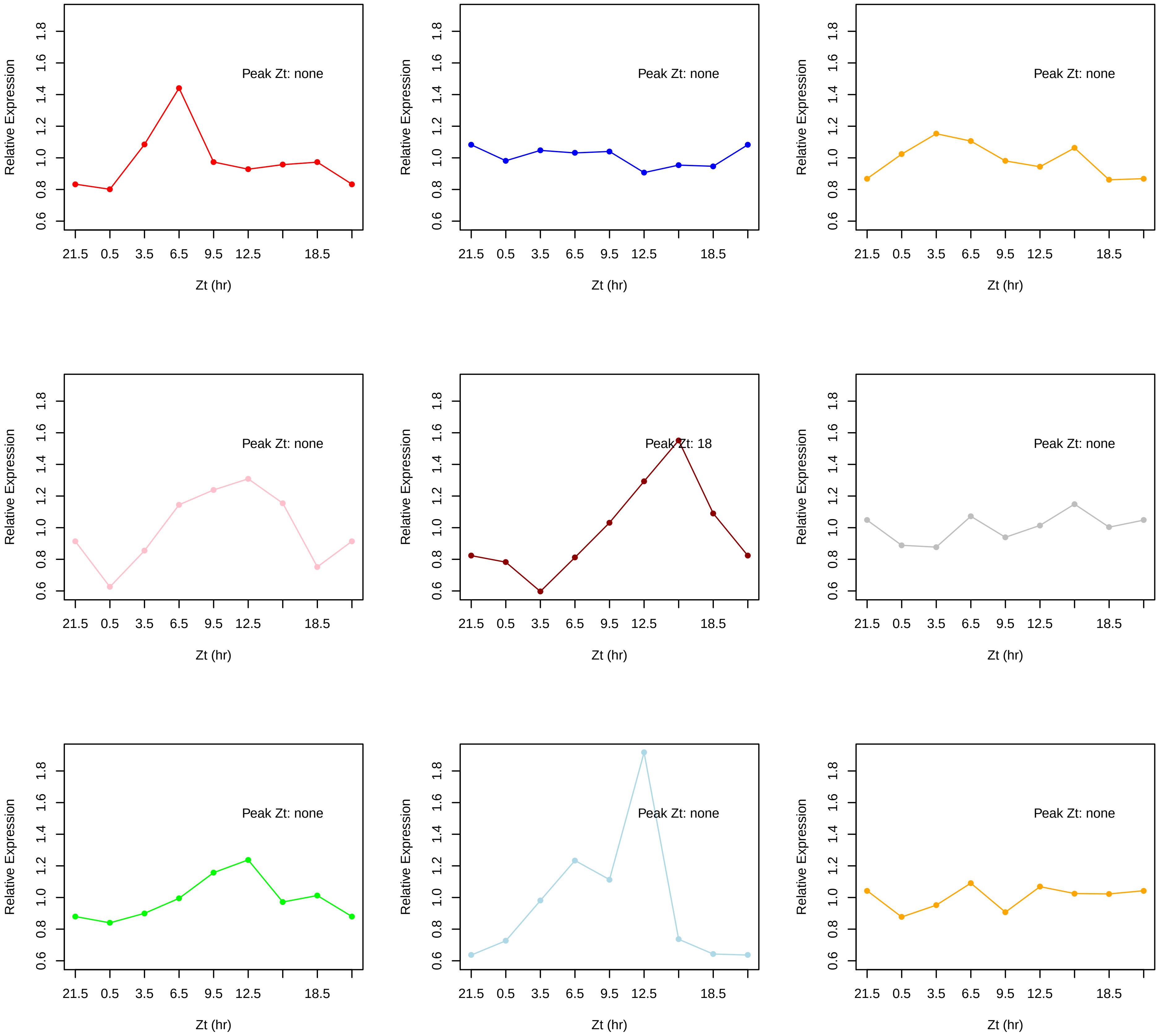

Singeleton: ENSXMAG00000000504 crebrf

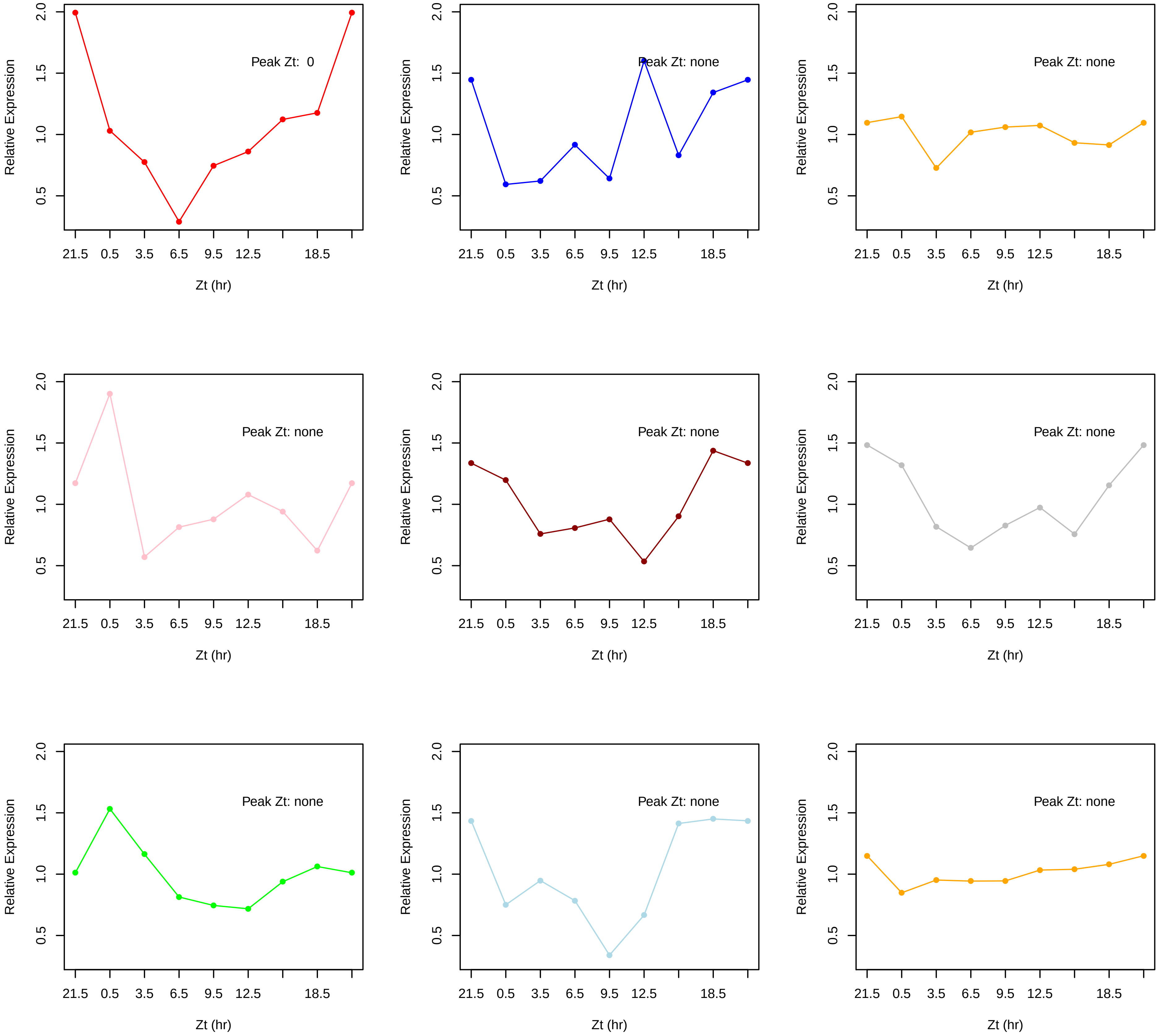

Singleton: ENSXMAG00000014561 hkdc1

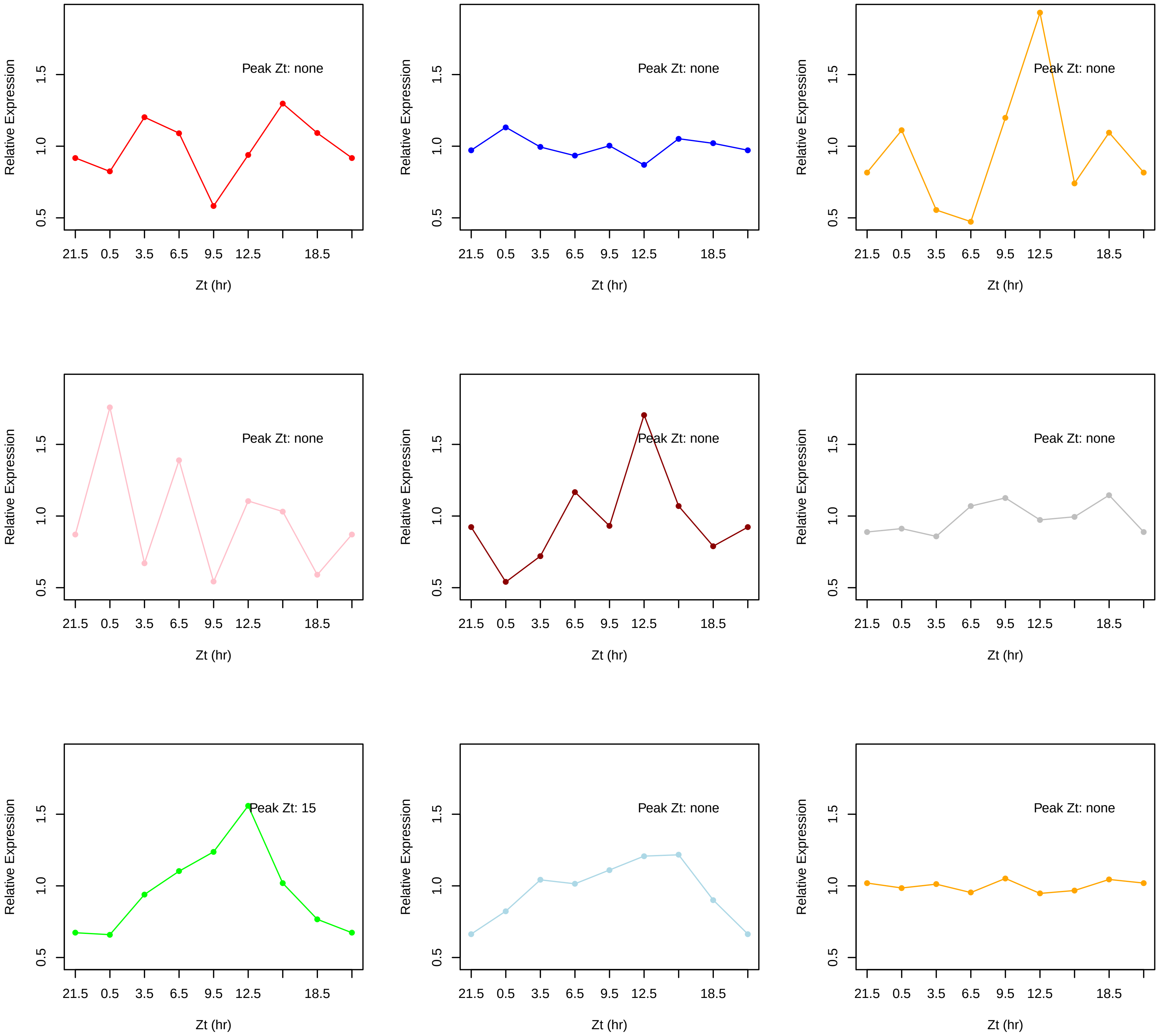

Singleton: ENSXMAG00000010825 deptor

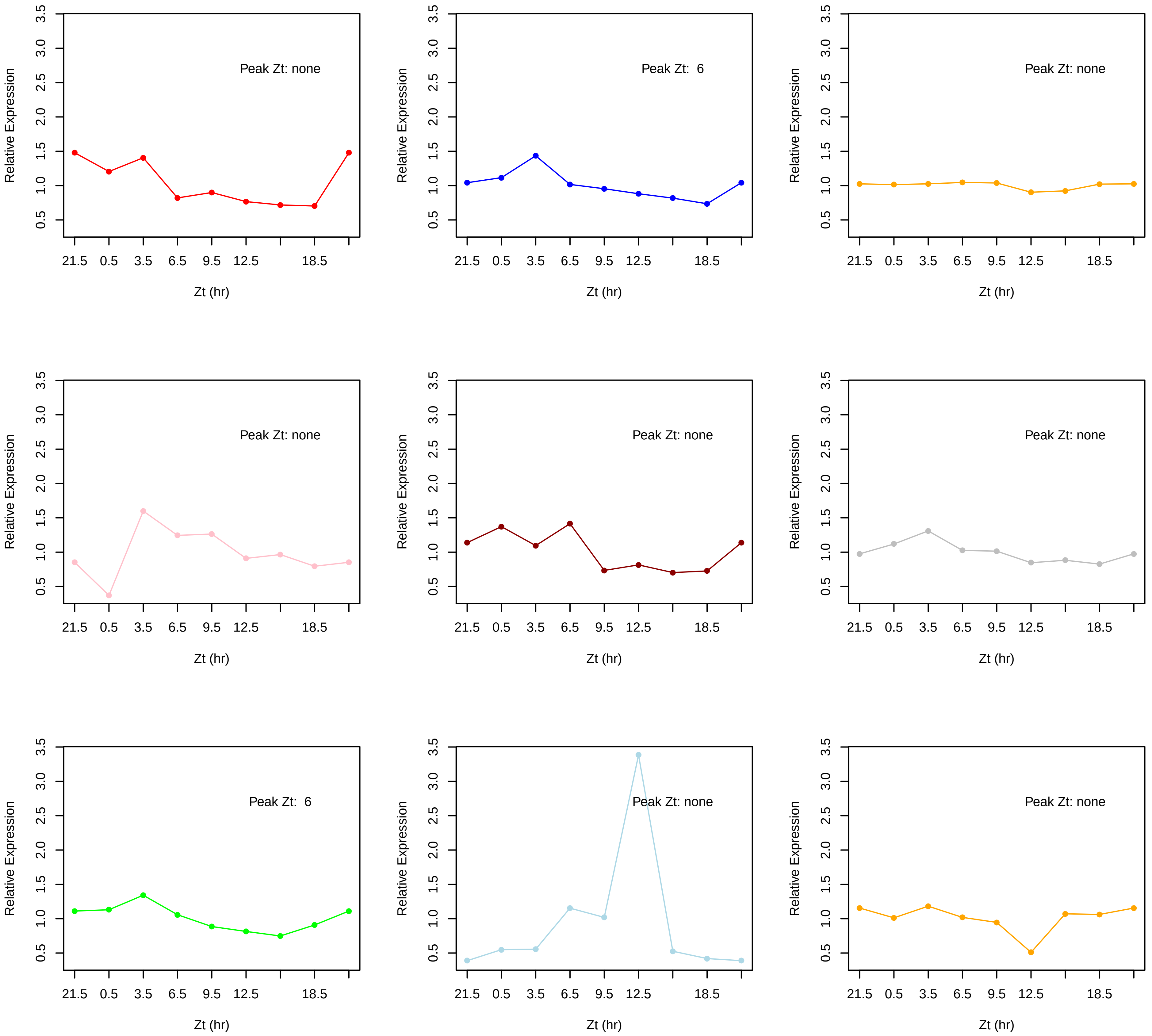

Singeleton: ENSXMAG00000018432

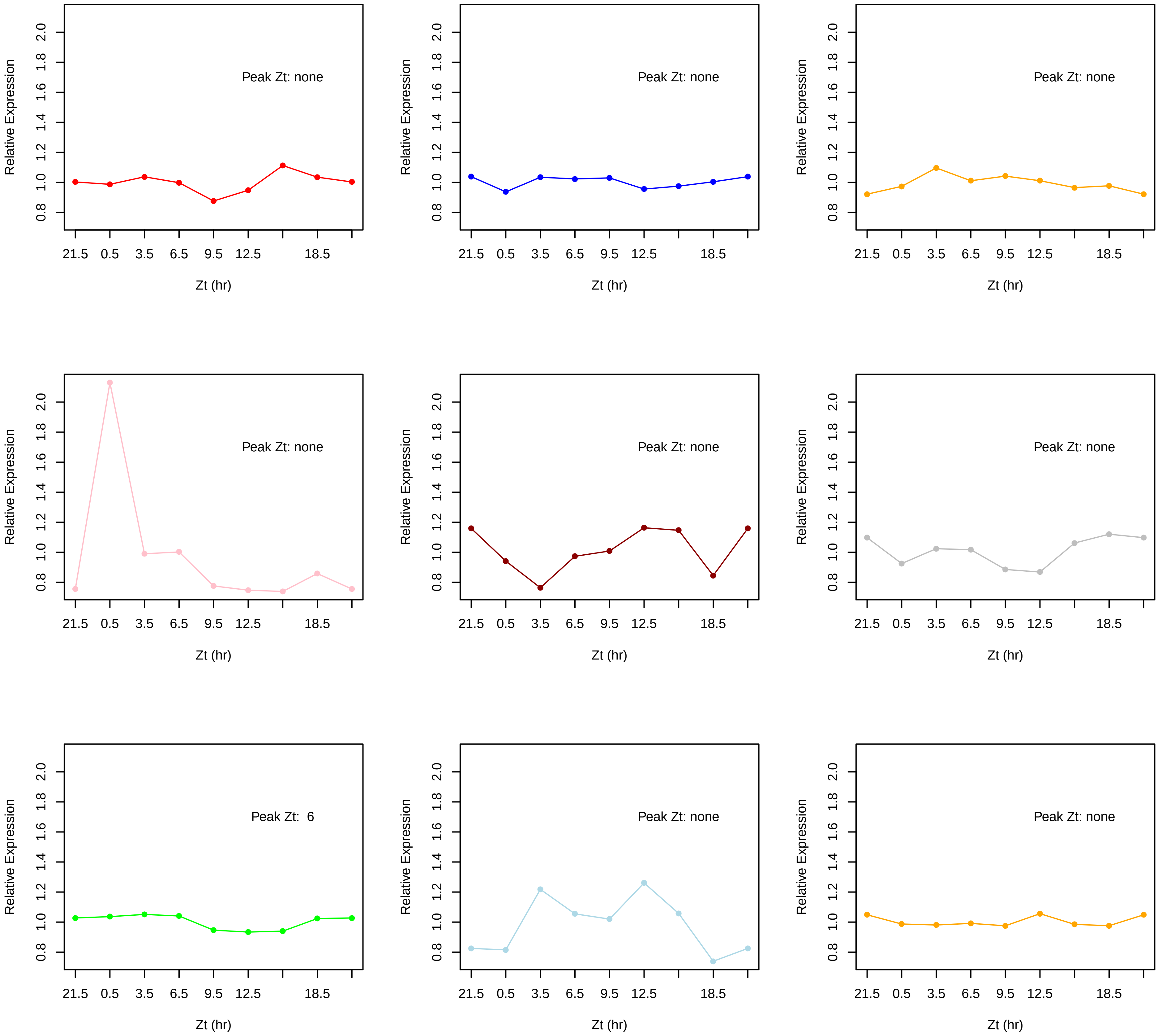

Singeleton: ENSXMAG00000010281 rpl26

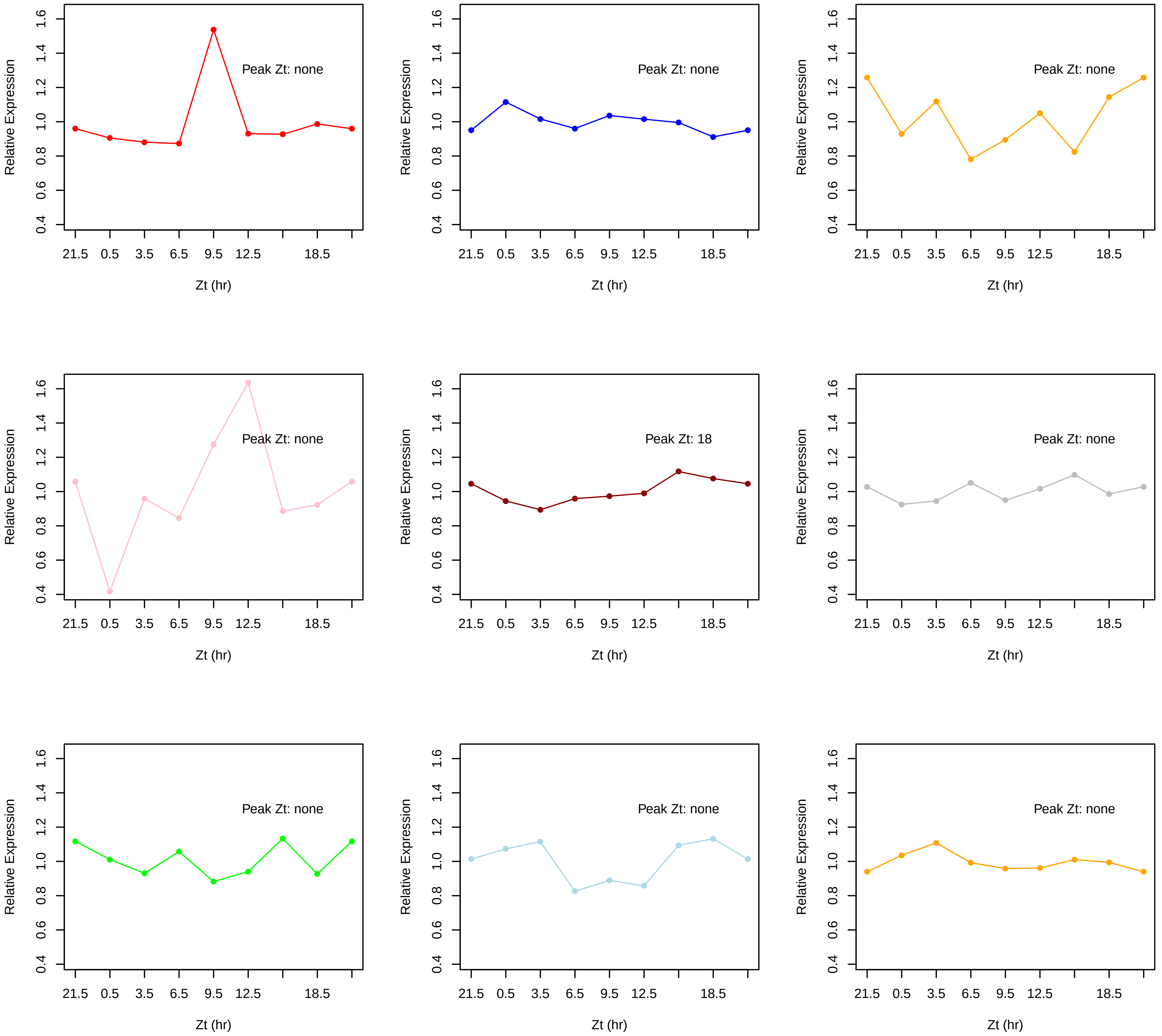

Singleton: ENSXMAG00000001458

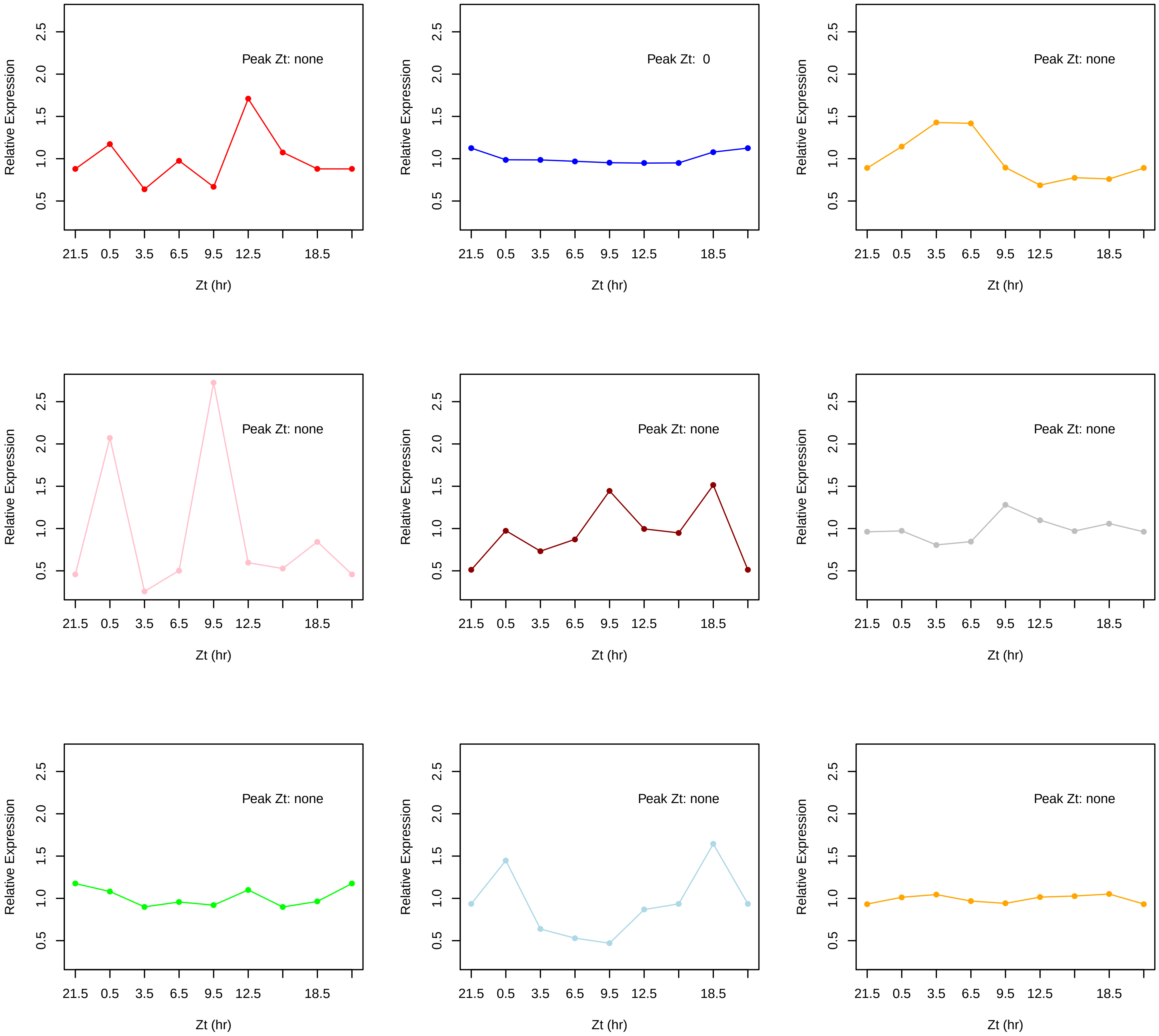

Singleton: ENSXMAG00000015986 HABP4

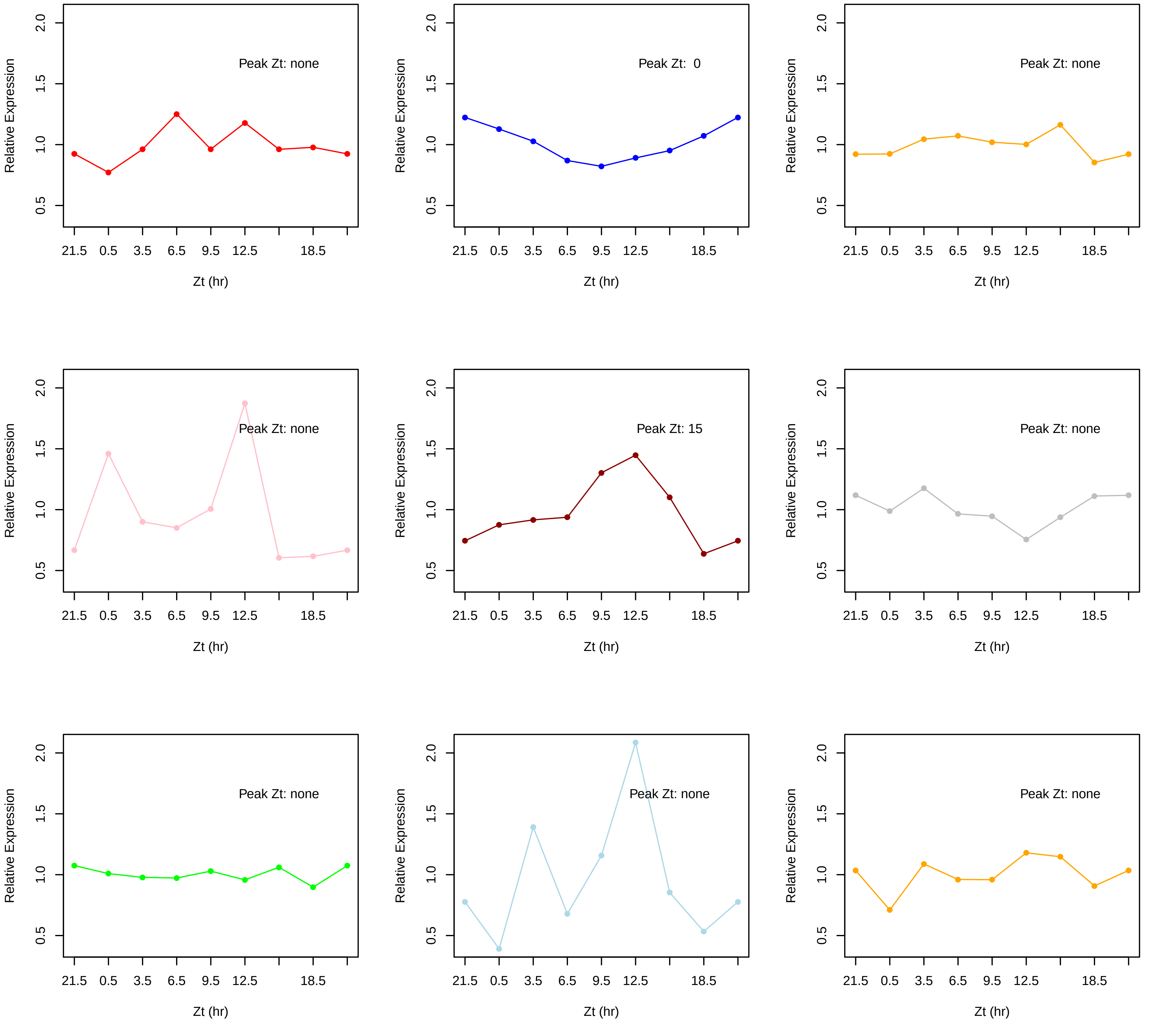

Singeleton: ENSXMAG00000030083 fbxo33

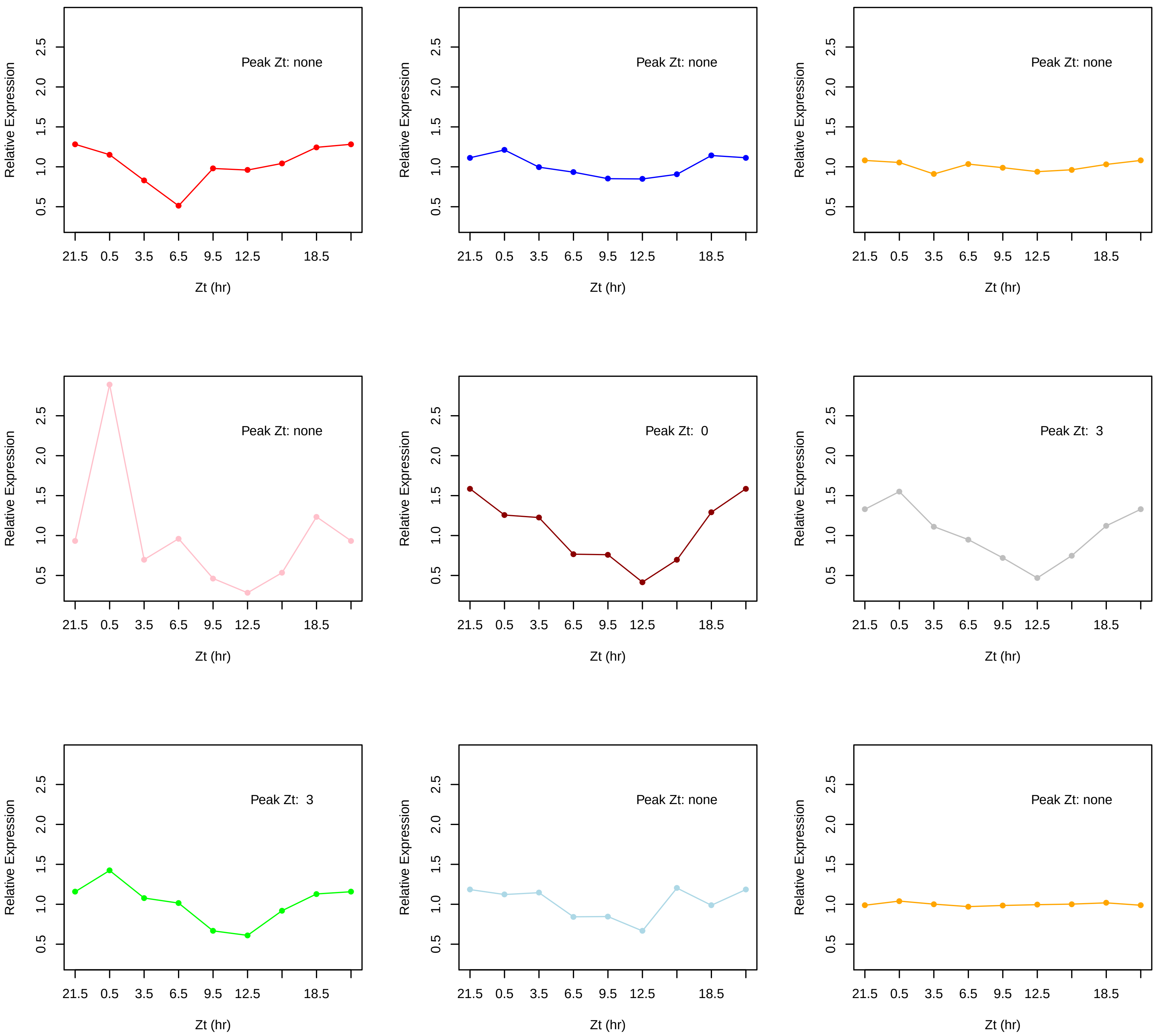

Singleton: ENSXMAG00000012154 efhd1

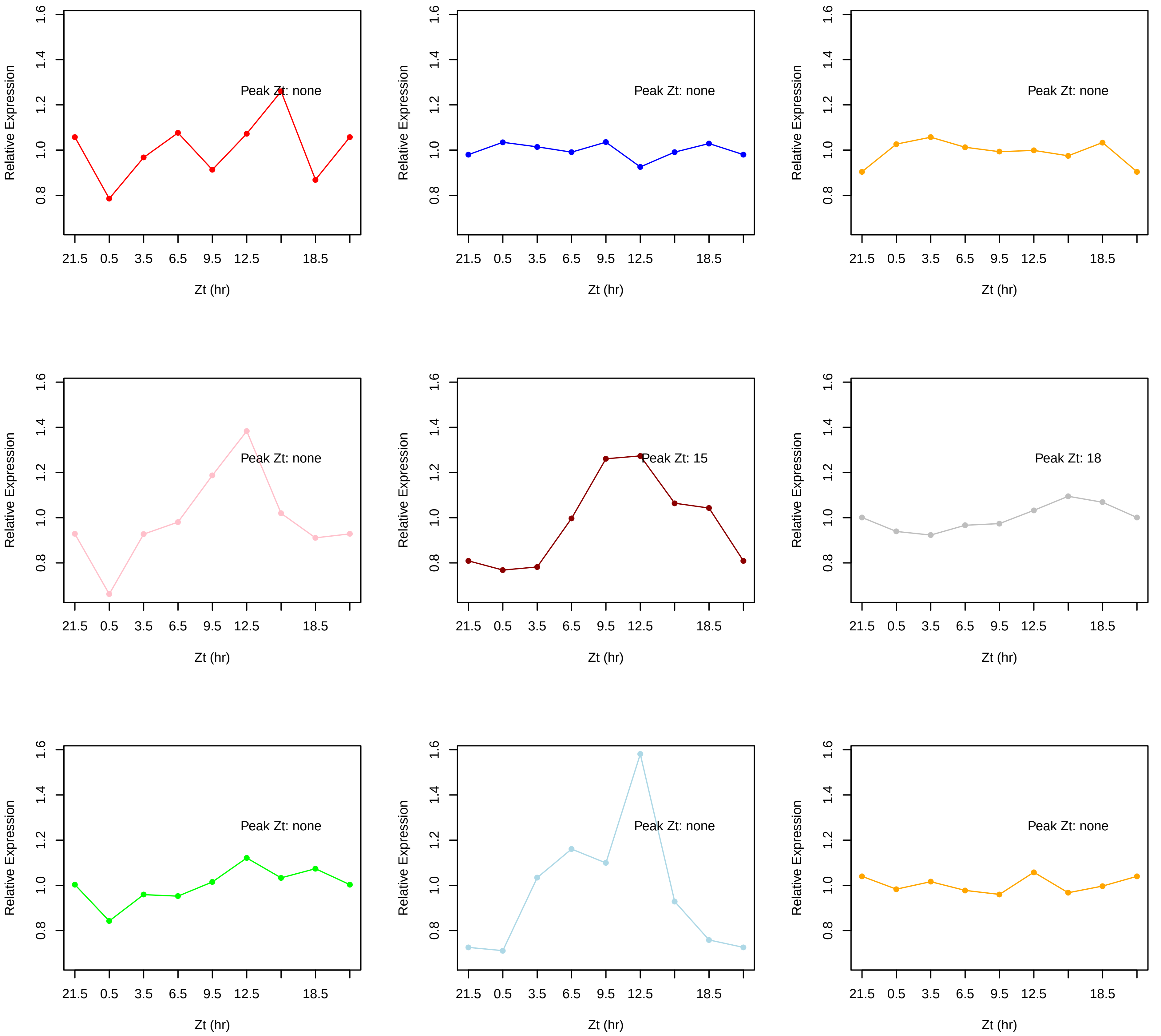

Singeleton: ENSXMAG00000017334 cab39l

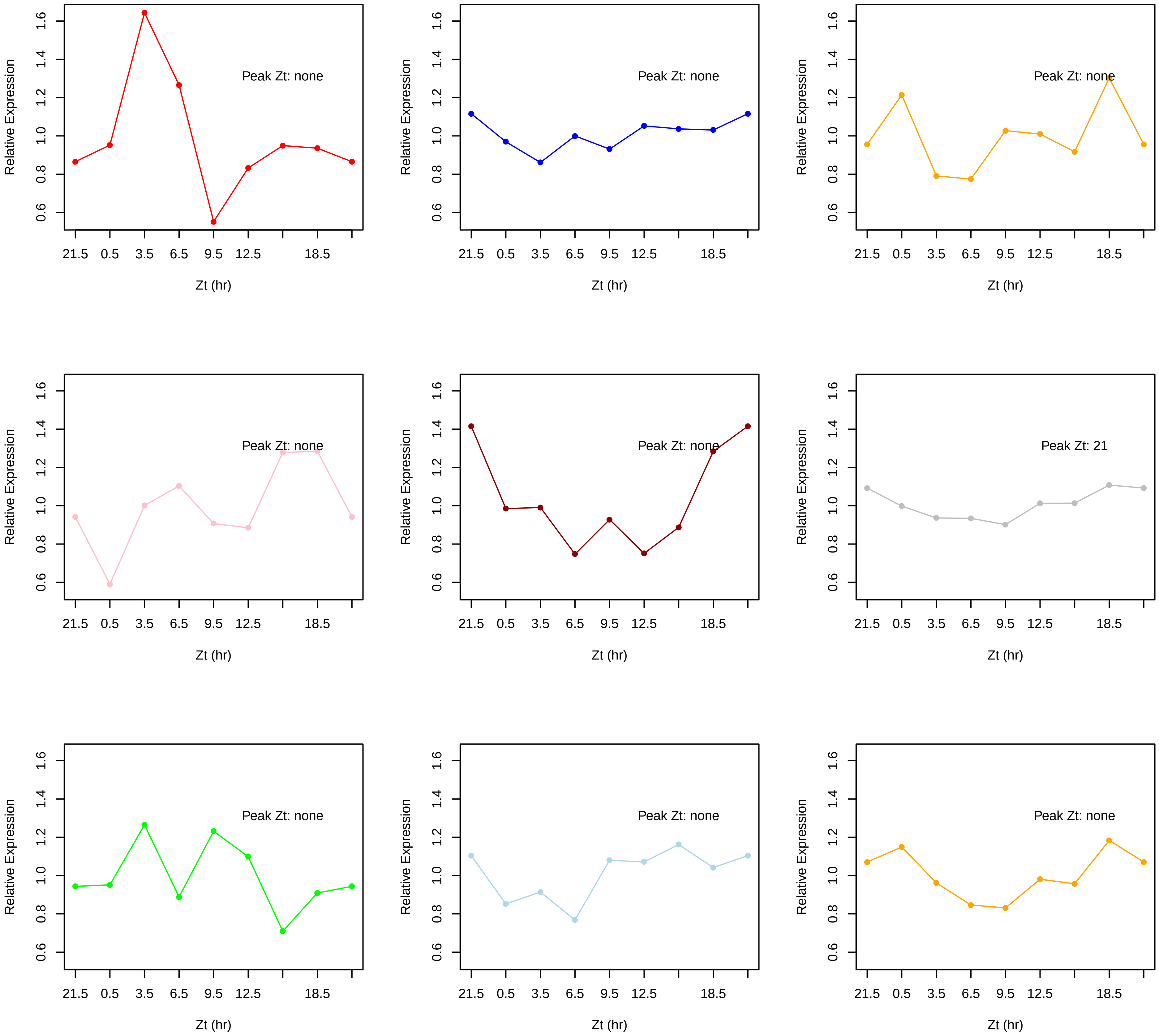

Singleton: ENSXMAG00000027639 PCYOX1L

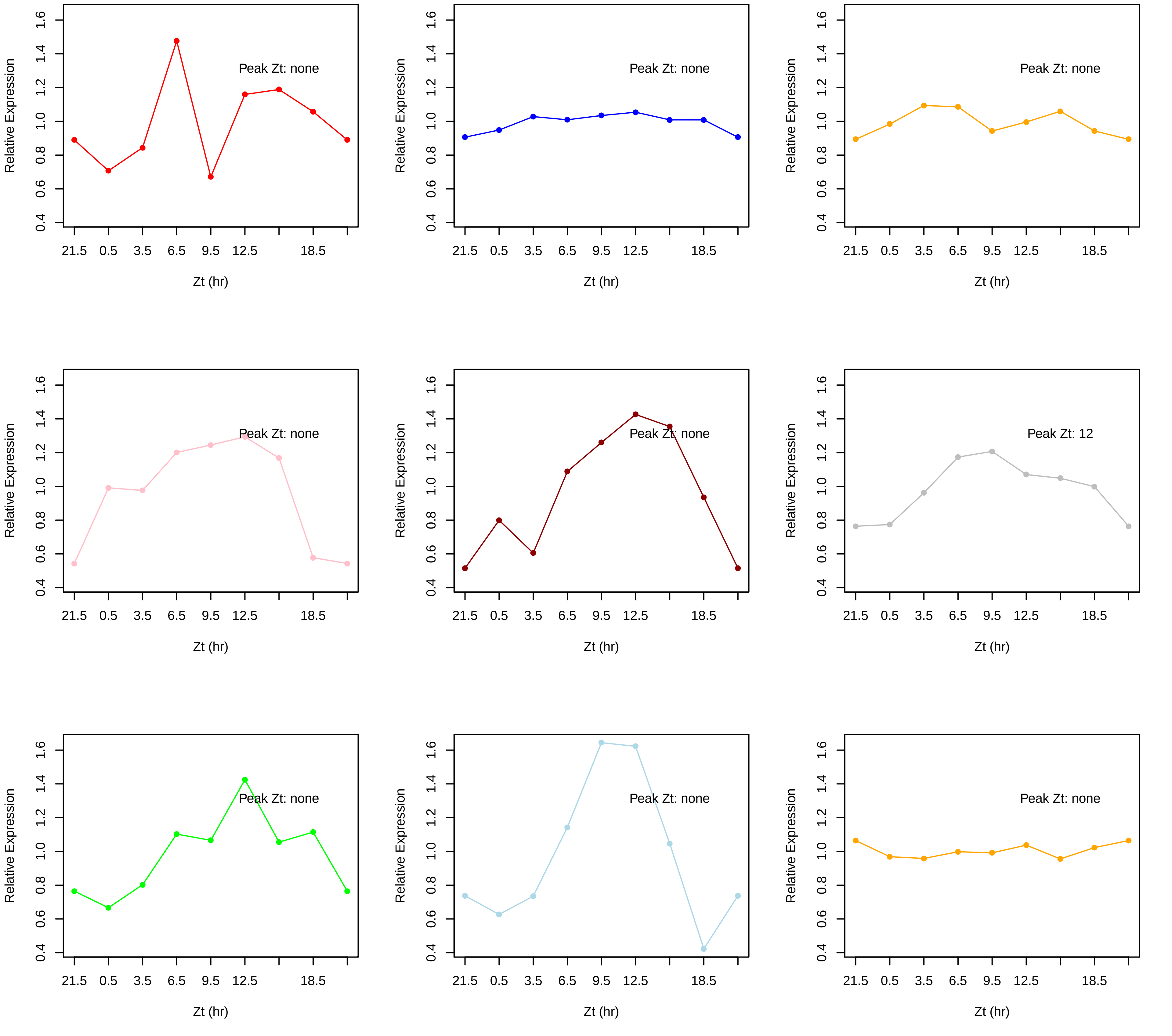

Singeleton: ENSXMAG00000018529

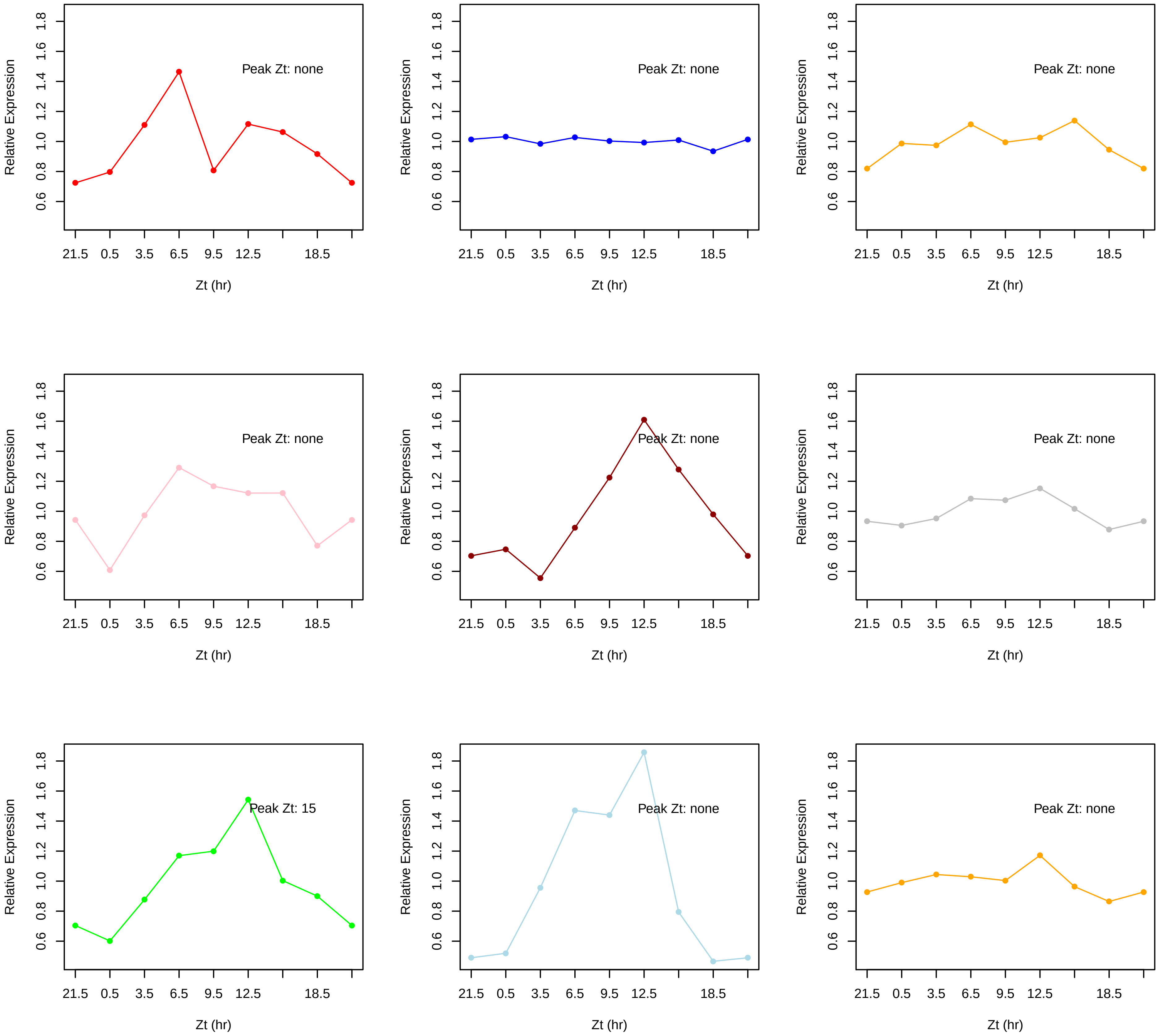

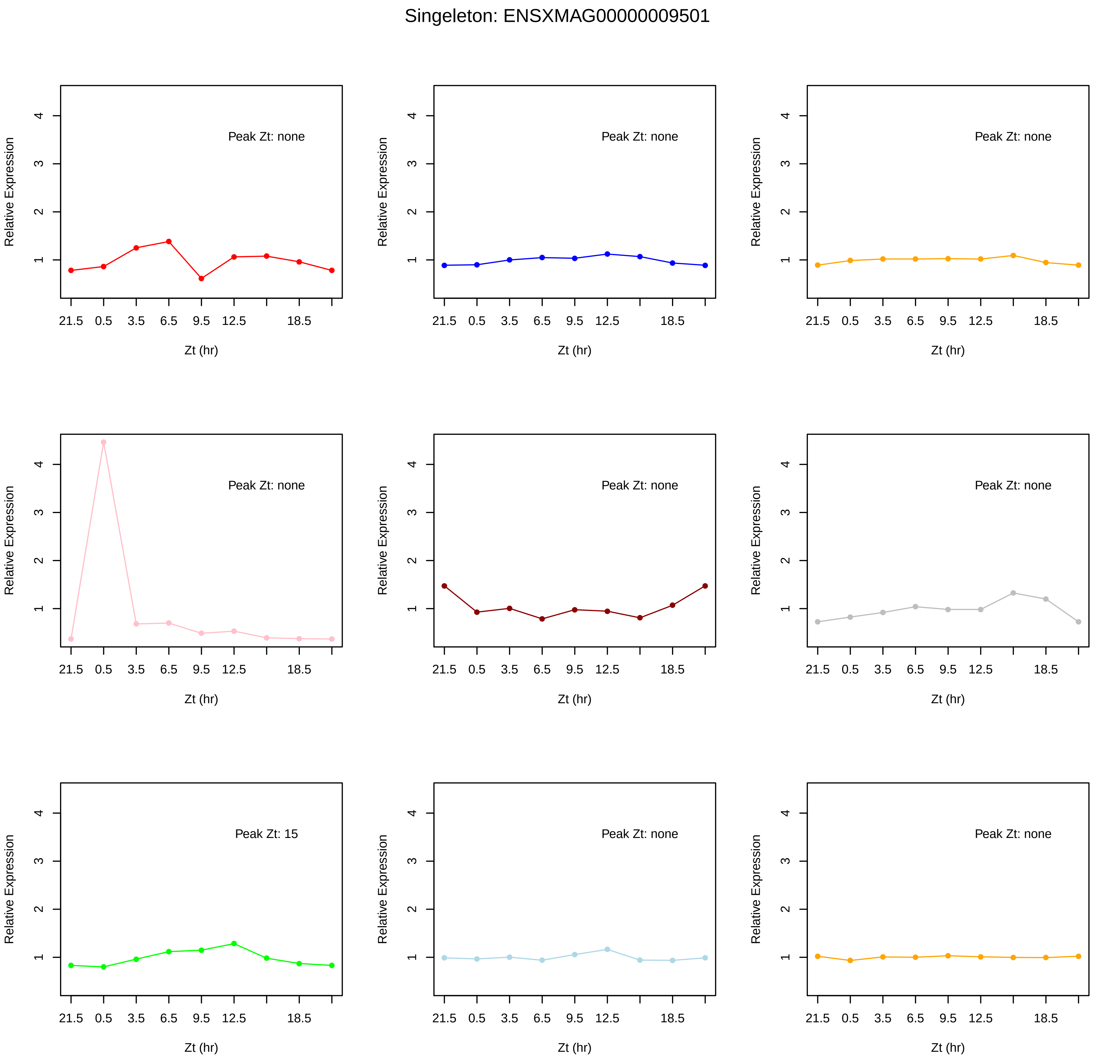
Singeleton: ENSXMAG00000009501
Peak Zt: none
Peak Zt: none
Peak Zt: none
Peak Zt: none
Peak Zt: none
Peak Zt: none
Peak Zt: 15
Peak Zt: none
Peak Zt: none
Relative Expression
Zt (hr)
21.5
0.5
3.5
6.5
9.5
12.5
18.5

Singeleton: ENSXMAG00000029074 ppp2r2ca

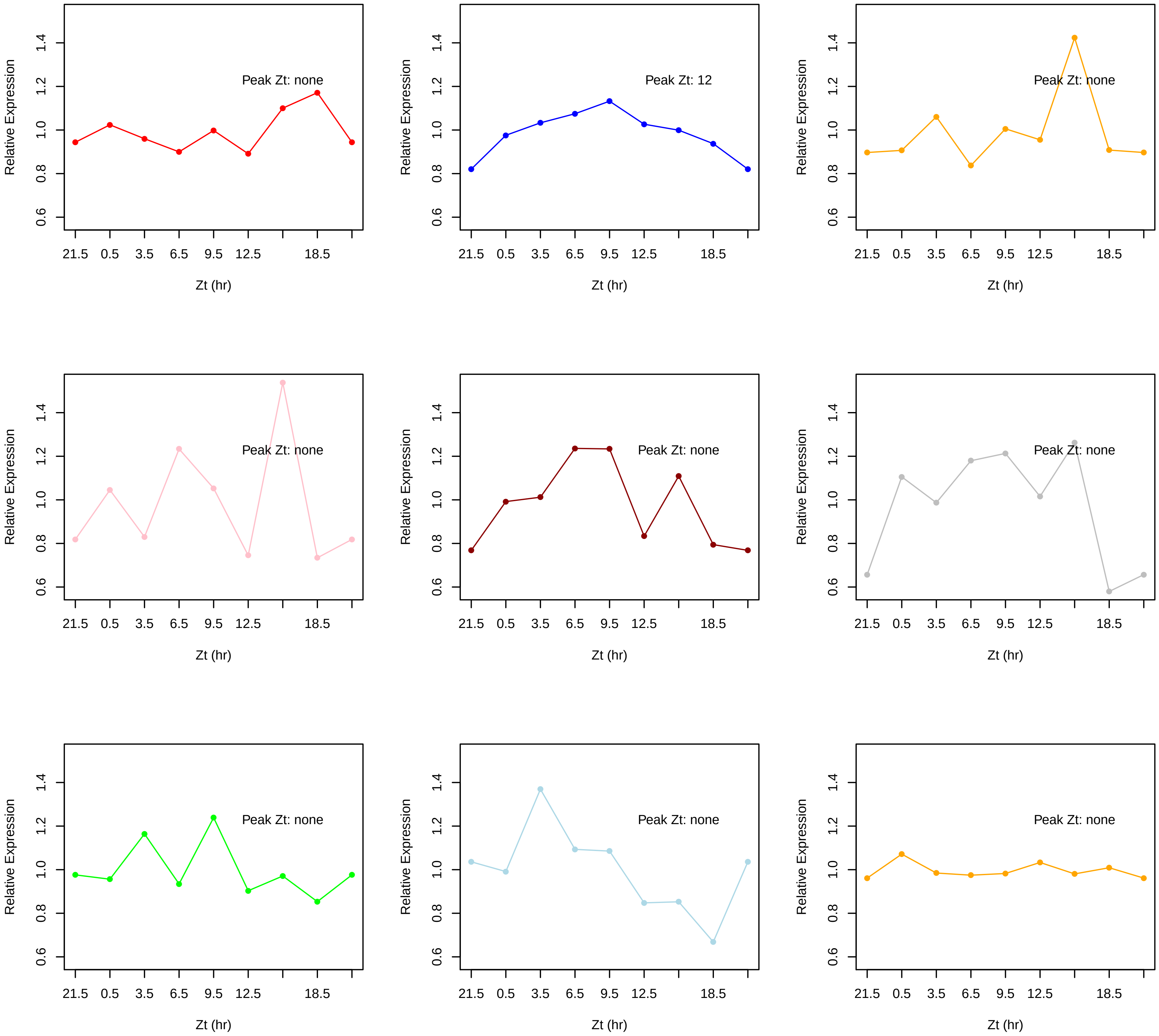

Singeleton: ENSXMAG00000014703 tacr2

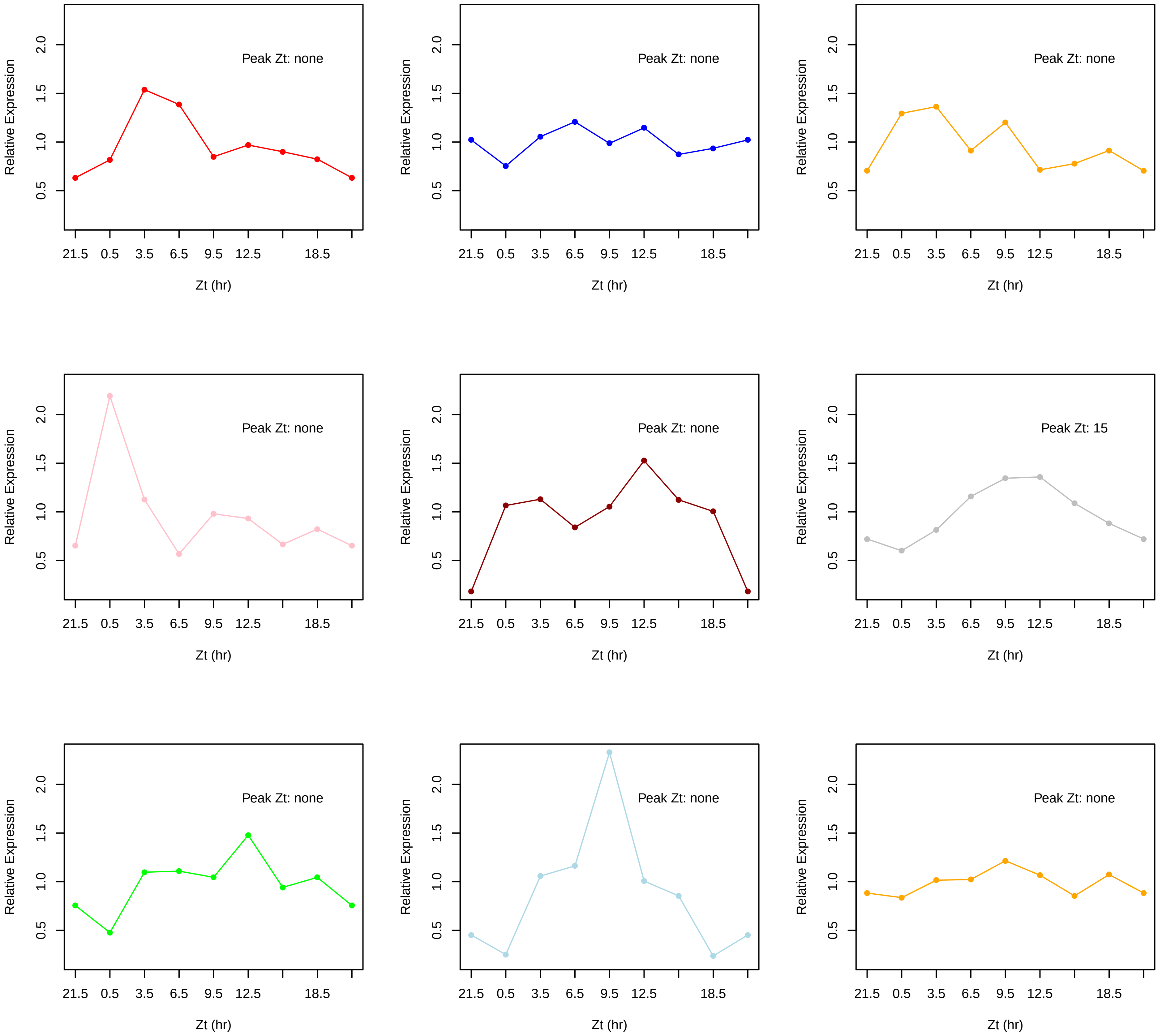

Singeleton: ENSXMAG00000017571 cfap298

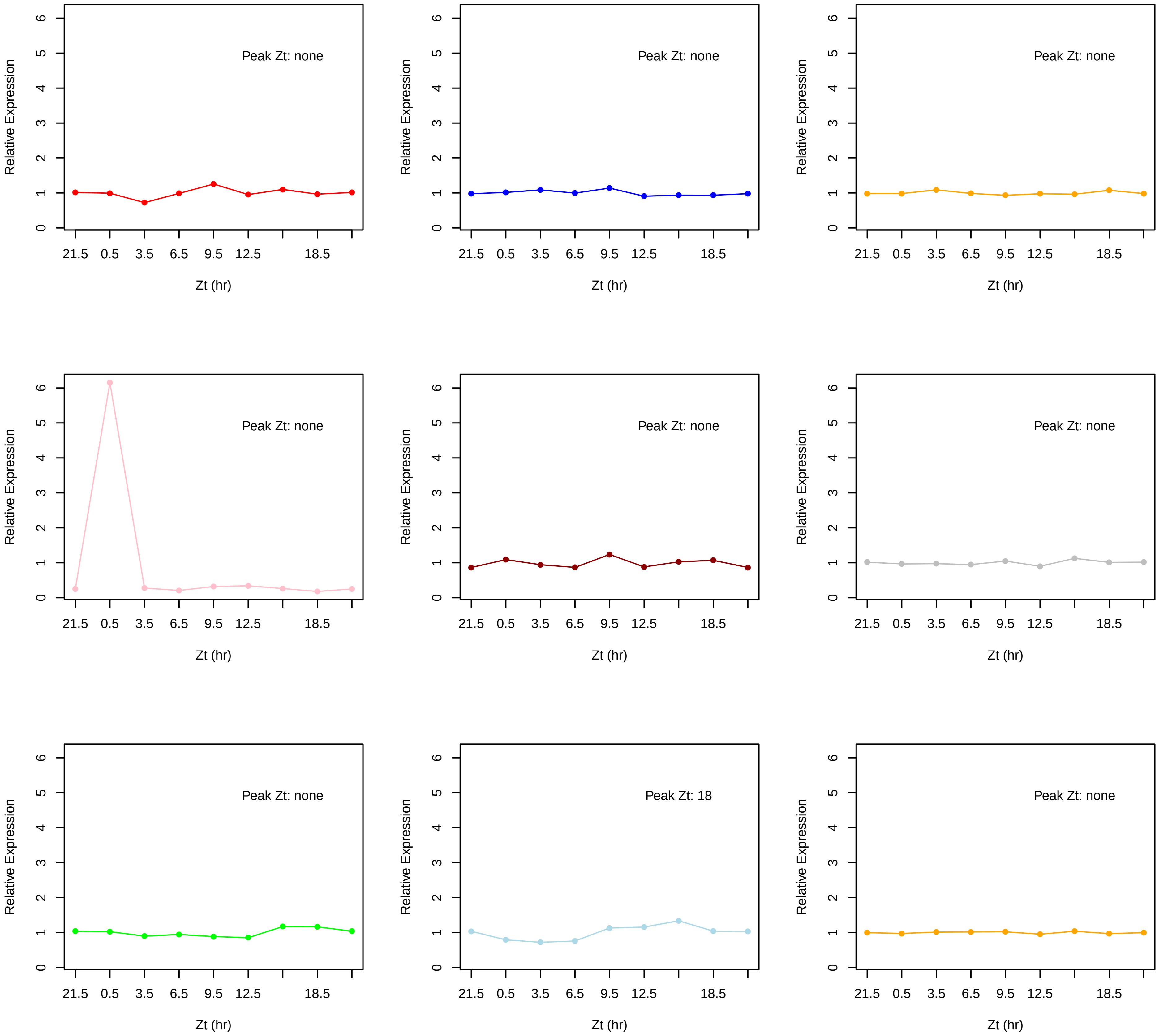

Singeleton: ENSXMAG00000009532 gemin2

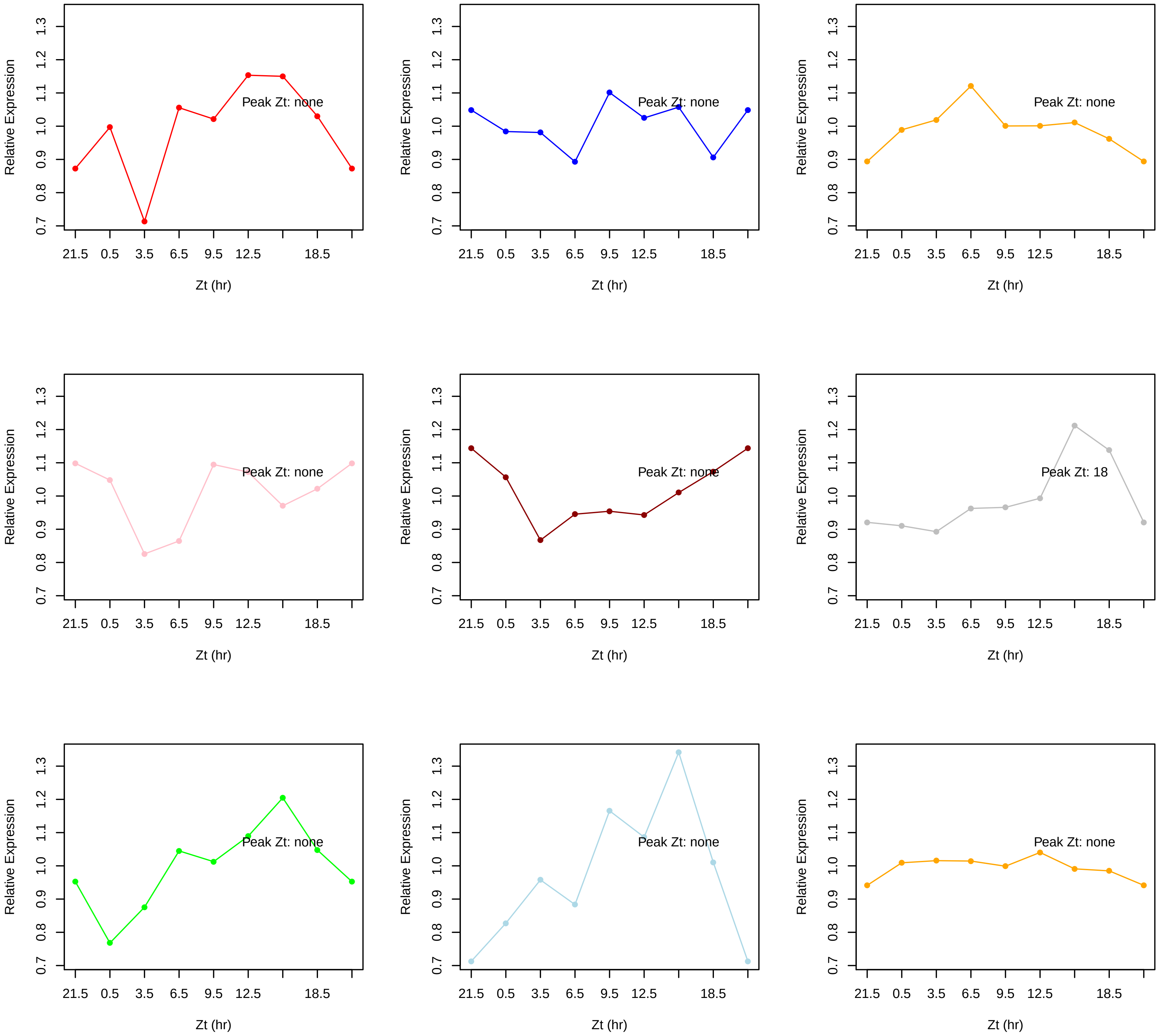

Singleton: ENSXMAG00000011008 sntb1

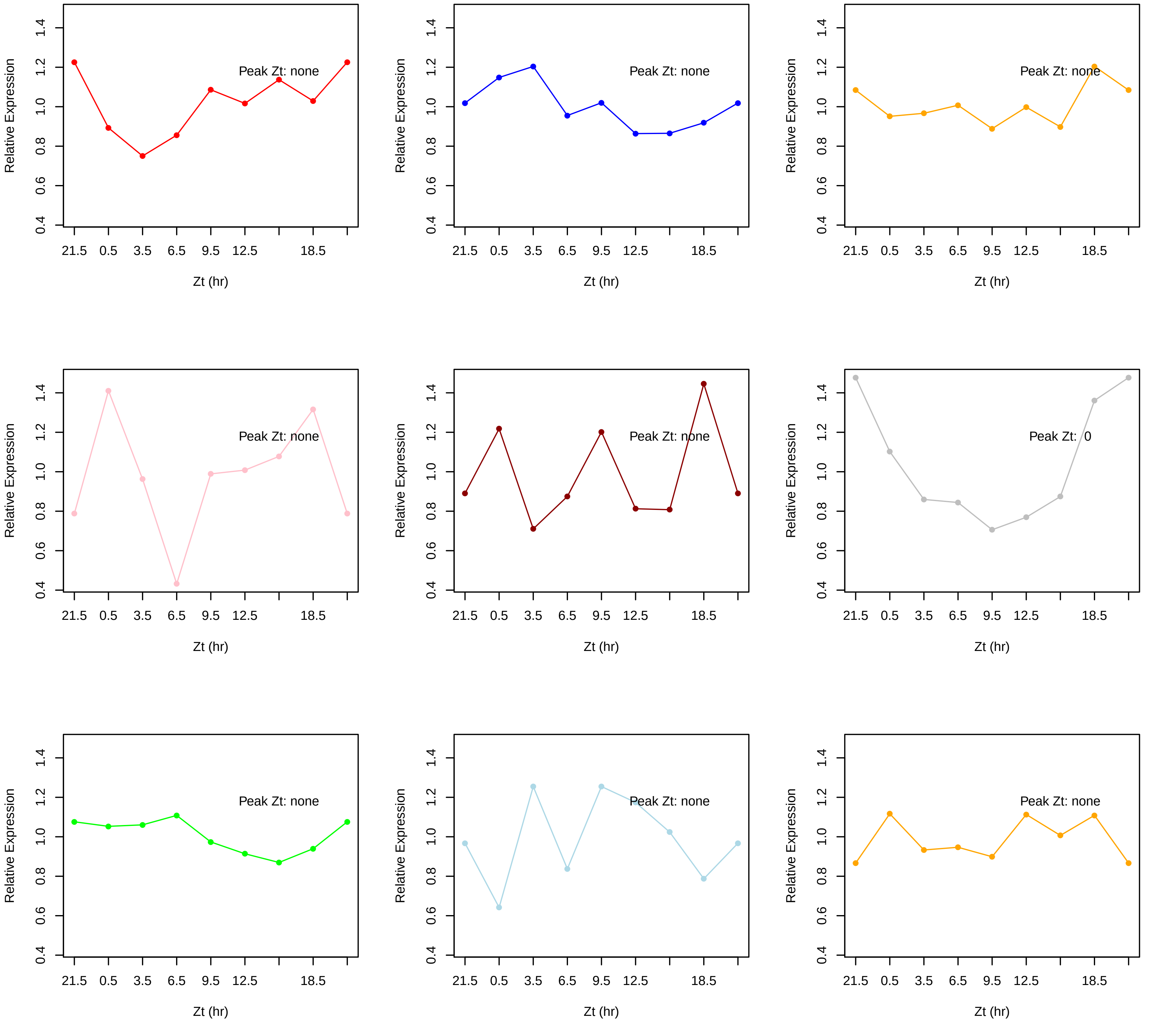

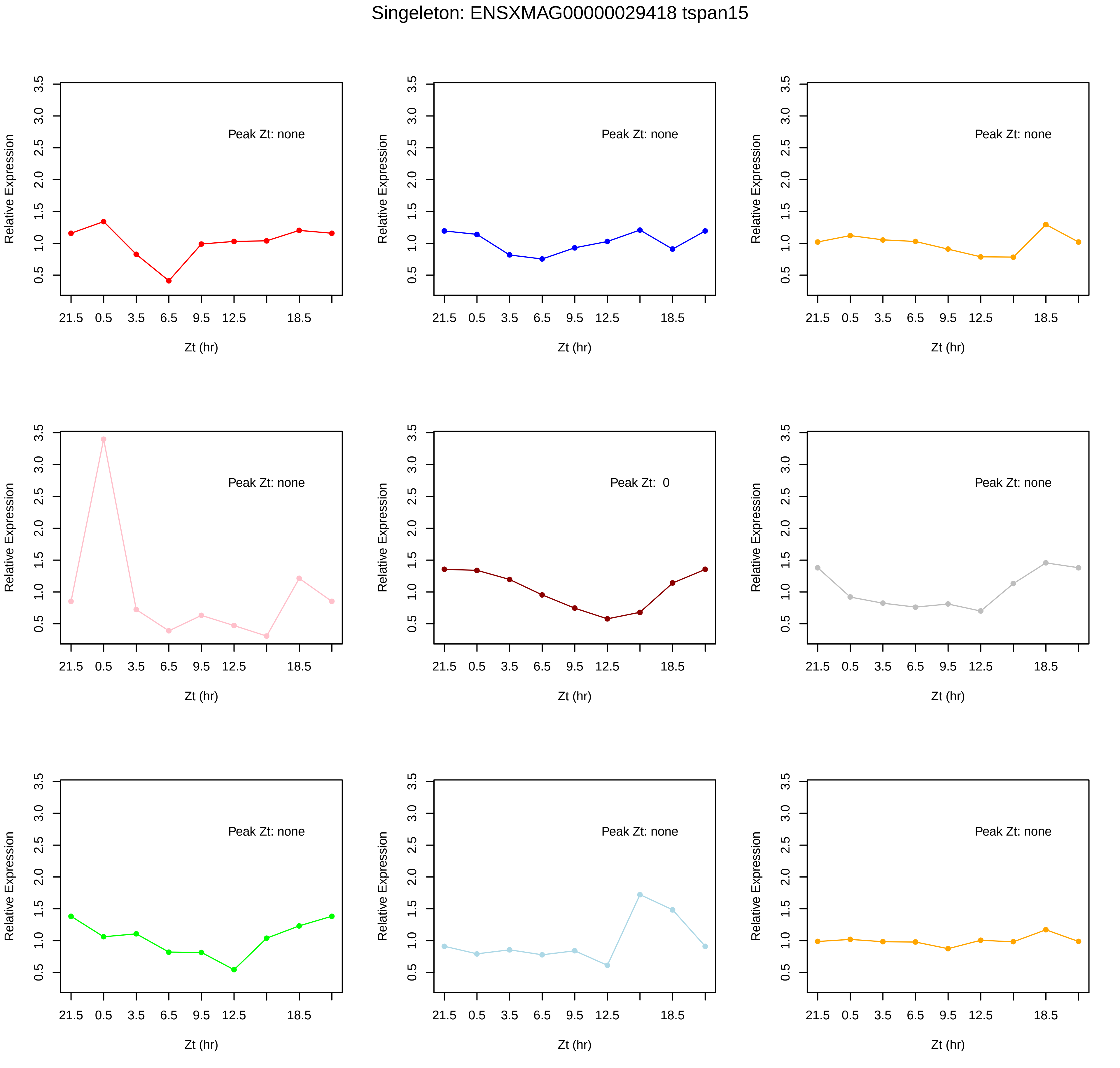

Singeleton: ENSXMAG00000029418 tspan15
Peak Zt: none
Peak Zt: none
Peak Zt: none
Peak Zt: none
Peak Zt: 0
Peak Zt: none
Peak Zt: none
Peak Zt: none
Peak Zt: none
Relative Expression
Zt (hr)
0.5 1.0 1.5 2.0 2.5 3.0 3.5
21.5 0.5 3.5 6.5 9.5 12.5 18.5

Singleton: ENSXMAG00000018958 acbd6

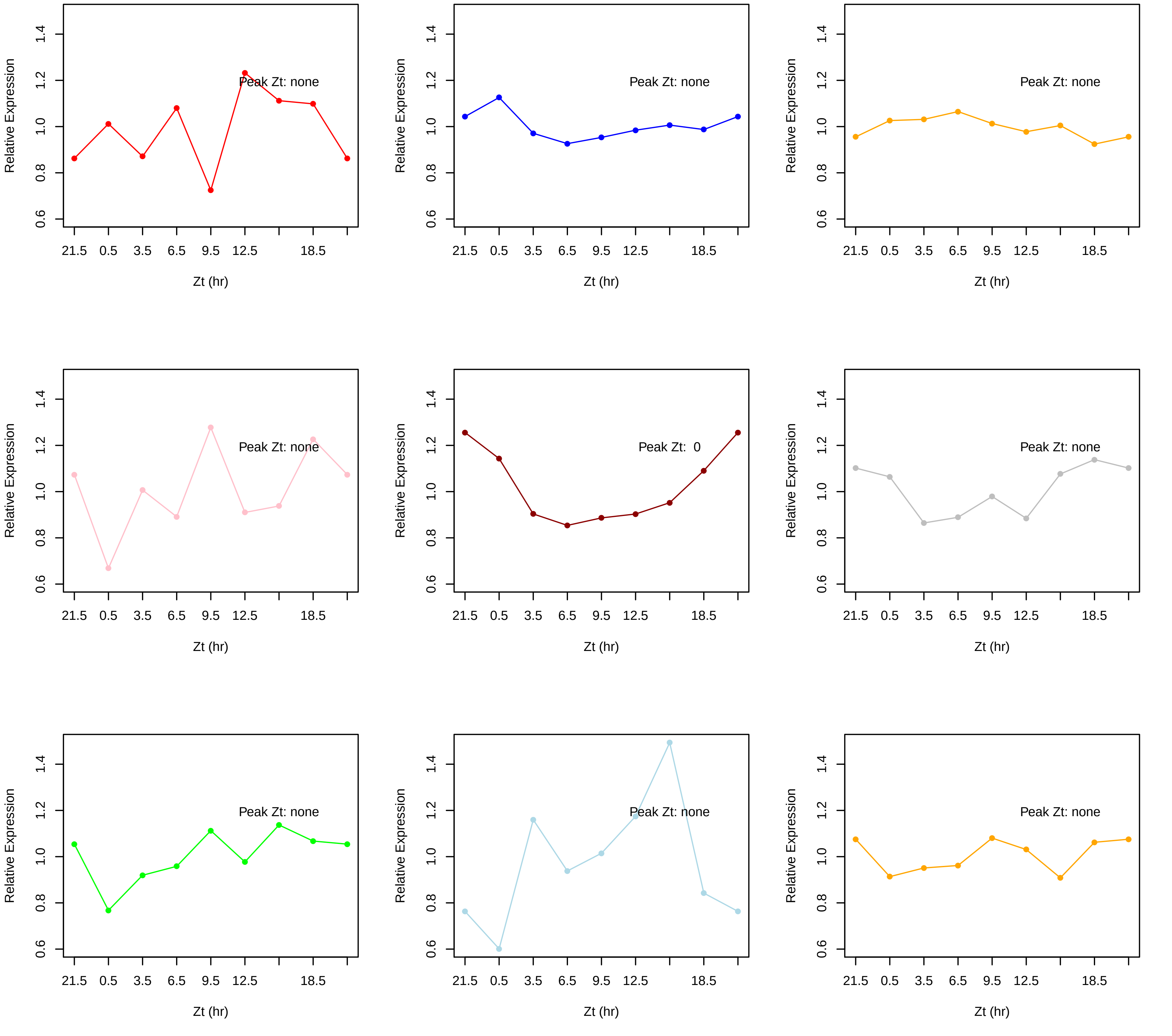

Singleton: ENSXMAG00000006572 slc4a3

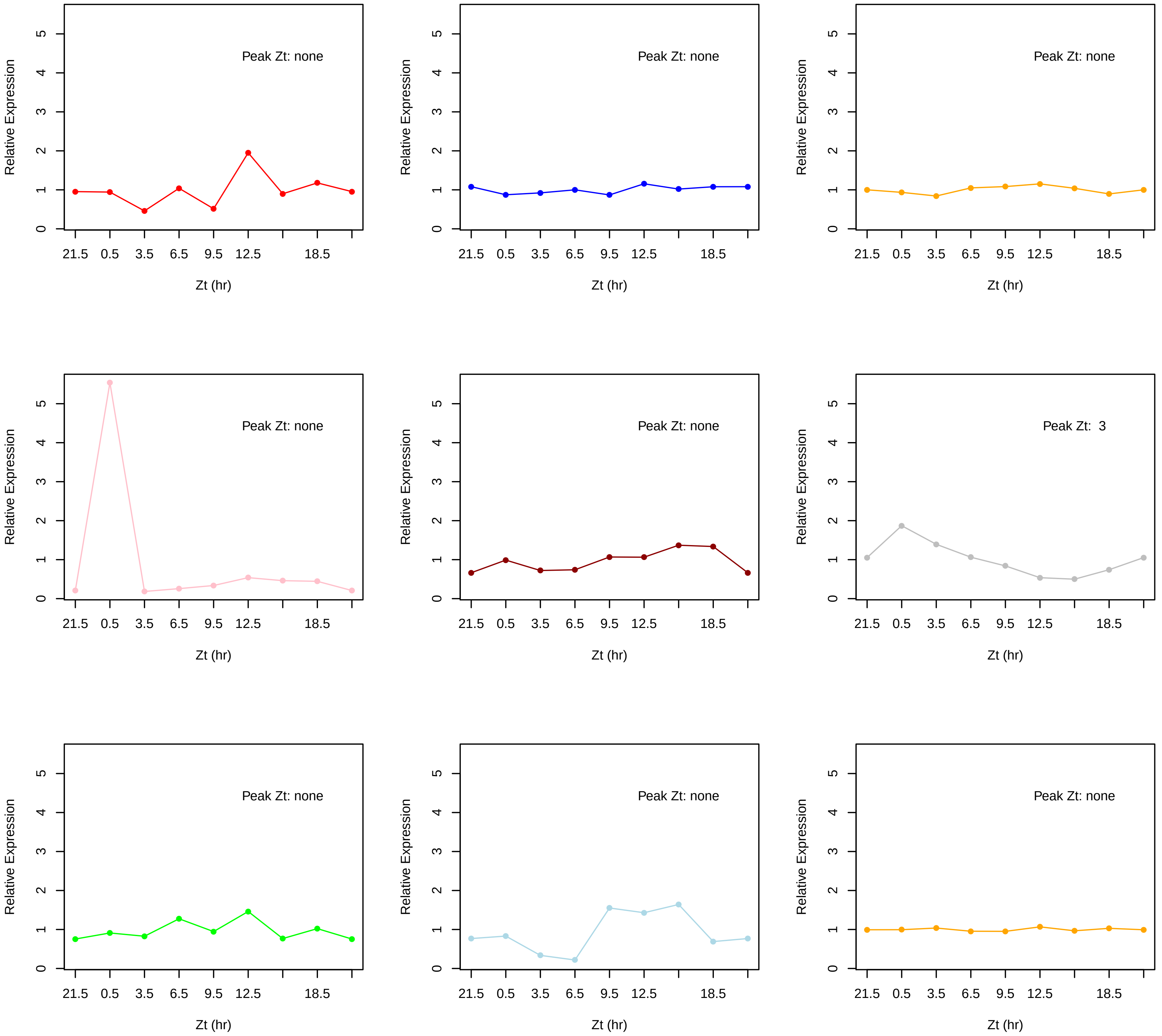

Singeleton: ENSXMAG00000028952 jmjd4

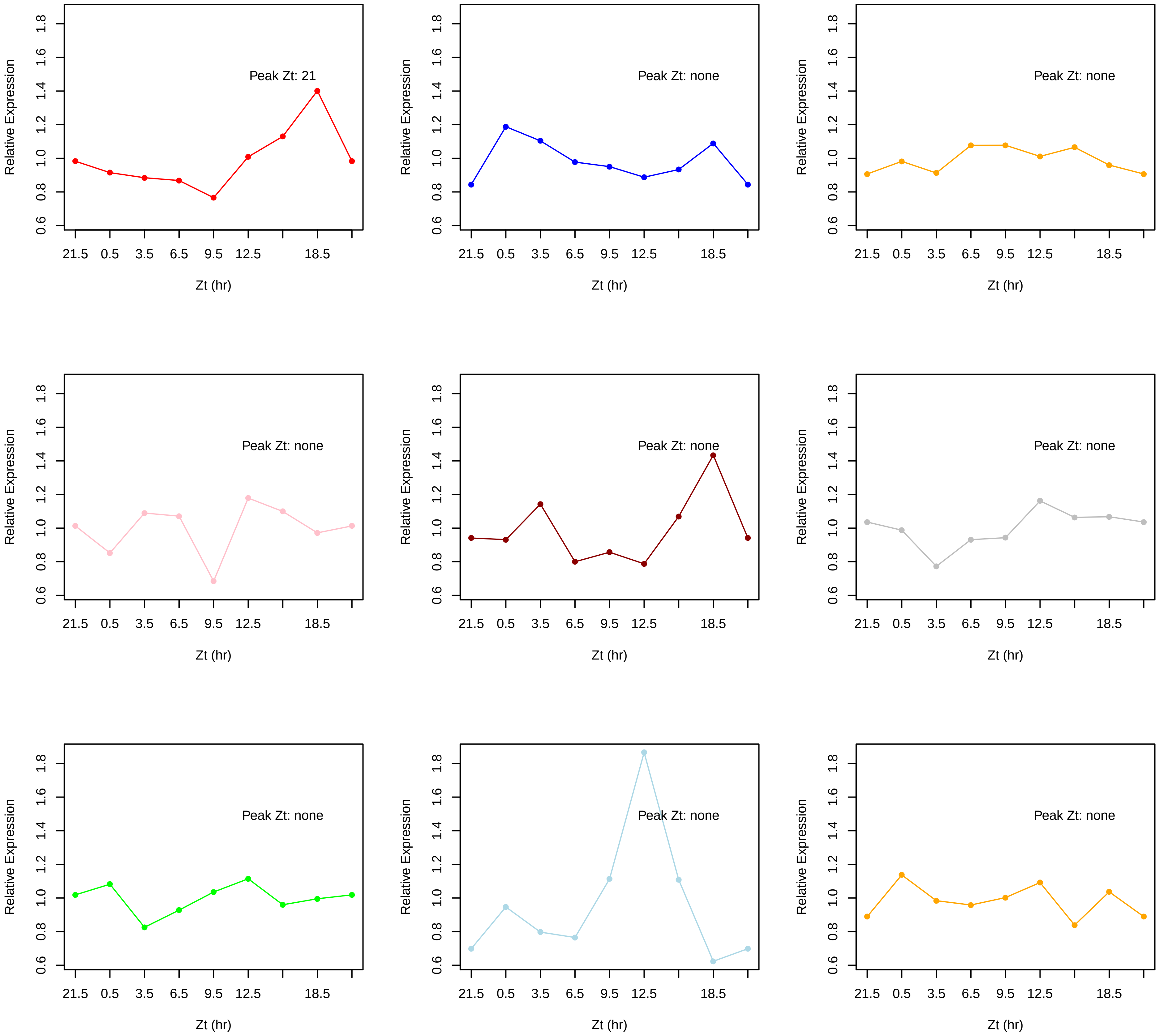

Singleton: ENSXMAG00000011285 kpna3

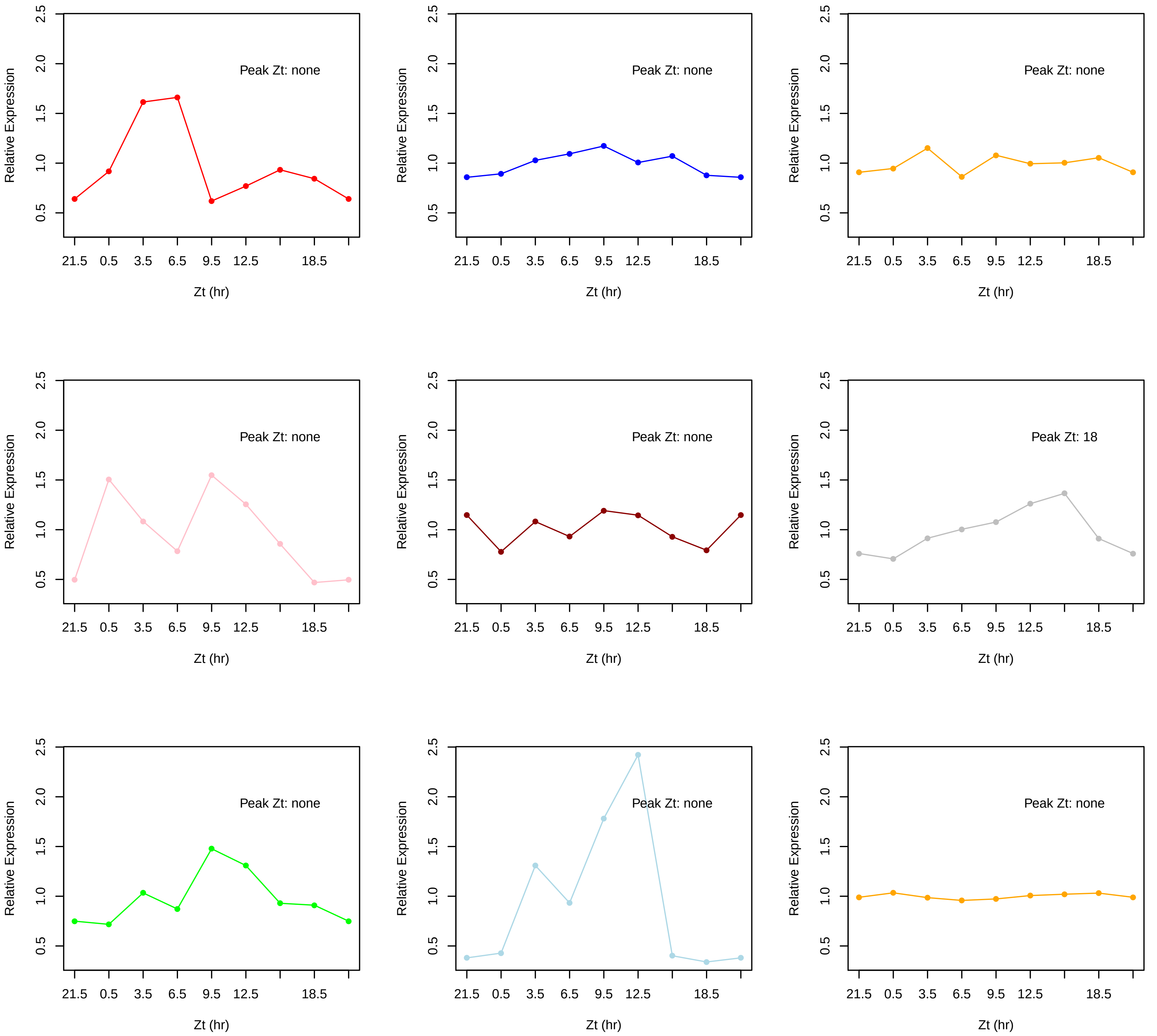

Singleton: ENSXMAG00000014775 HNRNPH3

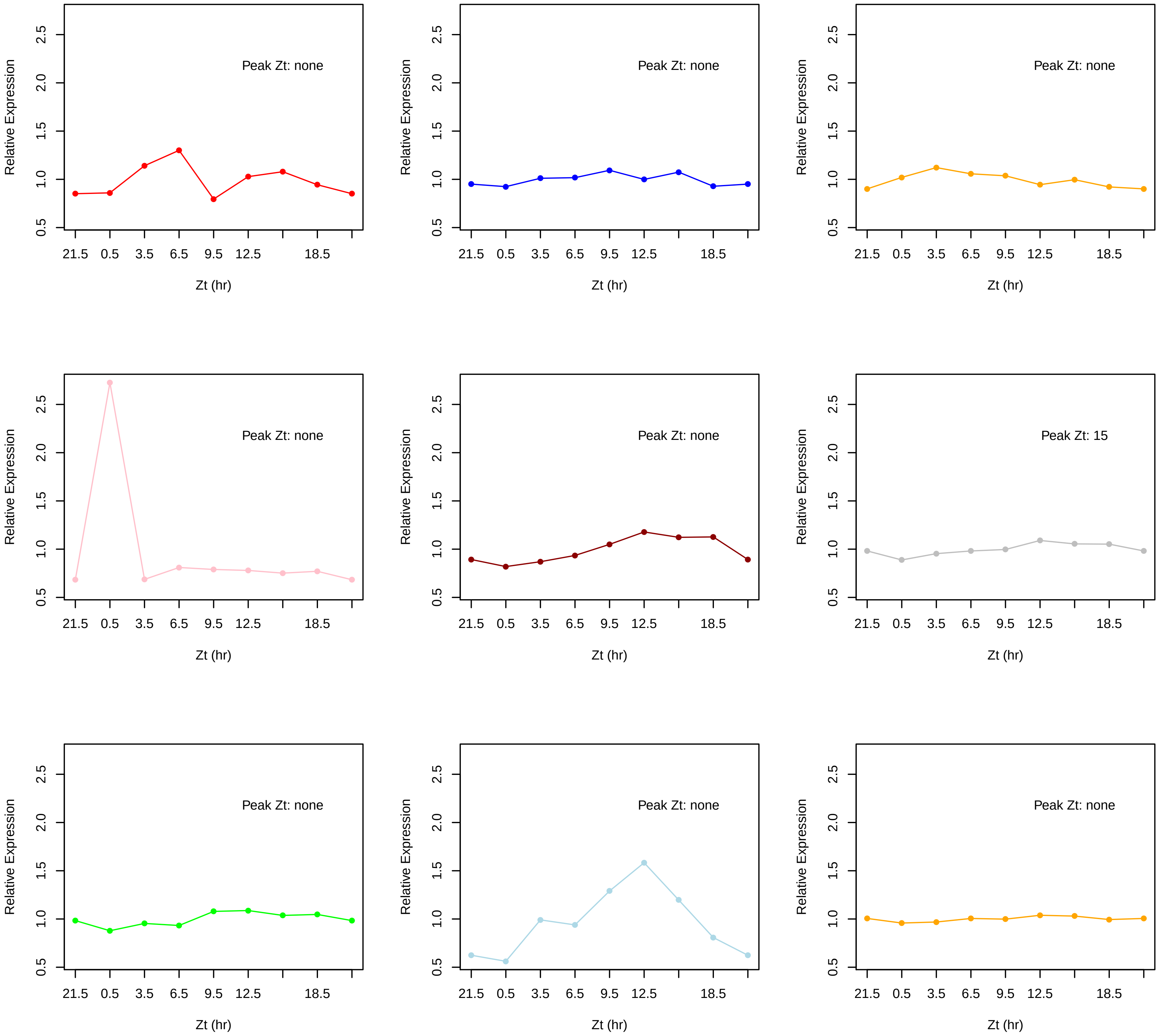

Singeleton: ENSXMAG00000018978 qsox1

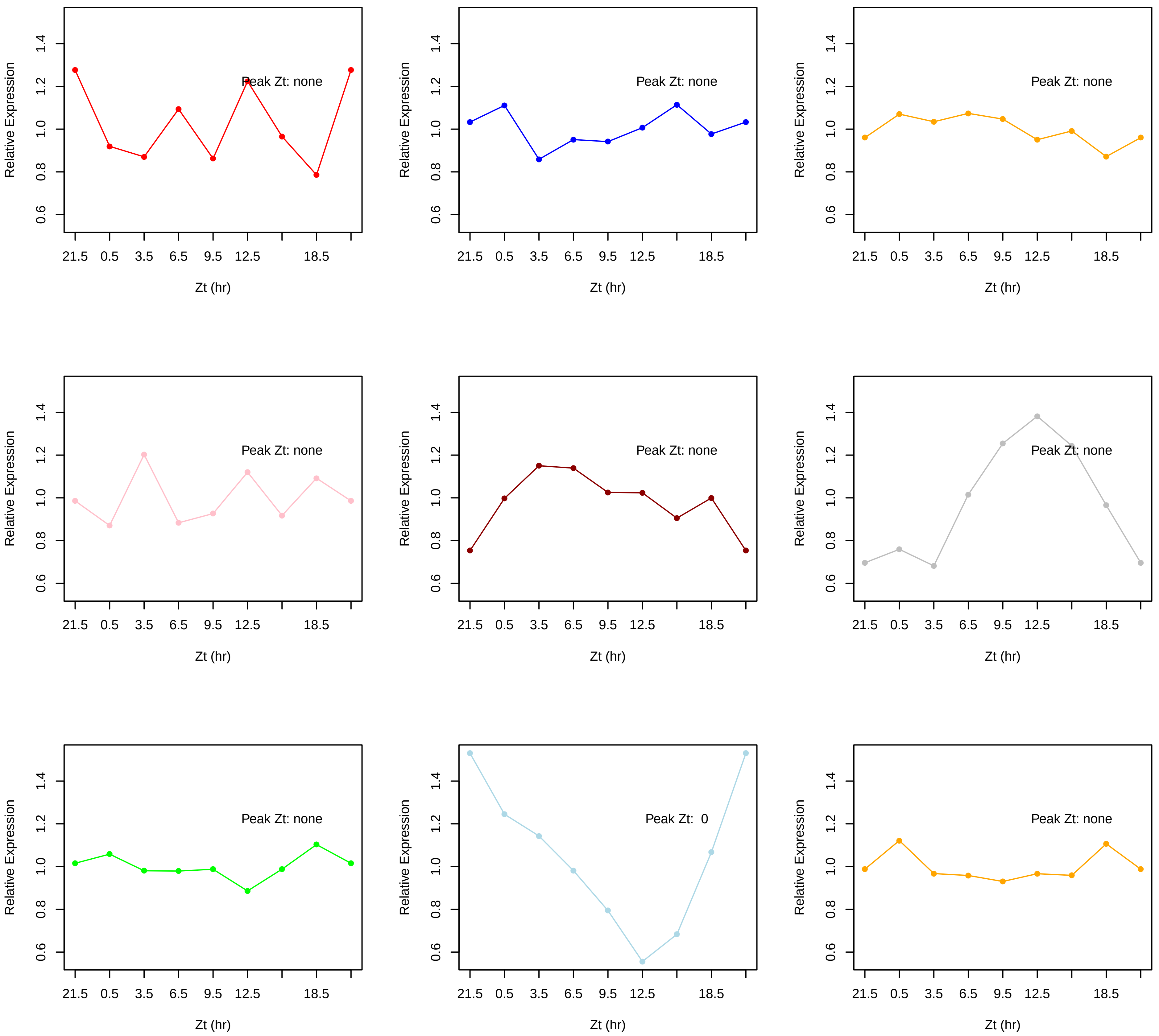

Singeleton: ENSXMAG00000029049 stk11ip

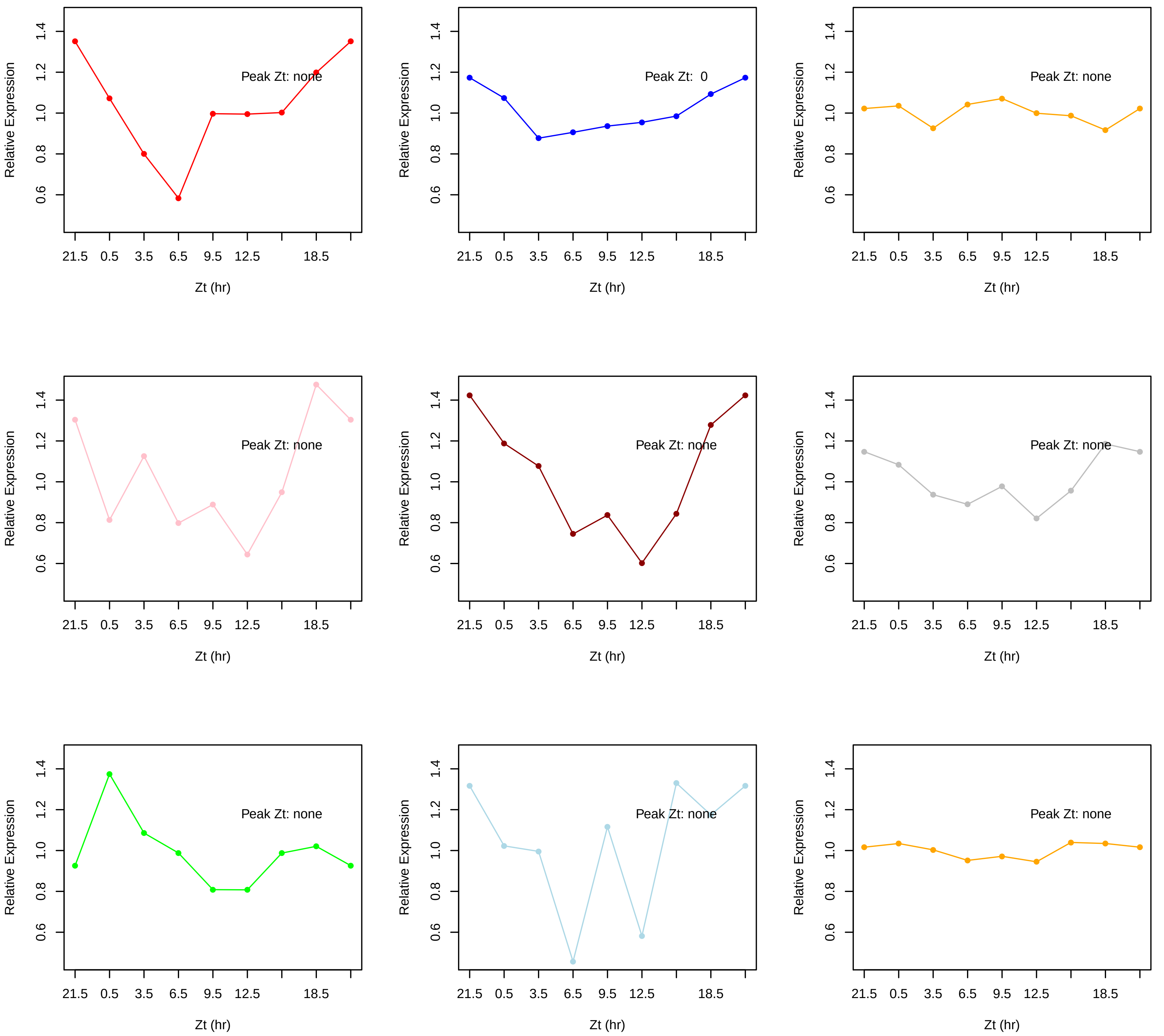

Singeleton: ENSXMAG00000008240 thg1l

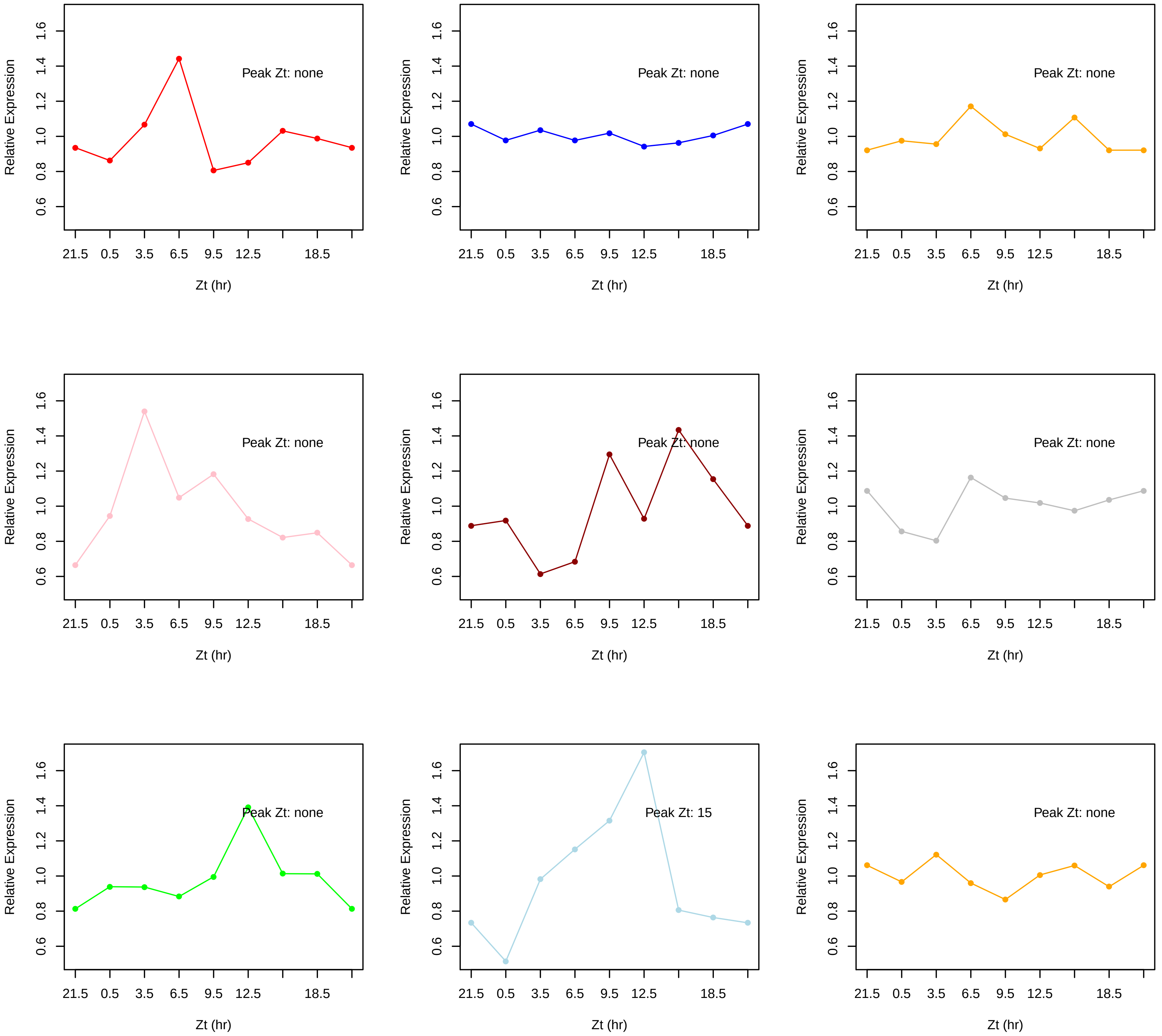

Singeleton: ENSXMAG00000011274 sprydb7b

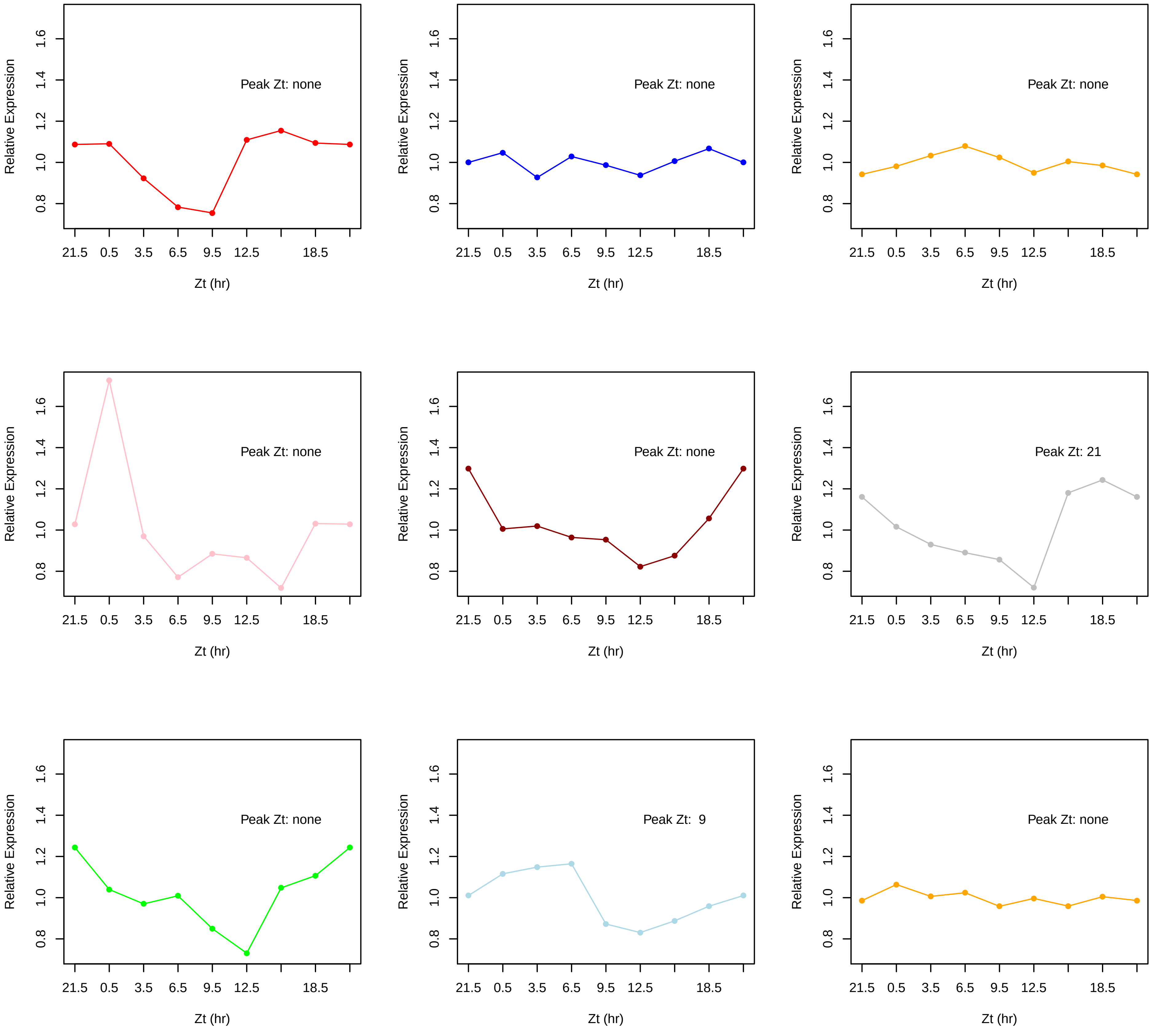

Singeleton: ENSXMAG00000005722 atg9b

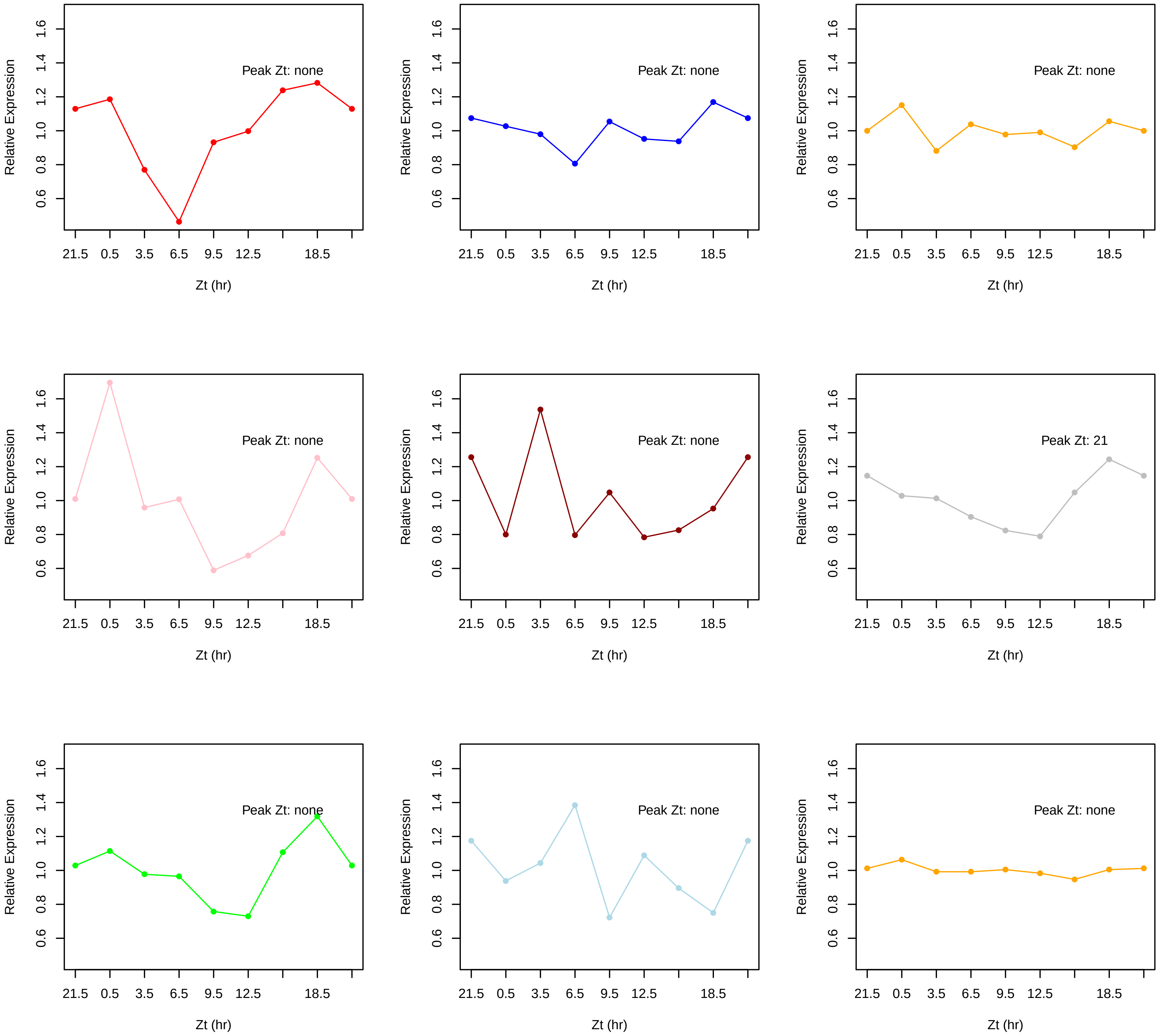

Singeleton: ENSXMAG00000014754 pbld

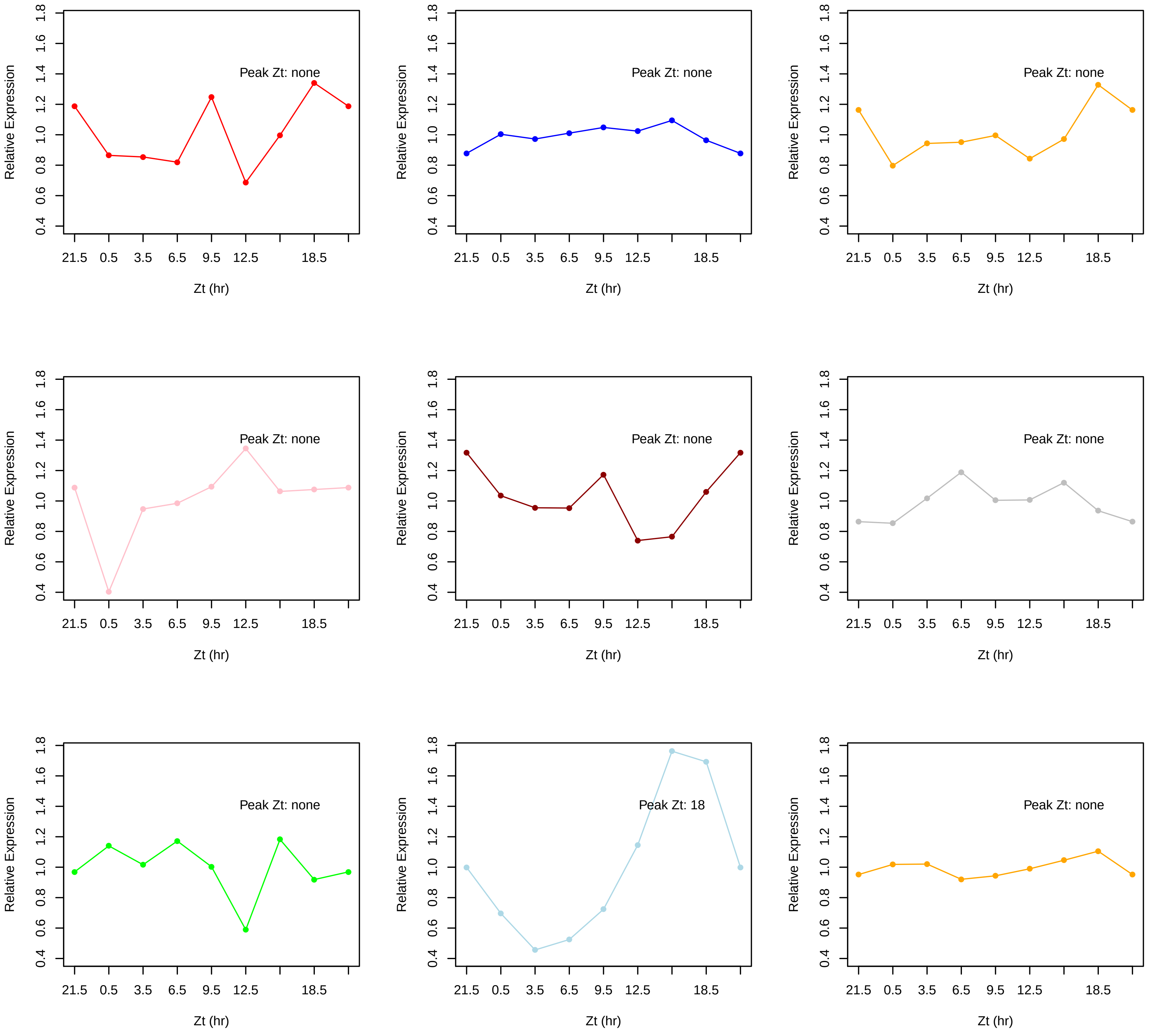

Singeleton: ENSXMAG00000000829 atg16l1

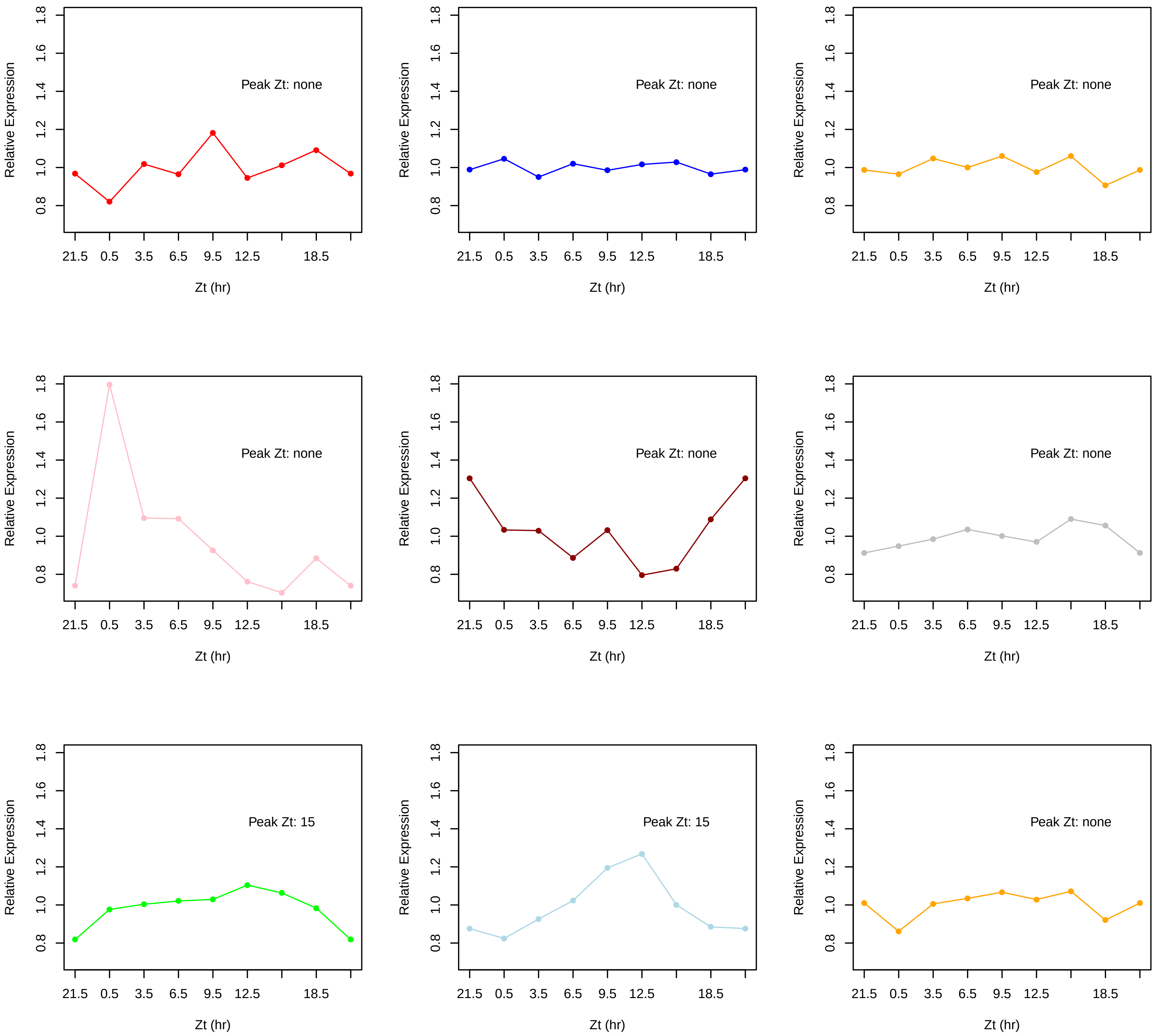

Singeleton: ENSXMAG00000003836 daam1b

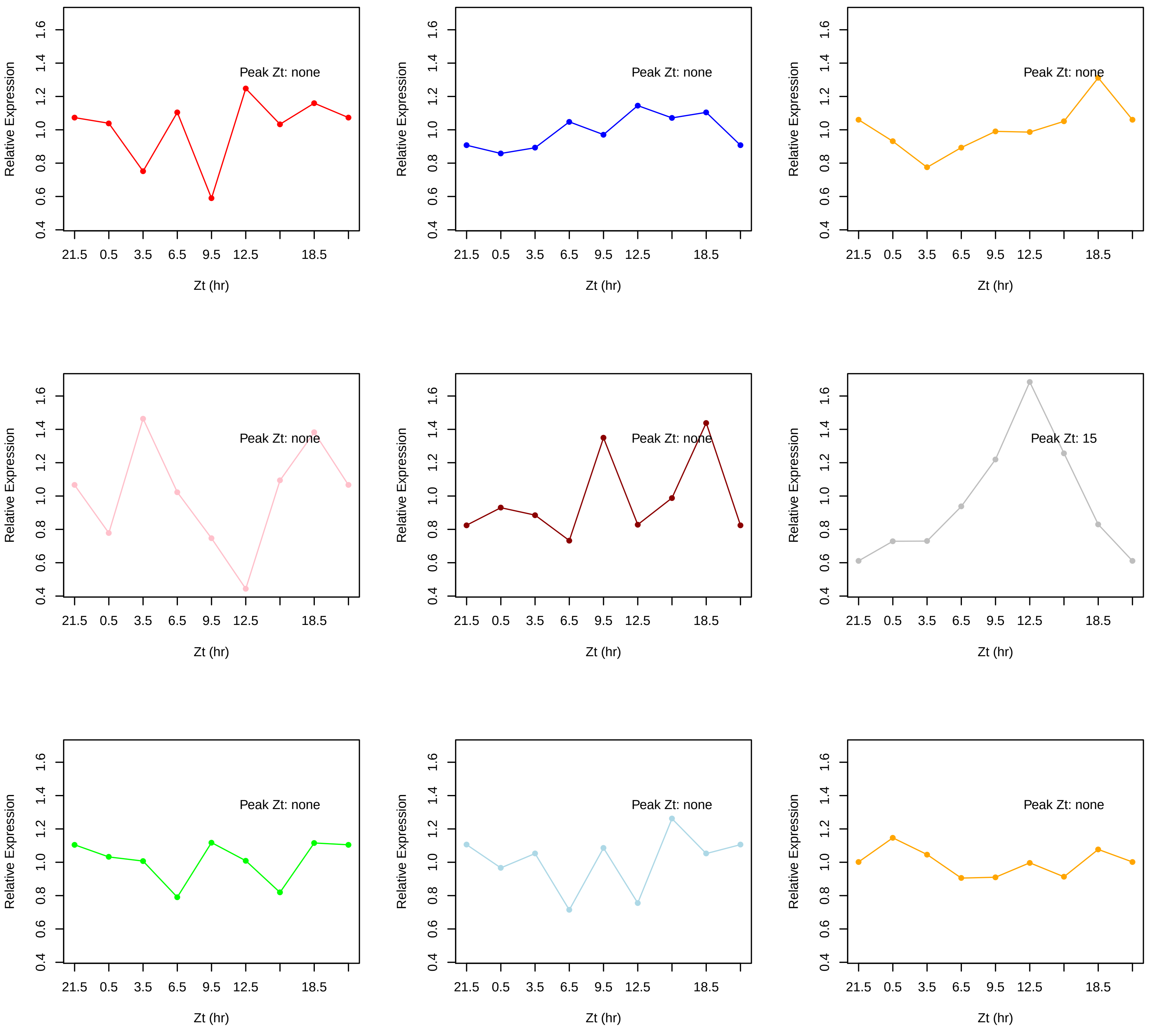

Singeleton: ENSXMAG00000000480 rnf145b

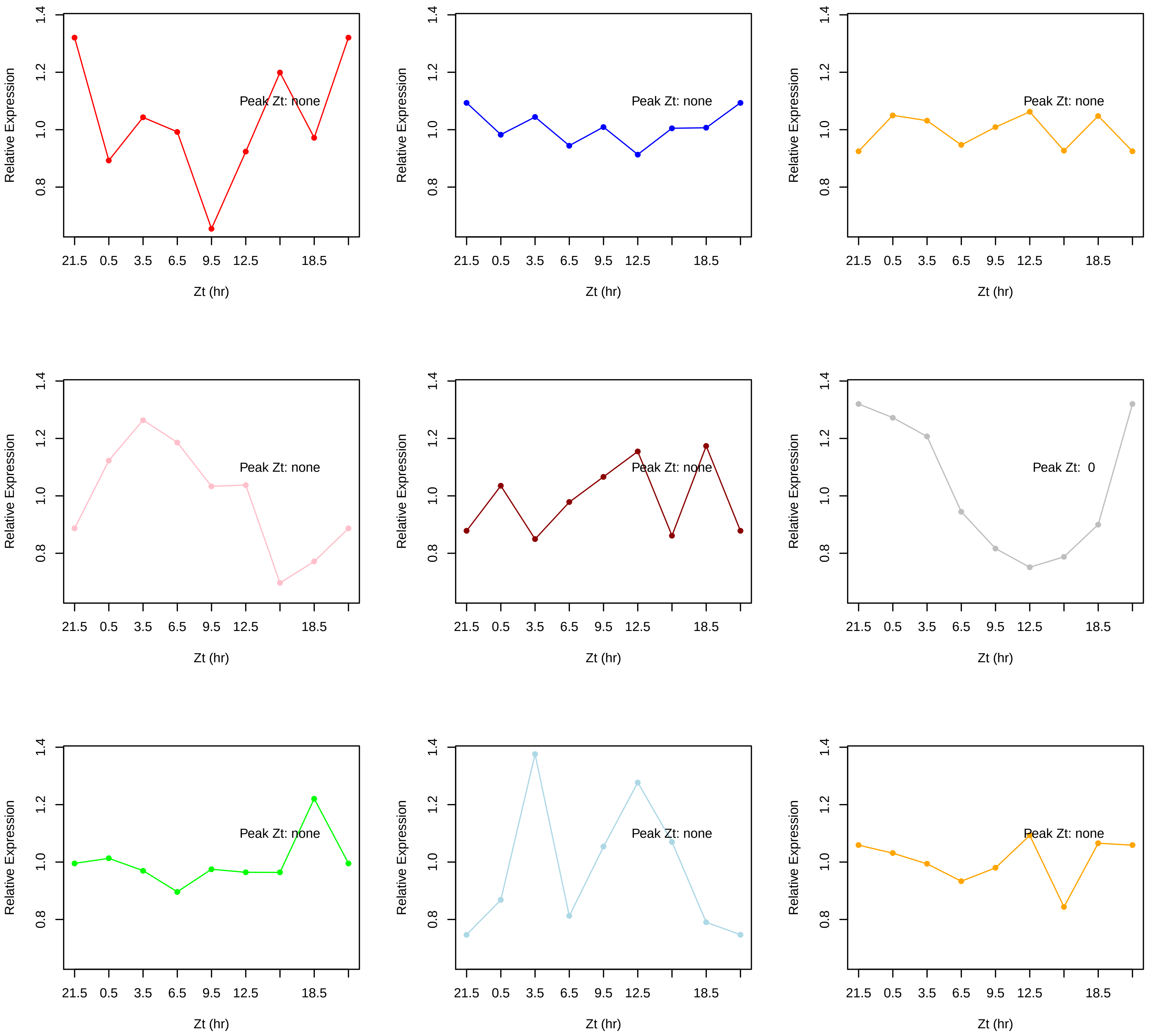

Singeleton: ENSXMAG00000022554 rnaseh2b

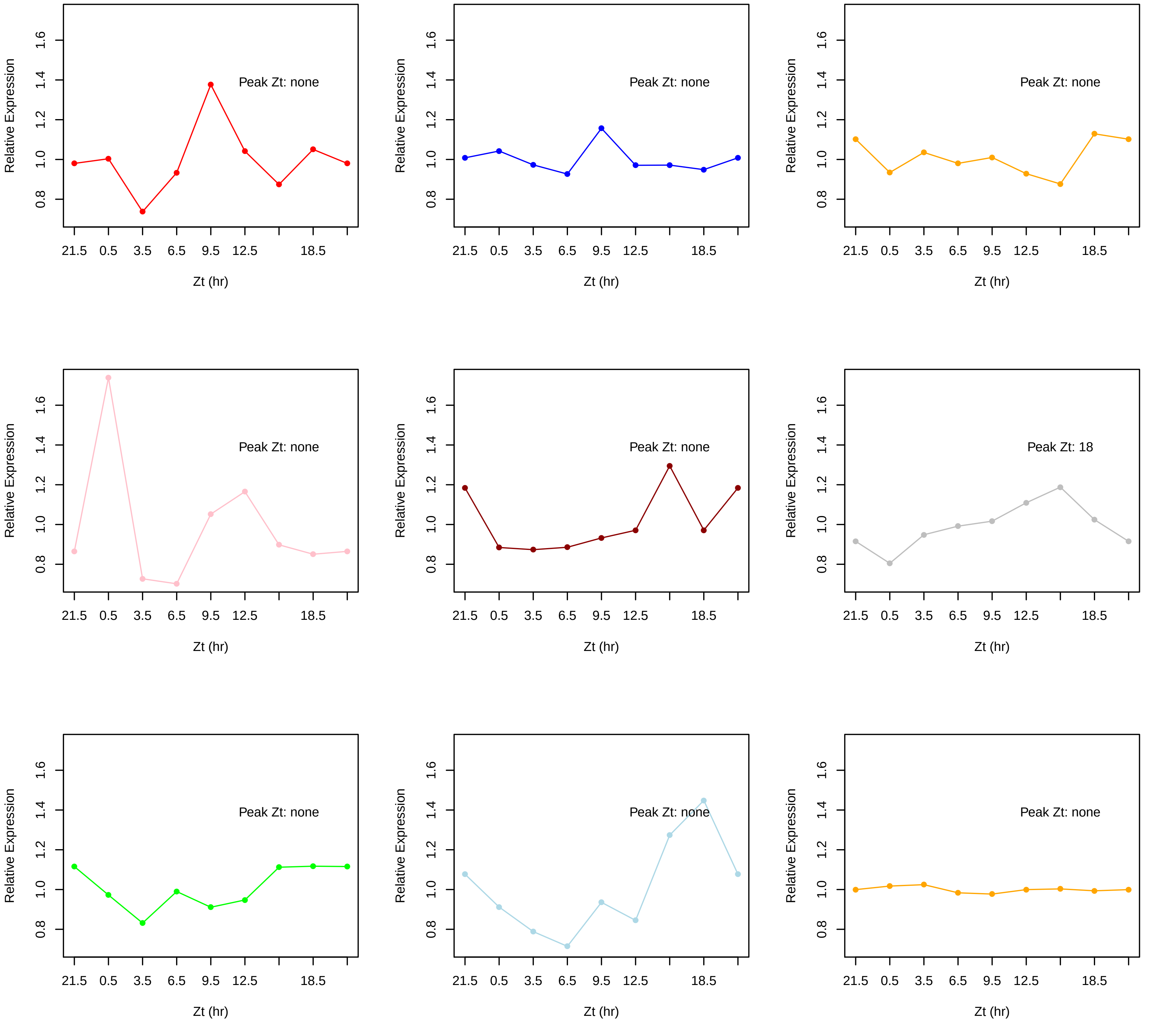

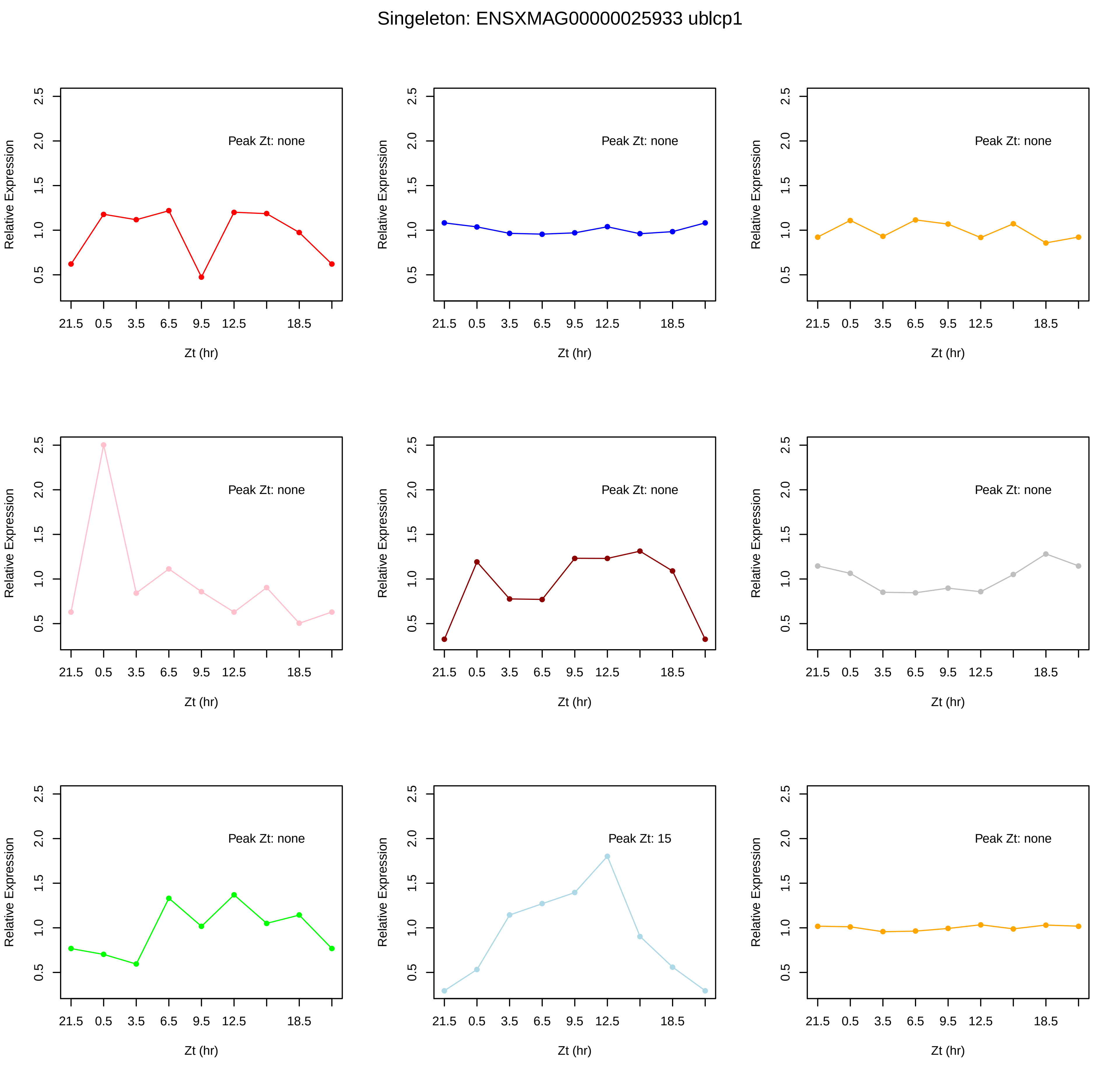
Singeleton: ENSXMAG00000025933 ublcp1
Relative Expression
Zt (hr)
Peak Zt: none
Peak Zt: none
Peak Zt: none
Peak Zt: none
Peak Zt: none
Peak Zt: none
Peak Zt: none
Peak Zt: 15
Peak Zt: none

Singeleton: ENSXMAG00000022157 psma2

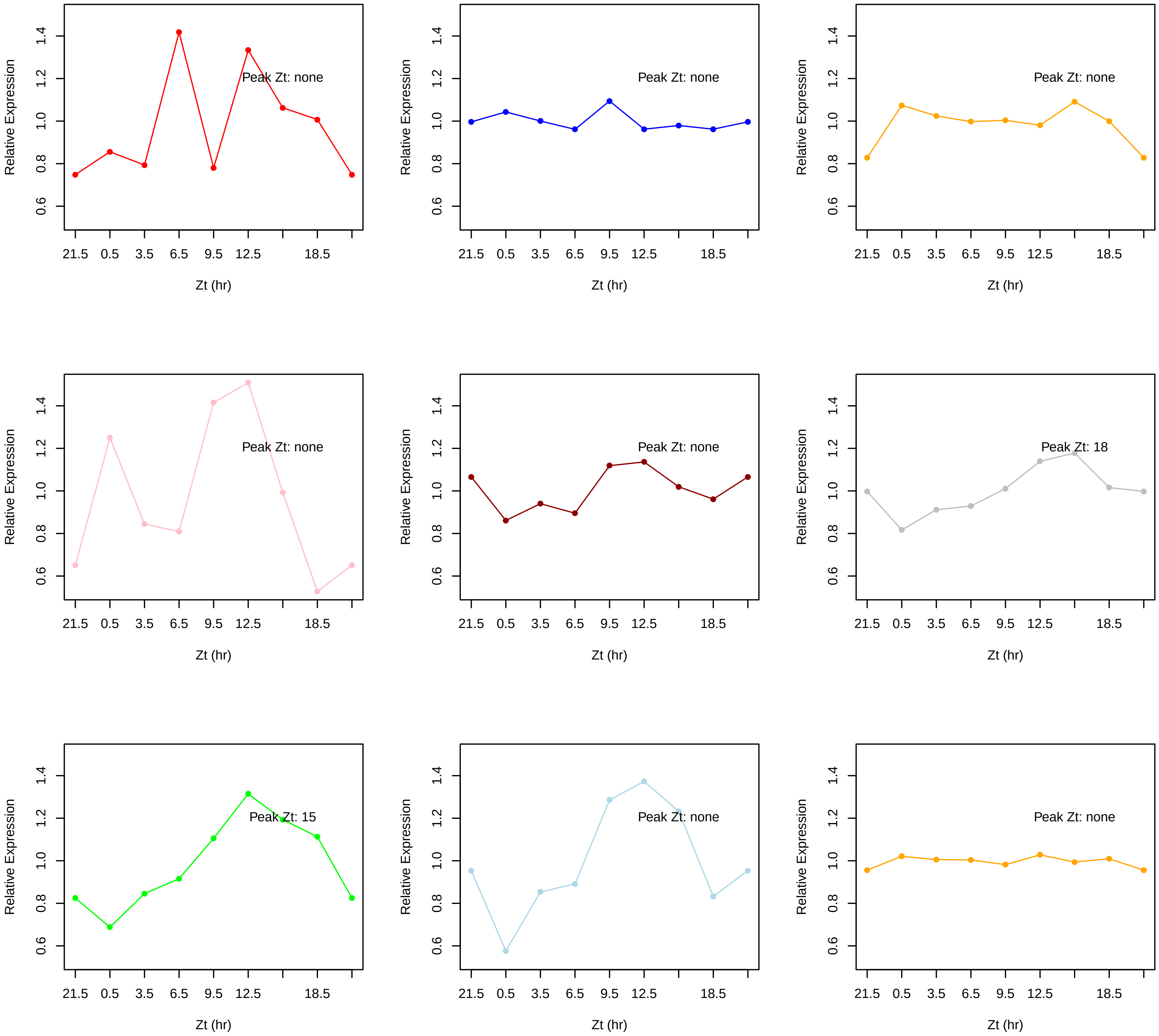

Singeleton: ENSXMAG00000003345

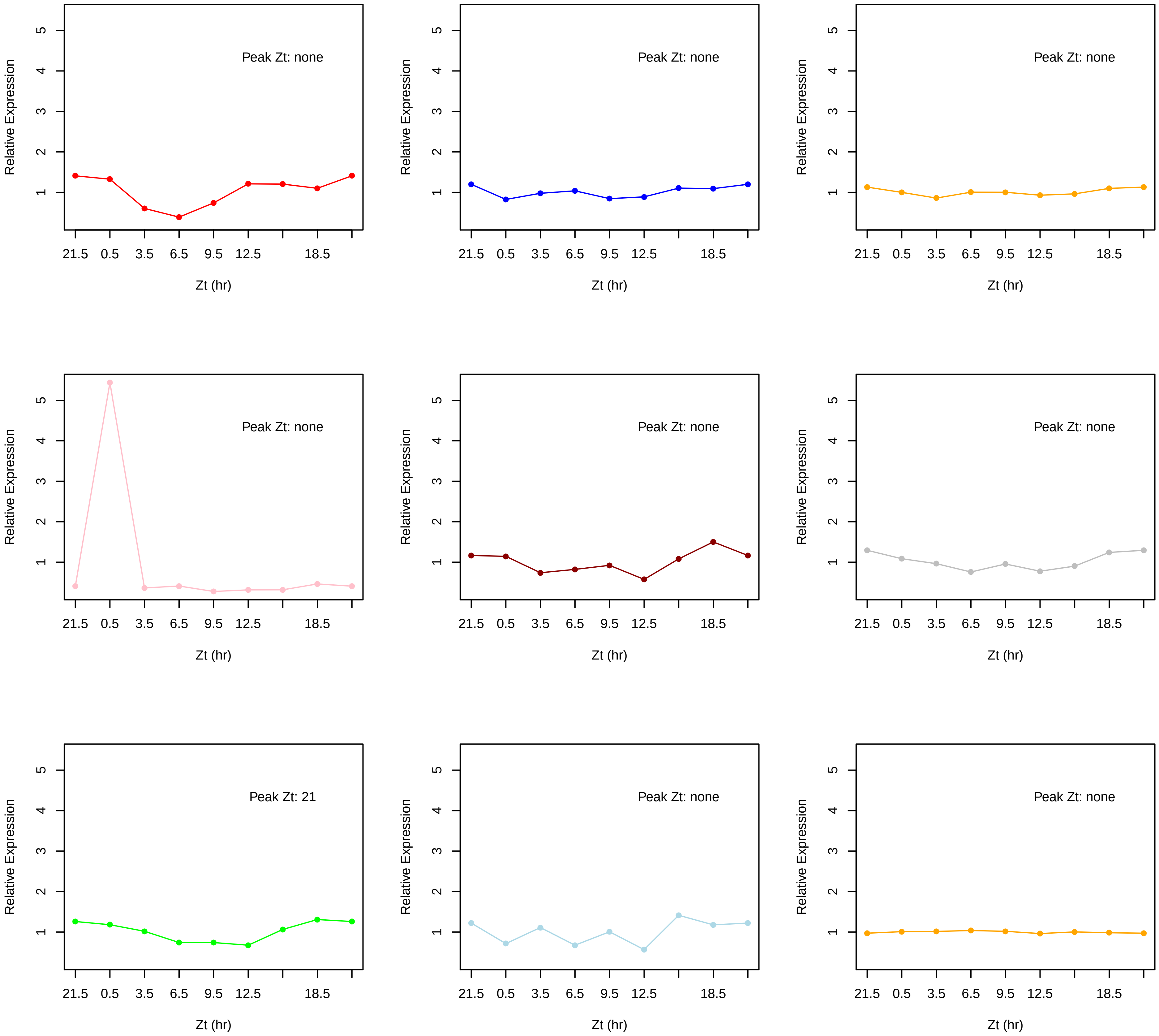

Singeleton: ENSXMAG00000028172 khnyn

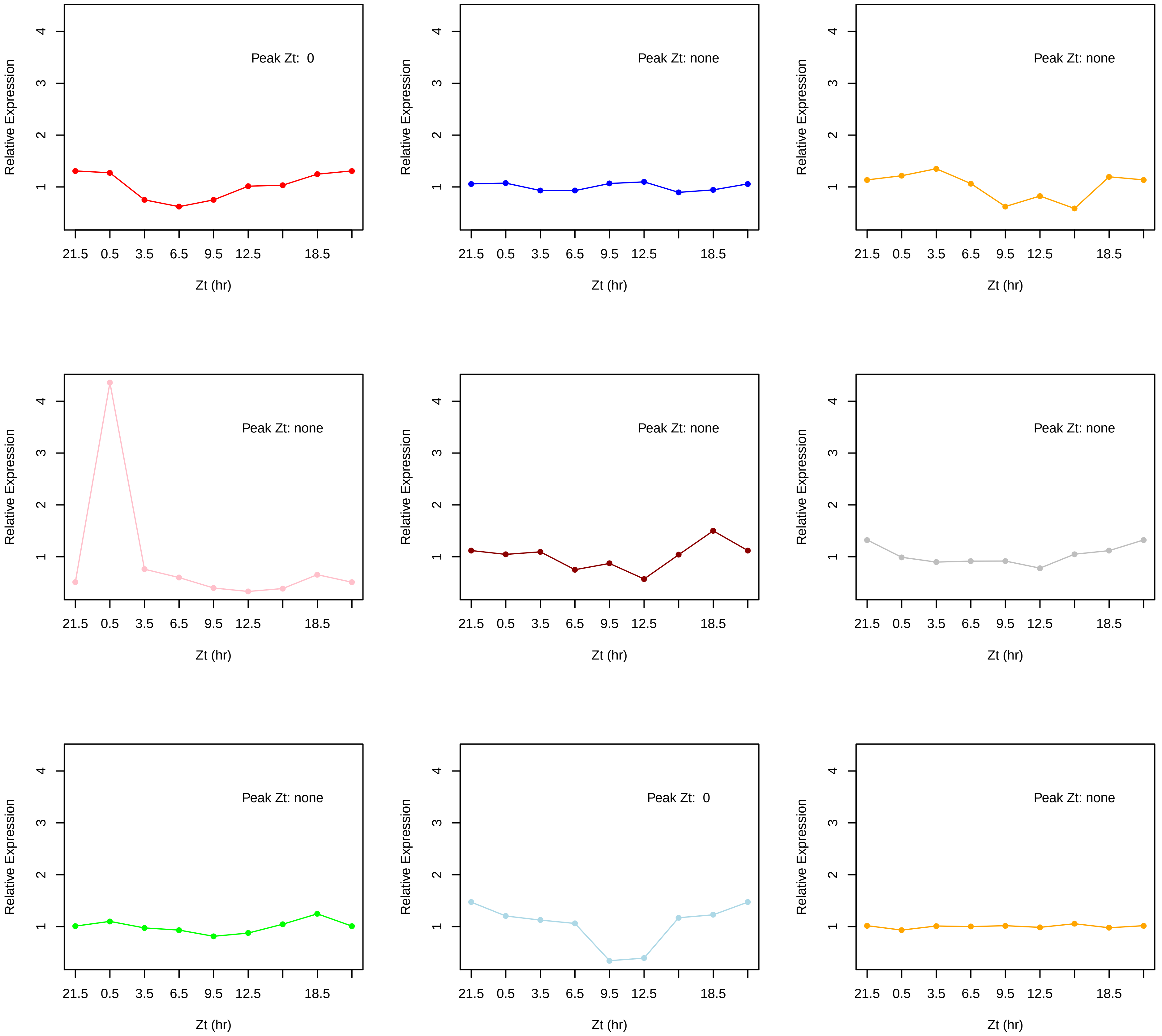

Singleton: ENSXMAG00000016117 sfxn5b

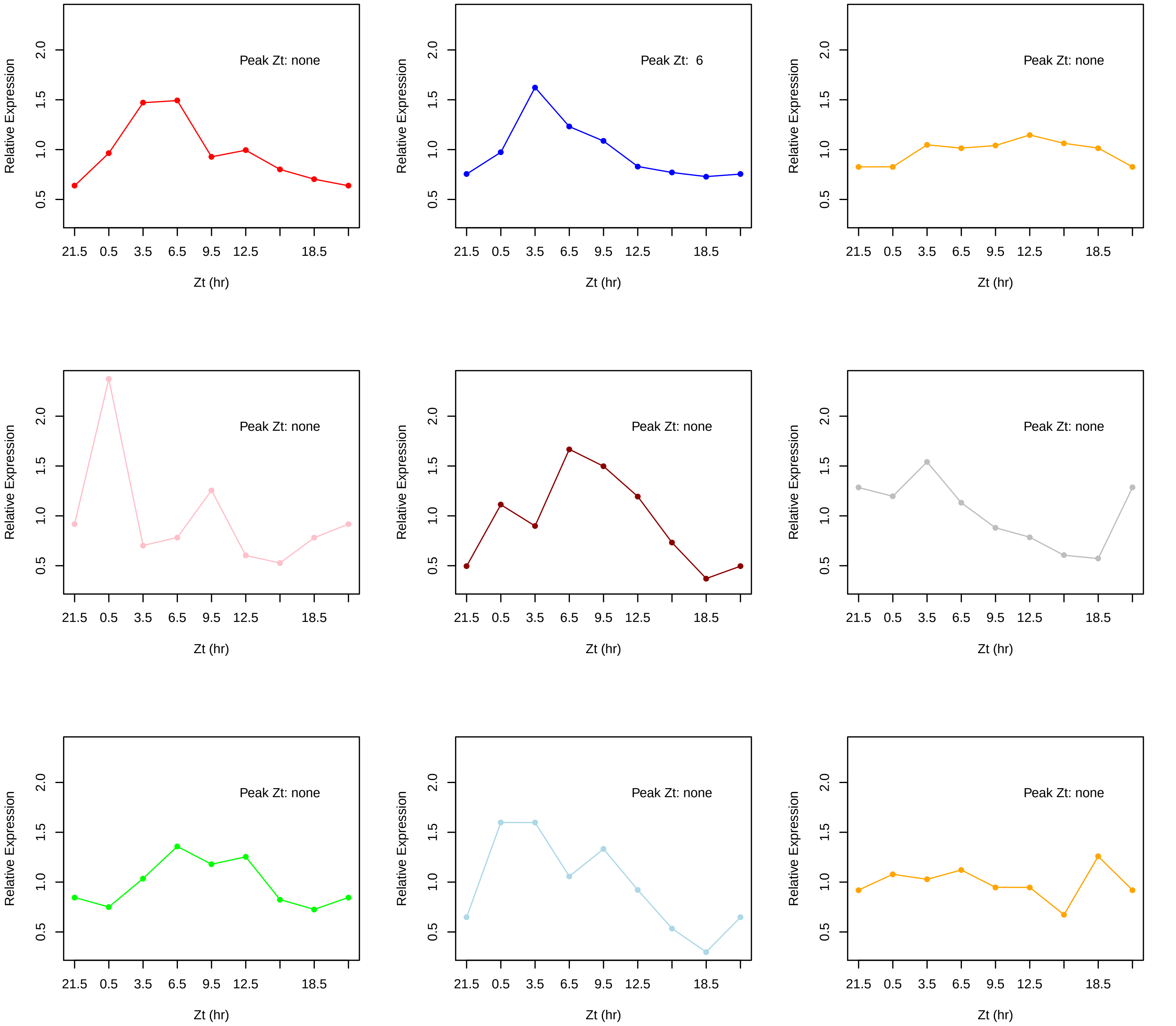

Singeleton: ENSXMAG00000023567 eif4e1c

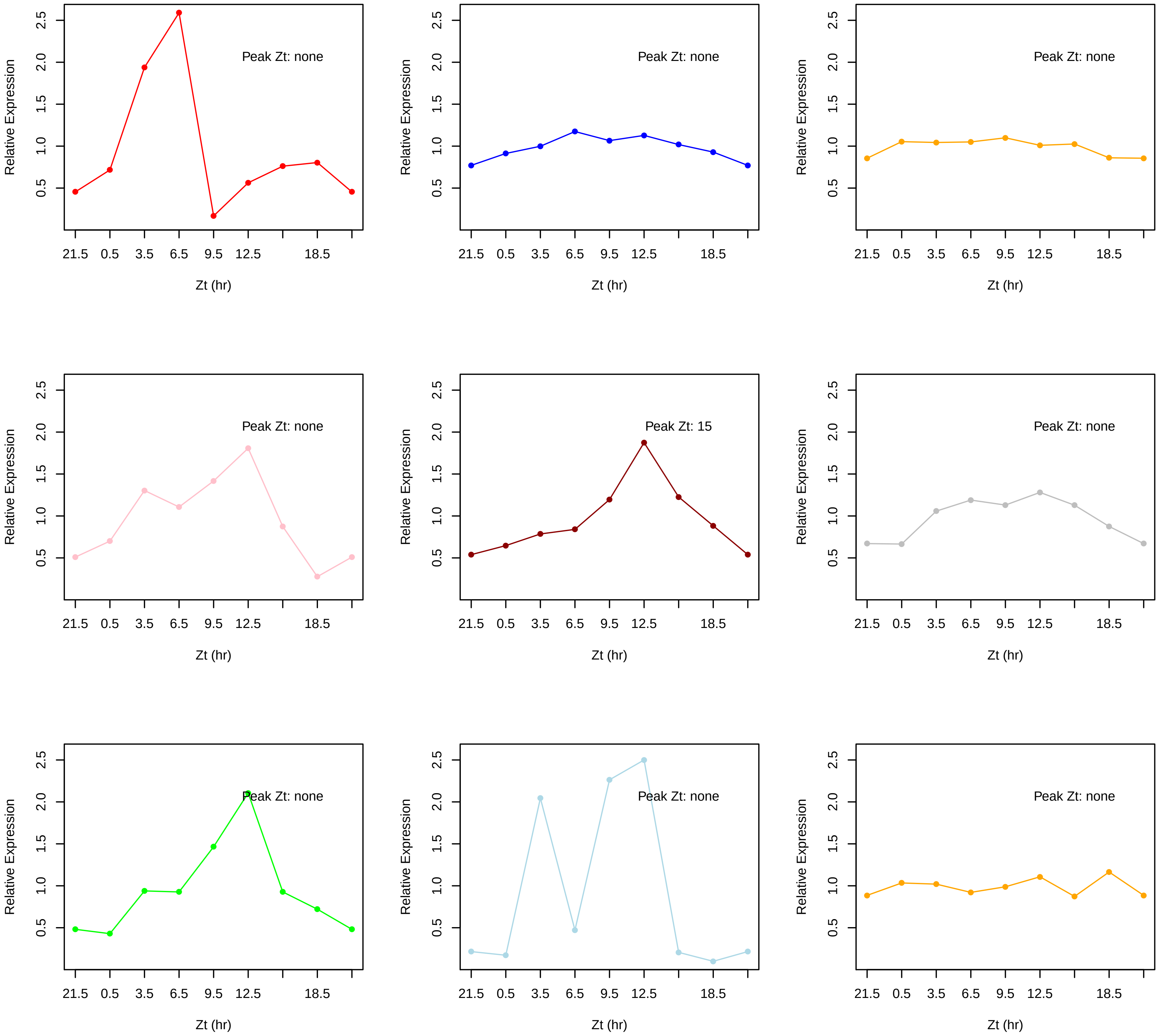

Singeleton: ENSXMAG00000011261 NR0B1

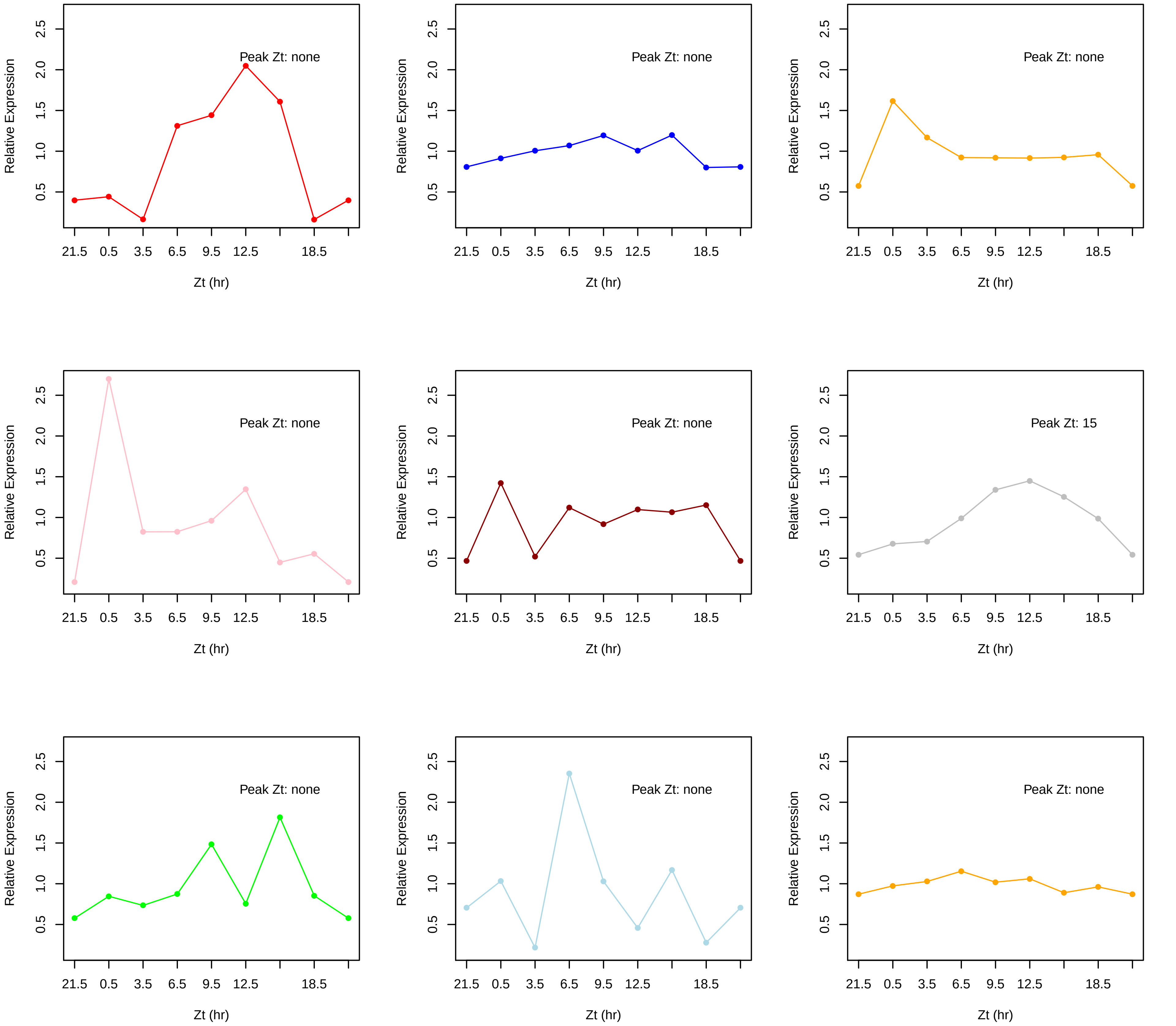

Singleton: ENSXMAG00000018883 asb10

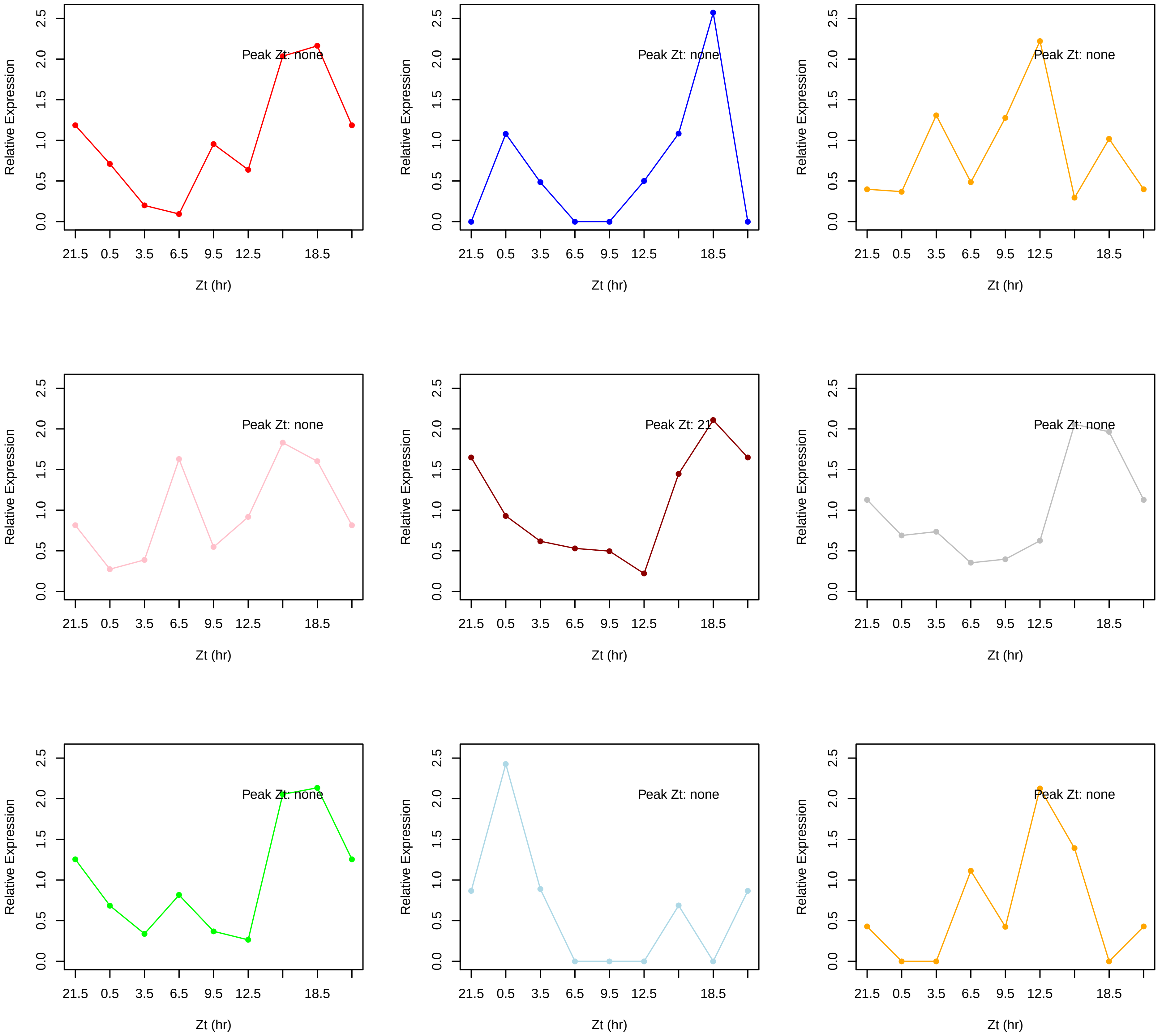

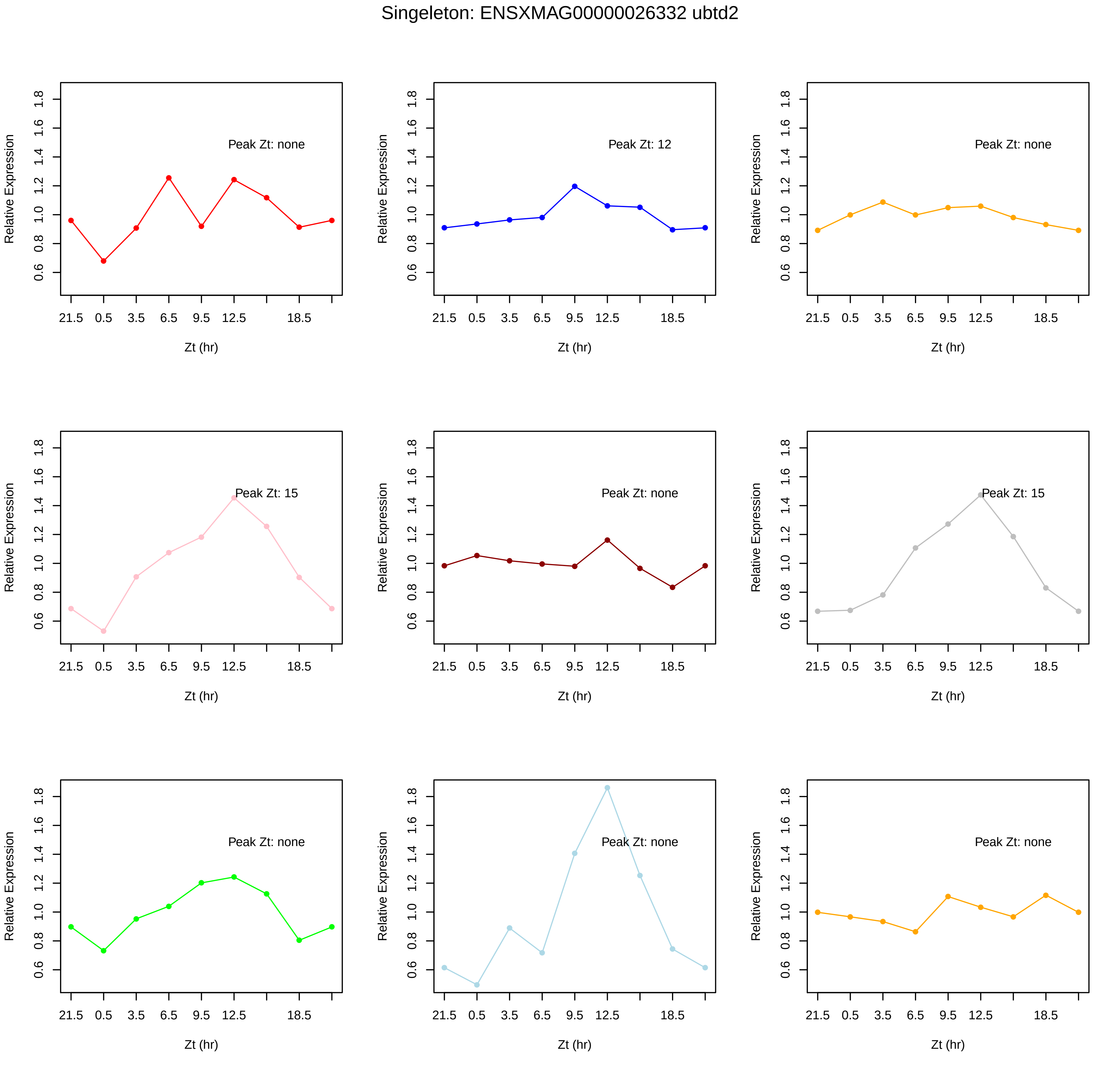

Singleton: ENSXMAG00000026332 ubtd2
Peak Zt: none
Peak Zt: 12
Peak Zt: none
Peak Zt: 15
Peak Zt: none
Peak Zt: 15
Peak Zt: none
Peak Zt: none
Peak Zt: none
Relative Expression
Zt (hr)
21.5
0.5
3.5
6.5
9.5
12.5
18.5
0.6
0.8
1.0
1.2
1.4
1.6
1.8

Singeleton: ENSXMAG00000021487 tet1

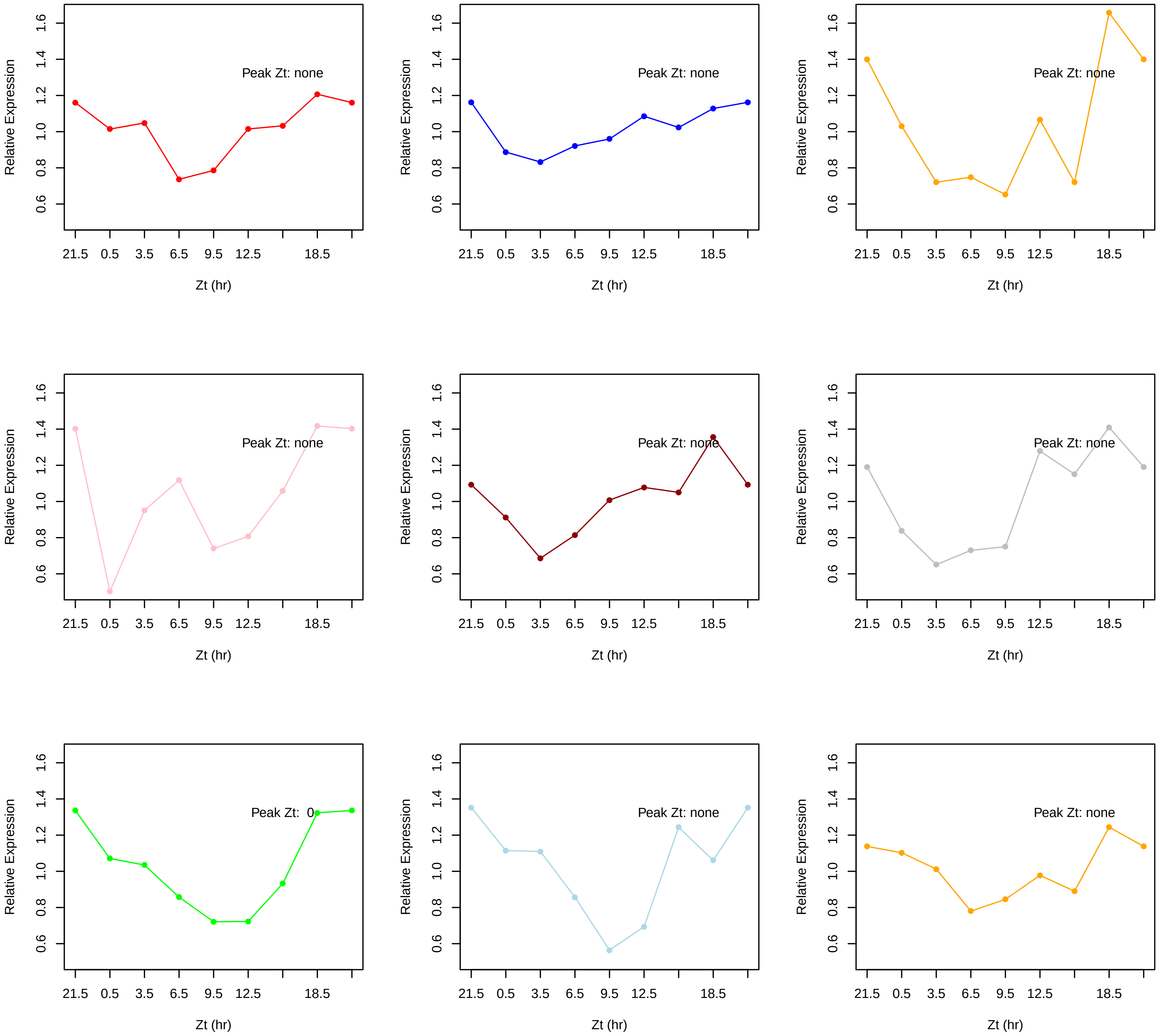

Singleton: ENSXMAG00000029156 timm9

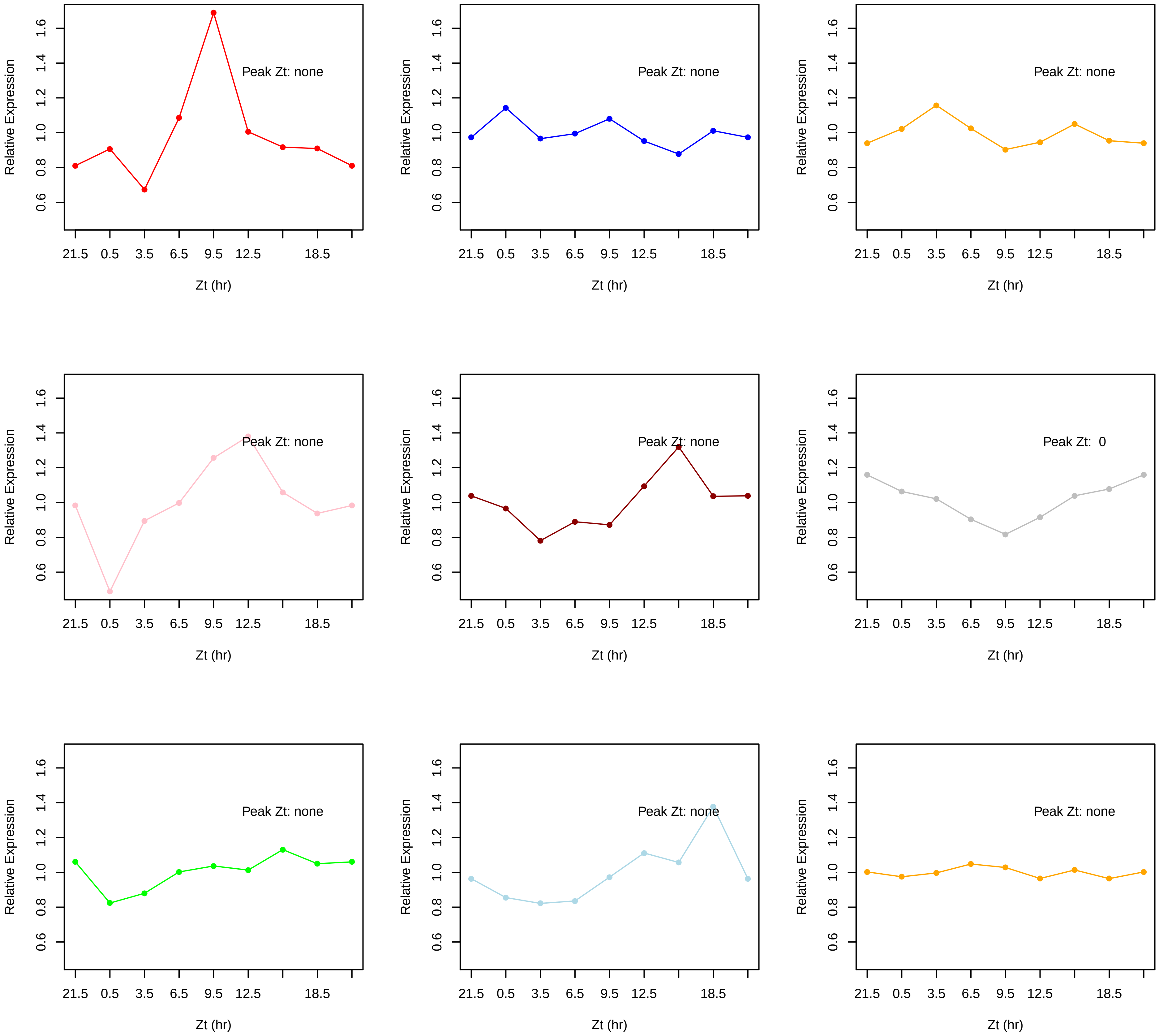

Singeleton: ENSXMAG00000008900 sdha

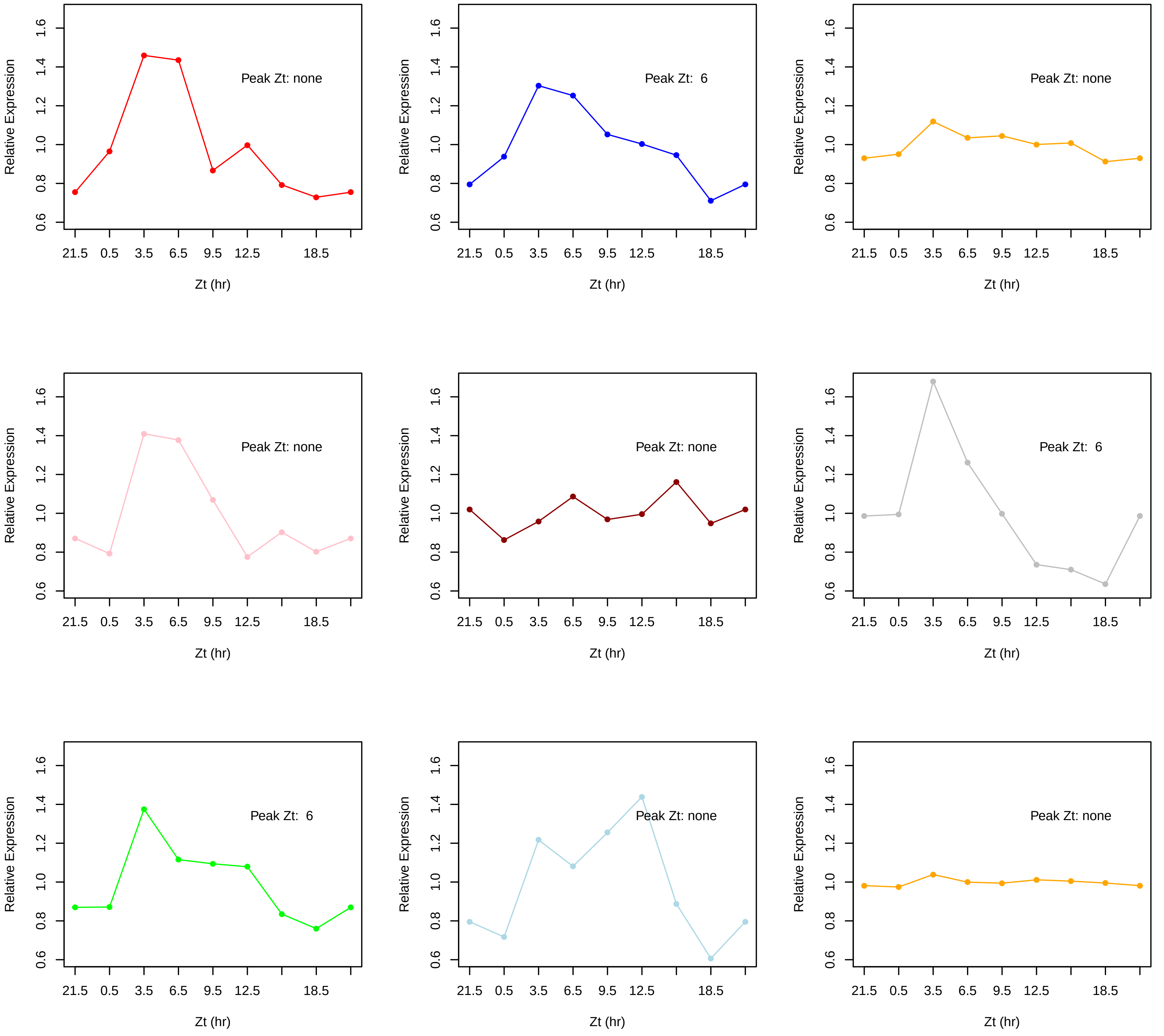

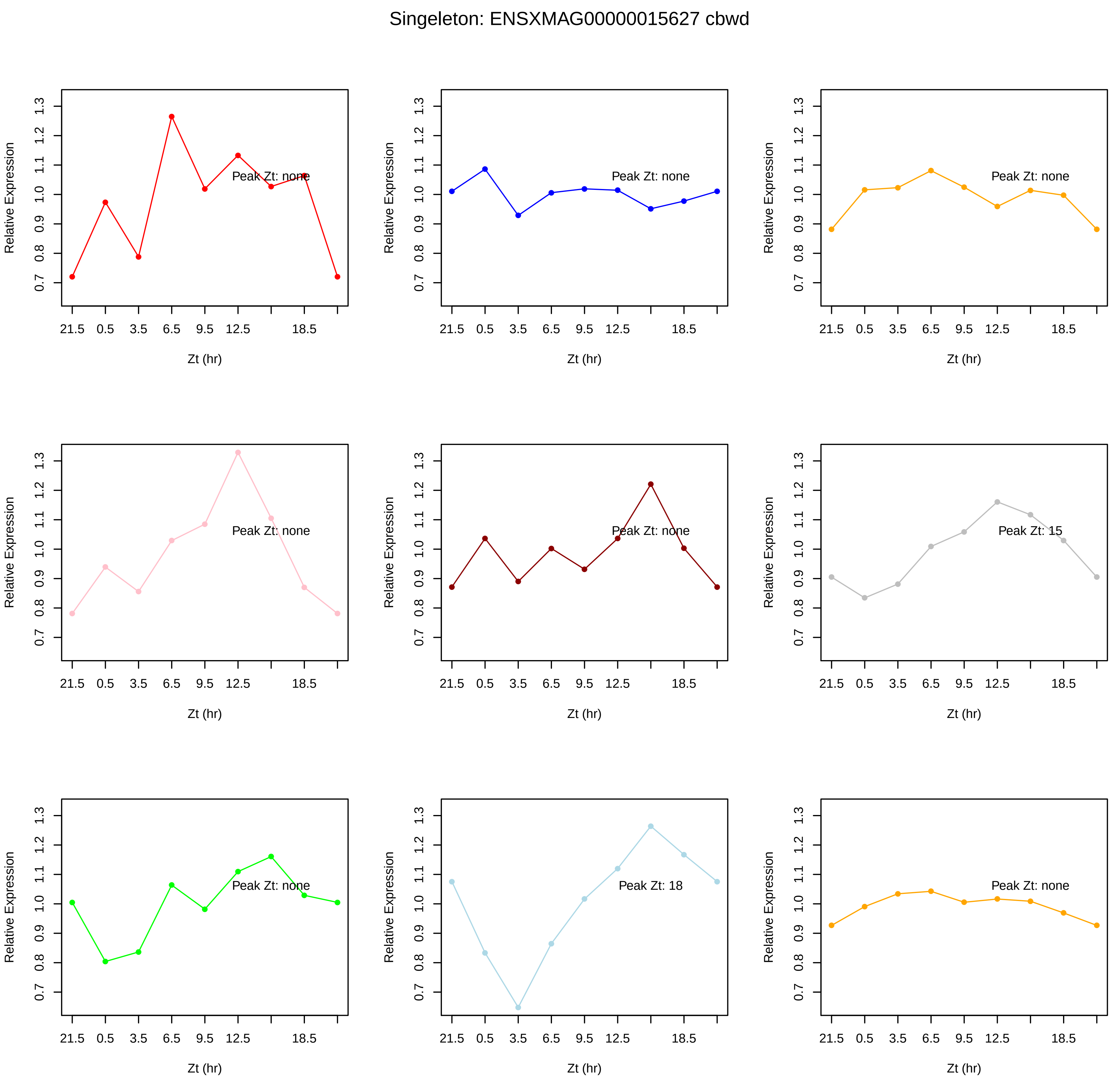
Singeleton: ENSXMAG00000015627 cbwd
Relative Expression
Zt (hr)
21.5
0.5
3.5
6.5
9.5
12.5
18.5
0.7
0.8
0.9
1.0
1.1
1.2
1.3
Peak Zt: none
Peak Zt: 15
Peak Zt: 18

Singleton: ENSXMAG00000018898 agap3

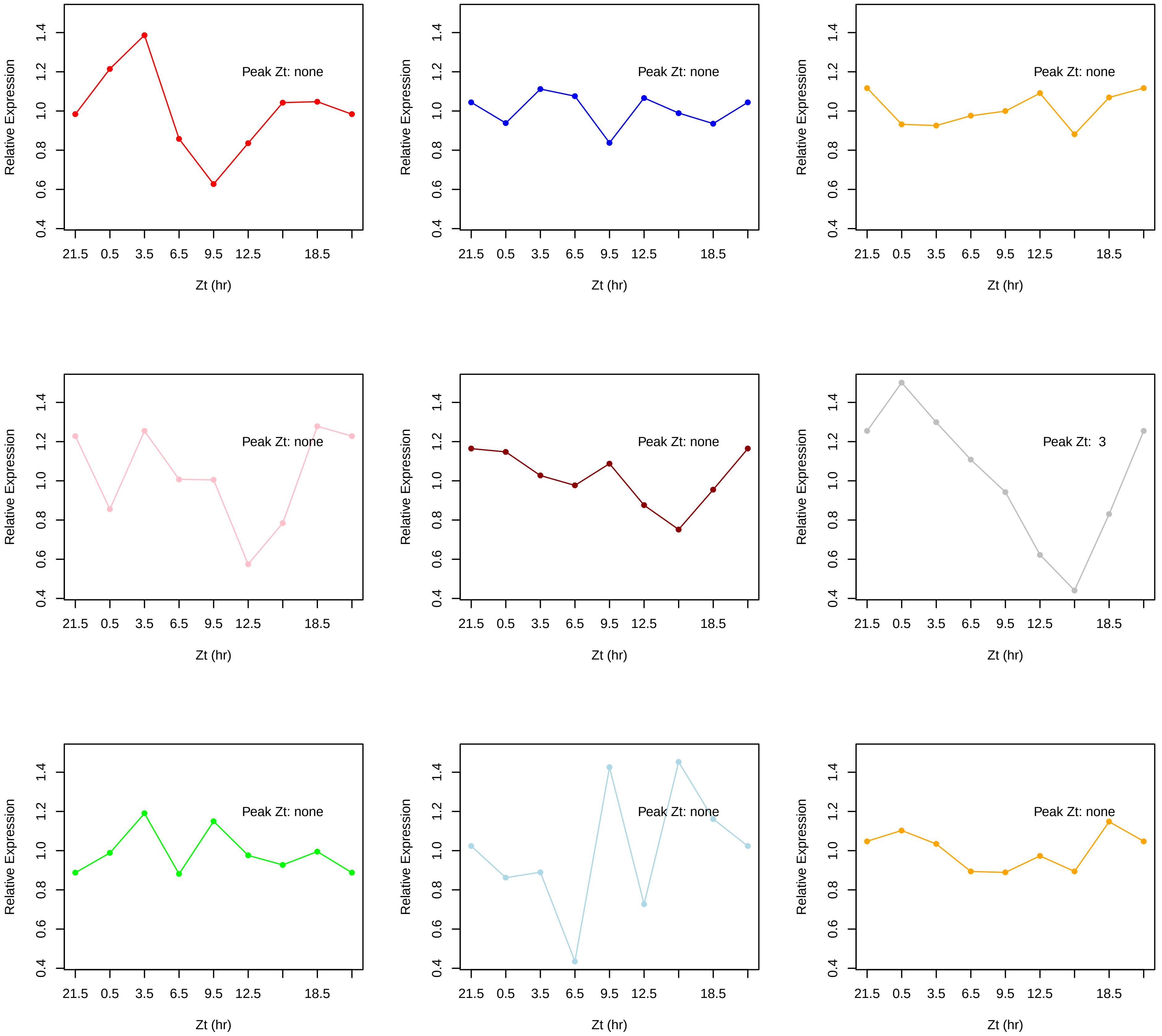

Singeleton: ENSXMAG00000008087 slc19a1

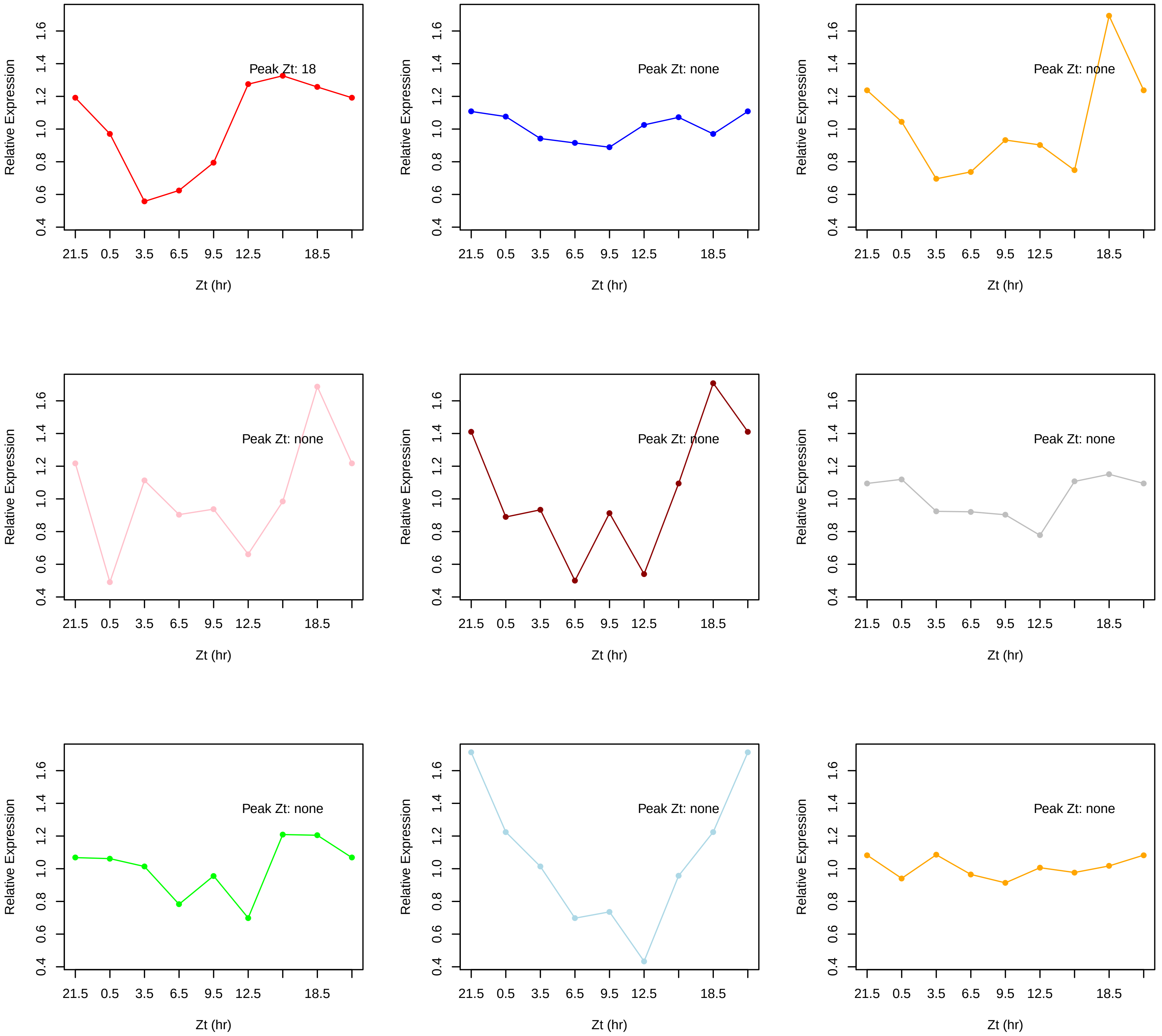

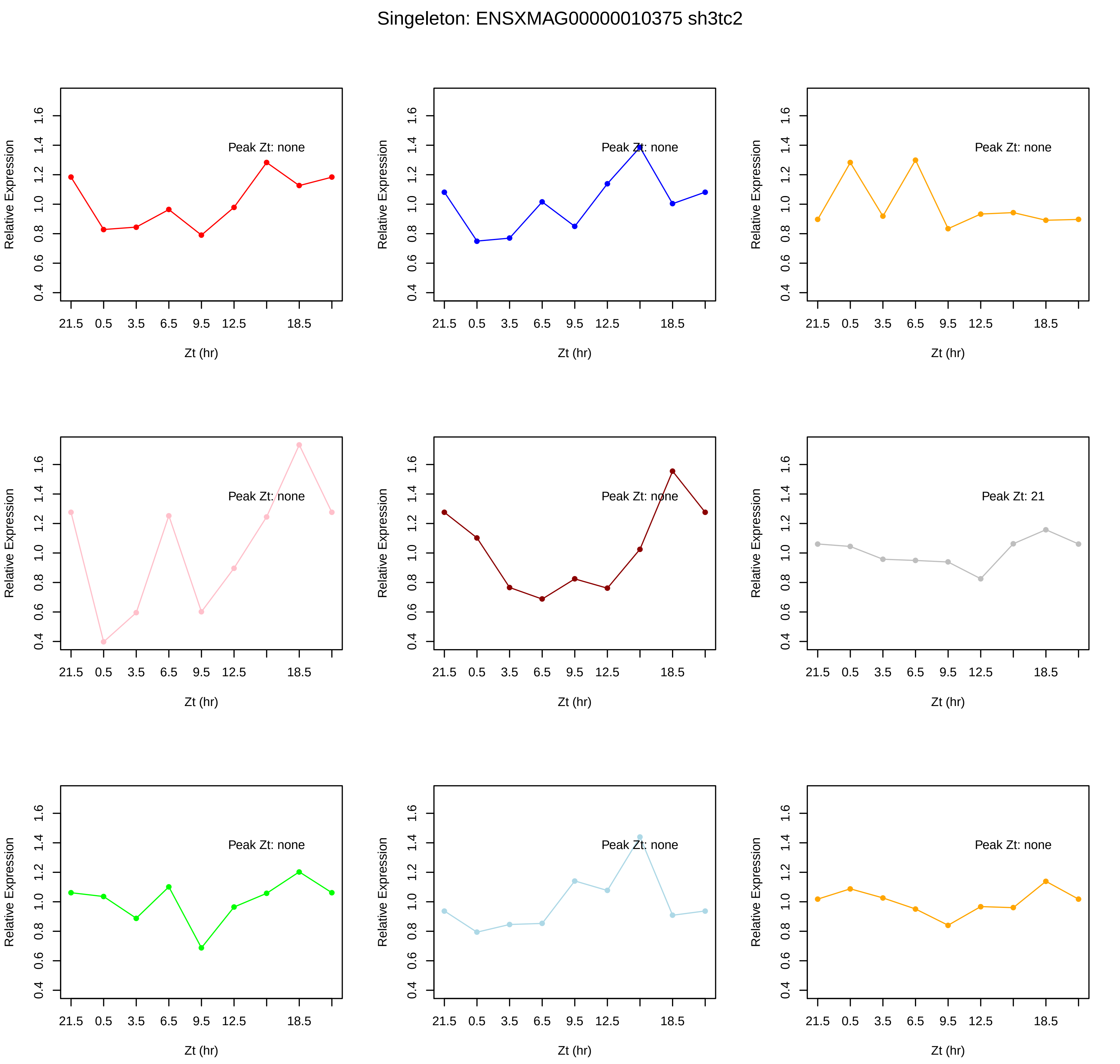
Singeleton: ENSXMAG00000010375 sh3tc2
Peak Zt: none
Peak Zt: none
Peak Zt: none
Peak Zt: none
Peak Zt: none
Peak Zt: 21
Peak Zt: none
Peak Zt: none
Peak Zt: none
Relative Expression
Zt (hr)
21.5
0.5
3.5
6.5
9.5
12.5
18.5
0.4
0.6
0.8
1.0
1.2
1.4
1.6

Singleton: ENSXMAG00000025683 elavl4

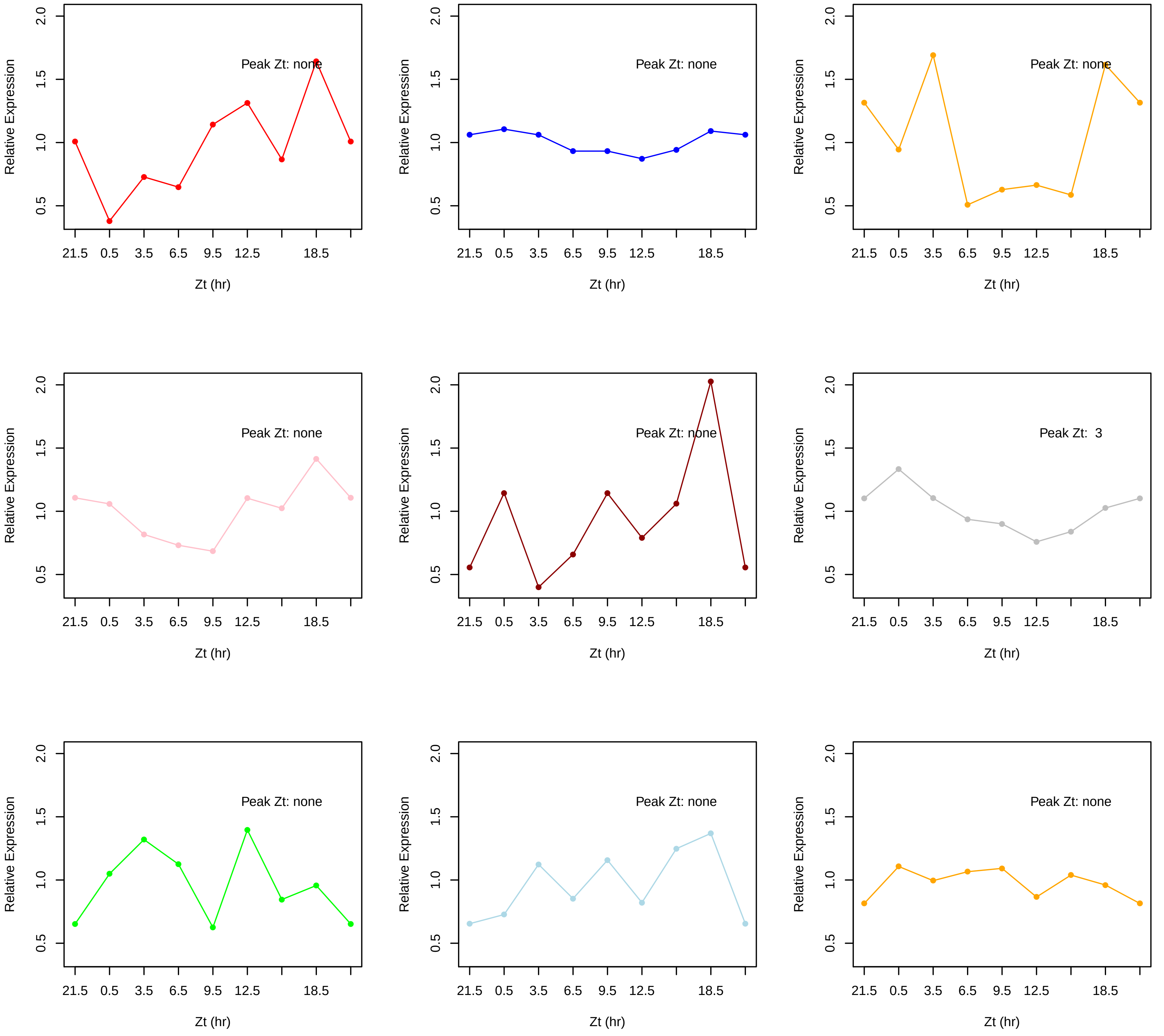

Singeleton: ENSXMAG00000004893 psma3

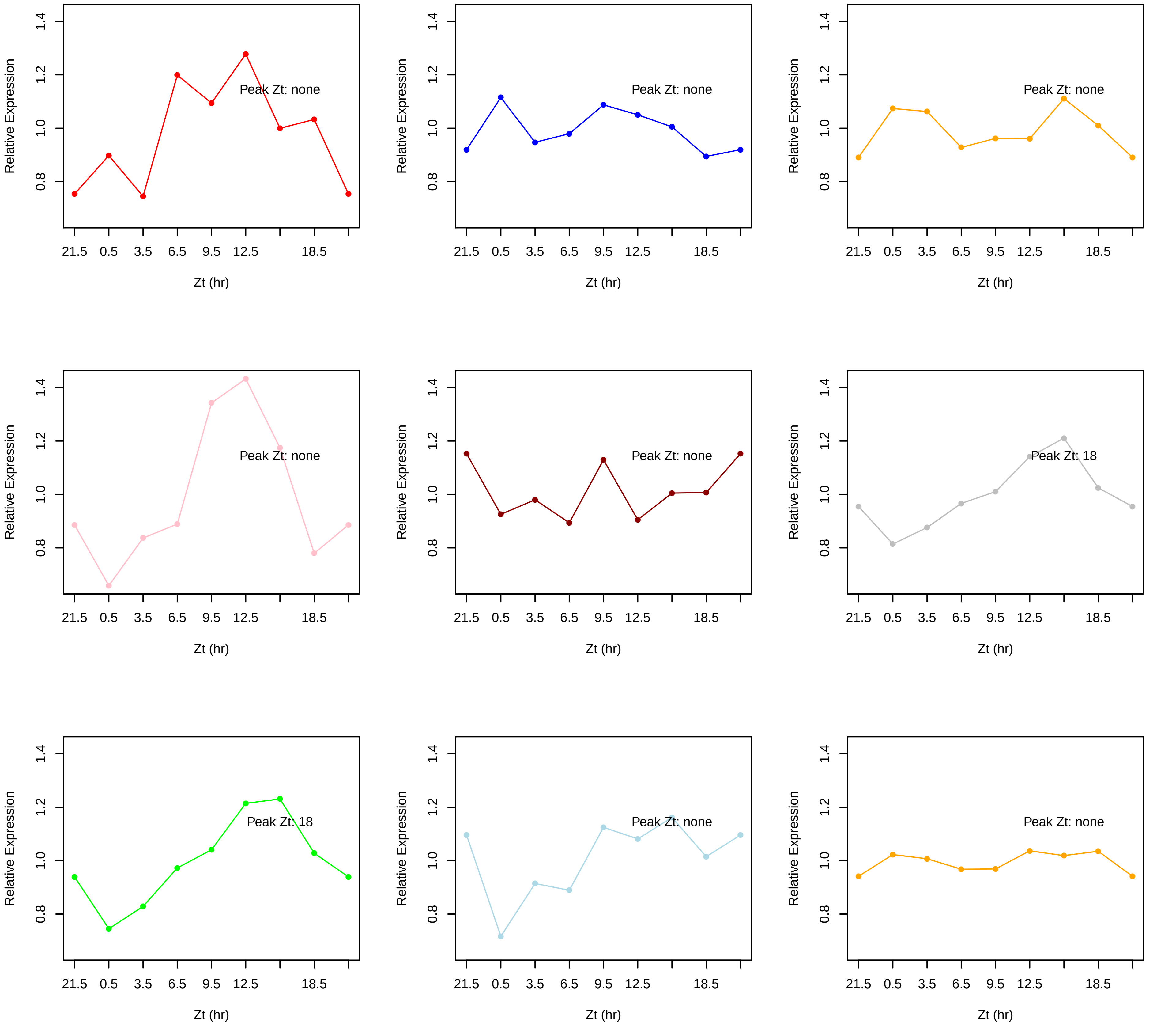

Singeleton: ENSXMAG00000002407 eif2ak3

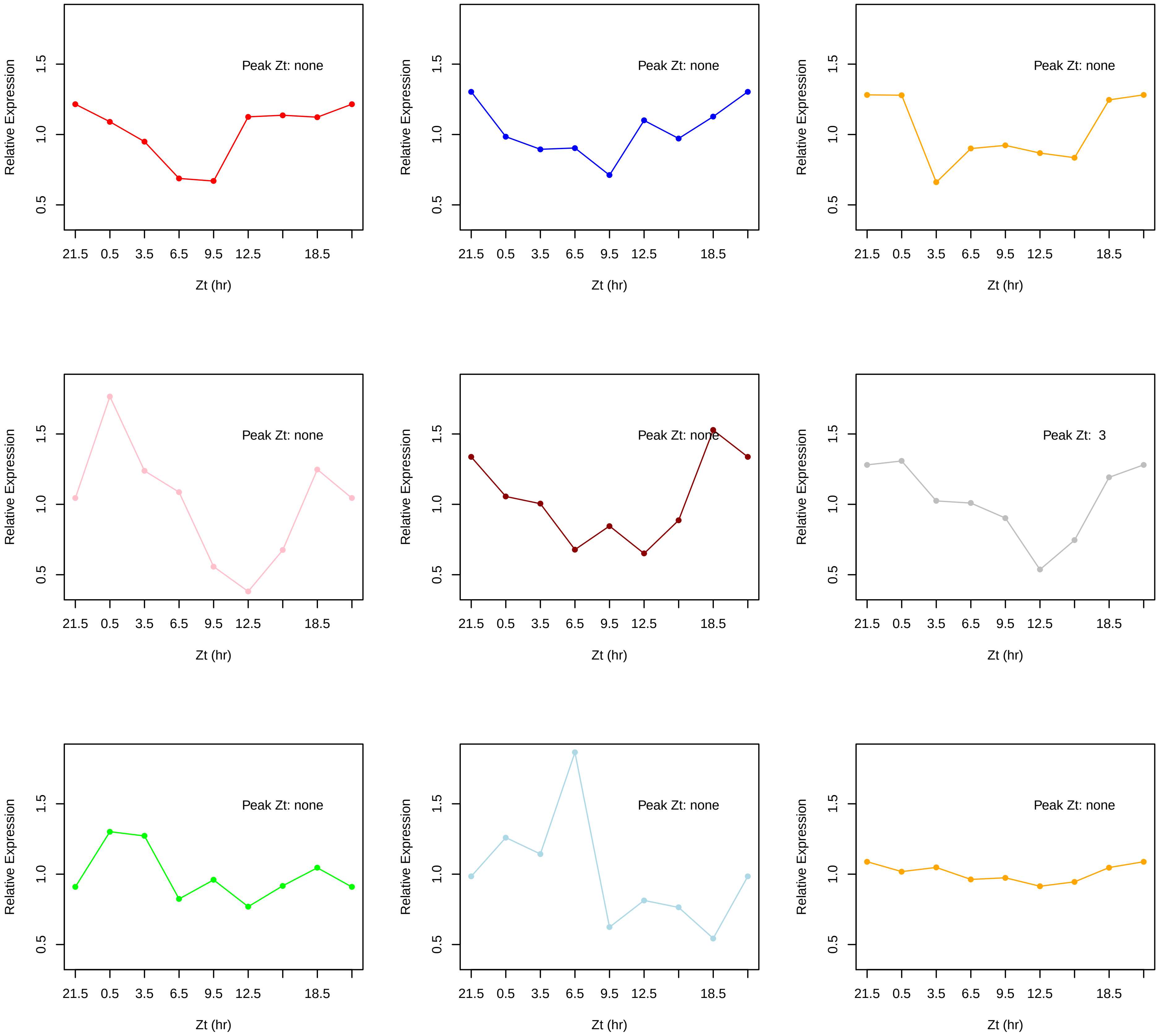

Singeleton: ENSXMAG00000022380 rnf144b

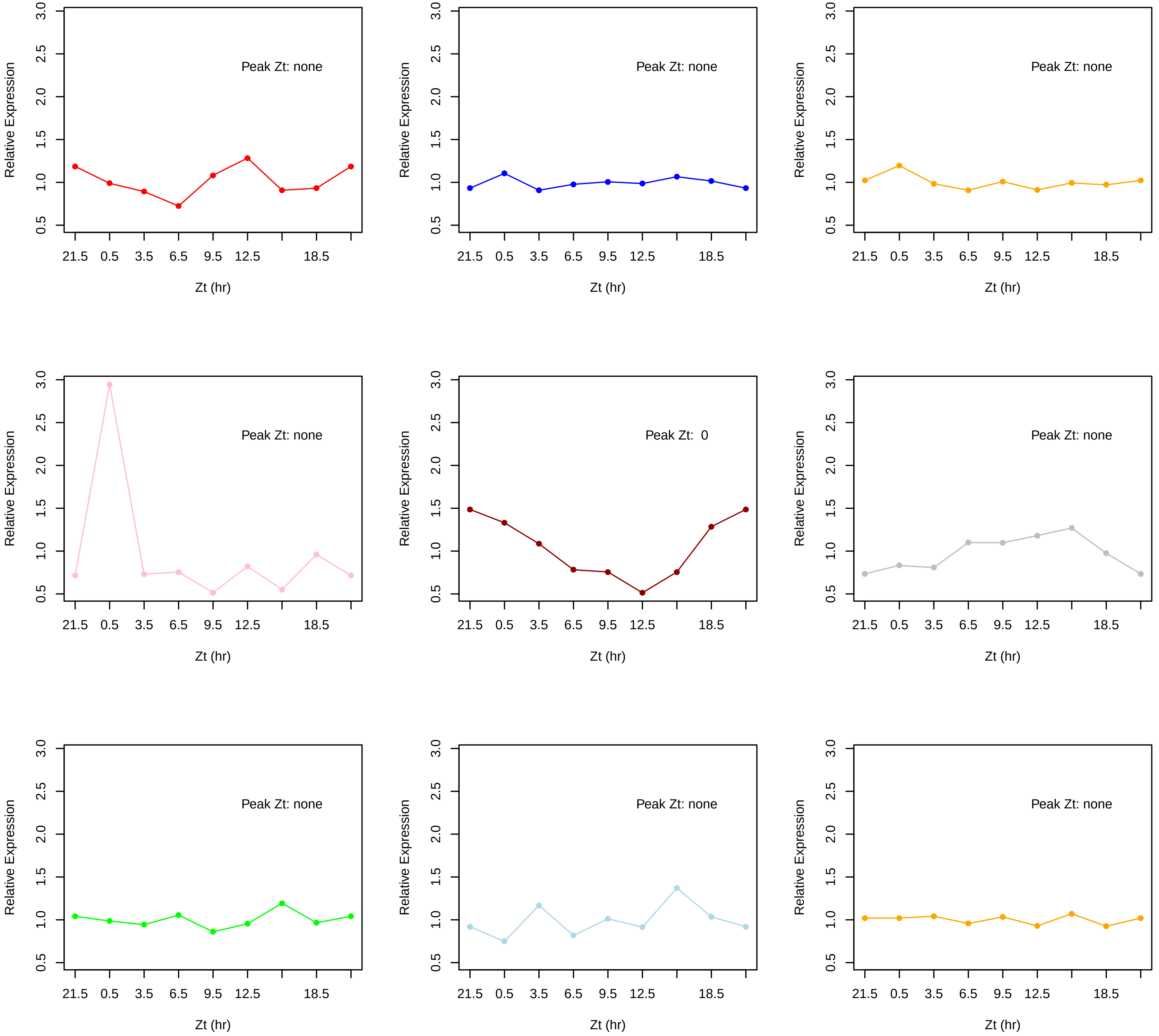

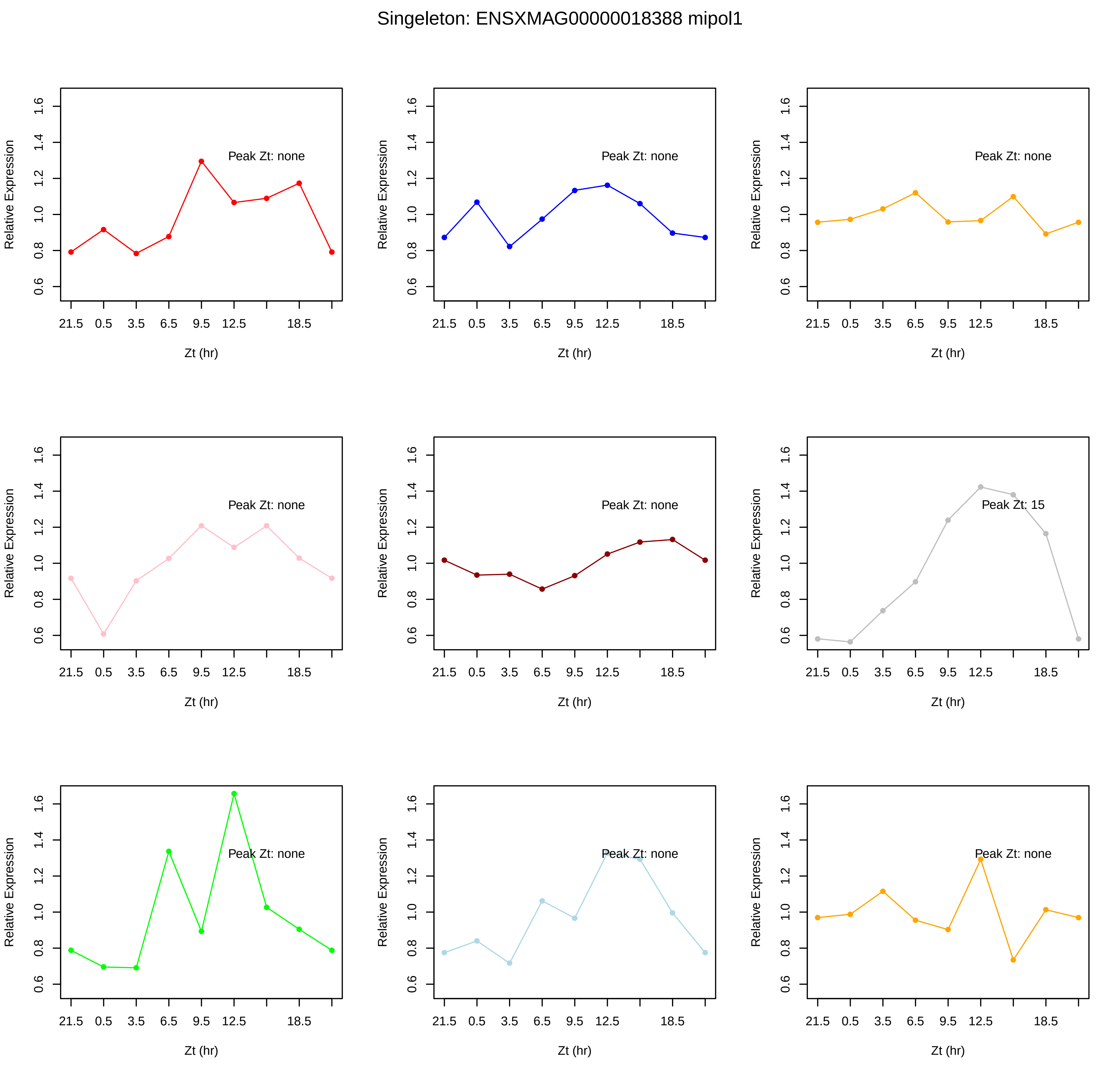
Singeleton: ENSXMAG00000018388 mipol1
Peak Zt: none
Peak Zt: none
Peak Zt: none
Peak Zt: none
Peak Zt: none
Peak Zt: 15
Peak Zt: none
Peak Zt: none
Peak Zt: none
Relative Expression
Zt (hr)
21.5
0.5
3.5
6.5
9.5
12.5
18.5
0.6
0.8
1.0
1.2
1.4
1.6

Singeleton: ENSXMAG00000014781

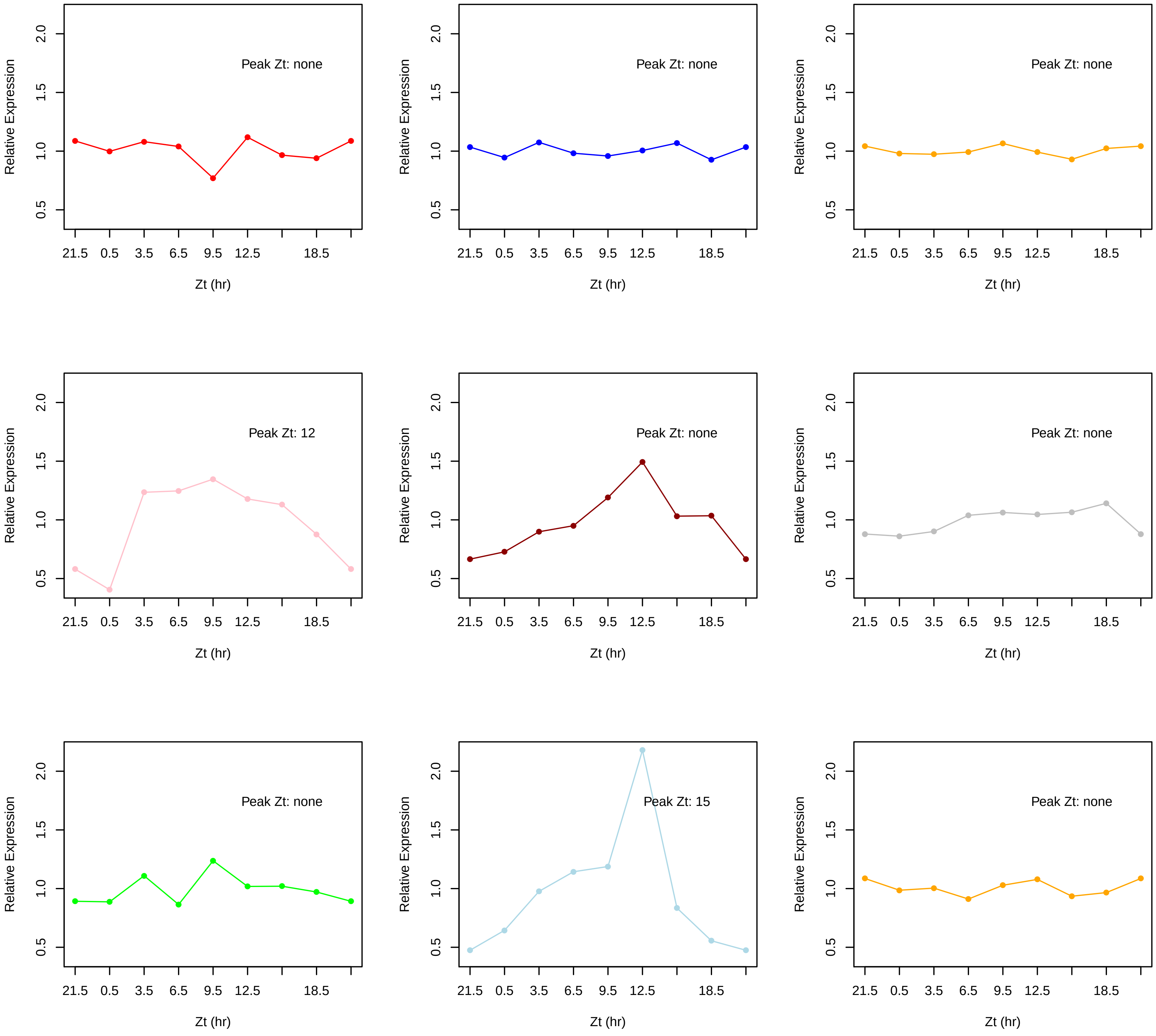

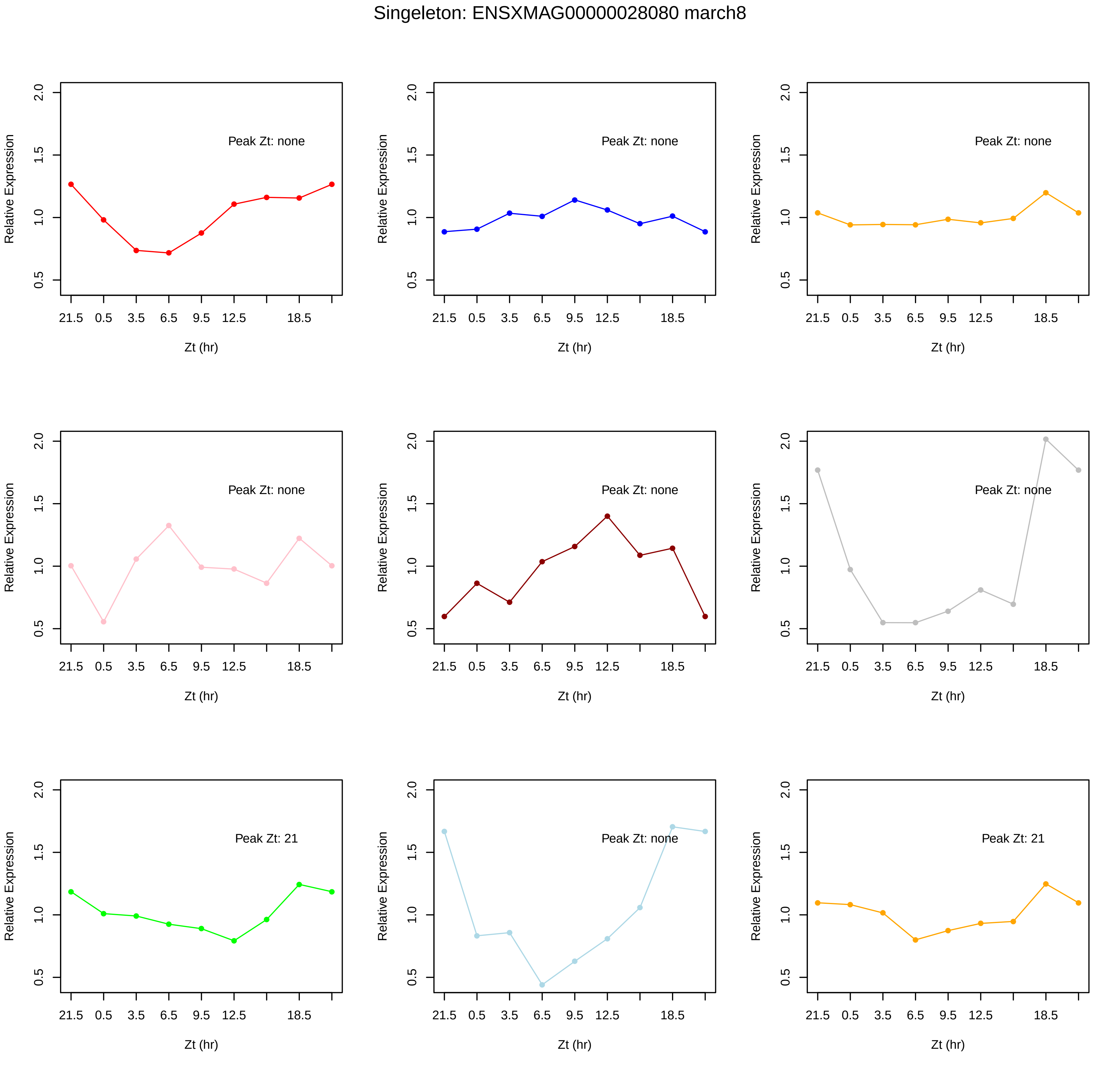
Singeleton: ENSXMAG00000028080 march8
Peak Zt: none
Peak Zt: none
Peak Zt: none
Peak Zt: none
Peak Zt: none
Peak Zt: none
Peak Zt: 21
Peak Zt: none
Peak Zt: 21
Relative Expression
Zt (hr)
21.5 0.5 3.5 6.5 9.5 12.5 18.5
0.5 1.0 1.5 2.0

Singeleton: ENSXMAG00000007077 unc45b

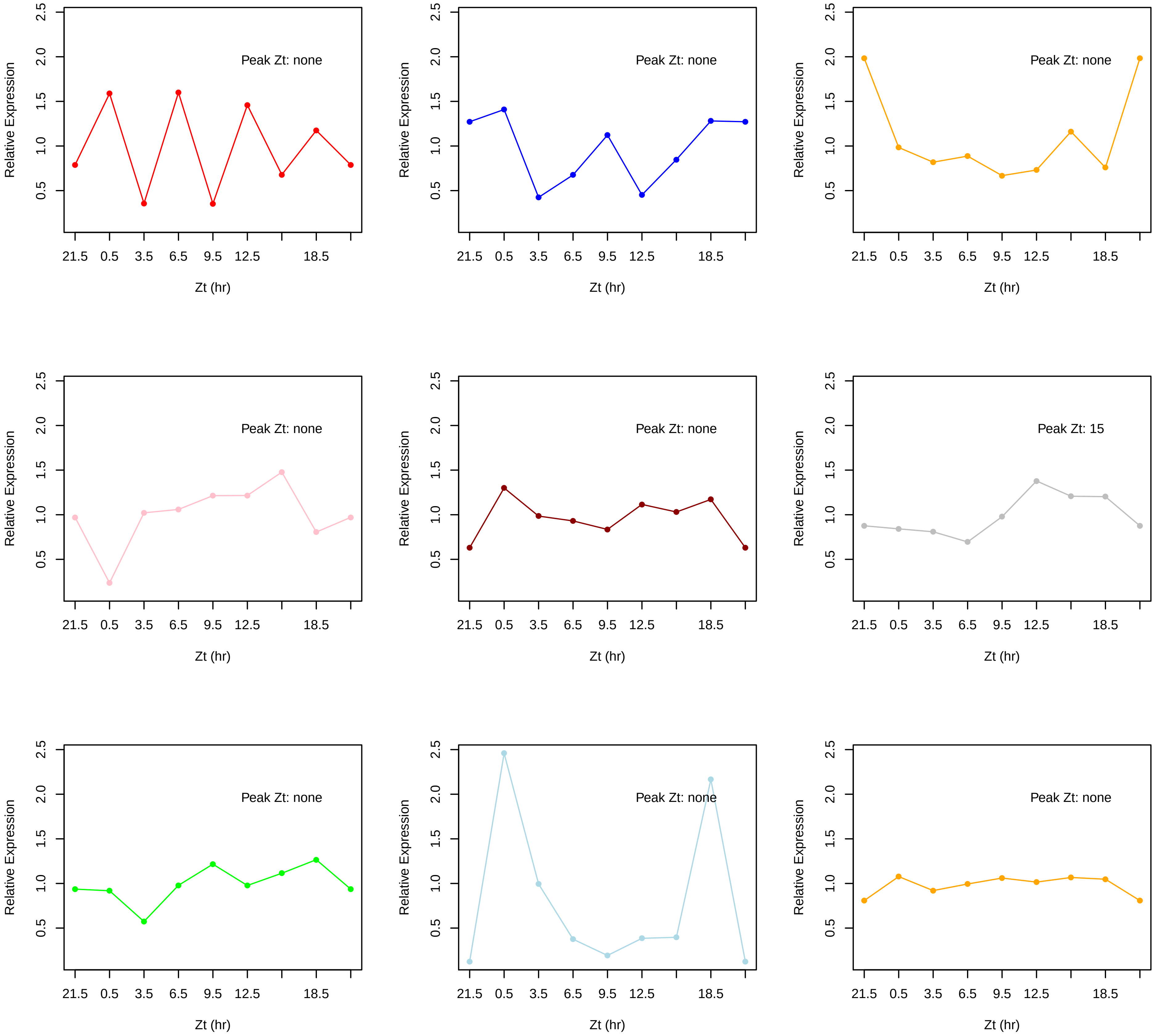

Singeleton: ENSXMAG00000011243 cmss1

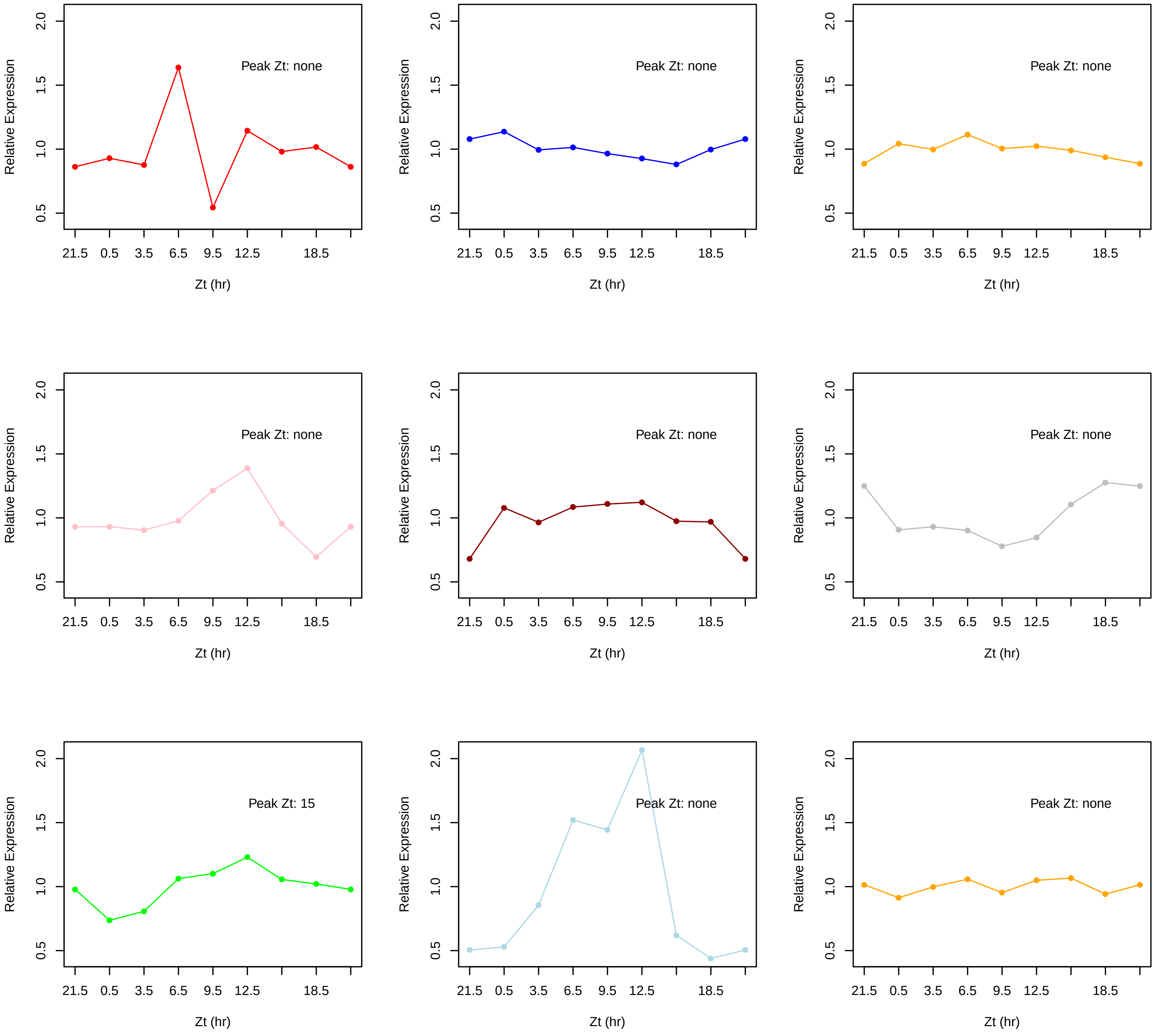

Singleton: ENSXMAG00000003868 OXR1

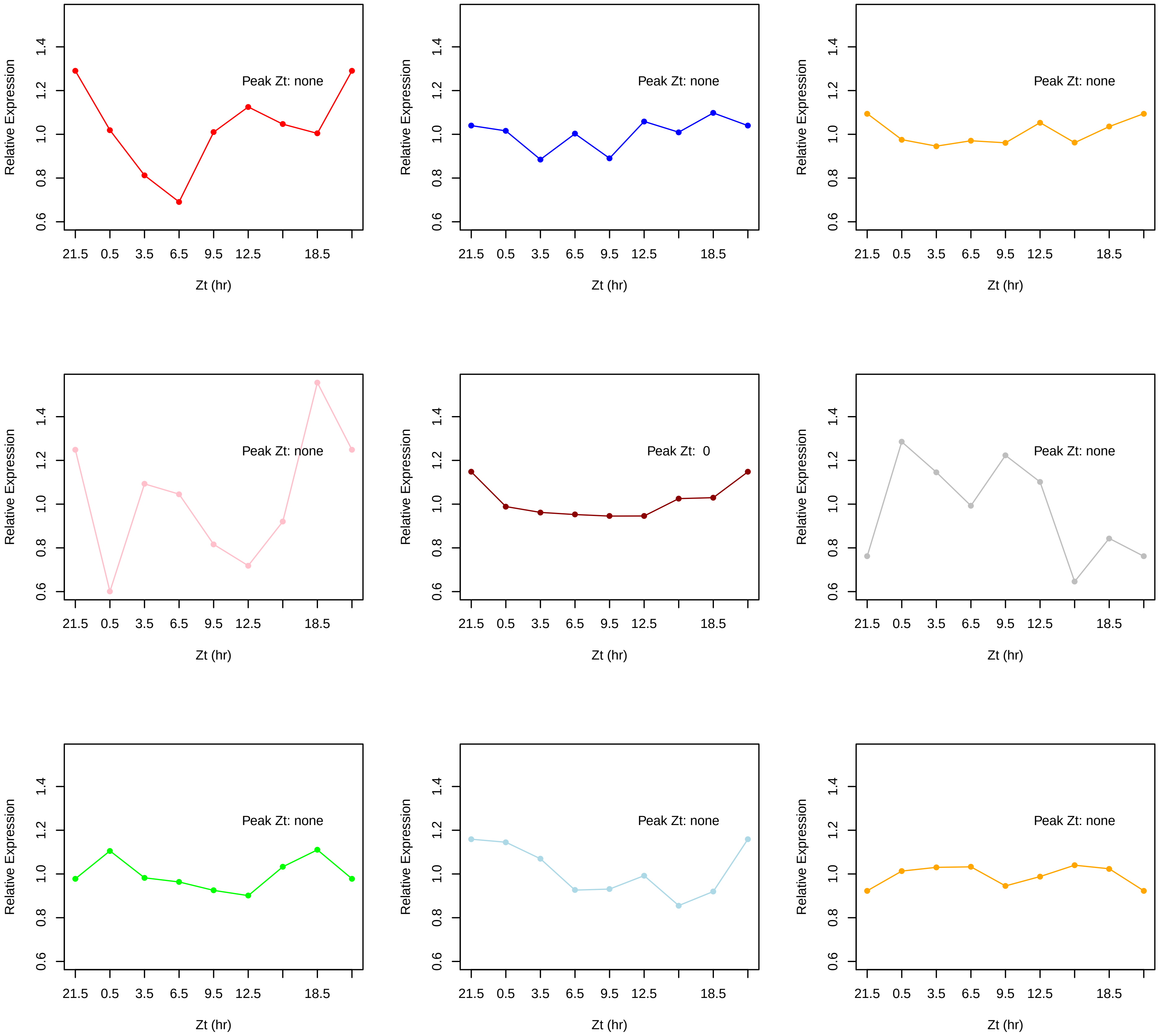

Singeleton: ENSXMAG00000009007 slu7

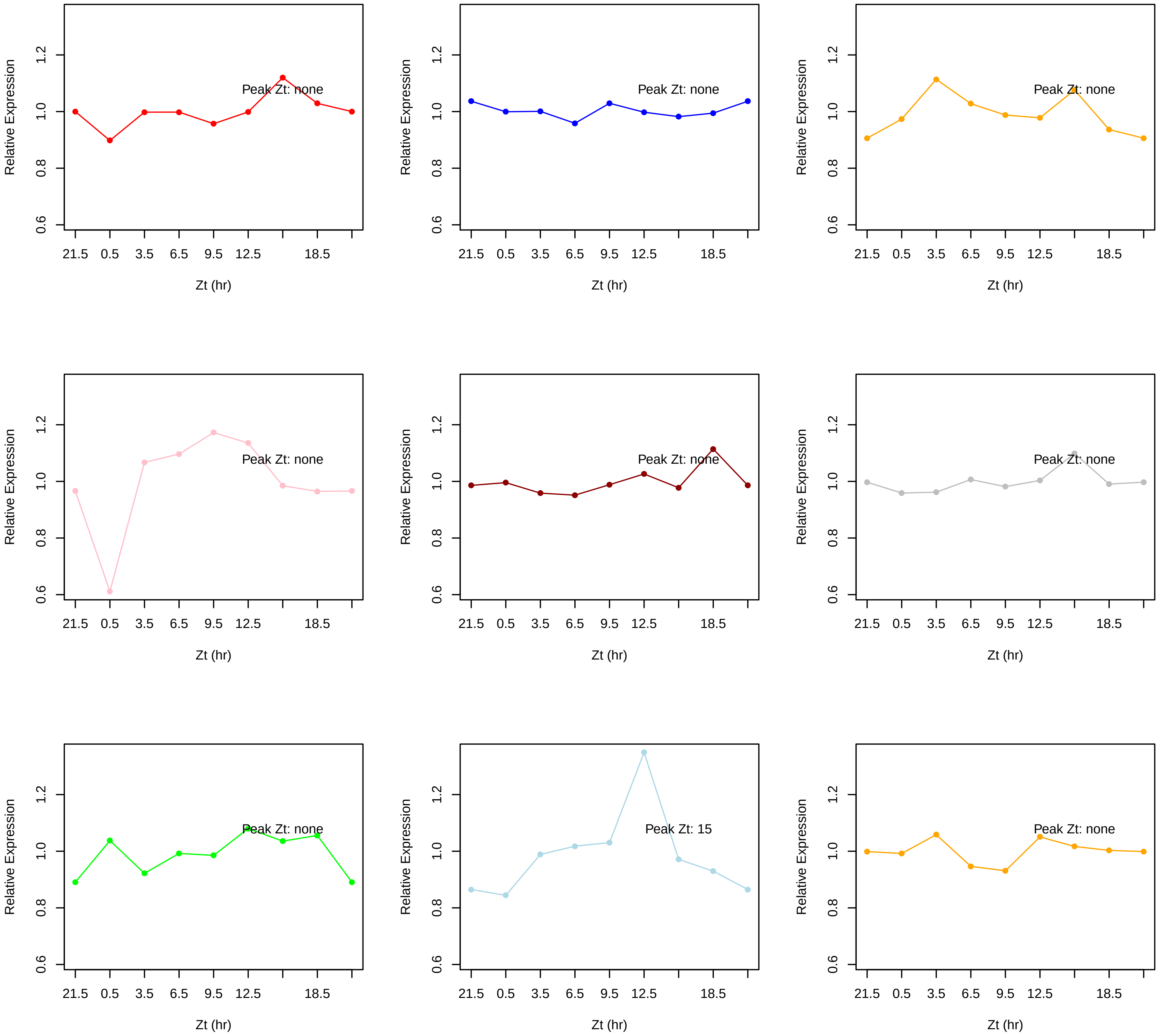

Singeleton: ENSXMAG00000011228 tmem30c

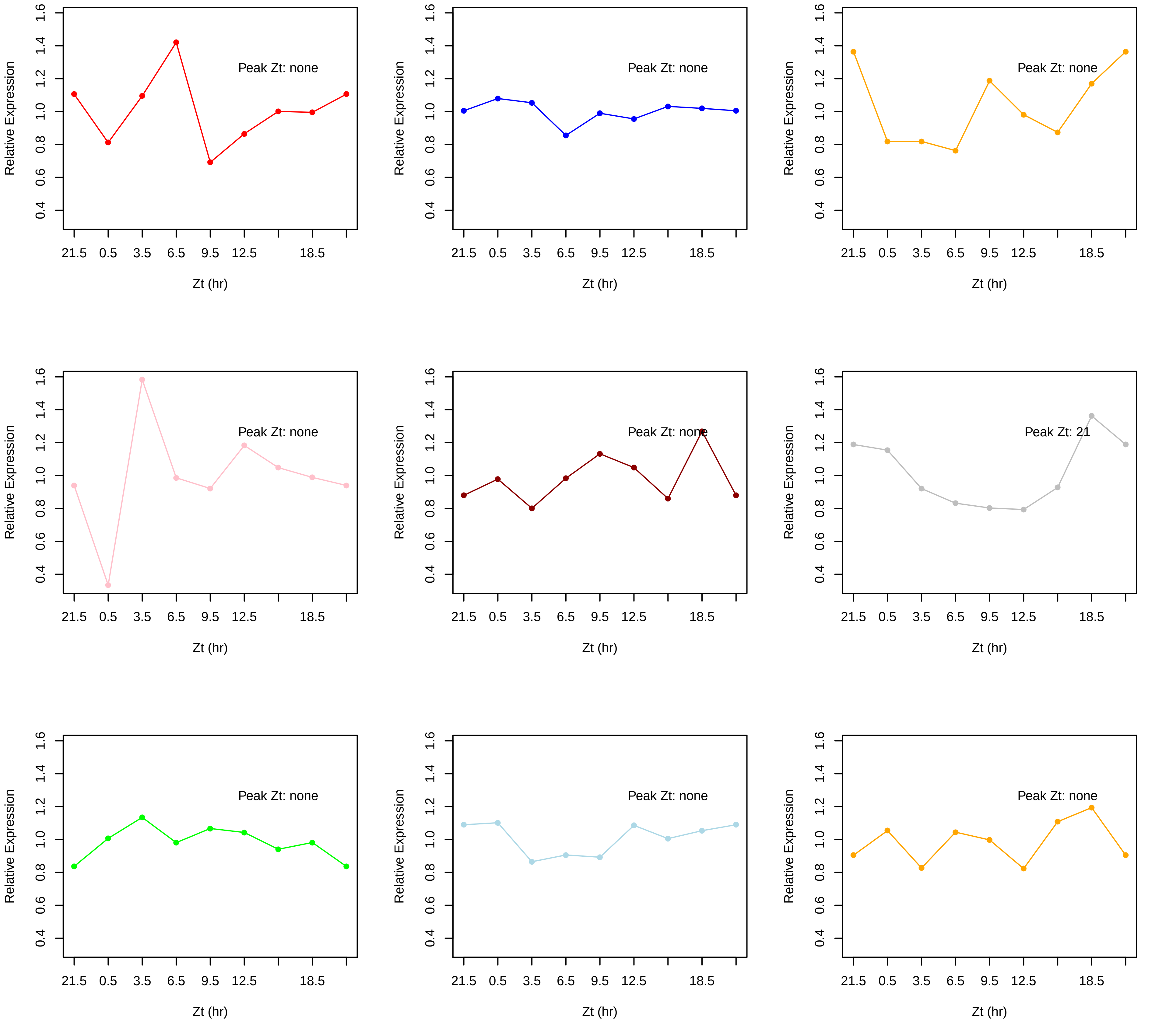

Singleton: ENSXMAG00000022219 si:dkey−122a22.2

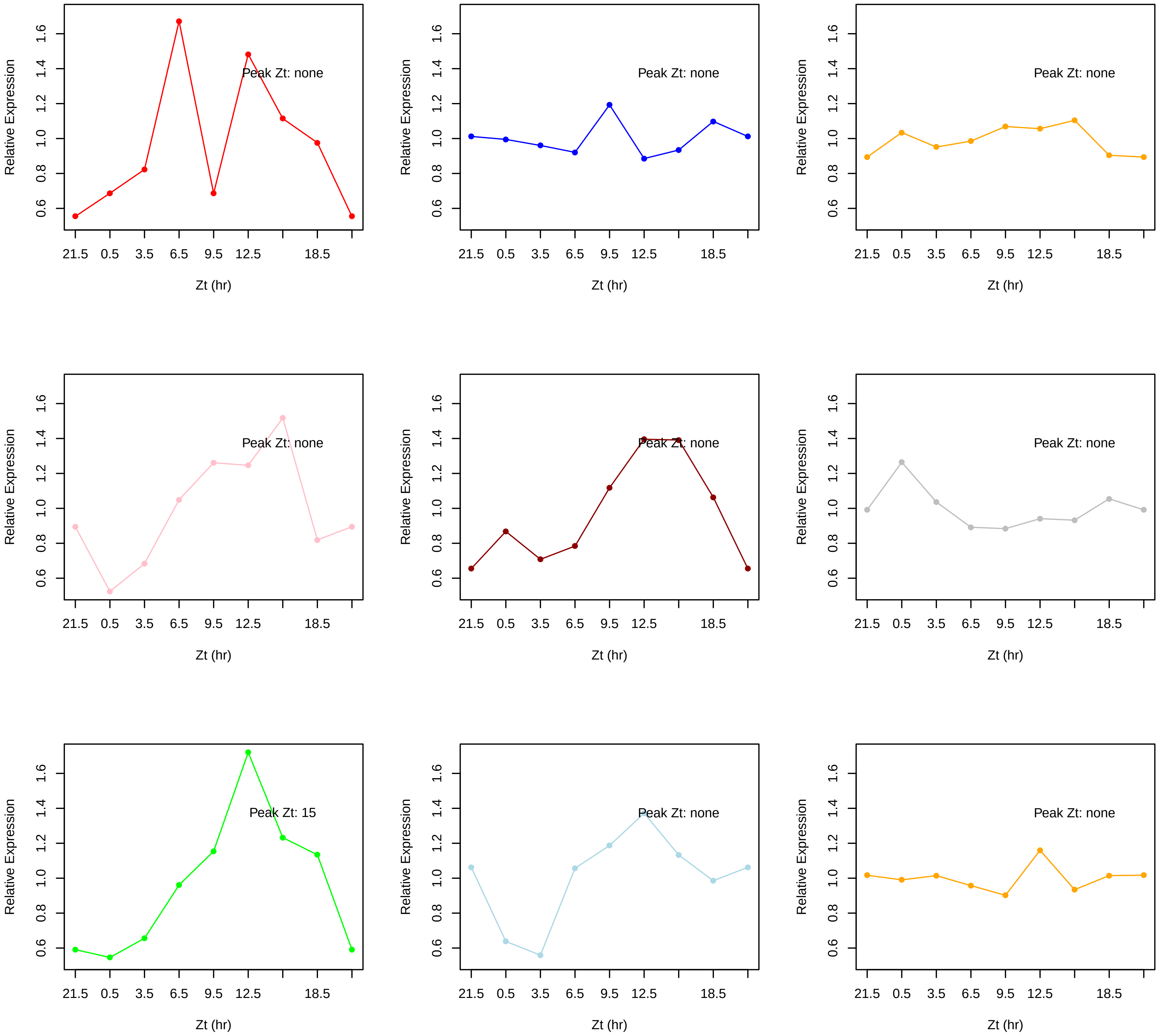

Singeleton: ENSXMAG00000005817 HIGD1A

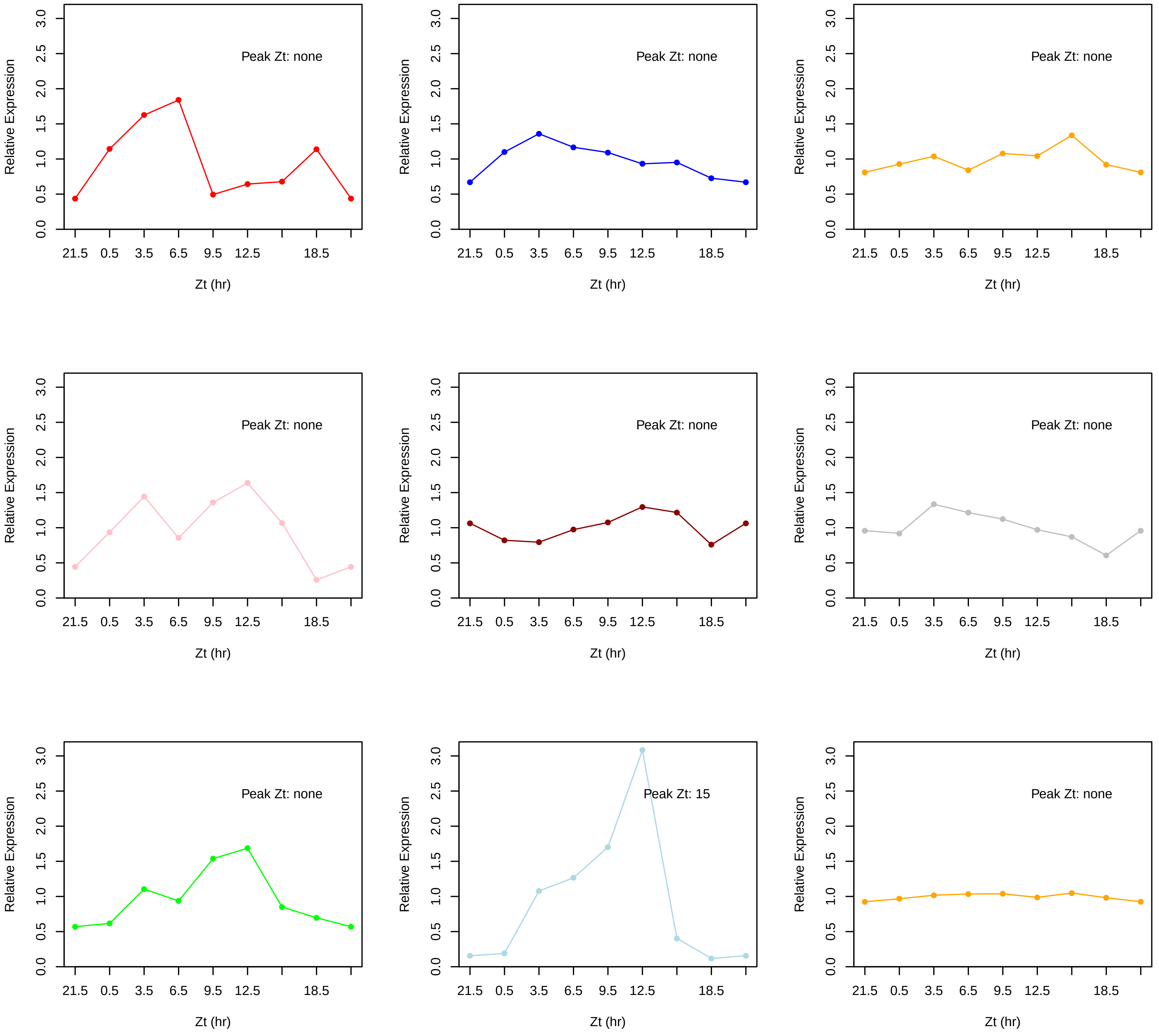

Singeleton: ENSXMAG00000001845 gba2

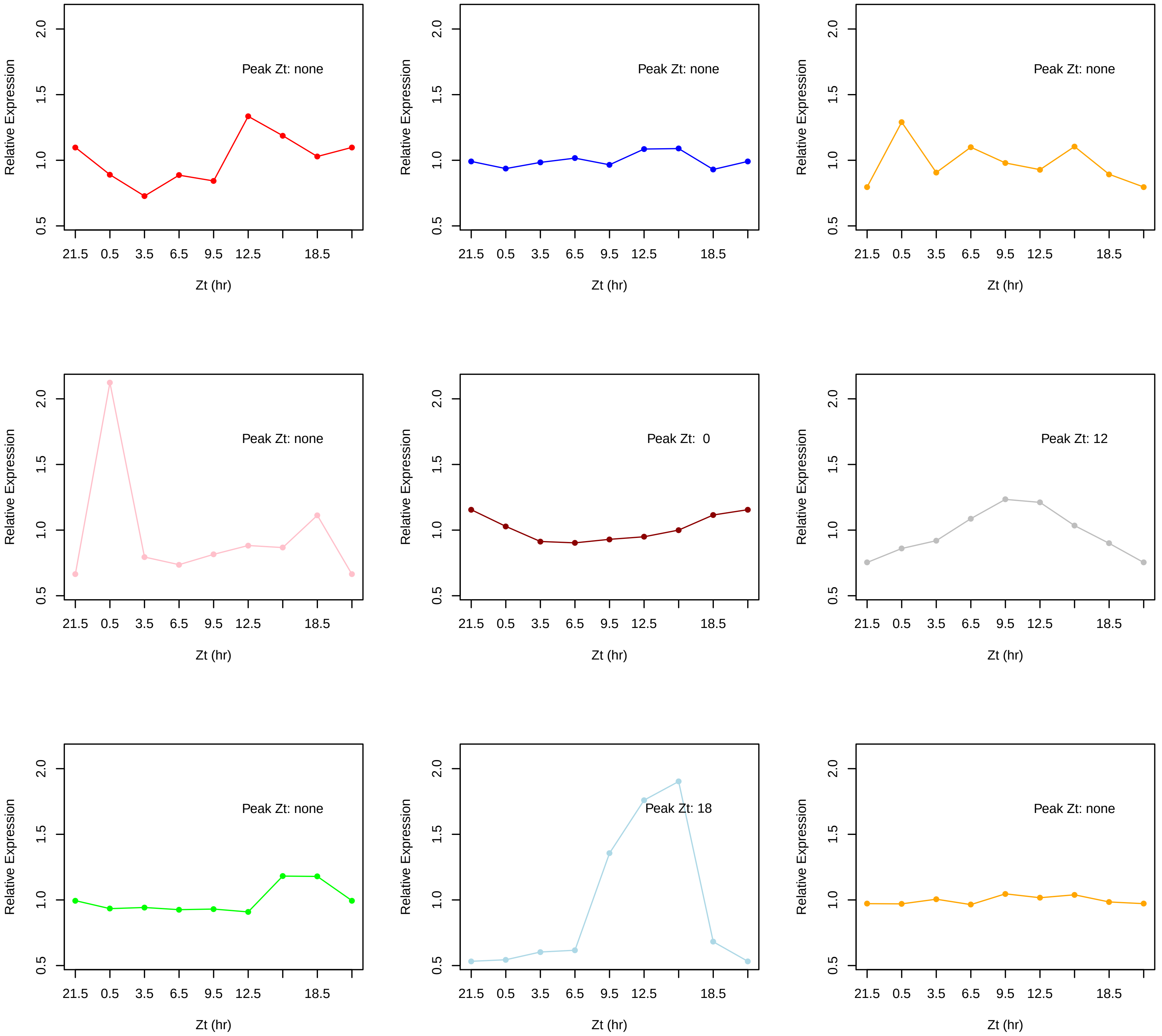

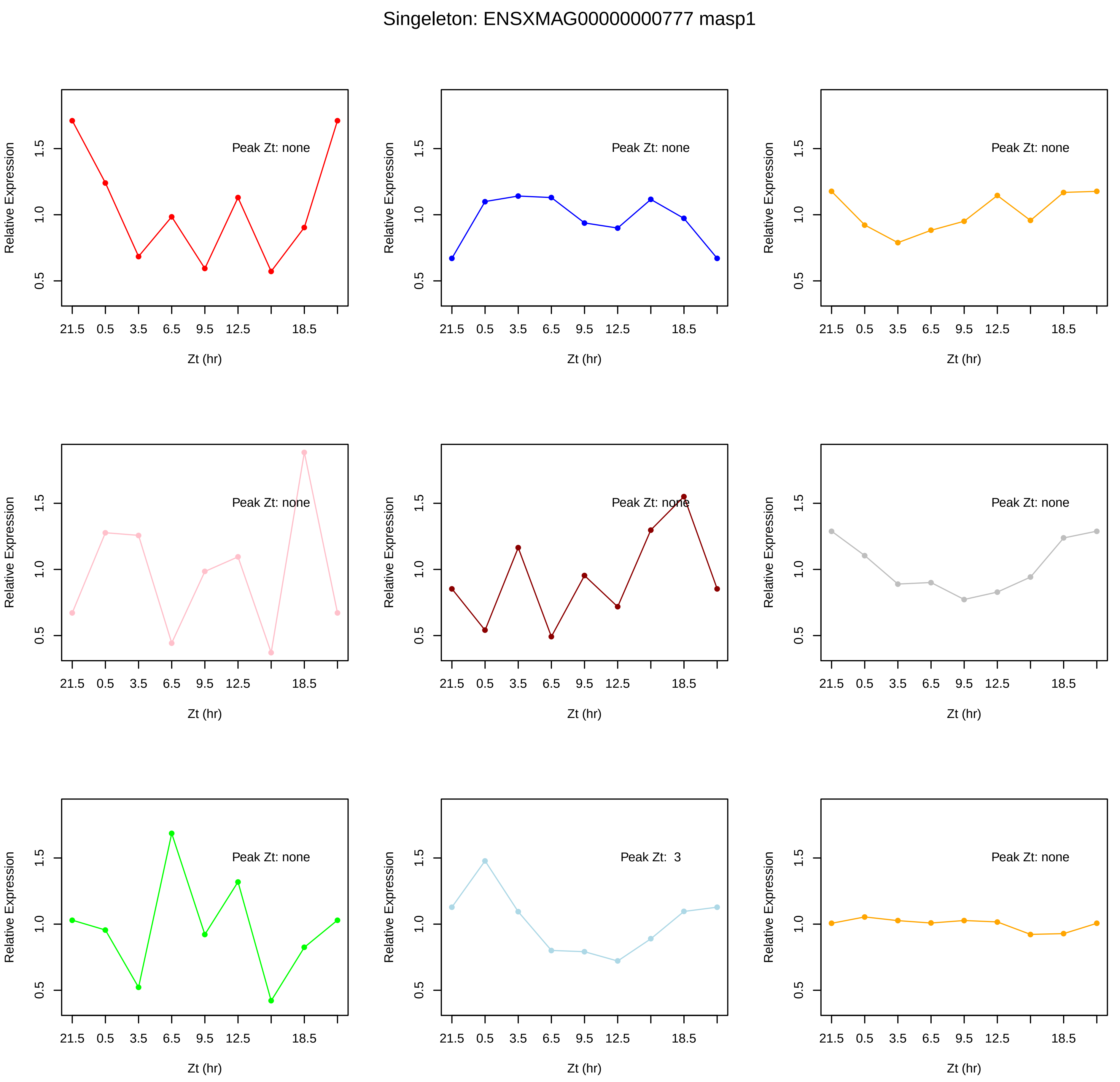

Singeleton: ENSXMAG00000000777 masp1
Peak Zt: none
Peak Zt: none
Peak Zt: none
Peak Zt: none
Peak Zt: none
Peak Zt: none
Peak Zt: none
Peak Zt: 3
Peak Zt: none
Relative Expression
Zt (hr)
21.5 0.5 3.5 6.5 9.5 12.5 18.5
0.5 1.0 1.5

Singleton: ENSXMAG00000003295 ttc39a

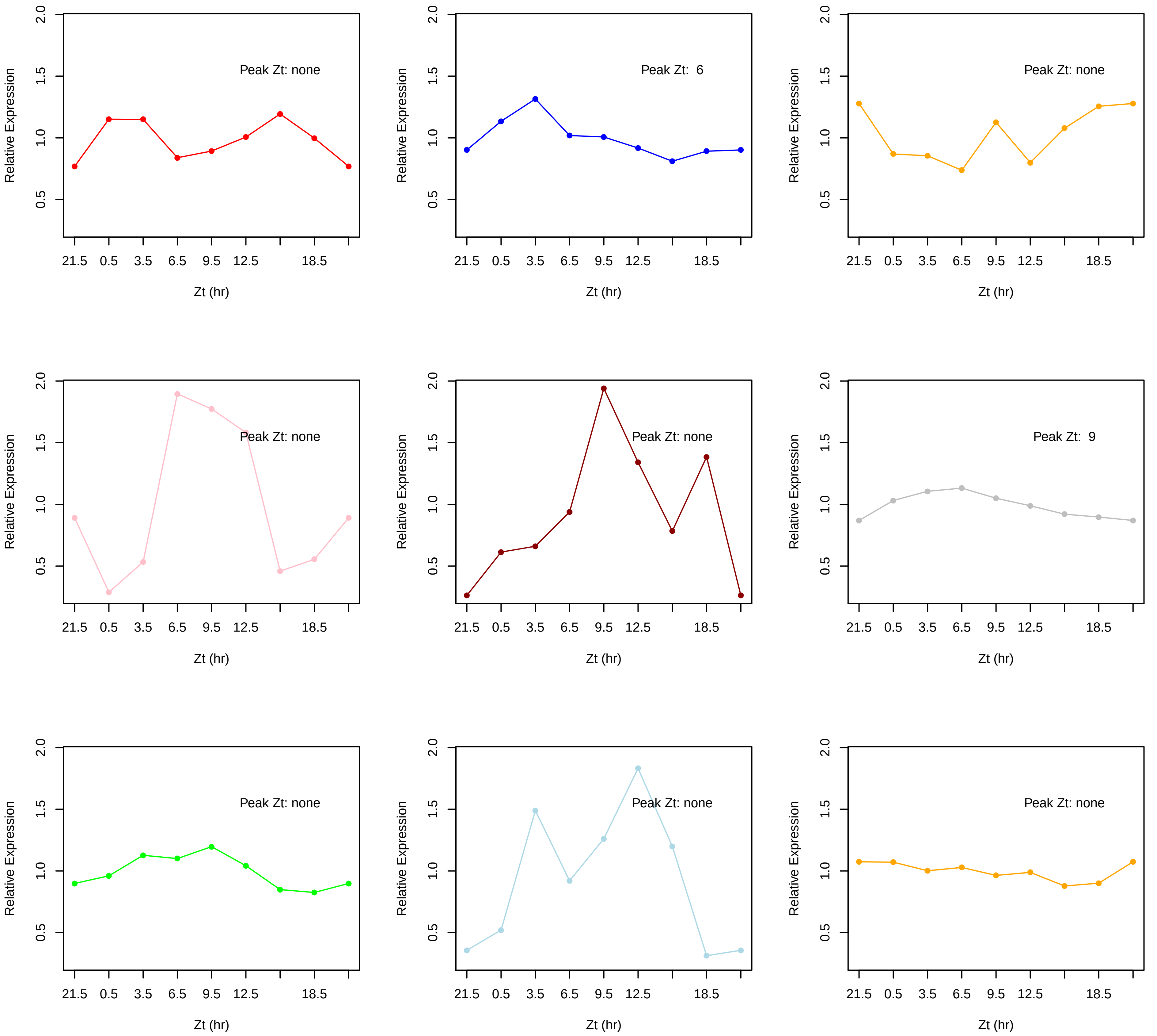

Singleton: ENSXMAG00000018315 srp54

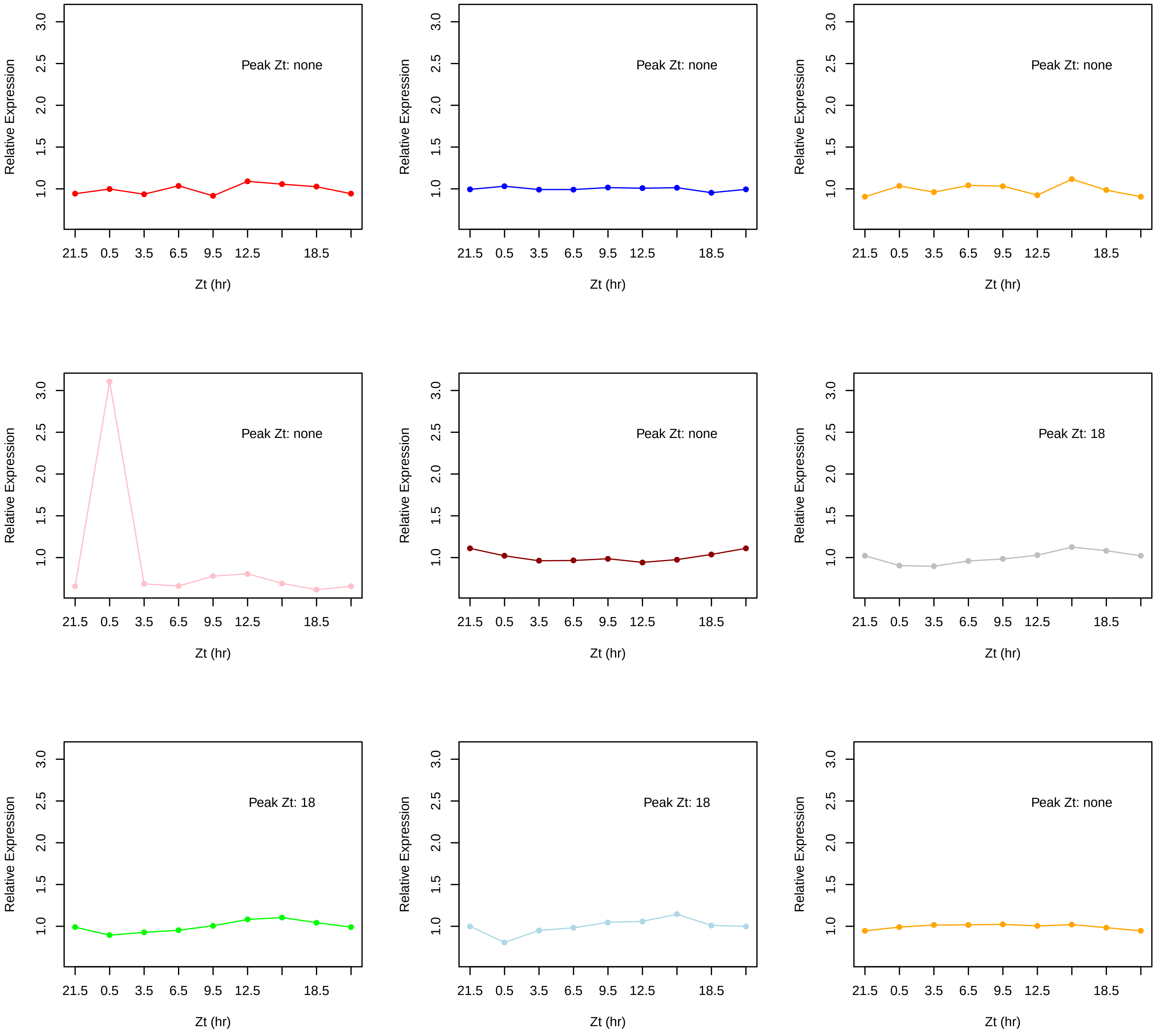

Singeleton: ENSXMAG00000017639

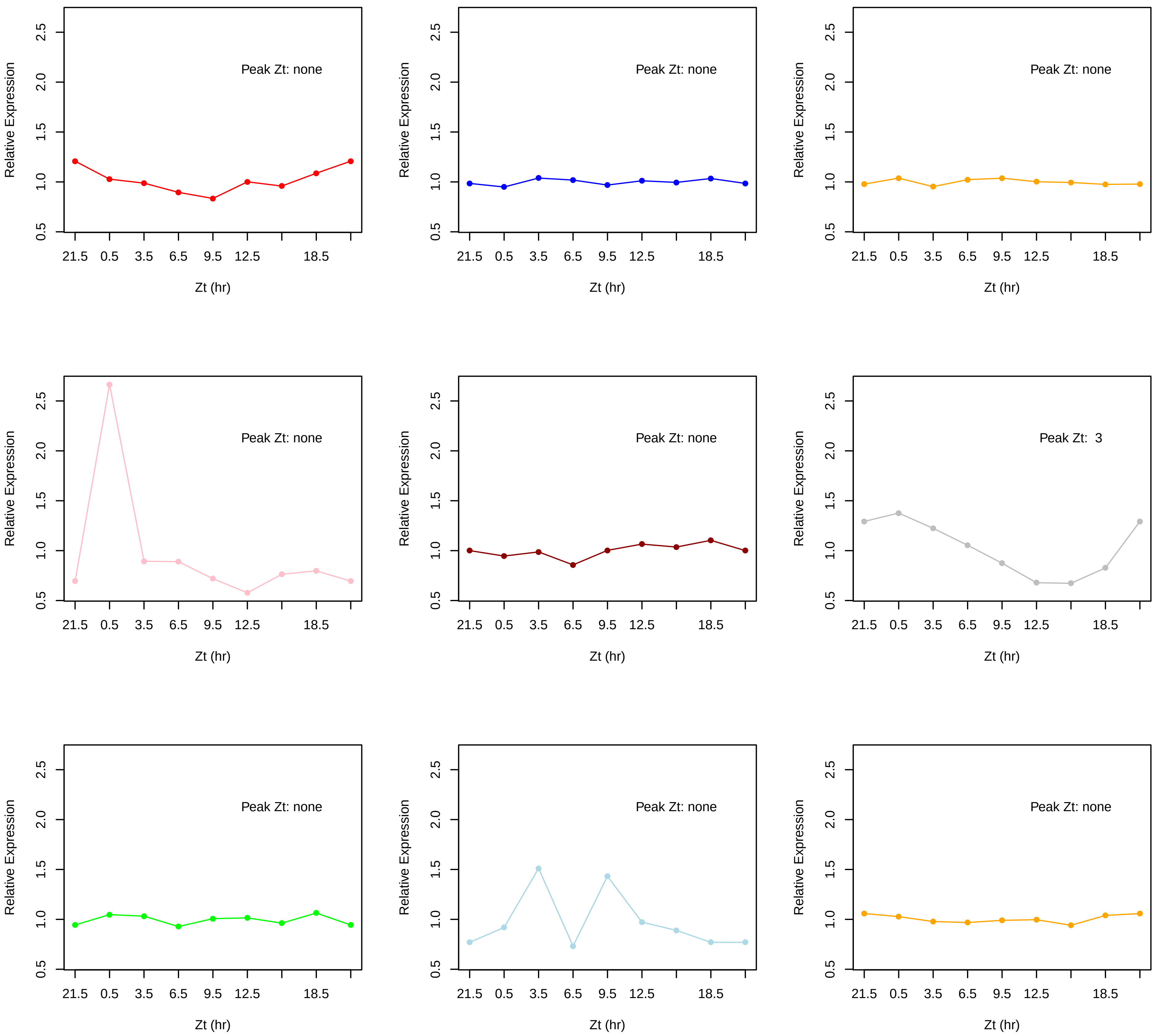

Singleton: ENSXMAG00000004820 BAZ1A

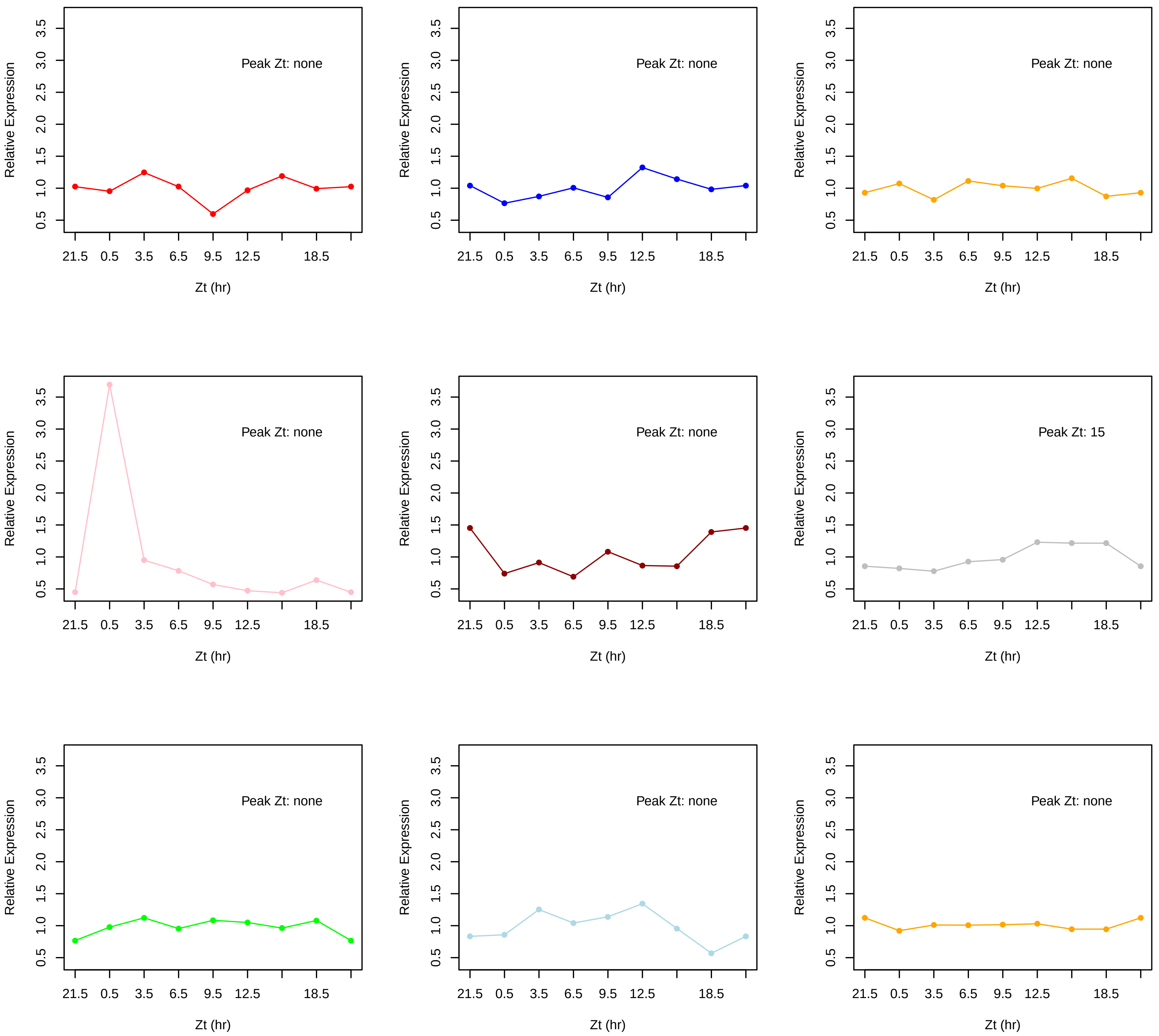

Singleton: ENSXMAG00000006333

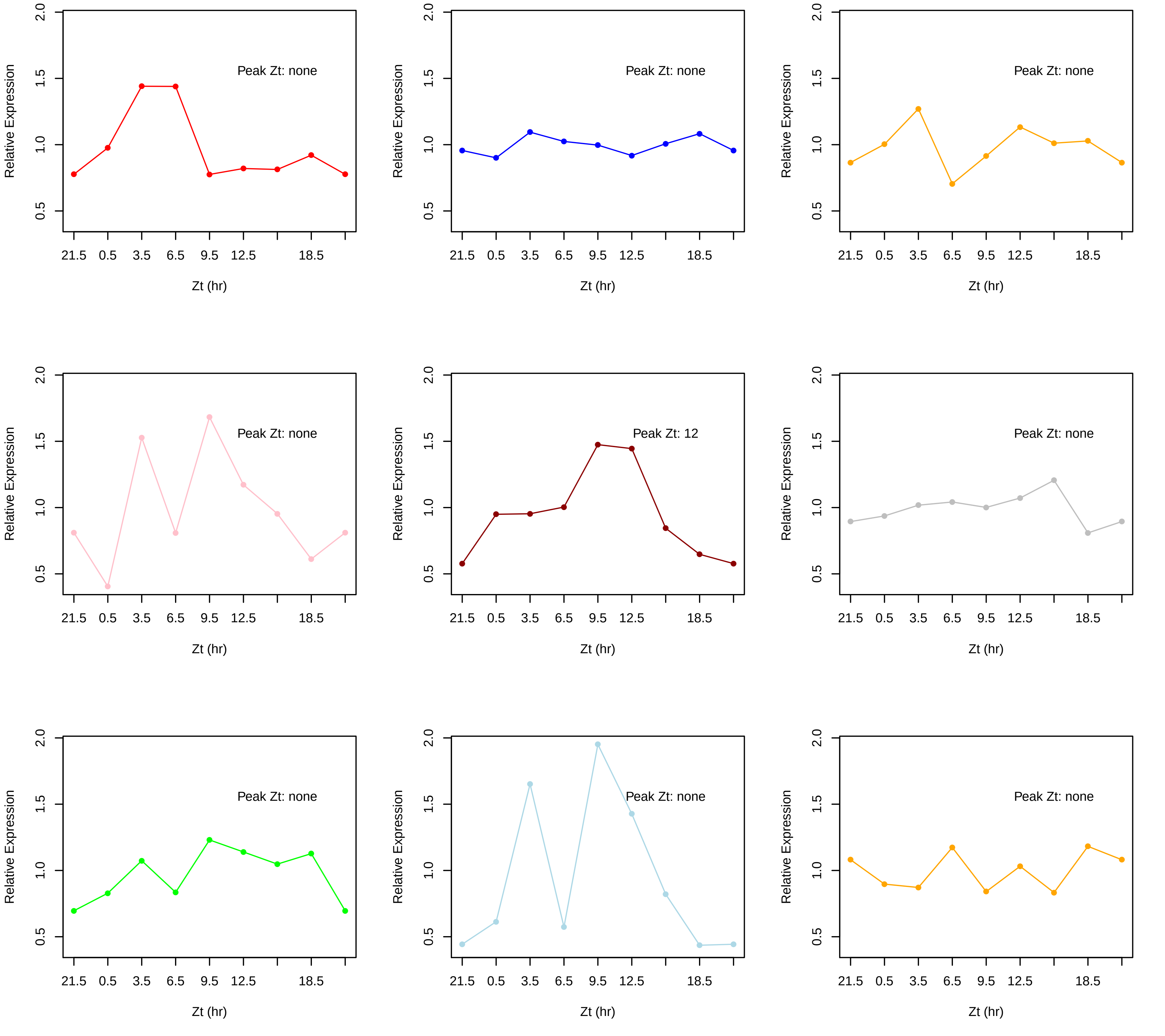

Singeleton: ENSXMAG00000028653 cfl2

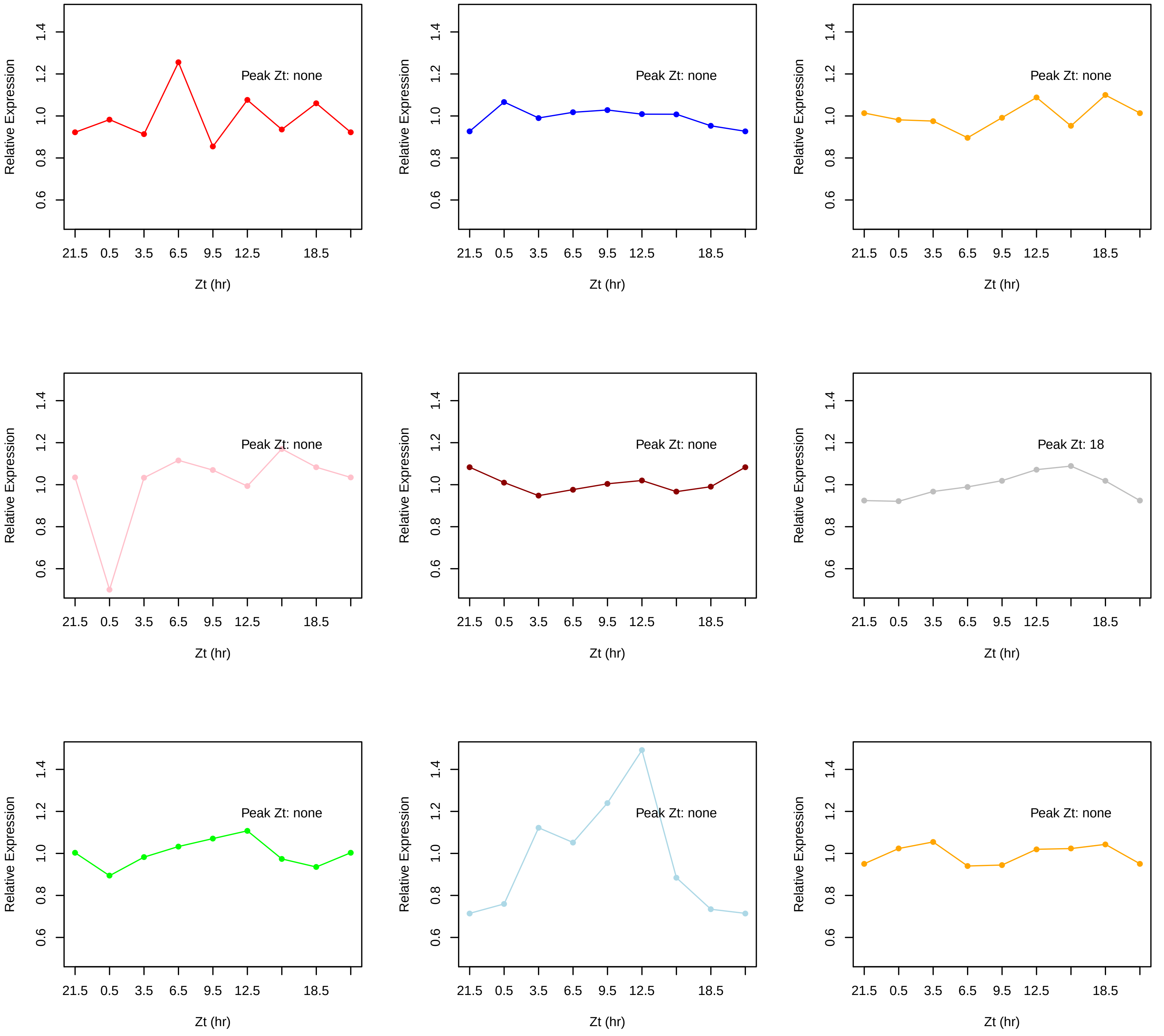

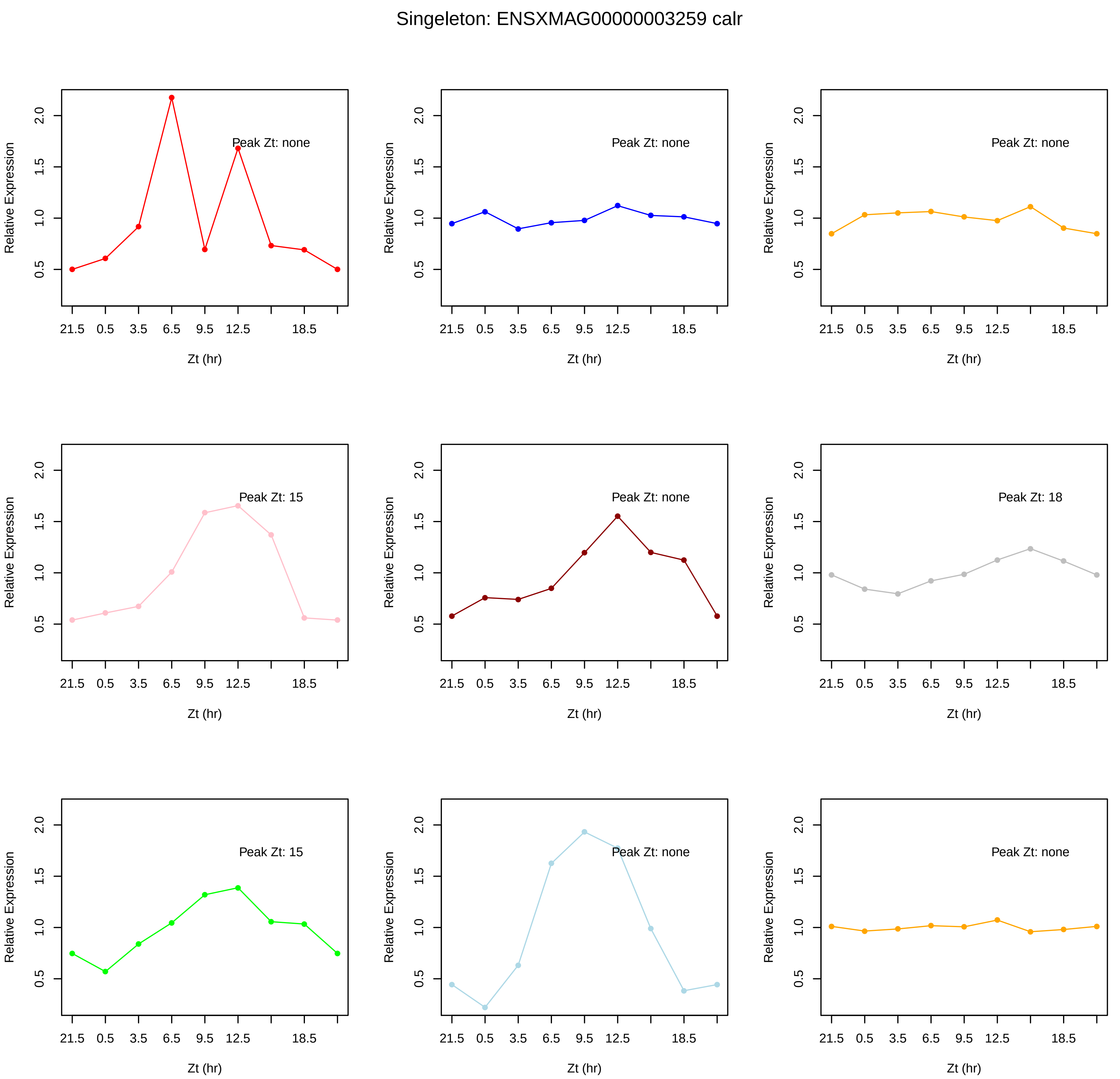

Singeleton: ENSXMAG00000003259 calr
Peak Zt: none
Peak Zt: none
Peak Zt: none
Peak Zt: 15
Peak Zt: none
Peak Zt: 18
Peak Zt: 15
Peak Zt: none
Peak Zt: none
Relative Expression
Zt (hr)

Singeleton: ENSXMAG00000003924 RIMS2

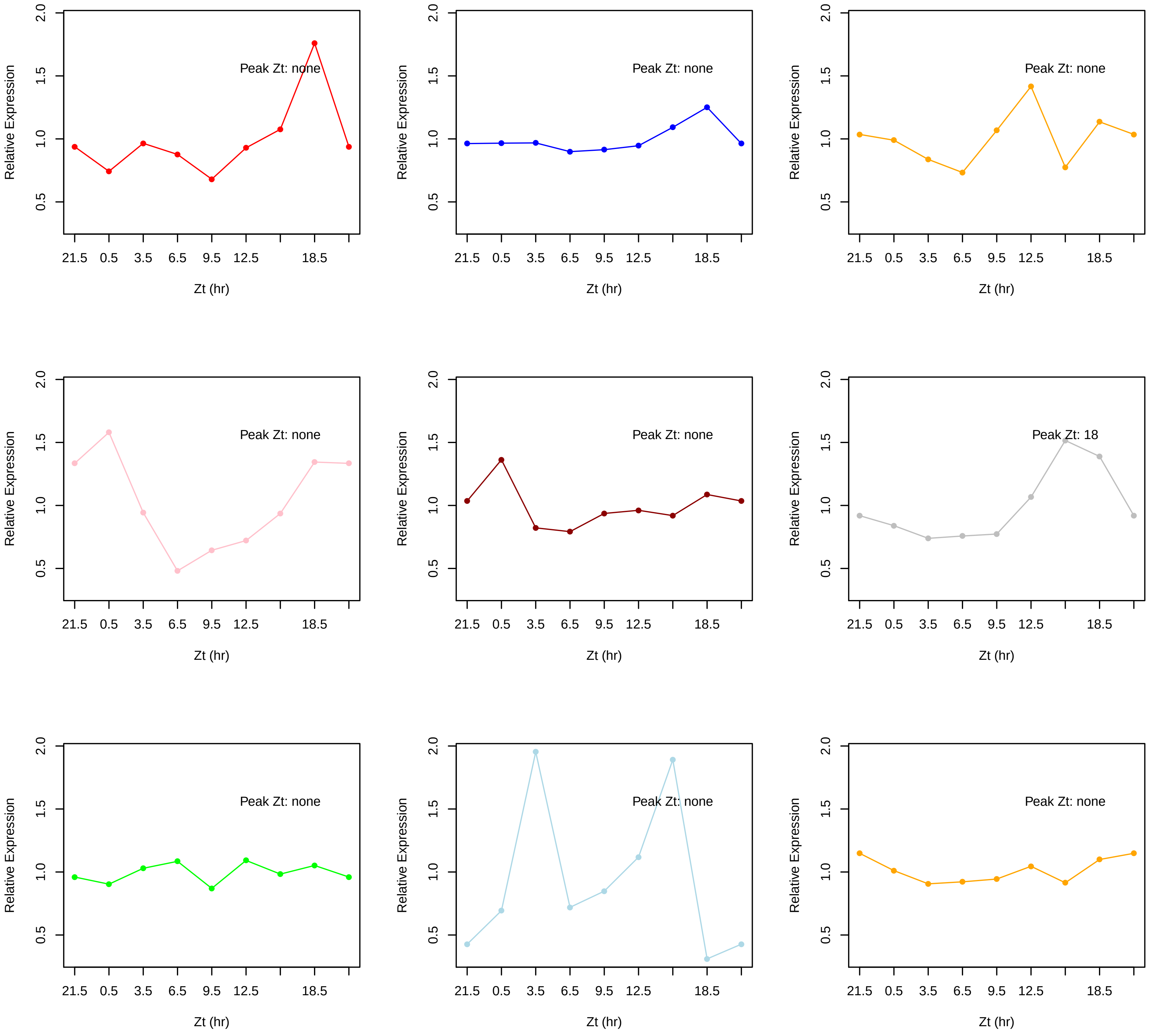

Singleton: ENSXMAG00000010046 robo2

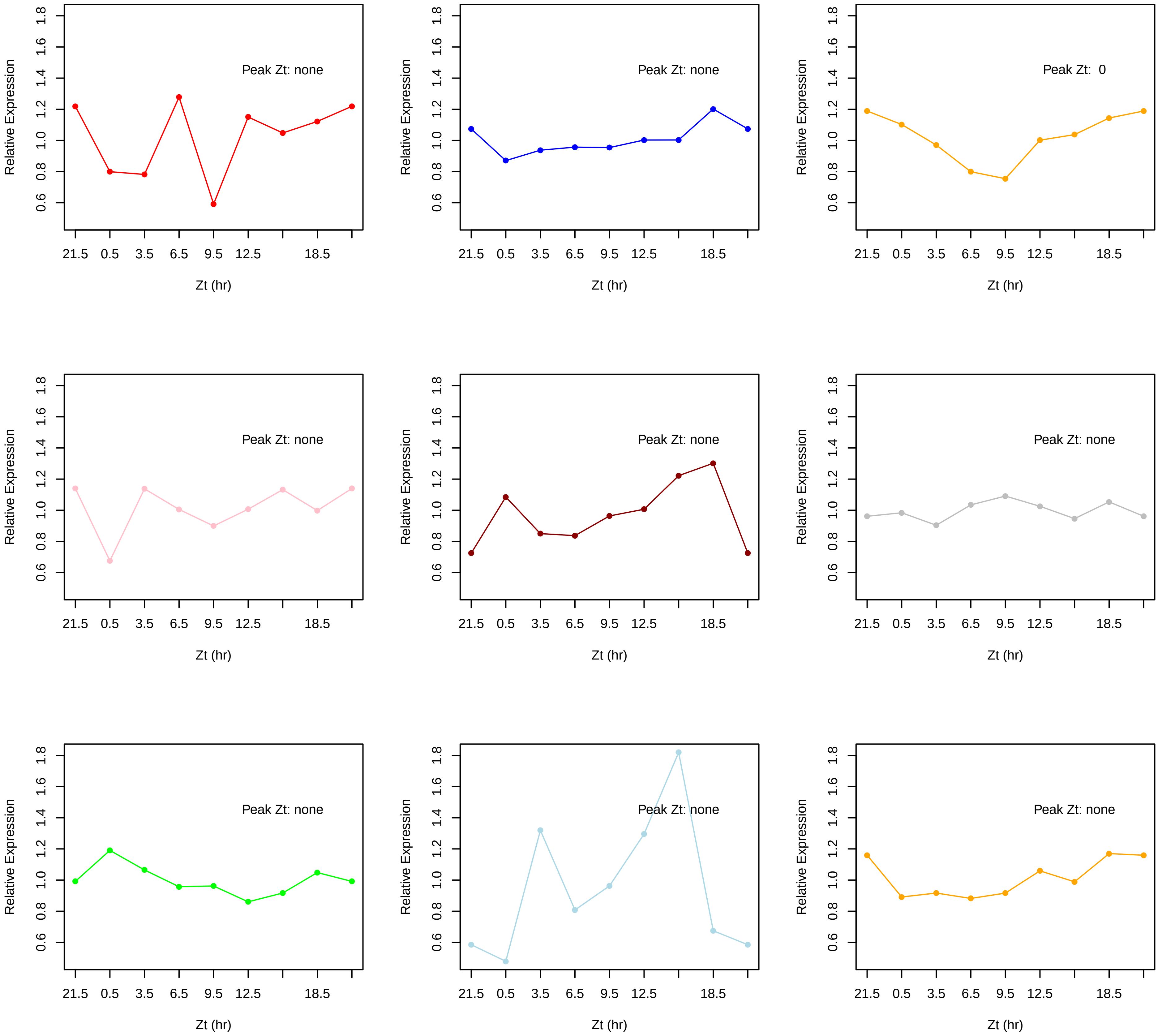

Singleton: ENSXMAG00000005830 pth1ra

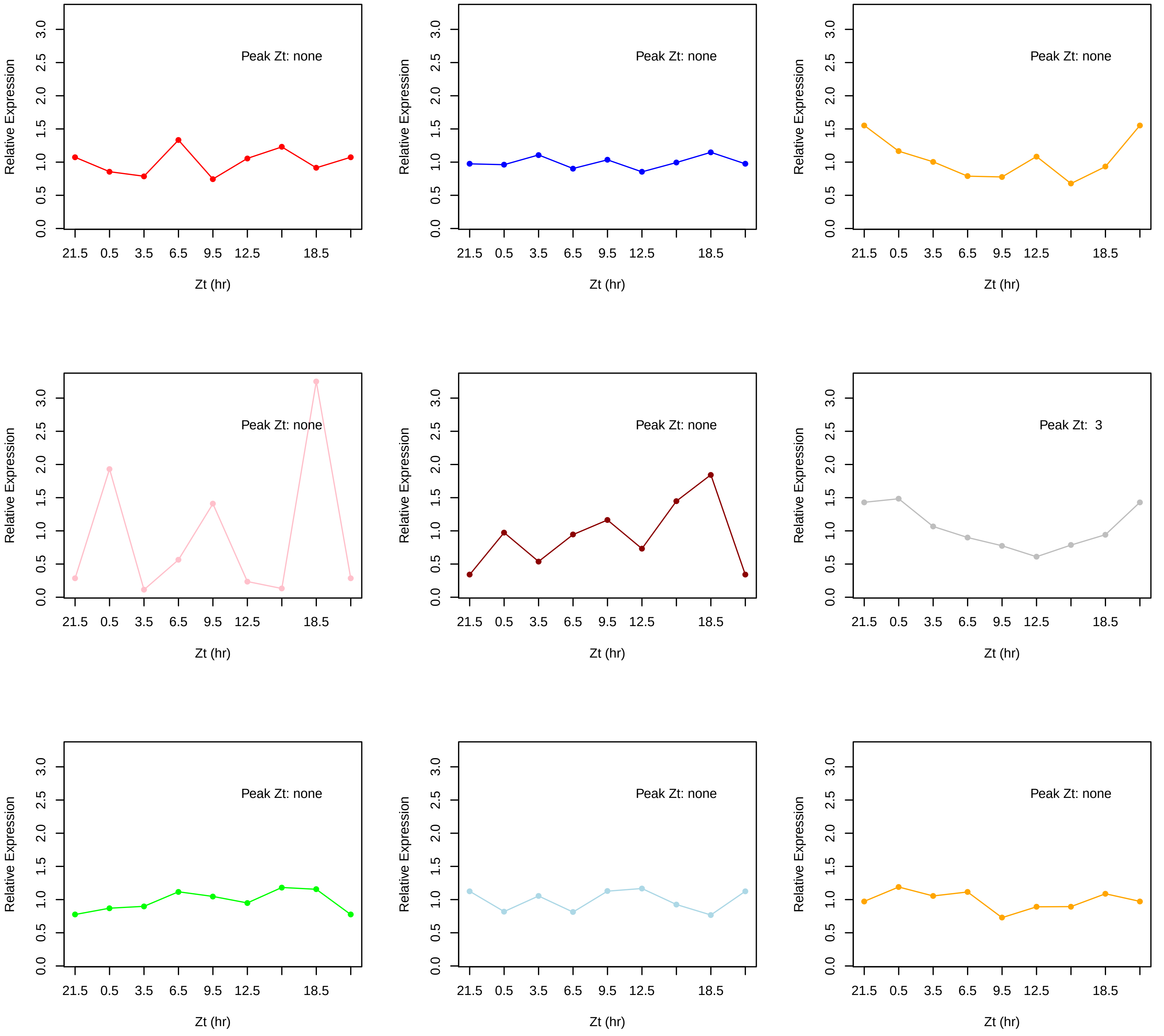

Singeleton: ENSXMAG00000028528 eapp

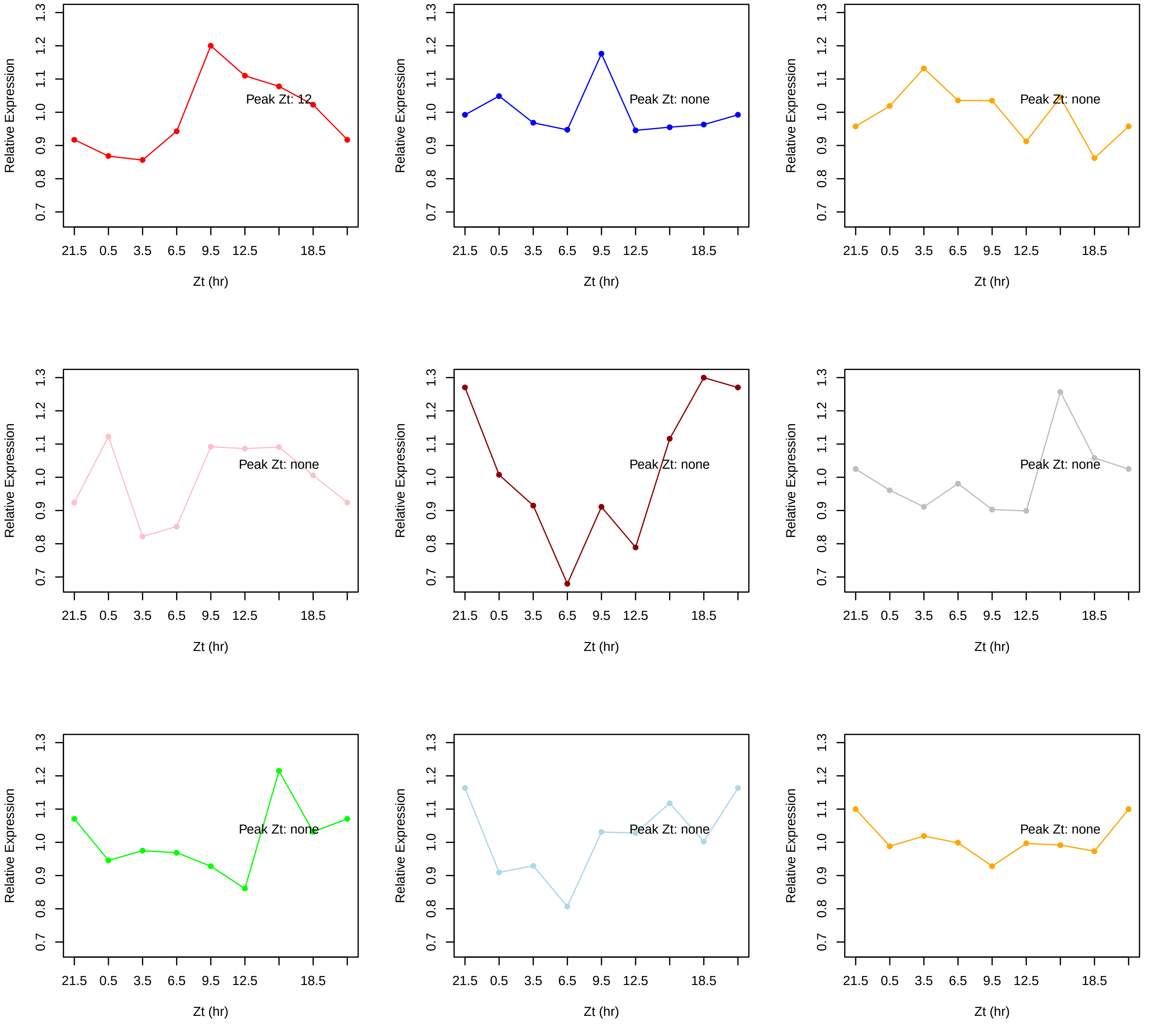

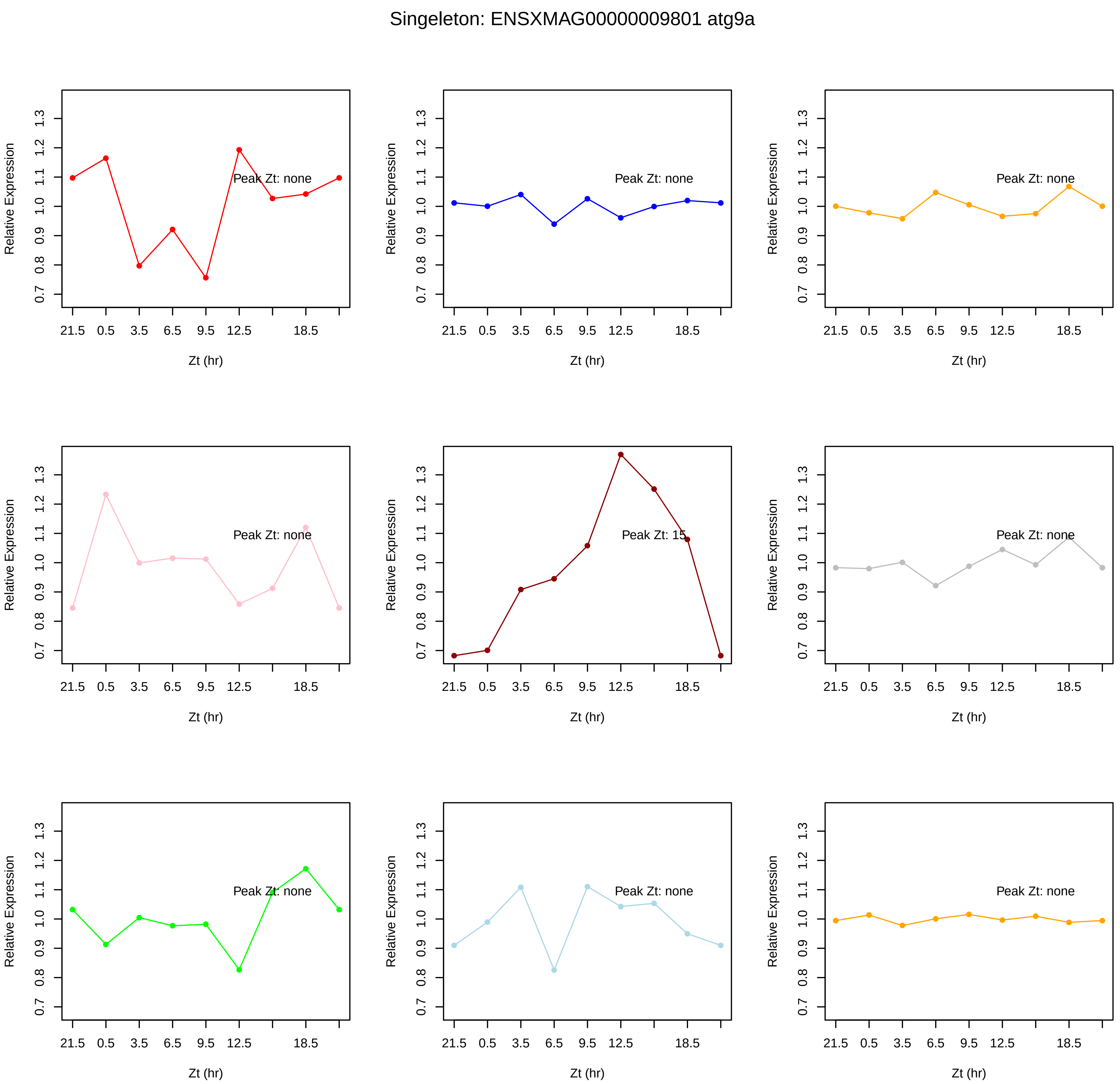
Singeleton: ENSXMAG00000009801 atg9a
Relative Expression
0.7 0.8 0.9 1.0 1.1 1.2 1.3
Zt (hr)
21.5 0.5 3.5 6.5 9.5 12.5 18.5
Peak Zt: none
Peak Zt: 15

Singeleton: ENSXMAG00000000707

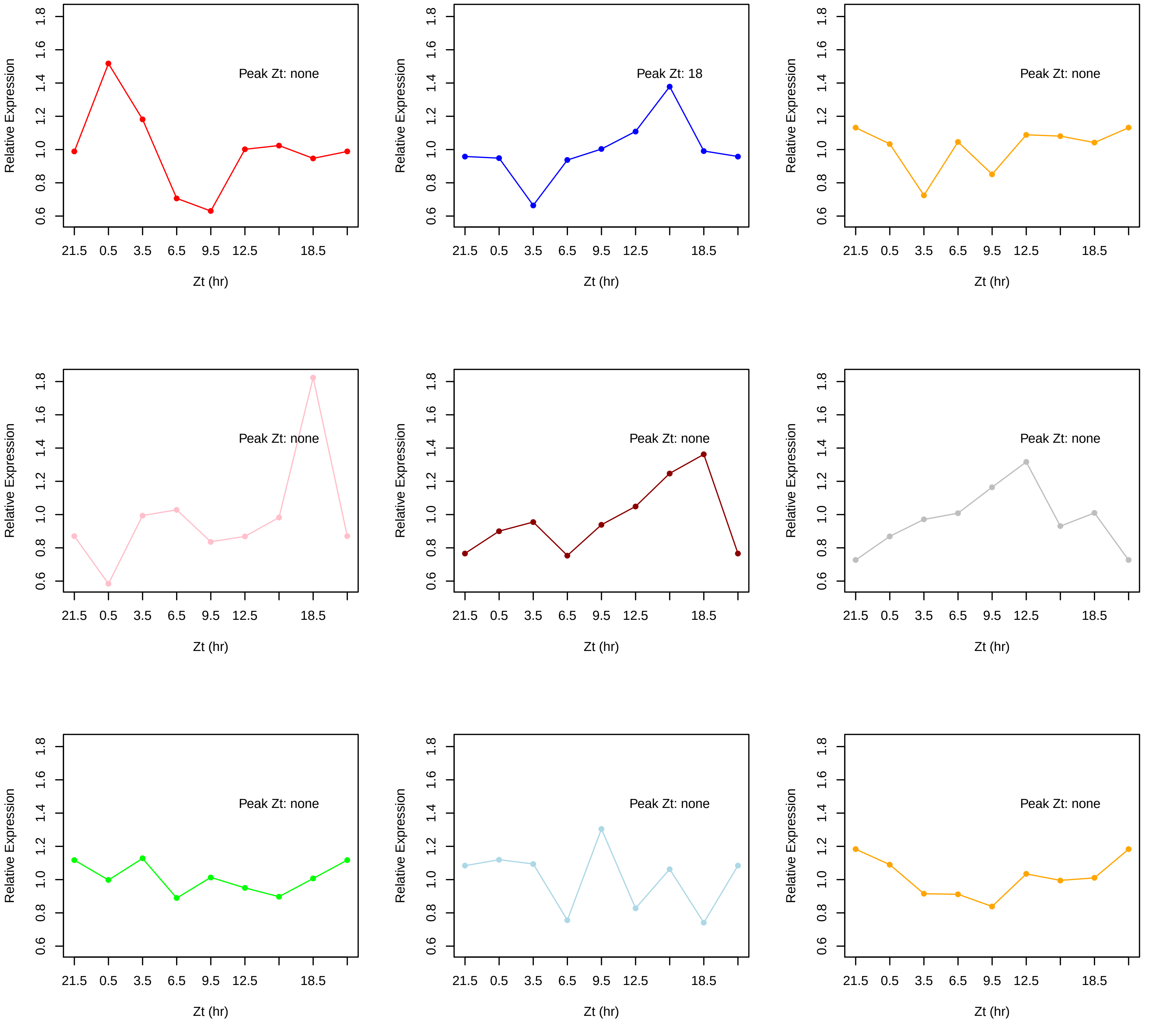

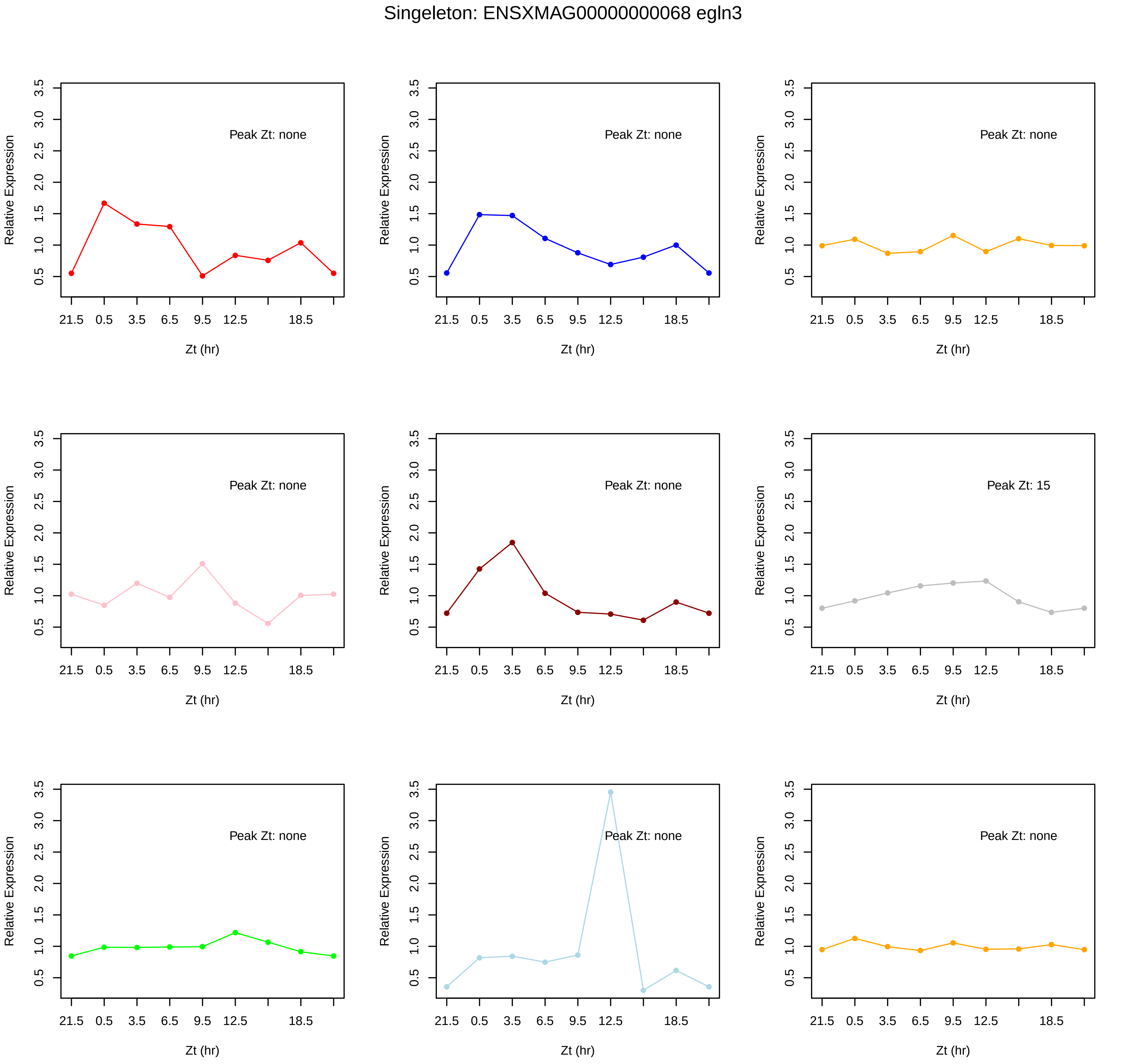
Singeleton: ENSXMAG00000000068 egln3
Peak Zt: none
Peak Zt: none
Peak Zt: none
Peak Zt: none
Peak Zt: none
Peak Zt: 15
Peak Zt: none
Peak Zt: none
Peak Zt: none
Relative Expression
Zt (hr)
21.5
0.5
3.5
6.5
9.5
12.5
18.5
0.5
1.0
1.5
2.0
2.5
3.0
3.5

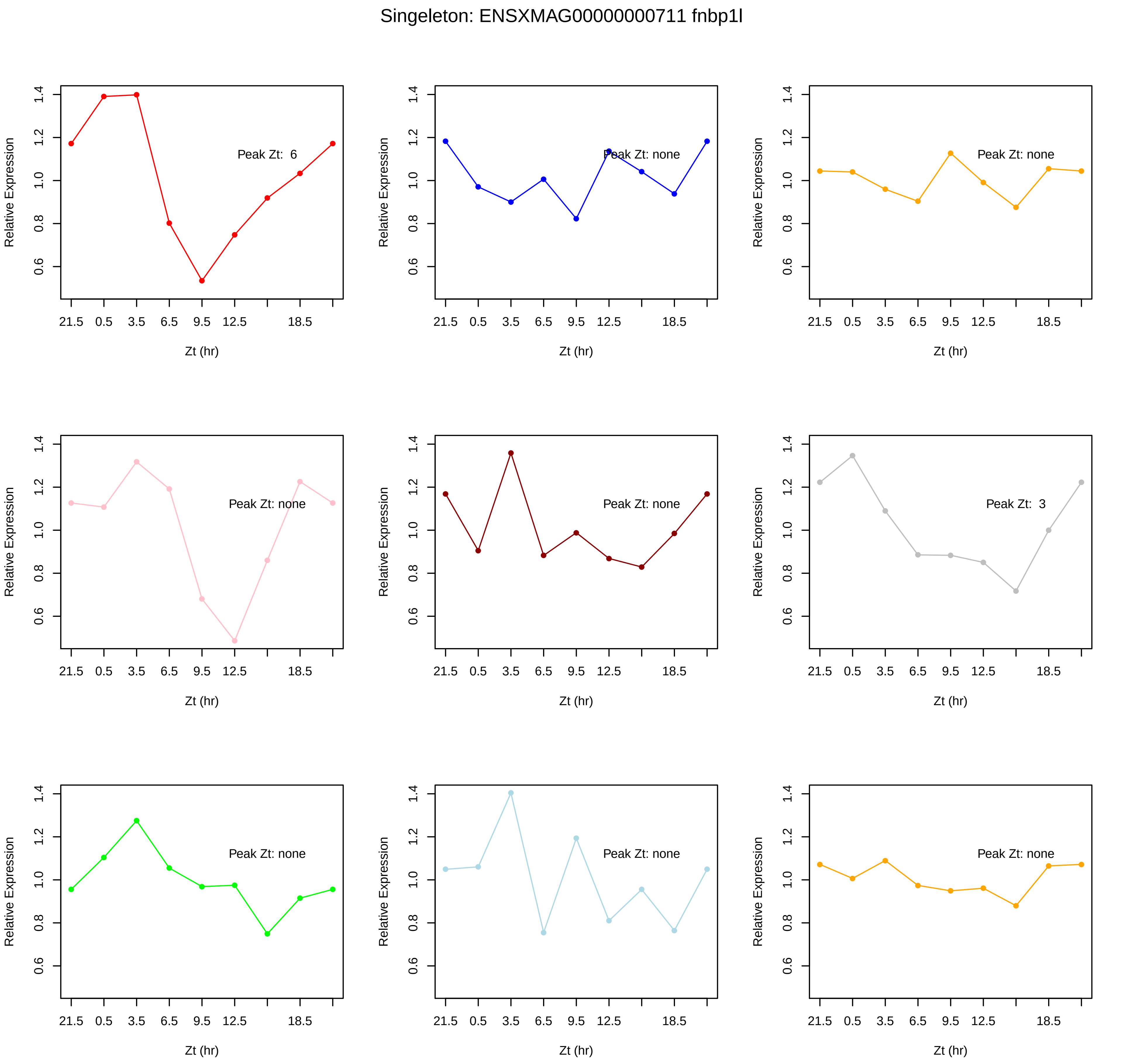
Singleton: ENSXMAG00000000711 fnbp1l
Peak Zt: 6
Peak Zt: none
Peak Zt: none
Peak Zt: none
Peak Zt: none
Peak Zt: 3
Peak Zt: none
Peak Zt: none
Peak Zt: none
Relative Expression
Zt (hr)
21.5 0.5 3.5 6.5 9.5 12.5 18.5
0.6 0.8 1.0 1.2 1.4

Singeleton: ENSXMAG00000001428 klf9

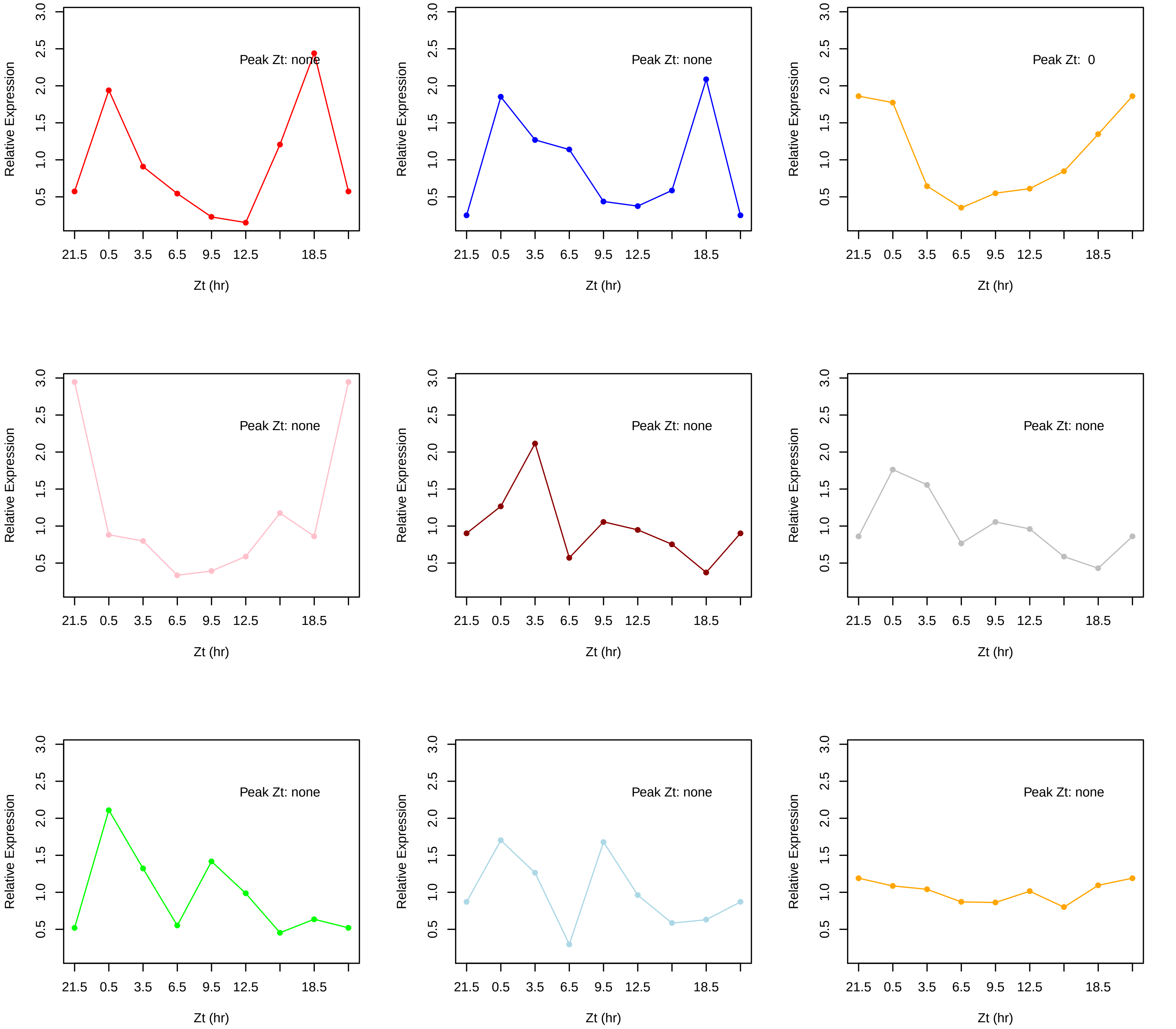

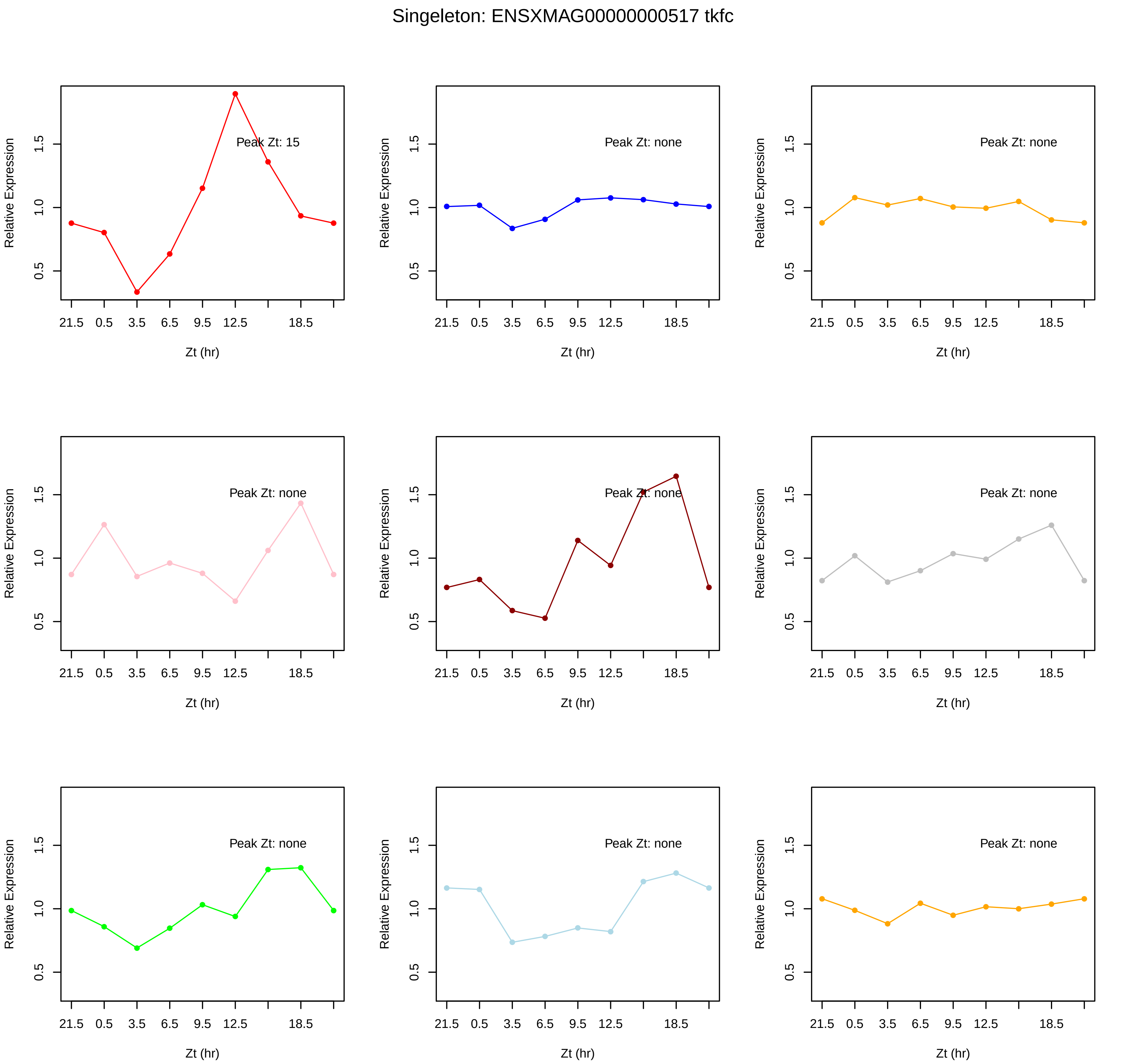
Singeleton: ENSXMAG00000000517 tkfc
Peak Zt: 15
Peak Zt: none
Peak Zt: none
Peak Zt: none
Peak Zt: none
Peak Zt: none
Peak Zt: none
Peak Zt: none
Peak Zt: none
Relative Expression
Zt (hr)
21.5 0.5 3.5 6.5 9.5 12.5 18.5
0.5 1.0 1.5

Singeleton: ENSXMAG00000027269 CTHRC1

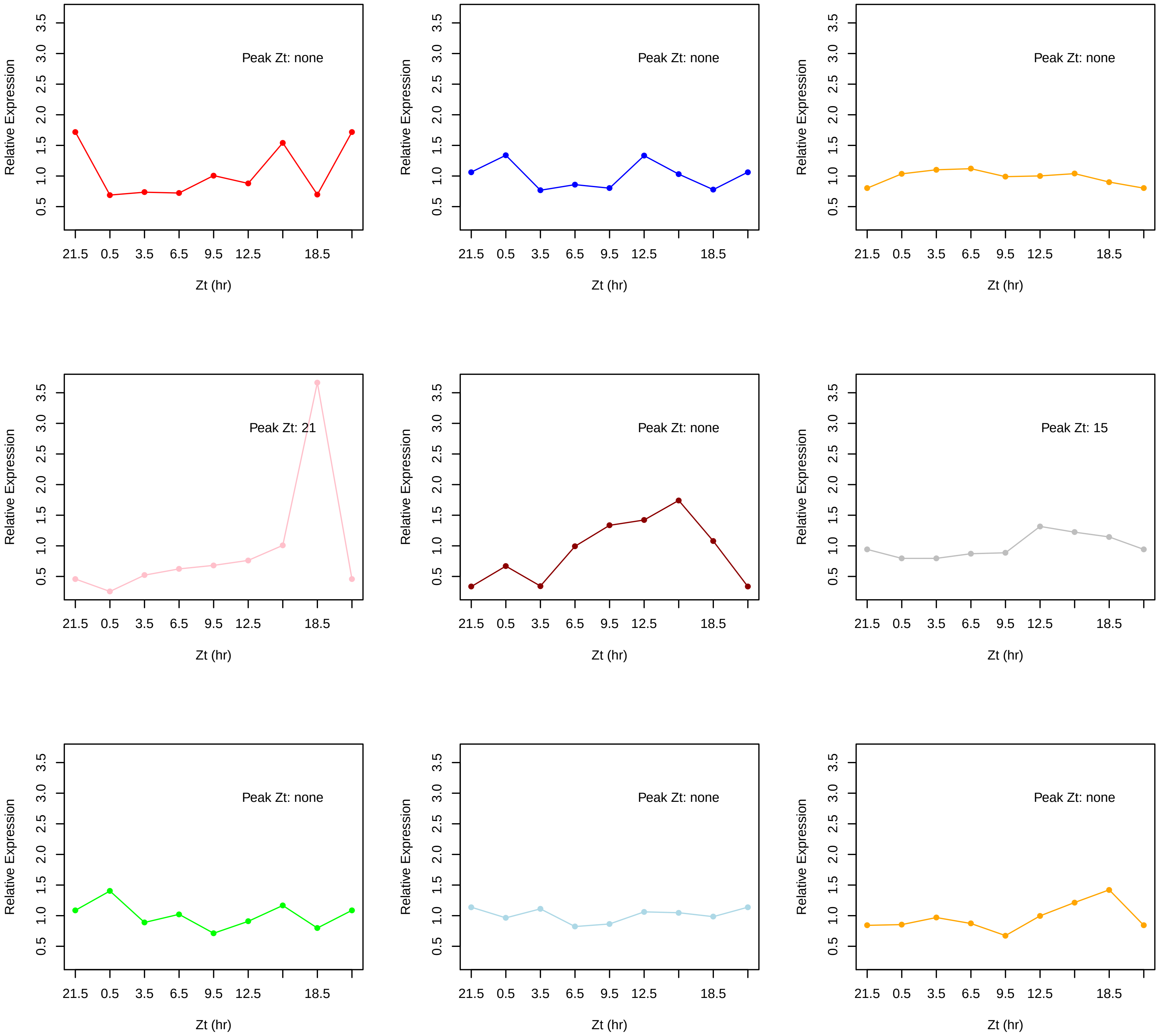

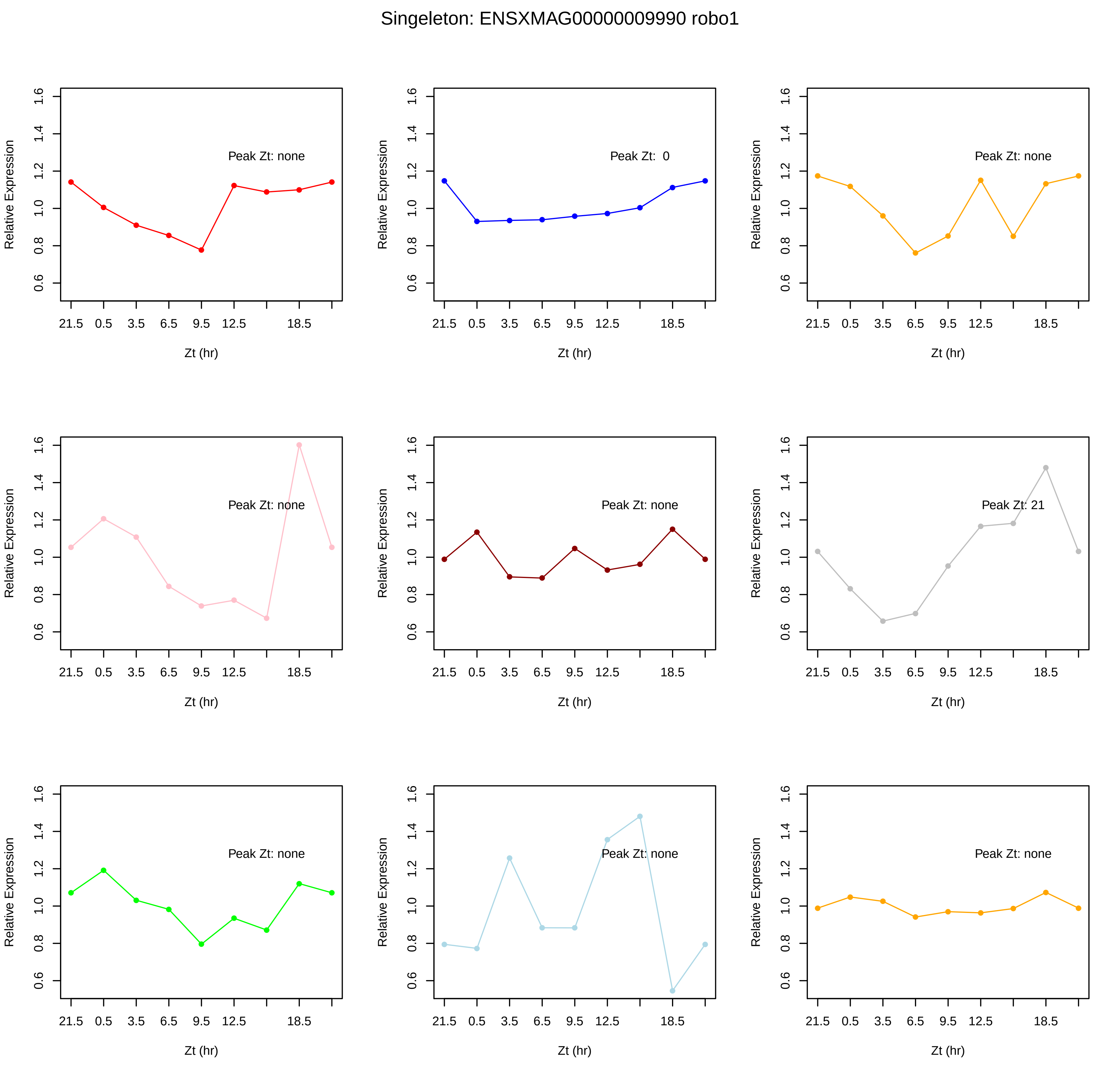
Singleton: ENSXMAG00000009990 robo1
Peak Zt: none
Peak Zt: 0
Peak Zt: none
Peak Zt: none
Peak Zt: none
Peak Zt: 21
Peak Zt: none
Peak Zt: none
Peak Zt: none
Relative Expression
Zt (hr)
21.5
0.5
3.5
6.5
9.5
12.5
18.5

Singleton: ENSXMAG00000018424 si:dkey−30h22.11

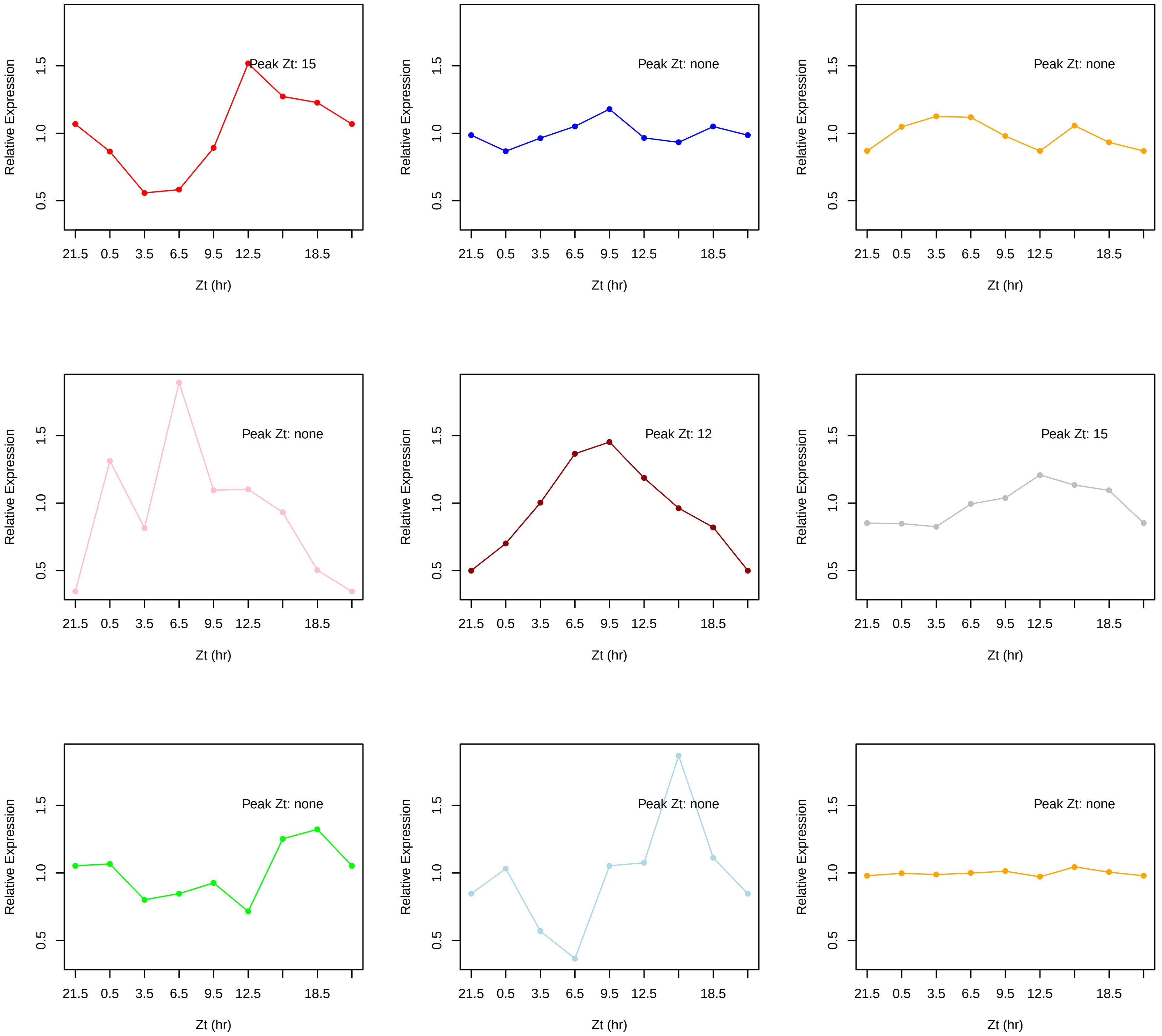

Singeleton: ENSXMAG00000000716 si:ch211−198n5.11

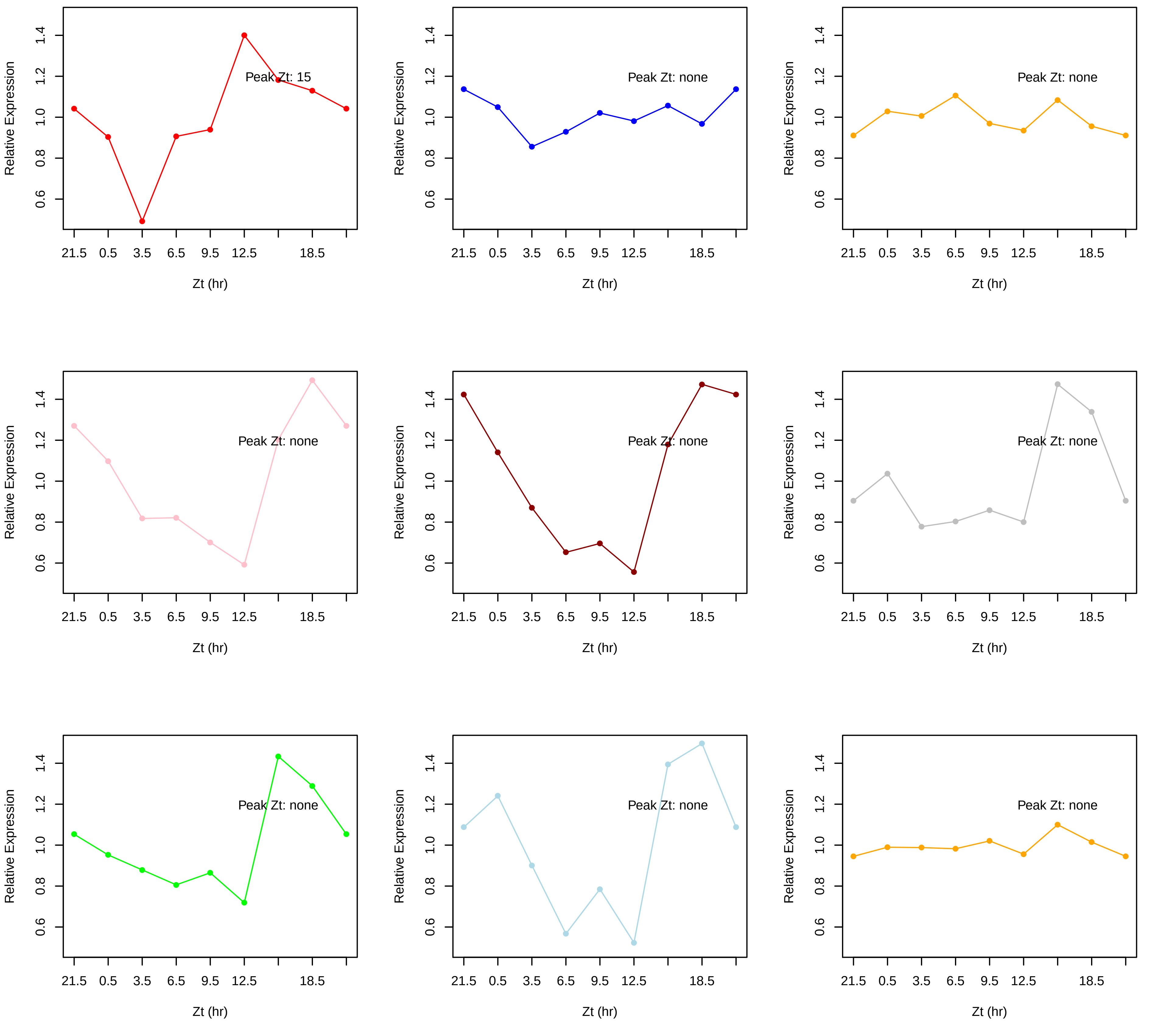

Singeleton: ENSXMAG00000022215 aqp1a.1

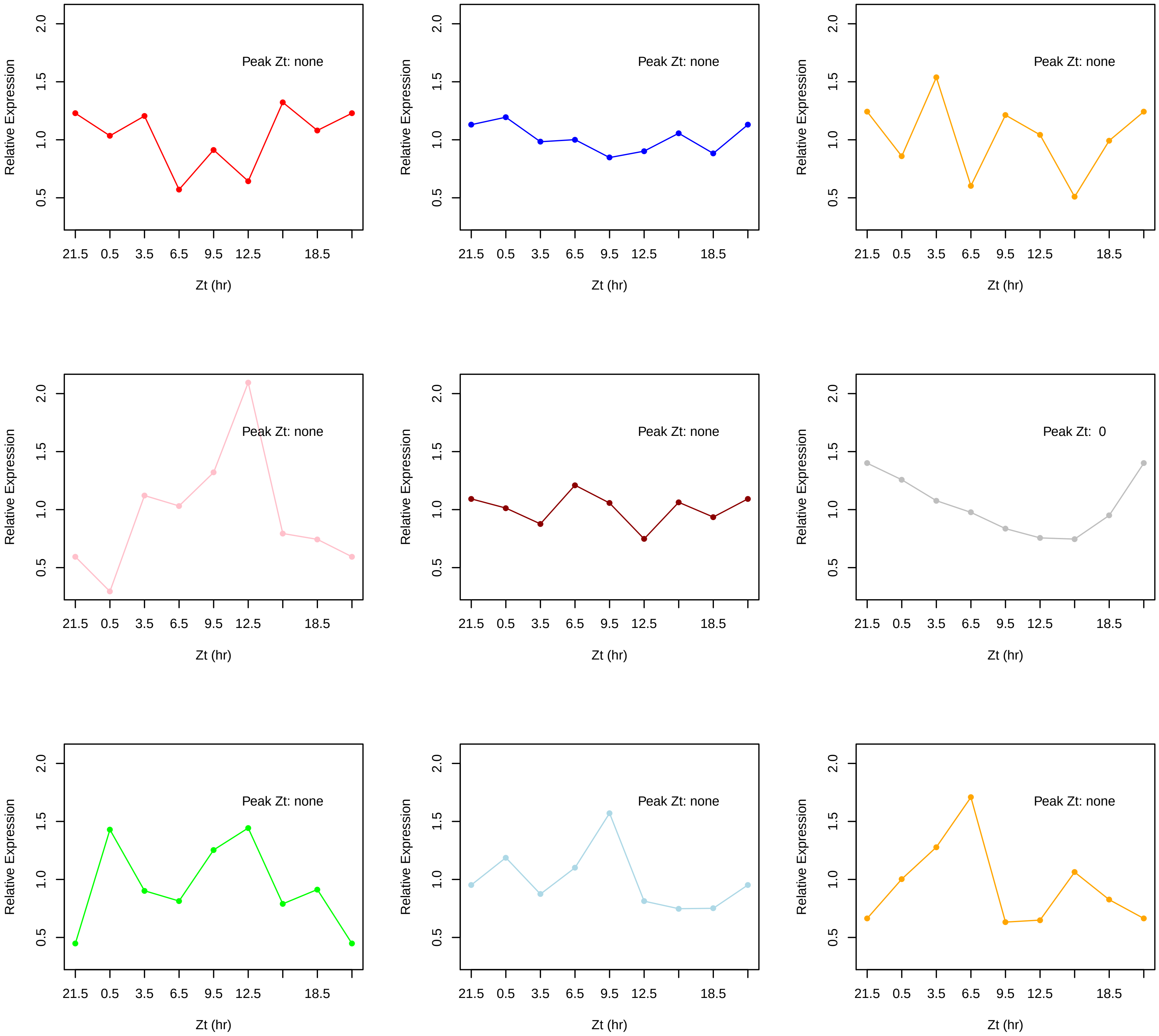

Singleton: ENSXMAG00000019193 txnl4b

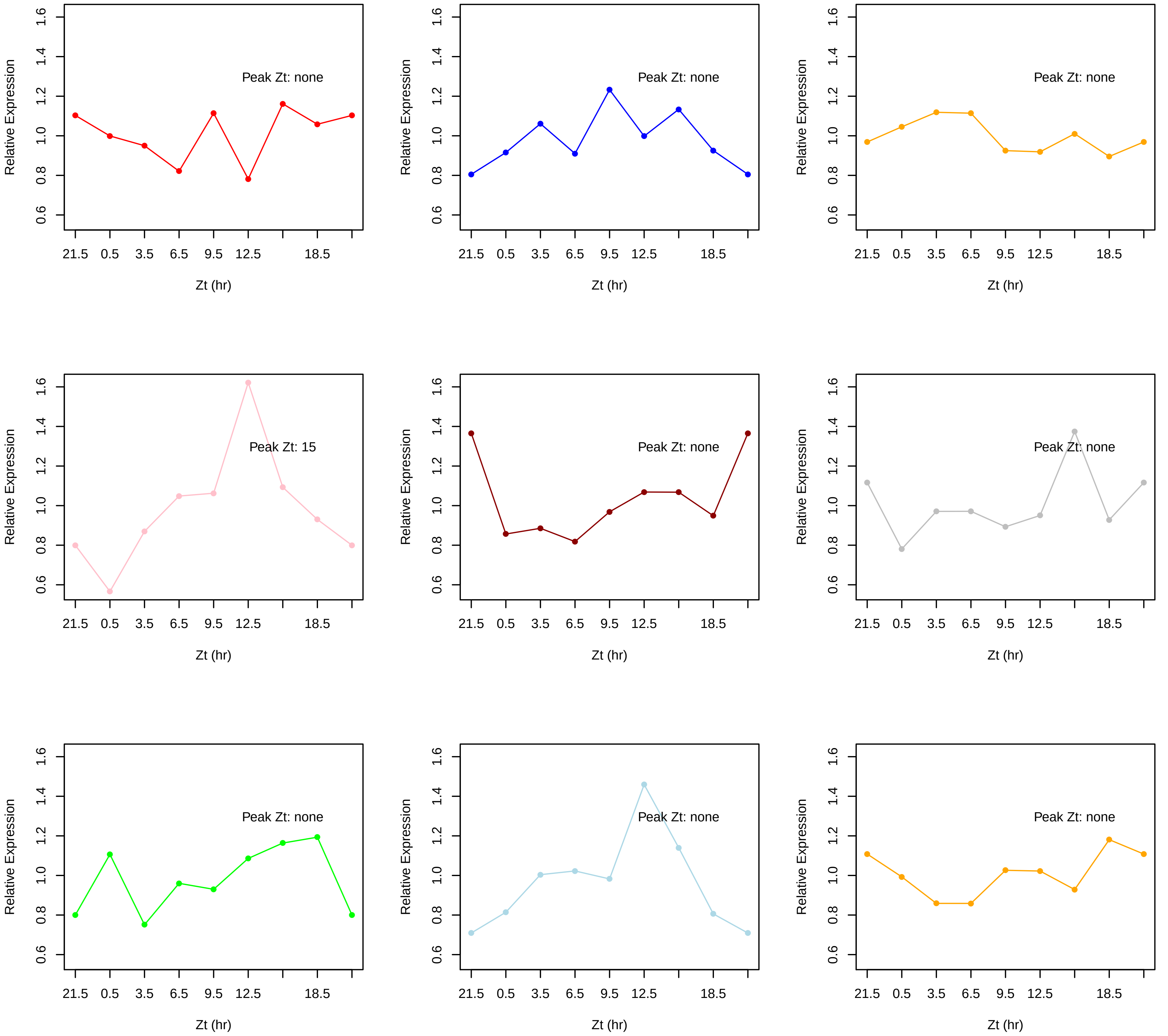

Singeleton: ENSXMAG00000017748

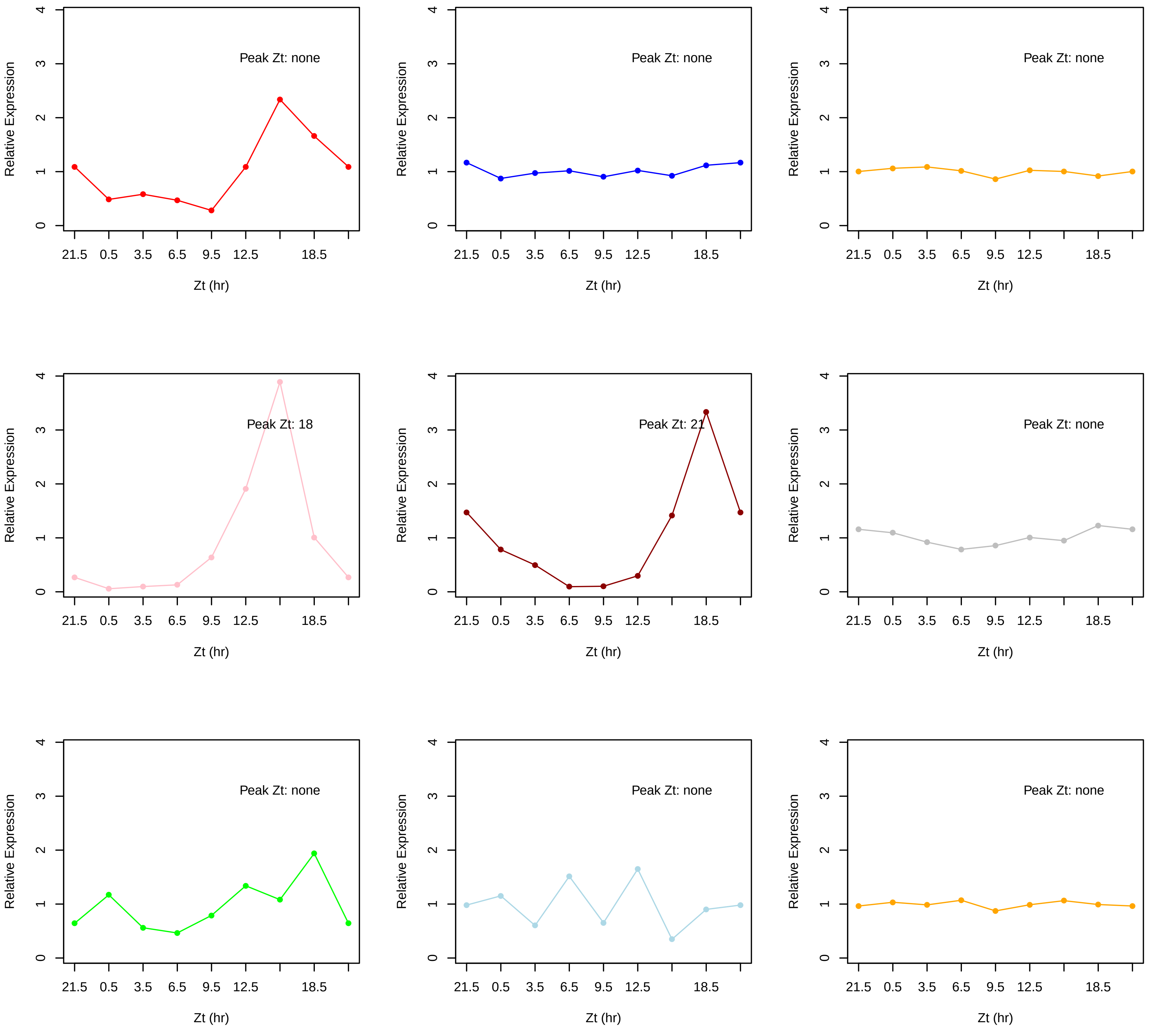

Singeleton: ENSXMAG00000016418 zbtb11

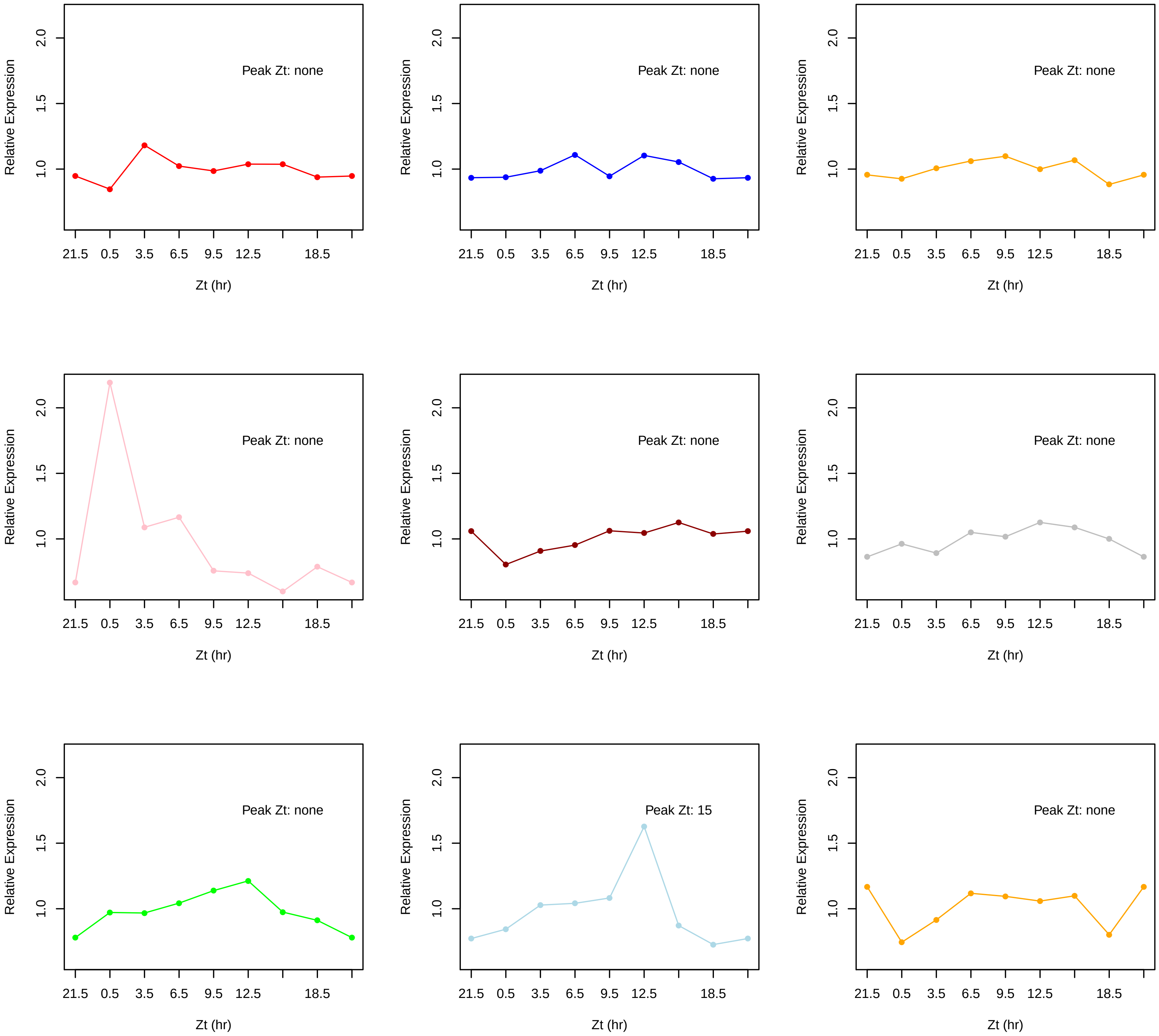

Singeleton: ENSXMAG00000029781 rpl24

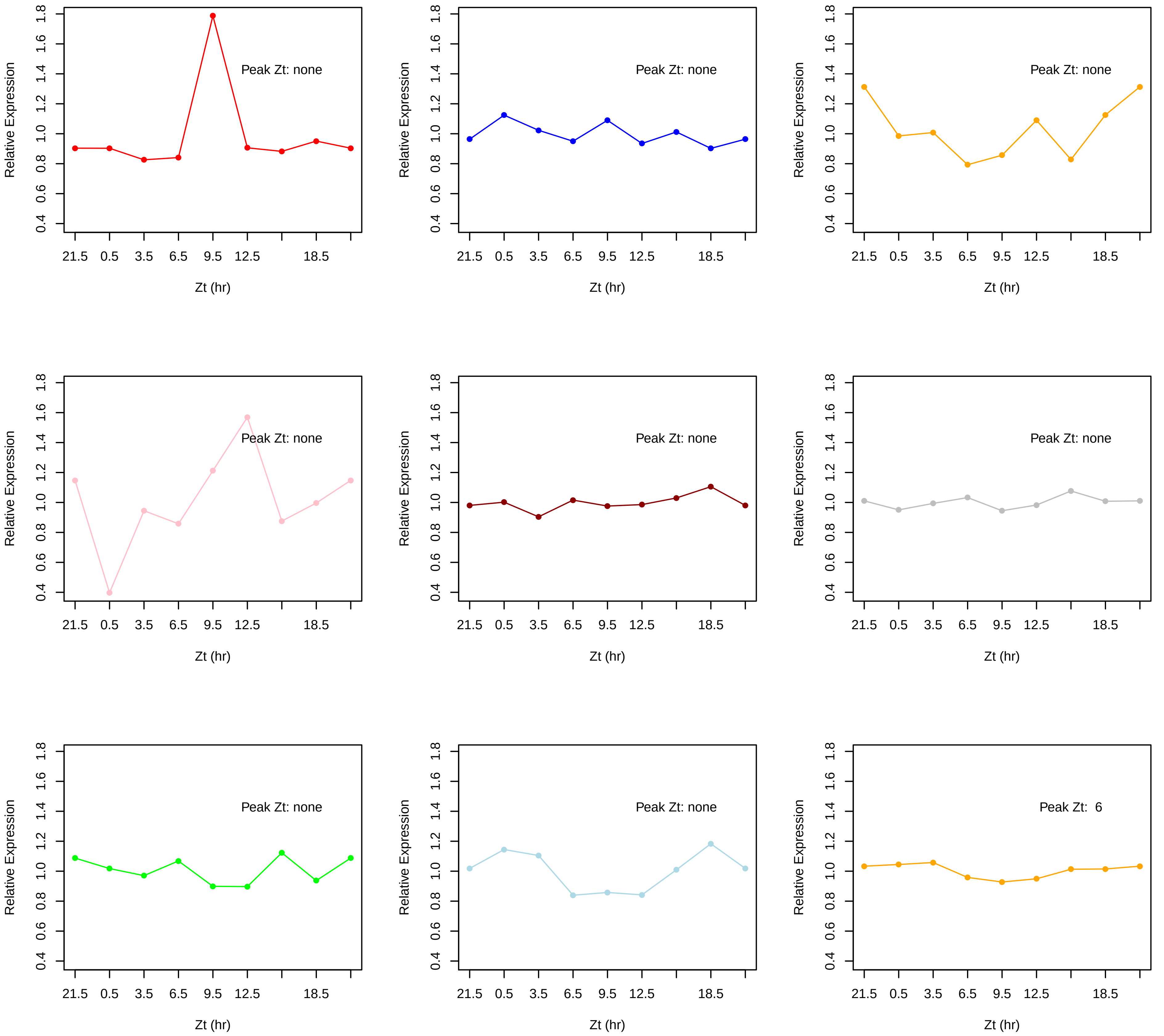

Singleton: ENSXMAG00000009451 nubpl

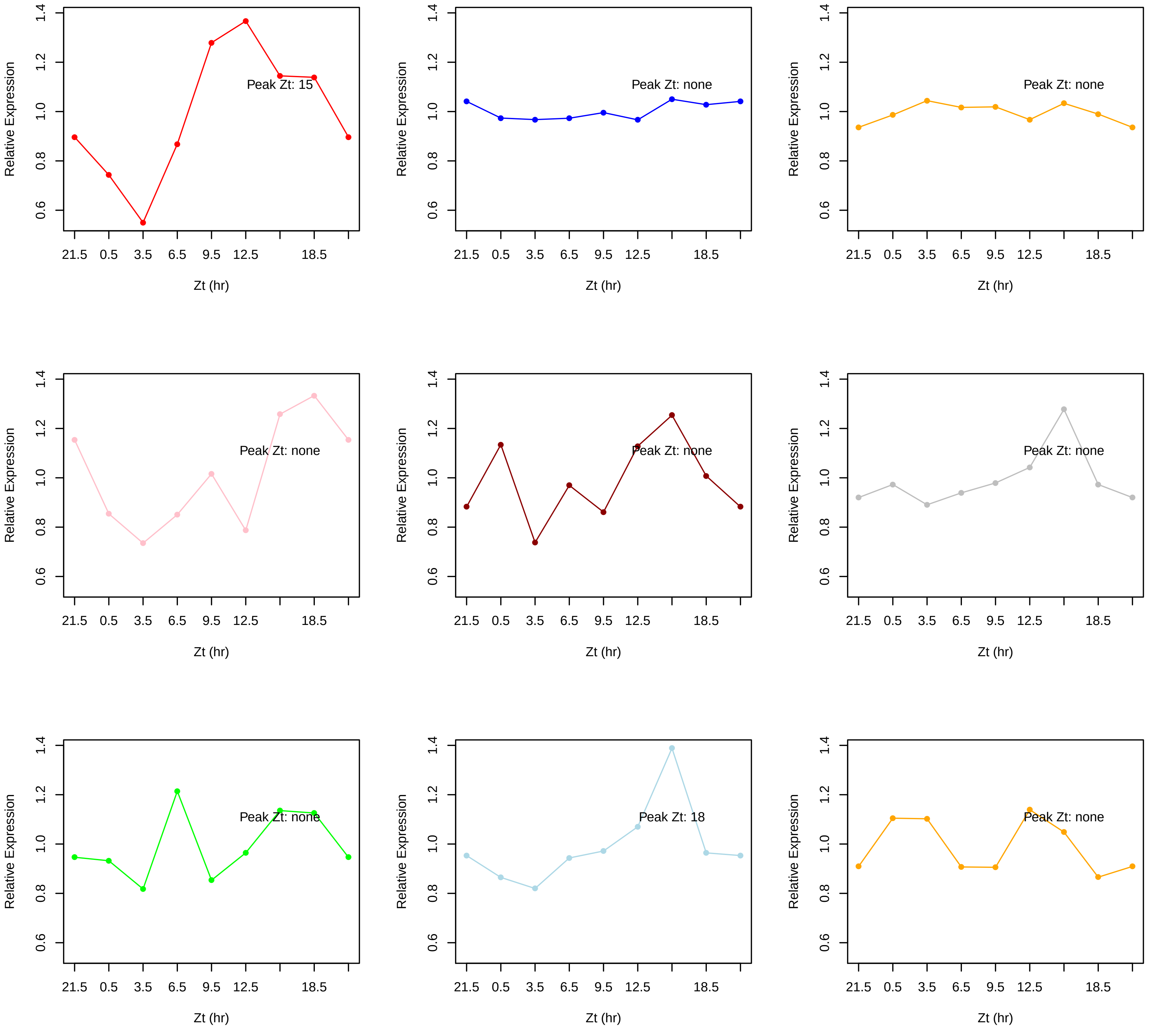

Singeleton: ENSXMAG00000024855 abhd17b

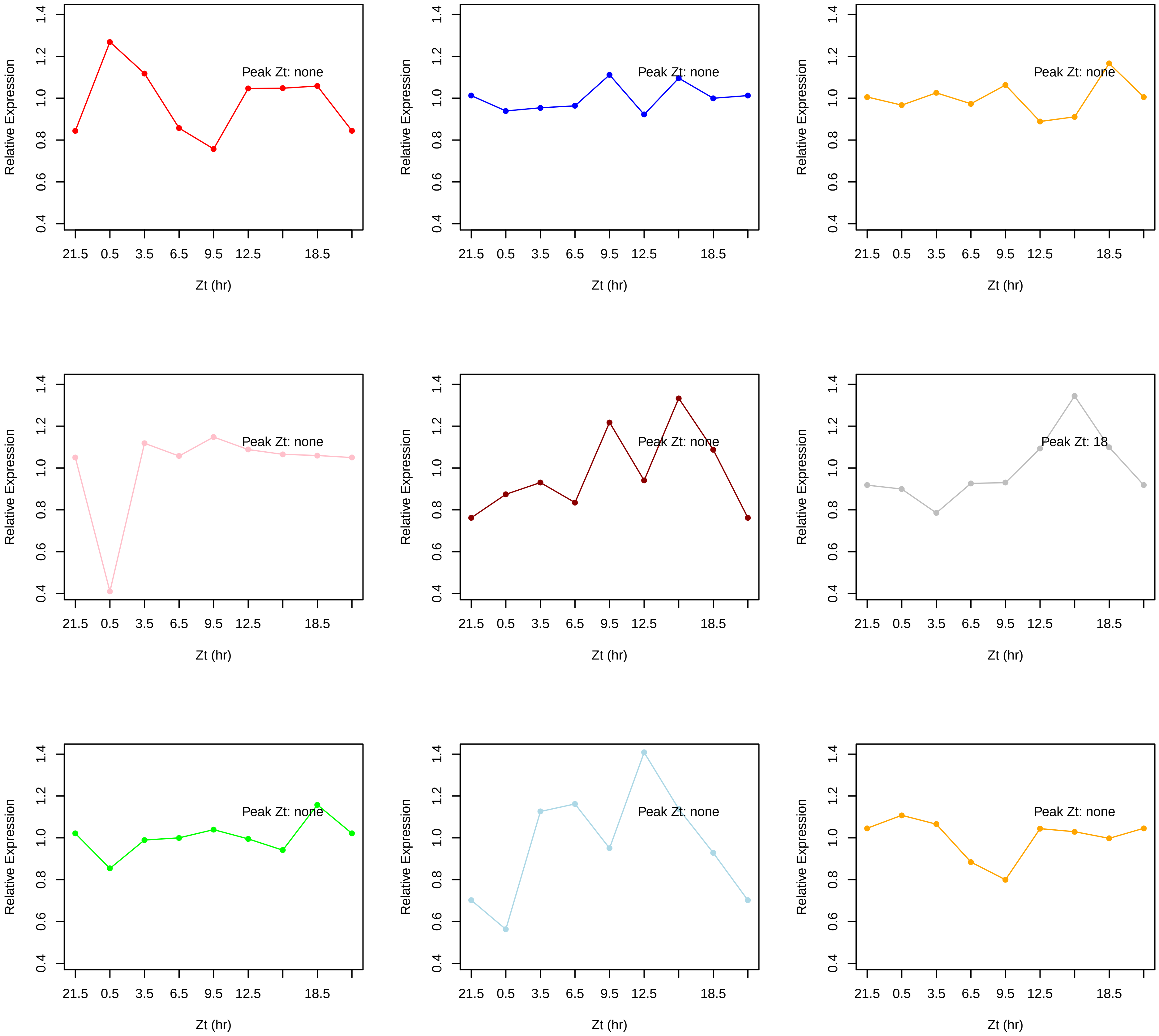

Singeleton: ENSXMAG00000008985 spon2a

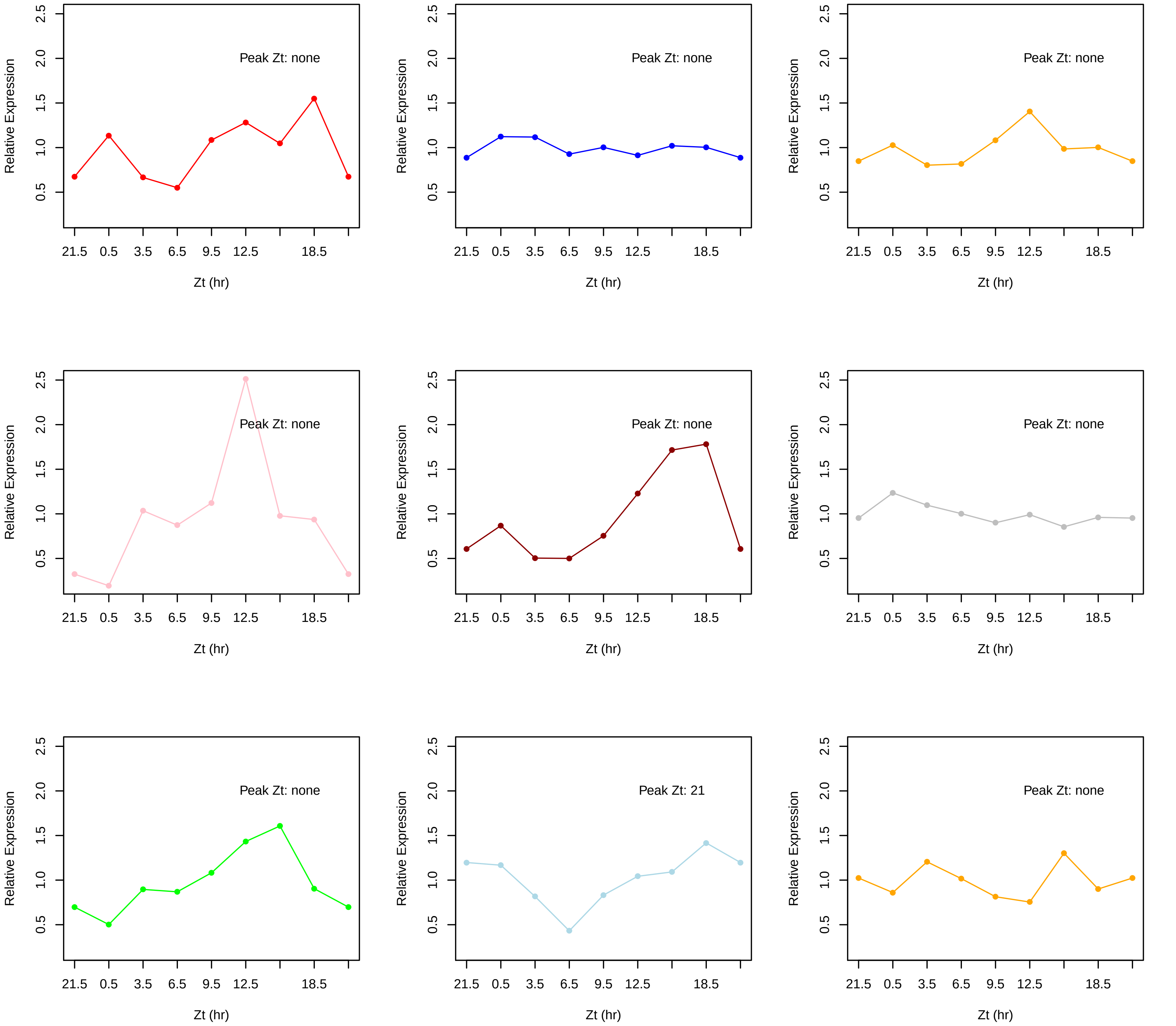

Singeleton: ENSXMAG00000019126 dtd2

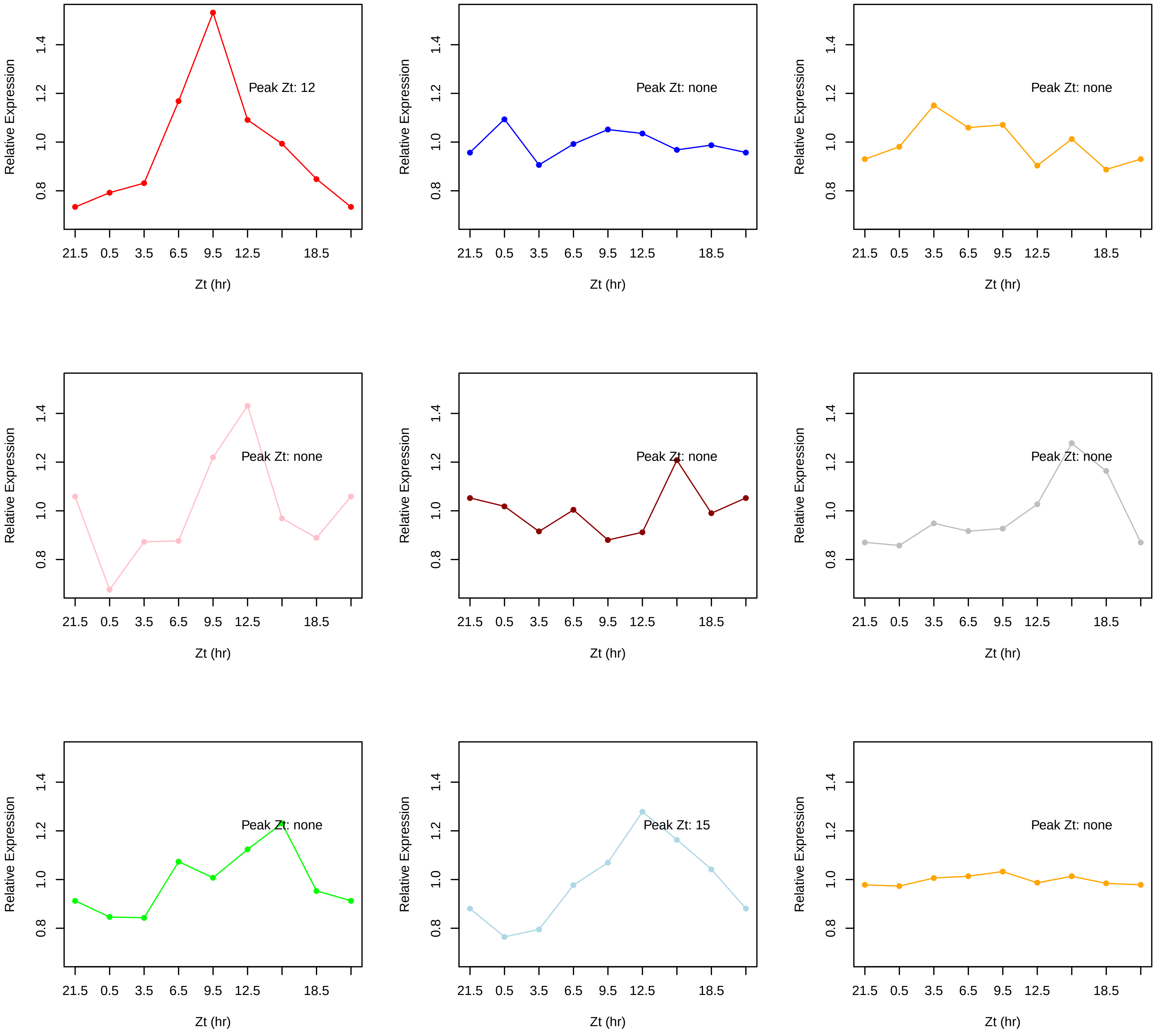

Singeleton: ENSXMAG00000017775 smpd1

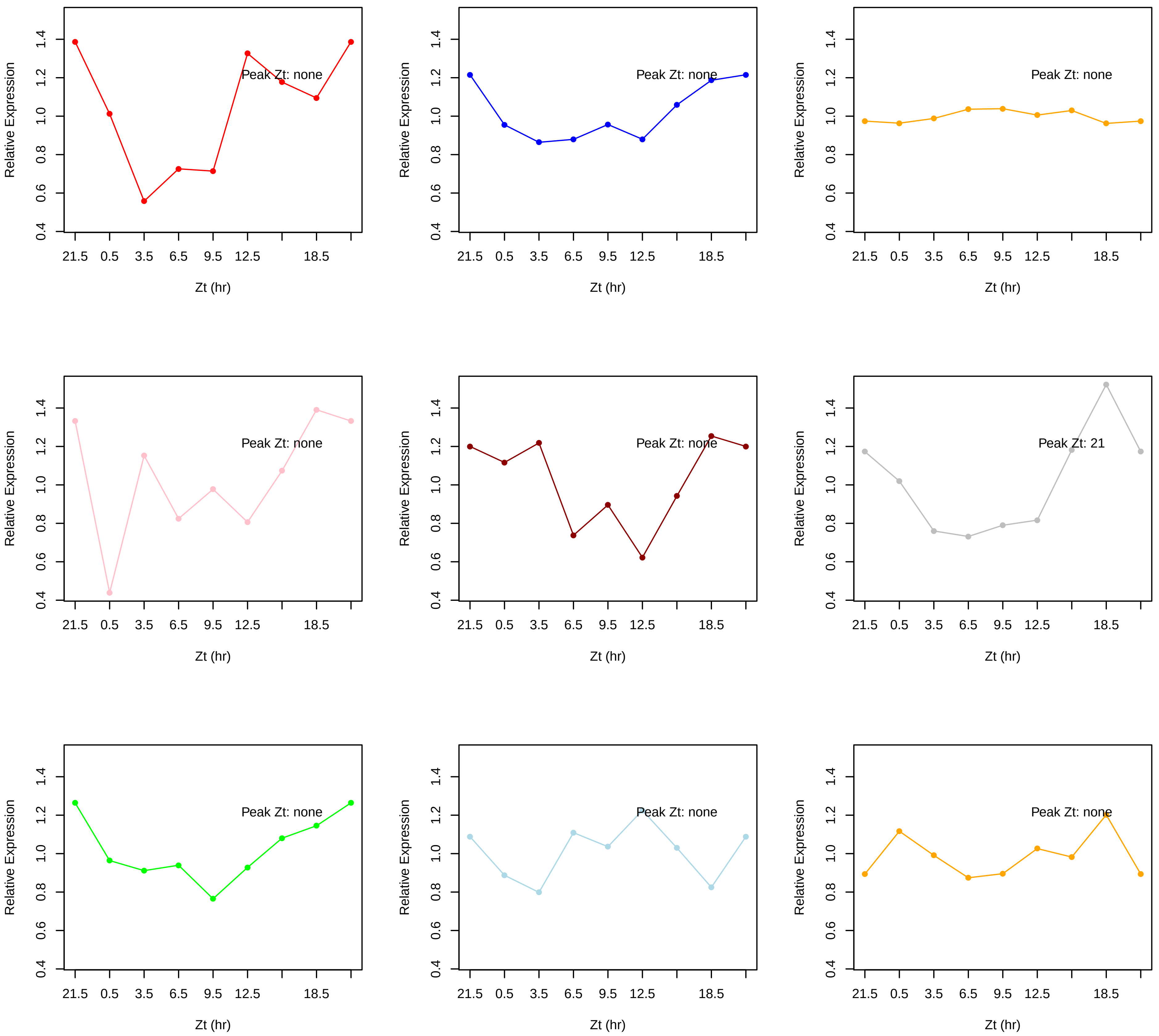

Singeleton: ENSXMAG00000005921 pitx3

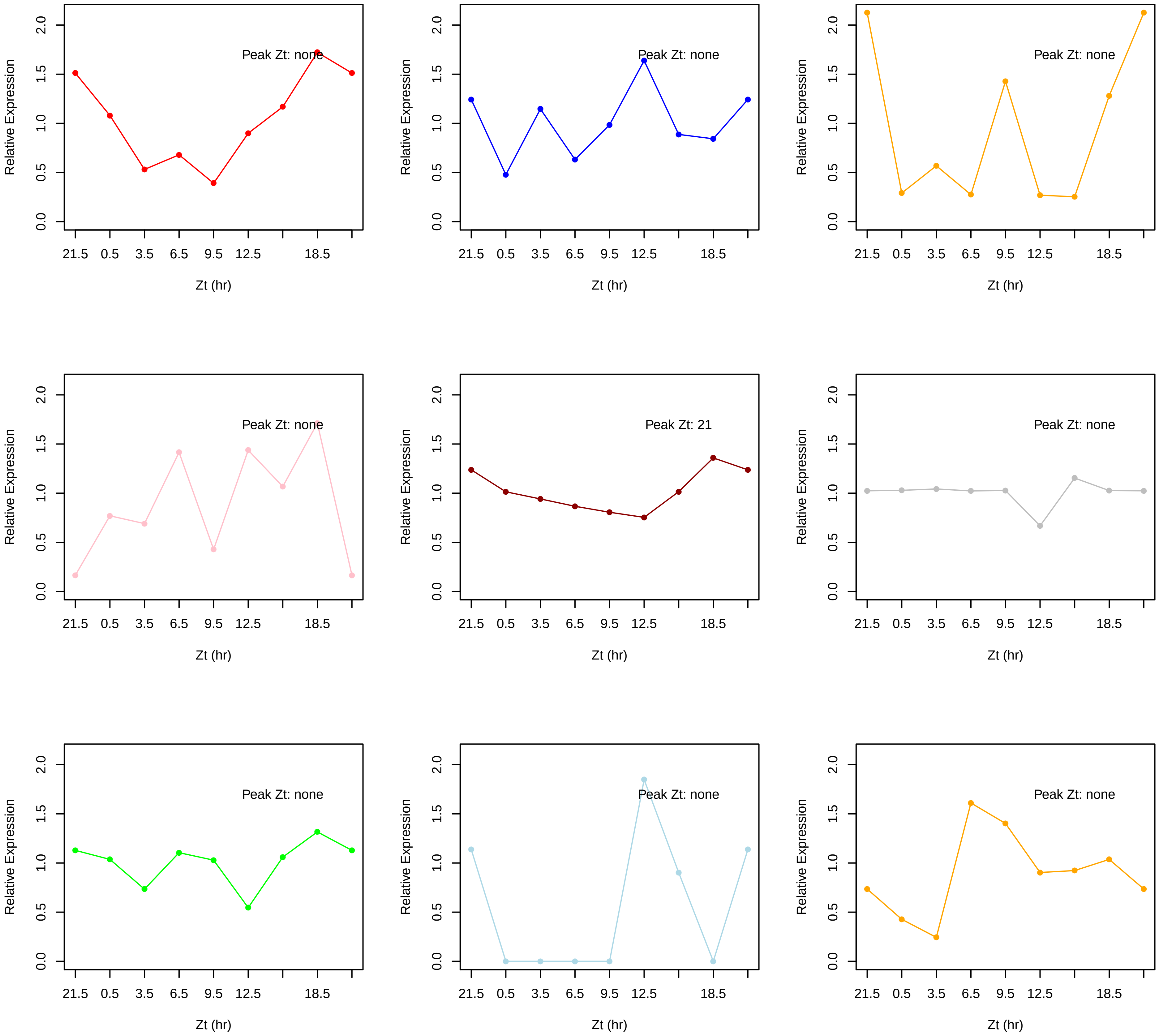

Singeleton: ENSXMAG00000010755 zgc:158328

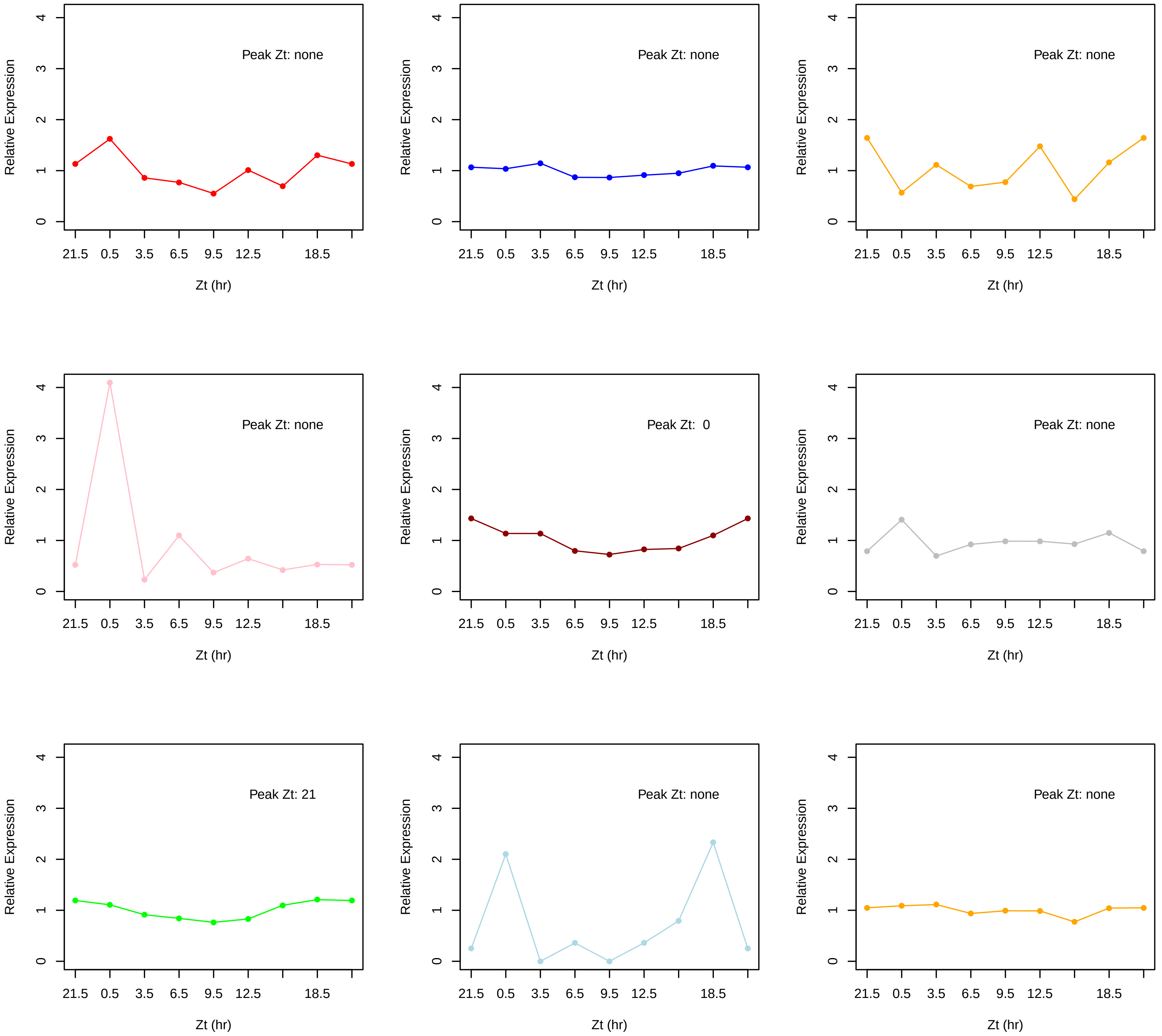

Singleton: ENSXMAG00000018720

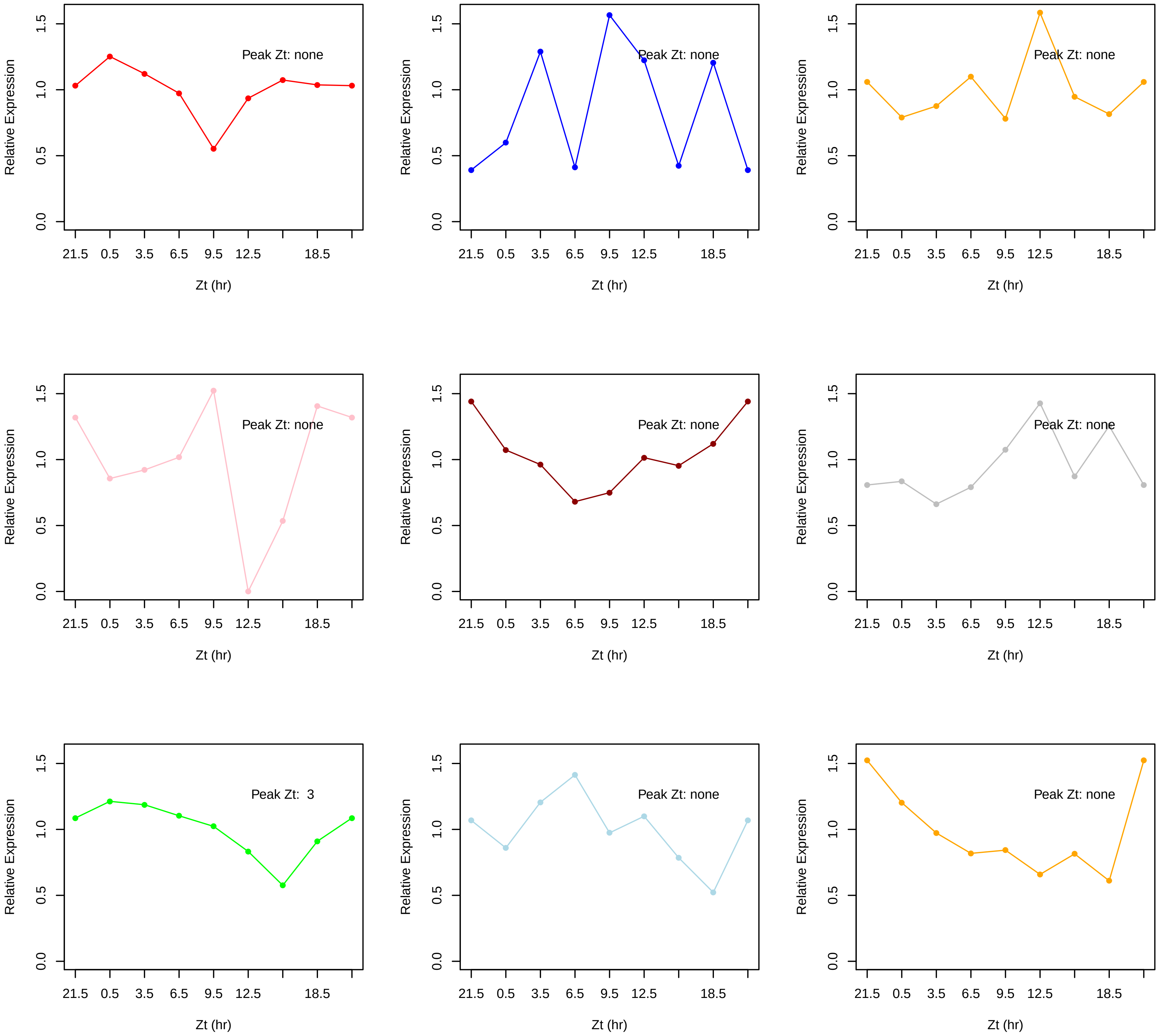

Singeleton: ENSXMAG00000008149 eed

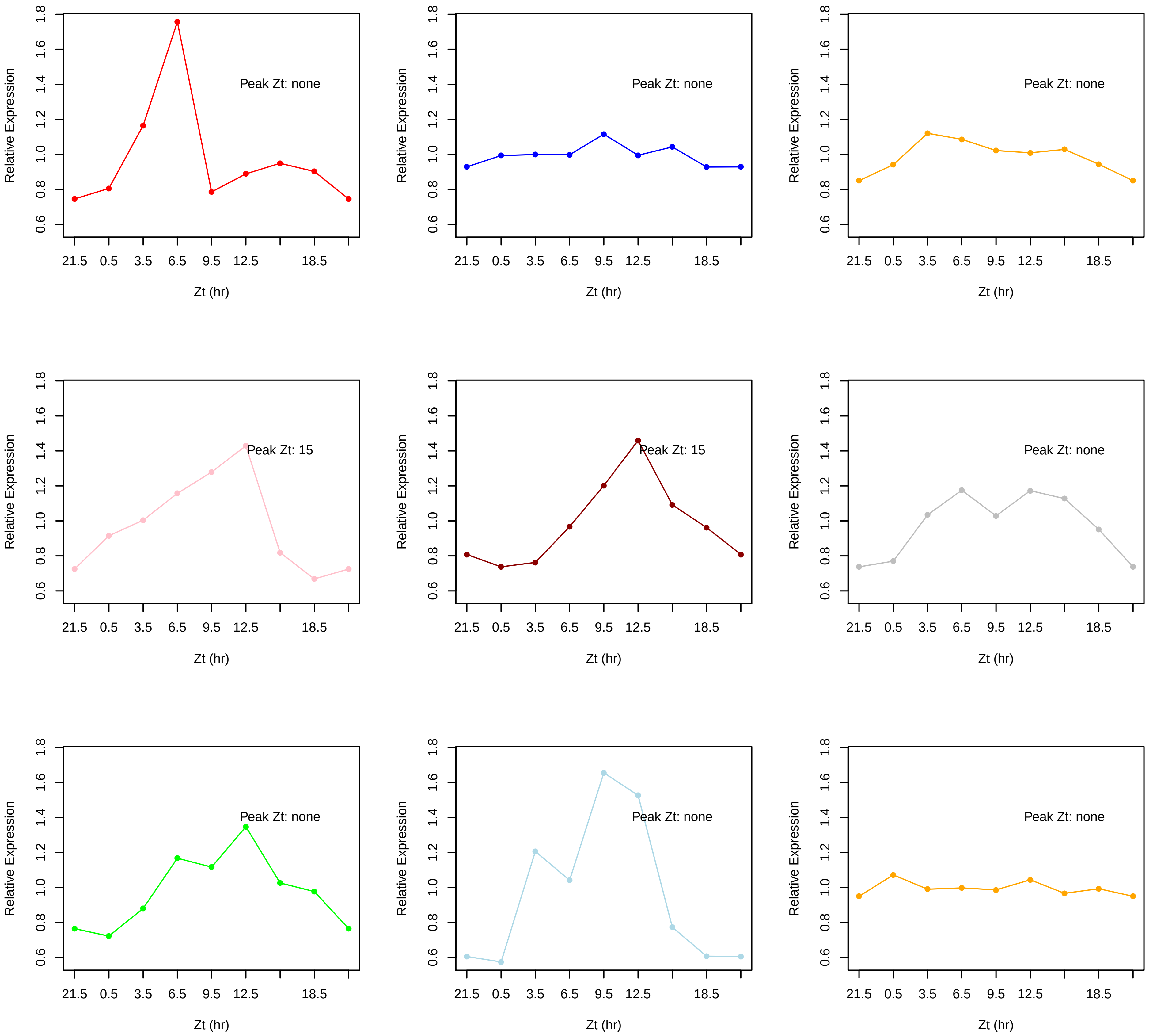

Singleton: ENSXMAG00000028922 cdc23

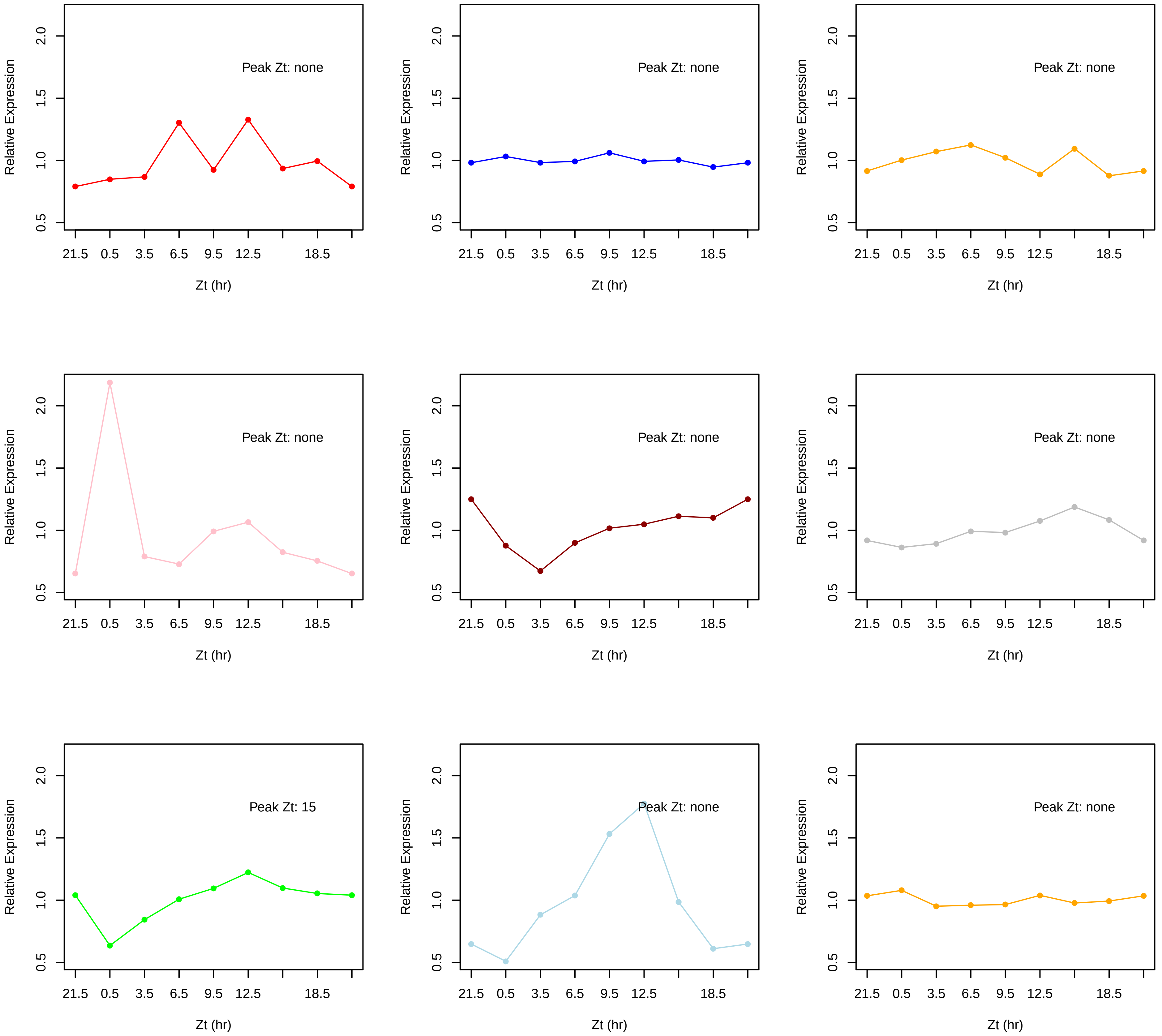

Singleton: ENSXMAG00000000782 shtn1

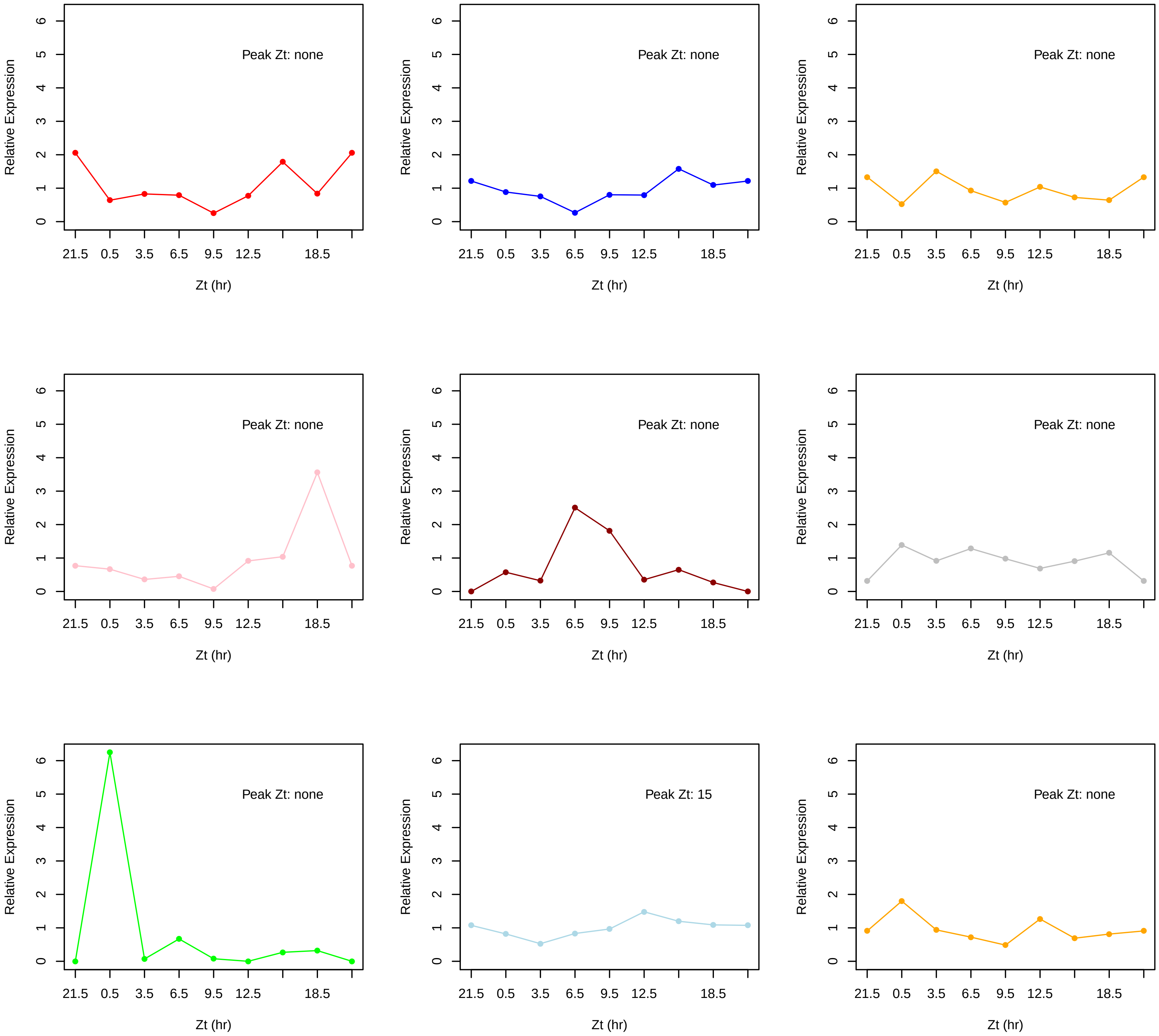

Singleton: ENSXMAG00000009513 vipr1b

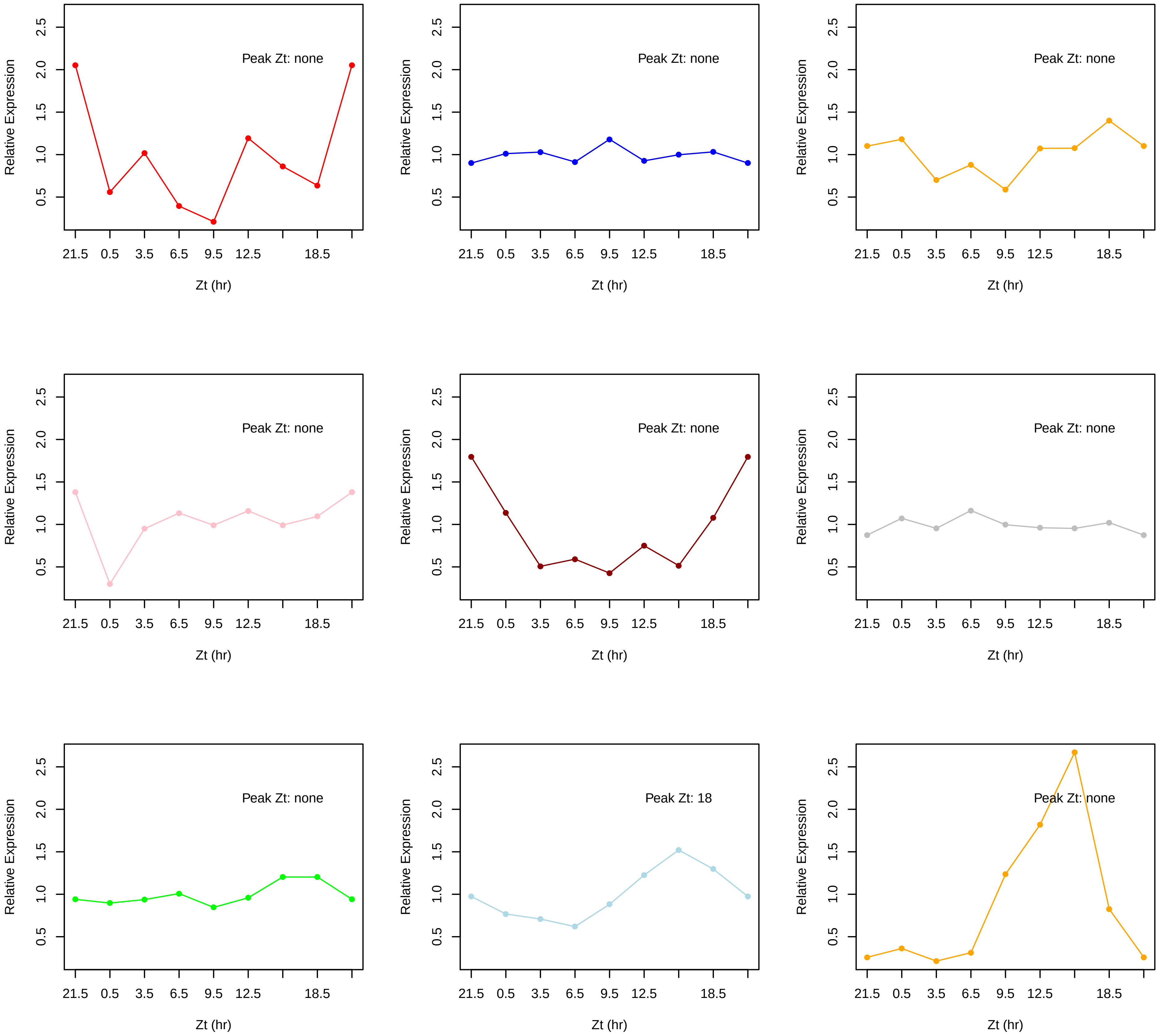

Singeleton: ENSXMAG00000009504 sec22c

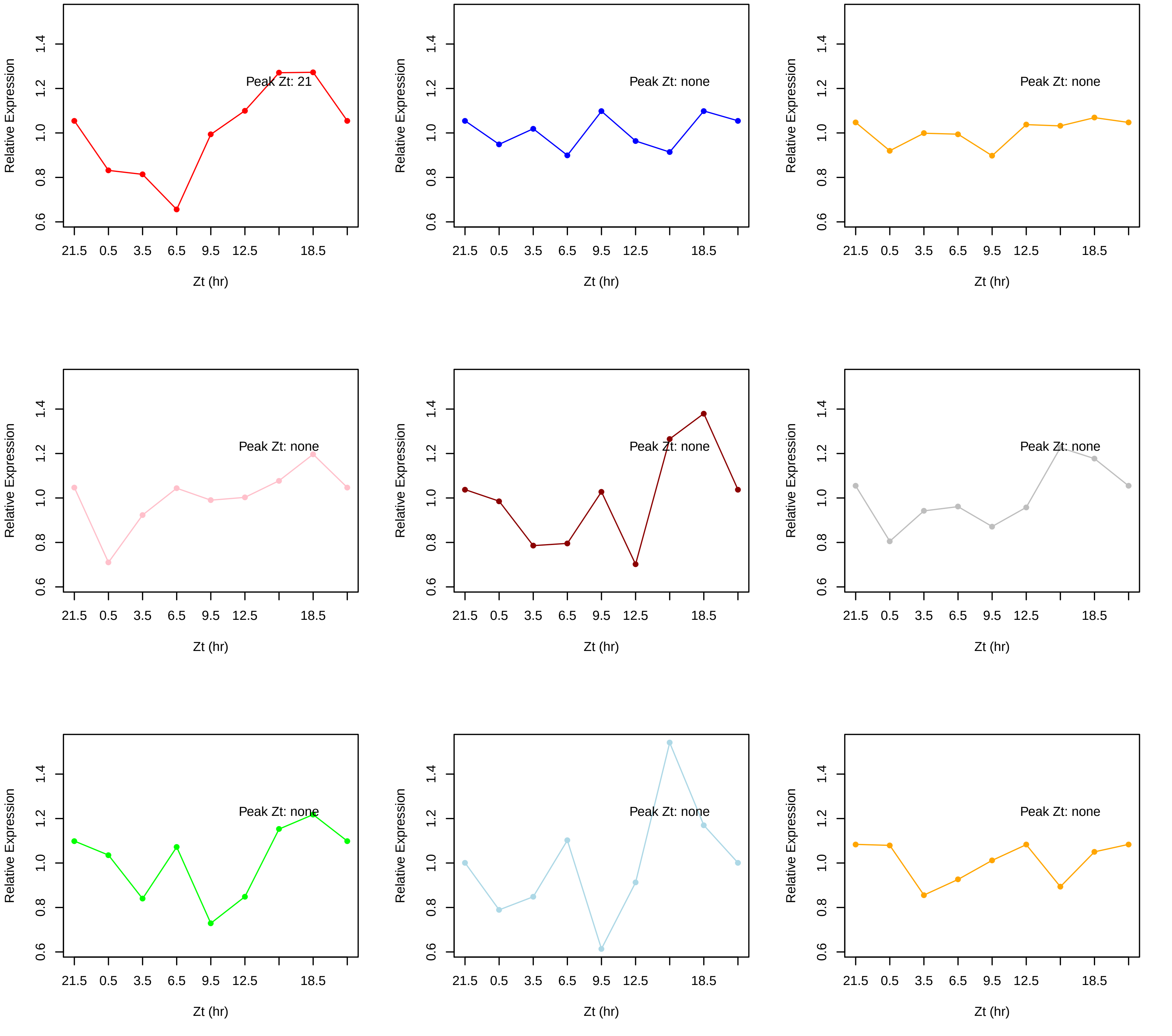

Singleton: ENSXMAG00000015556 nme8

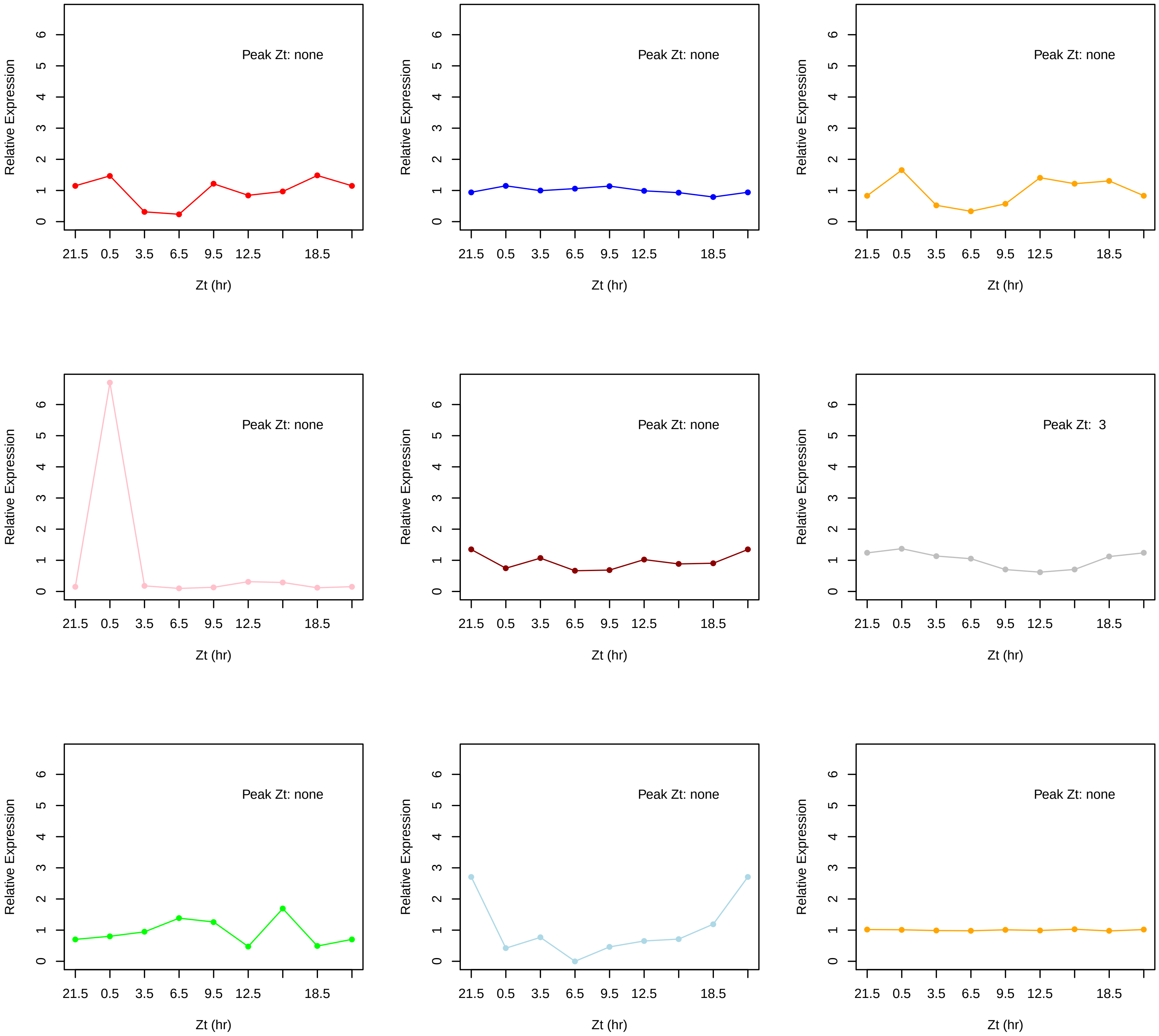

Singeleton: ENSXMAG00000007031 nhp2

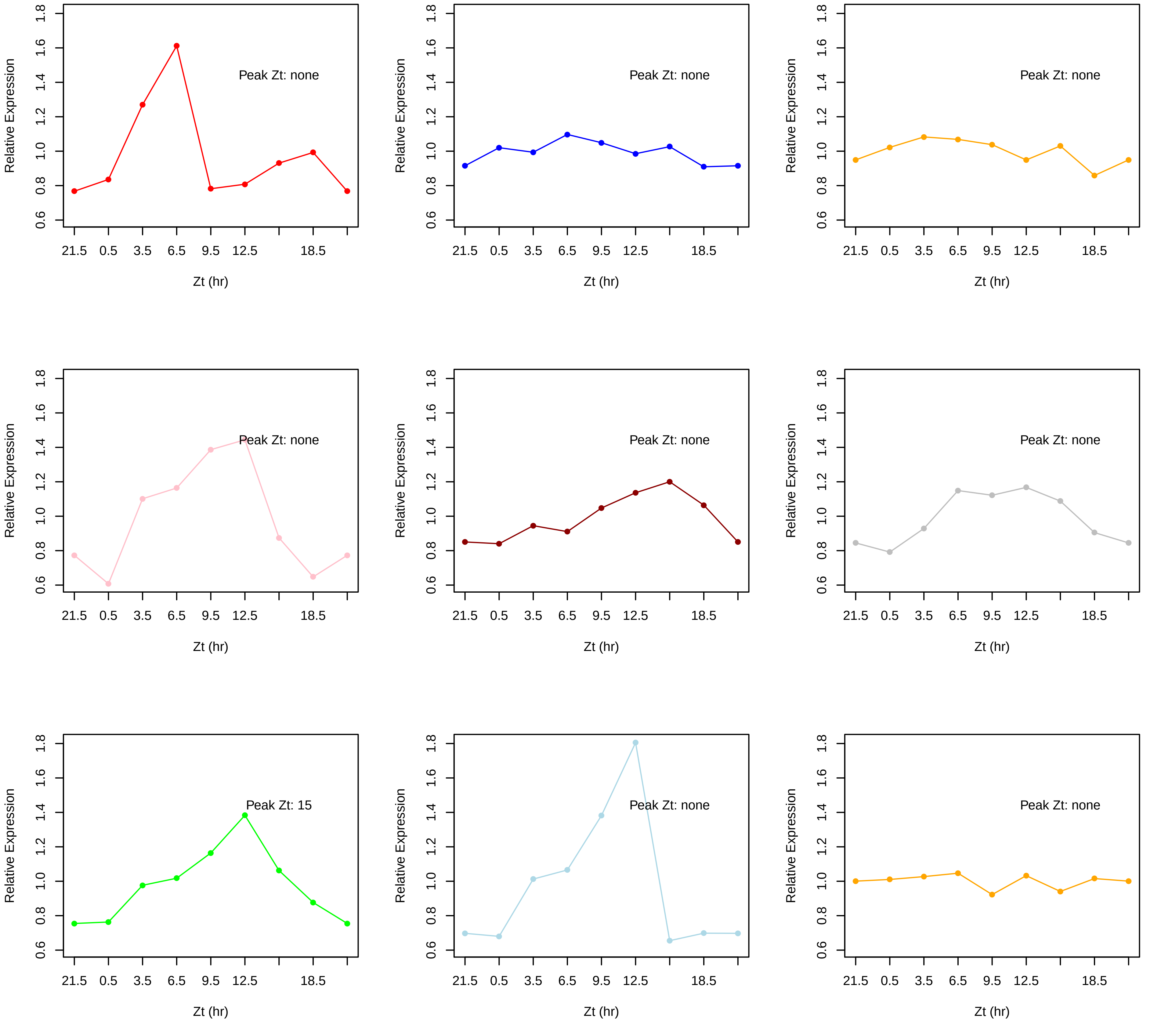

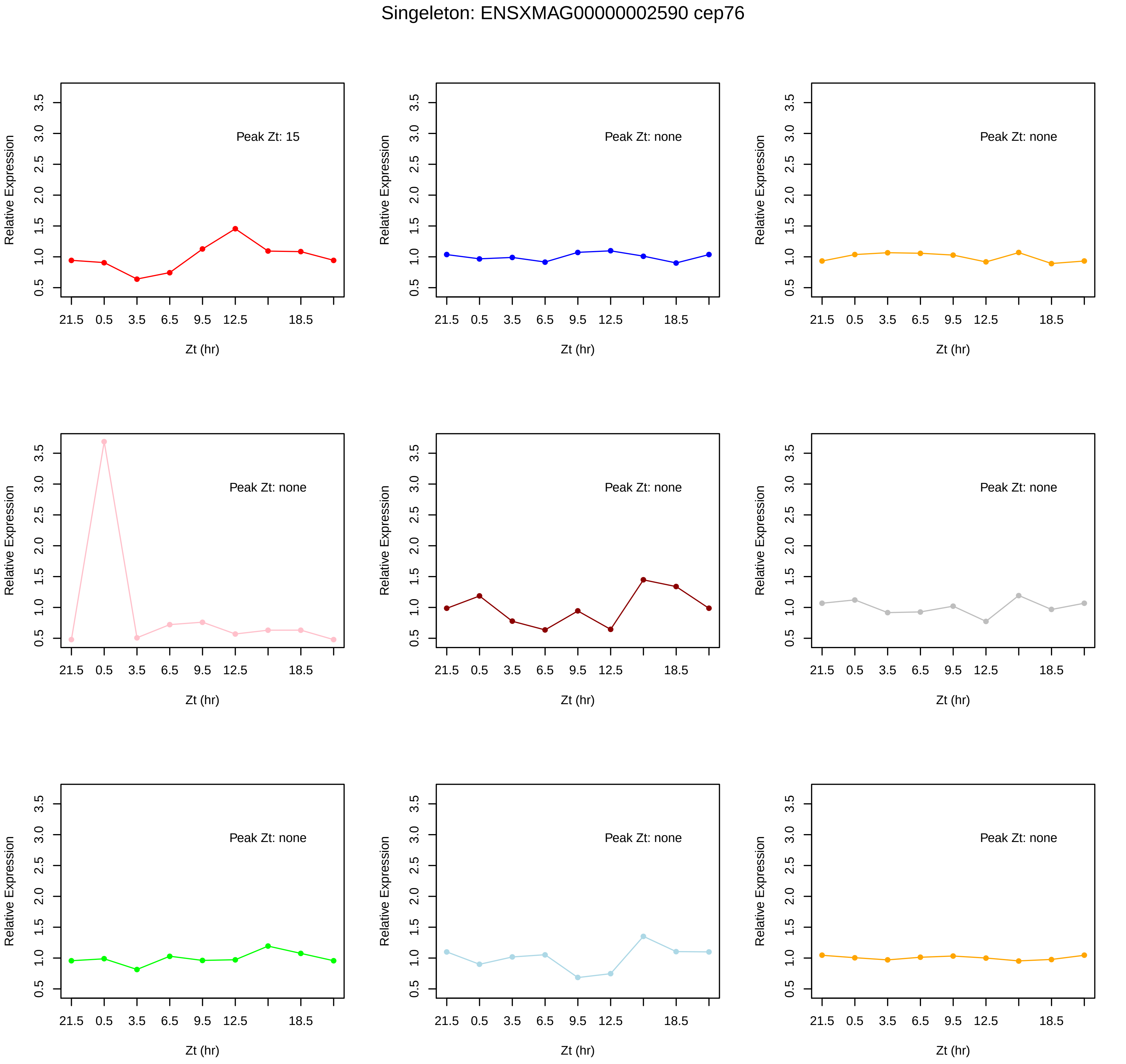
Singleton: ENSXMAG00000002590 cep76
Peak Zt: 15
Peak Zt: none
Peak Zt: none
Peak Zt: none
Peak Zt: none
Peak Zt: none
Peak Zt: none
Peak Zt: none
Peak Zt: none
Relative Expression
Zt (hr)
21.5 0.5 3.5 6.5 9.5 12.5 18.5
0.5 1.0 1.5 2.0 2.5 3.0 3.5

Singeleton: ENSXMAG00000009479 nktr

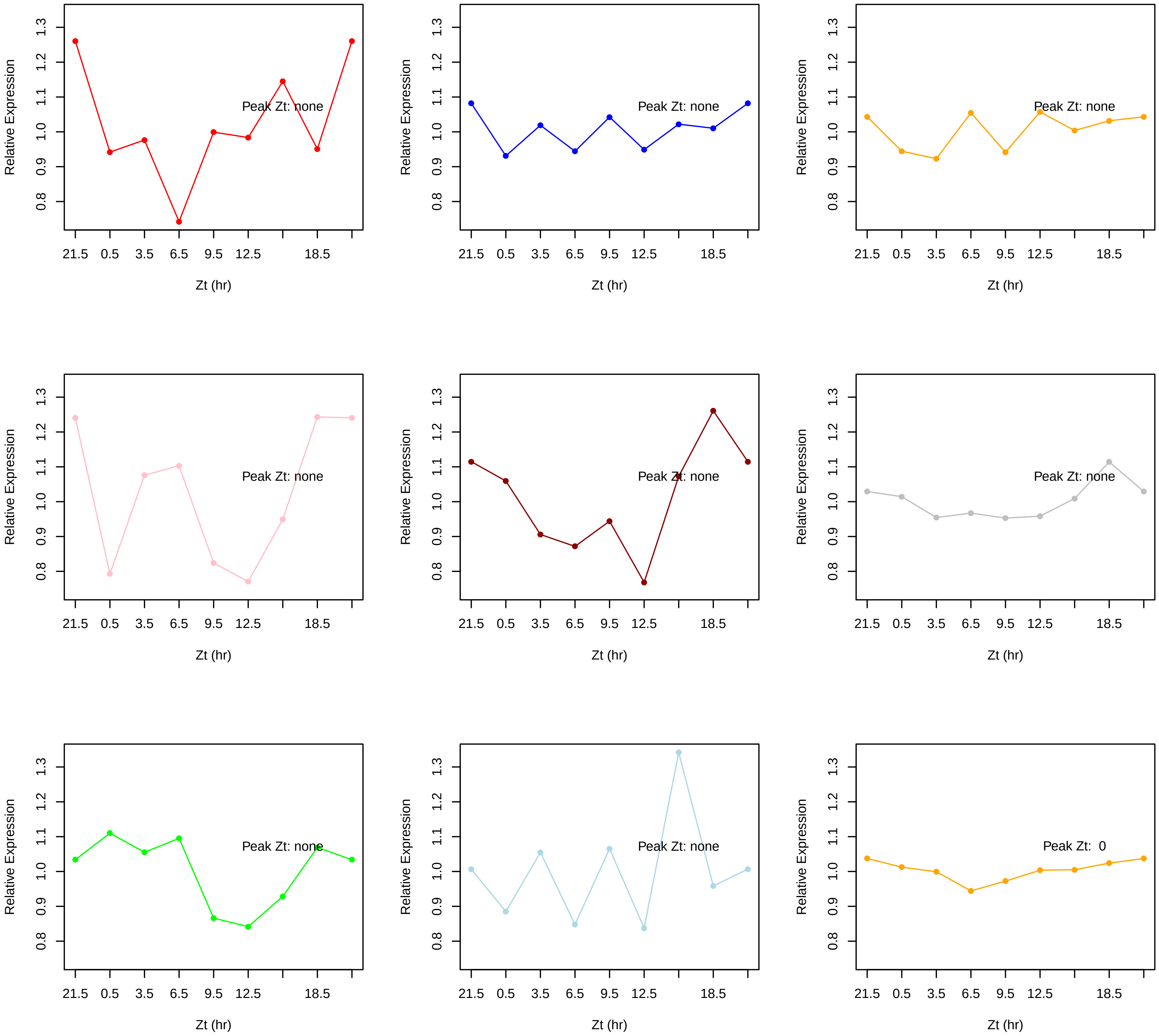

Singeleton: ENSXMAG00000001902 tmem39a

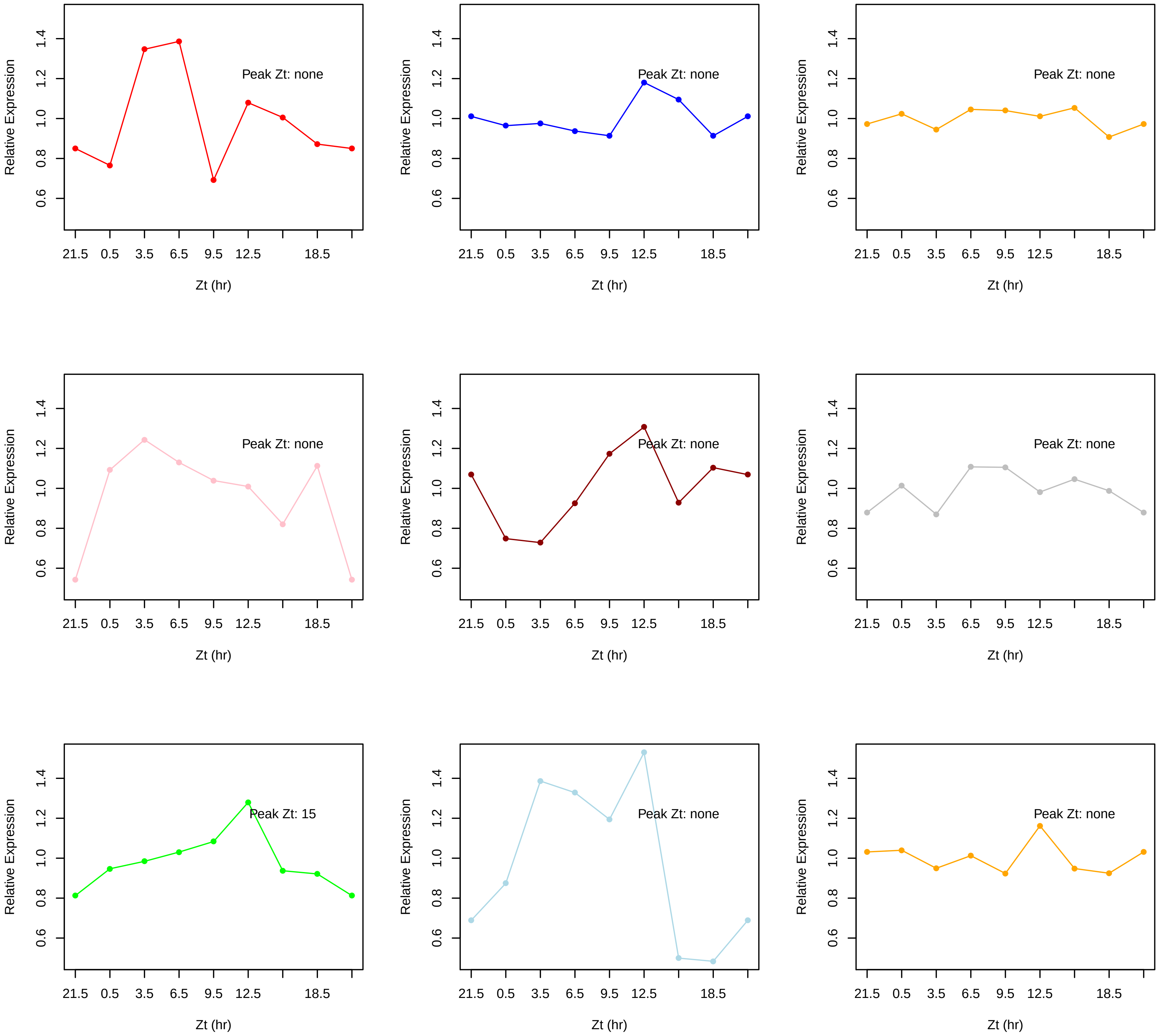

Singleton: ENSXMAG00000010787 mrpl44

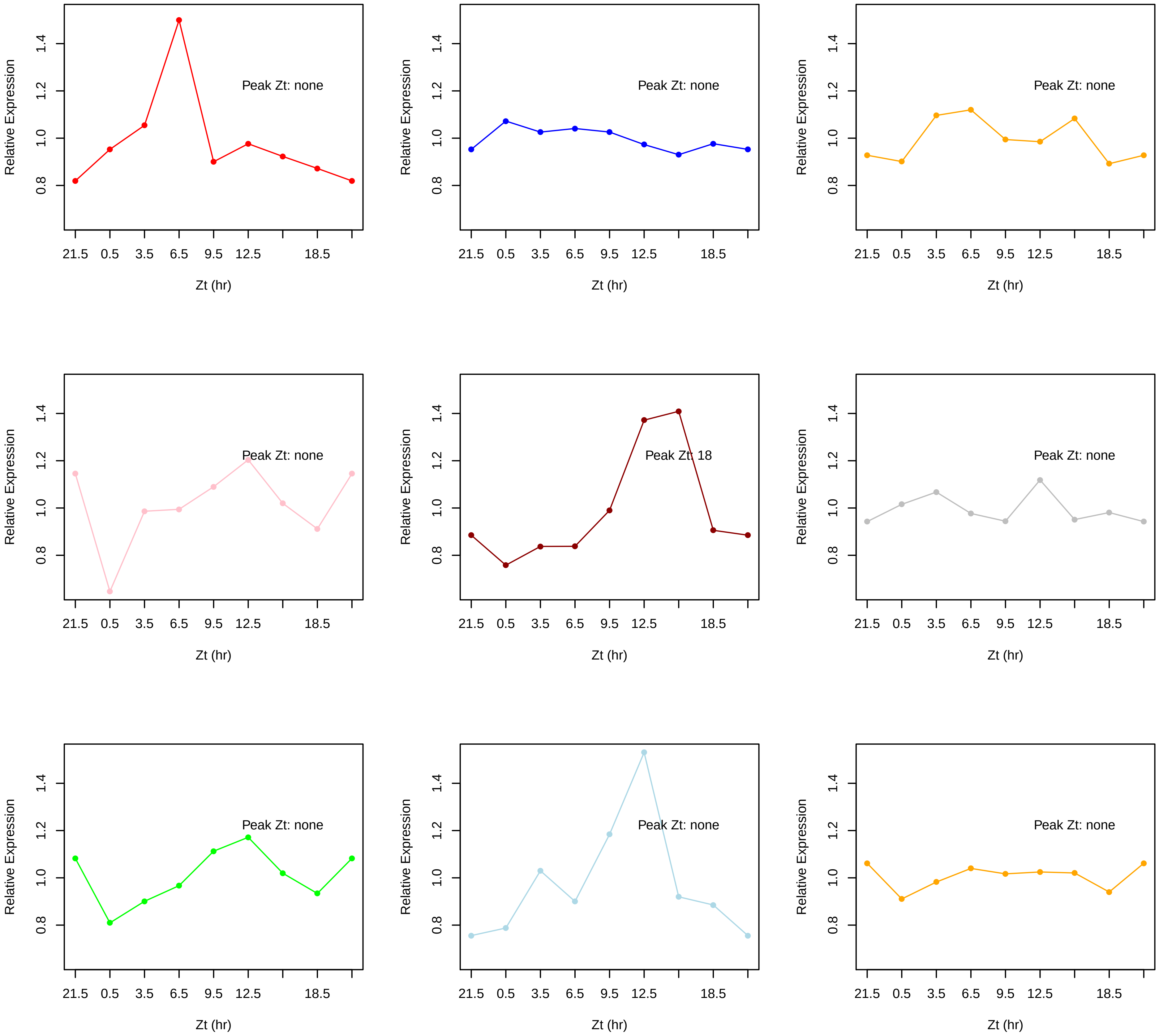

Singeleton: ENSXMAG00000027976 id4

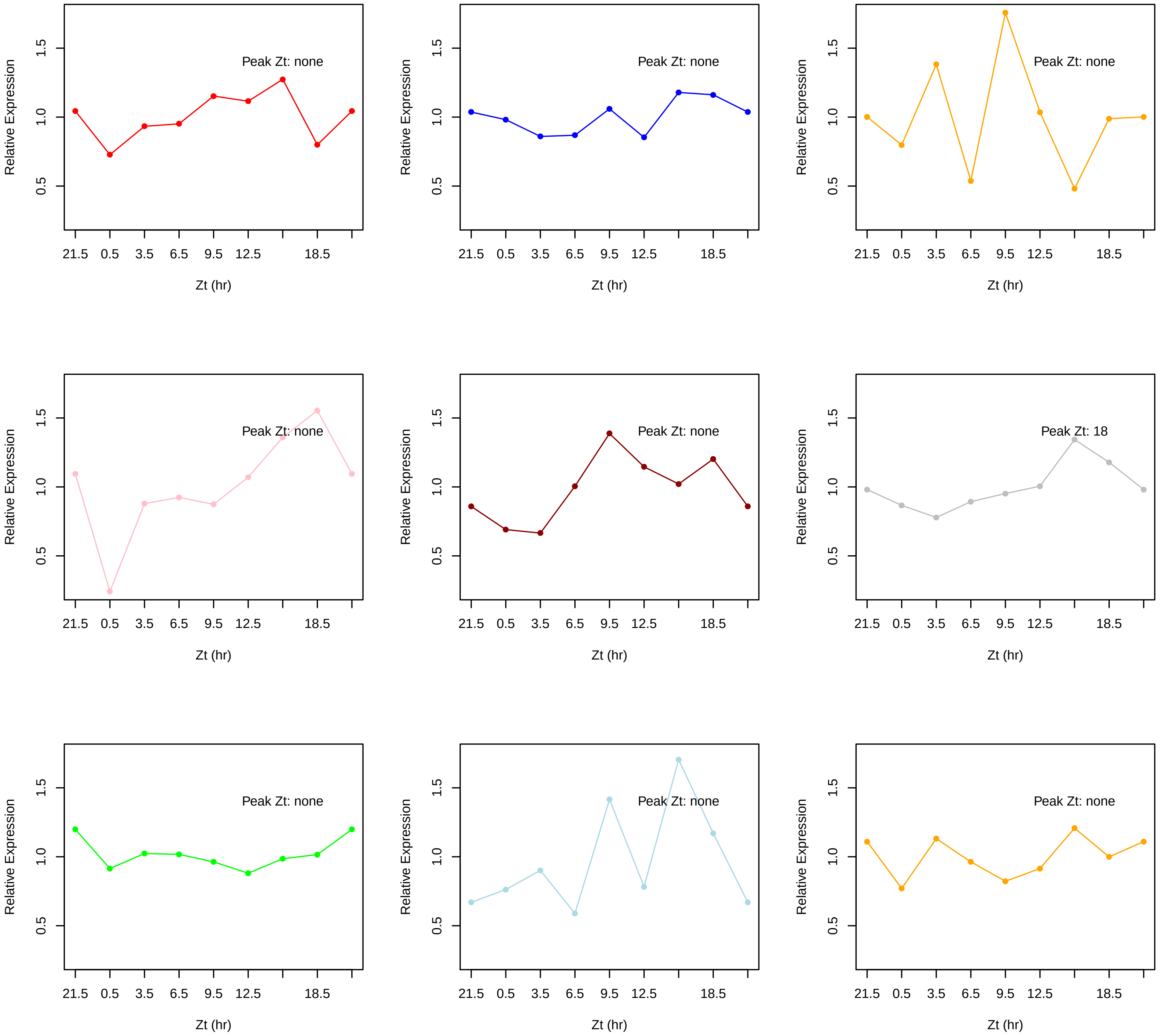

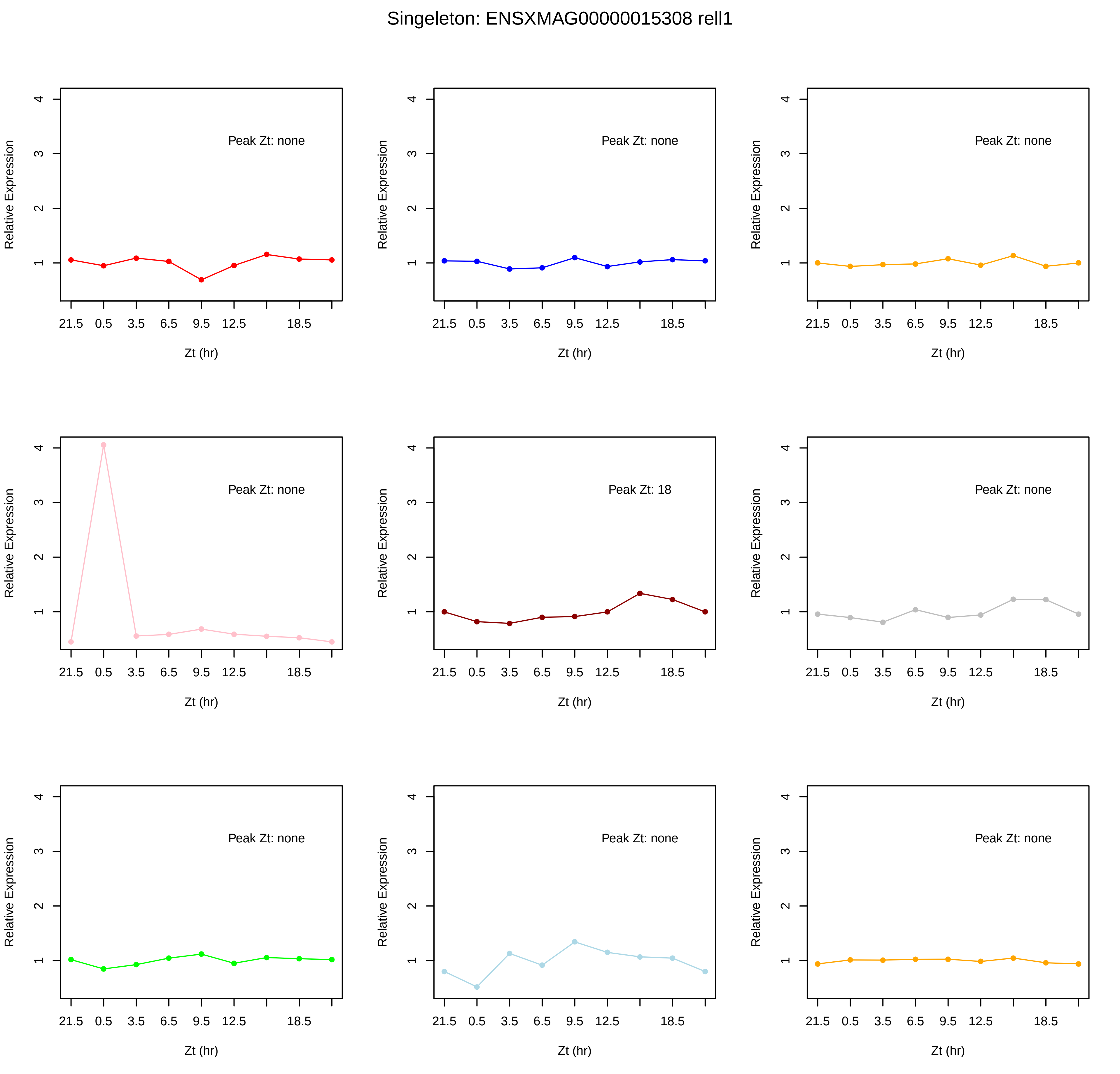
Singeleton: ENSXMAG00000015308 rell1
Peak Zt: none
Peak Zt: none
Peak Zt: none
Peak Zt: none
Peak Zt: 18
Peak Zt: none
Peak Zt: none
Peak Zt: none
Peak Zt: none
Relative Expression
Zt (hr)
21.5 0.5 3.5 6.5 9.5 12.5 18.5

Singeleton: ENSXMAG00000024227 ncalda

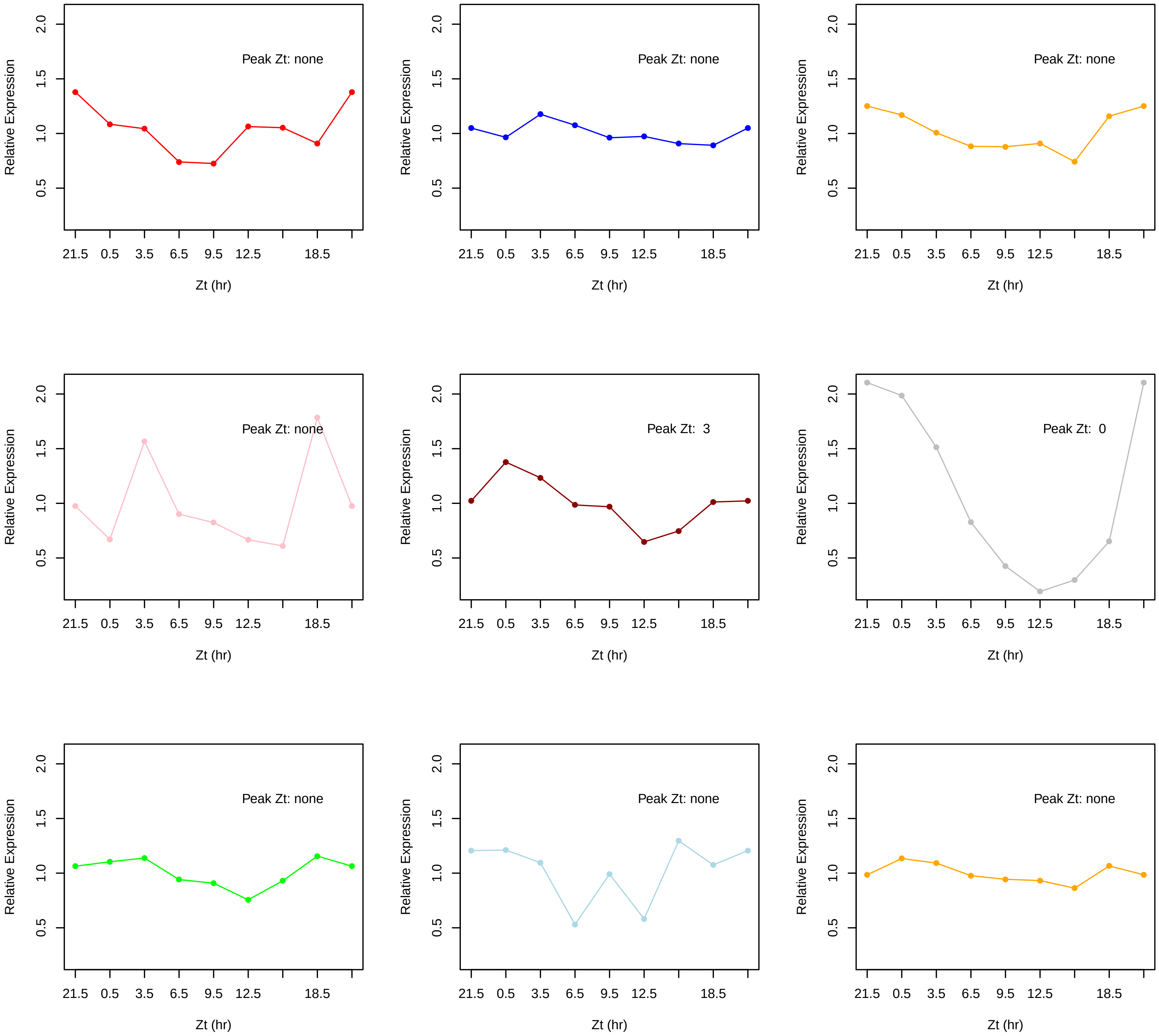

Singeleton: ENSXMAG00000029989 nwd2

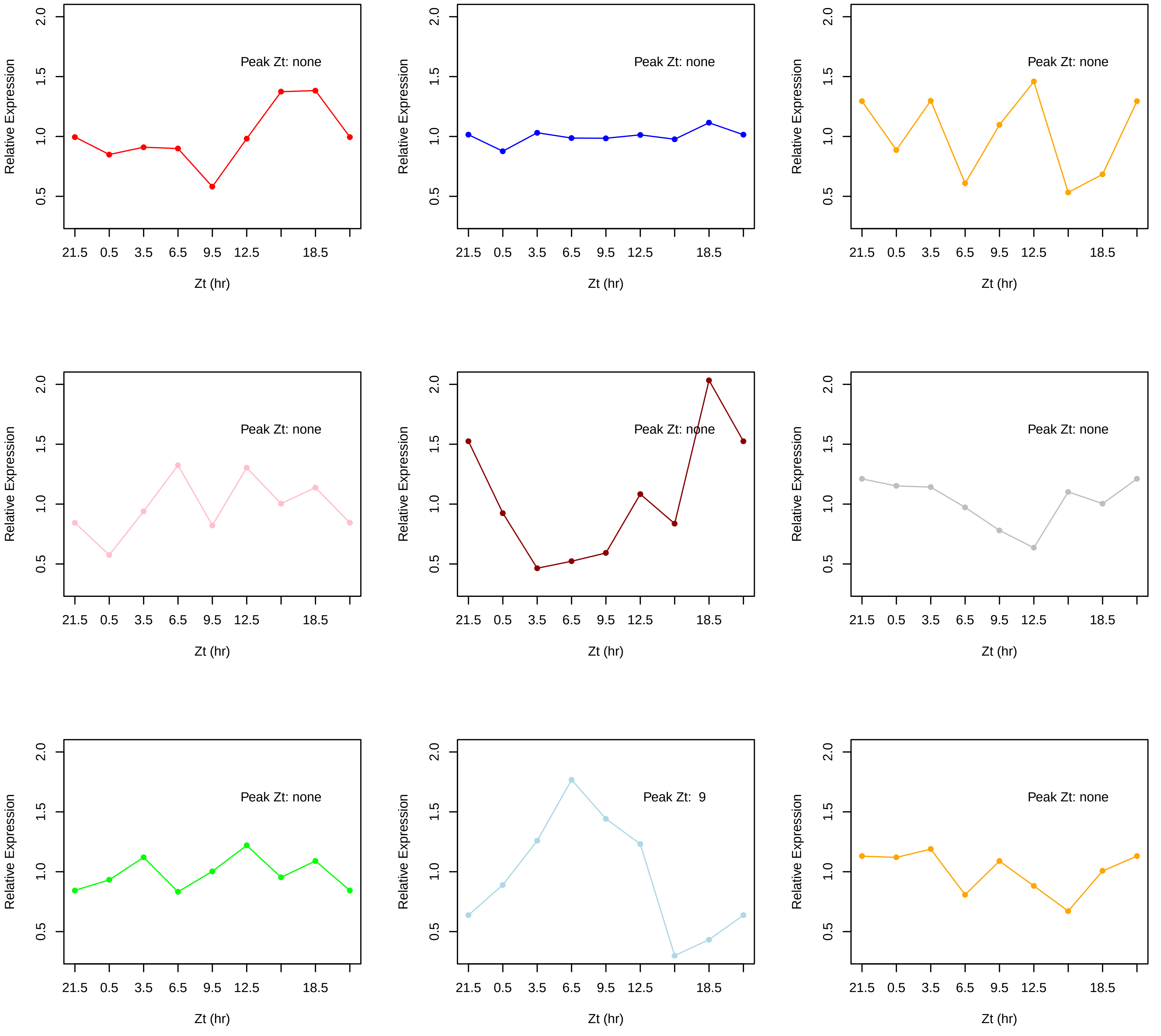

Singeleton: ENSXMAG00000003736 ZNF706

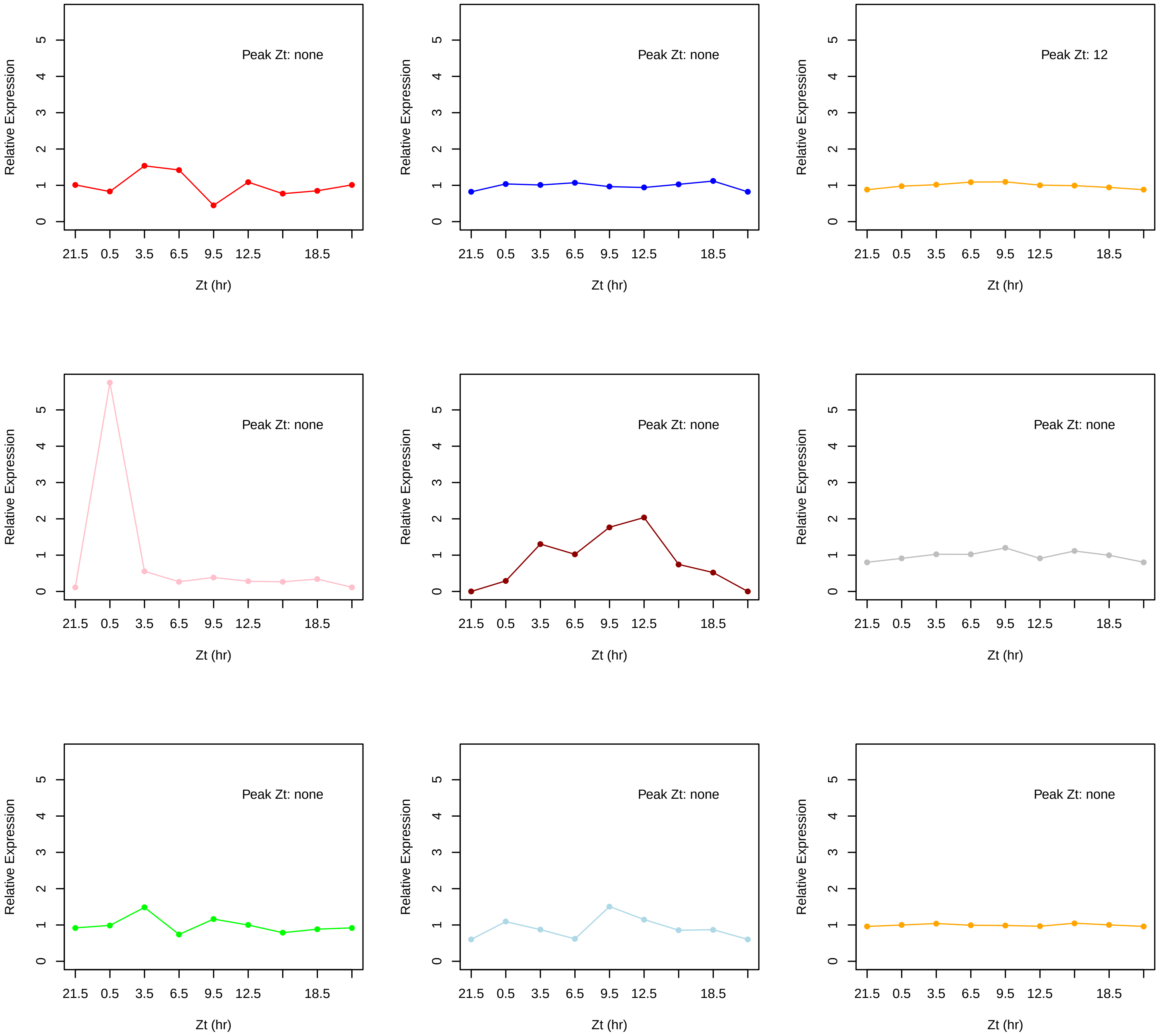

Singleton: ENSXMAG00000029757 scfd1

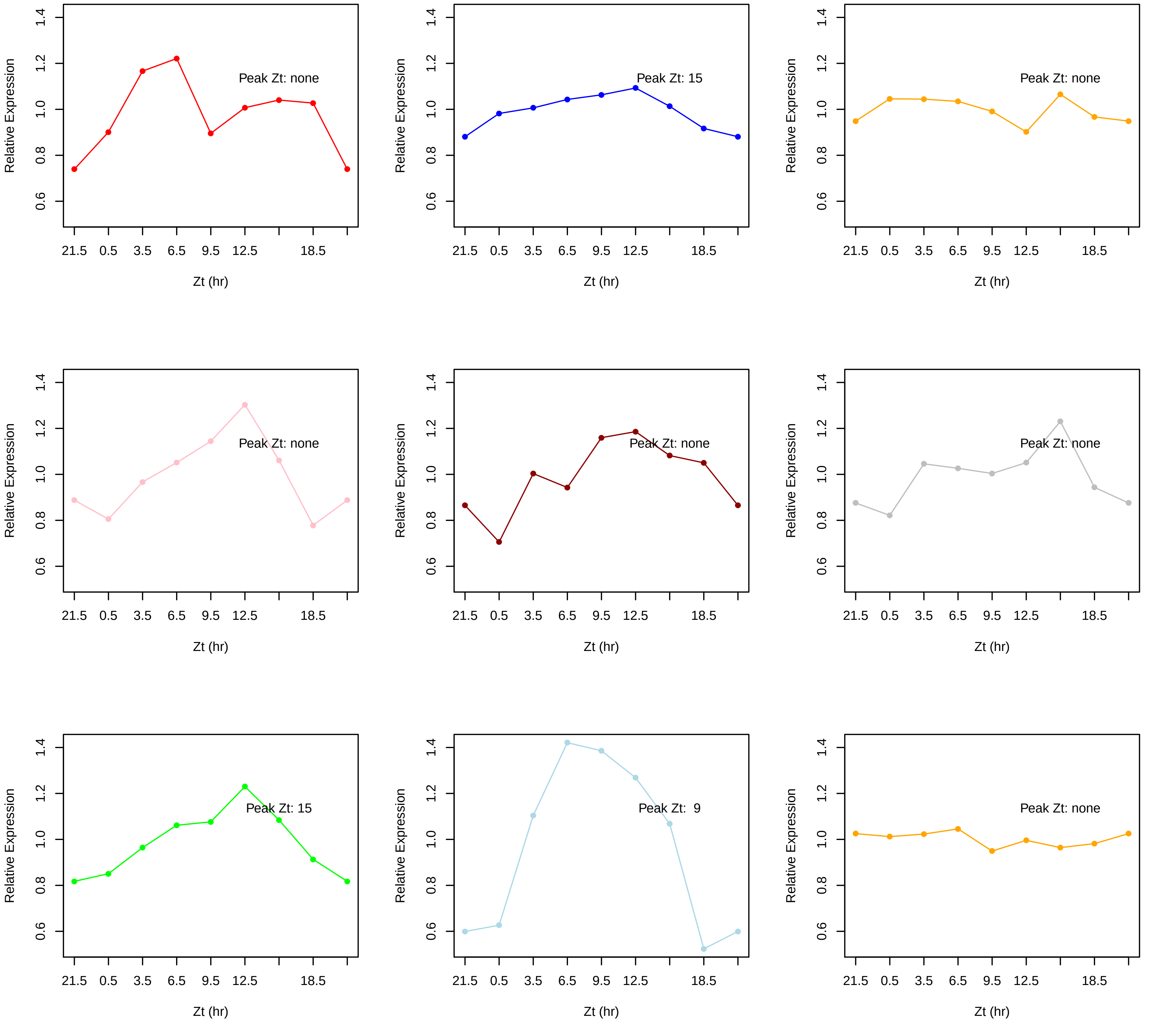

Singleton: ENSXMAG00000011666 col6a1

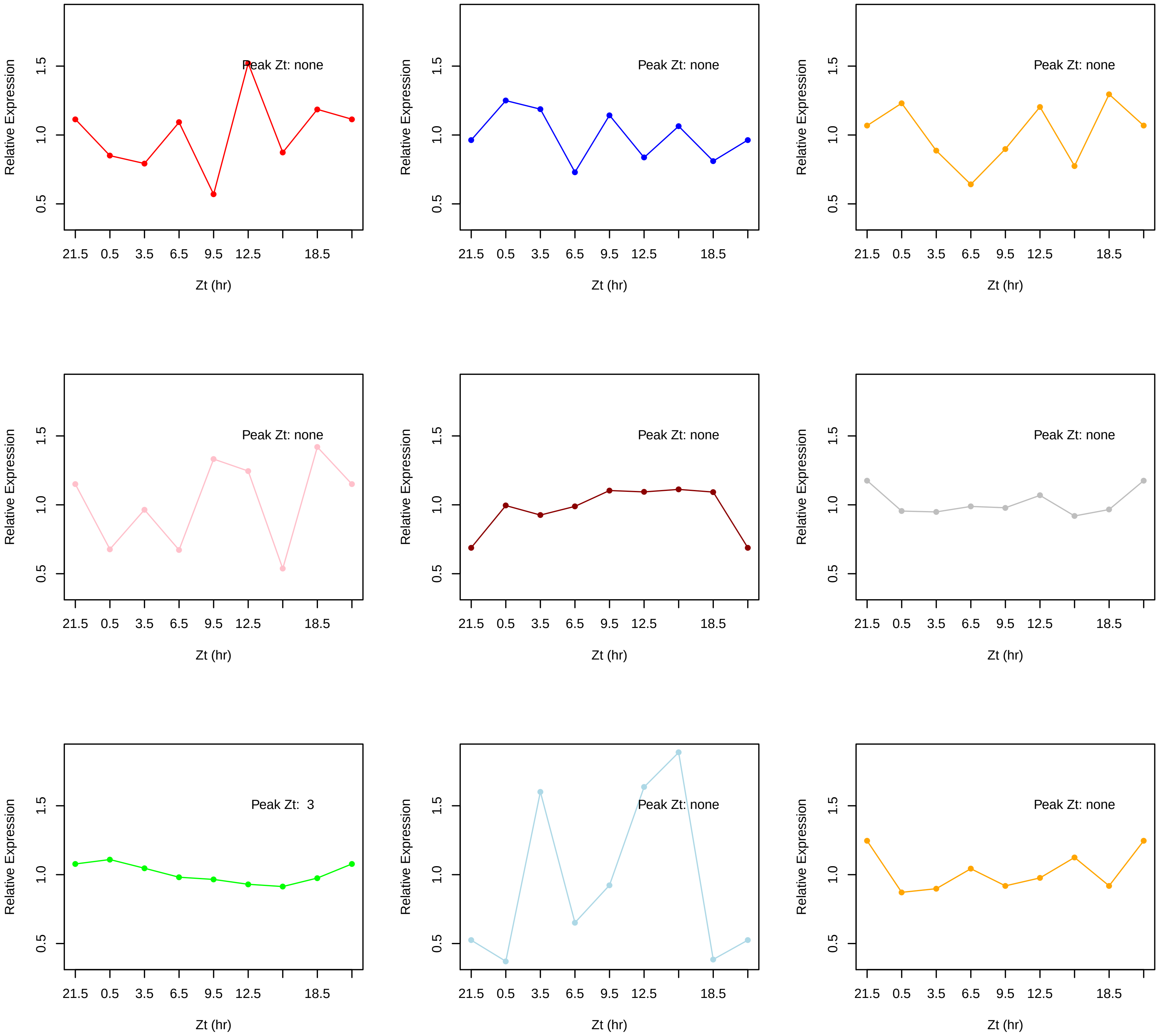

Singeleton: ENSXMAG00000009948 pcdh7b

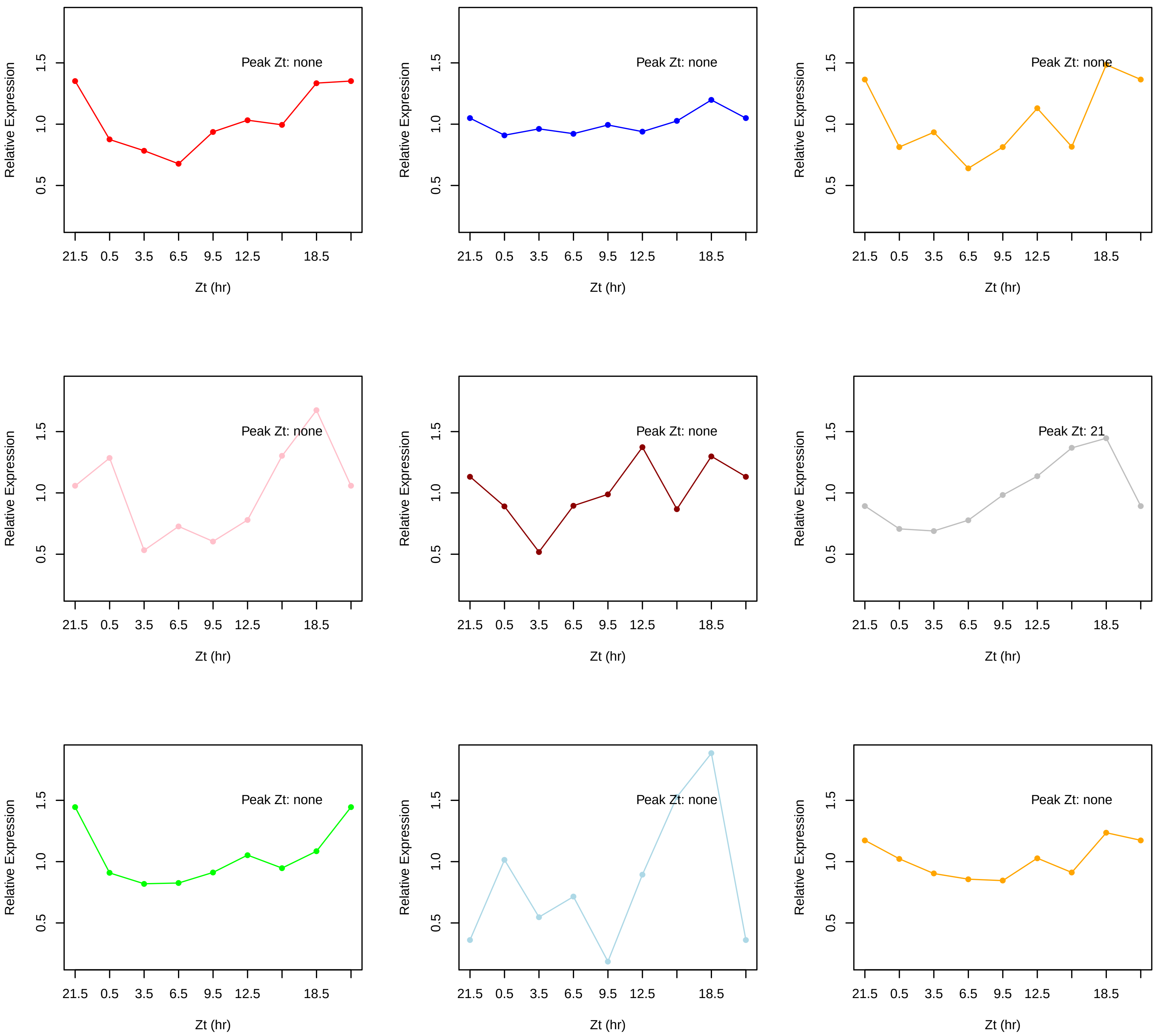

Singeleton: ENSXMAG00000009402 gars

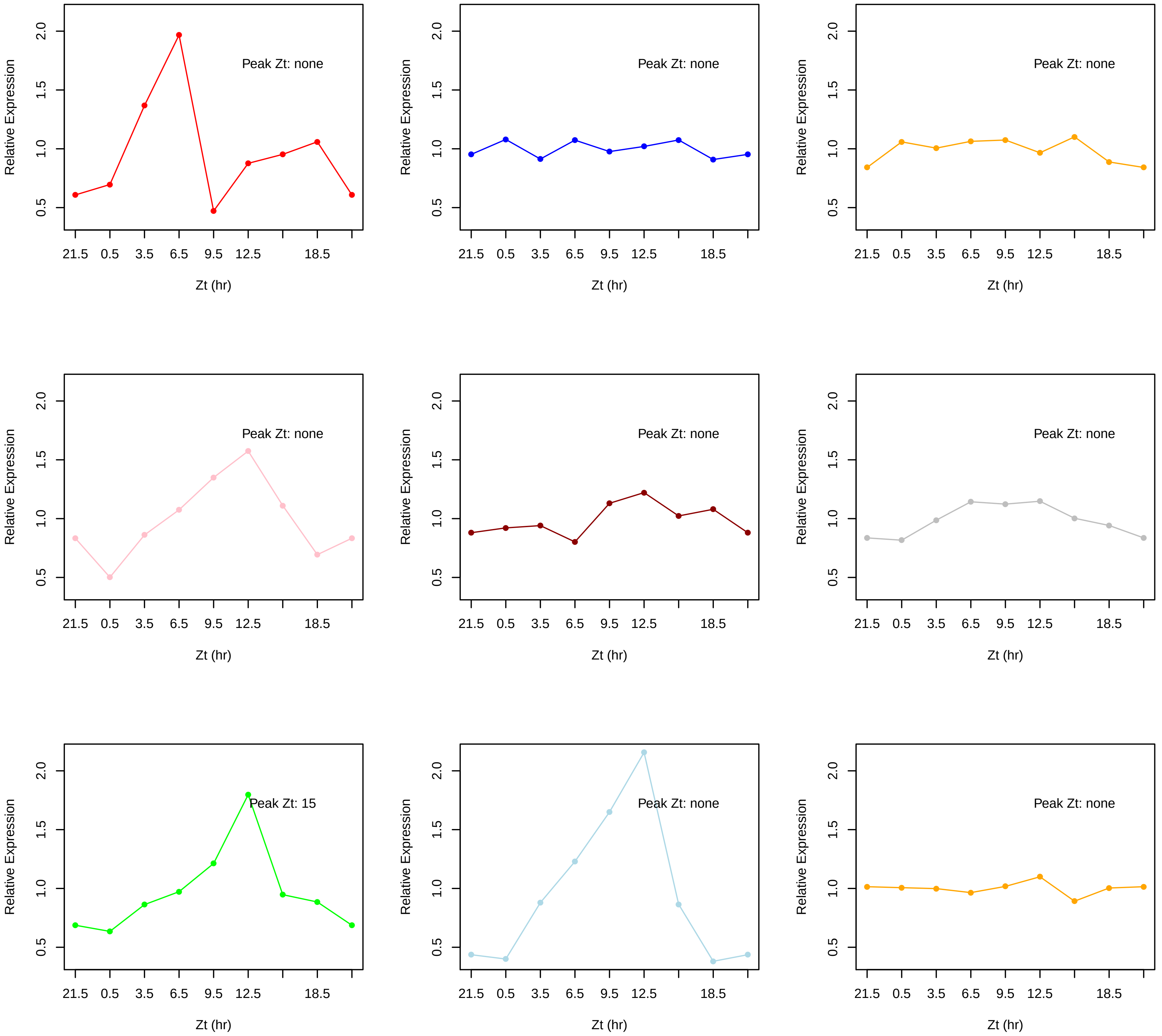

Singeleton: ENSXMAG00000023792

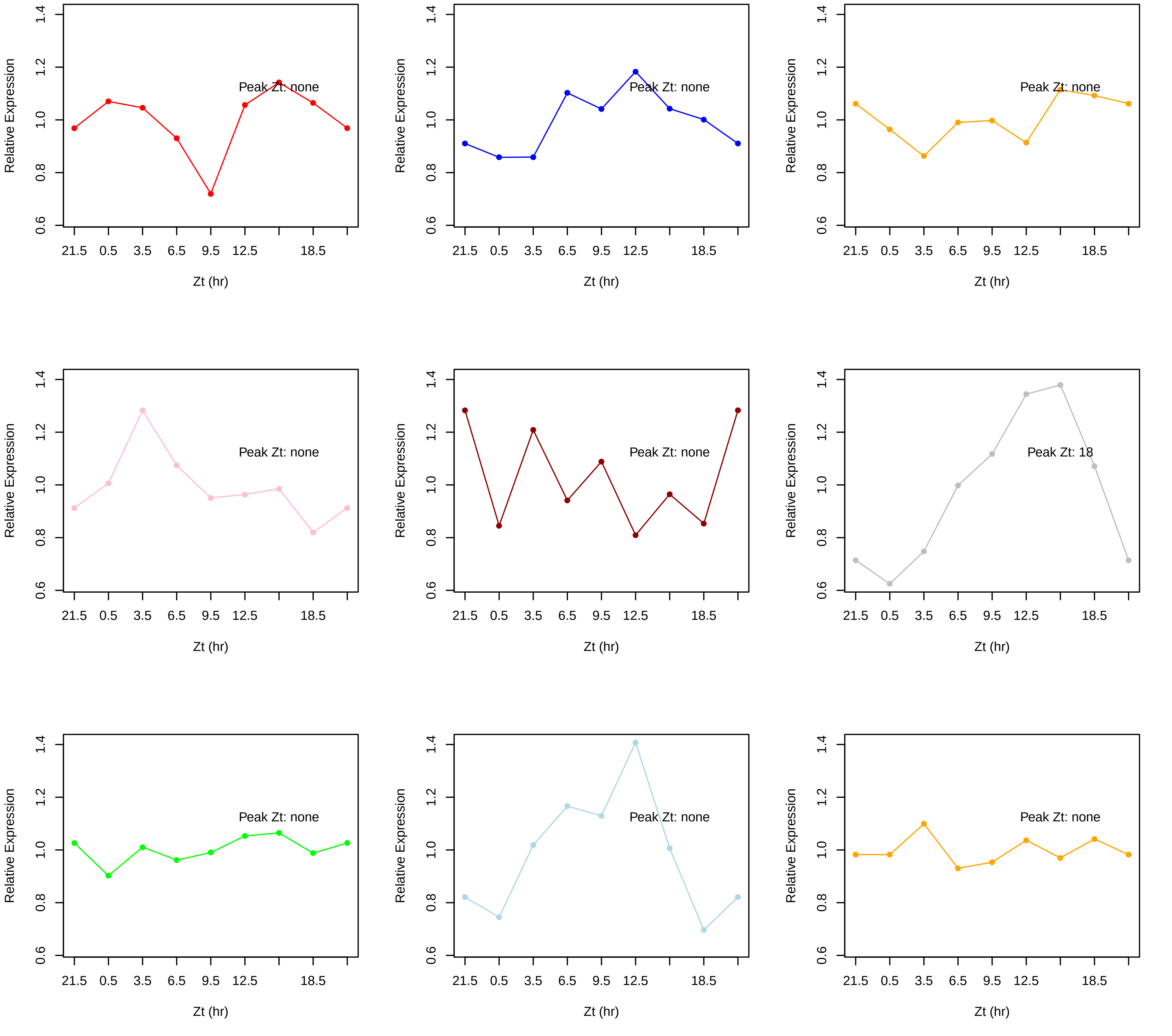

Singeleton: ENSXMAG00000025639 emx2

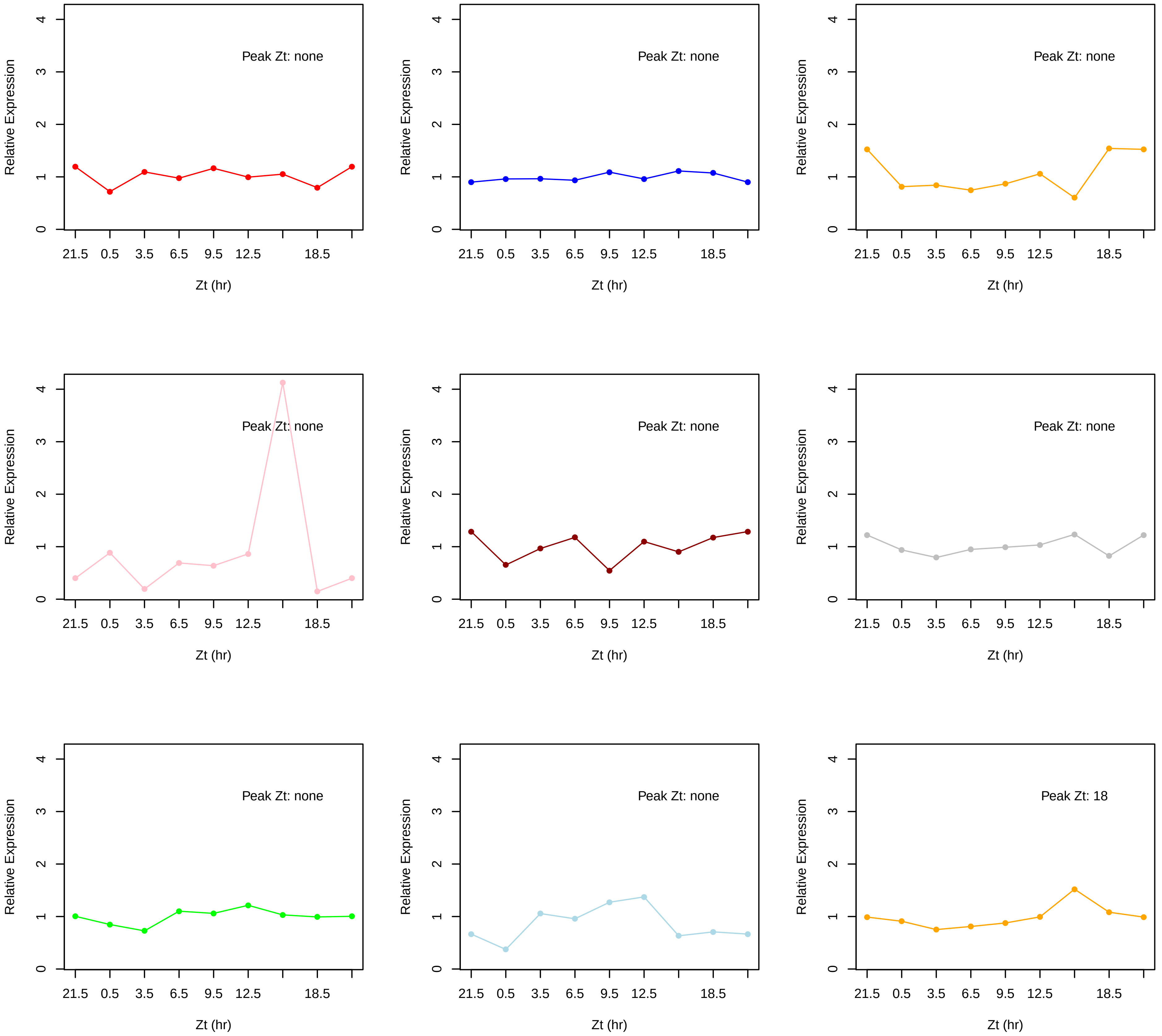

Singeleton: ENSXMAG00000002145 si:dkey−229d2.4

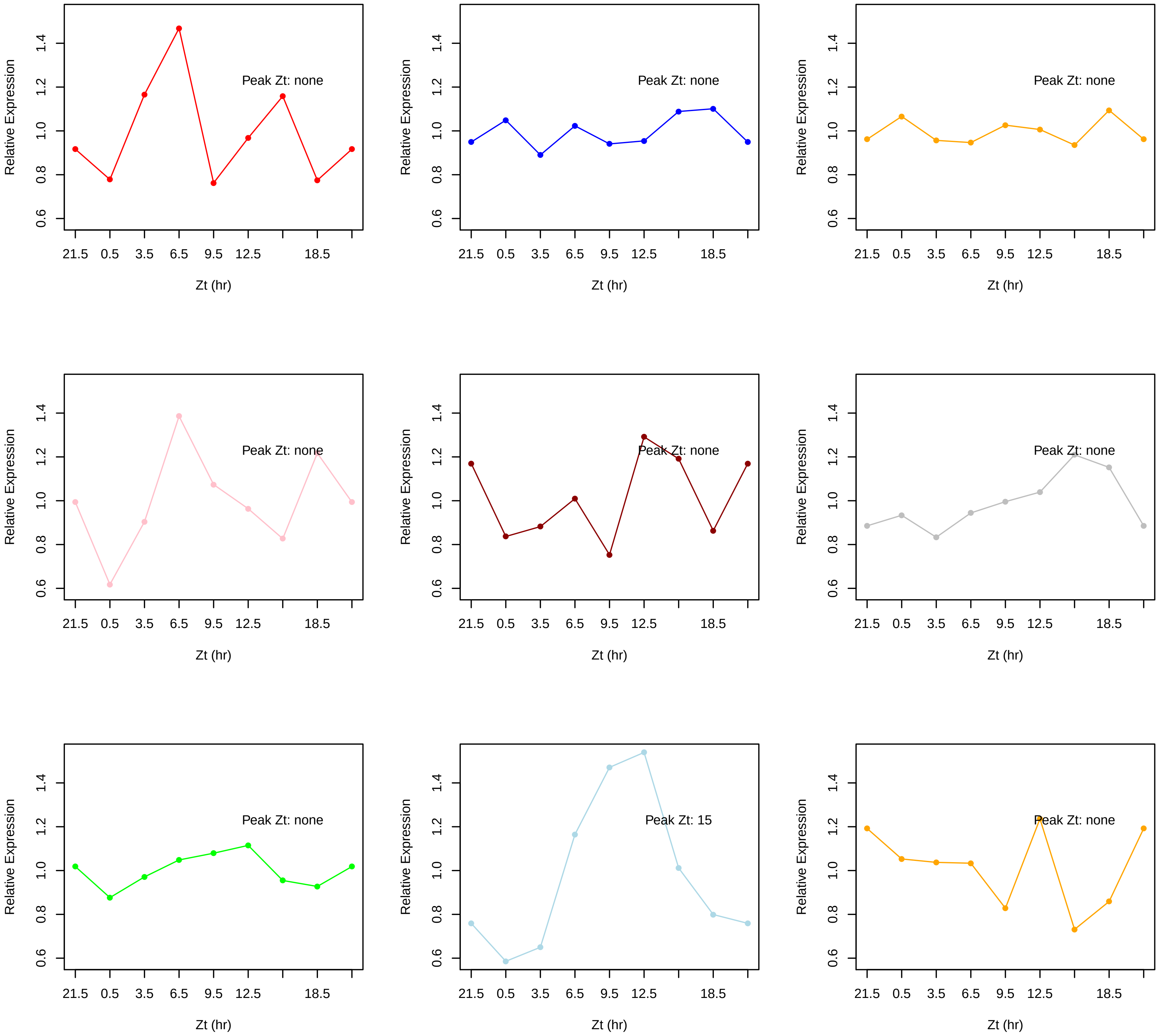

Singeleton: ENSXMAG00000002545 rnf19a

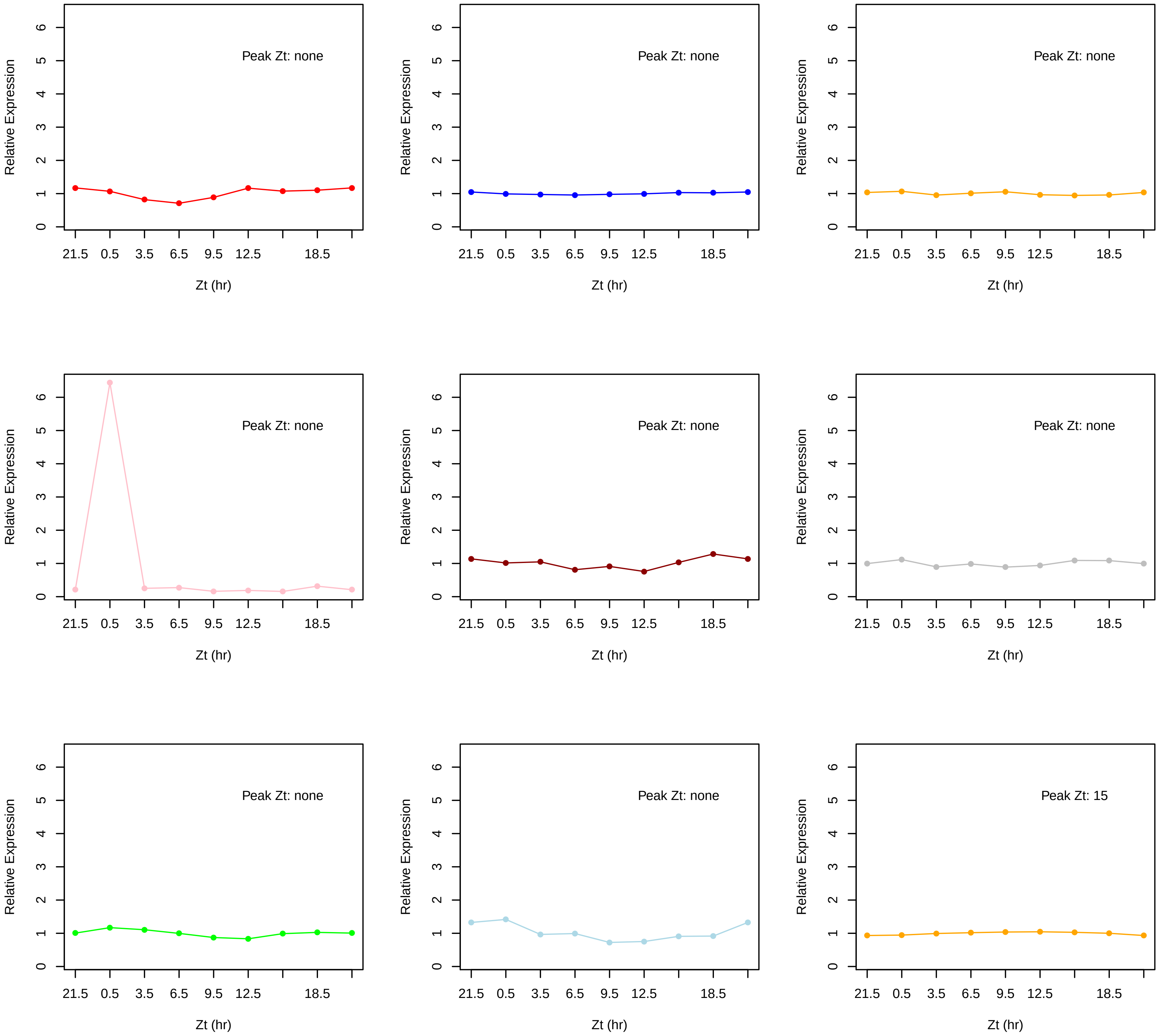

Singeleton: ENSXMAG00000015567 rorb

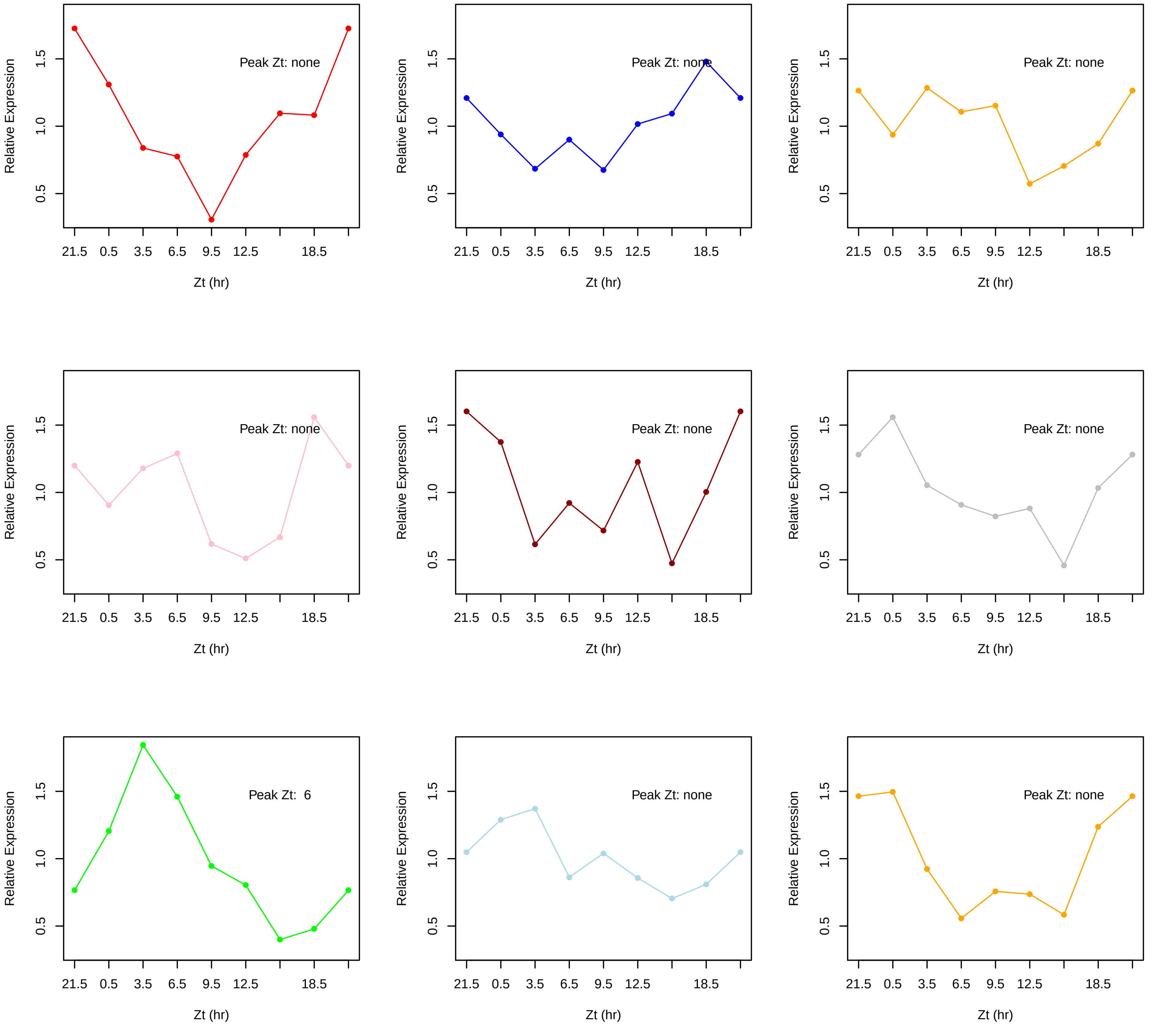

Singeleton: ENSXMAG00000024476 fam204a

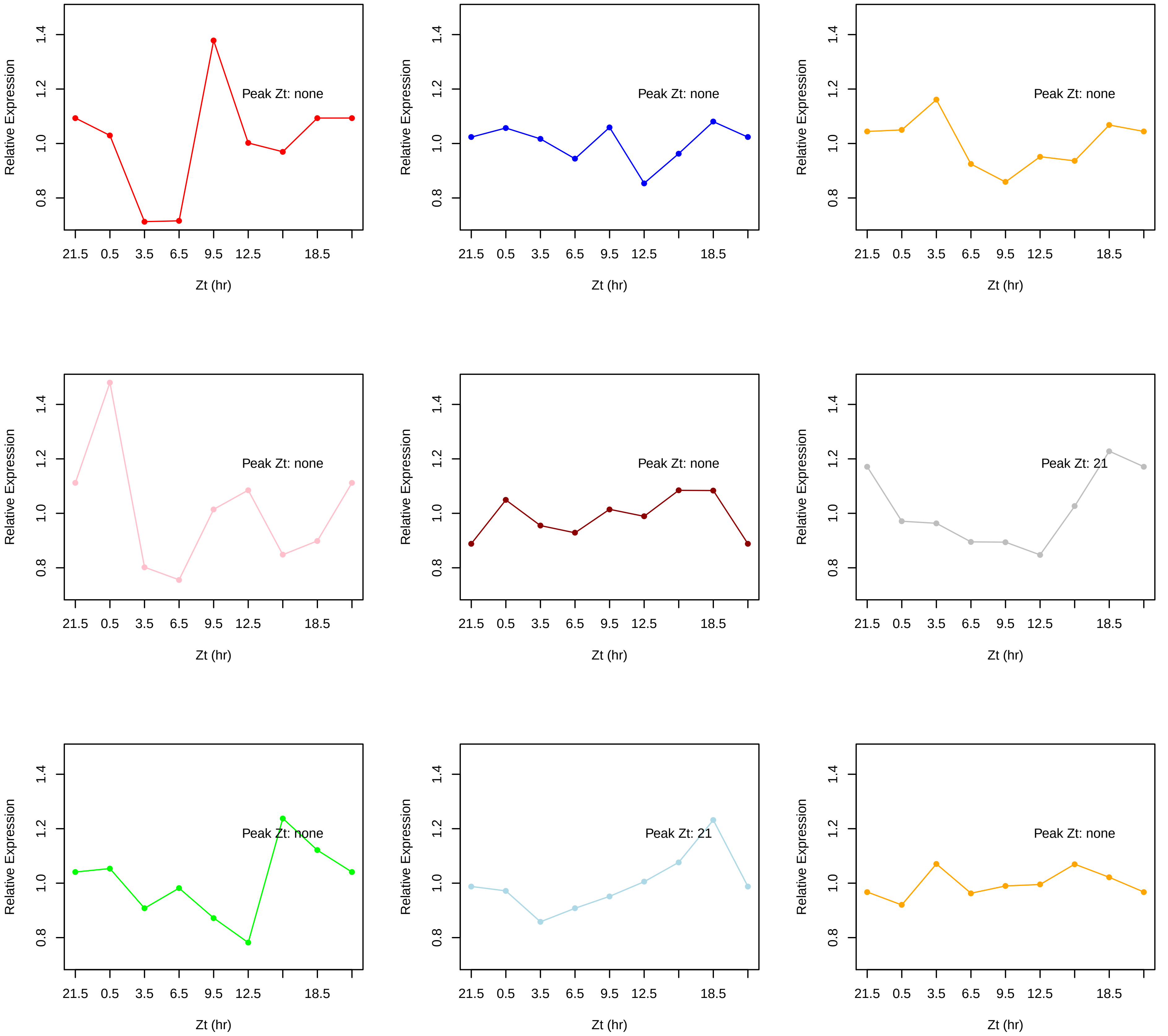

Singeleton: ENSXMAG00000011762 zgc:162816

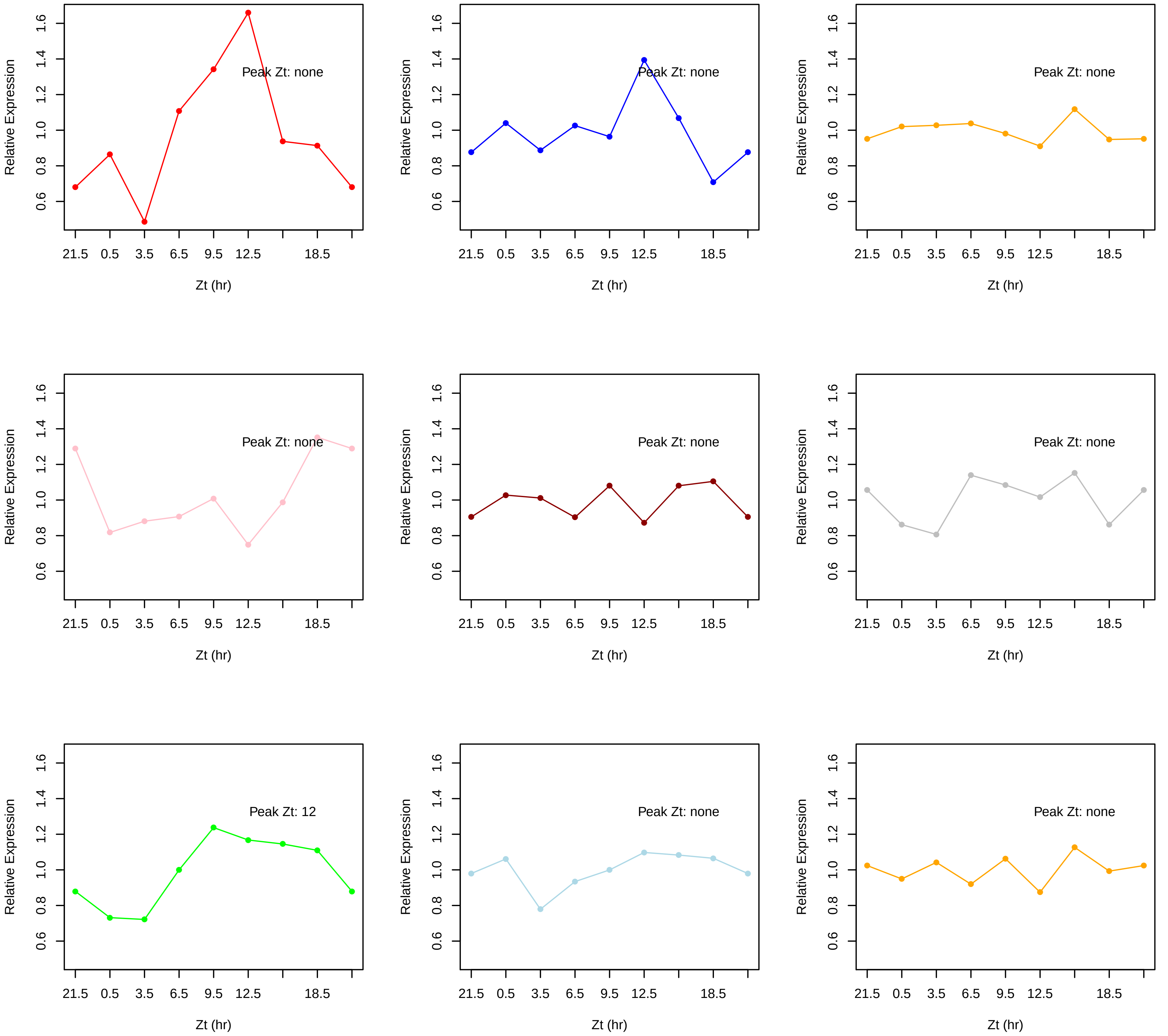

Singeleton: ENSXMAG00000009353 vill

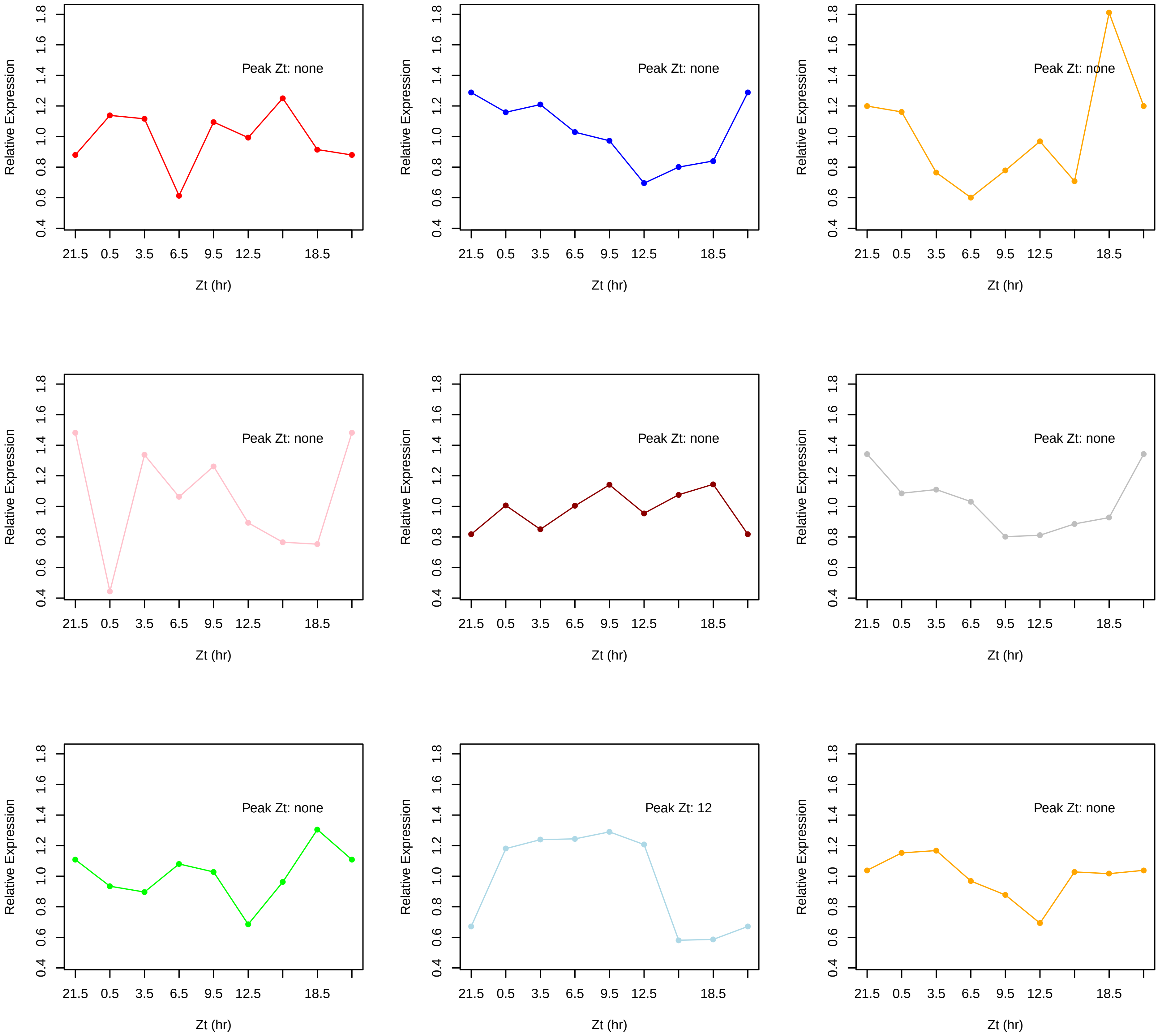

Singleton: ENSXMAG00000015525 trpm6

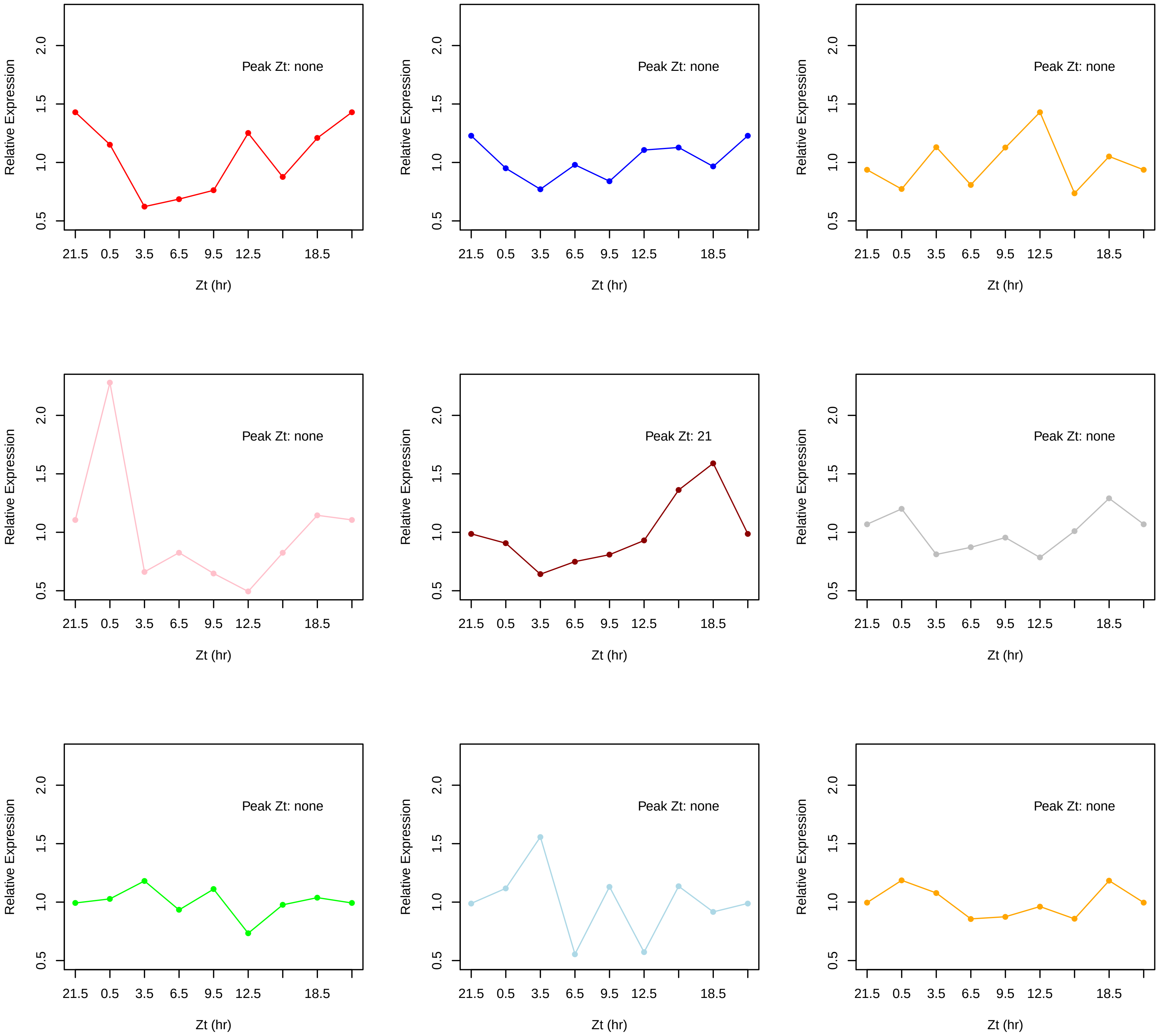

Singeleton: ENSXMAG00000029692 CCKAR

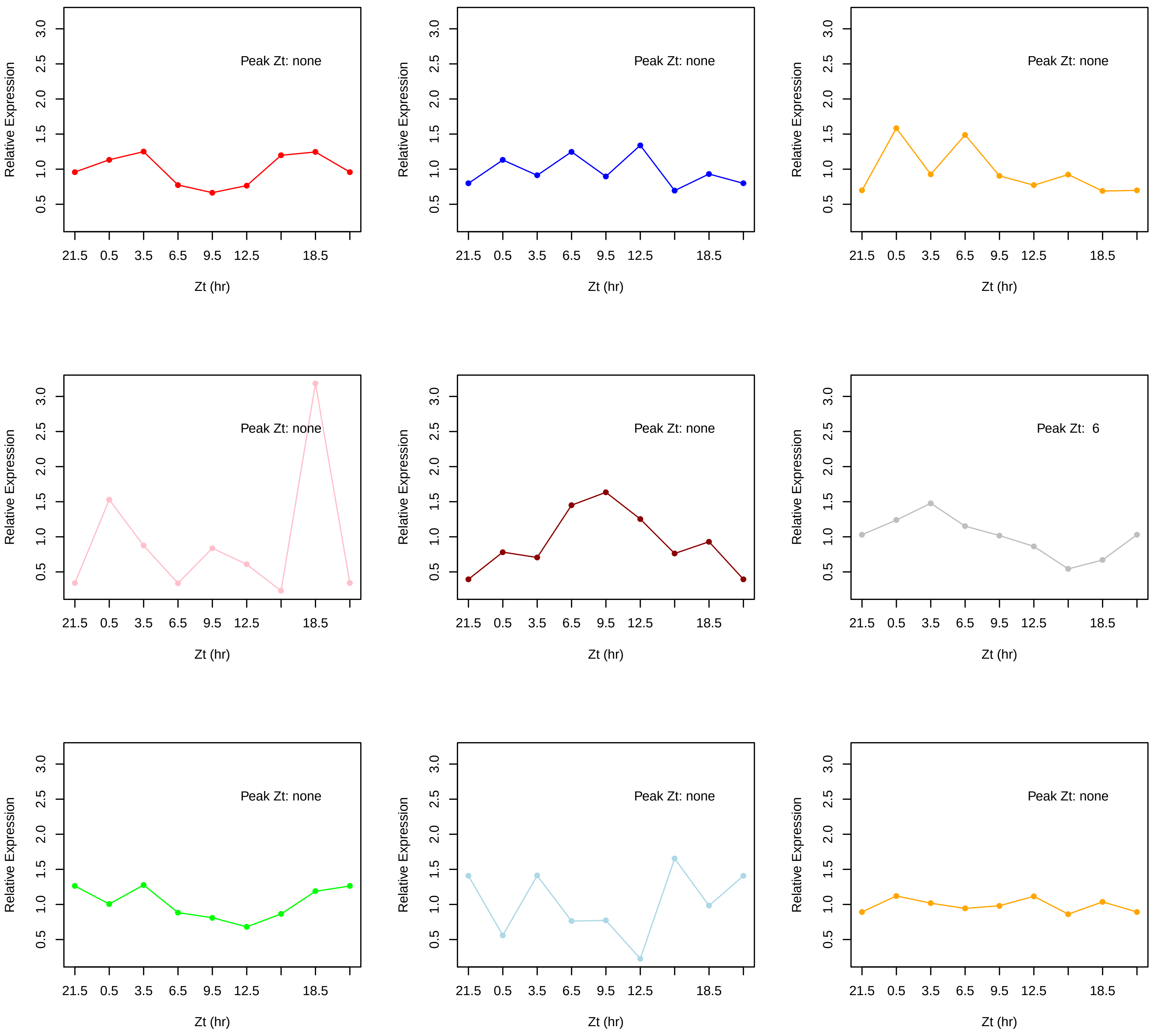

Singeleton: ENSXMAG00000006395 fam168b

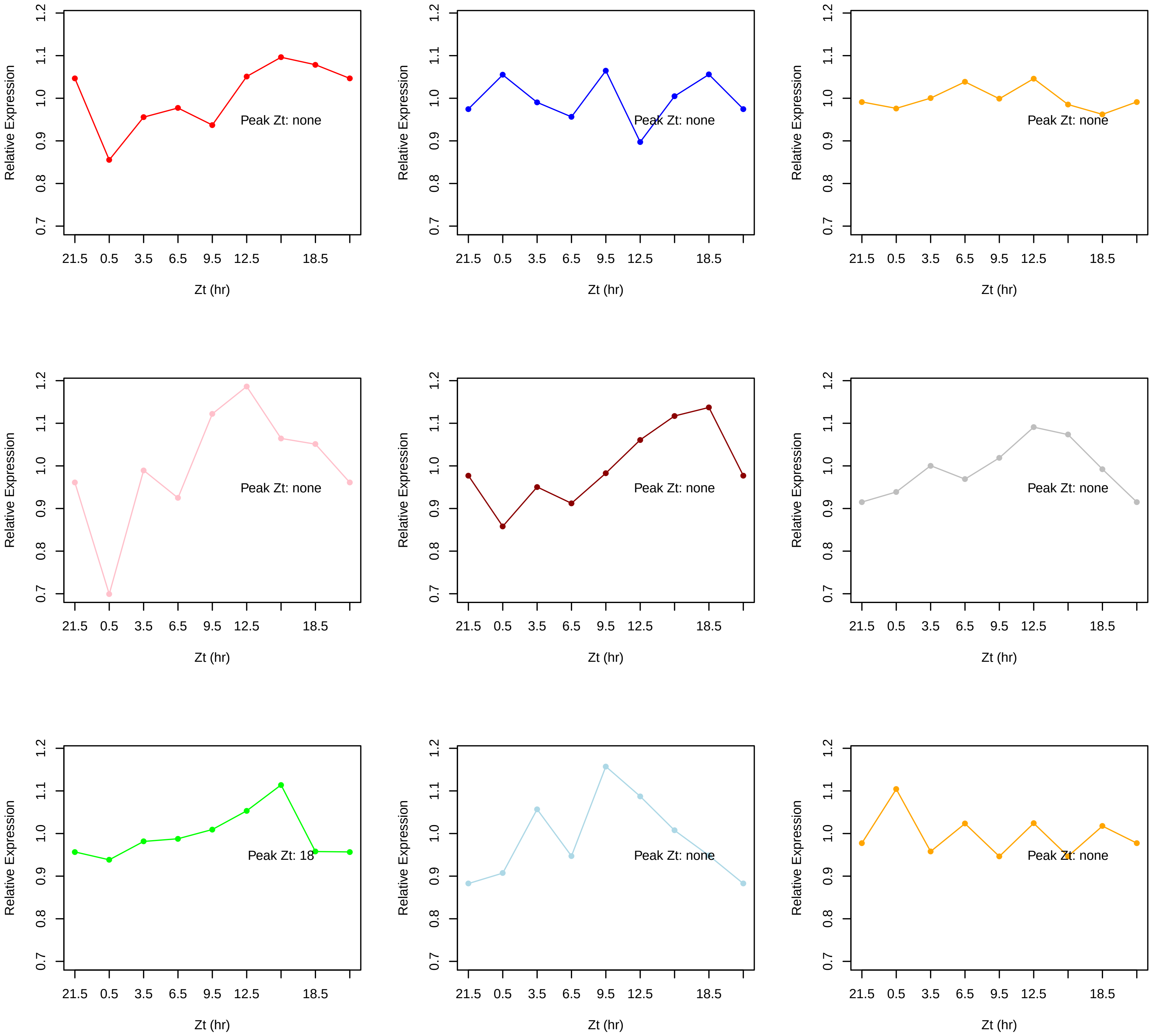

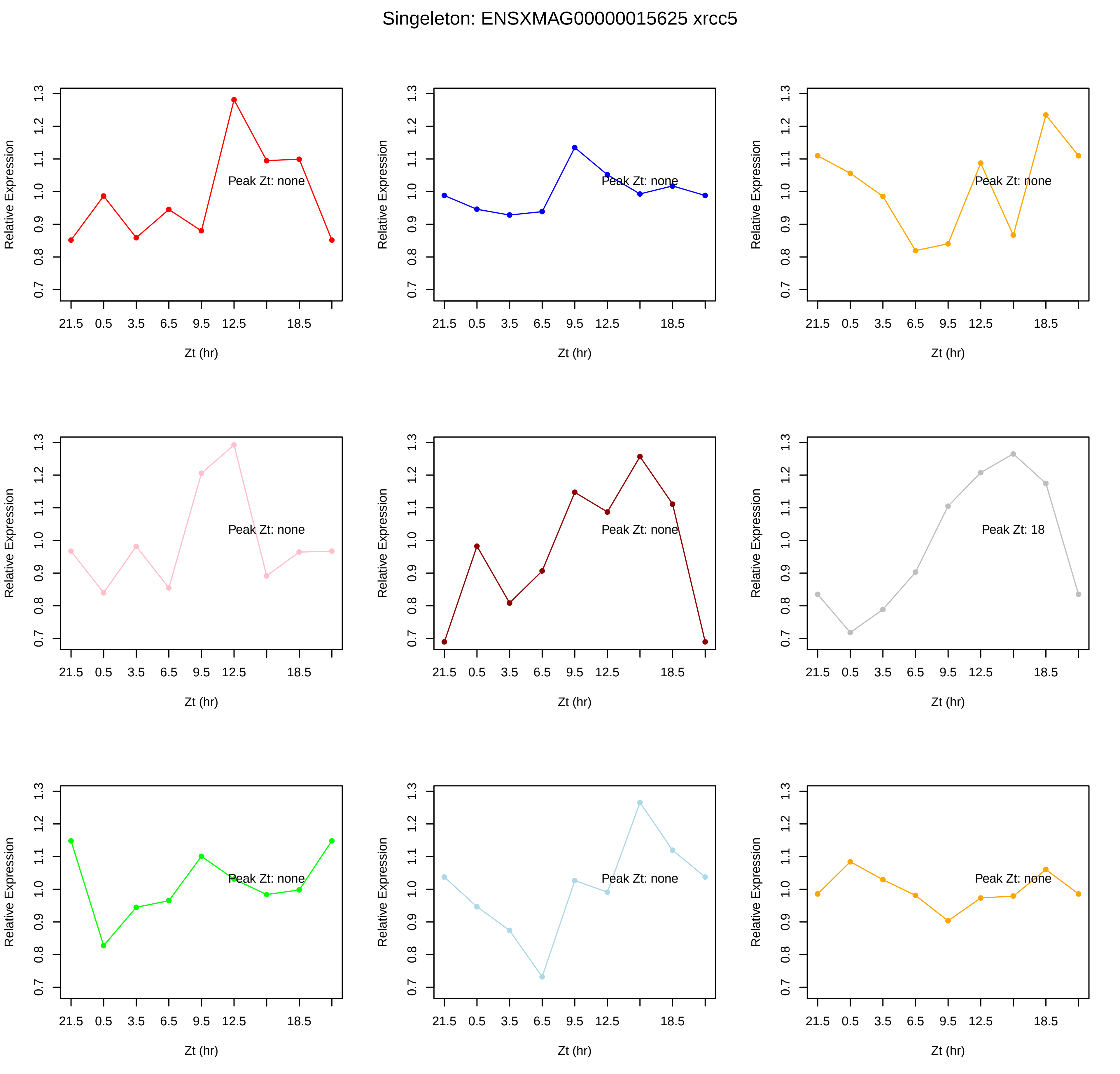

Singeleton: ENSXMAG00000015625 xrcc5
Peak Zt: none
Peak Zt: none
Peak Zt: none
Peak Zt: none
Peak Zt: none
Peak Zt: 18
Peak Zt: none
Peak Zt: none
Peak Zt: none
Relative Expression
Zt (hr)
21.5
0.5
3.5
6.5
9.5
12.5
18.5
0.7
0.8
0.9
1.0
1.1
1.2
1.3

Singeleton: ENSXMAG00000011315 DOCK2

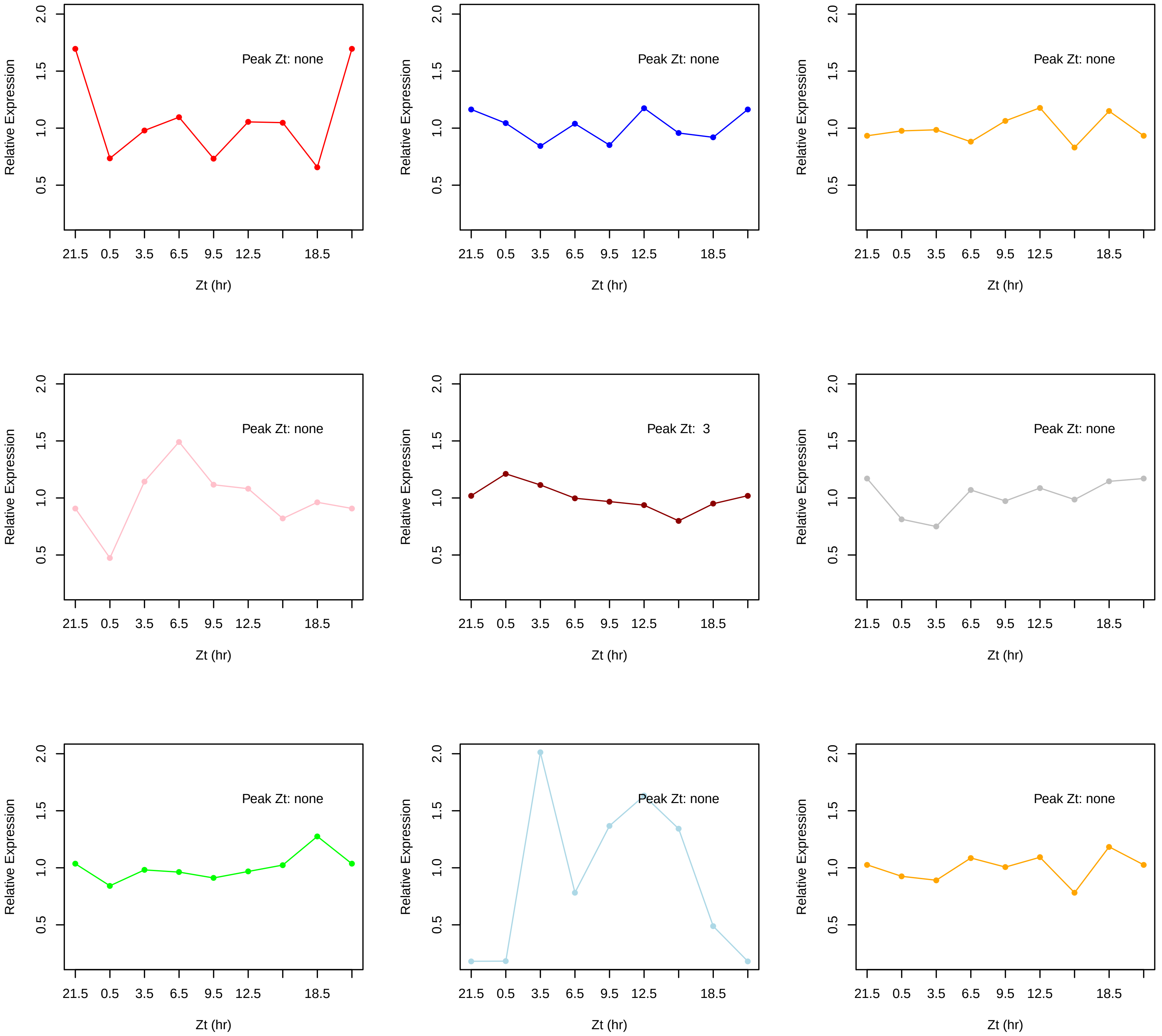

Singeleton: ENSXMAG00000009877 rbpjb

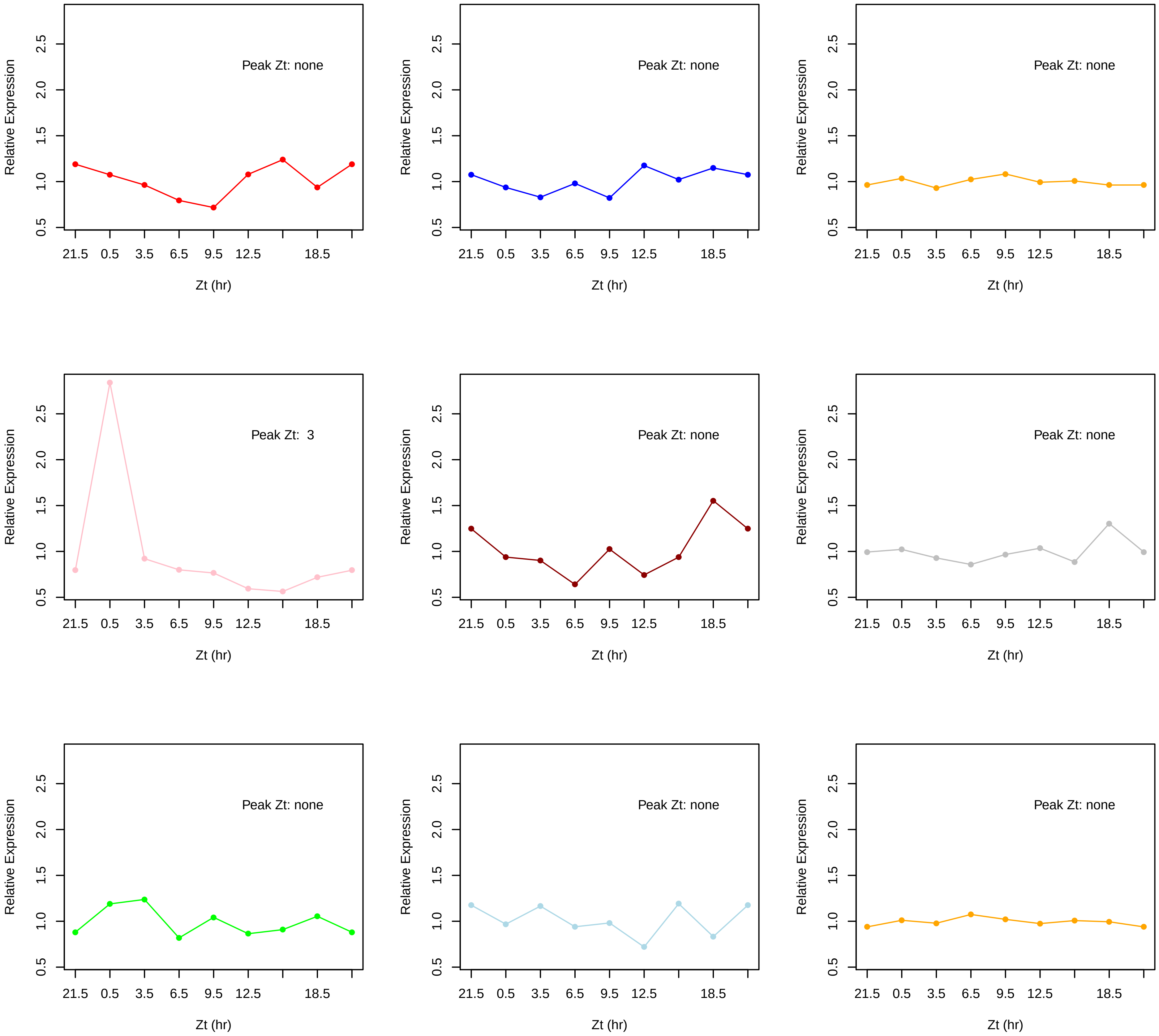

Singleton: ENSXMAG00000023838 creg1

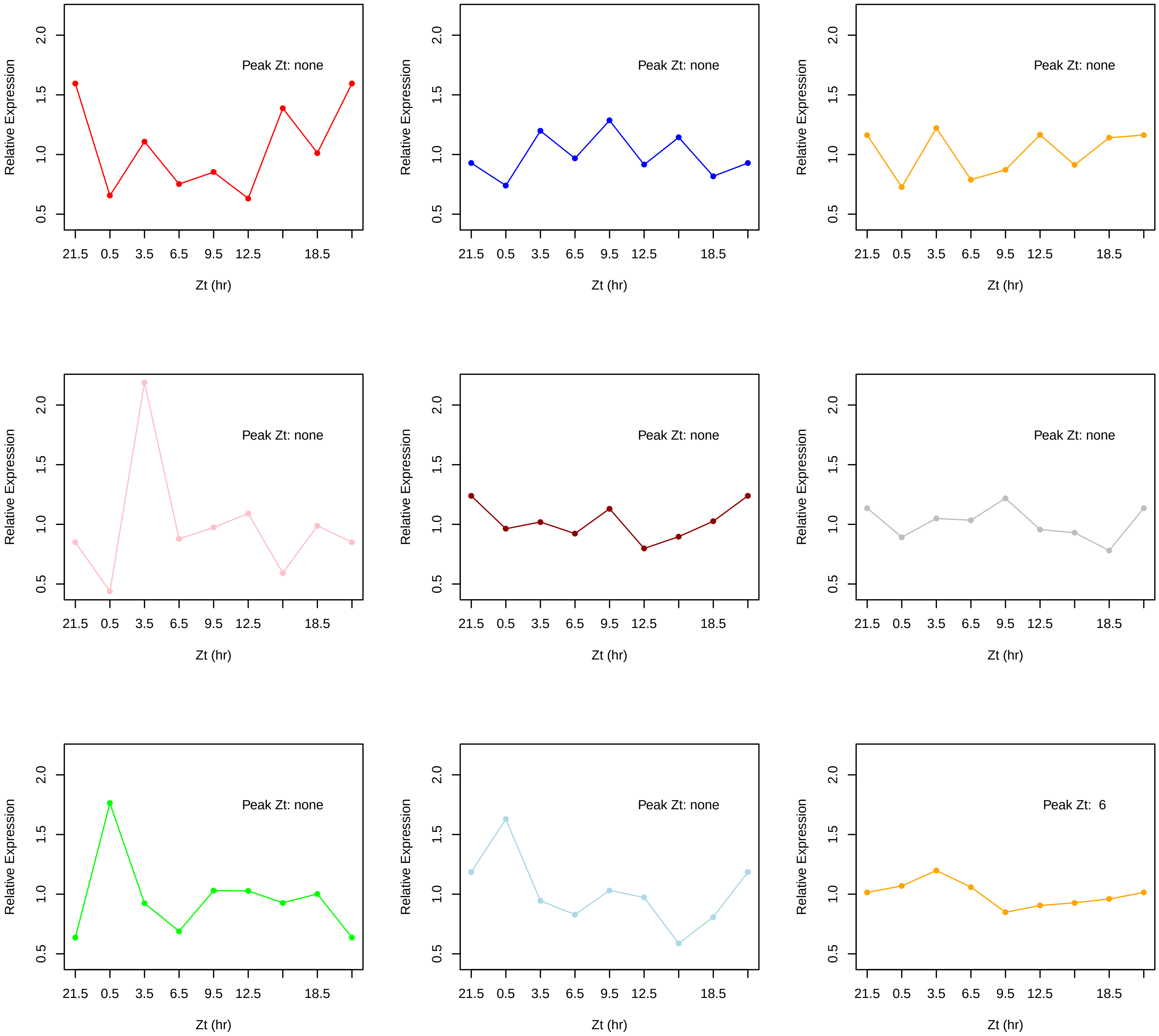

Singeleton: ENSXMAG00000026331 smim20

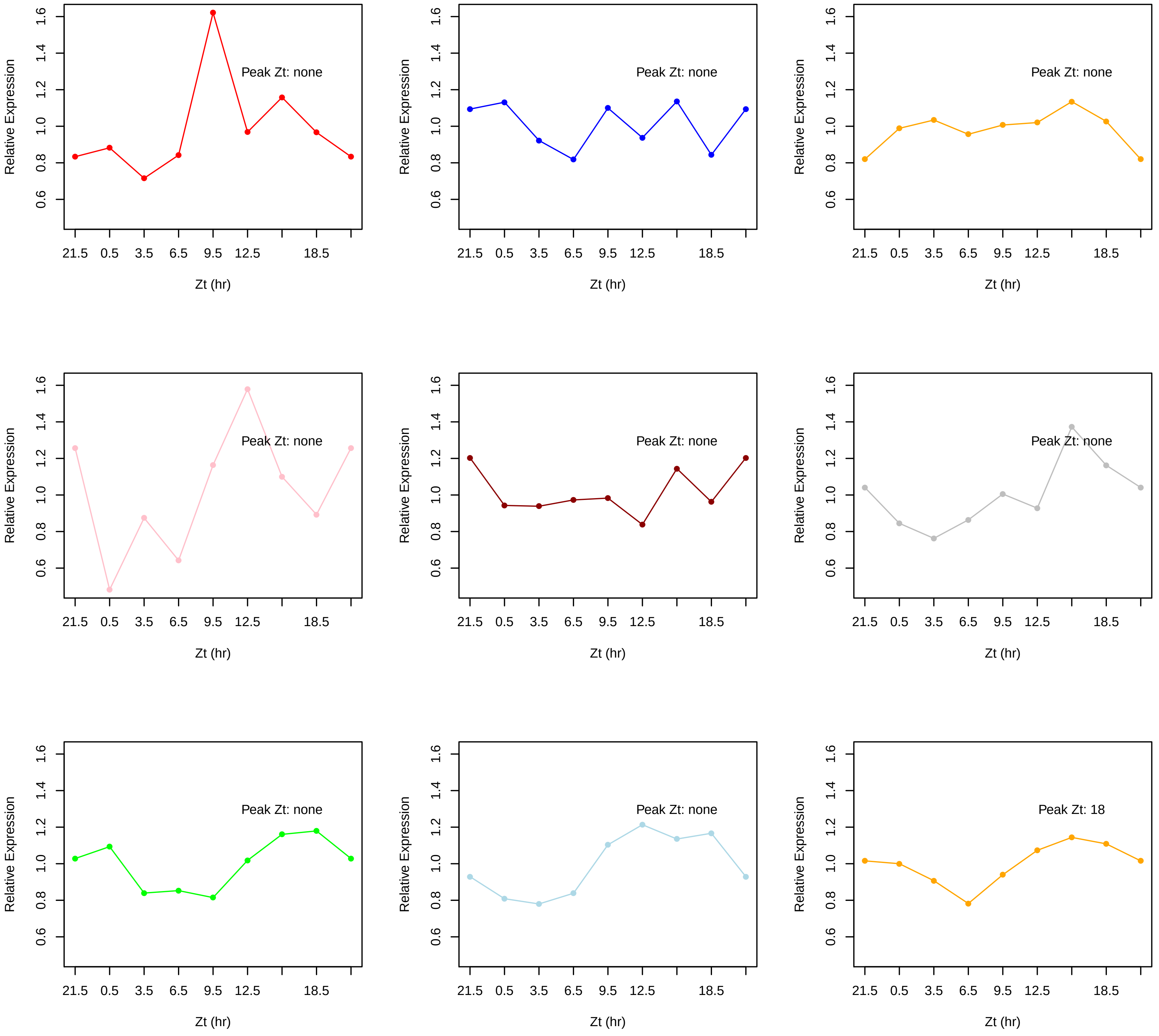

Singeleton: ENSXMAG00000015642 tmem169b

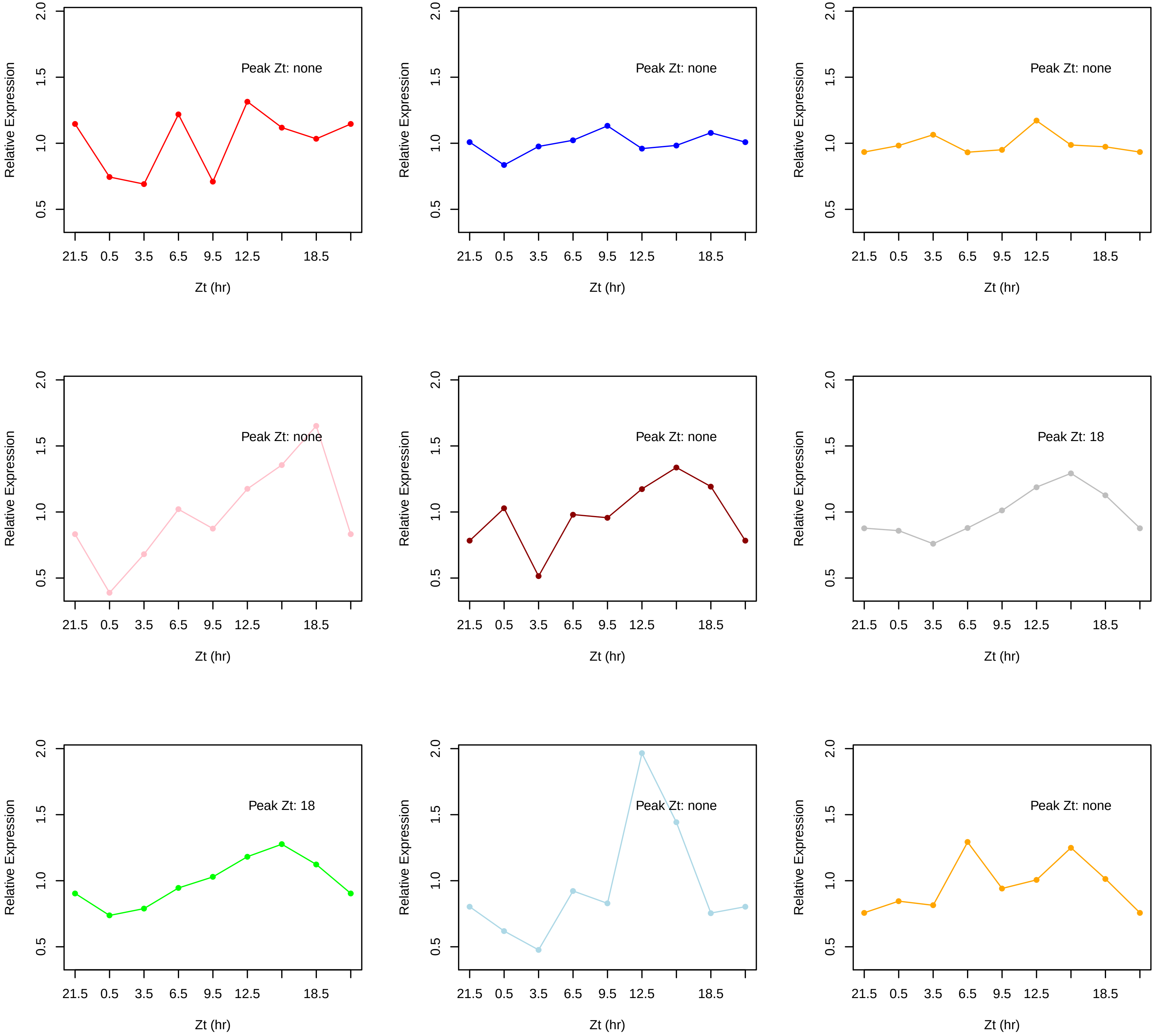

Singeleton: ENSXMAG00000007970 fam45a

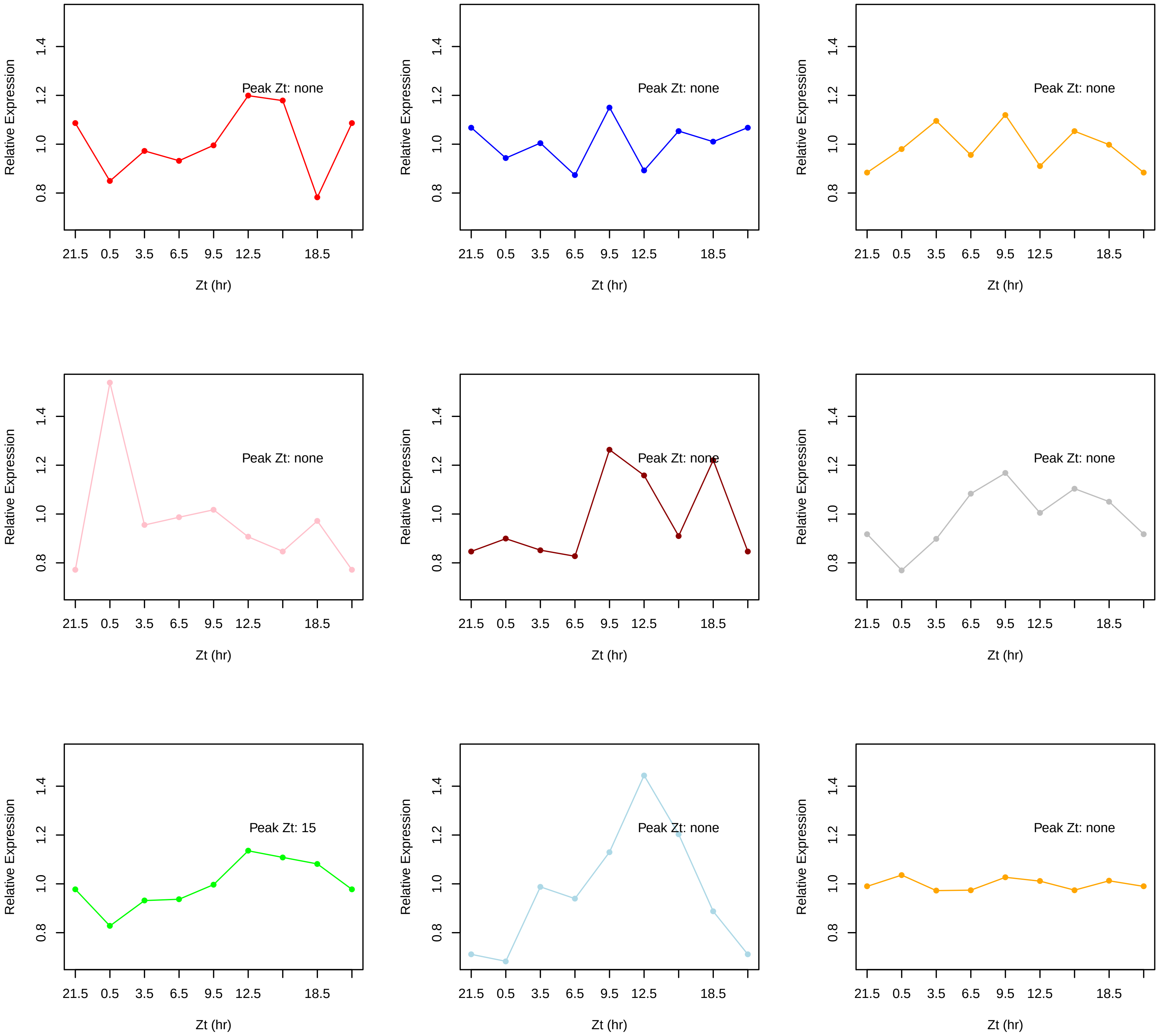

Singleton: ENSXMAG00000003187 soat1

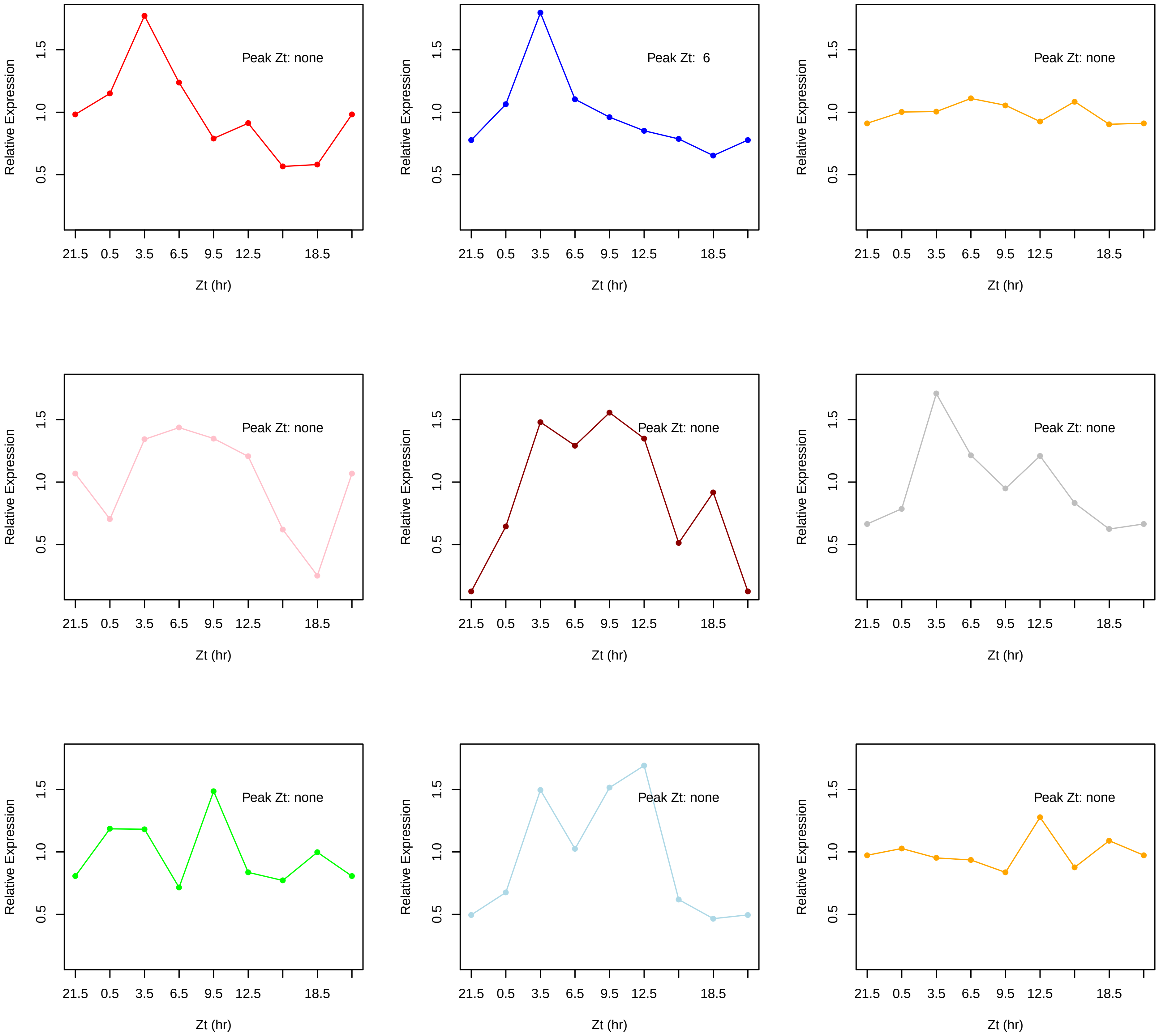

Singleton: ENSXMAG00000021228 EHD4

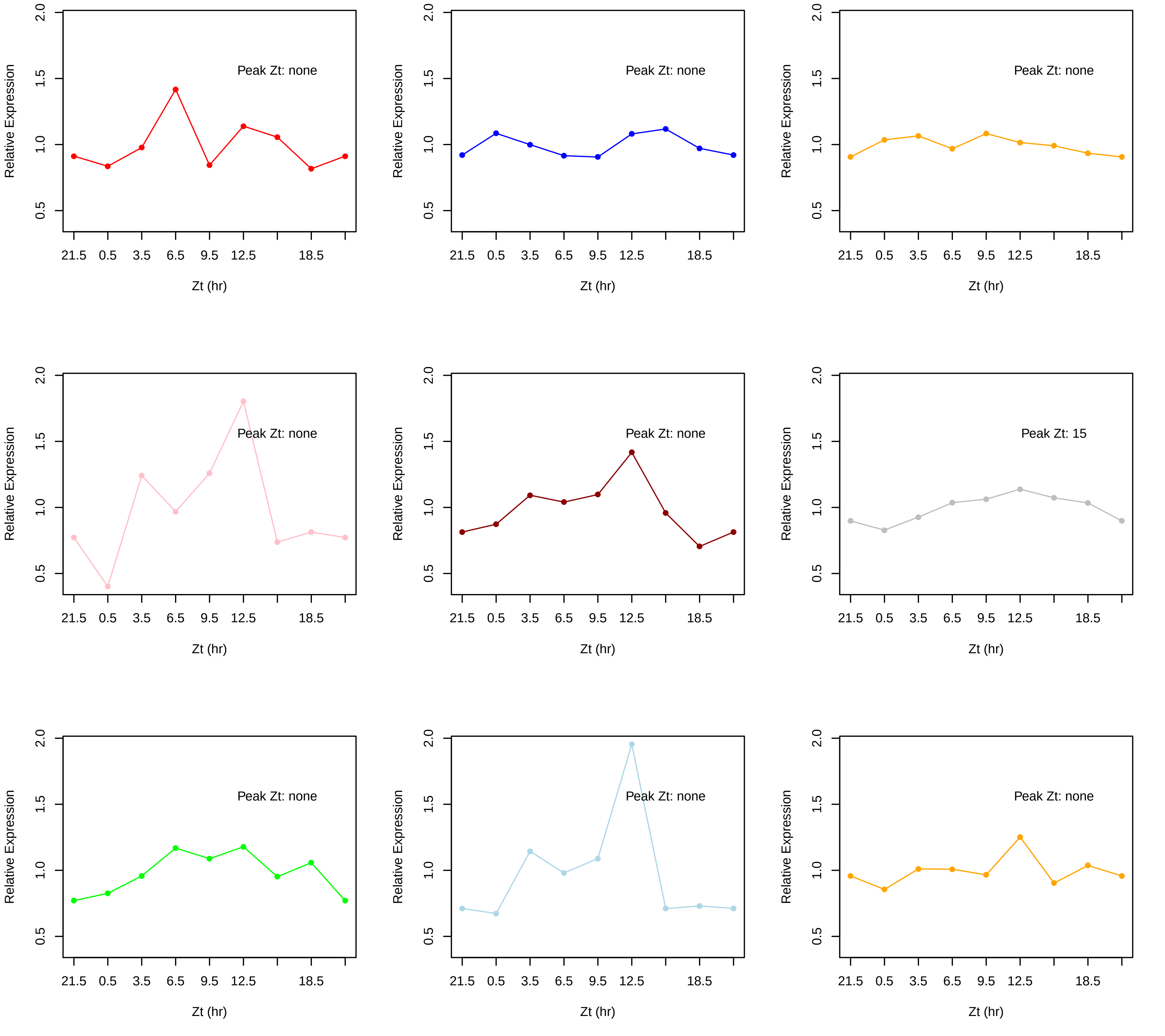

Singeleton: ENSXMAG00000015646 pfkla

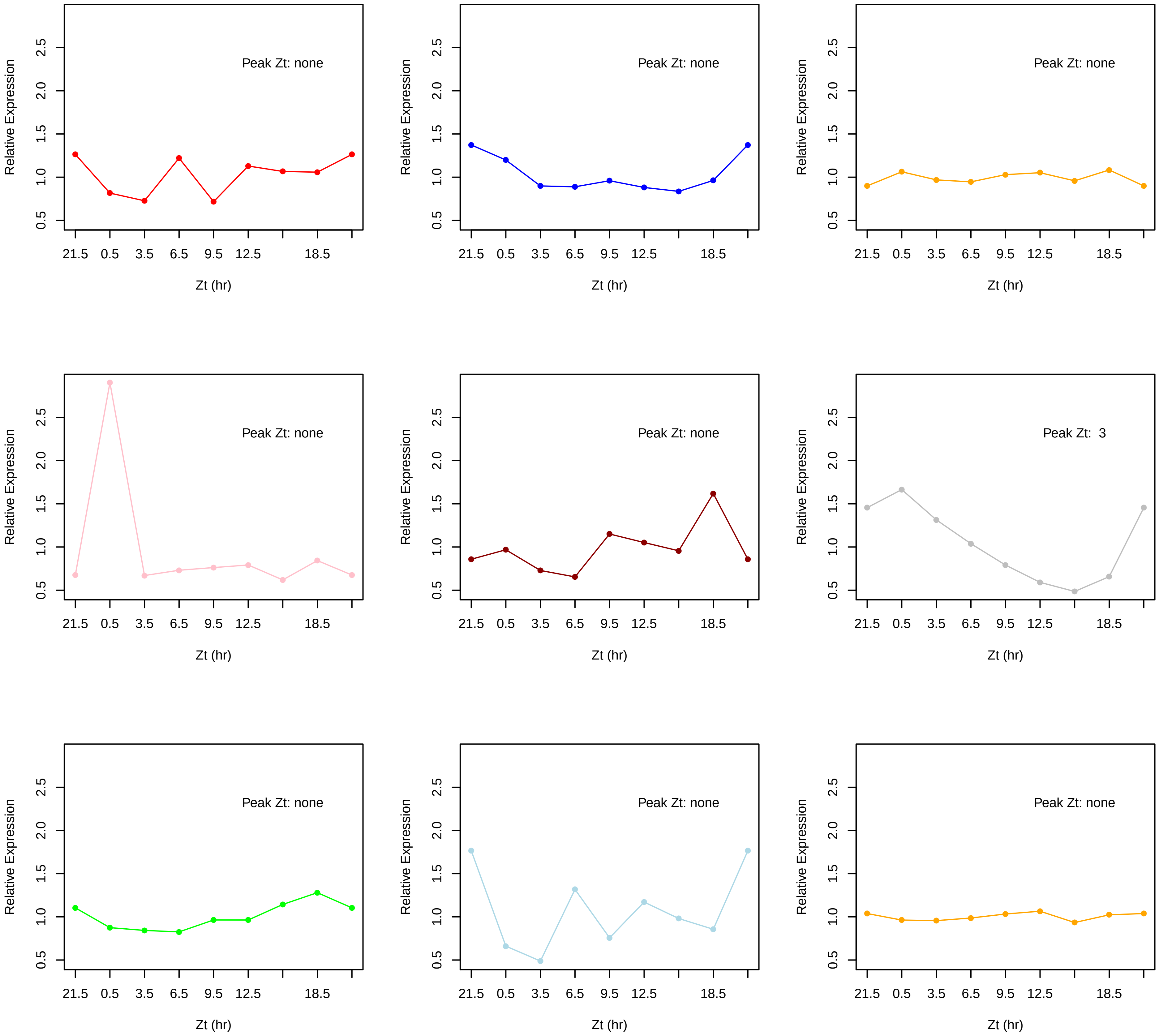

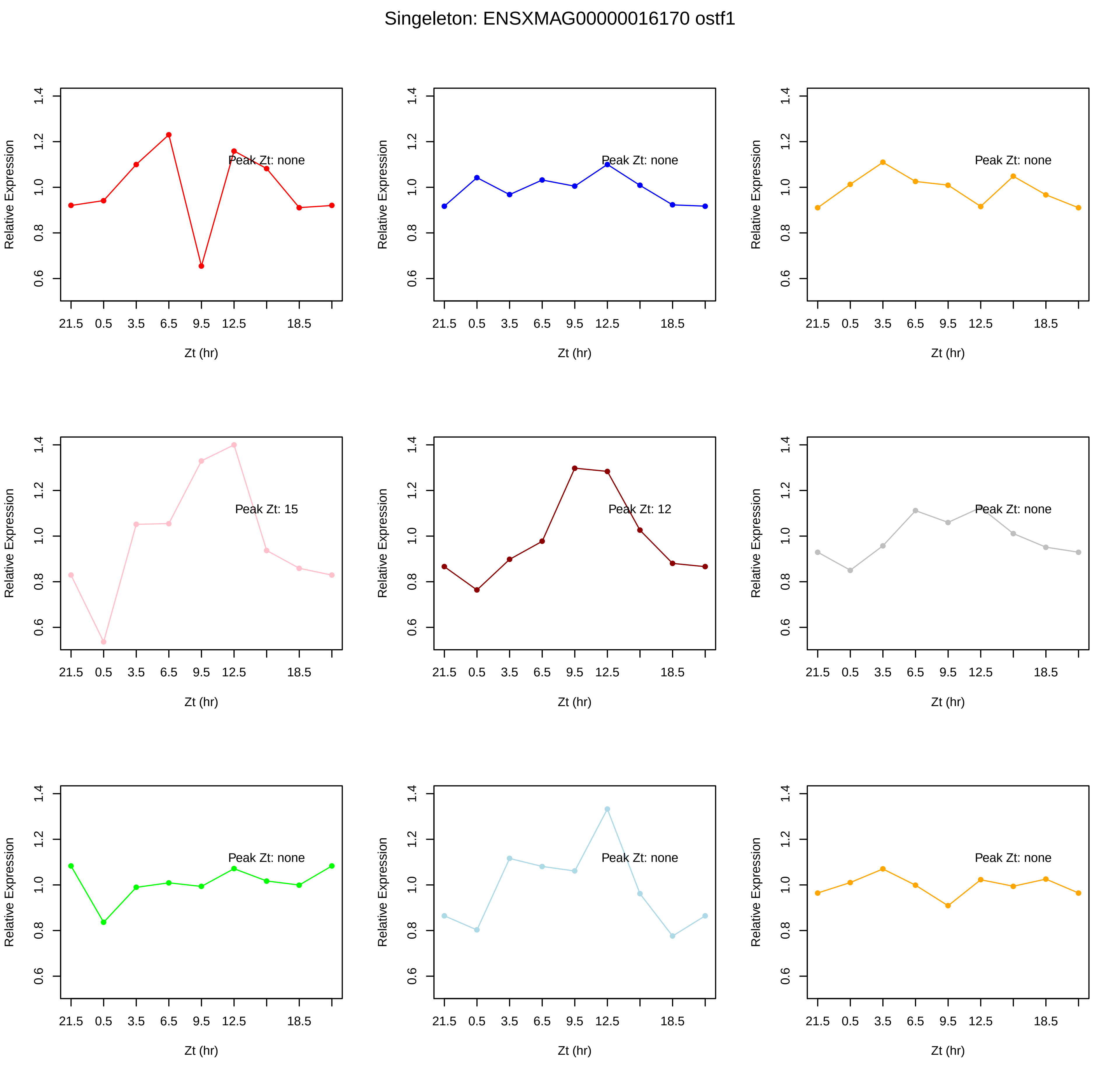

Singeleton: ENSXMAG00000016170 ostf1
Relative Expression
Zt (hr)
21.5
0.5
3.5
6.5
9.5
12.5
18.5
0.6
0.8
1.0
1.2
1.4
Peak Zt: none
Peak Zt: none
Peak Zt: none
Peak Zt: 15
Peak Zt: 12
Peak Zt: none
Peak Zt: none
Peak Zt: none
Peak Zt: none

Singleton: ENSXMAG00000029096

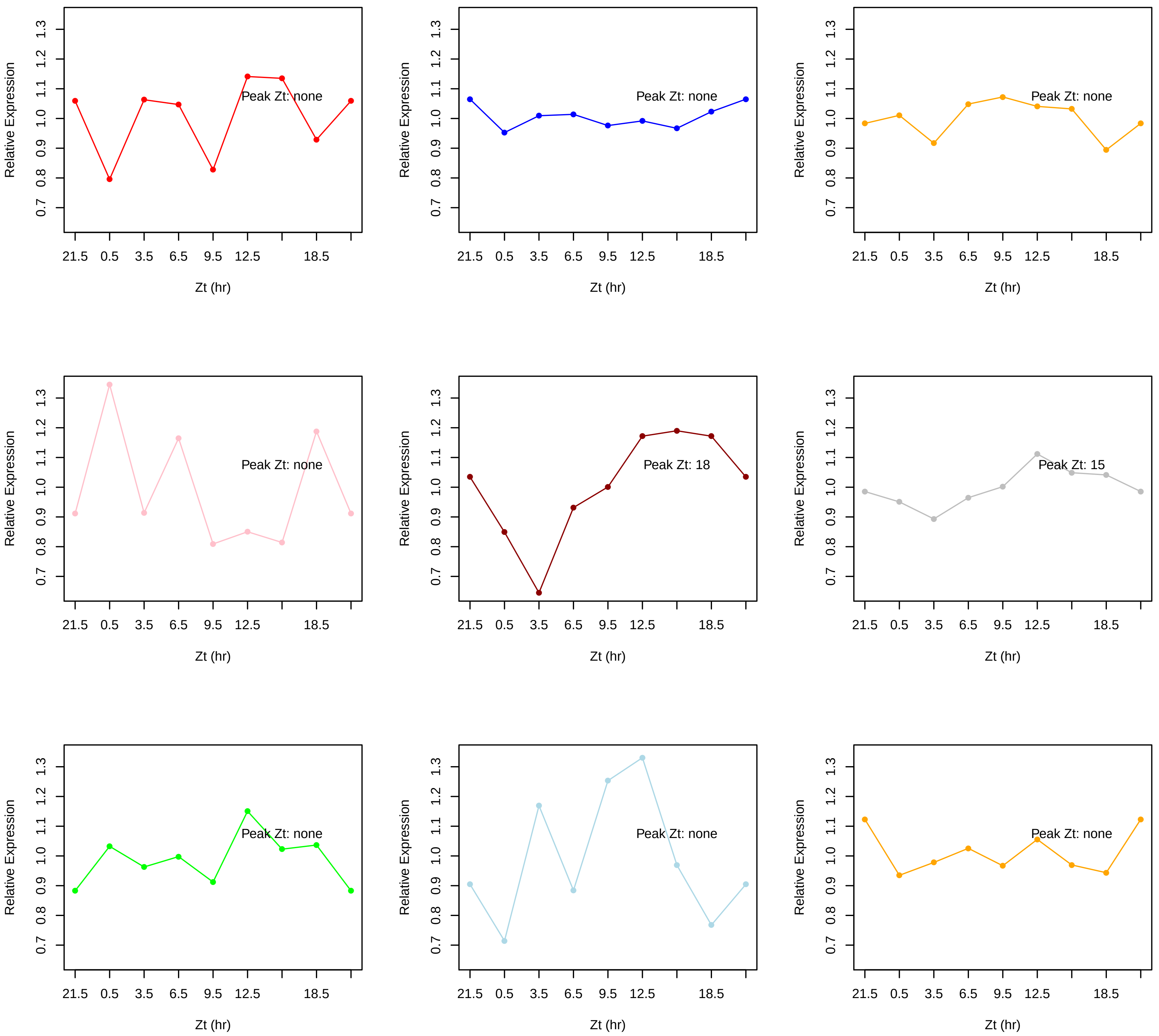

Singeleton: ENSXMAG00000008961 phykpl

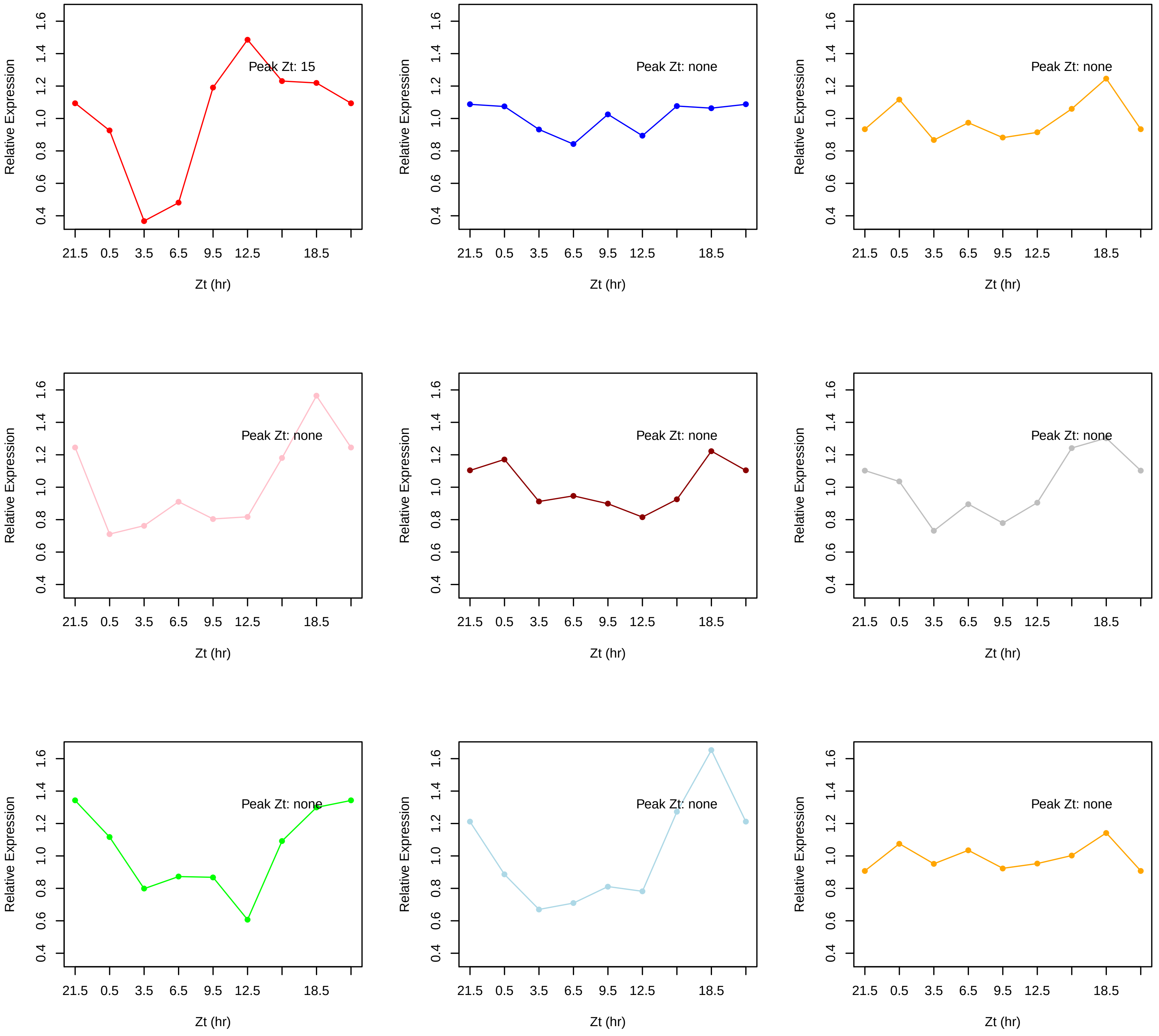

Singeleton: ENSXMAG00000004931 cinp

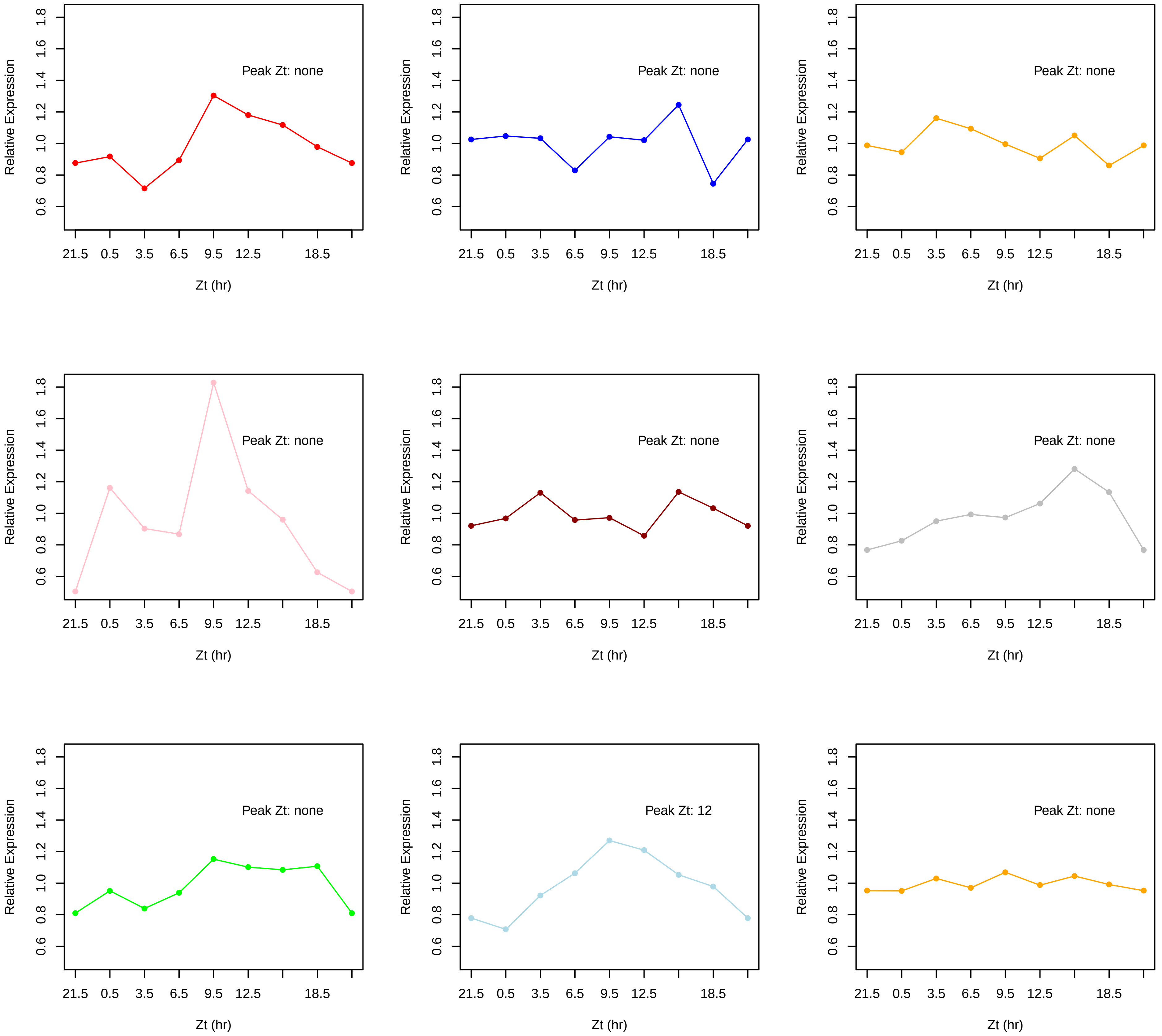

Singeleton: ENSXMAG00000013814 ndnf

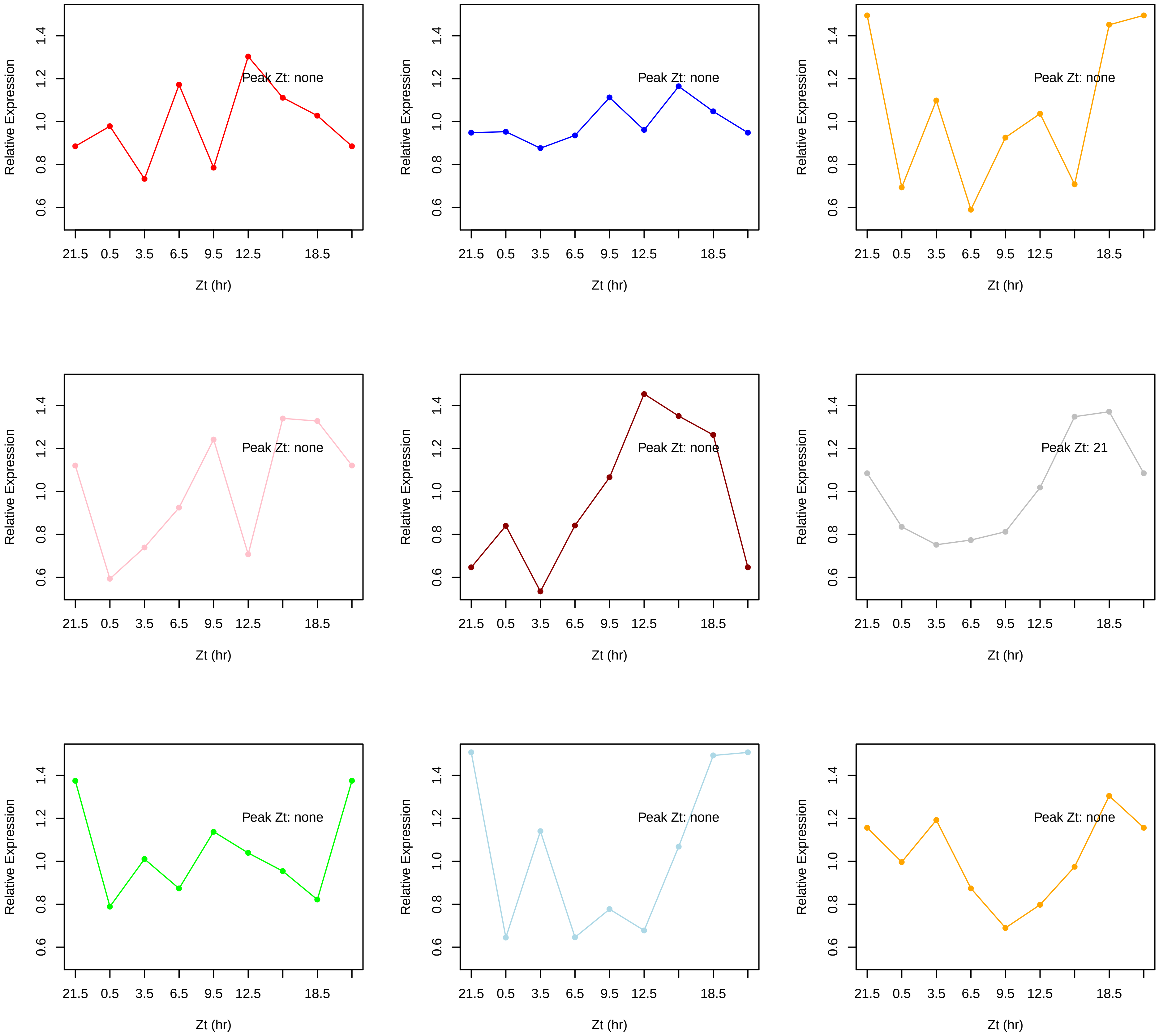

Singleton: ENSXMAG00000015692

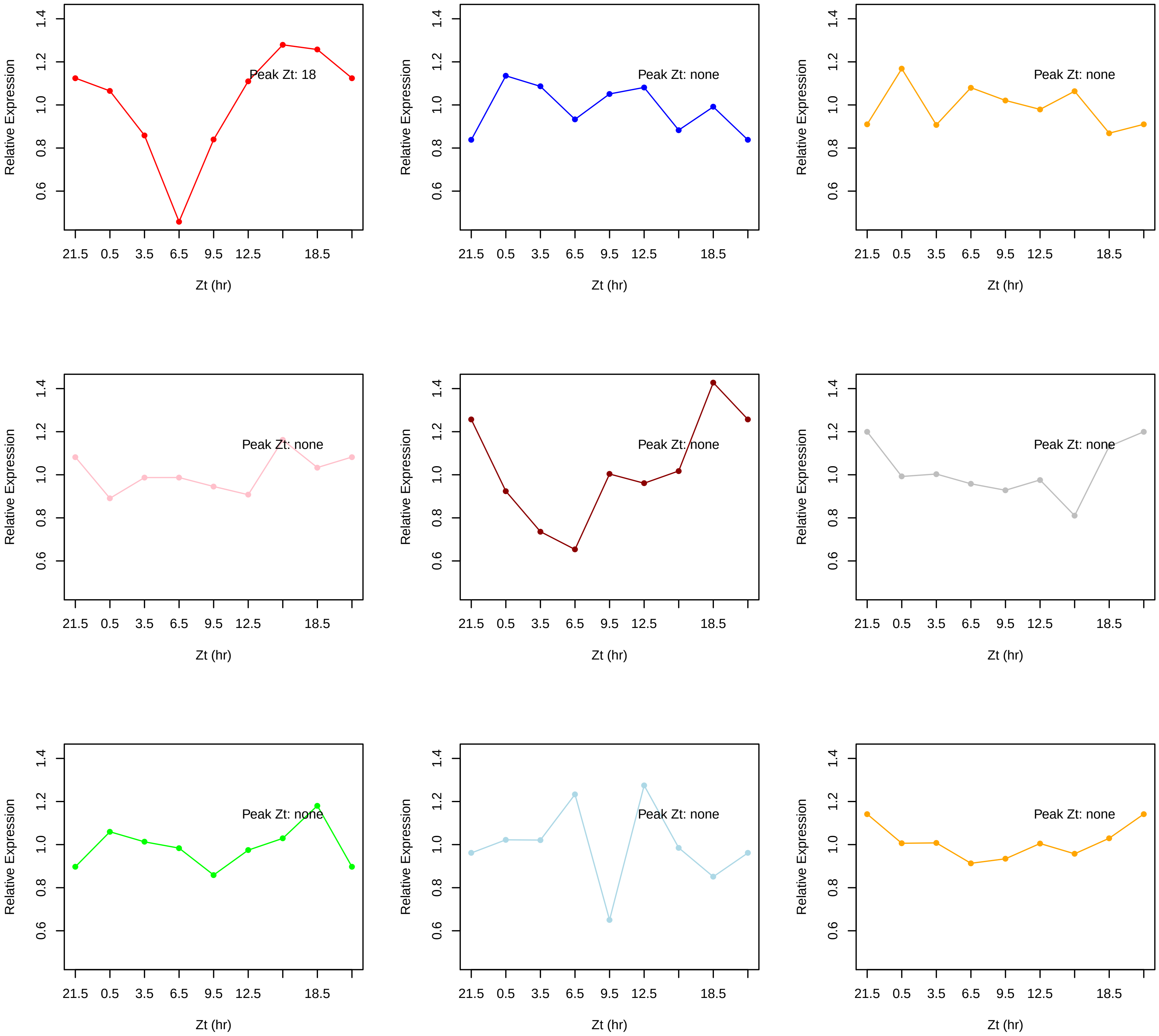

Singeleton: ENSXMAG00000021565 NIPAL2

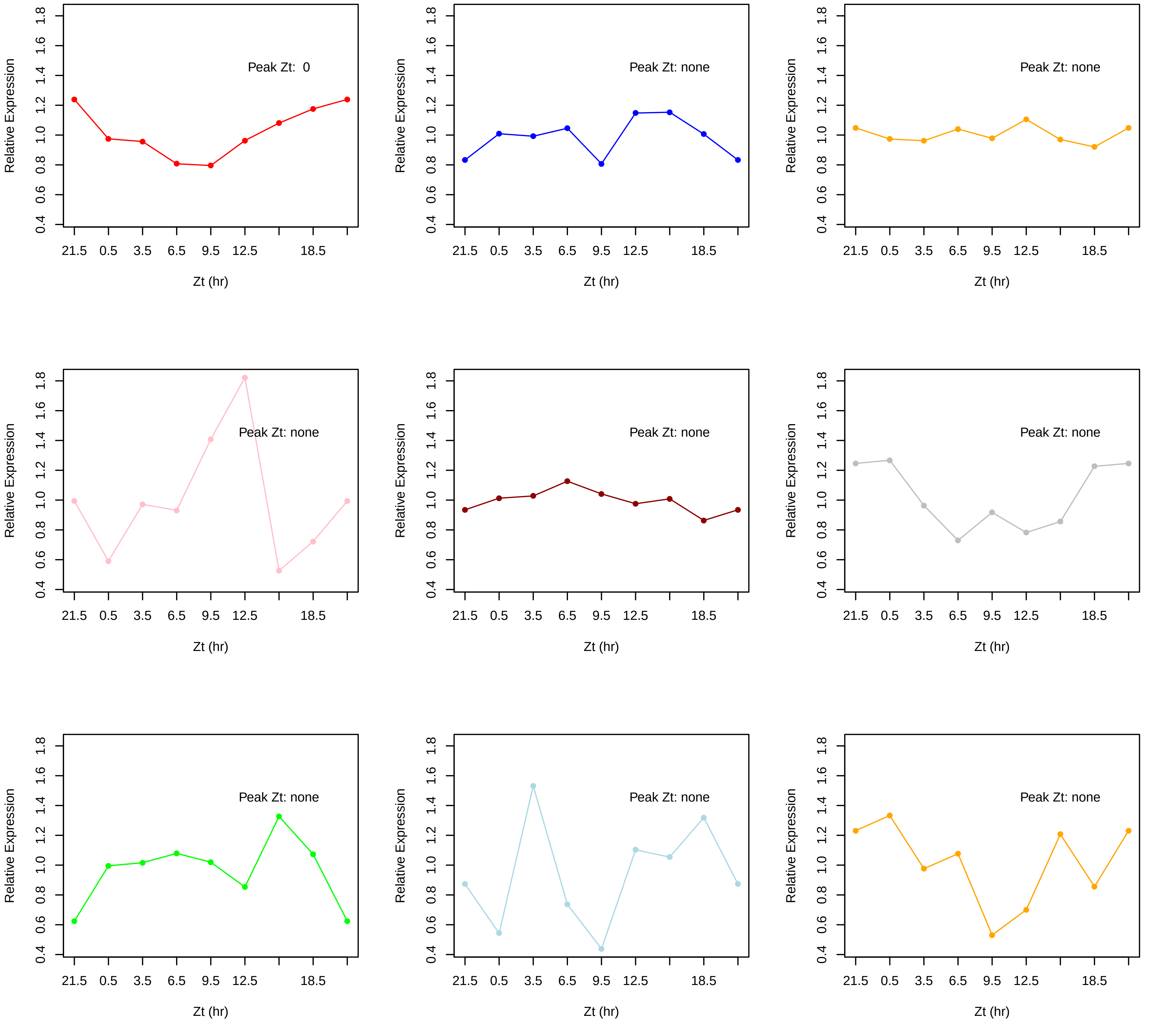

Singeleton: ENSXMAG00000021546 myd88

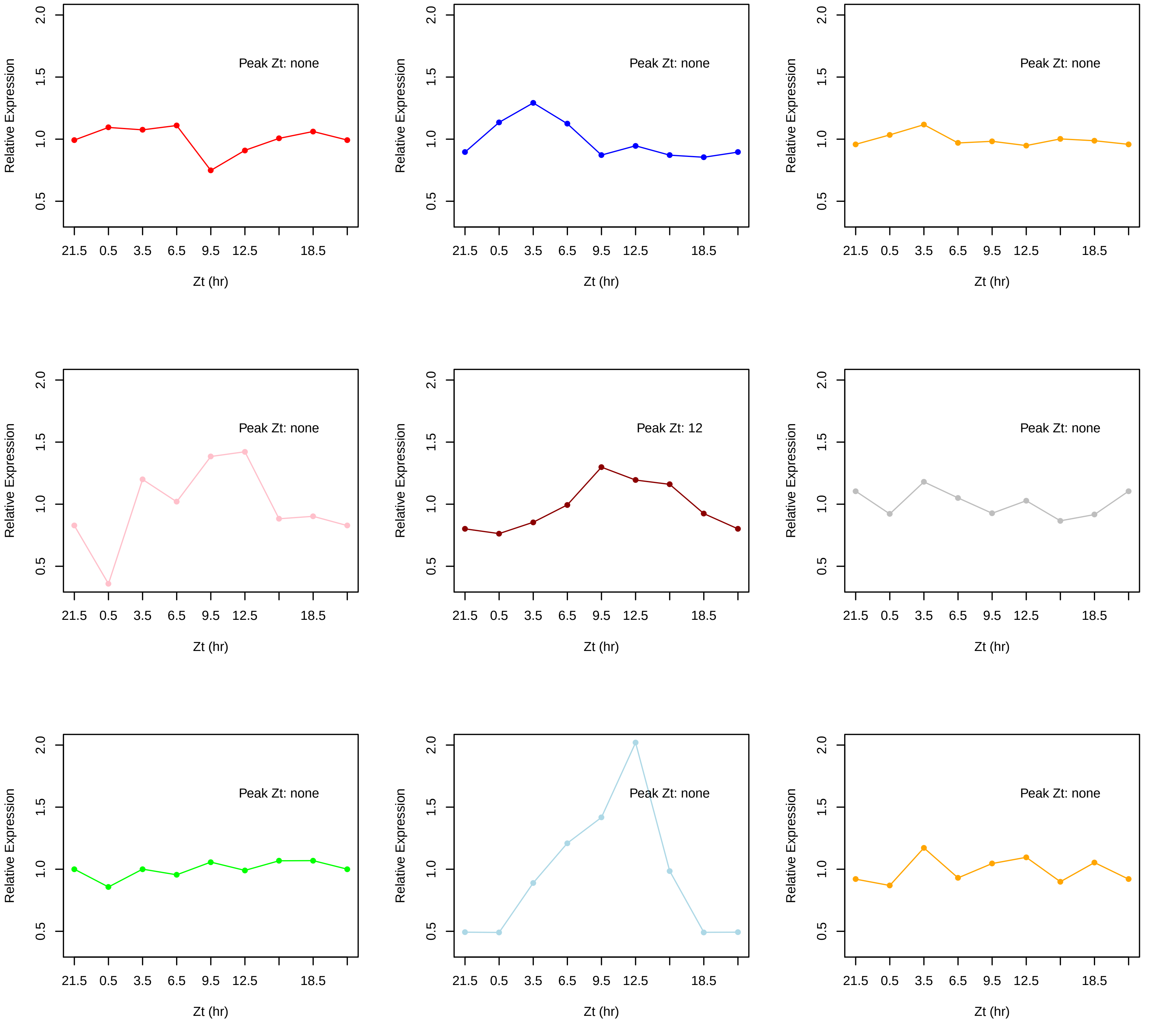

Singeleton: ENSXMAG00000019288 cul4a

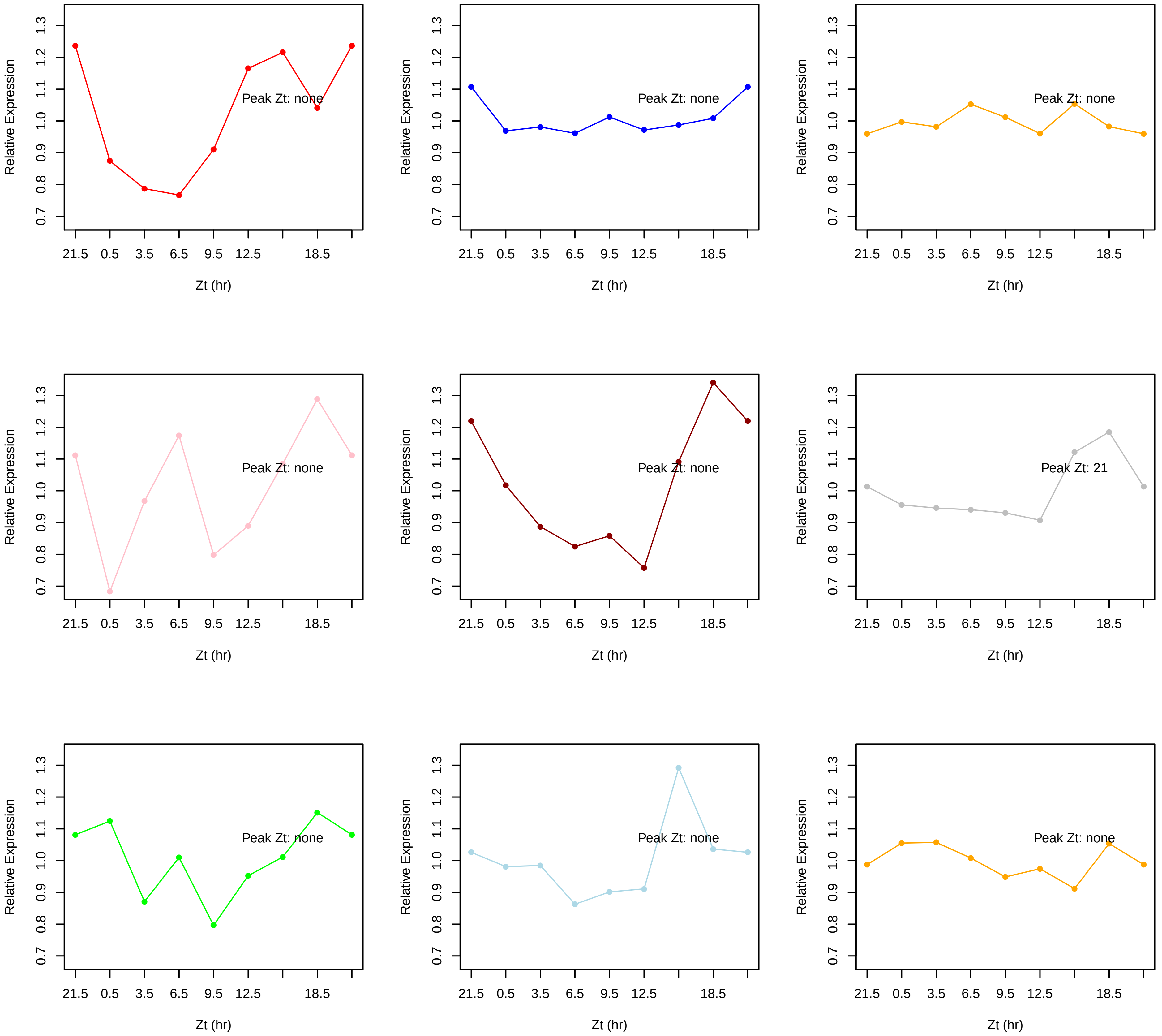

Singeleton: ENSXMAG00000018422 ankrd9

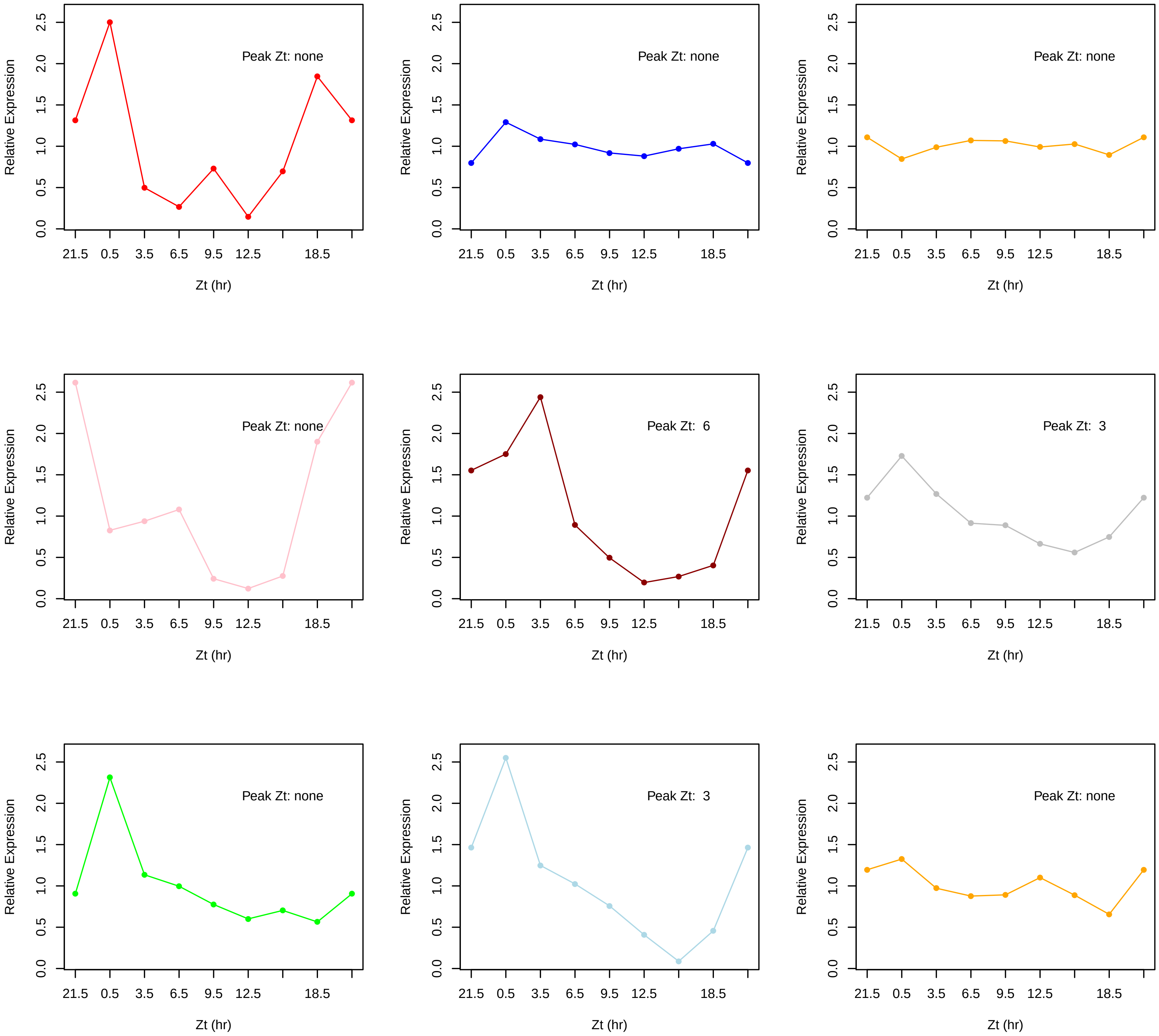

Singleton: ENSXMAG00000010505 lonrf1

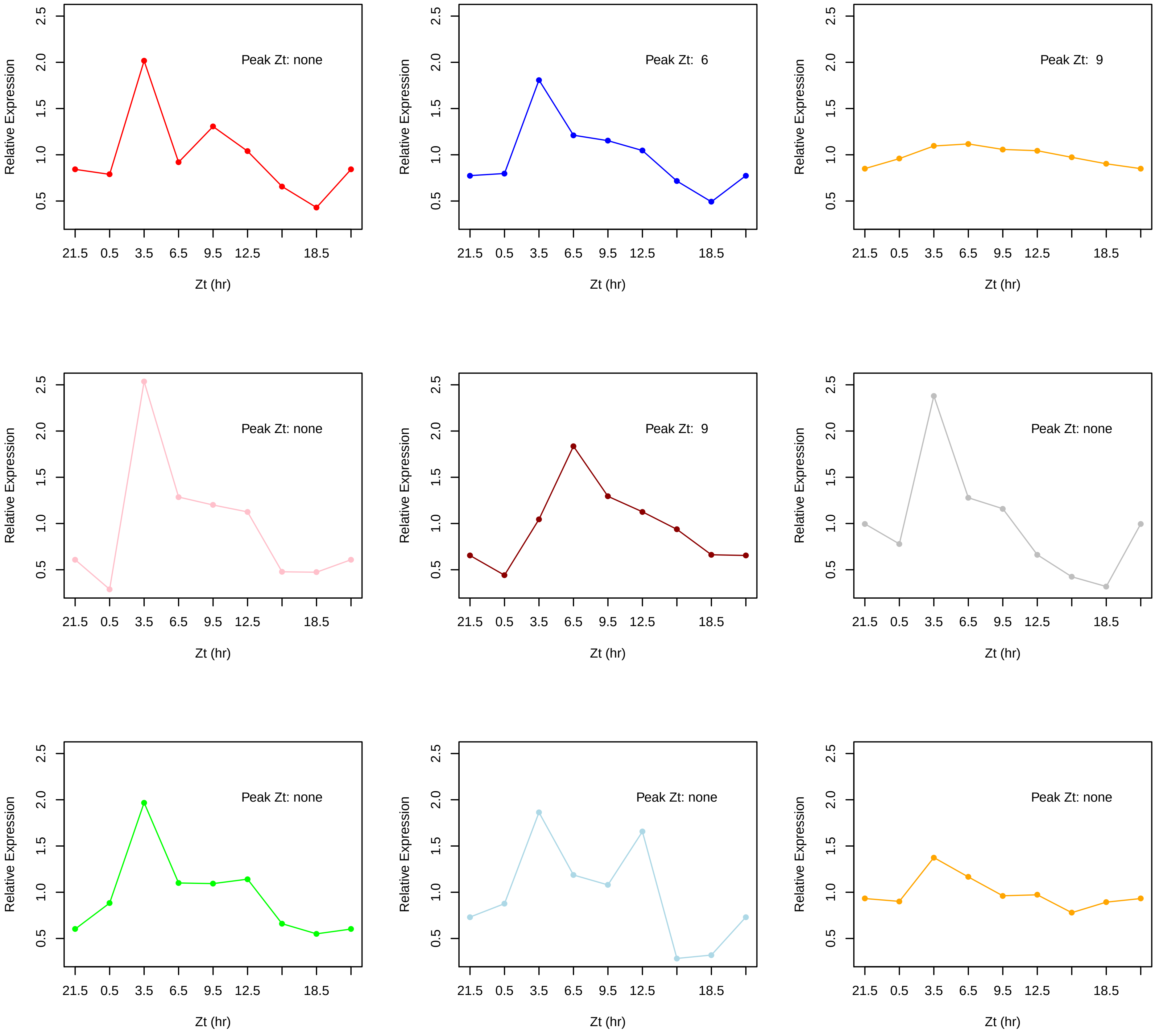

Singeleton: ENSXMAG00000027050 rfk

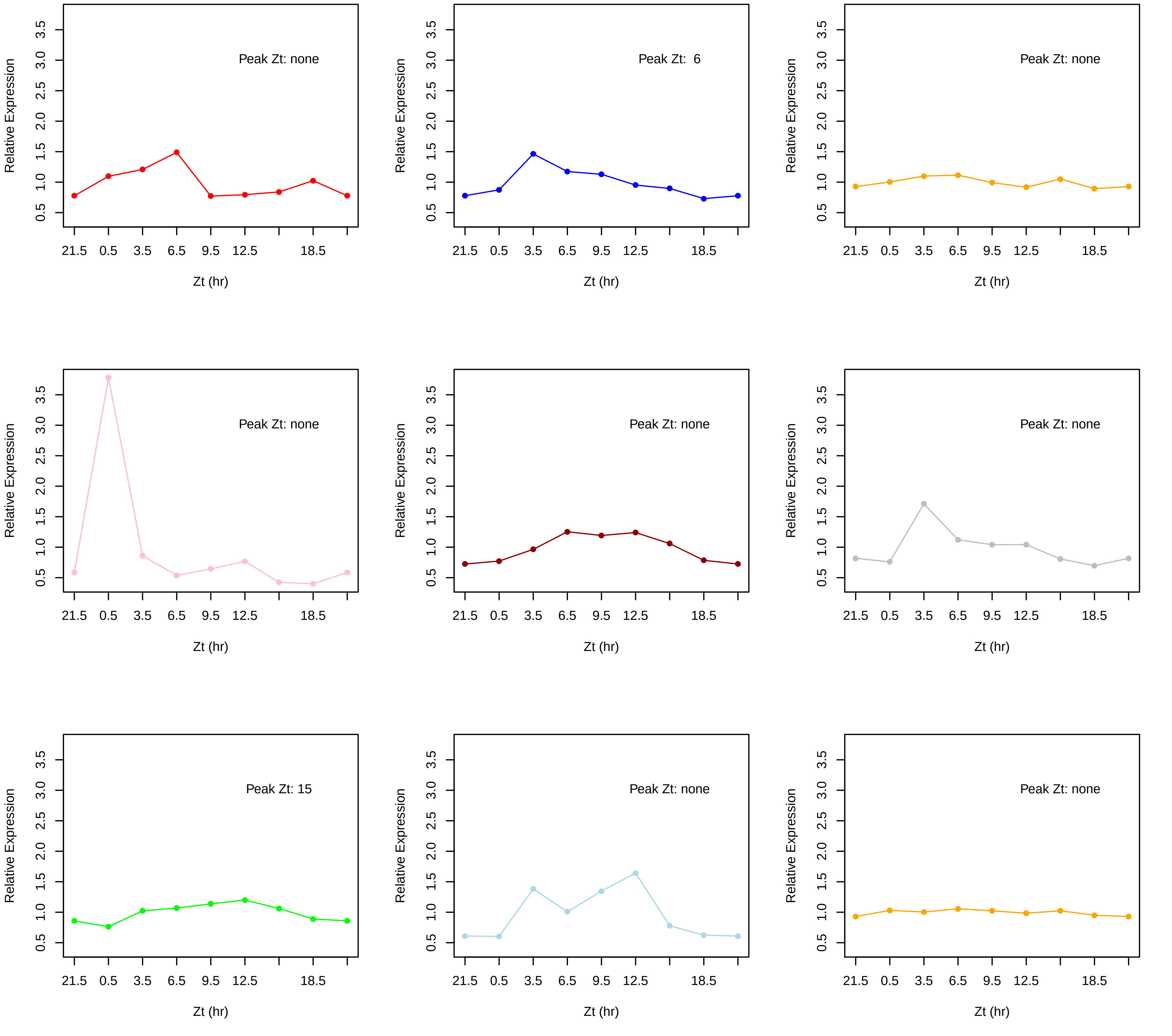

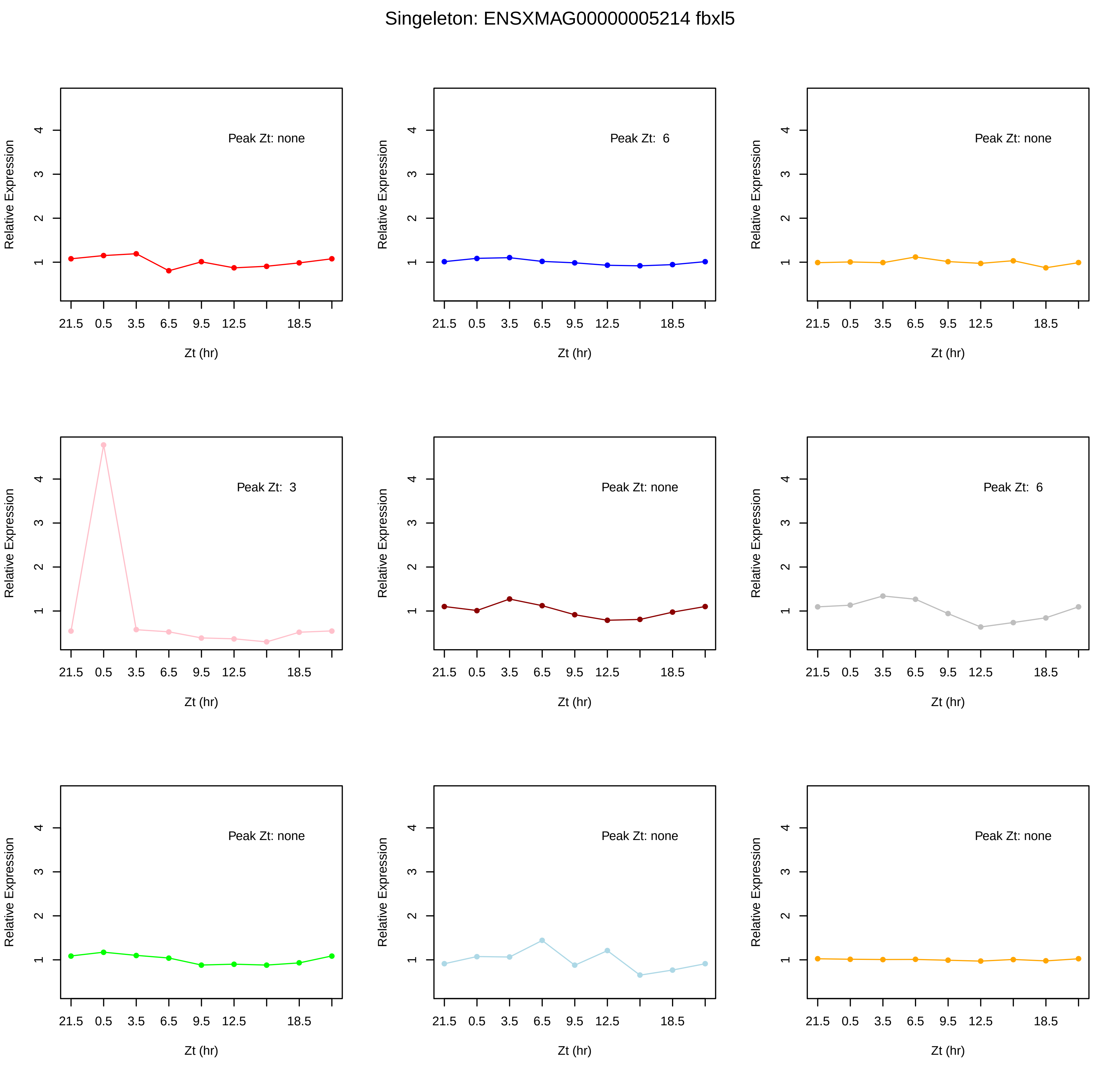
Singeleton: ENSXMAG00000005214 fbxl5
Peak Zt: none
Peak Zt: 6
Peak Zt: none
Peak Zt: 3
Peak Zt: none
Peak Zt: 6
Peak Zt: none
Peak Zt: none
Peak Zt: none
Relative Expression
Zt (hr)
21.5 0.5 3.5 6.5 9.5 12.5 18.5

Singeleton: ENSXMAG00000026292 prune2

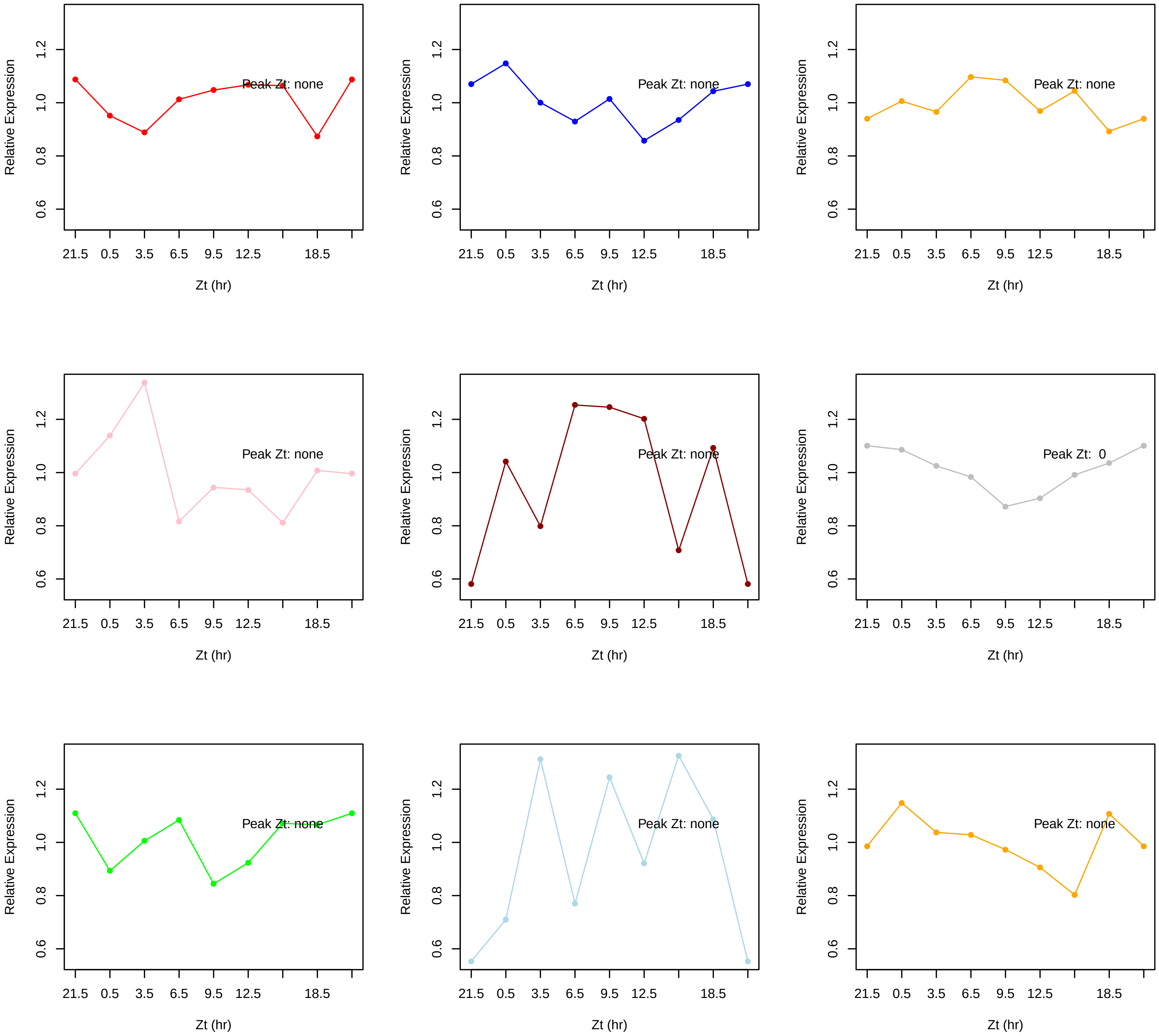

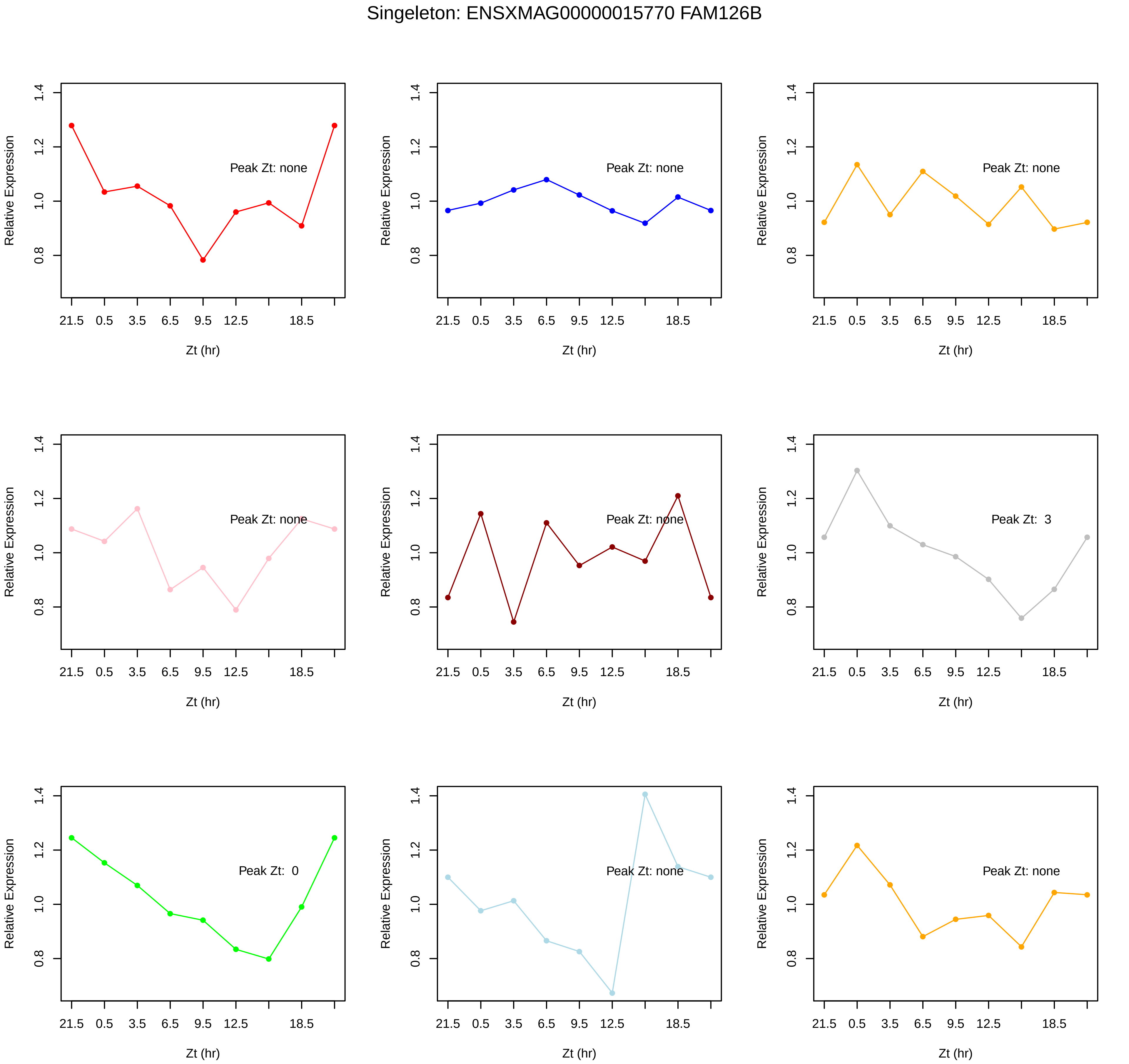
Singeleton: ENSXMAG00000015770 FAM126B
Relative Expression
Zt (hr)
Peak Zt: none
Peak Zt: 3
Peak Zt: 0
21.5 0.5 3.5 6.5 9.5 12.5 18.5
0.8 1.0 1.2 1.4

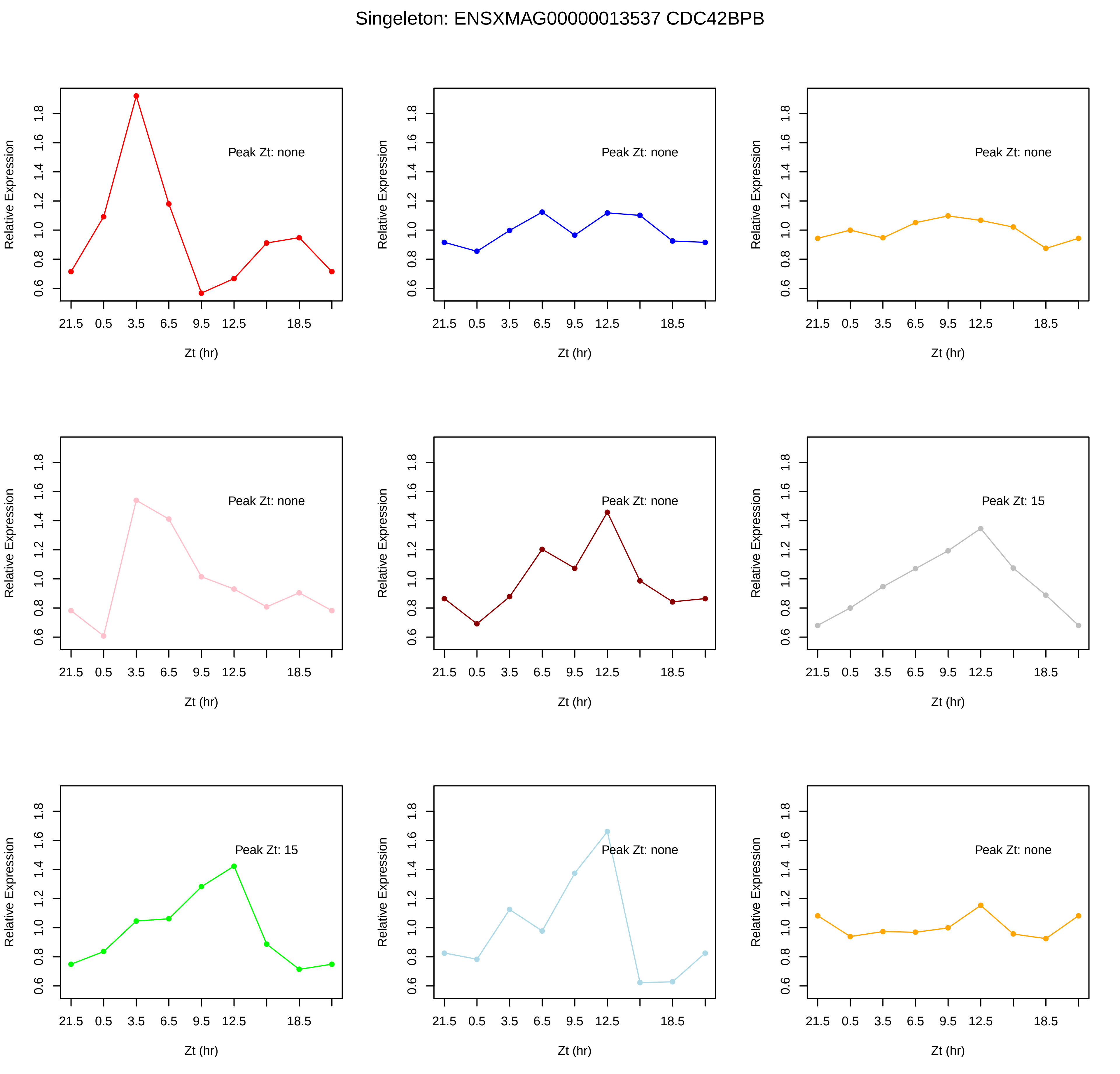

Singeleton: ENSXMAG00000013537 CDC42BPB
Peak Zt: none
Peak Zt: none
Peak Zt: none
Peak Zt: none
Peak Zt: none
Peak Zt: 15
Peak Zt: 15
Peak Zt: none
Peak Zt: none
Relative Expression
Zt (hr)
21.5 0.5 3.5 6.5 9.5 12.5 18.5
0.6 0.8 1.0 1.2 1.4 1.6 1.8

Singleton: ENSXMAG00000004446 cyfip2

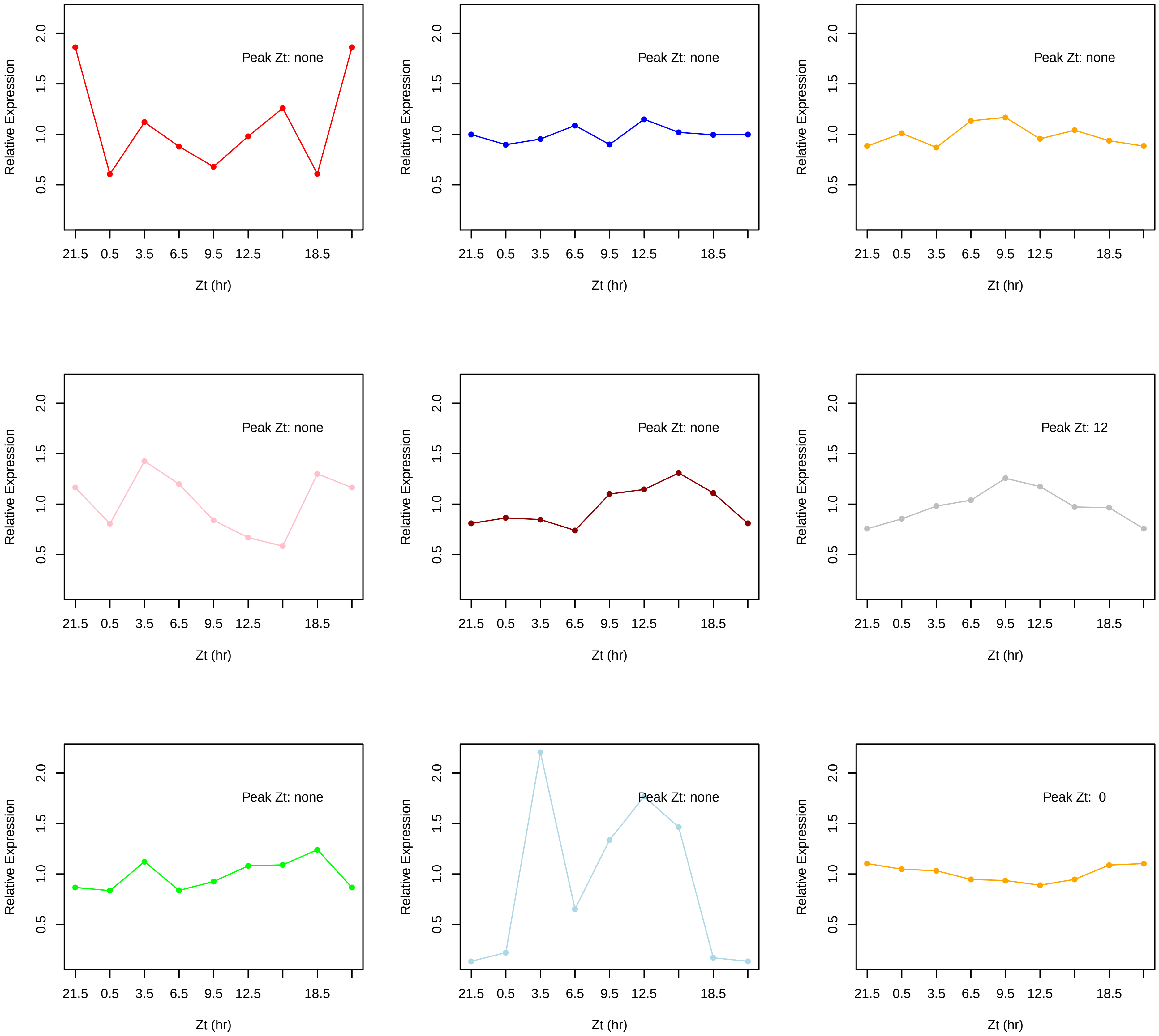

Singleton: ENSXMAG00000003683 mtdha

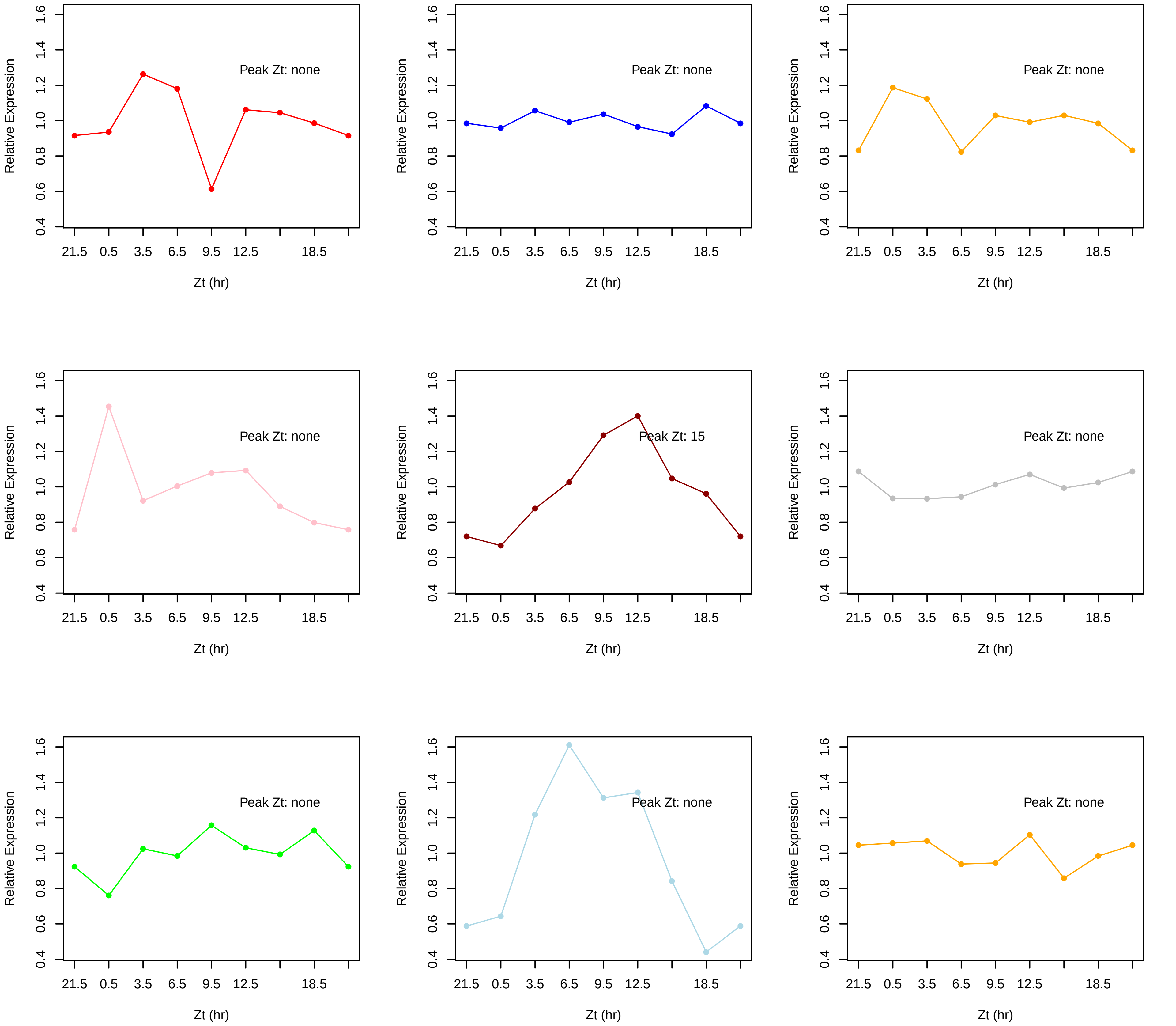

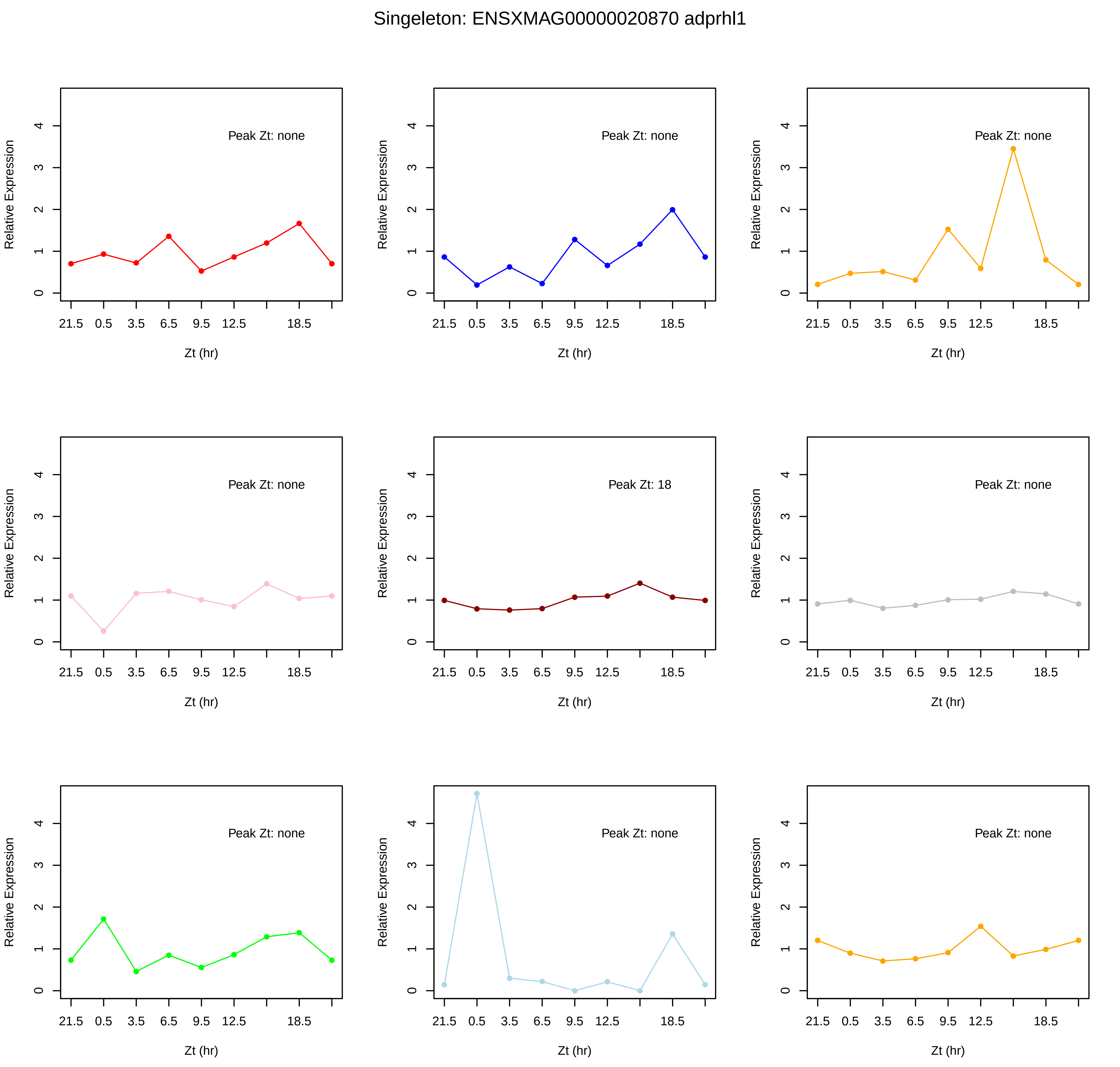

Singeleton: ENSXMAG00000020870 adprhl1
Peak Zt: none
Peak Zt: none
Peak Zt: none
Peak Zt: none
Peak Zt: 18
Peak Zt: none
Peak Zt: none
Peak Zt: none
Peak Zt: none
Relative Expression
Zt (hr)
21.5
0.5
3.5
6.5
9.5
12.5
18.5

Singleton: ENSXMAG00000003661 rrm2b

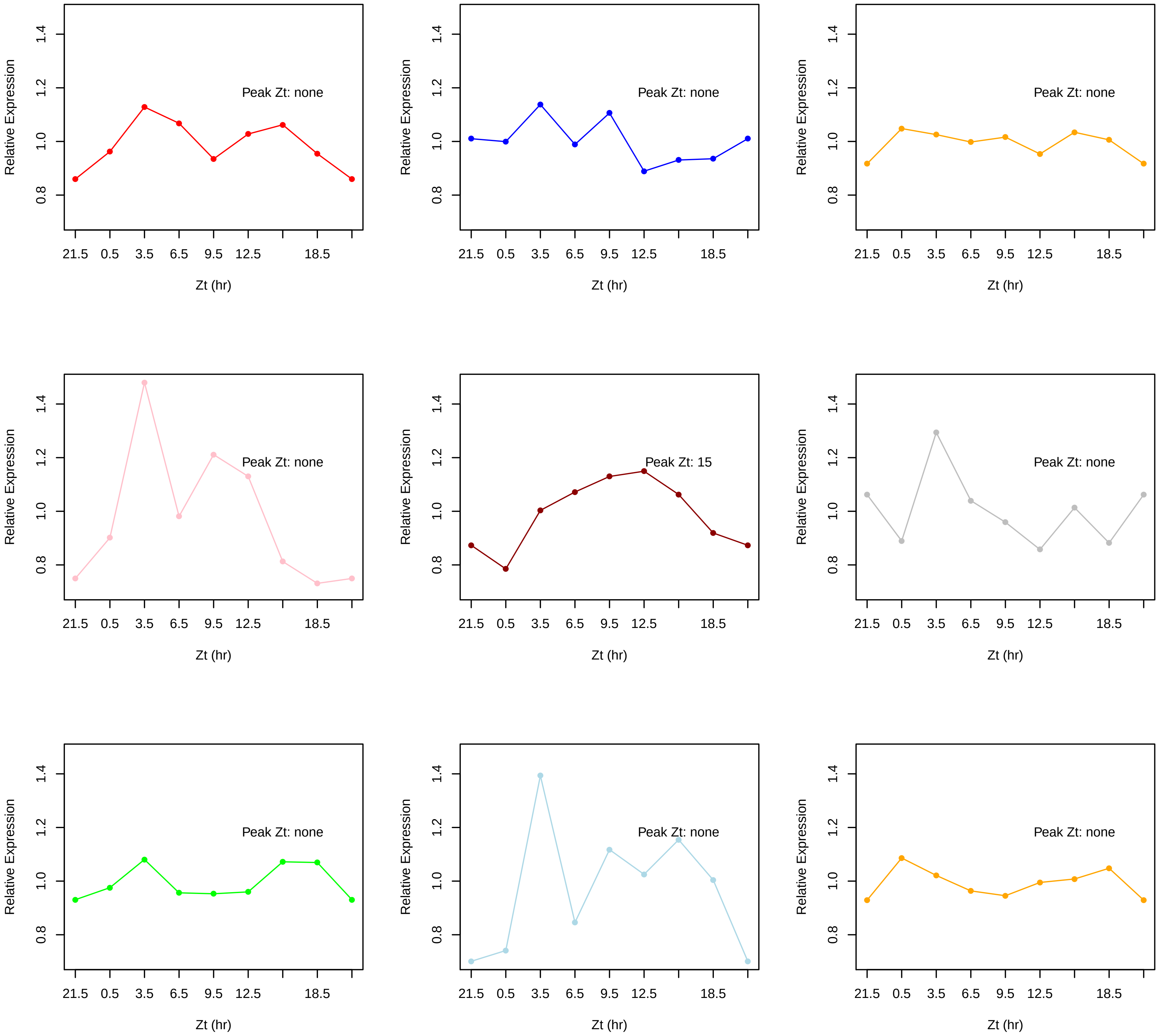

Singleton: ENSXMAG00000016486 cep78

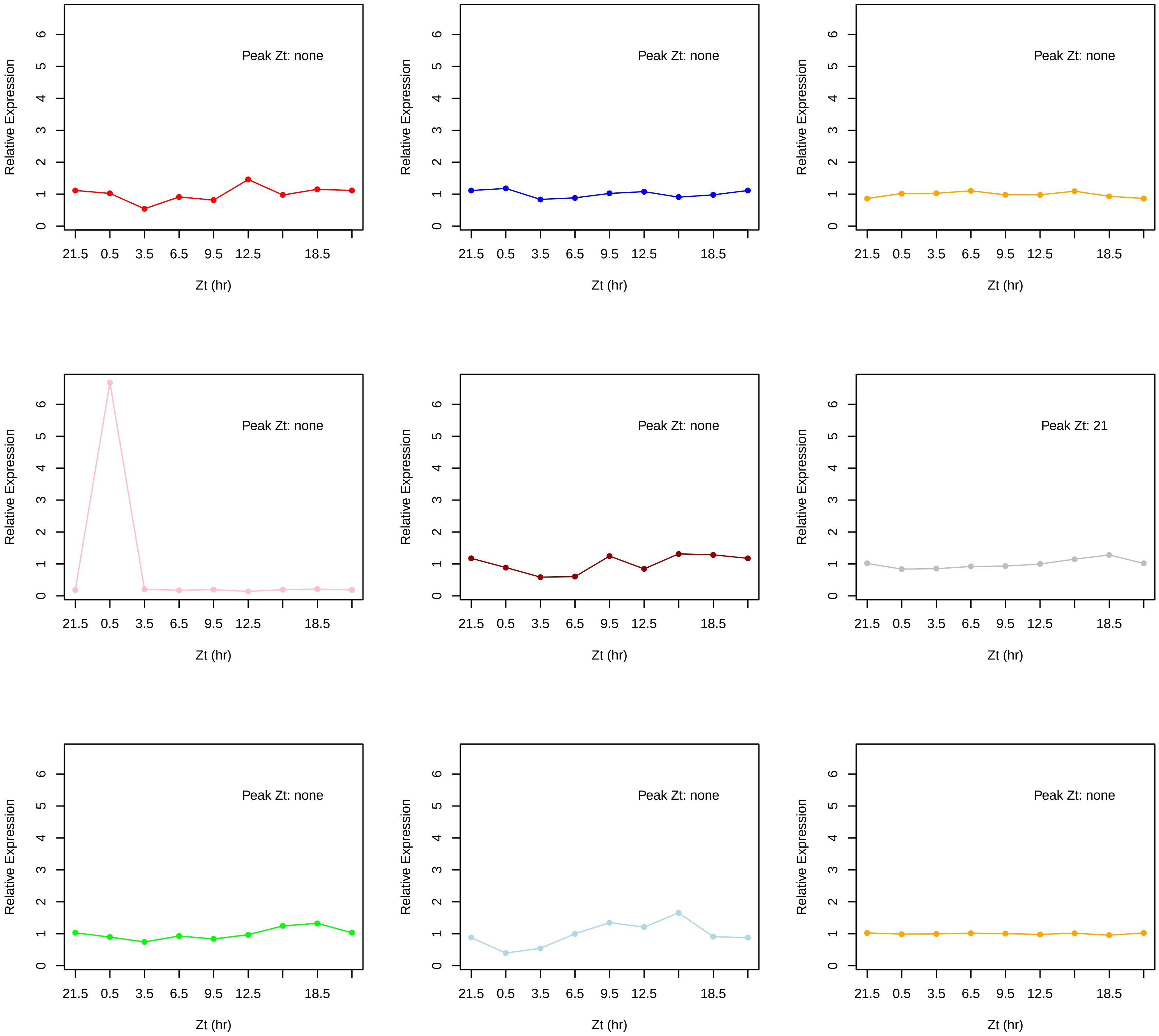

Singleton: ENSXMAG00000002128 cpeb2

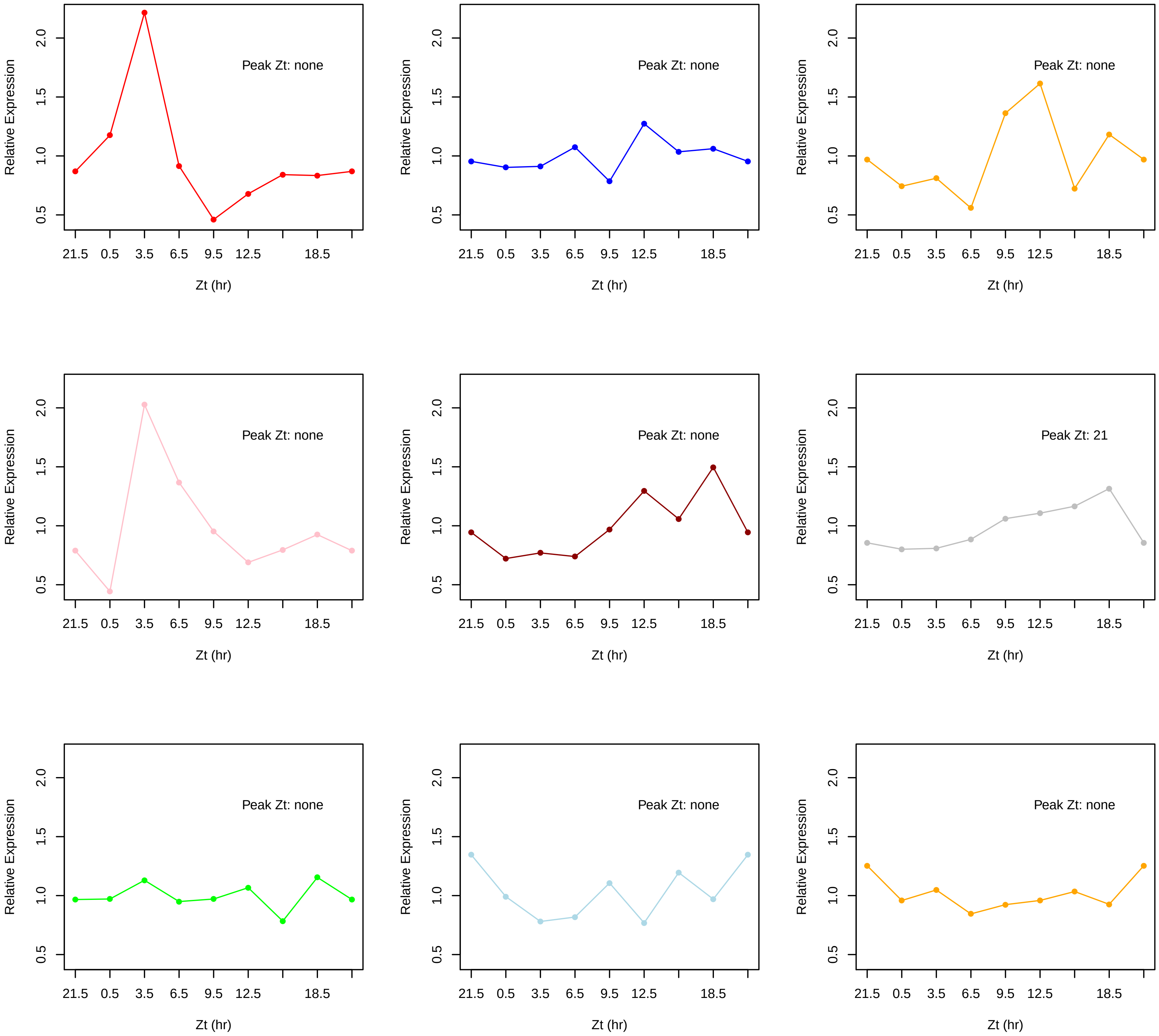

Singeleton: ENSXMAG00000016253 dip2a

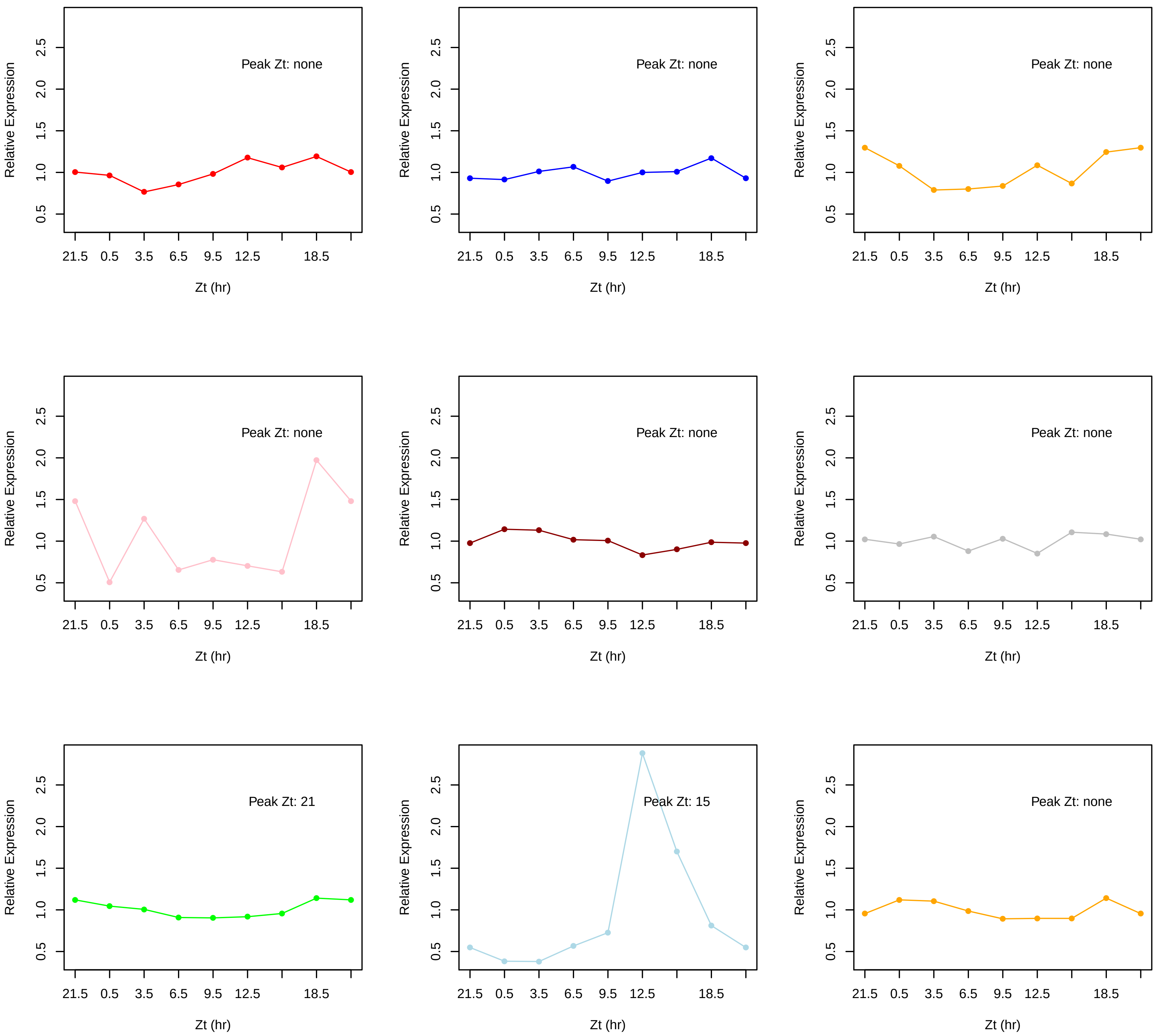

Singleton: ENSXMAG00000025924 eif5

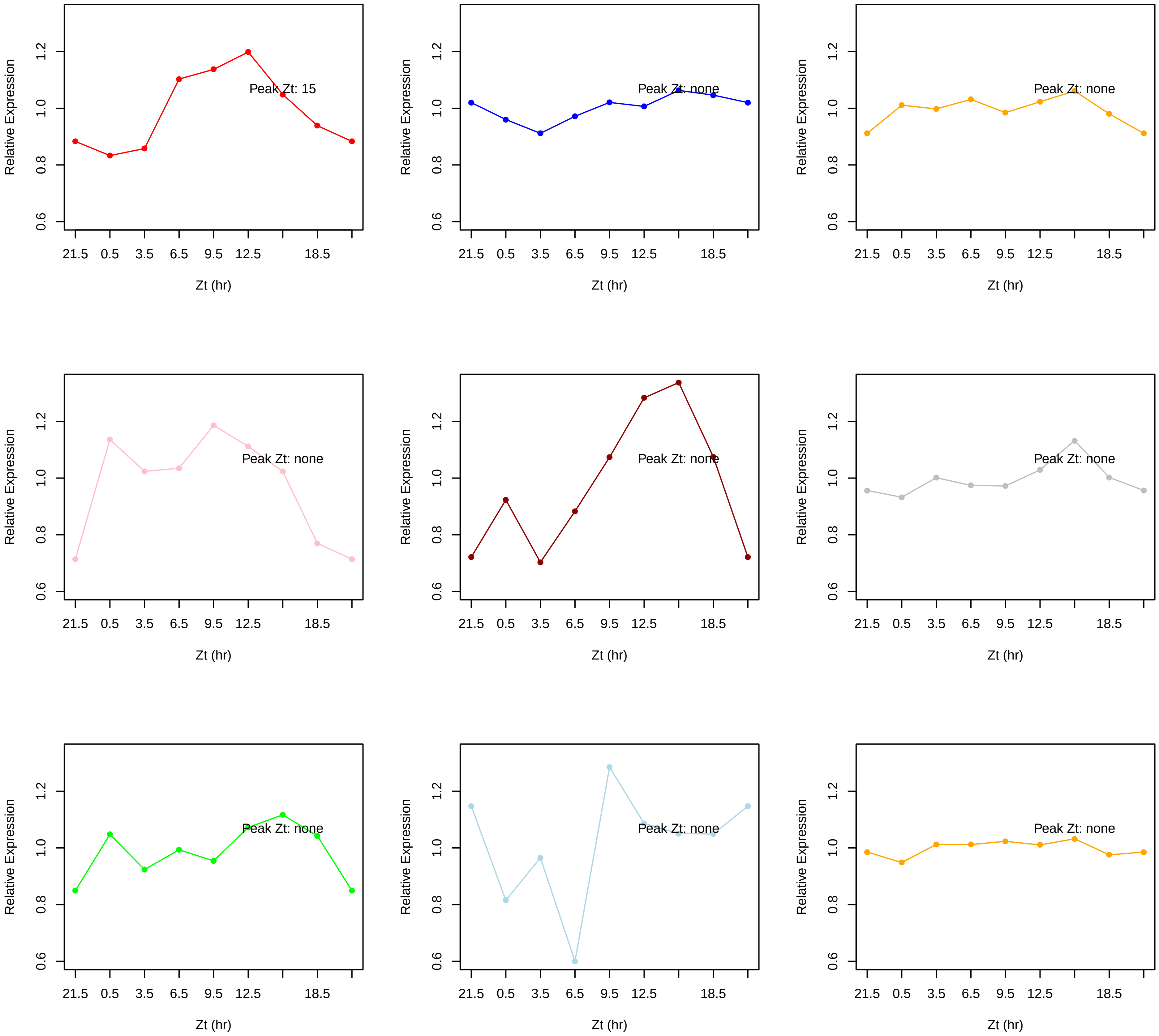

Singleton: ENSXMAG00000010512 hmmr

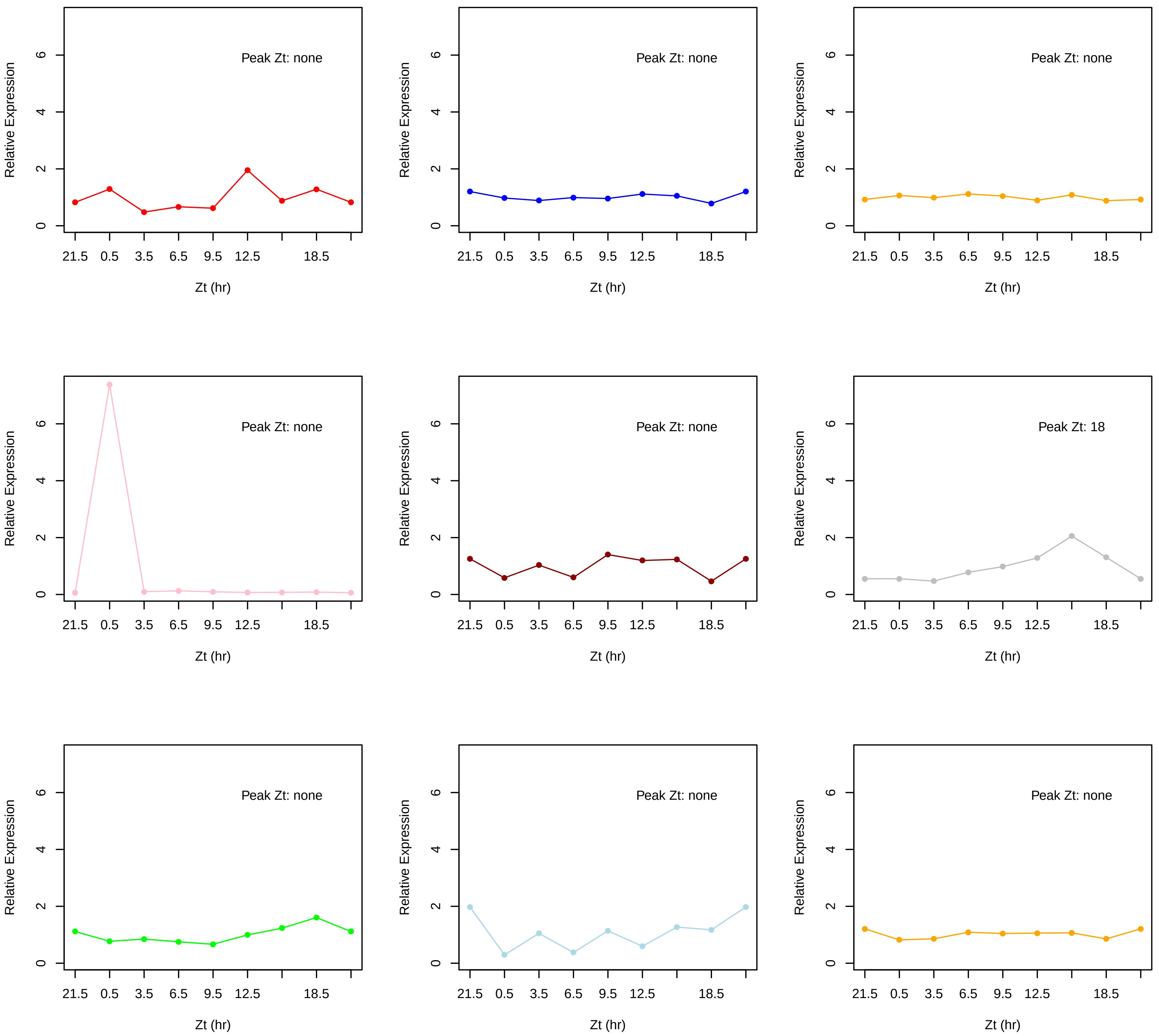

Singleton: ENSXMAG00000002786 psat1

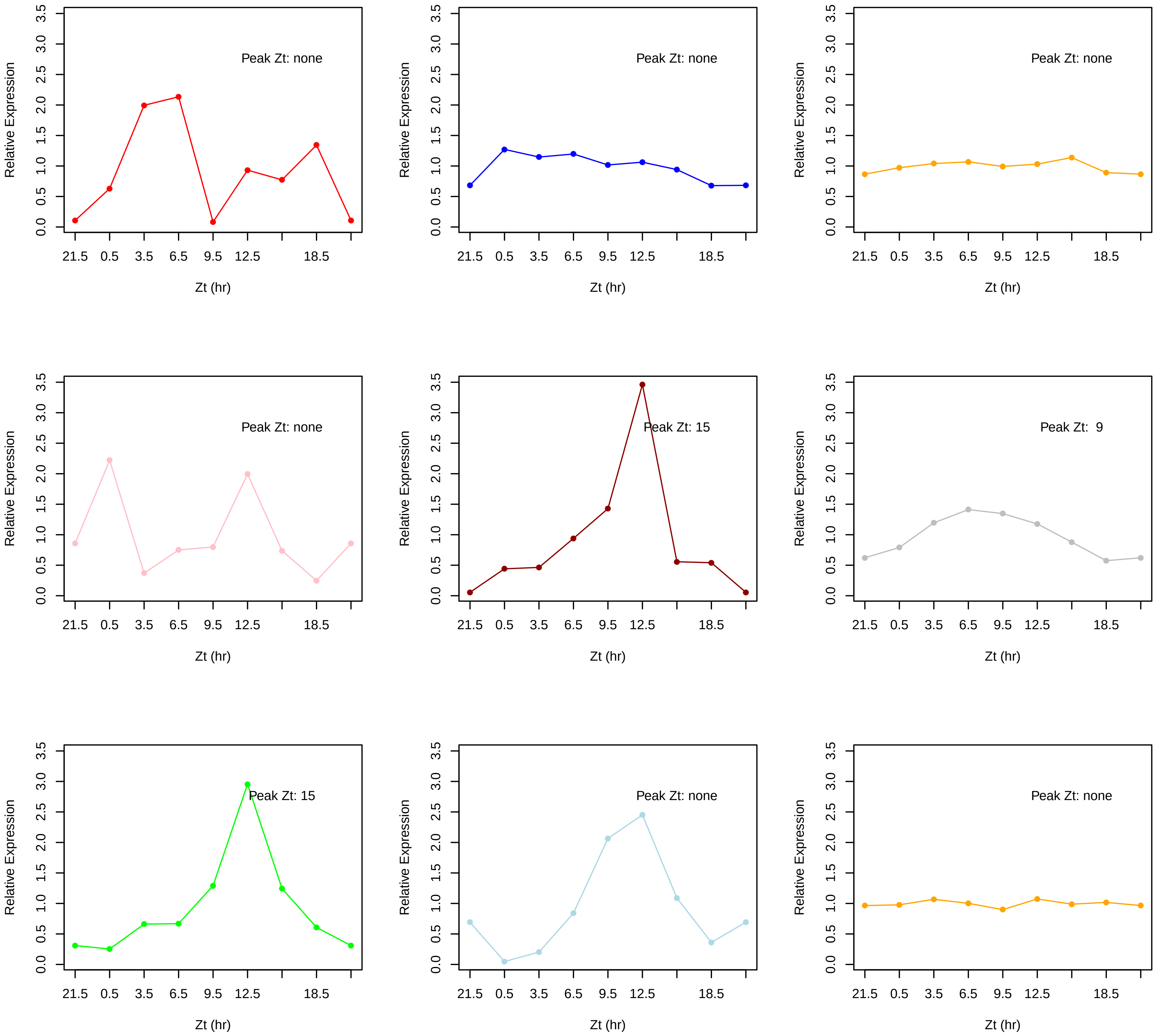

Singleton: ENSXMAG00000019274 tmco3

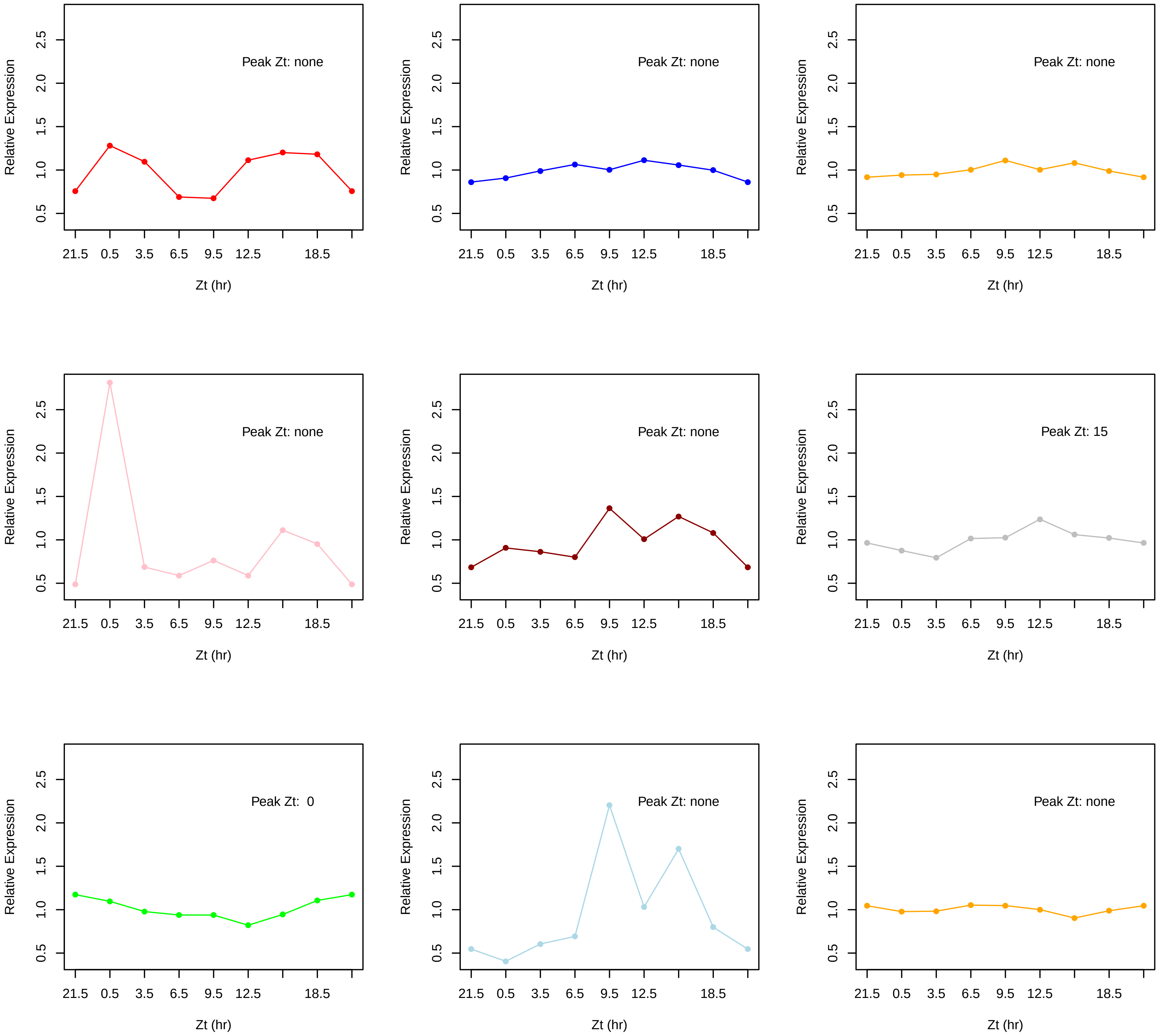

Singeleton: ENSXMAG00000013988 frem1b

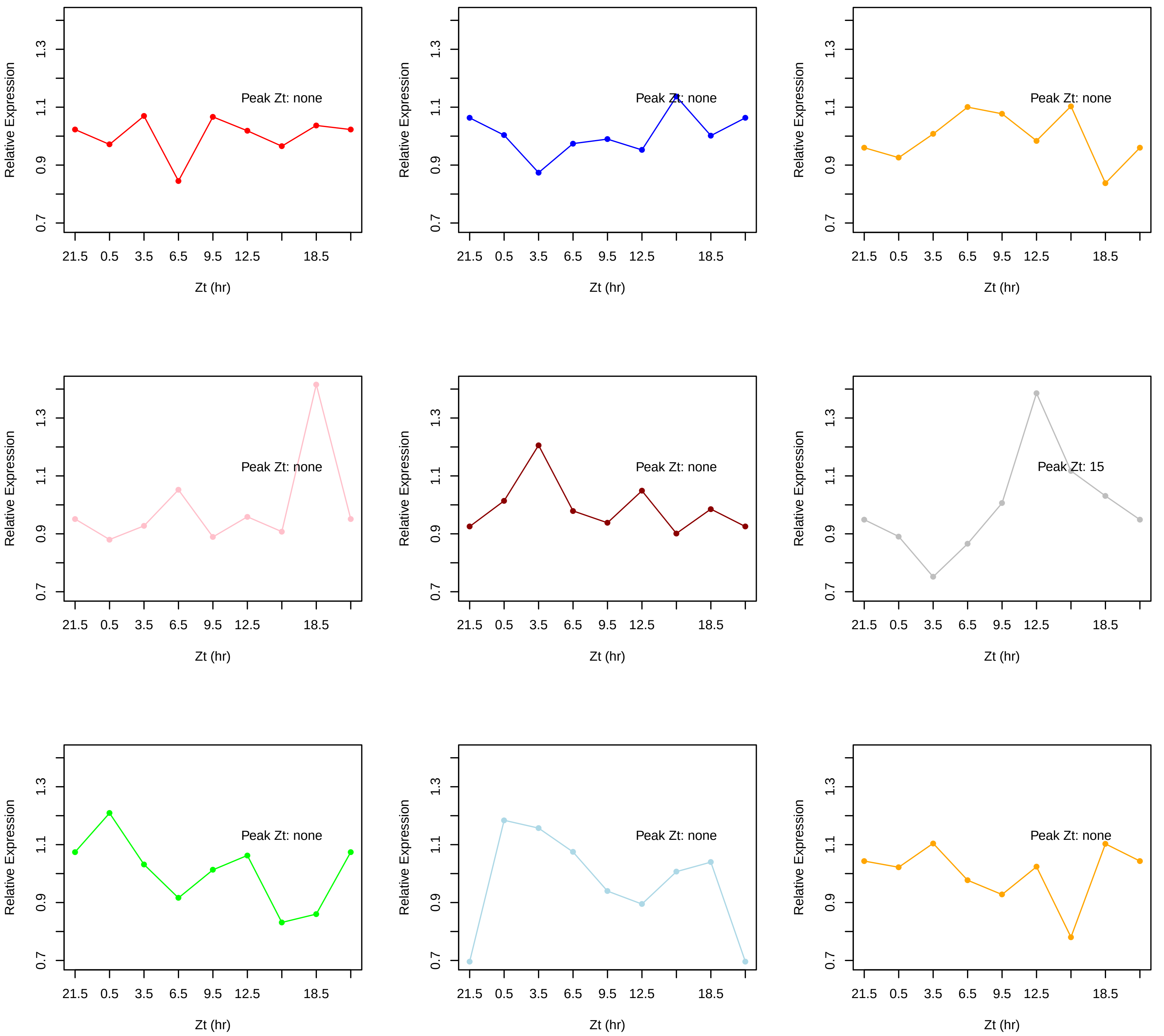

Singeleton: ENSXMAG00000005896 cntn3b

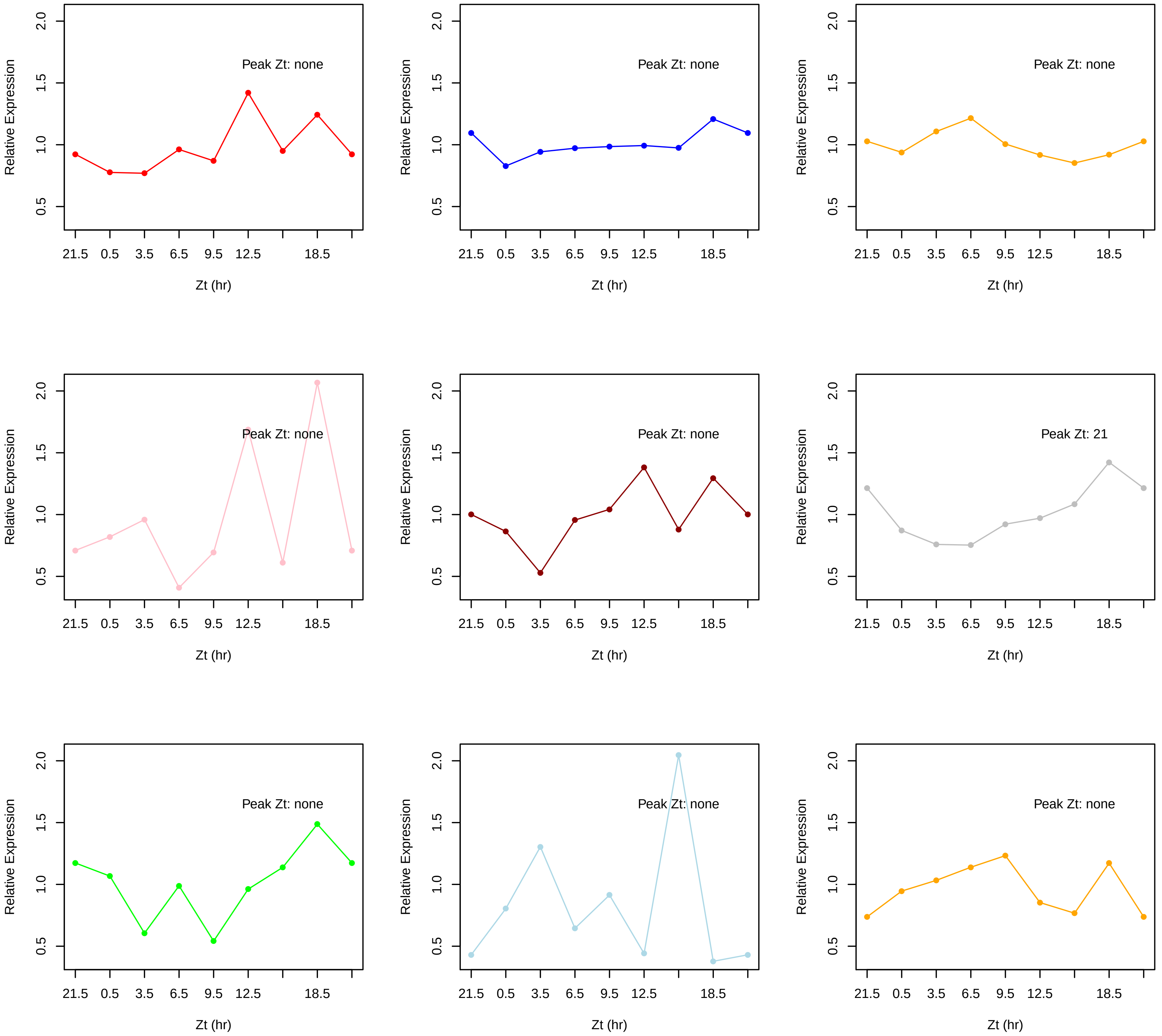

Singeleton: ENSXMAG00000006694 ckbb

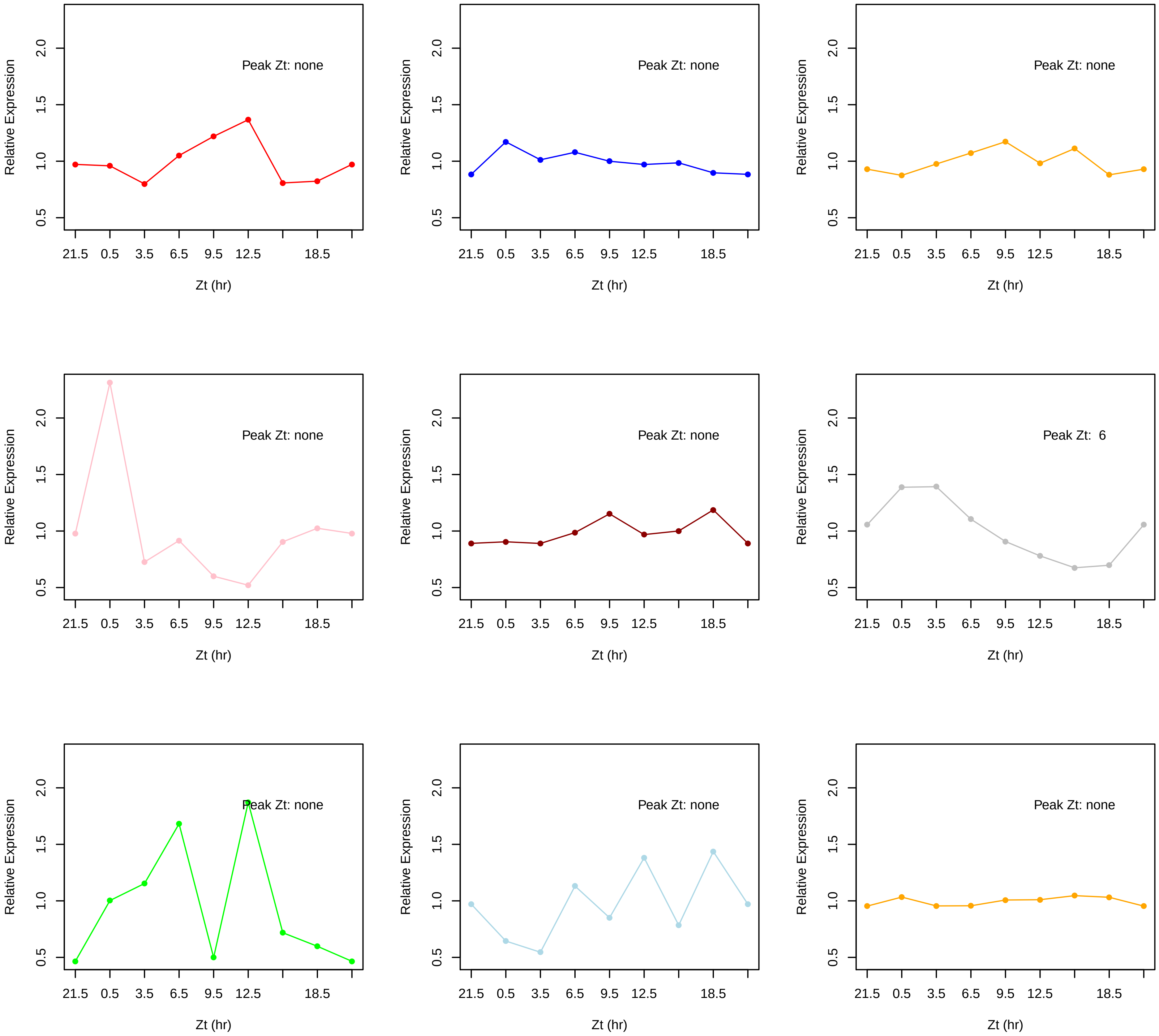

Singeleton: ENSXMAG00000002092

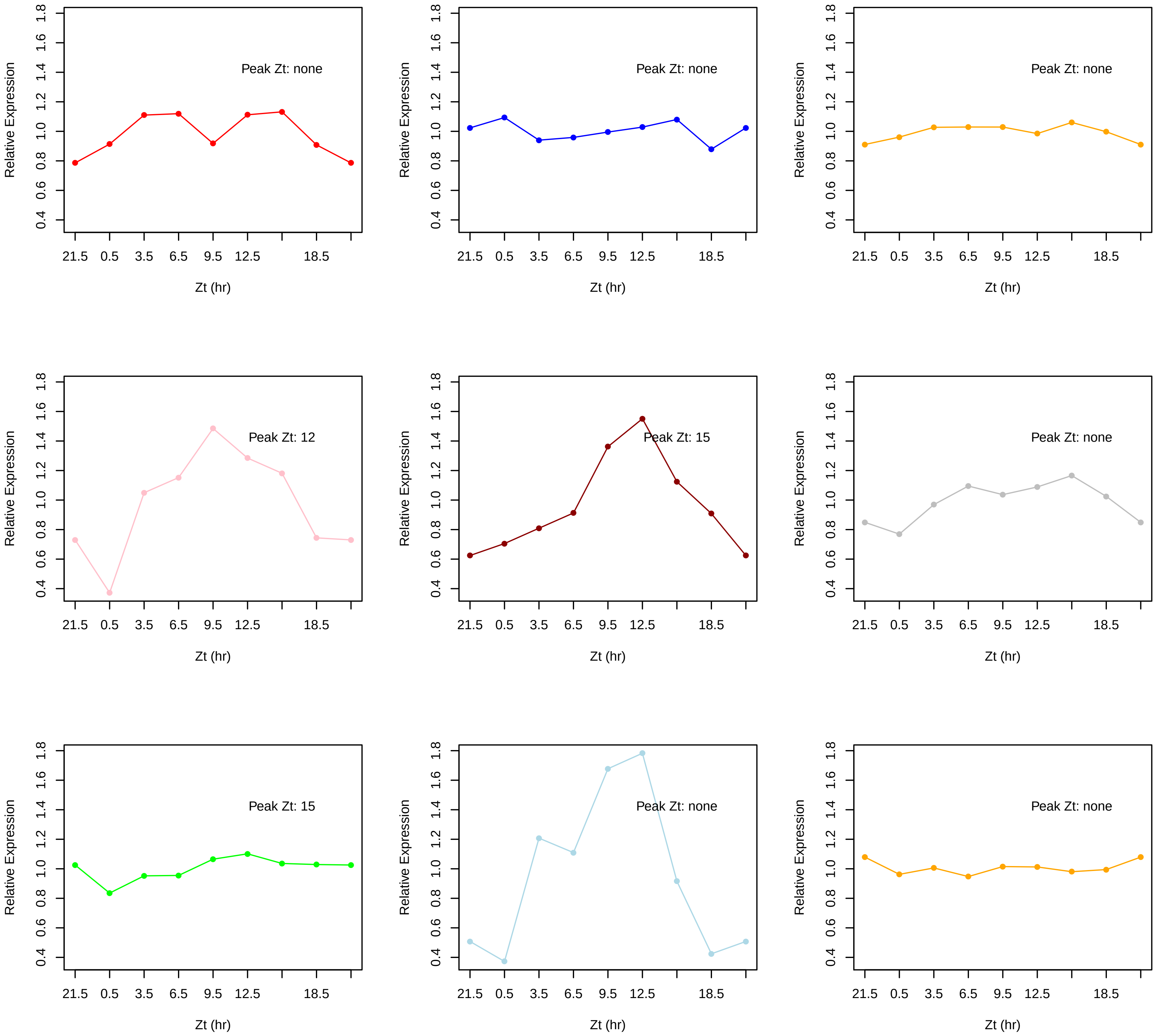

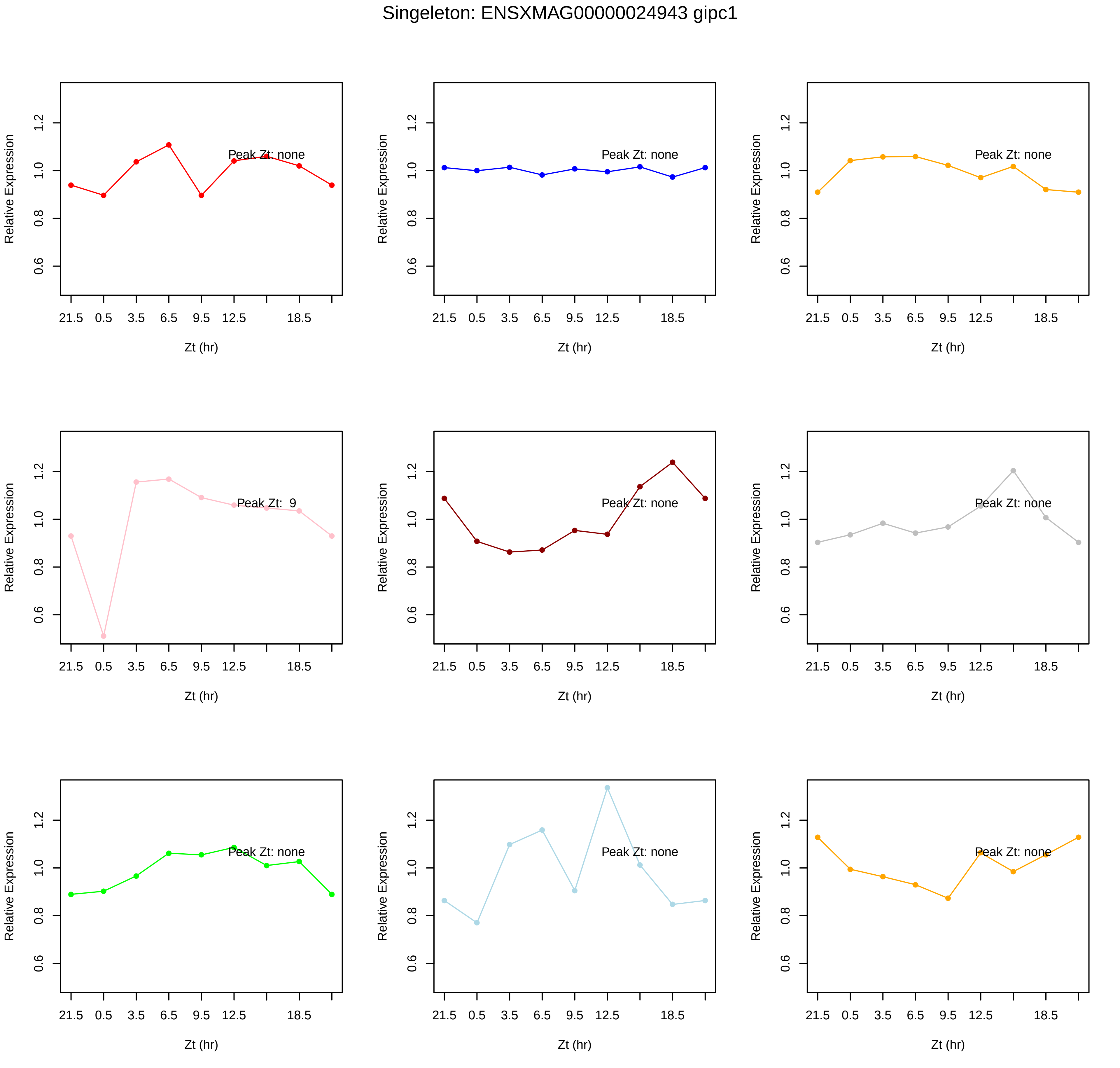
Singleton: ENSXMAG00000024943 gipc1
Relative Expression
Zt (hr)
21.5
0.5
3.5
6.5
9.5
12.5
18.5
0.6
0.8
1.0
1.2
Peak Zt: none
Peak Zt: 9

Singeleton: ENSXMAG00000022708 tm2d1

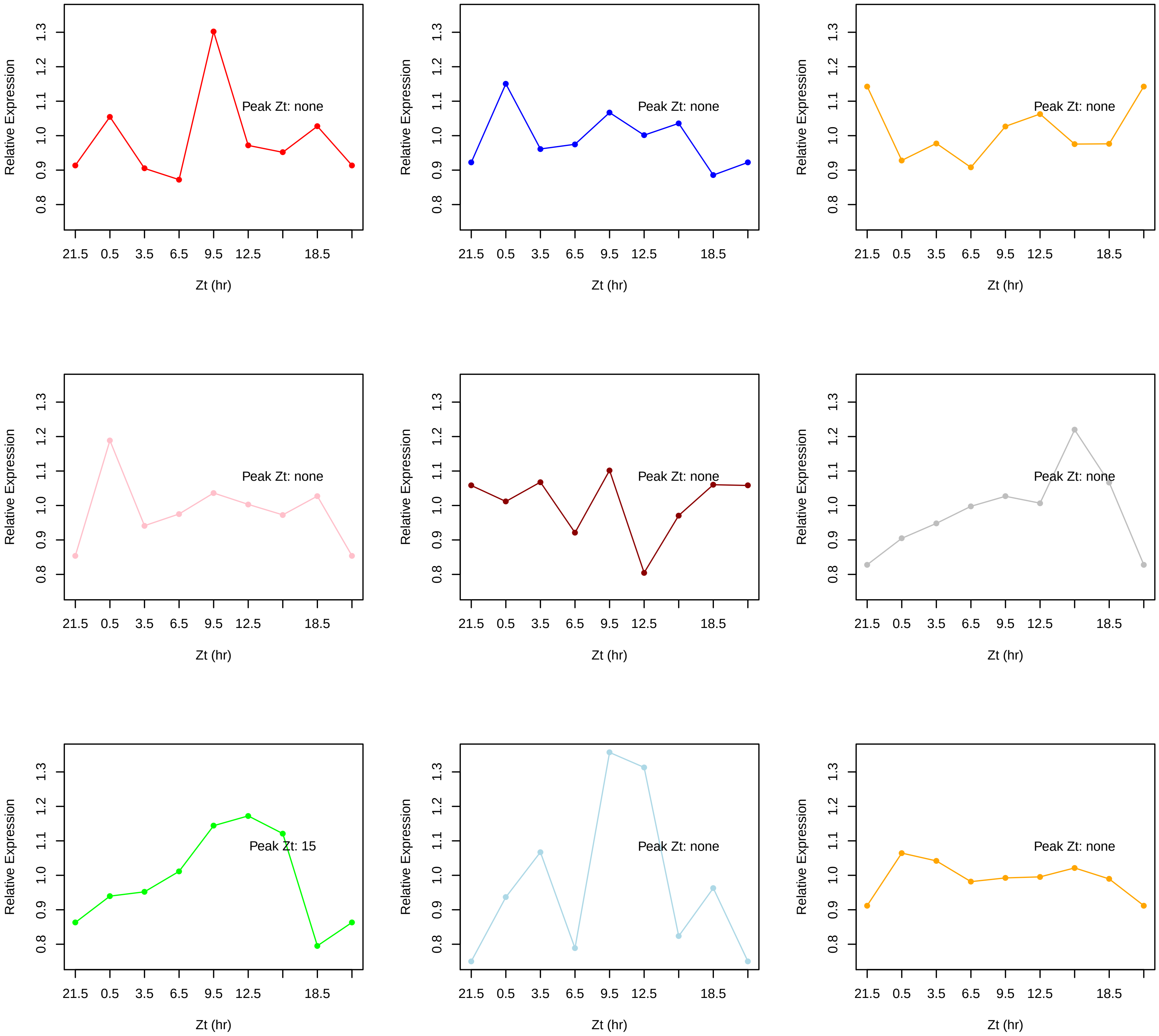

Singeleton: ENSXMAG00000018455 klc1a

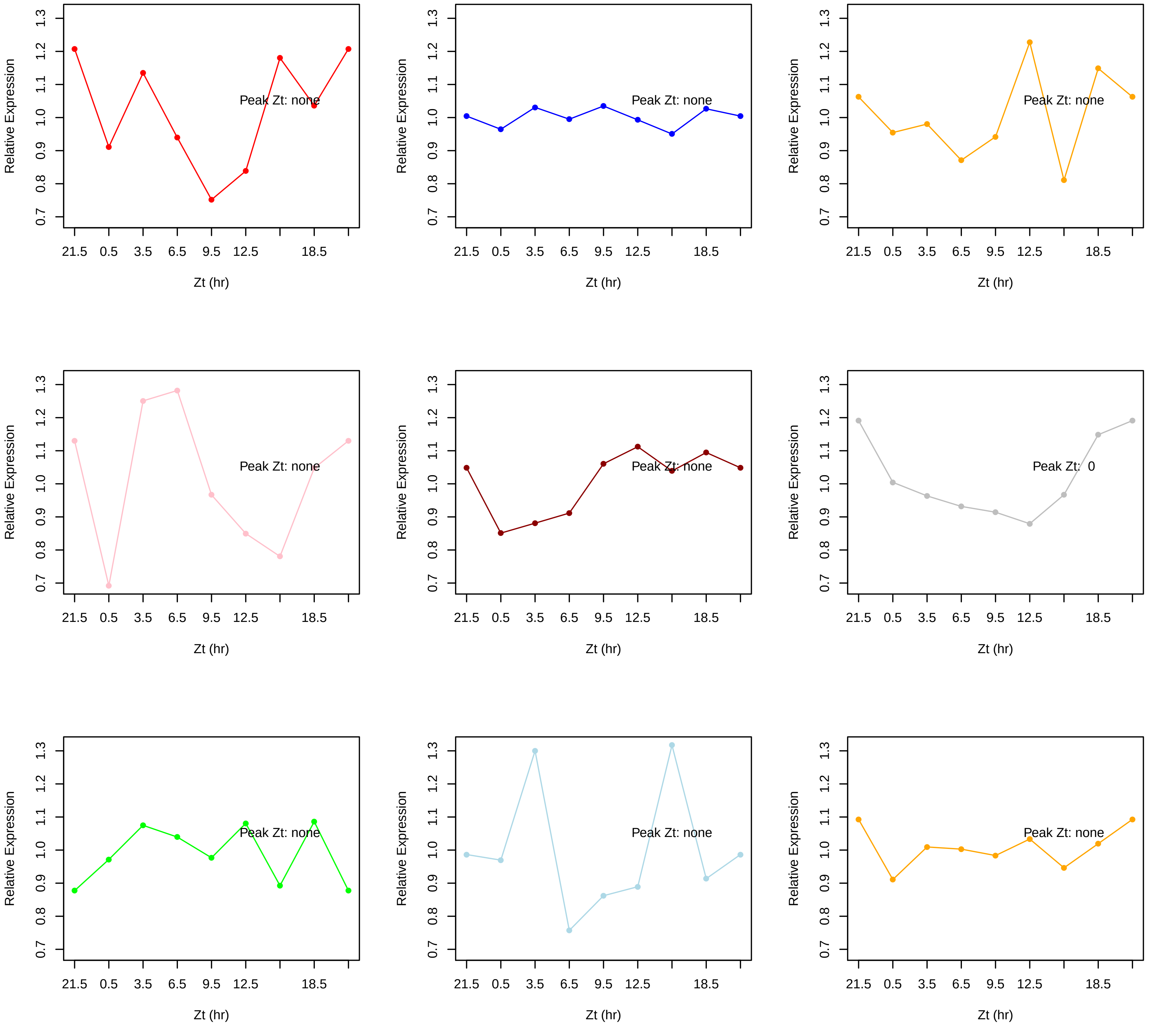

Singeleton: ENSXMAG00000002385 atp6v1c1a

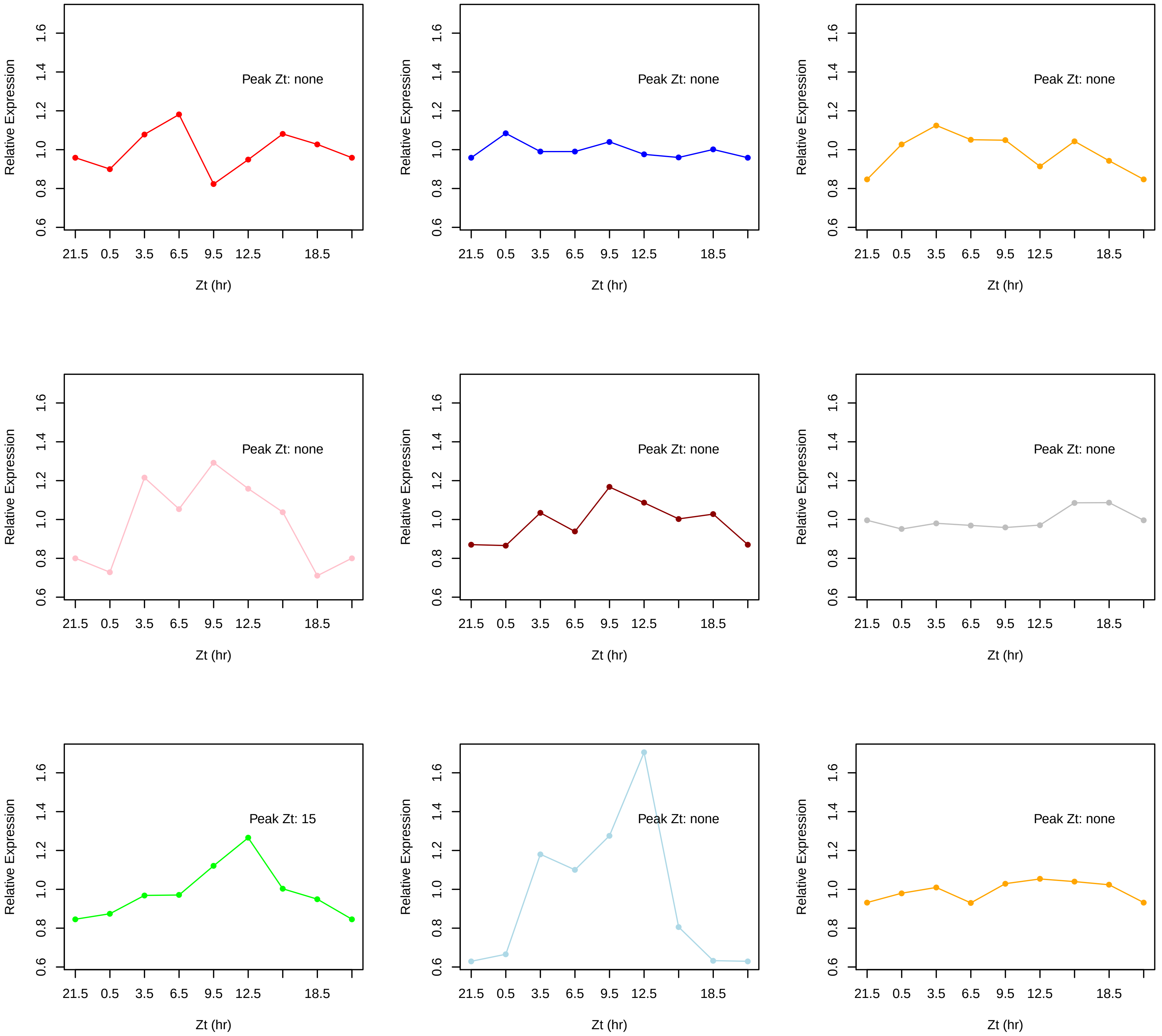

Singeleton: ENSXMAG00000007538 xrcc3

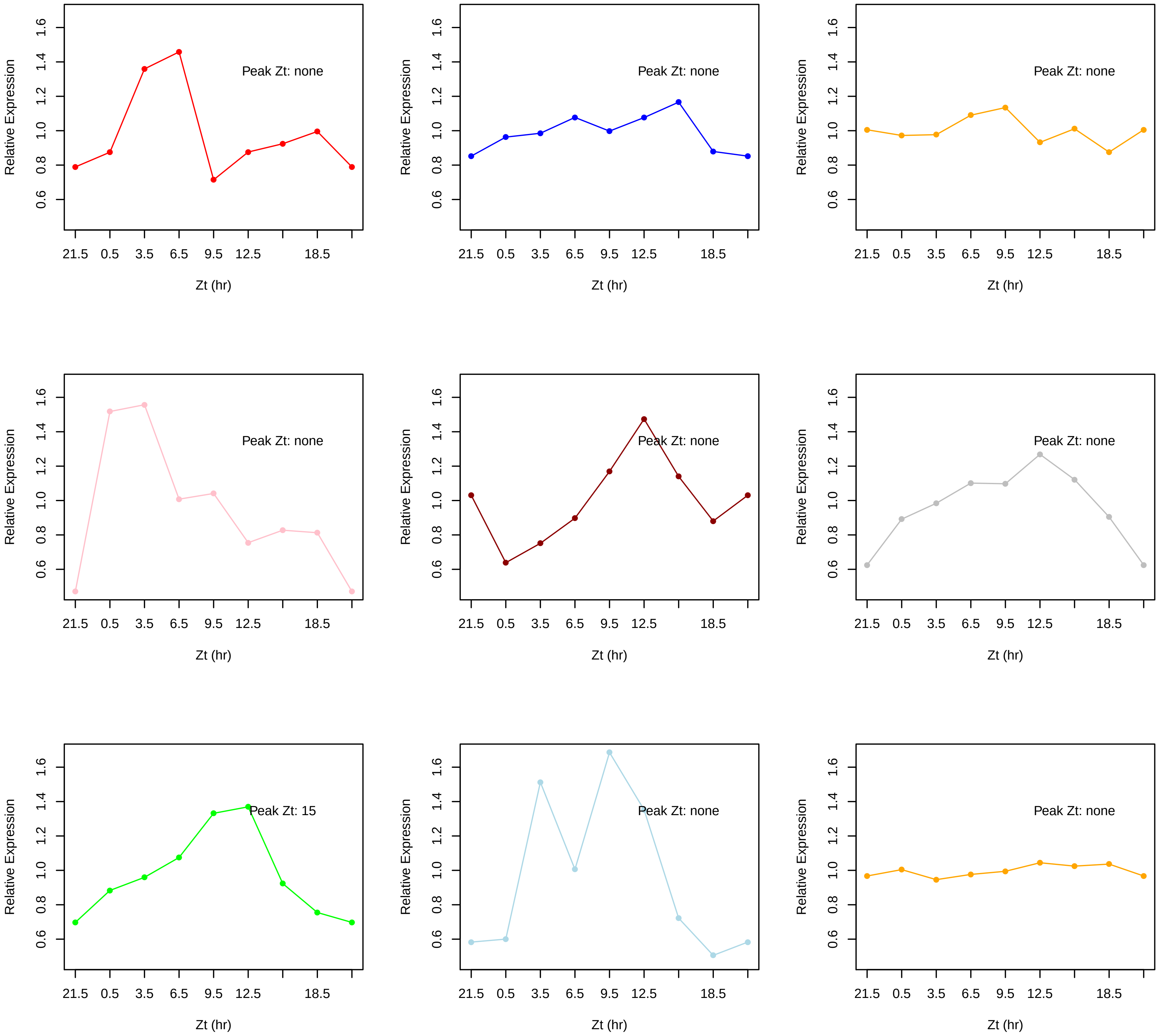

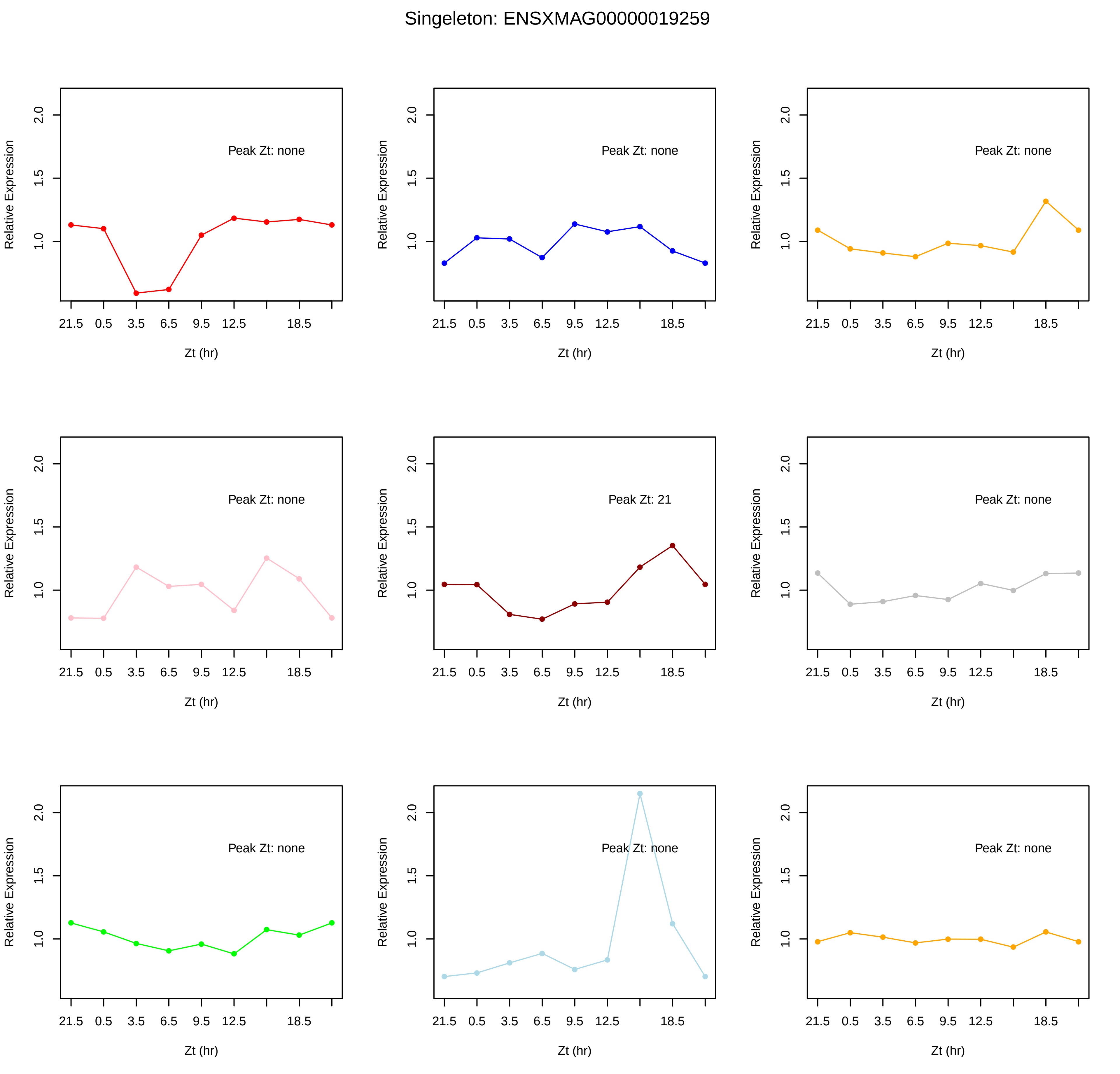

Singeleton: ENSXMAG00000019259
Peak Zt: none
Peak Zt: none
Peak Zt: none
Peak Zt: none
Peak Zt: 21
Peak Zt: none
Peak Zt: none
Peak Zt: none
Peak Zt: none
Relative Expression
Zt (hr)

Singeleton: ENSXMAG00000026106 tmem107

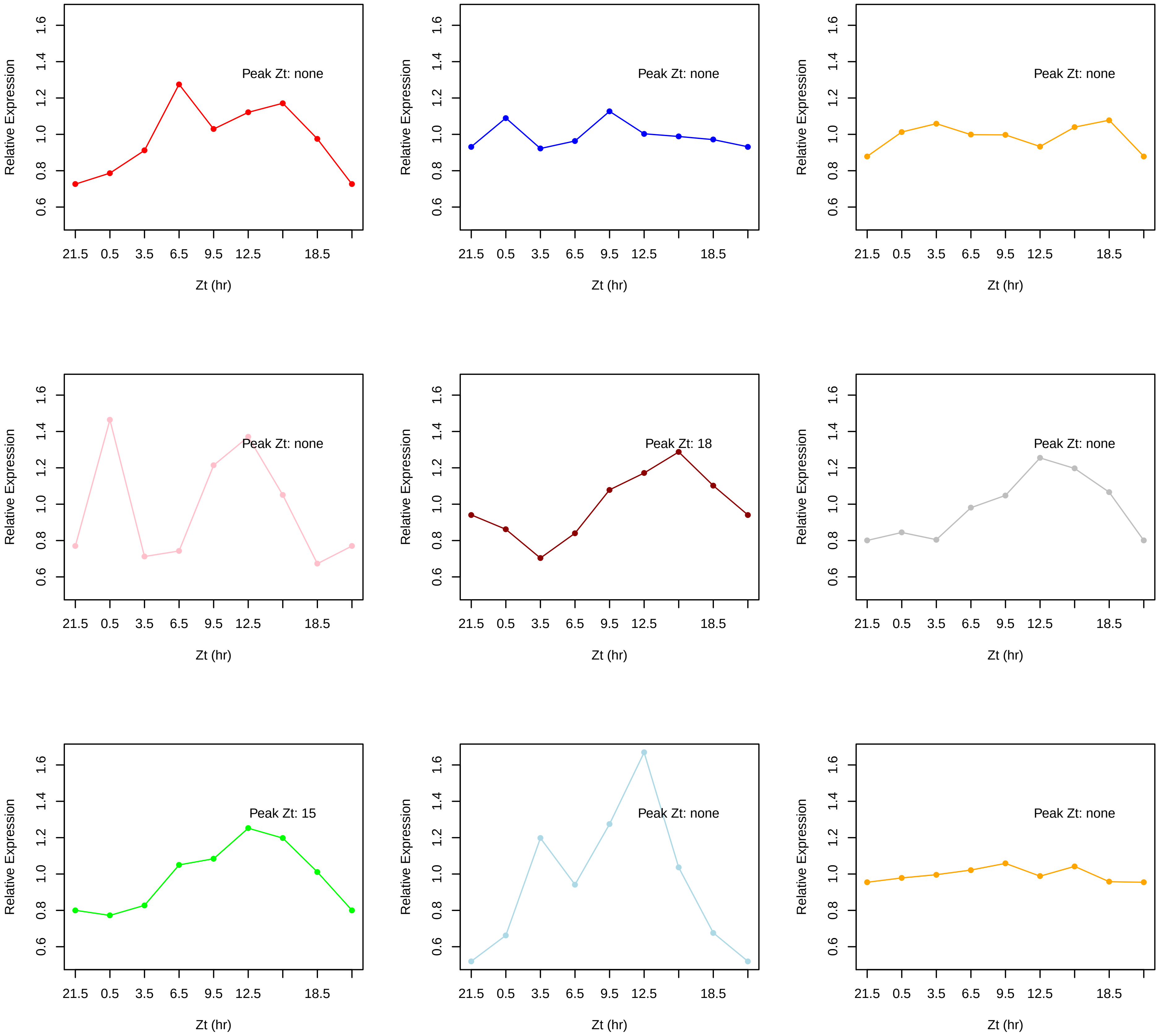

Singeleton: ENSXMAG00000025110 zfyve21

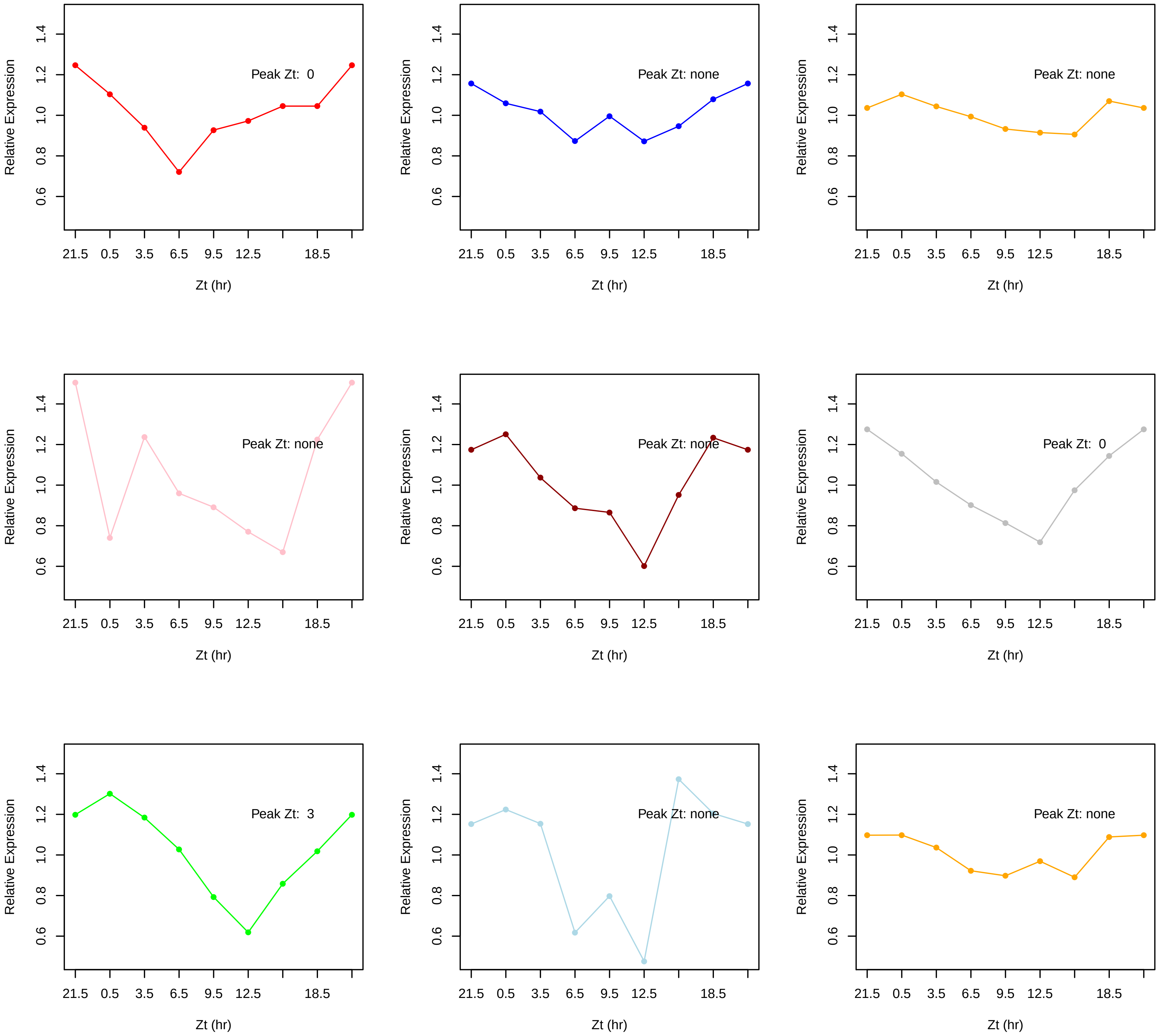

Singleton: ENSXMAG00000028872 rps10

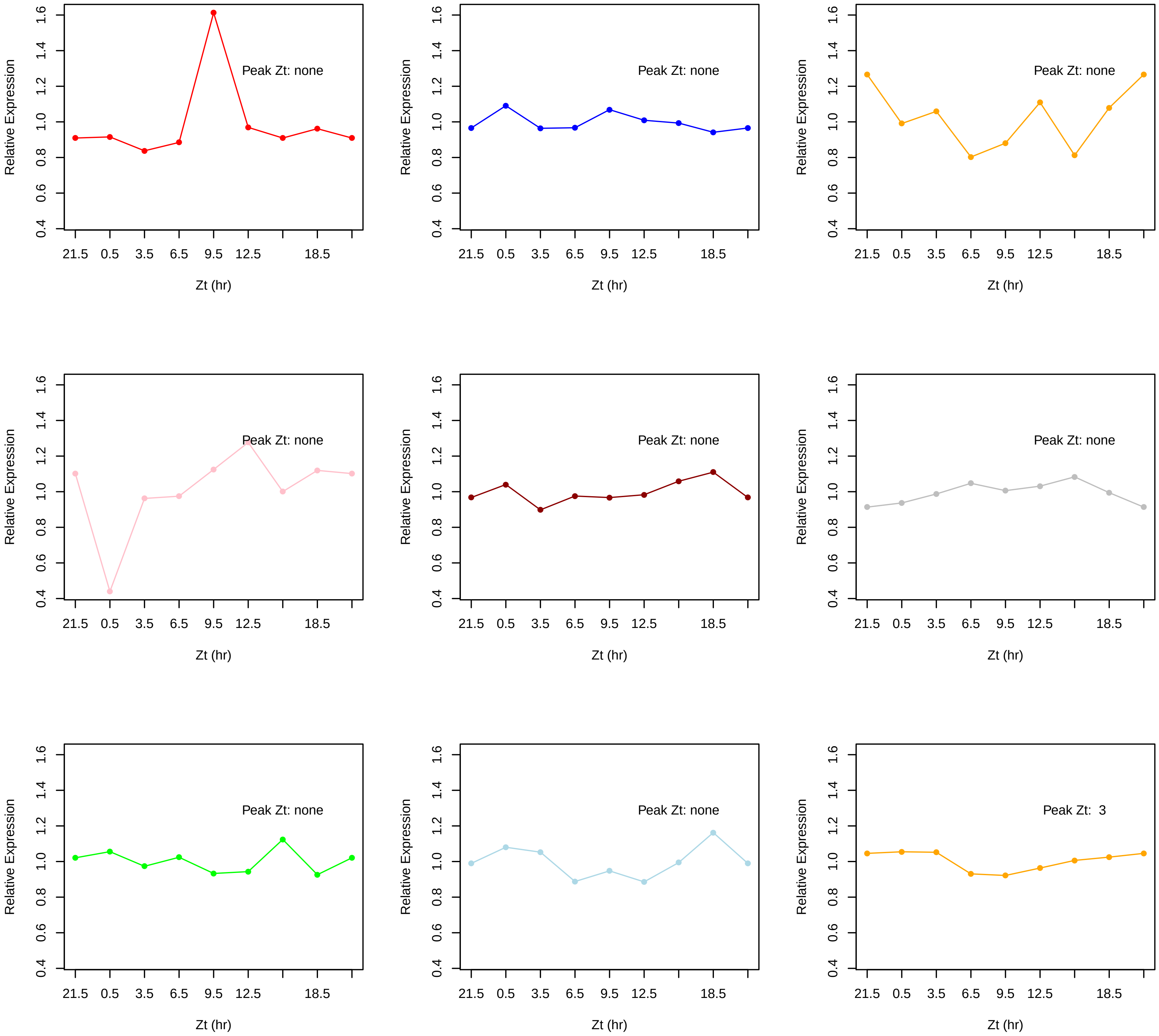

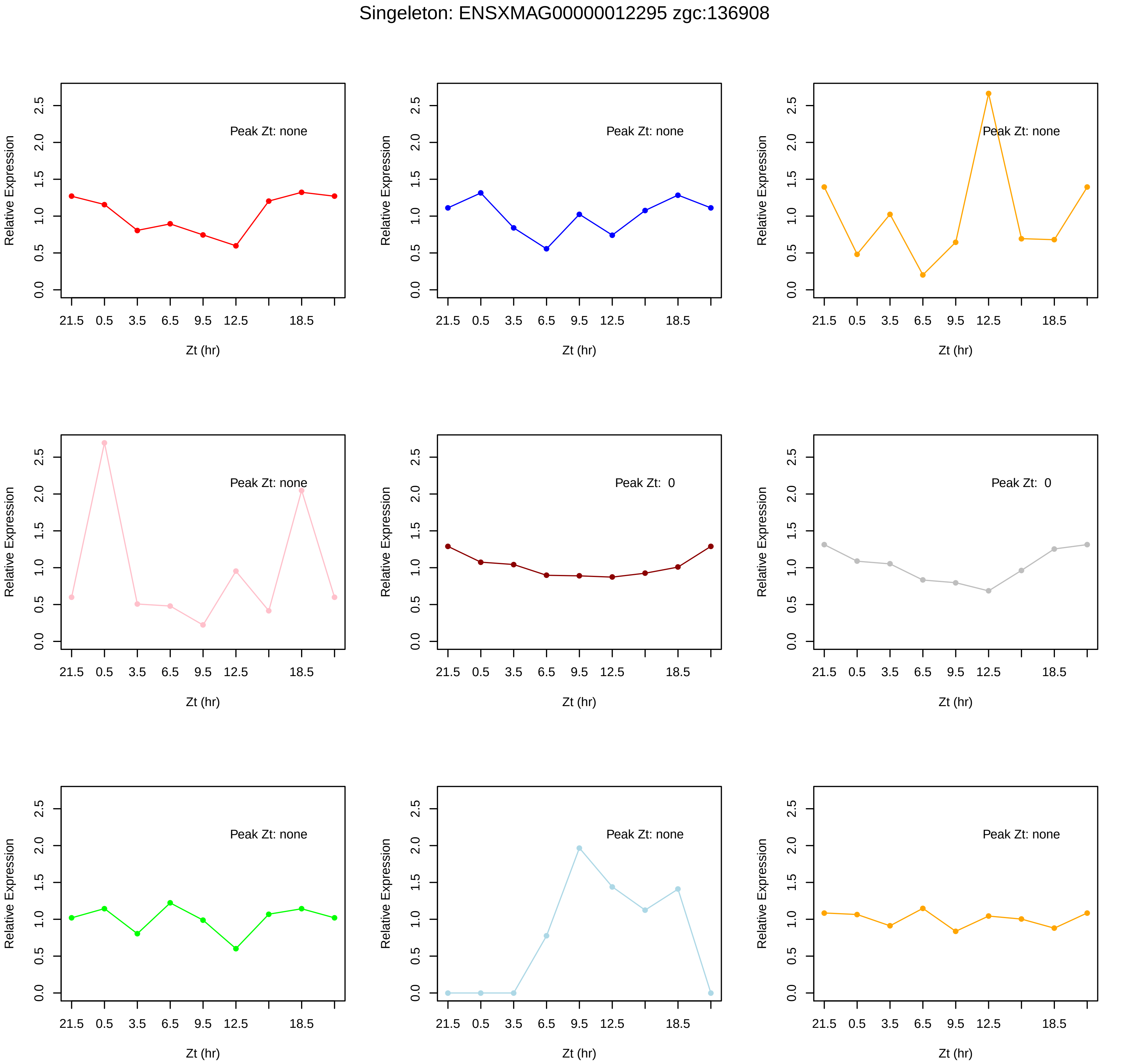
Singeleton: ENSXMAG00000012295 zgc:136908
Peak Zt: none
Peak Zt: none
Peak Zt: none
Peak Zt: none
Peak Zt: 0
Peak Zt: 0
Peak Zt: none
Peak Zt: none
Peak Zt: none
Relative Expression
Zt (hr)
21.5 0.5 3.5 6.5 9.5 12.5 18.5
0.0 0.5 1.0 1.5 2.0 2.5

Singeleton: ENSXMAG00000025238 tal1

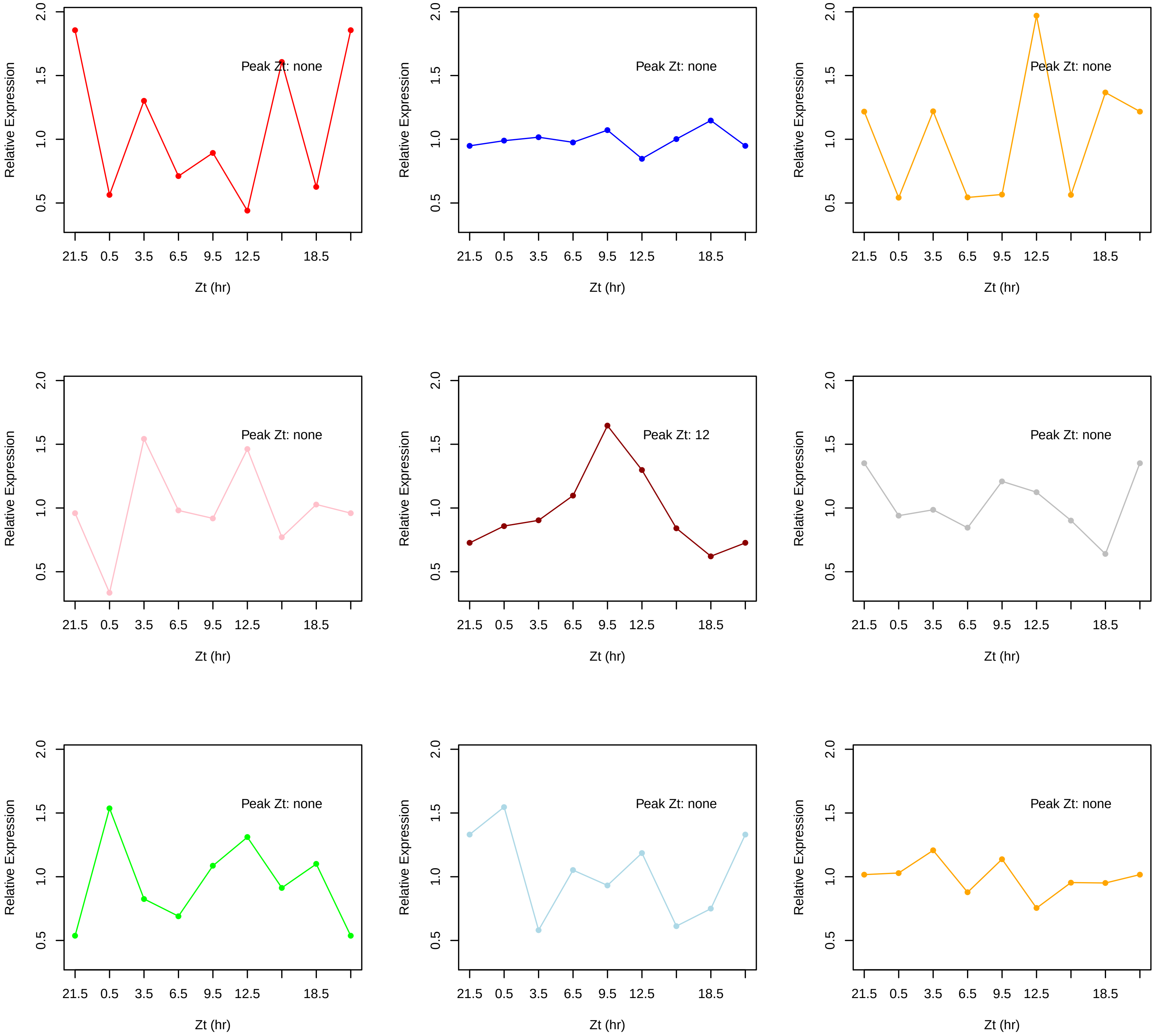

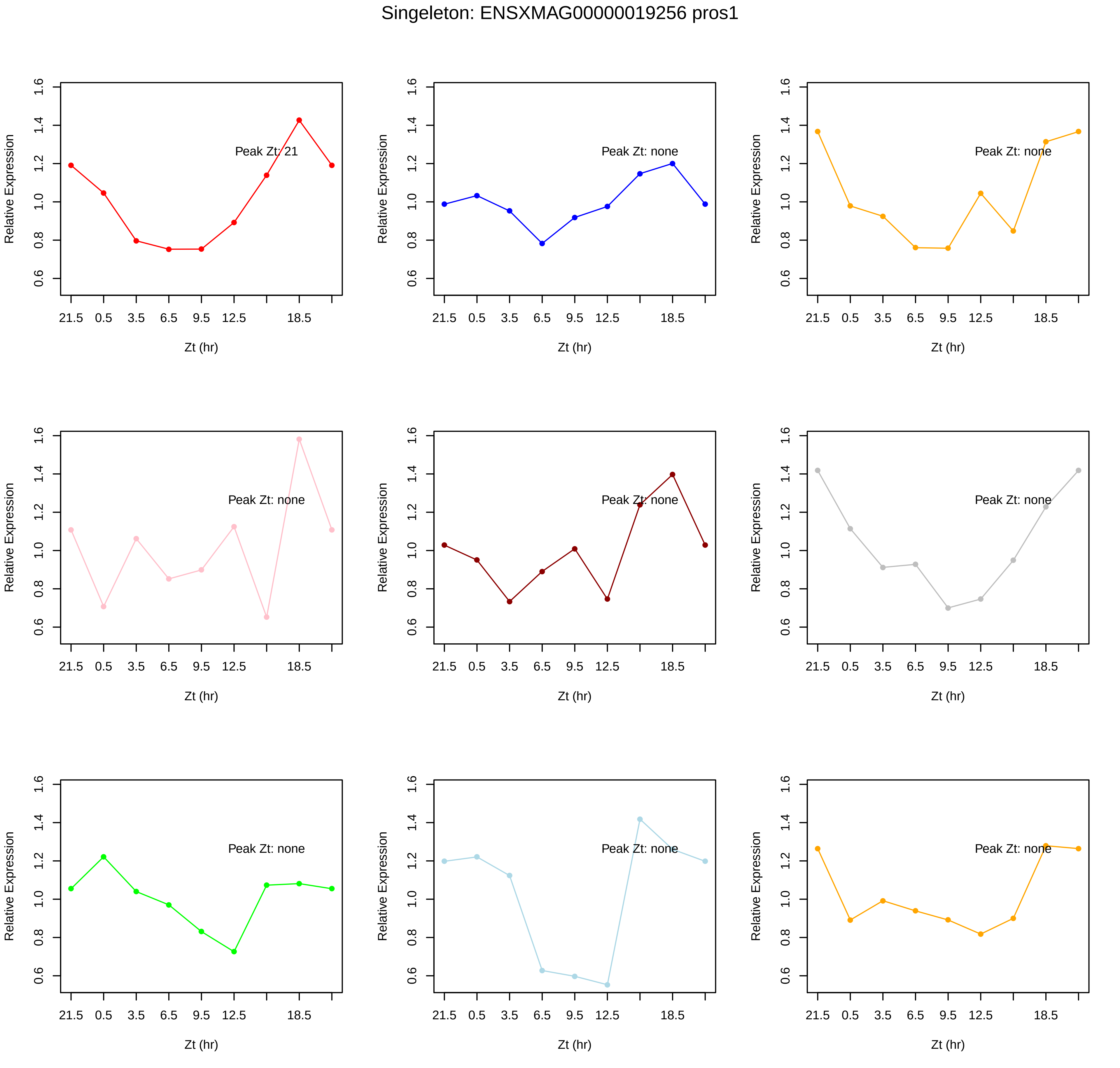
Singeleton: ENSXMAG00000019256 pros1
Peak Zt: 21
Peak Zt: none
Relative Expression
Zt (hr)
21.5
0.5
3.5
6.5
9.5
12.5
18.5
0.6
0.8
1.0
1.2
1.4
1.6

Singeleton: ENSXMAG00000021595 zbtb49

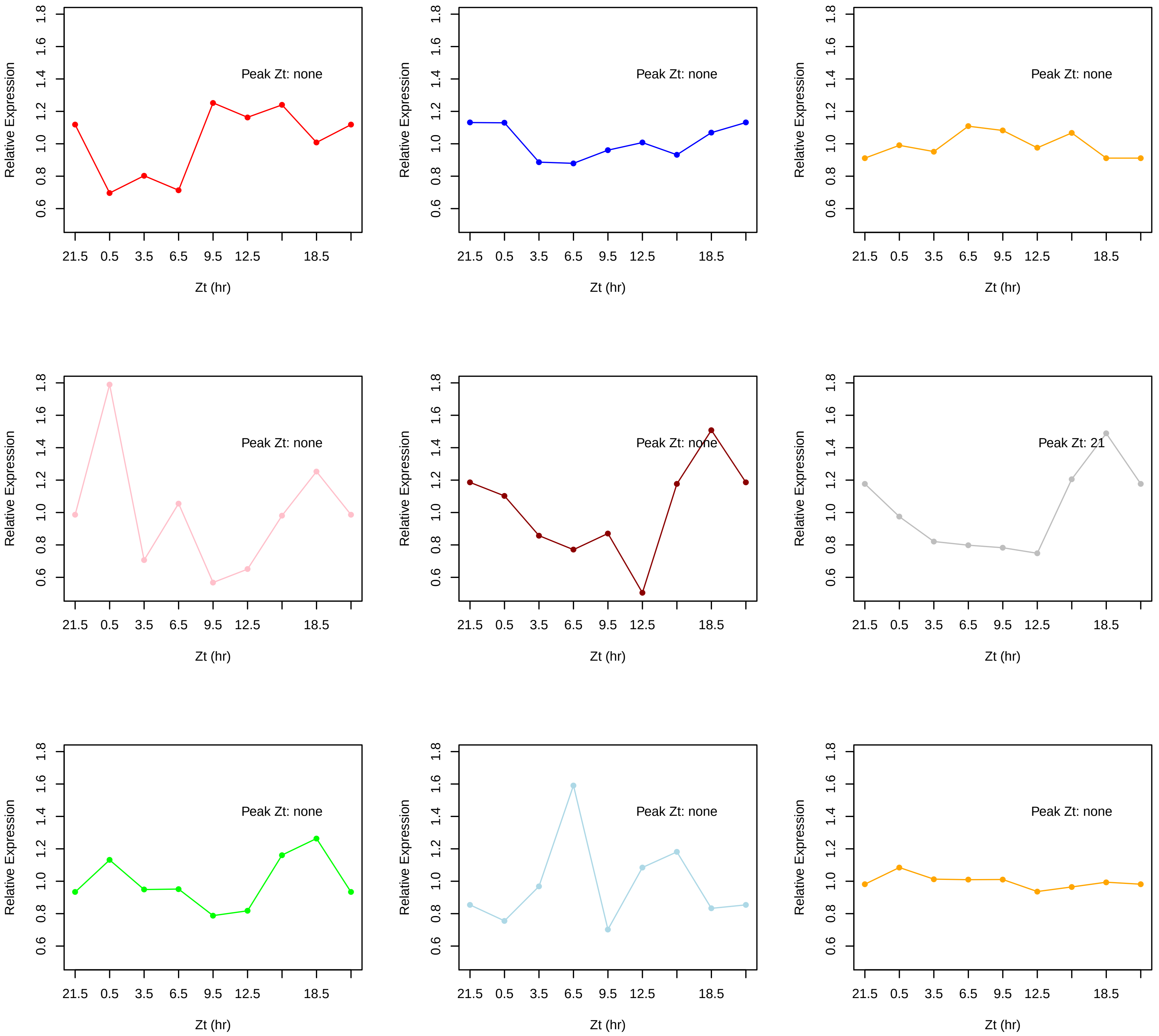

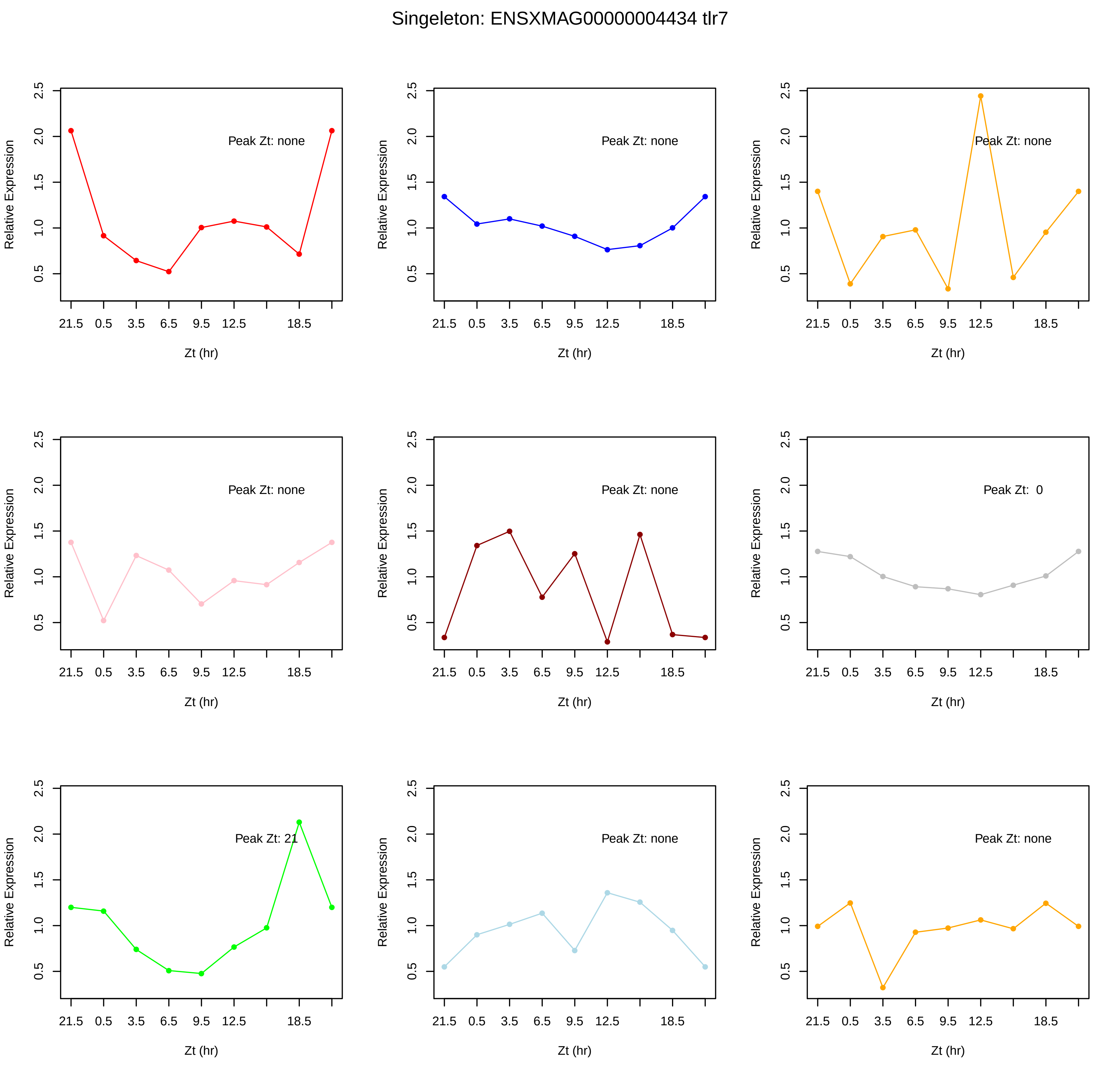
Singeleton: ENSXMAG00000004434 tlr7
Peak Zt: none
Peak Zt: none
Peak Zt: none
Peak Zt: none
Peak Zt: none
Peak Zt: 0
Peak Zt: 21
Peak Zt: none
Peak Zt: none
Relative Expression
Zt (hr)
21.5
0.5
3.5
6.5
9.5
12.5
18.5

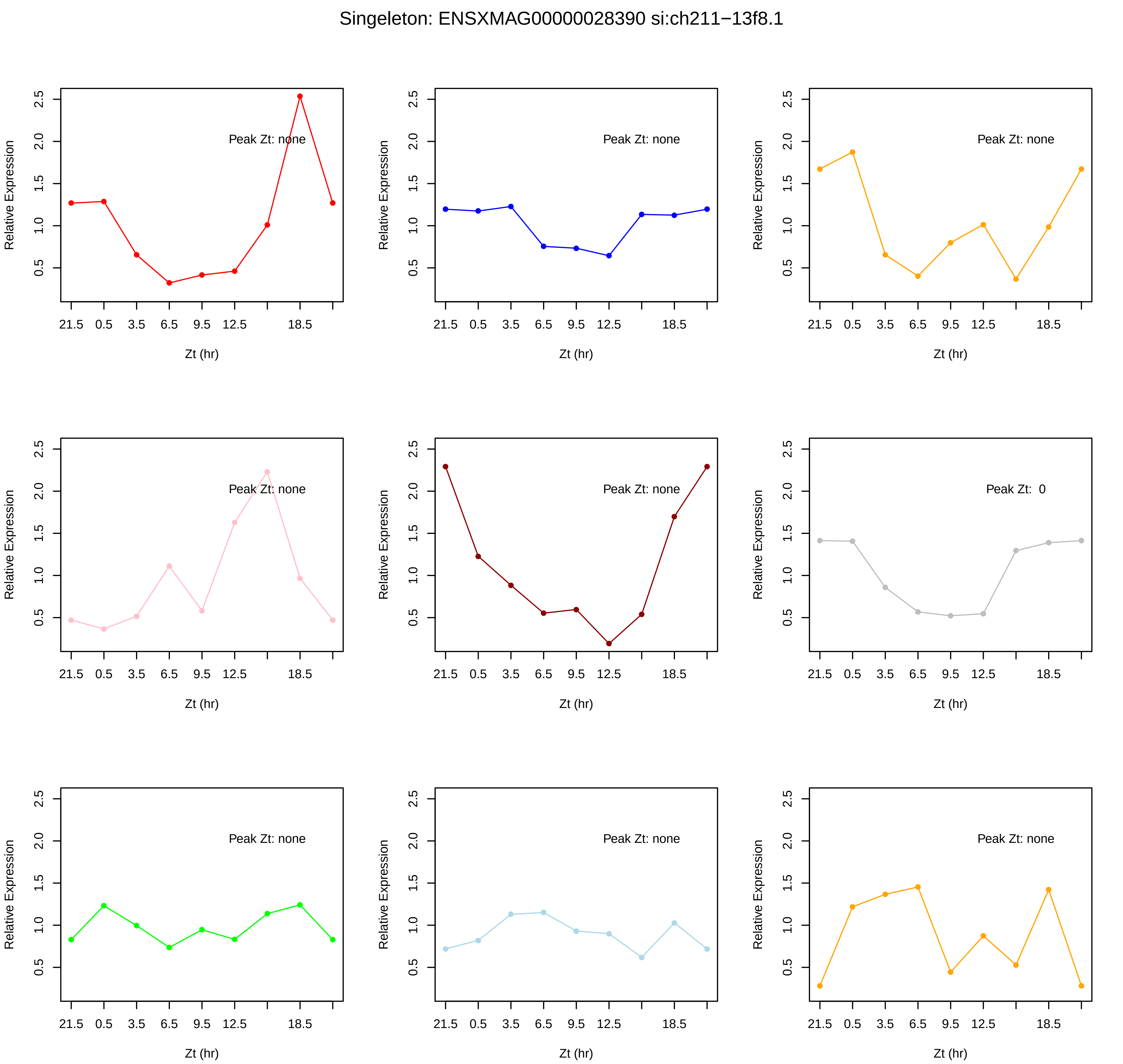

Singeleton: ENSXMAG00000028390 si:ch211−13f8.1
Peak Zt: none
Peak Zt: none
Peak Zt: none
Peak Zt: none
Peak Zt: none
Peak Zt: 0
Peak Zt: none
Peak Zt: none
Peak Zt: none
Relative Expression
Zt (hr)
21.5
0.5
3.5
6.5
9.5
12.5
18.5

Singeleton: ENSXMAG00000022645 prkacaa

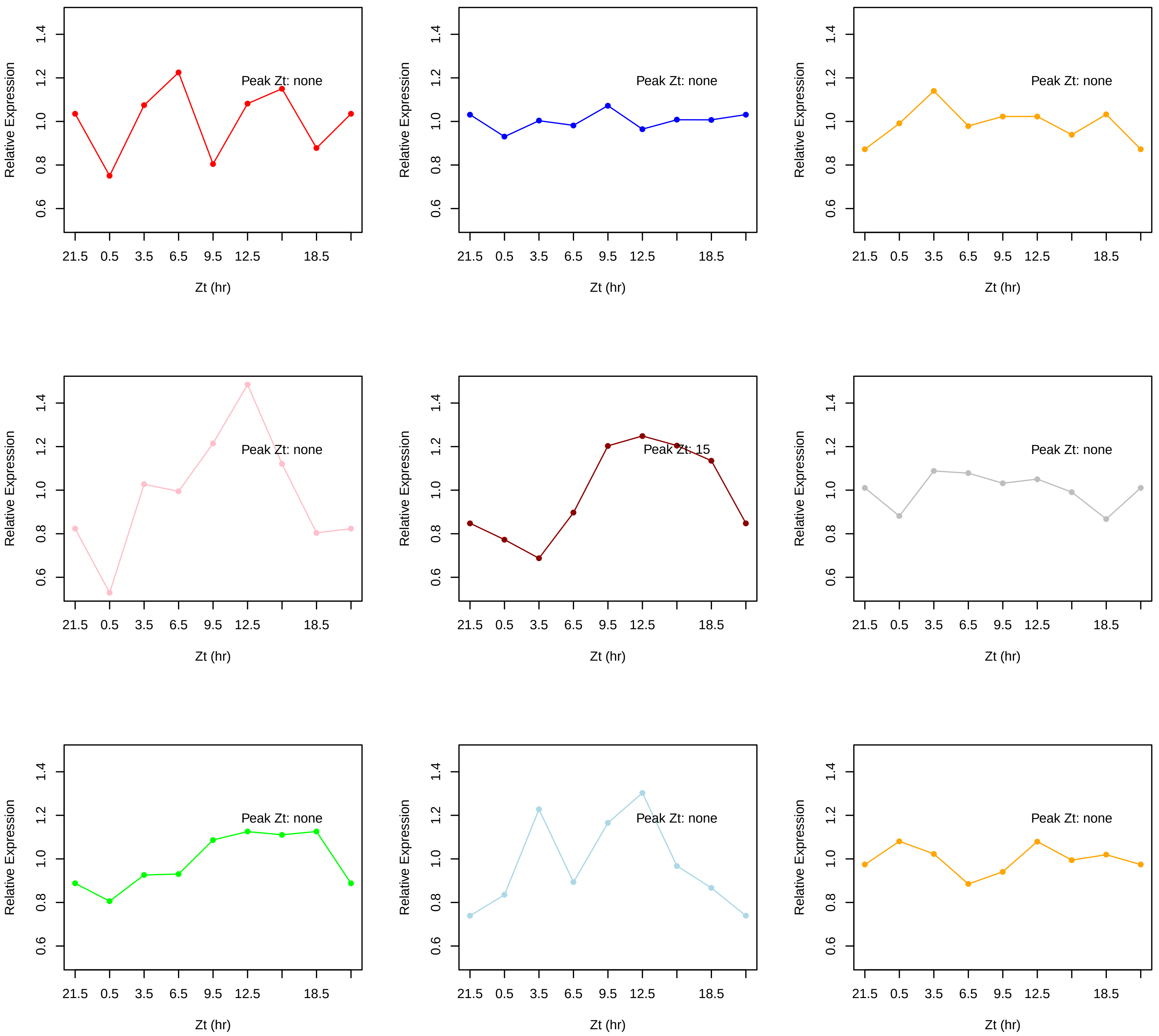

Singeleton: ENSXMAG00000007417

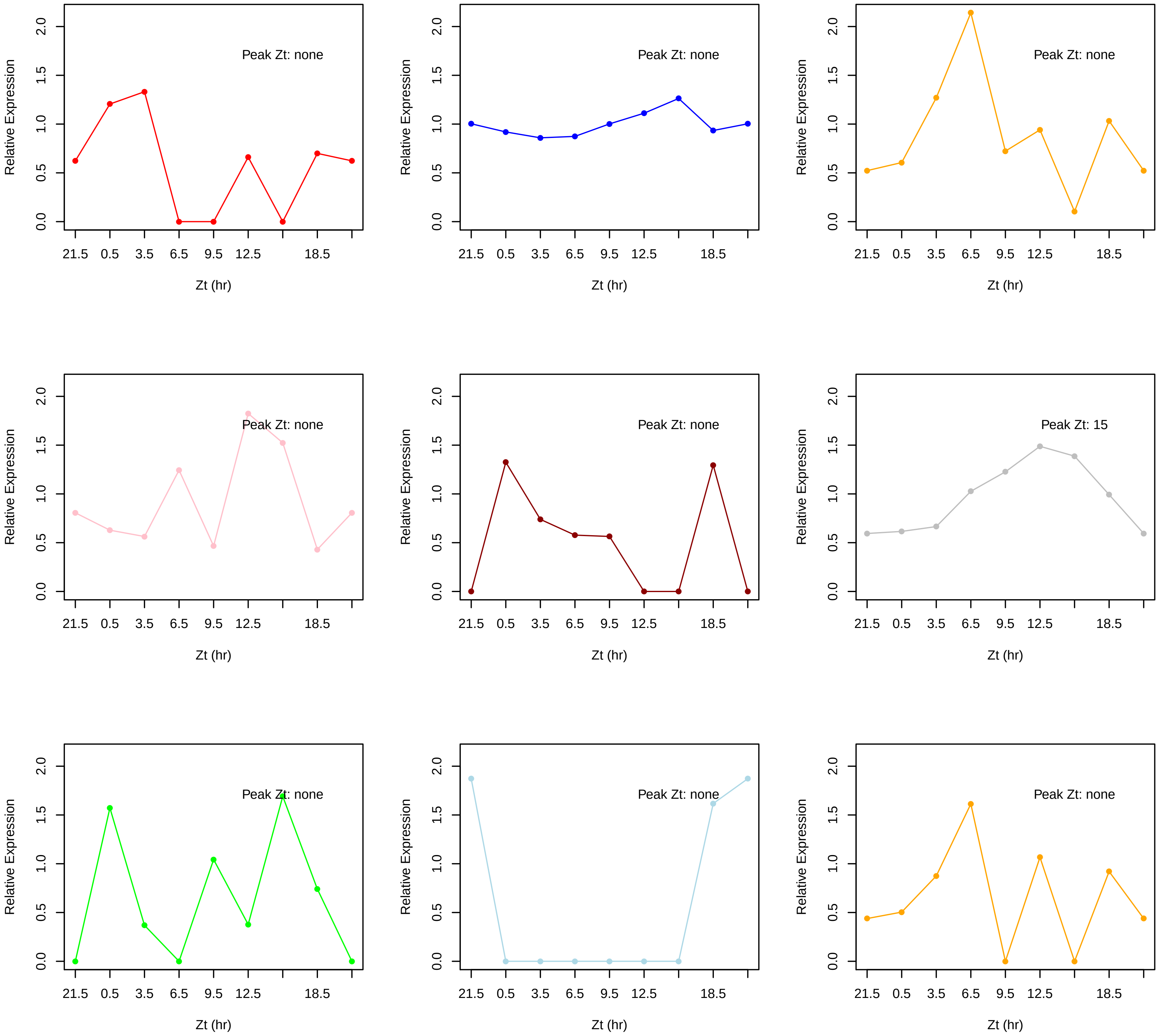

Singeleton: ENSXMAG00000008663 fbxo32

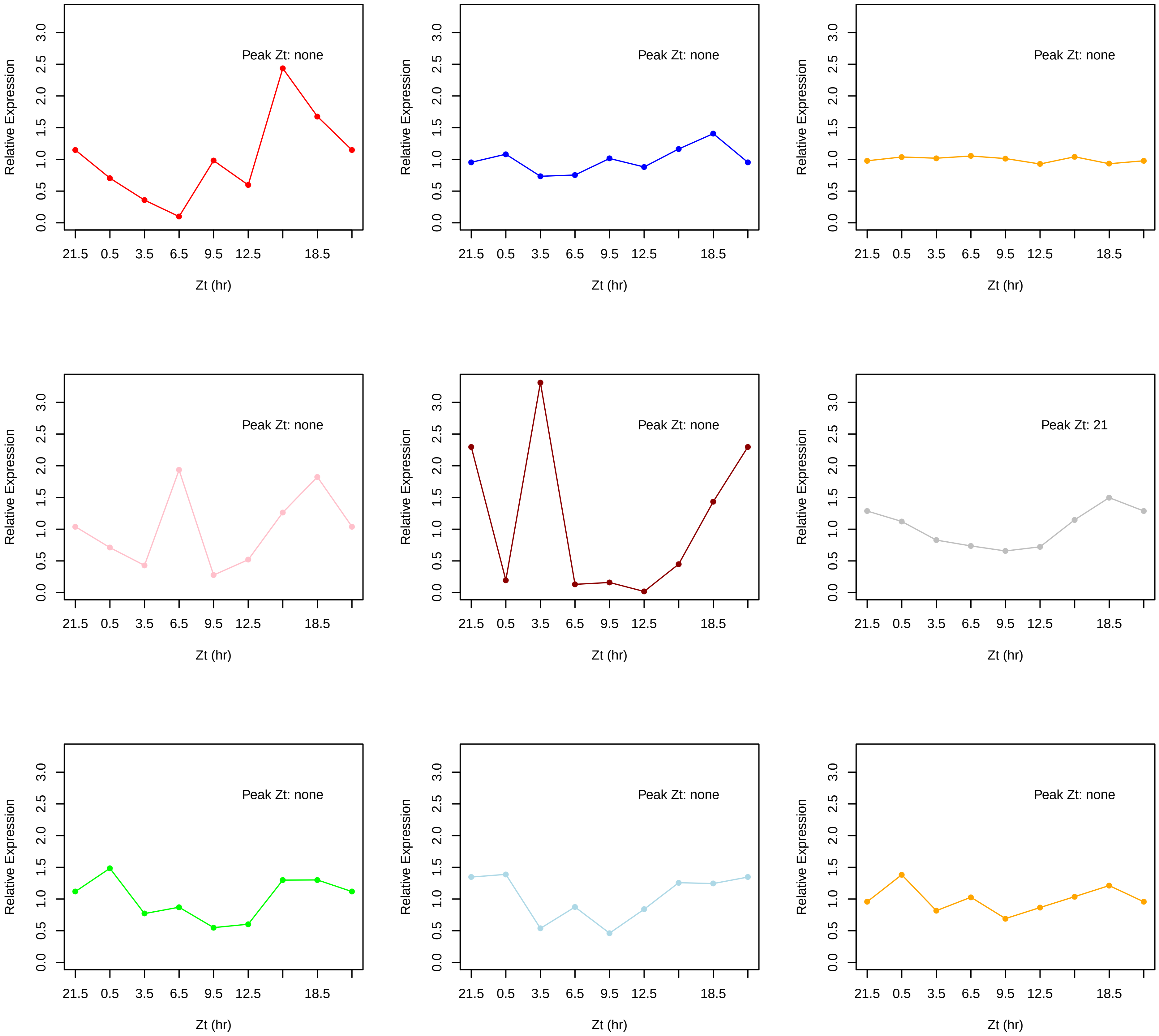

Singleton: ENSXMAG00000021261 stx18

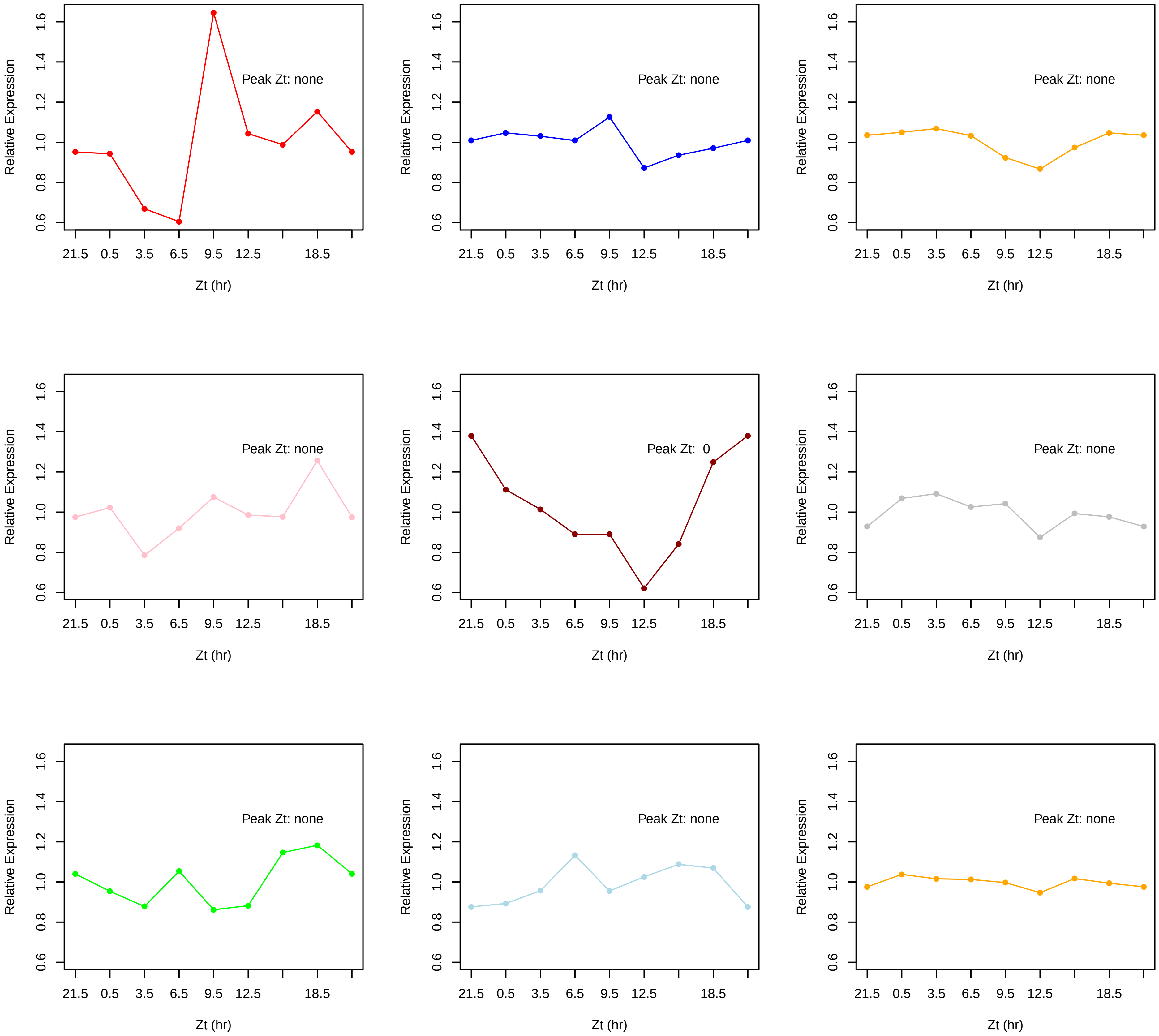

Singeleton: ENSXMAG00000001870 atic

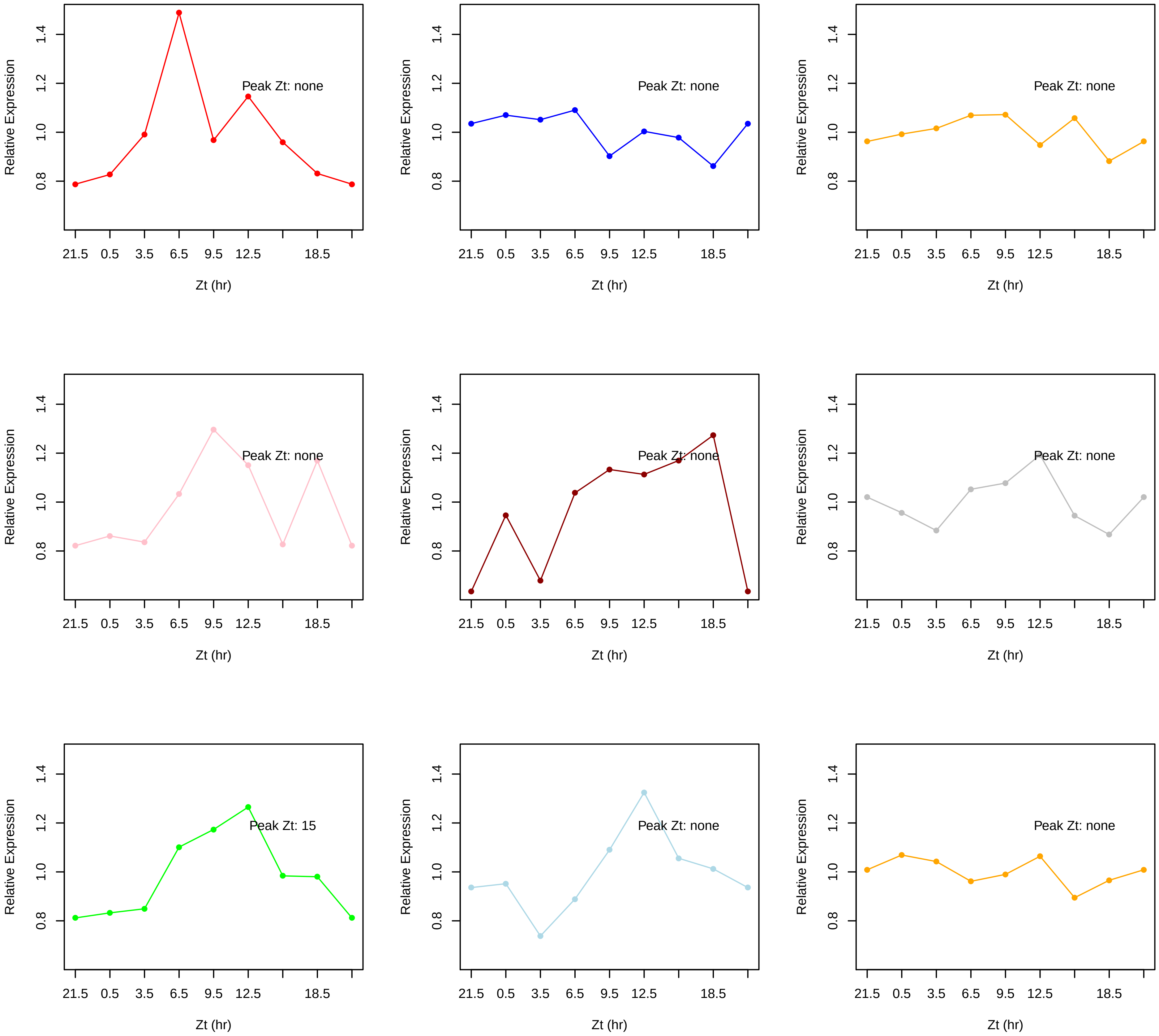

Singeleton: ENSXMAG00000016608 uhrf1bp1

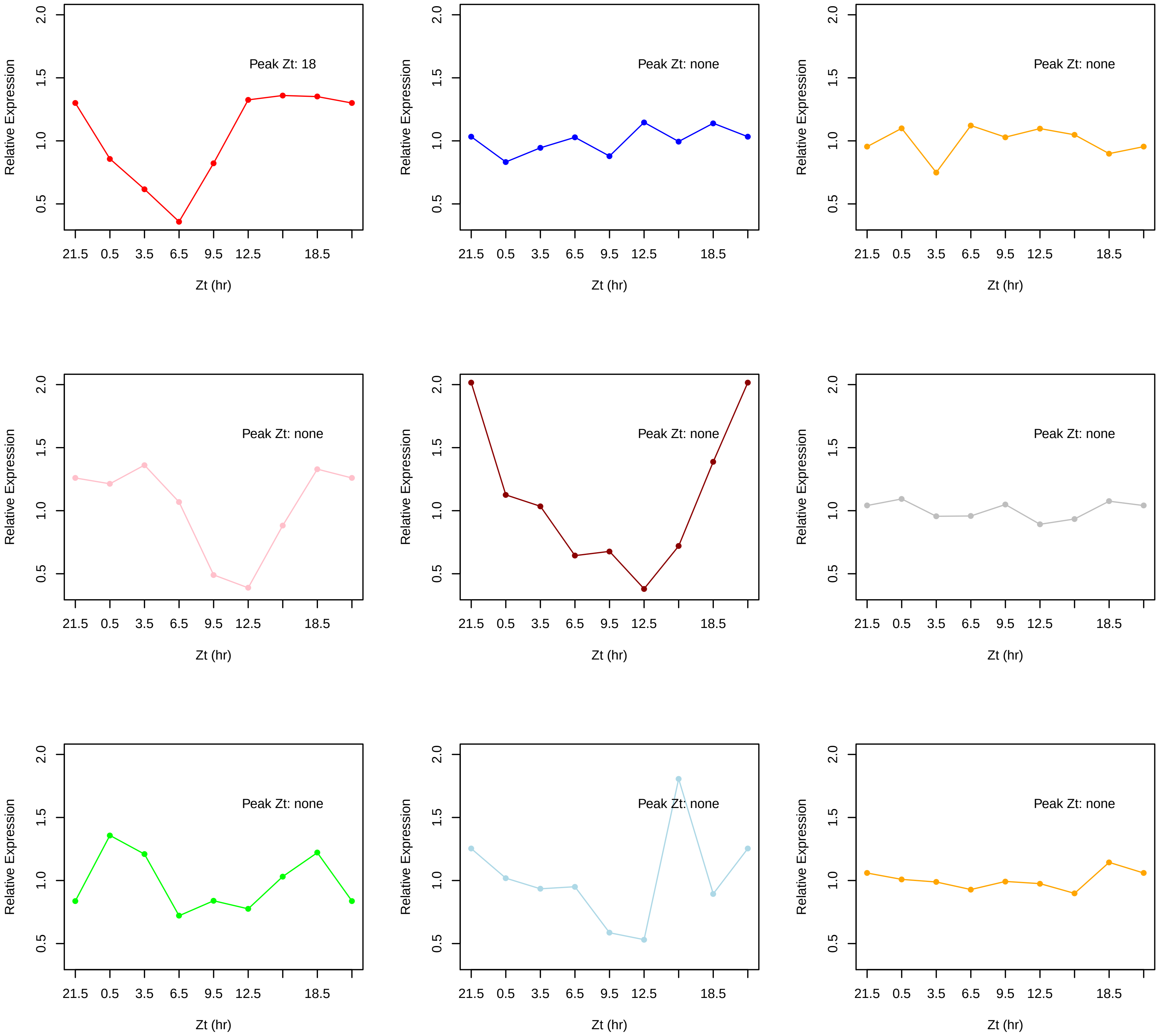

Singeleton: ENSXMAG00000004164 foxp1b

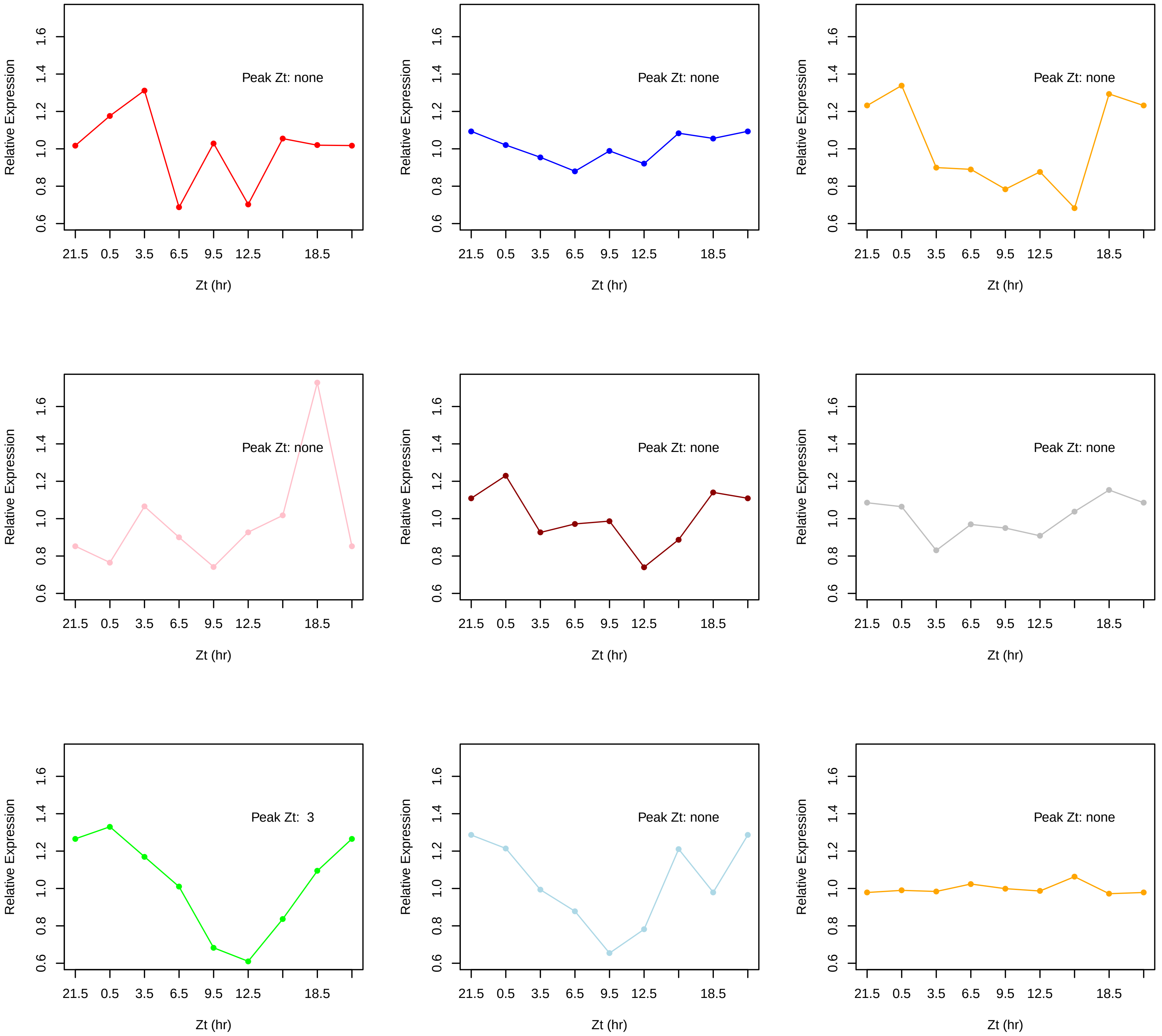

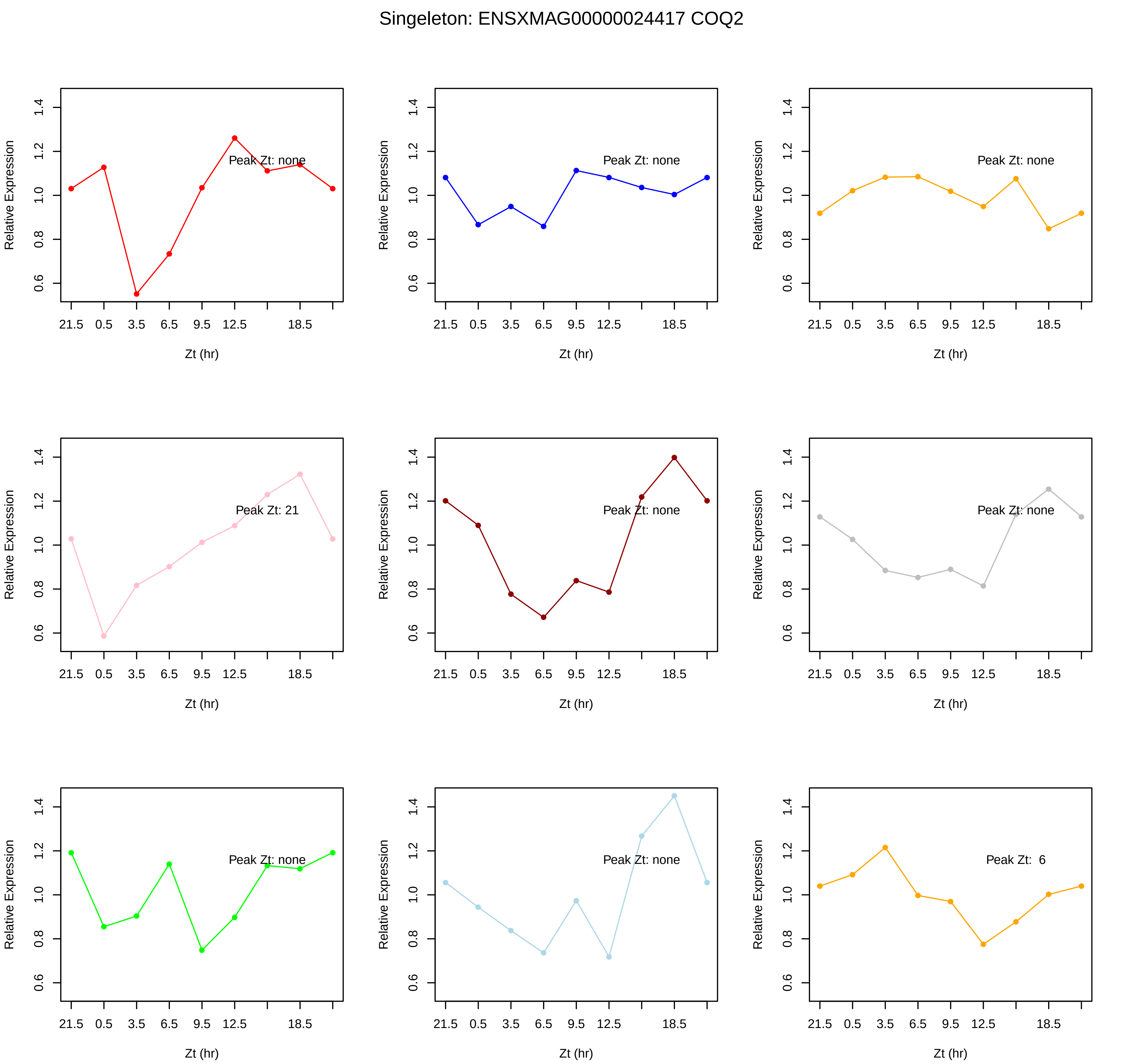
Singeleton: ENSXMAG00000024417 COQ2
Relative Expression
Zt (hr)
21.5 0.5 3.5 6.5 9.5 12.5 18.5
0.6 0.8 1.0 1.2 1.4
Peak Zt: none
Peak Zt: none
Peak Zt: none
Peak Zt: 21
Peak Zt: none
Peak Zt: none
Peak Zt: none
Peak Zt: none
Peak Zt: 6

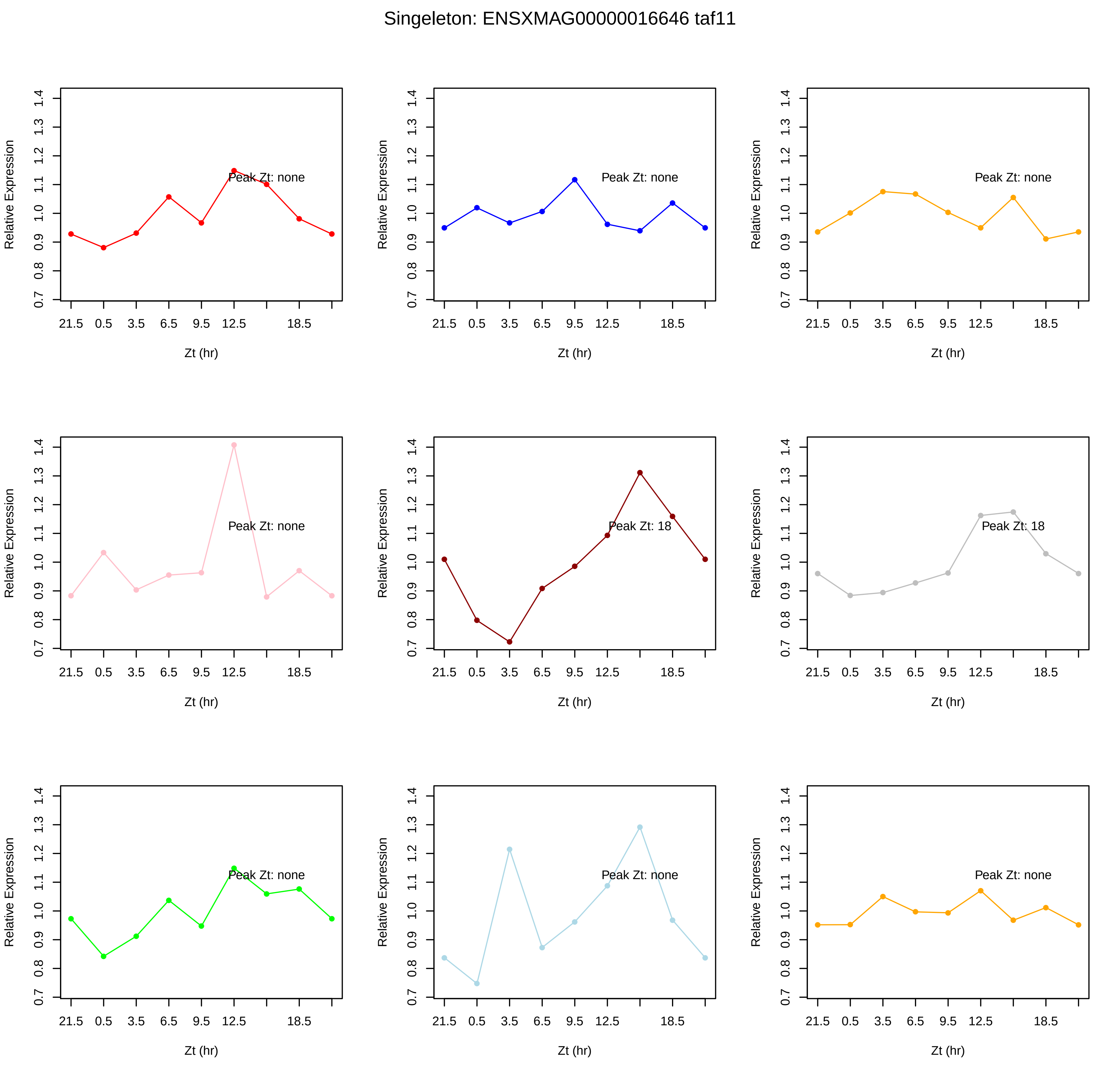

Singeleton: ENSXMAG00000016646 taf11
Relative Expression
Zt (hr)
Peak Zt: none
Peak Zt: none
Peak Zt: none
Peak Zt: none
Peak Zt: 18
Peak Zt: 18
Peak Zt: none
Peak Zt: none
Peak Zt: none

Singeleton: ENSXMAG00000005461 cthl

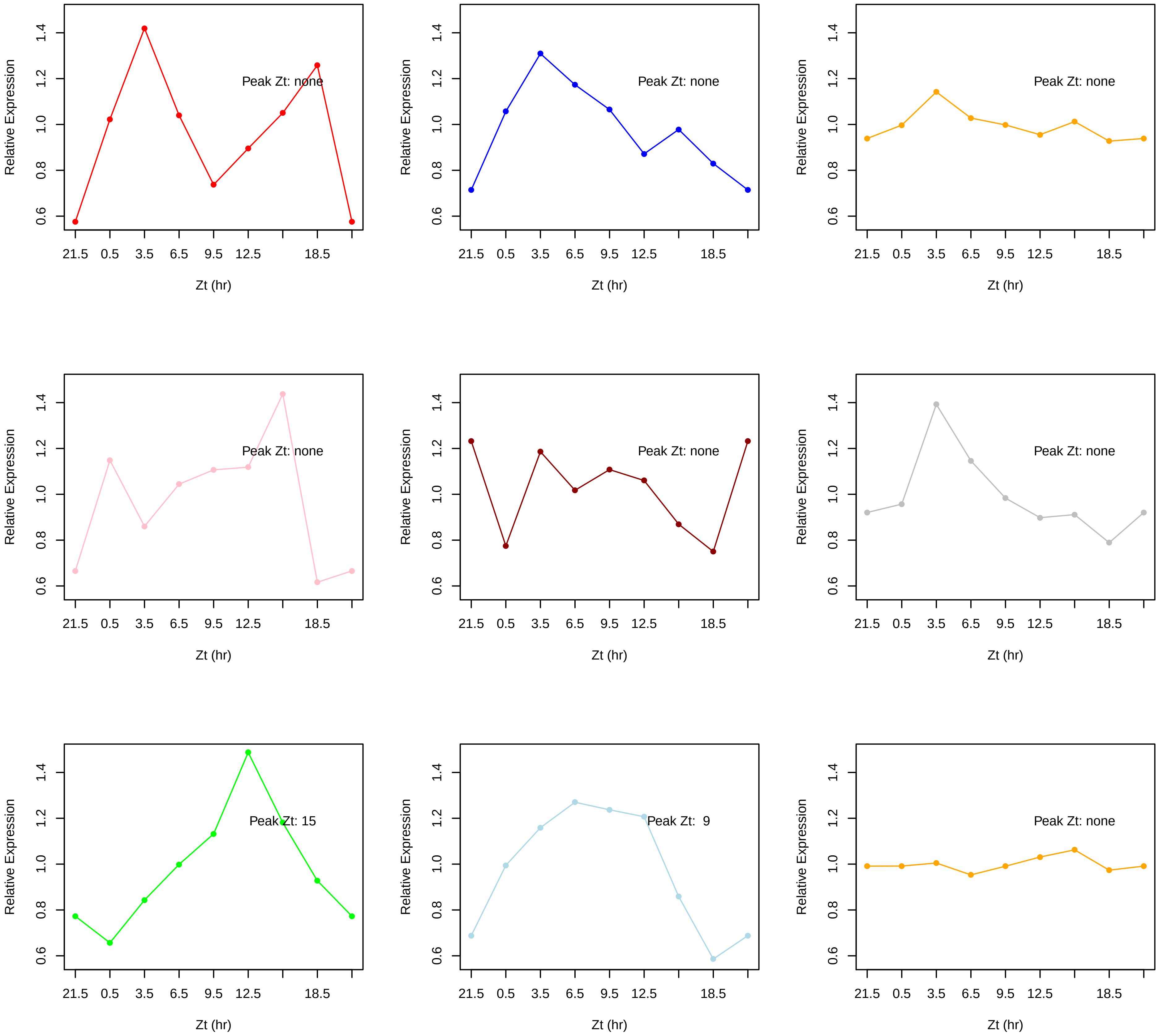

Singleton: ENSXMAG00000028044 oaz1b

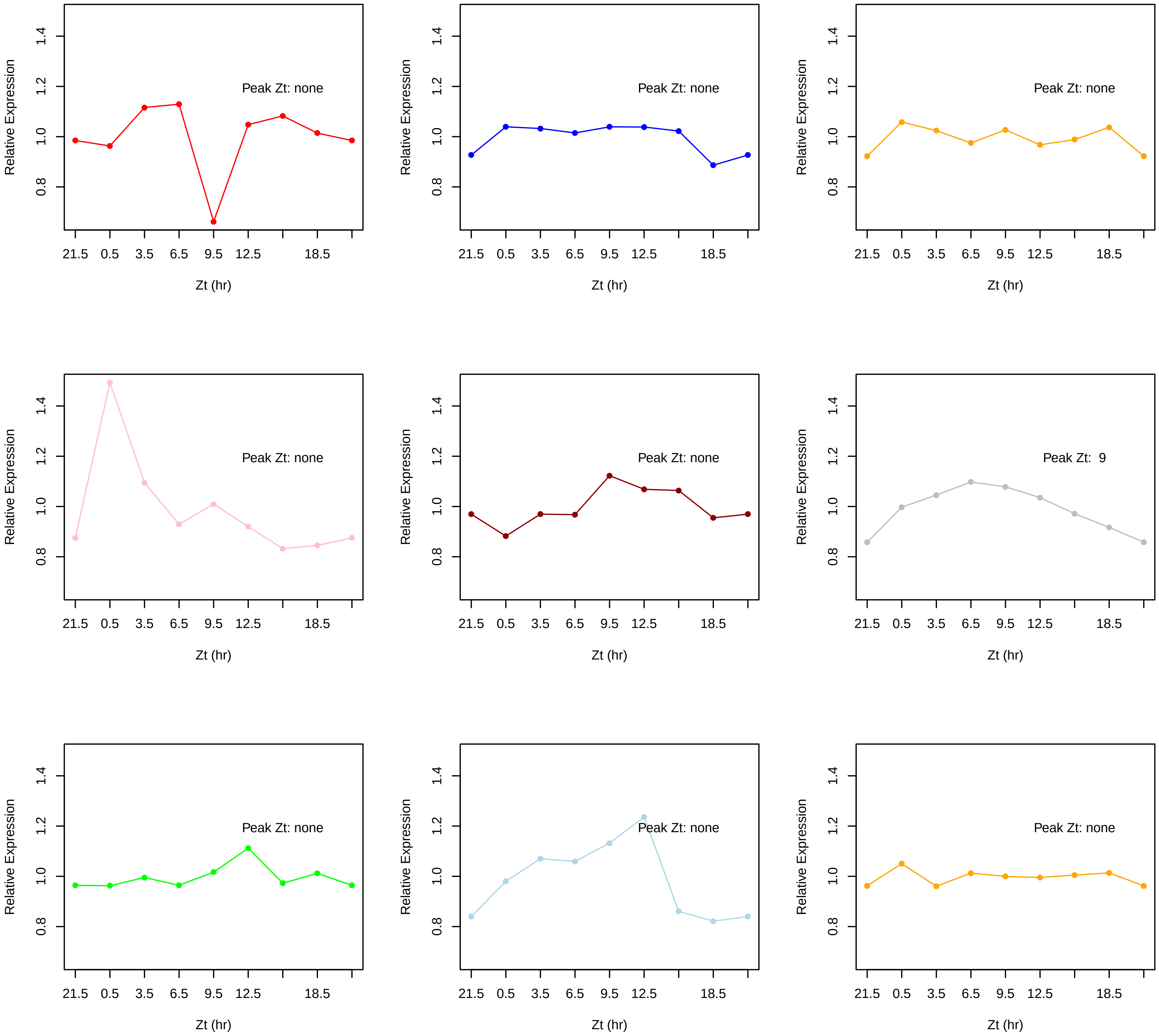

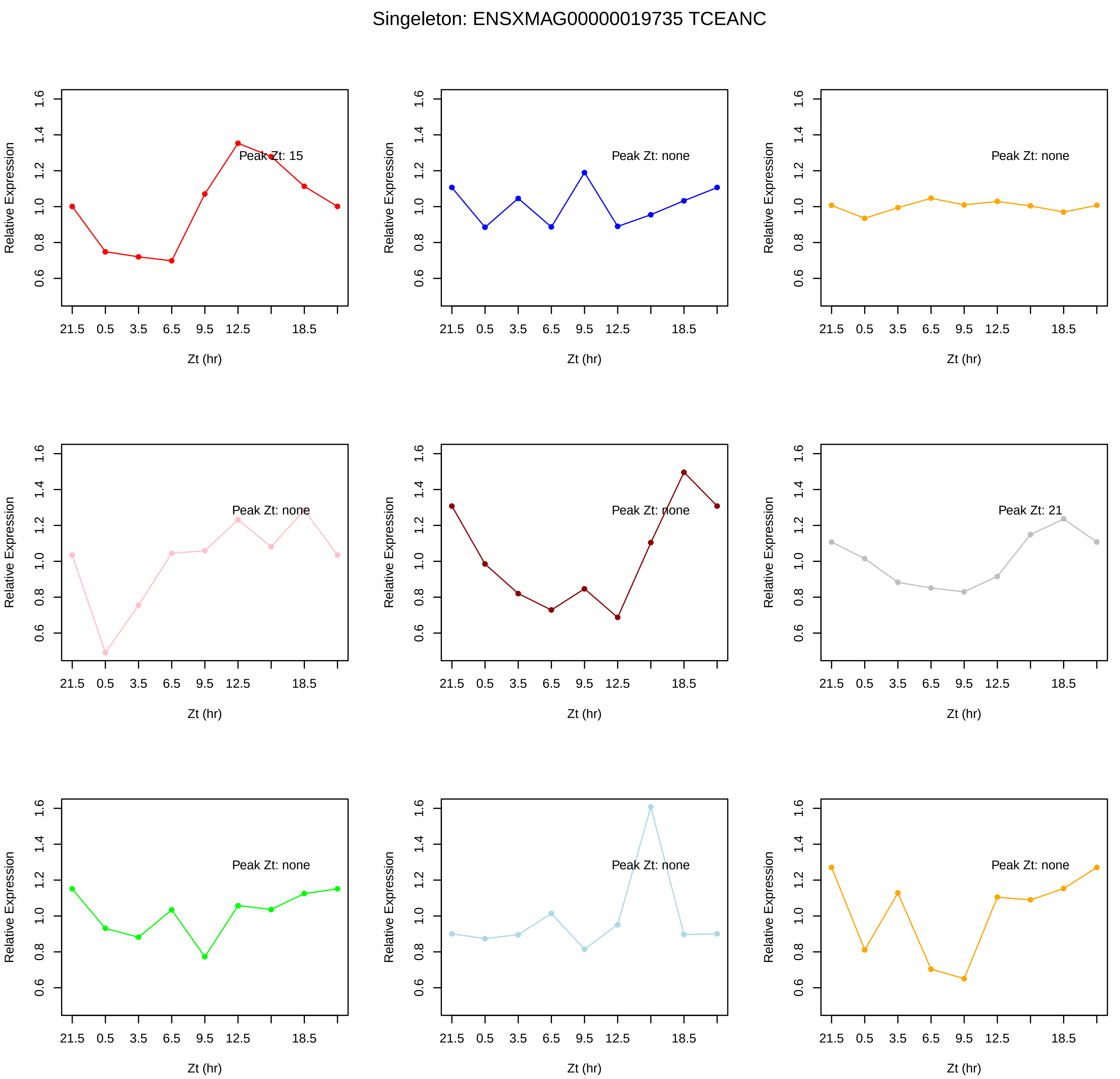
Singeleton: ENSXMAG00000019735 TCEANC
Peak Zt: 15
Peak Zt: none
Peak Zt: none
Peak Zt: none
Peak Zt: none
Peak Zt: 21
Peak Zt: none
Peak Zt: none
Peak Zt: none
Relative Expression
Zt (hr)
21.5
0.5
3.5
6.5
9.5
12.5
18.5

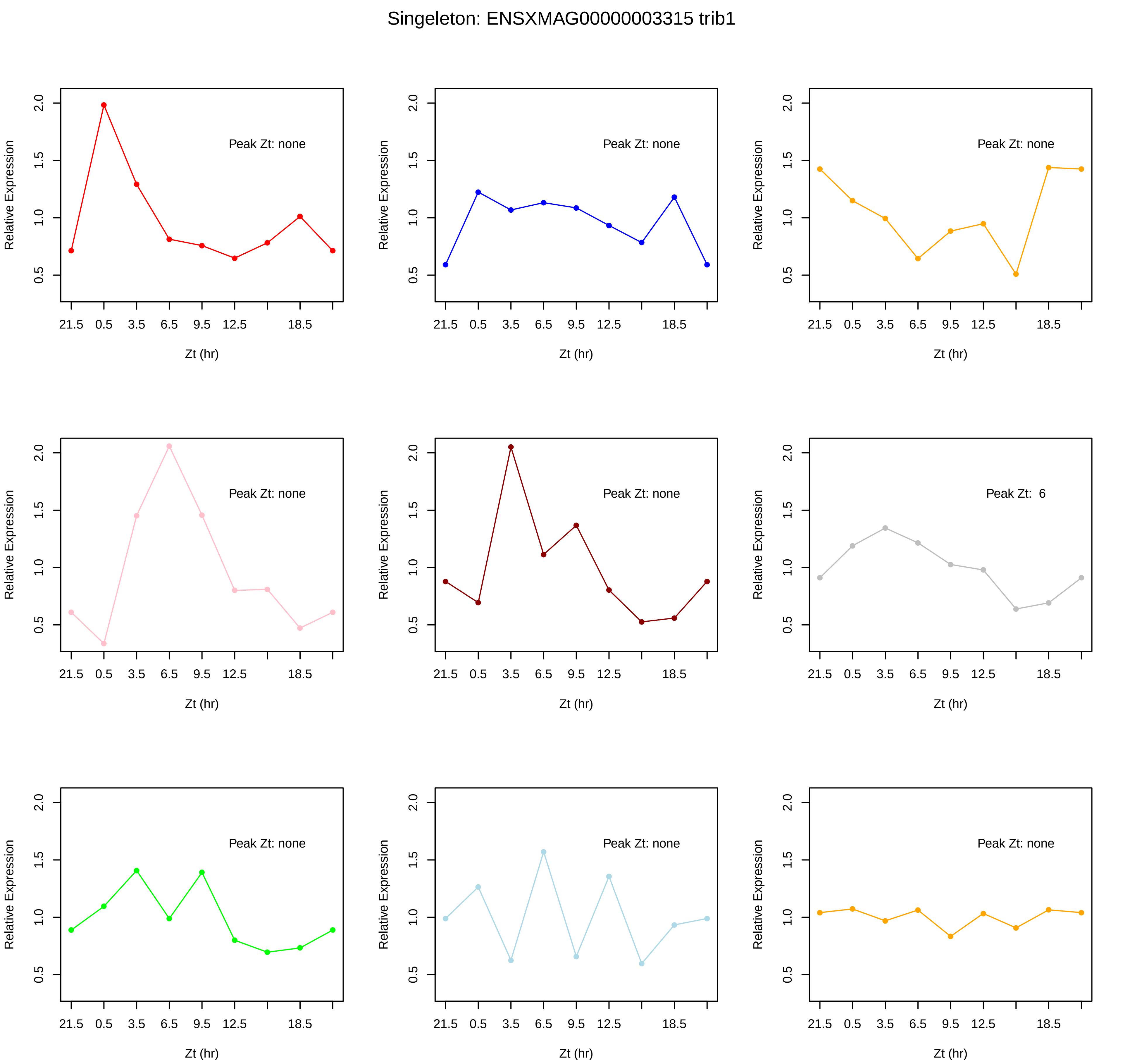
Singeleton: ENSXMAG00000003315 trib1
Peak Zt: none
Peak Zt: none
Peak Zt: none
Peak Zt: none
Peak Zt: none
Peak Zt: 6
Peak Zt: none
Peak Zt: none
Peak Zt: none
Relative Expression
Zt (hr)
21.5
0.5
3.5
6.5
9.5
12.5
18.5
0.5
1.0
1.5
2.0

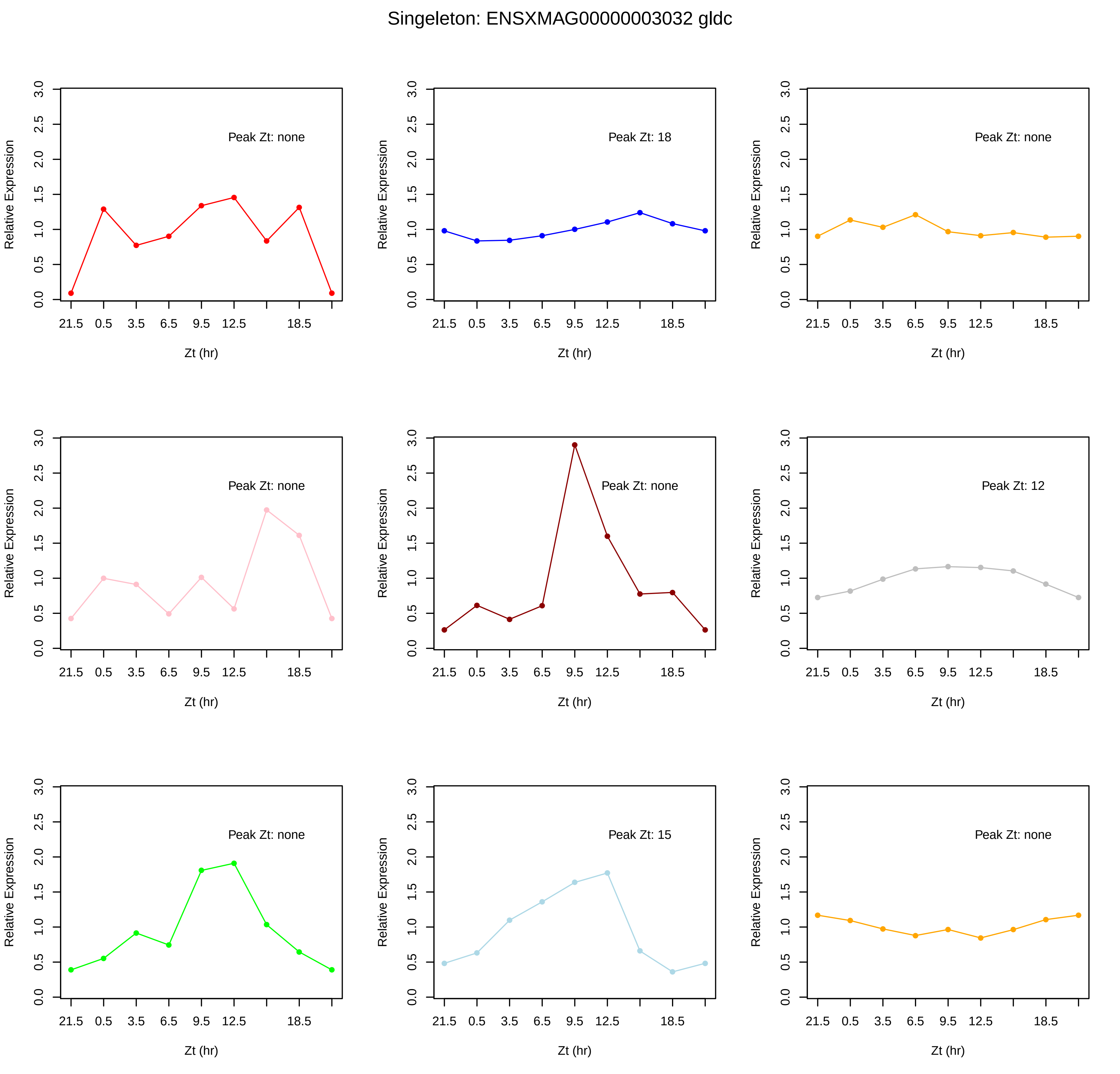
Singeleton: ENSXMAG00000003032 gldc
Peak Zt: none
Peak Zt: 18
Peak Zt: none
Peak Zt: none
Peak Zt: none
Peak Zt: 12
Peak Zt: none
Peak Zt: 15
Peak Zt: none
Relative Expression
Zt (hr)
21.5
0.5
3.5
6.5
9.5
12.5
18.5
0.0
0.5
1.0
1.5
2.0
2.5
3.0

Singeleton: ENSXMAG00000013518 LRRC9

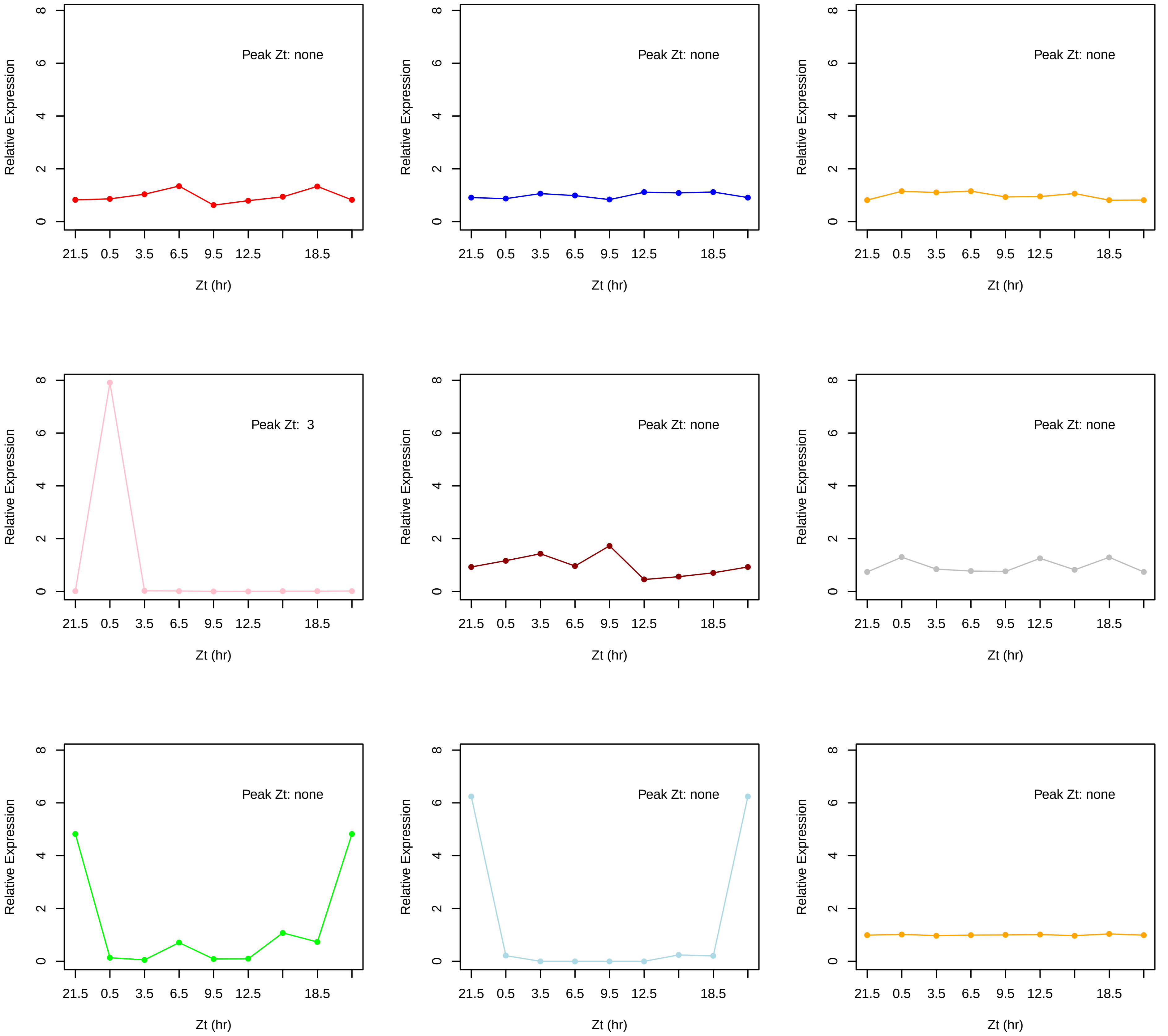

Singeleton: ENSXMAG00000008878 clk4a

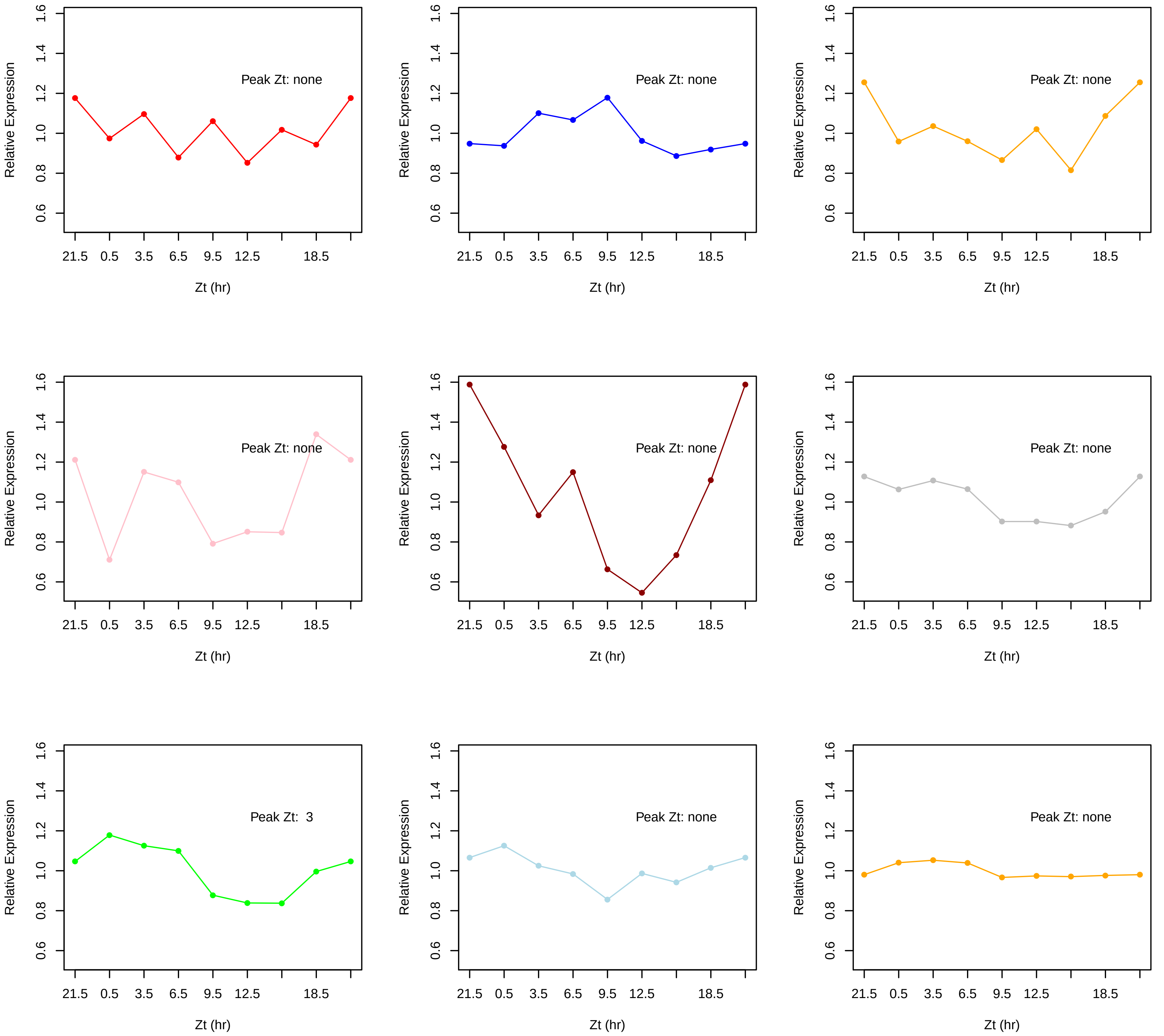

Singeleton: ENSXMAG00000004394 ofd1

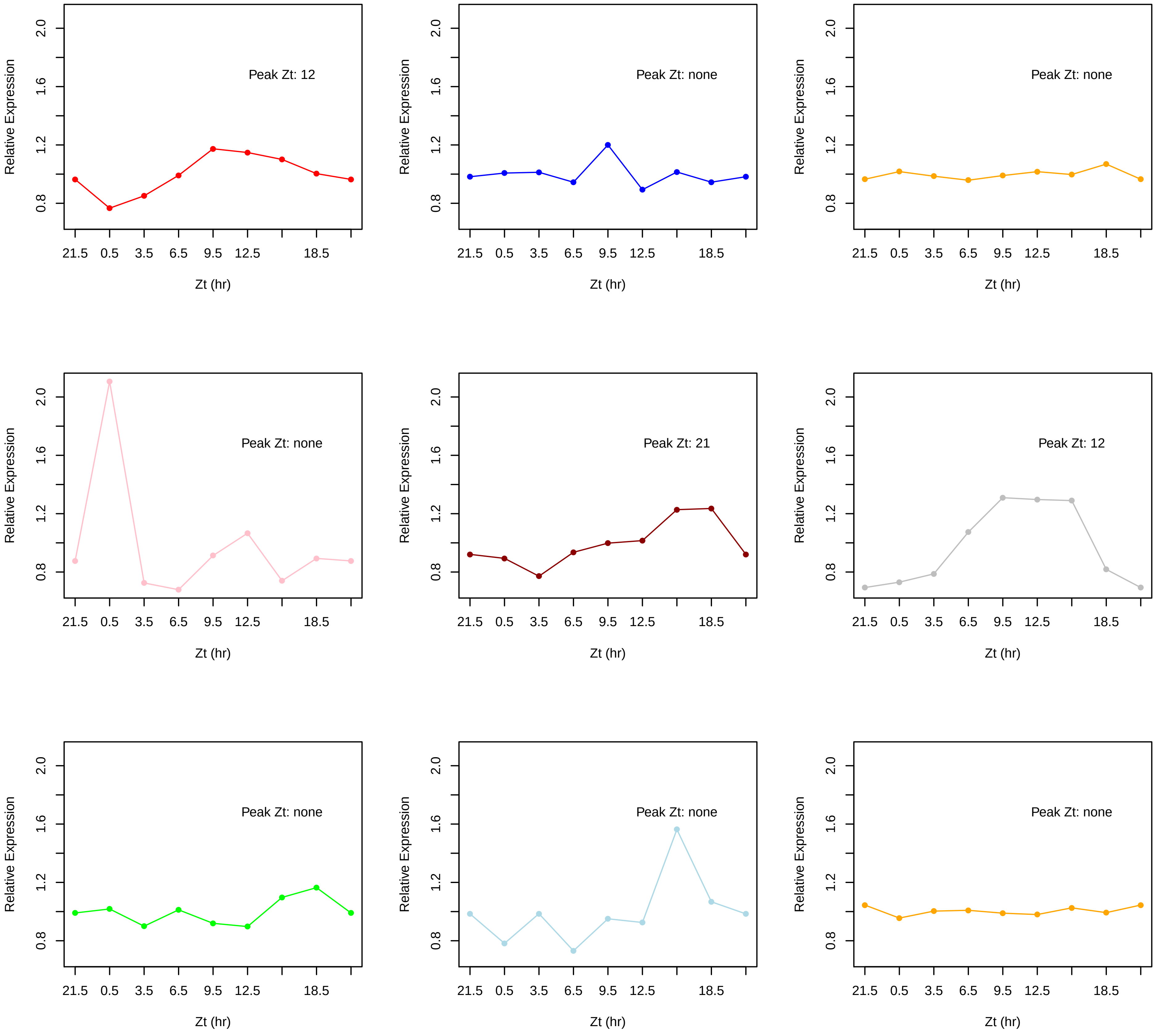

Singeleton: ENSXMAG00000017919

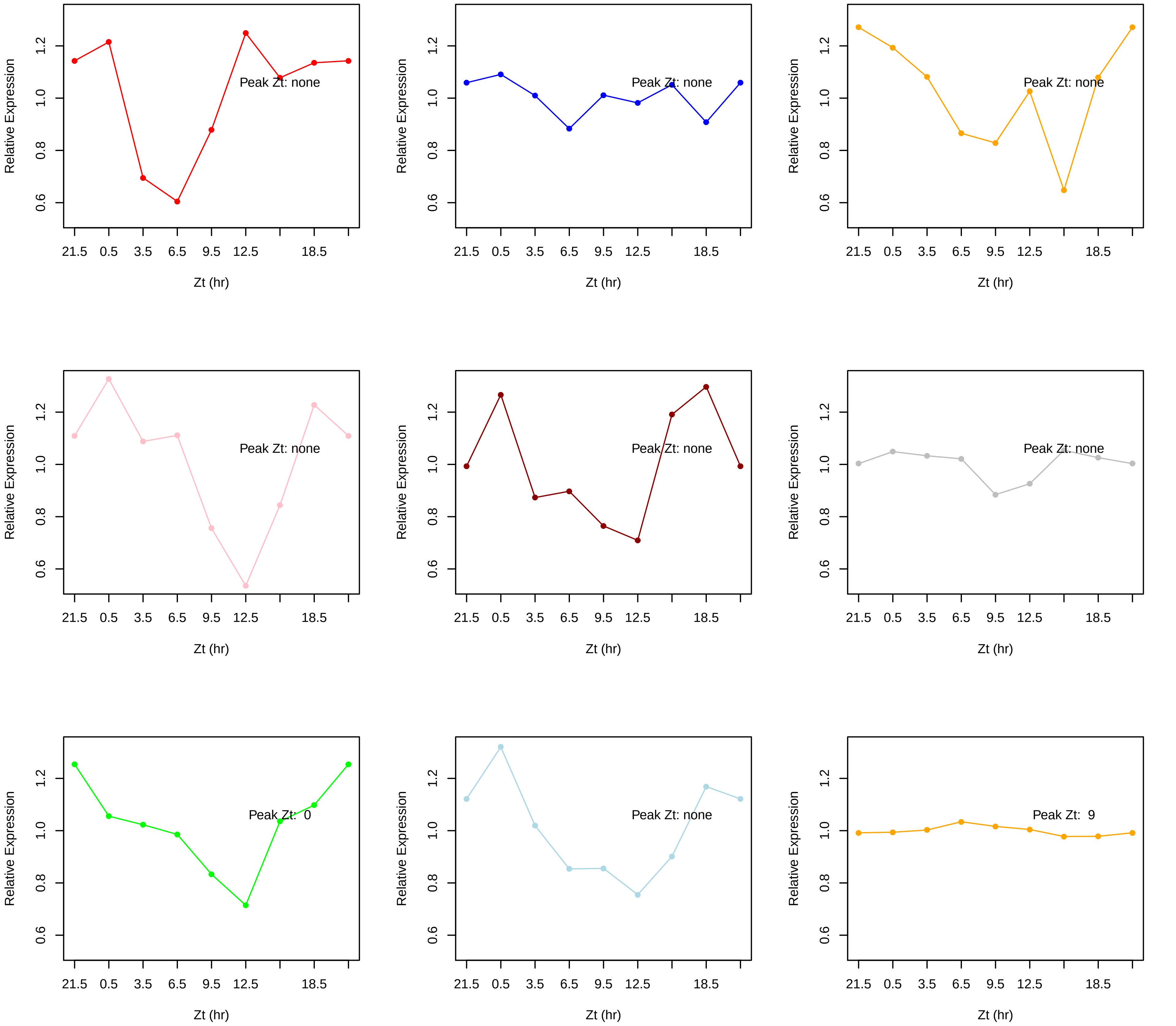

Singeleton: ENSXMAG00000005486 ankrd13c

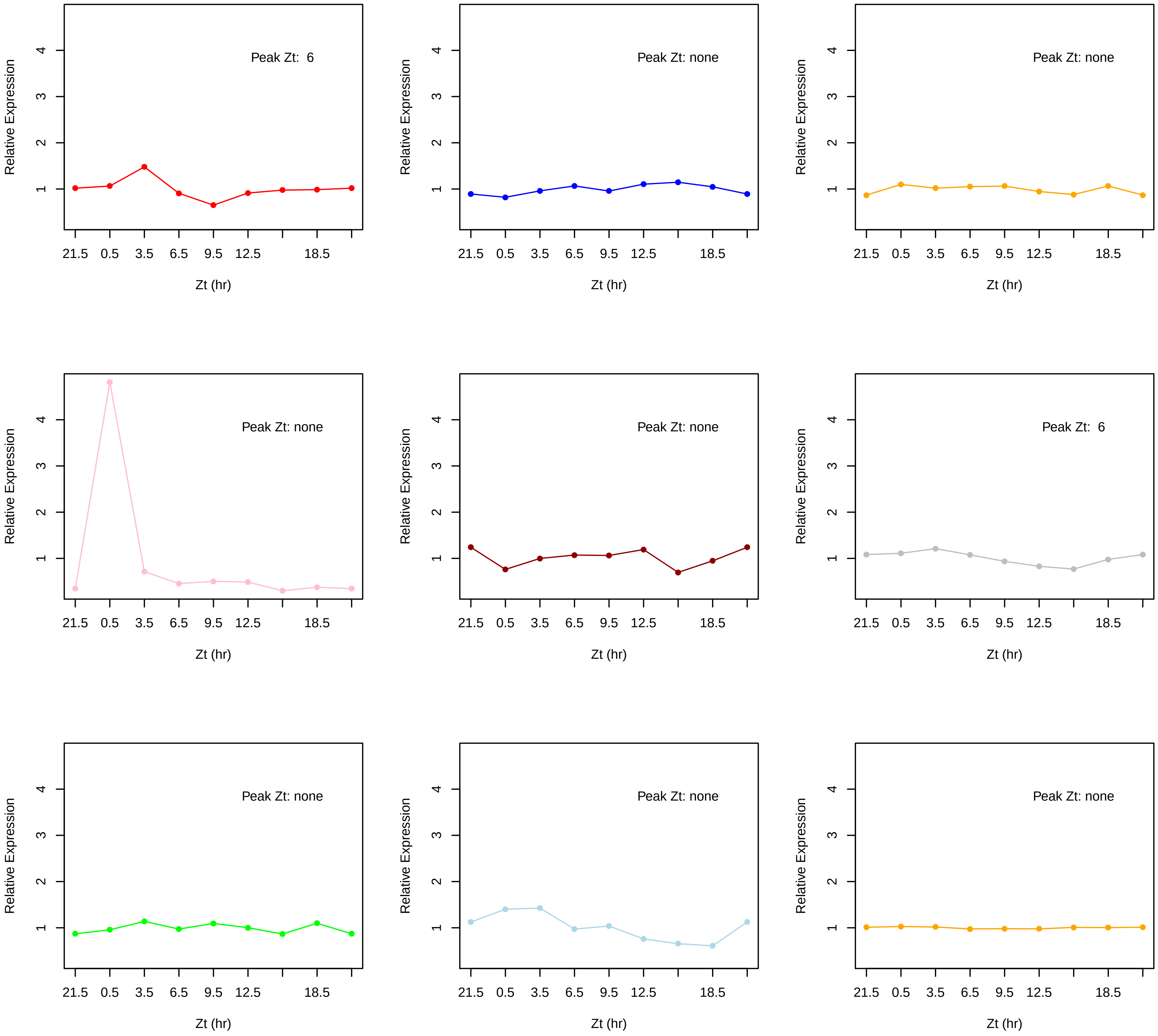

Singeleton: ENSXMAG00000005174 UHRF2

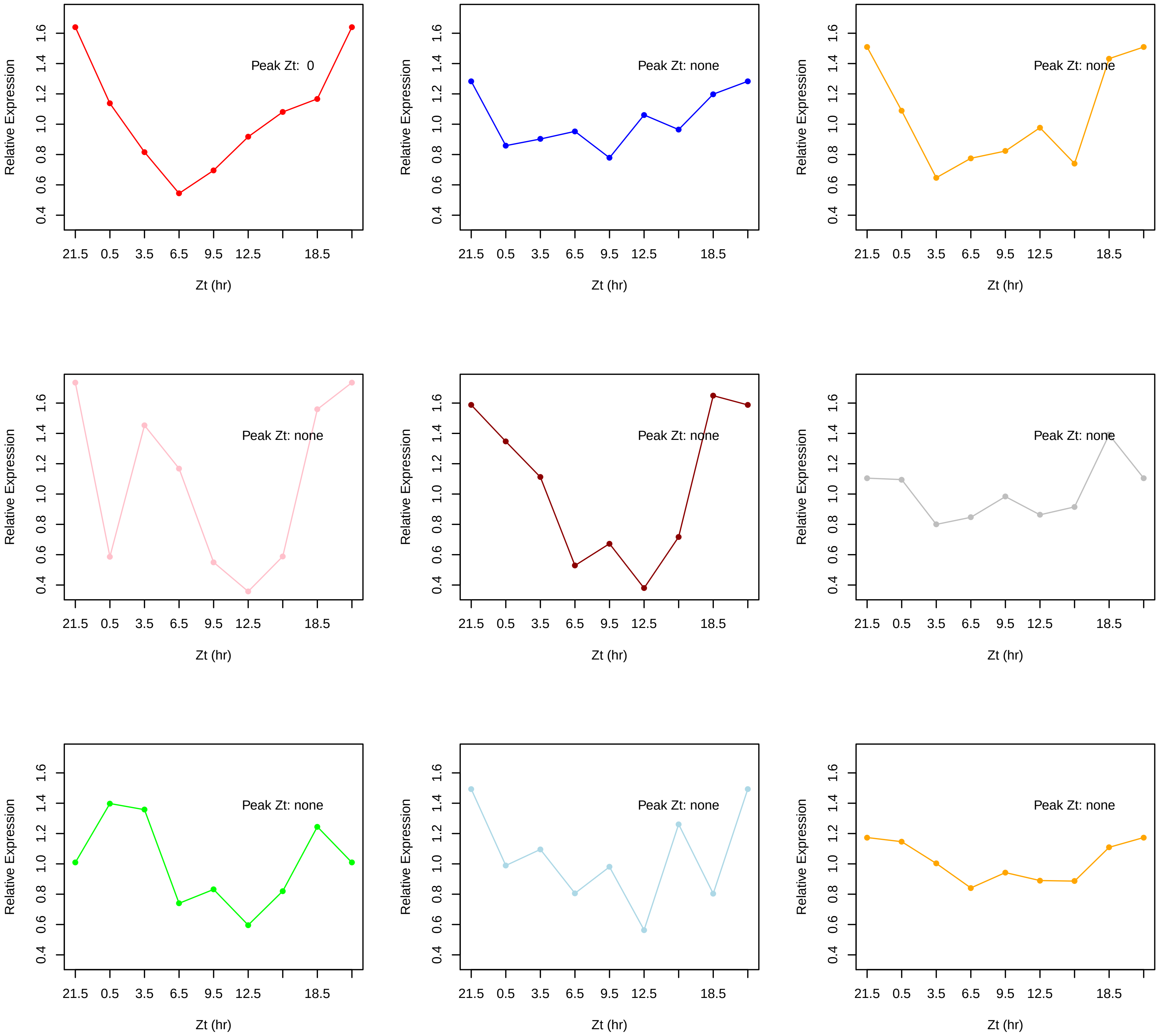

Singeleton: ENSXMAG00000017969 rpf1

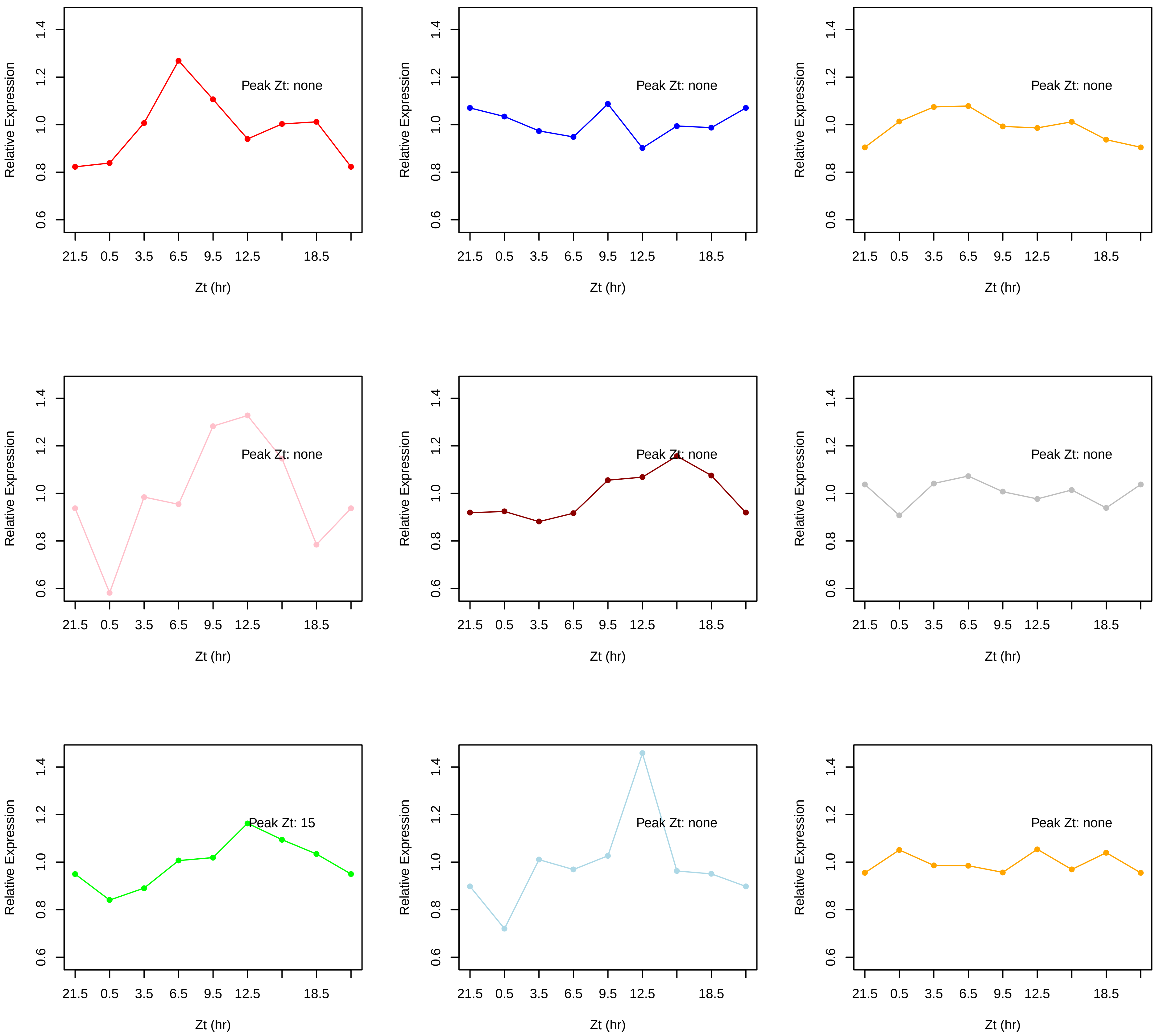

Singeleton: ENSXMAG00000027265 RASGEF1C

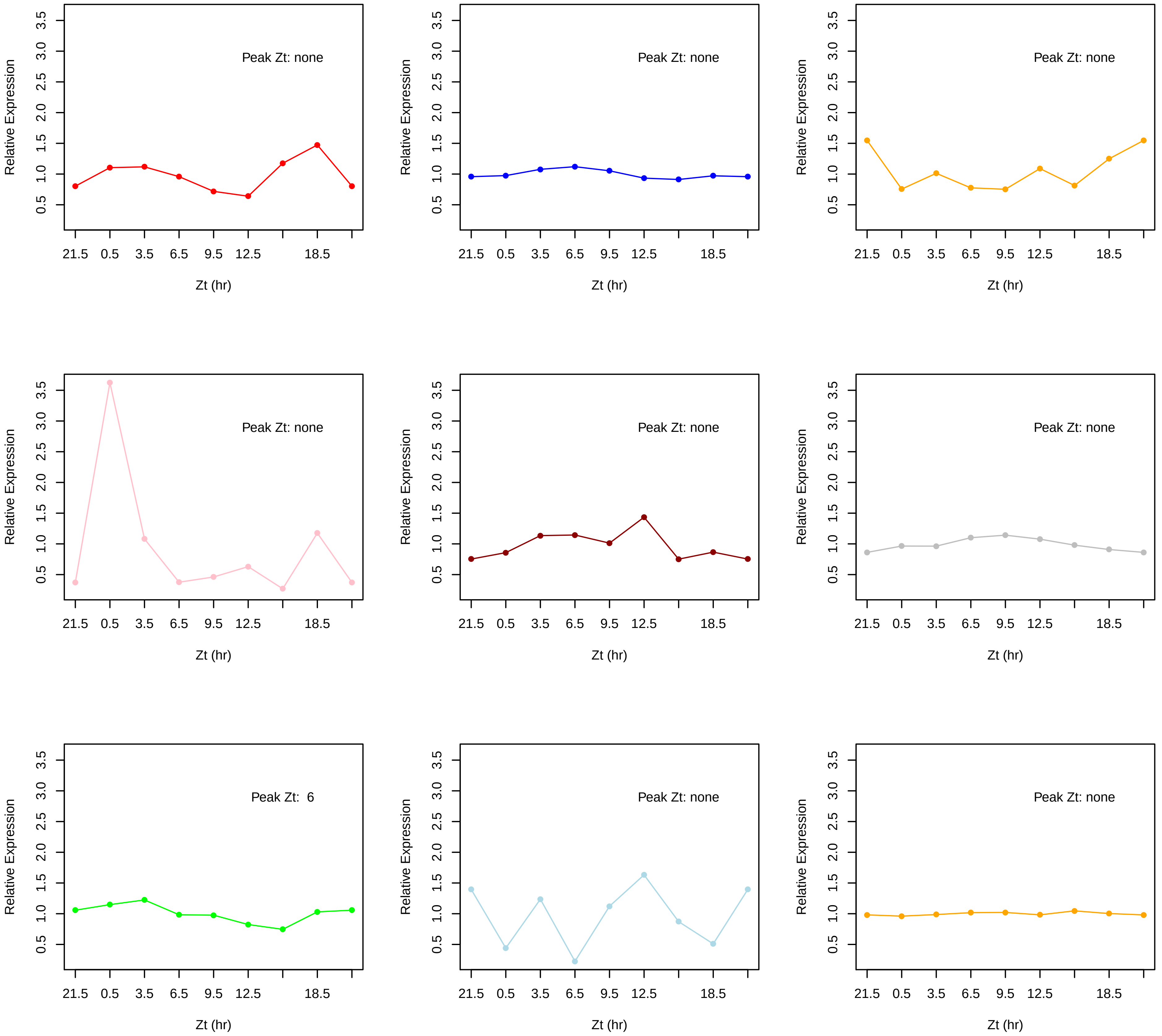

Singeleton: ENSXMAG00000018457

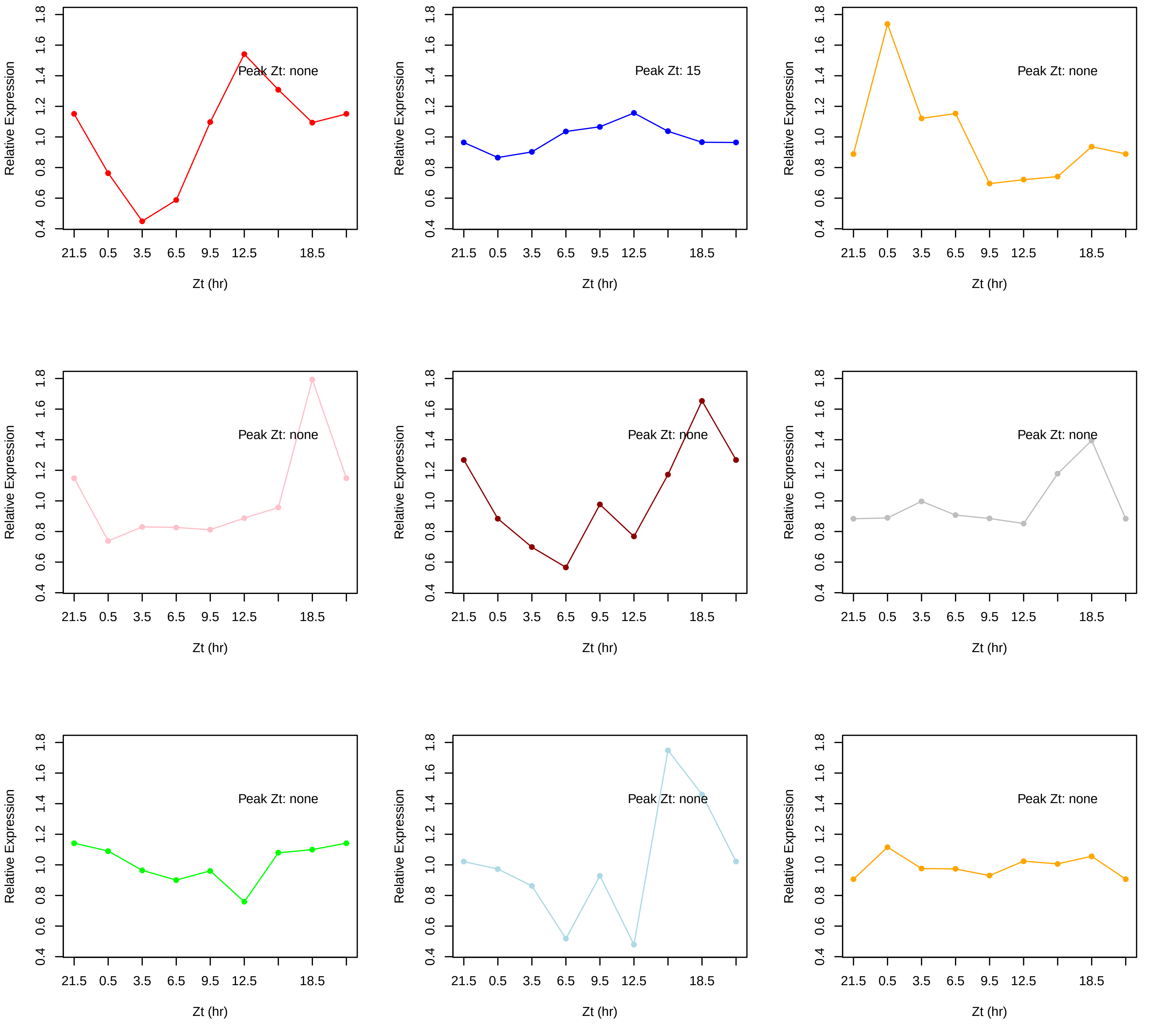

Singeleton: ENSXMAG00000005170

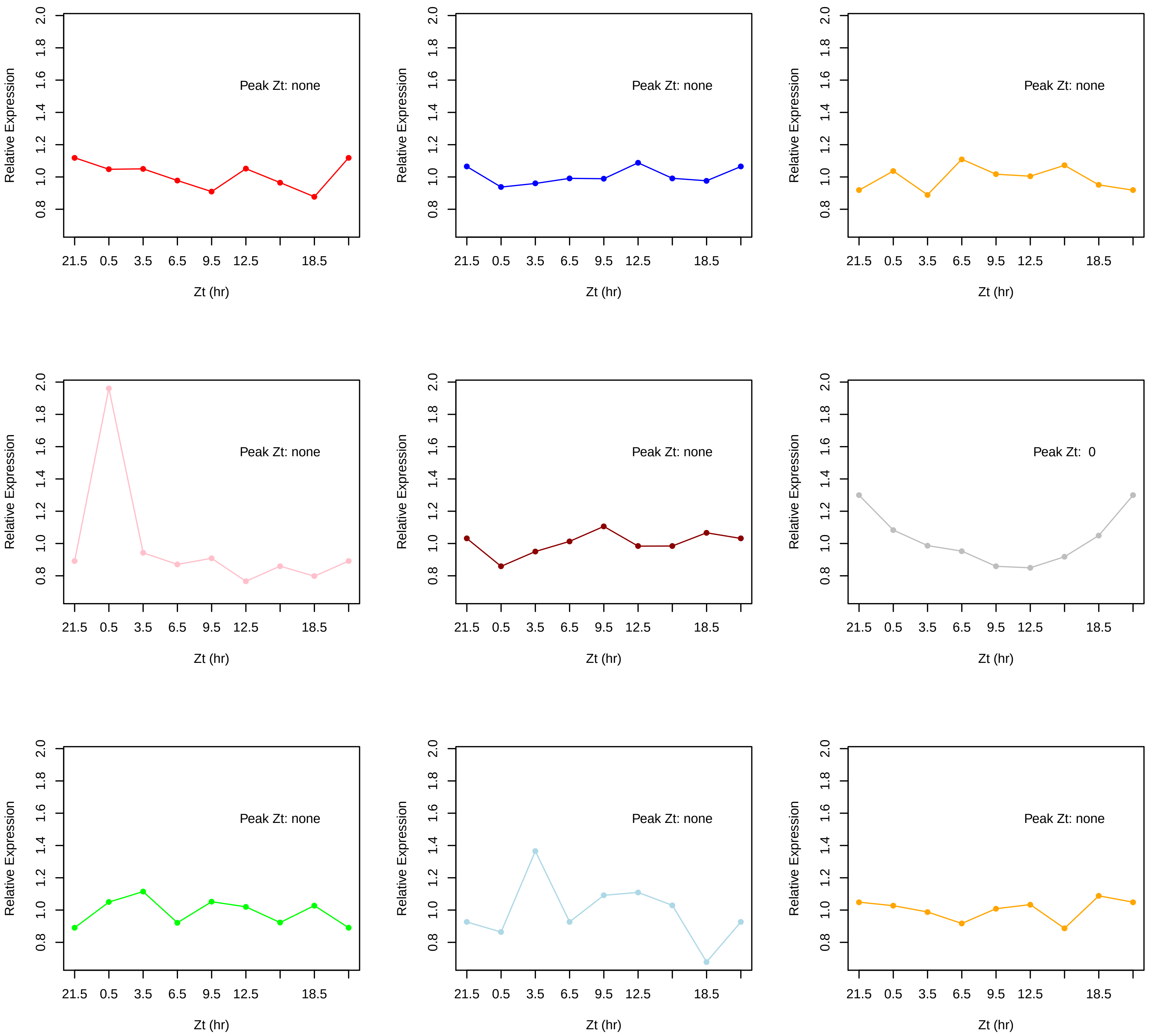

Singeleton: ENSXMAG00000004370 glra2

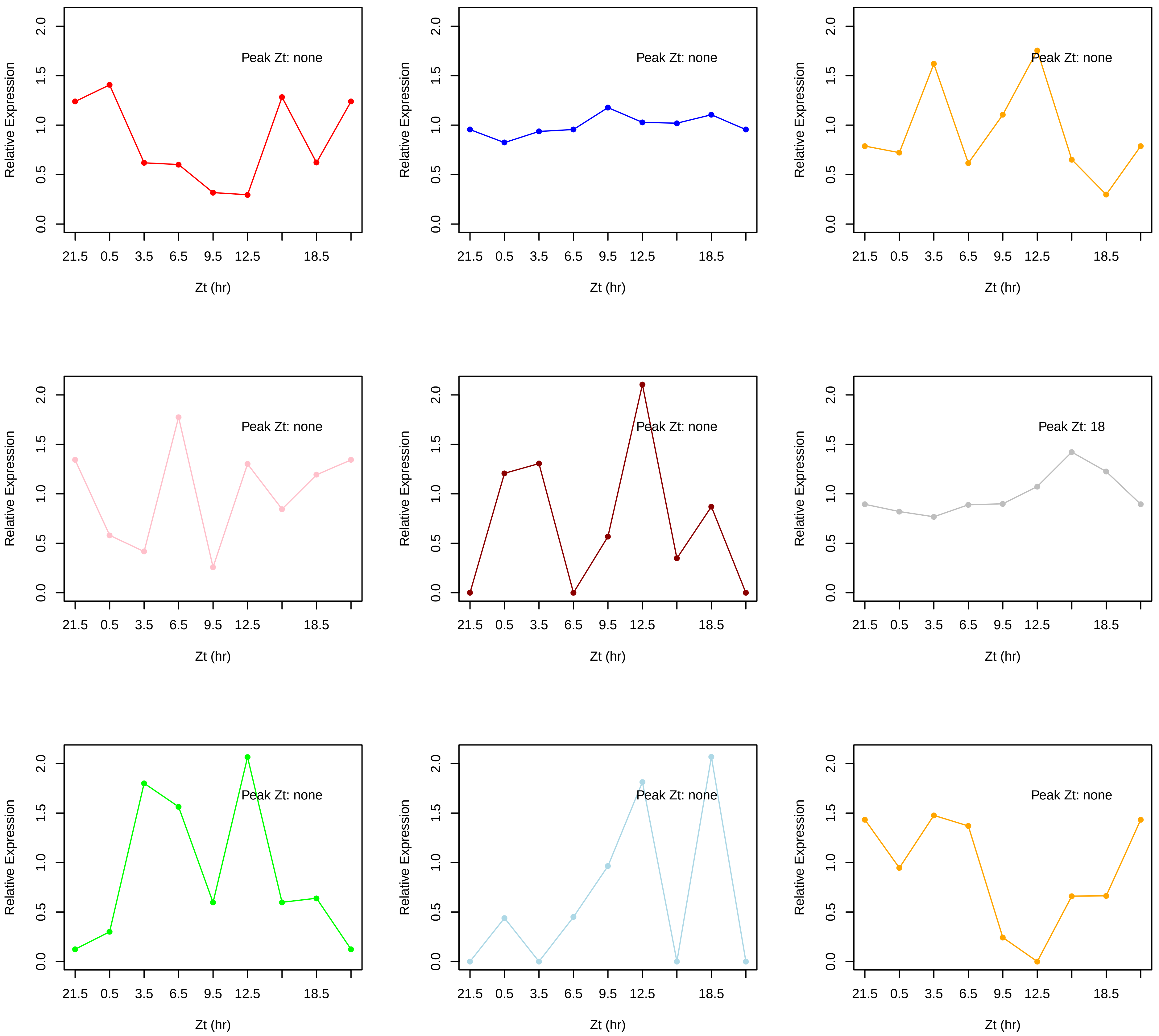

Singeleton: ENSXMAG00000008807 mapk9

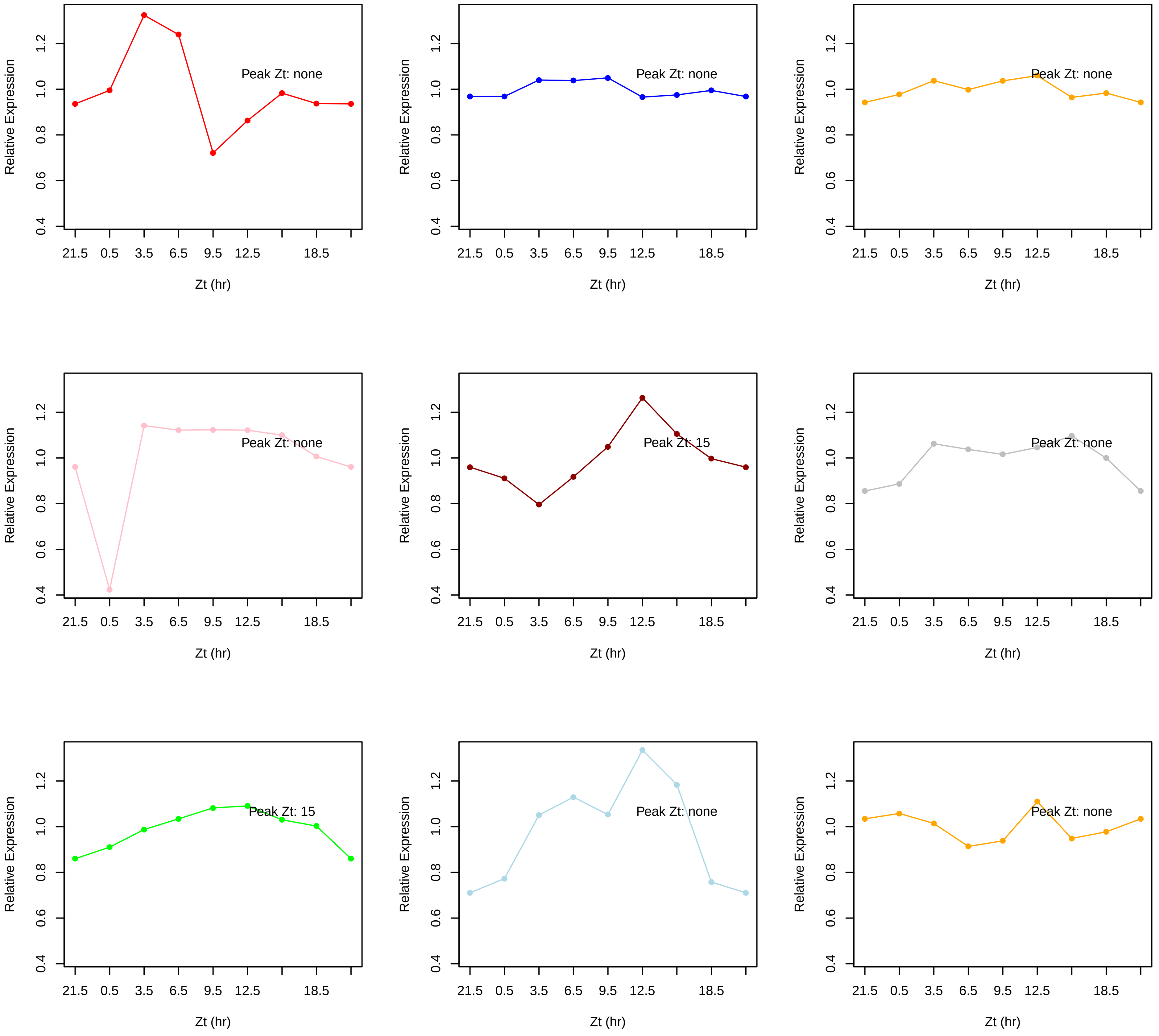

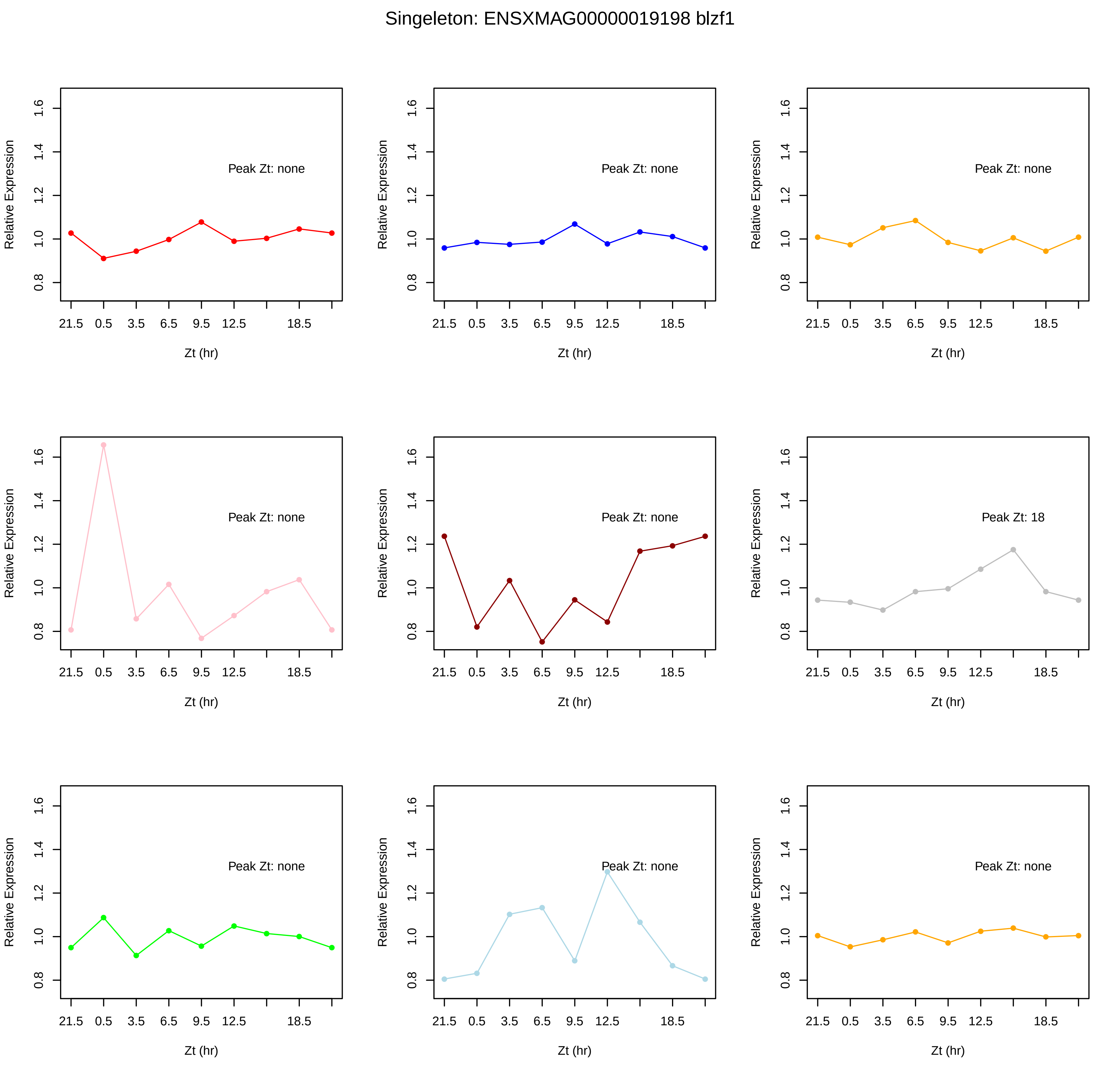
Singeleton: ENSXMAG00000019198 blzf1
Relative Expression
Zt (hr)
21.5
0.5
3.5
6.5
9.5
12.5
18.5
0.8
1.0
1.2
1.4
1.6
Peak Zt: none
Peak Zt: 18

Singleton: ENSXMAG00000012278 crmp1

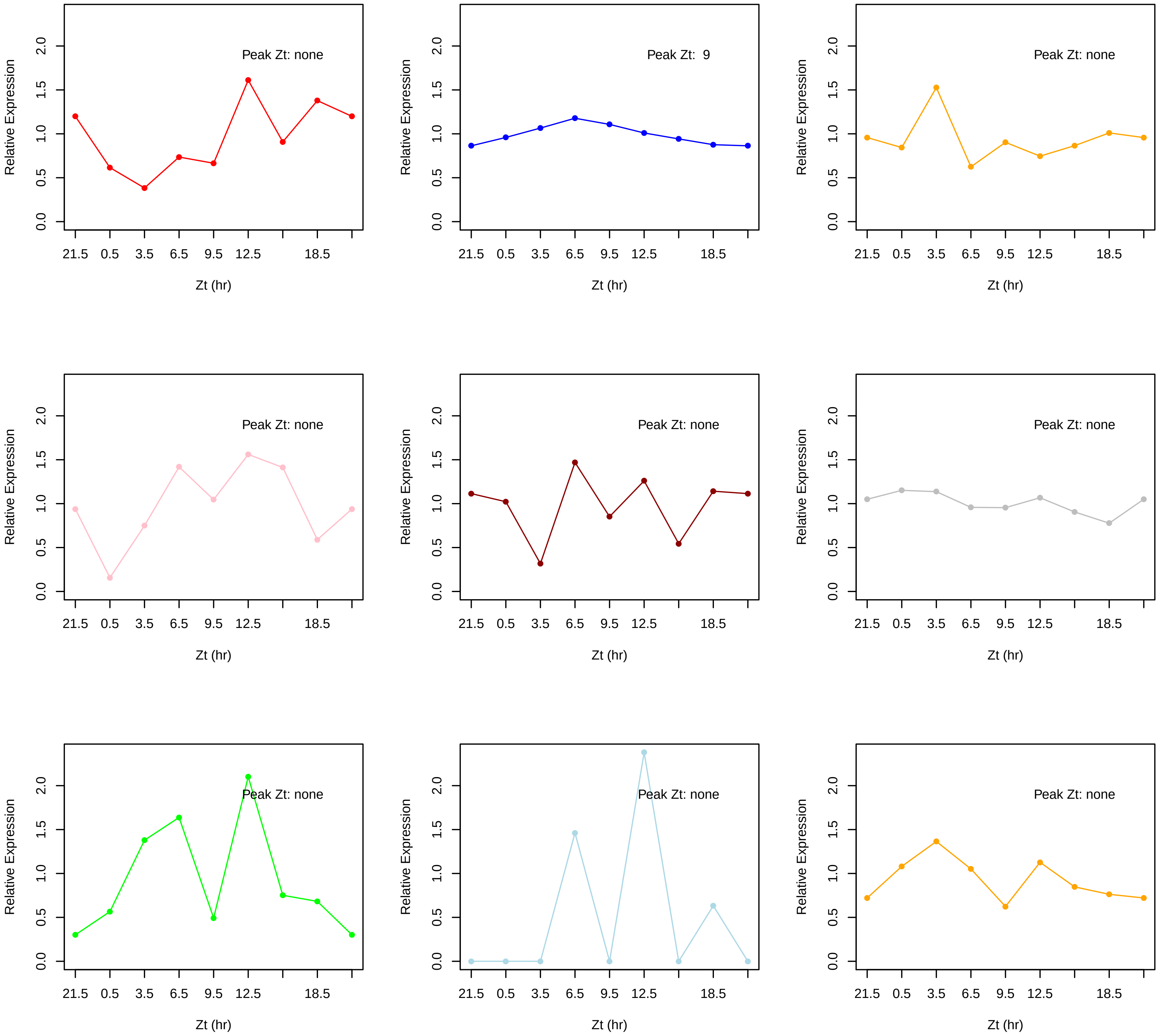

Singeleton: ENSXMAG00000001135 ndufa10

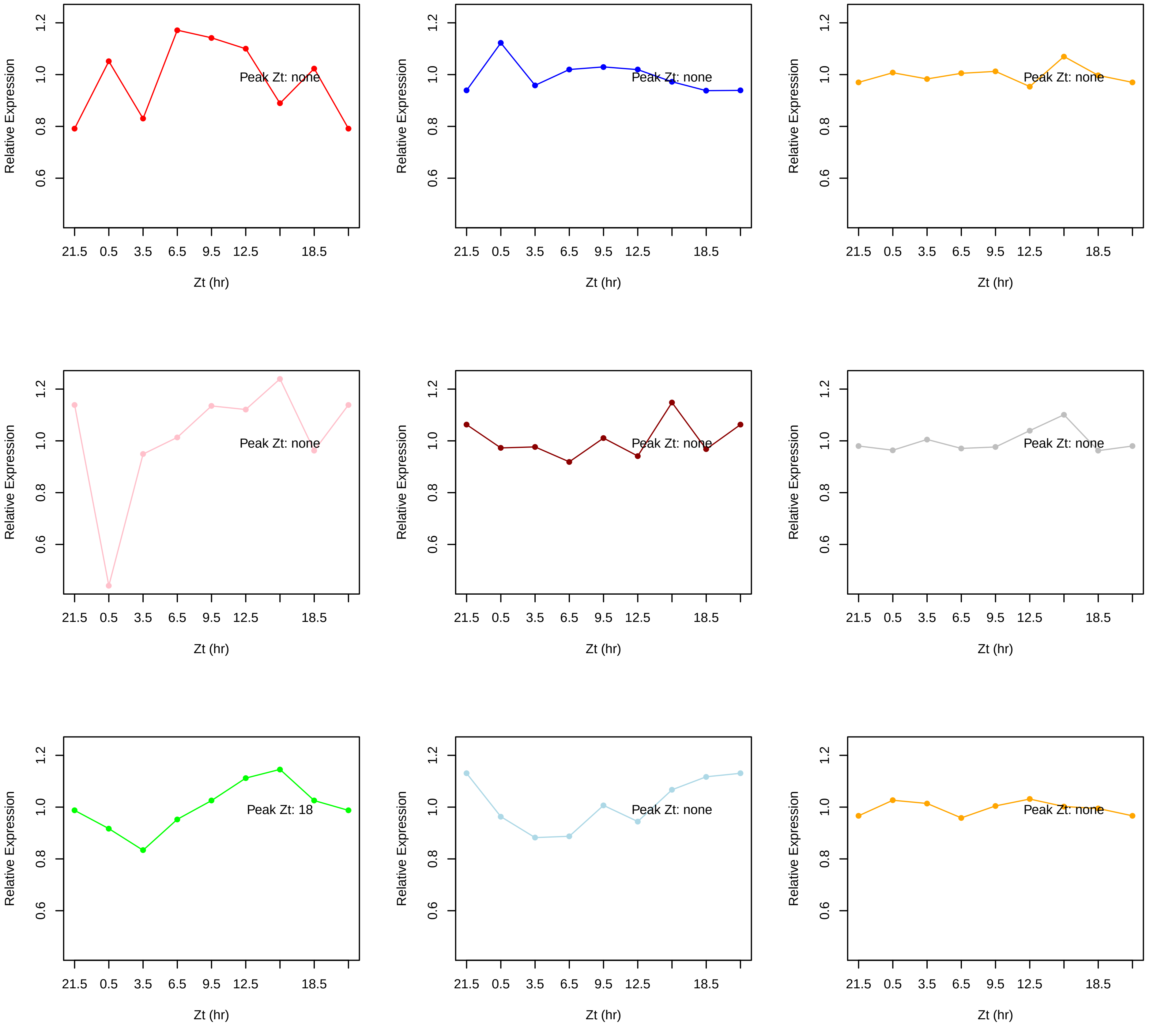

Singeleton: ENSXMAG00000025521 six6a

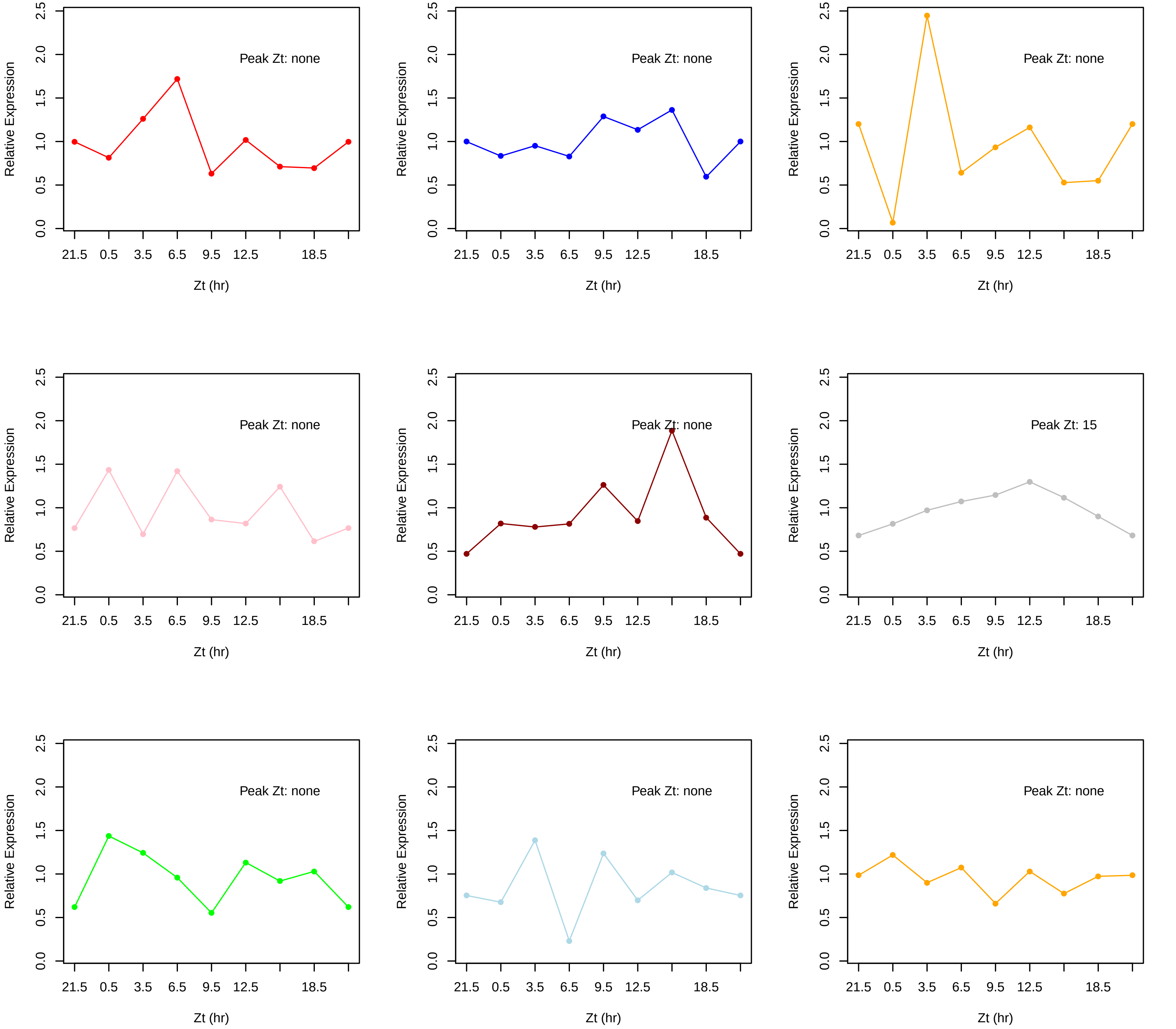

Singleton: ENSXMAG00000026312 six1b

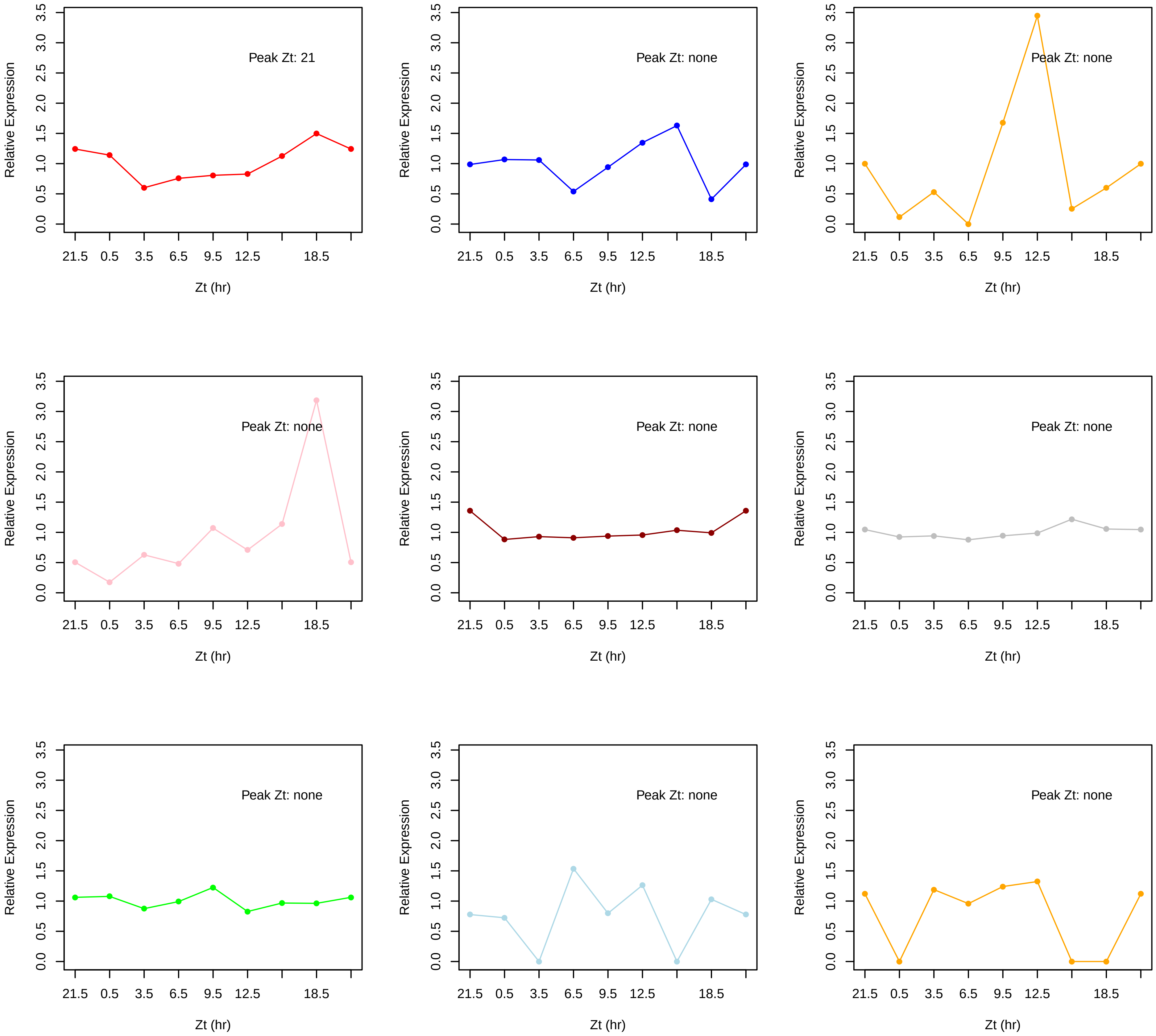

Singeleton: ENSXMAG00000004358 mospd2

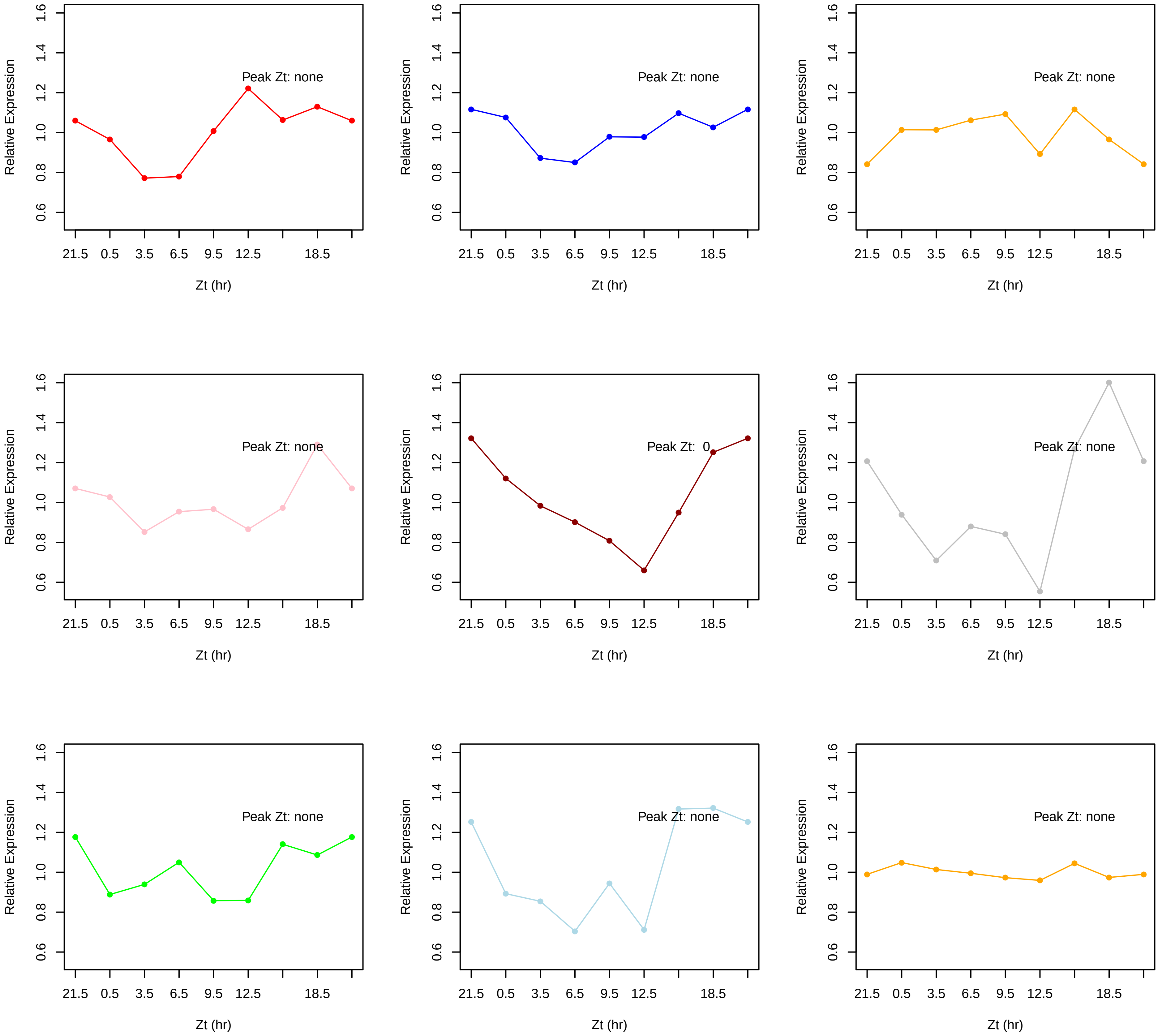

Singeleton: ENSXMAG00000002223 arl6ip5a

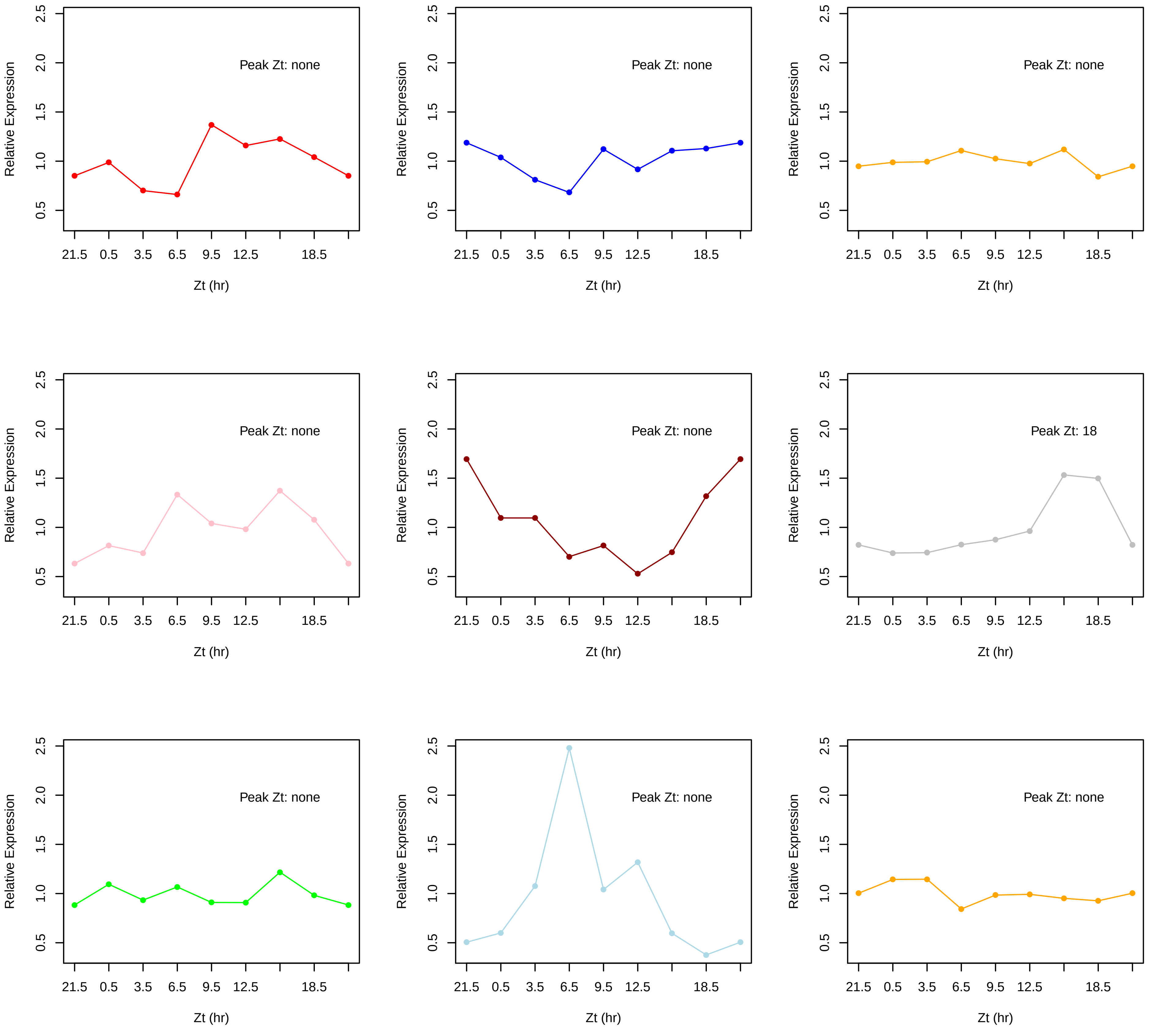

Singleton: ENSXMAG00000013476 mnat1

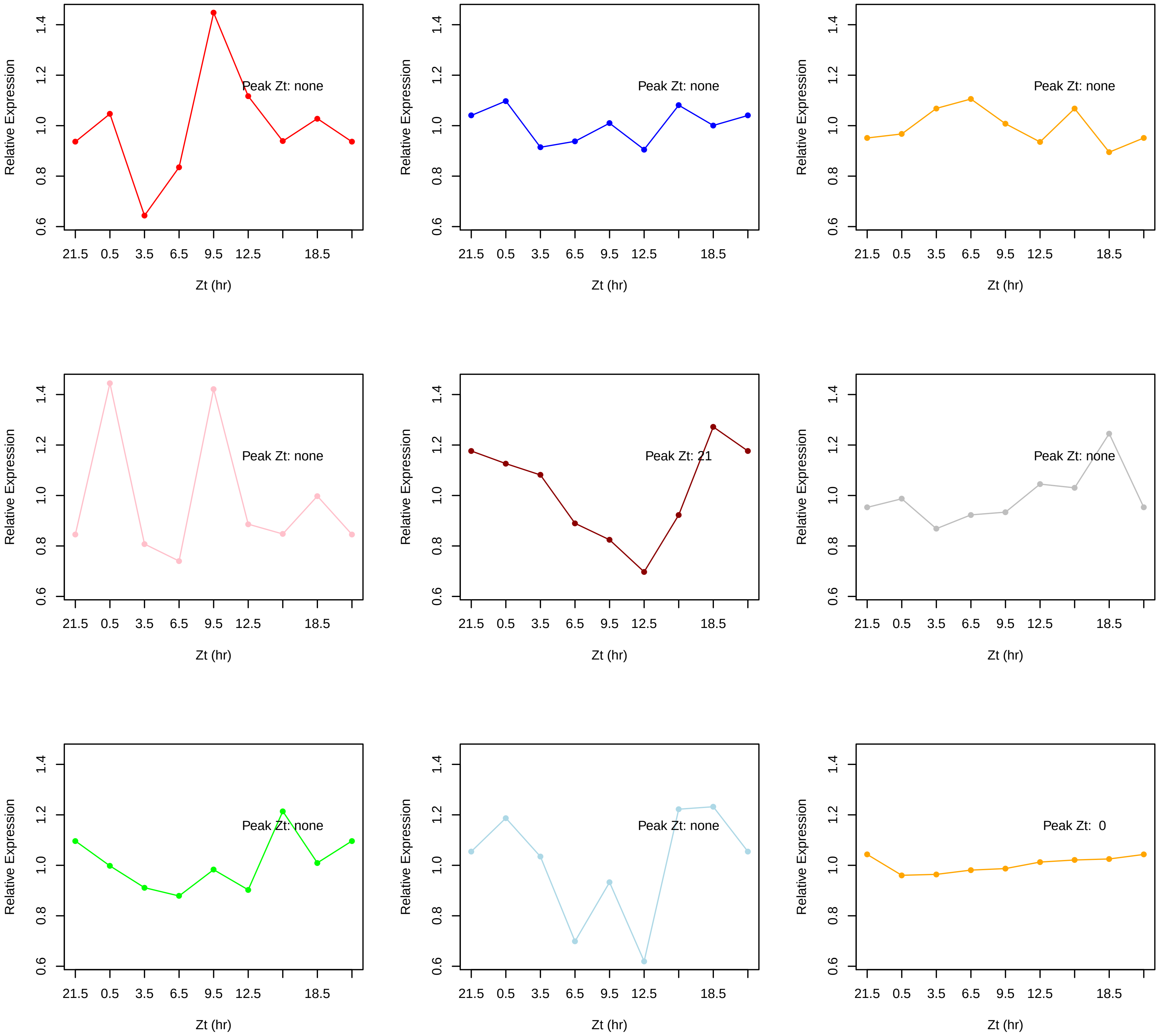

Singeleton: ENSXMAG00000001862 puf60b

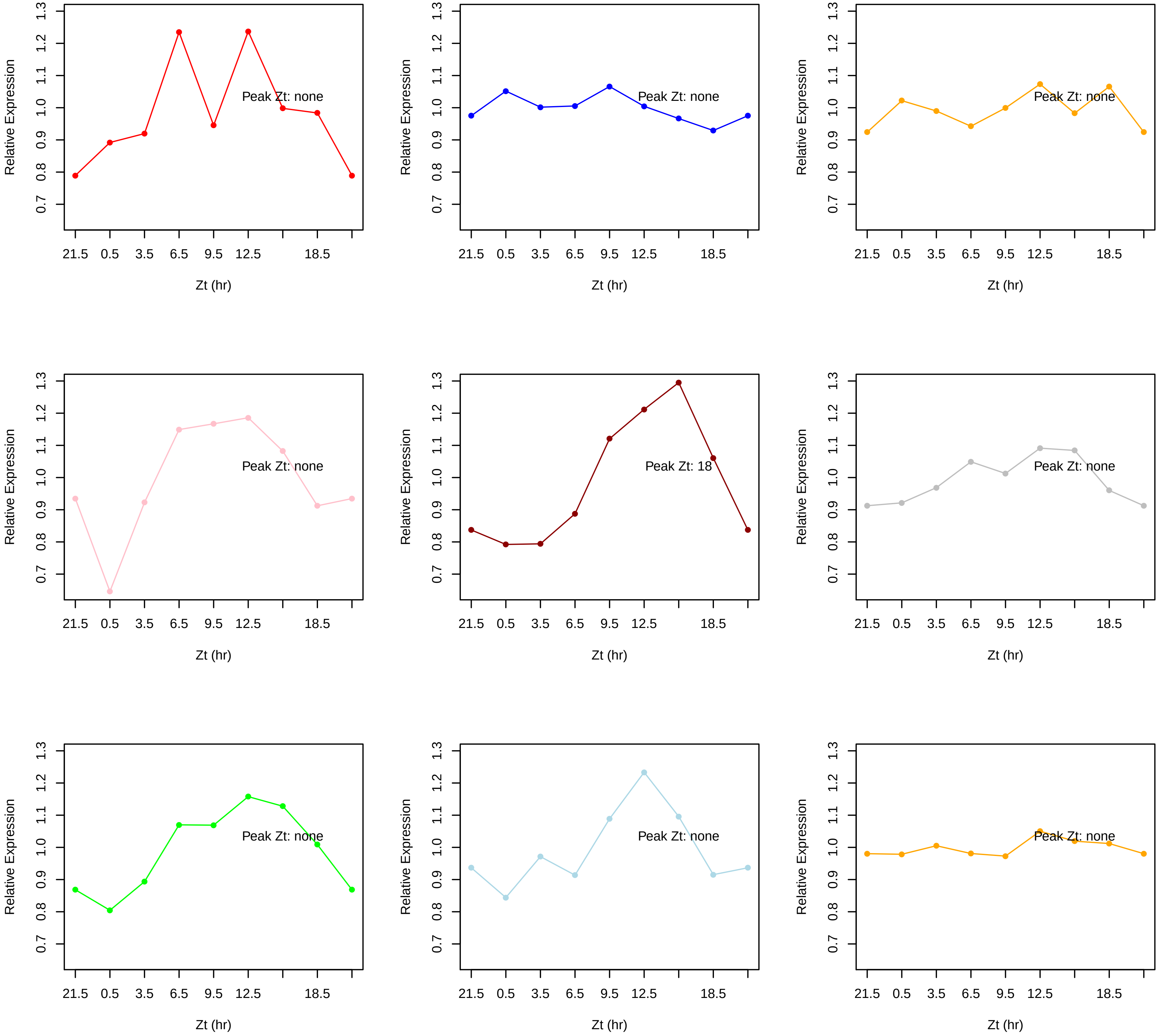

Singeleton: ENSXMAG00000006013 ipo9

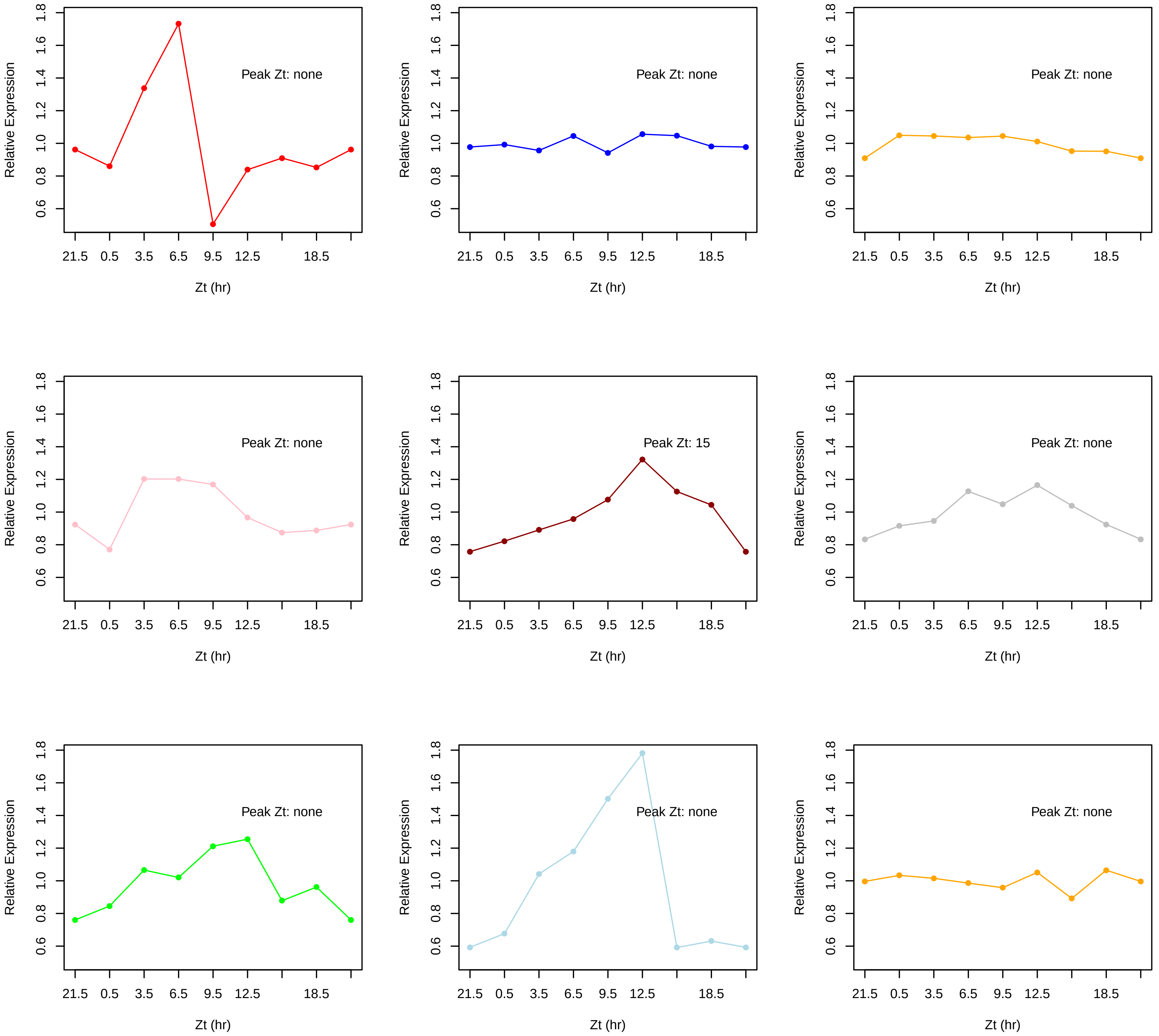

Singleton: ENSXMAG00000011447 JAKMIP1

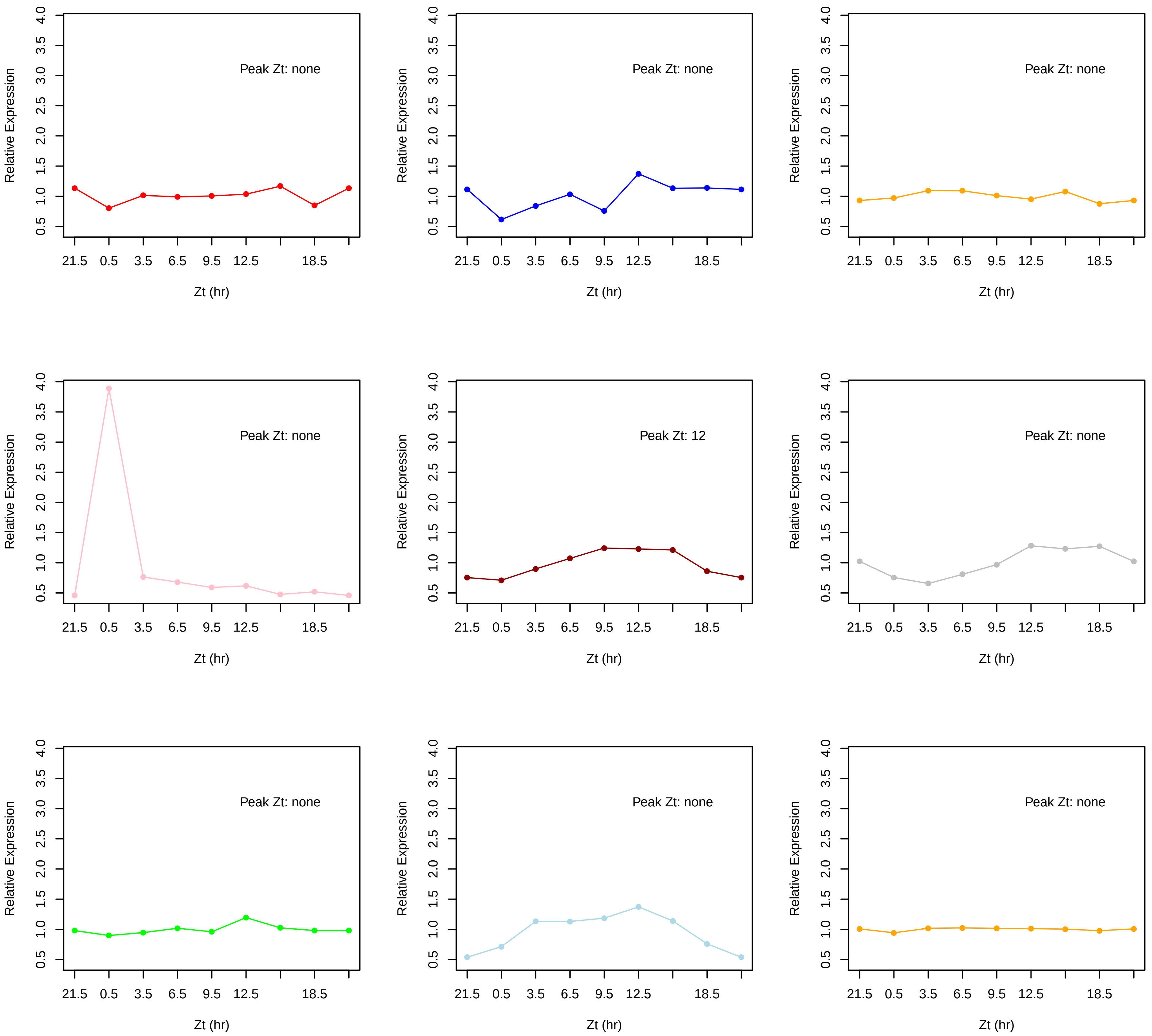

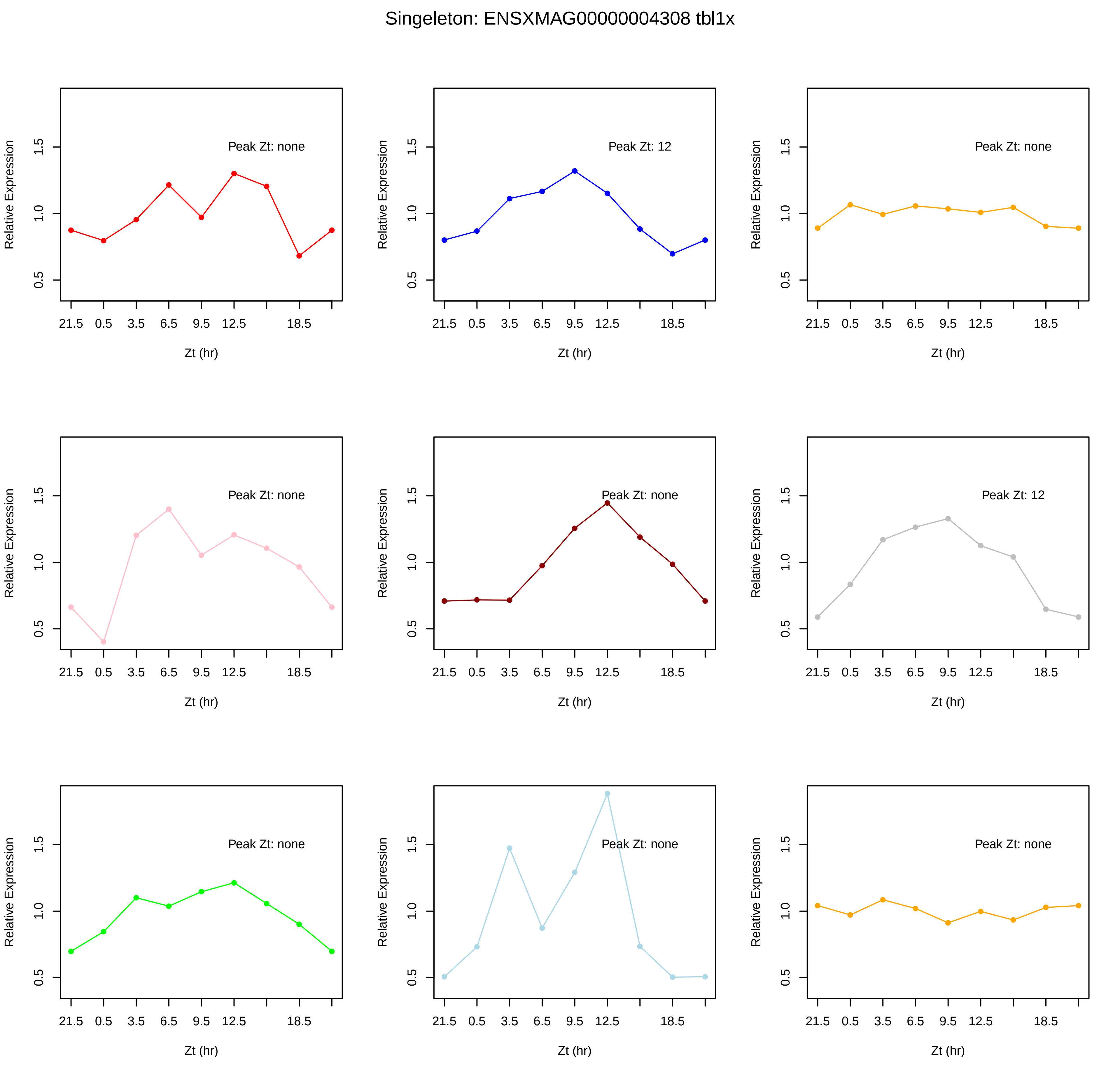
Singeleton: ENSXMAG00000004308 tbl1x
Peak Zt: none
Peak Zt: 12
Peak Zt: none
Peak Zt: none
Peak Zt: none
Peak Zt: 12
Peak Zt: none
Peak Zt: none
Peak Zt: none
Relative Expression
Zt (hr)
21.5 0.5 3.5 6.5 9.5 12.5 18.5
0.5 1.0 1.5

Singeleton: ENSXMAG00000013147 gem

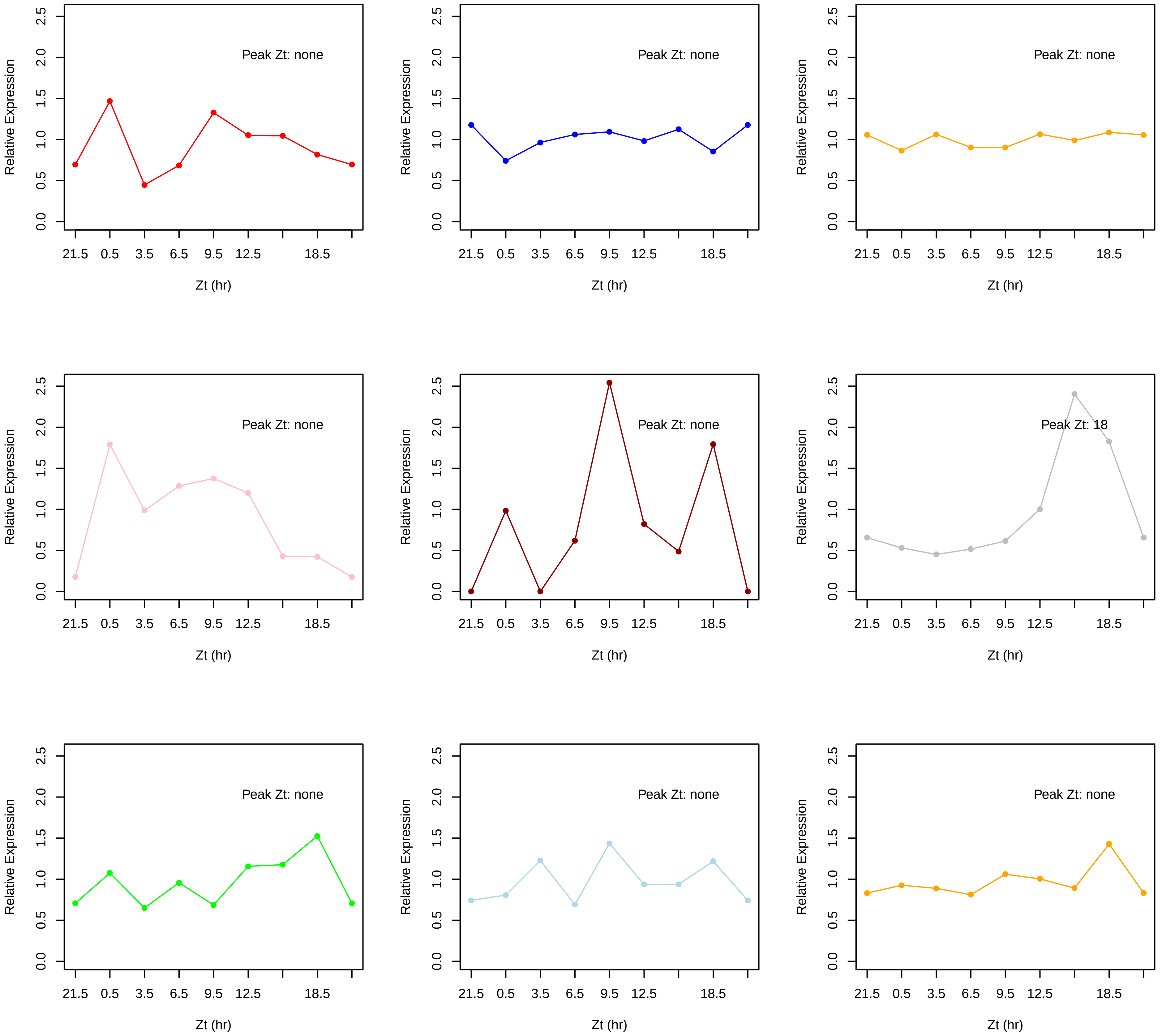

Singeleton: ENSXMAG00000010496 tmf1

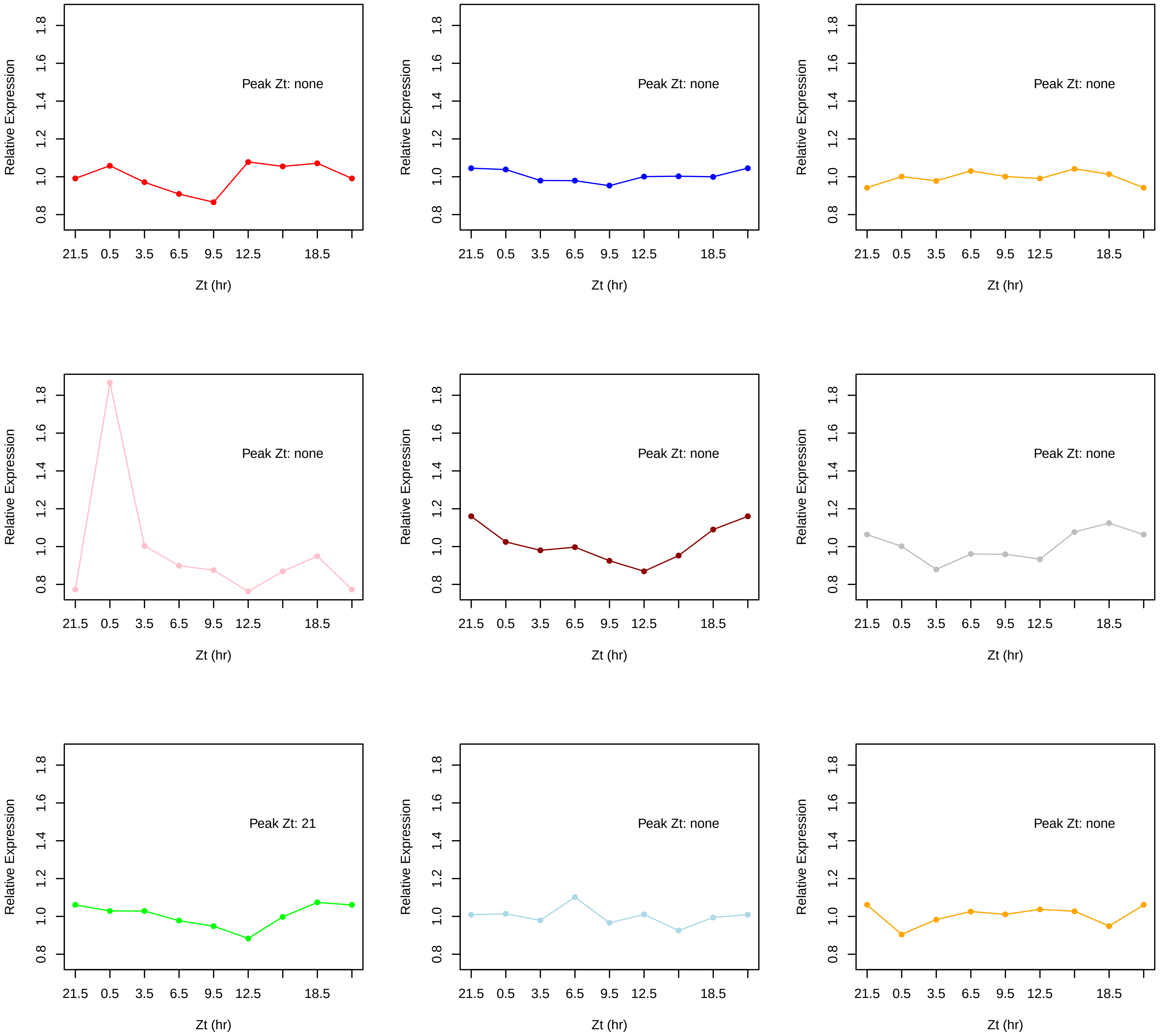

Singeleton: ENSXMAG00000013474 trmt5

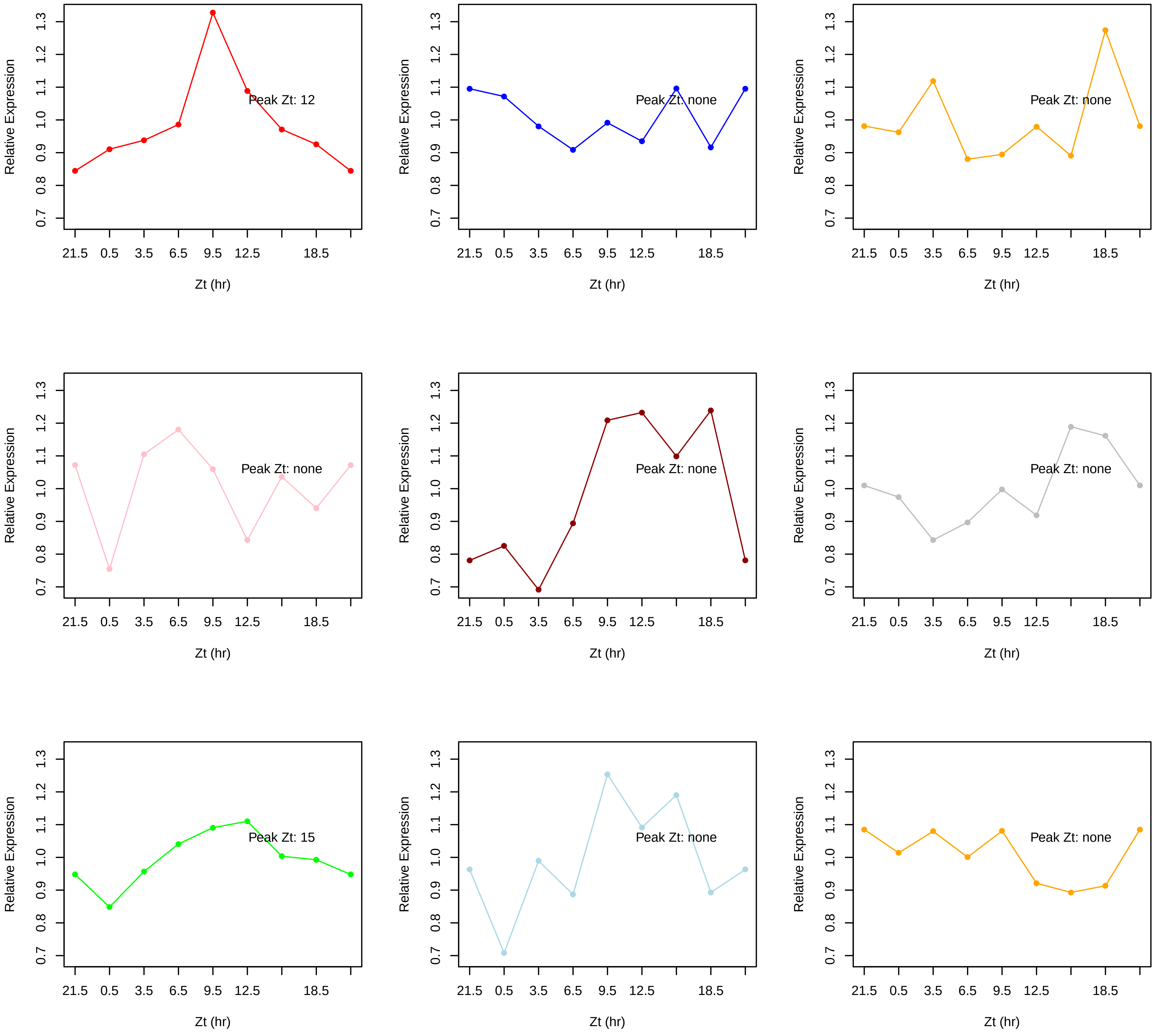

Singeleton: ENSXMAG00000010501 eogt

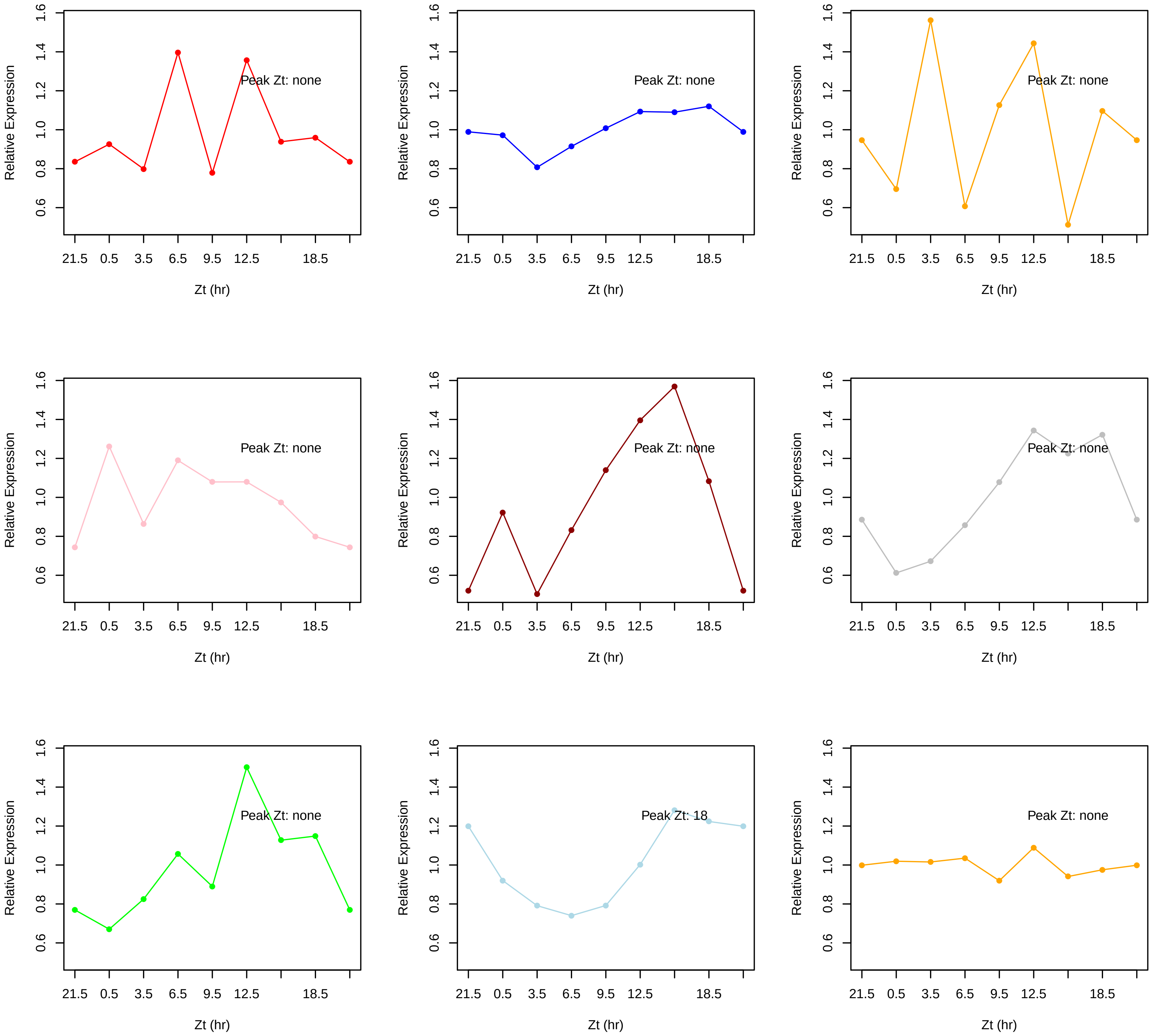

Singeleton: ENSXMAG00000001864 scrib

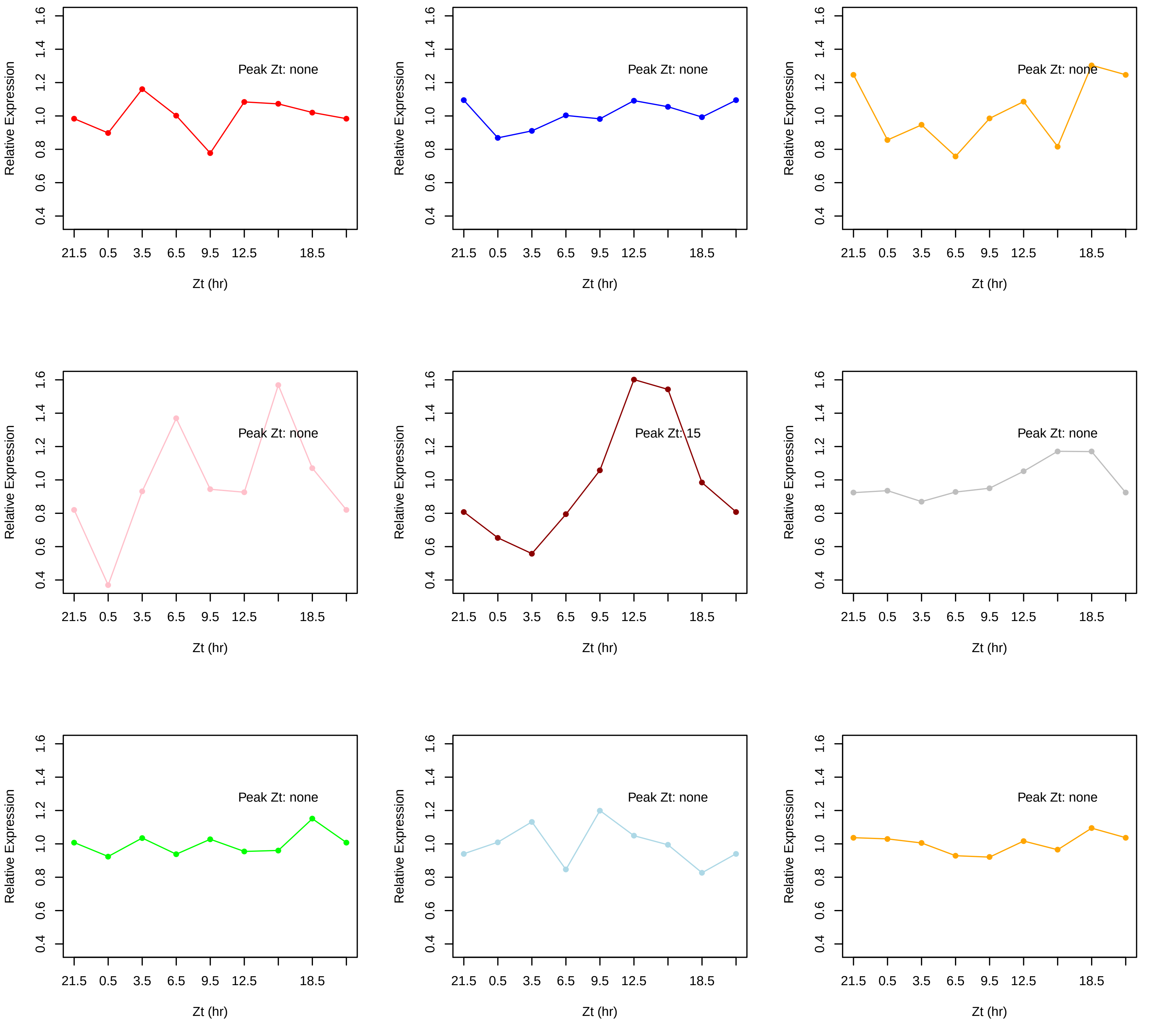

Singeleton: ENSXMAG00000023532 rabif

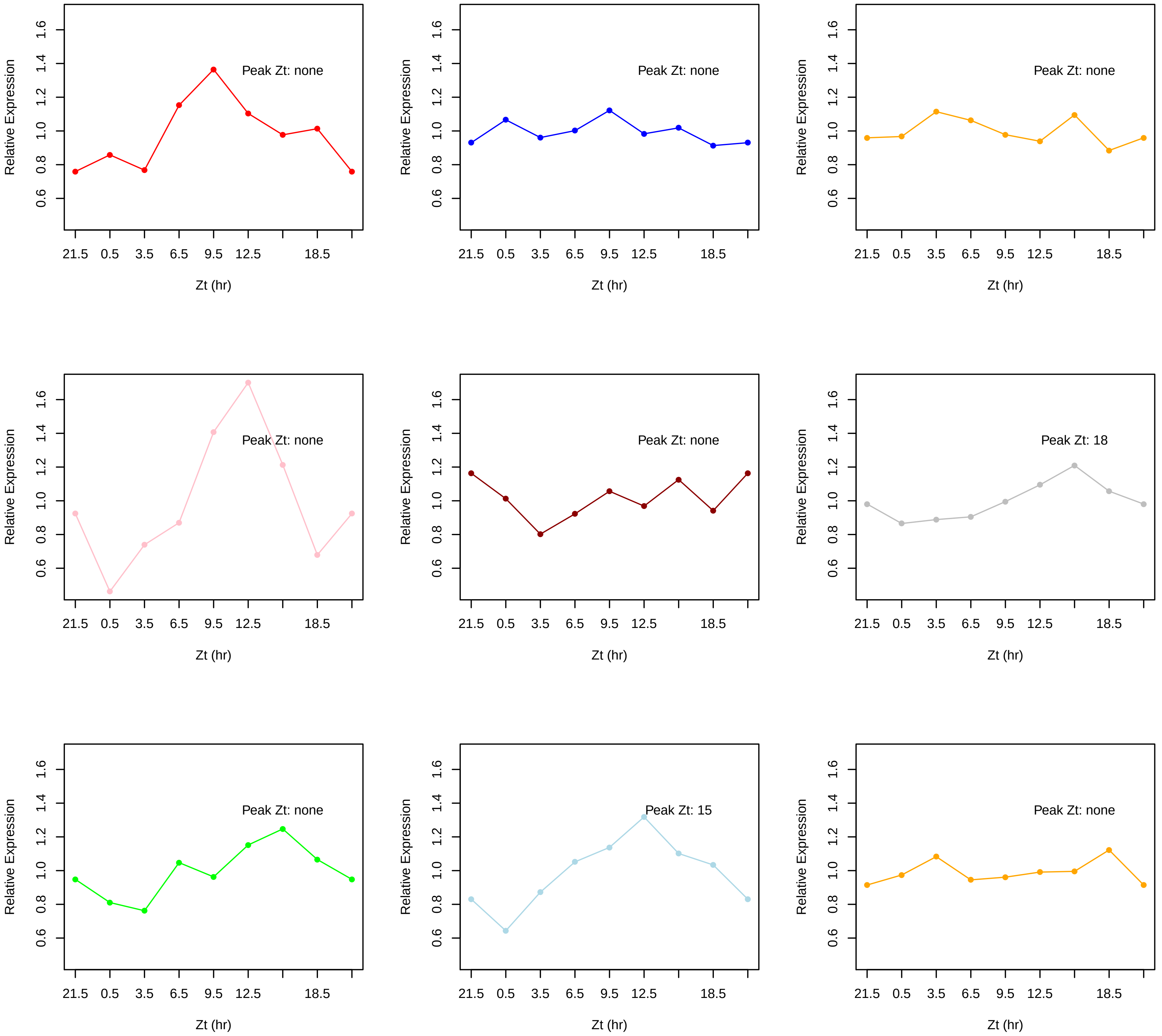

Singleton: ENSXMAG00000013448 hif1aa

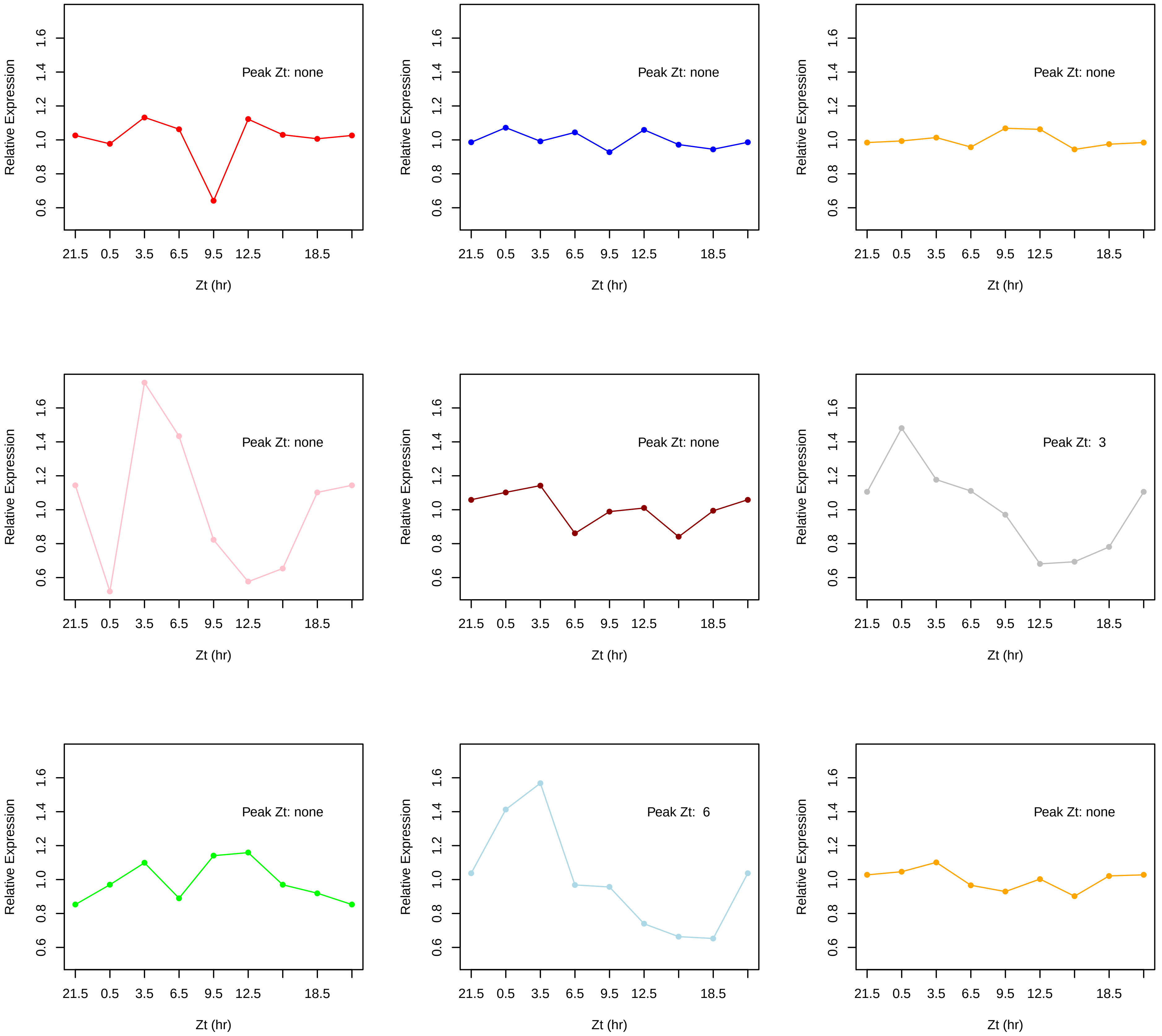

Singeleton: ENSXMAG00000027609 triqk

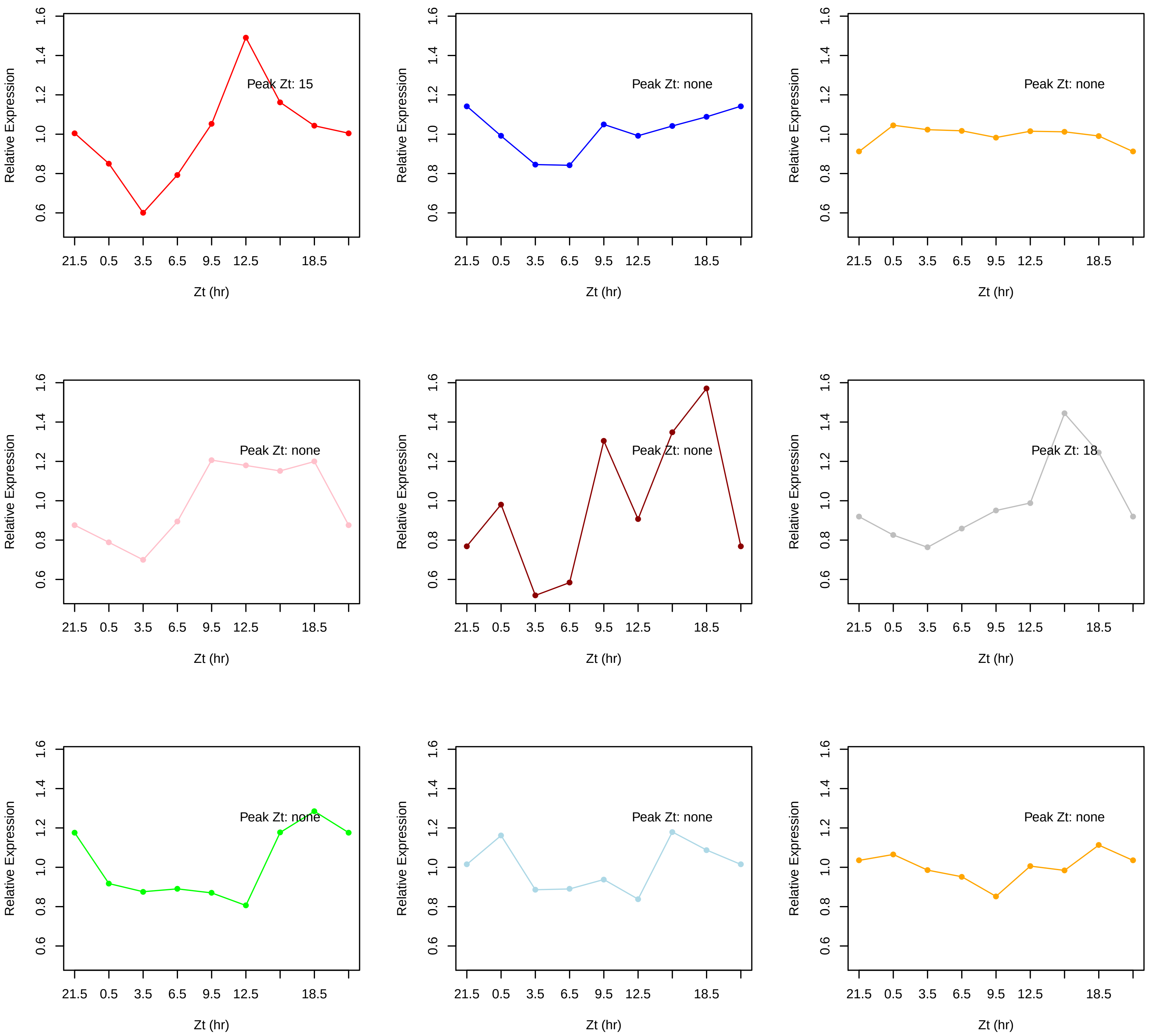

Singeleton: ENSXMAG00000011484

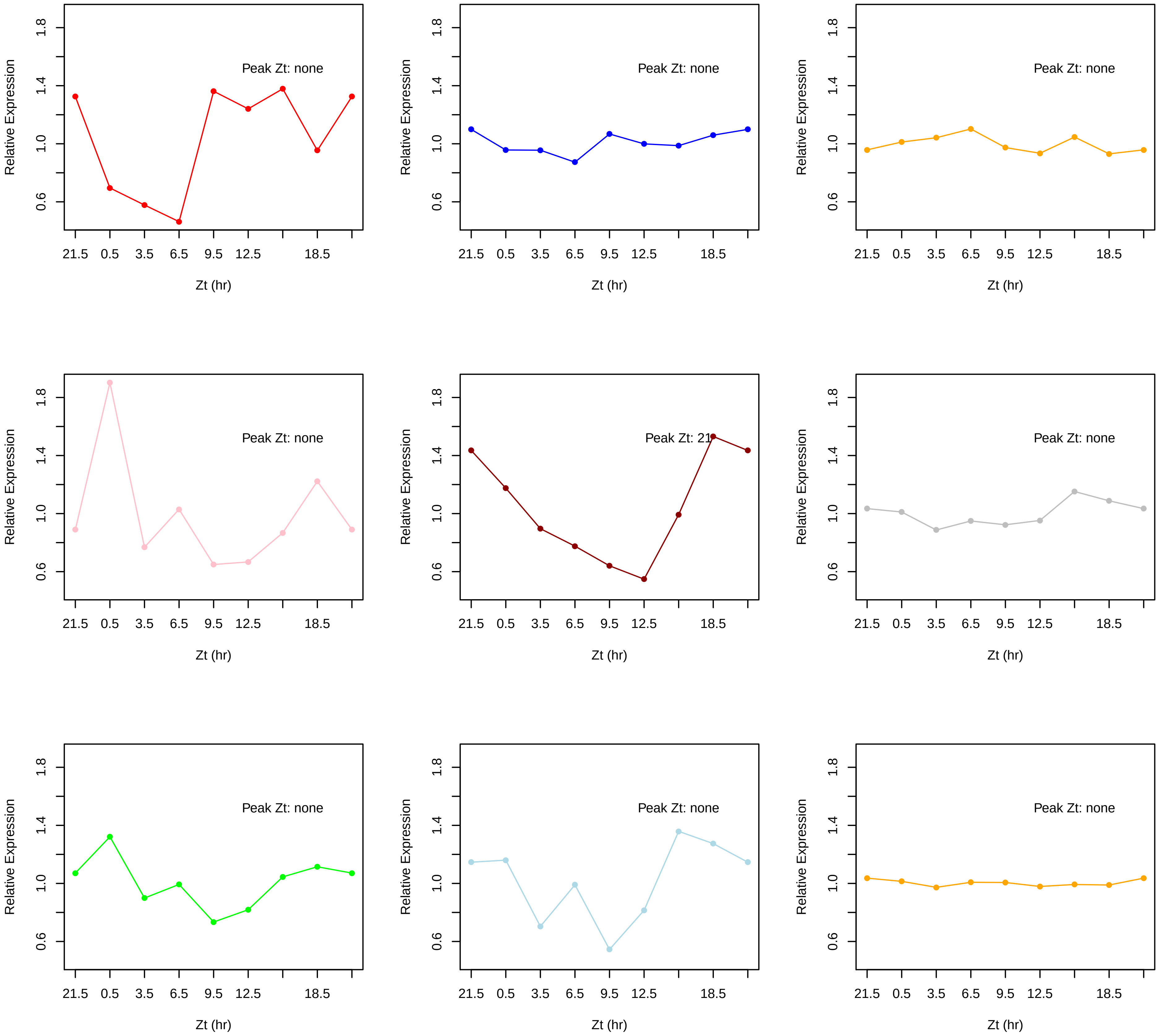

Singeleton: ENSXMAG00000010534 suclg2

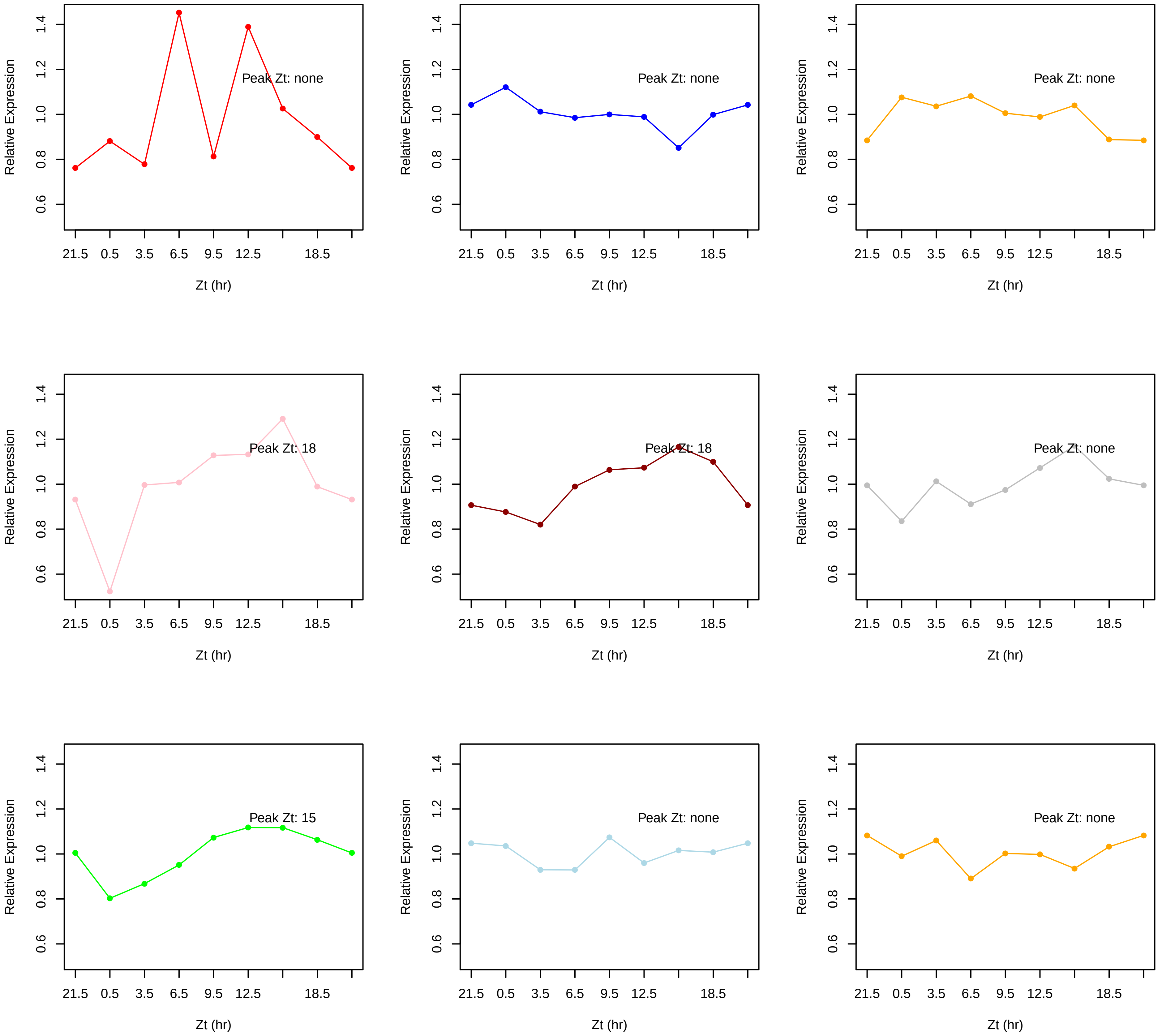

Singleton: ENSXMAG00000004847 dhx29

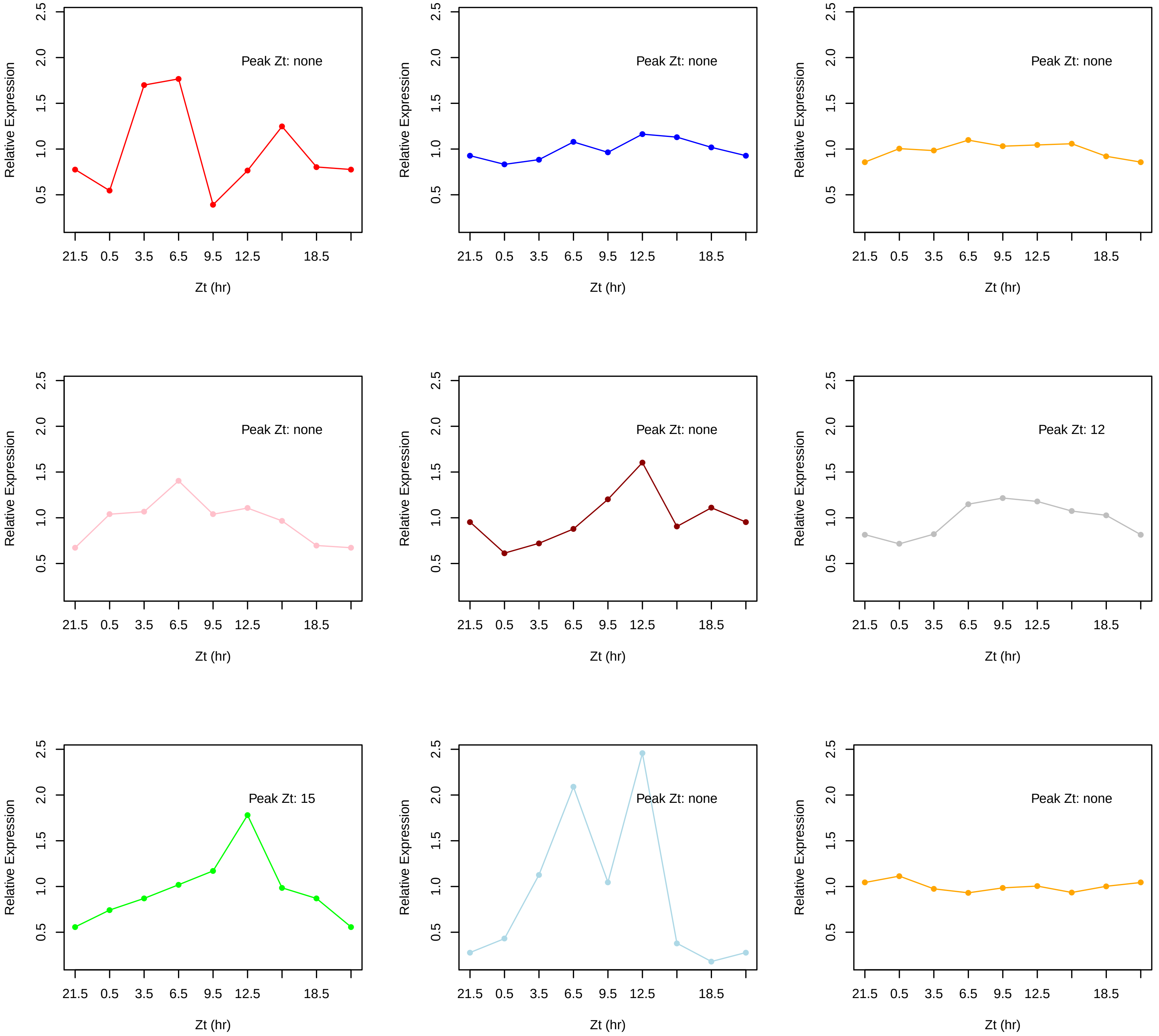

Singeleton: ENSXMAG00000008318

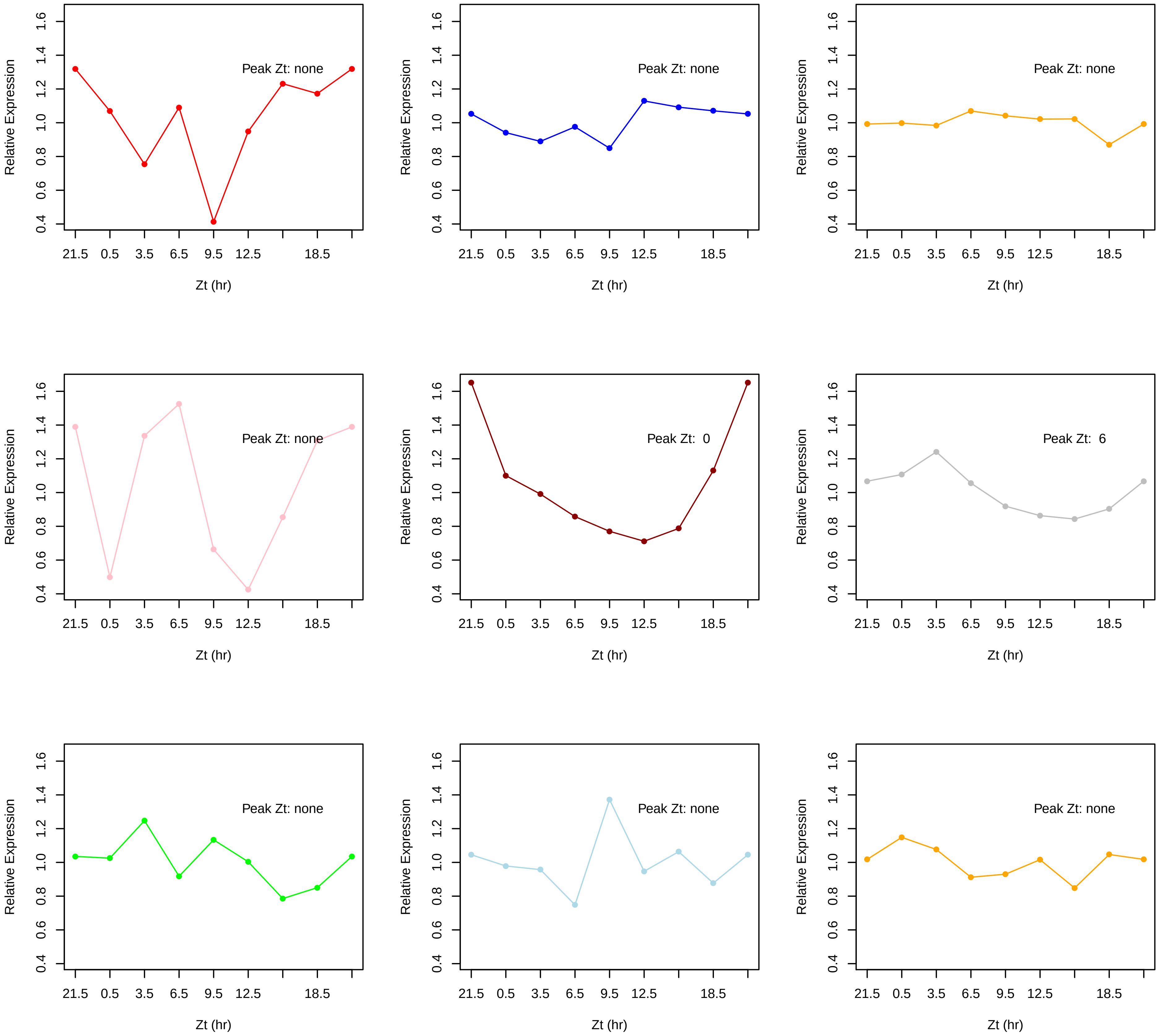

Singleton: ENSXMAG00000008312 ddx46

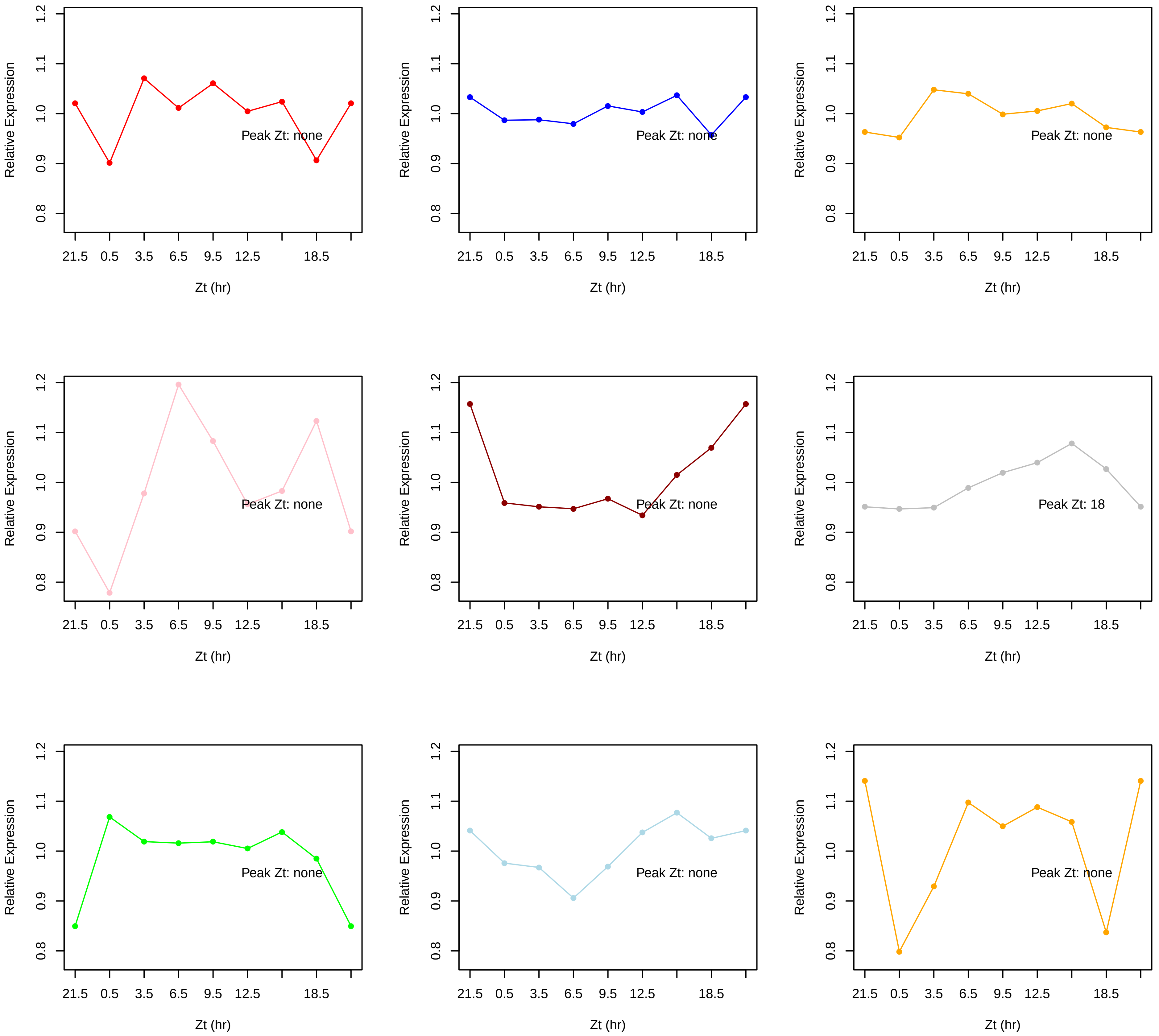

Singleton: ENSXMAG00000025726 PUDP

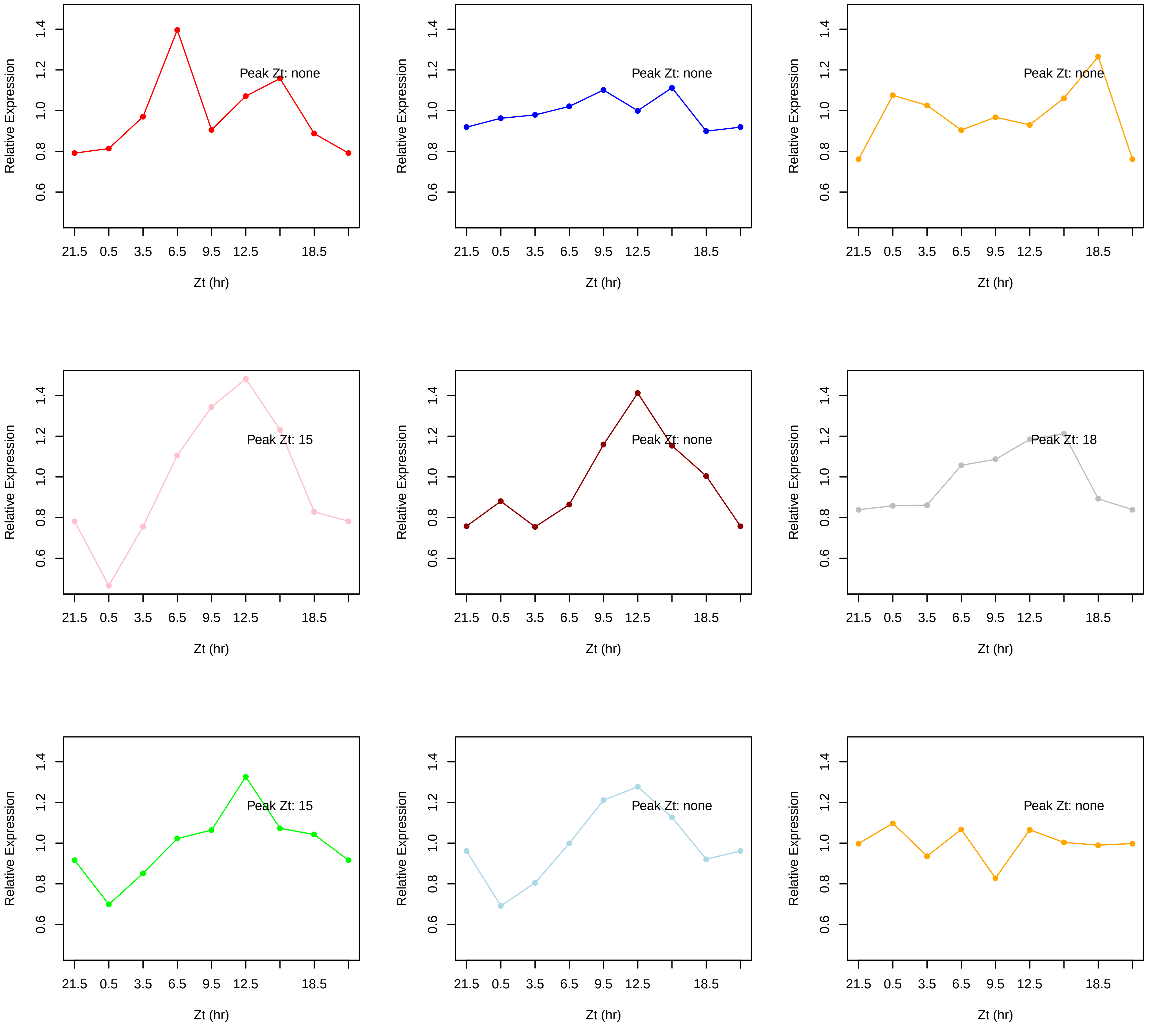

Singleton: ENSXMAG00000011390 grpel1

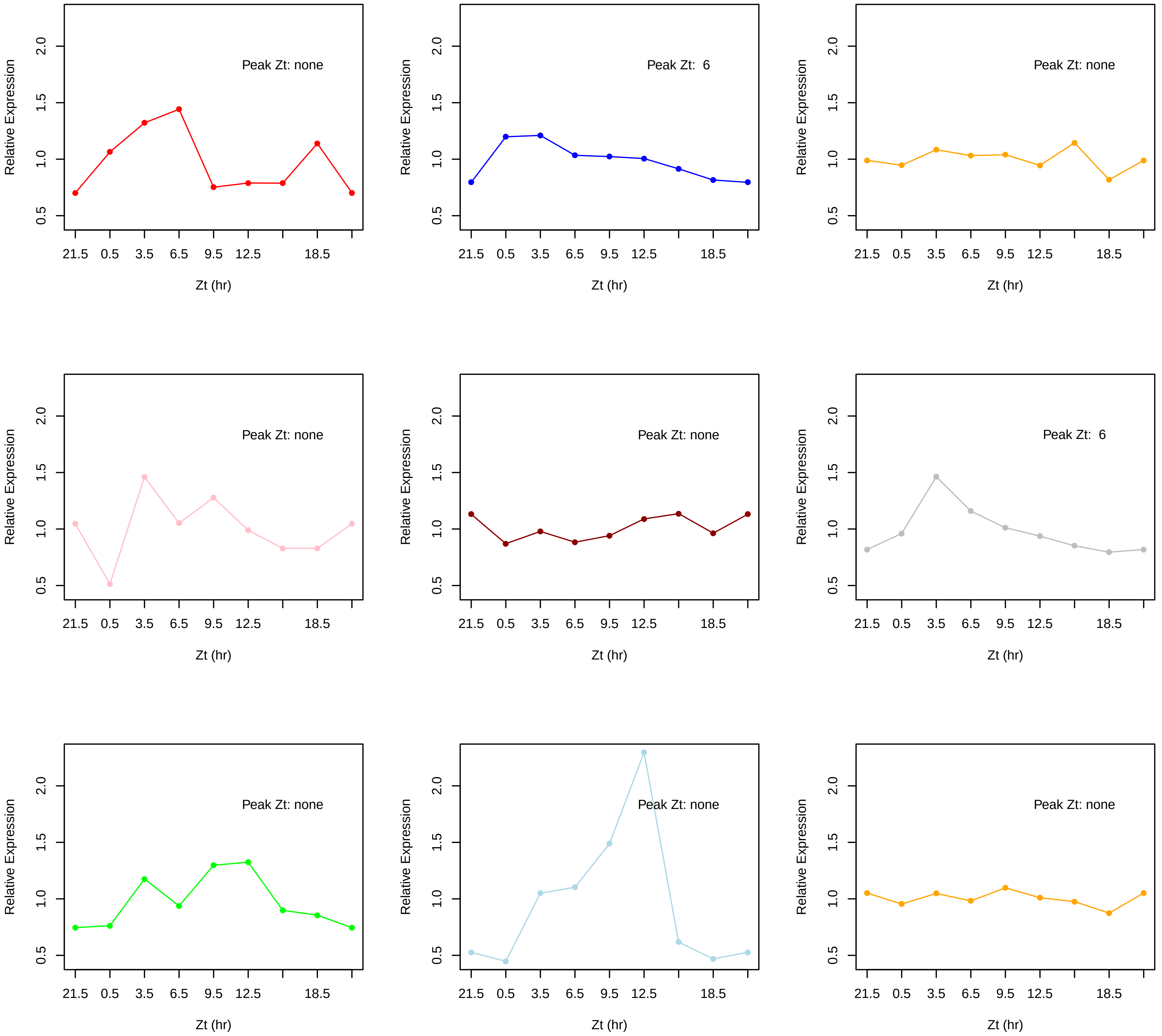

Singeleton: ENSXMAG00000003276 otud6b

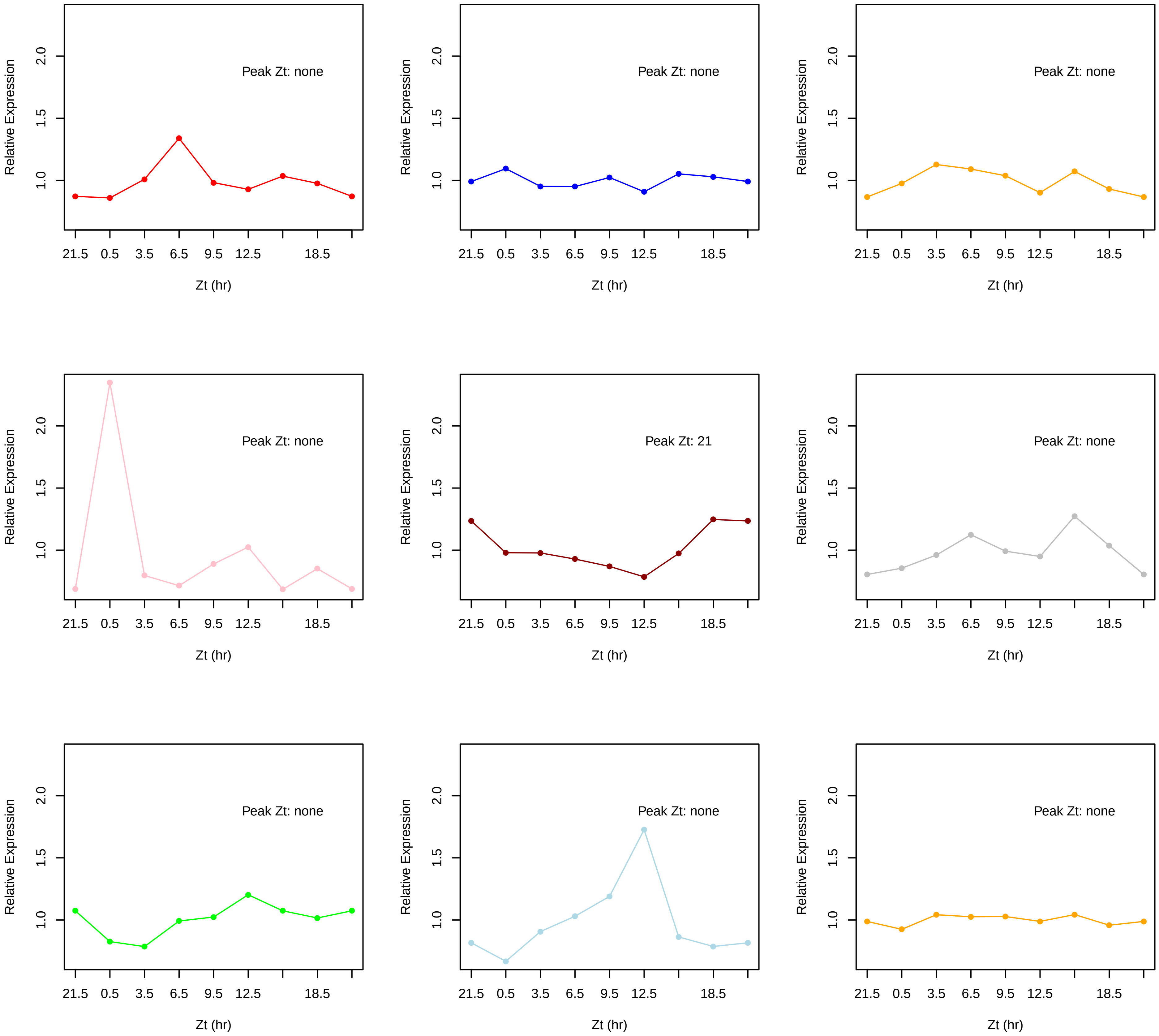

Singeleton: ENSXMAG00000004470 cmpk

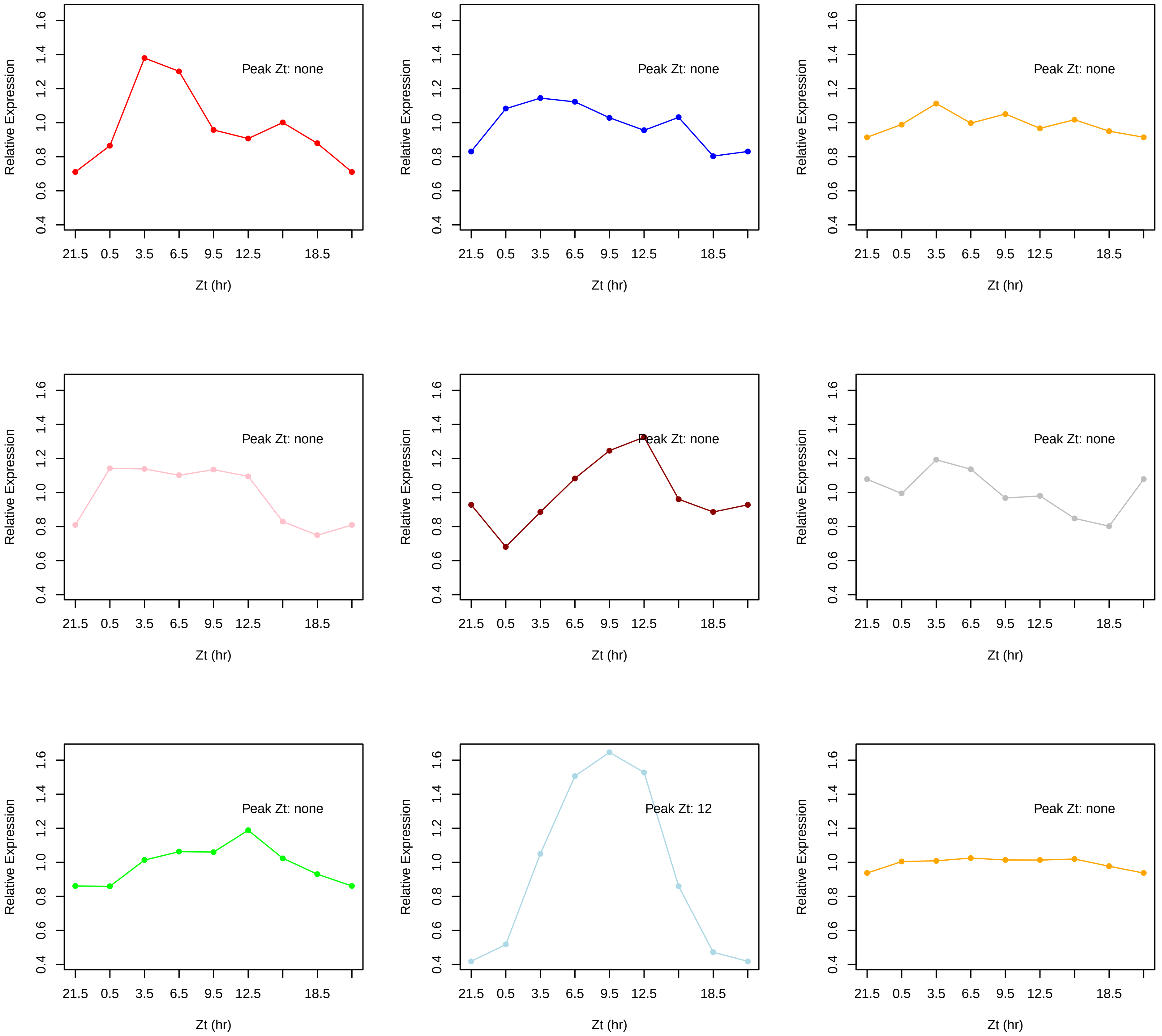

Singeleton: ENSXMAG00000008298 sec24a

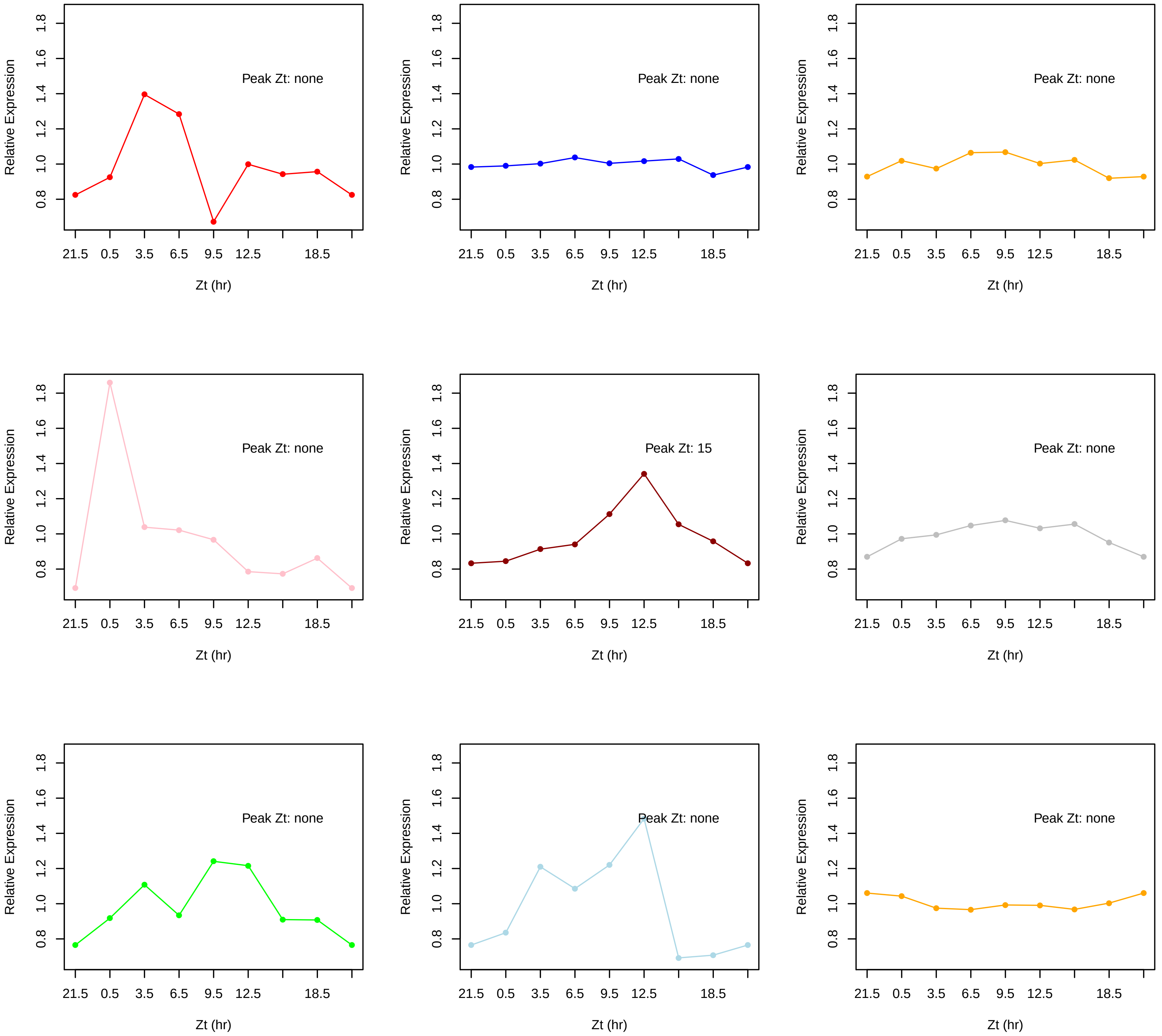

Singeleton: ENSXMAG00000018900 prkx

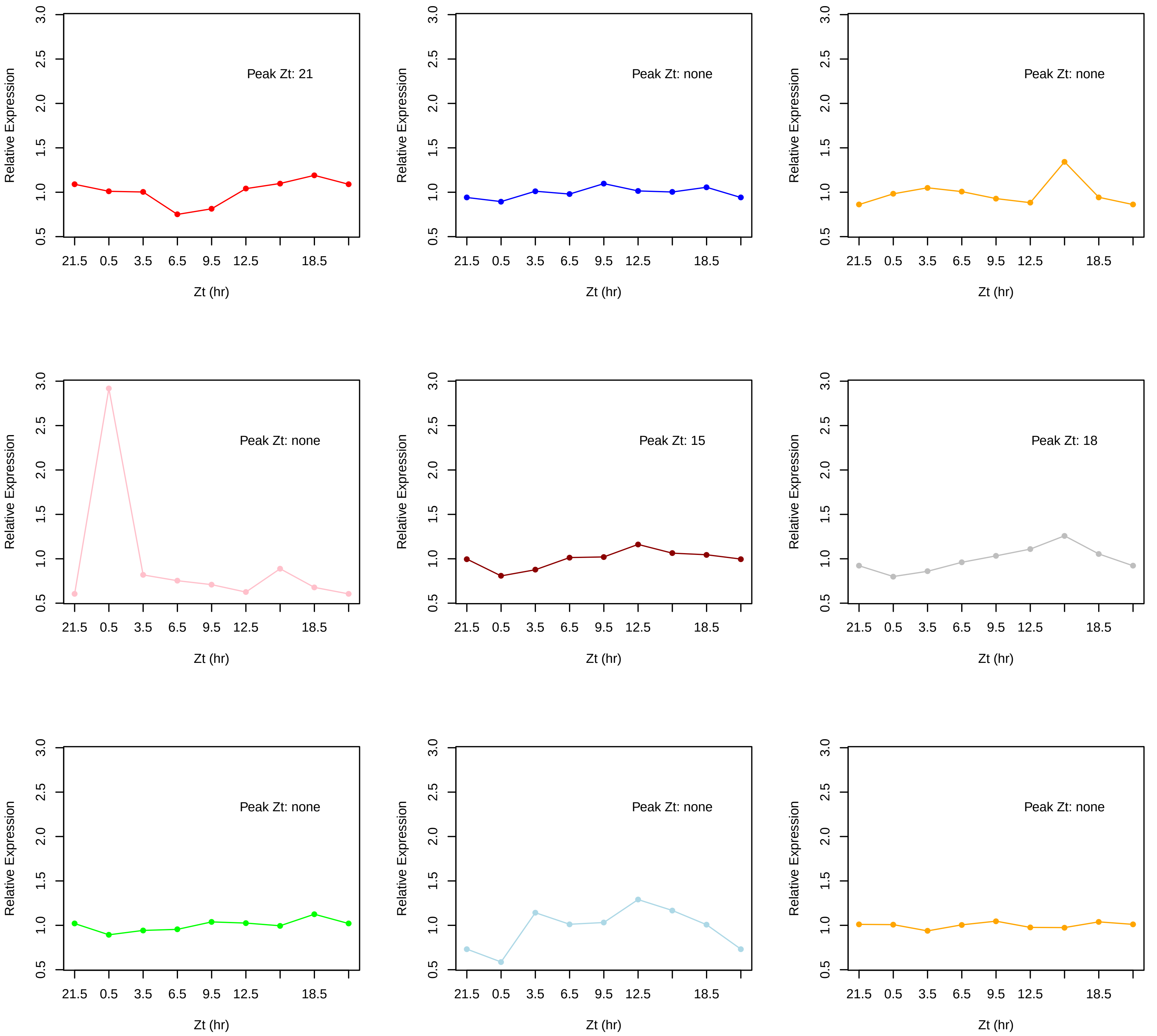

Singleton: ENSXMAG00000003494 pip4p2

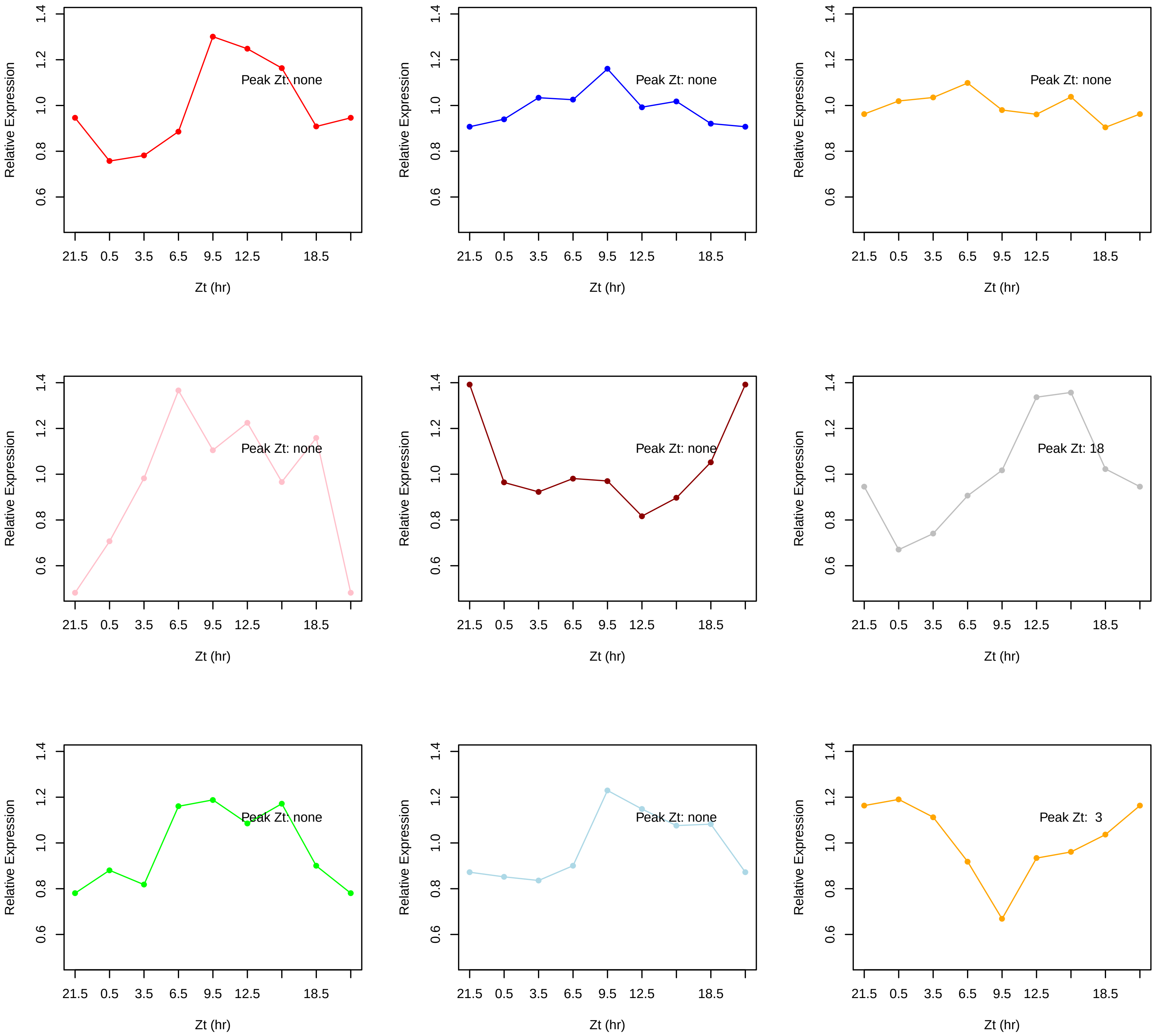

Singeleton: ENSXMAG00000013705 ical1

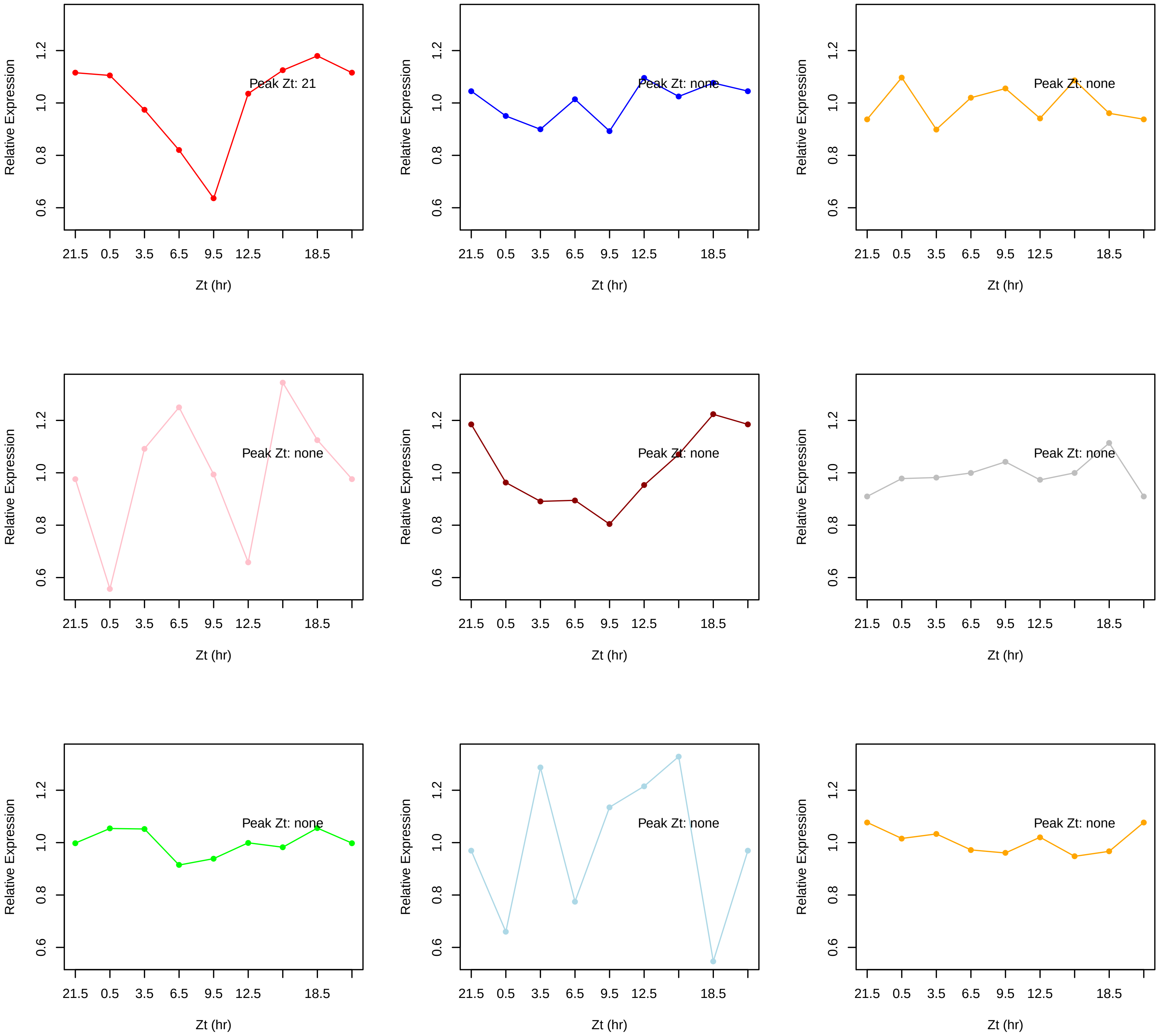

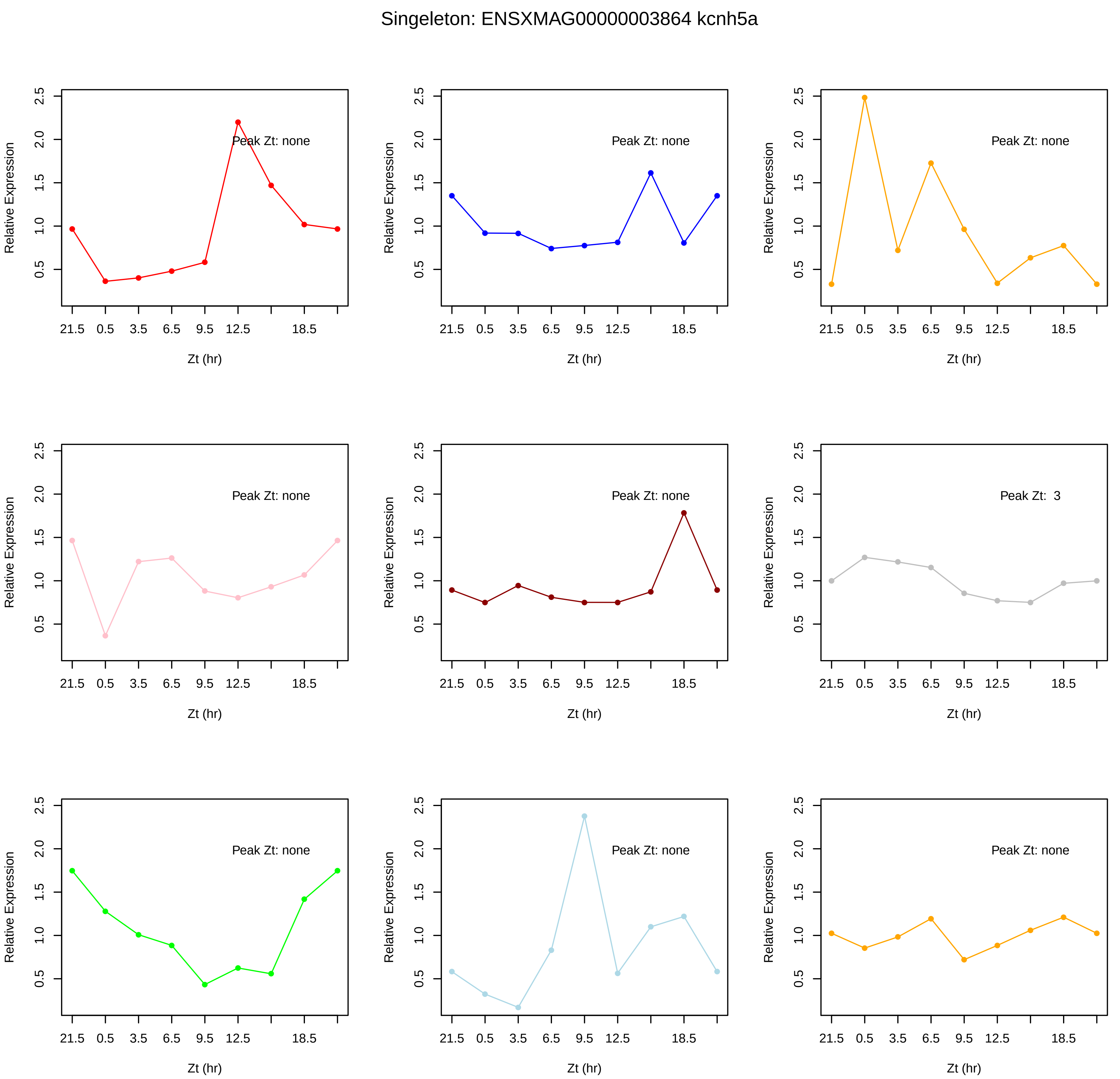
Singeleton: ENSXMAG00000003864 kcnh5a
Peak Zt: none
Peak Zt: none
Peak Zt: none
Peak Zt: none
Peak Zt: none
Peak Zt: 3
Peak Zt: none
Peak Zt: none
Peak Zt: none
Relative Expression
Zt (hr)
21.5
0.5
3.5
6.5
9.5
12.5
18.5
0.5
1.0
1.5
2.0
2.5

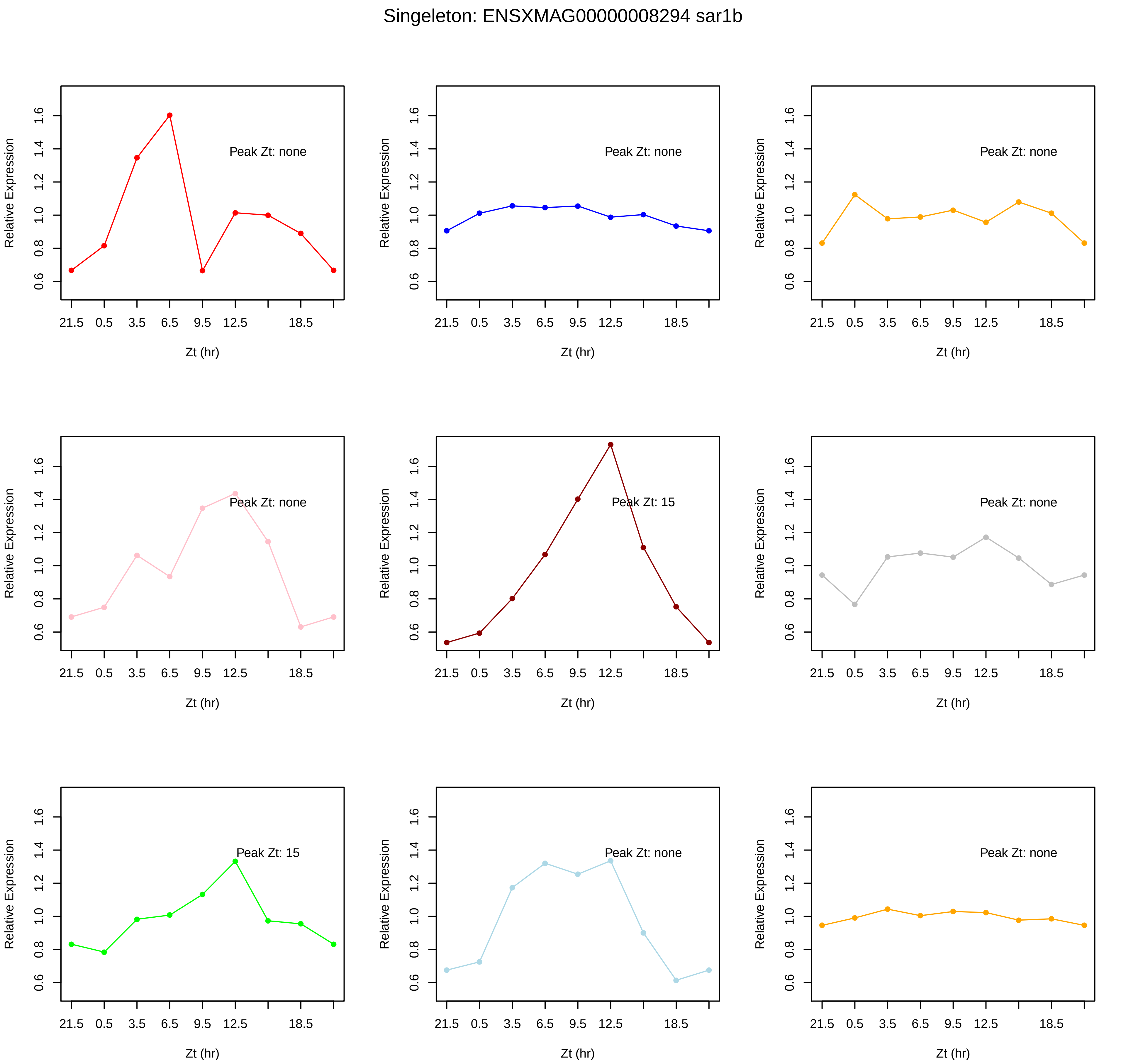
Singleton: ENSXMAG00000008294 sar1b
Peak Zt: none
Peak Zt: none
Peak Zt: none
Peak Zt: none
Peak Zt: 15
Peak Zt: none
Peak Zt: 15
Peak Zt: none
Peak Zt: none
Relative Expression
Zt (hr)
21.5
0.5
3.5
6.5
9.5
12.5
18.5
0.6
0.8
1.0
1.2
1.4
1.6

Singeleton: ENSXMAG00000010362 si:ch211−163l21.8

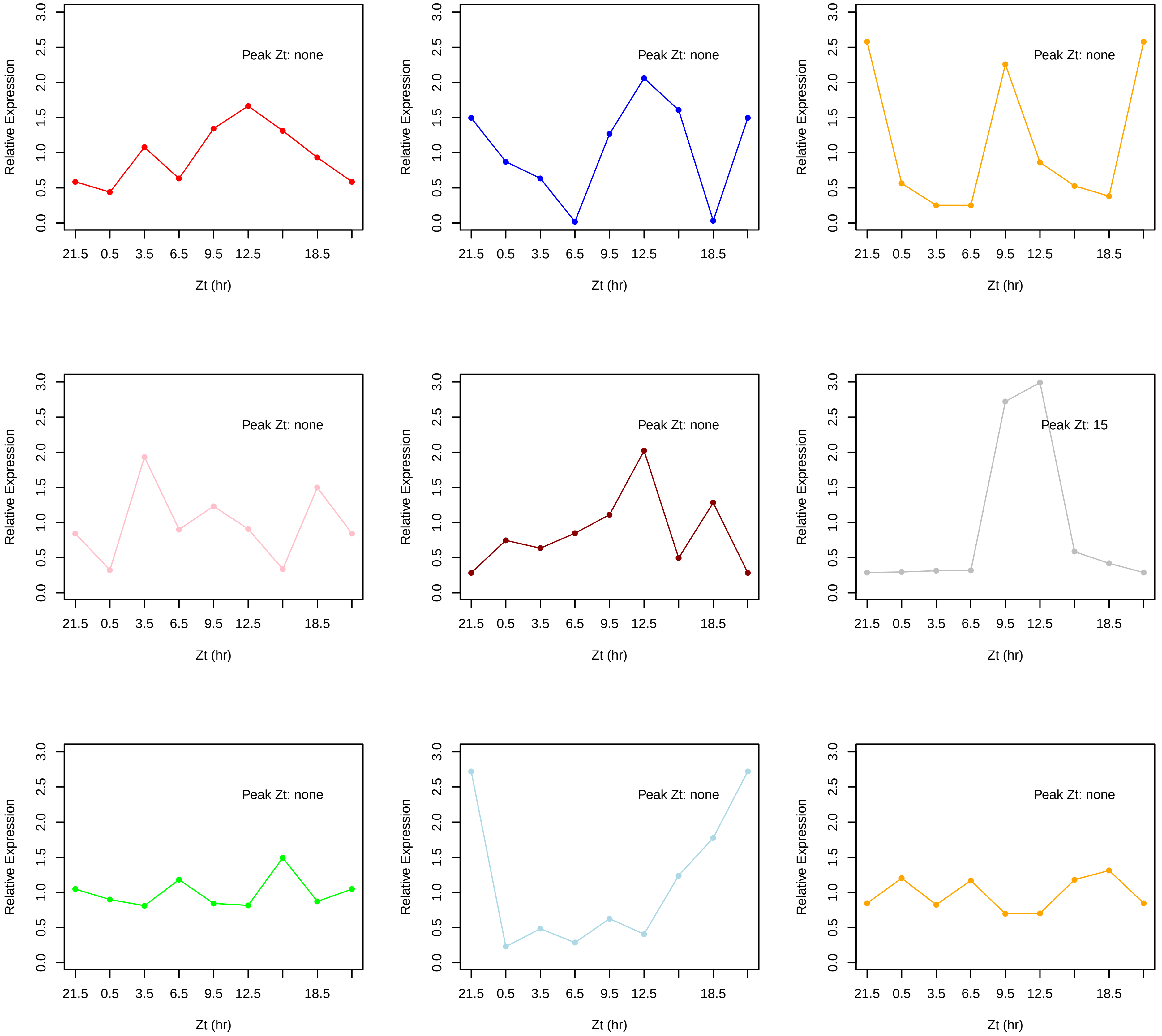

Singeleton: ENSXMAG00000003206 mxra5a

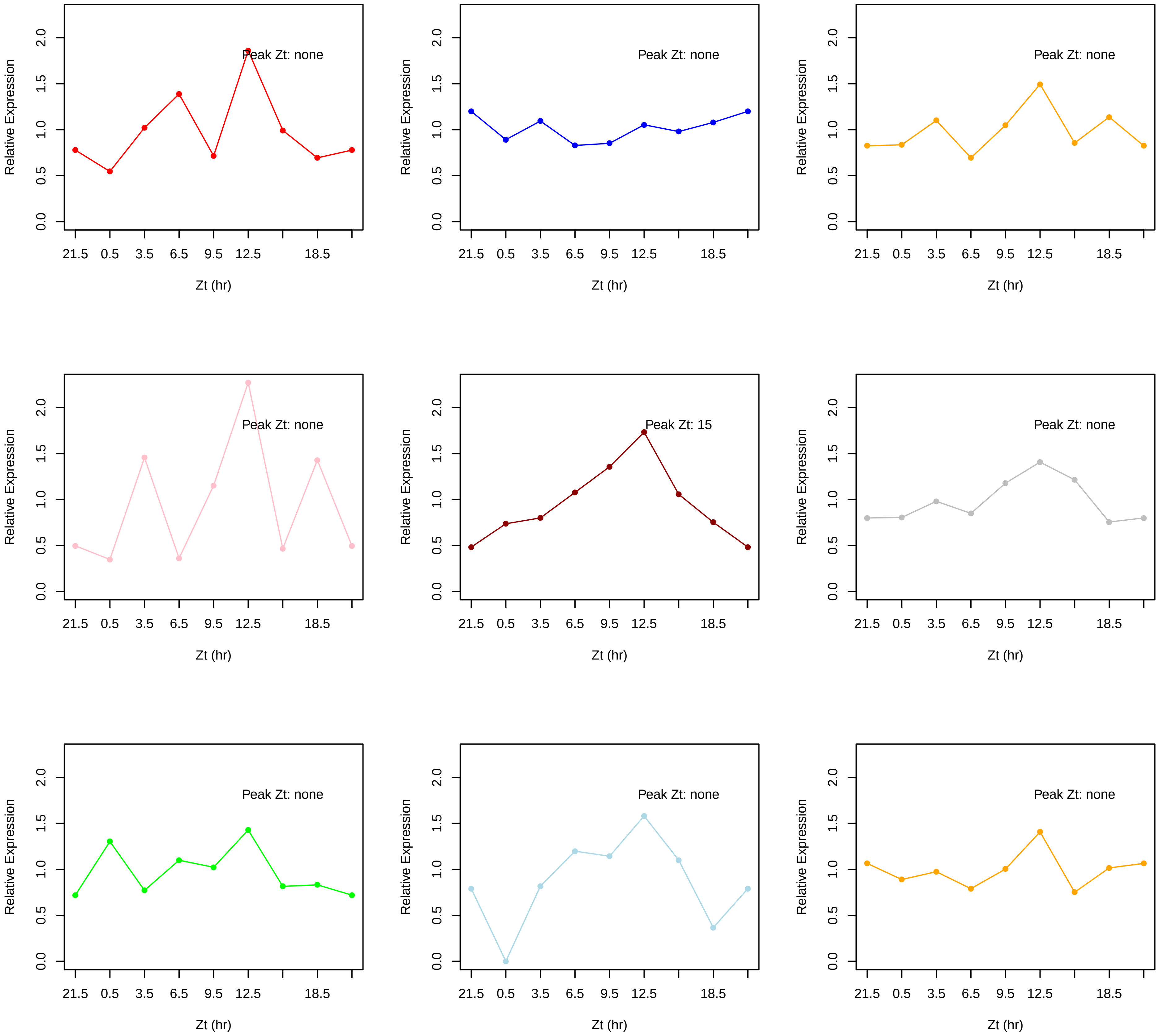

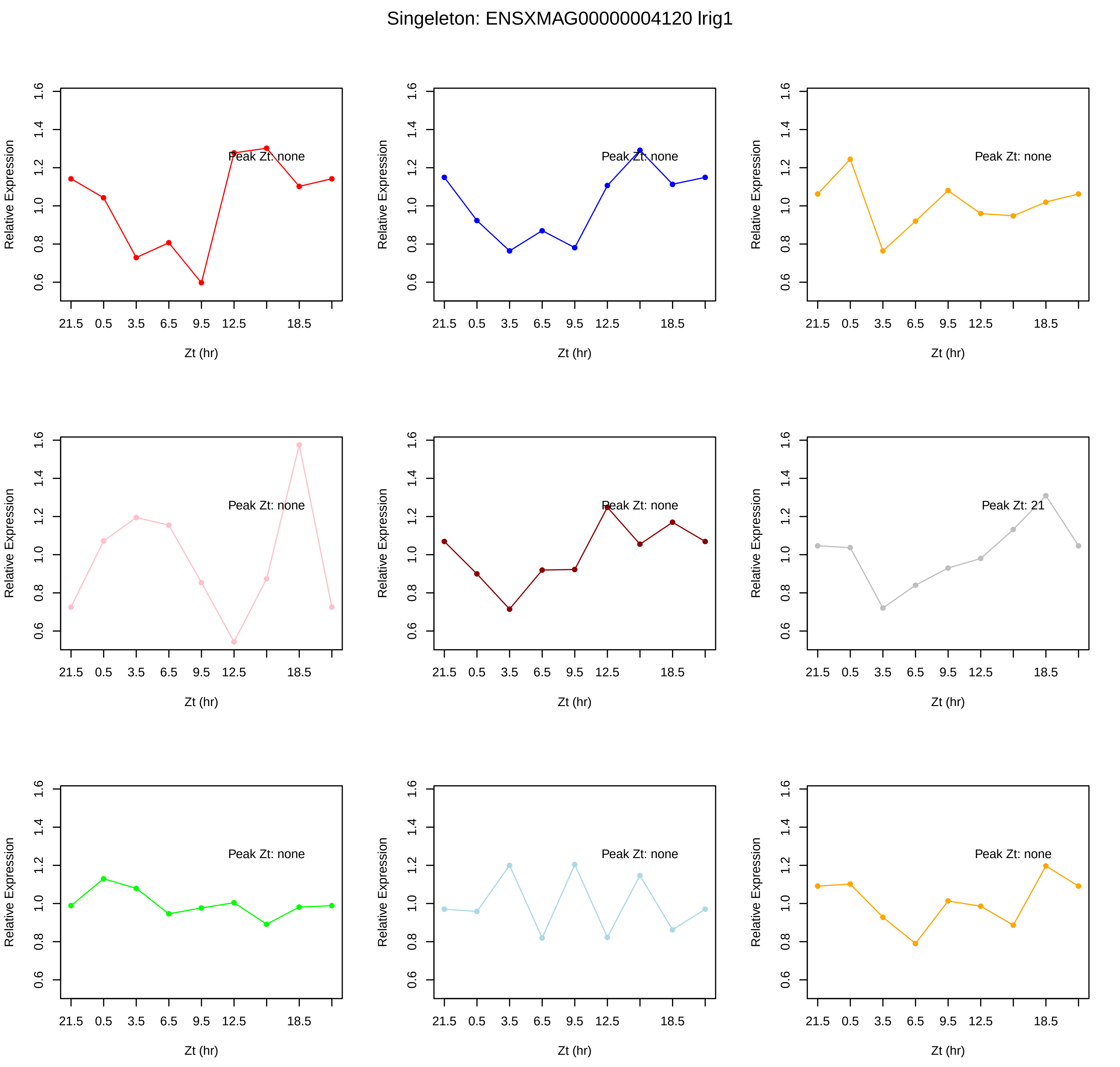

Singeleton: ENSXMAG00000004120 lrig1
Peak Zt: none
Peak Zt: none
Peak Zt: none
Peak Zt: none
Peak Zt: none
Peak Zt: 21
Peak Zt: none
Peak Zt: none
Peak Zt: none
Relative Expression
Zt (hr)
21.5 0.5 3.5 6.5 9.5 12.5 18.5
0.6 0.8 1.0 1.2 1.4 1.6

Singeleton: ENSXMAG00000012822

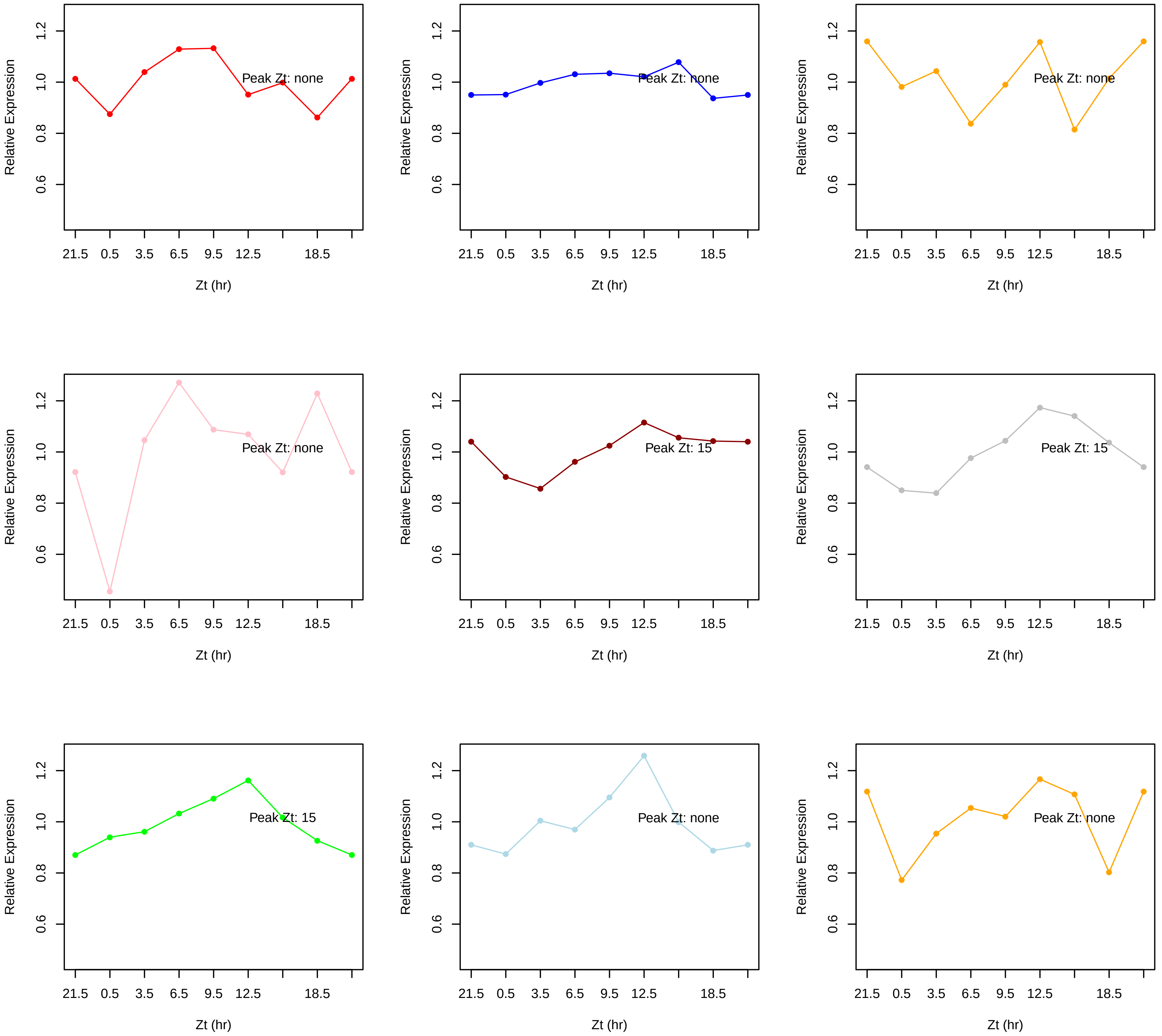

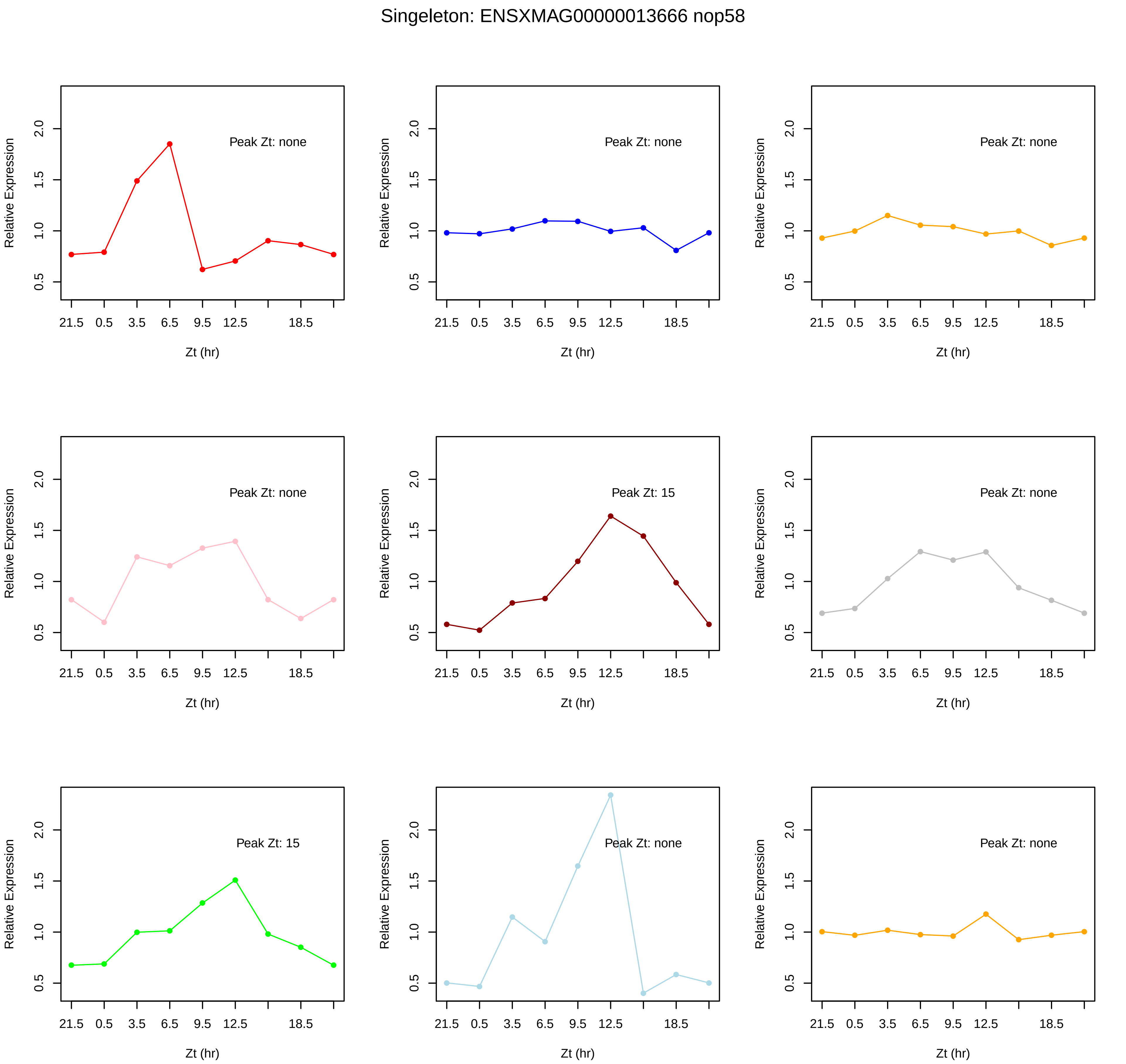
Singeleton: ENSXMAG00000013666 nop58
Peak Zt: none
Peak Zt: none
Peak Zt: none
Peak Zt: none
Peak Zt: 15
Peak Zt: none
Peak Zt: 15
Peak Zt: none
Peak Zt: none
Relative Expression
Zt (hr)
21.5
0.5
3.5
6.5
9.5
12.5
18.5
0.5
1.0
1.5
2.0

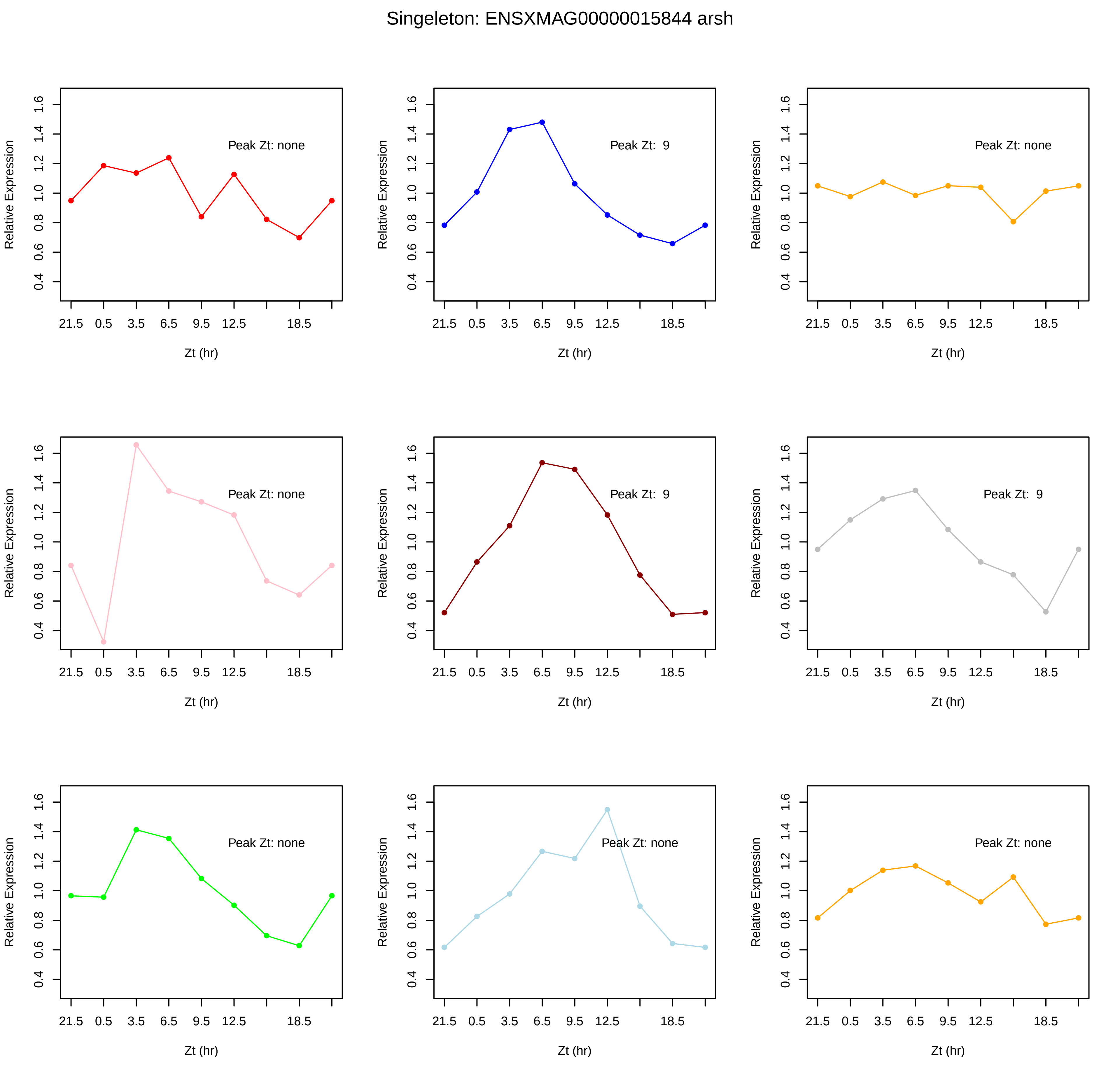

Singeleton: ENSXMAG00000015844 arsh
Peak Zt: none
Peak Zt: 9
Peak Zt: none
Peak Zt: none
Peak Zt: 9
Peak Zt: 9
Peak Zt: none
Peak Zt: none
Peak Zt: none
Relative Expression
Zt (hr)

Singeleton: ENSXMAG00000004145 slc25a26

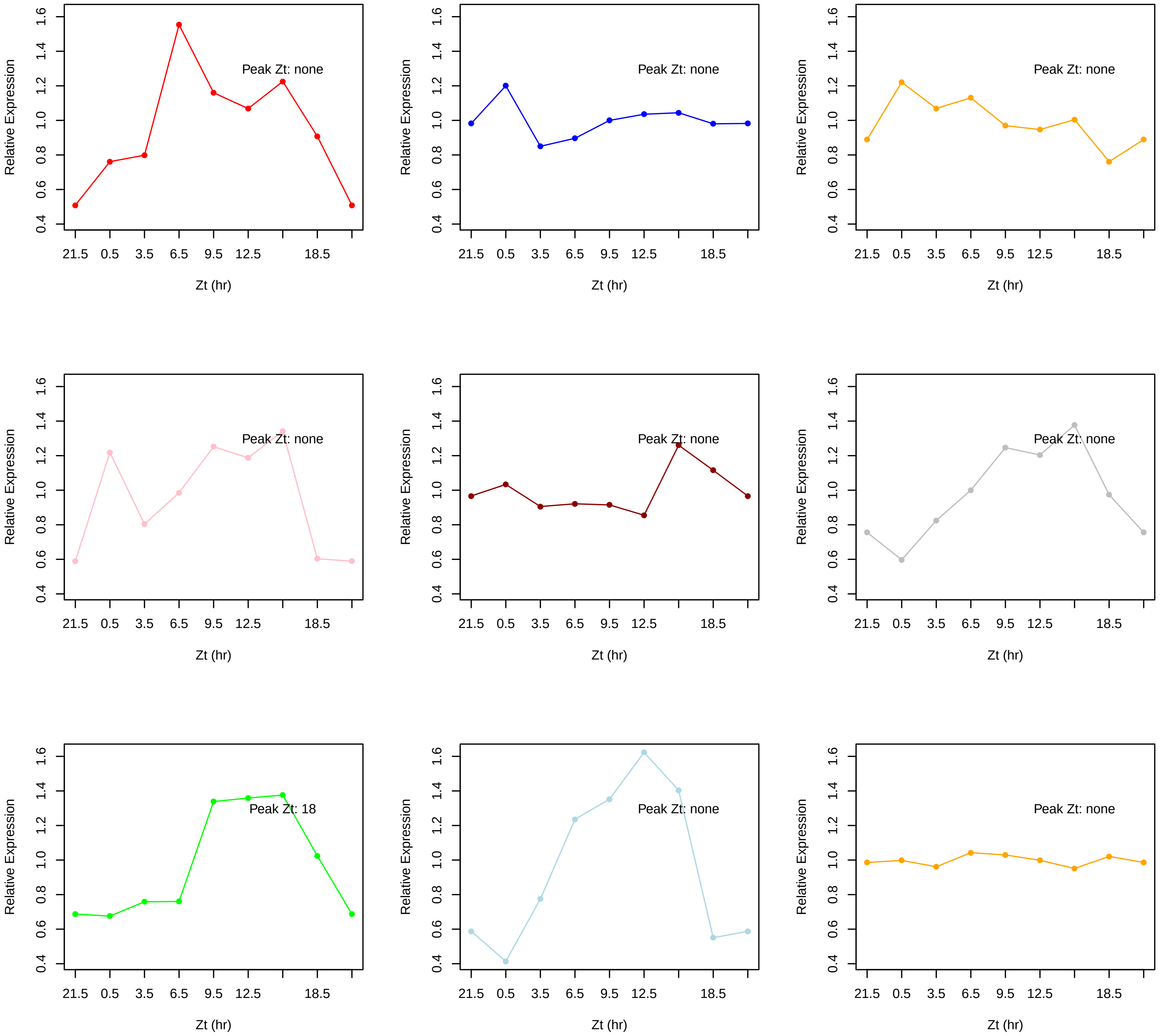

Singeleton: ENSXMAG00000006306

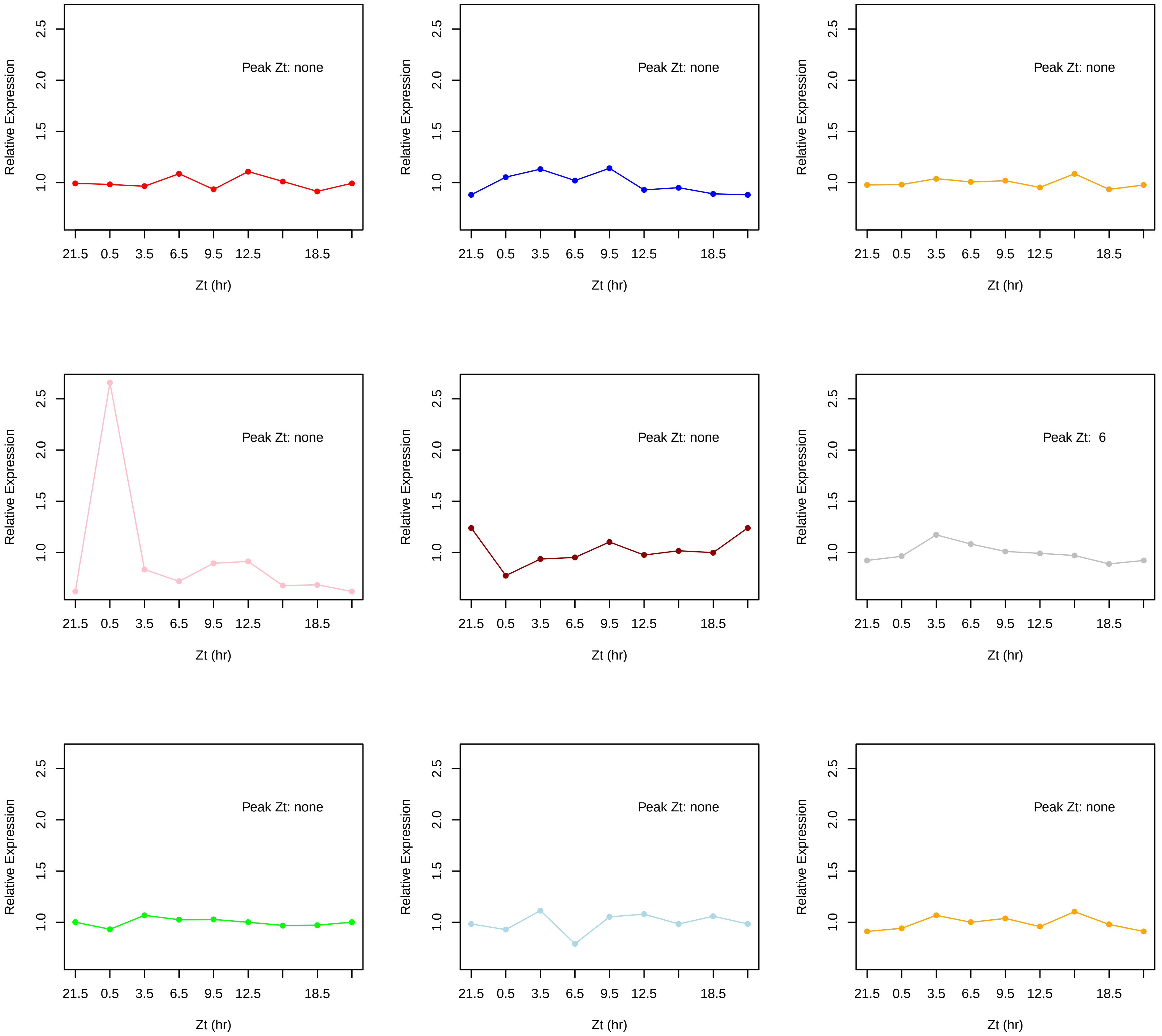

Singeleton: ENSXMAG00000023049 imp3

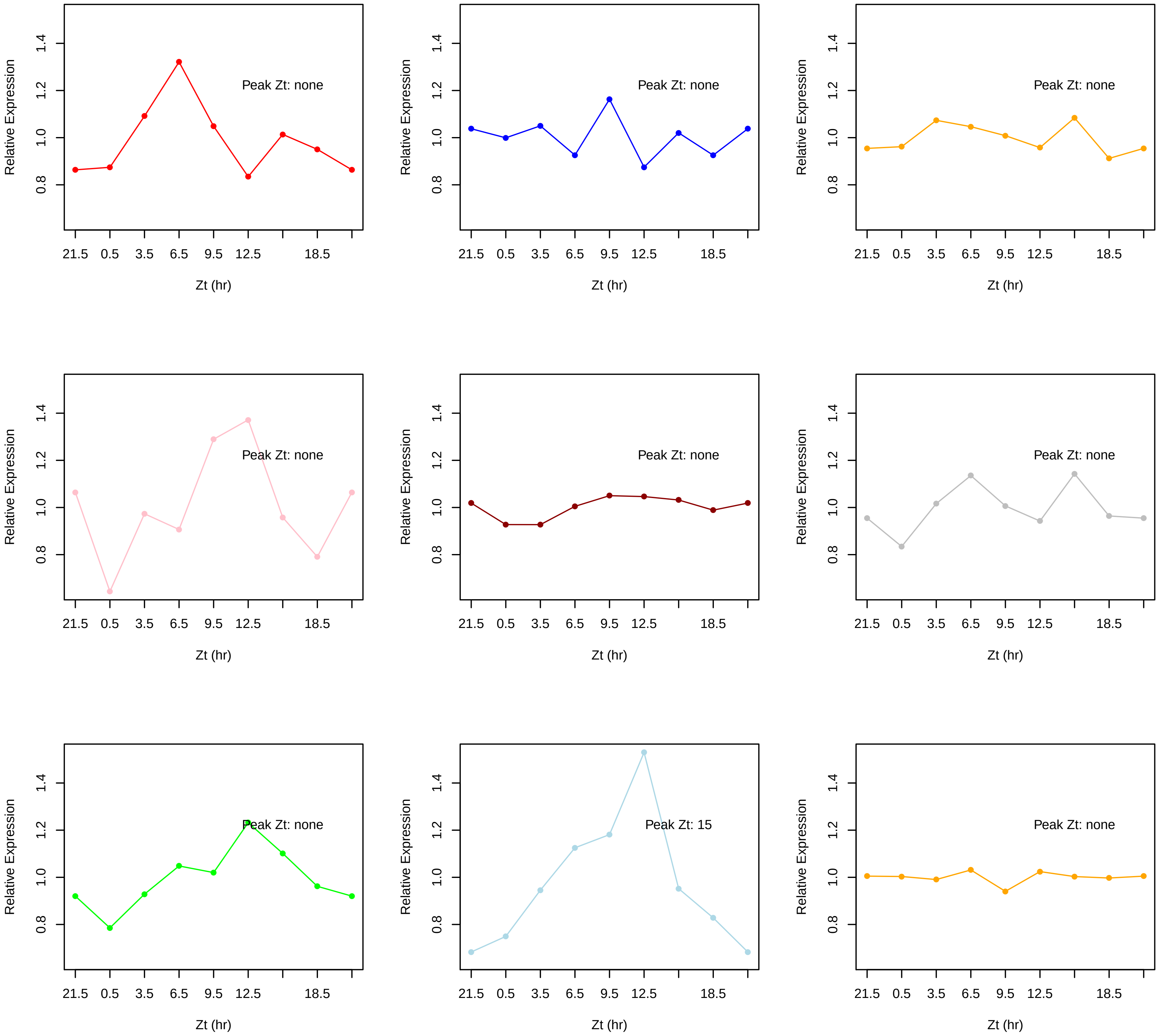

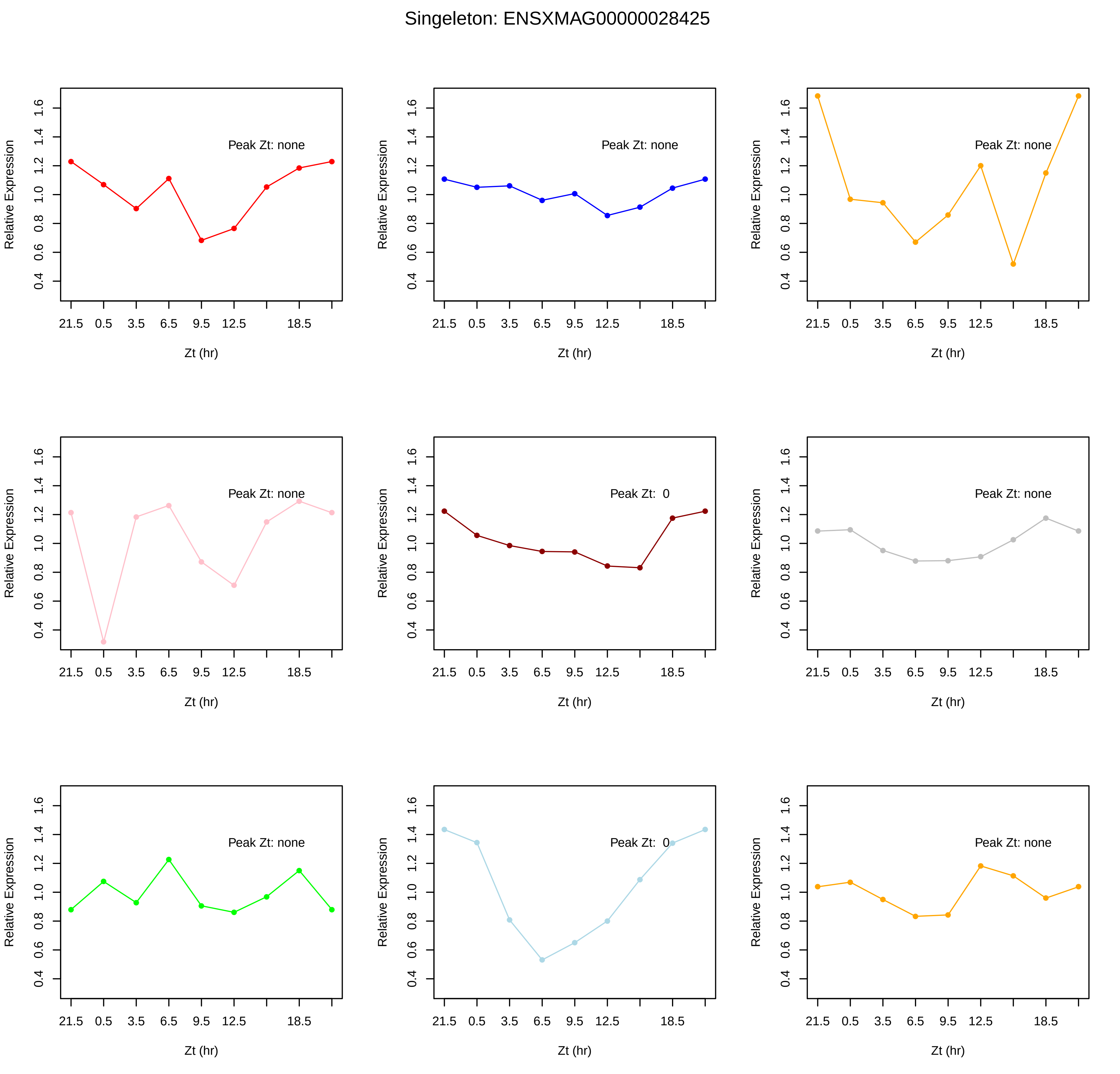
Singleton: ENSXMAG00000028425
Peak Zt: none
Peak Zt: none
Peak Zt: none
Peak Zt: none
Peak Zt: 0
Peak Zt: none
Peak Zt: none
Peak Zt: 0
Peak Zt: none
Relative Expression
Zt (hr)
21.5
0.5
3.5
6.5
9.5
12.5
18.5
0.4
0.6
0.8
1.0
1.2
1.4
1.6

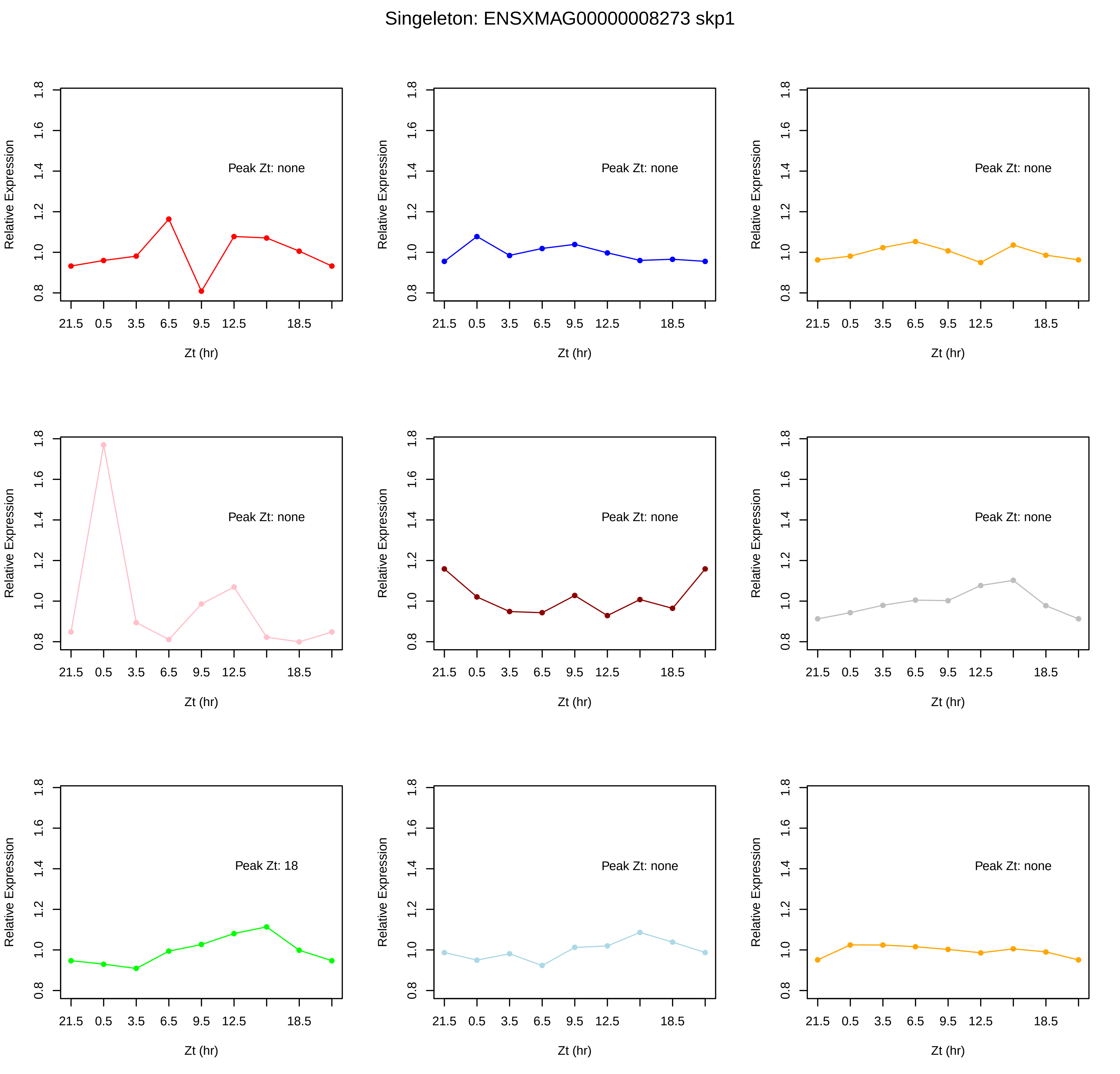
Singeleton: ENSXMAG00000008273 skp1
Peak Zt: none
Peak Zt: none
Peak Zt: none
Peak Zt: none
Peak Zt: none
Peak Zt: none
Peak Zt: 18
Peak Zt: none
Peak Zt: none
Relative Expression
Zt (hr)
21.5
0.5
3.5
6.5
9.5
12.5
18.5
0.8
1.0
1.2
1.4
1.6
1.8

Singleton: ENSXMAG00000004378 ap4b1

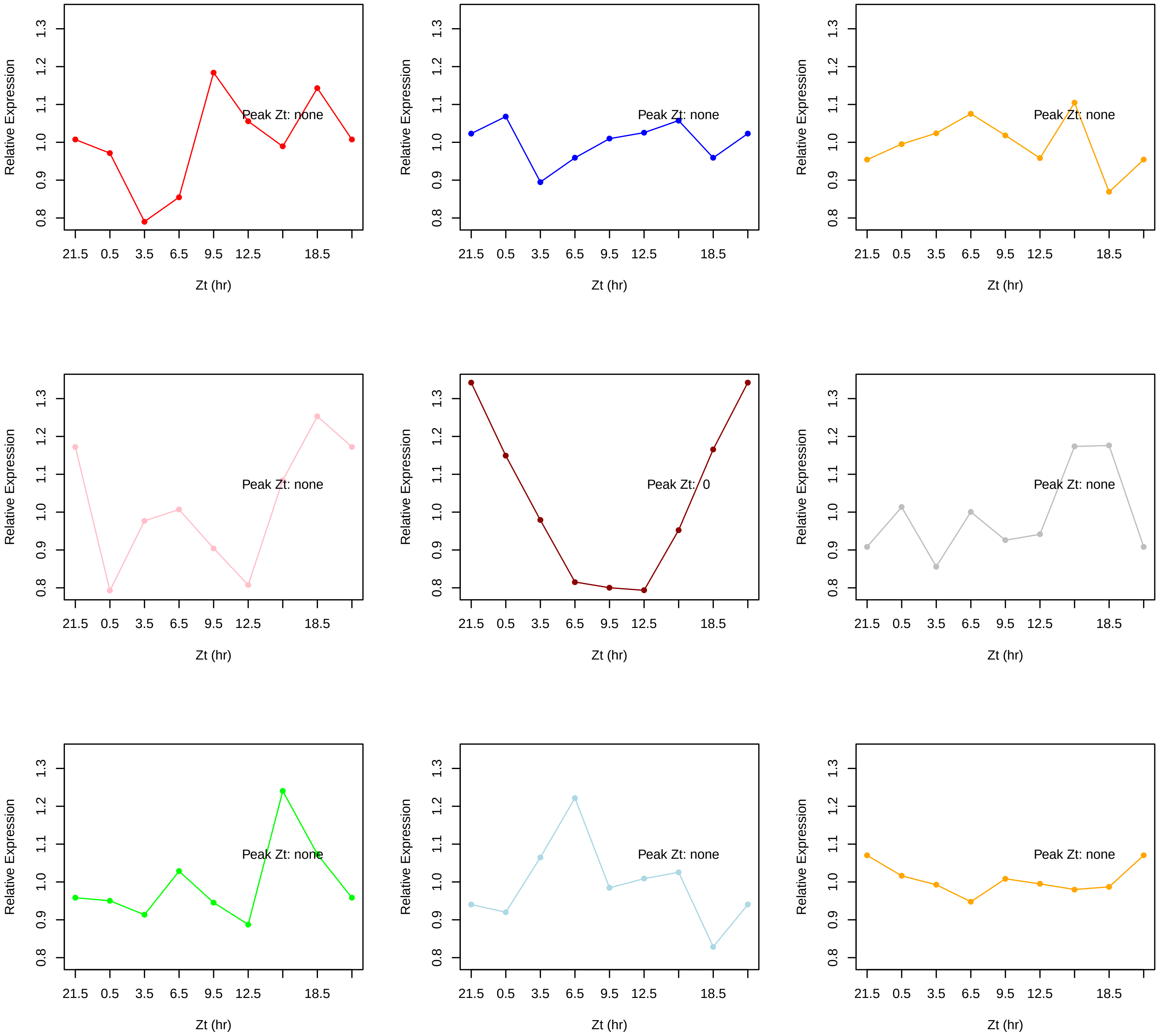

Singeleton: ENSXMAG00000014228

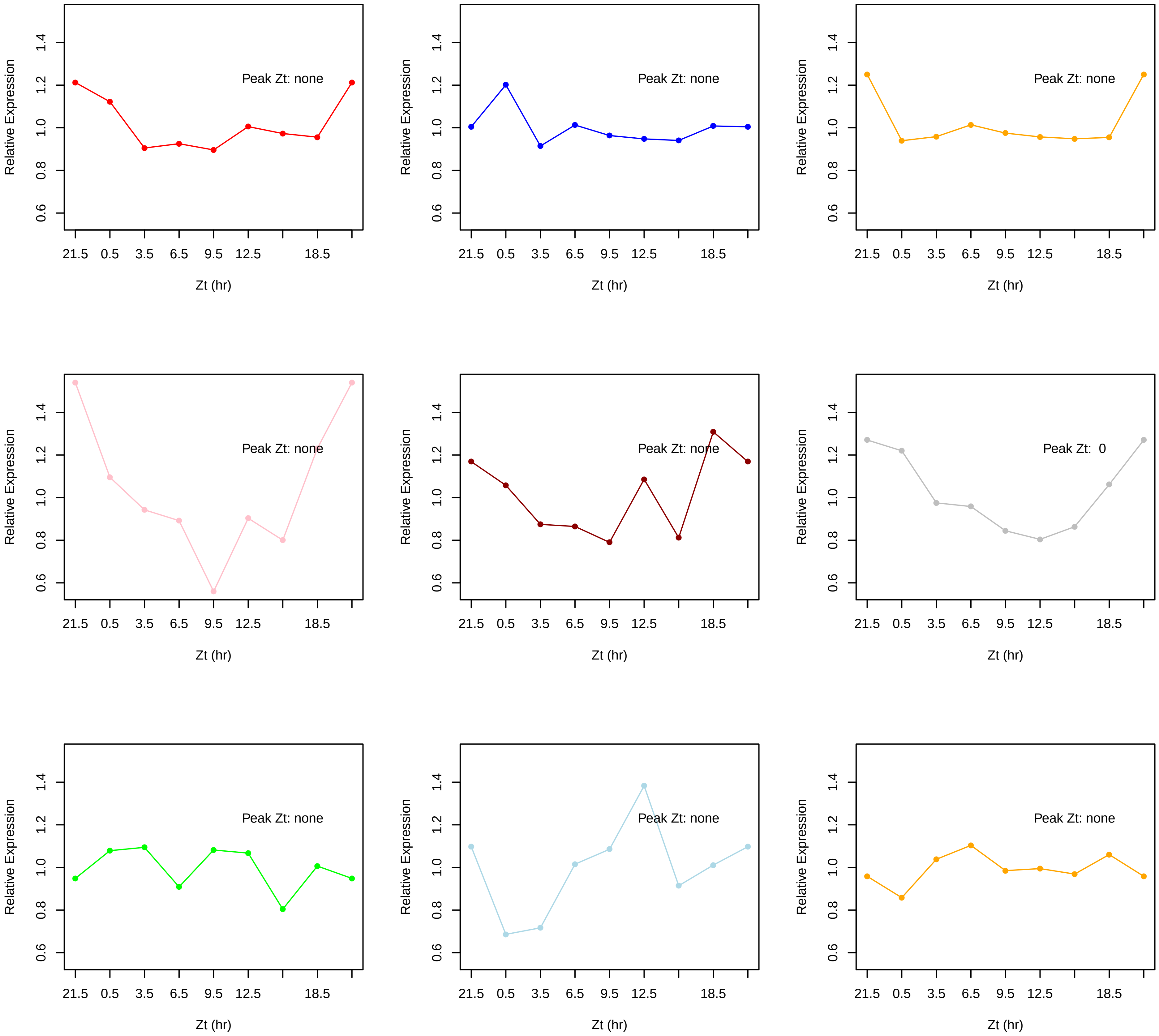

Singeleton: ENSXMAG00000016211 asmt

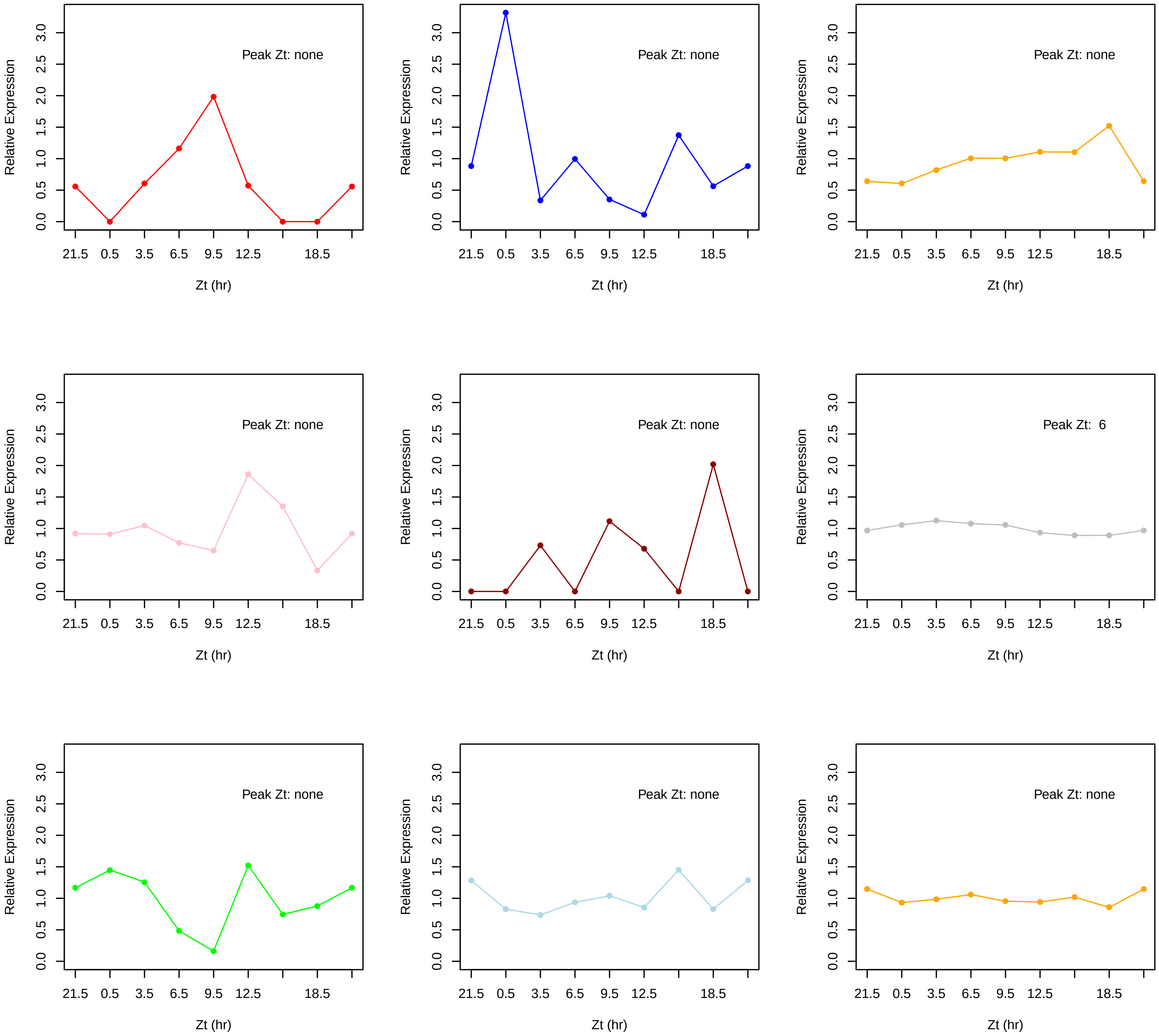

Singeleton: ENSXMAG00000018402 tab3

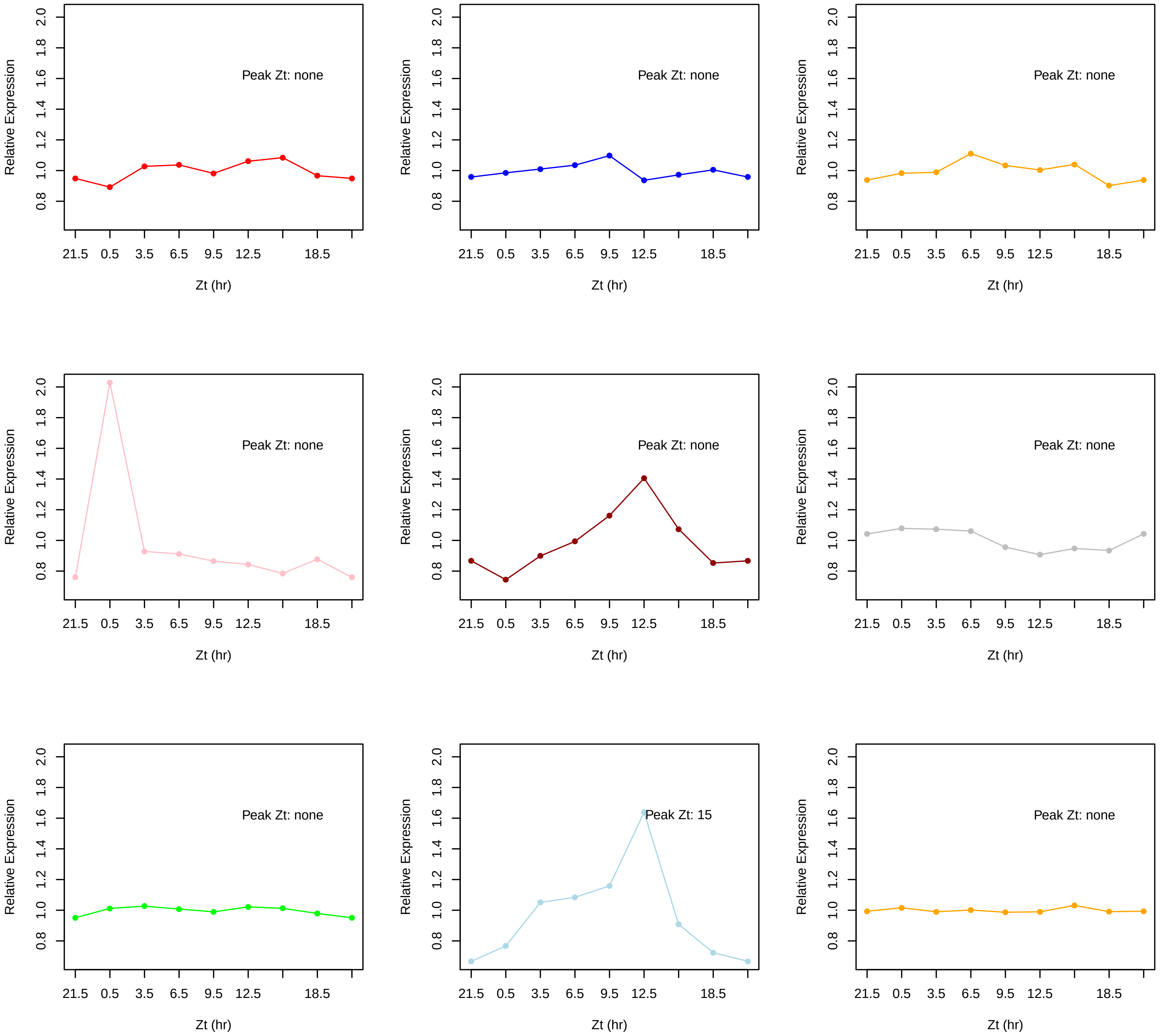

Singeleton: ENSXMAG00000012953 edem3

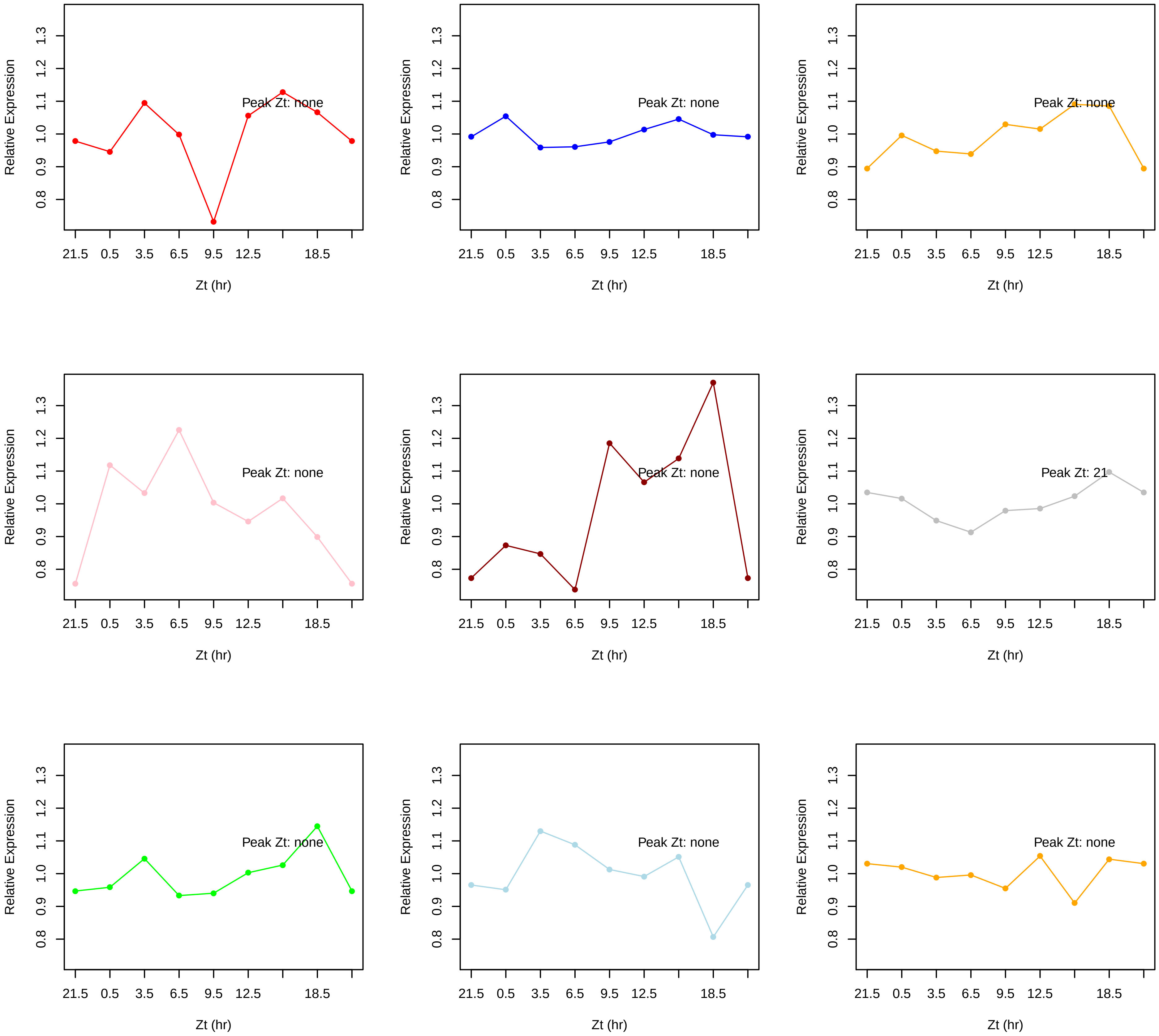

Singeleton: ENSXMAG00000007418 vdac1

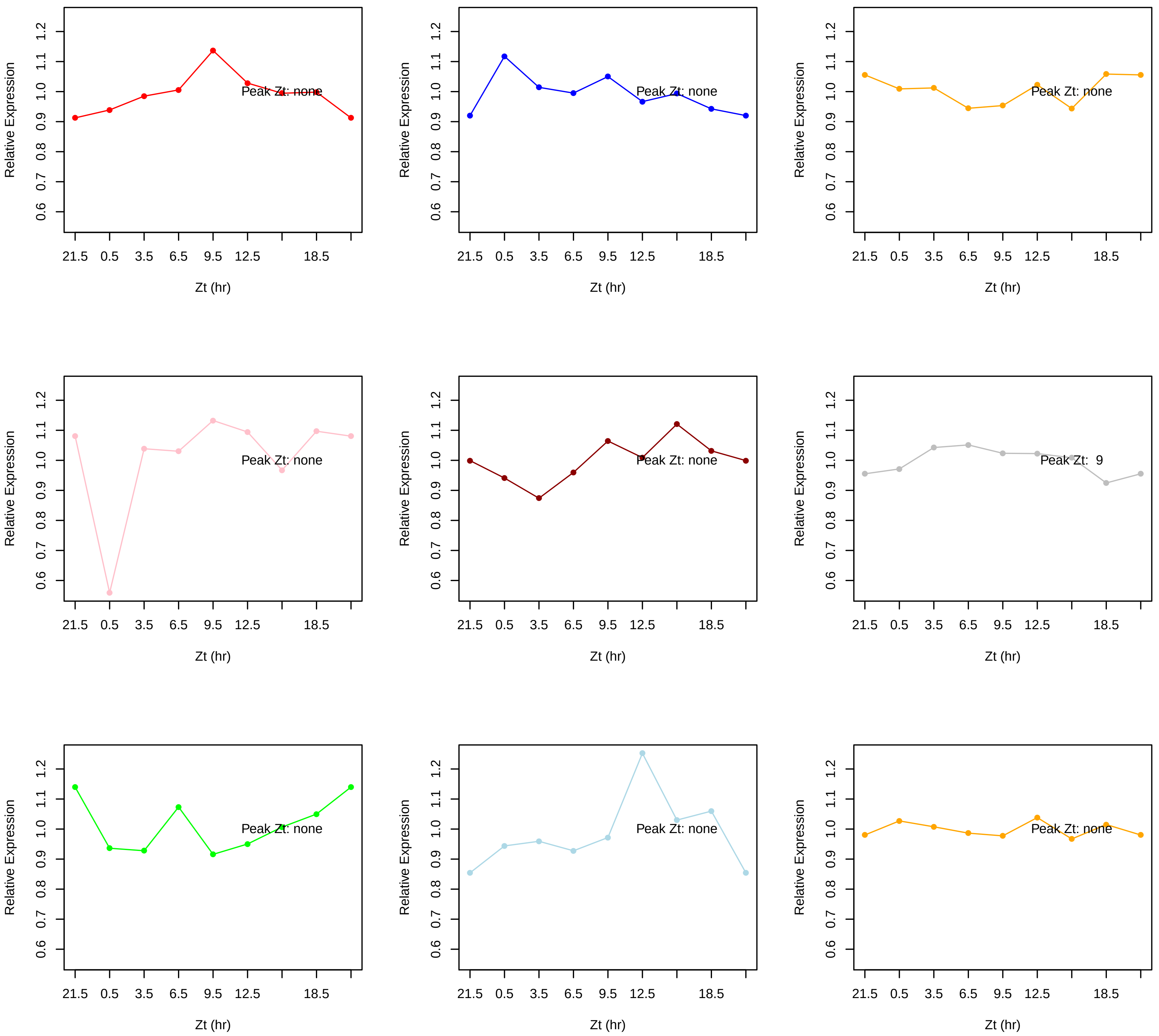

Singeleton: ENSXMAG00000016225 akap17a

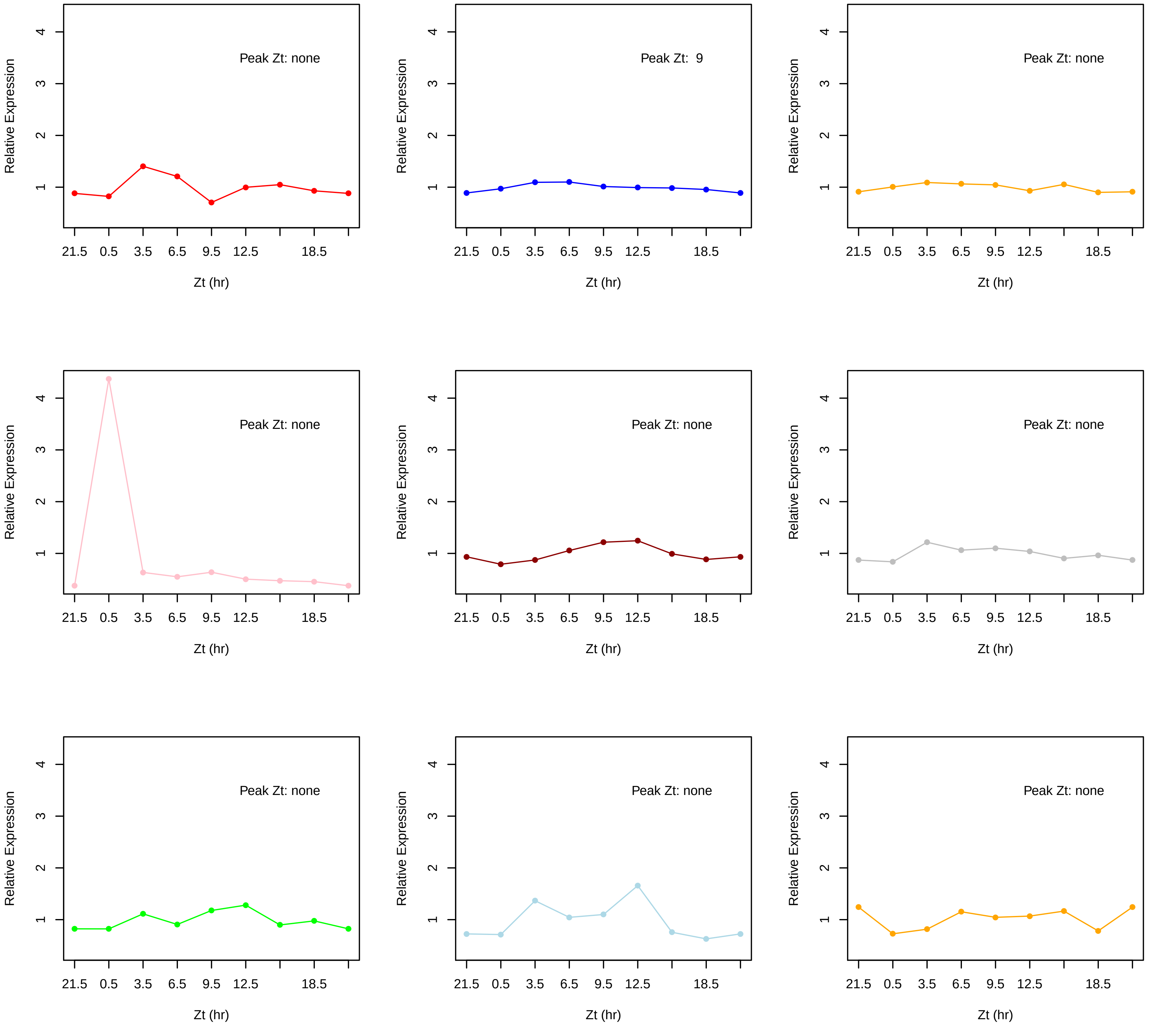

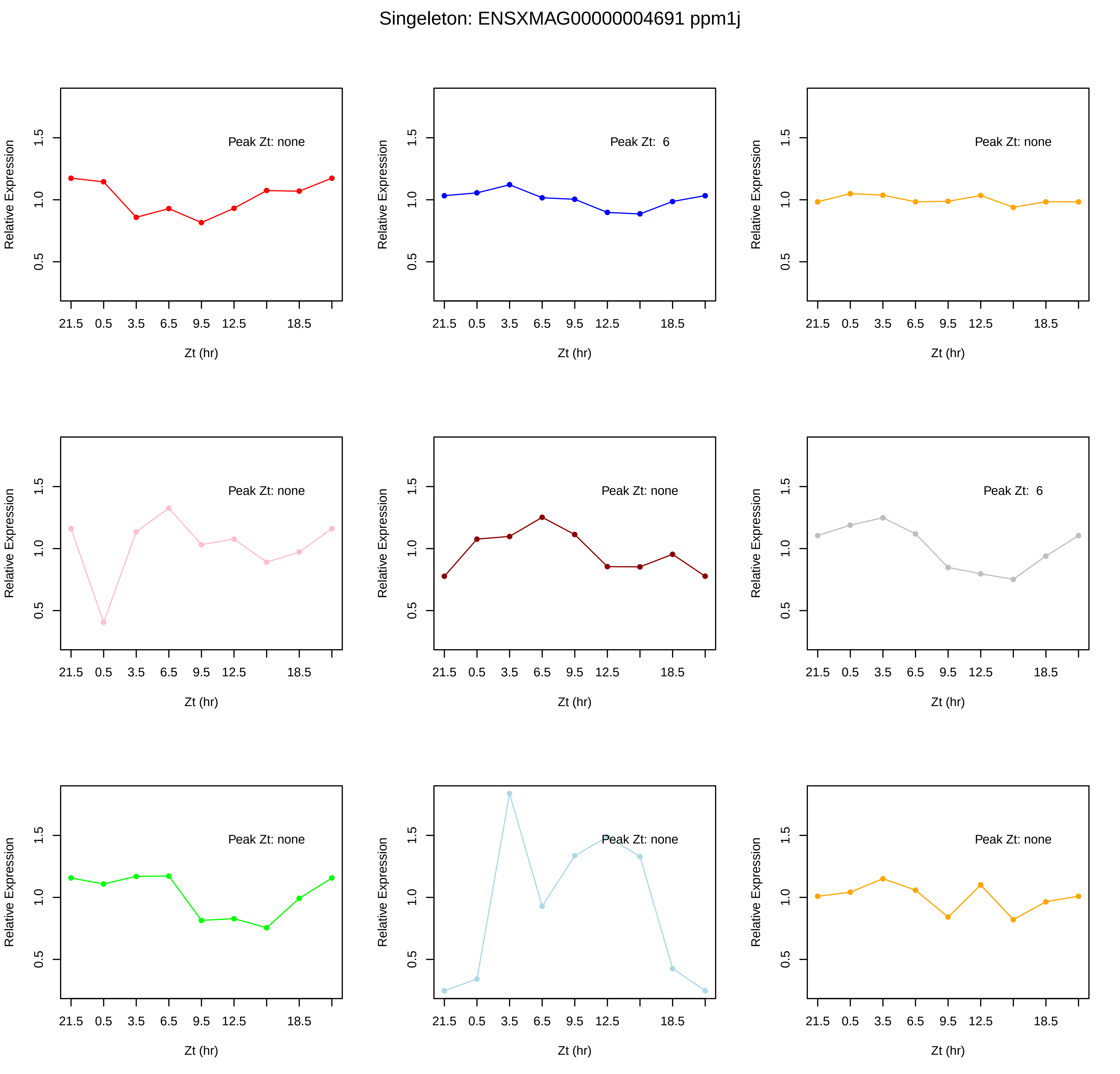

Singeleton: ENSXMAG00000004691 ppm1j
Peak Zt: none
Peak Zt: 6
Peak Zt: none
Peak Zt: none
Peak Zt: none
Peak Zt: 6
Peak Zt: none
Peak Zt: none
Peak Zt: none
Relative Expression
Zt (hr)
0.5
1.0
1.5
21.5
0.5
3.5
6.5
9.5
12.5
18.5

Singeleton: ENSXMAG00000008746 adamts9

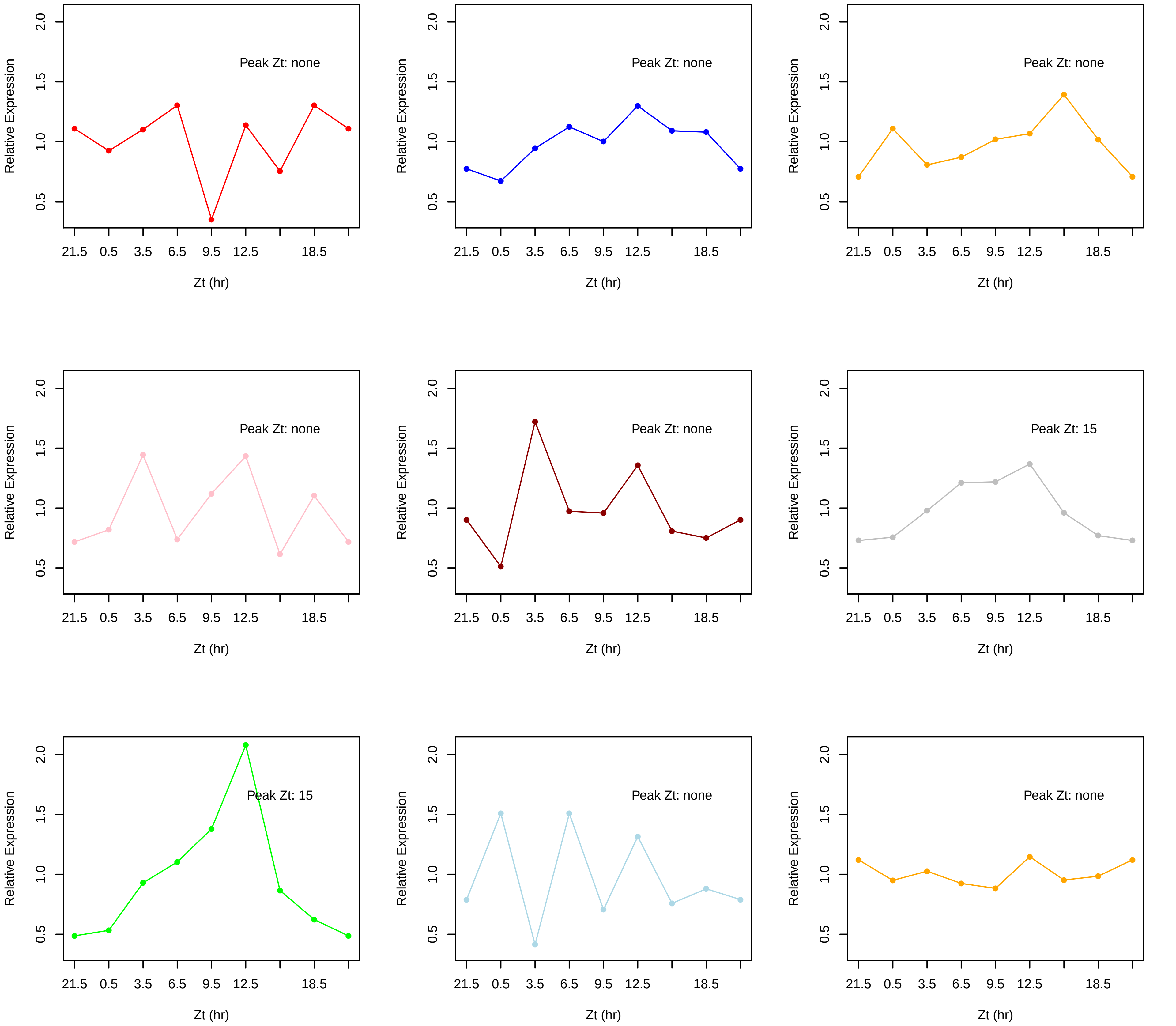

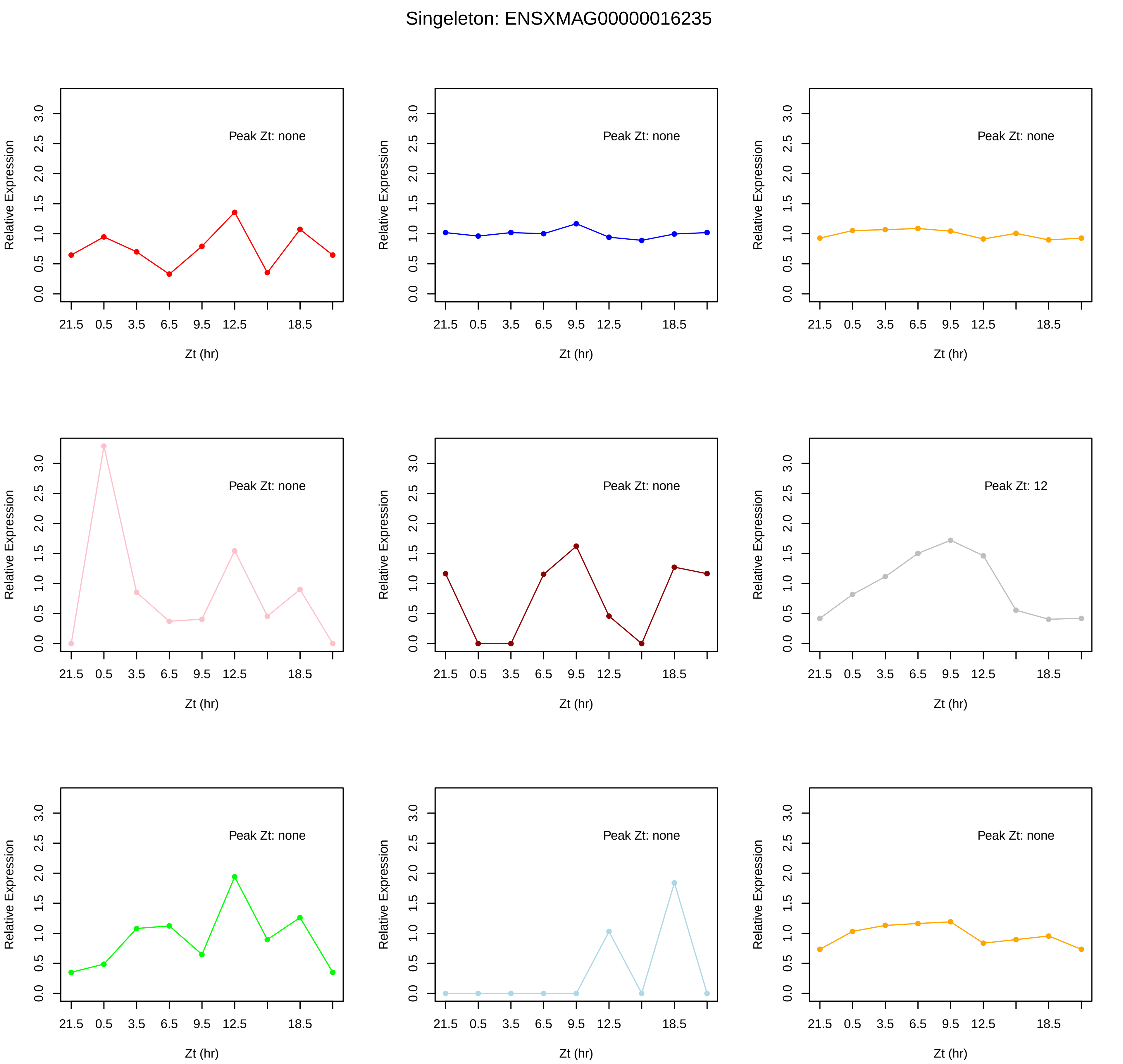
Singleton: ENSXMAG00000016235
Peak Zt: none
Peak Zt: none
Peak Zt: none
Peak Zt: none
Peak Zt: none
Peak Zt: 12
Peak Zt: none
Peak Zt: none
Peak Zt: none
Relative Expression
Zt (hr)
21.5 0.5 3.5 6.5 9.5 12.5 18.5
0.0 0.5 1.0 1.5 2.0 2.5 3.0

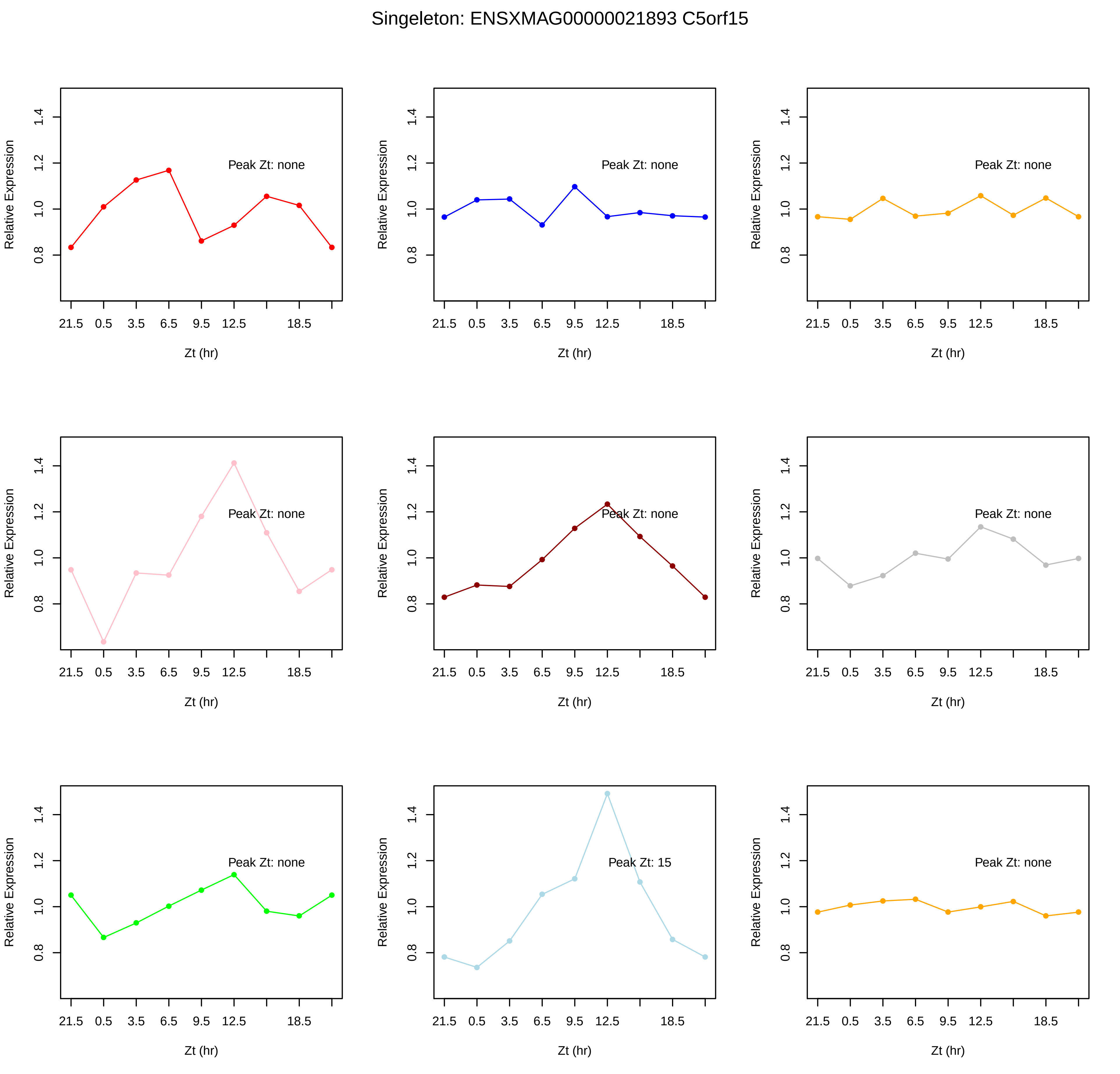
Singeleton: ENSXMAG00000021893 C5orf15
Relative Expression
Zt (hr)
21.5
0.5
3.5
6.5
9.5
12.5
18.5
0.8
1.0
1.2
1.4
Peak Zt: none
Peak Zt: 15

Singeleton: ENSXMAG00000016336

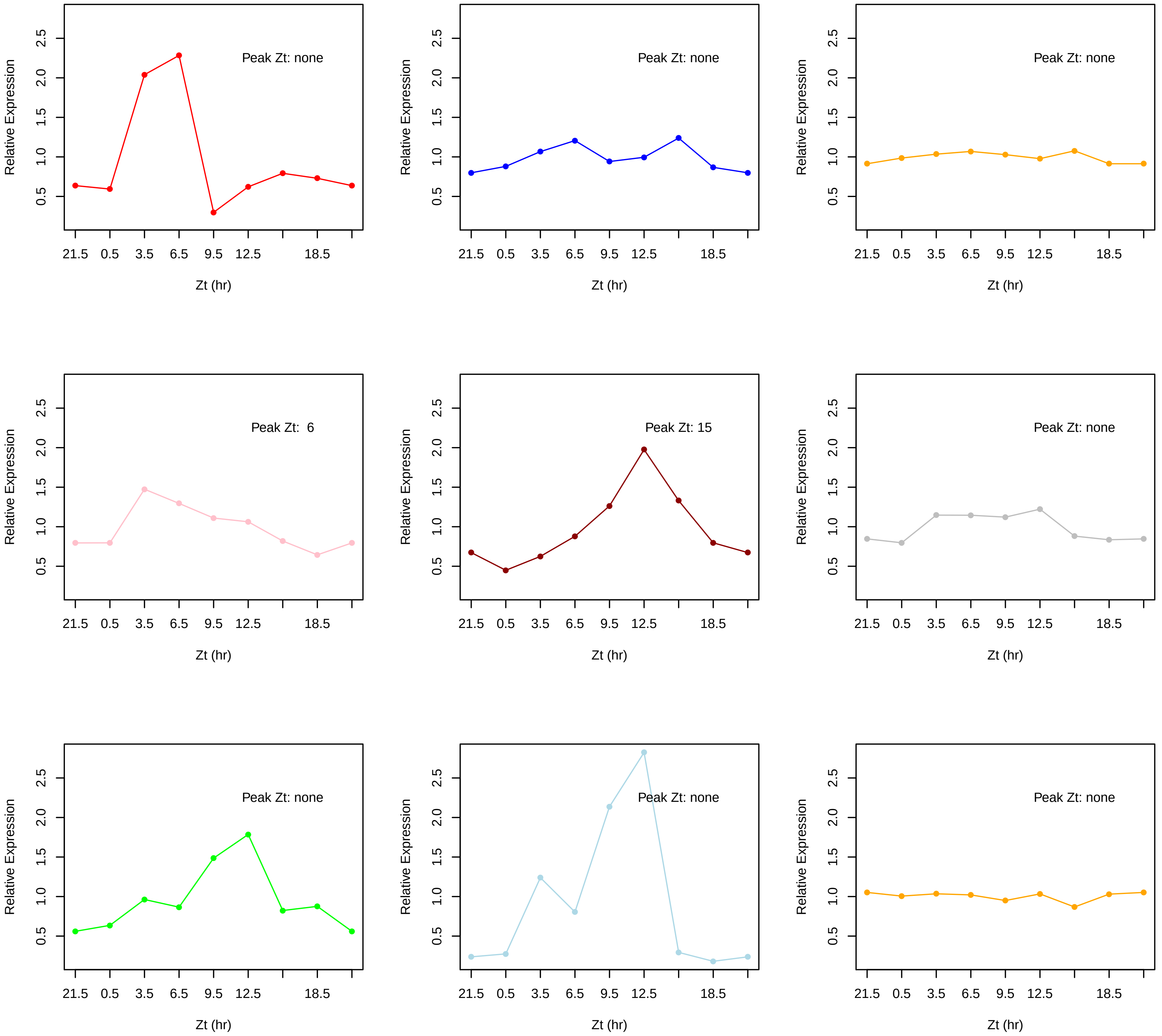

Singeleton: ENSXMAG00000015001 LRRC2

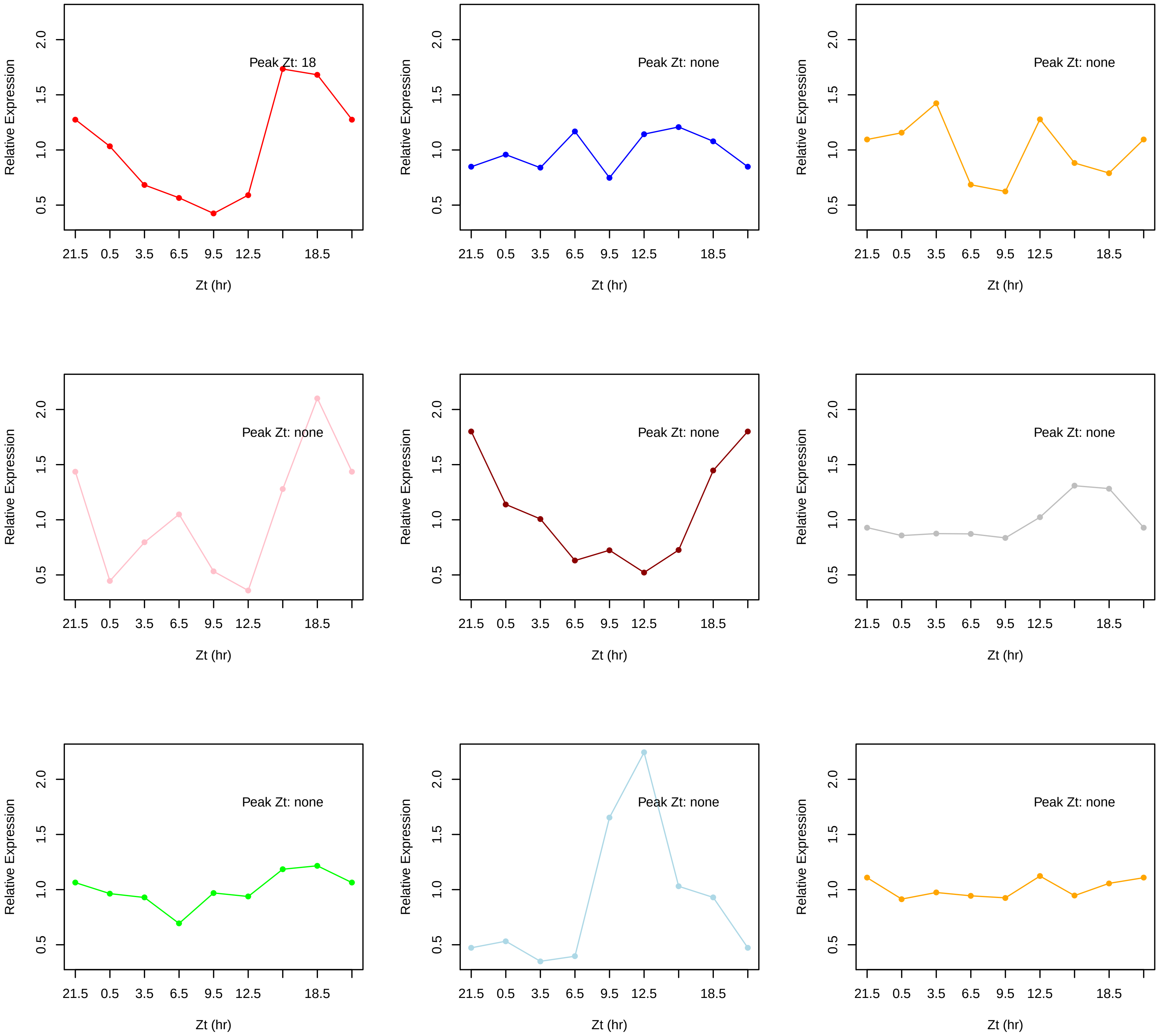

Singeleton: ENSXMAG00000008227

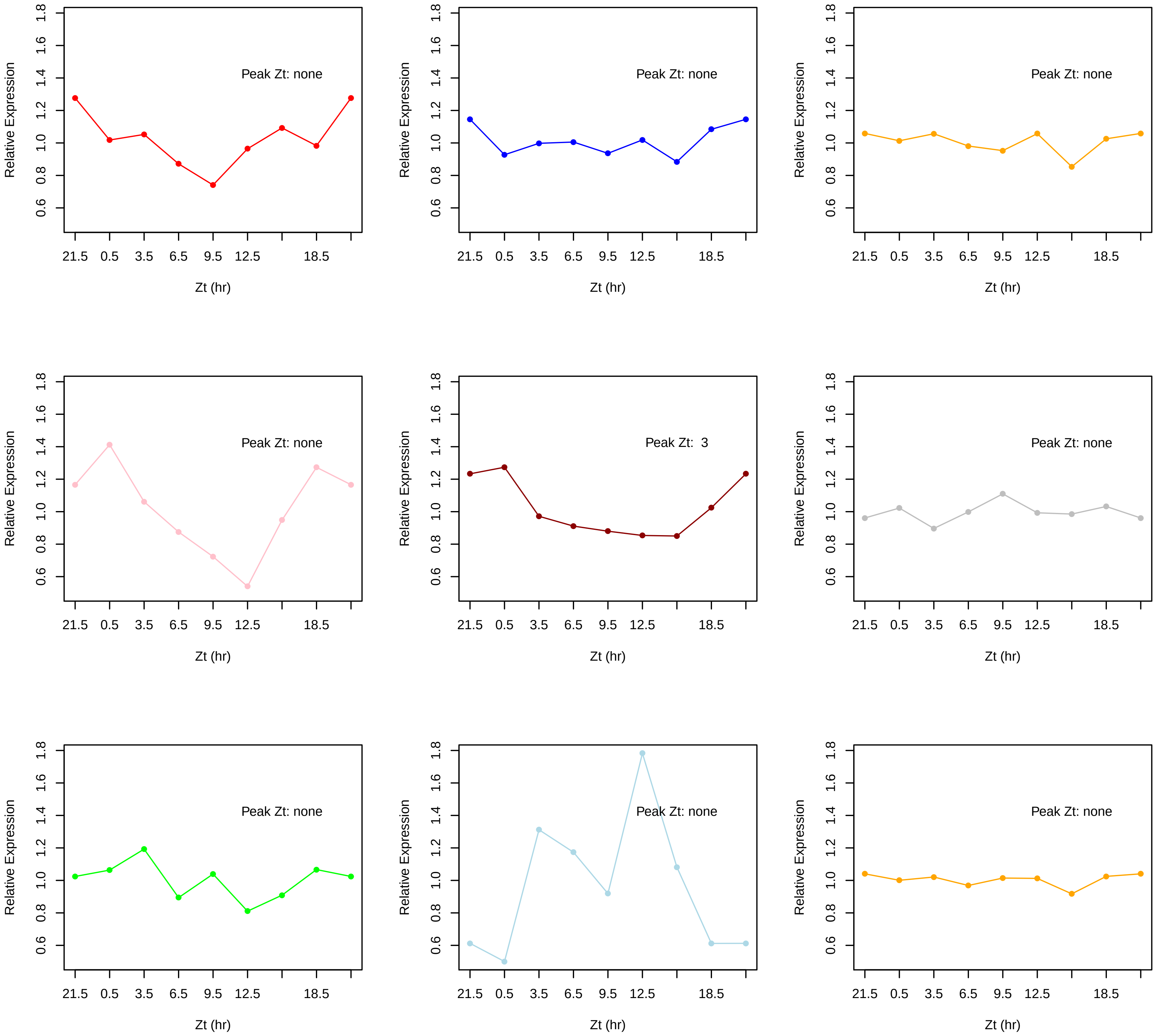

Singeleton: ENSXMAG00000012963 colgalt2

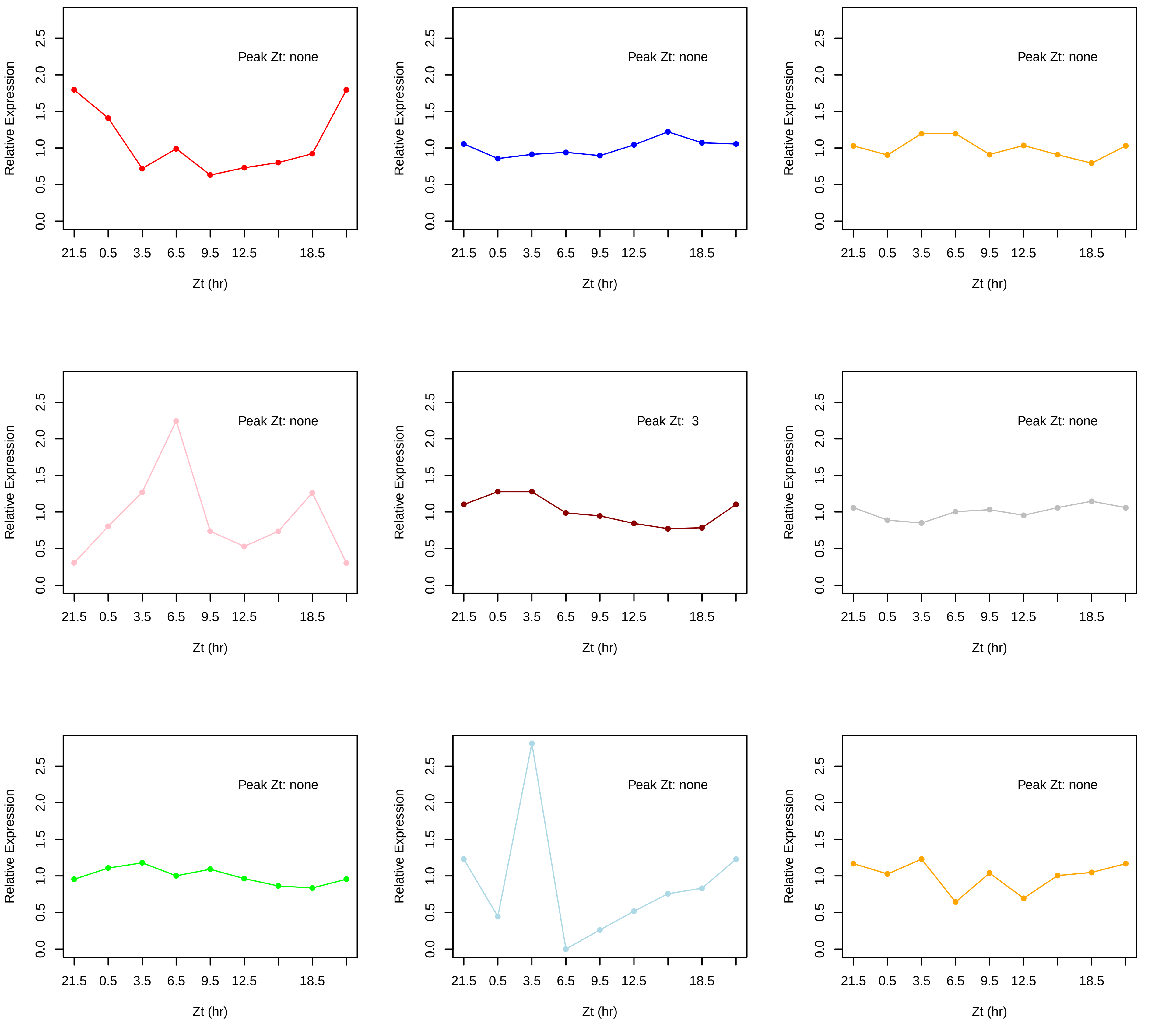

Singeleton: ENSXMAG00000028967 FAM124A

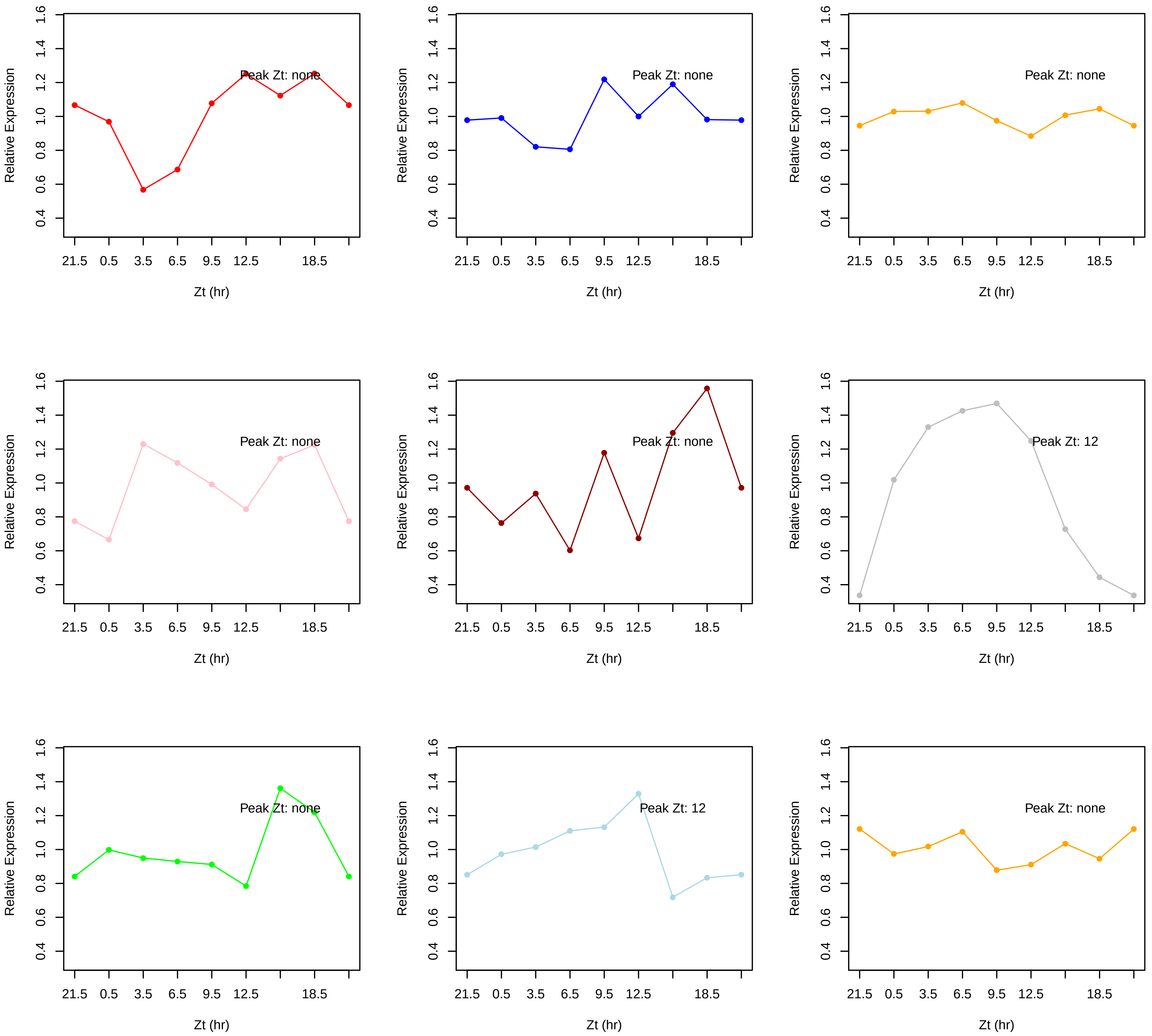

Singeleton: ENSXMAG00000014129 sh3rf1

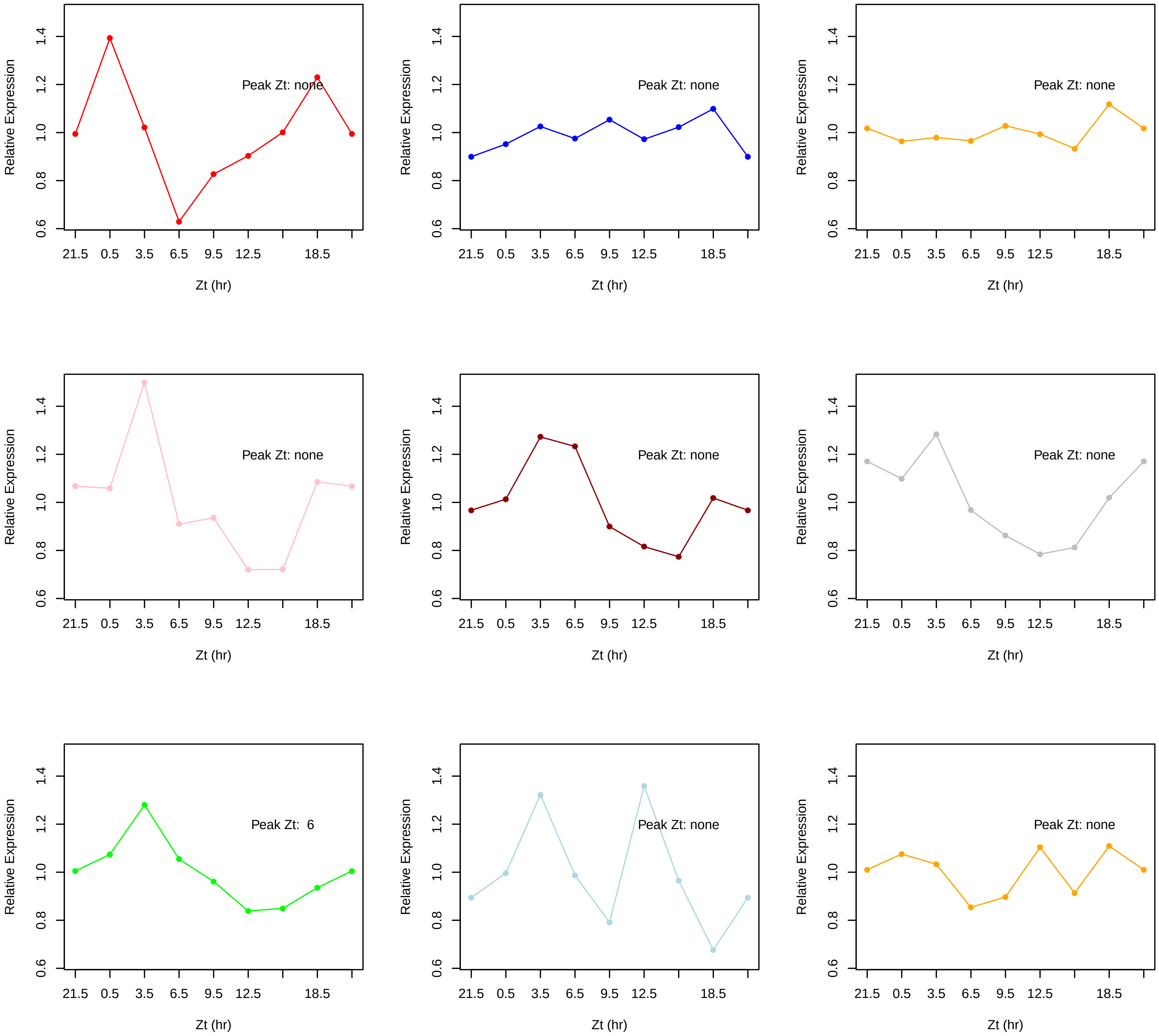

Singeleton: ENSXMAG00000029788

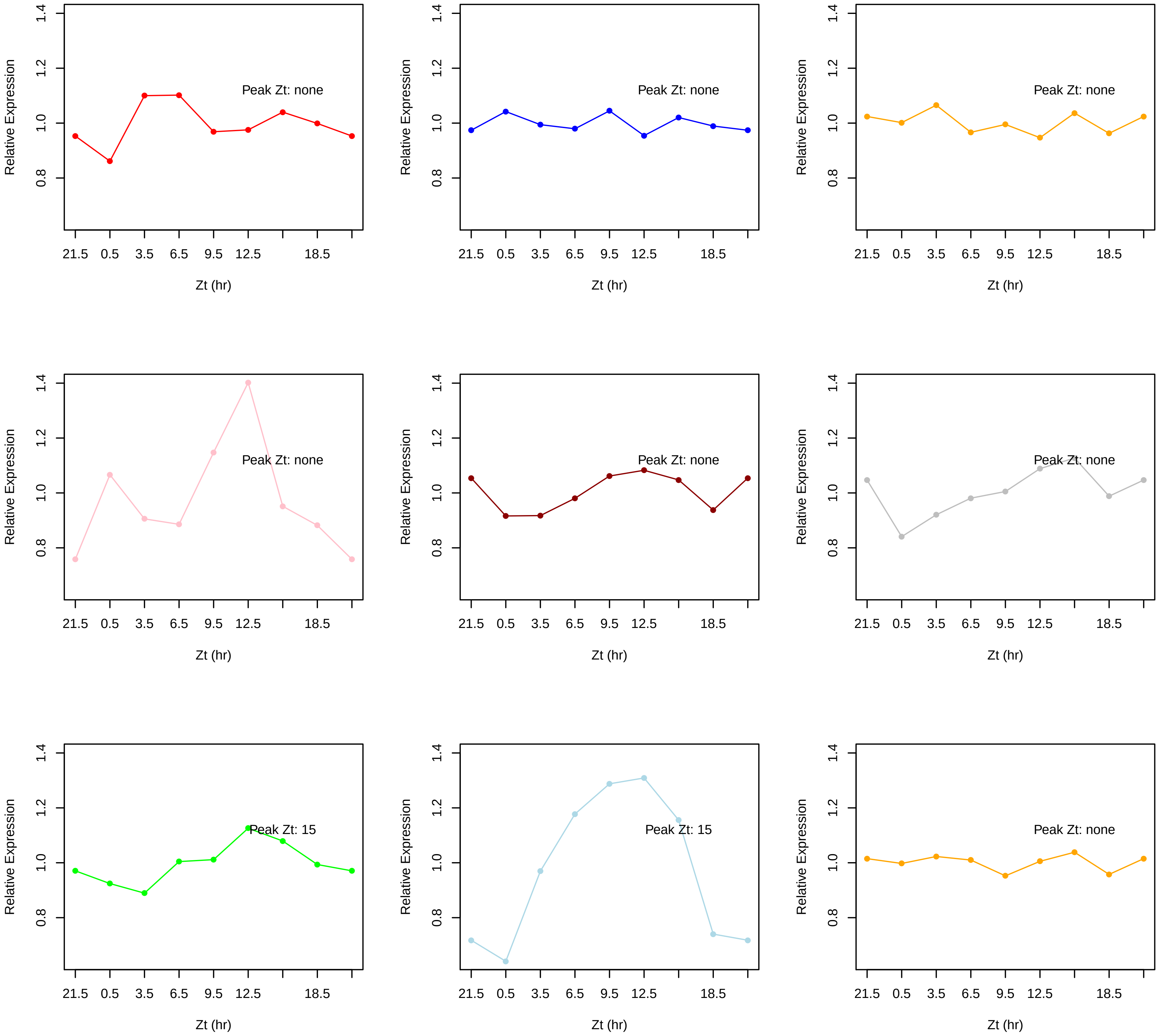

Singeleton: ENSXMAG00000007593 capza1b

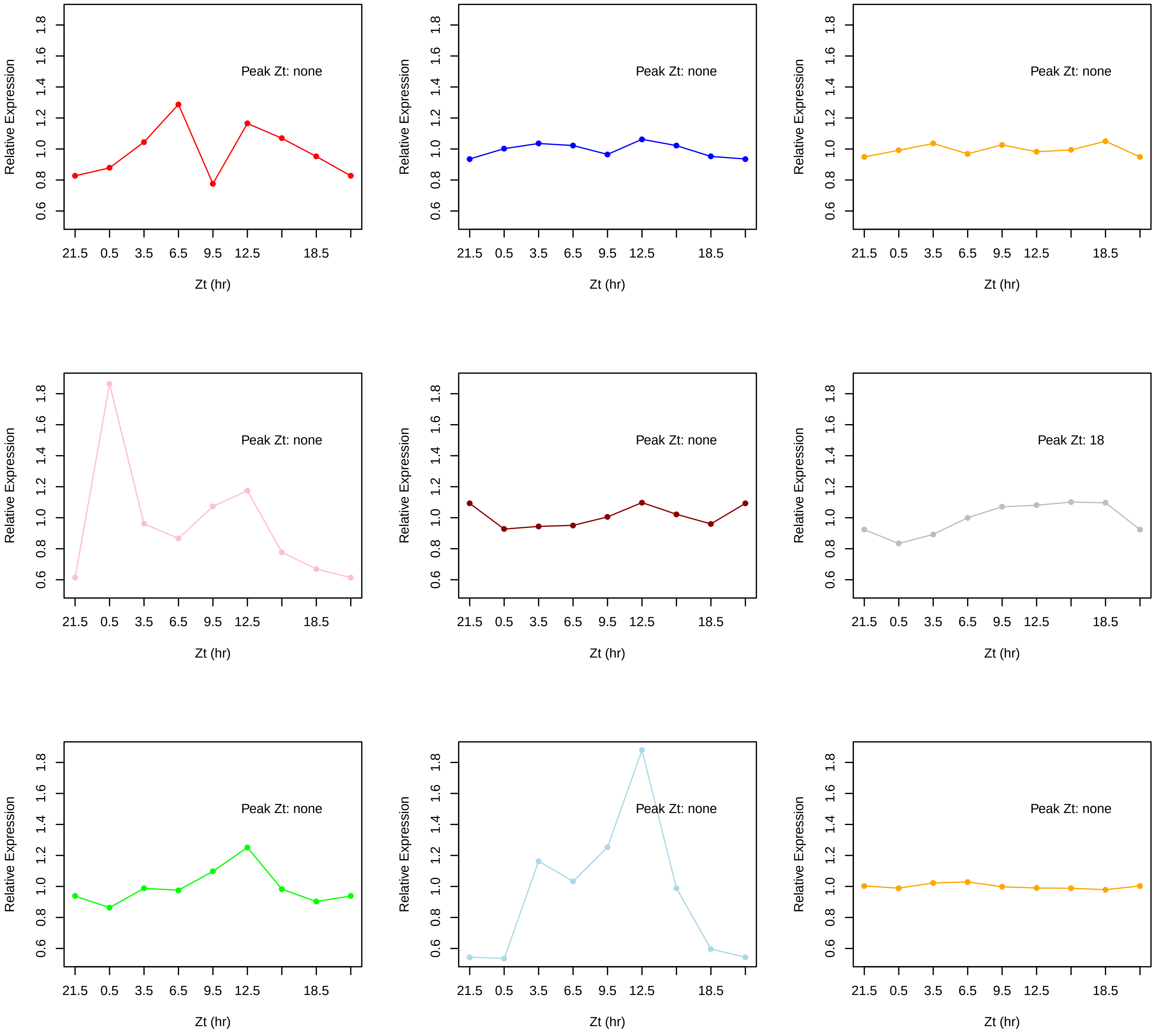

Singeleton: ENSXMAG00000012969 rgl1

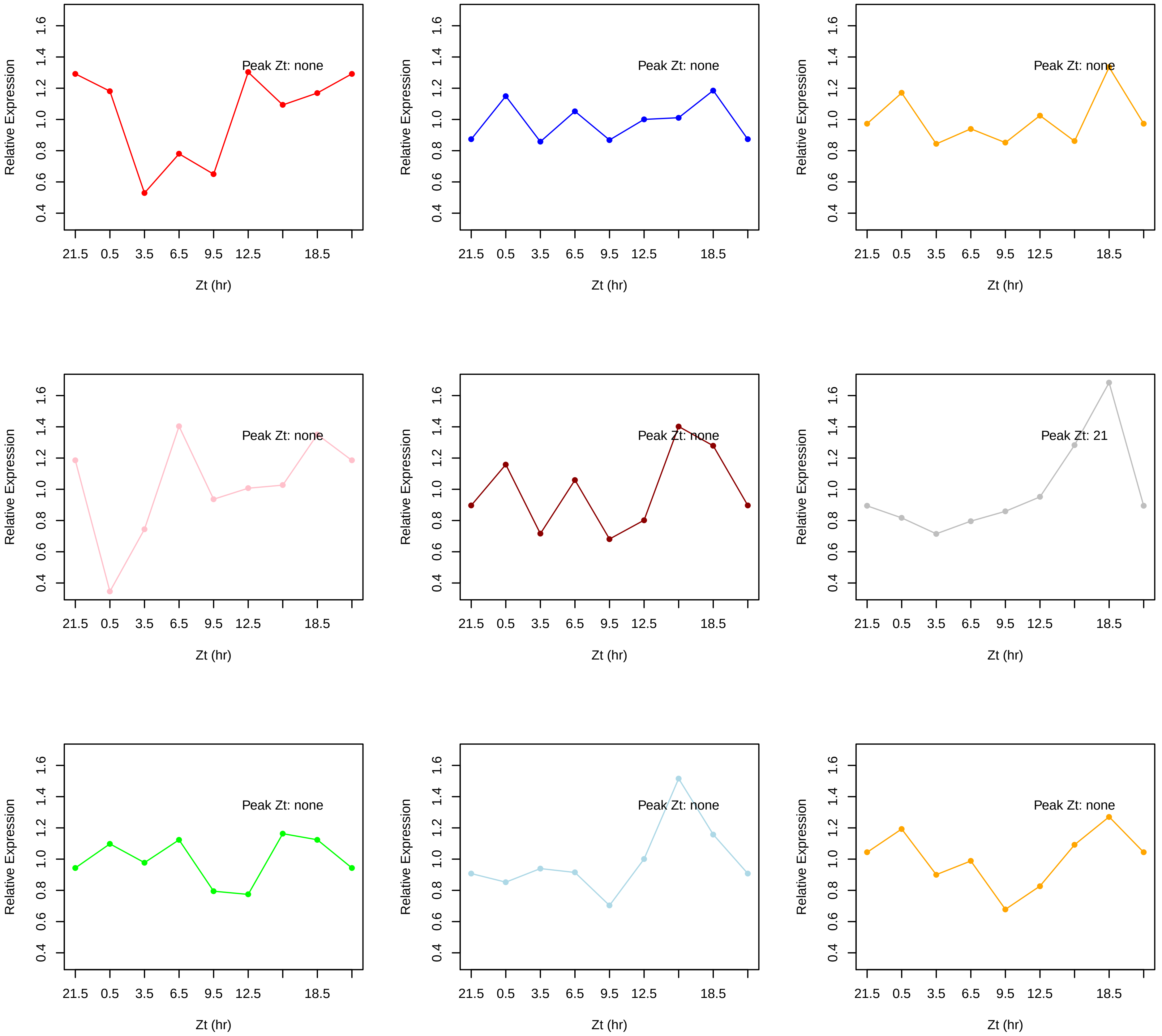

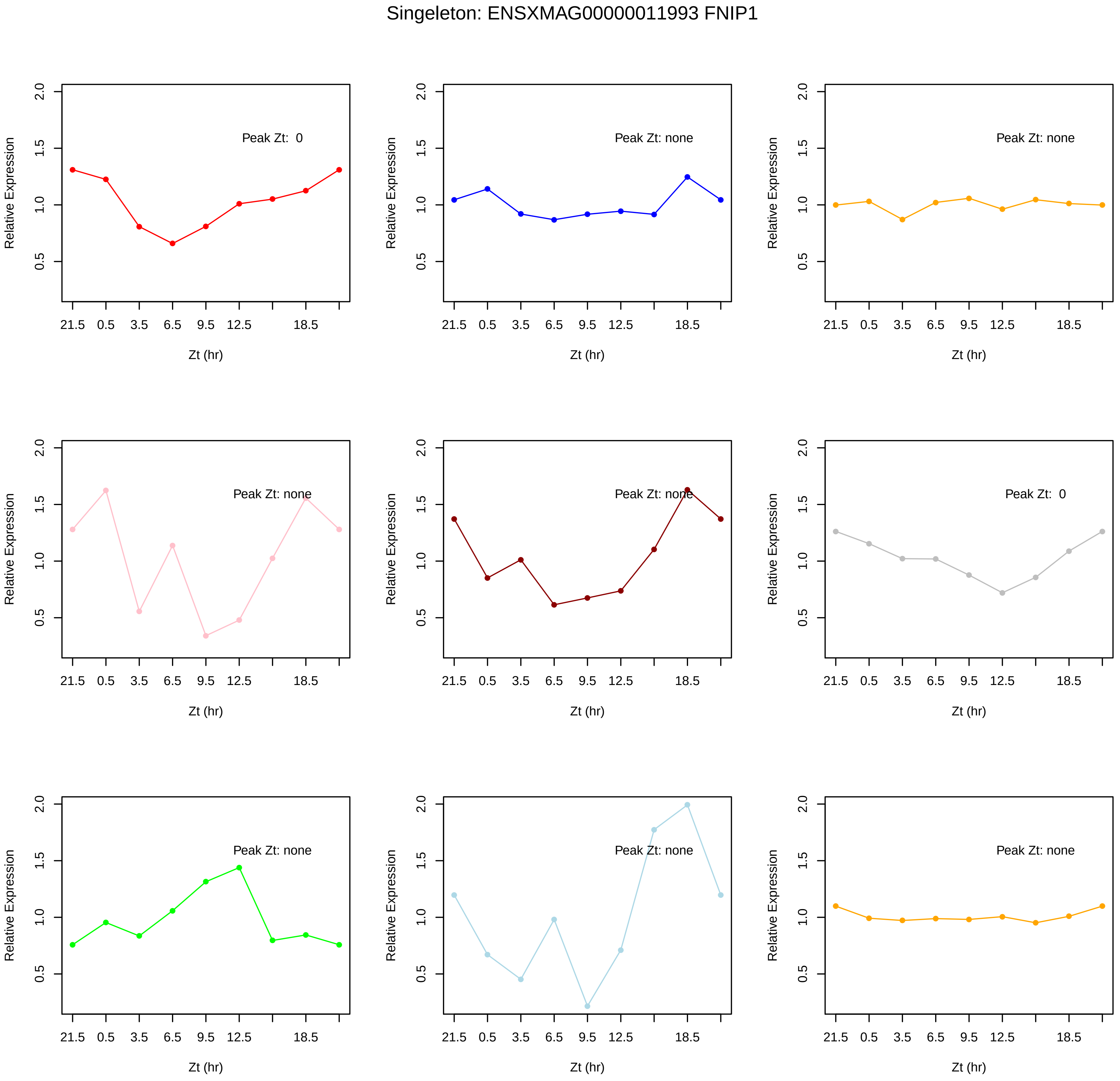
Singeleton: ENSXMAG00000011993 FNIP1
Peak Zt: 0
Peak Zt: none
Peak Zt: none
Peak Zt: none
Peak Zt: none
Peak Zt: 0
Peak Zt: none
Peak Zt: none
Peak Zt: none
Relative Expression
Zt (hr)

Singeleton: ENSXMAG00000001995 psmd6

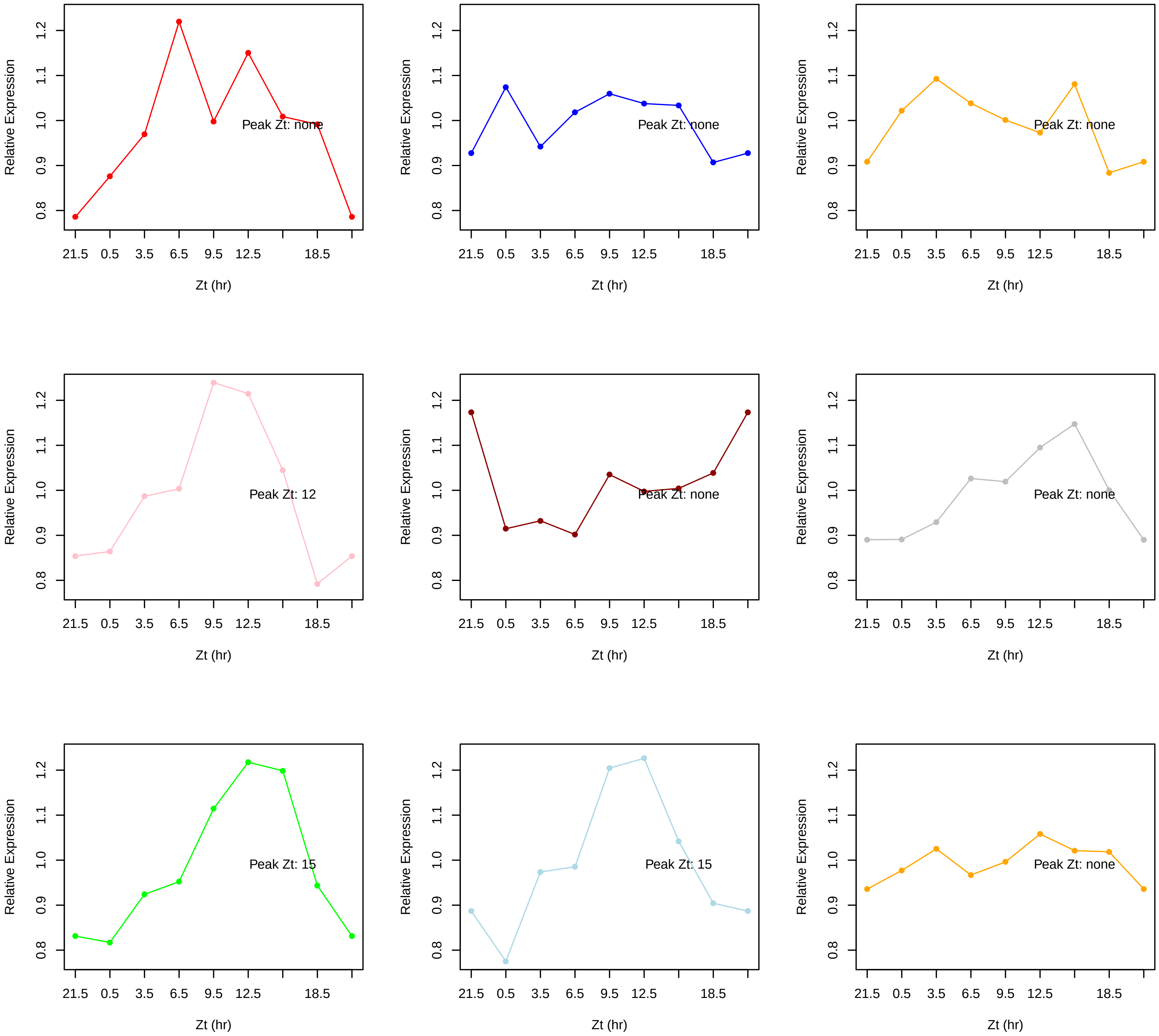

Singeleton: ENSXMAG00000006881 gtpbp10

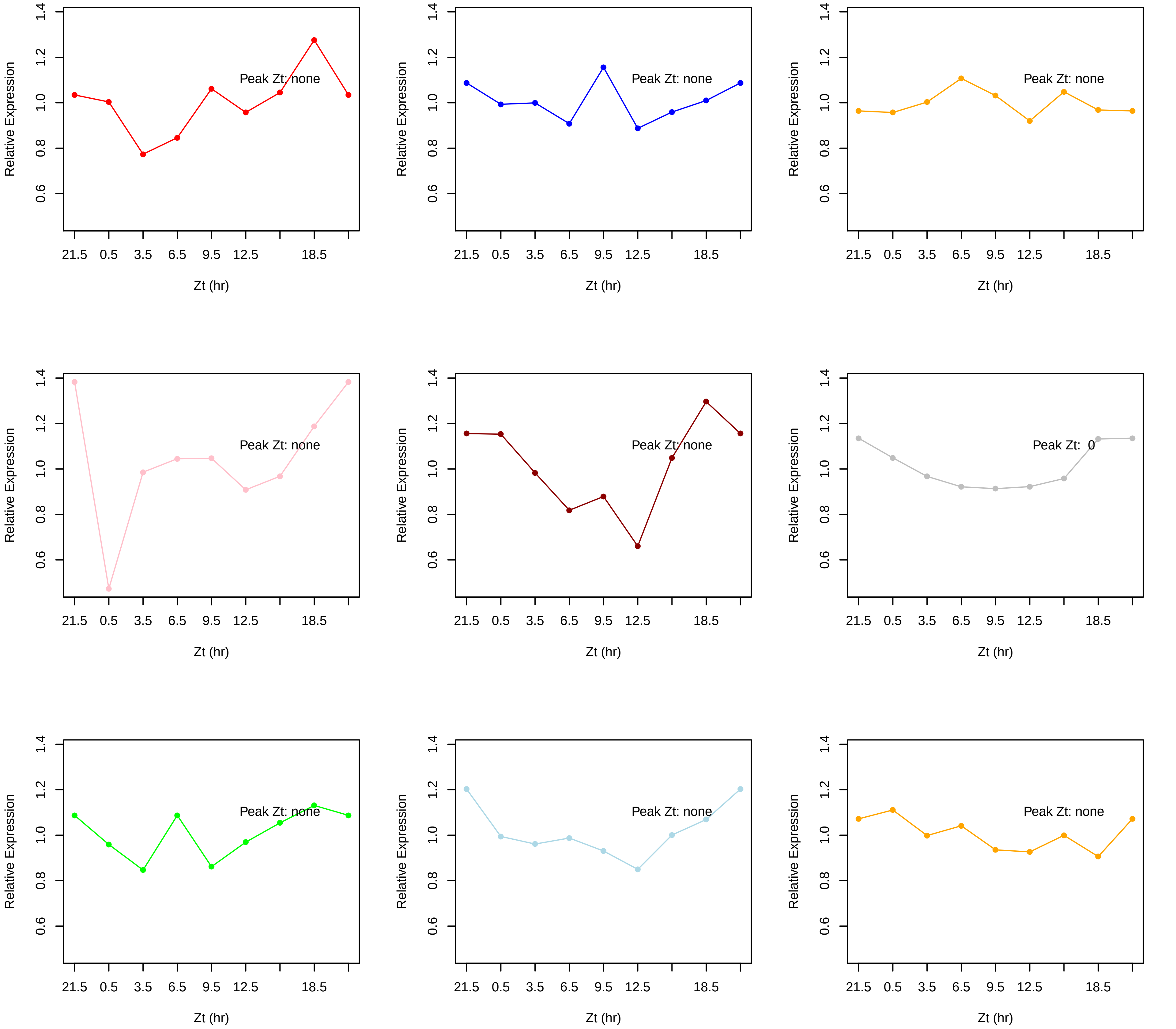

Singeleton: ENSXMAG00000015817 trmt44

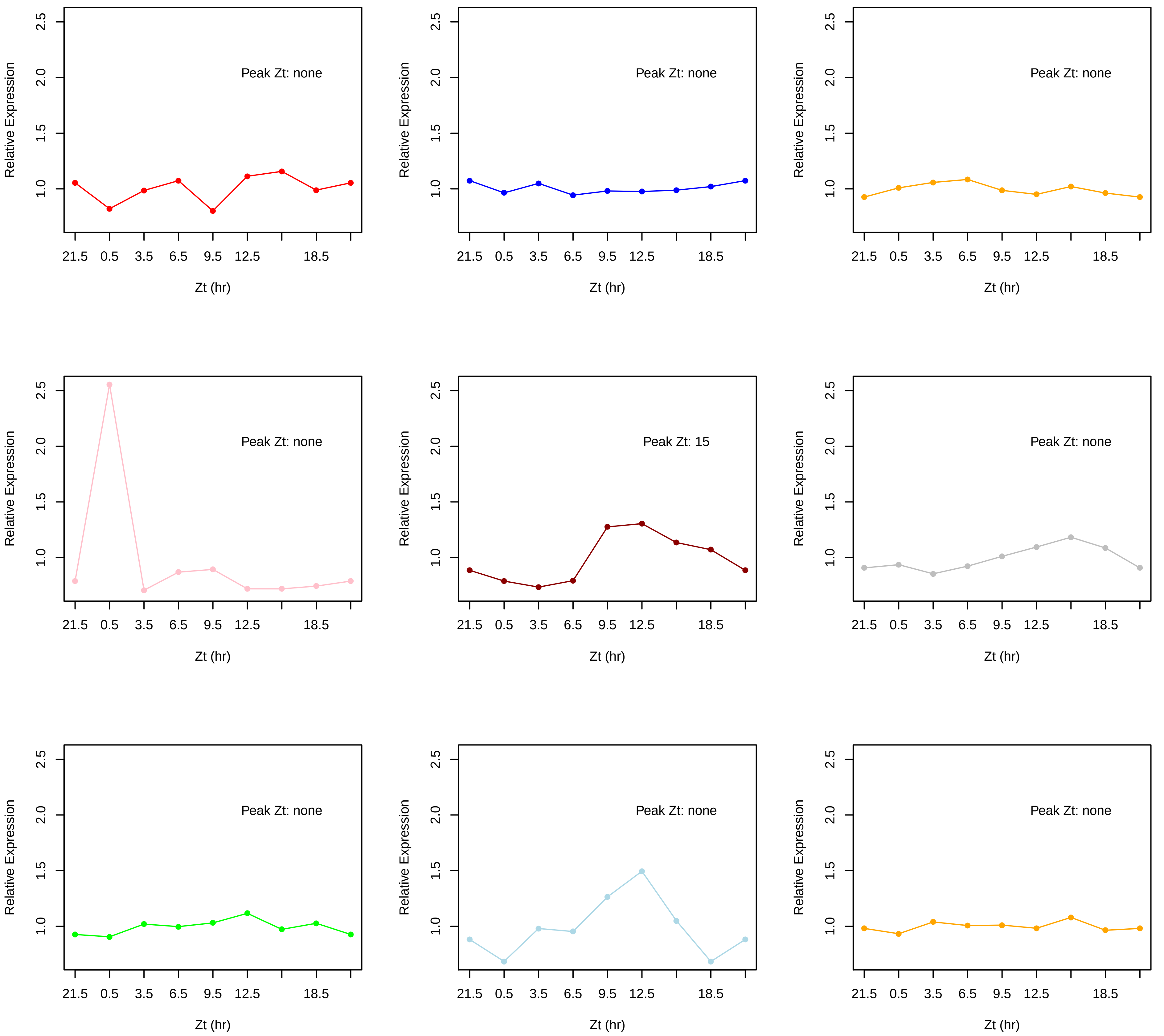

Singeleton: ENSXMAG00000012038

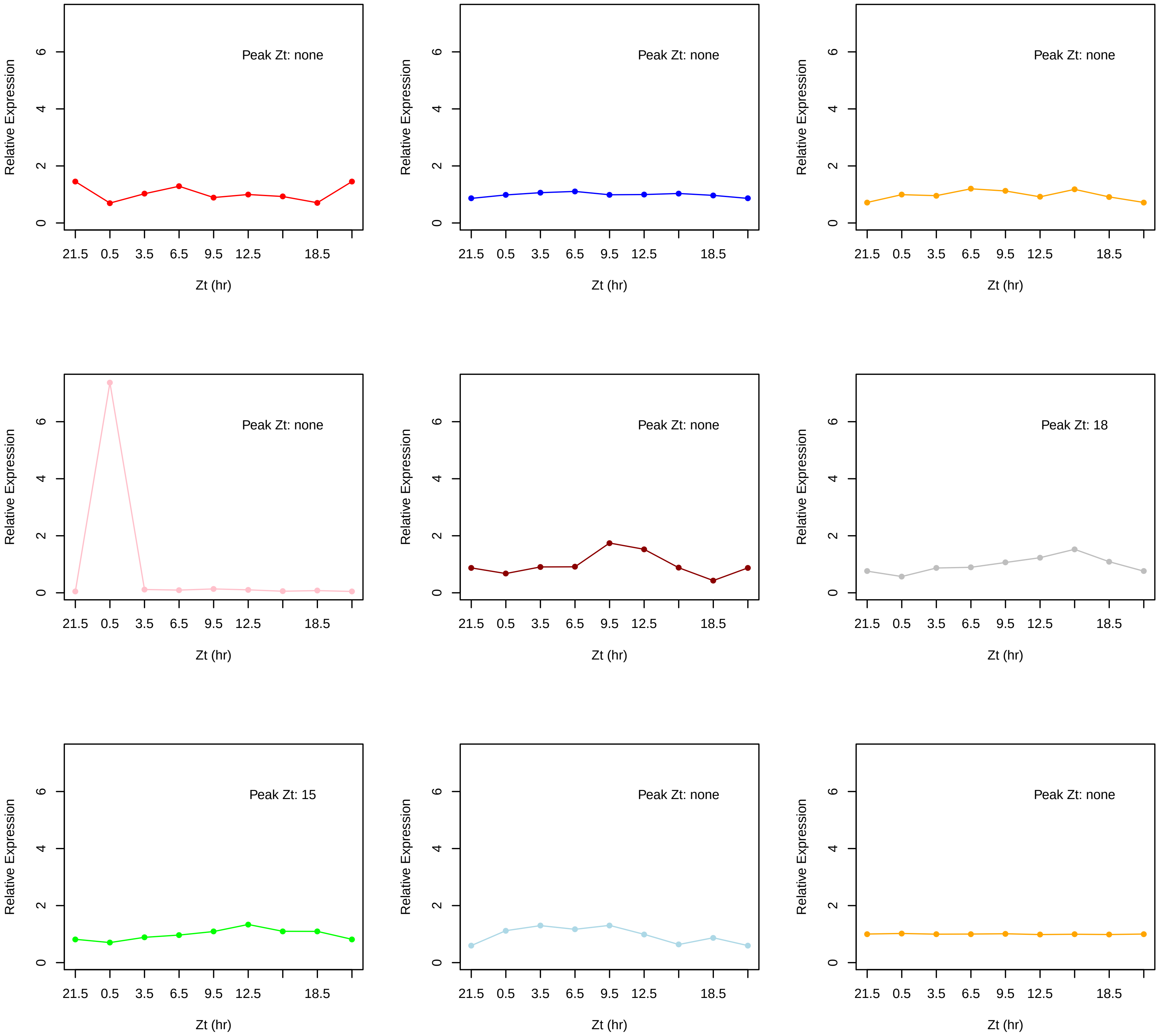

Singeleton: ENSXMAG00000005934 st7l

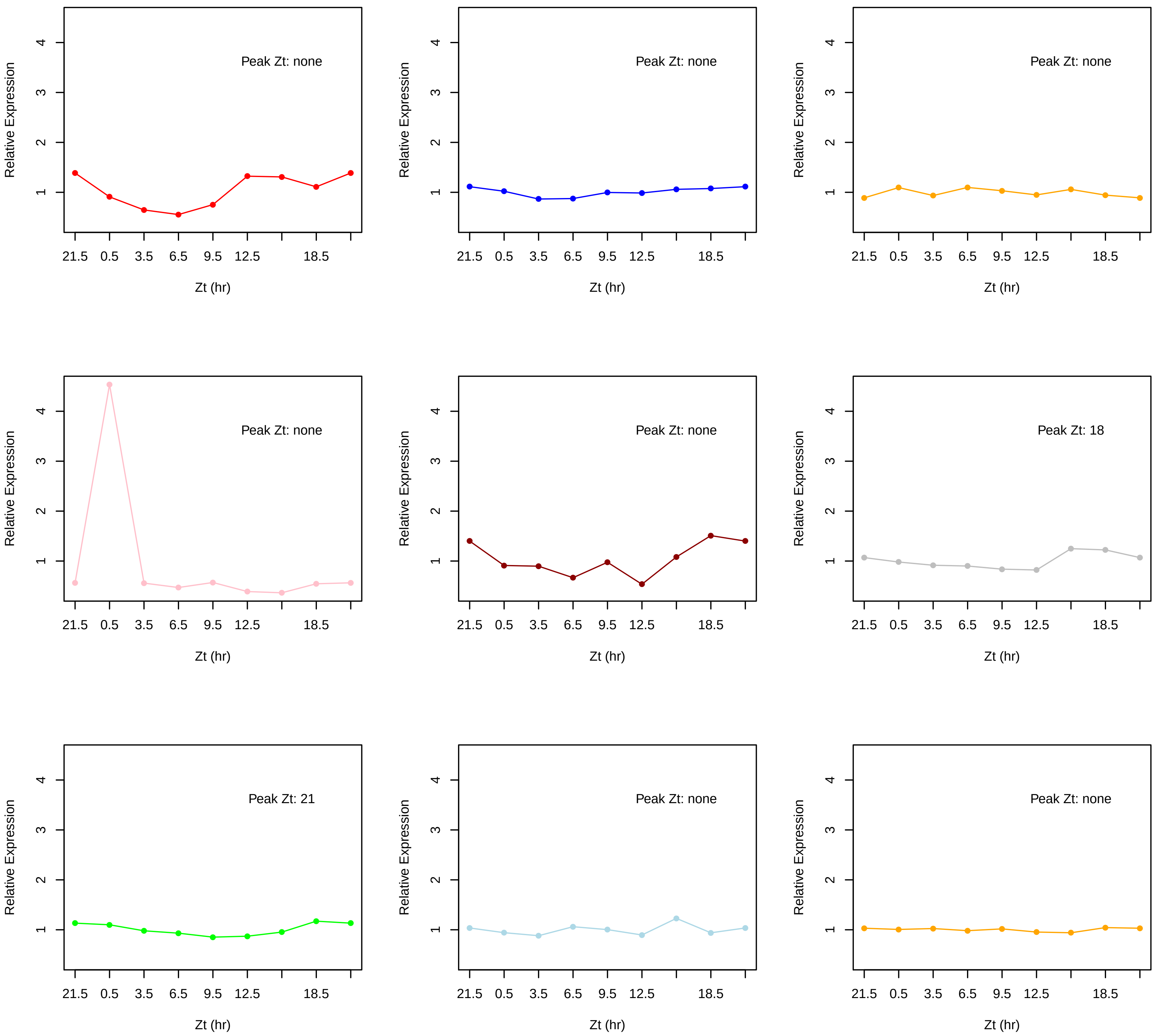

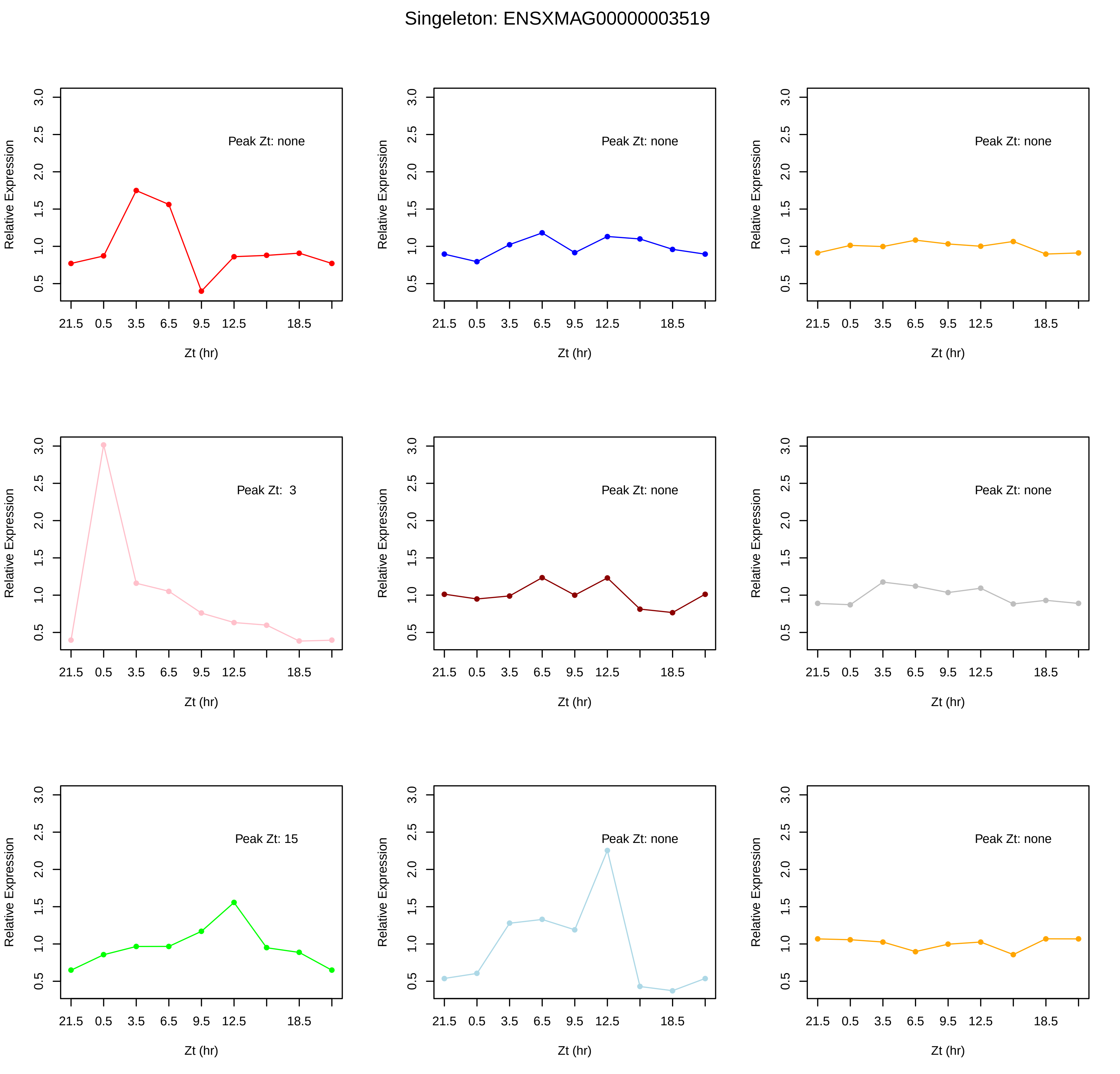
Singeleton: ENSXMAG00000003519
Peak Zt: none
Peak Zt: none
Peak Zt: none
Peak Zt: 3
Peak Zt: none
Peak Zt: none
Peak Zt: 15
Peak Zt: none
Peak Zt: none
Relative Expression
Zt (hr)
21.5
0.5
3.5
6.5
9.5
12.5
18.5
0.5
1.0
1.5
2.0
2.5
3.0

Singleton: ENSXMAG00000004345 wwp1

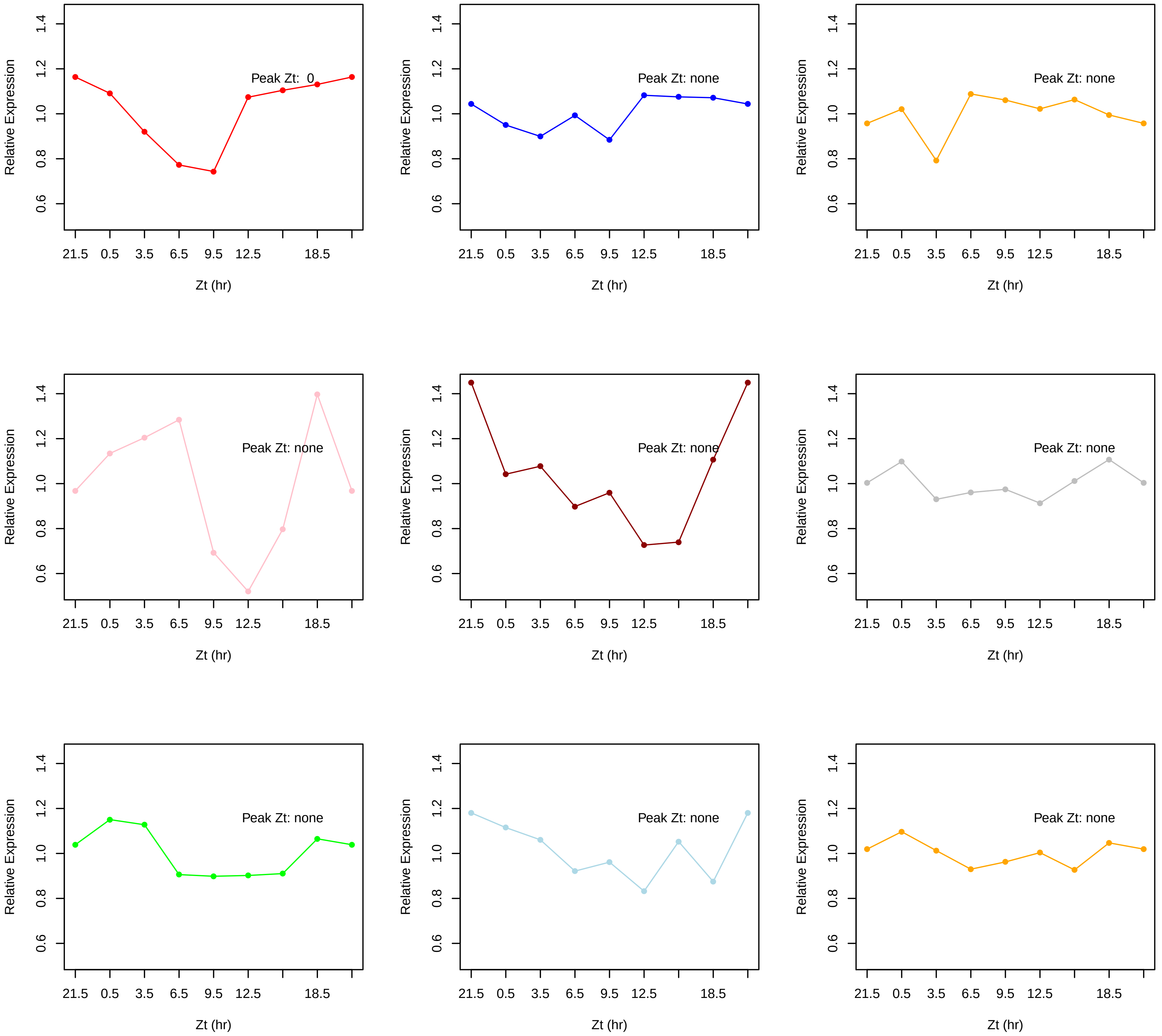

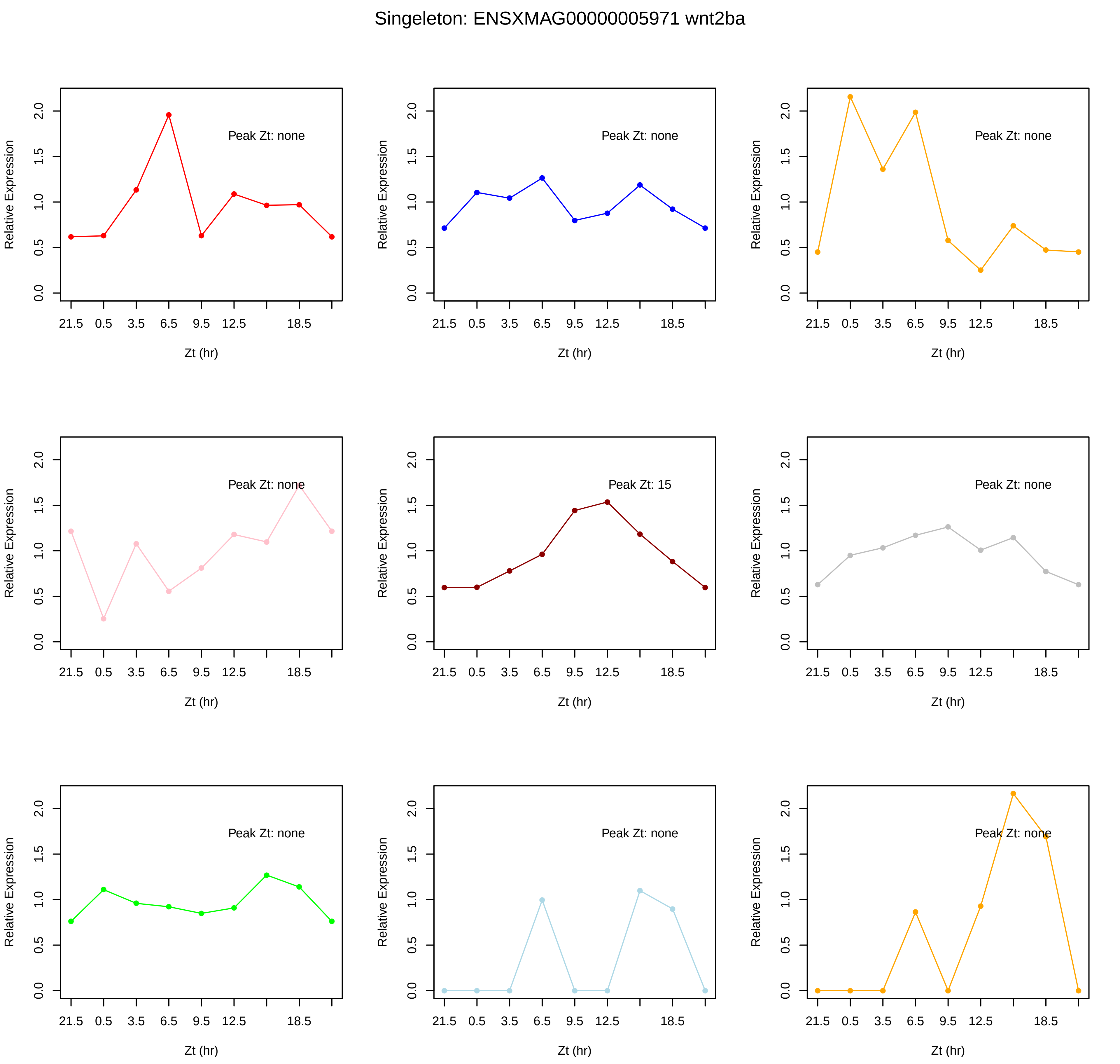
Singeleton: ENSXMAG00000005971 wnt2ba
Peak Zt: none
Peak Zt: none
Peak Zt: none
Peak Zt: none
Peak Zt: 15
Peak Zt: none
Peak Zt: none
Peak Zt: none
Peak Zt: none
Relative Expression
Zt (hr)
21.5
0.5
3.5
6.5
9.5
12.5
18.5
0.0
0.5
1.0
1.5
2.0

Singeleton: ENSXMAG00000024224 RMDN1

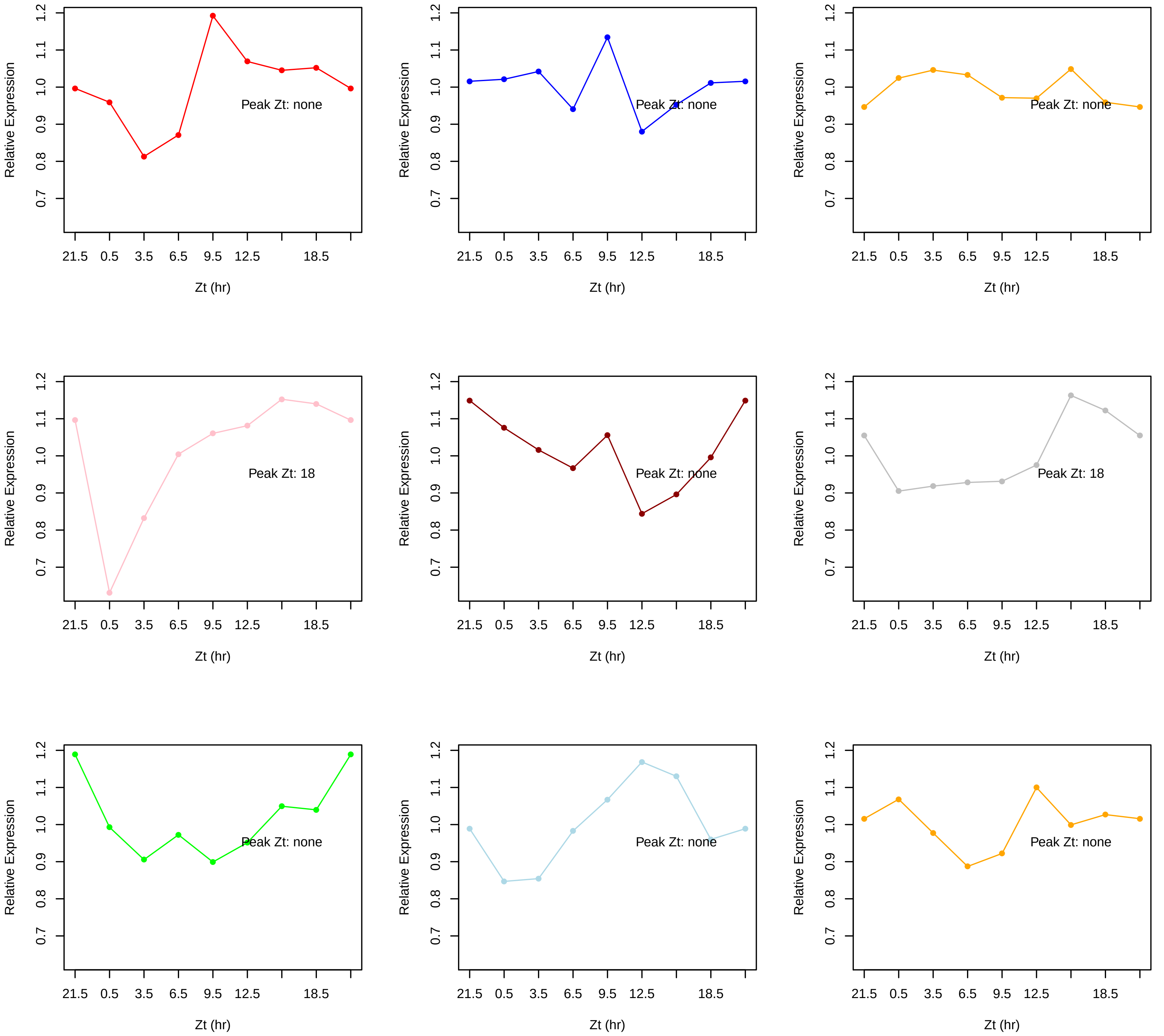

Singeleton: ENSXMAG00000021931 SYNPR

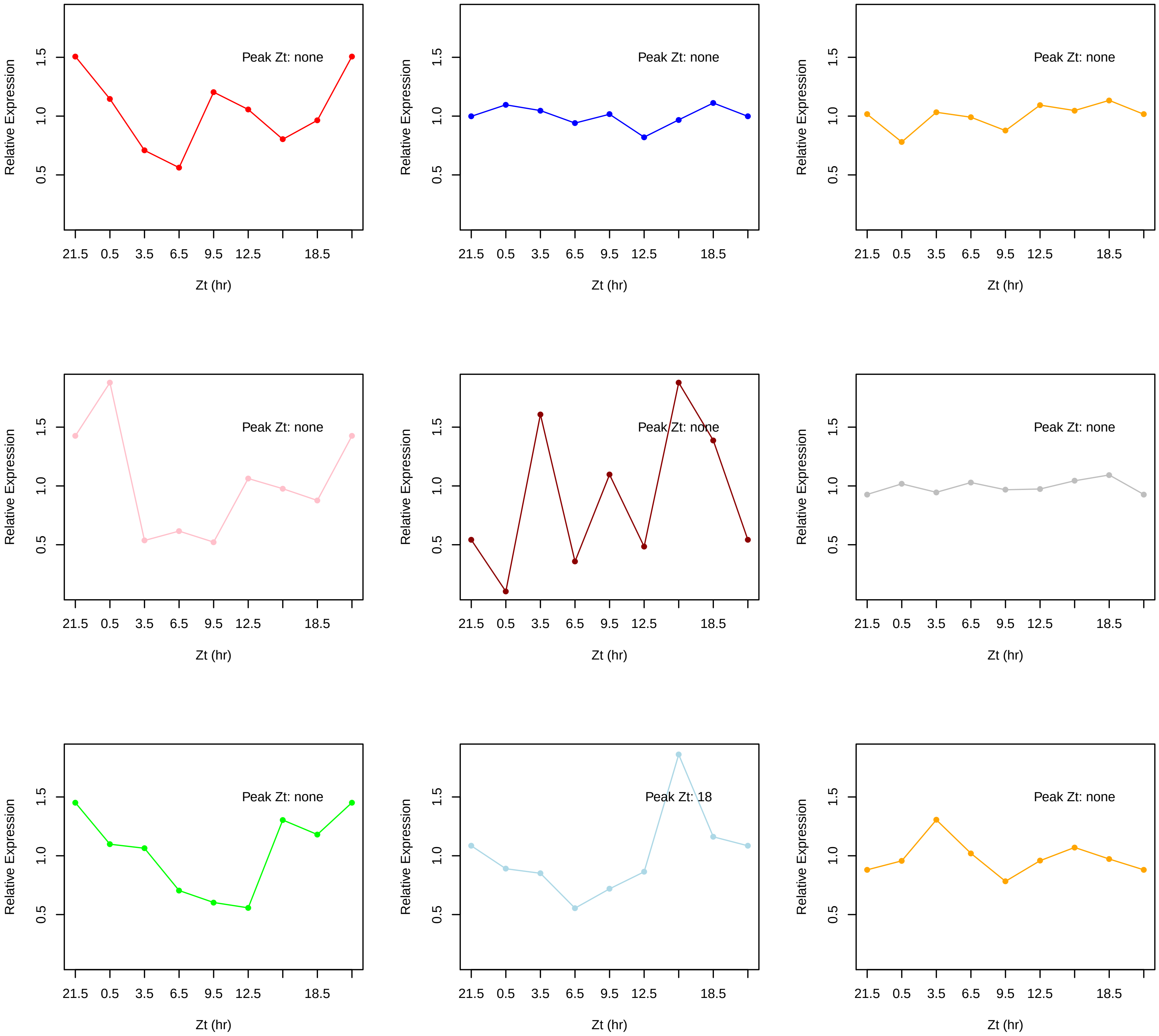

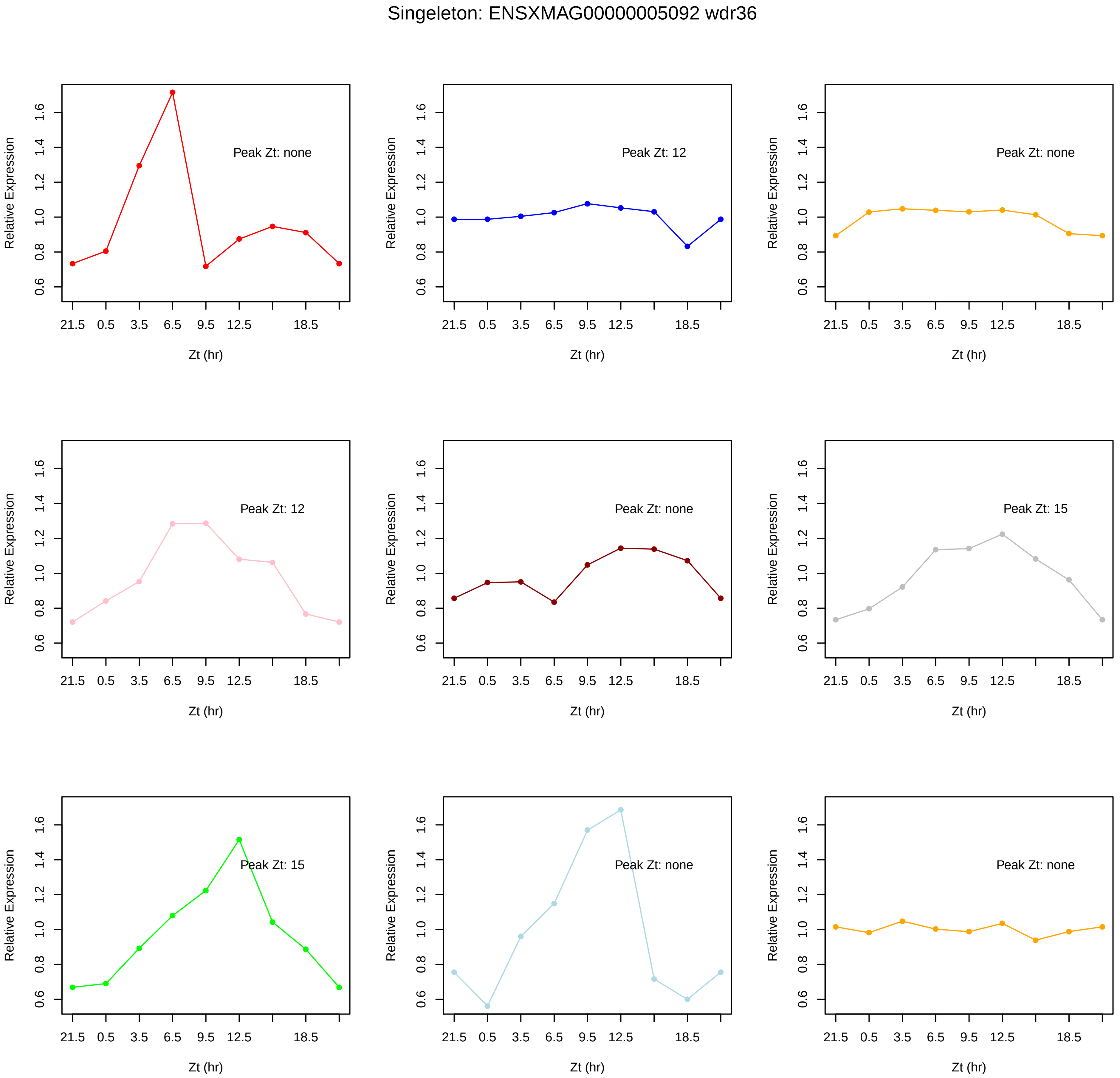

Singleton: ENSXMAG00000005092 wdr36
Relative Expression
Zt (hr)
21.5
0.5
3.5
6.5
9.5
12.5
18.5
0.6
0.8
1.0
1.2
1.4
1.6
Peak Zt: none
Peak Zt: 12
Peak Zt: none
Peak Zt: 12
Peak Zt: none
Peak Zt: 15
Peak Zt: 15
Peak Zt: none
Peak Zt: none

Singleton: ENSXMAG00000015836 DOK7

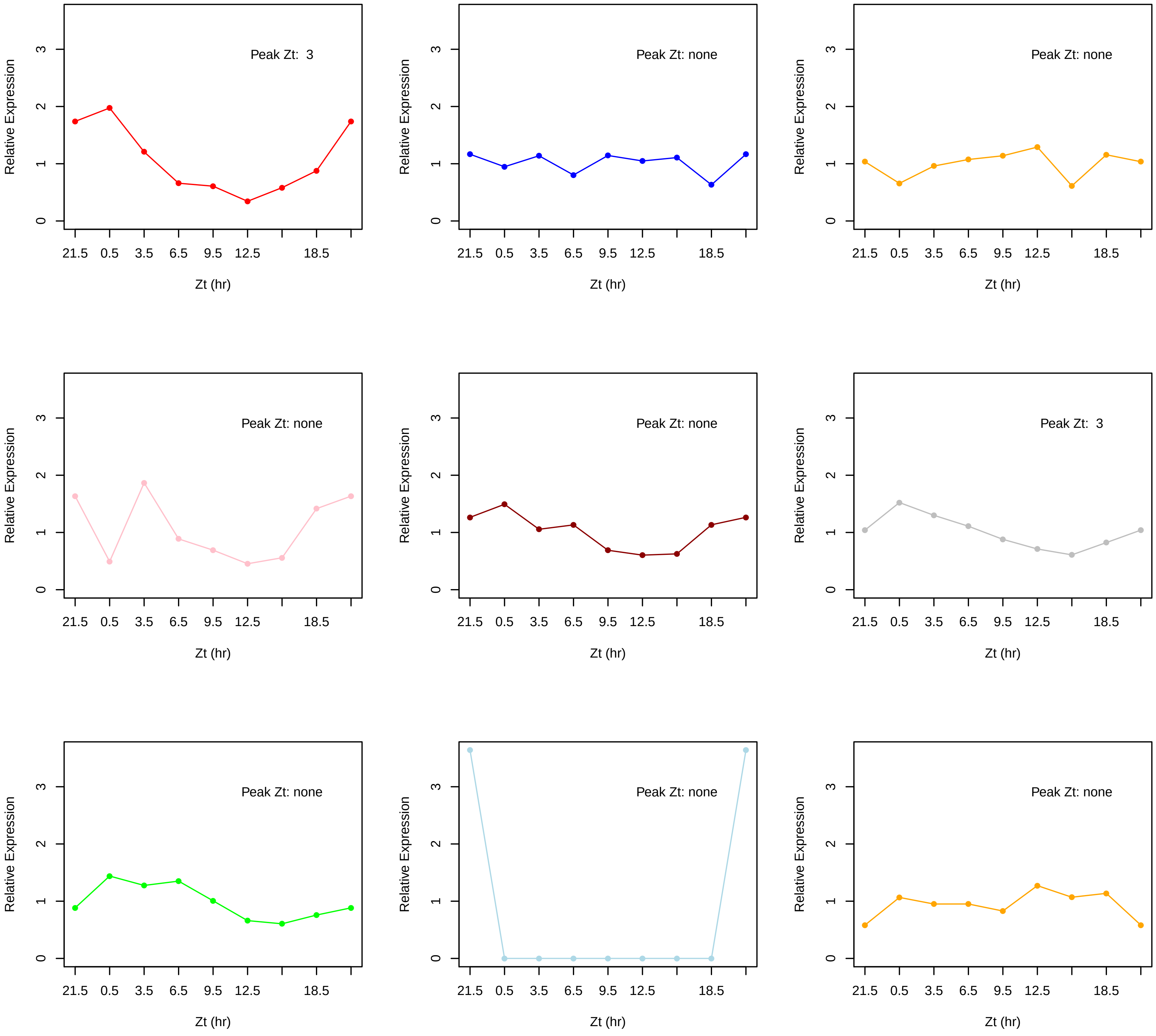

Singeleton: ENSXMAG00000007783 namptb

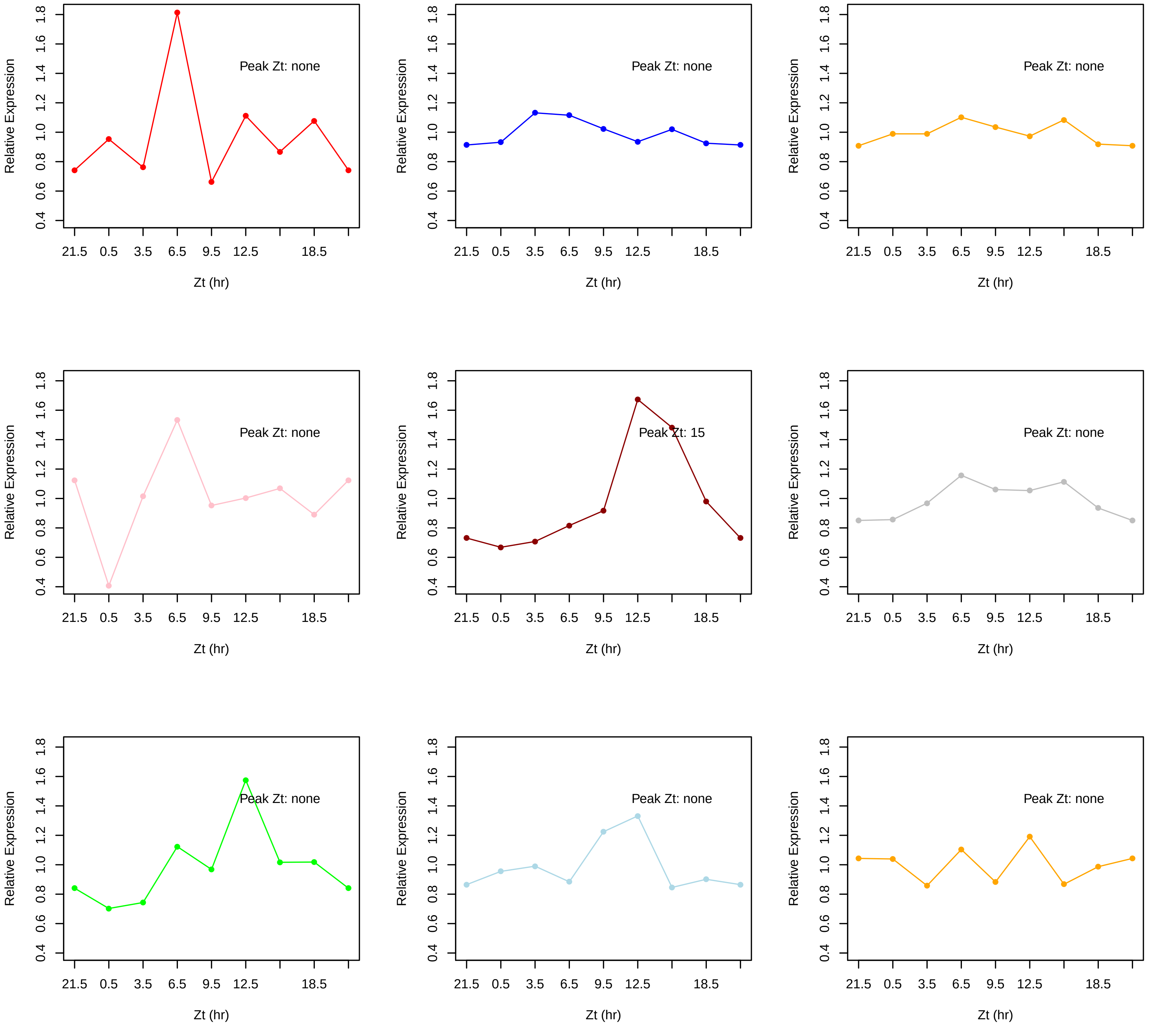

Singeleton: ENSXMAG00000005474 rgs12b

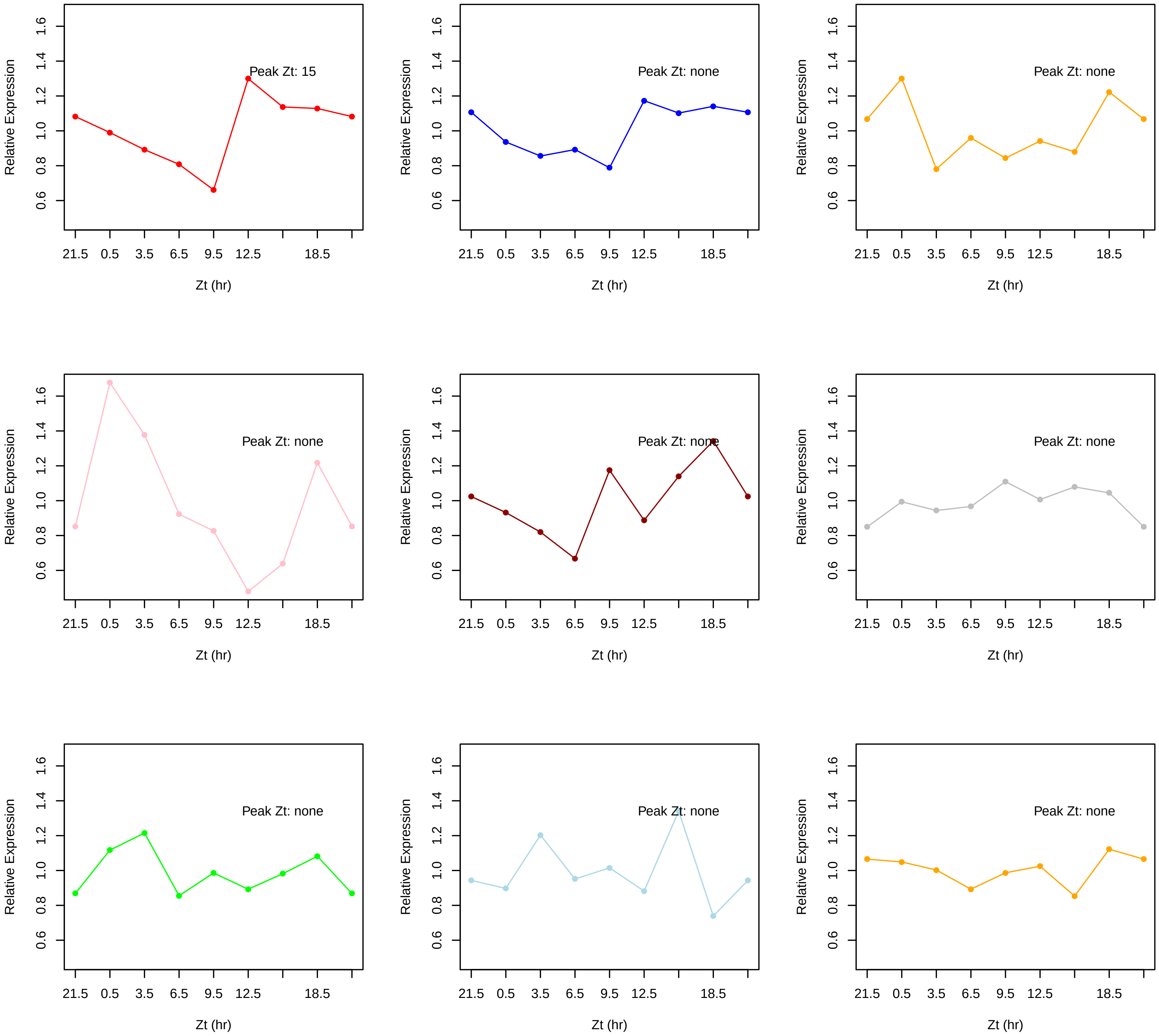

Singeleton: ENSXMAG00000005123 slc25a46

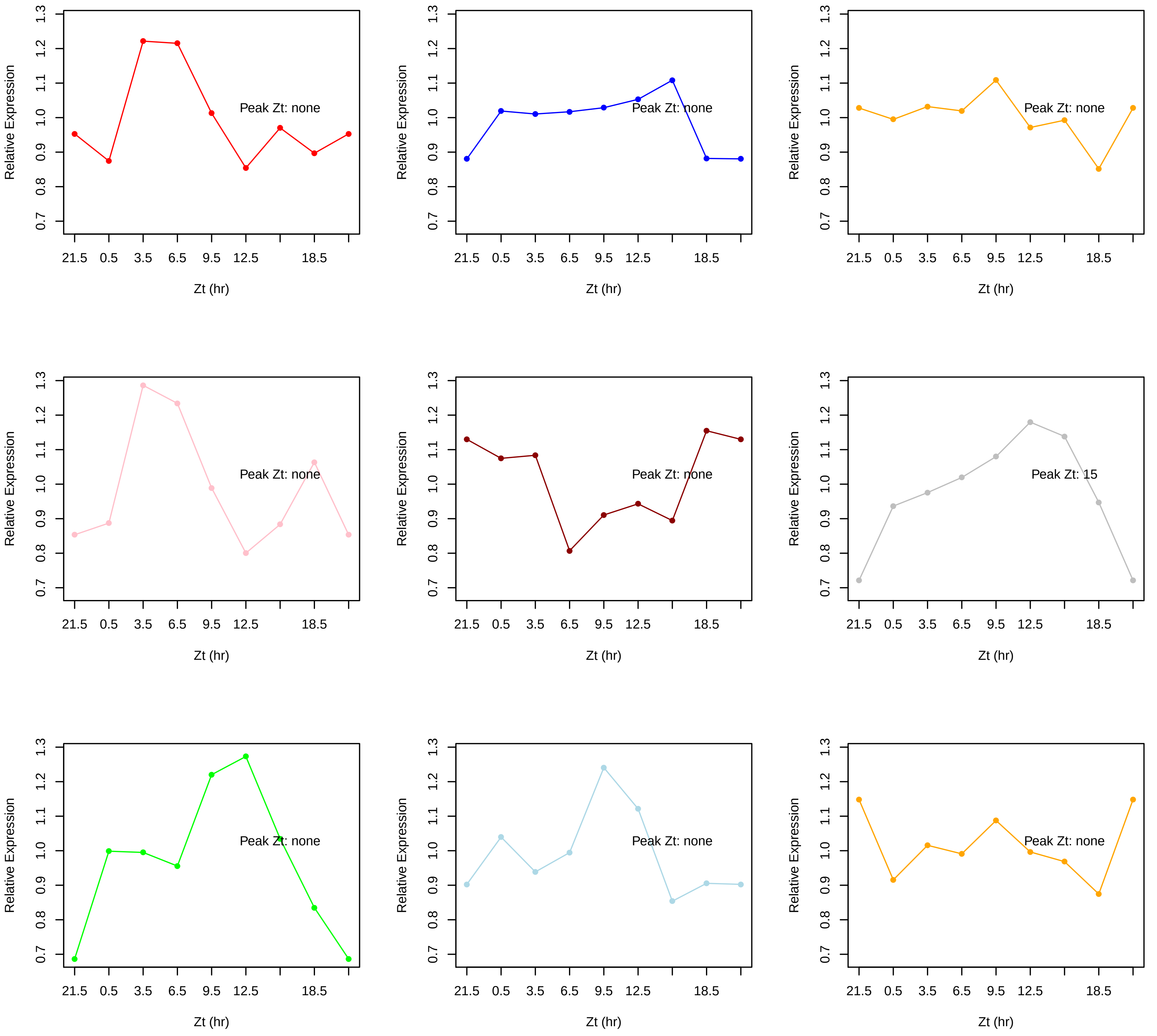

Singeleton: ENSXMAG00000012494 mdh1b

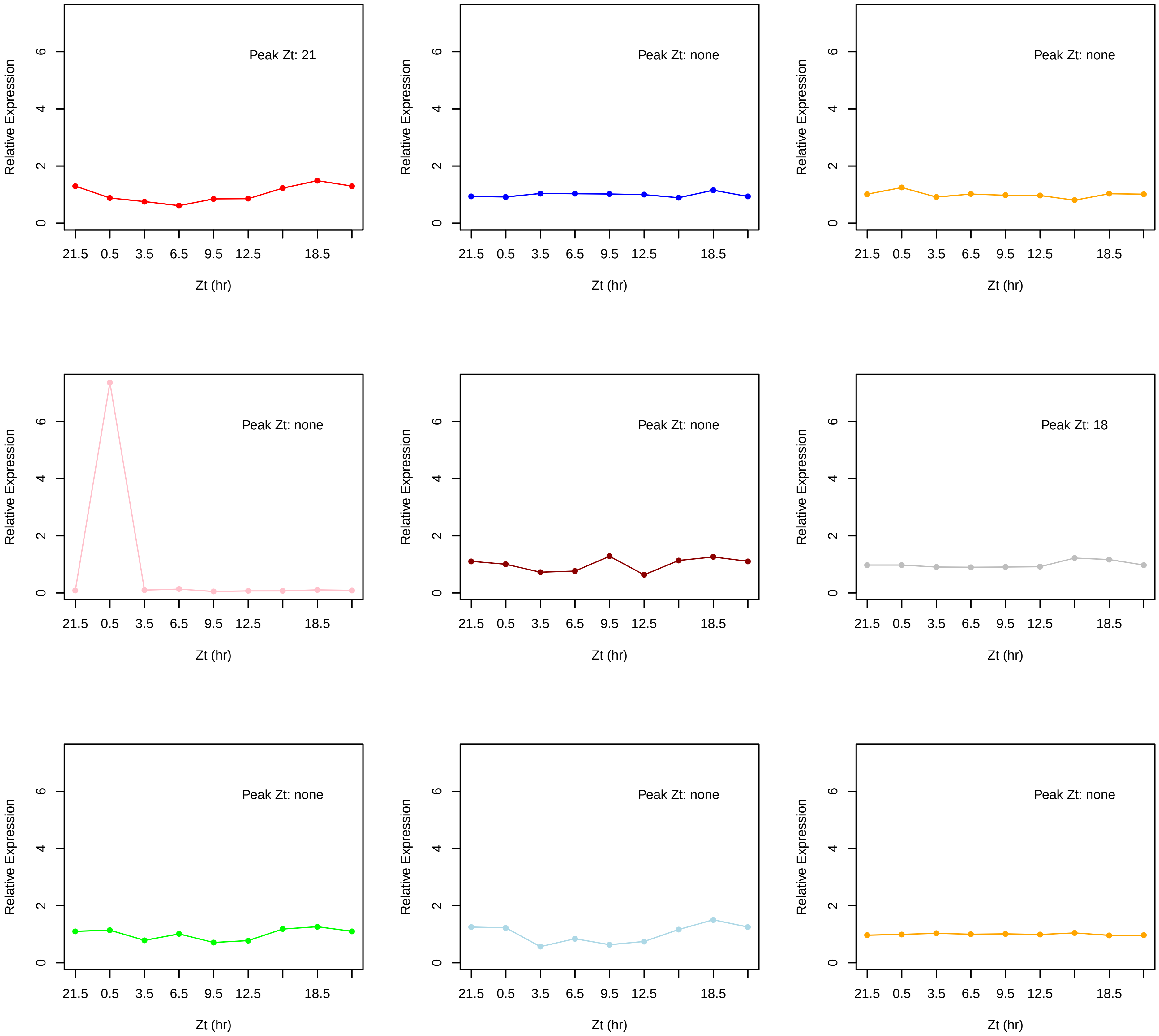

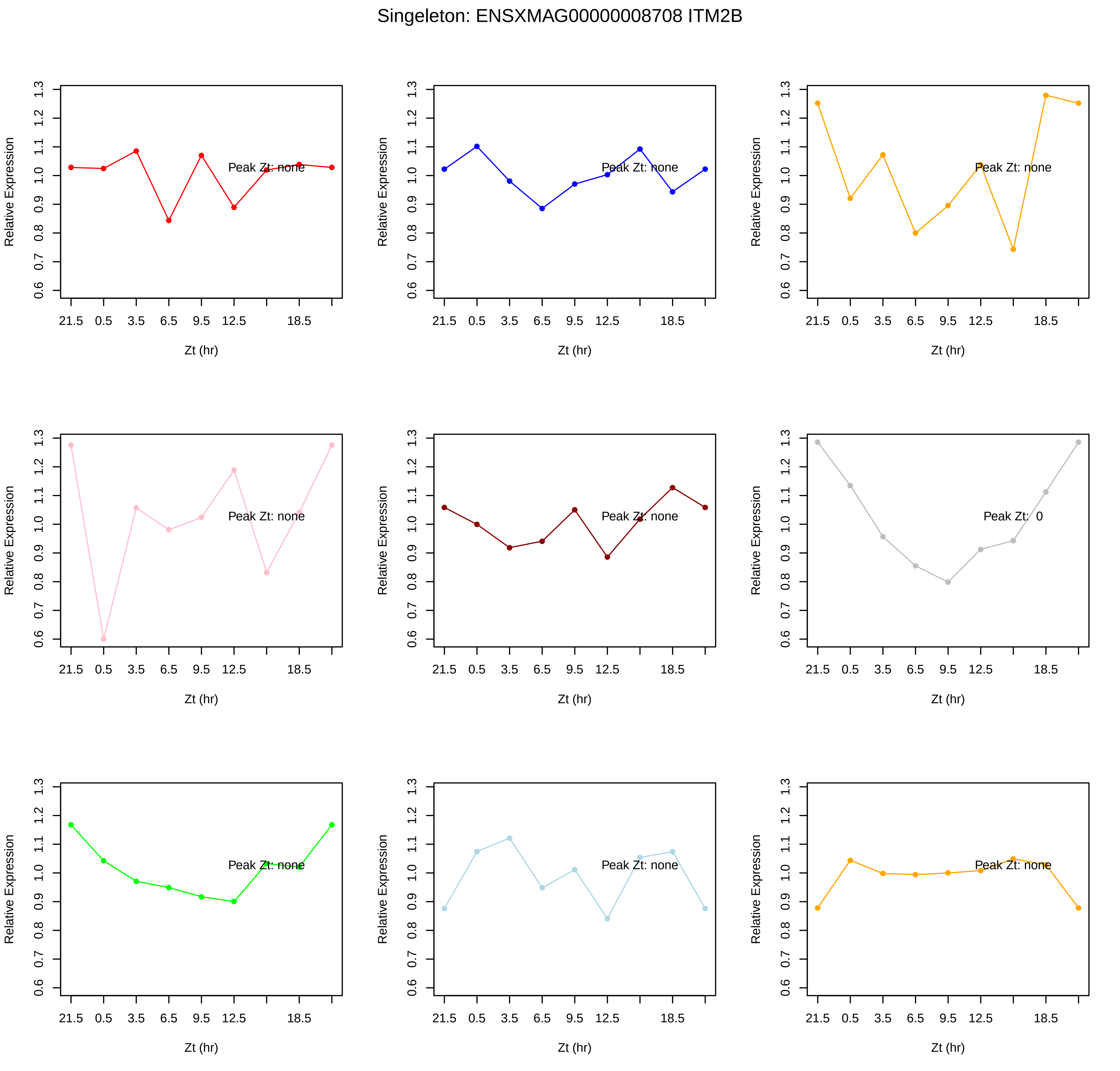
Singeleton: ENSXMAG00000008708 ITM2B
Relative Expression
Zt (hr)
21.5
0.5
3.5
6.5
9.5
12.5
18.5
0.6
0.7
0.8
0.9
1.0
1.1
1.2
1.3
Peak Zt: none
Peak Zt: 0

Singeleton: ENSXMAG00000009145 kif3a

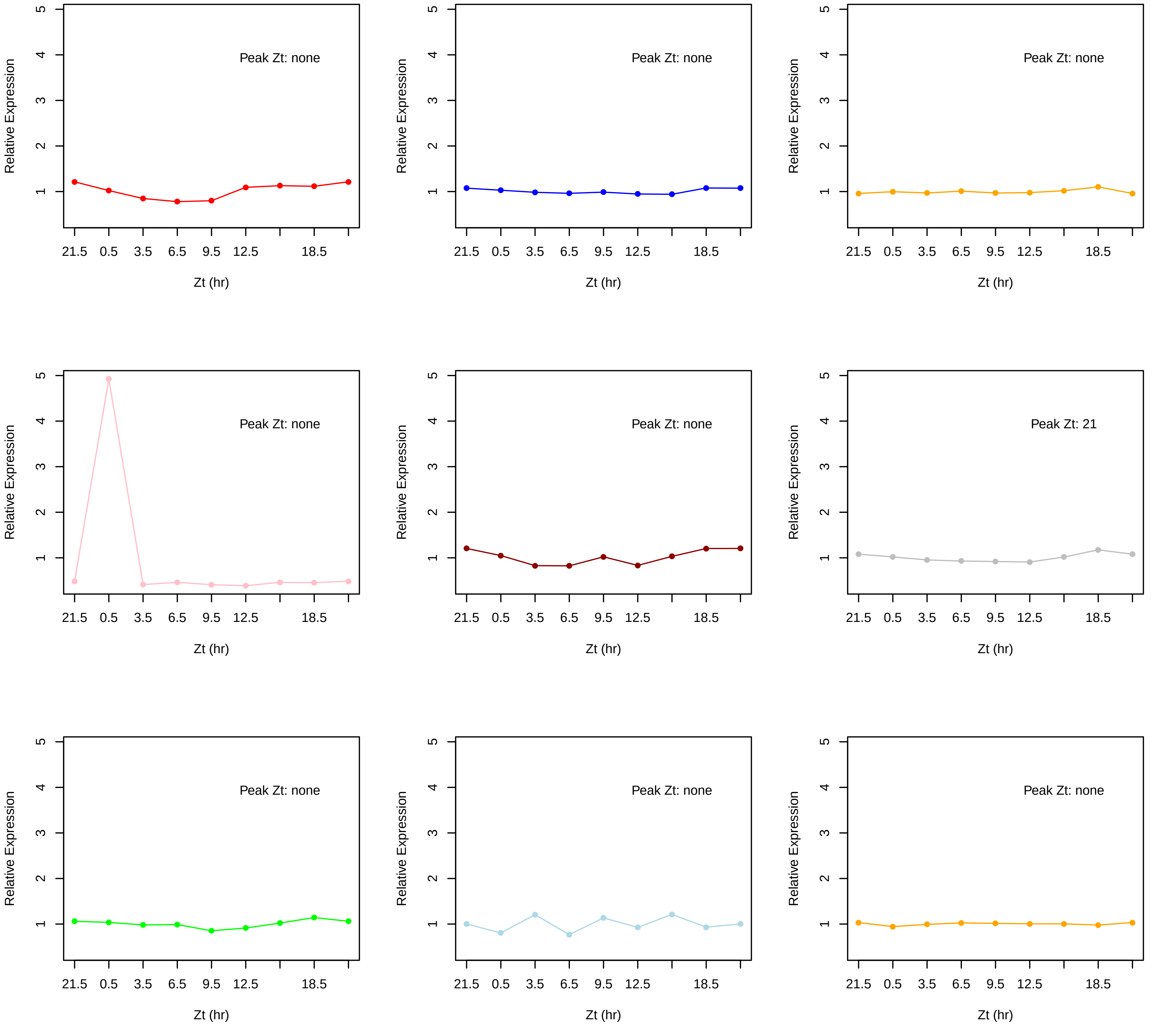

Singleton: ENSXMAG00000014899 EML5

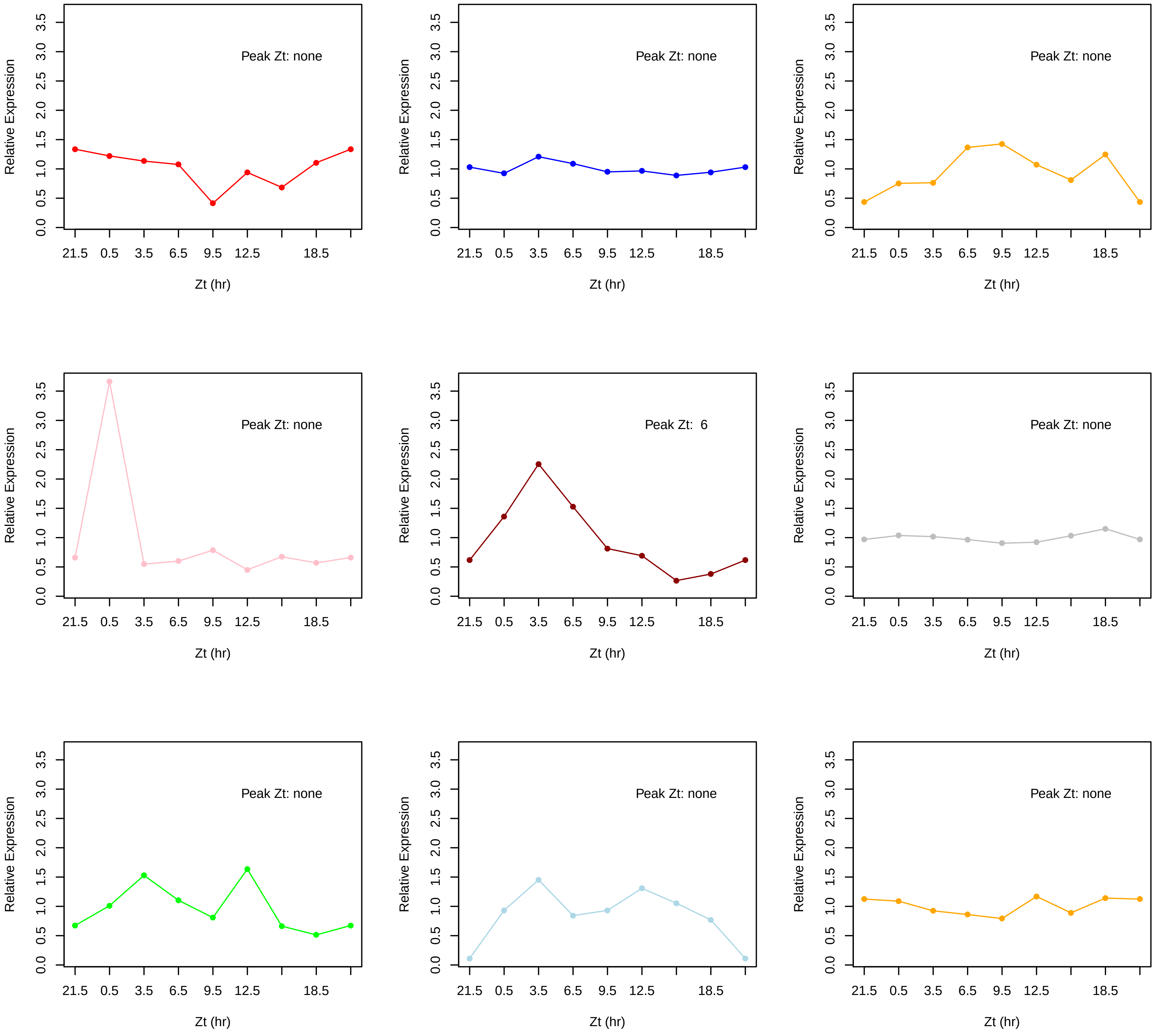

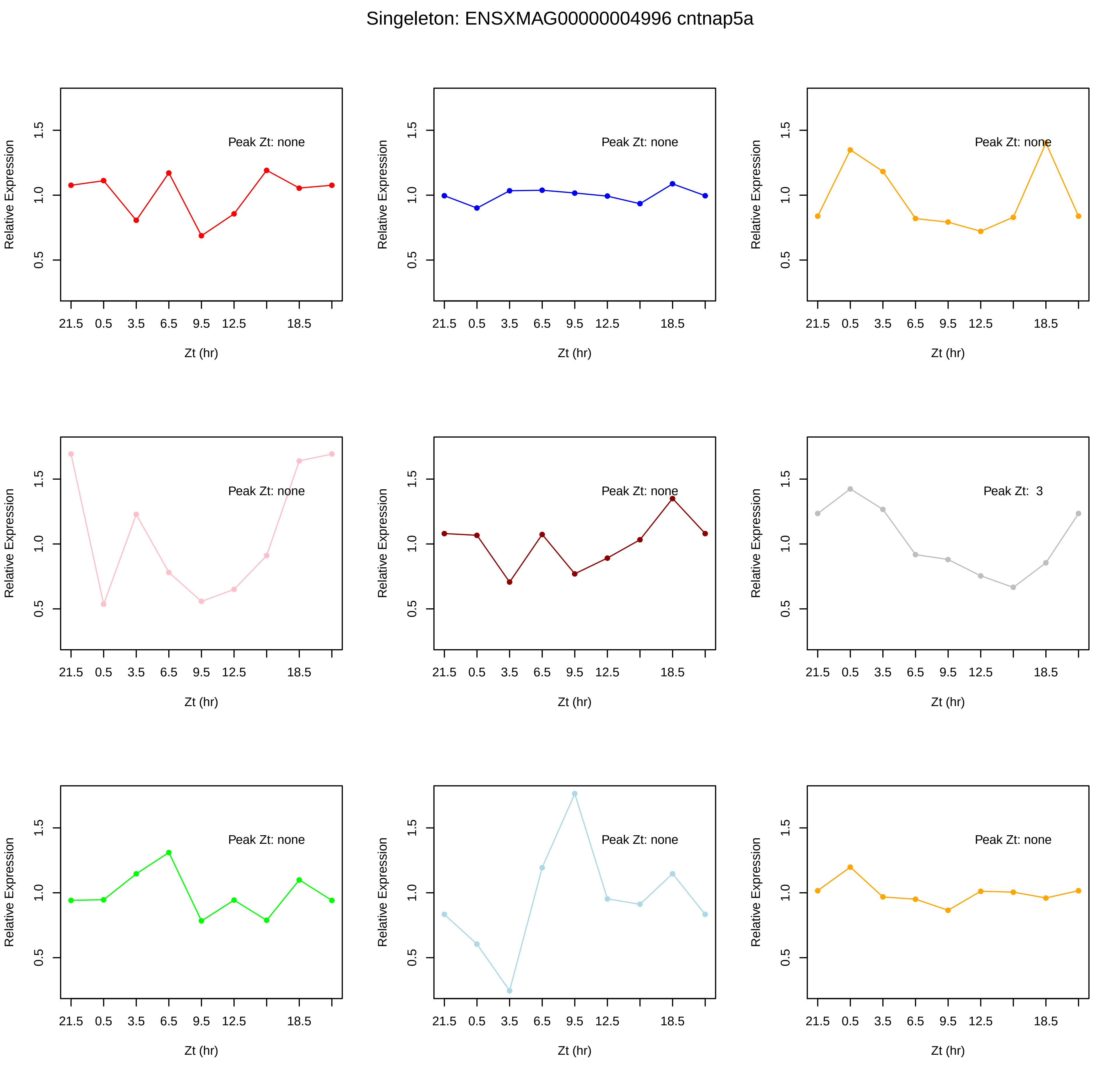
Singeleton: ENSXMAG00000004996 cntnap5a
Peak Zt: none
Peak Zt: none
Peak Zt: none
Peak Zt: none
Peak Zt: none
Peak Zt: 3
Peak Zt: none
Peak Zt: none
Peak Zt: none
Relative Expression
Zt (hr)
21.5
0.5
3.5
6.5
9.5
12.5
18.5

Singeleton: ENSXMAG00000006691 crot

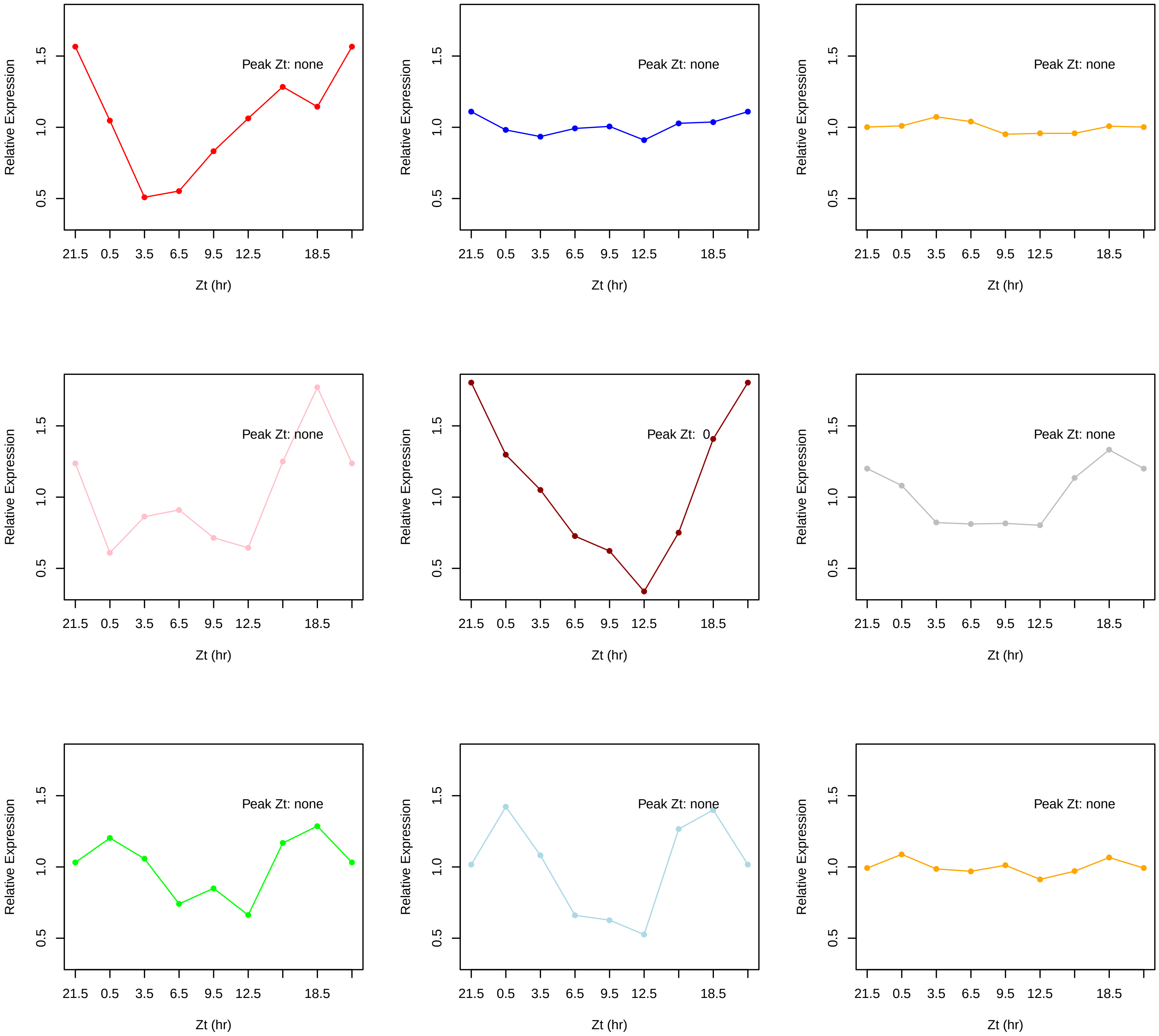

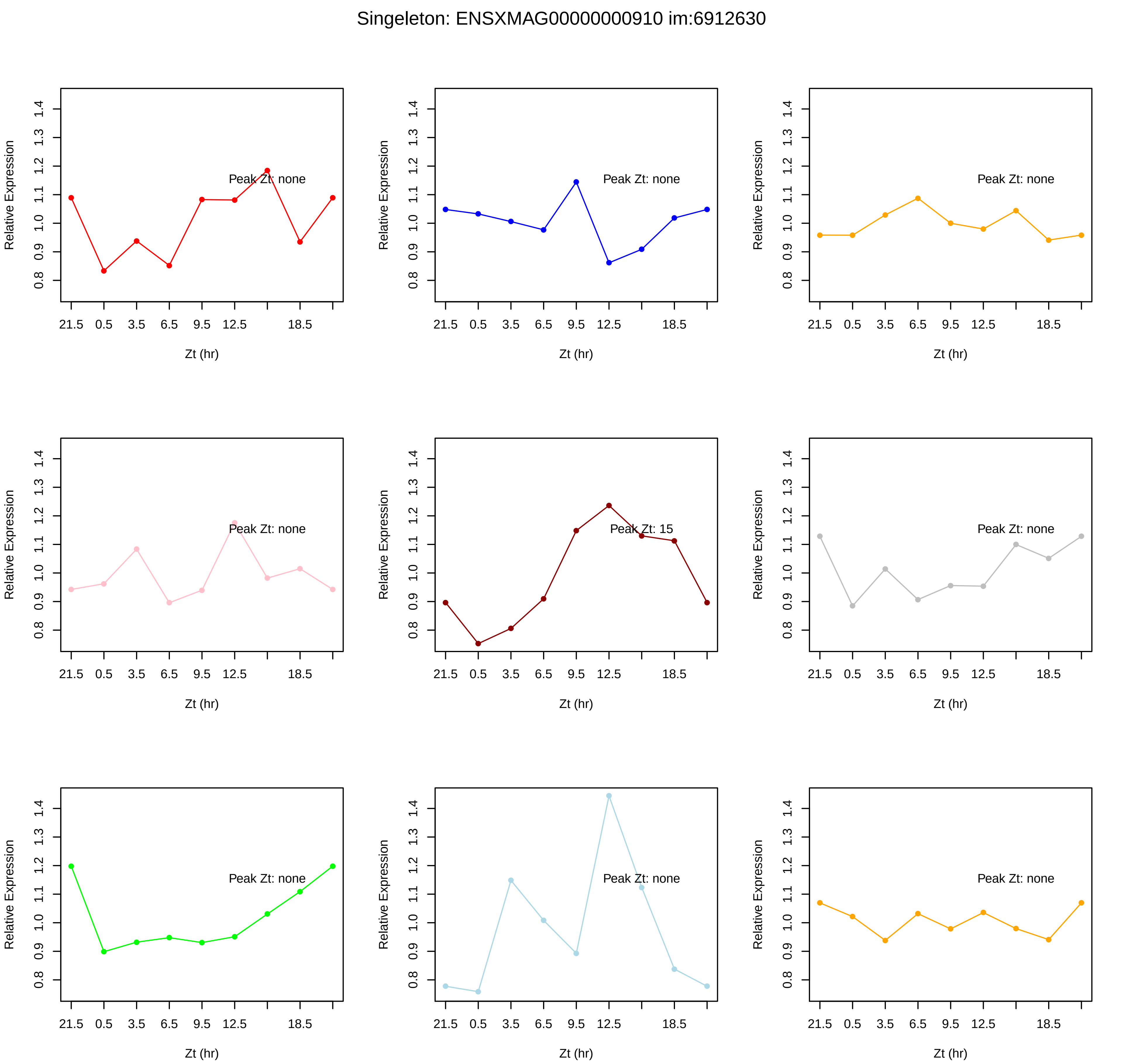
Singeleton: ENSXMAG00000000910 im:6912630
Peak Zt: none
Peak Zt: none
Peak Zt: none
Peak Zt: none
Peak Zt: 15
Peak Zt: none
Peak Zt: none
Peak Zt: none
Peak Zt: none
Relative Expression
Zt (hr)
0.8
0.9
1.0
1.1
1.2
1.3
1.4
21.5
0.5
3.5
6.5
9.5
12.5
18.5

Singeleton: ENSXMAG00000011300 itpa

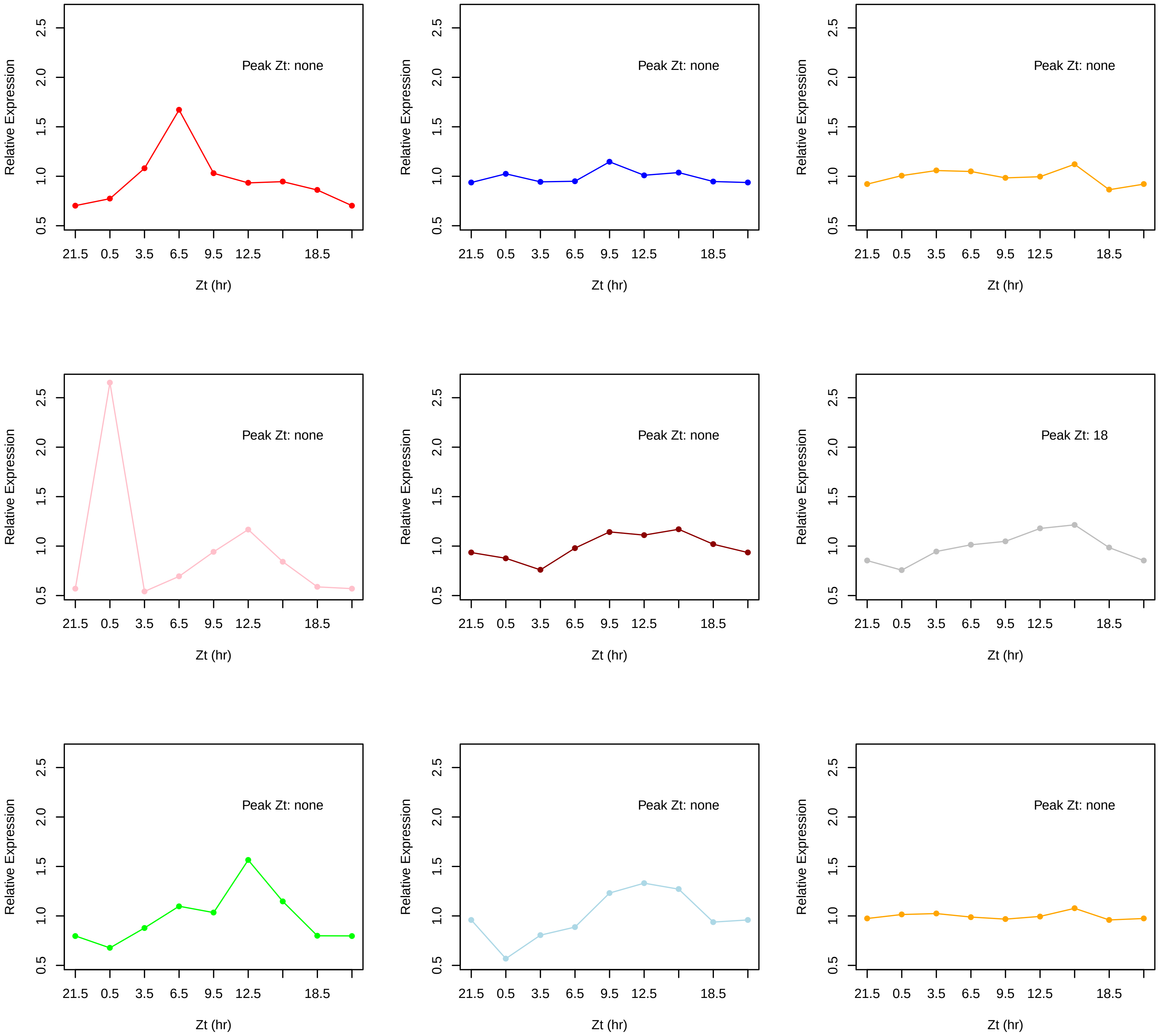

Singeleton: ENSXMAG00000015874 msantd1

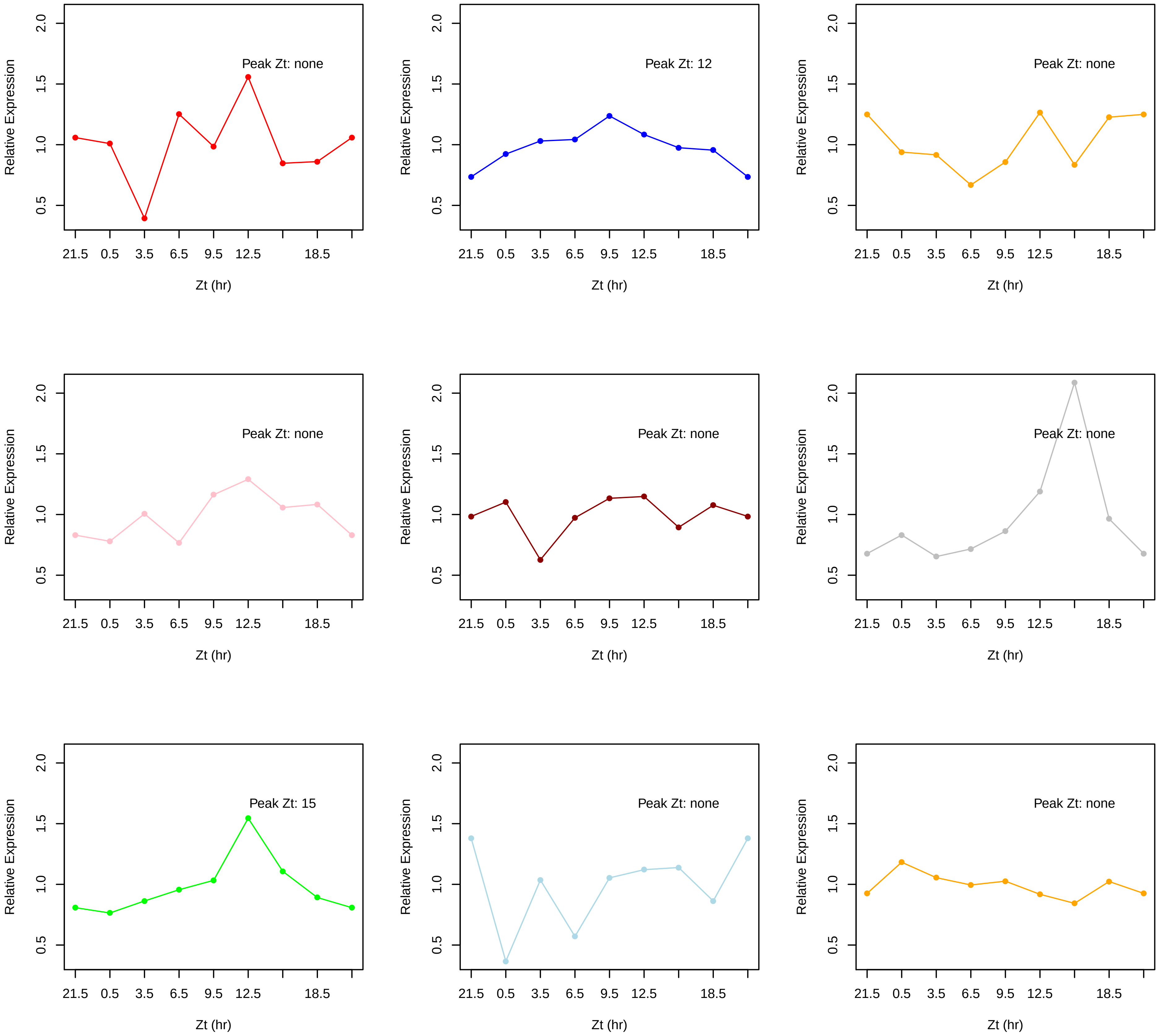

Singleton: ENSXMAG00000009080 csde1

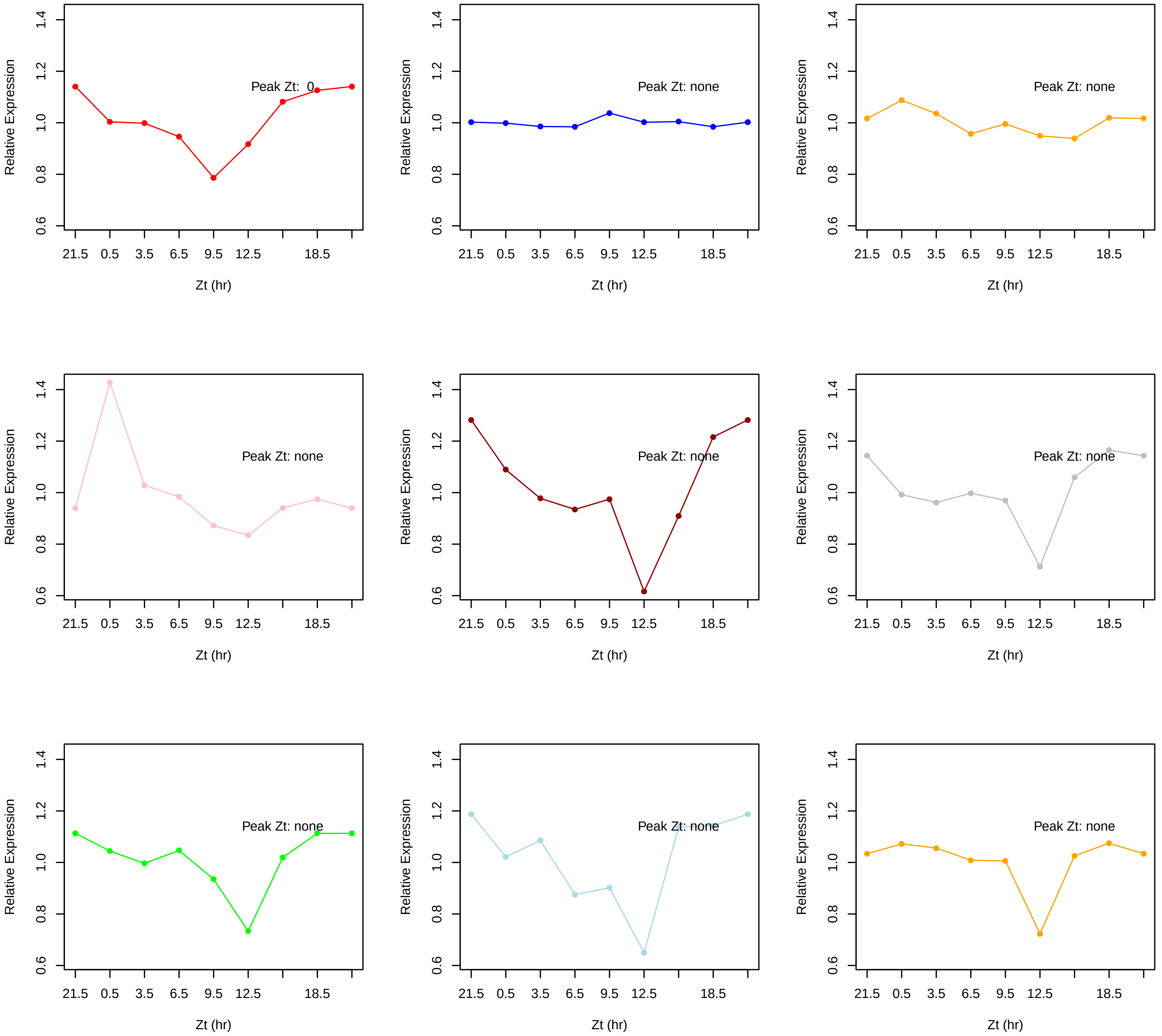

Singeleton: ENSXMAG00000021202 arl6

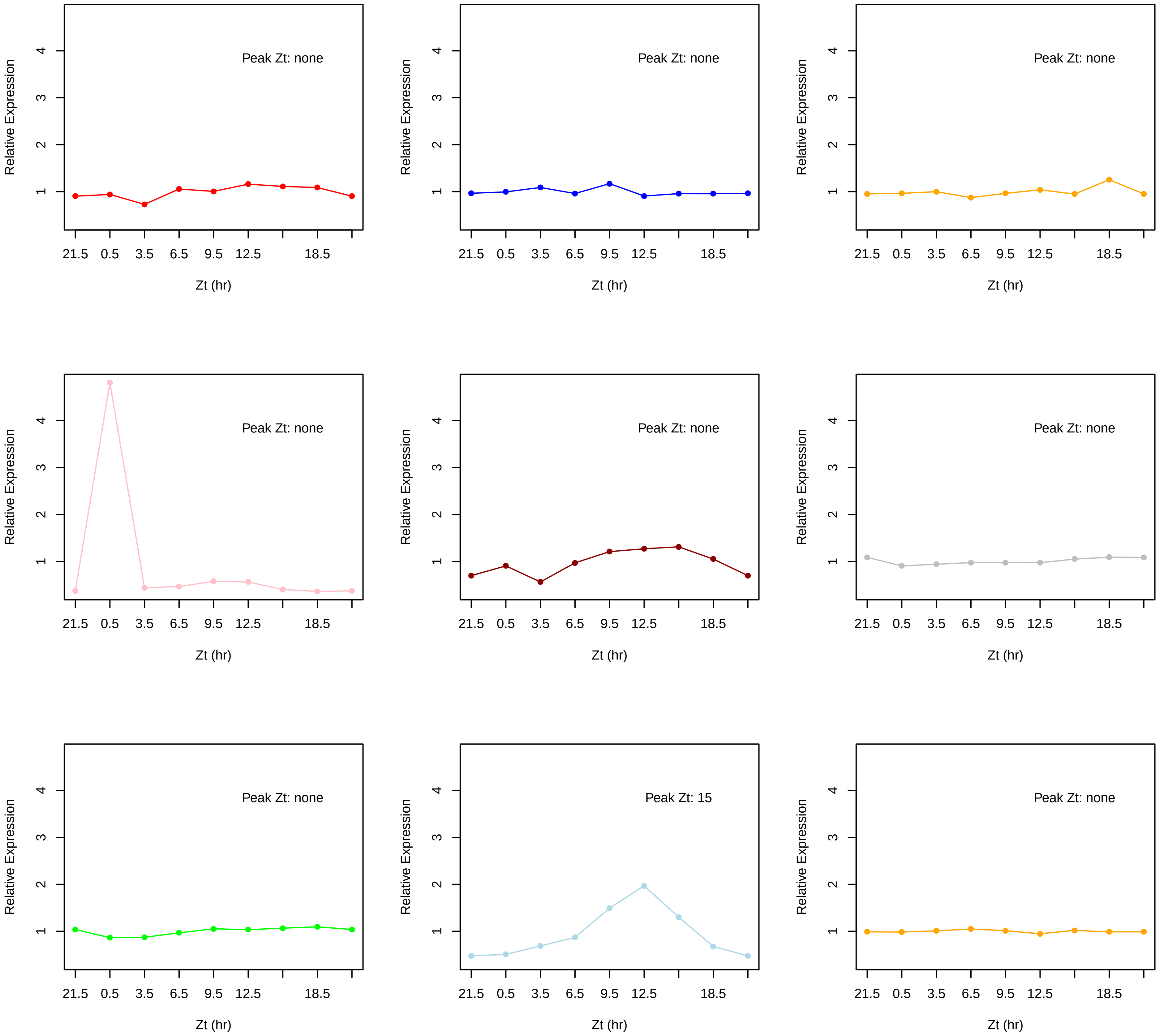

Singeleton: ENSXMAG00000022988 ripk2

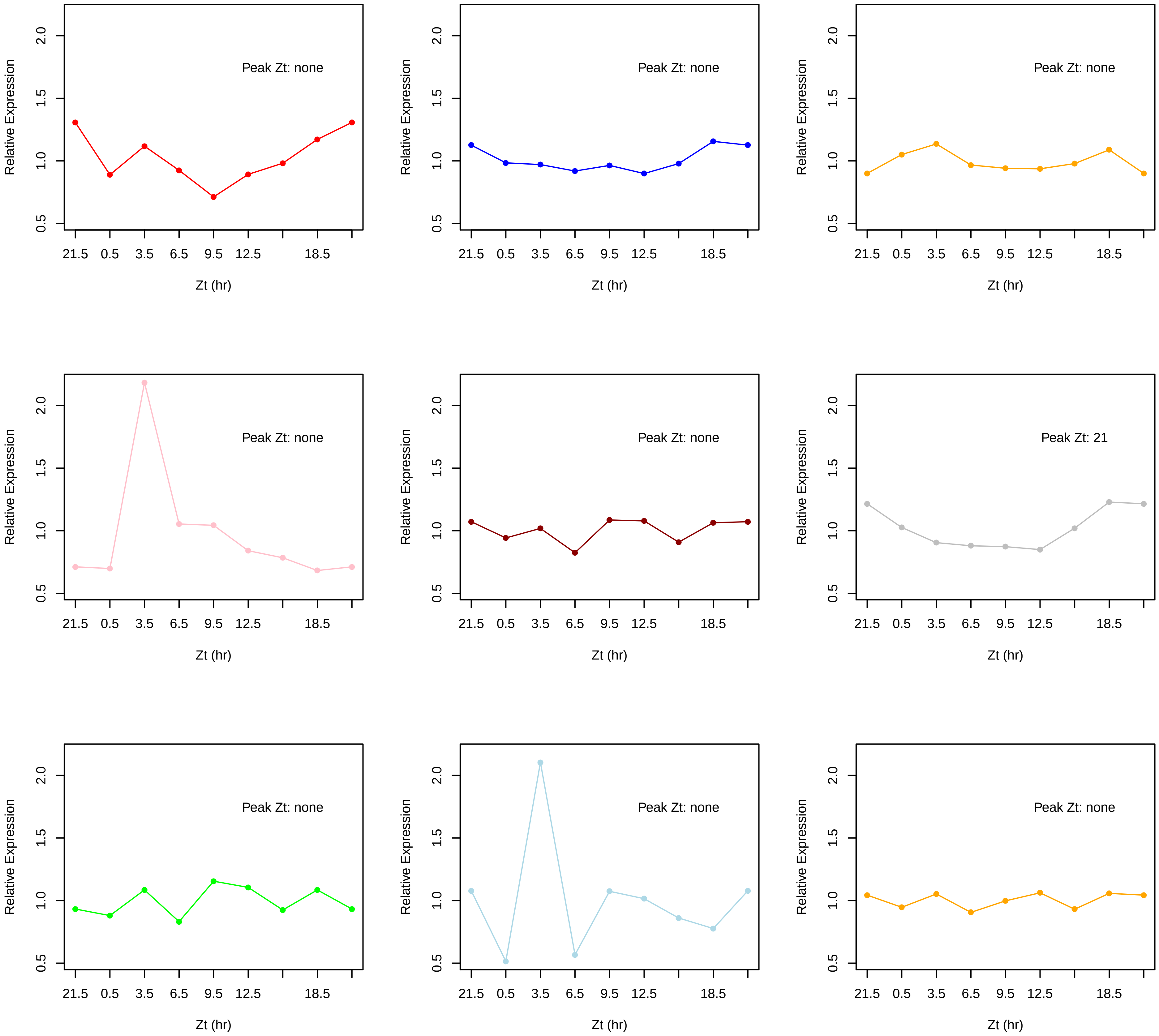

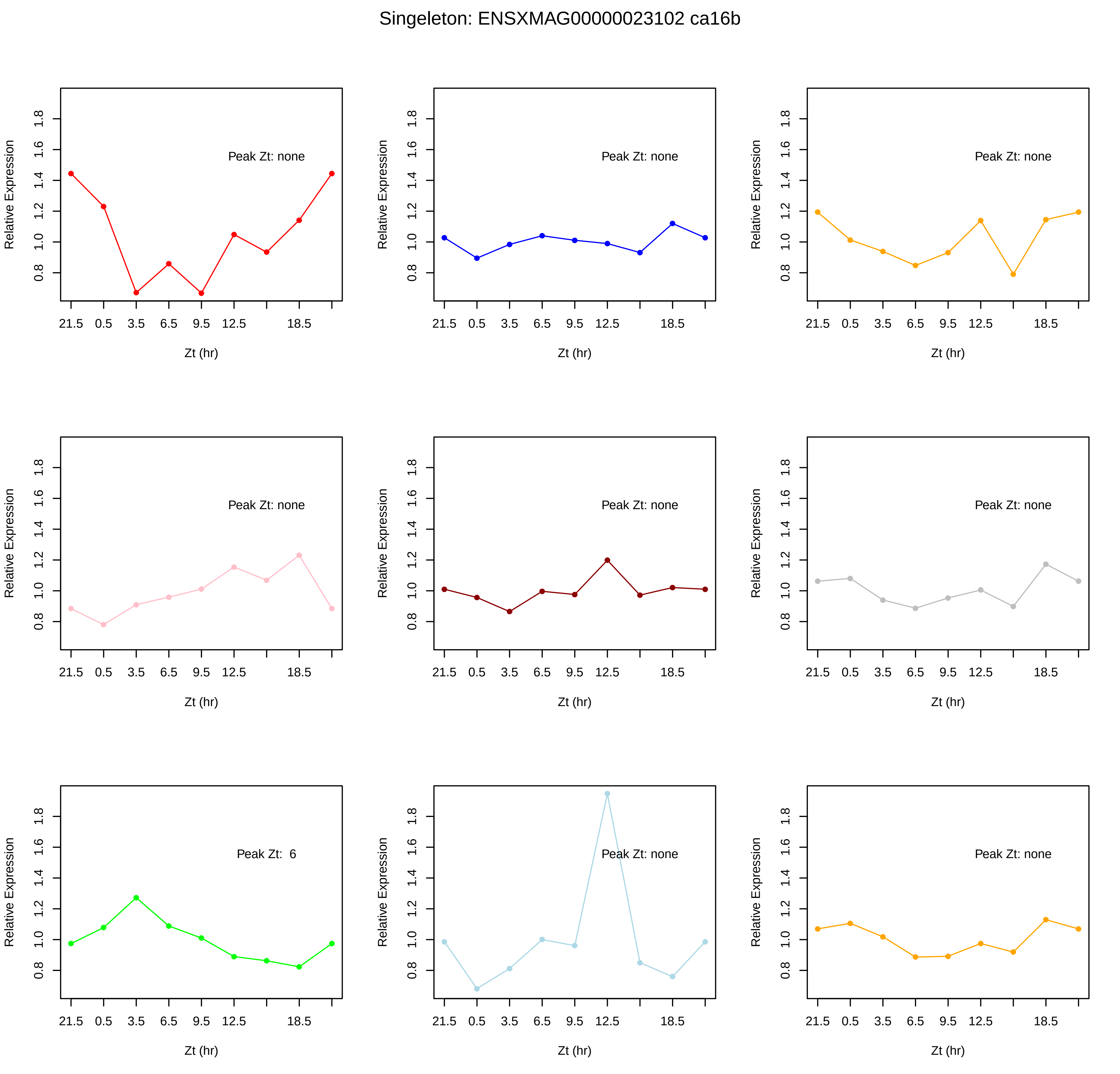
Singleton: ENSXMAG00000023102 ca16b
Peak Zt: none
Peak Zt: none
Peak Zt: none
Peak Zt: none
Peak Zt: none
Peak Zt: none
Peak Zt: 6
Peak Zt: none
Peak Zt: none
Relative Expression
Zt (hr)
21.5
0.5
3.5
6.5
9.5
12.5
18.5
0.8
1.0
1.2
1.4
1.6
1.8

Singeleton: ENSXMAG00000011288 mettl13

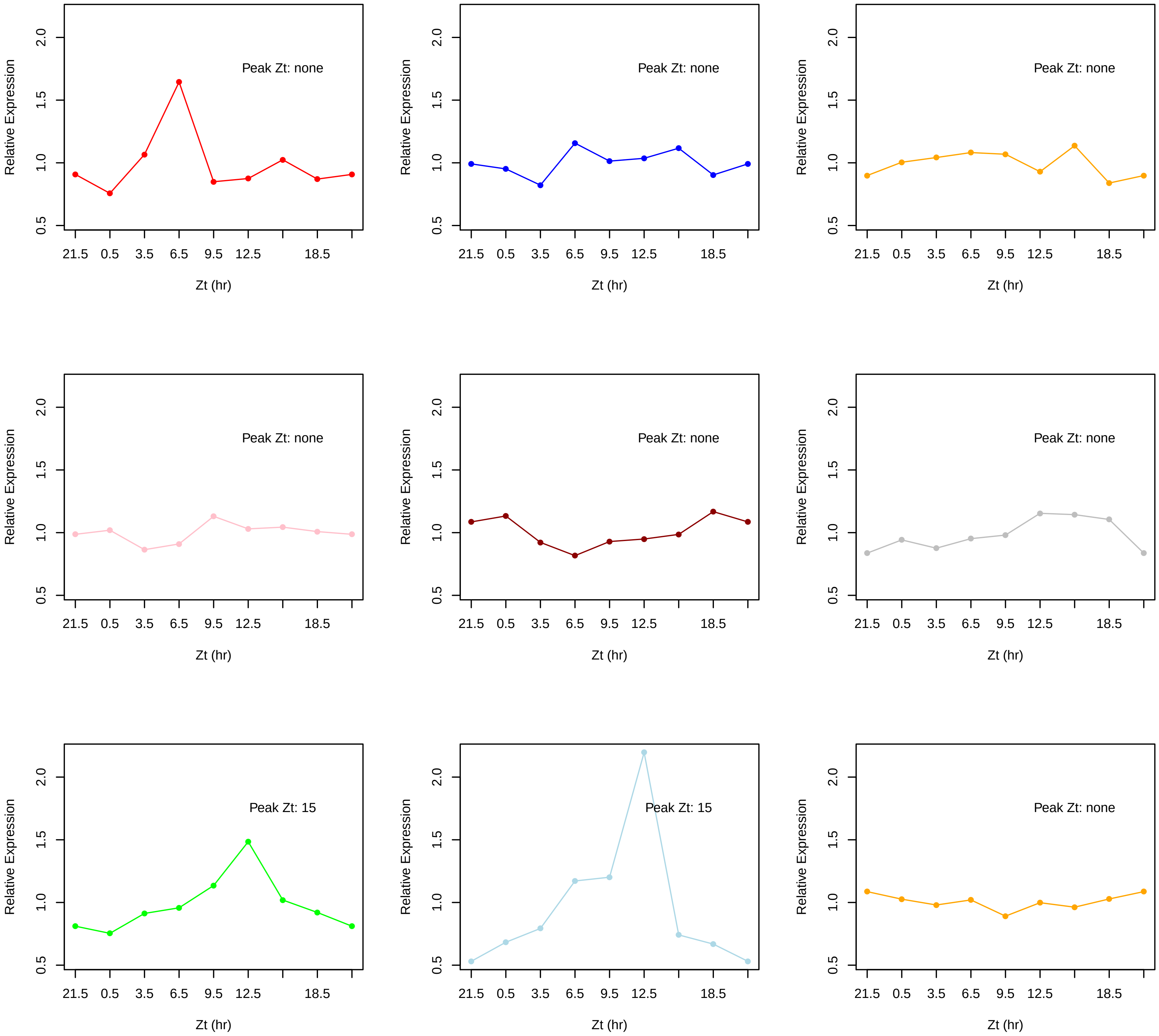

Singeleton: ENSXMAG00000001454 larsb

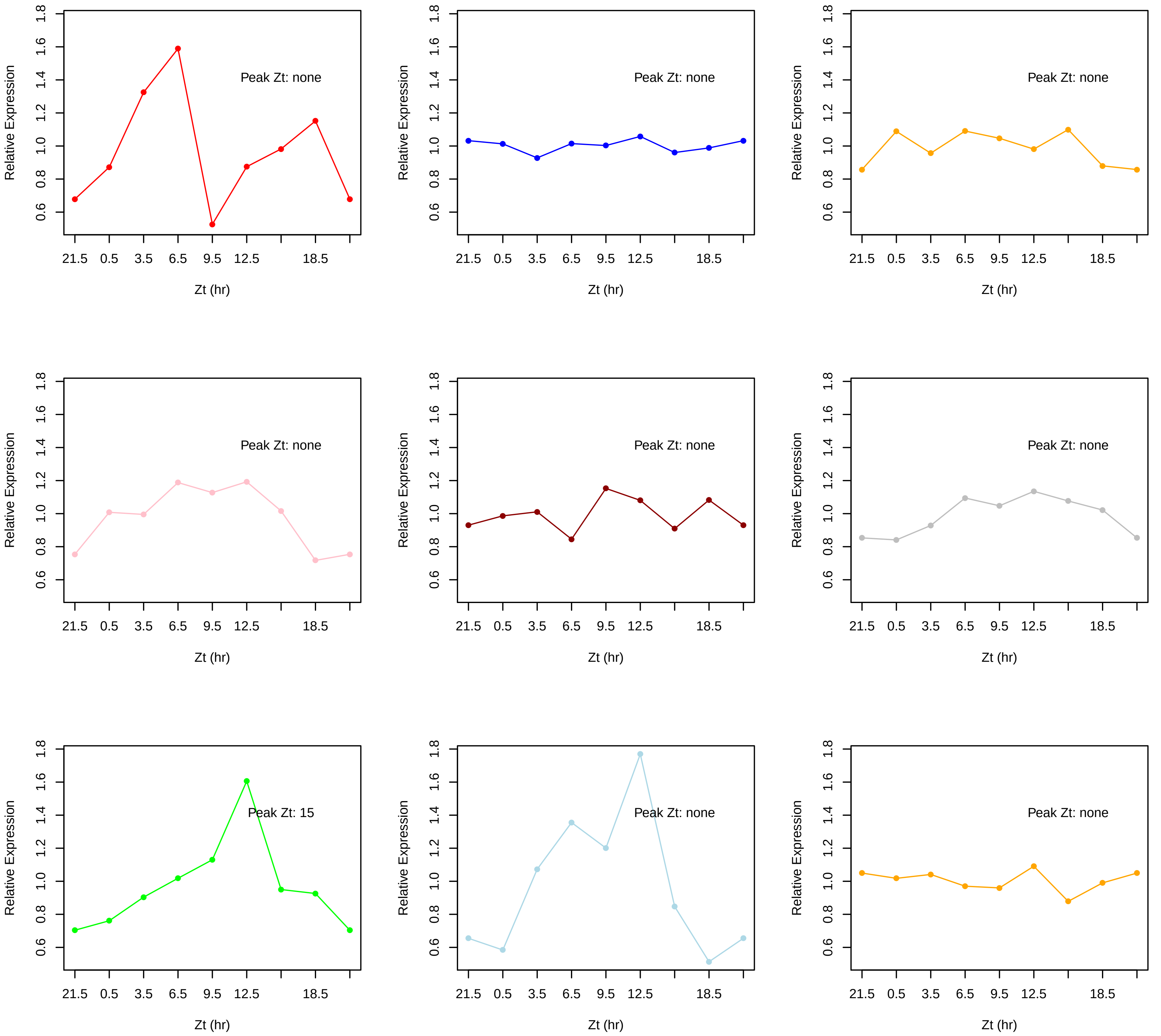

Singeleton: ENSXMAG00000011620 fer

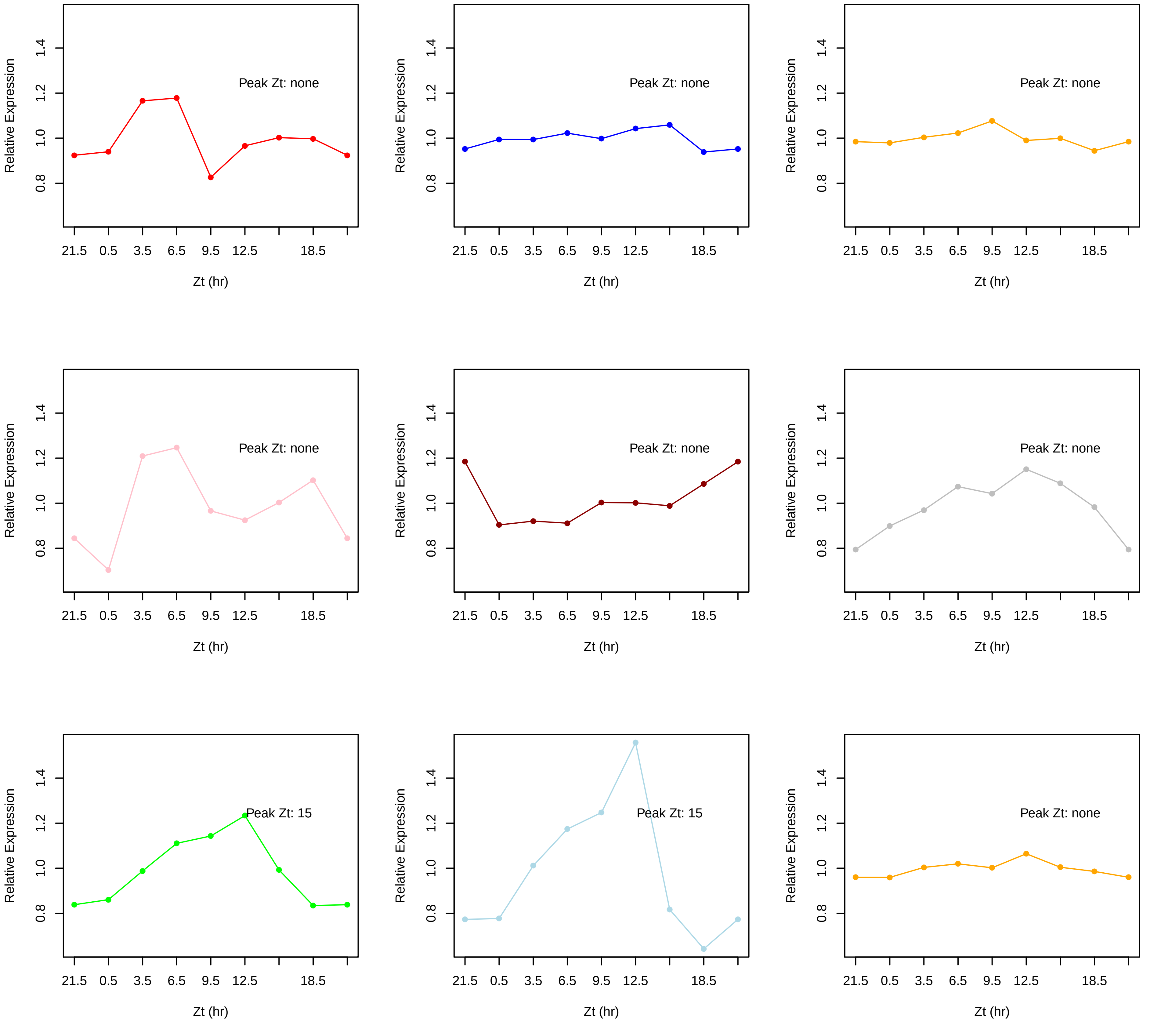

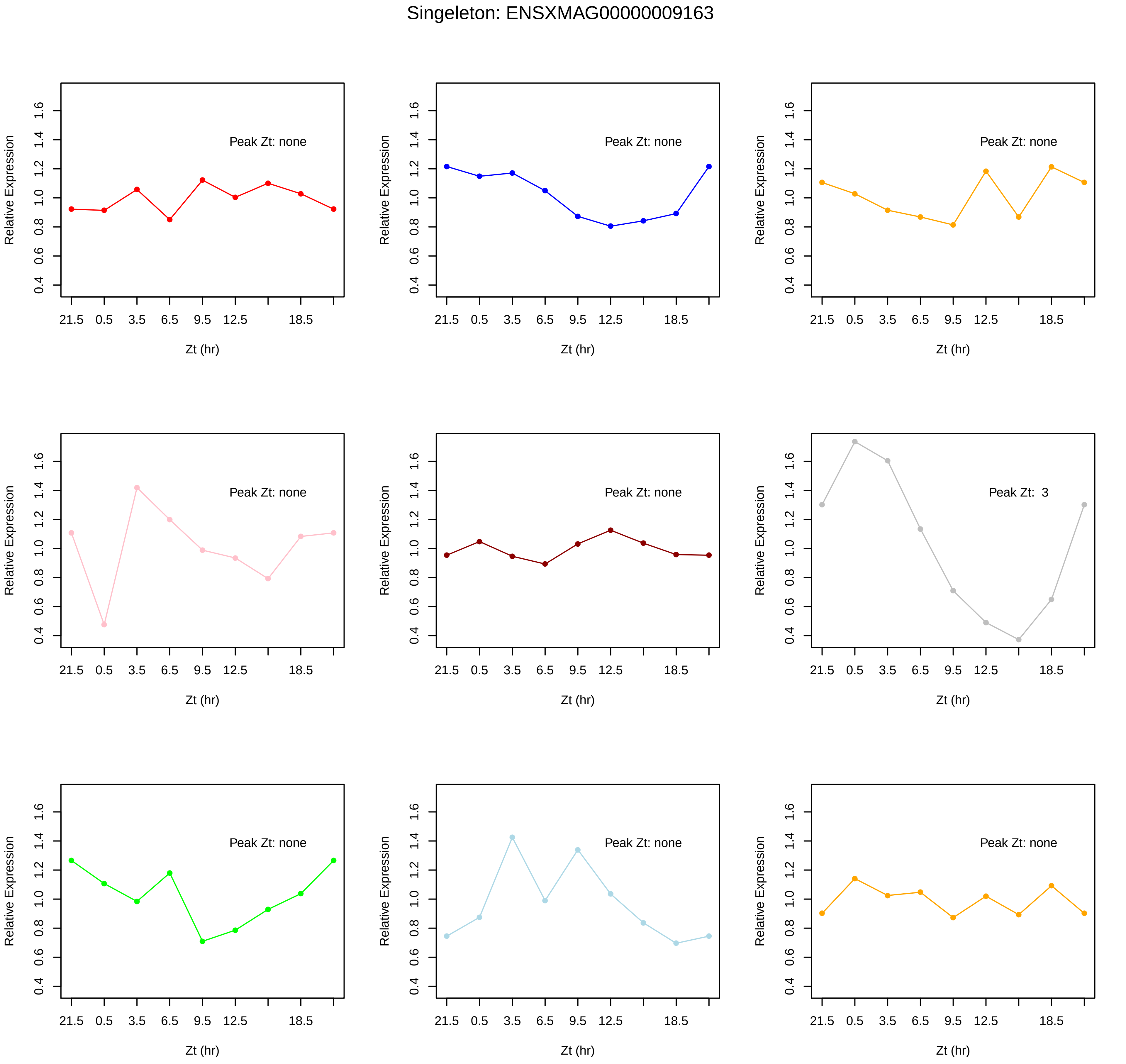

Singeleton: ENSXMAG00000009163
Peak Zt: none
Peak Zt: none
Peak Zt: none
Peak Zt: none
Peak Zt: none
Peak Zt: 3
Peak Zt: none
Peak Zt: none
Peak Zt: none
Relative Expression
Zt (hr)
21.5
0.5
3.5
6.5
9.5
12.5
18.5
0.4
0.6
0.8
1.0
1.2
1.4
1.6

Singeleton: ENSXMAG00000026573 dennd2c

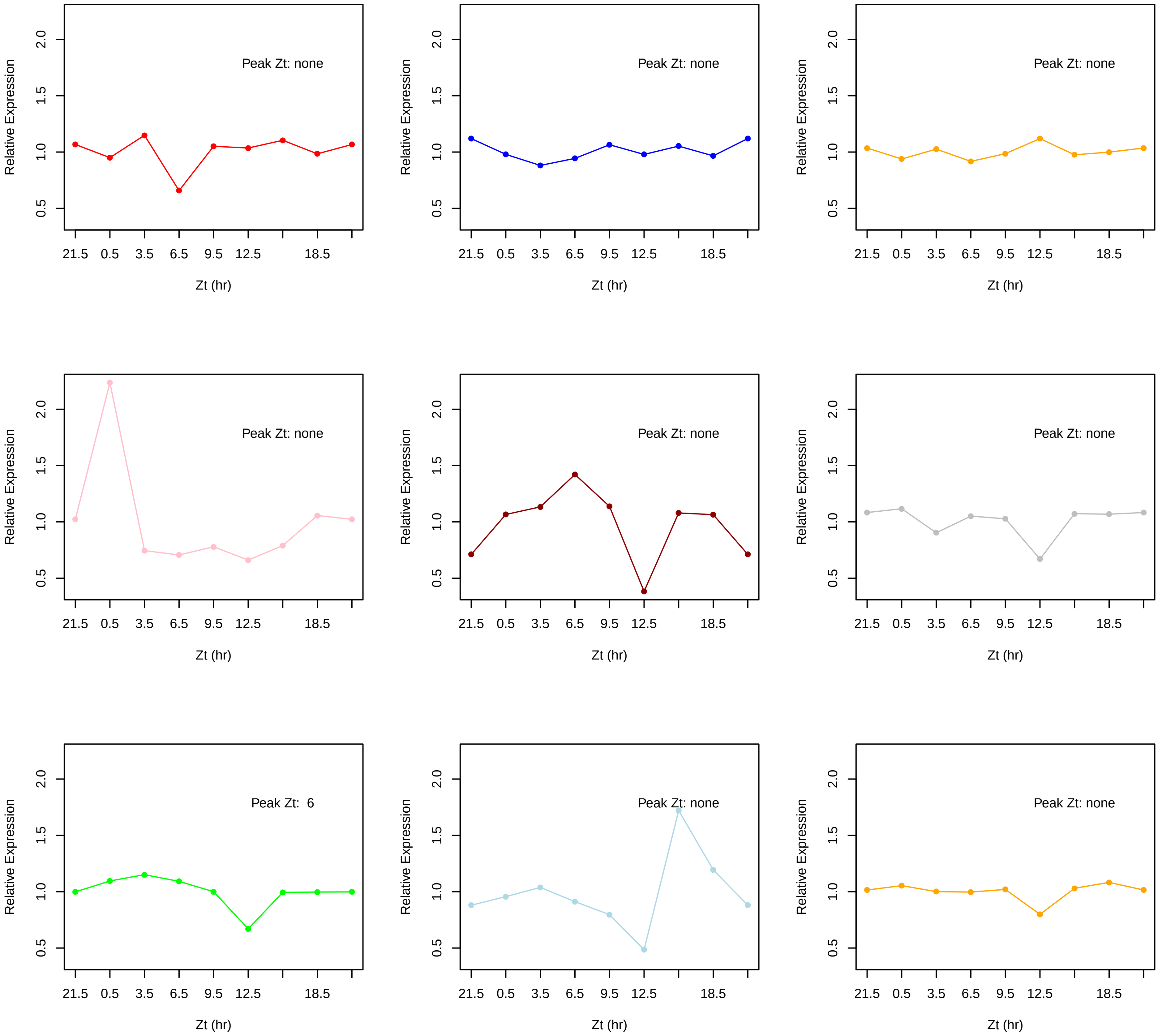

Singeleton: ENSXMAG00000010446 aox1

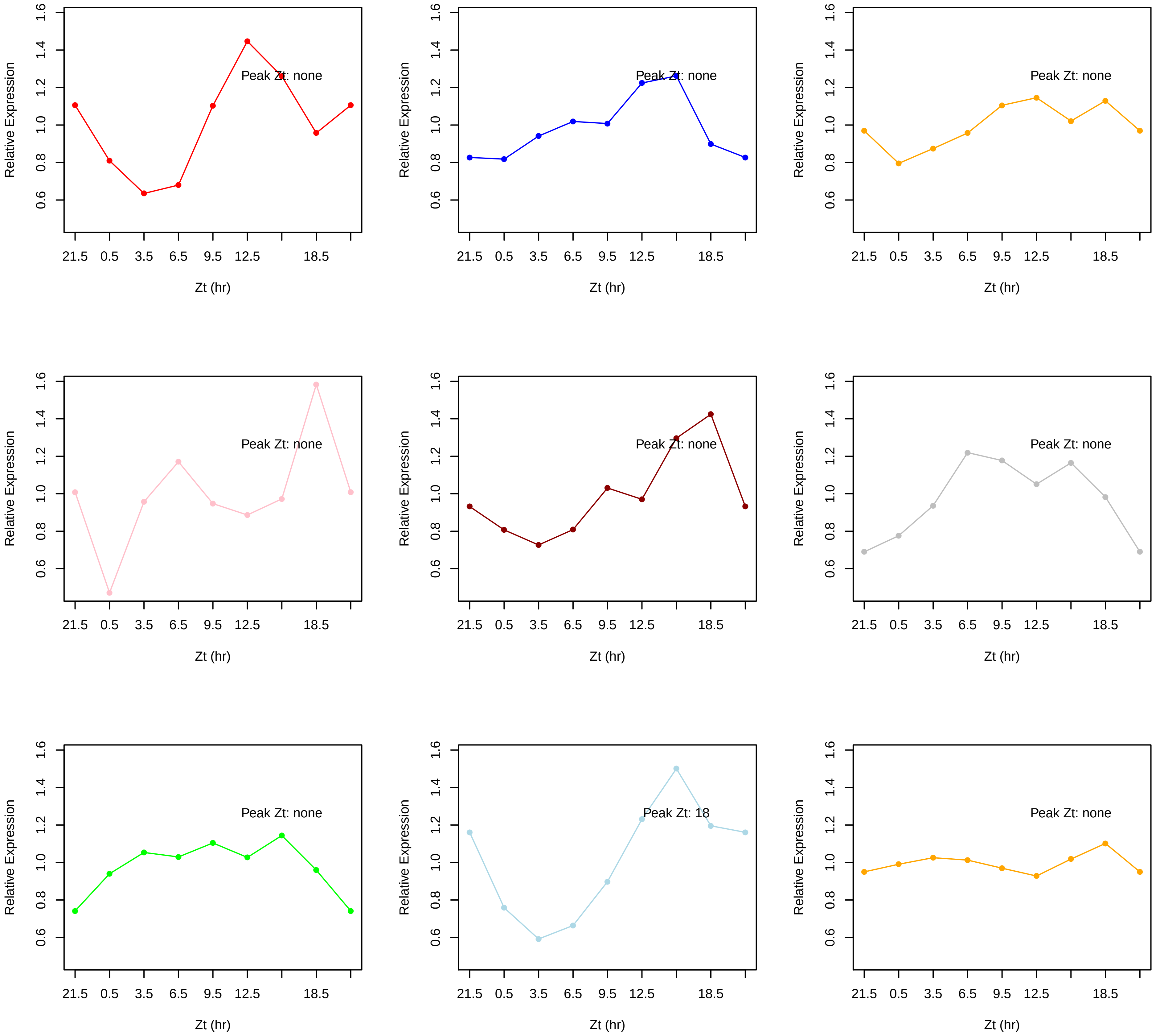

Singeleton: ENSXMAG00000024925

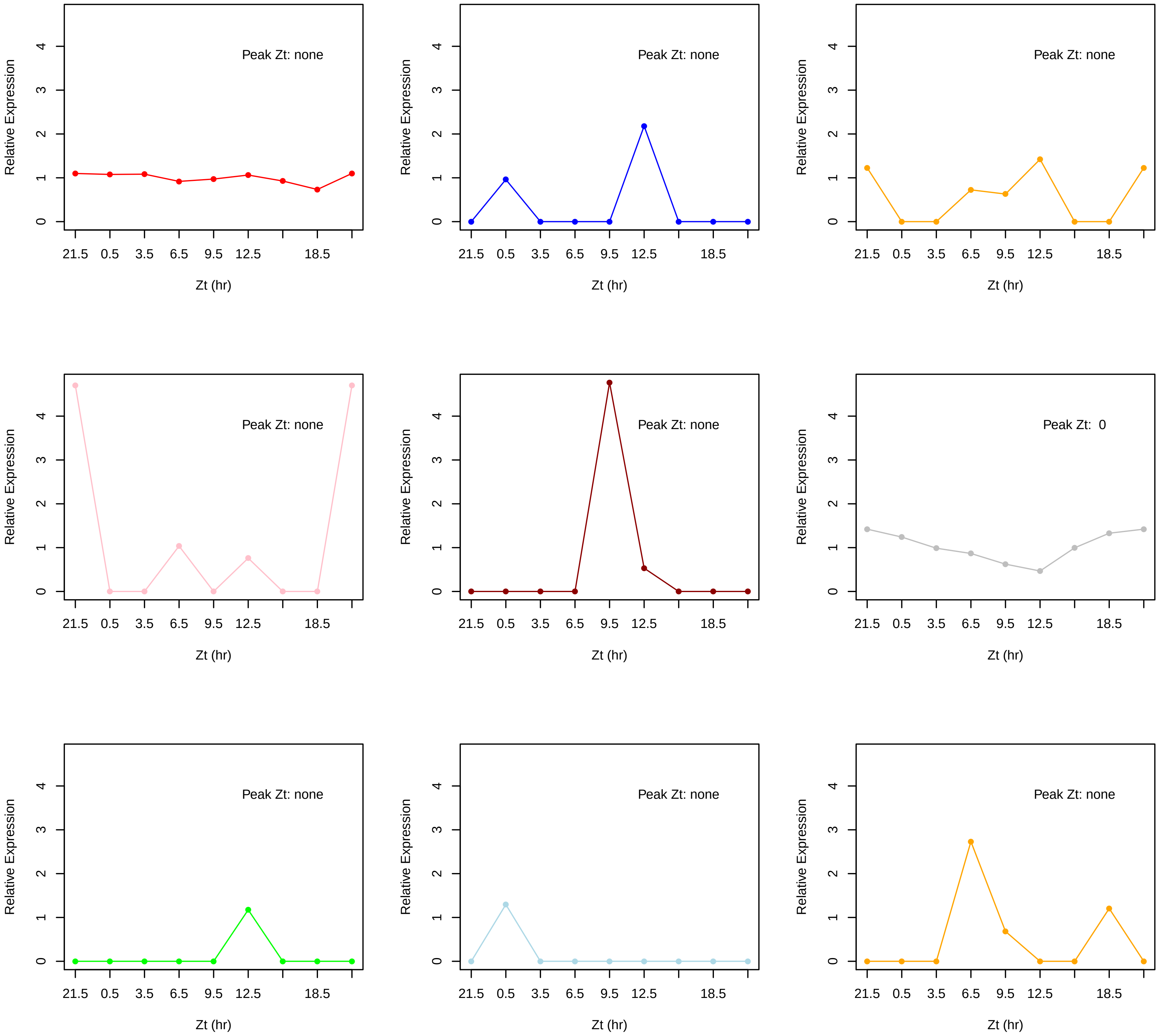

Singeleton: ENSXMAG00000011229 pigc

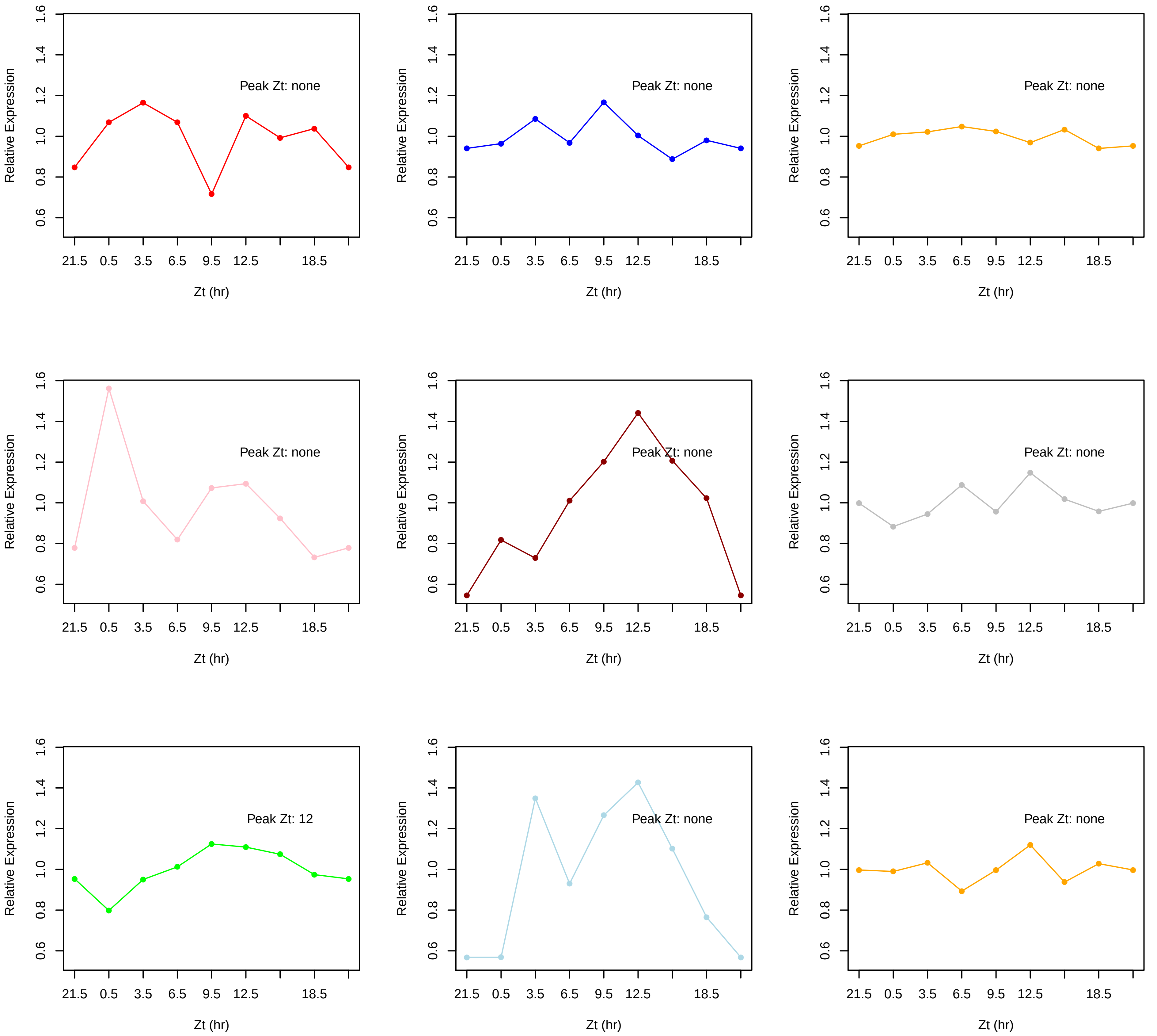

Singleton: ENSXMAG00000002089 arl13b

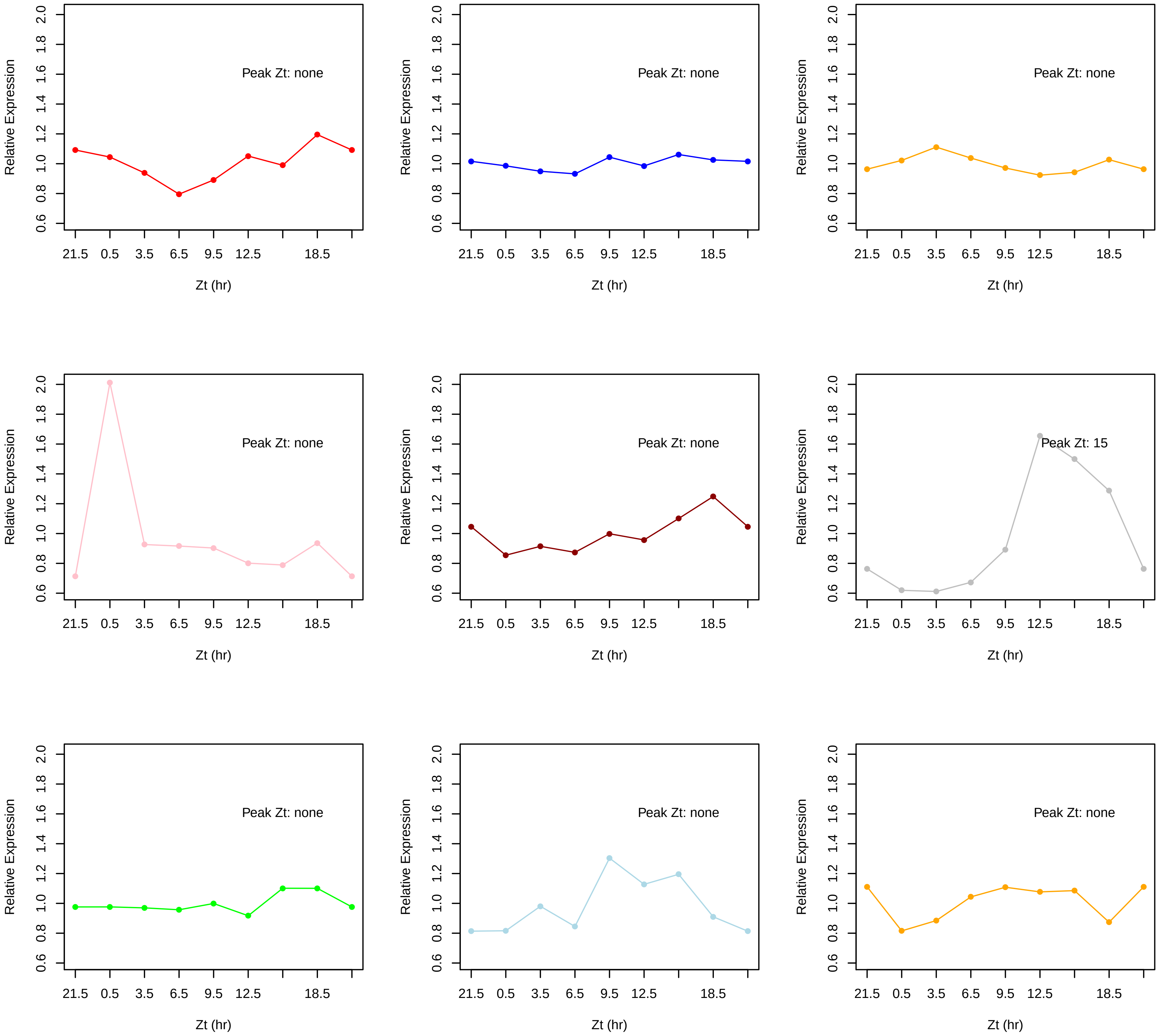

Singeleton: ENSXMAG00000011222 tmed5

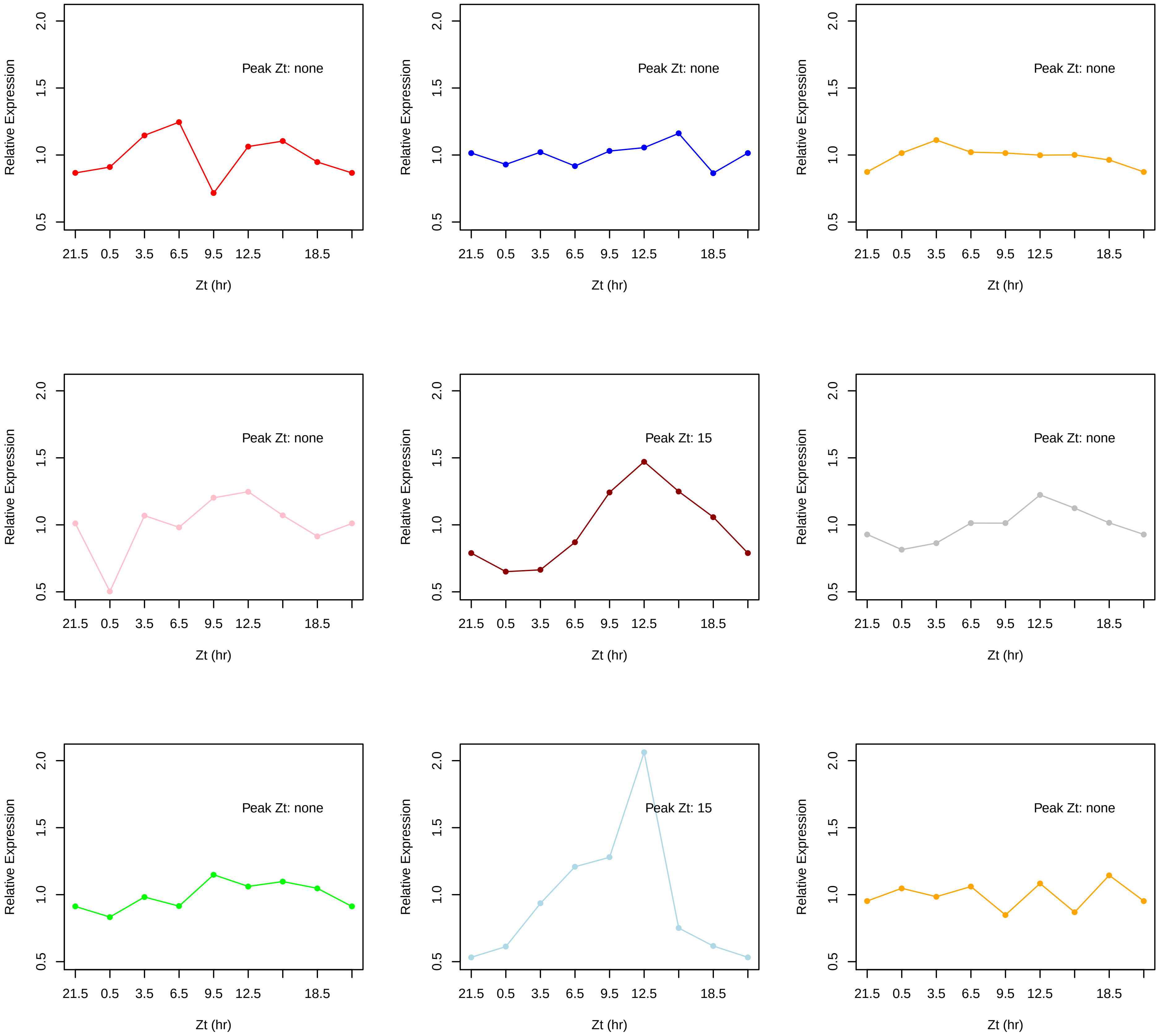

Singeleton: ENSXMAG00000028685

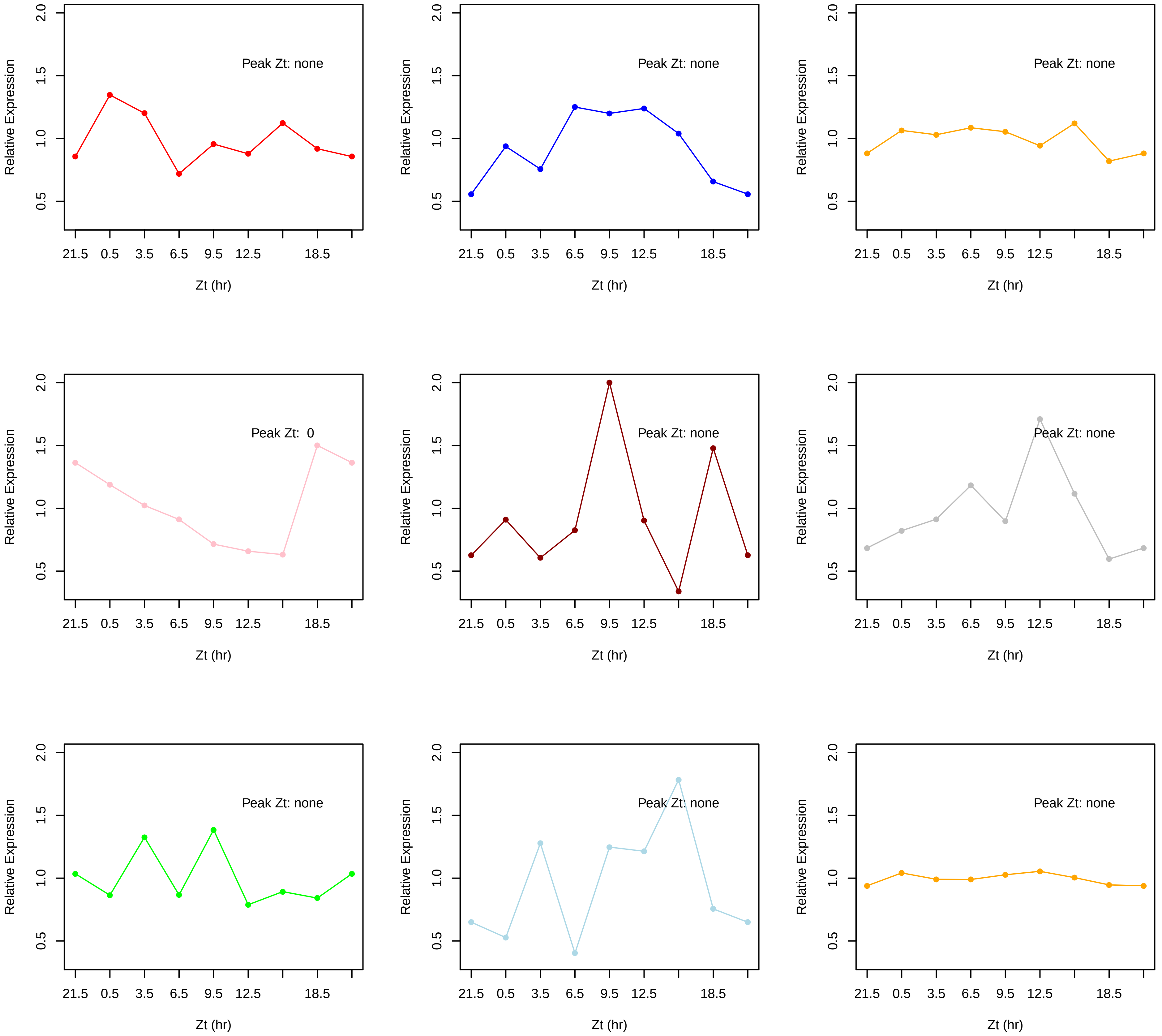

Singeleton: ENSXMAG00000011212 suco

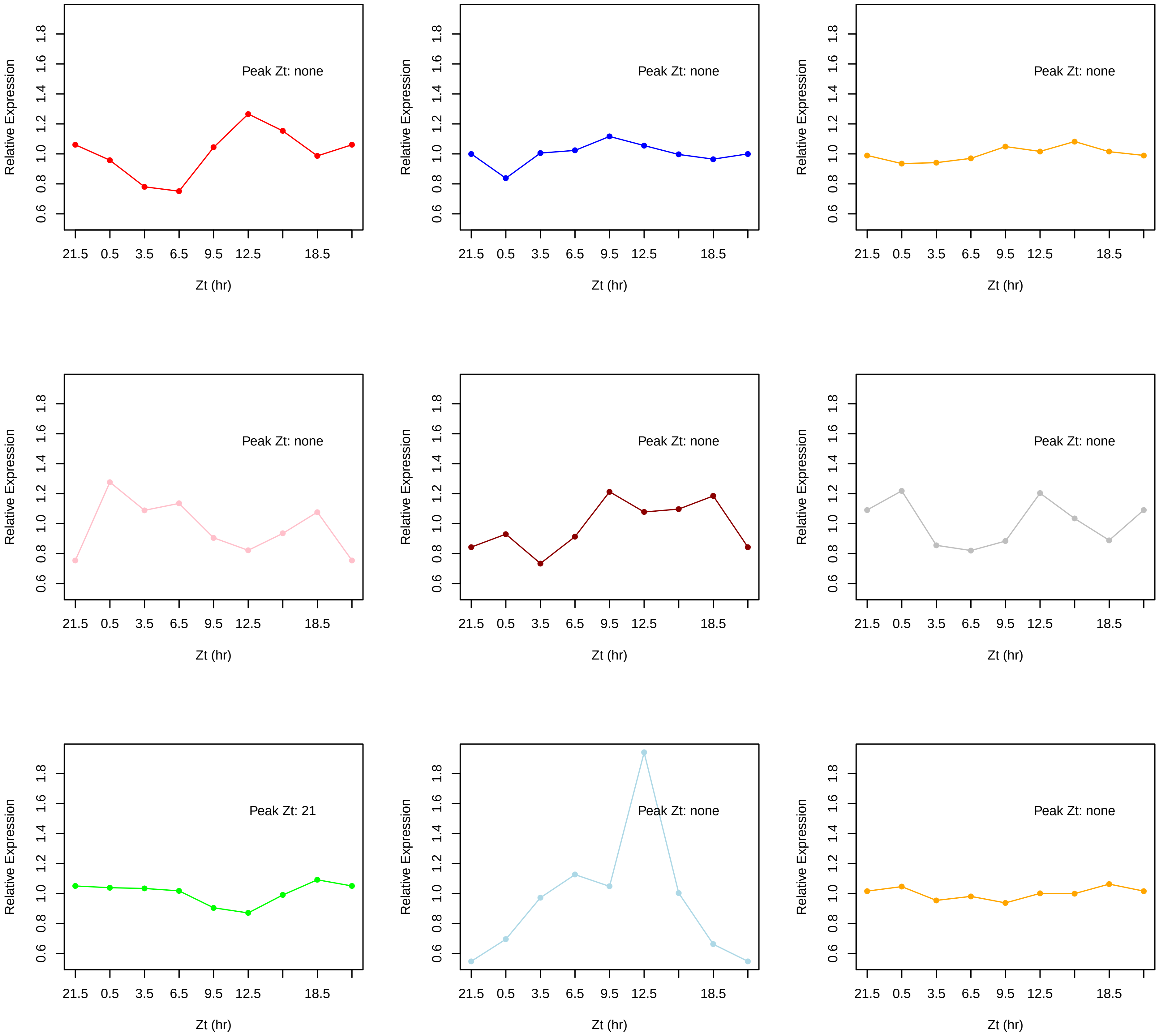

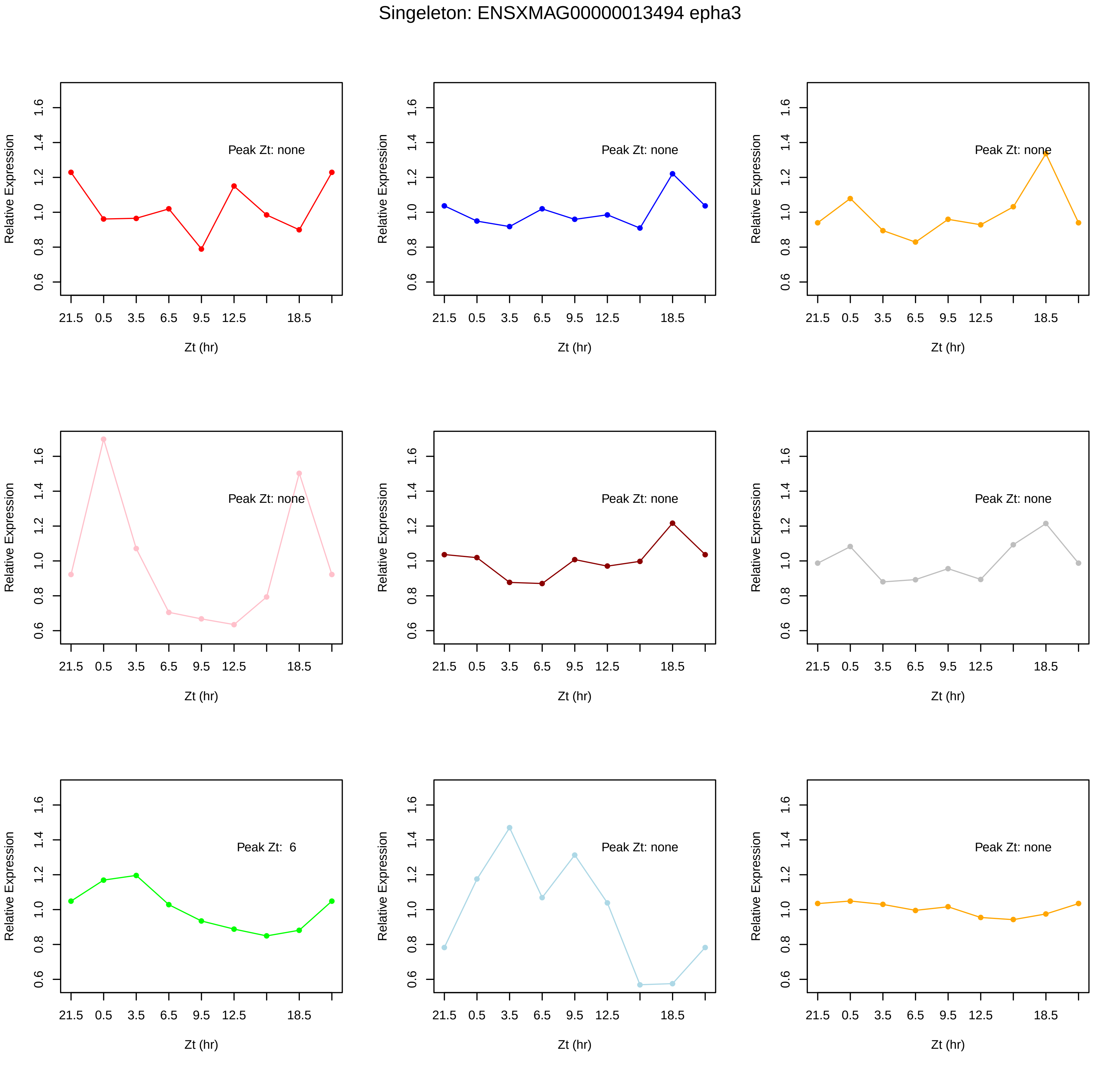

Singleton: ENSXMAG00000013494 epha3
Peak Zt: none
Peak Zt: none
Peak Zt: none
Peak Zt: none
Peak Zt: none
Peak Zt: none
Peak Zt: 6
Peak Zt: none
Peak Zt: none
Relative Expression
Zt (hr)
0.6 0.8 1.0 1.2 1.4 1.6
21.5 0.5 3.5 6.5 9.5 12.5 18.5

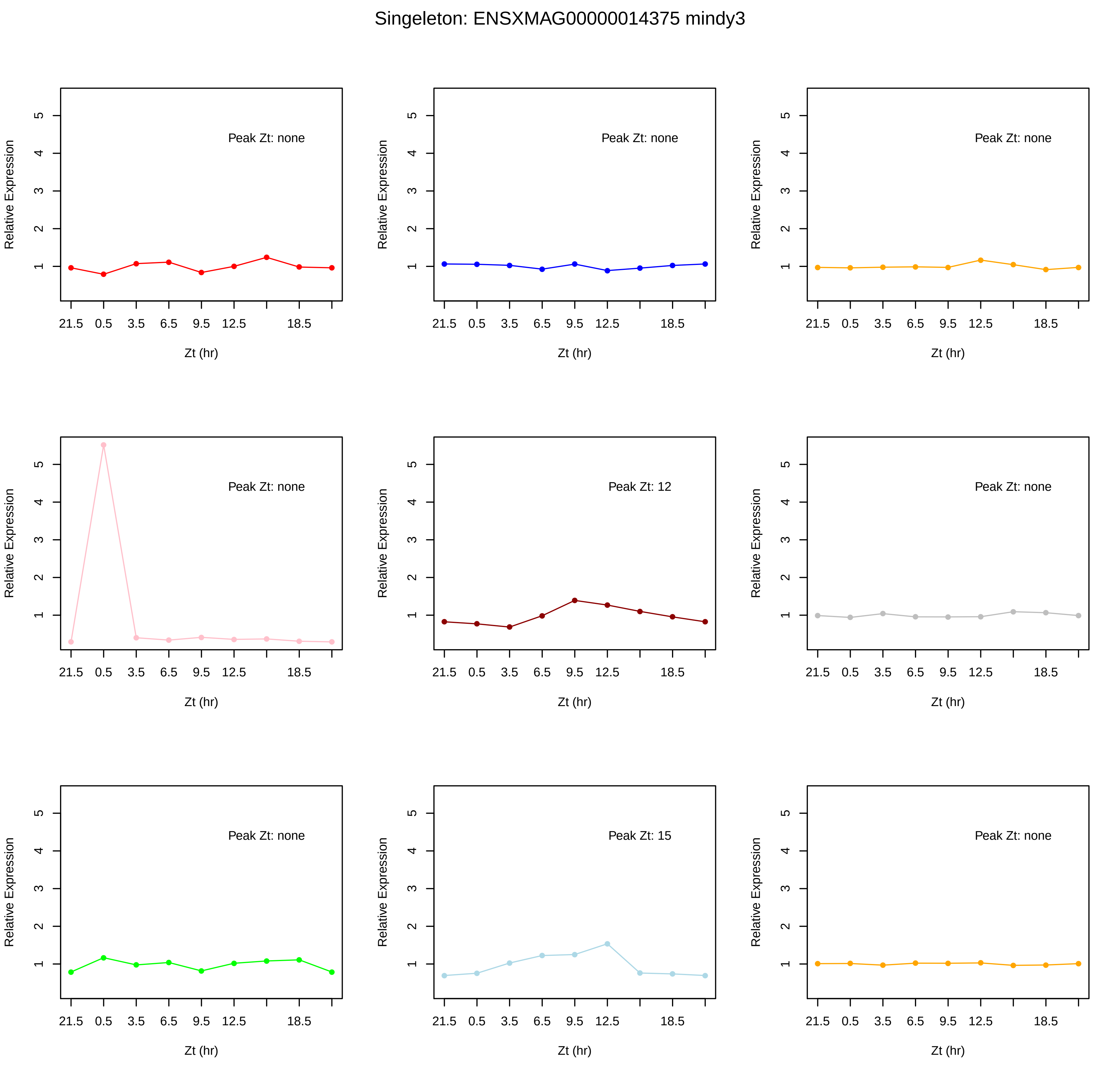

Singeleton: ENSXMAG00000014375 mindy3
Peak Zt: none
Peak Zt: none
Peak Zt: none
Peak Zt: none
Peak Zt: 12
Peak Zt: none
Peak Zt: none
Peak Zt: 15
Peak Zt: none
Relative Expression
Zt (hr)
21.5 0.5 3.5 6.5 9.5 12.5 18.5
1 2 3 4 5

Singleton: ENSXMAG00000012634 trim33

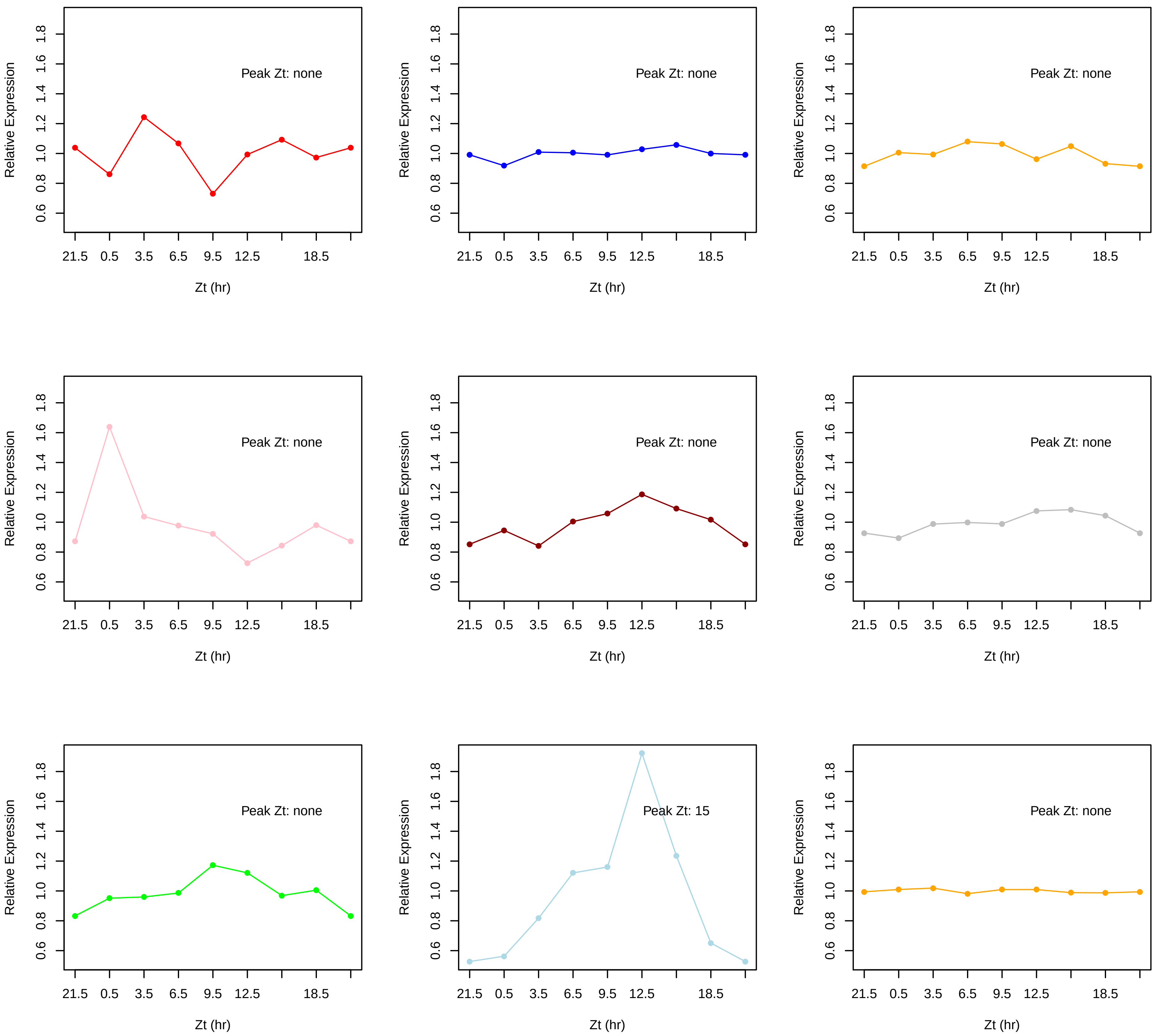

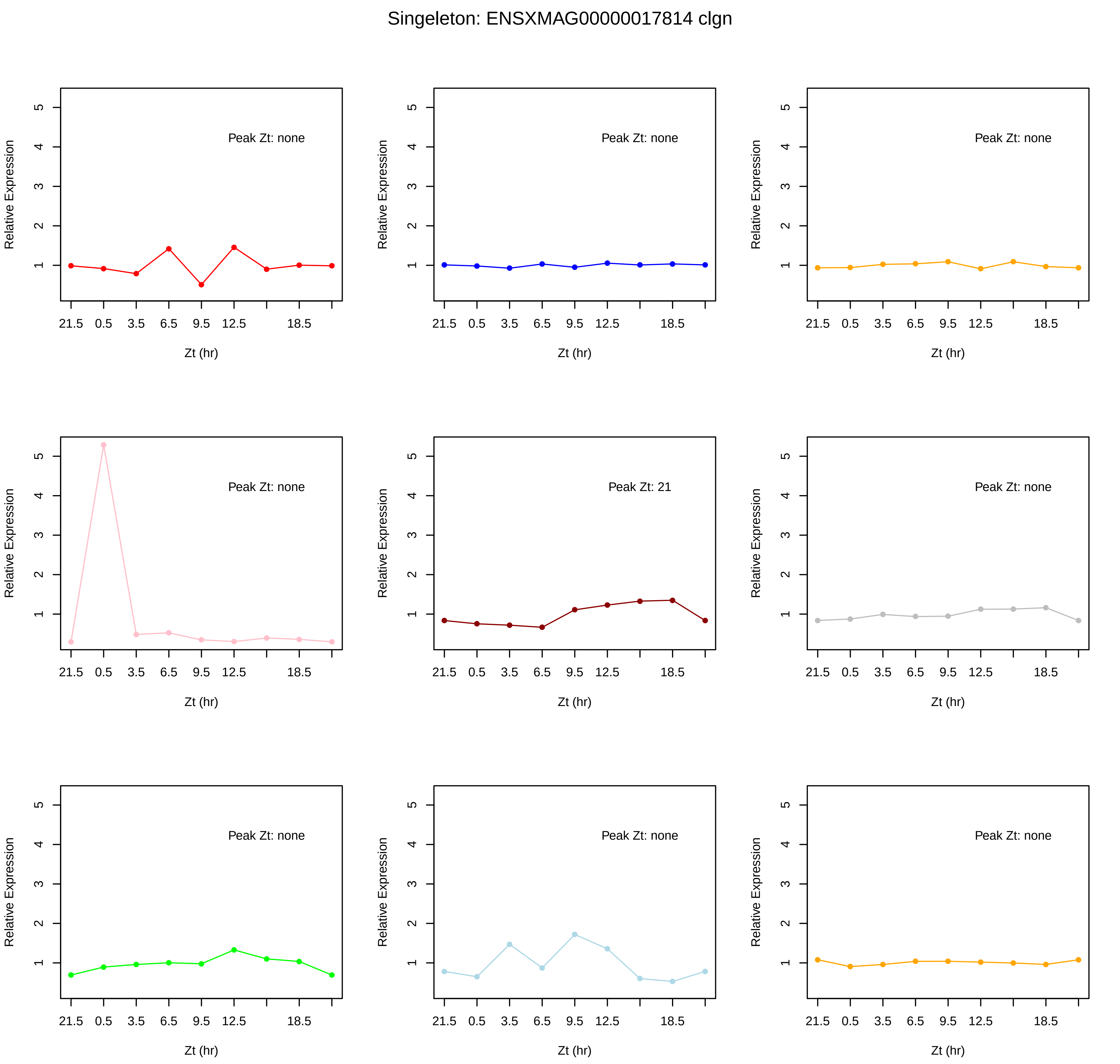
Singeleton: ENSXMAG00000017814 clgn
Peak Zt: none
Peak Zt: none
Peak Zt: none
Peak Zt: none
Peak Zt: 21
Peak Zt: none
Peak Zt: none
Peak Zt: none
Peak Zt: none
Relative Expression
Zt (hr)
21.5
0.5
3.5
6.5
9.5
12.5
18.5
1
2
3
4
5

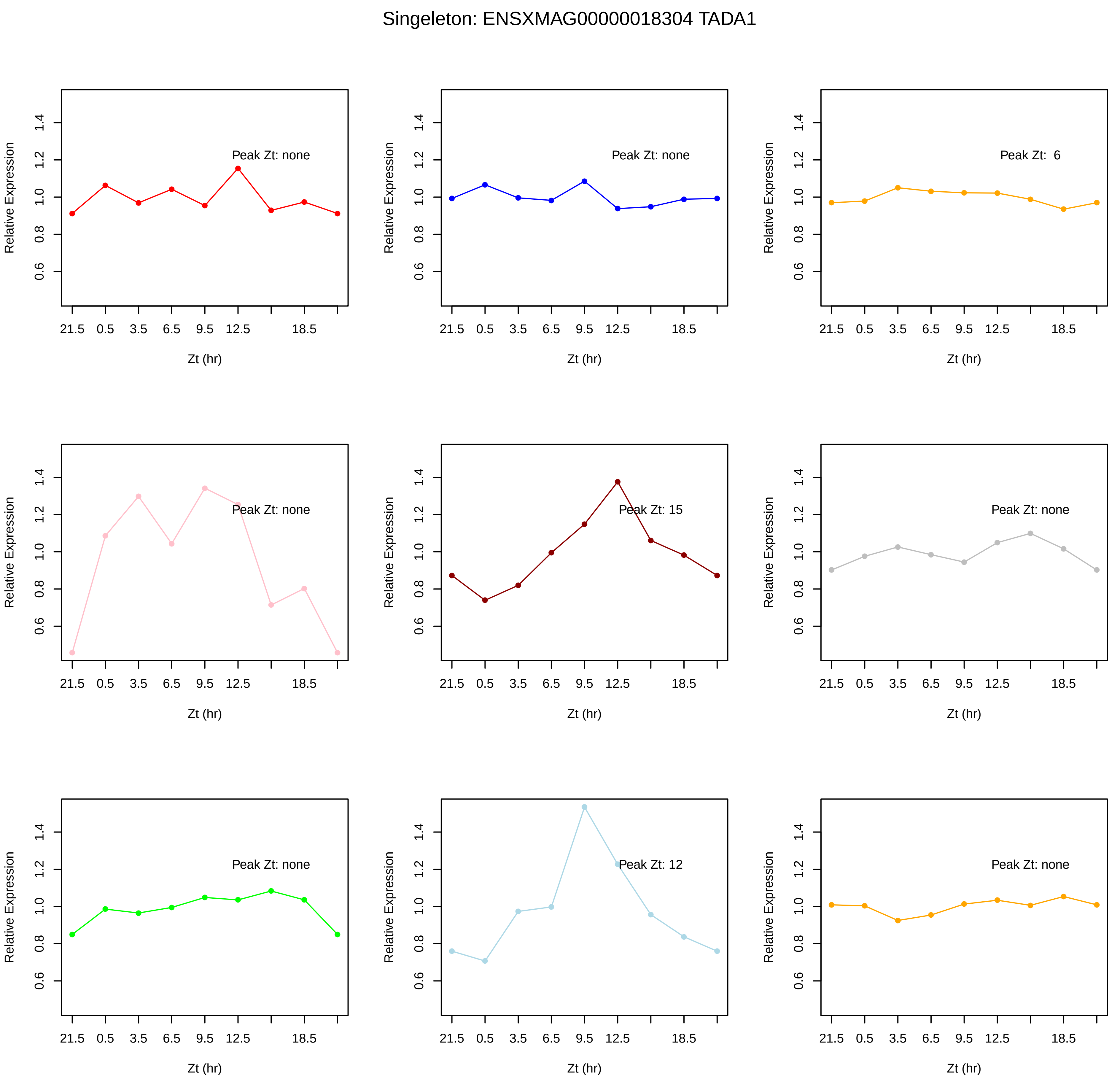
Singeleton: ENSXMAG00000018304 TADA1
Peak Zt: none
Peak Zt: none
Peak Zt: 6
Peak Zt: none
Peak Zt: 15
Peak Zt: none
Peak Zt: none
Peak Zt: 12
Peak Zt: none
Relative Expression
Zt (hr)
21.5
0.5
3.5
6.5
9.5
12.5
18.5
0.6
0.8
1.0
1.2
1.4

Singeleton: ENSXMAG00000001514 yipf5

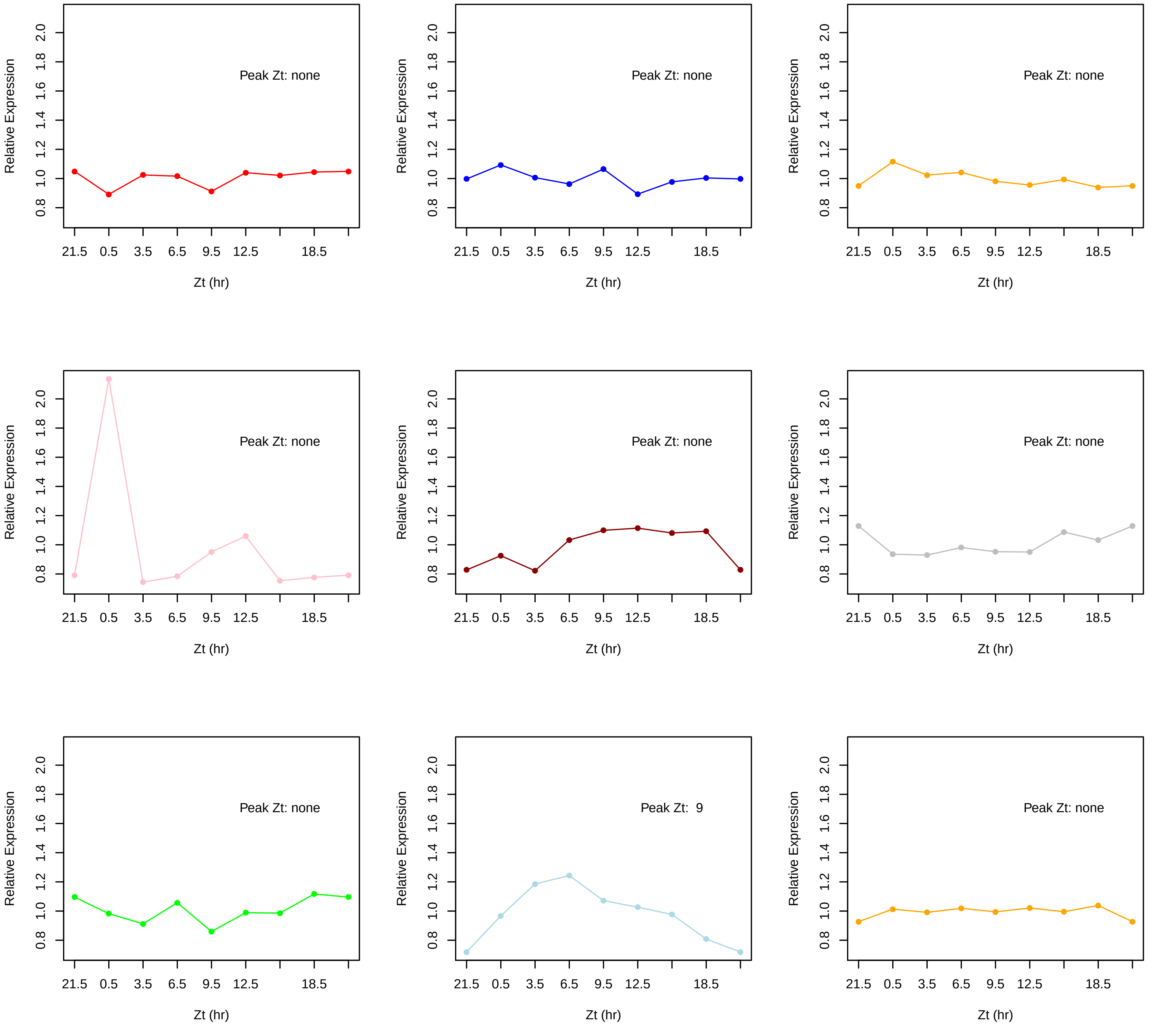

Singeleton: ENSXMAG00000005076 slc30a5

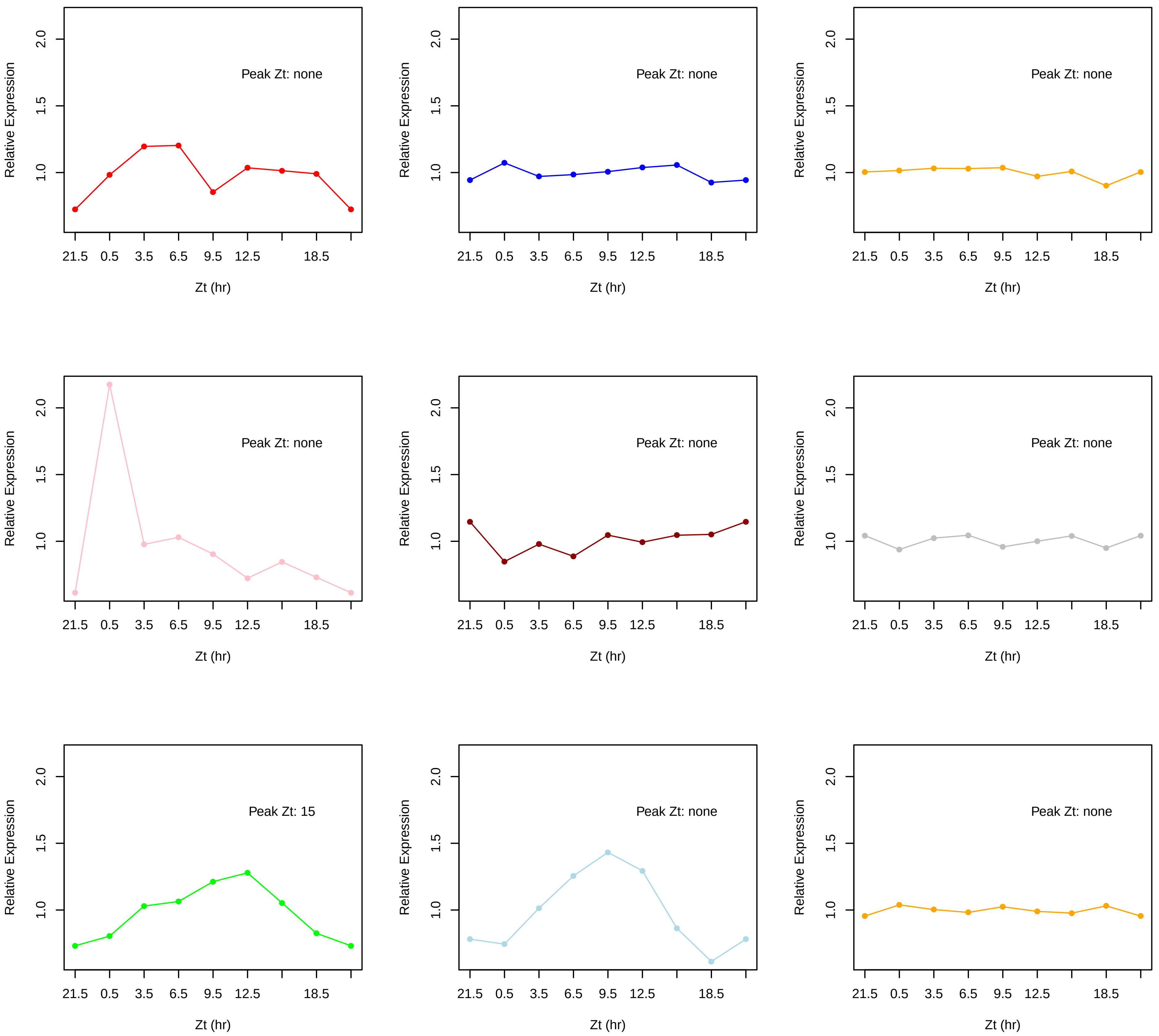

Singeleton: ENSXMAG00000026284

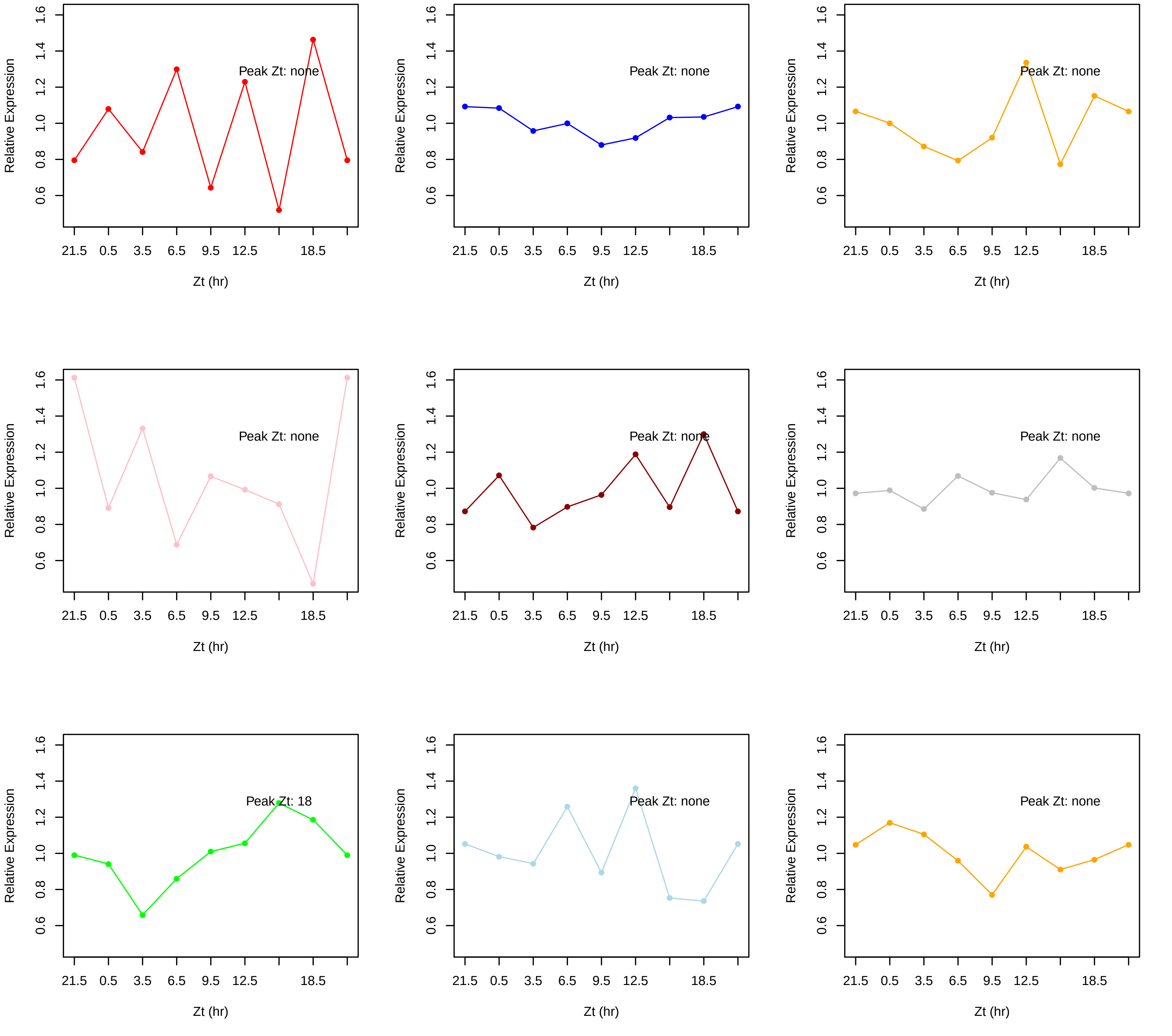

Singeleton: ENSXMAG00000011408 fam20b

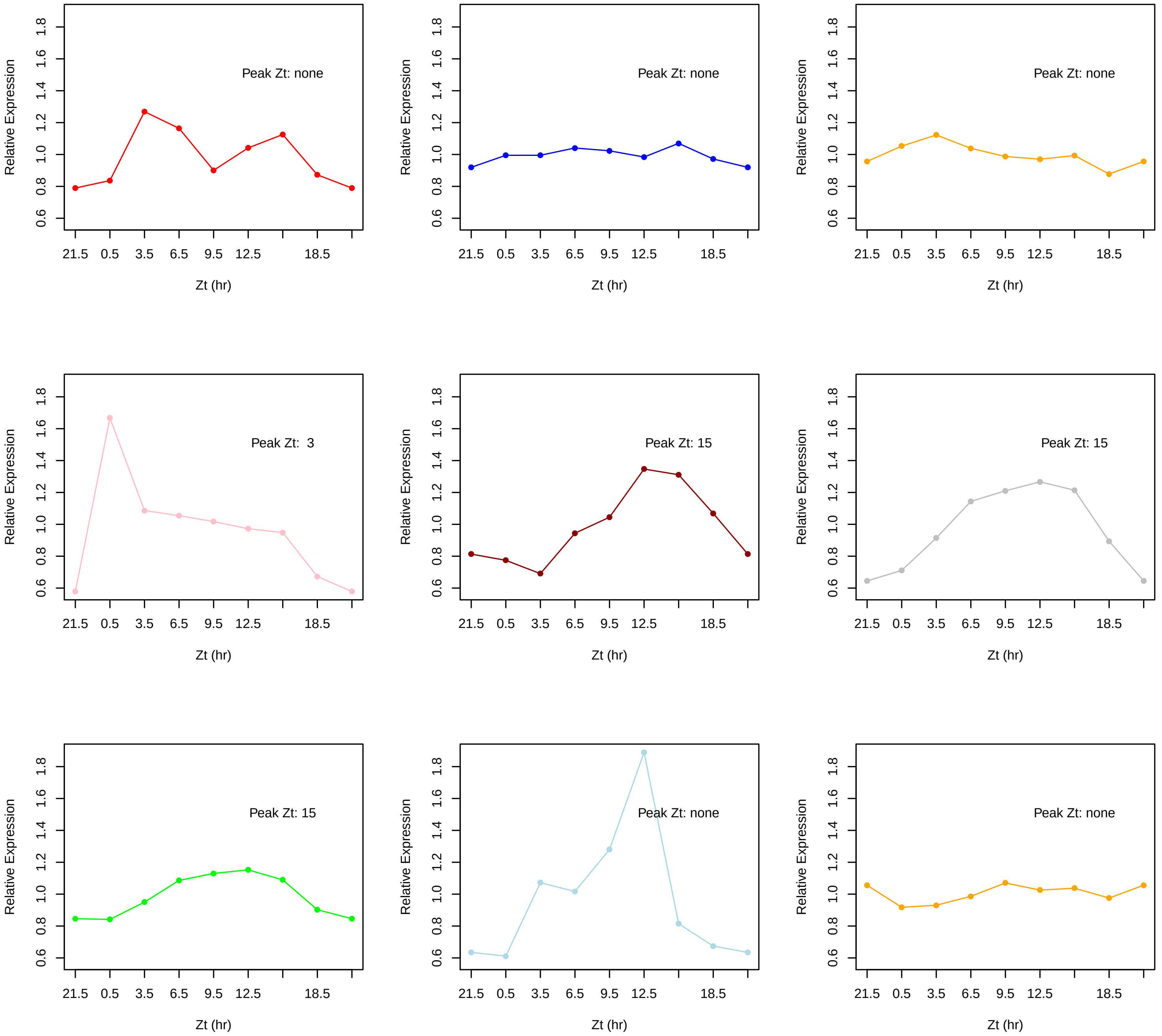

Singeleton: ENSXMAG00000024941 maml3

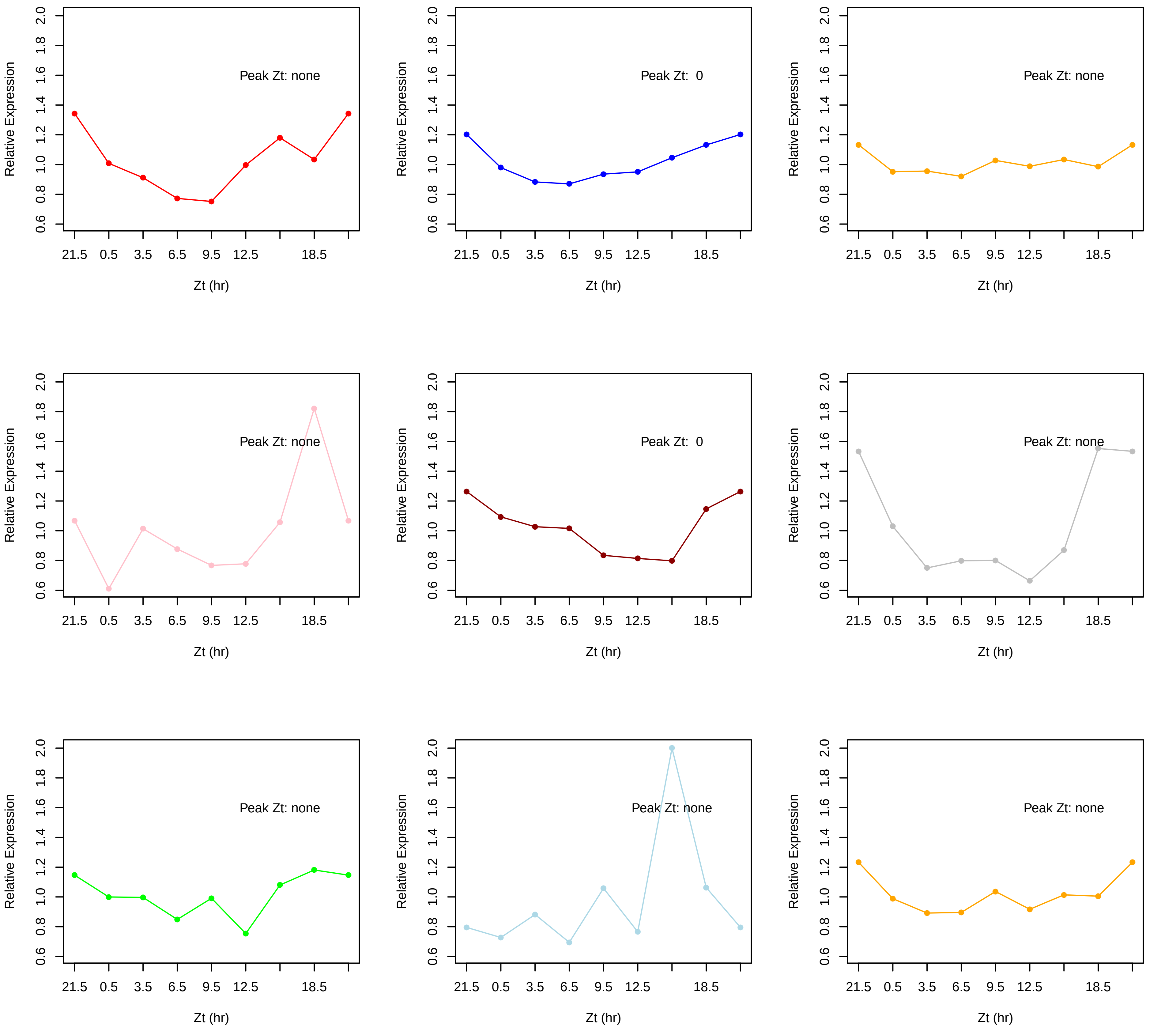

Singeleton: ENSXMAG00000011415 ralgps2

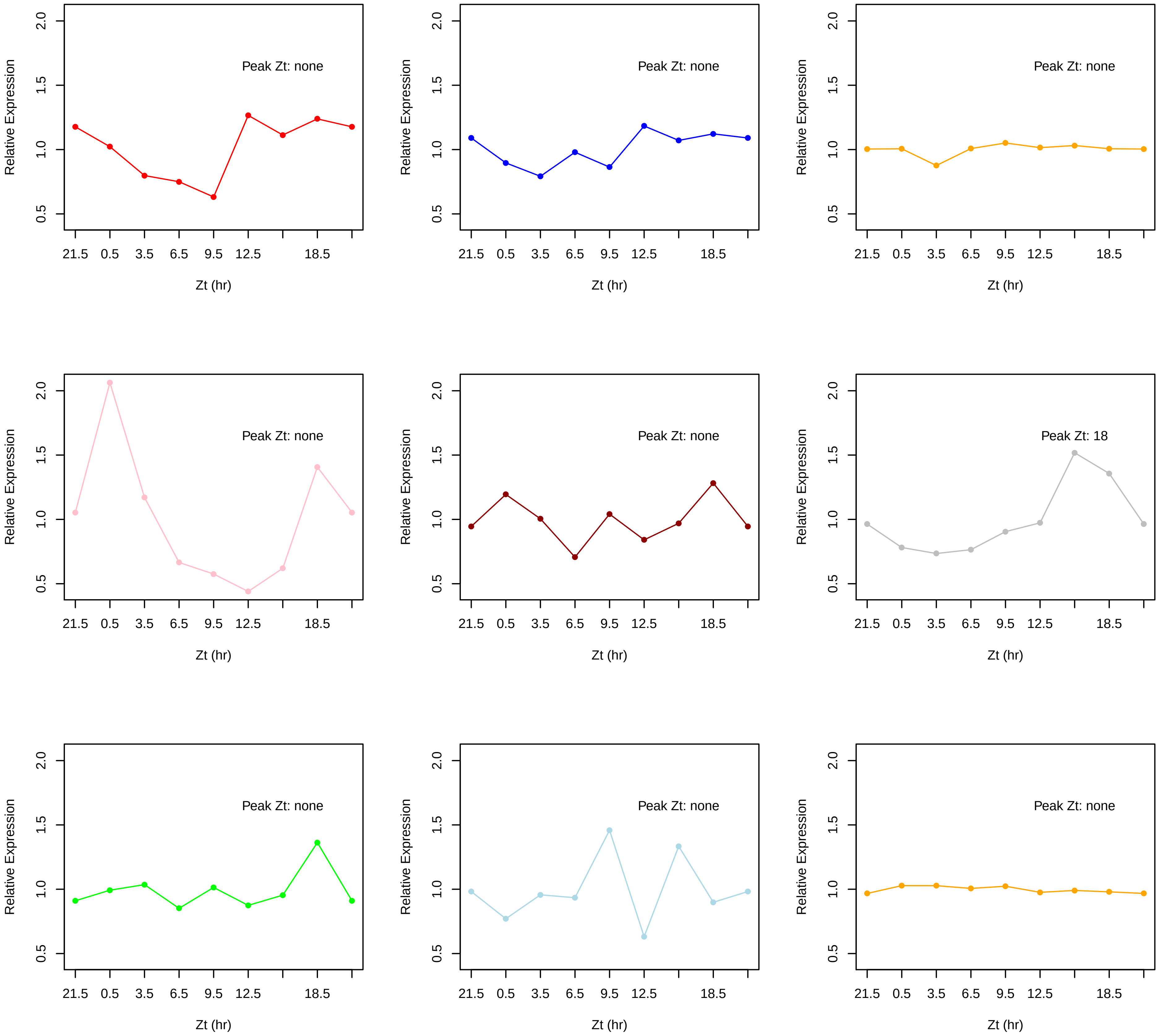

Singeleton: ENSXMAG00000004025 nek4

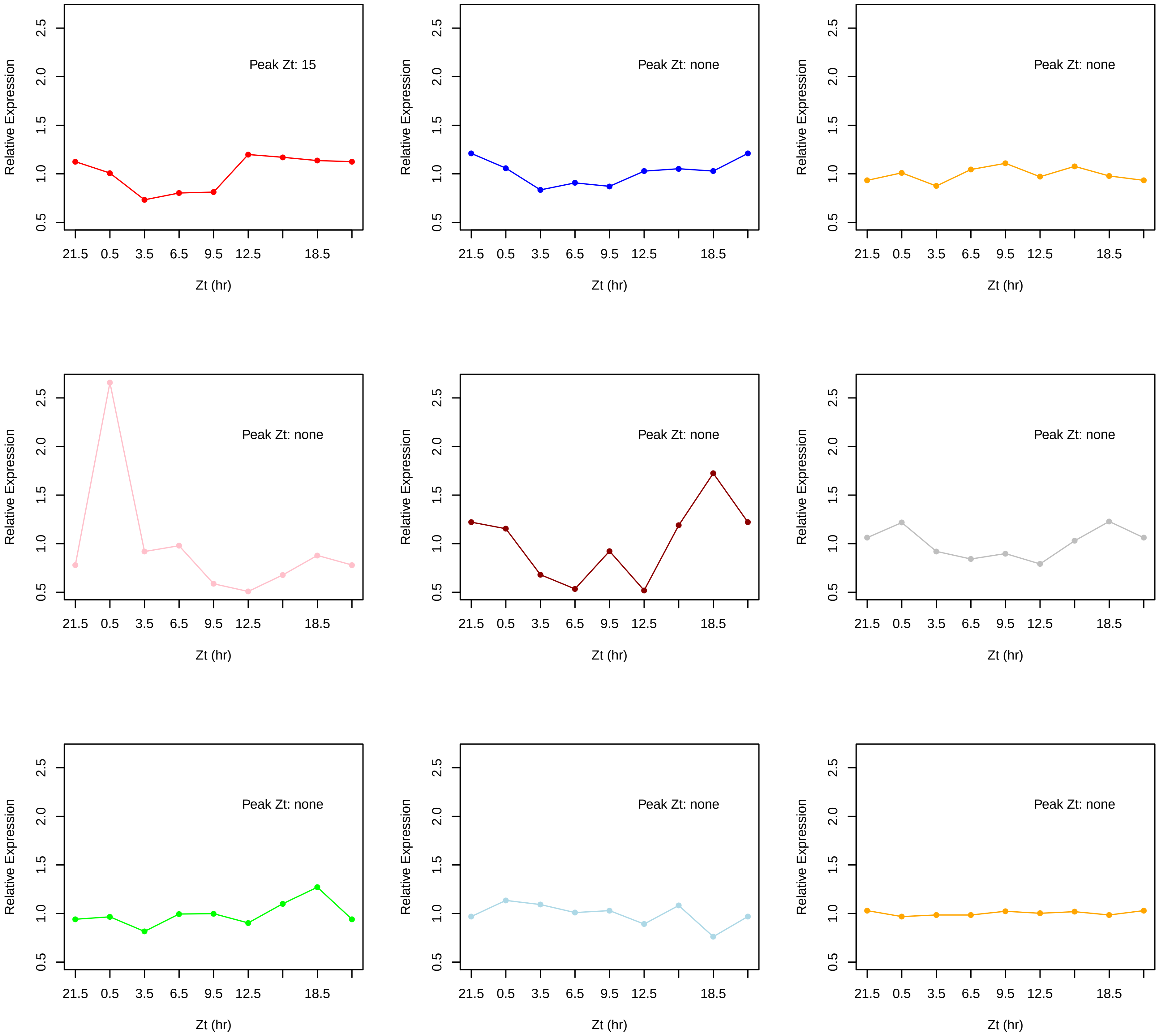

Singeleton: ENSXMAG00000001526 ARHGAP26

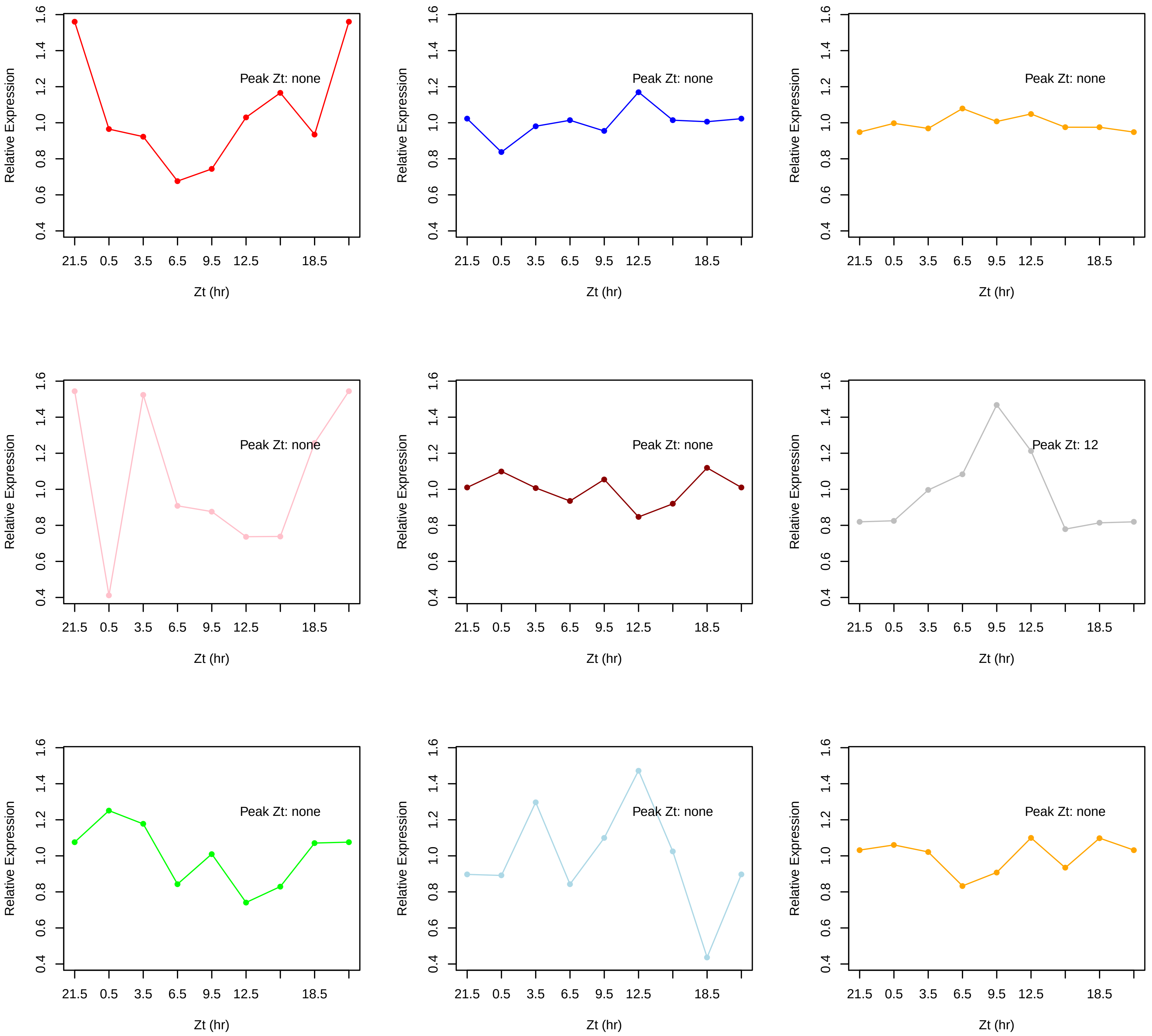

Singleton: ENSXMAG00000015252 dus3l

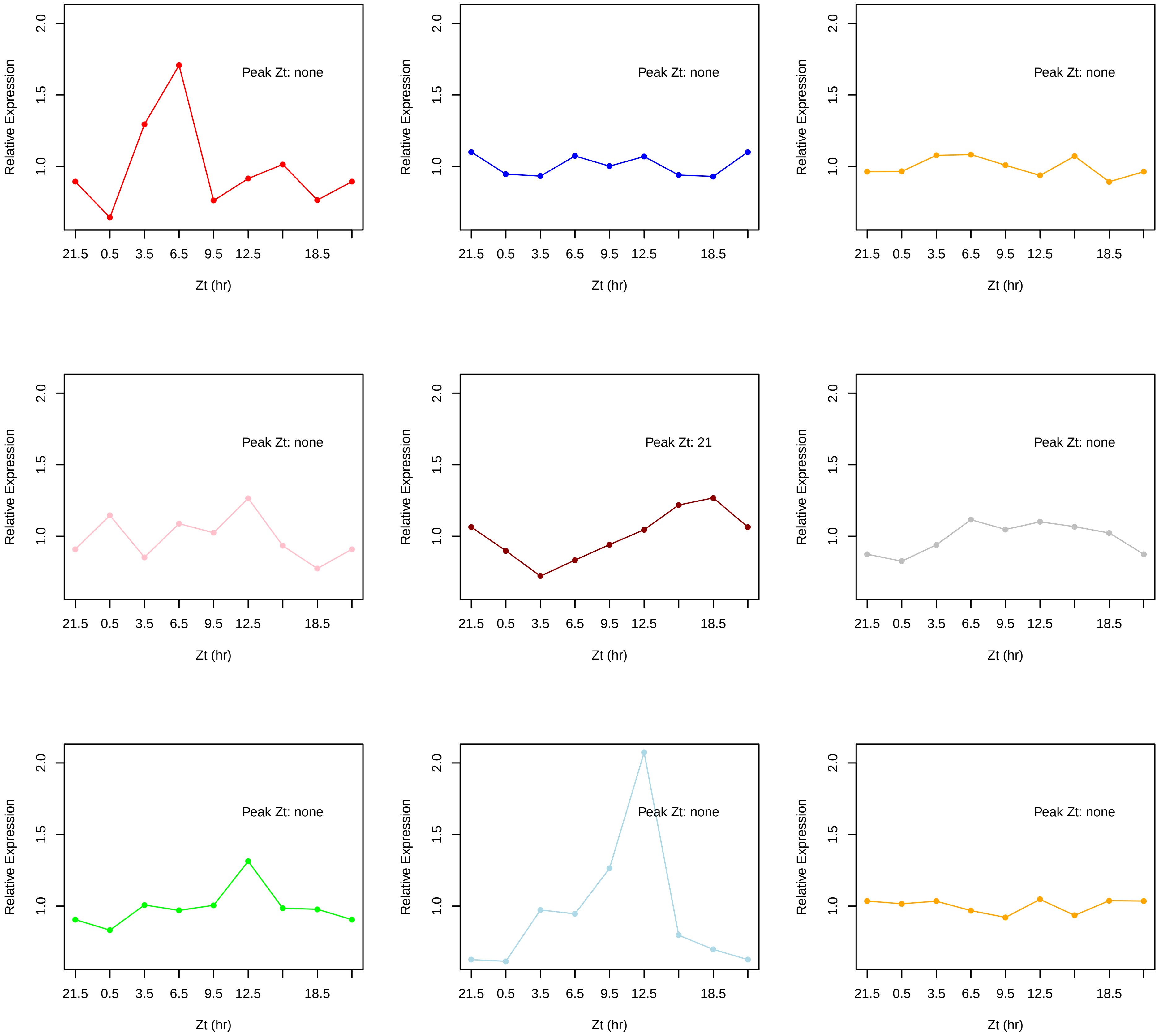

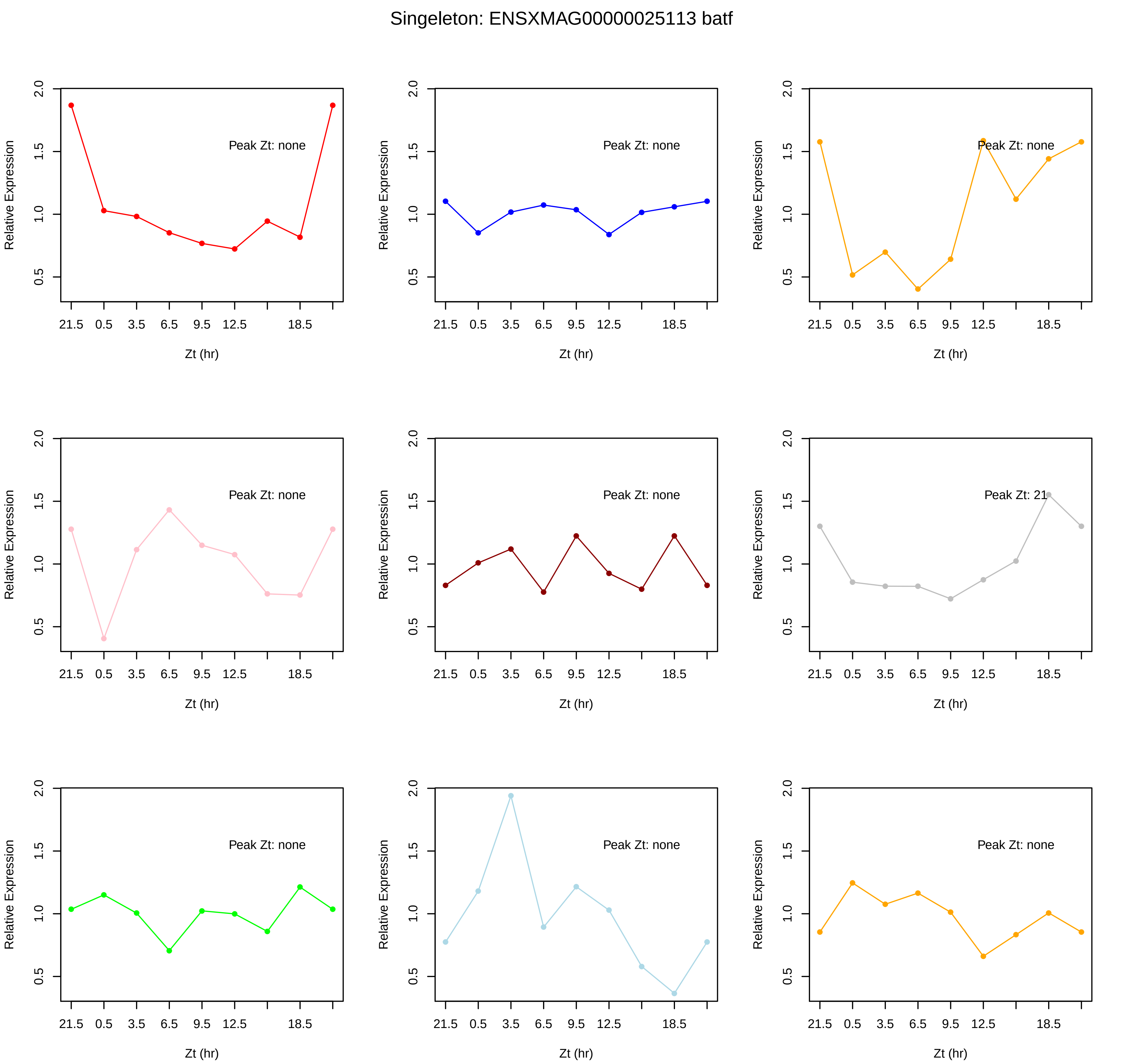

Singeleton: ENSXMAG00000025113 batf
Peak Zt: none
Peak Zt: none
Peak Zt: none
Peak Zt: none
Peak Zt: none
Peak Zt: 21
Peak Zt: none
Peak Zt: none
Peak Zt: none
Relative Expression
Zt (hr)
21.5
0.5
3.5
6.5
9.5
12.5
18.5
0.5
1.0
1.5
2.0

Singeleton: ENSXMAG00000011448 coa7

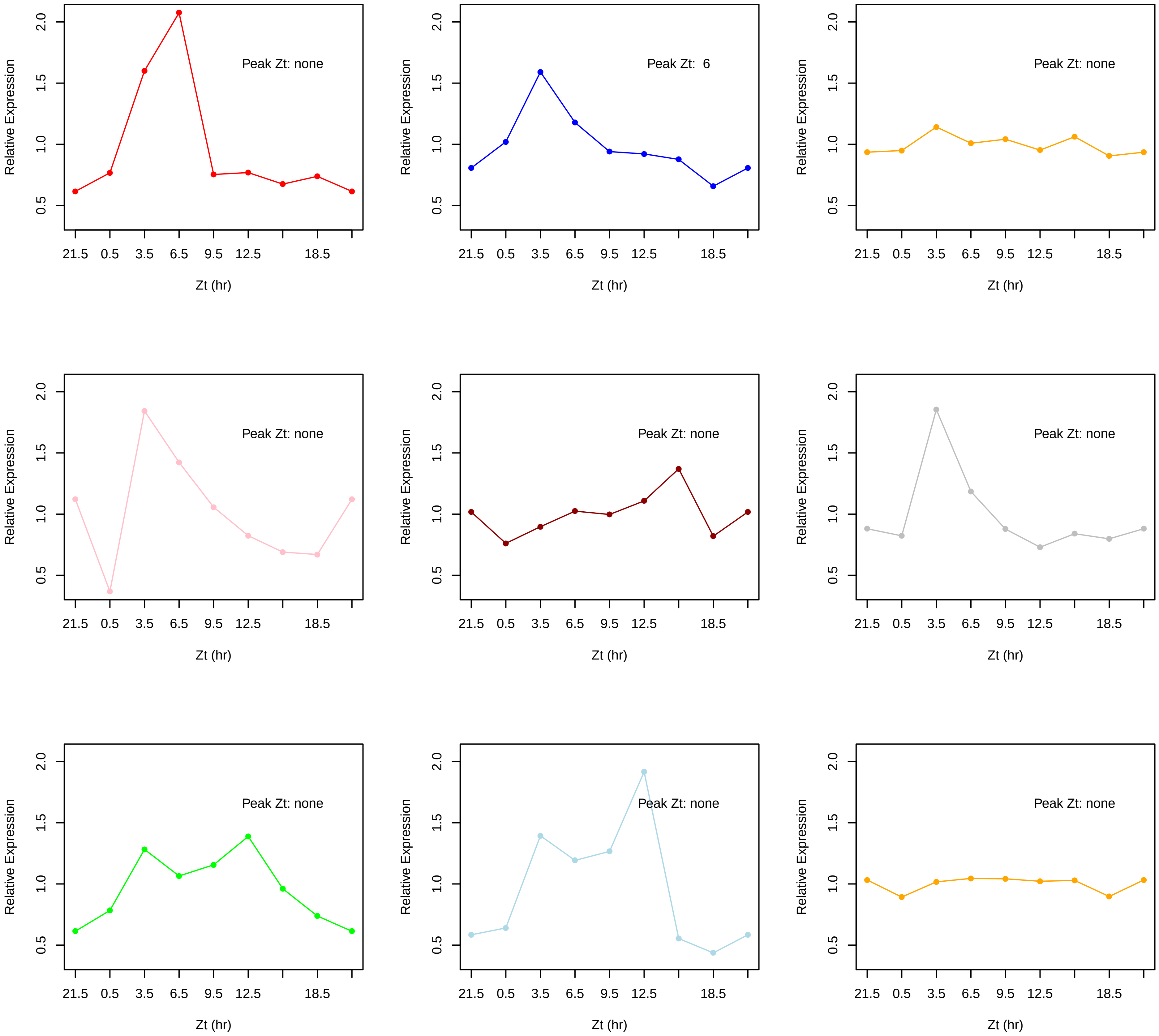

Singleton: ENSXMAG00000011451 zyg11

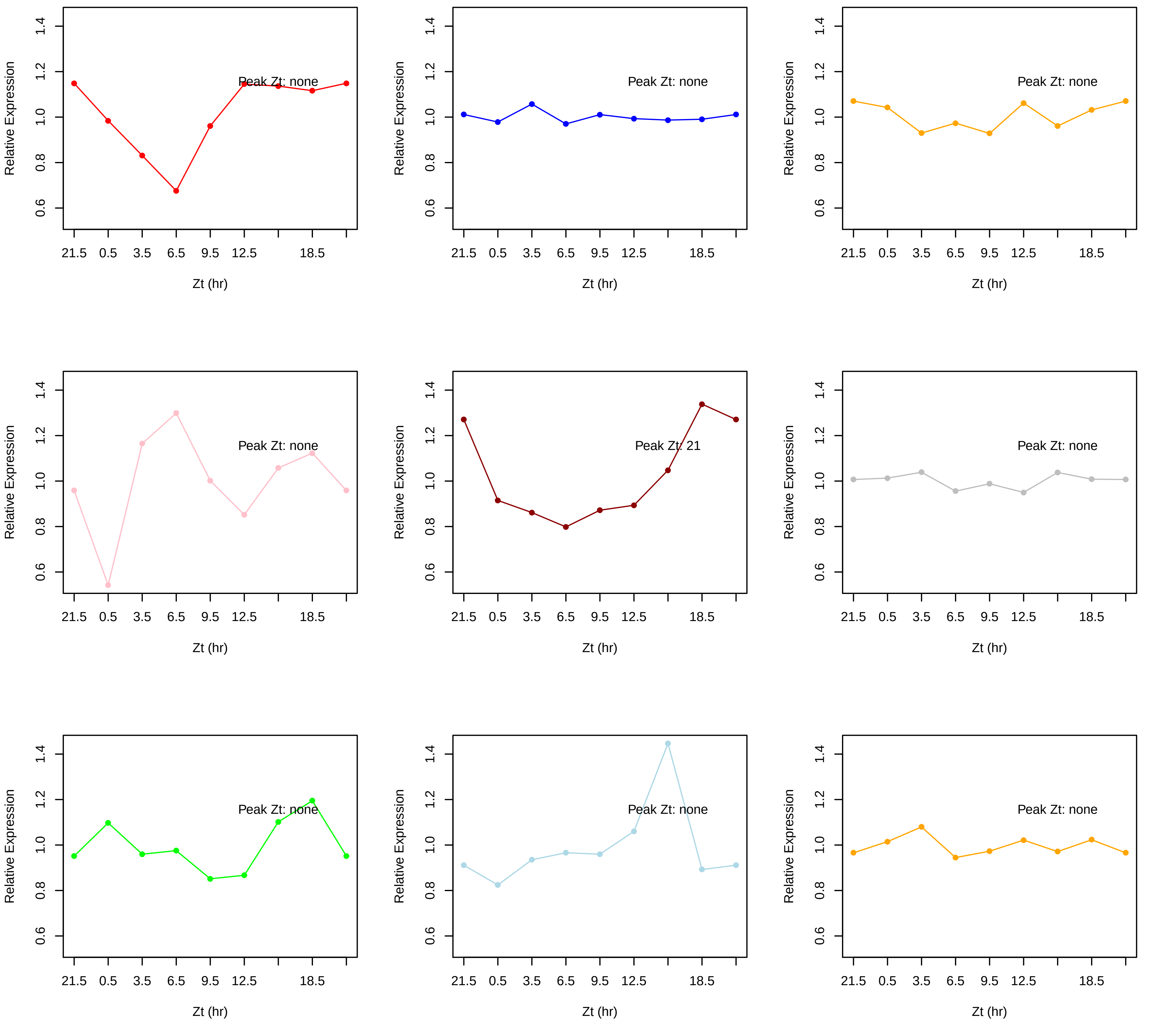

Singleton: ENSXMAG00000001386 cdk14

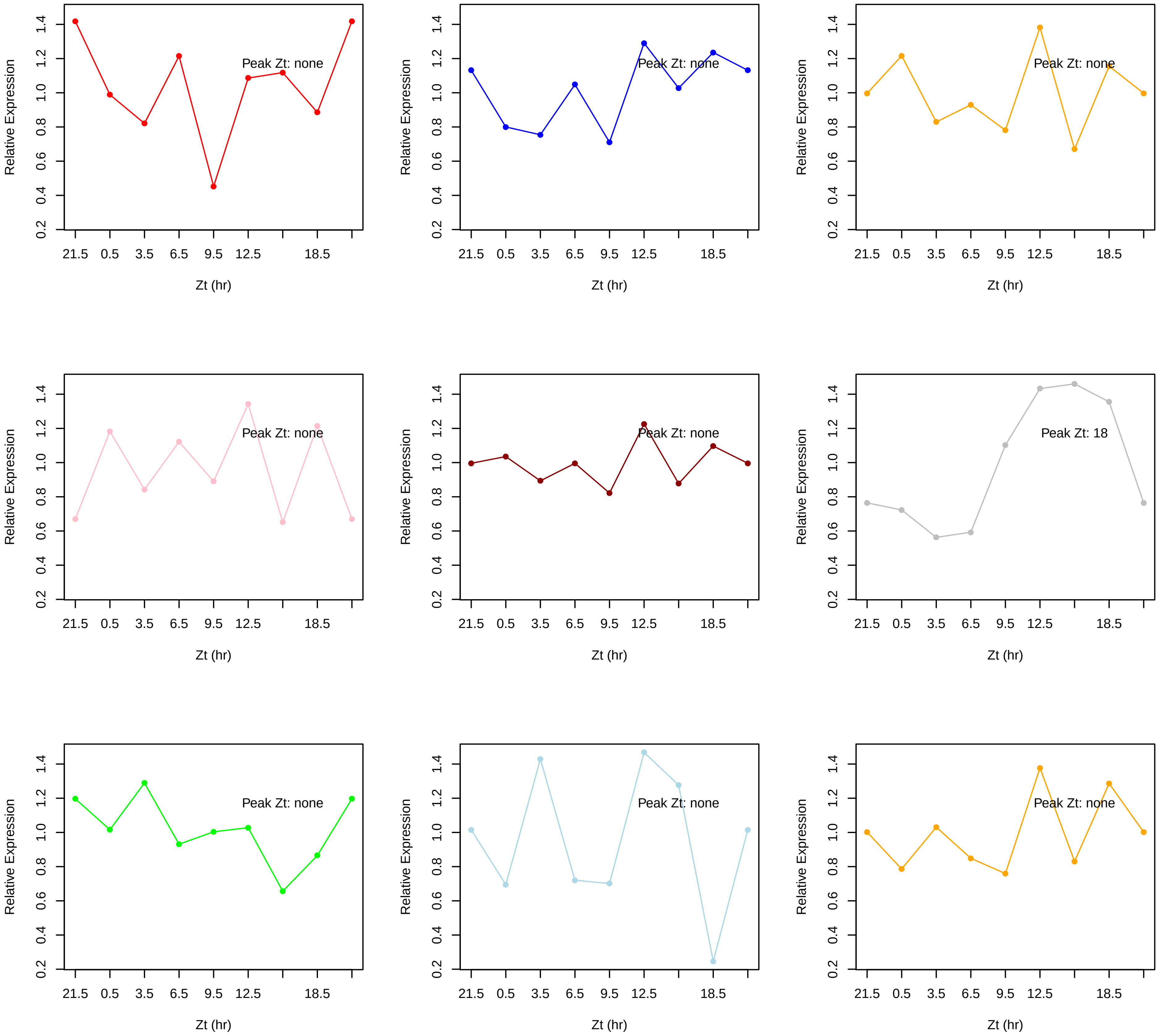

Singeleton: ENSXMAG00000014737 ttll5

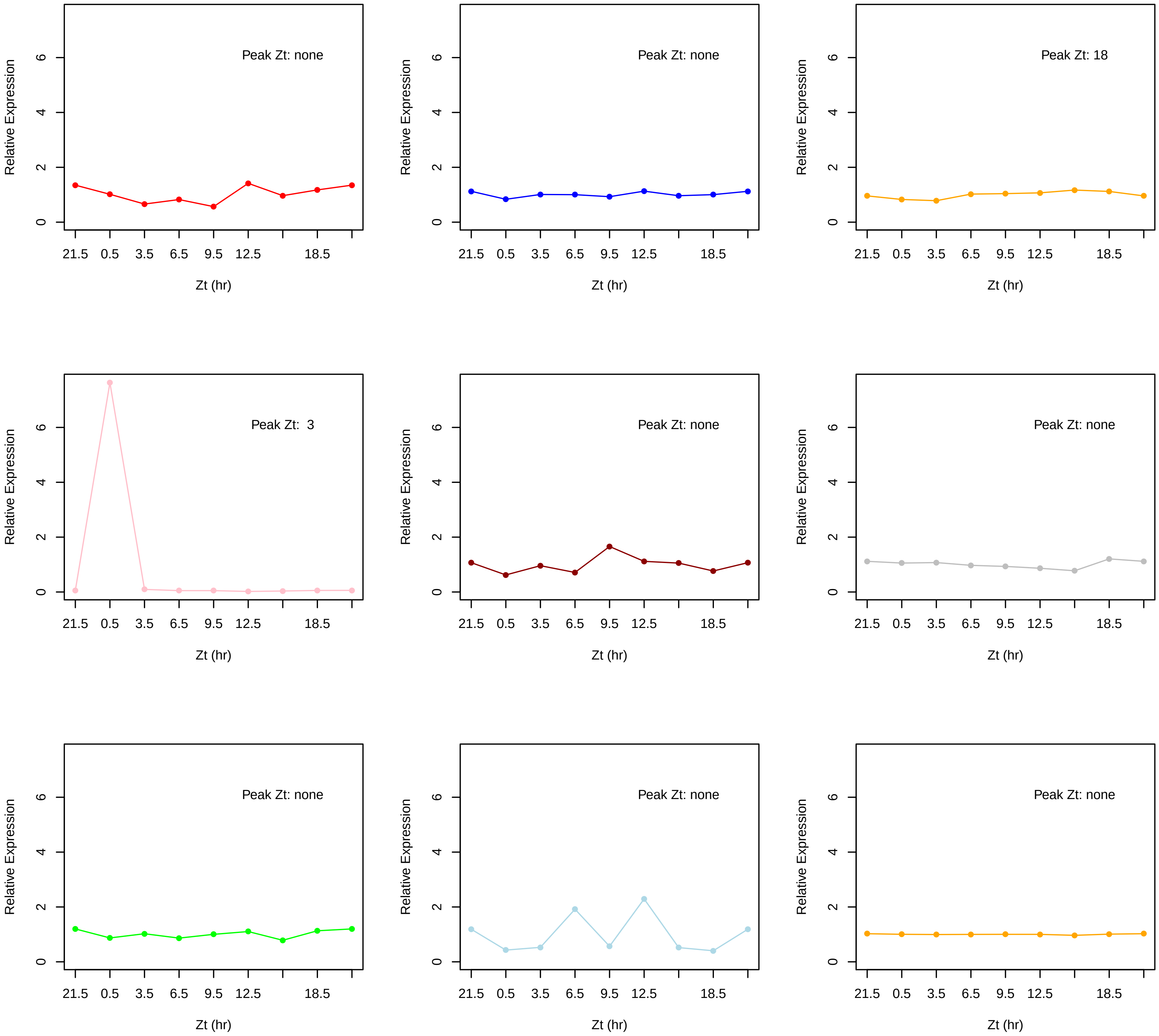

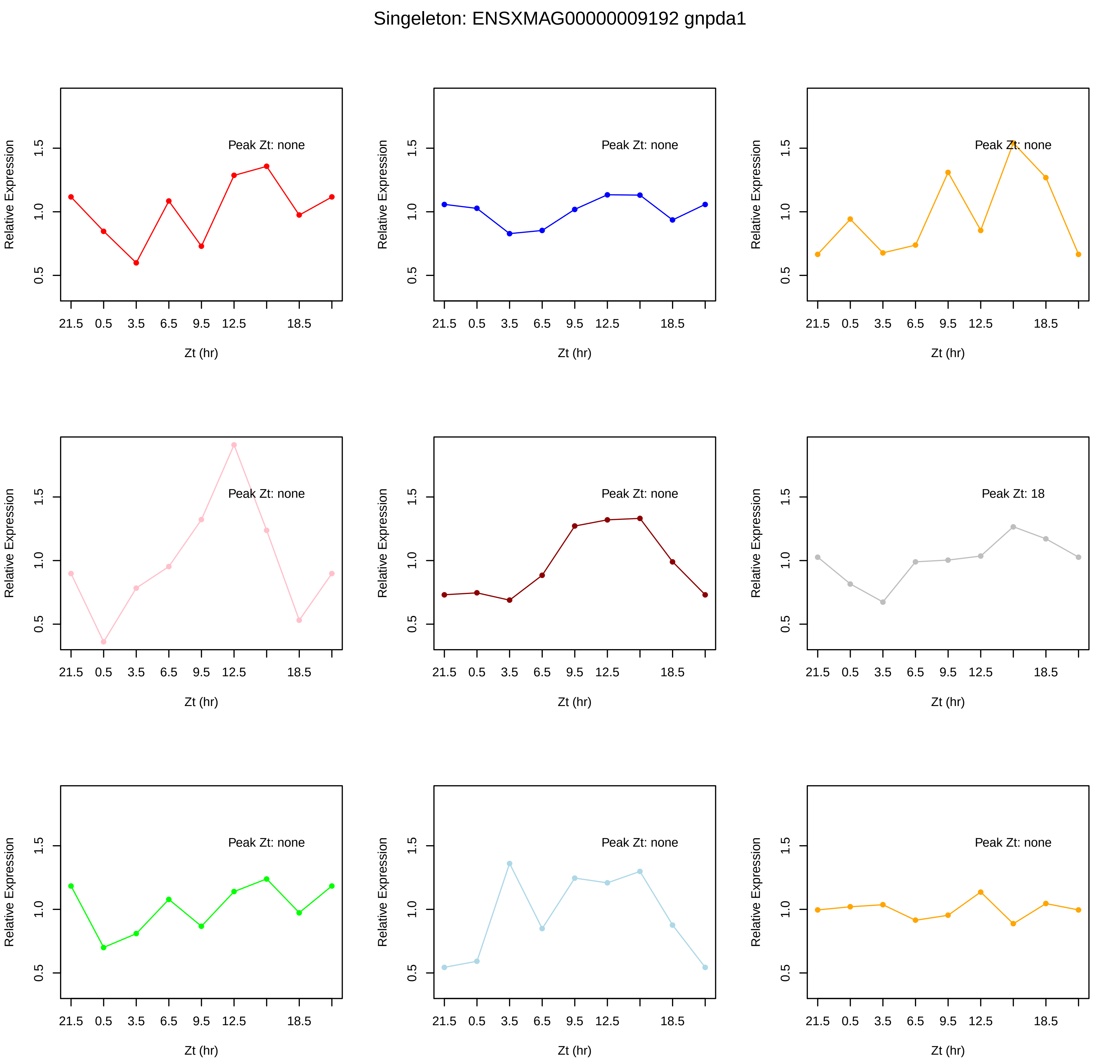
Singeleton: ENSXMAG00000009192 gnpda1
Peak Zt: none
Peak Zt: none
Peak Zt: none
Peak Zt: none
Peak Zt: none
Peak Zt: 18
Peak Zt: none
Peak Zt: none
Peak Zt: none
Relative Expression
Zt (hr)
21.5
0.5
3.5
6.5
9.5
12.5
18.5
0.5
1.0
1.5

Singeleton: ENSXMAG00000015898 chd1l

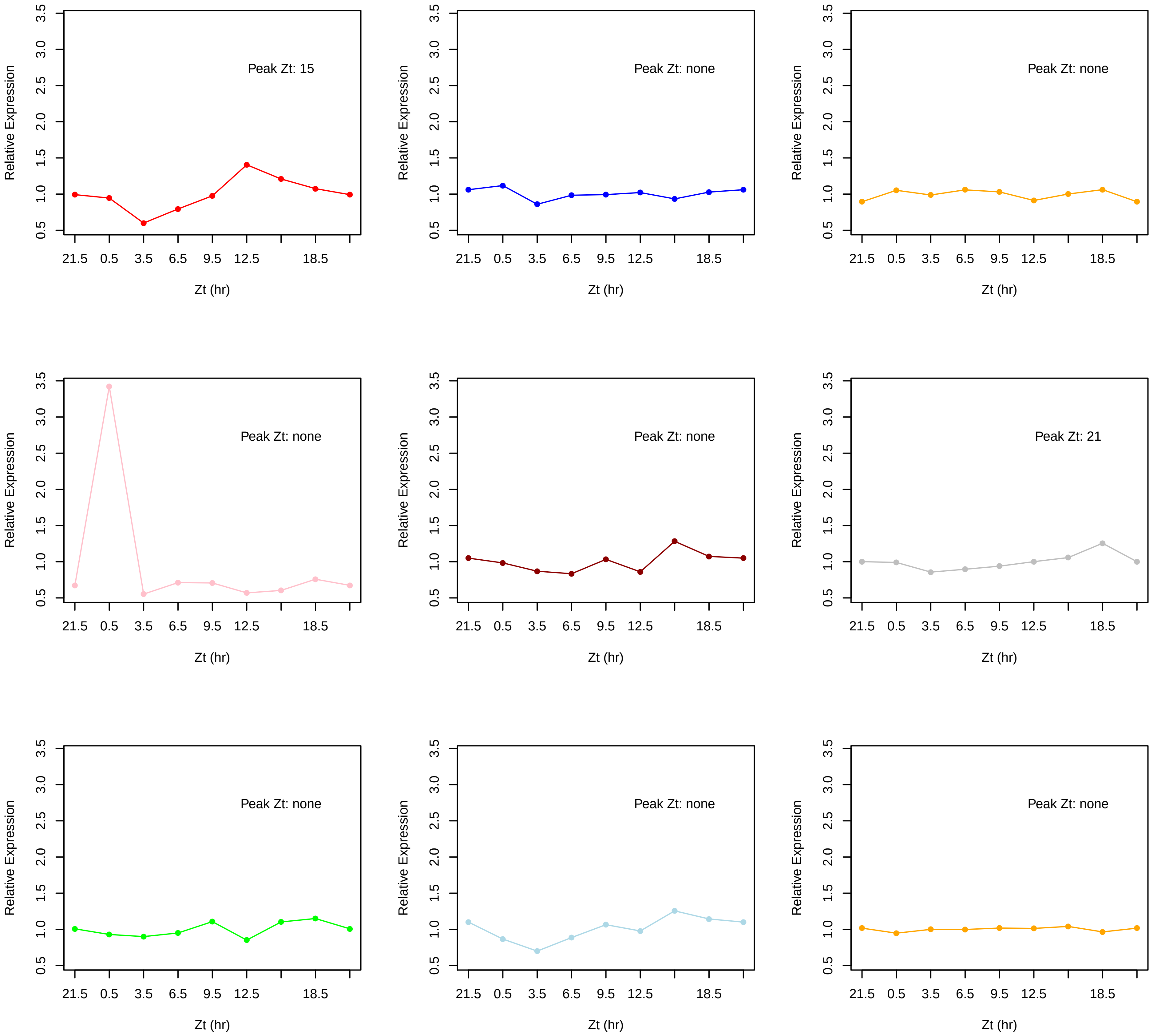

Singeleton: ENSXMAG00000028954 trappc13

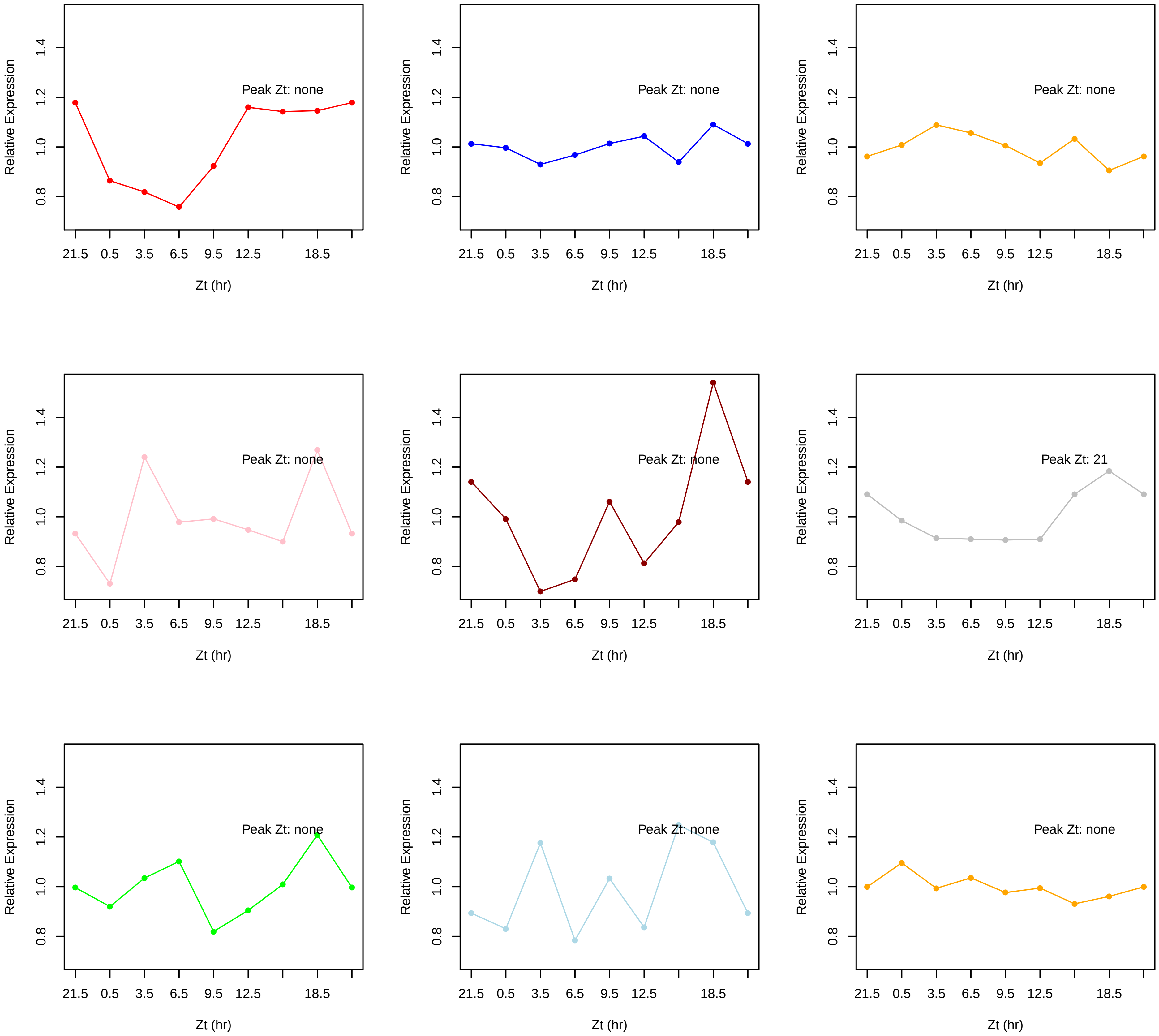

Singeleton: ENSXMAG00000008671 cnot11

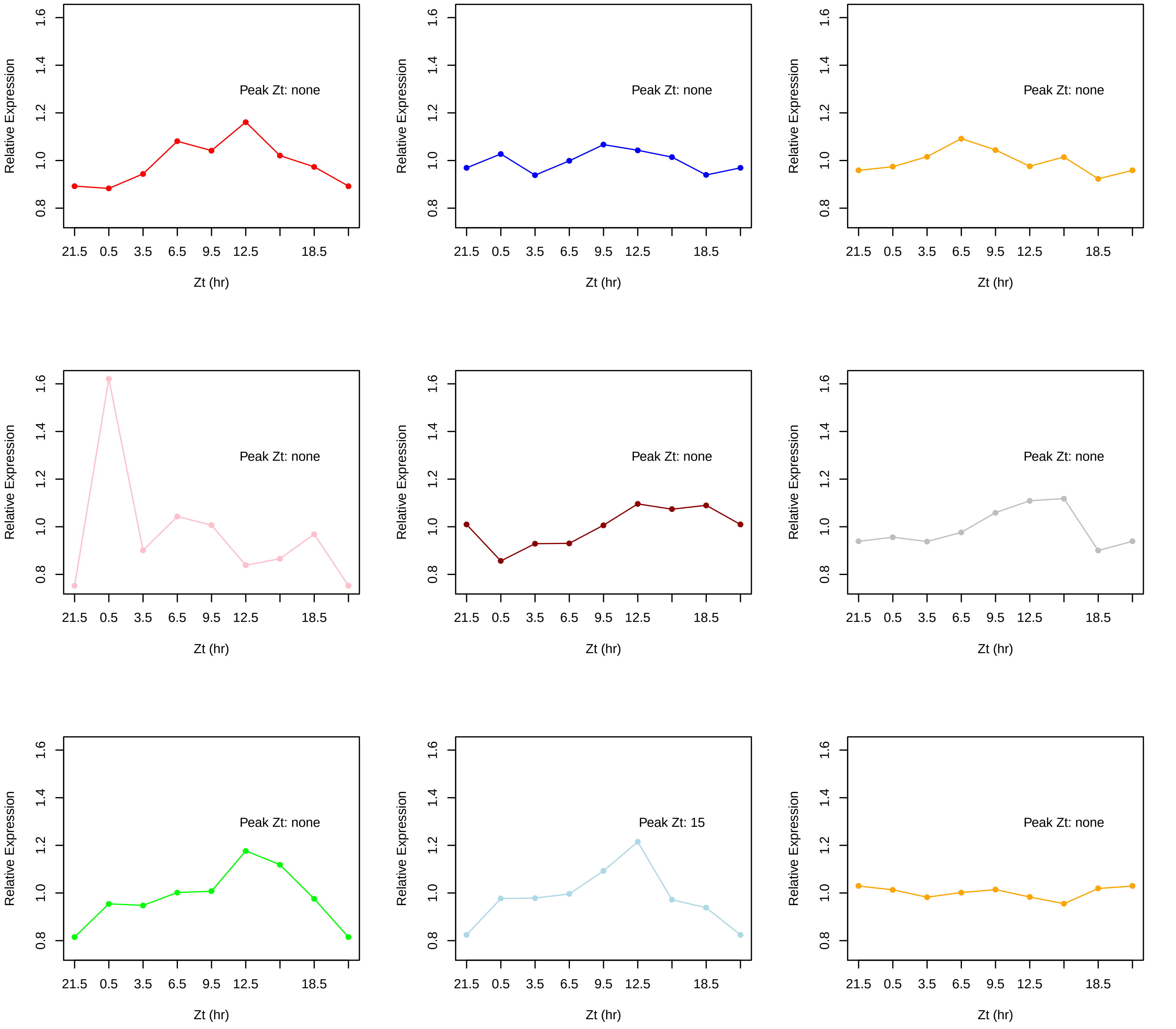

Singleton: ENSXMAG00000017764 ELF2

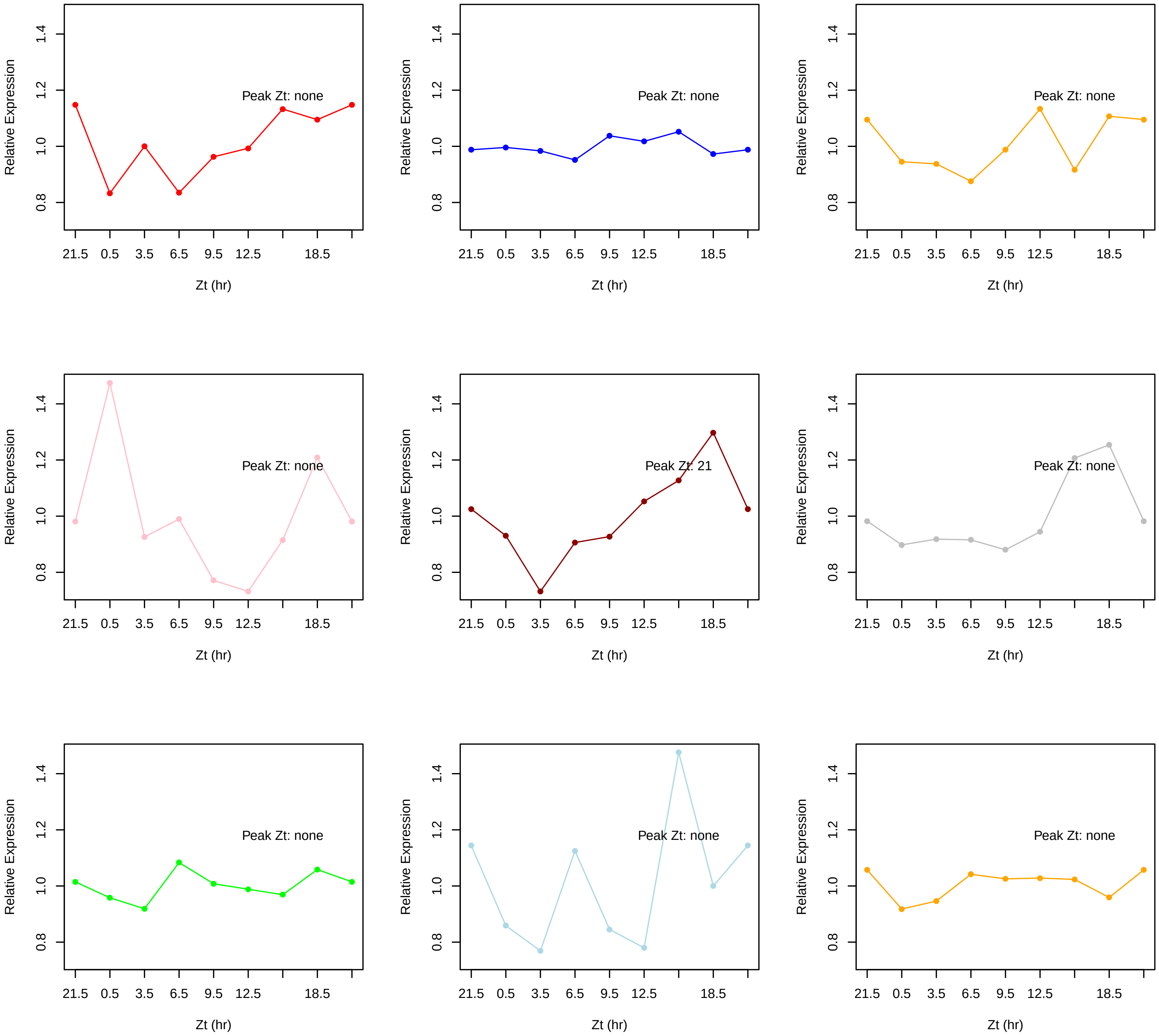

Singleton: ENSXMAG00000019005 trim23

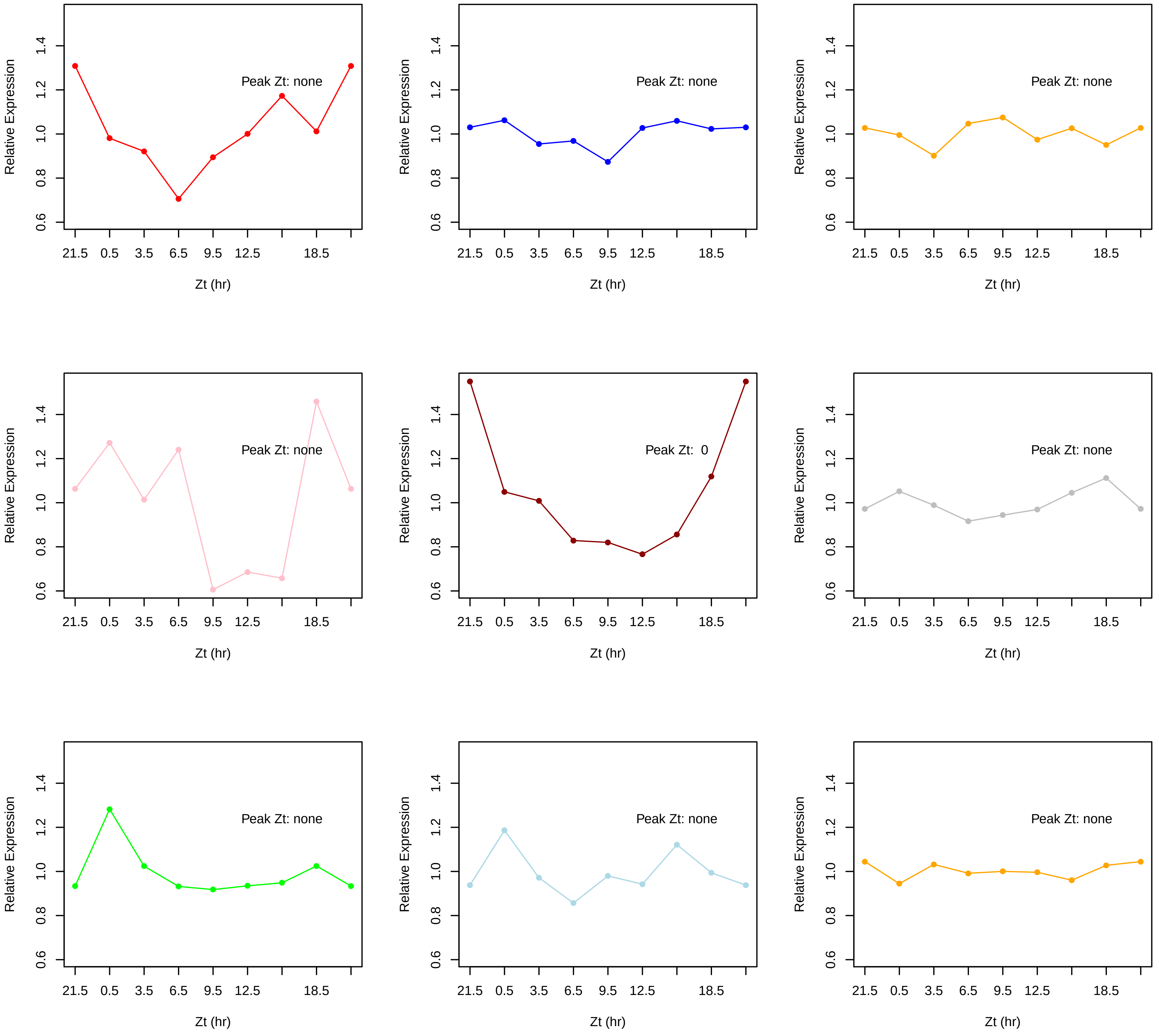

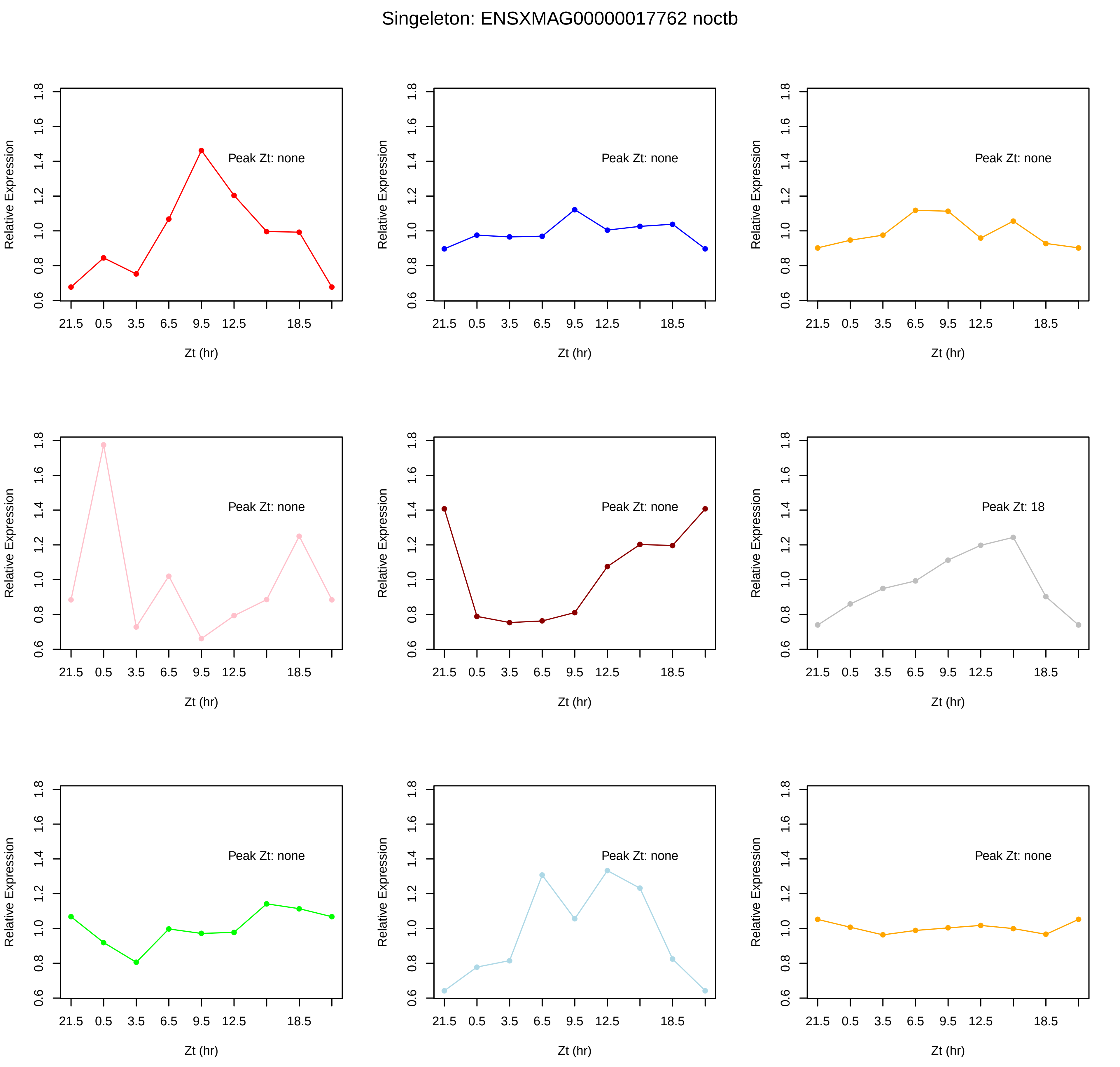

Singeleton: ENSXMAG00000017762 noctb
Relative Expression
Zt (hr)
Peak Zt: none
Peak Zt: none
Peak Zt: none
Peak Zt: none
Peak Zt: none
Peak Zt: 18
Peak Zt: none
Peak Zt: none
Peak Zt: none

Singeleton: ENSXMAG00000019191 mgst3a

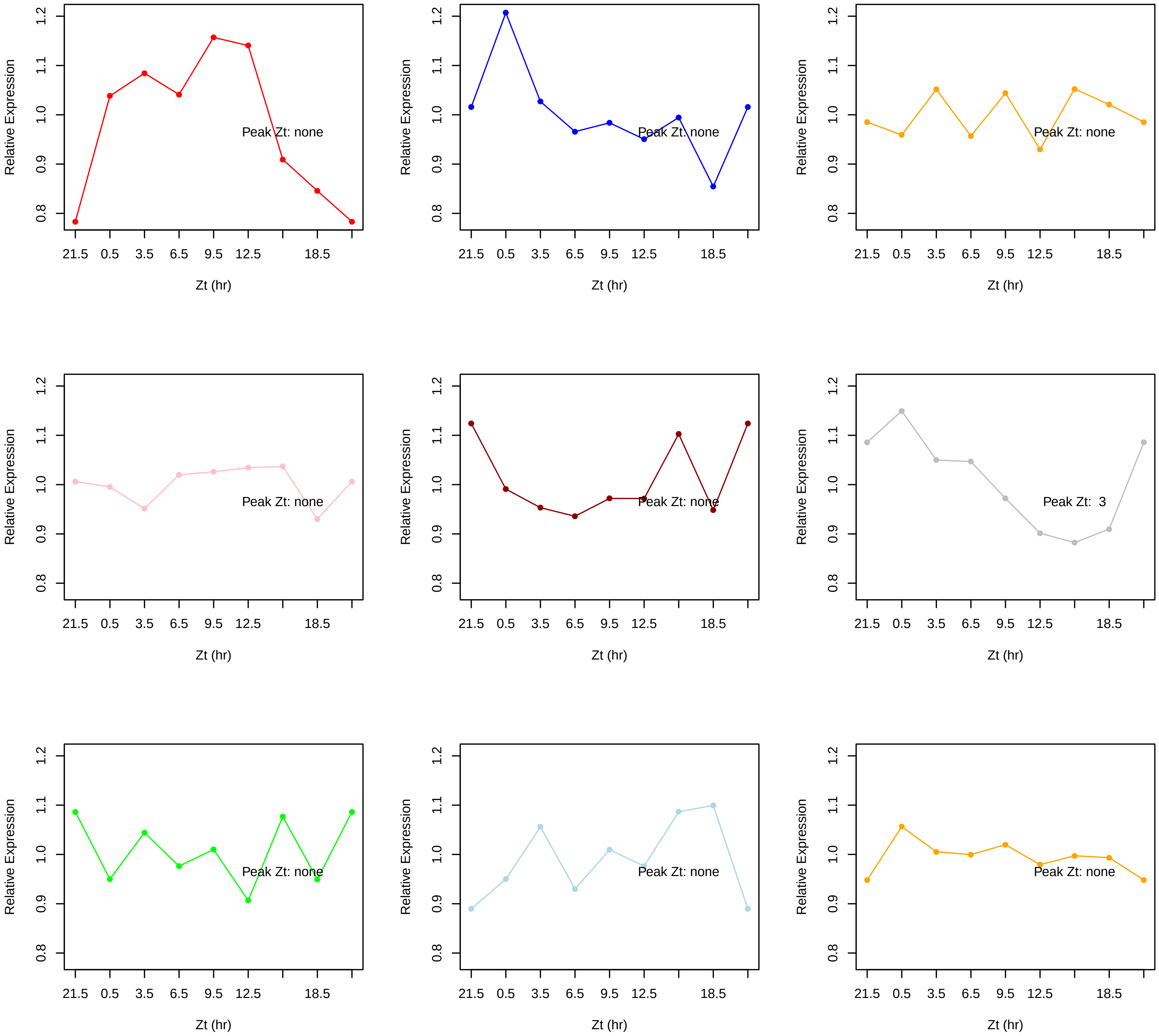

Singleton: ENSXMAG00000011479 MMACHC

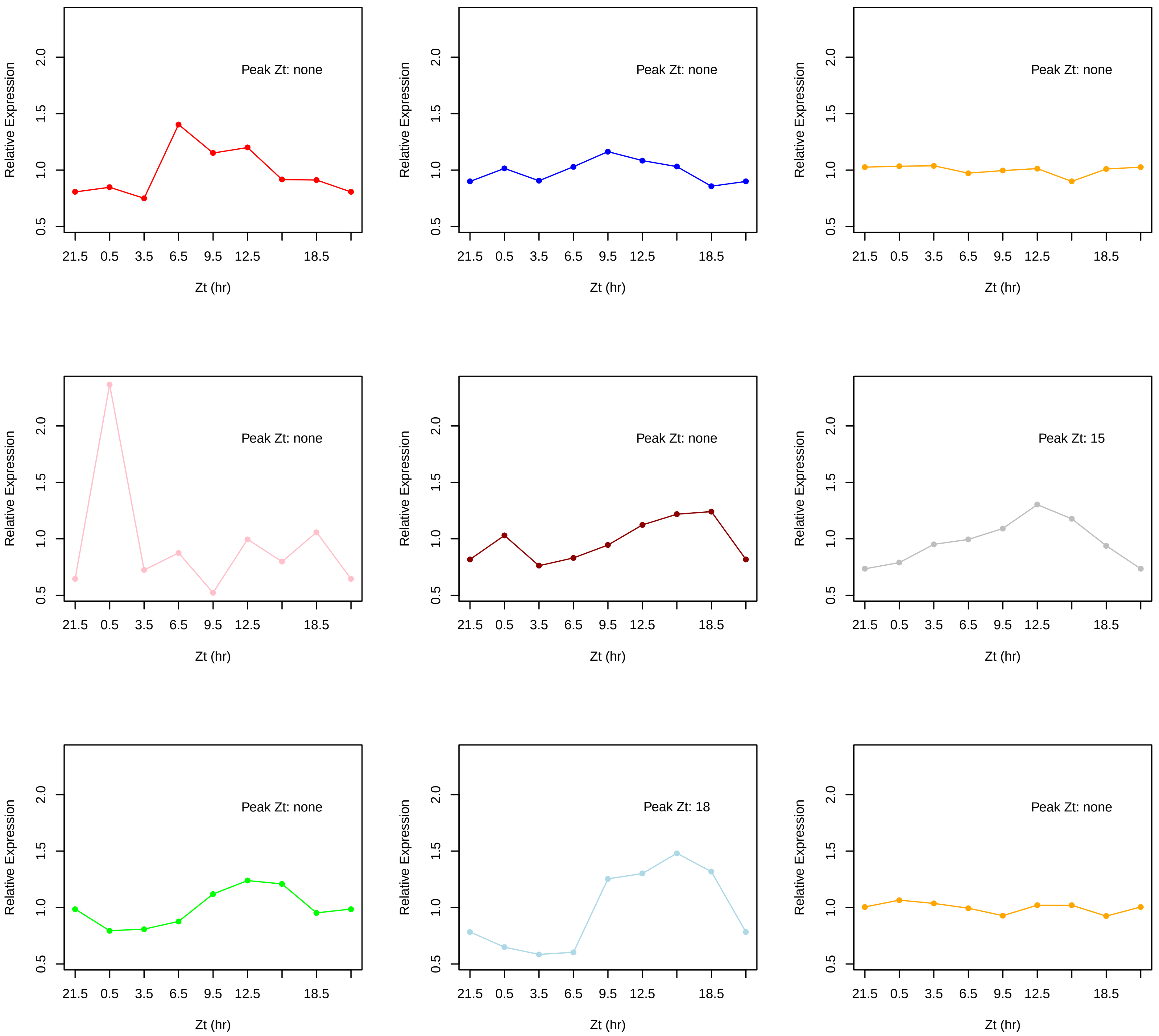

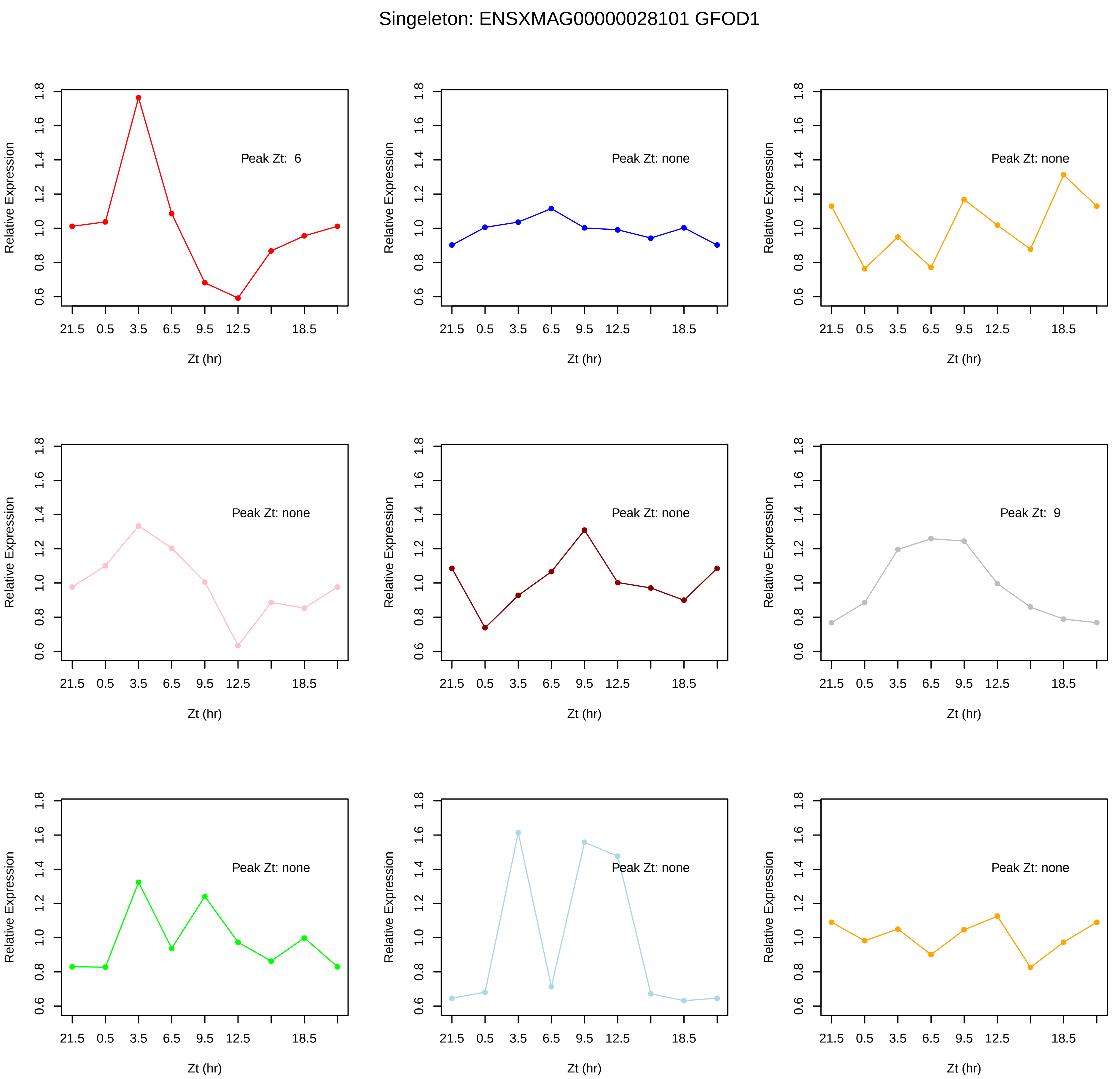

Singeleton: ENSXMAG00000028101 GFOD1
Peak Zt: 6
Peak Zt: none
Peak Zt: none
Peak Zt: none
Peak Zt: none
Peak Zt: 9
Peak Zt: none
Peak Zt: none
Peak Zt: none
Relative Expression
Zt (hr)

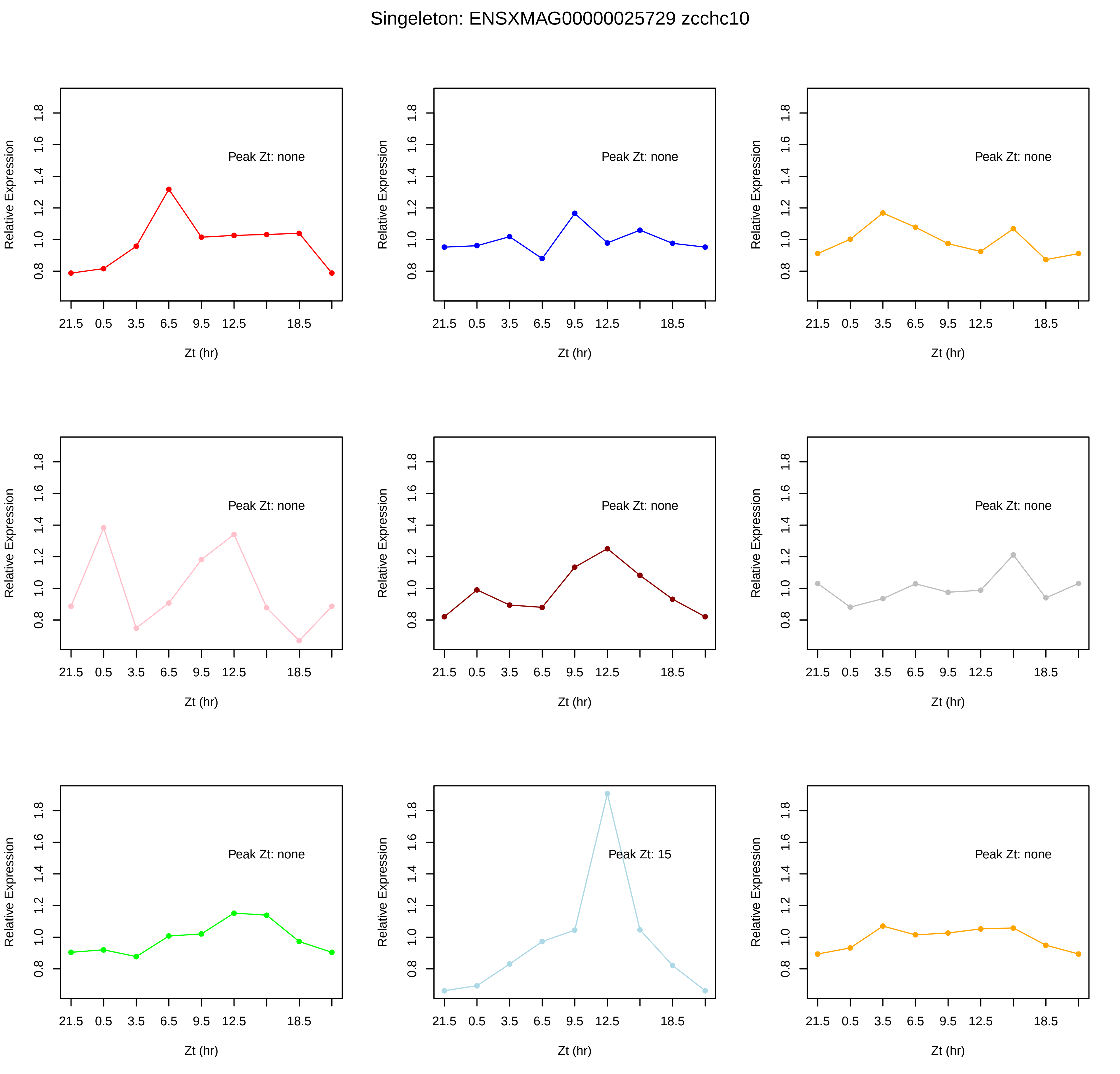
Singeleton: ENSXMAG00000025729 zcchc10
Peak Zt: none
Peak Zt: none
Peak Zt: none
Peak Zt: none
Peak Zt: none
Peak Zt: none
Peak Zt: none
Peak Zt: 15
Peak Zt: none
Relative Expression
Zt (hr)
21.5
0.5
3.5
6.5
9.5
12.5
18.5
0.8
1.0
1.2
1.4
1.6
1.8

Singeleton: ENSXMAG00000000318 nisch

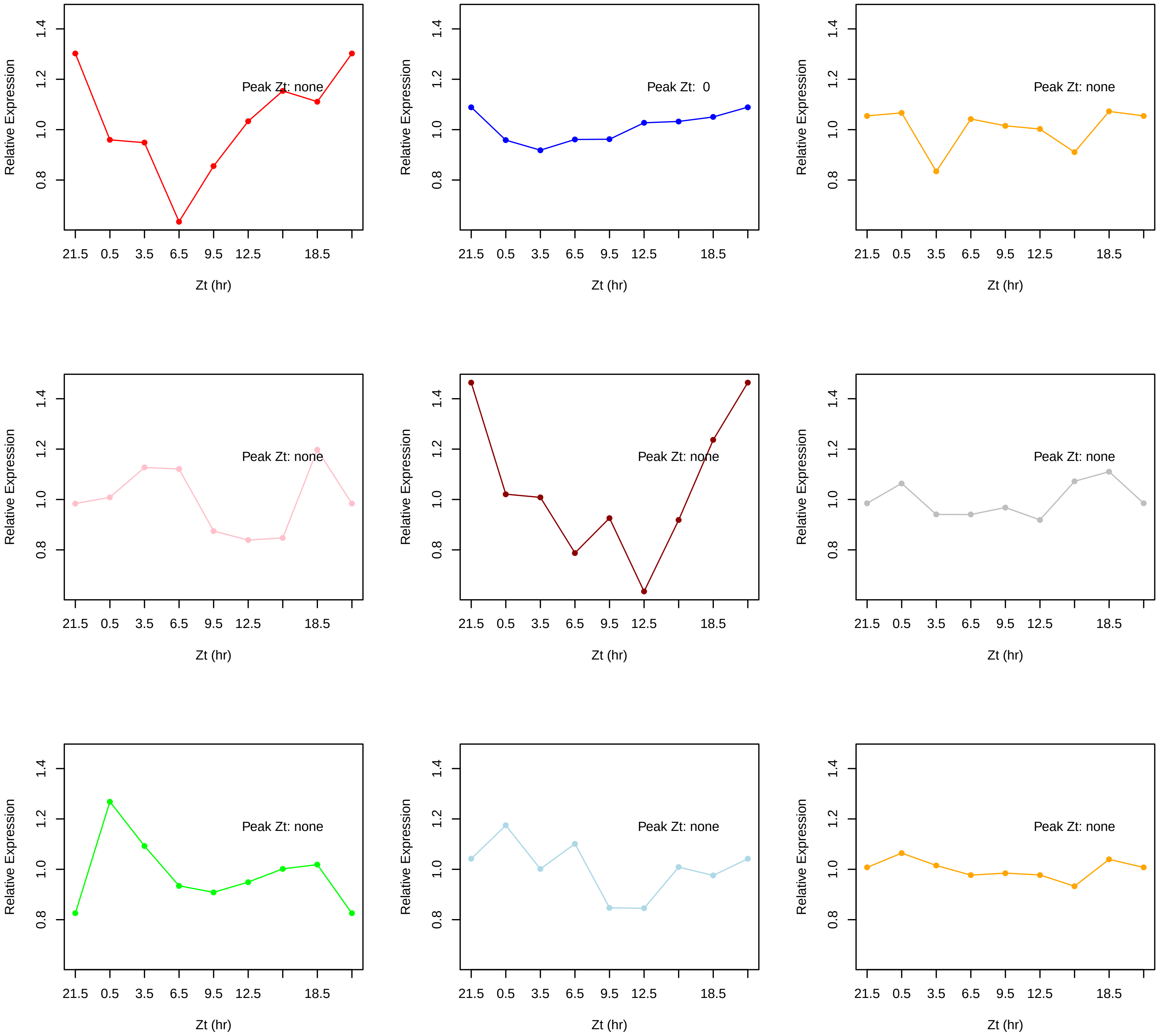

Singleton: ENSXMAG00000001642 aff4

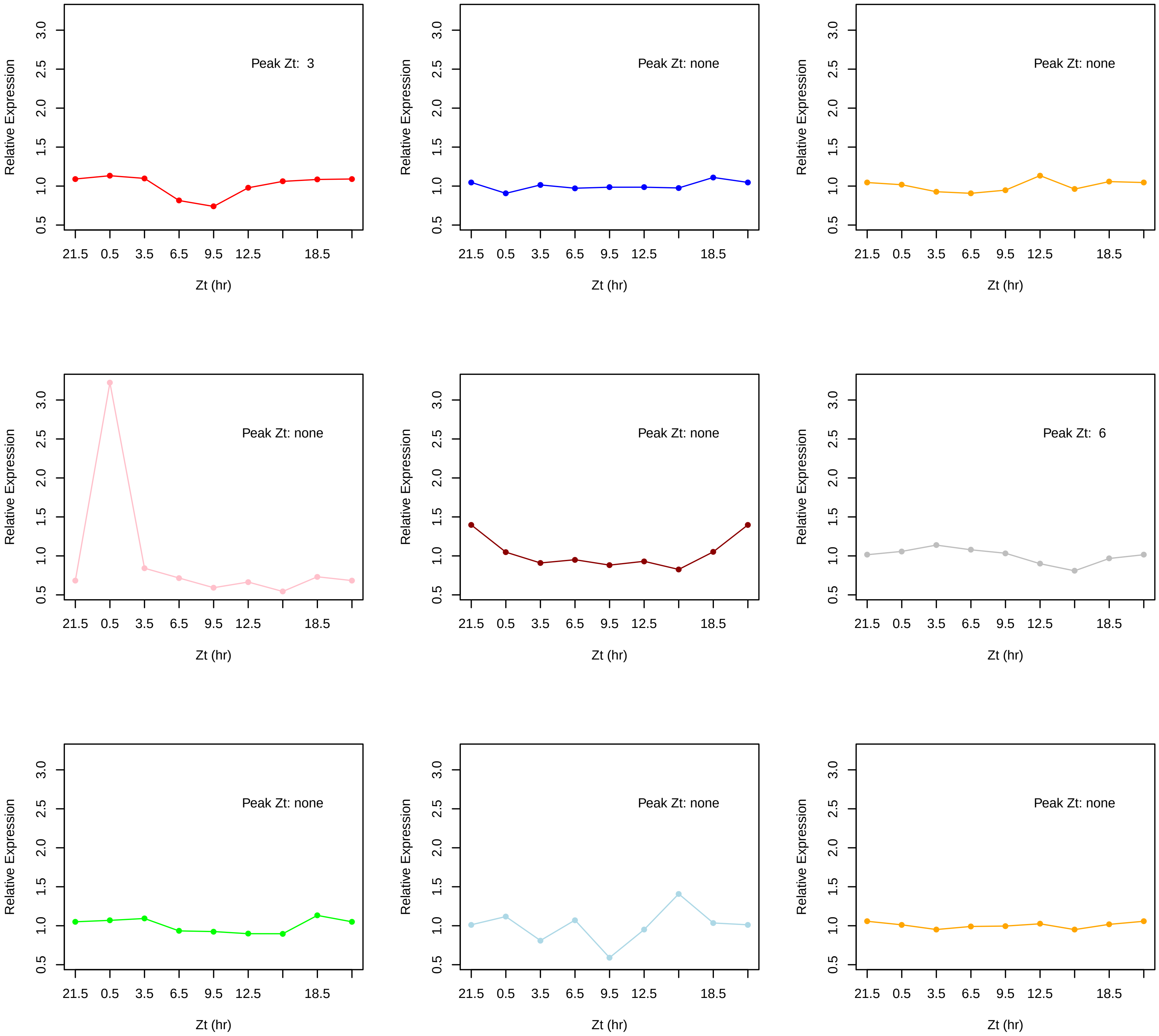

Singeleton: ENSXMAG00000019046 adamts6

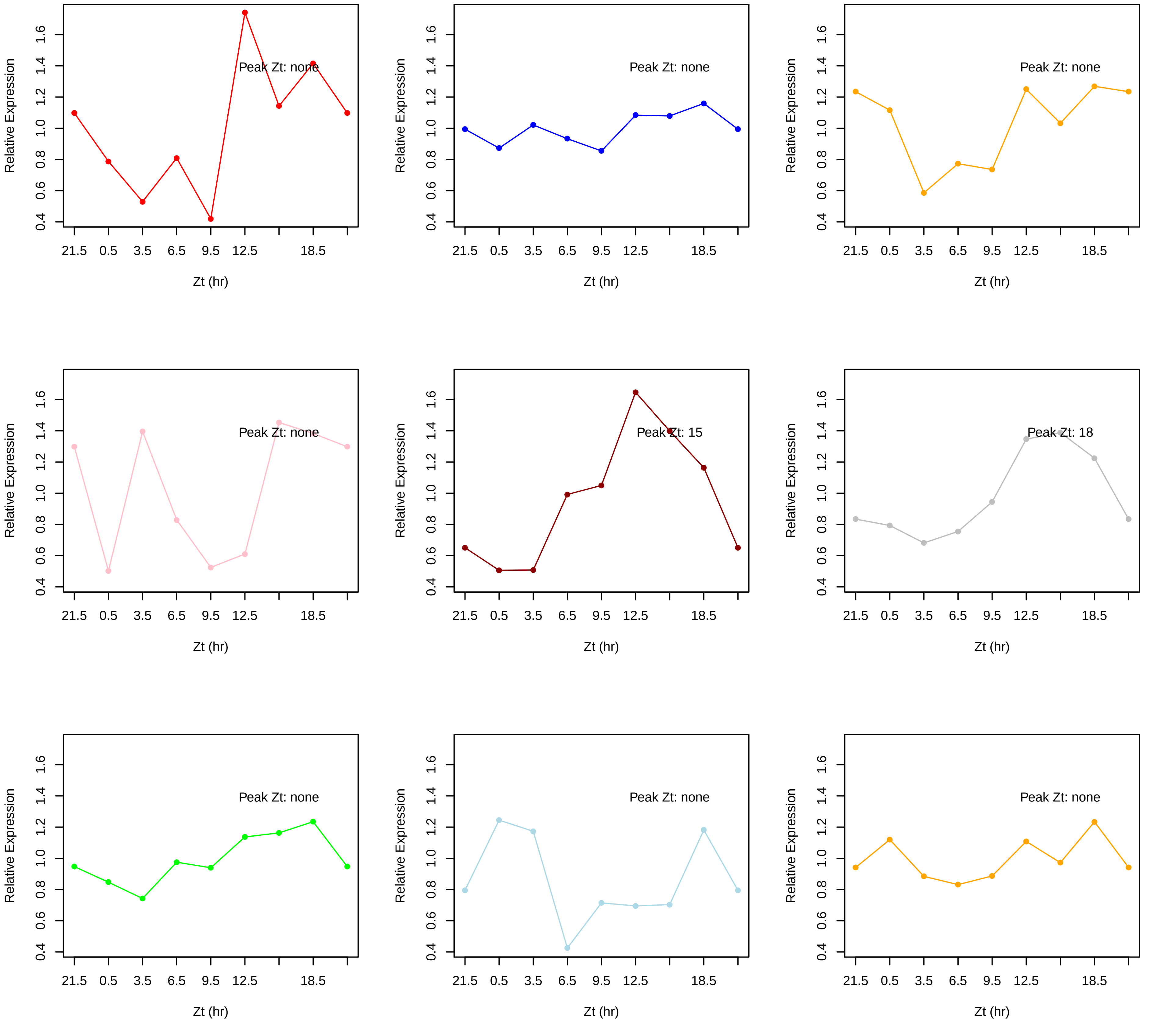

Singeleton: ENSXMAG00000029501 GPATCH2L

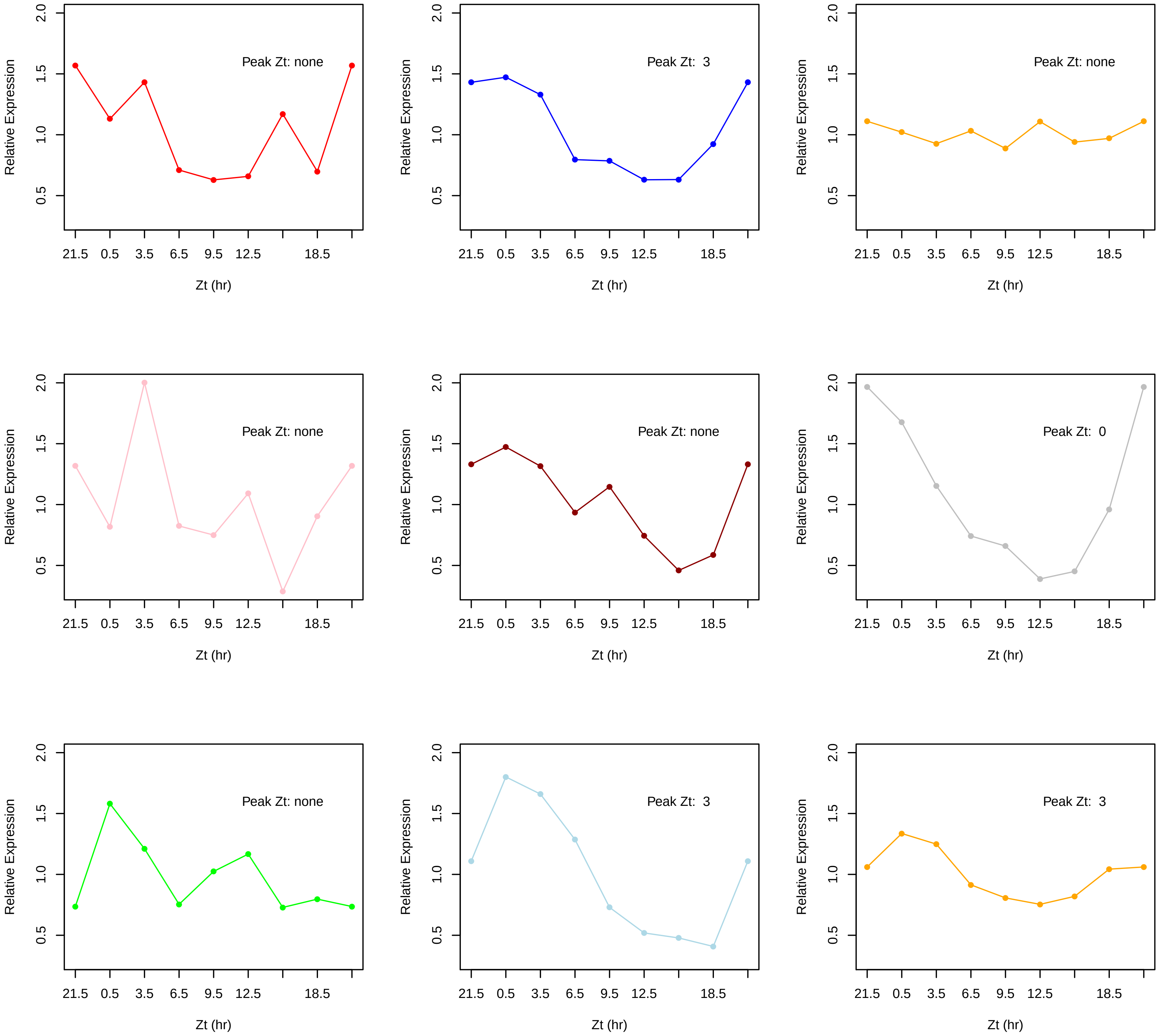

Singeleton: ENSXMAG00000015127 krit1

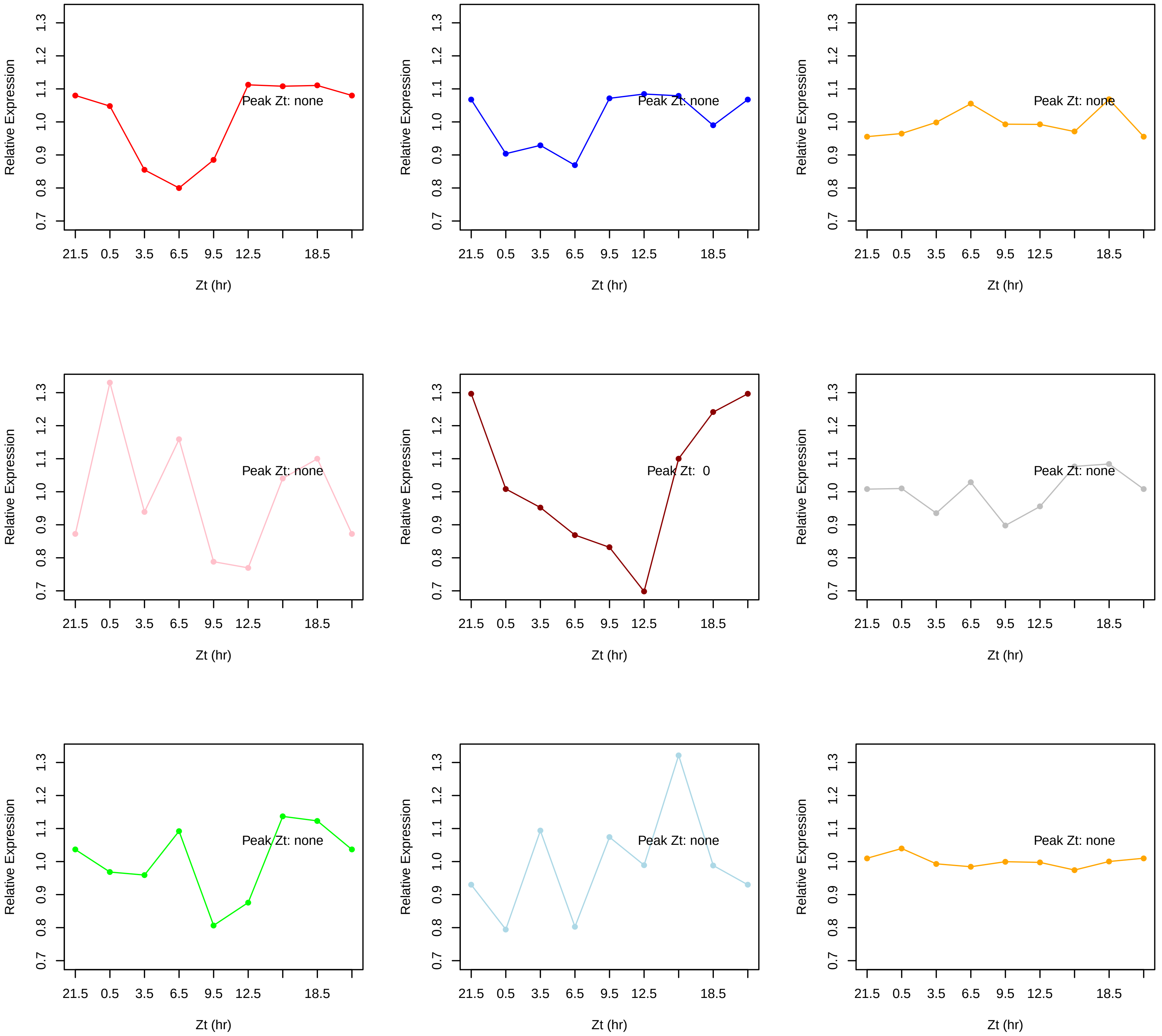

Singeleton: ENSXMAG00000015930 tomm70a

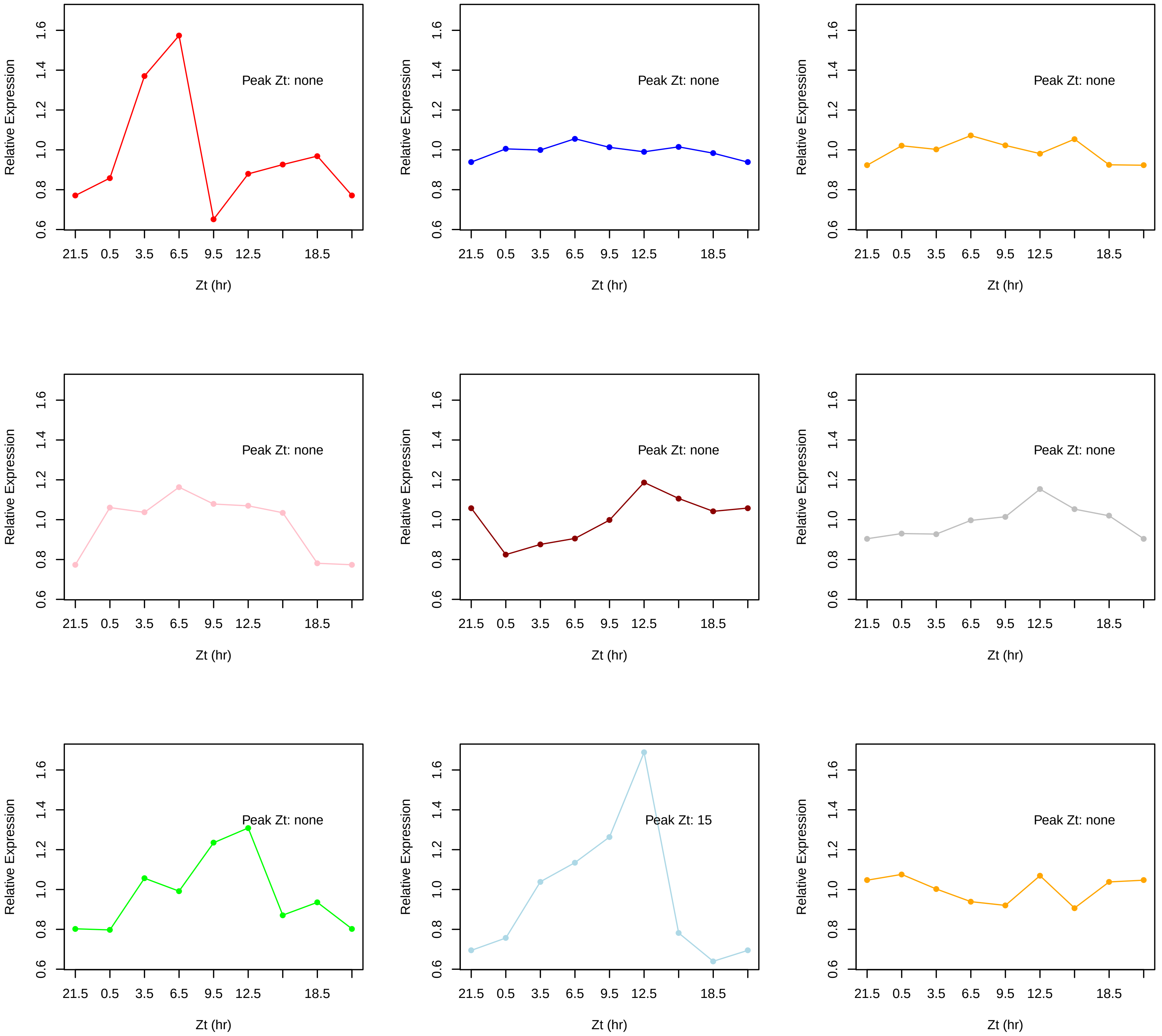

Singeleton: ENSXMAG00000014672

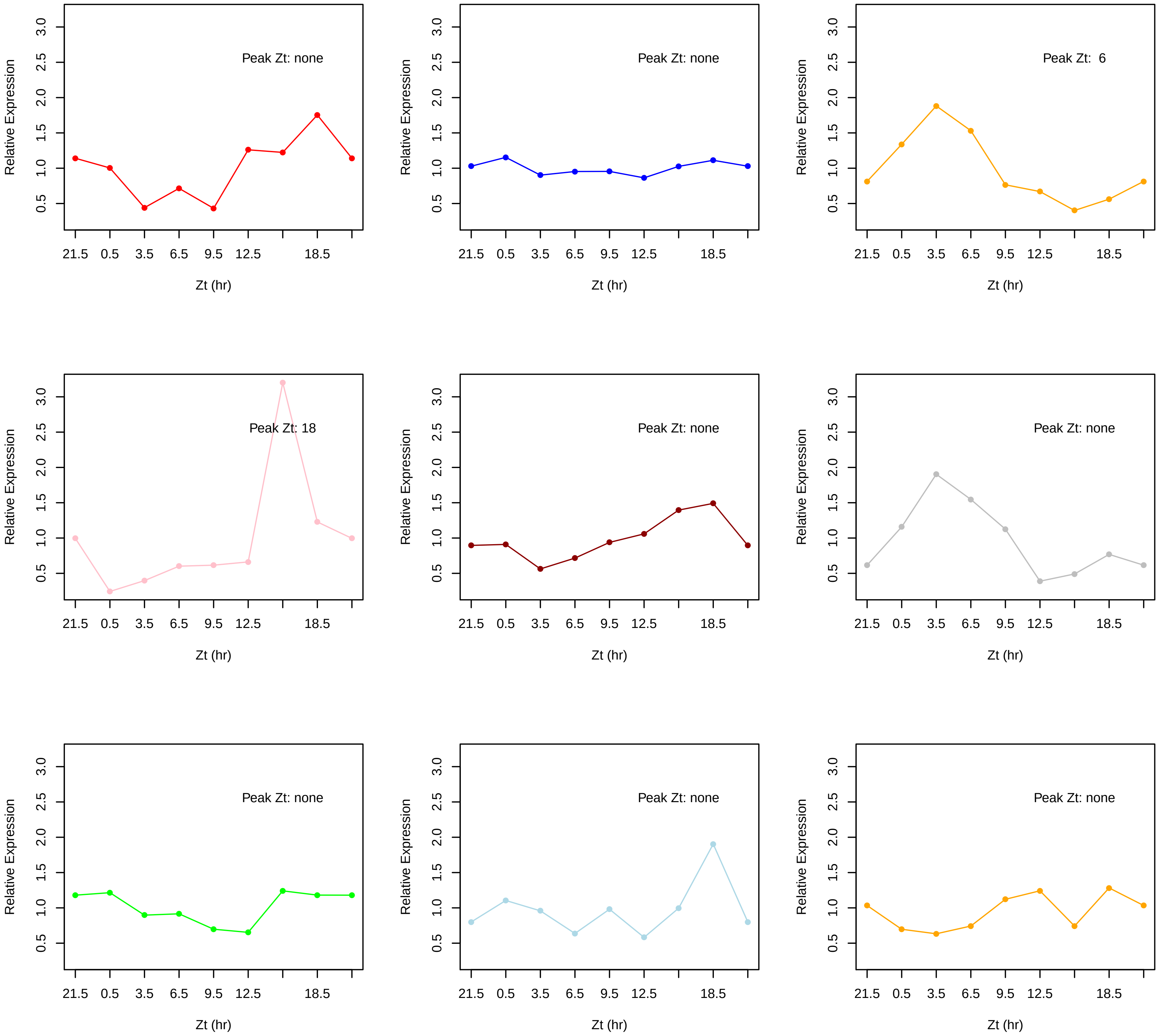

Singeleton: ENSXMAG00000019028 cwc27

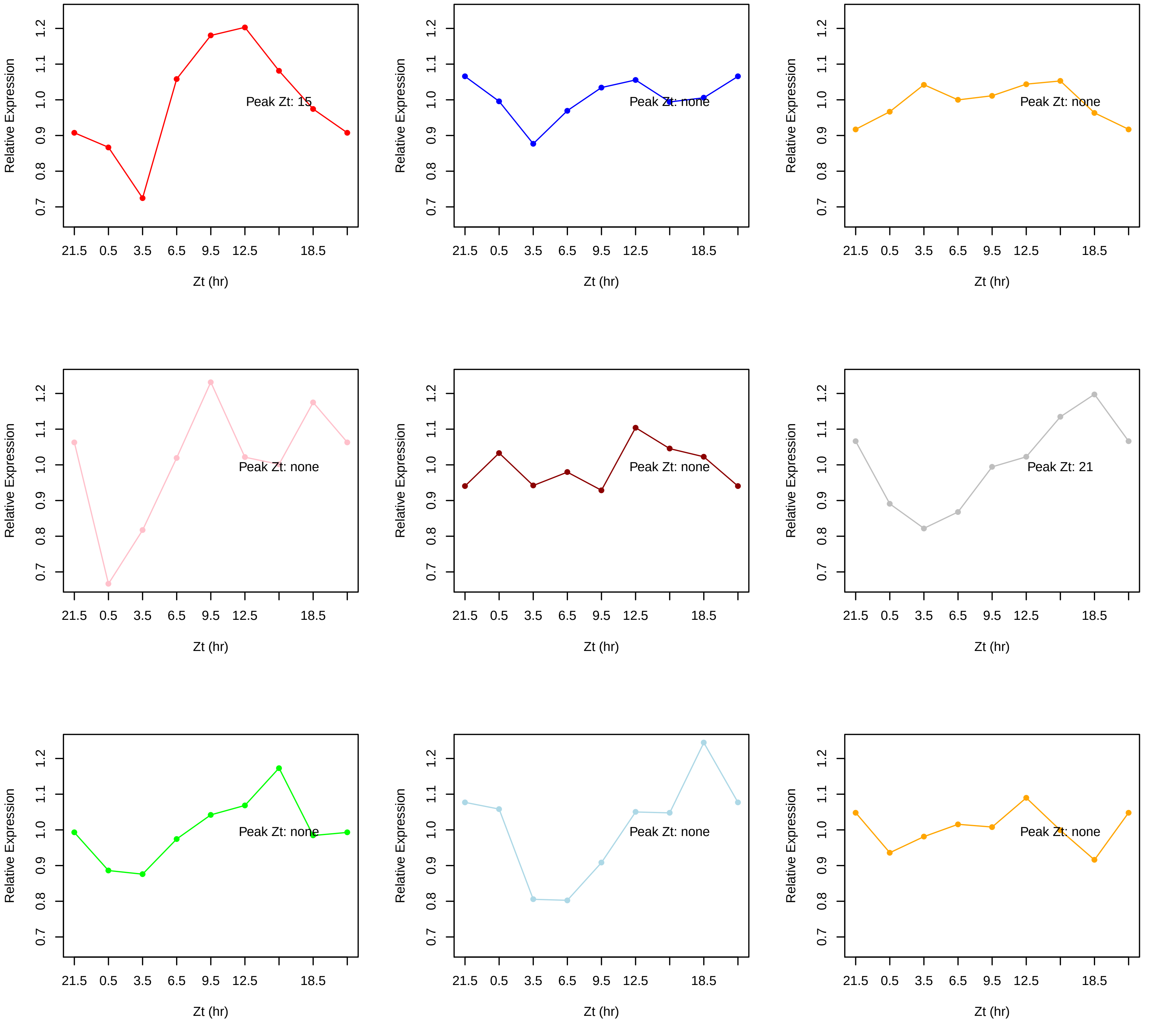

Singleton: ENSXMAG00000019107 si:ch1073−440b2.1

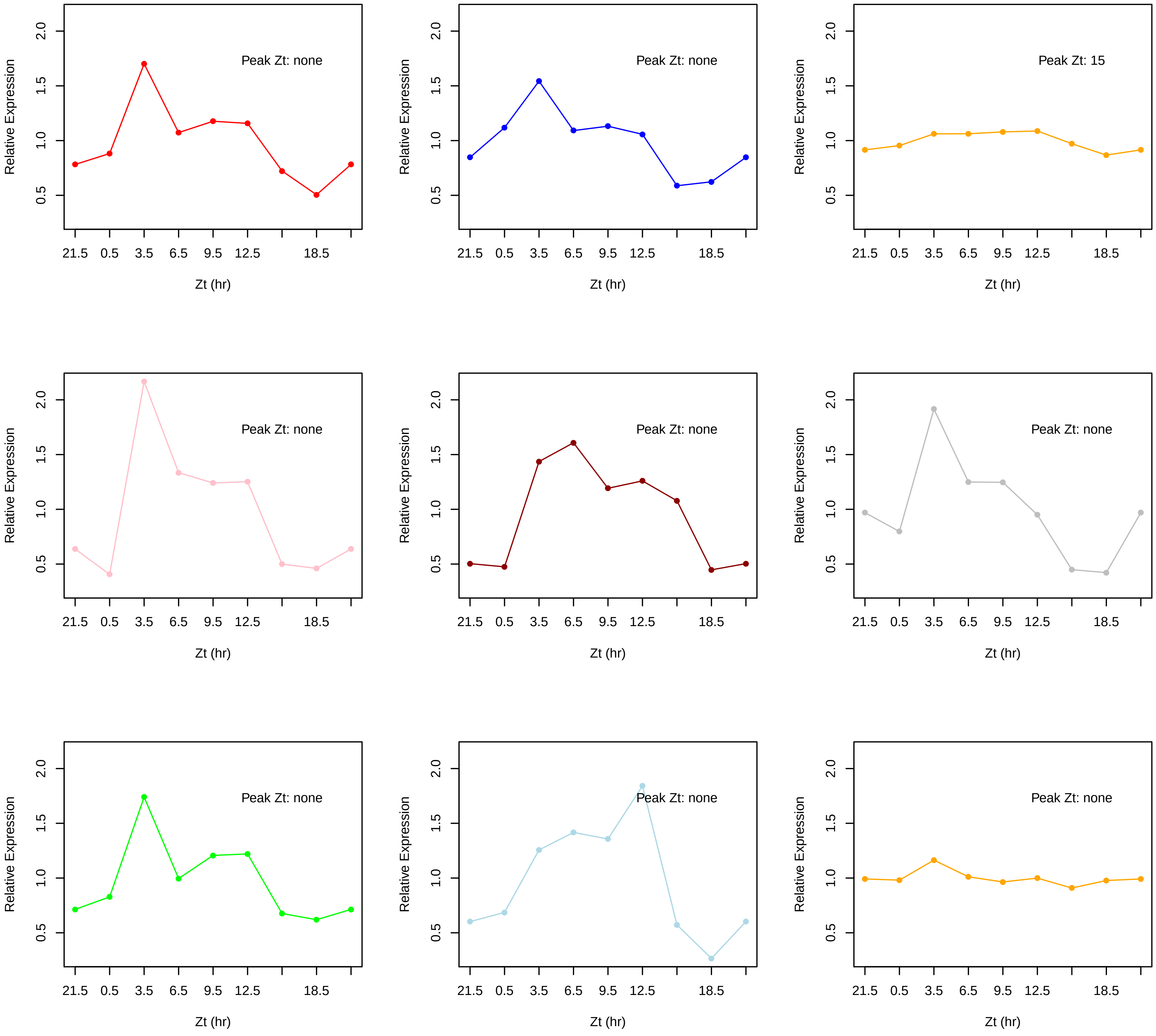

Singeleton: ENSXMAG00000015938 tbc1d23

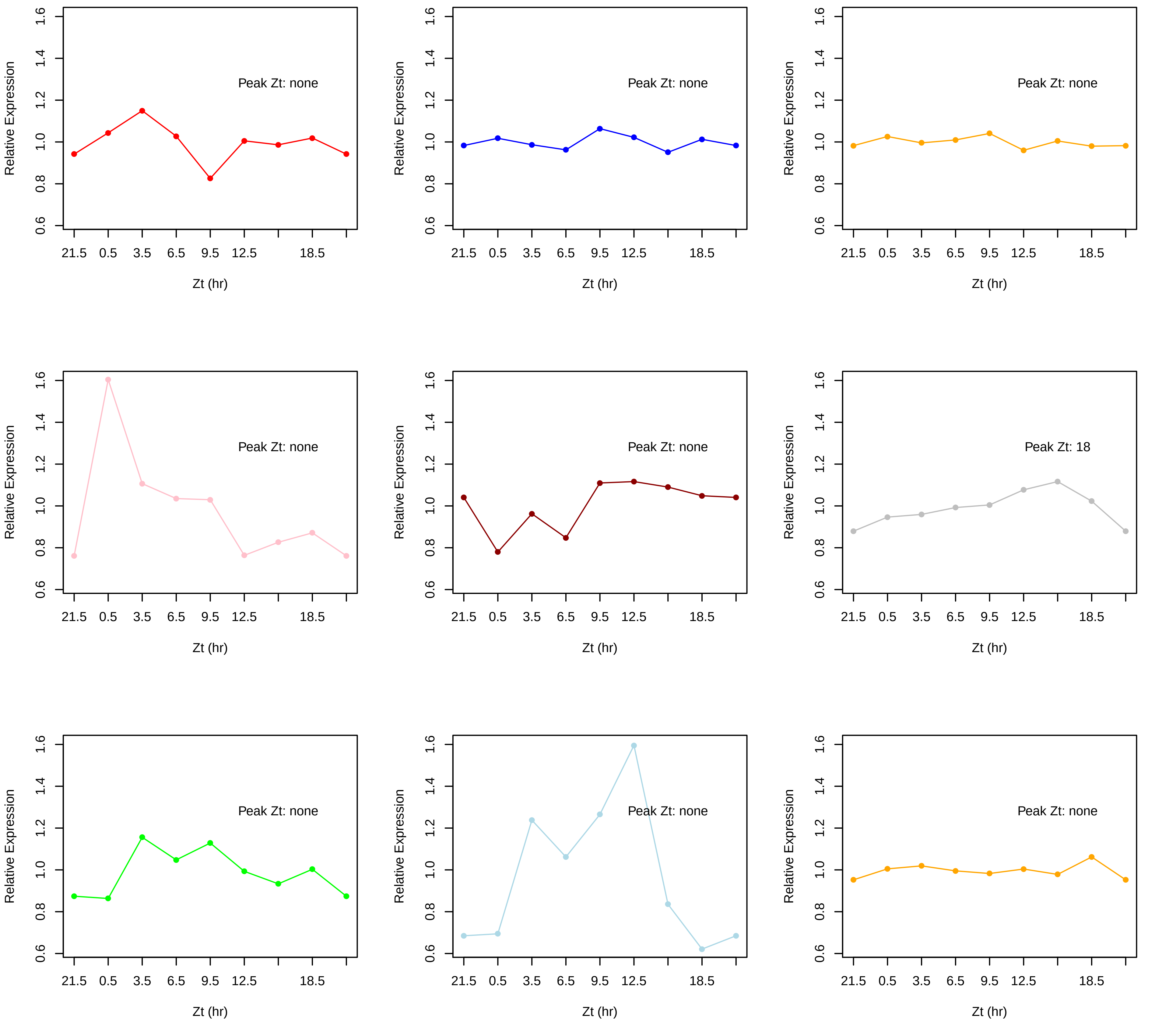

Singeleton: ENSXMAG00000001683 shroom1

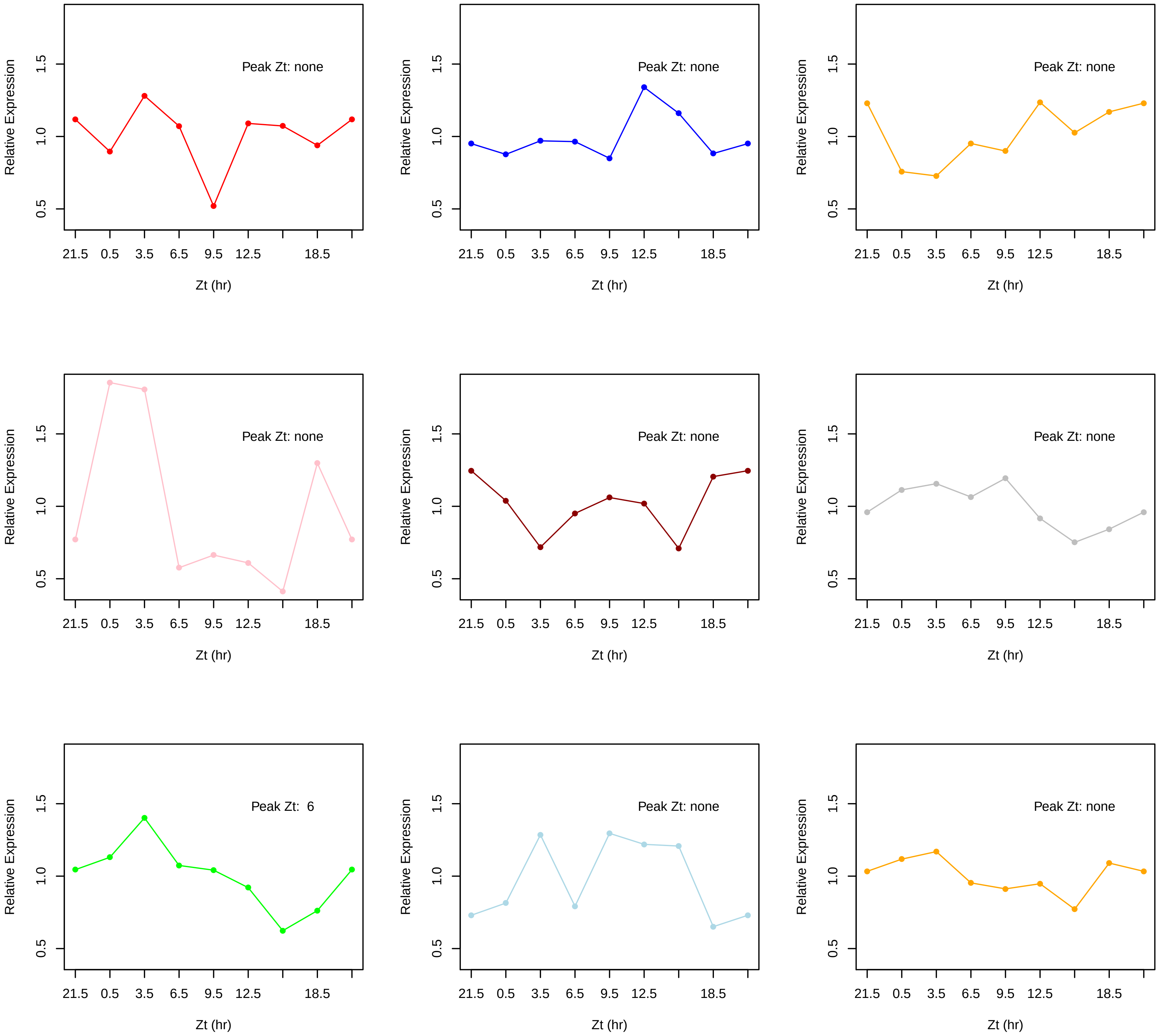

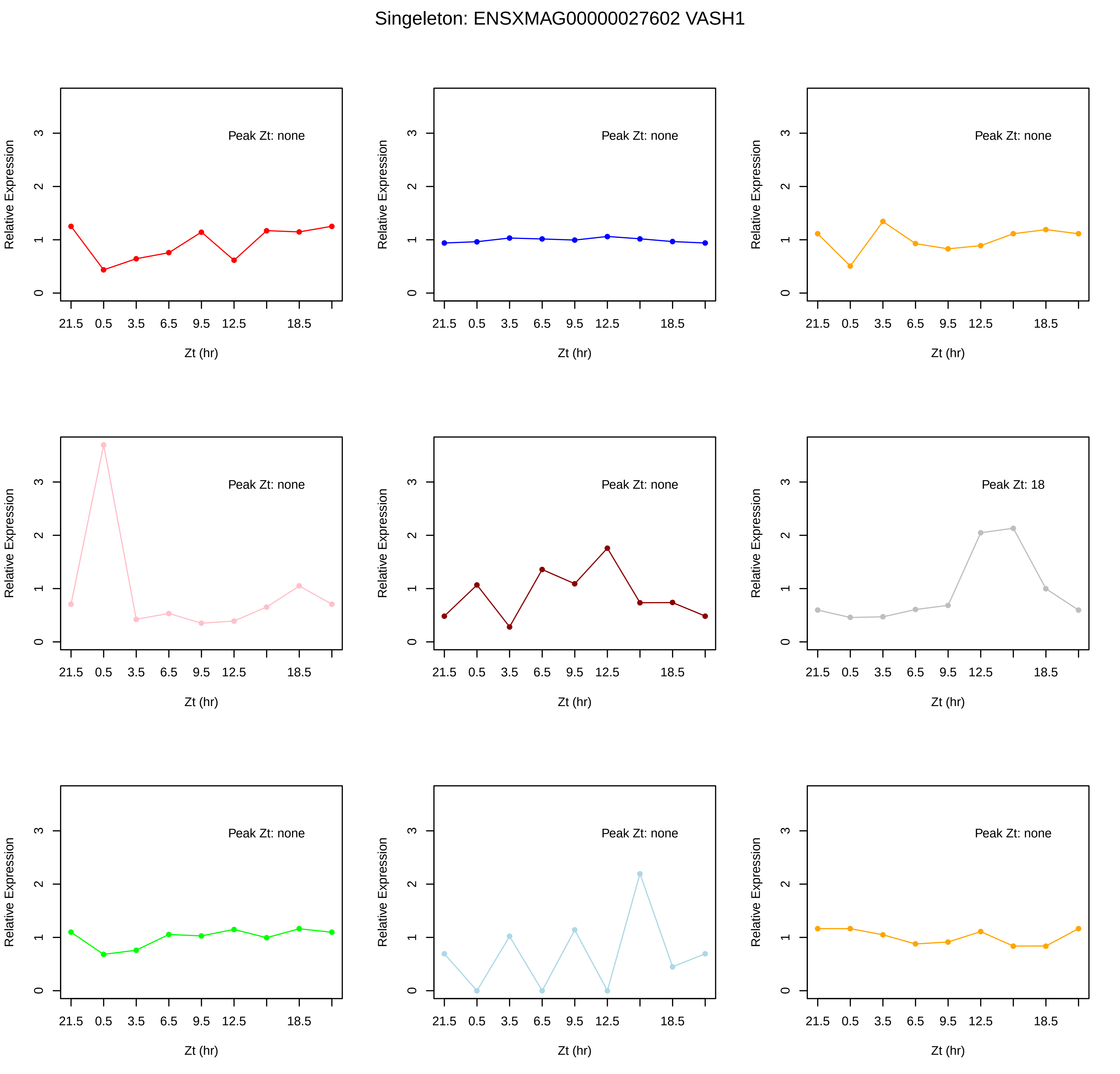
Singeleton: ENSXMAG00000027602 VASH1
Peak Zt: none
Peak Zt: none
Peak Zt: none
Peak Zt: none
Peak Zt: none
Peak Zt: 18
Peak Zt: none
Peak Zt: none
Peak Zt: none
Relative Expression
Zt (hr)
0
1
2
3
21.5
0.5
3.5
6.5
9.5
12.5
18.5

Singeleton: ENSXMAG00000008136 jade1

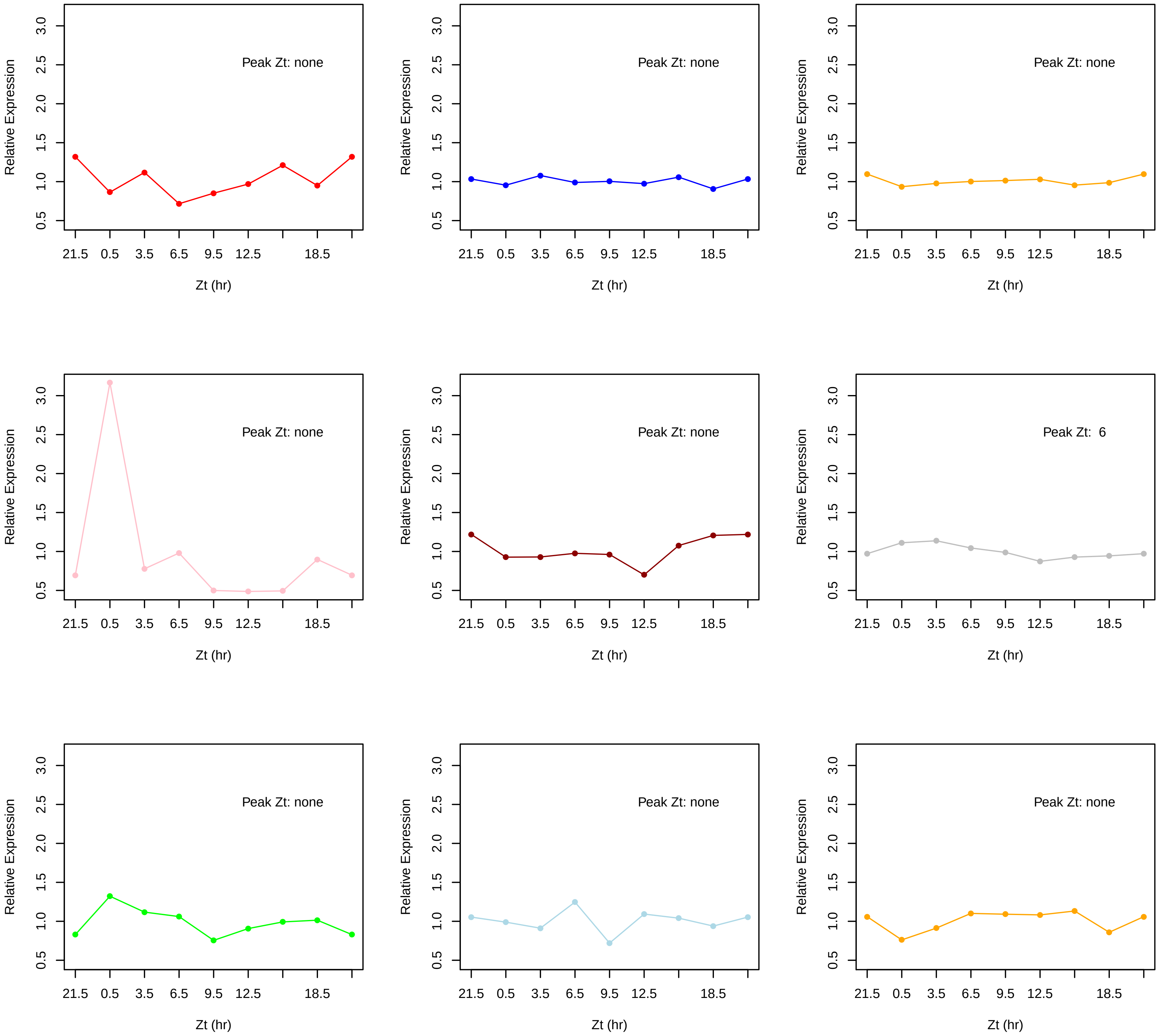

Singeleton: ENSXMAG00000014605 angel1

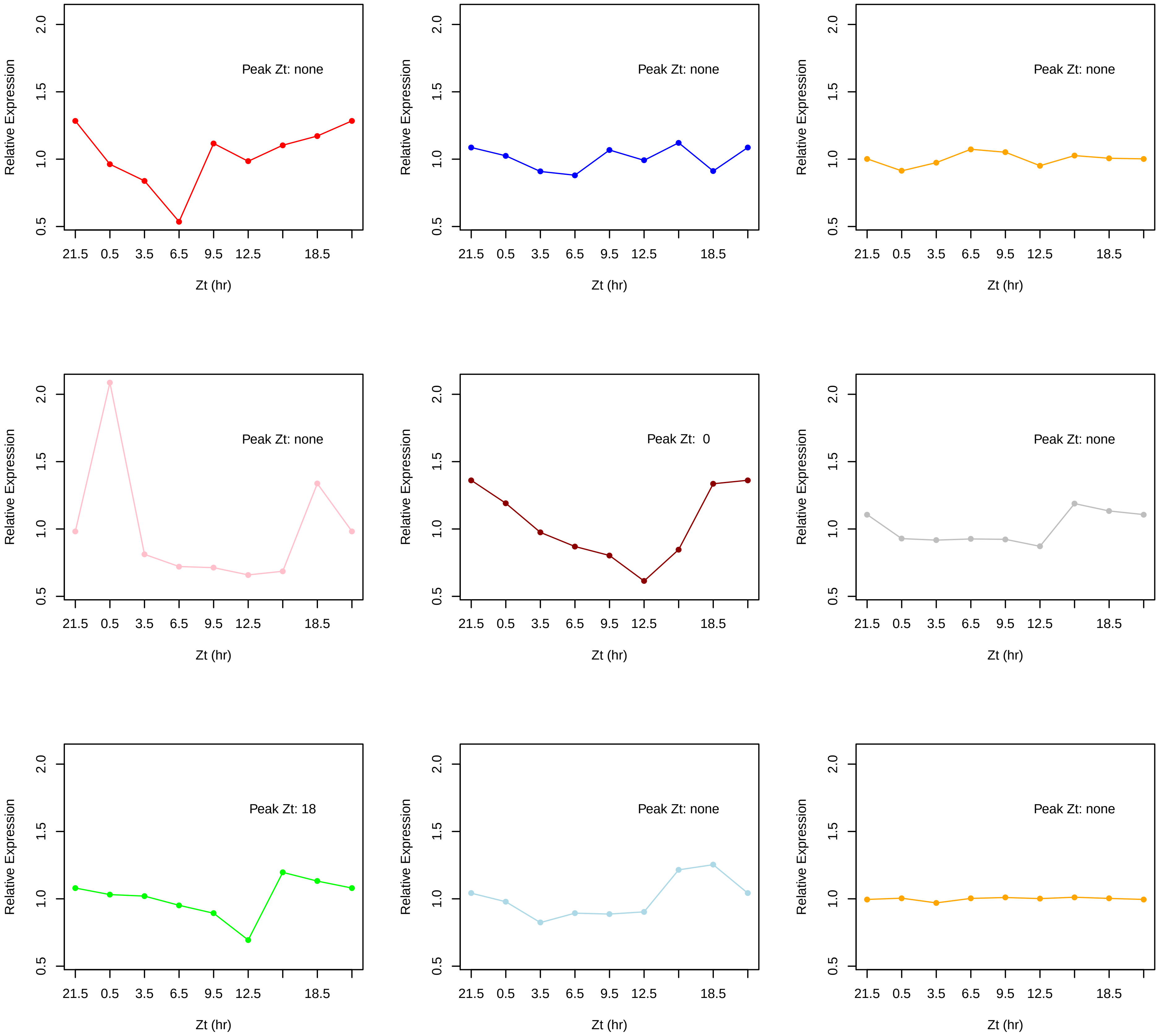

Singeleton: ENSXMAG00000001535 cav3

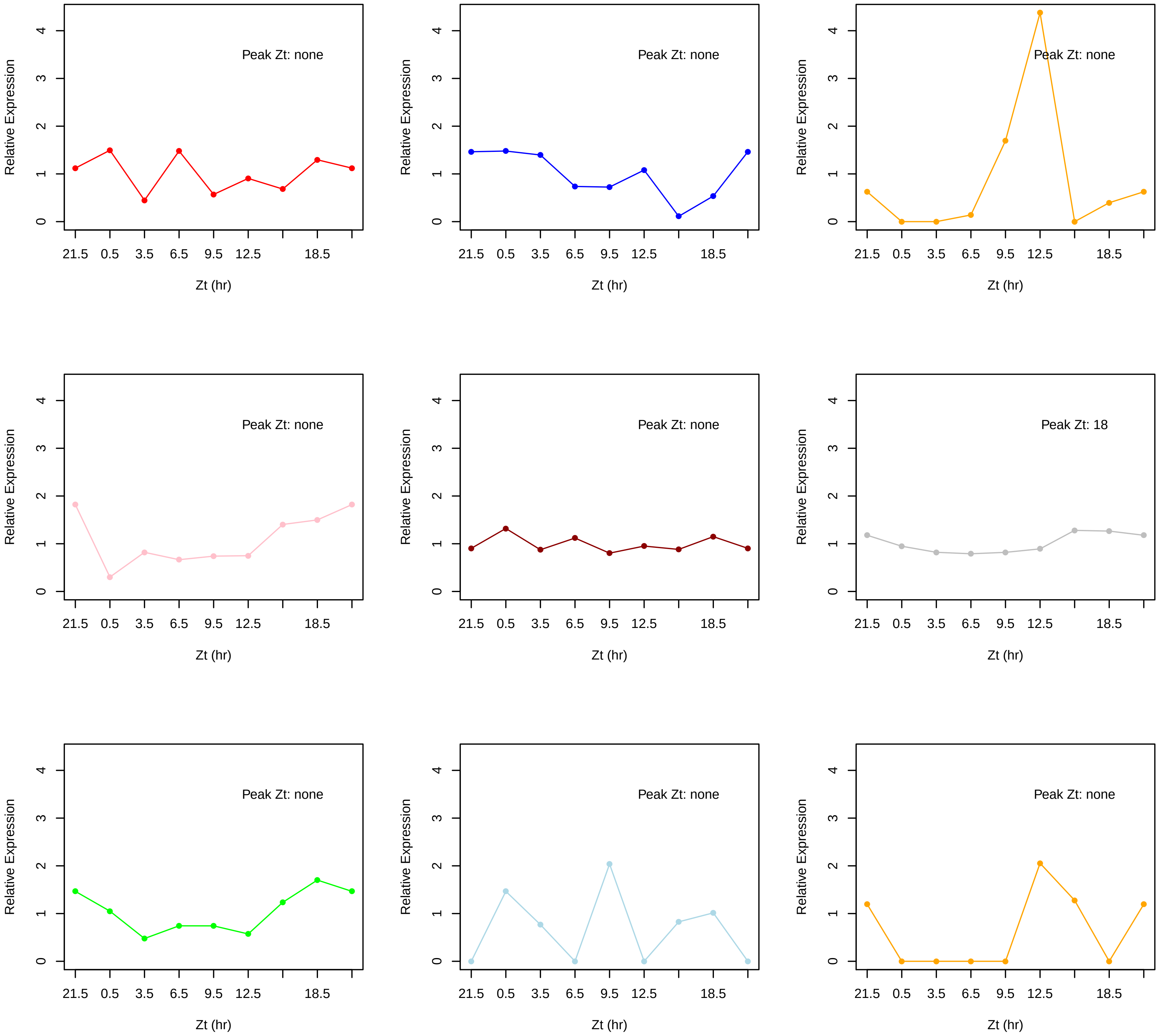

Singeleton: ENSXMAG00000017308 reck

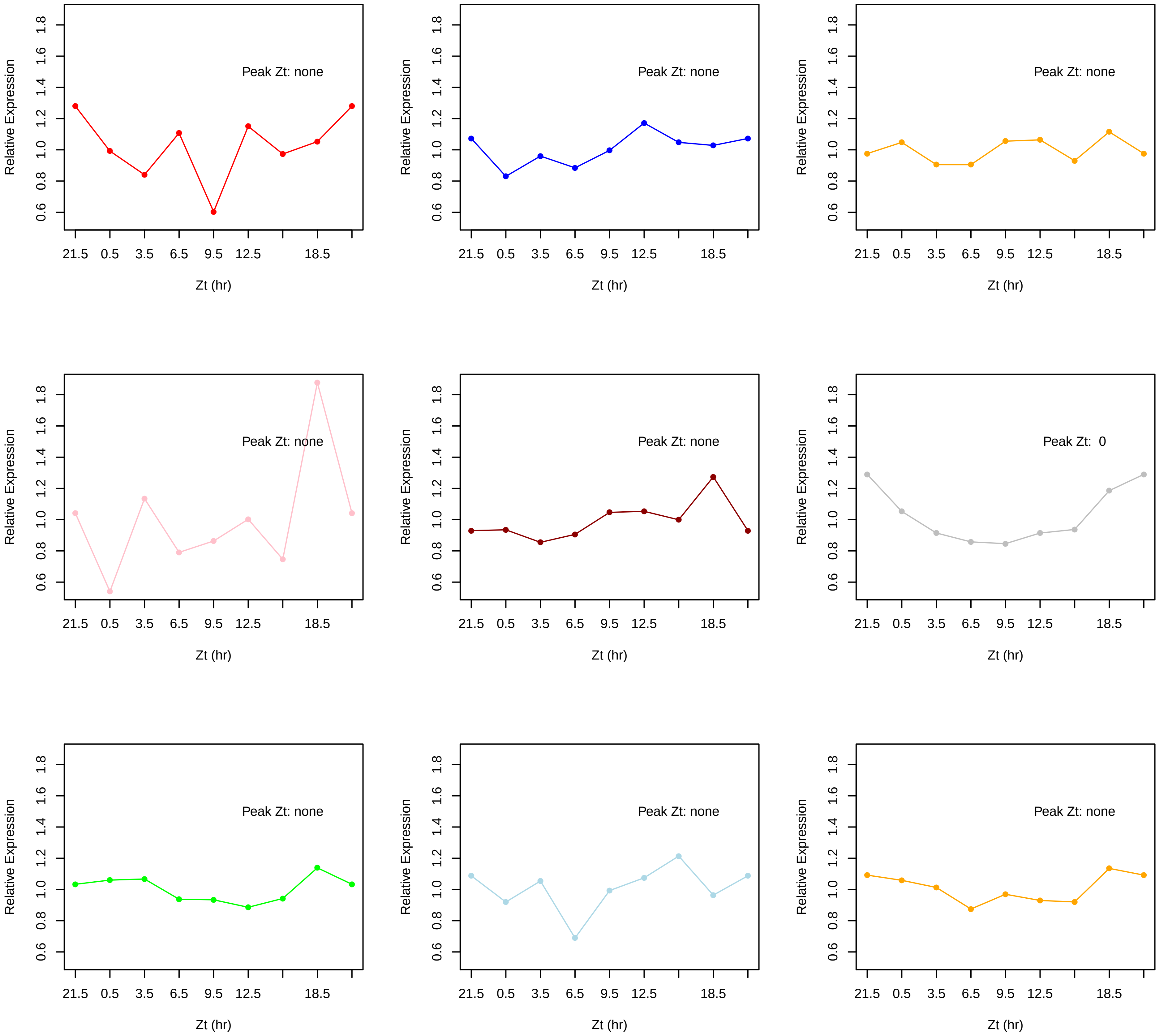

Singeleton: ENSXMAG00000015972 nit2

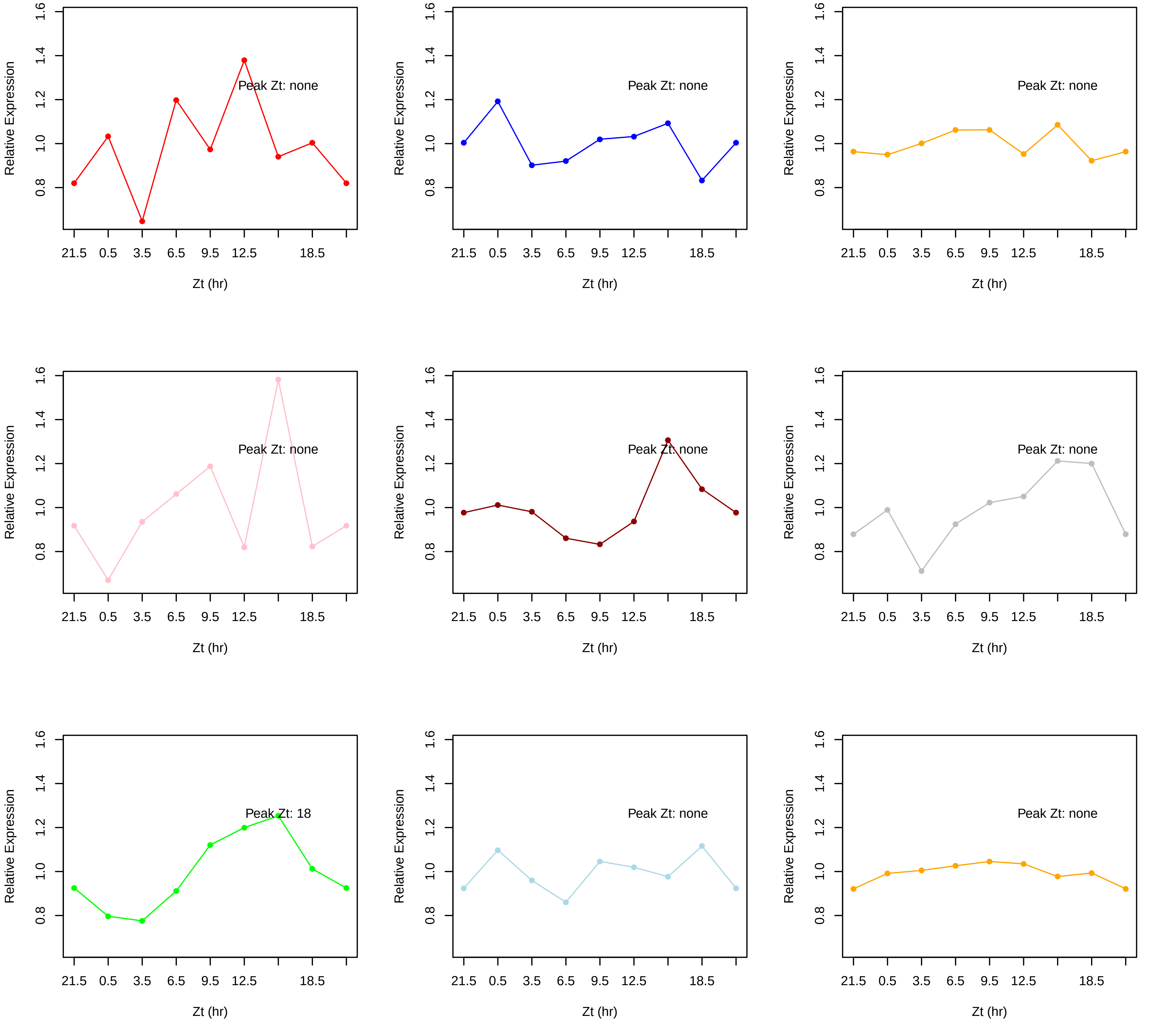

Singleton: ENSXMAG00000009925 asmt2

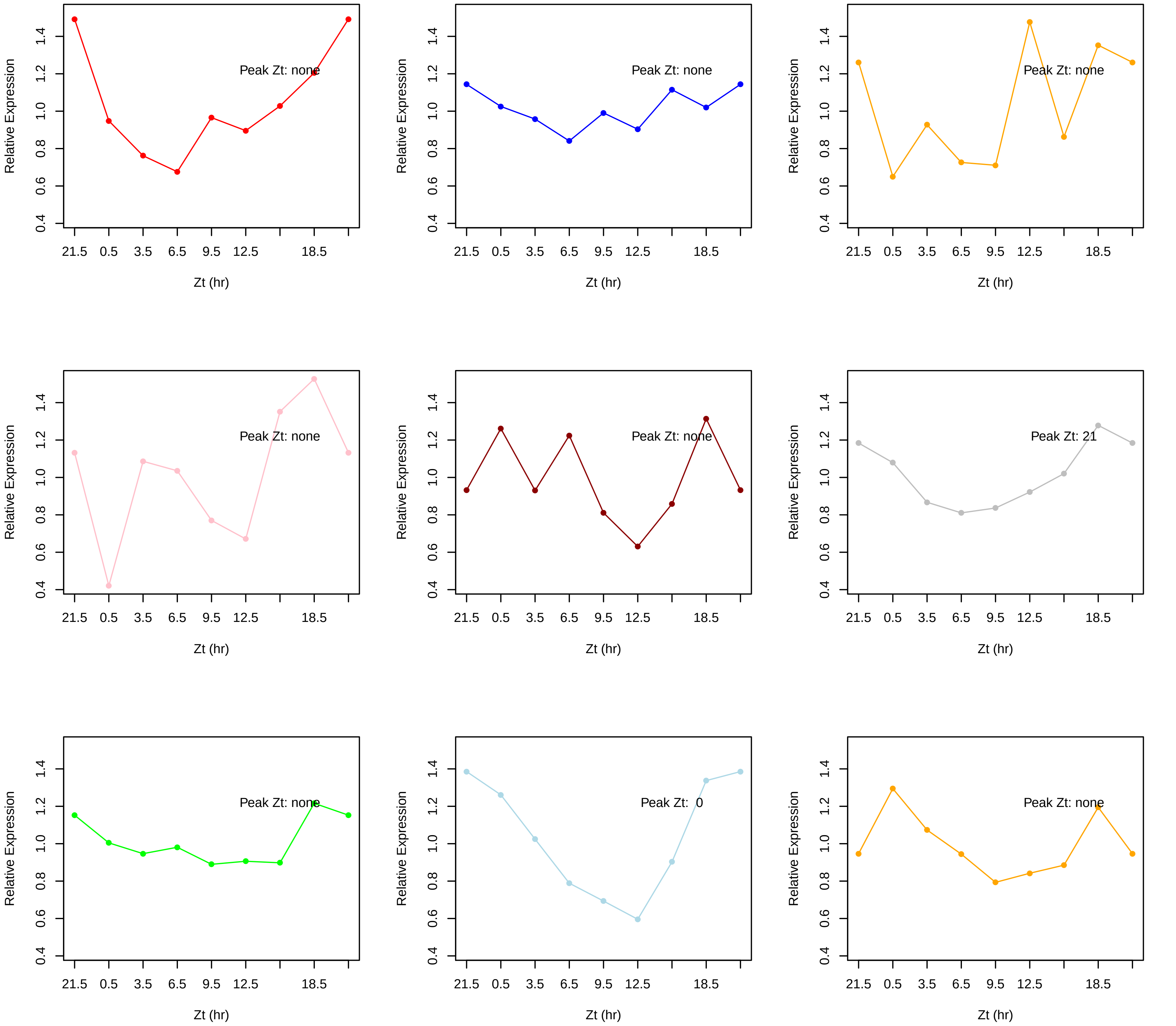

Singeleton: ENSXMAG00000001709 ccni2

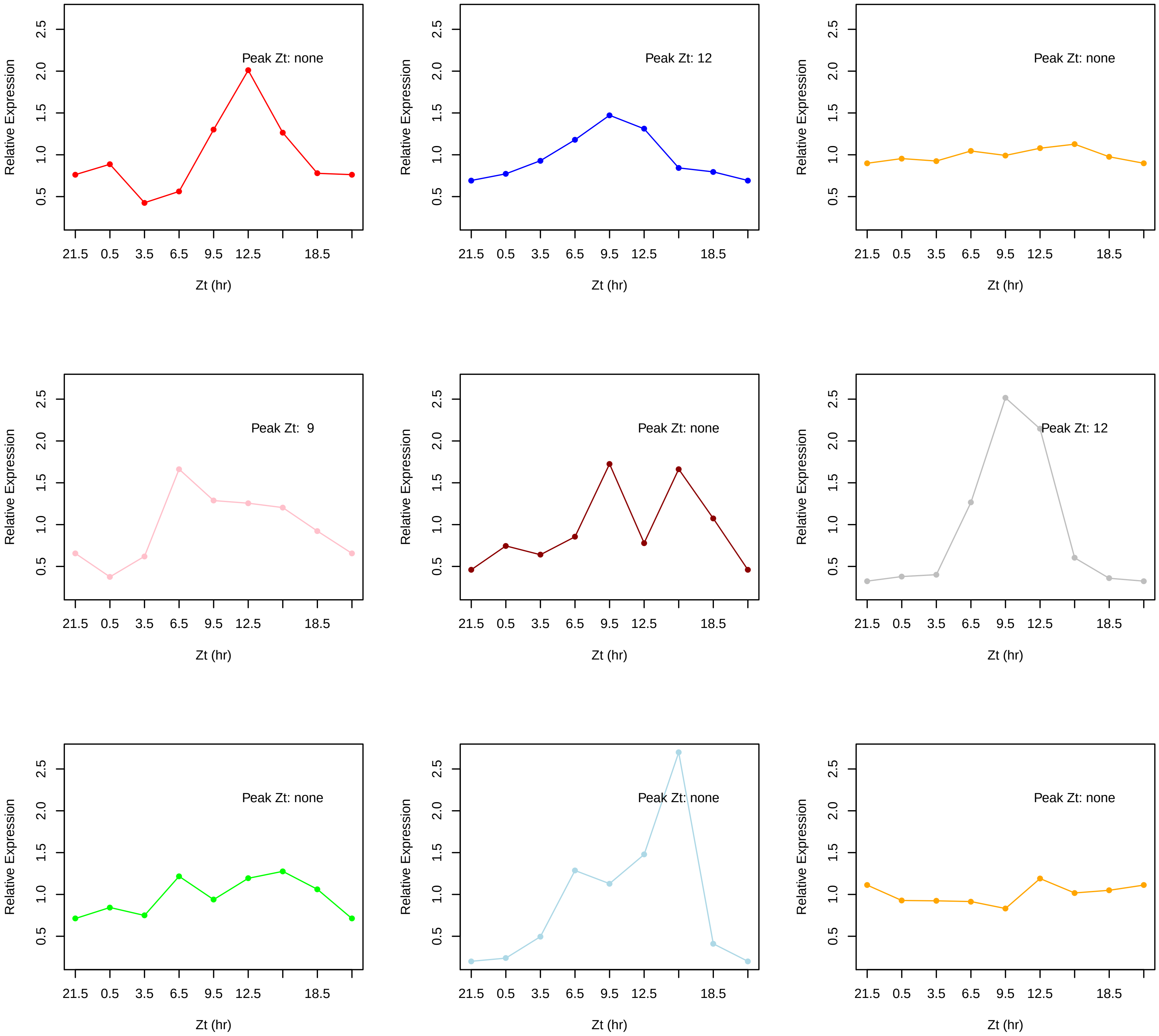

Singeleton: ENSXMAG00000016308 rrp9

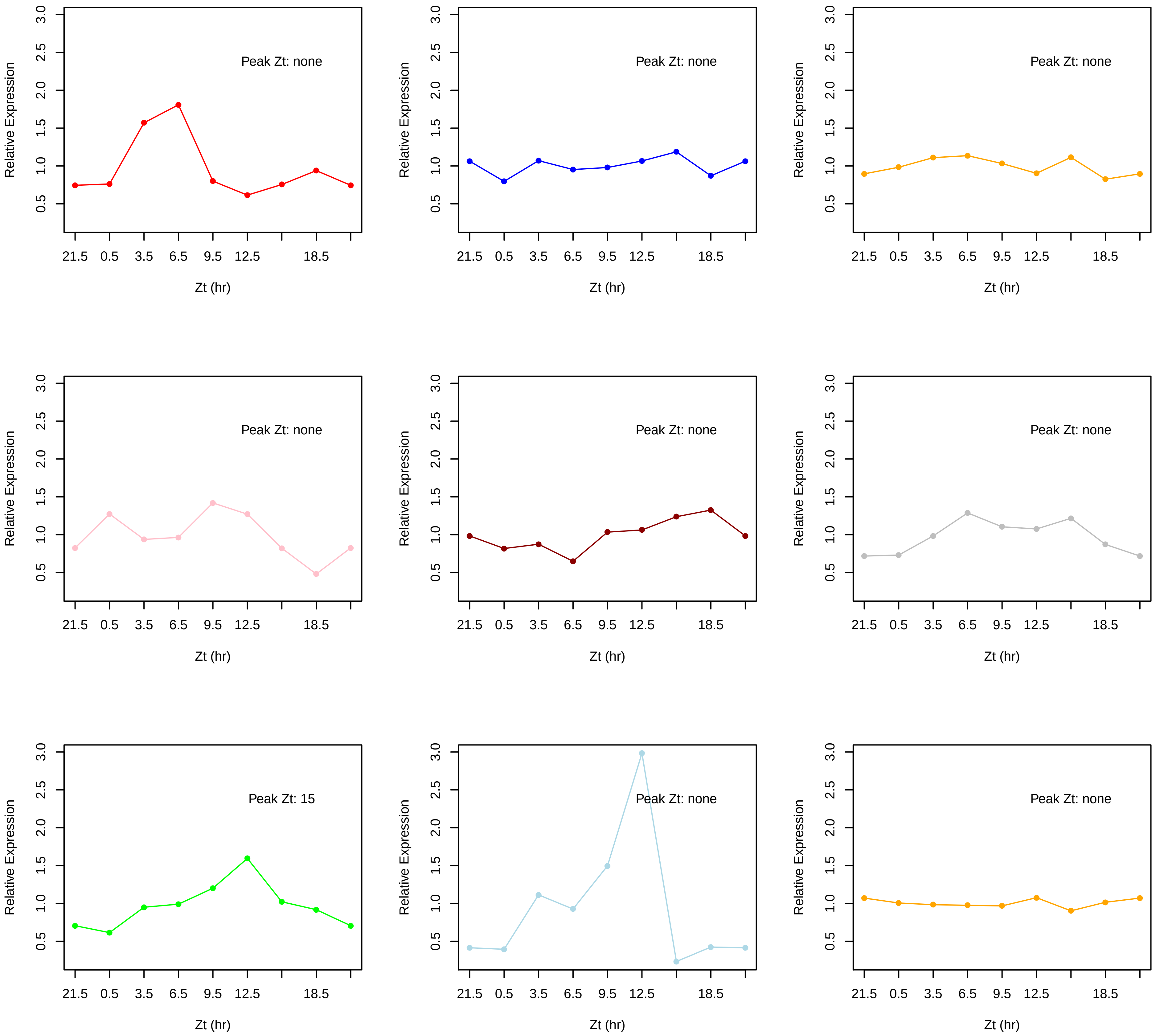

Singleton: ENSXMAG00000009248 ccng1

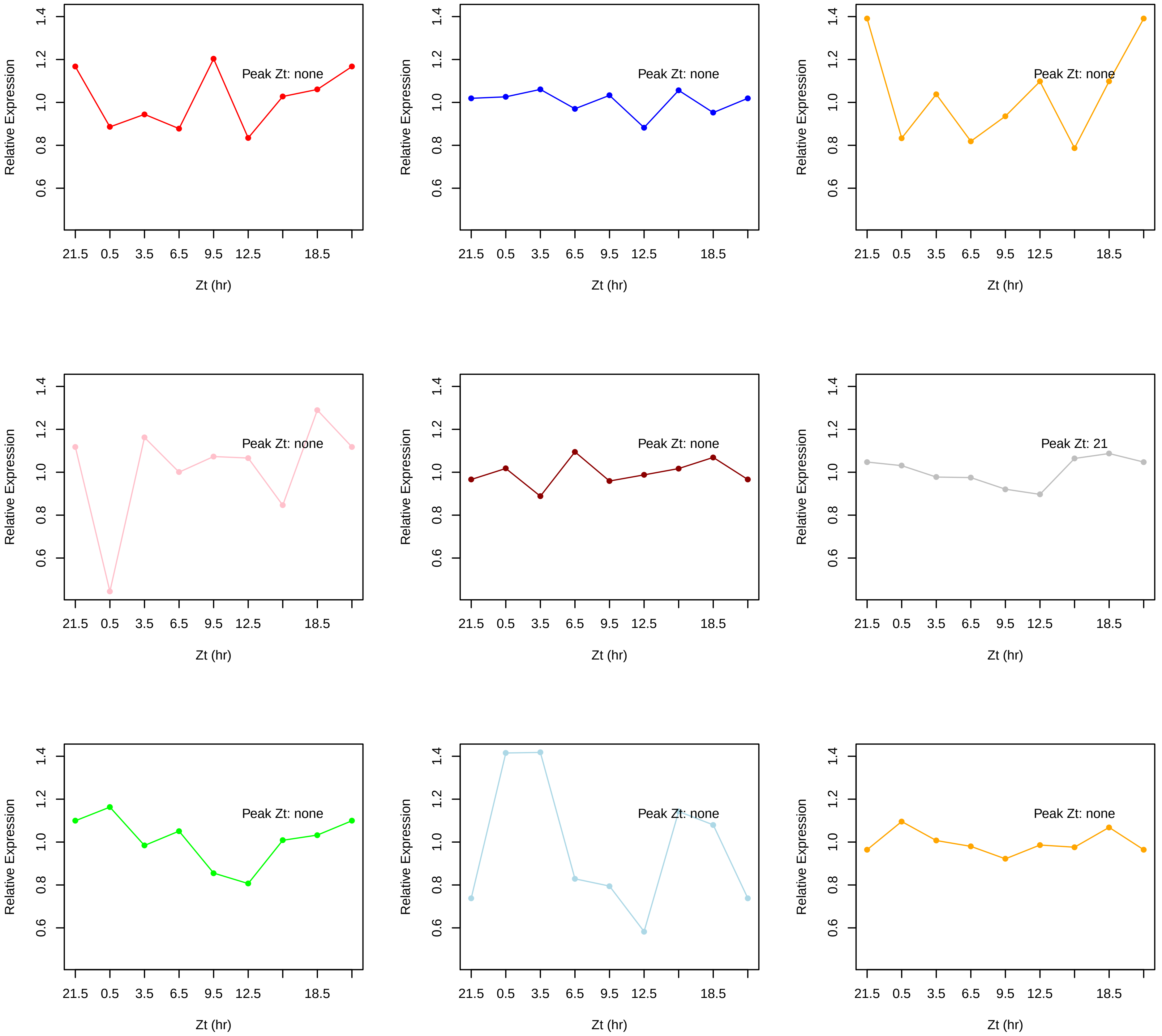

Singleton: ENSXMAG00000003874 tmem183a

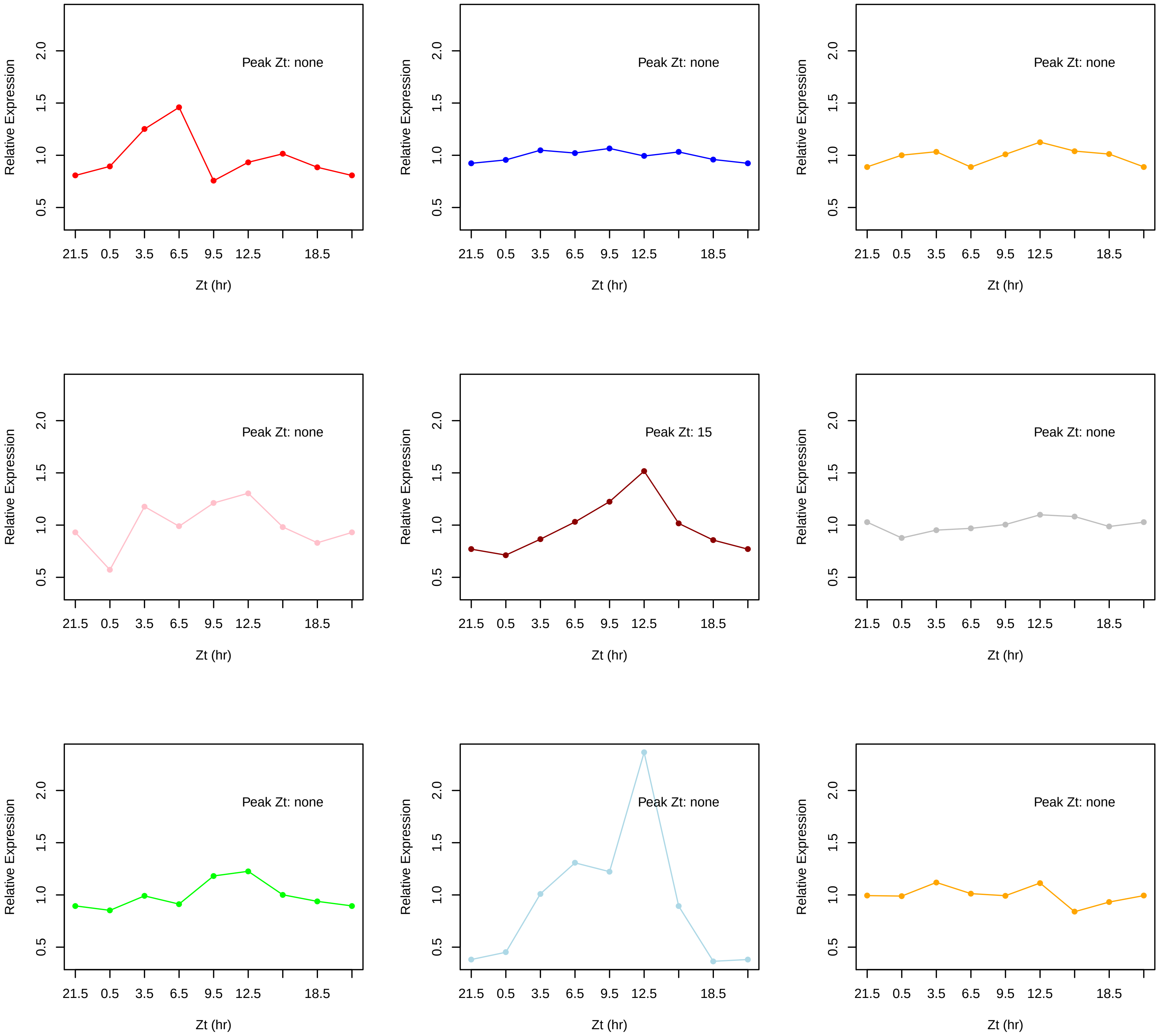

Singleton: ENSXMAG00000003883 ppfia4

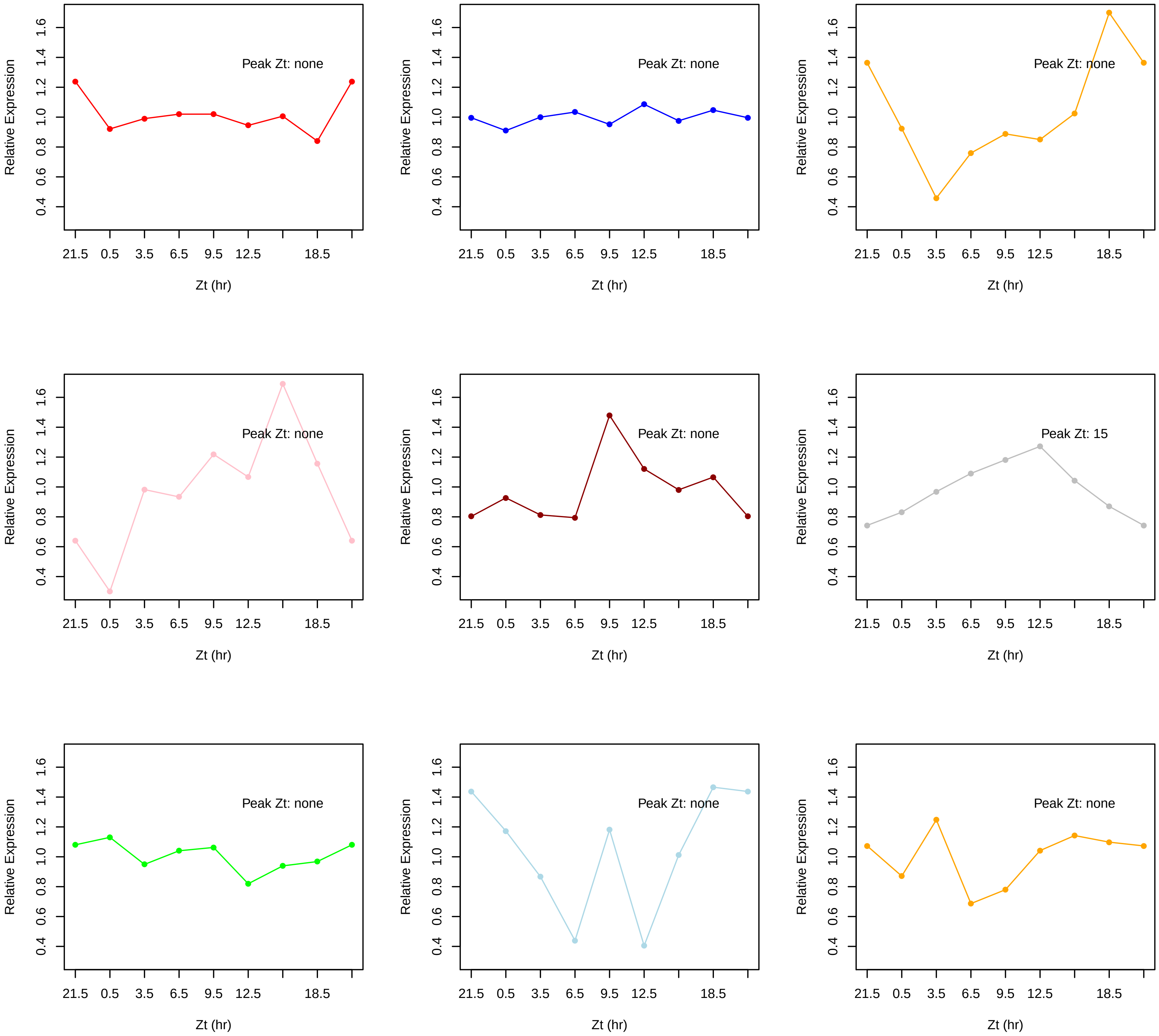

Singleton: ENSXMAG00000001711 GABRG2

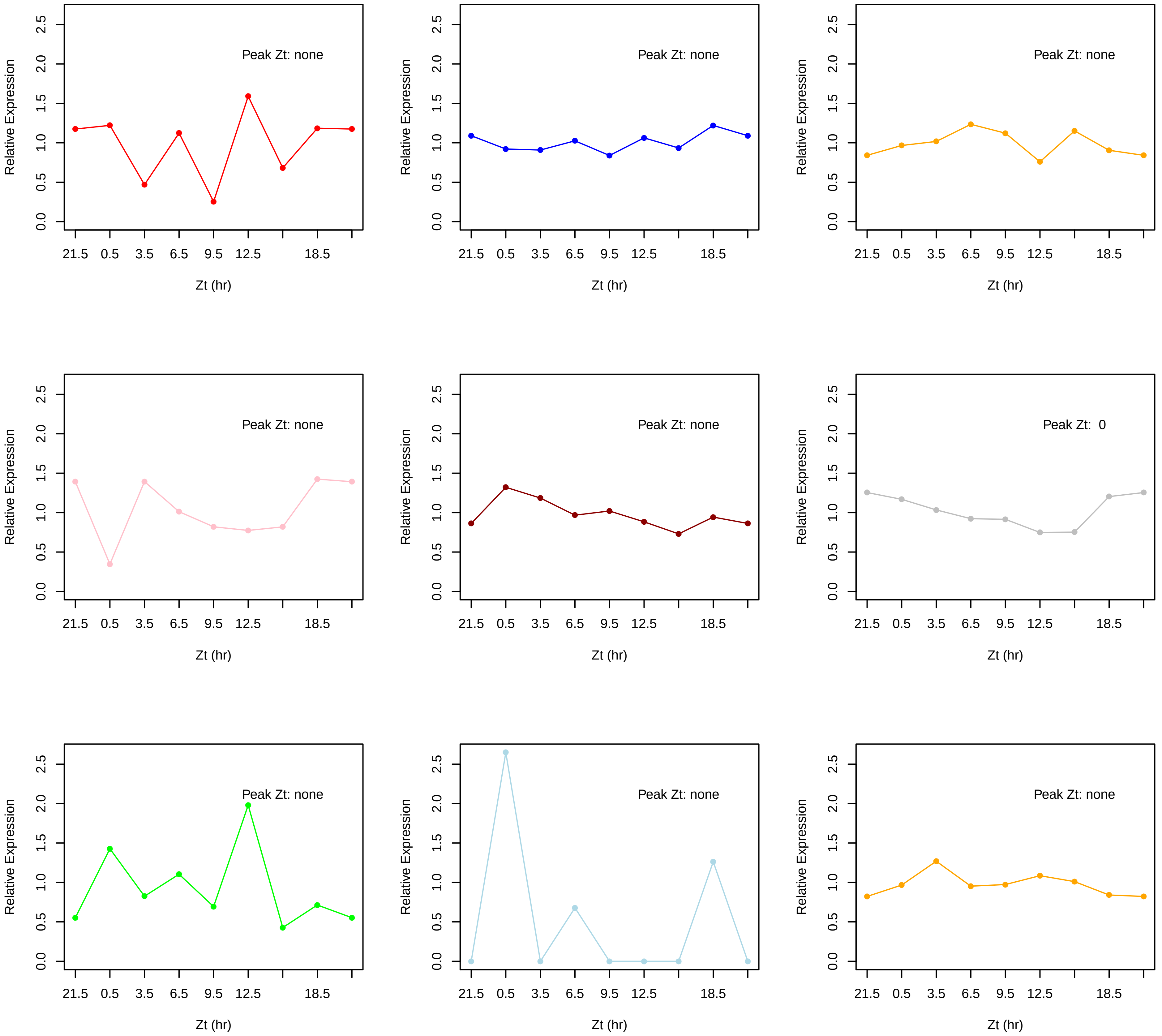

Singeleton: ENSXMAG00000019078

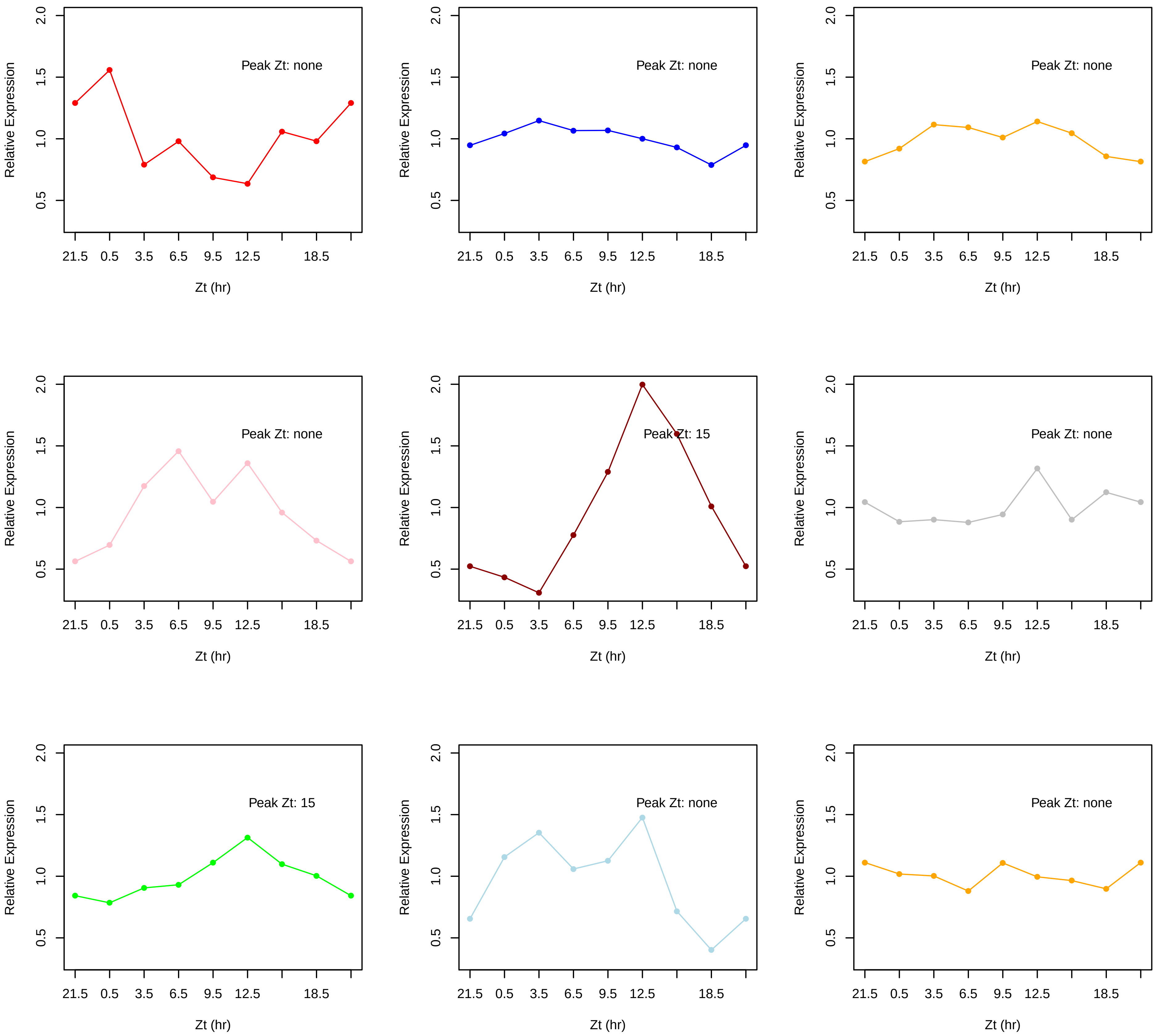

Singeleton: ENSXMAG00000012556 mfsd8

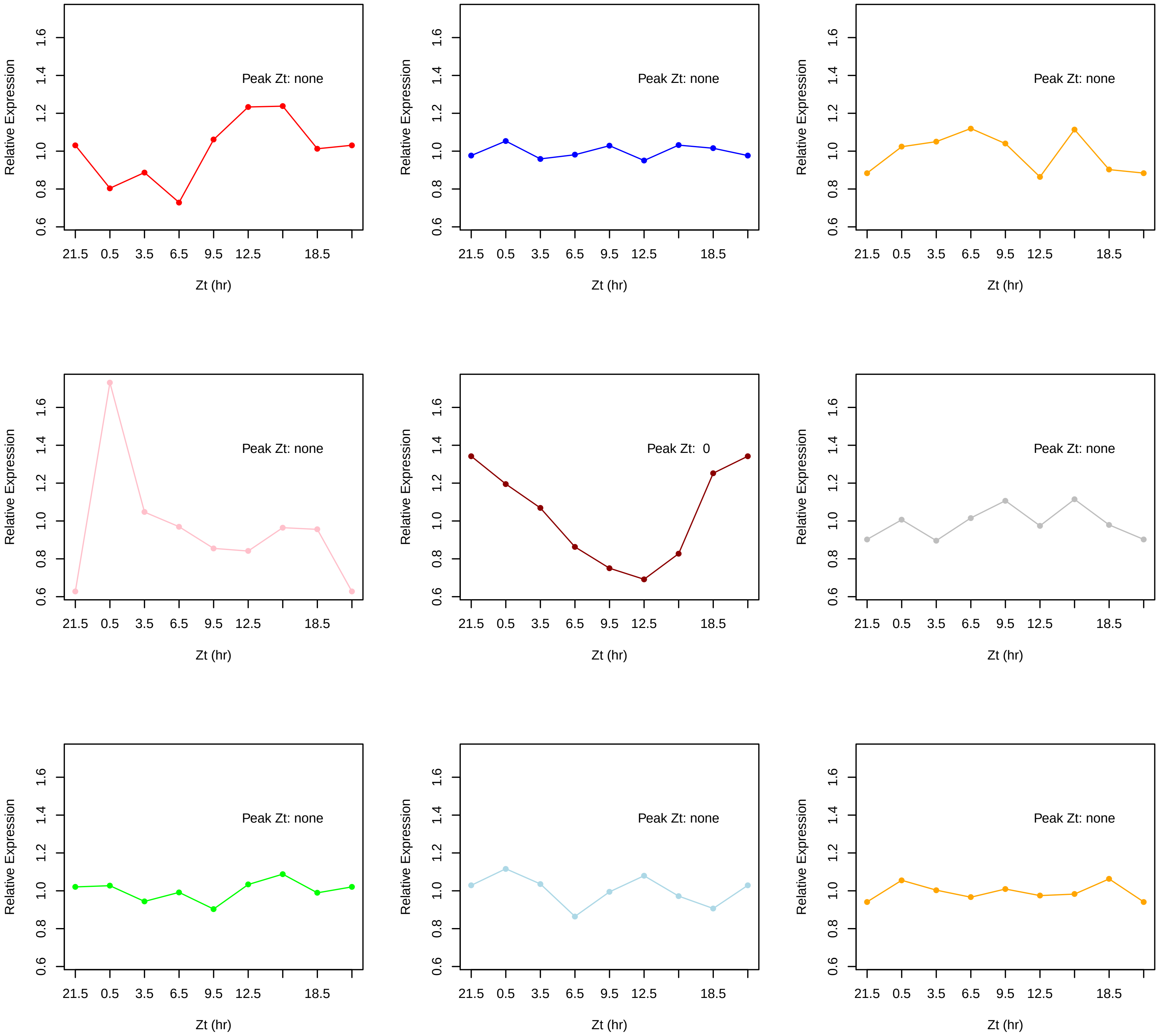

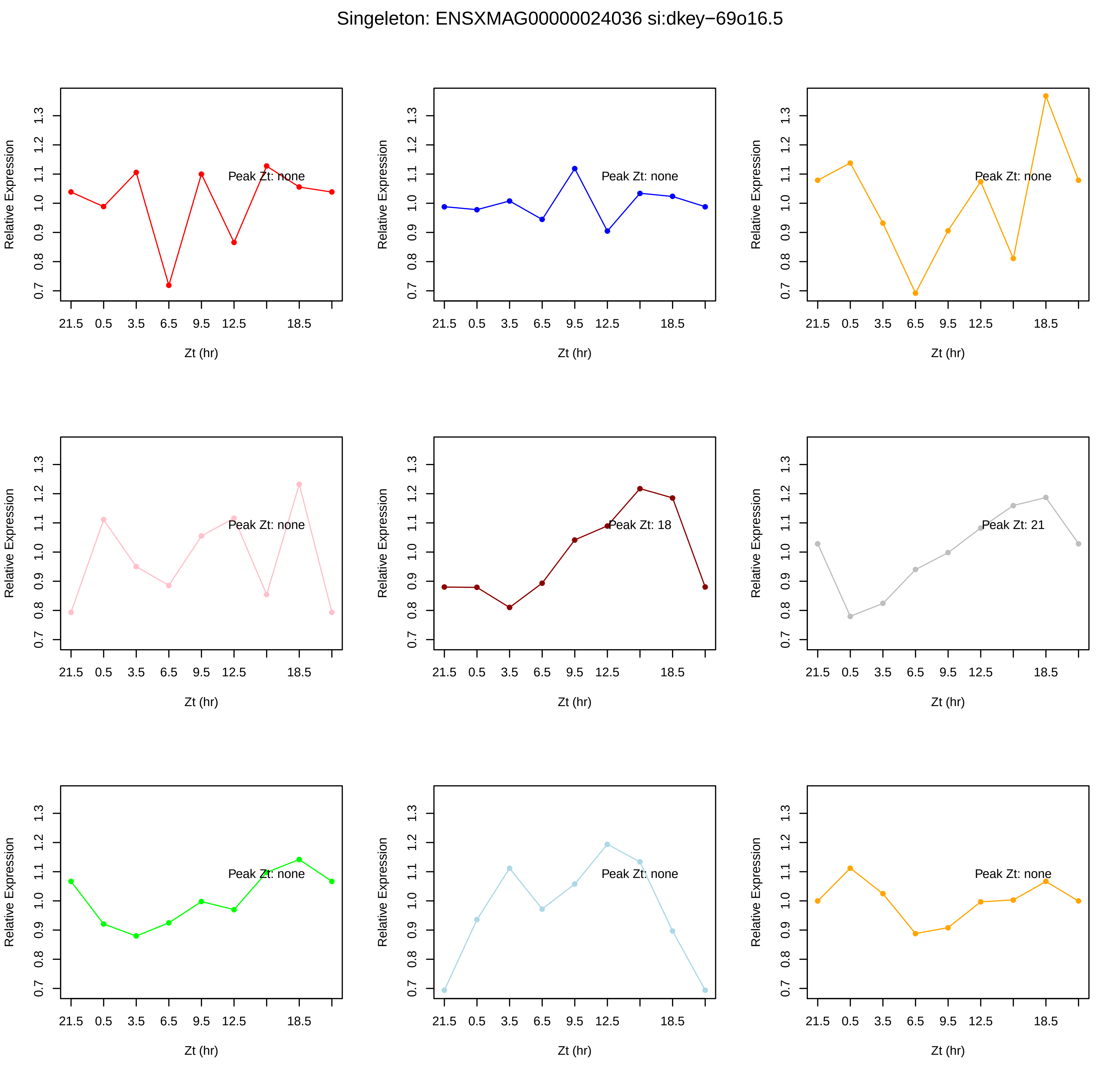

Singeleton: ENSXMAG00000024036 si:dkey−69o16.5
Peak Zt: none
Peak Zt: none
Peak Zt: none
Peak Zt: none
Peak Zt: 18
Peak Zt: 21
Peak Zt: none
Peak Zt: none
Peak Zt: none
Relative Expression
Zt (hr)
21.5
0.5
3.5
6.5
9.5
12.5
18.5

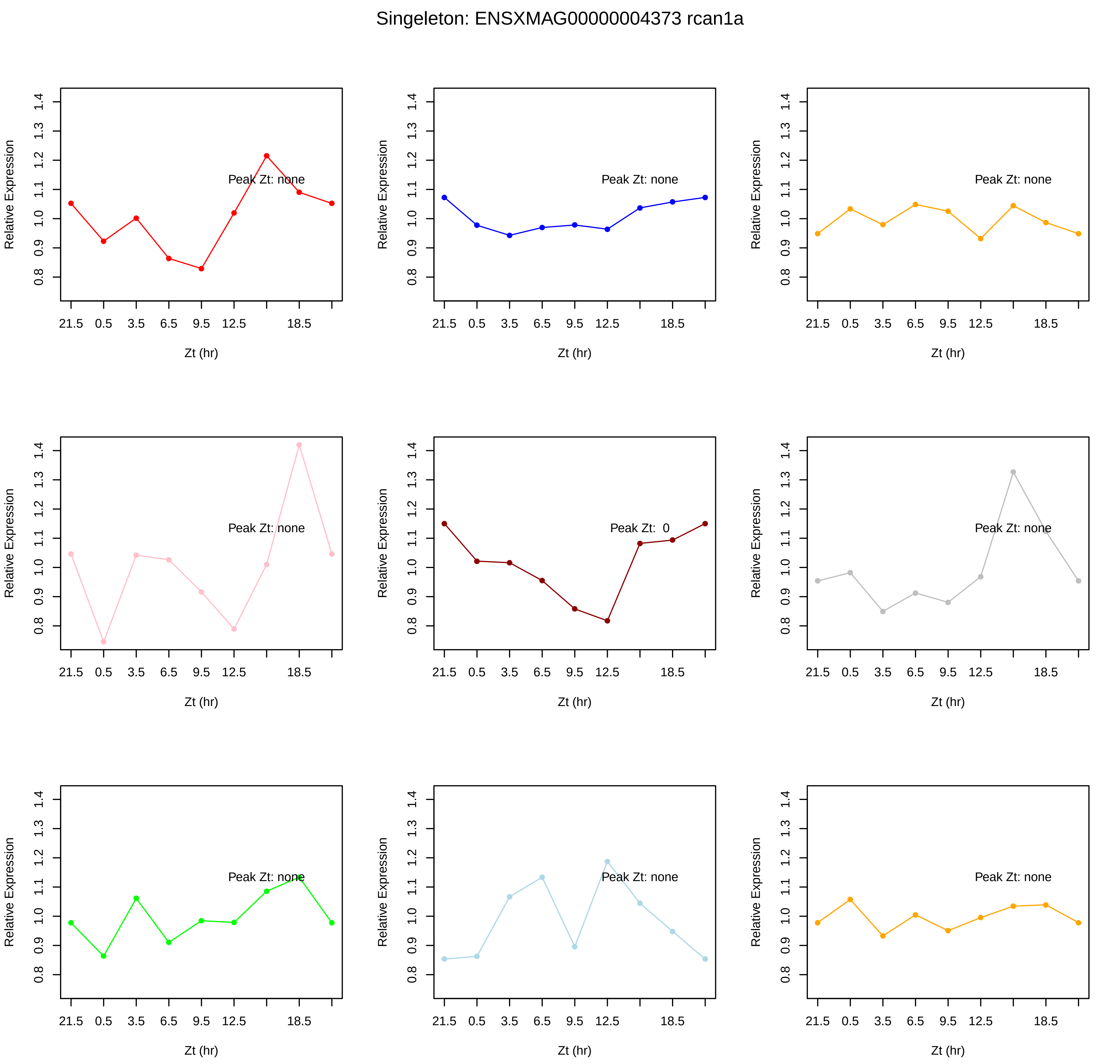
Singeleton: ENSXMAG00000004373 rcan1a
Relative Expression
Zt (hr)
21.5
0.5
3.5
6.5
9.5
12.5
18.5
0.8
0.9
1.0
1.1
1.2
1.3
1.4
Peak Zt: none
Peak Zt: 0

Singeleton: ENSXMAG00000012643 mrpl39

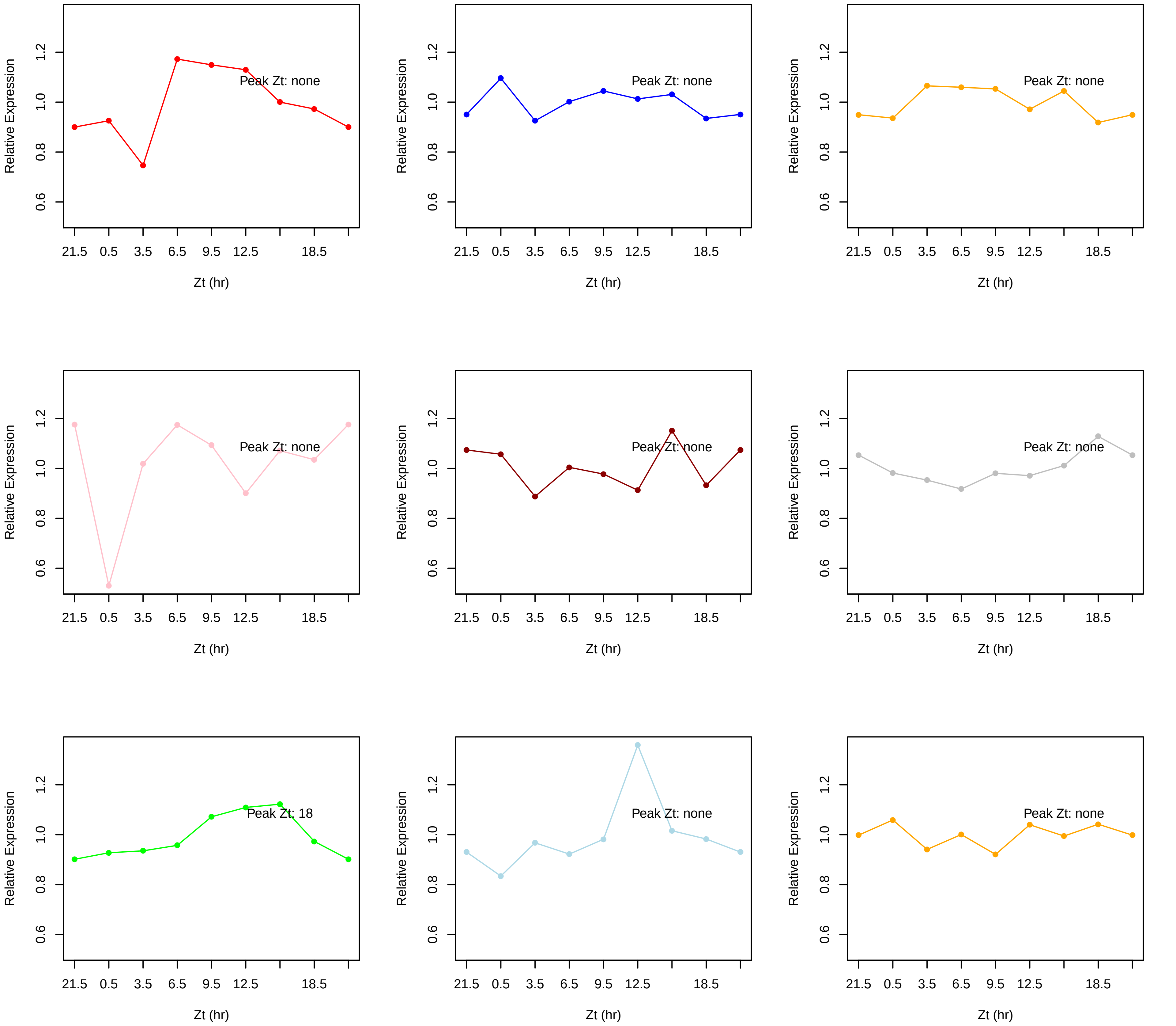

Singleton: ENSXMAG00000003988 ylpm1

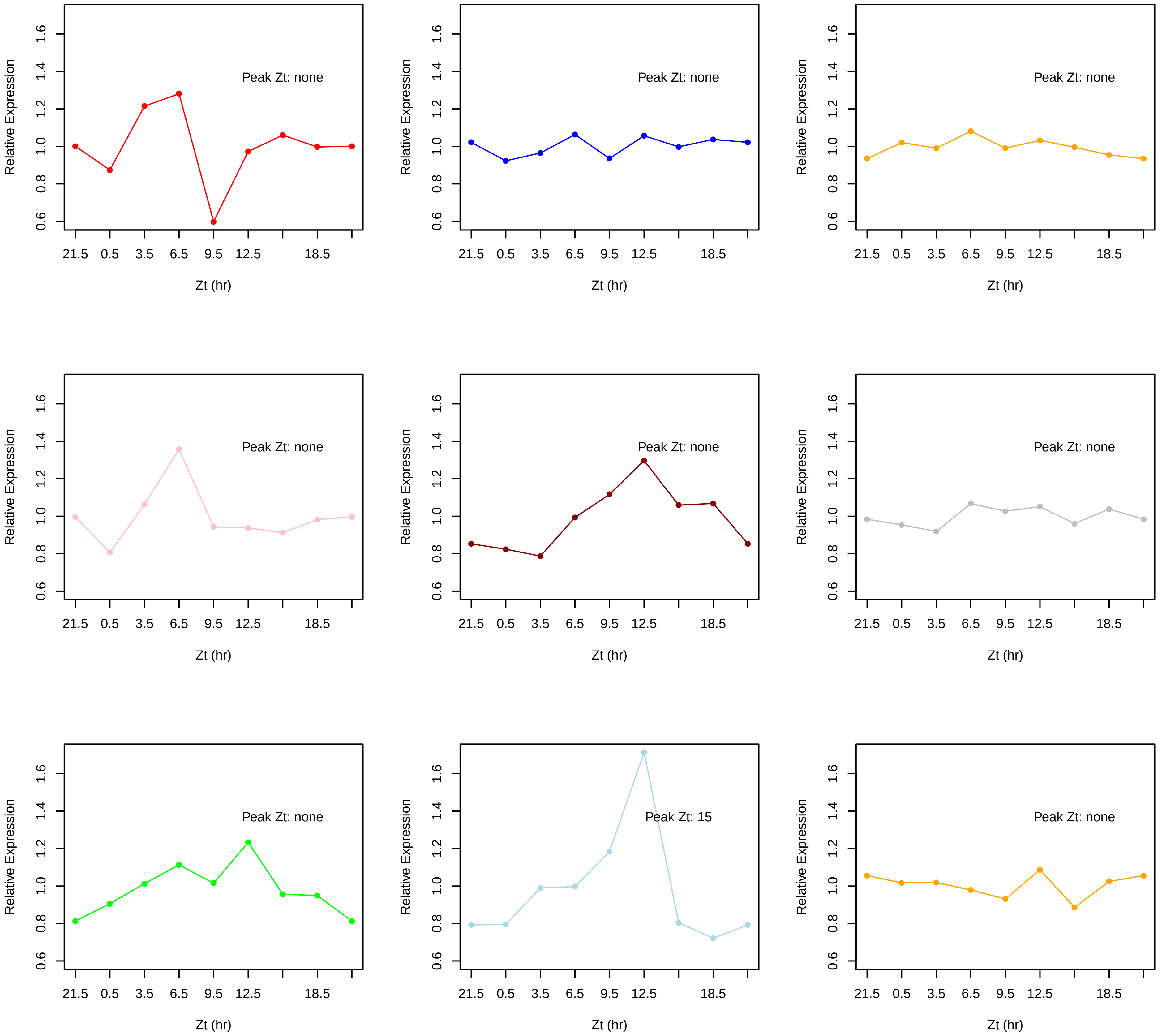

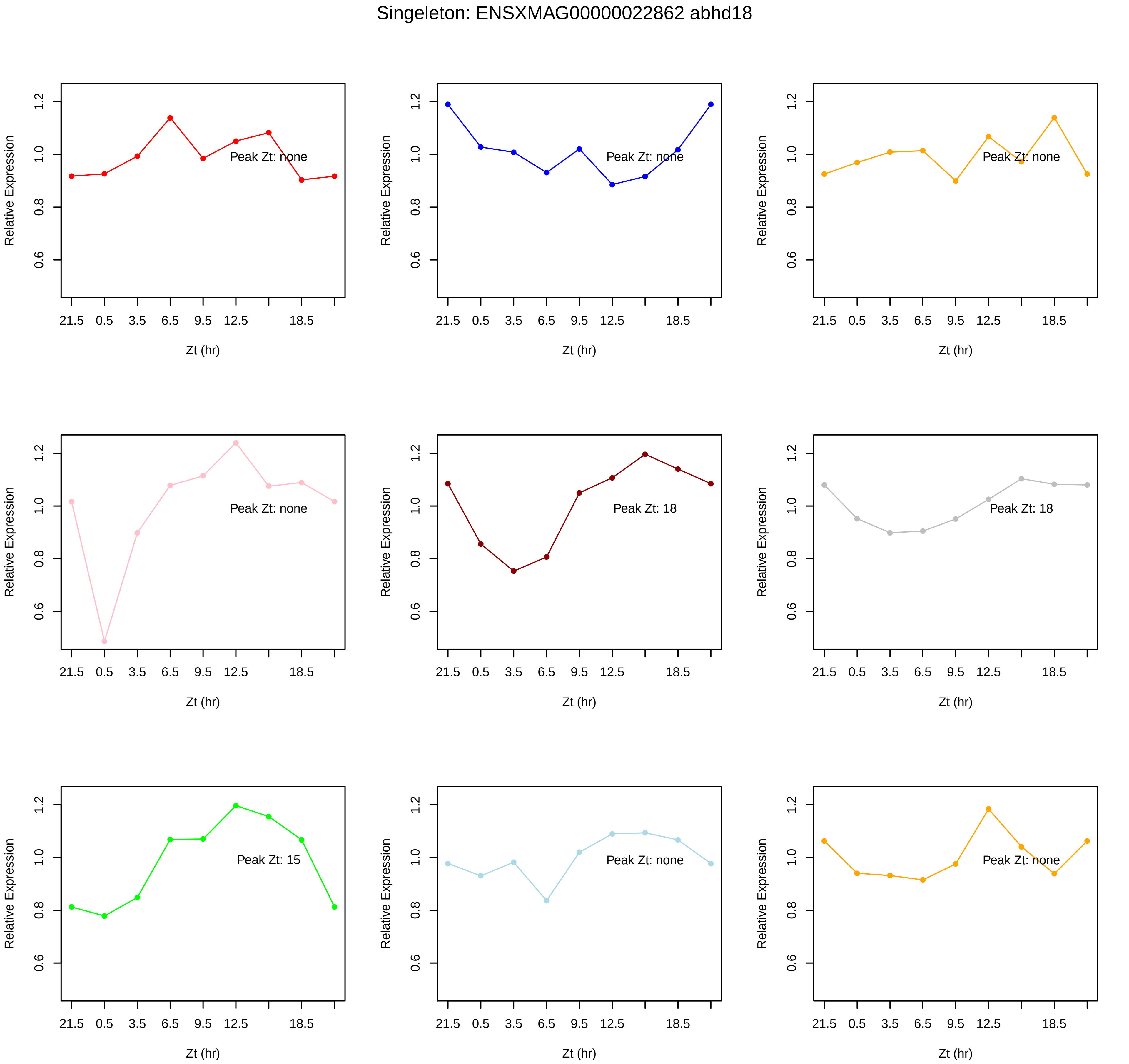
Singeleton: ENSXMAG00000022862 abhd18
Peak Zt: none
Peak Zt: none
Peak Zt: none
Peak Zt: none
Peak Zt: 18
Peak Zt: 18
Peak Zt: 15
Peak Zt: none
Peak Zt: none
Relative Expression
Zt (hr)

Singeleton: ENSXMAG00000017140 spata20

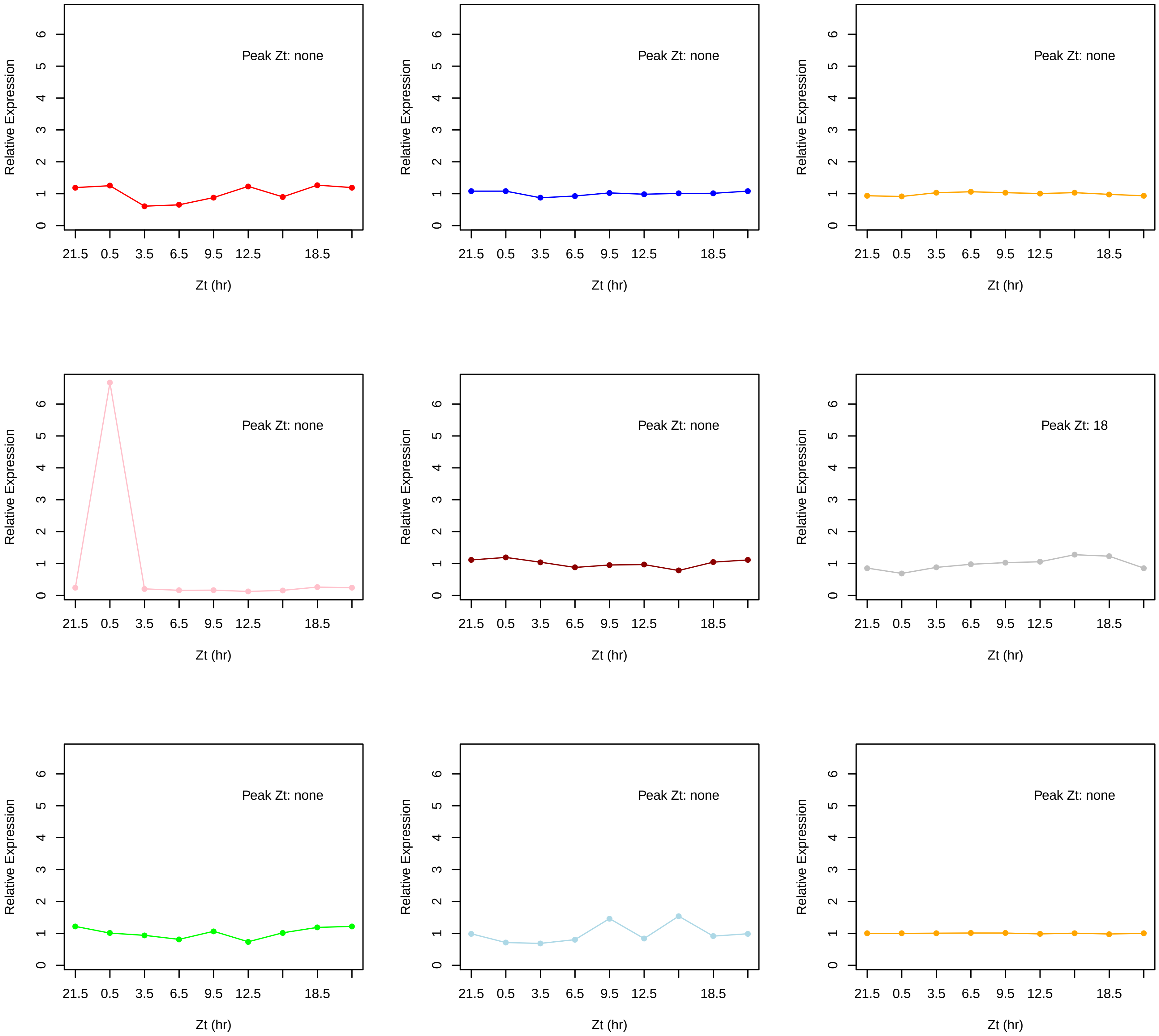

Singeleton: ENSXMAG00000026042 fam133b

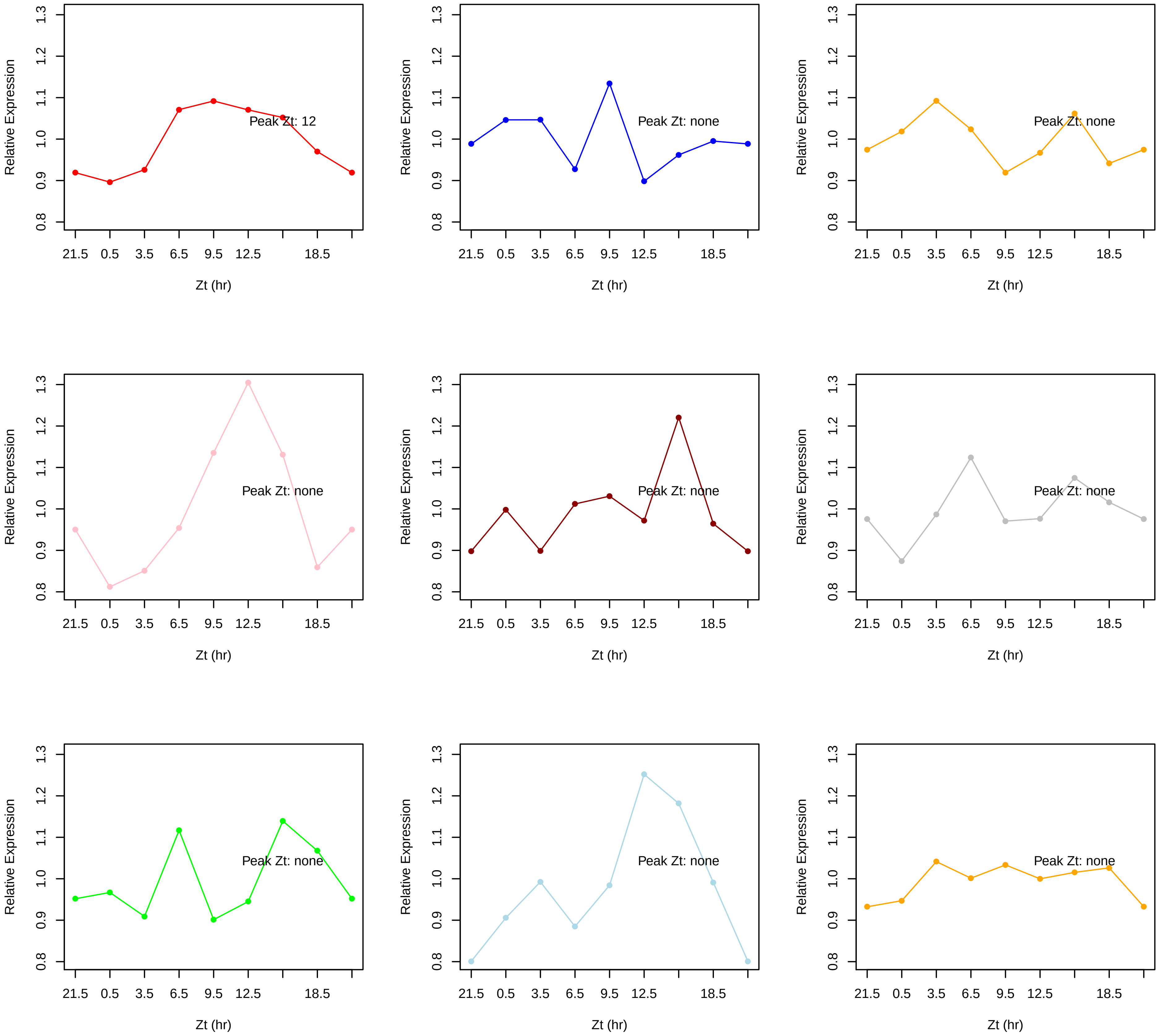

Singeleton: ENSXMAG00000001019 blvra

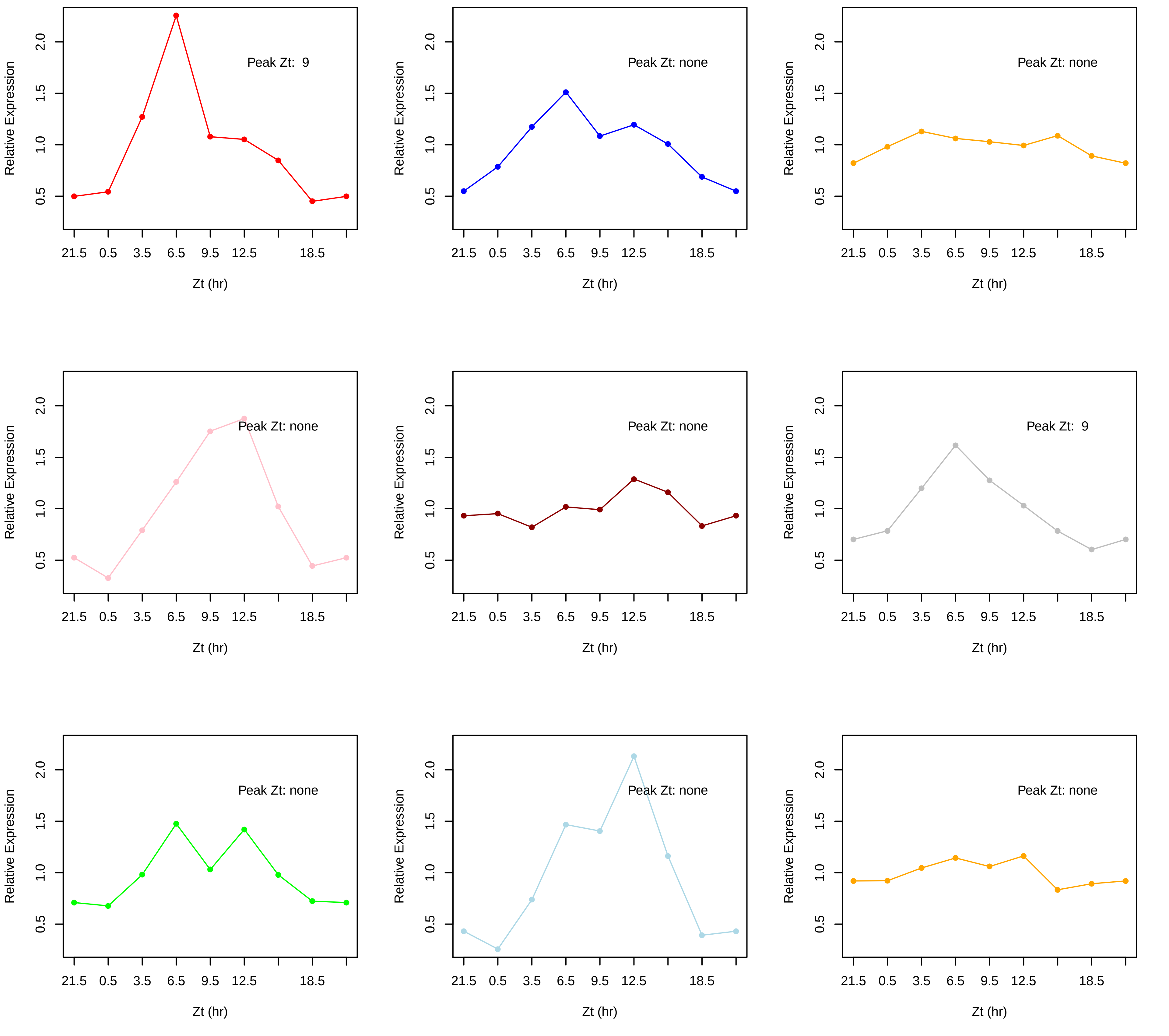

Singeleton: ENSXMAG00000029119 tmem150c

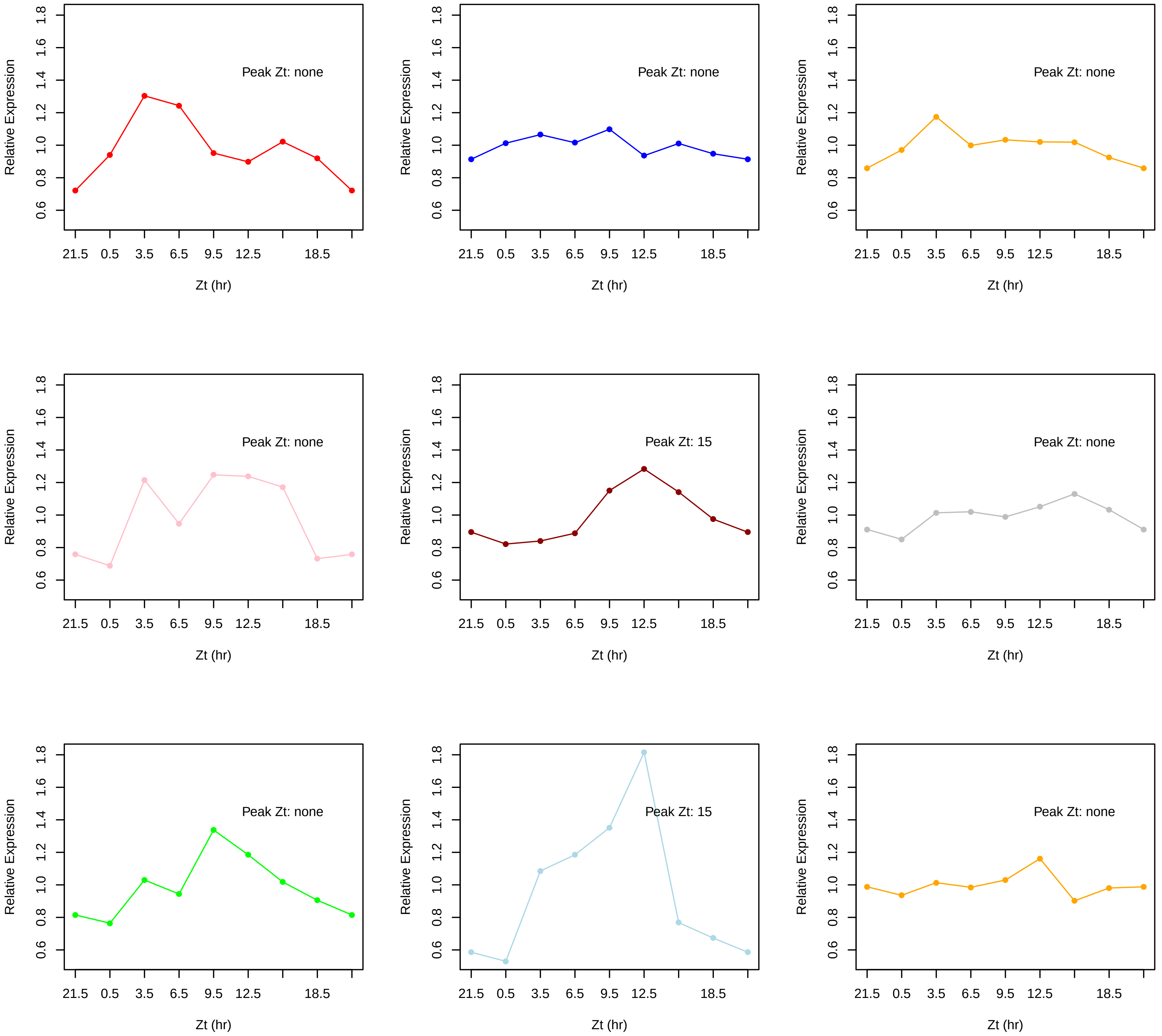

Singleton: ENSXMAG00000001657 emc3

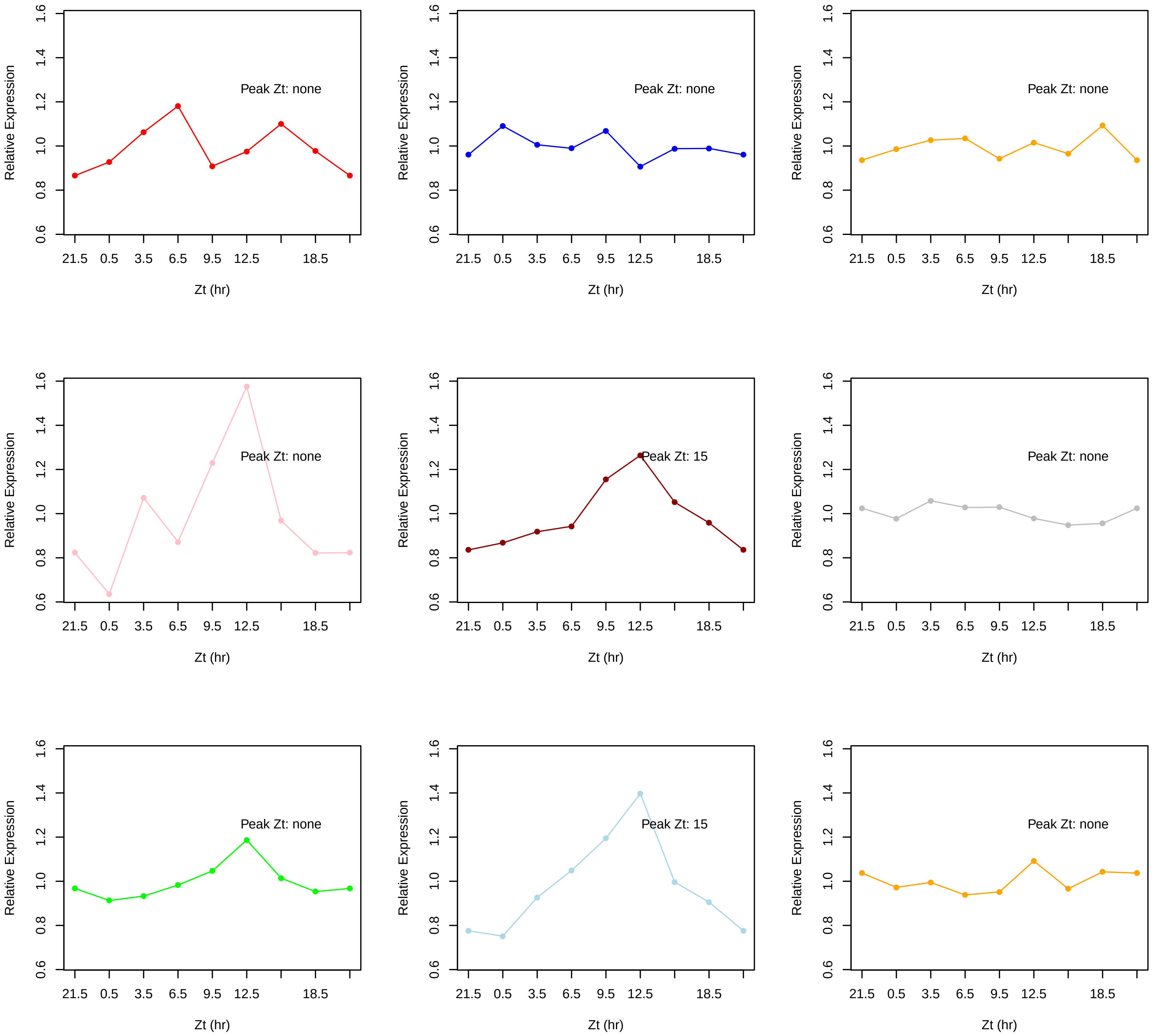

Singeleton: ENSXMAG00000014576 fcf1

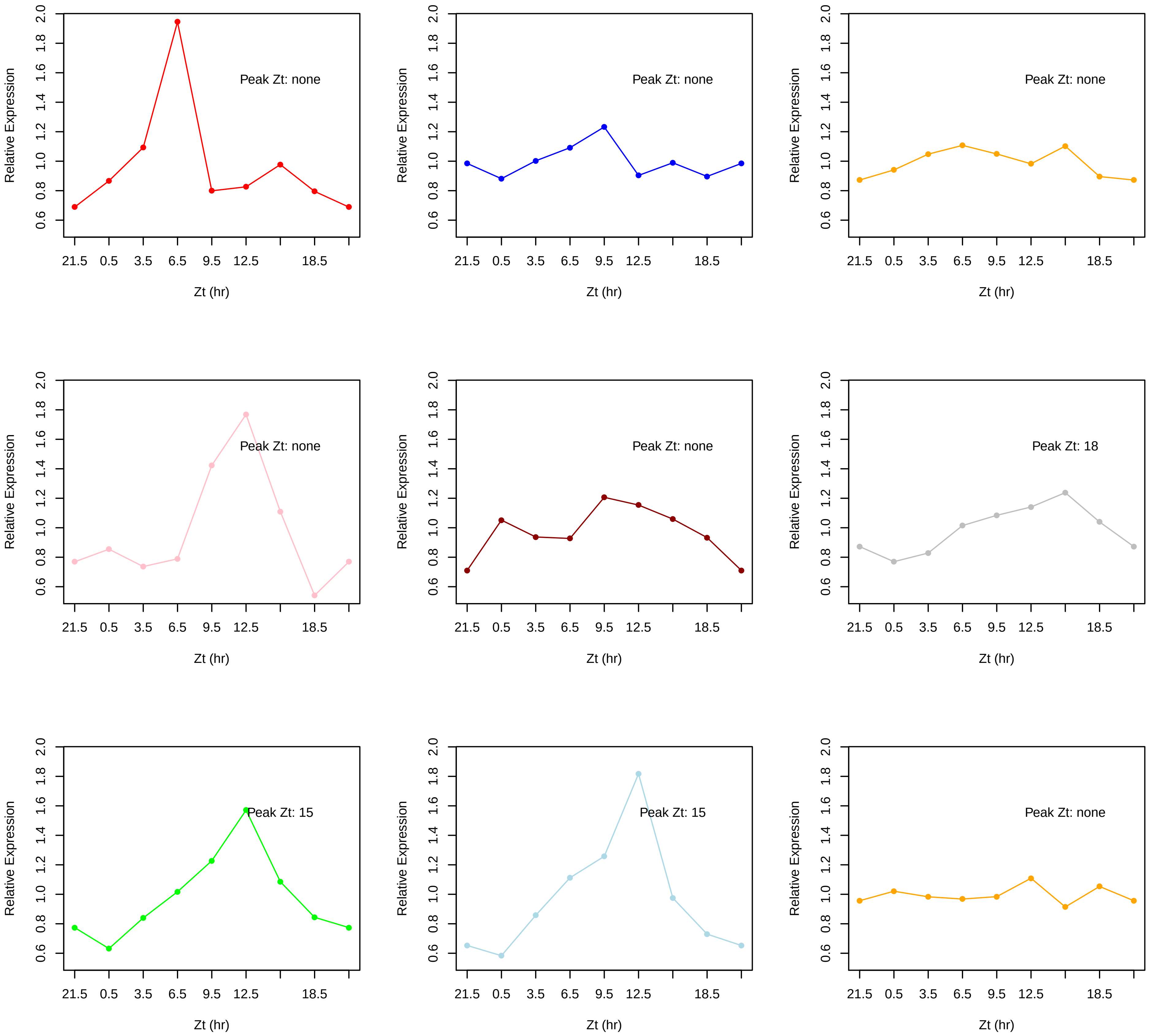

Singeleton: ENSXMAG00000013565 plk4

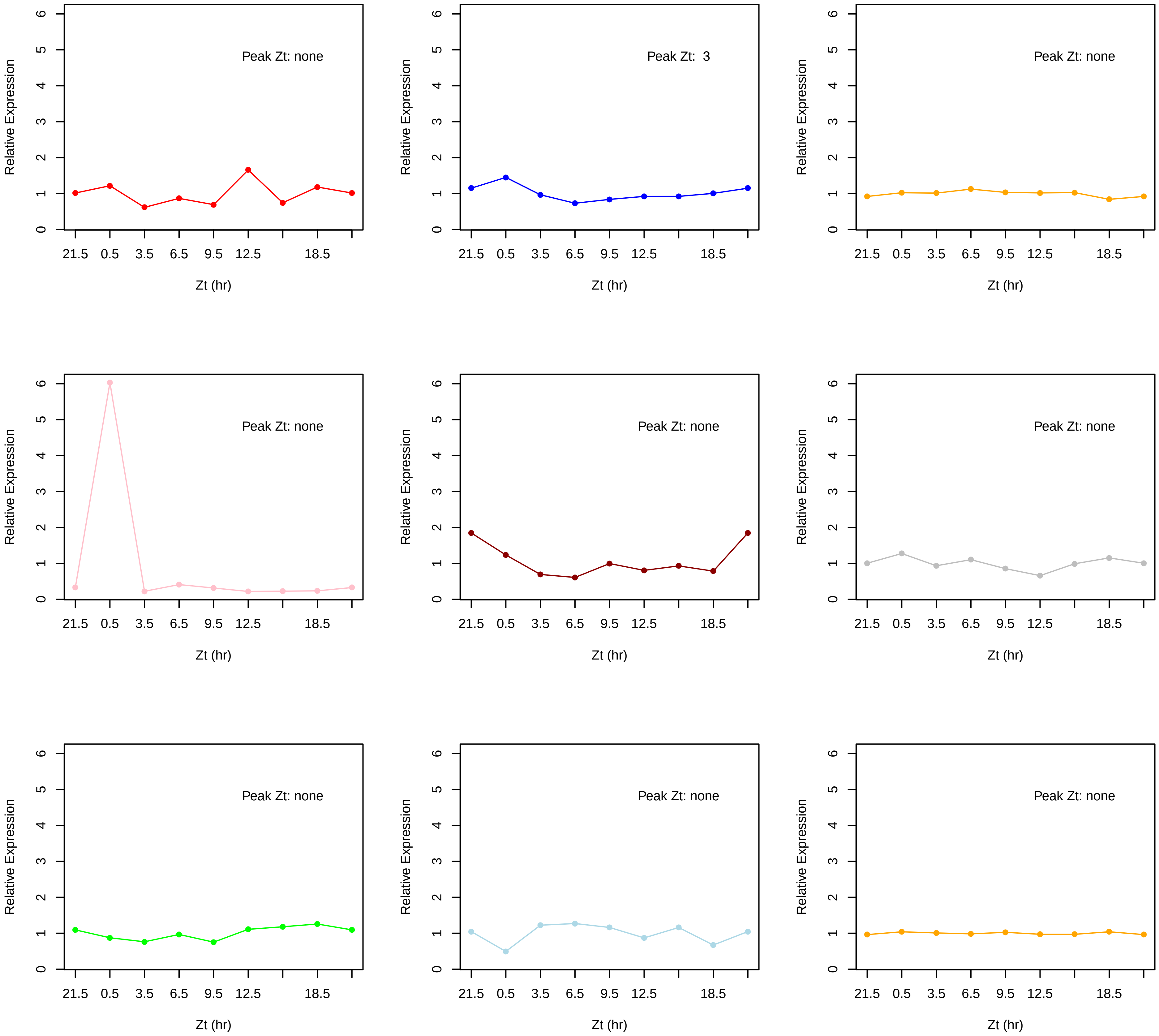

Singeleton: ENSXMAG00000009322 atp10b

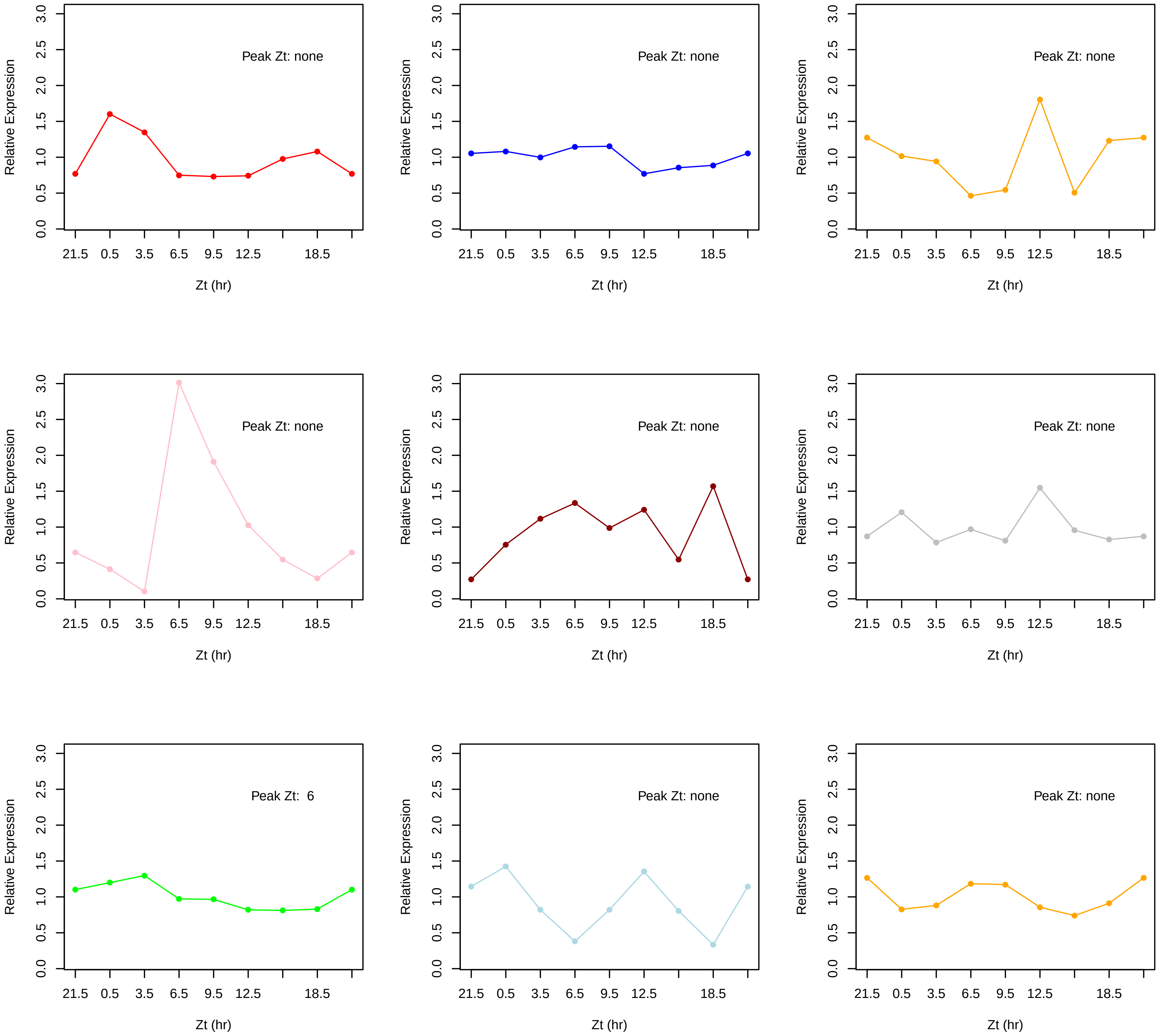

Singleton: ENSXMAG00000014558 AREL1

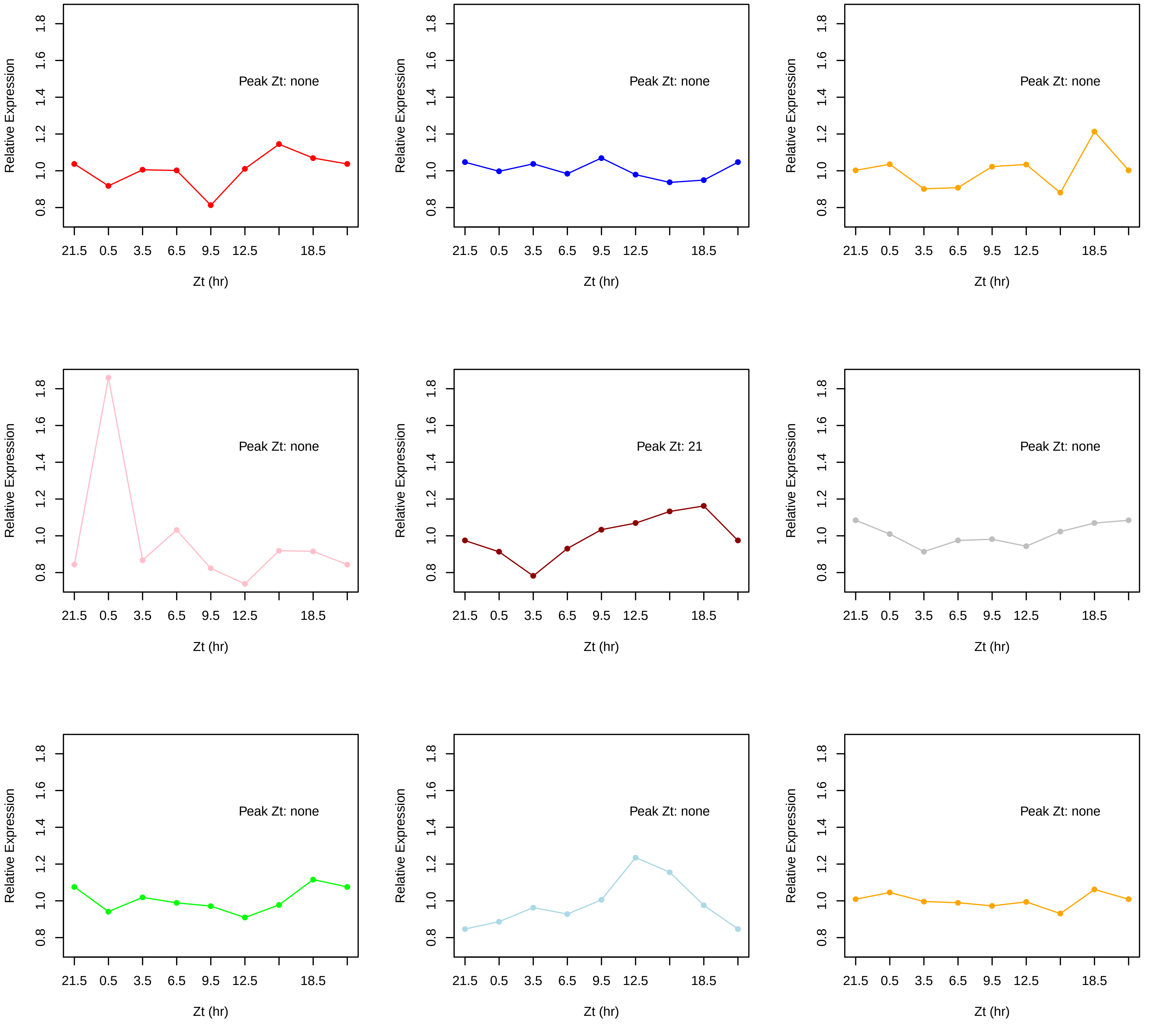

Singeleton: ENSXMAG00000019151 hnrnpd

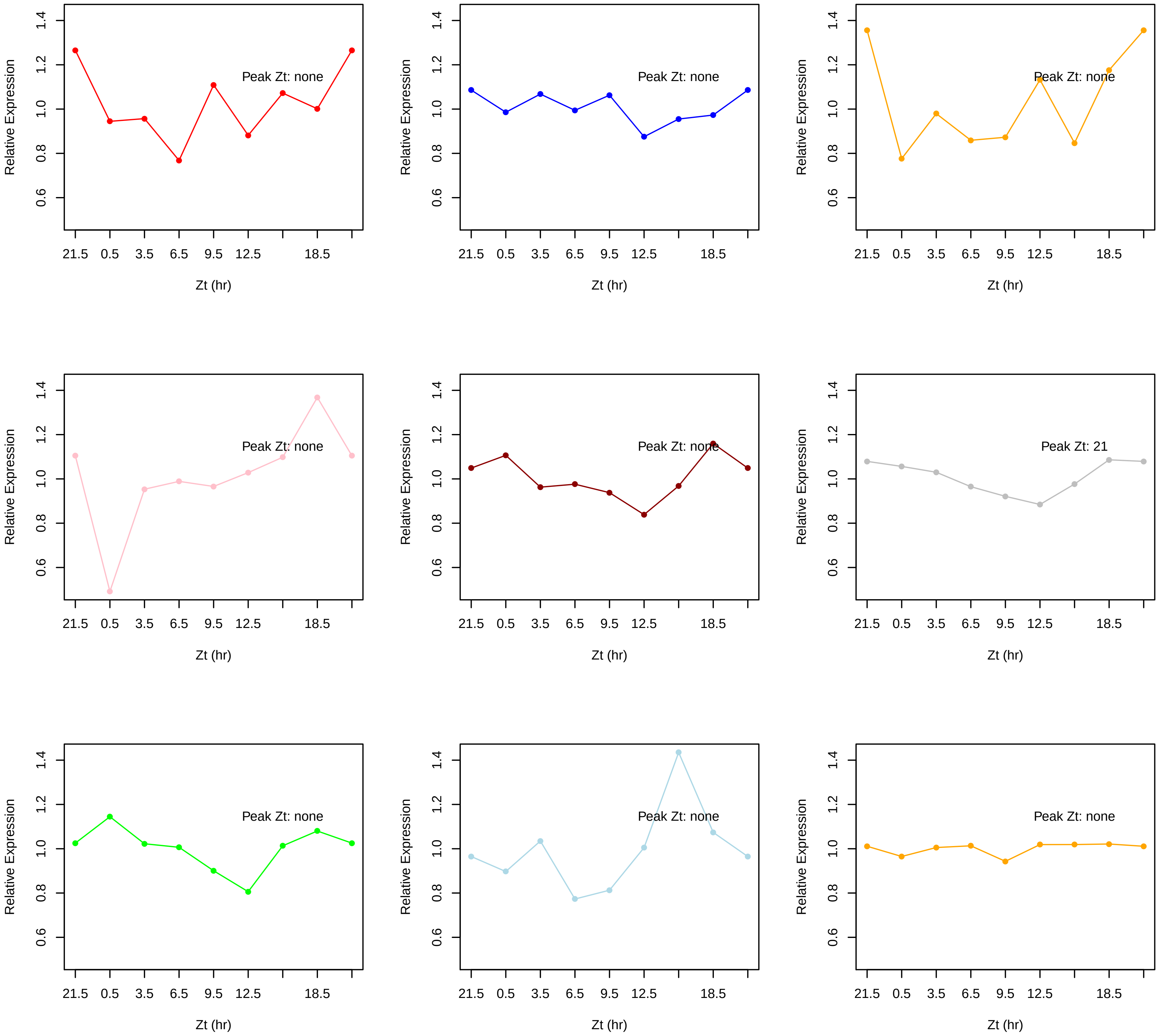

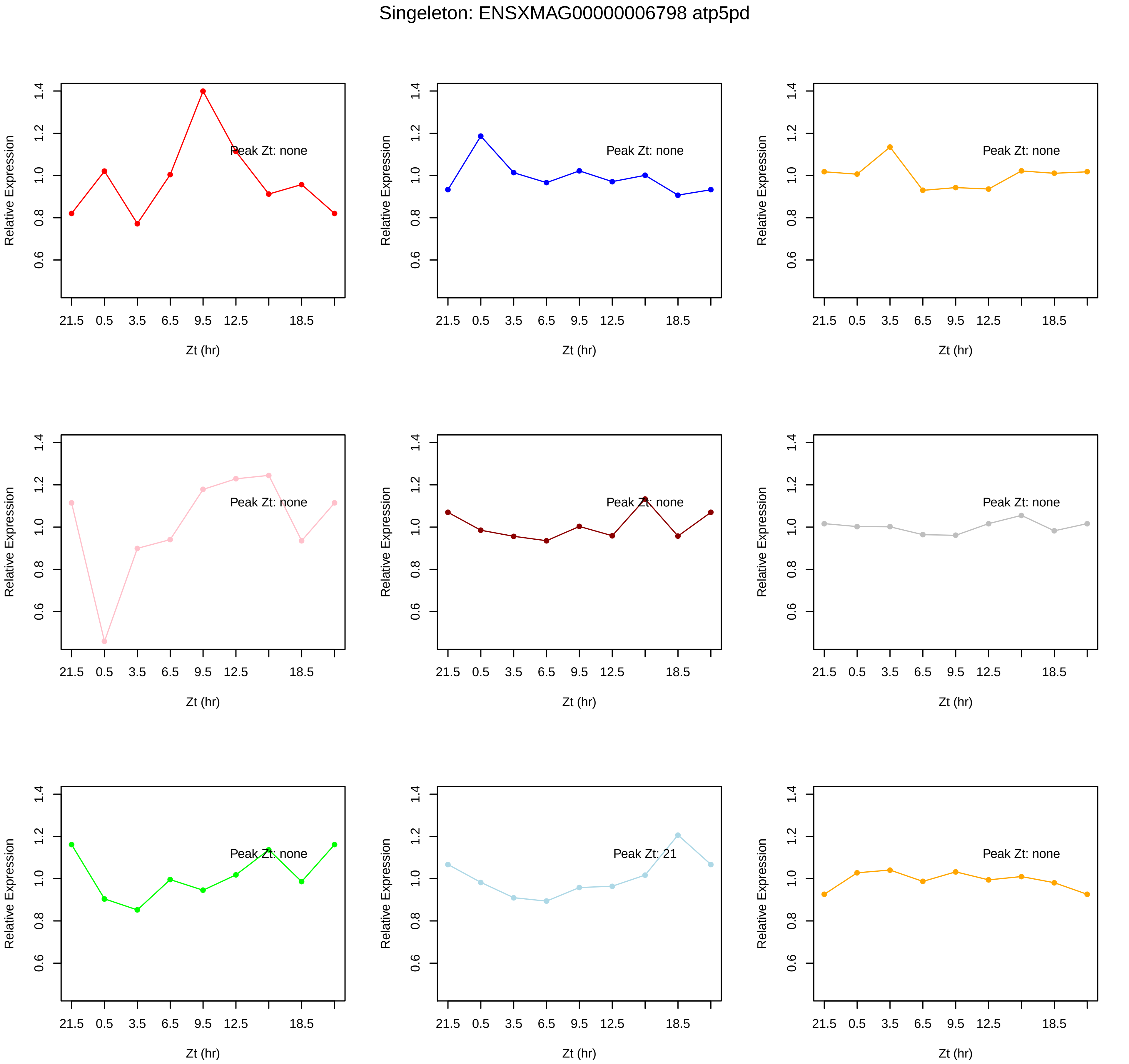
Singeleton: ENSXMAG00000006798 atp5pd
Peak Zt: none
Peak Zt: none
Peak Zt: none
Peak Zt: none
Peak Zt: none
Peak Zt: none
Peak Zt: none
Peak Zt: 21
Peak Zt: none
Relative Expression
Zt (hr)
21.5
0.5
3.5
6.5
9.5
12.5
18.5
0.6
0.8
1.0
1.2
1.4

Singleton: ENSXMAG00000026774

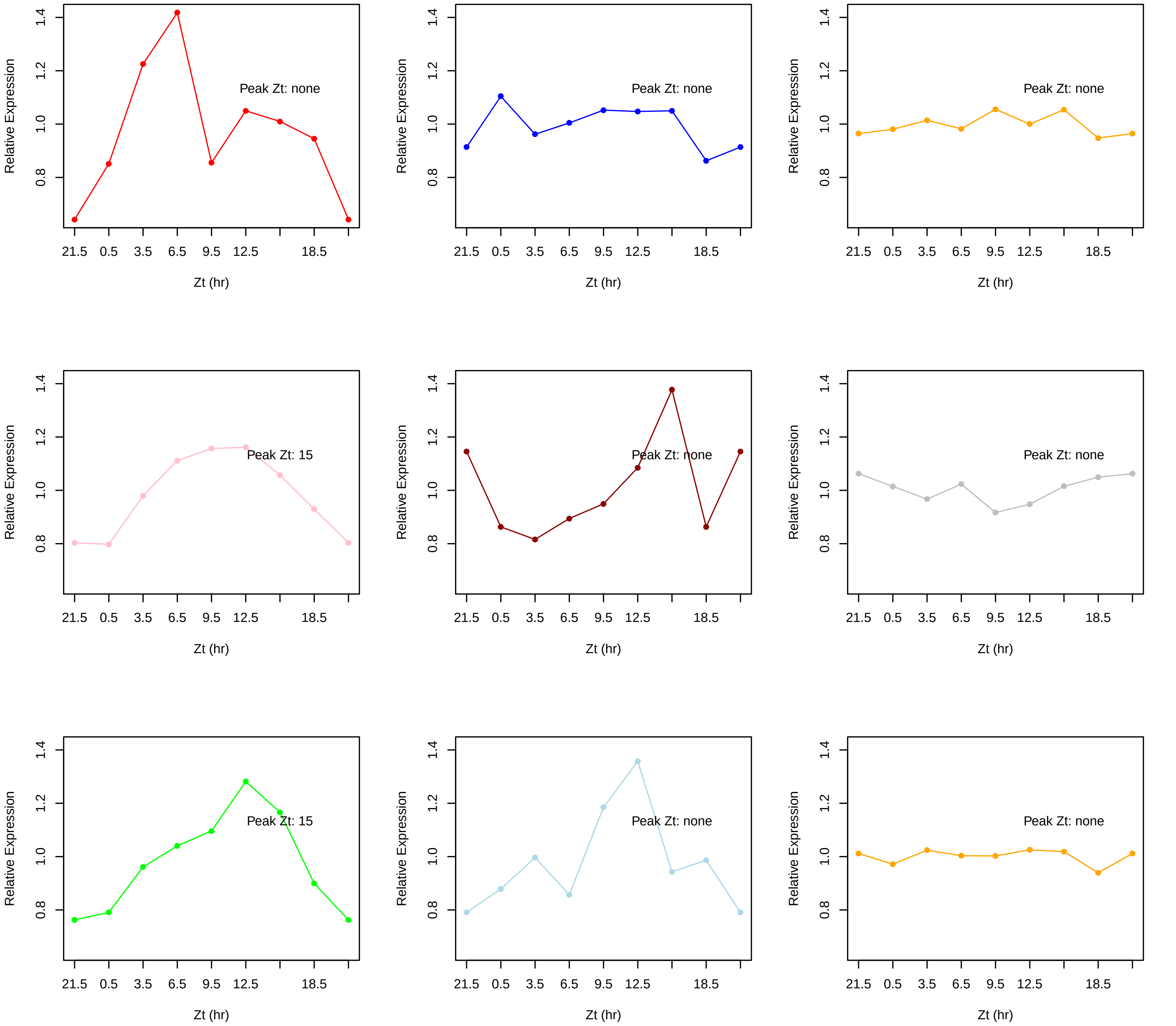

Singeleton: ENSXMAG00000009345 fbxl3l

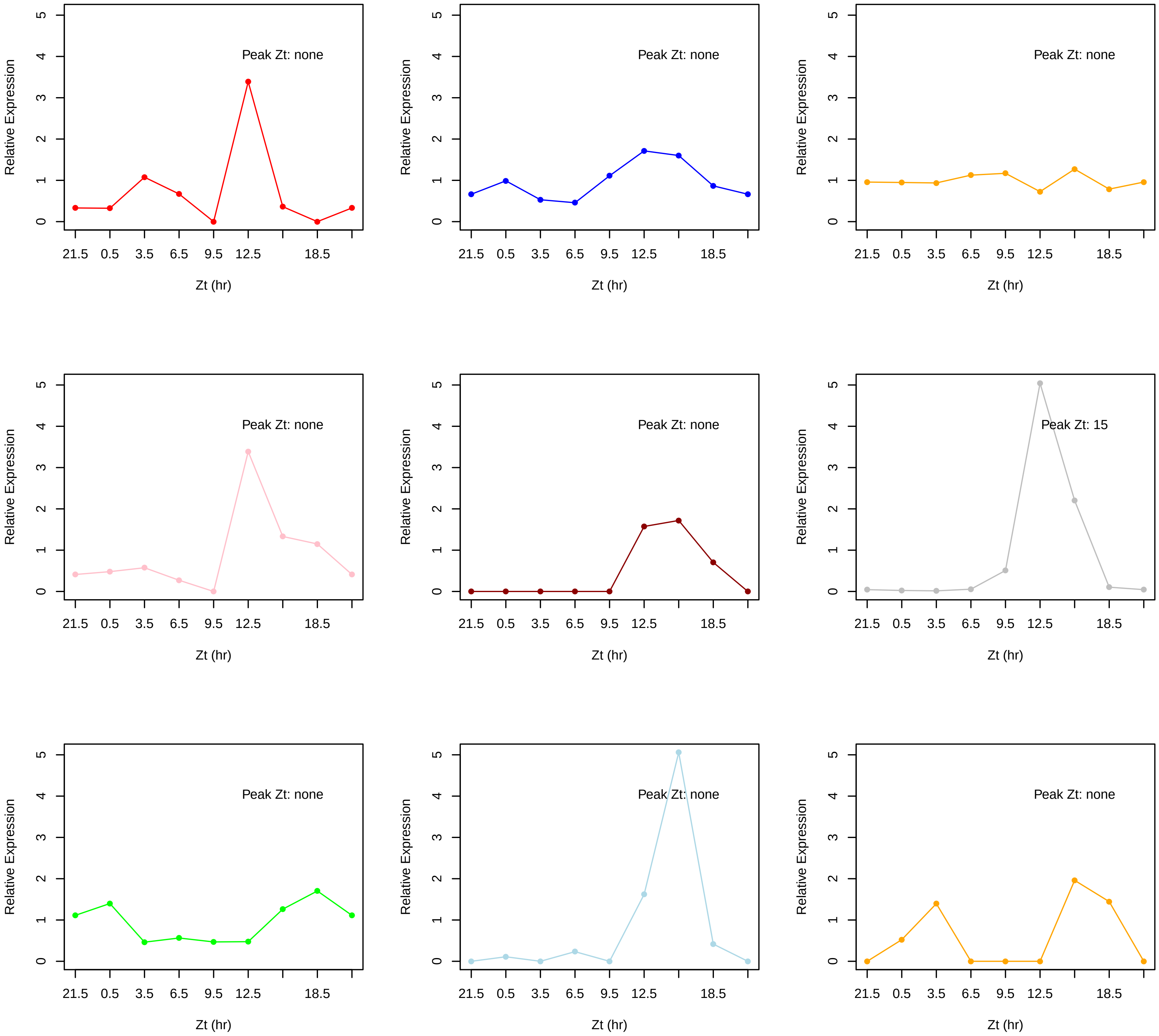

Singleton: ENSXMAG00000014515 pomt2

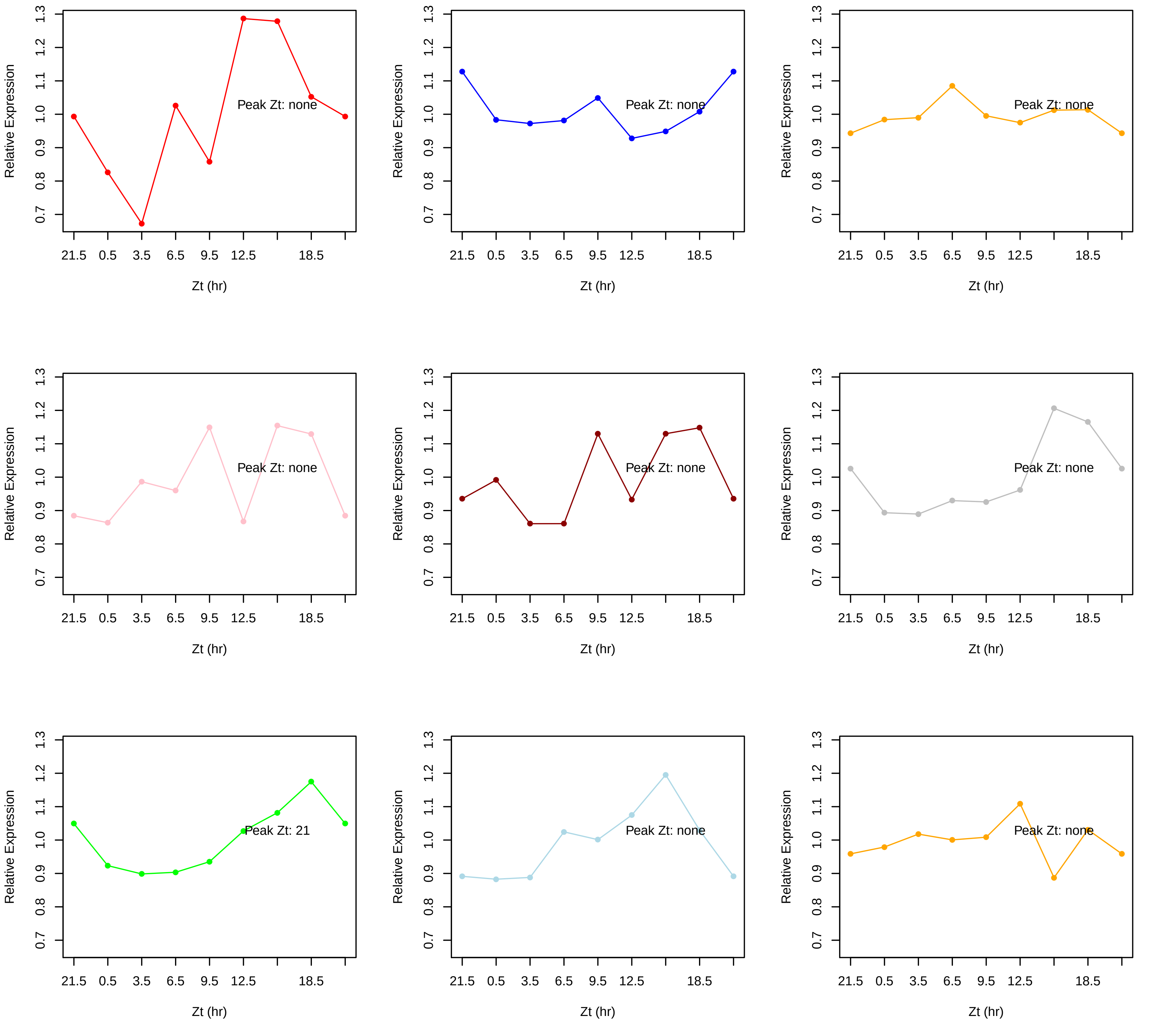

Singleleton: ENSXMAG00000026895 sh3pxd2b

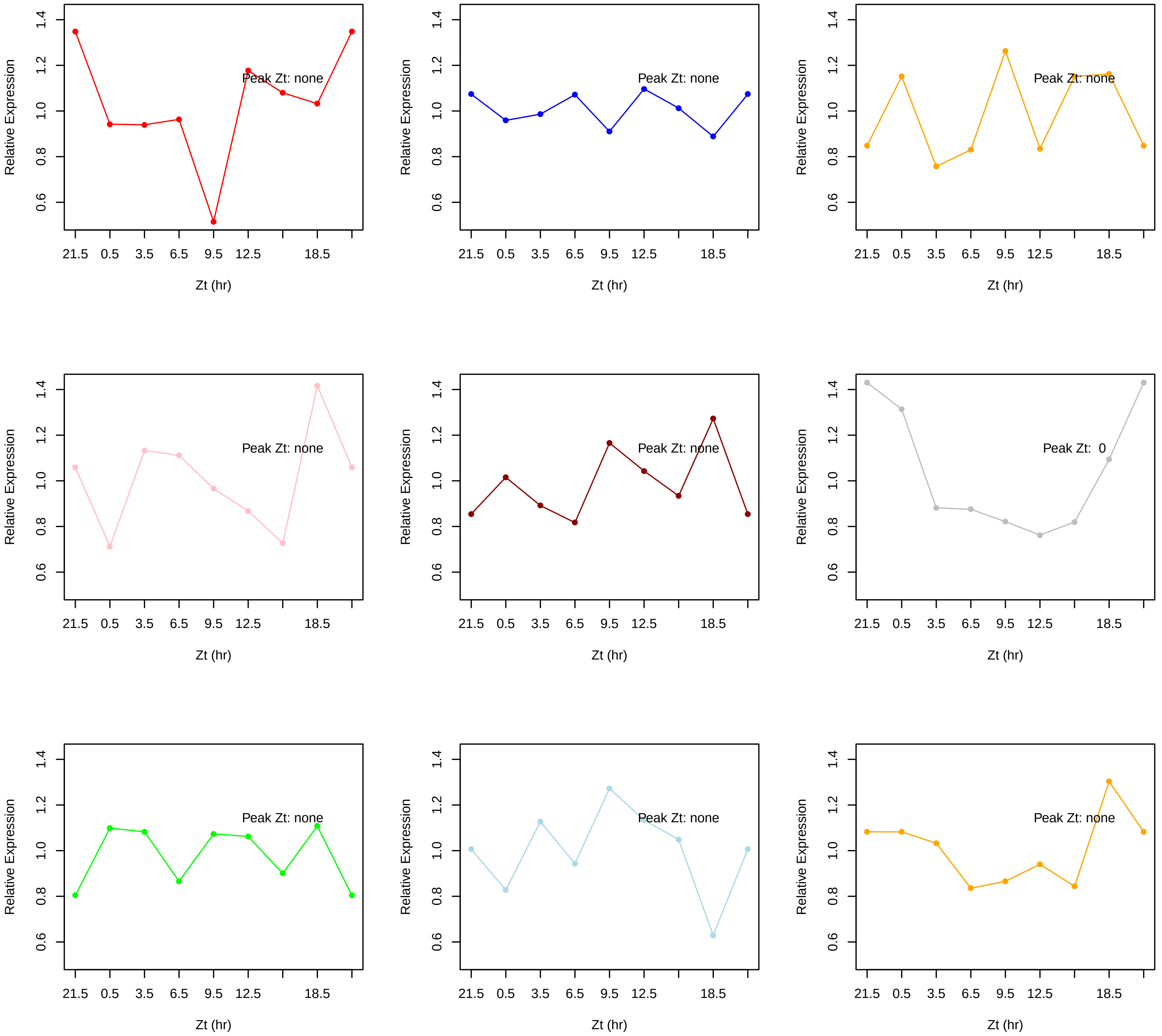

Singeleton: ENSXMAG00000013555 hspa4l

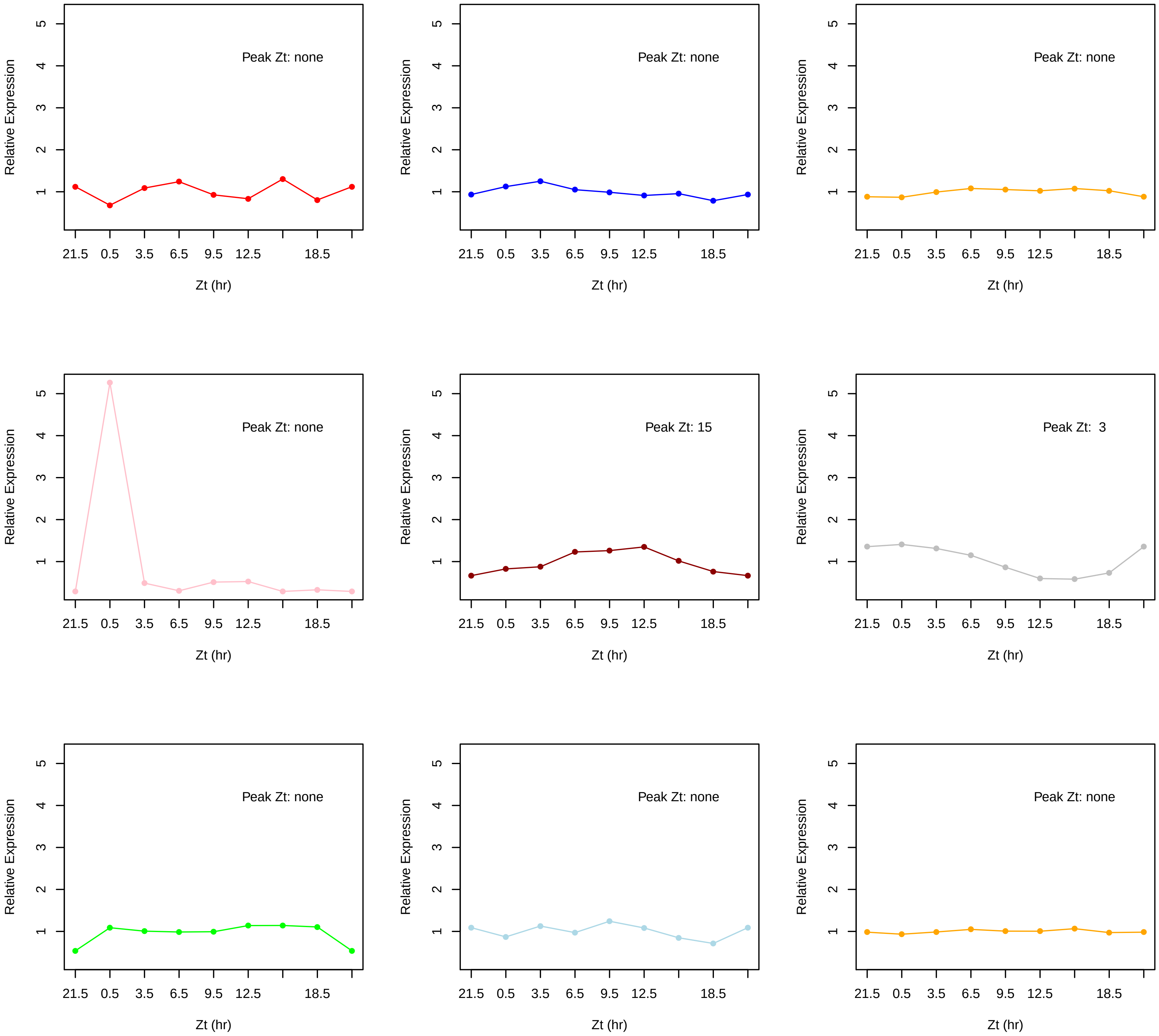

Singeleton: ENSXMAG00000016369 rbm5

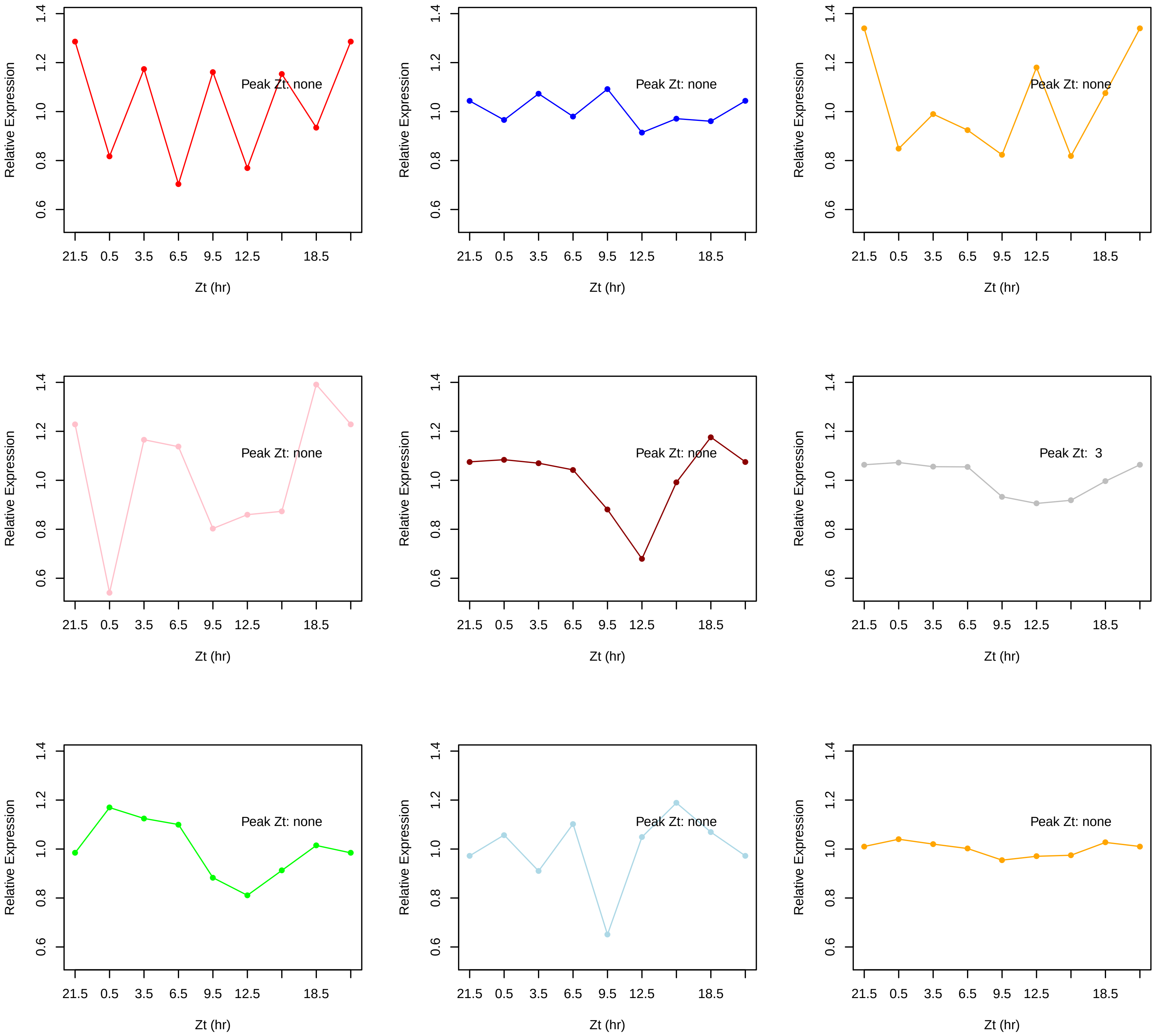

Singeleton: ENSXMAG00000014803 armc7

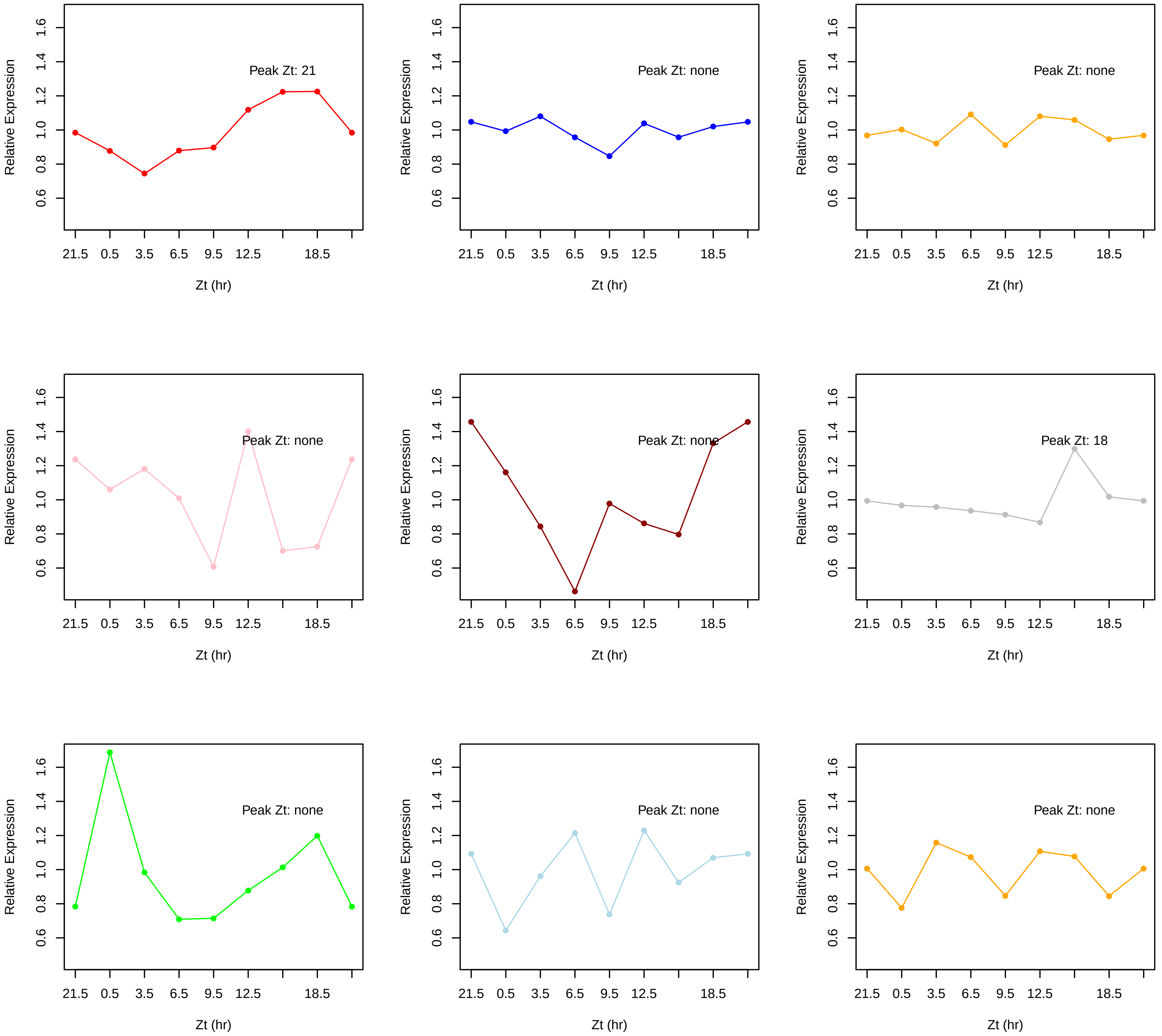

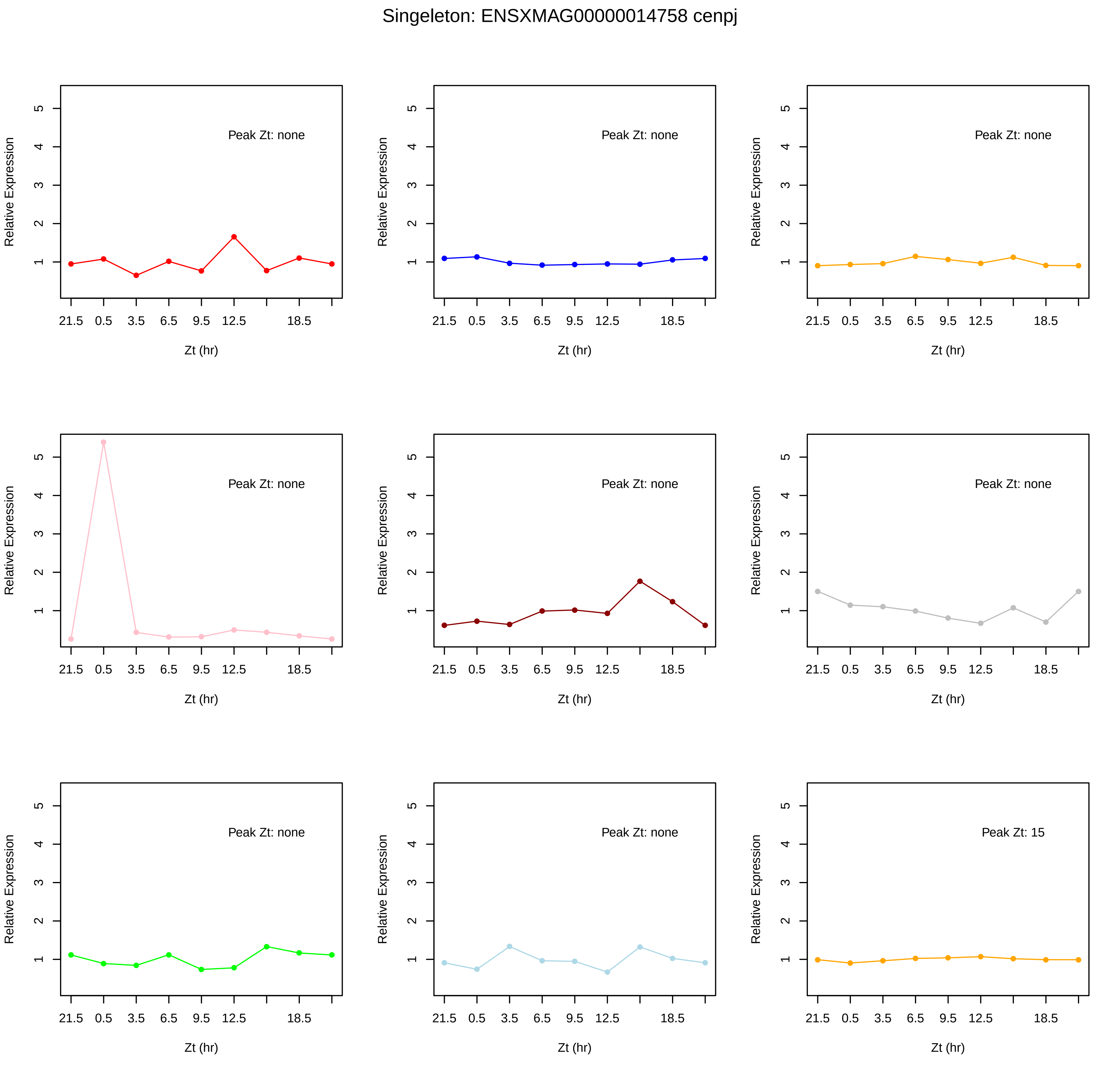
Singeleton: ENSXMAG00000014758 cenpj
Peak Zt: none
Peak Zt: none
Peak Zt: none
Peak Zt: none
Peak Zt: none
Peak Zt: none
Peak Zt: none
Peak Zt: none
Peak Zt: 15
Relative Expression
Zt (hr)
21.5
0.5
3.5
6.5
9.5
12.5
18.5

Singeleton: ENSXMAG00000015074 gstz1

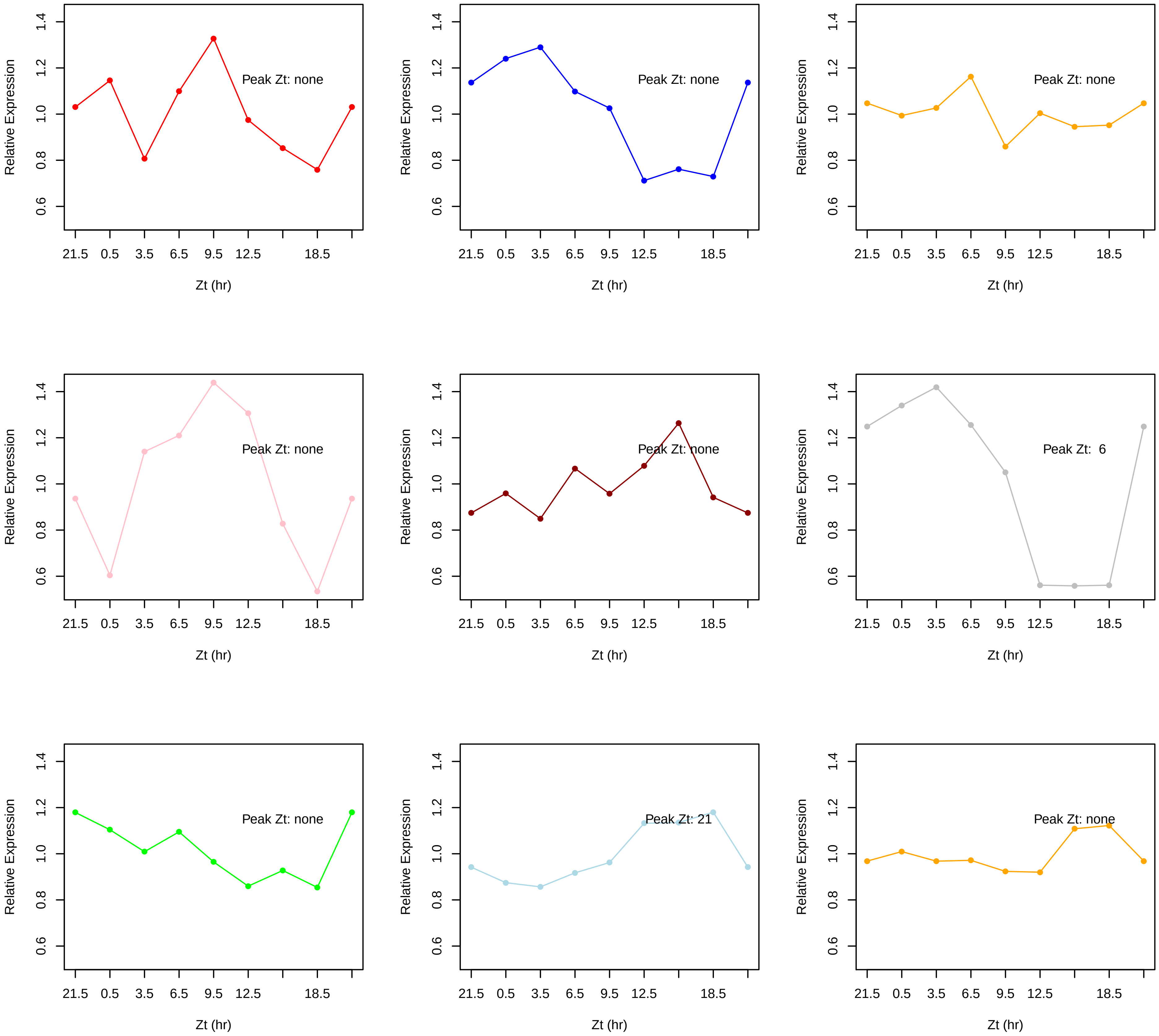

Singeleton: ENSXMAG00000009137 adamts1

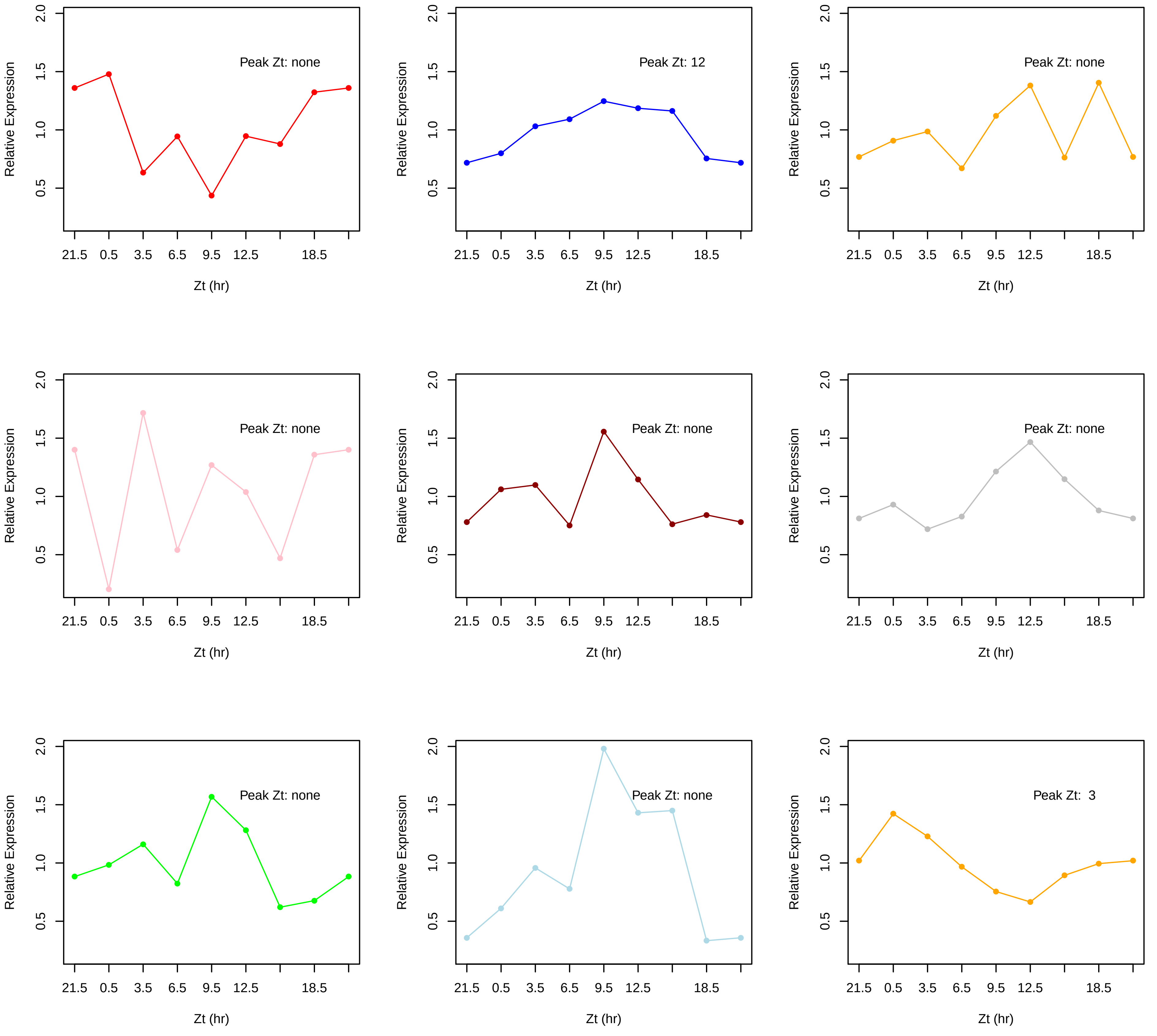

Singeleton: ENSXMAG00000000680 calcr

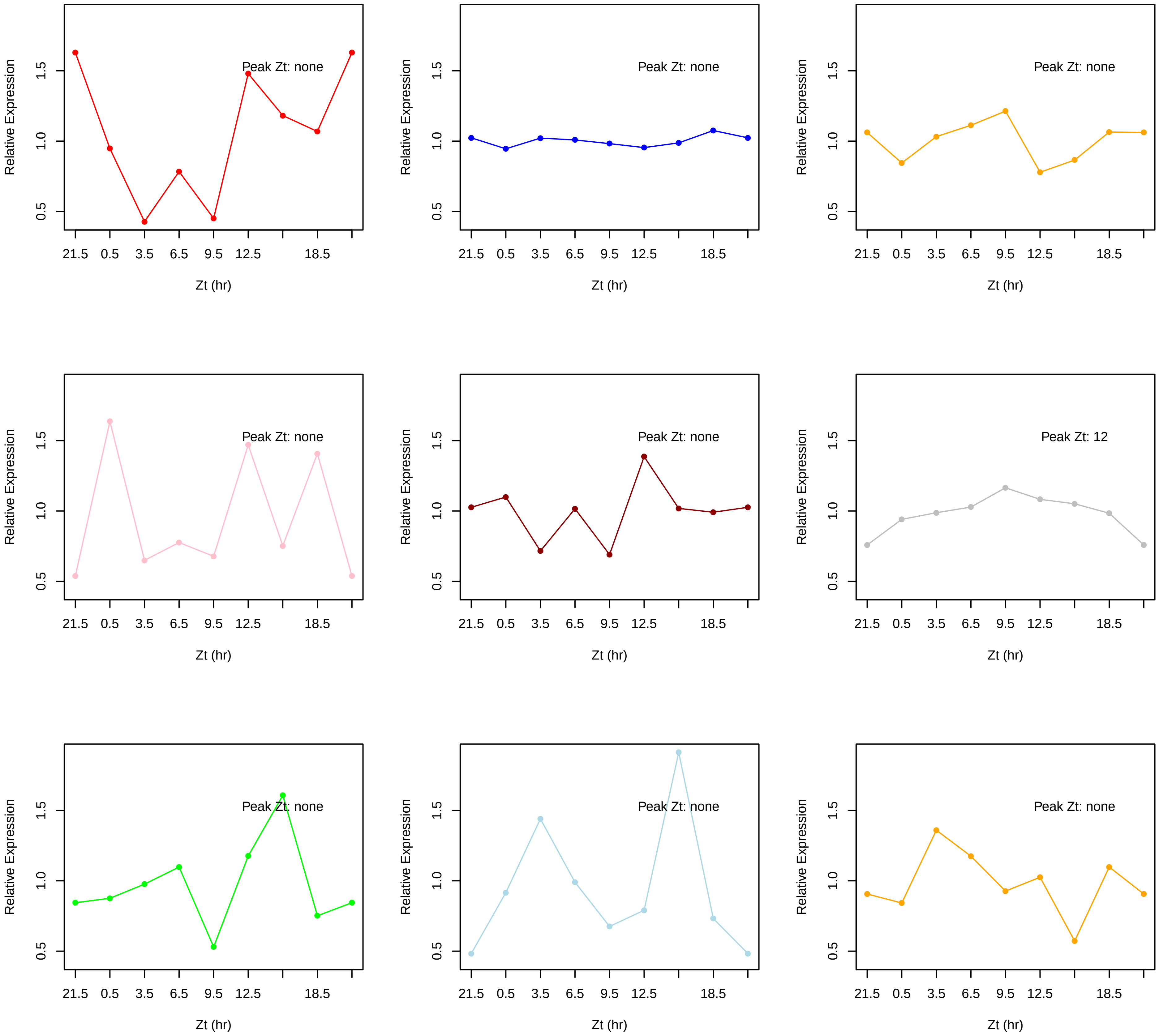

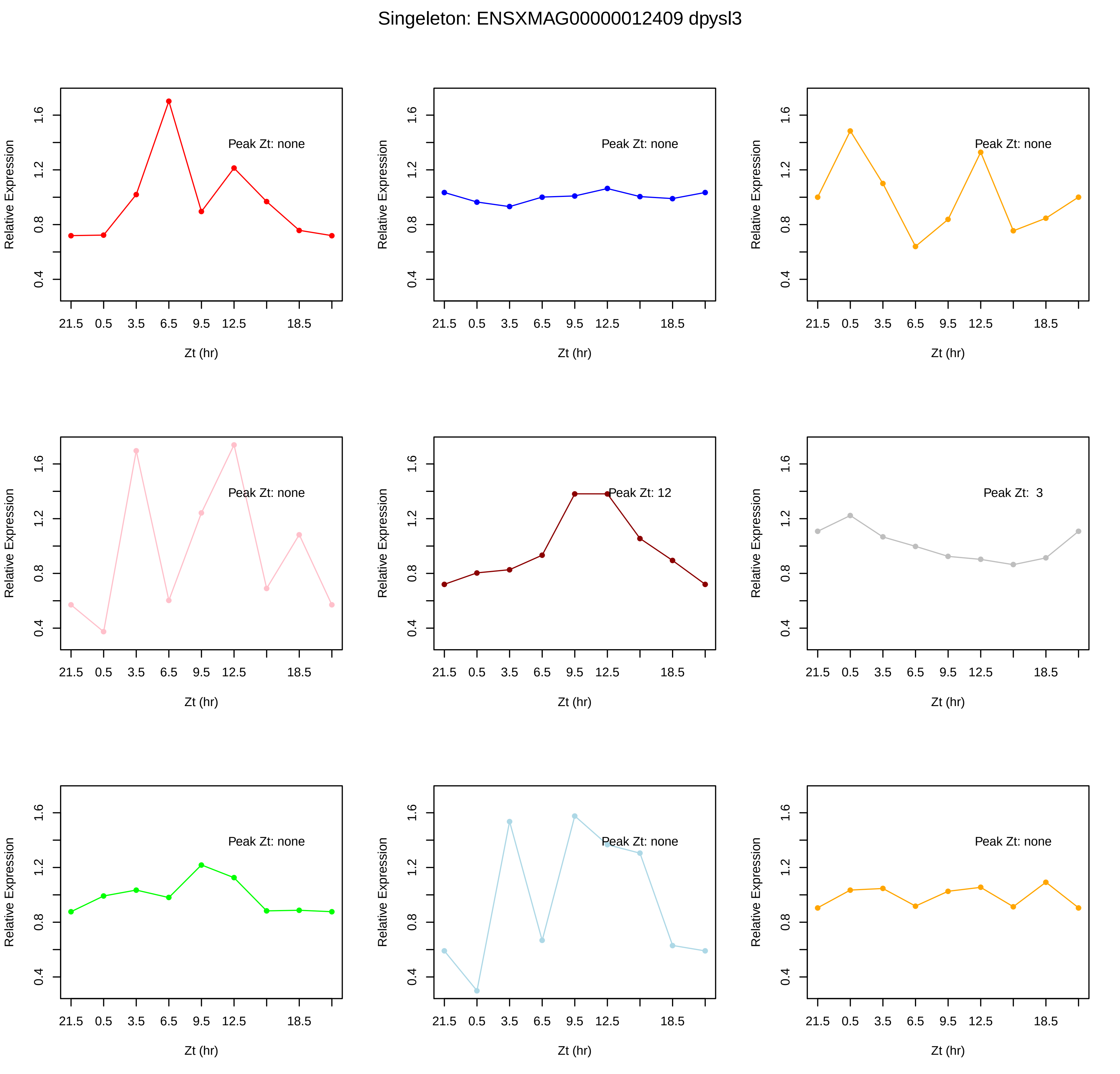

Singleton: ENSXMAG00000012409 dpysl3
Peak Zt: none
Peak Zt: none
Peak Zt: none
Peak Zt: none
Peak Zt: 12
Peak Zt: 3
Peak Zt: none
Peak Zt: none
Peak Zt: none
Relative Expression
Zt (hr)
21.5
0.5
3.5
6.5
9.5
12.5
18.5
0.4
0.8
1.2
1.6

Singleton: ENSXMAG00000020886 ADAMTS5

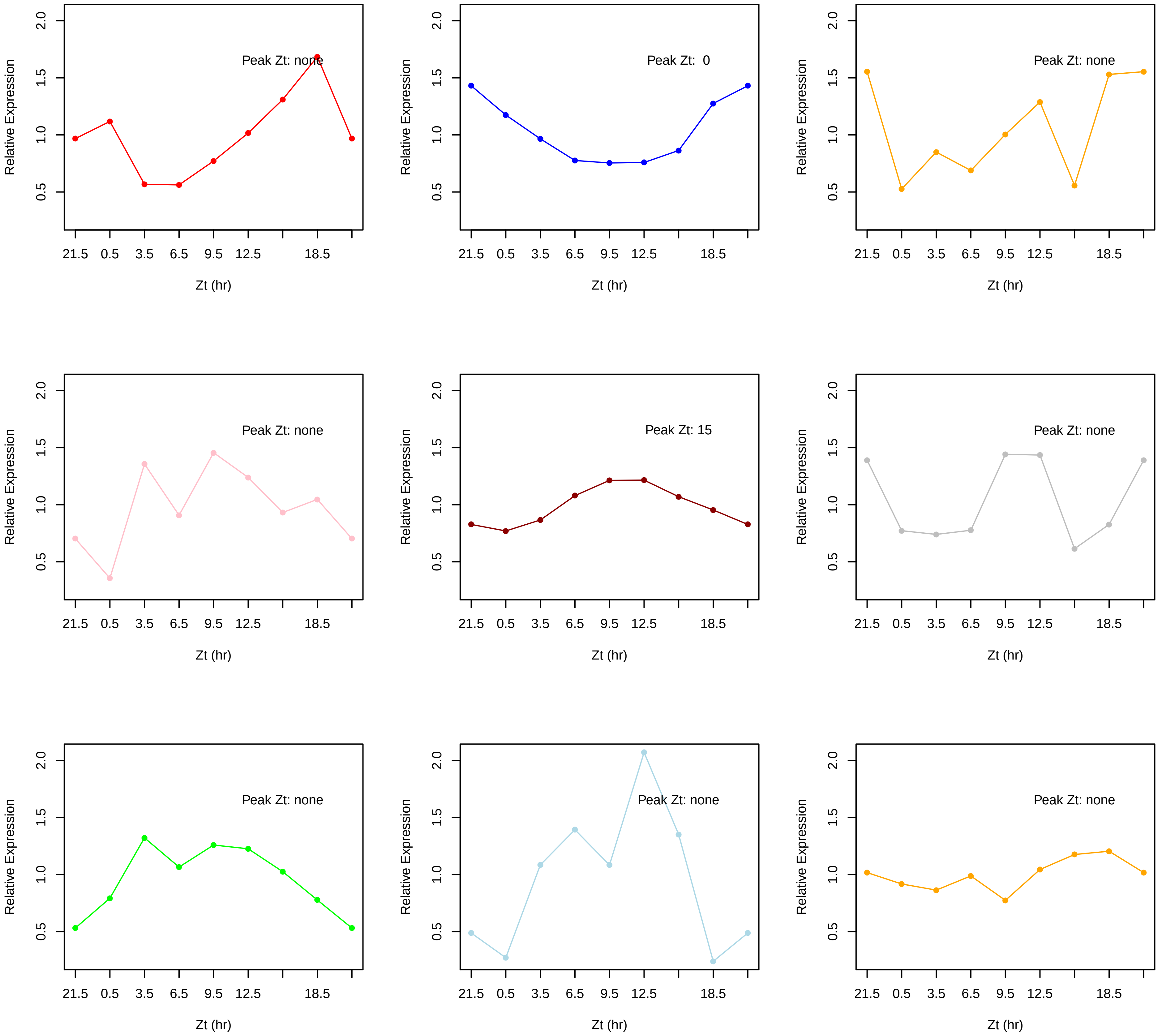

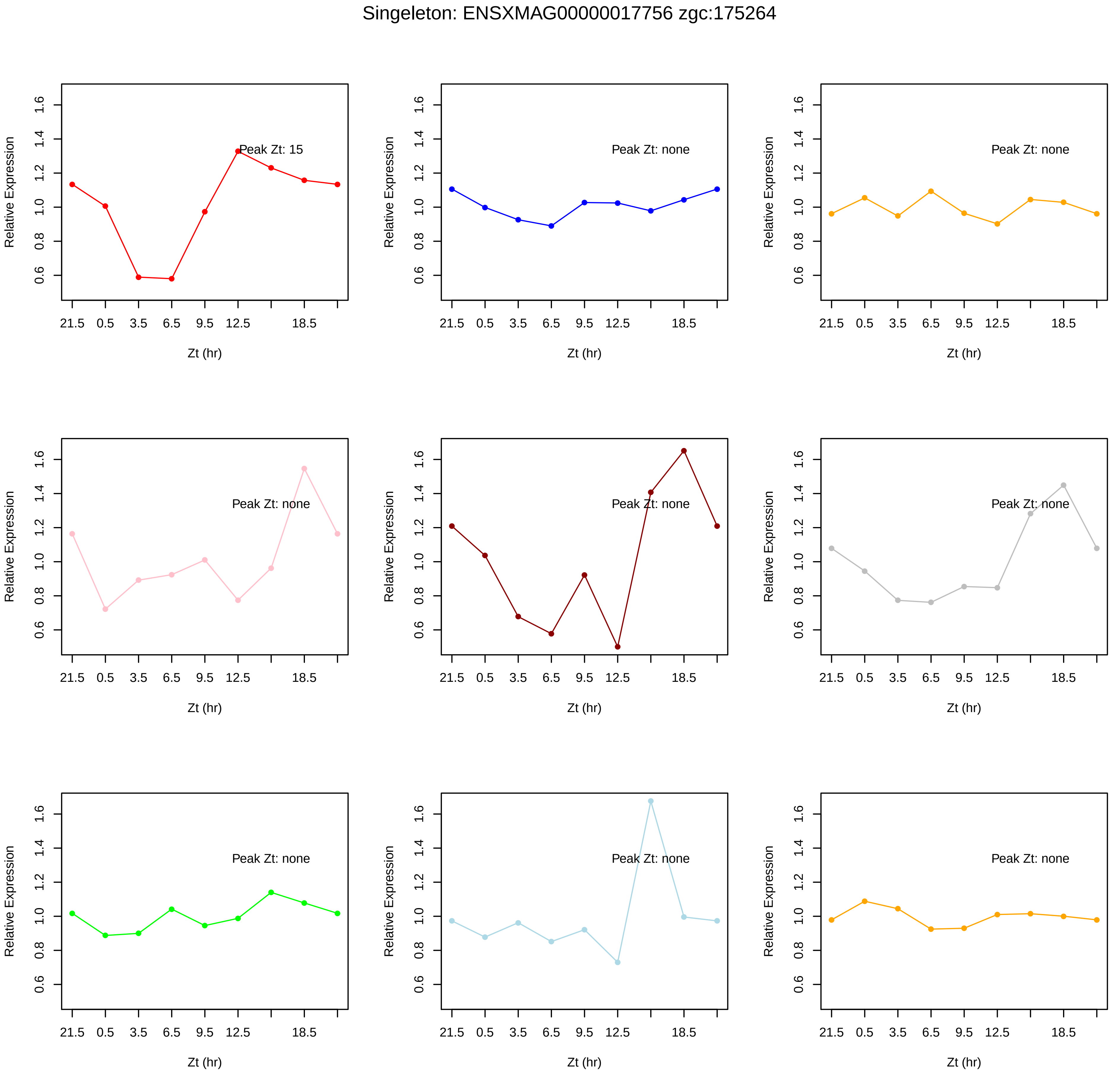
Singeleton: ENSXMAG00000017756 zgc:175264
Peak Zt: 15
Peak Zt: none
Peak Zt: none
Peak Zt: none
Peak Zt: none
Peak Zt: none
Peak Zt: none
Peak Zt: none
Peak Zt: none
Relative Expression
Zt (hr)
21.5
0.5
3.5
6.5
9.5
12.5
18.5
0.6
0.8
1.0
1.2
1.4
1.6

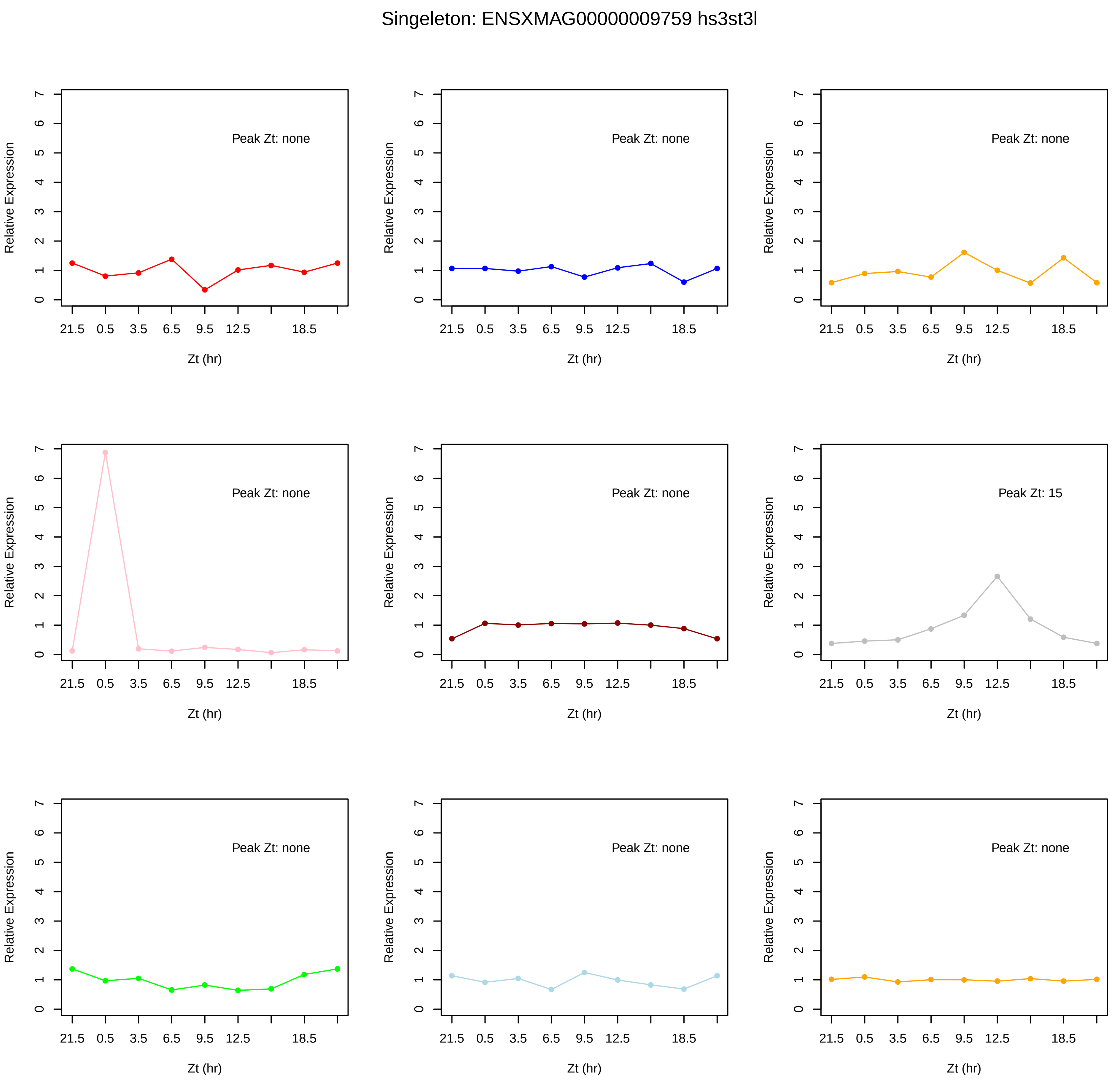
Singeleton: ENSXMAG00000009759 hs3st3l
Peak Zt: none
Peak Zt: none
Peak Zt: none
Peak Zt: none
Peak Zt: none
Peak Zt: 15
Peak Zt: none
Peak Zt: none
Peak Zt: none
Relative Expression
Zt (hr)
21.5
0.5
3.5
6.5
9.5
12.5
18.5

Singeleton: ENSXMAG00000002875 n6amt1

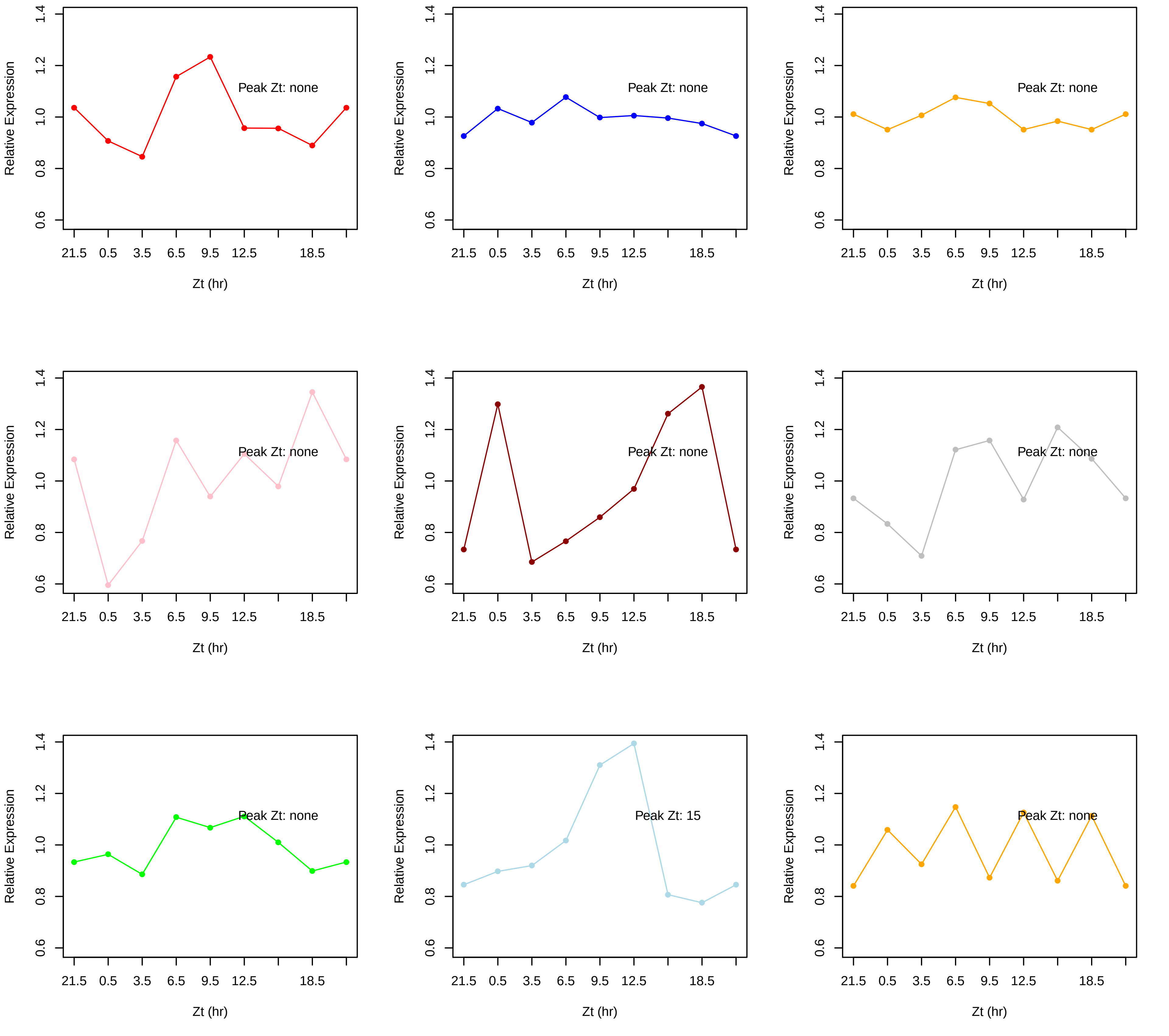

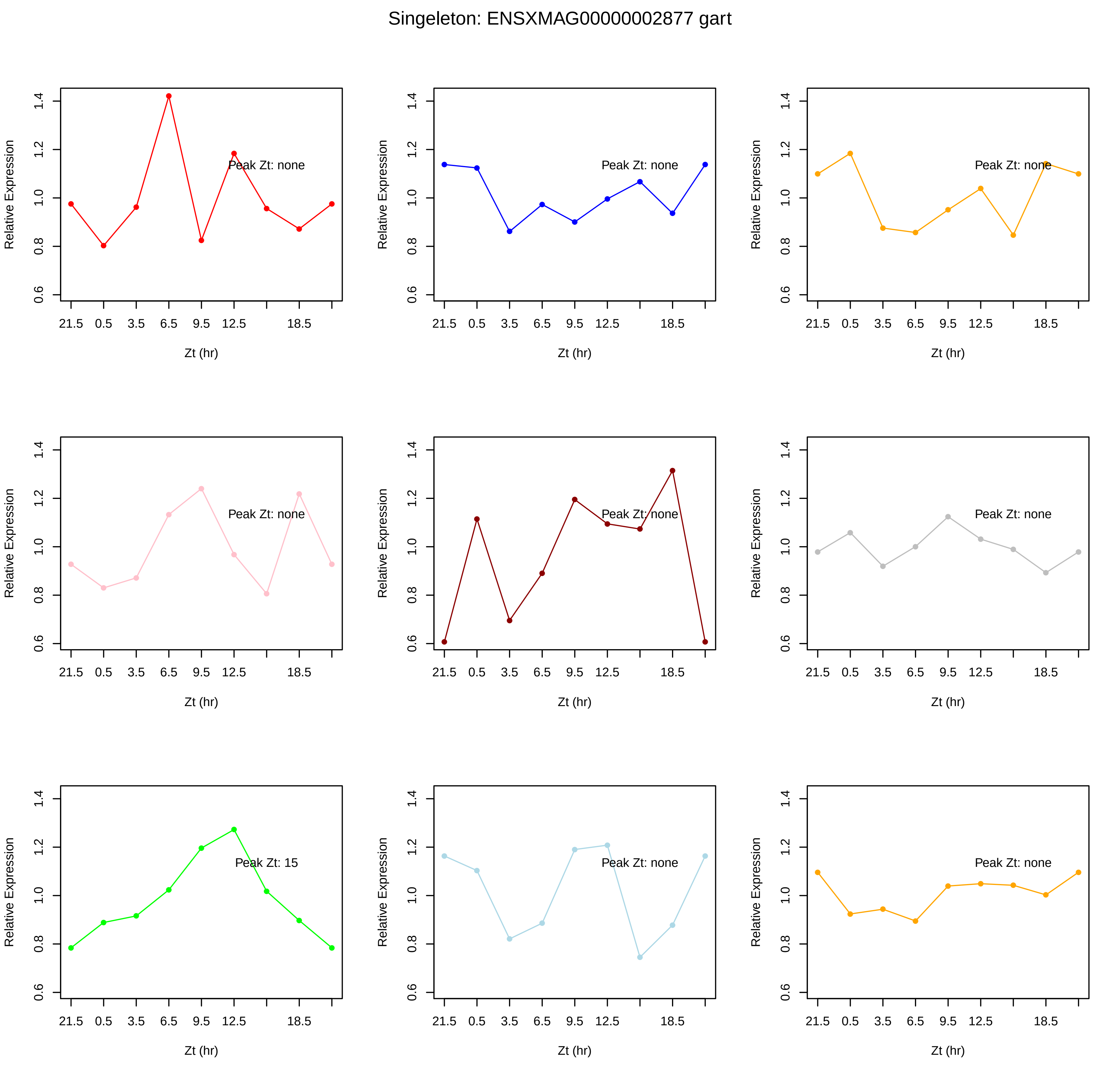
Singeleton: ENSXMAG00000002877 gart
Peak Zt: none
Peak Zt: none
Peak Zt: none
Peak Zt: none
Peak Zt: none
Peak Zt: none
Peak Zt: 15
Peak Zt: none
Peak Zt: none
Relative Expression
Zt (hr)
21.5
0.5
3.5
6.5
9.5
12.5
18.5
0.6
0.8
1.0
1.2
1.4

Singeleton: ENSXMAG00000006813 slco2b1

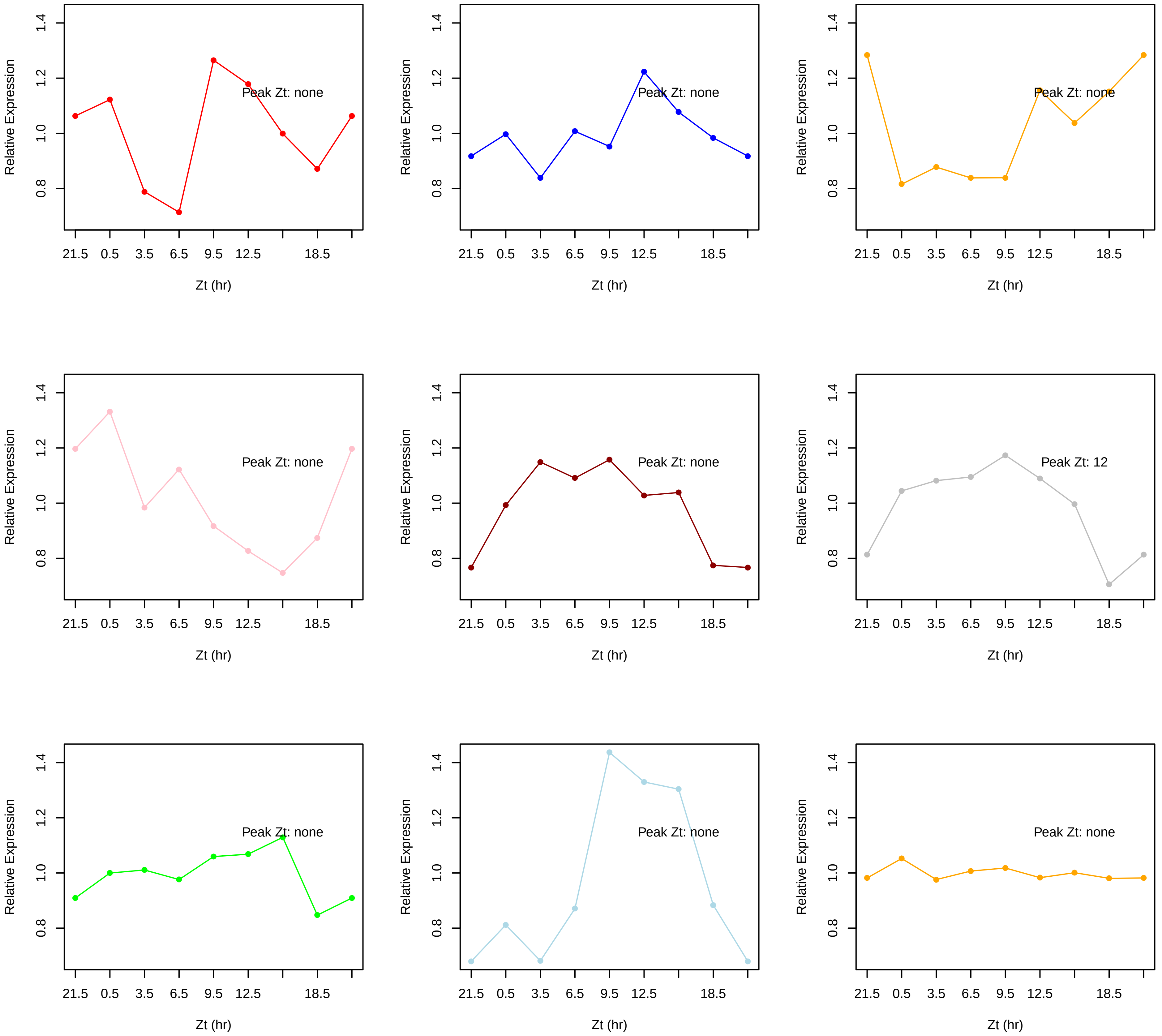

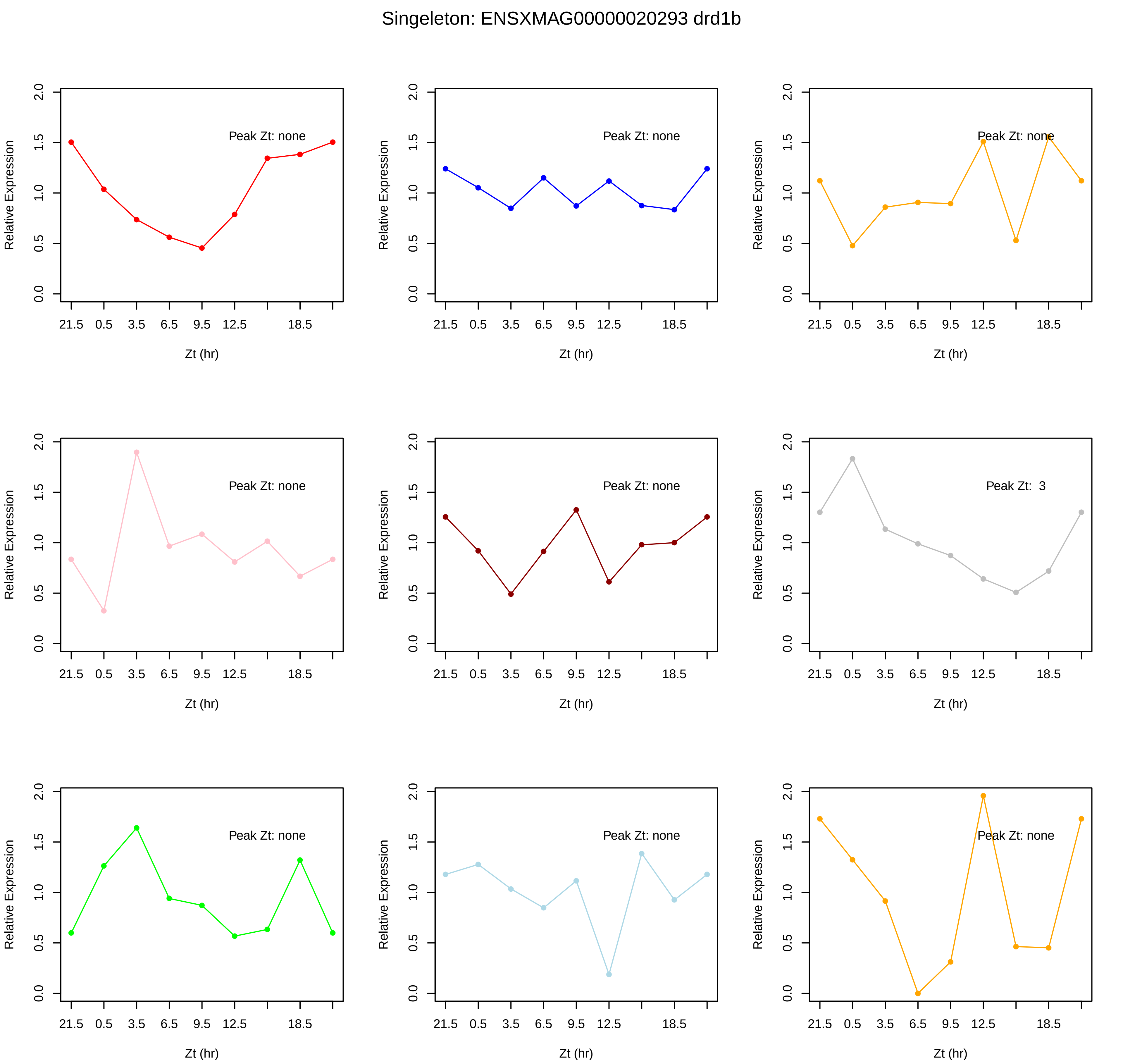
Singleton: ENSXMAG00000020293 drd1b
Peak Zt: none
Peak Zt: none
Peak Zt: none
Peak Zt: none
Peak Zt: none
Peak Zt: 3
Peak Zt: none
Peak Zt: none
Peak Zt: none
Relative Expression
Zt (hr)
21.5
0.5
3.5
6.5
9.5
12.5
18.5
0.0
0.5
1.0
1.5
2.0

Singleton: ENSXMAG00000013551 reep1

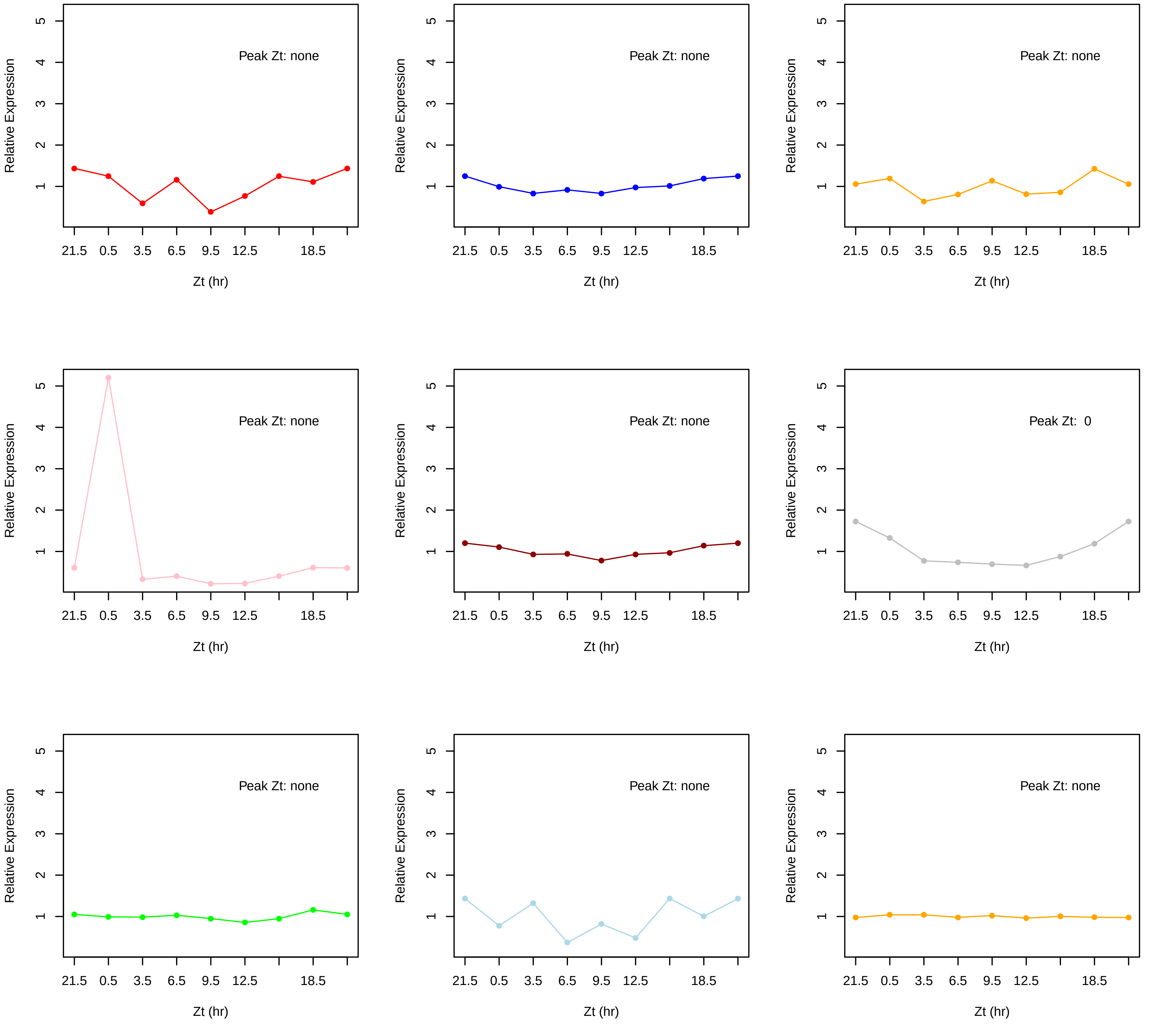

Singeleton: ENSXMAG00000000768

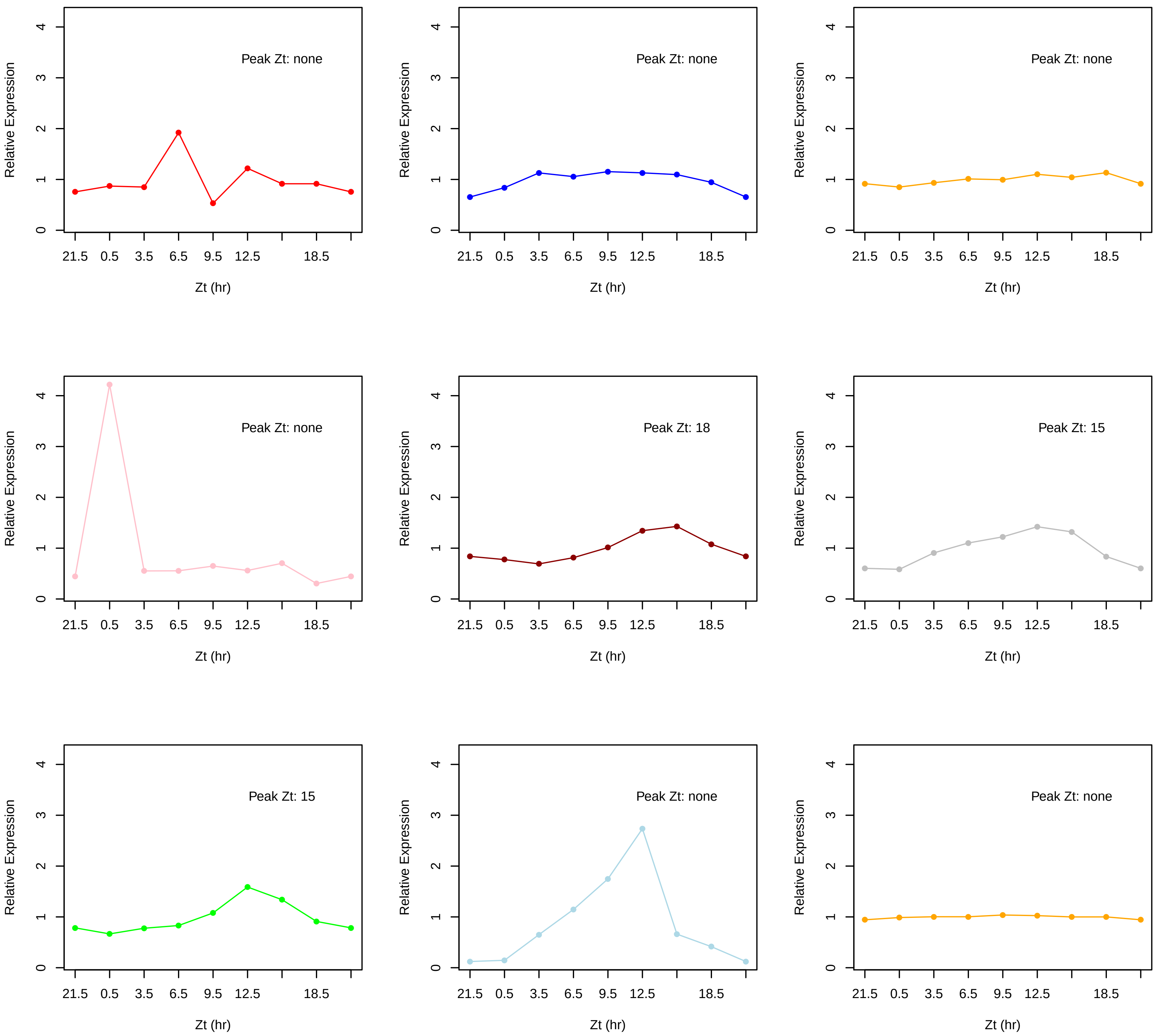

Singeleton: ENSXMAG00000013548 mrpl35

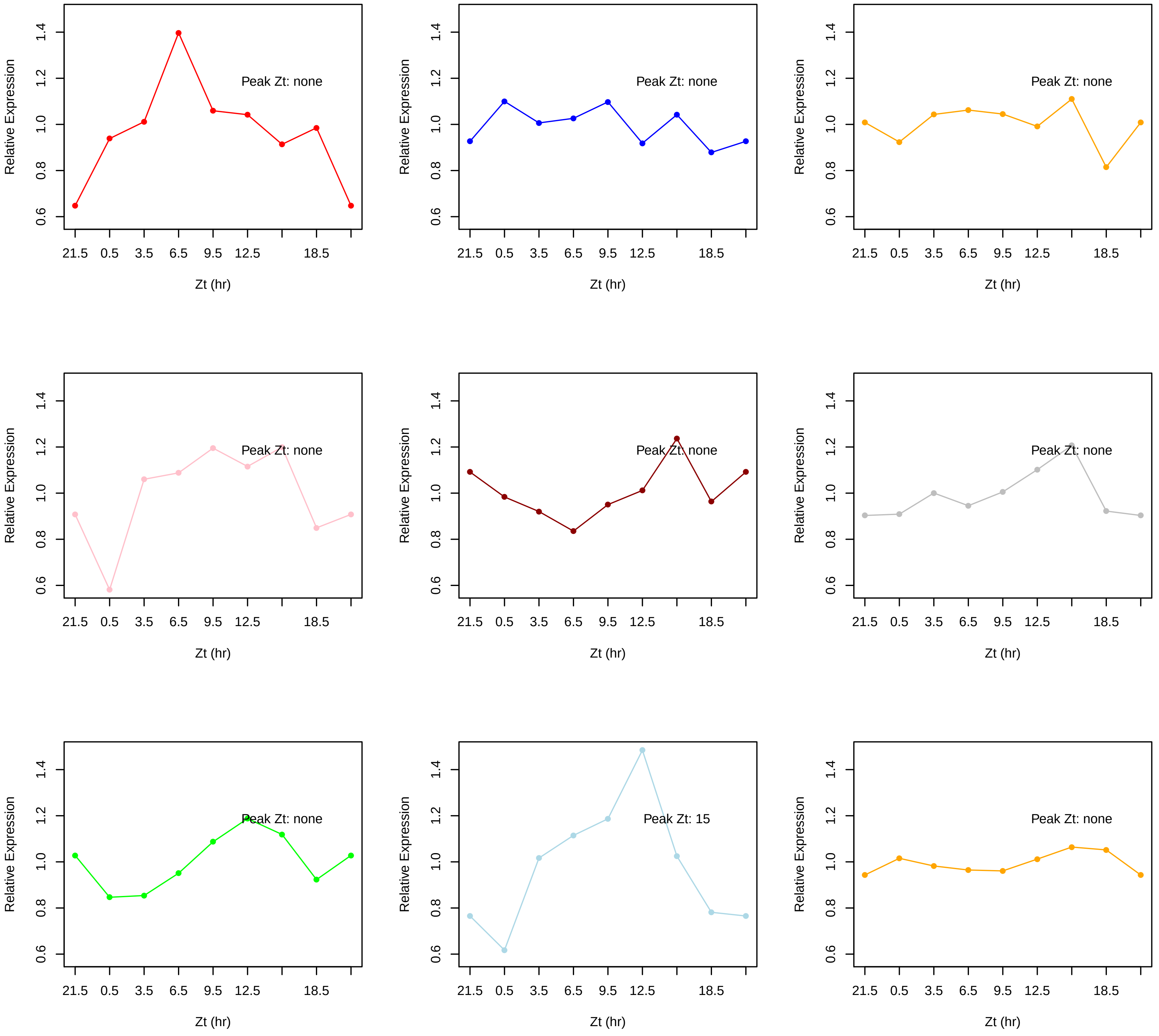

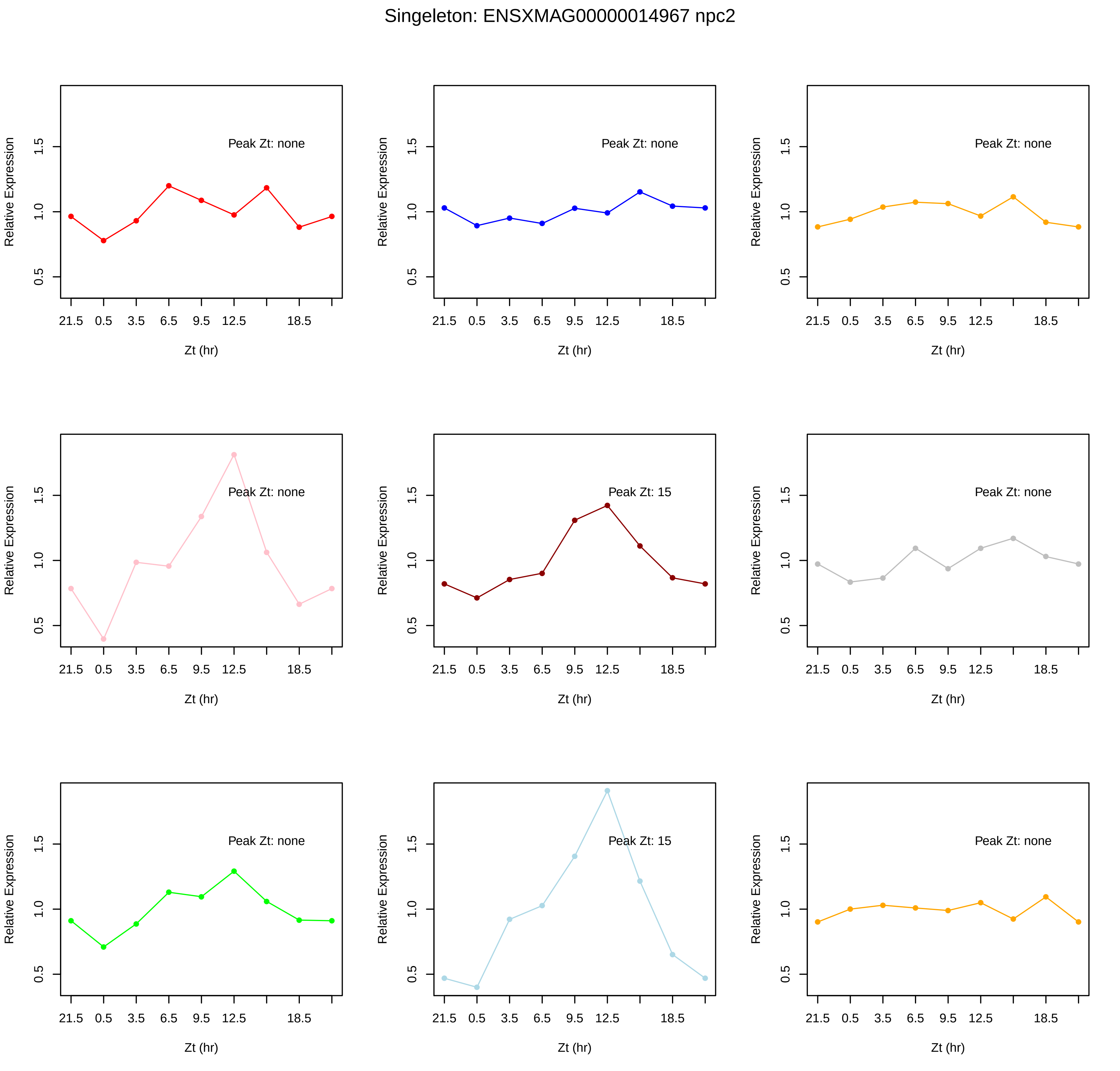
Singeleton: ENSXMAG00000014967 npc2
Peak Zt: none
Peak Zt: none
Peak Zt: none
Peak Zt: none
Peak Zt: 15
Peak Zt: none
Peak Zt: none
Peak Zt: 15
Peak Zt: none
Relative Expression
Zt (hr)
21.5
0.5
3.5
6.5
9.5
12.5
18.5
0.5
1.0
1.5

Singeleton: ENSXMAG00000010606 ino80da

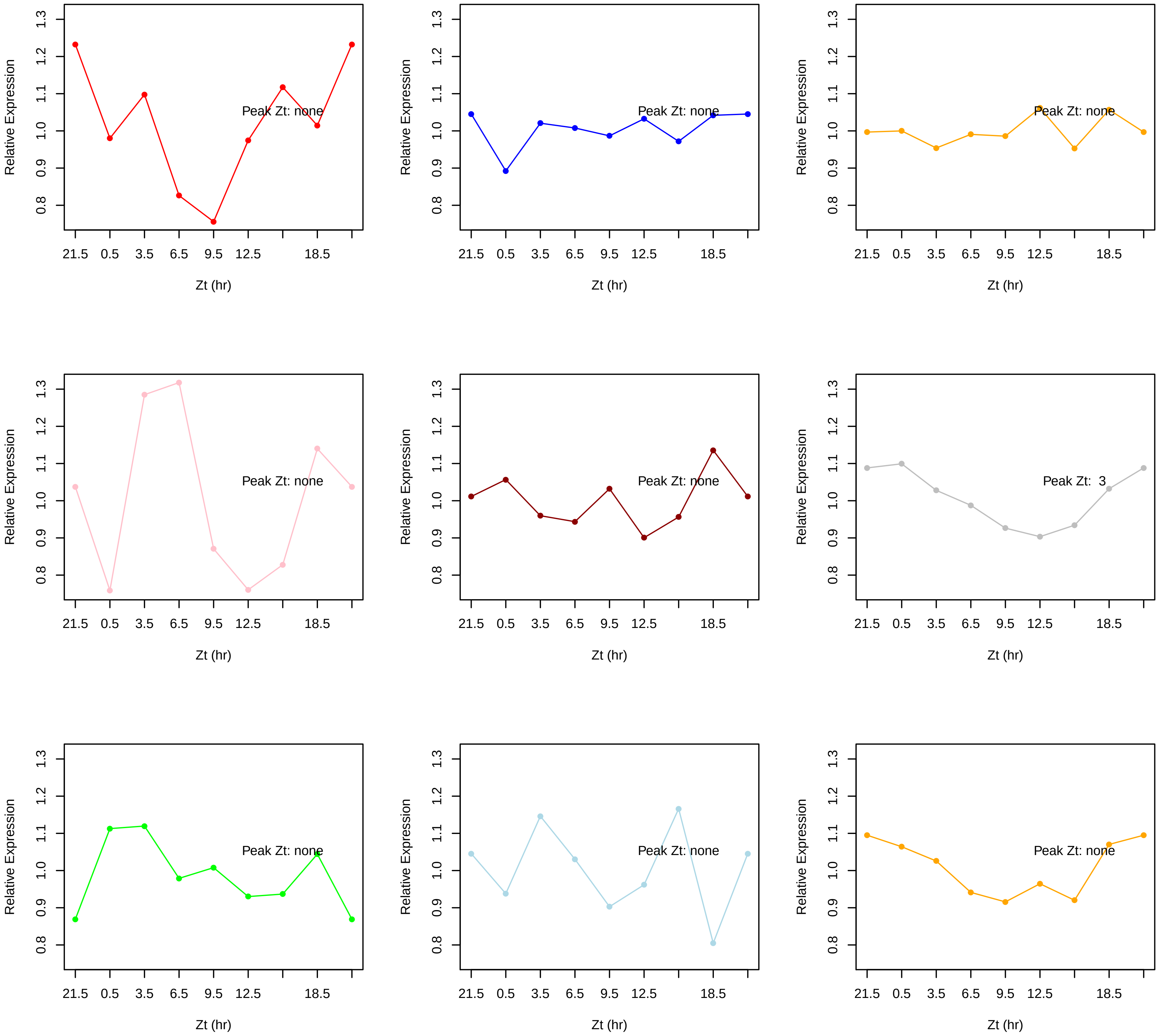

Singeleton: ENSXMAG00000000291 nsg2

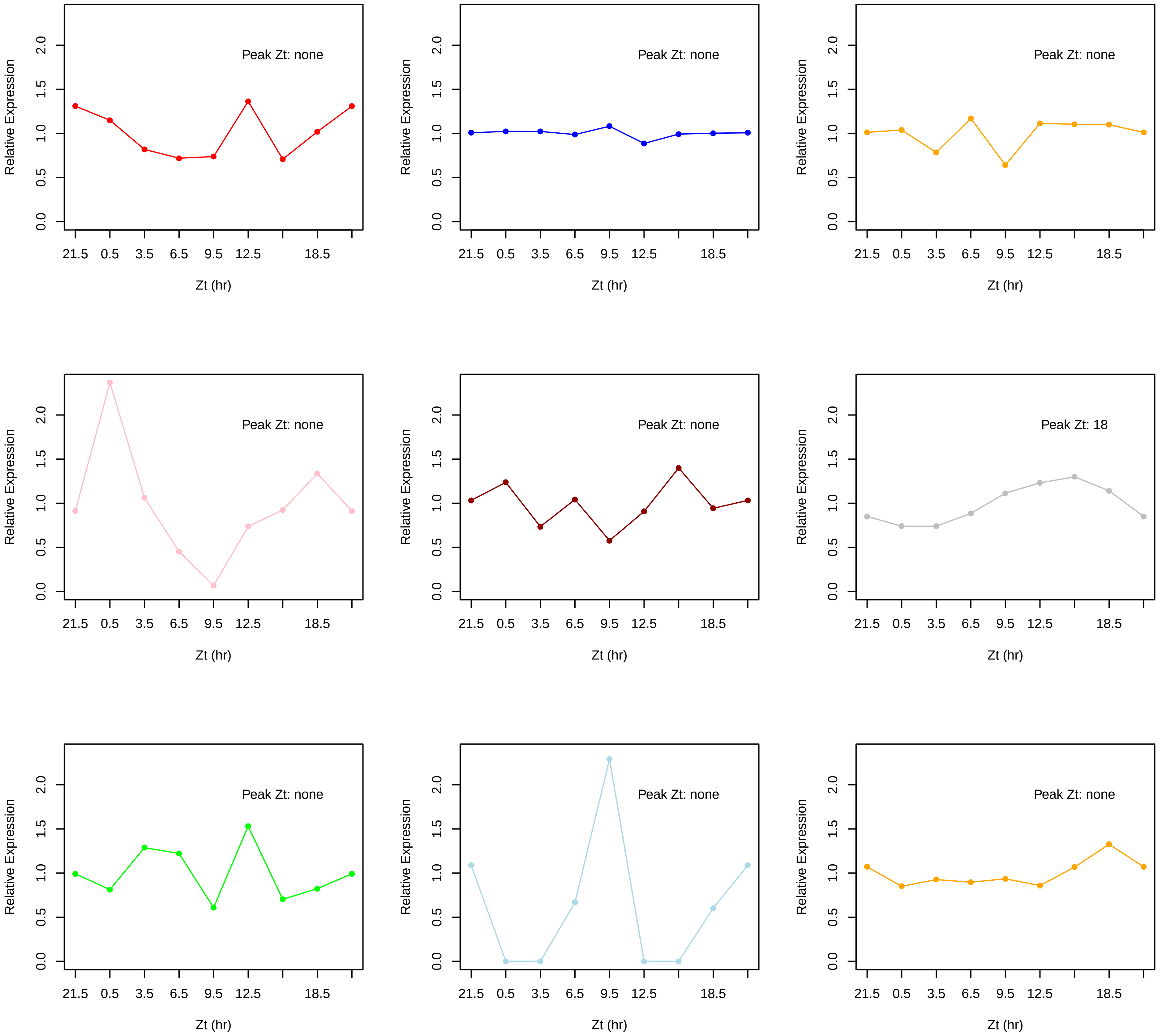

Singleton: ENSXMAG00000021924 mprip

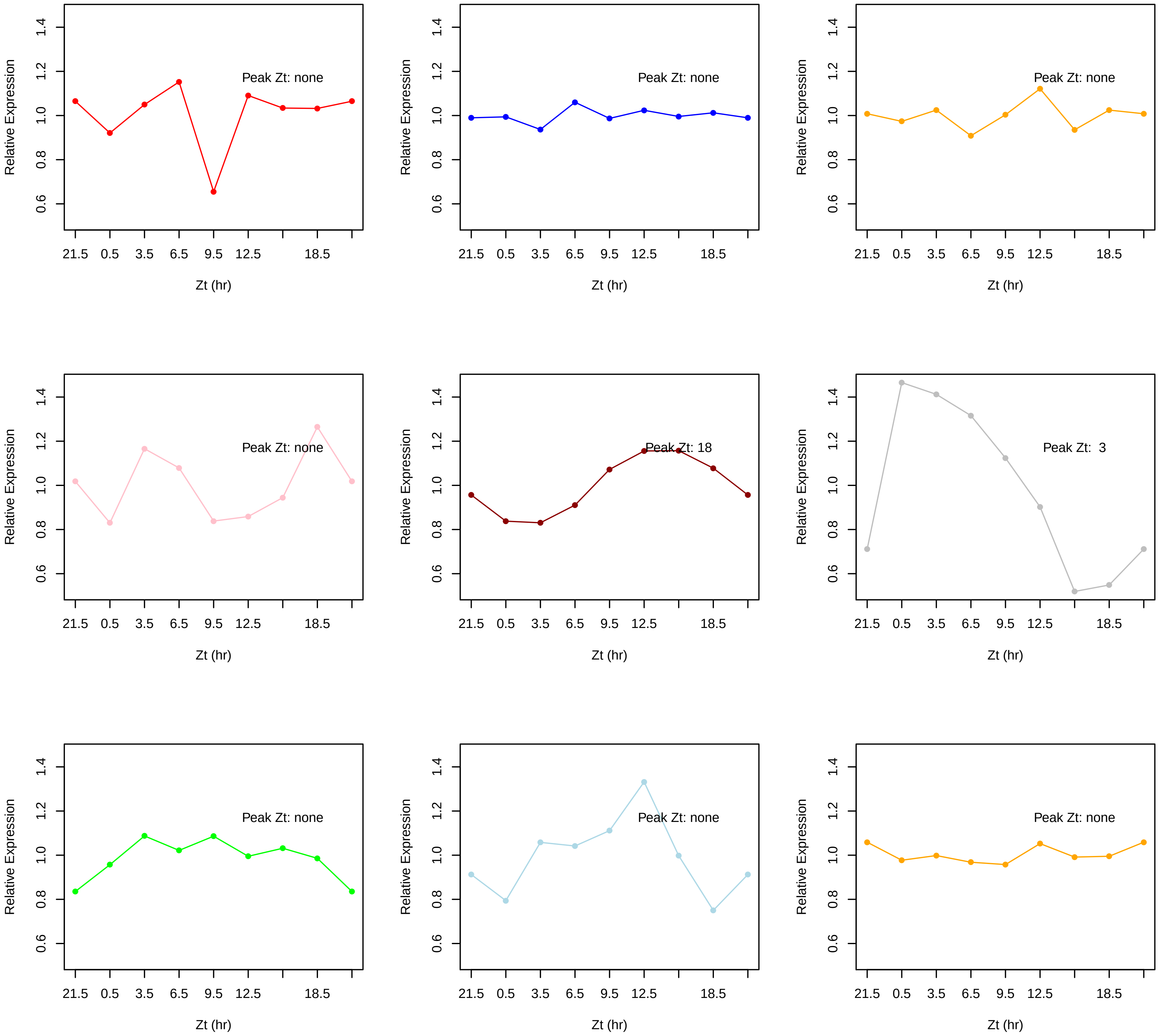

Singeleton: ENSXMAG00000012834 immt

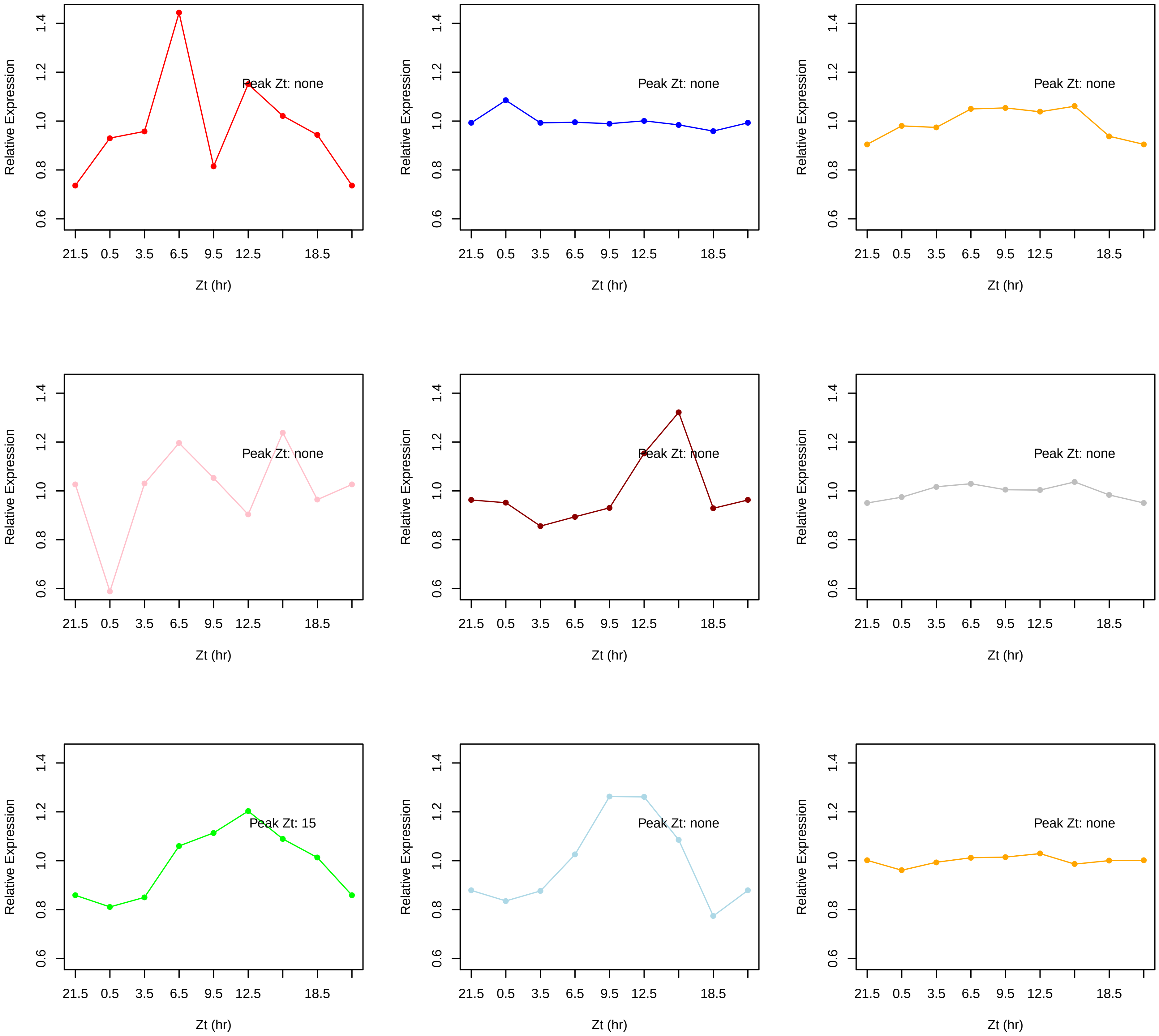

Singeleton: ENSXMAG00000014962 isca2

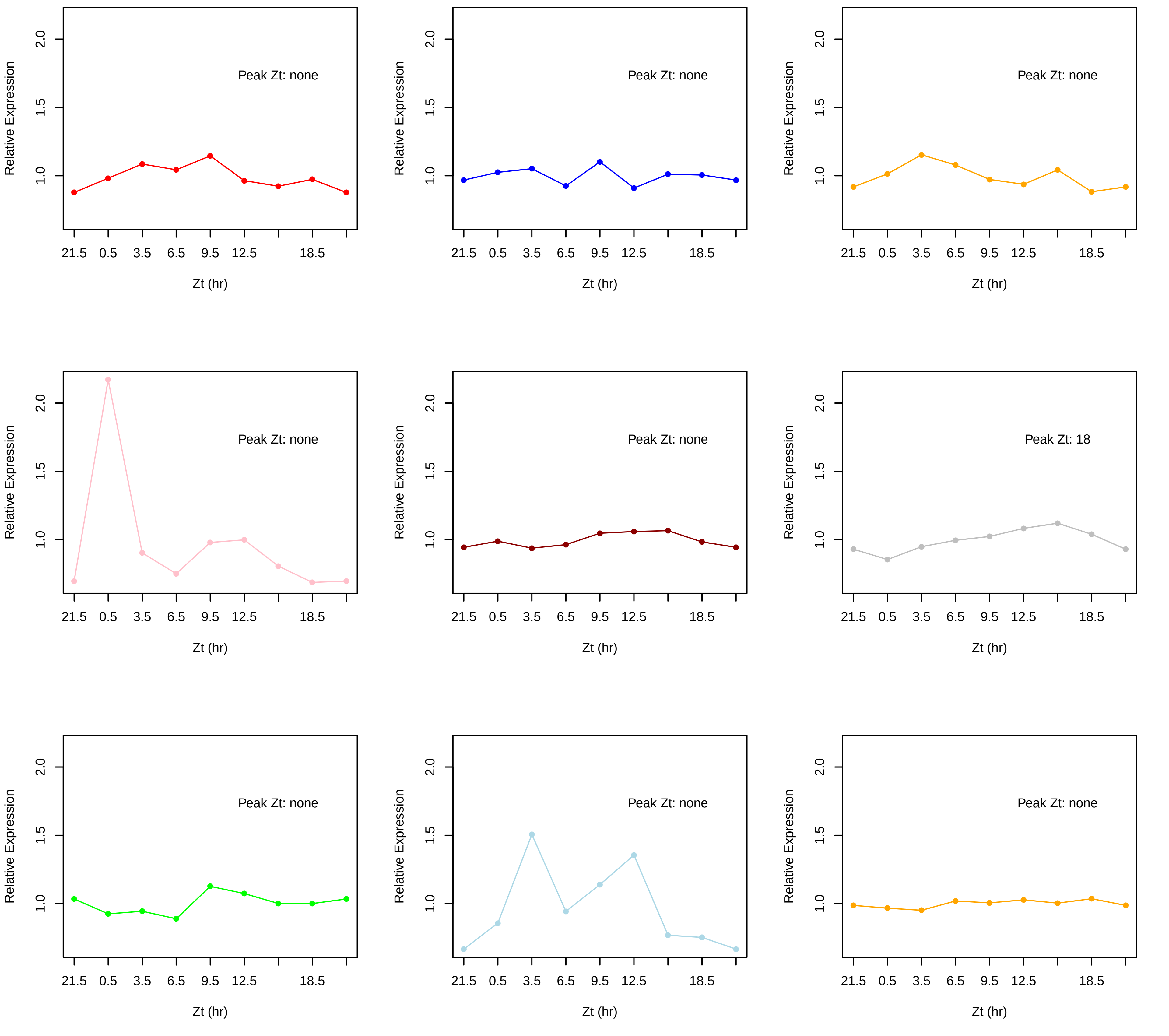

Singleton: ENSXMAG00000010700 nhej1

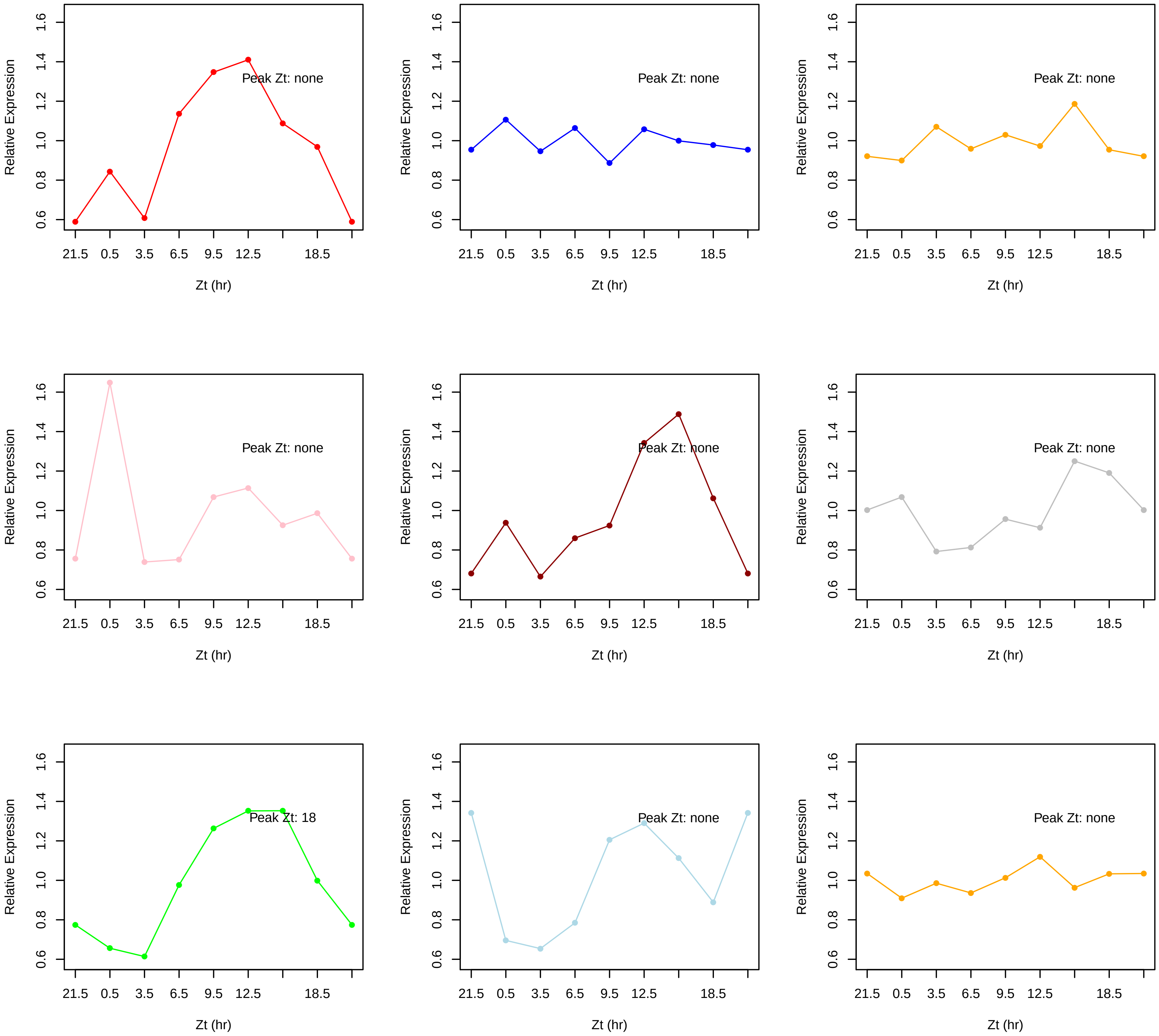

Singleton: ENSXMAG00000002289 NSUN6

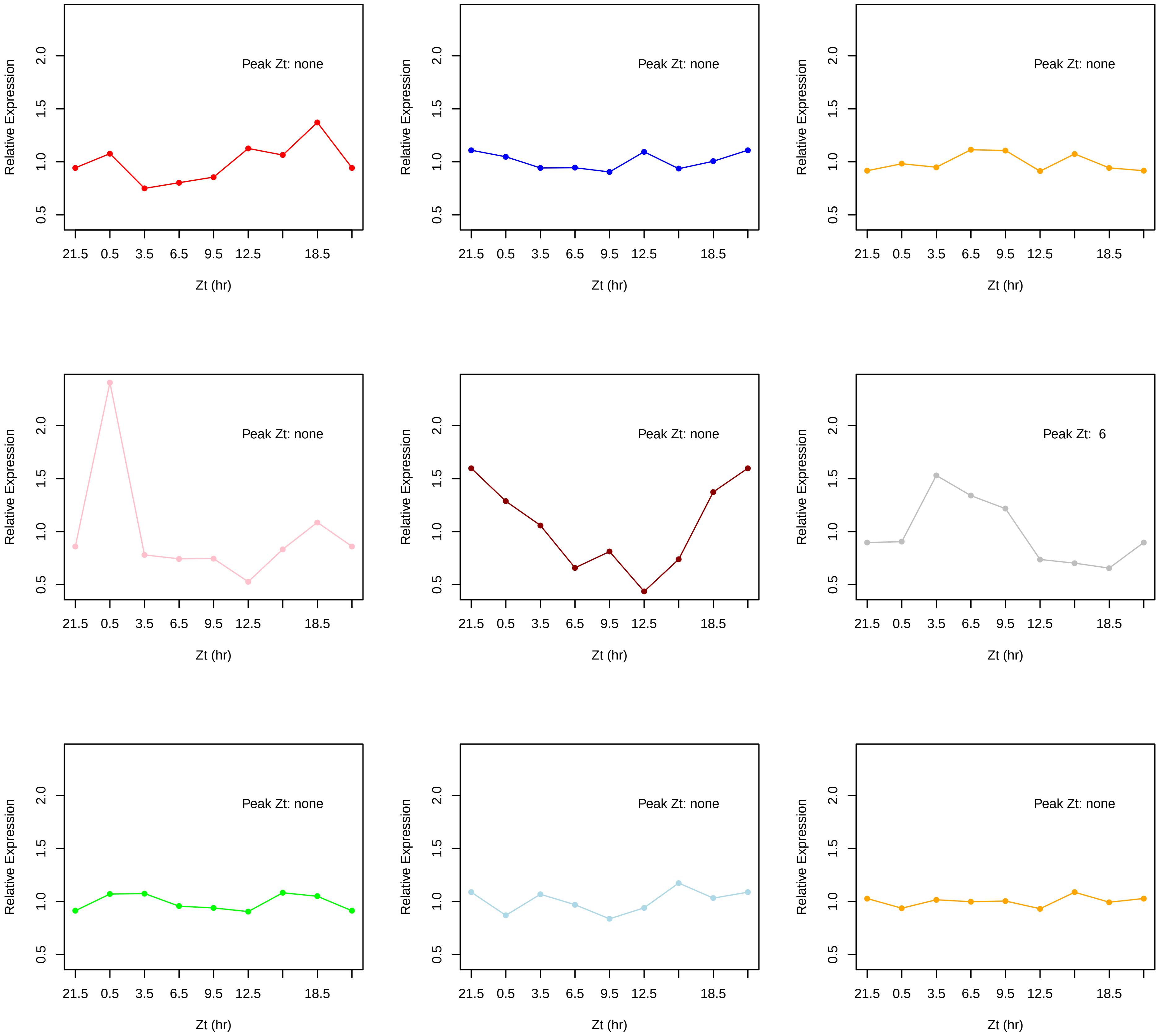

Singeleton: ENSXMAG00000028814 cryl1

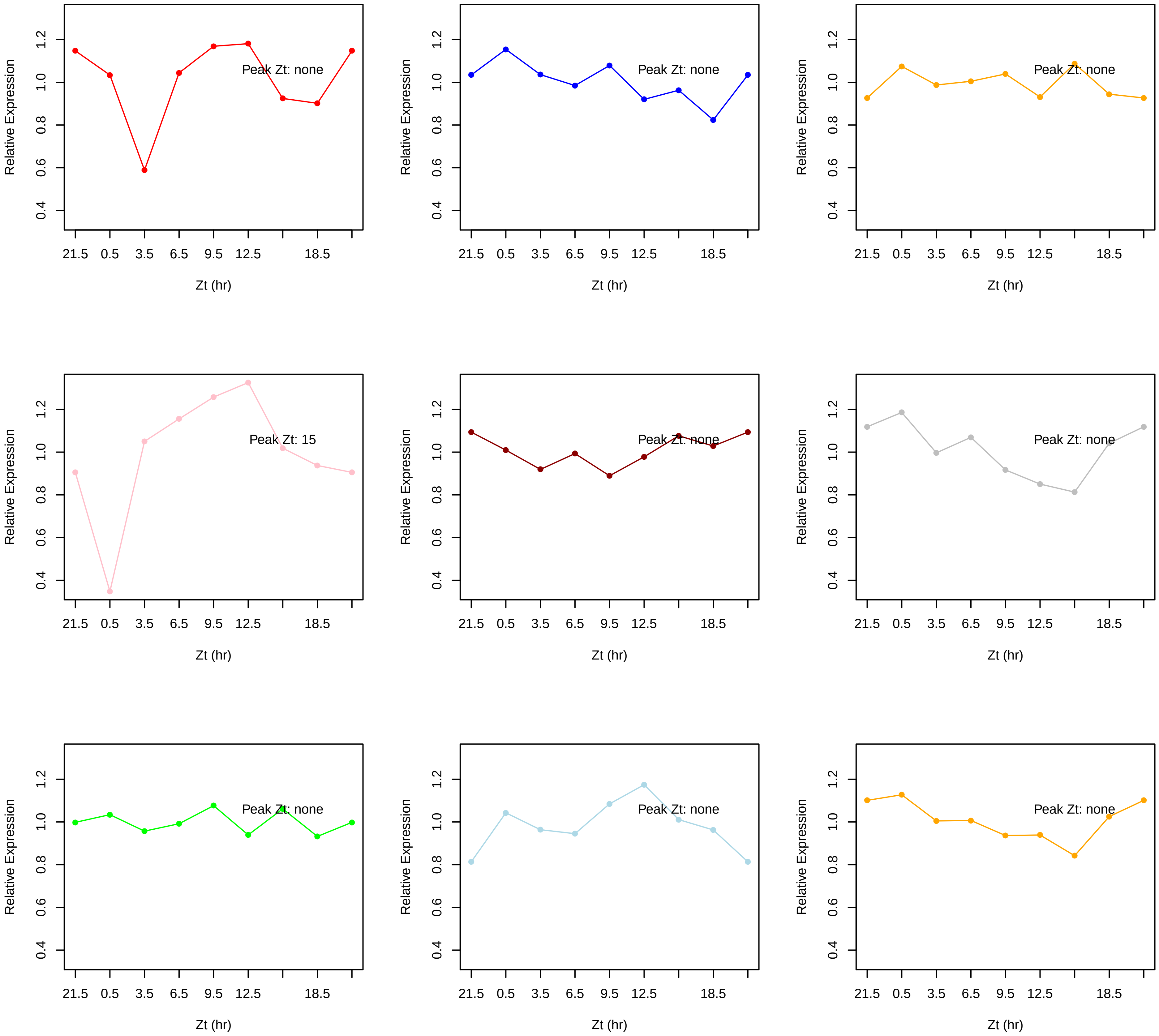

Singeleton: ENSXMAG00000010526 TENM2

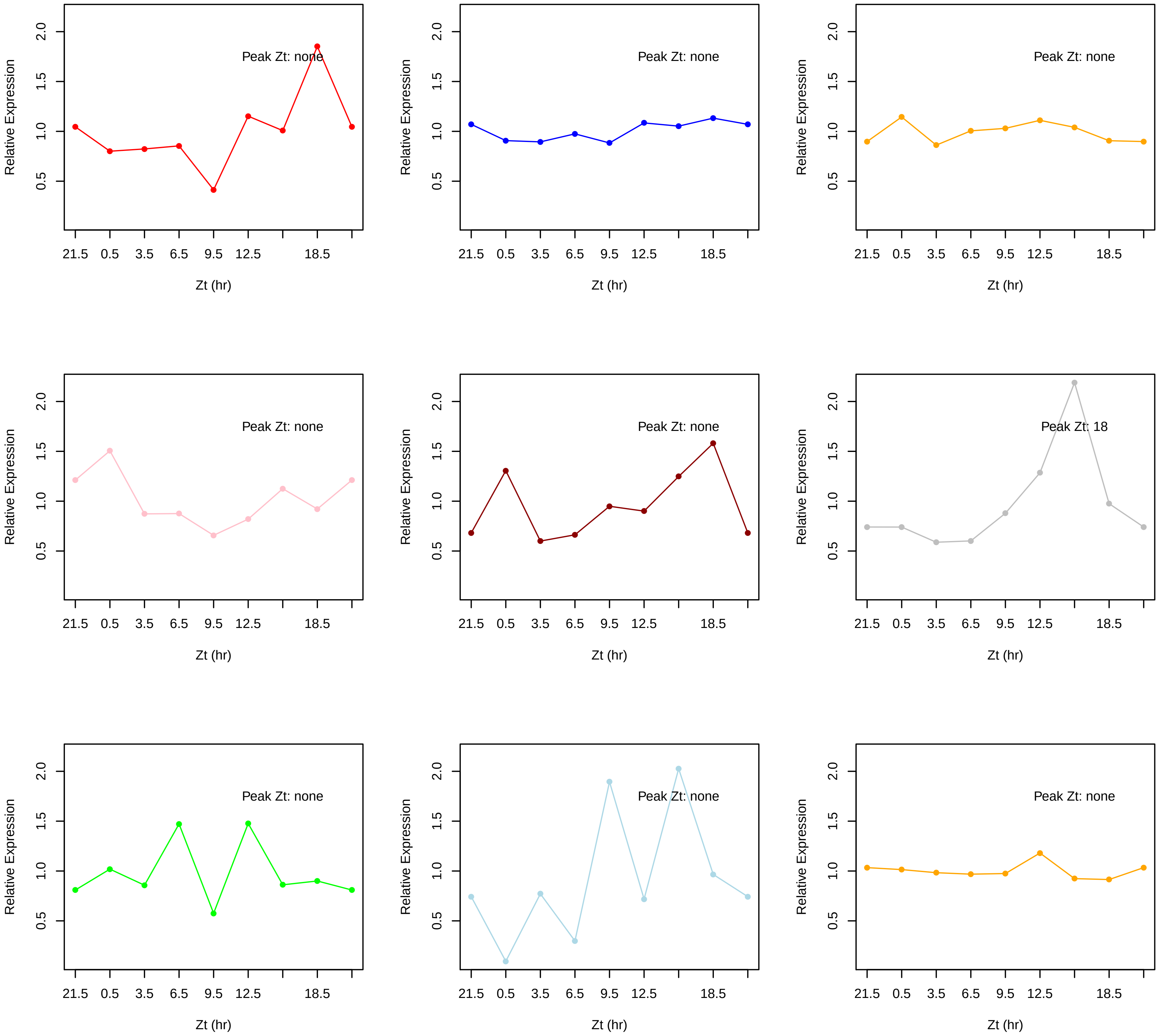

Singeleton: ENSXMAG00000004008 ptcd3

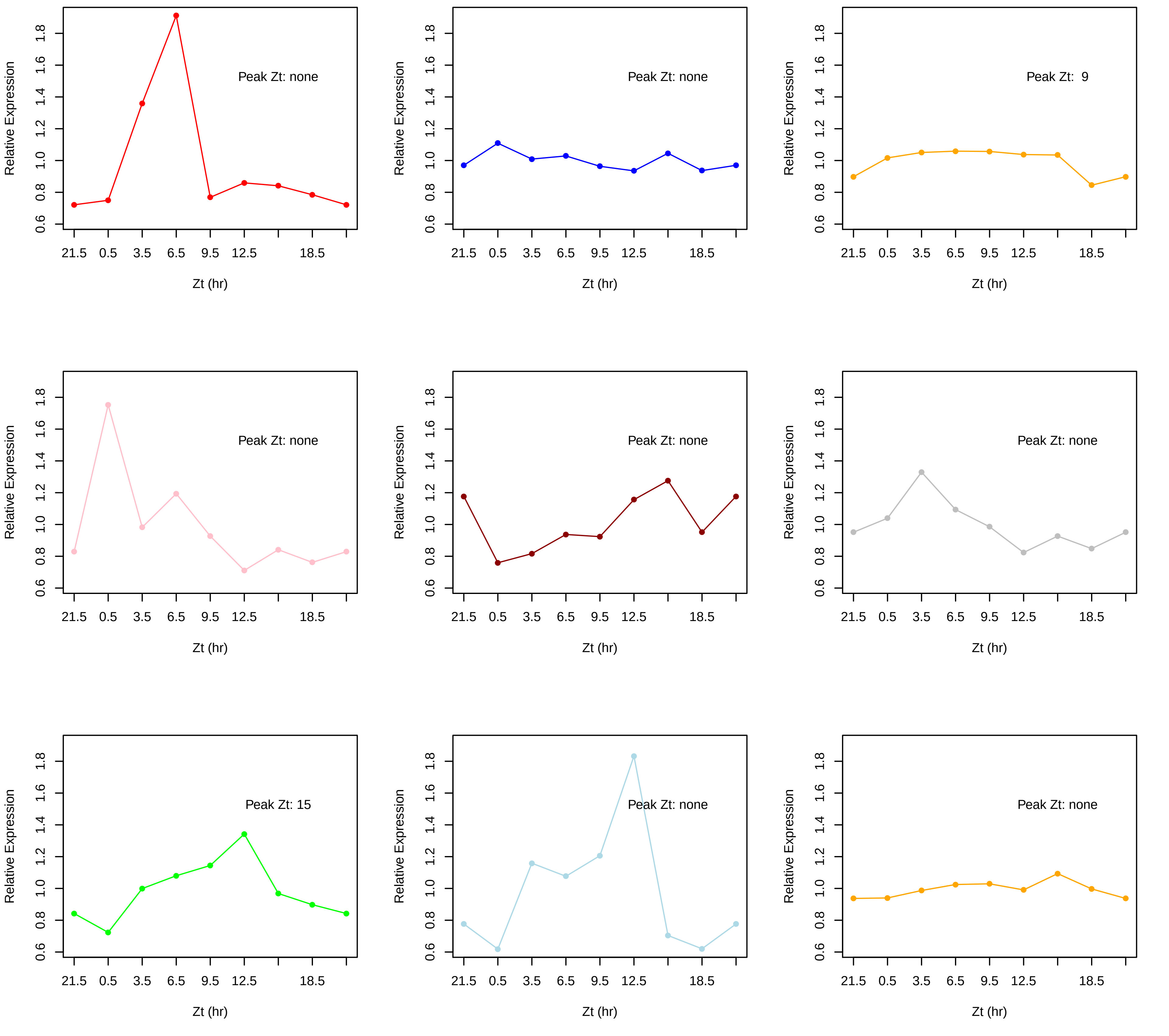

Singeleton: ENSXMAG00000005403 bsk146

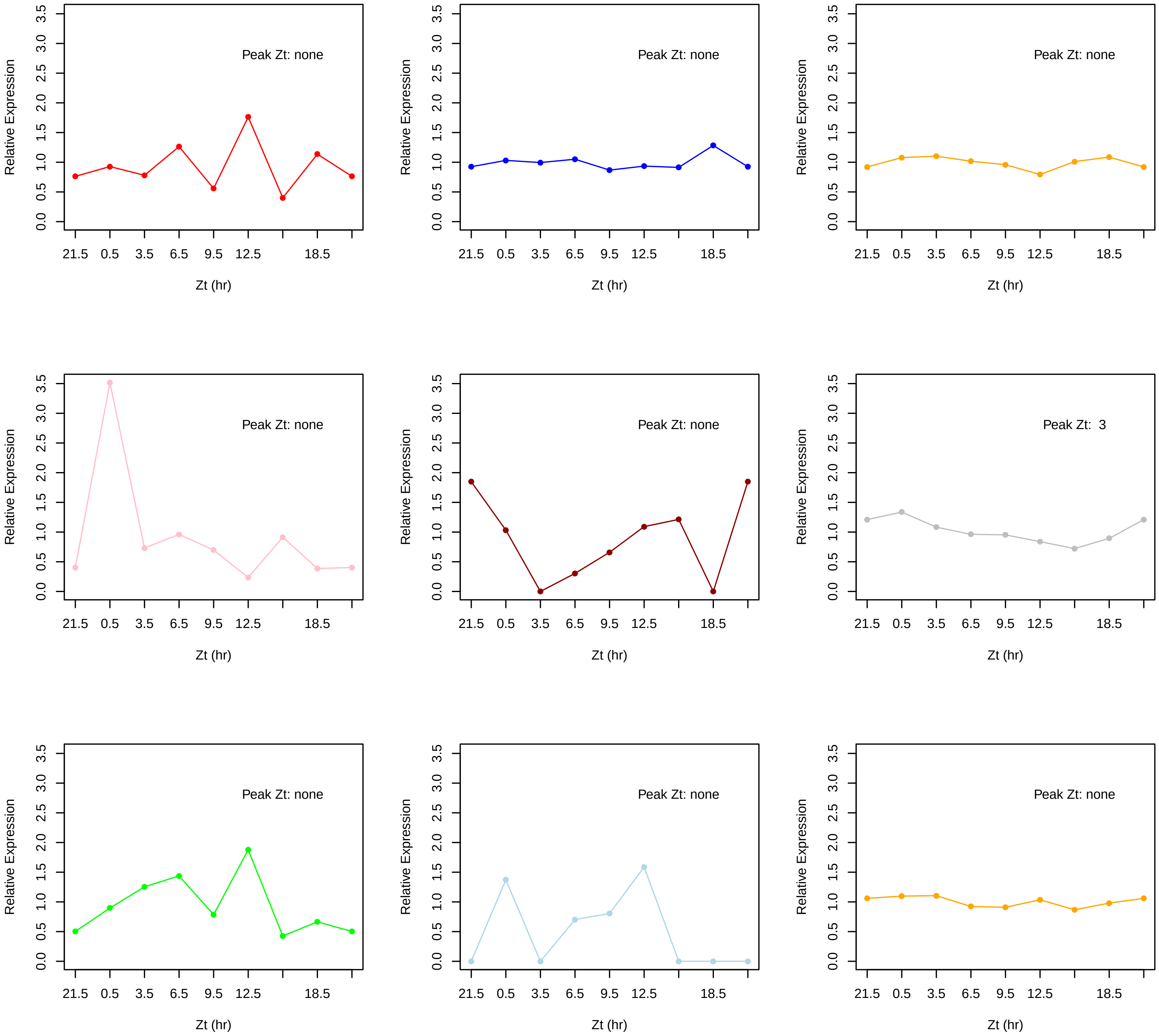

Singleton: ENSXMAG00000002274 SLC39A12

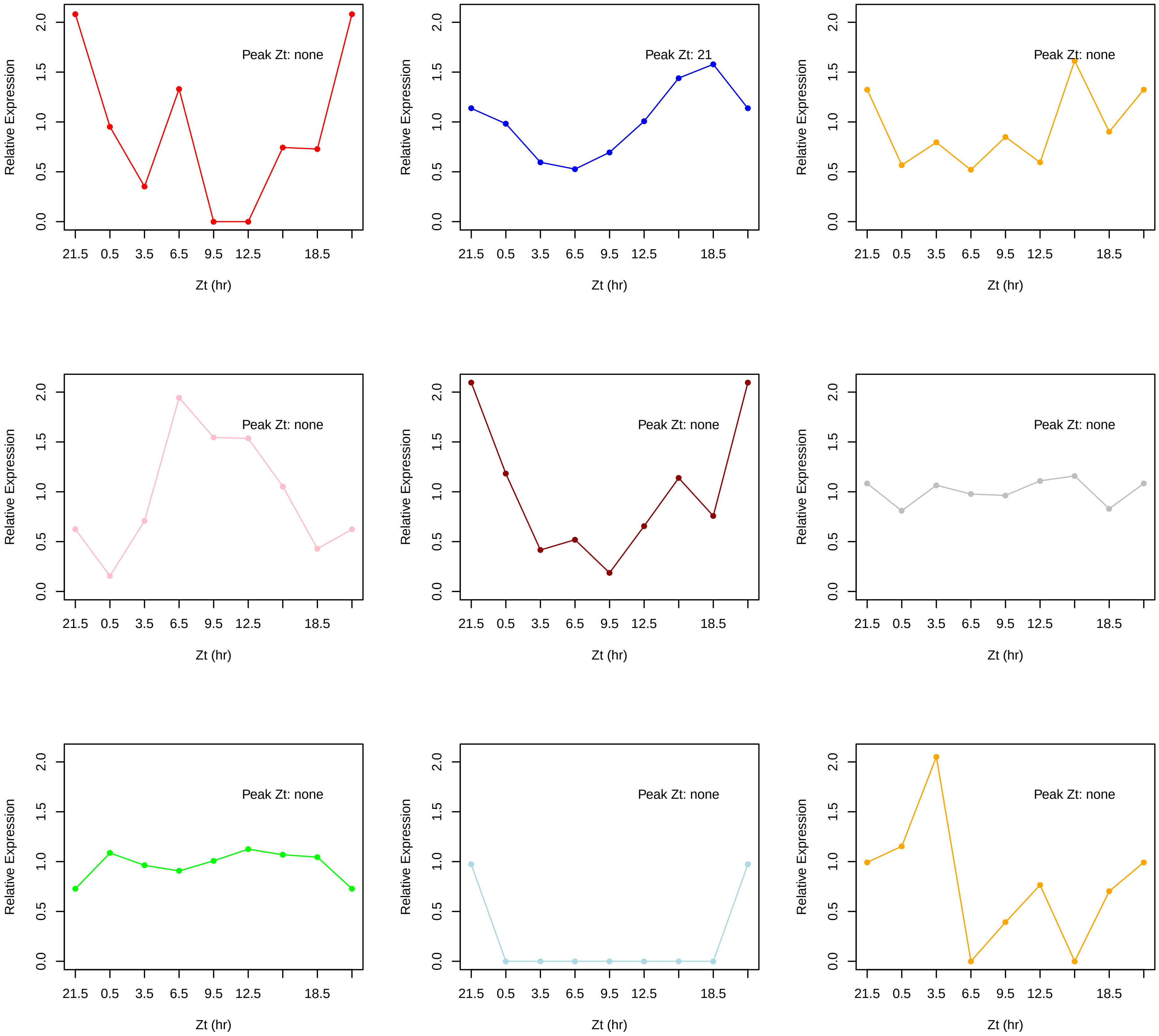

Singleton: ENSXMAG00000029444 eef1akmt1

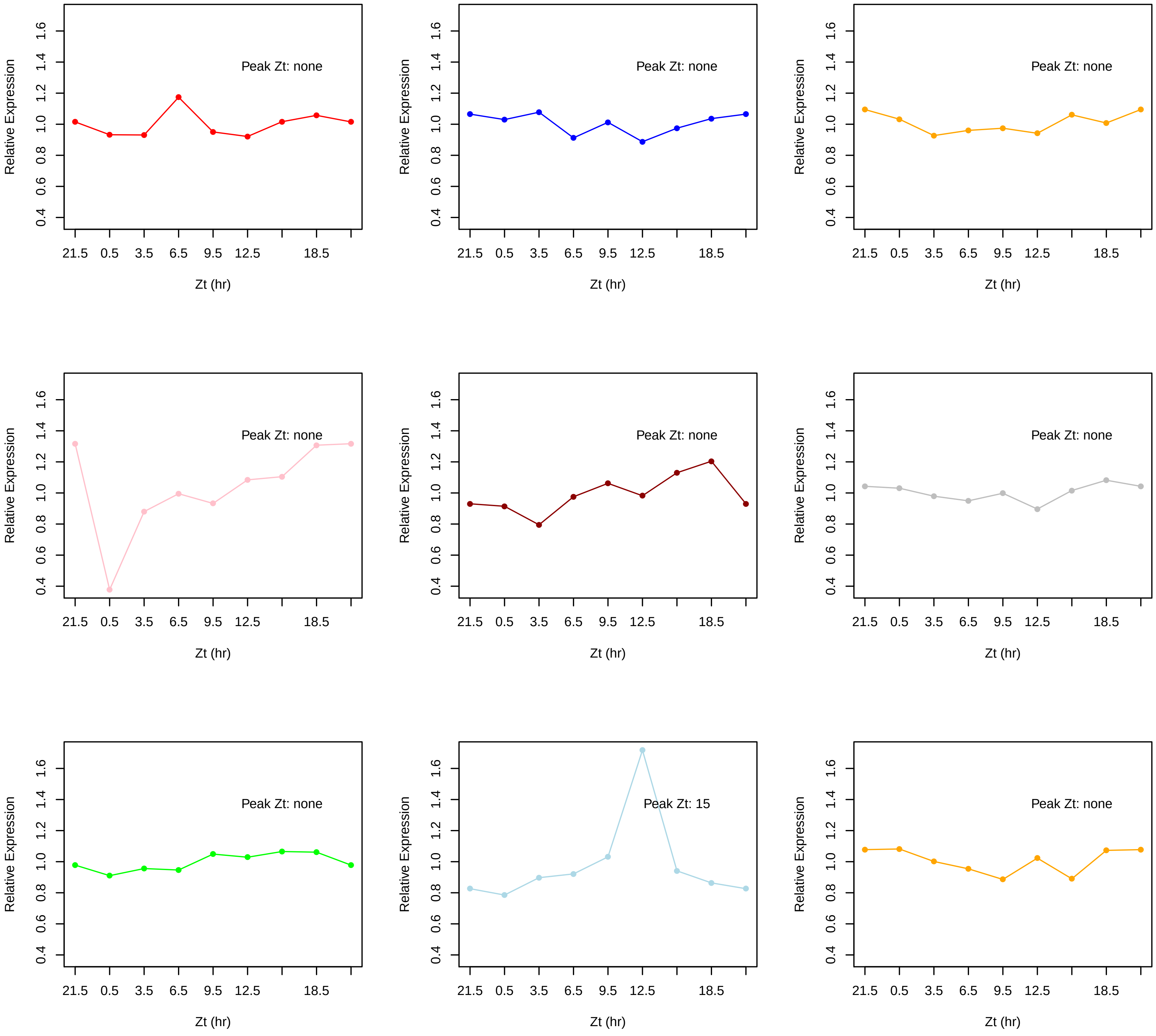

Singleton: ENSXMAG00000002252

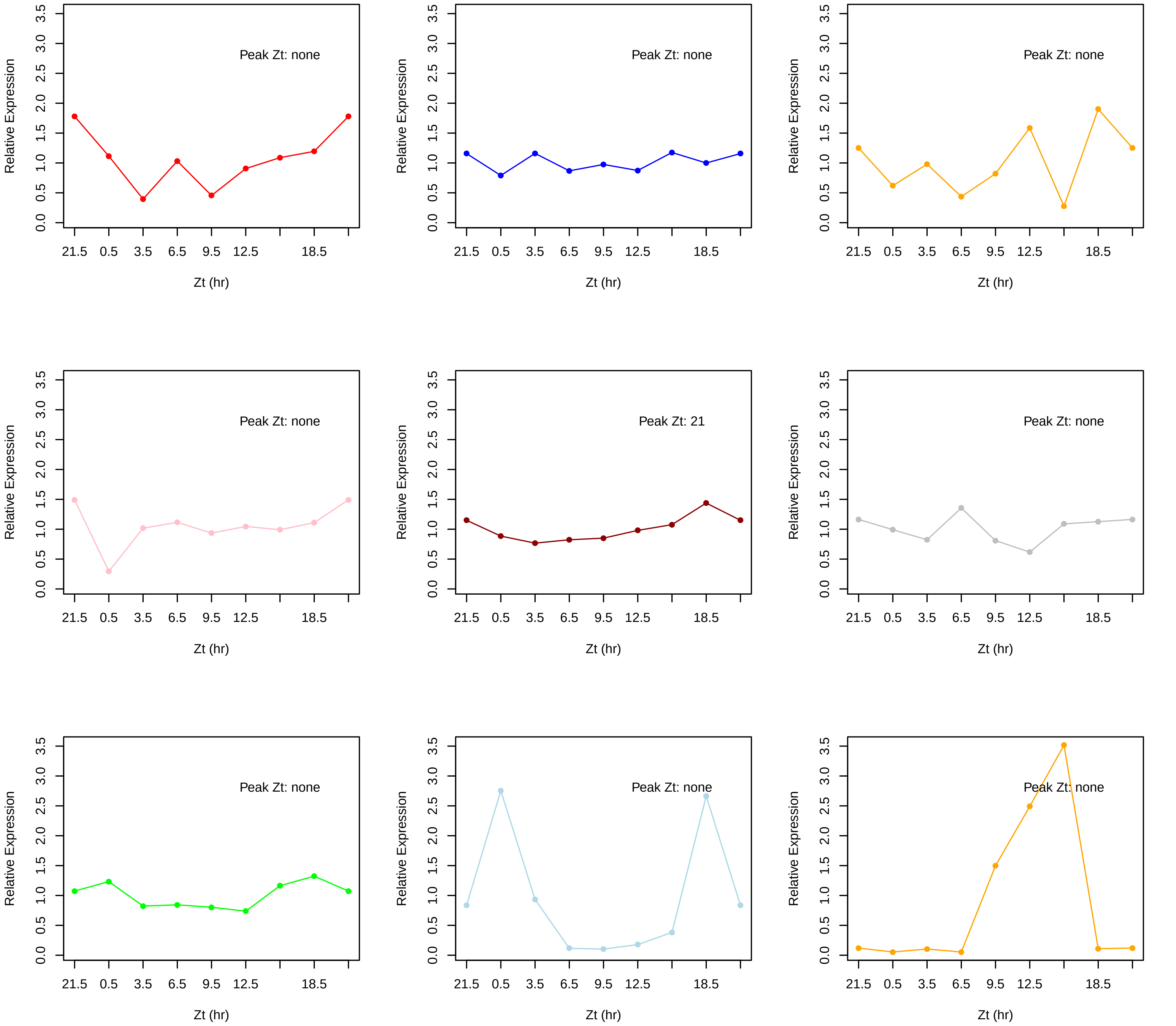

Singeleton: ENSXMAG00000029801 ACSS1

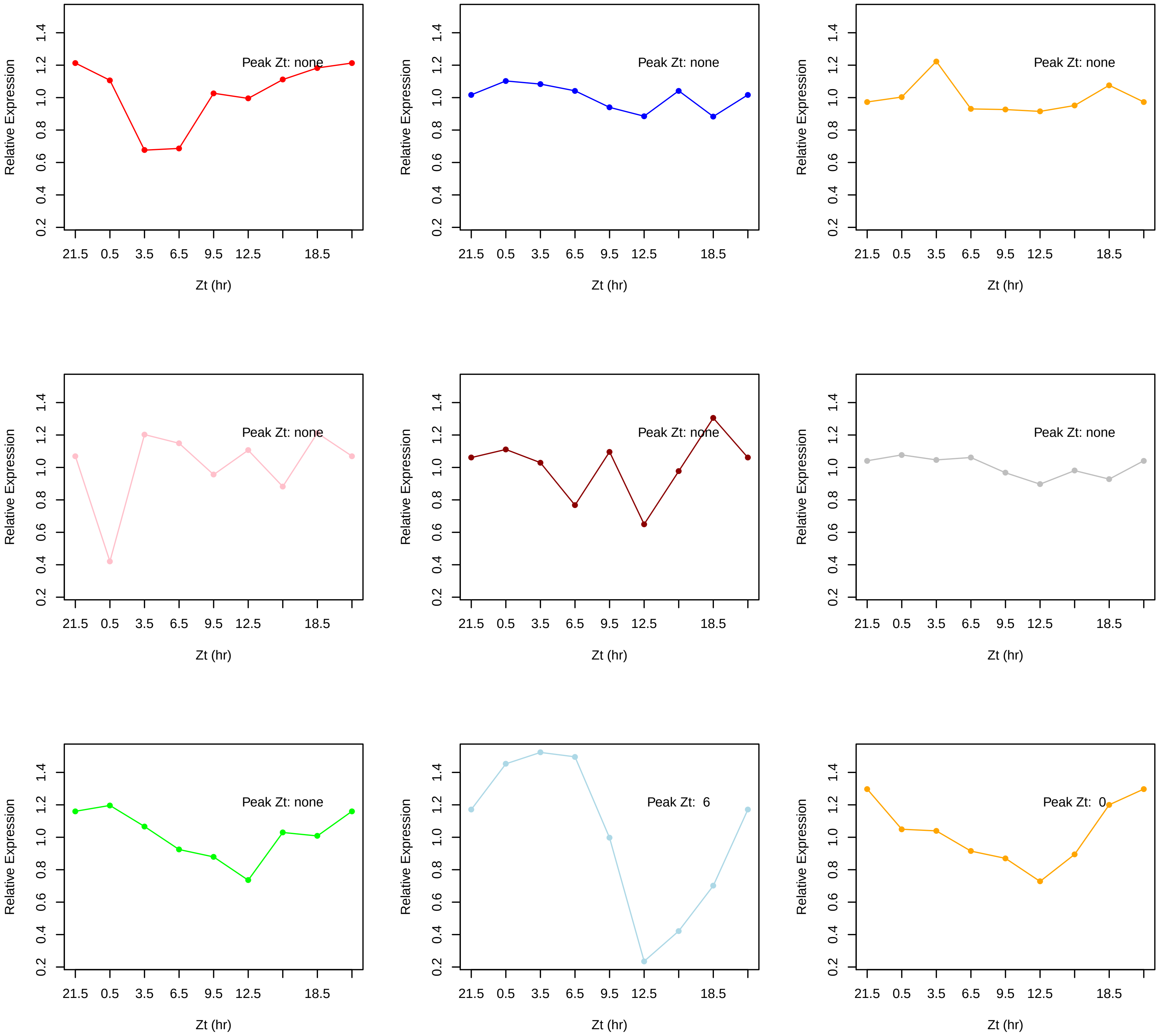

Singeleton: ENSXMAG00000000712 ppp1r9a

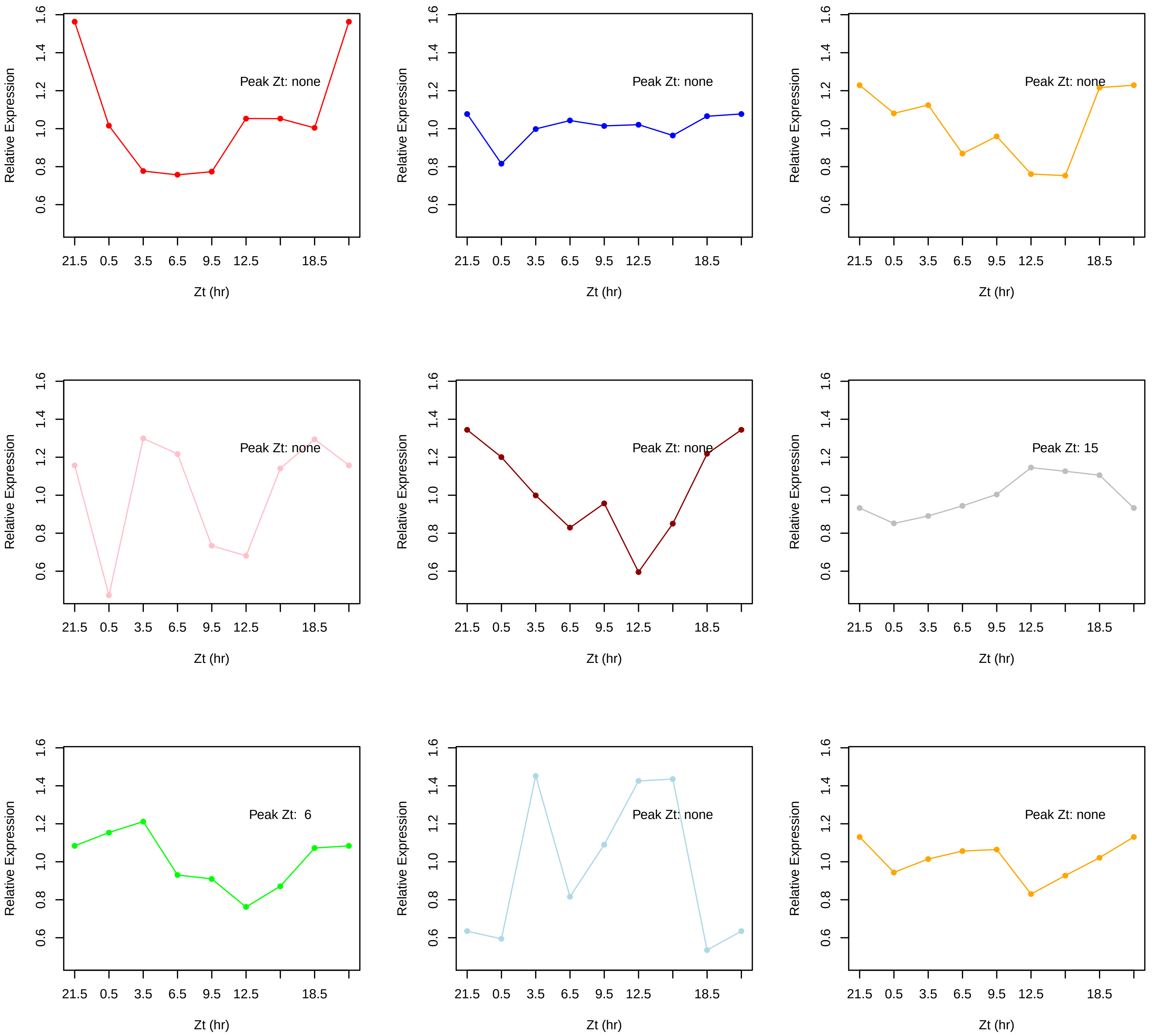

Singleton: ENSXMAG00000012203 abcc3

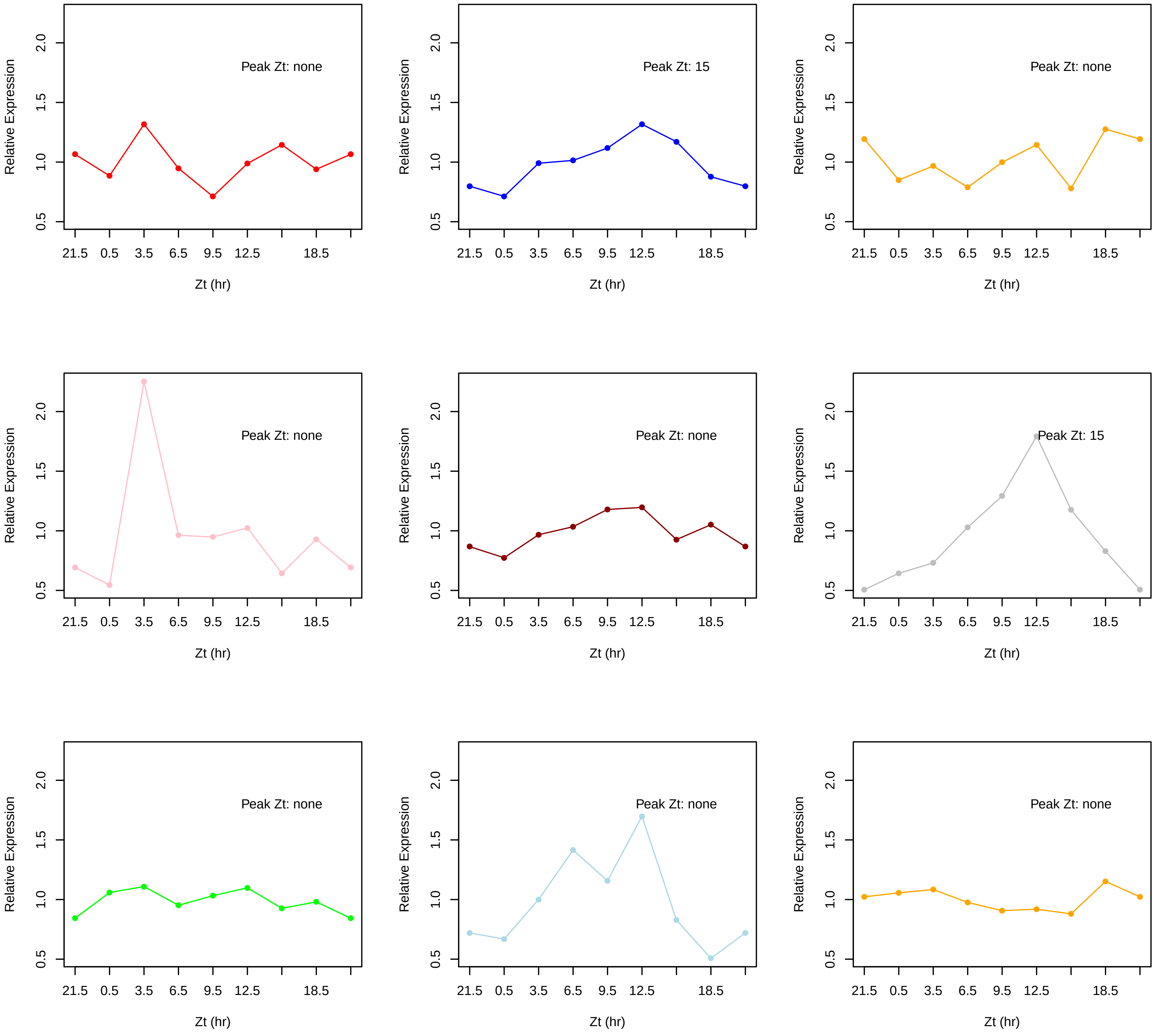

Singeleton: ENSXMAG00000019488 adra2da

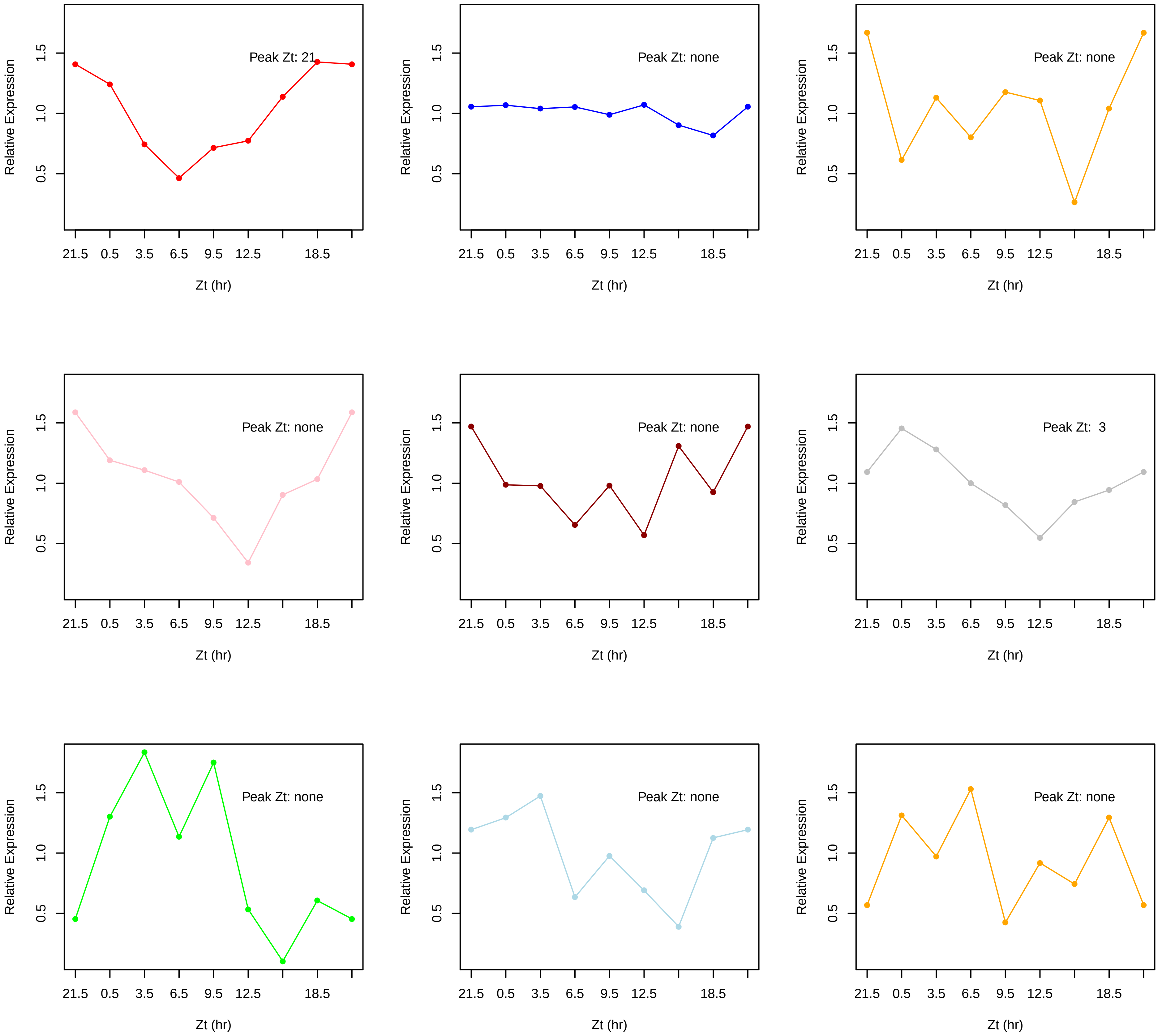

Singeleton: ENSXMAG00000018767 lats2

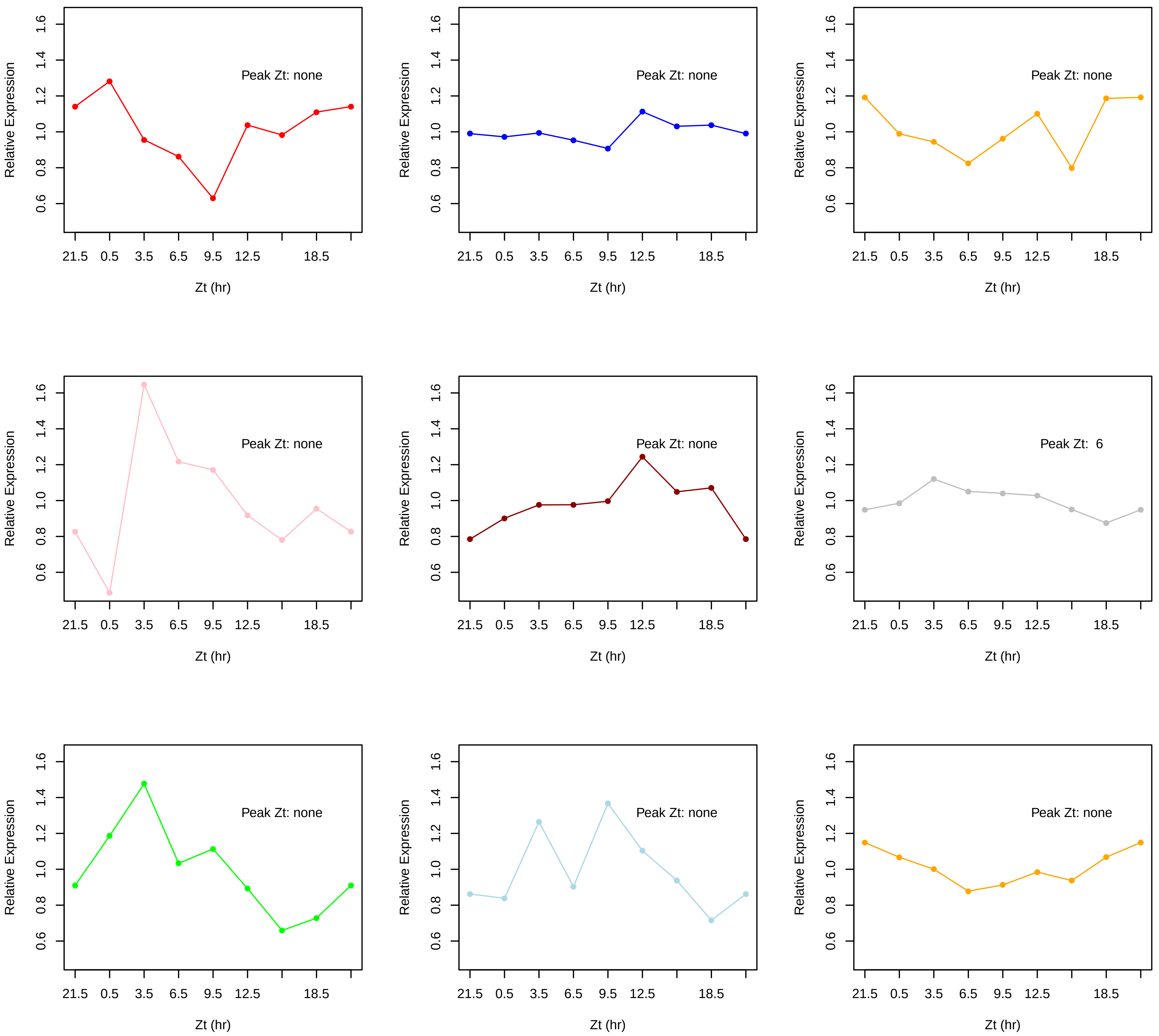

Singeleton: ENSXMAG00000028873 lin52

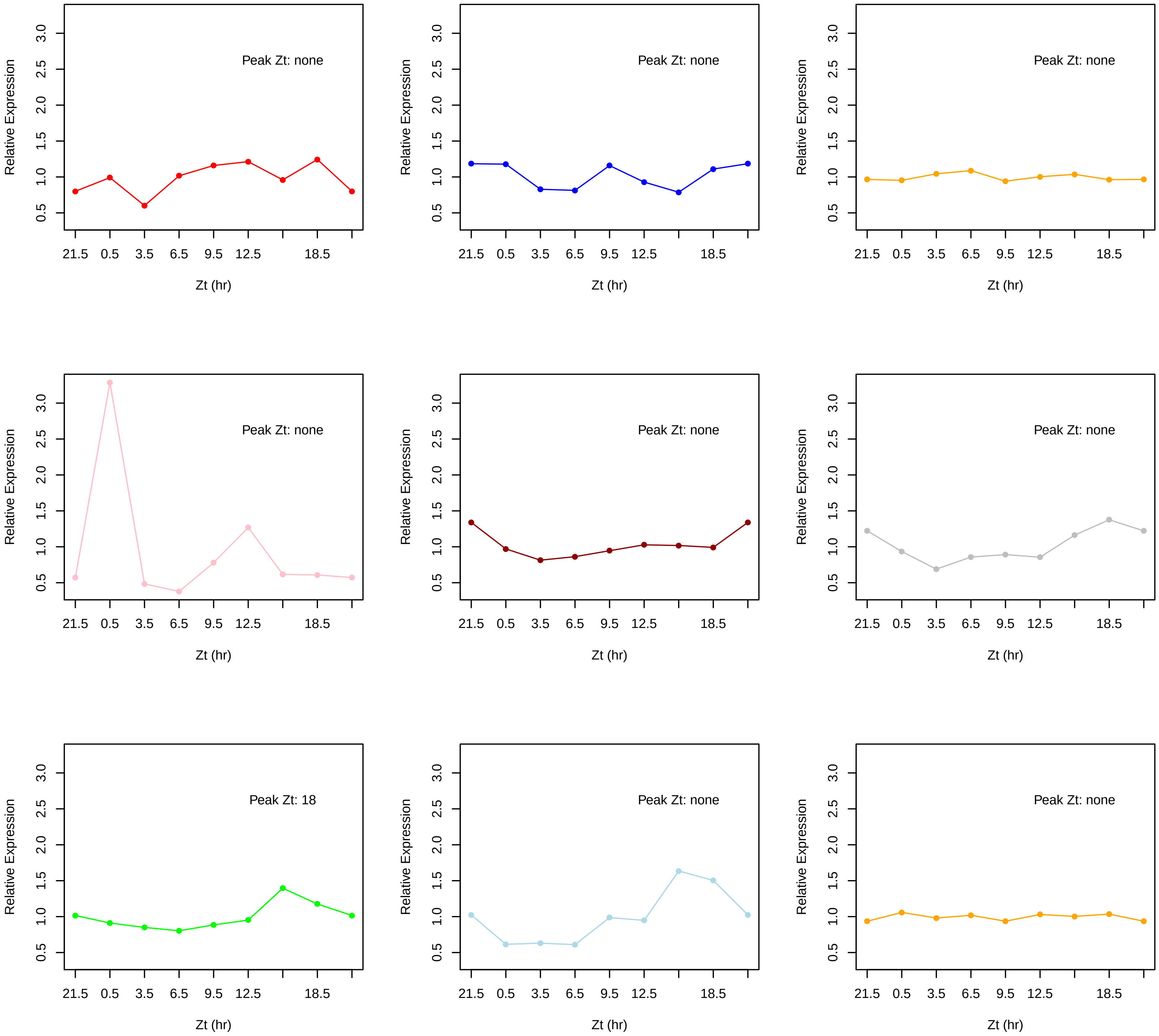

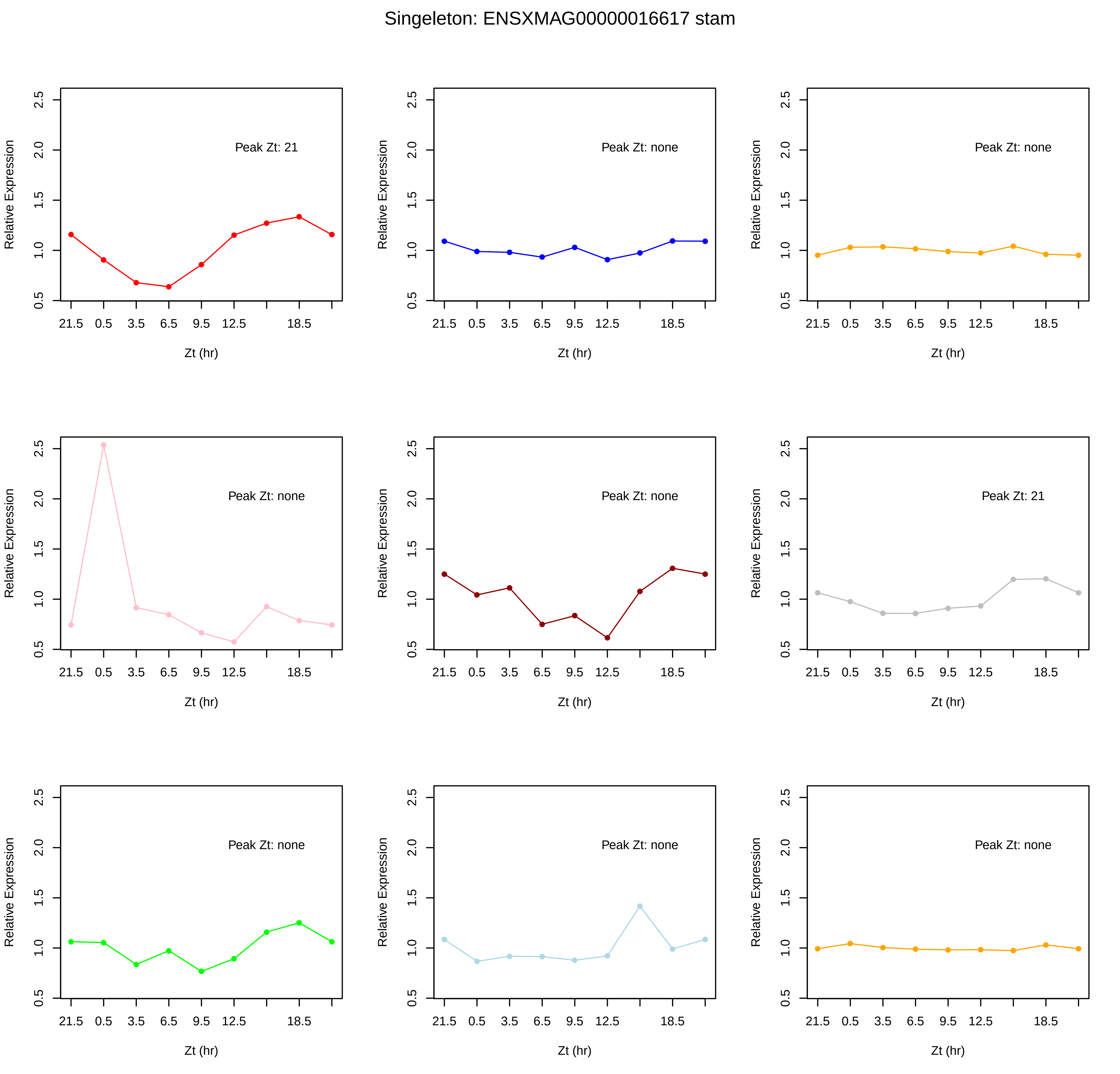
Singeleton: ENSXMAG00000016617 stam
Peak Zt: 21
Peak Zt: none
Peak Zt: none
Peak Zt: none
Peak Zt: none
Peak Zt: 21
Peak Zt: none
Peak Zt: none
Peak Zt: none
Relative Expression
Zt (hr)
21.5
0.5
3.5
6.5
9.5
12.5
18.5

Singleton: ENSXMAG00000028308 sap18

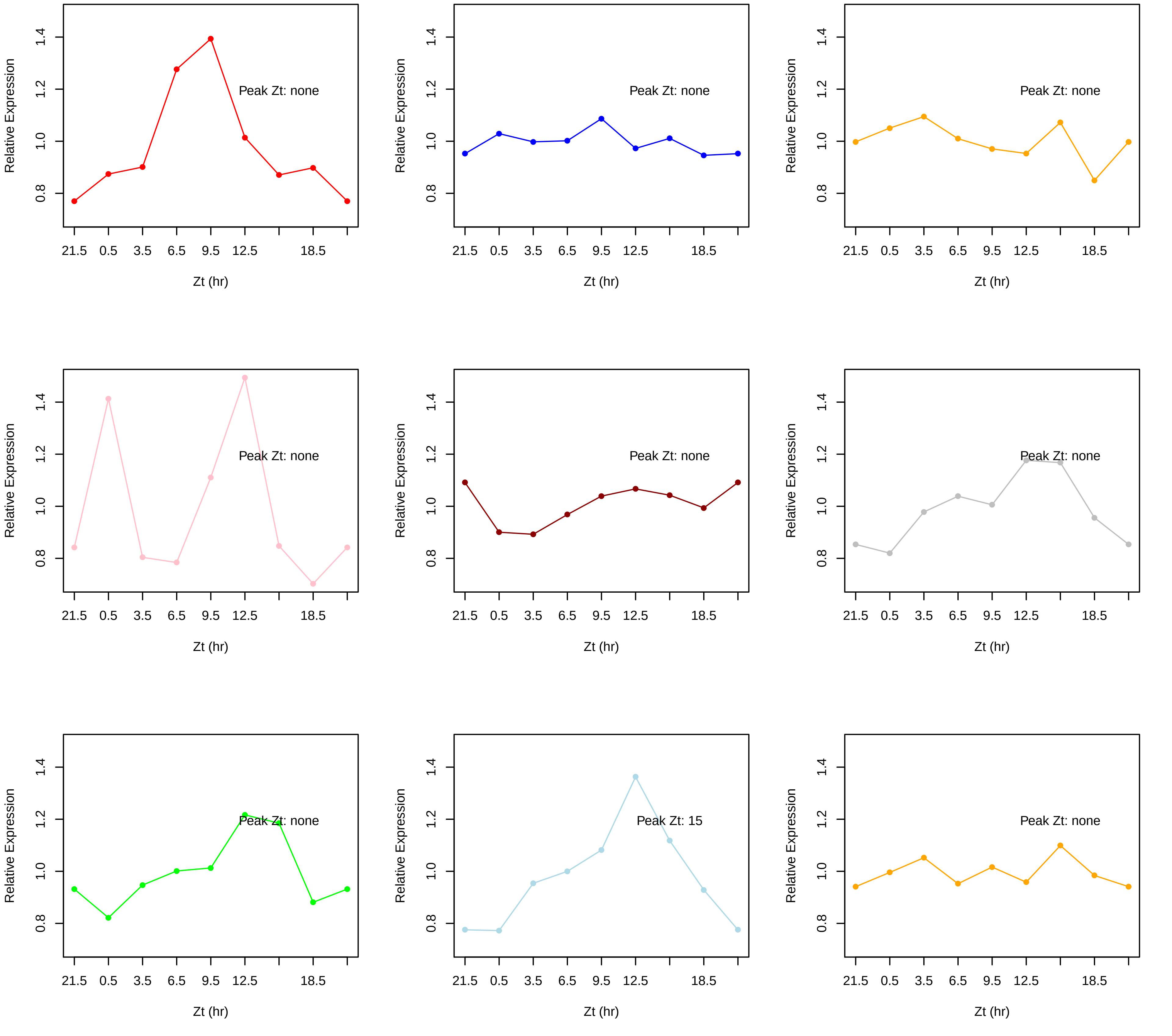

Singeleton: ENSXMAG00000001880 stk16

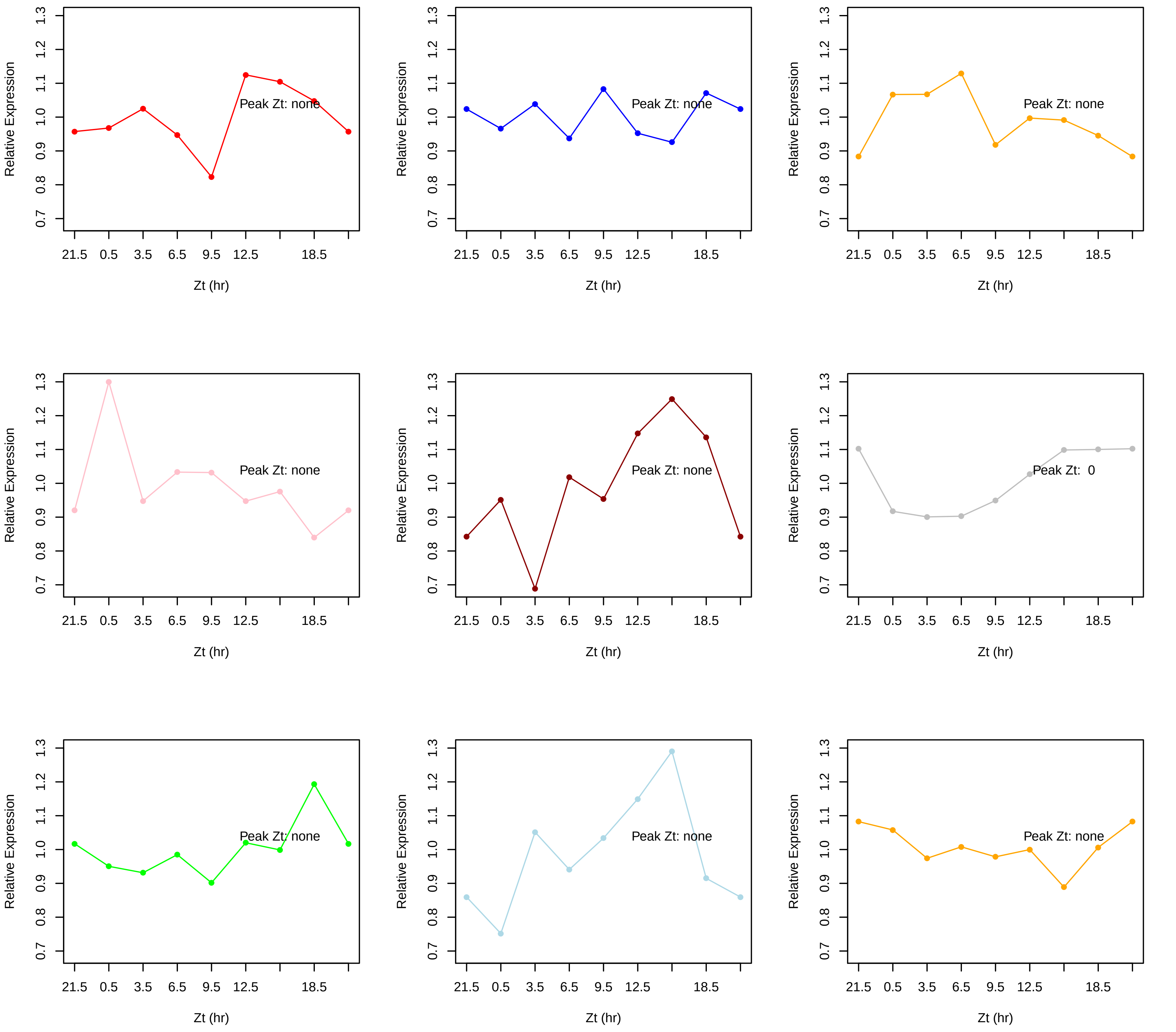

Singleton: ENSXMAG00000016611 gnat1

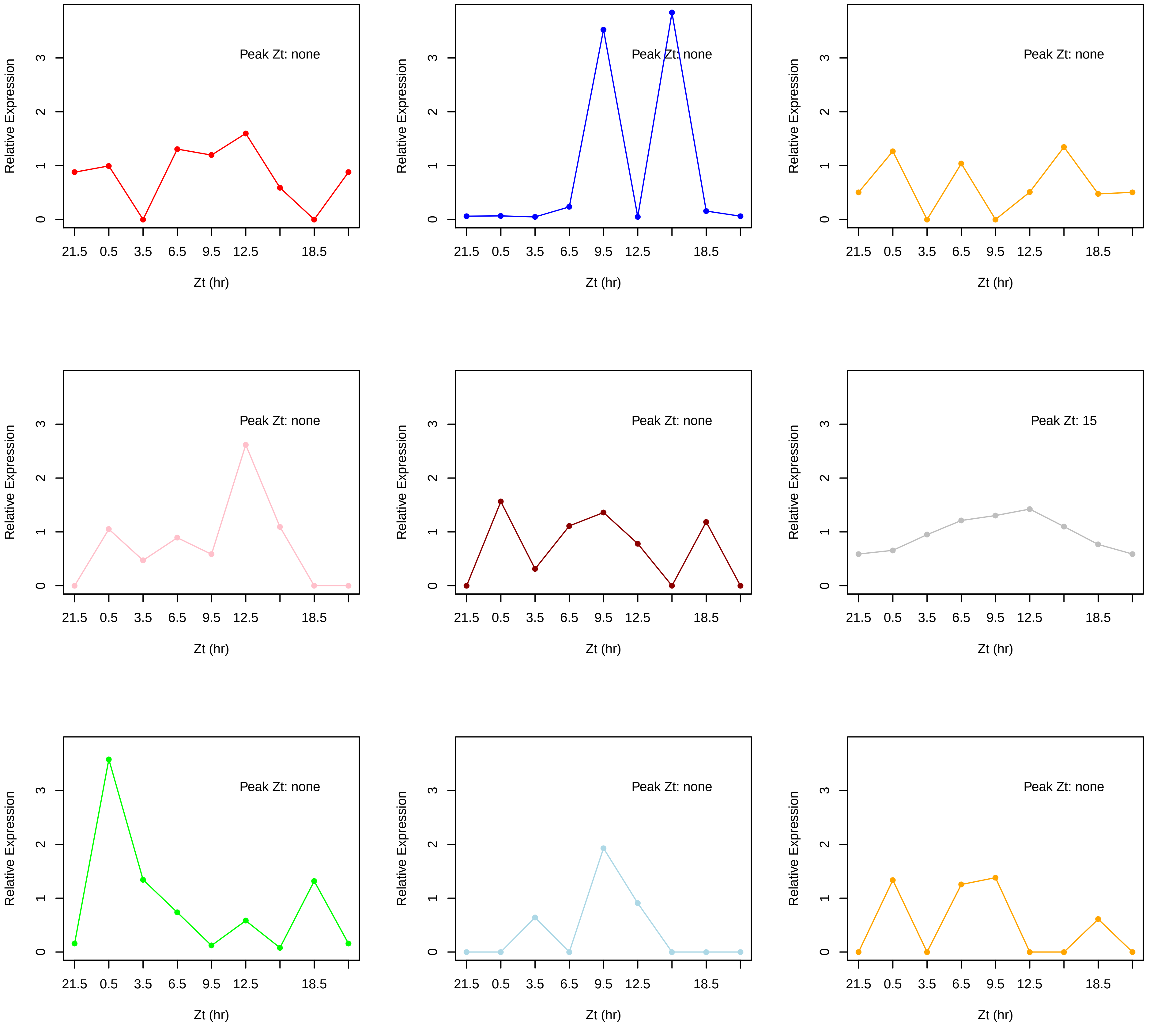

Singeleton: ENSXMAG00000006091 hmgcs1

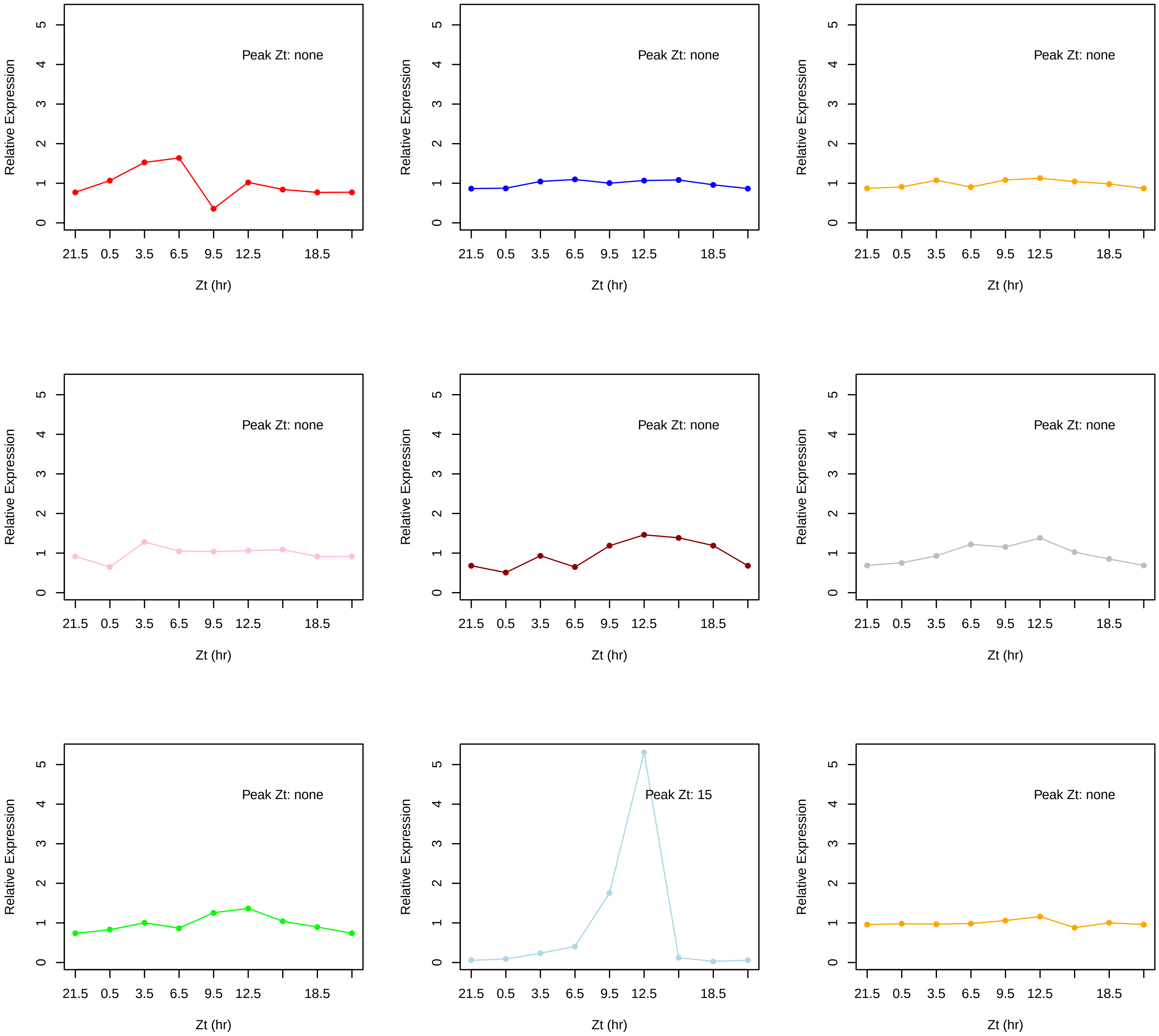

Singeleton: ENSXMAG00000014096 ankrd40

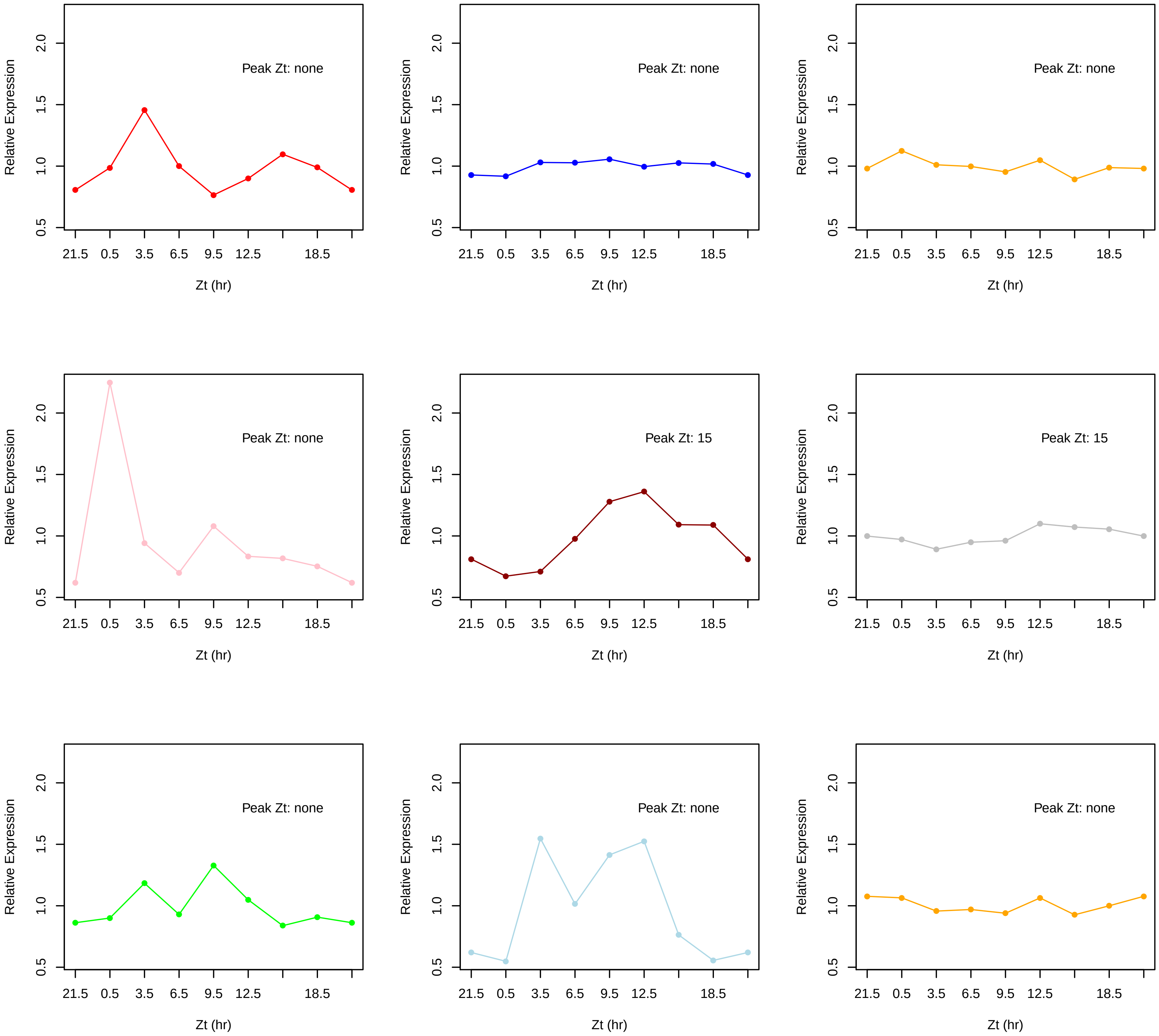

Singleton: ENSXMAG00000008700 arl3l2

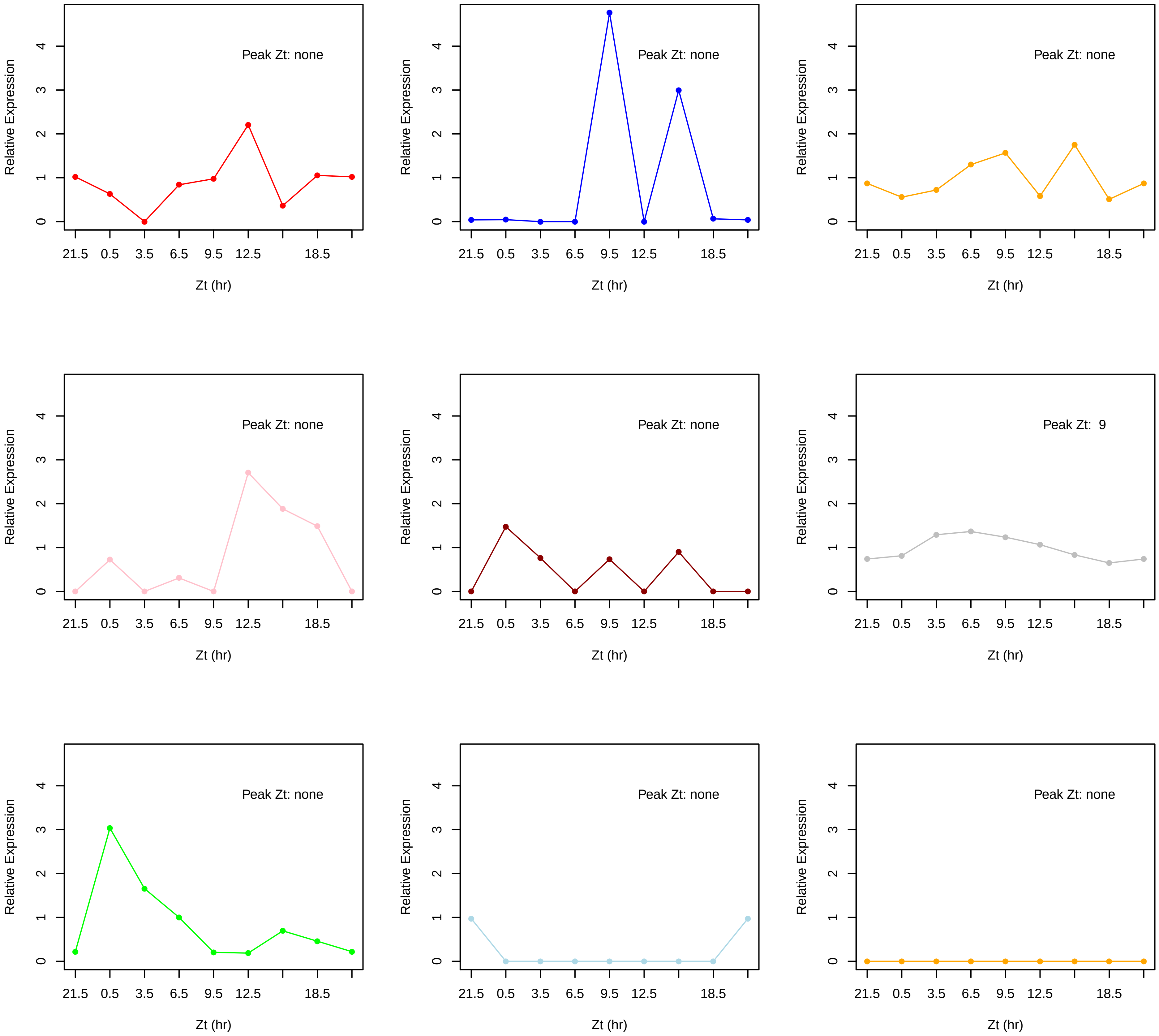

Singeleton: ENSXMAG00000000718 pdk4

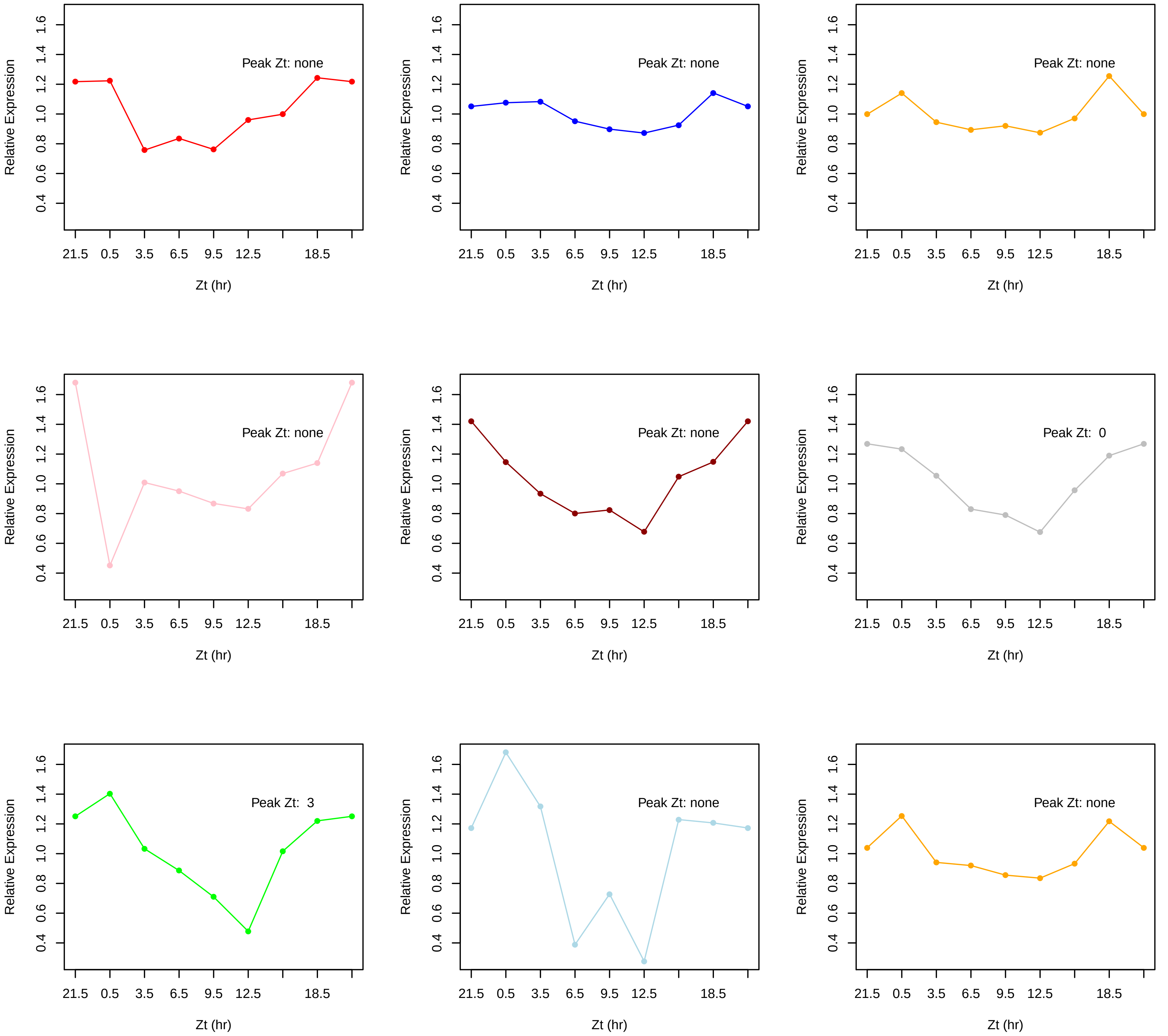

Singeleton: ENSXMAG00000006454 zgc:136929

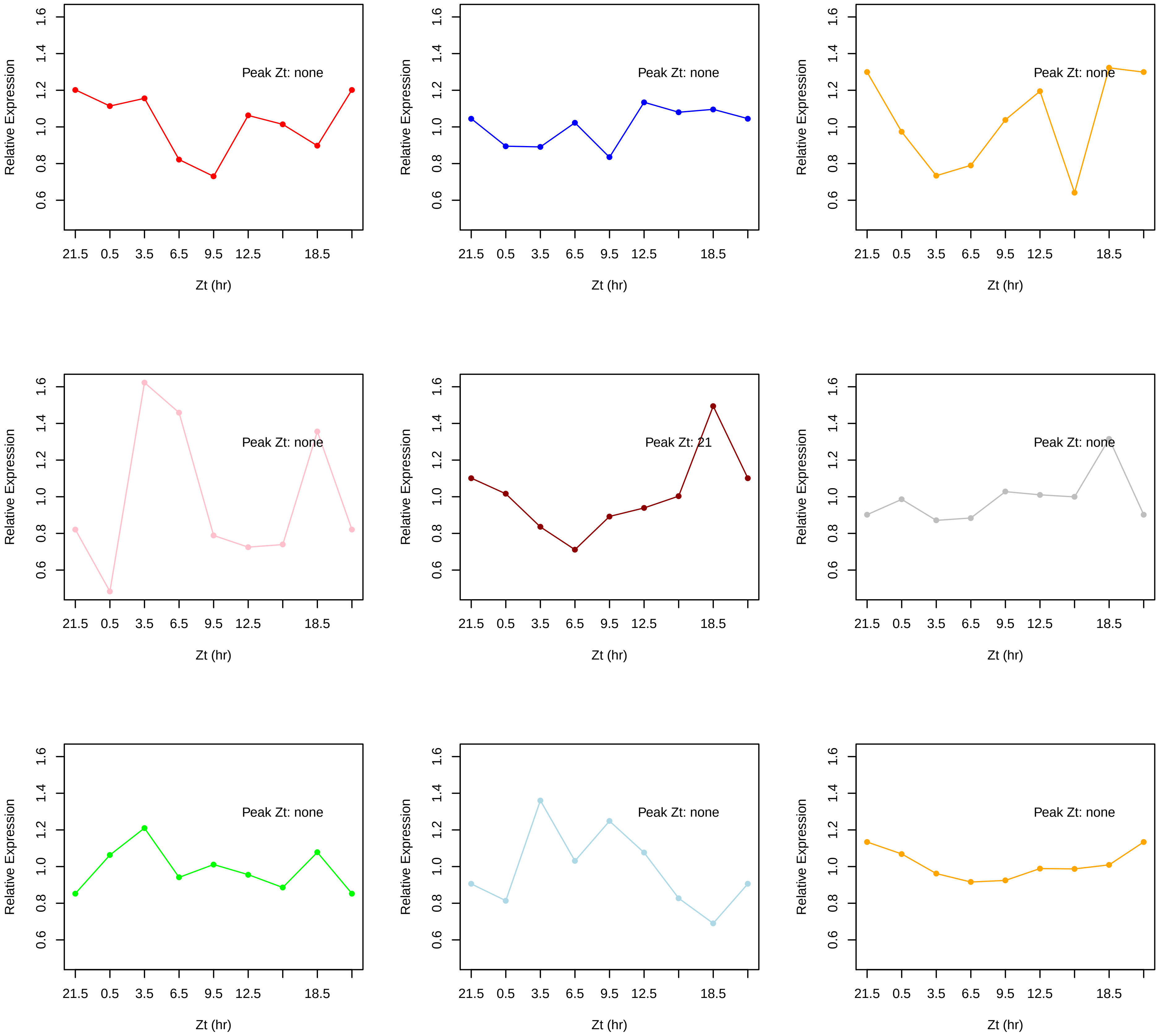

Singeleton: ENSXMAG00000019187 mrpl16

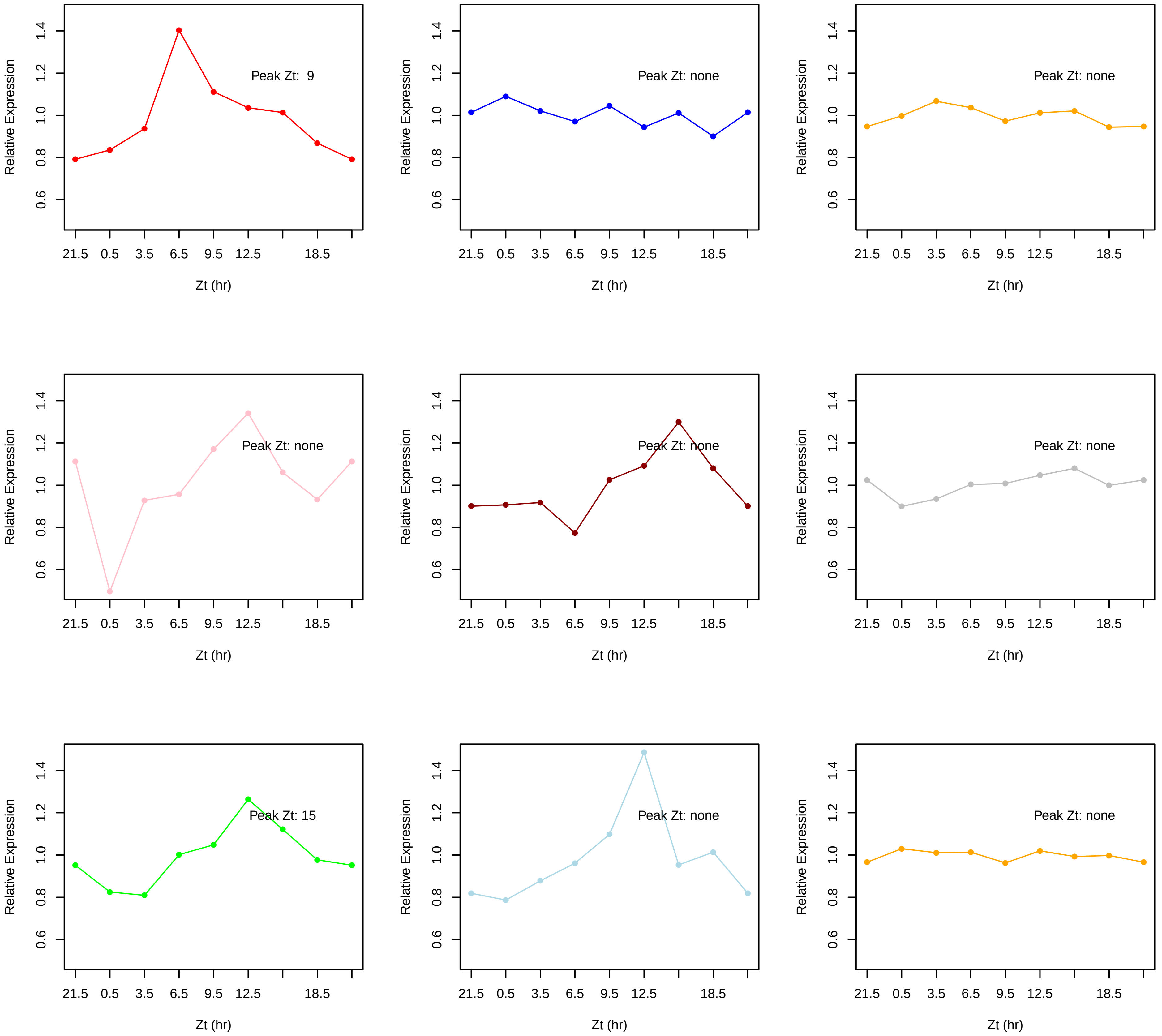

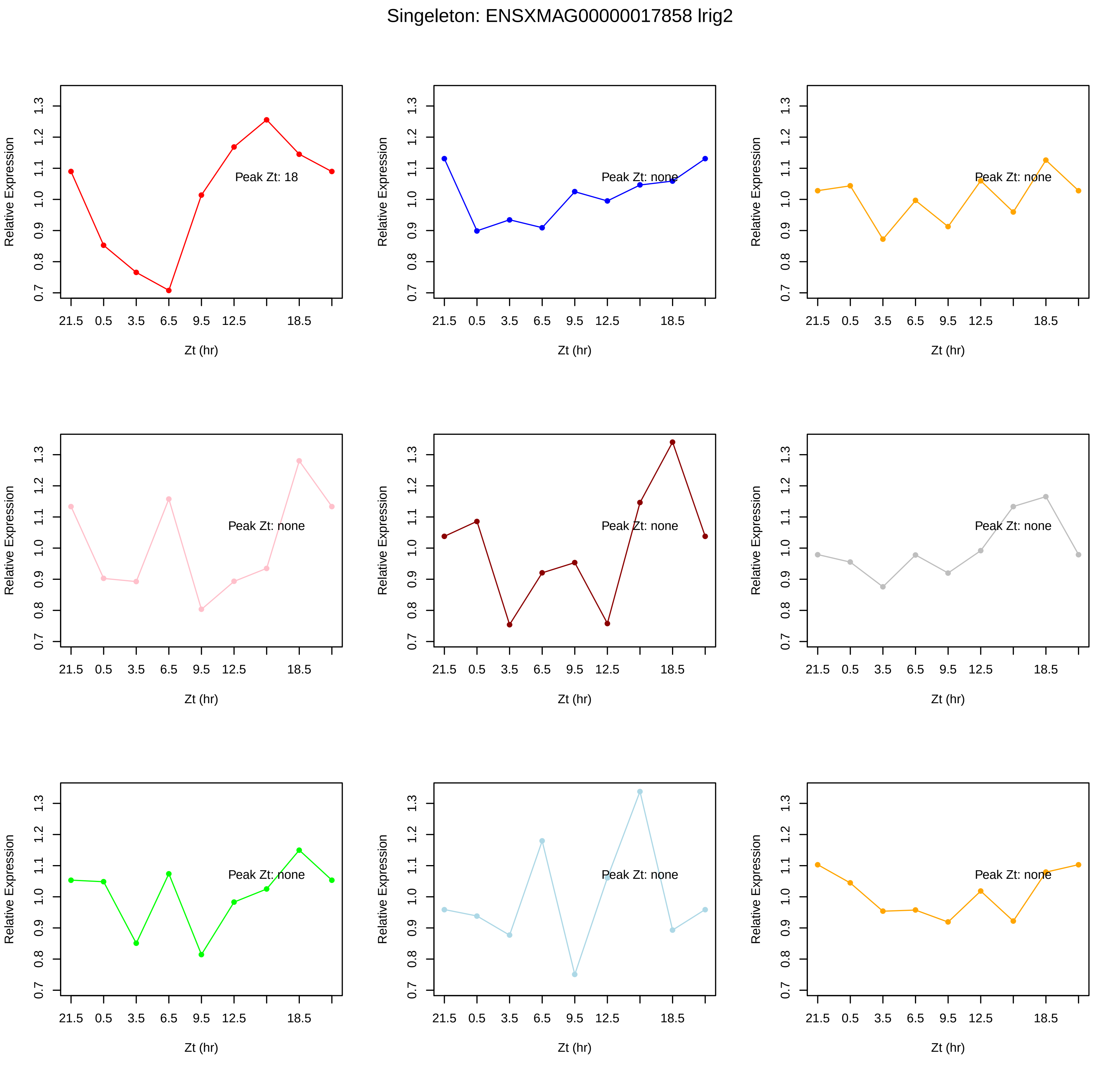
Singeleton: ENSXMAG00000017858 lrig2
Relative Expression
Zt (hr)
21.5
0.5
3.5
6.5
9.5
12.5
18.5
0.7
0.8
0.9
1.0
1.1
1.2
1.3
Peak Zt: 18
Peak Zt: none

Singeleton: ENSXMAG00000008681 rbm22

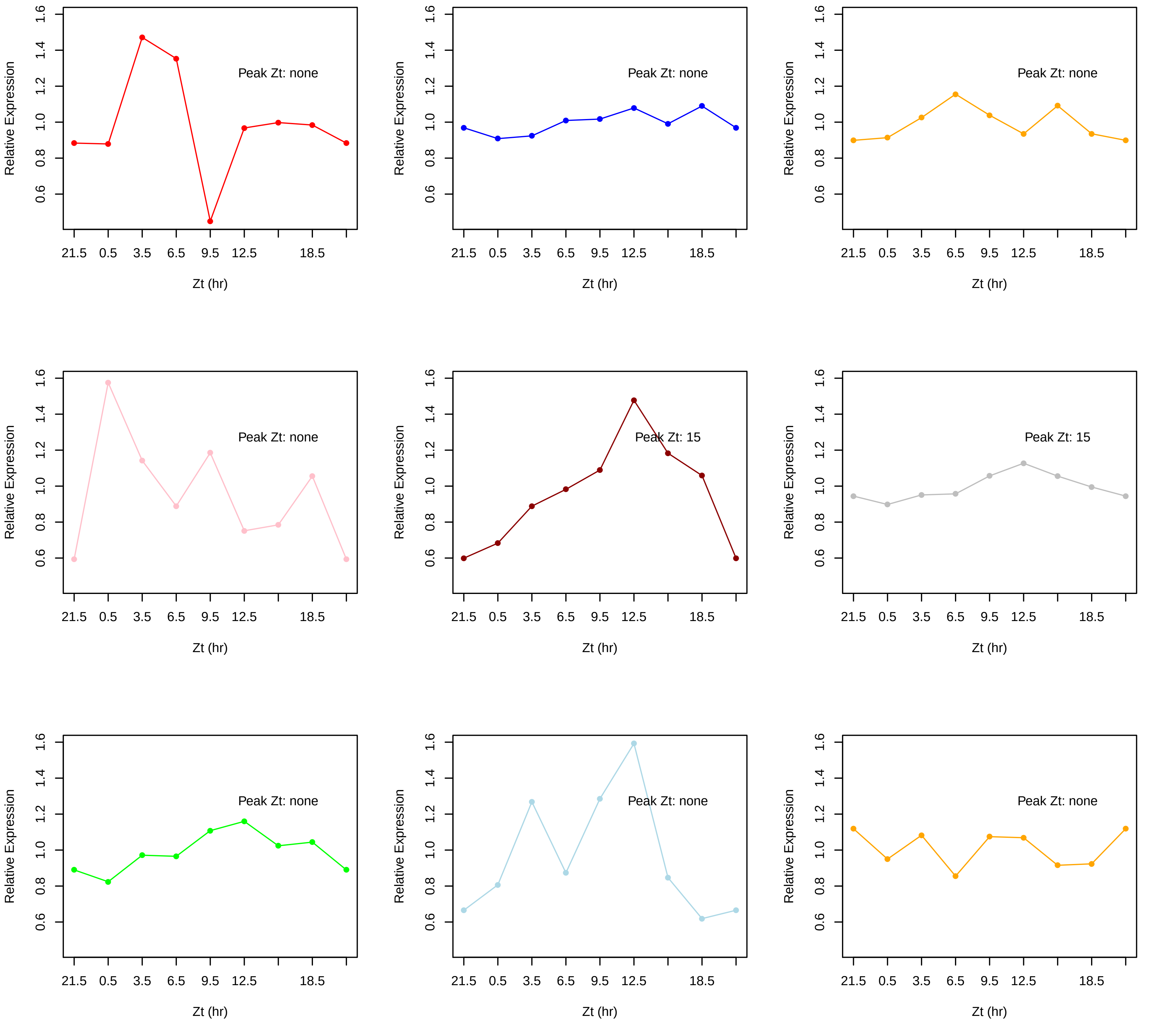

Singeleton: ENSXMAG00000010972 uvssa

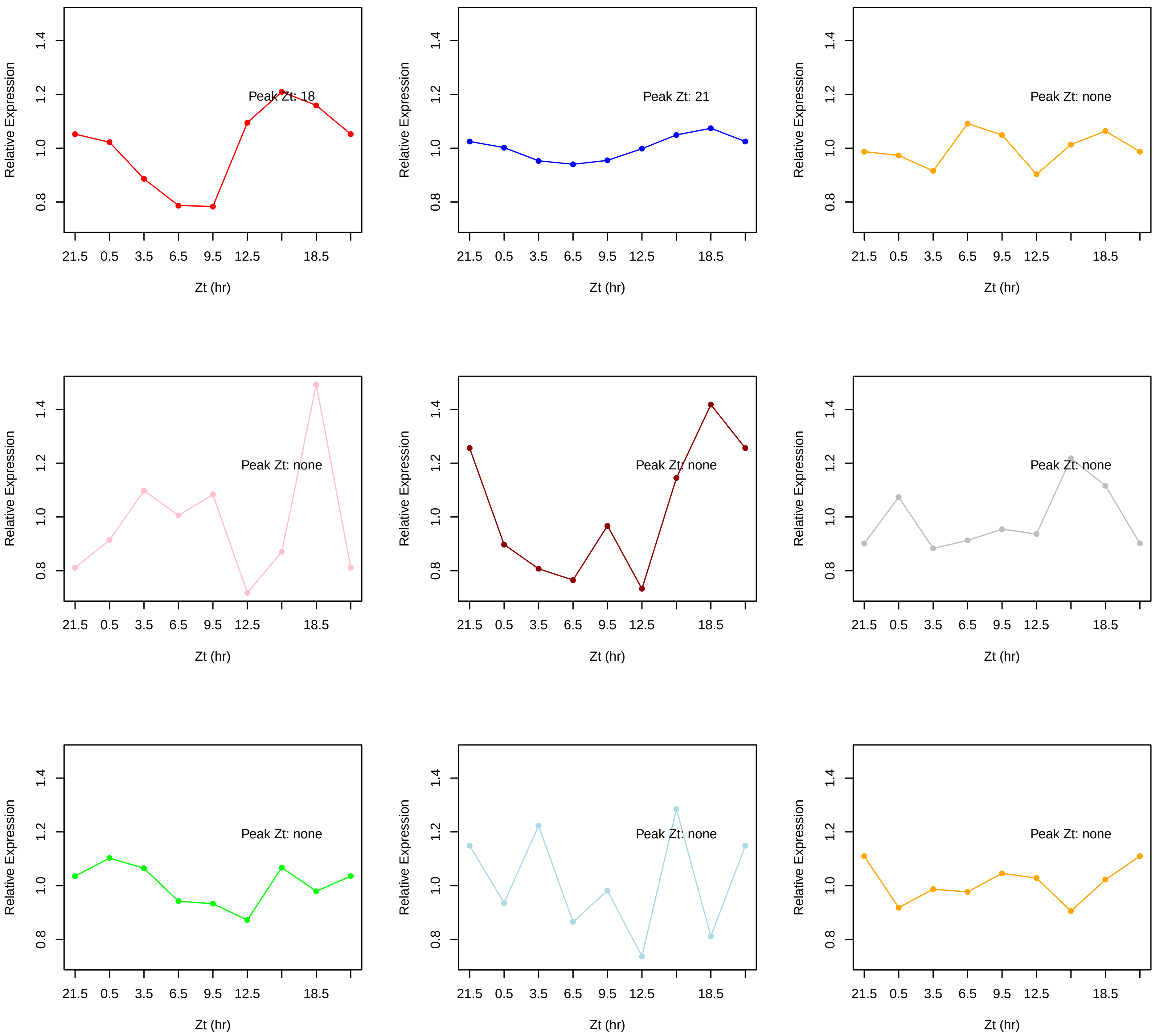

Singeleton: ENSXMAG00000008661 ndst1a

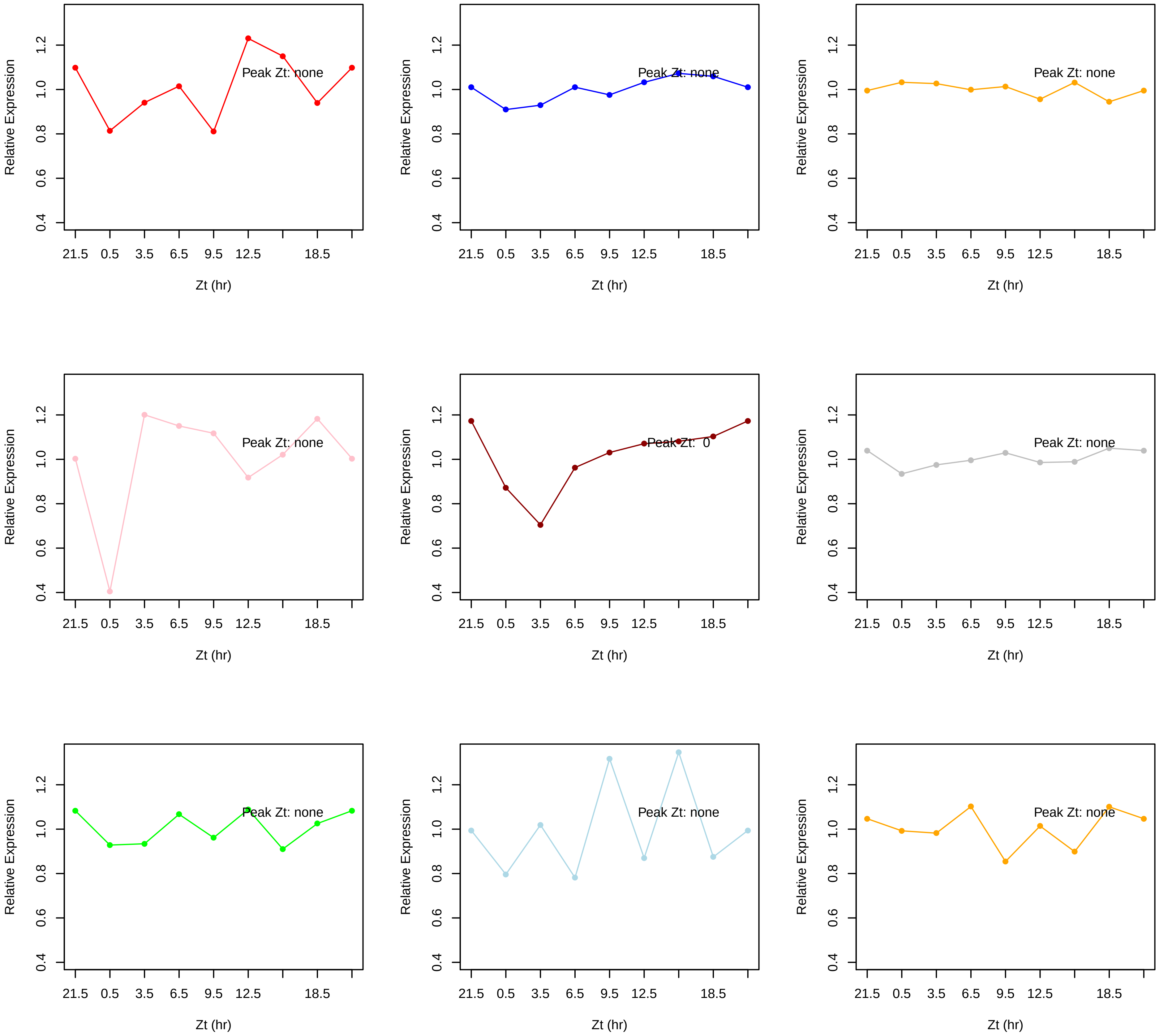

Singleton: ENSXMAG00000025935 retreg2

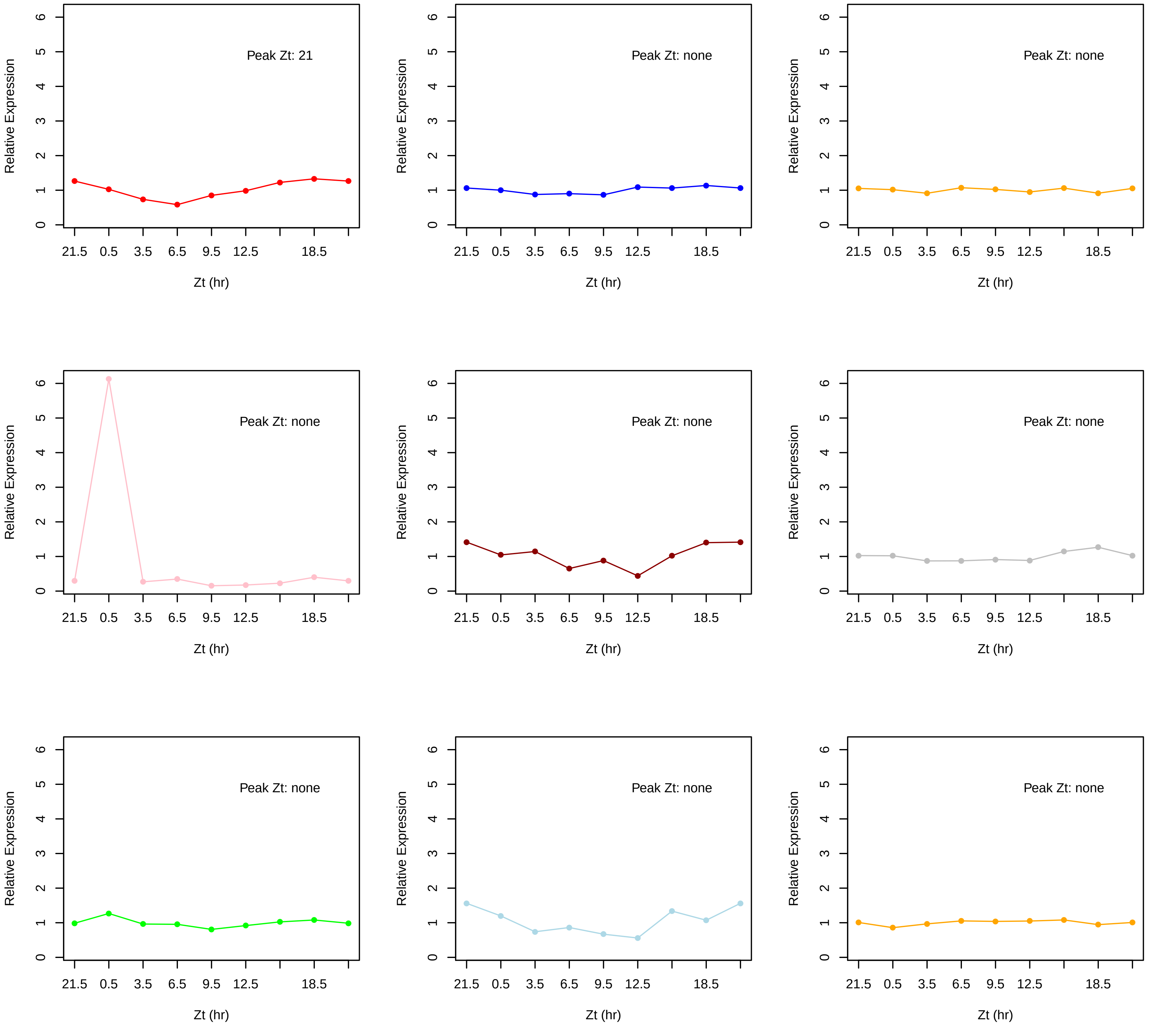

Singeleton: ENSXMAG00000021151 magi3a

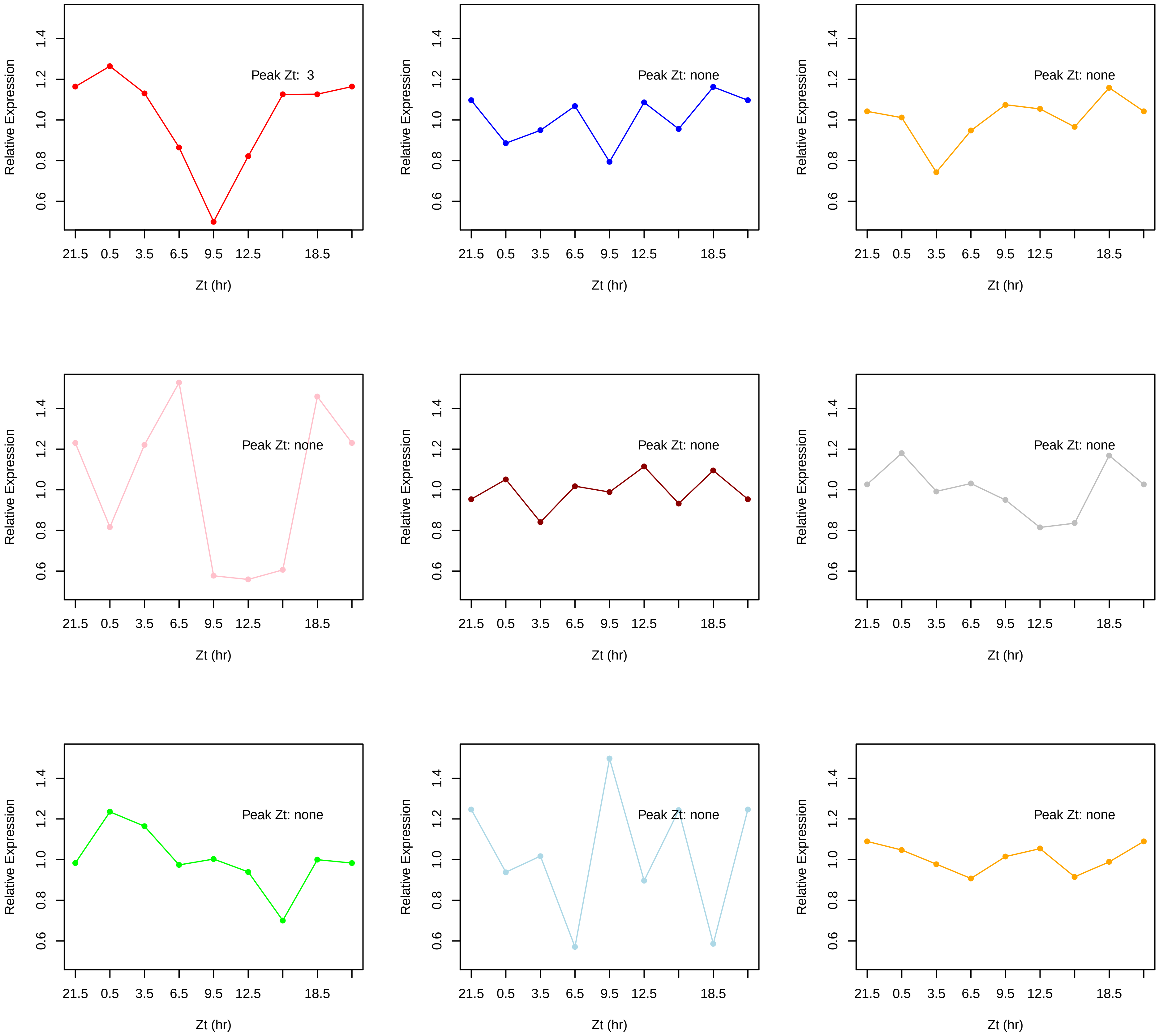

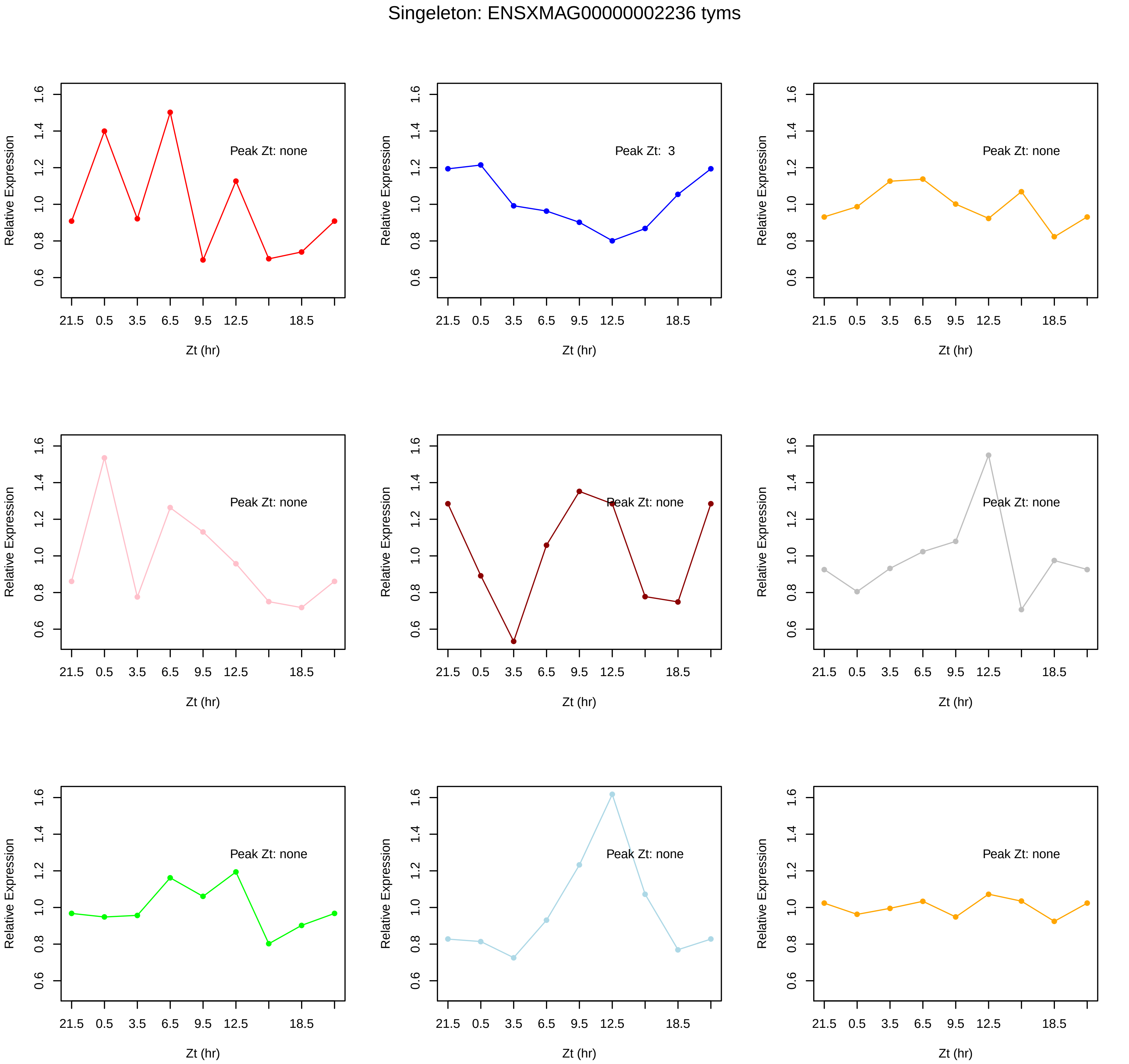
Singeleton: ENSXMAG00000002236 tyms
Peak Zt: none
Peak Zt: 3
Peak Zt: none
Peak Zt: none
Peak Zt: none
Peak Zt: none
Peak Zt: none
Peak Zt: none
Peak Zt: none
Relative Expression
Zt (hr)
21.5
0.5
3.5
6.5
9.5
12.5
18.5
0.6
0.8
1.0
1.2
1.4
1.6

Singeleton: ENSXMAG00000000752 sem1

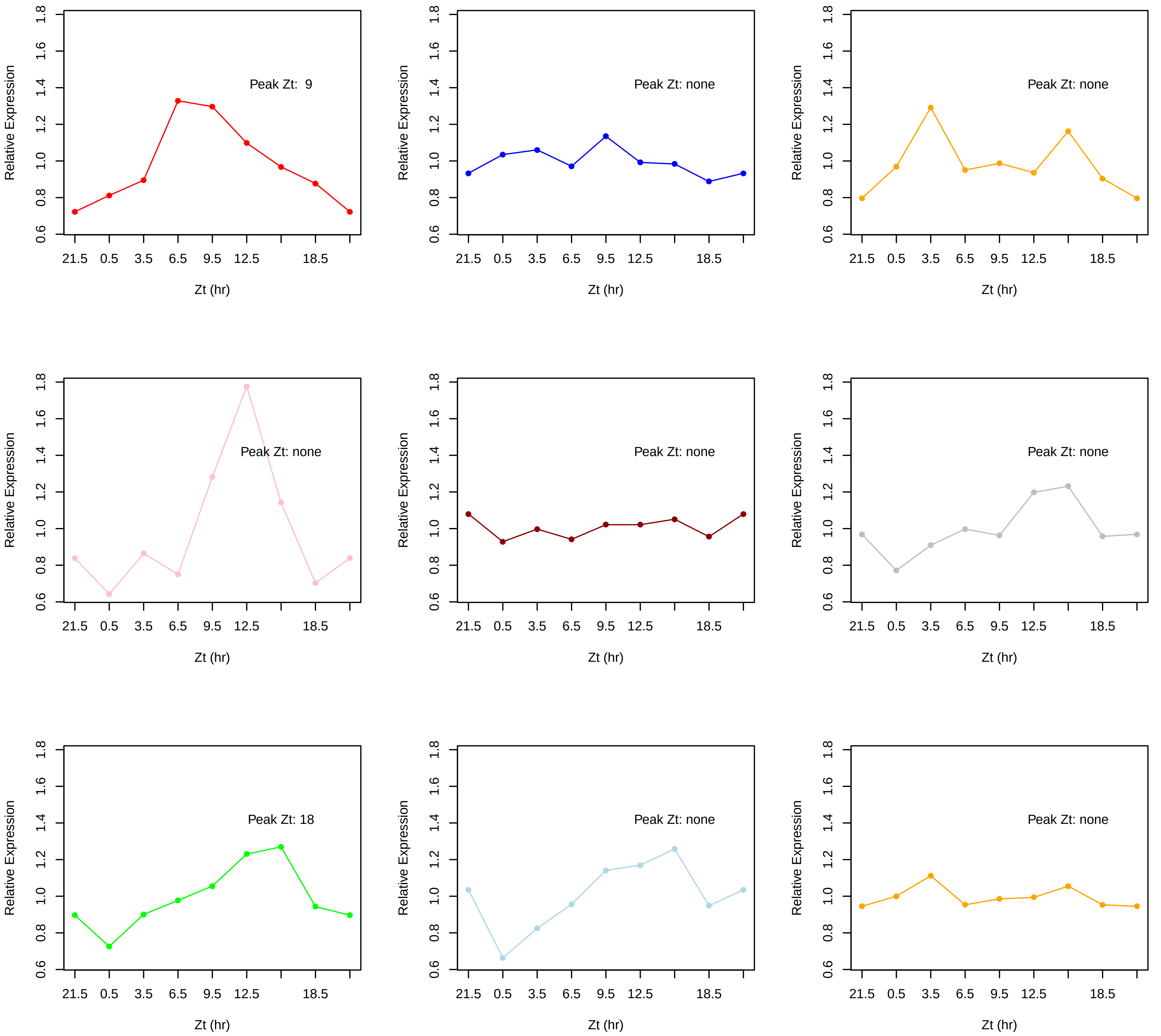

Singeleton: ENSXMAG00000012327

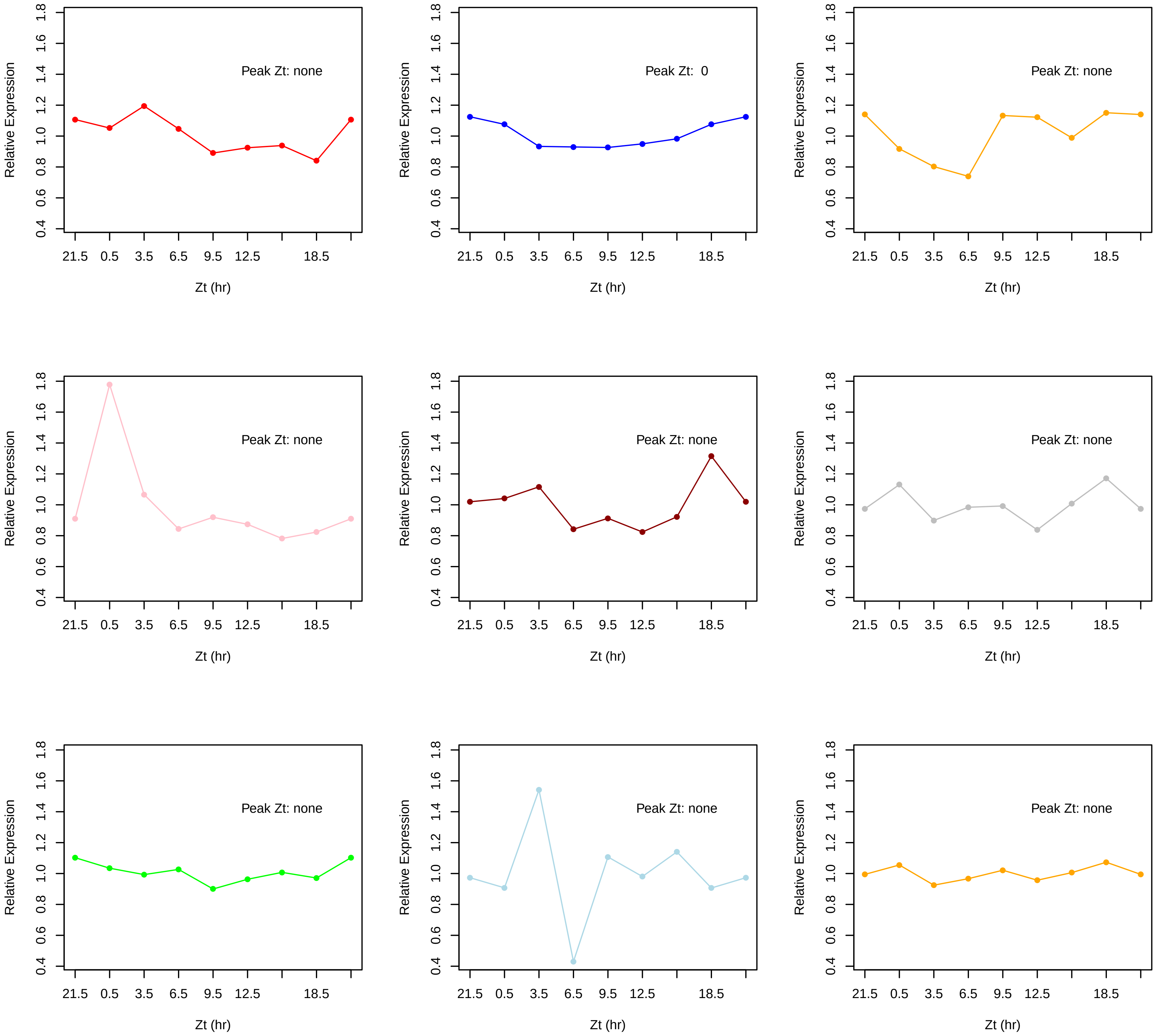

Singeleton: ENSXMAG00000008410 znf410

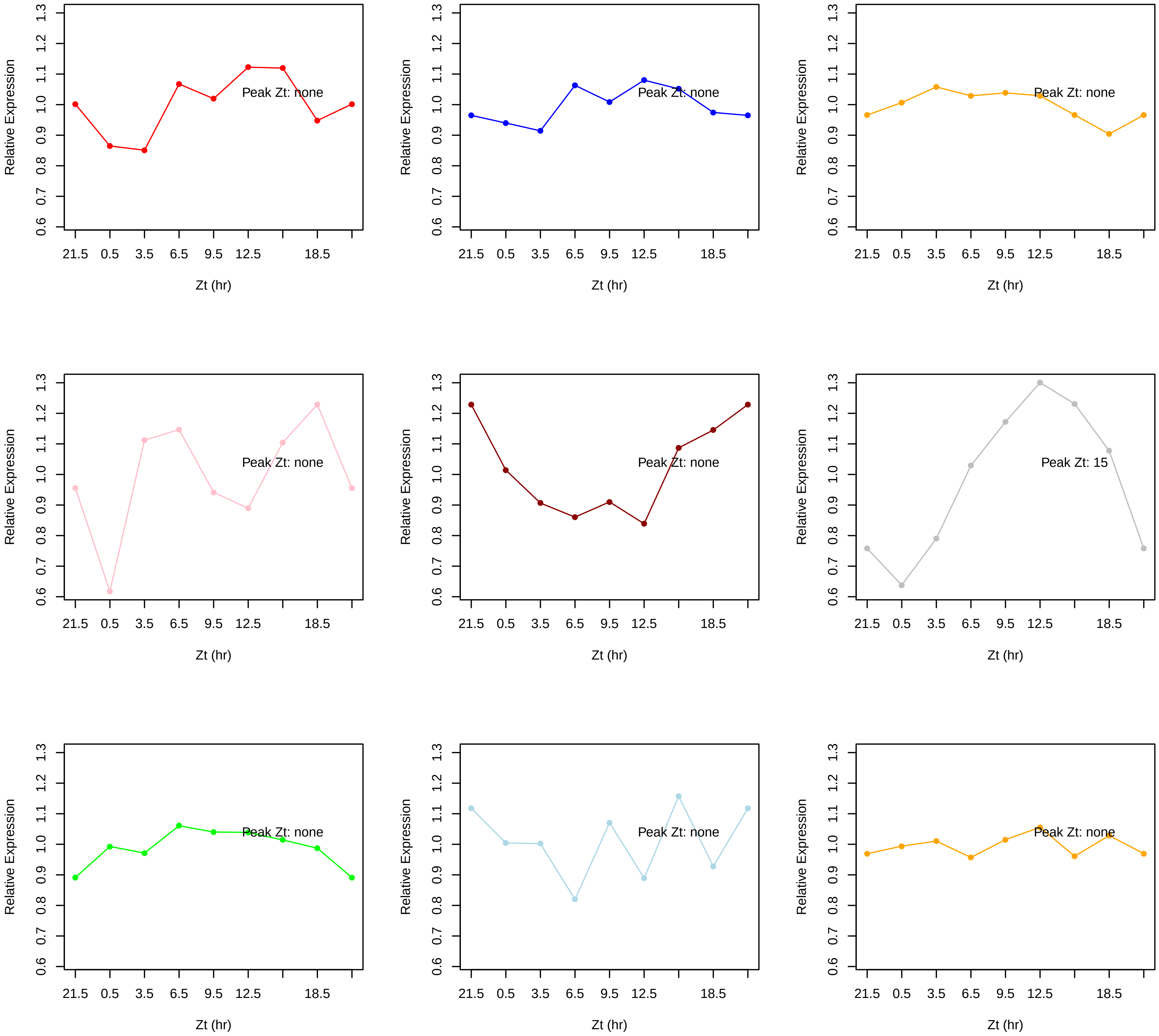

Singeleton: ENSXMAG00000002221 yes1

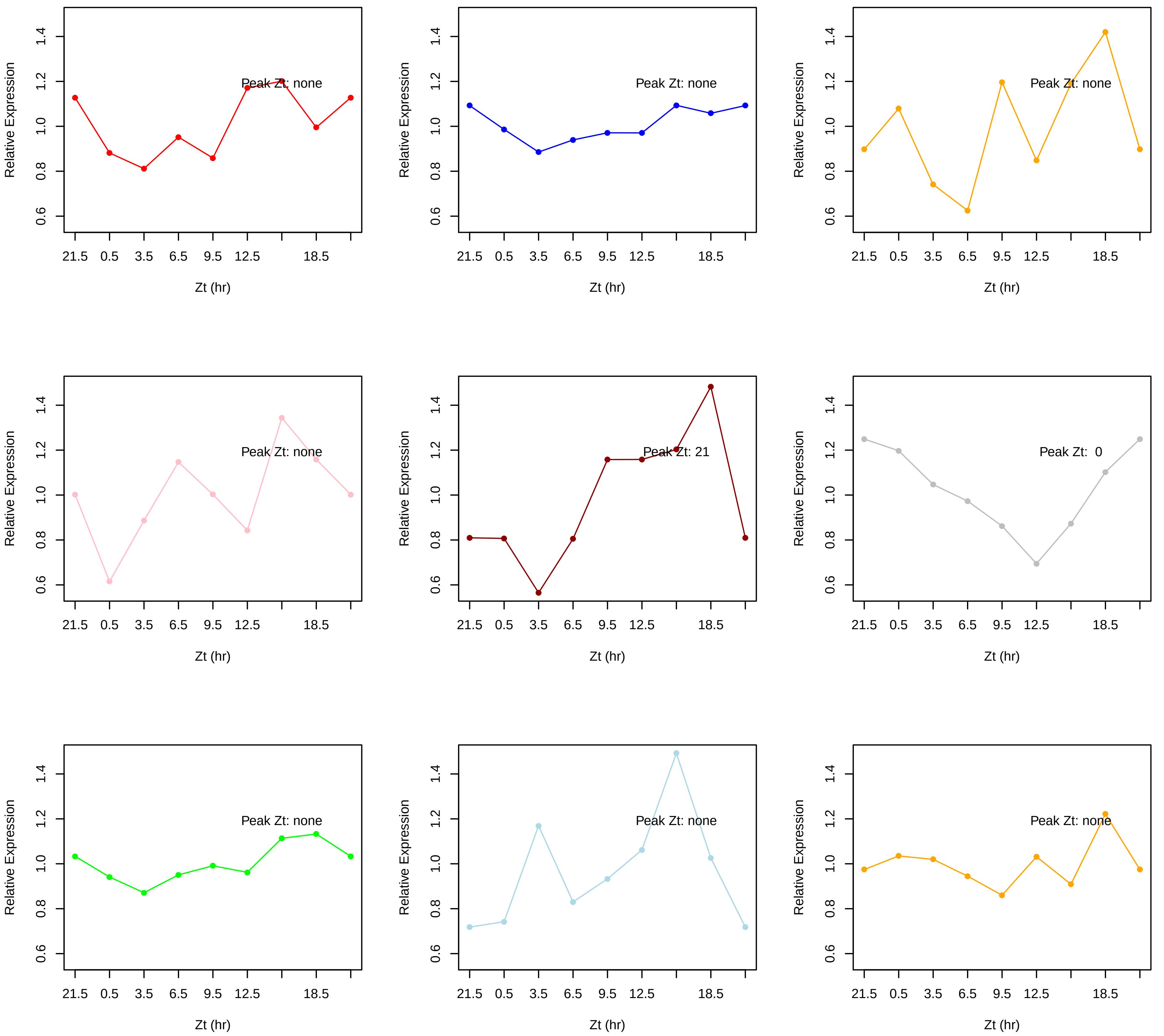

Singeleton: ENSXMAG00000027084

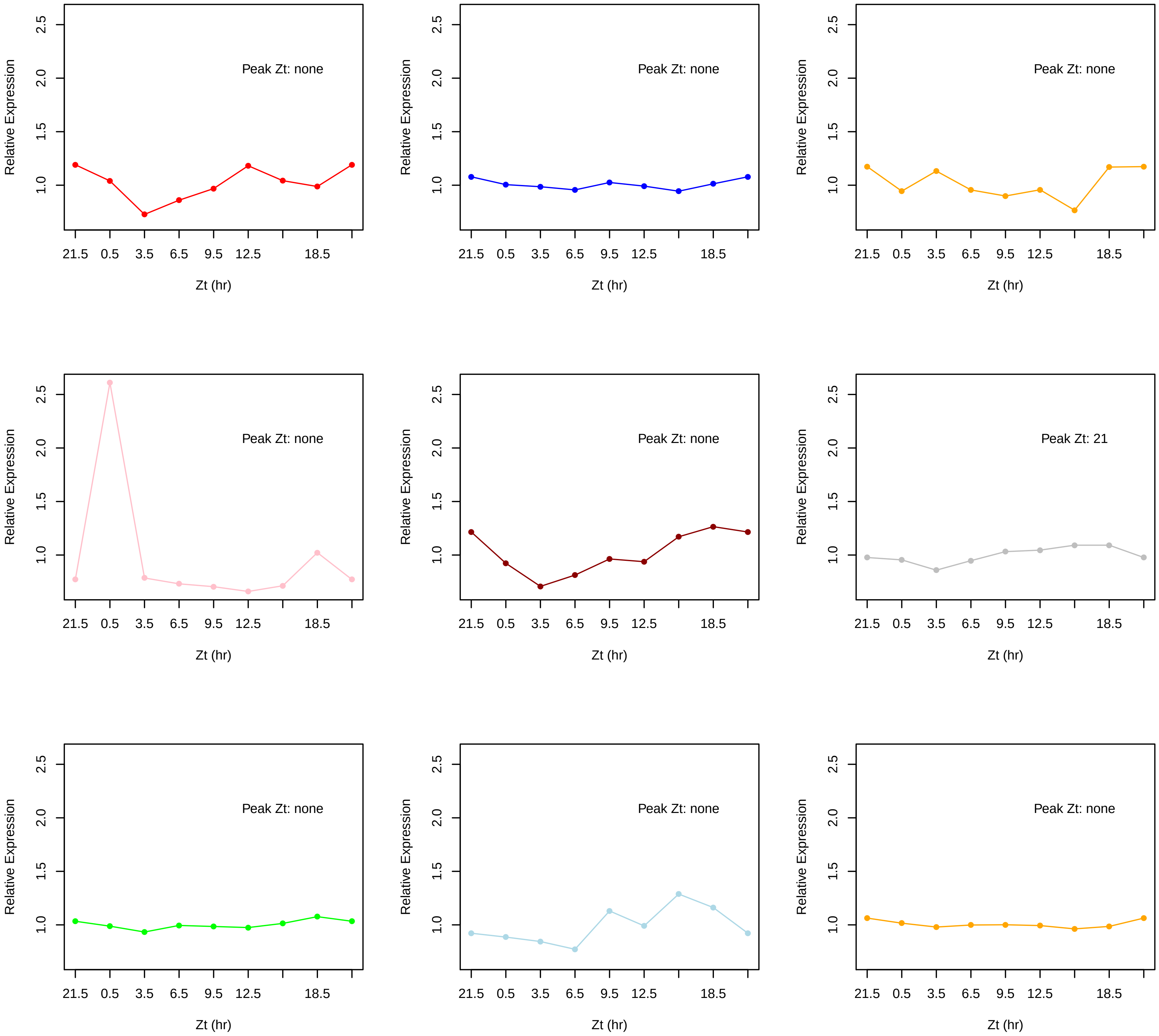

Singleton: ENSXMAG00000021908 tbx15

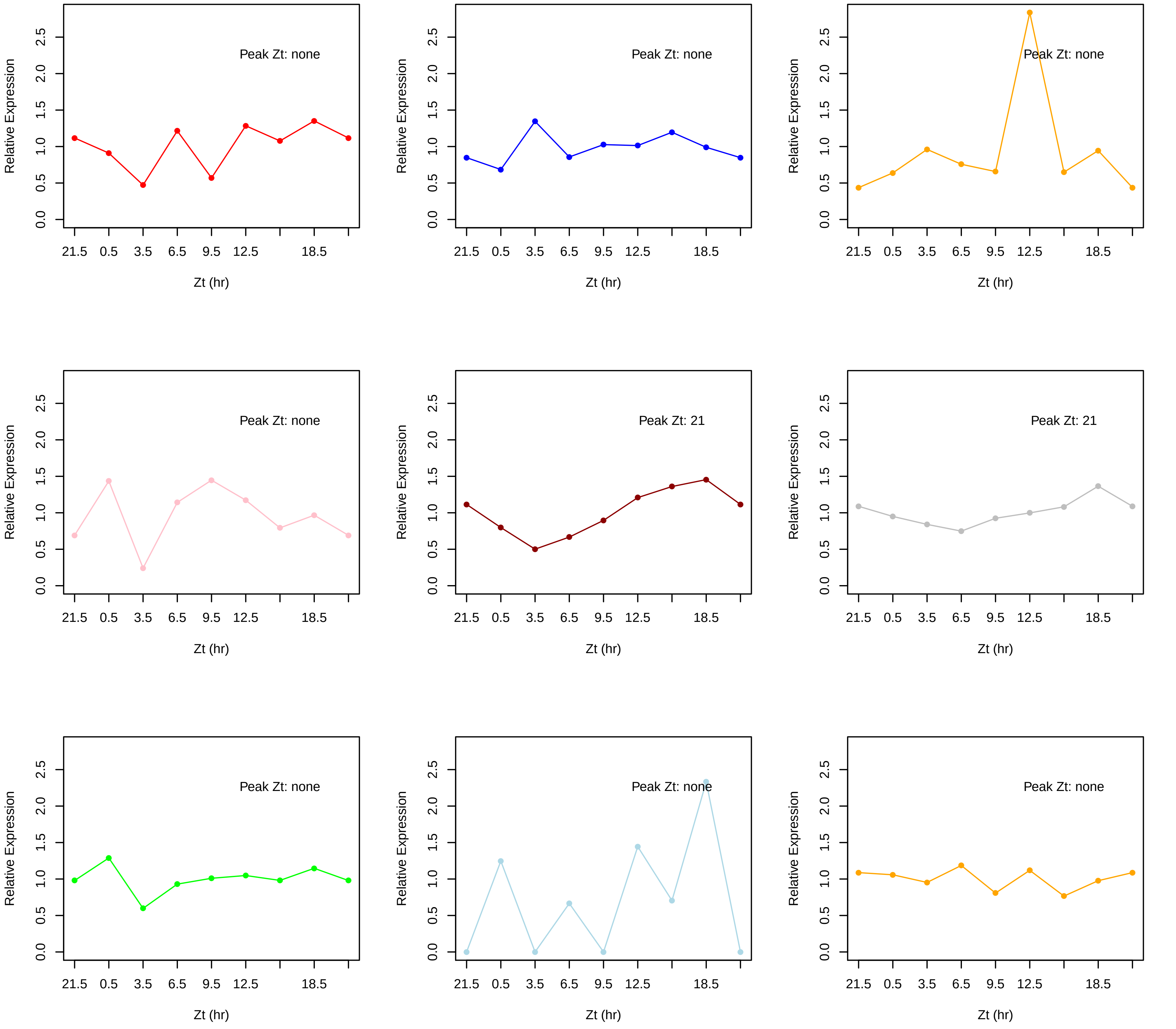

Singeleton: ENSXMAG00000001331

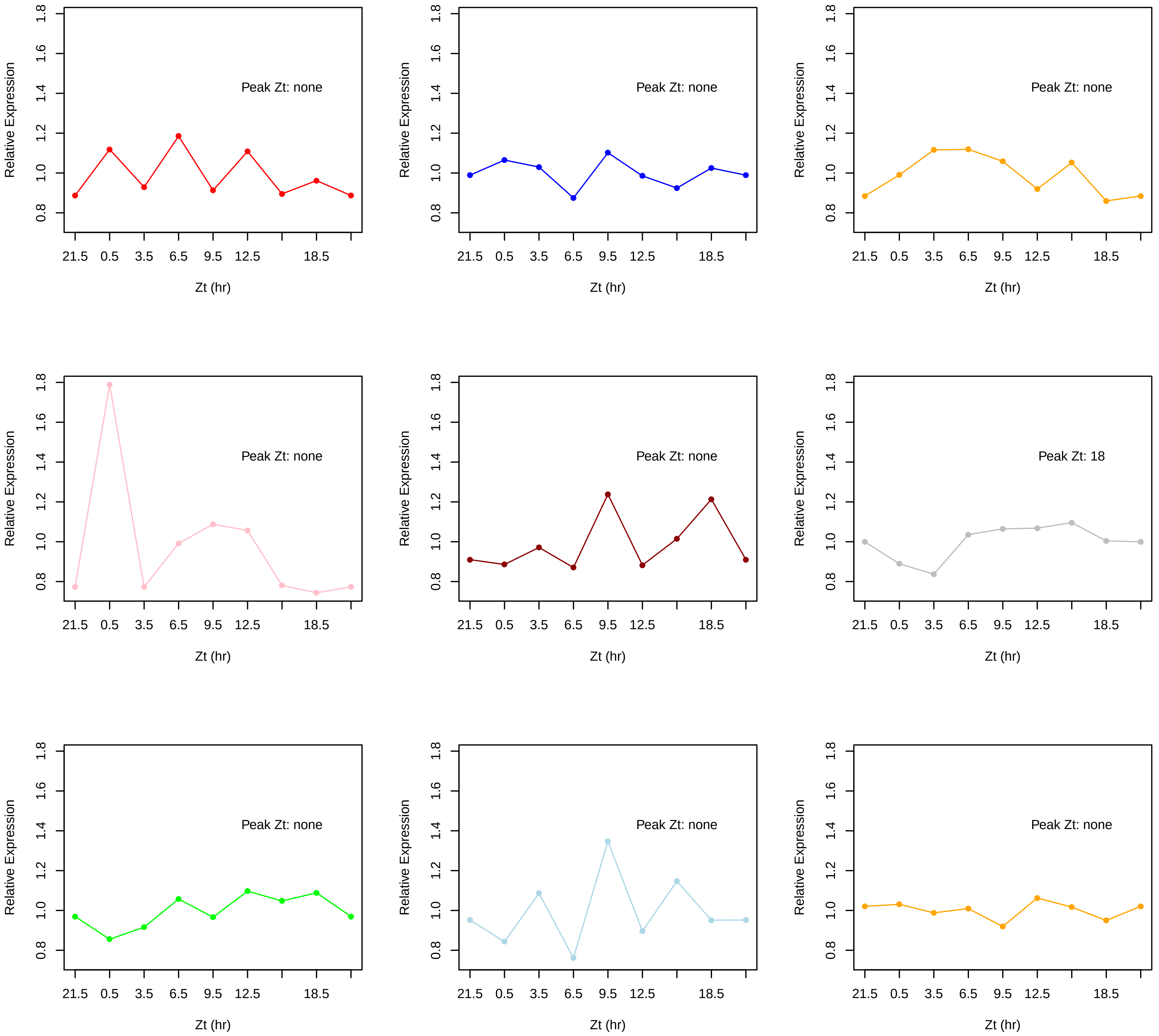

Singeleton: ENSXMAG00000006600

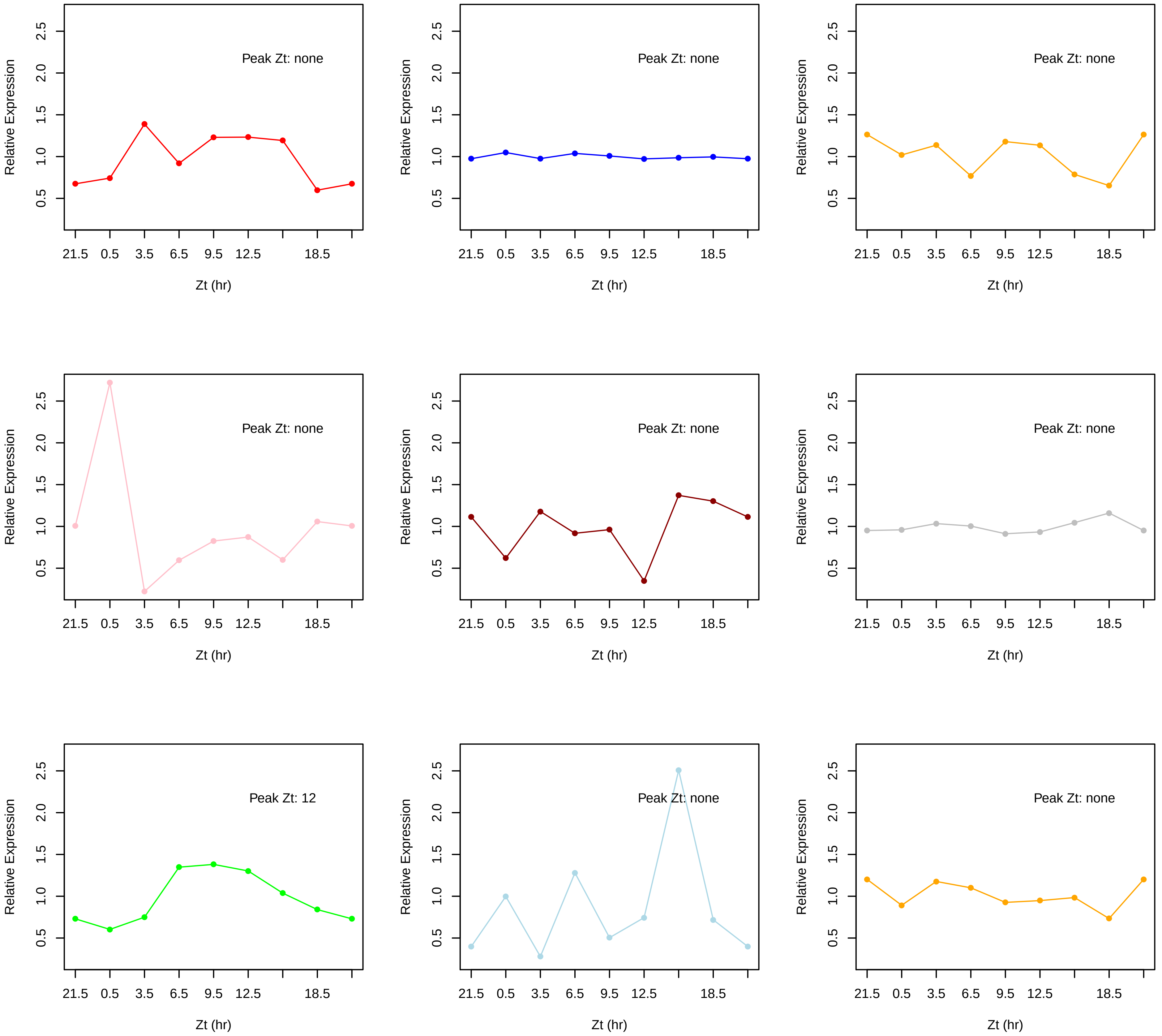

Singeleton: ENSXMAG00000014206 utp15

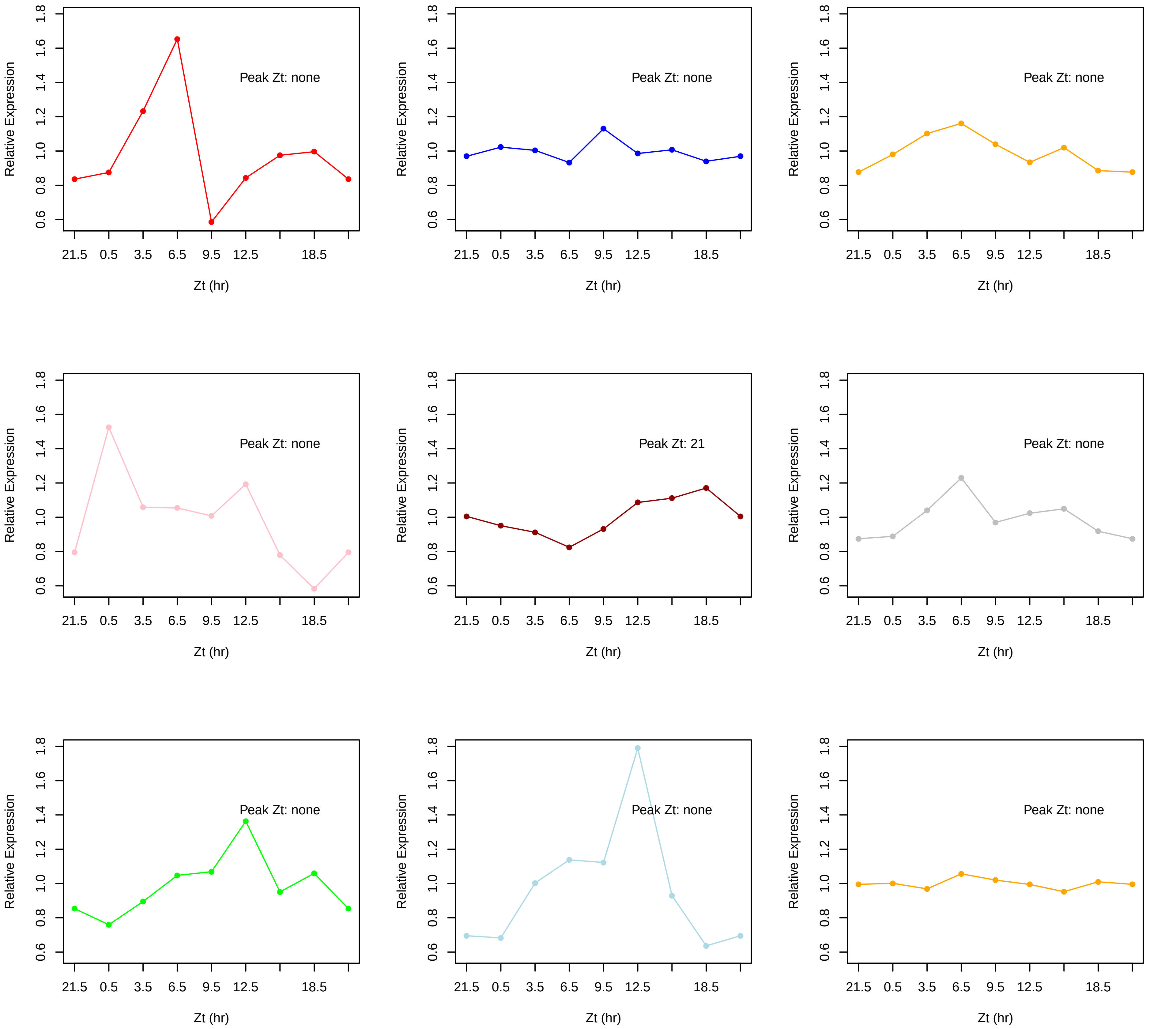

Singeleton: ENSXMAG00000009827 wdr3

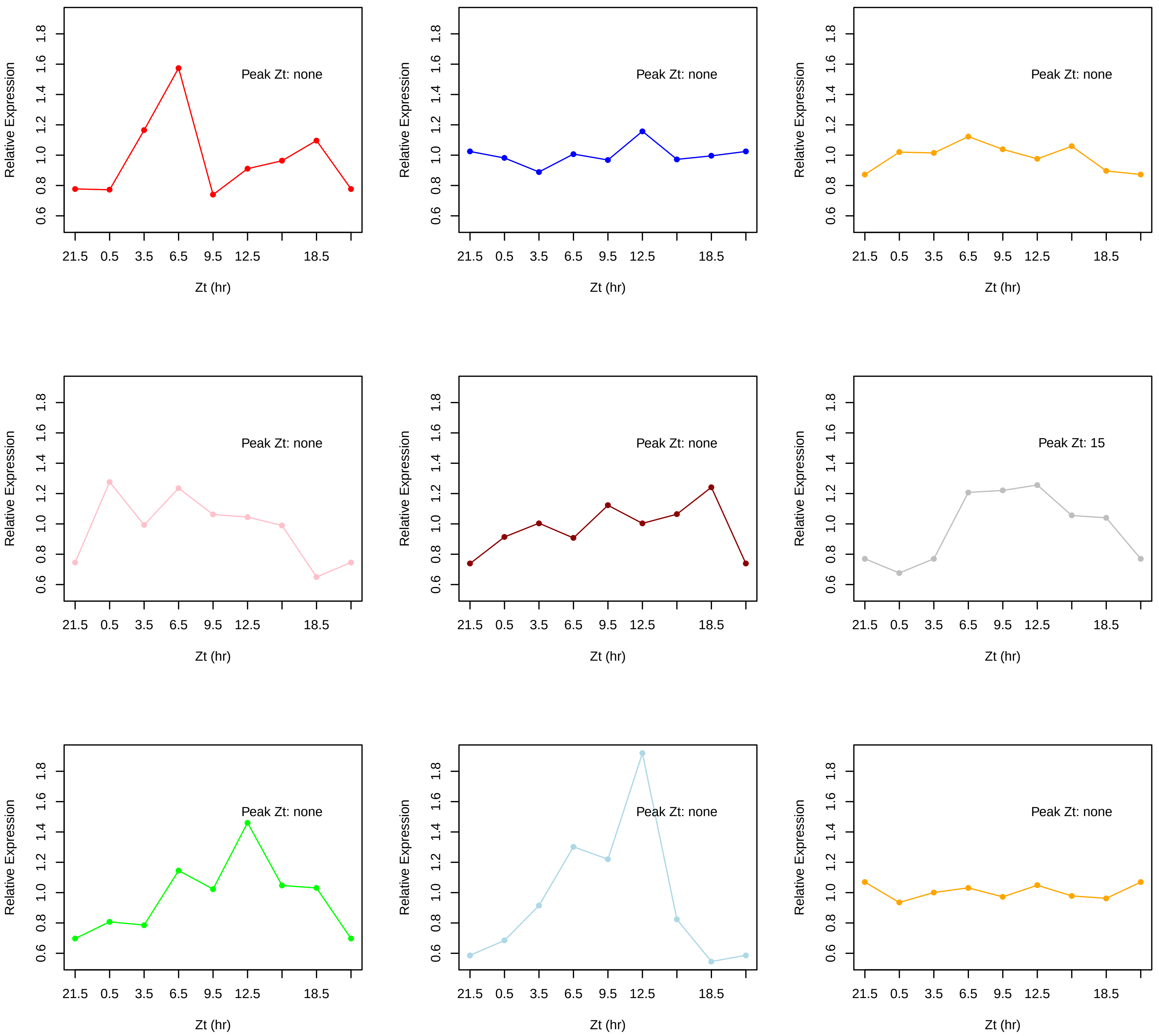

Singeleton: ENSXMAG00000016868 asns

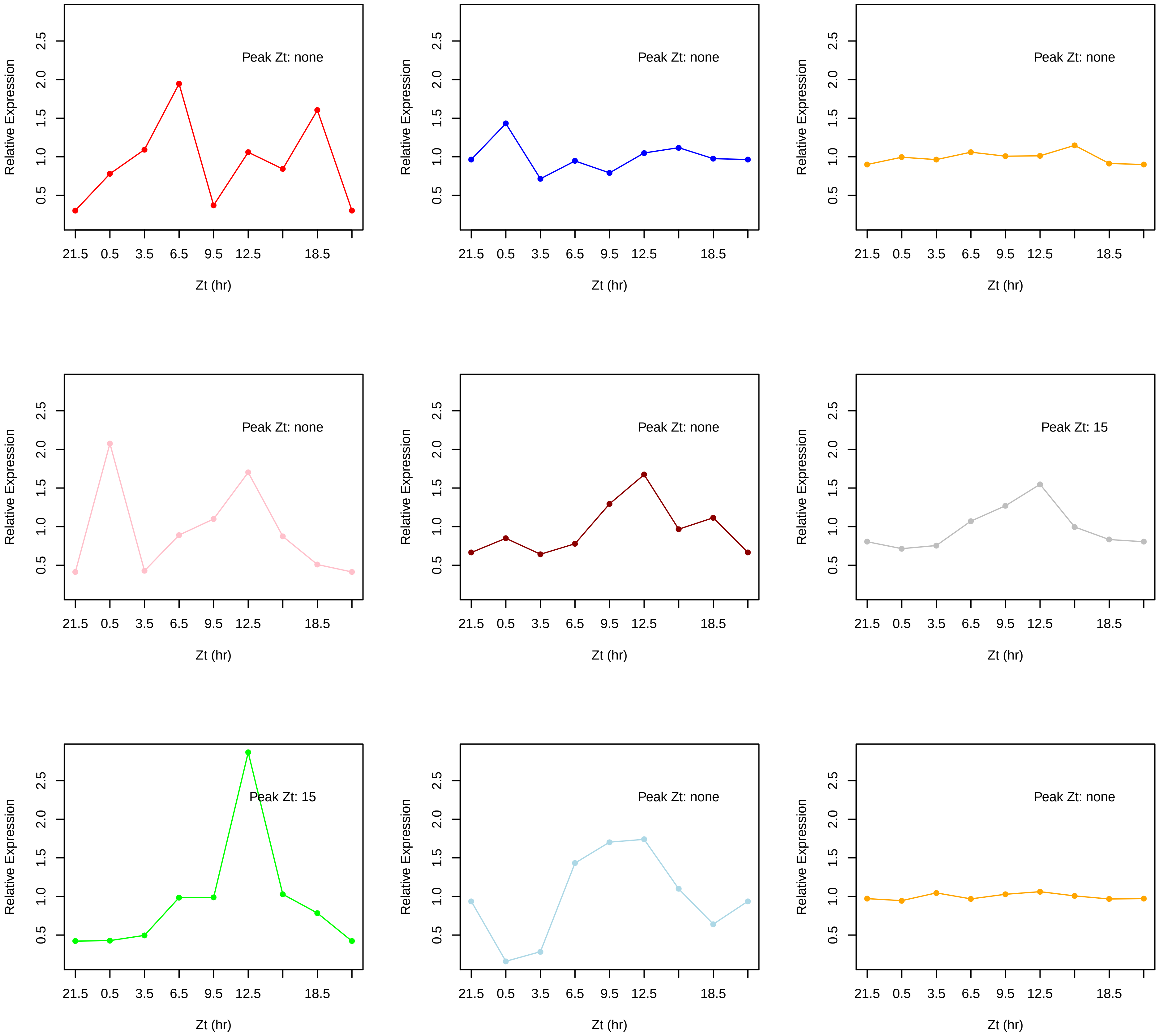

Singeleton: ENSXMAG00000004320 tbc1d22b

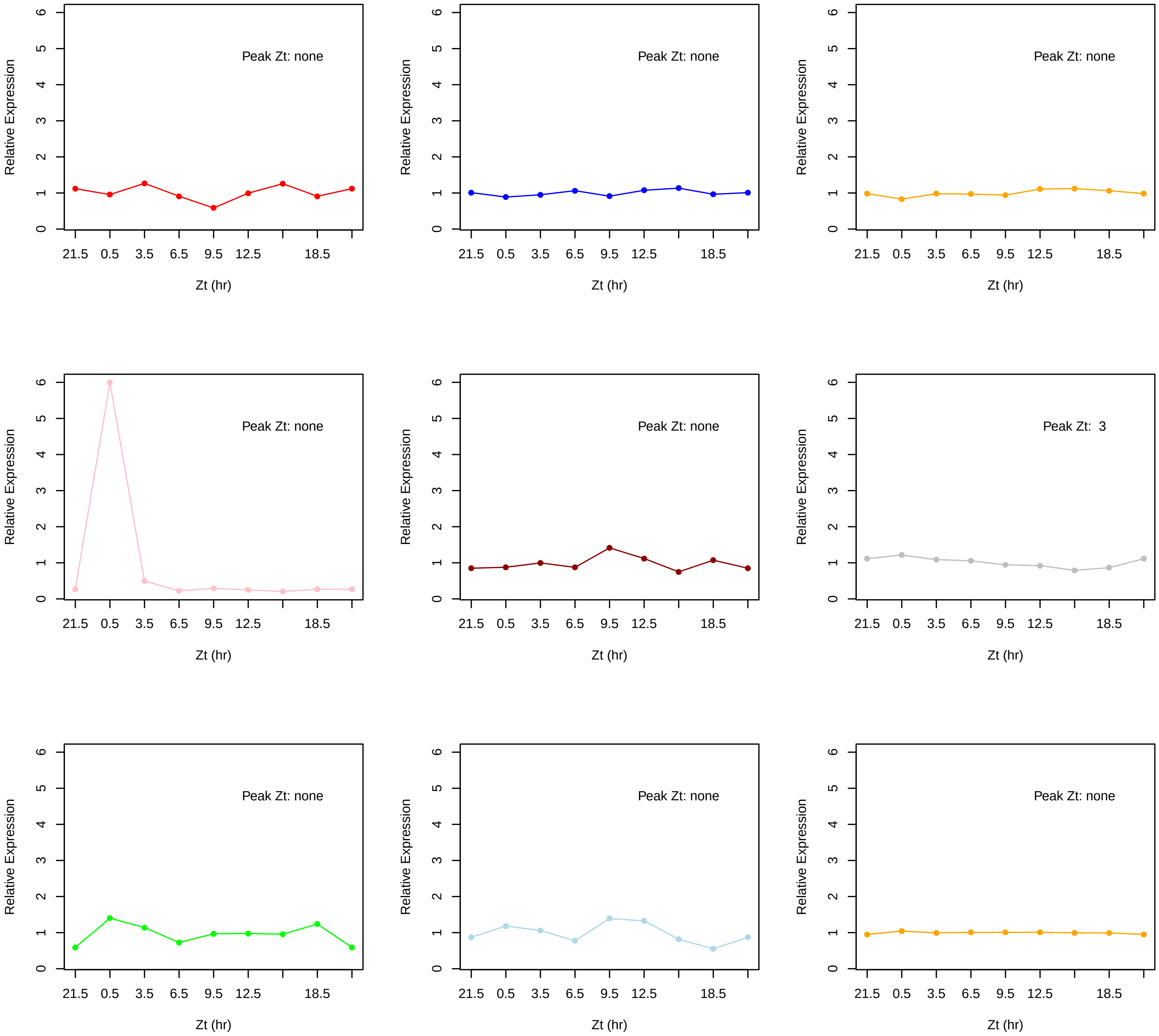

Singleton: ENSXMAG00000024410

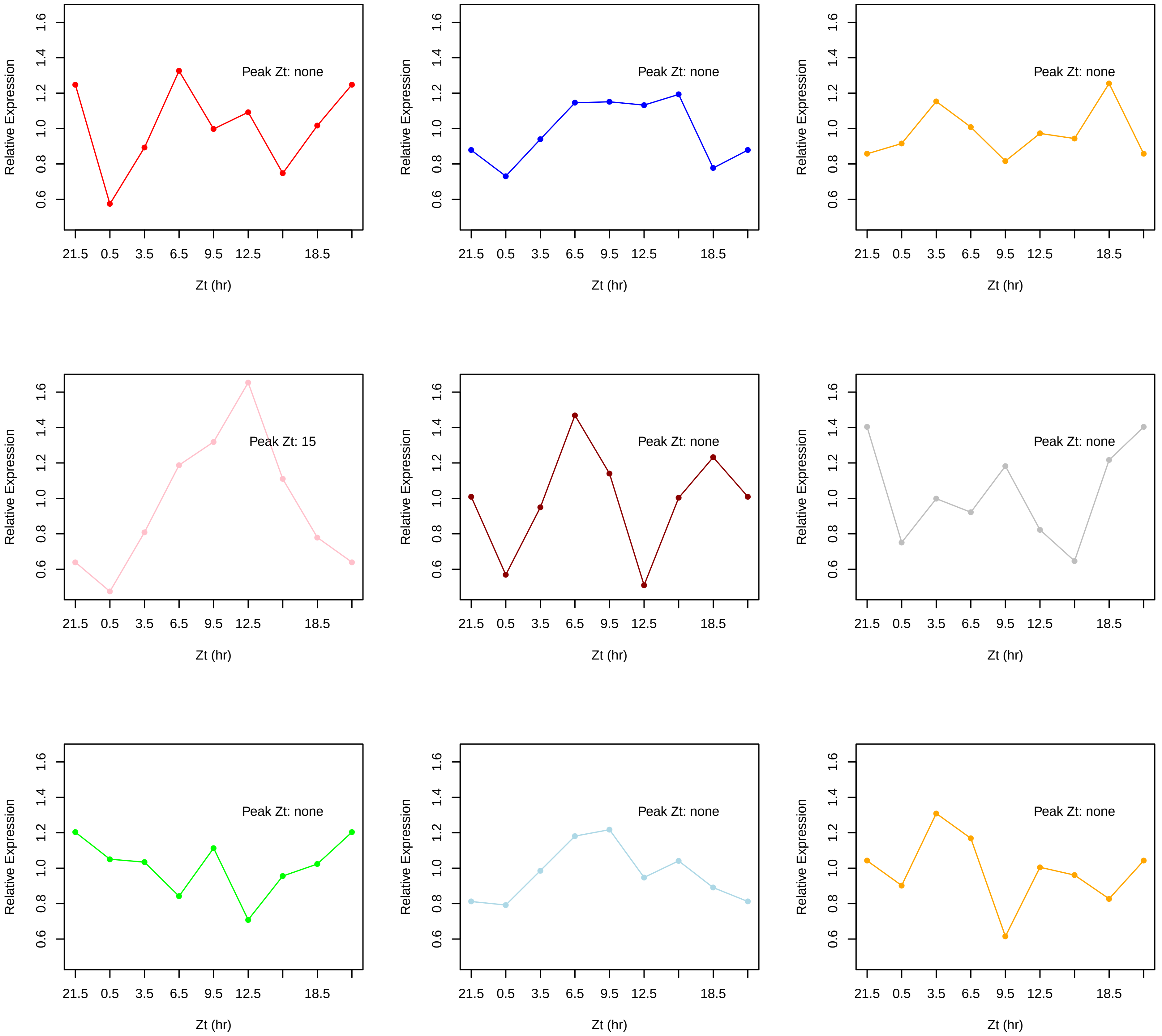

Singeleton: ENSXMAG00000018902

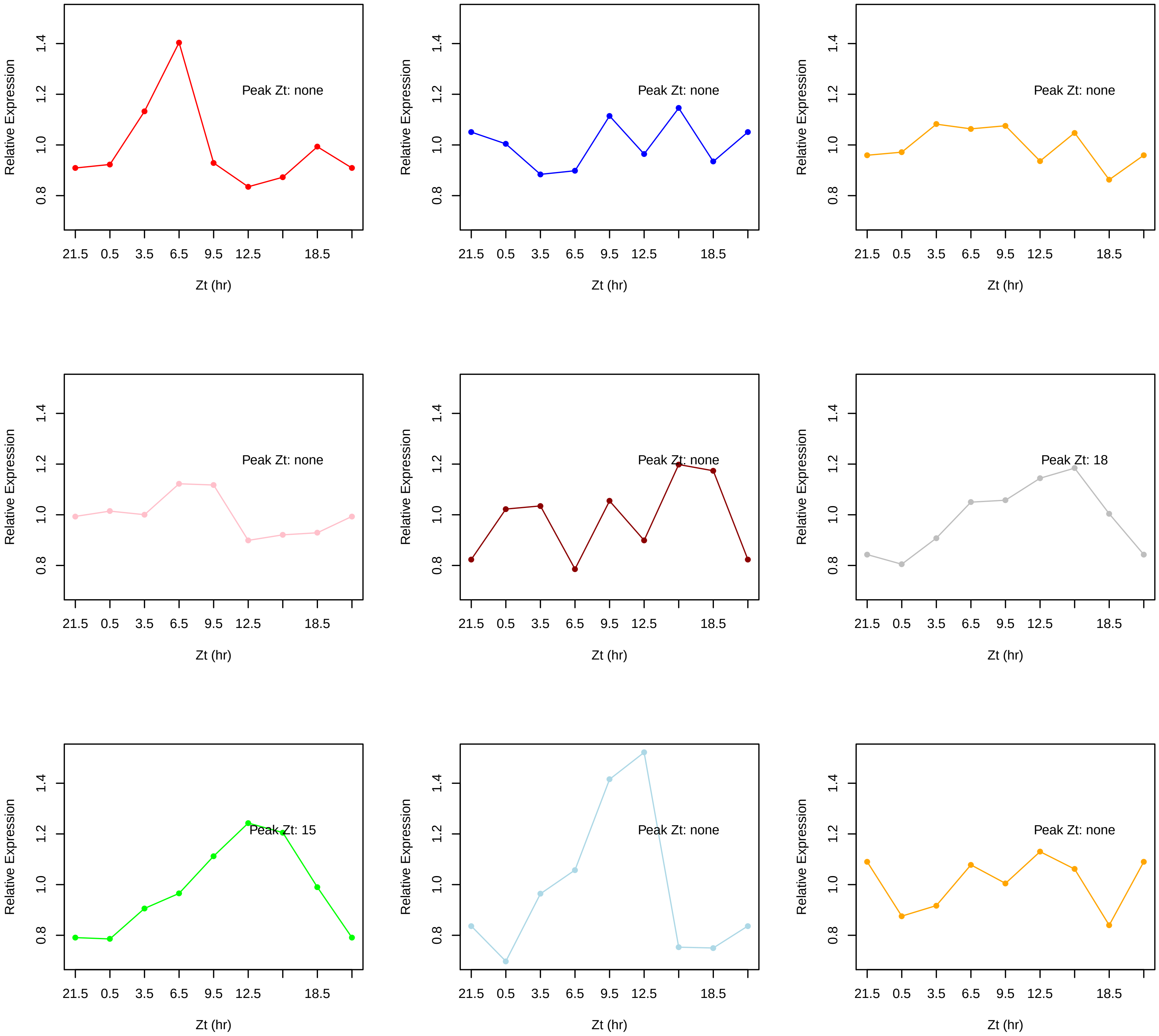

Singeleton: ENSXMAG00000008049

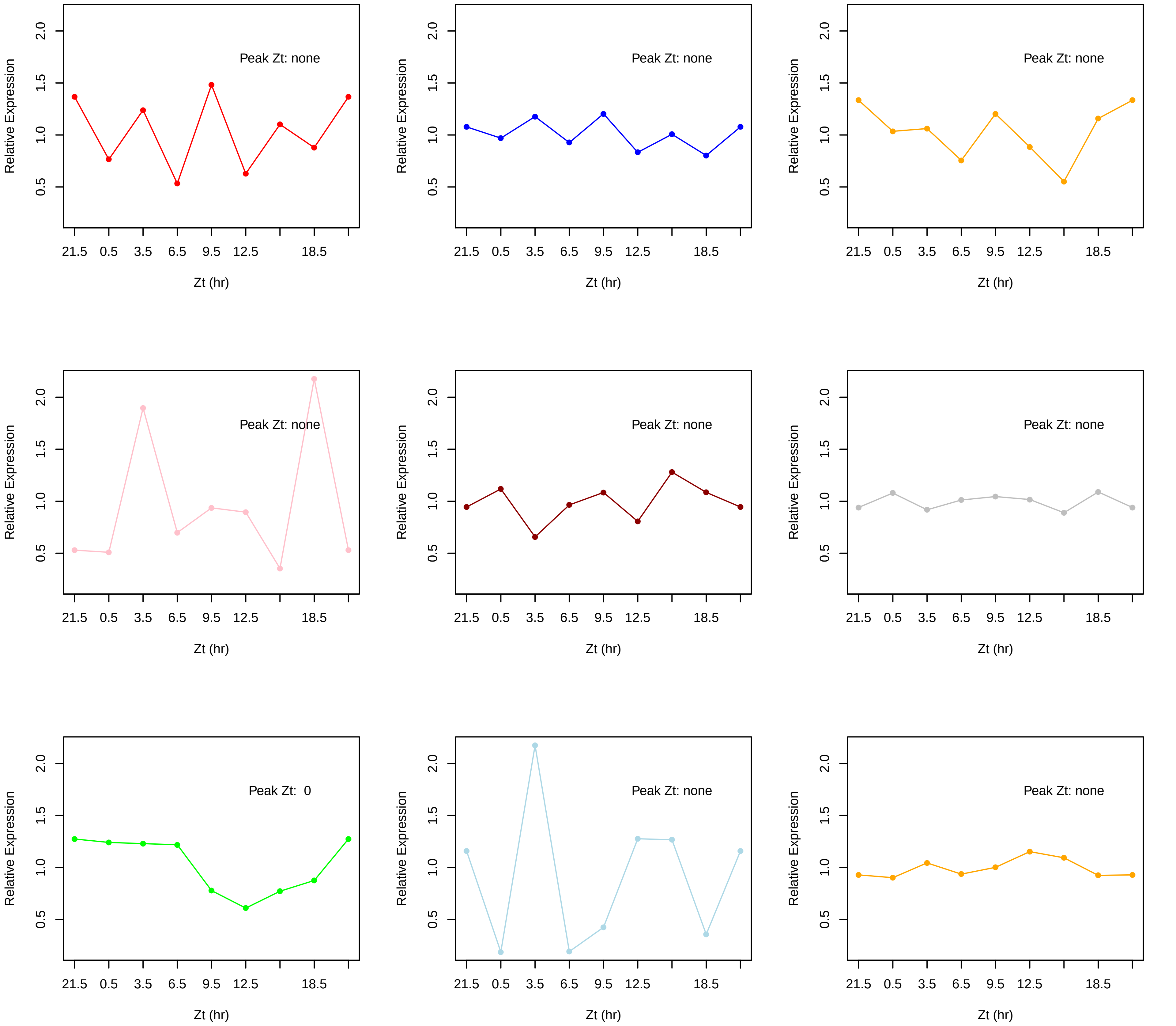

Singeleton: ENSXMAG00000023603 aco1

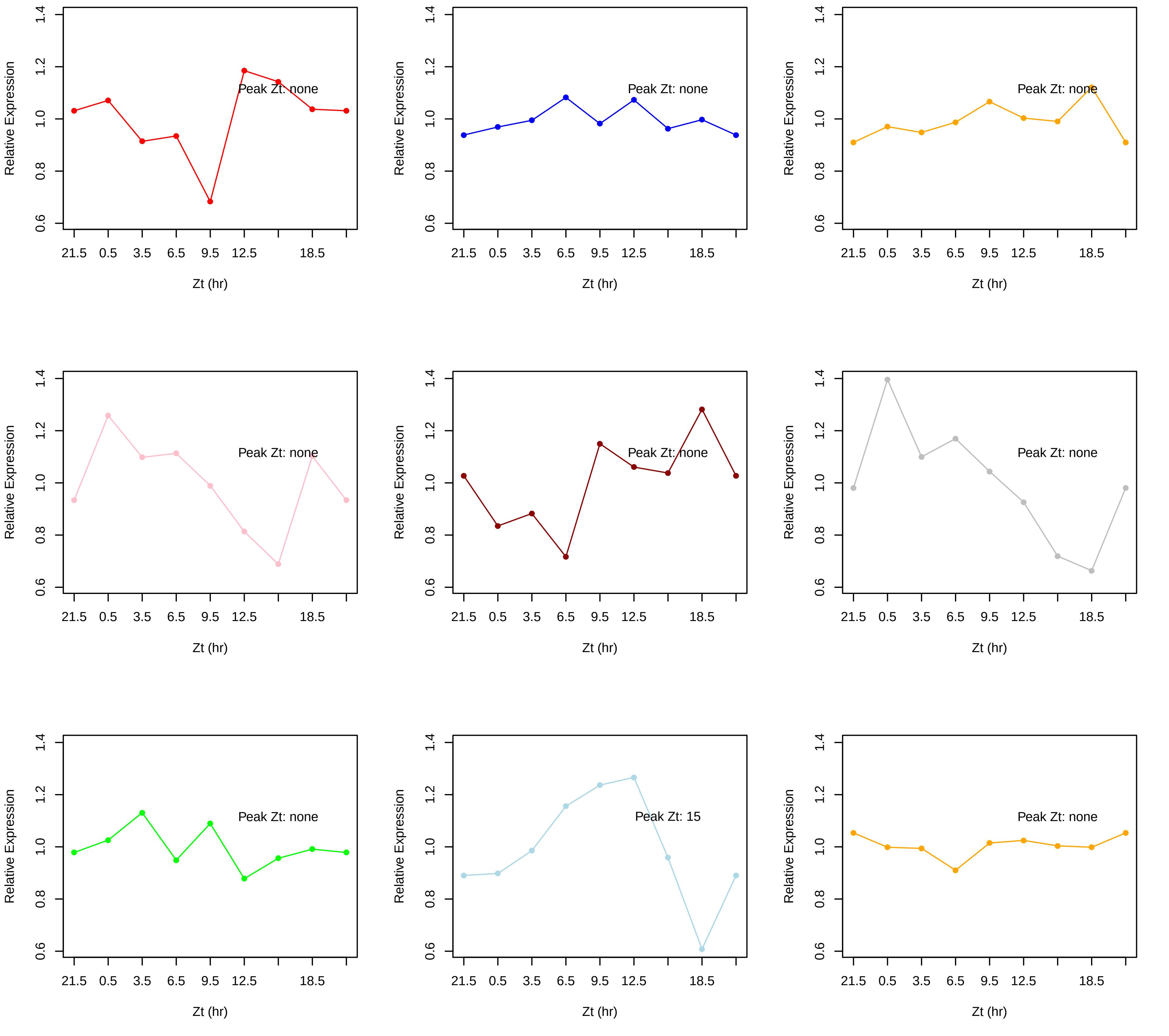

Singeleton: ENSXMAG00000027517 crbn

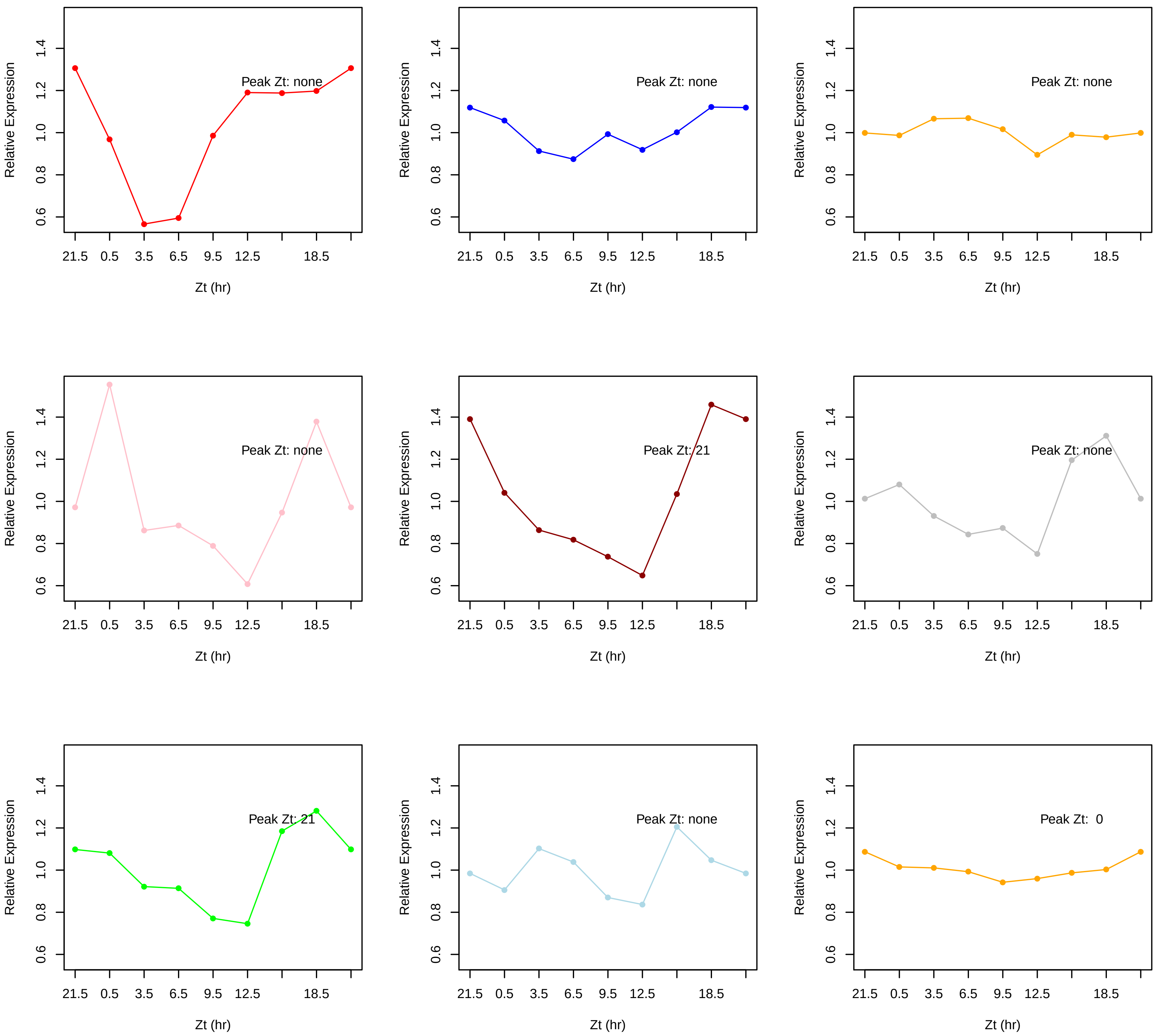

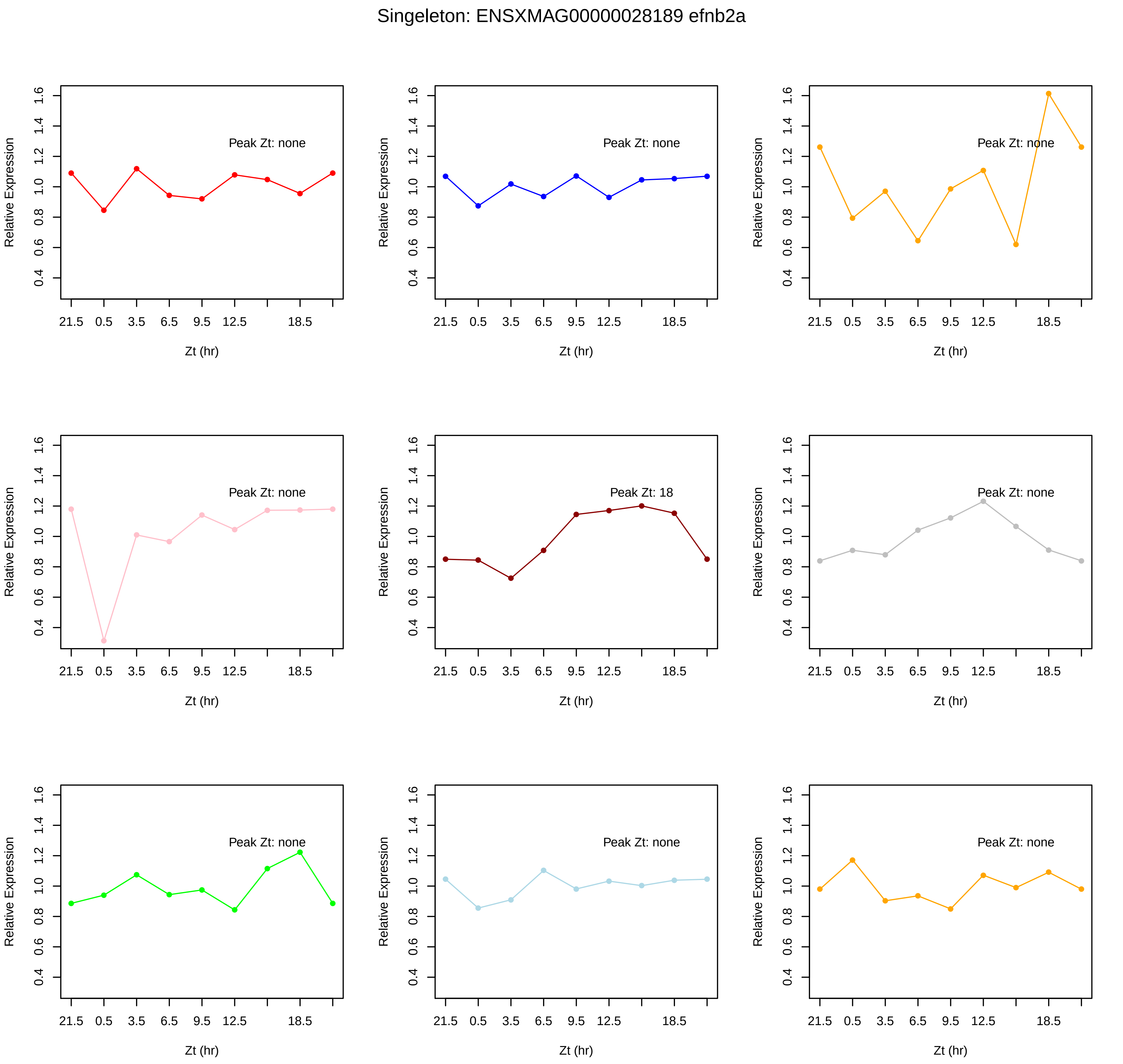

Singeleton: ENSXMAG00000028189 efnb2a
Peak Zt: none
Peak Zt: none
Peak Zt: none
Peak Zt: none
Peak Zt: 18
Peak Zt: none
Peak Zt: none
Peak Zt: none
Peak Zt: none
Relative Expression
Zt (hr)
21.5
0.5
3.5
6.5
9.5
12.5
18.5
0.4
0.6
0.8
1.0
1.2
1.4
1.6

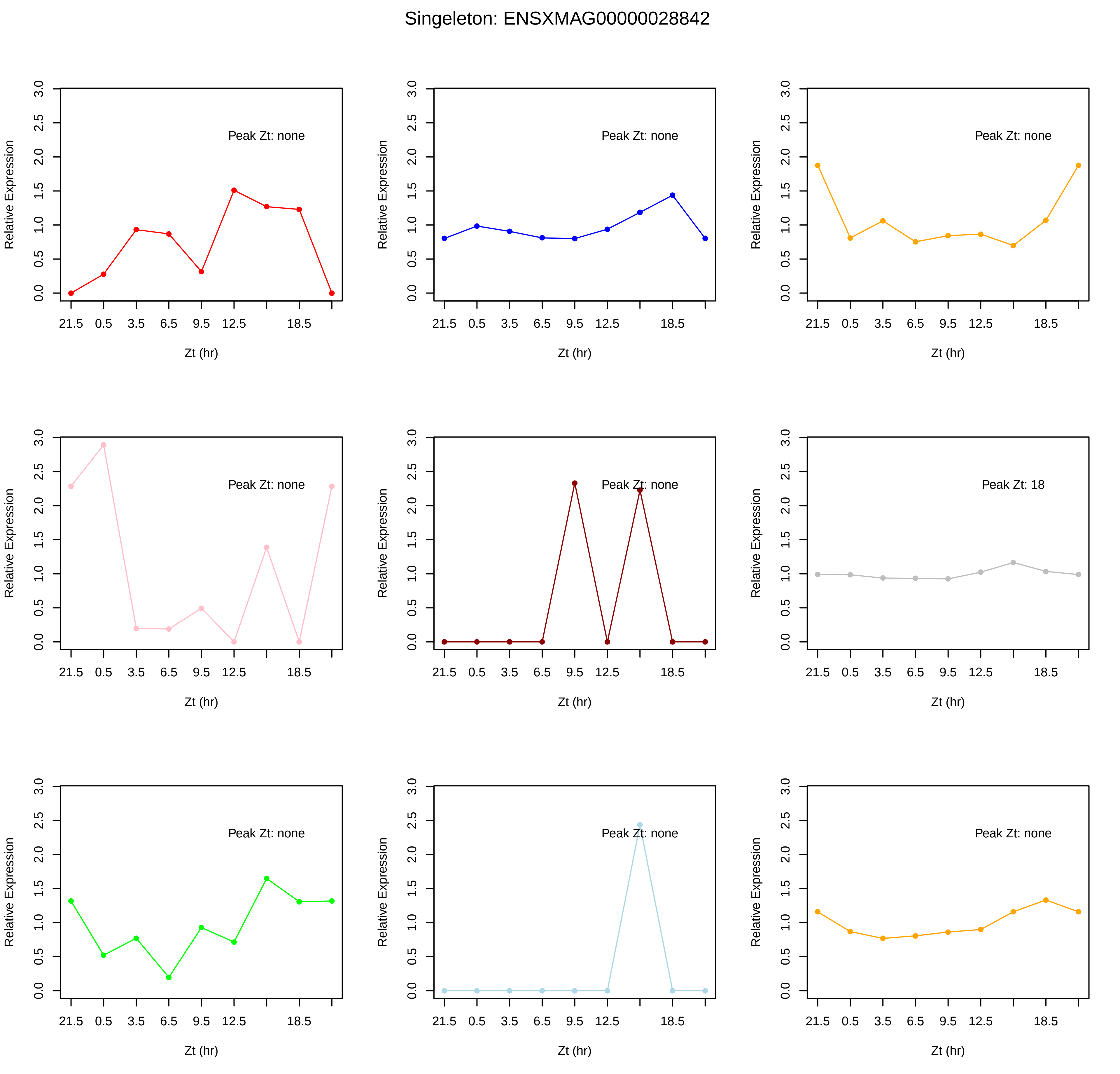

Singeleton: ENSXMAG00000028842
Peak Zt: none
Peak Zt: none
Peak Zt: none
Peak Zt: none
Peak Zt: none
Peak Zt: 18
Peak Zt: none
Peak Zt: none
Peak Zt: none
Relative Expression
Zt (hr)
21.5
0.5
3.5
6.5
9.5
12.5
18.5

Singeleton: ENSXMAG00000007389 arglu1a

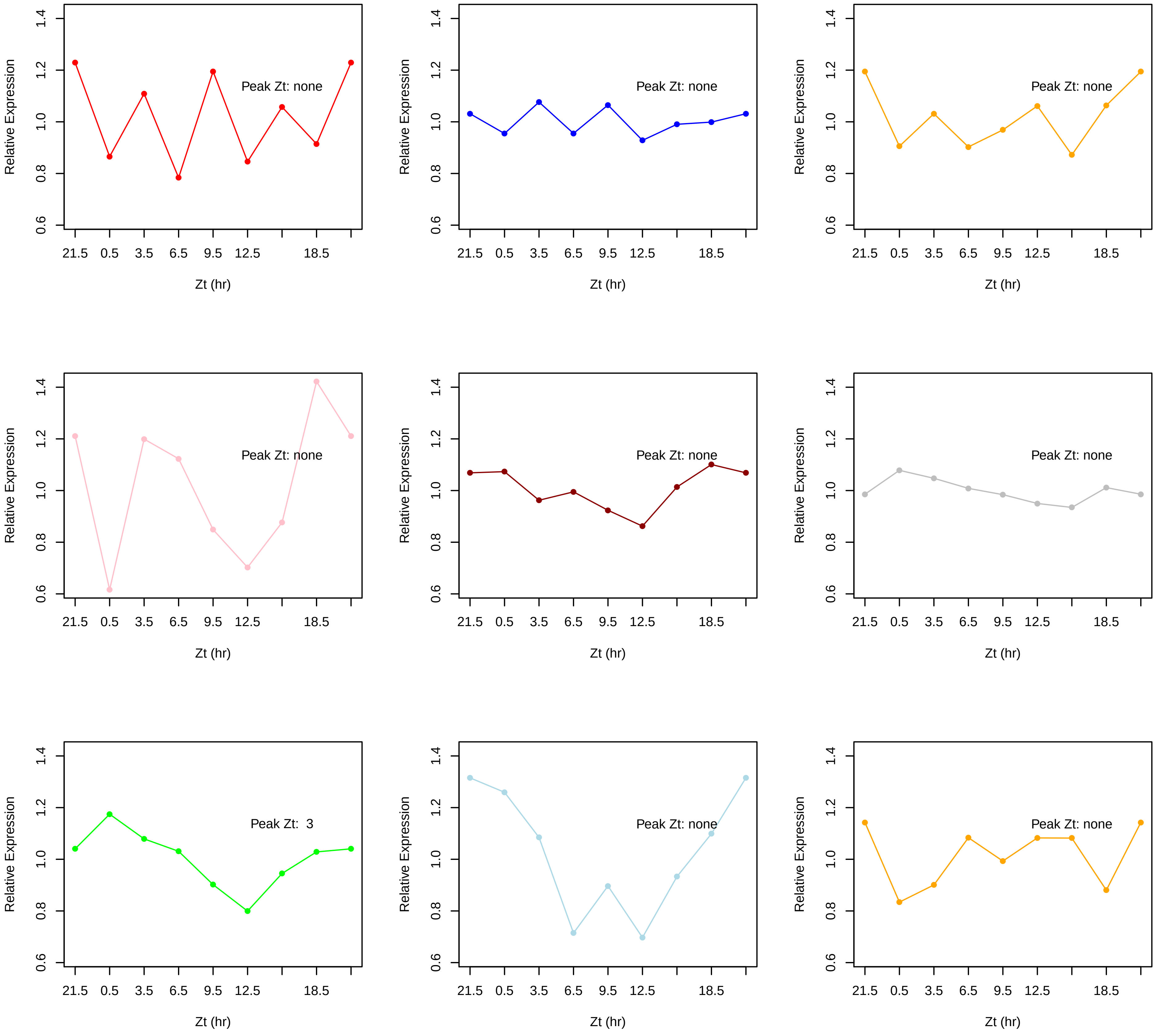

Singeleton: ENSXMAG00000008391 actr10

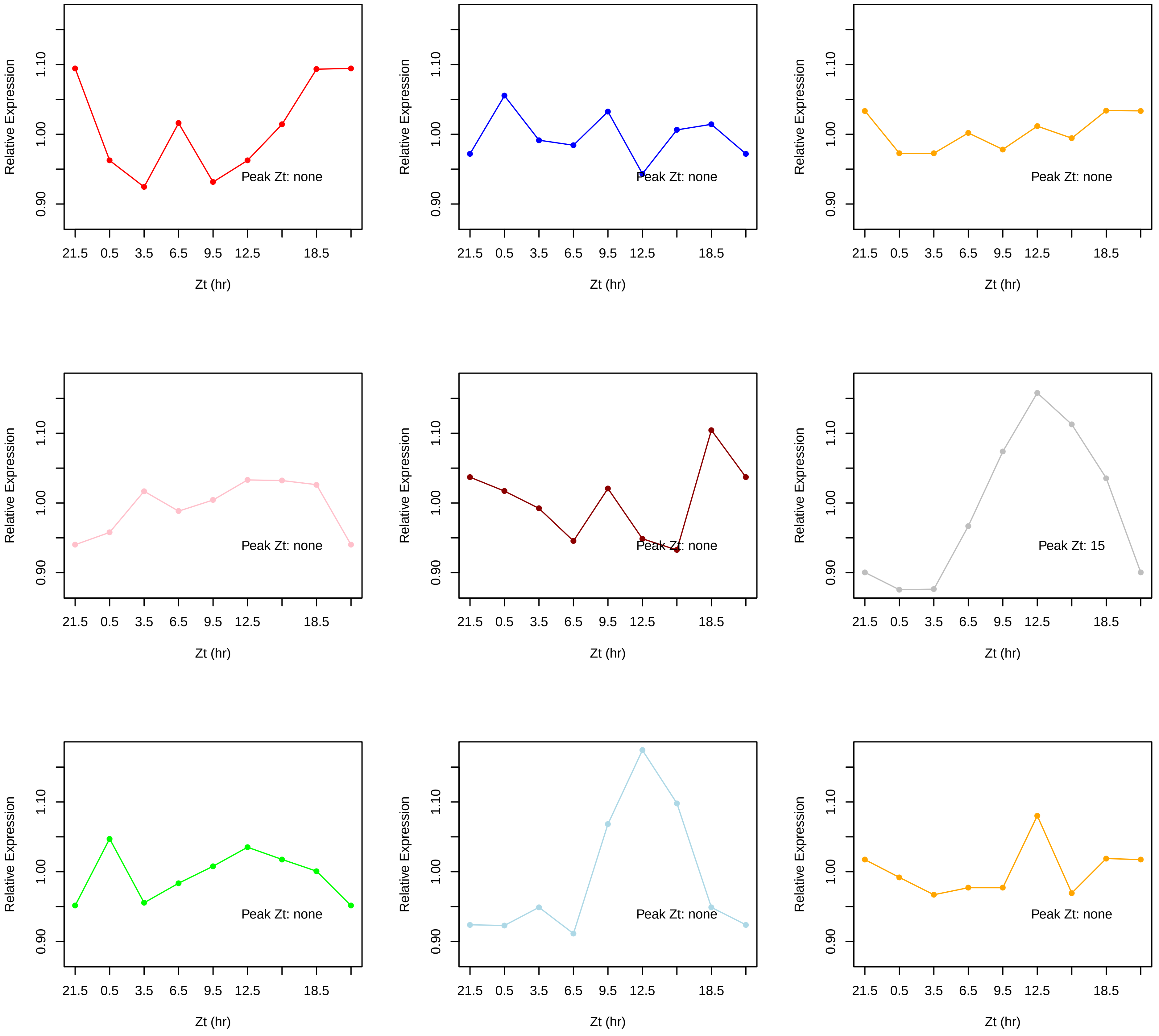

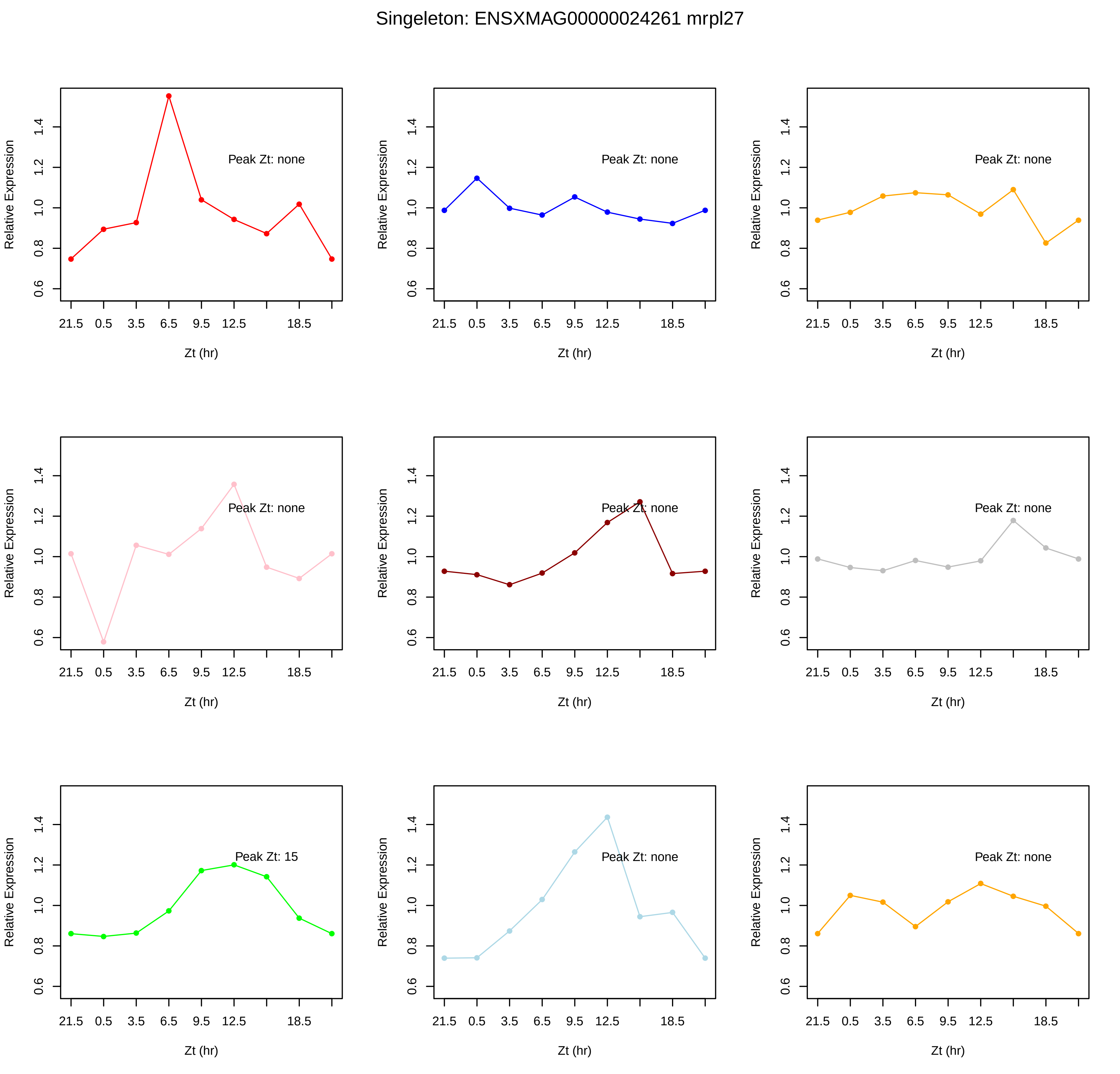
Singeleton: ENSXMAG00000024261 mrpl27
Relative Expression
Zt (hr)
Peak Zt: none
Peak Zt: 15
21.5
0.5
3.5
6.5
9.5
12.5
18.5
0.6
0.8
1.0
1.2
1.4

Singleton: ENSXMAG00000009820 trim45

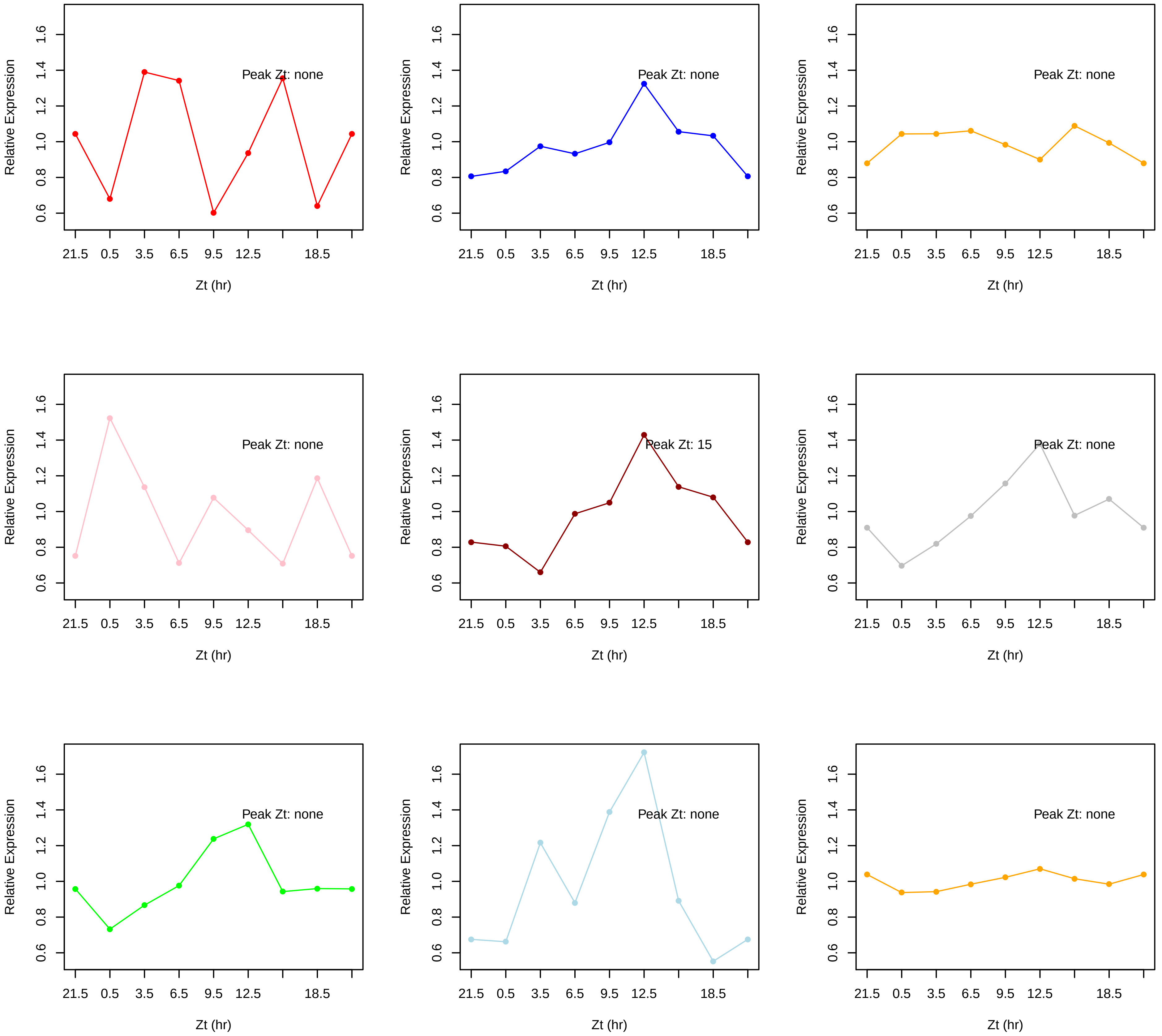

Singleton: ENSXMAG00000005820 acer2

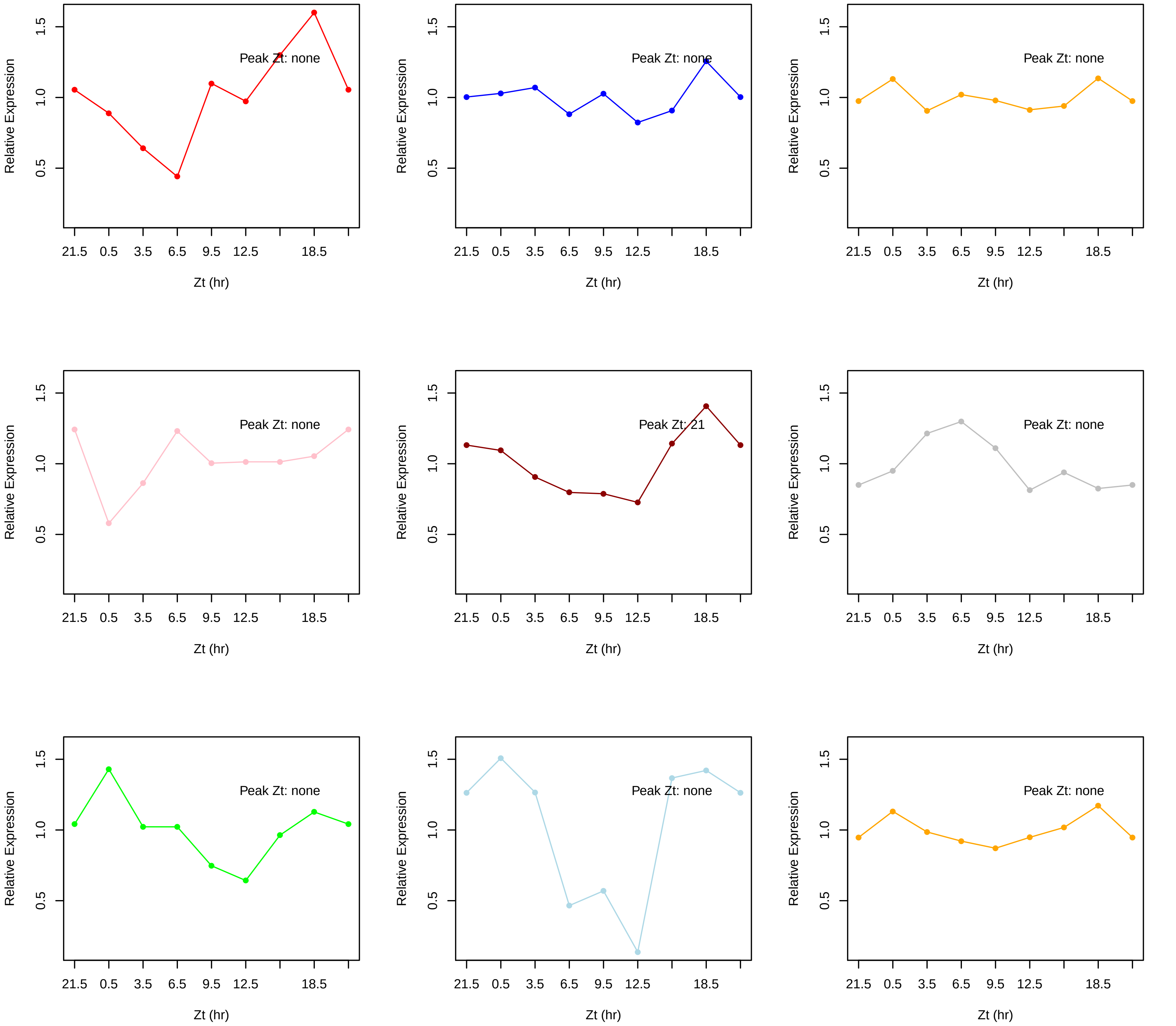

Singeleton: ENSXMAG00000018827 mios

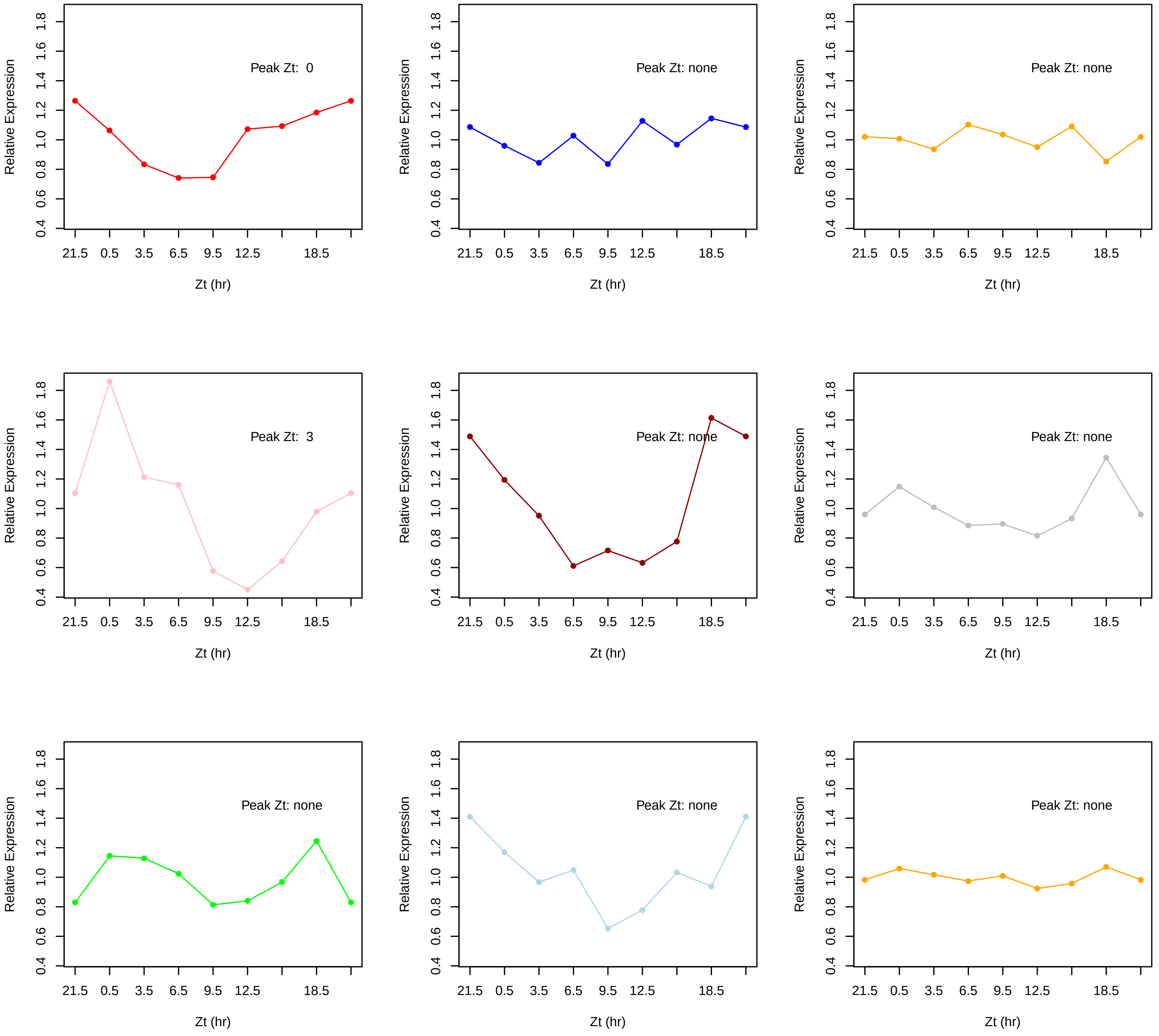

Singeleton: ENSXMAG00000012699 mapk13

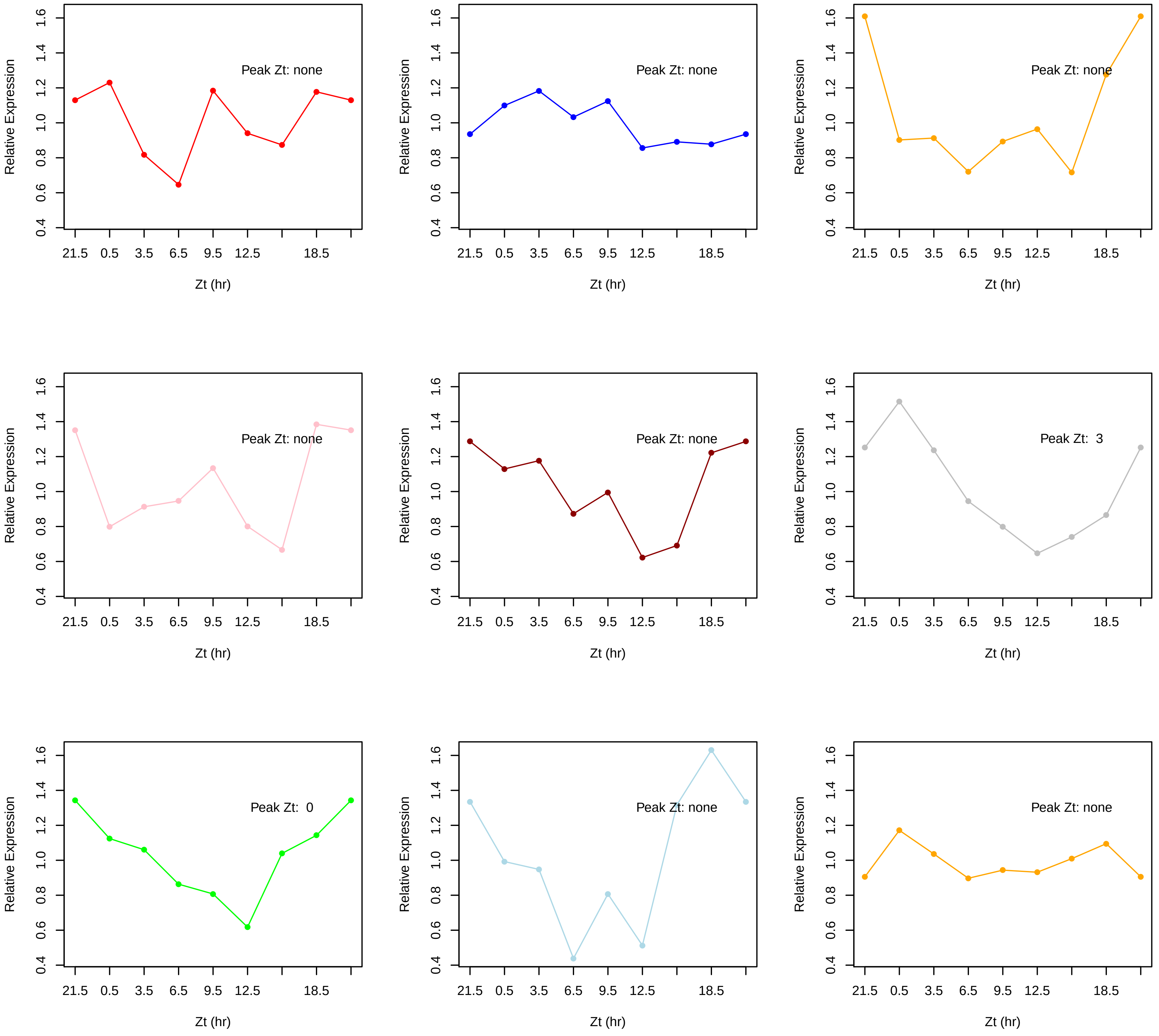

Singeleton: ENSXMAG00000002227 trnt1

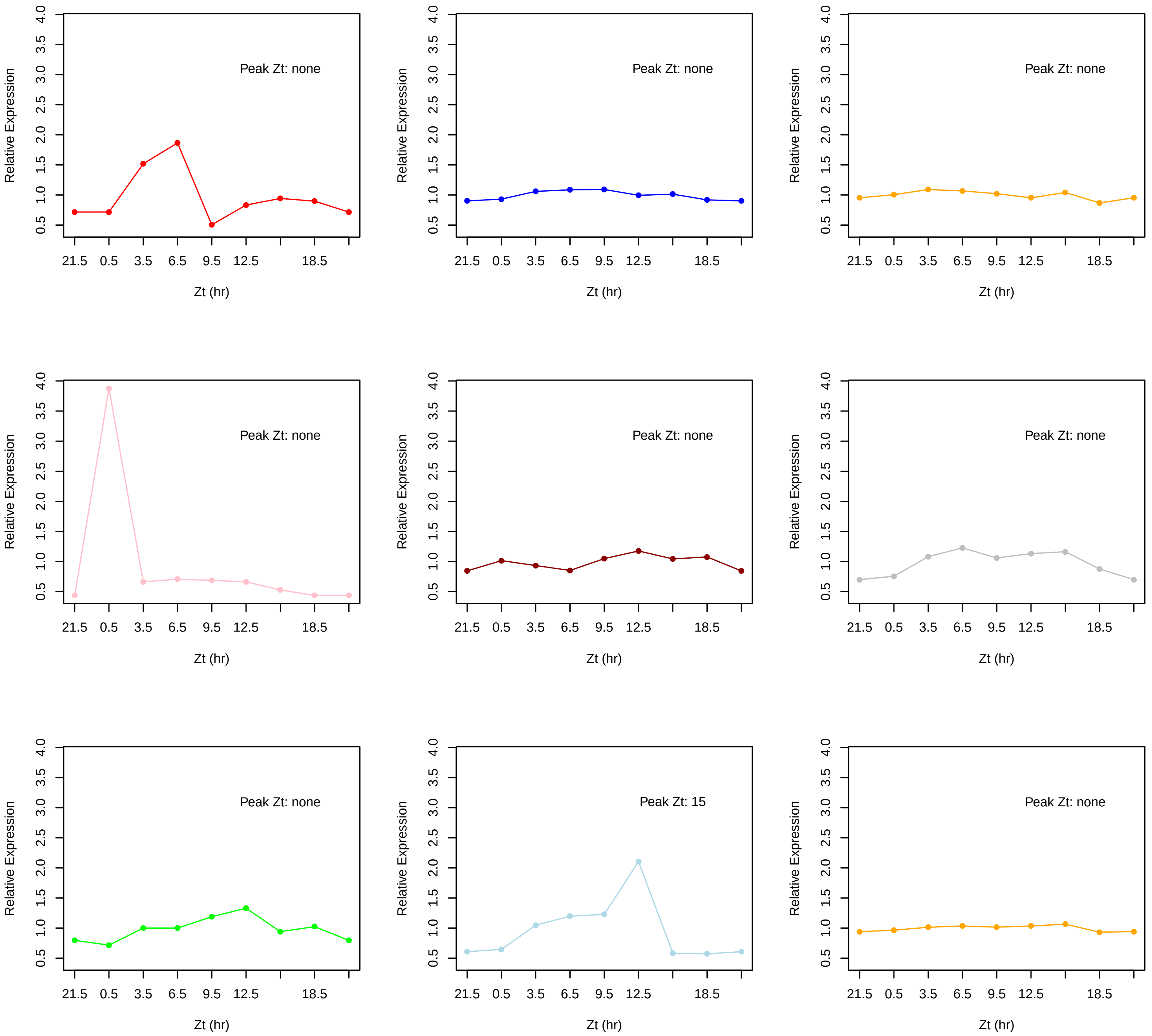

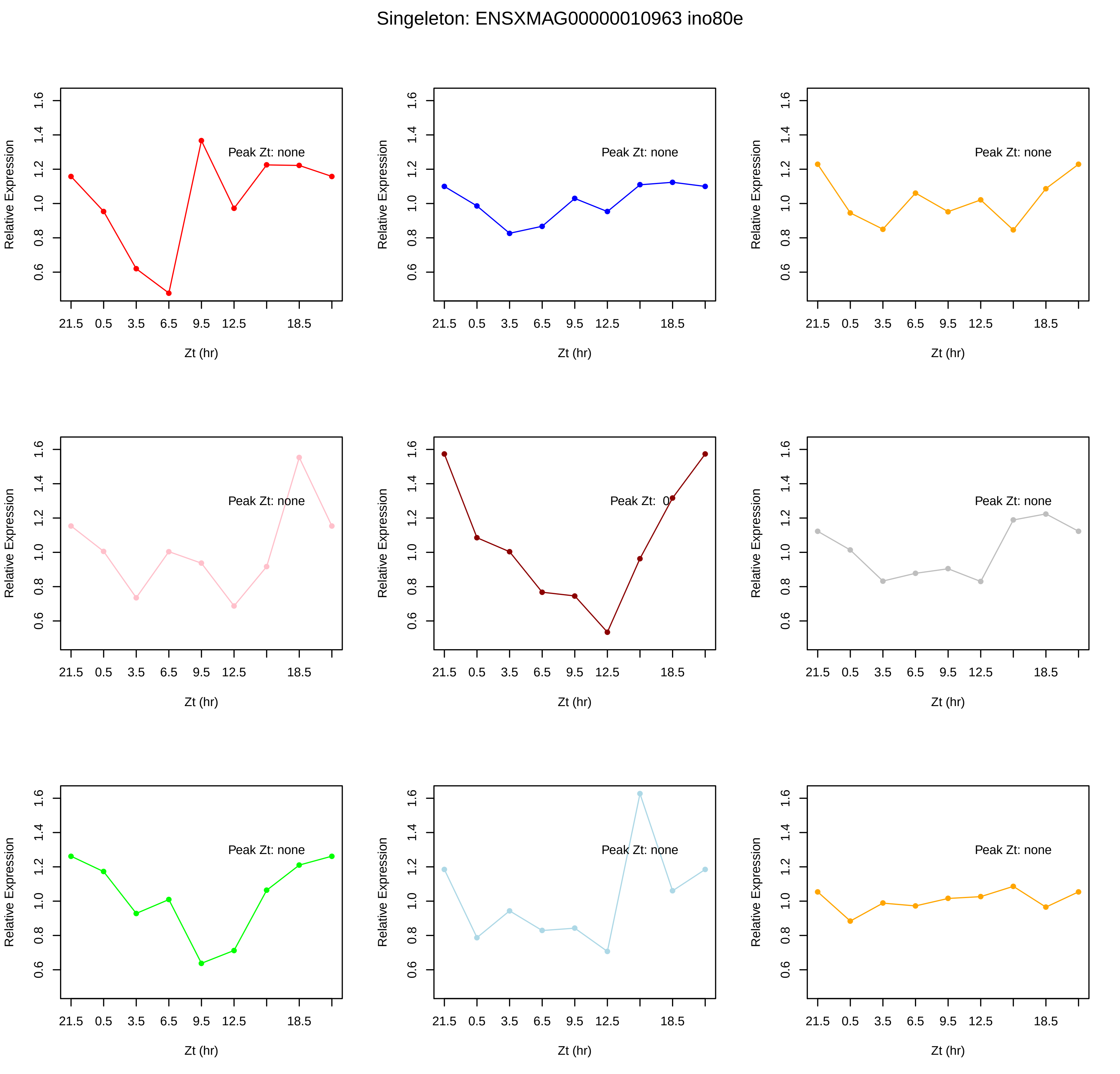
Singeleton: ENSXMAG00000010963 ino80e
Peak Zt: none
Peak Zt: none
Peak Zt: none
Peak Zt: none
Peak Zt: 0
Peak Zt: none
Peak Zt: none
Peak Zt: none
Peak Zt: none
Relative Expression
Zt (hr)

Singeleton: ENSXMAG00000016840 rpa3

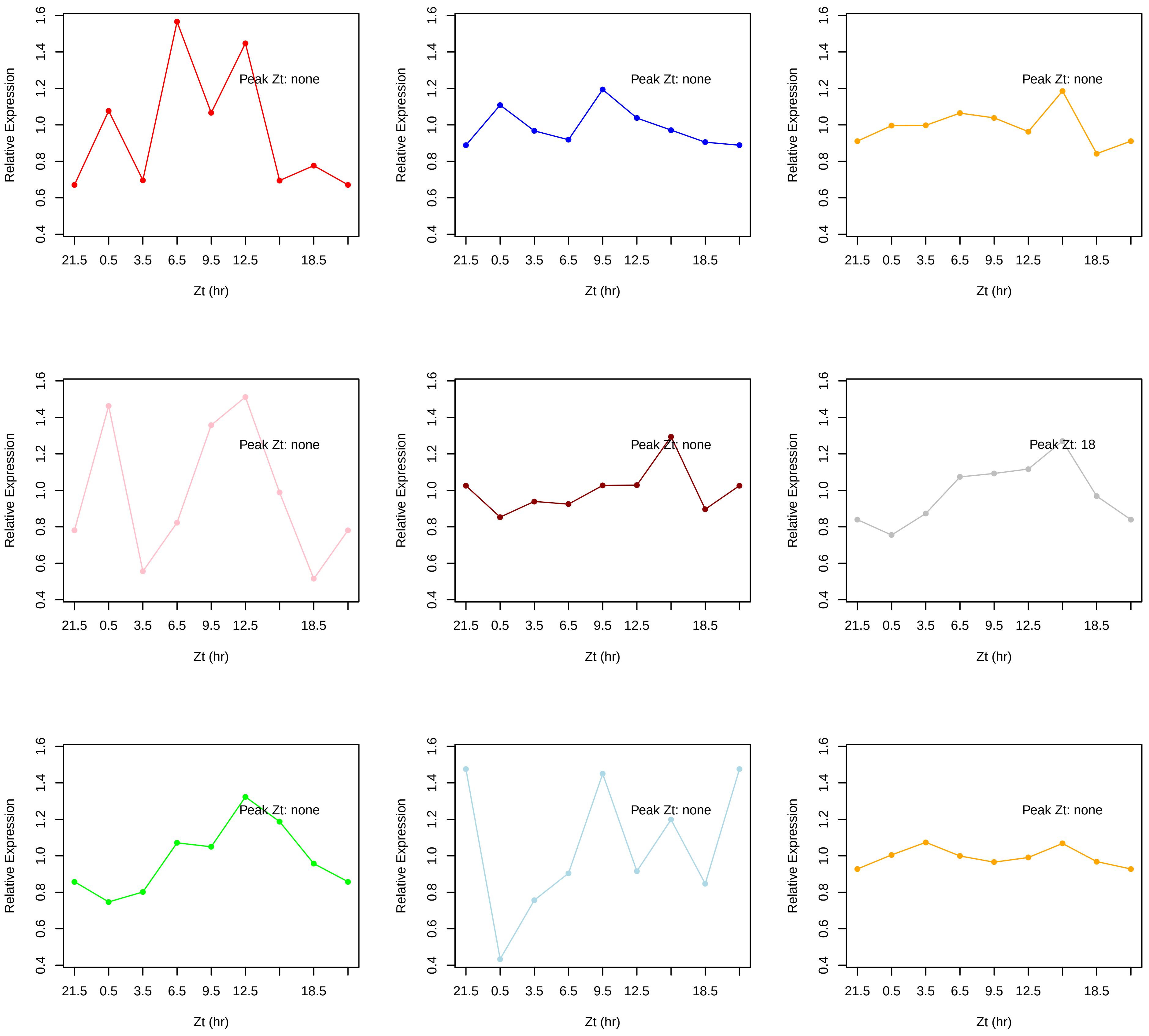

Singeleton: ENSXMAG00000012122 xylt2

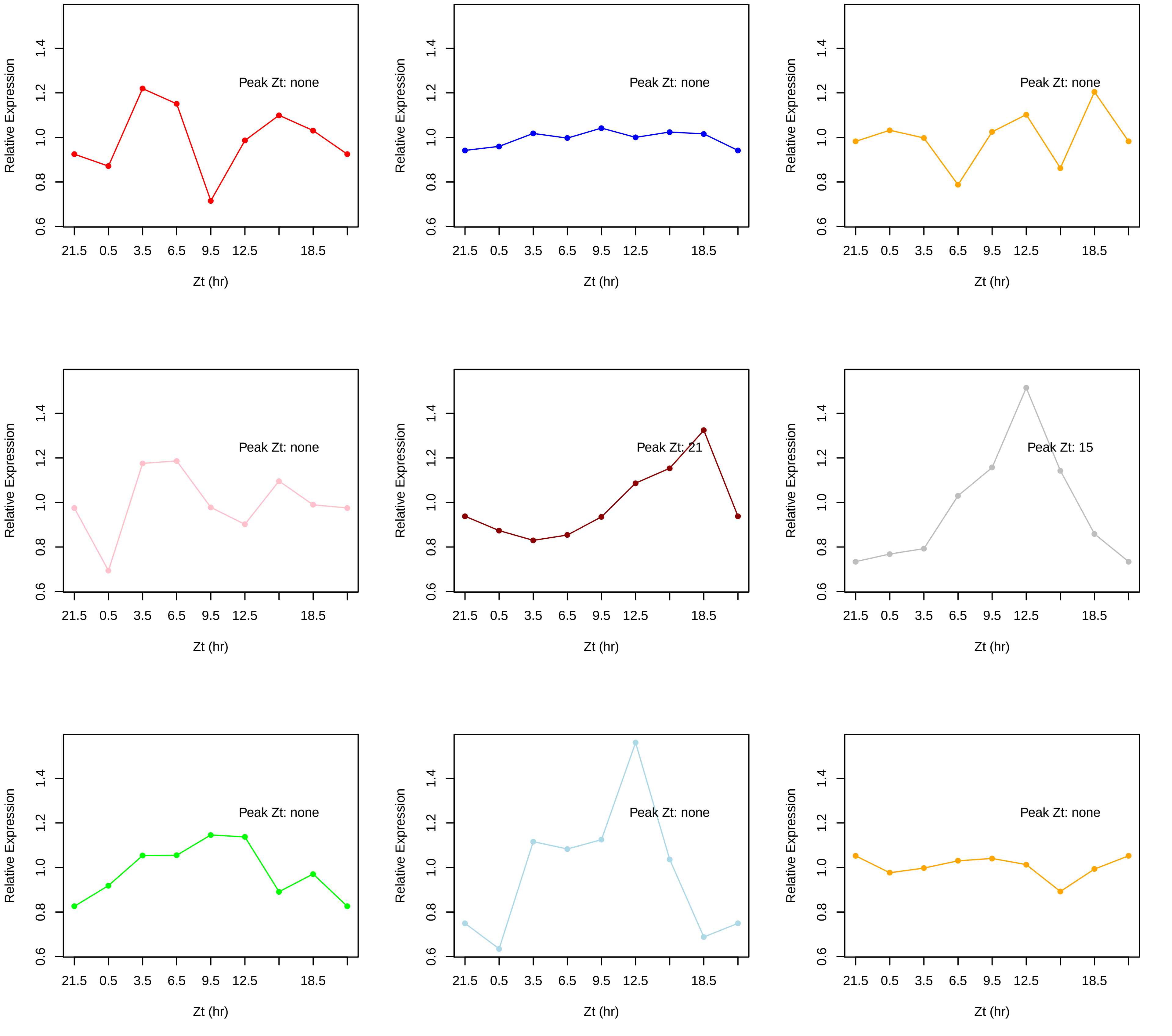

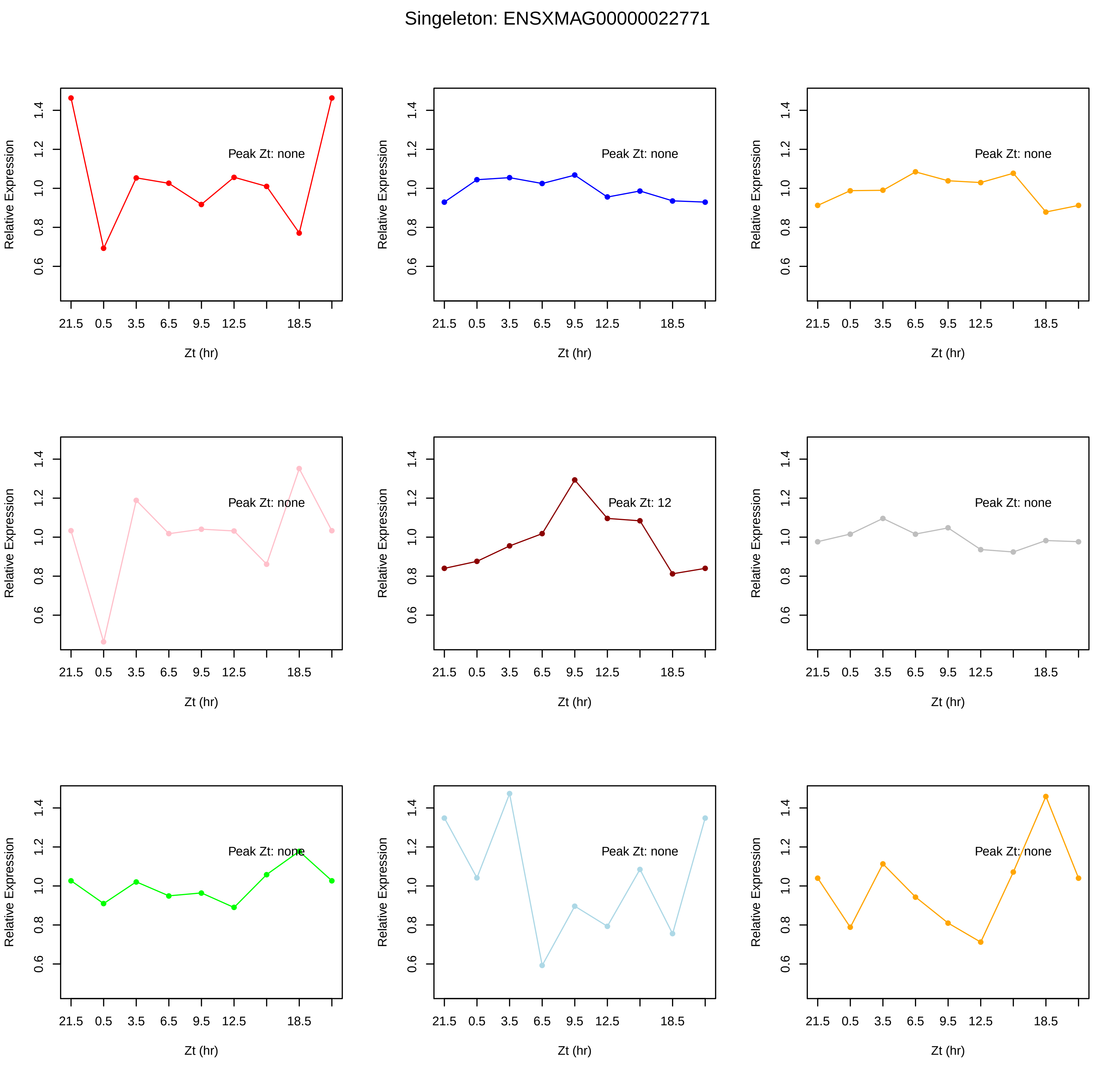

Singeleton: ENSXMAG00000022771
Peak Zt: none
Peak Zt: none
Peak Zt: none
Peak Zt: none
Peak Zt: 12
Peak Zt: none
Peak Zt: none
Peak Zt: none
Peak Zt: none
Relative Expression
Zt (hr)

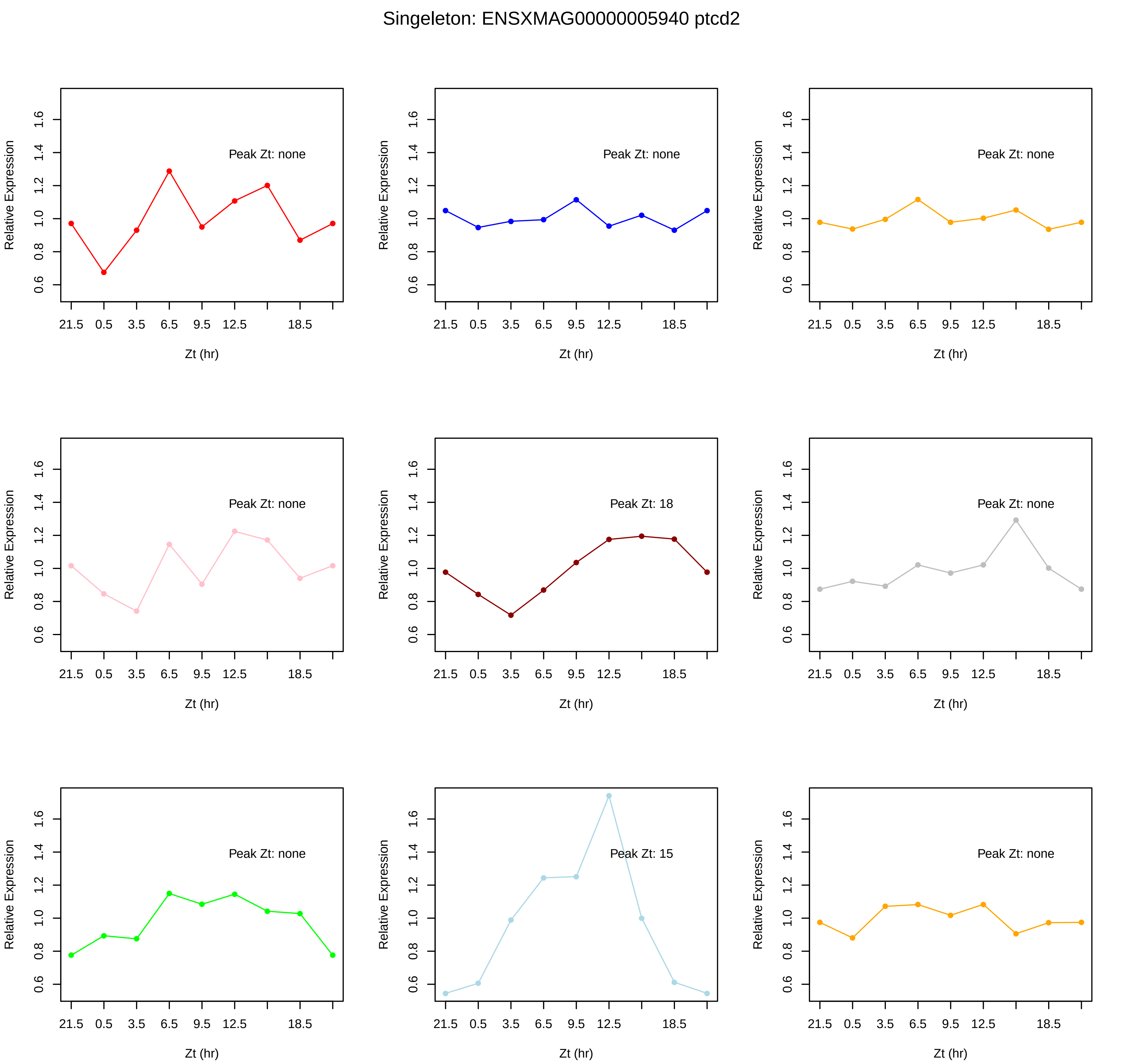
Singeleton: ENSXMAG00000005940 ptcd2
Peak Zt: none
Peak Zt: none
Peak Zt: none
Peak Zt: none
Peak Zt: 18
Peak Zt: none
Peak Zt: none
Peak Zt: 15
Peak Zt: none
Relative Expression
Zt (hr)
21.5
0.5
3.5
6.5
9.5
12.5
18.5
0.6
0.8
1.0
1.2
1.4
1.6

Singeleton: ENSXMAG00000015242 slc35f4

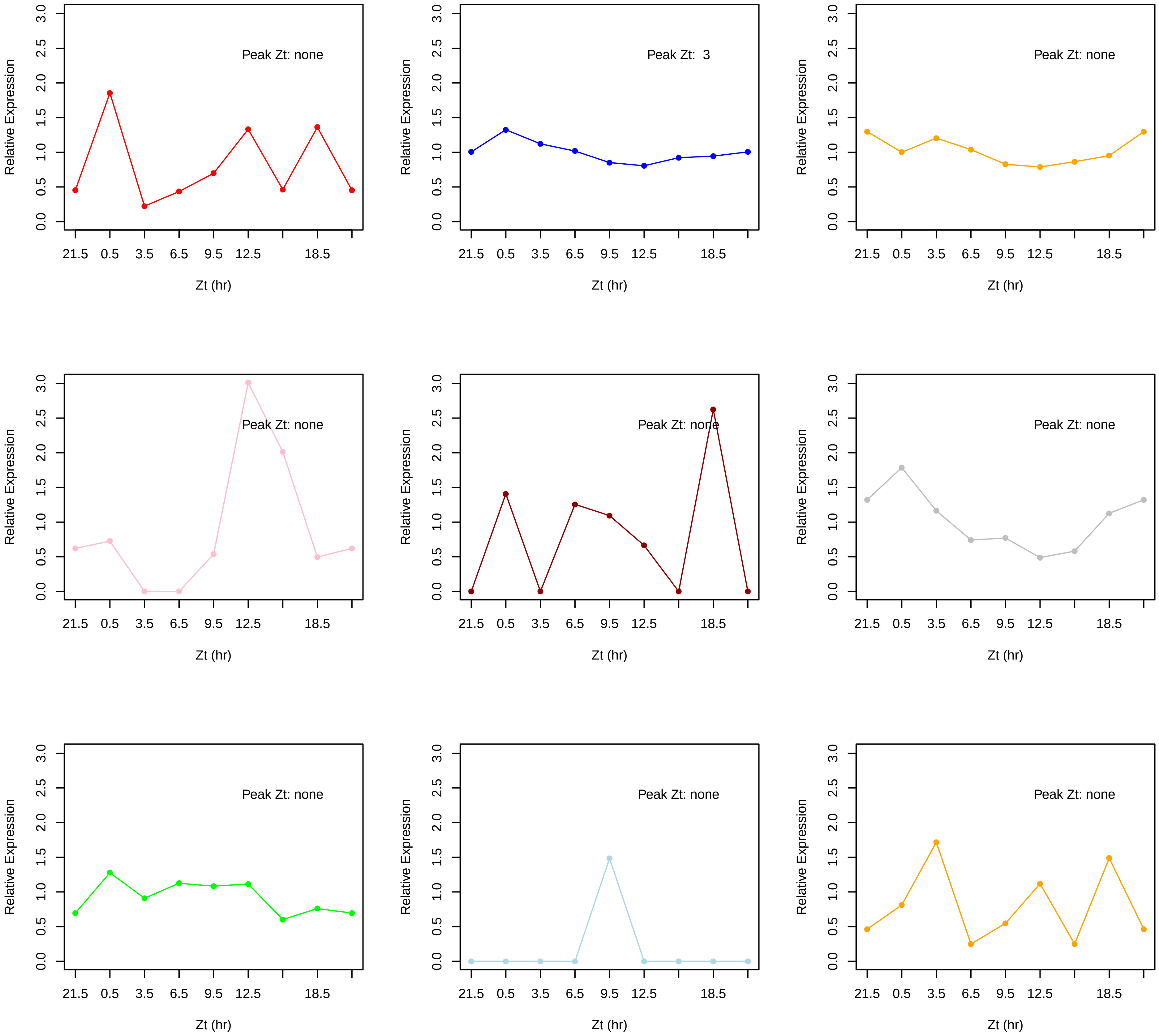

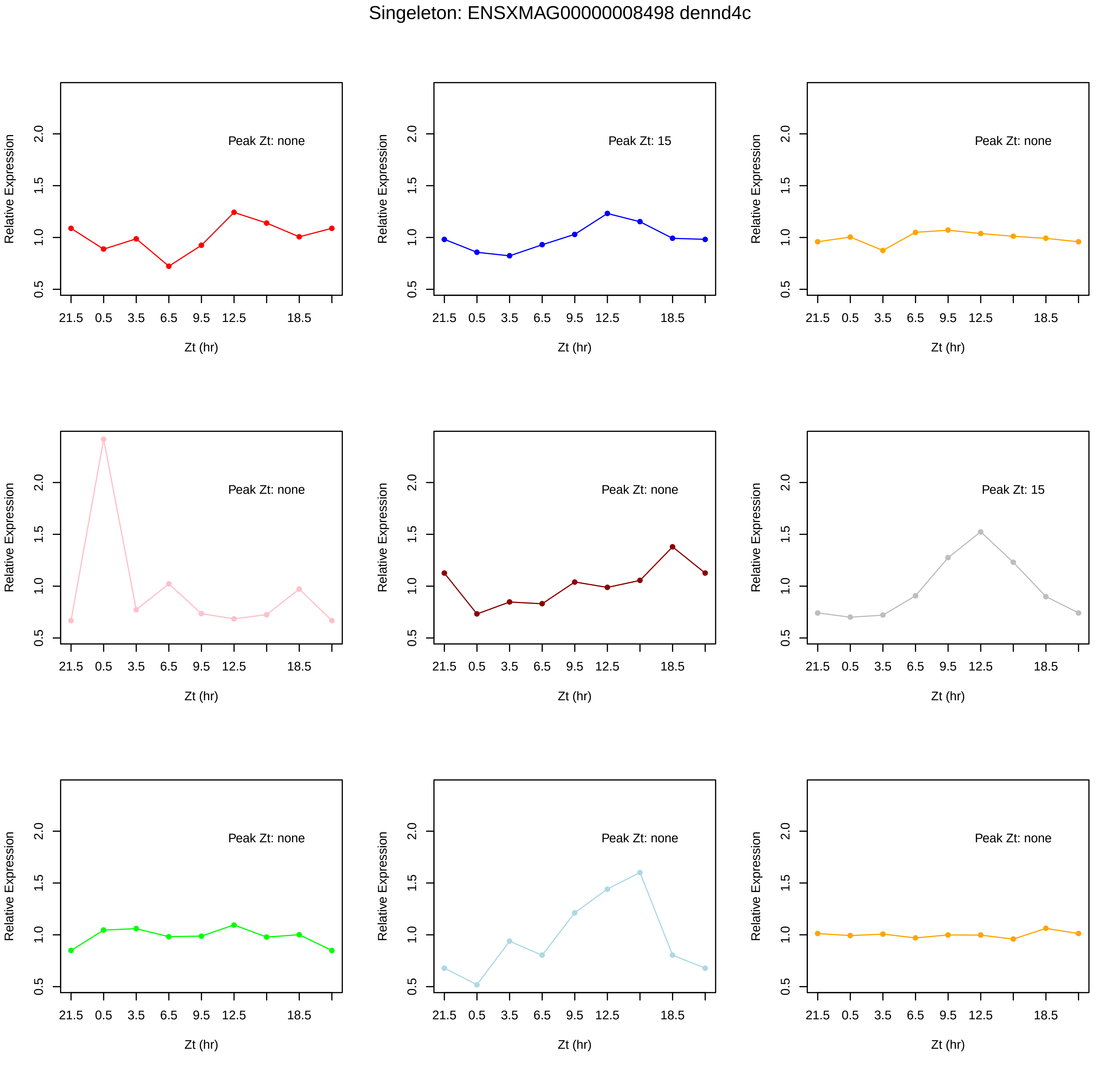
Singeleton: ENSXMAG00000008498 dennd4c
Peak Zt: none
Peak Zt: 15
Peak Zt: none
Peak Zt: none
Peak Zt: none
Peak Zt: 15
Peak Zt: none
Peak Zt: none
Peak Zt: none
Relative Expression
Zt (hr)
21.5
0.5
3.5
6.5
9.5
12.5
18.5
0.5
1.0
1.5
2.0

Singleton: ENSXMAG00000002065

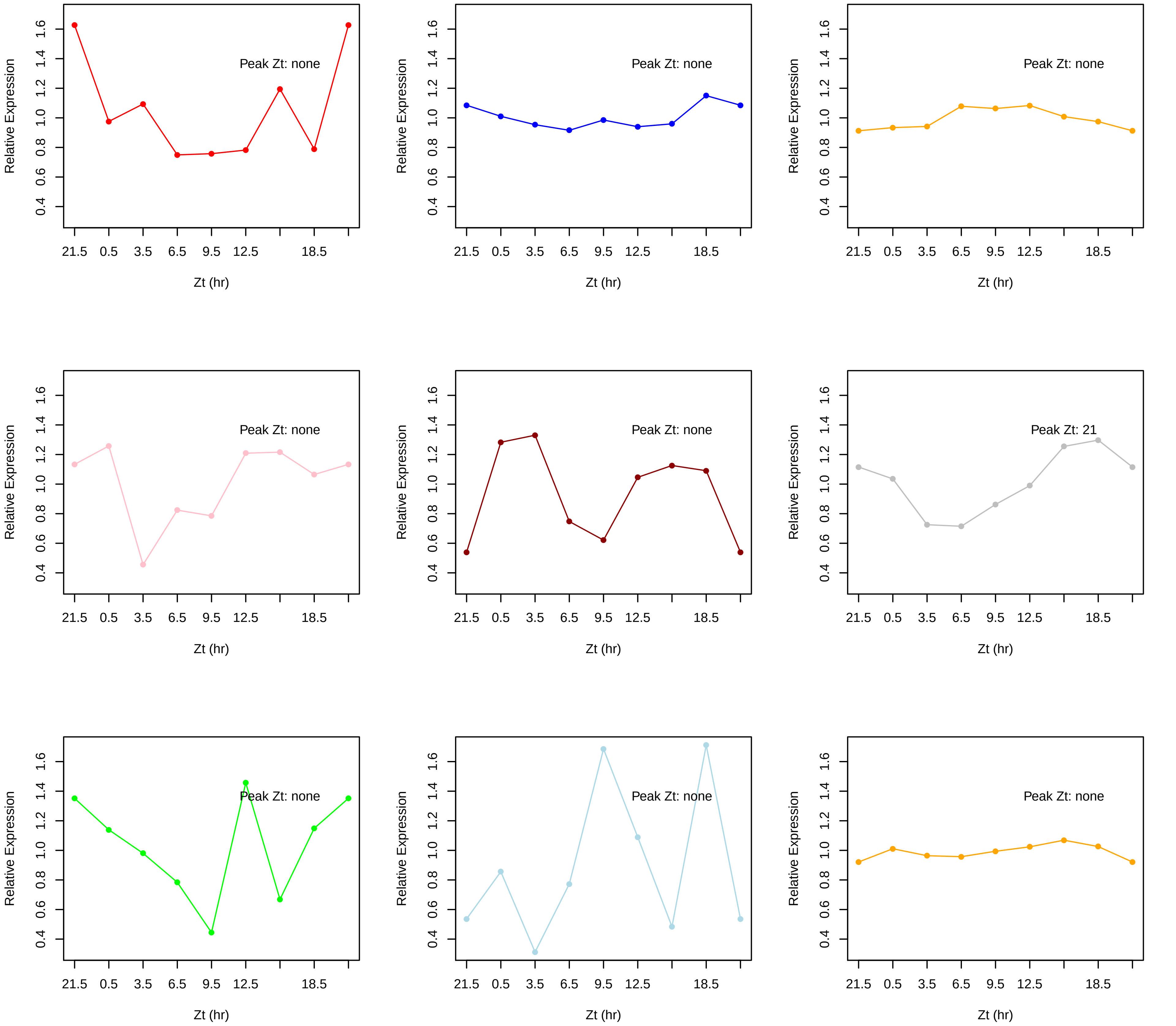

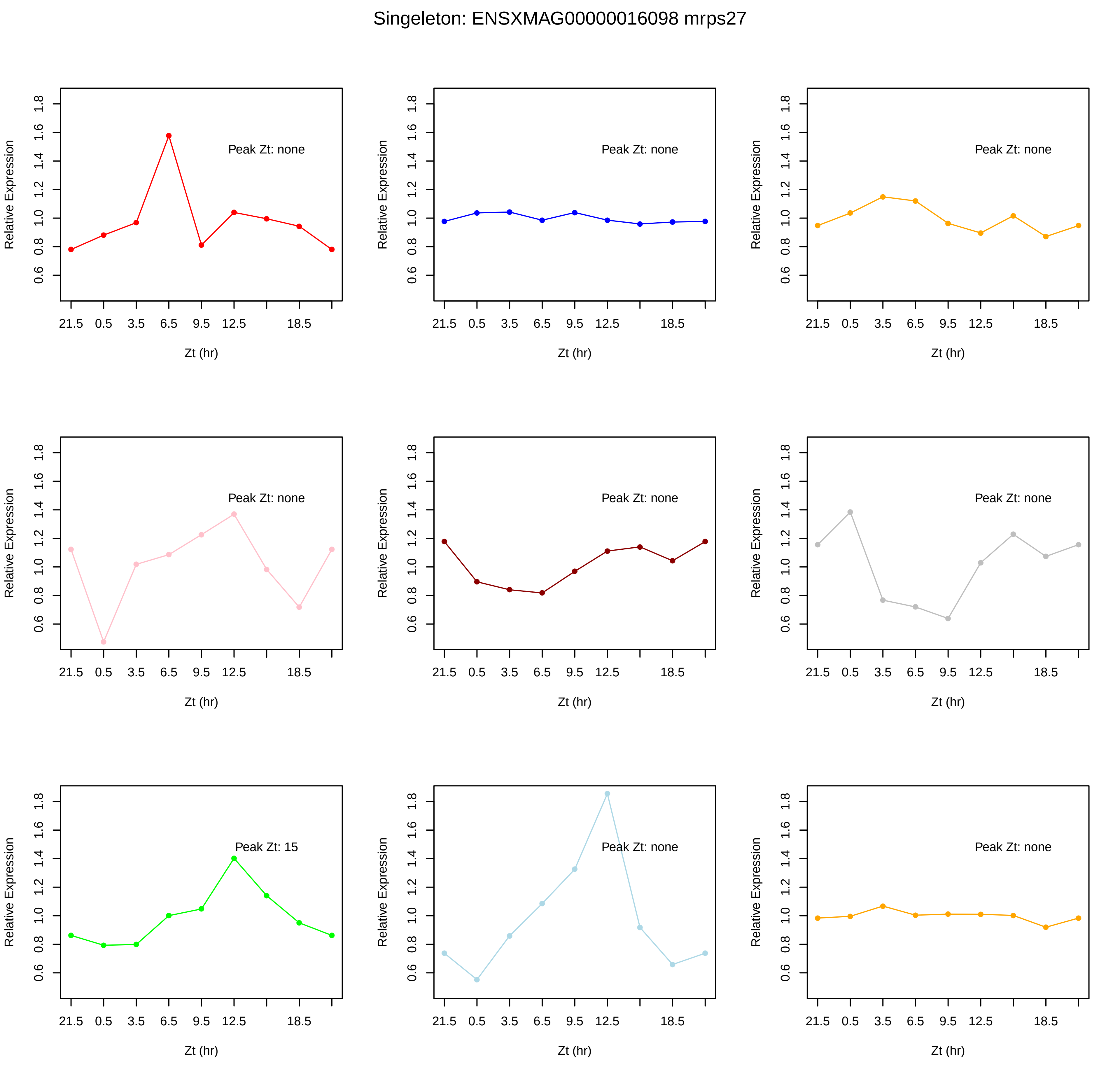
Singeleton: ENSXMAG00000016098 mrps27
Peak Zt: none
Peak Zt: none
Peak Zt: none
Peak Zt: none
Peak Zt: none
Peak Zt: none
Peak Zt: 15
Peak Zt: none
Peak Zt: none
Relative Expression
Zt (hr)

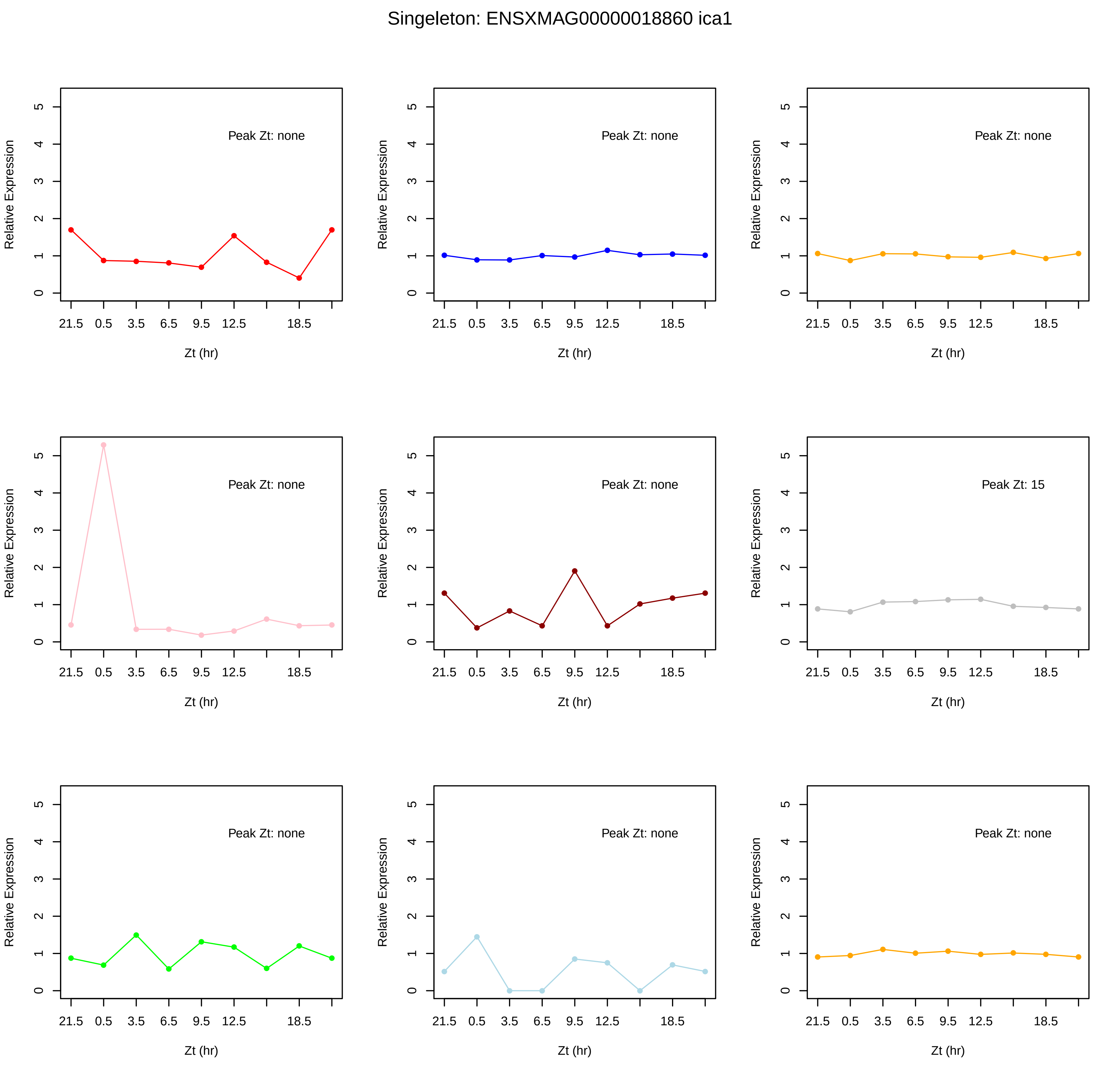
Singeleton: ENSXMAG00000018860 ica1
Peak Zt: none
Peak Zt: none
Peak Zt: none
Peak Zt: none
Peak Zt: none
Peak Zt: 15
Peak Zt: none
Peak Zt: none
Peak Zt: none
Relative Expression
Zt (hr)
21.5
0.5
3.5
6.5
9.5
12.5
18.5

Singeleton: ENSXMAG00000025297 rps19bp1

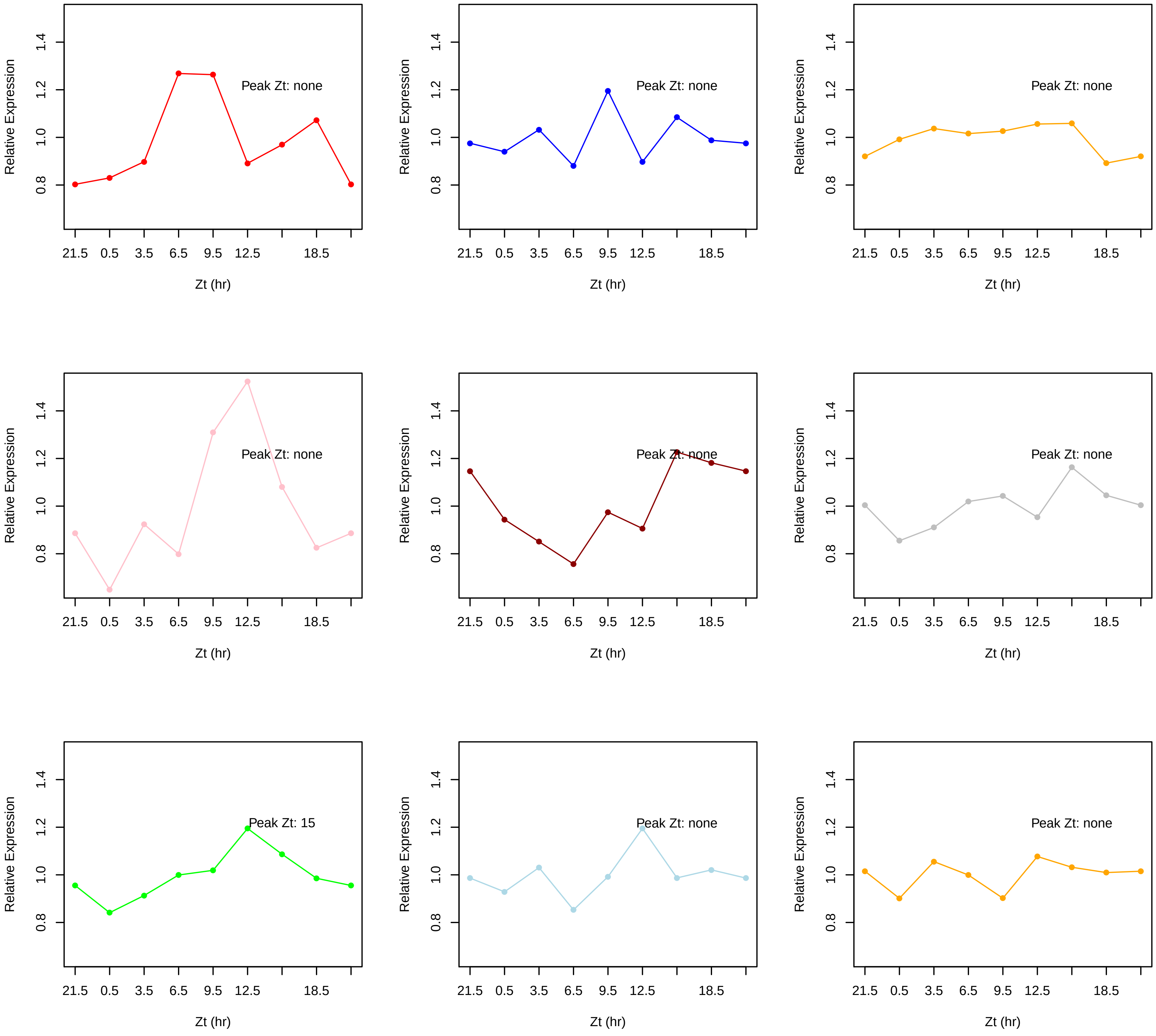

Singleton: ENSXMAG00000005956 map1b

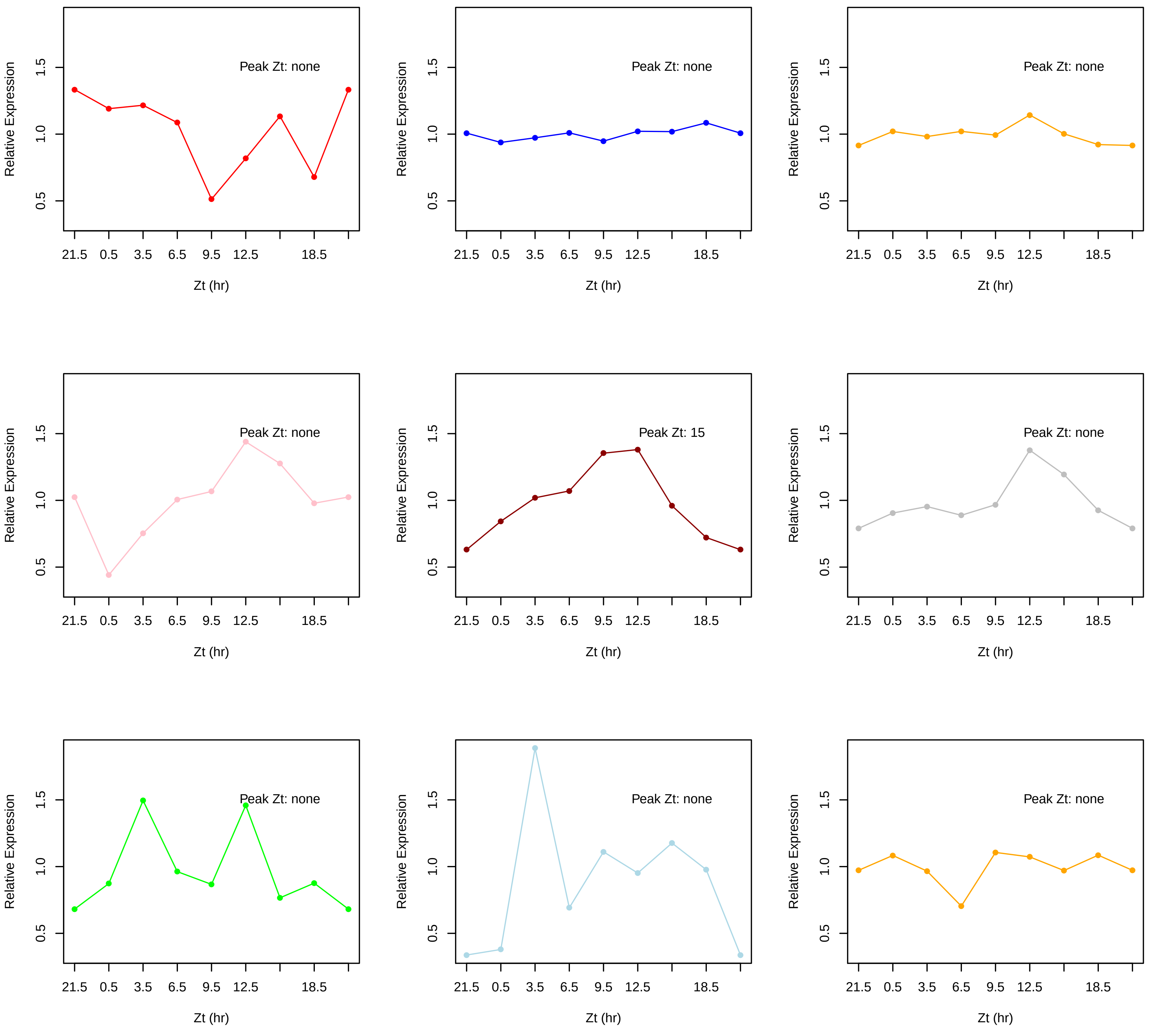

Singeleton: ENSXMAG00000026327 tmem104

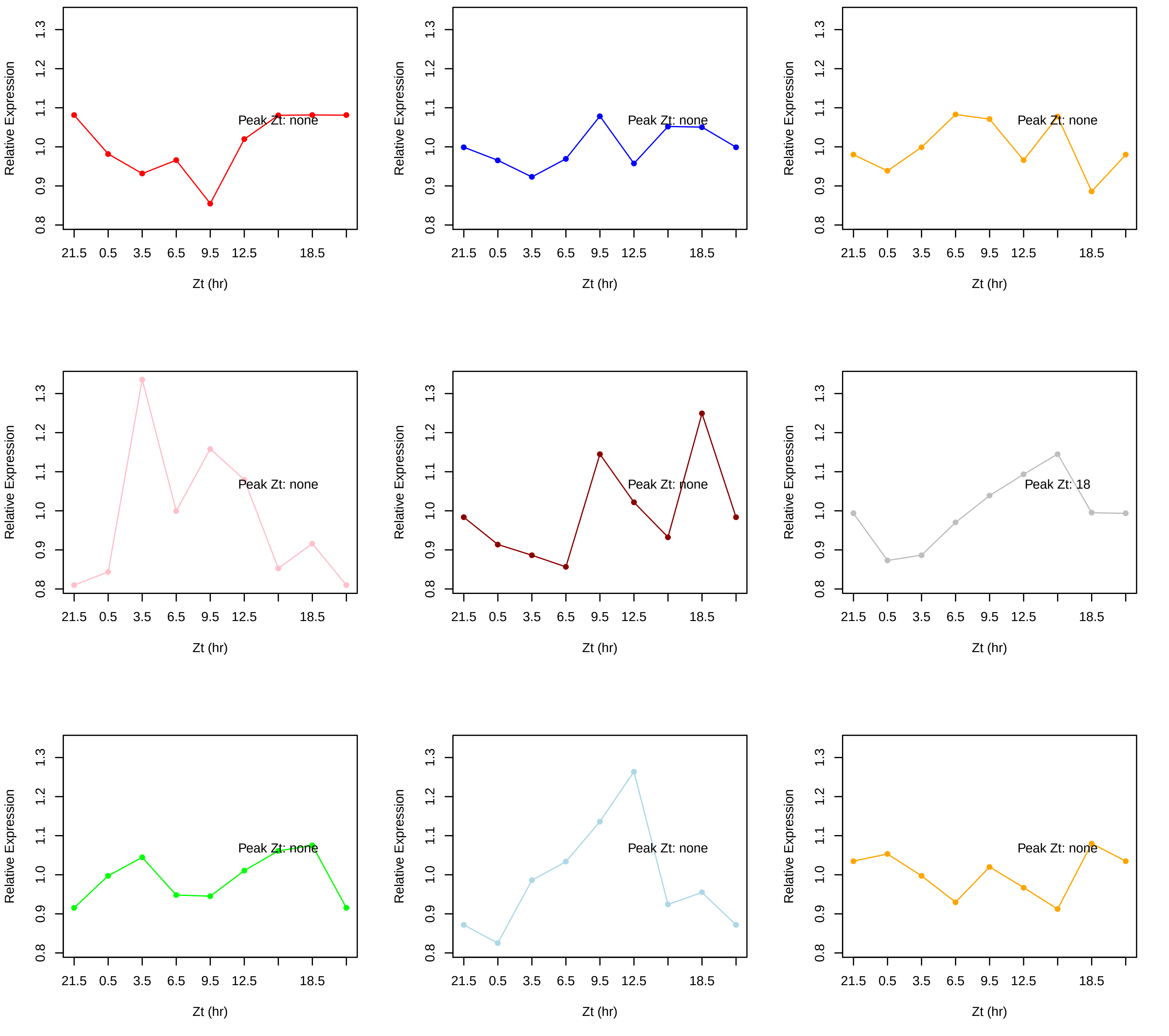

Singeleton: ENSXMAG00000021856 clta

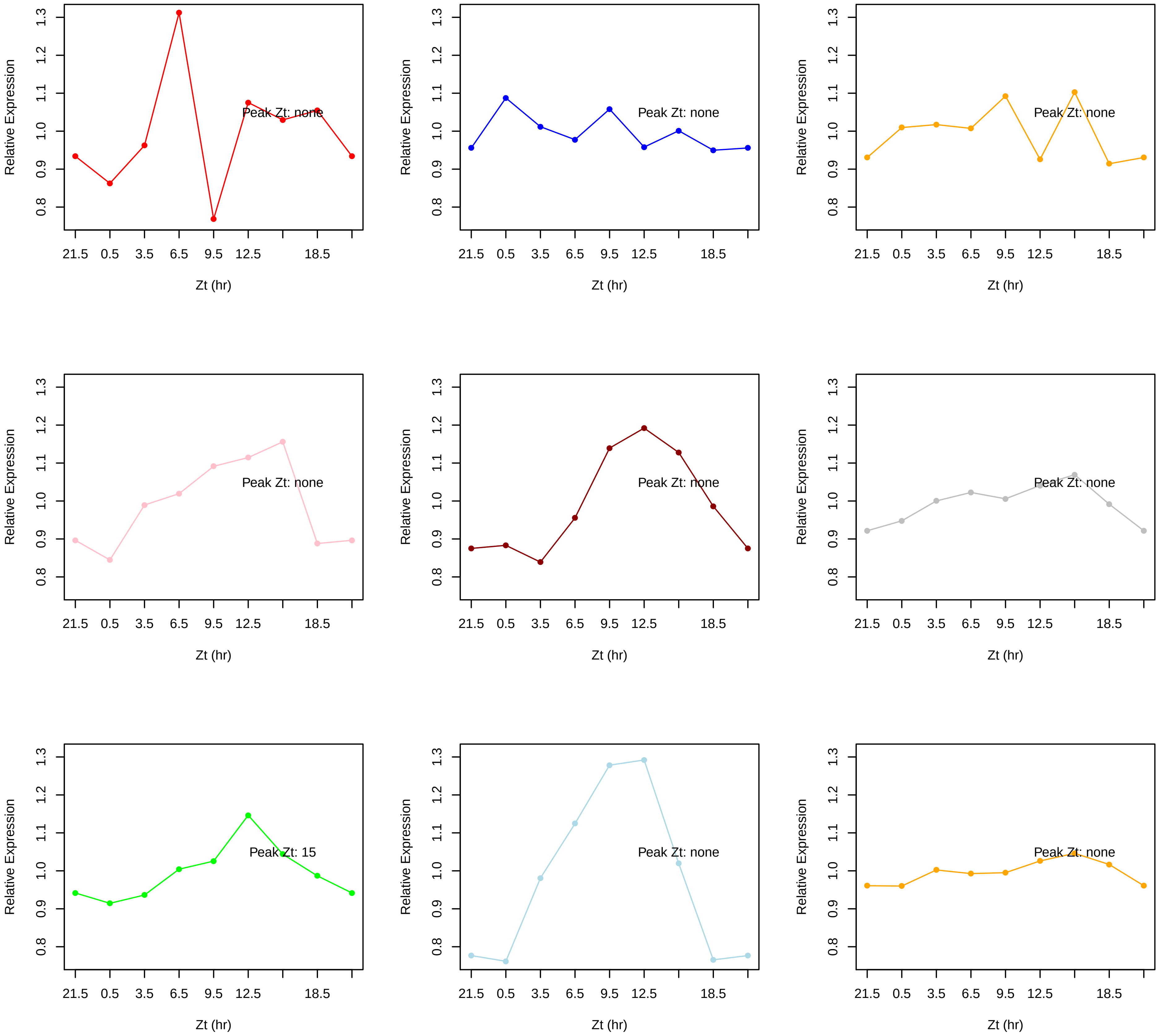

Singeleton: ENSXMAG00000004255

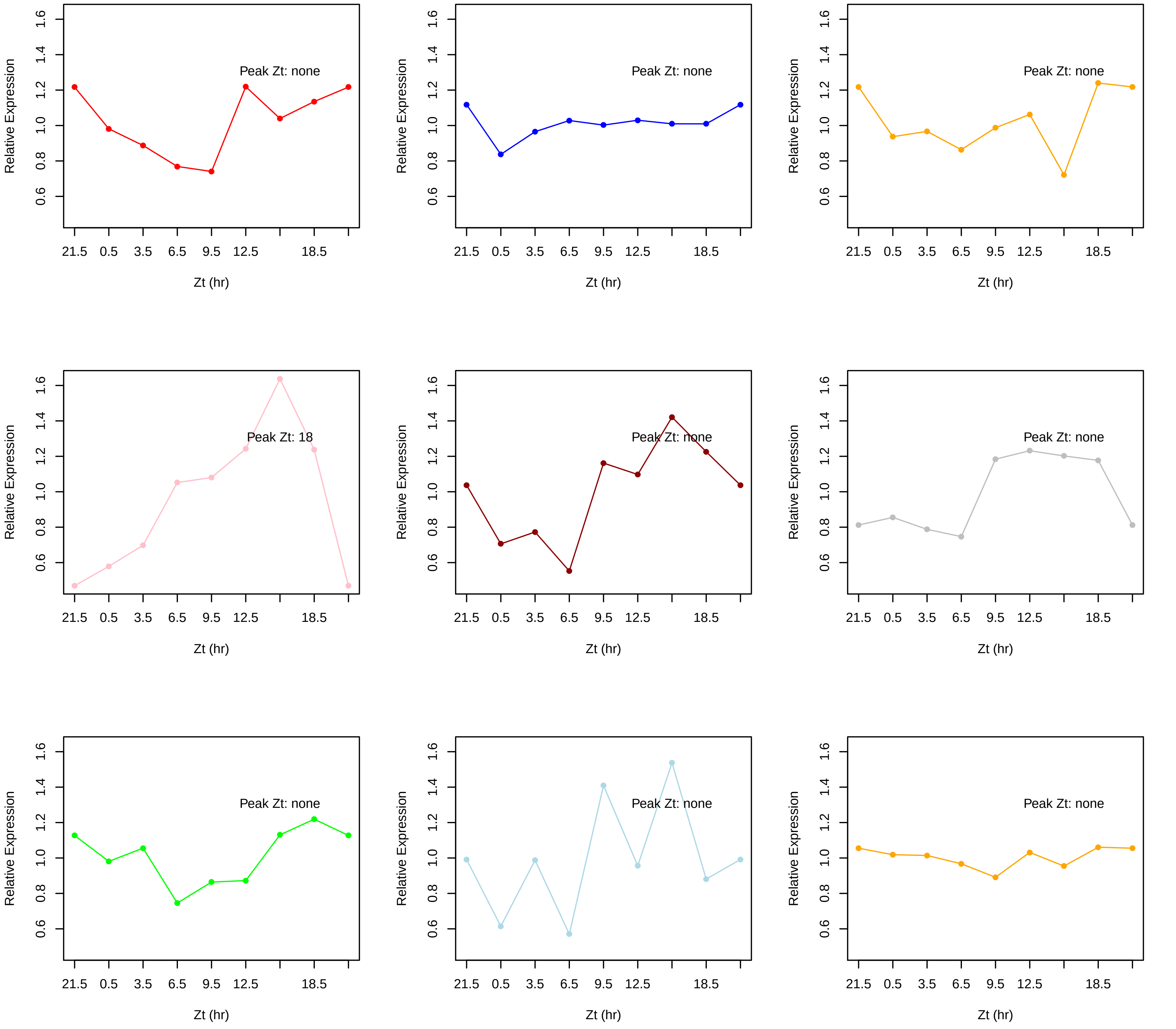

Singeleton: ENSXMAG00000027147 srpk1b

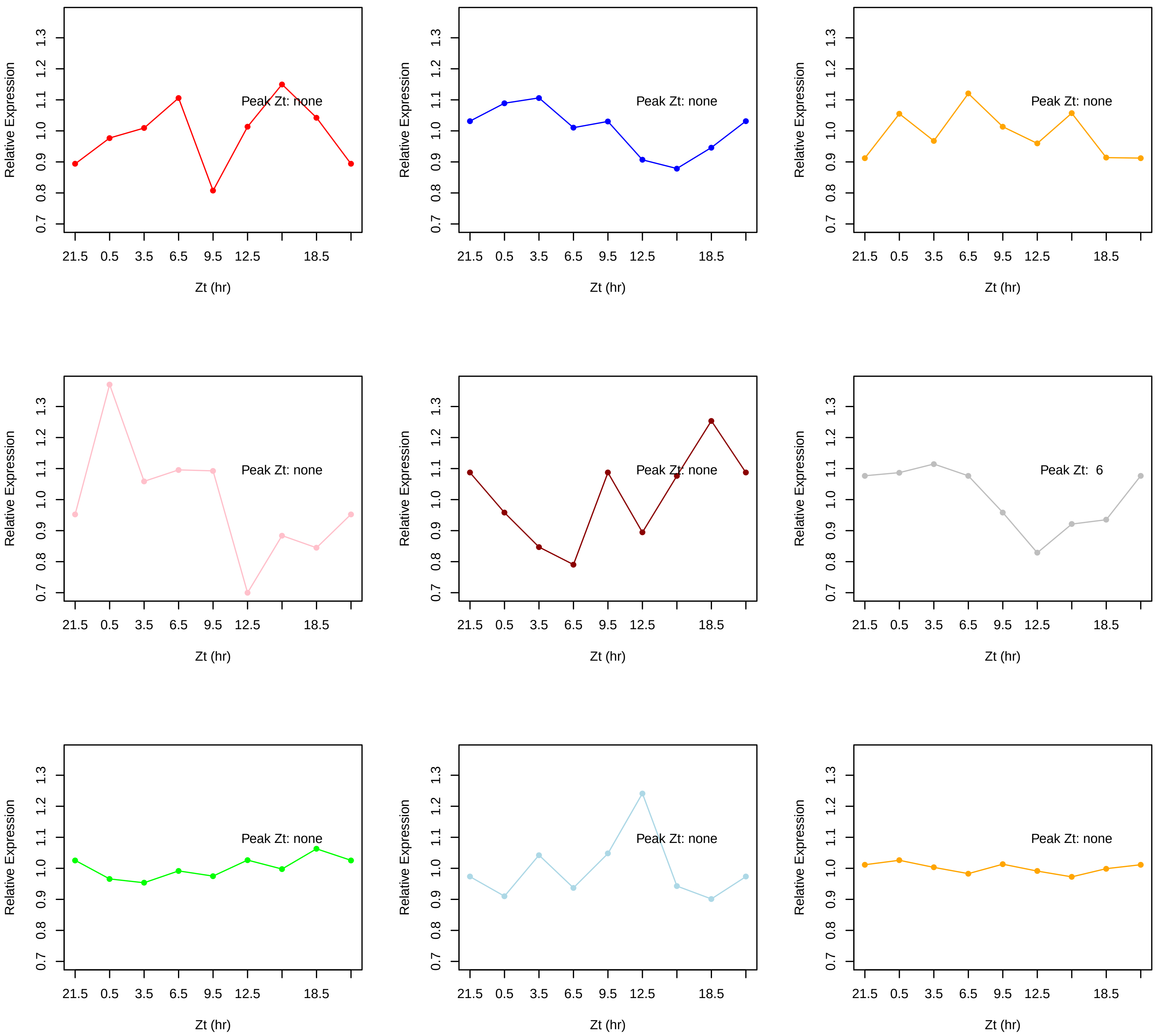

Singleton: ENSXMAG00000004264

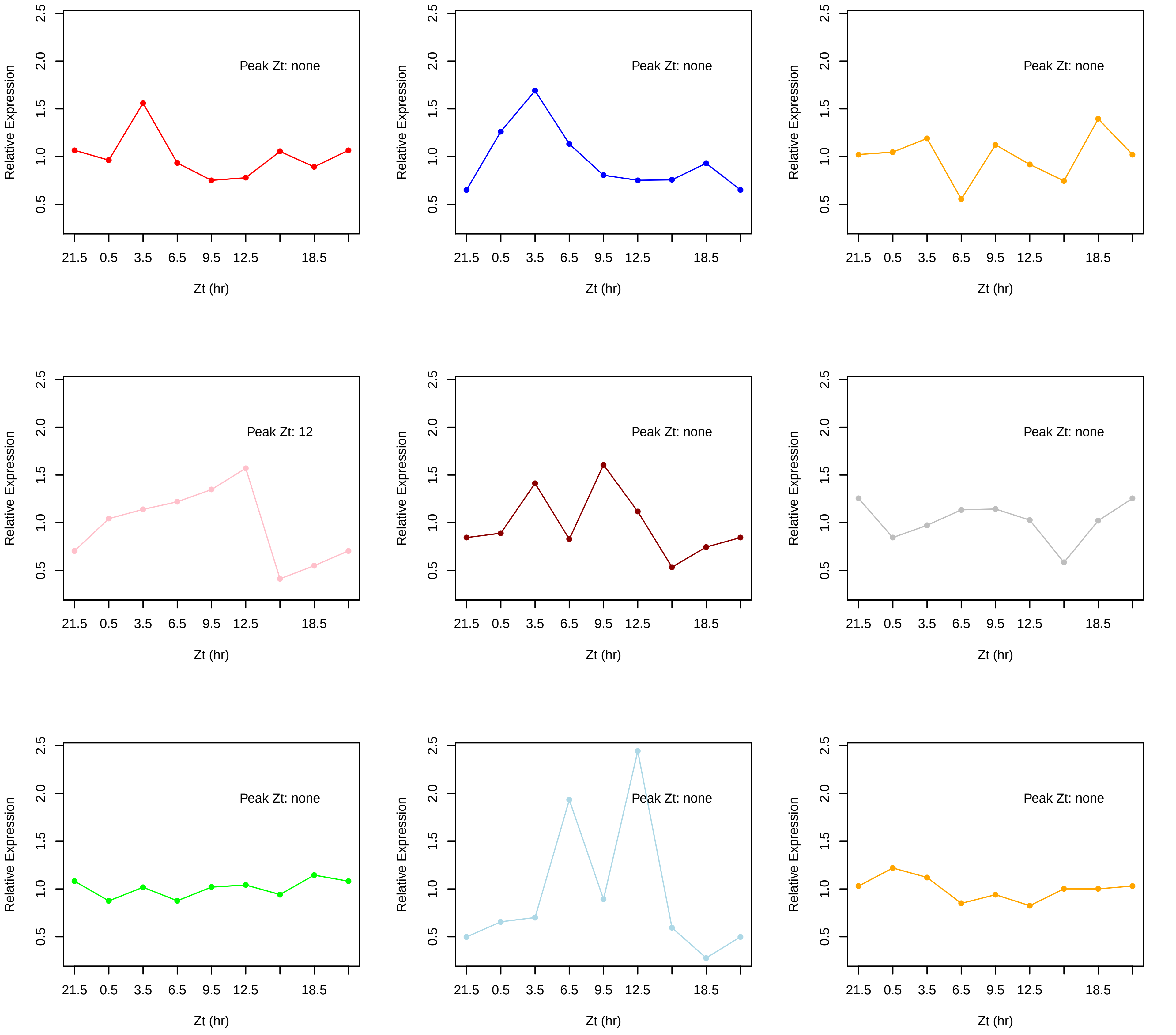

Singeleton: ENSXMAG00000023324 mrps25

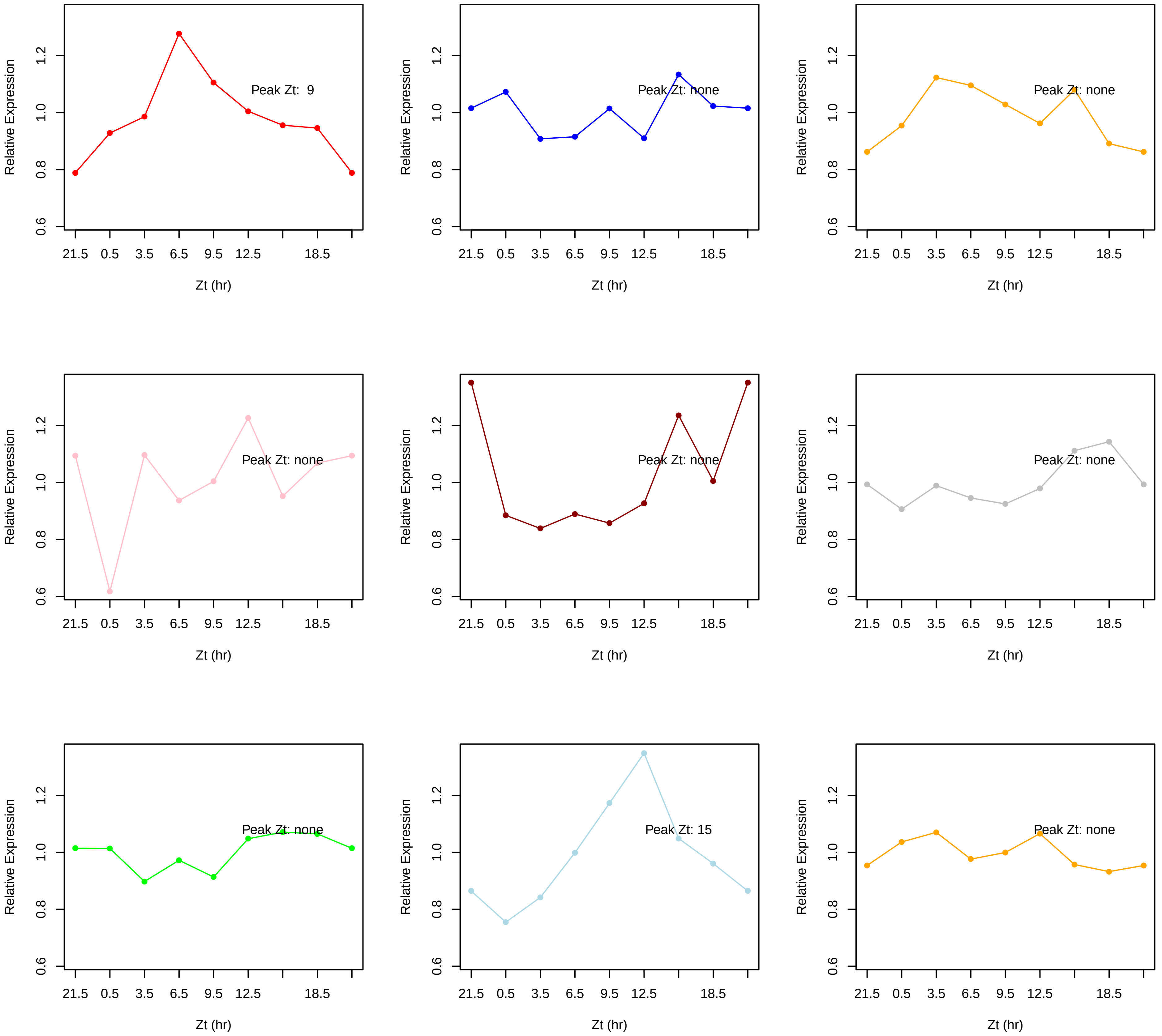

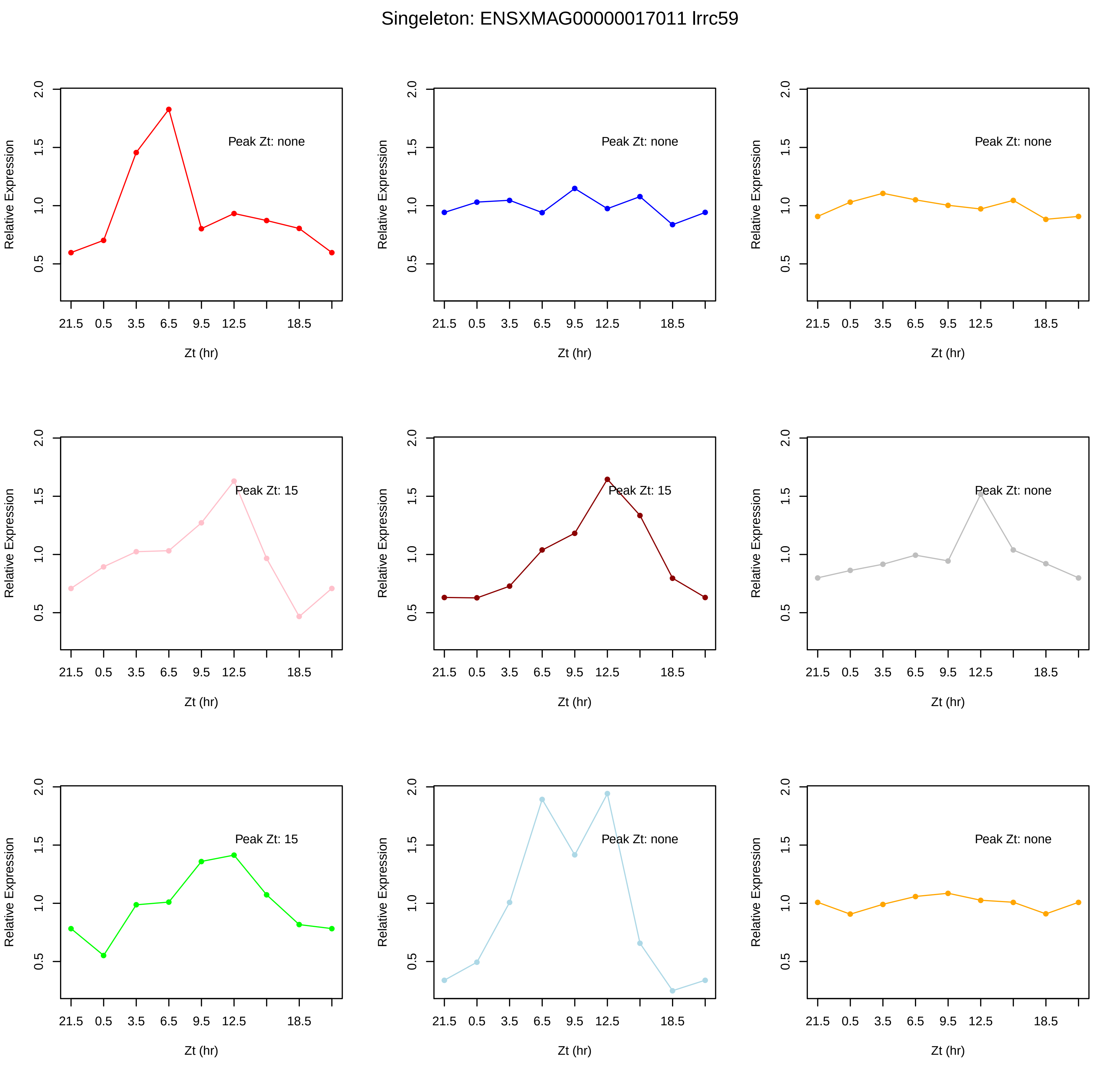
Singleton: ENSXMAG00000017011 lrrc59
Relative Expression
Zt (hr)
Peak Zt: none
Peak Zt: none
Peak Zt: none
Peak Zt: 15
Peak Zt: 15
Peak Zt: none
Peak Zt: 15
Peak Zt: none
Peak Zt: none
21.5
0.5
3.5
6.5
9.5
12.5
18.5
0.5
1.0
1.5
2.0

Singeleton: ENSXMAG00000006628 nansa

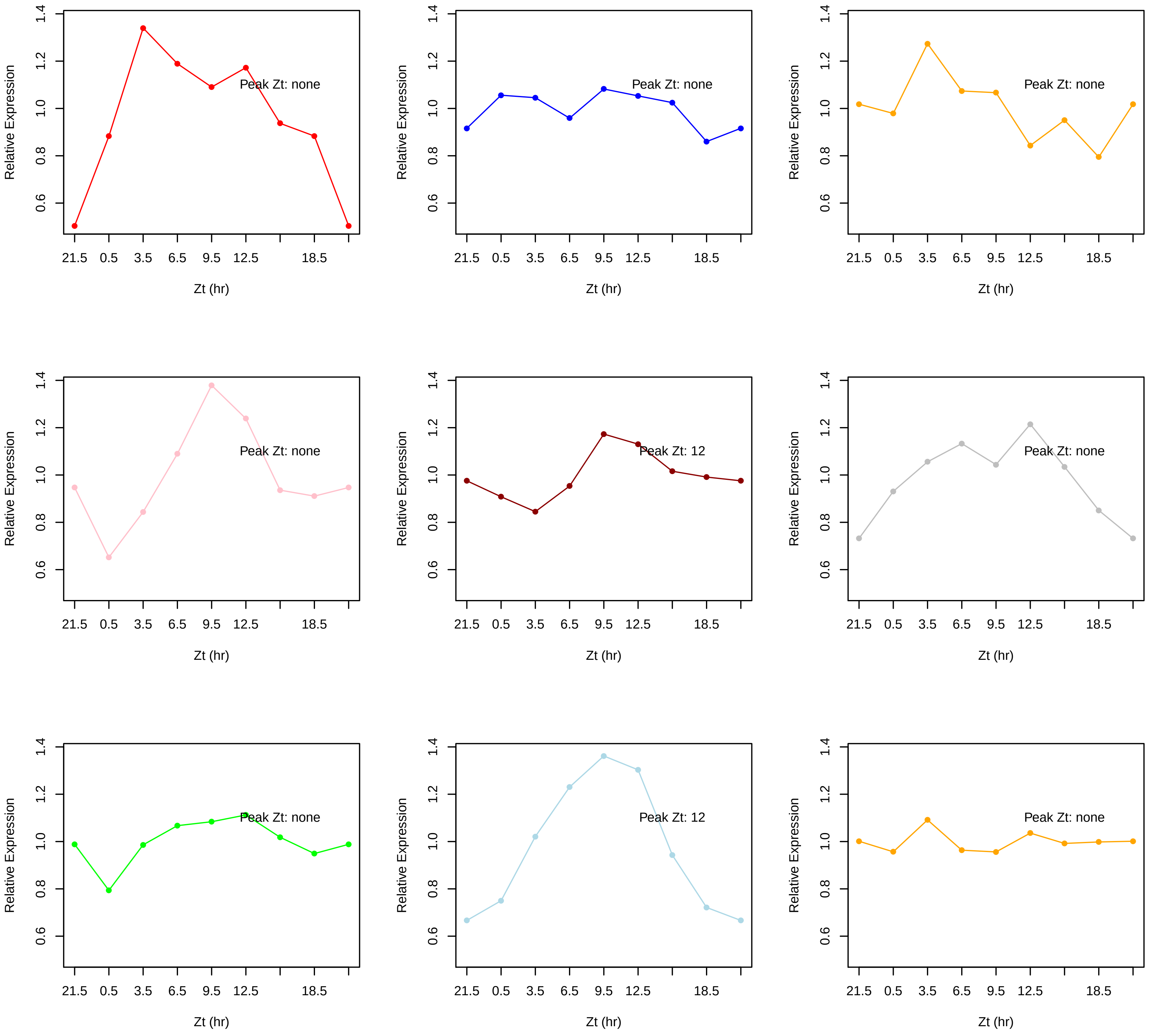

Singleton: ENSXMAG00000008048

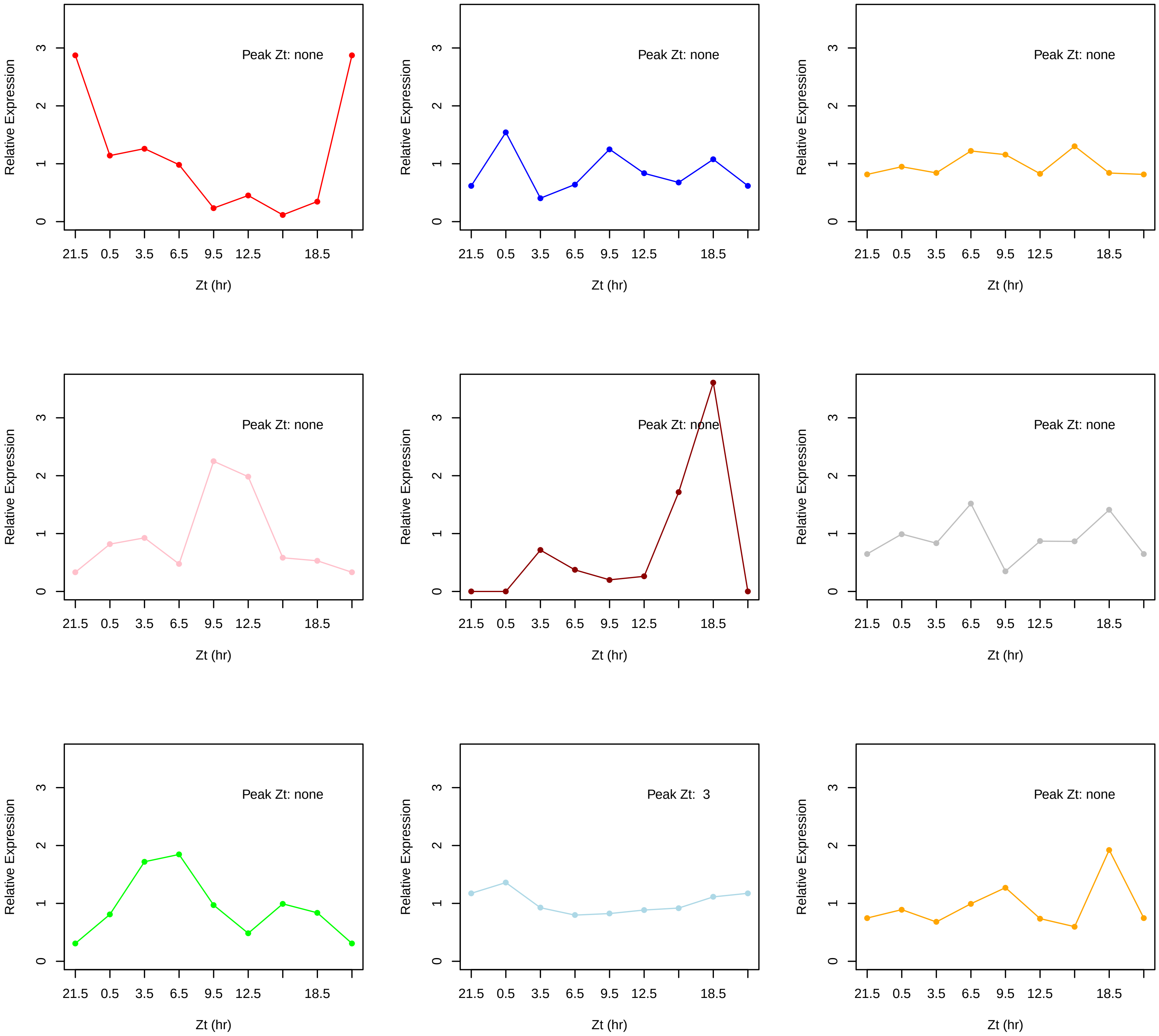

Singeleton: ENSXMAG00000004148

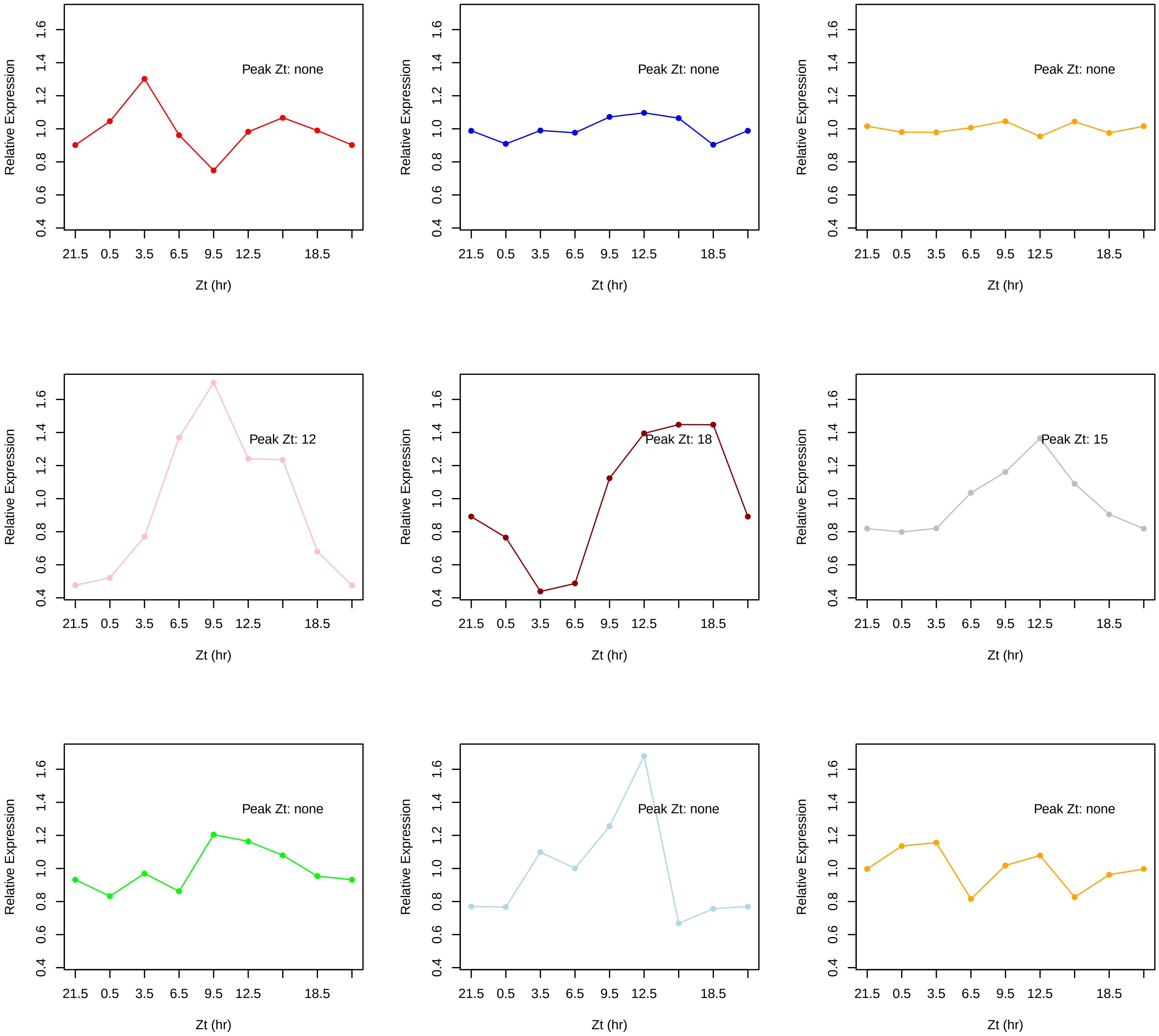

Singeleton: ENSXMAG00000018818

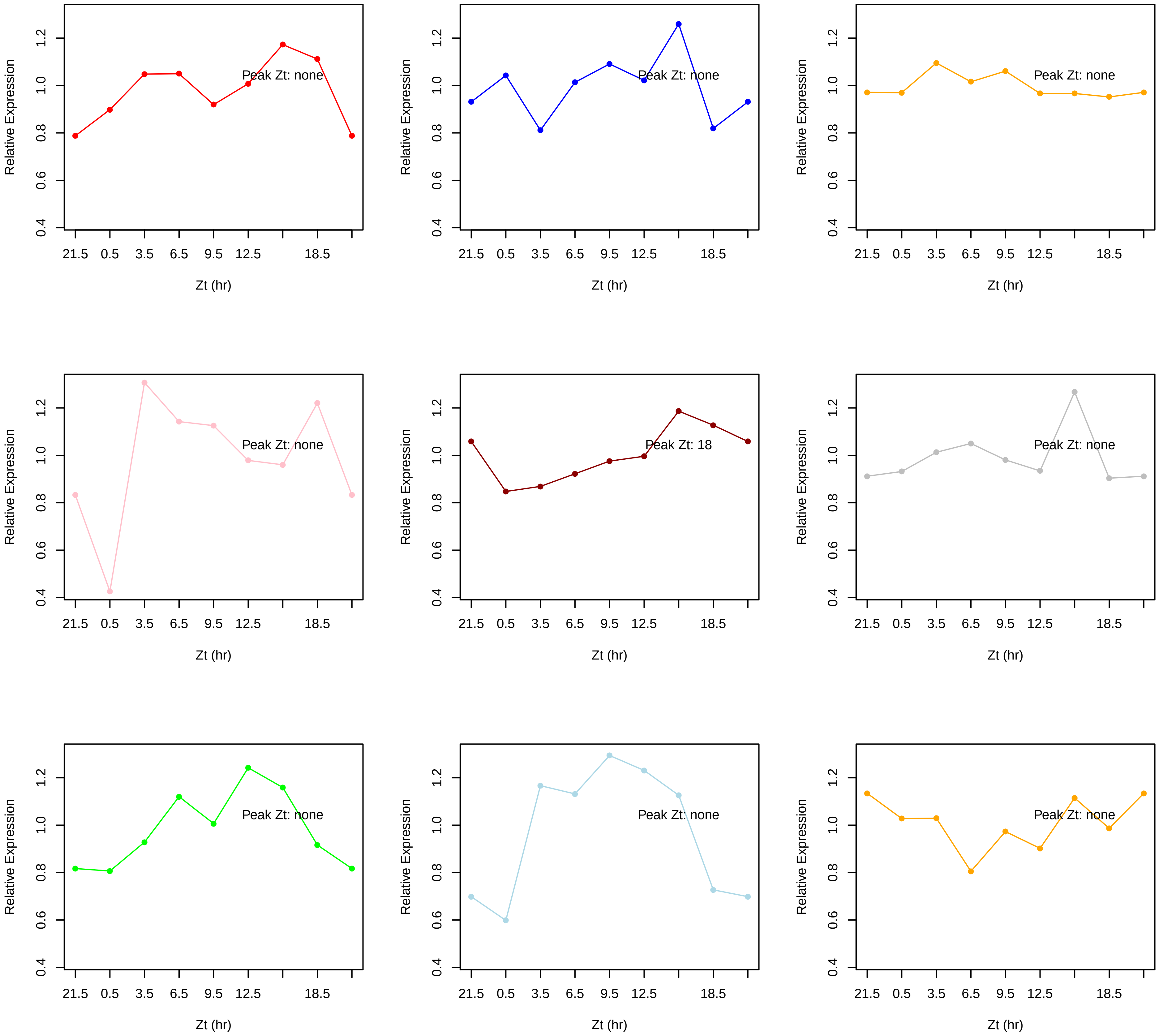

Singeleton: ENSXMAG00000001125 c1qtnf6a

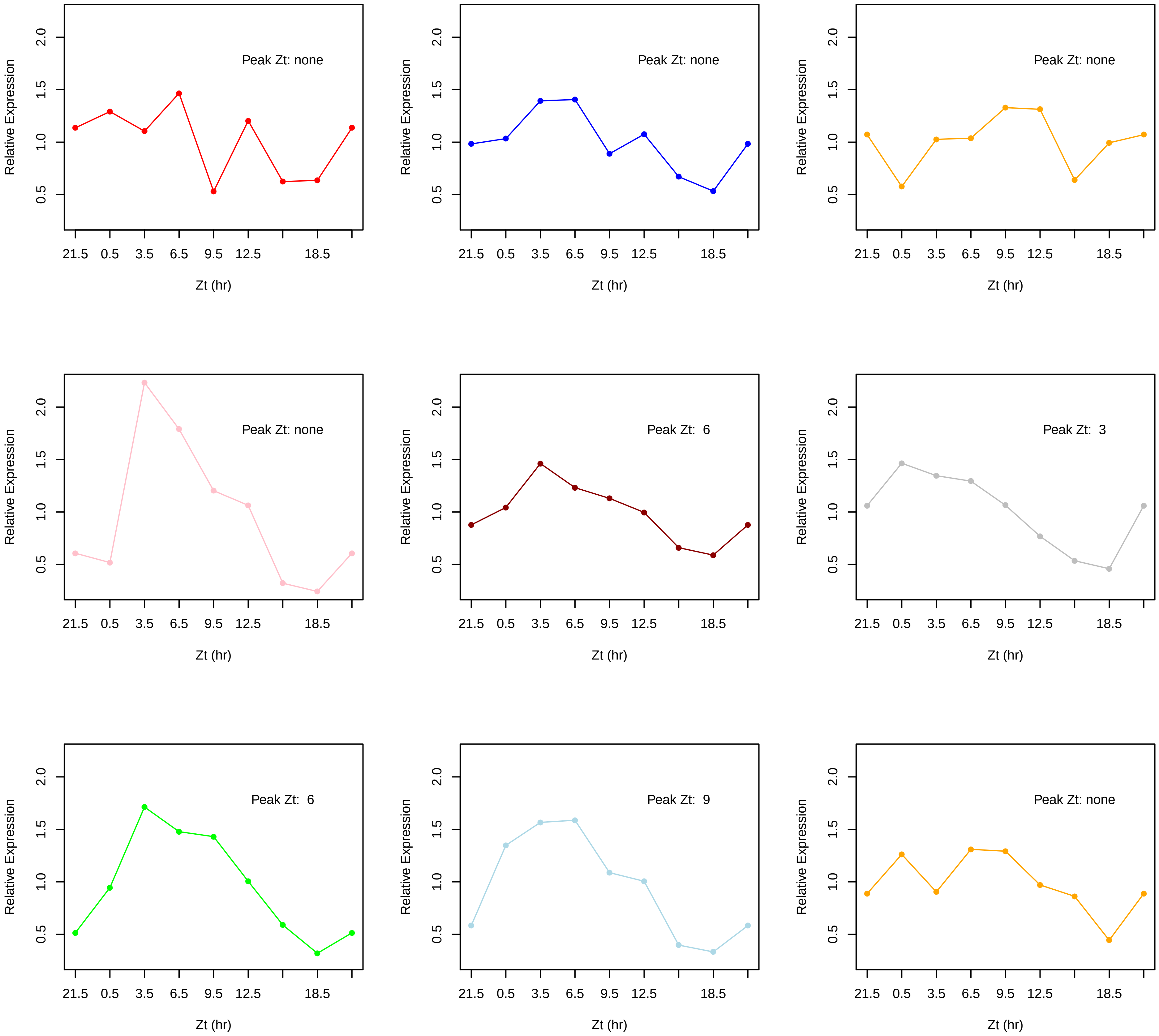

Singeleton: ENSXMAG00000006681 mgat3b

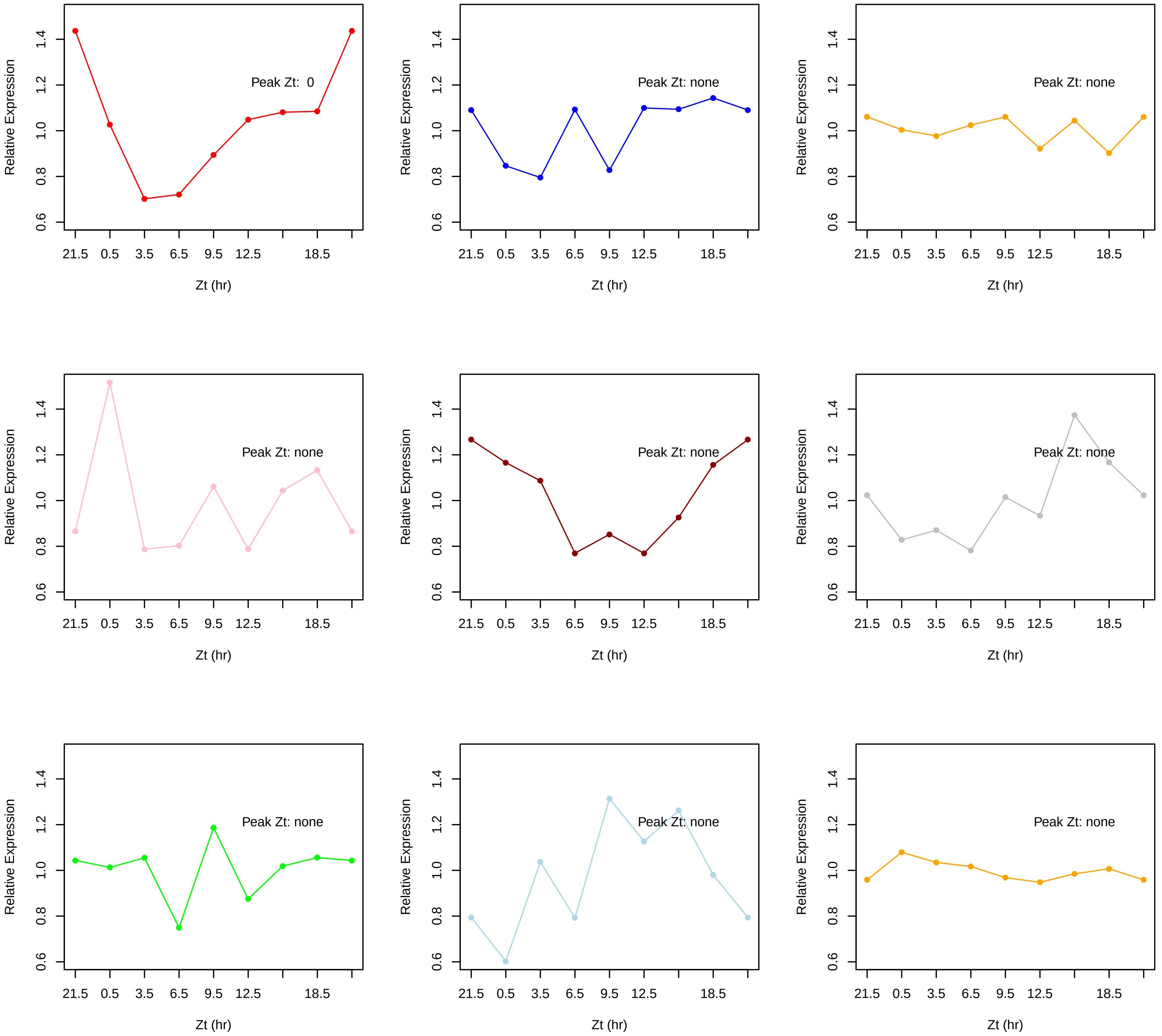

Singeleton: ENSXMAG00000010938 hars

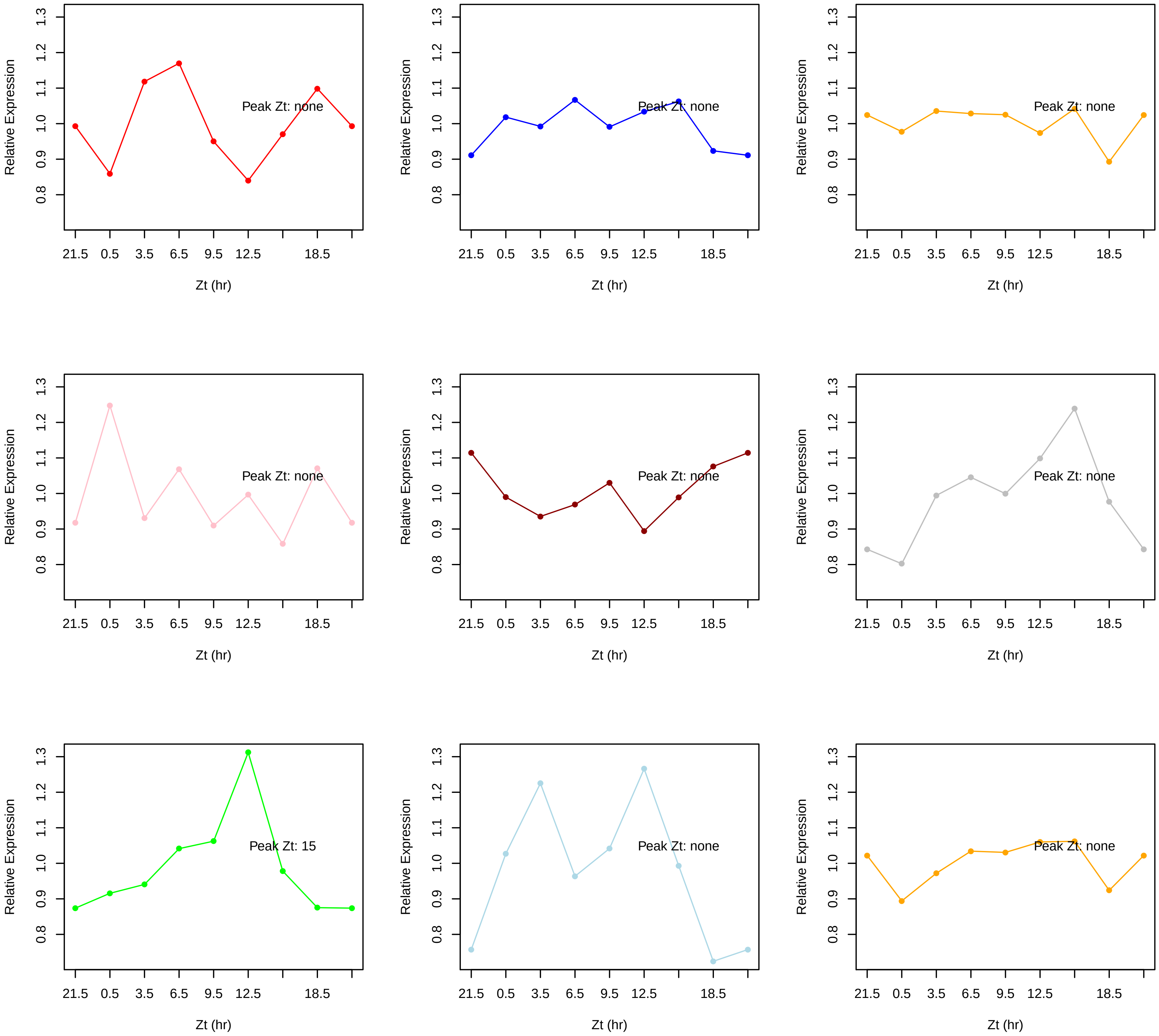

Singeleton: ENSXMAG00000015321 peli2

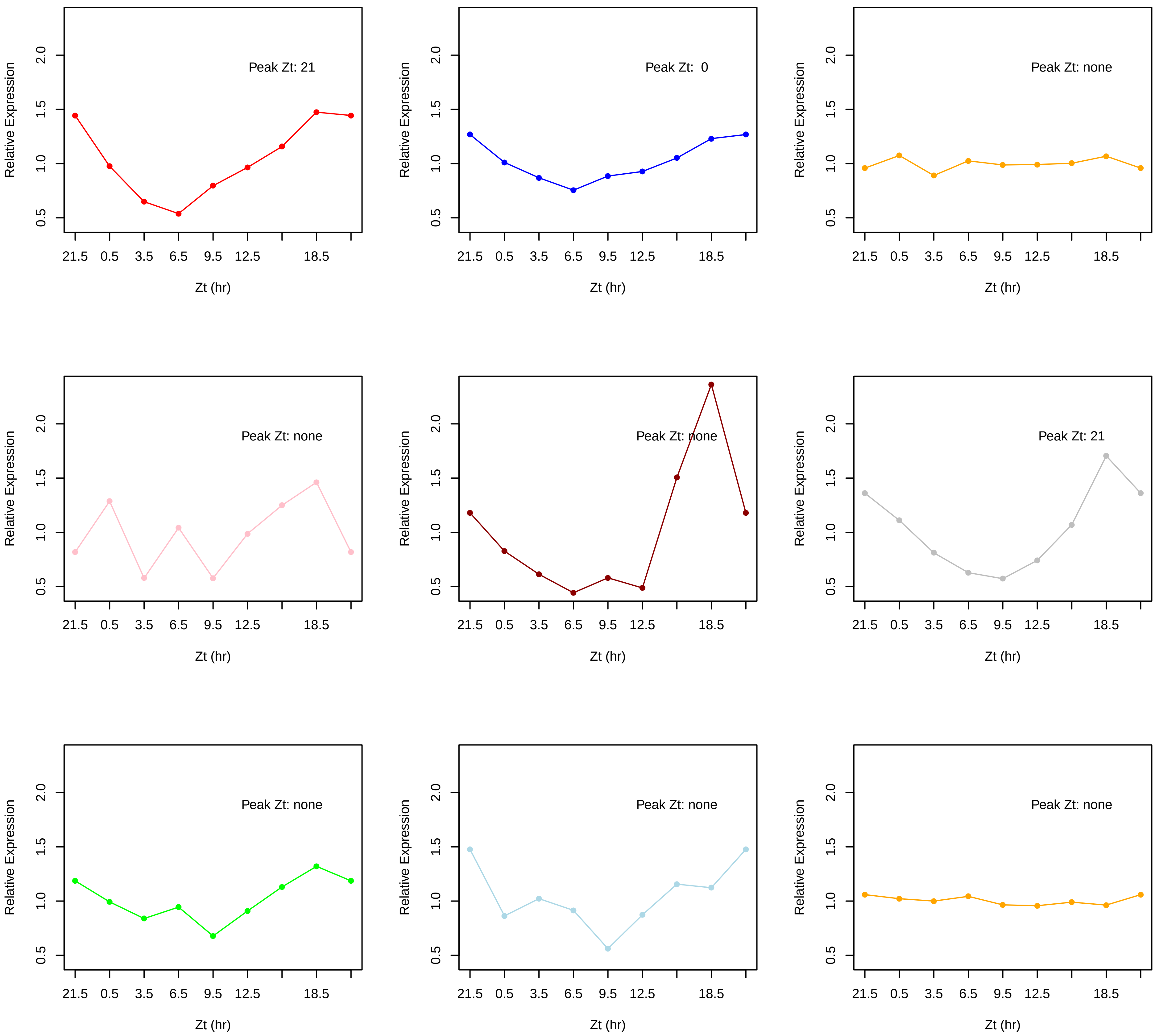

Singeleton: ENSXMAG00000025801 st8sia5

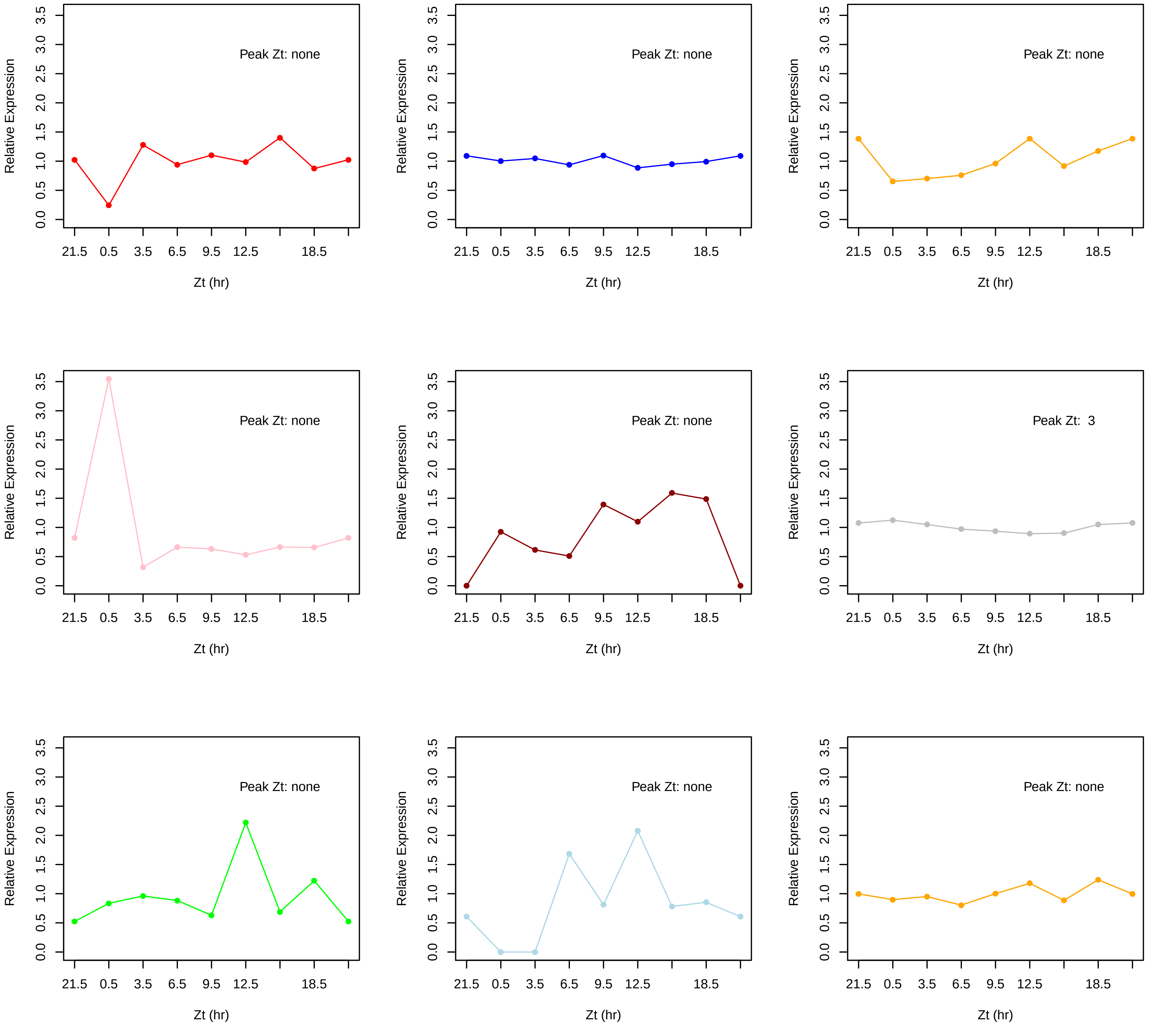

Singeleton: ENSXMAG00000014001 rpl3

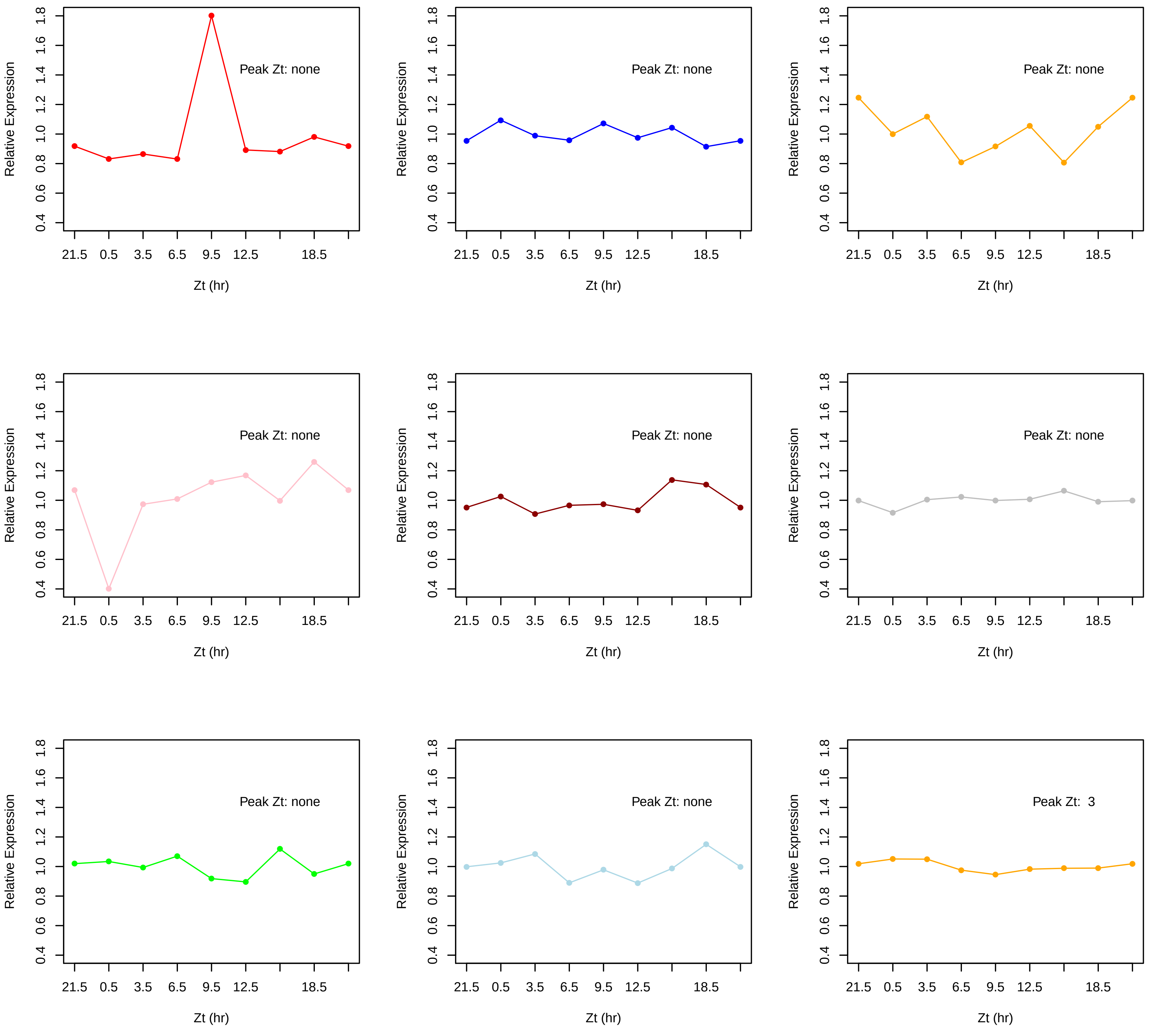

Singeleton: ENSXMAG00000004290

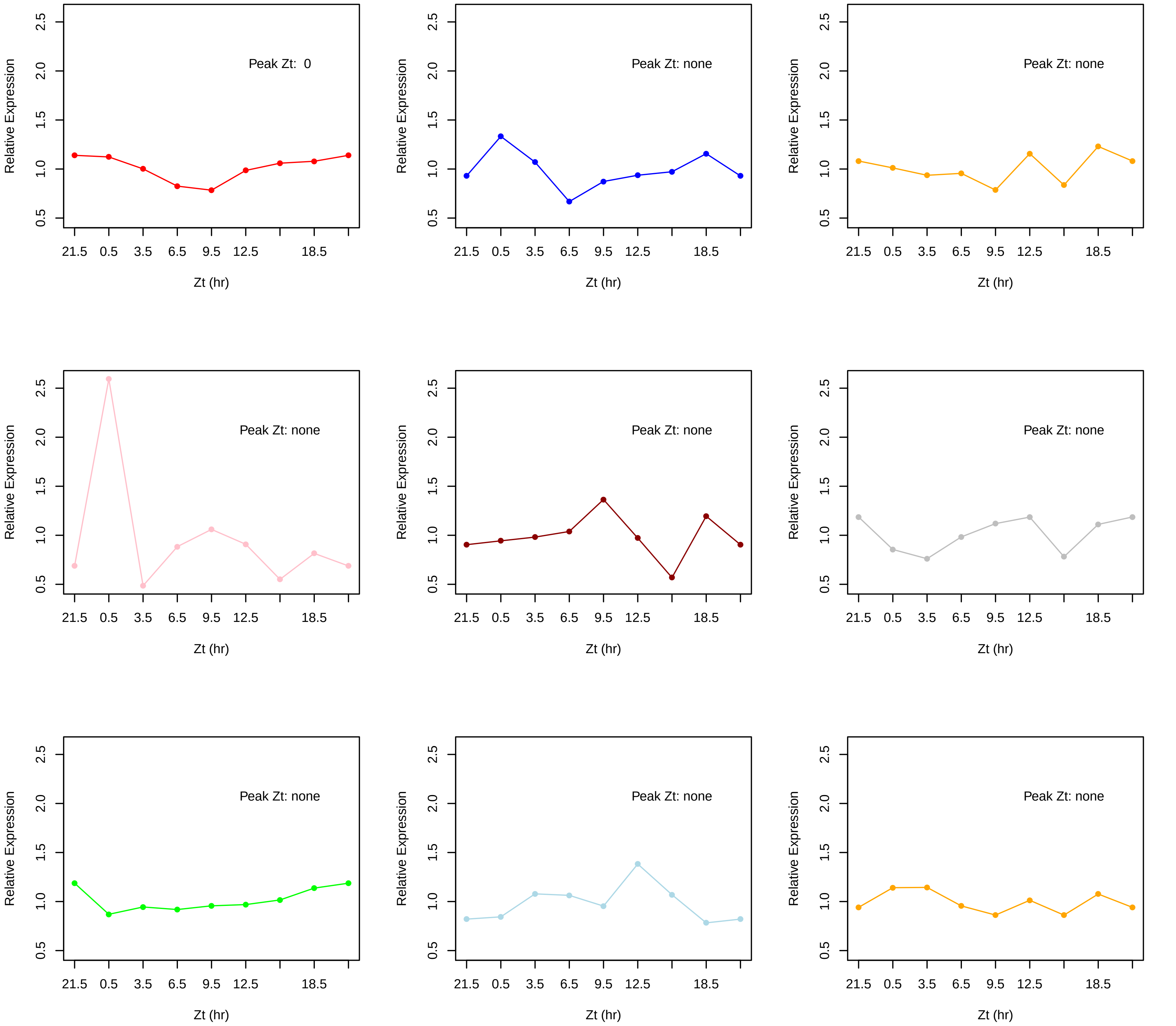

Singeleton: ENSXMAG00000015330 ktn1

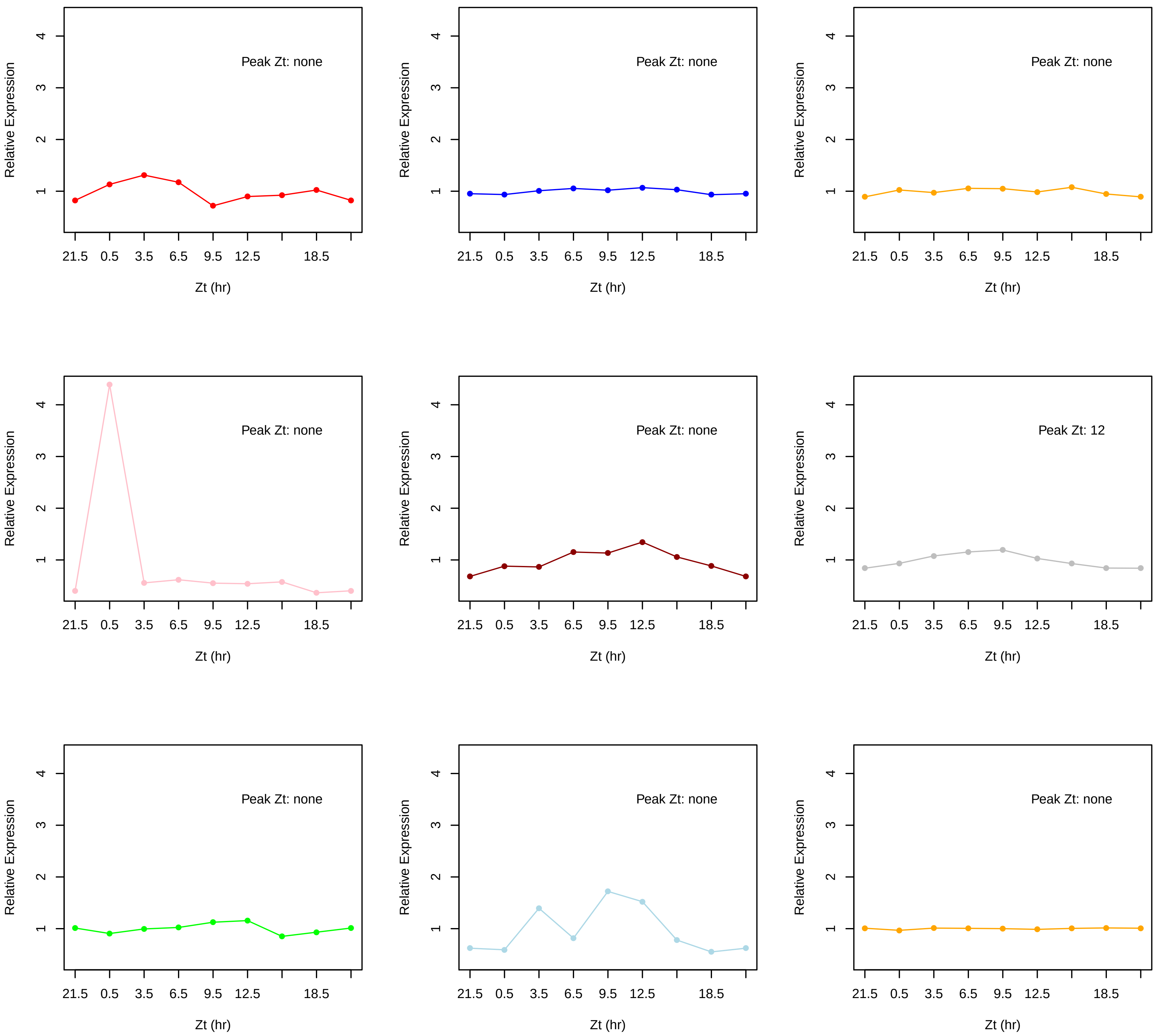

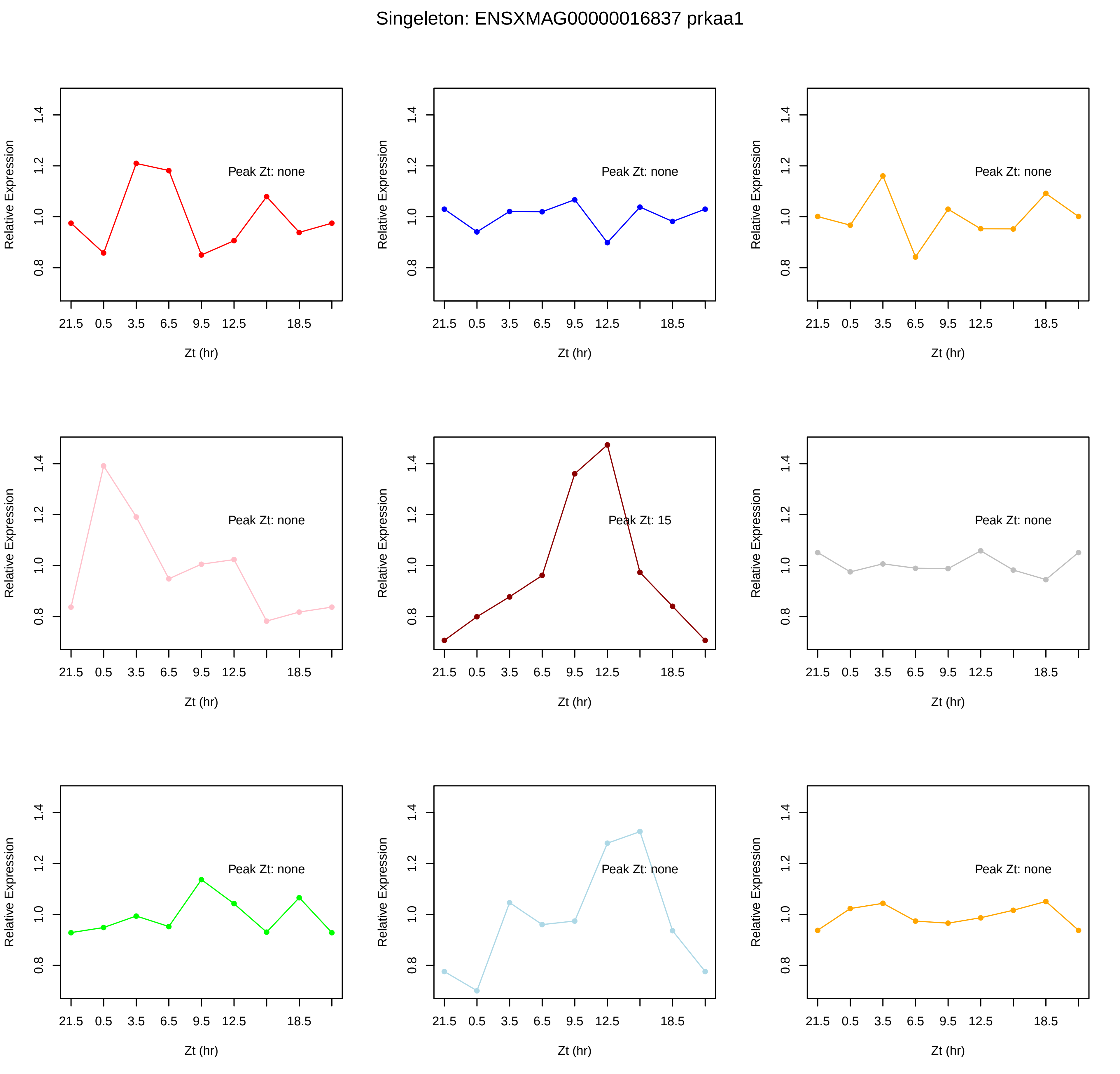
Singeleton: ENSXMAG00000016837 prkaa1
Peak Zt: none
Peak Zt: none
Peak Zt: none
Peak Zt: none
Peak Zt: 15
Peak Zt: none
Peak Zt: none
Peak Zt: none
Peak Zt: none
Relative Expression
Zt (hr)
21.5
0.5
3.5
6.5
9.5
12.5
18.5
0.8
1.0
1.2
1.4

Singeleton: ENSXMAG00000007602 mfsd11

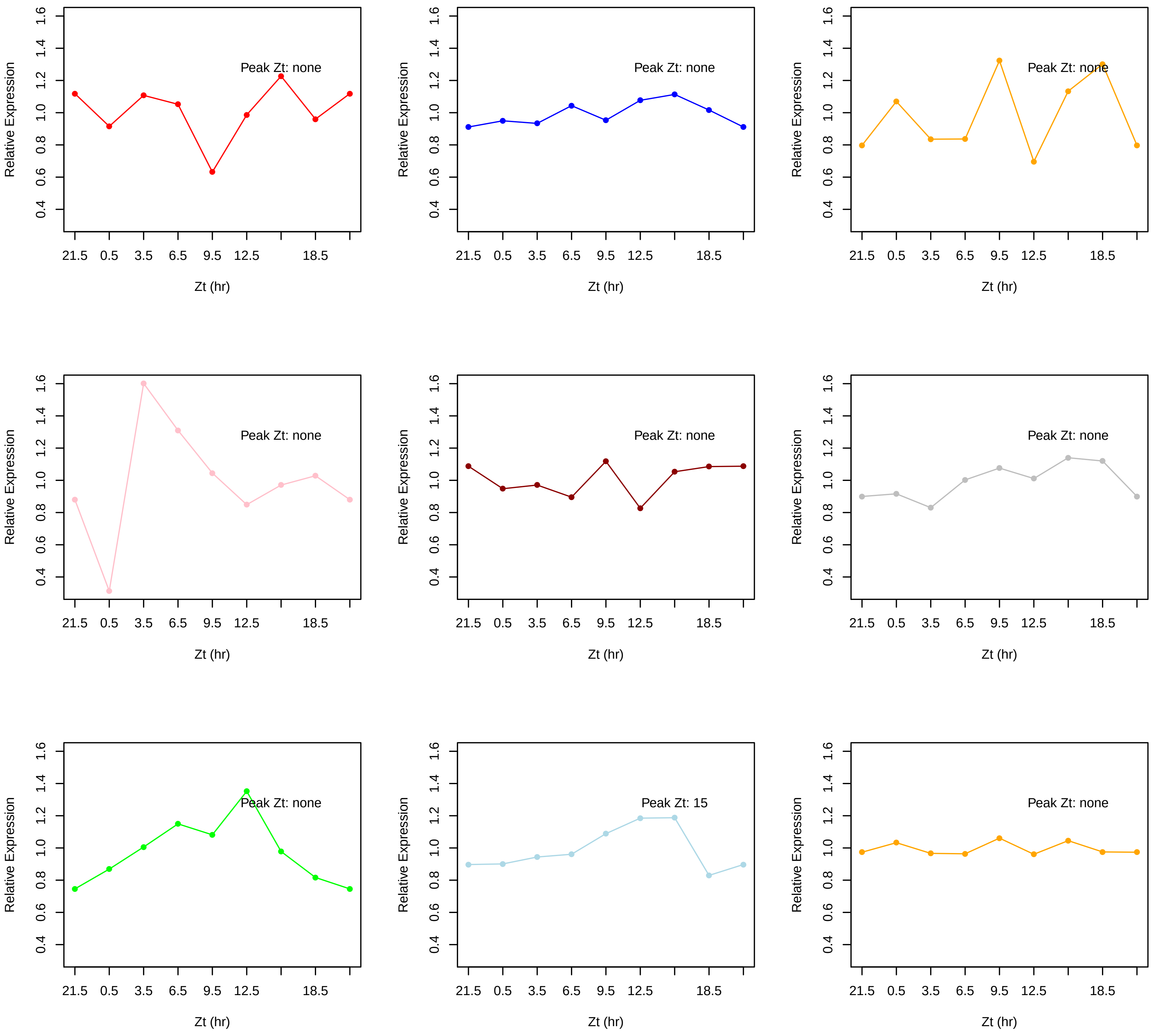

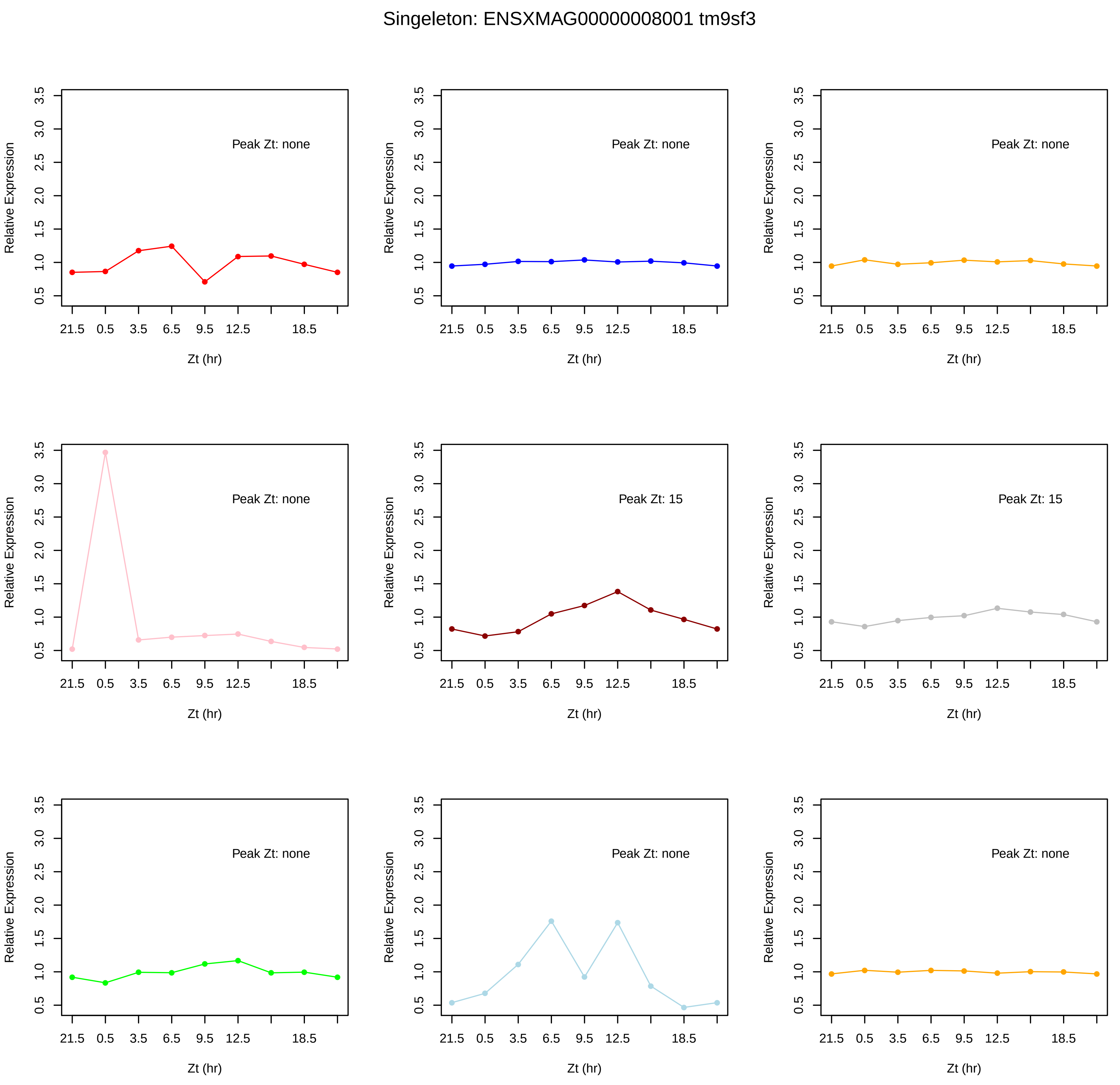

Singeleton: ENSXMAG00000008001 tm9sf3
Peak Zt: none
Peak Zt: none
Peak Zt: none
Peak Zt: none
Peak Zt: 15
Peak Zt: 15
Peak Zt: none
Peak Zt: none
Peak Zt: none
Relative Expression
Zt (hr)
21.5
0.5
3.5
6.5
9.5
12.5
18.5

Singeleton: ENSXMAG00000001998 si:dkey−37o8.1

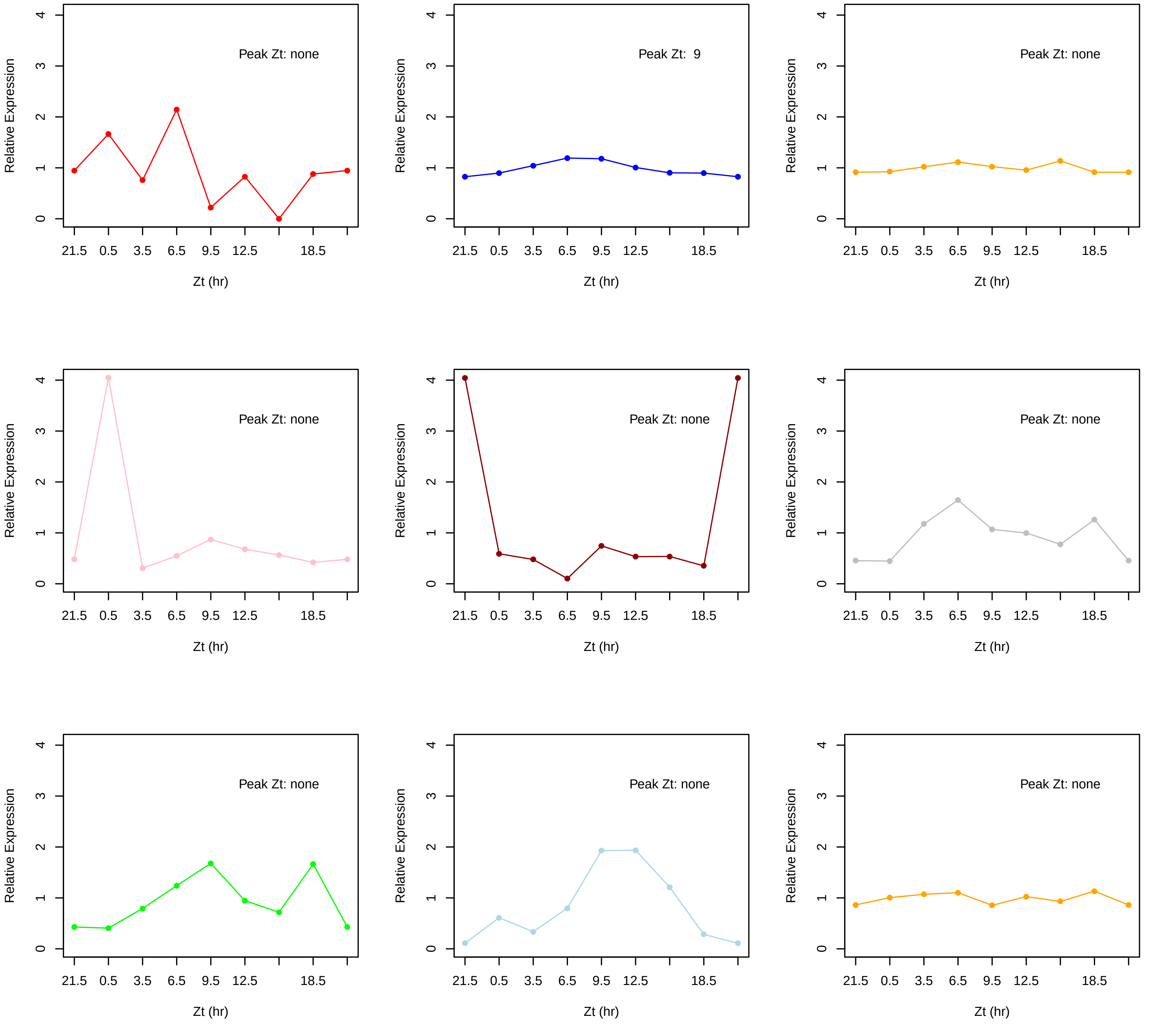

Singeleton: ENSXMAG00000015676 SRSF2

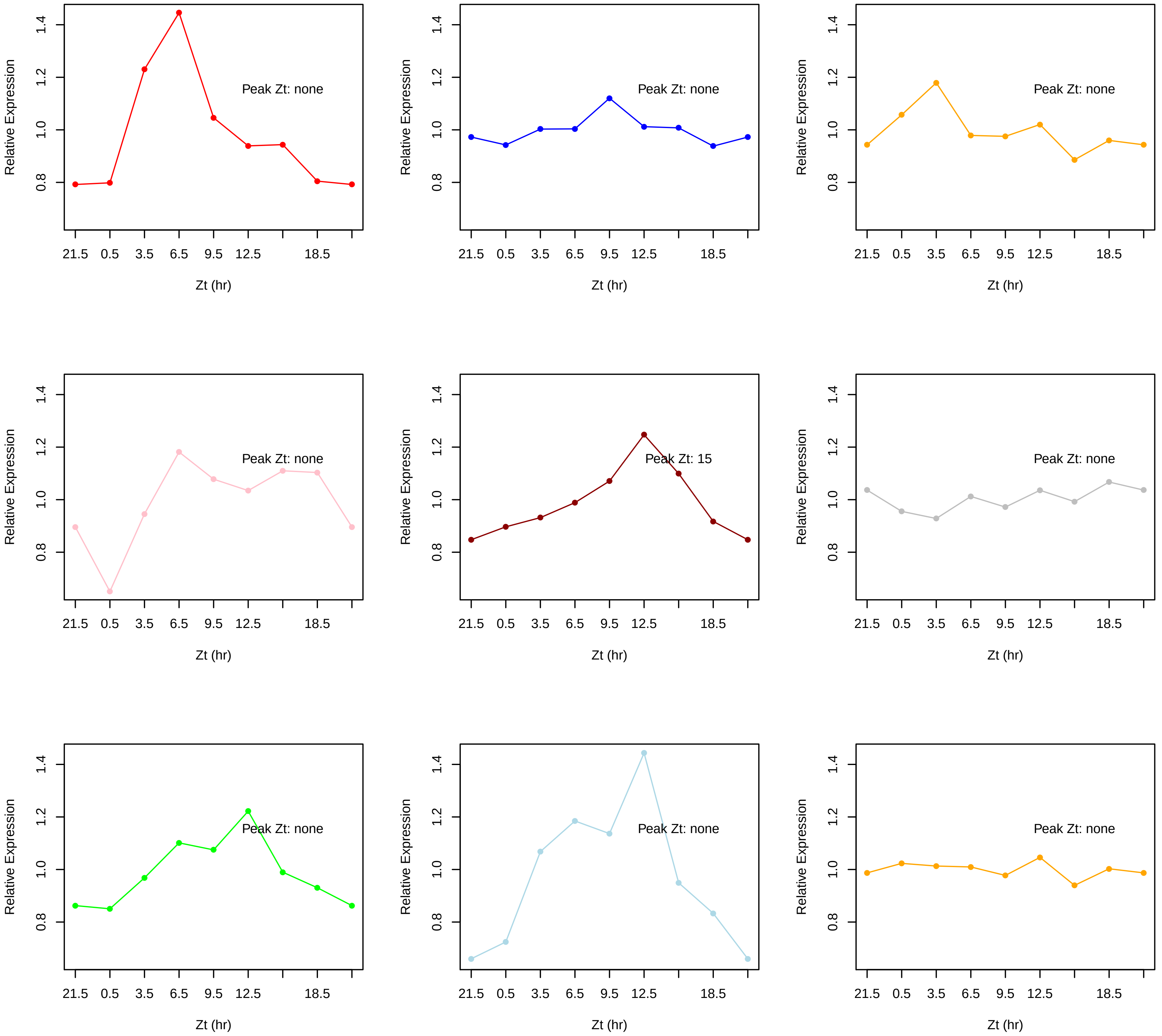

Singeleton: ENSXMAG00000019003 agmo

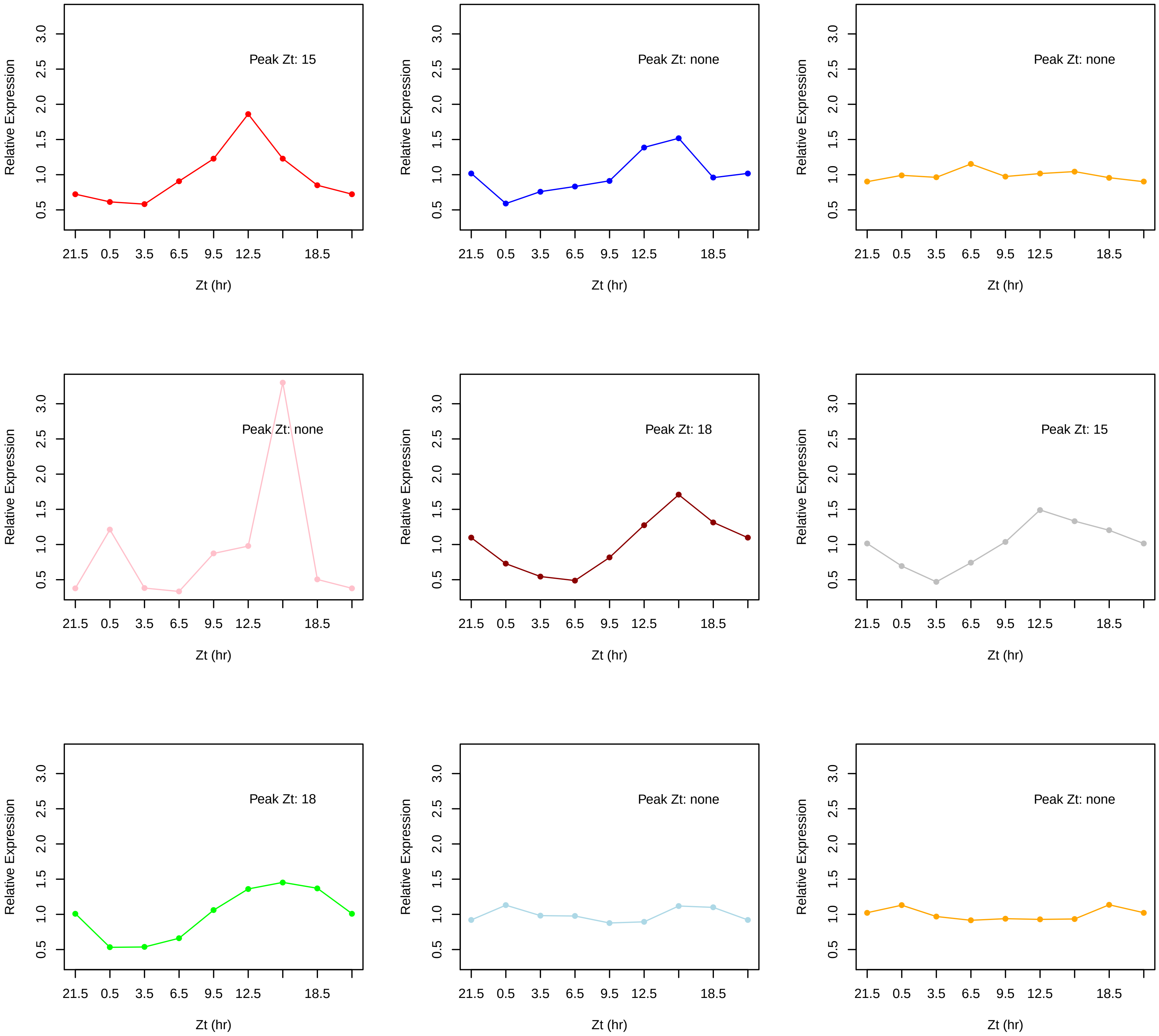

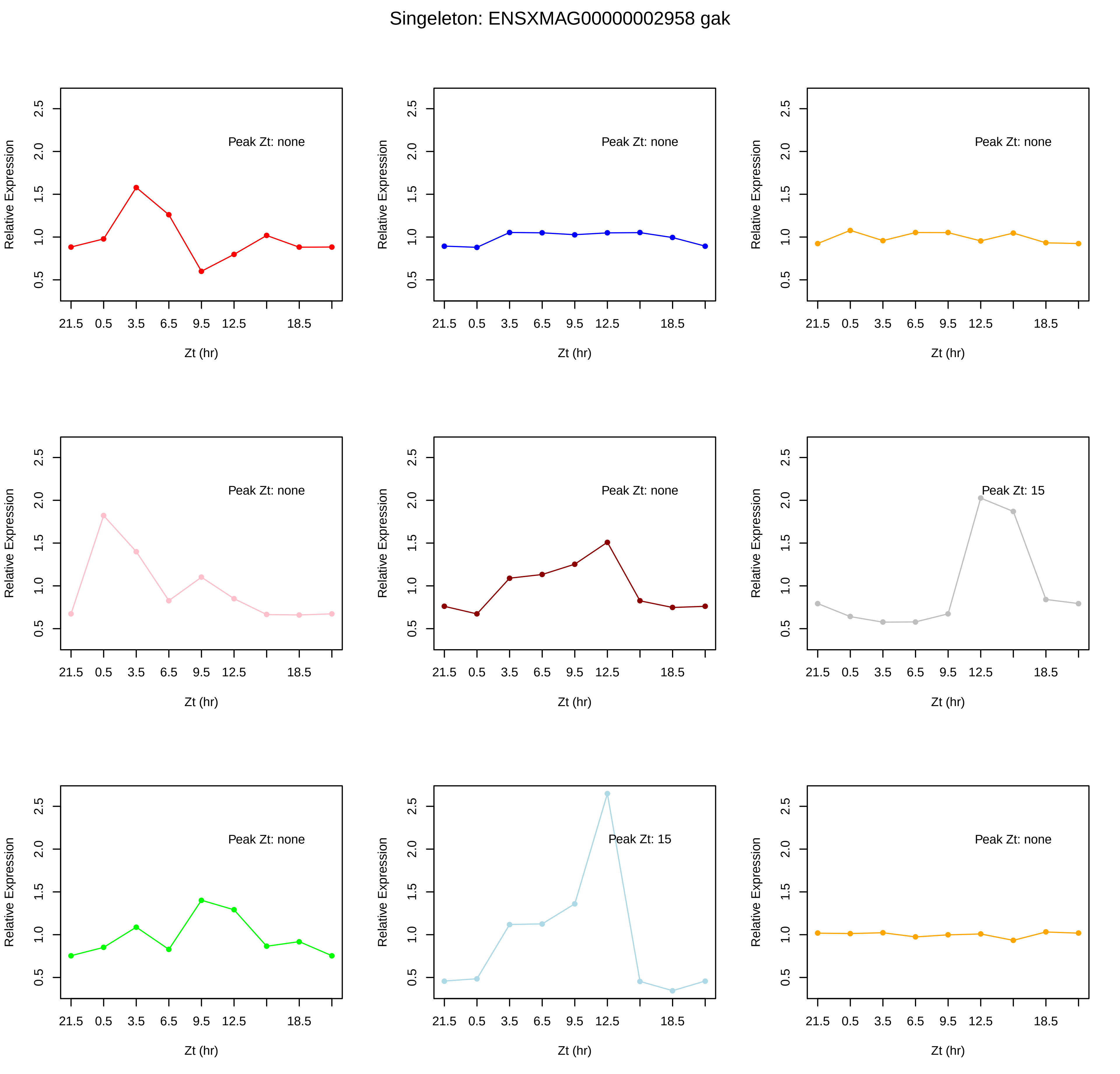
Singeleton: ENSXMAG00000002958 gak
Peak Zt: none
Peak Zt: none
Peak Zt: none
Peak Zt: none
Peak Zt: none
Peak Zt: 15
Peak Zt: none
Peak Zt: 15
Peak Zt: none
Relative Expression
Zt (hr)
0.5
1.0
1.5
2.0
2.5
21.5
0.5
3.5
6.5
9.5
12.5
18.5

Singleton: ENSXMAG00000027107 jmjd6

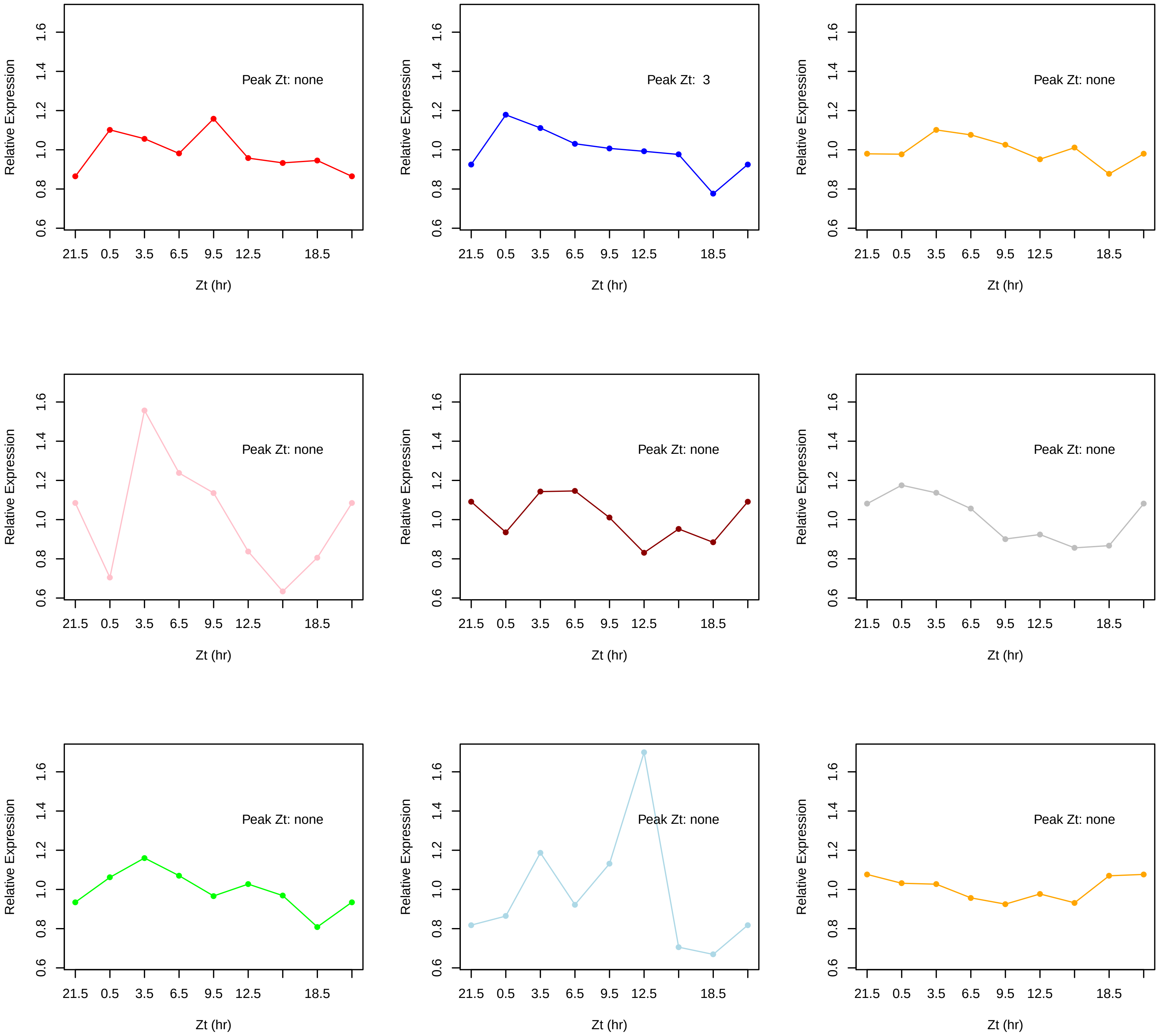

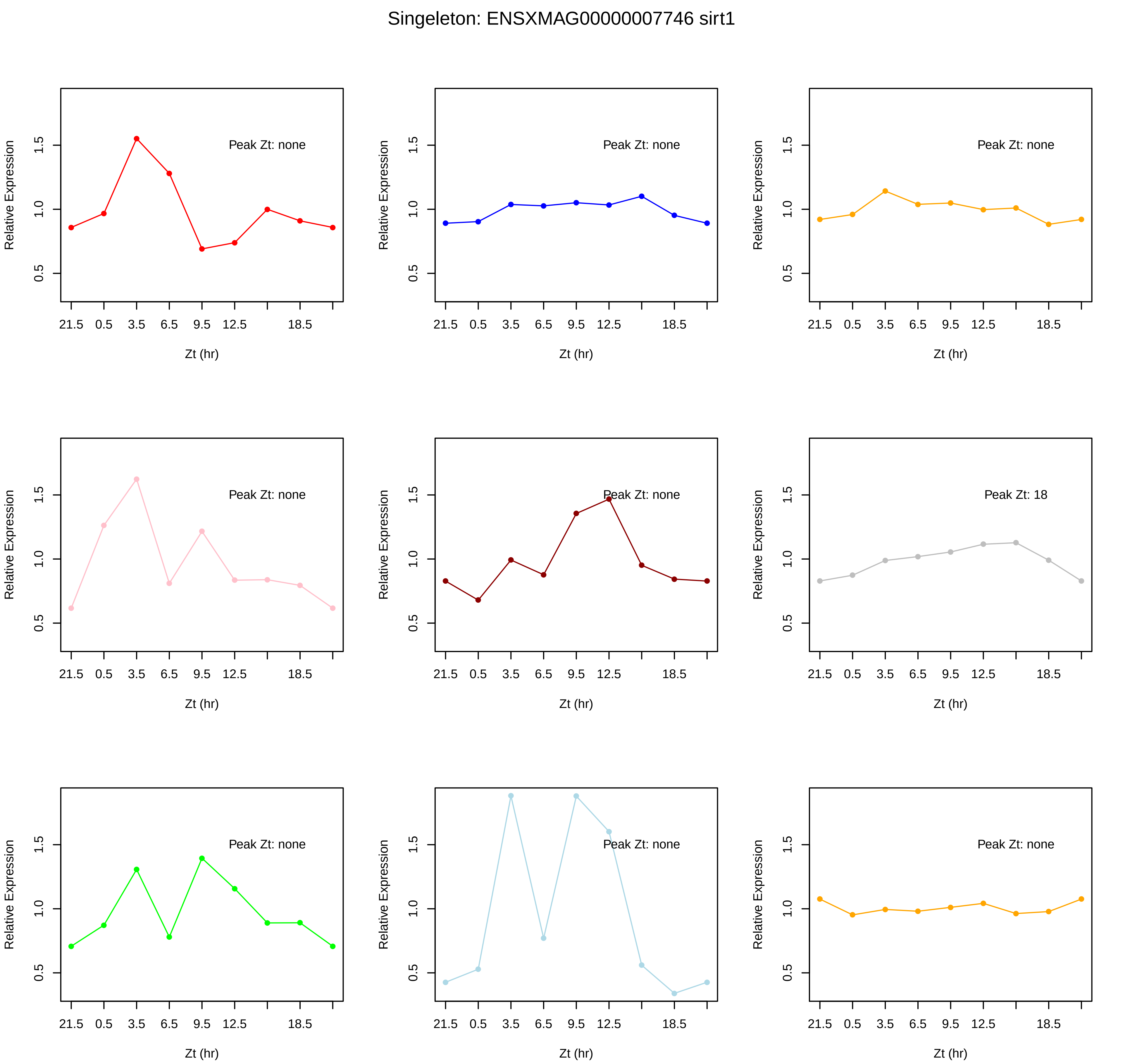
Singeleton: ENSXMAG00000007746 sirt1
Peak Zt: none
Peak Zt: none
Peak Zt: none
Peak Zt: none
Peak Zt: none
Peak Zt: 18
Peak Zt: none
Peak Zt: none
Peak Zt: none
Relative Expression
Zt (hr)
21.5
0.5
3.5
6.5
9.5
12.5
18.5
0.5
1.0
1.5

Singleton: ENSXMAG00000007658

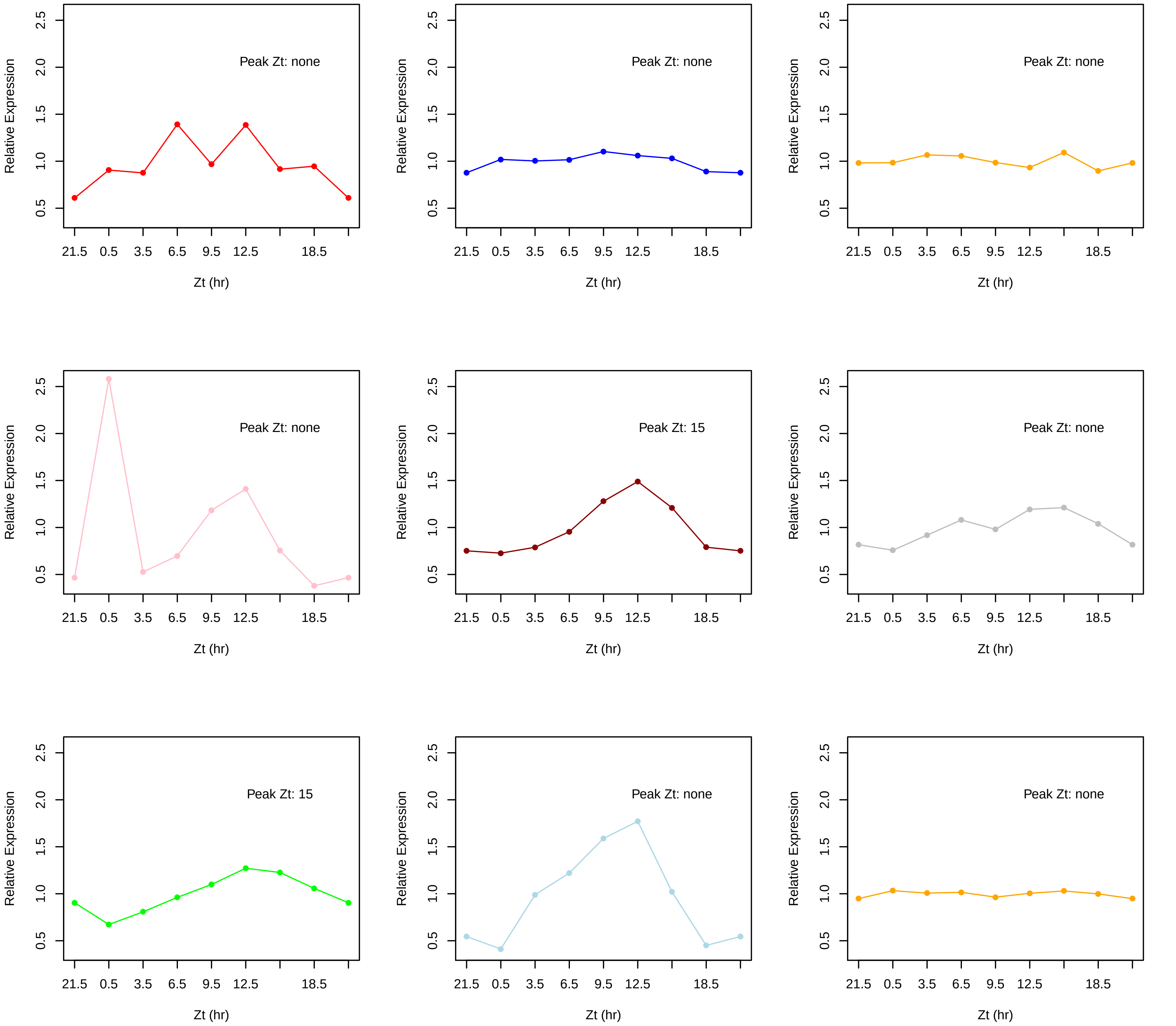

Singeleton: ENSXMAG00000009883

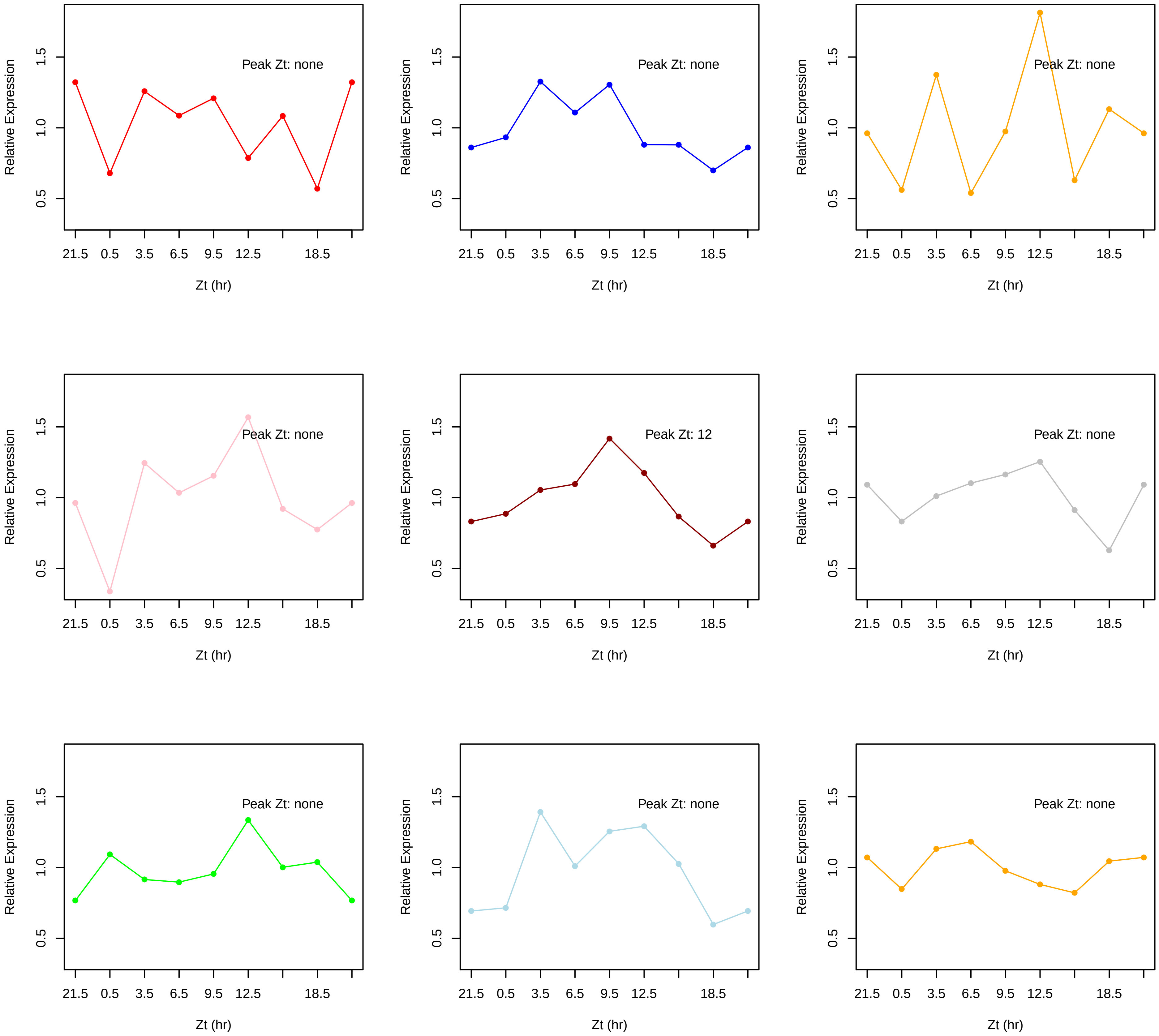

Singeleton: ENSXMAG00000019015 ispd

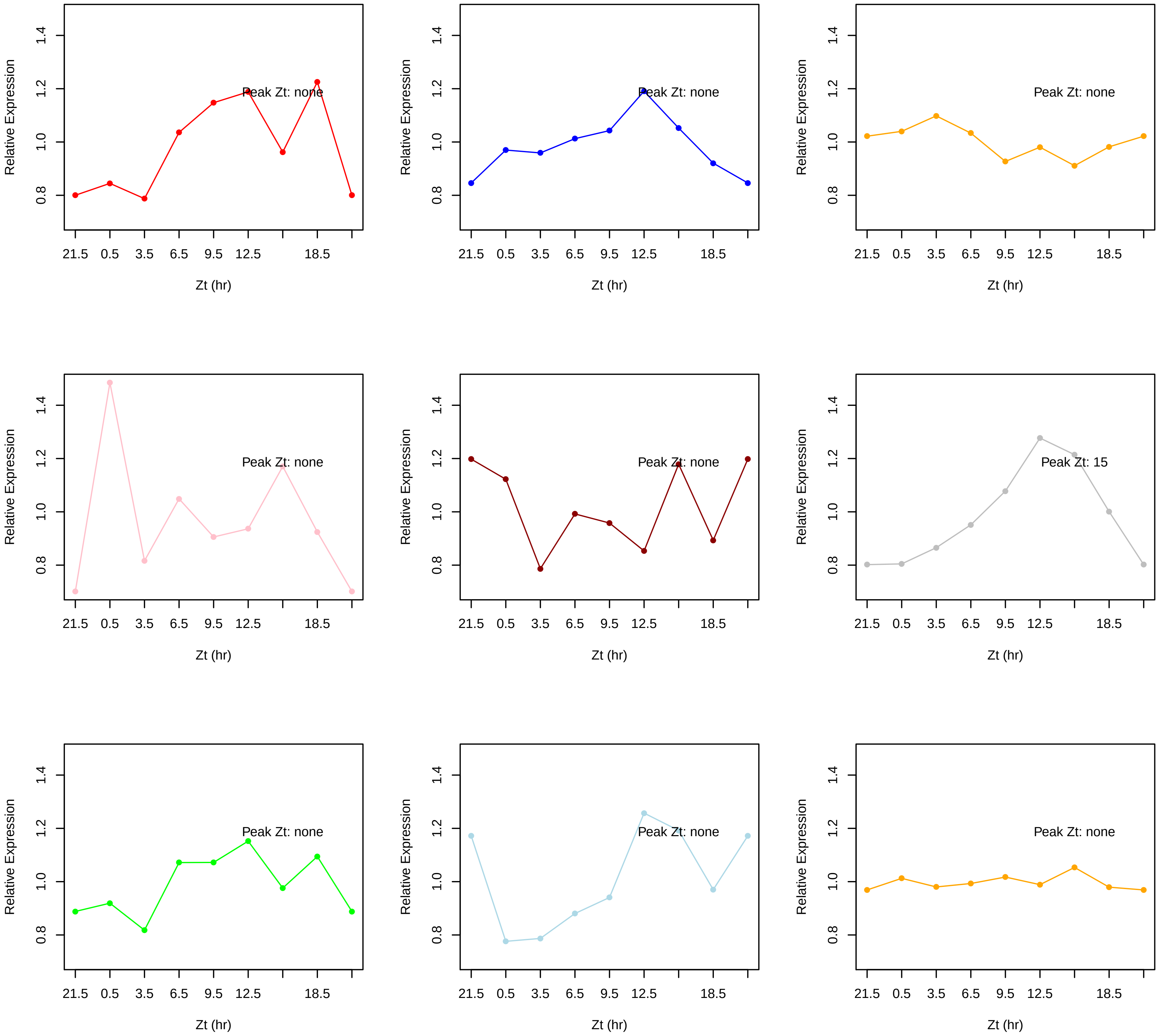

Singleton: ENSXMAG00000002027 ankha

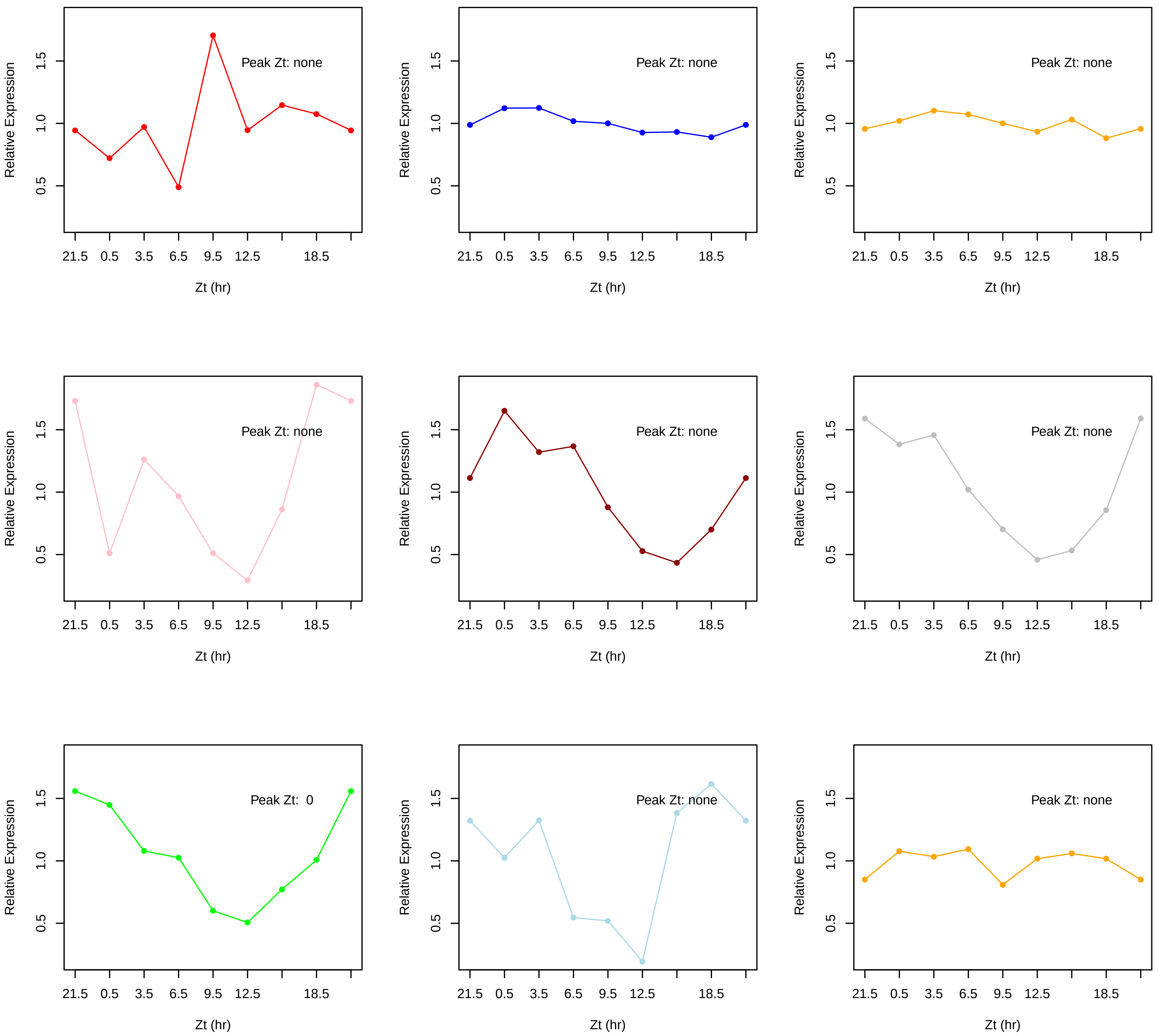

Singeleton: ENSXMAG00000014579 acaa2

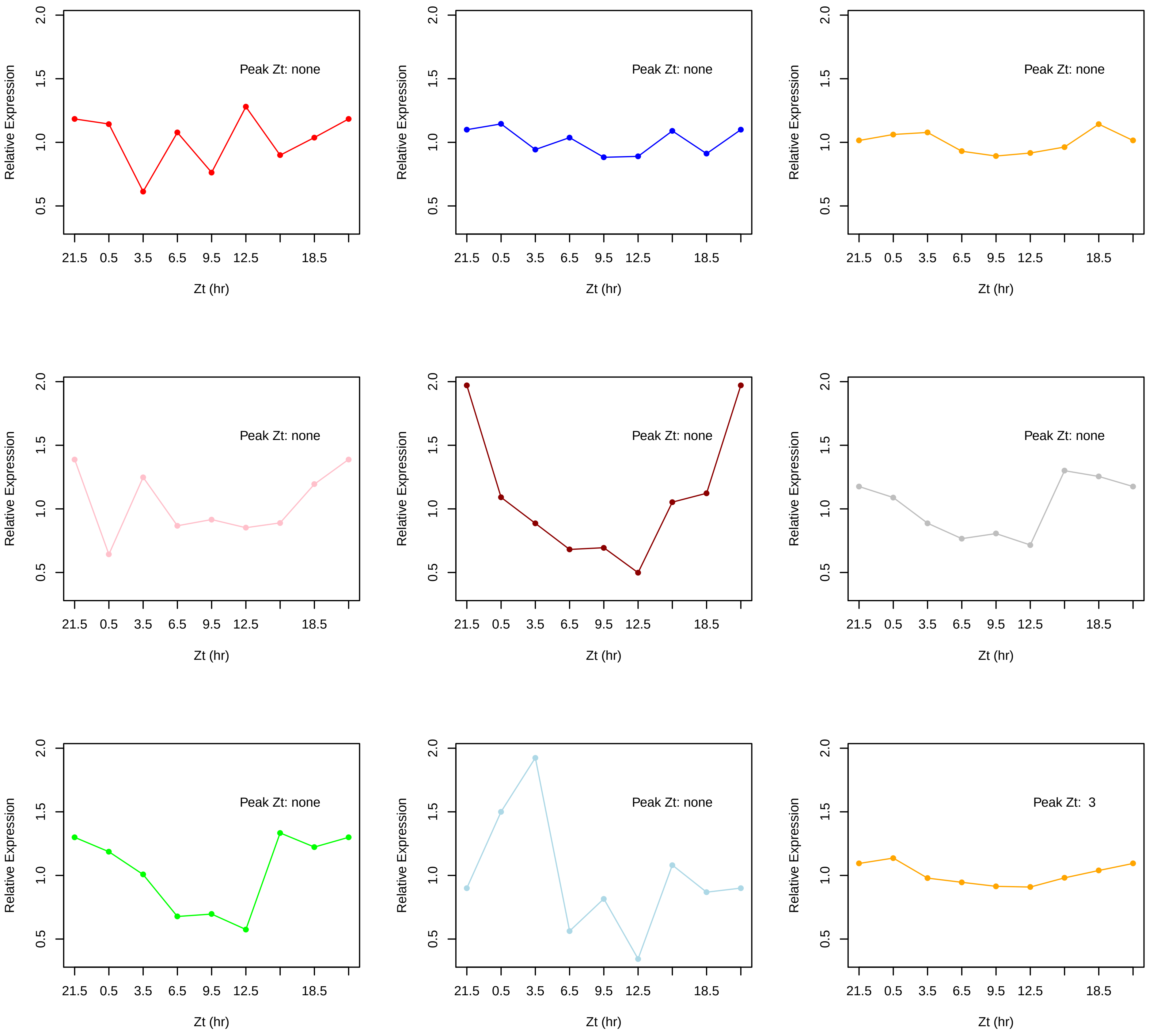

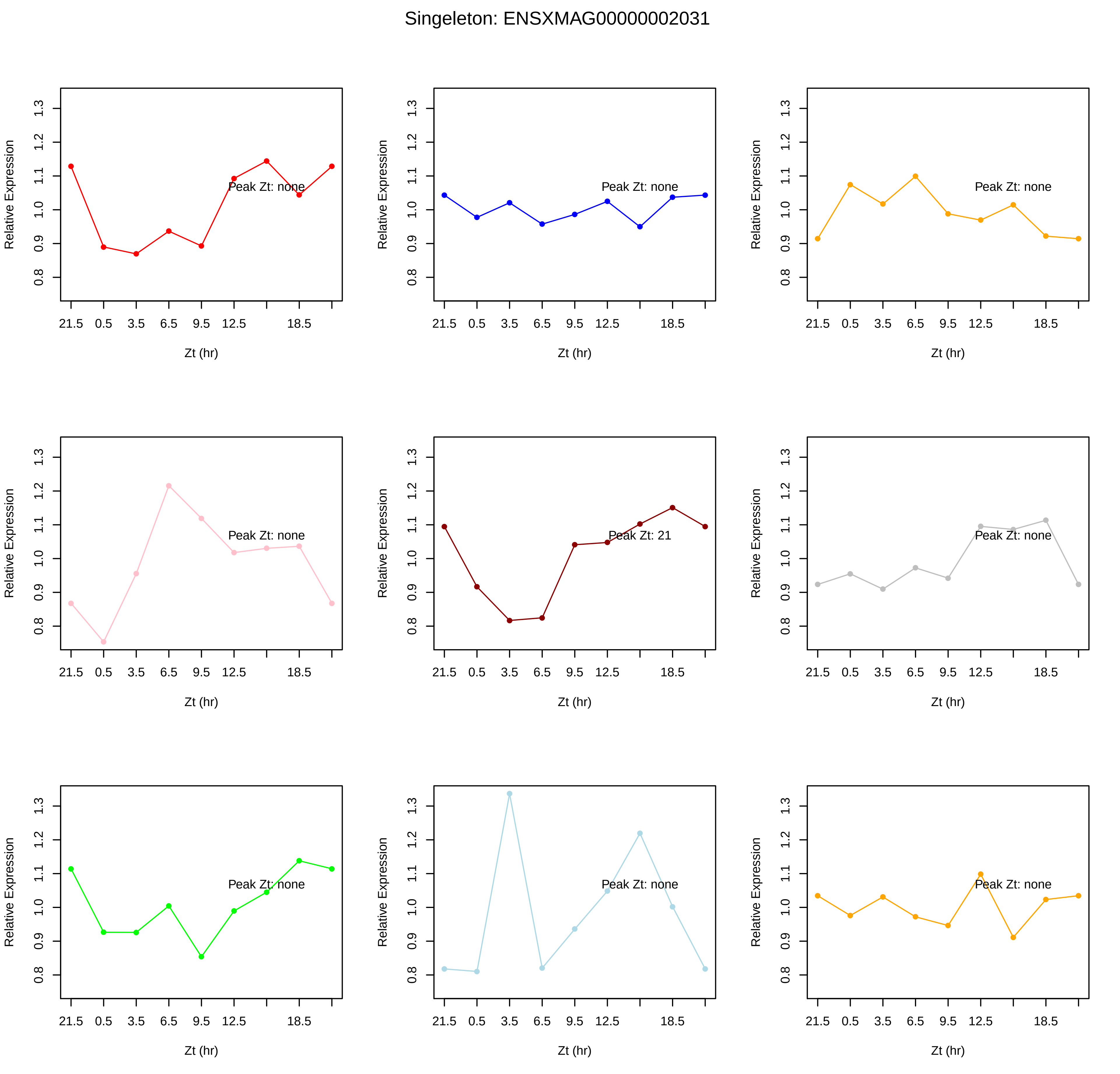

Singeleton: ENSXMAG00000002031
Peak Zt: none
Peak Zt: none
Peak Zt: none
Peak Zt: none
Peak Zt: 21
Peak Zt: none
Peak Zt: none
Peak Zt: none
Peak Zt: none
Relative Expression
Zt (hr)
21.5
0.5
3.5
6.5
9.5
12.5
18.5
0.8
0.9
1.0
1.1
1.2
1.3

Singleton: ENSXMAG00000018208 MZT1

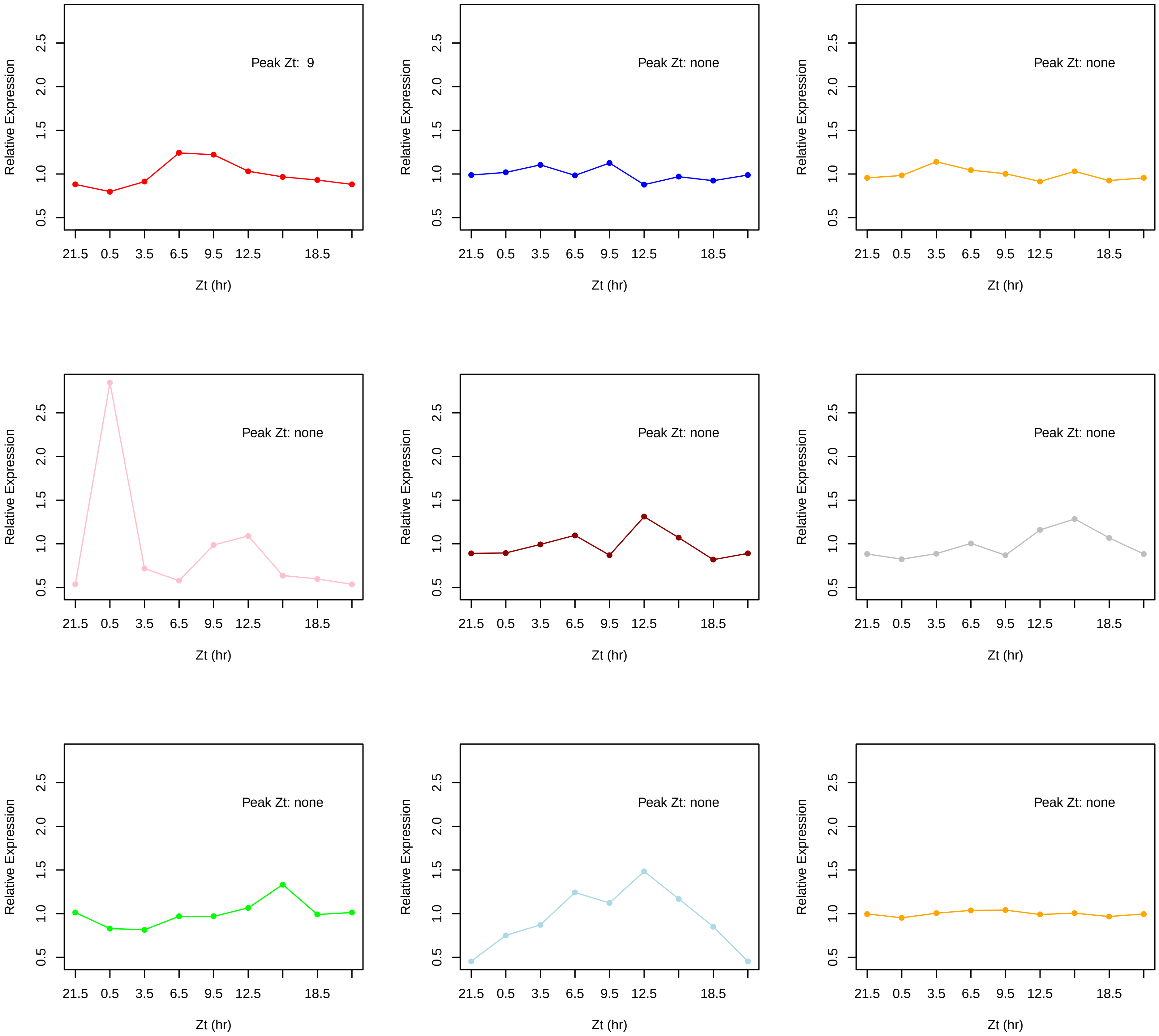

Singeleton: ENSXMAG00000010221 lipg

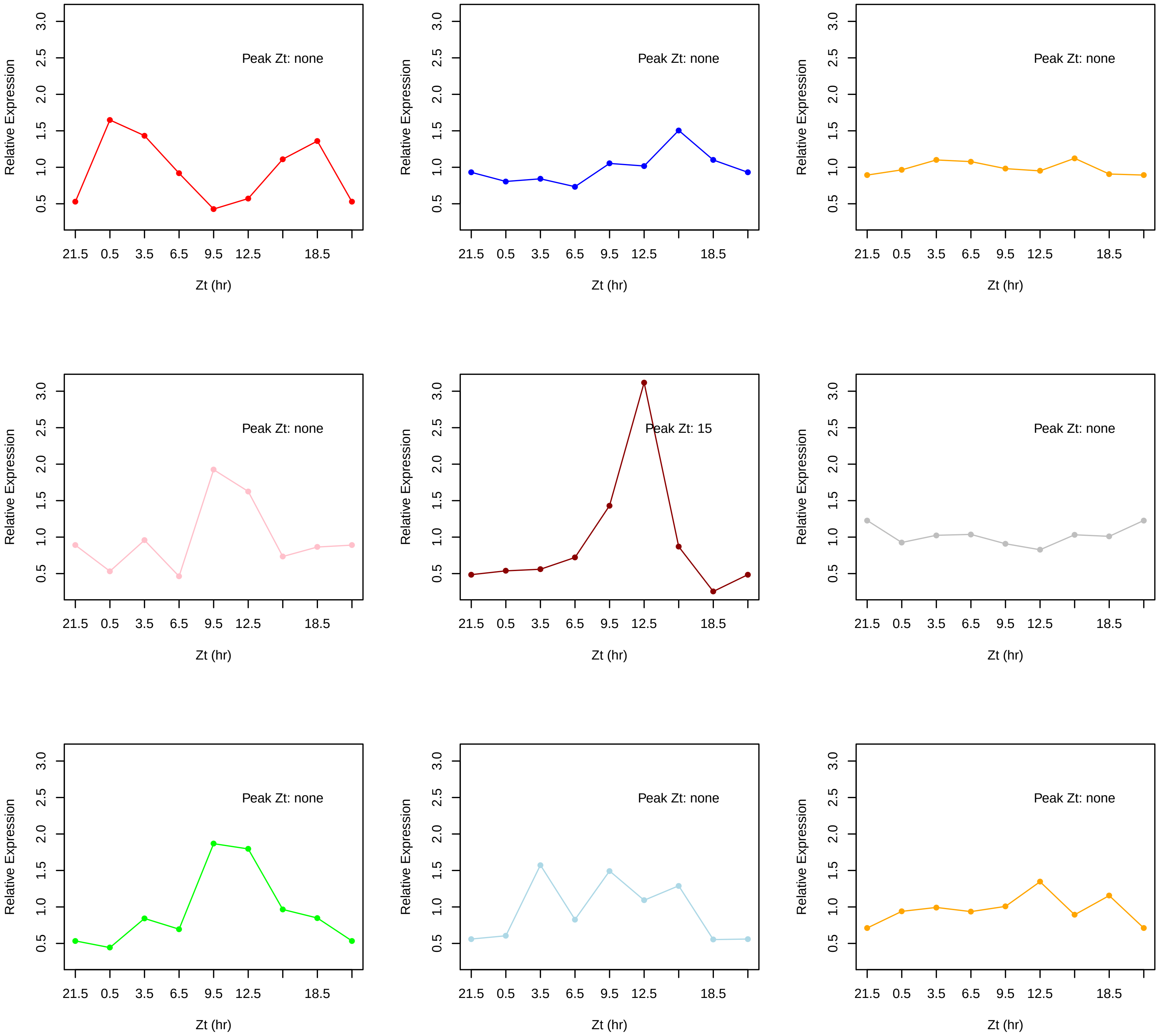

Singeleton: ENSXMAG00000008560 PPP3CA

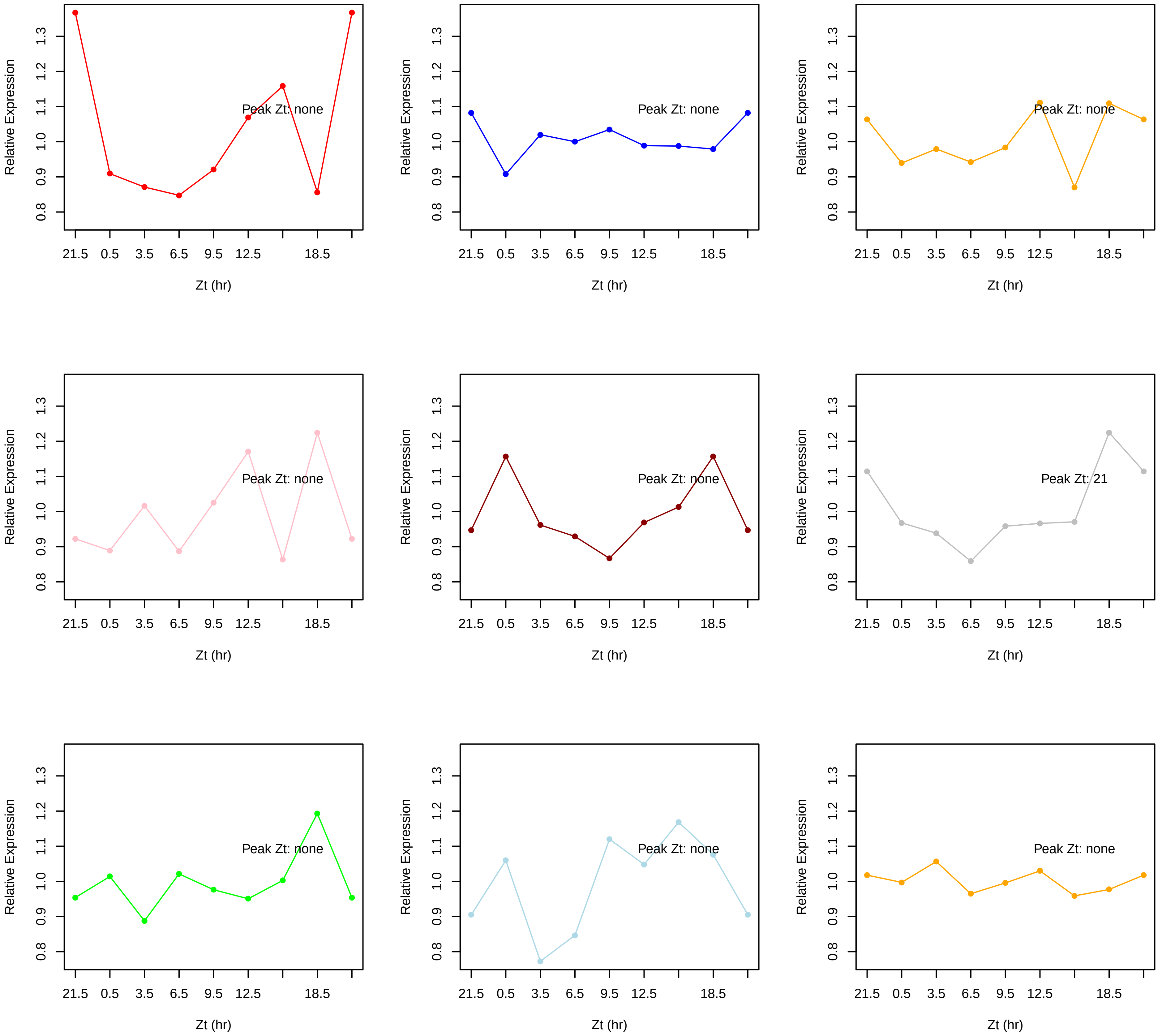

Singeleton: ENSXMAG00000021215 otulina

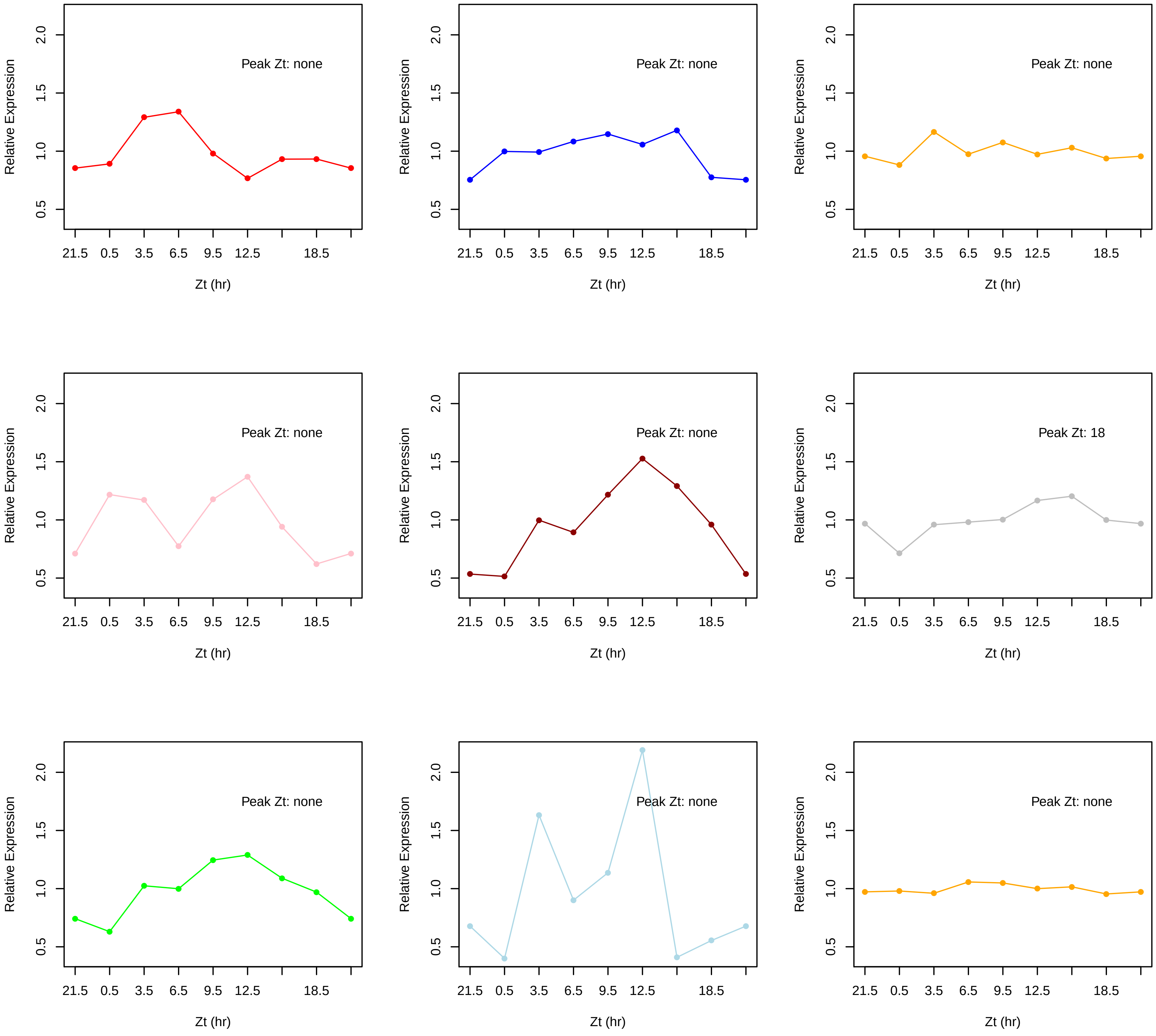

Singeleton: ENSXMAG00000022127 guca1b

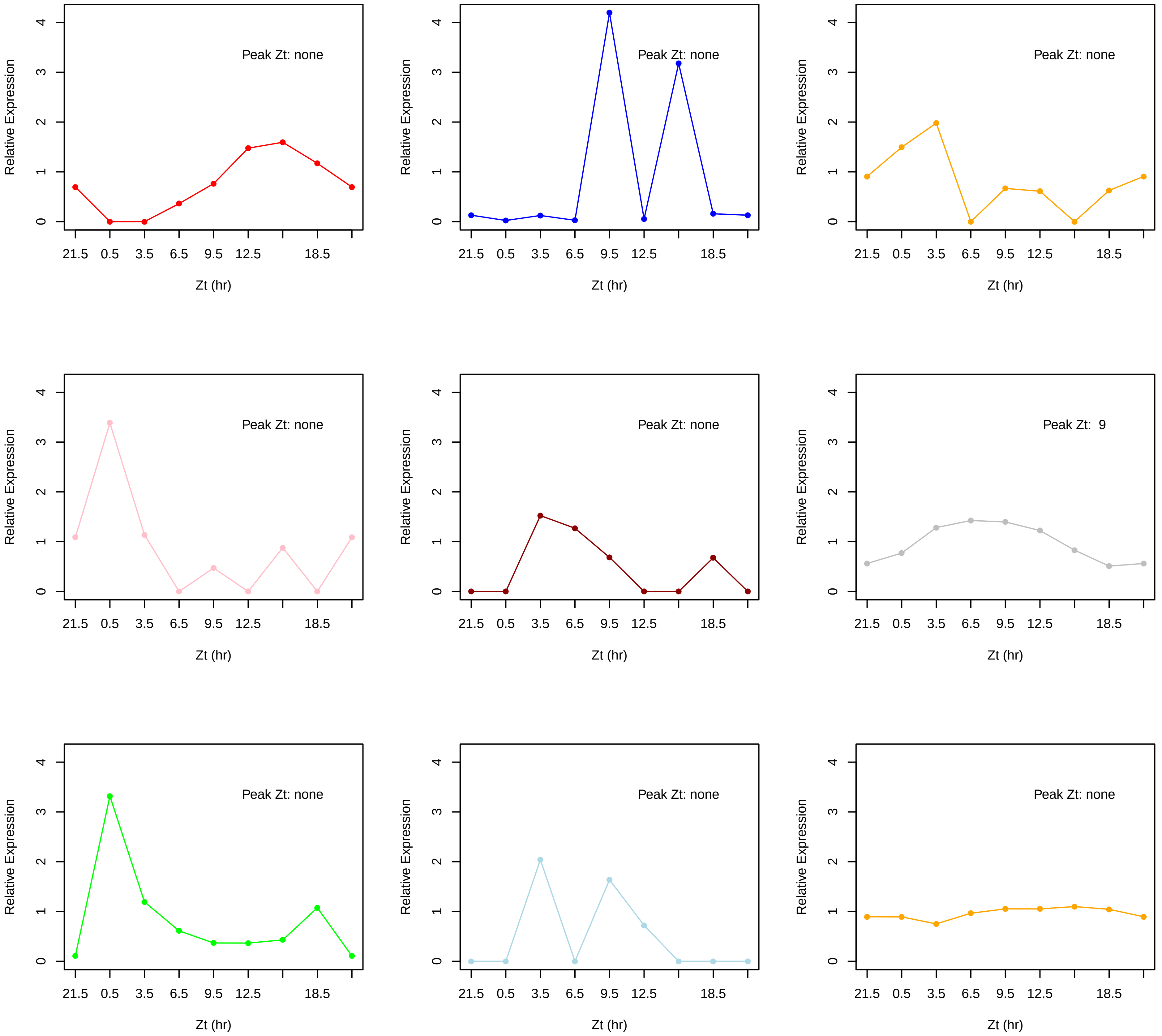

Singeleton: ENSXMAG00000008349 fkbp3

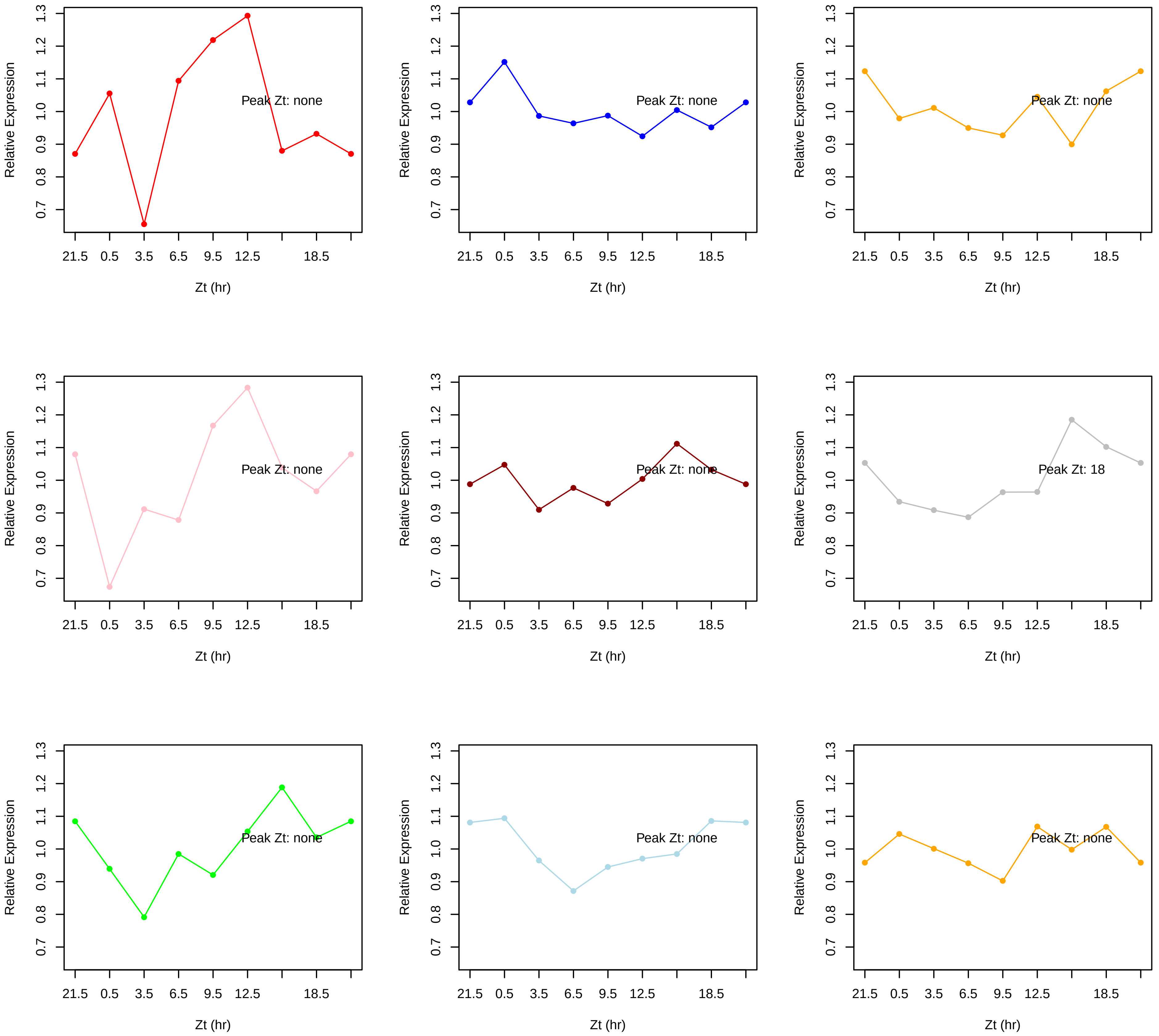

Singeleton: ENSXMAG00000019024 ankmy2a

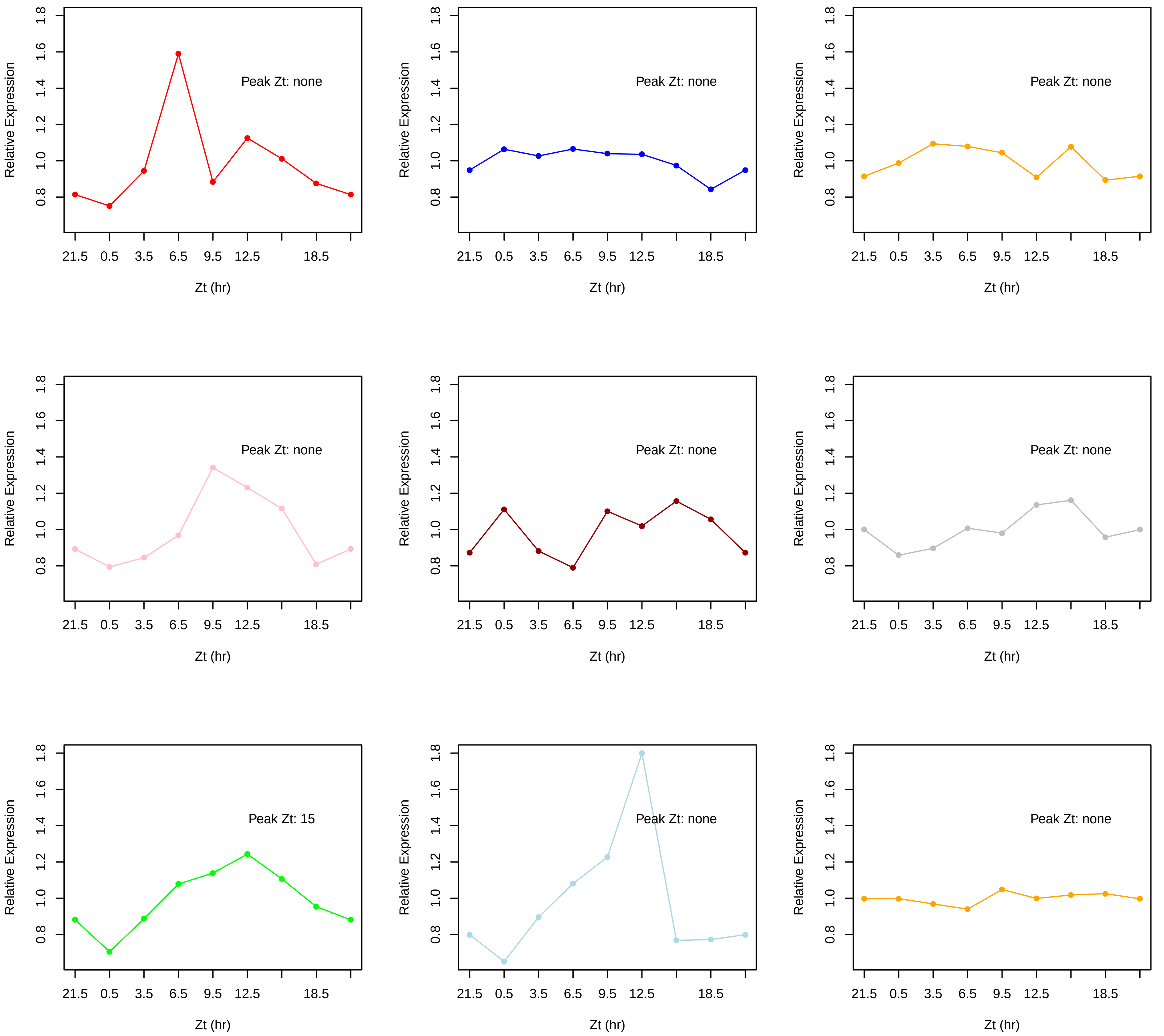

Singleton: ENSXMAG00000028250 pcdh1g32

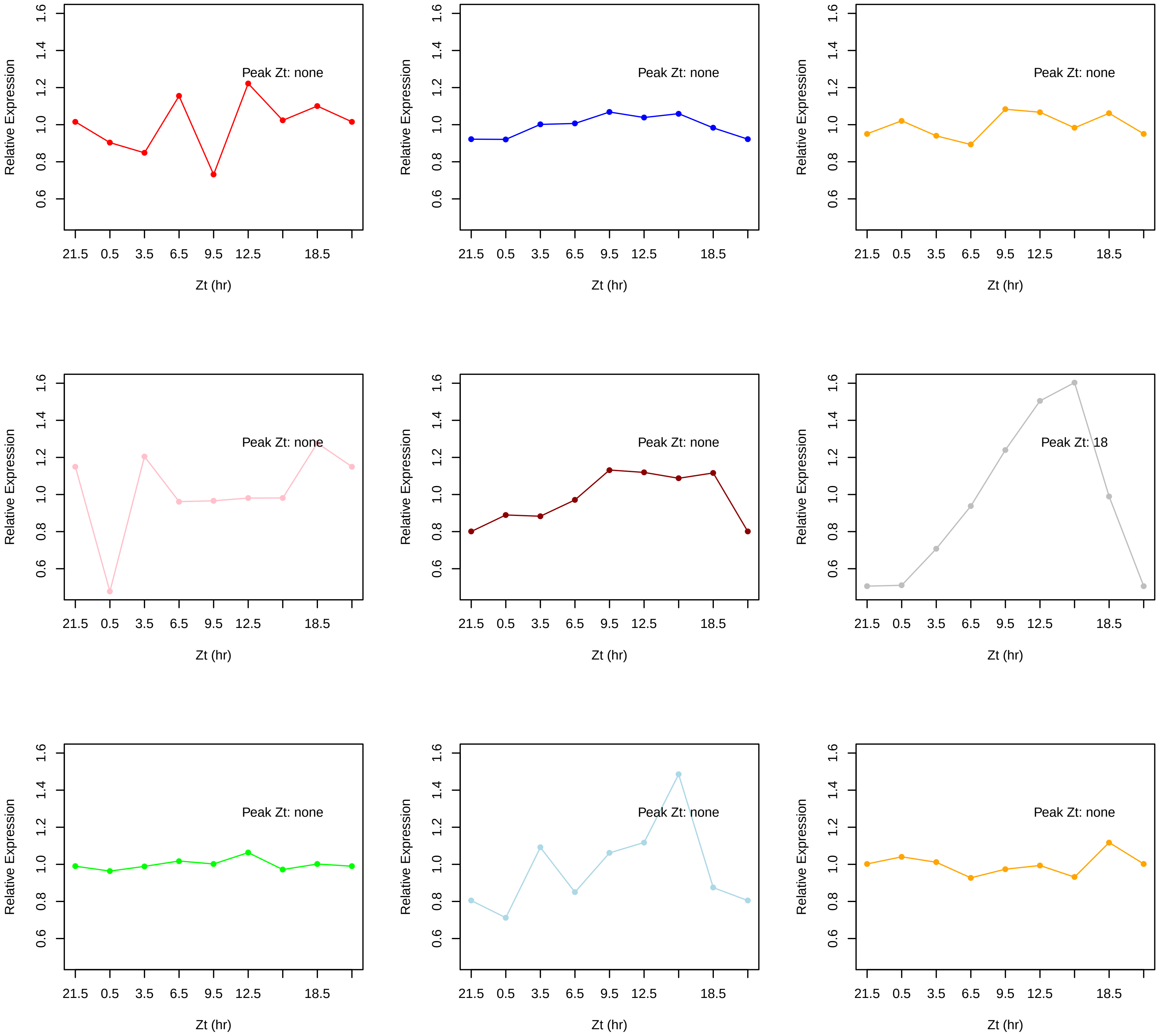

Singeleton: ENSXMAG00000007702

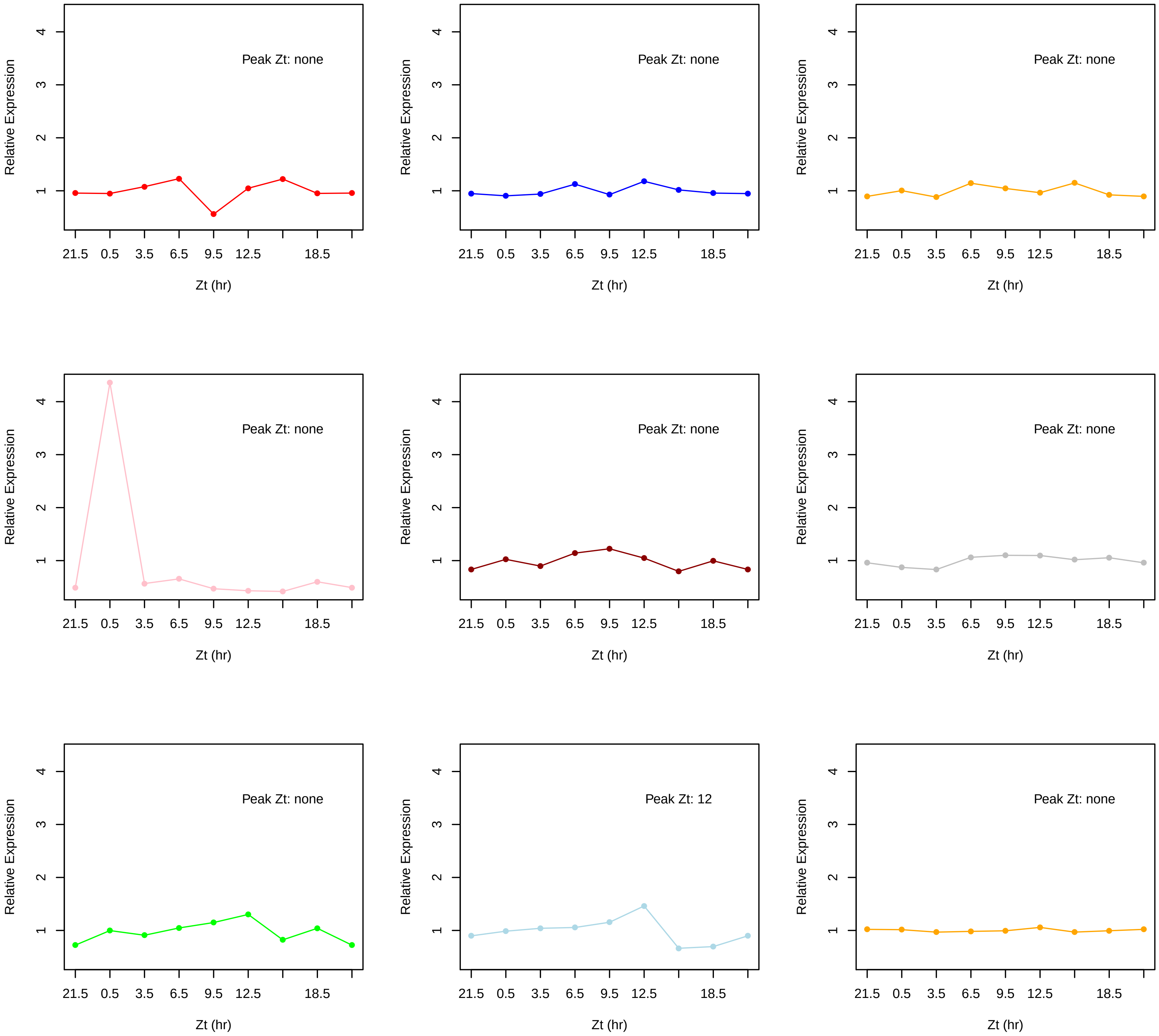

Singleton: ENSXMAG00000008538 NFIB

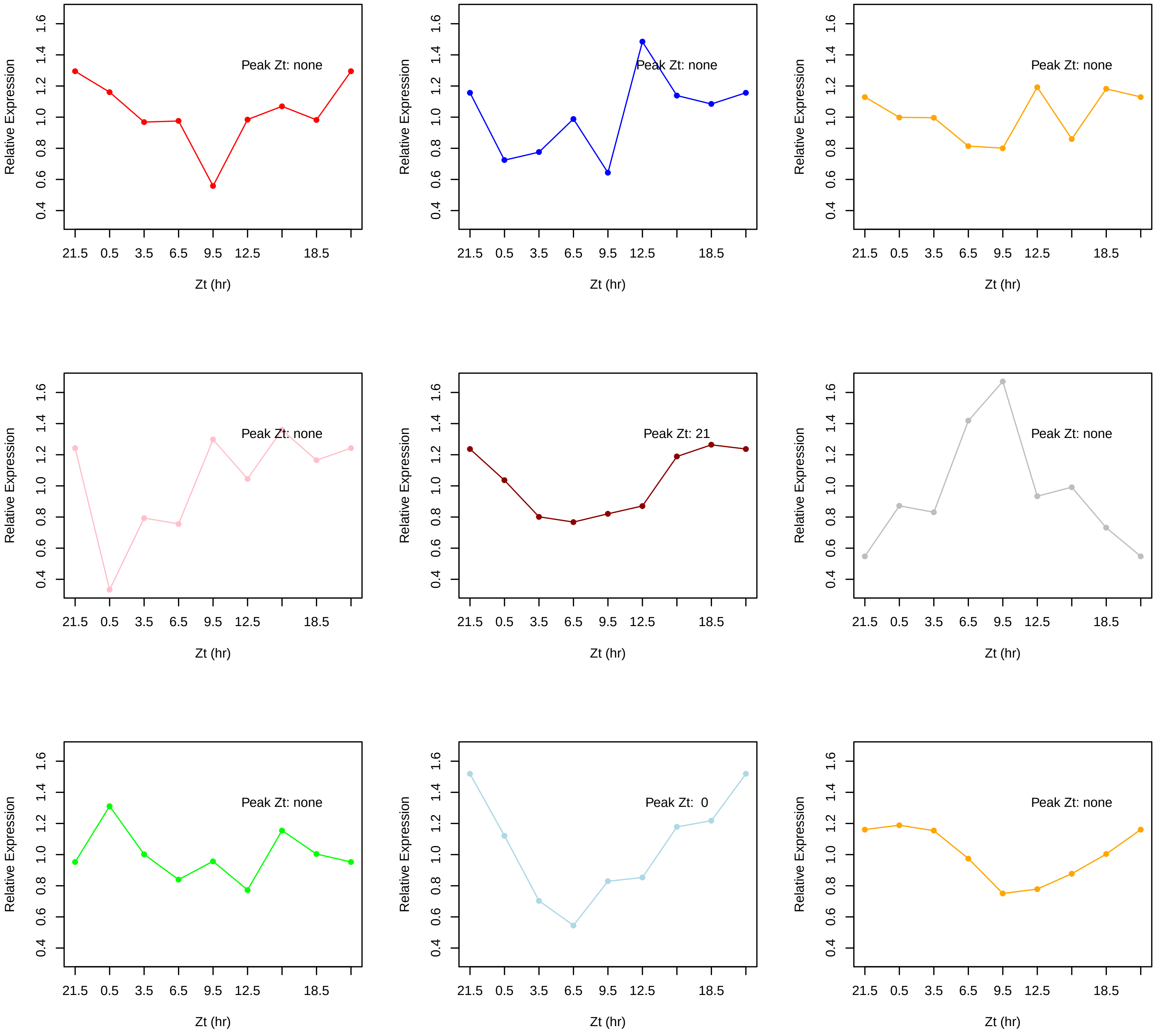

Singeleton: ENSXMAG00000007759 MYPN

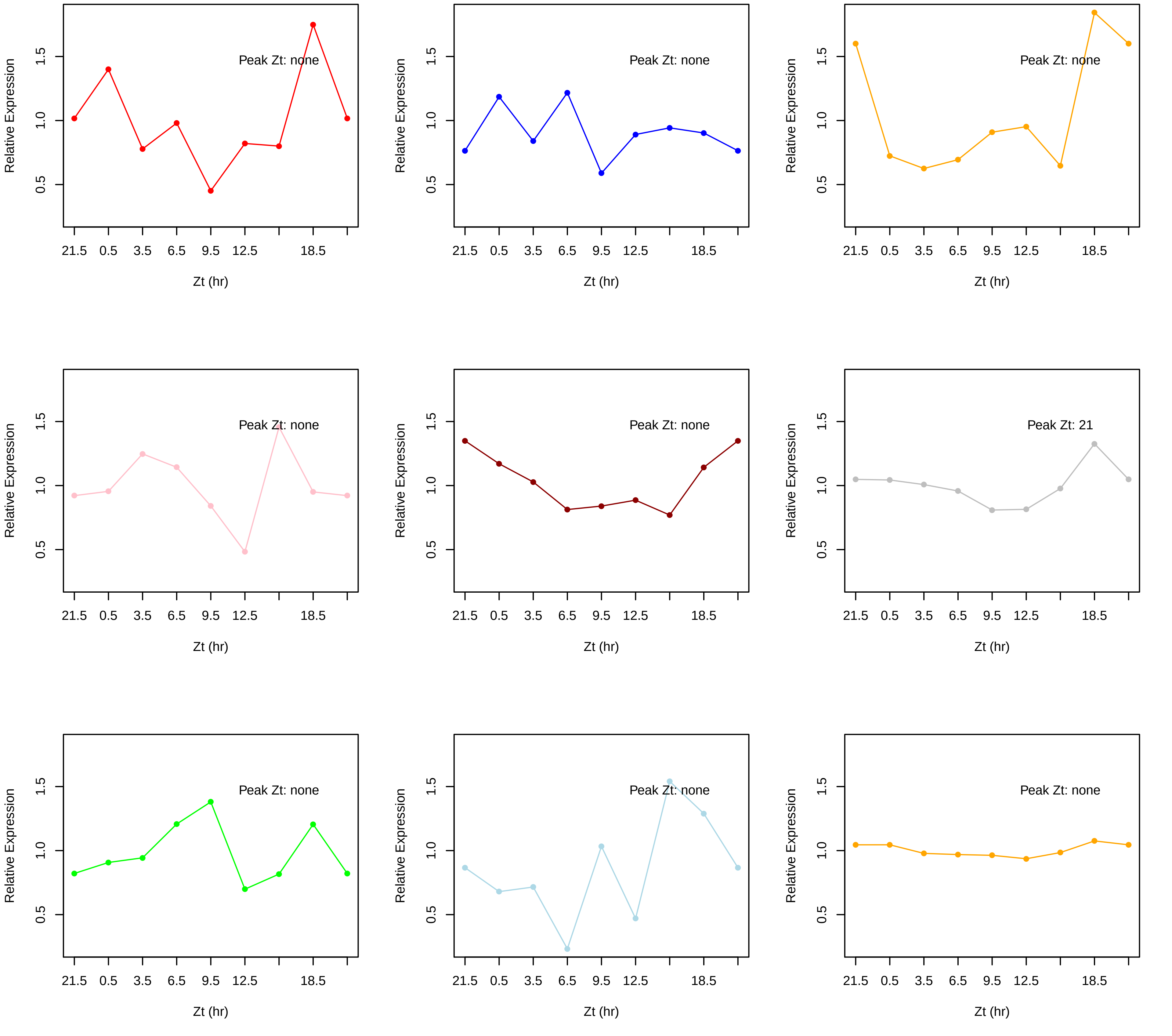

Singeleton: ENSXMAG00000007160

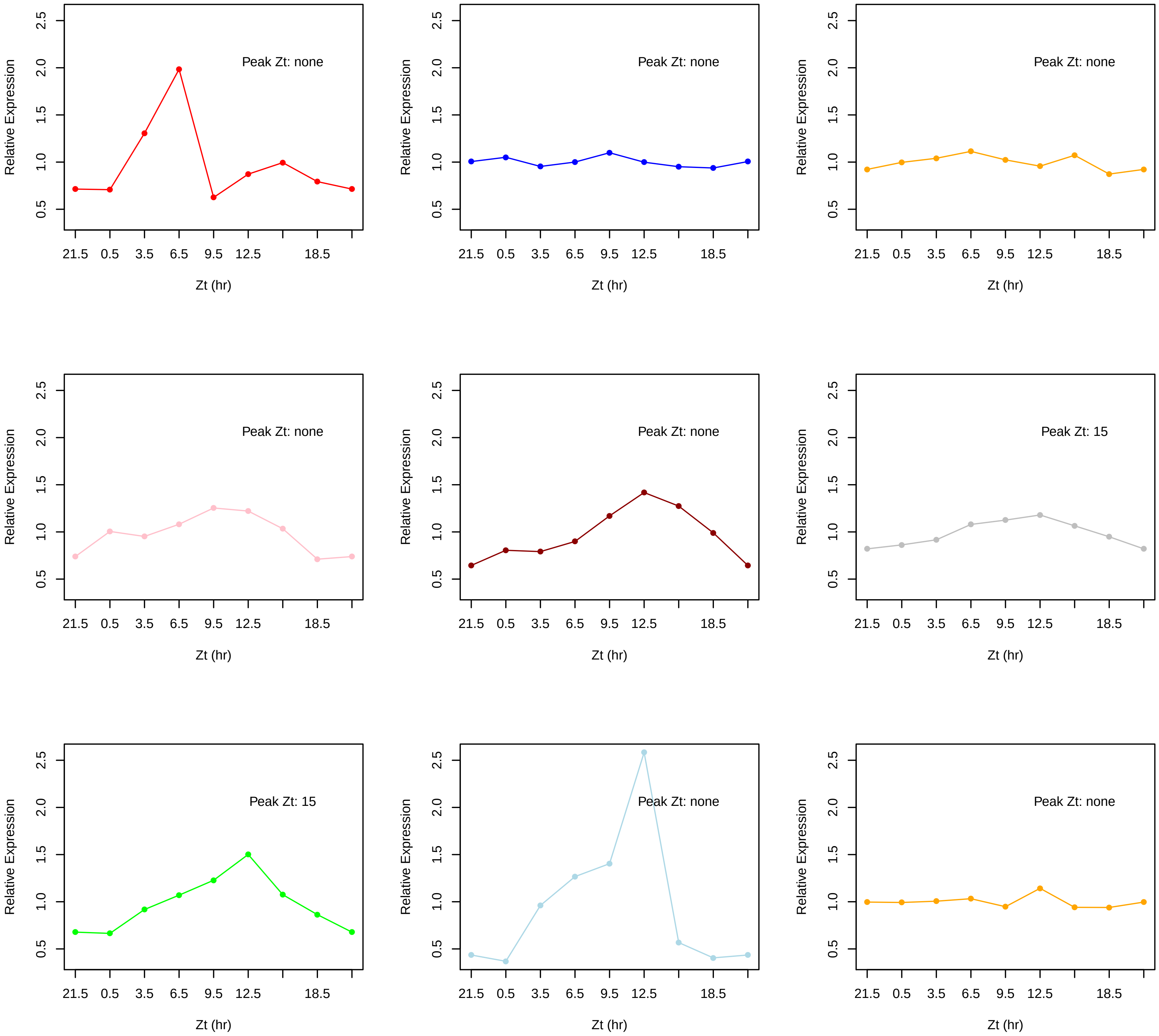

Singeleton: ENSXMAG00000007713 soul3

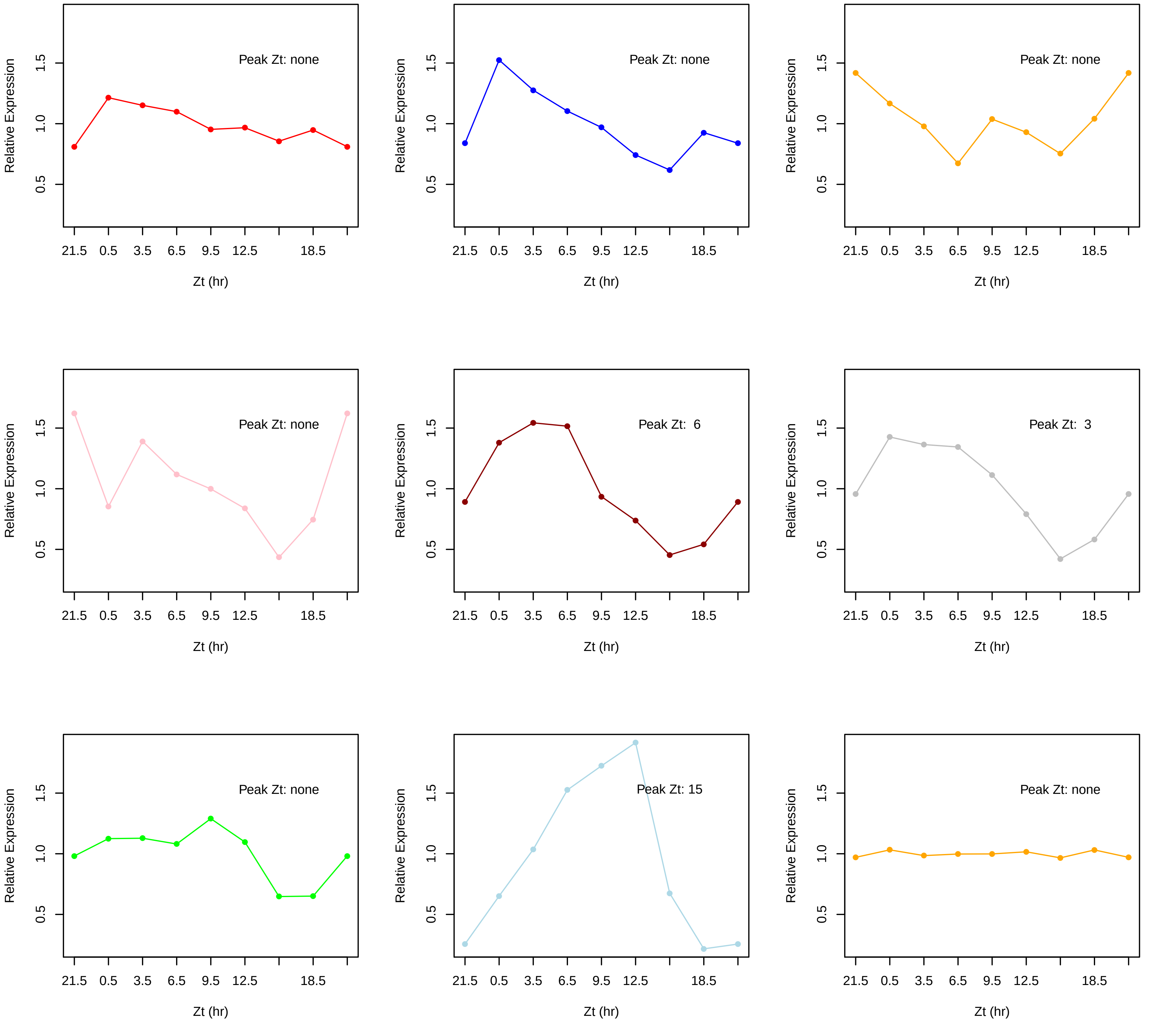

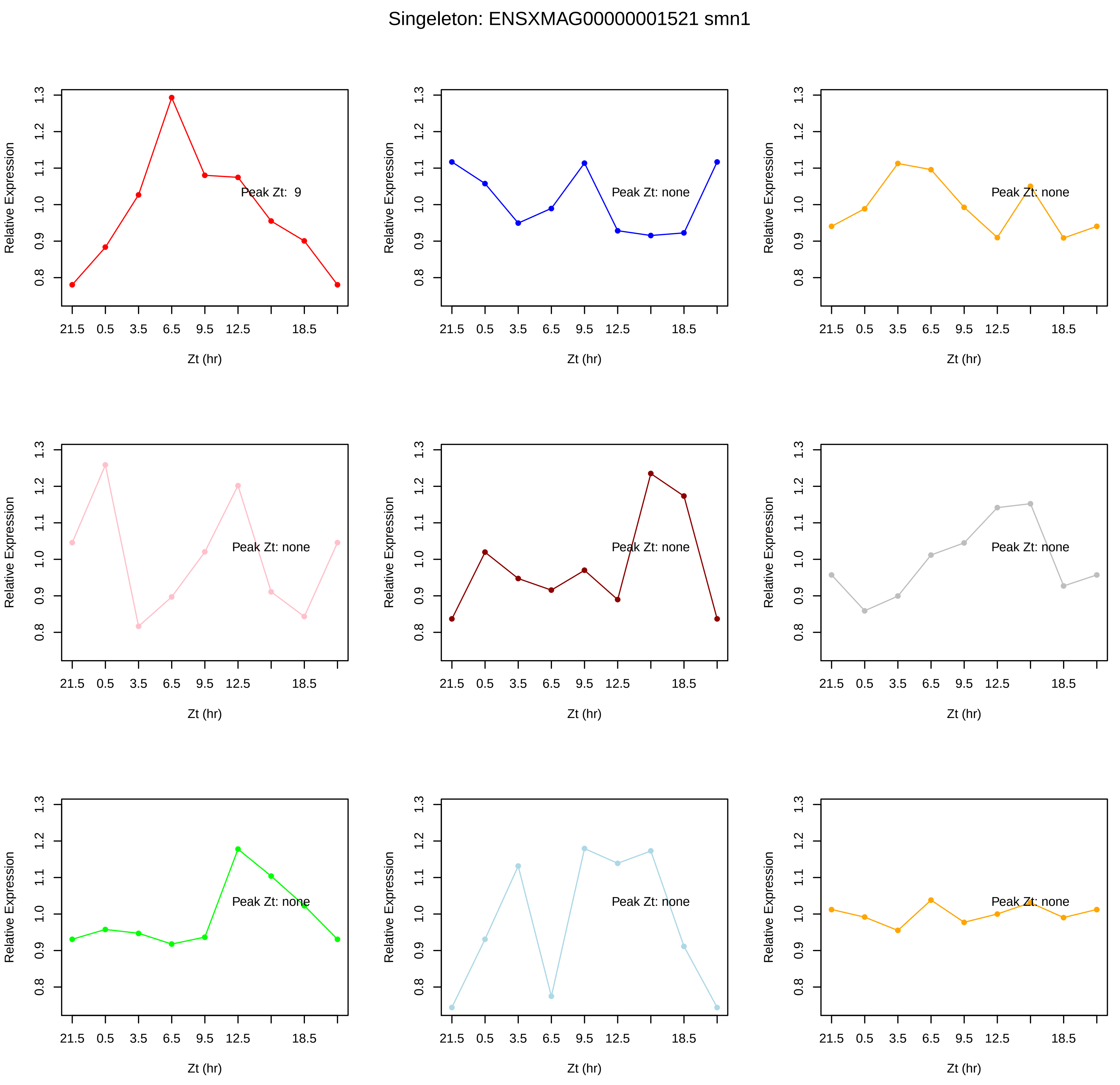
Singeleton: ENSXMAG00000001521 smn1
Relative Expression
Zt (hr)
21.5
0.5
3.5
6.5
9.5
12.5
18.5
0.8
0.9
1.0
1.1
1.2
1.3
Peak Zt: 9
Peak Zt: none

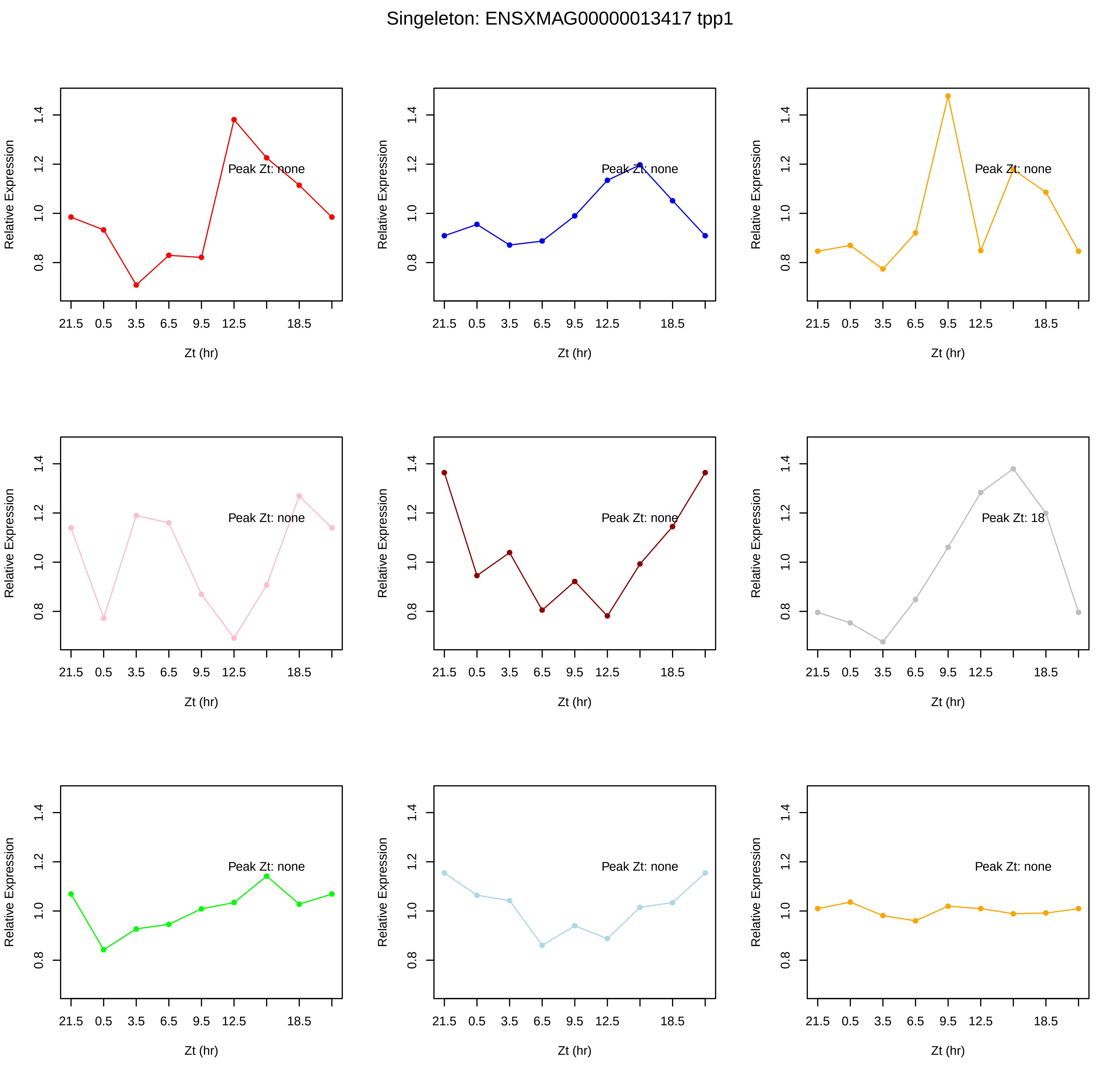
Singeleton: ENSXMAG00000013417 tpp1
Peak Zt: none
Peak Zt: none
Peak Zt: none
Peak Zt: none
Peak Zt: none
Peak Zt: 18
Peak Zt: none
Peak Zt: none
Peak Zt: none
Relative Expression
Zt (hr)
21.5
0.5
3.5
6.5
9.5
12.5
18.5
0.8
1.0
1.2
1.4

Singleton: ENSXMAG00000011014 klf12b

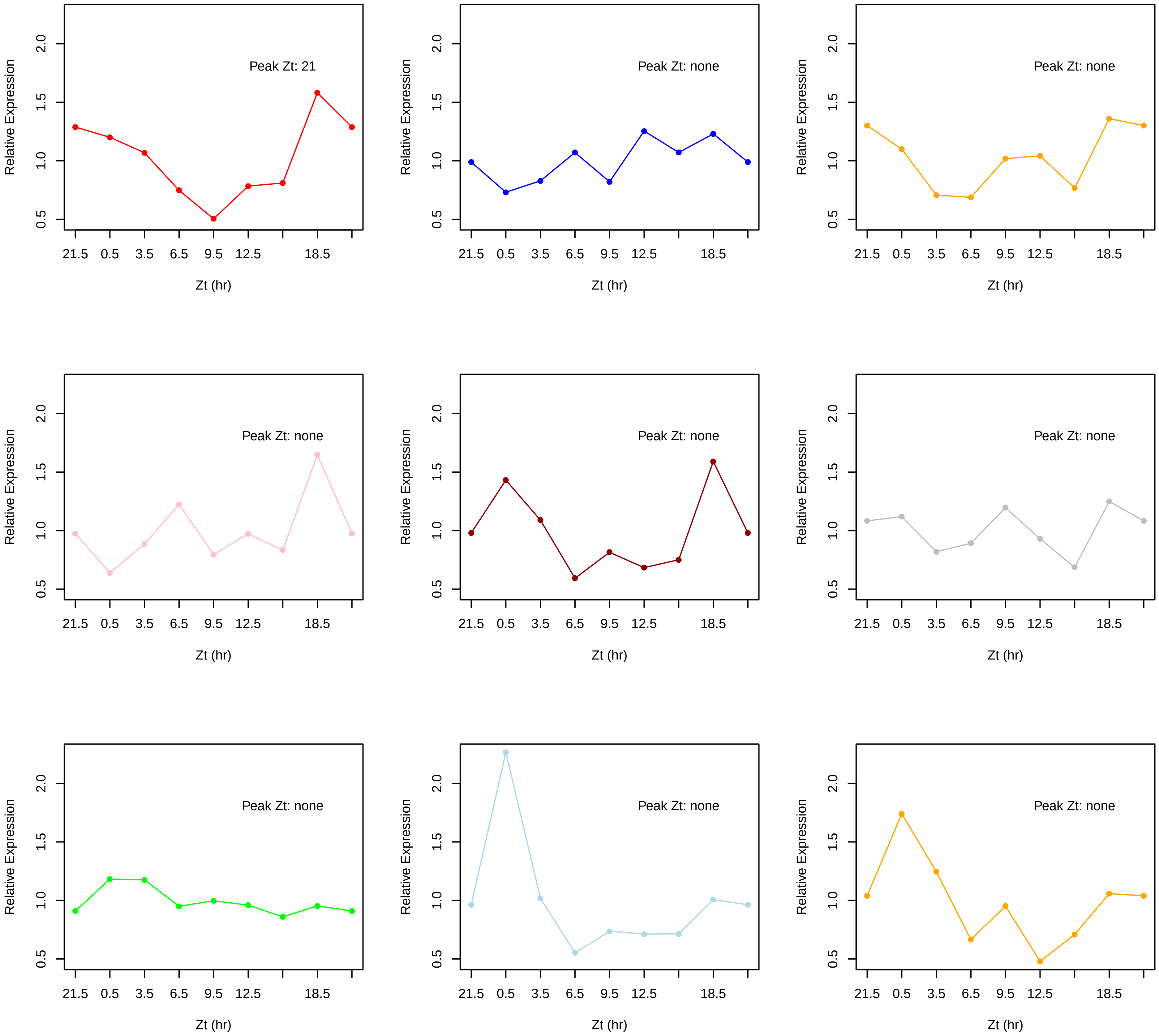

Singeleton: ENSXMAG00000008356 spock1

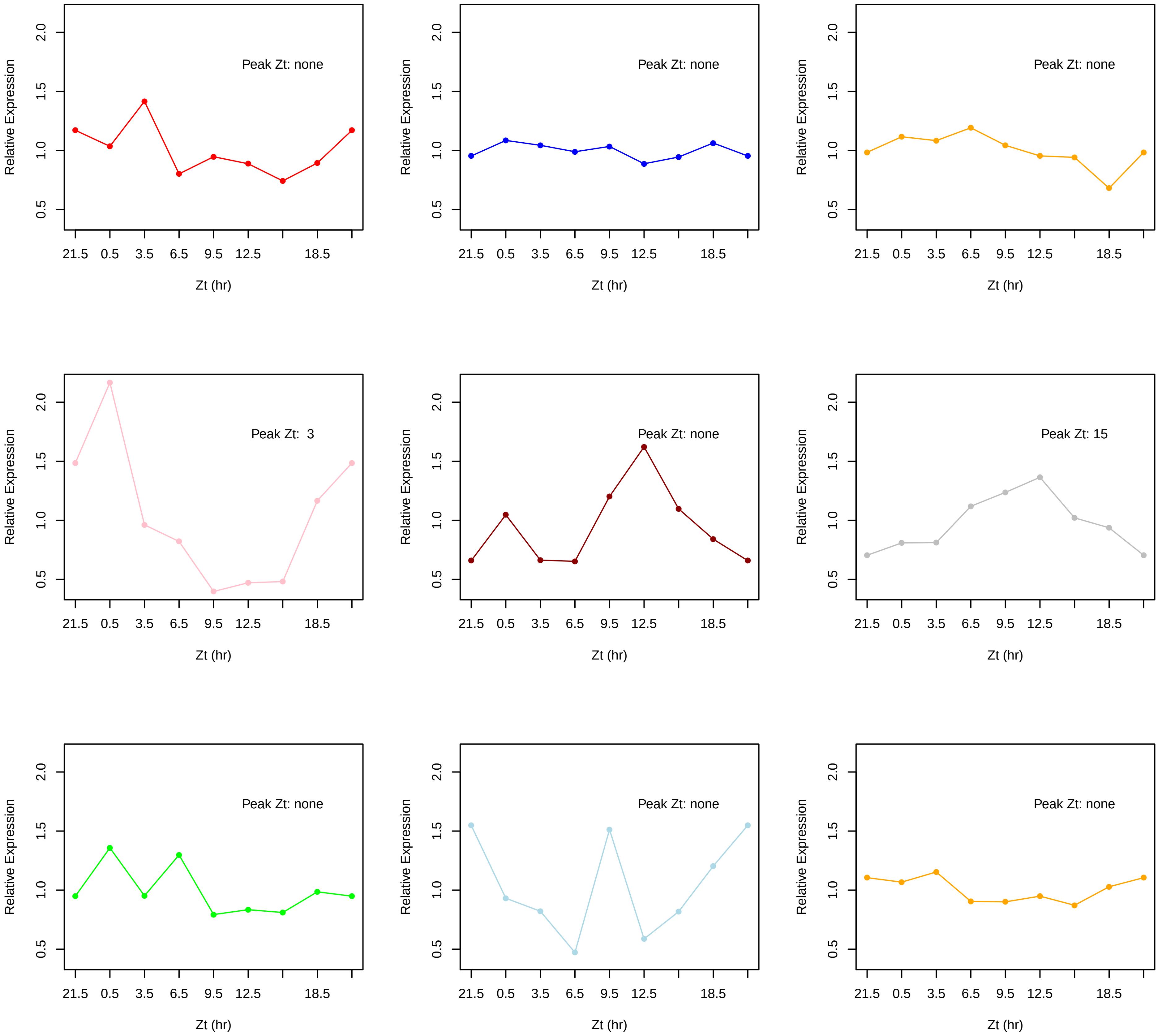

Singeleton: ENSXMAG00000011020 tbc1d4

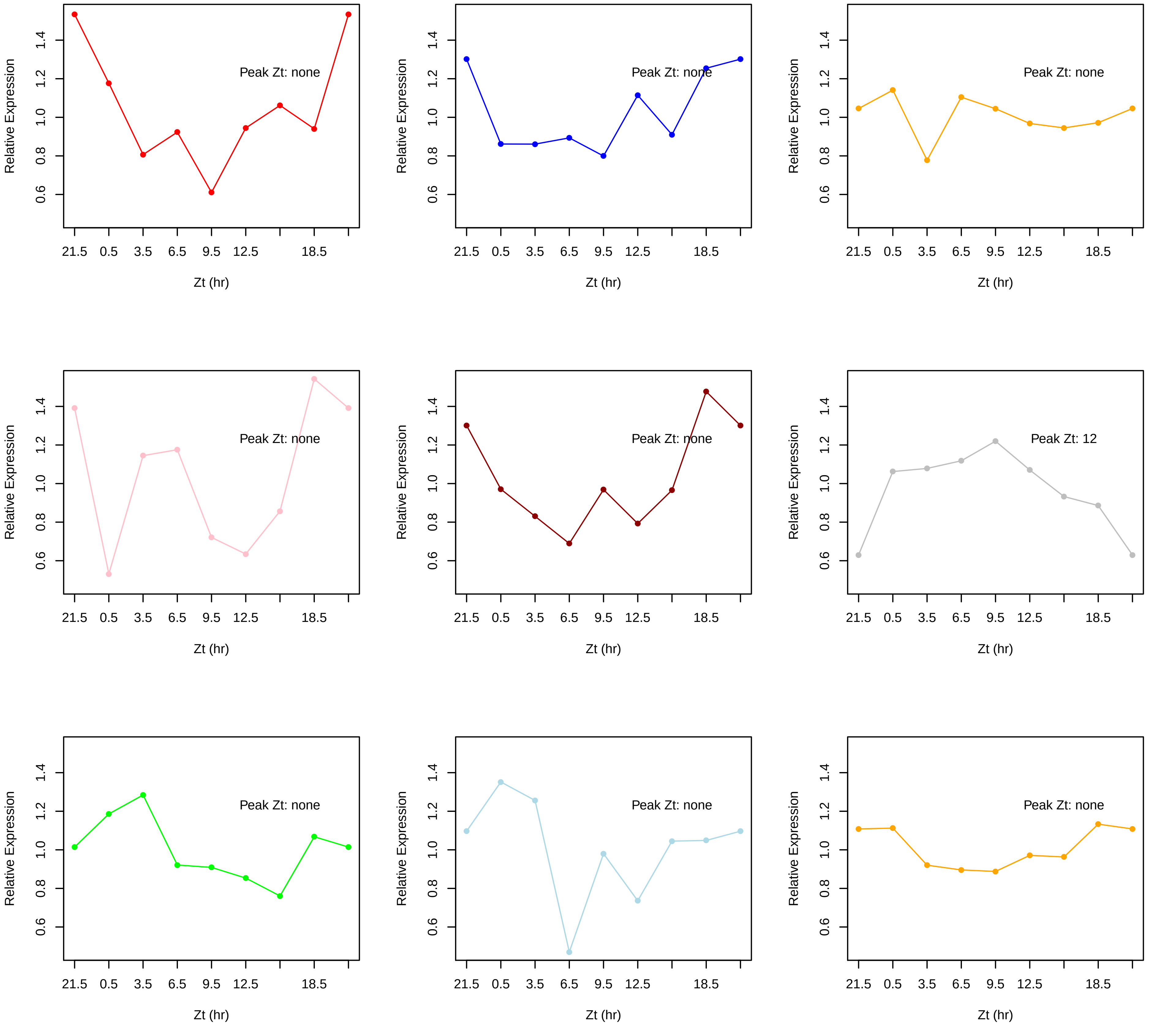

Singleton: ENSXMAG00000000631 snx13

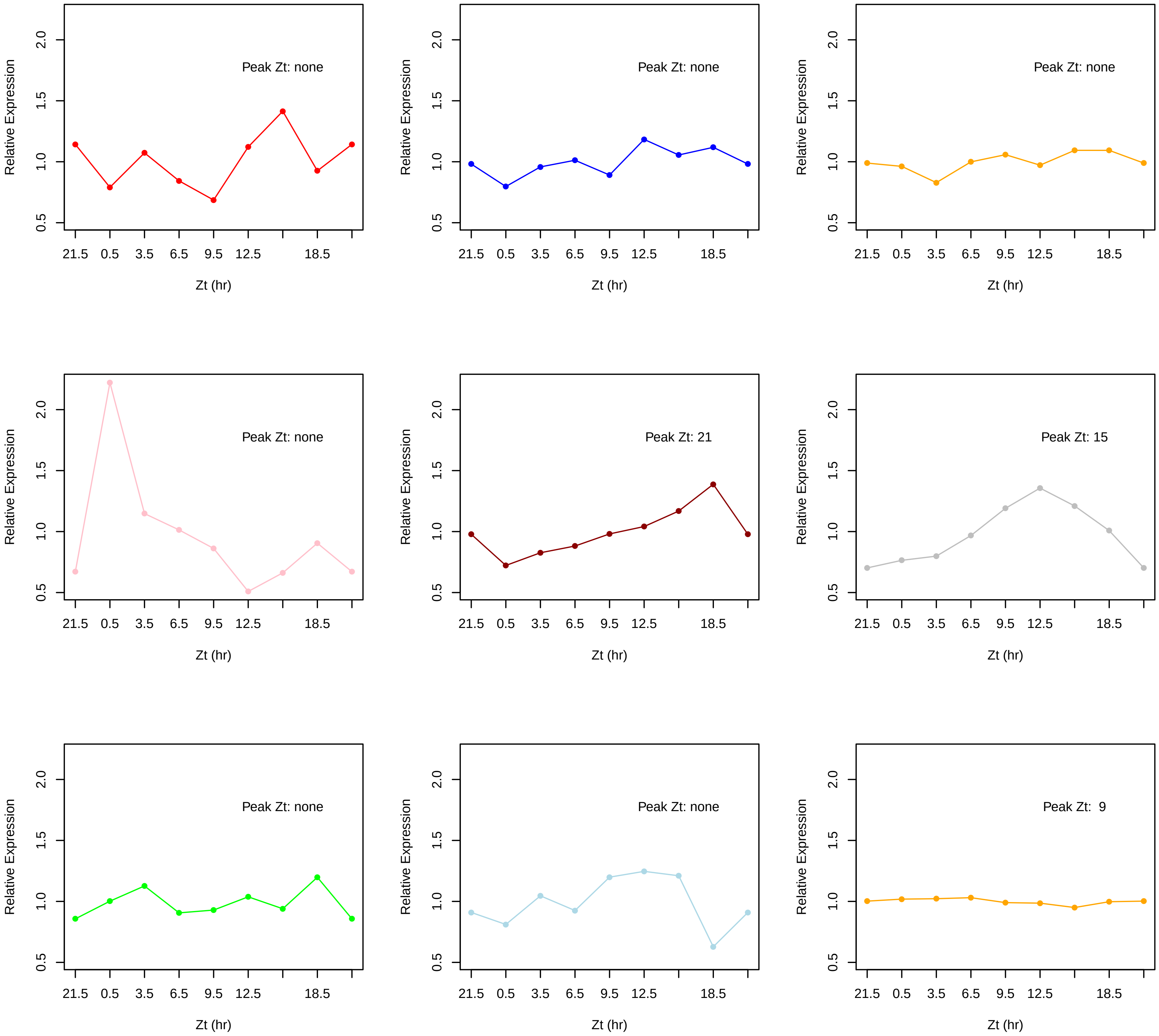

Singleton: ENSXMAG00000009956 becn1

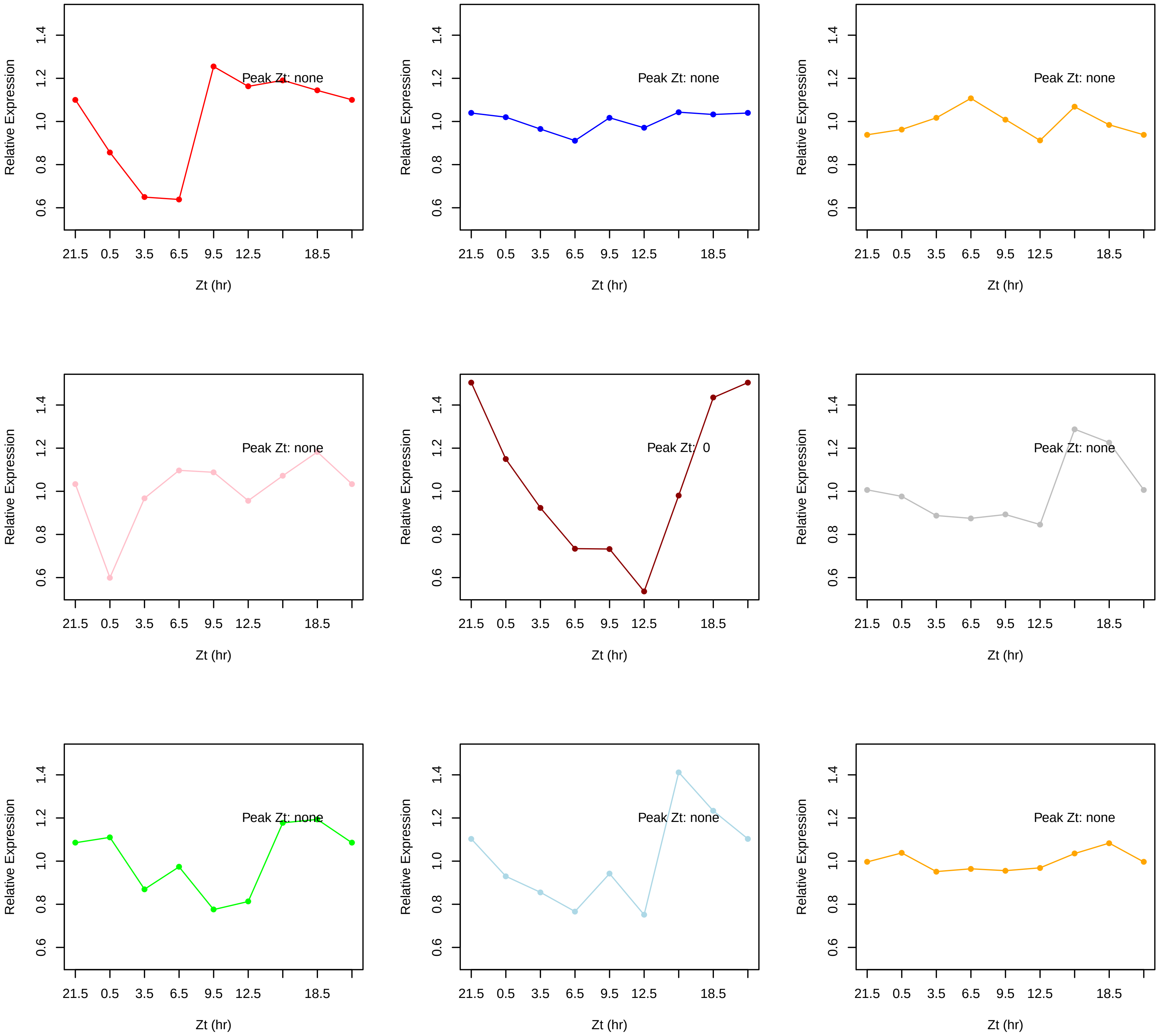

Singeleton: ENSXMAG00000005287 dab2

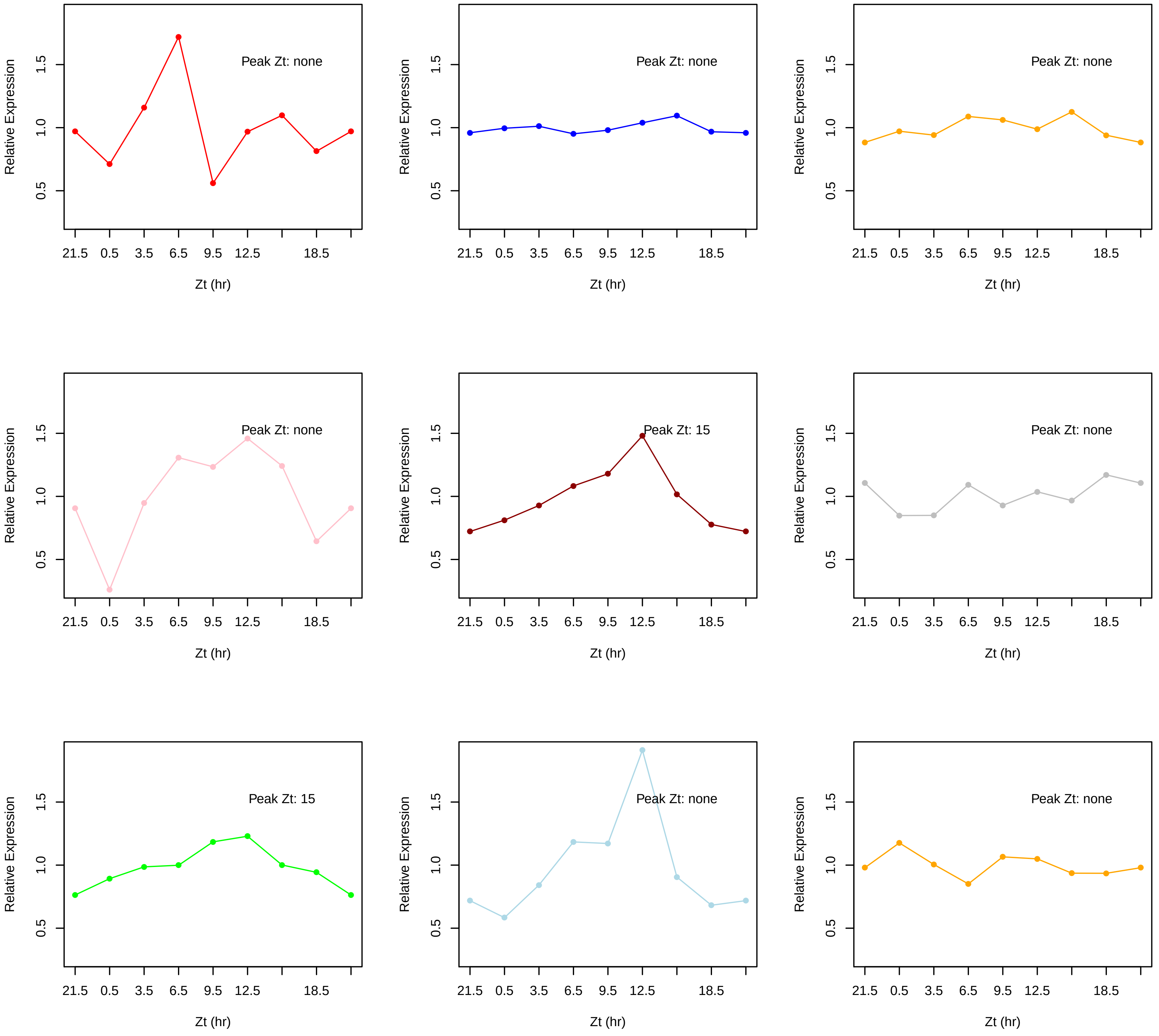

Singeleton: ENSXMAG00000011568 si:ch211−119c20.2

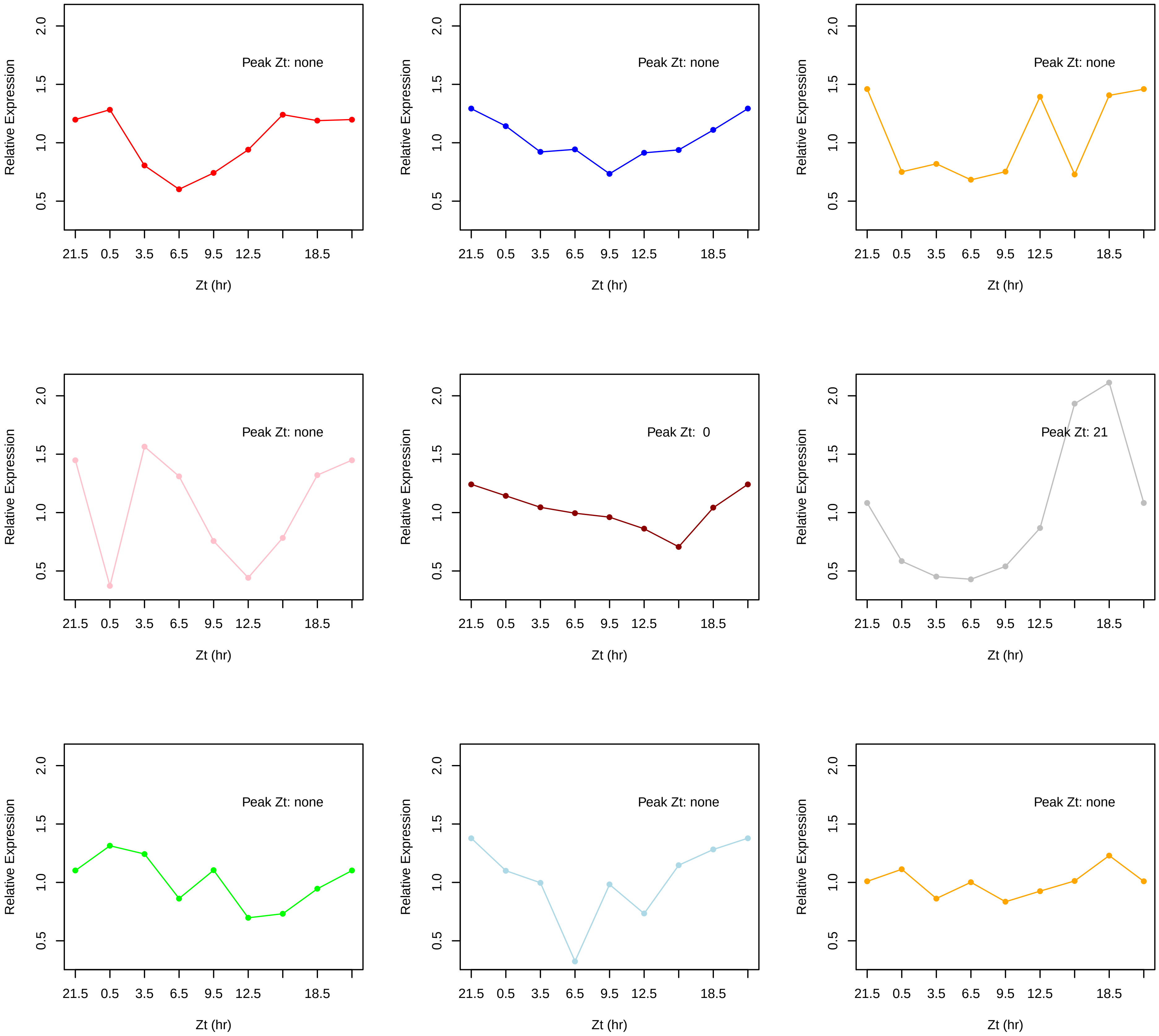

Singleton: ENSXMAG00000014756 tdrkh

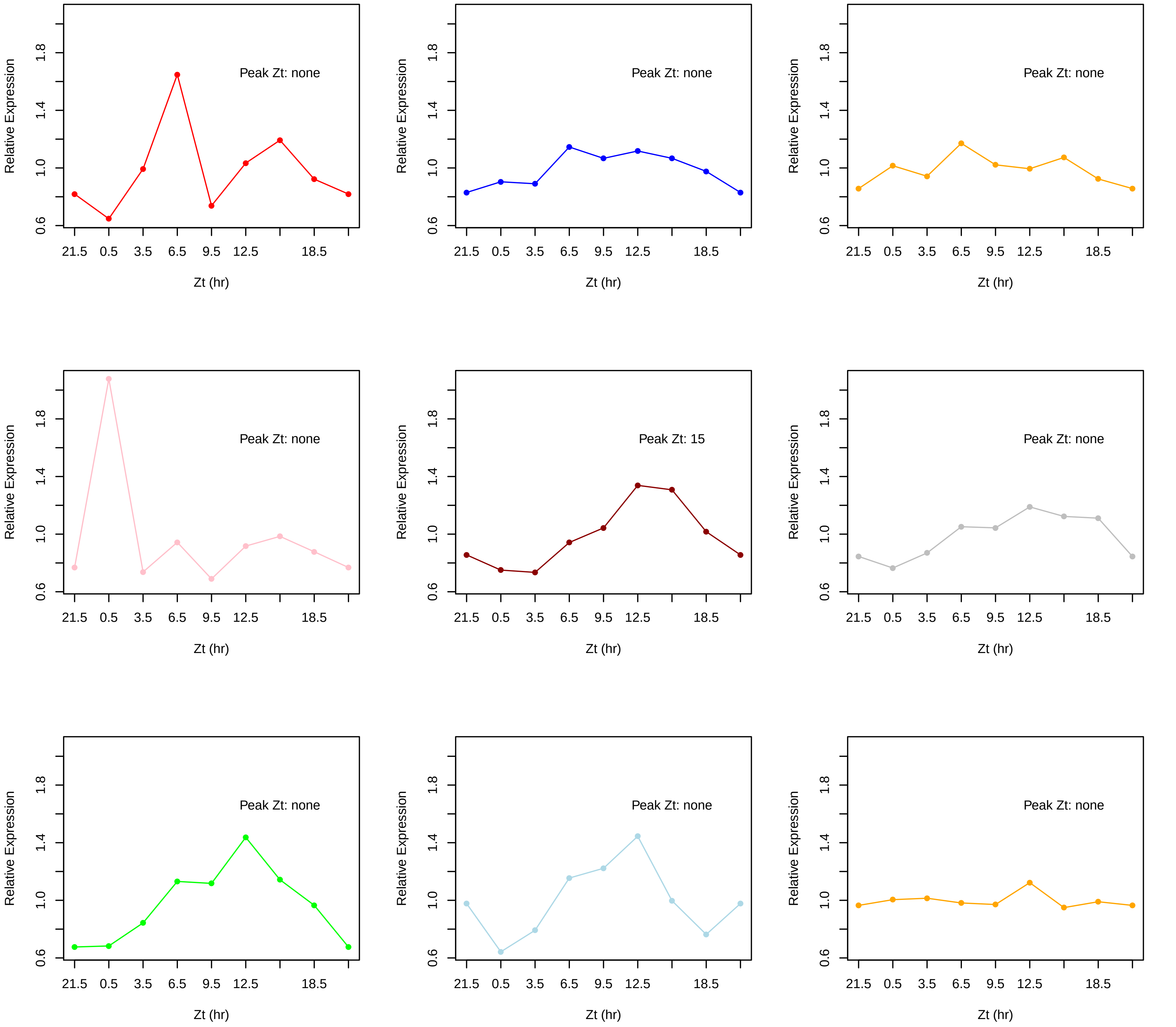

Singeleton: ENSXMAG00000011048 commd6

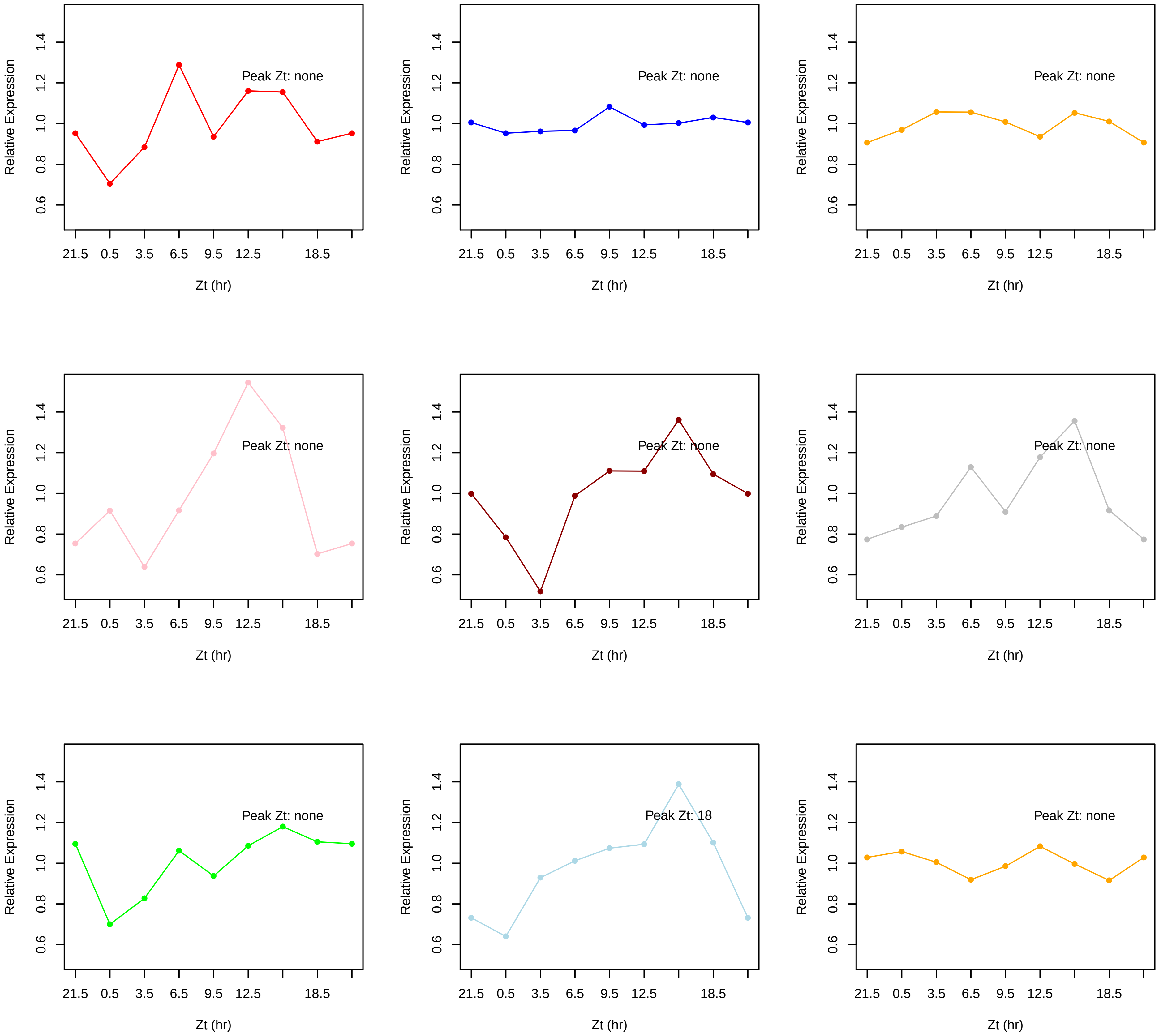

Singleton: ENSXMAG00000004113 rbbp5

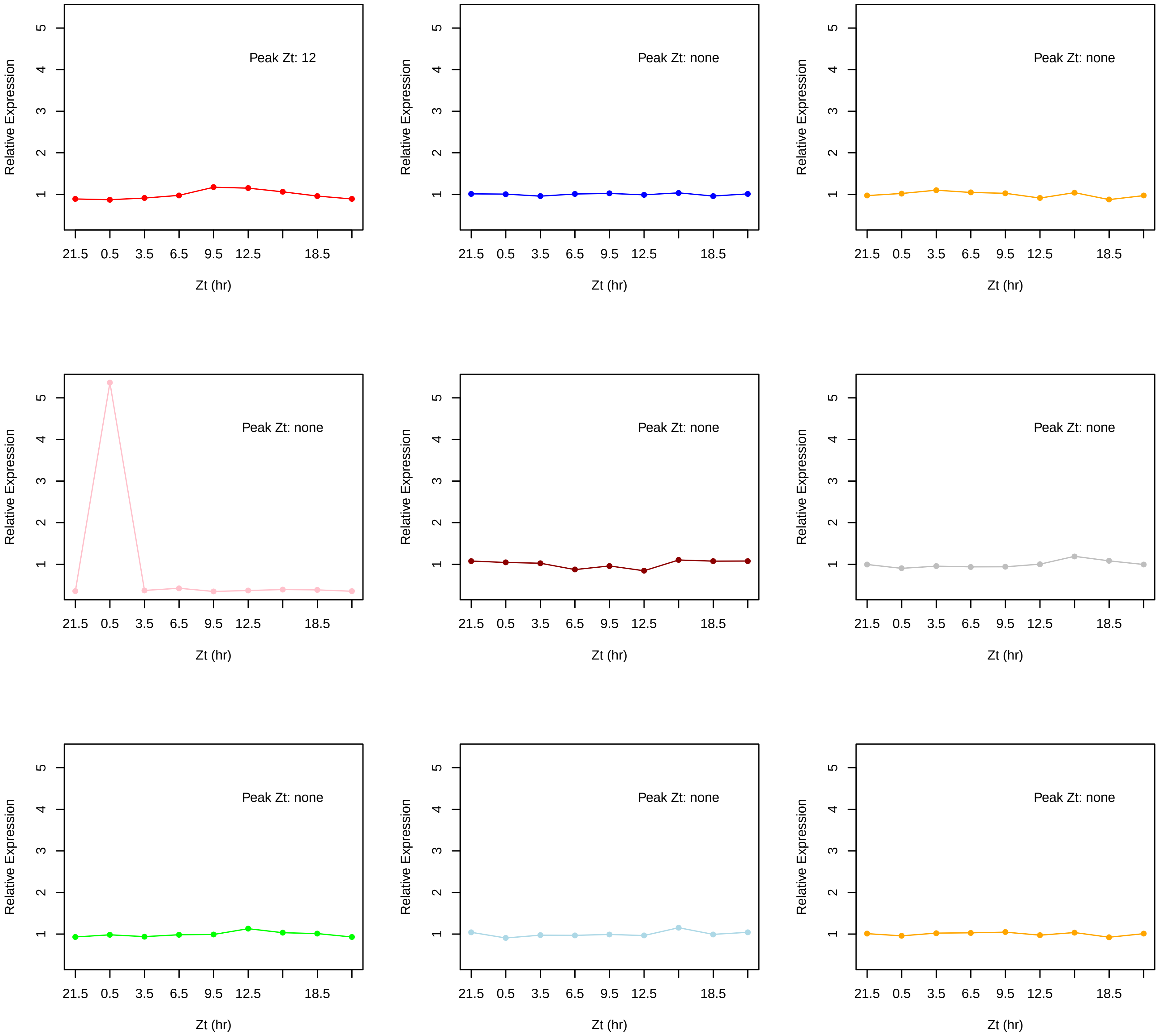

Singeleton: ENSXMAG00000002868 si:dkey−84j12.1

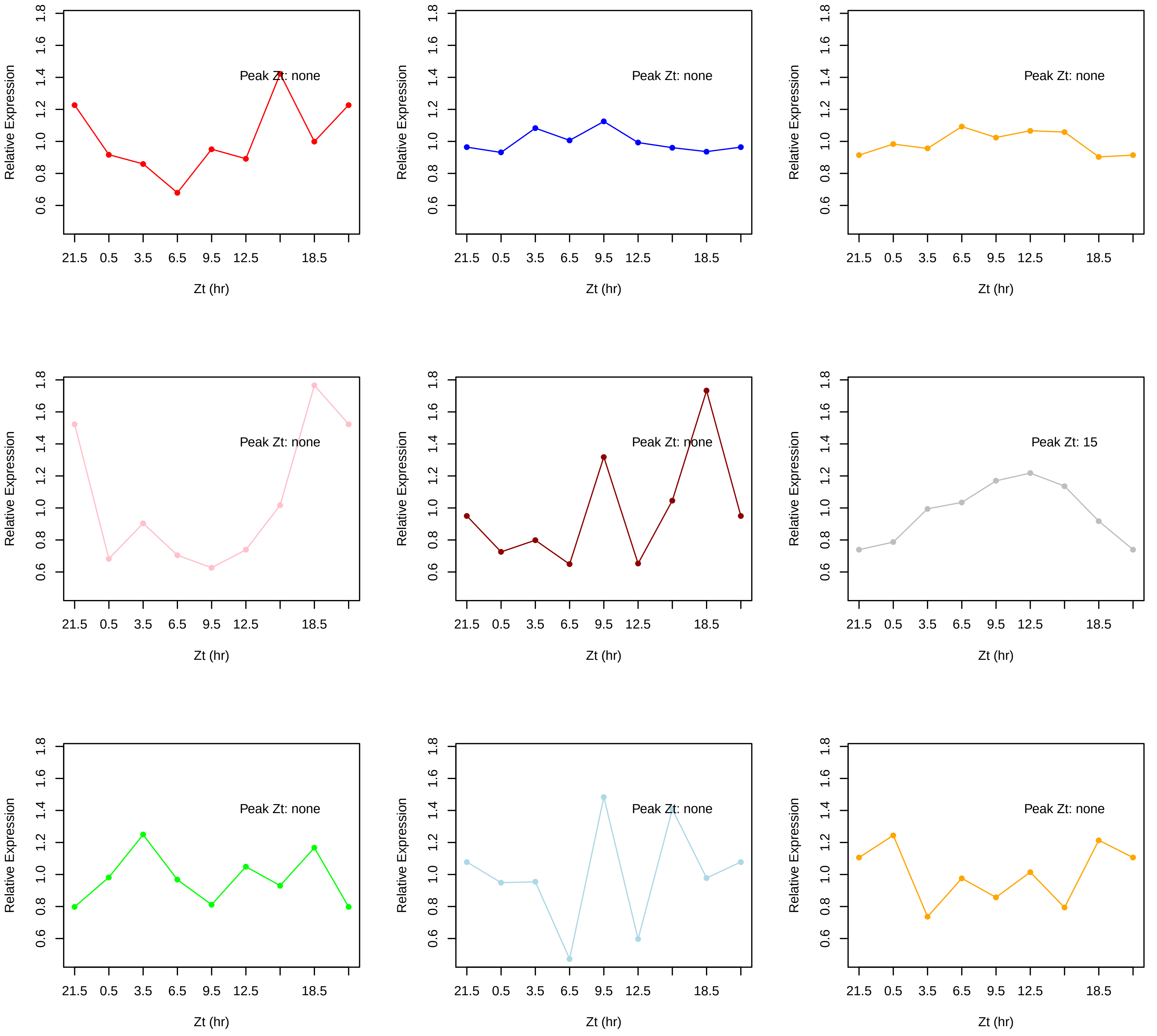

Singleton: ENSXMAG00000013617

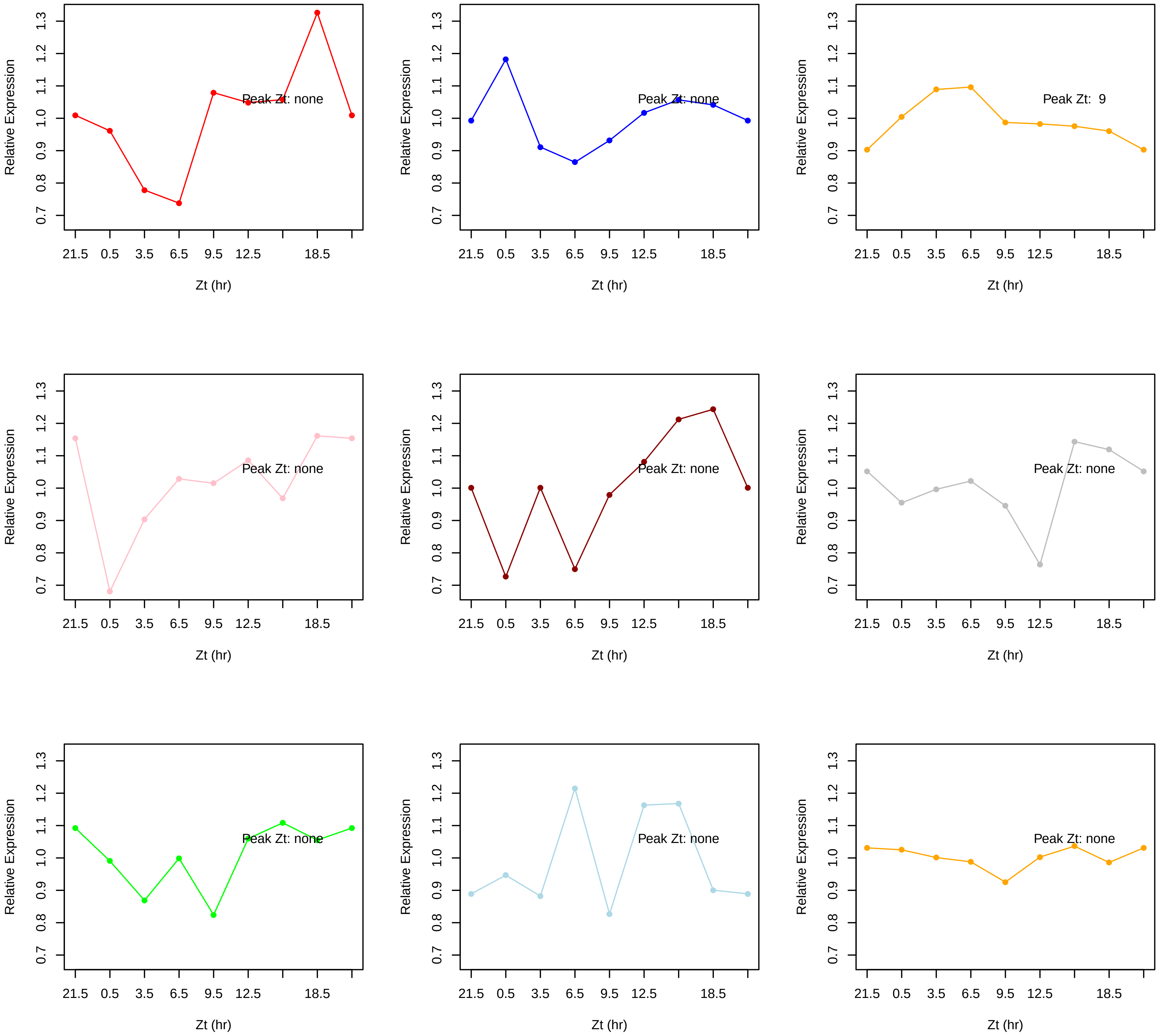

Singleton: ENSXMAG00000011049 uchl3

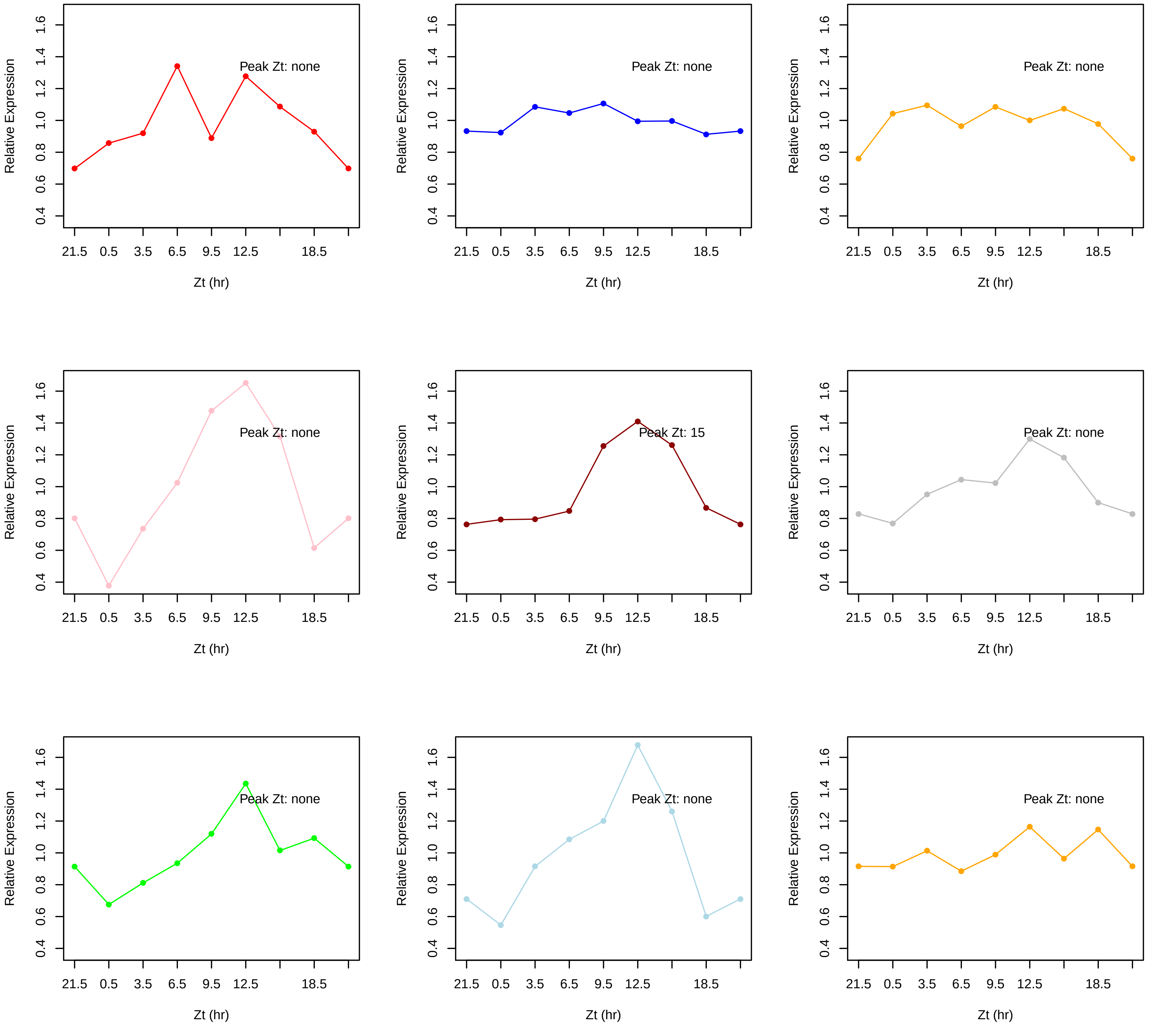

Singeleton: ENSXMAG00000002878 tars

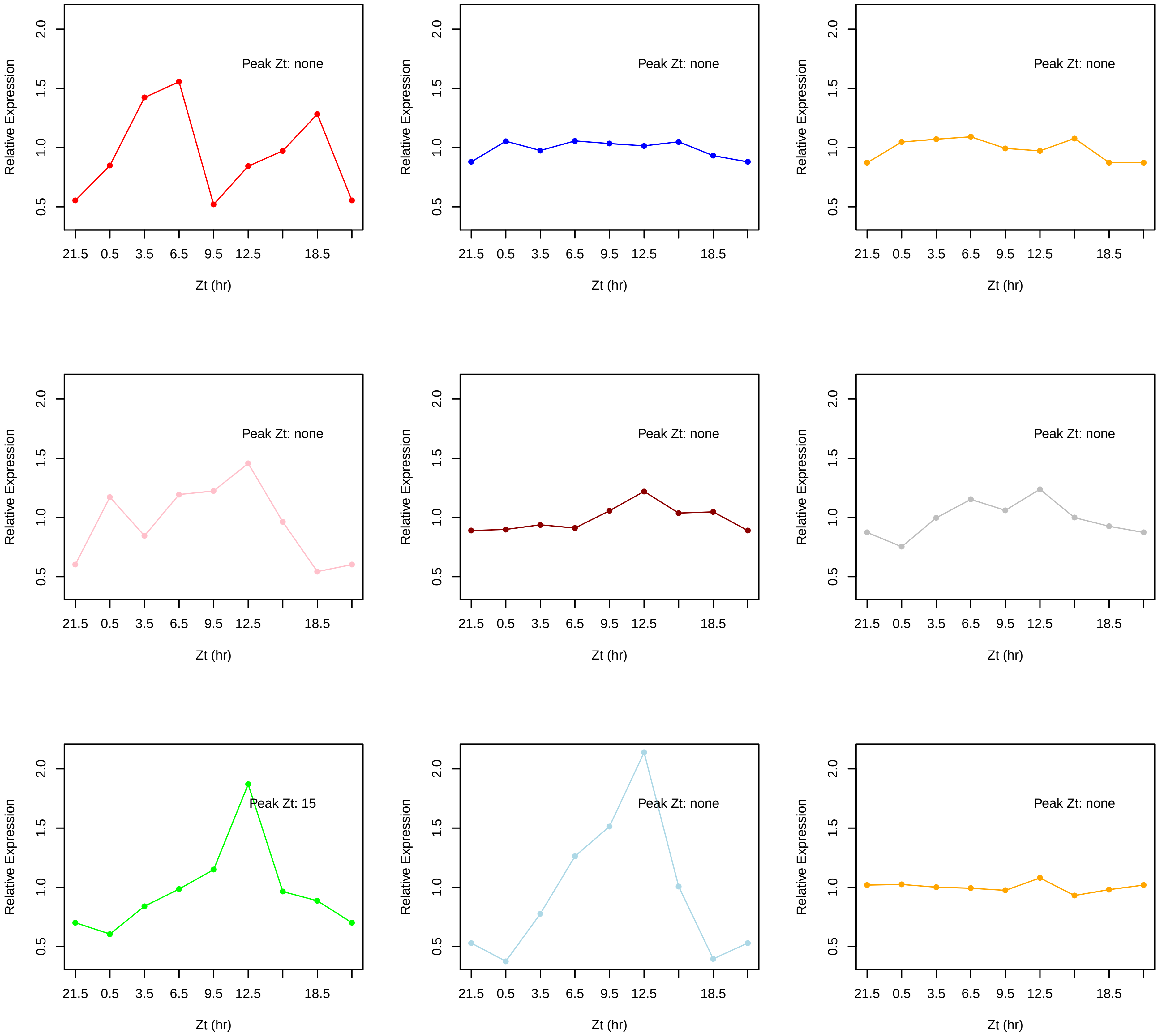

Singleton: ENSXMAG00000015150 cox18

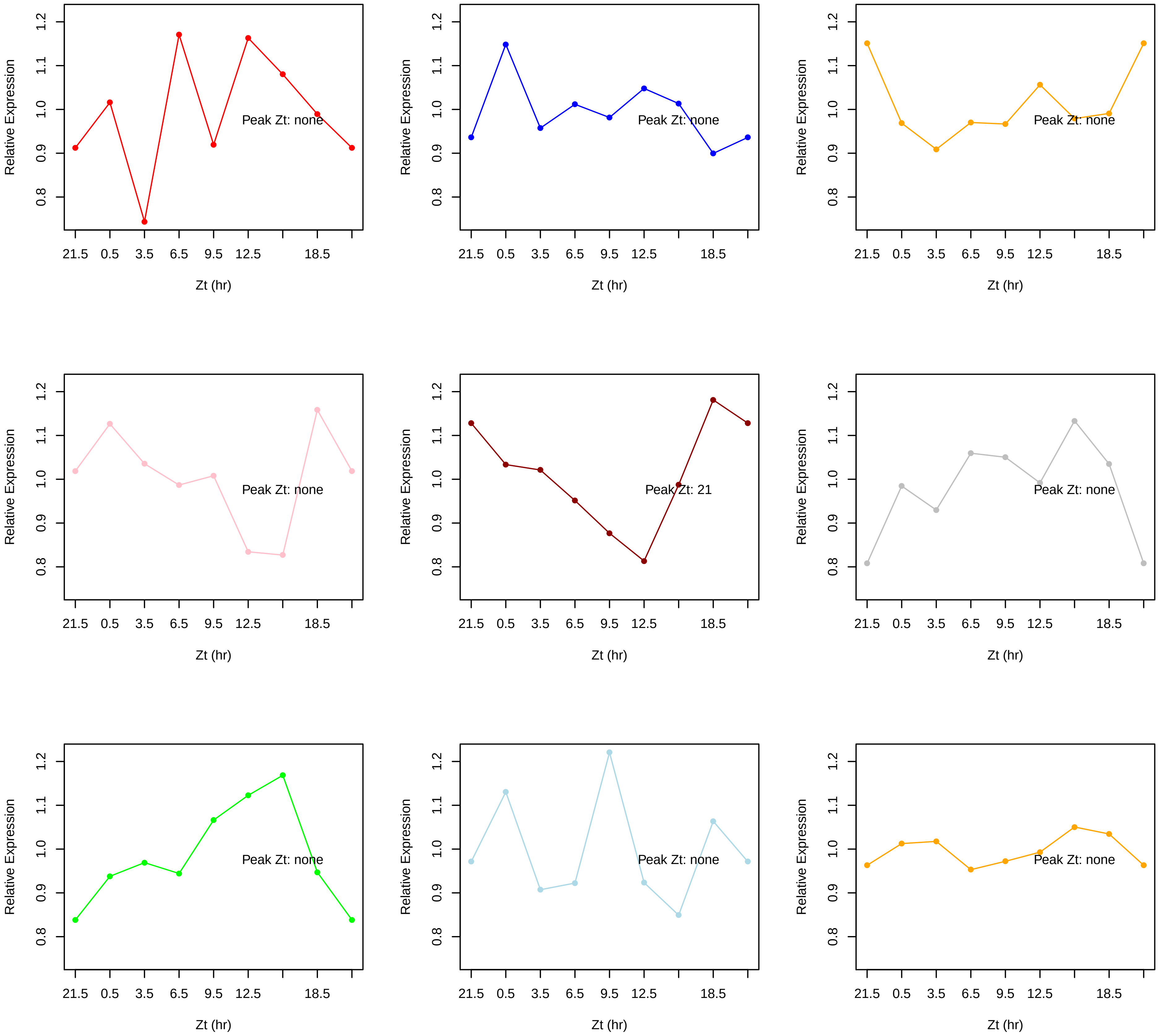

Singeleton: ENSXMAG00000027915 tial1

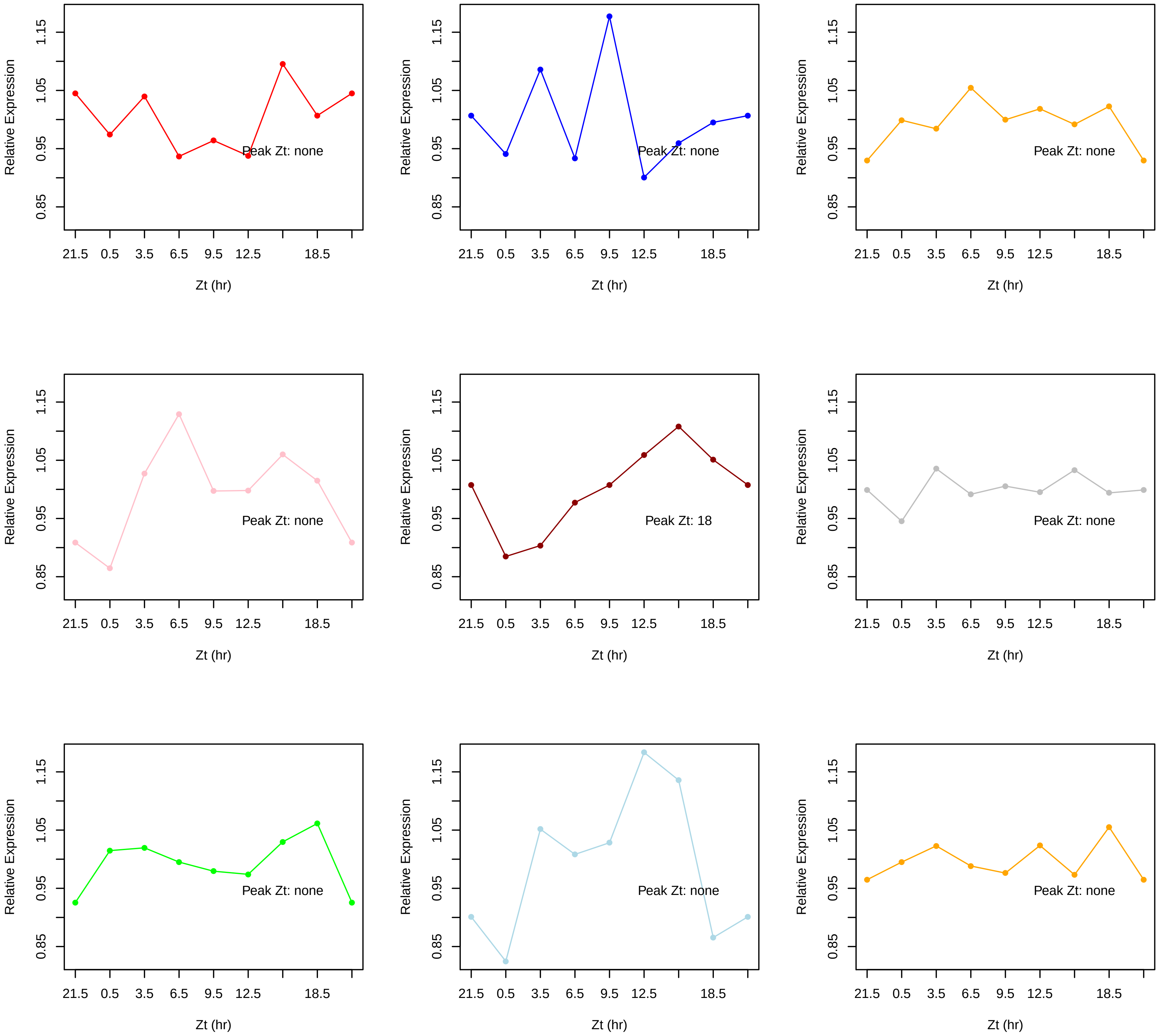

Singeleton: ENSXMAG00000003252 pcdh12

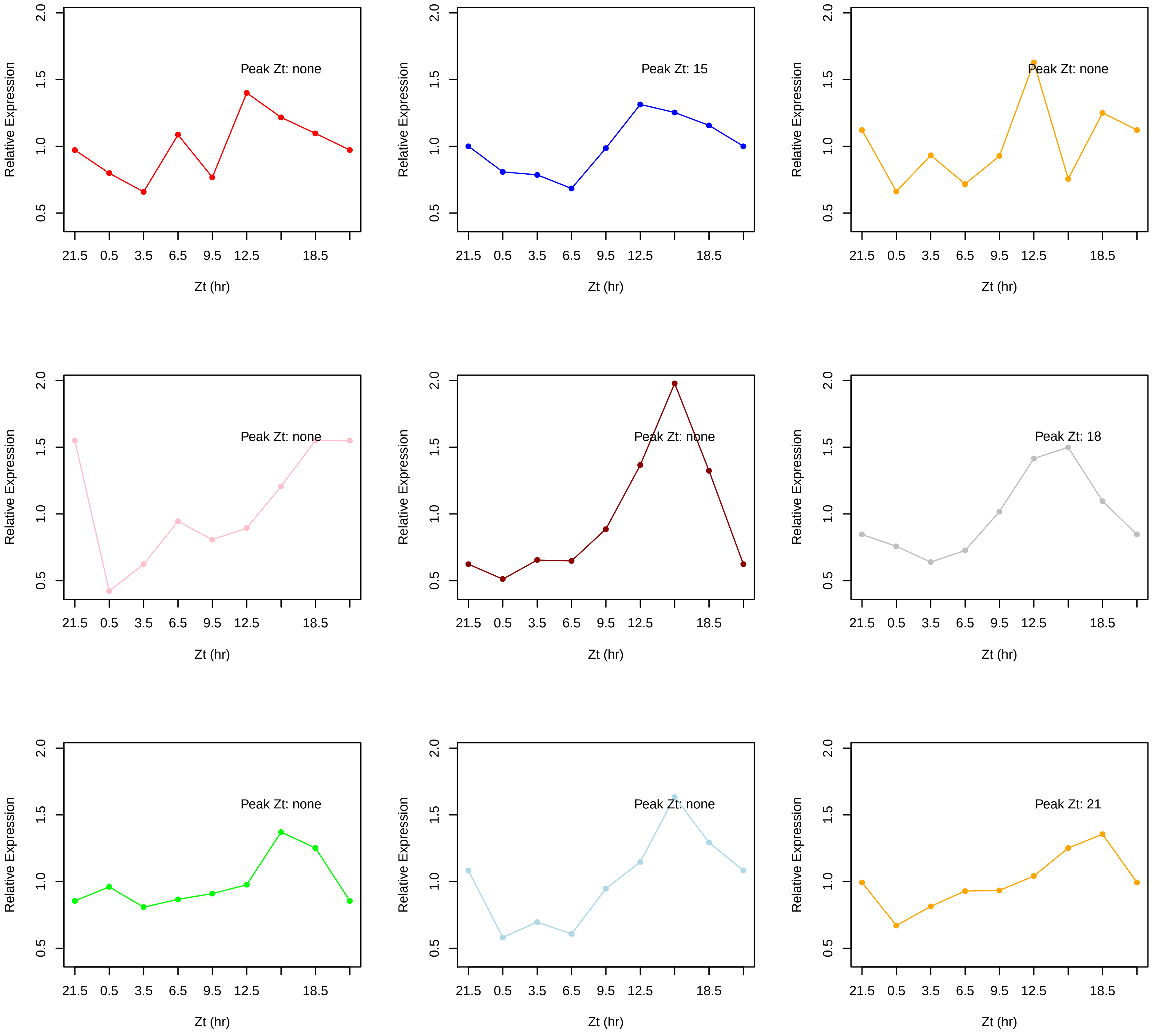

Singeleton: ENSXMAG00000016063 ltc4s

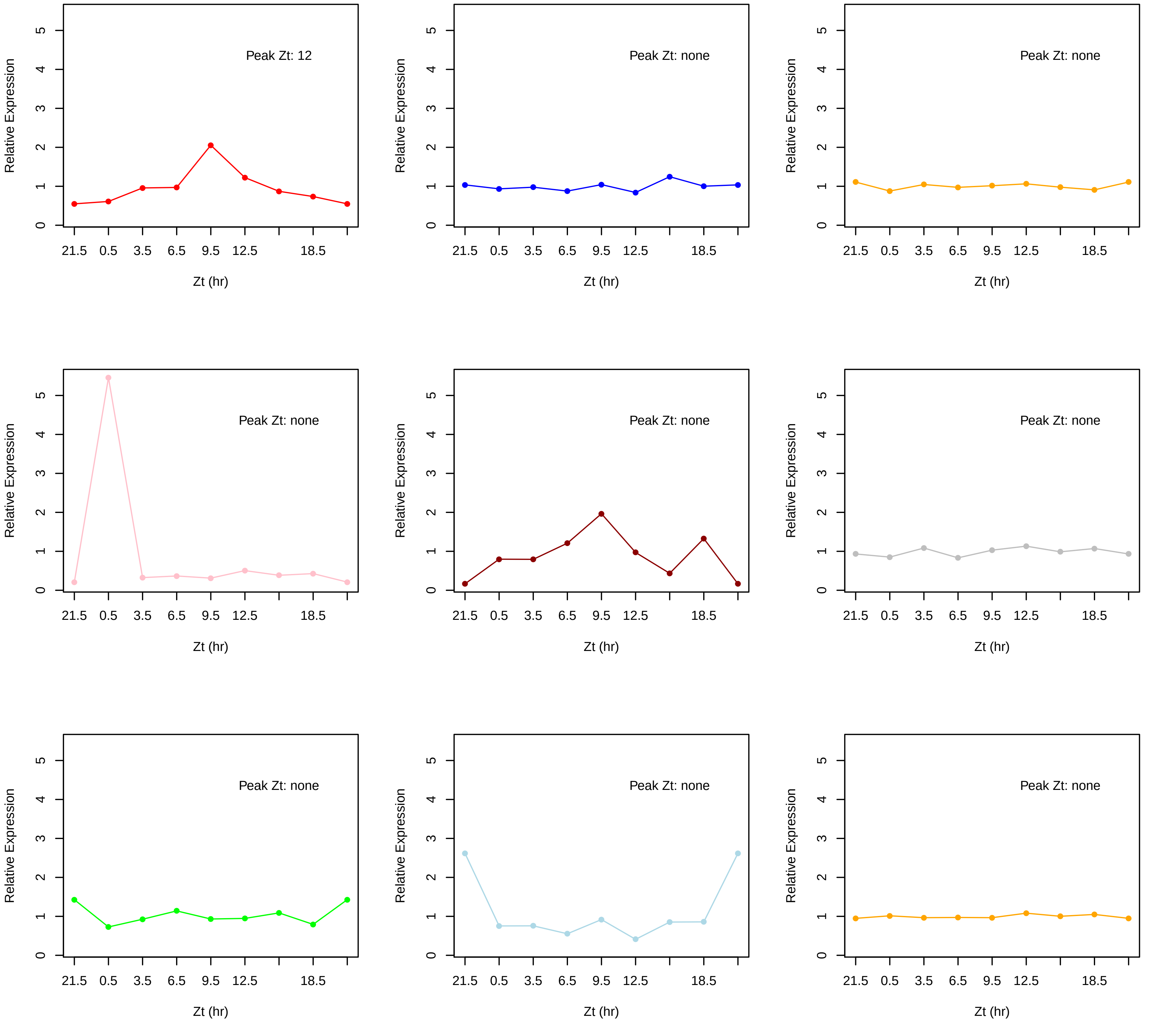

Singeleton: ENSXMAG00000009278 cry1ba

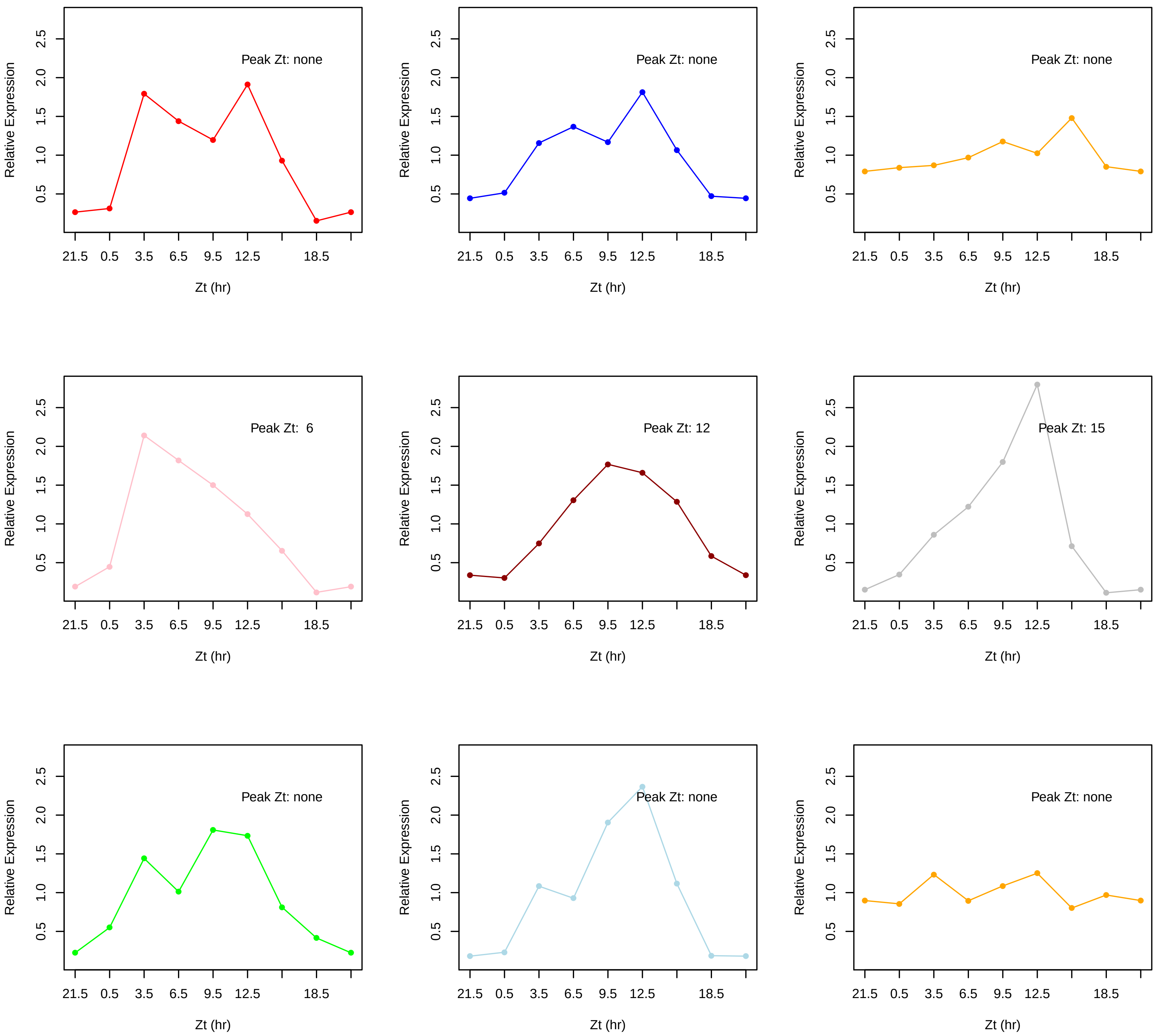

Singleton: ENSXMAG00000015491 dapk1

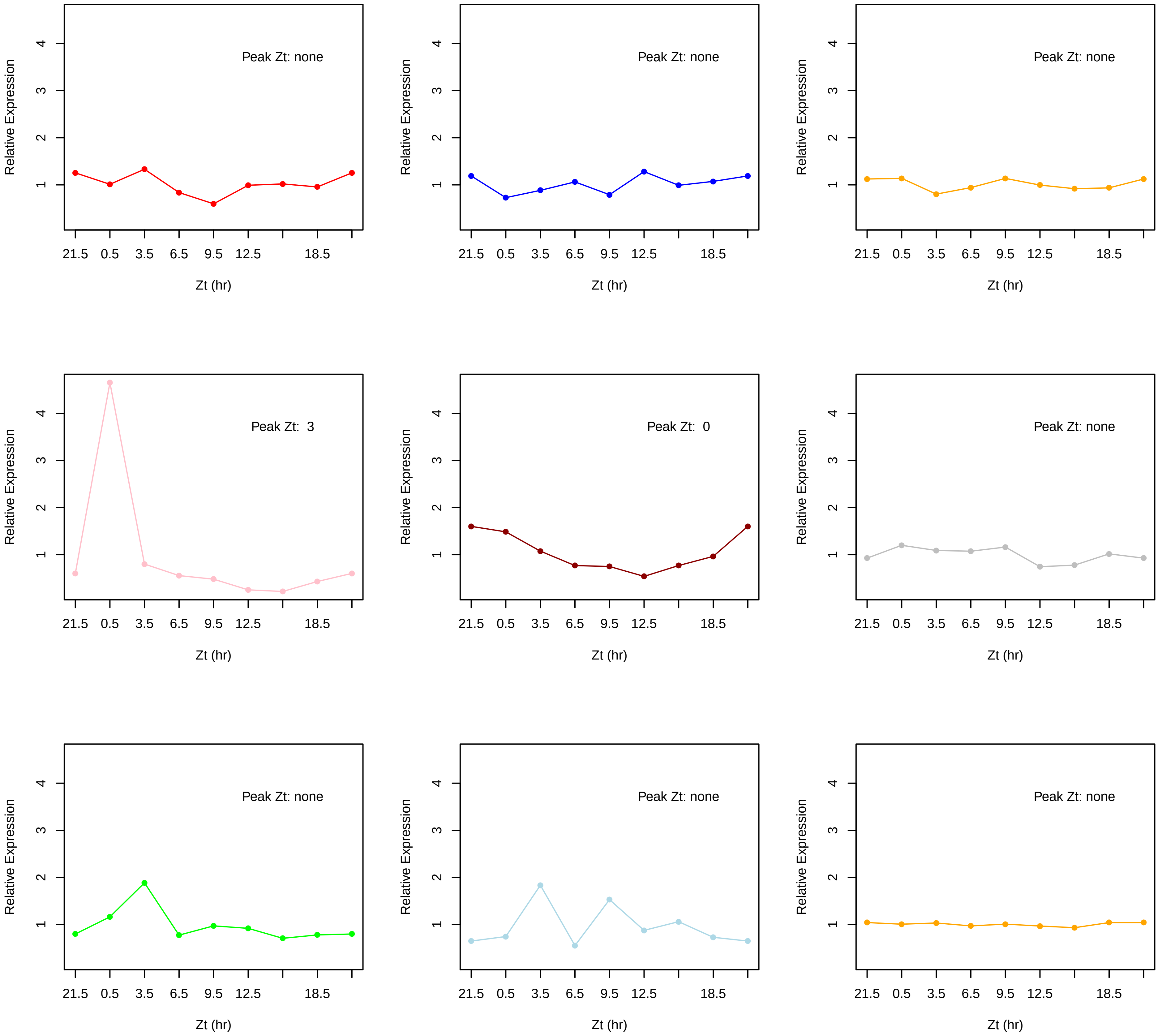

Singeleton: ENSXMAG00000015282 polr2b

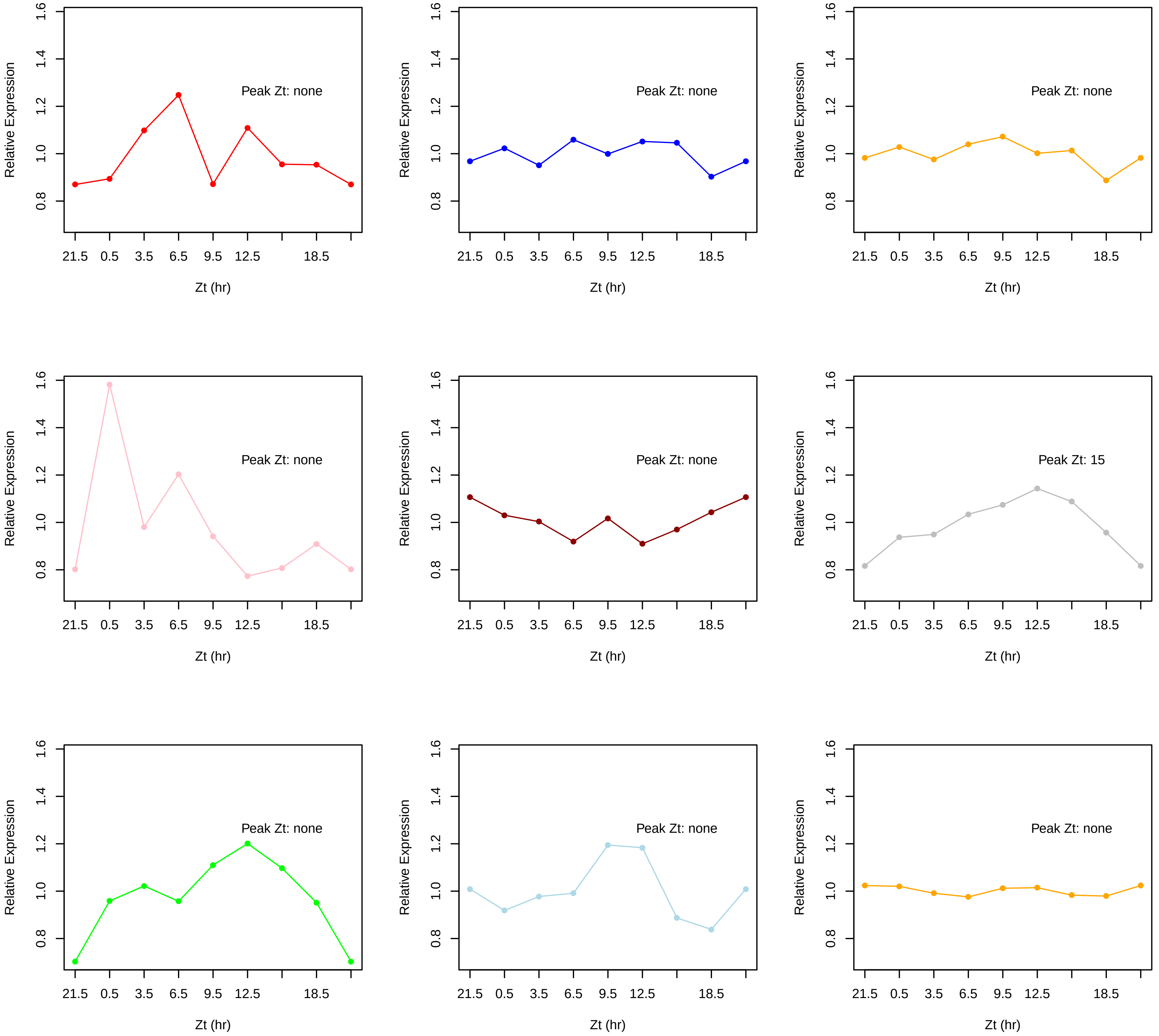

Singeleton: ENSXMAG00000022860

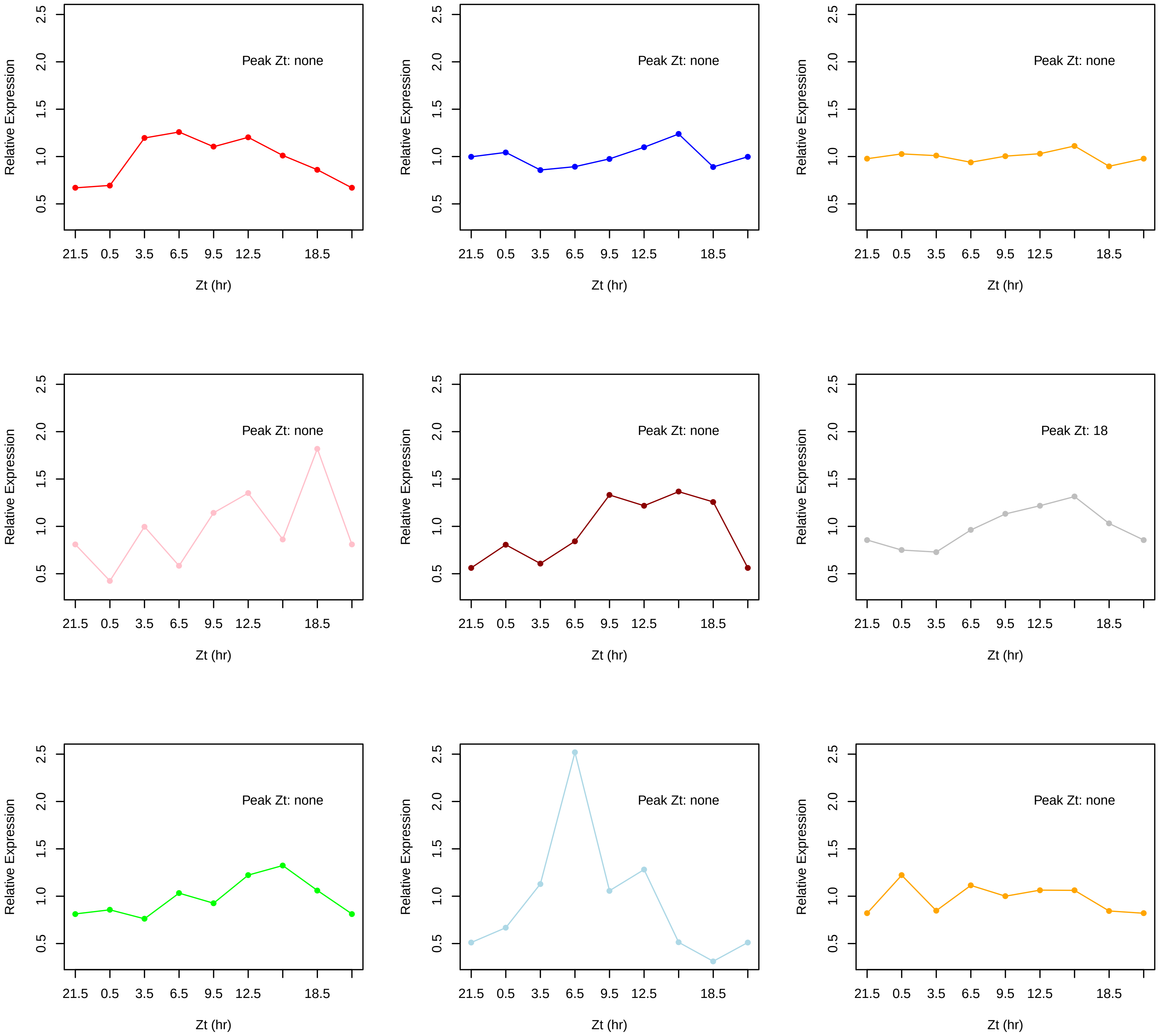

Singleton: ENSXMAG00000006780 epdr1

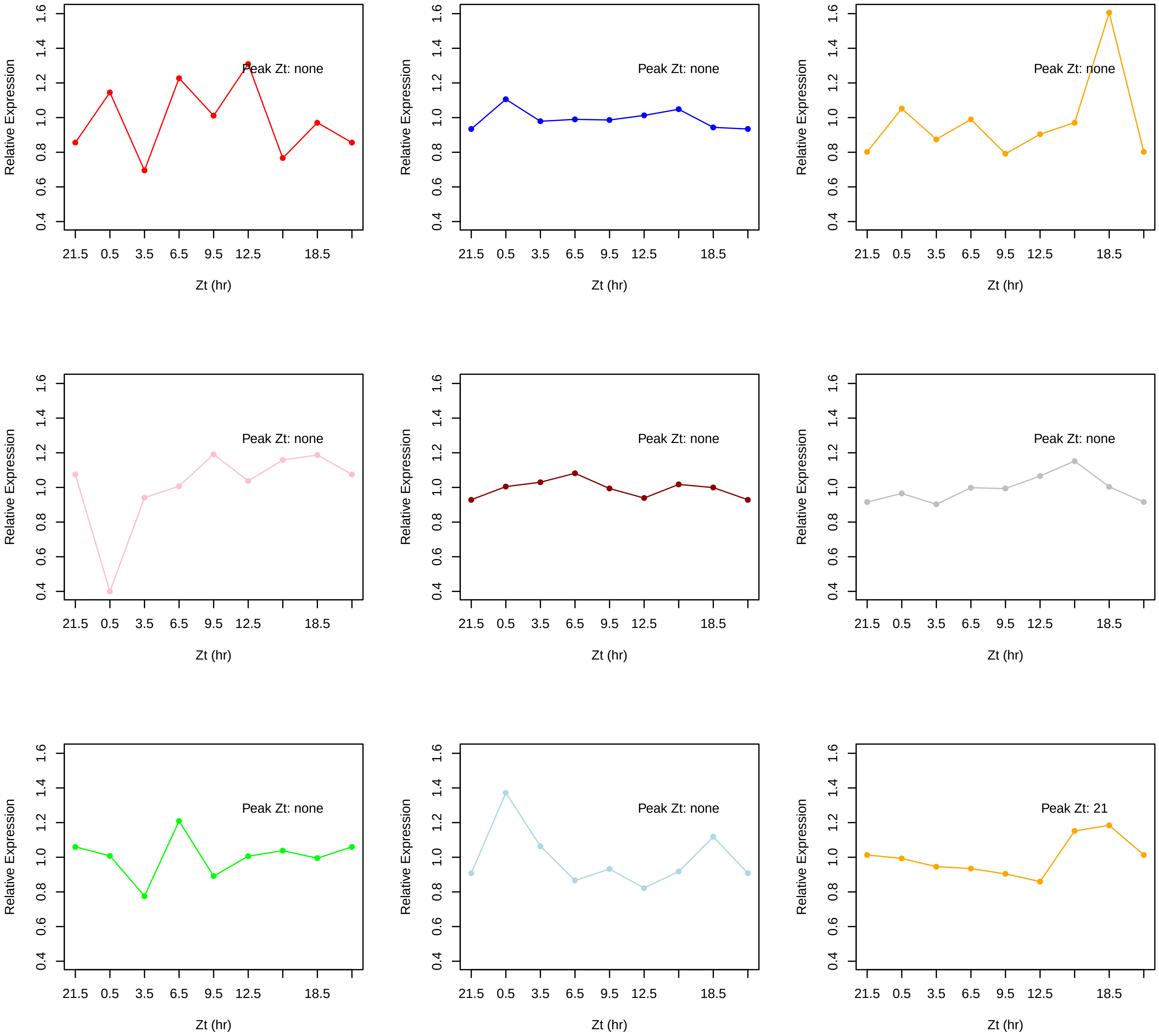

Singeleton: ENSXMAG00000016067 canx

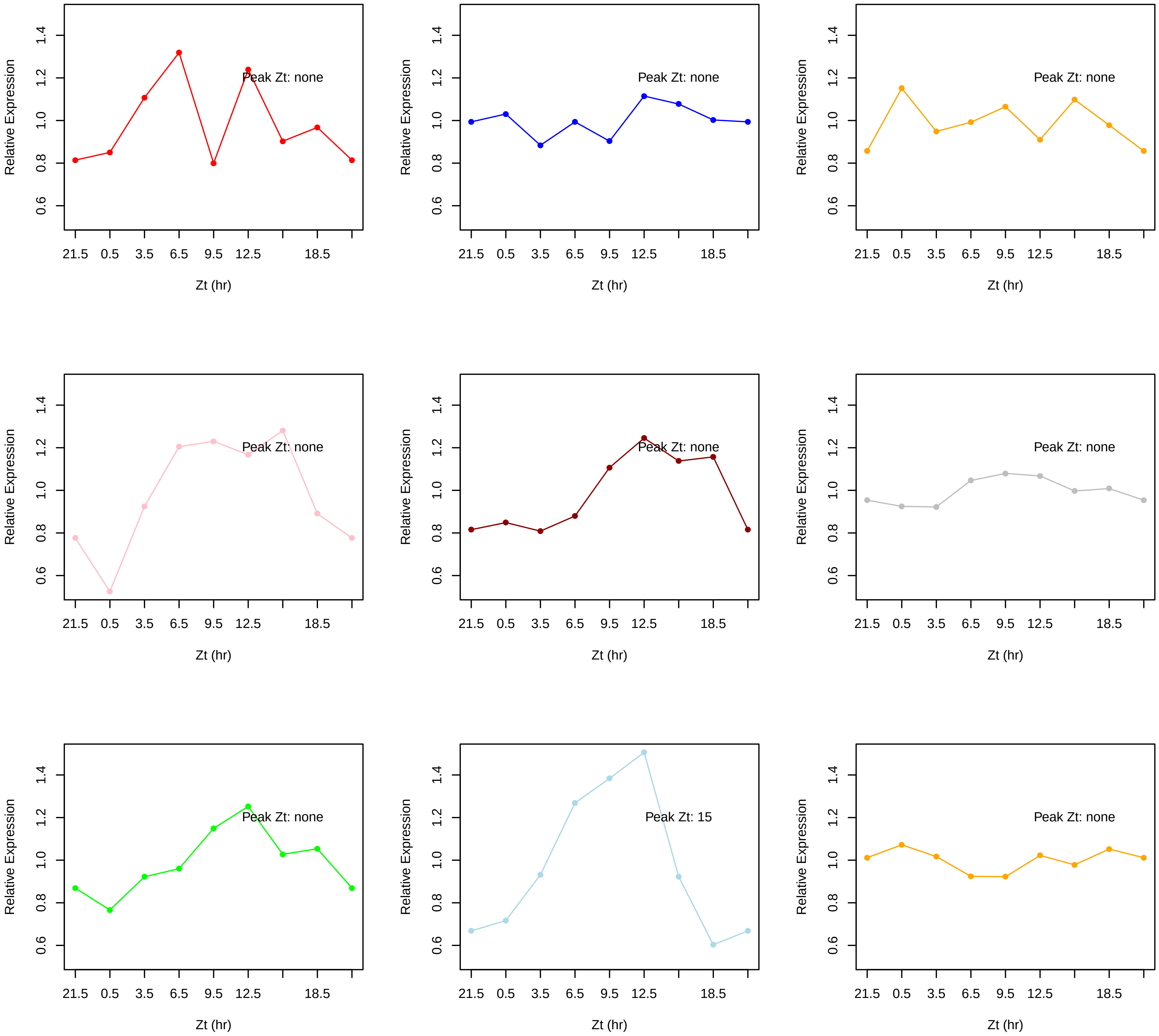

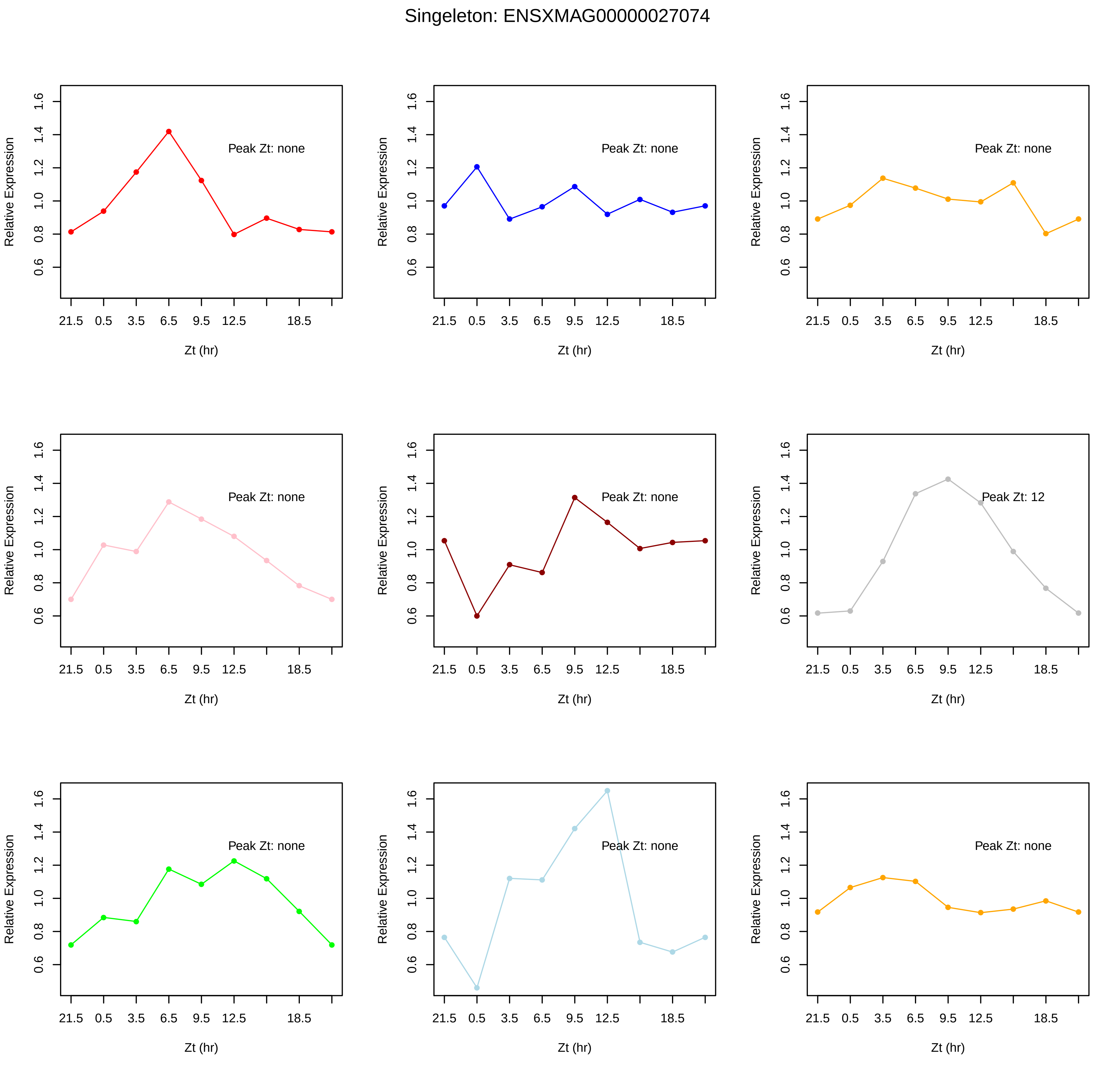
Singeleton: ENSXMAG00000027074
Peak Zt: none
Peak Zt: none
Peak Zt: none
Peak Zt: none
Peak Zt: none
Peak Zt: 12
Peak Zt: none
Peak Zt: none
Peak Zt: none
Relative Expression
Zt (hr)
21.5
0.5
3.5
6.5
9.5
12.5
18.5
0.6
0.8
1.0
1.2
1.4
1.6

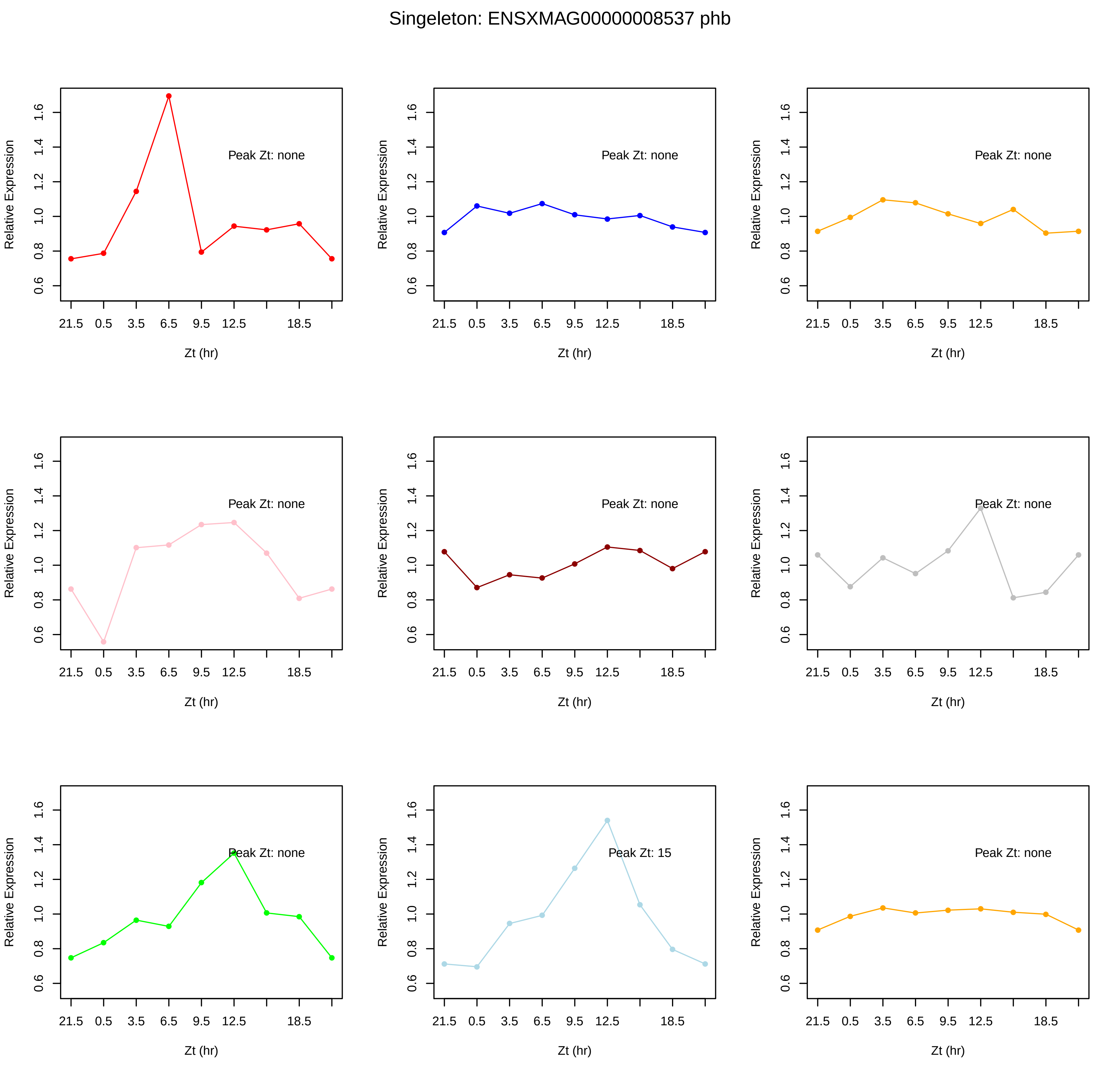
Singeleton: ENSXMAG00000008537 phb
Relative Expression
0.6
0.8
1.0
1.2
1.4
1.6
Zt (hr)
21.5
0.5
3.5
6.5
9.5
12.5
18.5
Peak Zt: none
Peak Zt: 15

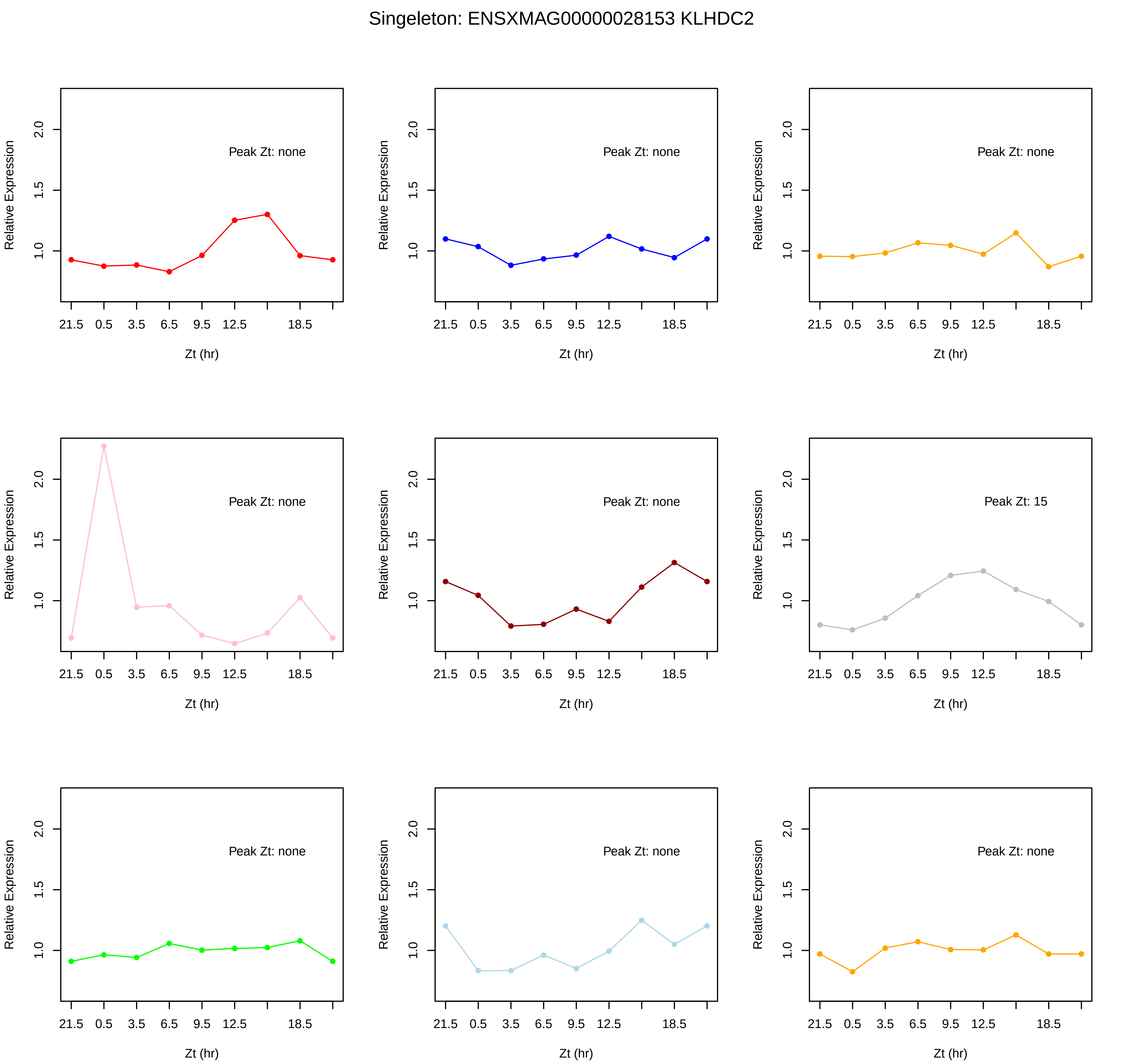
Singeleton: ENSXMAG00000028153 KLHDC2
Peak Zt: none
Peak Zt: none
Peak Zt: none
Peak Zt: none
Peak Zt: none
Peak Zt: 15
Peak Zt: none
Peak Zt: none
Peak Zt: none
Relative Expression
Zt (hr)
21.5
0.5
3.5
6.5
9.5
12.5
18.5
1.0
1.5
2.0

Singeleton: ENSXMAG00000008011 ncf4

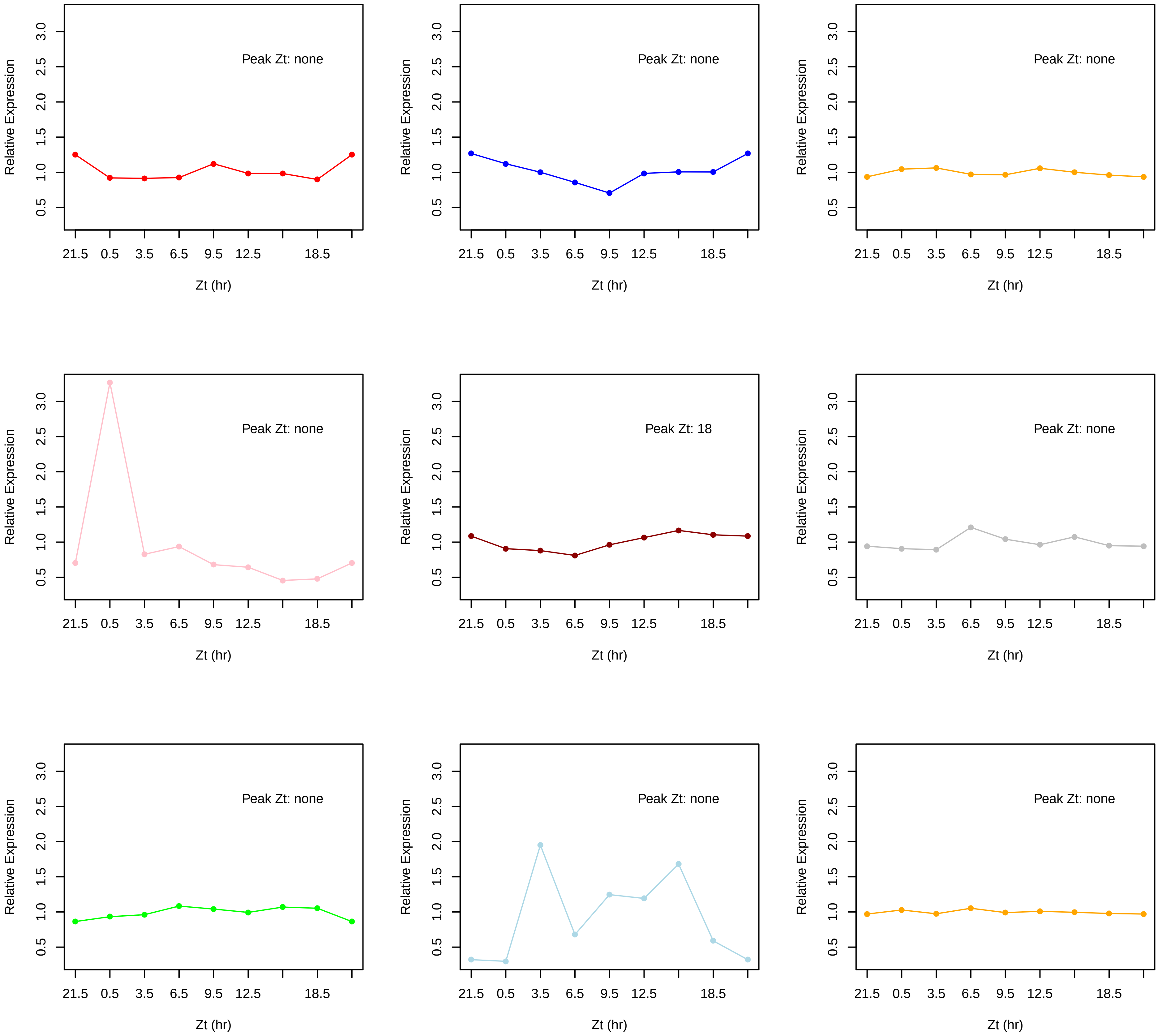

Singeleton: ENSXMAG00000015489 mgat4b

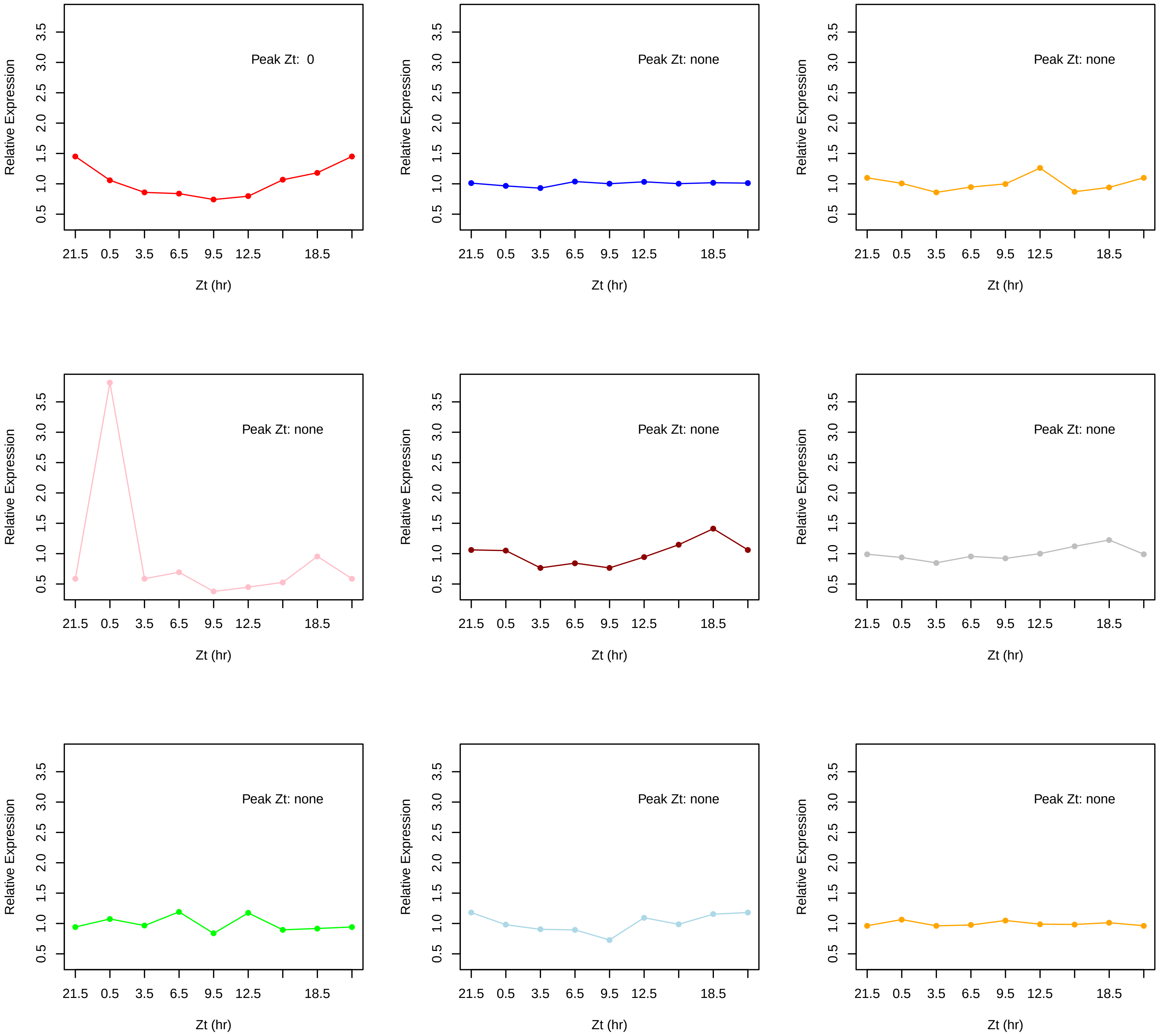

Singeleton: ENSXMAG00000008515 znf652

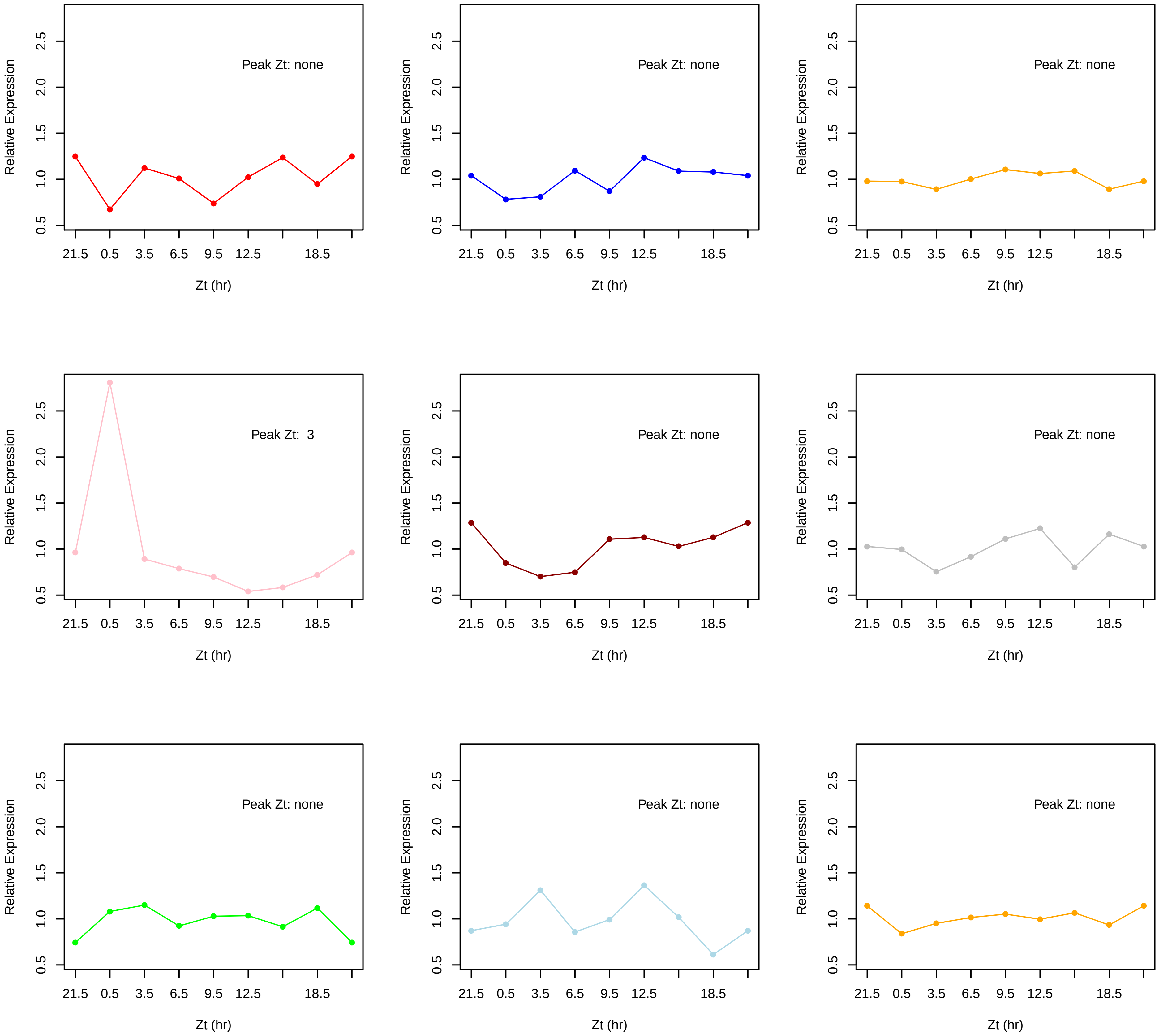

Singleton: ENSXMAG00000009024 NEMF

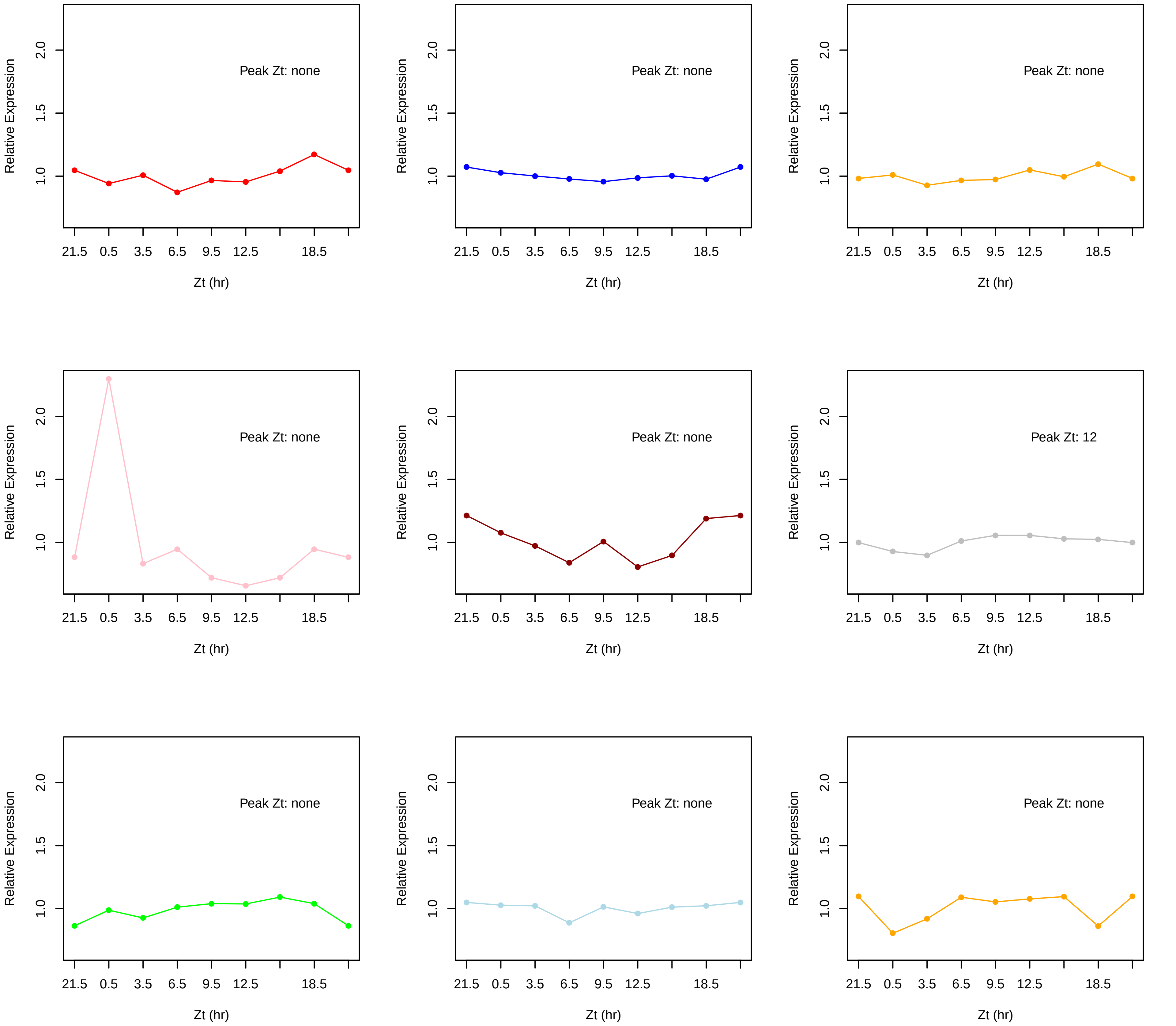

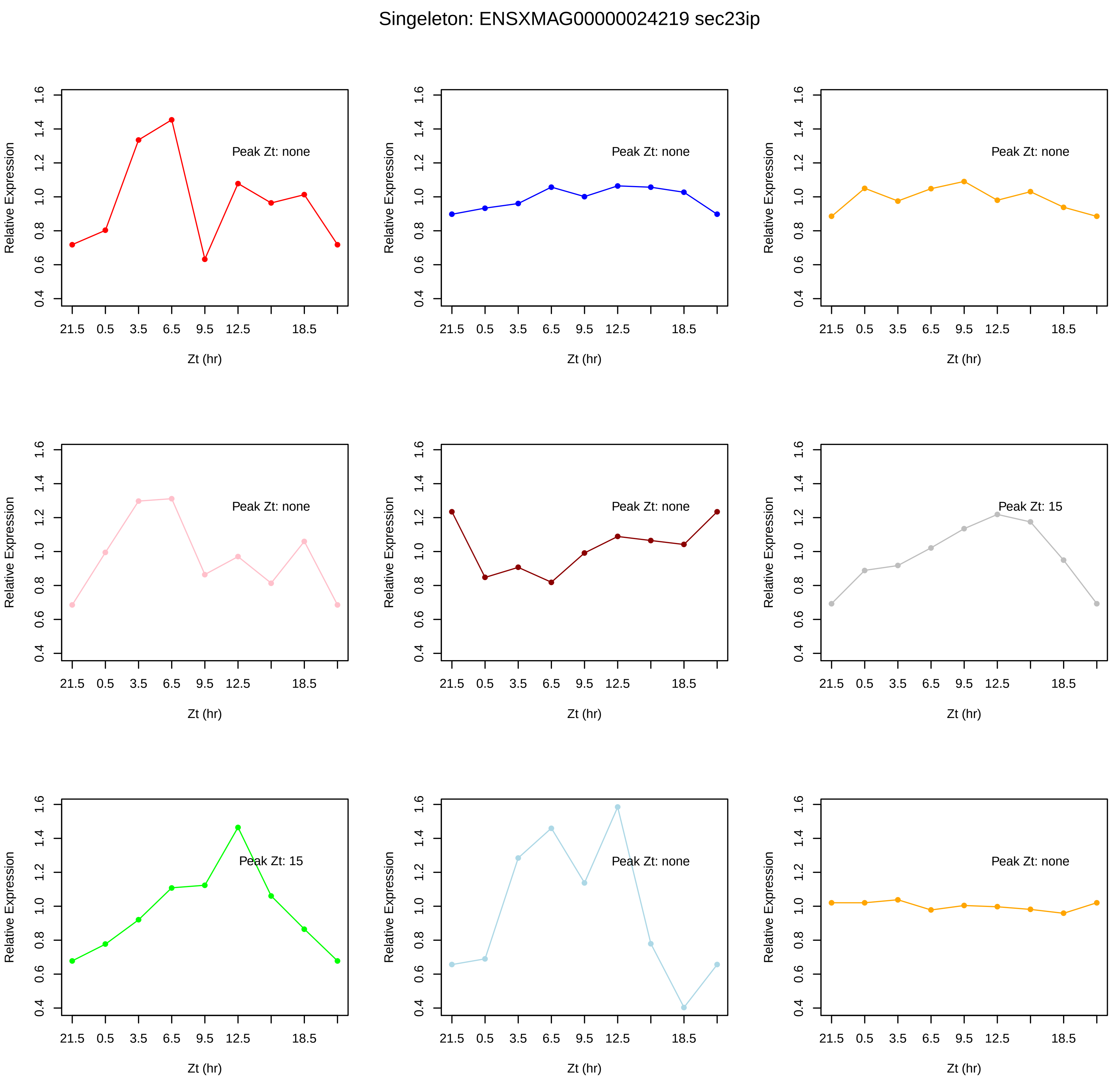
Singeleton: ENSXMAG00000024219 sec23ip
Peak Zt: none
Peak Zt: none
Peak Zt: none
Peak Zt: none
Peak Zt: none
Peak Zt: 15
Peak Zt: 15
Peak Zt: none
Peak Zt: none
Relative Expression
Zt (hr)
21.5
0.5
3.5
6.5
9.5
12.5
18.5
0.4
0.6
0.8
1.0
1.2
1.4
1.6

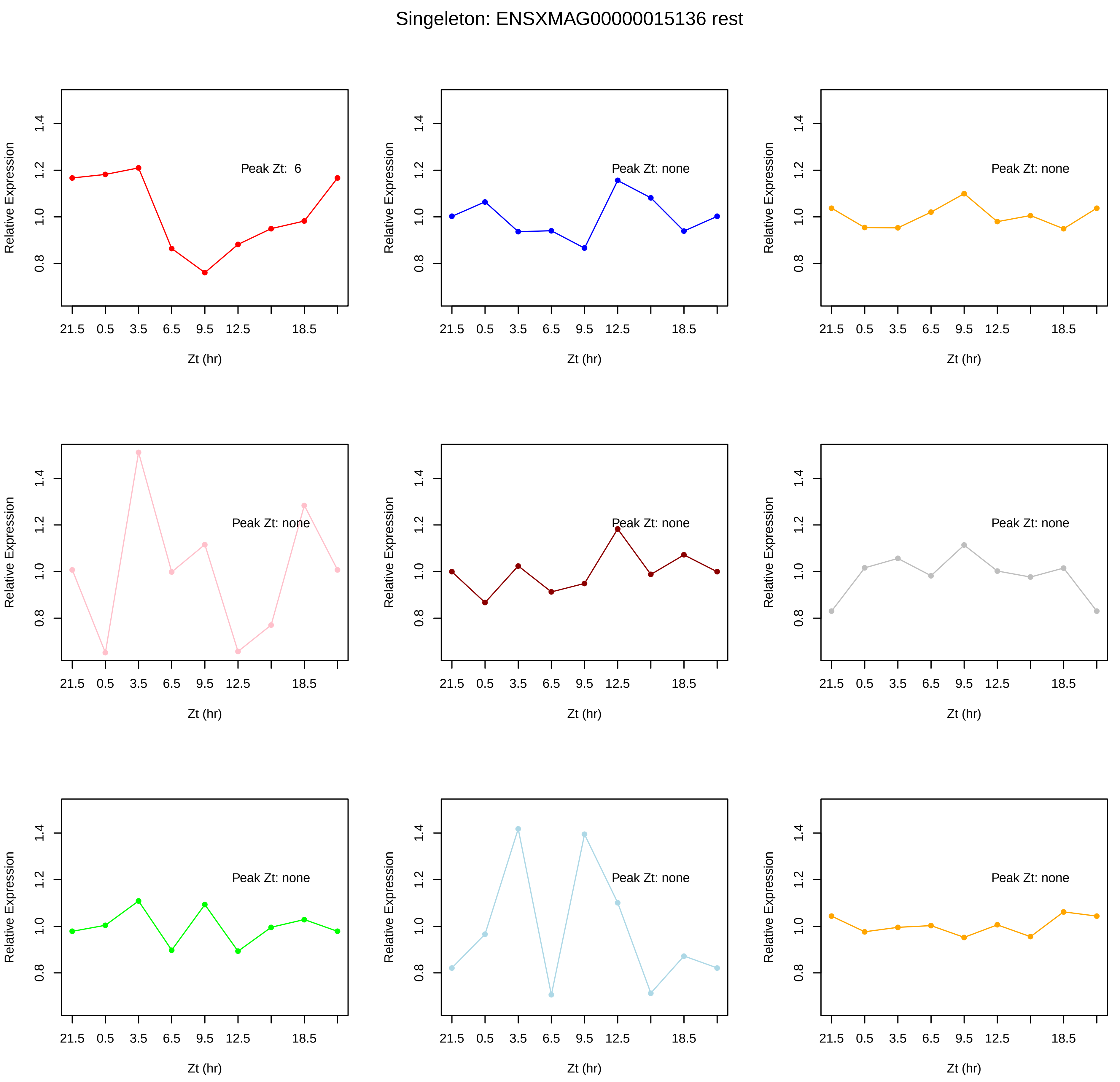
Singeleton: ENSXMAG00000015136 rest
Peak Zt: 6
Peak Zt: none
Peak Zt: none
Peak Zt: none
Peak Zt: none
Peak Zt: none
Peak Zt: none
Peak Zt: none
Peak Zt: none
Relative Expression
Zt (hr)
21.5
0.5
3.5
6.5
9.5
12.5
18.5
0.8
1.0
1.2
1.4

Singeleton: ENSXMAG00000015579 kank1a

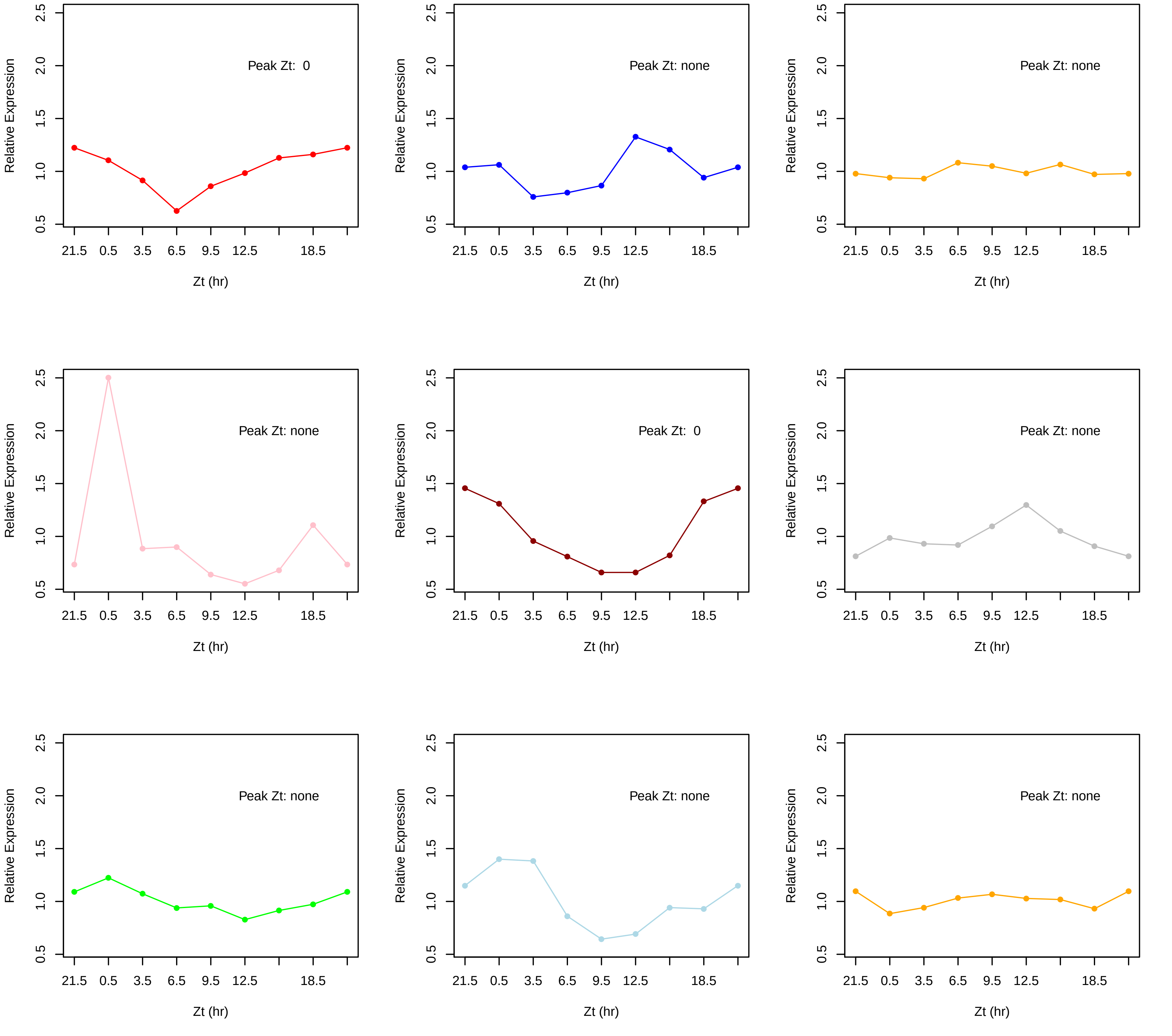

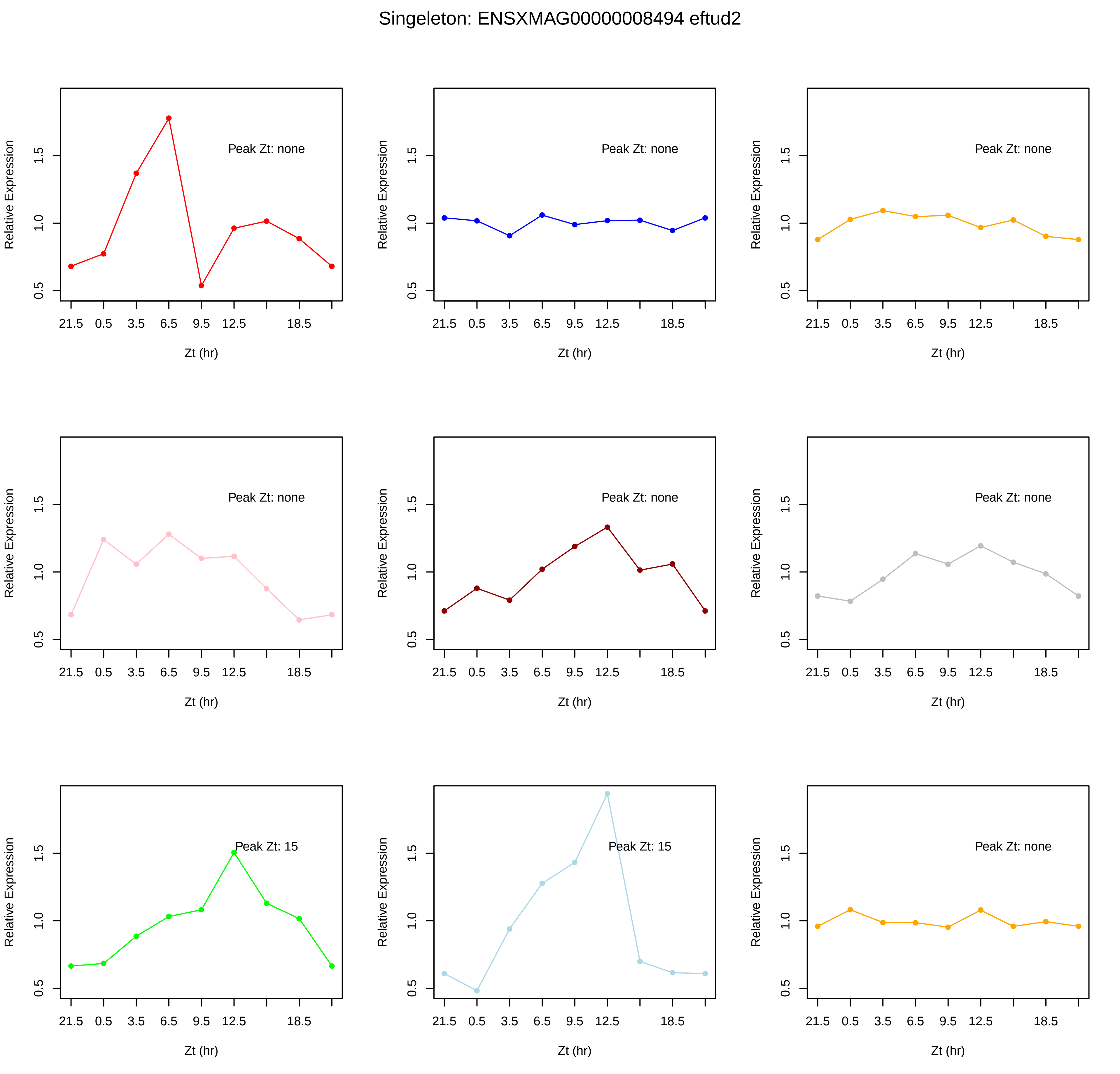
Singleton: ENSXMAG00000008494 eftud2
Peak Zt: none
Peak Zt: none
Peak Zt: none
Peak Zt: none
Peak Zt: none
Peak Zt: none
Peak Zt: 15
Peak Zt: 15
Peak Zt: none
Relative Expression
Zt (hr)
21.5
0.5
3.5
6.5
9.5
12.5
18.5
0.5
1.0
1.5

Singeleton: ENSXMAG00000007617 galk1

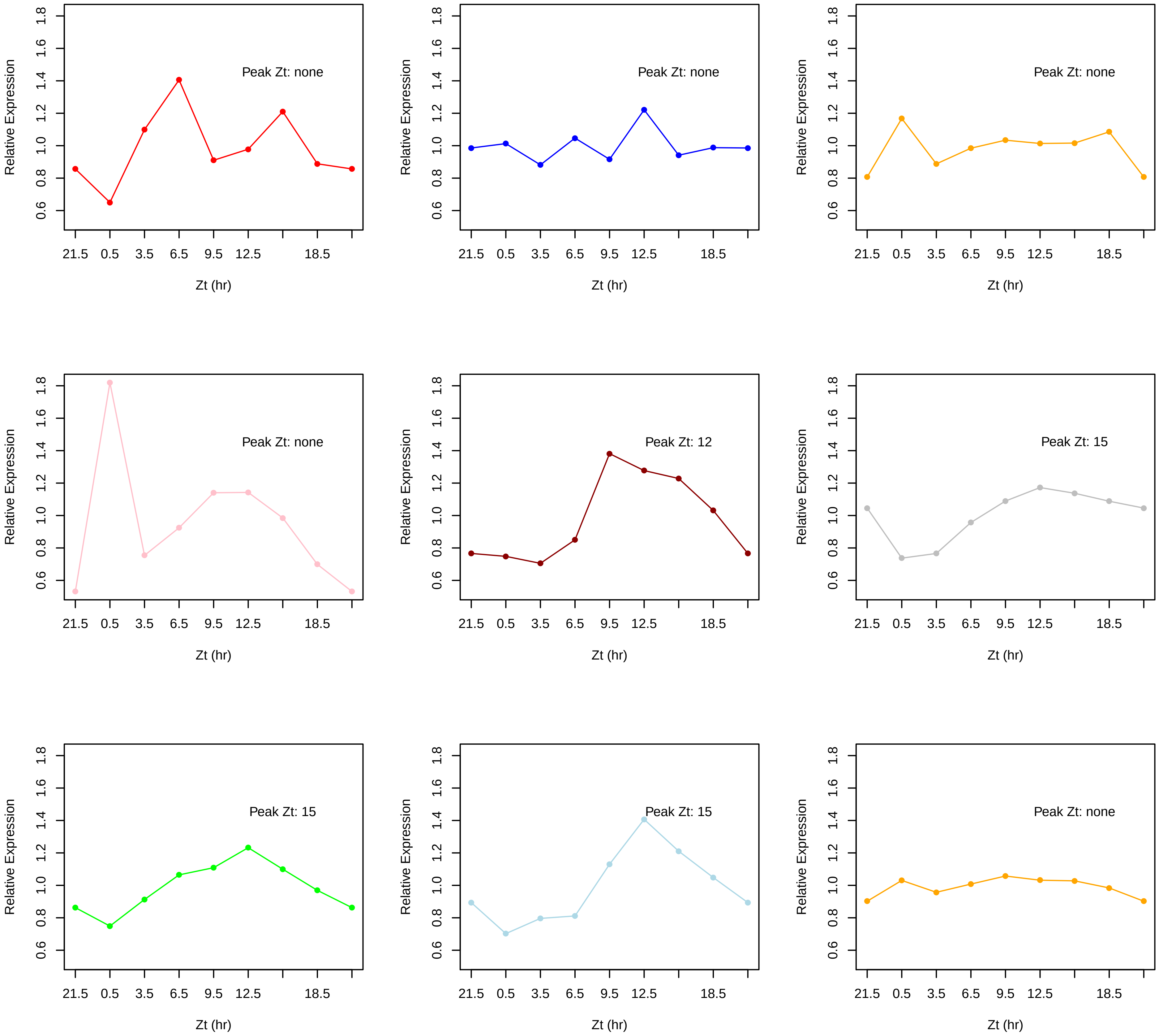

Singleton: ENSXMAG00000004839

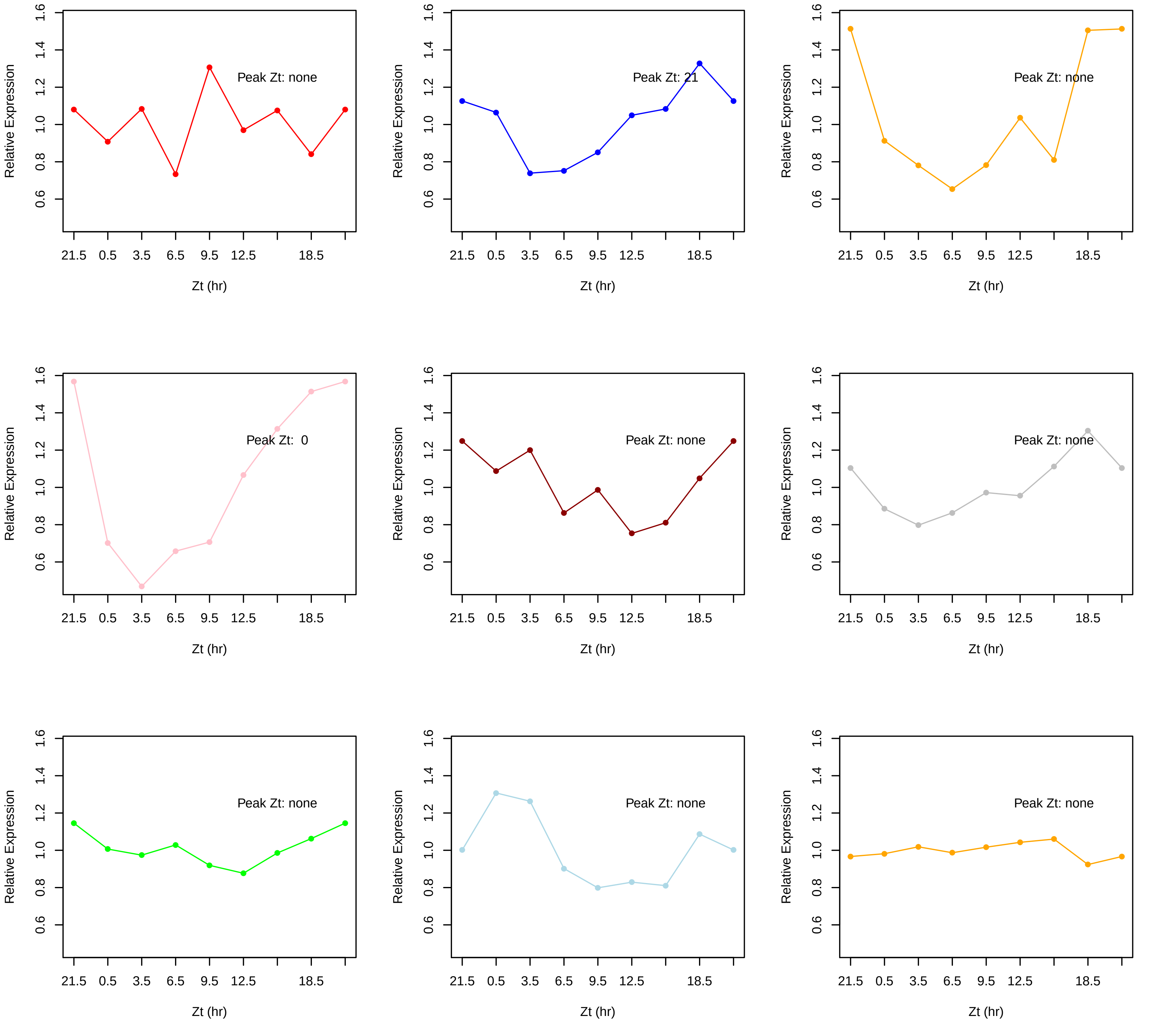

Singeleton: ENSXMAG00000015600 dmrt3a

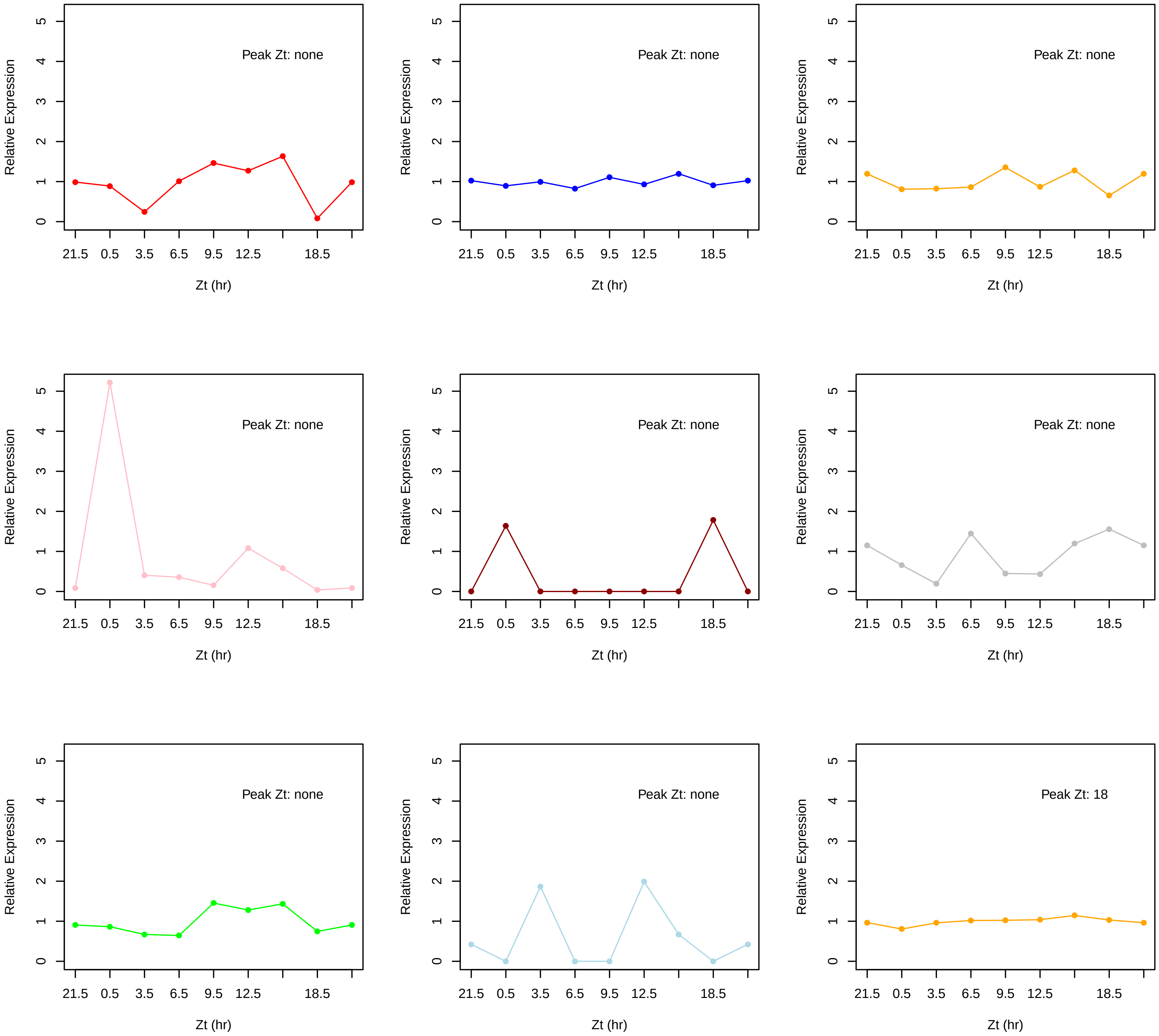

Singeleton: ENSXMAG00000015265

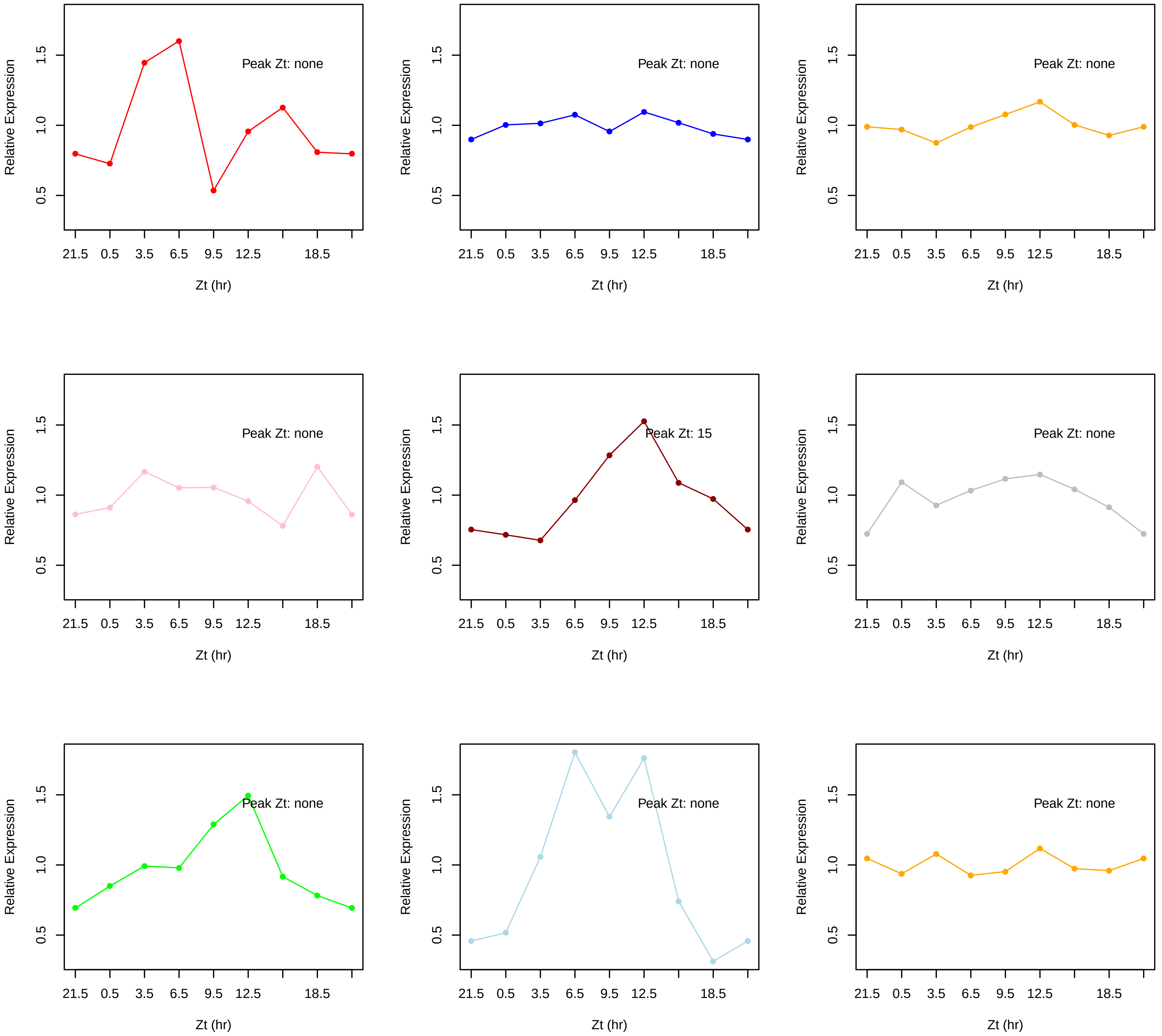

Singleton: ENSXMAG00000012133 recql5

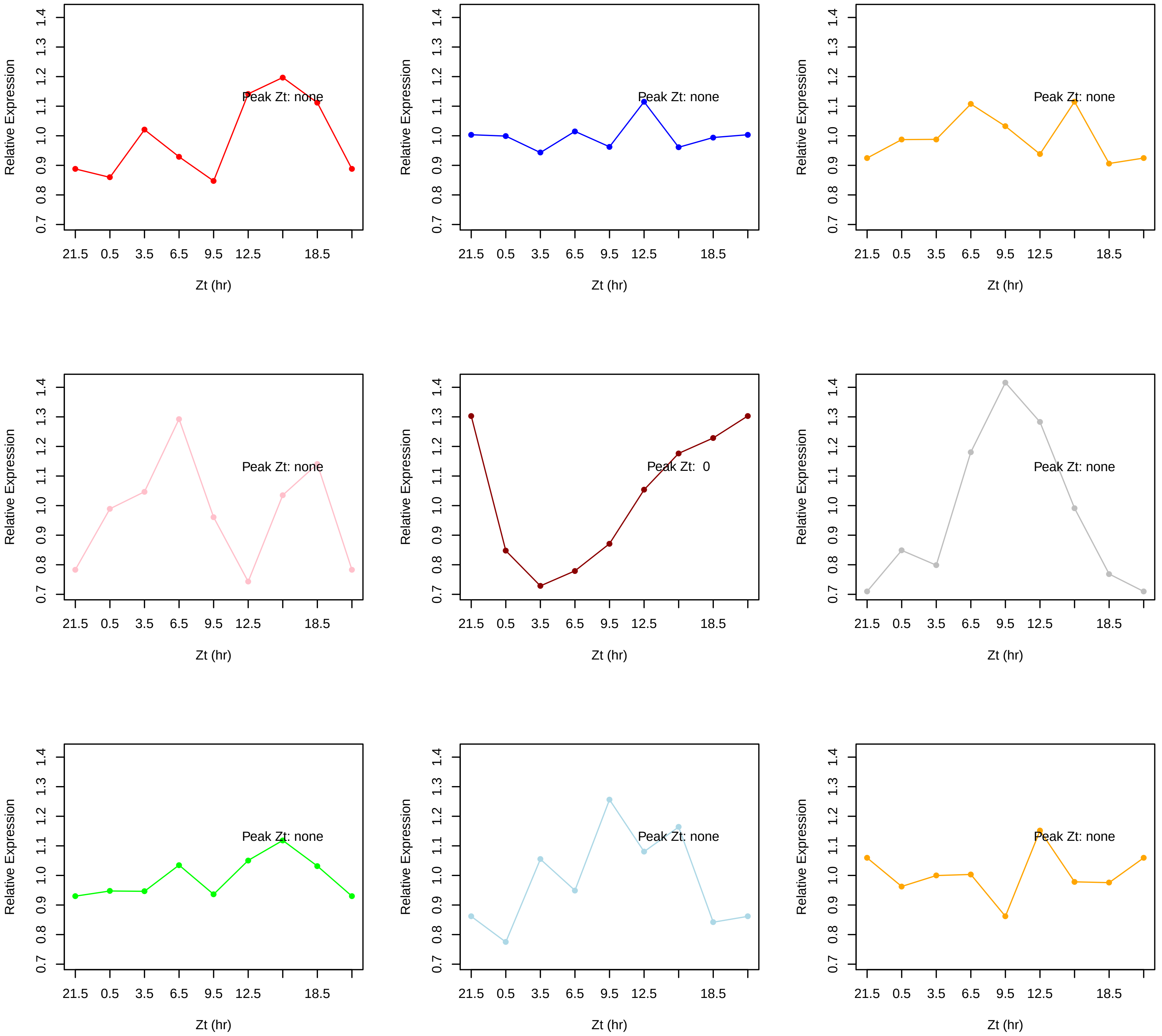

Singeleton: ENSXMAG00000027397 csgalnact2

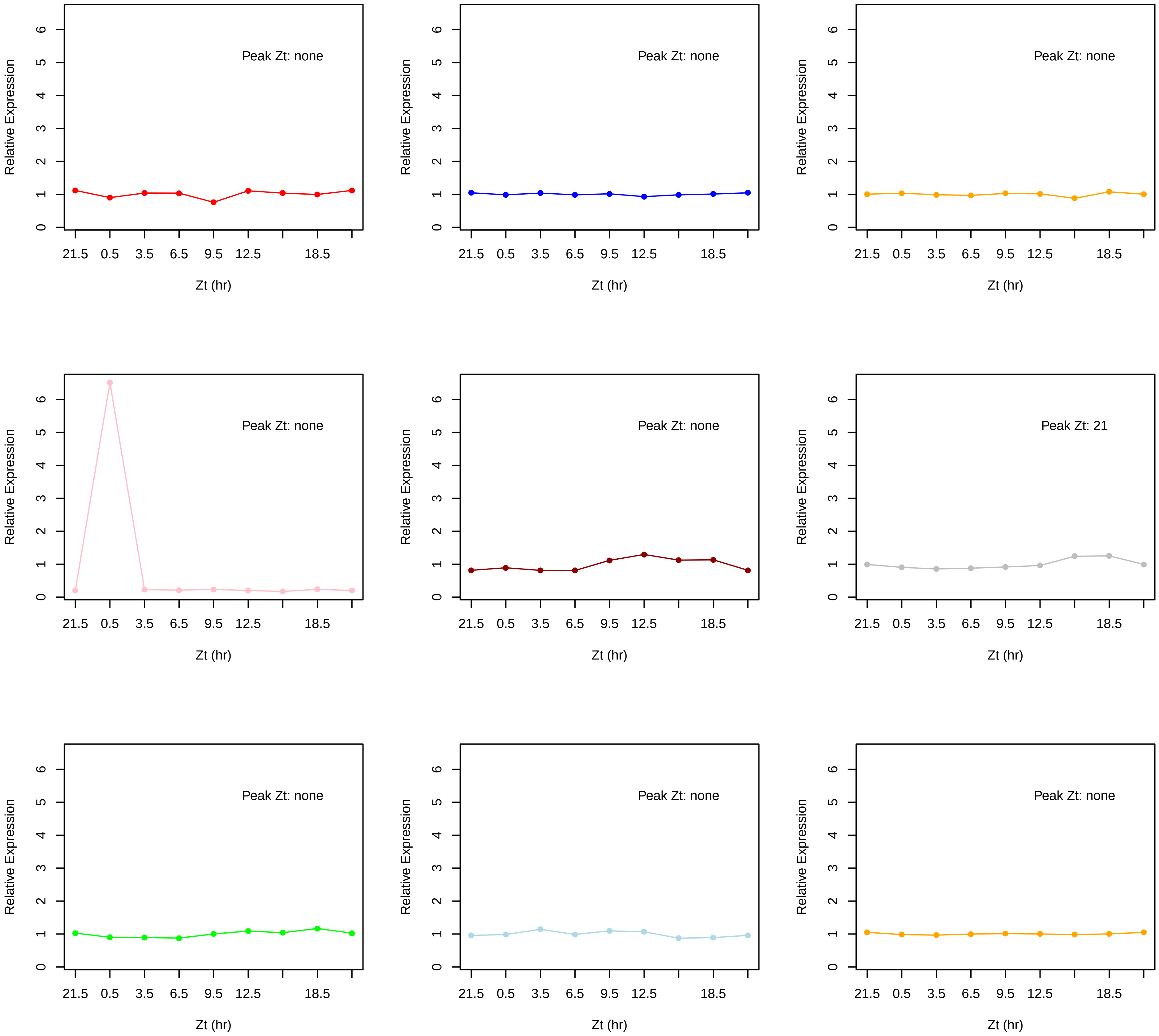

Singeleton: ENSXMAG00000001478

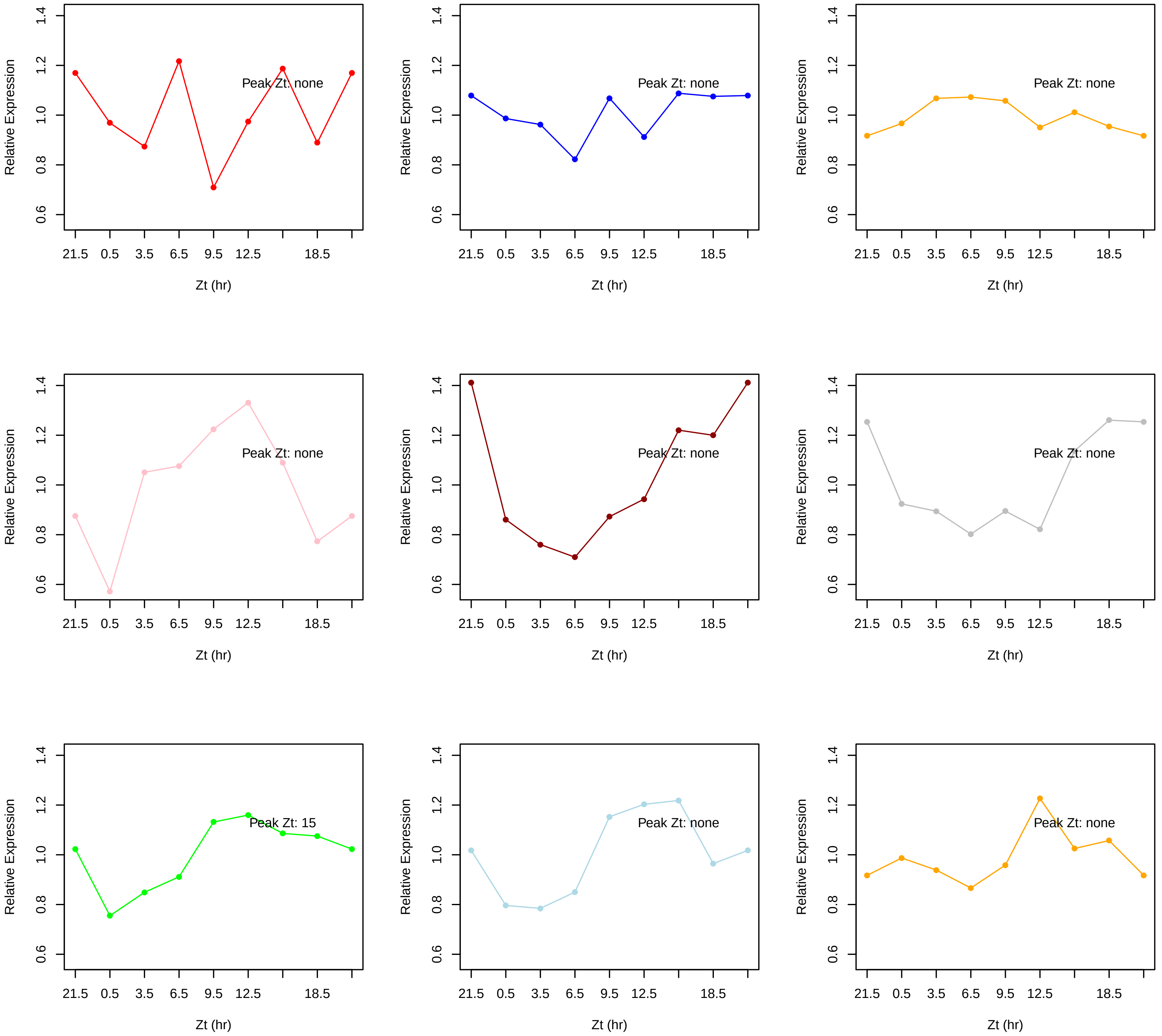

Singeleton: ENSXMAG00000009671 mrtfab

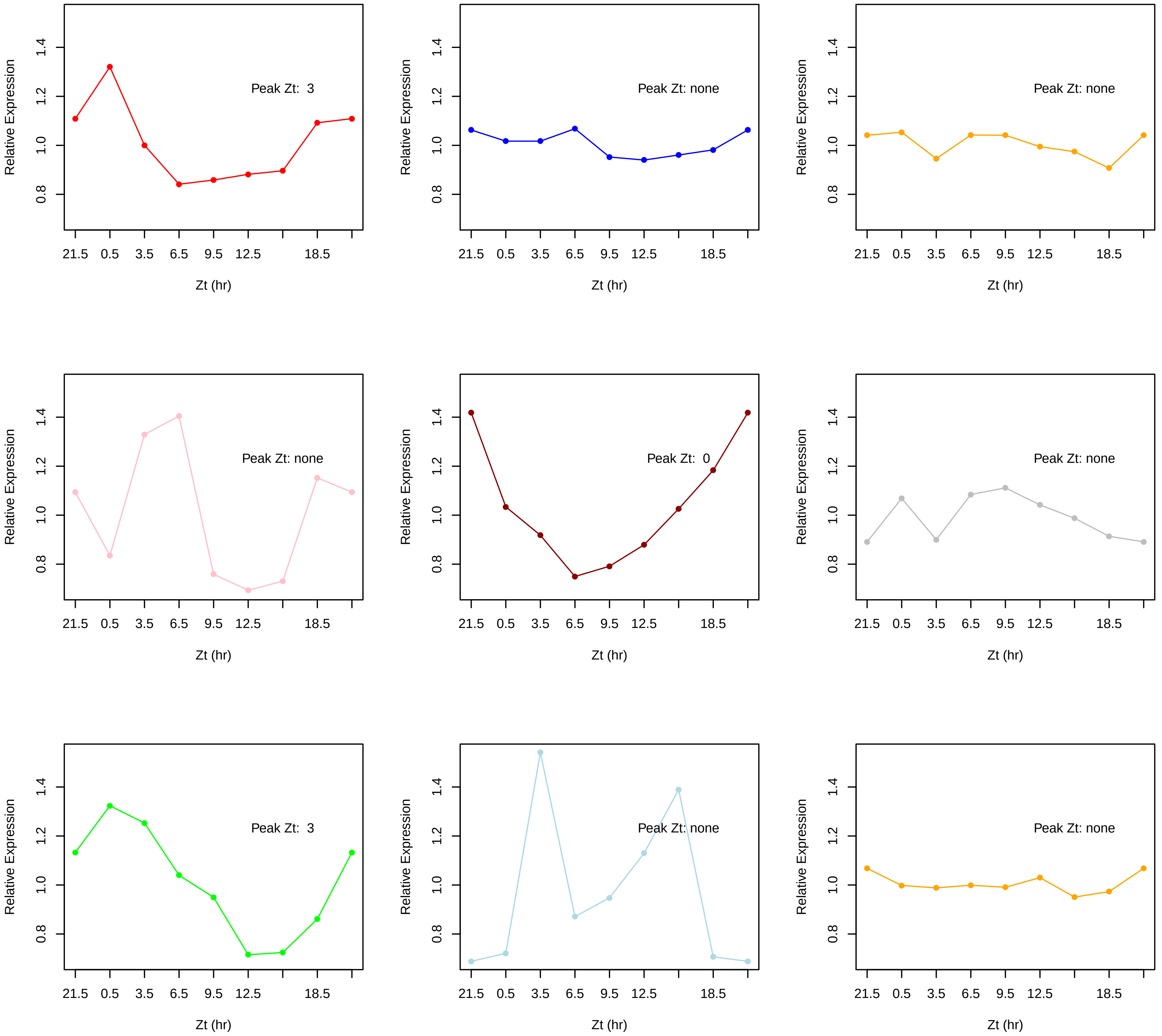

Singeleton: ENSXMAG00000014398 ret

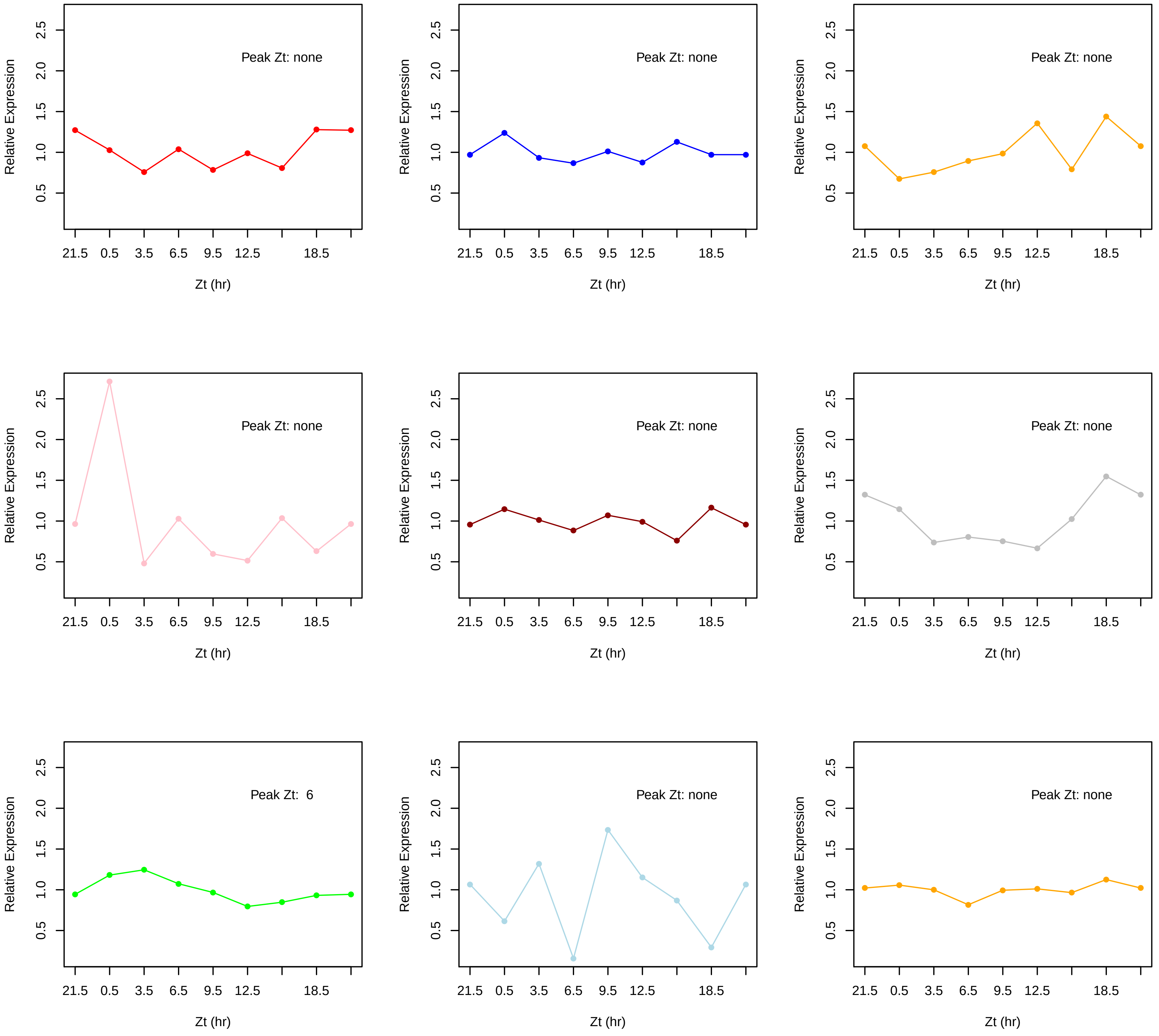

Singeleton: ENSXMAG00000015611 smarca2

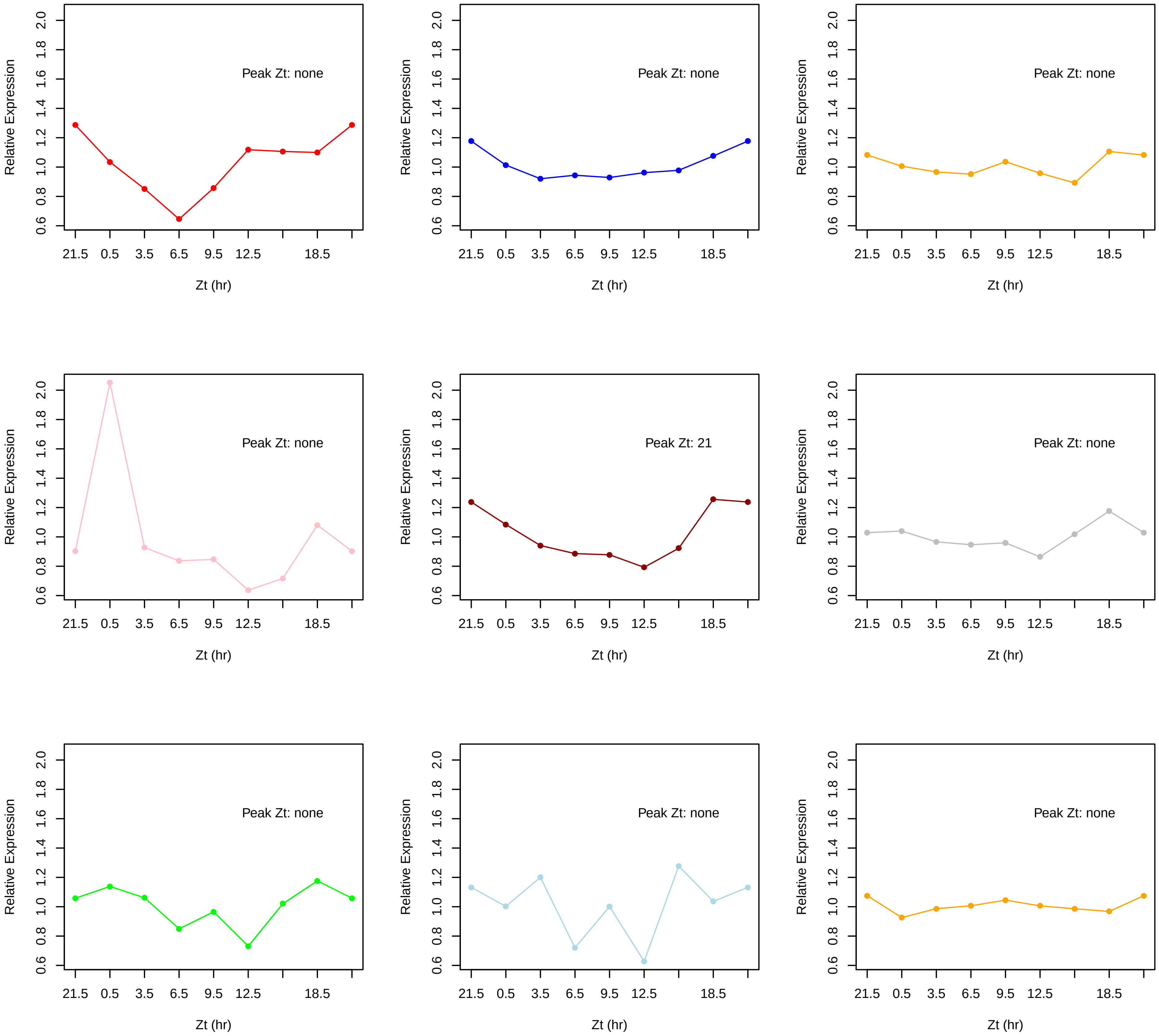

Singleton: ENSXMAG00000006646 sycp3

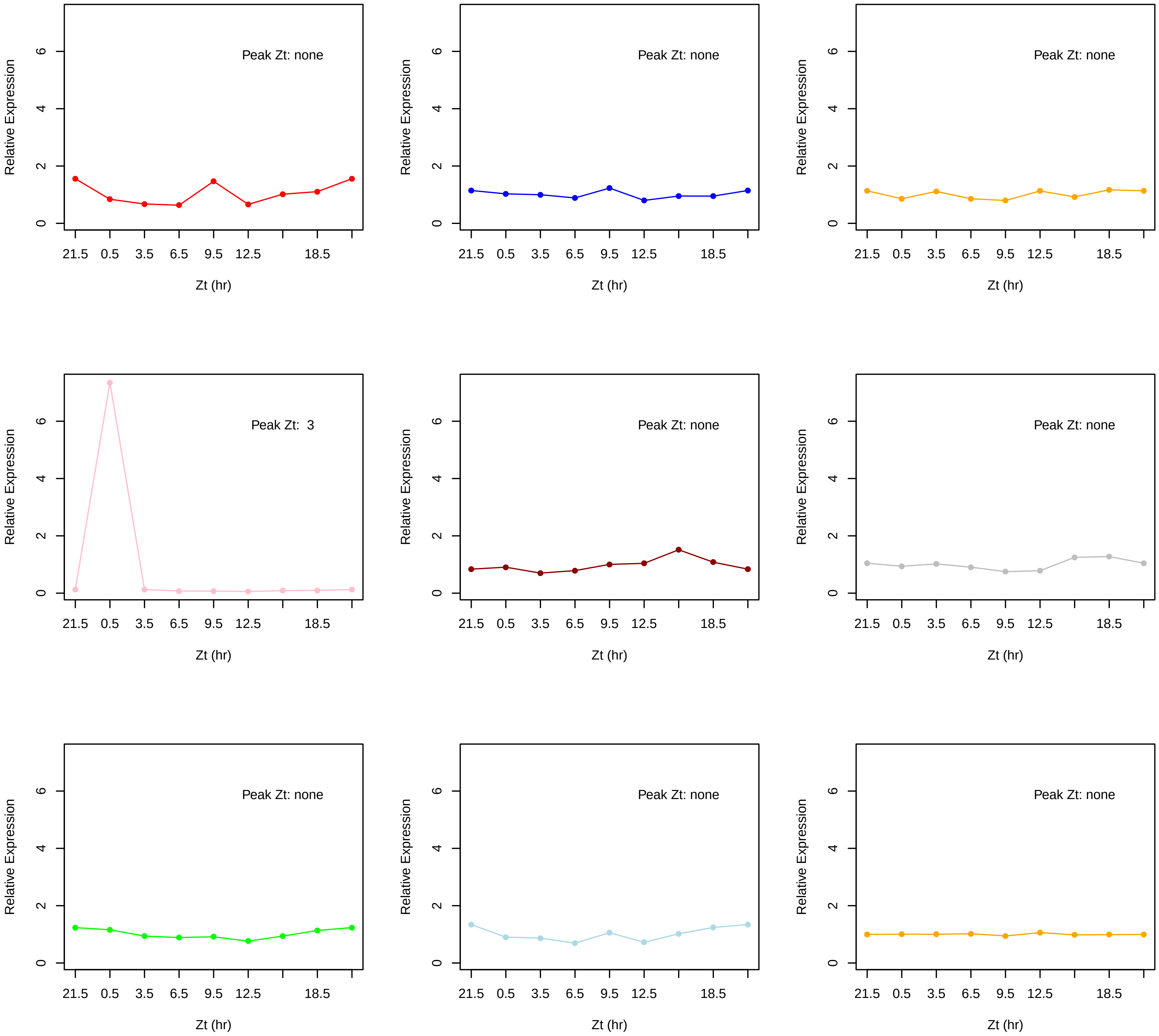

Singeleton: ENSXMAG00000008315 hsp90aa1.1

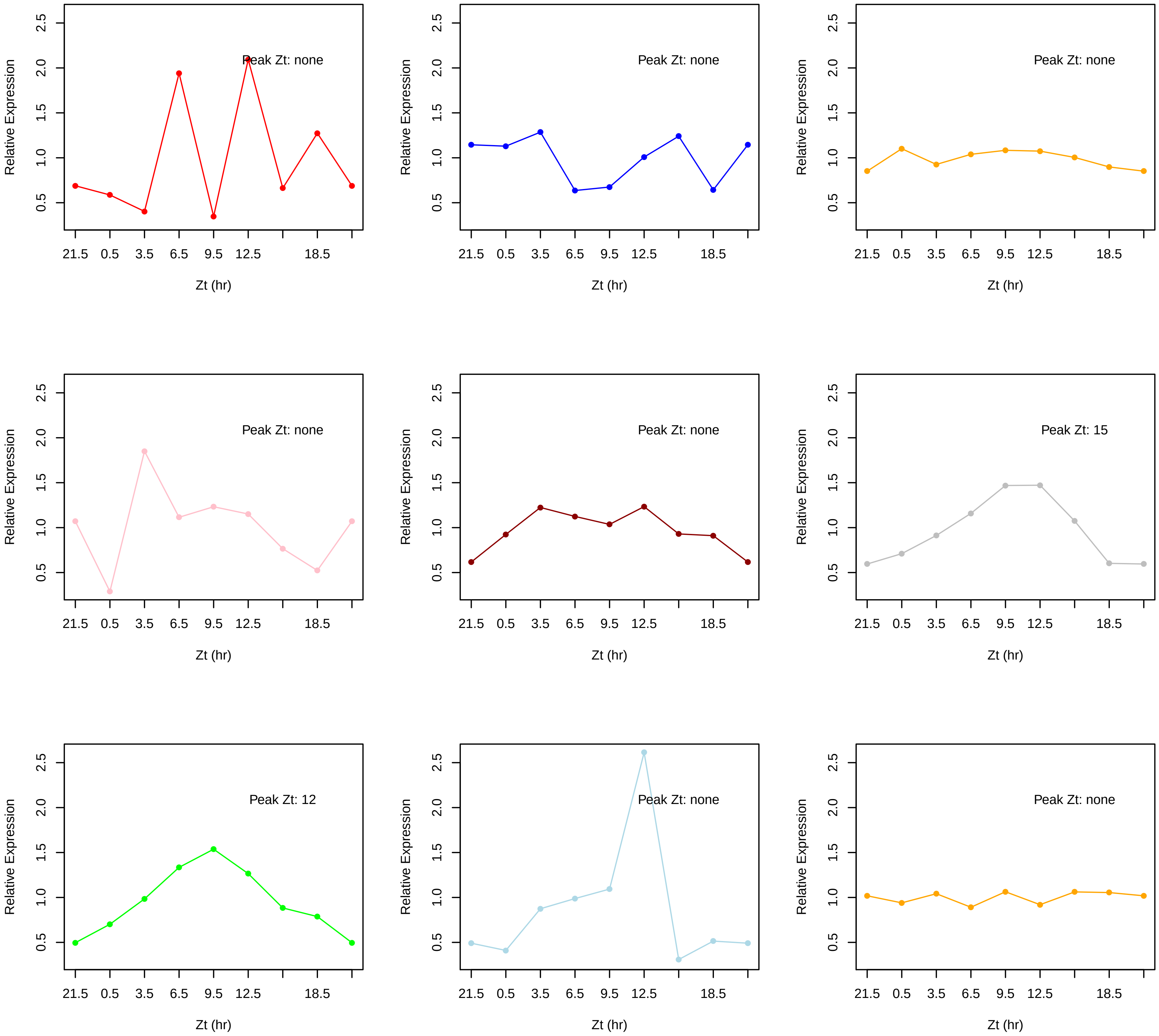

Singeleton: ENSXMAG00000020878 nol7

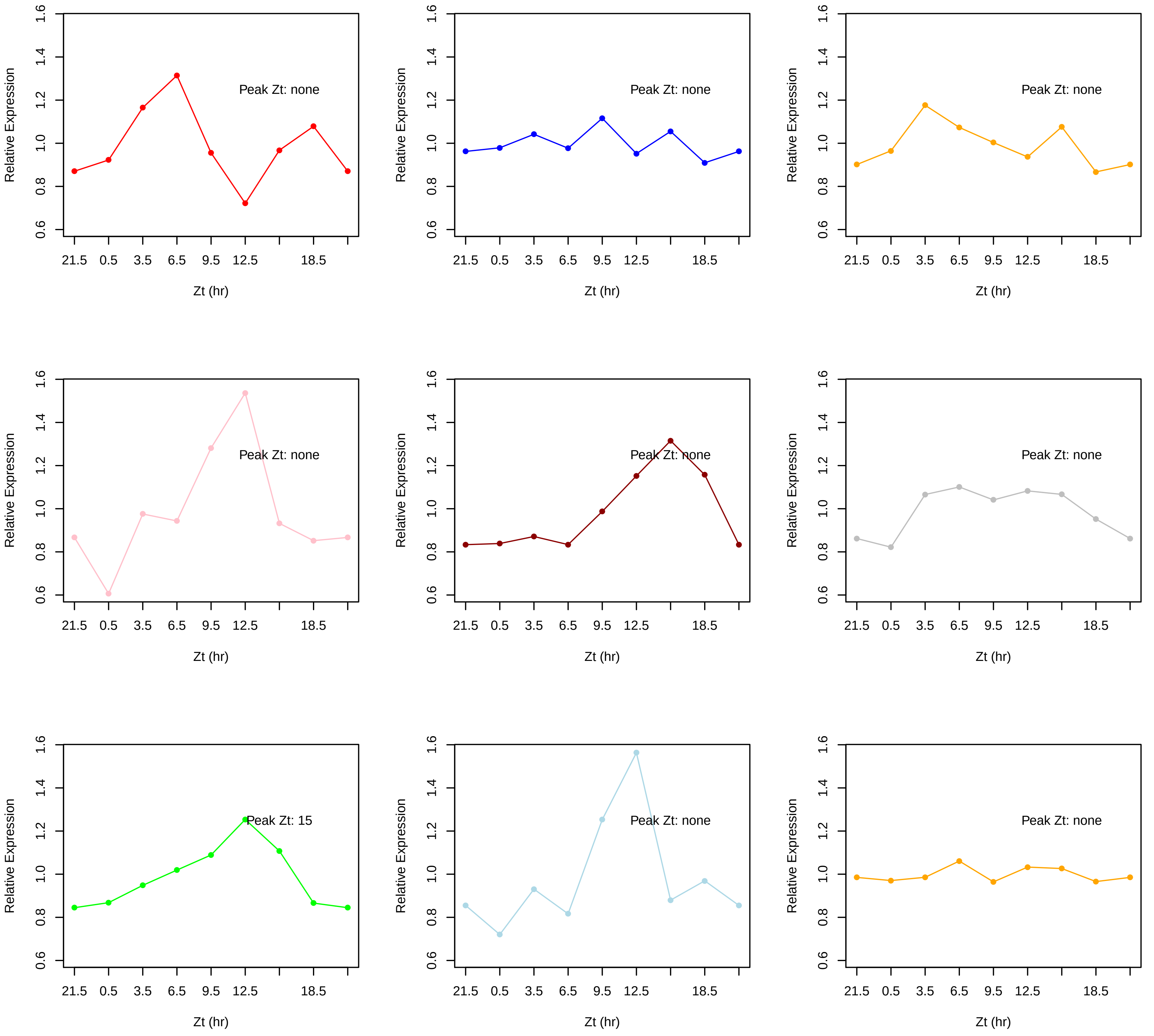

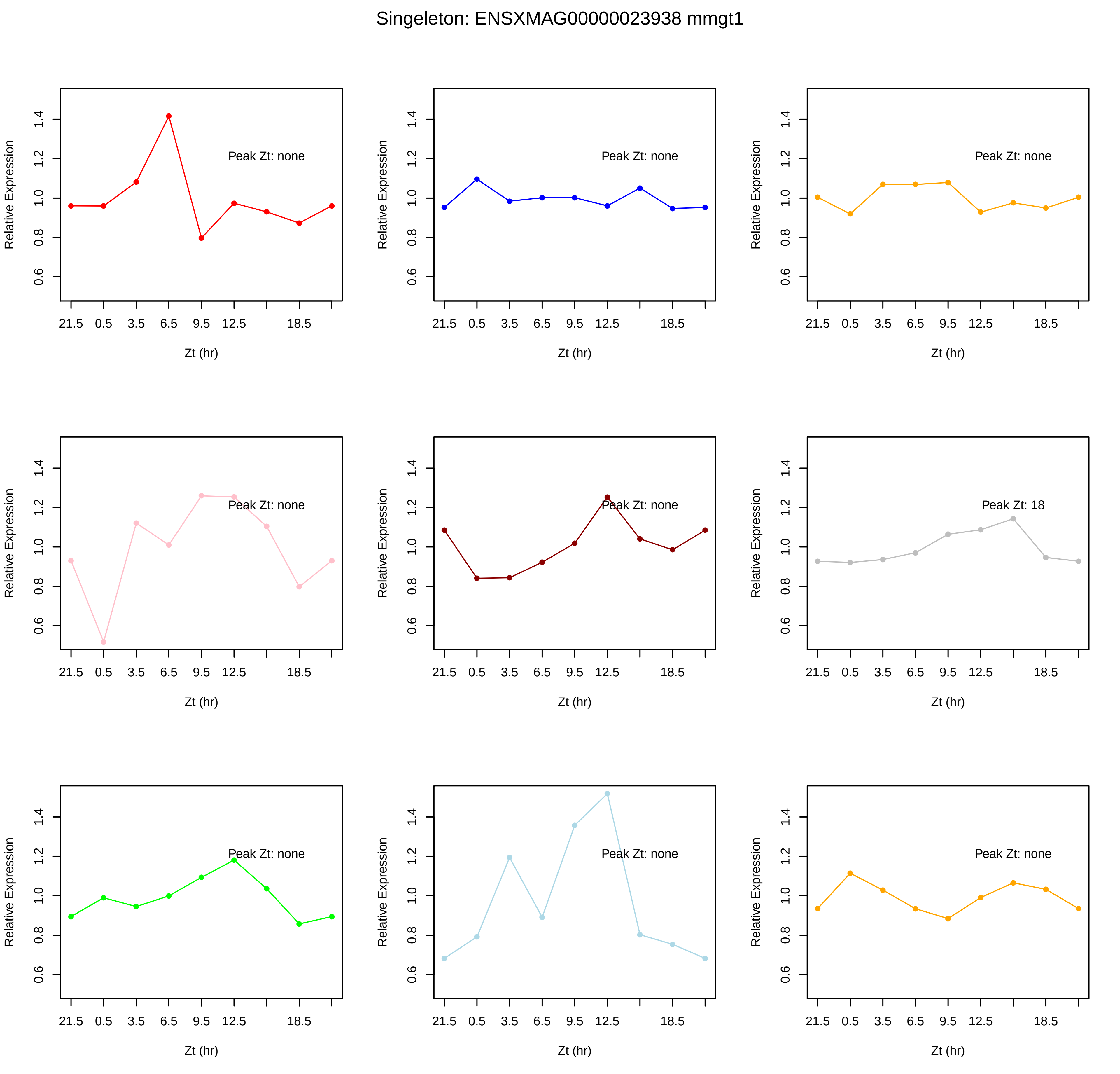
Singeleton: ENSXMAG00000023938 mmgt1
Relative Expression
Zt (hr)
Peak Zt: none
Peak Zt: 18
21.5
0.5
3.5
6.5
9.5
12.5
18.5
0.6
0.8
1.0
1.2
1.4

Singeleton: ENSXMAG00000012425 sap30bp

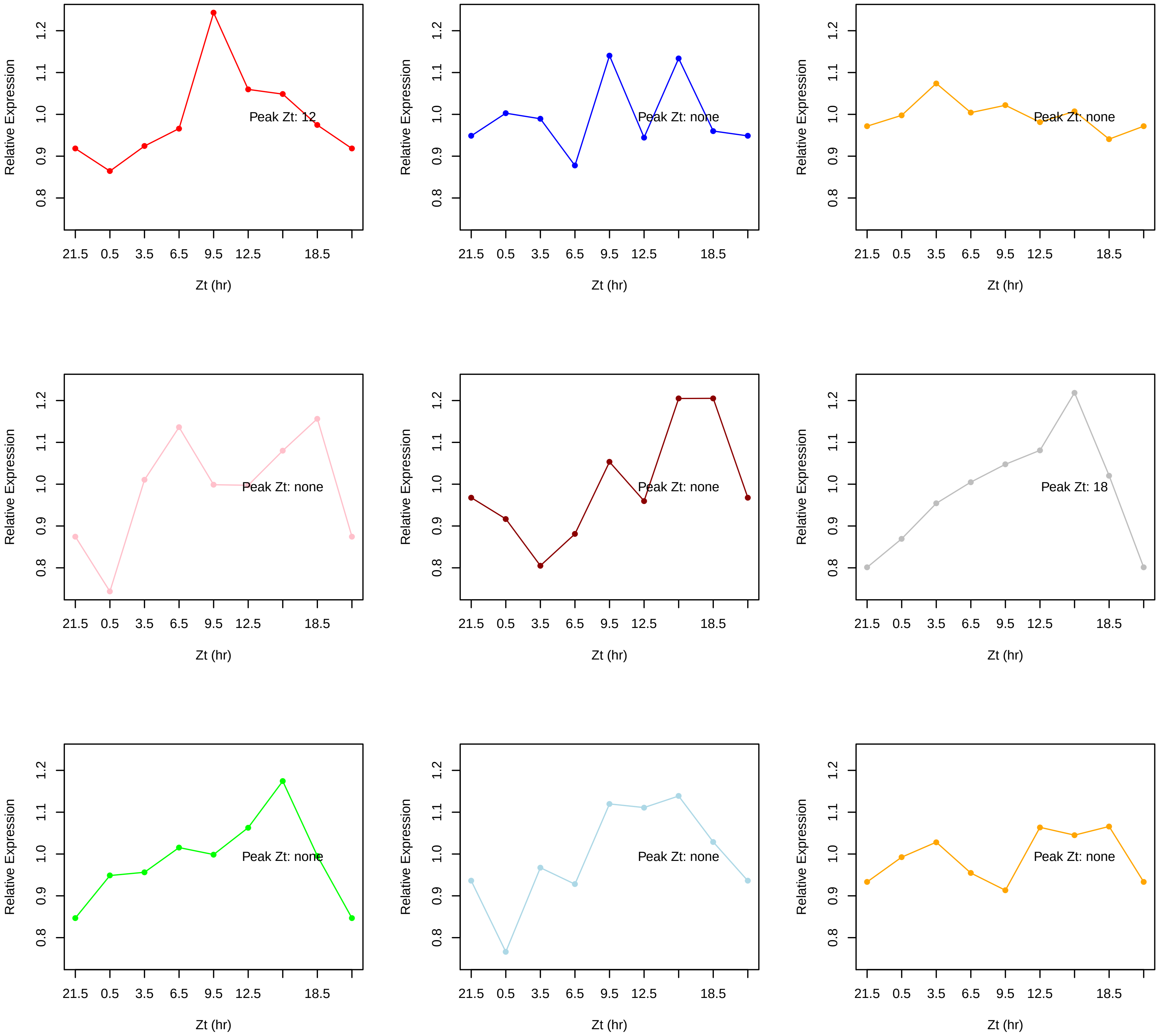

Singeleton: ENSXMAG00000006783 ranbp9

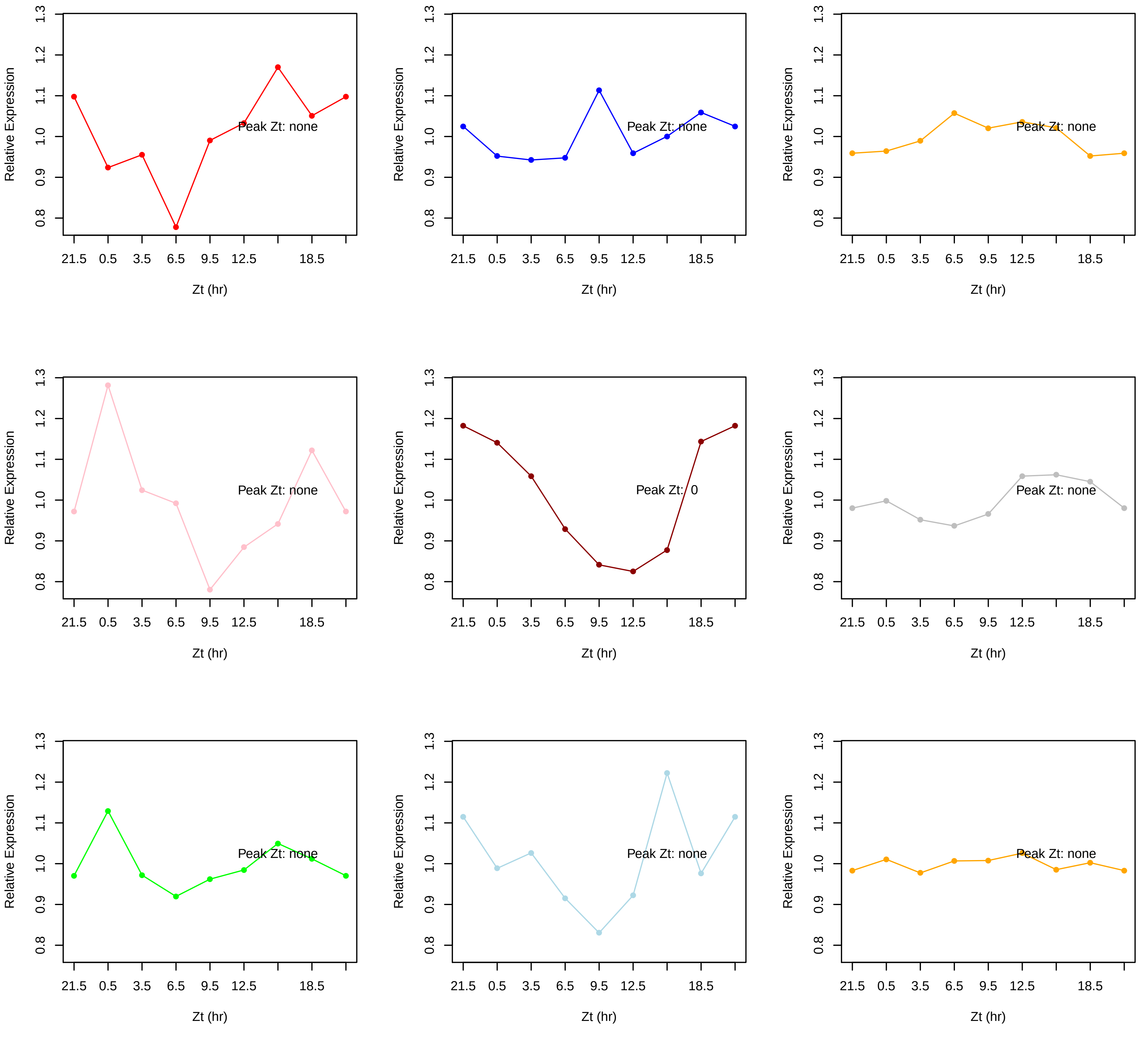

Singeleton: ENSXMAG00000007880 sgpl1

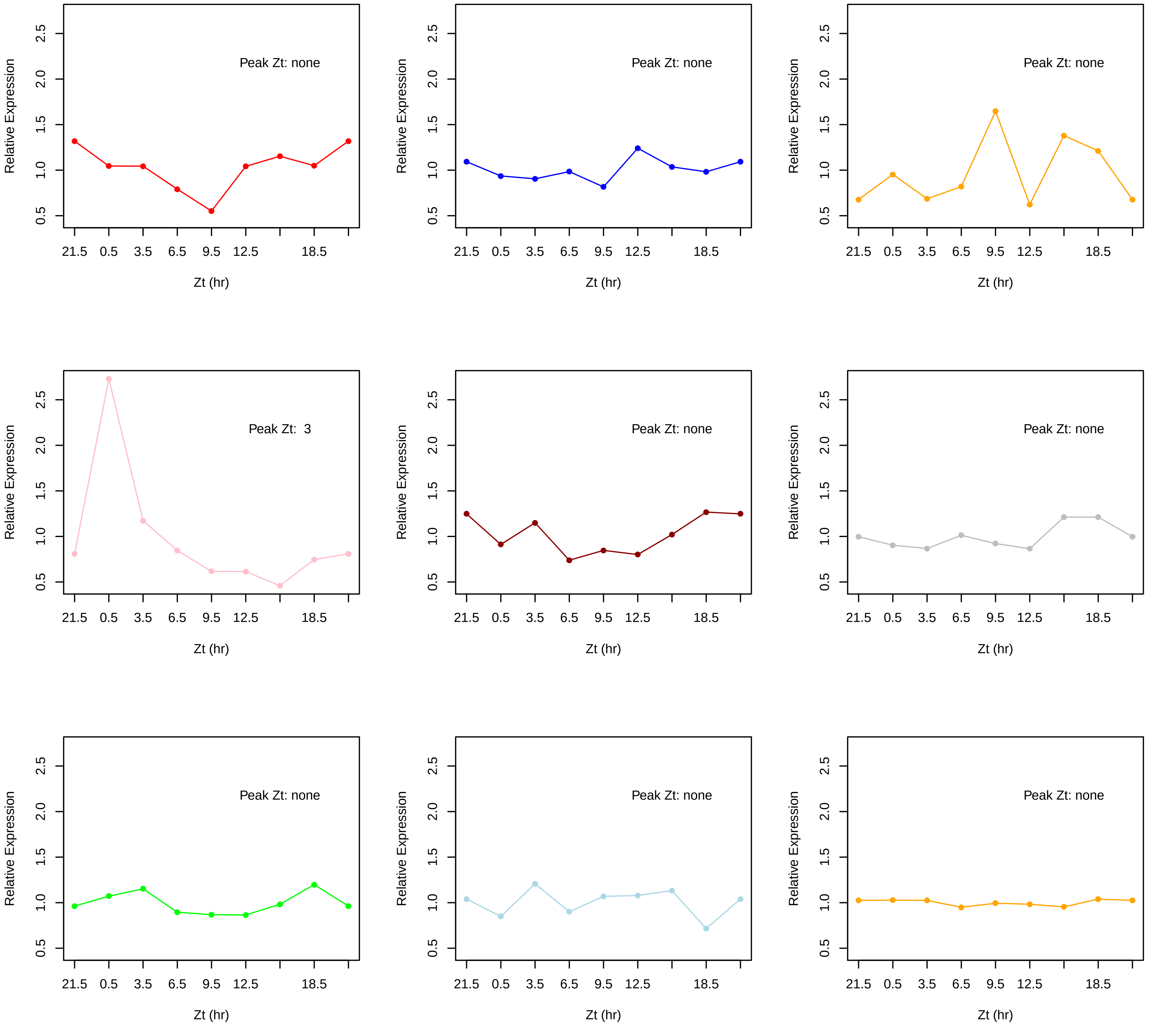

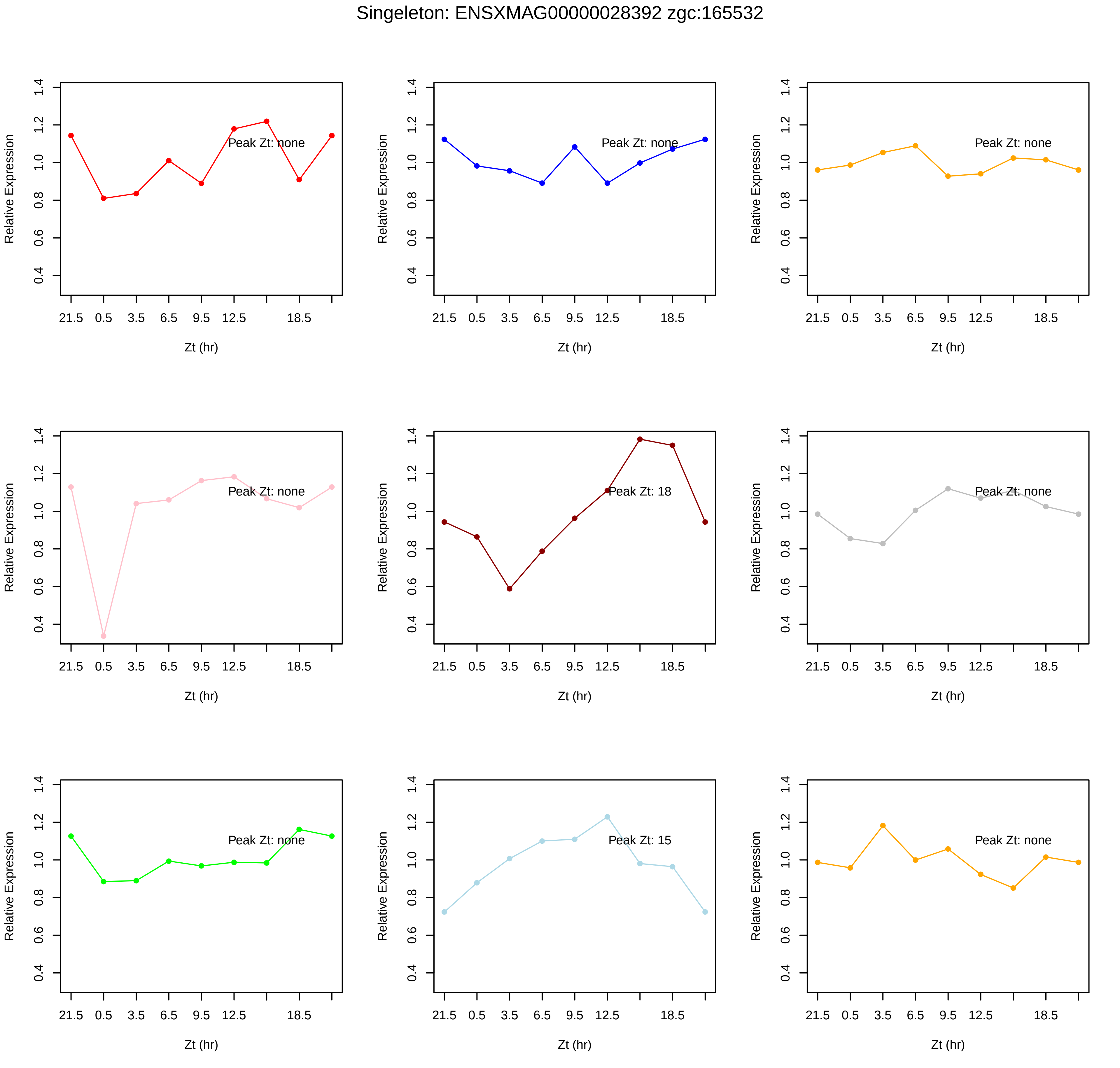
Singeleton: ENSXMAG00000028392 zgc:165532
Peak Zt: none
Peak Zt: none
Peak Zt: none
Peak Zt: none
Peak Zt: 18
Peak Zt: none
Peak Zt: none
Peak Zt: 15
Peak Zt: none
Relative Expression
Zt (hr)
21.5 0.5 3.5 6.5 9.5 12.5 18.5
0.4 0.6 0.8 1.0 1.2 1.4

Singeleton: ENSXMAG00000028912 rgs9bp

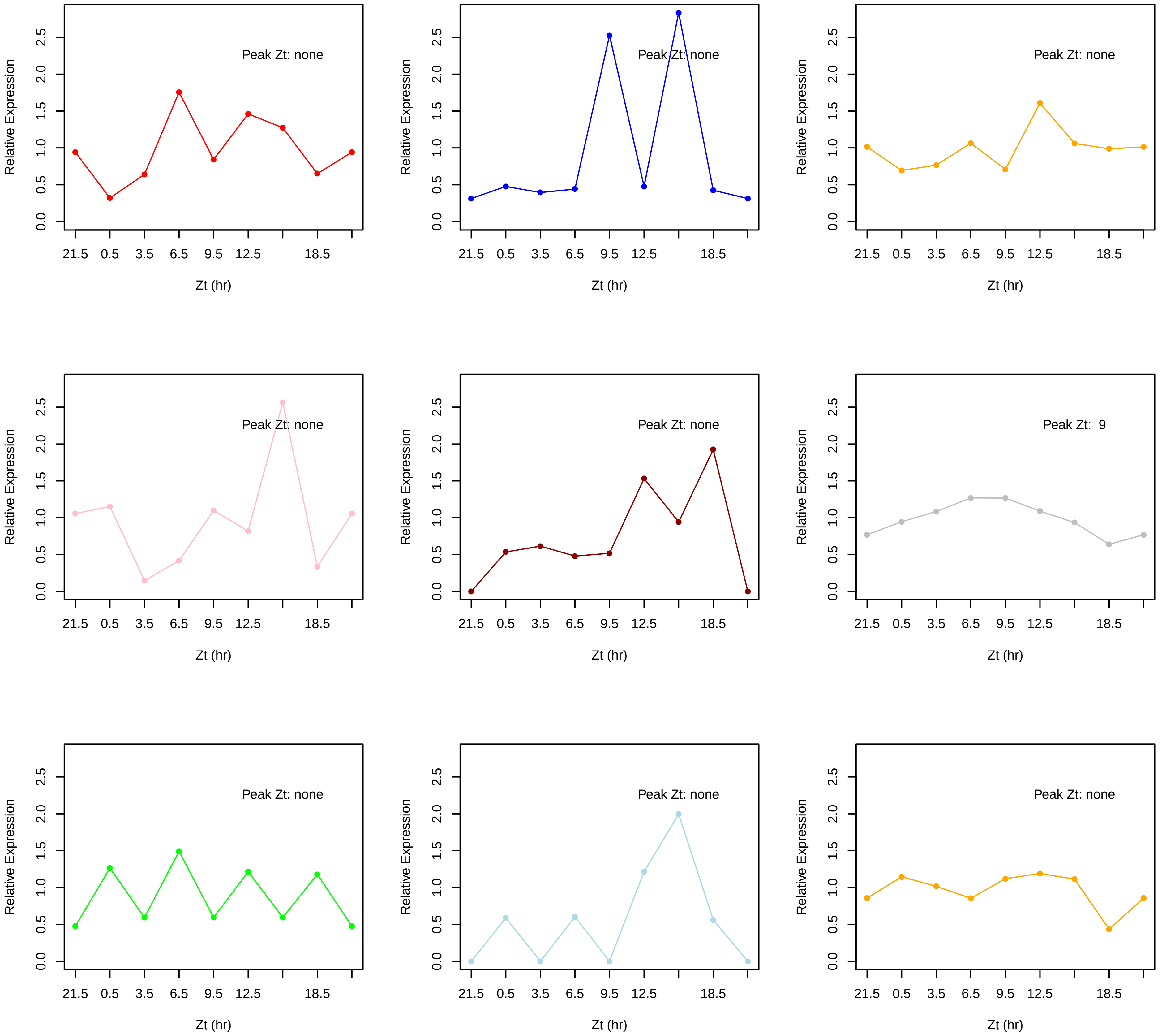

Singeleton: ENSXMAG00000009691 xrcc6

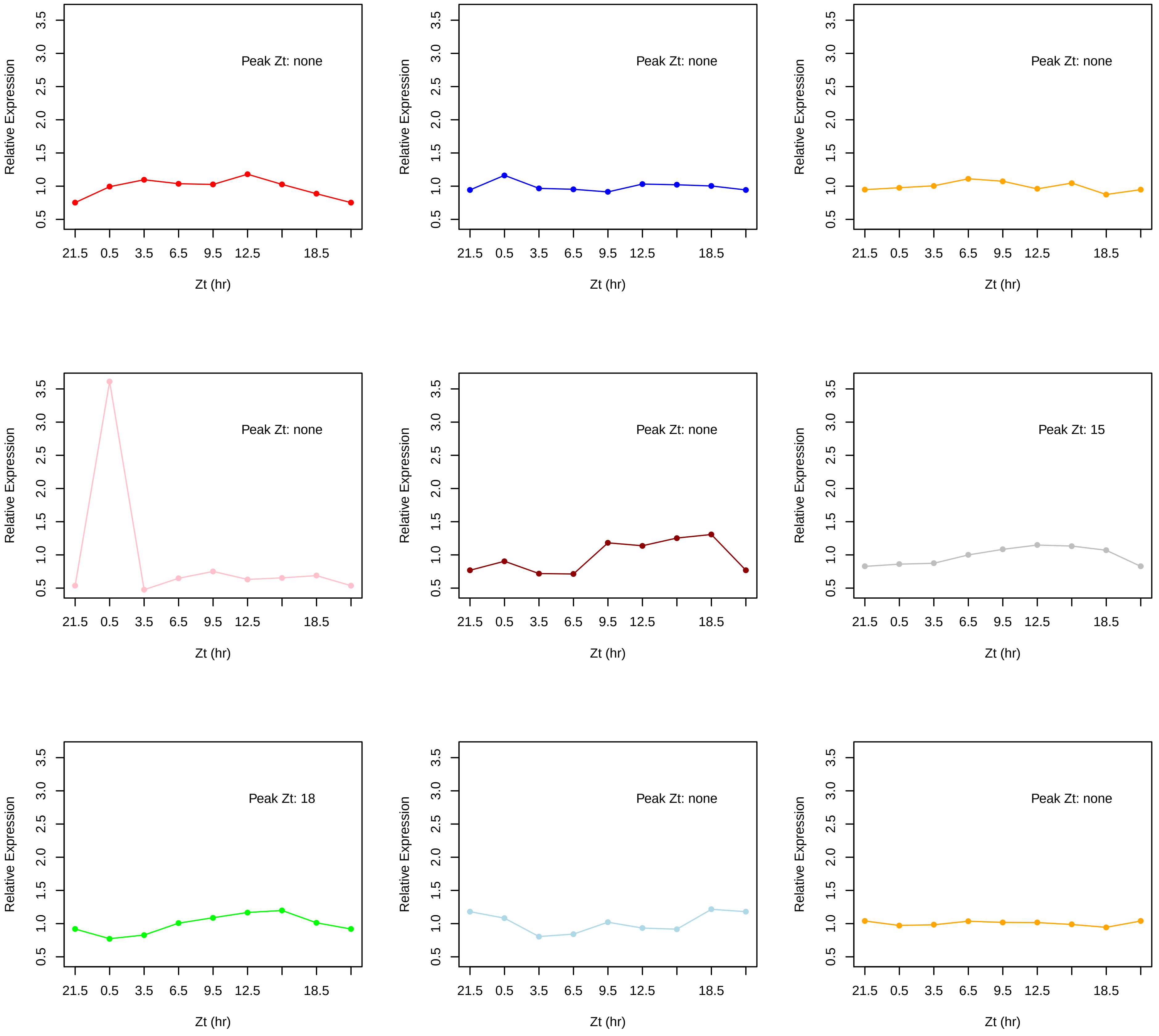

Singleton: ENSXMAG00000014421 acbd4

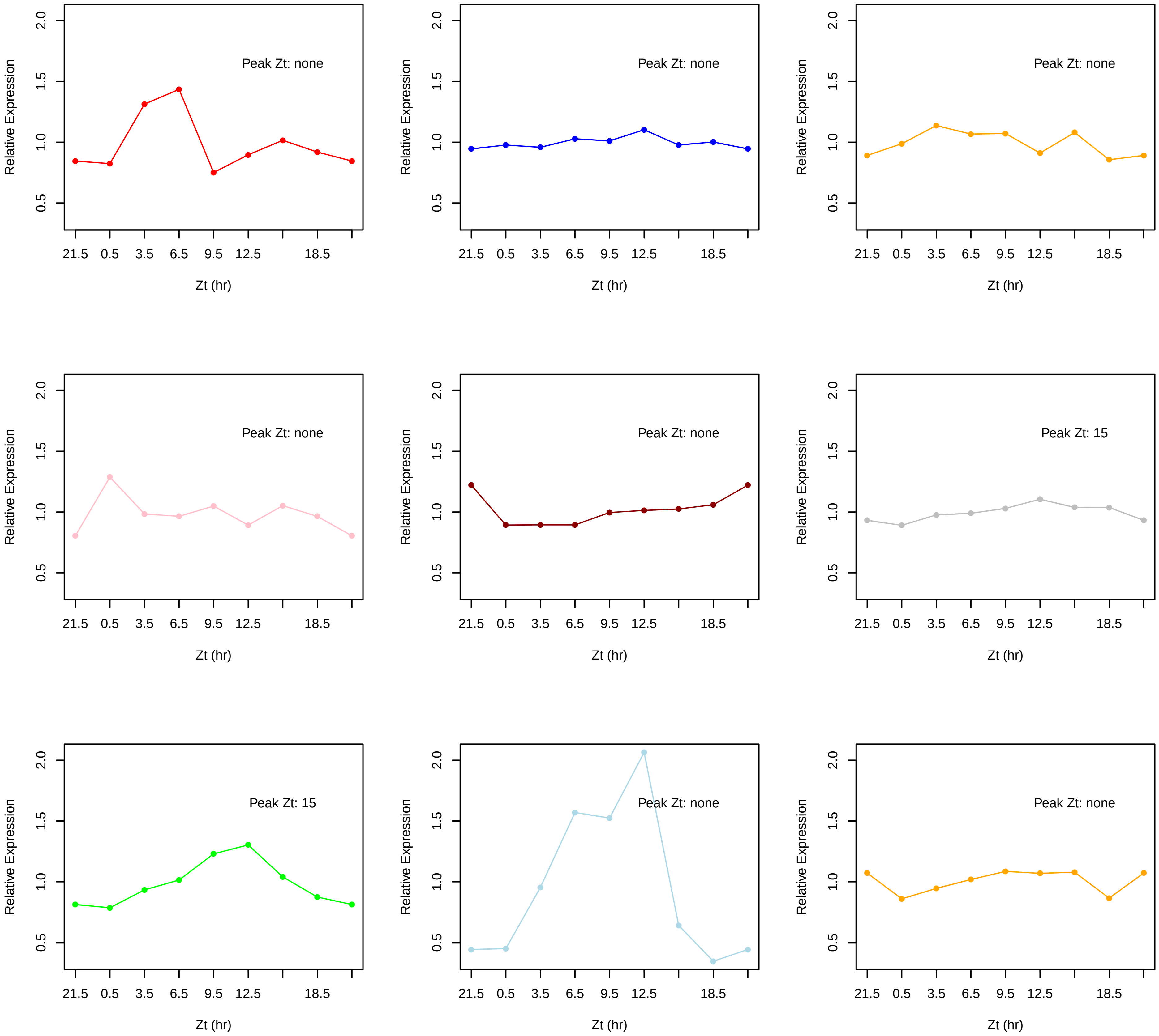

Singeleton: ENSXMAG00000016482 egfra

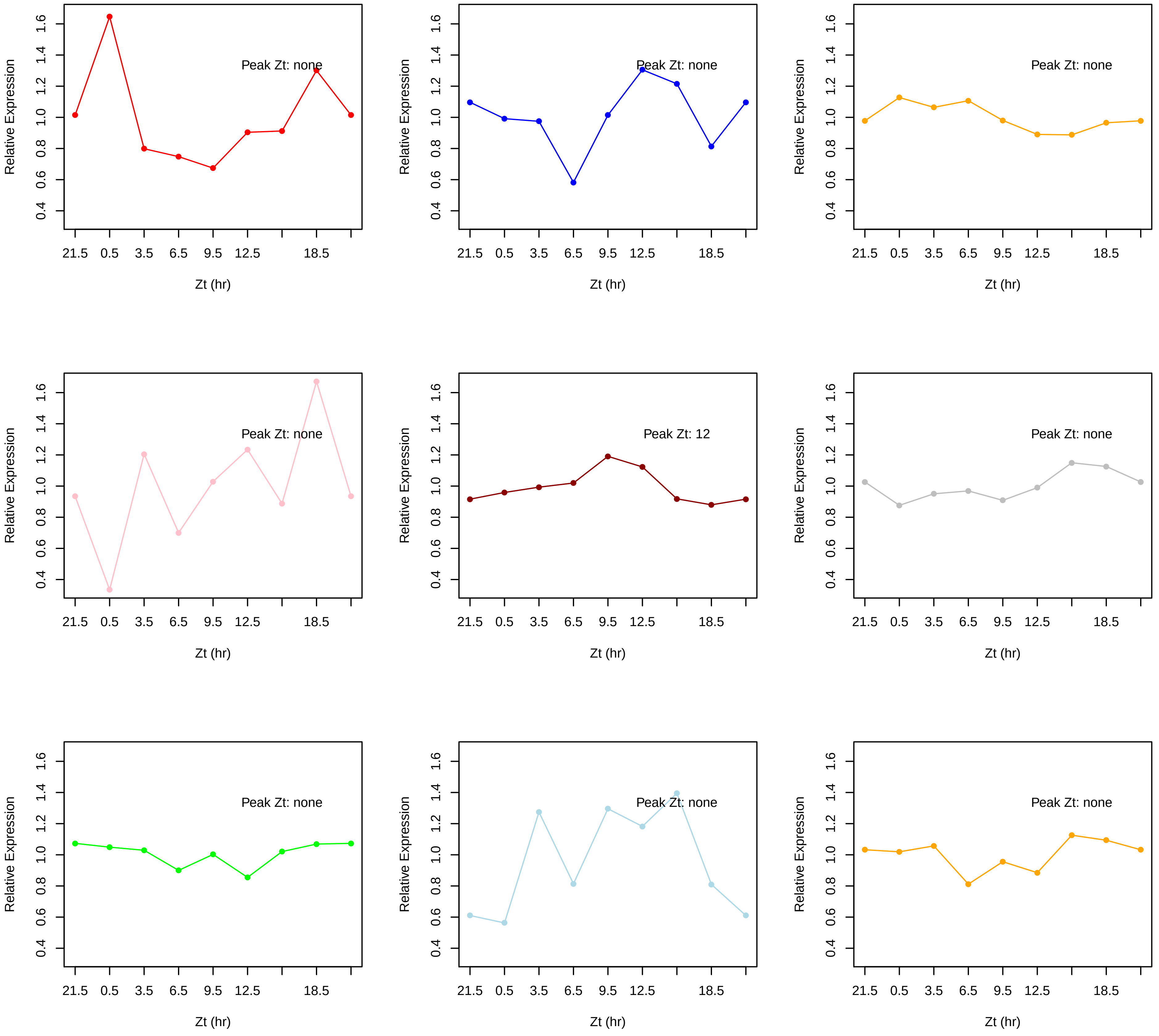

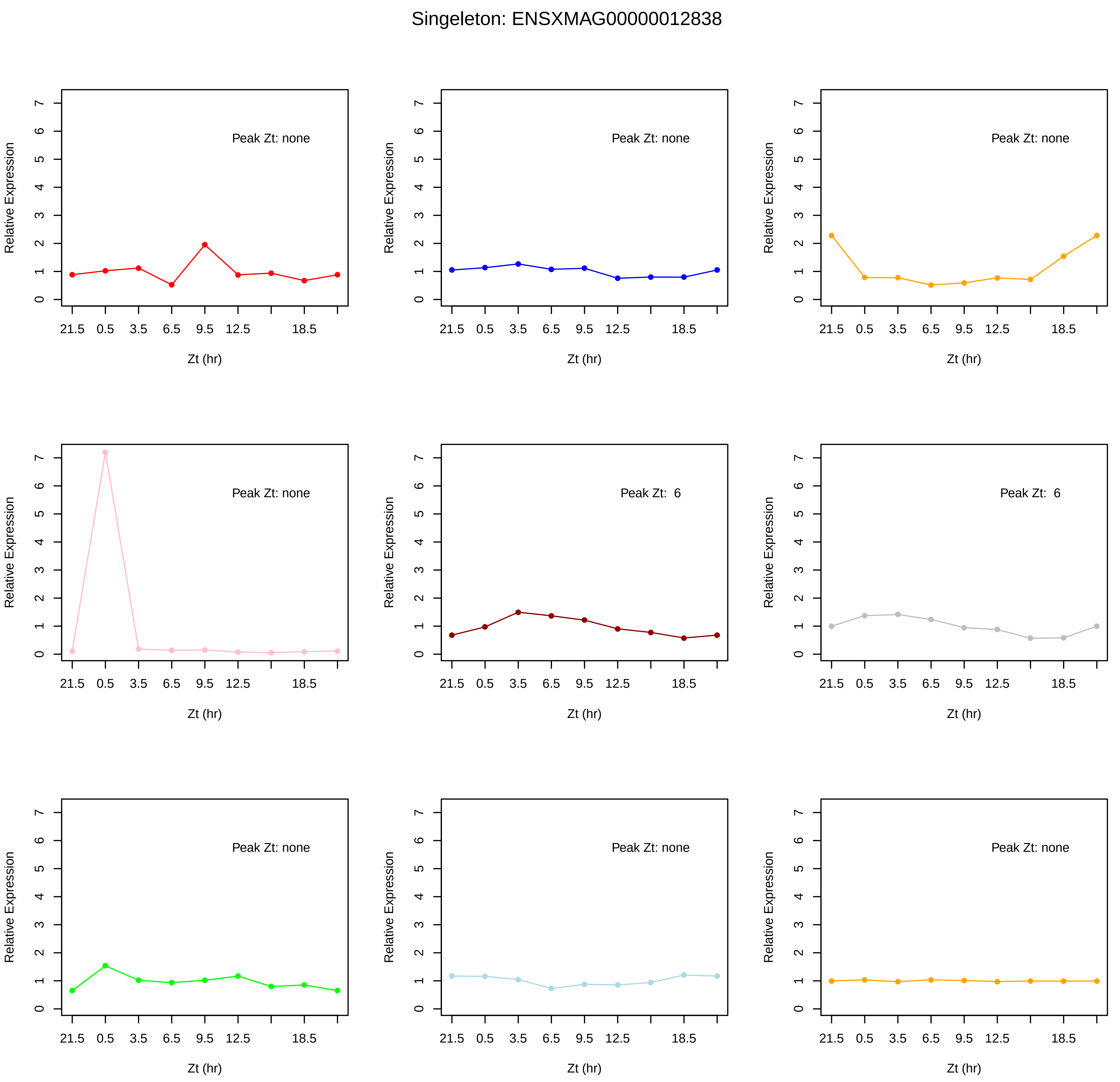
Singleton: ENSXMAG00000012838
Peak Zt: none
Peak Zt: none
Peak Zt: none
Peak Zt: none
Peak Zt: 6
Peak Zt: 6
Peak Zt: none
Peak Zt: none
Peak Zt: none
Relative Expression
Zt (hr)
21.5
0.5
3.5
6.5
9.5
12.5
18.5

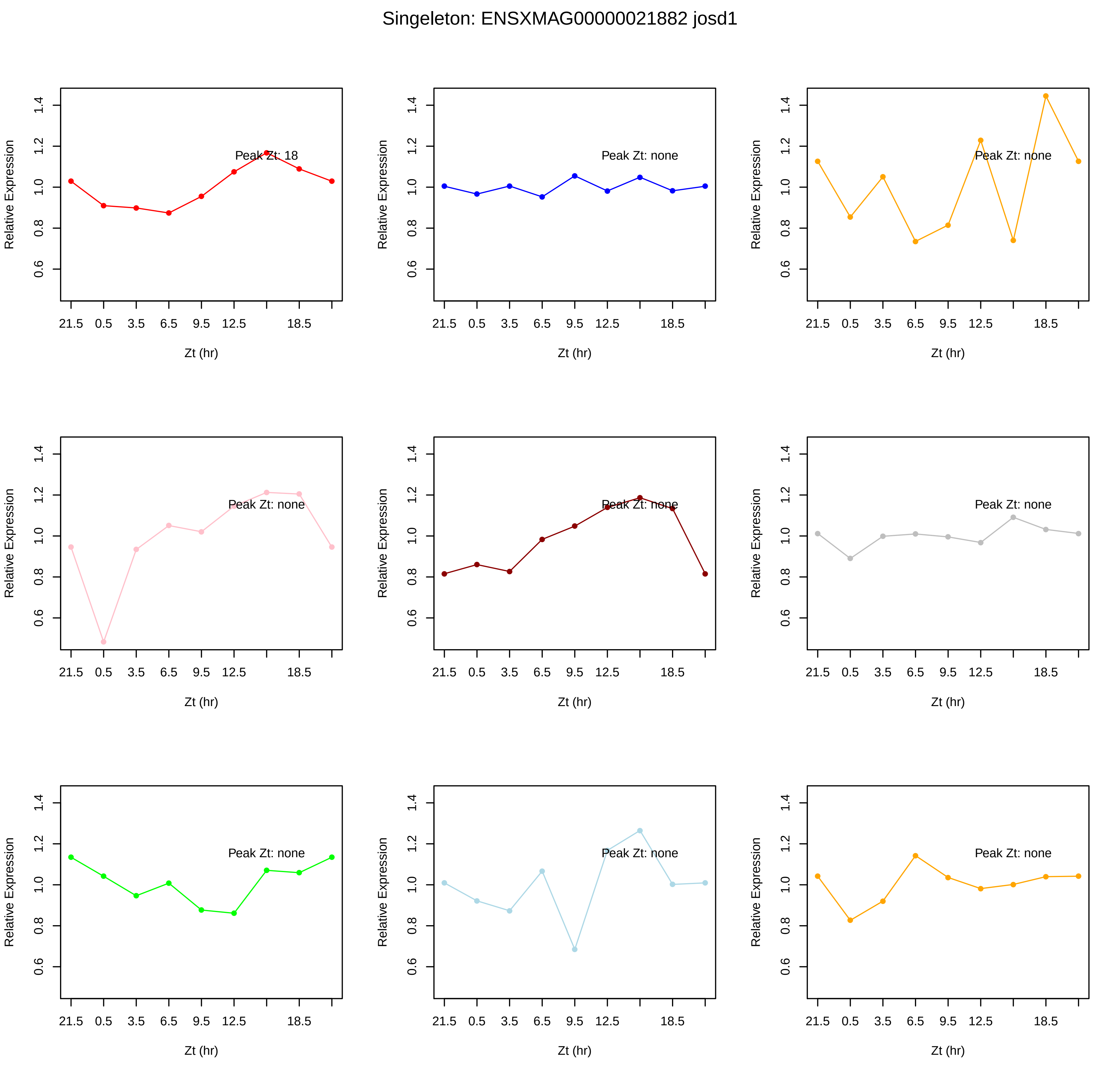
Singeleton: ENSXMAG00000021882 josd1
Peak Zt: 18
Peak Zt: none
Relative Expression
Zt (hr)

Singeleton: ENSXMAG00000007912

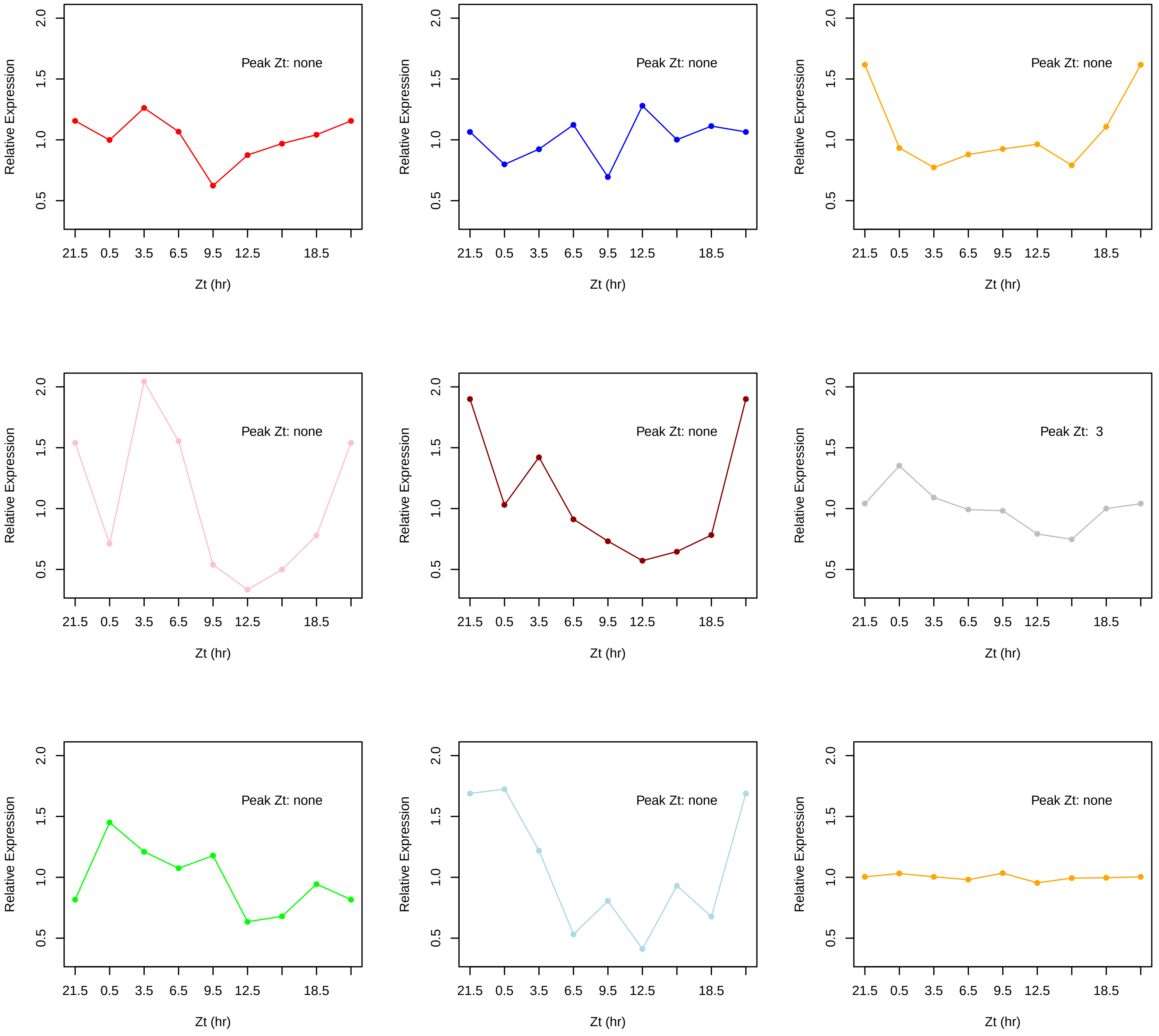

Singeleton: ENSXMAG00000022086 aptx

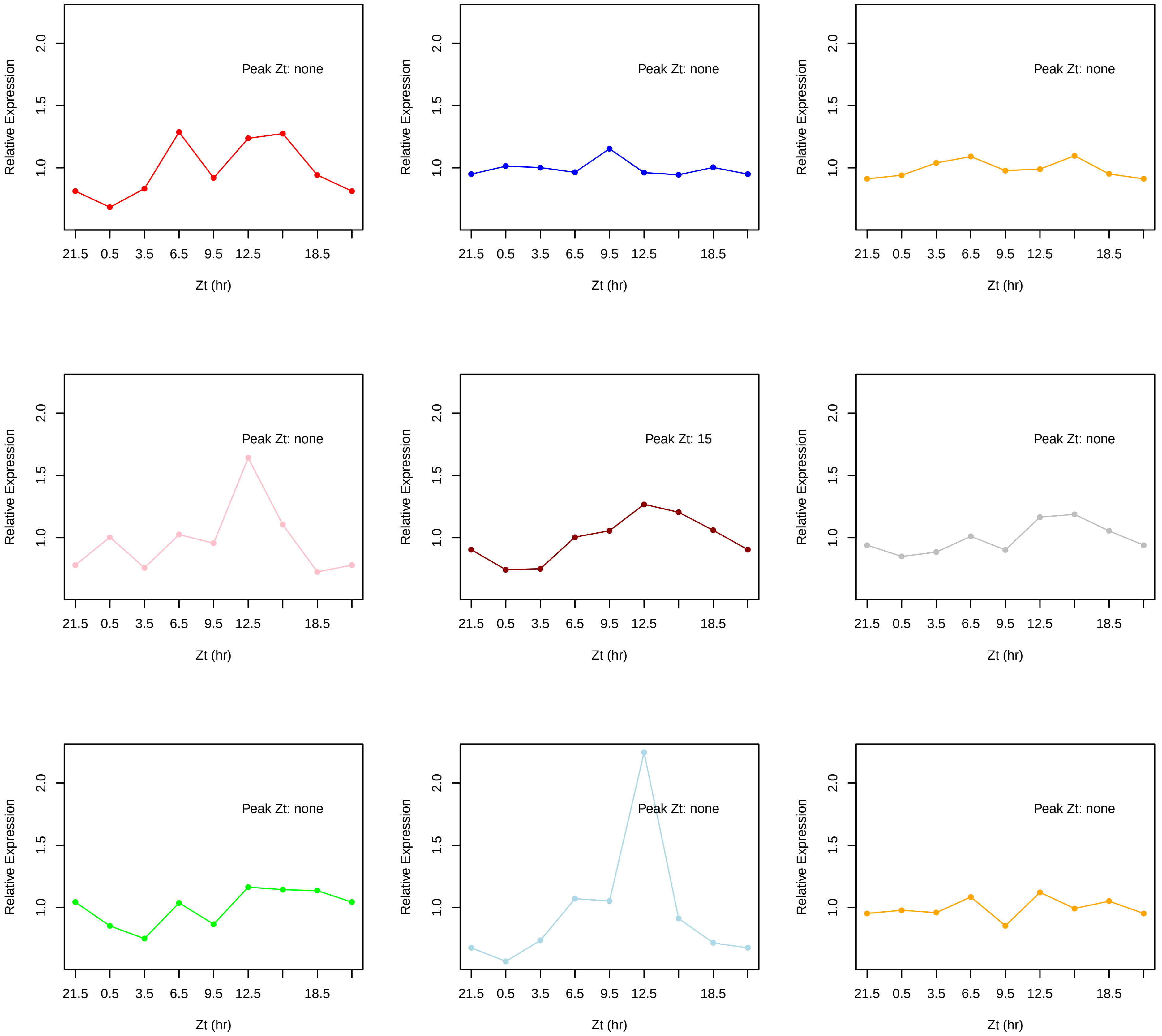

Singeleton: ENSXMAG00000007797 si:dkeyp−94b4.1

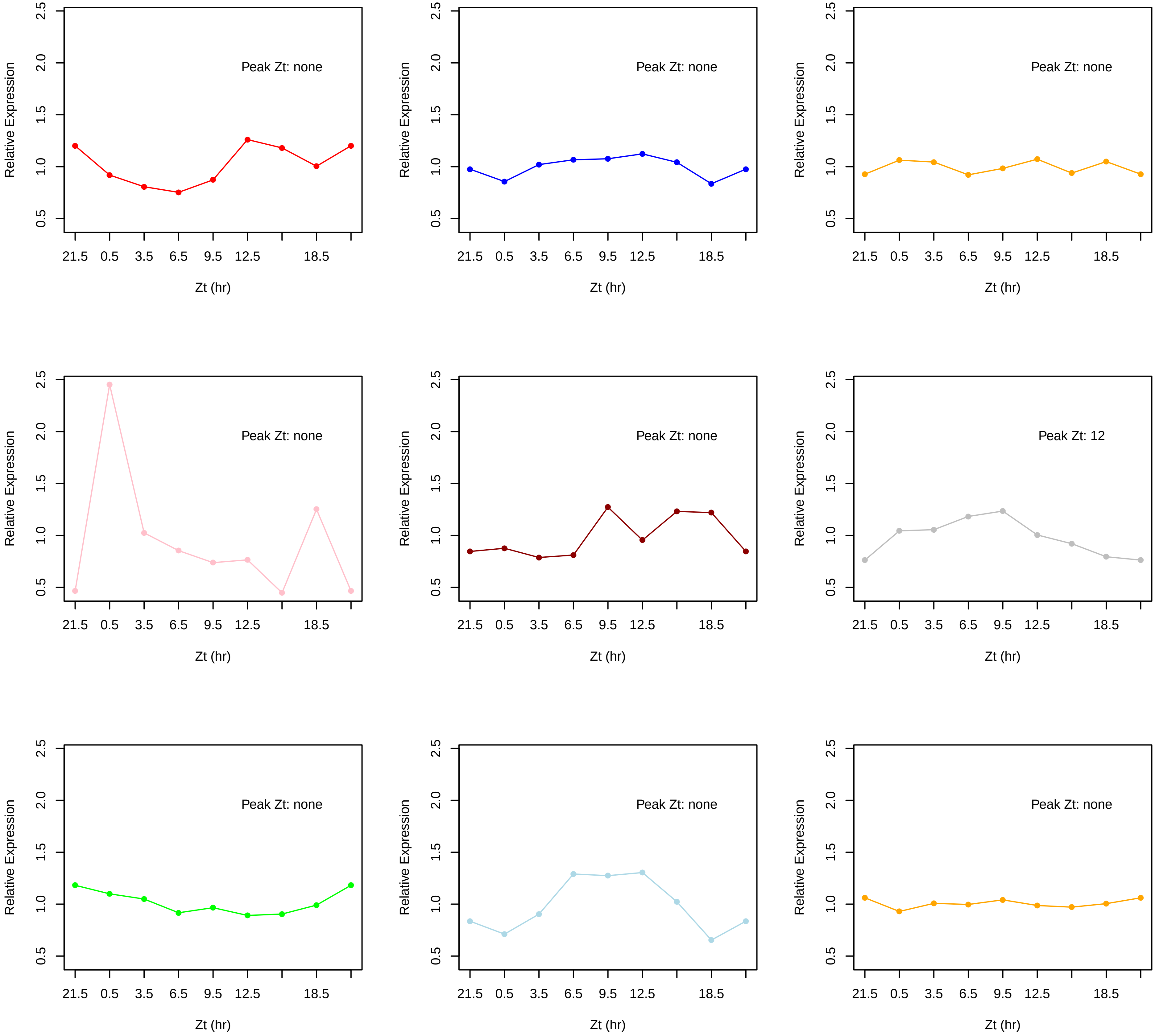

Singeleton: ENSXMAG00000005855 dnaja1

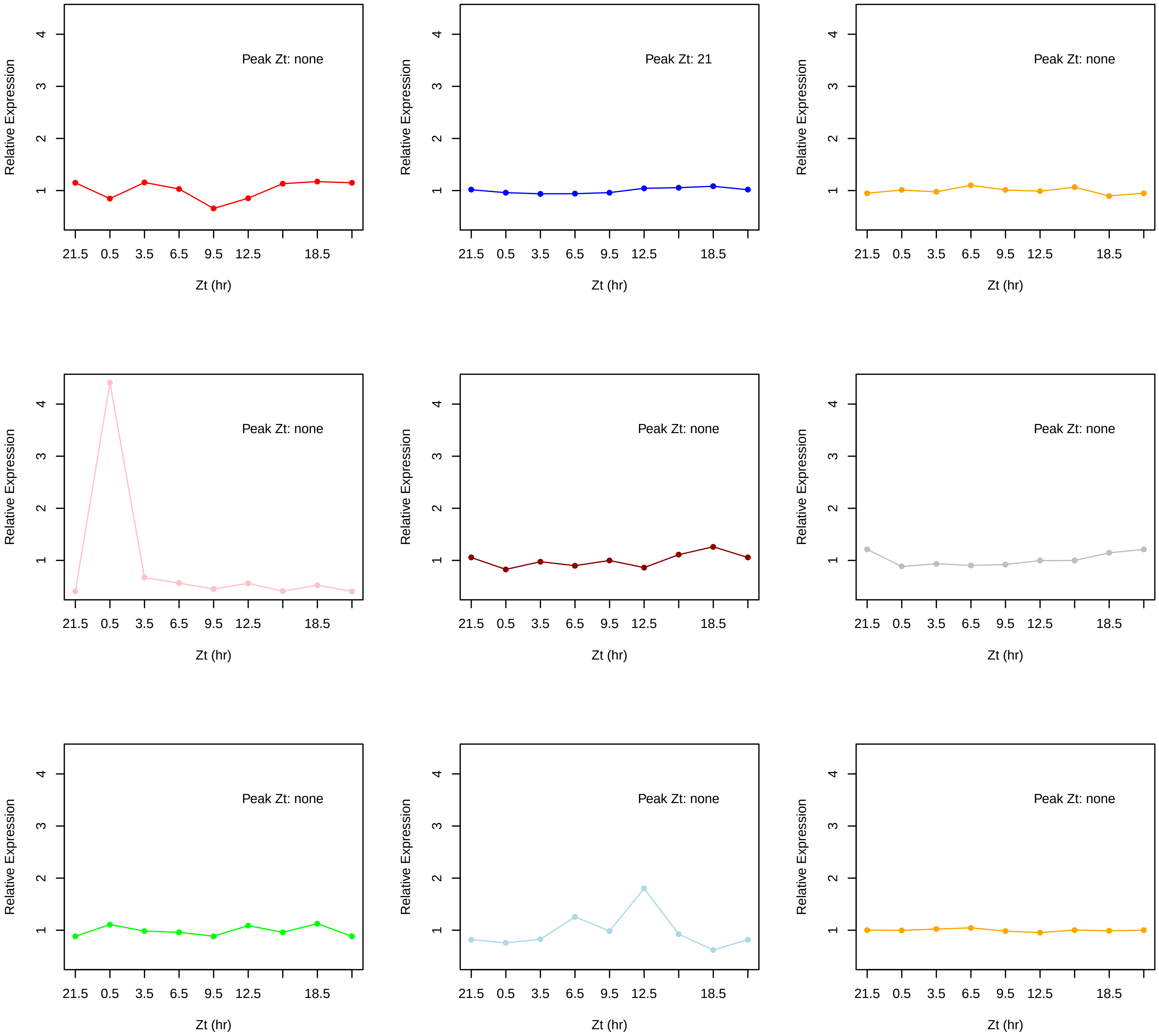

Singeleton: ENSXMAG00000003707 xpnpep3

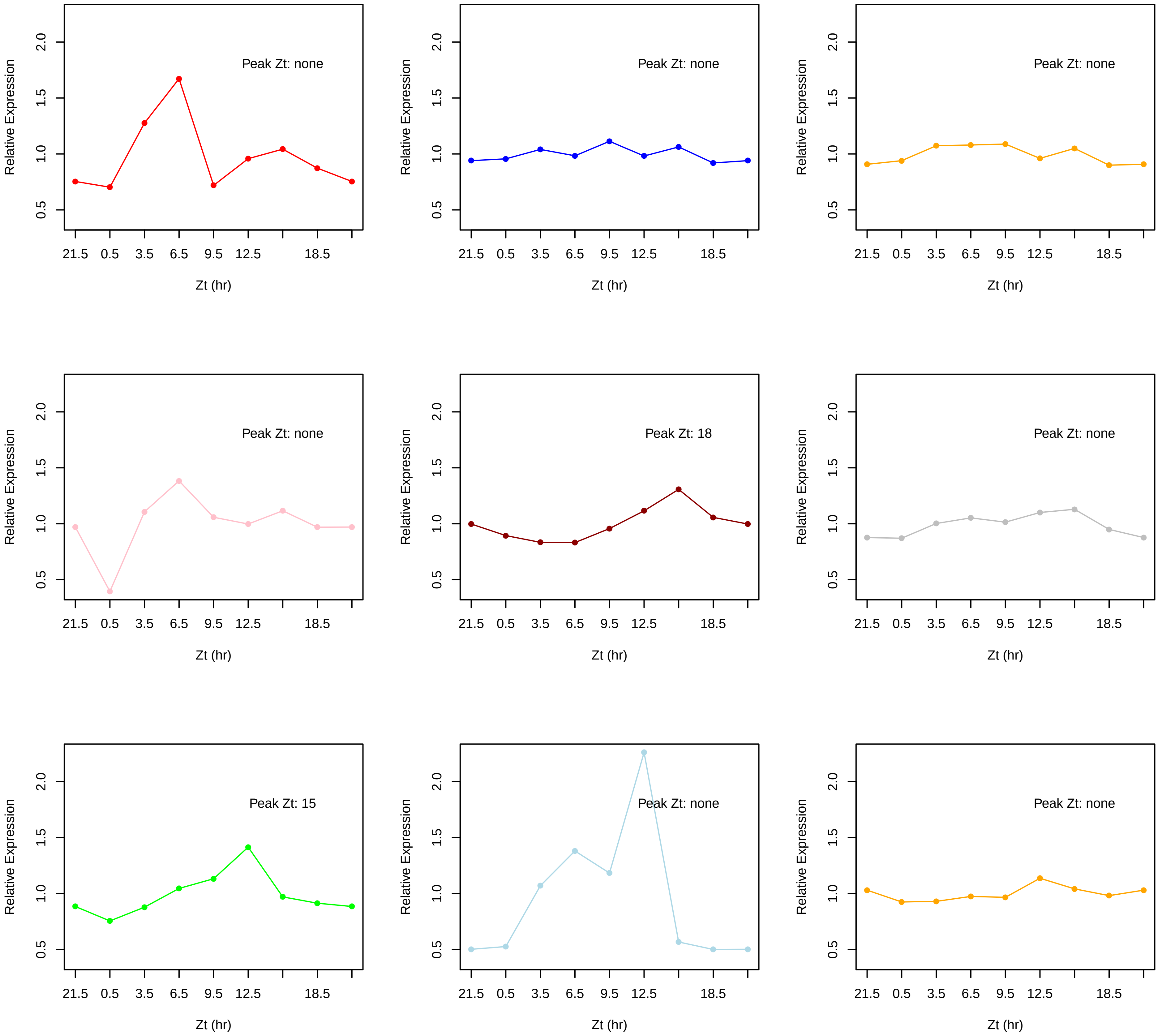

Singeleton: ENSXMAG00000005427 mob1bb

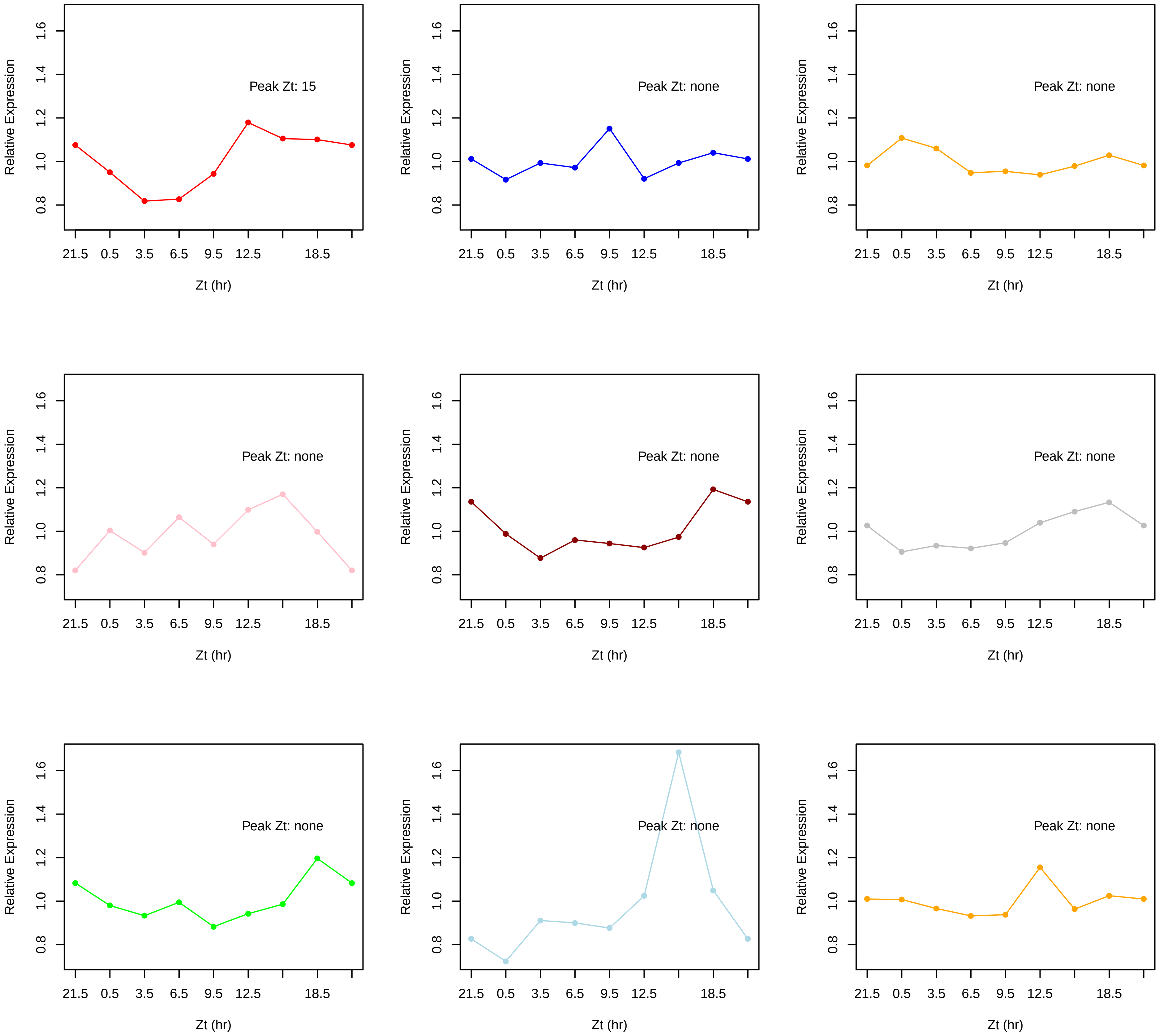

Singeleton: ENSXMAG00000012562 unk

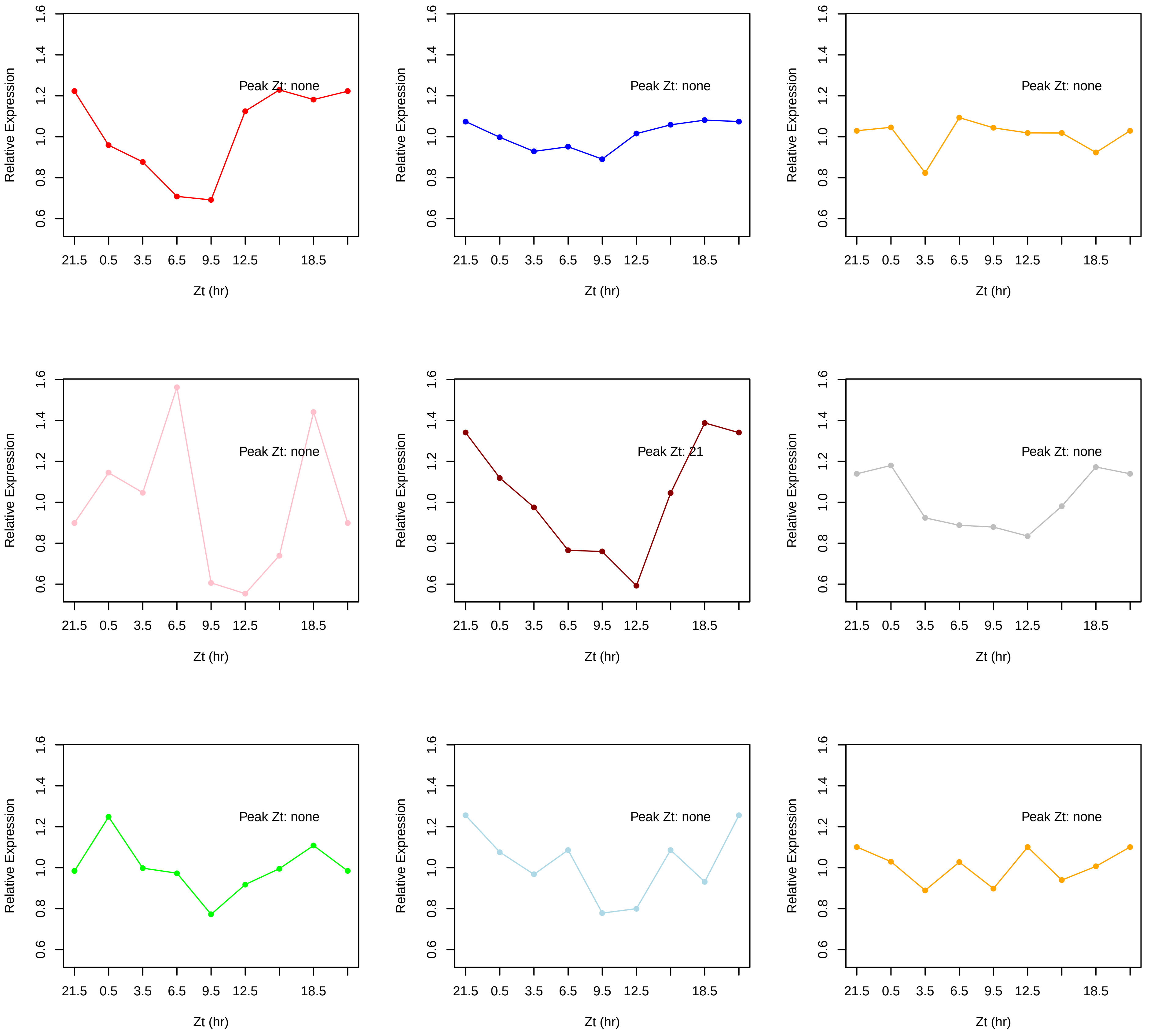

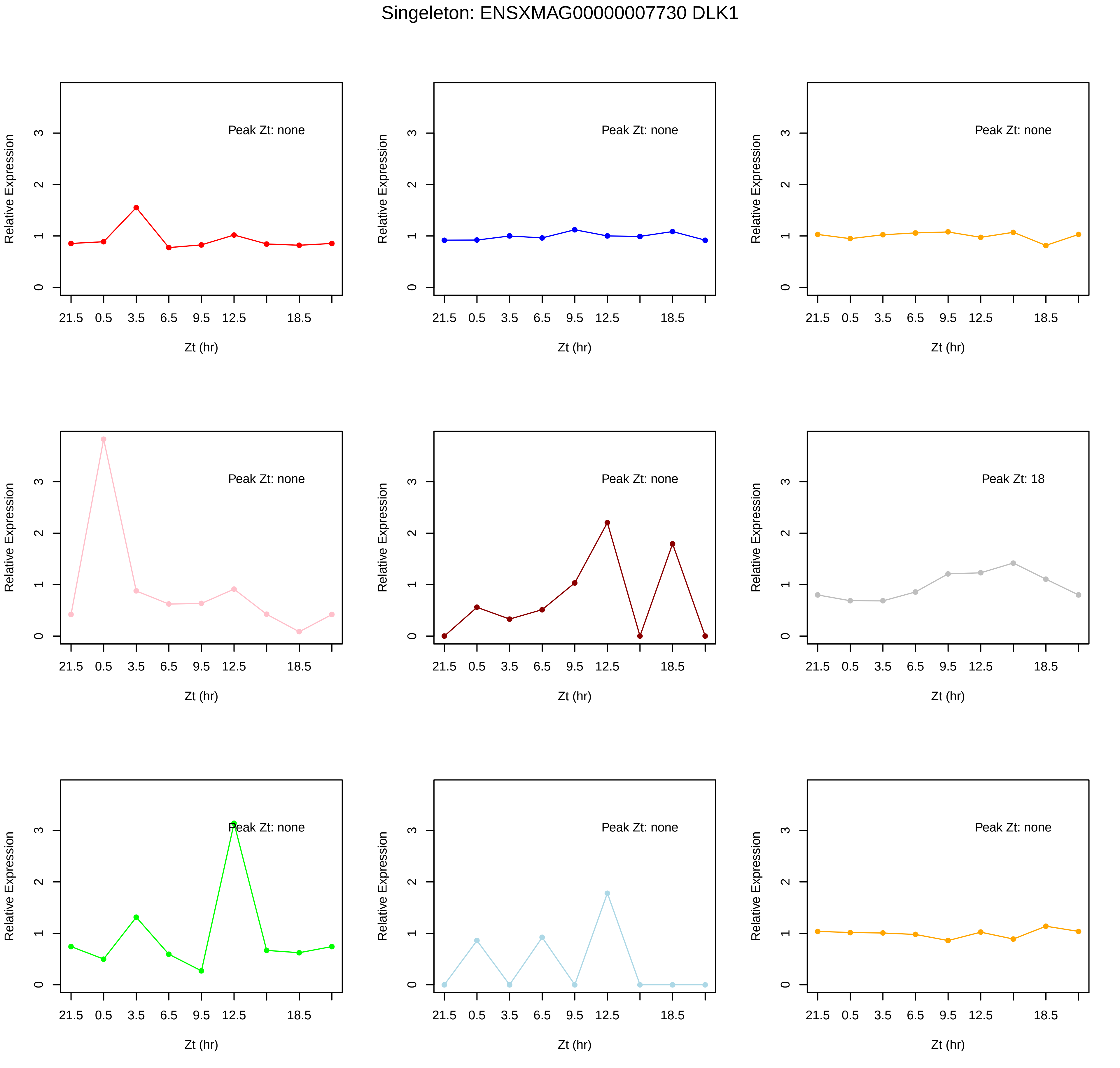

Singleton: ENSXMAG00000007730 DLK1
Peak Zt: none
Peak Zt: none
Peak Zt: none
Peak Zt: none
Peak Zt: none
Peak Zt: 18
Peak Zt: none
Peak Zt: none
Peak Zt: none
Relative Expression
Zt (hr)
21.5
0.5
3.5
6.5
9.5
12.5
18.5

Singeleton: ENSXMAG00000002591 acad11

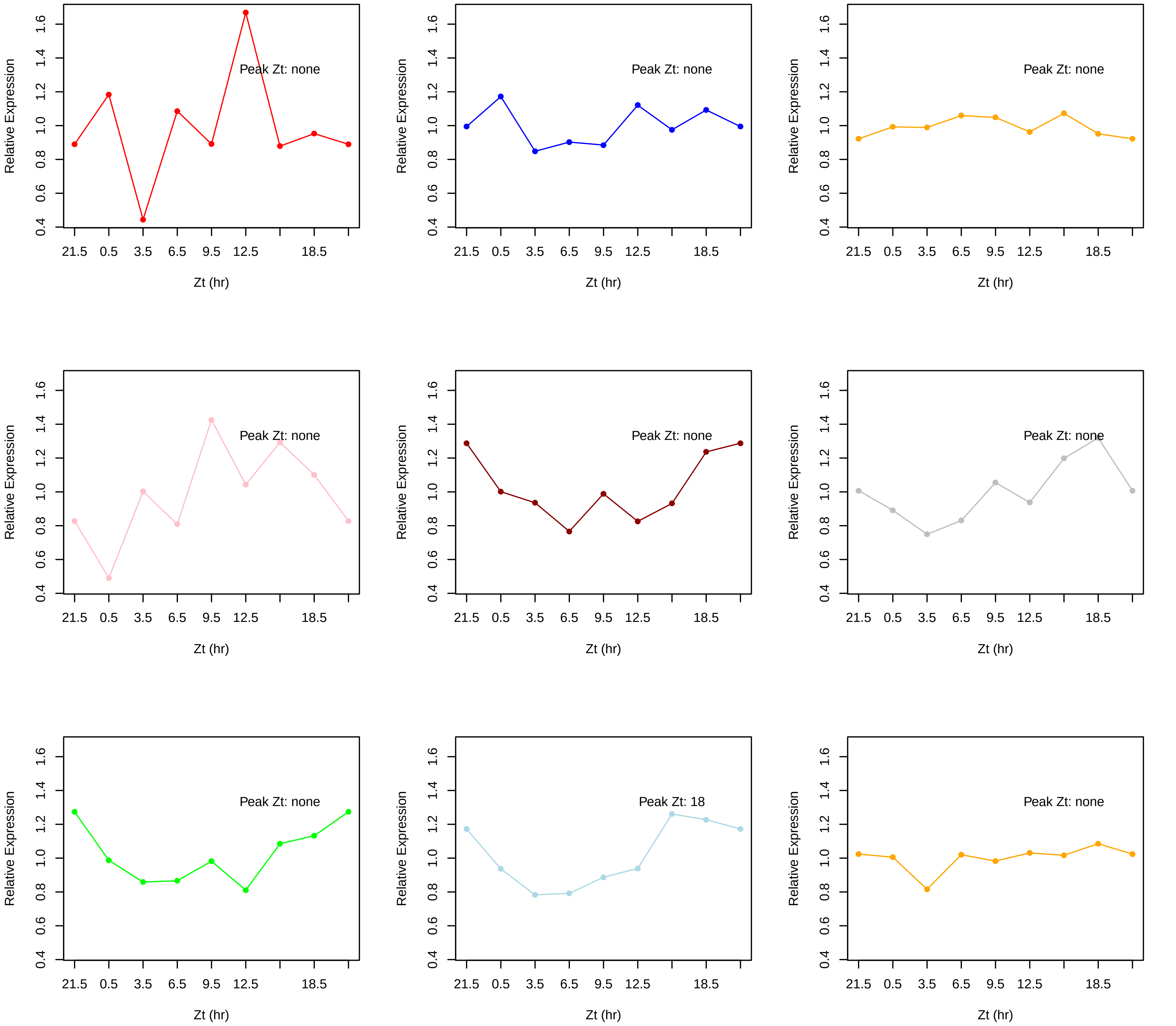

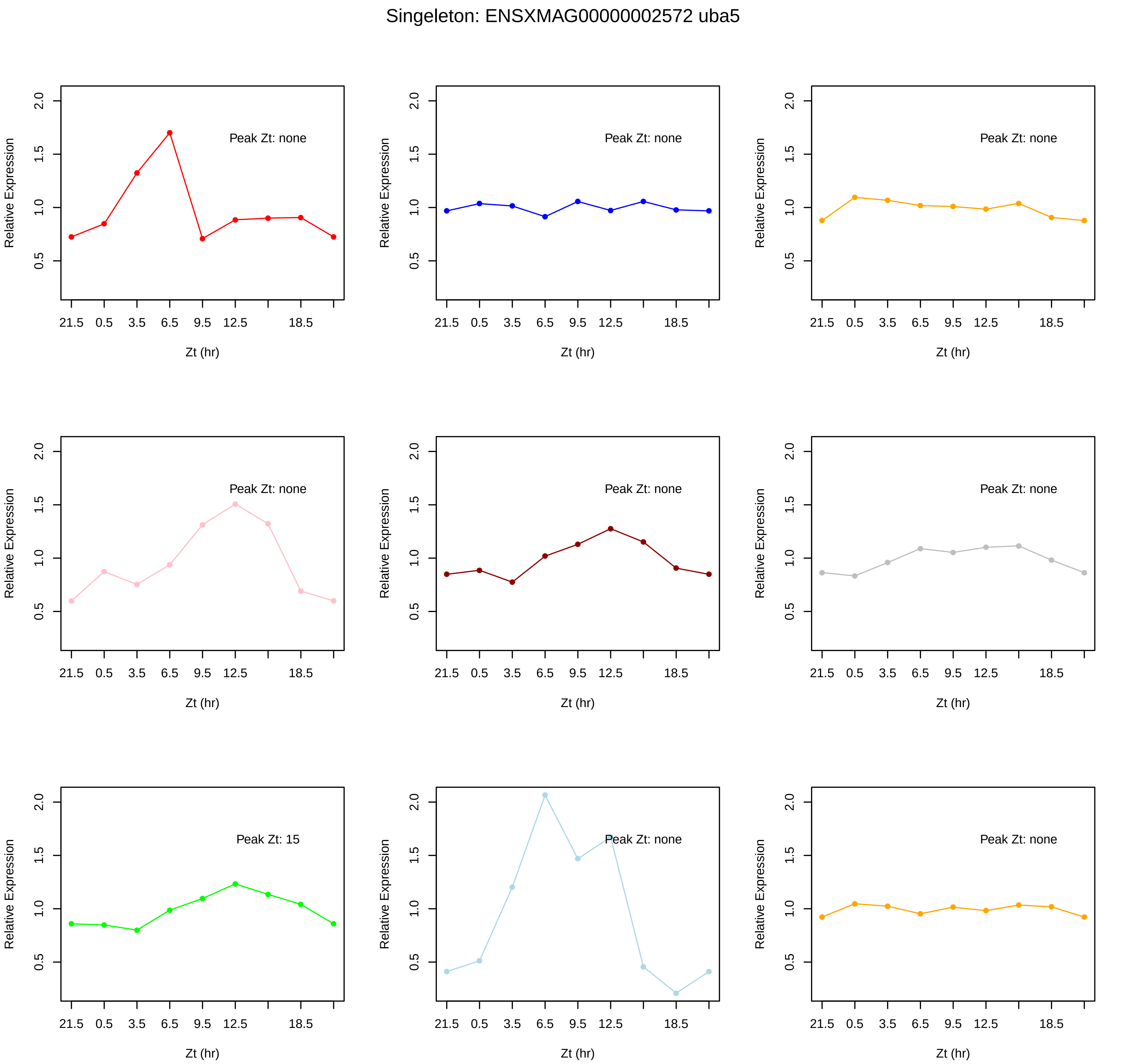
Singeleton: ENSXMAG00000002572 uba5
Peak Zt: none
Peak Zt: none
Peak Zt: none
Peak Zt: none
Peak Zt: none
Peak Zt: none
Peak Zt: 15
Peak Zt: none
Peak Zt: none
Relative Expression
Zt (hr)
21.5 0.5 3.5 6.5 9.5 12.5 18.5
0.5 1.0 1.5 2.0

Singeleton: ENSXMAG00000029086 WARS

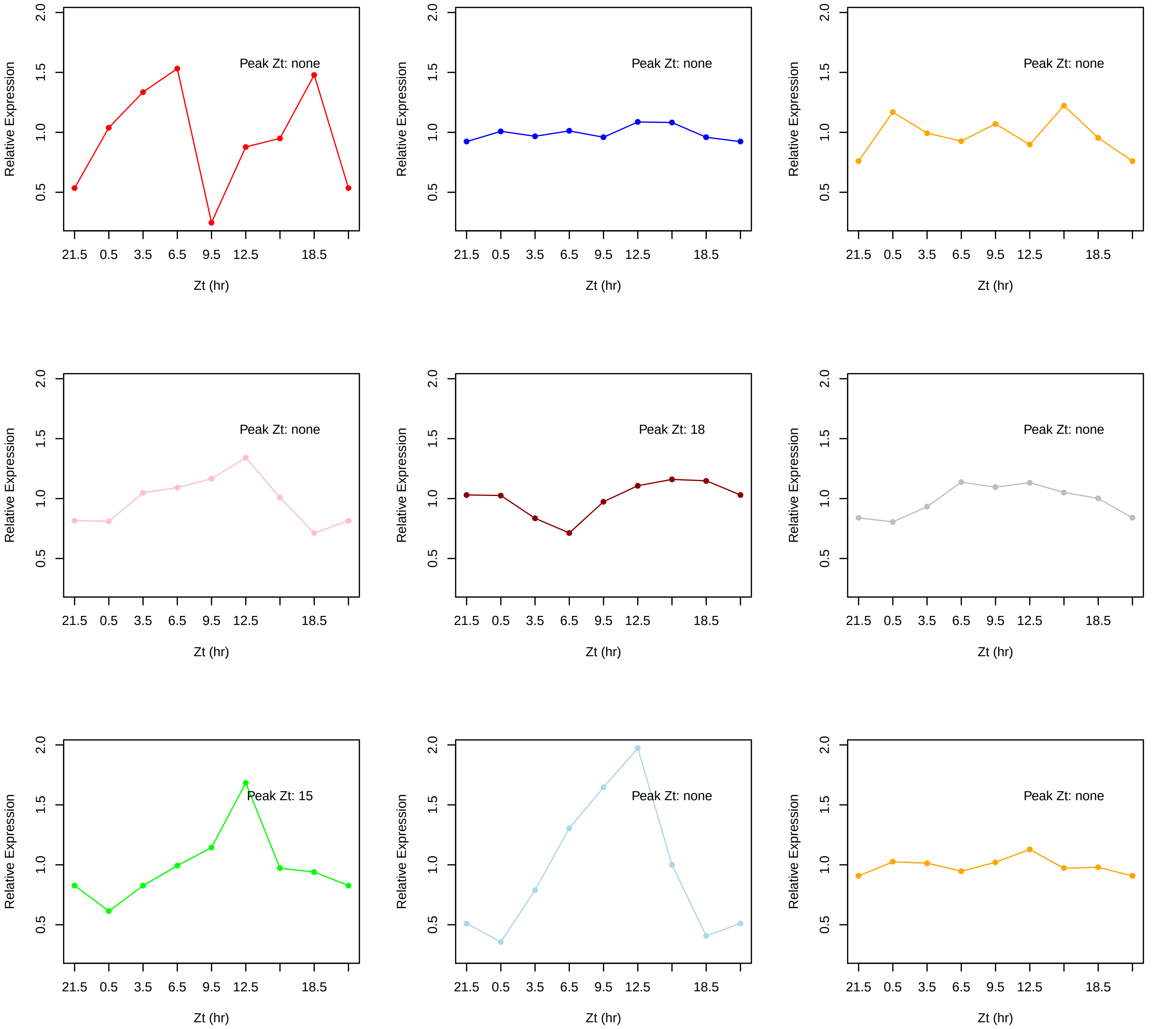

Singleton: ENSXMAG00000021999 TOB2

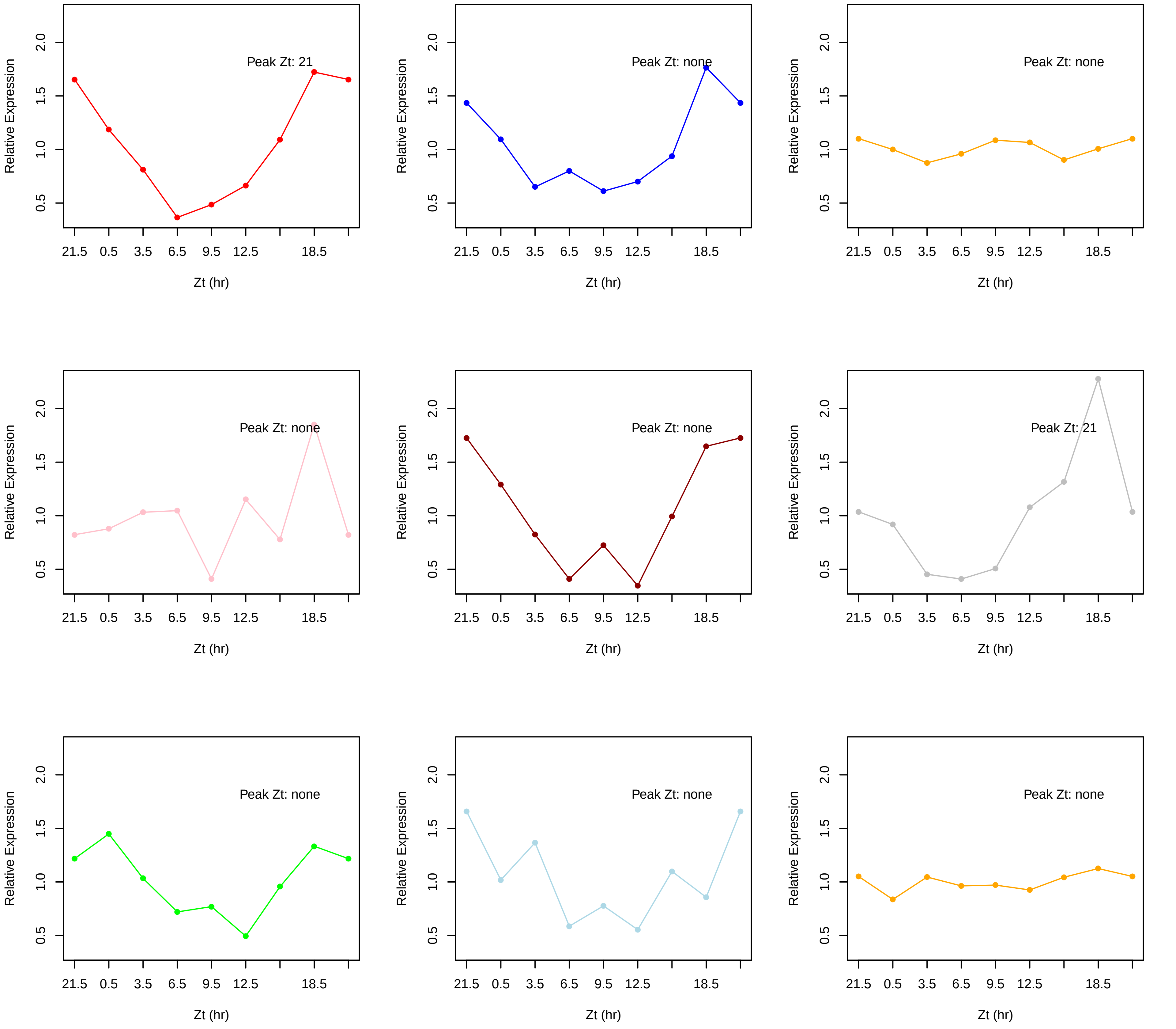

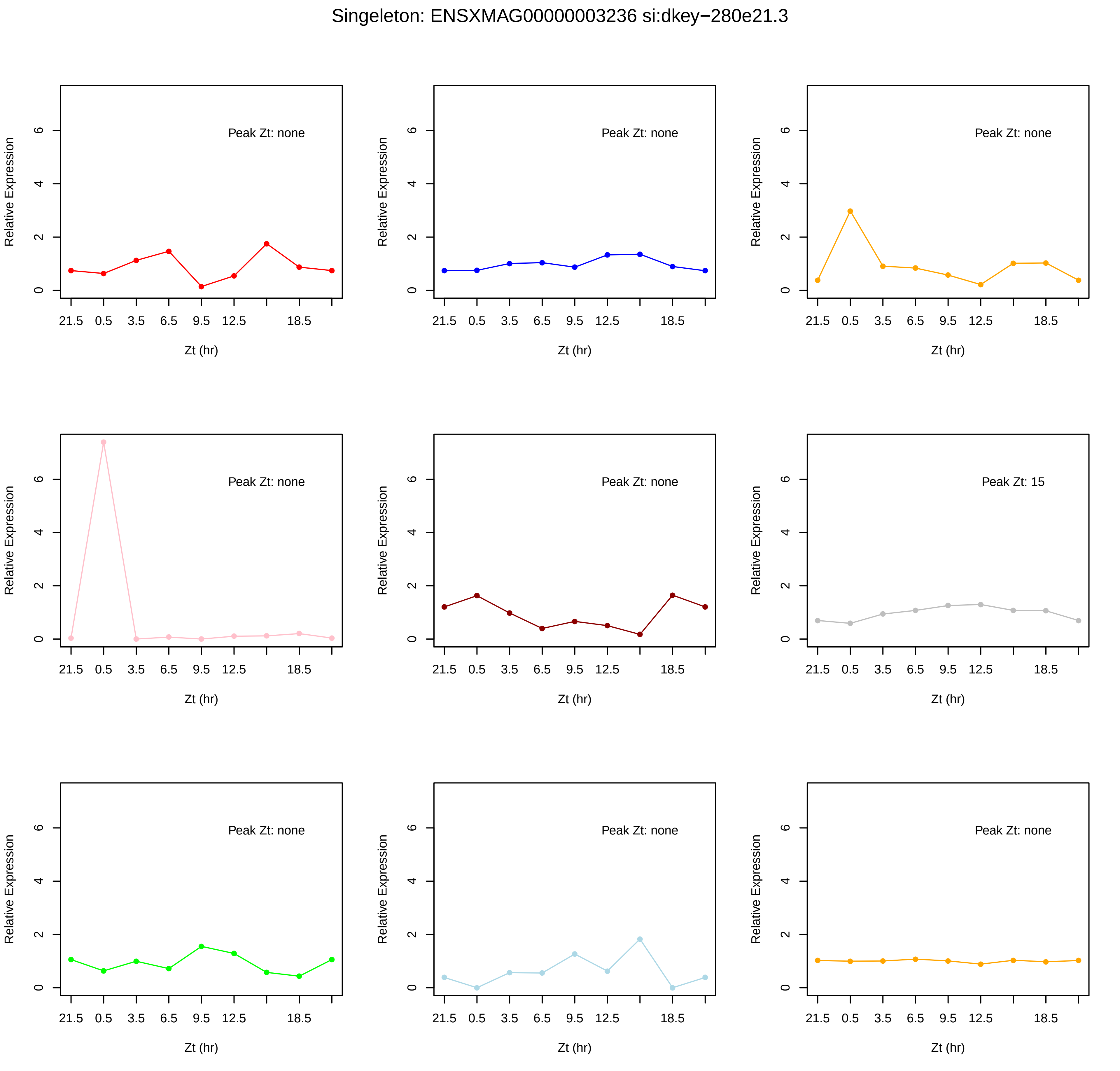
Singeleton: ENSXMAG00000003236 si:dkey−280e21.3
Peak Zt: none
Peak Zt: none
Peak Zt: none
Peak Zt: none
Peak Zt: none
Peak Zt: 15
Peak Zt: none
Peak Zt: none
Peak Zt: none
Relative Expression
Zt (hr)
21.5
0.5
3.5
6.5
9.5
12.5
18.5
0
2
4
6

Singeleton: ENSXMAG00000013521 slc25a47a

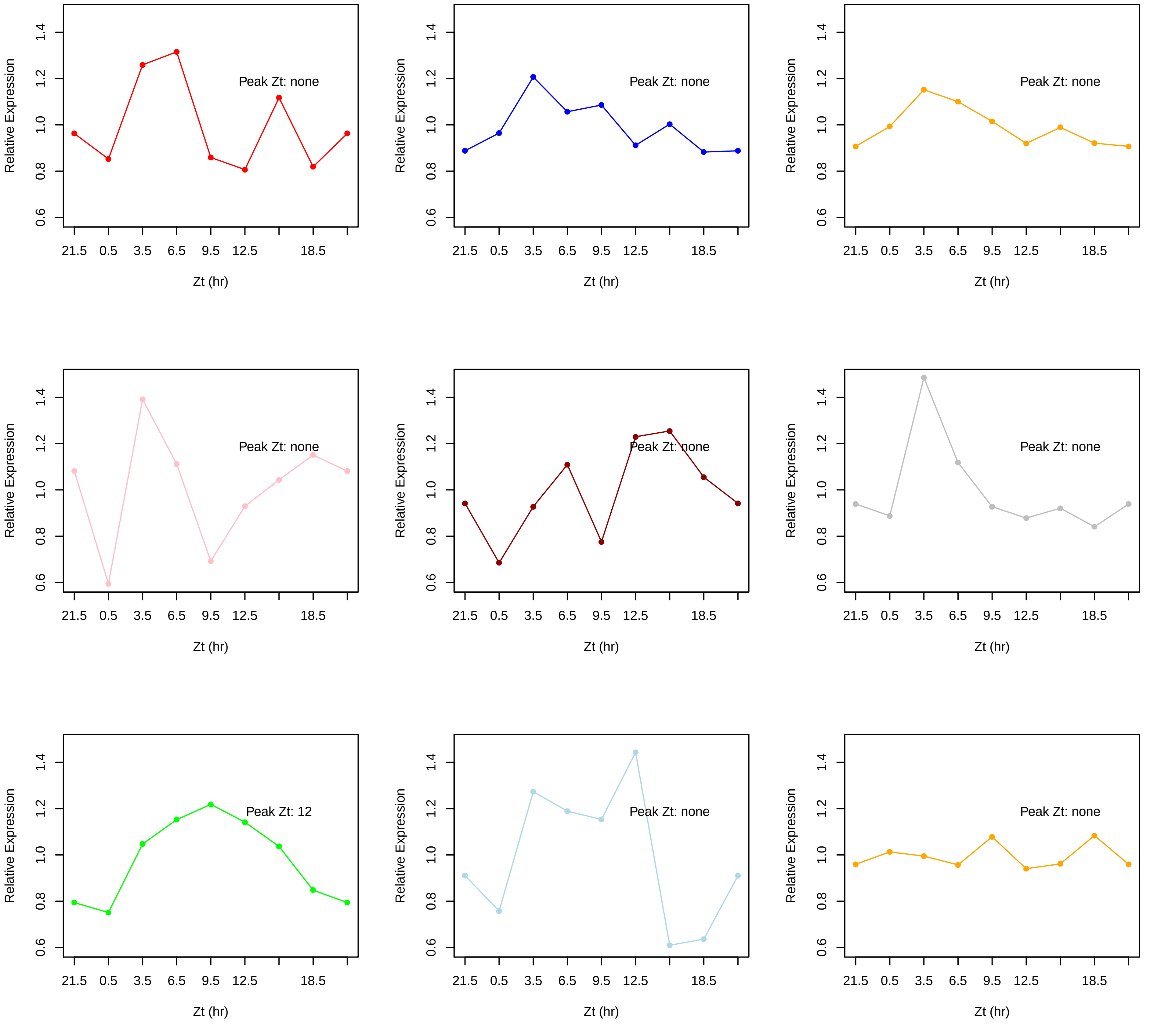

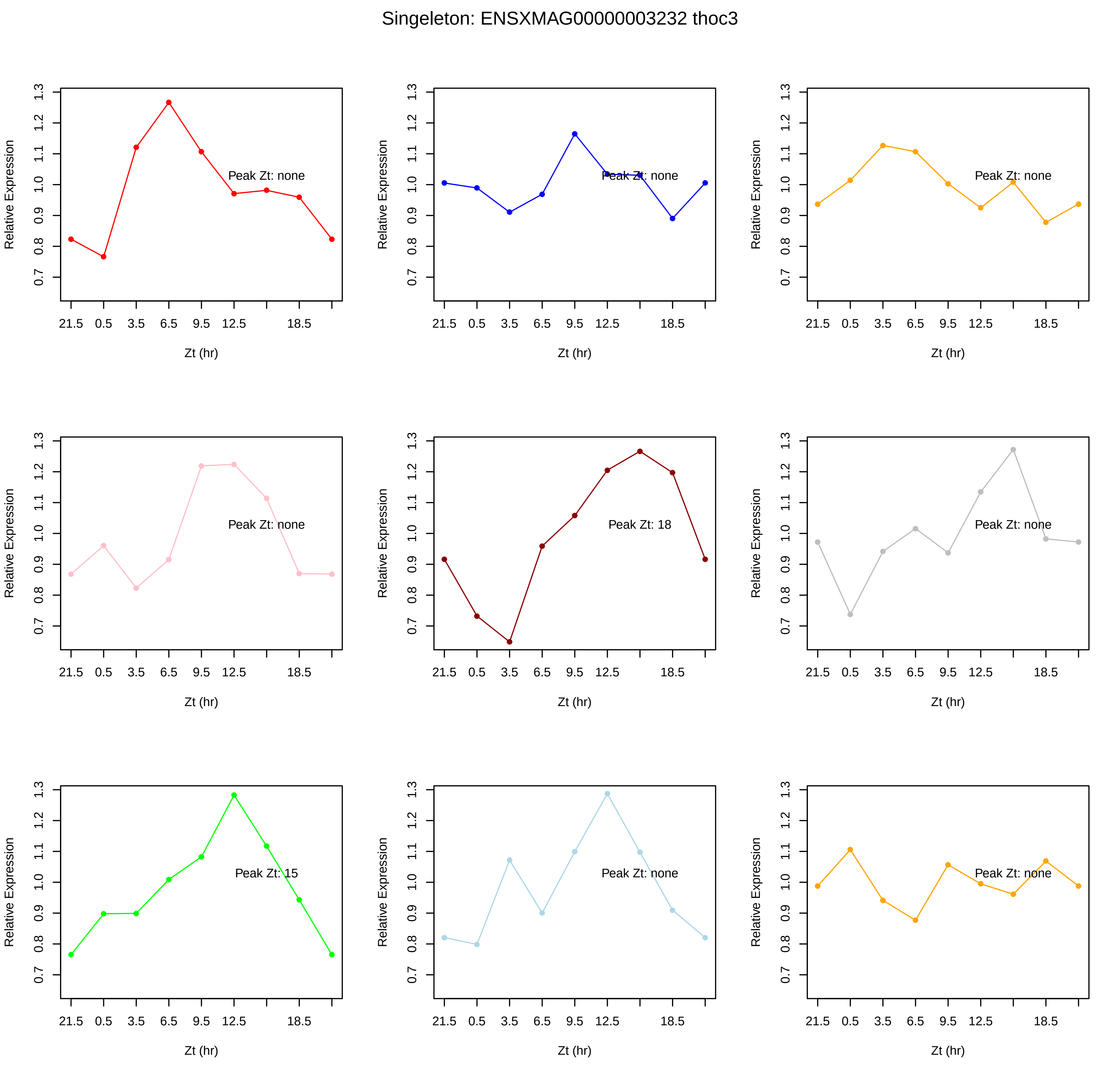
Singeleton: ENSXMAG00000003232 thoc3
Relative Expression
Zt (hr)
21.5 0.5 3.5 6.5 9.5 12.5 18.5
0.7 0.8 0.9 1.0 1.1 1.2 1.3
Peak Zt: none
Peak Zt: none
Peak Zt: none
Peak Zt: none
Peak Zt: 18
Peak Zt: none
Peak Zt: 15
Peak Zt: none
Peak Zt: none

Singleton: ENSXMAG00000006921 polr3h

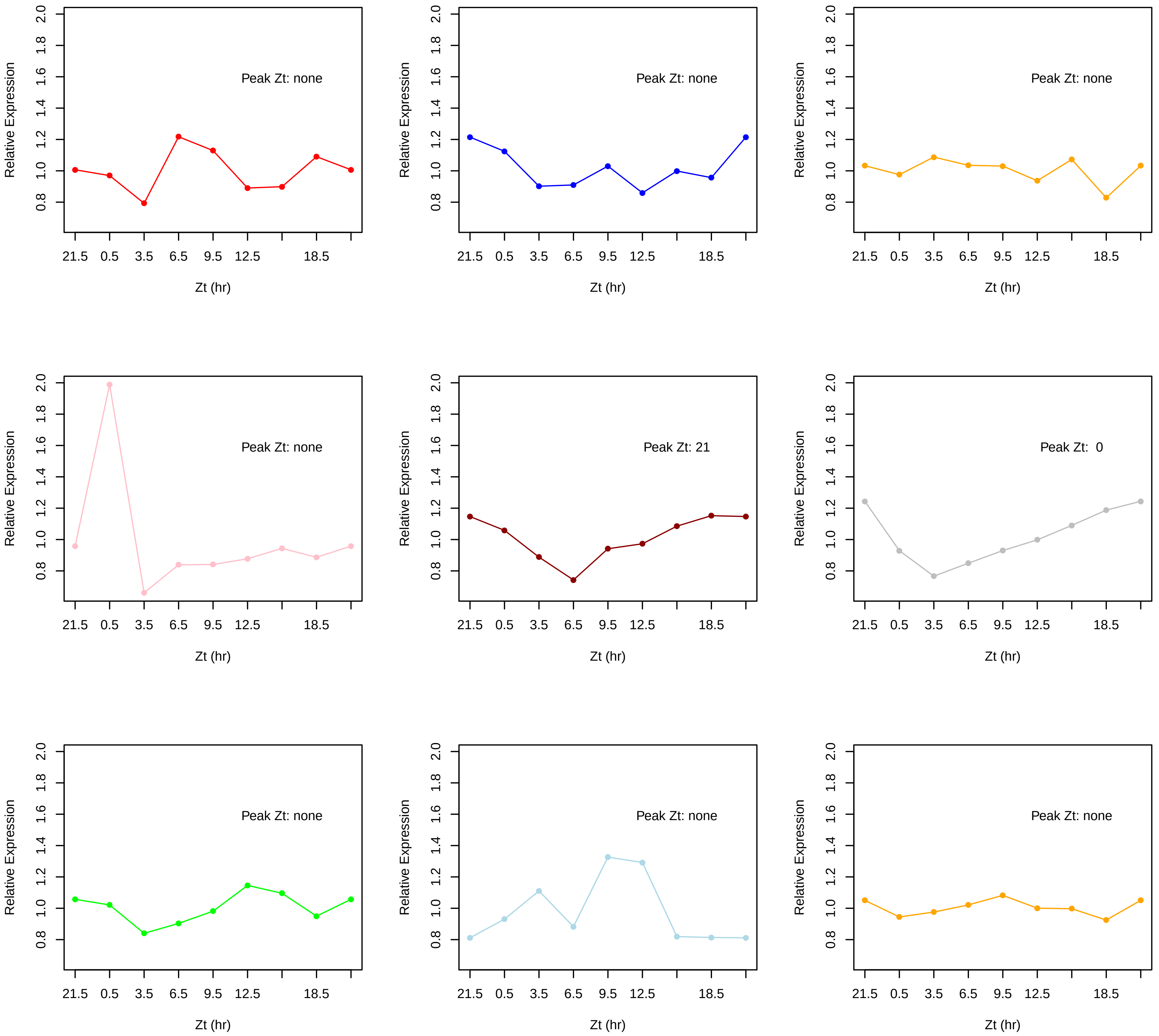

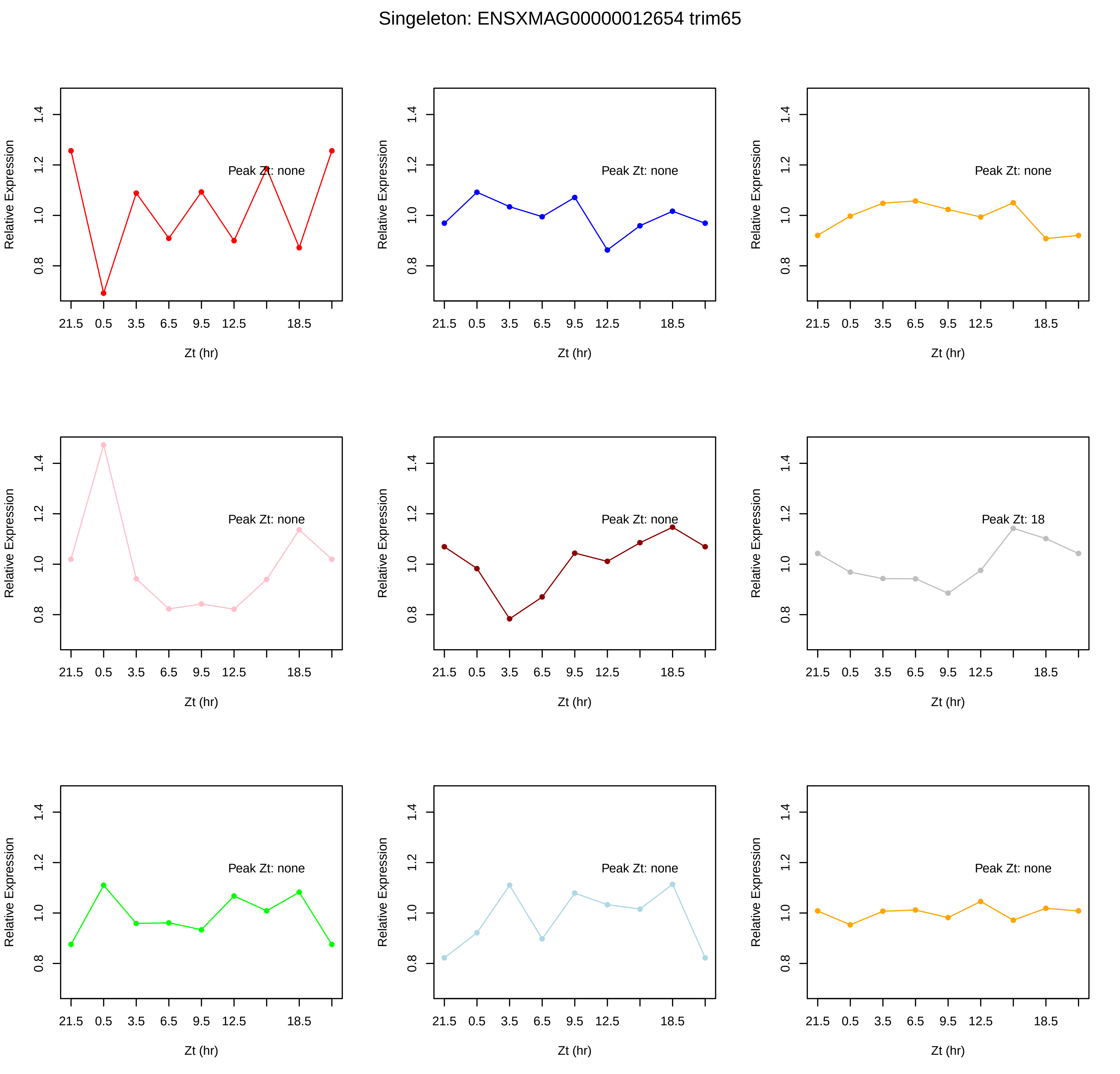
Singeleton: ENSXMAG00000012654 trim65
Peak Zt: none
Peak Zt: none
Peak Zt: none
Peak Zt: none
Peak Zt: none
Peak Zt: 18
Peak Zt: none
Peak Zt: none
Peak Zt: none
Relative Expression
Zt (hr)
21.5 0.5 3.5 6.5 9.5 12.5 18.5
0.8 1.0 1.2 1.4

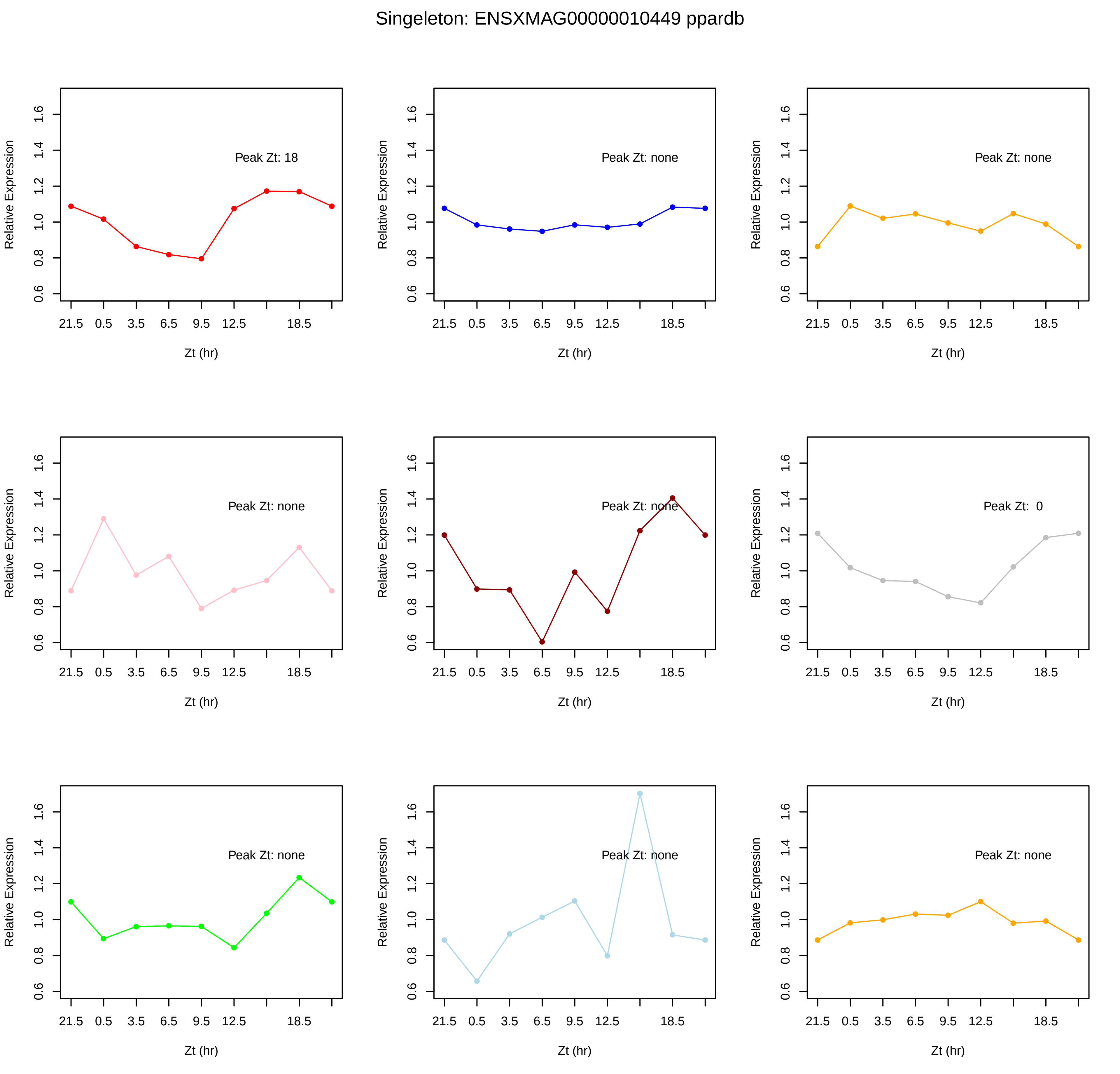

Singeleton: ENSXMAG00000010449 ppardb
Peak Zt: 18
Peak Zt: none
Peak Zt: none
Peak Zt: none
Peak Zt: none
Peak Zt: 0
Peak Zt: none
Peak Zt: none
Peak Zt: none
Relative Expression
Zt (hr)
21.5
0.5
3.5
6.5
9.5
12.5
18.5
0.6
0.8
1.0
1.2
1.4
1.6

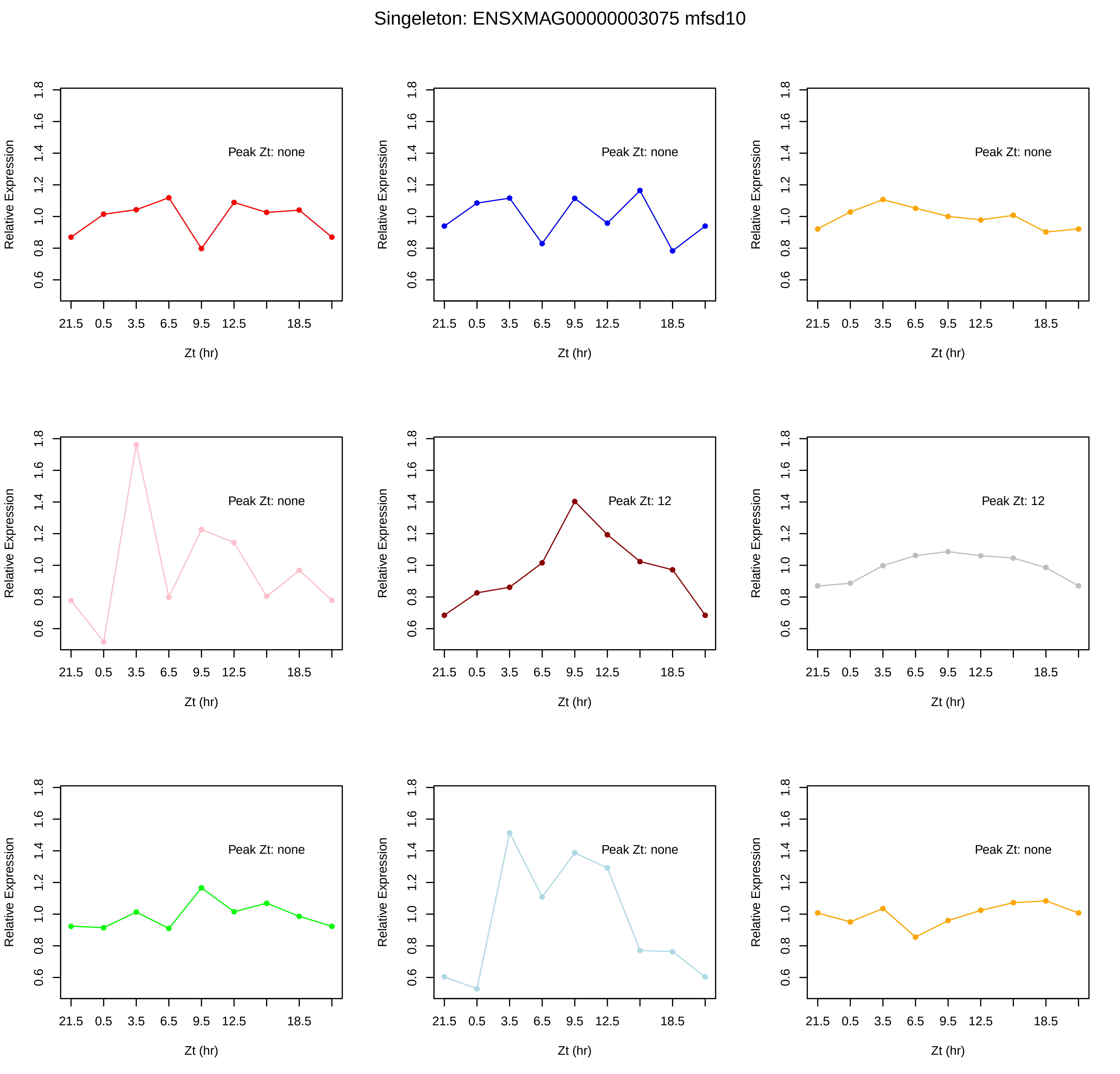

Singeleton: ENSXMAG00000003075 mfsd10
Peak Zt: none
Peak Zt: none
Peak Zt: none
Peak Zt: none
Peak Zt: 12
Peak Zt: 12
Peak Zt: none
Peak Zt: none
Peak Zt: none
Relative Expression
Zt (hr)
21.5
0.5
3.5
6.5
9.5
12.5
18.5
0.6
0.8
1.0
1.2
1.4
1.6
1.8

Singeleton: ENSXMAG00000006944 PMM1

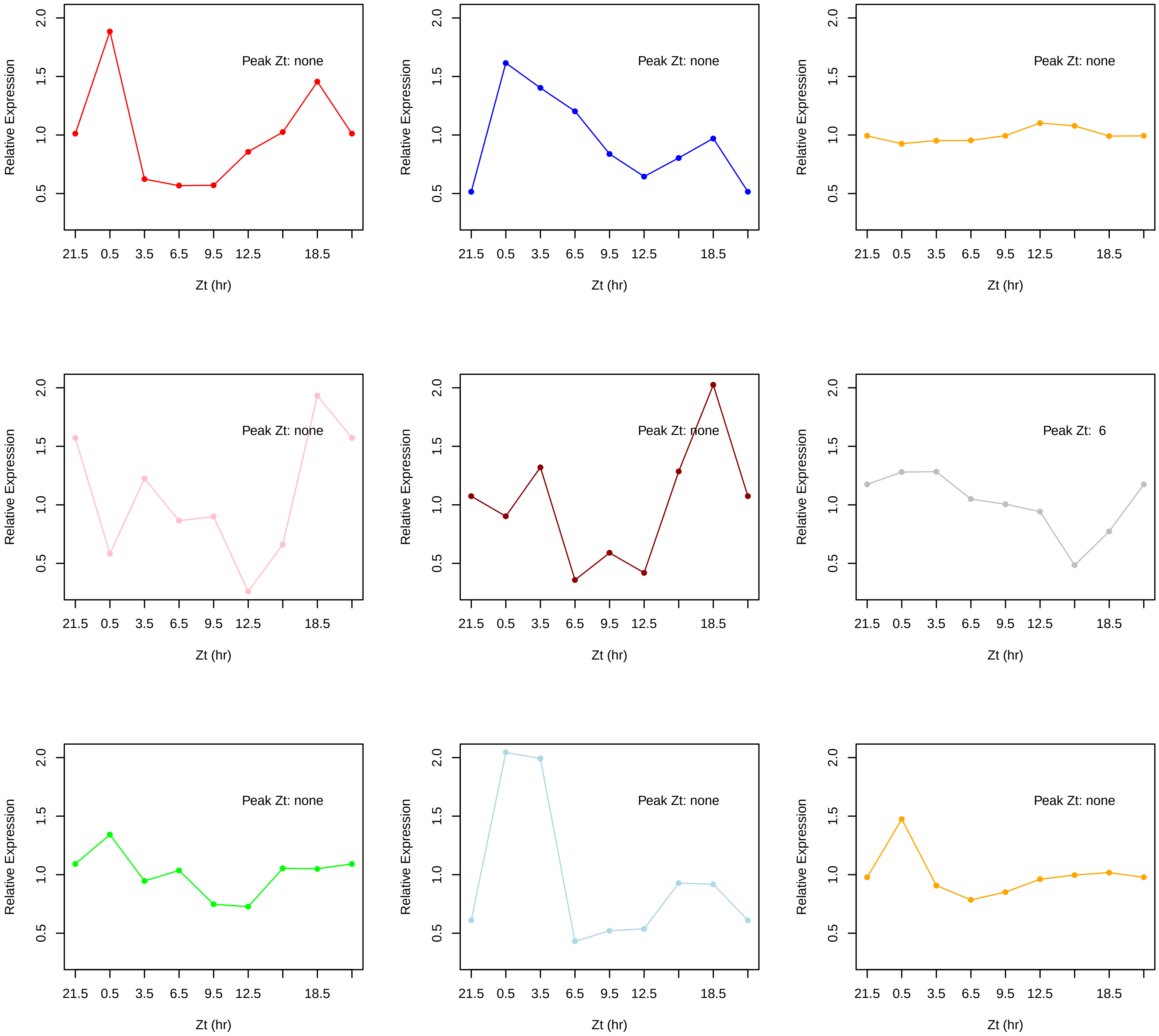

Singleton: ENSXMAG00000028318 CDYL

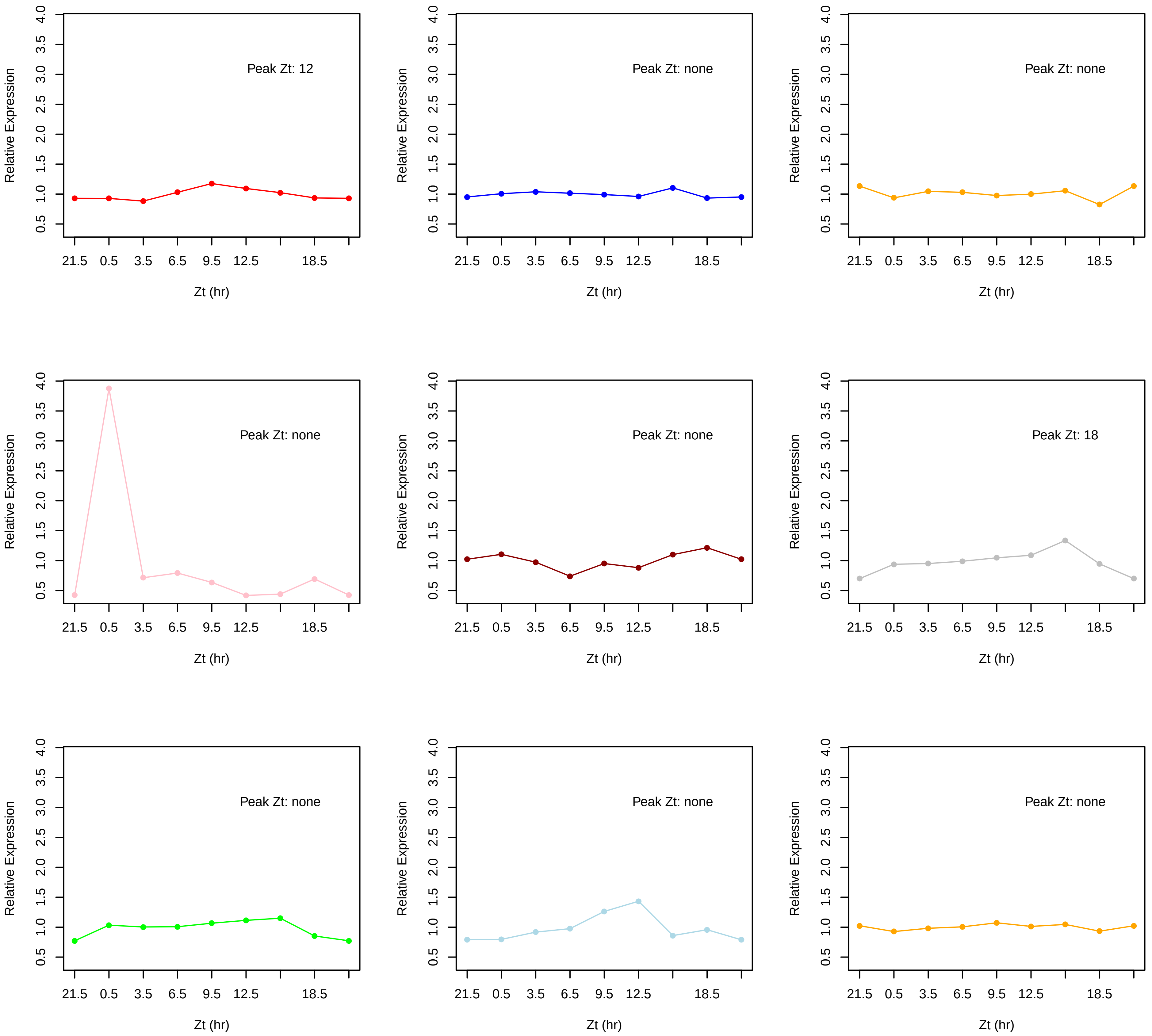

Singeleton: ENSXMAG00000005769 tlx1

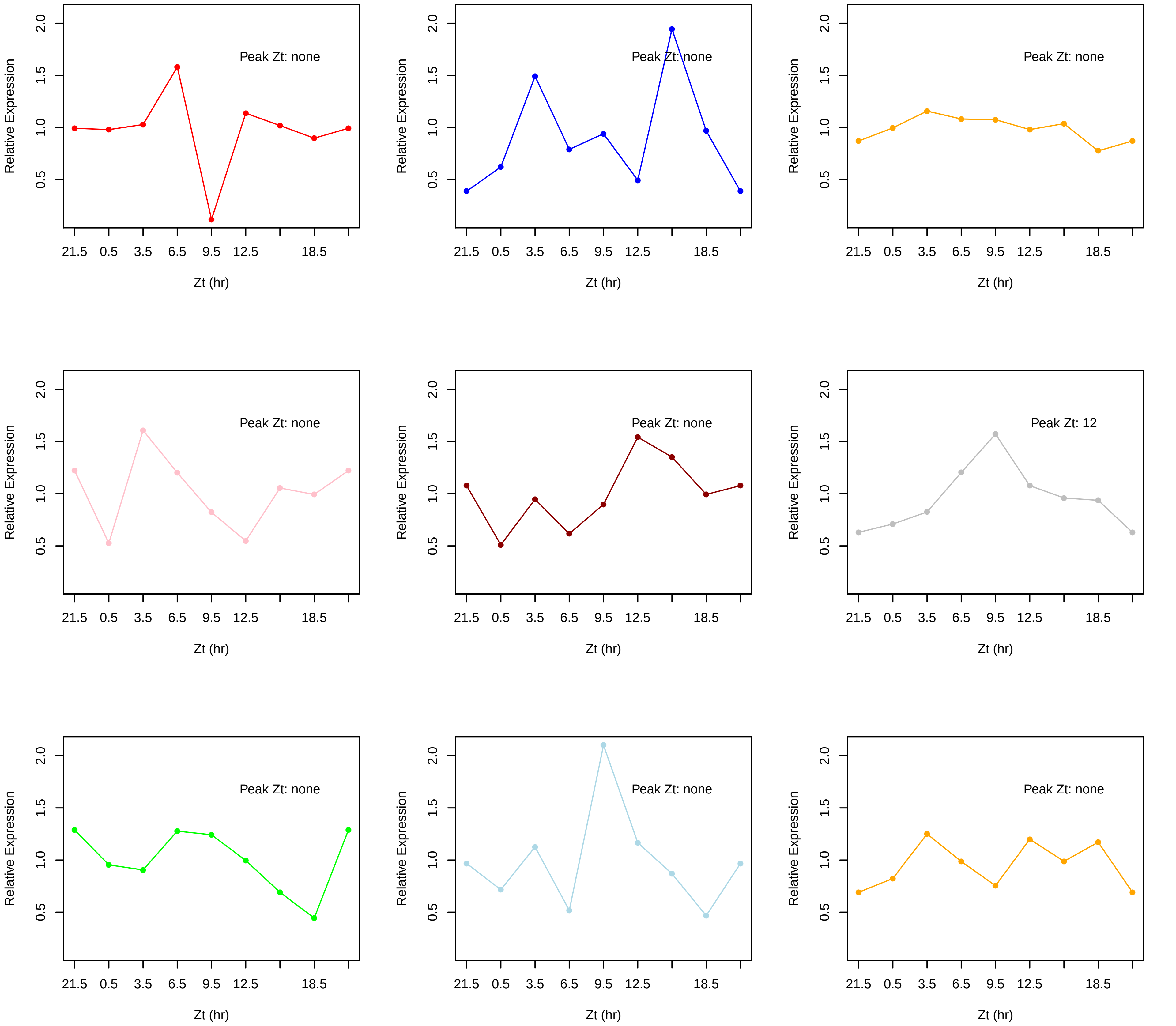

Singeleton: ENSXMAG00000024940

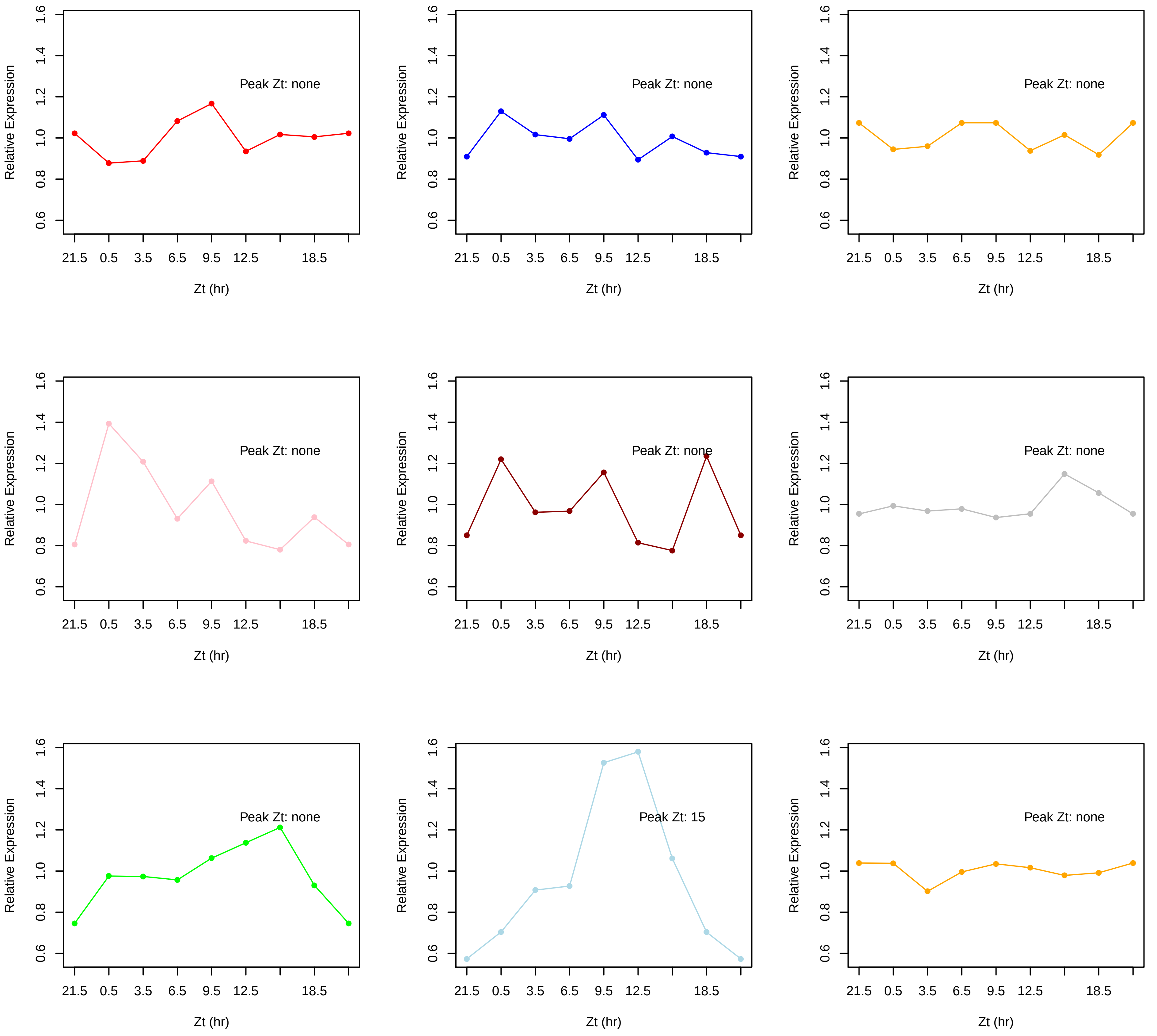

Singleton: ENSXMAG00000019255 g3bp1

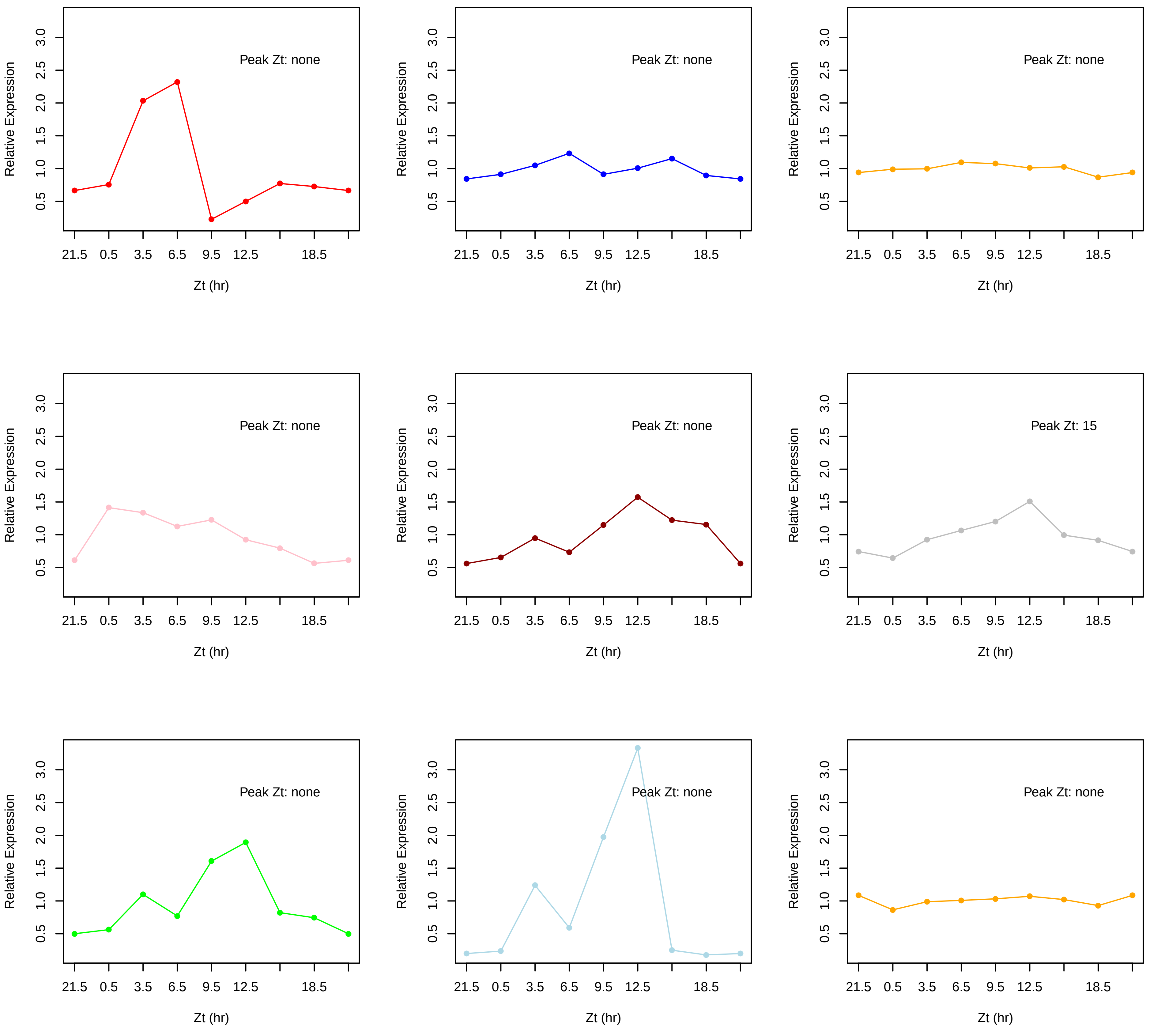

Singeleton: ENSXMAG00000015562 commd8

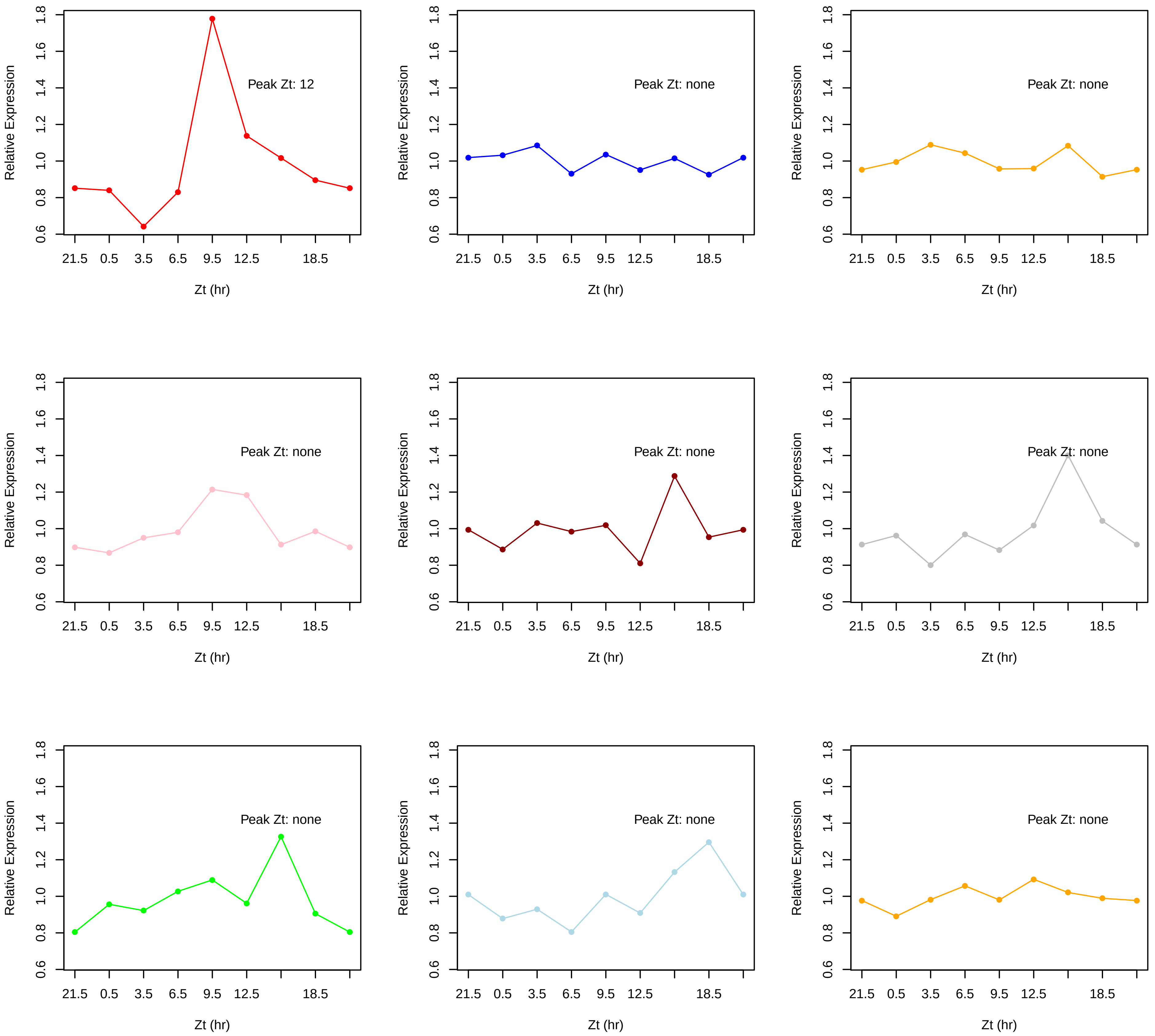

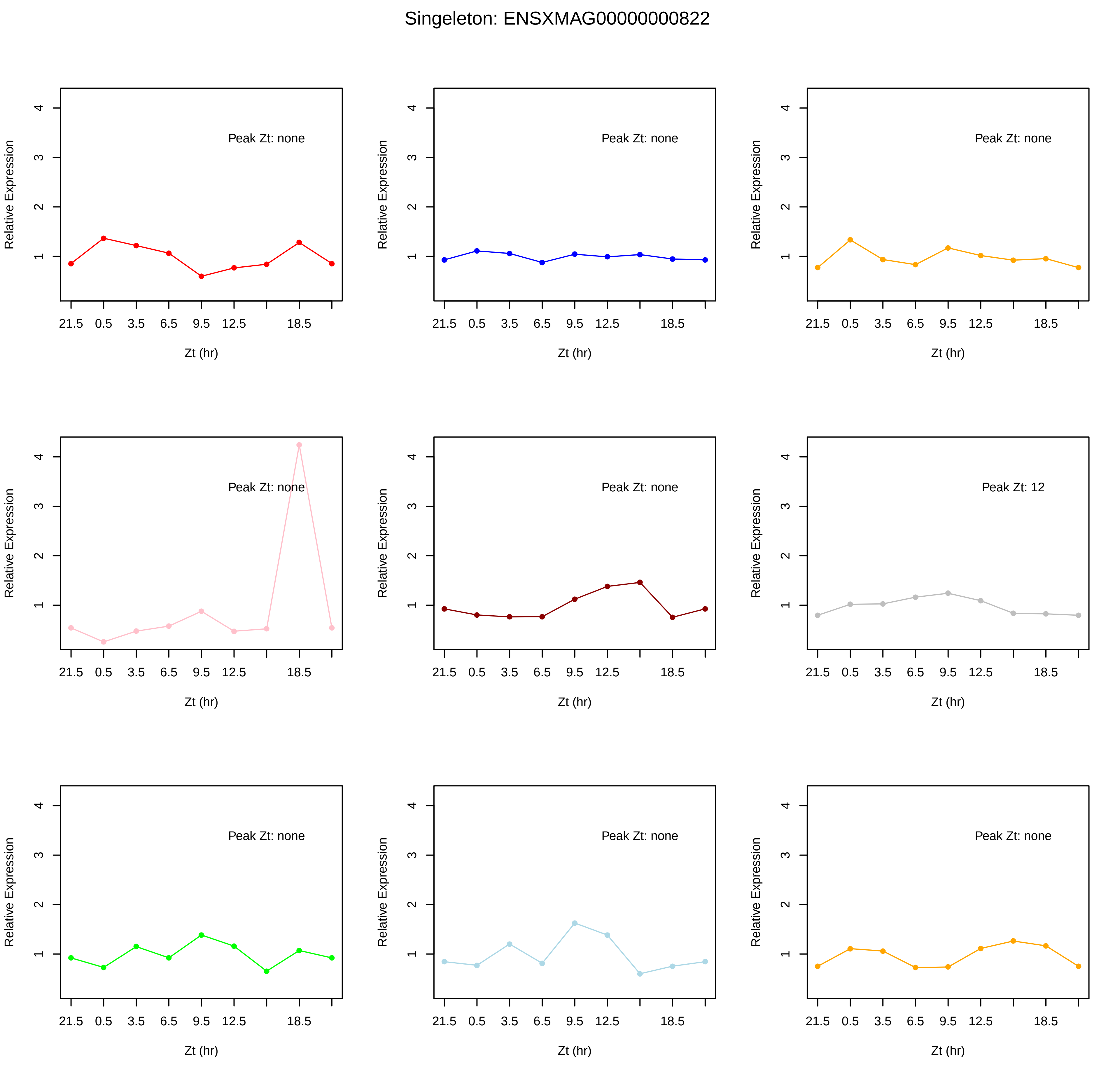

Singeleton: ENSXMAG00000000822
Peak Zt: none
Peak Zt: none
Peak Zt: none
Peak Zt: none
Peak Zt: none
Peak Zt: 12
Peak Zt: none
Peak Zt: none
Peak Zt: none
Relative Expression
Zt (hr)
21.5
0.5
3.5
6.5
9.5
12.5
18.5

Singleton: ENSXMAG00000018189 si:ch211−152c8.4

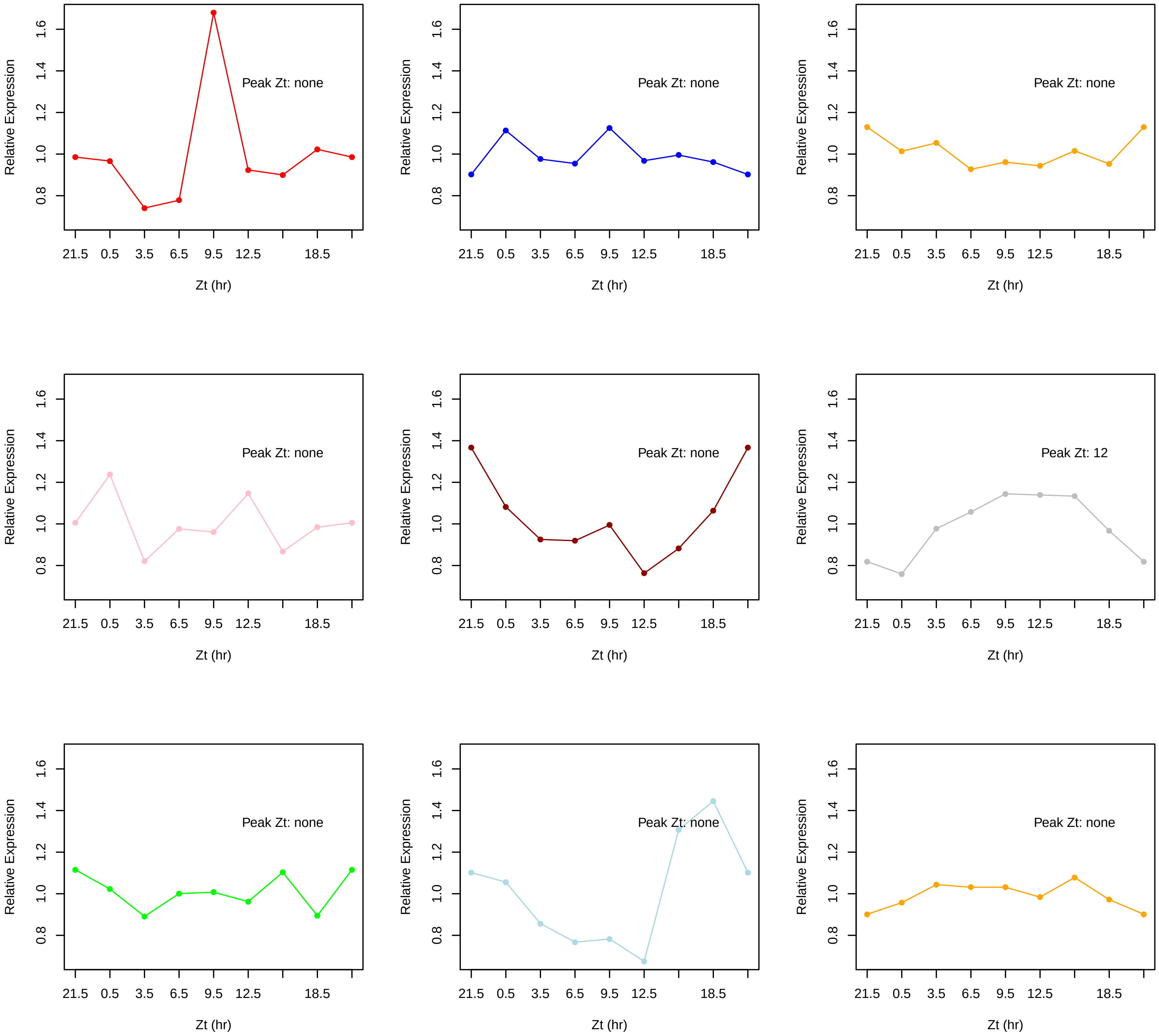

Singeleton: ENSXMAG00000025303 C18orf32

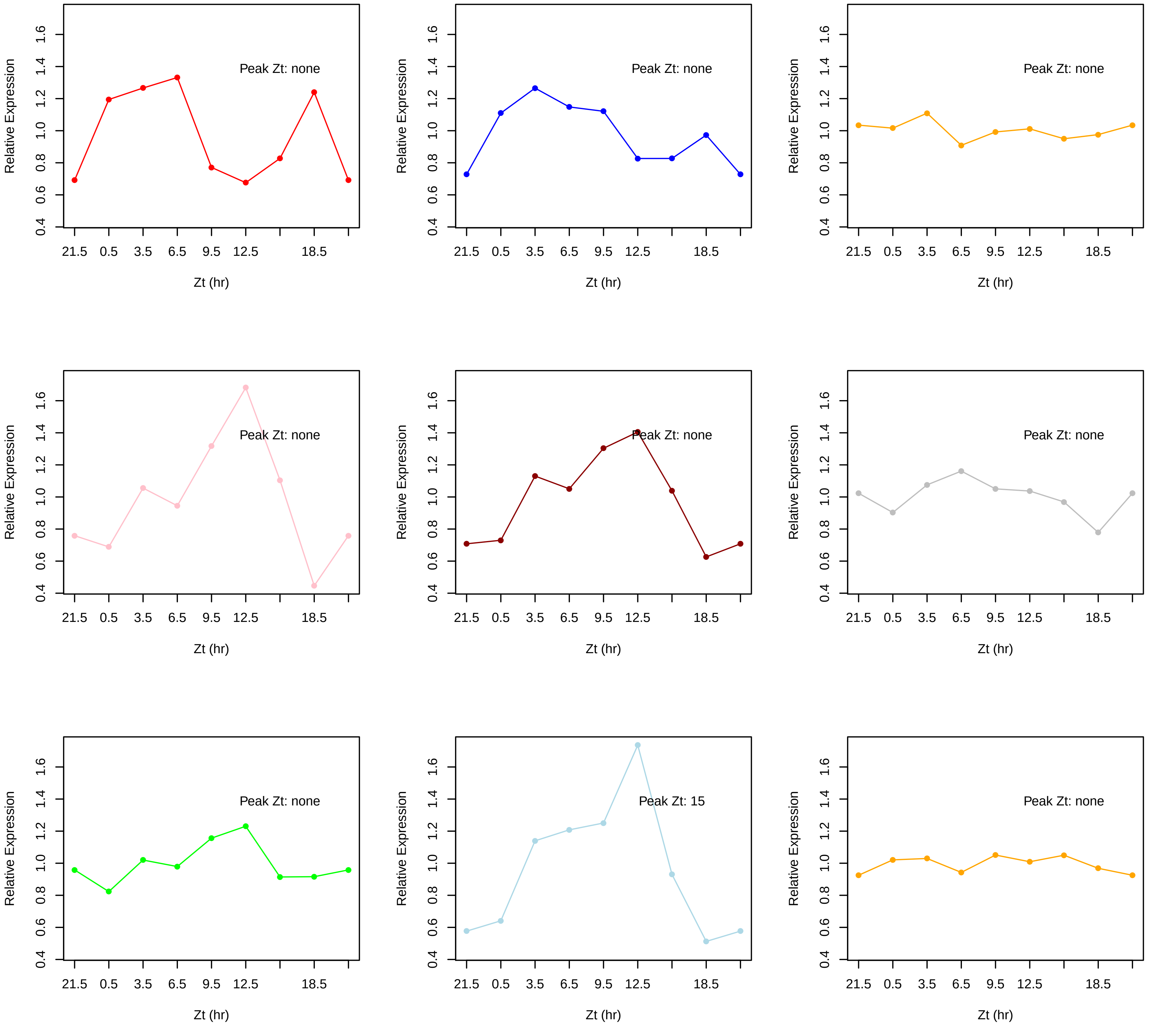

Singeleton: ENSXMAG00000015571 atp10d

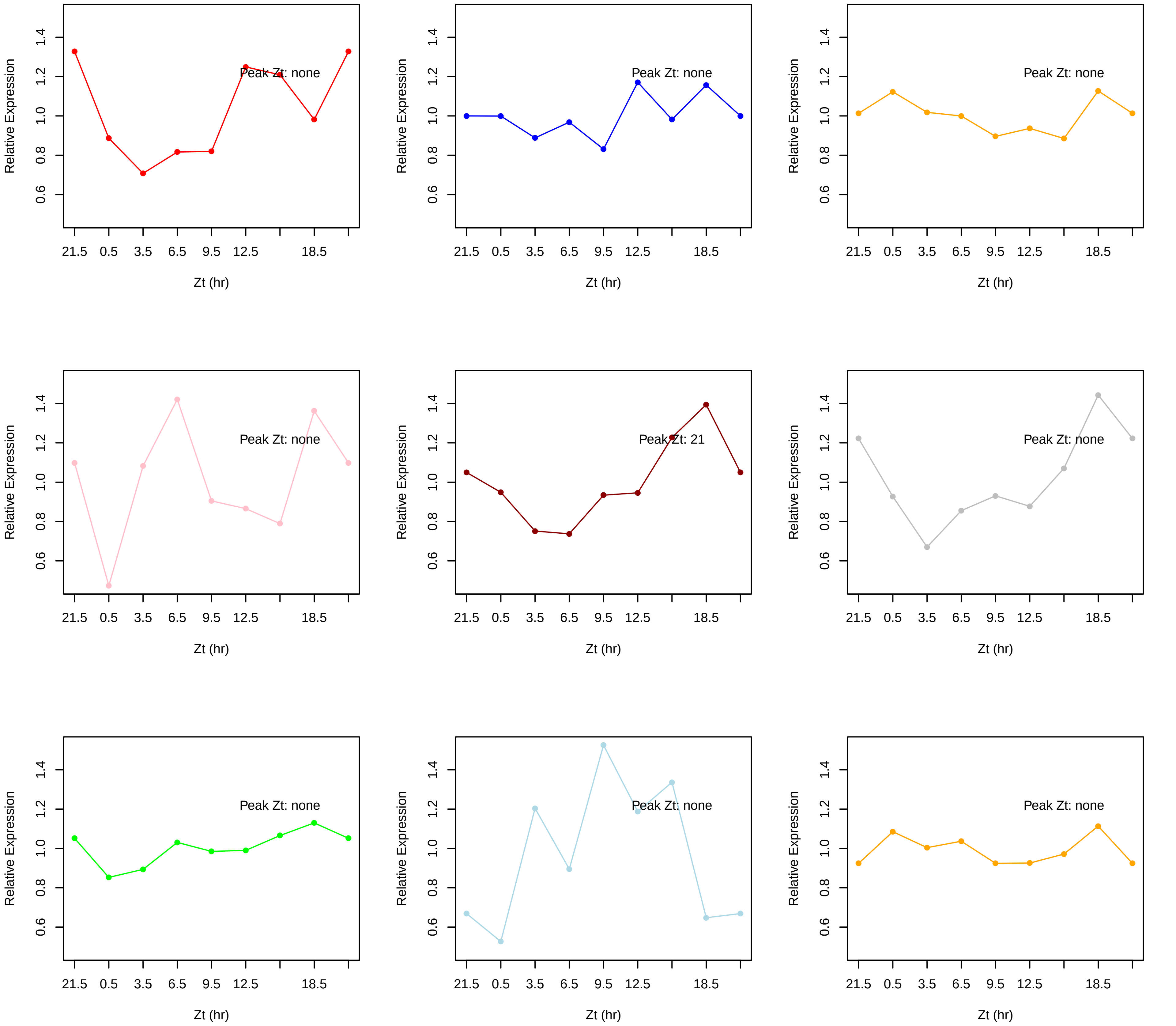

Singeleton: ENSXMAG00000019249 slc36a1

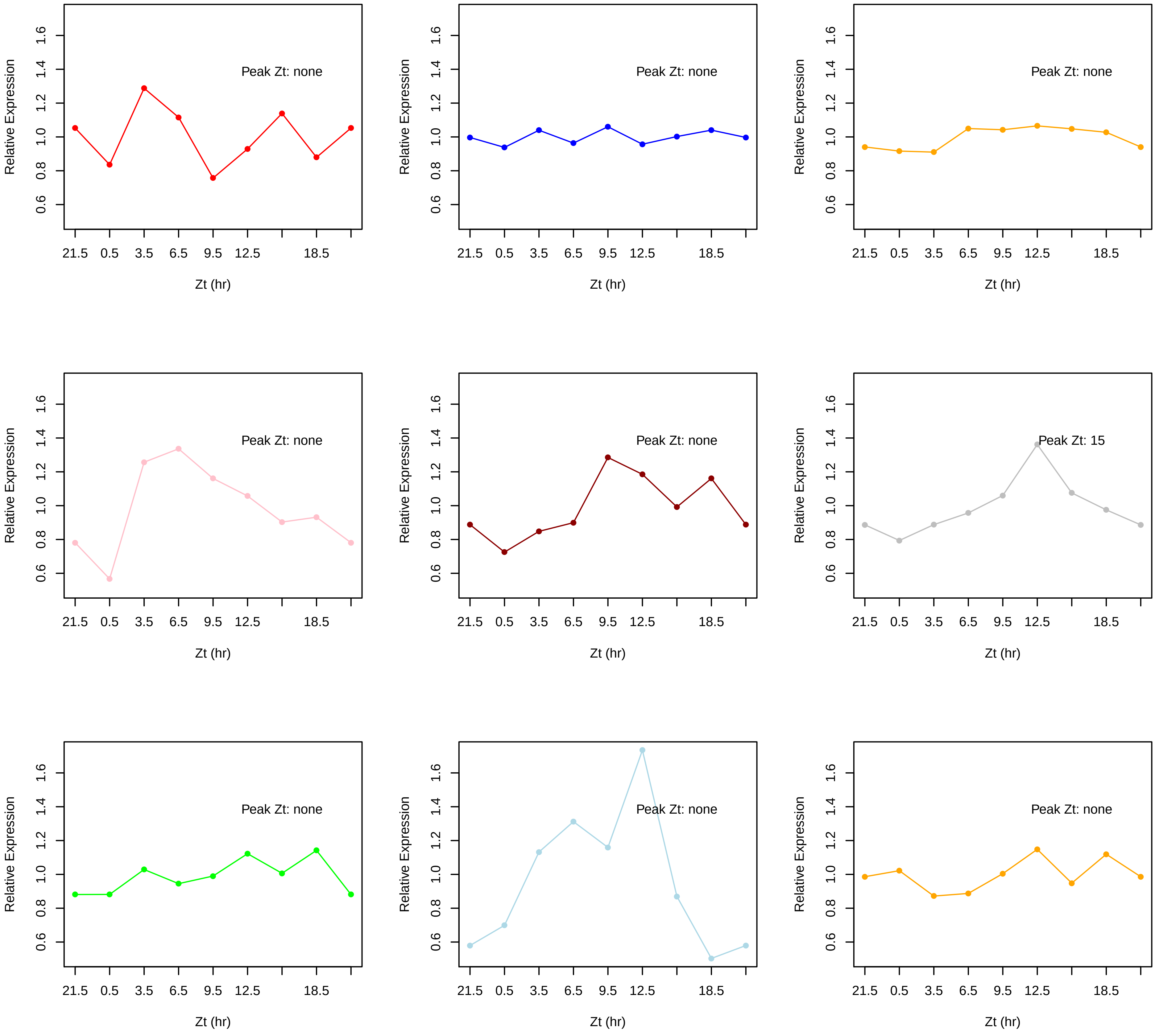

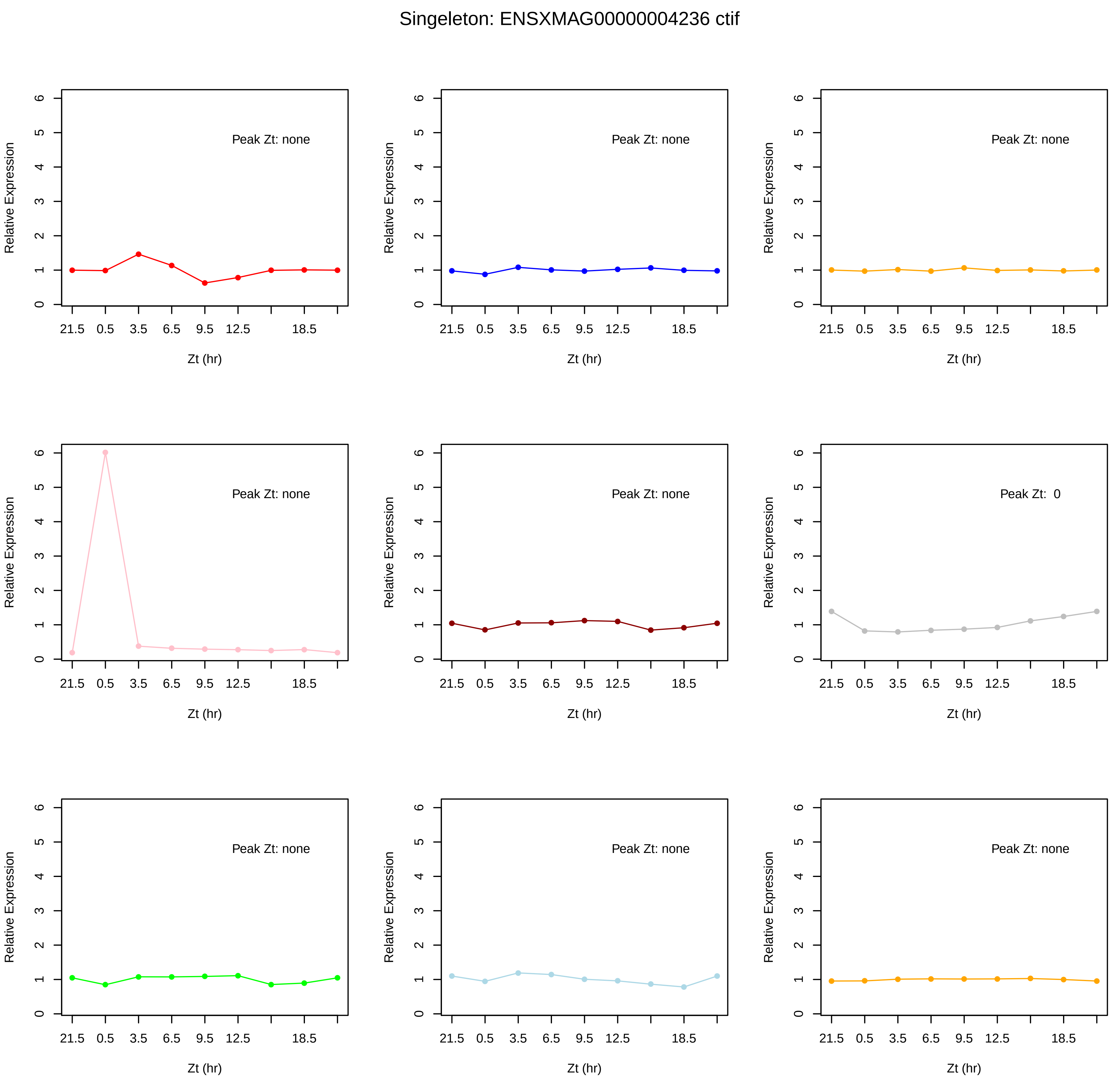
Singeleton: ENSXMAG00000004236 ctif
Peak Zt: none
Peak Zt: 0
Relative Expression
Zt (hr)
21.5
0.5
3.5
6.5
9.5
12.5
18.5
0
1
2
3
4
5
6

Singeleton: ENSXMAG00000028069 KCNC4

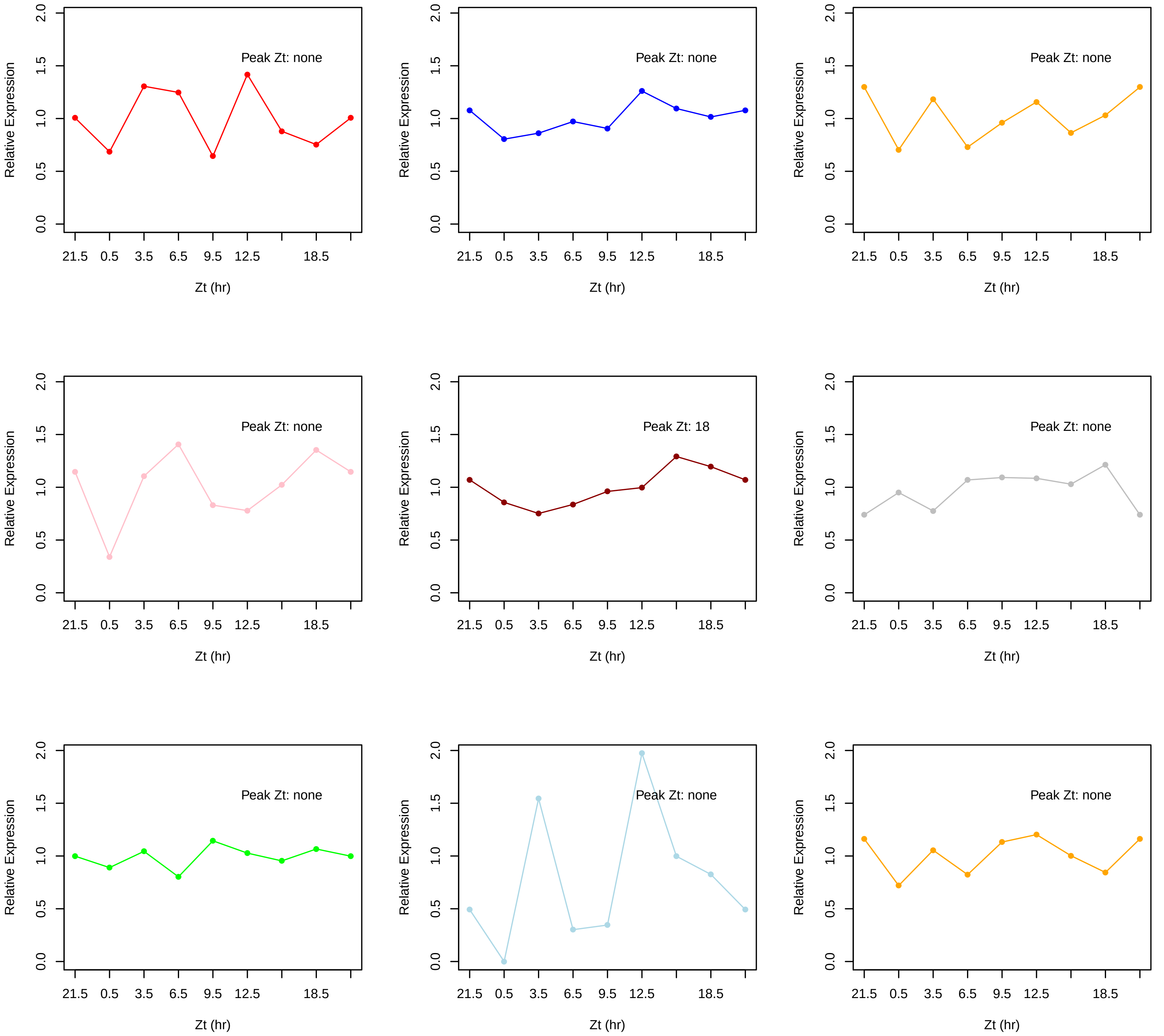

Singeleton: ENSXMAG00000025030 slc35b1

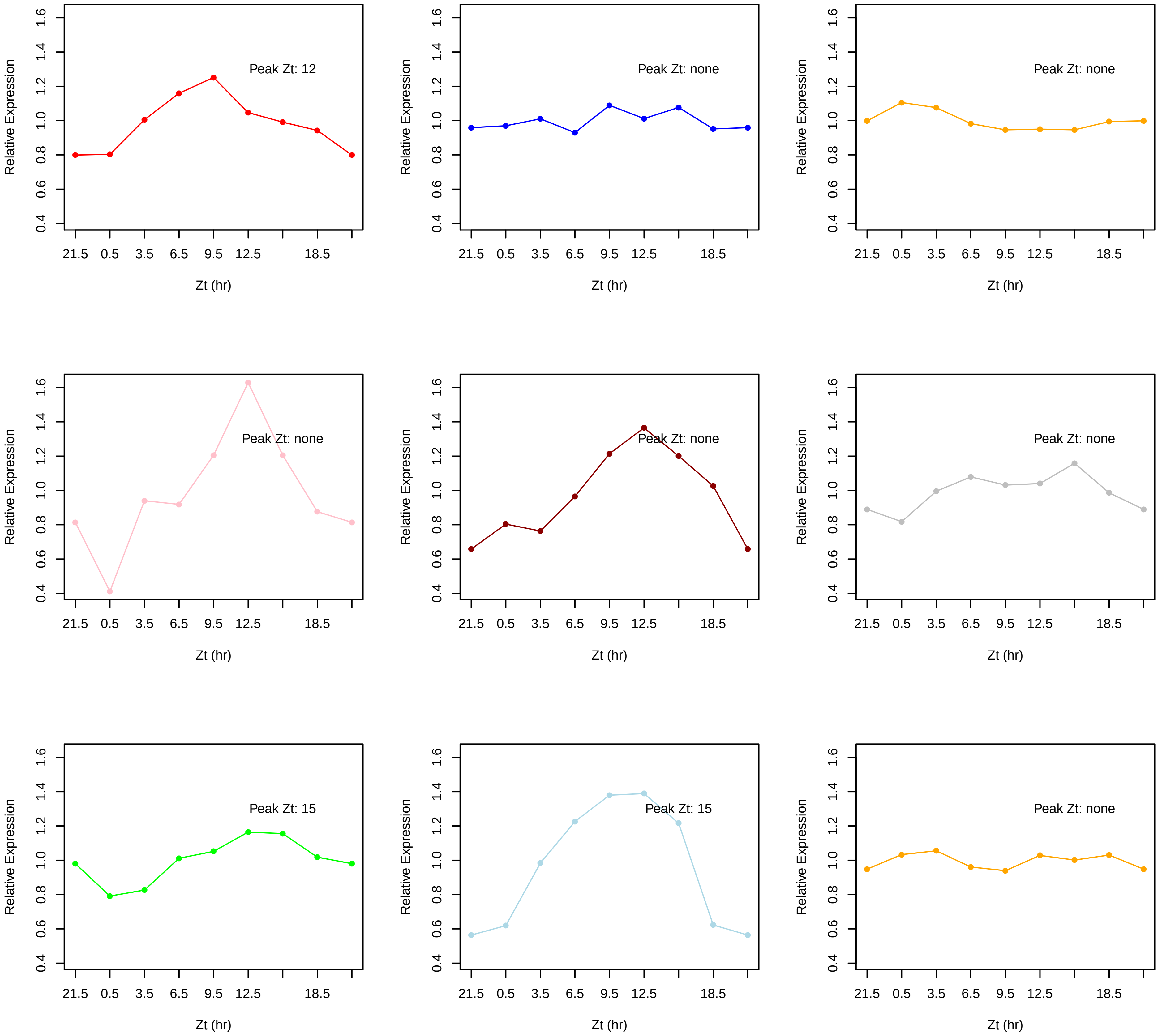

Singeleton: ENSXMAG00000010114 slc16a4

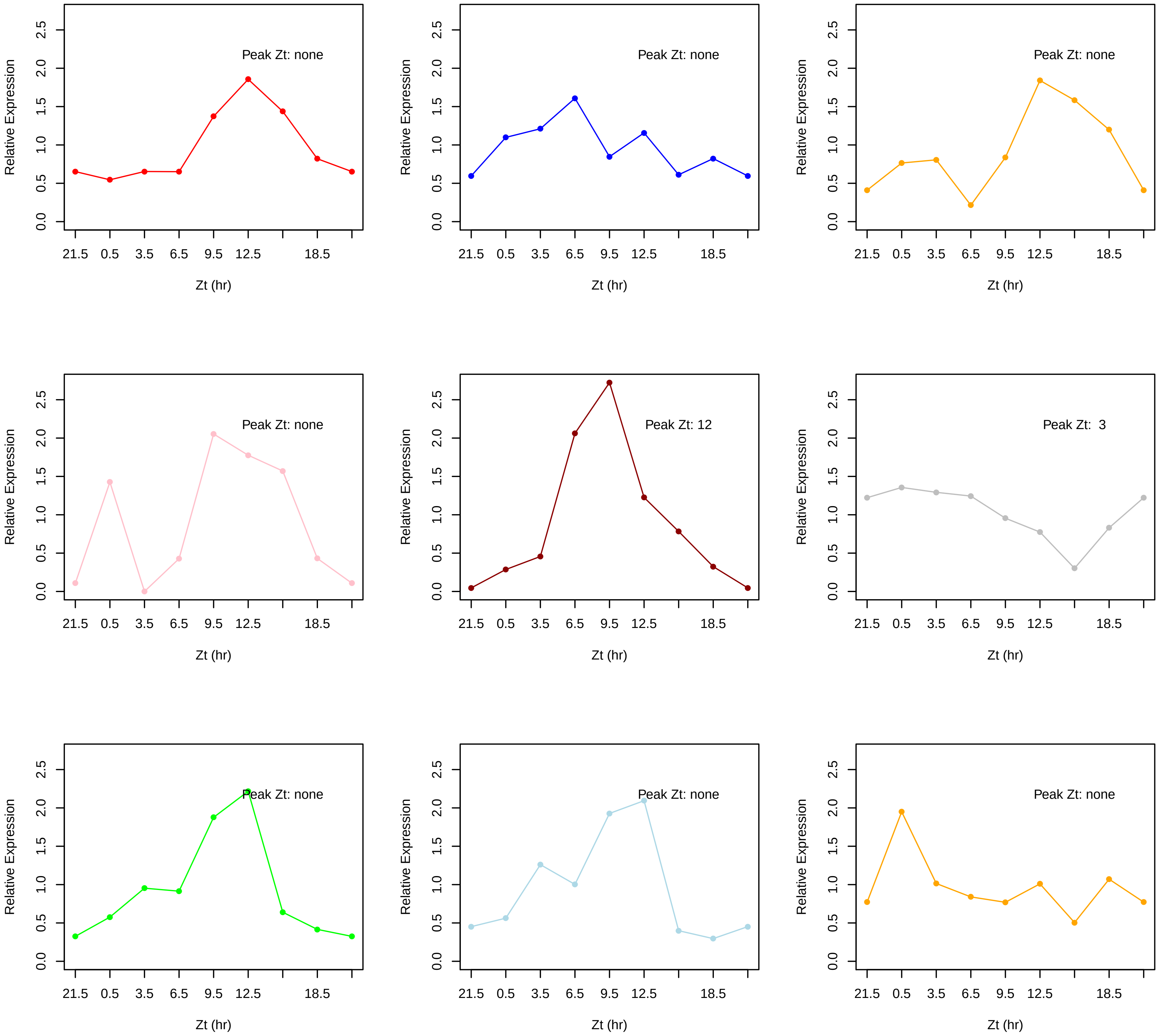

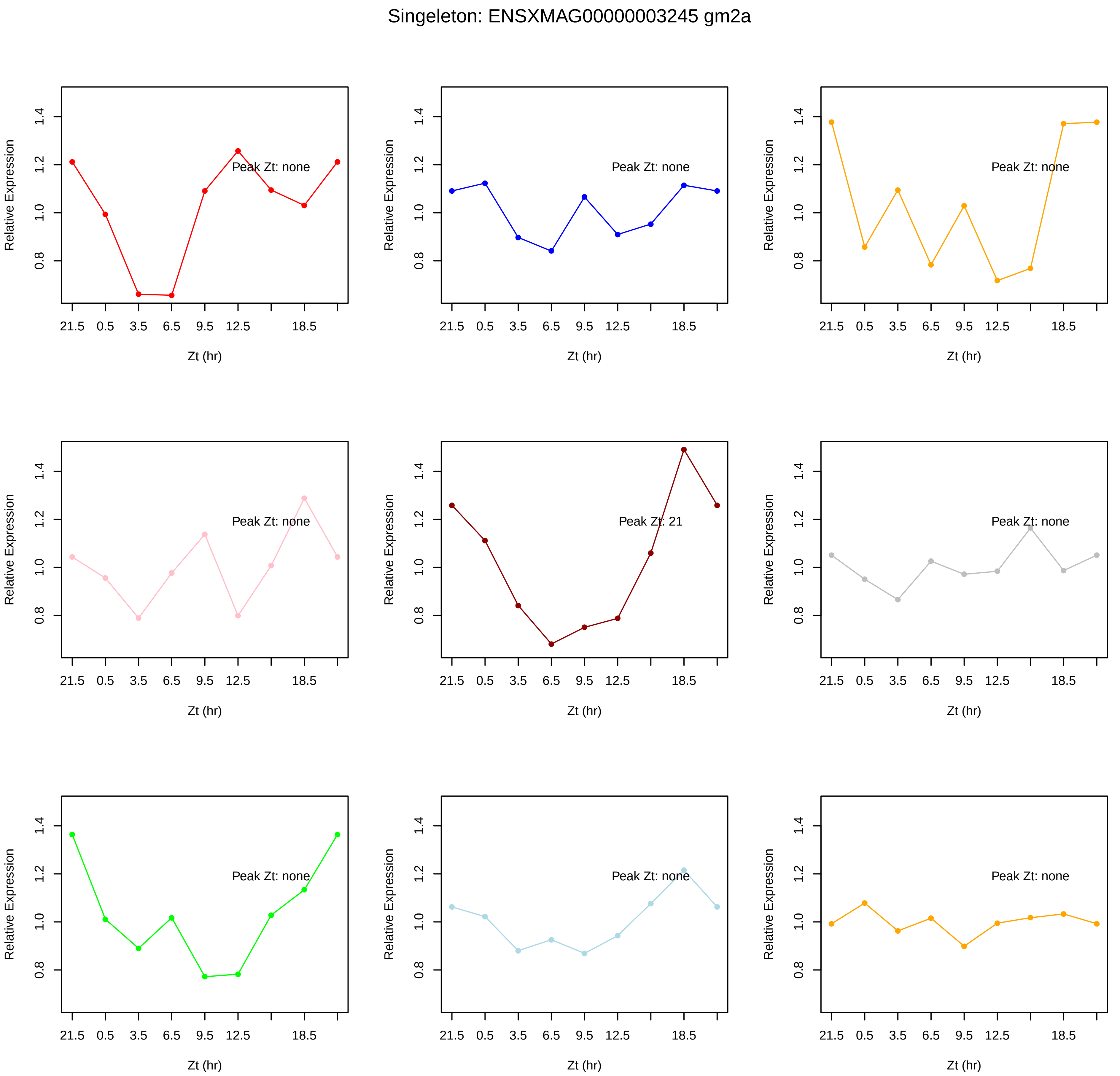

Singeleton: ENSXMAG00000003245 gm2a
Relative Expression
Zt (hr)
21.5
0.5
3.5
6.5
9.5
12.5
18.5
0.8
1.0
1.2
1.4
Peak Zt: none
Peak Zt: 21

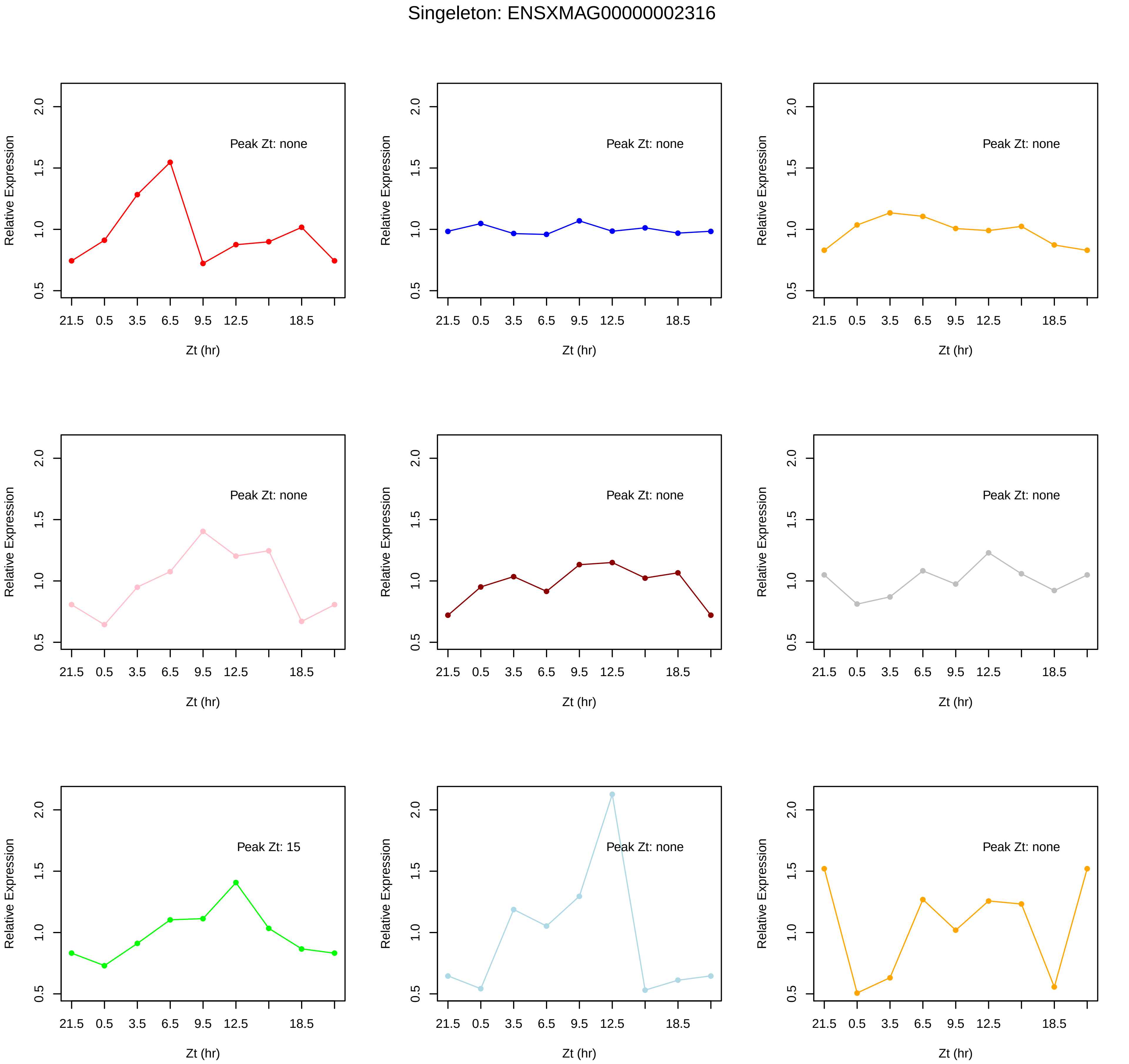
Singeleton: ENSXMAG00000002316
Peak Zt: none
Peak Zt: none
Peak Zt: none
Peak Zt: none
Peak Zt: none
Peak Zt: none
Peak Zt: 15
Peak Zt: none
Peak Zt: none
Relative Expression
Zt (hr)
21.5
0.5
3.5
6.5
9.5
12.5
18.5
0.5
1.0
1.5
2.0

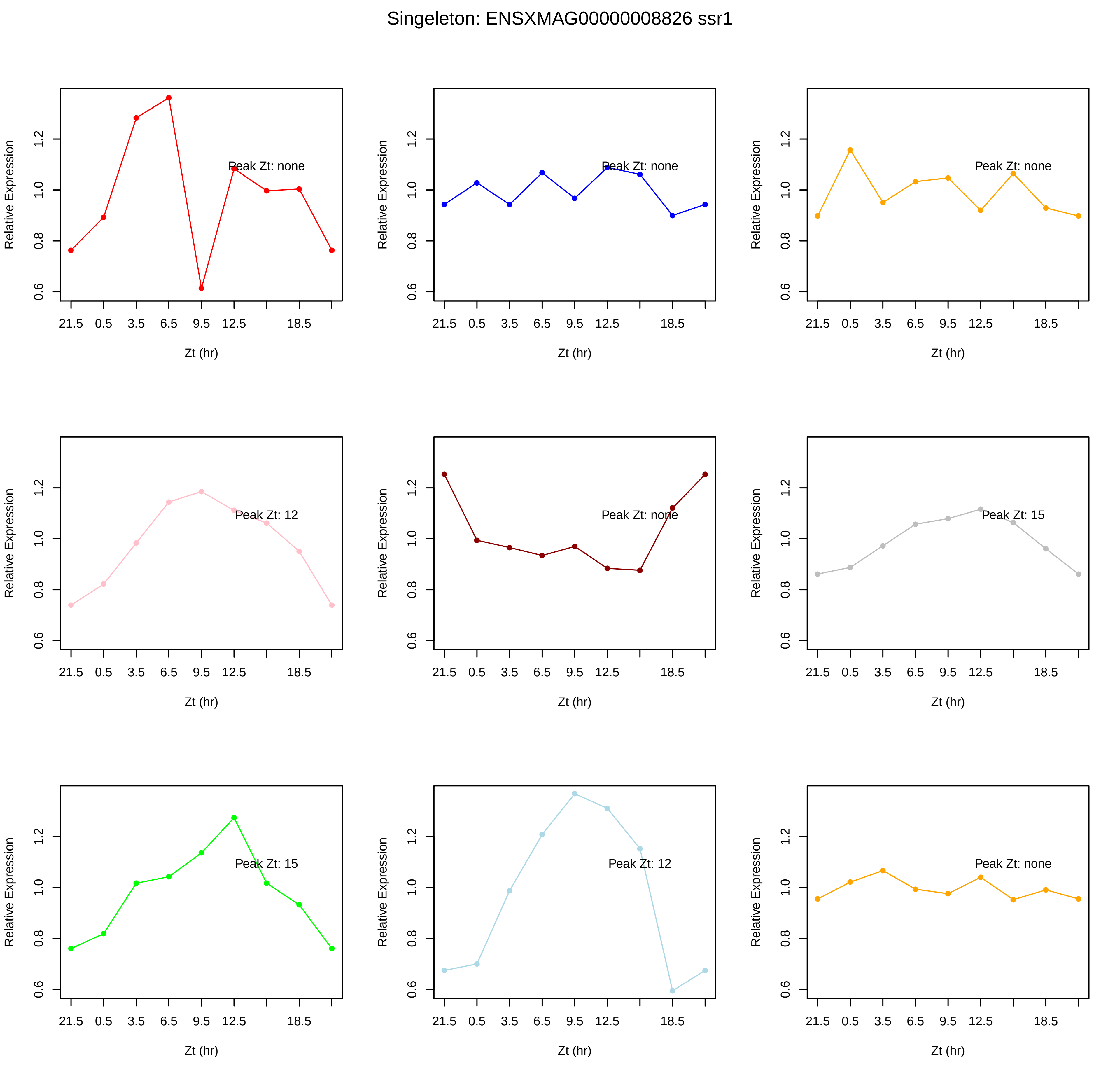
Singeleton: ENSXMAG00000008826 ssr1
Peak Zt: none
Peak Zt: none
Peak Zt: none
Peak Zt: 12
Peak Zt: none
Peak Zt: 15
Peak Zt: 15
Peak Zt: 12
Peak Zt: none
Relative Expression
Zt (hr)
21.5
0.5
3.5
6.5
9.5
12.5
18.5
0.6
0.8
1.0
1.2

Singeleton: ENSXMAG00000008111 map3k3

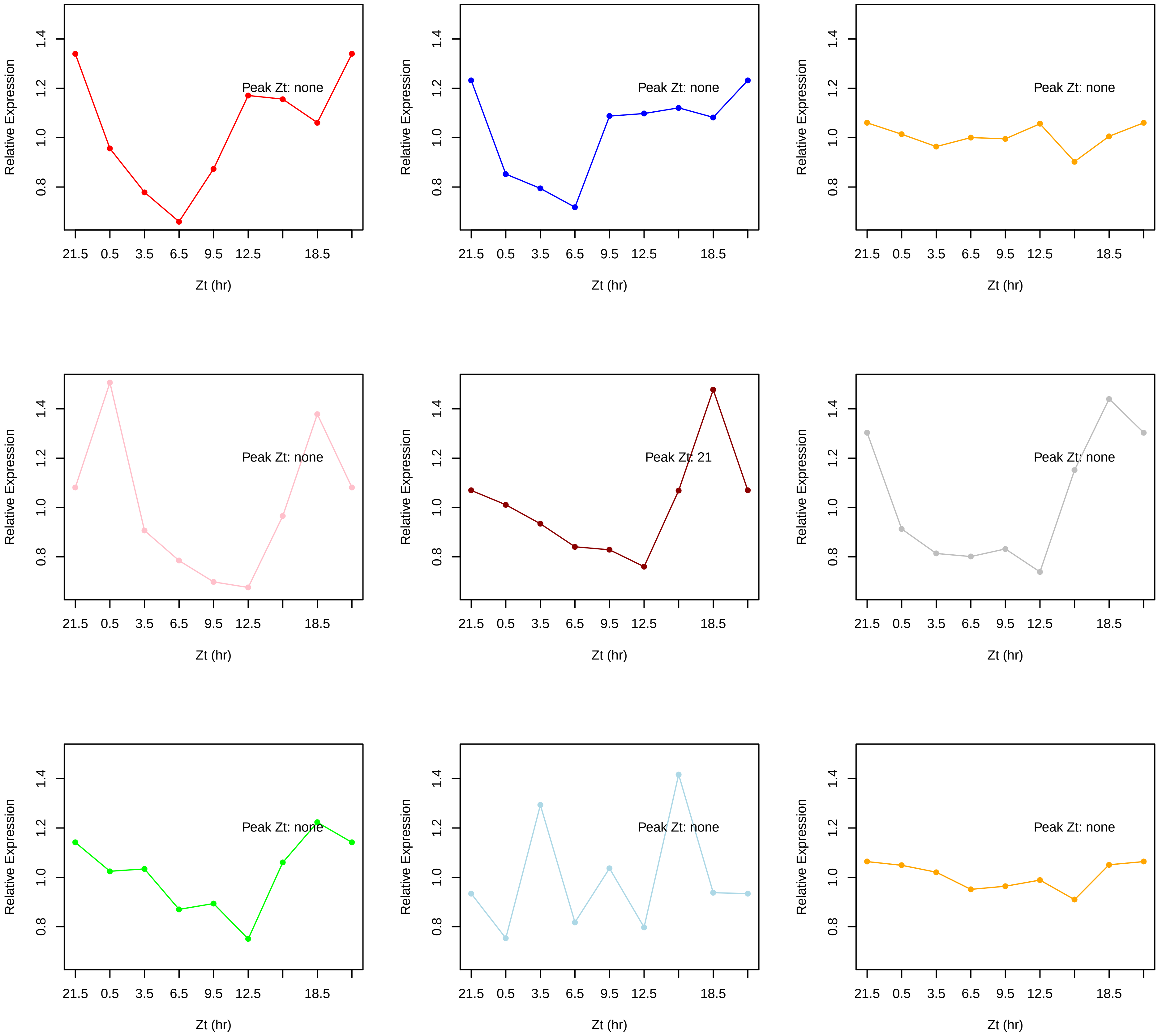

Singeleton: ENSXMAG00000014939 jmjd7

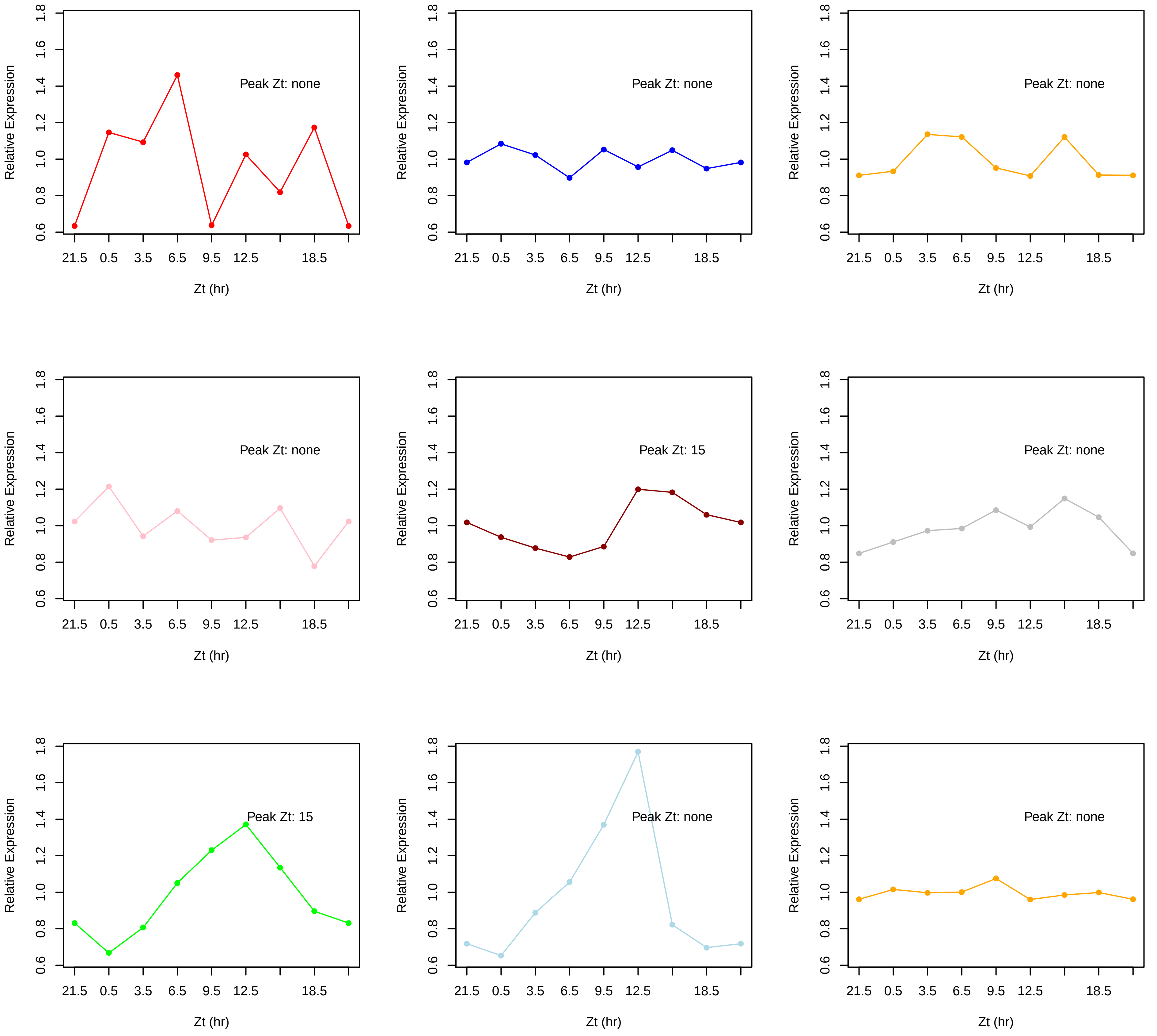

Singleton: ENSXMAG00000014572

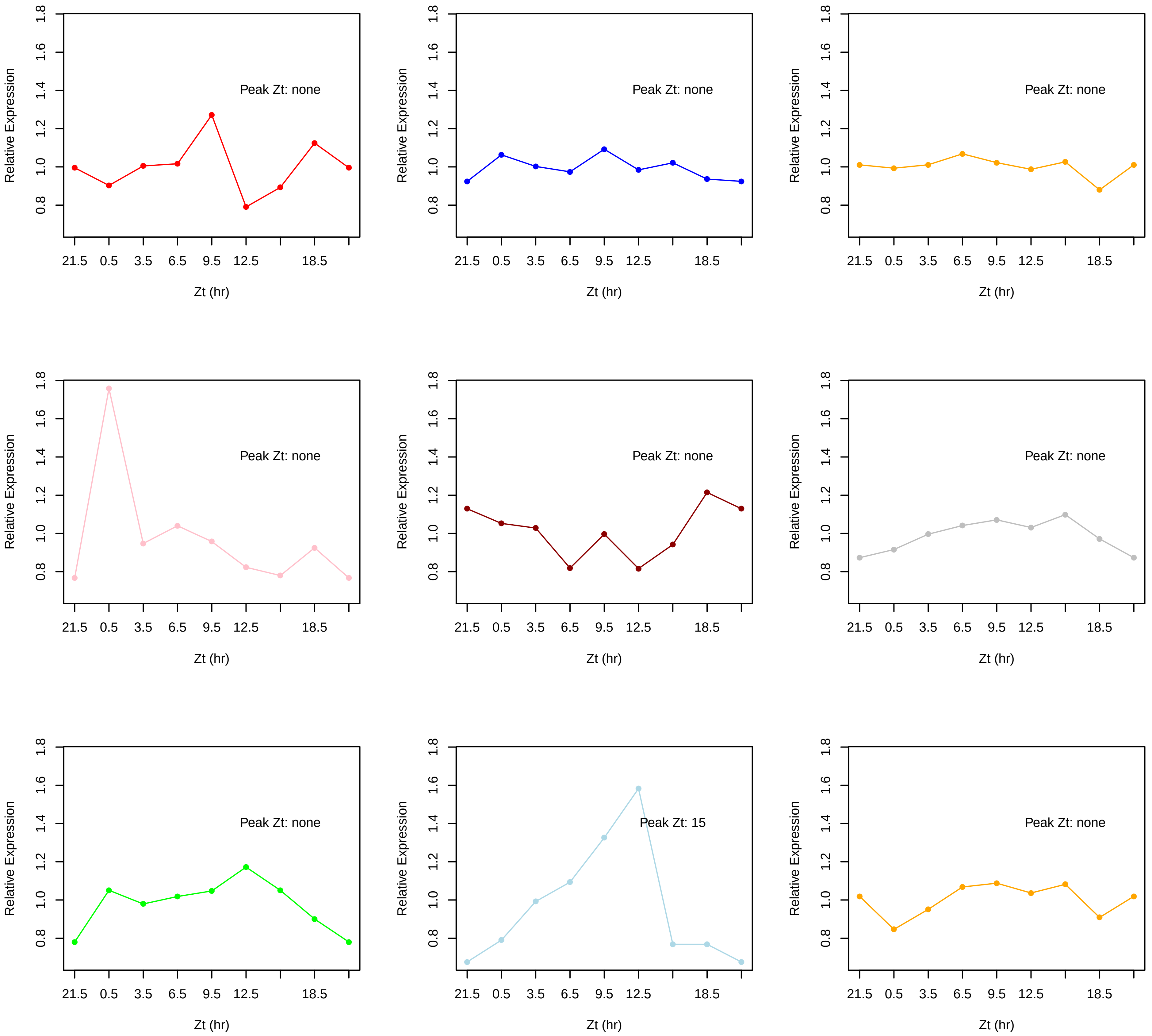

Singeleton: ENSXMAG00000012696 fdxr

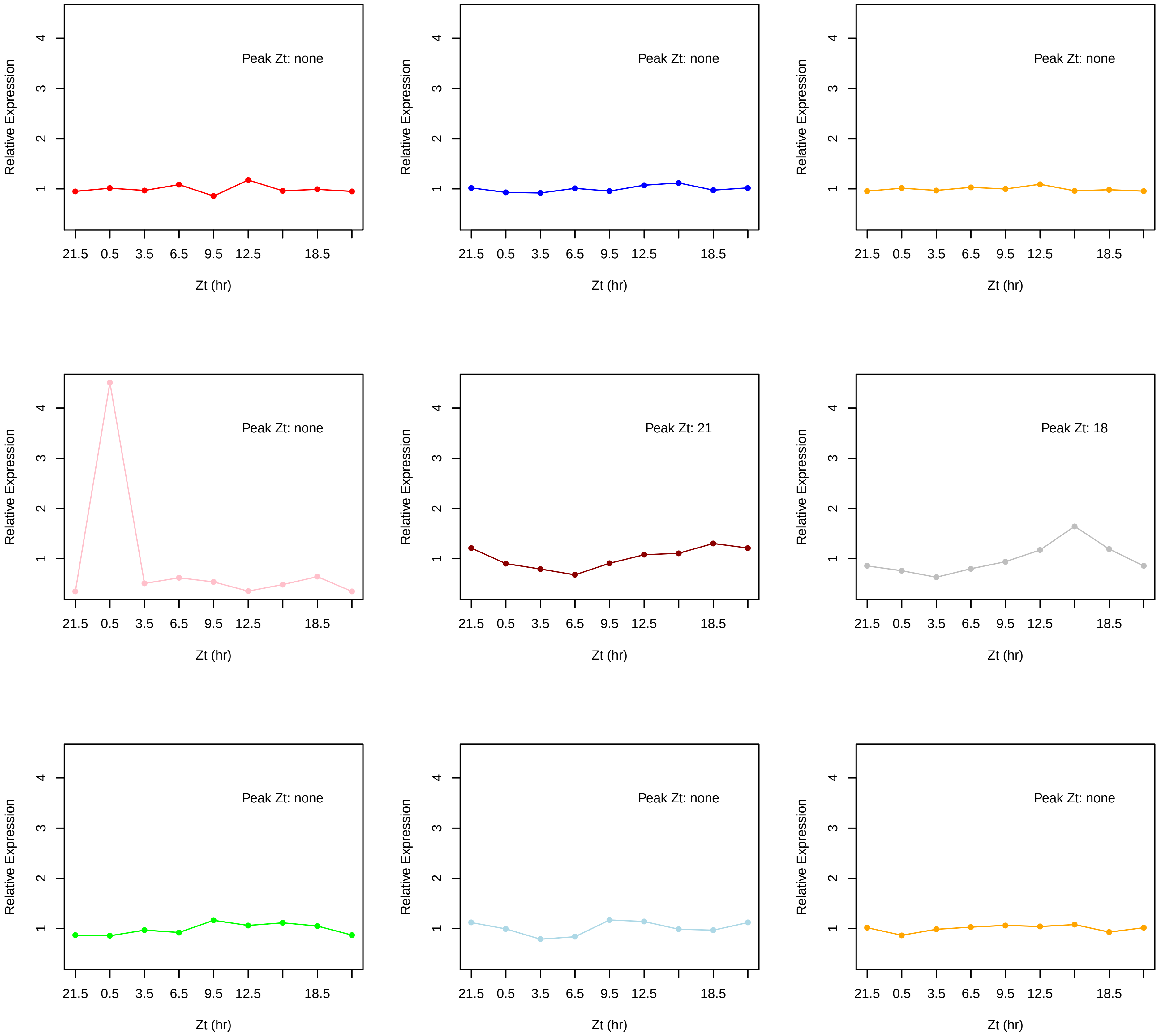

Singeleton: ENSXMAG00000027359

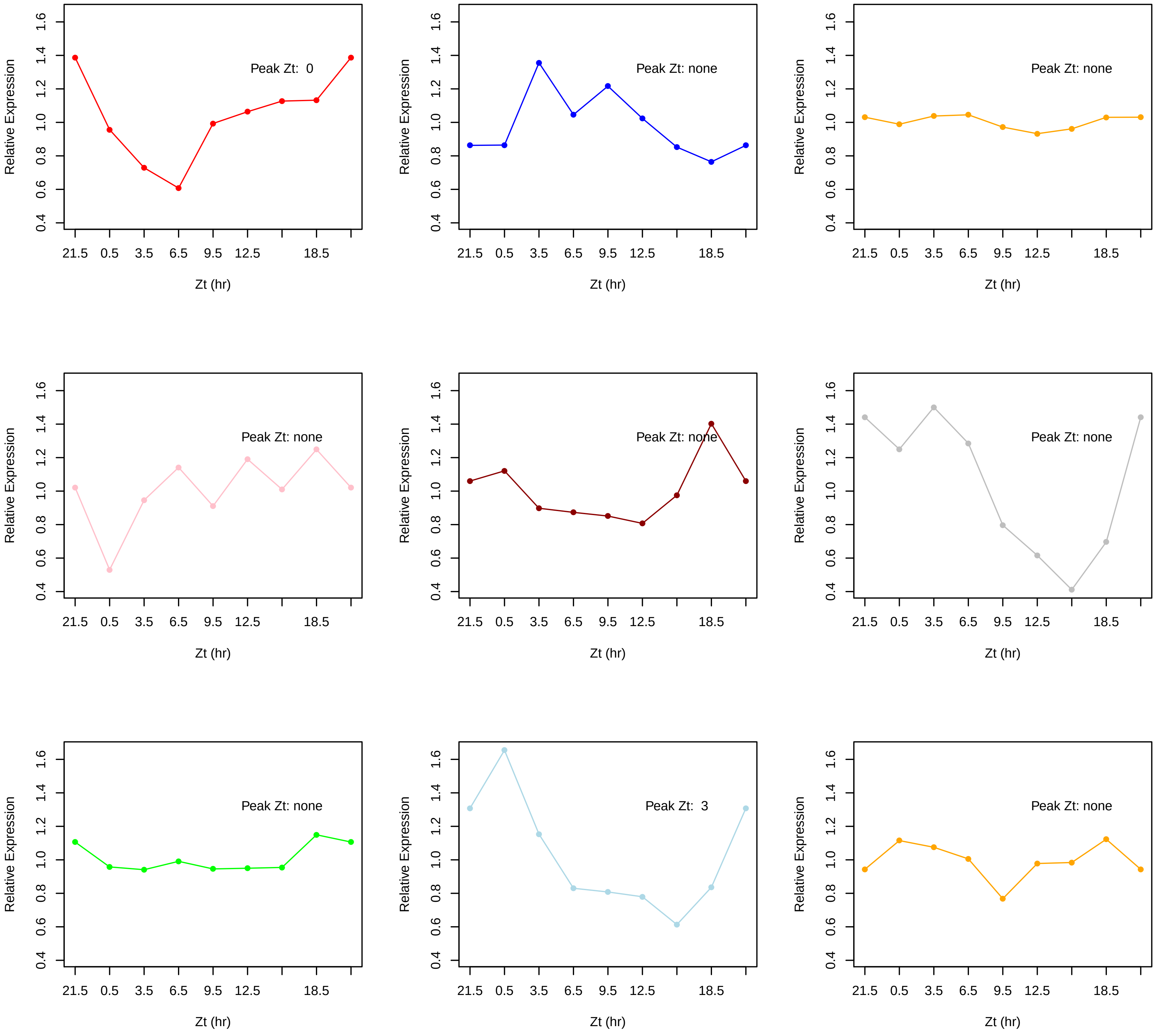

Singleton: ENSXMAG00000021020 nfe2l3

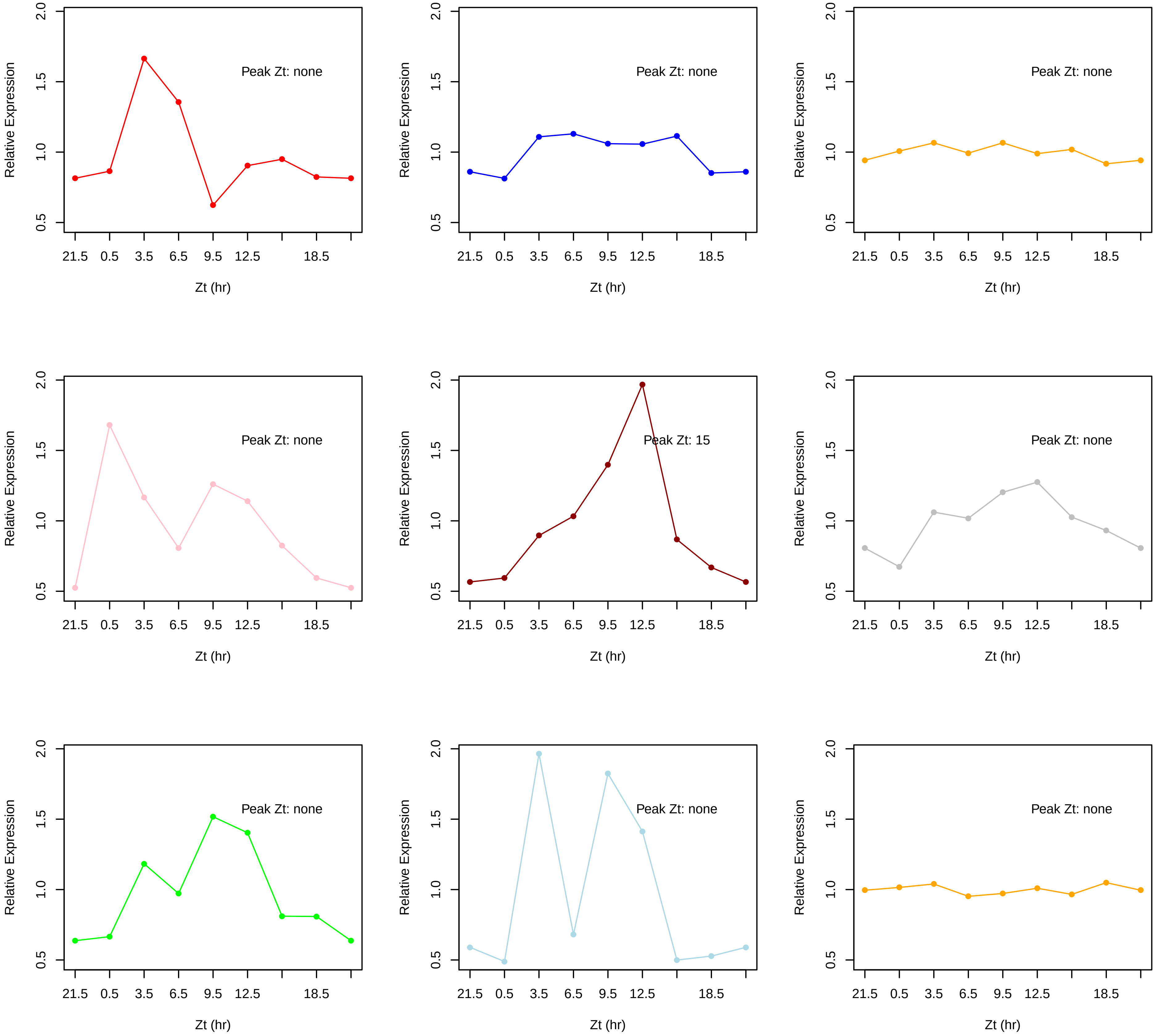

Singeleton: ENSXMAG00000019122 ATG14

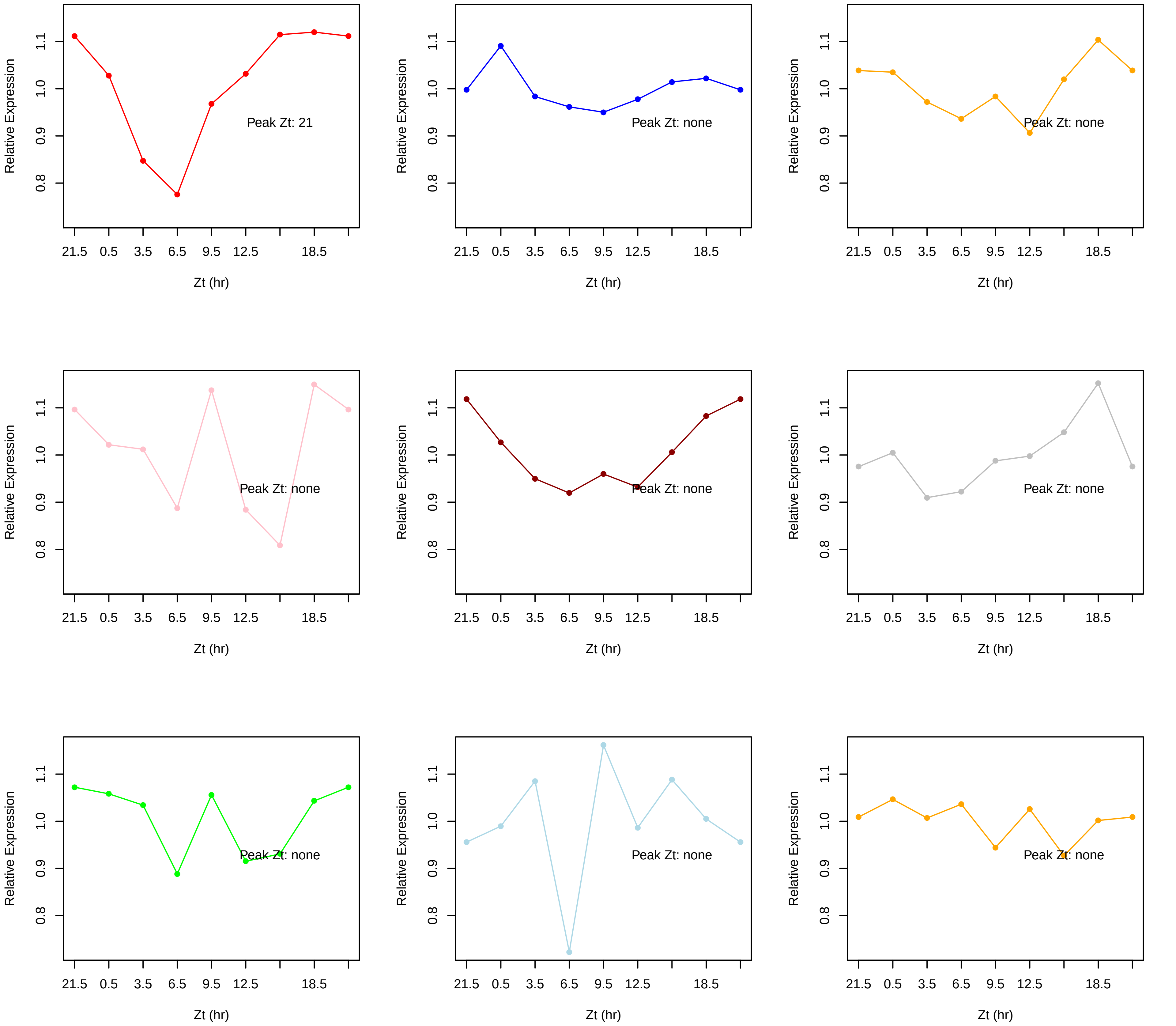

Singleton: ENSXMAG00000013706 enpep

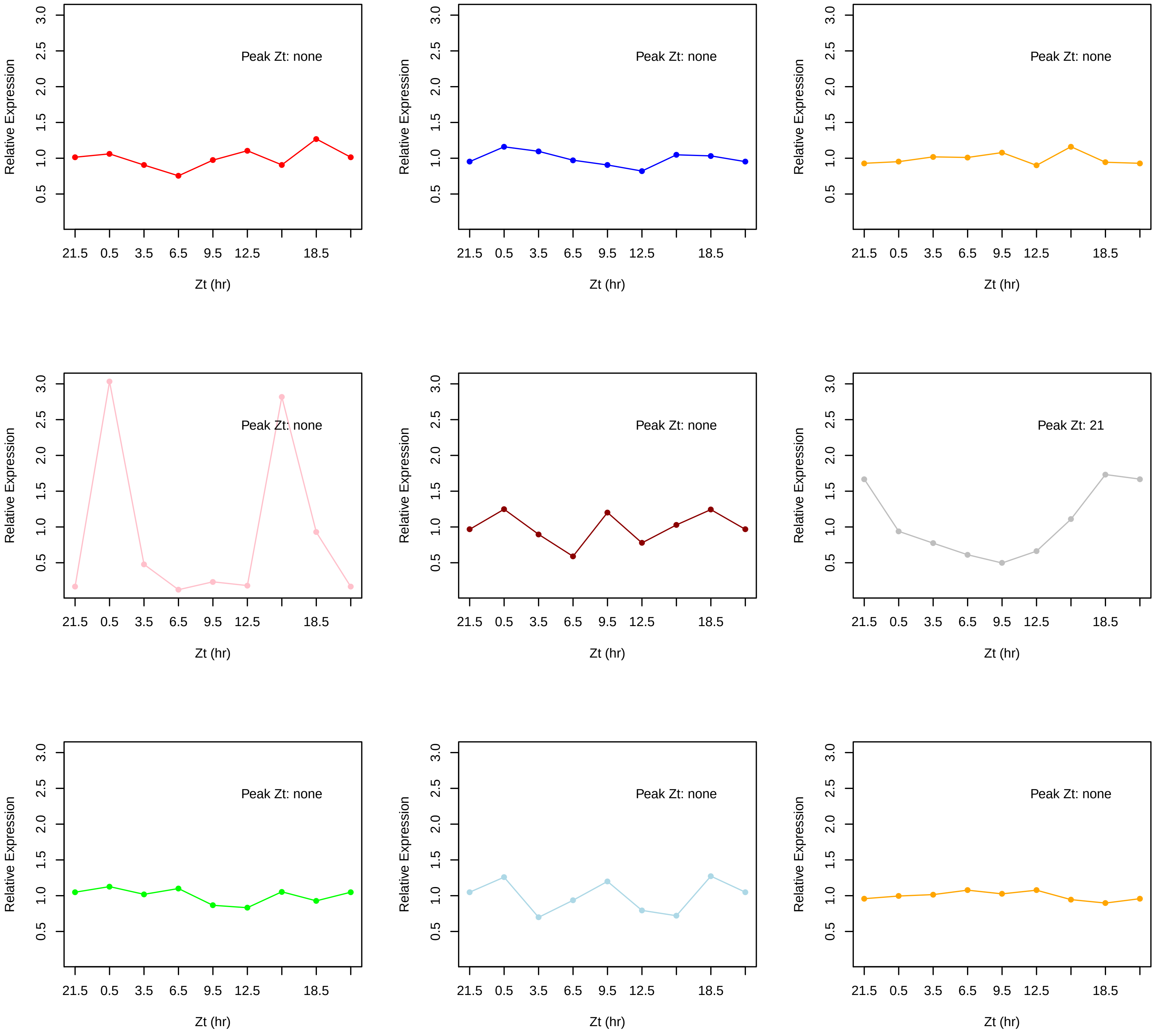

Singeleton: ENSXMAG00000010027

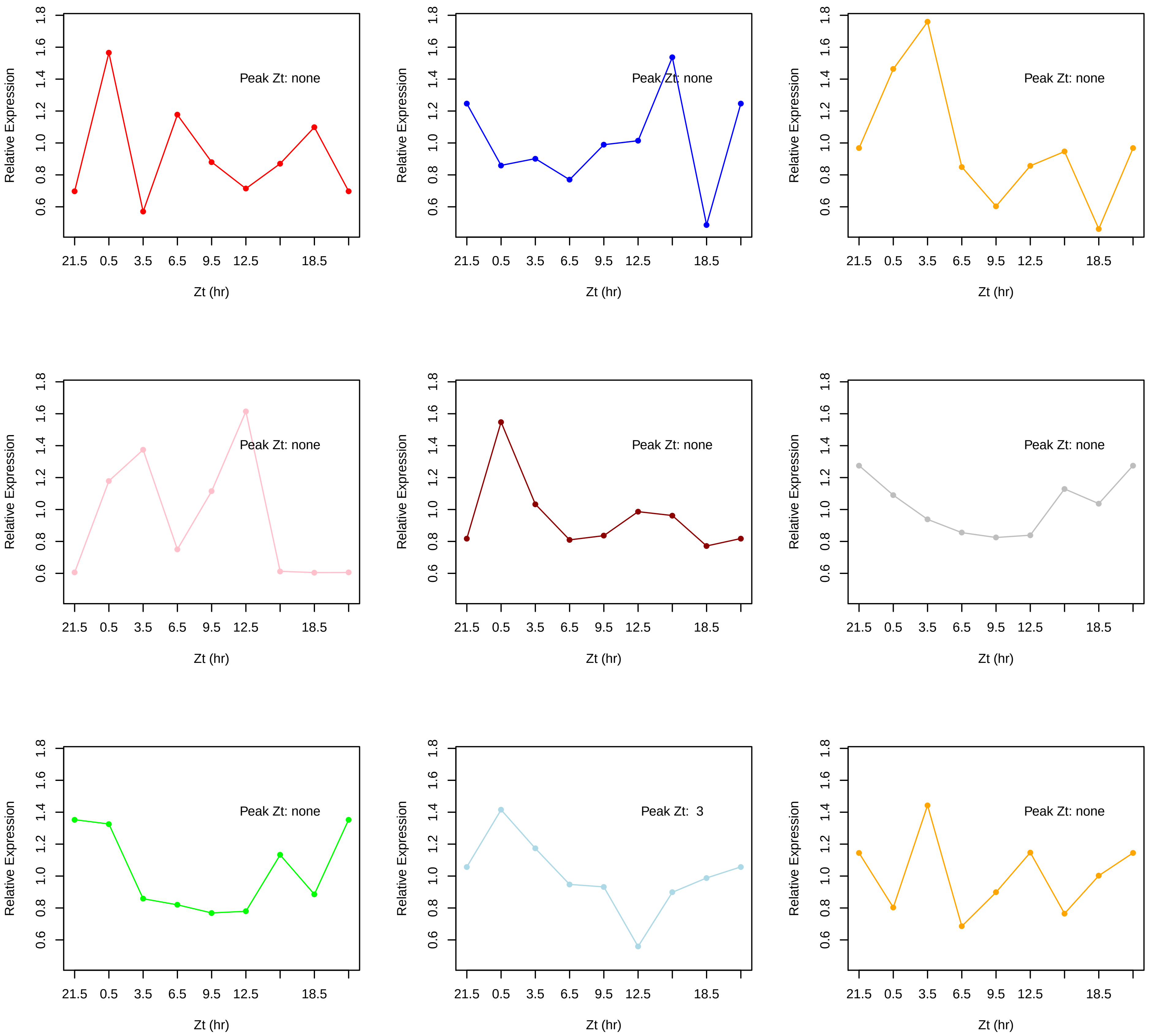

Singeleton: ENSXMAG00000001027 cbx3a

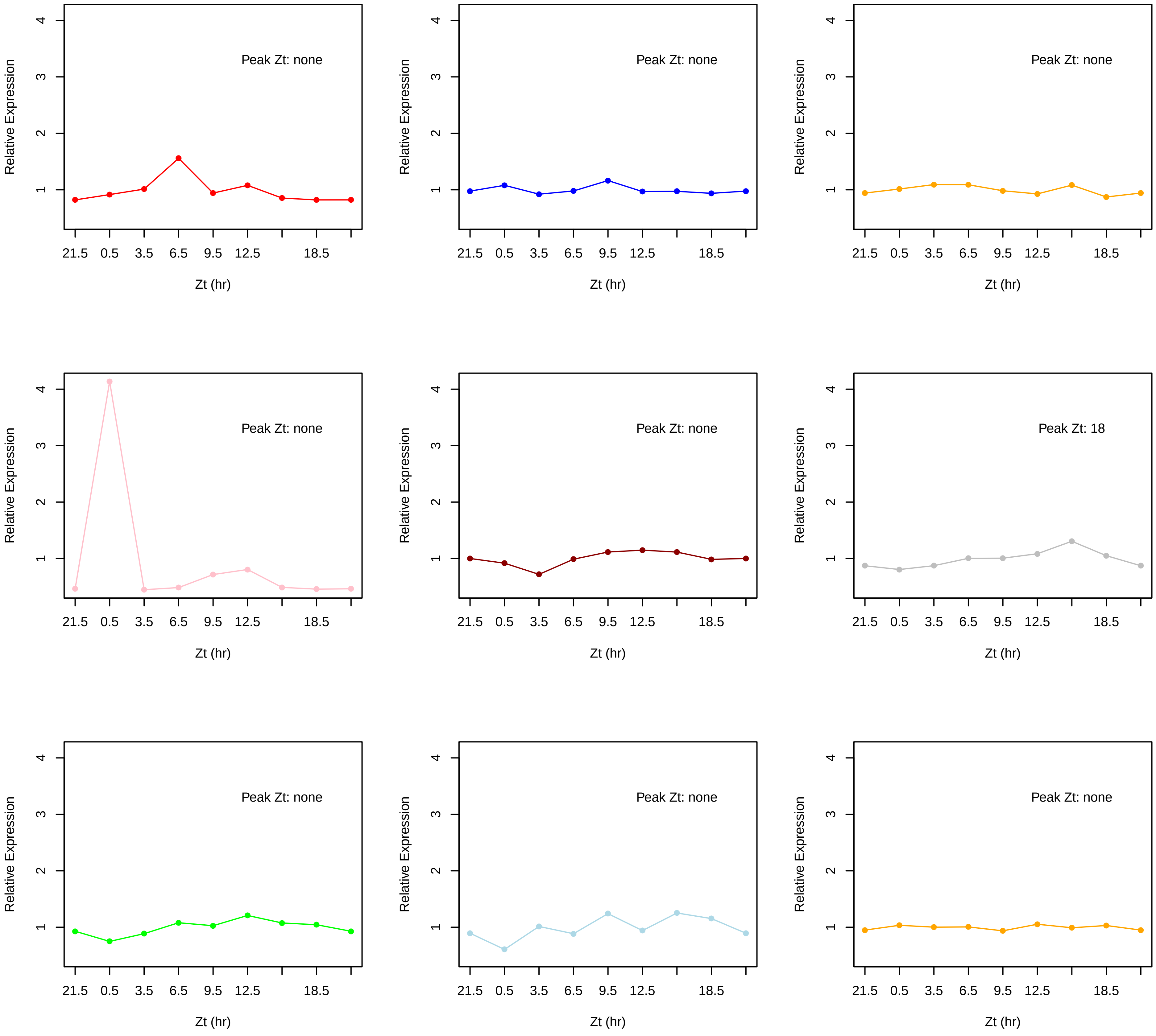

Singleton: ENSXMAG00000014192 SEMA4D

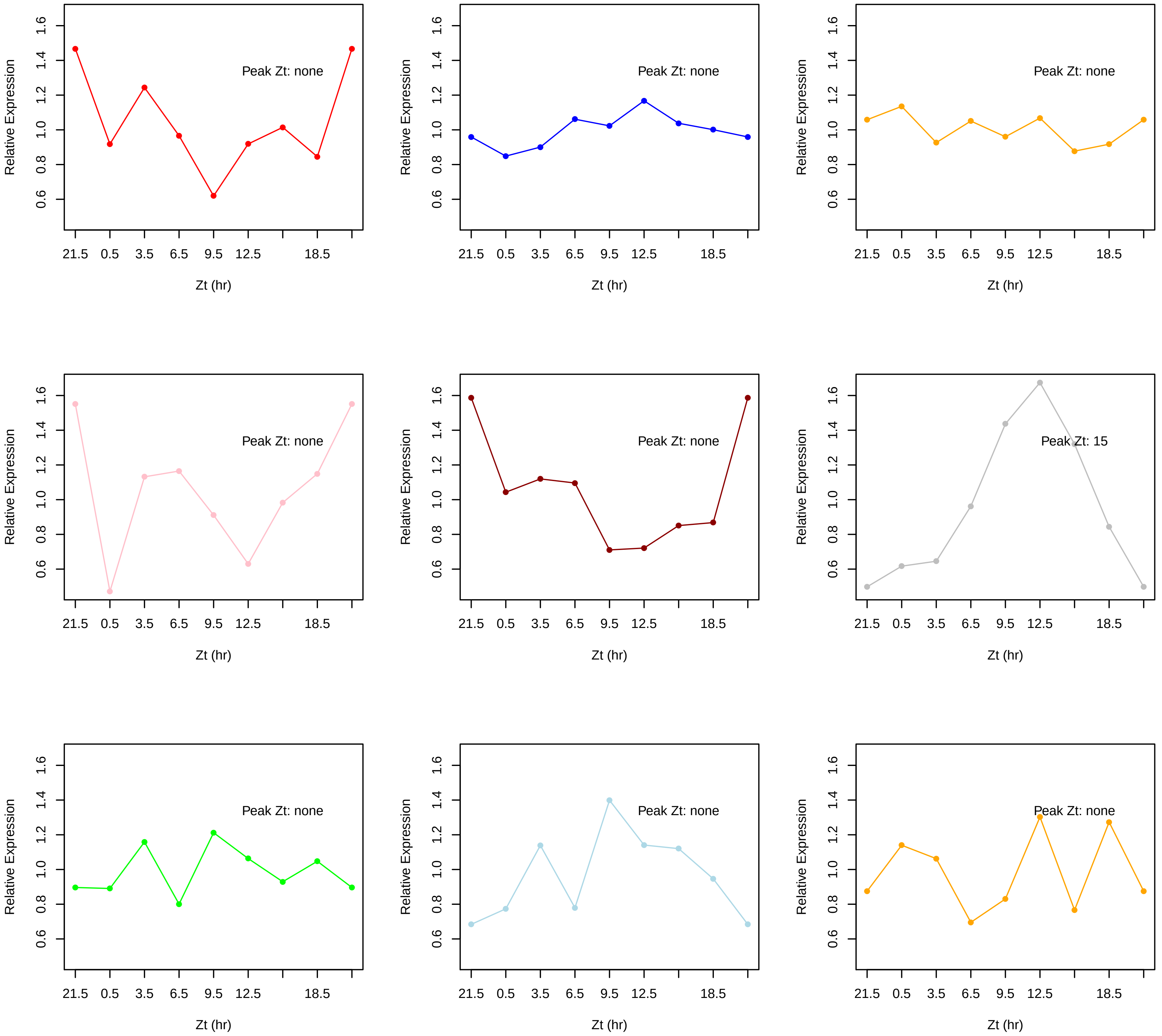

Singeleton: ENSXMAG00000000054 cnnm2b

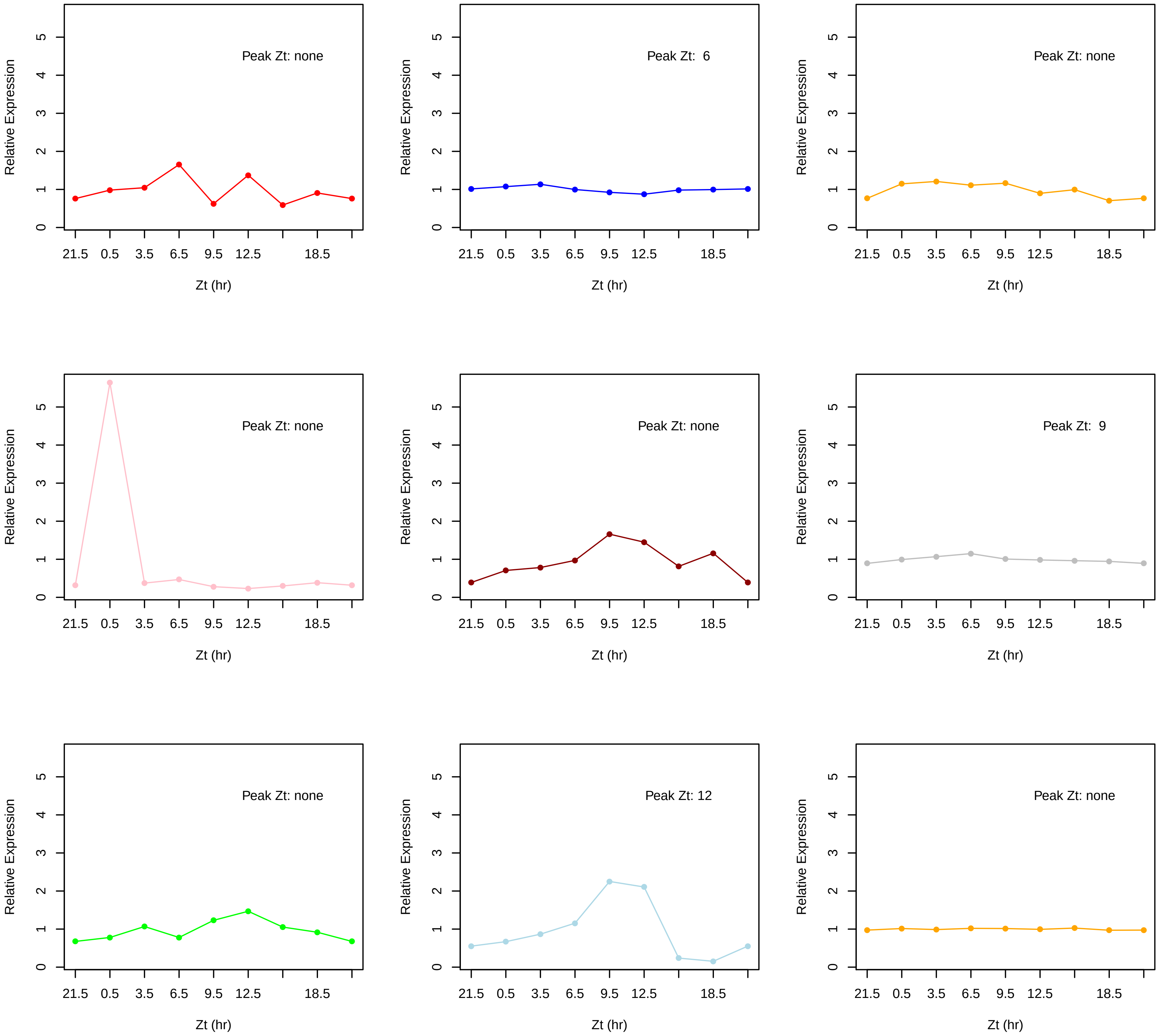

Singeleton: ENSXMAG00000025098

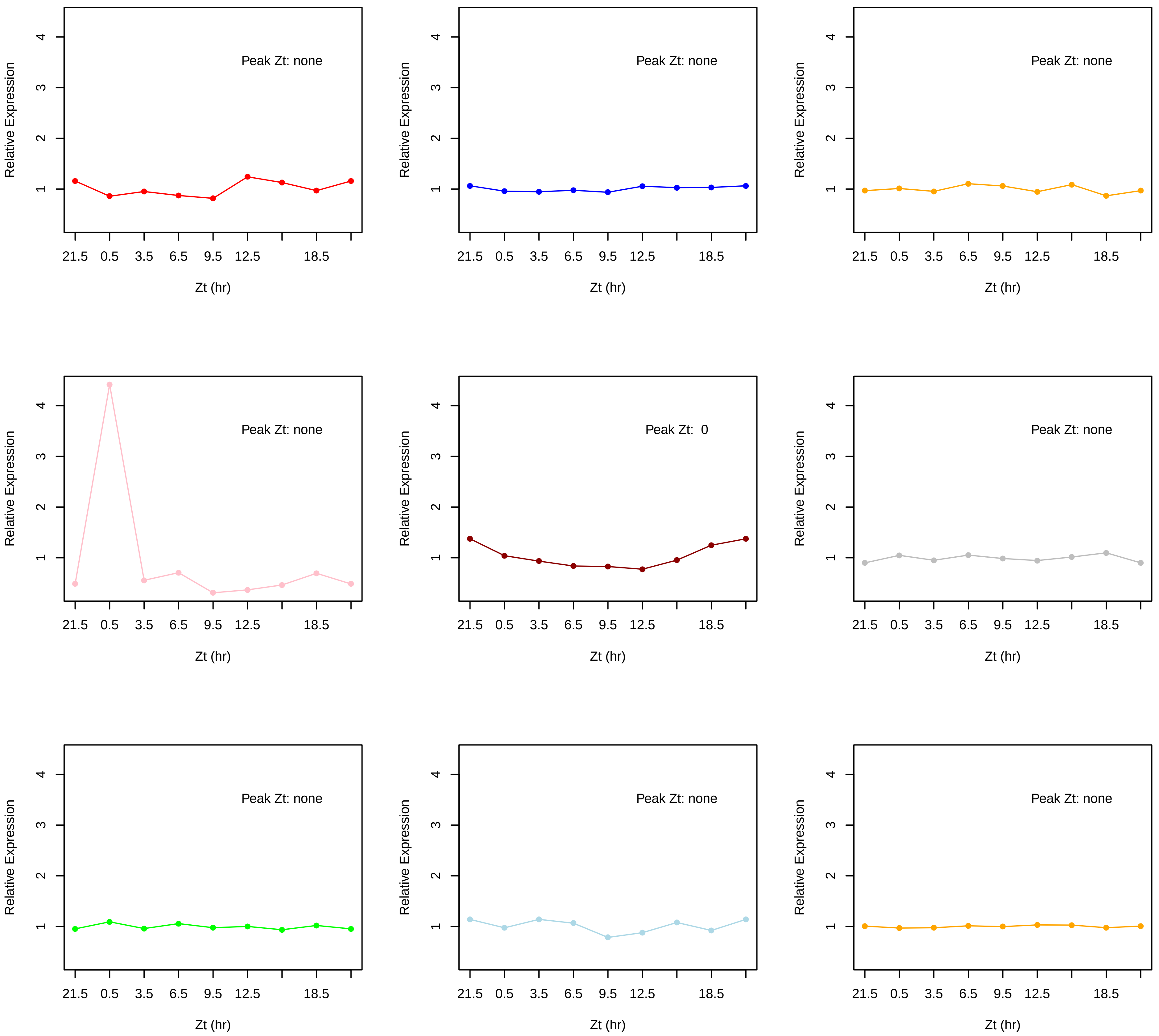

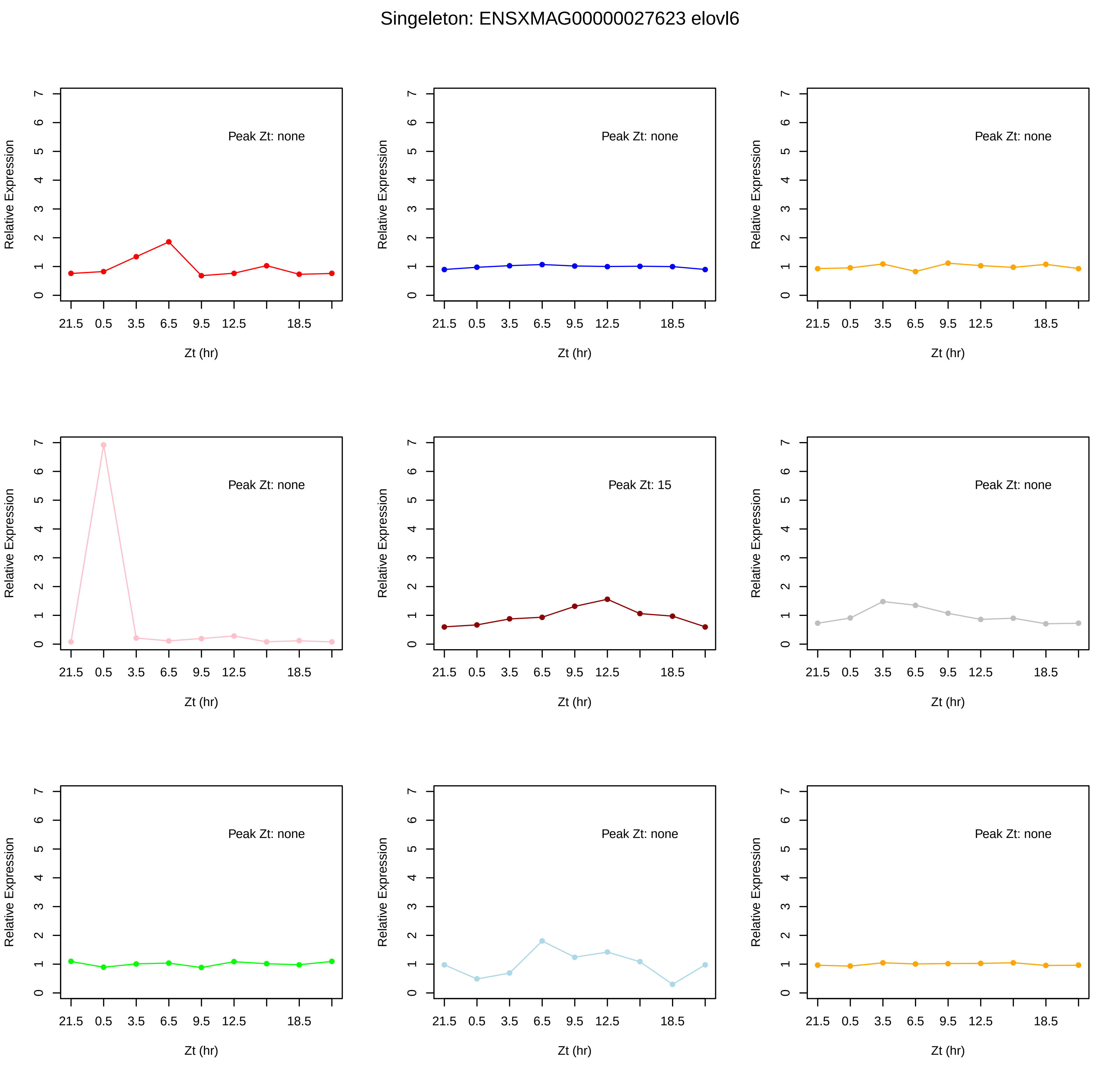

Singleton: ENSXMAG00000027623 elovl6
Peak Zt: none
Peak Zt: none
Peak Zt: none
Peak Zt: none
Peak Zt: 15
Peak Zt: none
Peak Zt: none
Peak Zt: none
Peak Zt: none
Relative Expression
Zt (hr)
21.5
0.5
3.5
6.5
9.5
12.5
18.5

Singeleton: ENSXMAG00000022324 narf

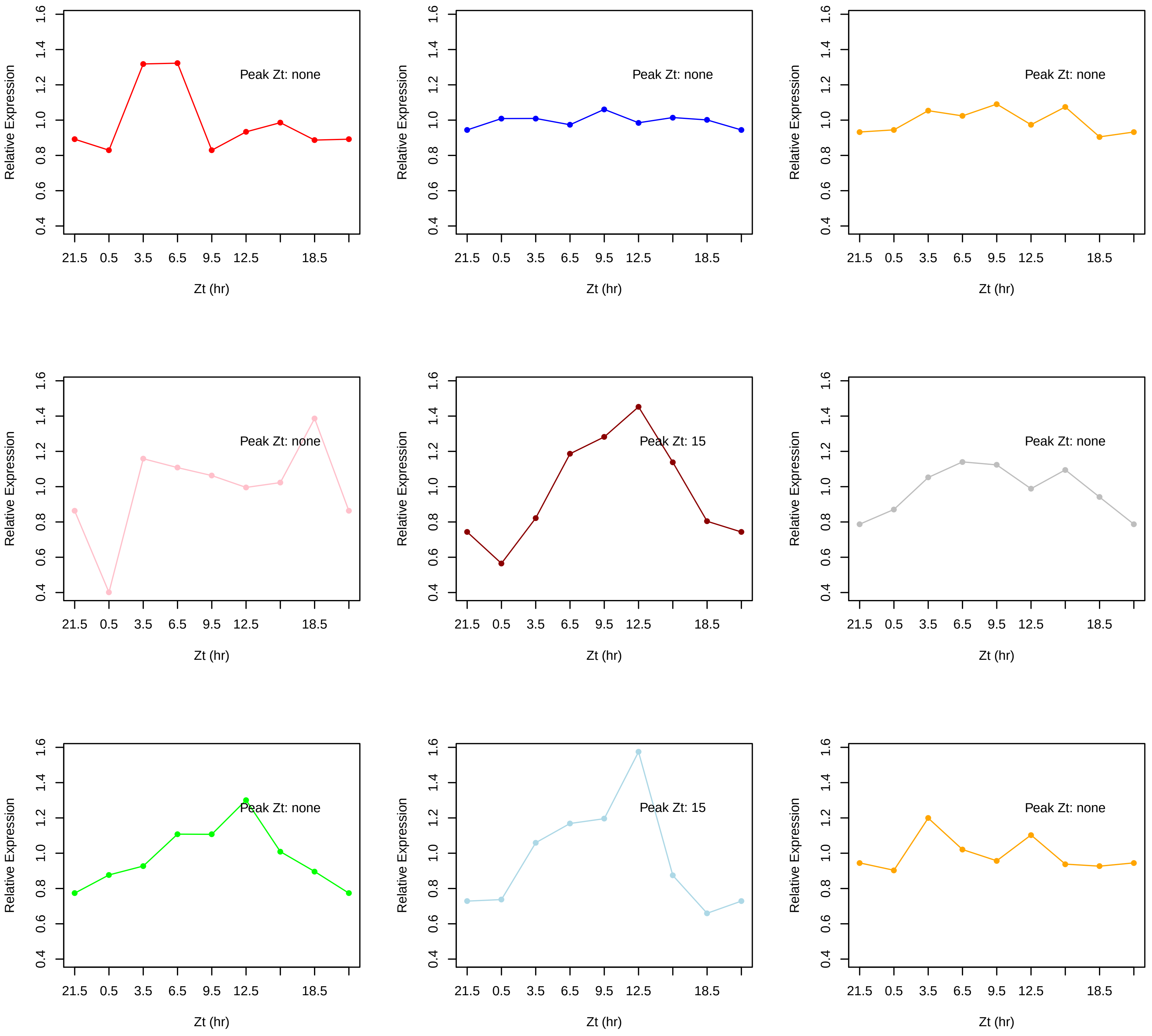

Singleton: ENSXMAG00000010058 anxa6

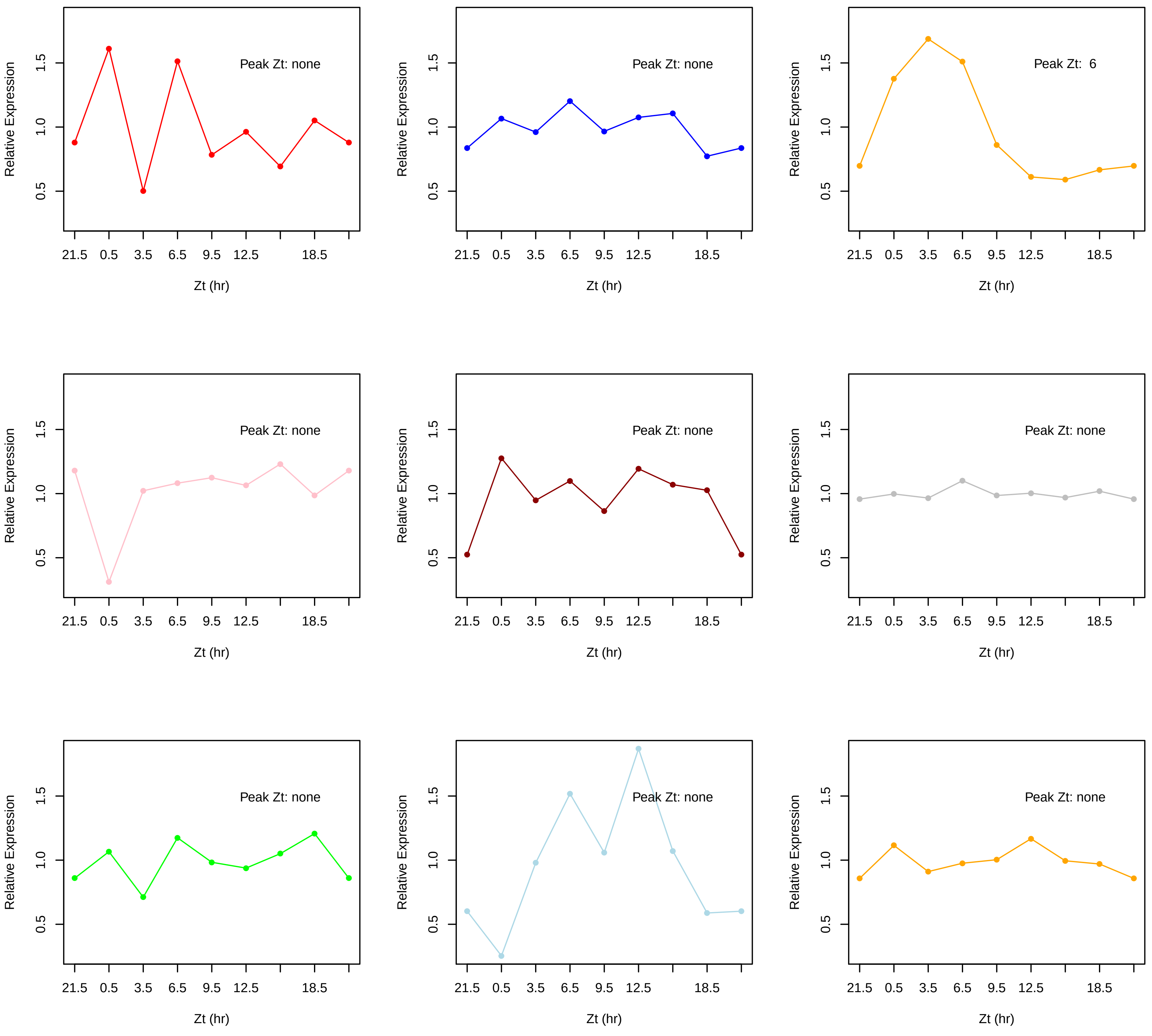

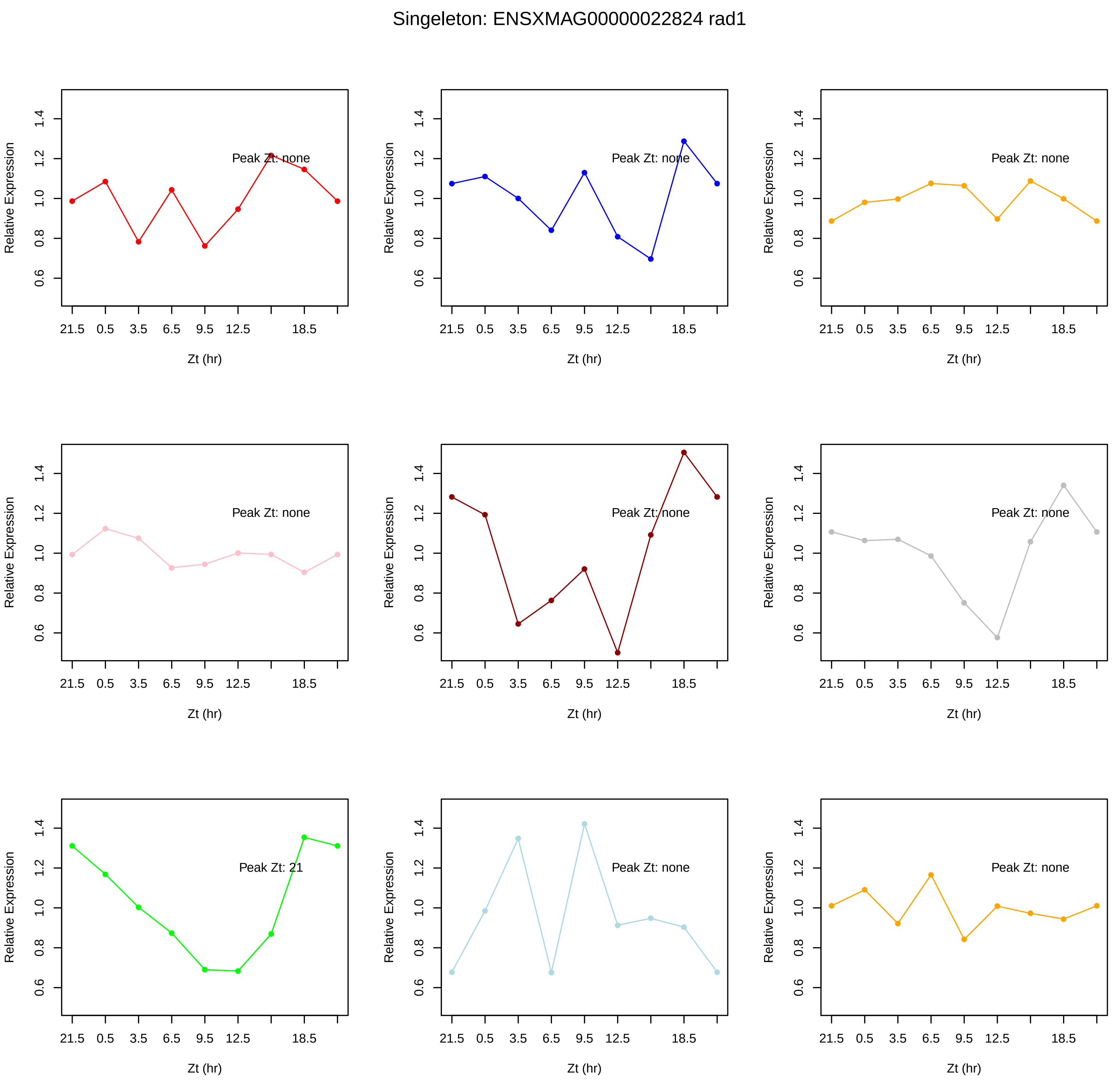
Singeleton: ENSXMAG00000022824 rad1
Relative Expression
Zt (hr)
Peak Zt: none
Peak Zt: 21
21.5 0.5 3.5 6.5 9.5 12.5 18.5
0.6 0.8 1.0 1.2 1.4

Singeleton: ENSXMAG00000026639 tomm22

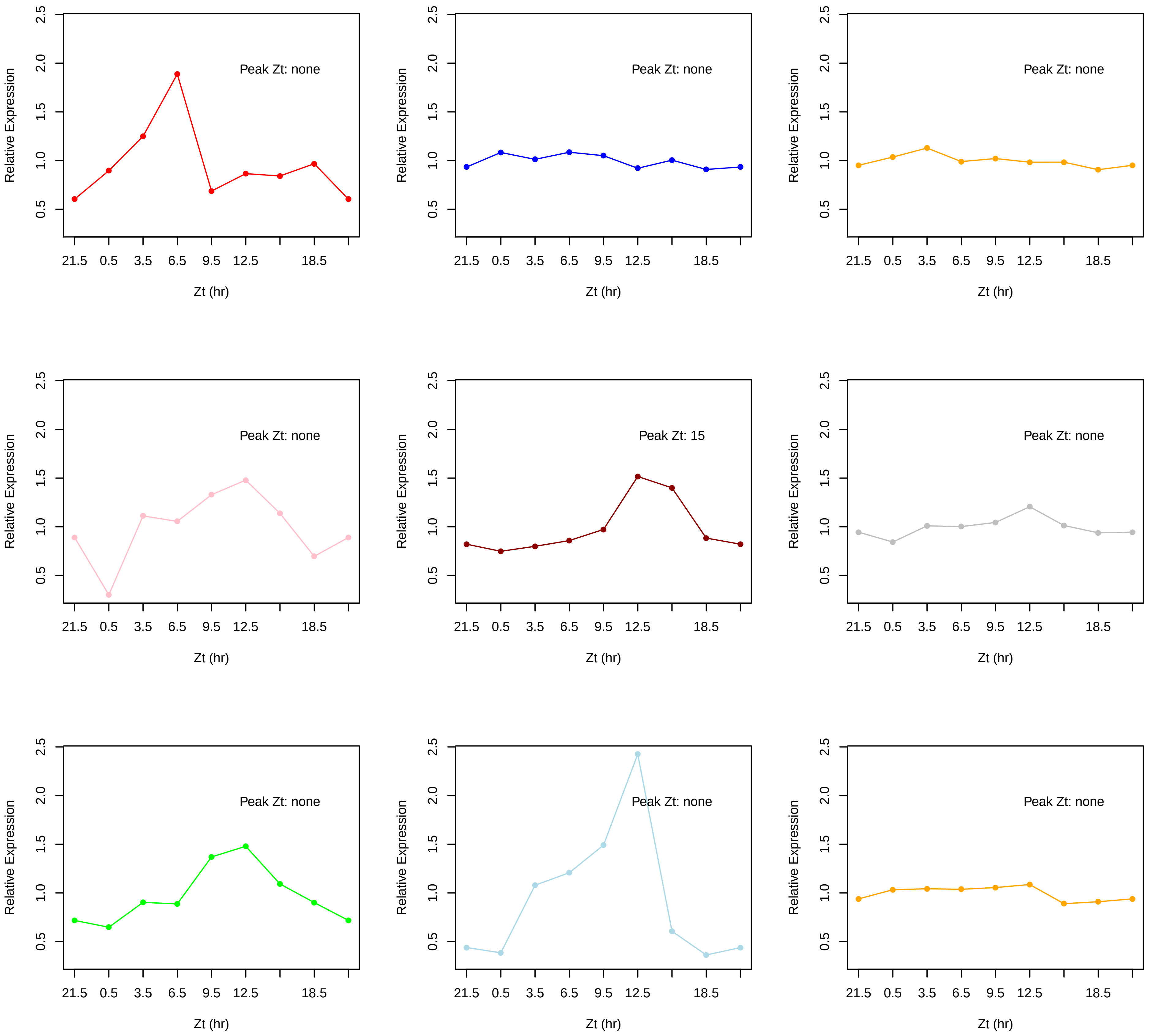

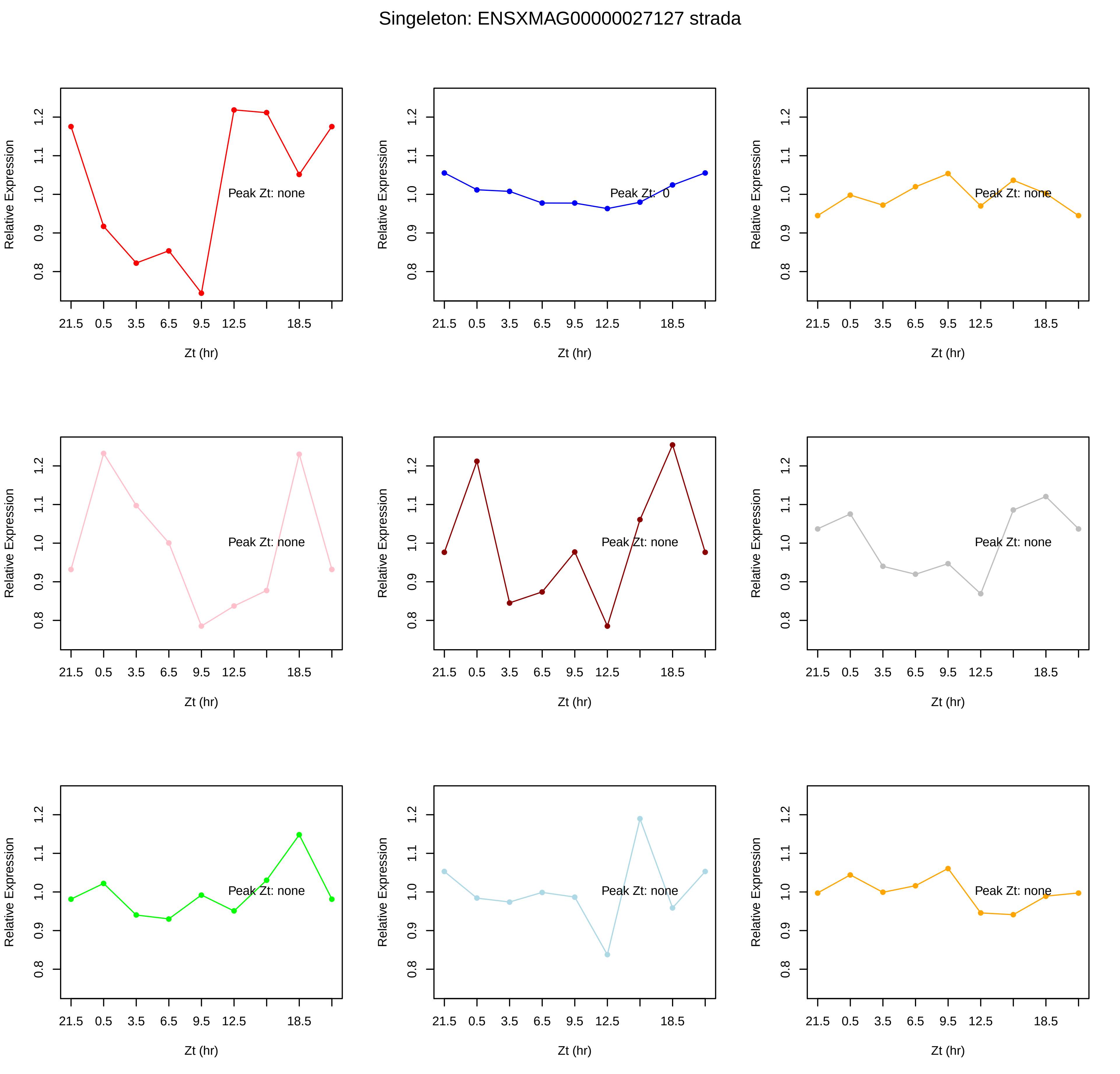

Singeleton: ENSXMAG00000027127 strada
Relative Expression
Zt (hr)
21.5
0.5
3.5
6.5
9.5
12.5
18.5
0.8
0.9
1.0
1.1
1.2
Peak Zt: none
Peak Zt: 0

Singeleton: ENSXMAG00000023968

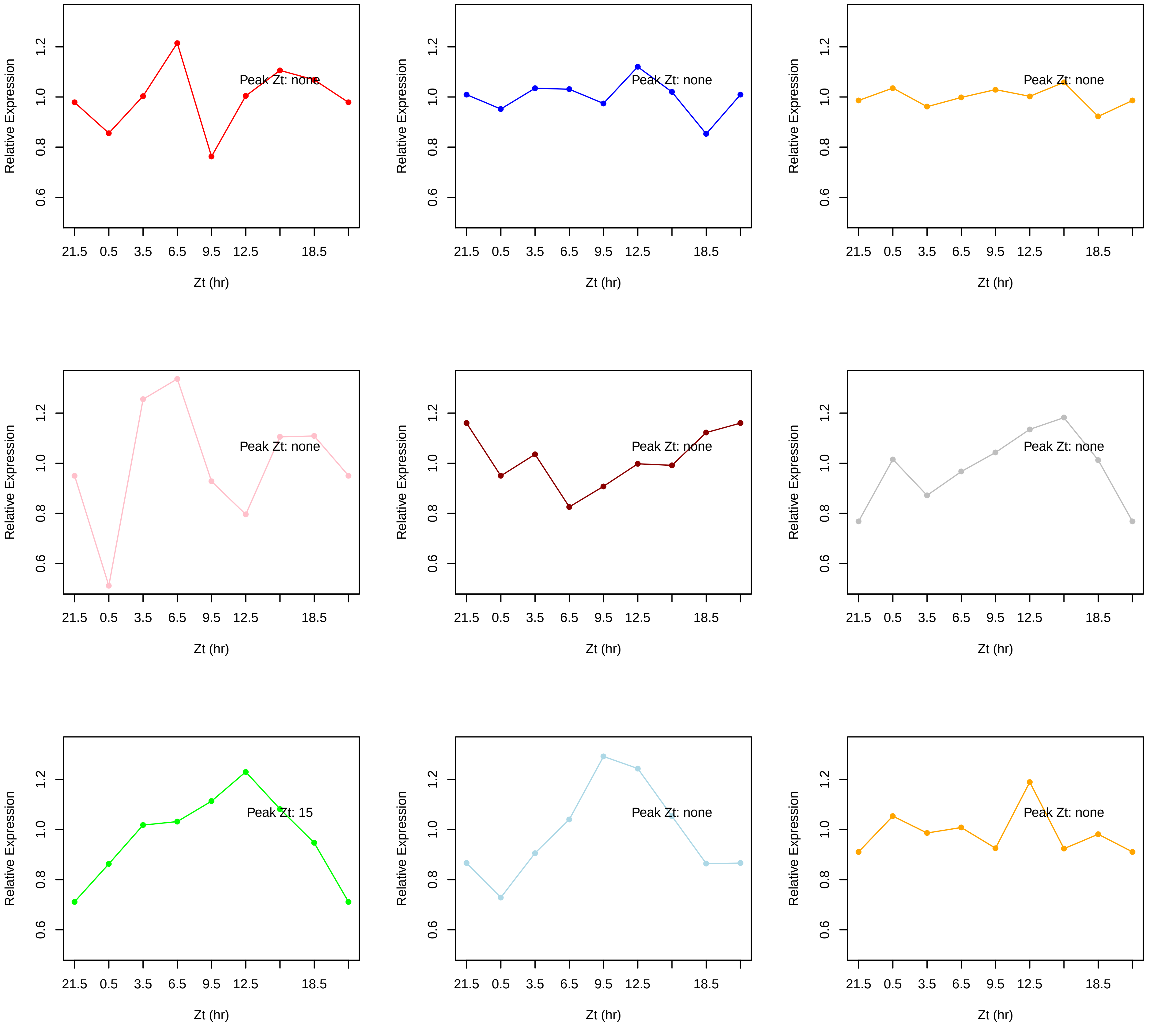

Singleton: ENSXMAG00000012742 hexdc

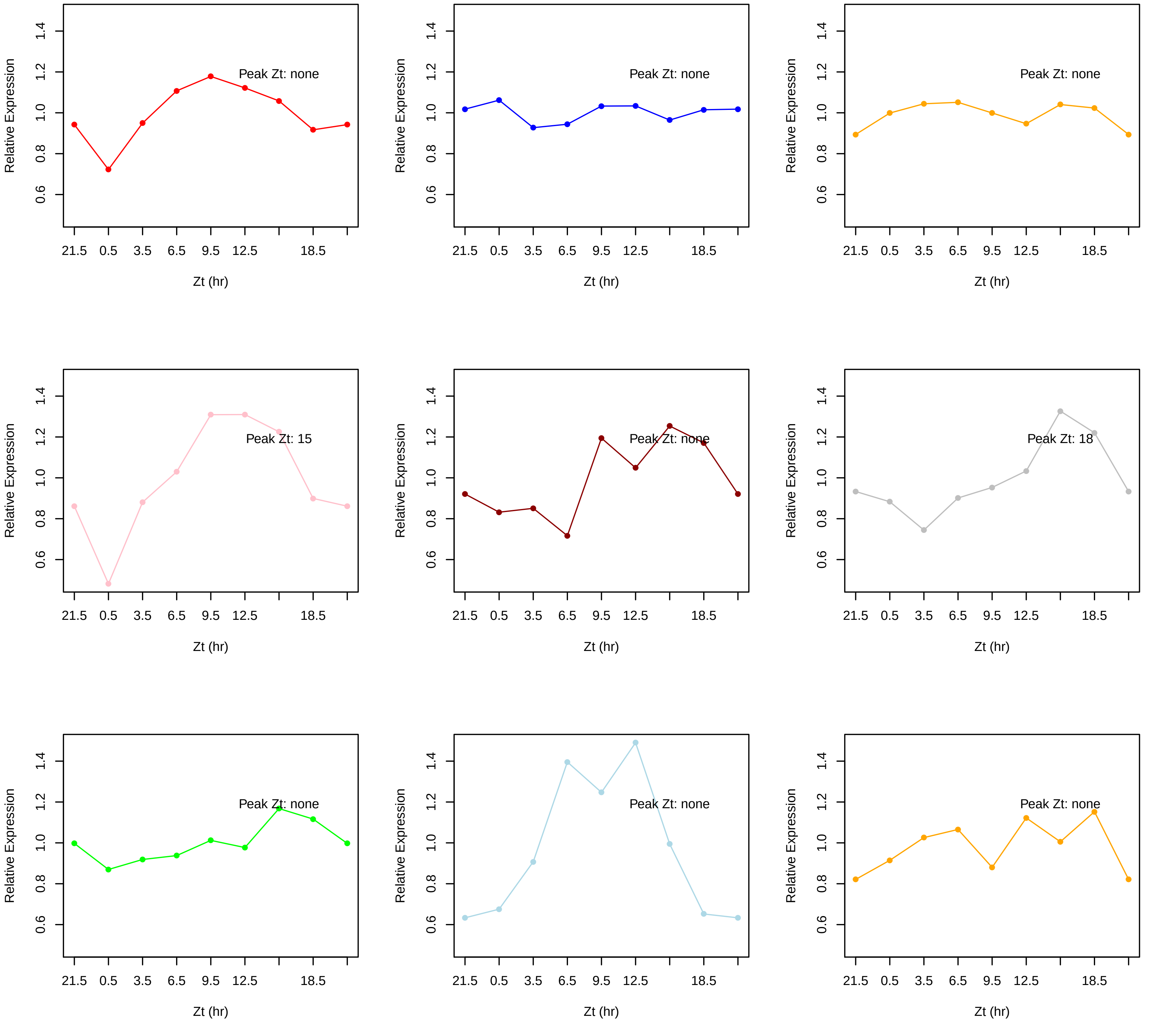

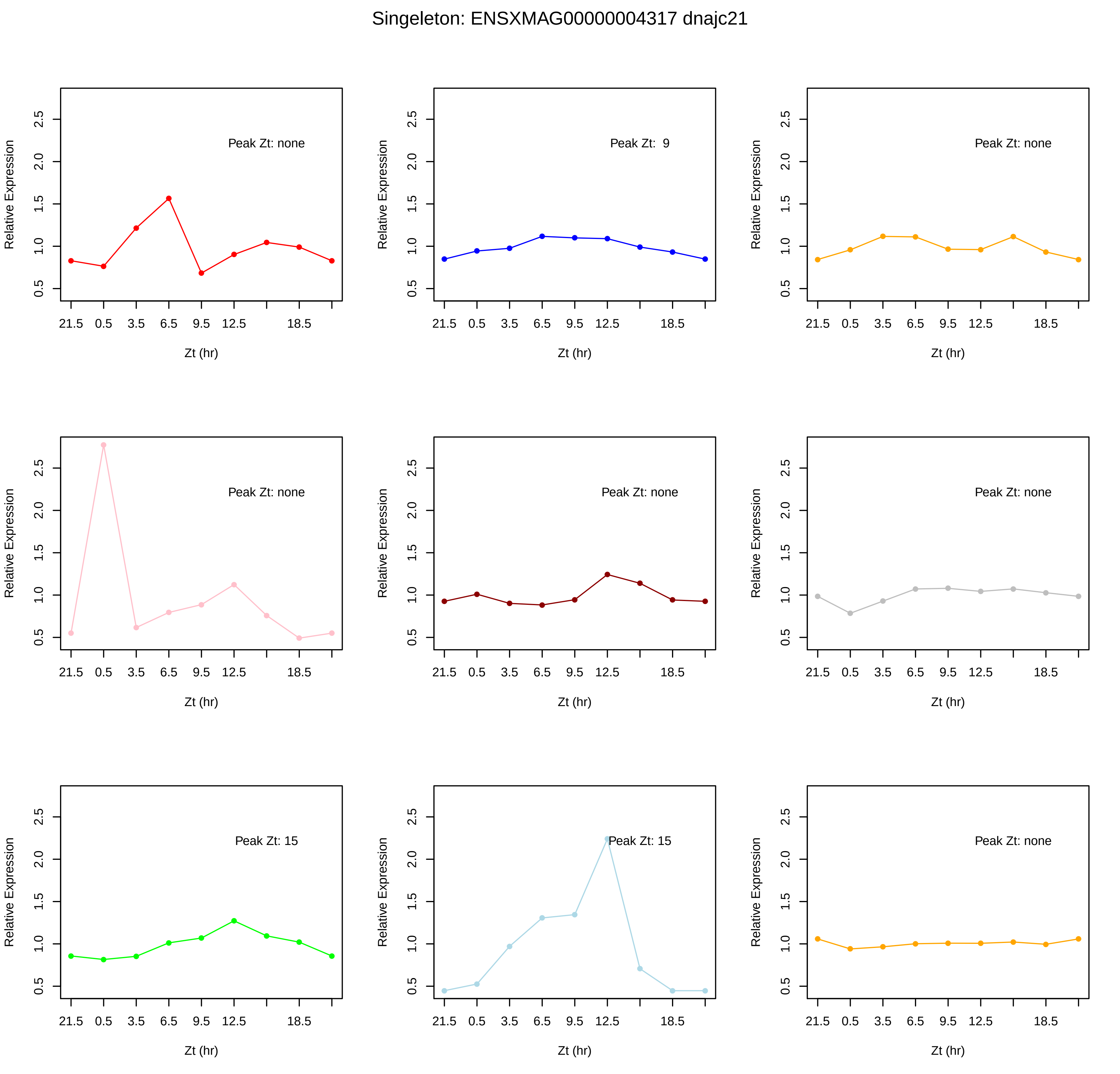
Singeleton: ENSXMAG00000004317 dnajc21
Peak Zt: none
Peak Zt: 9
Peak Zt: none
Peak Zt: none
Peak Zt: none
Peak Zt: none
Peak Zt: 15
Peak Zt: 15
Peak Zt: none
Relative Expression
Zt (hr)
21.5
0.5
3.5
6.5
9.5
12.5
18.5
0.5
1.0
1.5
2.0
2.5

Singeleton: ENSXMAG00000008169 rnf113a

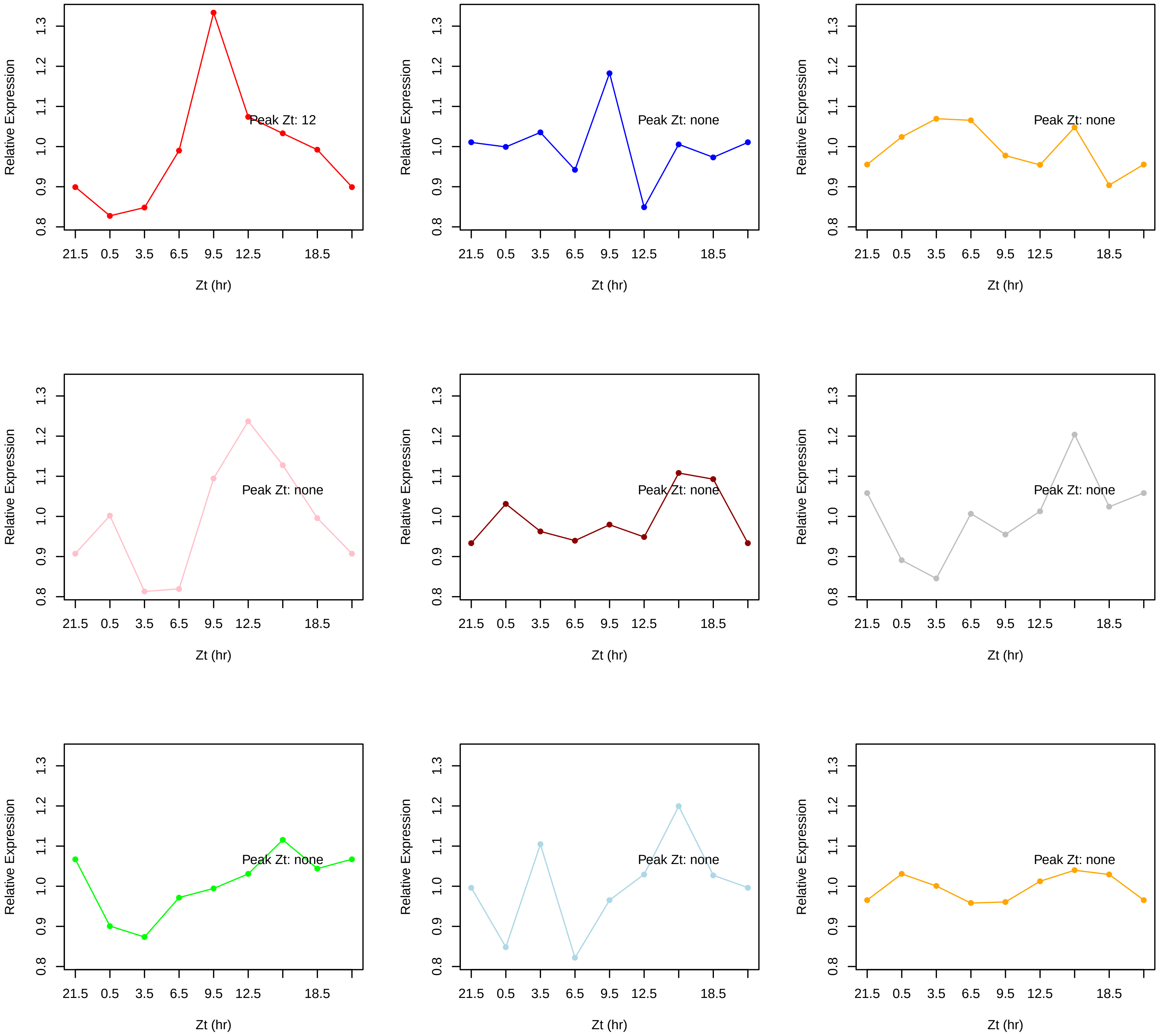

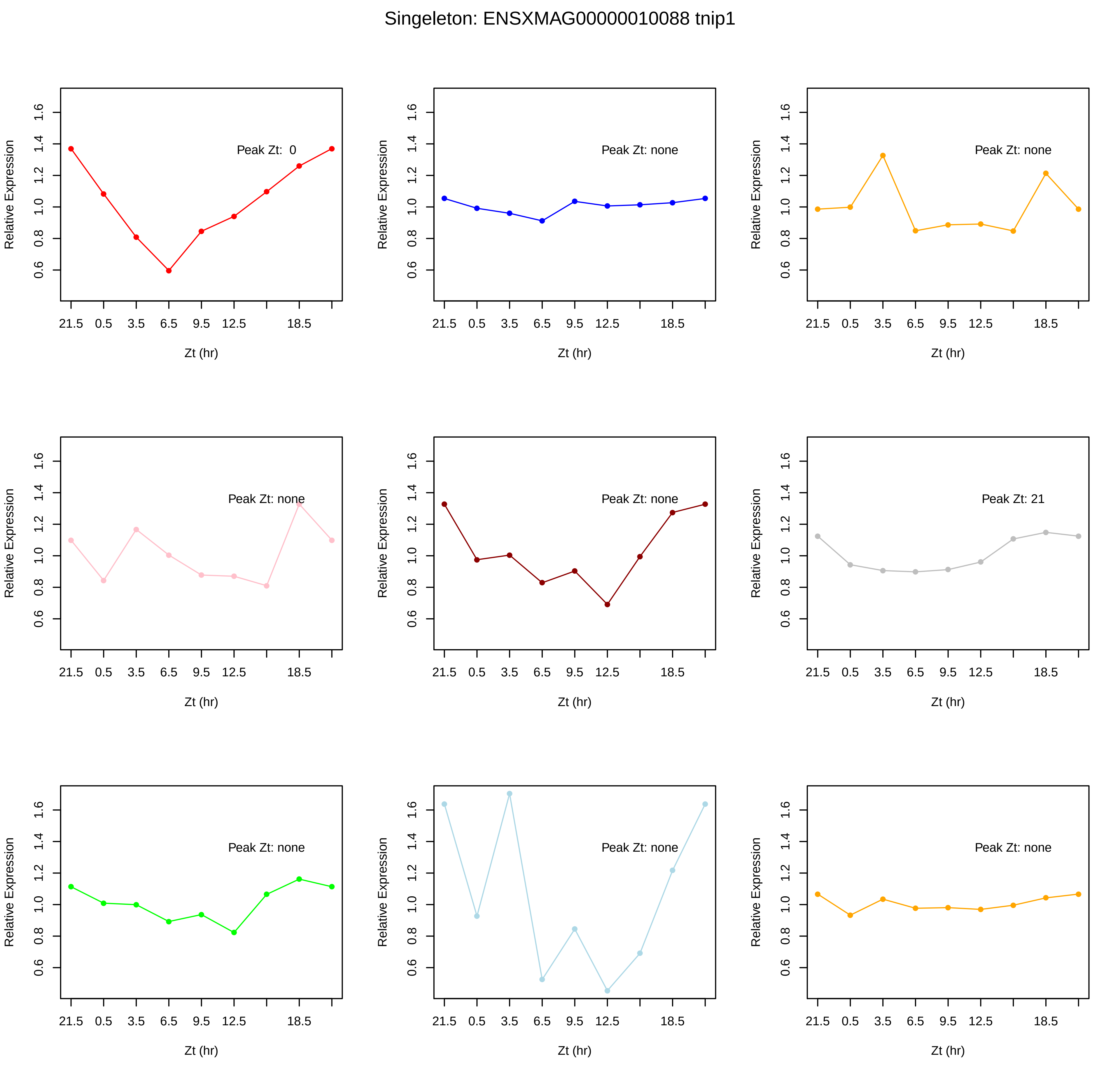
Singeleton: ENSXMAG00000010088 tnip1
Peak Zt: 0
Peak Zt: none
Peak Zt: none
Peak Zt: none
Peak Zt: none
Peak Zt: 21
Peak Zt: none
Peak Zt: none
Peak Zt: none
Relative Expression
Zt (hr)
21.5 0.5 3.5 6.5 9.5 12.5 18.5
0.6 0.8 1.0 1.2 1.4 1.6

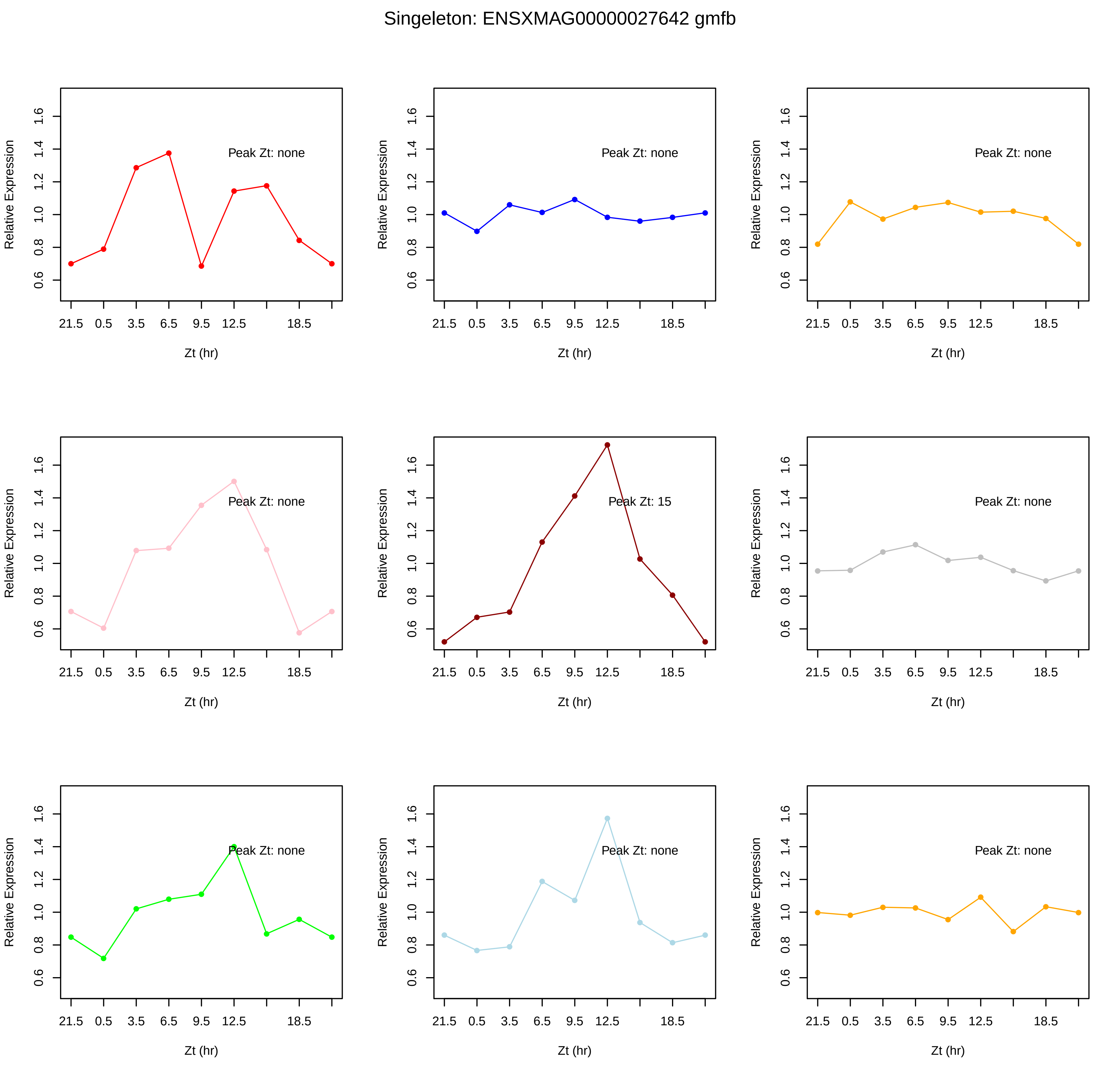
Singeleton: ENSXMAG00000027642 gmfb
Peak Zt: none
Peak Zt: none
Peak Zt: none
Peak Zt: none
Peak Zt: 15
Peak Zt: none
Peak Zt: none
Peak Zt: none
Peak Zt: none
Relative Expression
Zt (hr)
21.5
0.5
3.5
6.5
9.5
12.5
18.5
0.6
0.8
1.0
1.2
1.4
1.6

Singleton: ENSXMAG00000007347

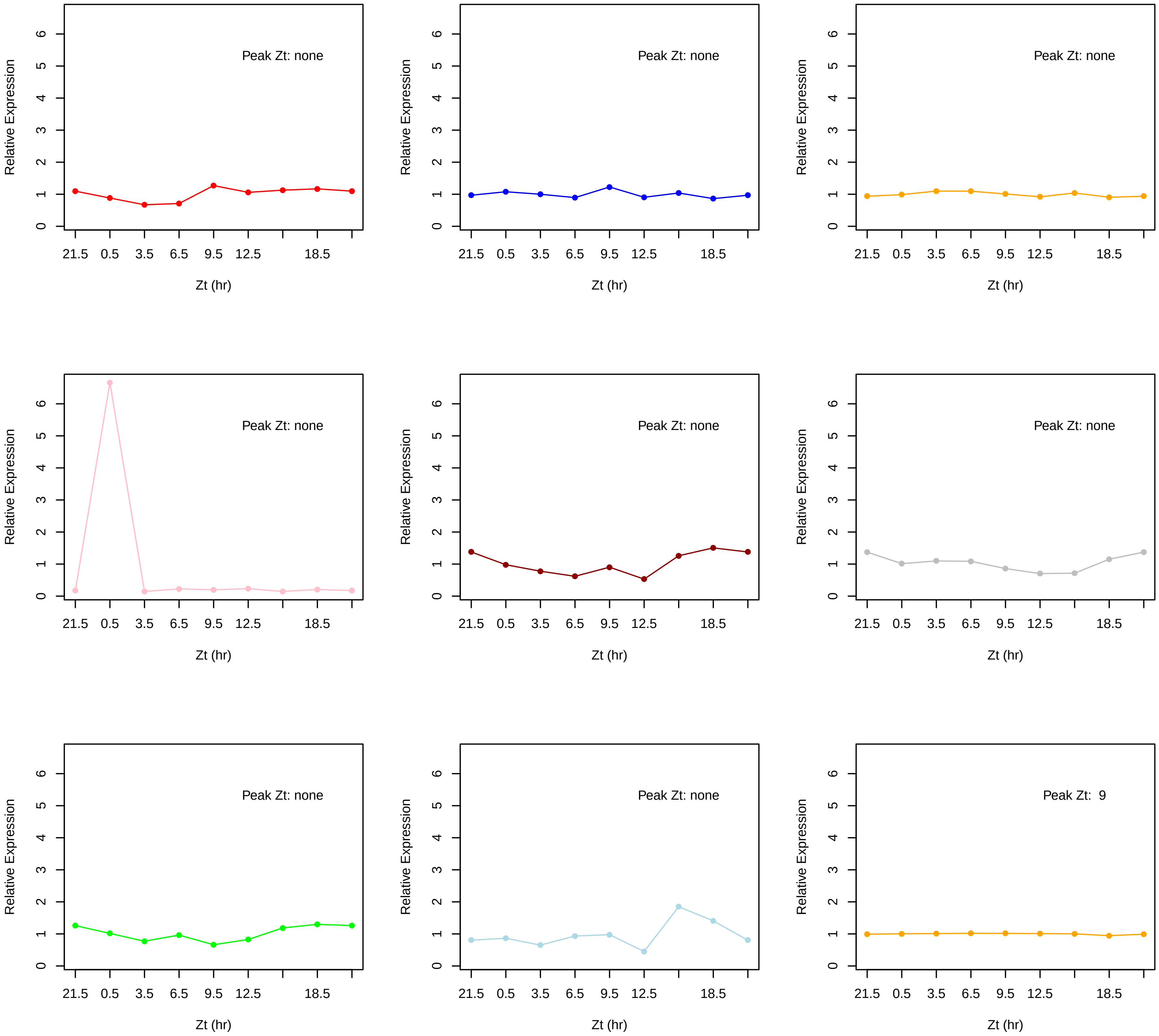

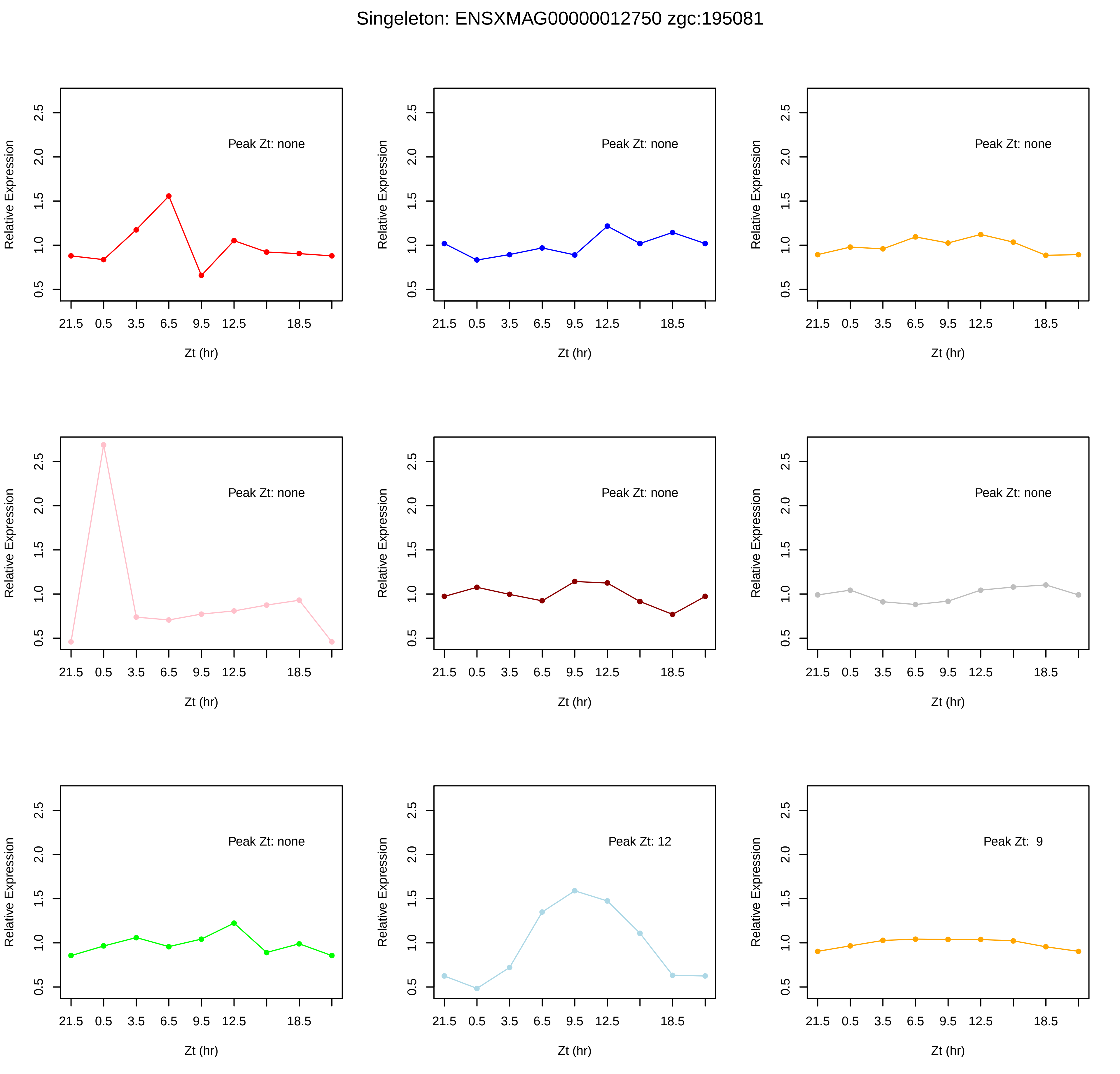

Singeleton: ENSXMAG00000012750 zgc:195081
Peak Zt: none
Peak Zt: none
Peak Zt: none
Peak Zt: none
Peak Zt: none
Peak Zt: none
Peak Zt: none
Peak Zt: 12
Peak Zt: 9
Relative Expression
Zt (hr)
21.5
0.5
3.5
6.5
9.5
12.5
18.5
0.5
1.0
1.5
2.0
2.5

Singleton: ENSXMAG00000027934 agxt2

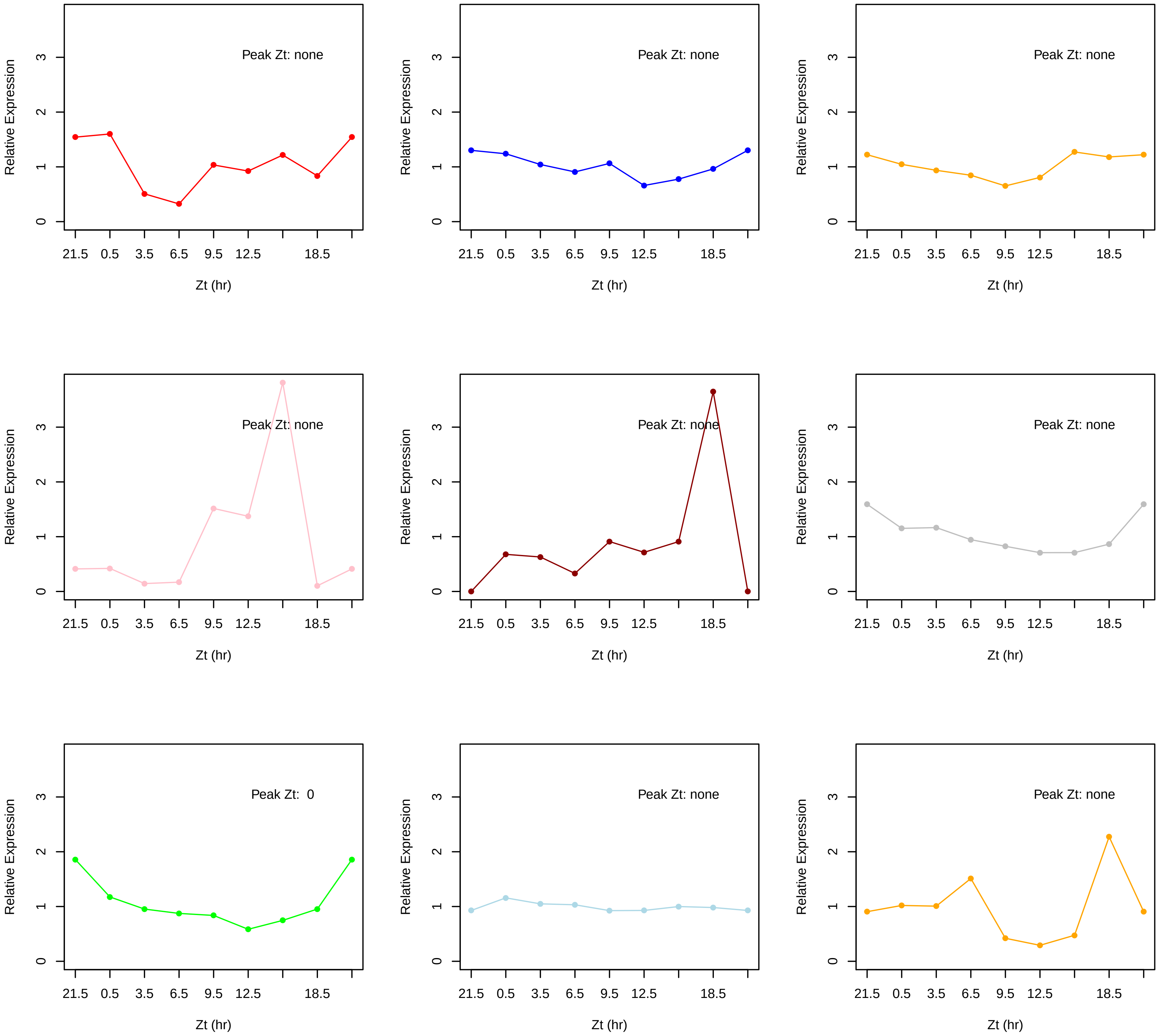

Singeleton: ENSXMAG00000002556 nucks1a

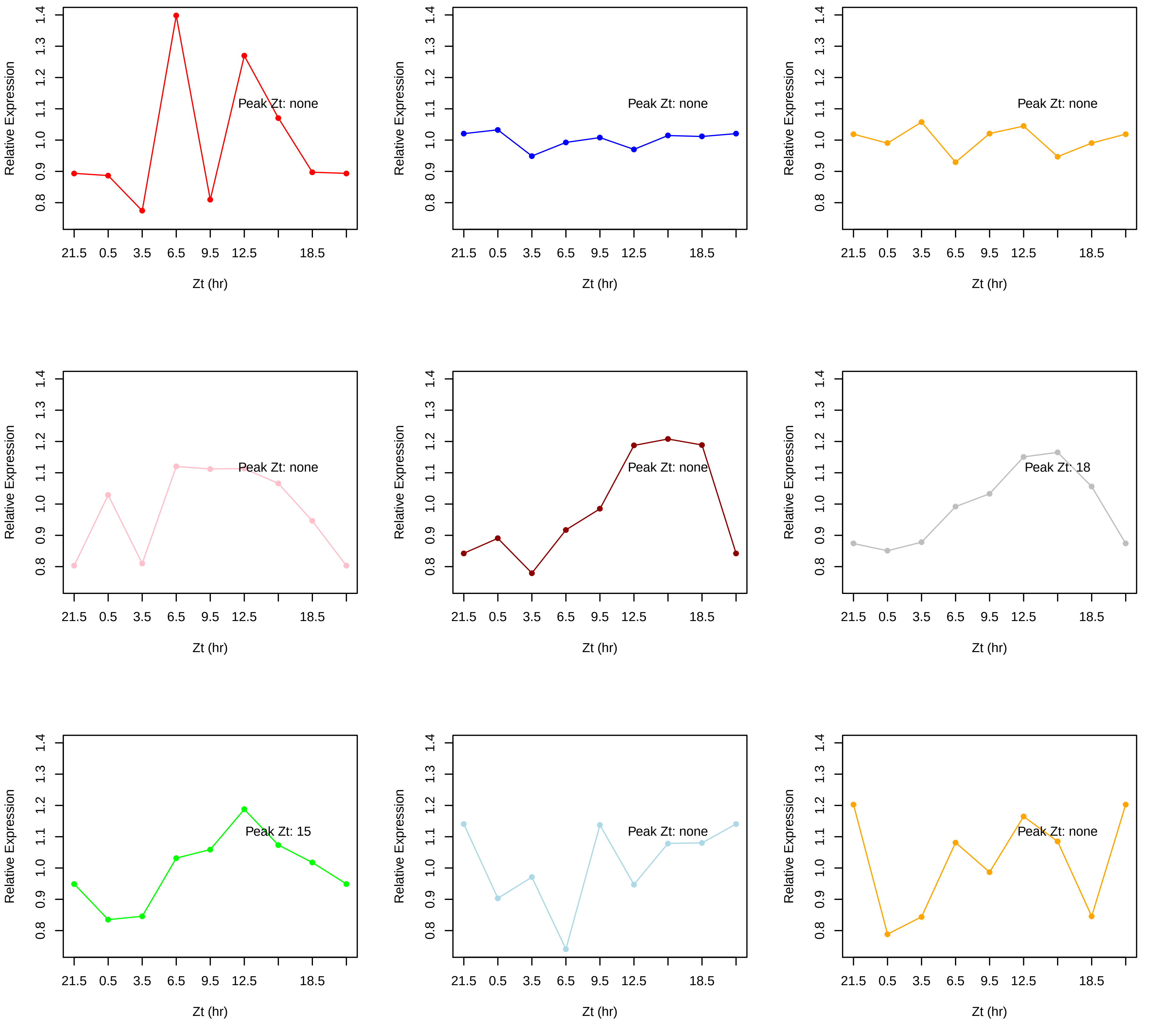

Singeleton: ENSXMAG00000007076 commd3

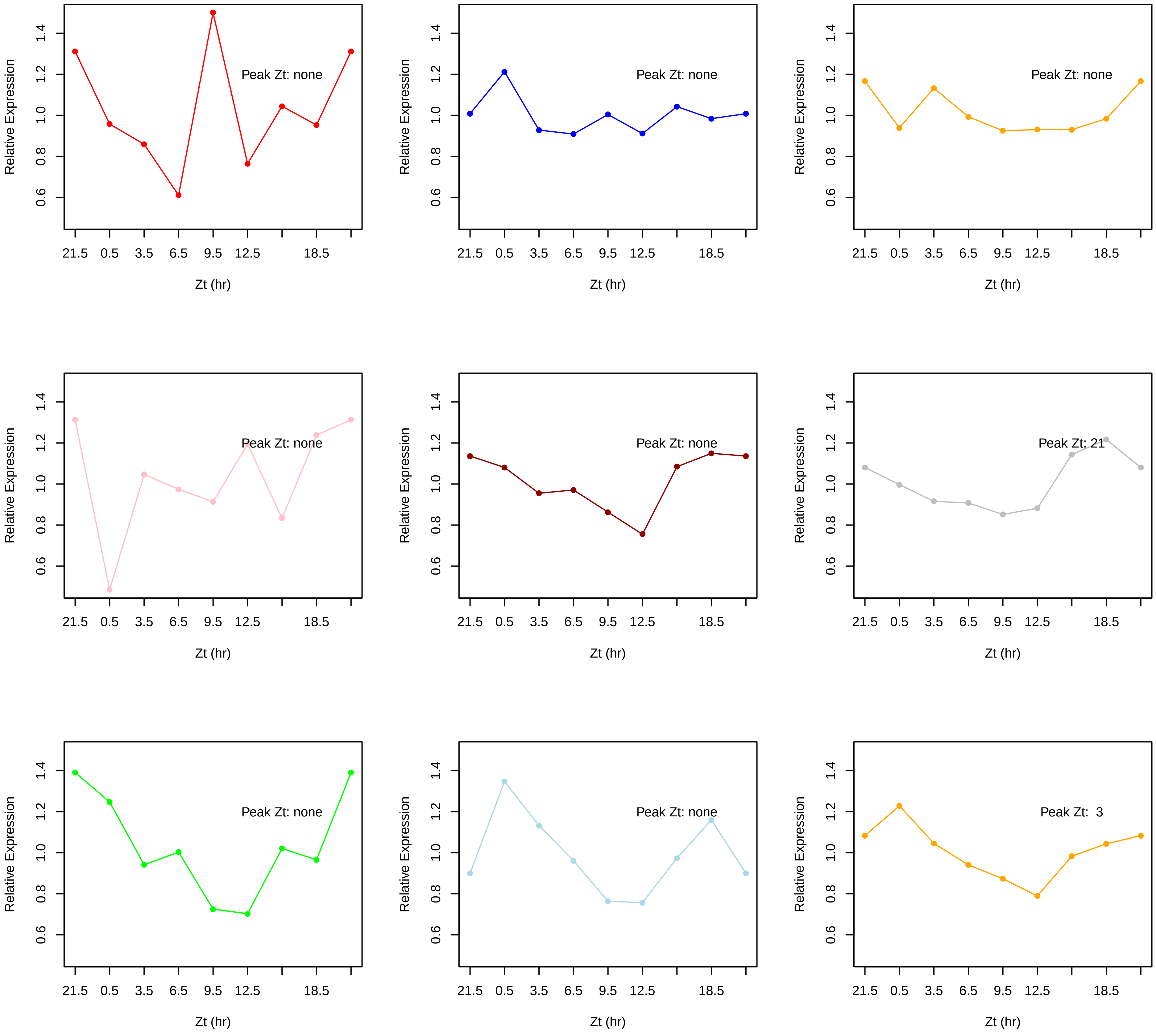

Singeleton: ENSXMAG00000005121

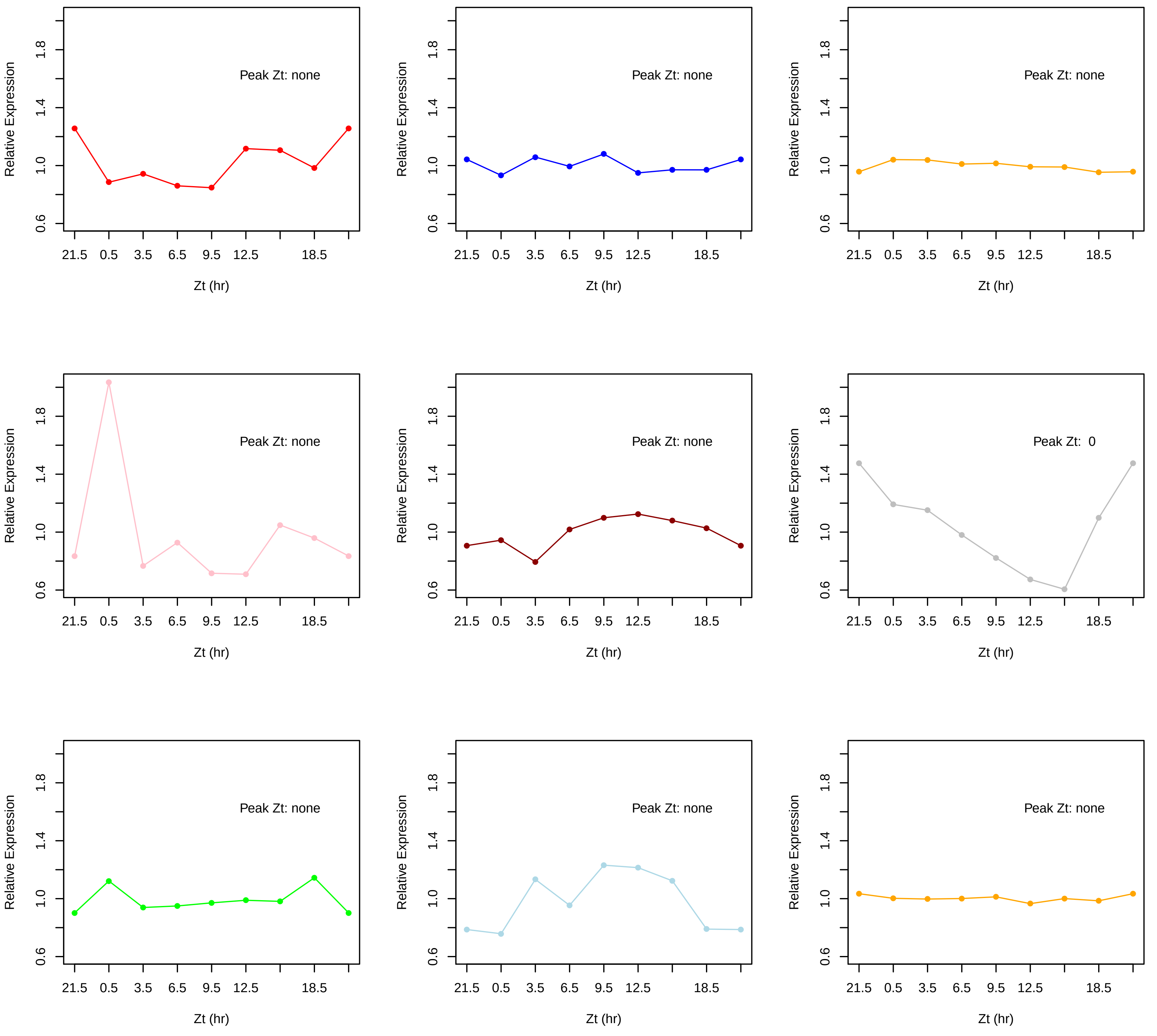

Singeleton: ENSXMAG00000030000 gfap

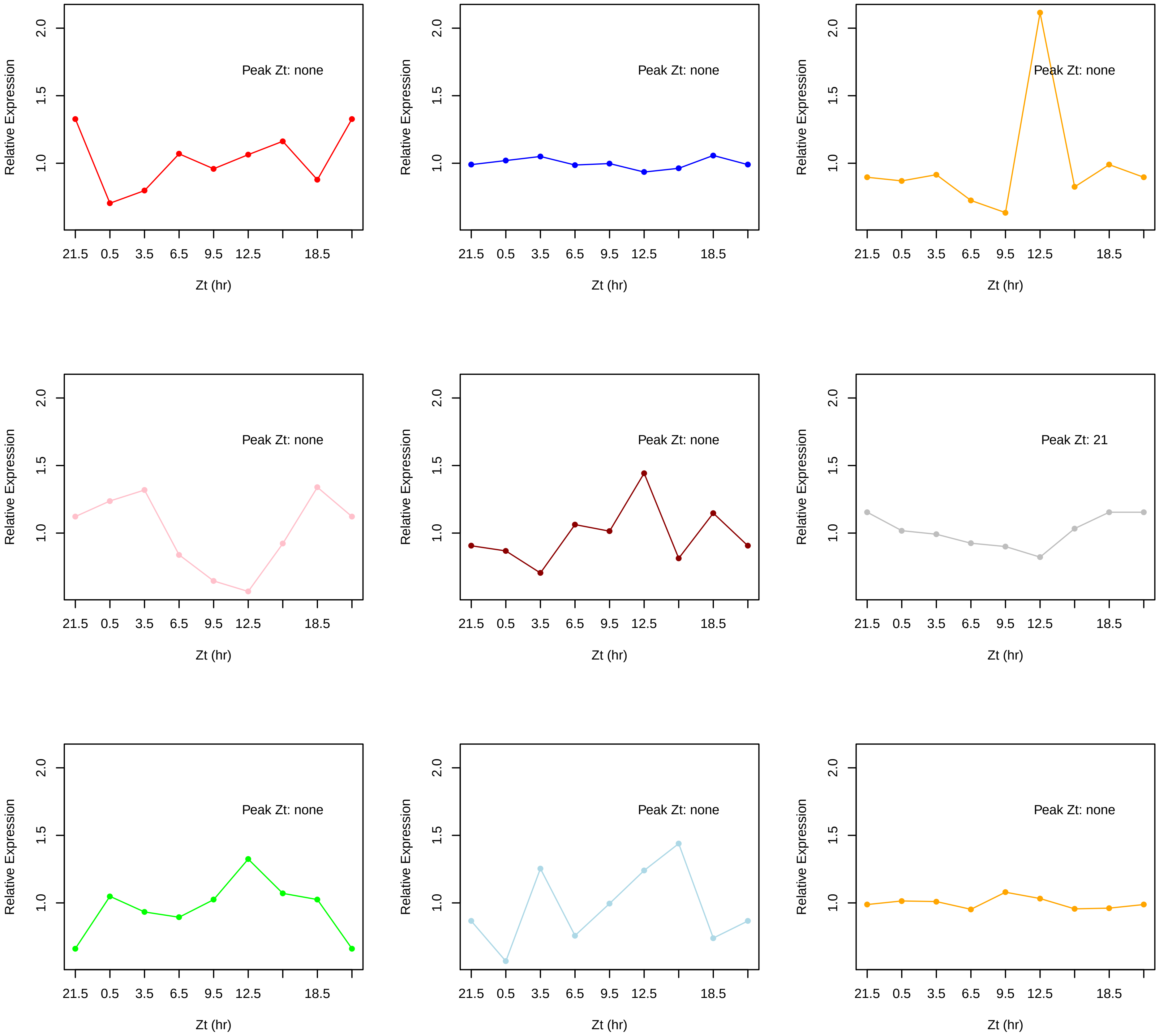

Singleton: ENSXMAG00000010102 dctn4

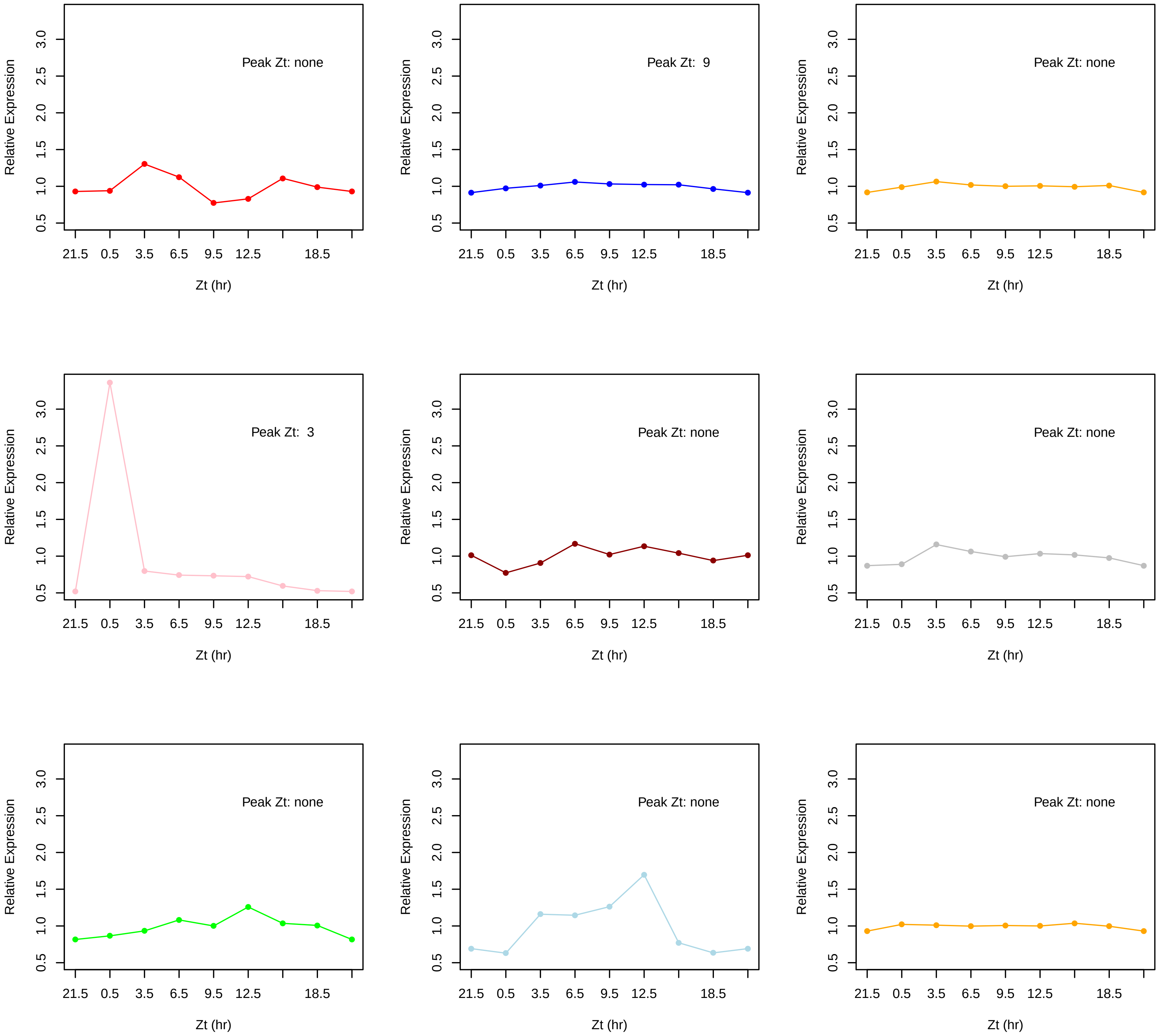

Singeleton: ENSXMAG00000001054 hoxa5a

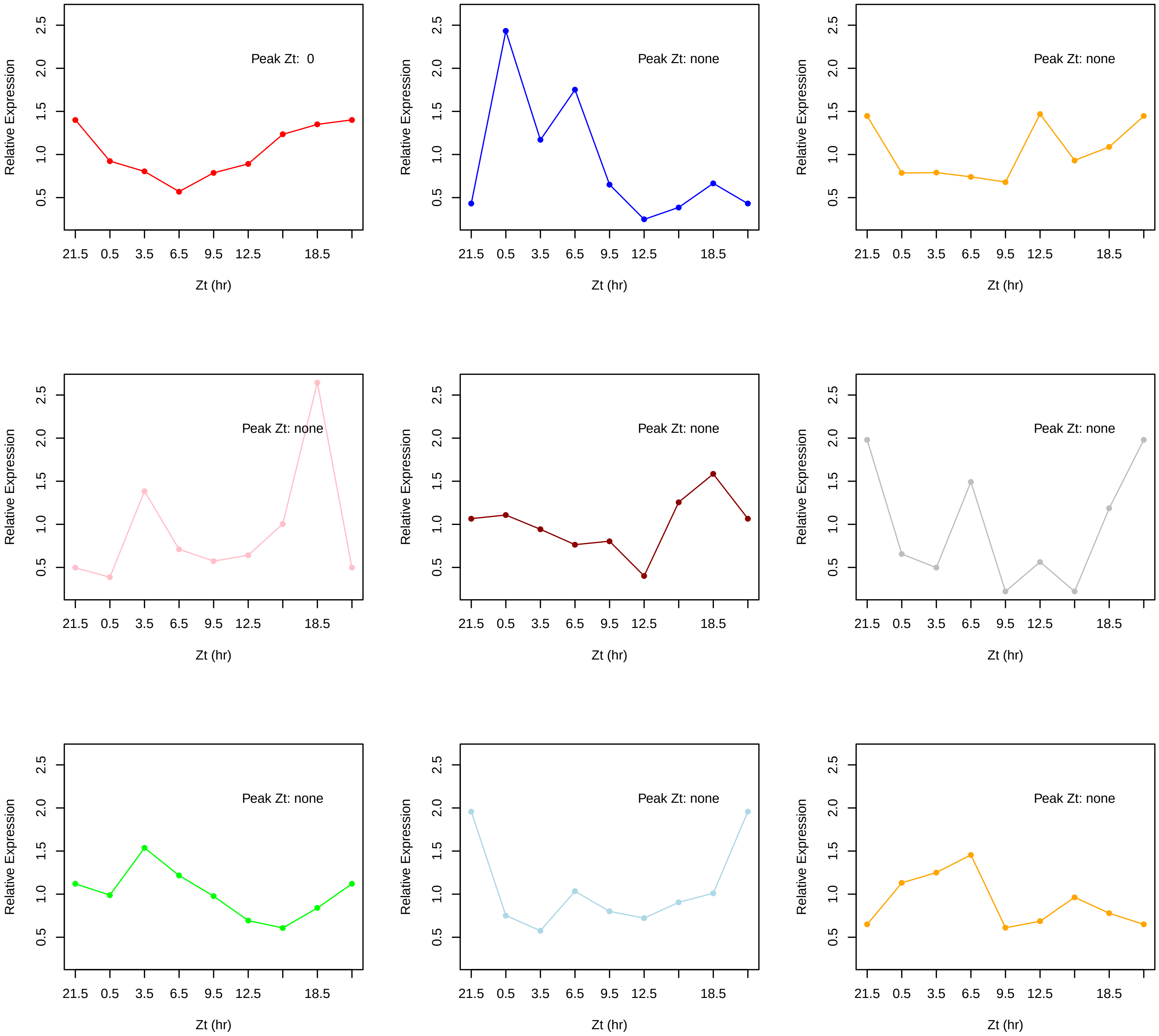

Singeleton: ENSXMAG00000005305

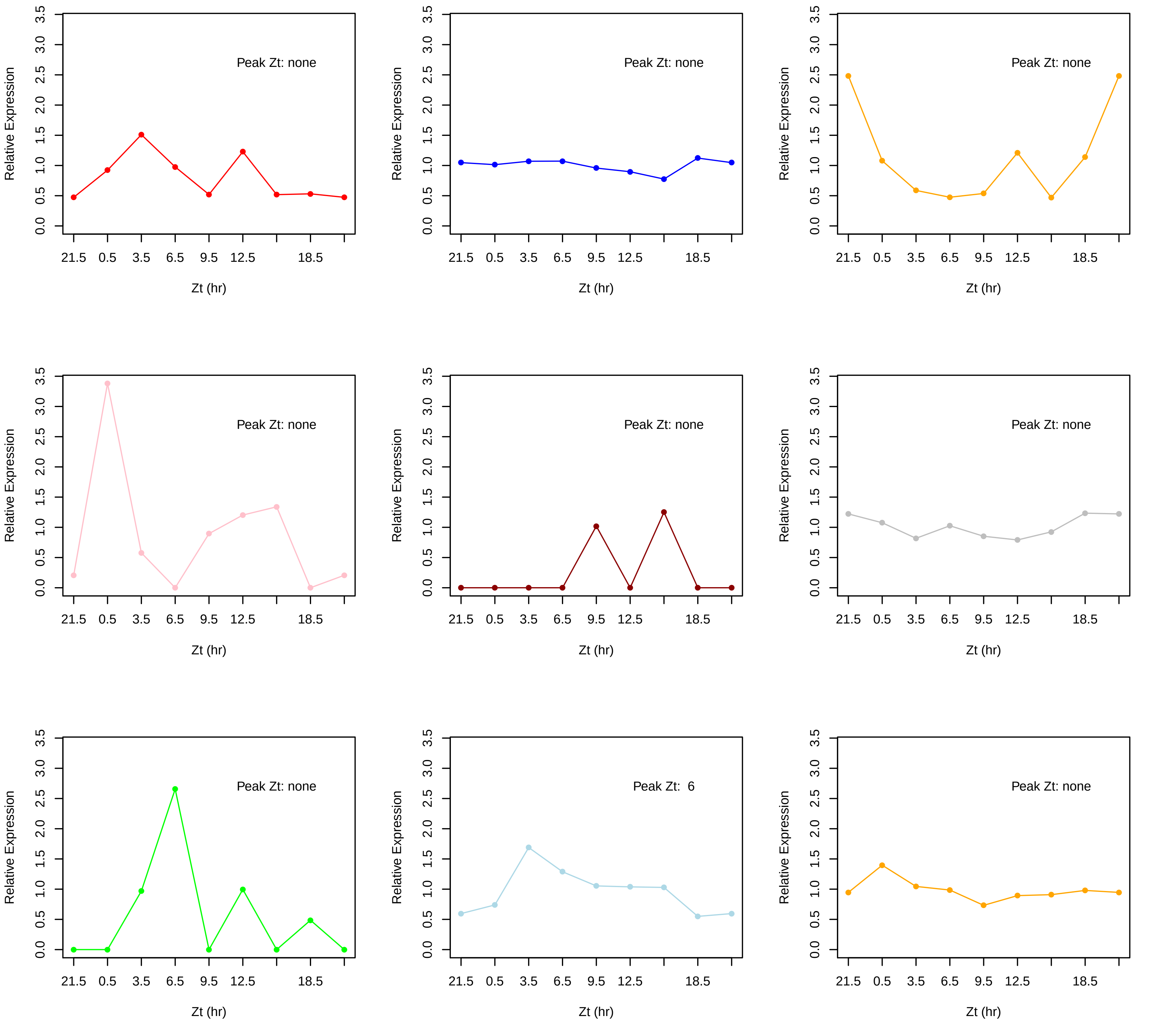

Singeleton: ENSXMAG00000010118 tspan17

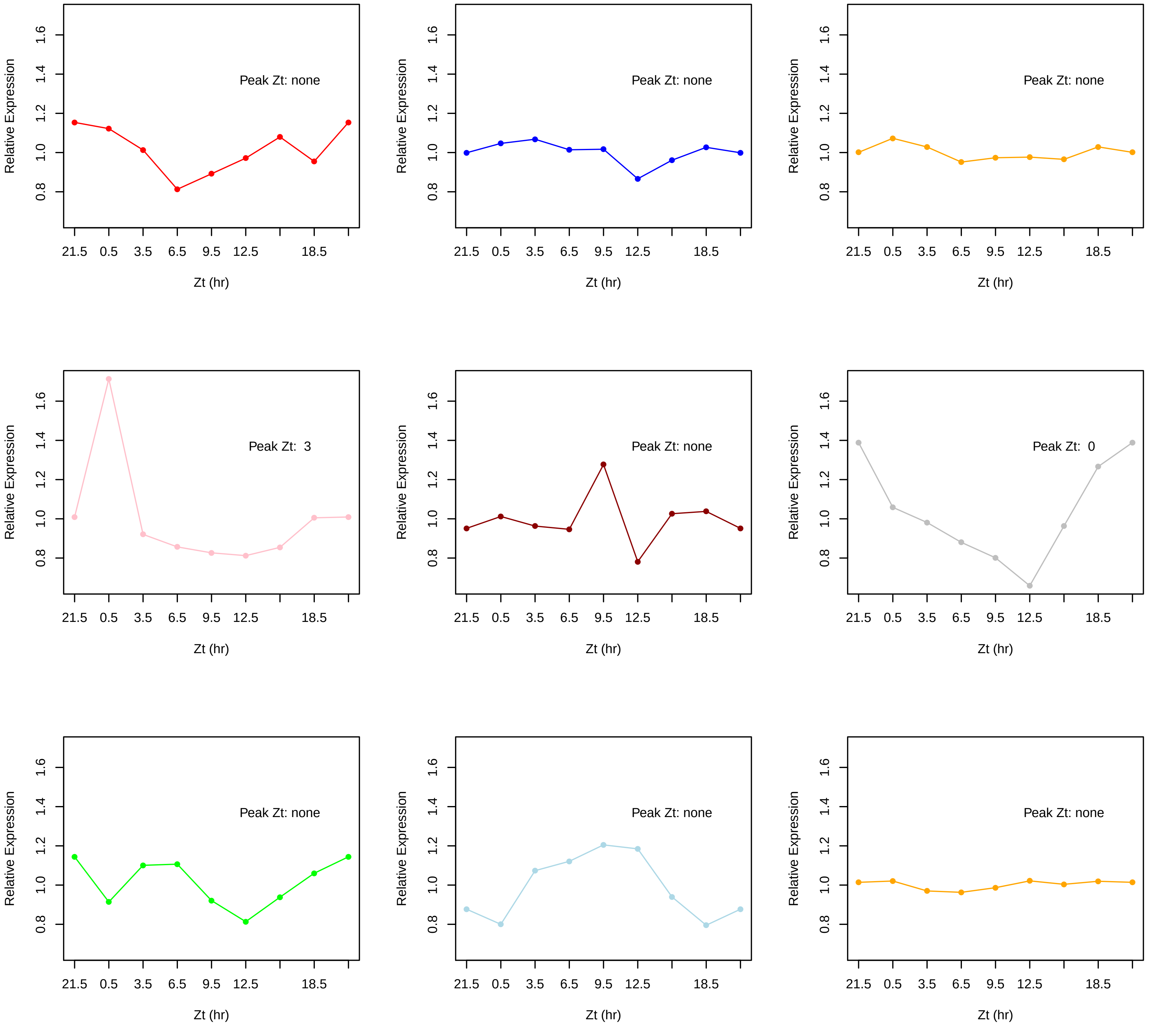

Singeleton: ENSXMAG00000004333 nedd4l

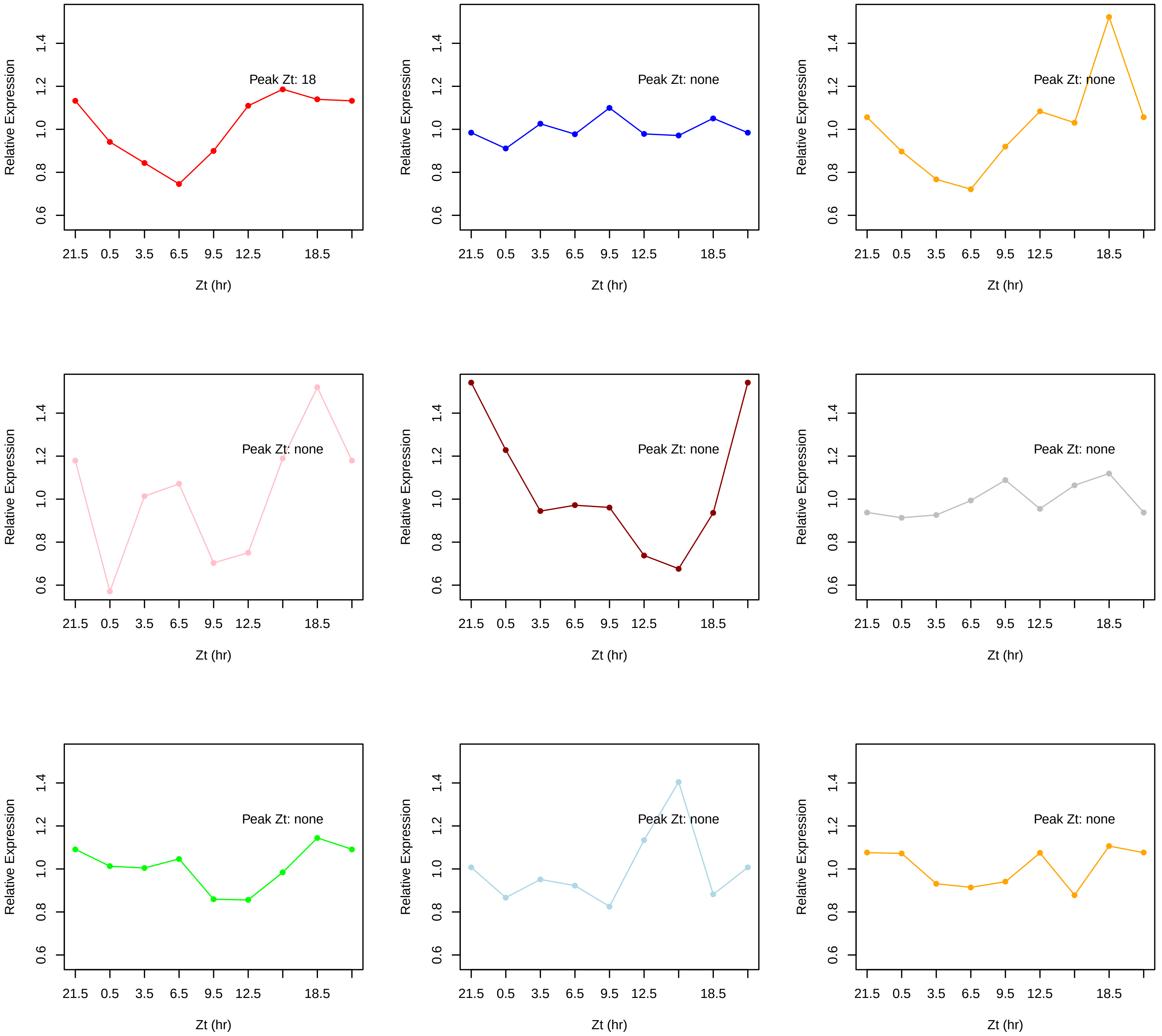

Singeleton: ENSXMAG00000014651 l2hgdh

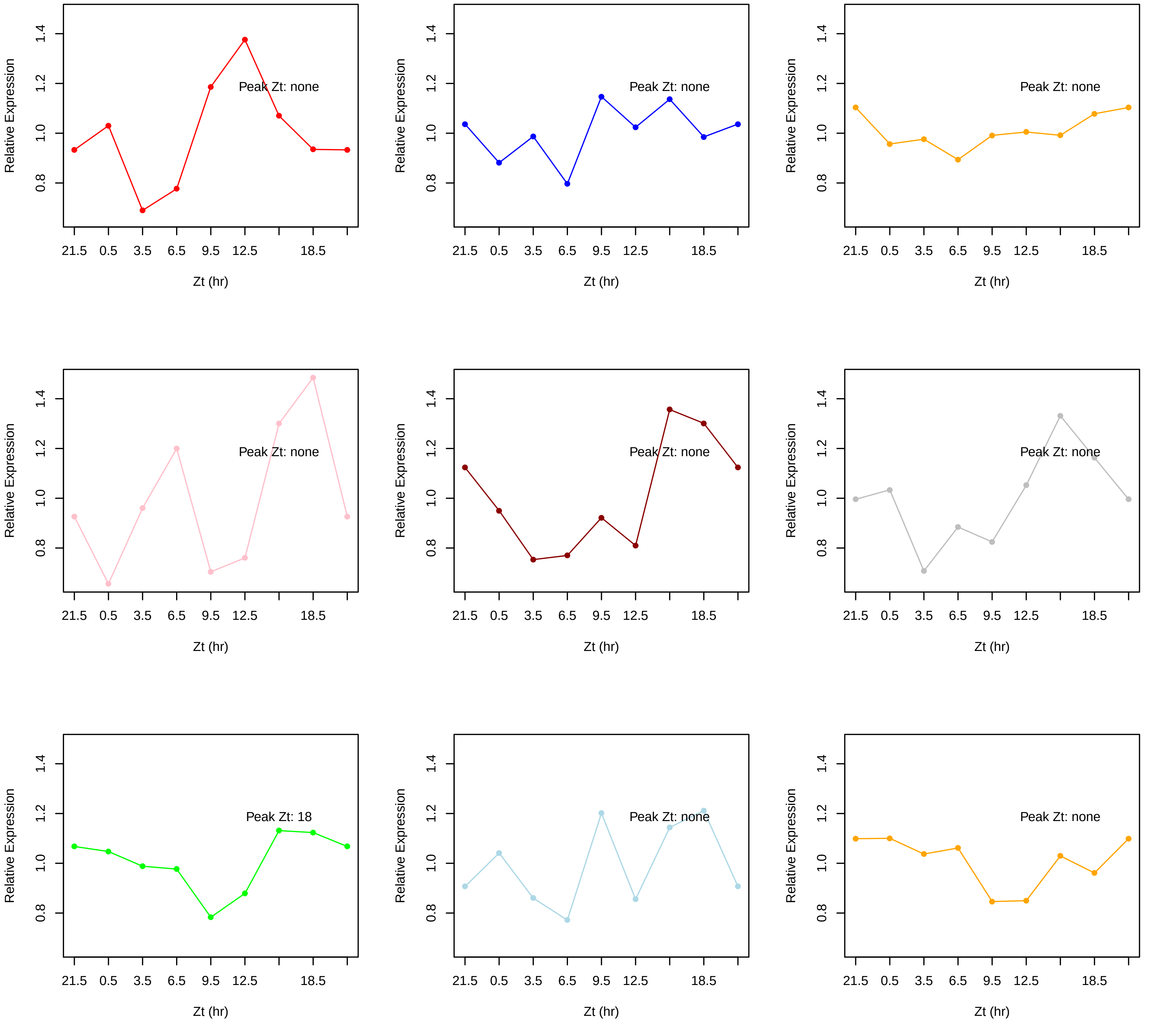

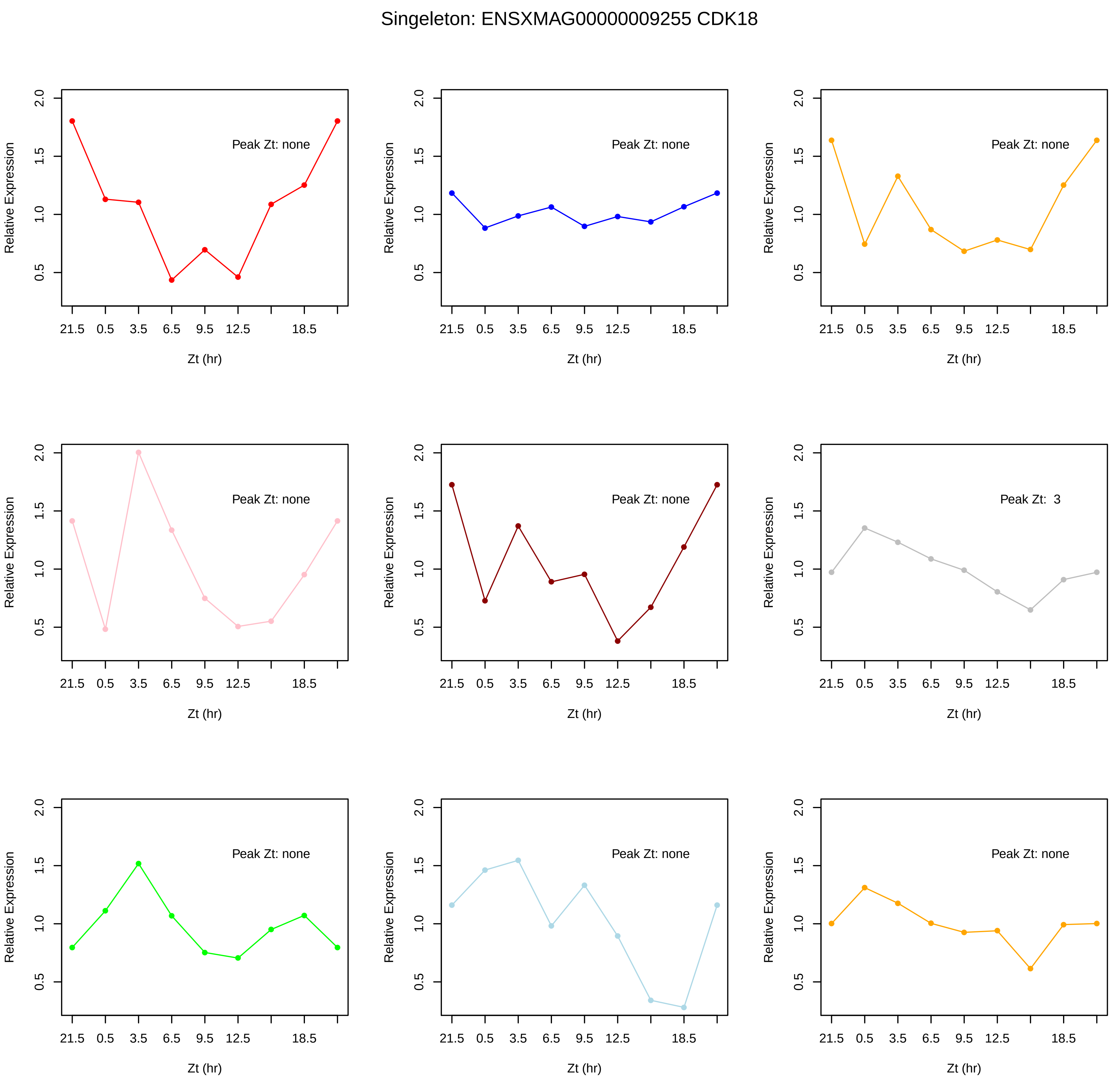

Singeleton: ENSXMAG00000009255 CDK18
Peak Zt: none
Peak Zt: none
Peak Zt: none
Peak Zt: none
Peak Zt: none
Peak Zt: 3
Peak Zt: none
Peak Zt: none
Peak Zt: none
Relative Expression
Zt (hr)
21.5 0.5 3.5 6.5 9.5 12.5 18.5
0.5 1.0 1.5 2.0

Singeleton: ENSXMAG00000010139 ctnna1

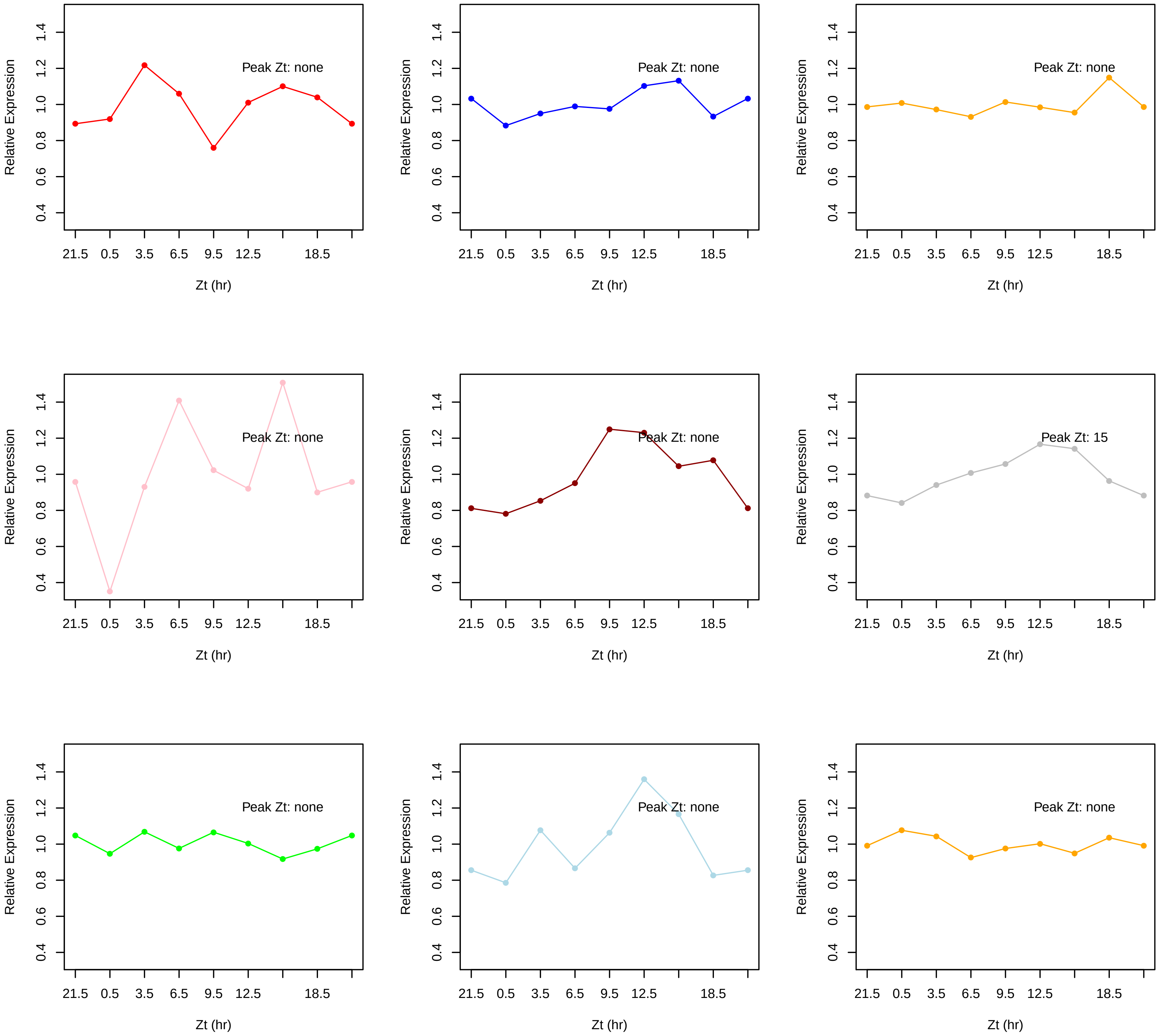

Singleton: ENSXMAG00000005292

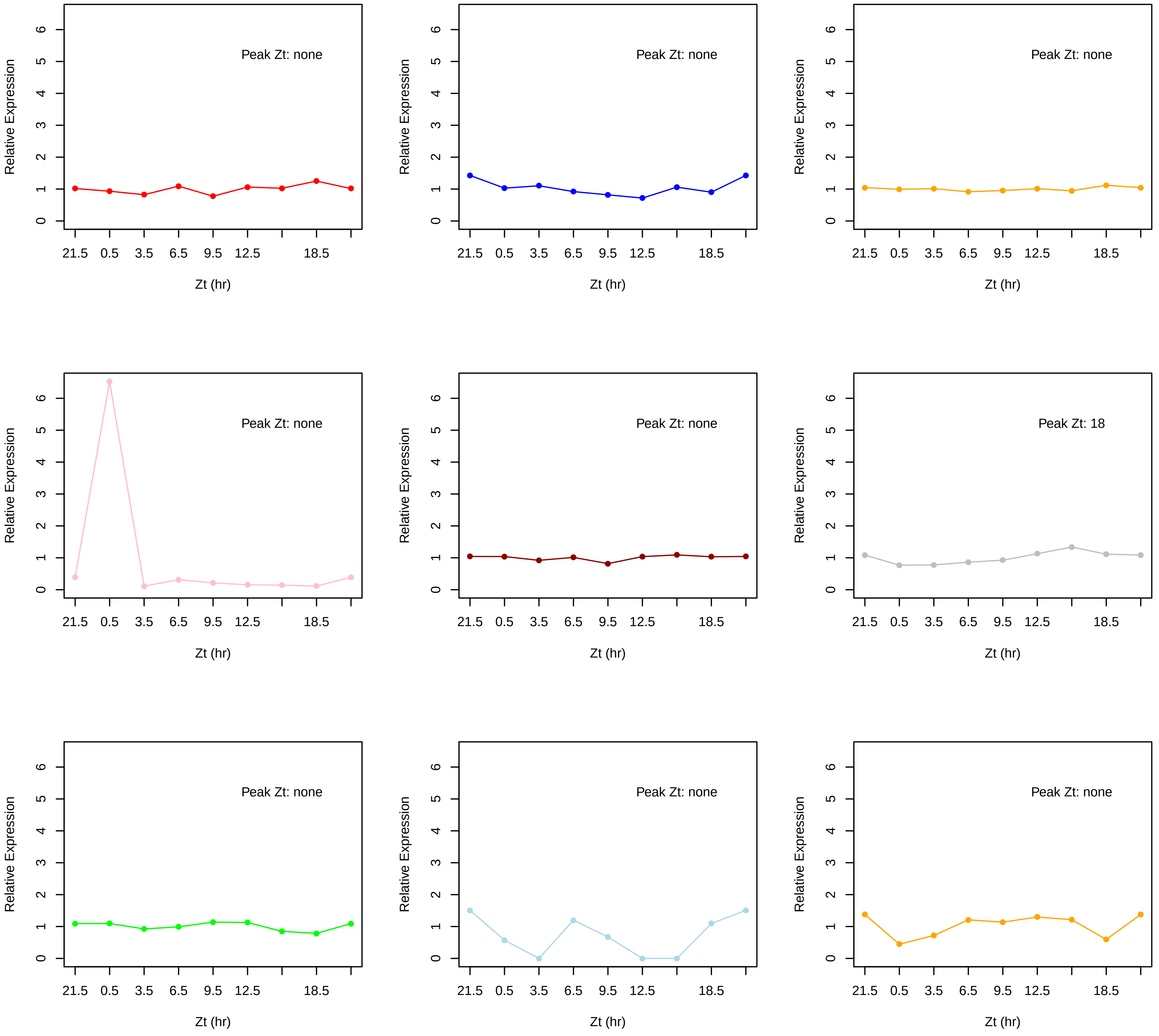

Singeleton: ENSXMAG00000010120 SNAPC3

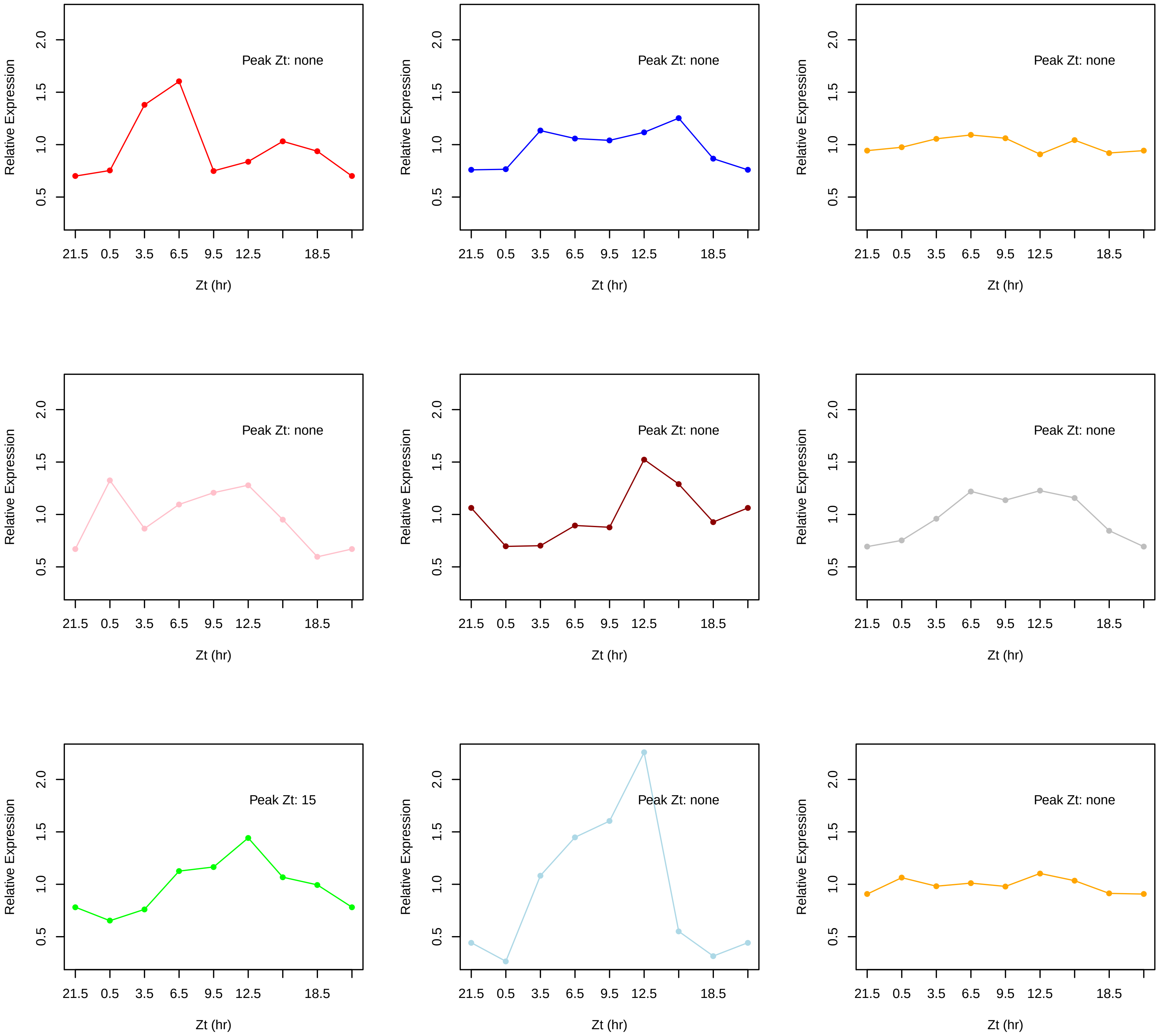

Singleton: ENSXMAG00000000556 msrb2

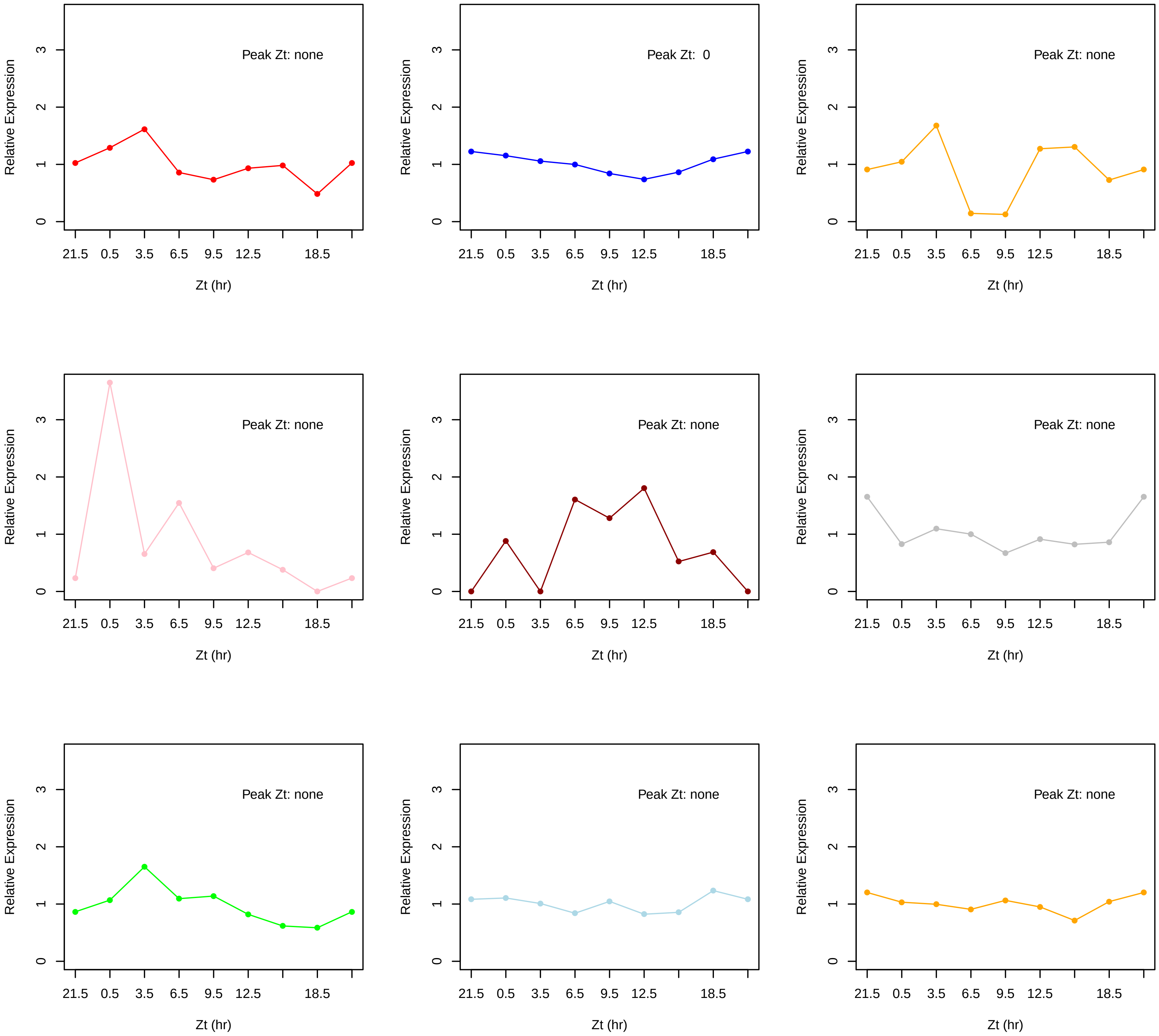

Singleton: ENSXMAG00000024444 si:dkey−167k11.5

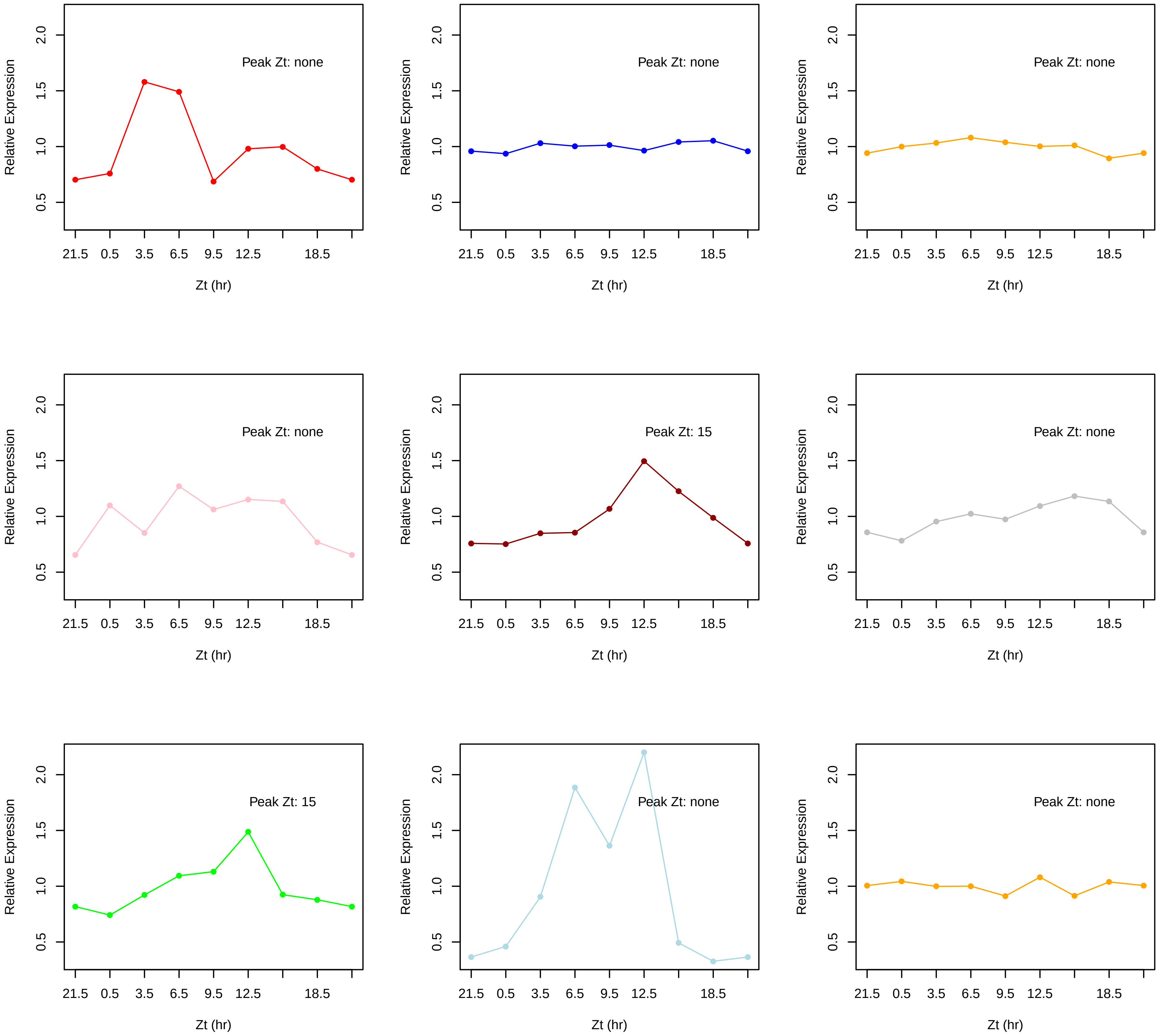

Singleton: ENSXMAG00000010112 PSIP1

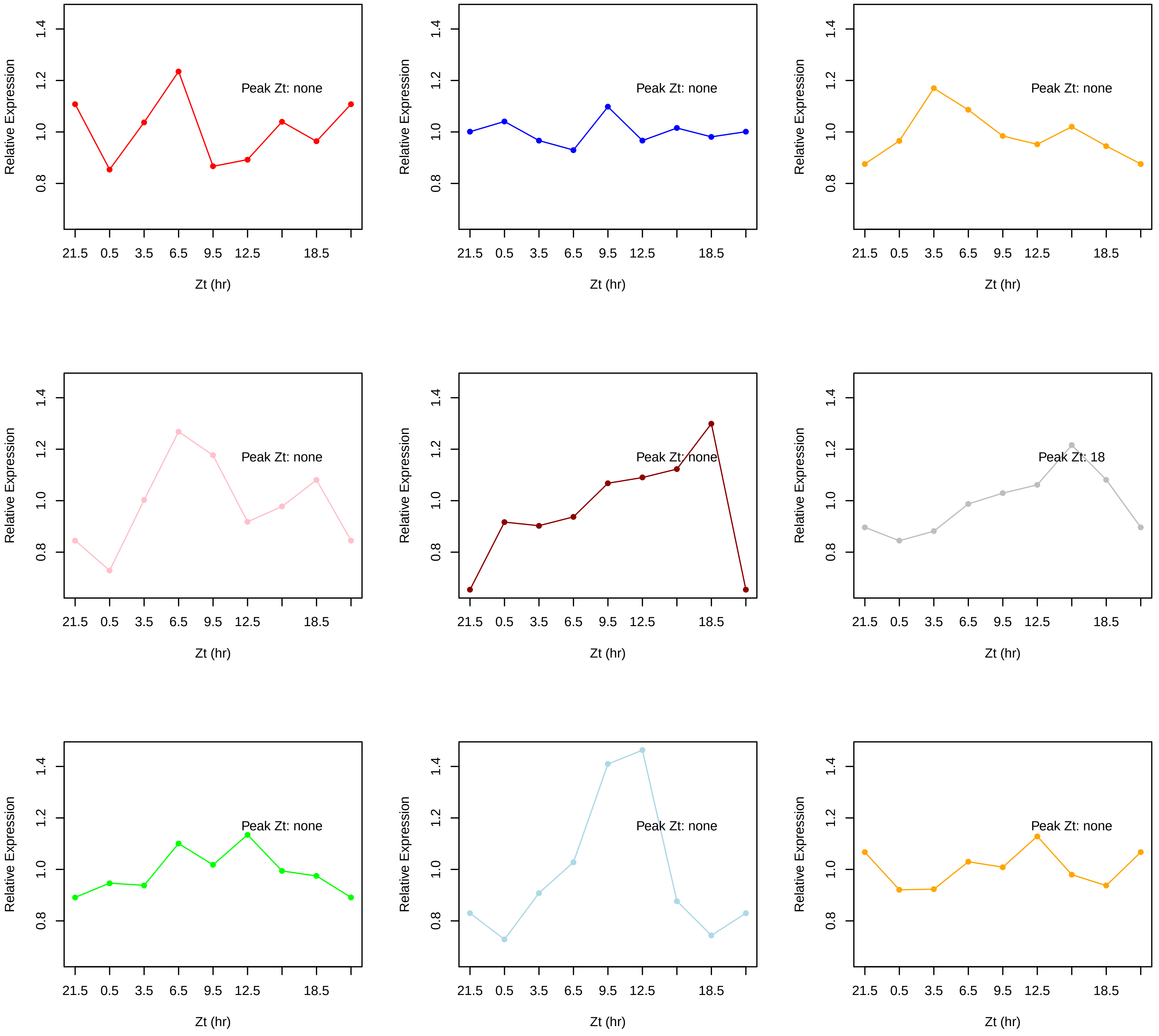

Singeleton: ENSXMAG00000013899 hibadhb

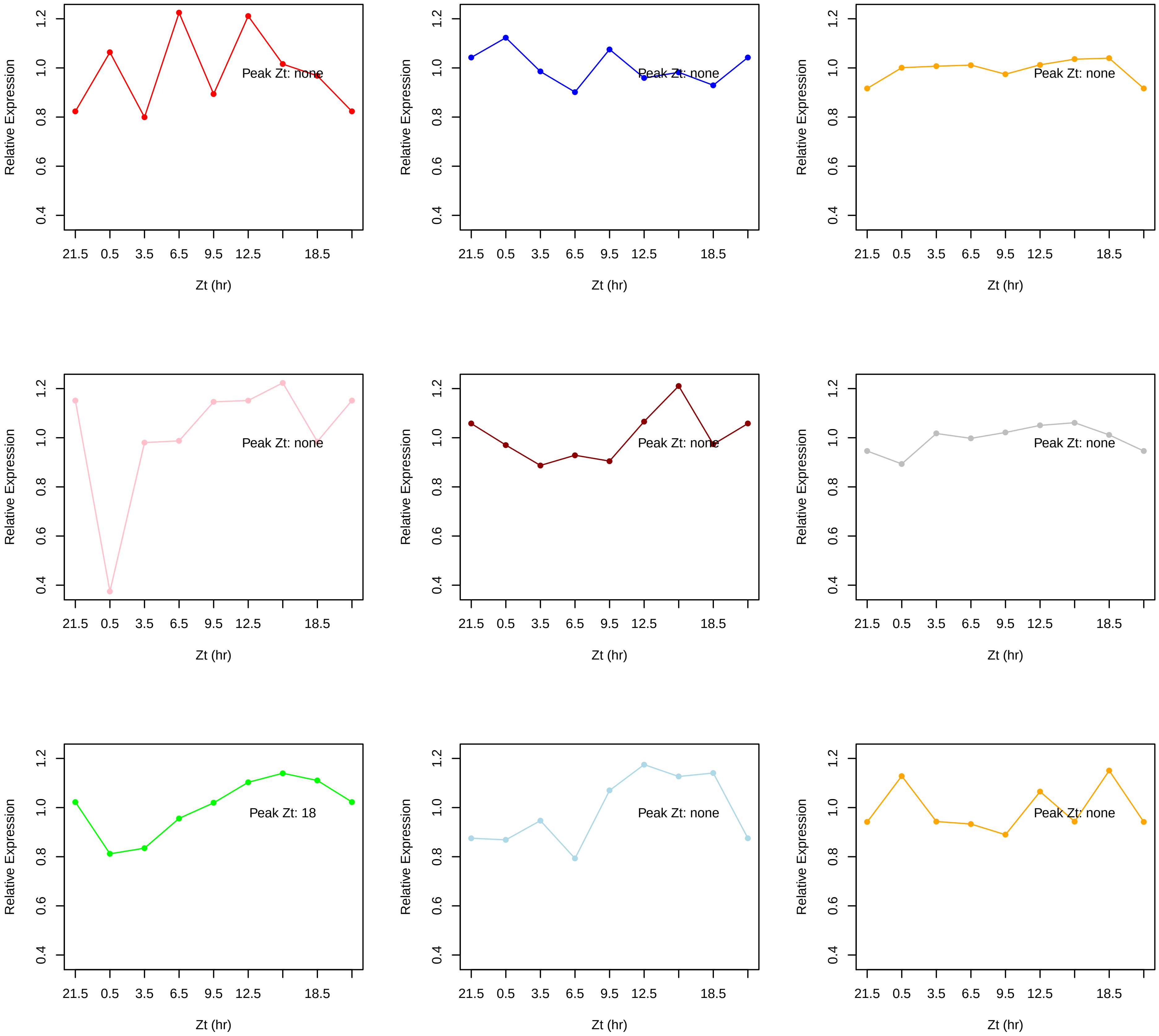

Singeleton: ENSXMAG00000026666 klhdc8a

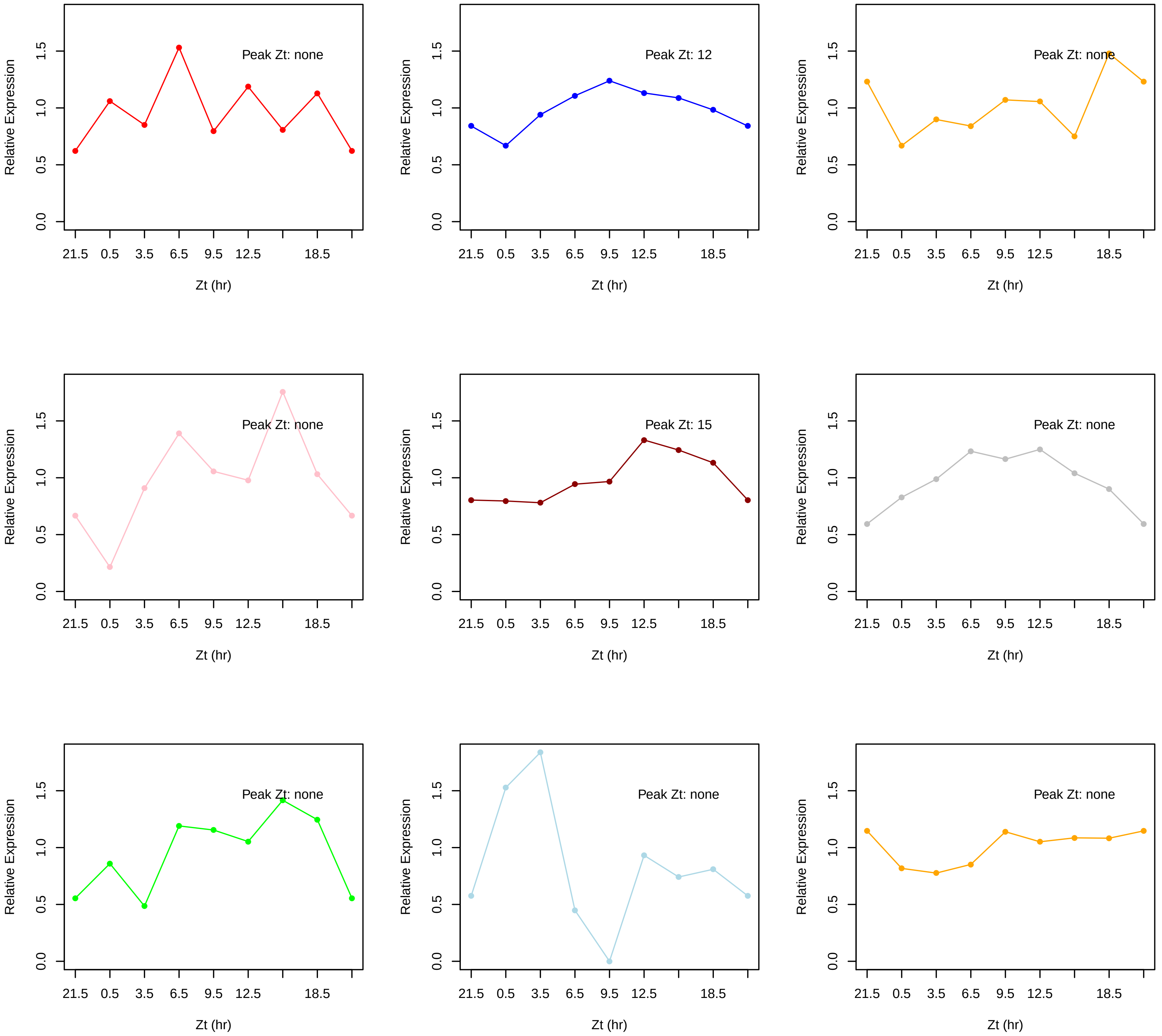

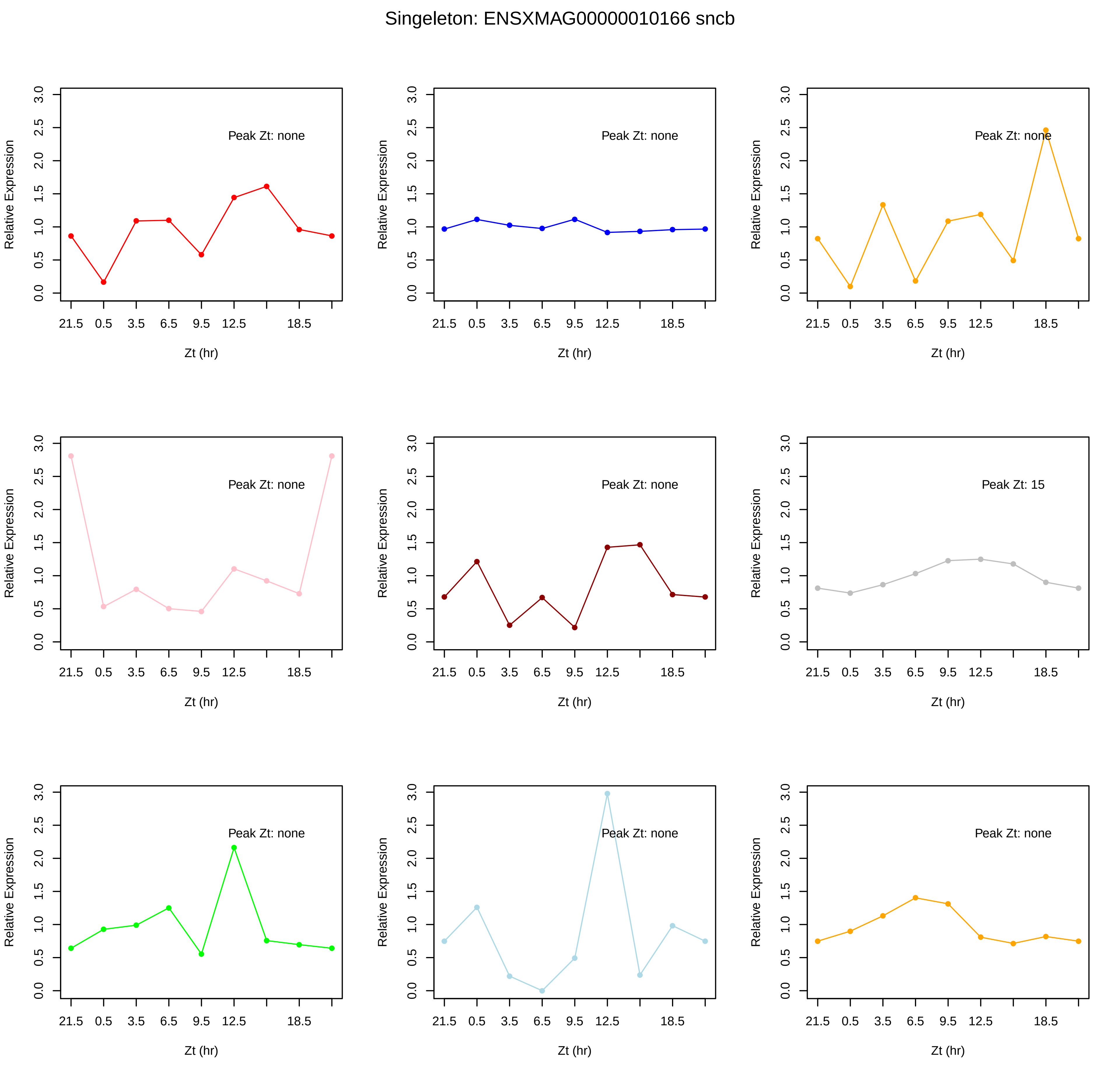
Singeleton: ENSXMAG00000010166 sncb
Peak Zt: none
Peak Zt: none
Peak Zt: none
Peak Zt: none
Peak Zt: none
Peak Zt: 15
Peak Zt: none
Peak Zt: none
Peak Zt: none
Relative Expression
Zt (hr)
21.5
0.5
3.5
6.5
9.5
12.5
18.5
0.0
0.5
1.0
1.5
2.0
2.5
3.0

Singeleton: ENSXMAG00000005768 pm20d1.2

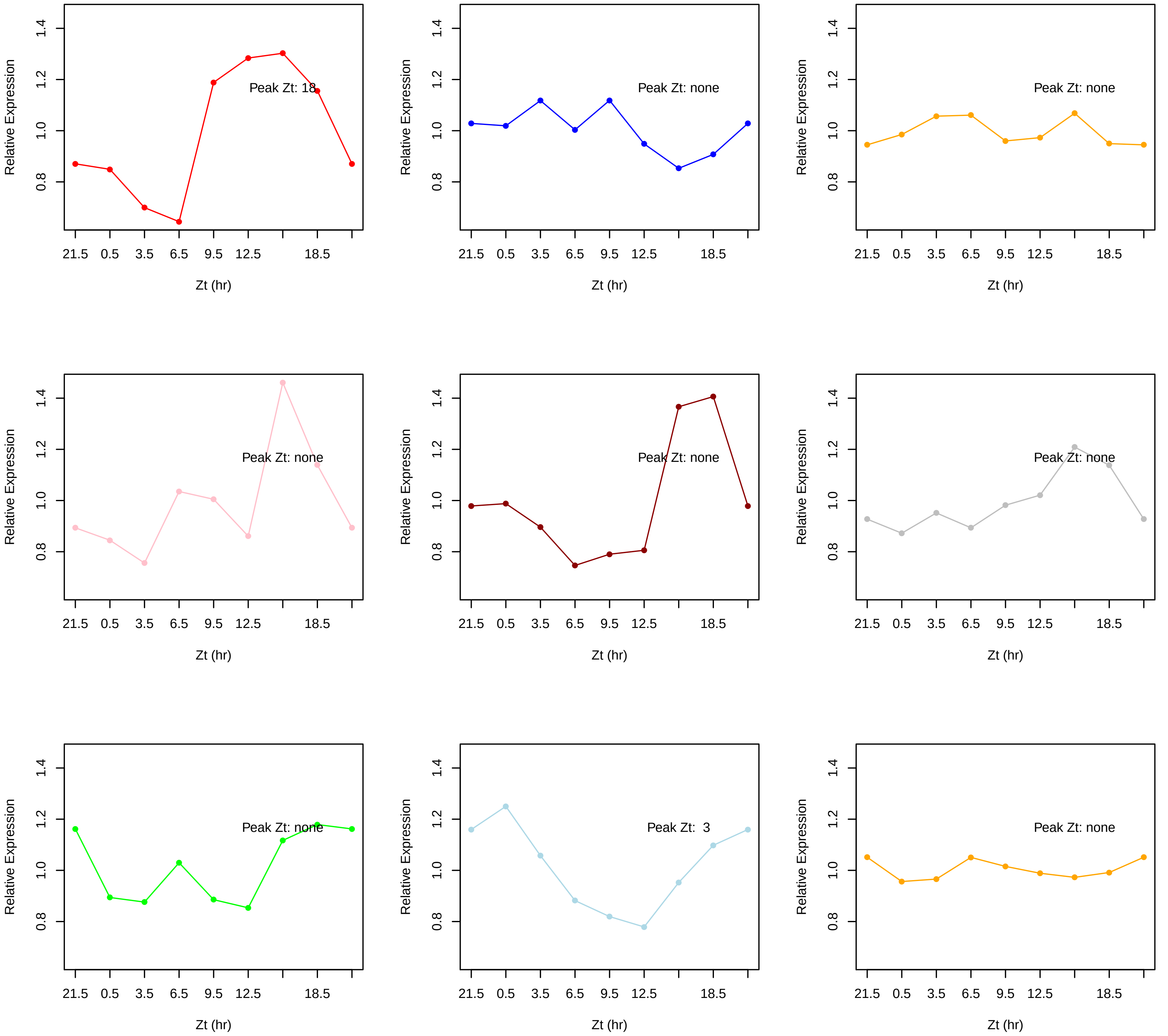

Singeleton: ENSXMAG00000012933 cfap43

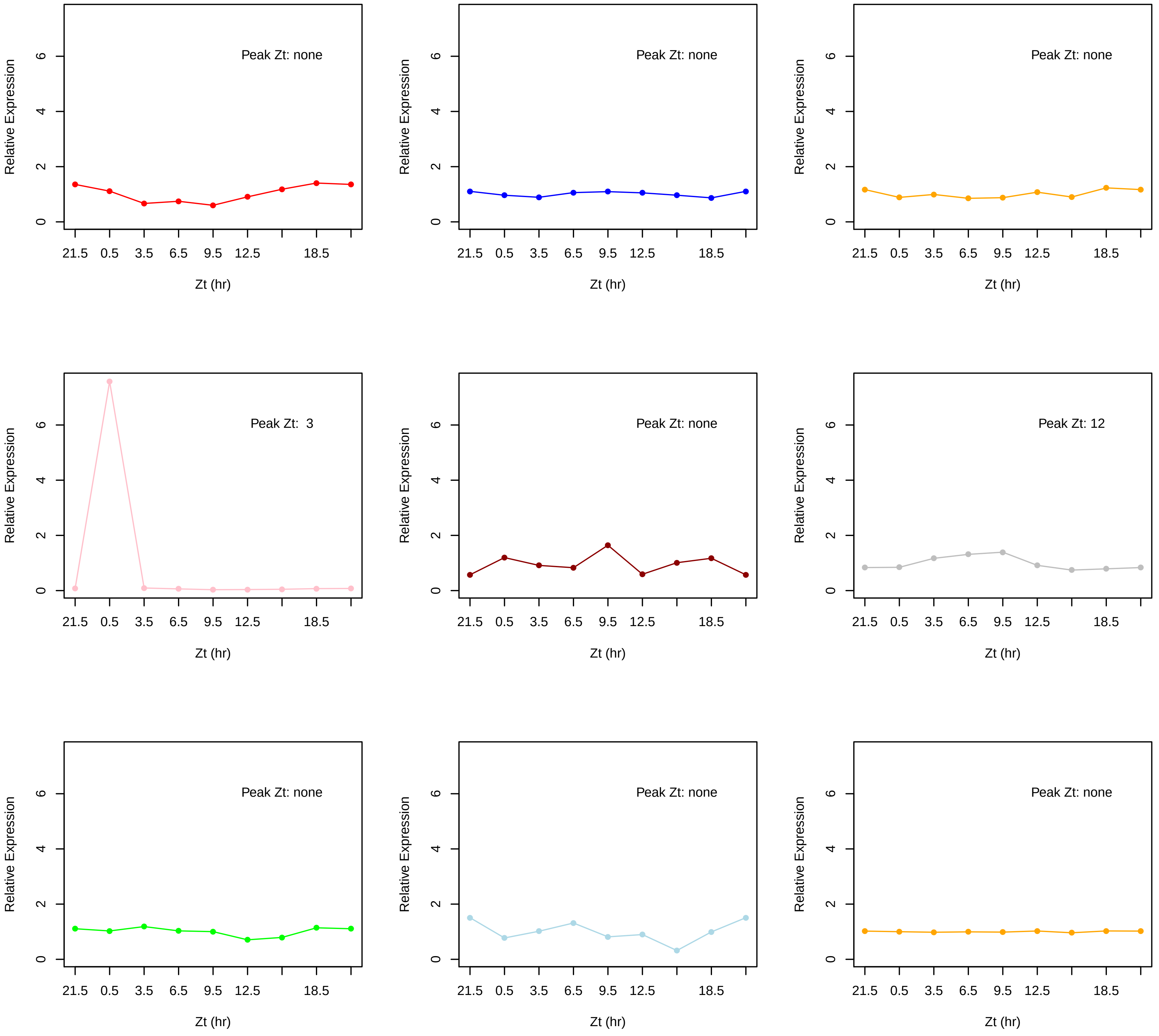

Singleton: ENSXMAG00000026713 srebf2

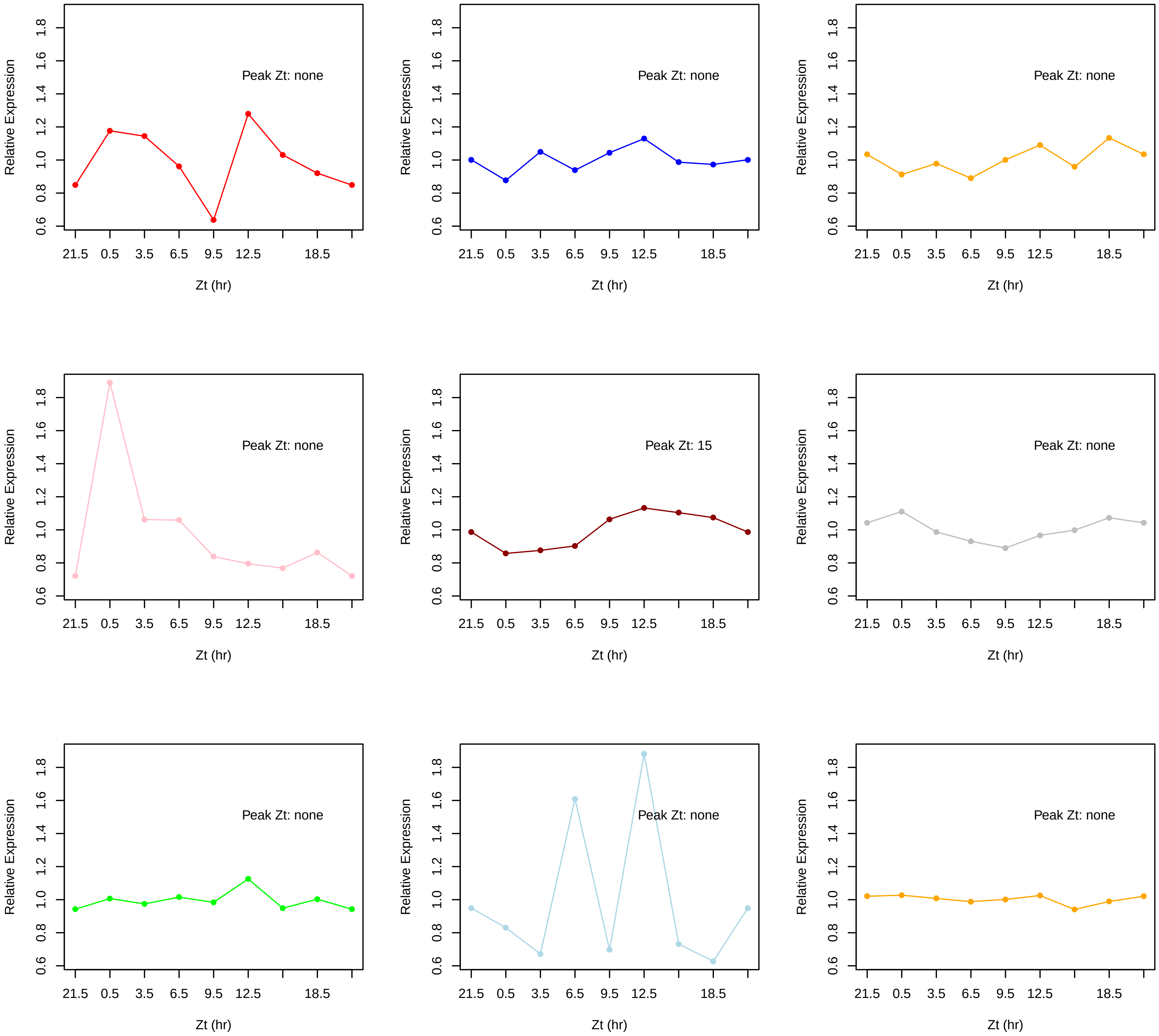

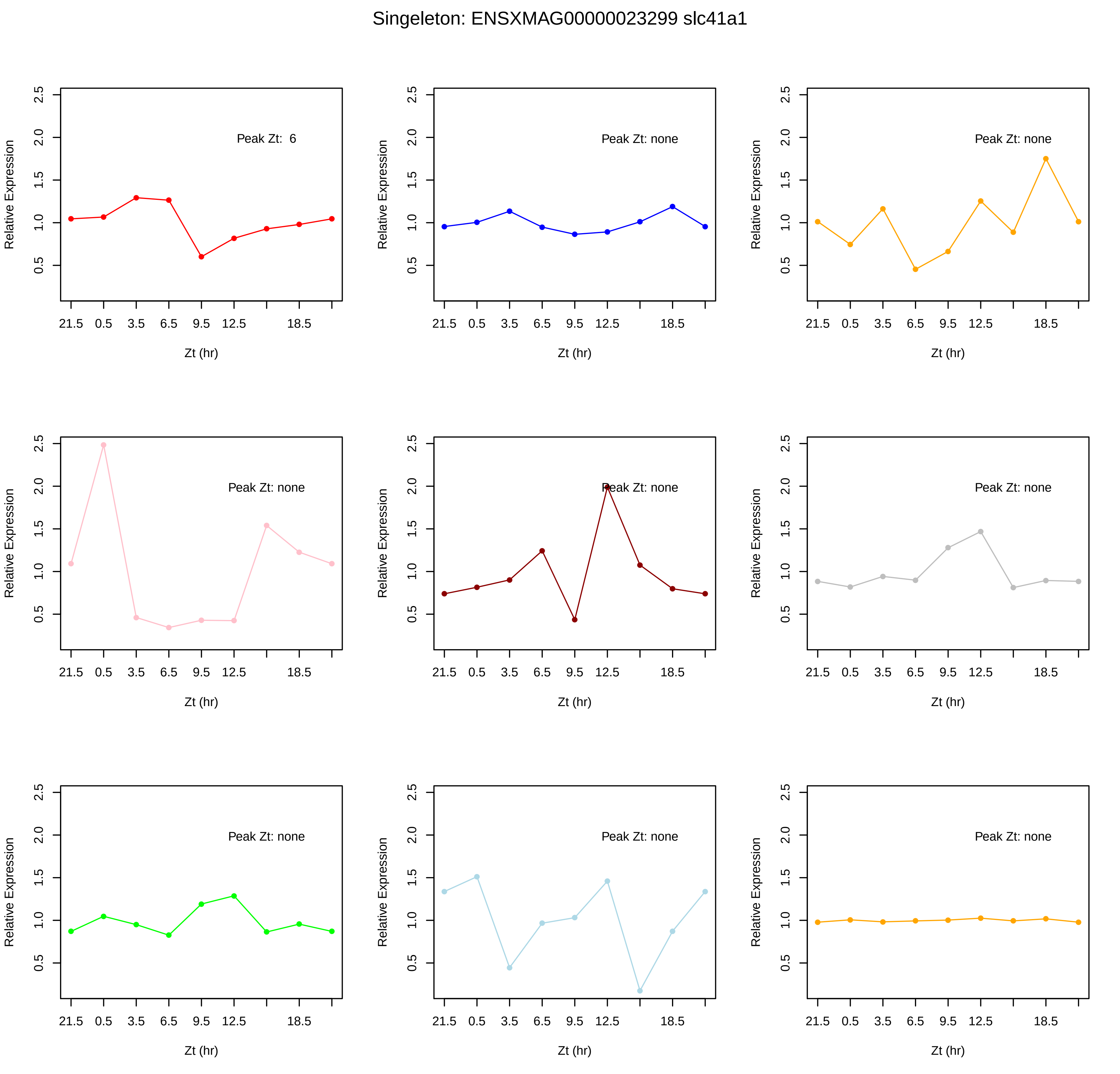
Singeleton: ENSXMAG00000023299 slc41a1
Relative Expression
Zt (hr)
Peak Zt: 6
Peak Zt: none
0.5
1.0
1.5
2.0
2.5
21.5
0.5
3.5
6.5
9.5
12.5
18.5

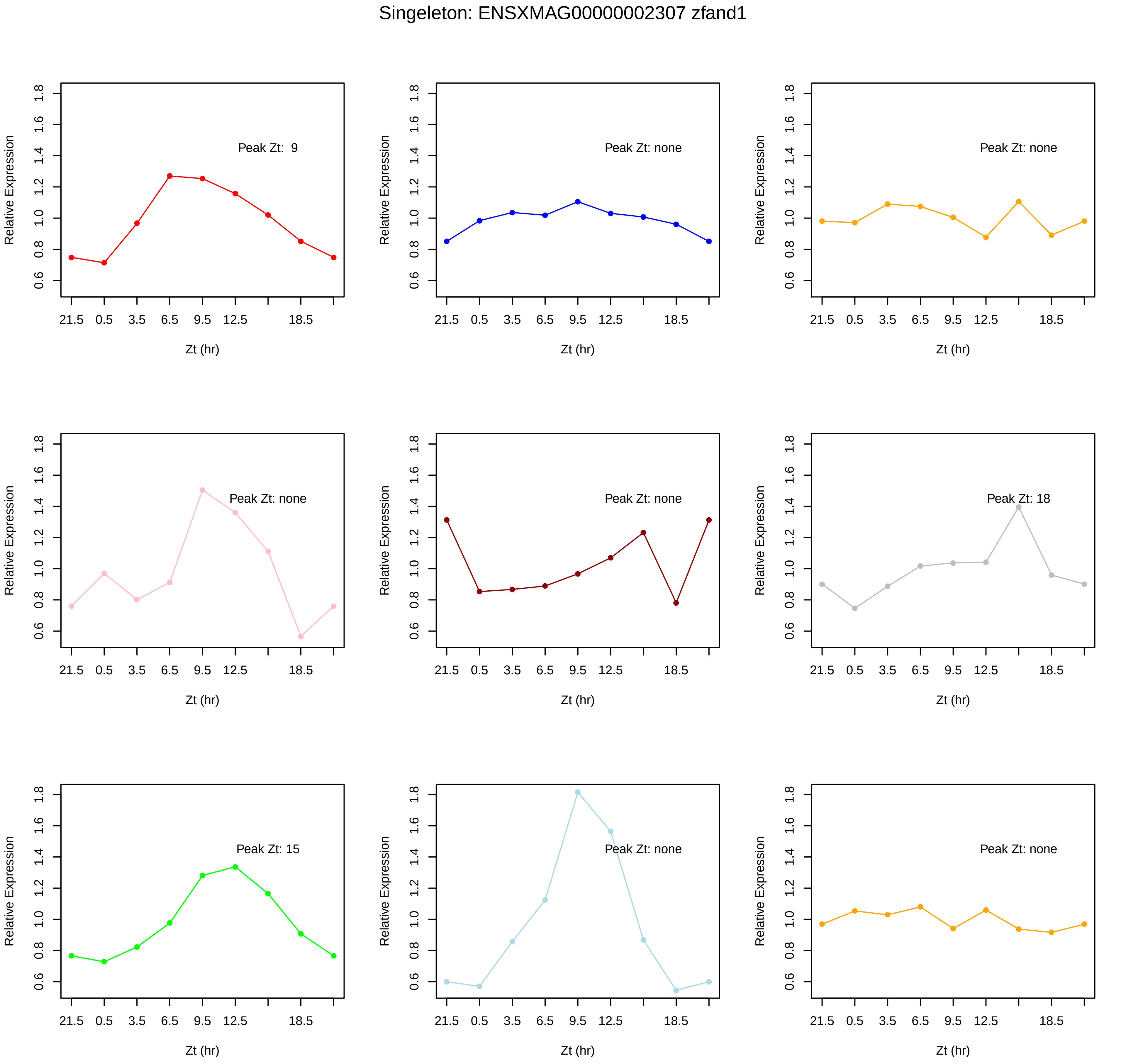
Singeleton: ENSXMAG00000002307 zfand1
Peak Zt: 9
Peak Zt: none
Peak Zt: none
Peak Zt: none
Peak Zt: none
Peak Zt: 18
Peak Zt: 15
Peak Zt: none
Peak Zt: none
Relative Expression
Zt (hr)
21.5 0.5 3.5 6.5 9.5 12.5 18.5
0.6 0.8 1.0 1.2 1.4 1.6 1.8

Singeleton: ENSXMAG00000027242 znf532

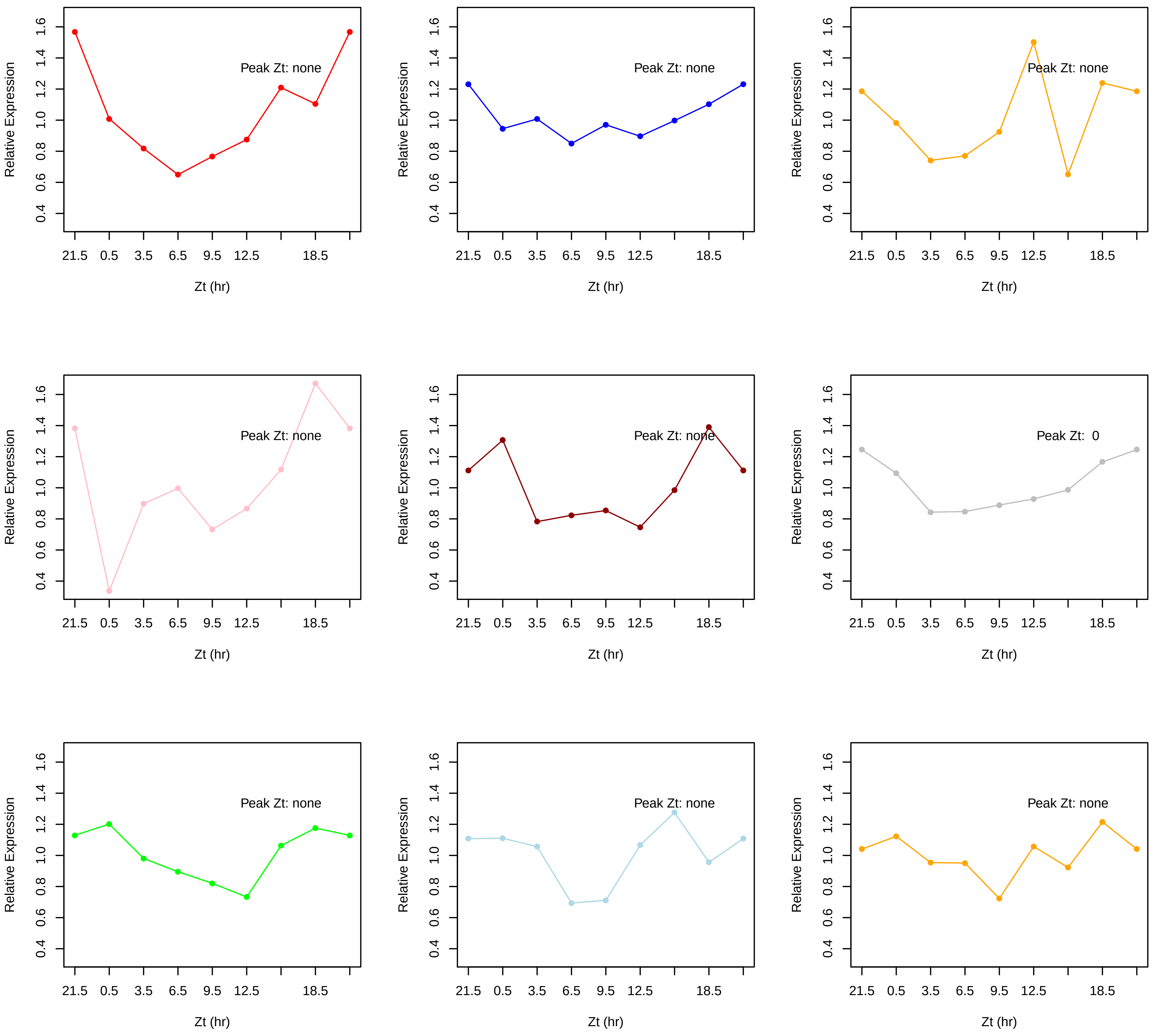

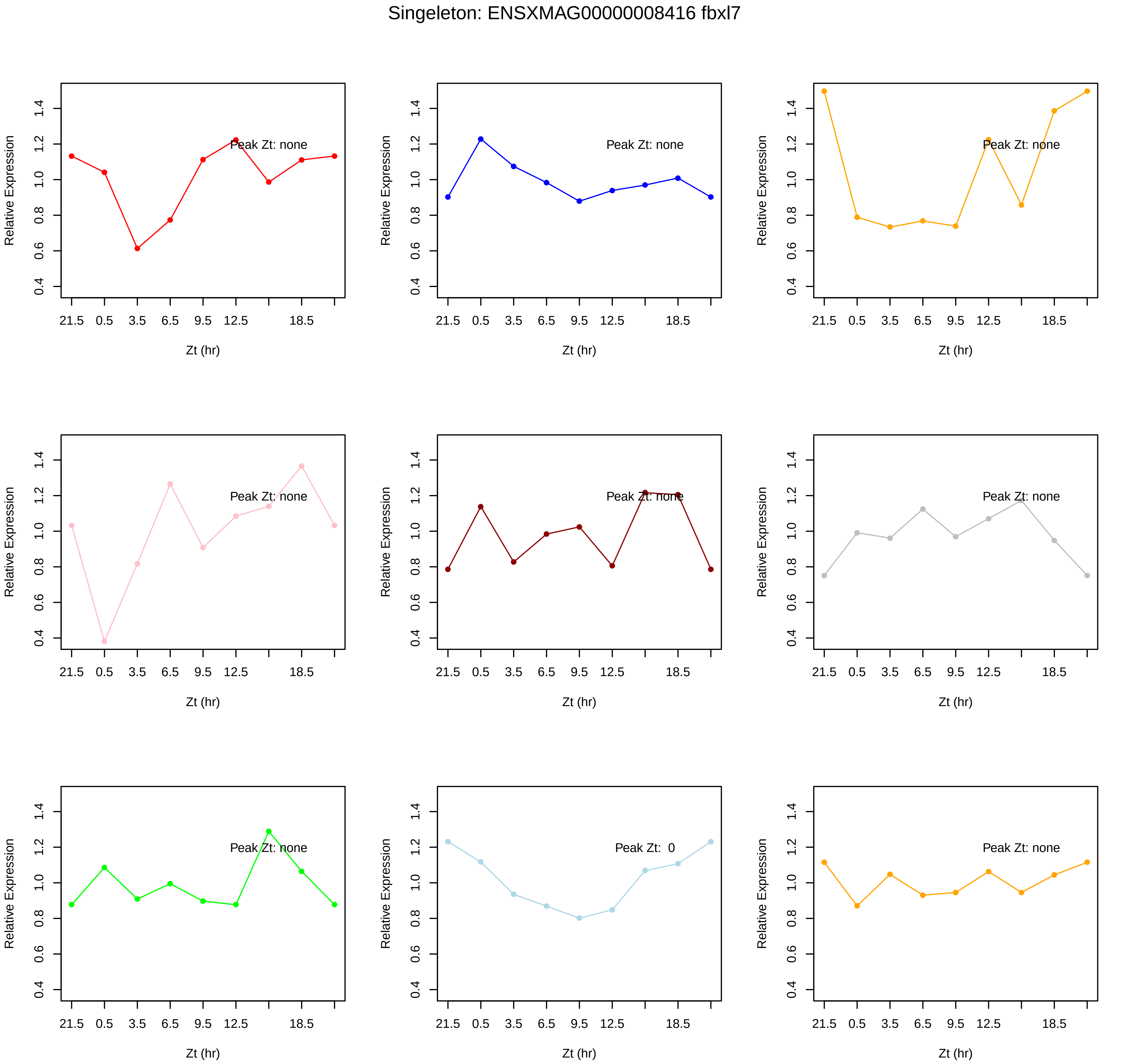

Singeleton: ENSXMAG00000008416 fbxl7
Peak Zt: none
Peak Zt: 0
Relative Expression
Zt (hr)

Singleton: ENSXMAG00000008855 sox13

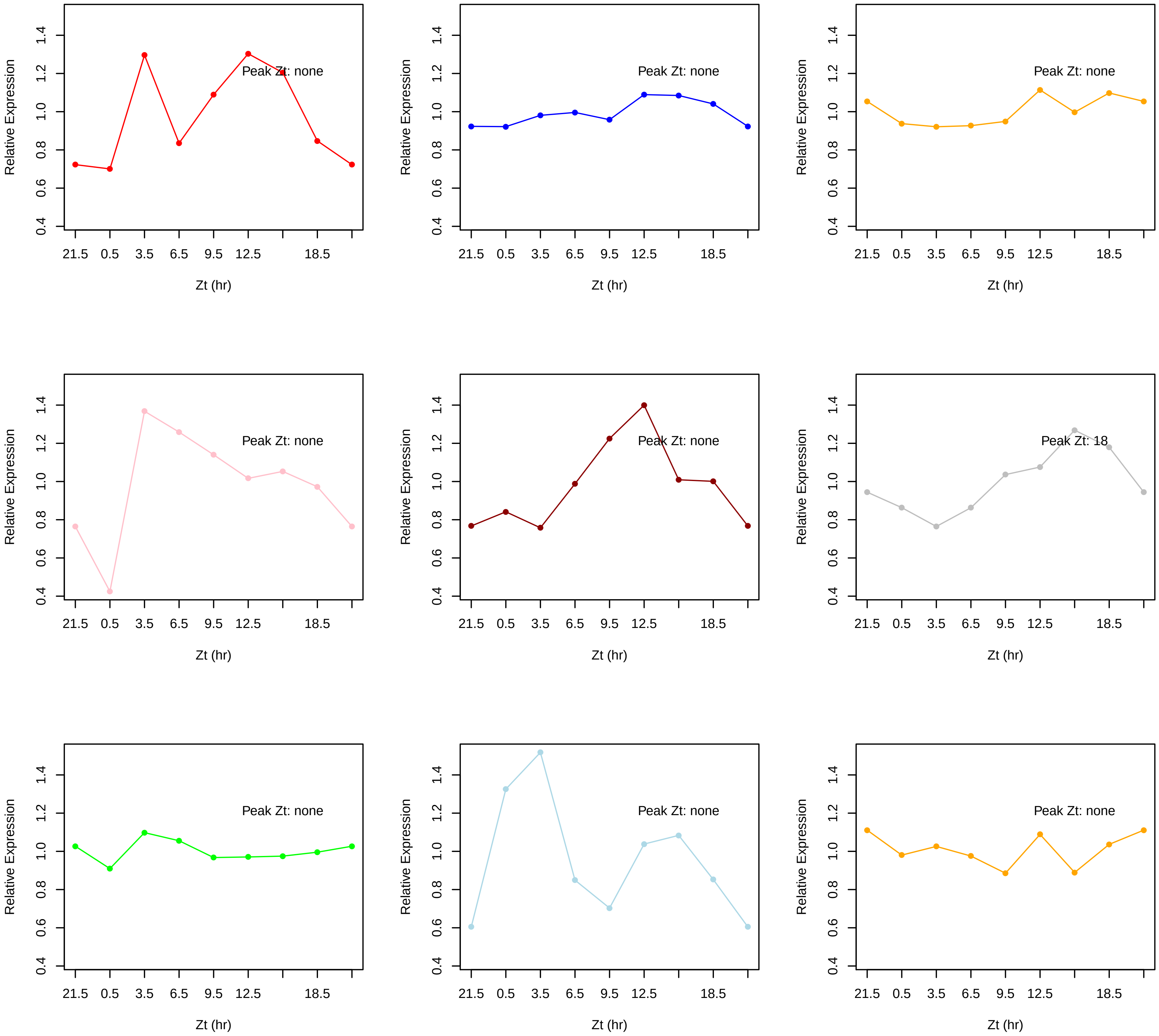

Singleton: ENSXMAG00000008850 snrpe

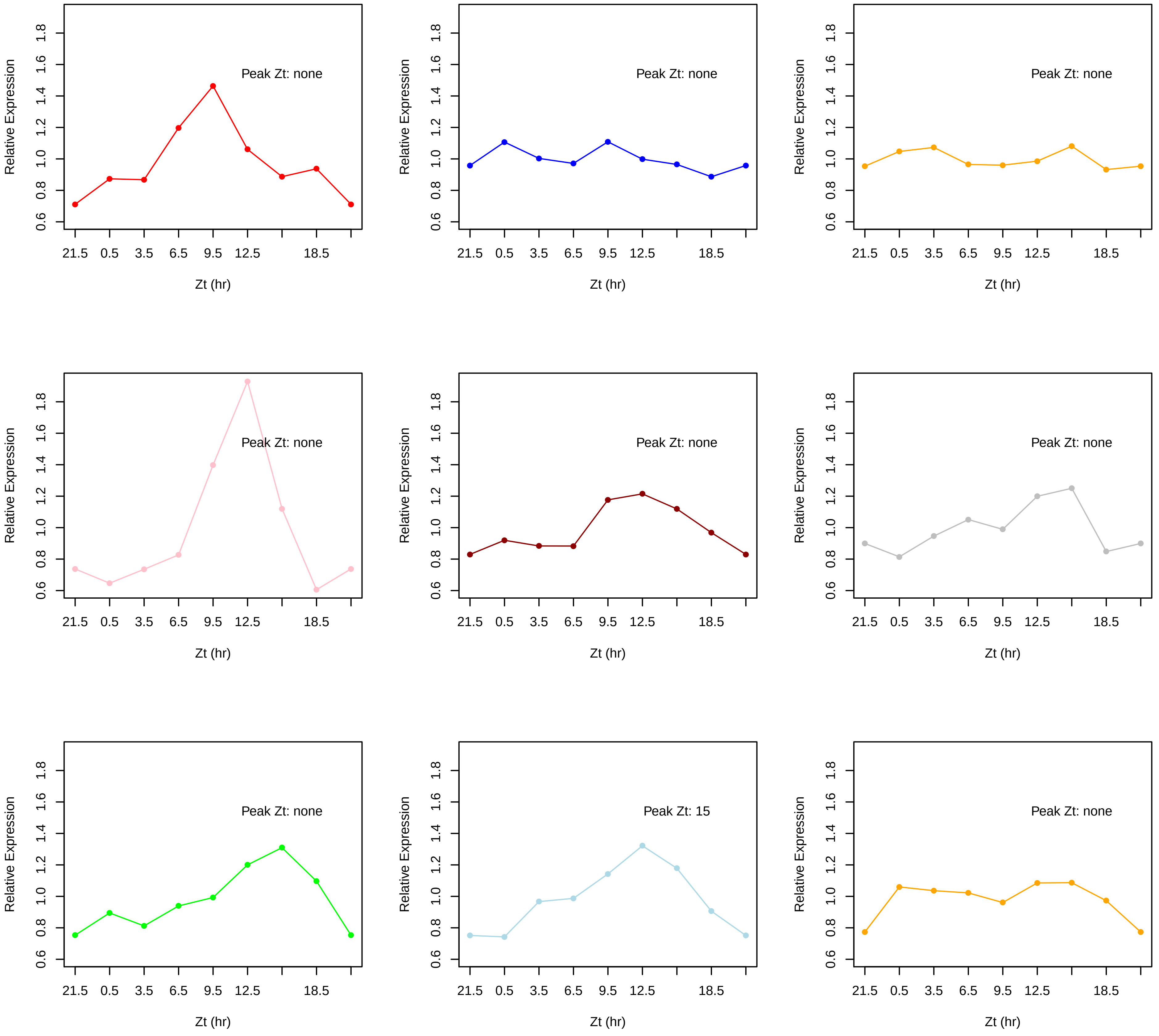

Singleton: ENSXMAG00000004398 lman1

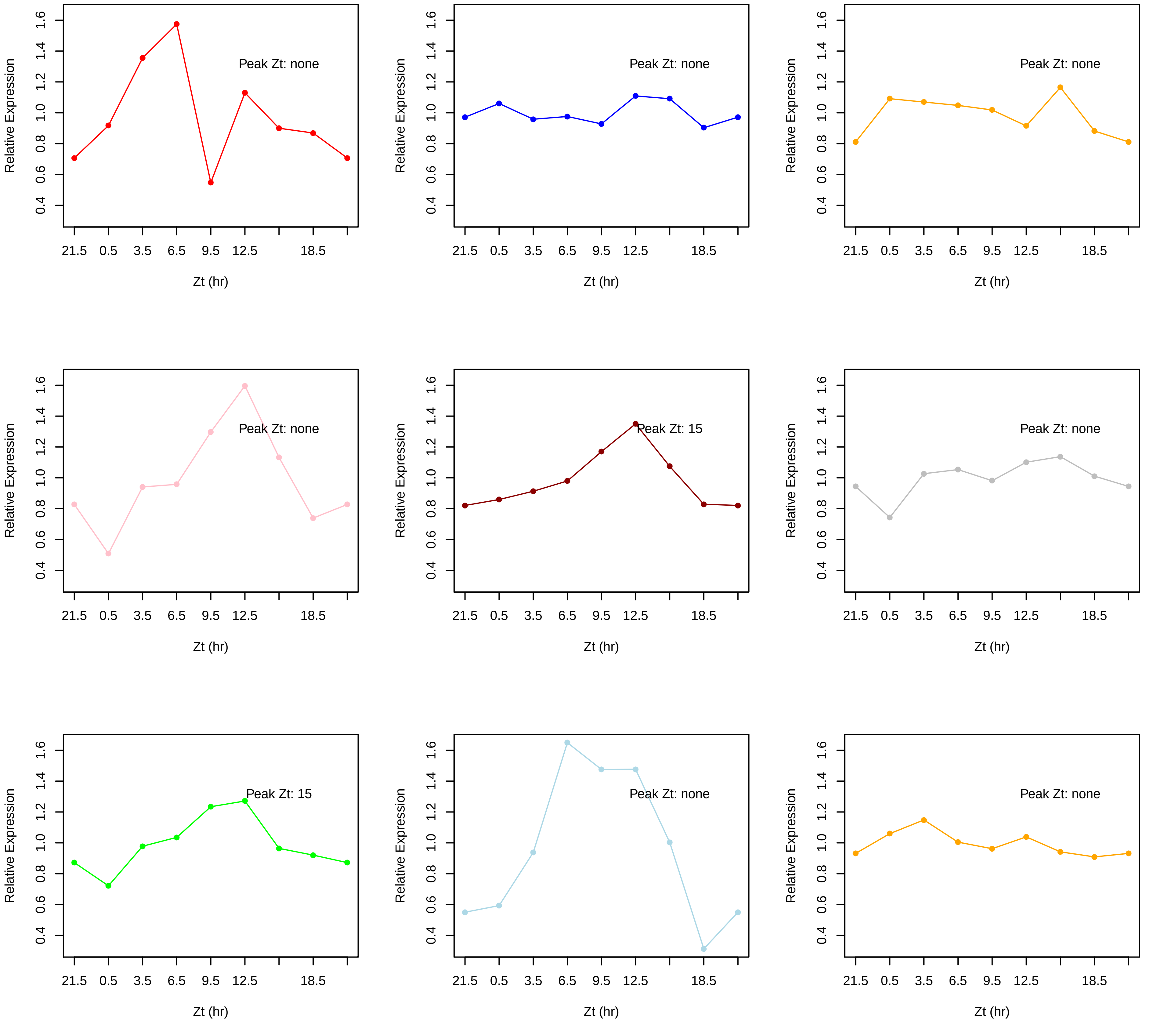

Singeleton: ENSXMAG00000007807 ccdc43

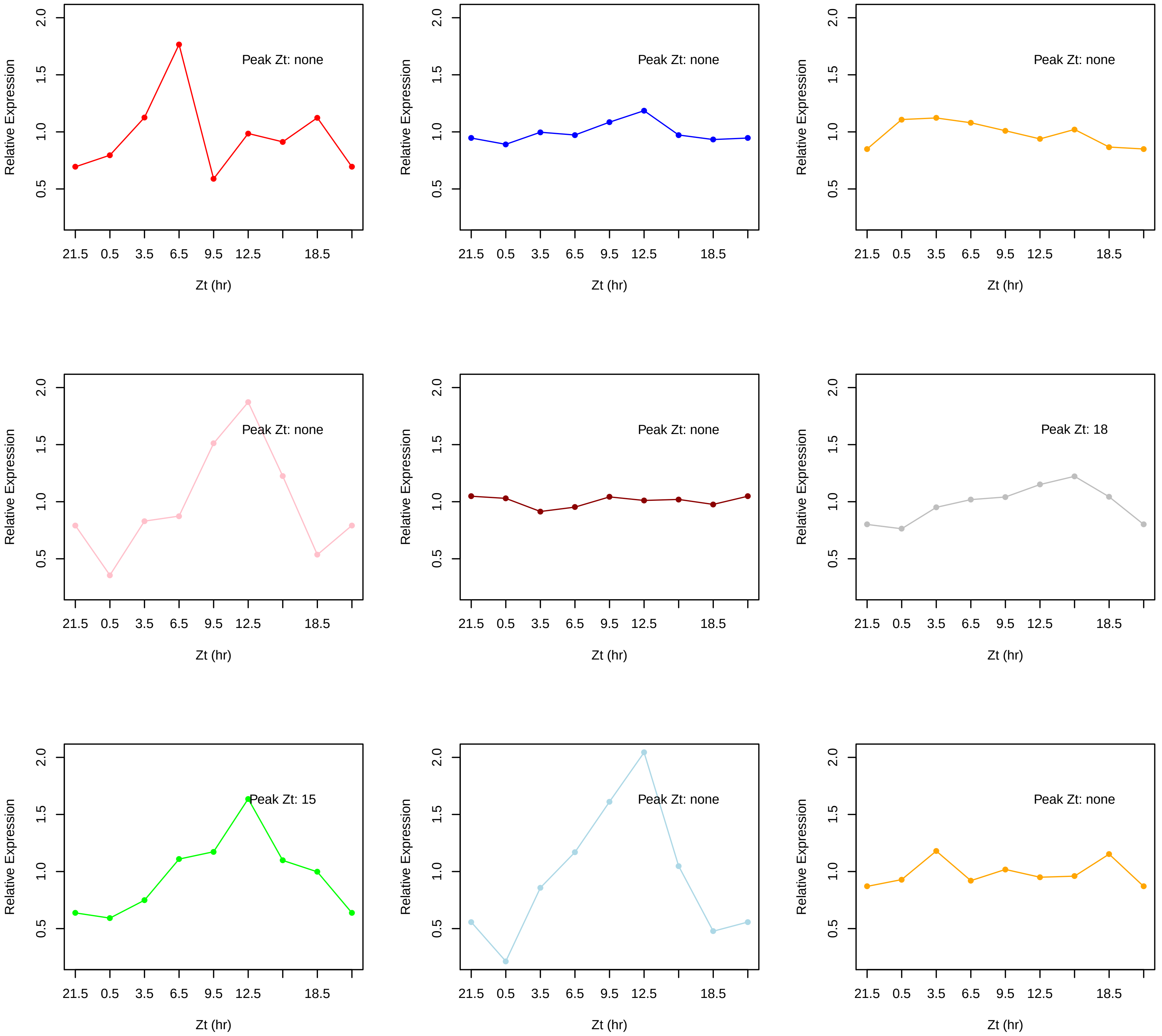

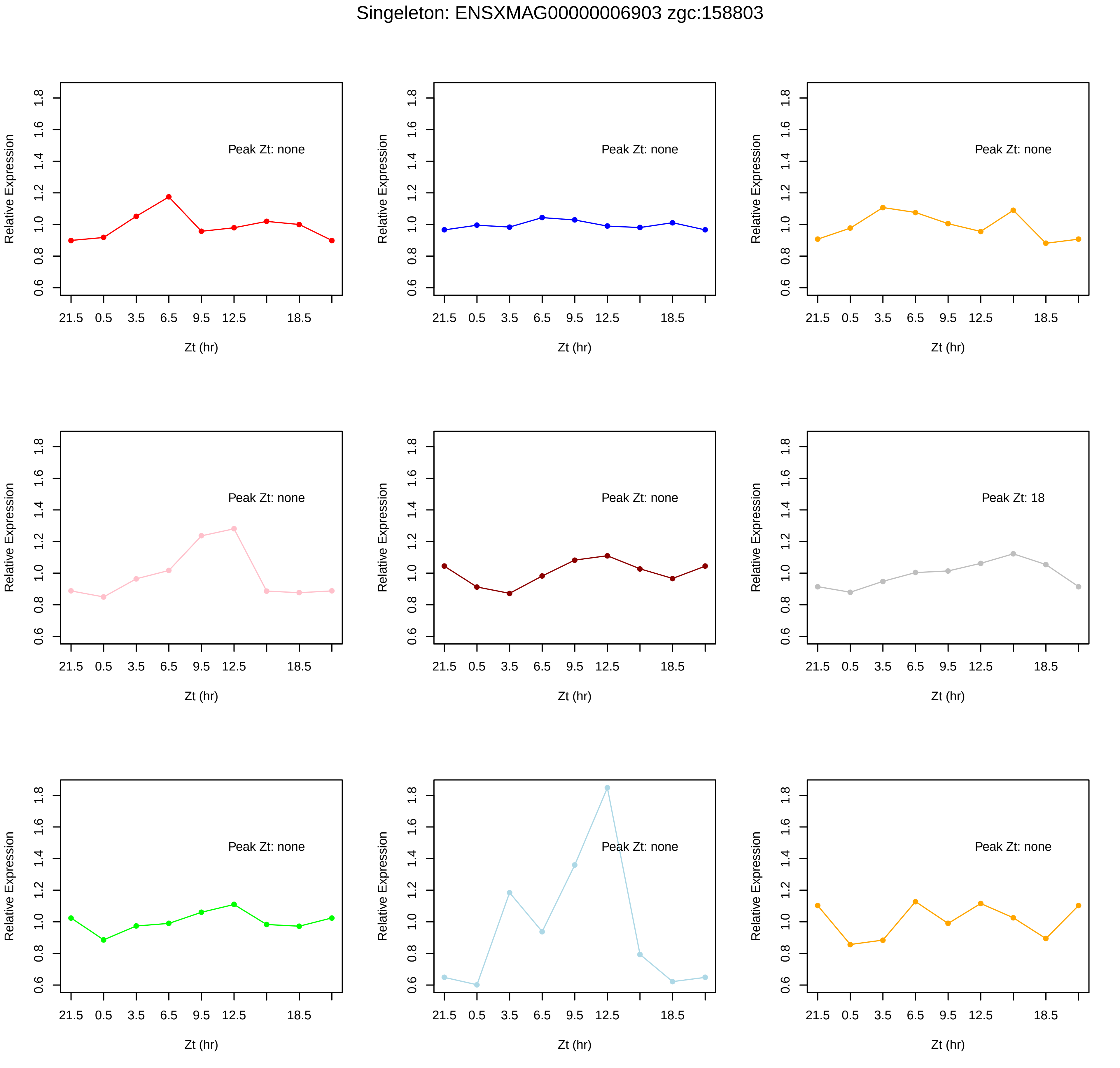
Singeleton: ENSXMAG00000006903 zgc:158803
Peak Zt: none
Peak Zt: none
Peak Zt: none
Peak Zt: none
Peak Zt: none
Peak Zt: 18
Peak Zt: none
Peak Zt: none
Peak Zt: none
Relative Expression
Zt (hr)

Singleton: ENSXMAG00000026845 ccbe1

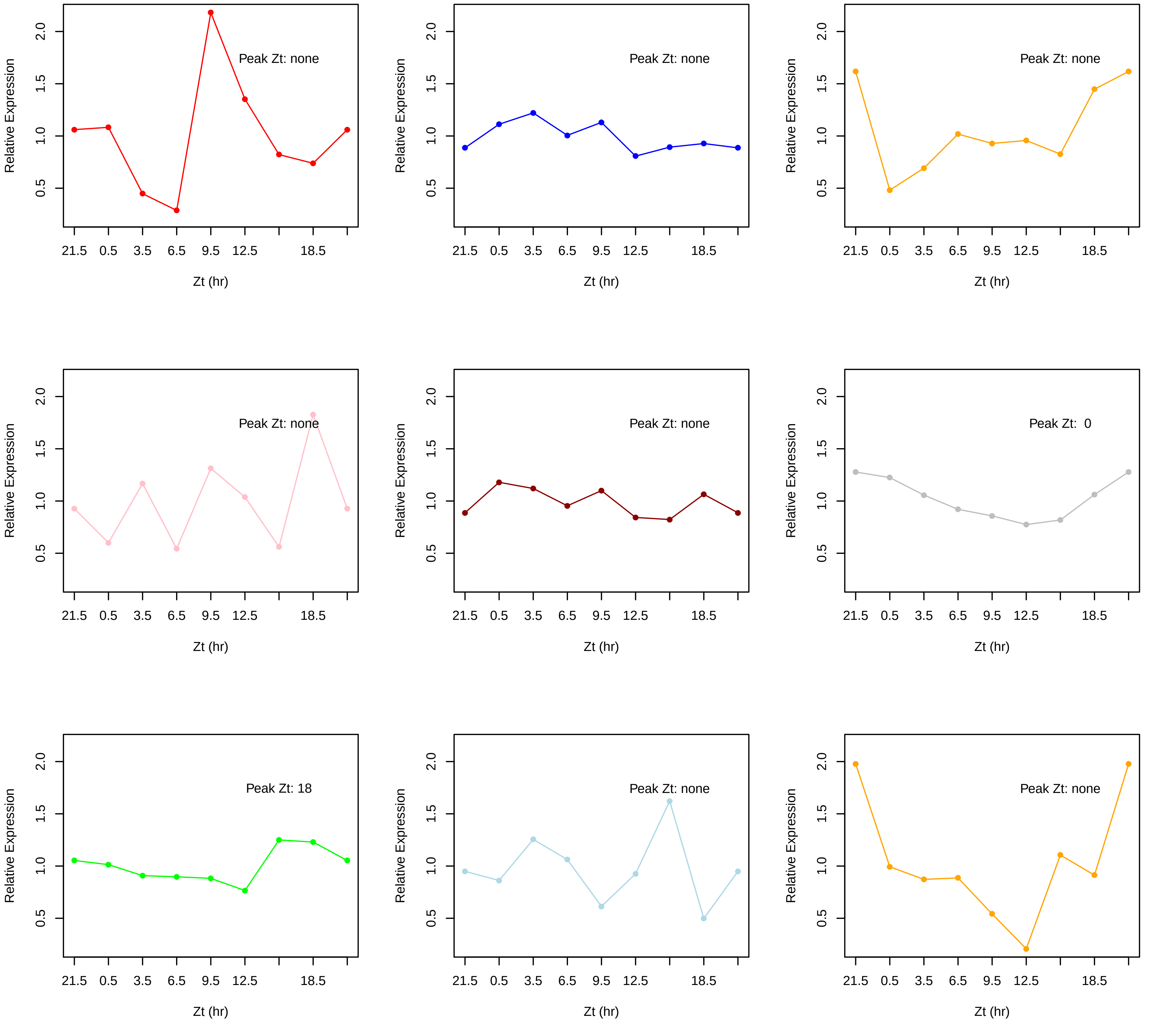

Singeleton: ENSXMAG00000014865 pygl

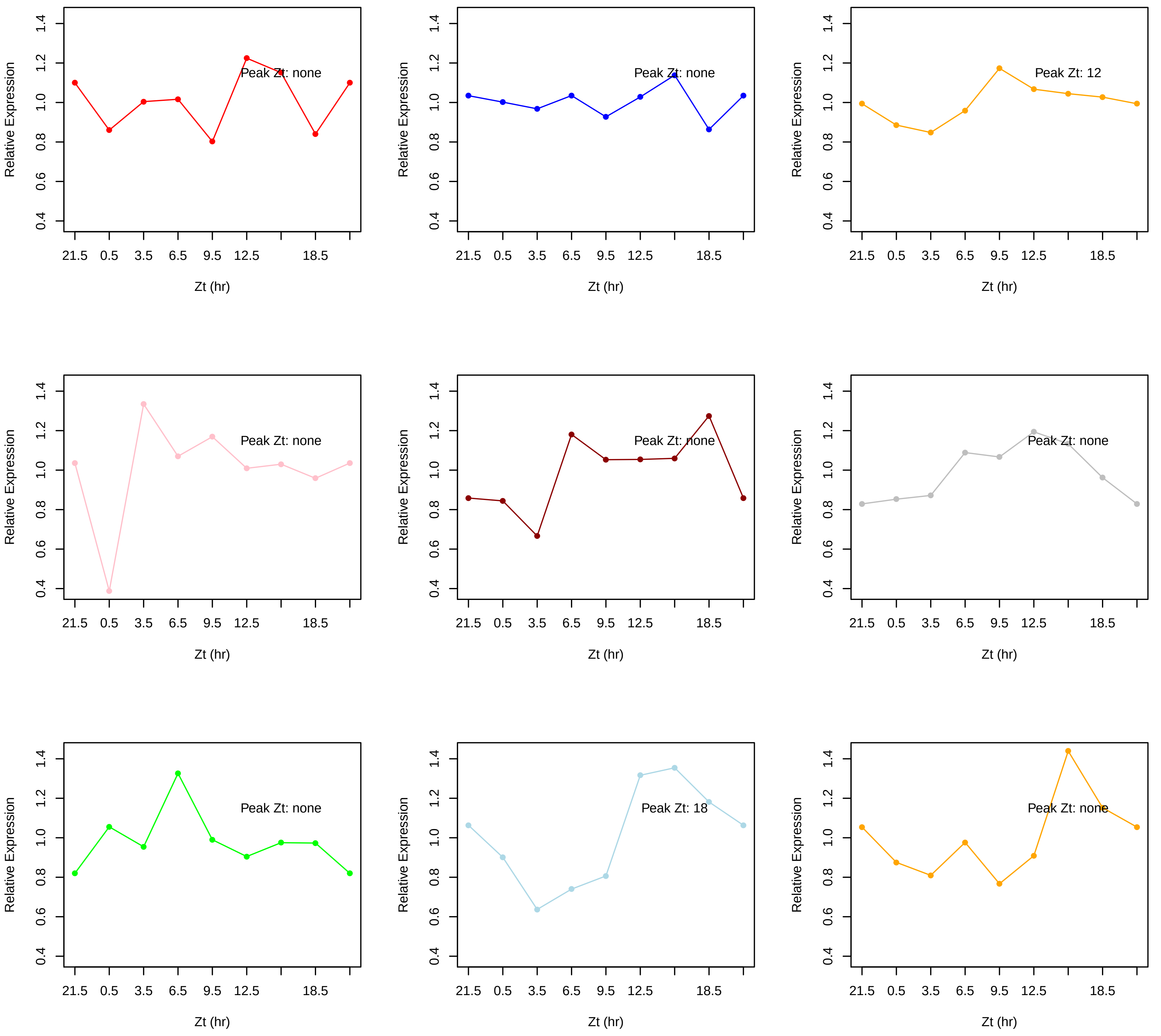

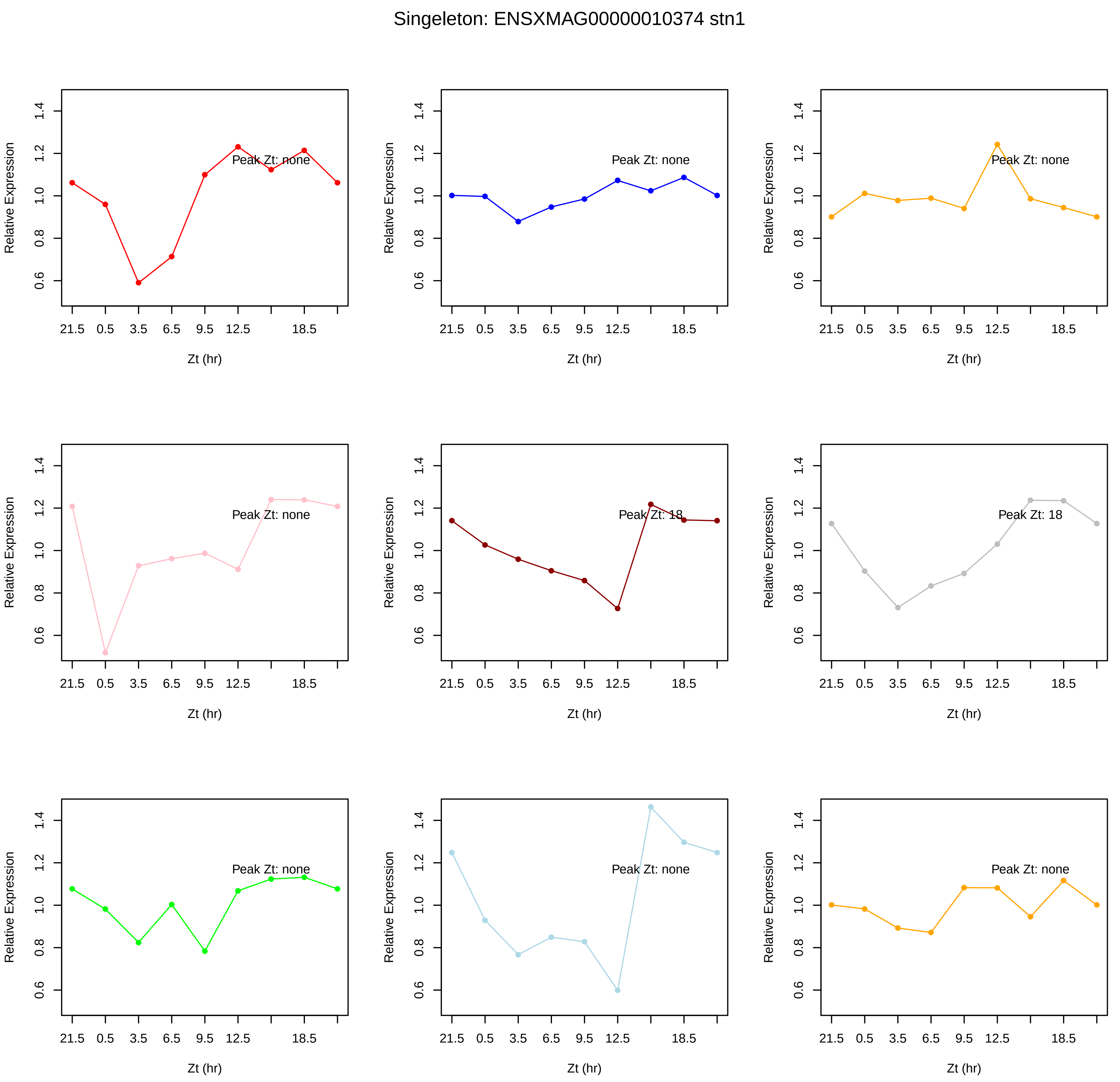
Singeleton: ENSXMAG00000010374 stn1
Relative Expression
Zt (hr)
21.5
0.5
3.5
6.5
9.5
12.5
18.5
0.6
0.8
1.0
1.2
1.4
Peak Zt: none
Peak Zt: none
Peak Zt: none
Peak Zt: none
Peak Zt: 18
Peak Zt: 18
Peak Zt: none
Peak Zt: none
Peak Zt: none

Singeleton: ENSXMAG00000006152 poldip3

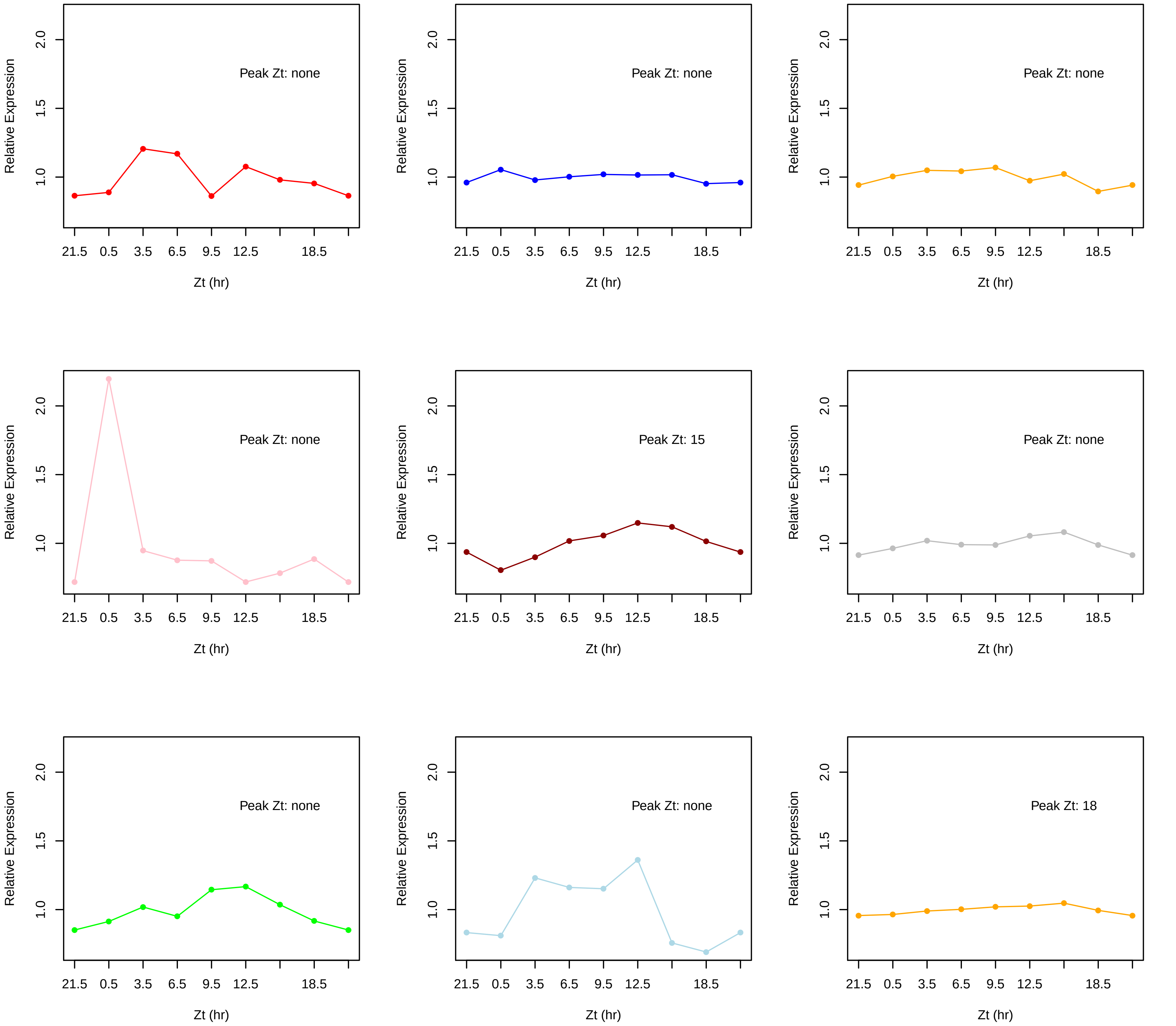

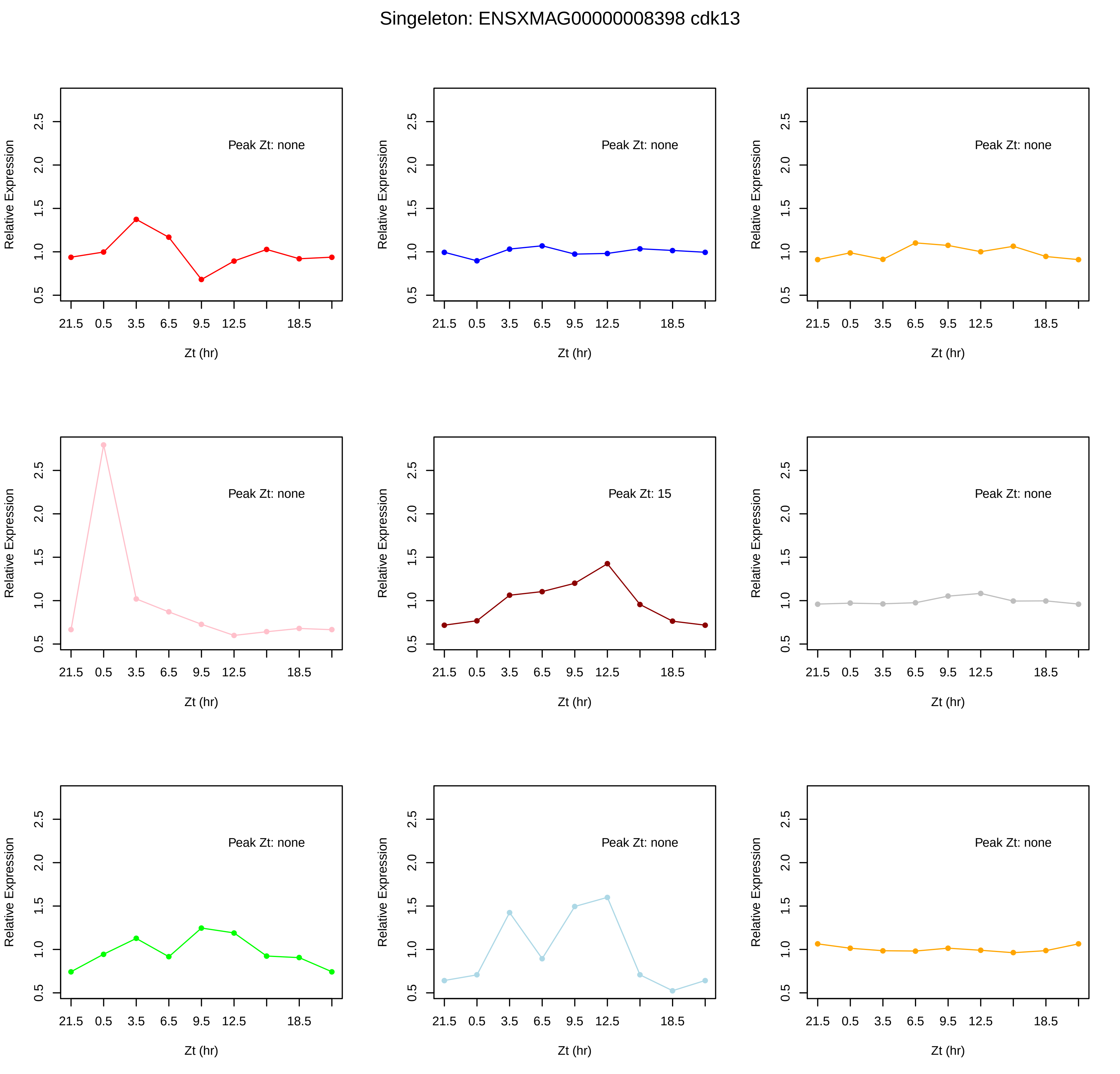
Singleton: ENSXMAG00000008398 cdk13
Peak Zt: none
Peak Zt: none
Peak Zt: none
Peak Zt: none
Peak Zt: 15
Peak Zt: none
Peak Zt: none
Peak Zt: none
Peak Zt: none
Relative Expression
Zt (hr)
21.5
0.5
3.5
6.5
9.5
12.5
18.5
0.5
1.0
1.5
2.0
2.5

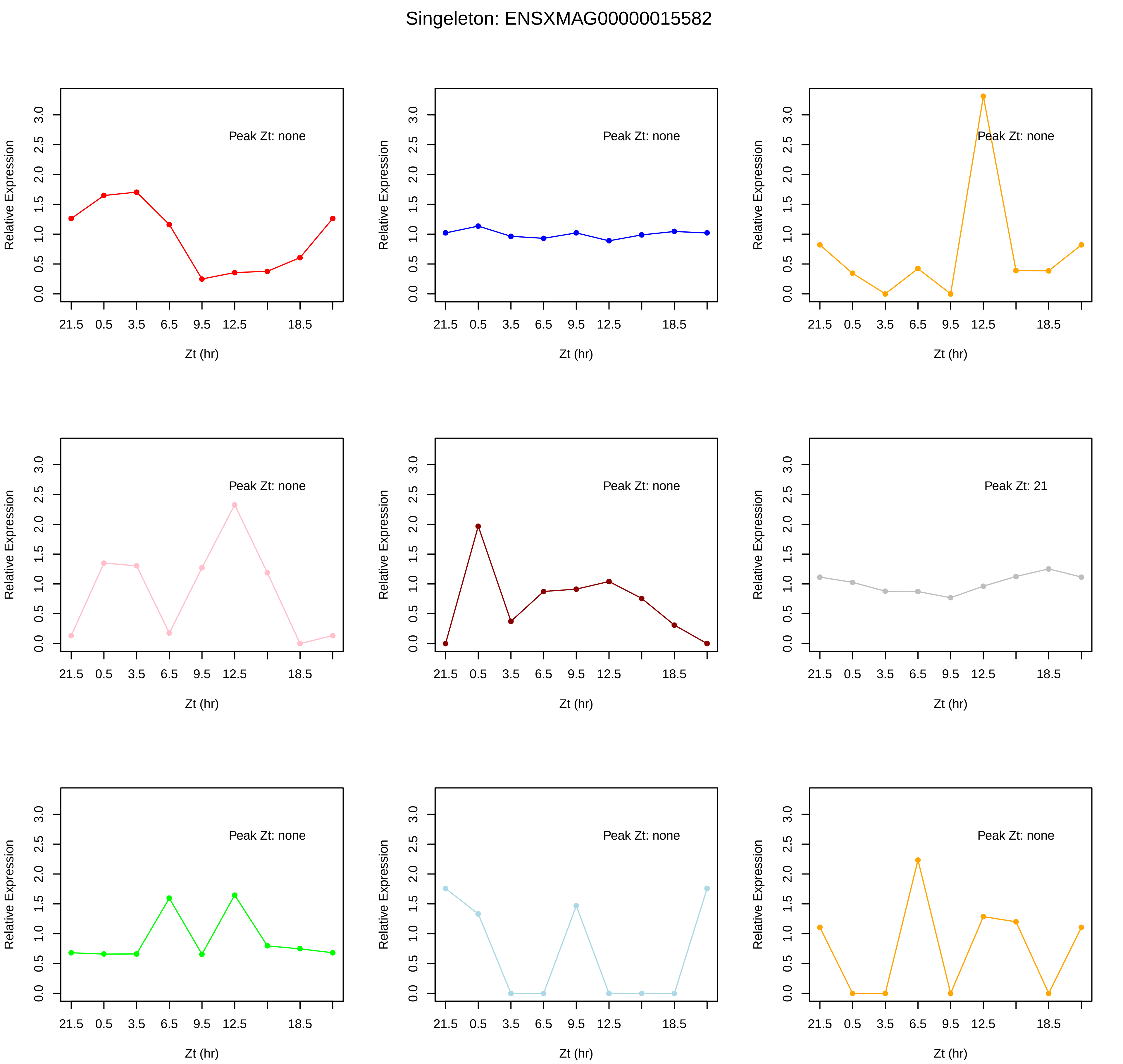
Singleton: ENSXMAG00000015582
Peak Zt: none
Peak Zt: none
Peak Zt: none
Peak Zt: none
Peak Zt: none
Peak Zt: 21
Peak Zt: none
Peak Zt: none
Peak Zt: none
Relative Expression
Zt (hr)
21.5 0.5 3.5 6.5 9.5 12.5 18.5
0.0 0.5 1.0 1.5 2.0 2.5 3.0

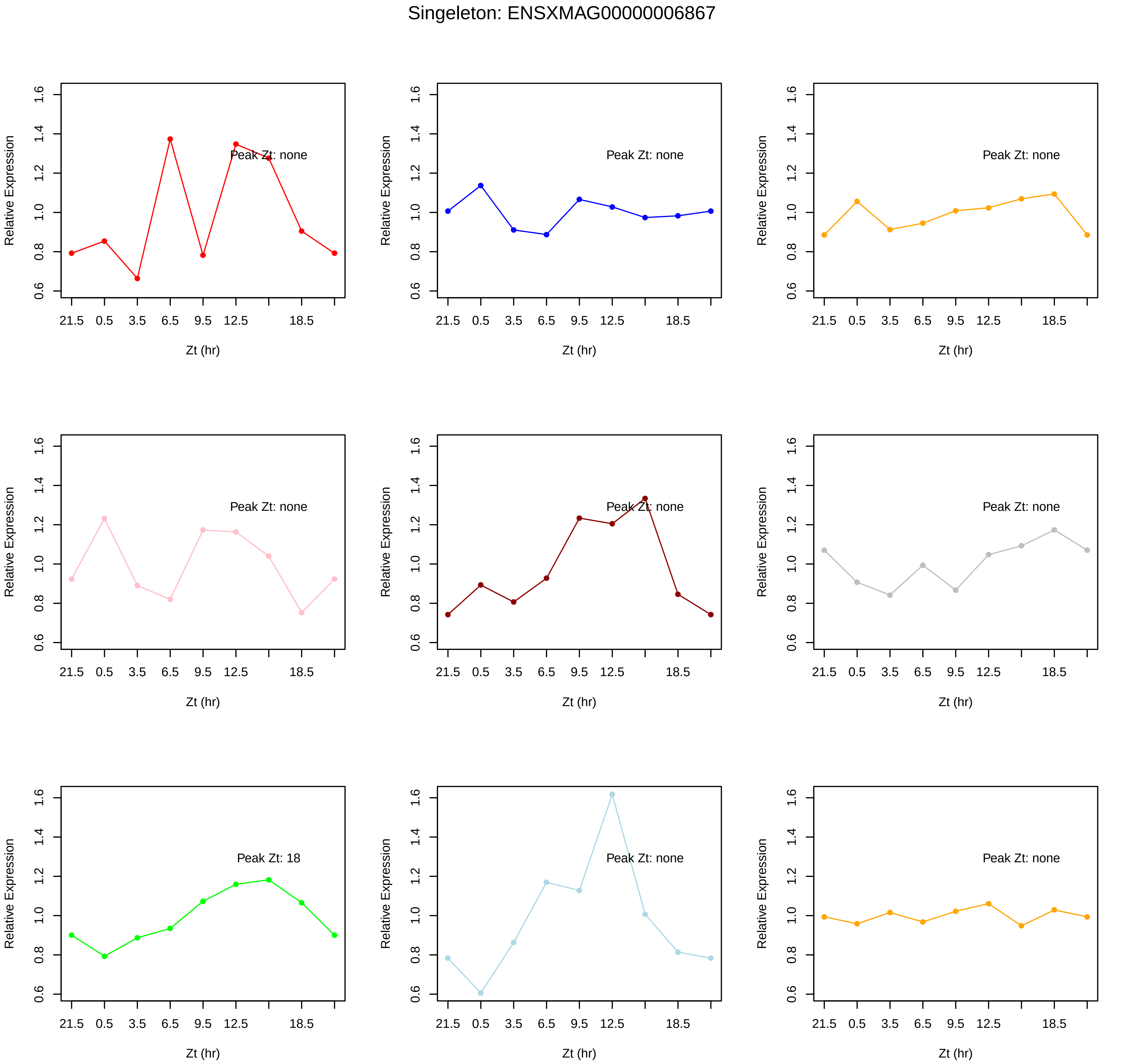
Singeleton: ENSXMAG00000006867
Peak Zt: none
Peak Zt: none
Peak Zt: none
Peak Zt: none
Peak Zt: none
Peak Zt: none
Peak Zt: 18
Peak Zt: none
Peak Zt: none
Relative Expression
Zt (hr)
21.5
0.5
3.5
6.5
9.5
12.5
18.5
0.6
0.8
1.0
1.2
1.4
1.6

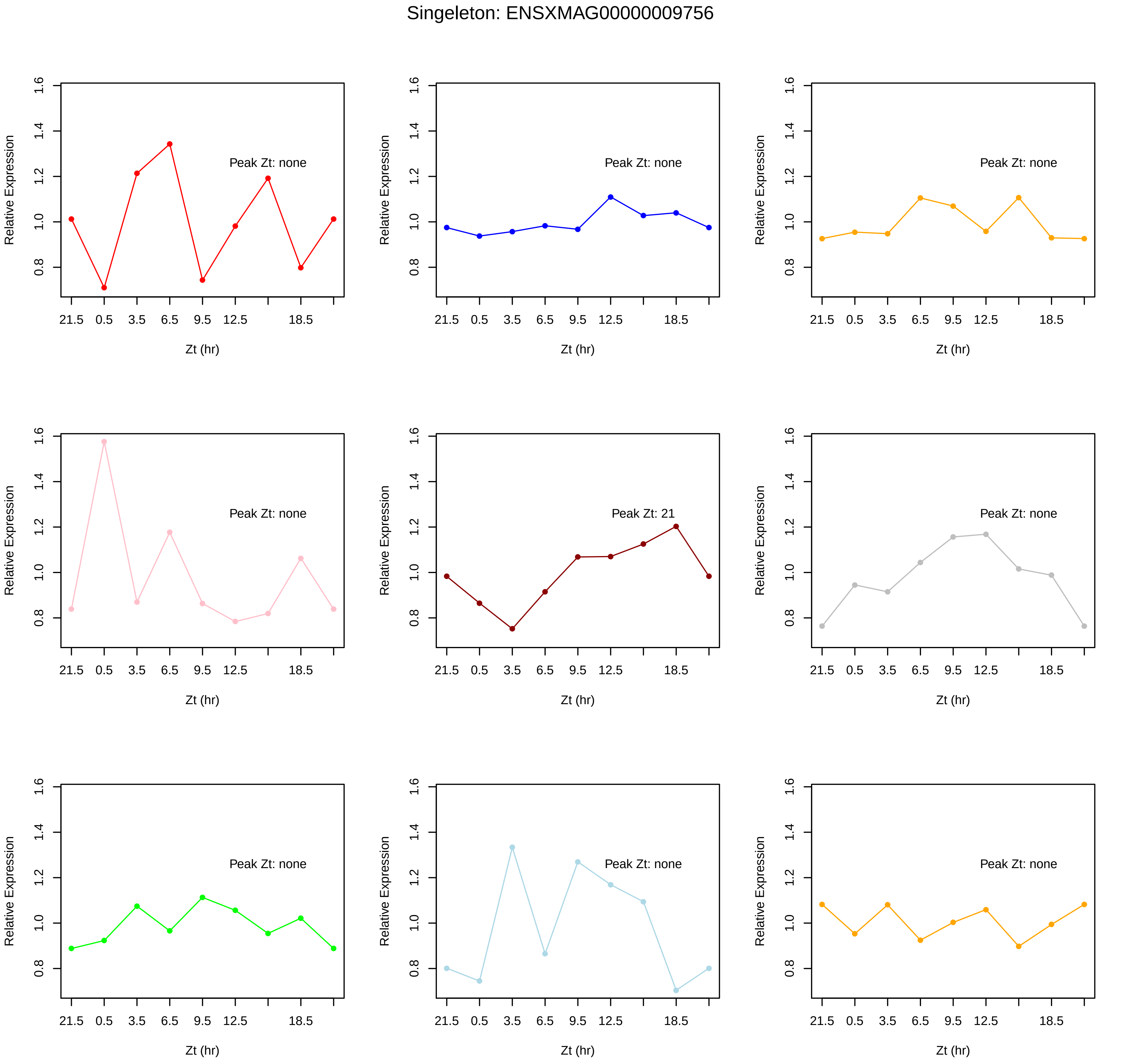
Singleton: ENSXMAG00000009756
Relative Expression
Zt (hr)
21.5
0.5
3.5
6.5
9.5
12.5
18.5
0.8
1.0
1.2
1.4
1.6
Peak Zt: none
Peak Zt: none
Peak Zt: none
Peak Zt: none
Peak Zt: 21
Peak Zt: none
Peak Zt: none
Peak Zt: none
Peak Zt: none

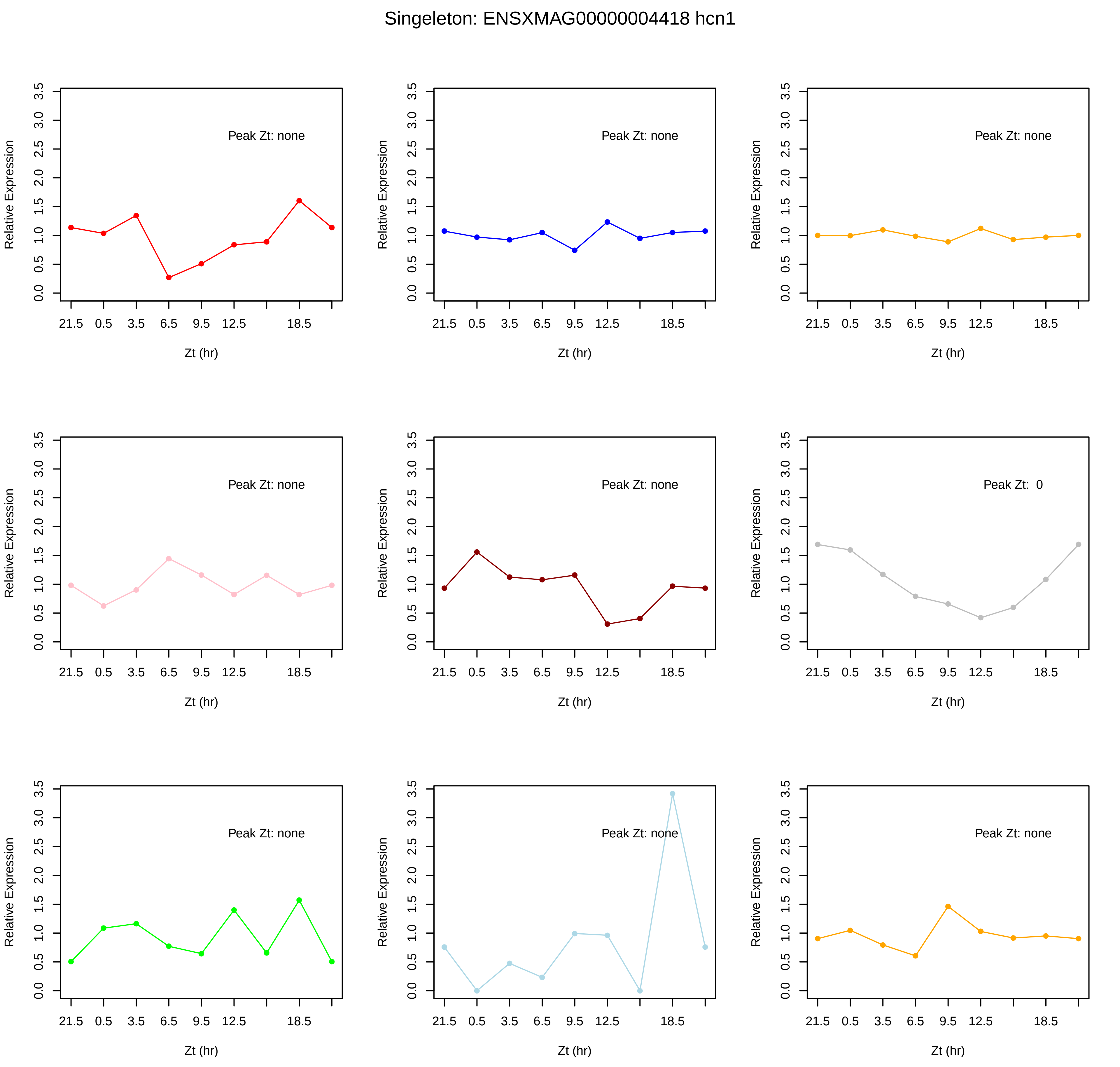
Singeleton: ENSXMAG00000004418 hcn1
Peak Zt: none
Peak Zt: none
Peak Zt: none
Peak Zt: none
Peak Zt: none
Peak Zt: 0
Peak Zt: none
Peak Zt: none
Peak Zt: none
Relative Expression
Zt (hr)
21.5 0.5 3.5 6.5 9.5 12.5 18.5
0.0 0.5 1.0 1.5 2.0 2.5 3.0 3.5

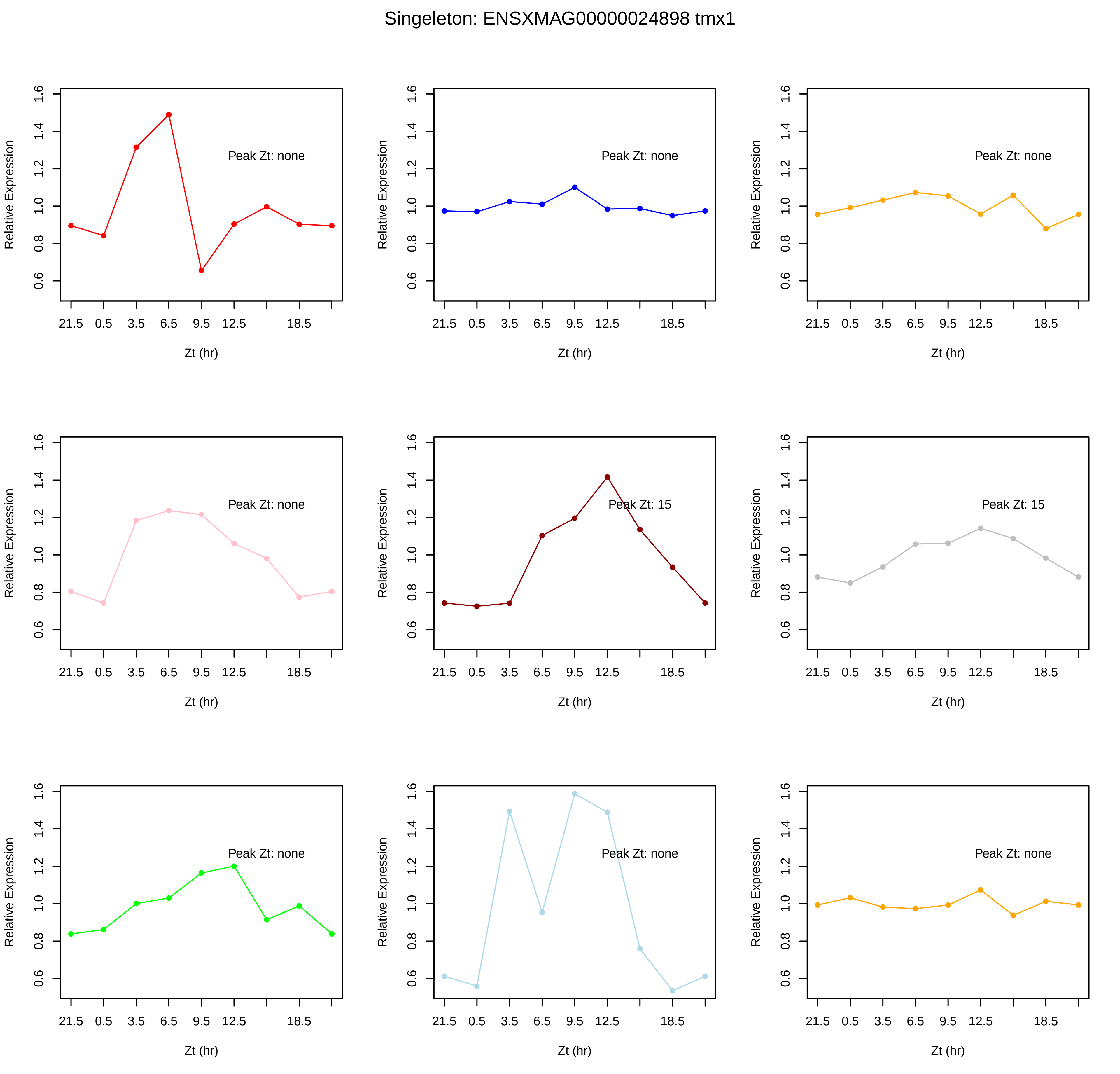
Singeleton: ENSXMAG00000024898 tmx1
Relative Expression
Zt (hr)
21.5 0.5 3.5 6.5 9.5 12.5 18.5
0.6 0.8 1.0 1.2 1.4 1.6
Peak Zt: none
Peak Zt: none
Peak Zt: none
Peak Zt: none
Peak Zt: 15
Peak Zt: 15
Peak Zt: none
Peak Zt: none
Peak Zt: none

Singeleton: ENSXMAG00000009739 mxd3

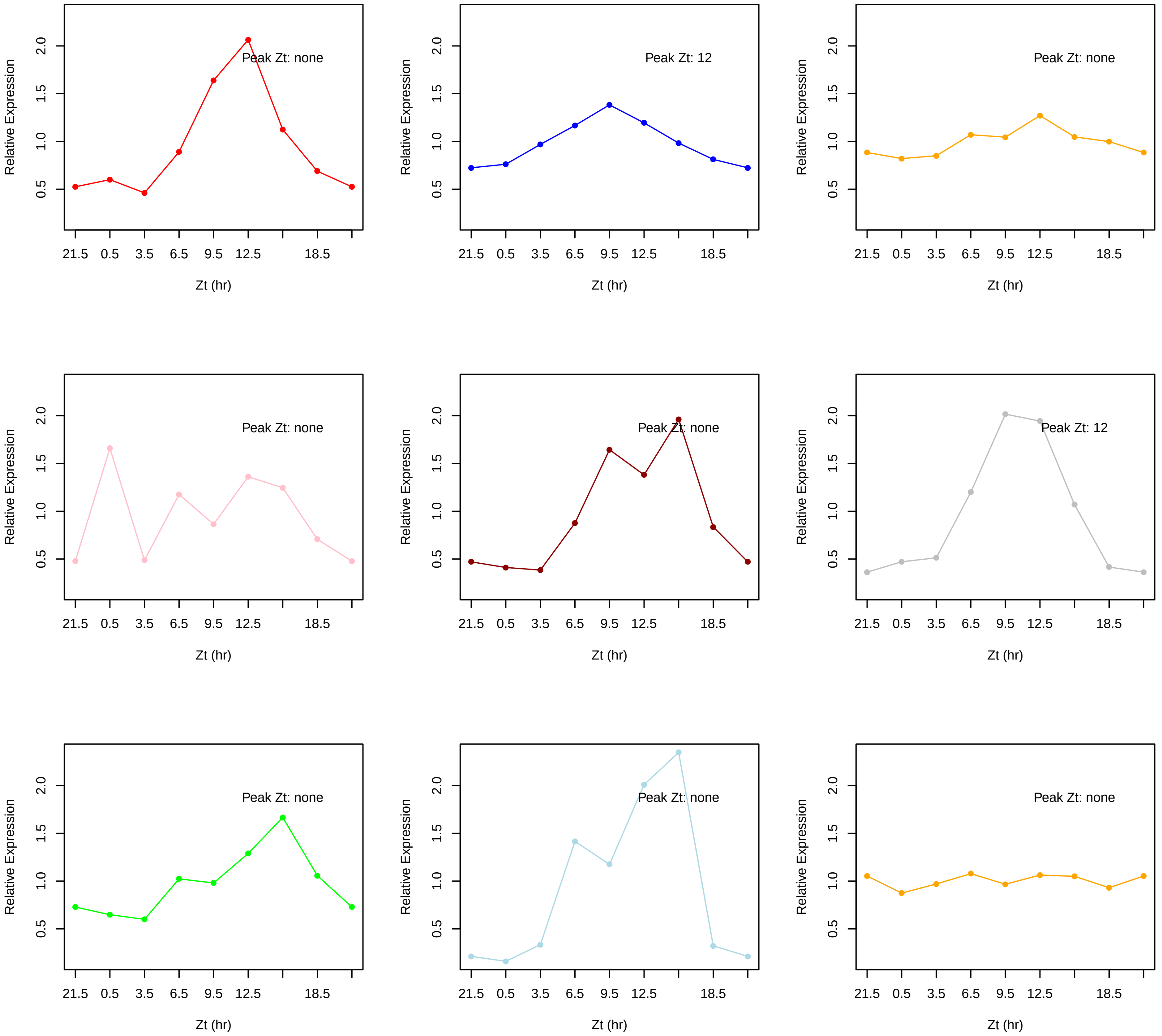

Singeleton: ENSXMAG00000026925 hint2

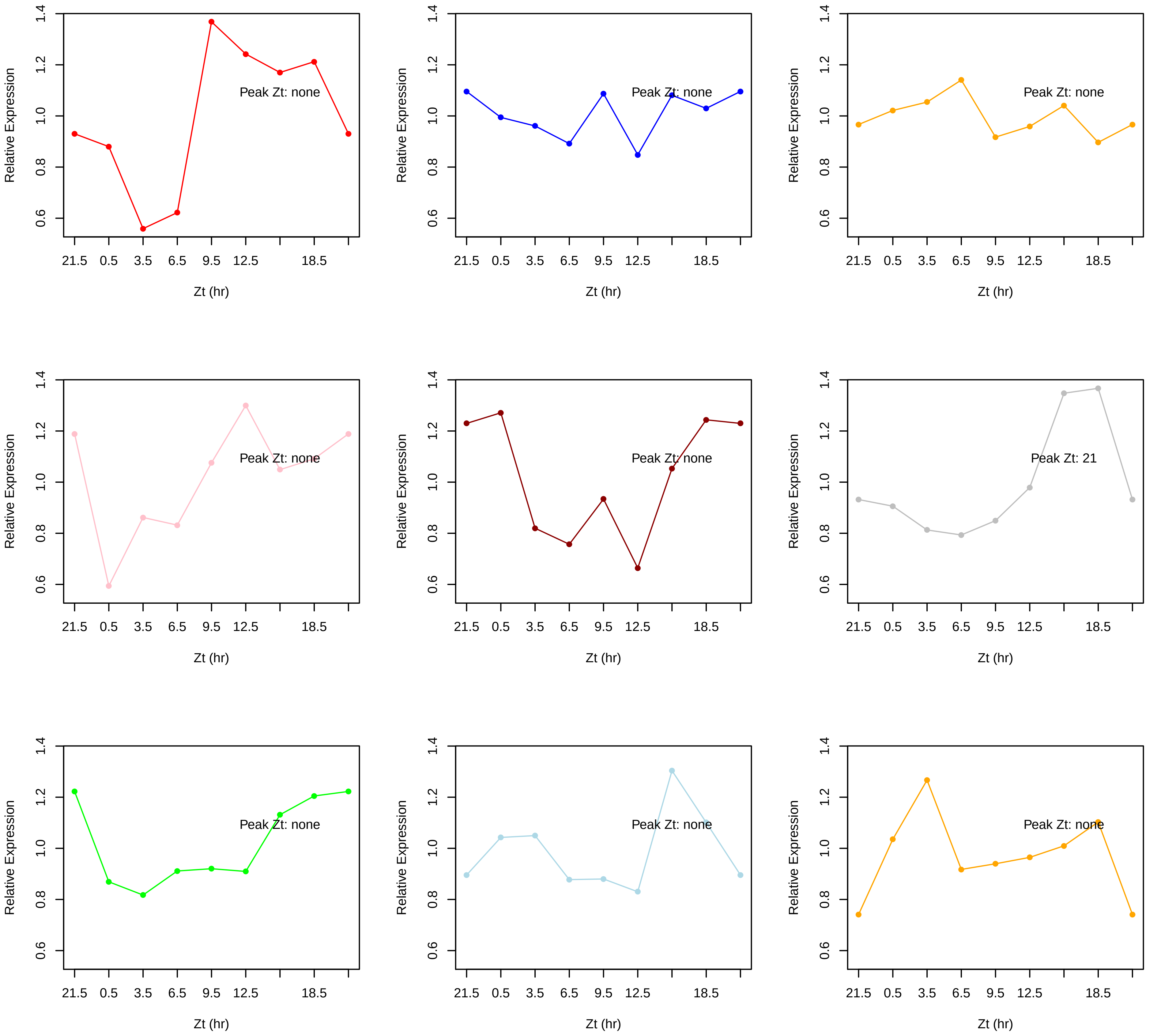

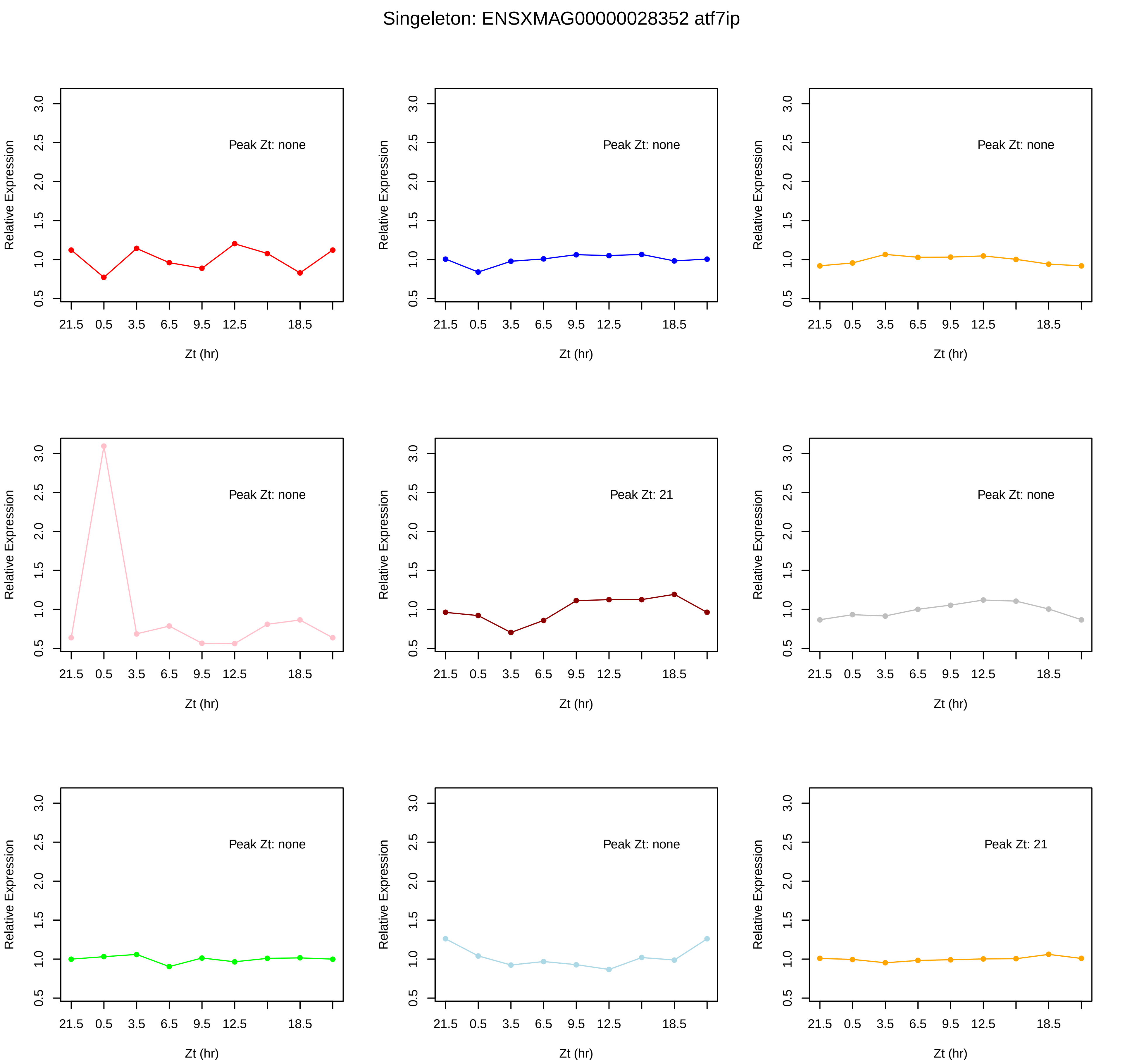
Singeleton: ENSXMAG00000028352 atf7ip
Peak Zt: none
Peak Zt: none
Peak Zt: none
Peak Zt: none
Peak Zt: 21
Peak Zt: none
Peak Zt: none
Peak Zt: none
Peak Zt: 21
Relative Expression
Zt (hr)
21.5 0.5 3.5 6.5 9.5 12.5 18.5
0.5 1.0 1.5 2.0 2.5 3.0

Singeleton: ENSXMAG00000009733 prelid1a

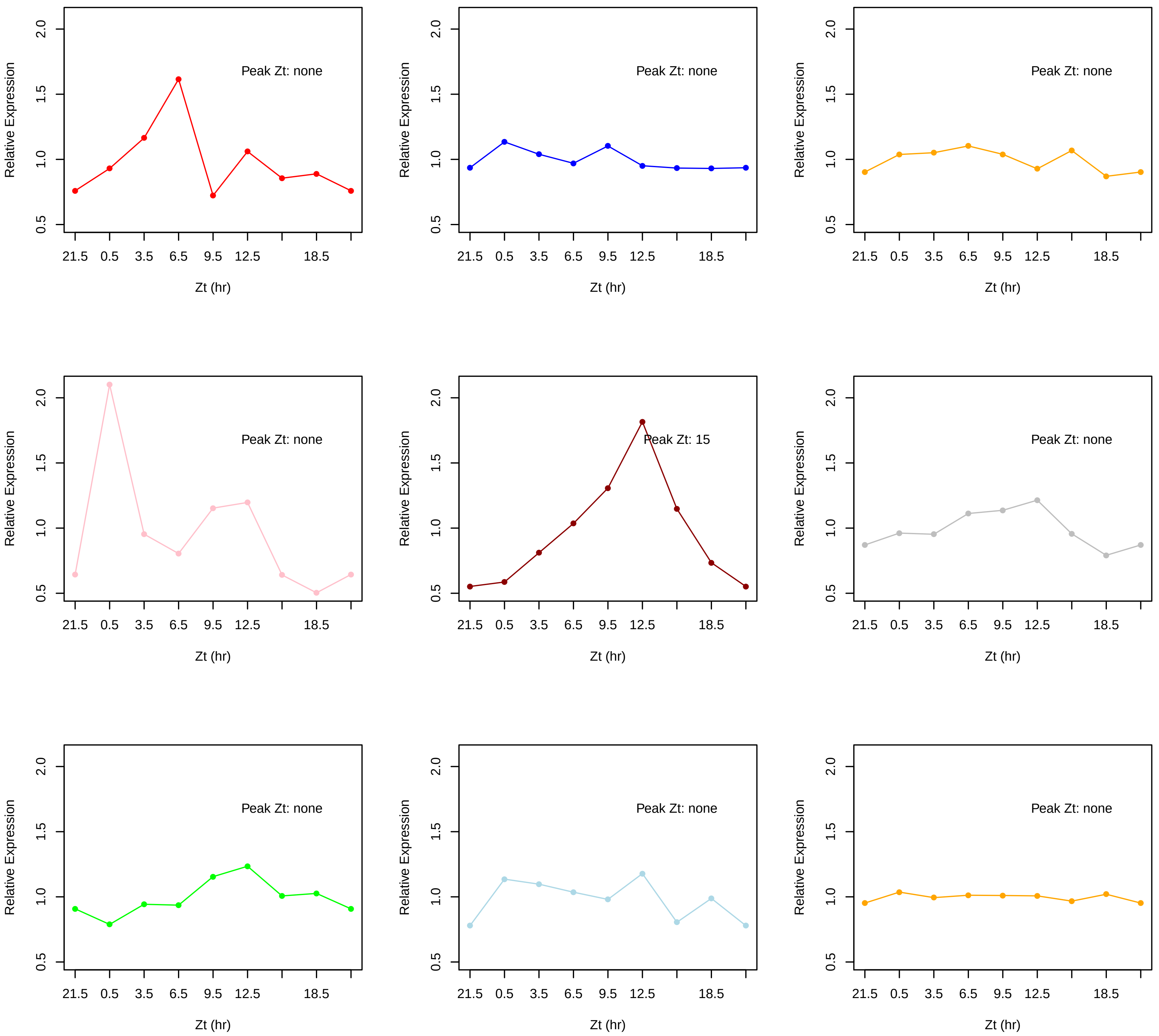

Singeleton: ENSXMAG00000016945 psmc5

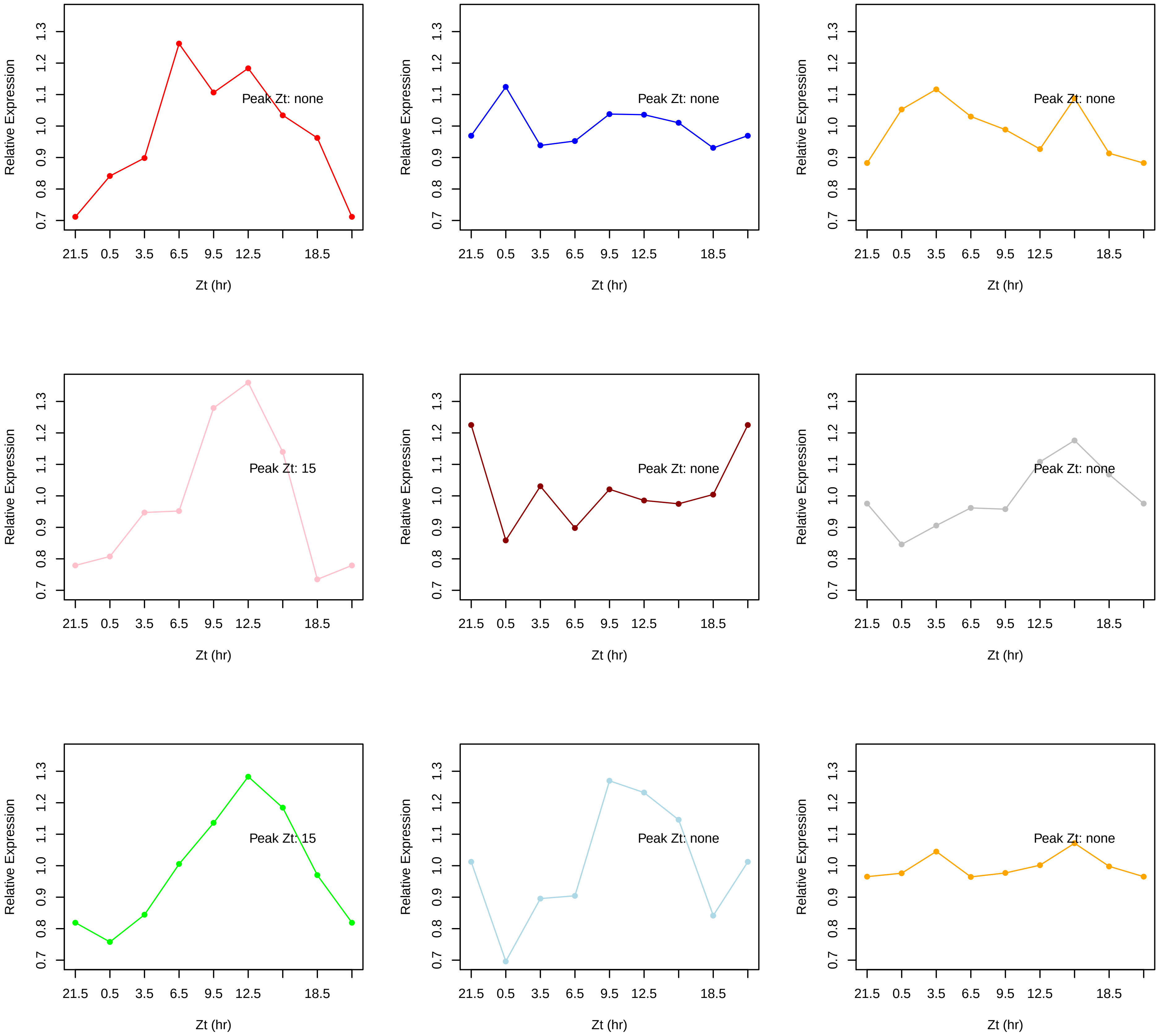

Singeleton: ENSXMAG00000015292 rtraf

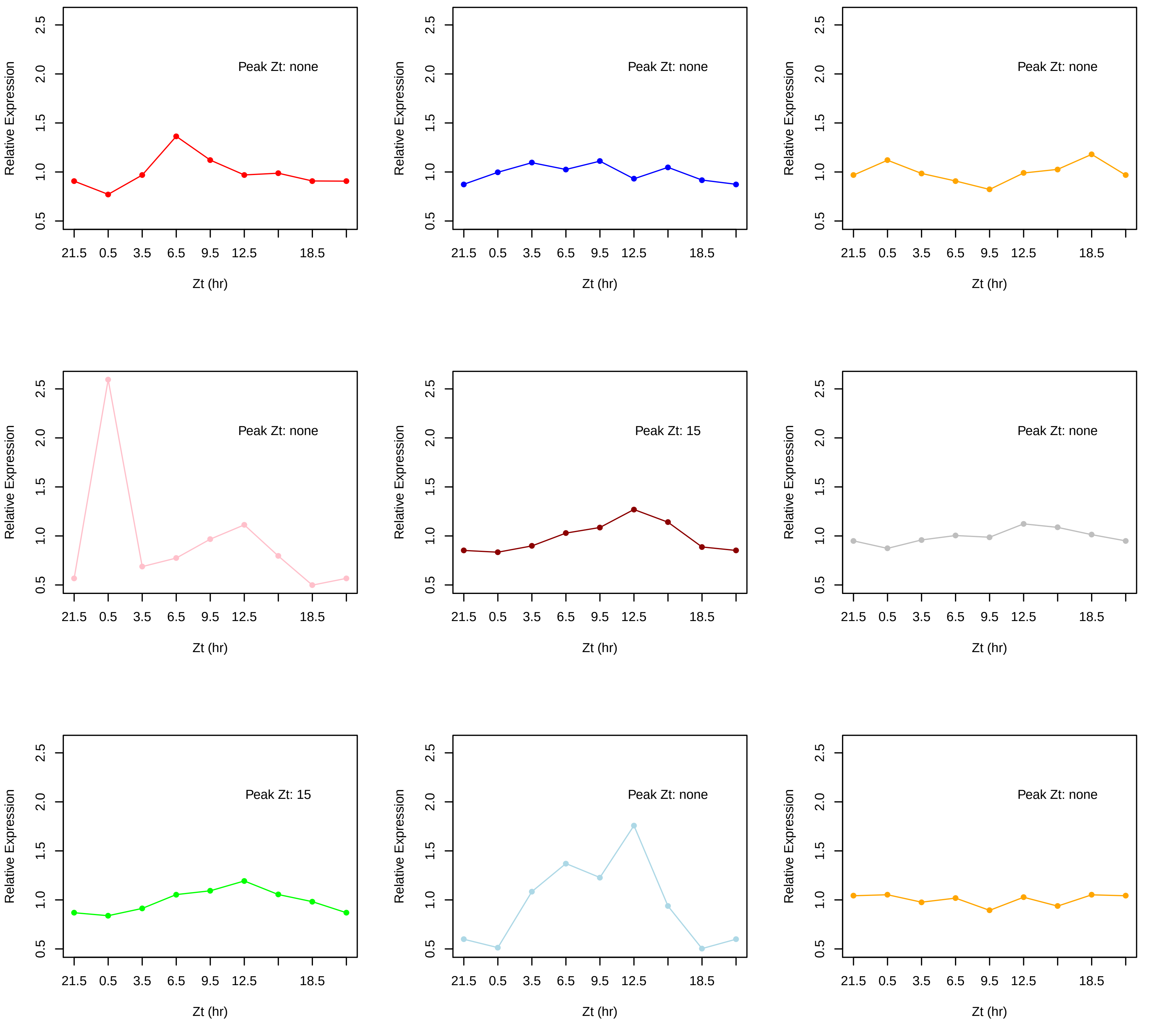

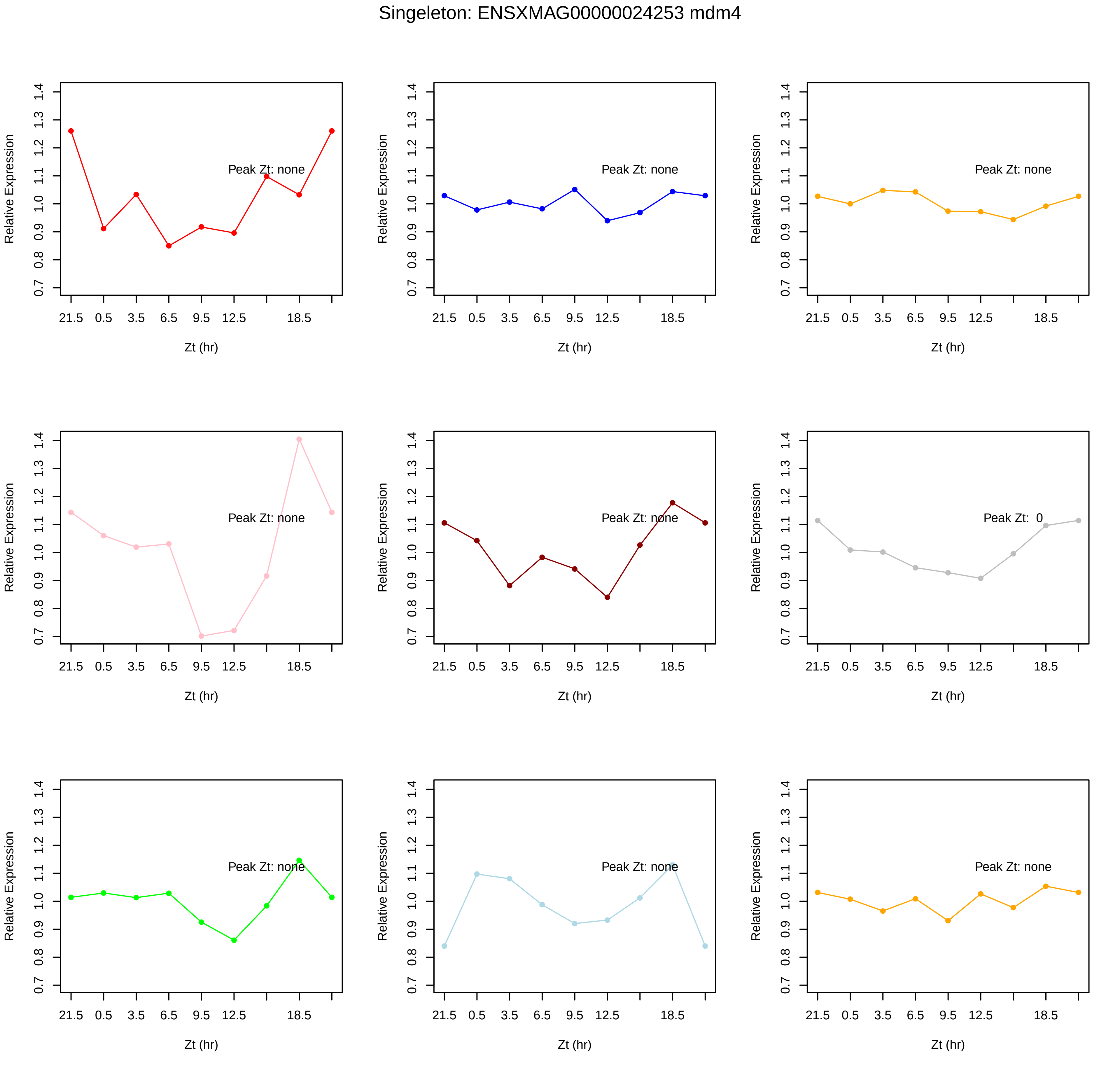

Singeleton: ENSXMAG00000024253 mdm4
Relative Expression
Zt (hr)
21.5
0.5
3.5
6.5
9.5
12.5
18.5
0.7
0.8
0.9
1.0
1.1
1.2
1.3
1.4
Peak Zt: none
Peak Zt: 0

Singeleton: ENSXMAG00000001791 DNAJC13

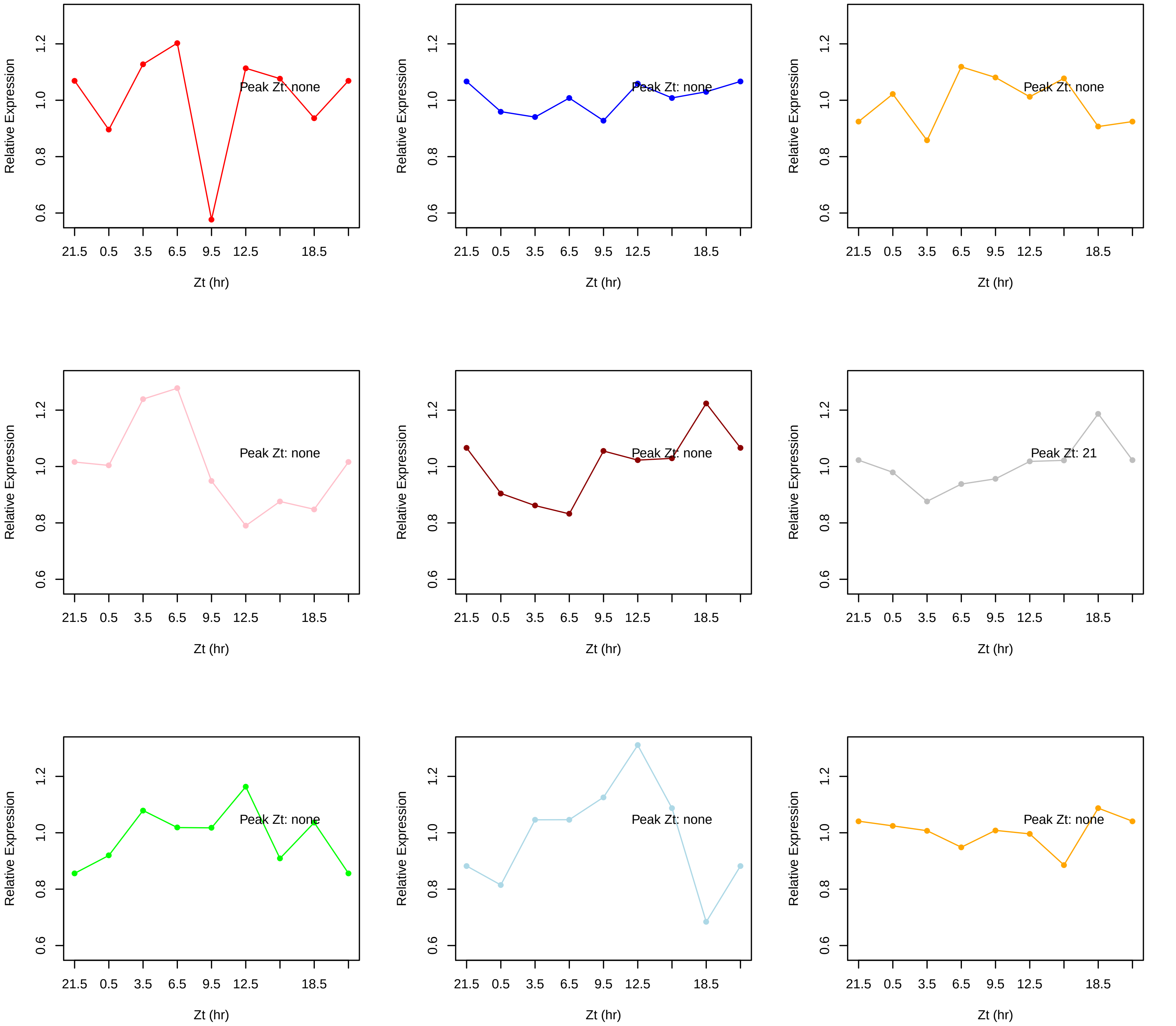

Singleton: ENSXMAG00000004465 esm1

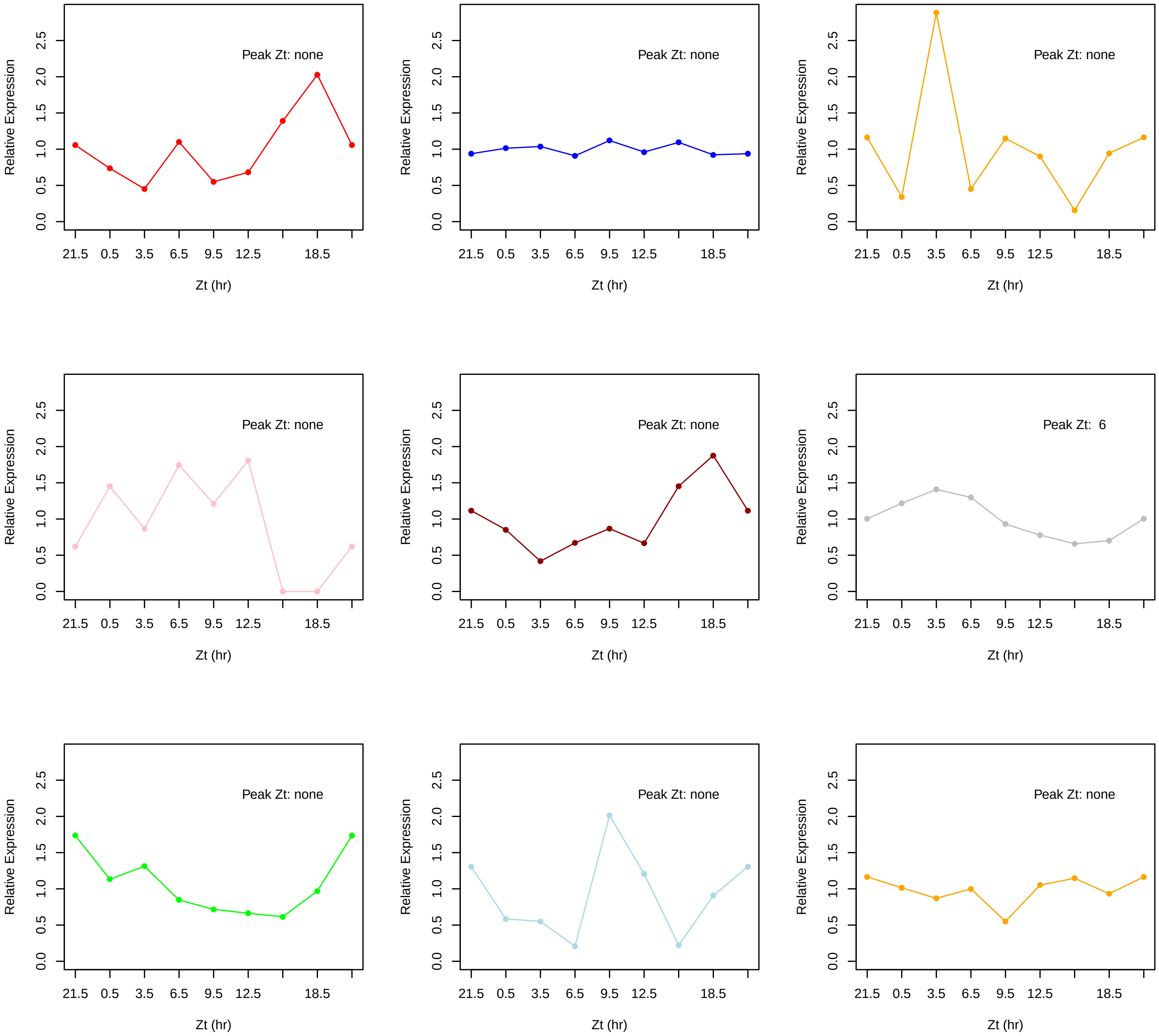

Singeleton: ENSXMAG00000005585 tacr3l

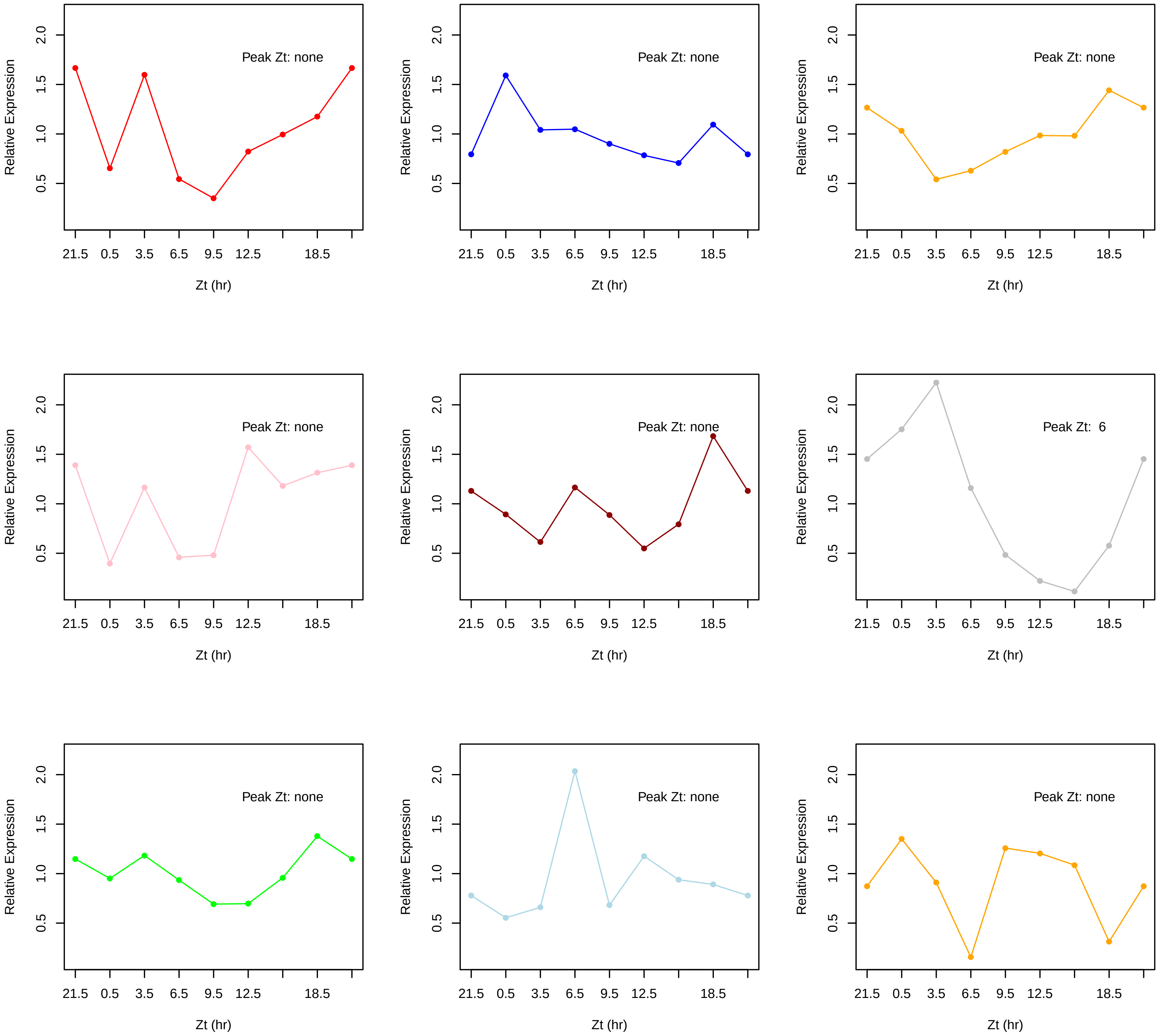

Singeleton: ENSXMAG00000008089 vwa2

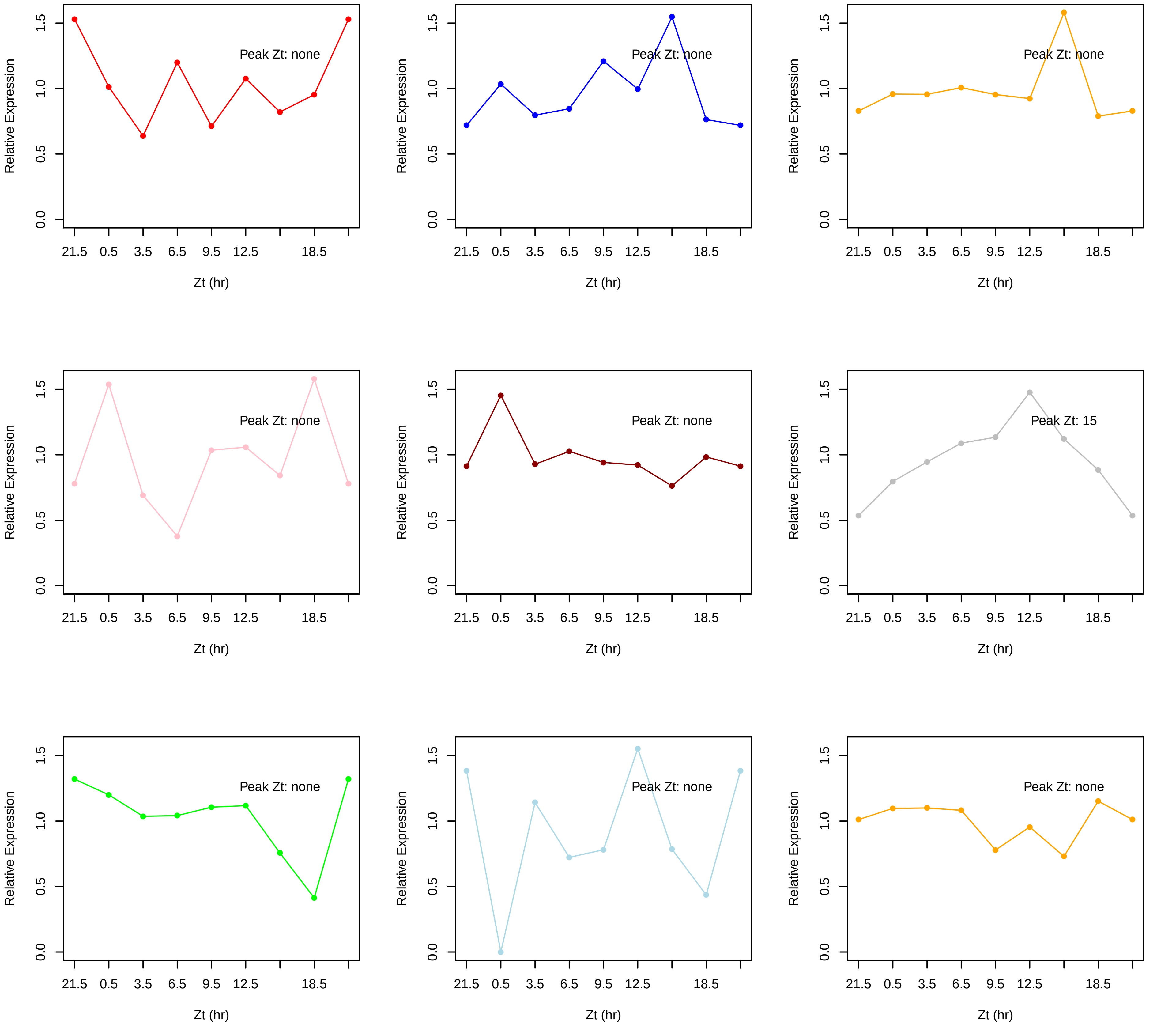

Singleton: ENSXMAG00000009666 ergic1

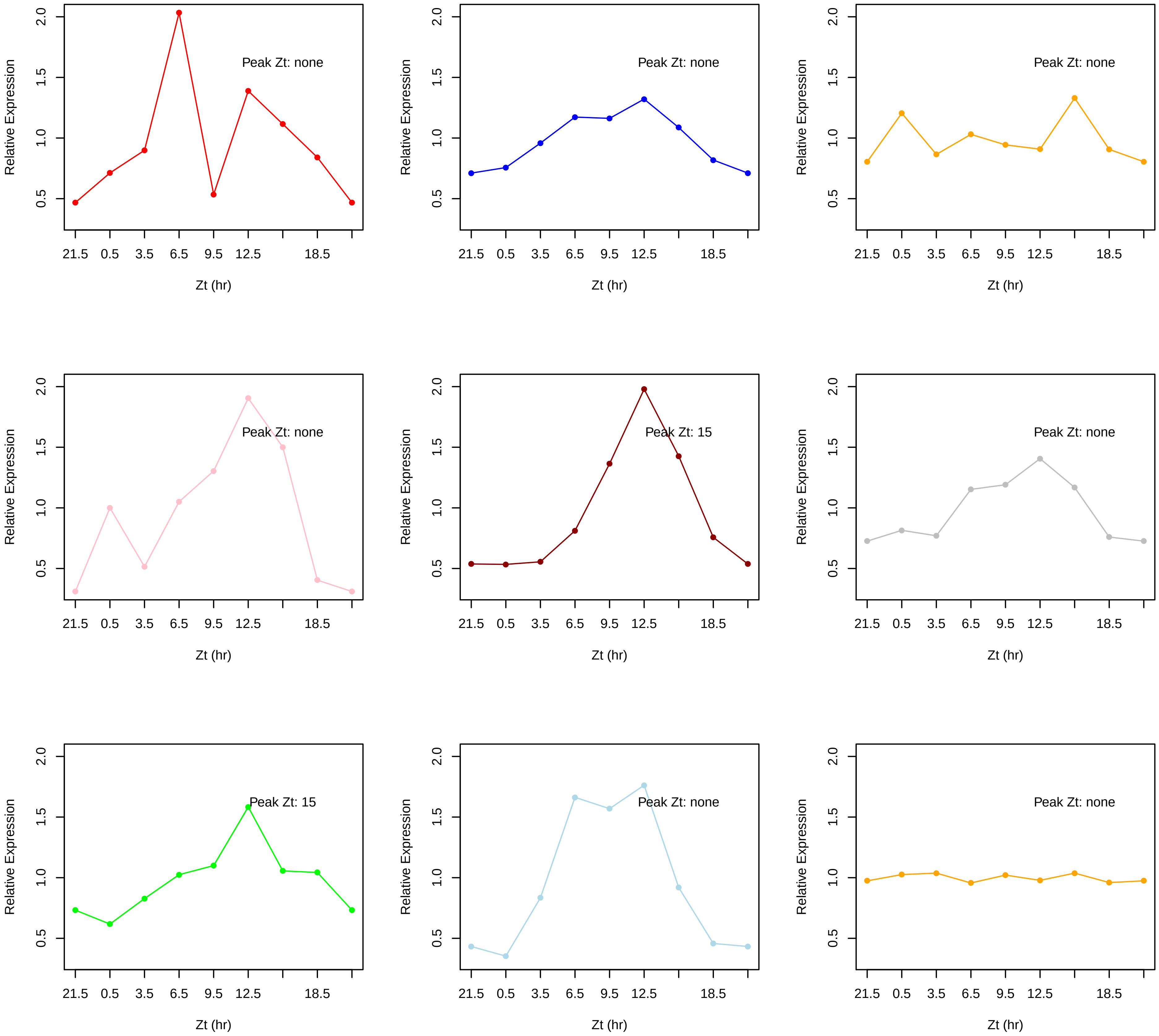

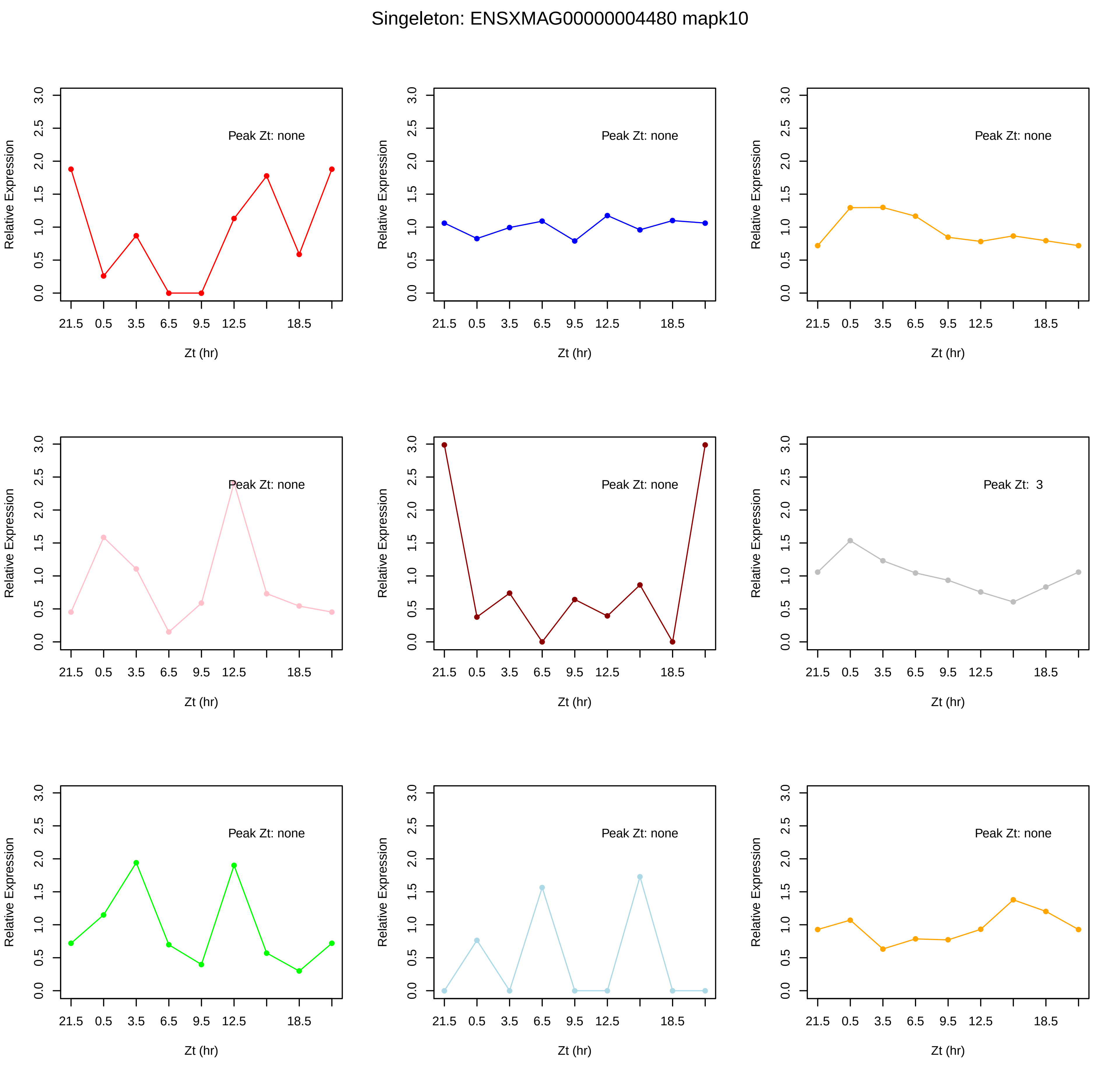
Singeleton: ENSXMAG00000004480 mapk10
Peak Zt: none
Peak Zt: none
Peak Zt: none
Peak Zt: none
Peak Zt: none
Peak Zt: 3
Peak Zt: none
Peak Zt: none
Peak Zt: none
Relative Expression
Zt (hr)
21.5
0.5
3.5
6.5
9.5
12.5
18.5
0.0
0.5
1.0
1.5
2.0
2.5
3.0

Singleton: ENSXMAG00000008279 asb16

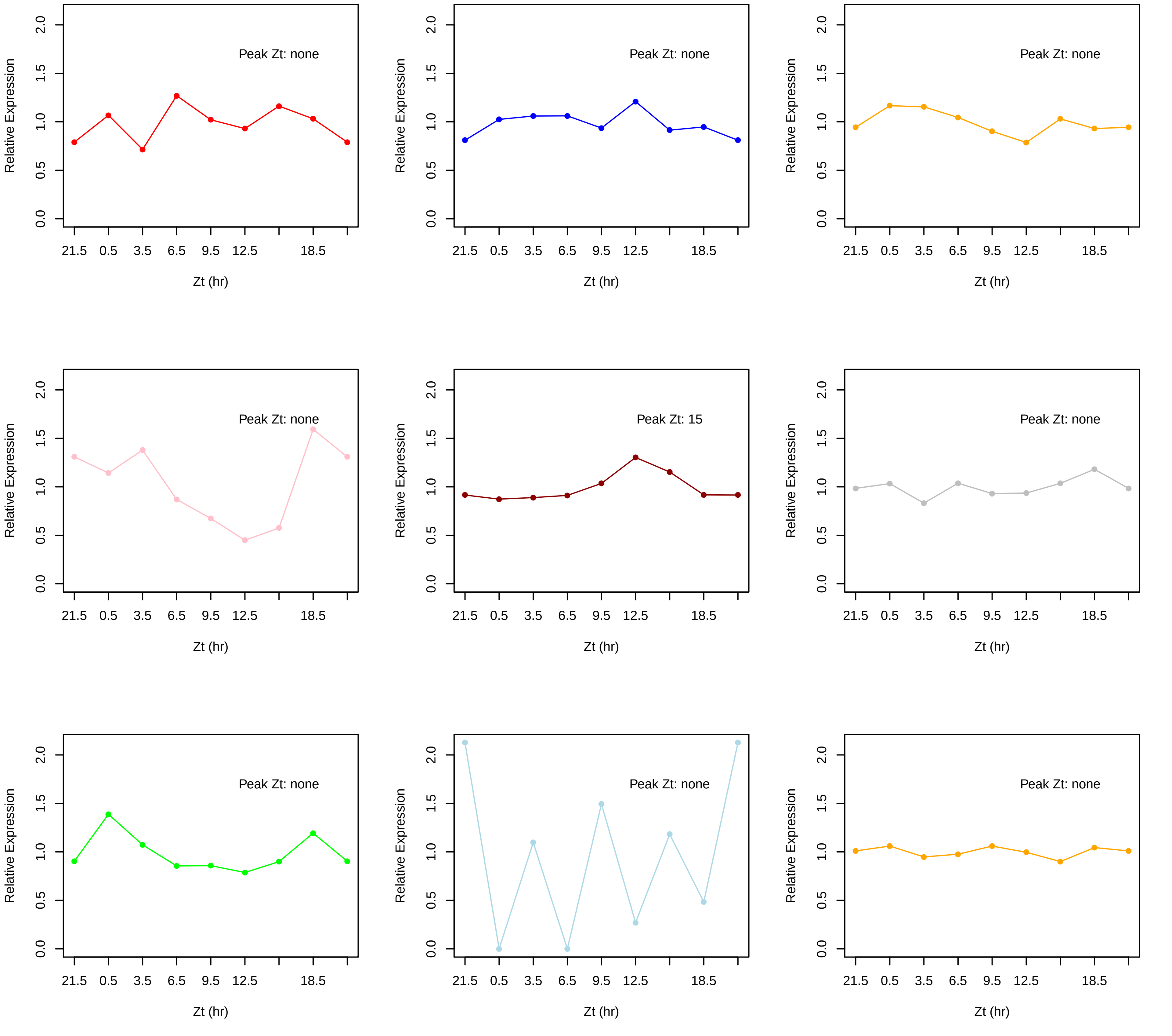

Singeleton: ENSXMAG00000004518 ptpn13

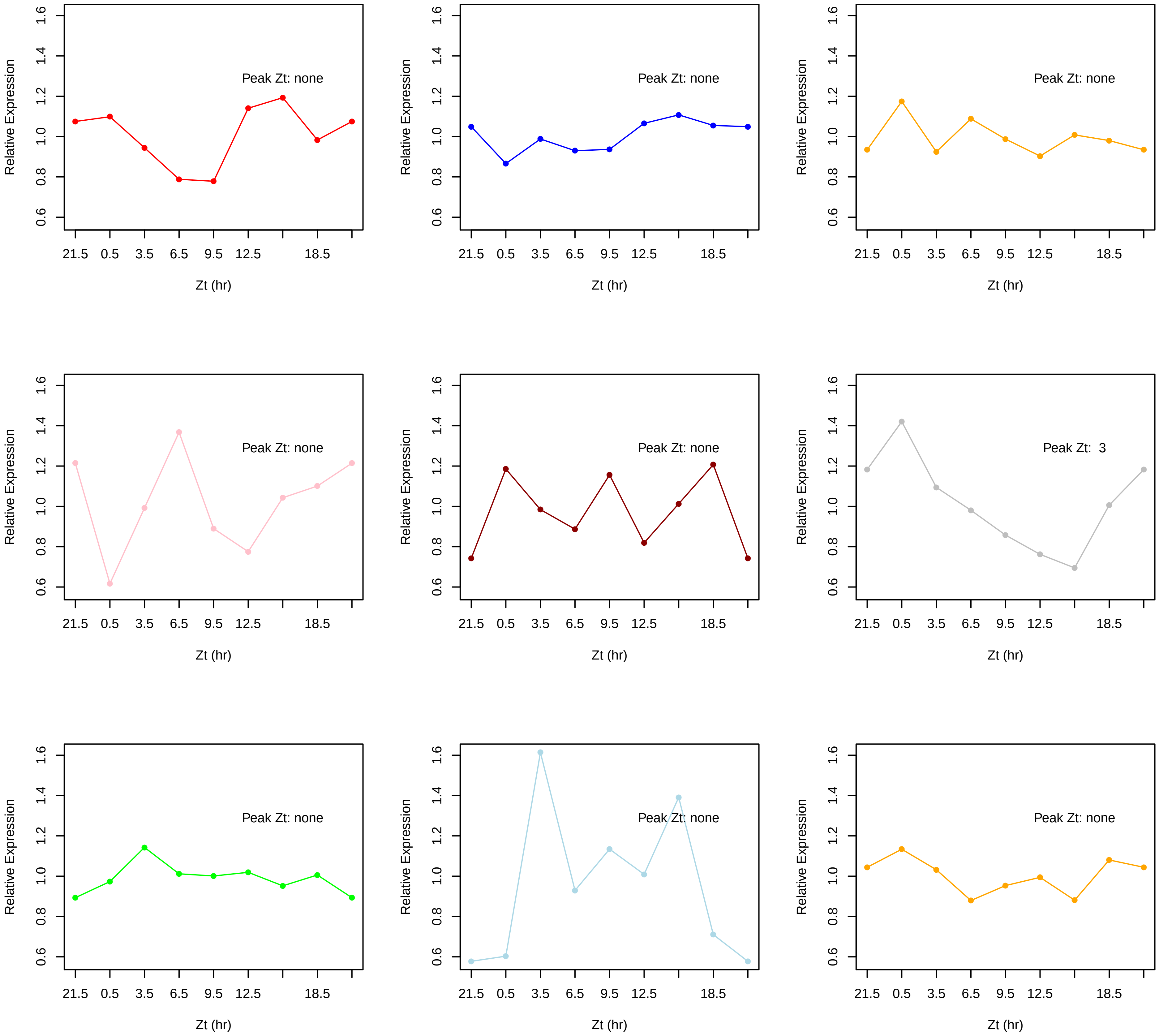

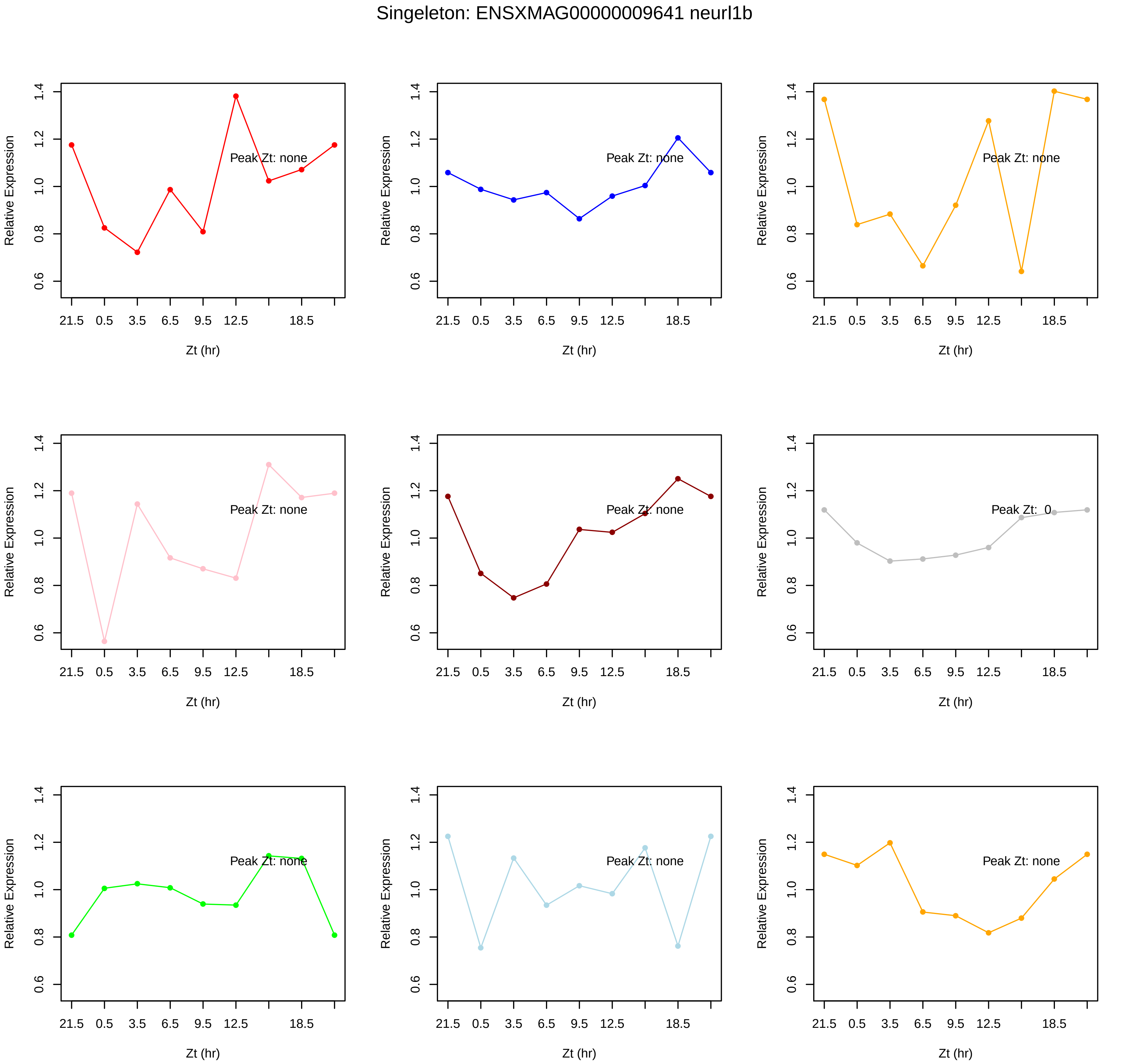
Singeleton: ENSXMAG00000009641 neurl1b
Relative Expression
Zt (hr)
21.5 0.5 3.5 6.5 9.5 12.5 18.5
0.6 0.8 1.0 1.2 1.4
Peak Zt: none
Peak Zt: none
Peak Zt: none
Peak Zt: none
Peak Zt: none
Peak Zt: 0
Peak Zt: none
Peak Zt: none
Peak Zt: none

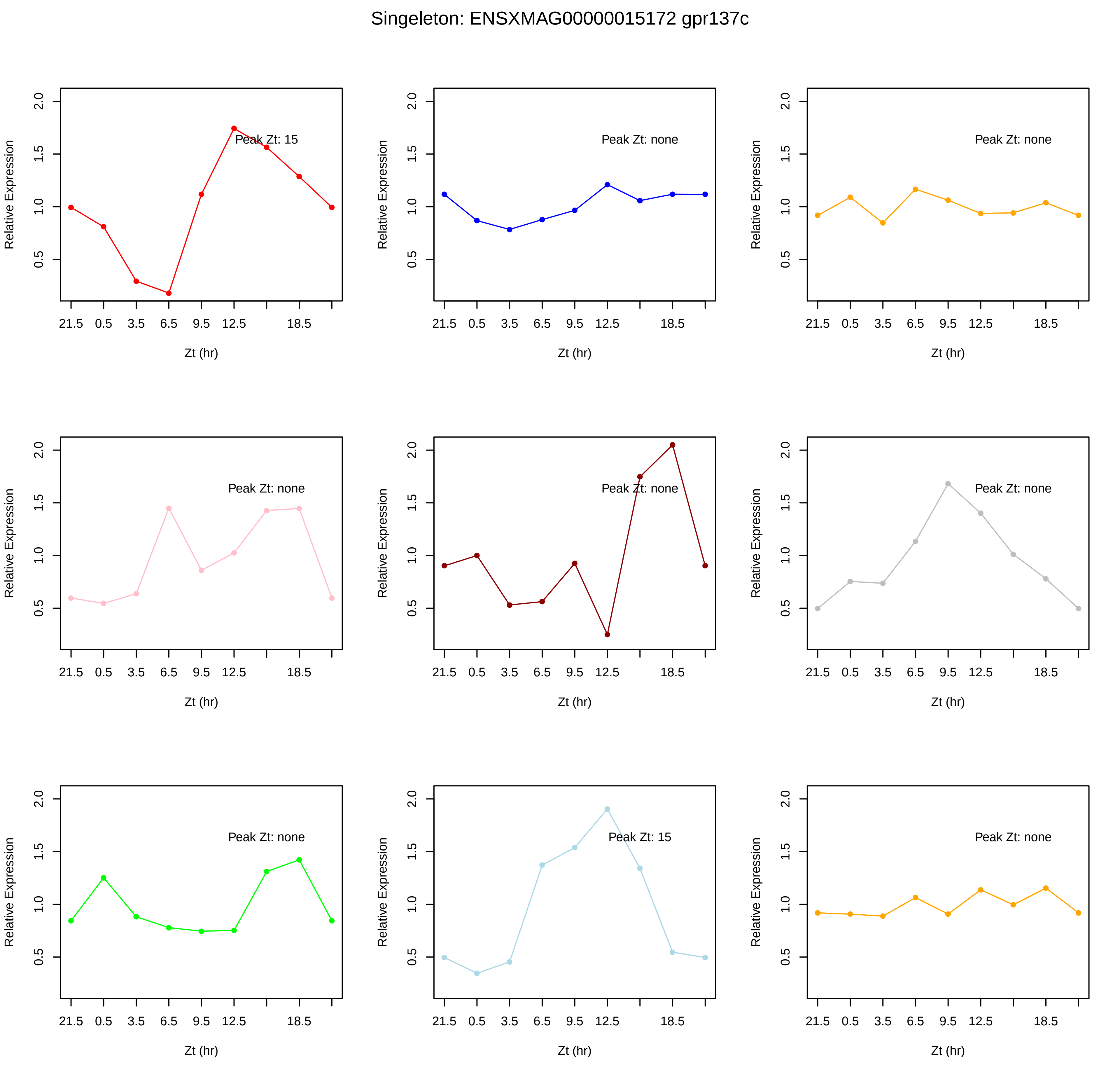
Singeleton: ENSXMAG00000015172 gpr137c
Peak Zt: 15
Peak Zt: none
Peak Zt: none
Peak Zt: none
Peak Zt: none
Peak Zt: none
Peak Zt: none
Peak Zt: 15
Peak Zt: none
Relative Expression
Zt (hr)
21.5
0.5
3.5
6.5
9.5
12.5
18.5
0.5
1.0
1.5
2.0

Singleton: ENSXMAG00000029457 ero1a

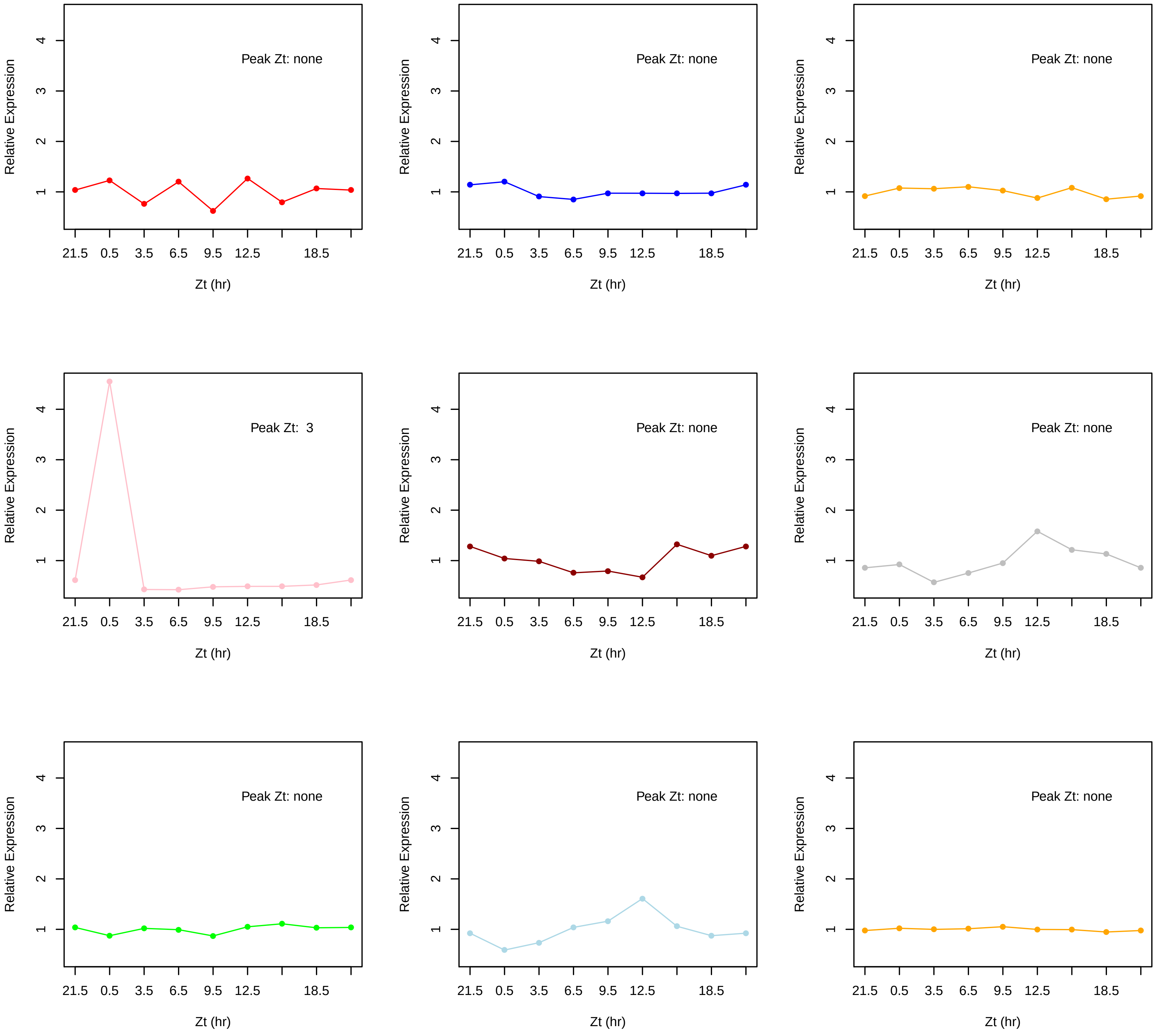

Singleton: ENSXMAG00000008126 nhlrc2

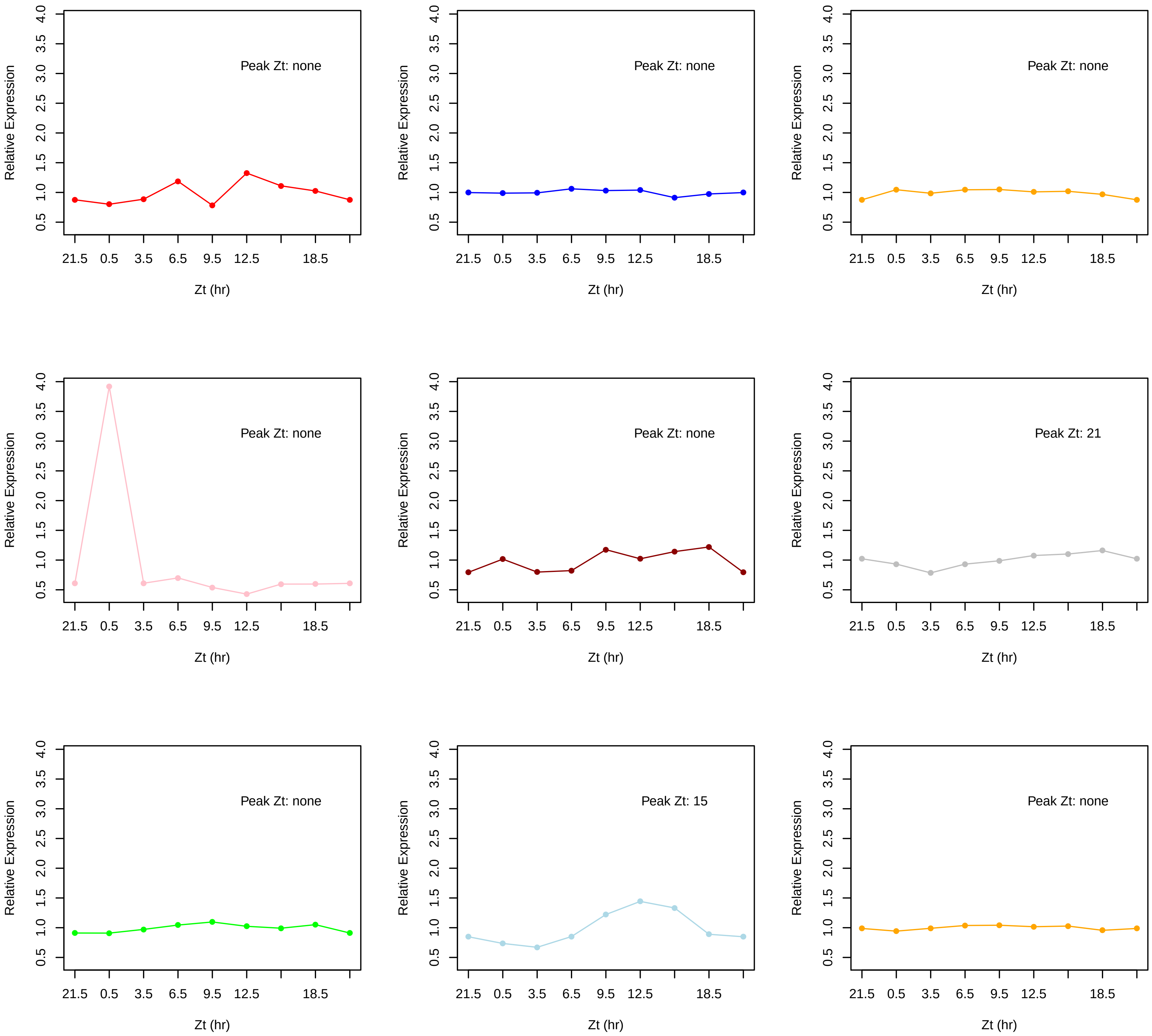

Singleton: ENSXMAG00000015357

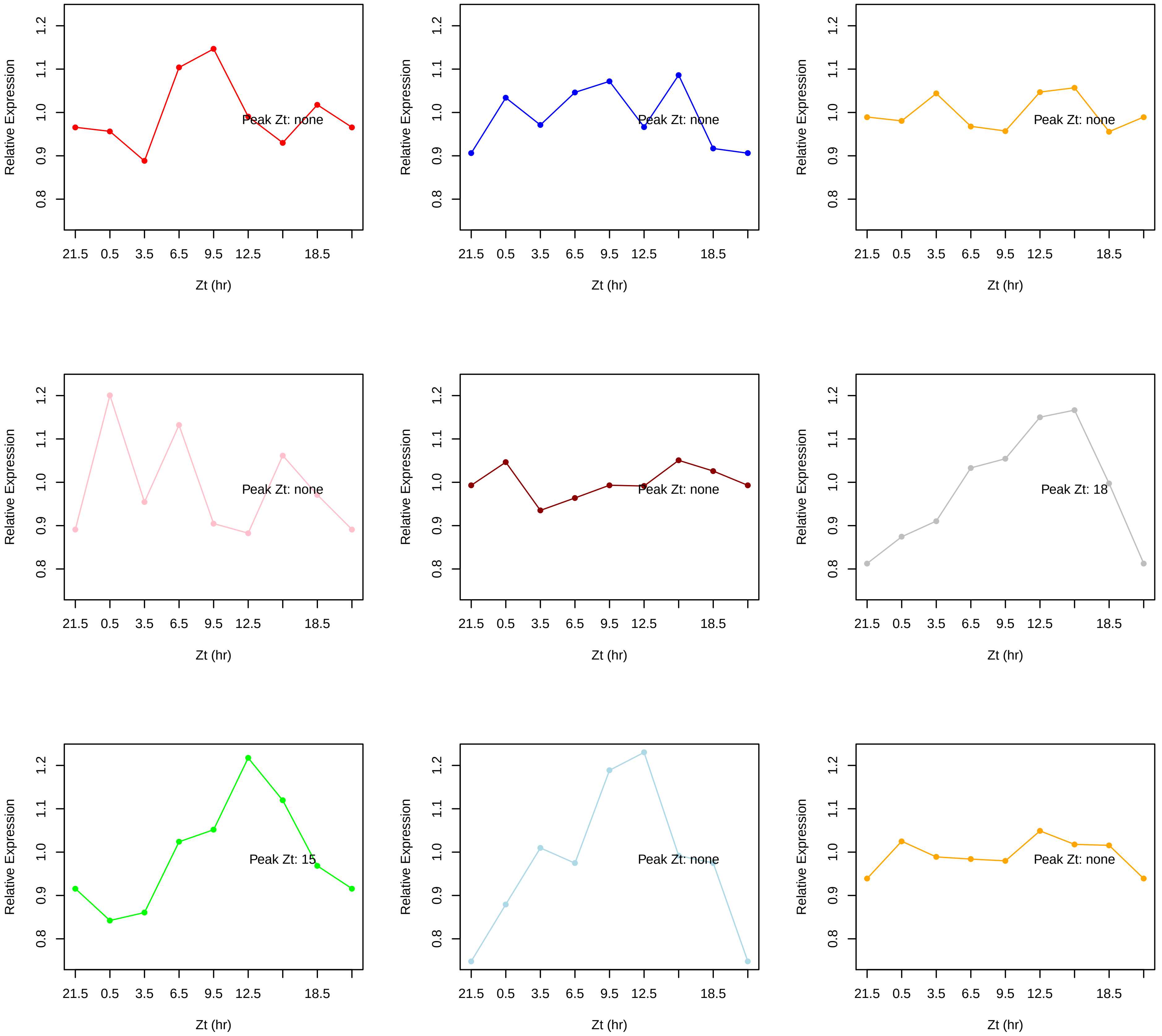

Singeleton: ENSXMAG00000023092

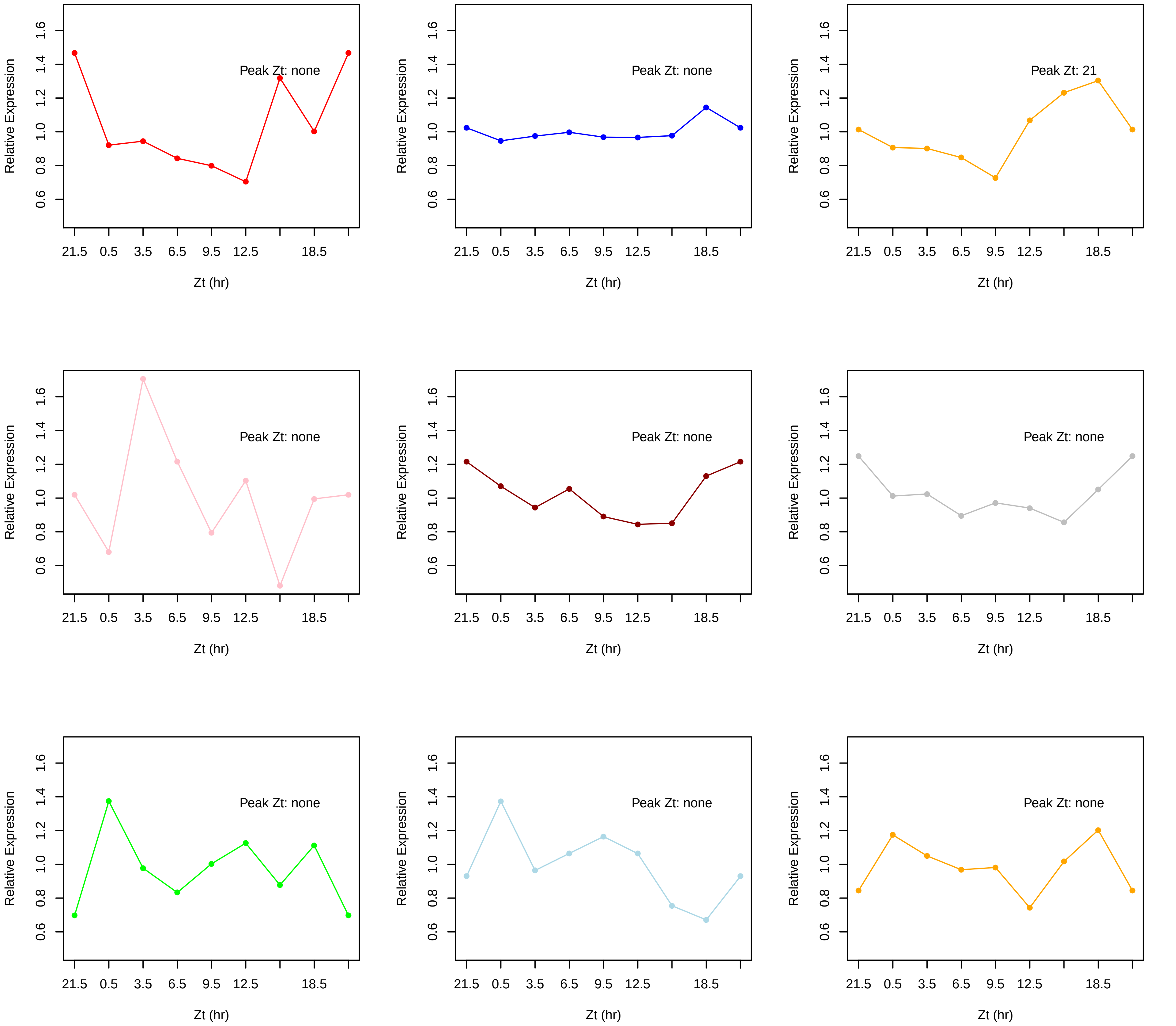

Singeleton: ENSXMAG00000023660 cdv3

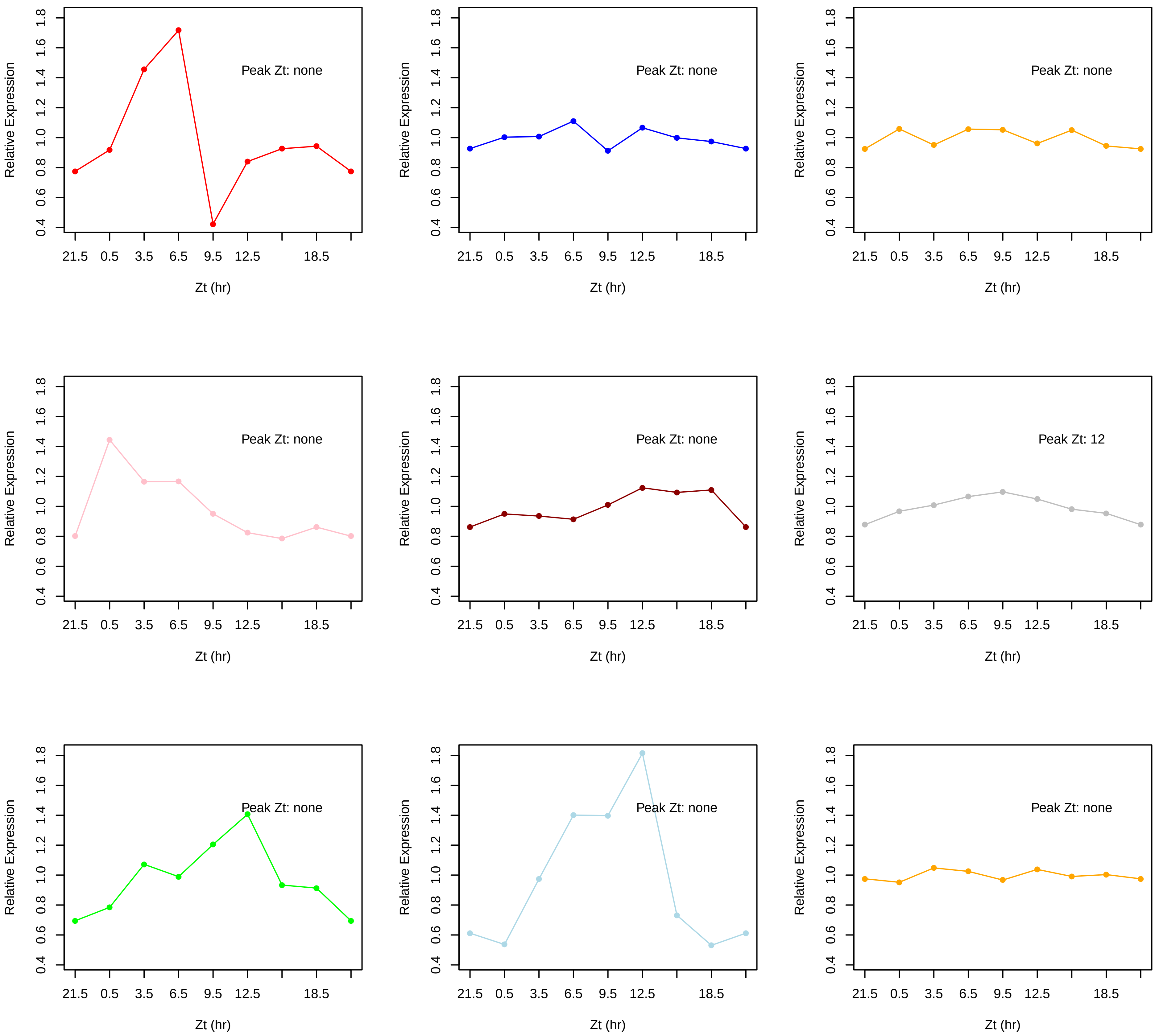

Singeleton: ENSXMAG00000005307 psmc6

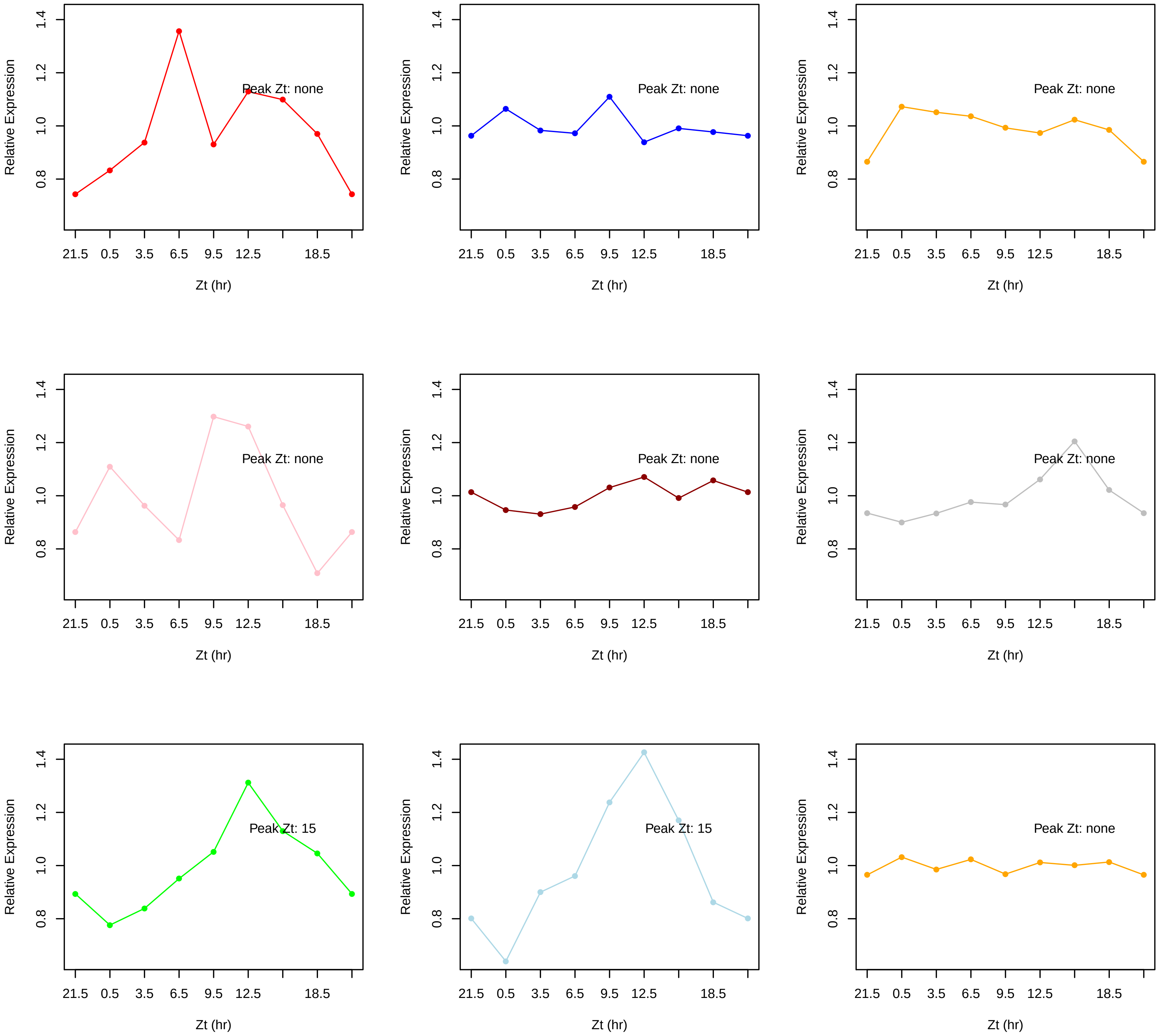

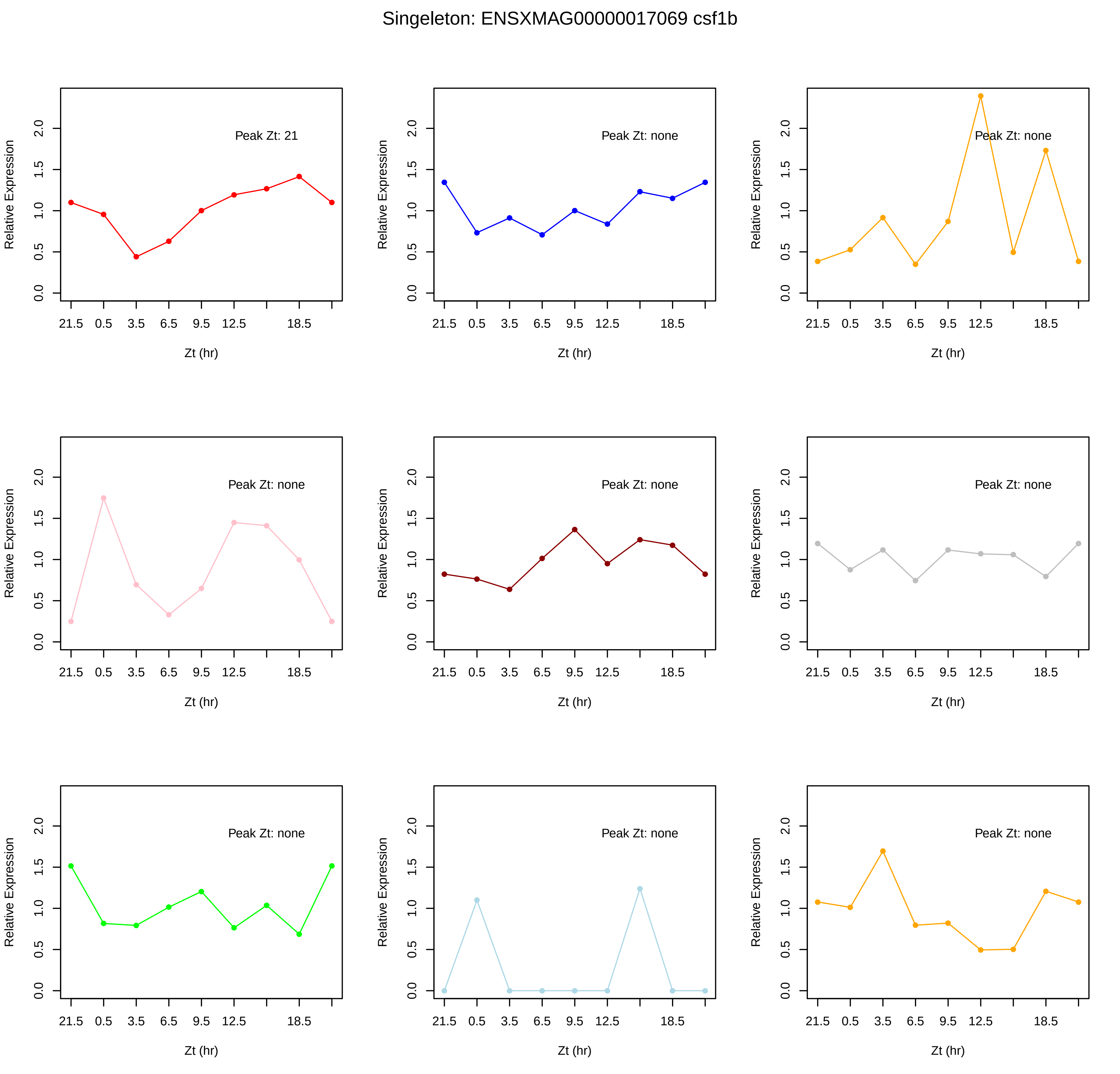
Singleton: ENSXMAG00000017069 csf1b
Peak Zt: 21
Peak Zt: none
Peak Zt: none
Peak Zt: none
Peak Zt: none
Peak Zt: none
Peak Zt: none
Peak Zt: none
Peak Zt: none
Relative Expression
Zt (hr)
0.0 0.5 1.0 1.5 2.0
21.5 0.5 3.5 6.5 9.5 12.5 18.5

Singeleton: ENSXMAG00000013640

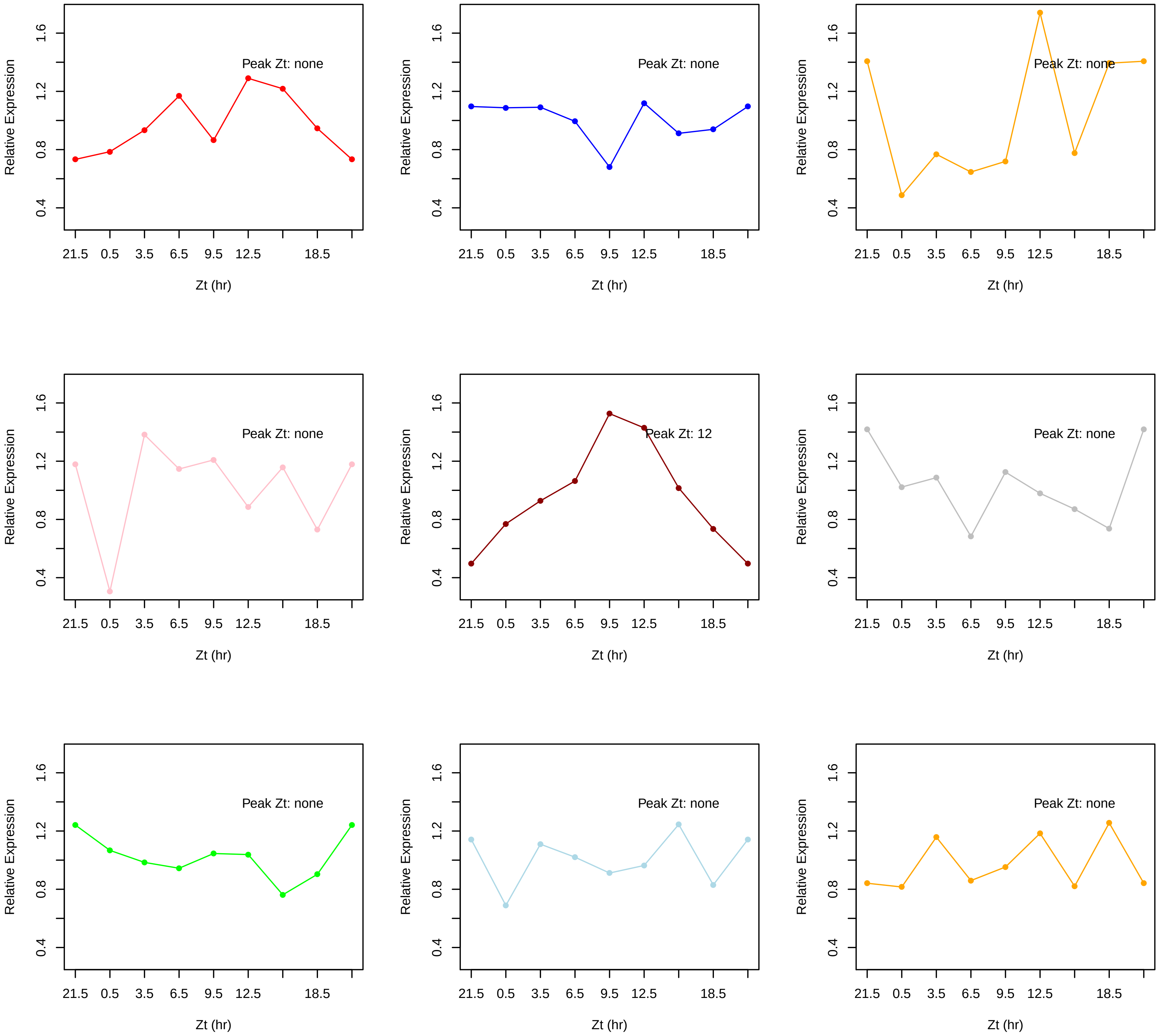

Singeleton: ENSXMAG00000011012

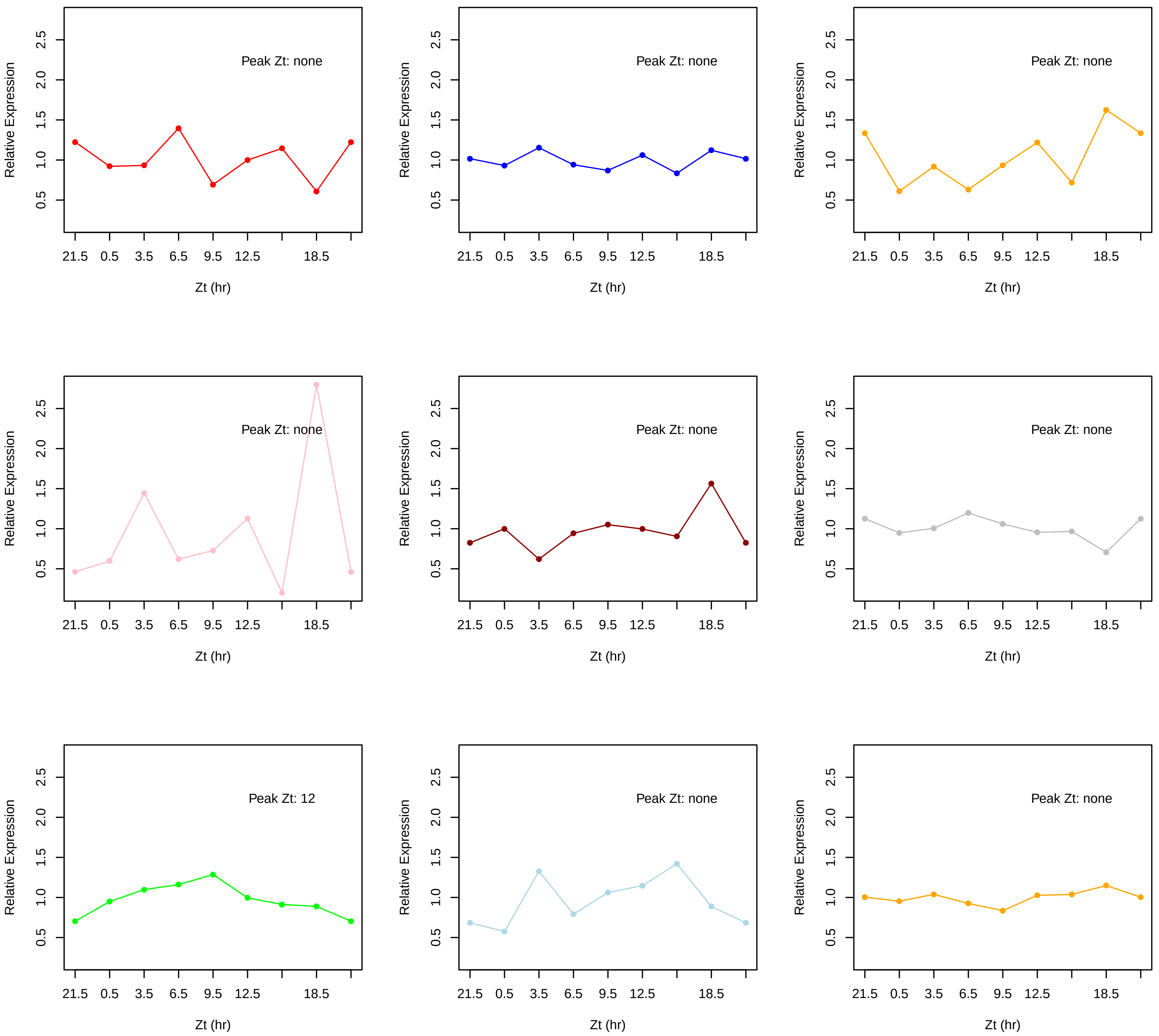

Singleton: ENSXMAG00000004114

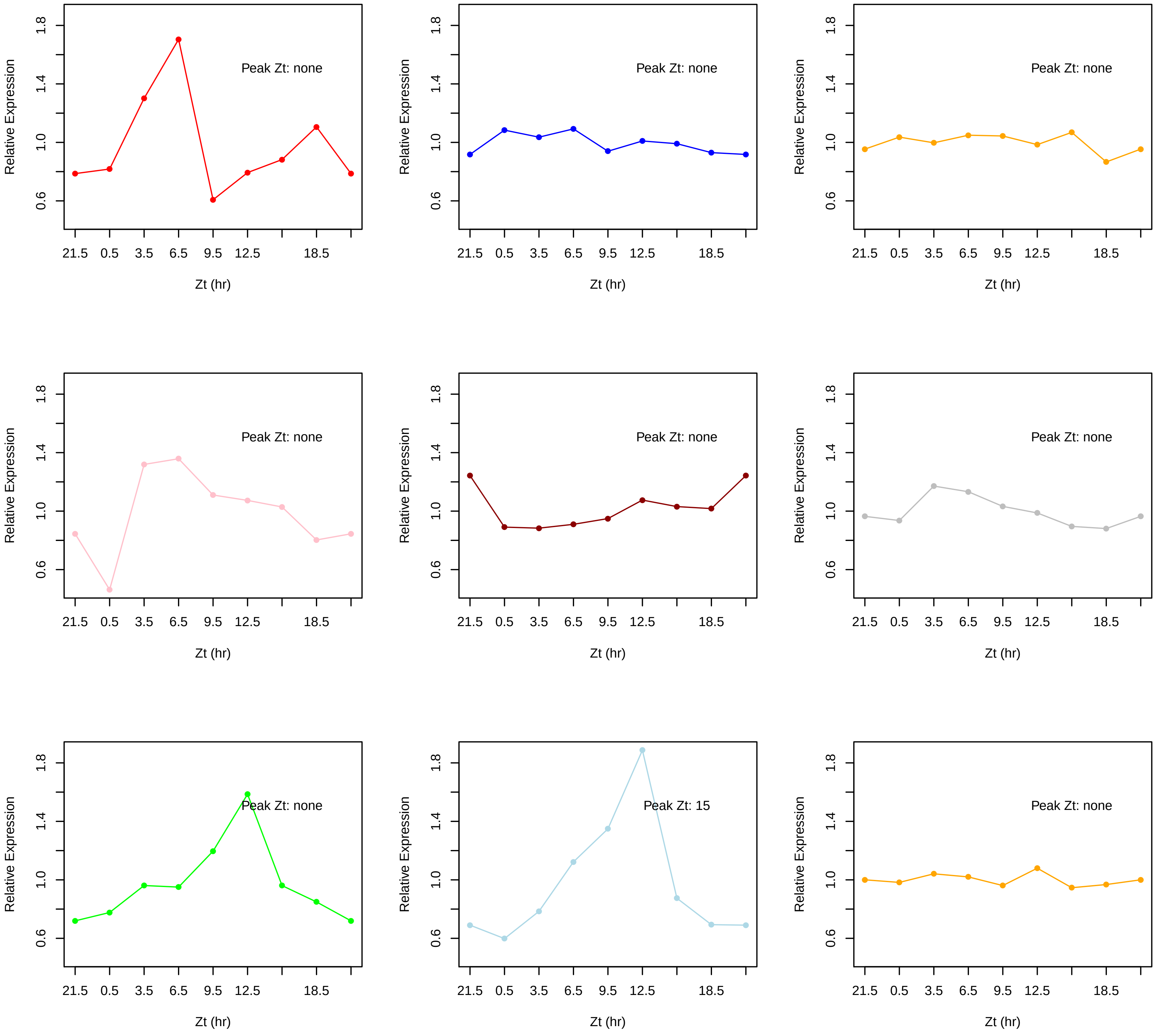

Singeleton: ENSXMAG00000024117 gnpnat1

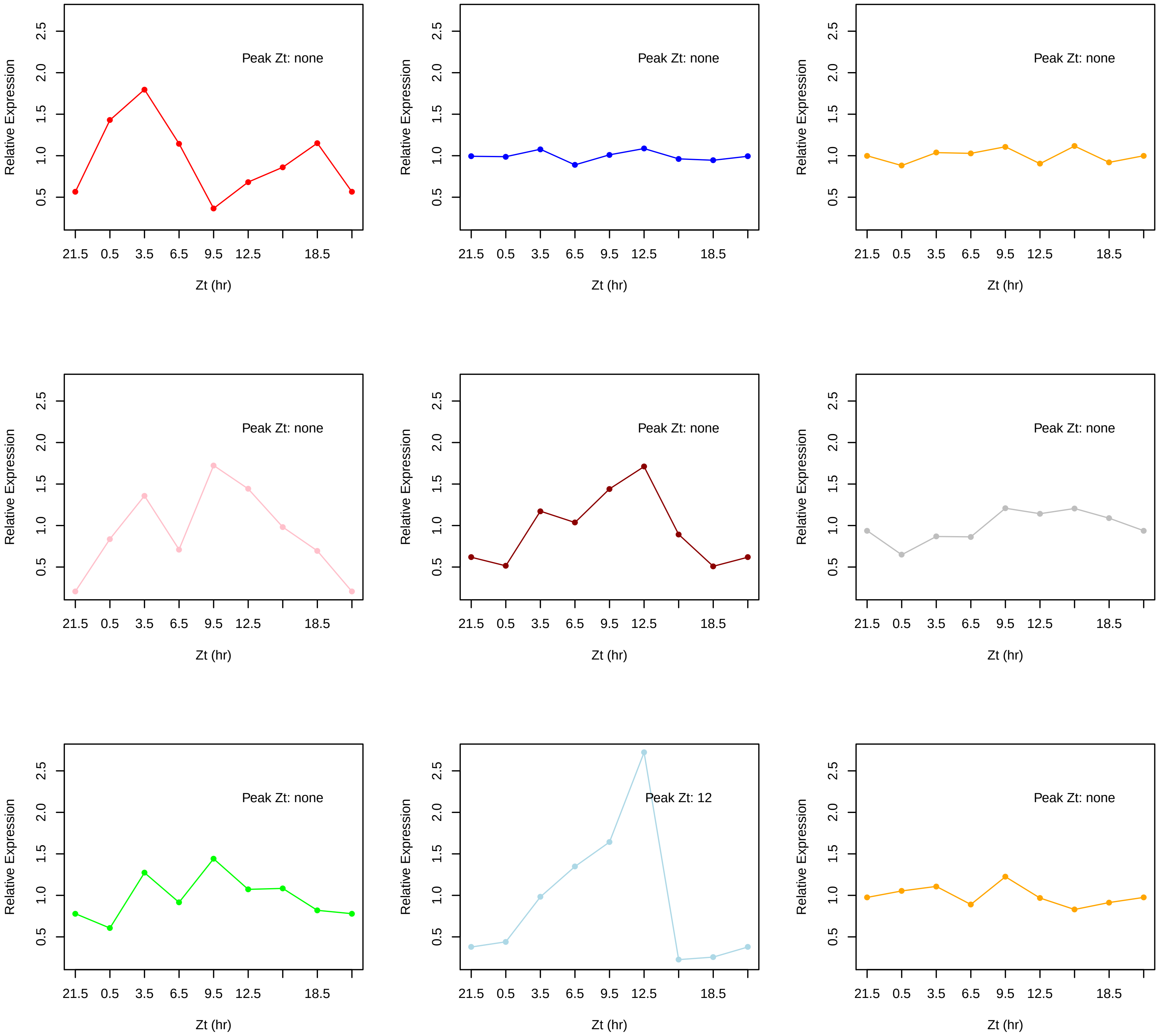

Singleton: ENSXMAG00000001579 cyb5a

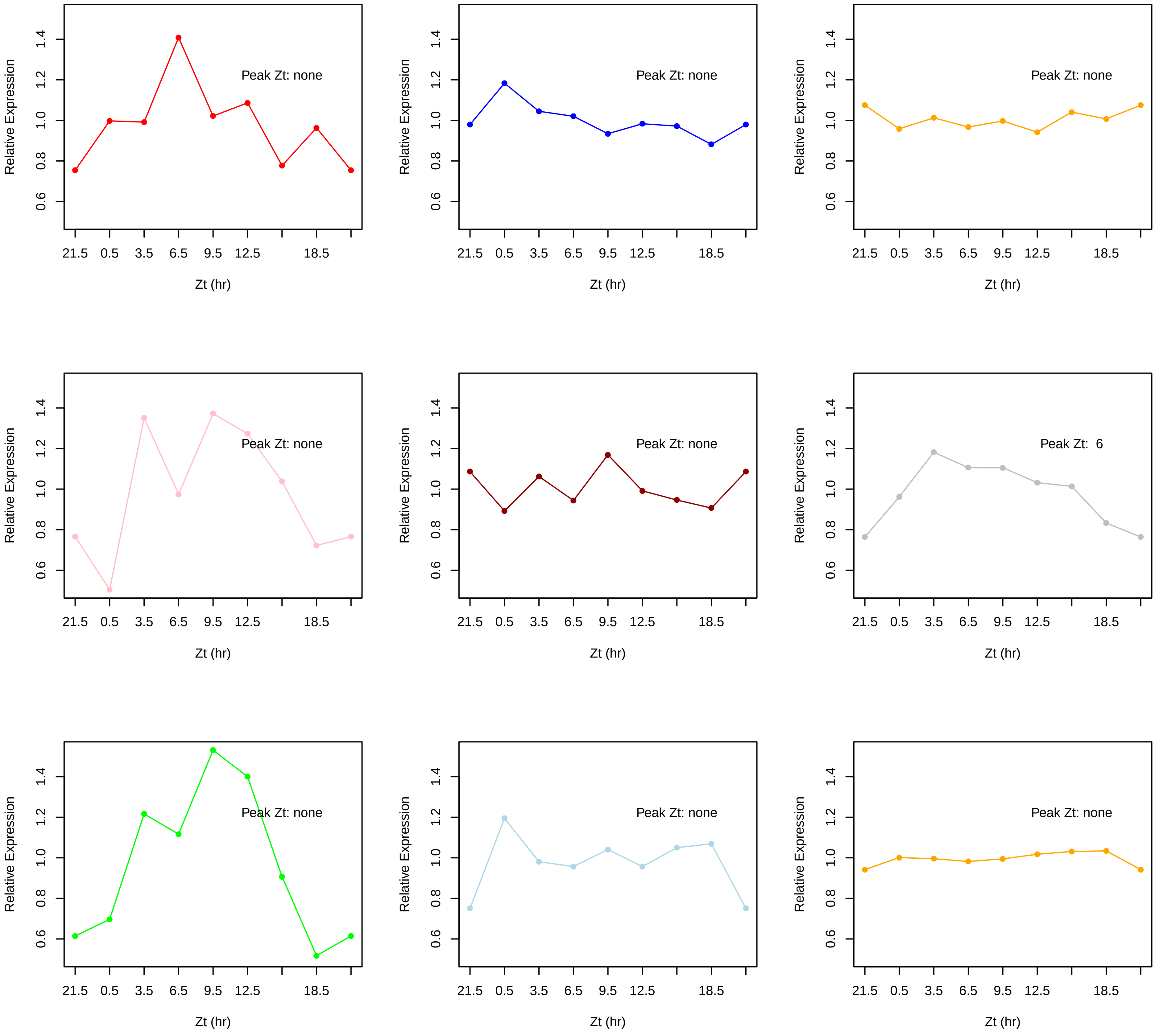

Singeleton: ENSXMAG00000024342 nrap

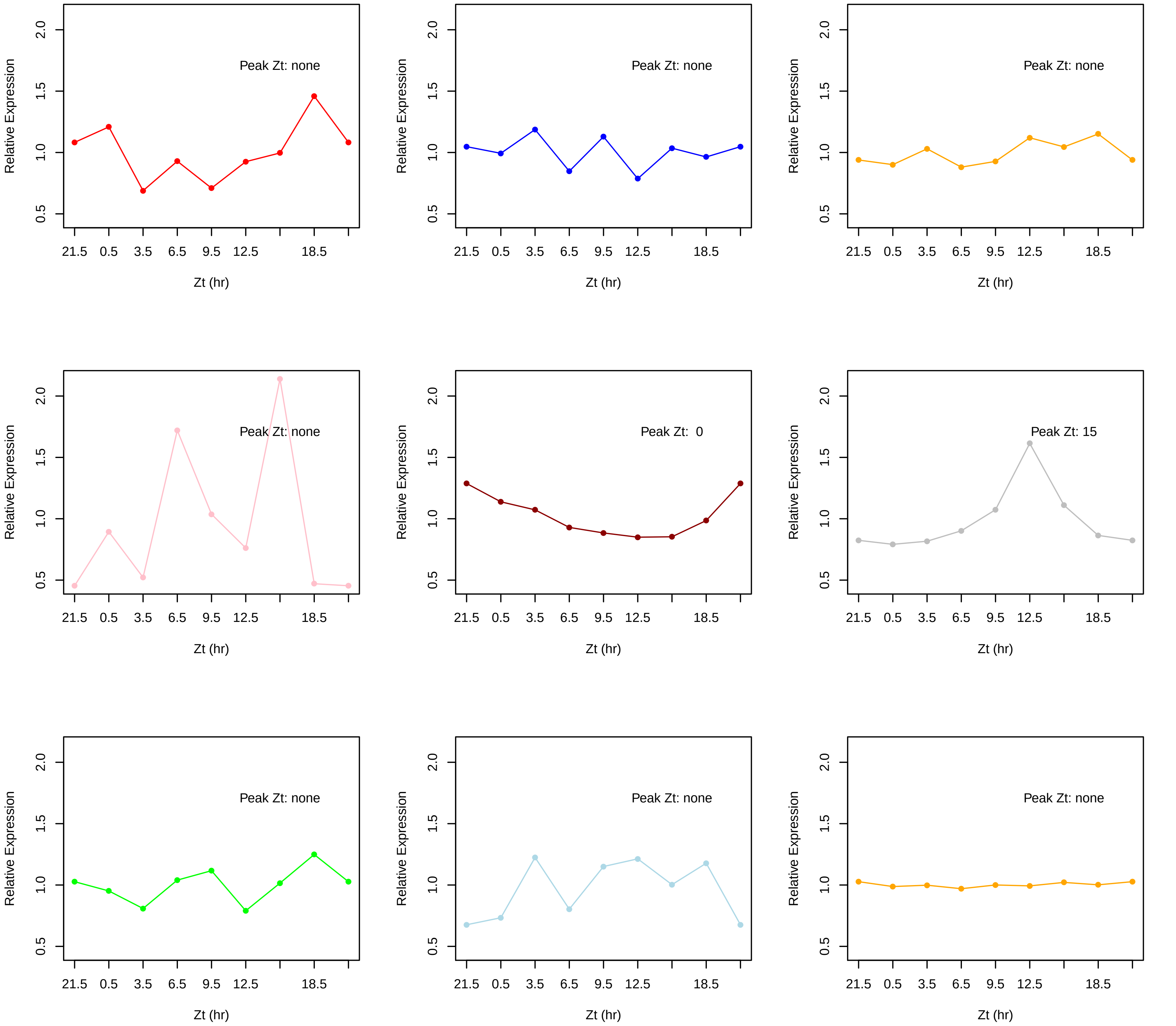

Singeleton: ENSXMAG00000009846 gch1

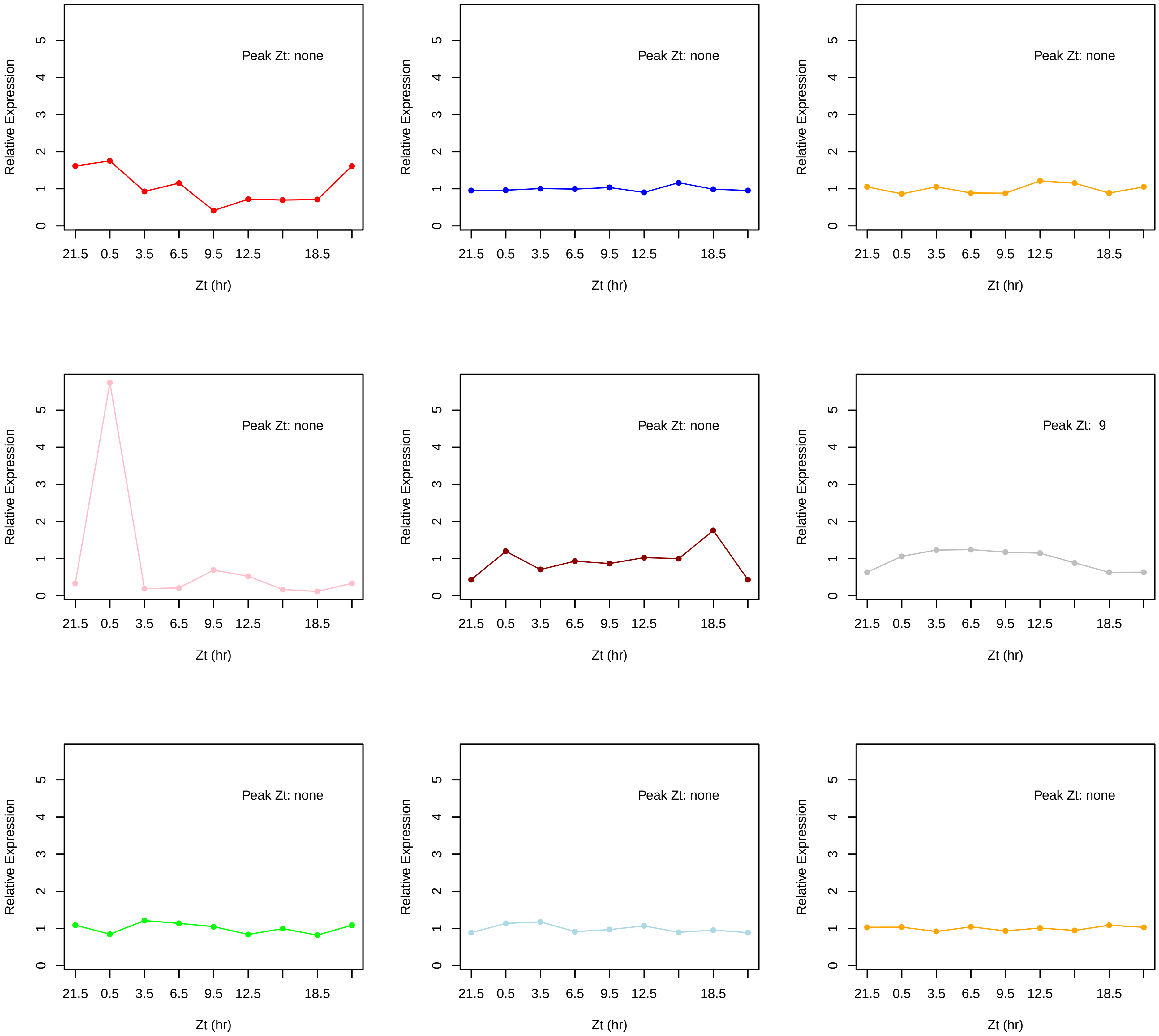

Singeleton: ENSXMAG00000025556 fam69c

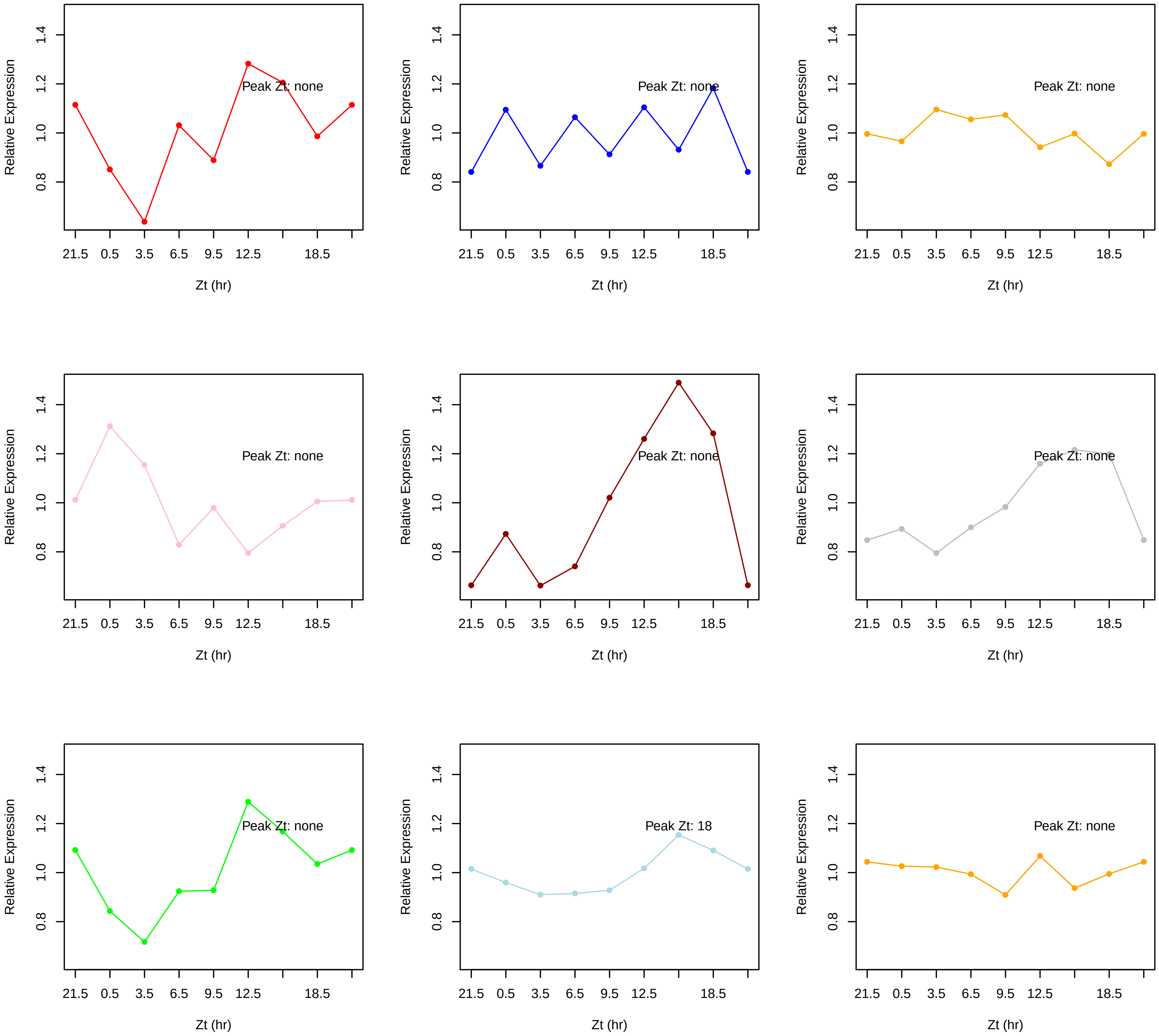

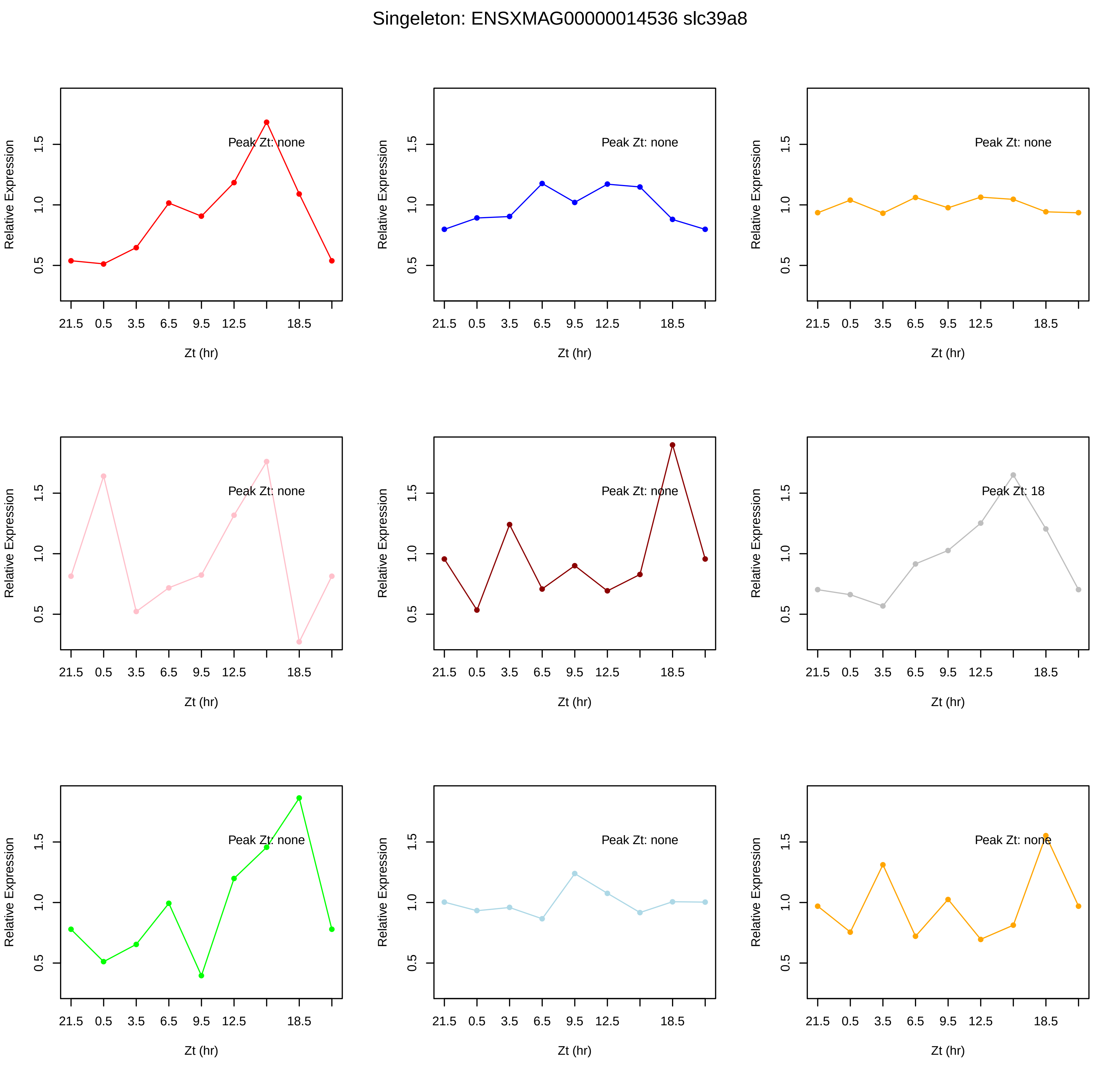

Singeleton: ENSXMAG00000014536 slc39a8
Peak Zt: none
Peak Zt: none
Peak Zt: none
Peak Zt: none
Peak Zt: none
Peak Zt: 18
Peak Zt: none
Peak Zt: none
Peak Zt: none
Relative Expression
Zt (hr)
21.5
0.5
3.5
6.5
9.5
12.5
18.5

Singeleton: ENSXMAG00000012563 helq

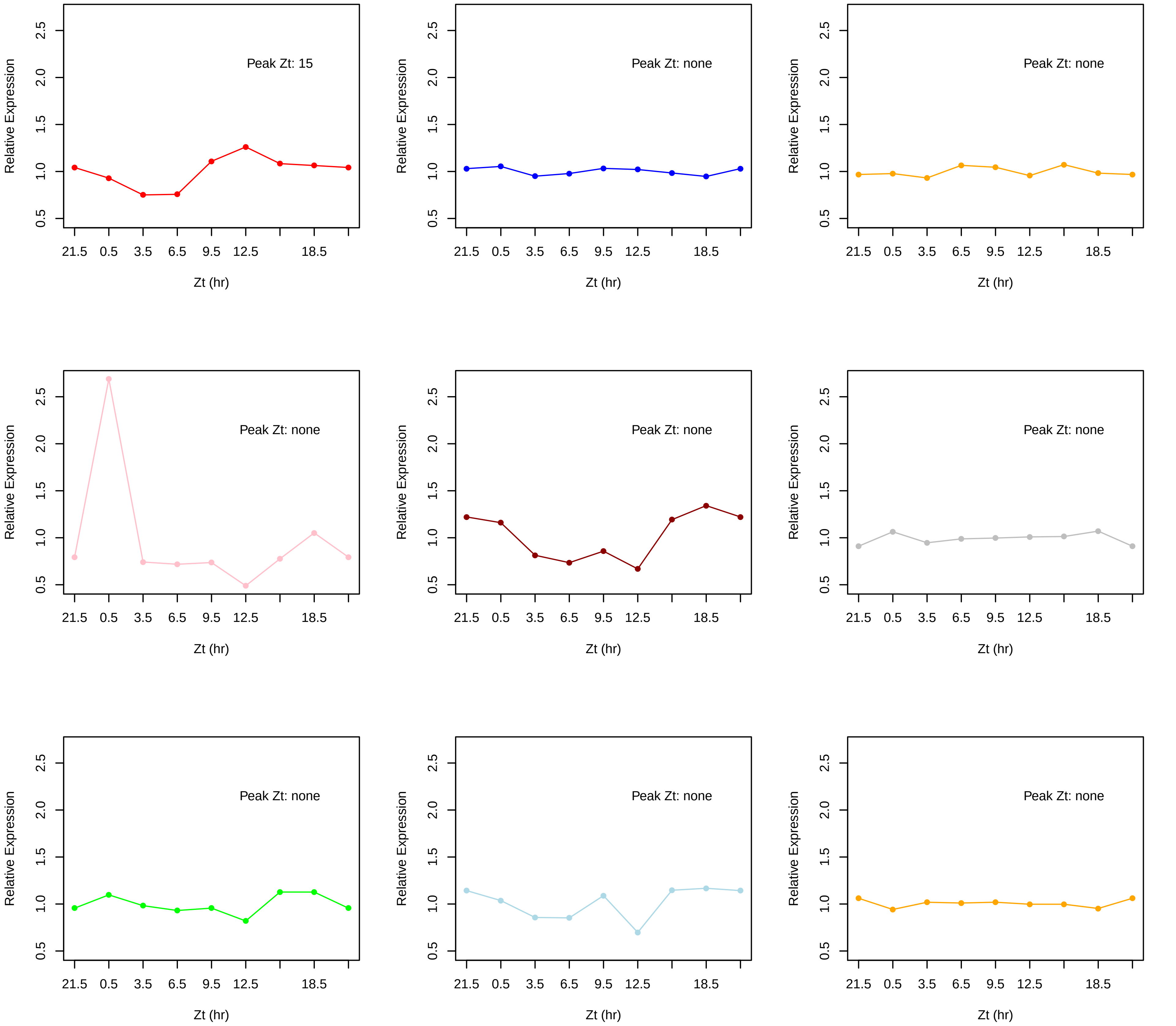

Singeleton: ENSXMAG00000012532 pik3c2b

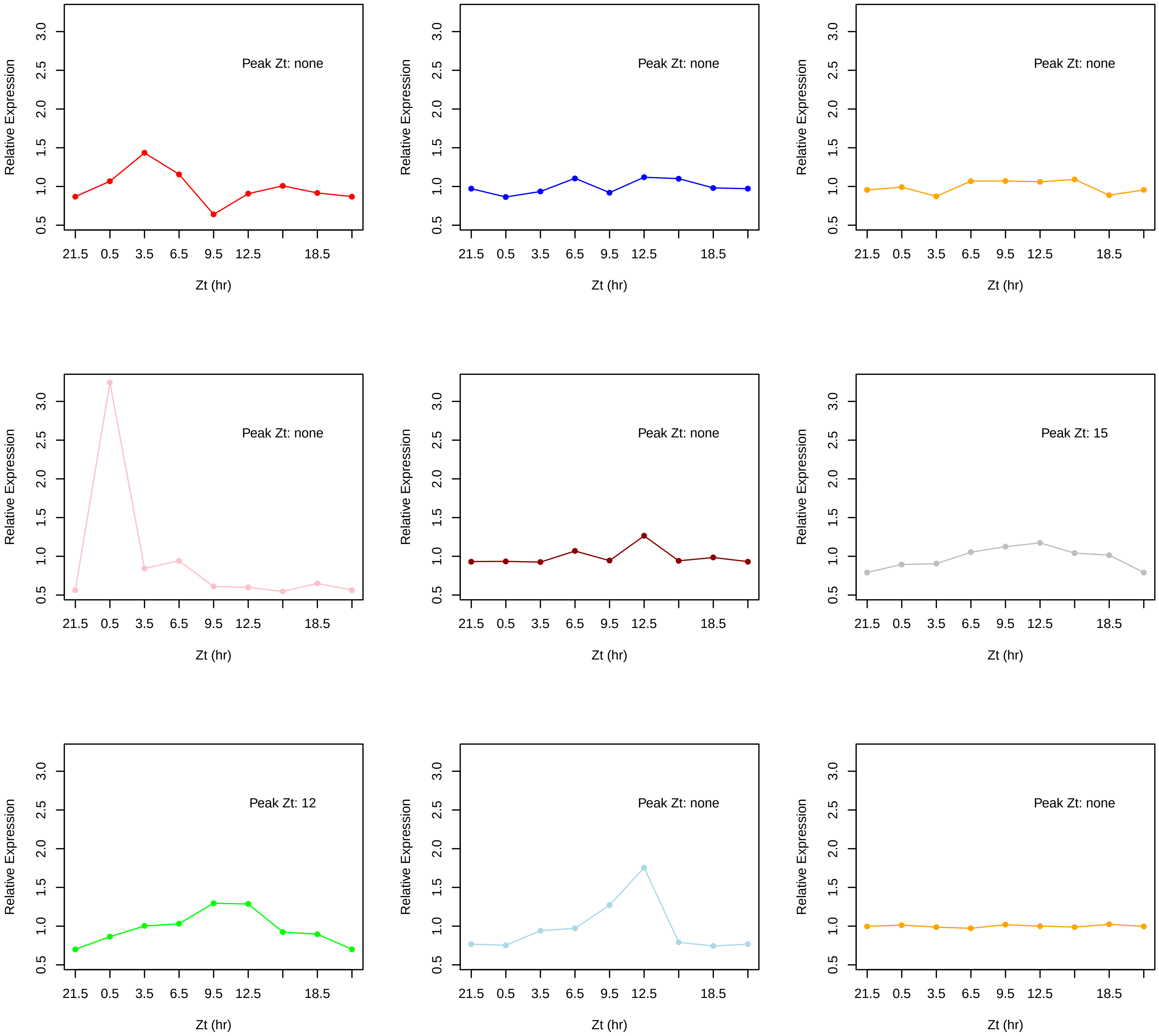

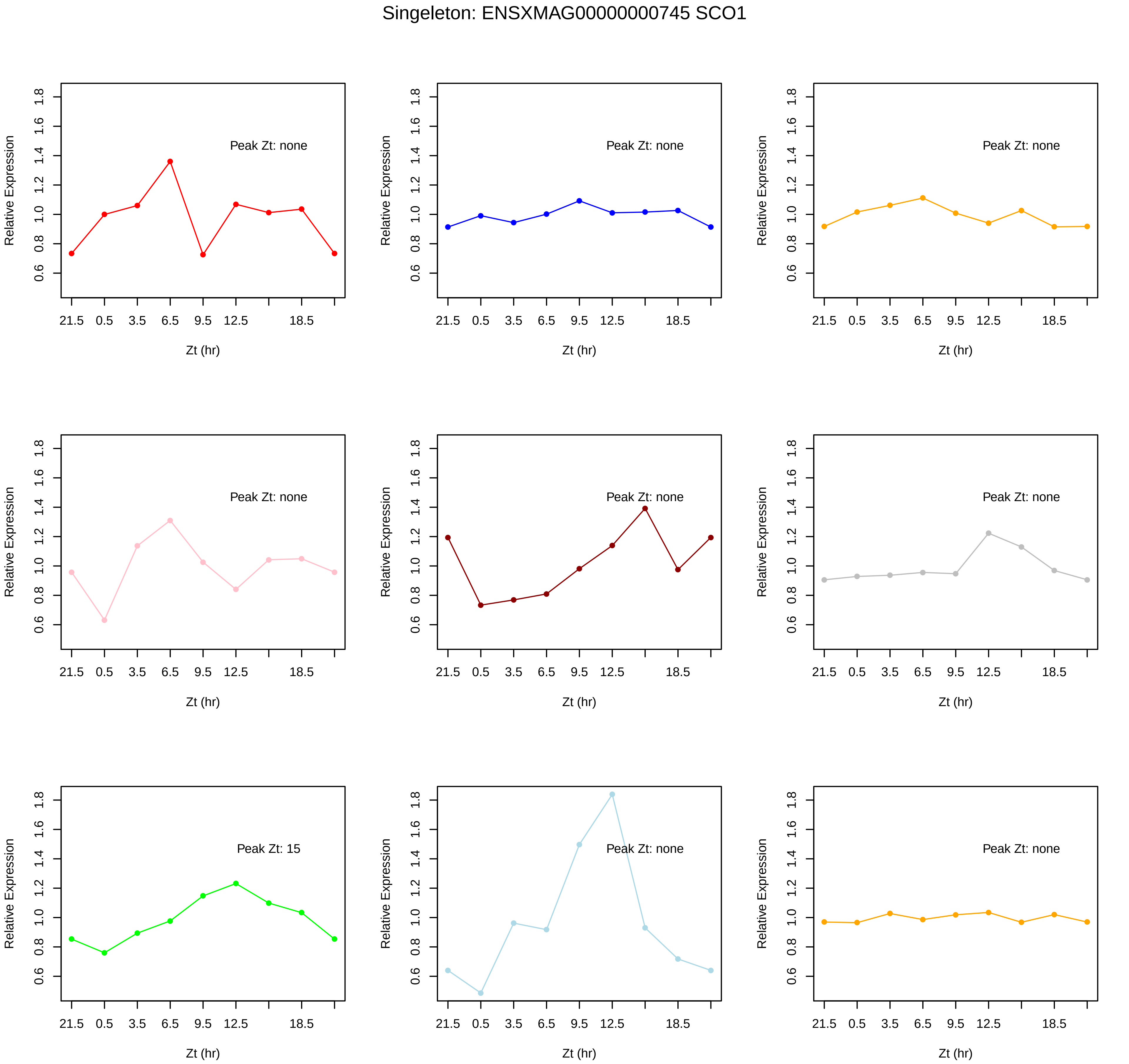

Singleton: ENSXMAG00000000745 SCO1
Relative Expression
Zt (hr)
Peak Zt: none
Peak Zt: none
Peak Zt: none
Peak Zt: none
Peak Zt: none
Peak Zt: none
Peak Zt: 15
Peak Zt: none
Peak Zt: none
0.6
0.8
1.0
1.2
1.4
1.6
1.8
21.5
0.5
3.5
6.5
9.5
12.5
18.5

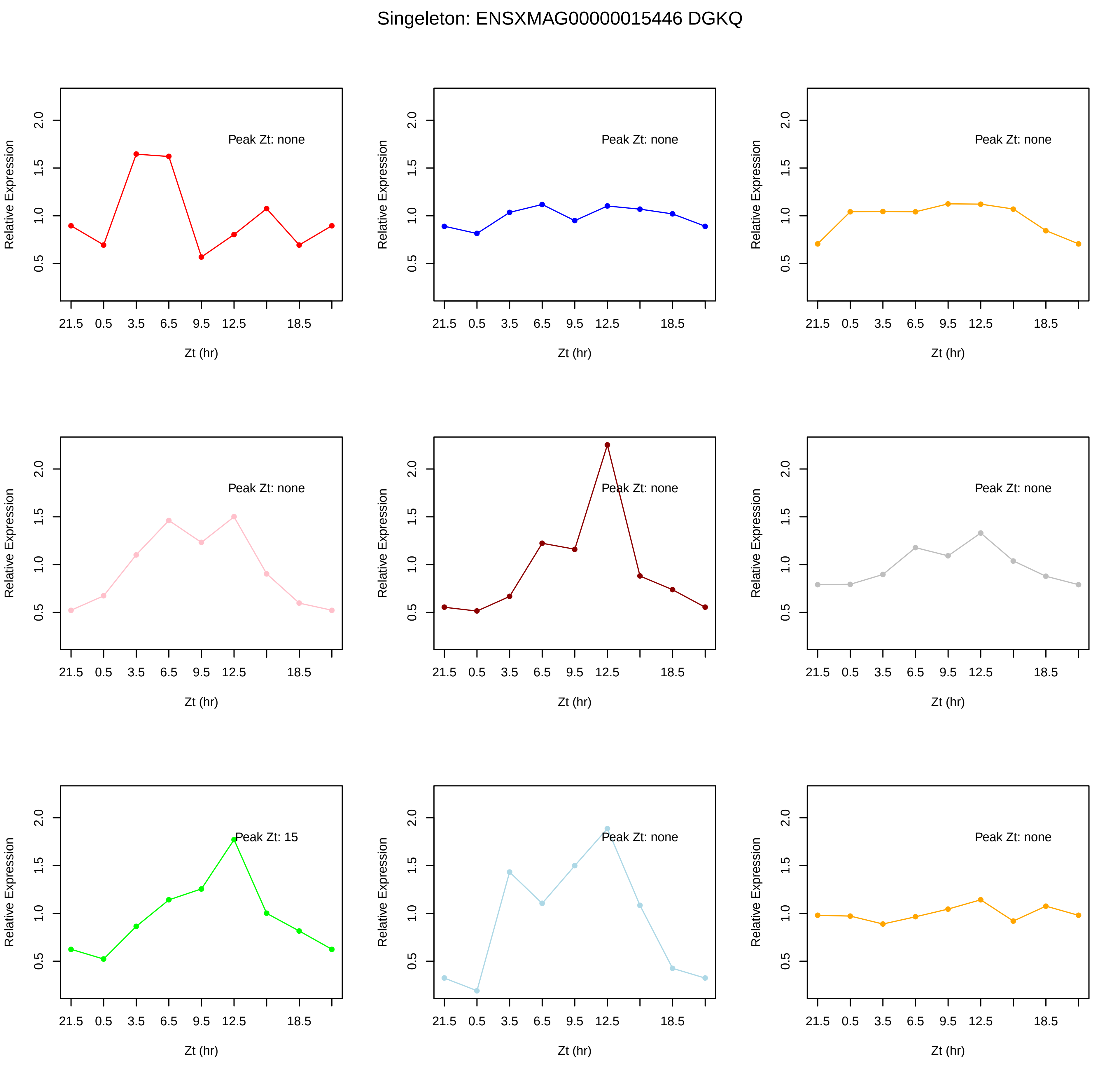

Singeleton: ENSXMAG00000015446 DGKQ
Peak Zt: none
Peak Zt: none
Peak Zt: none
Peak Zt: none
Peak Zt: none
Peak Zt: none
Peak Zt: 15
Peak Zt: none
Peak Zt: none
Relative Expression
Zt (hr)
21.5
0.5
3.5
6.5
9.5
12.5
18.5
0.5
1.0
1.5
2.0

Singeleton: ENSXMAG00000026522 adprm

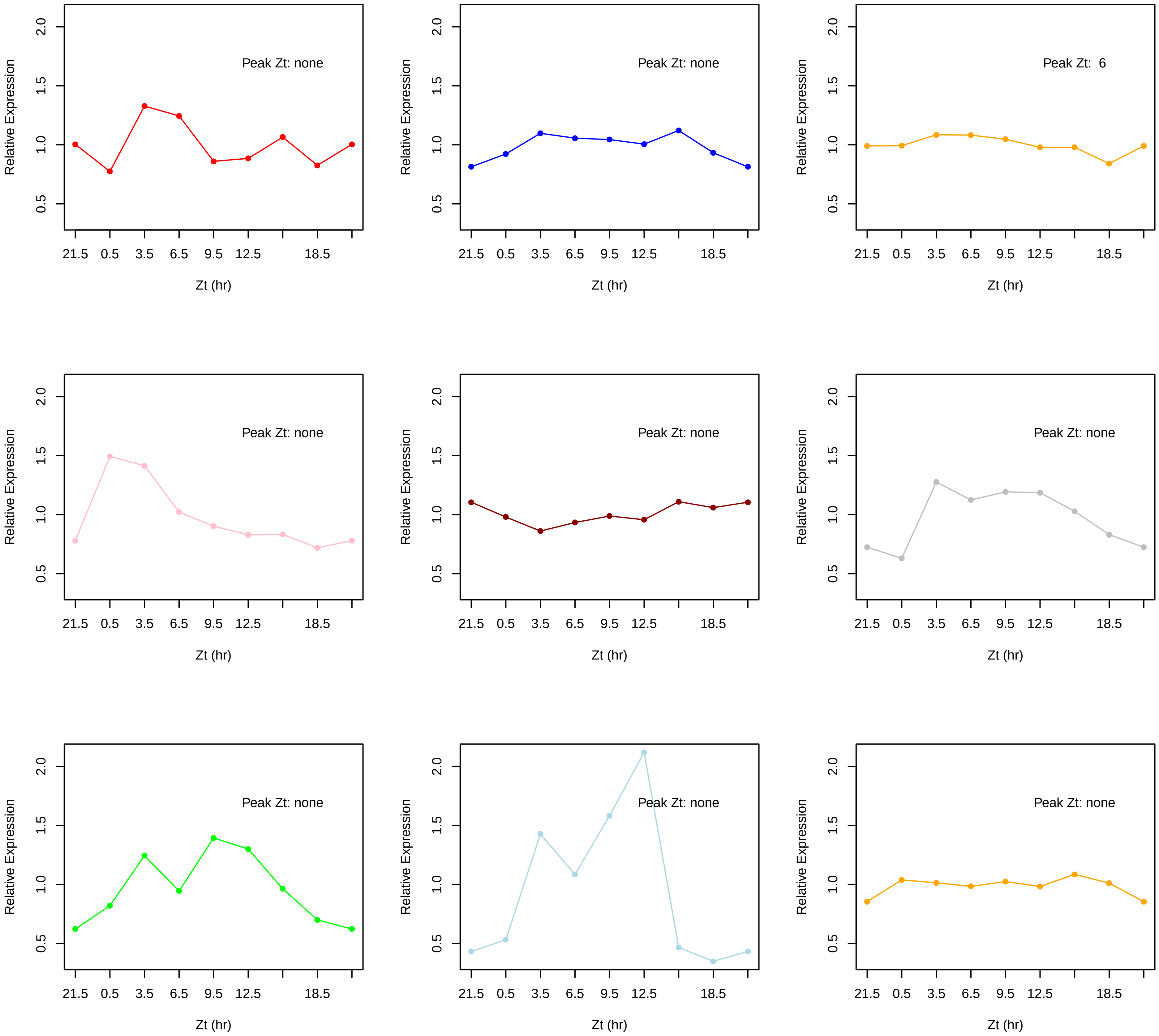

Singleton: ENSXMAG00000014624 wdr32

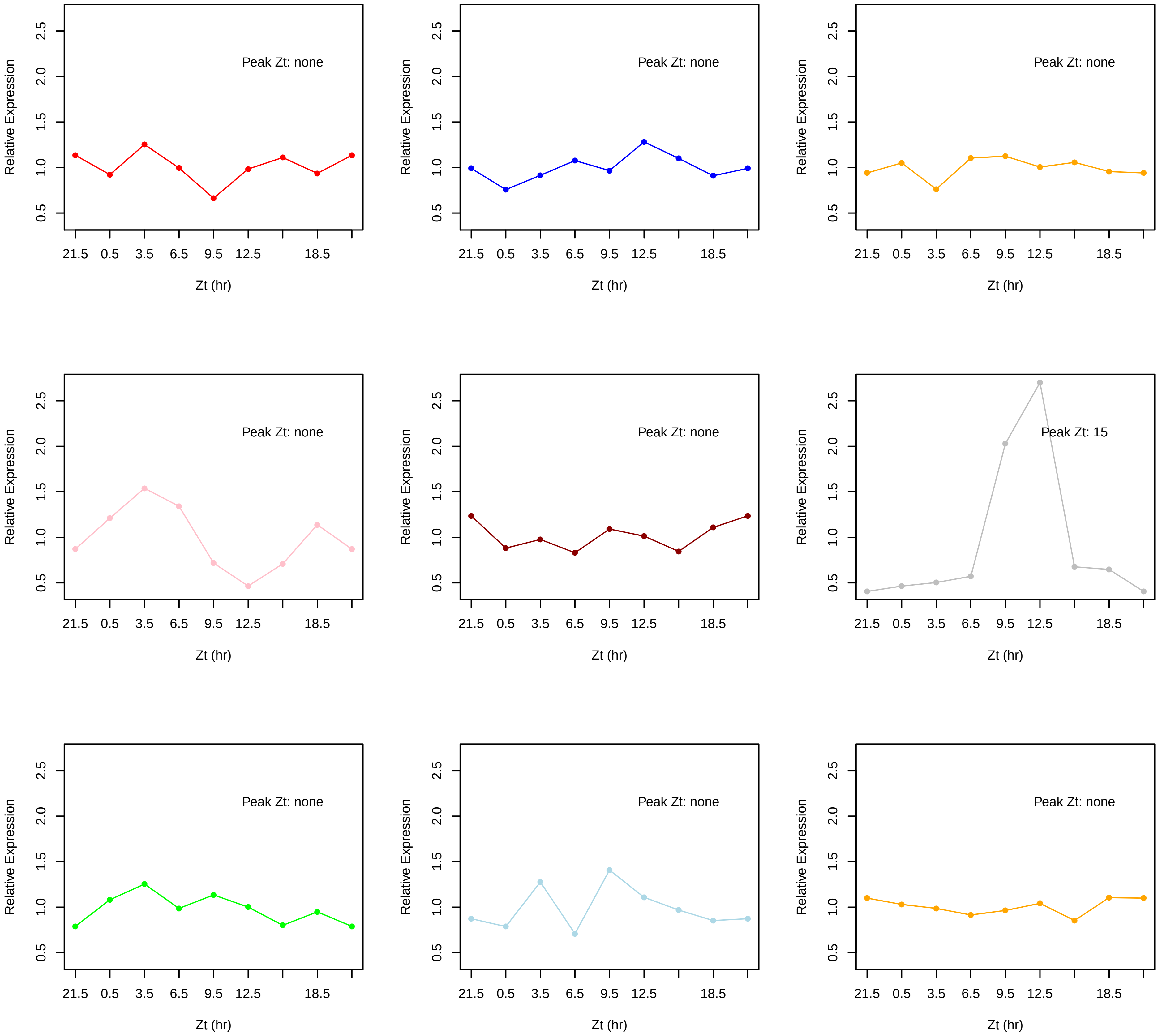

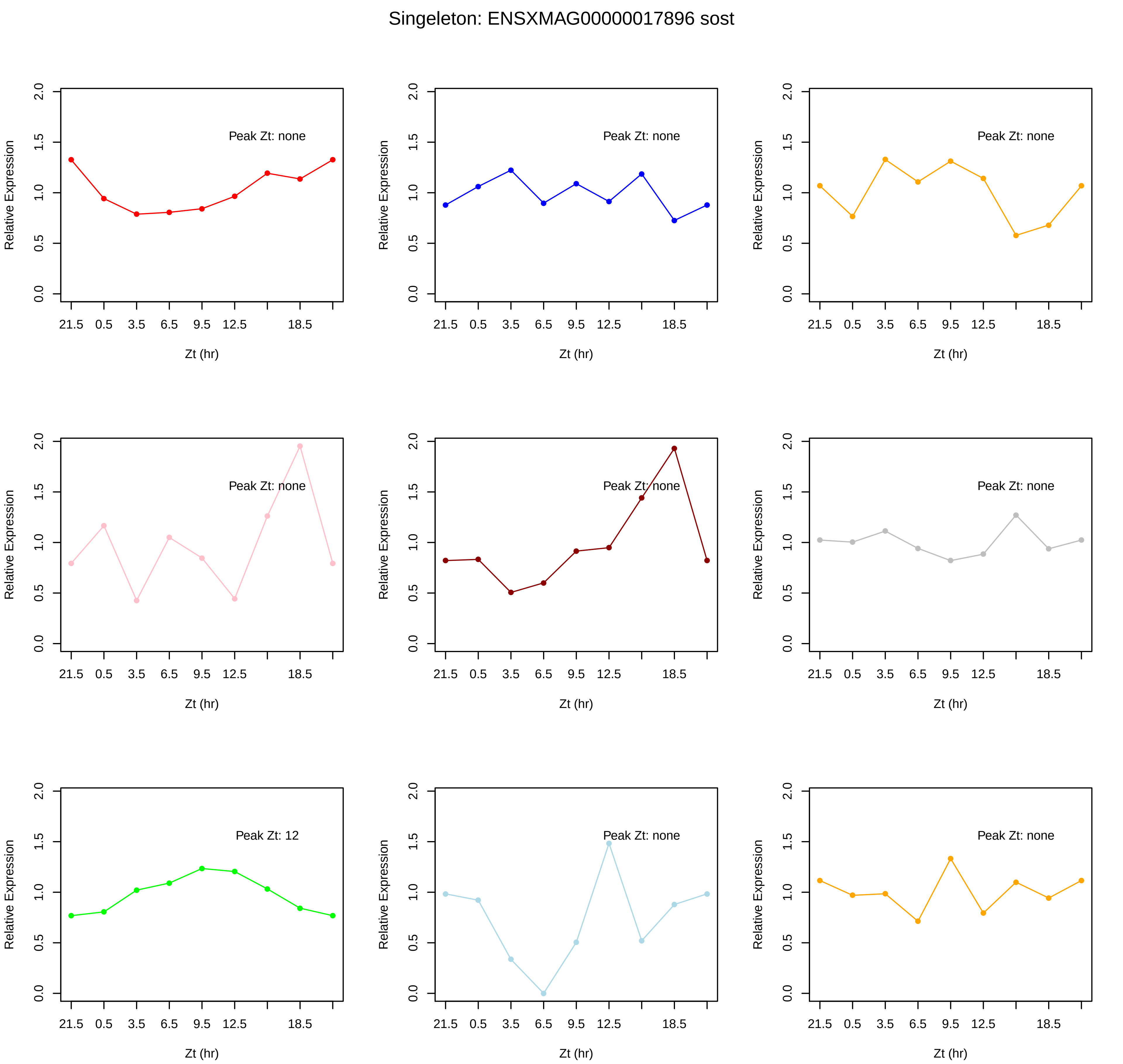
Singeleton: ENSXMAG00000017896 sost
Peak Zt: none
Peak Zt: none
Peak Zt: none
Peak Zt: none
Peak Zt: none
Peak Zt: none
Peak Zt: 12
Peak Zt: none
Peak Zt: none
Relative Expression
Zt (hr)

Singeleton: ENSXMAG00000005298 cgrrf1

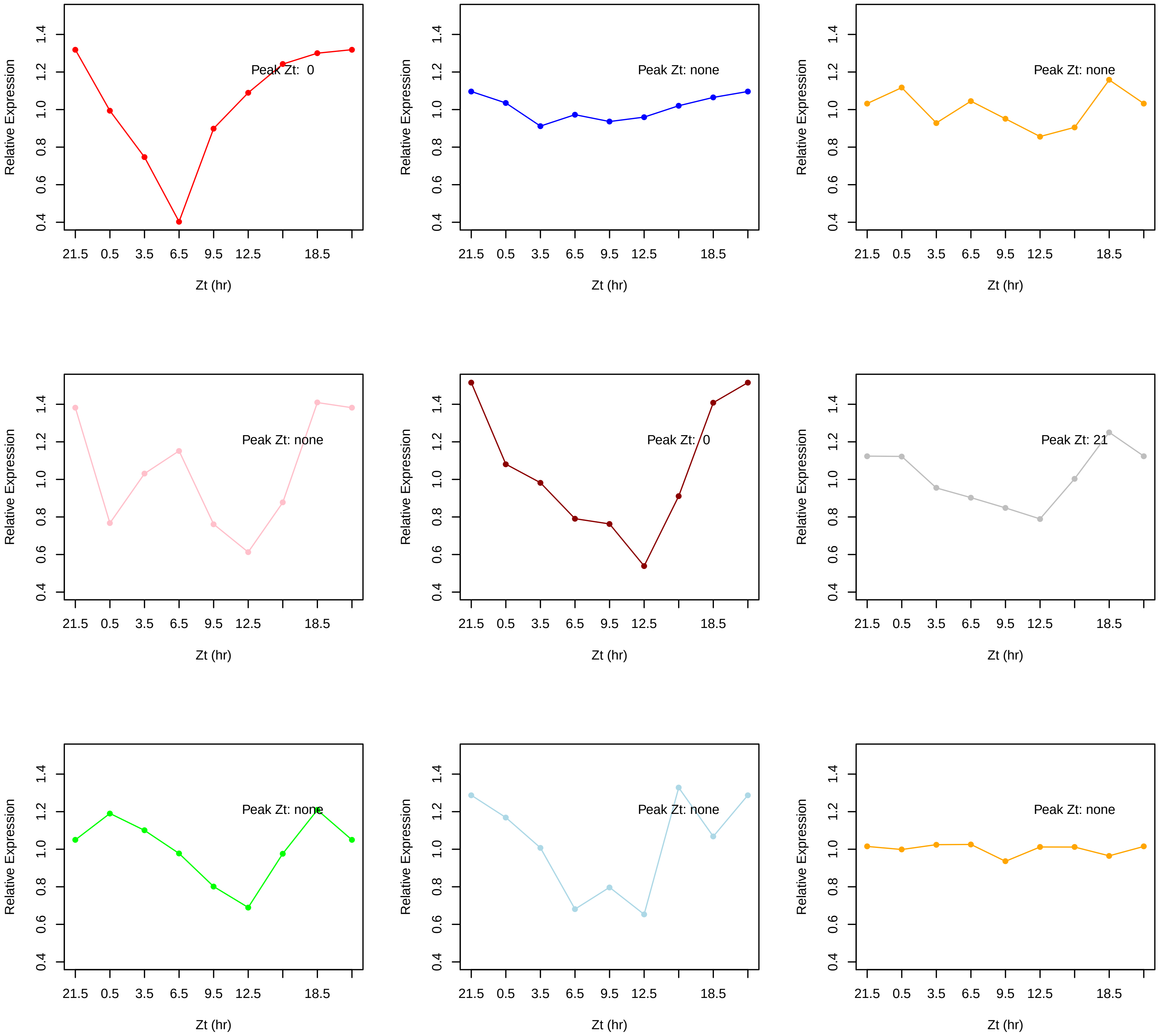

Singleton: ENSXMAG00000021451

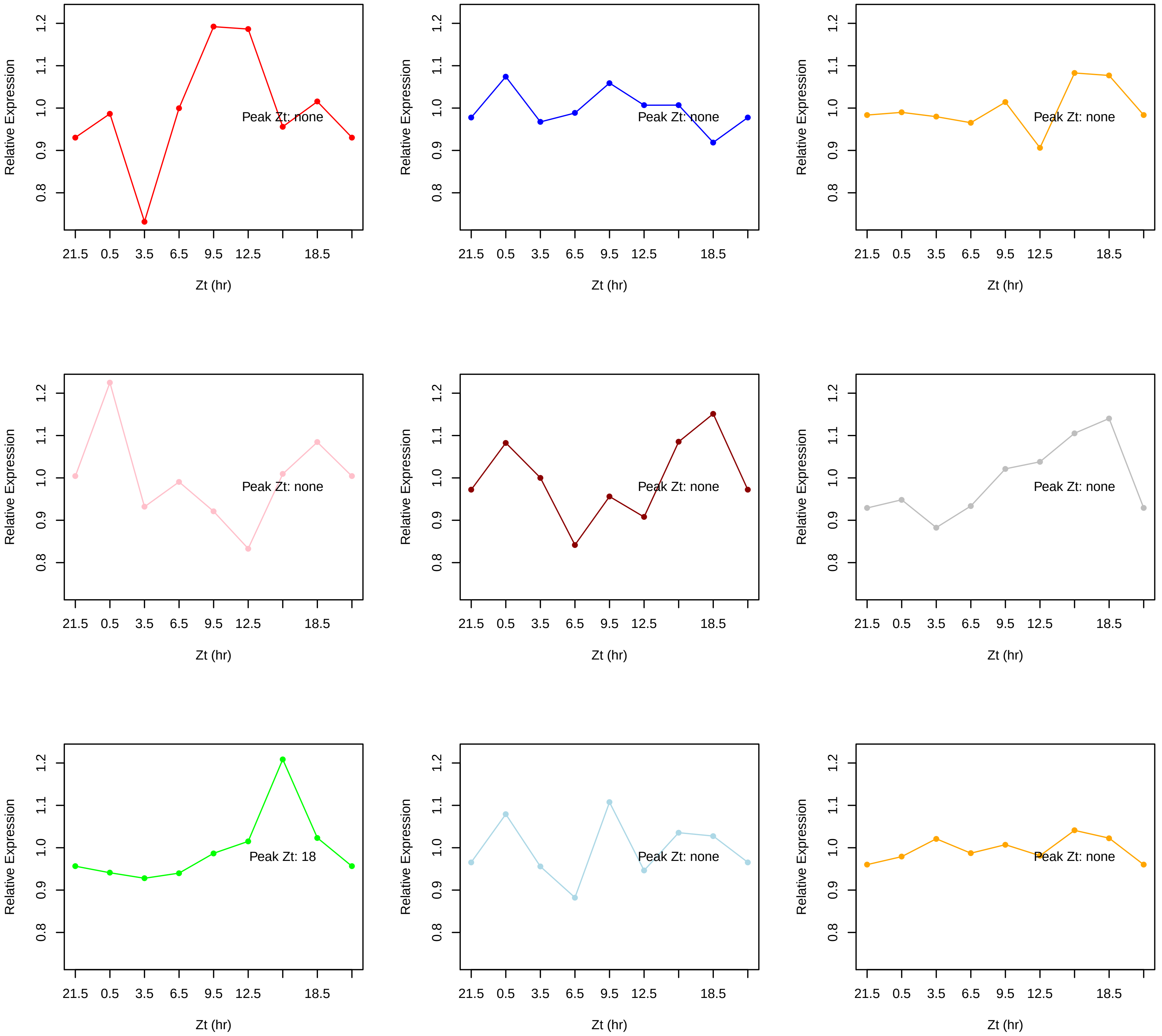

Singeleton: ENSXMAG00000017899 meox1

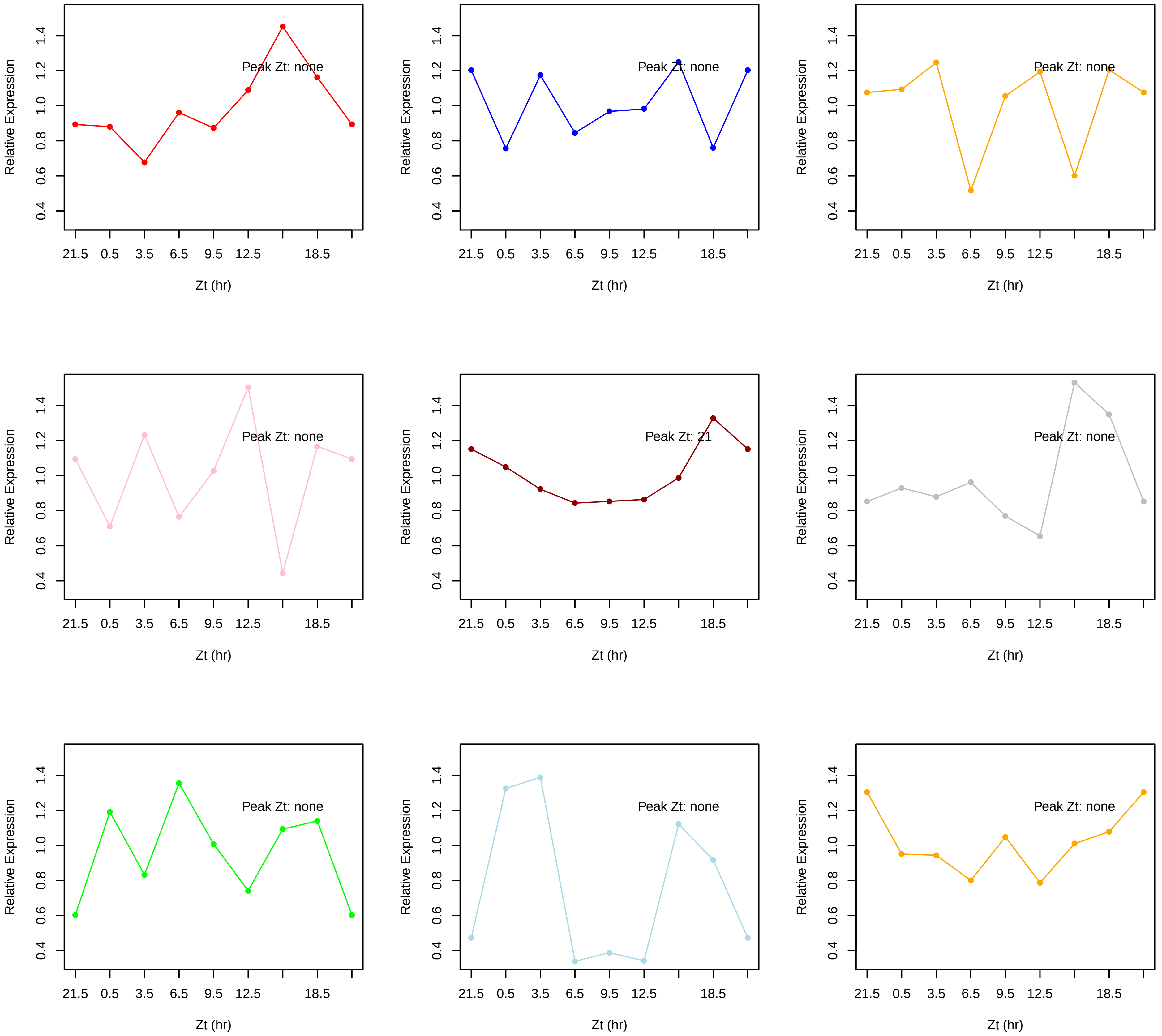

Singeleton: ENSXMAG00000000766 bmp4

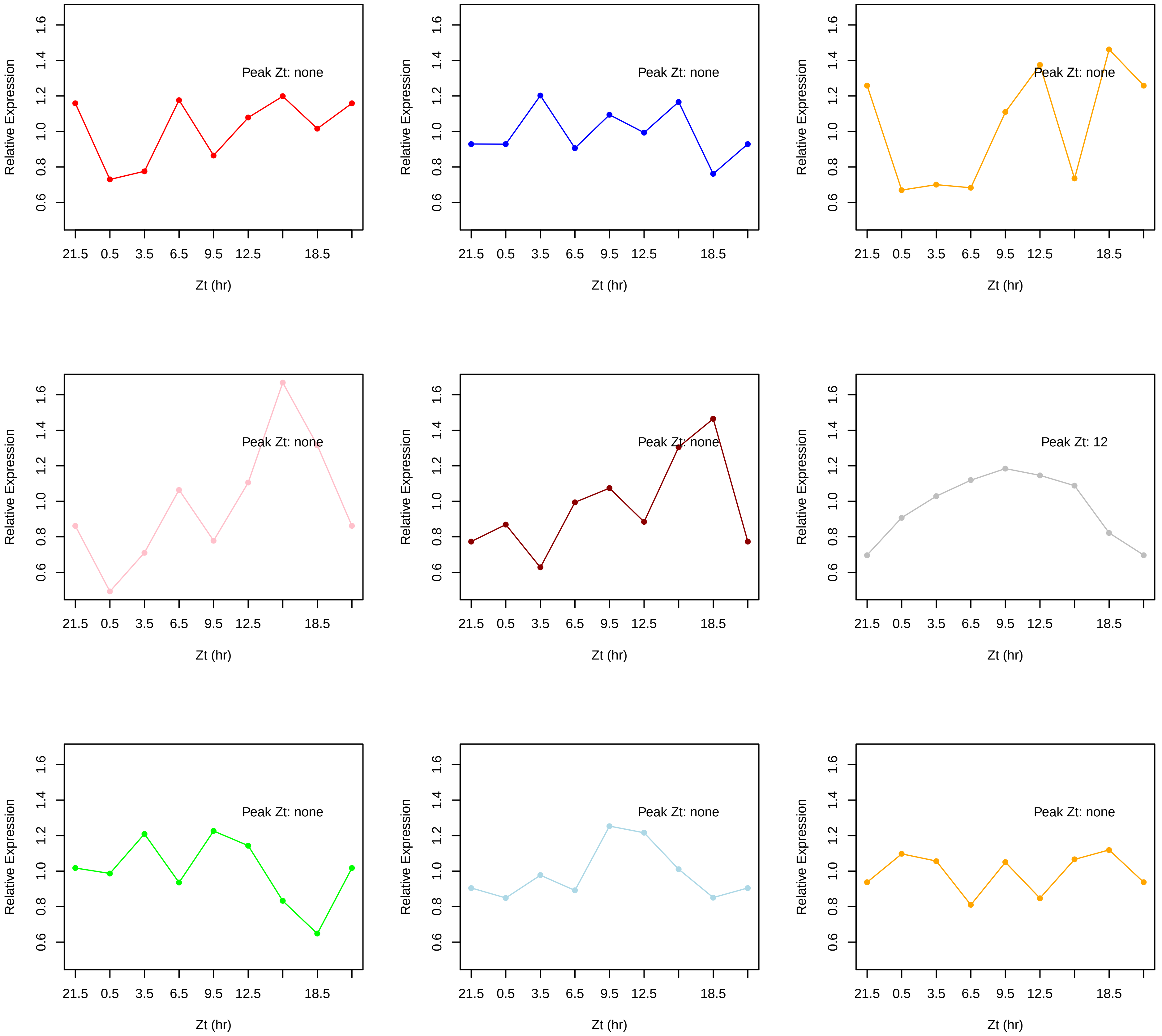

Singeleton: ENSXMAG00000003727 zgc:113307

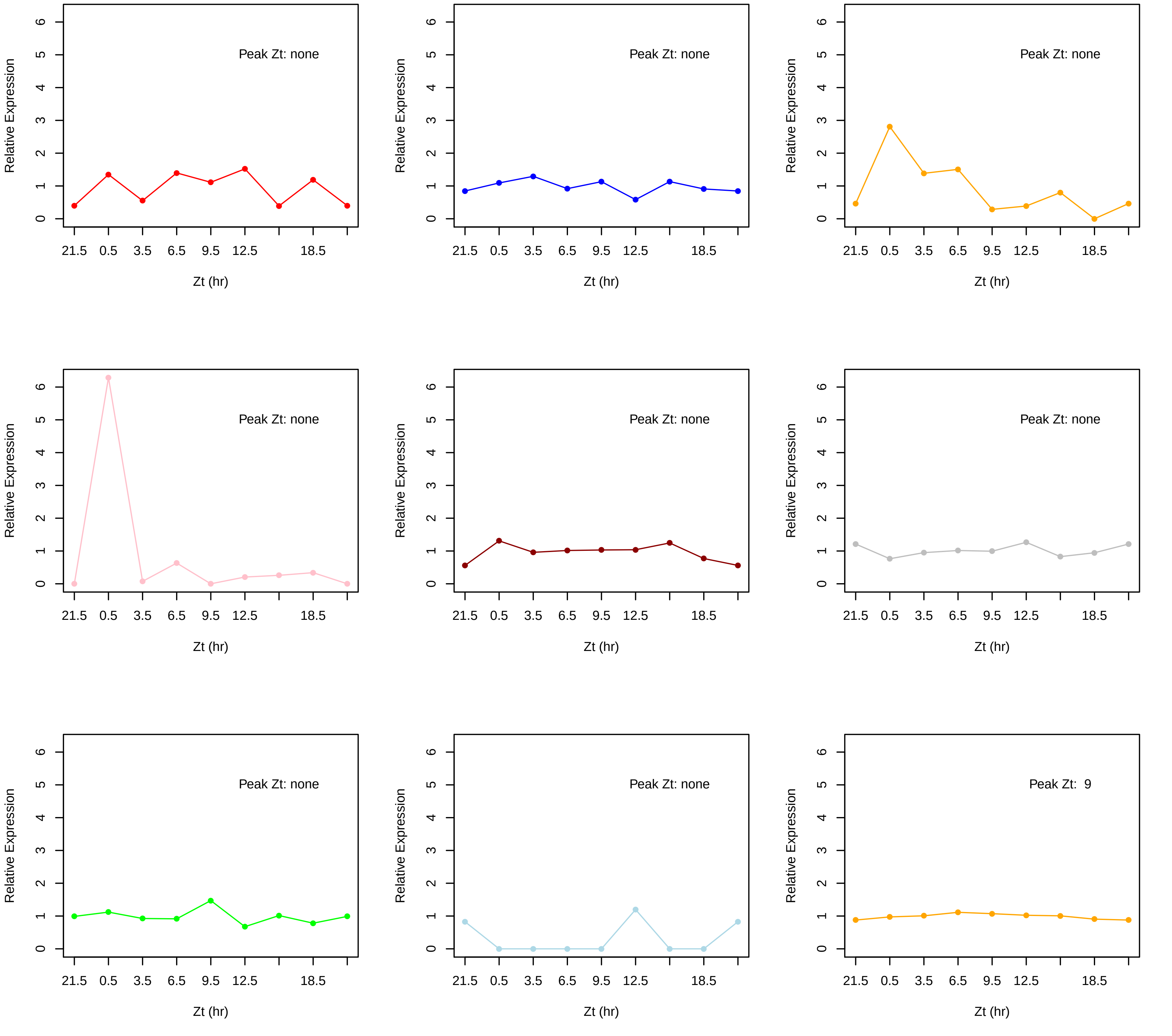

Singeleton: ENSXMAG00000001762 prpf4bb

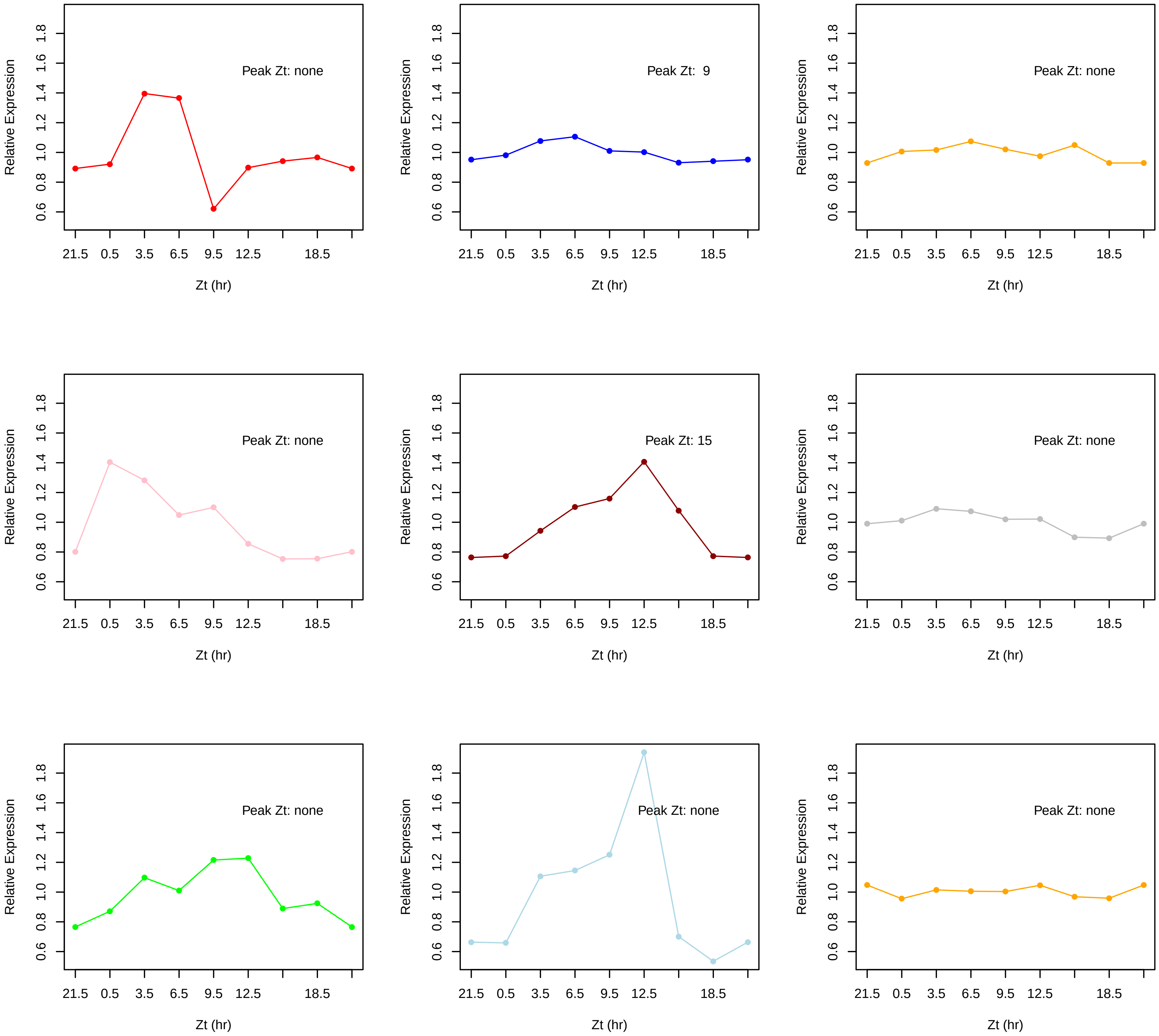

Singeleton: ENSXMAG00000005228

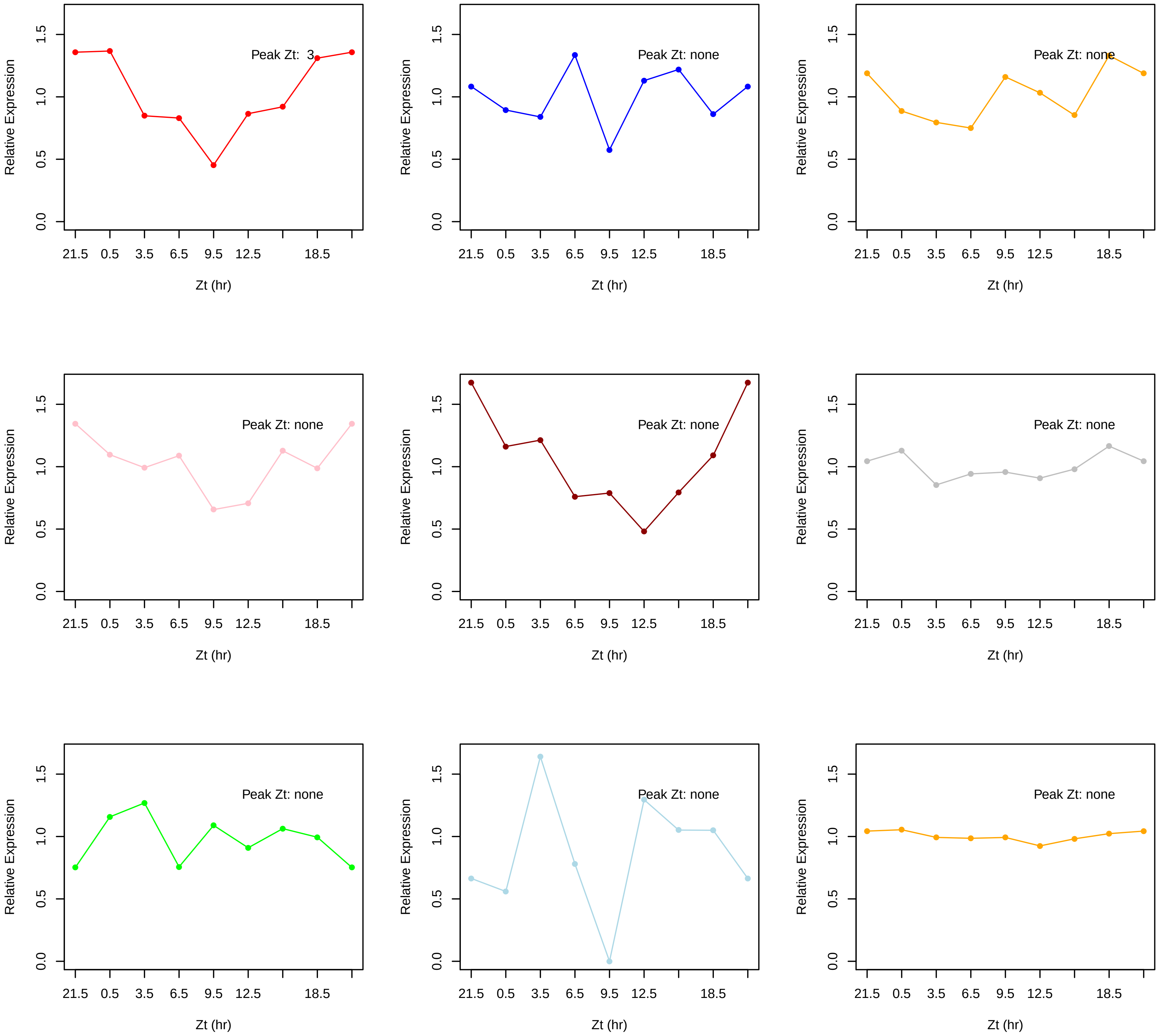

Singleton: ENSXMAG00000016330 pomk

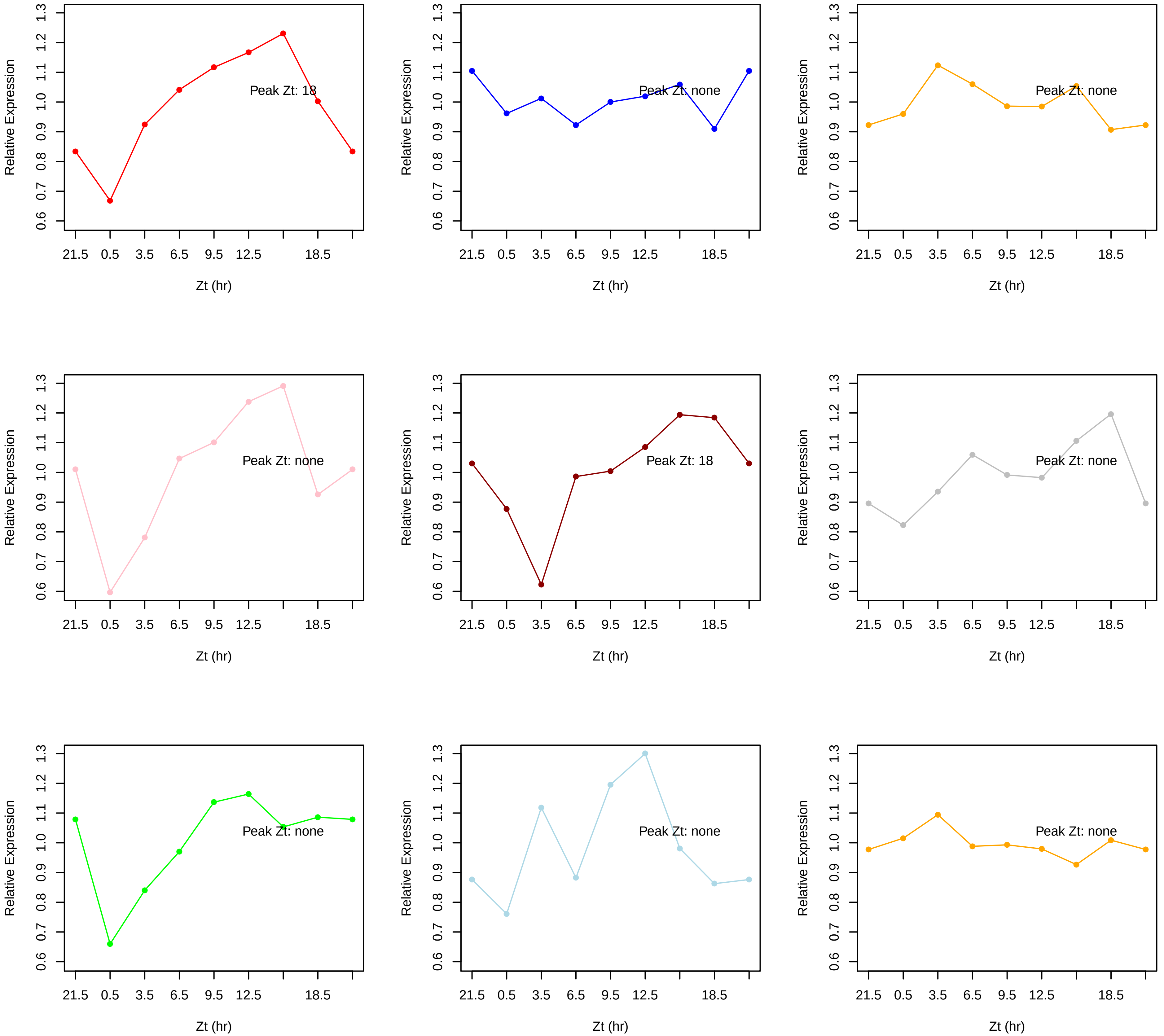

Singeleton: ENSXMAG00000022719 prelp

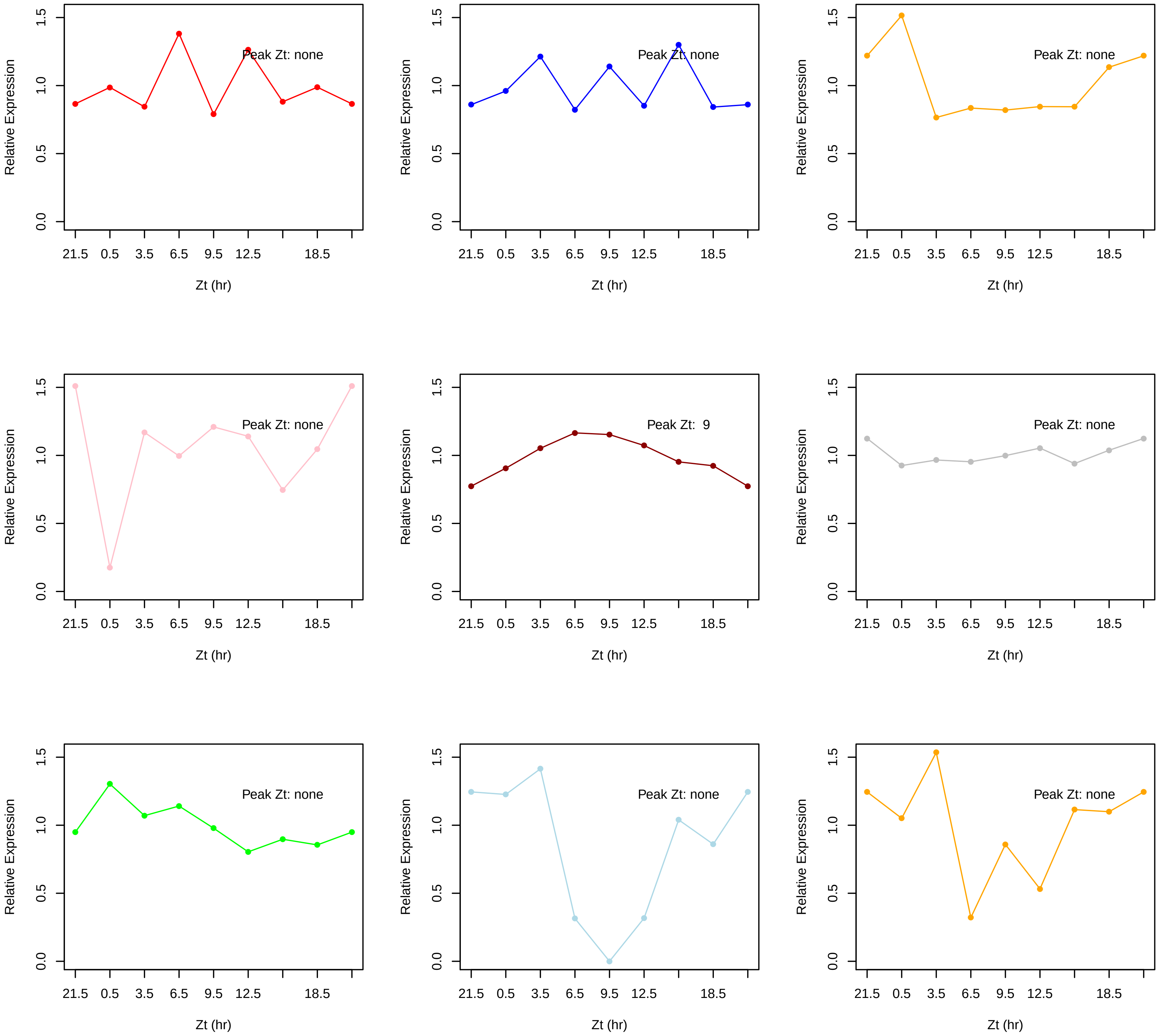

Singeleton: ENSXMAG00000010010 rmnd5b

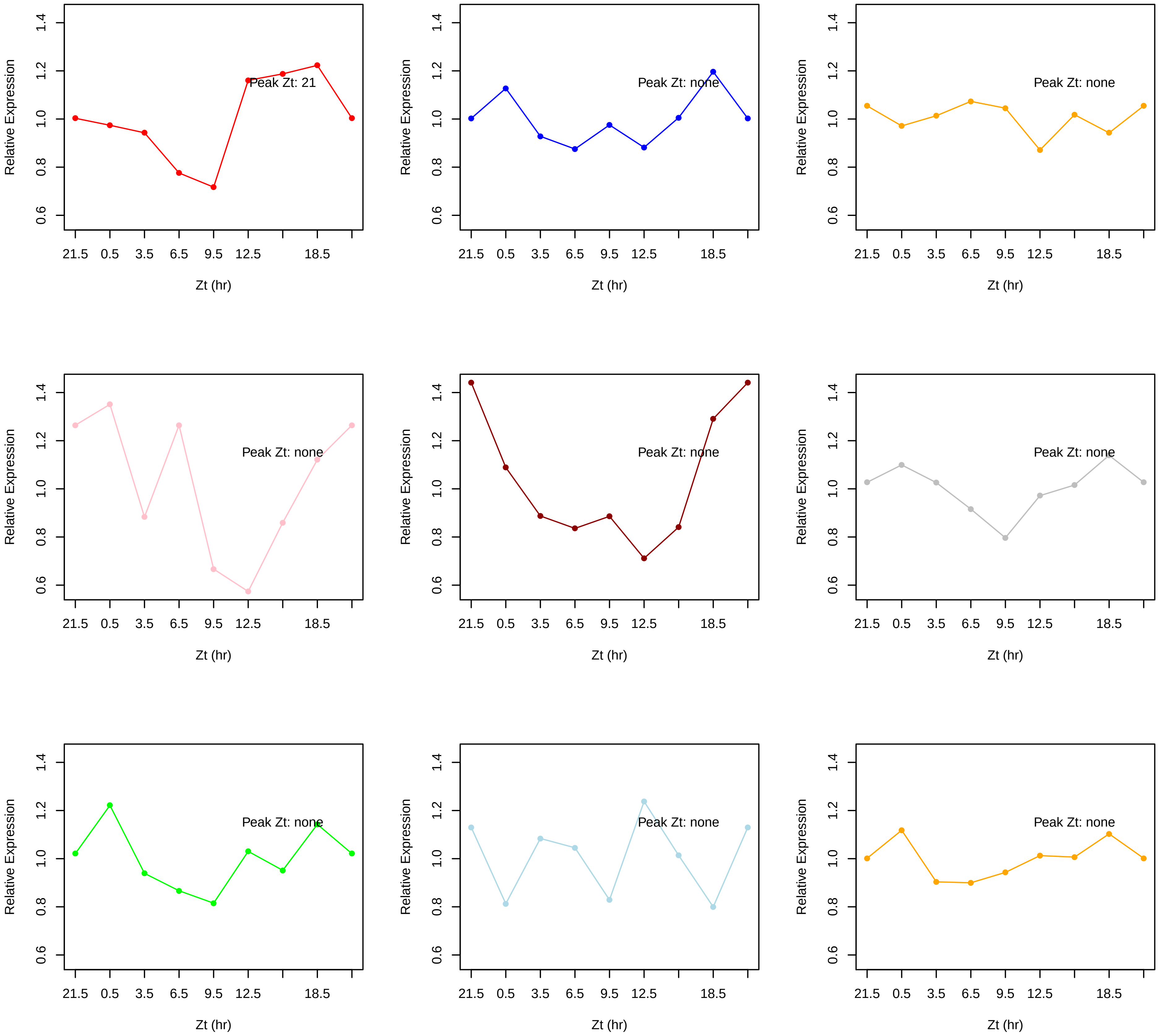

Singeleton: ENSXMAG00000021662

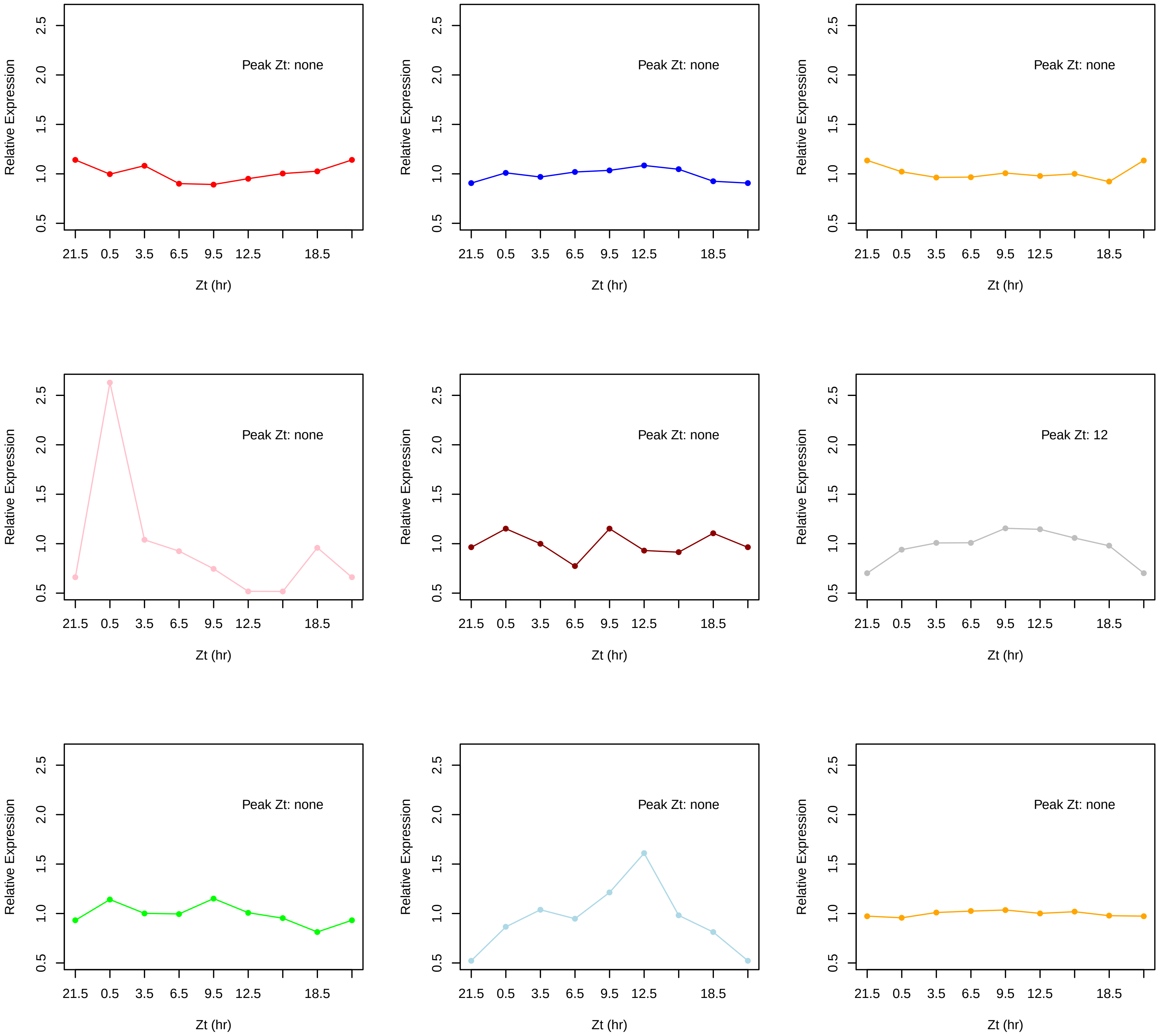

Singleton: ENSXMAG00000010022 n4bp3

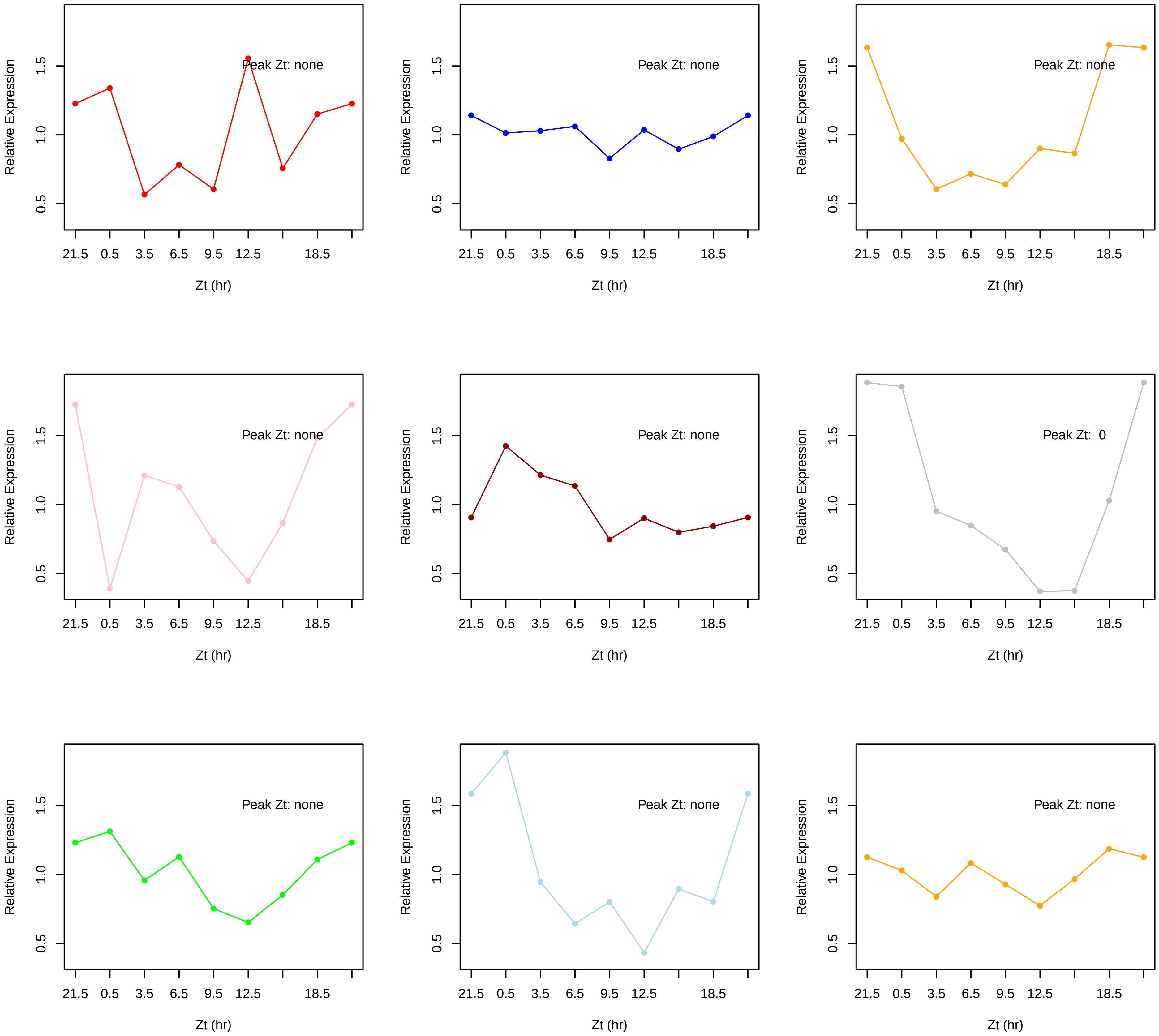

Singeleton: ENSXMAG00000009635 fermt2

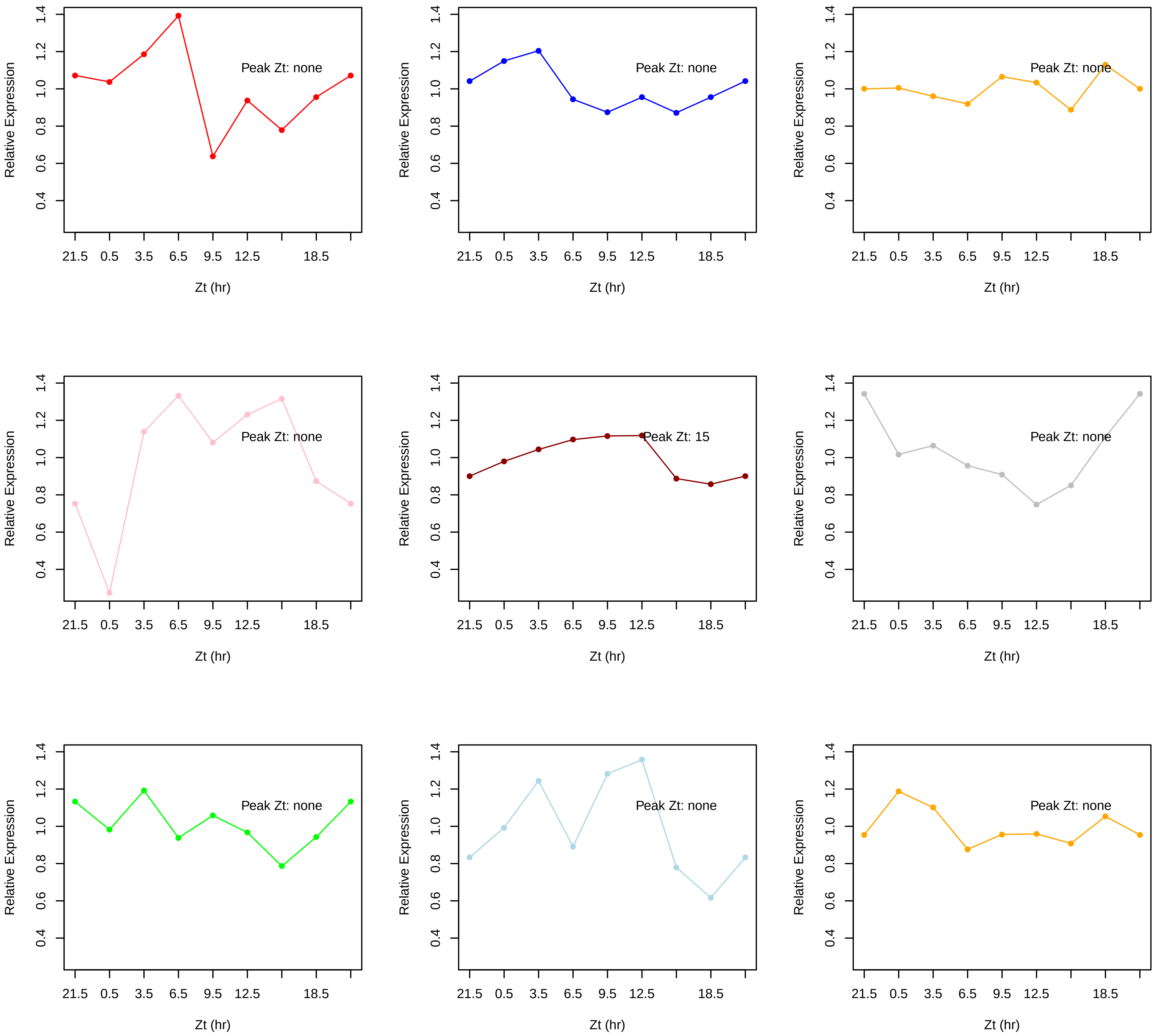

Singeleton: ENSXMAG00000000212 mfhas1

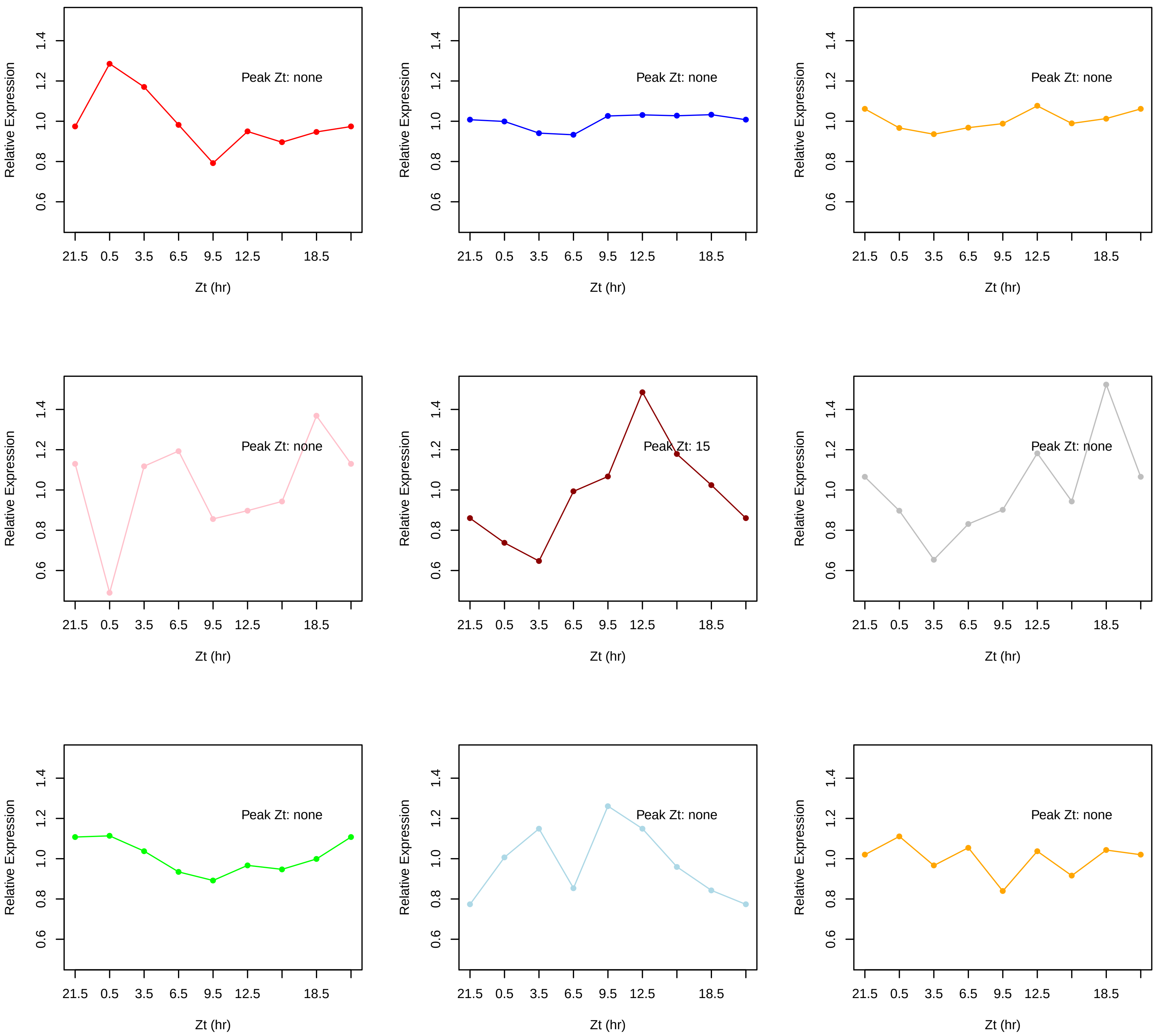

Singeleton: ENSXMAG00000001758 pxdc1b

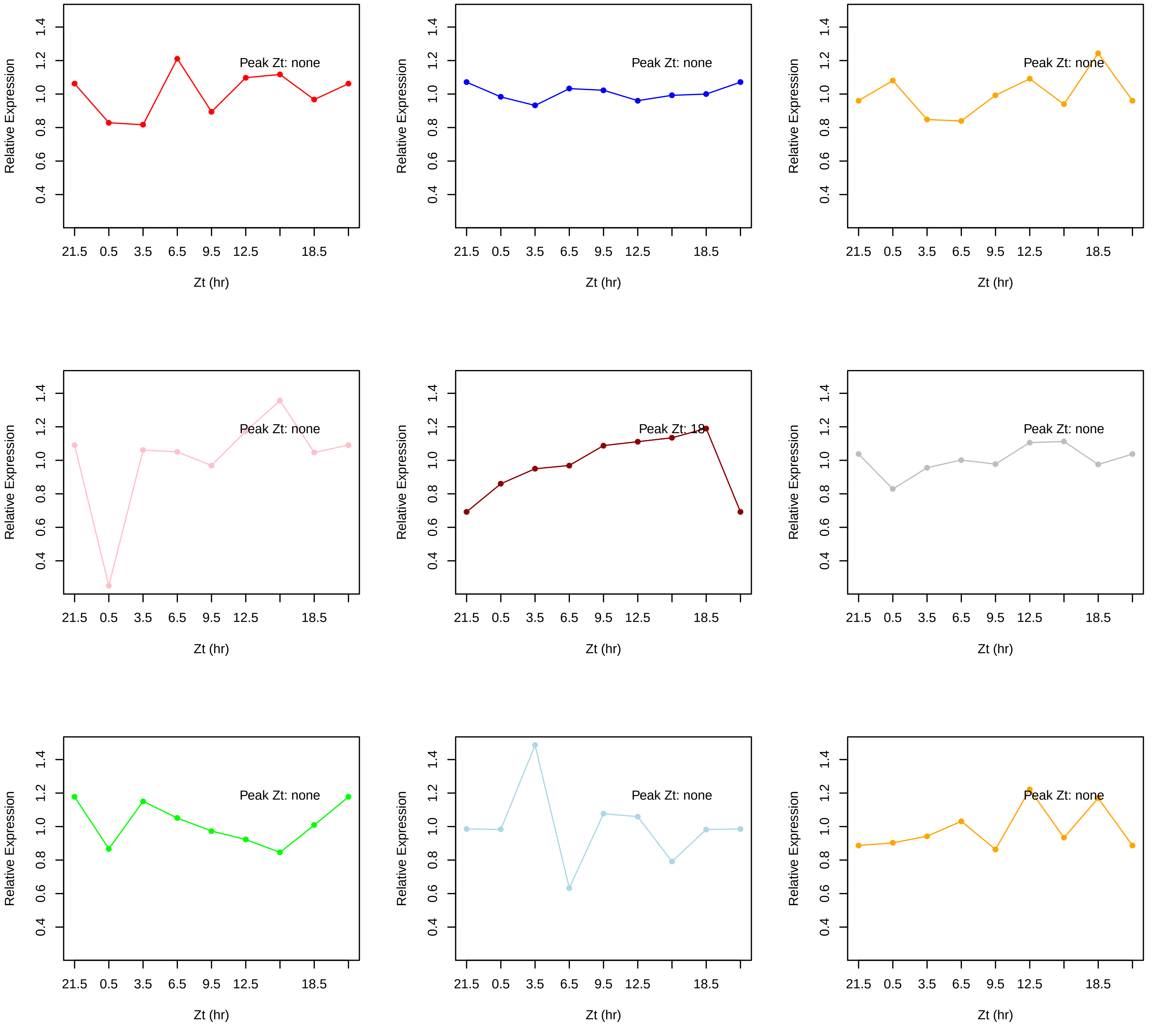

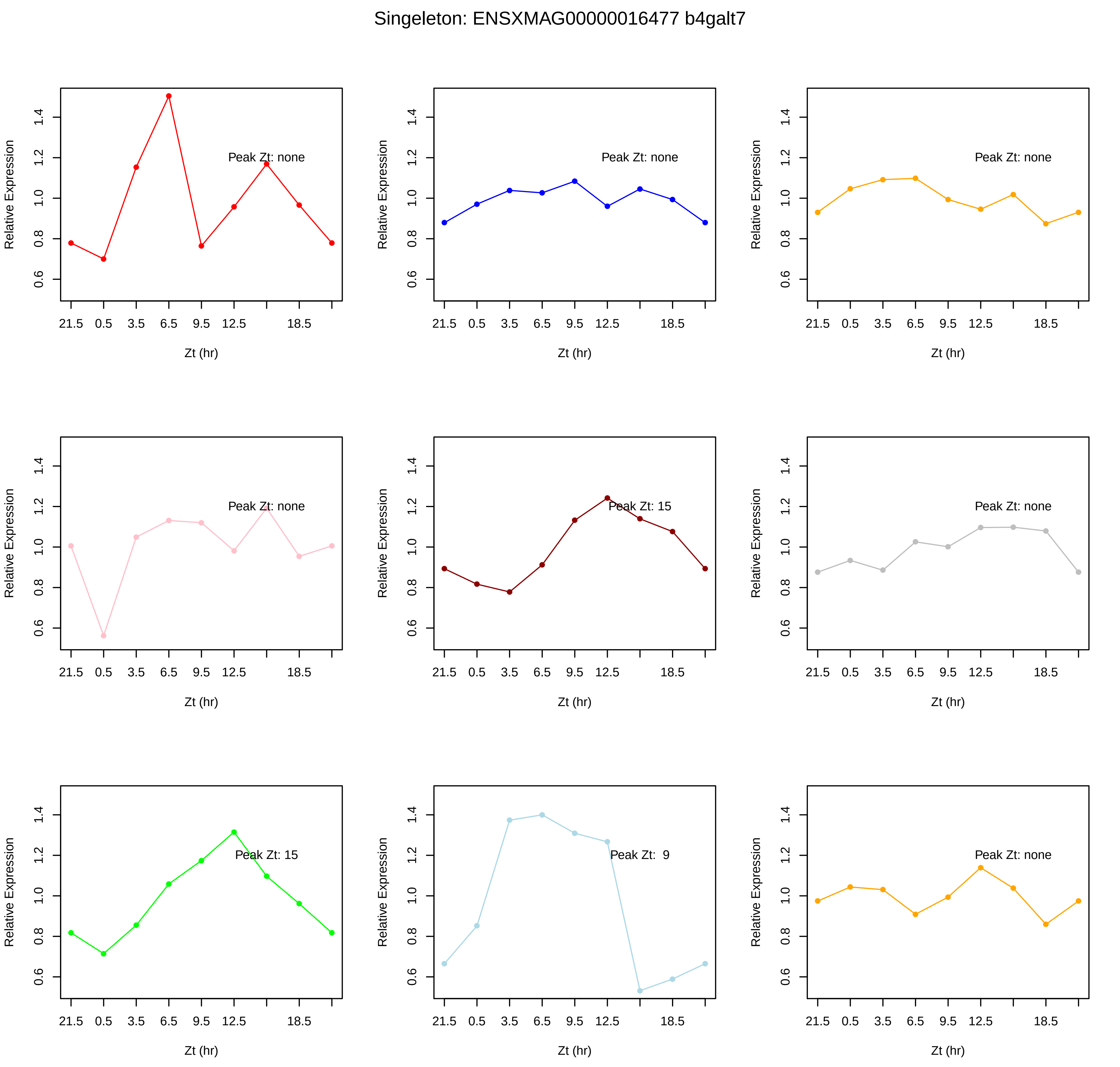
Singeleton: ENSXMAG00000016477 b4galt7
Peak Zt: none
Peak Zt: none
Peak Zt: none
Peak Zt: none
Peak Zt: 15
Peak Zt: none
Peak Zt: 15
Peak Zt: 9
Peak Zt: none
Relative Expression
Zt (hr)
21.5
0.5
3.5
6.5
9.5
12.5
18.5
0.6
0.8
1.0
1.2
1.4

Singleton: ENSXMAG00000008936 ERI1

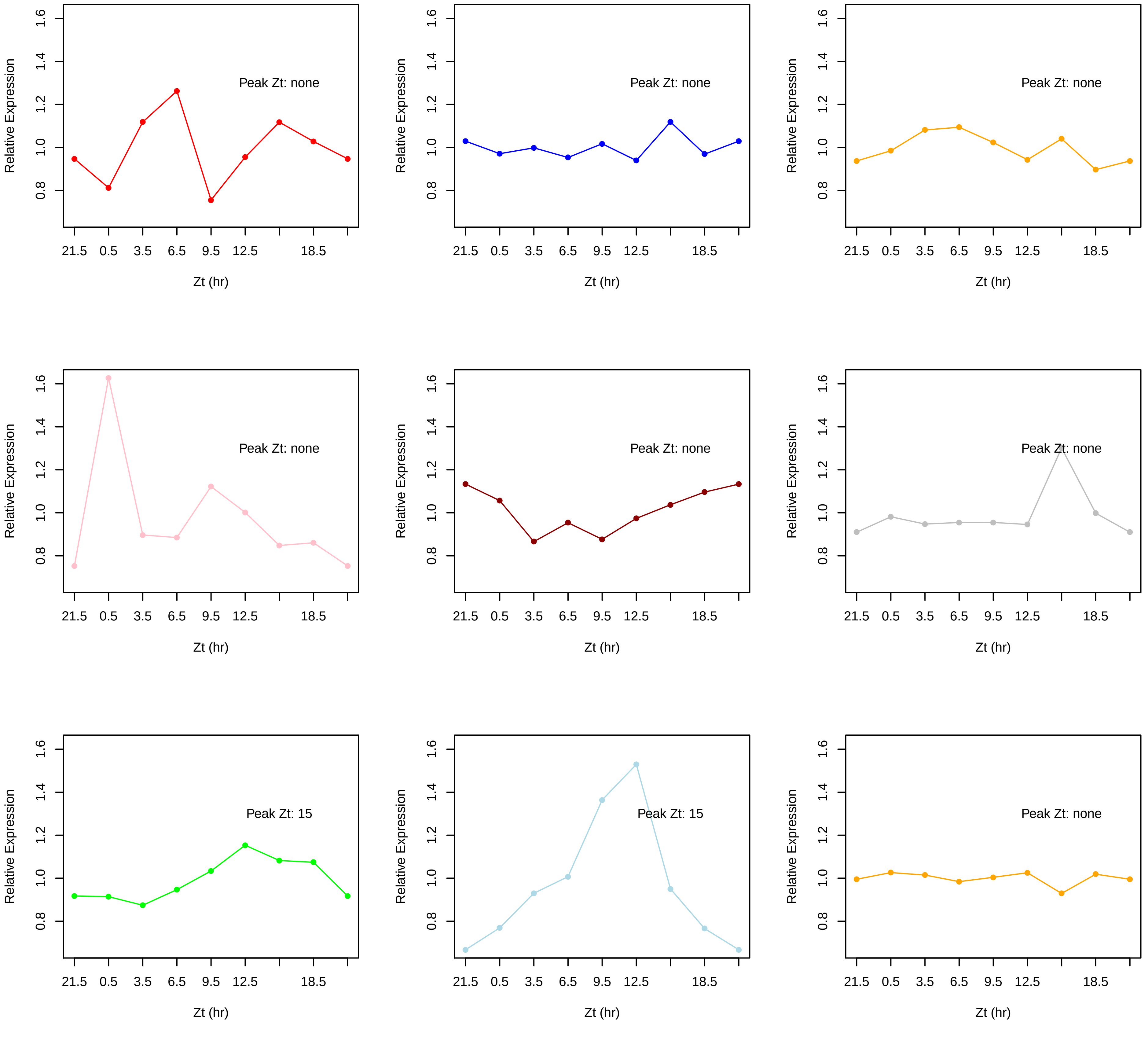

Singeleton: ENSXMAG00000017892 tmem106a

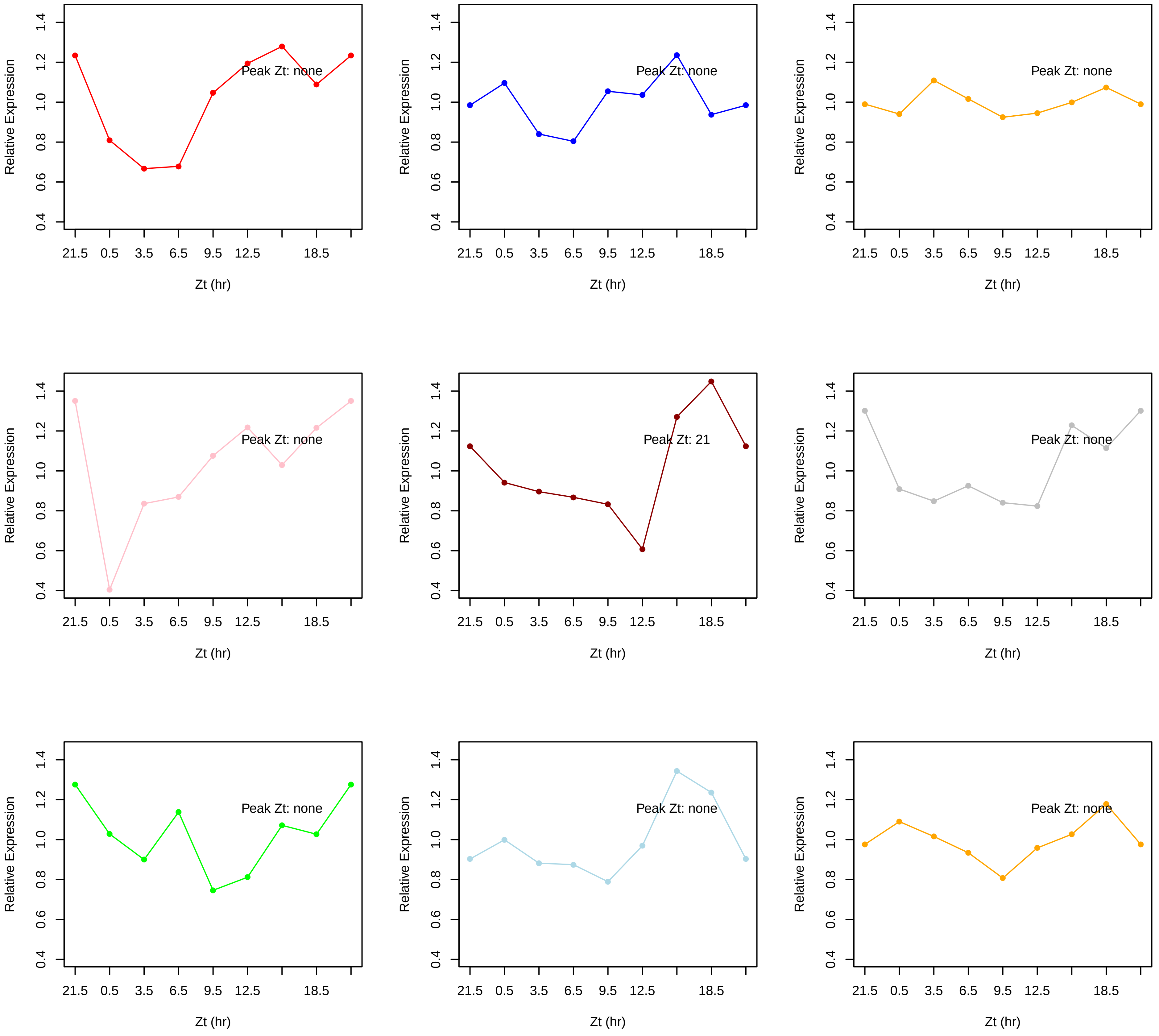

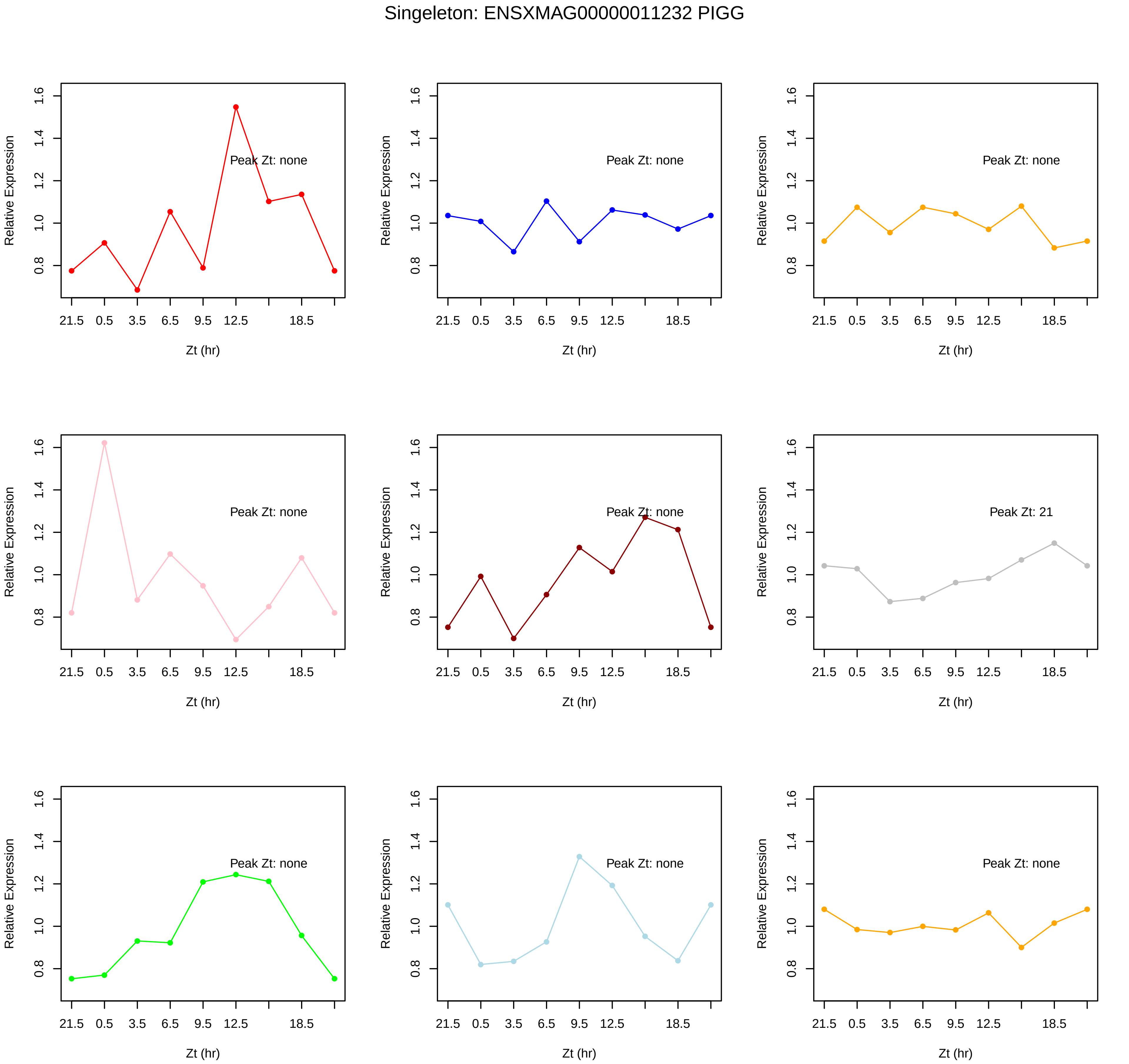

Singleton: ENSXMAG00000011232 PIGG
Peak Zt: none
Peak Zt: none
Peak Zt: none
Peak Zt: none
Peak Zt: none
Peak Zt: 21
Peak Zt: none
Peak Zt: none
Peak Zt: none
Relative Expression
Zt (hr)
21.5
0.5
3.5
6.5
9.5
12.5
18.5
0.8
1.0
1.2
1.4
1.6

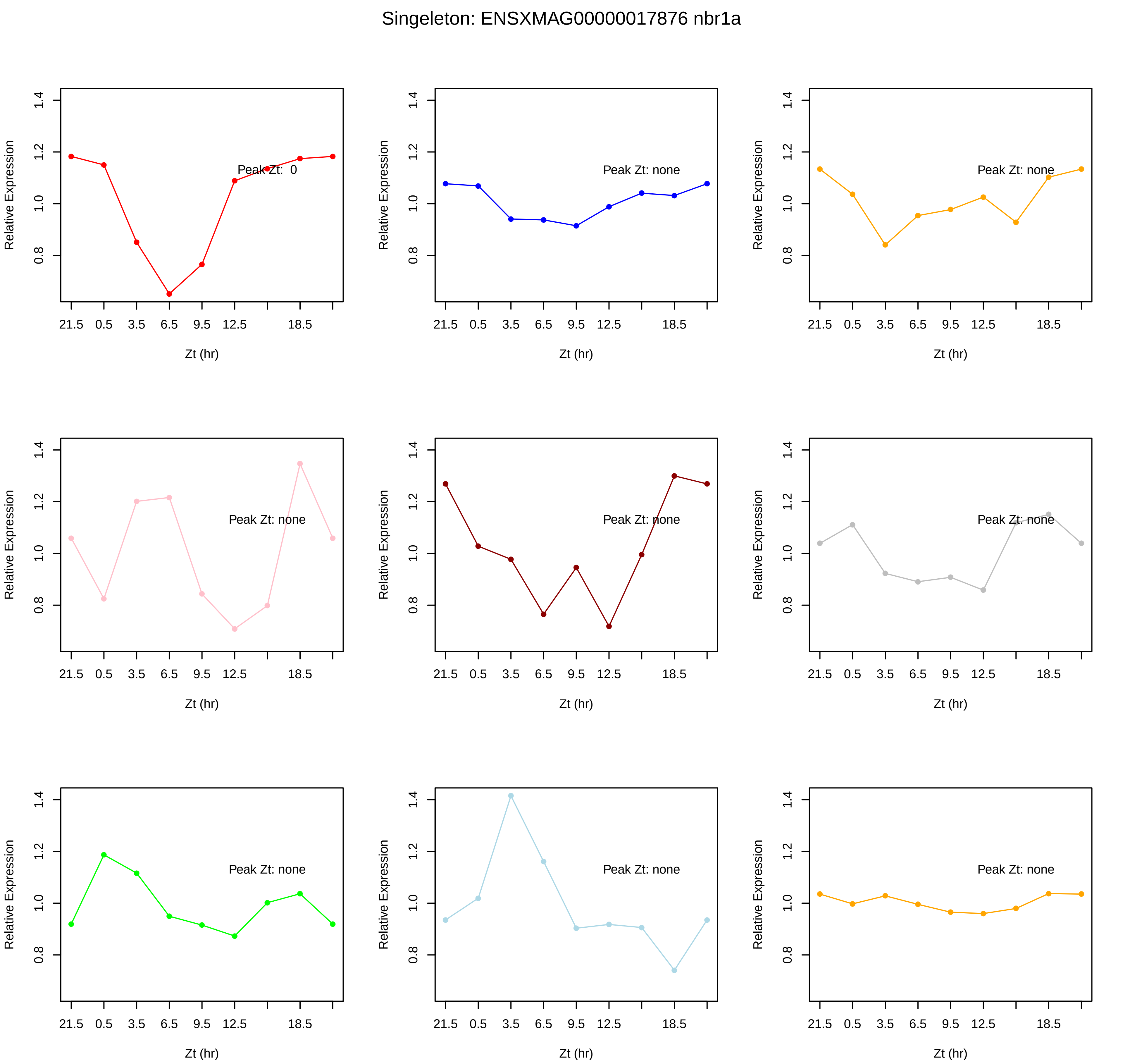
Singeleton: ENSXMAG00000017876 nbr1a
Relative Expression
Zt (hr)
21.5
0.5
3.5
6.5
9.5
12.5
18.5
0.8
1.0
1.2
1.4
Peak Zt: 0
Peak Zt: none

Singleton: ENSXMAG00000021989 GJD2

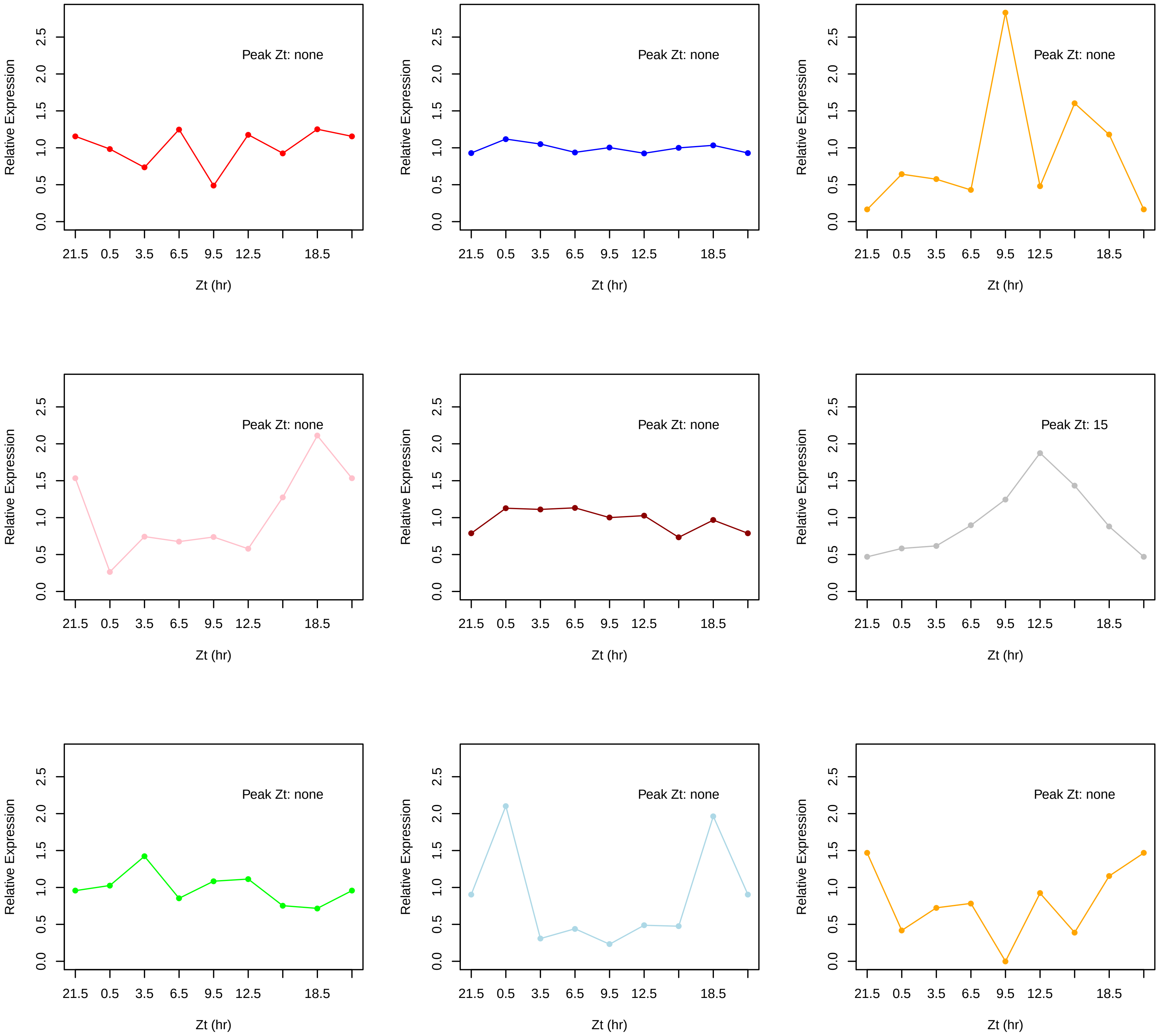

Singeleton: ENSXMAG00000019228

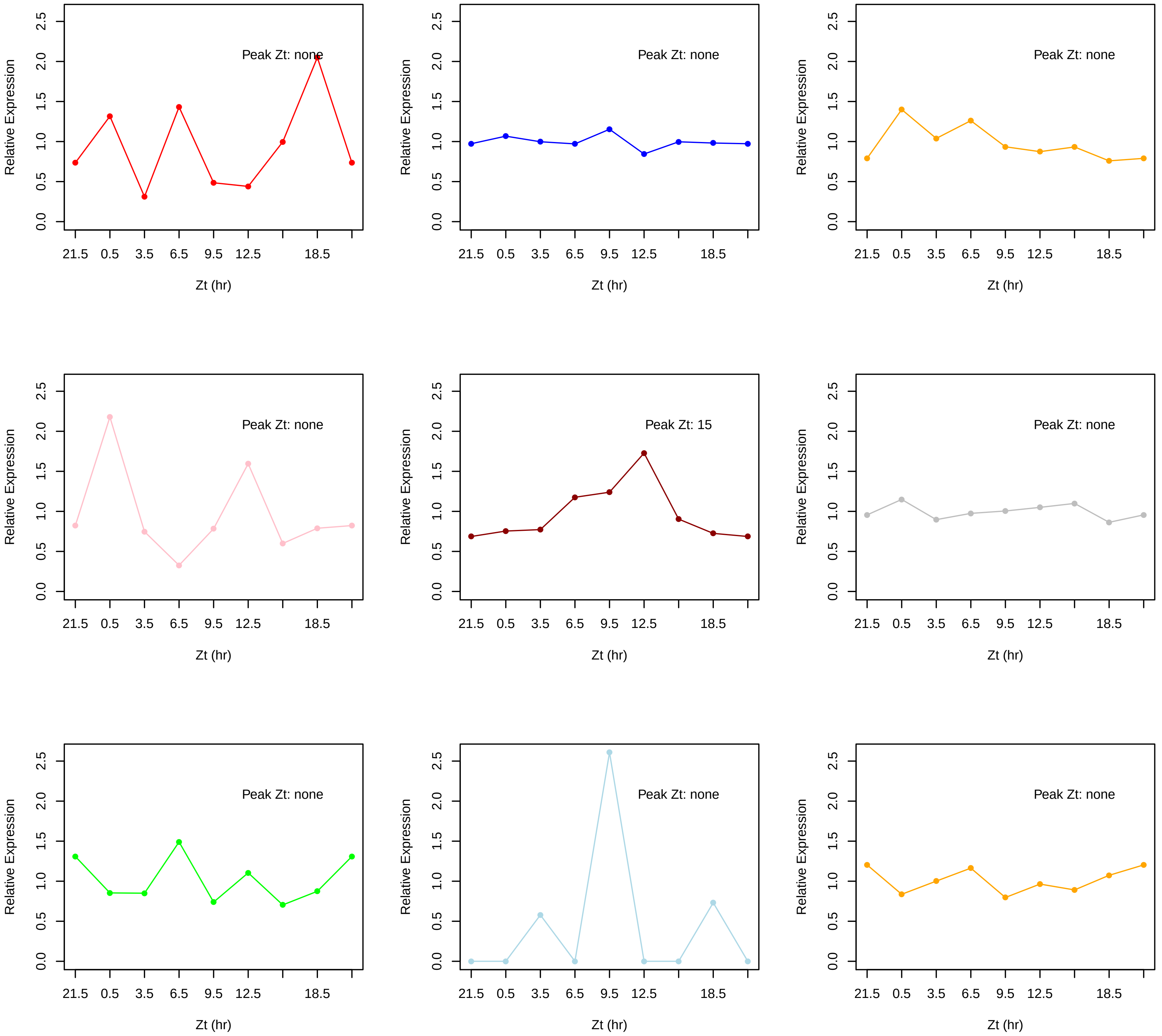

Singeleton: ENSXMAG00000009617 ACTC1

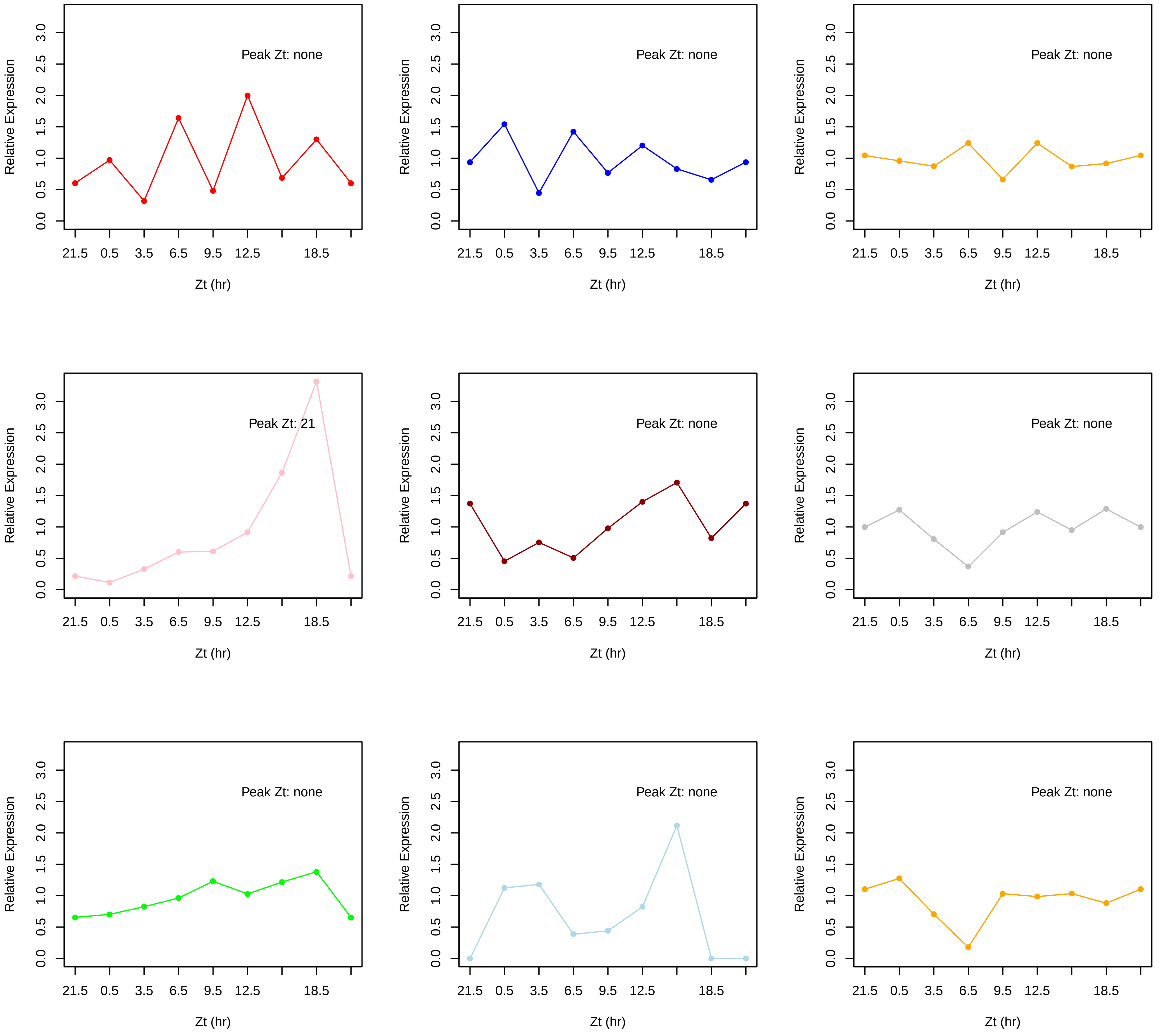

Singeleton: ENSXMAG00000003785 srgap2

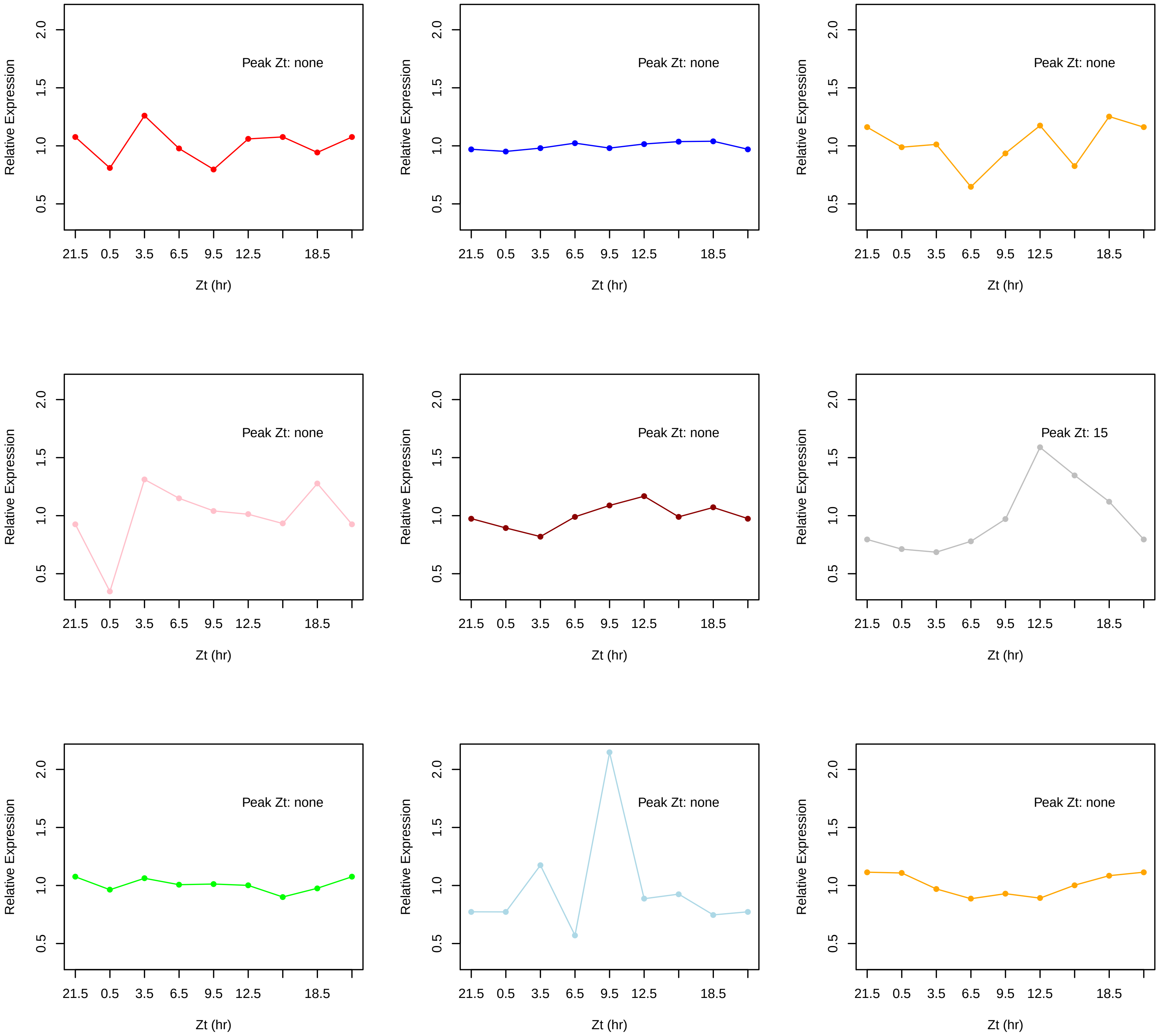

Singeleton: ENSXMAG00000027968 psme3

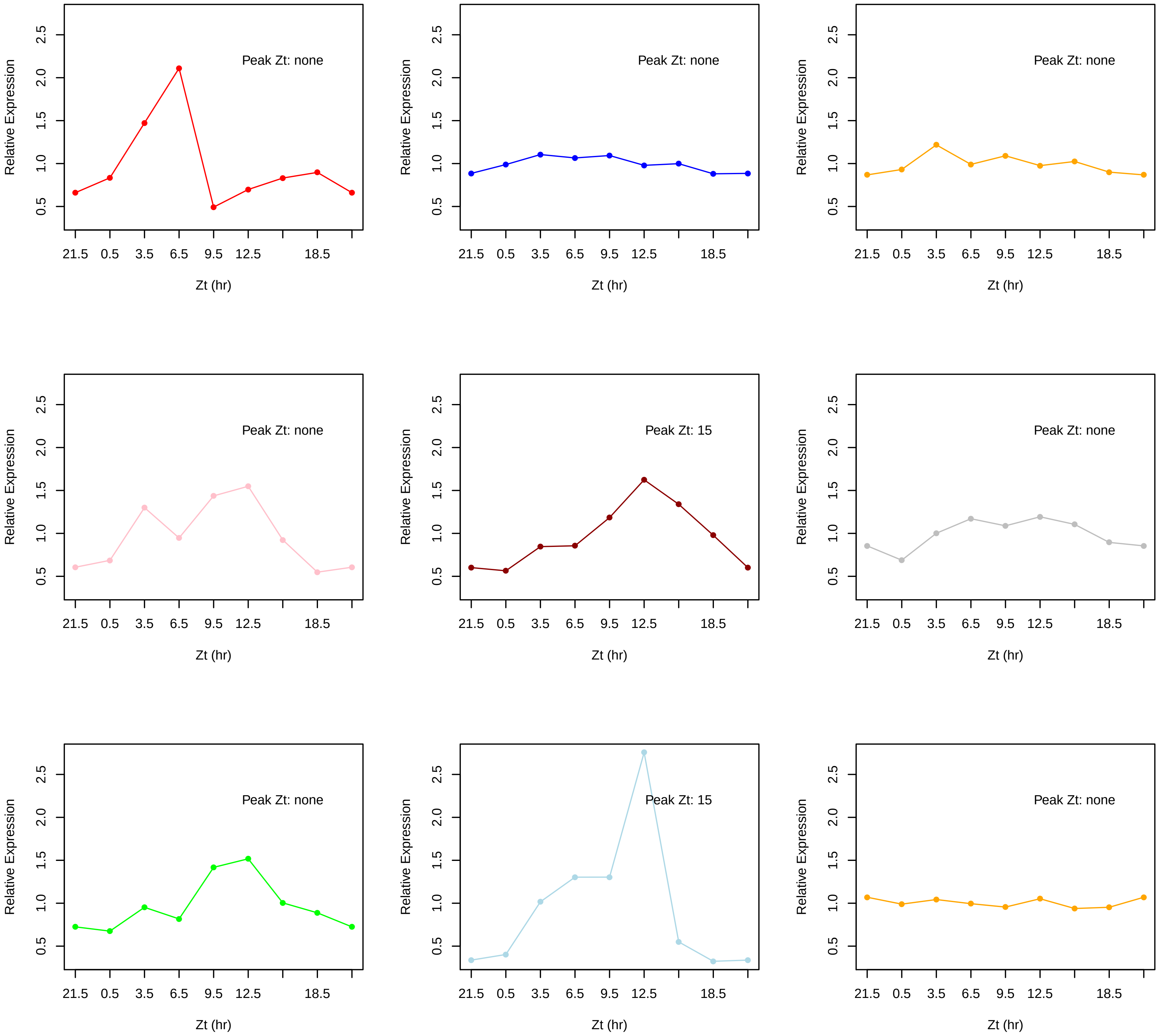

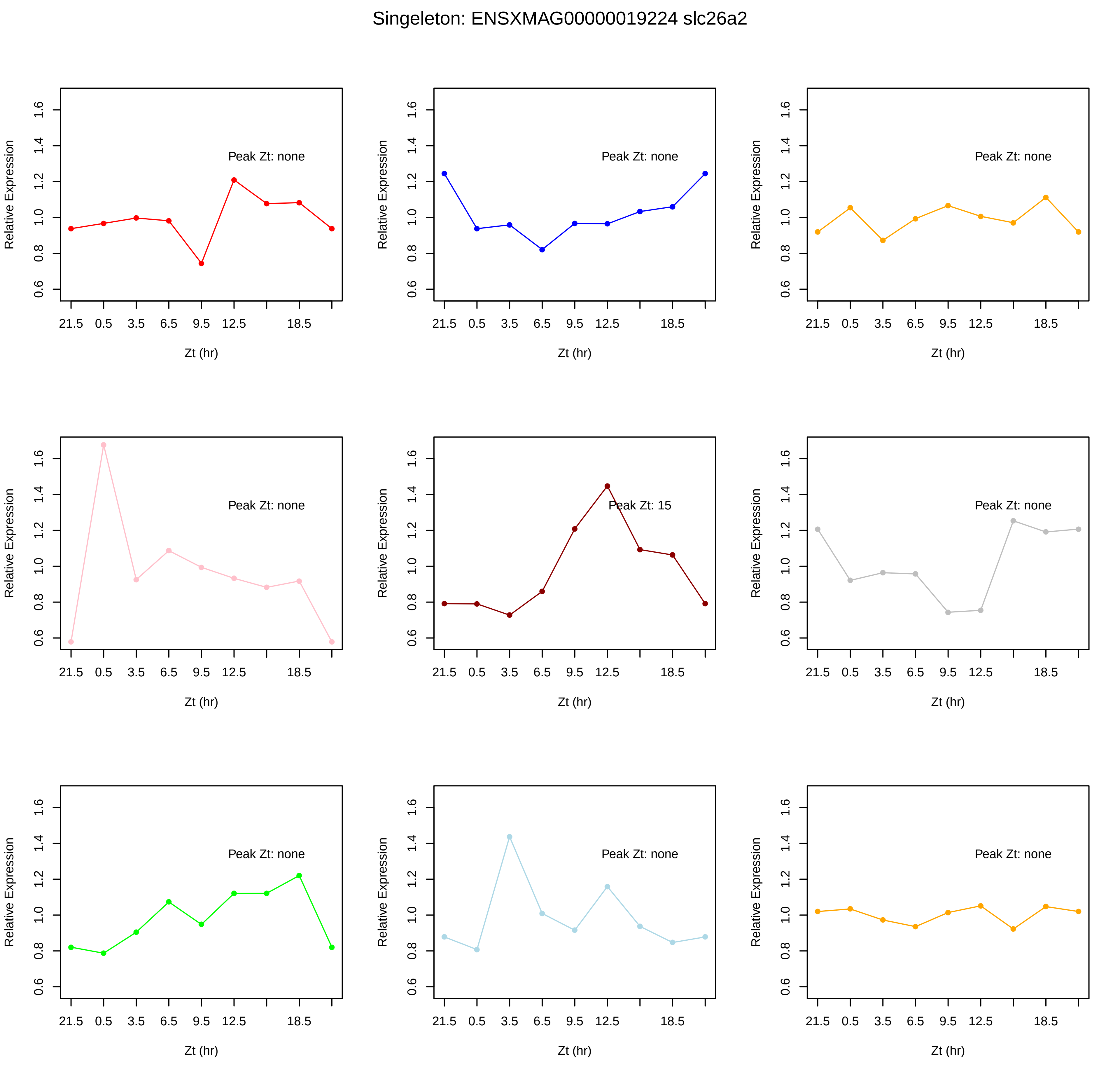
Singeleton: ENSXMAG00000019224 slc26a2
Peak Zt: none
Peak Zt: none
Peak Zt: none
Peak Zt: none
Peak Zt: 15
Peak Zt: none
Peak Zt: none
Peak Zt: none
Peak Zt: none
Relative Expression
Zt (hr)

Singeleton: ENSXMAG00000009596 aqr

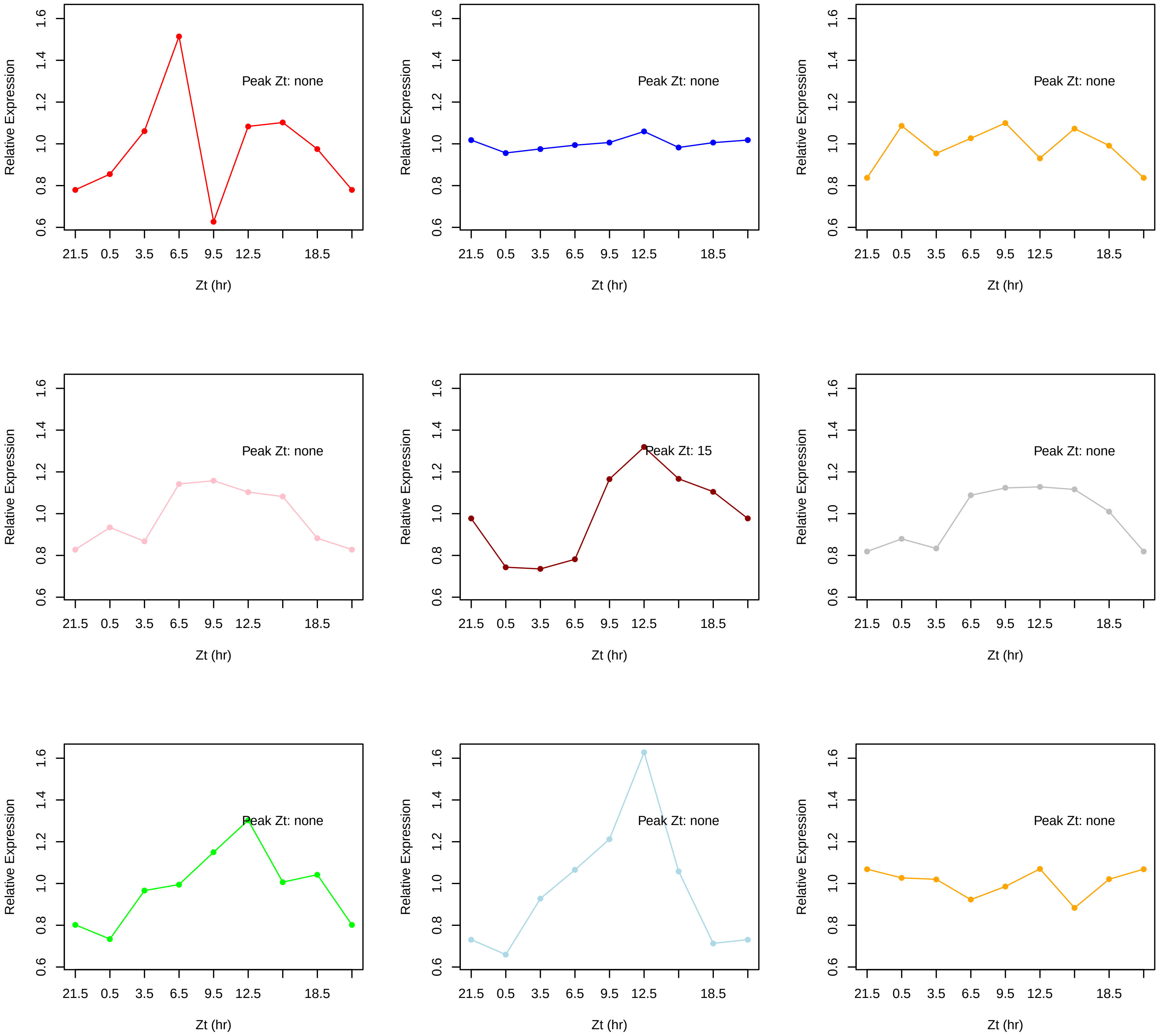

Singeleton: ENSXMAG00000019181 pde6a

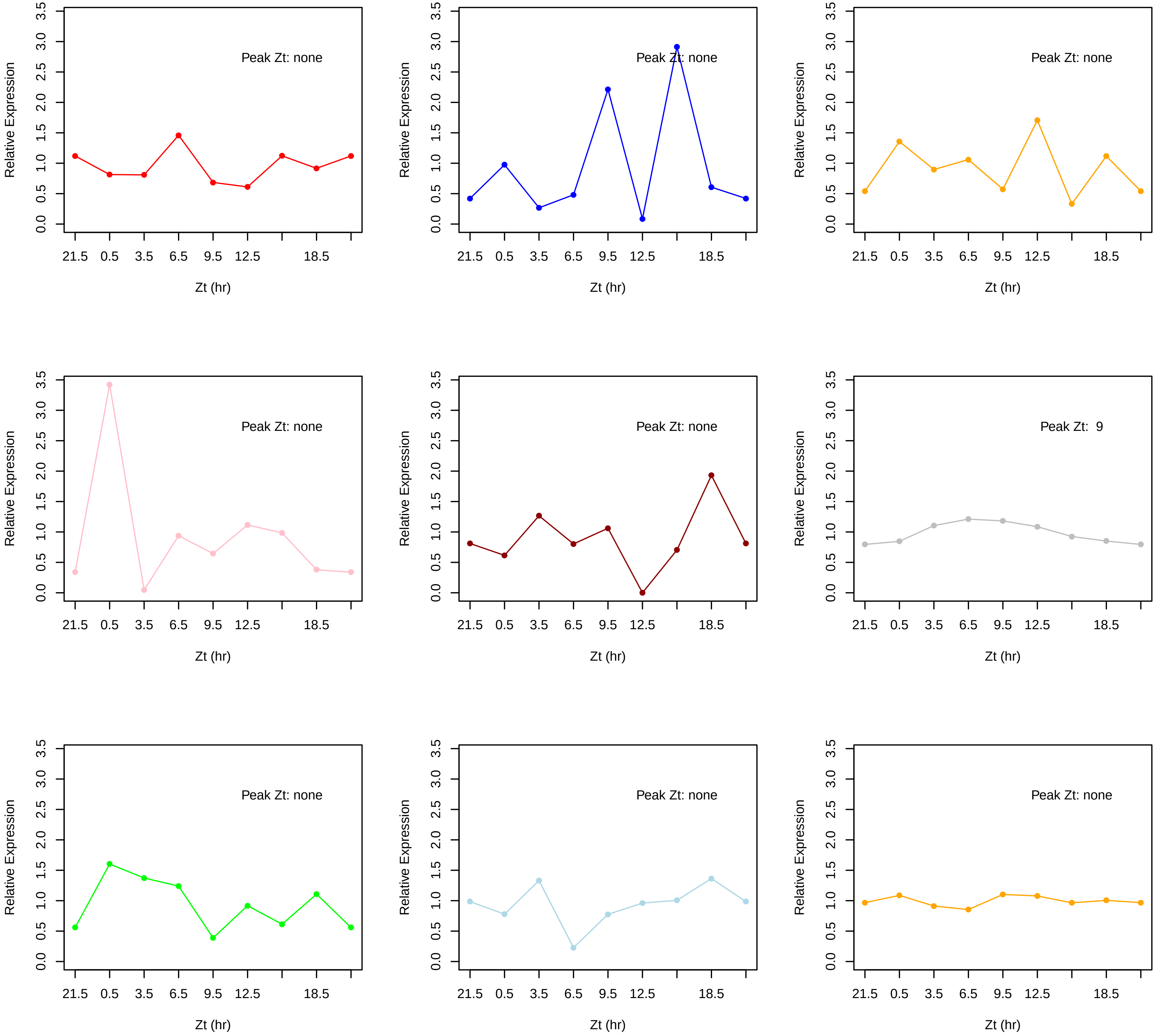

Singeleton: ENSXMAG00000002813 aoc2

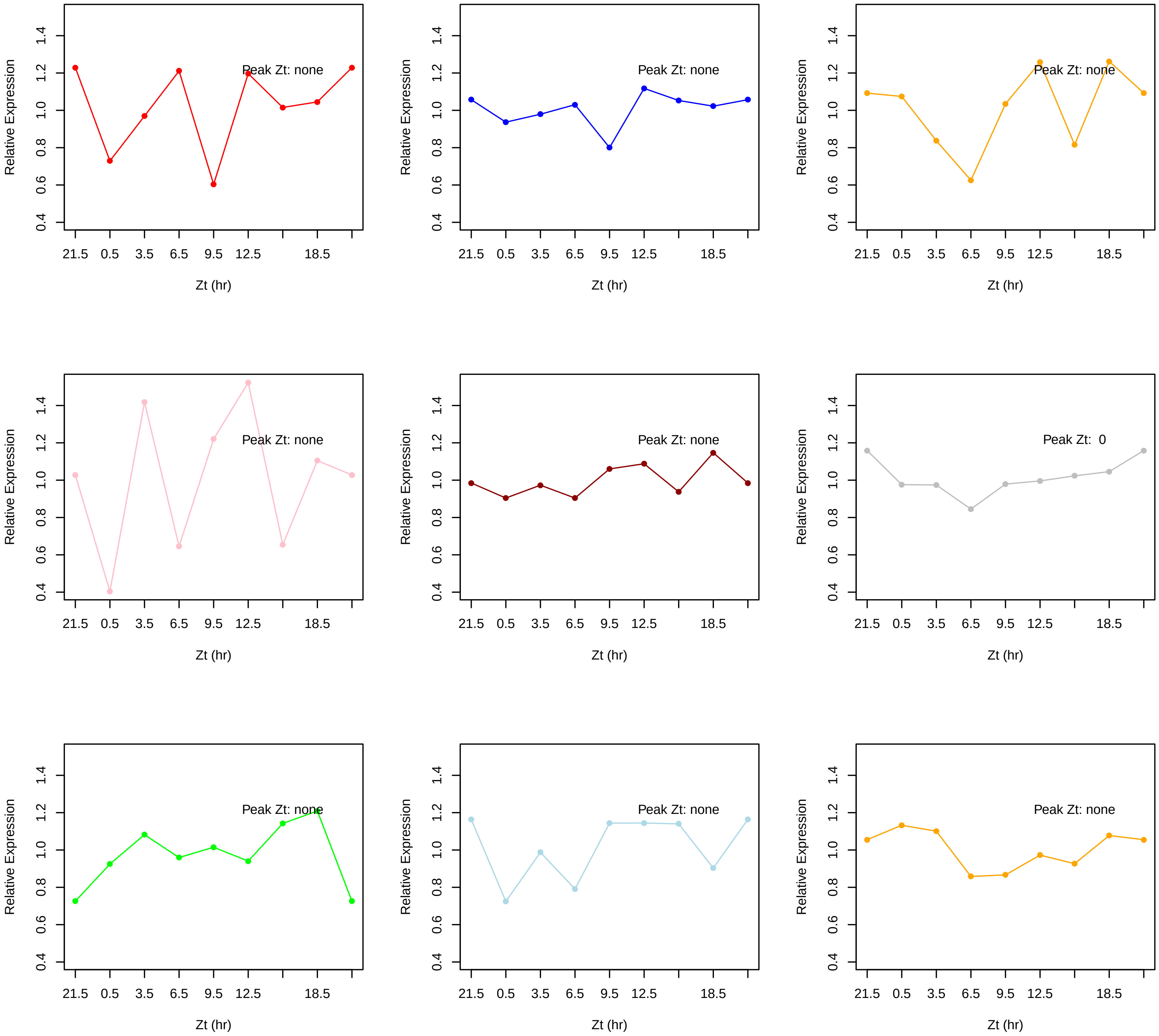

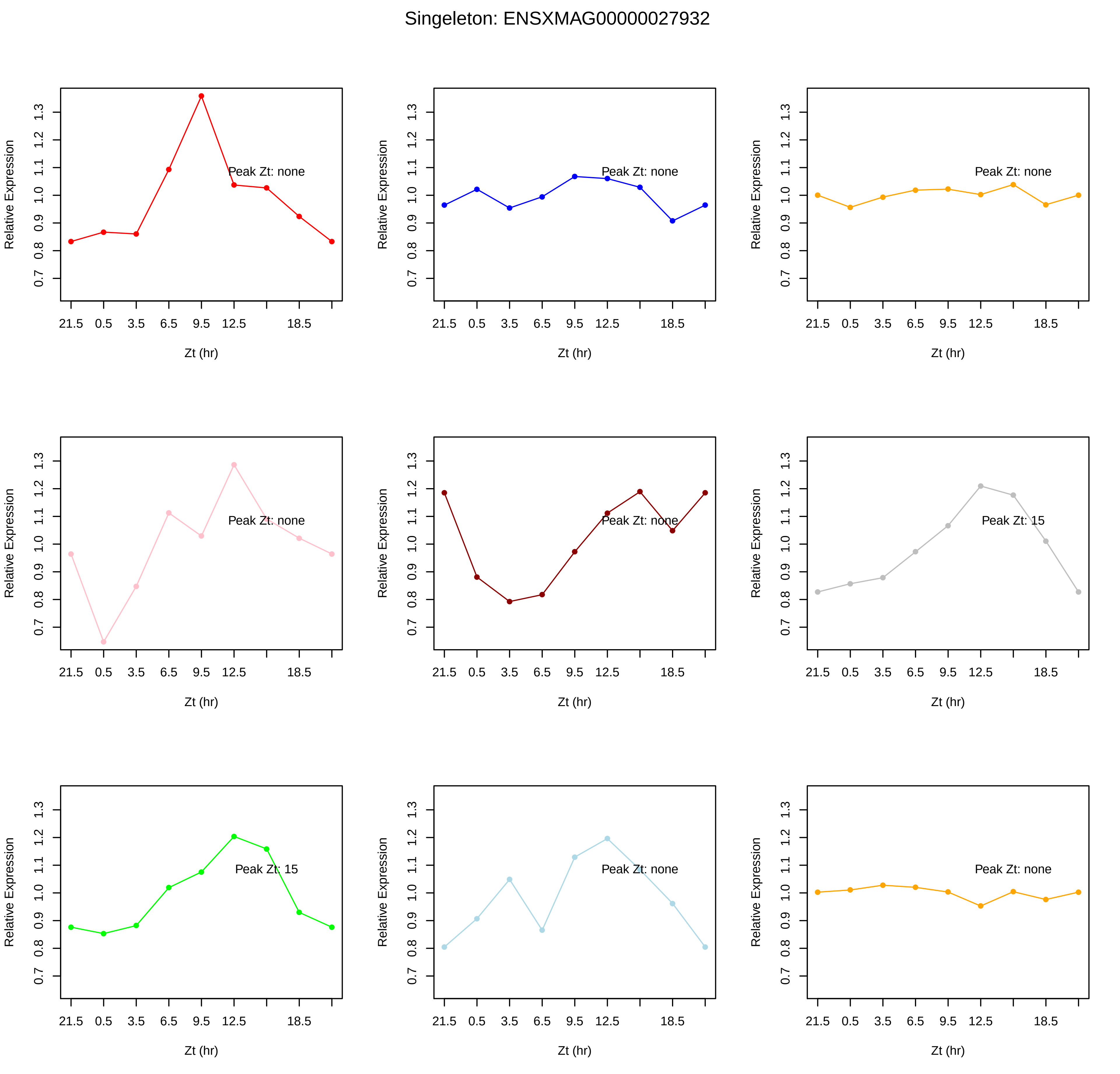
Singeleton: ENSXMAG00000027932
Peak Zt: none
Peak Zt: none
Peak Zt: none
Peak Zt: none
Peak Zt: none
Peak Zt: 15
Peak Zt: 15
Peak Zt: none
Peak Zt: none
Relative Expression
Zt (hr)
21.5
0.5
3.5
6.5
9.5
12.5
18.5
0.7
0.8
0.9
1.0
1.1
1.2
1.3

Singeleton: ENSXMAG00000019168 arhgef37

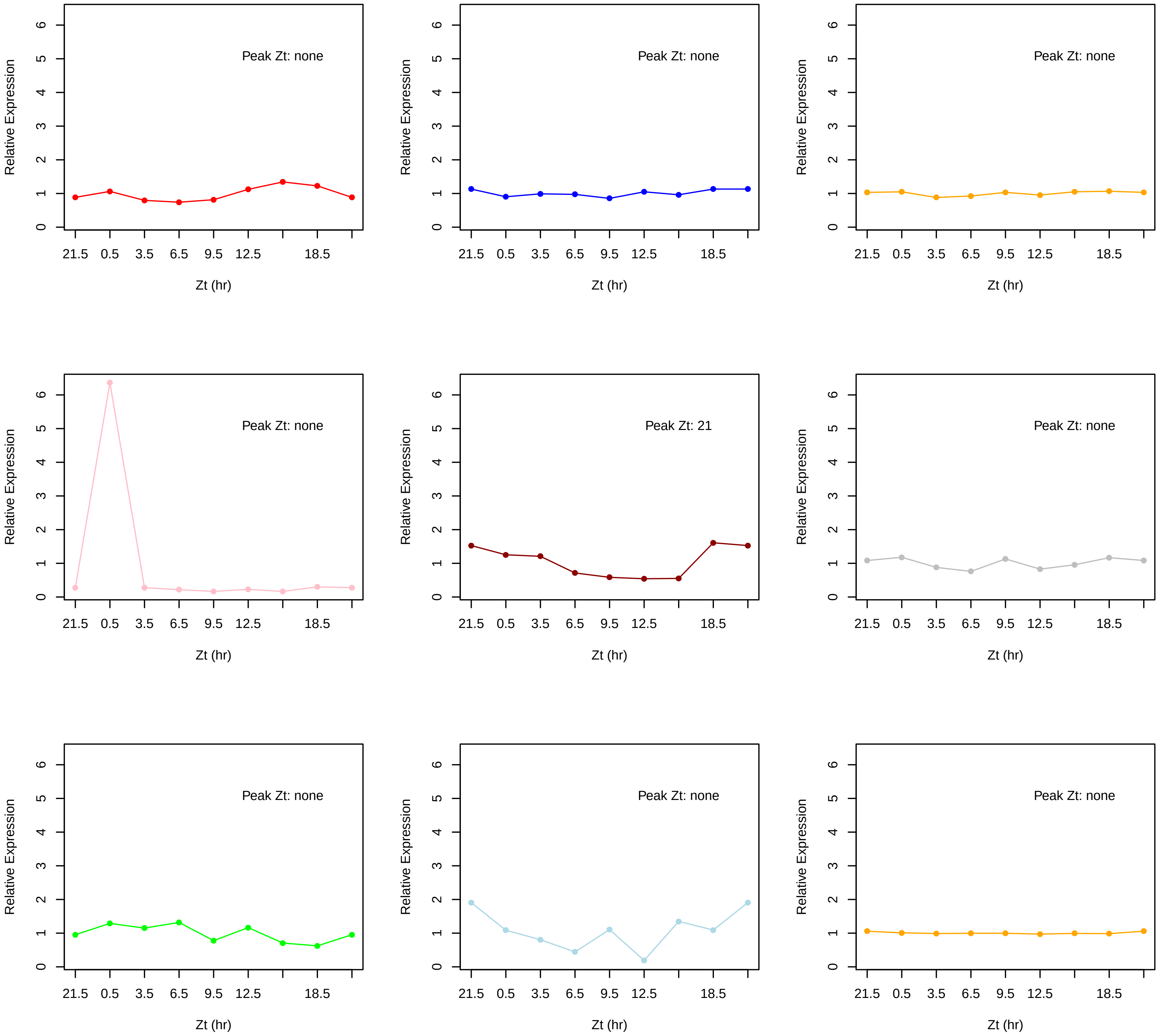

Singleton: ENSXMAG00000015316 metap1

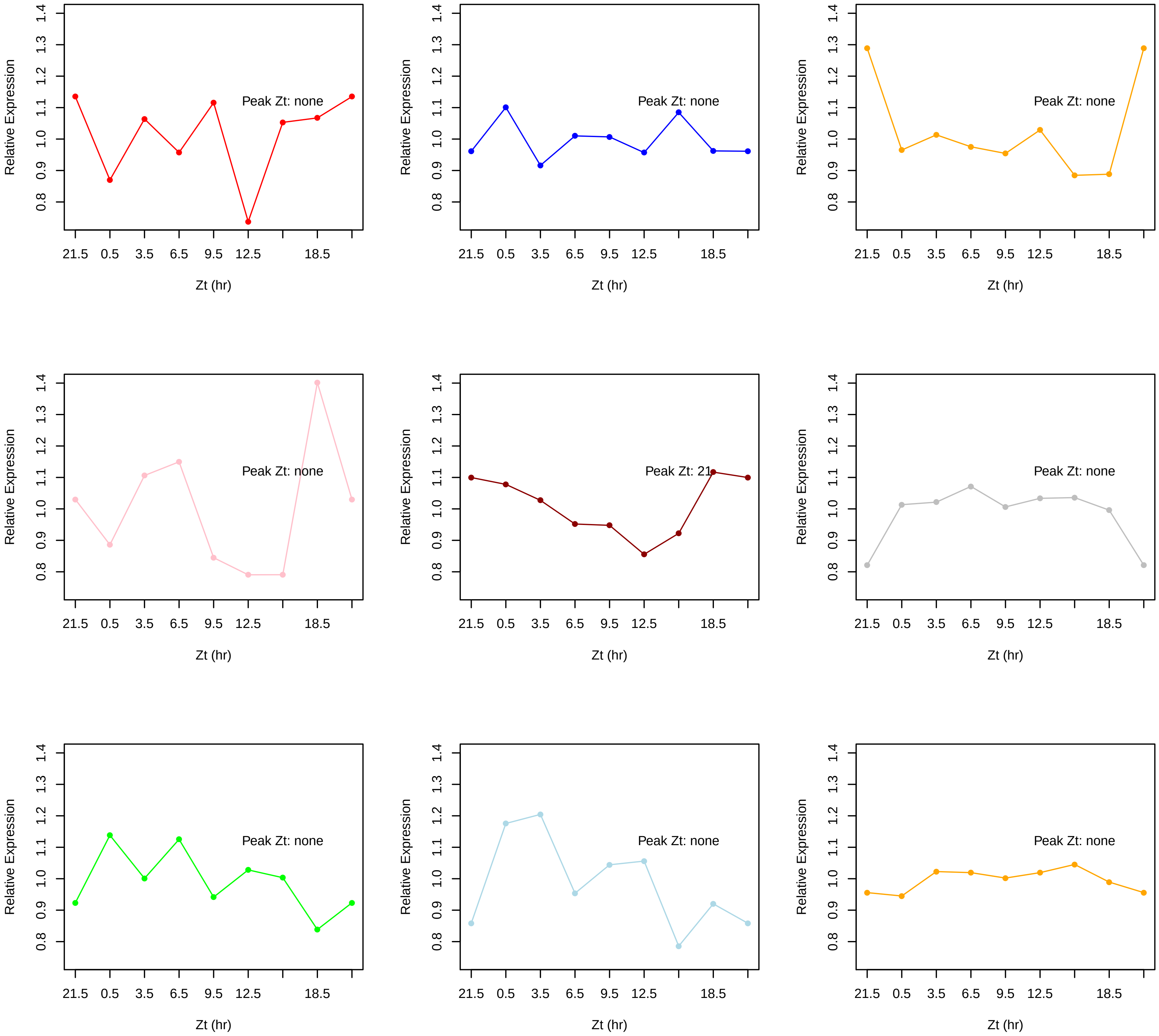

Singleton: ENSXMAG00000027765 tcf7l2

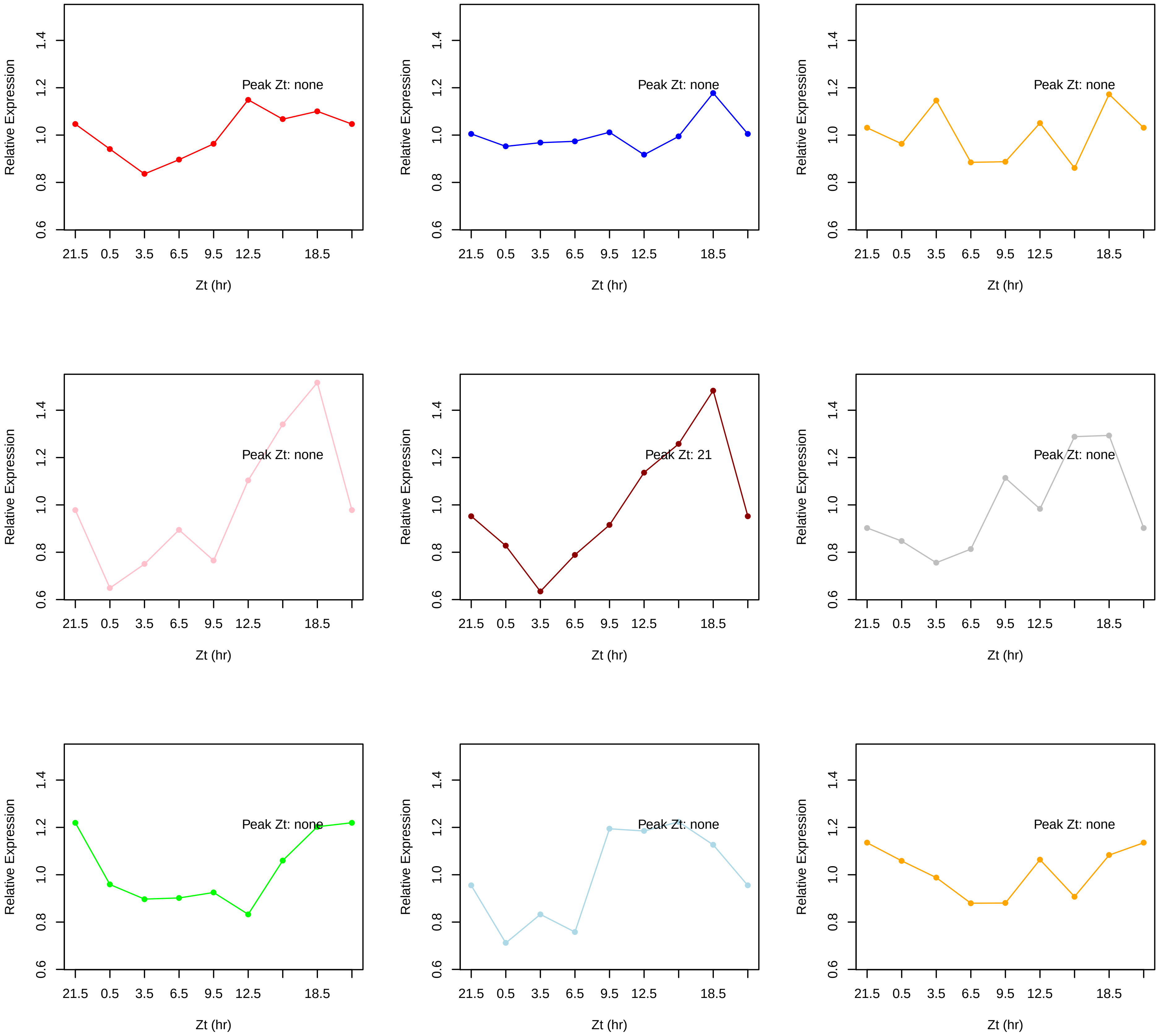

Singeleton: ENSXMAG00000026802 rab40b

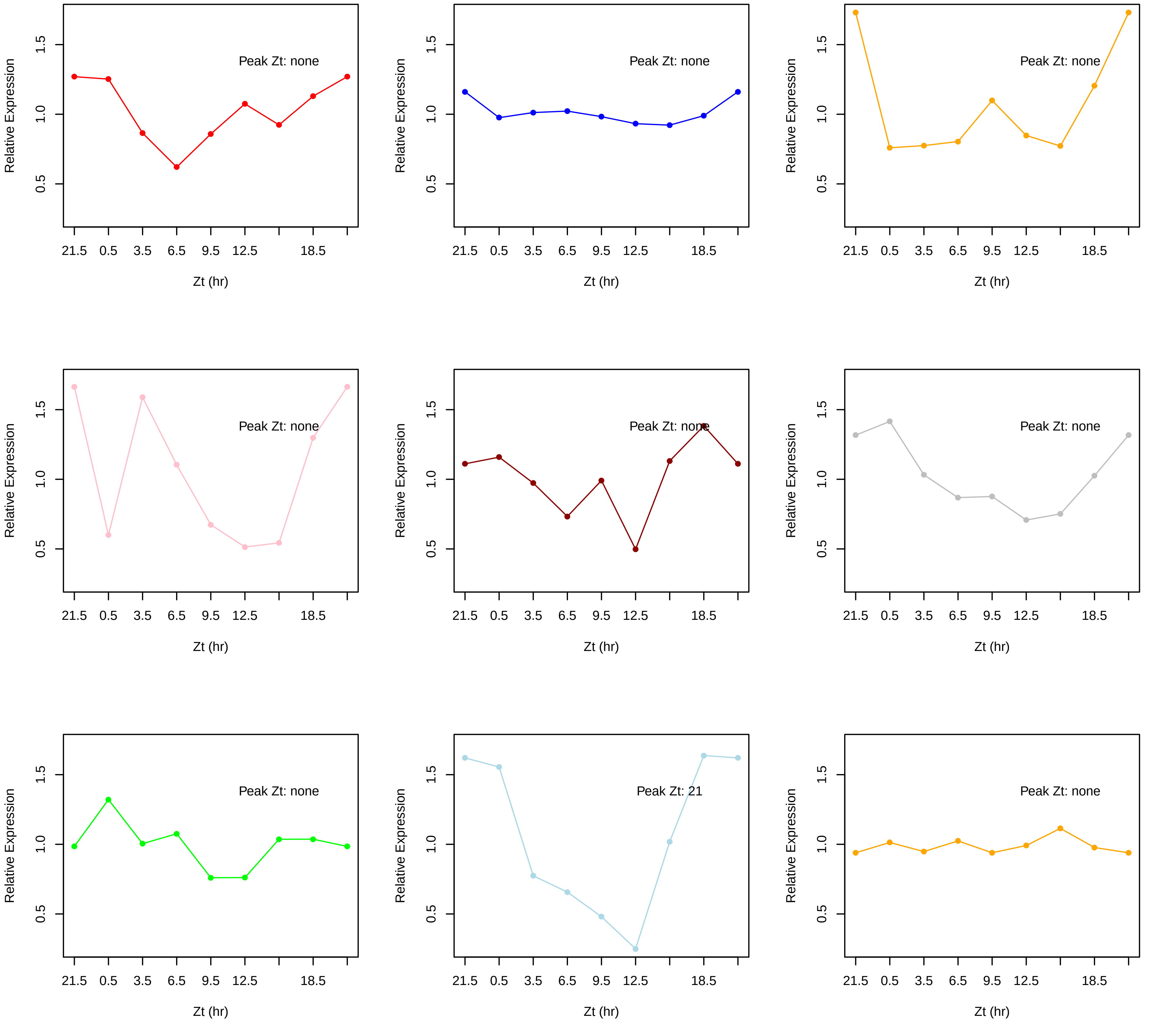

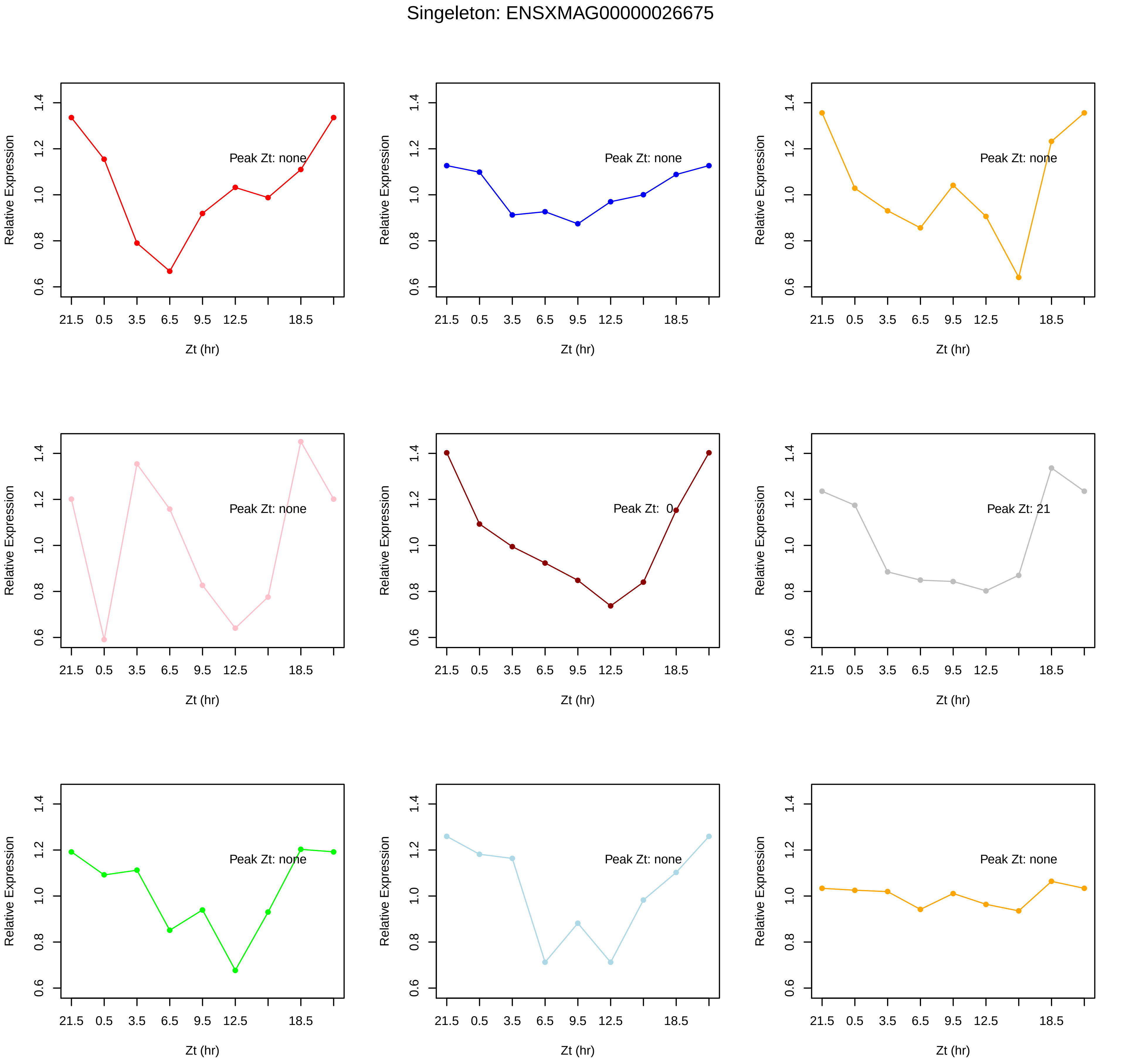
Singeleton: ENSXMAG00000026675
Relative Expression
Zt (hr)
21.5 0.5 3.5 6.5 9.5 12.5 18.5
0.6 0.8 1.0 1.2 1.4
Peak Zt: none
Peak Zt: none
Peak Zt: none
Peak Zt: none
Peak Zt: 0
Peak Zt: 21
Peak Zt: none
Peak Zt: none
Peak Zt: none

Singleton: ENSXMAG00000016918 cox11

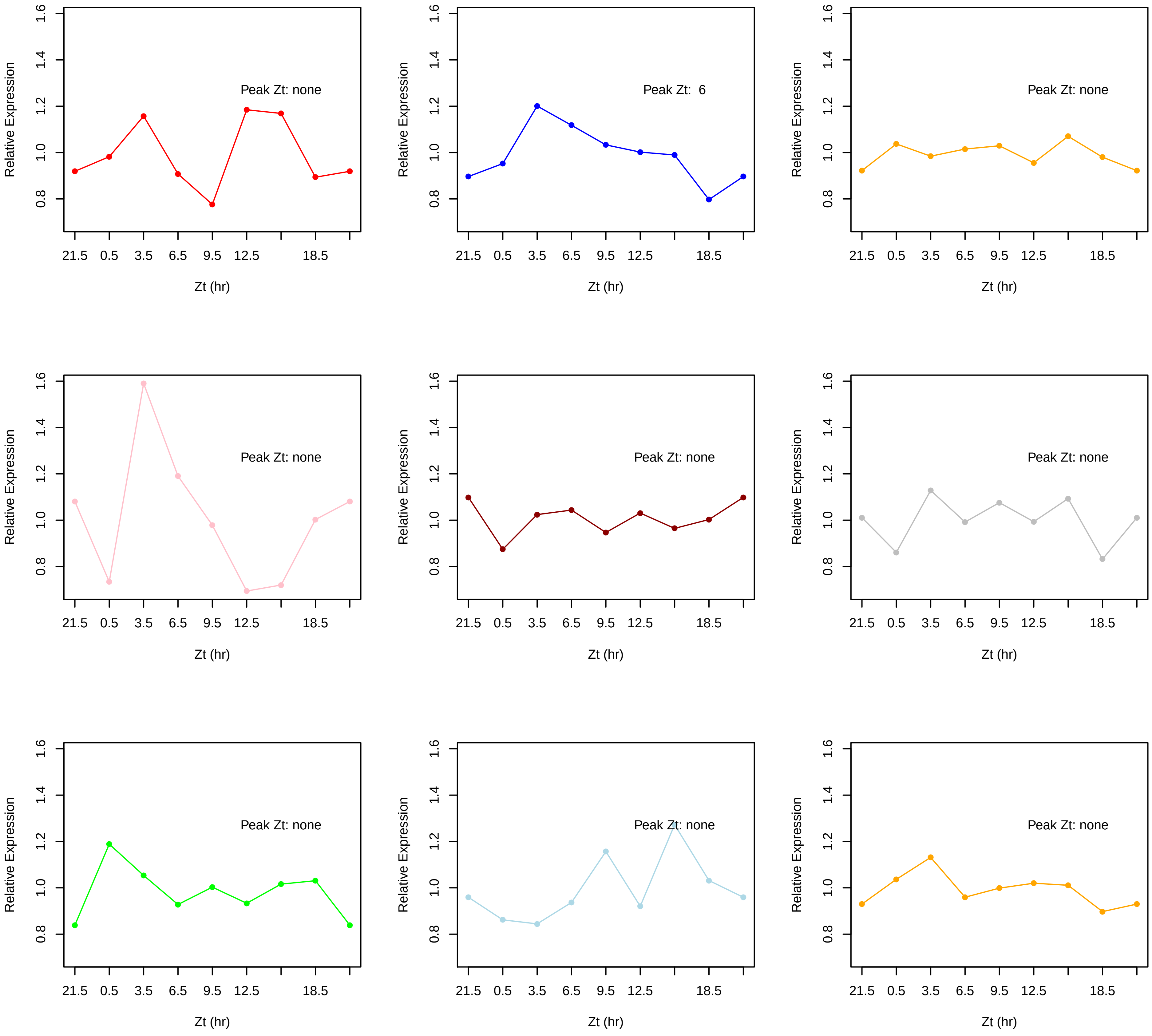

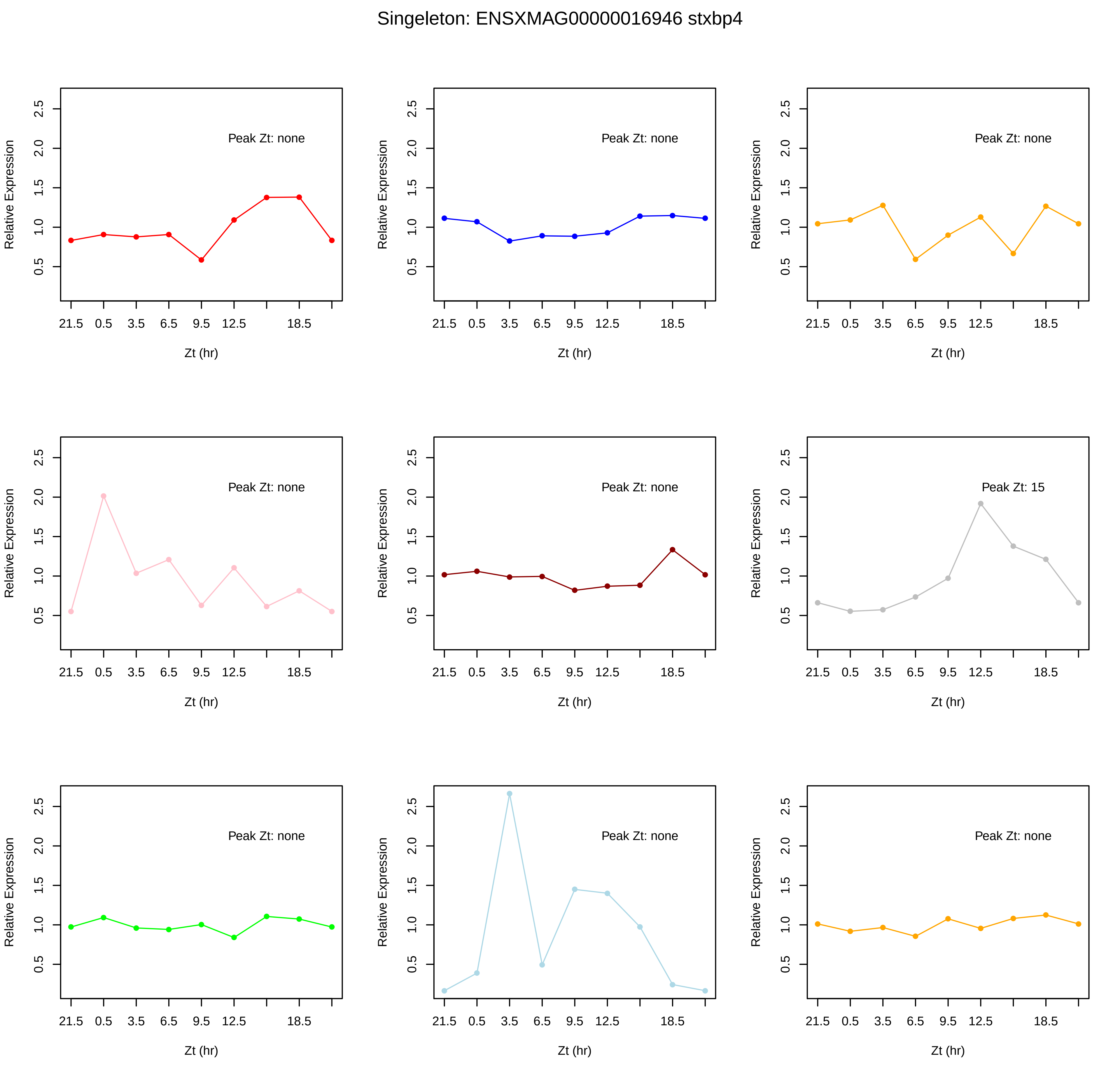
Singeleton: ENSXMAG00000016946 stxbp4
Peak Zt: none
Peak Zt: none
Peak Zt: none
Peak Zt: none
Peak Zt: none
Peak Zt: 15
Peak Zt: none
Peak Zt: none
Peak Zt: none
Relative Expression
Zt (hr)
21.5 0.5 3.5 6.5 9.5 12.5 18.5
0.5 1.0 1.5 2.0 2.5

Singeleton: ENSXMAG00000026378 vti1a

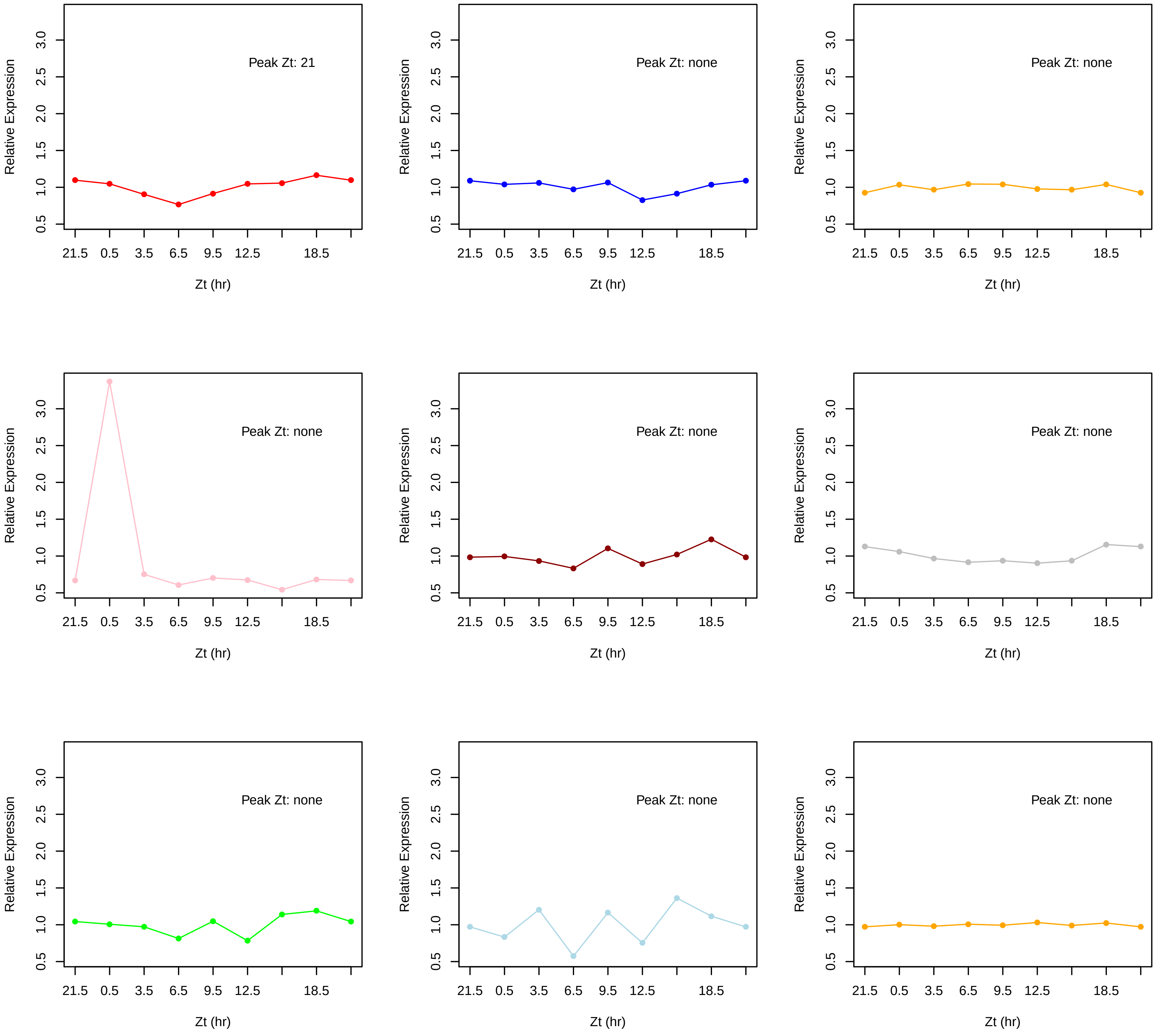

Singeleton: ENSXMAG00000009557 meis2a

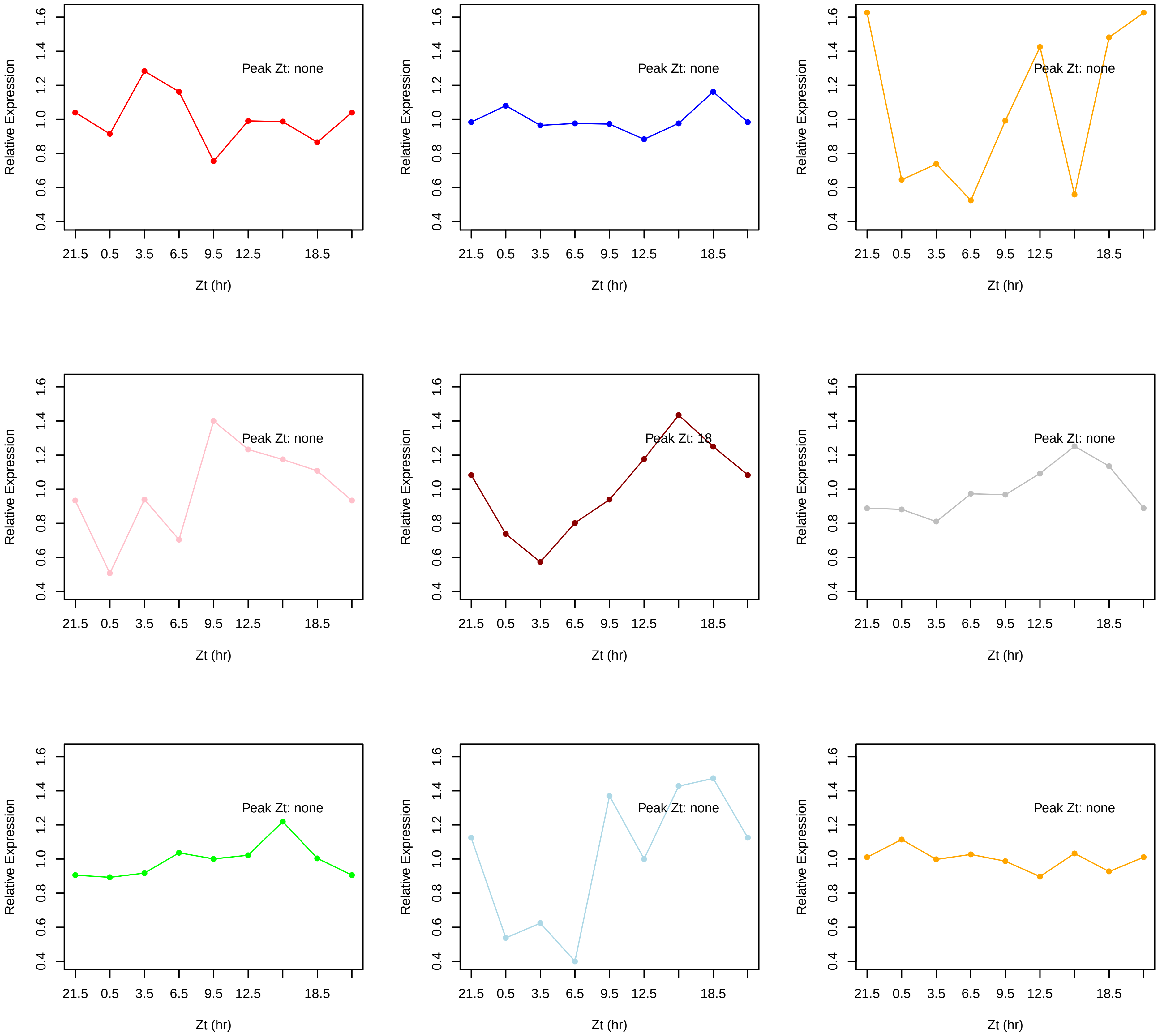

Singeleton: ENSXMAG00000014696 eif2d

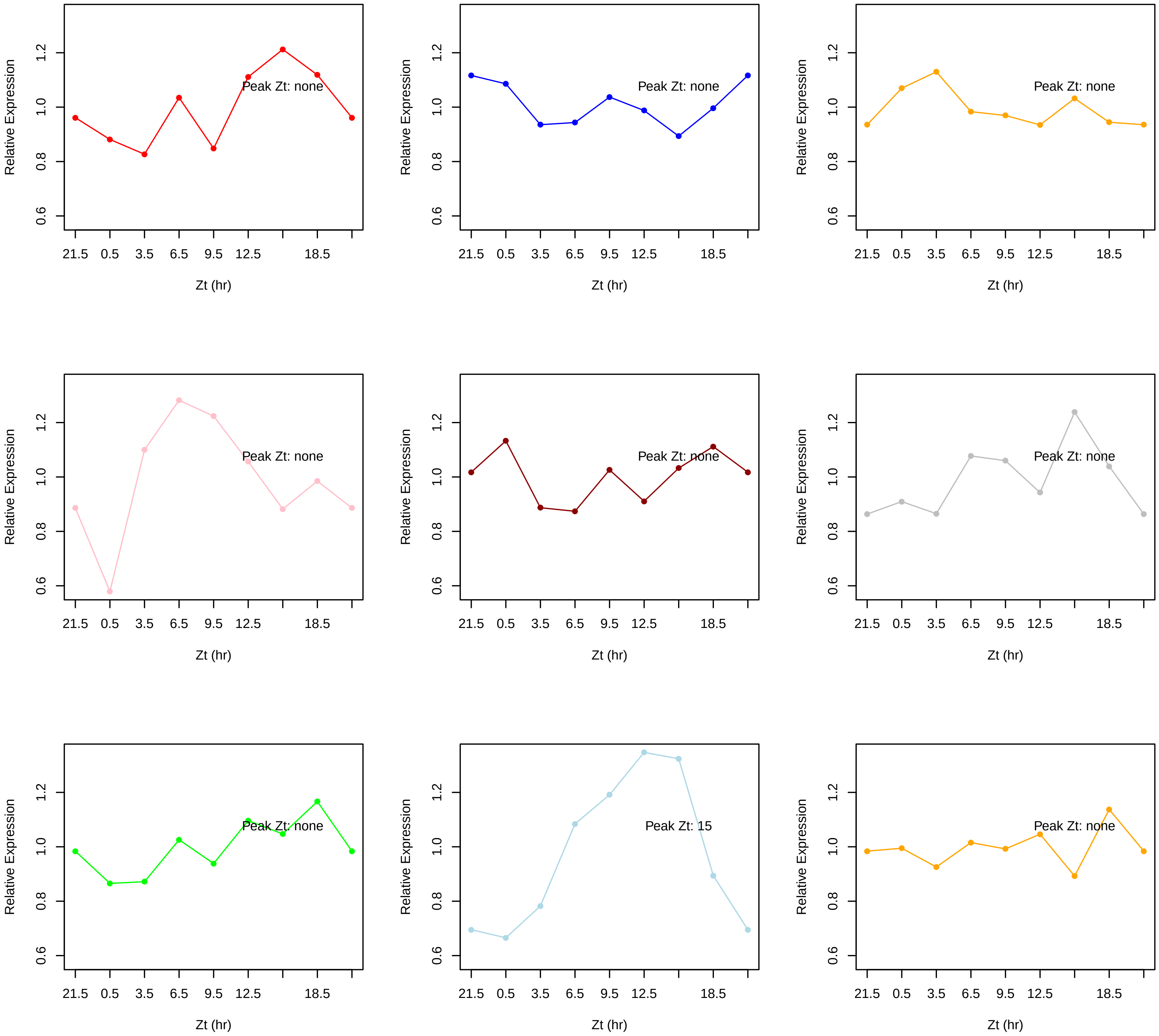

Singeleton: ENSXMAG00000028629 mmd

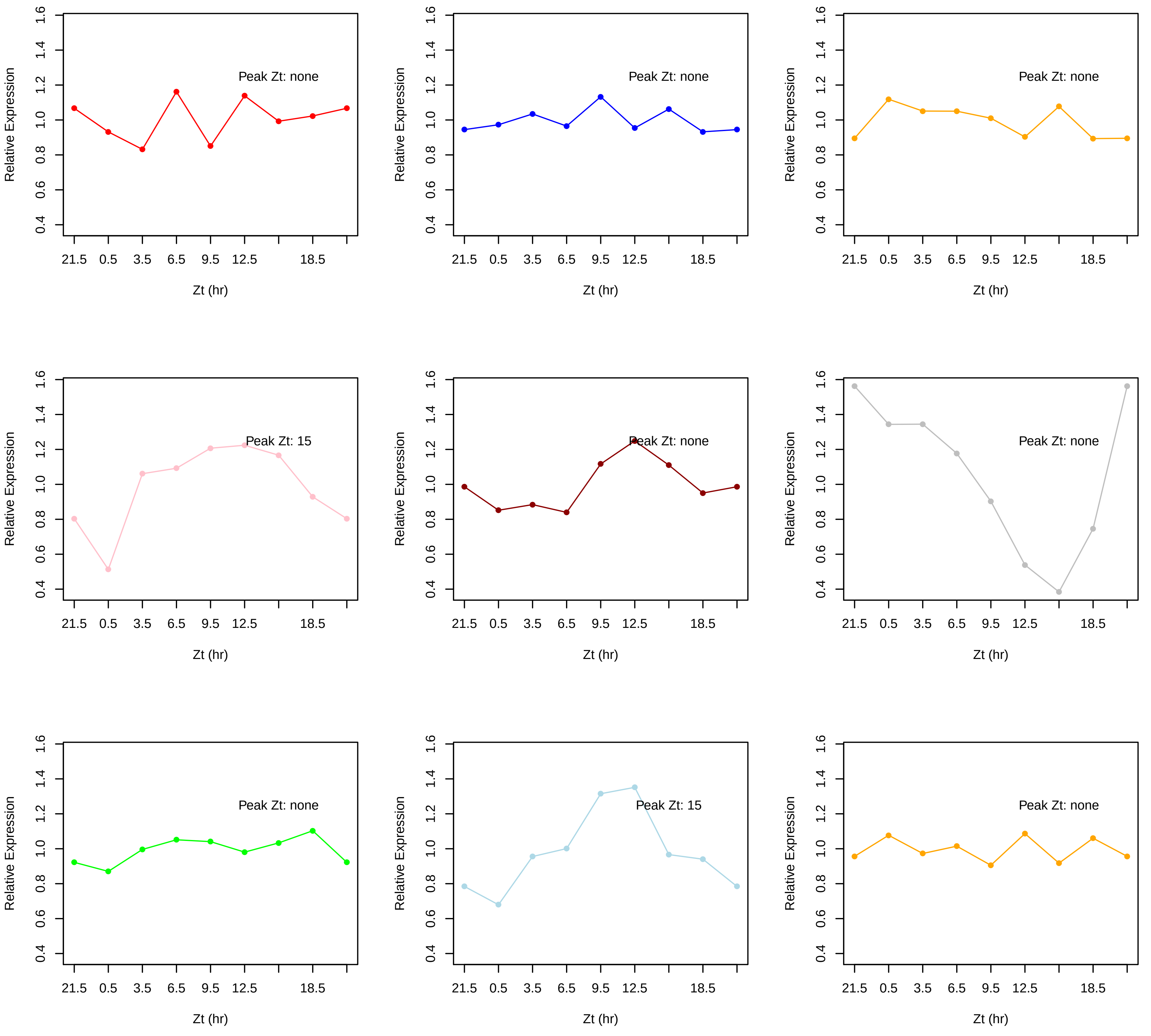

Singeleton: ENSXMAG00000003582 pde6b

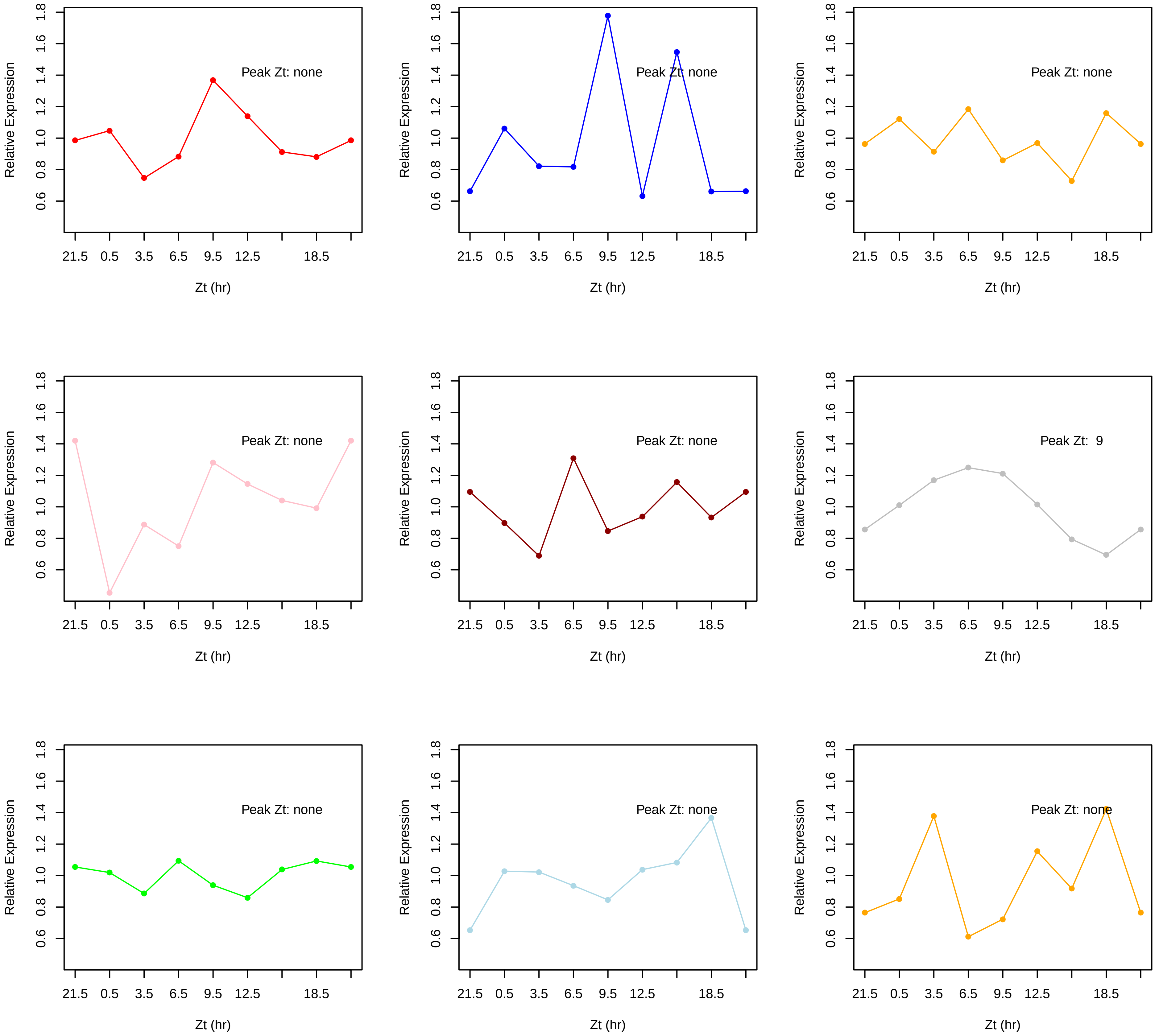

Singeleton: ENSXMAG00000014776 zdhhc6

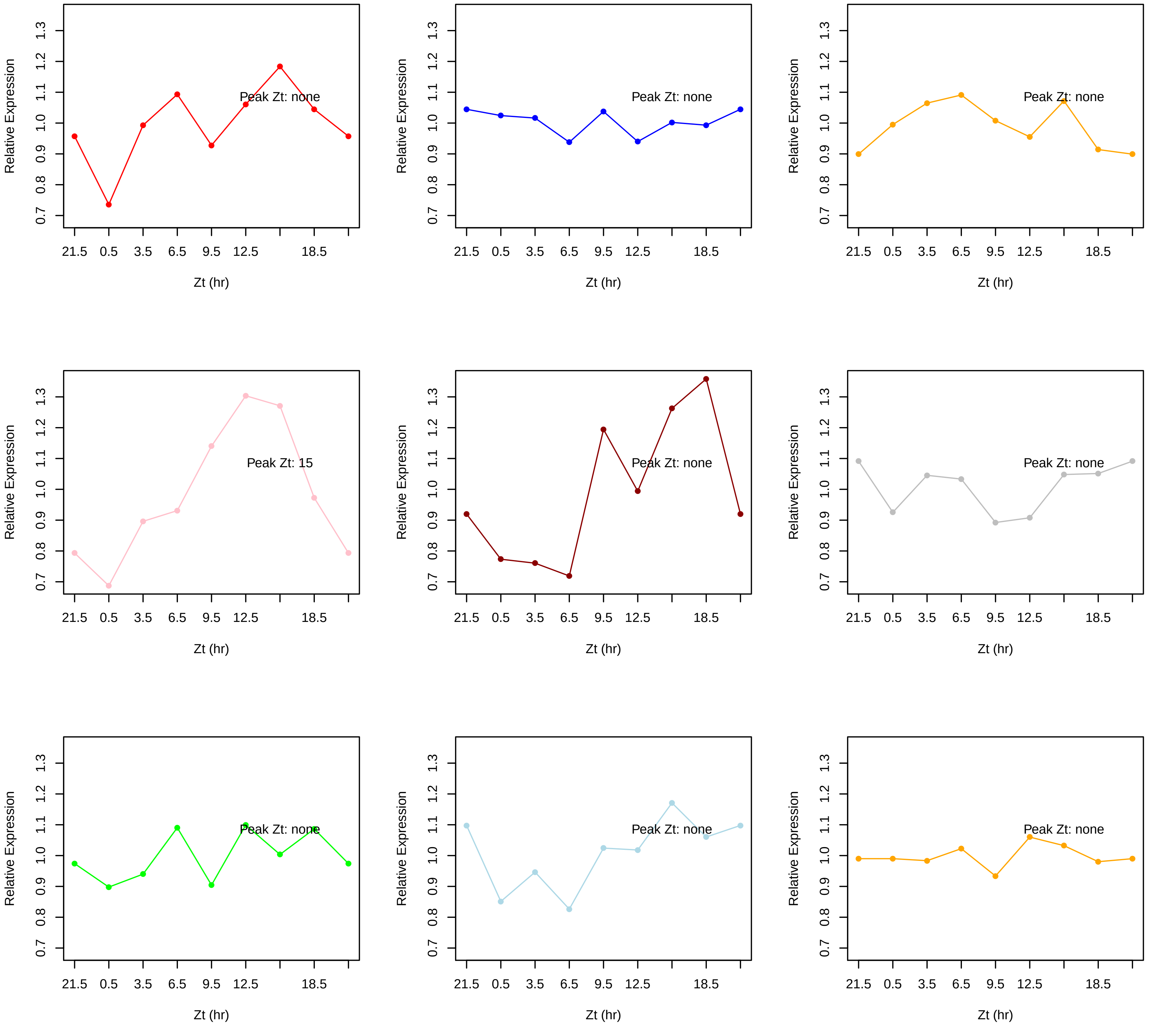

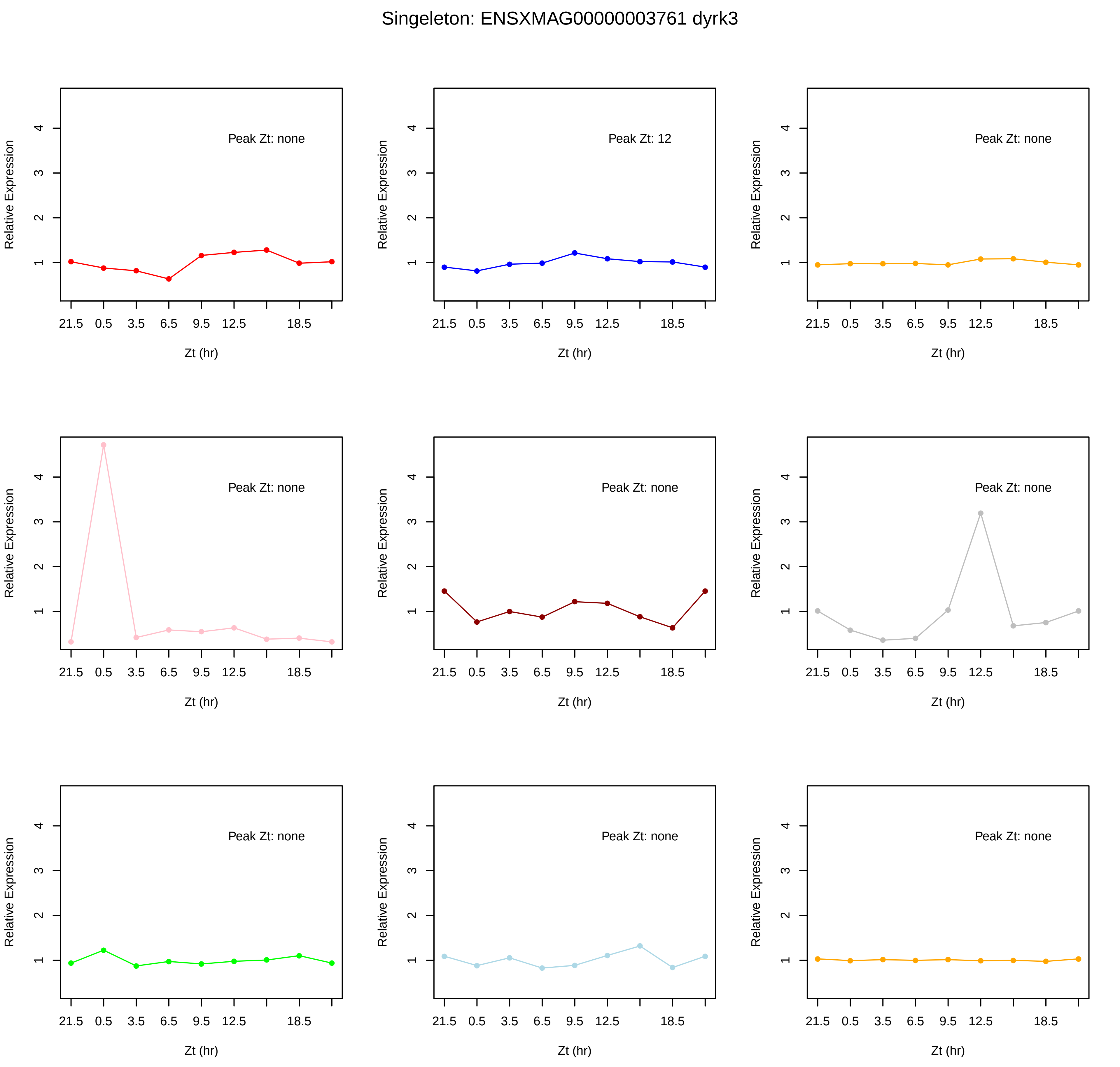
Singleton: ENSXMAG00000003761 dyrk3
Peak Zt: none
Peak Zt: 12
Peak Zt: none
Peak Zt: none
Peak Zt: none
Peak Zt: none
Peak Zt: none
Peak Zt: none
Peak Zt: none
Relative Expression
Zt (hr)
21.5
0.5
3.5
6.5
9.5
12.5
18.5
1
2
3
4

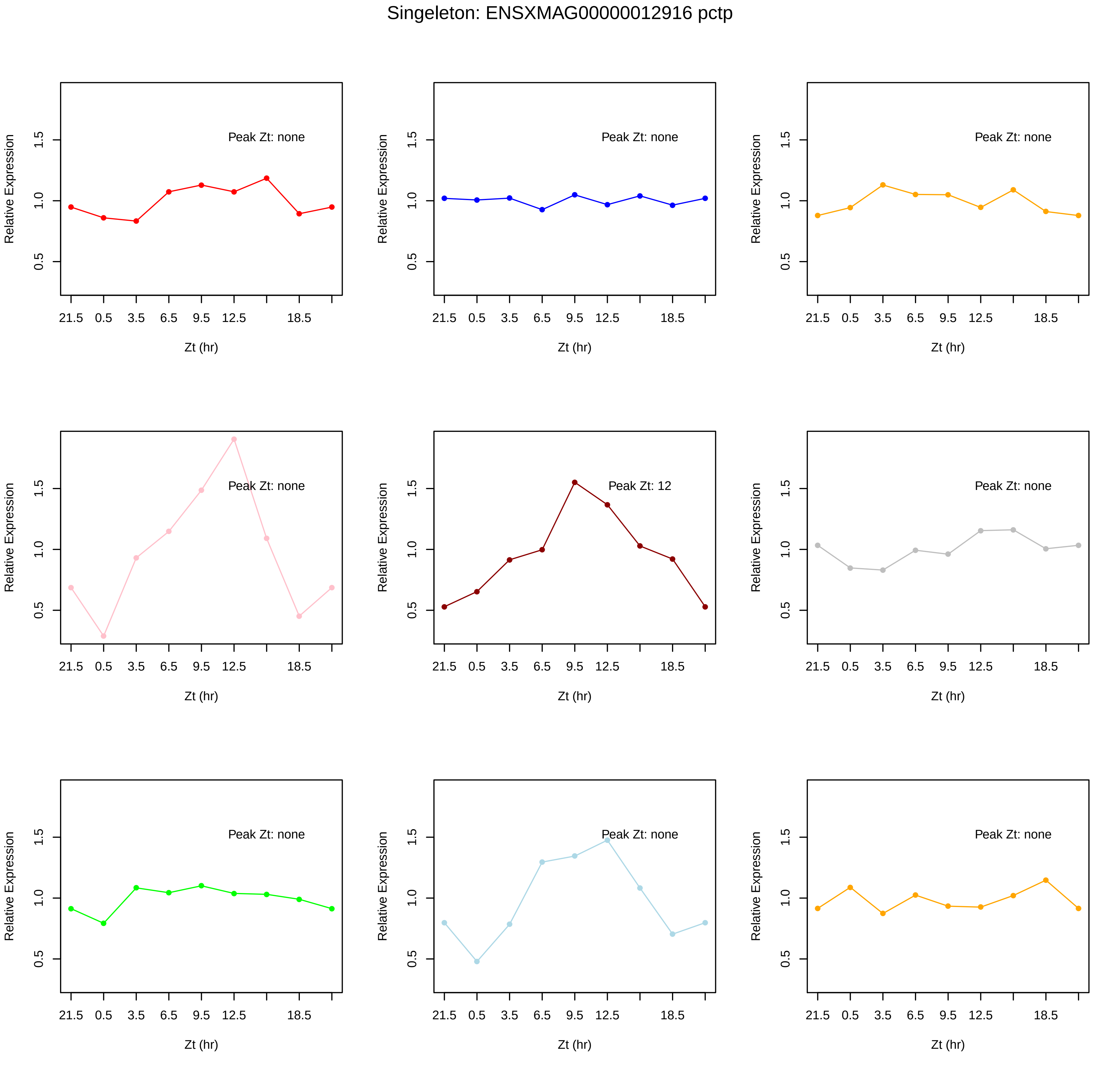
Singeleton: ENSXMAG00000012916 pctp
Peak Zt: none
Peak Zt: none
Peak Zt: none
Peak Zt: none
Peak Zt: 12
Peak Zt: none
Peak Zt: none
Peak Zt: none
Peak Zt: none
Relative Expression
Zt (hr)
21.5 0.5 3.5 6.5 9.5 12.5 18.5
0.5 1.0 1.5

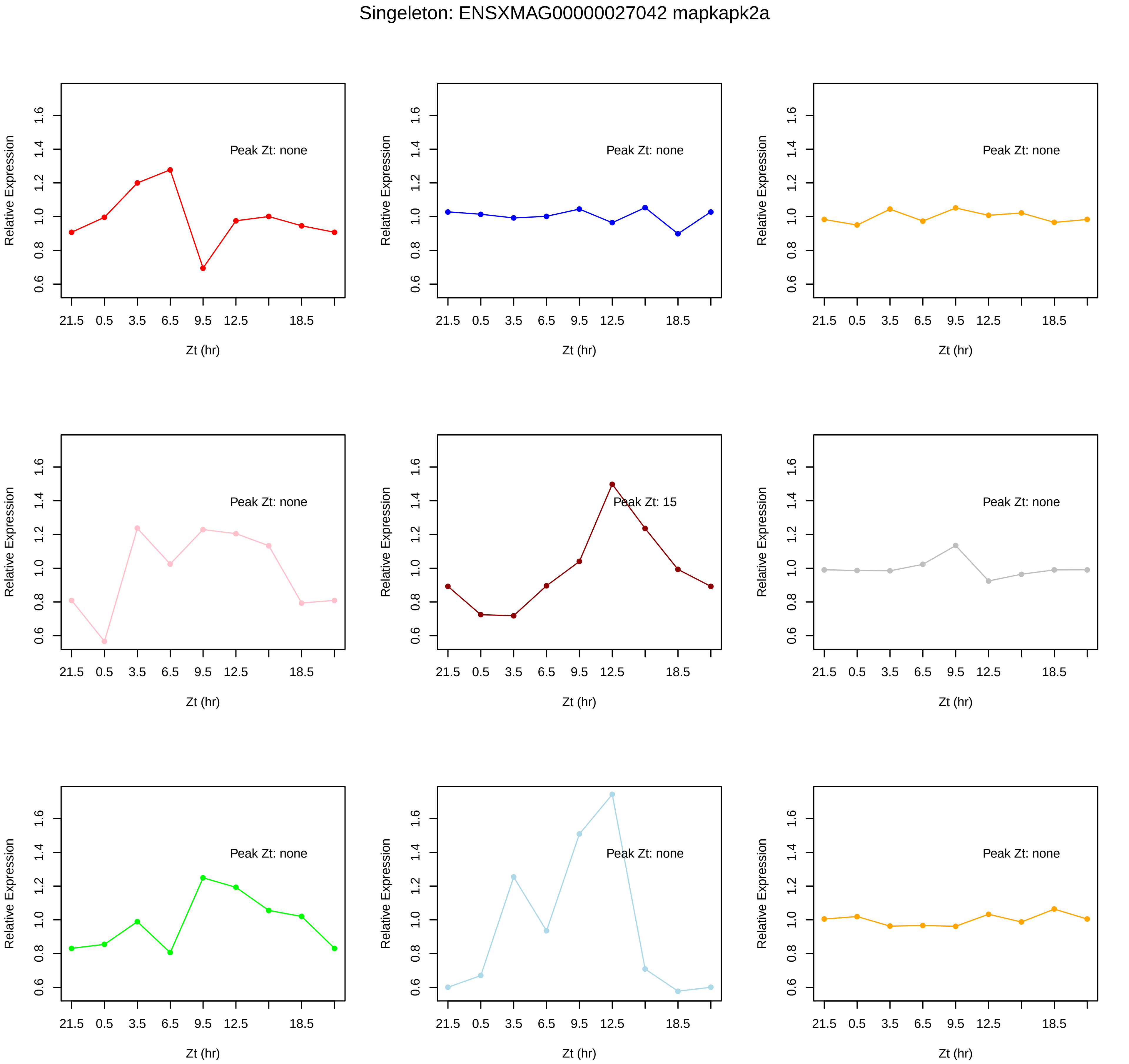
Singeleton: ENSXMAG00000027042 mapkapk2a
Peak Zt: none
Peak Zt: 15
Relative Expression
Zt (hr)
21.5 0.5 3.5 6.5 9.5 12.5 18.5
0.6 0.8 1.0 1.2 1.4 1.6

Singleton: ENSXMAG00000008516 cxcl14

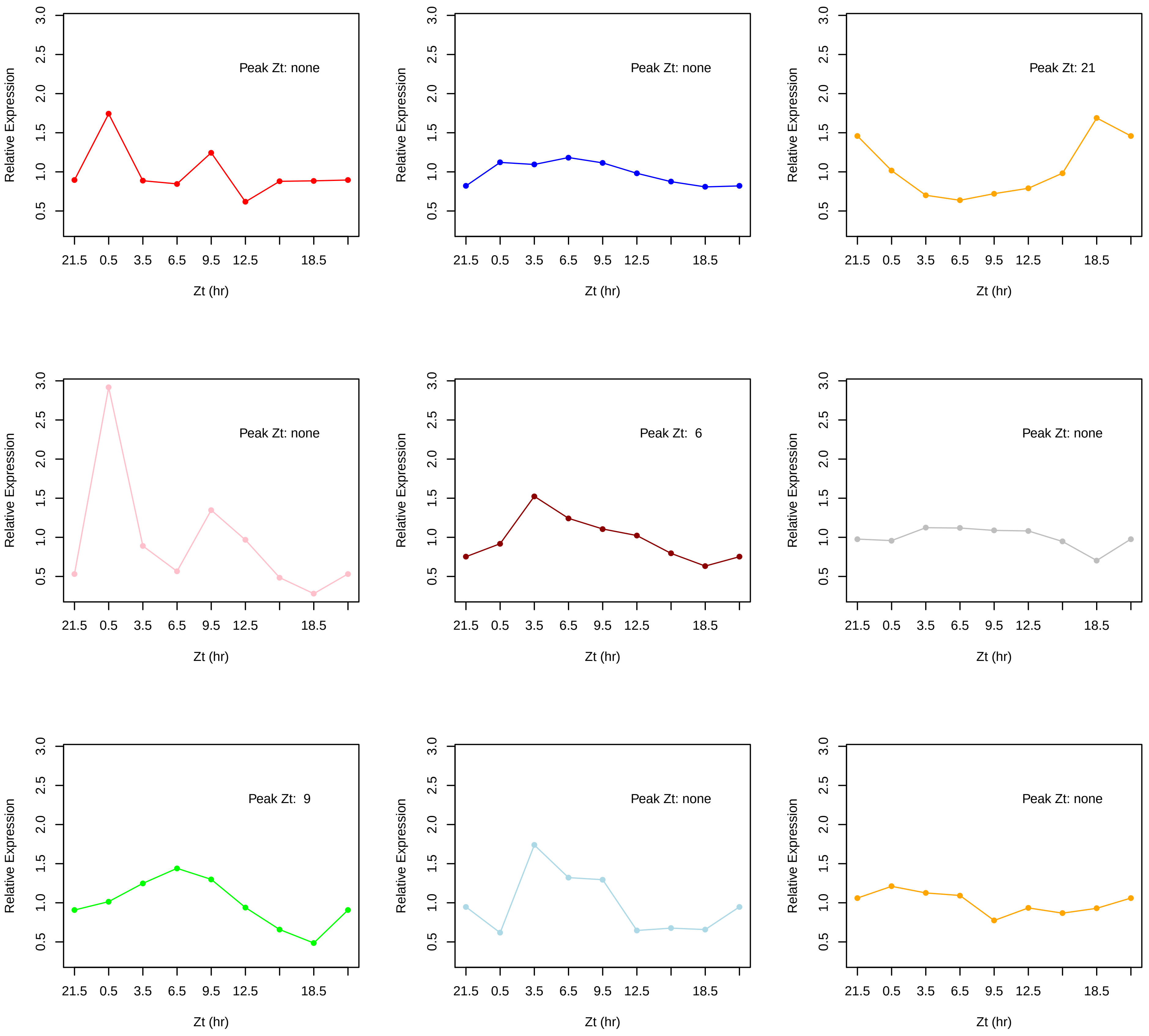

Singeleton: ENSXMAG00000010172 pla2g12a

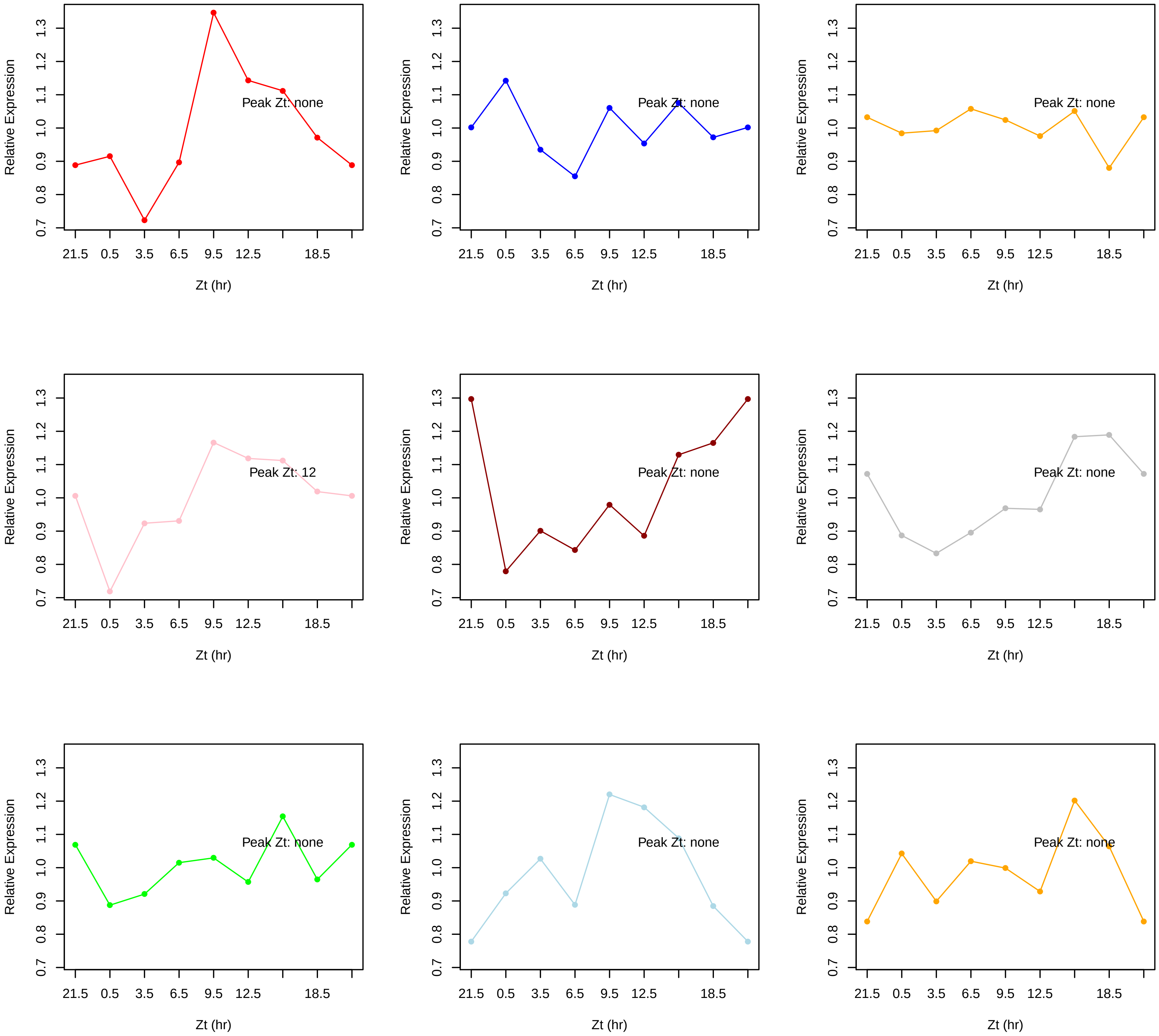

Singeleton: ENSXMAG00000017724 gucy2g

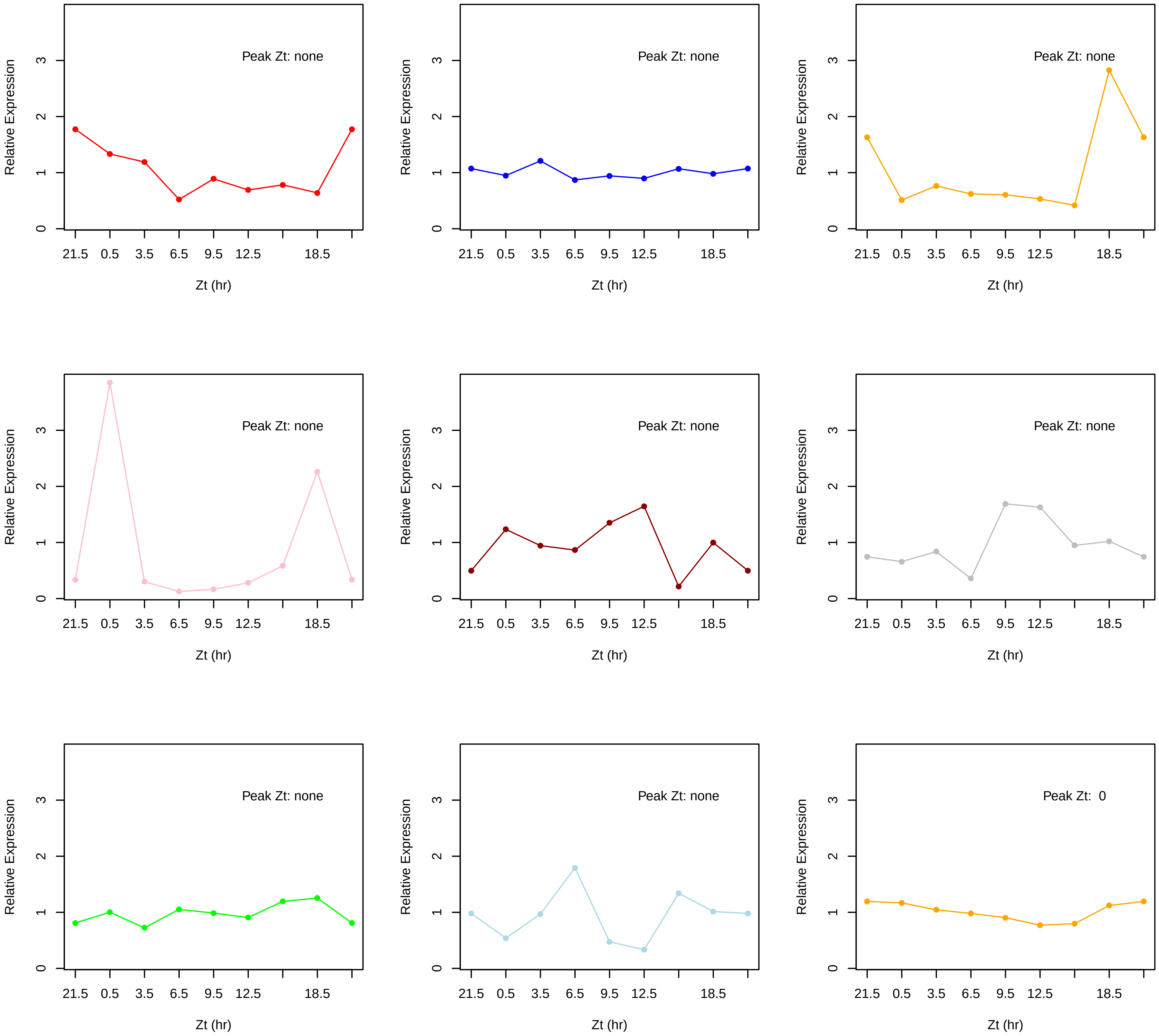

Singeleton: ENSXMAG00000008518 fam13b

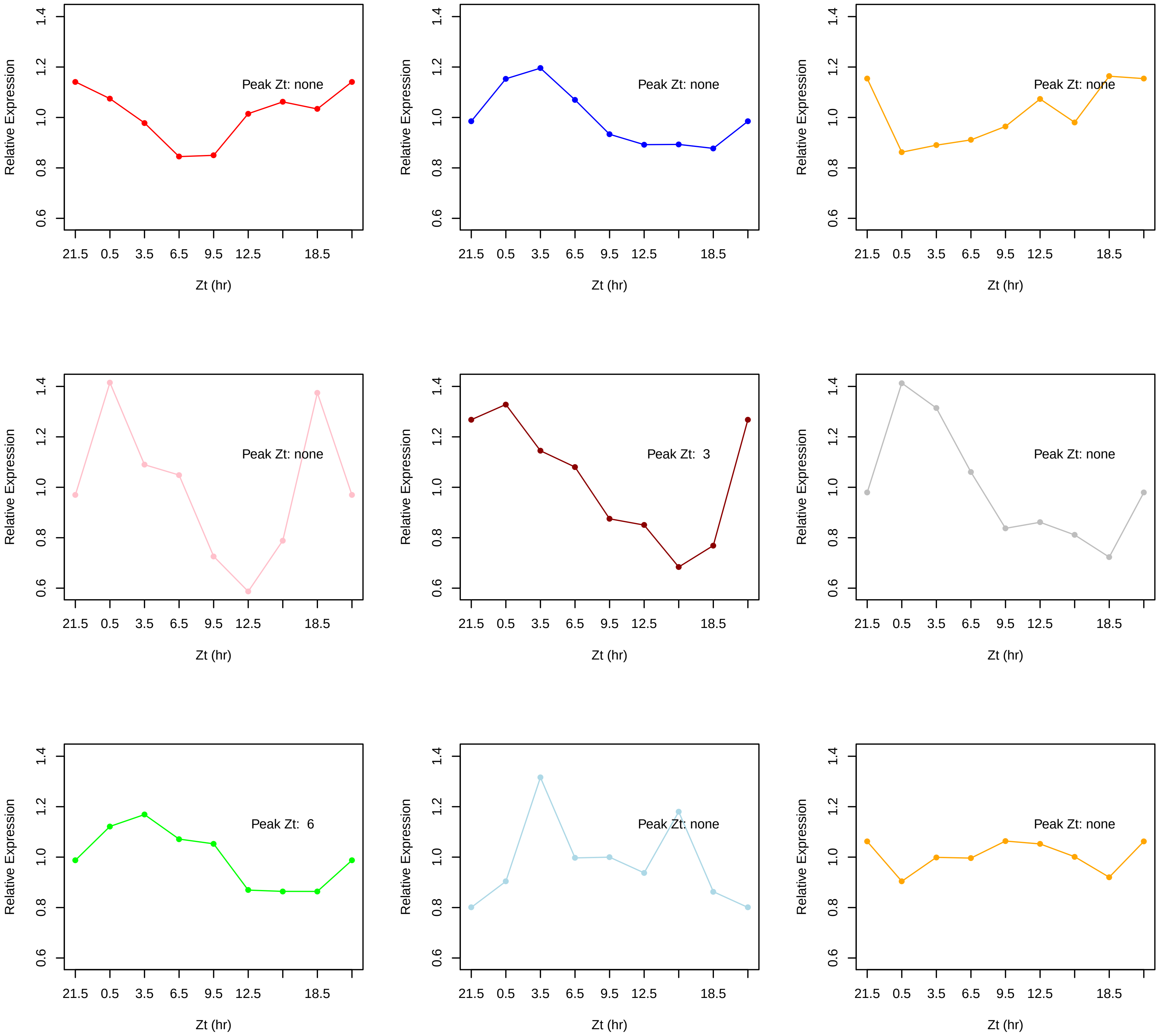

Singeleton: ENSXMAG00000014464

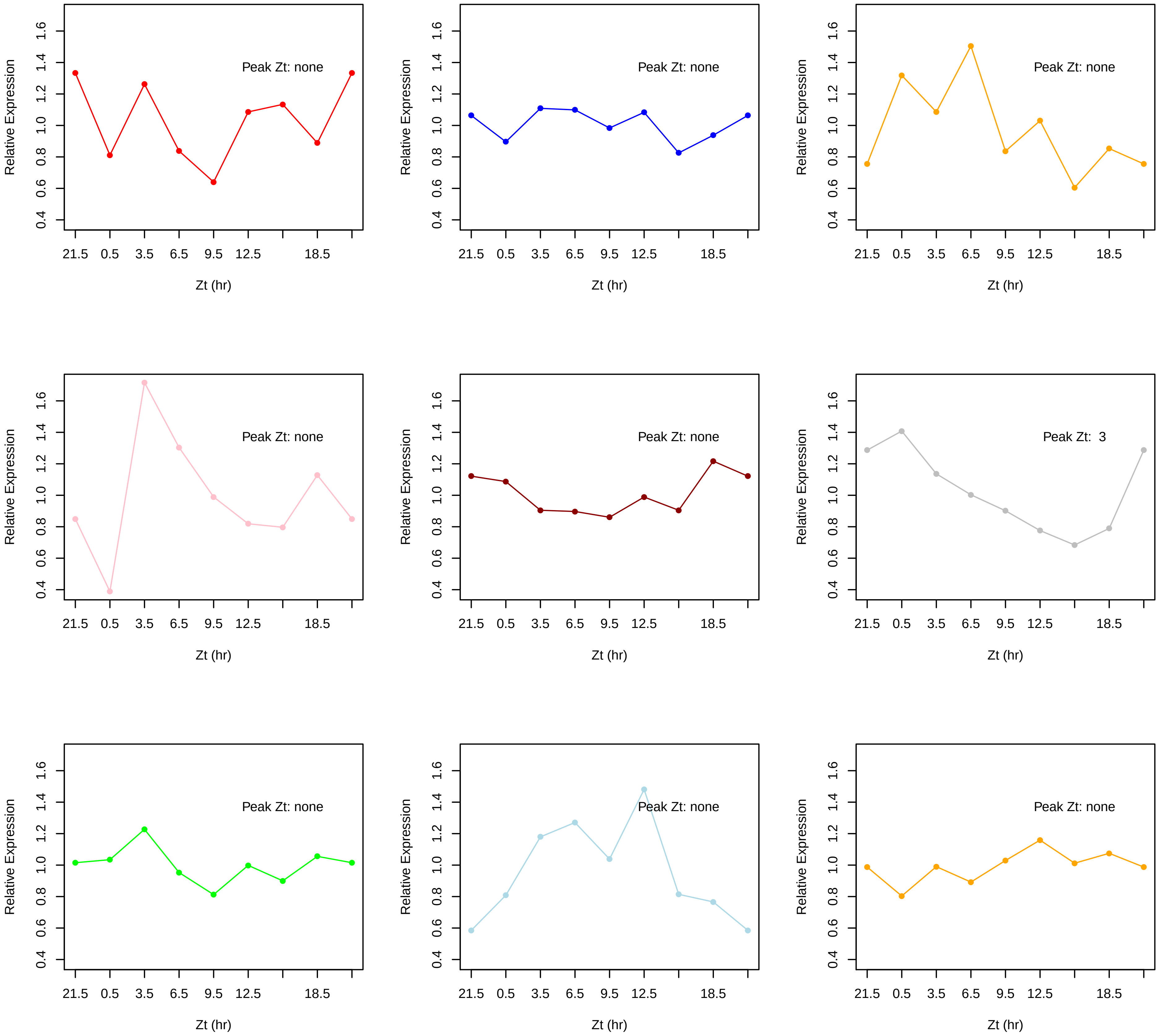

Singeleton: ENSXMAG00000010142 paip1

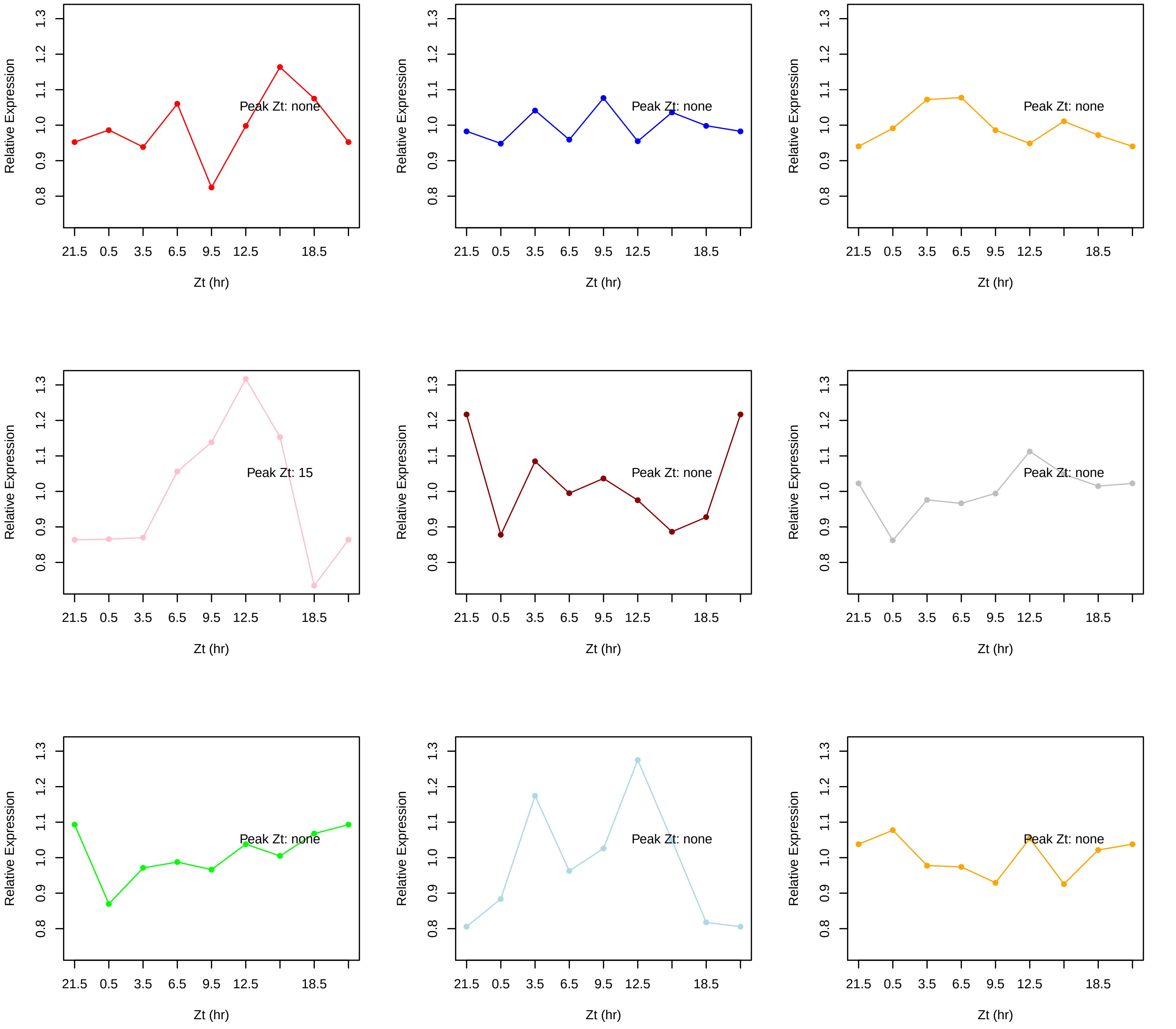

Singeleton: ENSXMAG00000004594 idi1

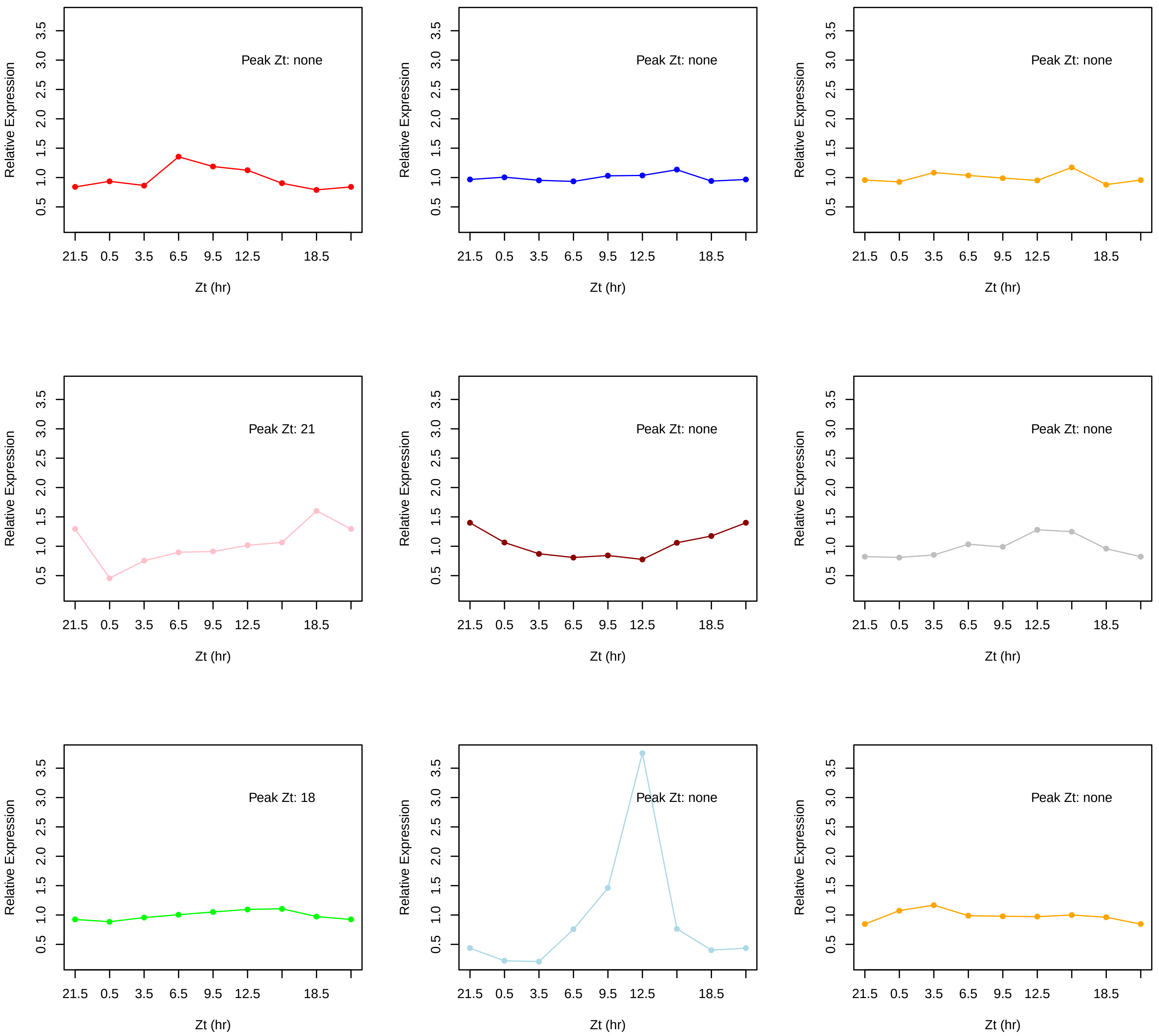

Singeleton: ENSXMAG00000010205 lef1

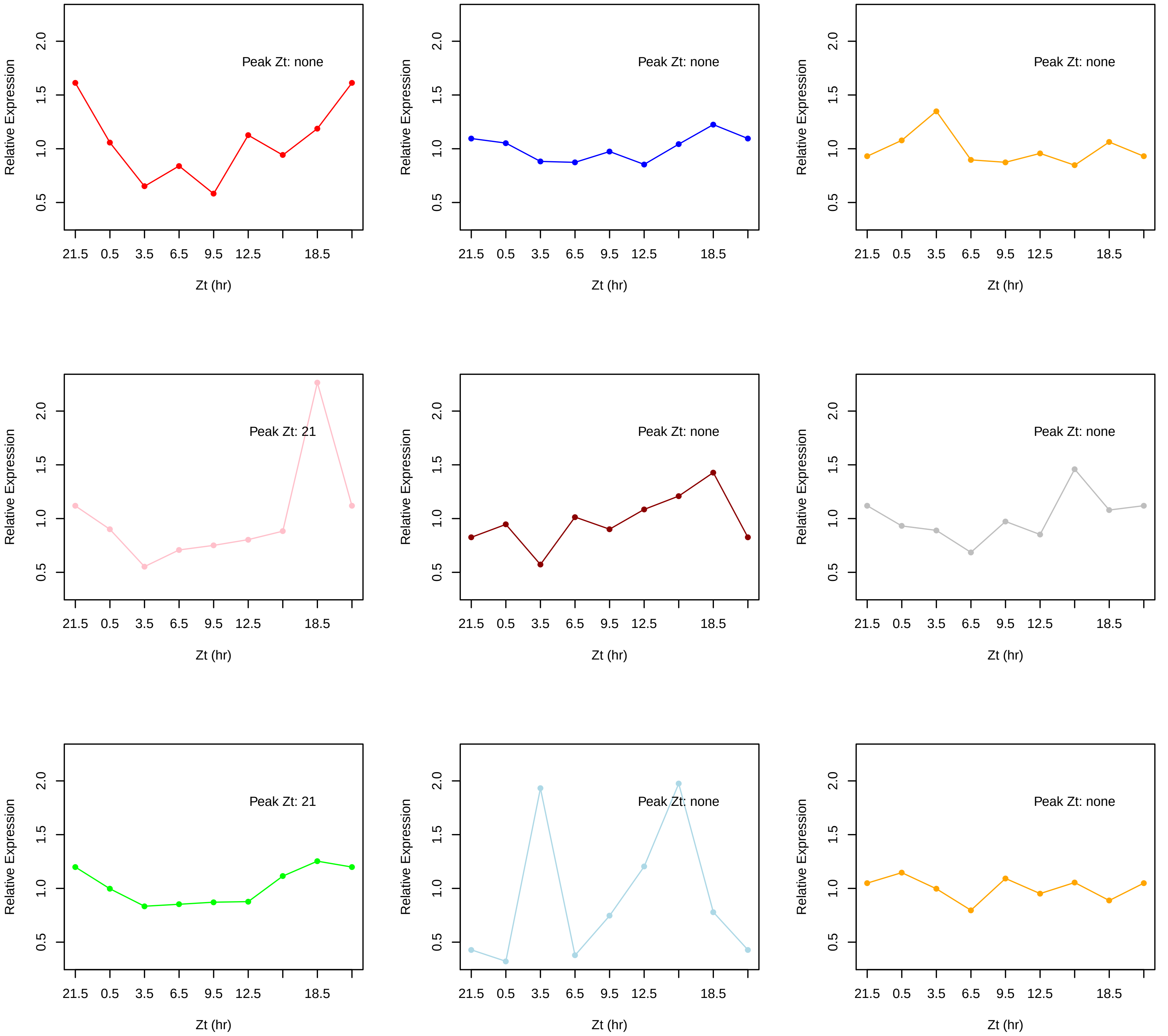

Singeleton: ENSXMAG00000003631 nnt

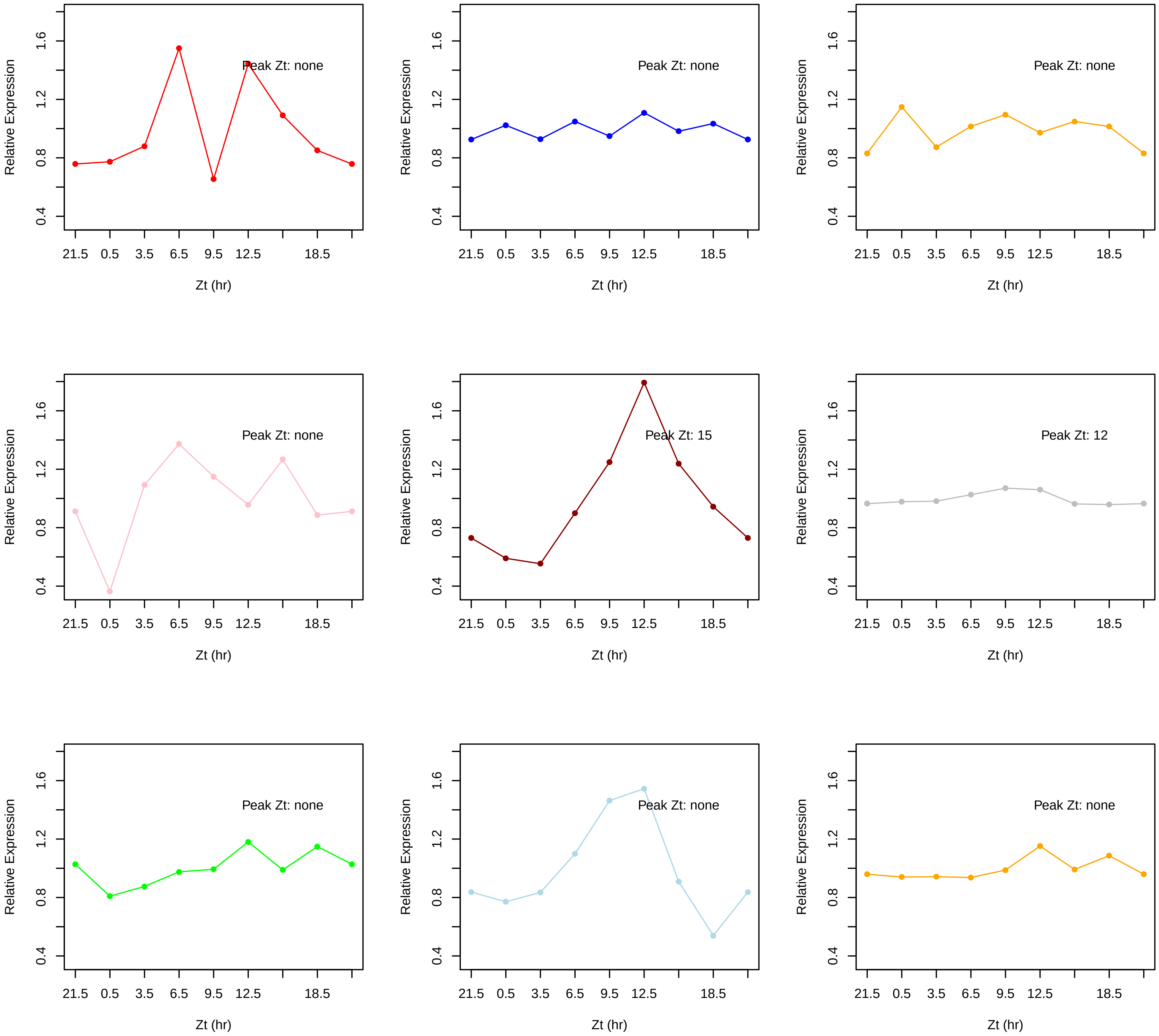

Singeleton: ENSXMAG00000017819 gpam

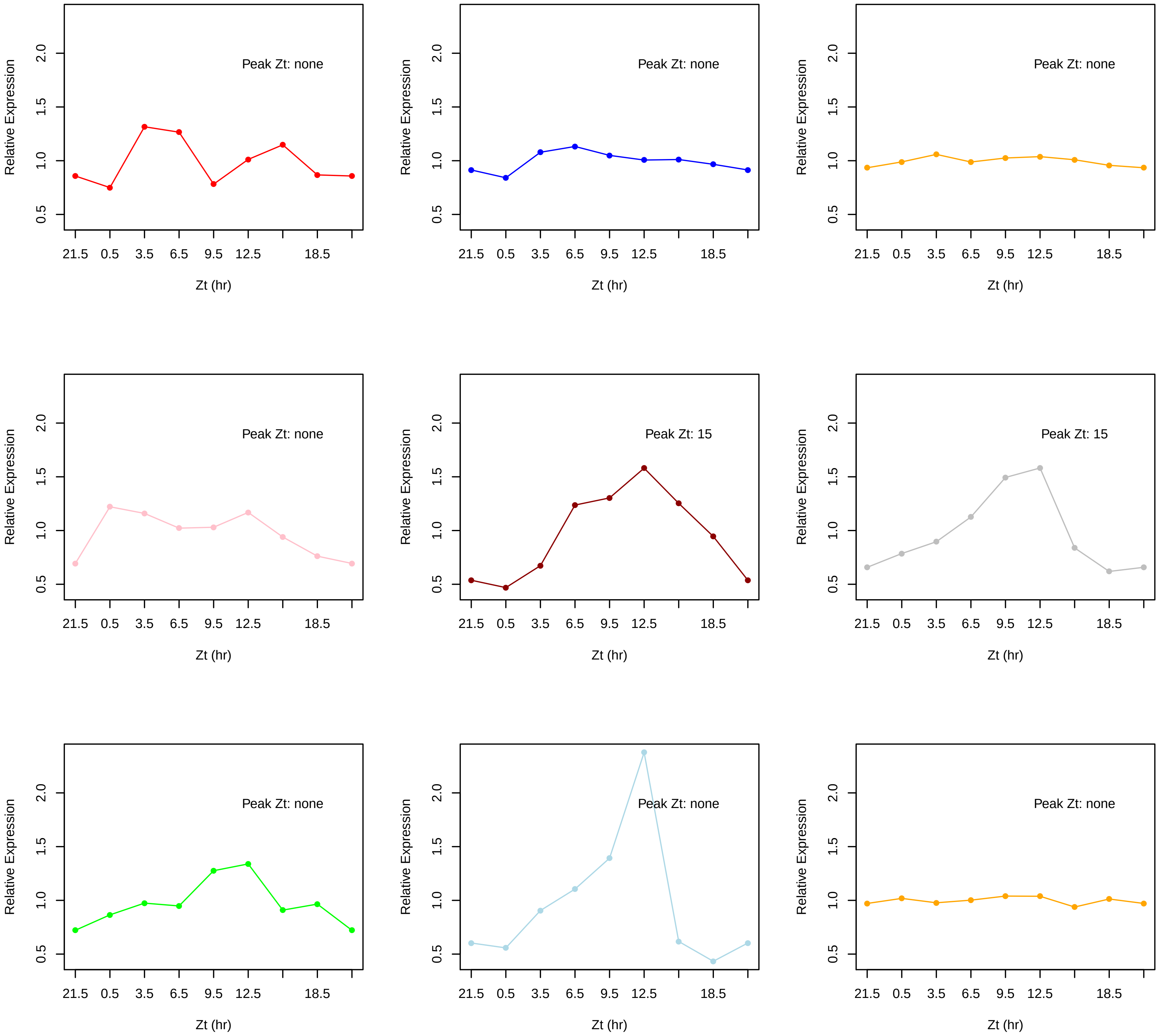

Singeleton: ENSXMAG00000010227 hadh

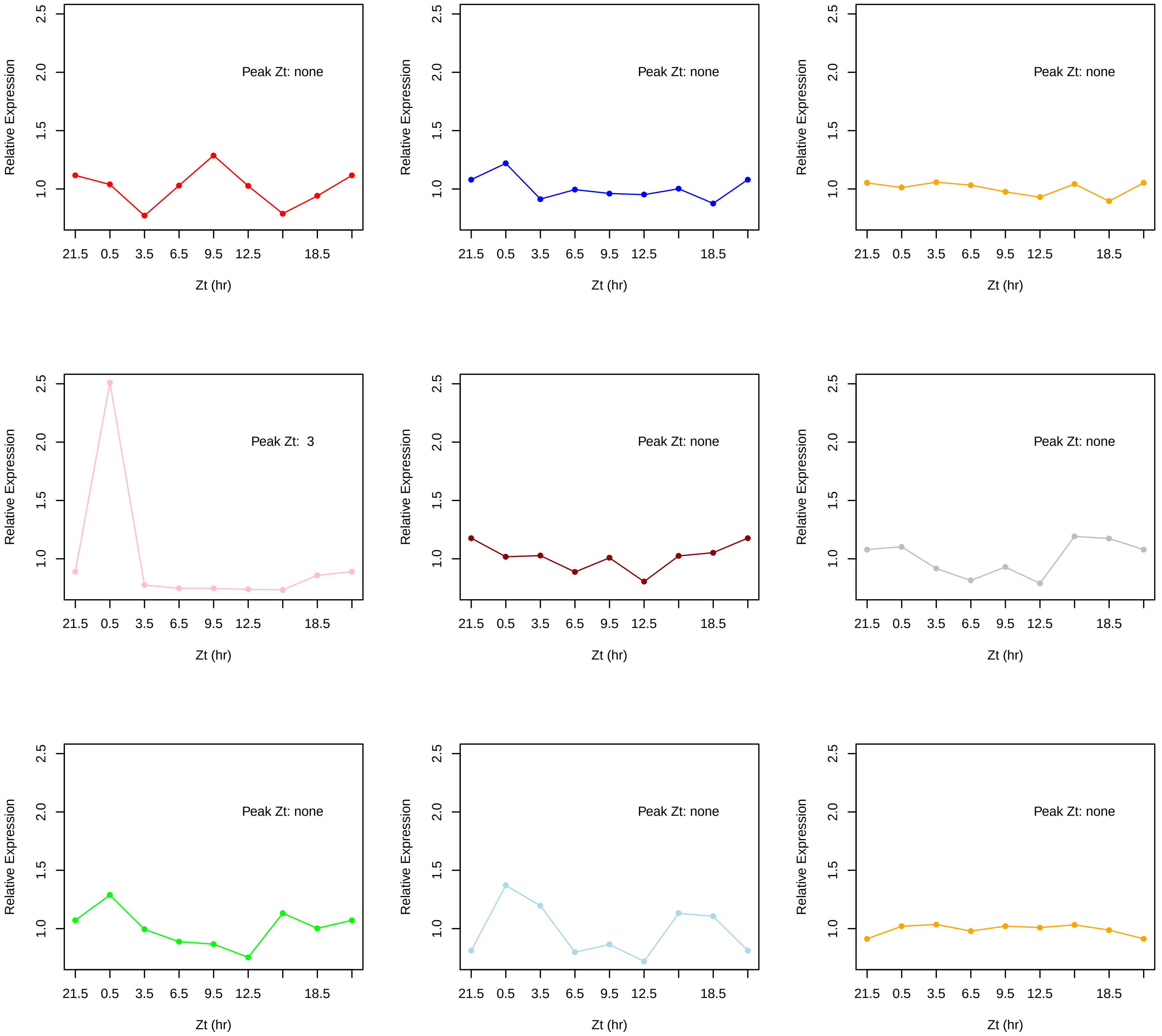

Singeleton: ENSXMAG00000010239 cyp2u1

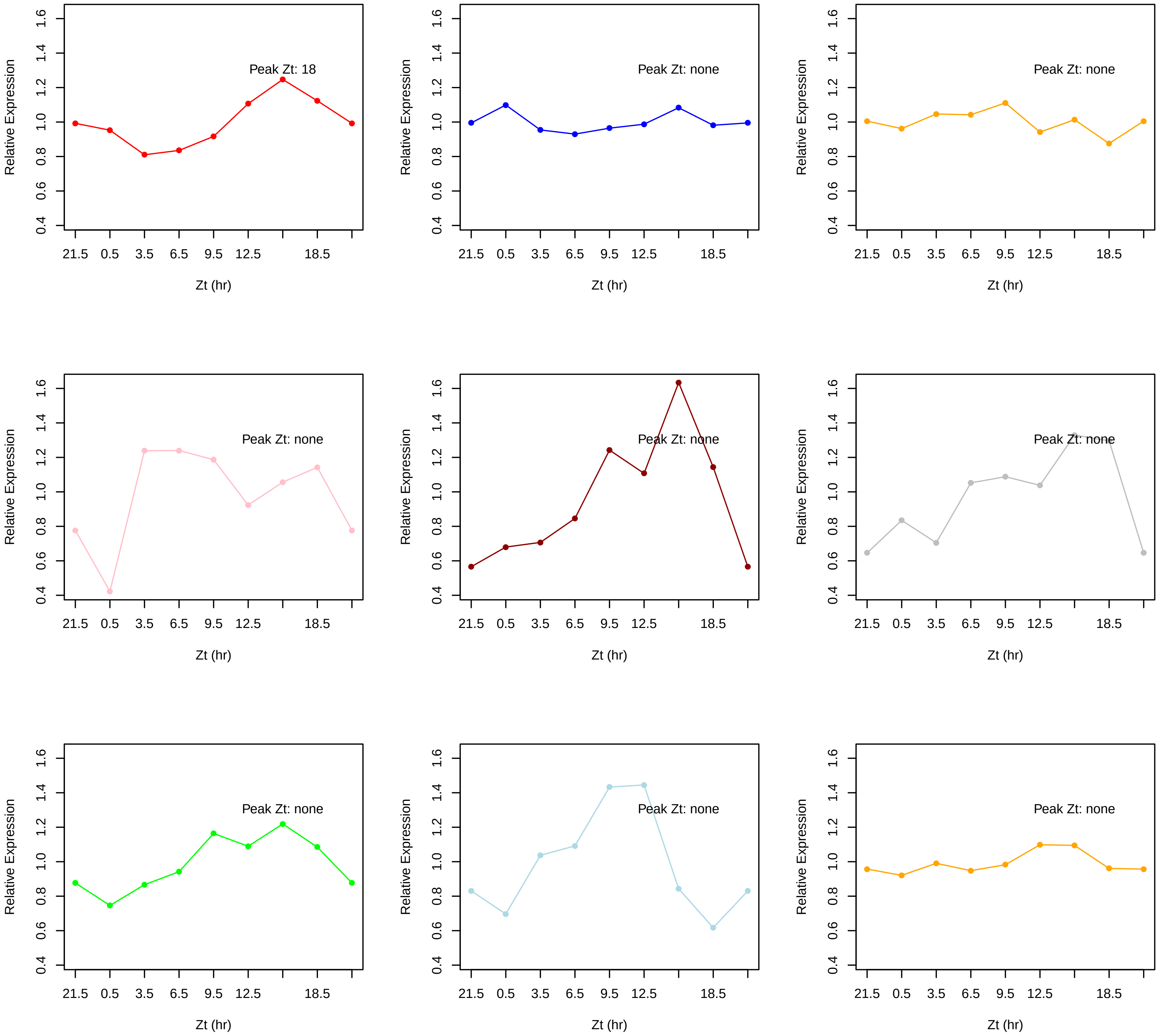

Singeleton: ENSXMAG00000003430 csnk1db

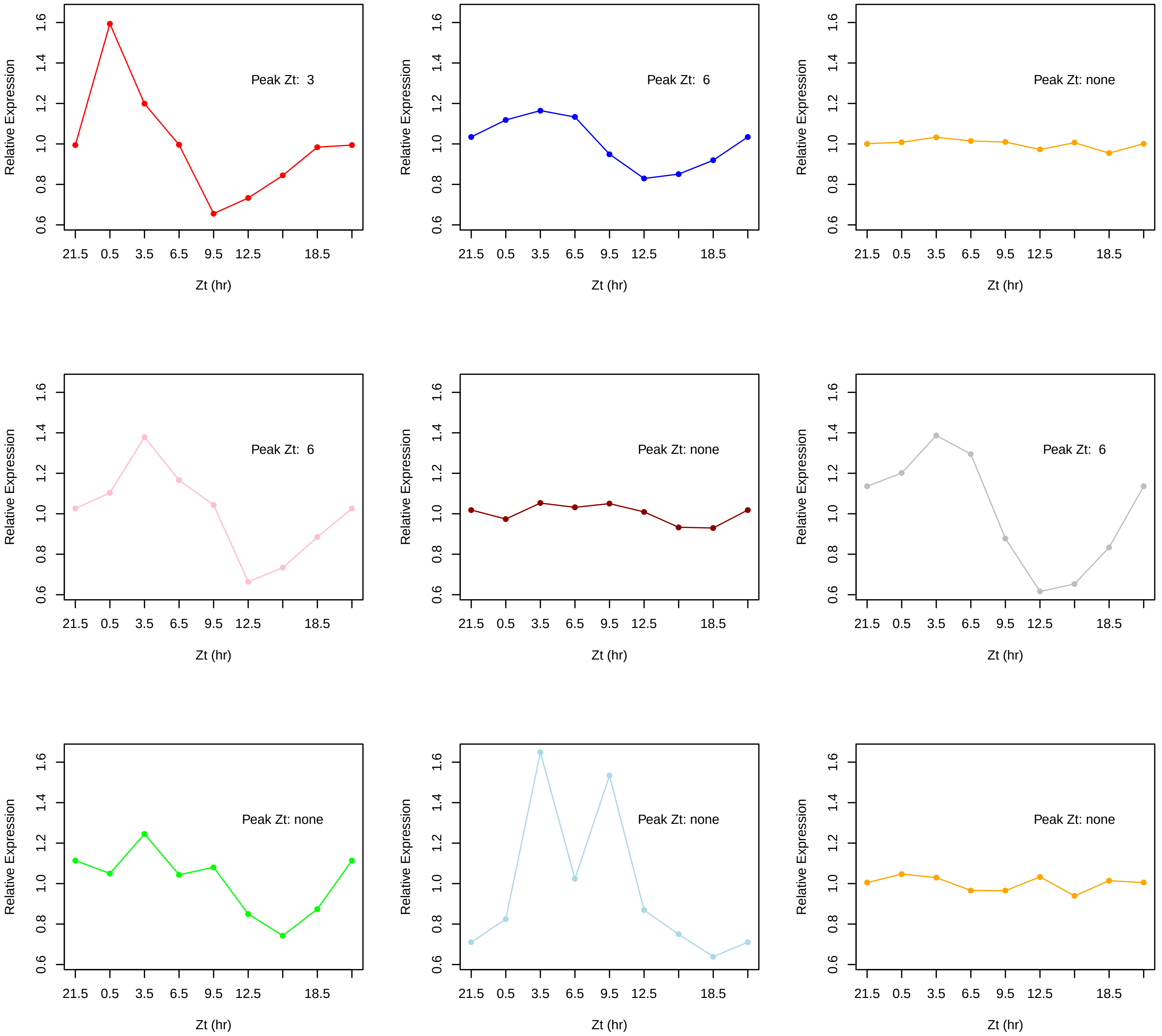

Singeleton: ENSXMAG00000003662 rai14

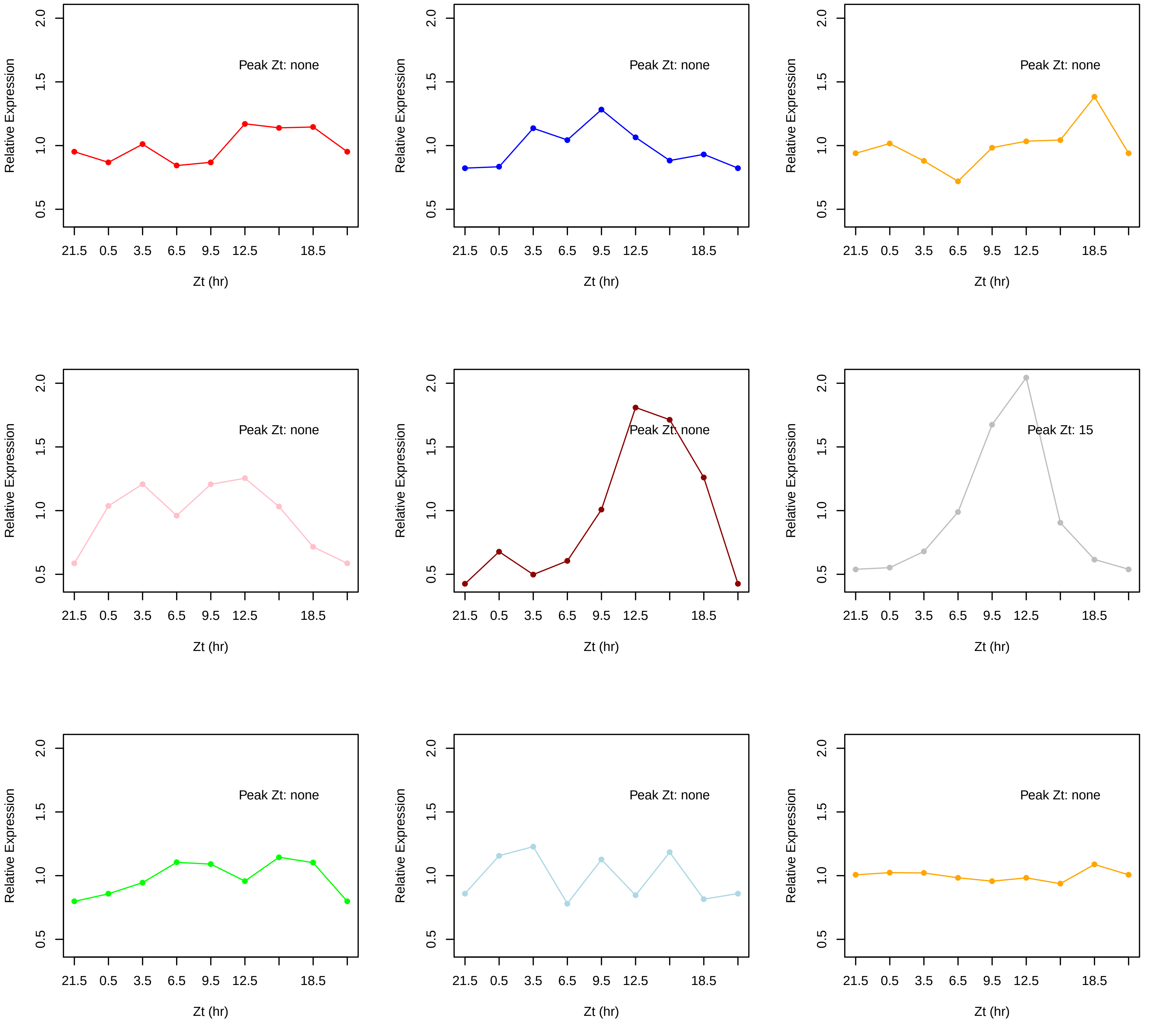

Singeleton: ENSXMAG00000029085 ngrn

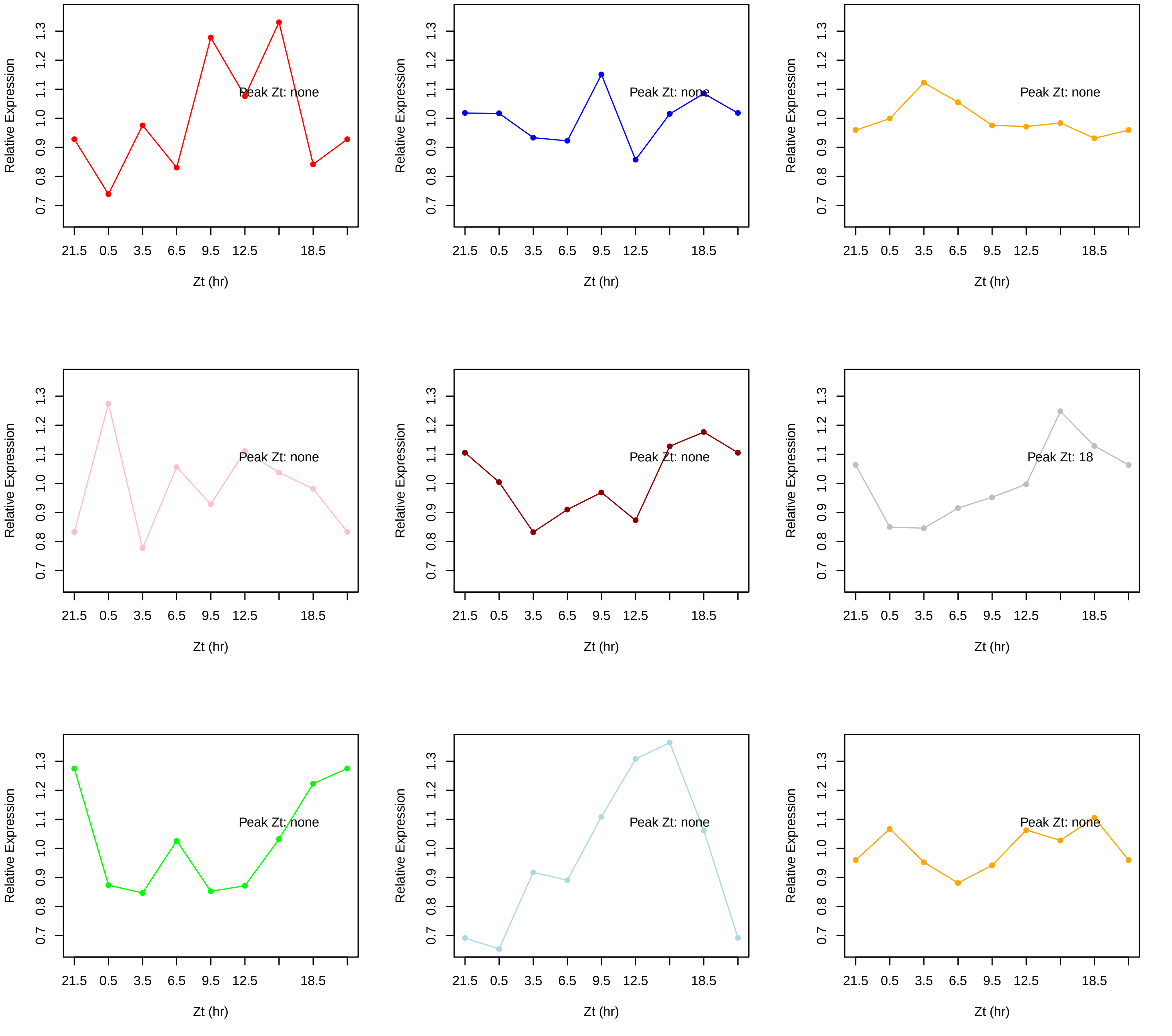

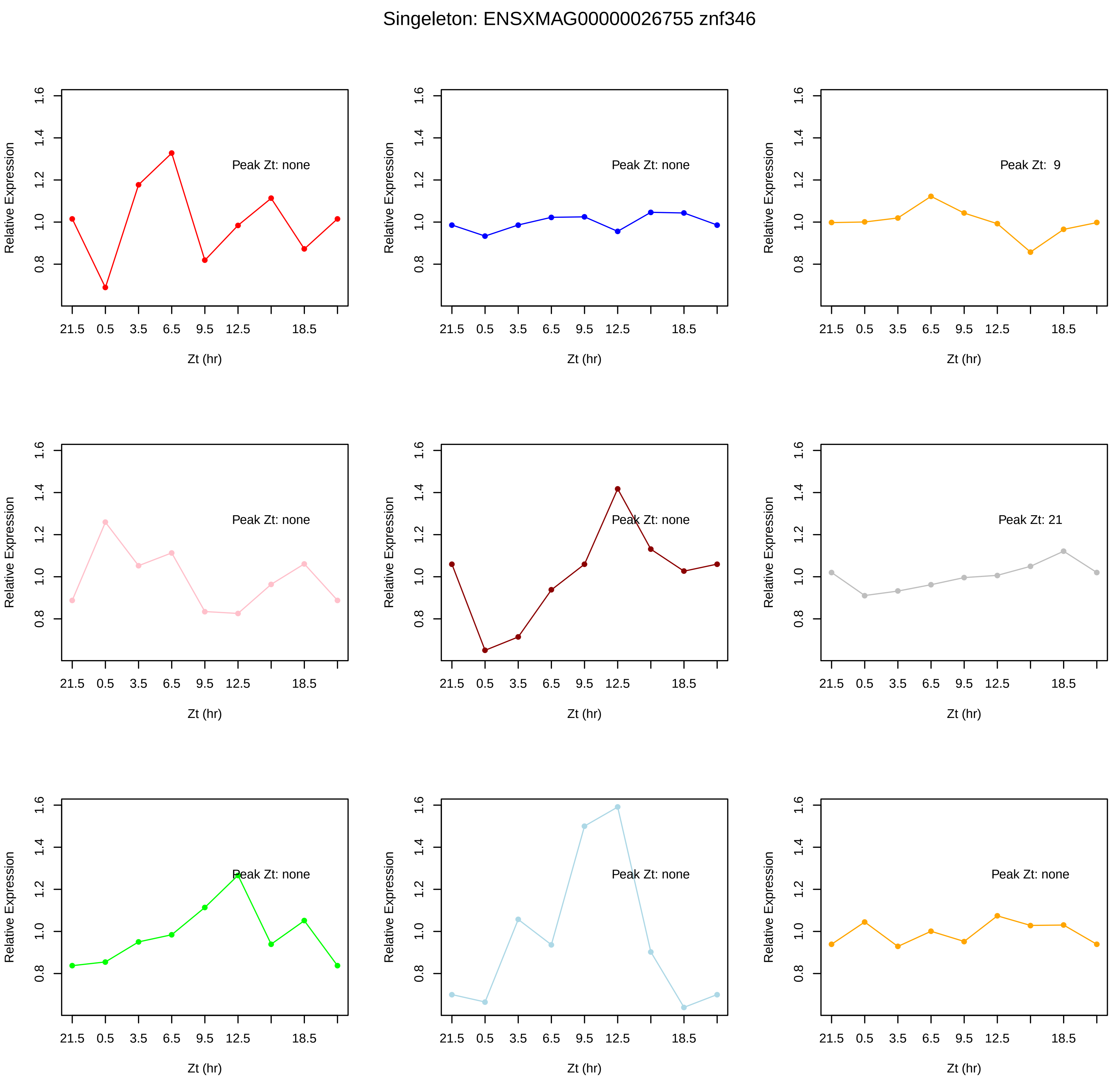
Singeleton: ENSXMAG00000026755 znf346
Peak Zt: none
Peak Zt: none
Peak Zt: 9
Peak Zt: none
Peak Zt: none
Peak Zt: 21
Peak Zt: none
Peak Zt: none
Peak Zt: none
Relative Expression
0.8
1.0
1.2
1.4
1.6
Zt (hr)
21.5
0.5
3.5
6.5
9.5
12.5
18.5

Singeleton: ENSXMAG00000005761 mttp

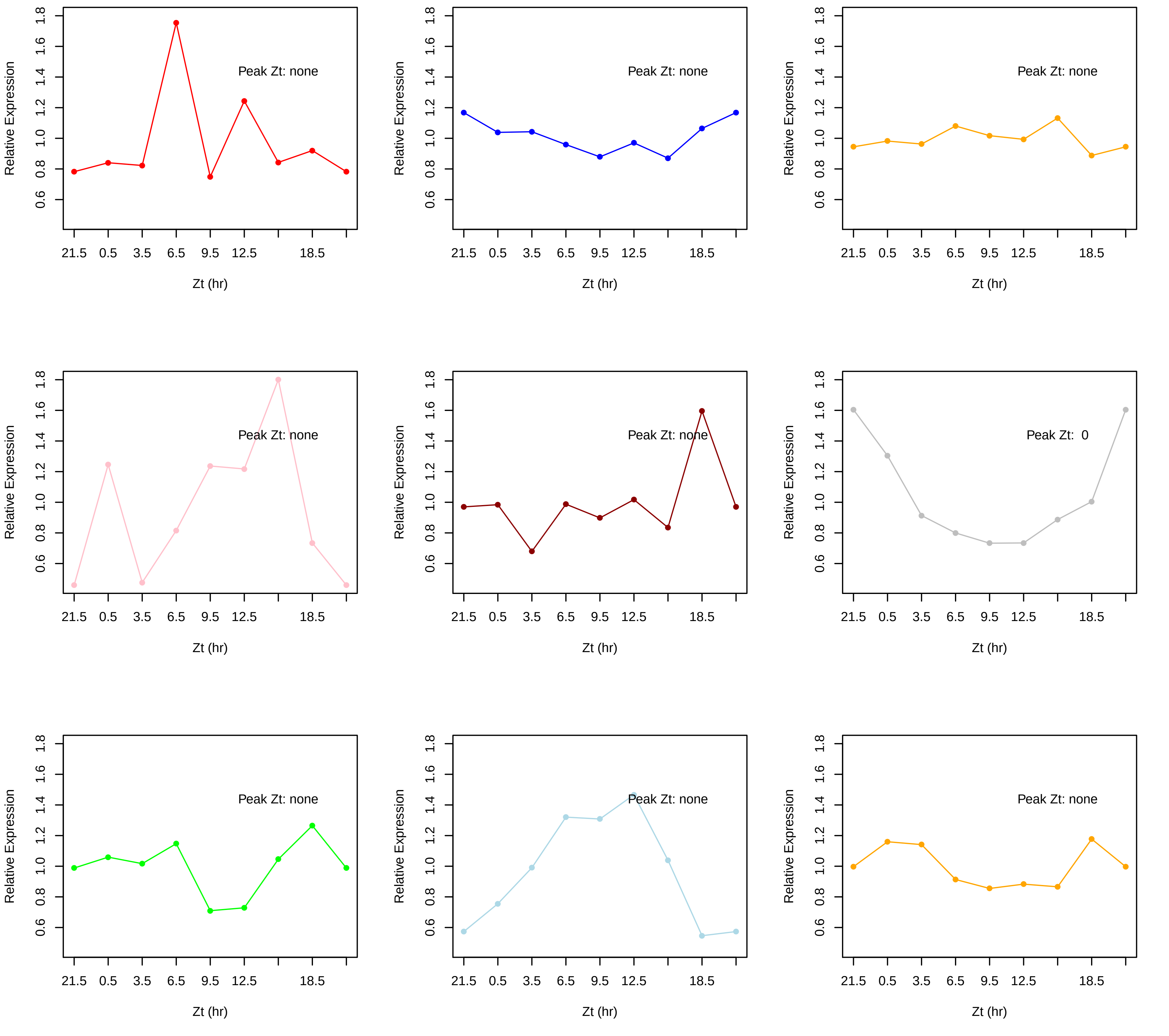

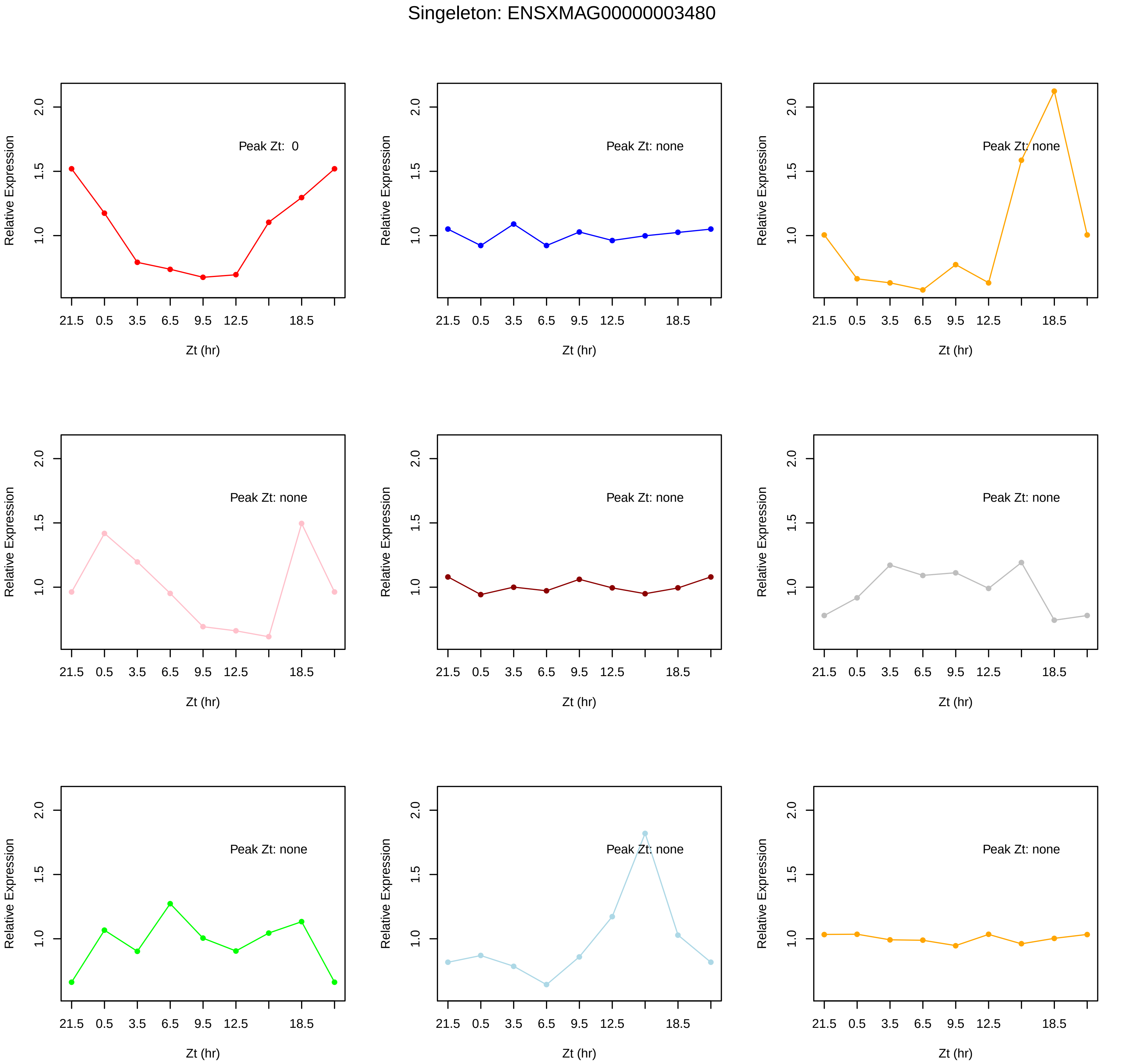
Singeleton: ENSXMAG00000003480
Peak Zt: 0
Peak Zt: none
Relative Expression
Zt (hr)
21.5
0.5
3.5
6.5
9.5
12.5
18.5
1.0
1.5
2.0

Singeleton: ENSXMAG00000006928 pdcd4b

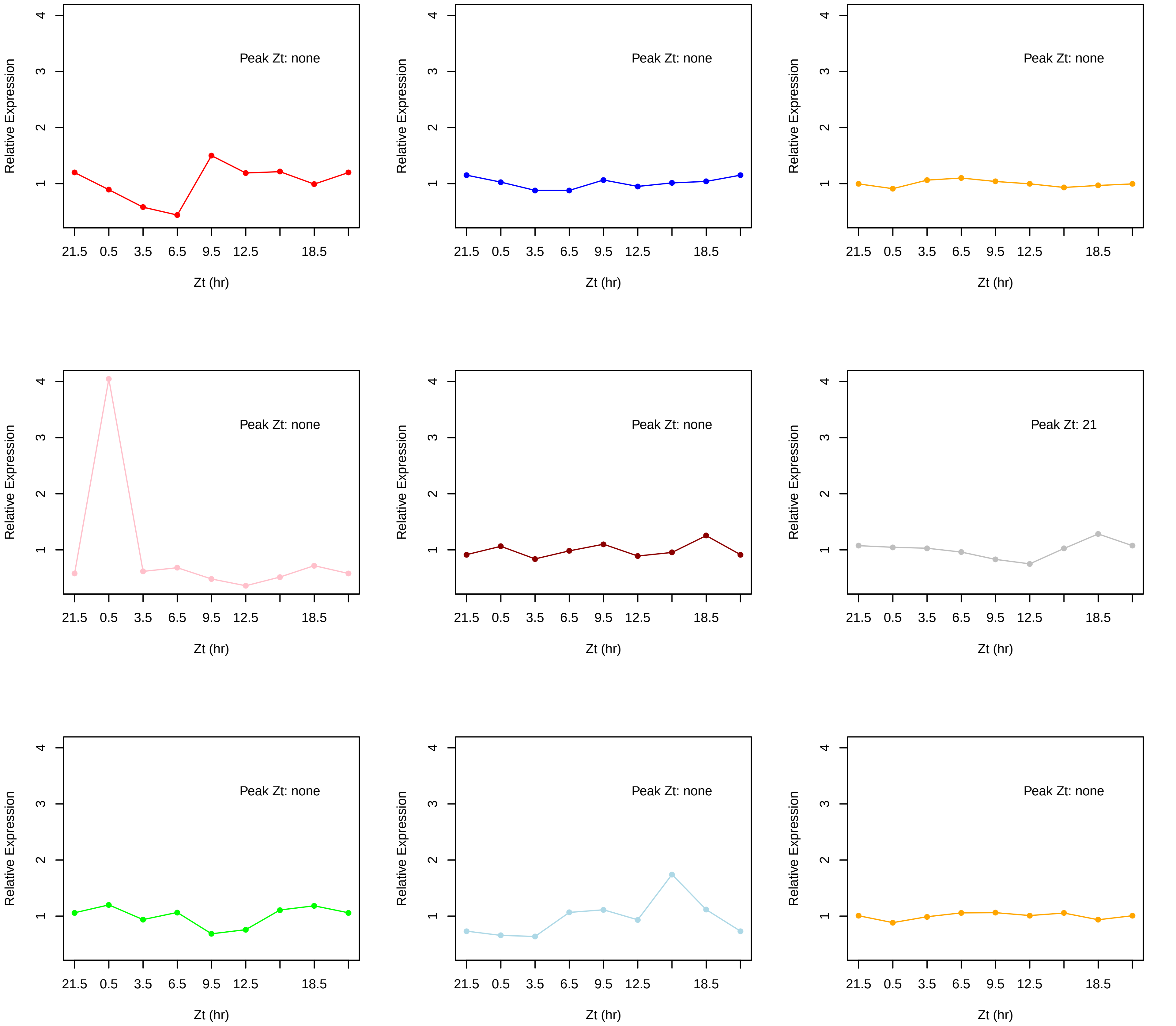

Singeleton: ENSXMAG00000006968 RBM20

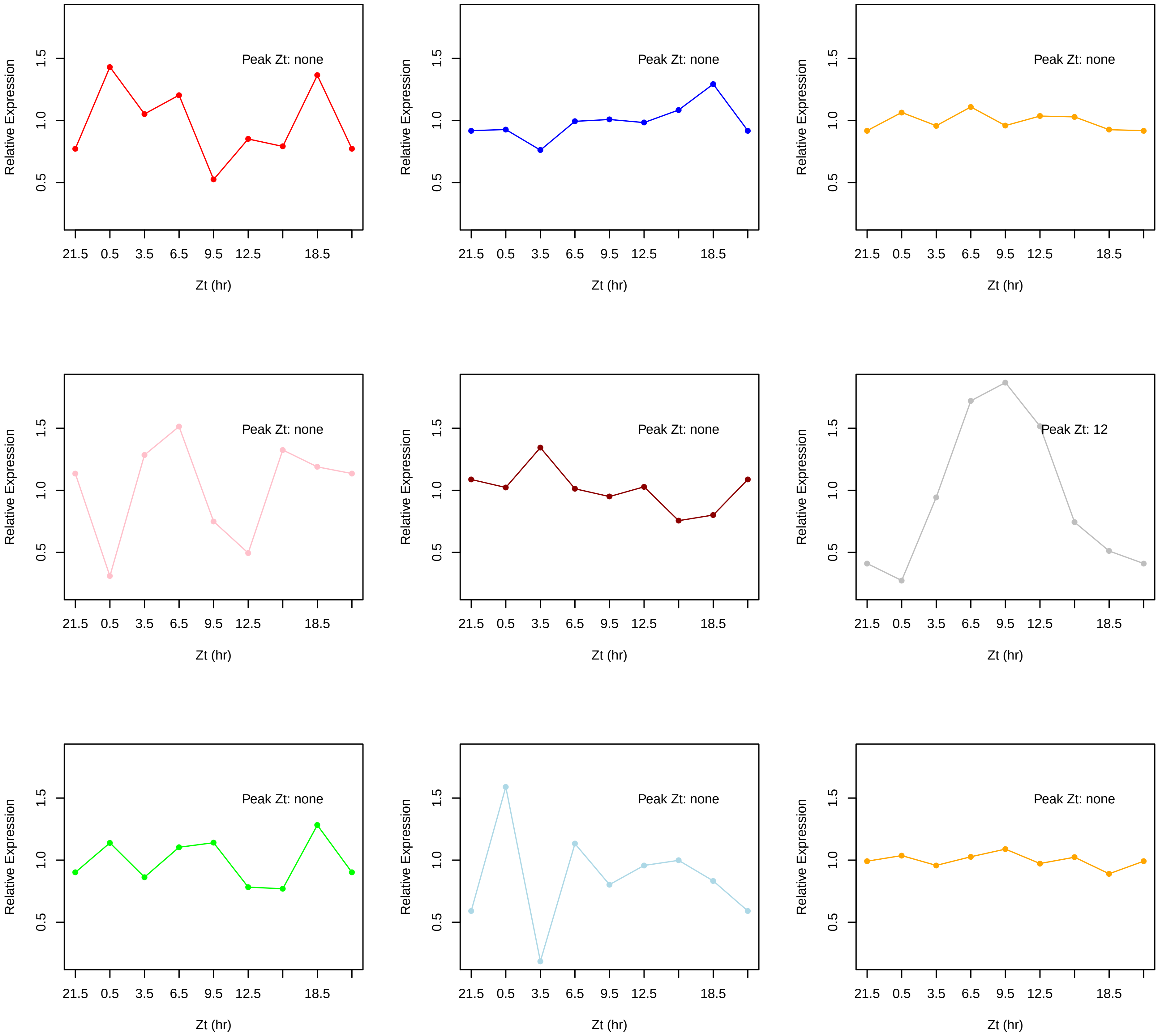

Singeleton: ENSXMAG00000023371 amacr

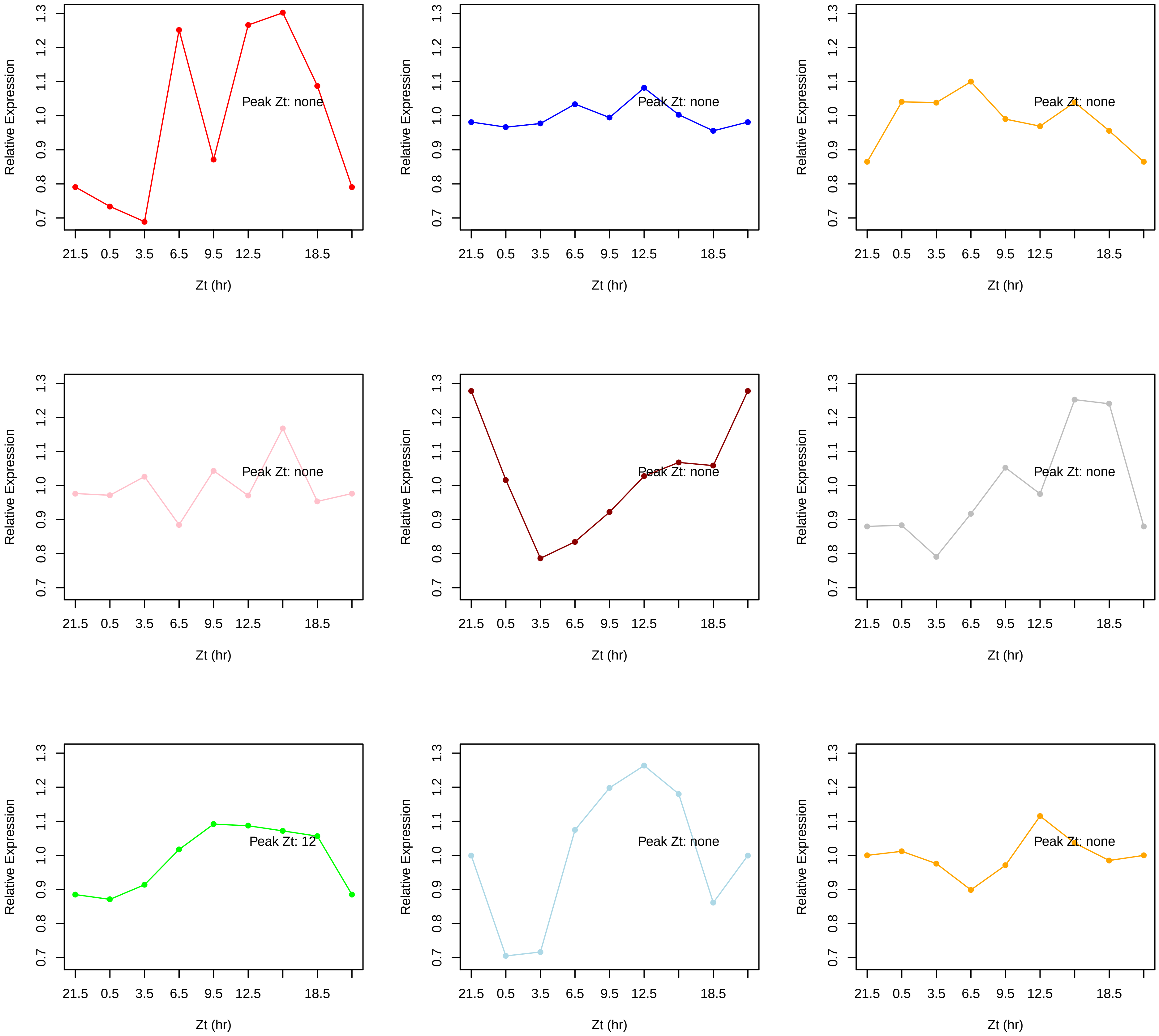

Singeleton: ENSXMAG00000004010 psma5

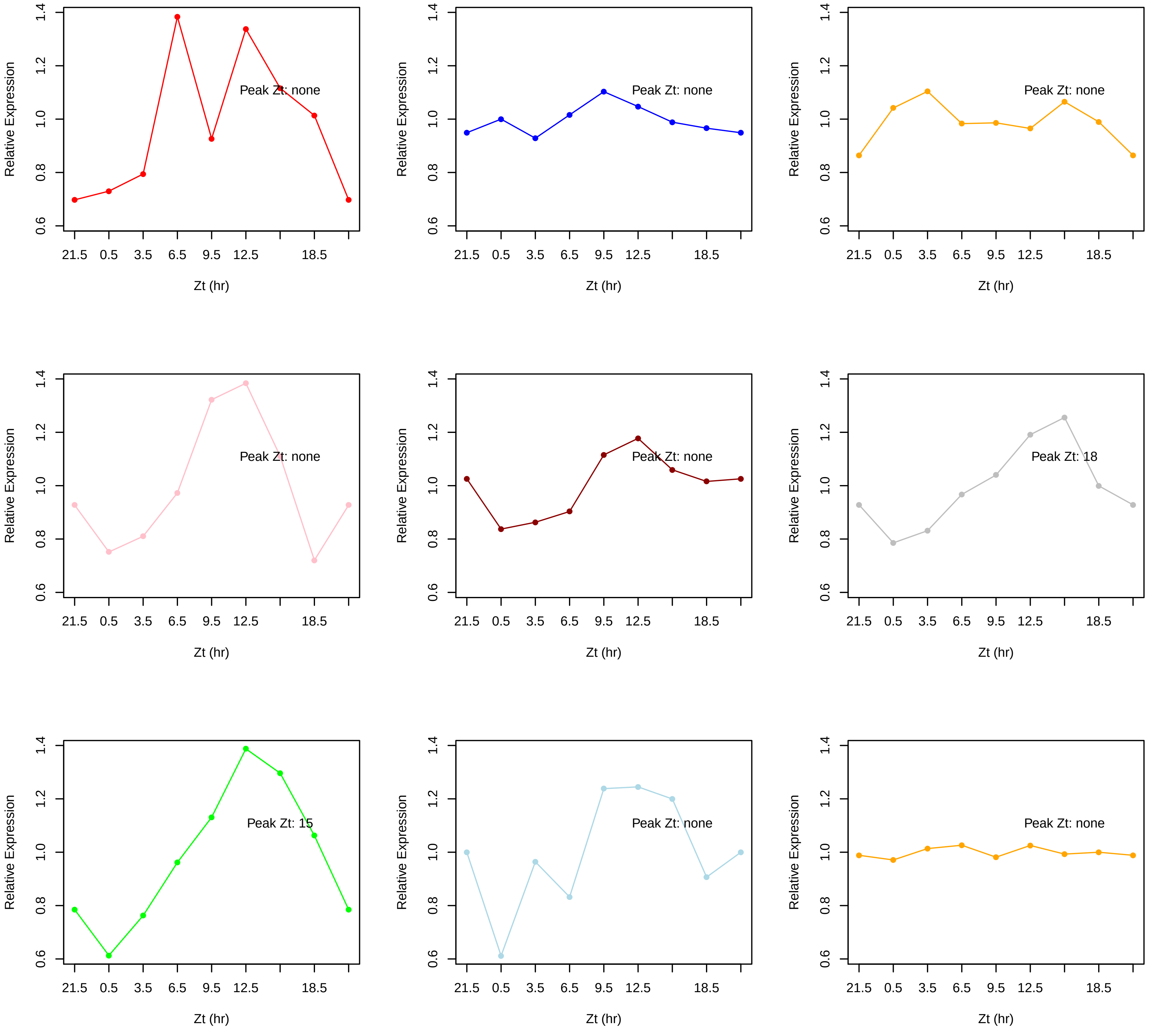

Singeleton: ENSXMAG00000014598 fasn

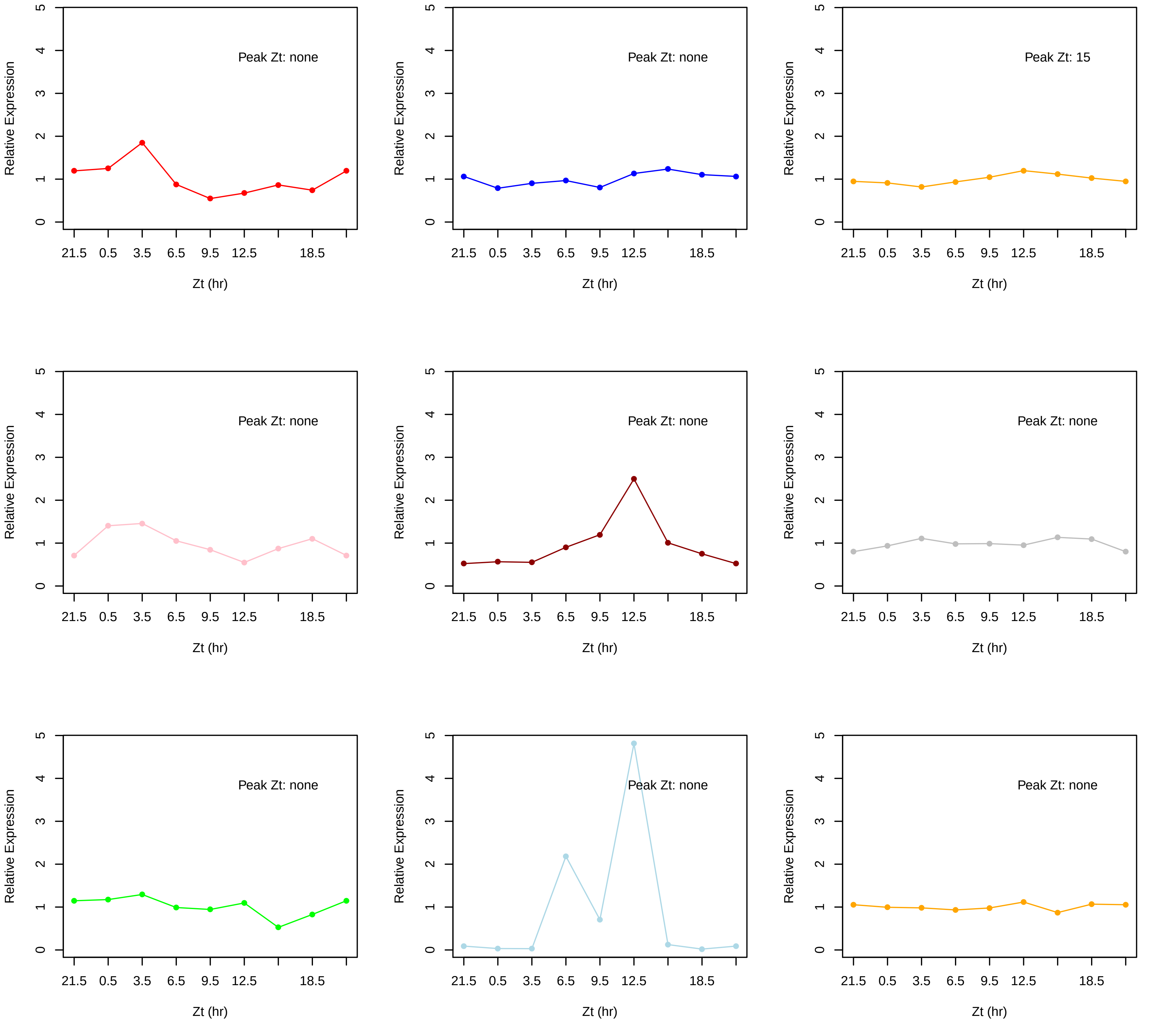

Singleton: ENSXMAG00000010104 adamts12

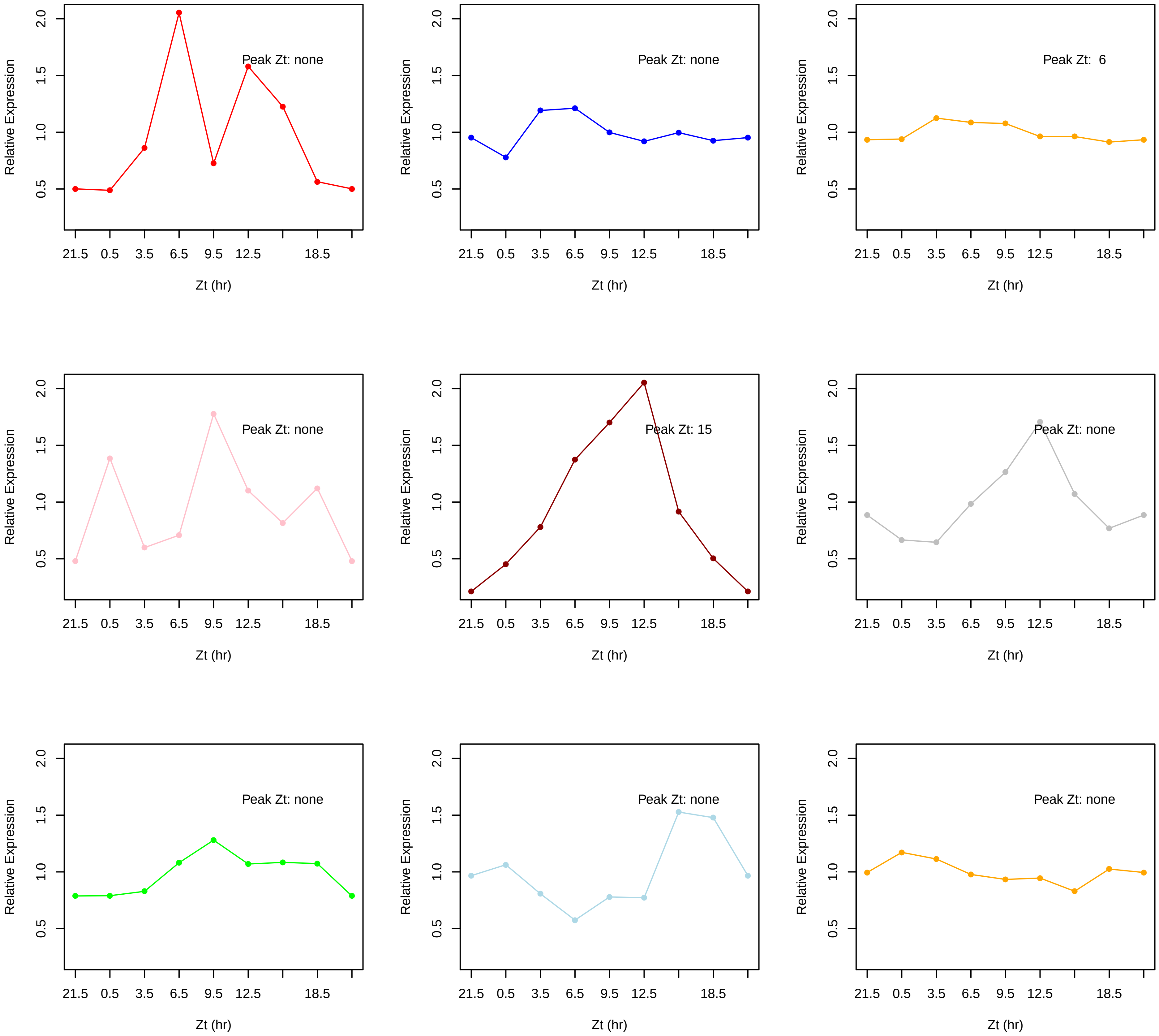

Singeleton: ENSXMAG00000004726 atxn7l2a

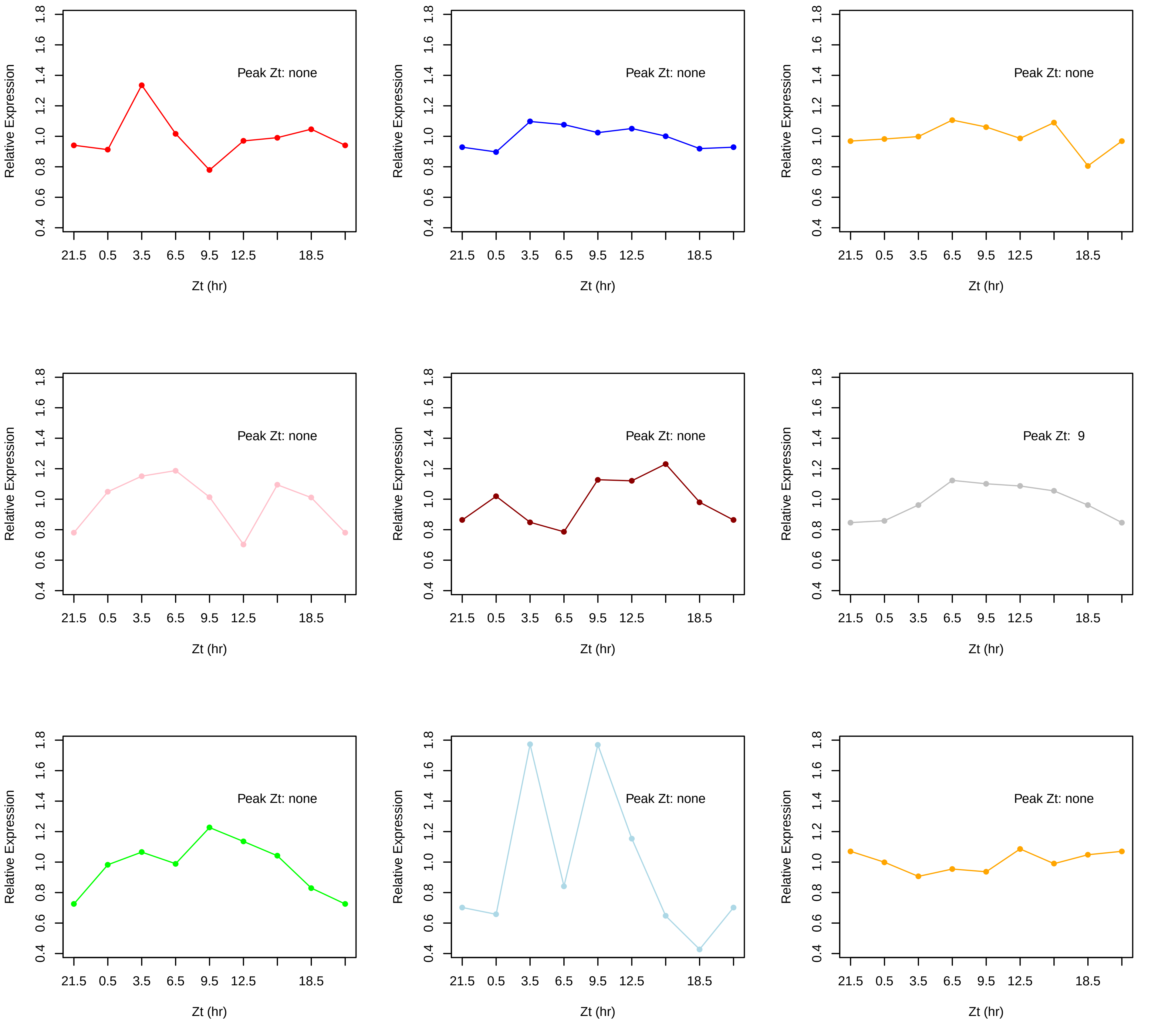

Singeleton: ENSXMAG00000006801 pigh

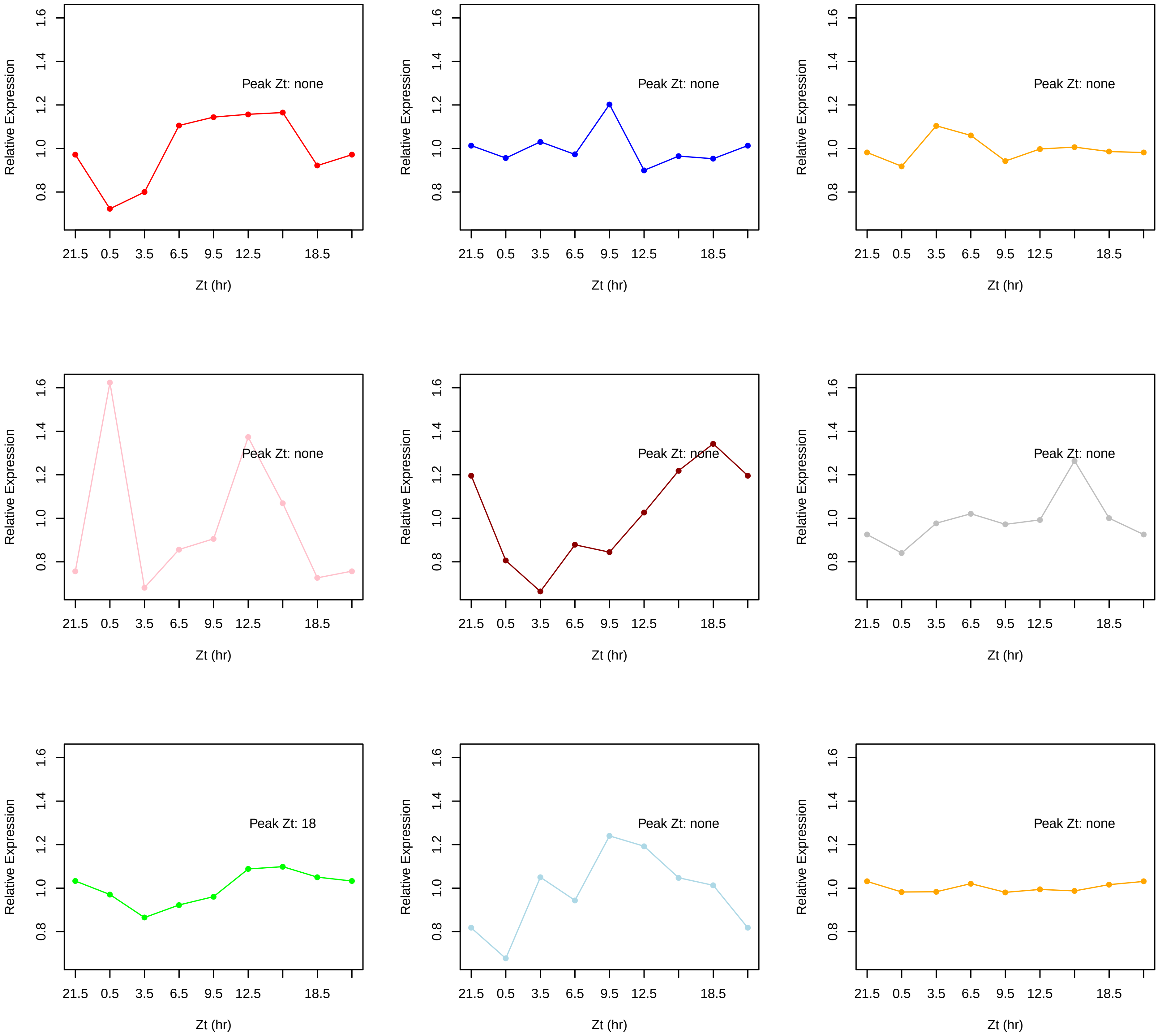

Singleton: ENSXMAG00000023246 cyb561d1

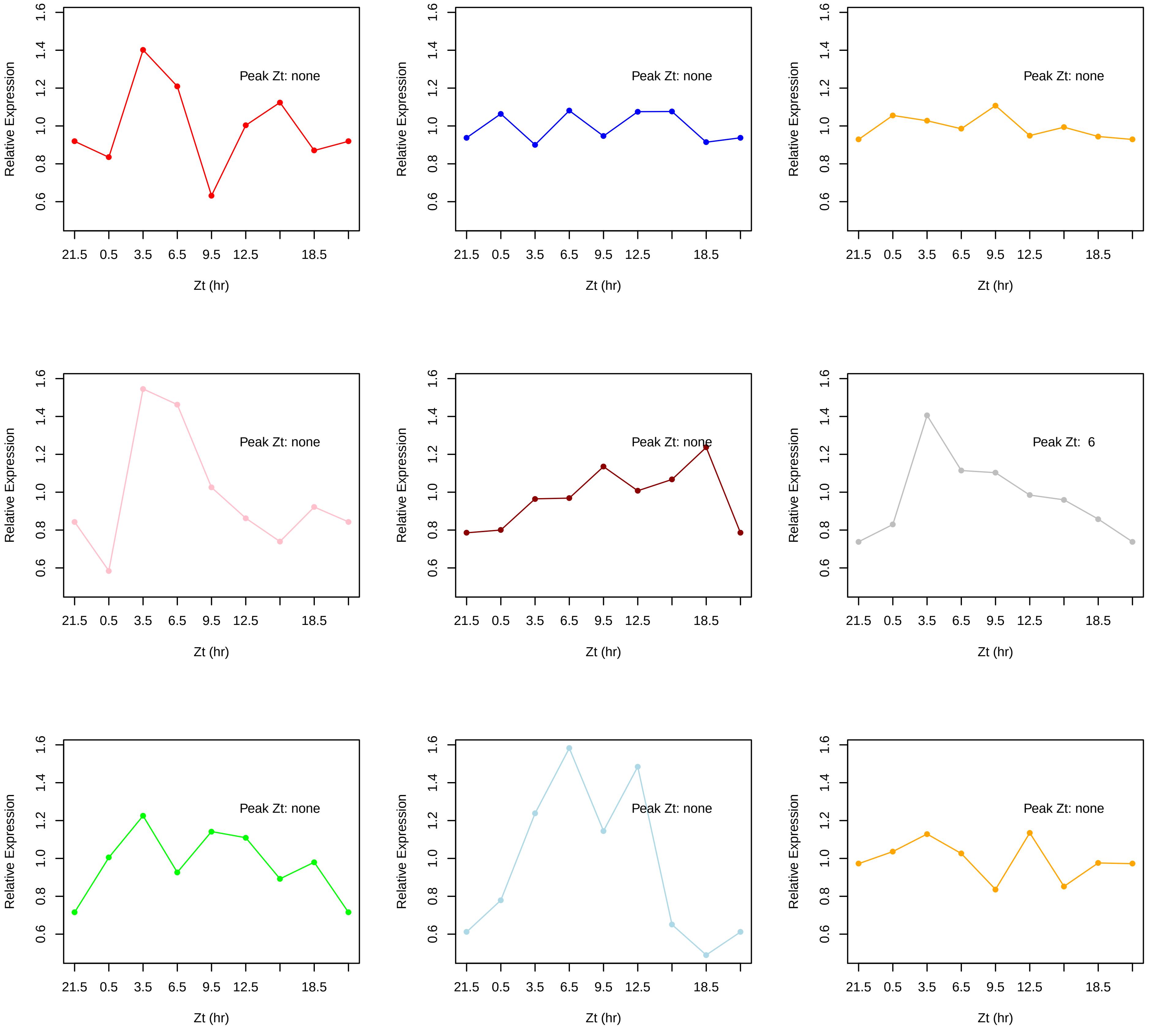

Singleton: ENSXMAG00000025465 dusp5

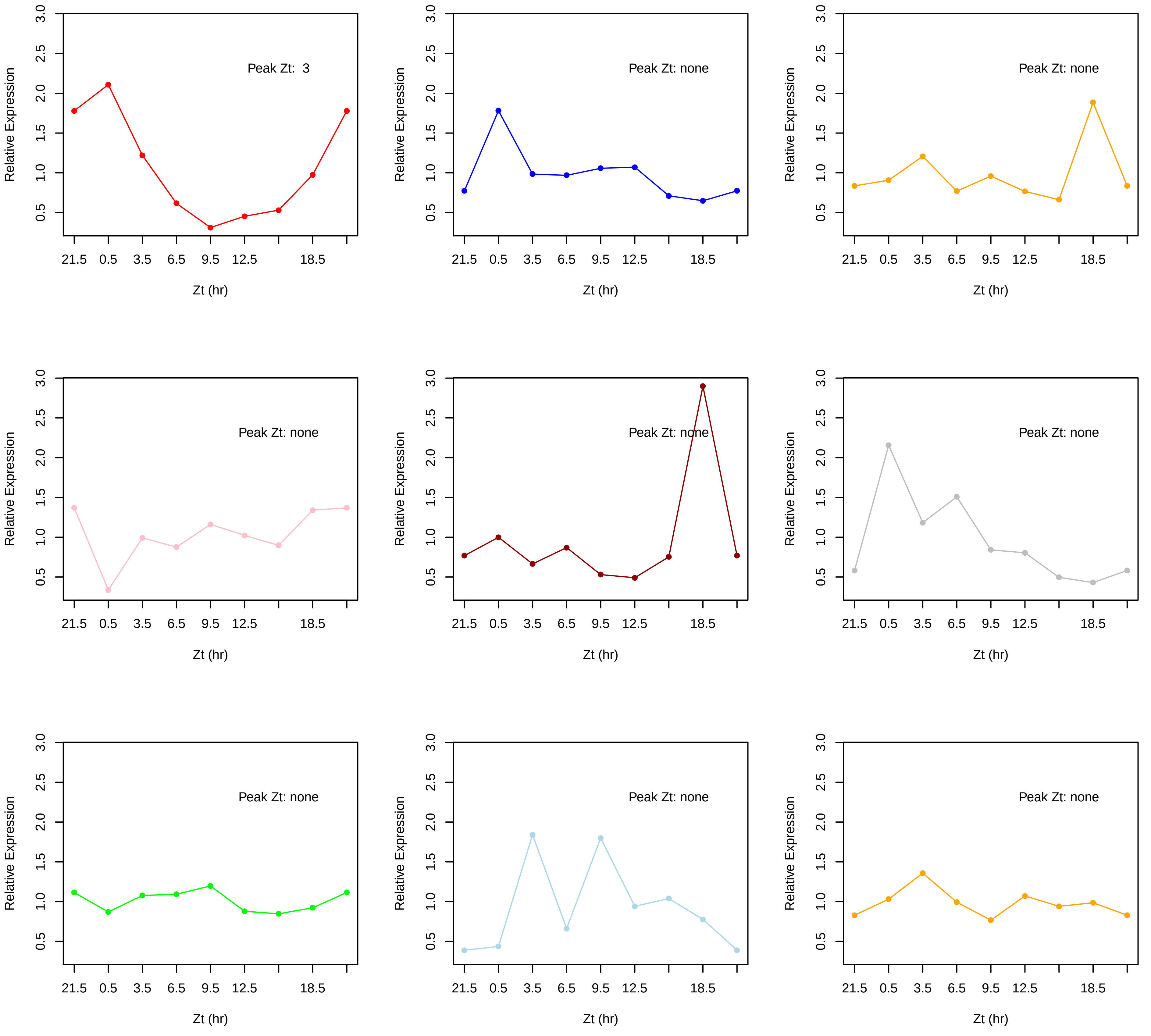

Singeleton: ENSXMAG00000005147 dus1l

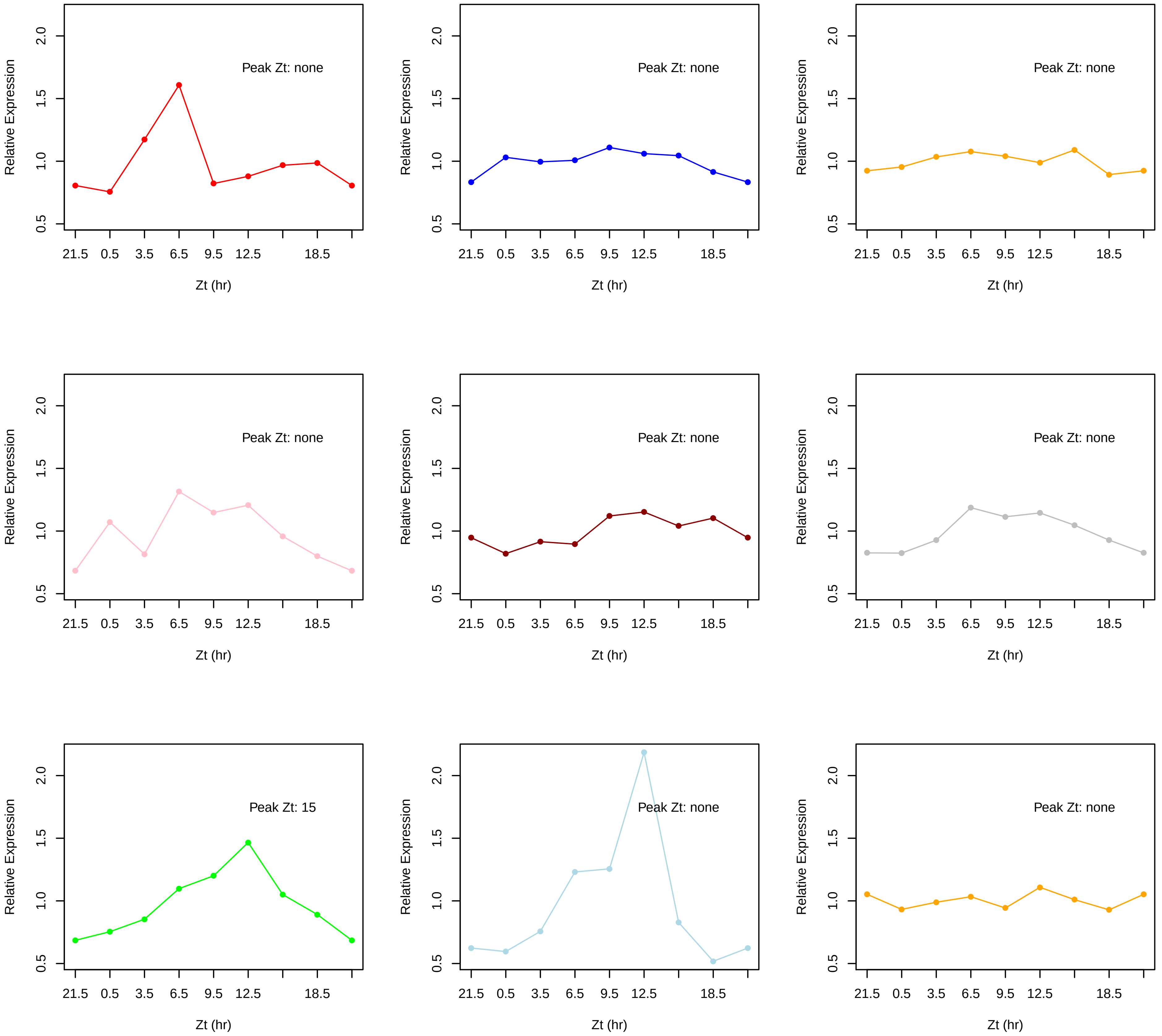

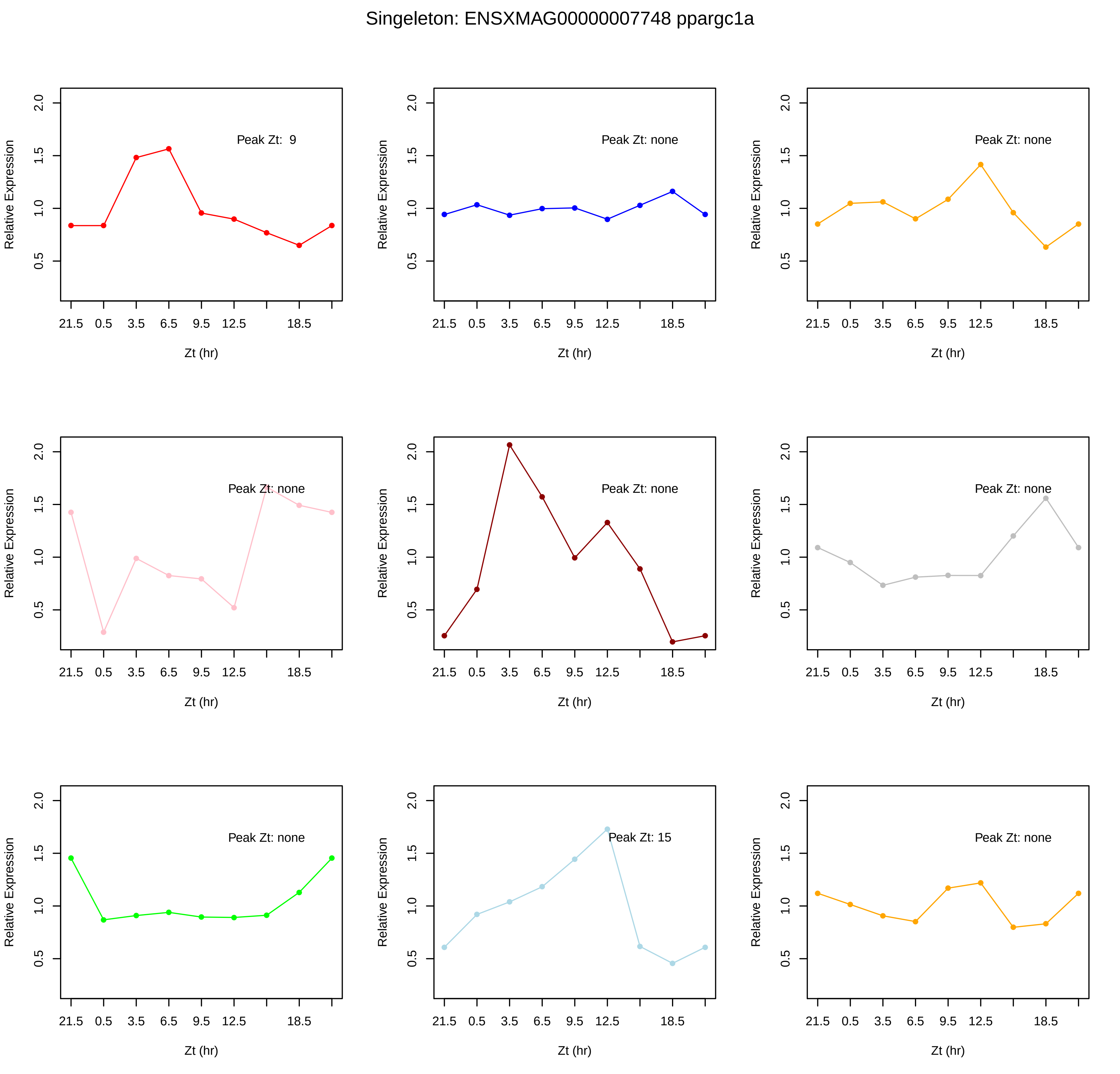
Singeleton: ENSXMAG00000007748 ppargc1a
Peak Zt: 9
Peak Zt: none
Peak Zt: none
Peak Zt: none
Peak Zt: none
Peak Zt: none
Peak Zt: none
Peak Zt: 15
Peak Zt: none
Relative Expression
Zt (hr)
21.5 0.5 3.5 6.5 9.5 12.5 18.5
0.5 1.0 1.5 2.0

Singeleton: ENSXMAG00000009964 ampd2b

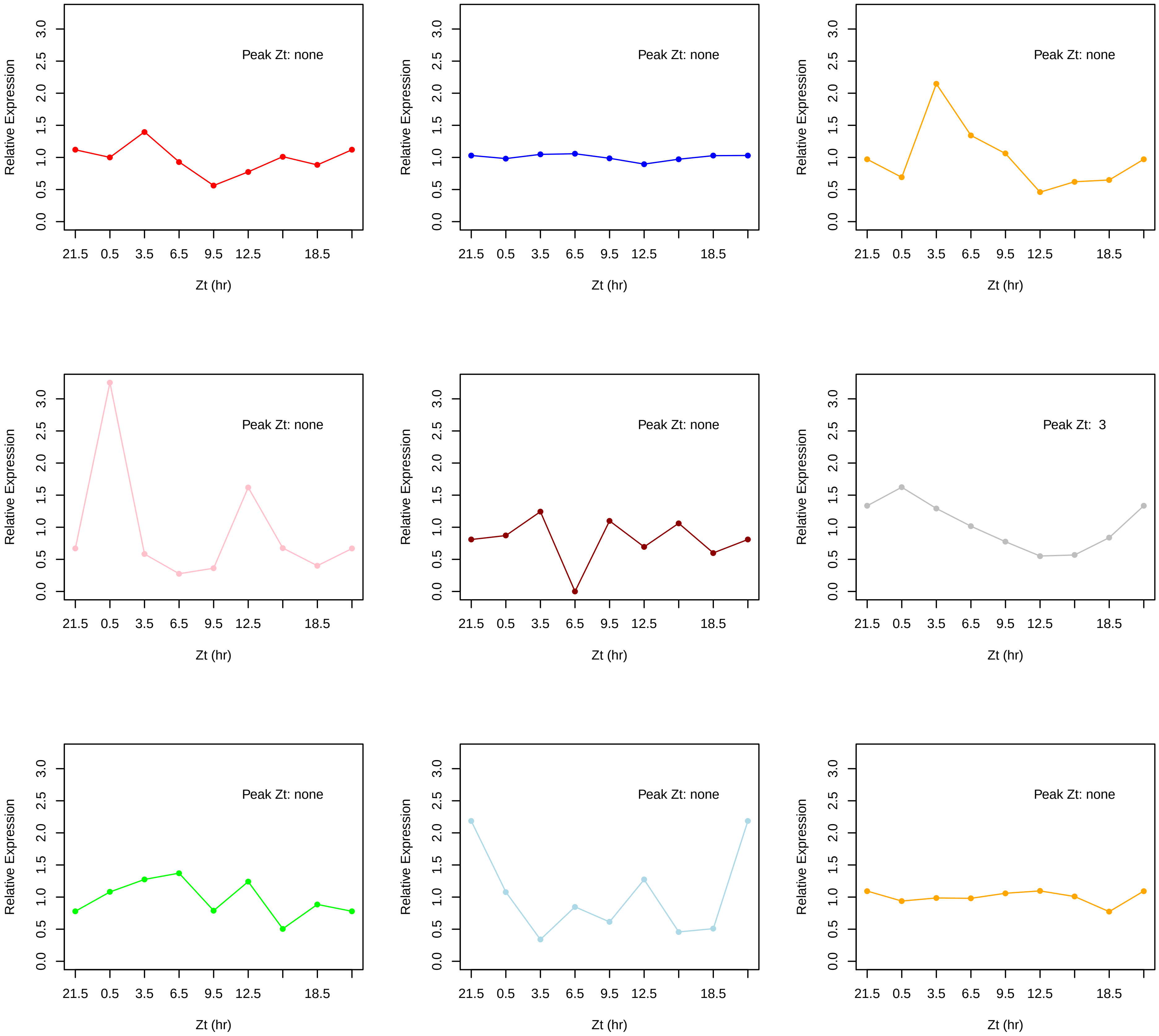

Singeleton: ENSXMAG00000015622

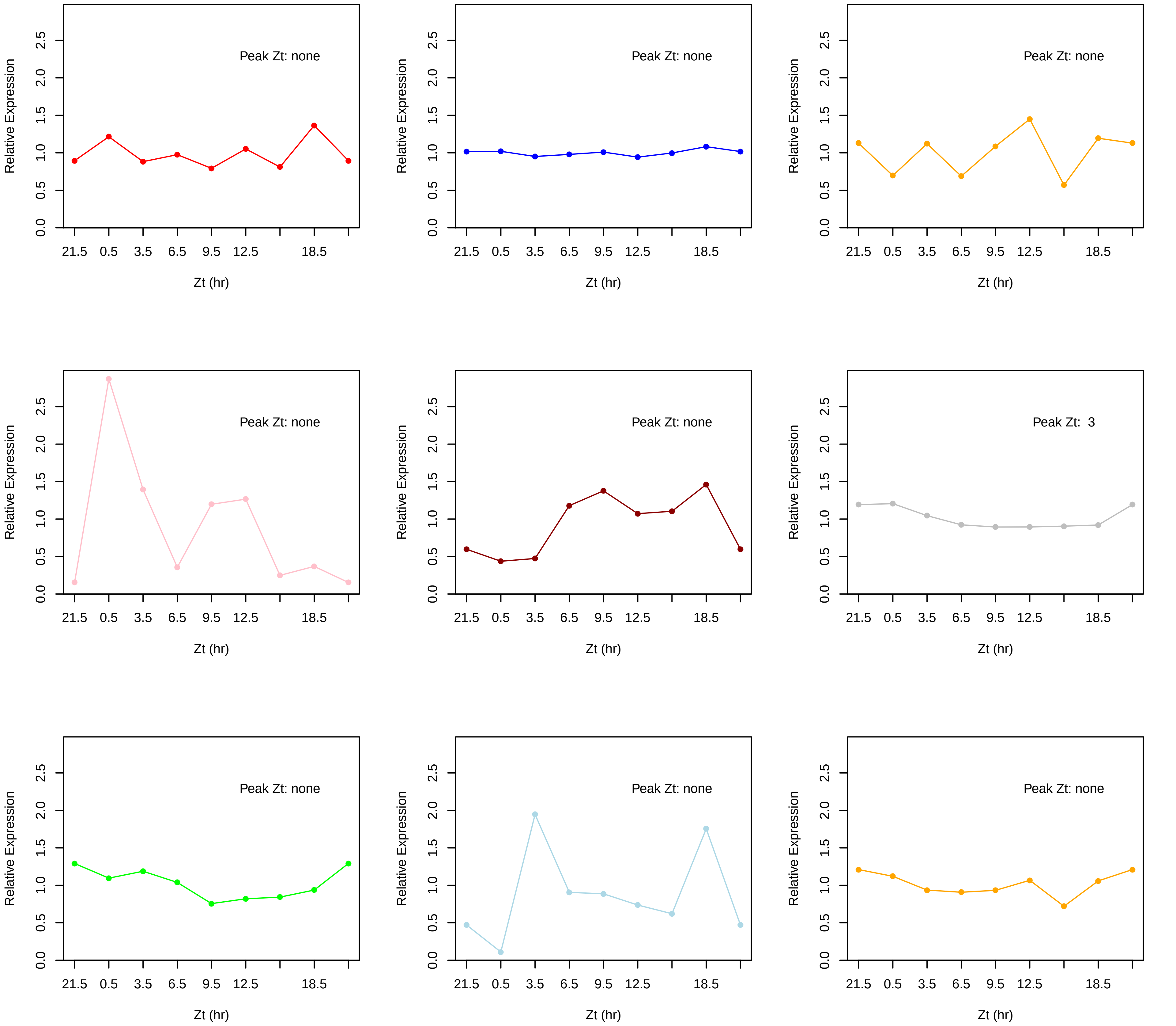

Singeleton: ENSXMAG00000025576 cntfr

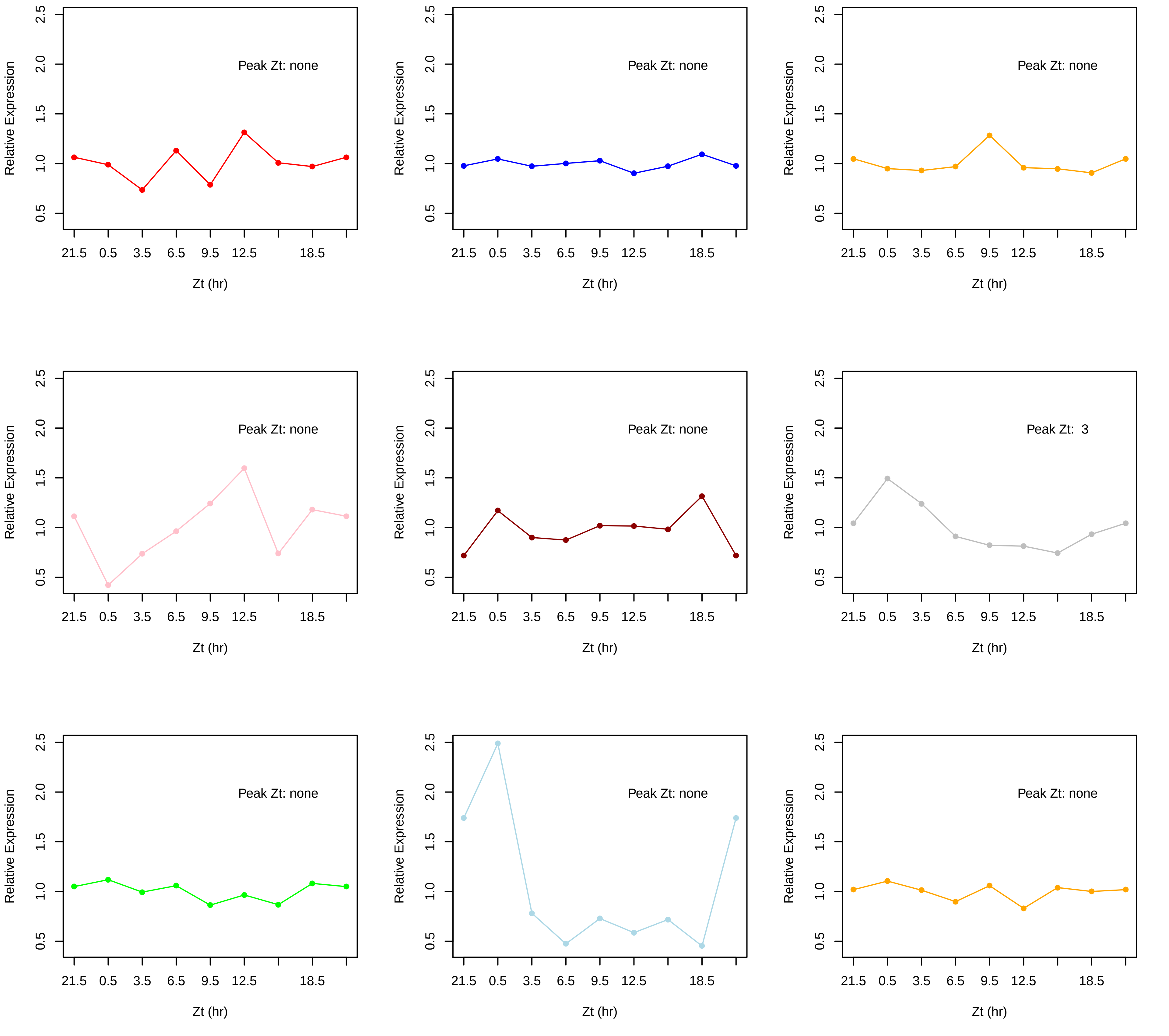

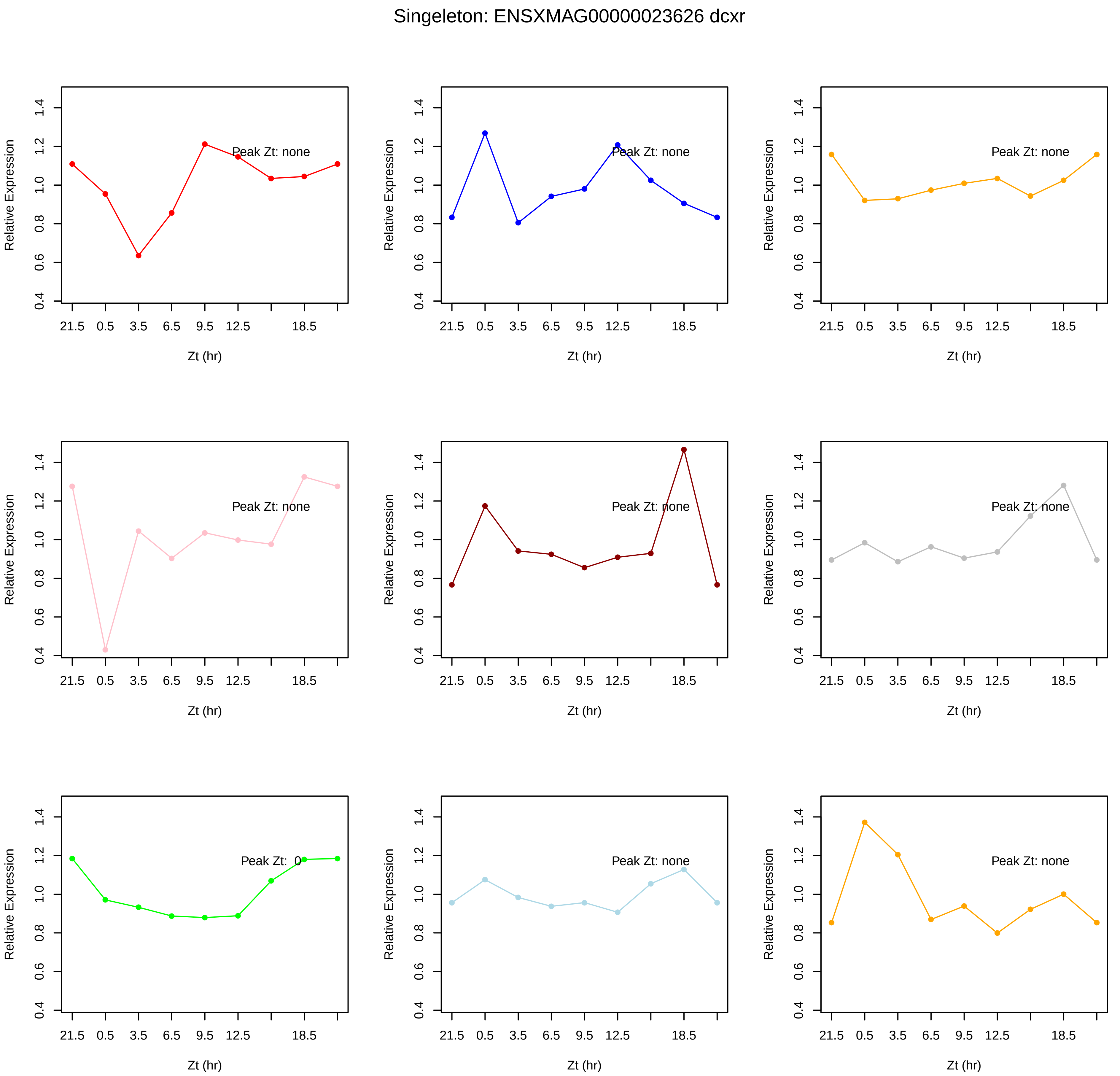
Singeleton: ENSXMAG00000023626 dcxr
Peak Zt: none
Peak Zt: none
Peak Zt: none
Peak Zt: none
Peak Zt: none
Peak Zt: none
Peak Zt: 0
Peak Zt: none
Peak Zt: none
Relative Expression
Zt (hr)
21.5
0.5
3.5
6.5
9.5
12.5
18.5
0.4
0.6
0.8
1.0
1.2
1.4

Singleton: ENSXMAG00000014734 rac3b

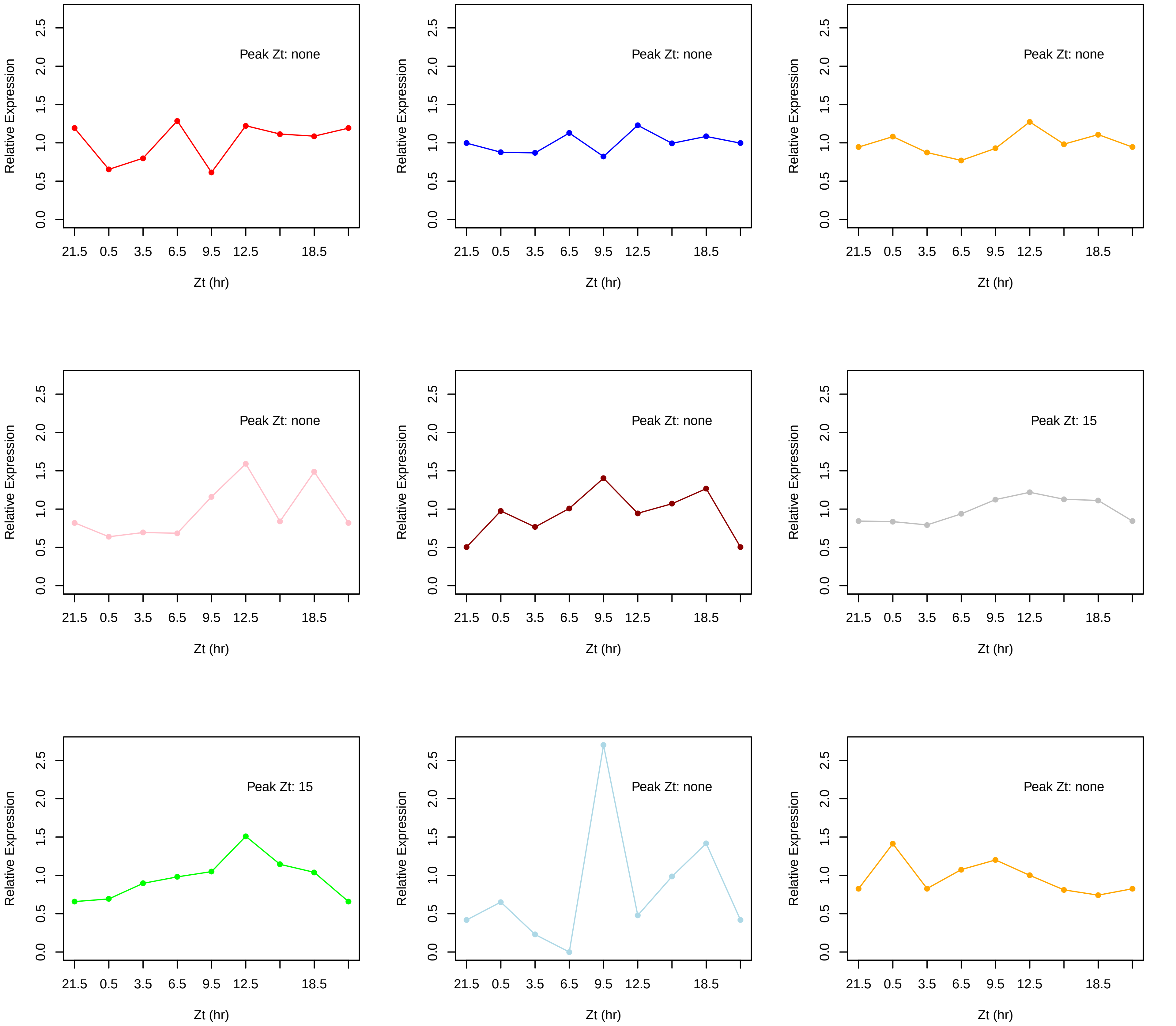

Singeleton: ENSXMAG00000015595 rars

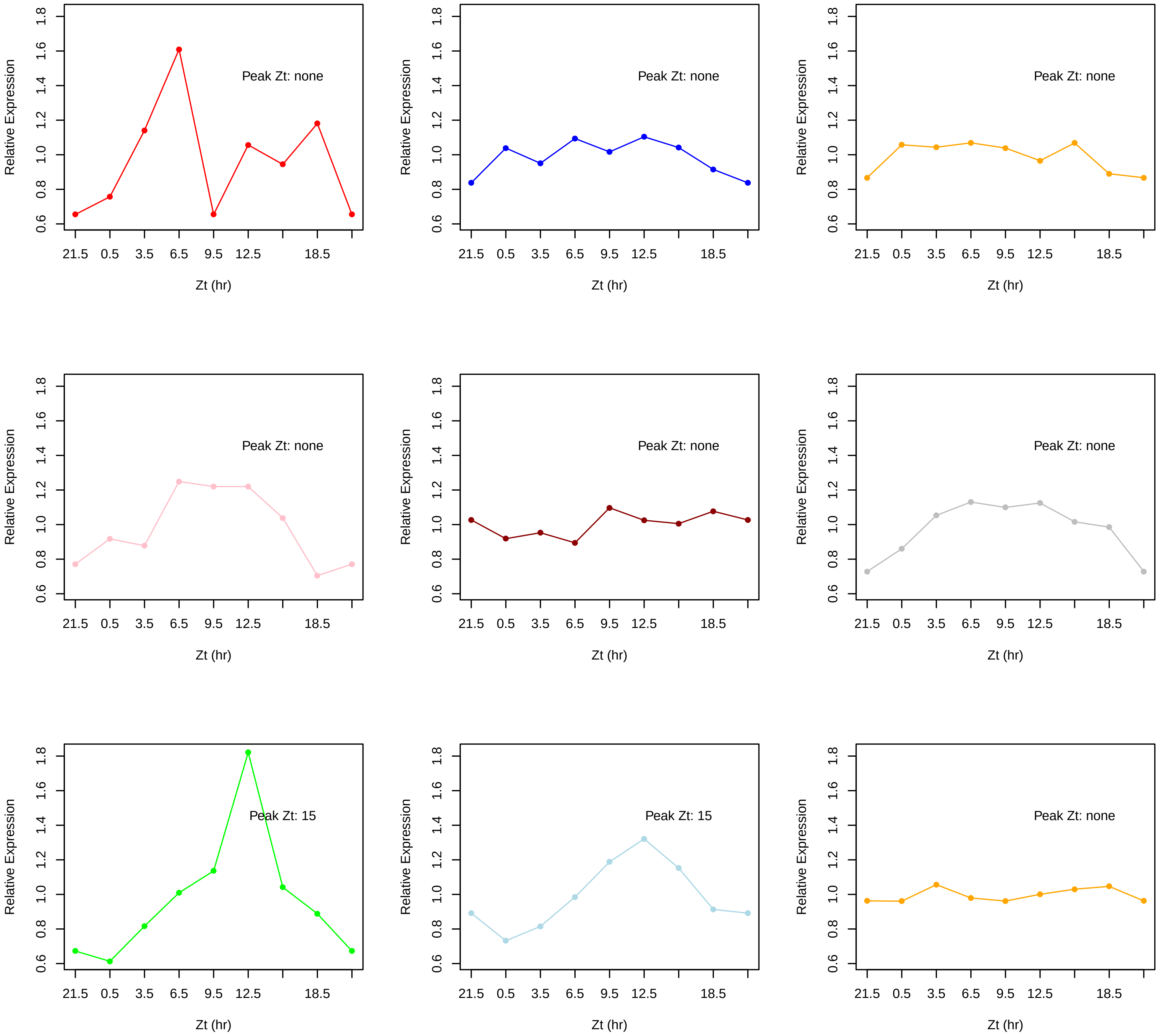

Singleton: ENSXMAG00000014712 lrrc45

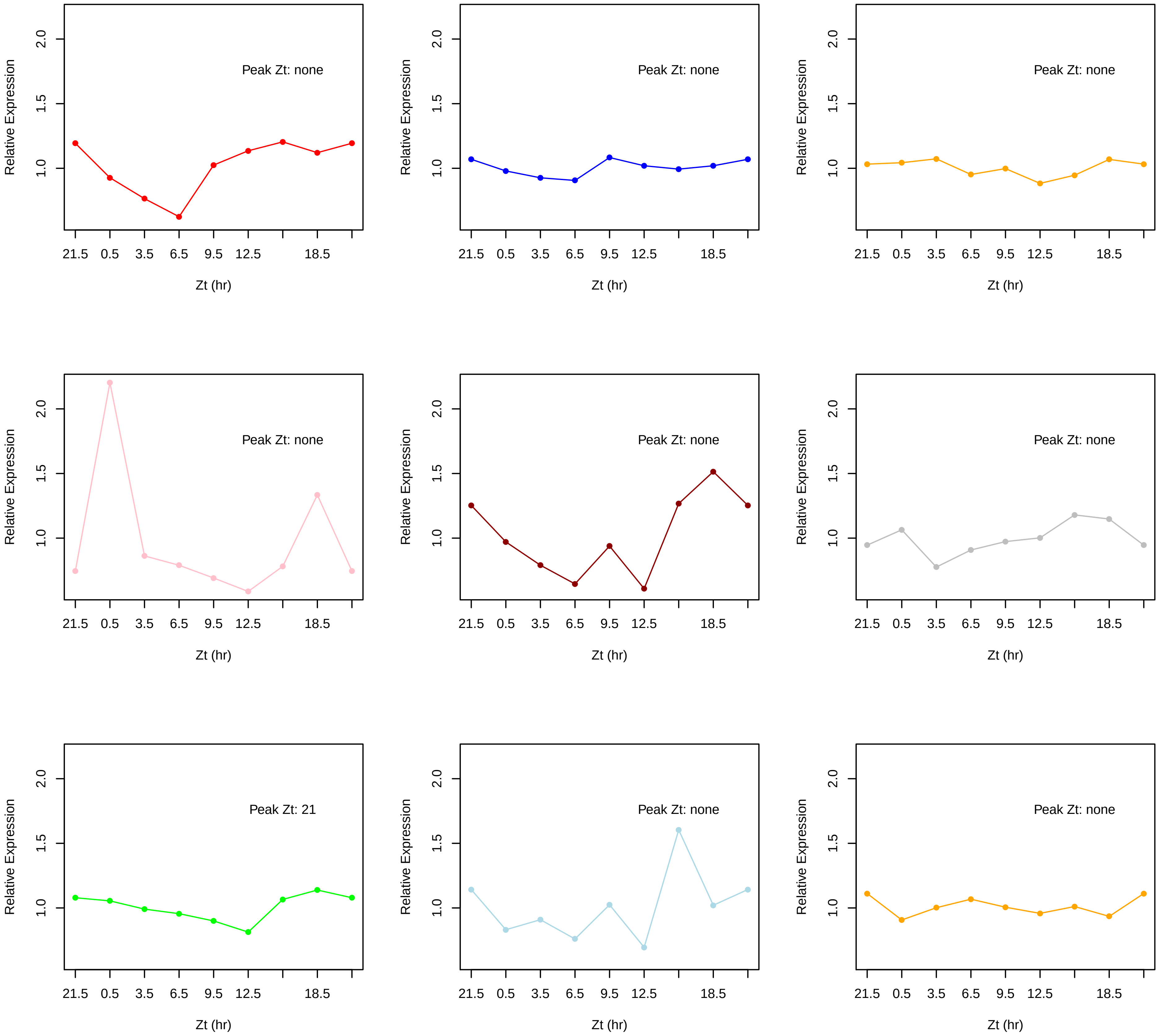

Singeleton: ENSXMAG00000015580 si:dkeyp−23e4.3

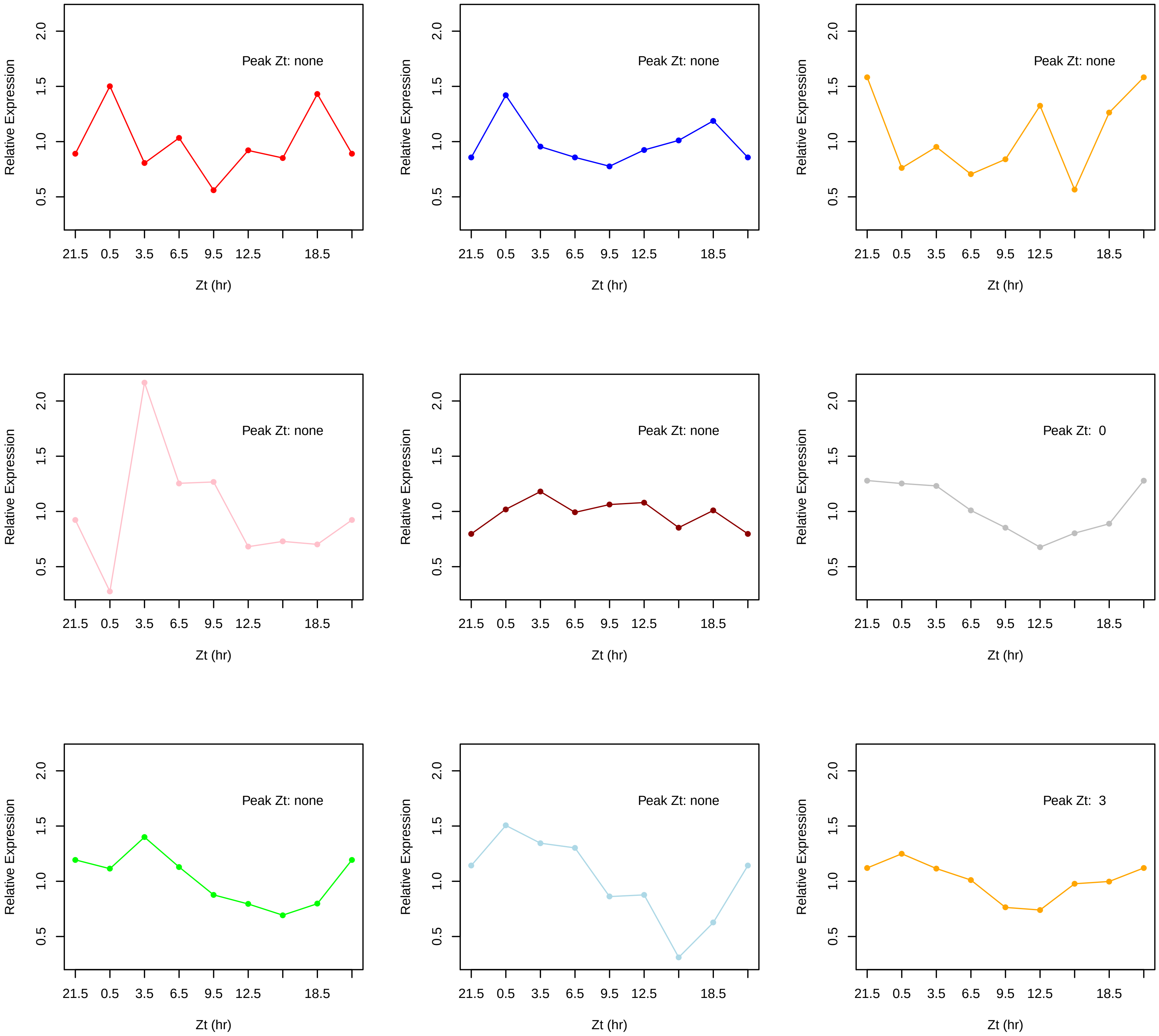

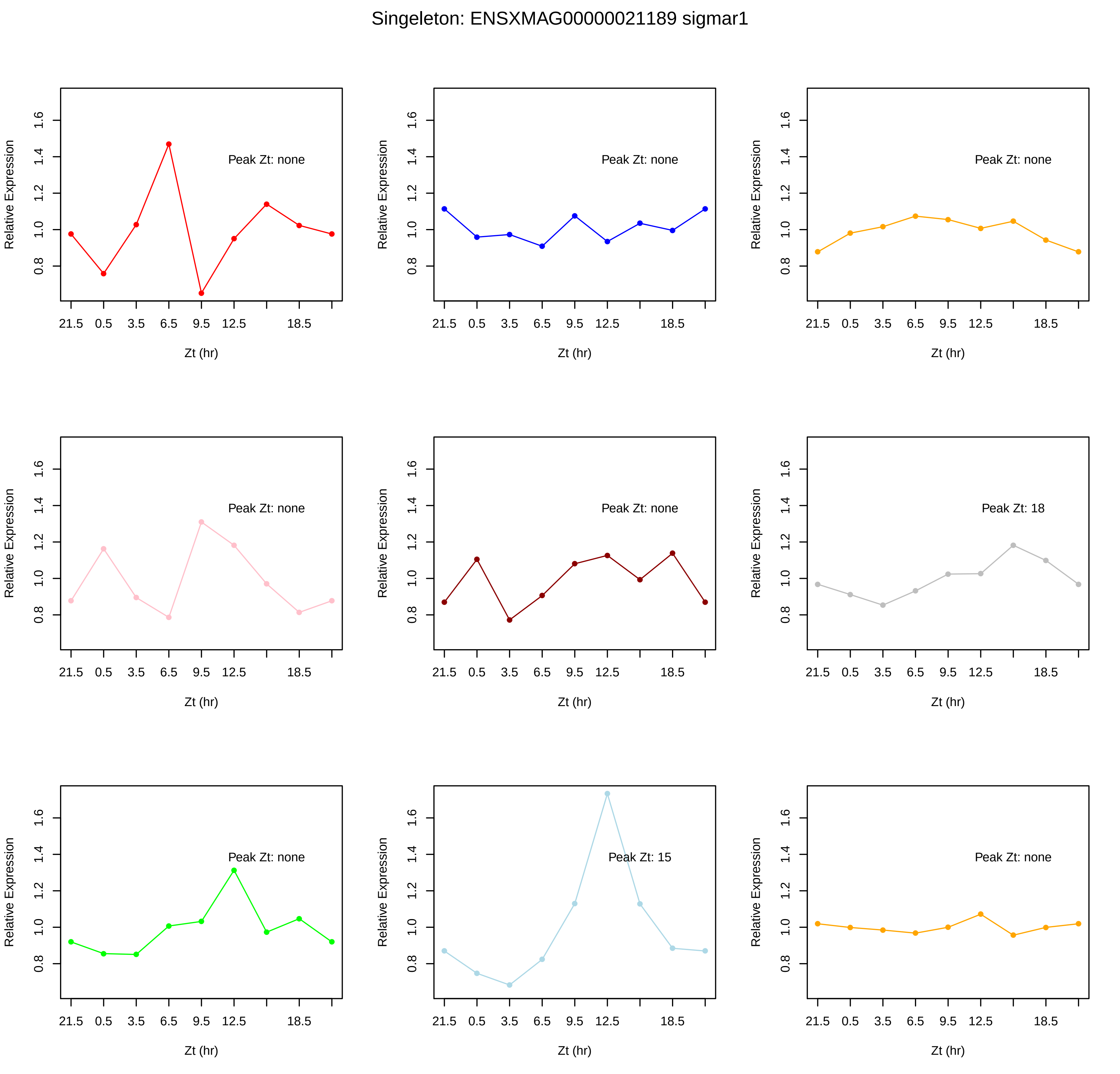
Singeleton: ENSXMAG00000021189 sigmar1
Peak Zt: none
Peak Zt: none
Peak Zt: none
Peak Zt: none
Peak Zt: none
Peak Zt: 18
Peak Zt: none
Peak Zt: 15
Peak Zt: none
Relative Expression
Zt (hr)

Singleton: ENSXMAG00000009128 ramp2

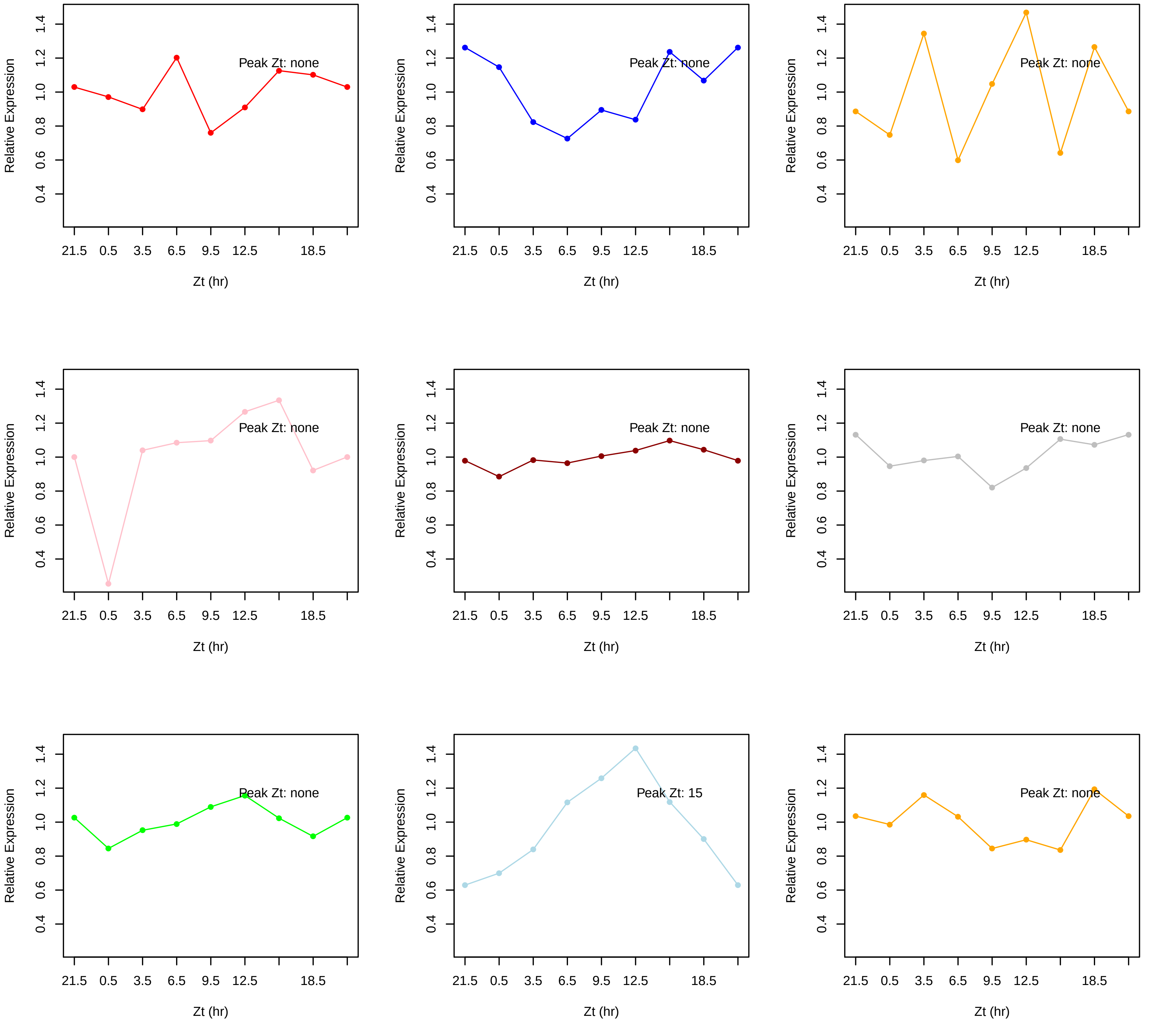

Singeleton: ENSXMAG00000016046 aifm2

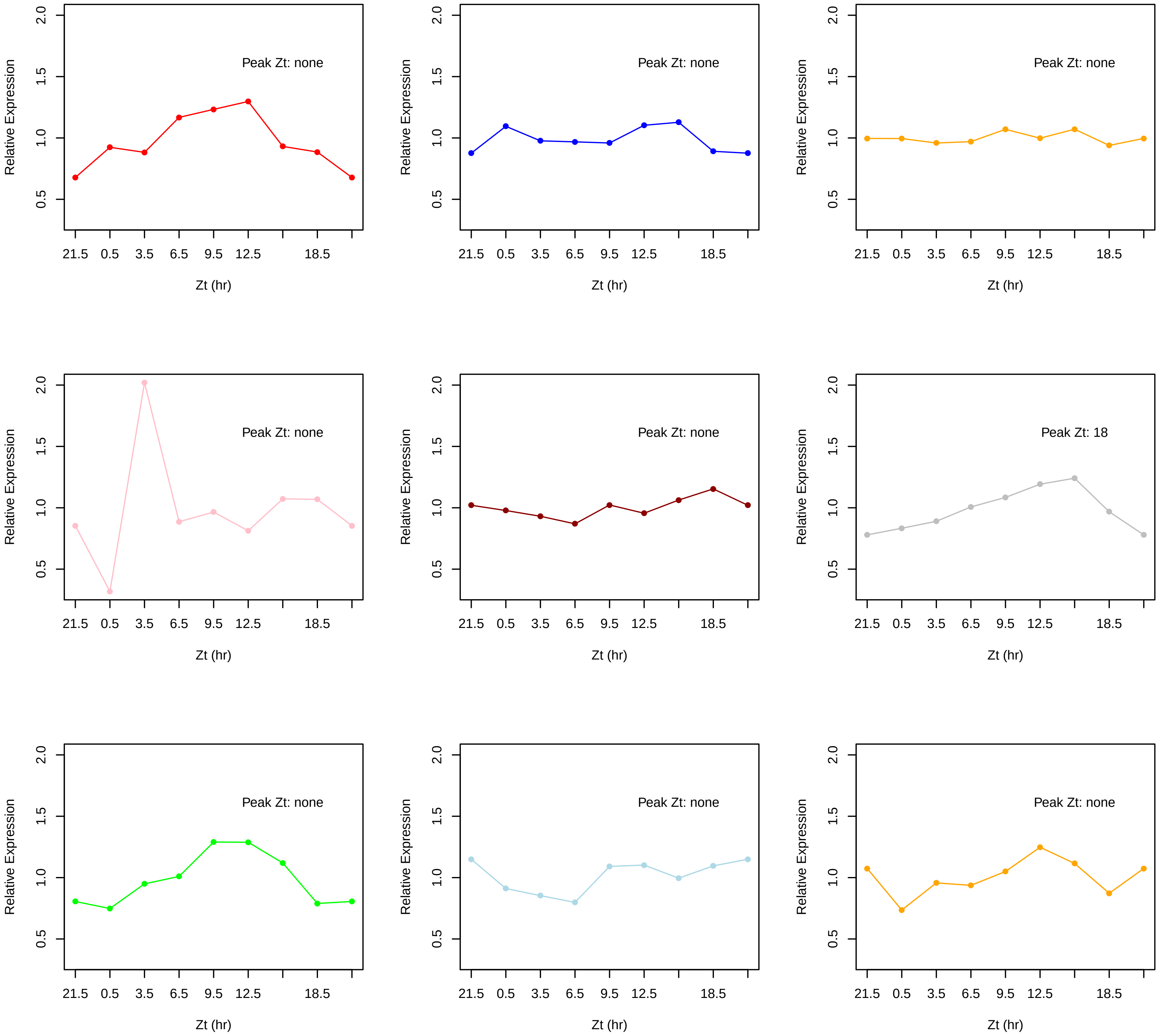

Singeleton: ENSXMAG00000023326

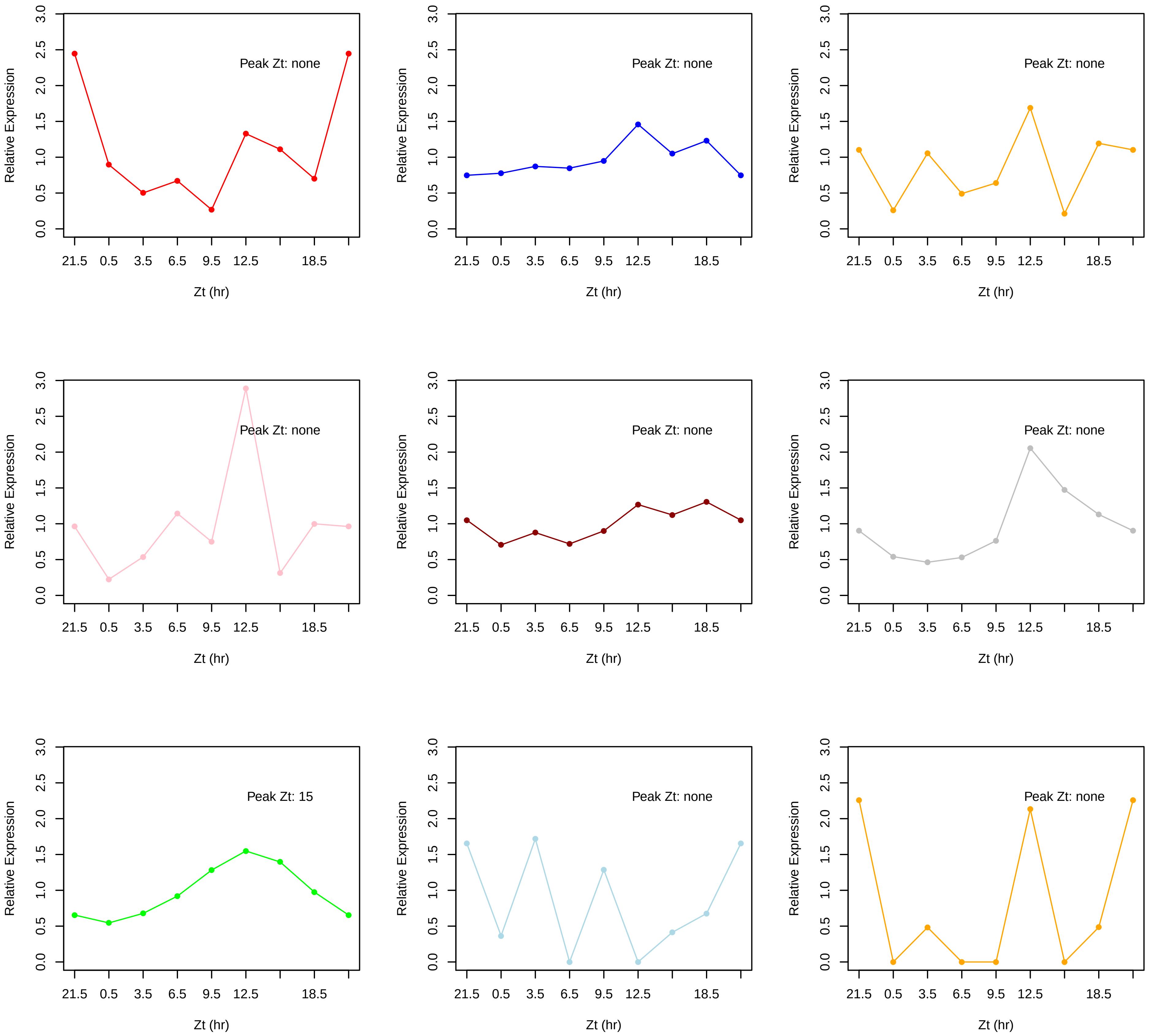

Singleton: ENSXMAG00000002205 hdhd2

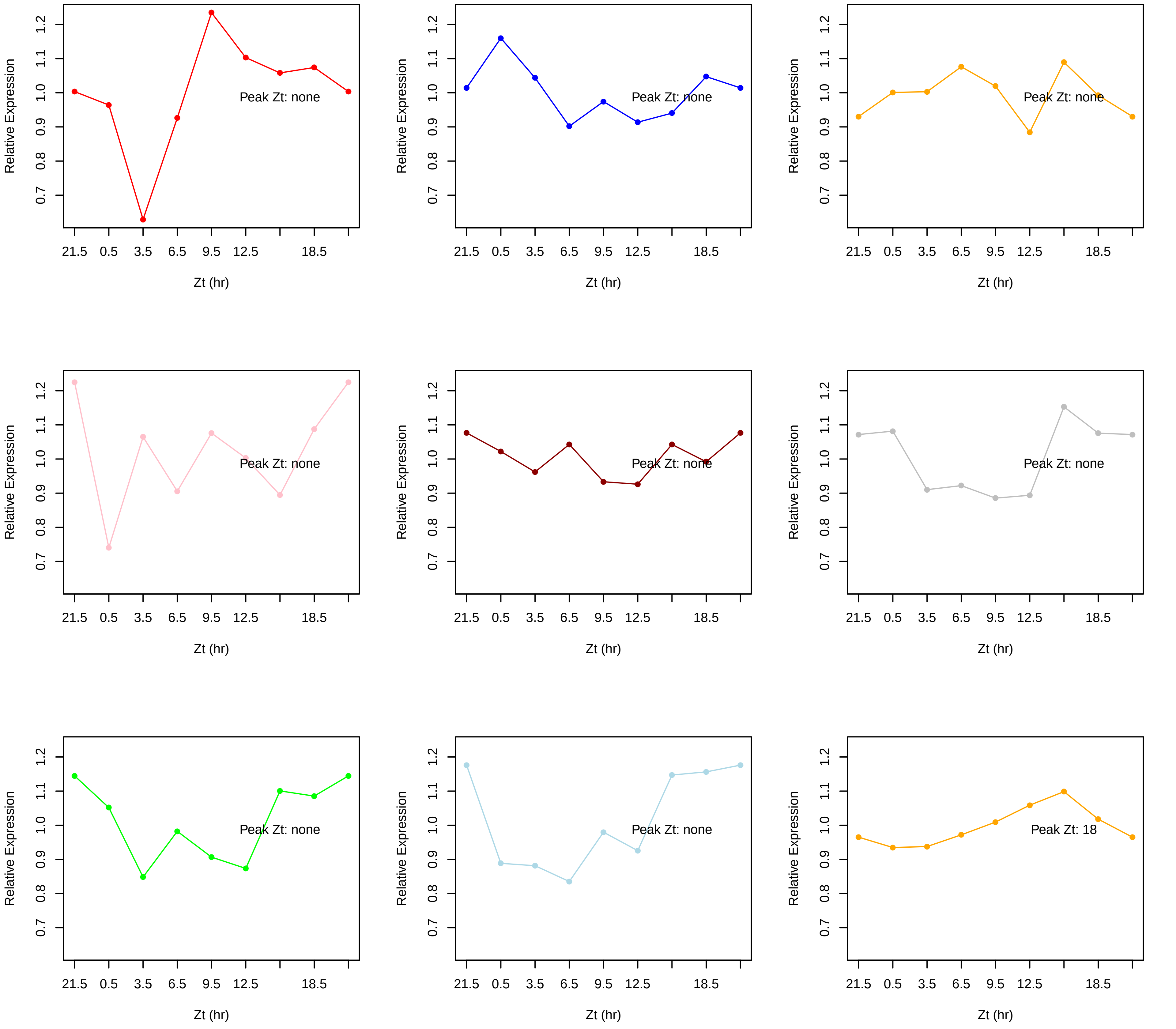

Singleton: ENSXMAG00000009130 EZH1

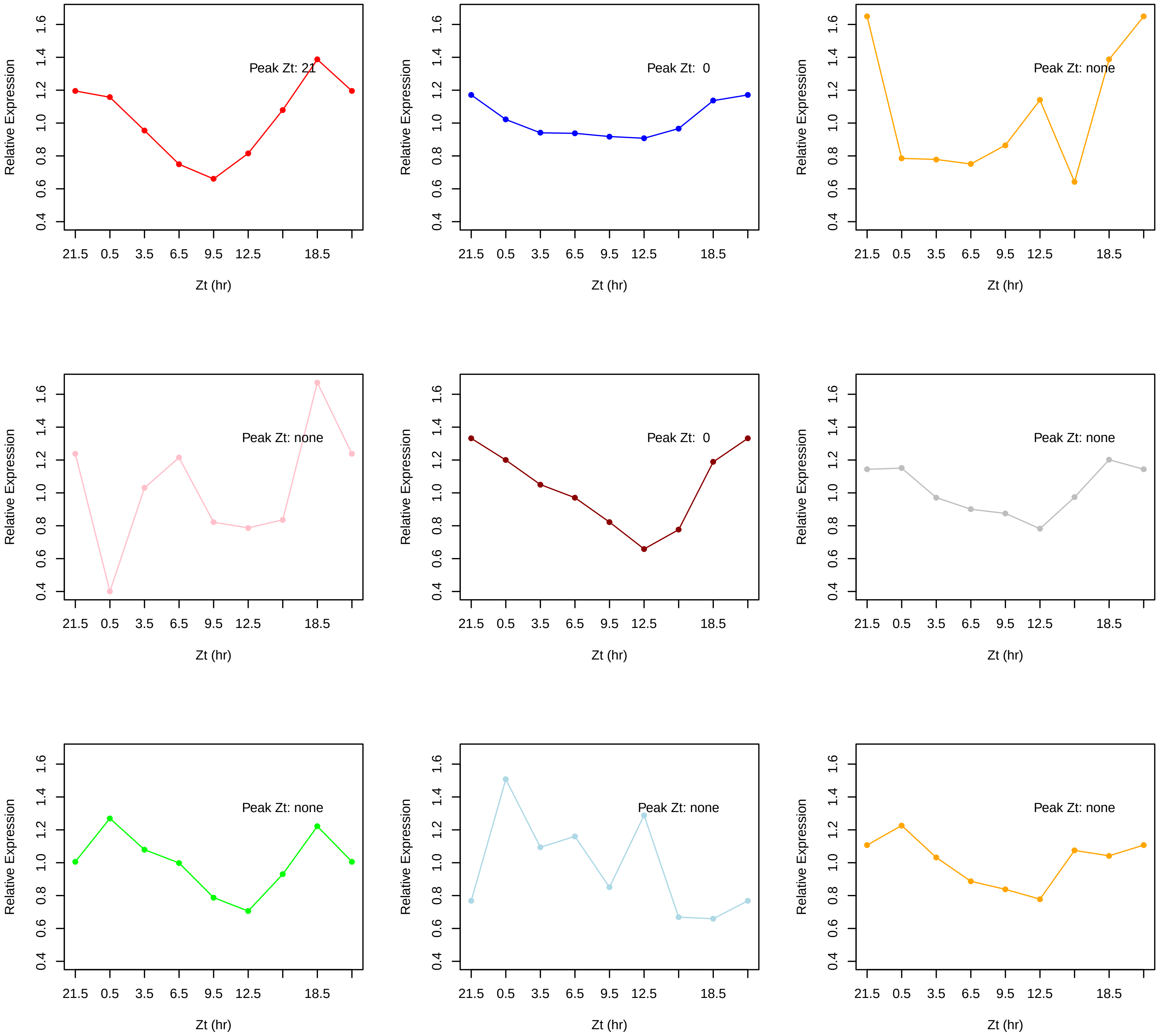

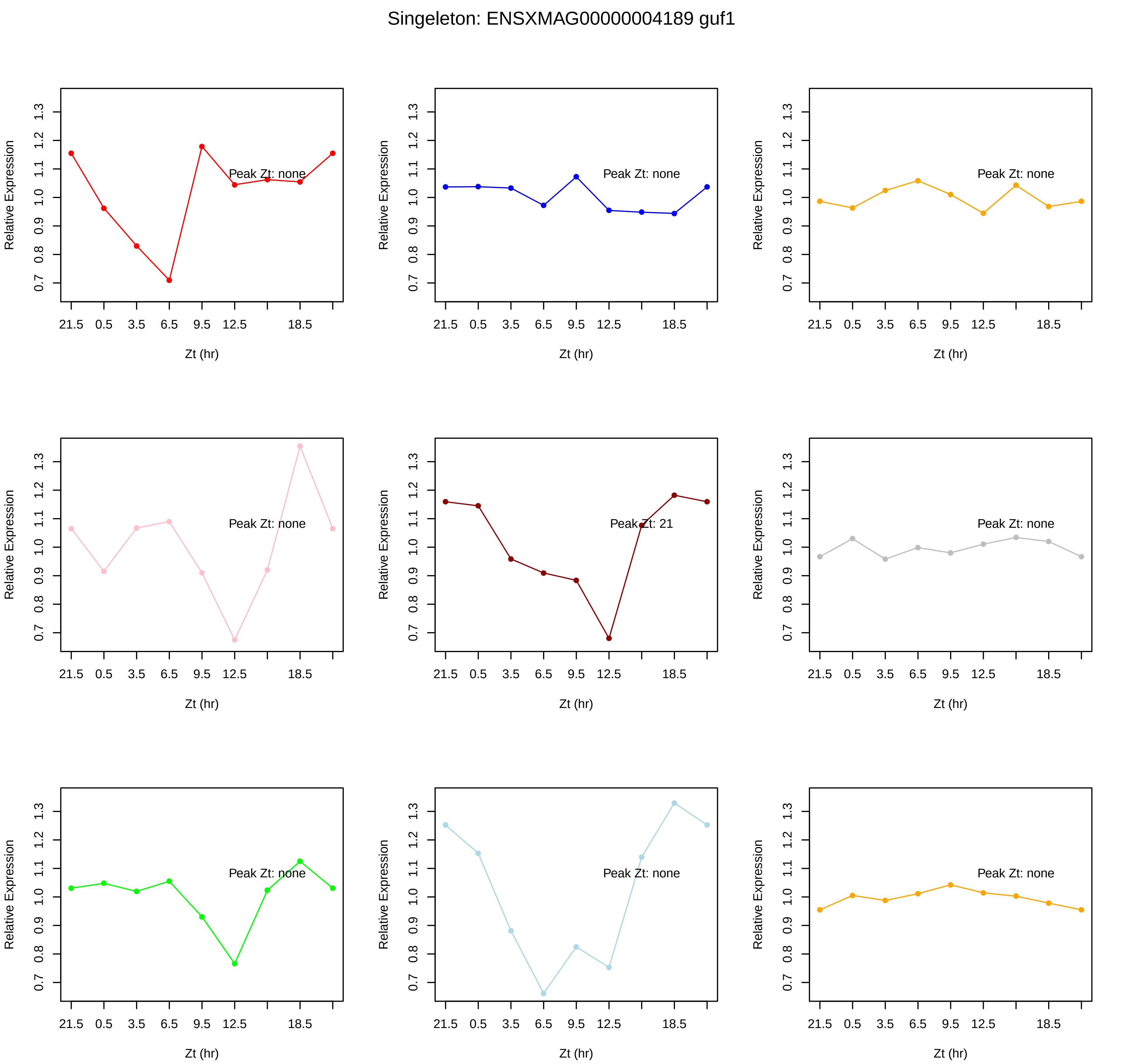
Singeleton: ENSXMAG00000004189 guf1
Relative Expression
Zt (hr)
Peak Zt: none
Peak Zt: 21
21.5
0.5
3.5
6.5
9.5
12.5
18.5
0.7
0.8
0.9
1.0
1.1
1.2
1.3

Singeleton: ENSXMAG00000015564 mrpl22

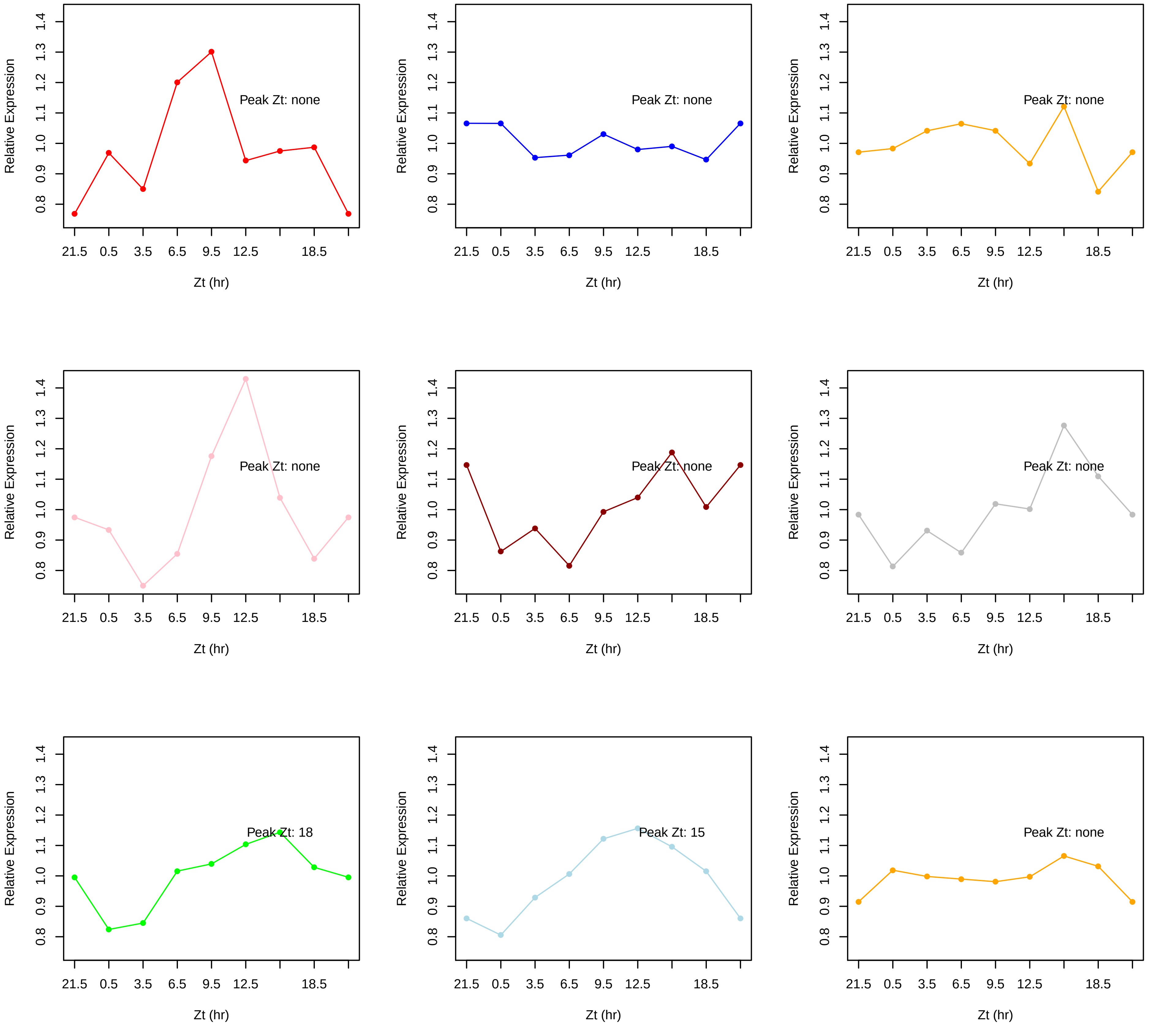

Singeleton: ENSXMAG00000015538 gemin5

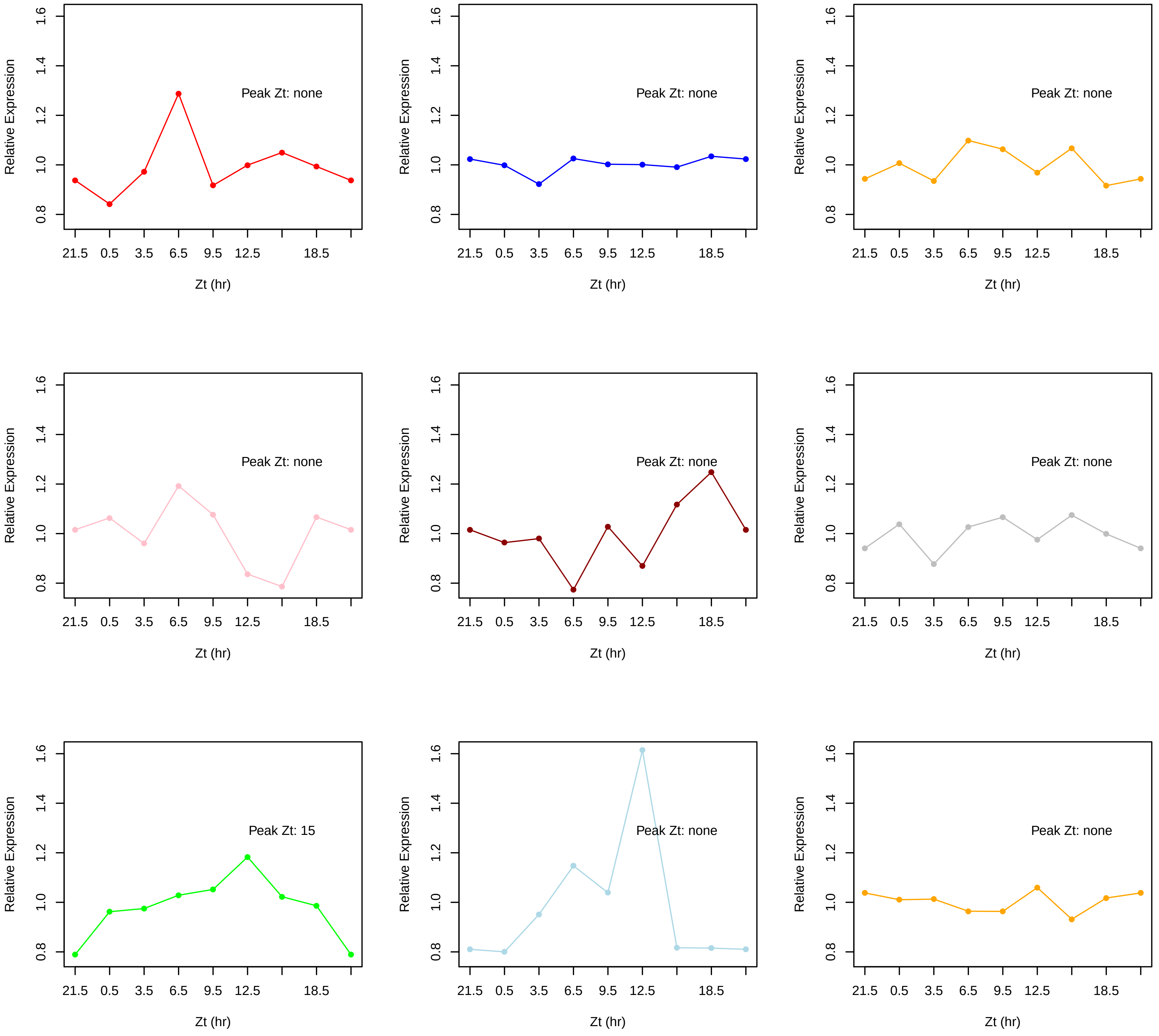

Singeleton: ENSXMAG00000016321 prkar1ab

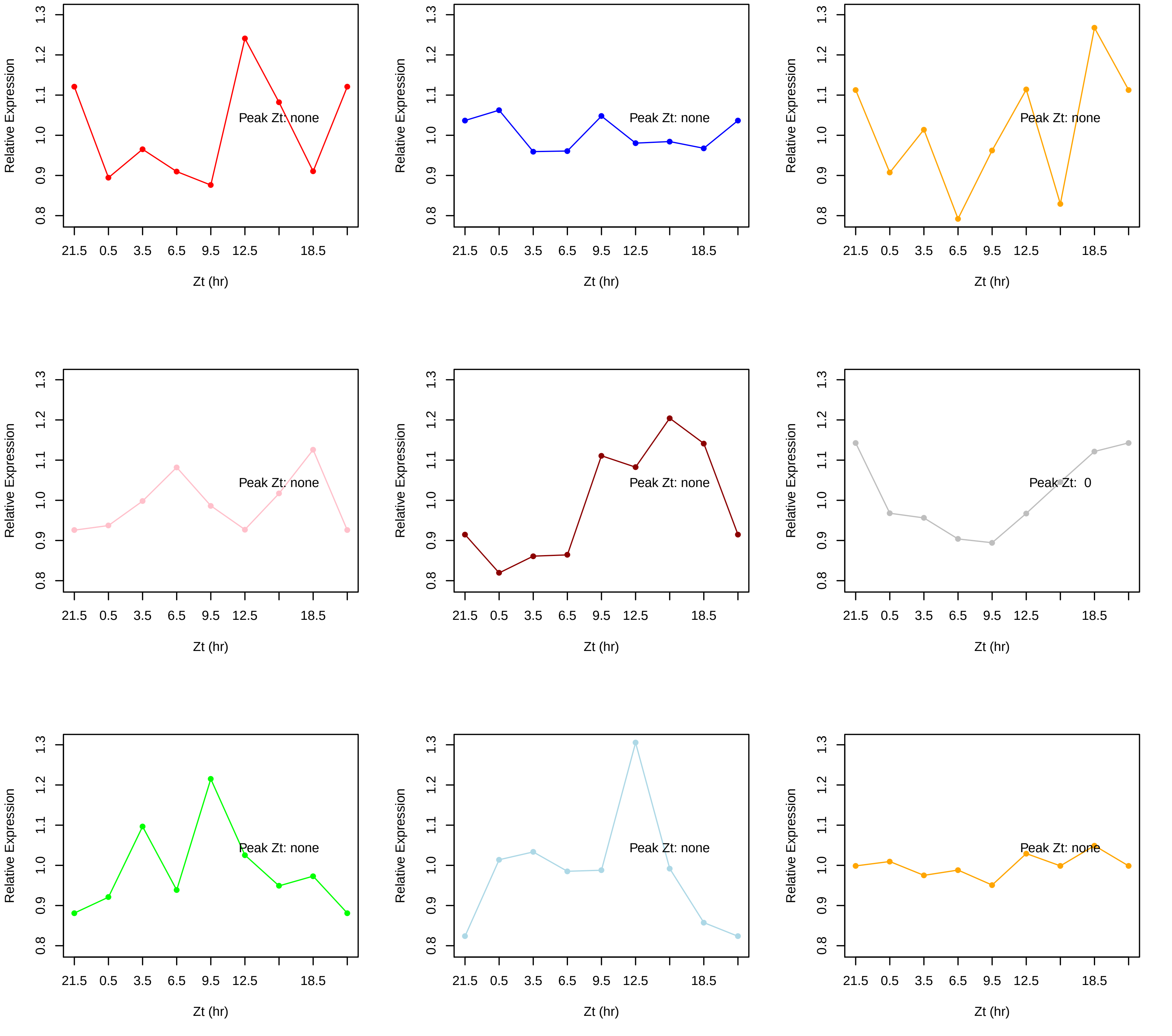

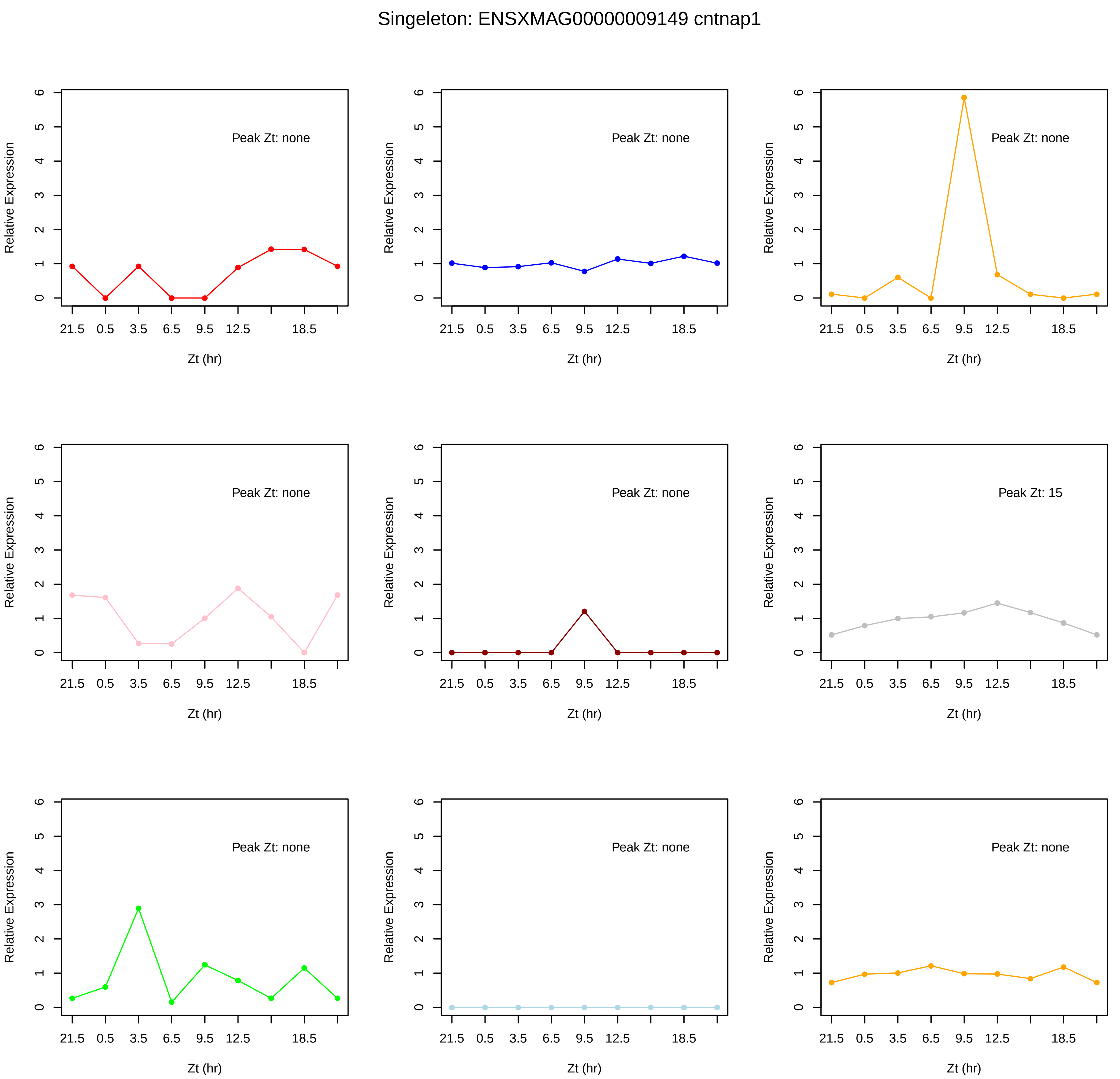
Singeleton: ENSXMAG00000009149 cntnap1
Peak Zt: none
Peak Zt: none
Peak Zt: none
Peak Zt: none
Peak Zt: none
Peak Zt: 15
Peak Zt: none
Peak Zt: none
Peak Zt: none
Relative Expression
Zt (hr)
21.5
0.5
3.5
6.5
9.5
12.5
18.5
0
1
2
3
4
5
6

Singleton: ENSXMAG00000015521 cnot8

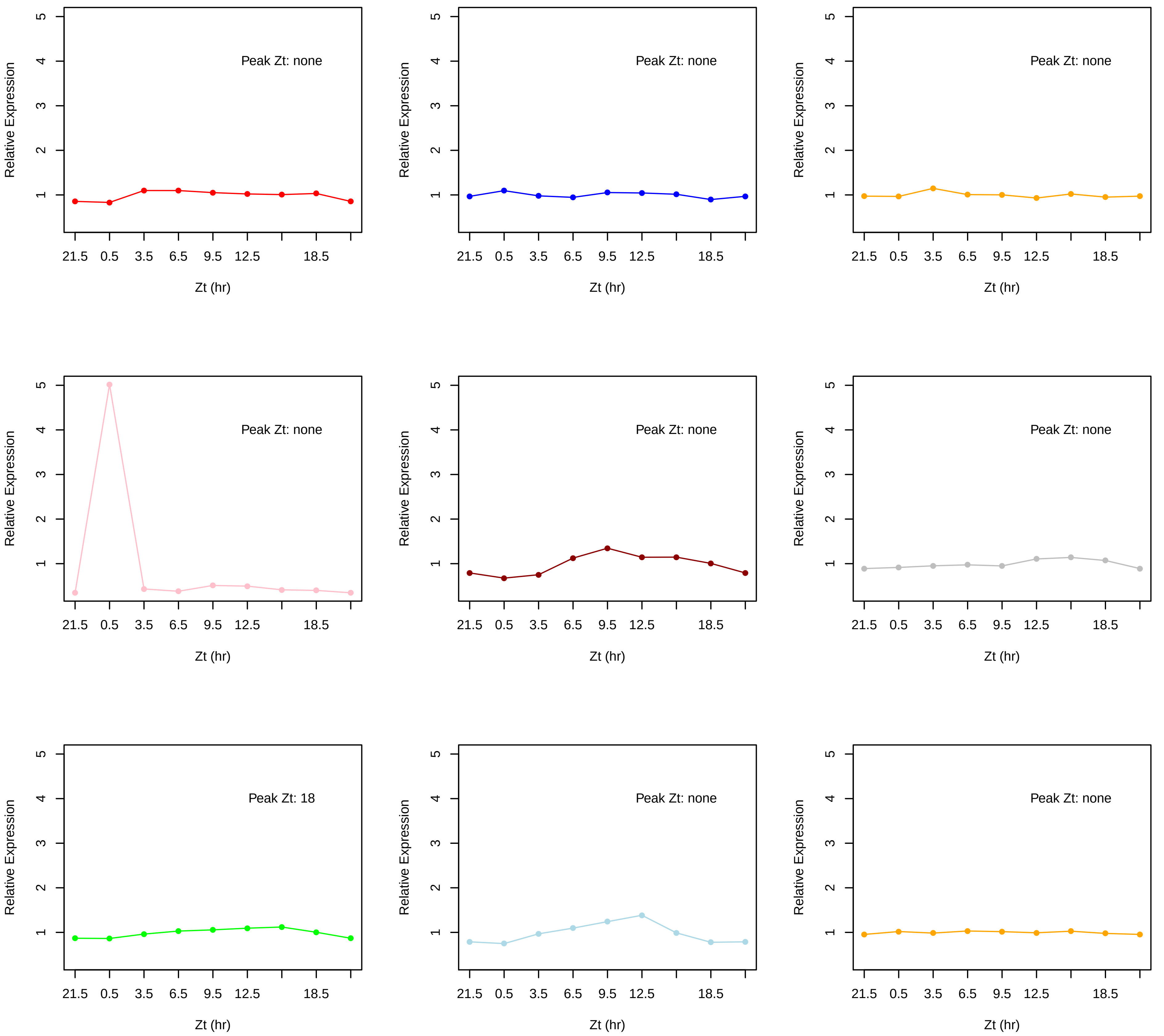

Singleton: ENSXMAG00000008134 apobec2b

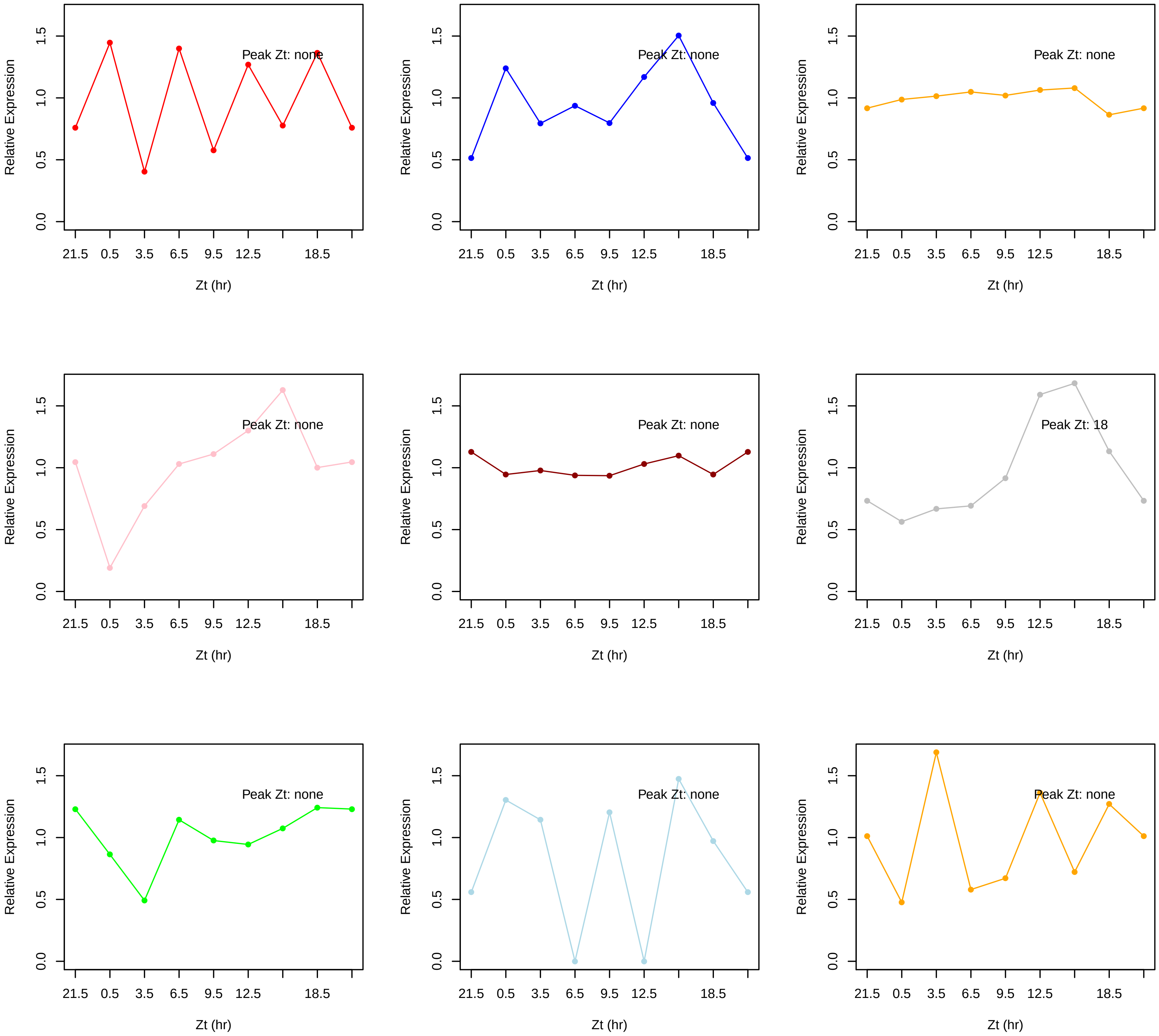

Singeleton: ENSXMAG00000015499 fam114a2

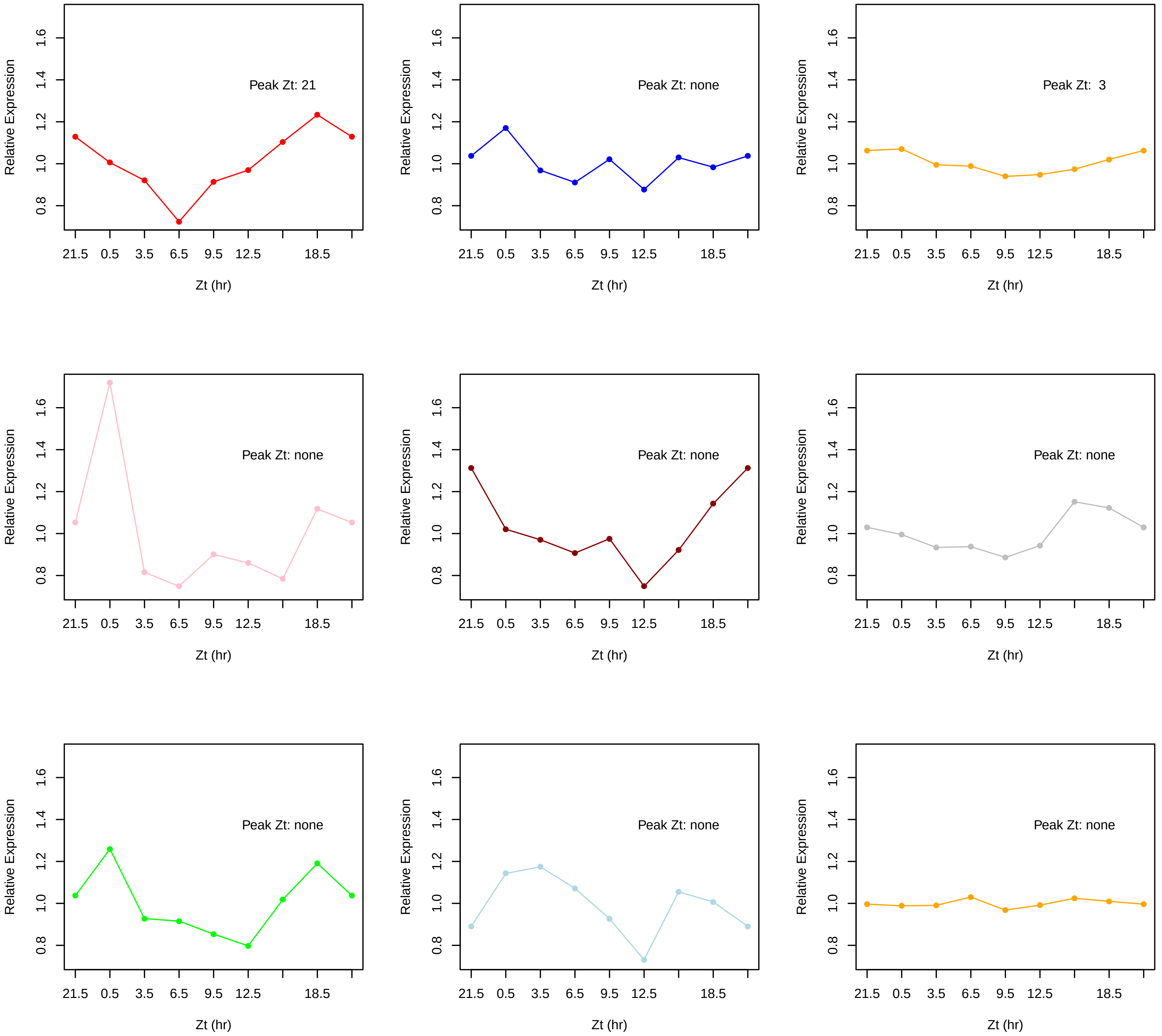

Singeleton: ENSXMAG00000000643 zgc:86896

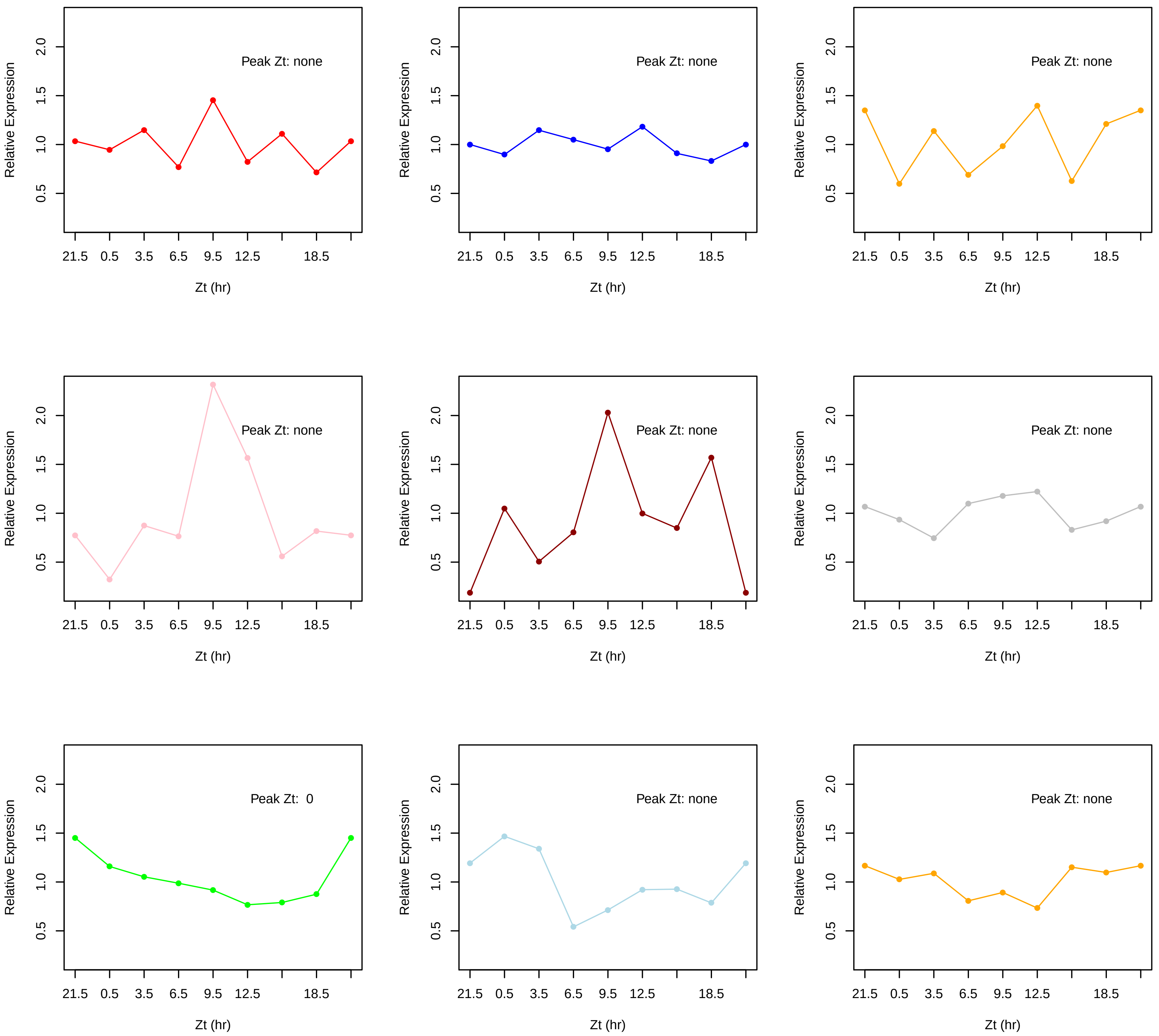

Singleton: ENSXMAG00000013854 PLEKHH3

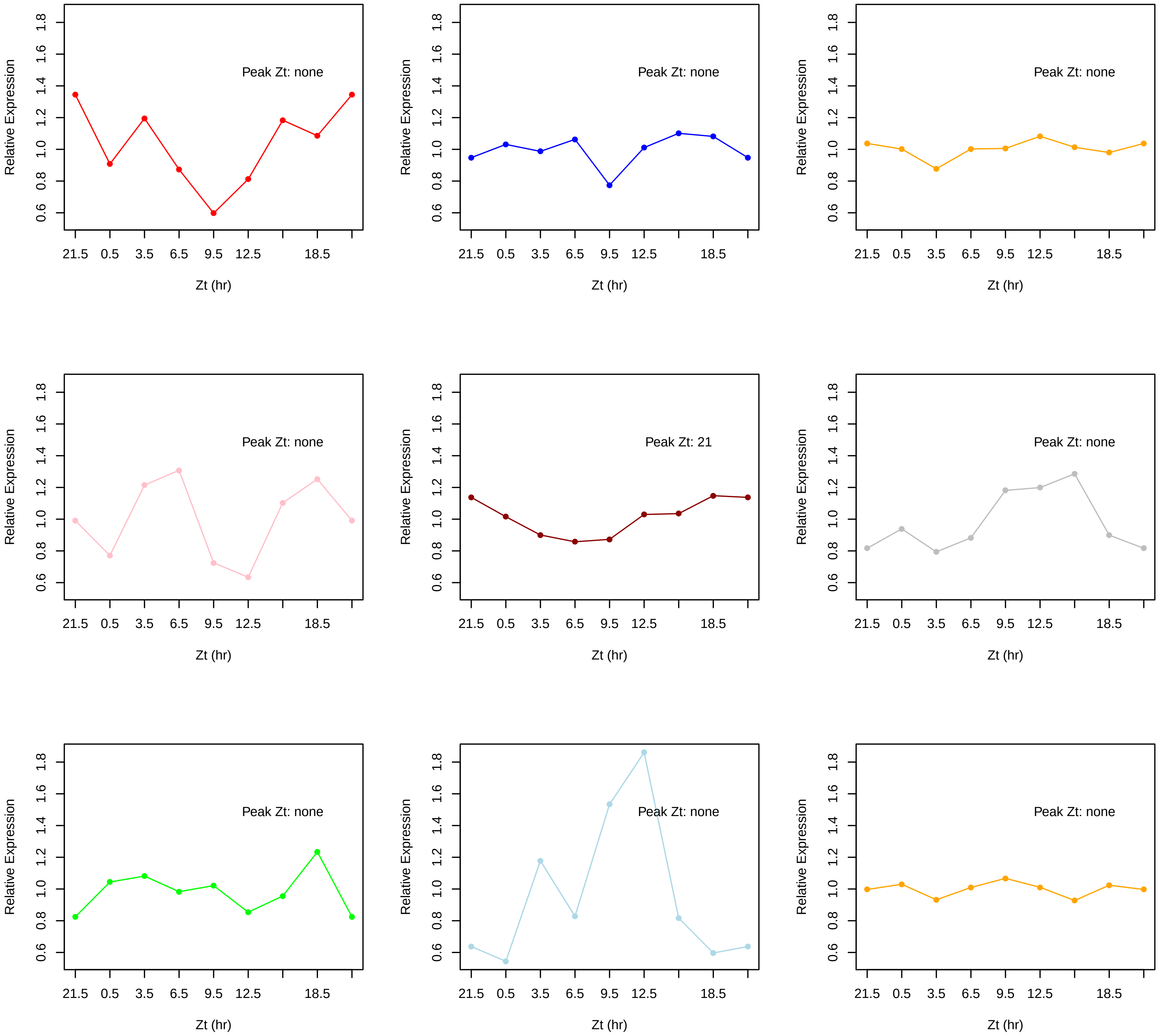

Singeleton: ENSXMAG00000013850 tubg1

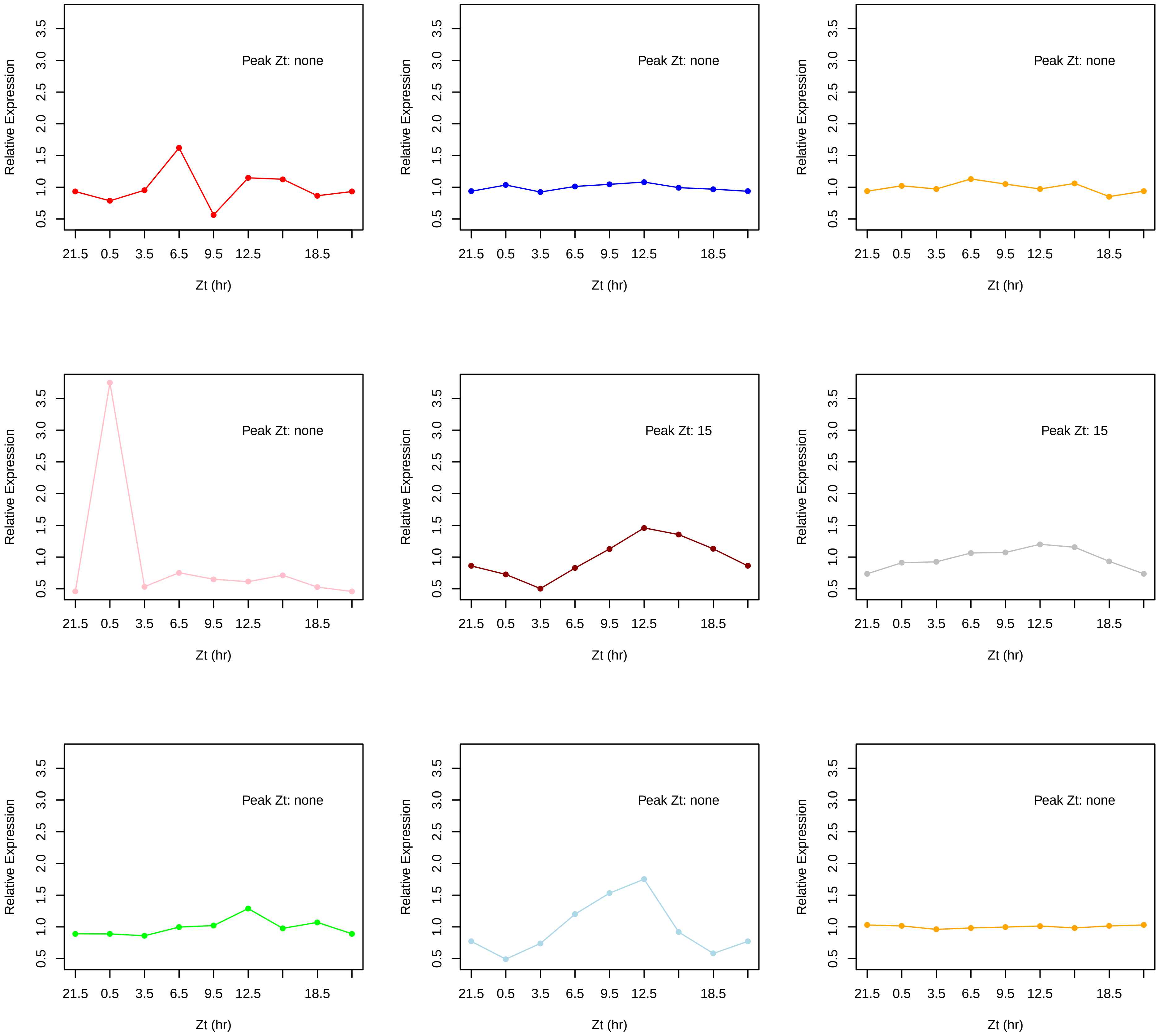

Singeleton: ENSXMAG00000016159 atp8a1

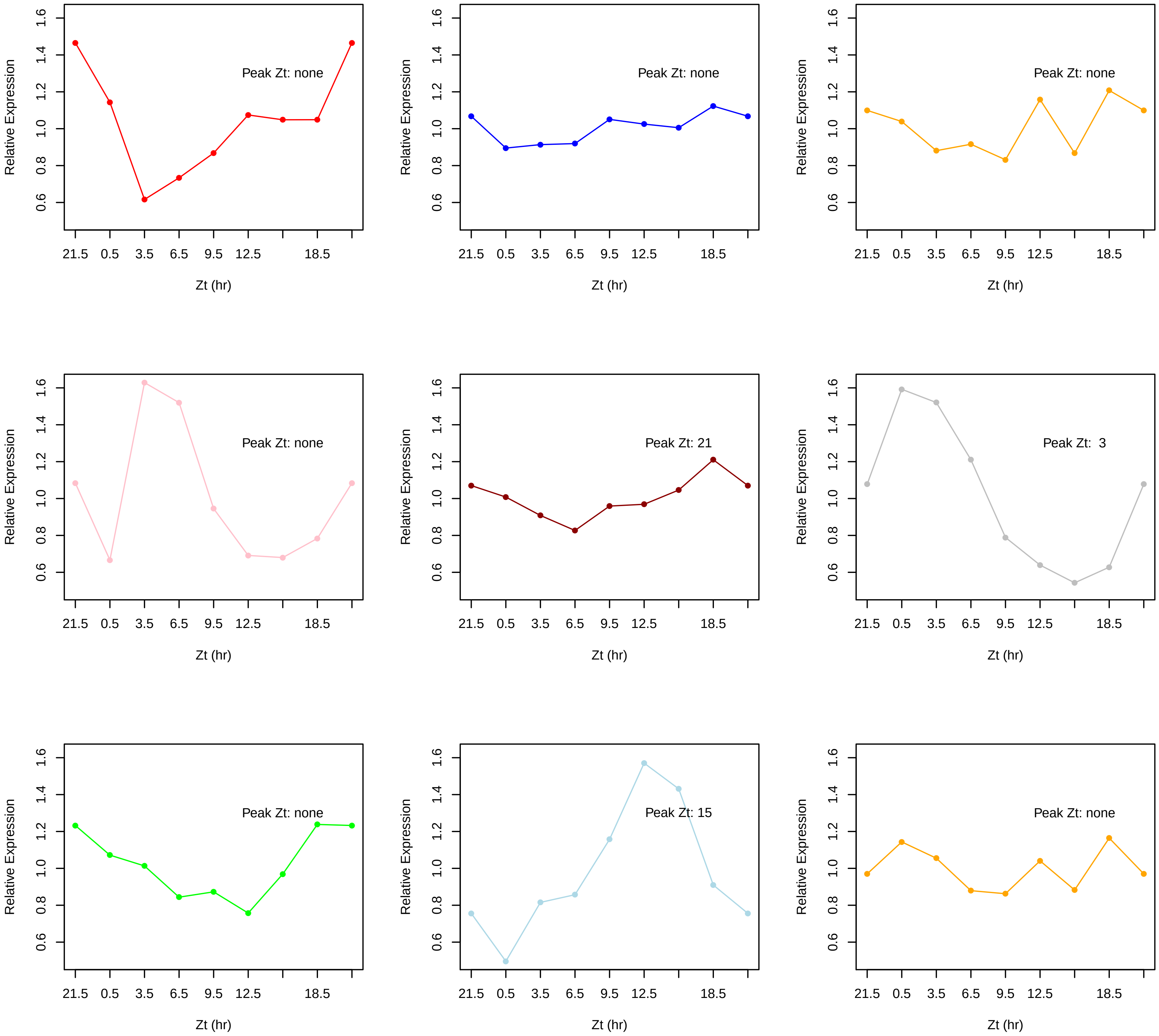

Singeleton: ENSXMAG00000015941 glud1a

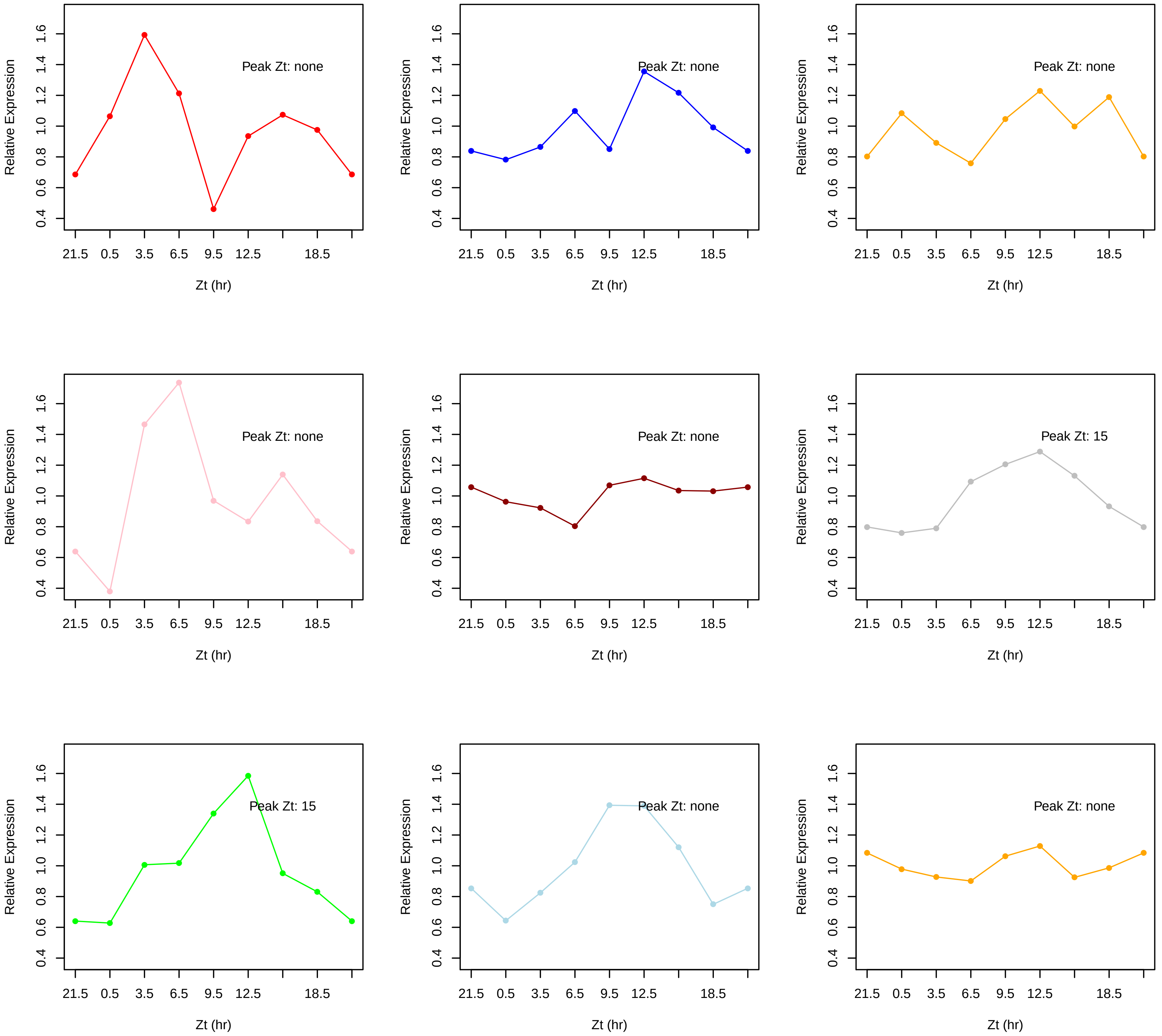

Singeleton: ENSXMAG00000025155 retreg3

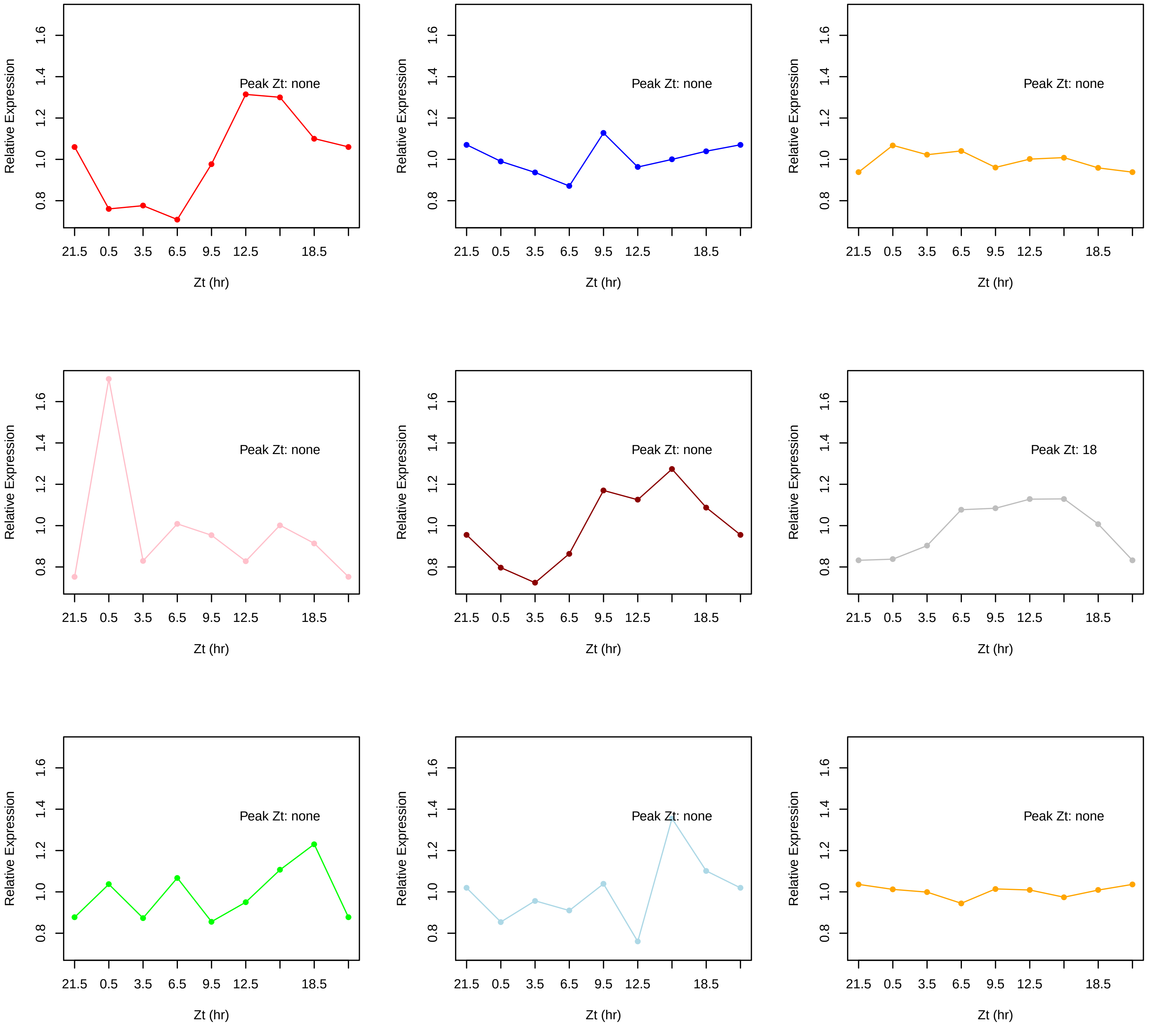

Singleton: ENSXMAG00000026416 srsf5b

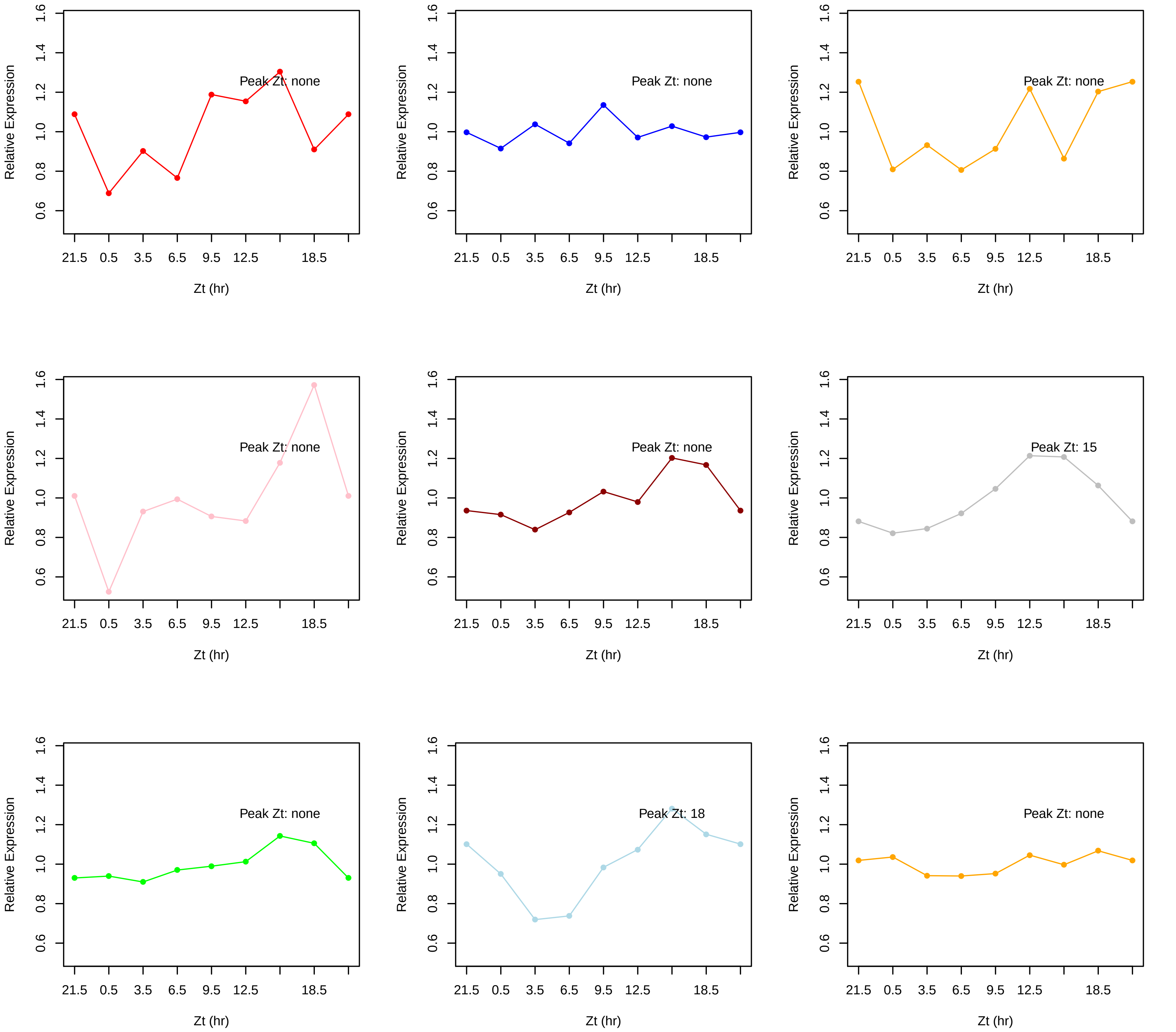

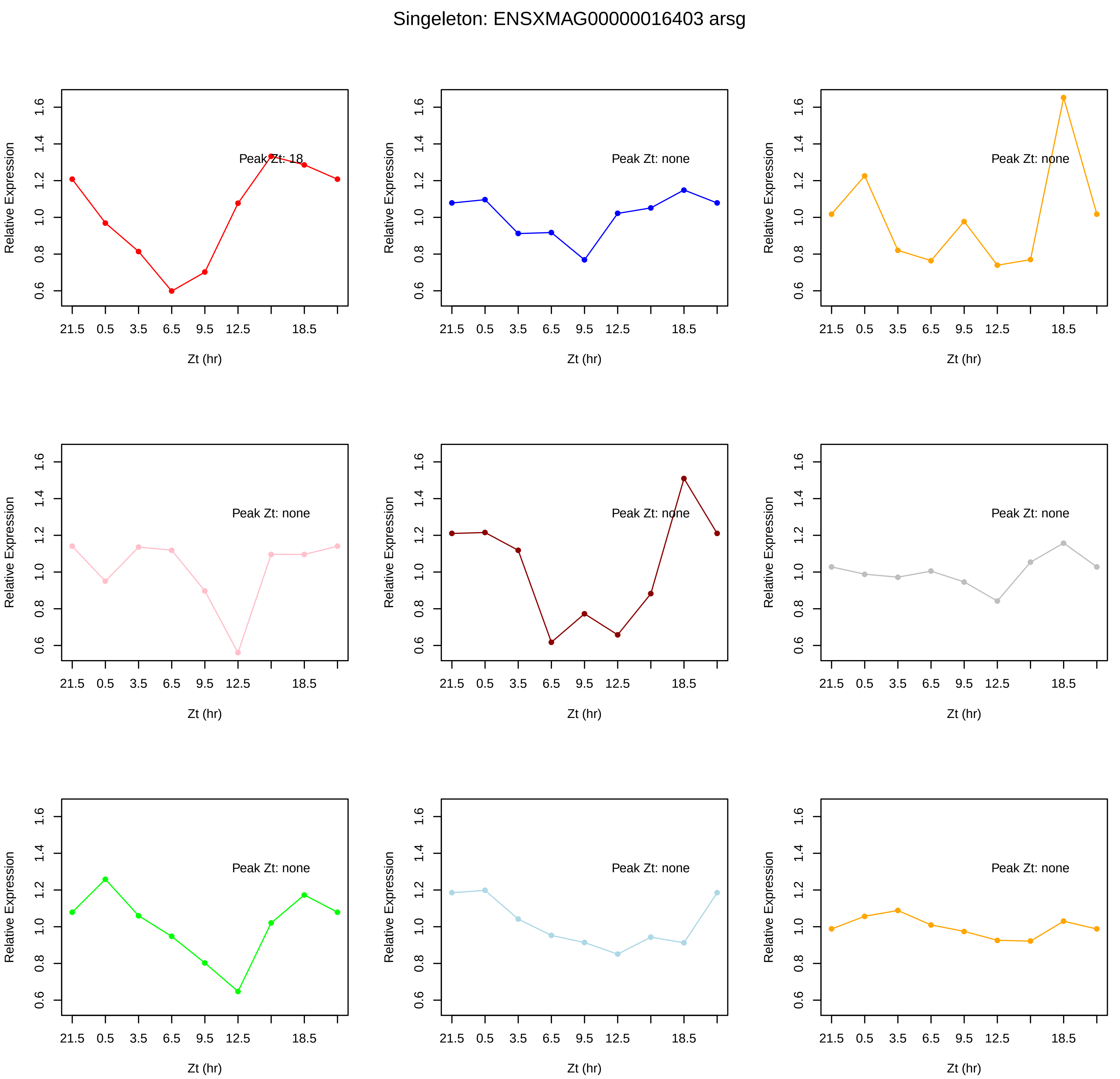

Singeleton: ENSXMAG00000016403 arsg
Peak Zt: 18
Peak Zt: none
Peak Zt: none
Peak Zt: none
Peak Zt: none
Peak Zt: none
Peak Zt: none
Peak Zt: none
Peak Zt: none
Relative Expression
Zt (hr)
21.5
0.5
3.5
6.5
9.5
12.5
18.5
0.6
0.8
1.0
1.2
1.4
1.6

Singeleton: ENSXMAG00000015434 sap30l

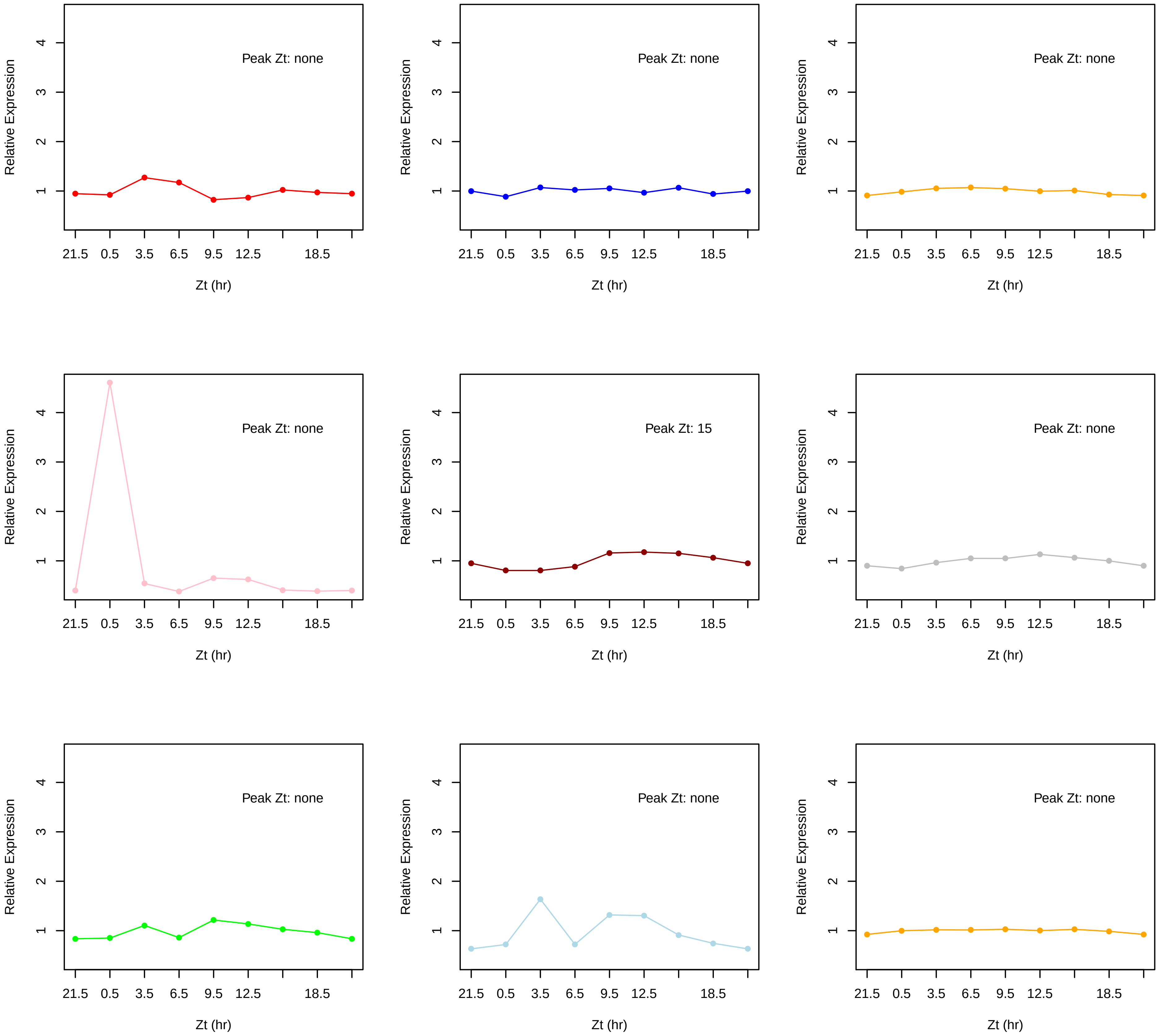

Singleton: ENSXMAG00000002625

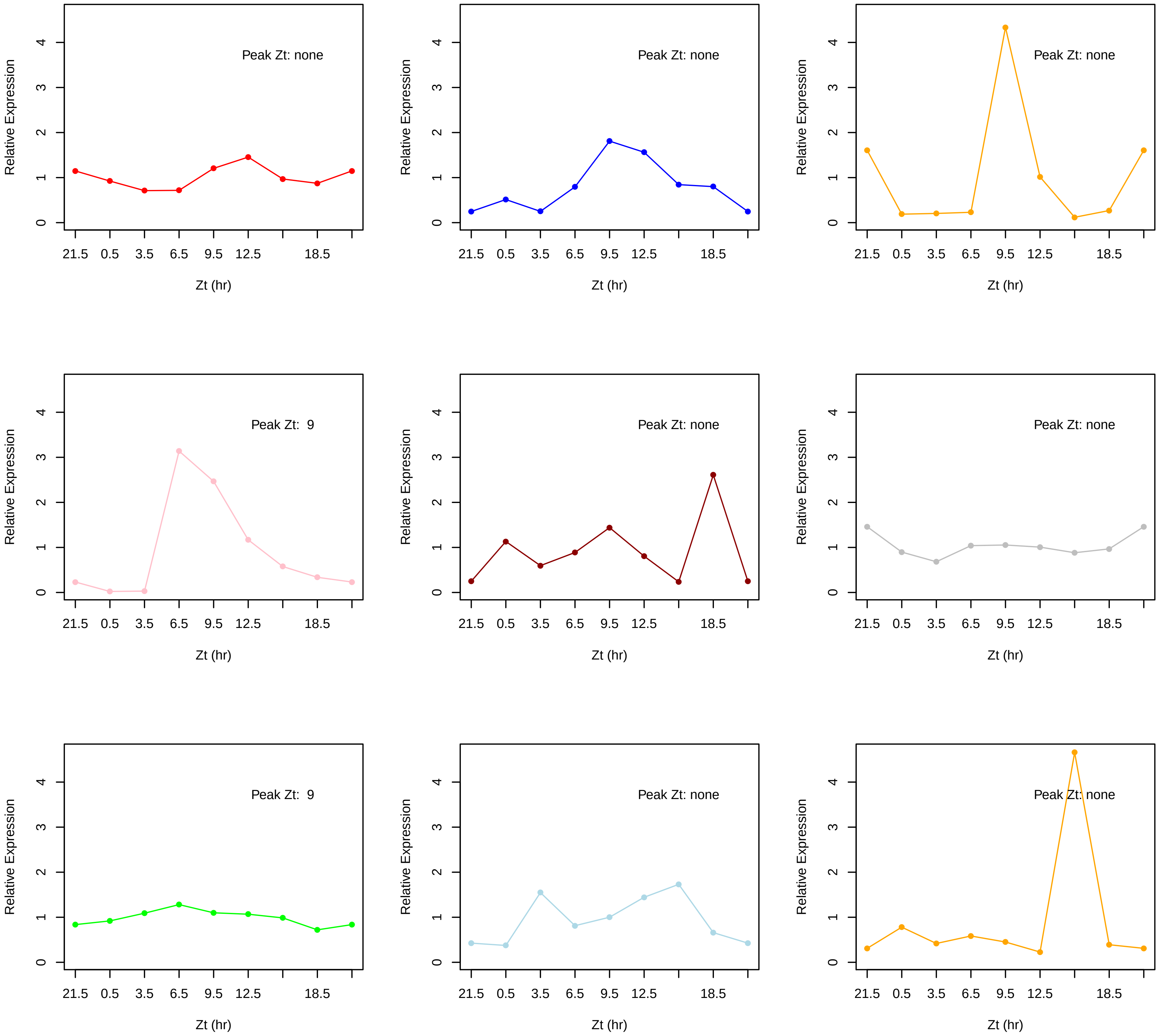

Singleton: ENSXMAG00000002451 nom1

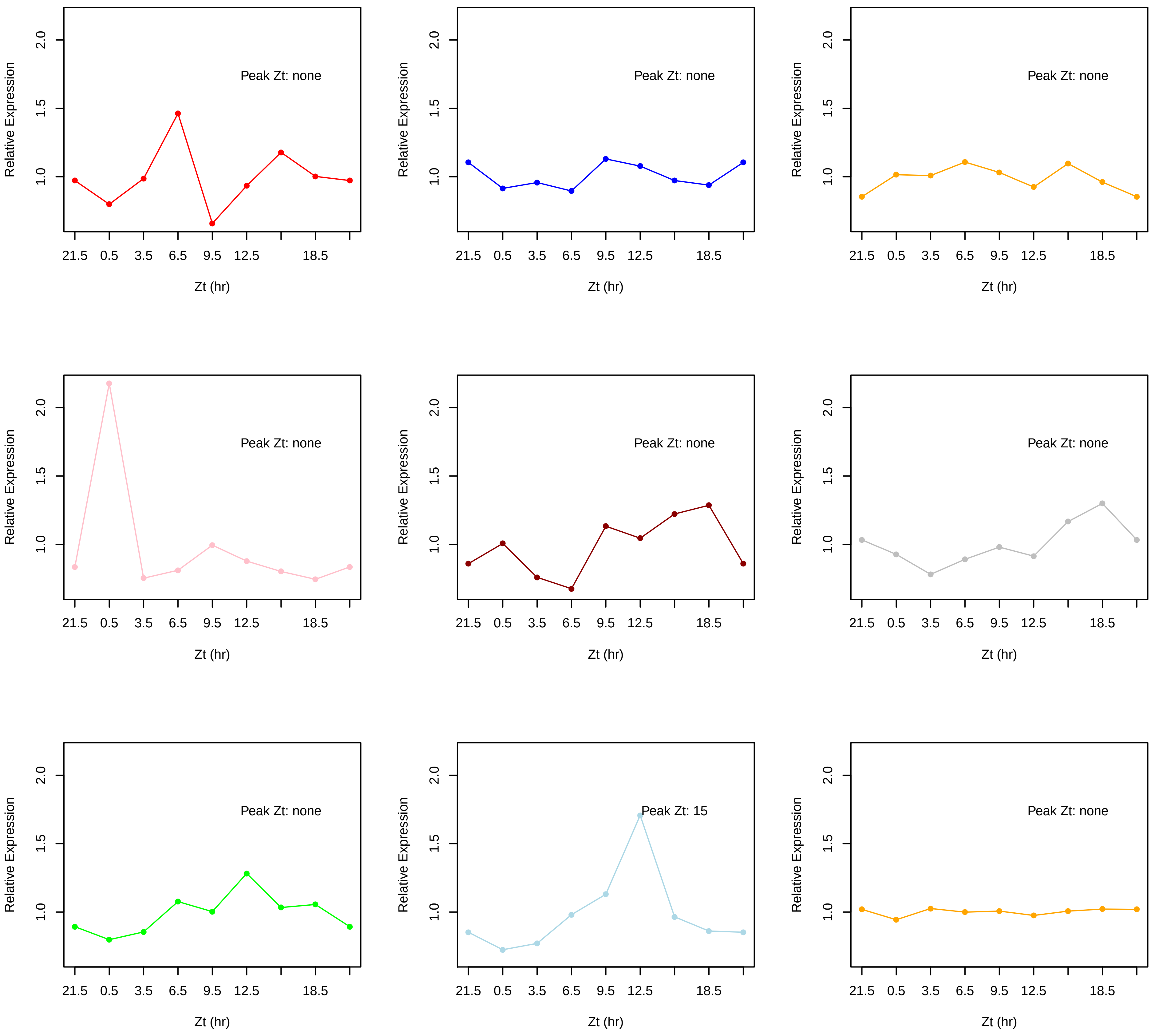

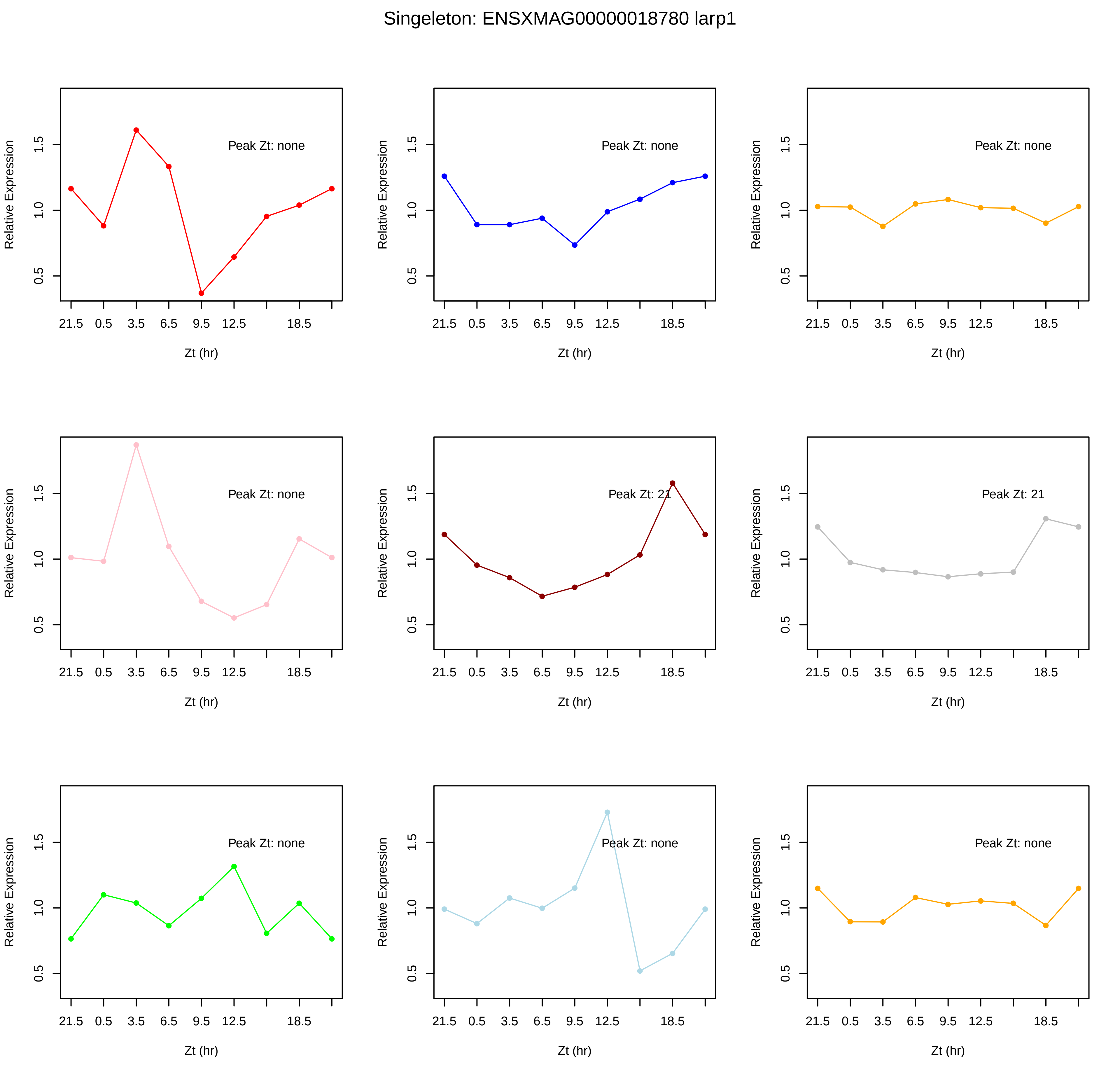
Singeleton: ENSXMAG00000018780 larp1
Peak Zt: none
Peak Zt: none
Peak Zt: none
Peak Zt: none
Peak Zt: 21
Peak Zt: 21
Peak Zt: none
Peak Zt: none
Peak Zt: none
Relative Expression
Zt (hr)
0.5
1.0
1.5
21.5
0.5
3.5
6.5
9.5
12.5
18.5

Singeleton: ENSXMAG00000022521 shisa3

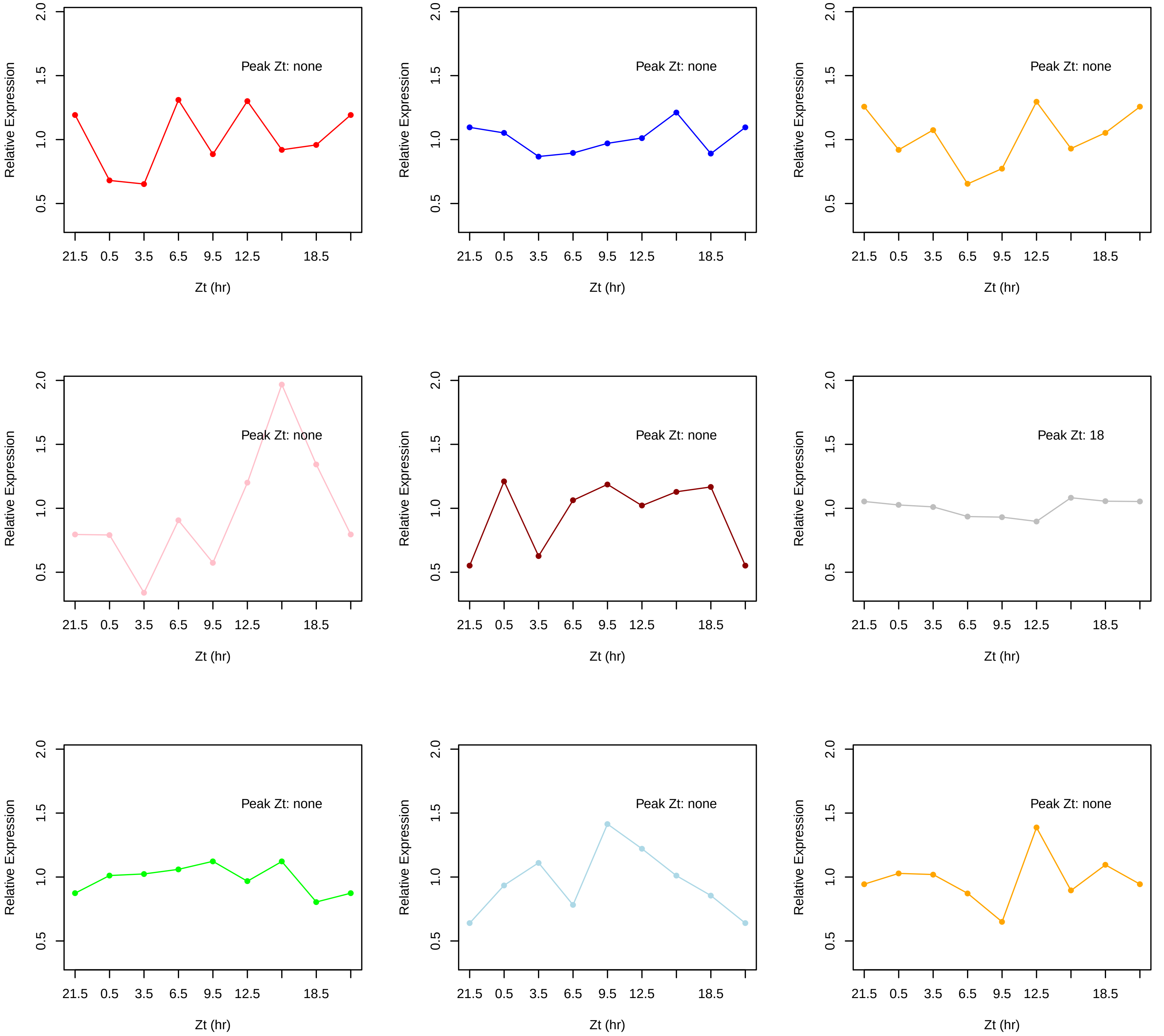

Singleton: ENSXMAG00000015601 coasy

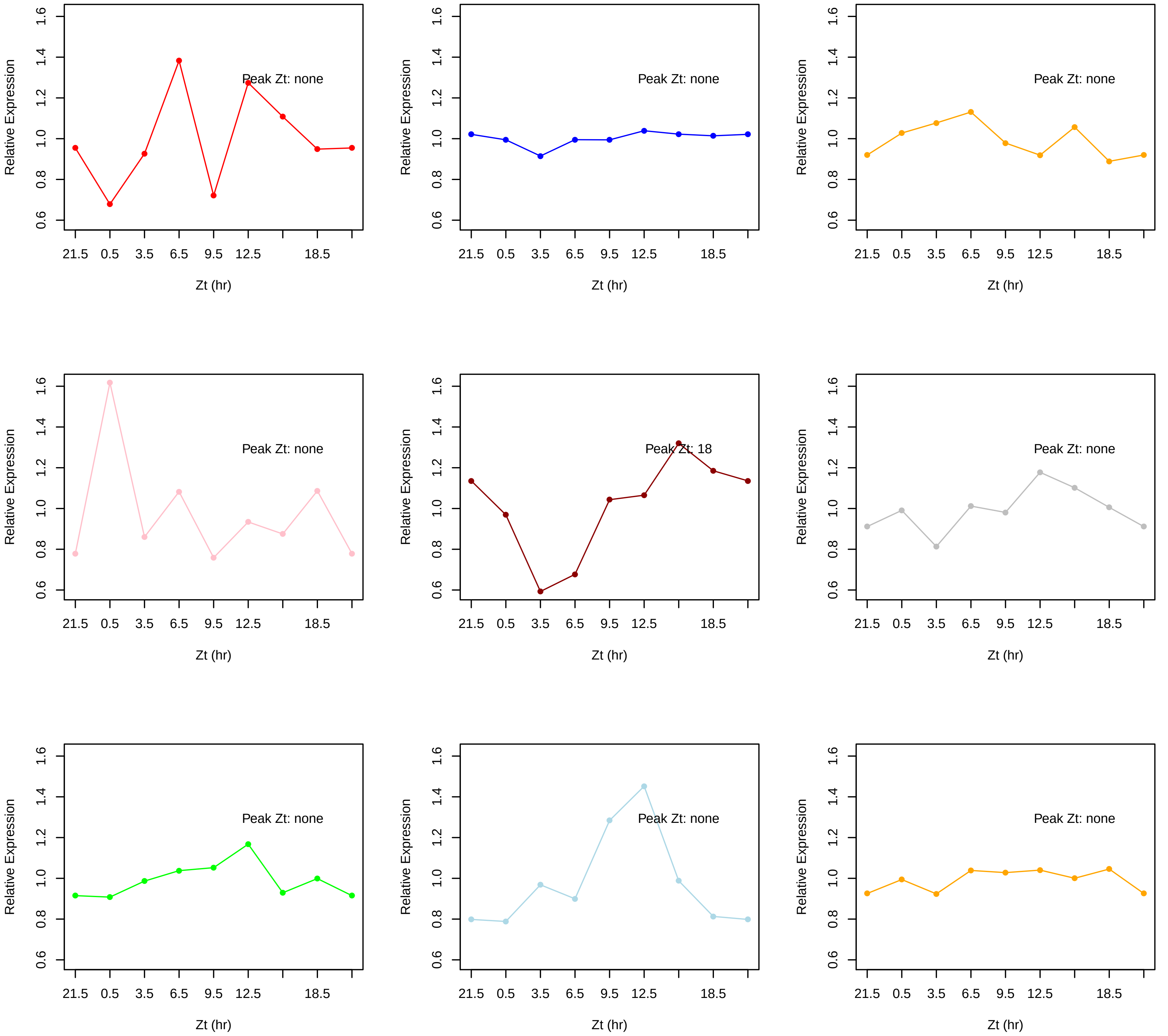

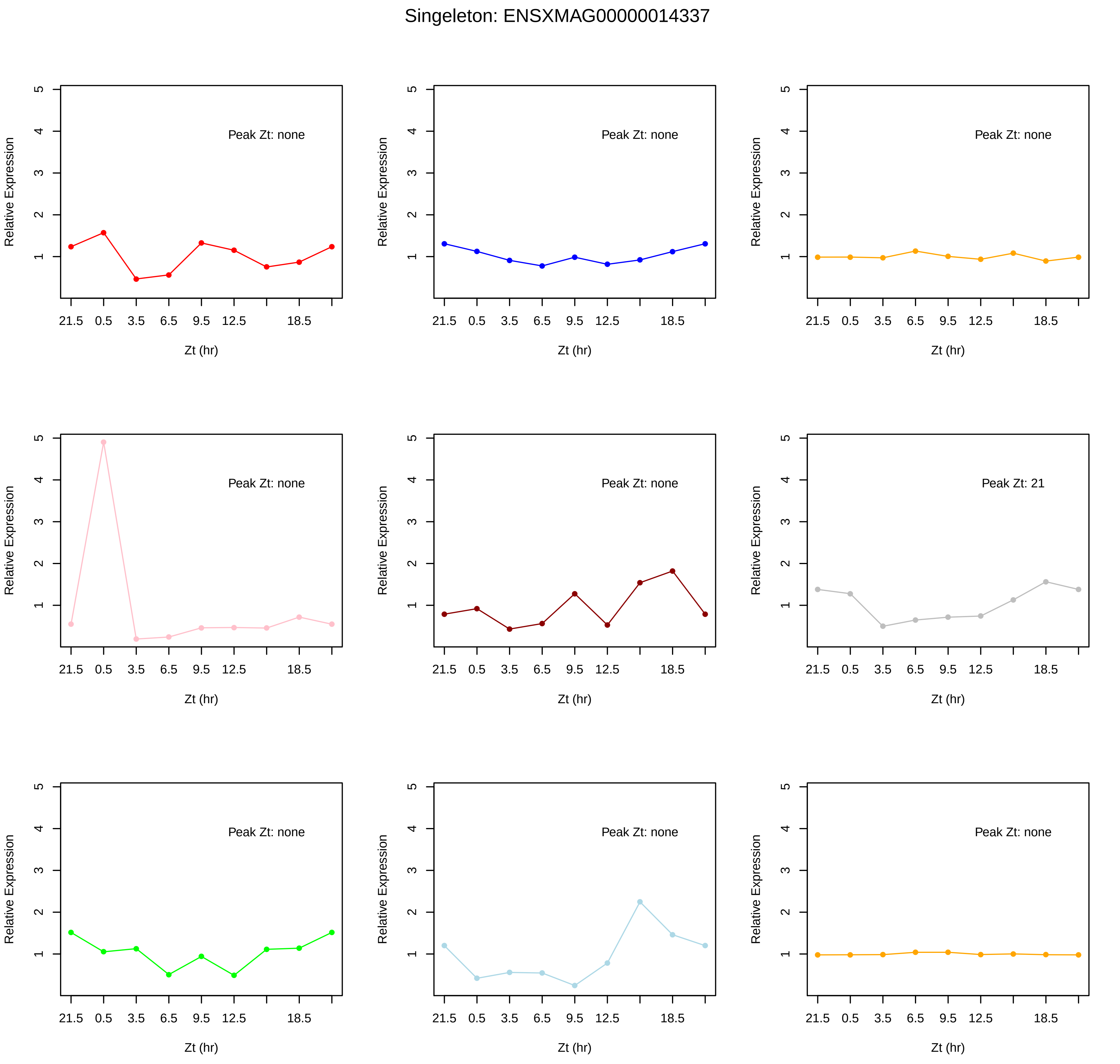
Singeleton: ENSXMAG00000014337
Peak Zt: none
Peak Zt: none
Peak Zt: none
Peak Zt: none
Peak Zt: none
Peak Zt: 21
Peak Zt: none
Peak Zt: none
Peak Zt: none
Relative Expression
Zt (hr)
21.5 0.5 3.5 6.5 9.5 12.5 18.5
1 2 3 4 5

Singeleton: ENSXMAG00000004259 bmpr1aa

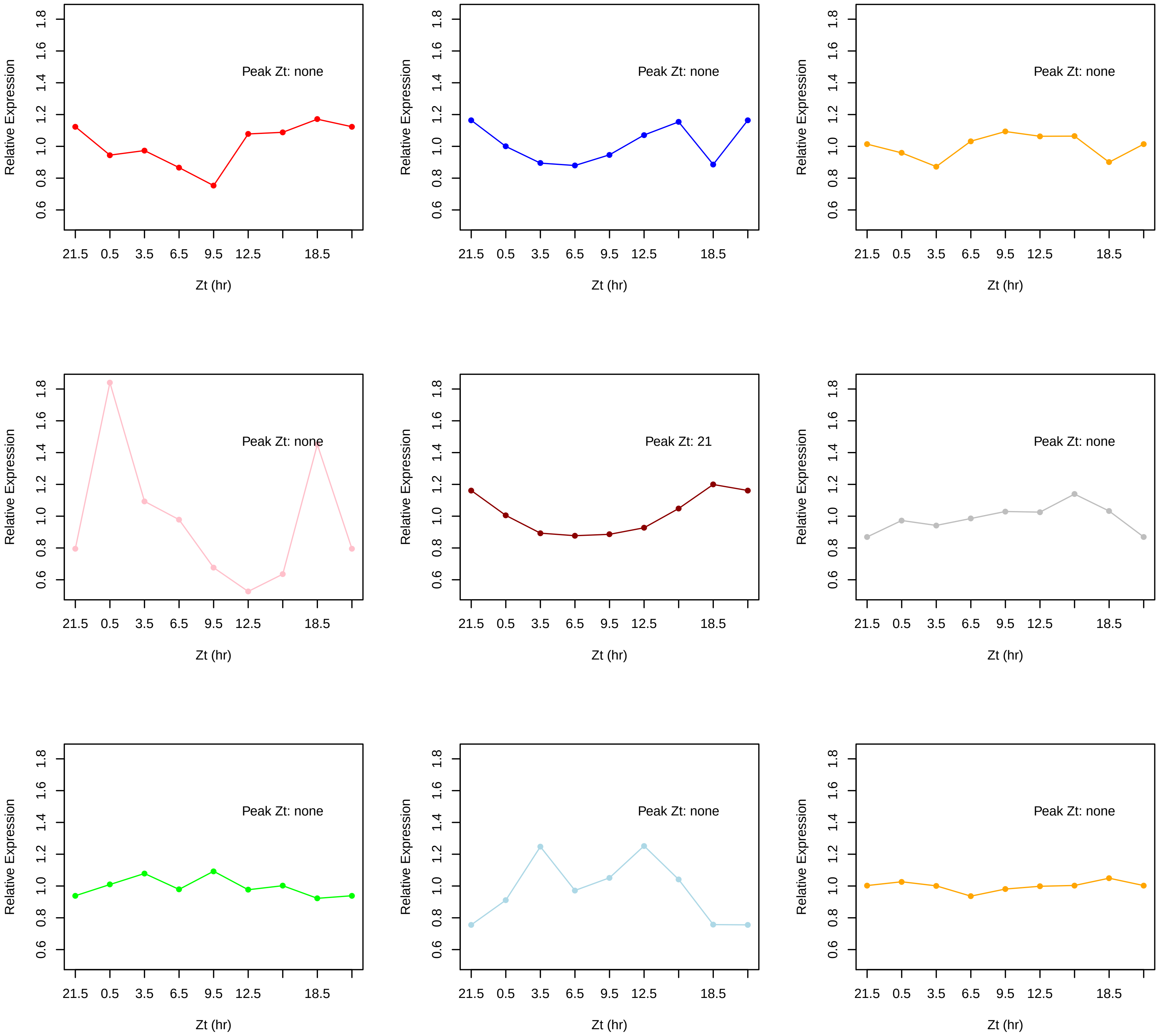

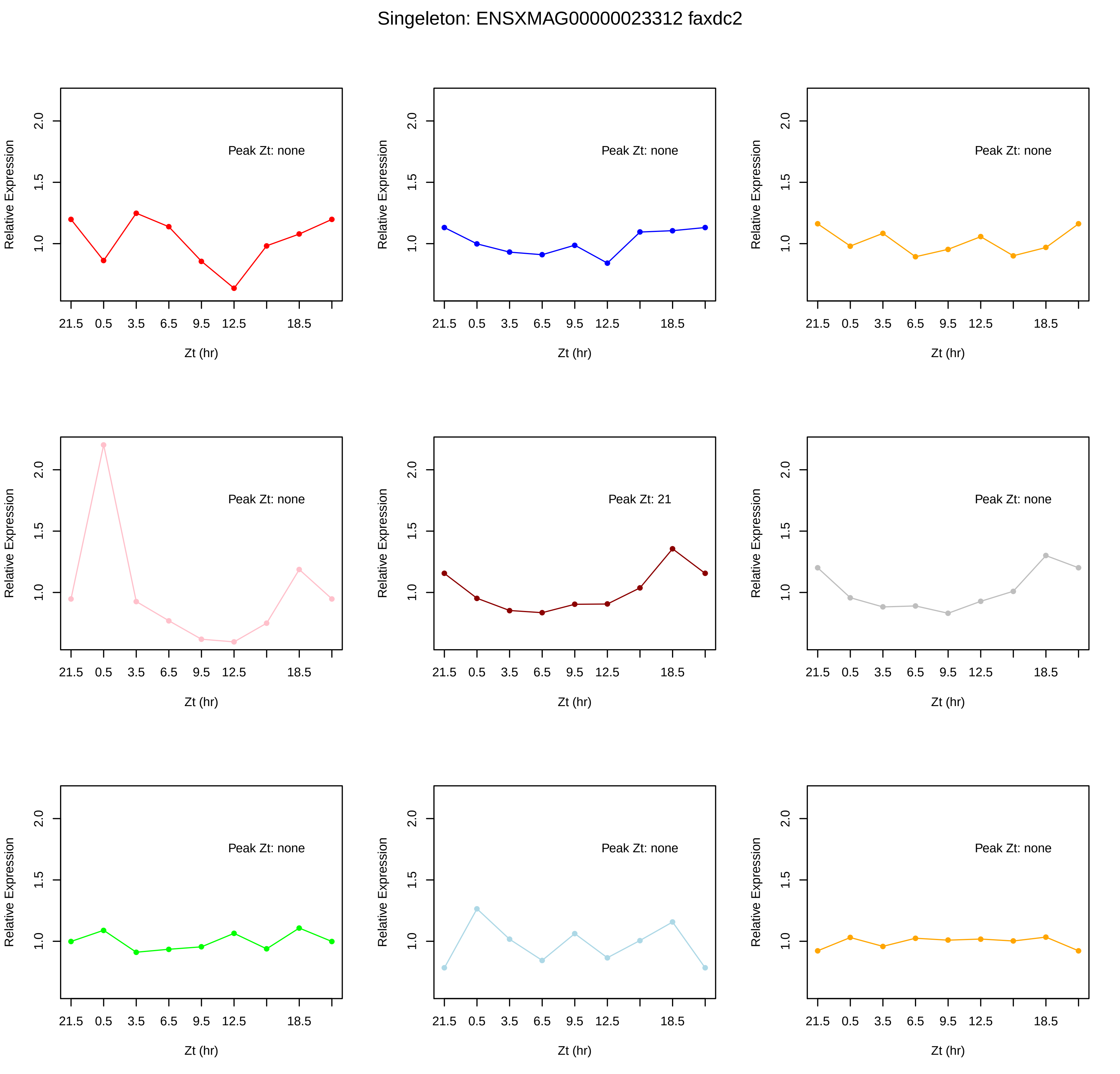

Singeleton: ENSXMAG00000023312 faxdc2
Peak Zt: none
Peak Zt: none
Peak Zt: none
Peak Zt: none
Peak Zt: 21
Peak Zt: none
Peak Zt: none
Peak Zt: none
Peak Zt: none
Relative Expression
Zt (hr)
21.5
0.5
3.5
6.5
9.5
12.5
18.5
1.0
1.5
2.0

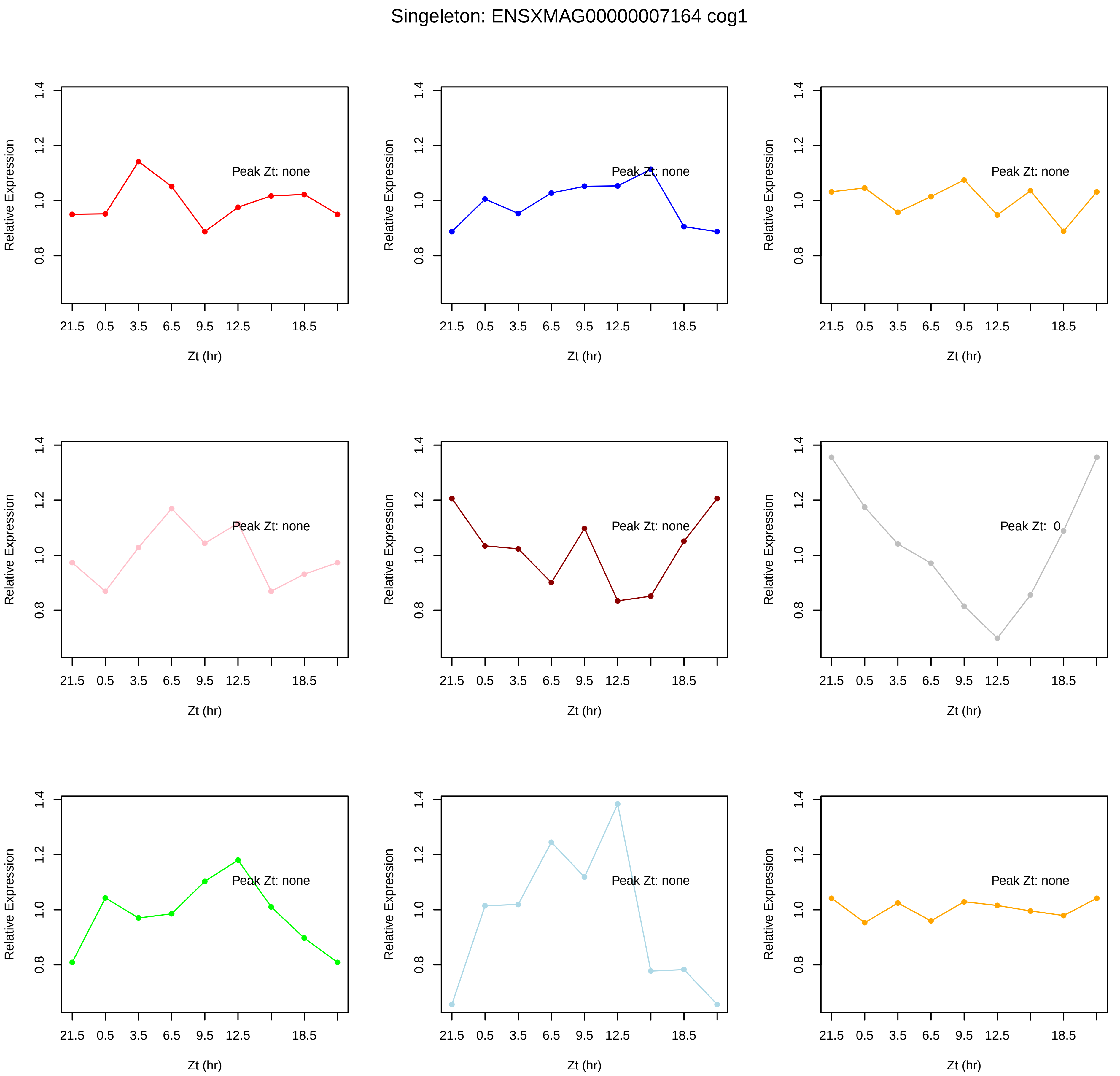
Singeleton: ENSXMAG00000007164 cog1
Peak Zt: none
Peak Zt: none
Peak Zt: none
Peak Zt: none
Peak Zt: none
Peak Zt: 0
Peak Zt: none
Peak Zt: none
Peak Zt: none
Relative Expression
Zt (hr)
21.5
0.5
3.5
6.5
9.5
12.5
18.5
0.8
1.0
1.2
1.4

Singeleton: ENSXMAG00000023477

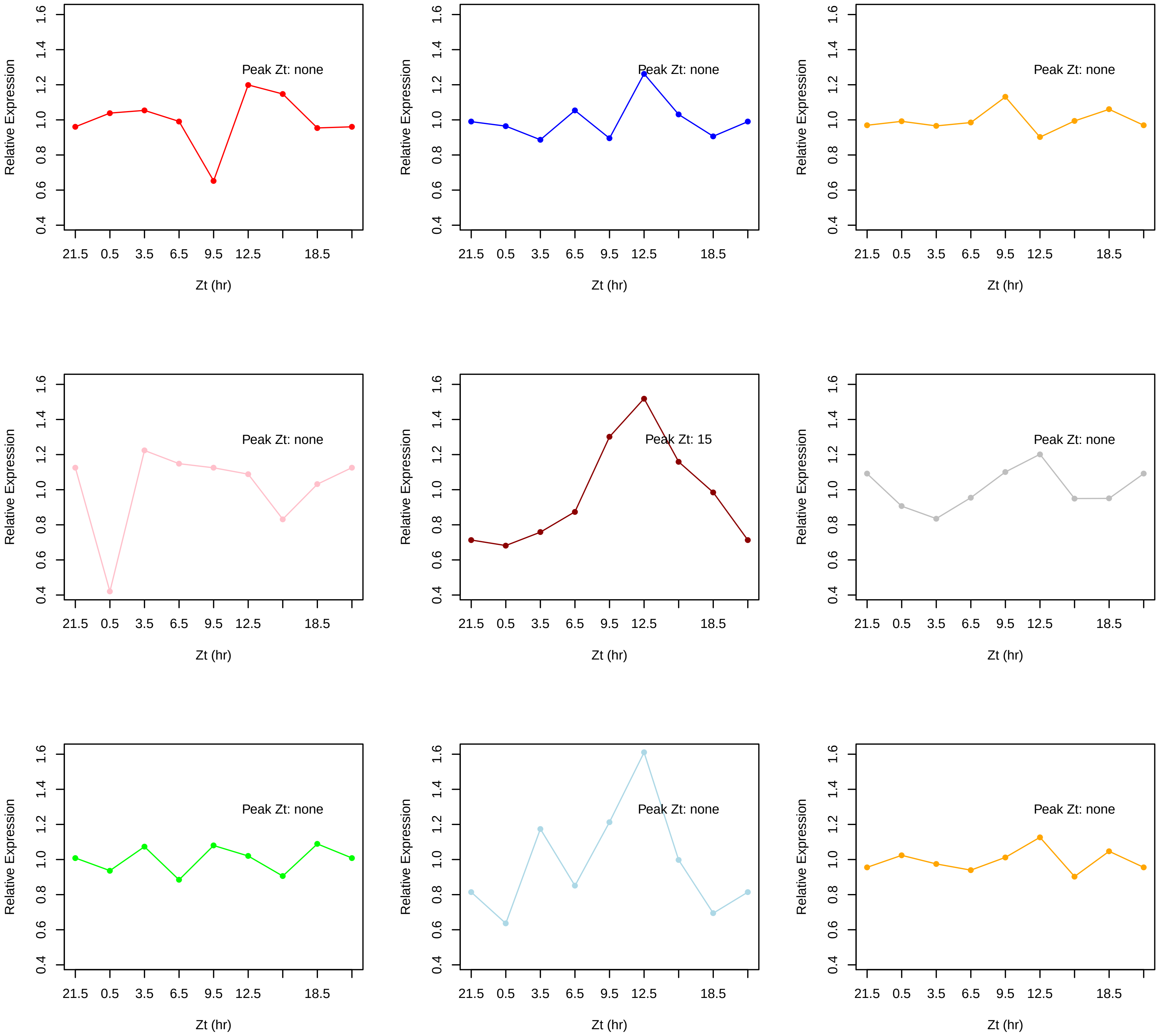

Singeleton: ENSXMAG00000009103 stk17al

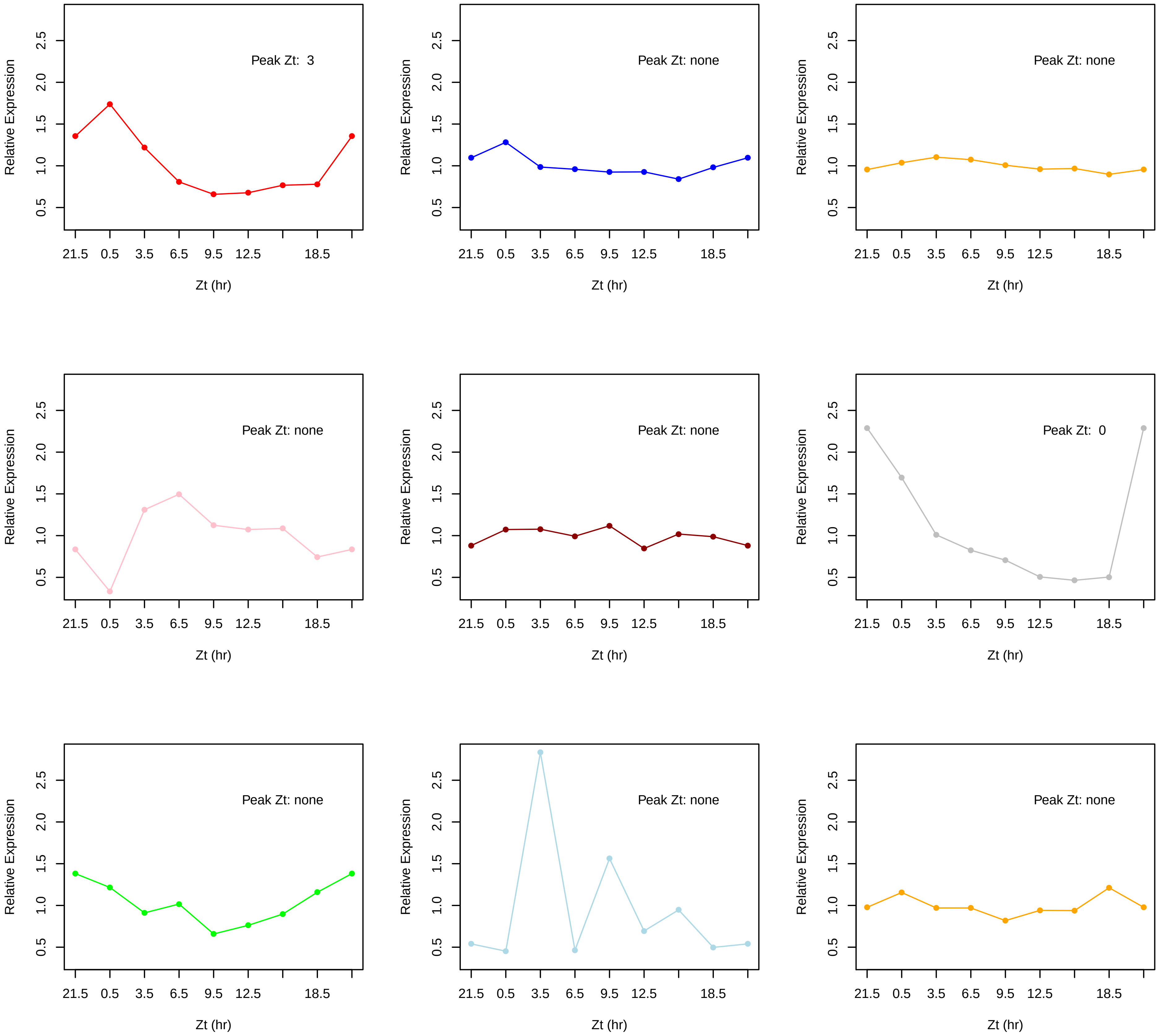

Singeleton: ENSXMAG00000029979 SLC39A11

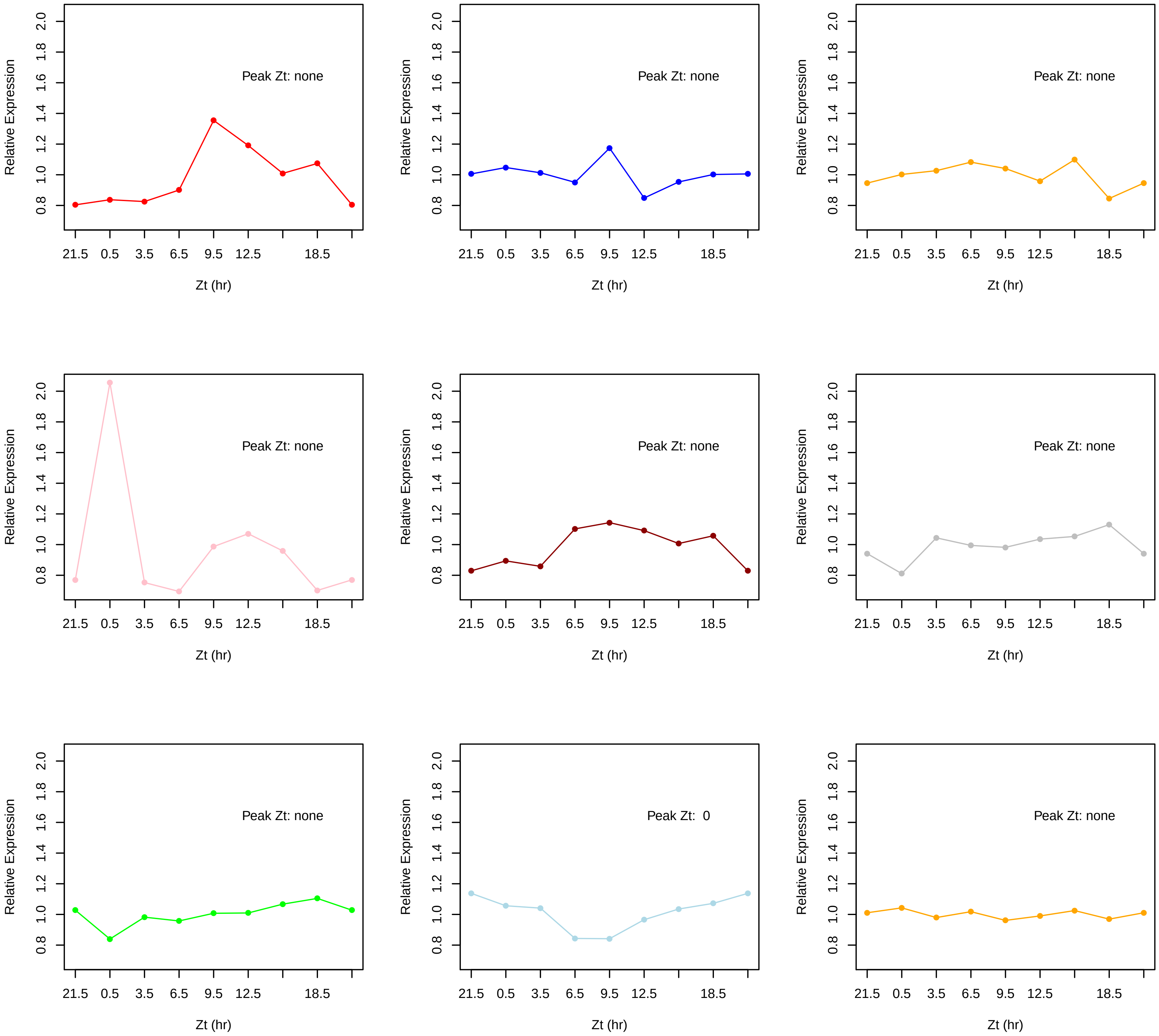

Singeleton: ENSXMAG00000012548 si:dkey−82j4.2

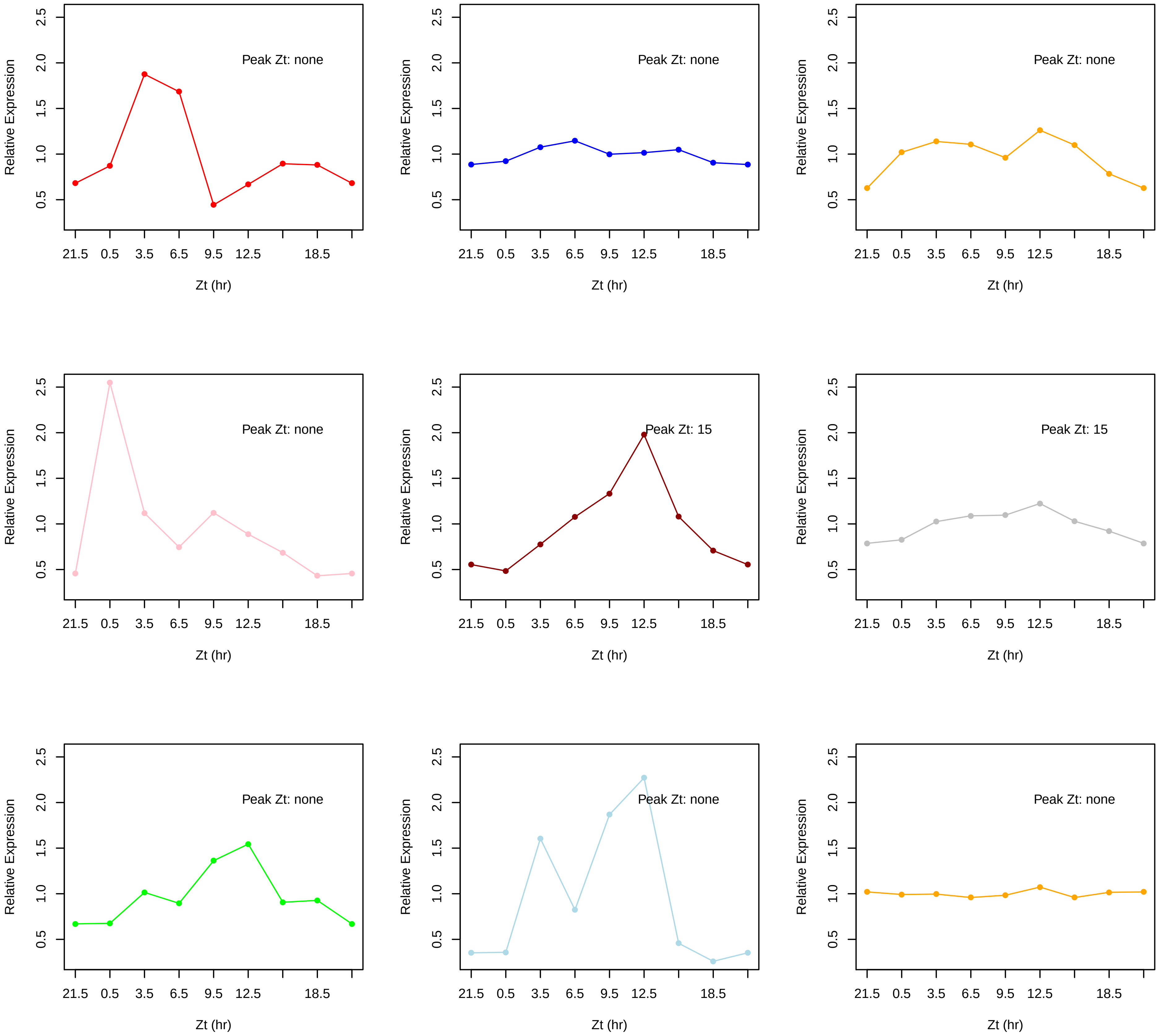

Singeleton: ENSXMAG00000028797

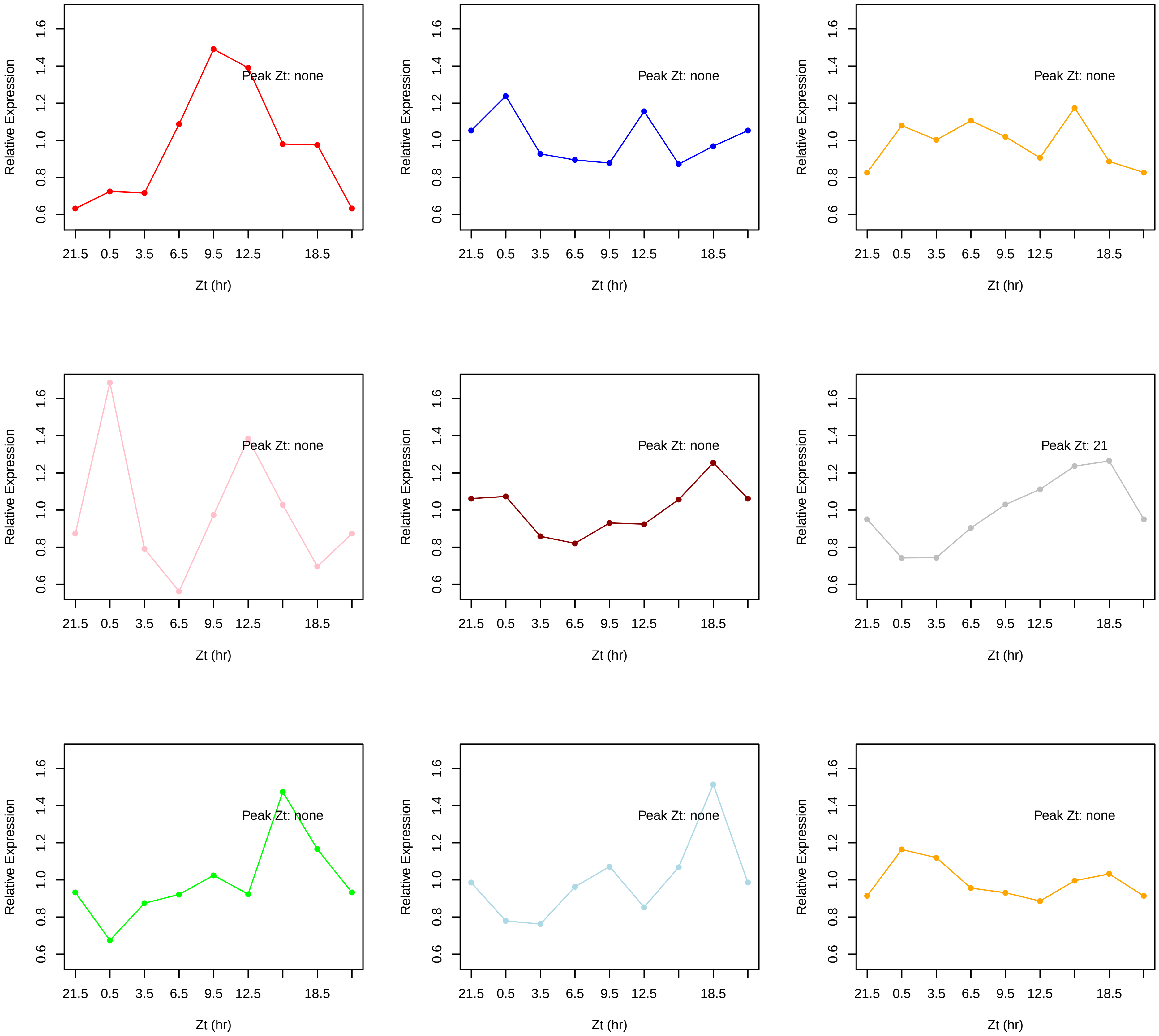

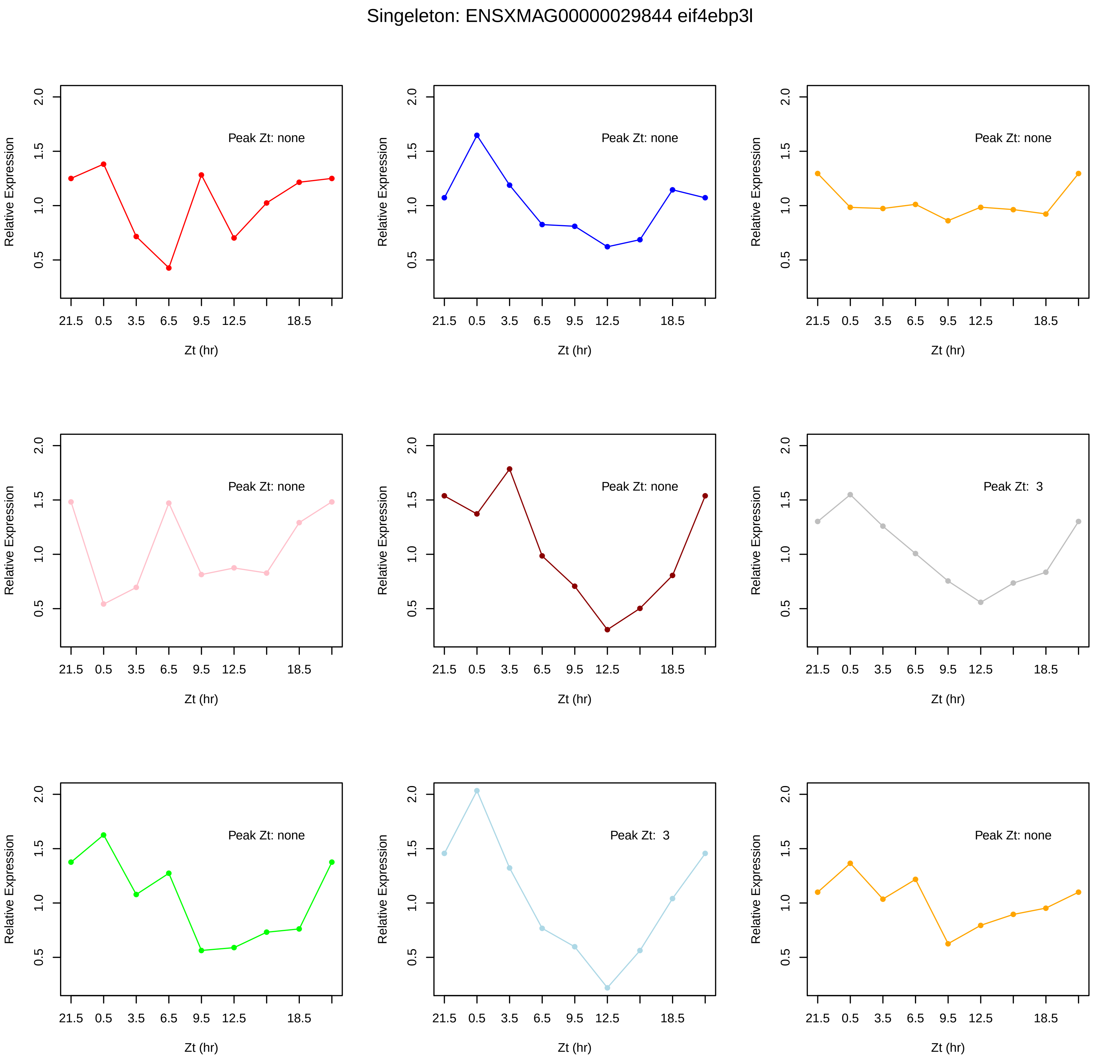
Singeleton: ENSXMAG00000029844 eif4ebp3l
Peak Zt: none
Peak Zt: none
Peak Zt: none
Peak Zt: none
Peak Zt: none
Peak Zt: 3
Peak Zt: none
Peak Zt: 3
Peak Zt: none
Relative Expression
Zt (hr)
21.5
0.5
3.5
6.5
9.5
12.5
18.5
0.5
1.0
1.5
2.0

Singeleton: ENSXMAG00000016312 vldlr

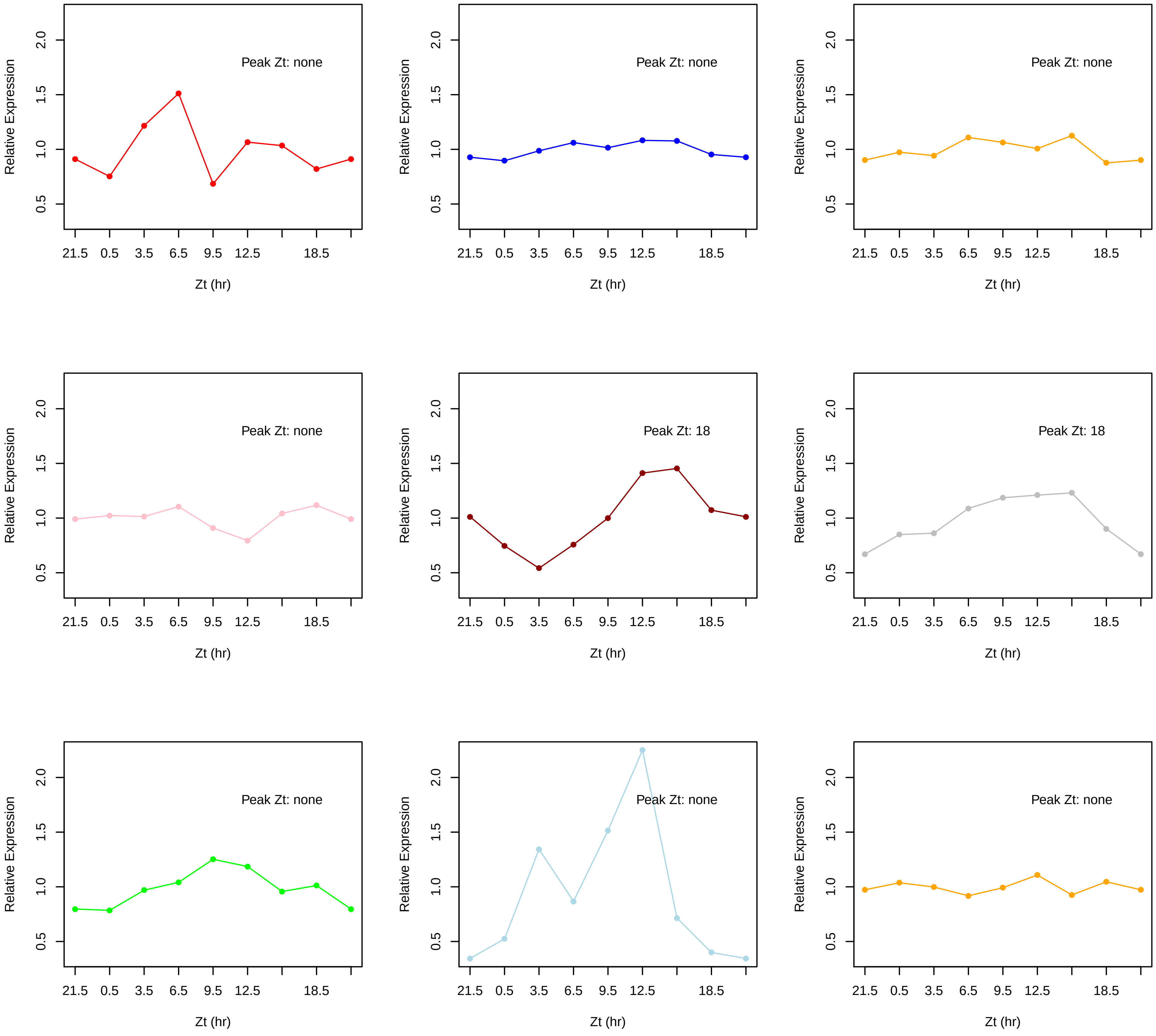

Singleton: ENSXMAG00000002501 insig1

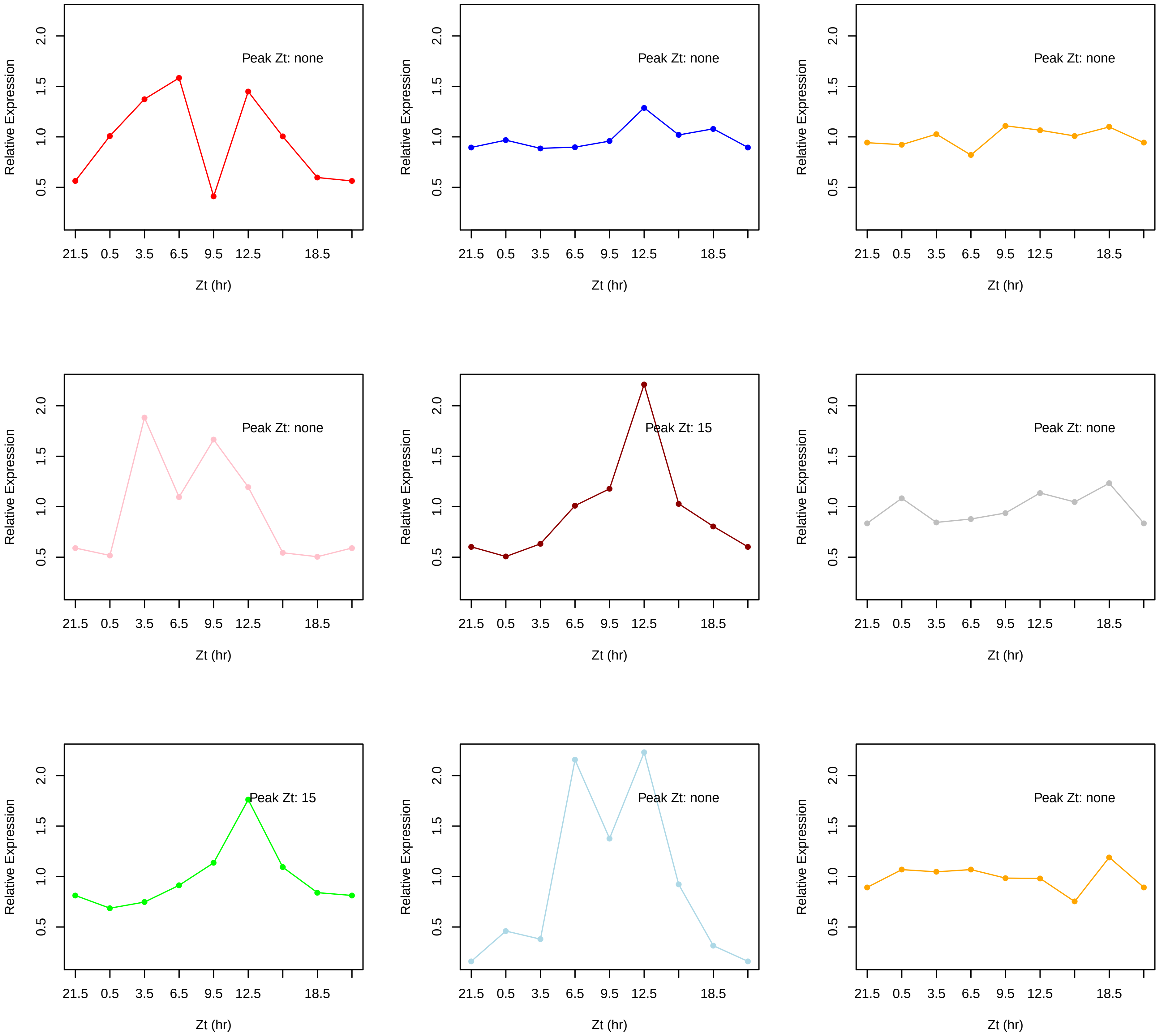

Singleton: ENSXMAG00000015416 fkbp5

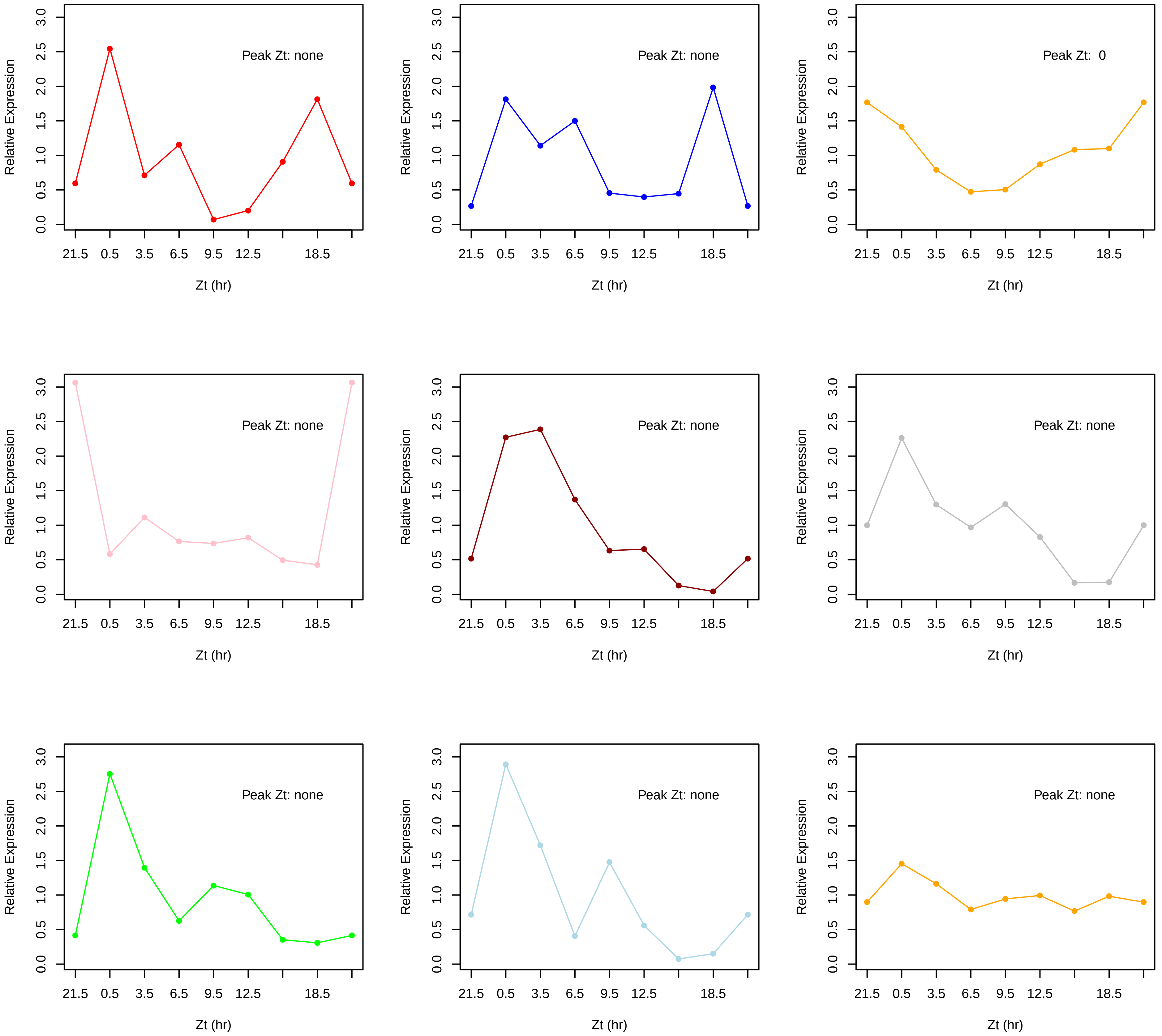

Singeleton: ENSXMAG00000021332 hnrnph1

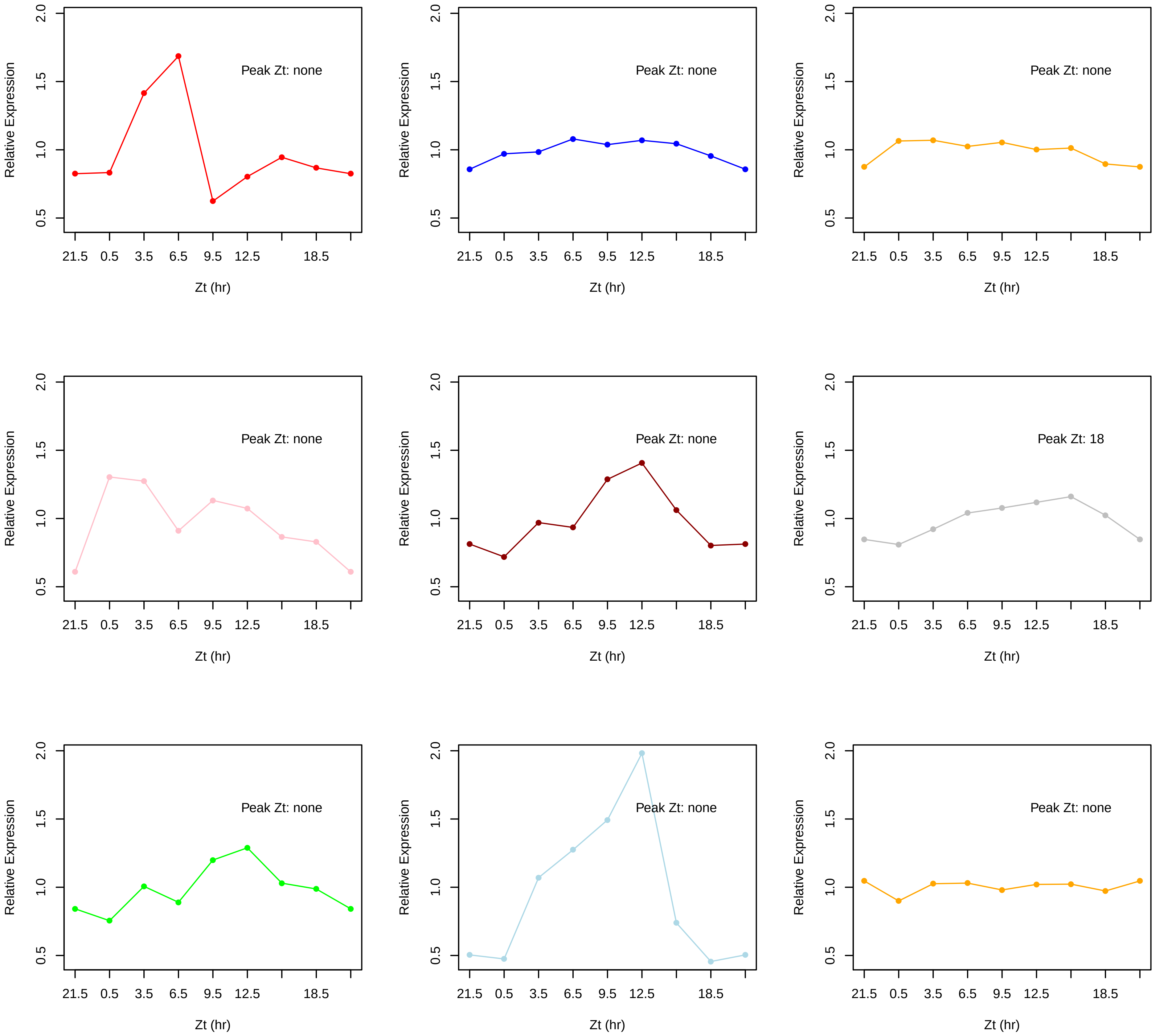

Singeleton: ENSXMAG00000009967 rufy1

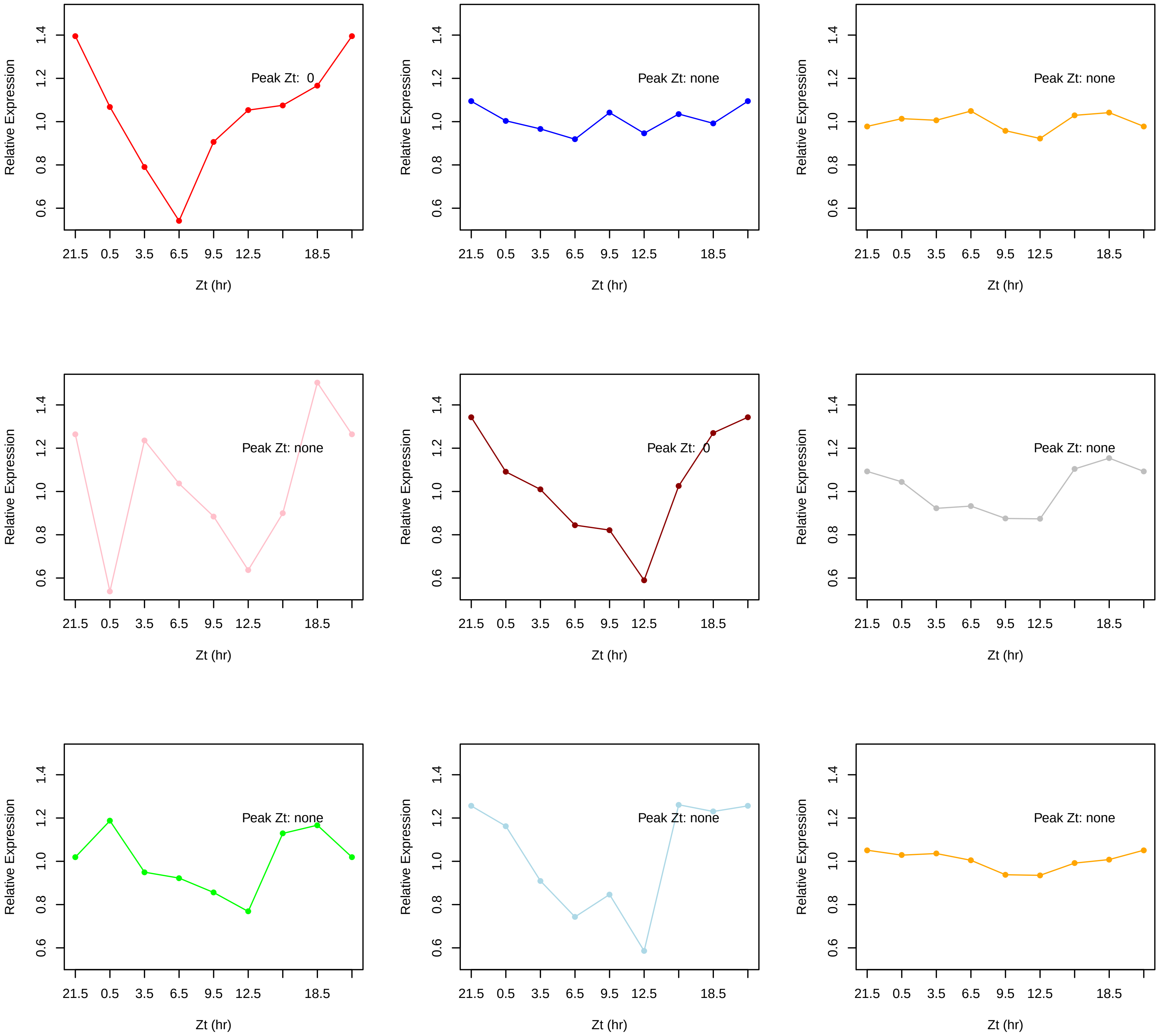

Singeleton: ENSXMAG00000001097 SRSF3

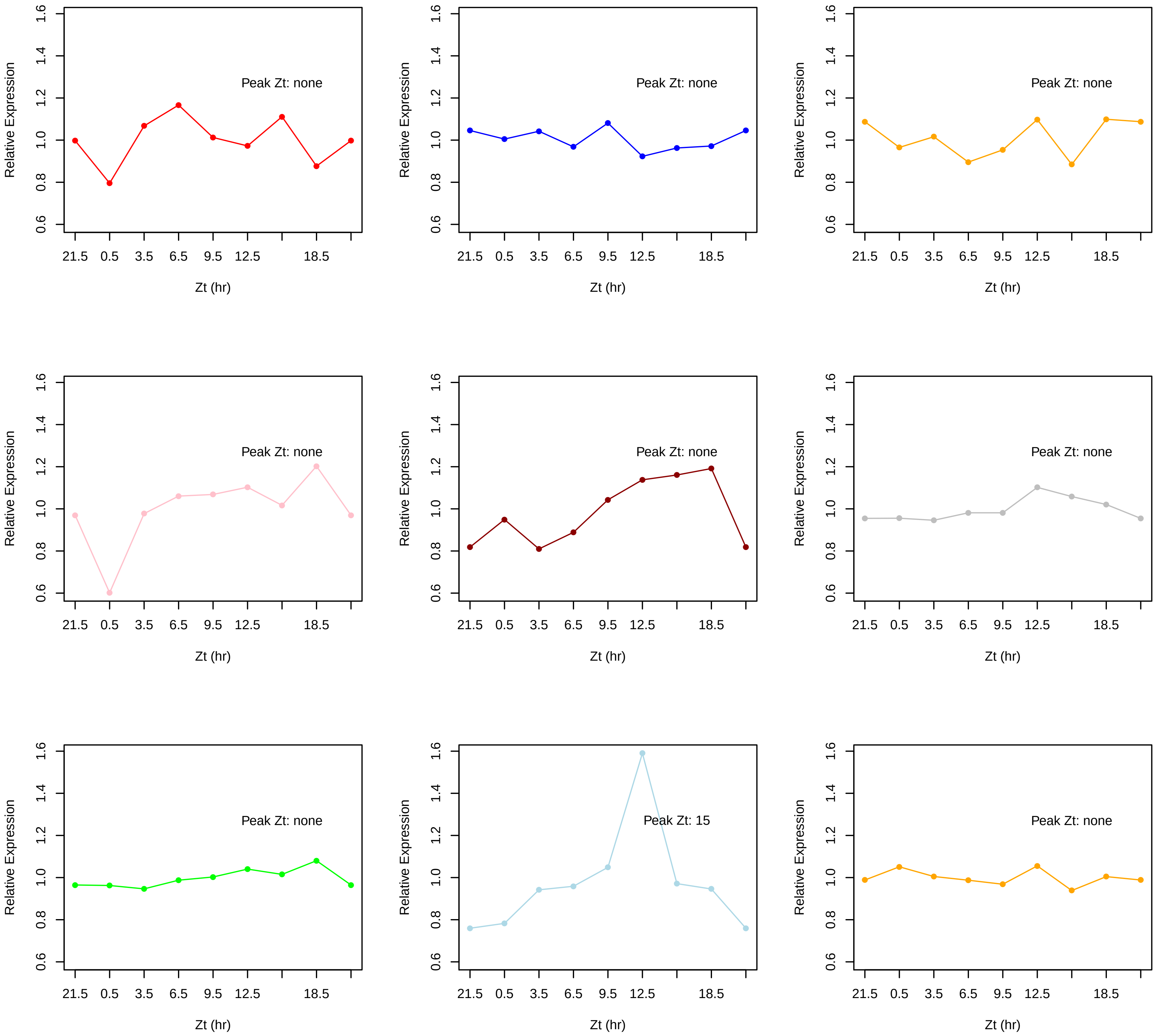

Singeleton: ENSXMAG00000001944 sec24d

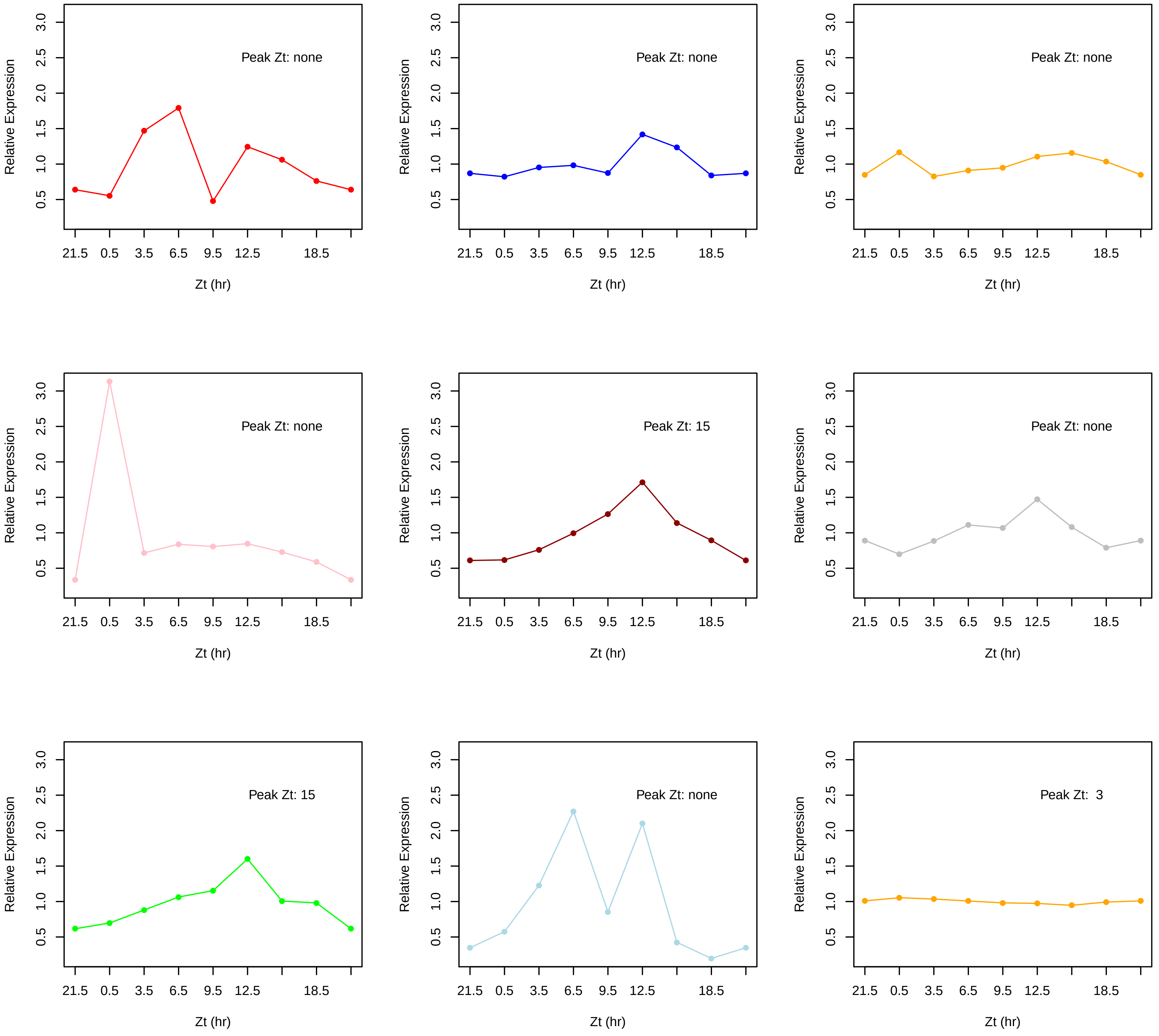

Singeleton: ENSXMAG00000001096 cdkn1a

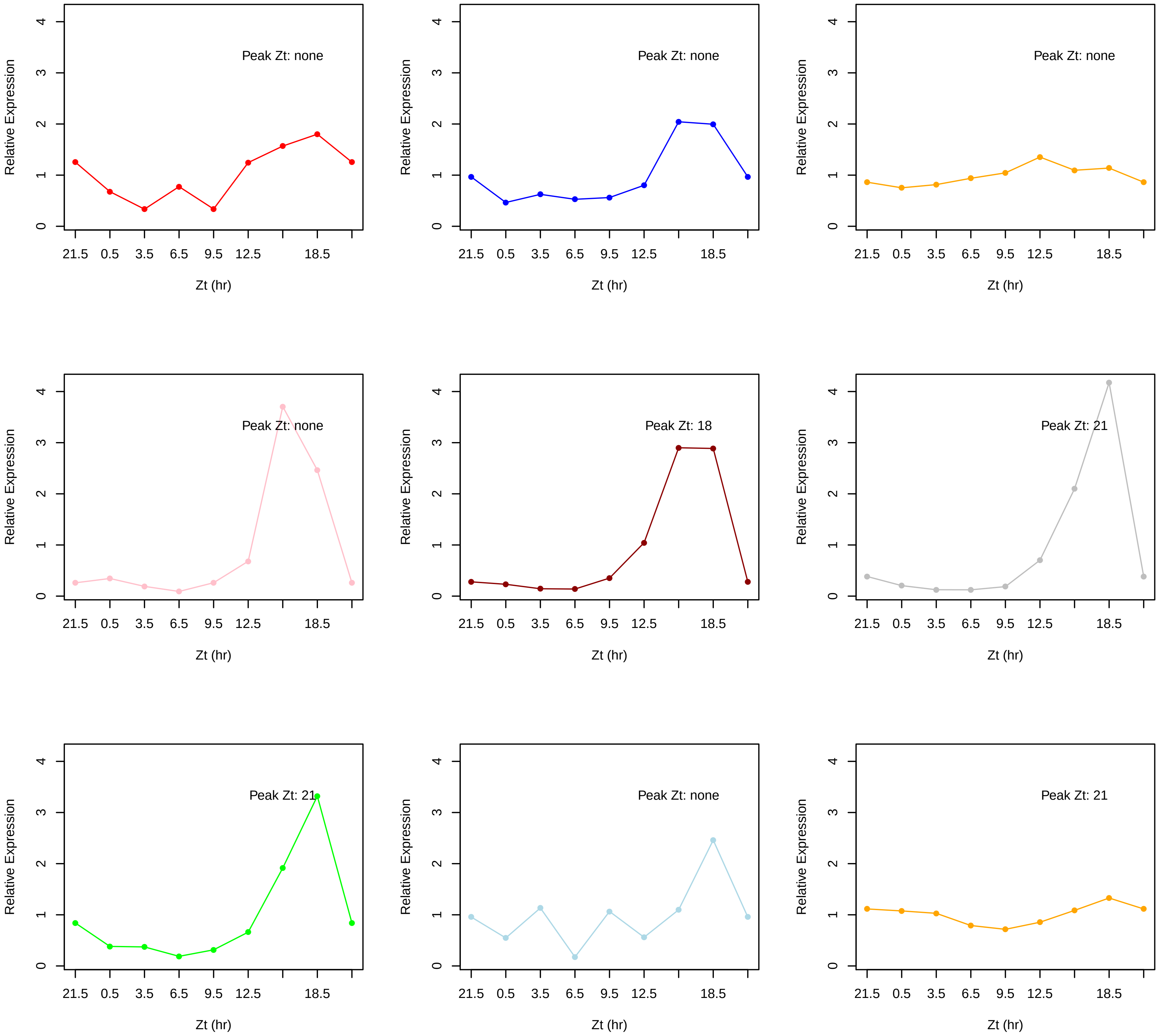

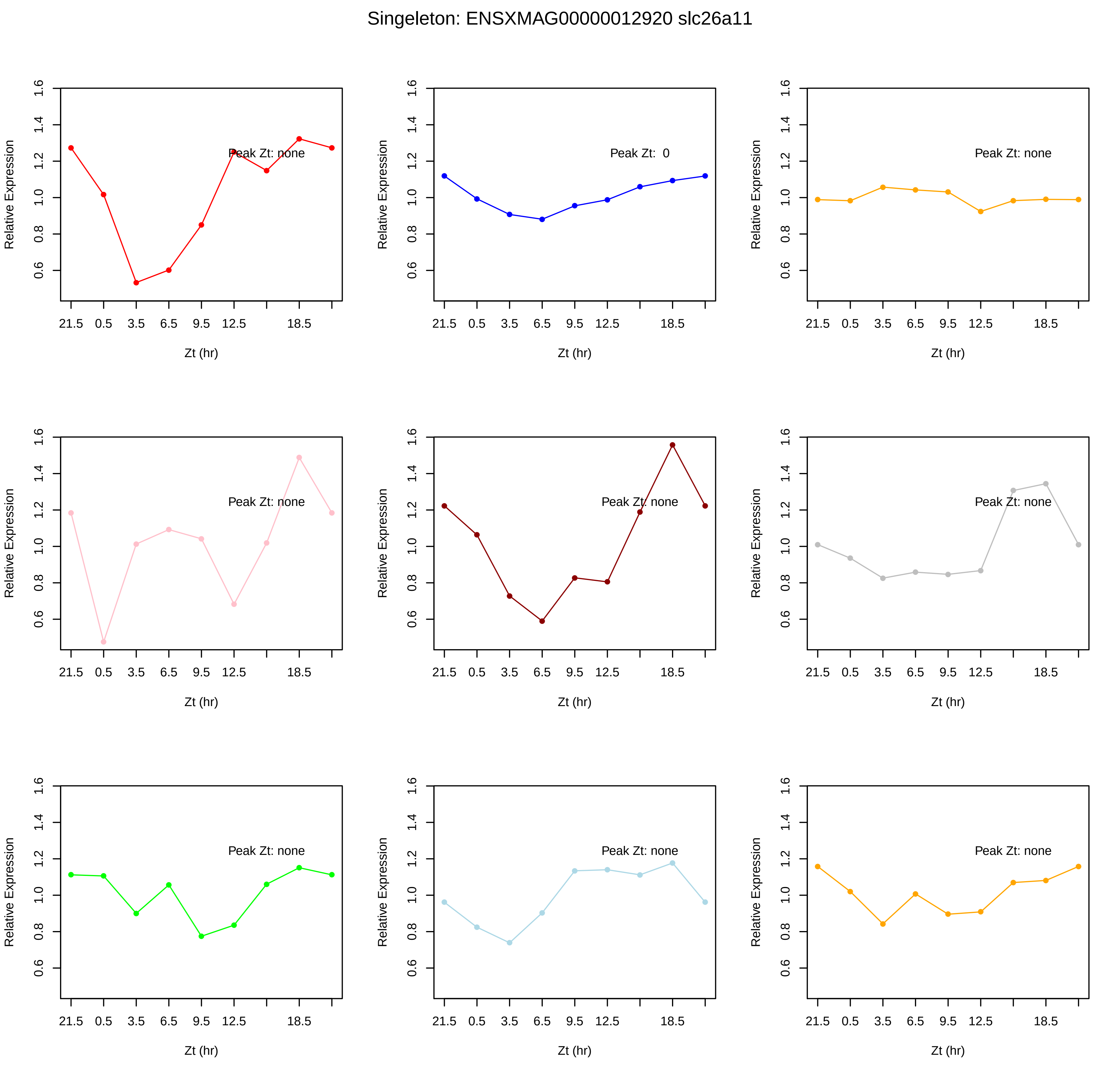

Singeleton: ENSXMAG00000012920 slc26a11
Peak Zt: none
Peak Zt: 0
Peak Zt: none
Peak Zt: none
Peak Zt: none
Peak Zt: none
Peak Zt: none
Peak Zt: none
Peak Zt: none
Relative Expression
Zt (hr)
0.6
0.8
1.0
1.2
1.4
1.6
21.5
0.5
3.5
6.5
9.5
12.5
18.5

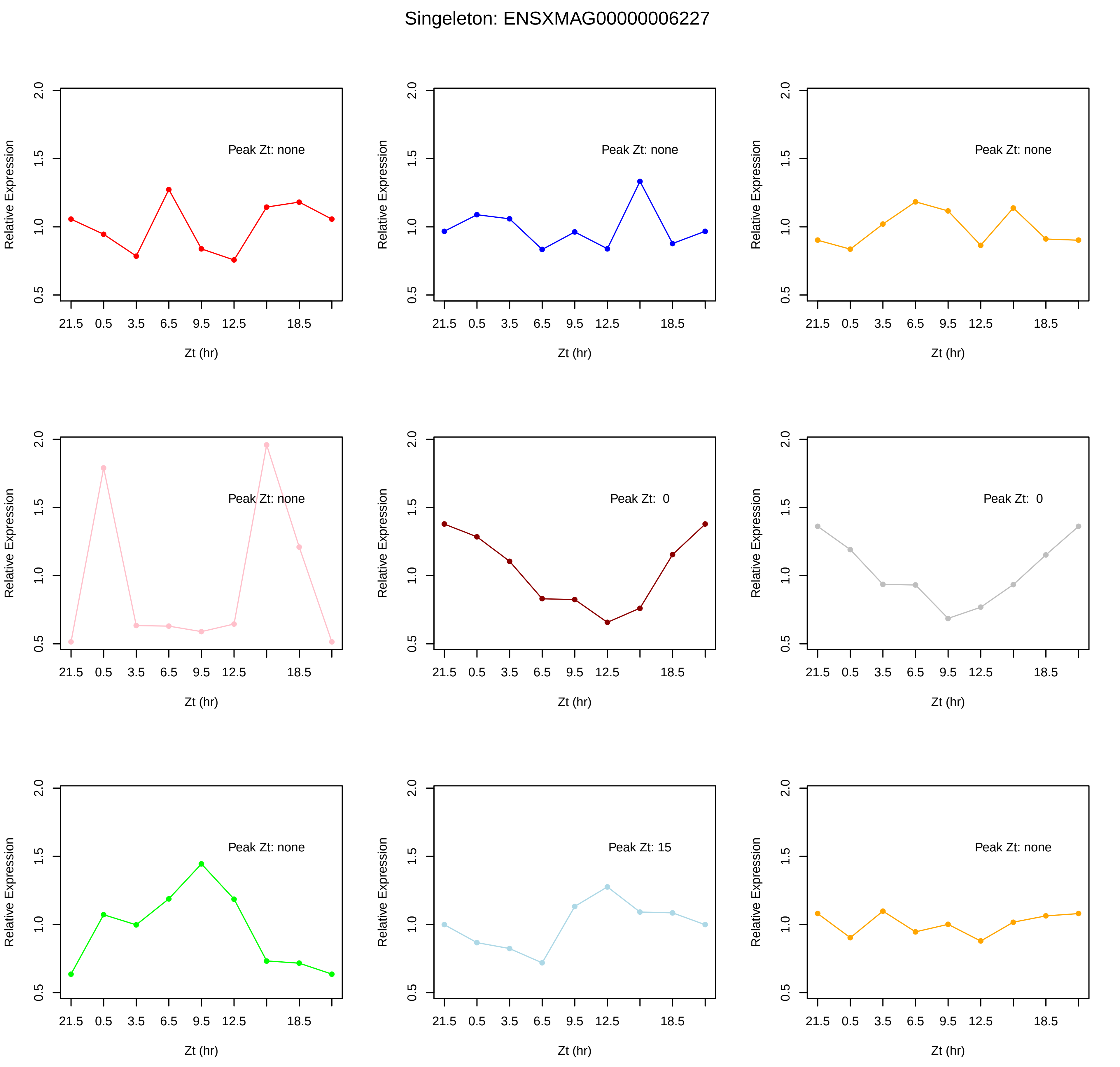
Singeleton: ENSXMAG00000006227
Relative Expression
Zt (hr)
21.5 0.5 3.5 6.5 9.5 12.5 18.5
0.5 1.0 1.5 2.0
Peak Zt: none
Peak Zt: none
Peak Zt: none
Peak Zt: none
Peak Zt: 0
Peak Zt: 0
Peak Zt: none
Peak Zt: 15
Peak Zt: none

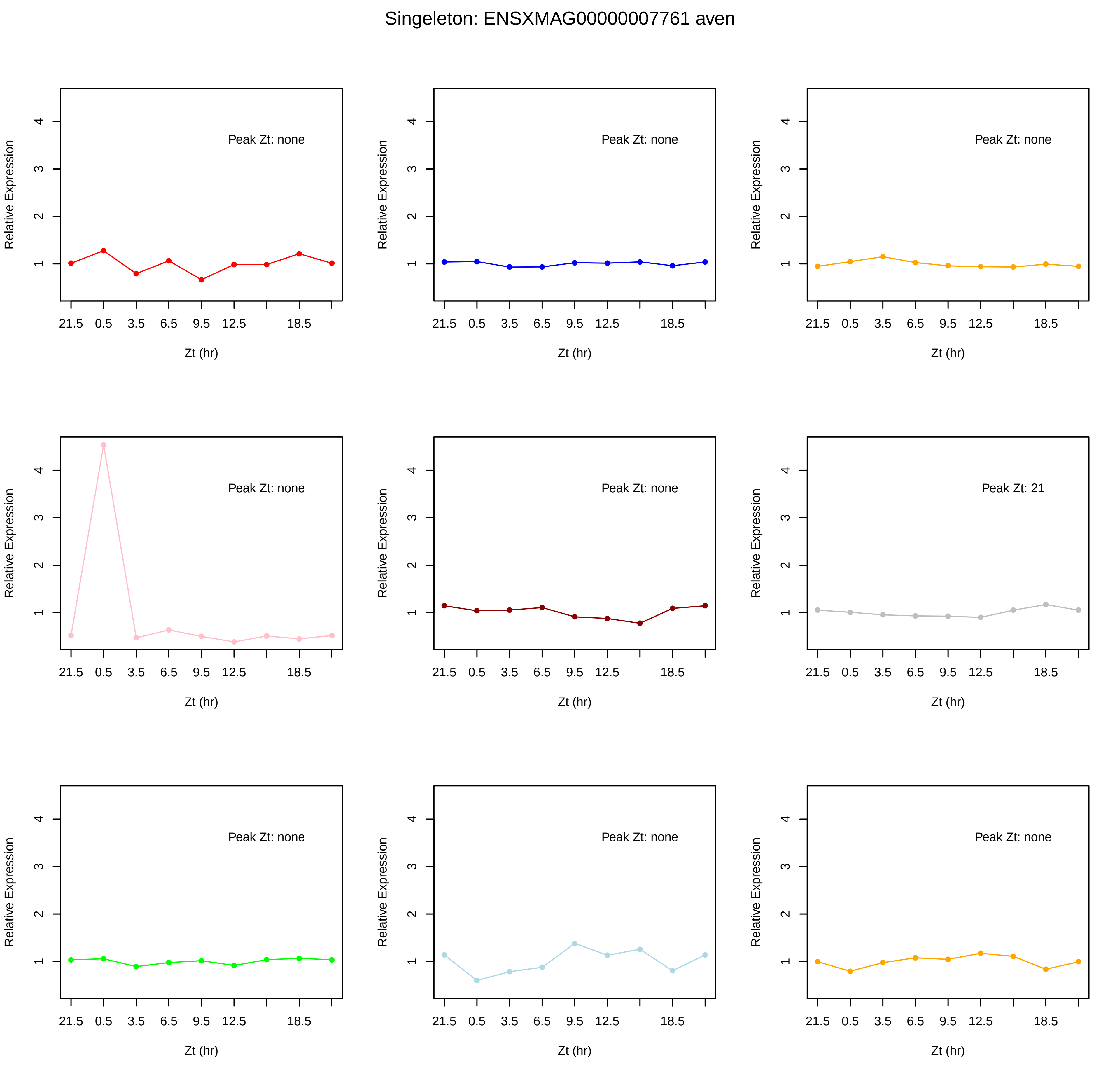
Singeleton: ENSXMAG00000007761 aven
Peak Zt: none
Peak Zt: none
Peak Zt: none
Peak Zt: none
Peak Zt: none
Peak Zt: 21
Peak Zt: none
Peak Zt: none
Peak Zt: none
Relative Expression
Zt (hr)
21.5
0.5
3.5
6.5
9.5
12.5
18.5

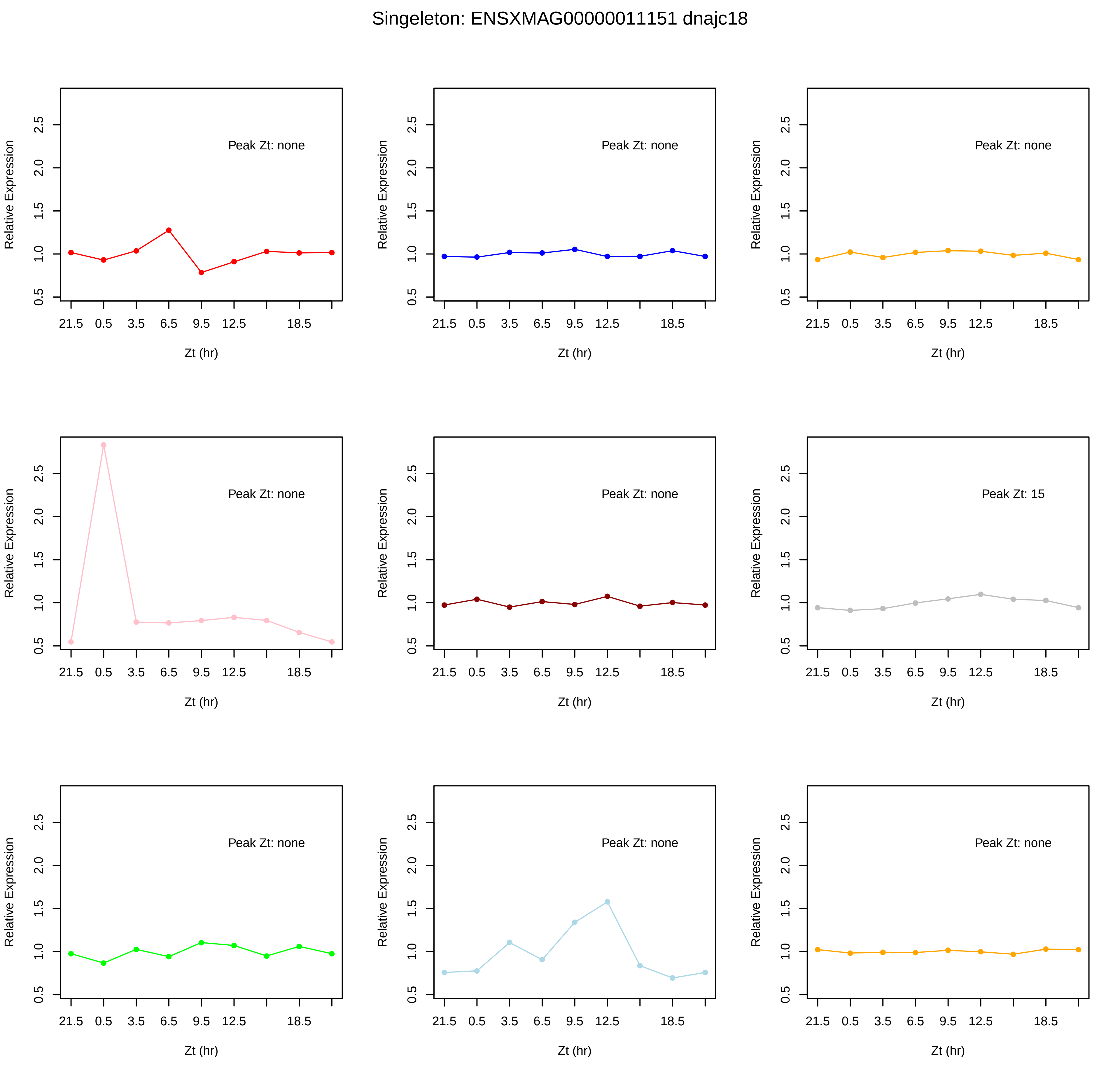
Singeleton: ENSXMAG00000011151 dnajc18
Peak Zt: none
Peak Zt: none
Peak Zt: none
Peak Zt: none
Peak Zt: none
Peak Zt: 15
Peak Zt: none
Peak Zt: none
Peak Zt: none
Relative Expression
Zt (hr)
21.5
0.5
3.5
6.5
9.5
12.5
18.5
0.5
1.0
1.5
2.0
2.5

Singeleton: ENSXMAG00000016138 sars

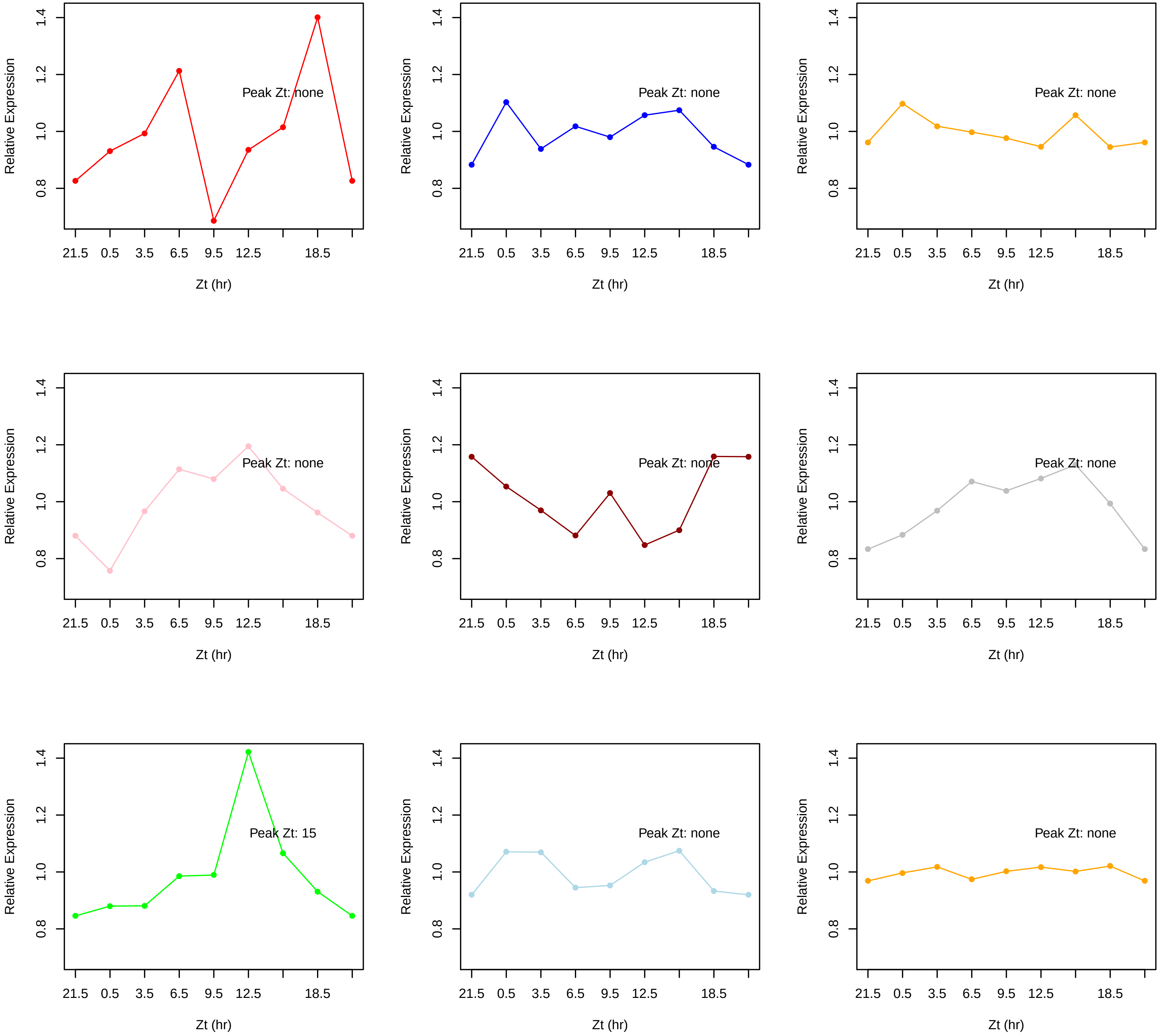

Singleton: ENSXMAG00000000990 celsr2

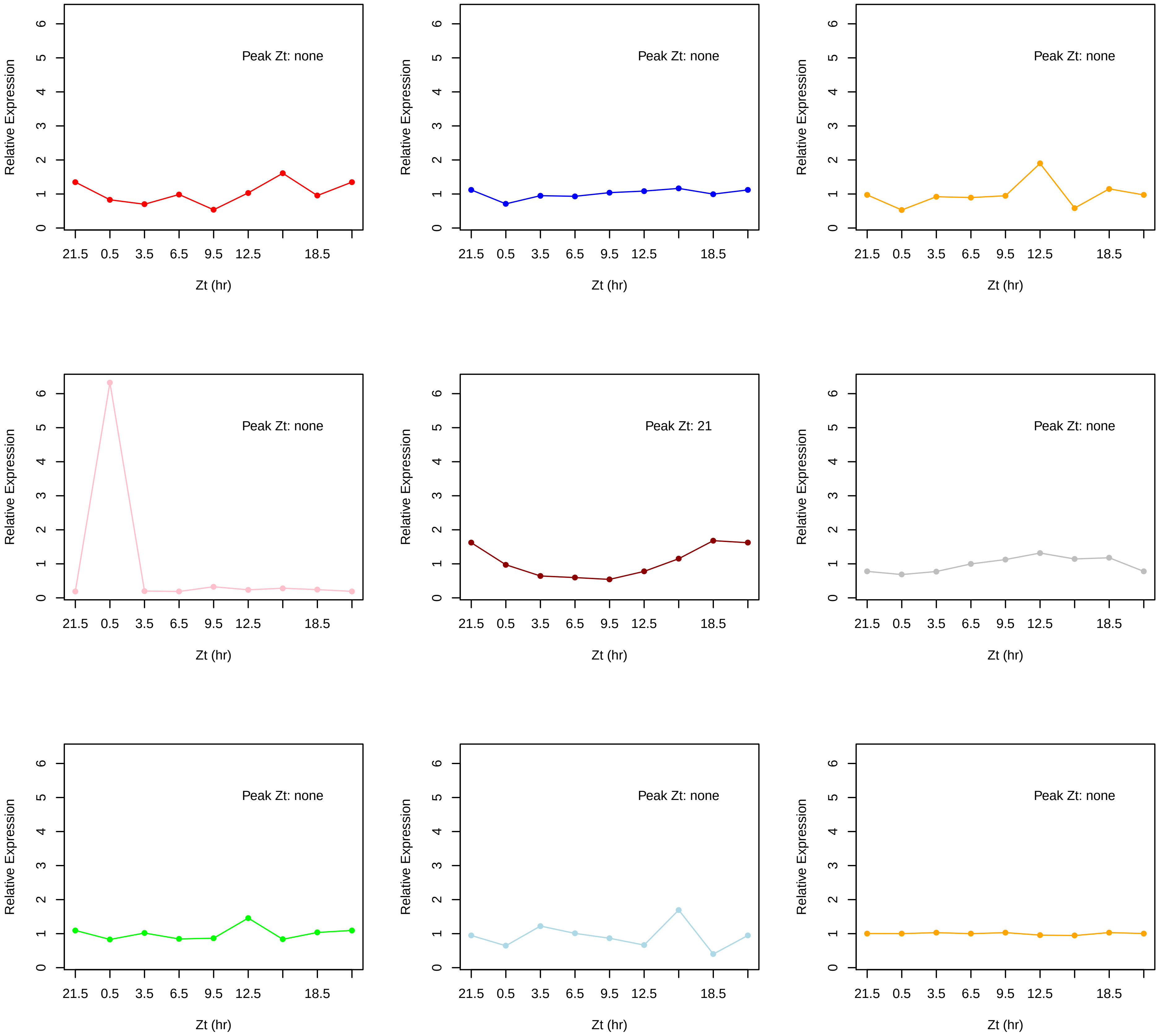

Singeleton: ENSXMAG00000008915 kat2a

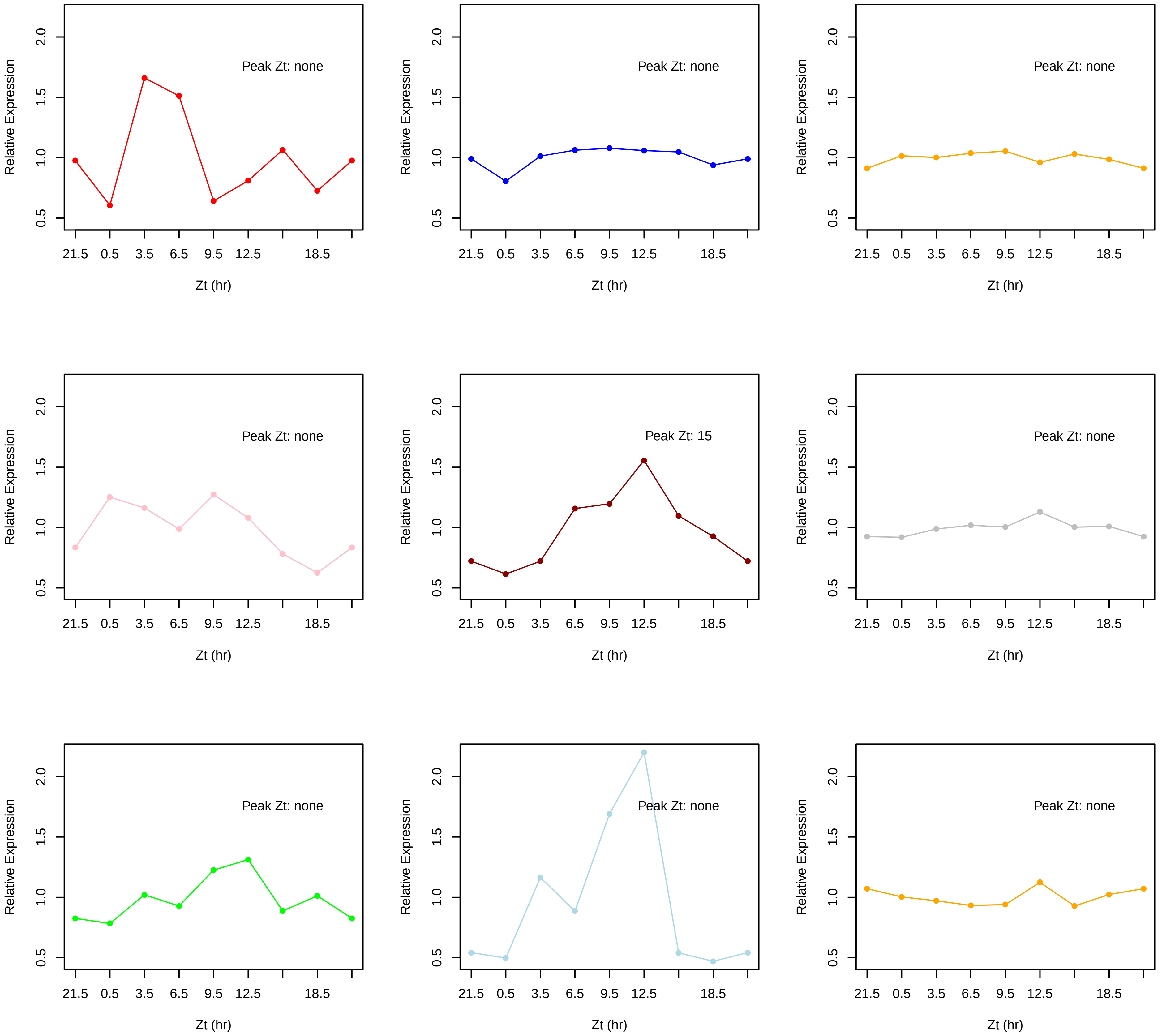

Singeleton: ENSXMAG00000012959 sirt7

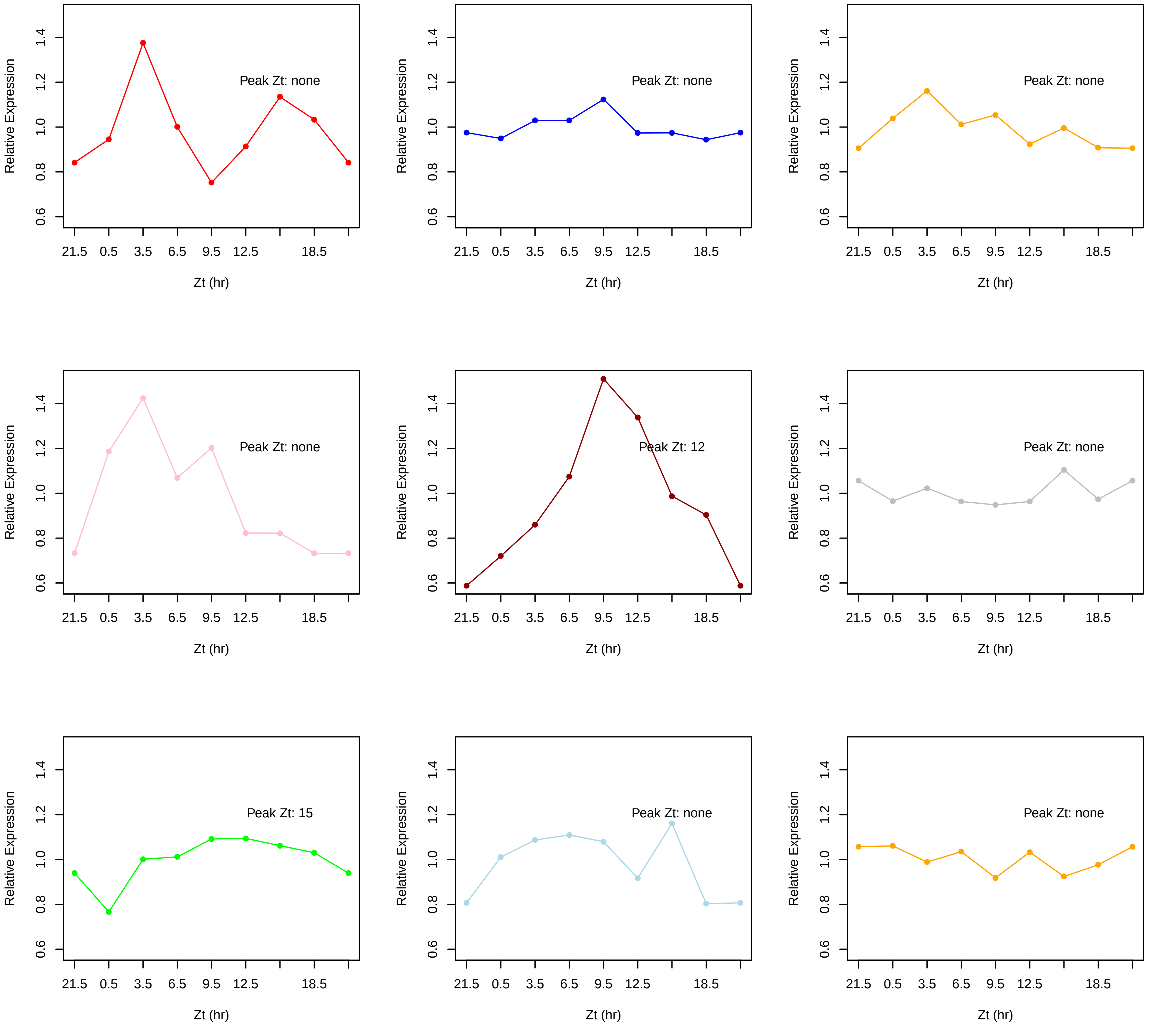

Singleton: ENSXMAG00000029211 elavl2

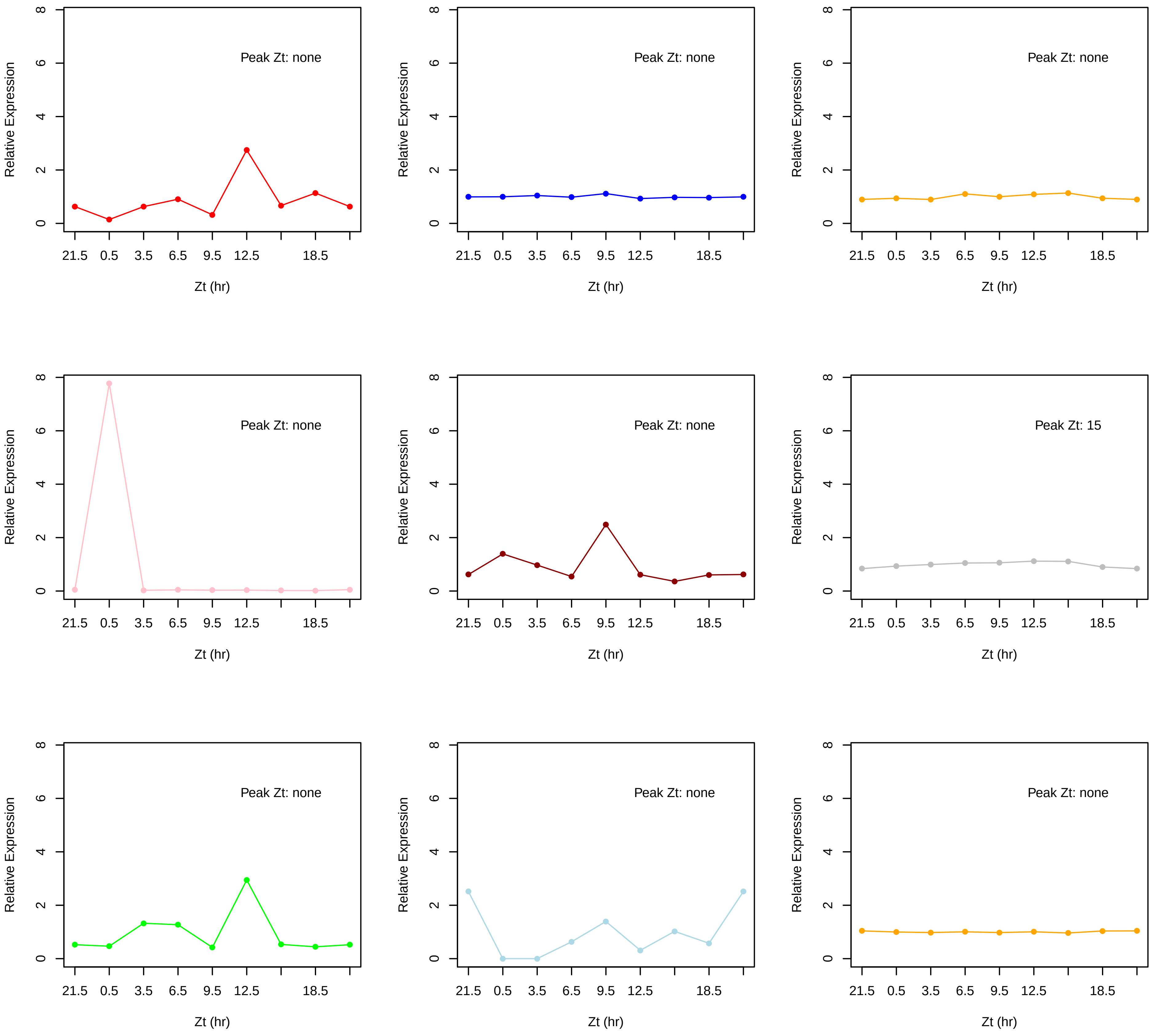

Singeleton: ENSXMAG00000026314

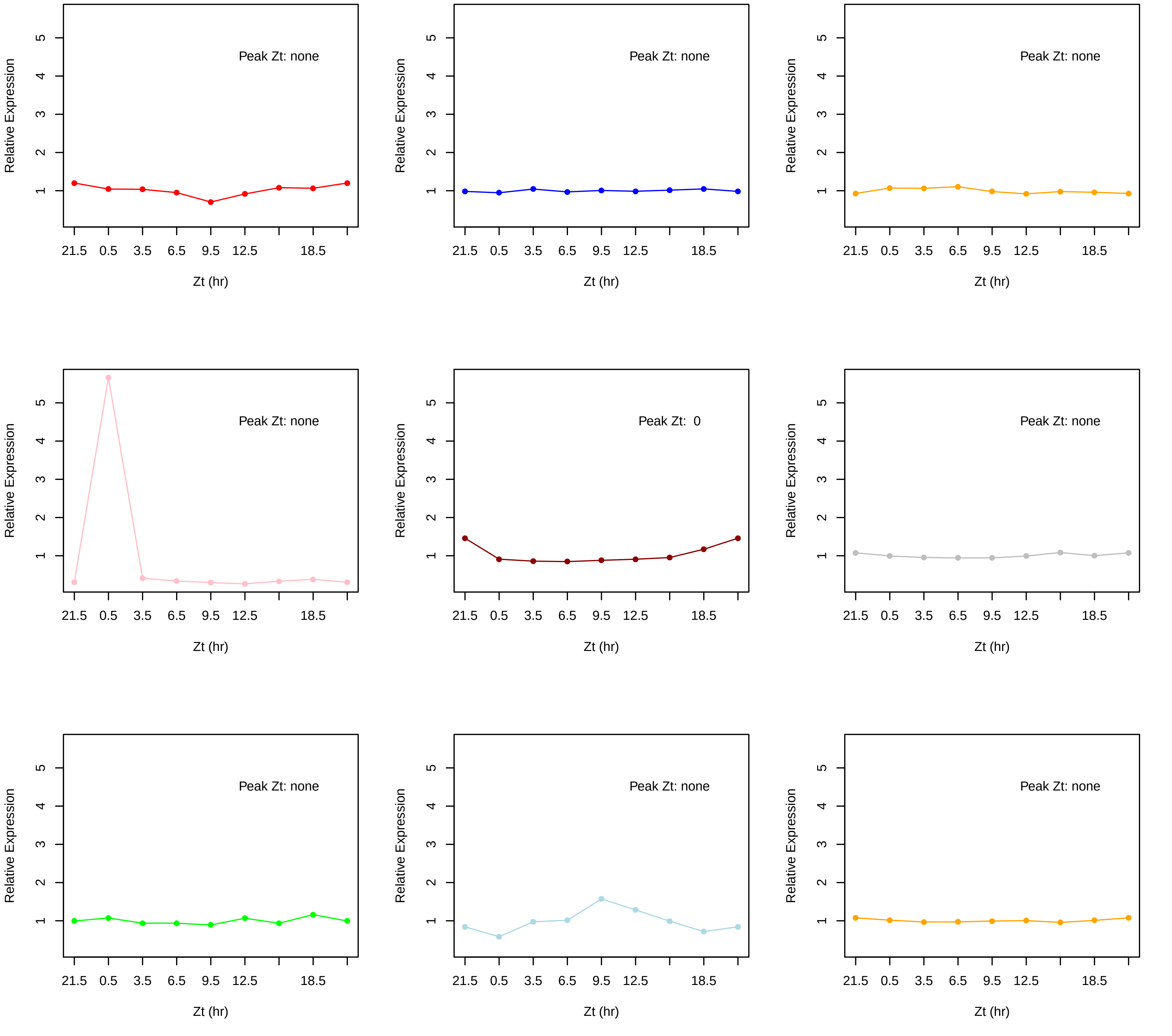

Singeleton: ENSXMAG00000012305 pycr1a

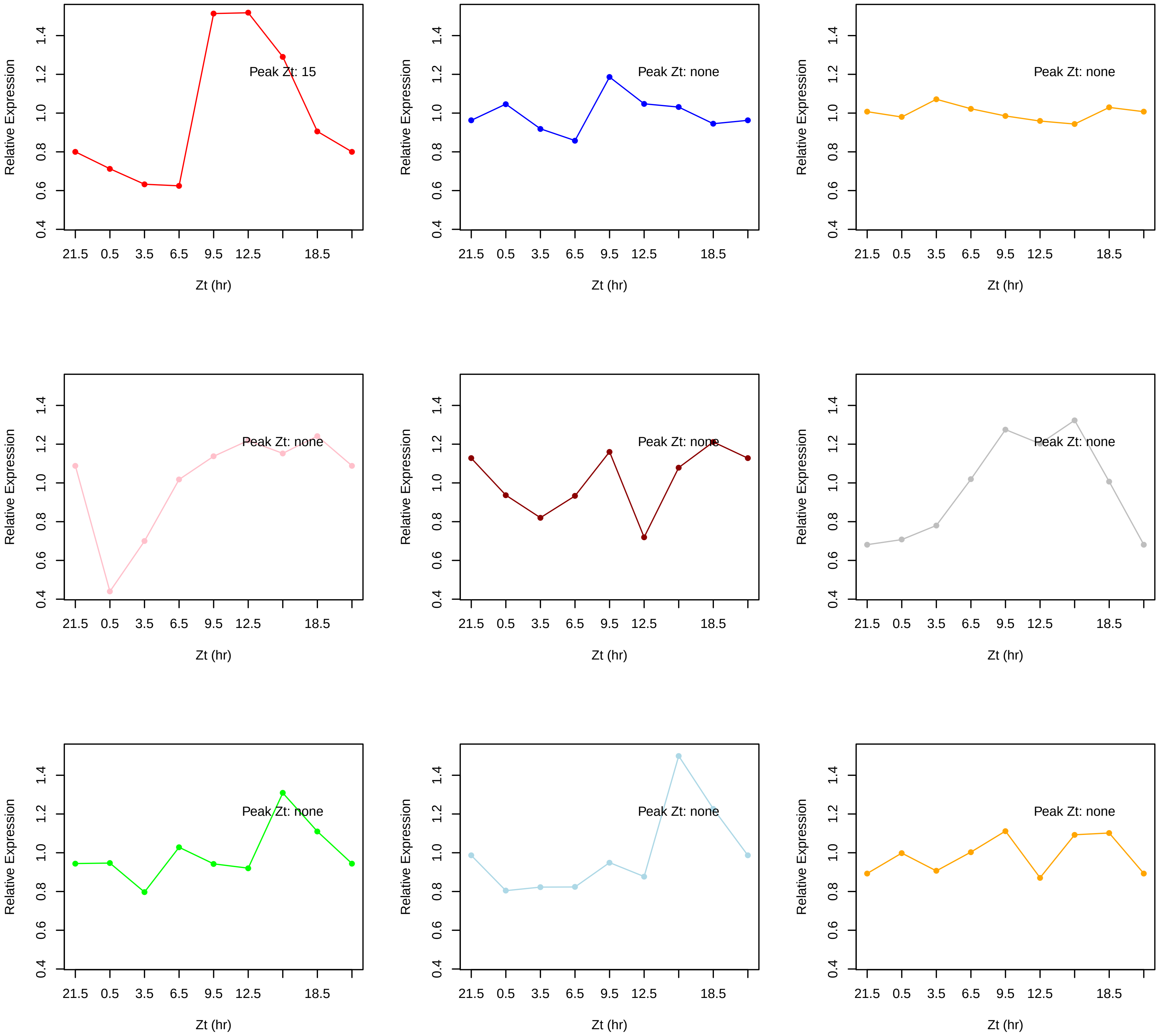

Singeleton: ENSXMAG00000022303 CAAP1

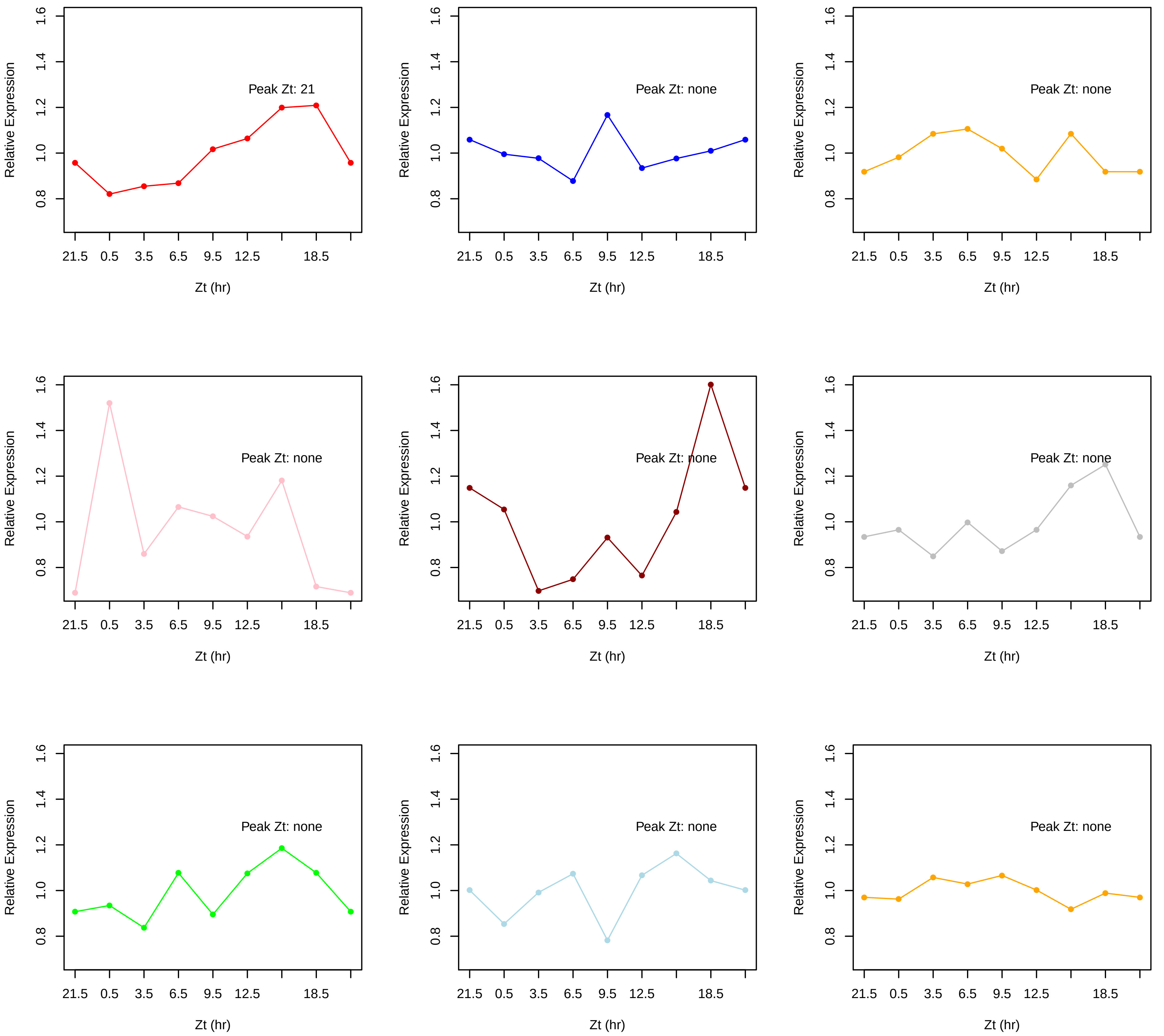

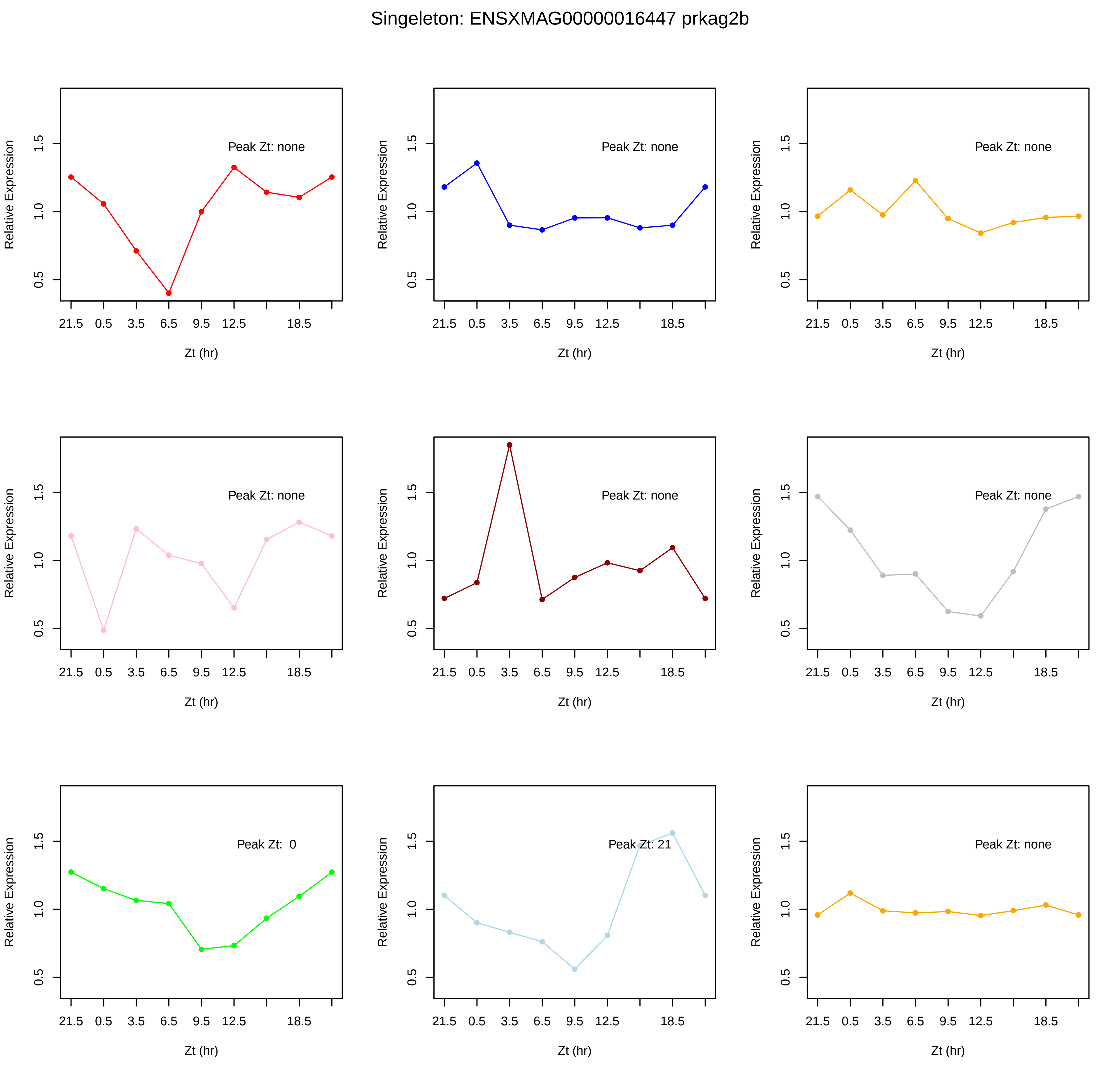
Singeleton: ENSXMAG00000016447 prkag2b
Peak Zt: none
Peak Zt: none
Peak Zt: none
Peak Zt: none
Peak Zt: none
Peak Zt: none
Peak Zt: 0
Peak Zt: 21
Peak Zt: none
Relative Expression
Zt (hr)
21.5
0.5
3.5
6.5
9.5
12.5
18.5
0.5
1.0
1.5

Singeleton: ENSXMAG00000029409 ppil1

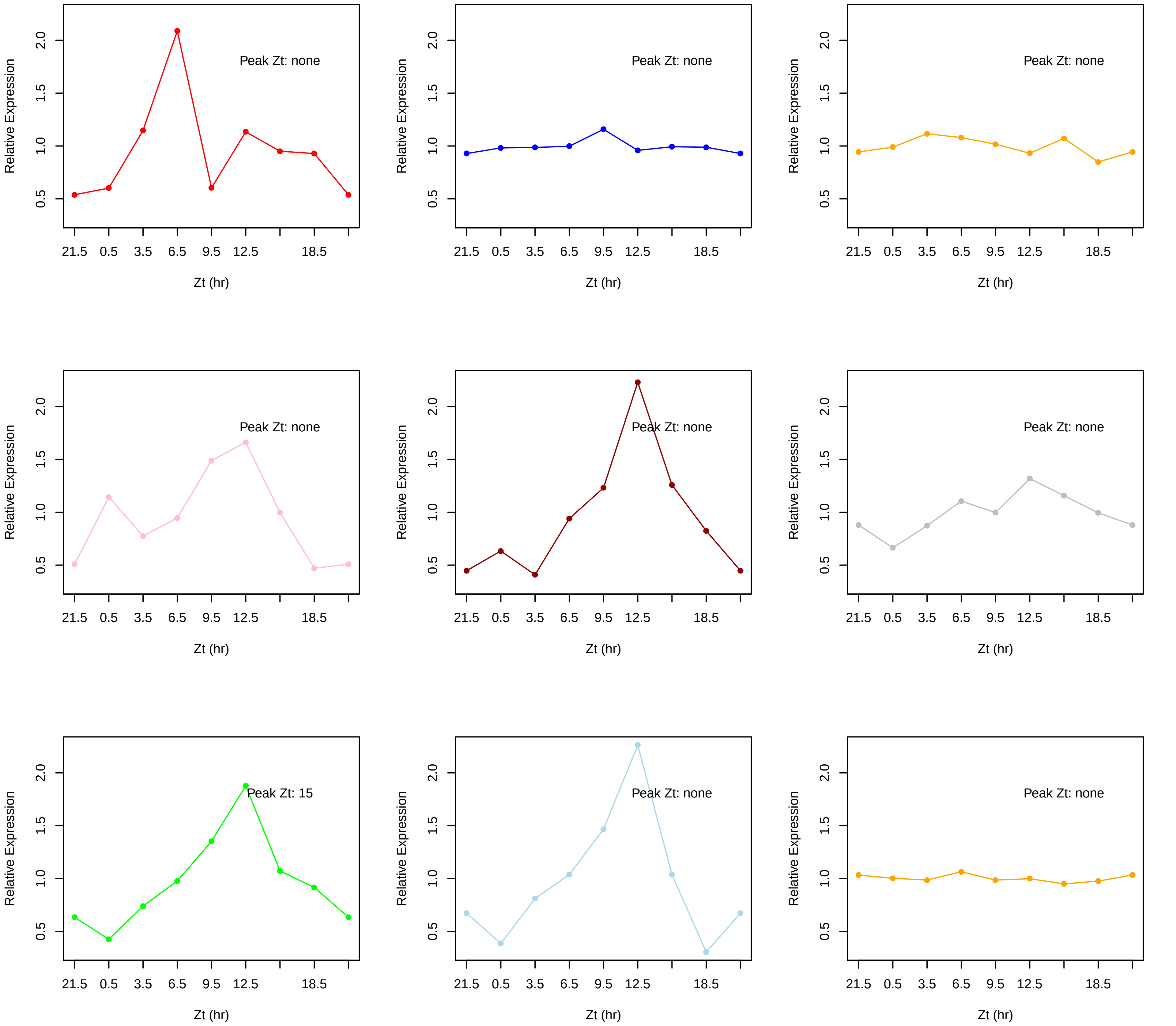

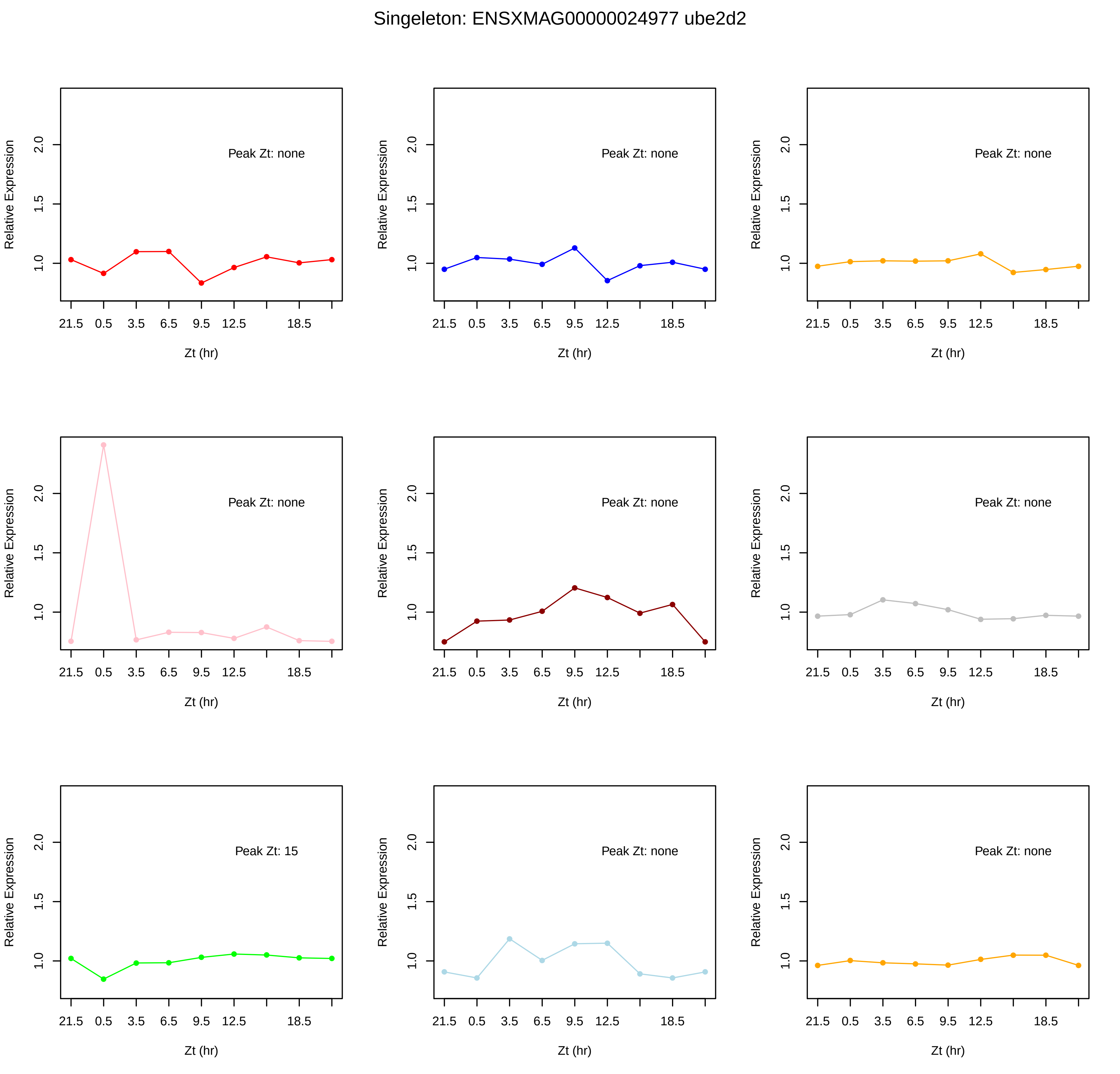
Singeleton: ENSXMAG00000024977 ube2d2
Peak Zt: none
Peak Zt: none
Peak Zt: none
Peak Zt: none
Peak Zt: none
Peak Zt: none
Peak Zt: 15
Peak Zt: none
Peak Zt: none
Relative Expression
Zt (hr)
21.5
0.5
3.5
6.5
9.5
12.5
18.5
1.0
1.5
2.0

Singeleton: ENSXMAG00000007696 scg5

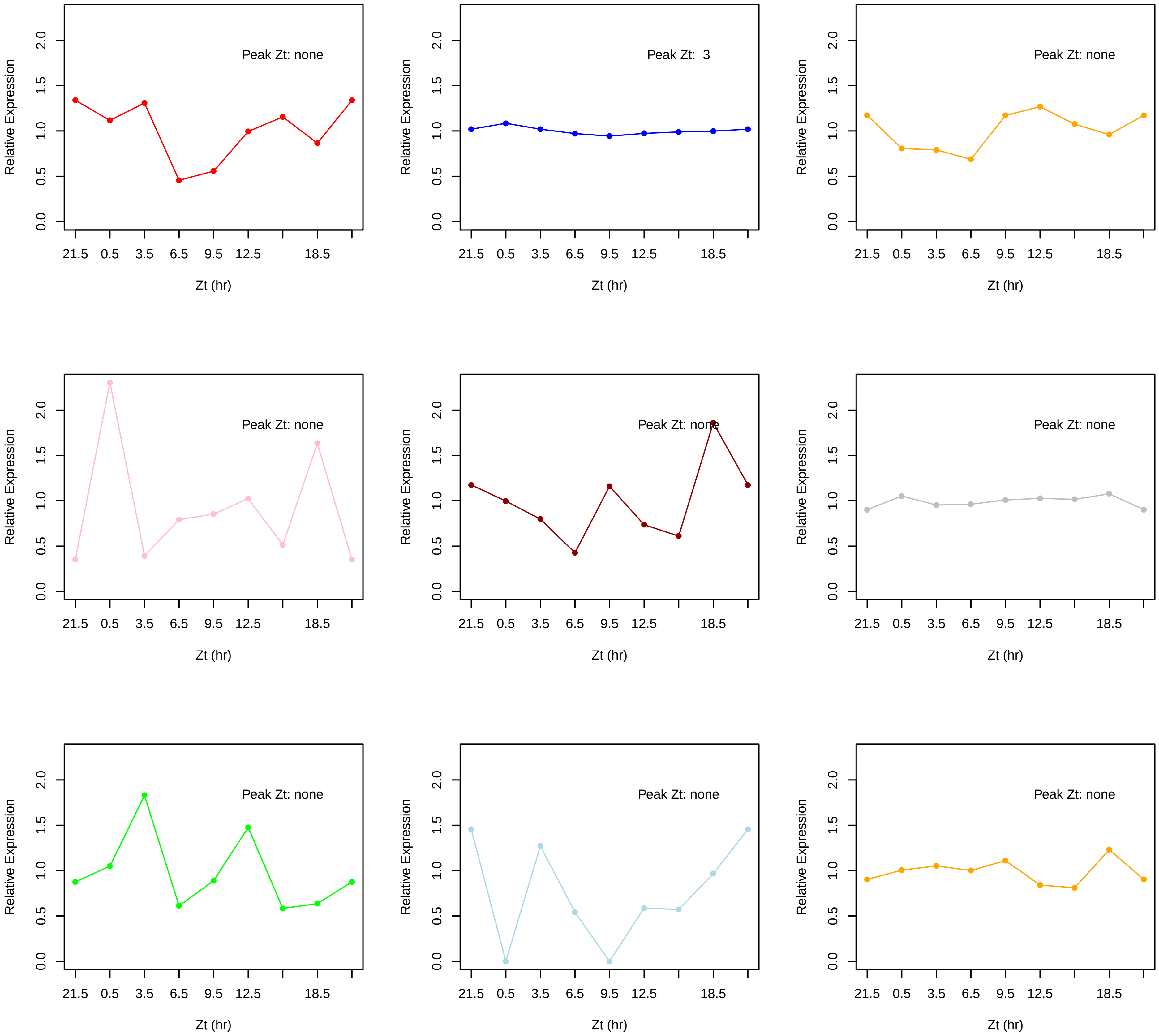

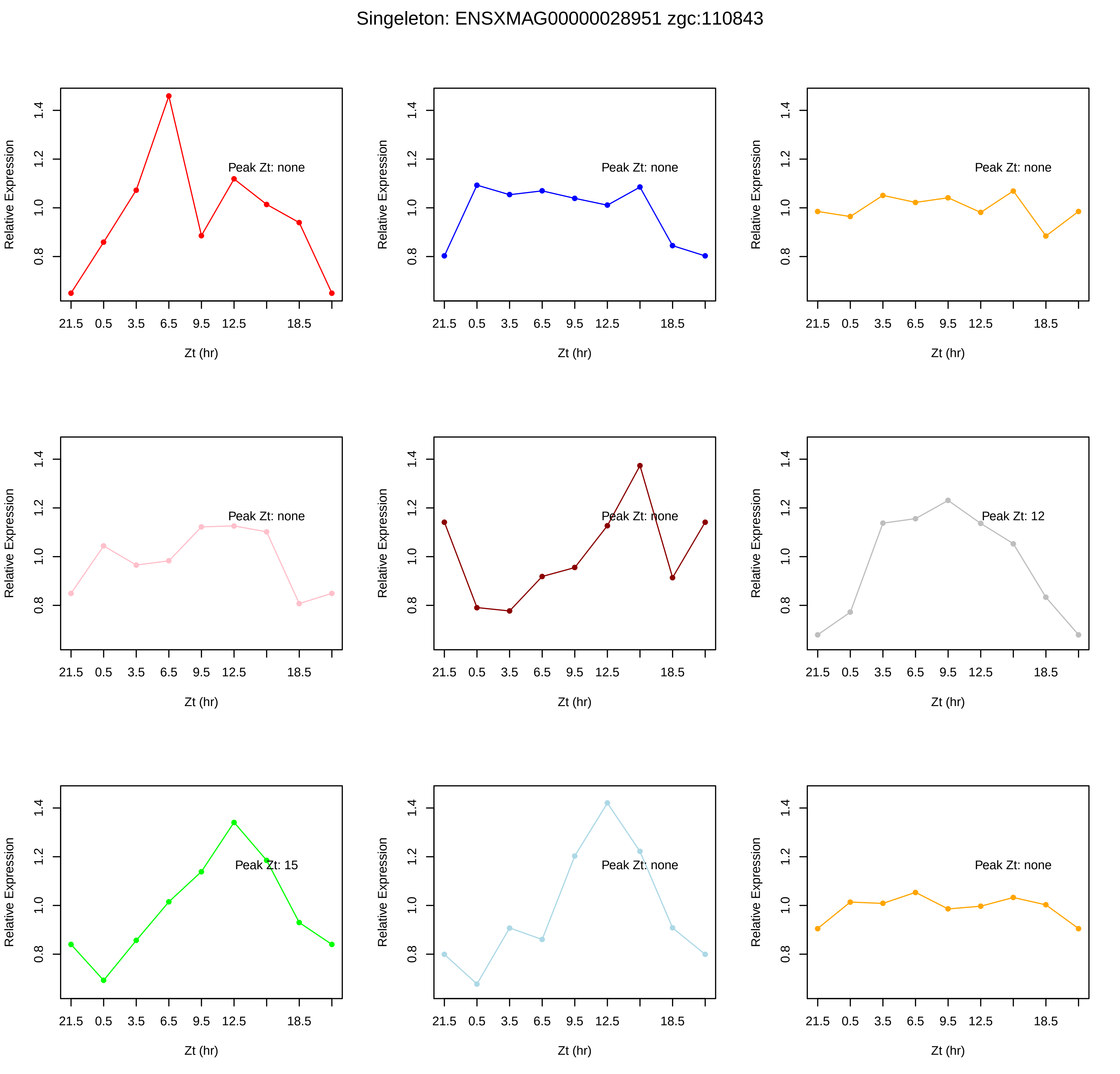

Singeleton: ENSXMAG00000028951 zgc:110843
Peak Zt: none
Peak Zt: none
Peak Zt: none
Peak Zt: none
Peak Zt: none
Peak Zt: 12
Peak Zt: 15
Peak Zt: none
Peak Zt: none
Relative Expression
Zt (hr)

Singeleton: ENSXMAG00000005399 ppa2

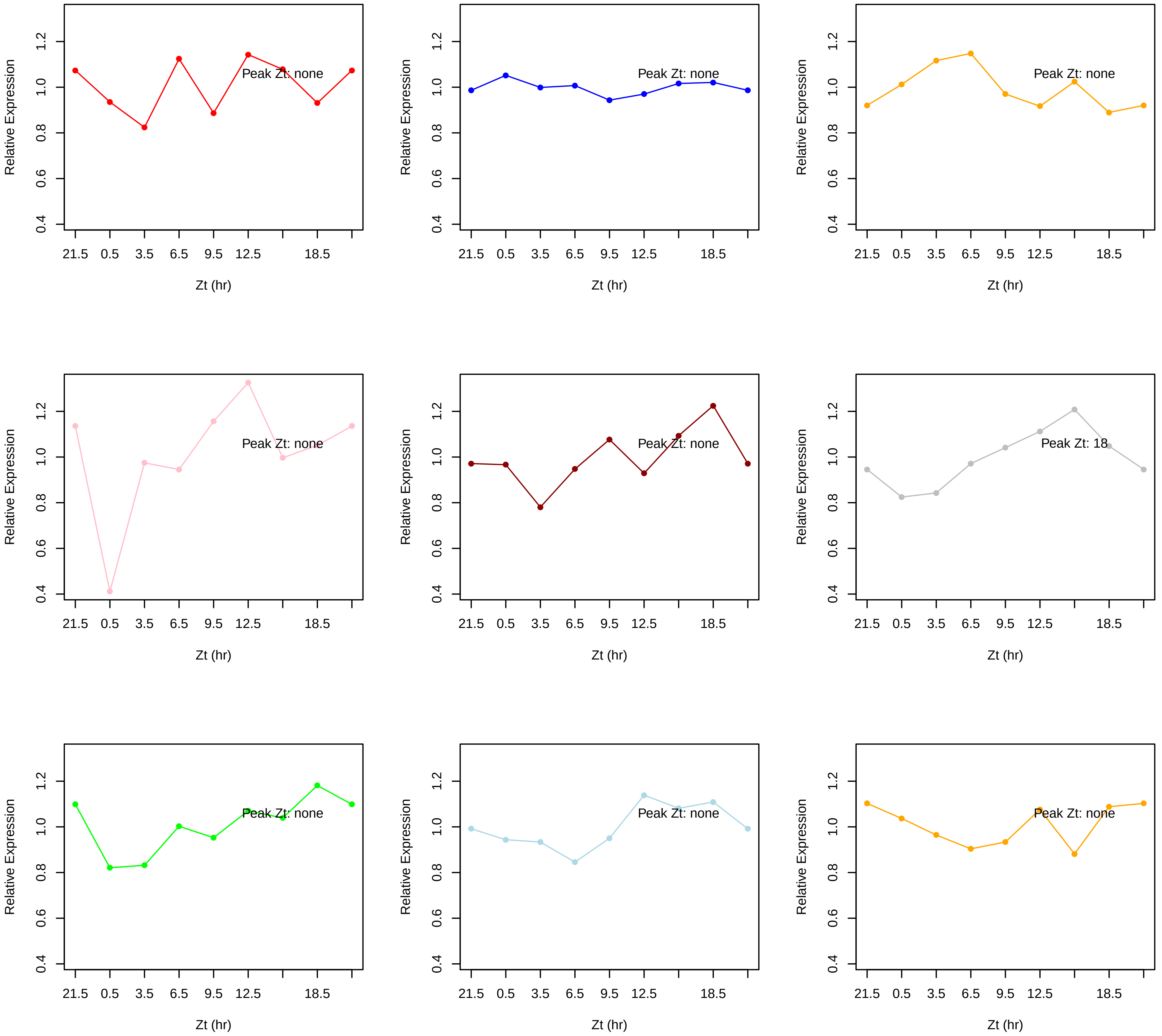

Singeleton: ENSXMAG00000011127 cxxc5a

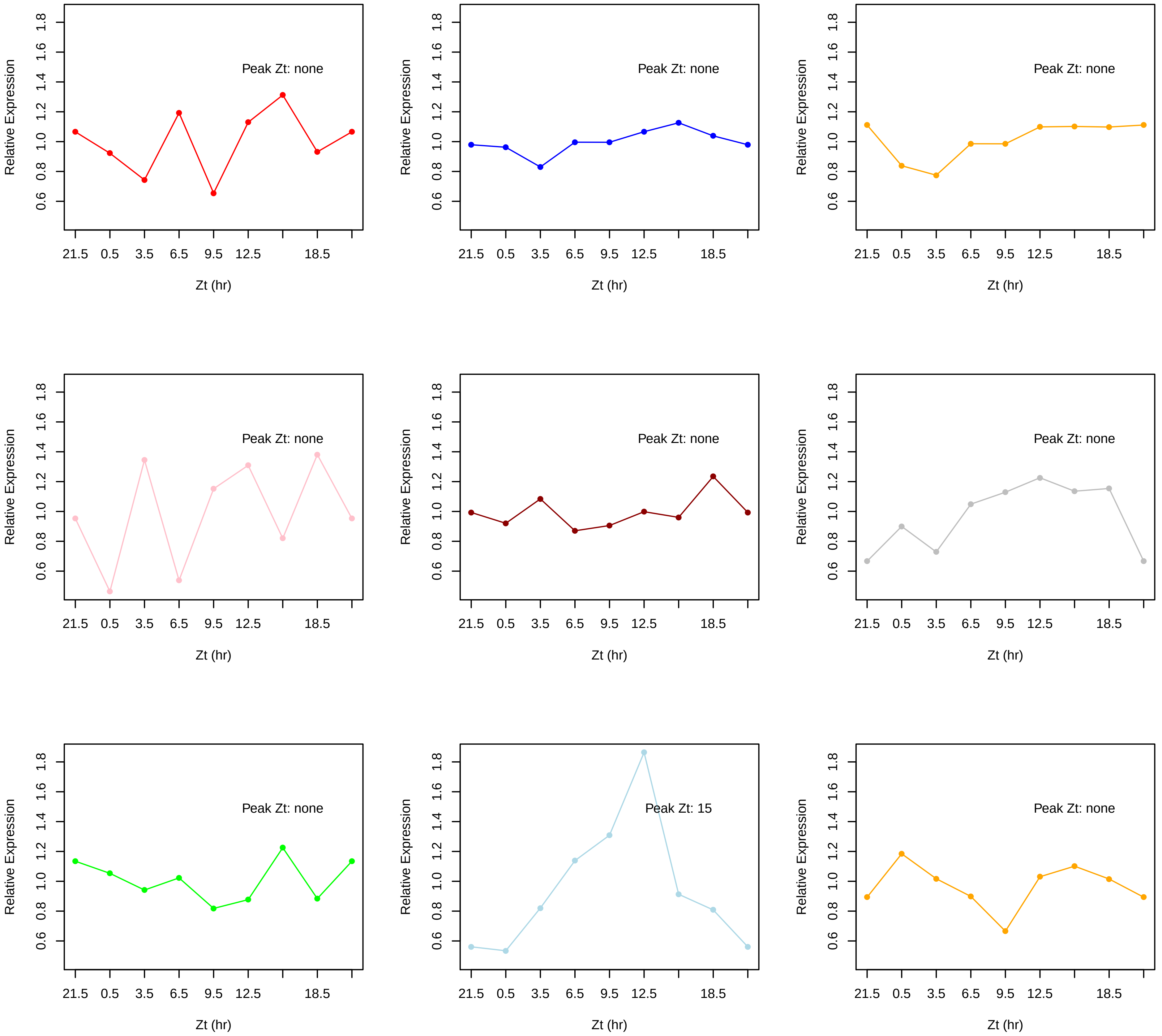

Singleton: ENSXMAG00000017655 lzts2b

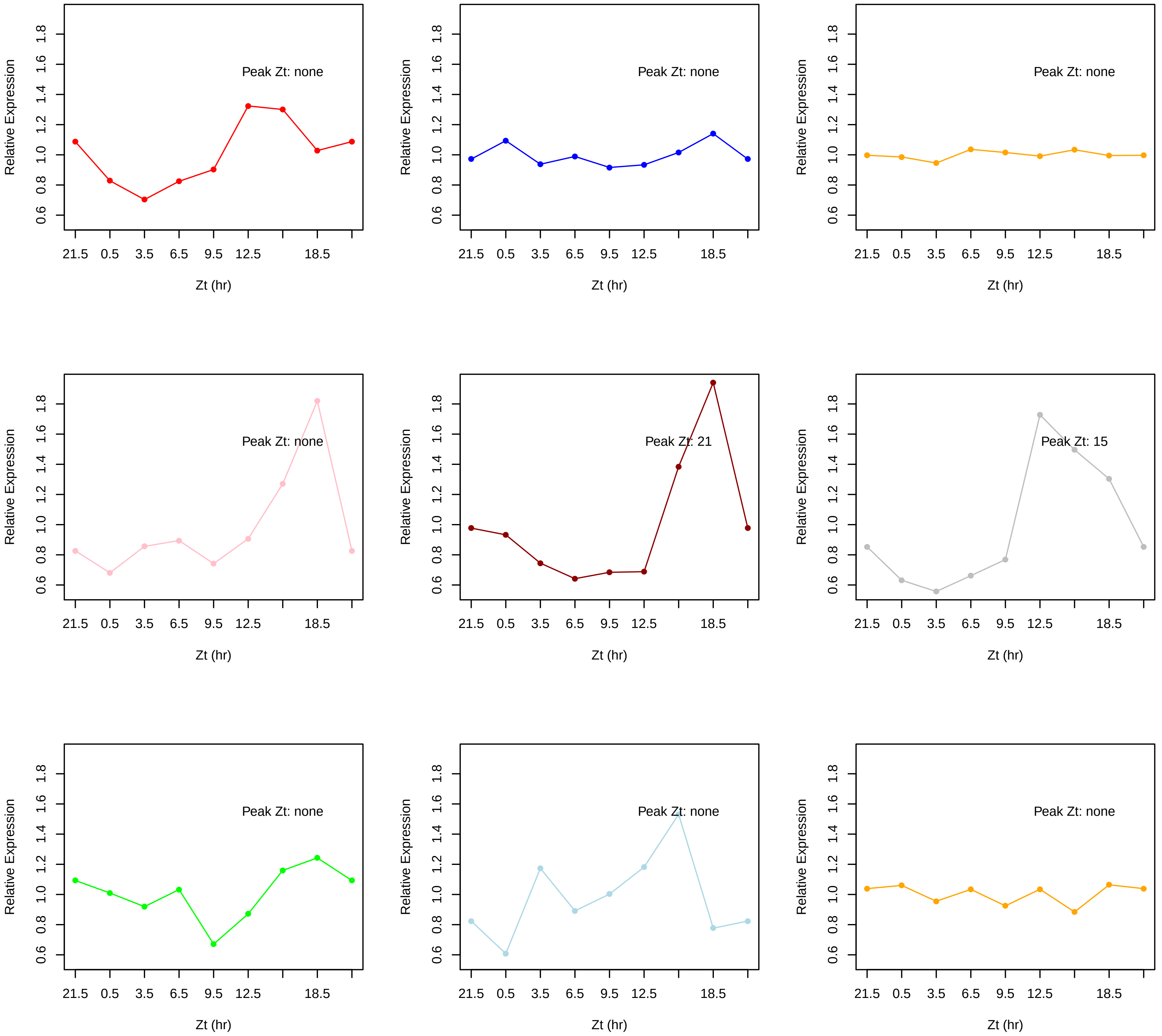

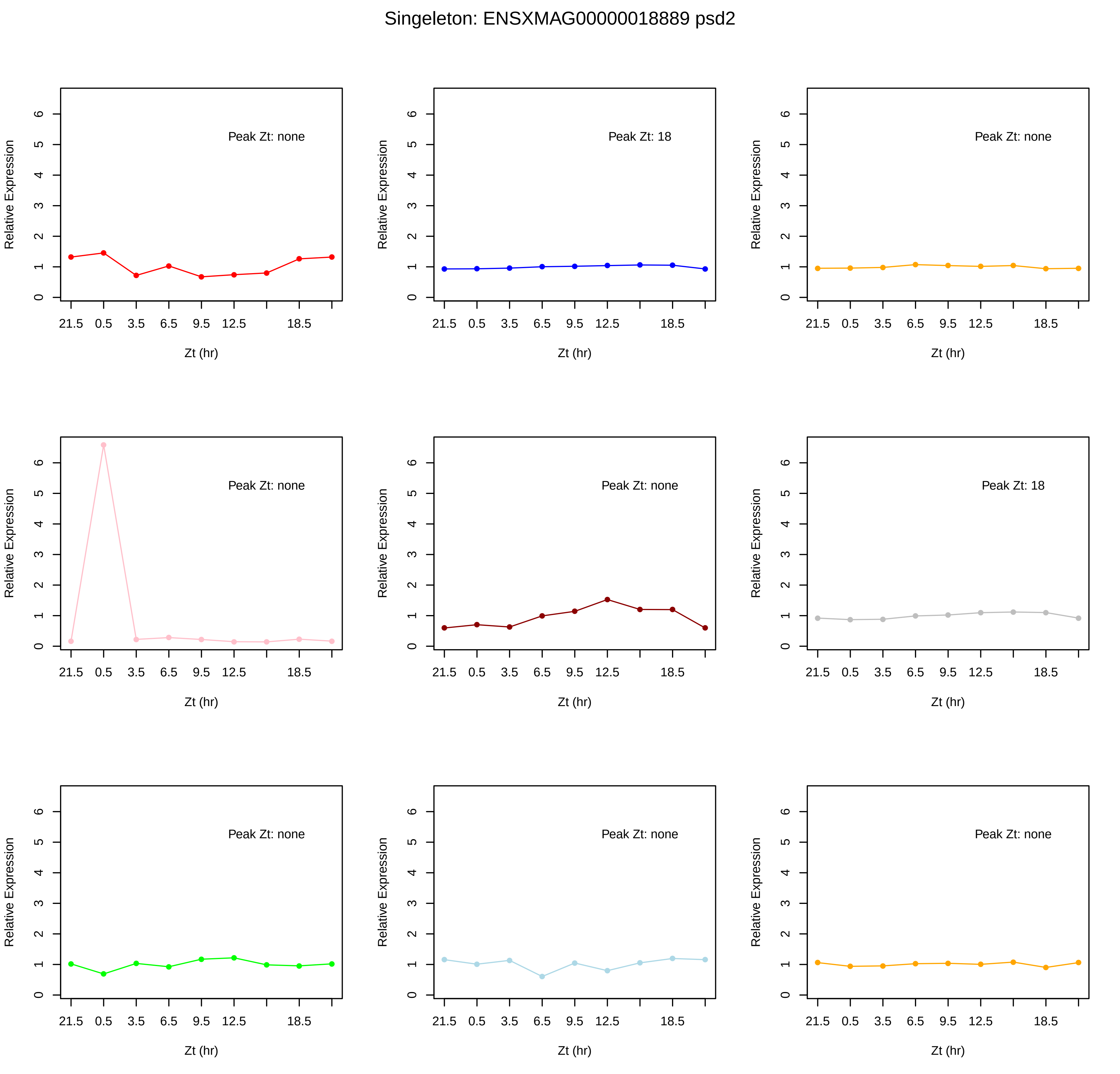
Singeleton: ENSXMAG00000018889 psd2
Peak Zt: none
Peak Zt: 18
Peak Zt: none
Peak Zt: none
Peak Zt: none
Peak Zt: 18
Peak Zt: none
Peak Zt: none
Peak Zt: none
Relative Expression
Zt (hr)
21.5 0.5 3.5 6.5 9.5 12.5 18.5

Singeleton: ENSXMAG00000025207 retreg1

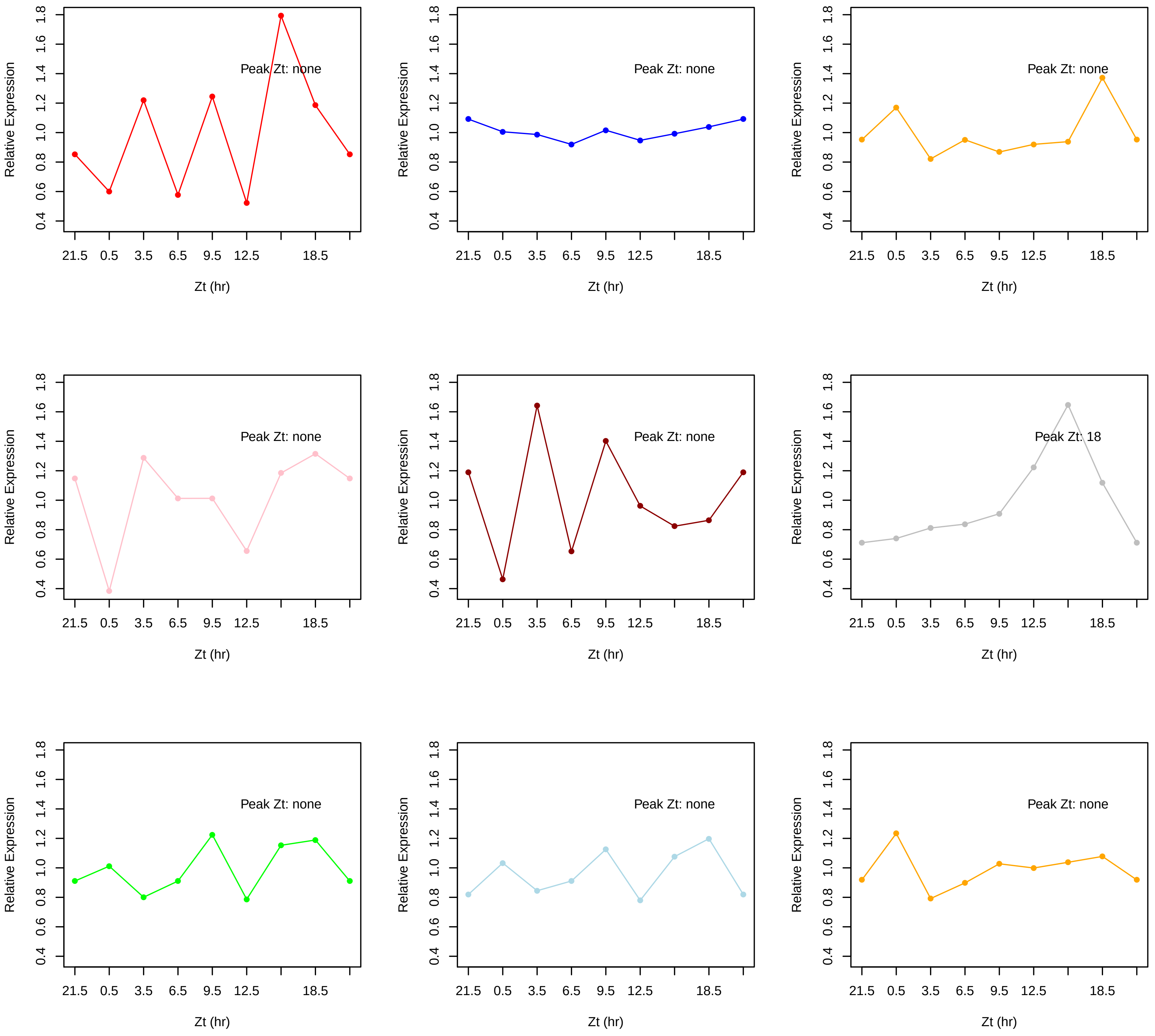

Singeleton: ENSXMAG00000008845

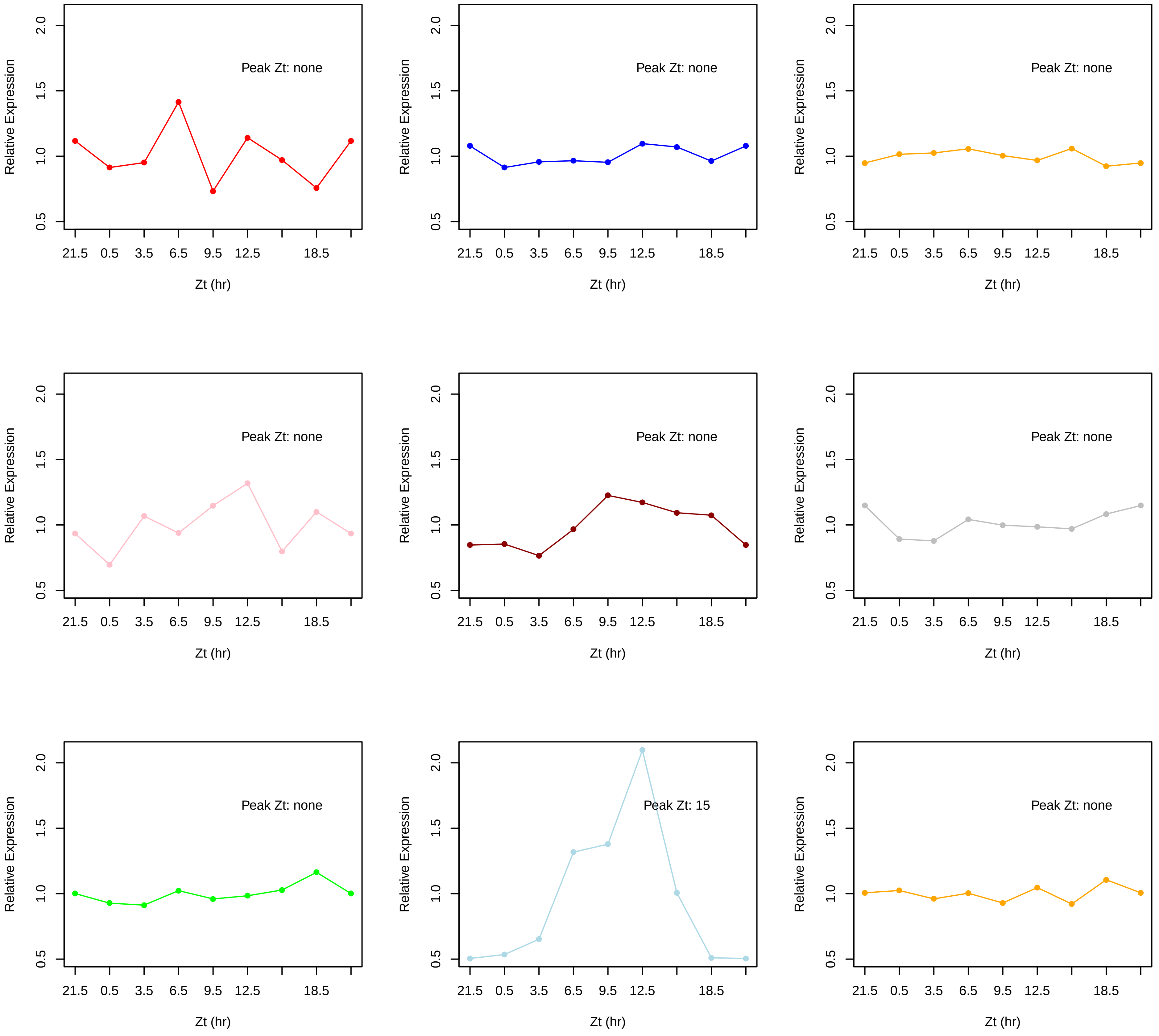

Singeleton: ENSXMAG00000017061 EGFLAM

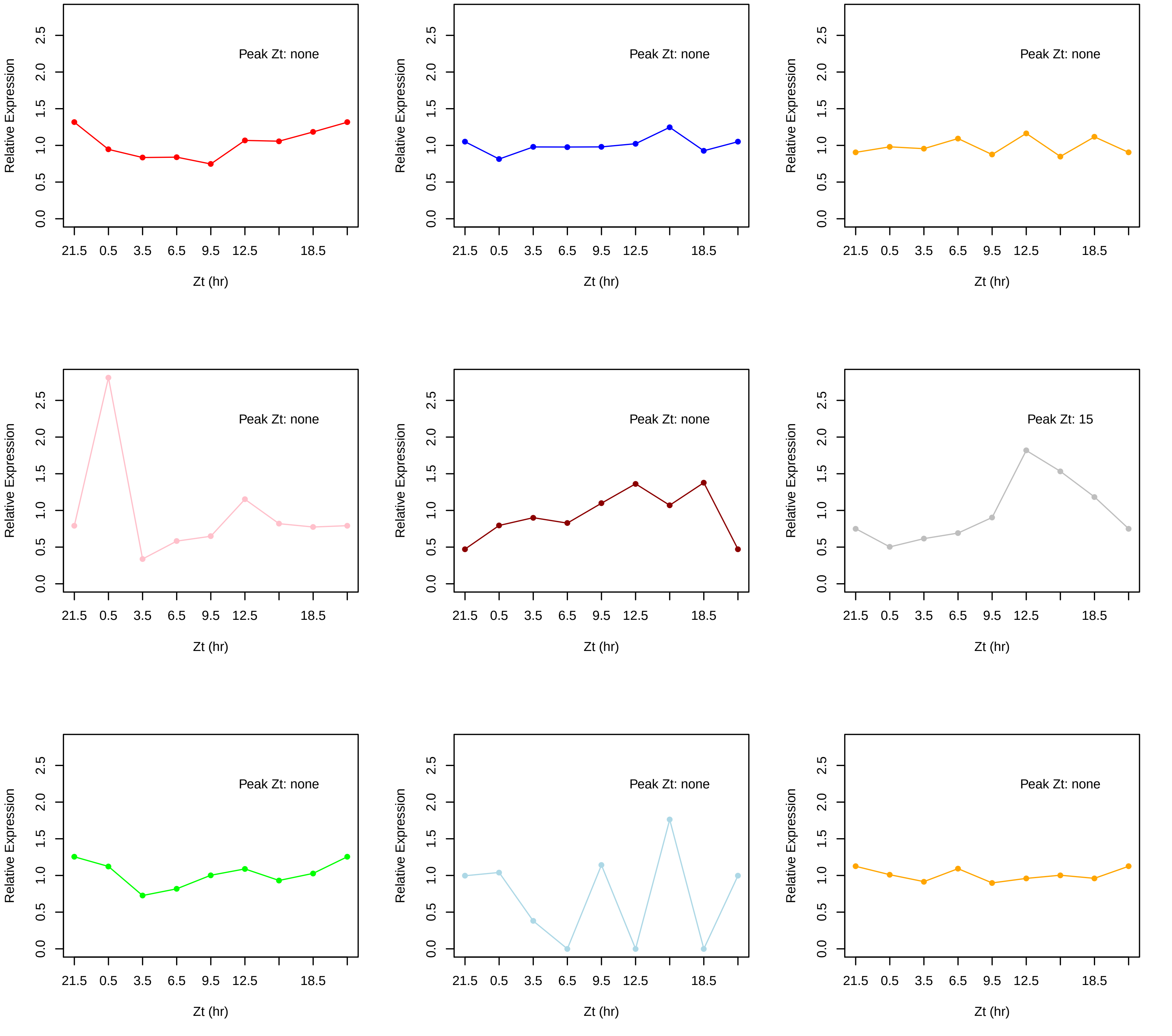

Singeleton: ENSXMAG00000012060 tbck

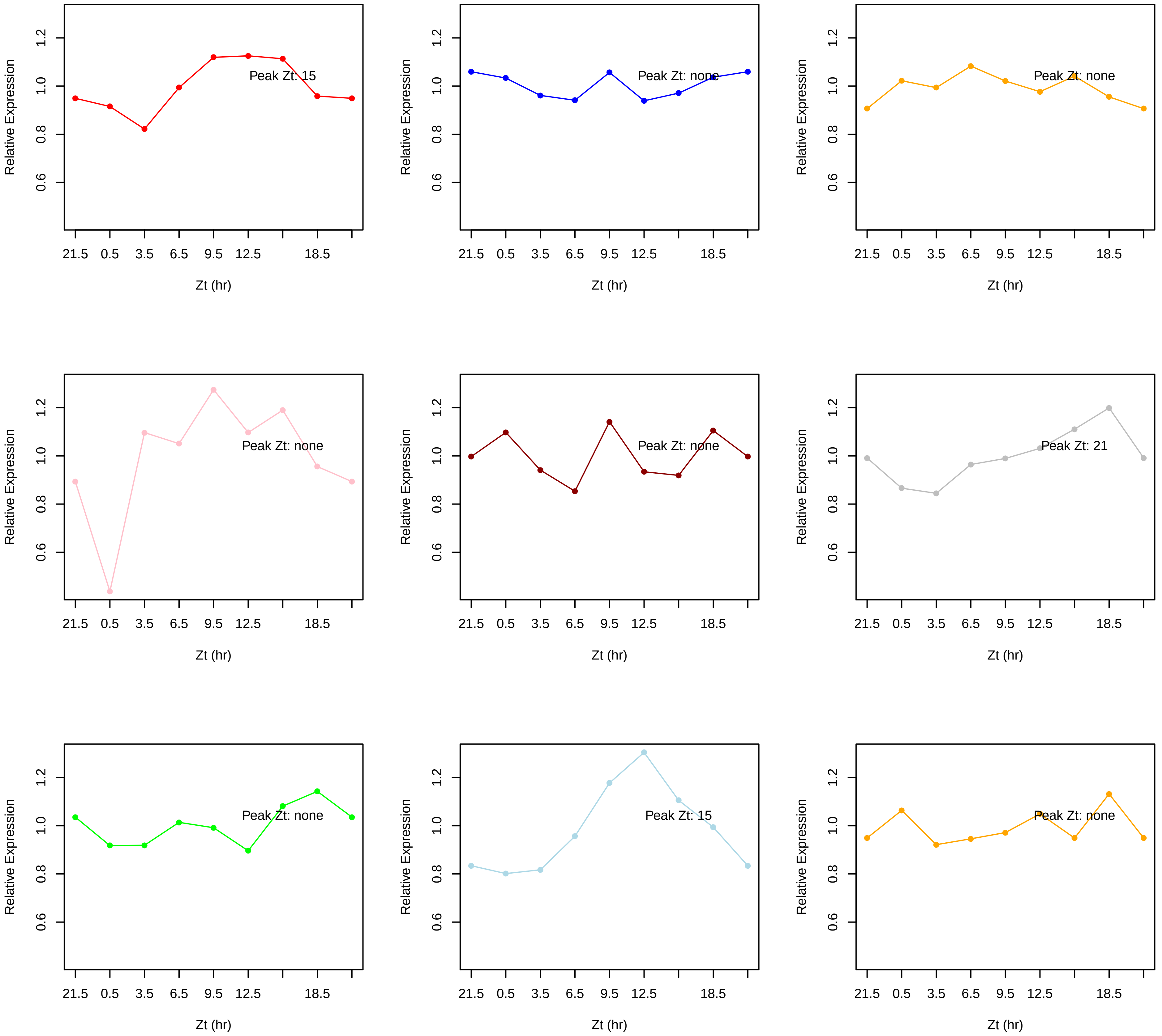

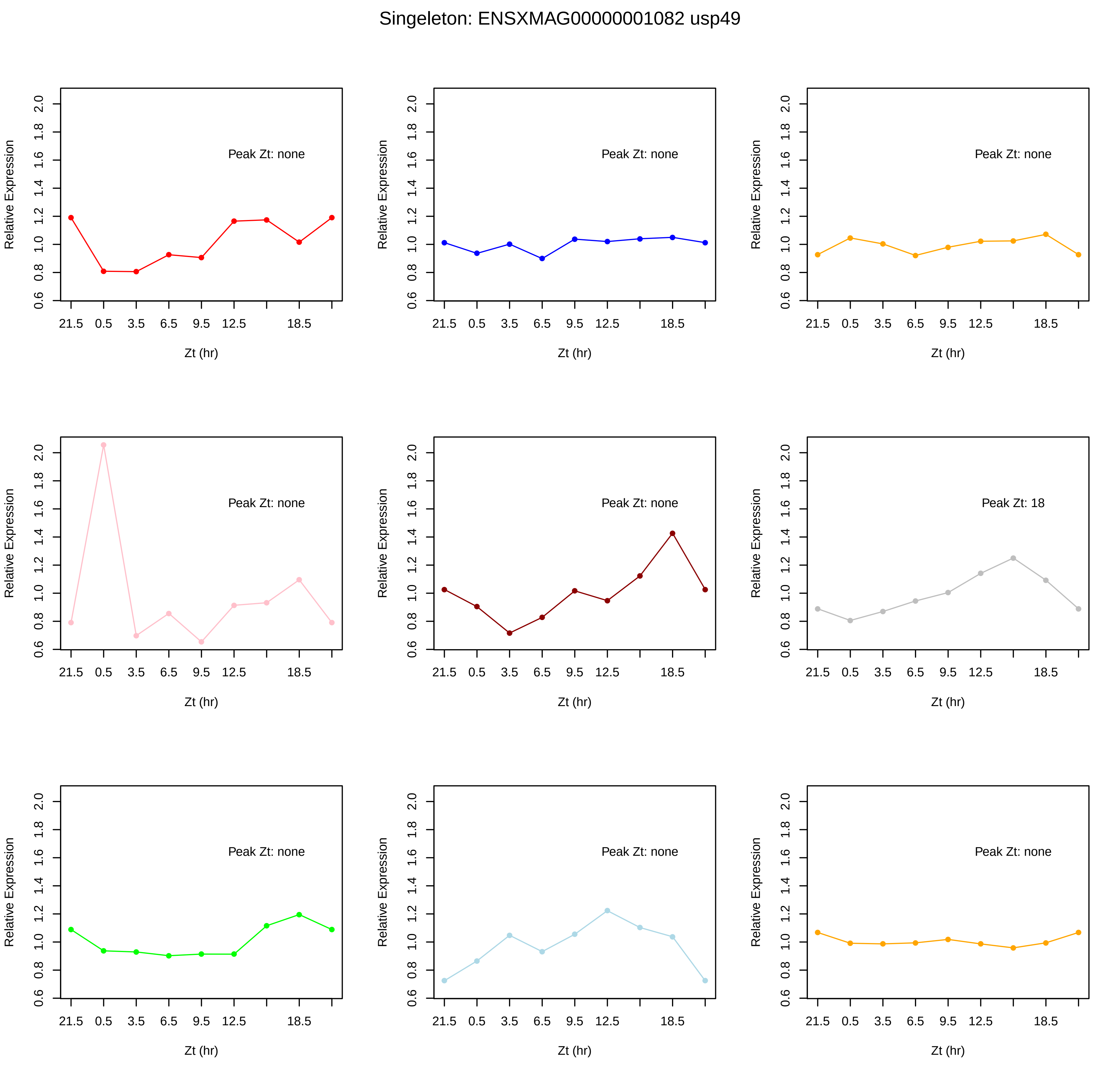
Singleton: ENSXMAG00000001082 usp49
Peak Zt: none
Peak Zt: none
Peak Zt: none
Peak Zt: none
Peak Zt: none
Peak Zt: 18
Peak Zt: none
Peak Zt: none
Peak Zt: none
Relative Expression
Zt (hr)
21.5
0.5
3.5
6.5
9.5
12.5
18.5
0.6
0.8
1.0
1.2
1.4
1.6
1.8
2.0

Singeleton: ENSXMAG00000001071 med20

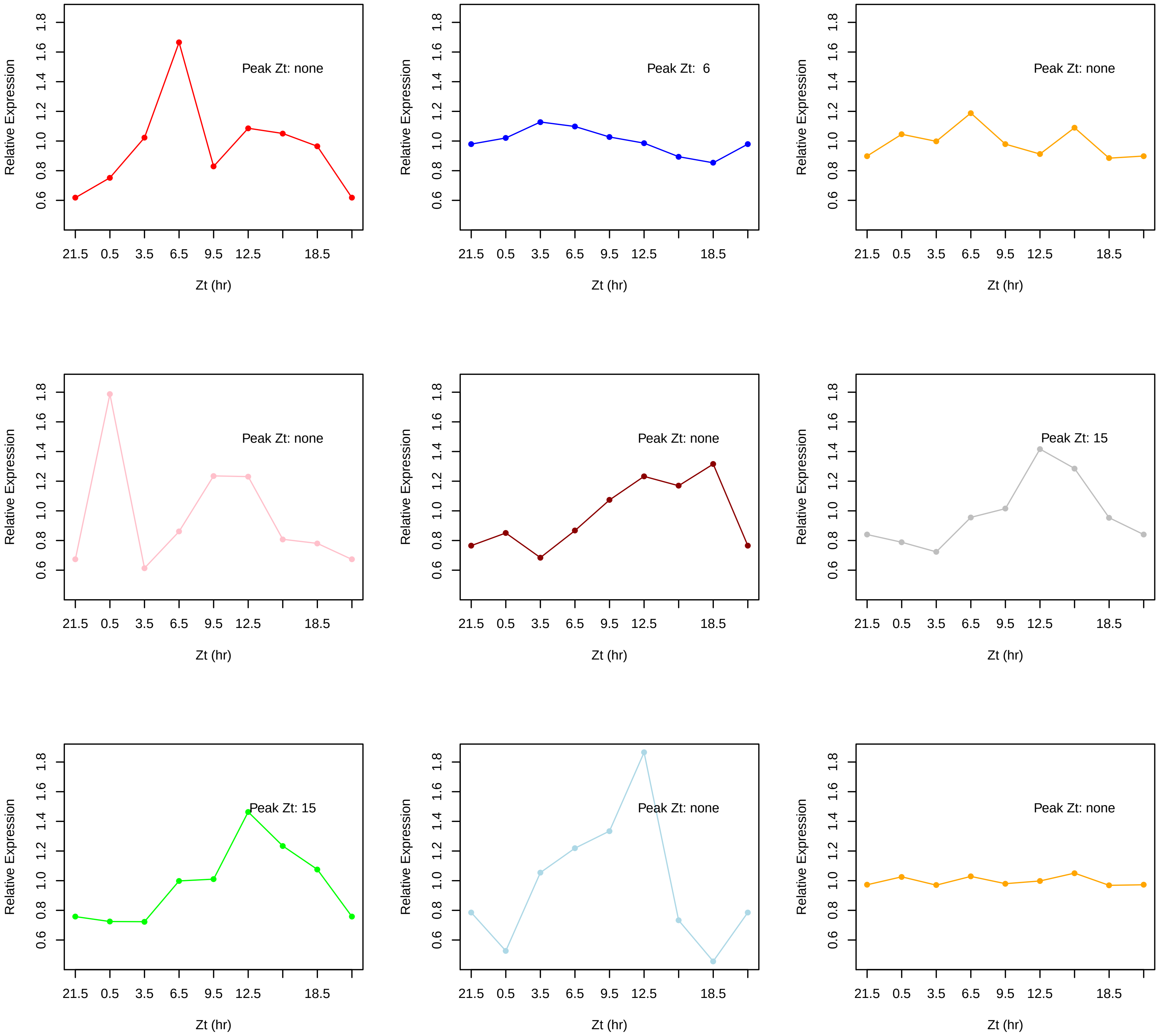

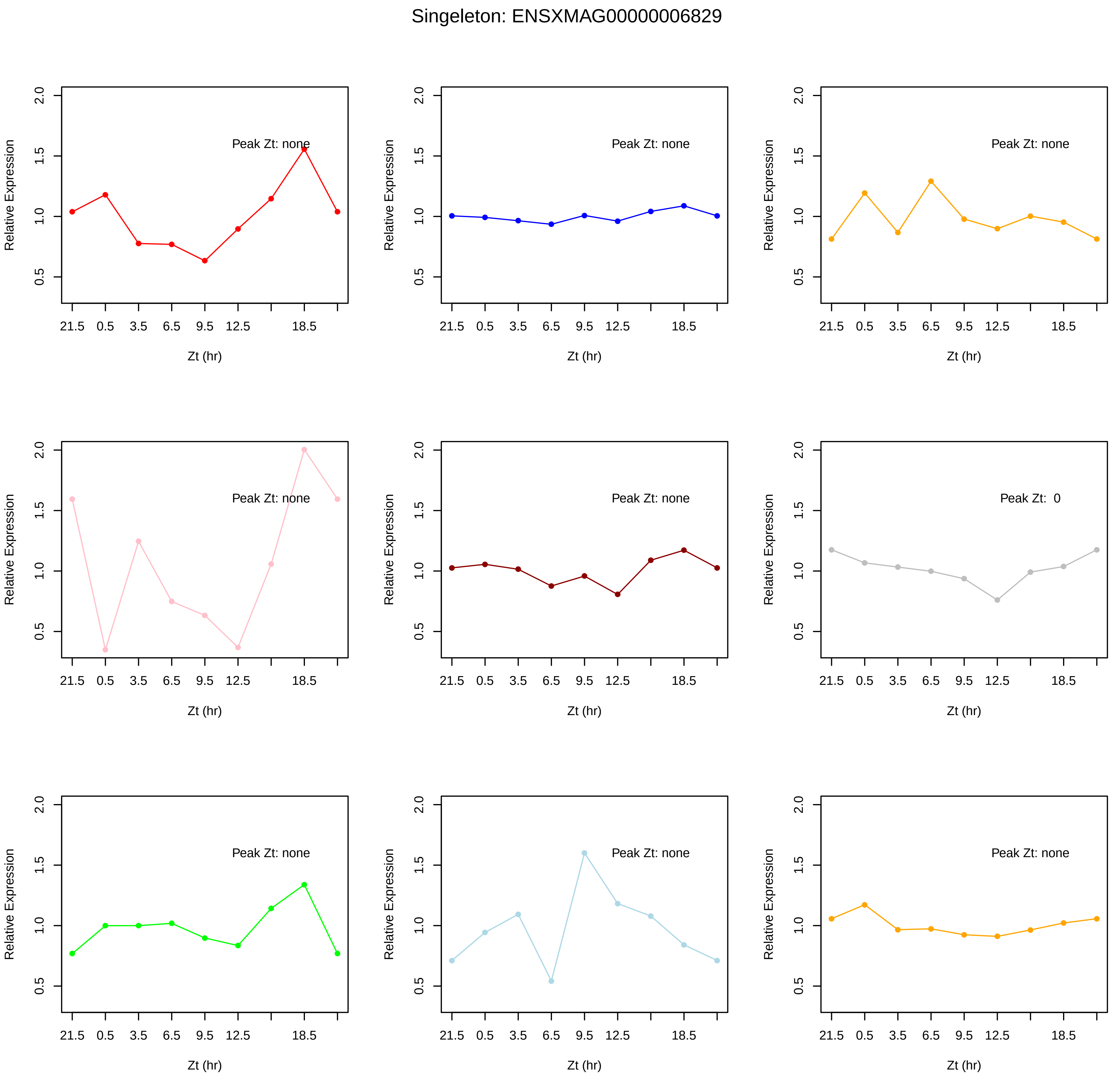
Singleton: ENSXMAG00000006829
Peak Zt: none
Peak Zt: none
Peak Zt: none
Peak Zt: none
Peak Zt: none
Peak Zt: 0
Peak Zt: none
Peak Zt: none
Peak Zt: none
Relative Expression
Zt (hr)
21.5 0.5 3.5 6.5 9.5 12.5 18.5
0.5 1.0 1.5 2.0

Singeleton: ENSXMAG00000001060 bysl

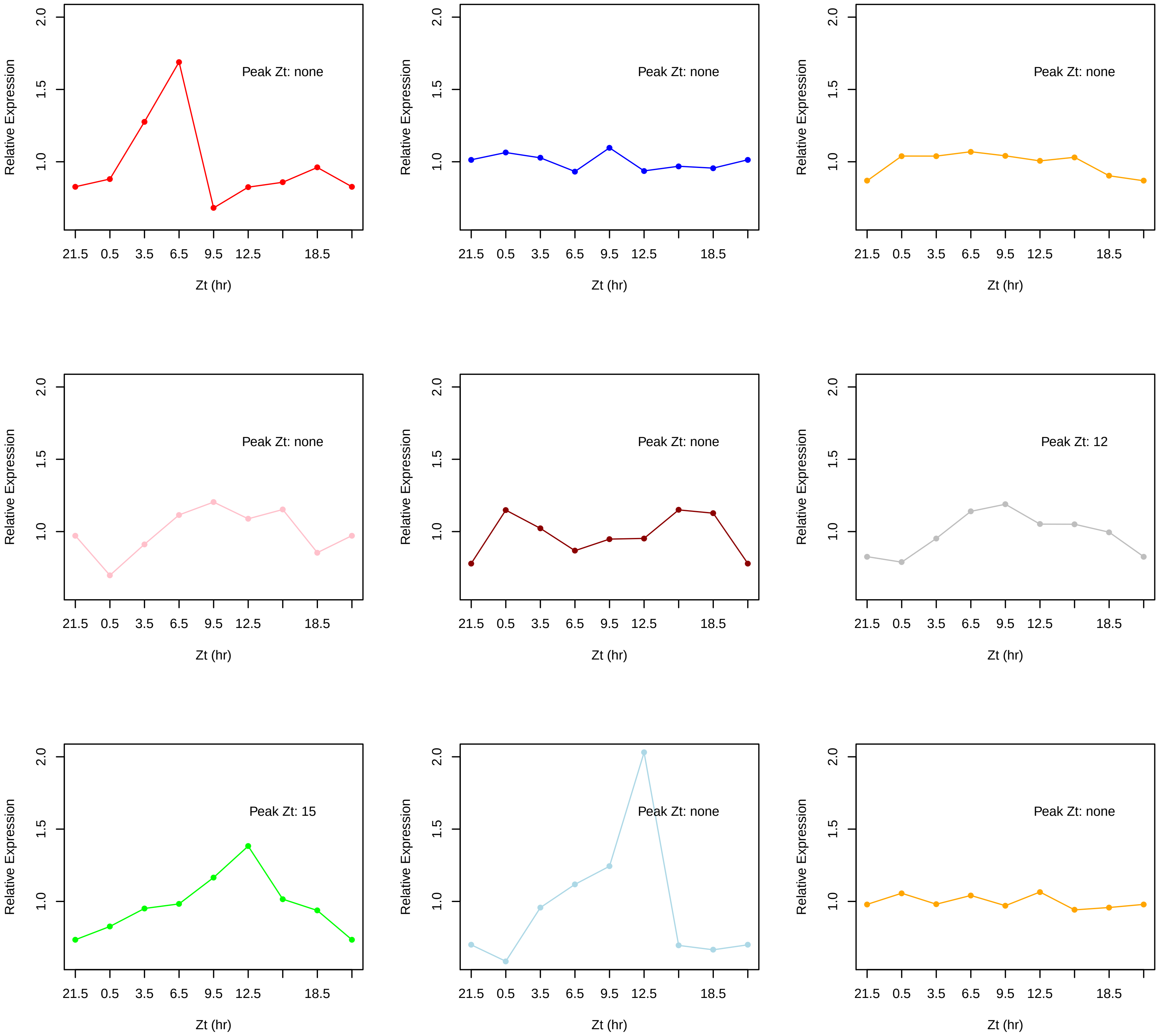

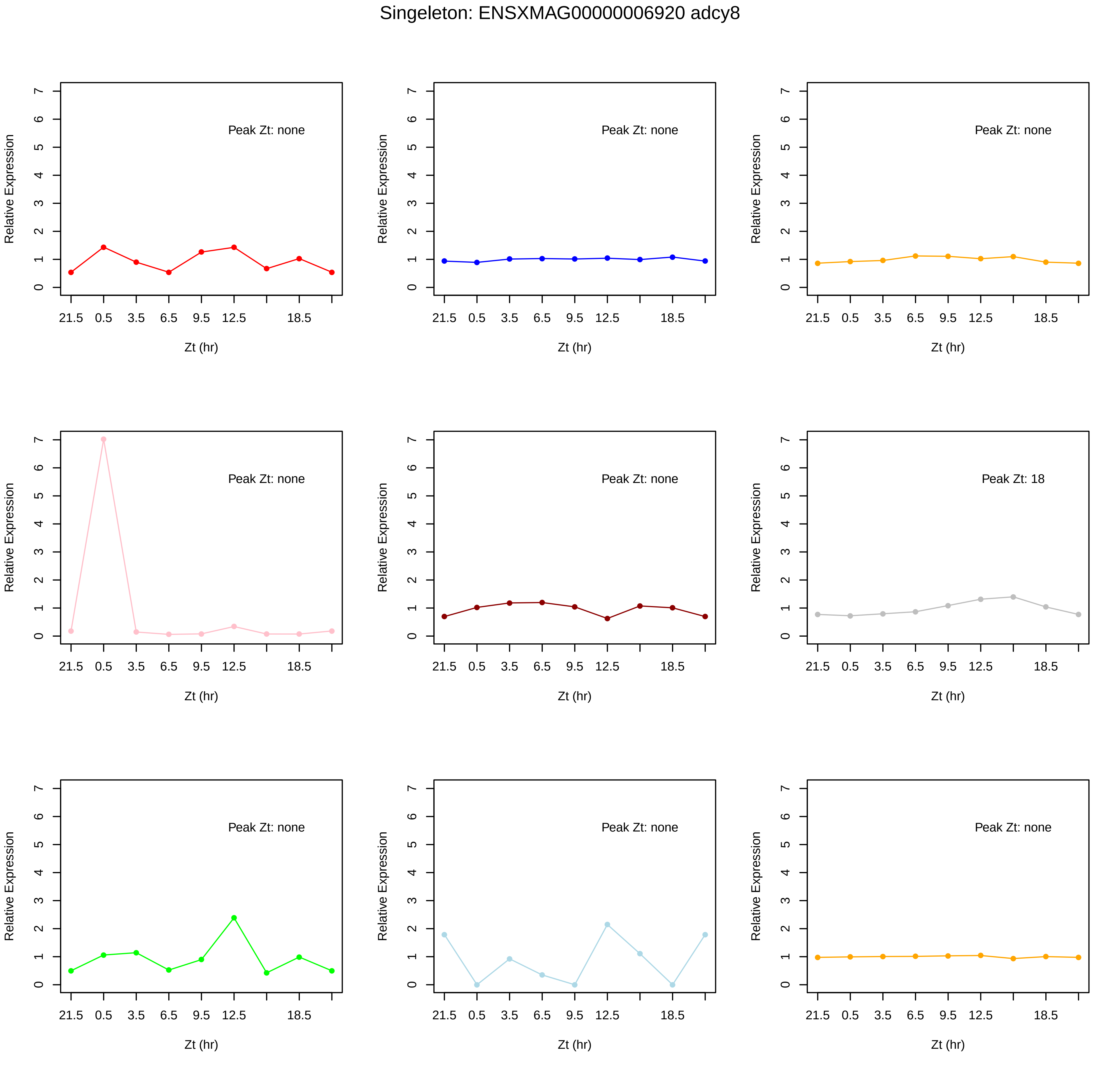

Singleton: ENSXMAG00000006920 adcy8
Relative Expression
Zt (hr)
21.5
0.5
3.5
6.5
9.5
12.5
18.5
Peak Zt: none
Peak Zt: none
Peak Zt: none
Peak Zt: none
Peak Zt: none
Peak Zt: 18
Peak Zt: none
Peak Zt: none
Peak Zt: none

Singeleton: ENSXMAG00000021736 papss1

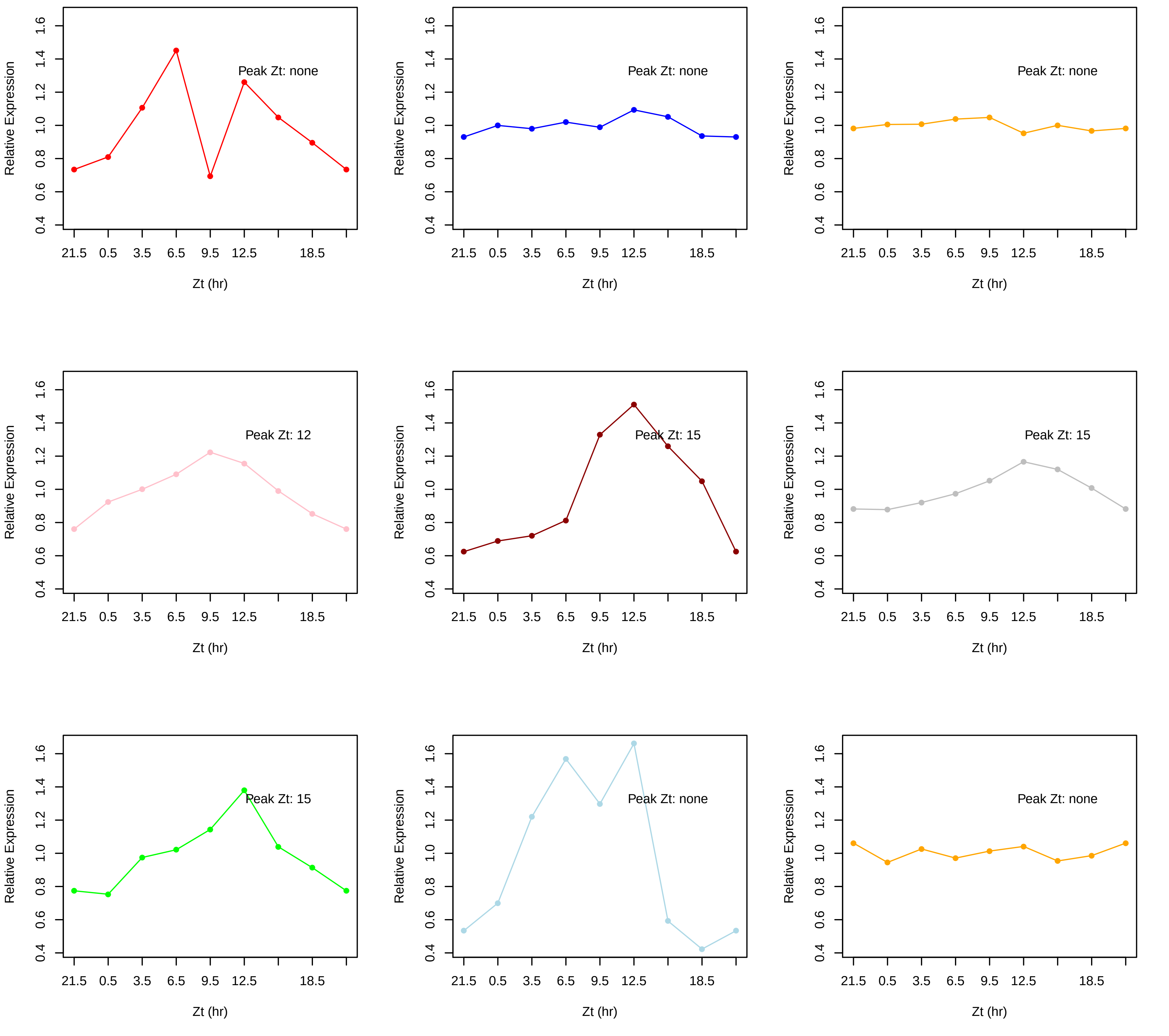

Singleton: ENSXMAG00000011016 efr3a

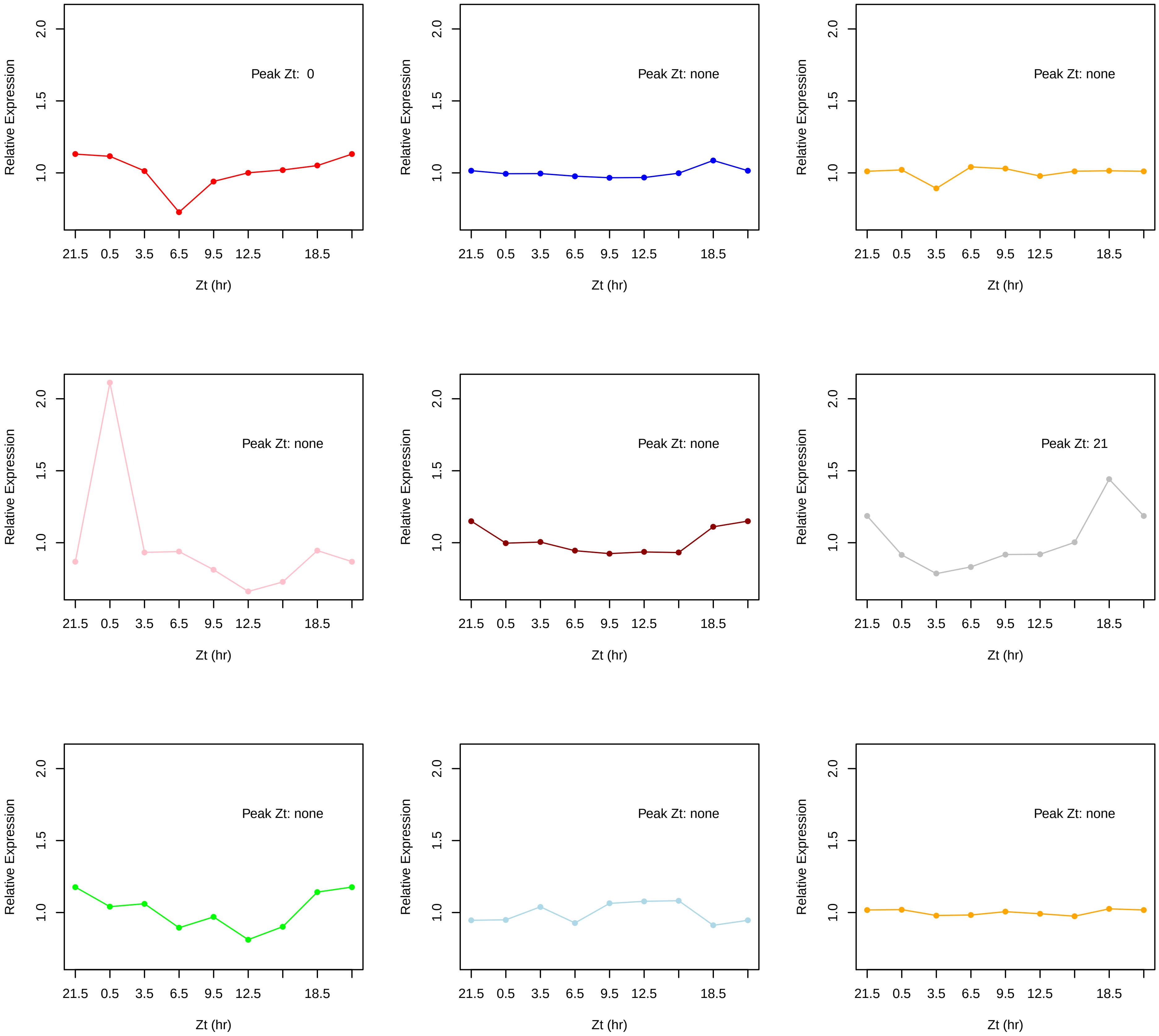

Singeleton: ENSXMAG00000013479 sufu

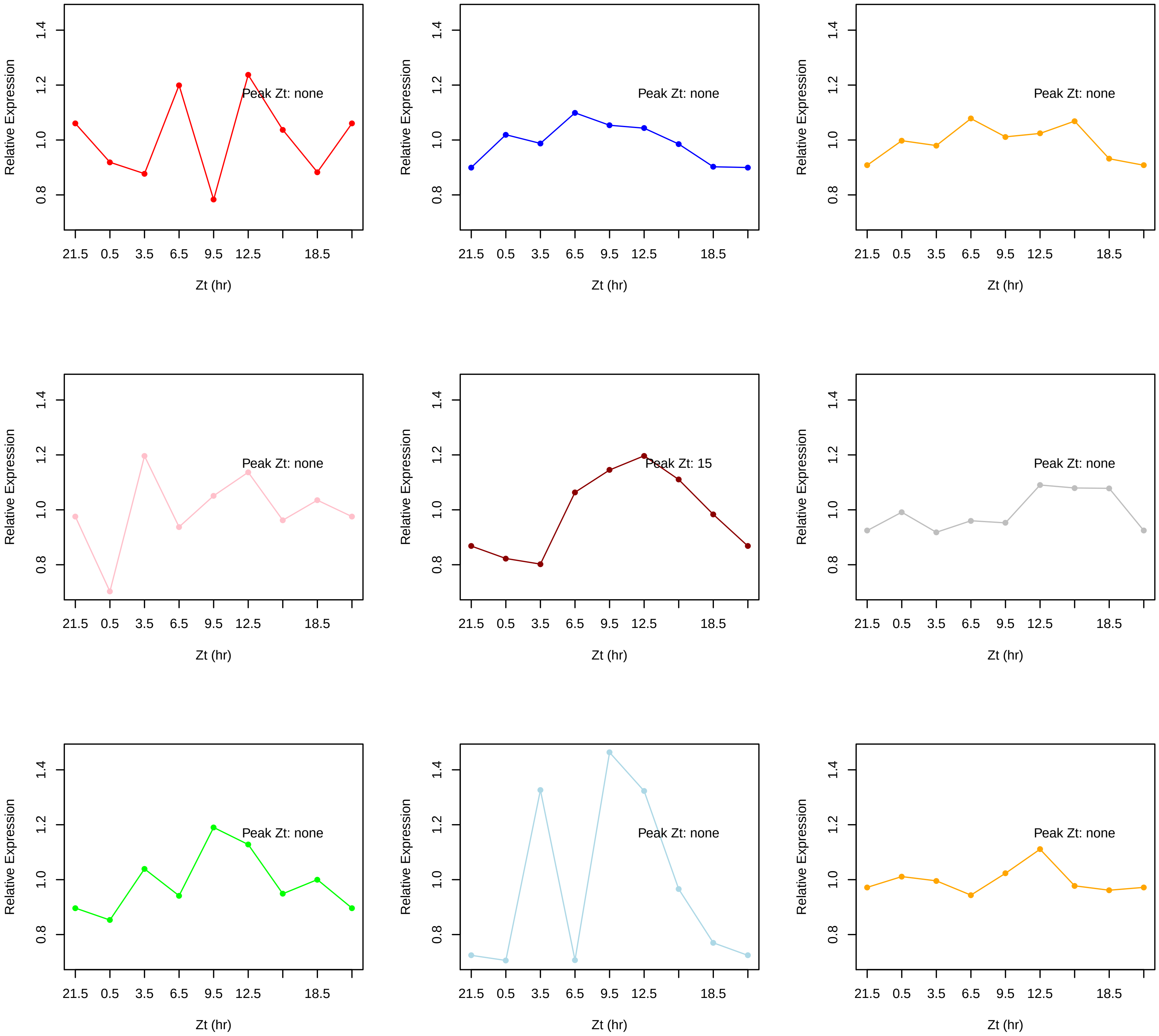

Singeleton: ENSXMAG00000005080 nup155

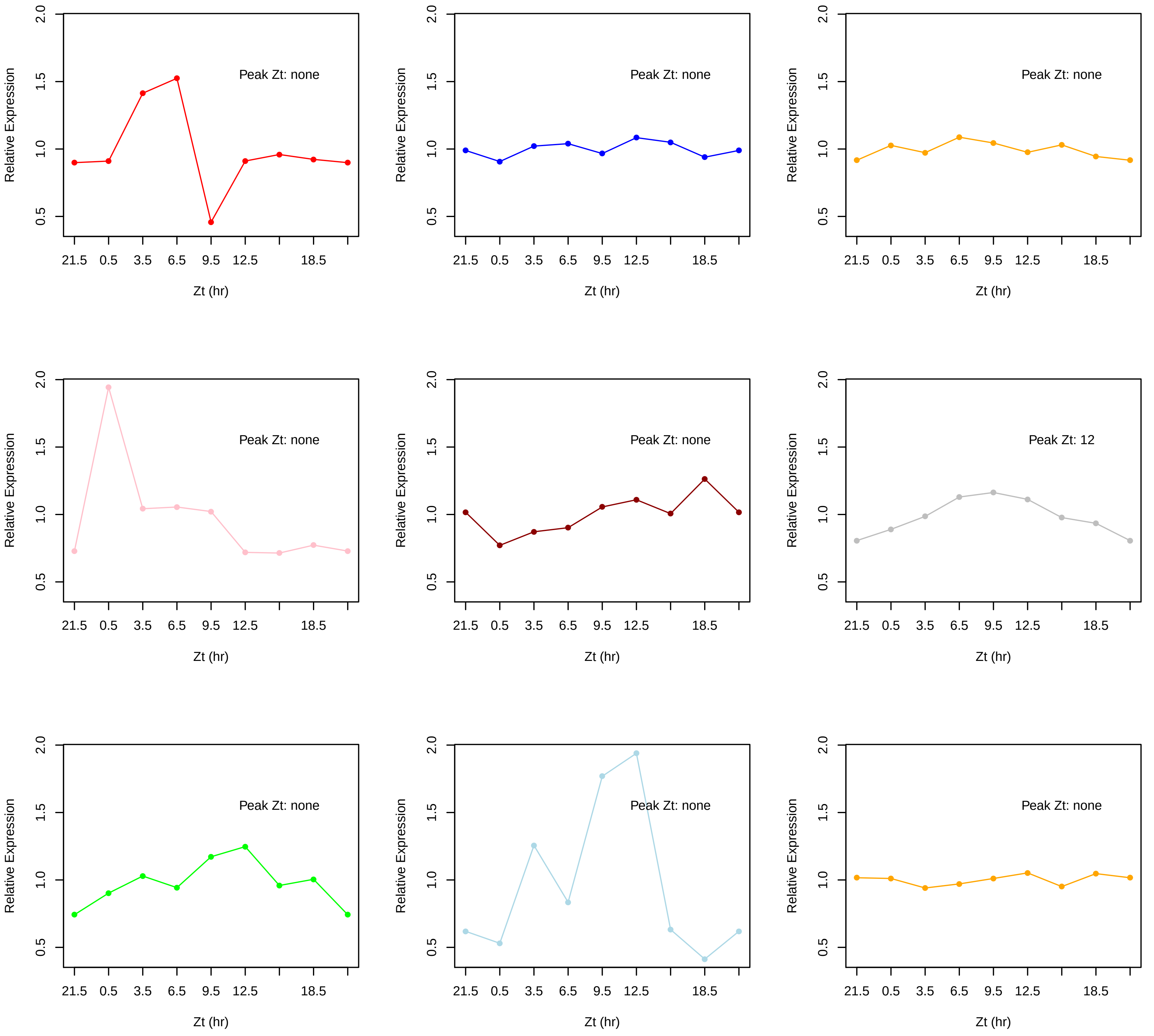

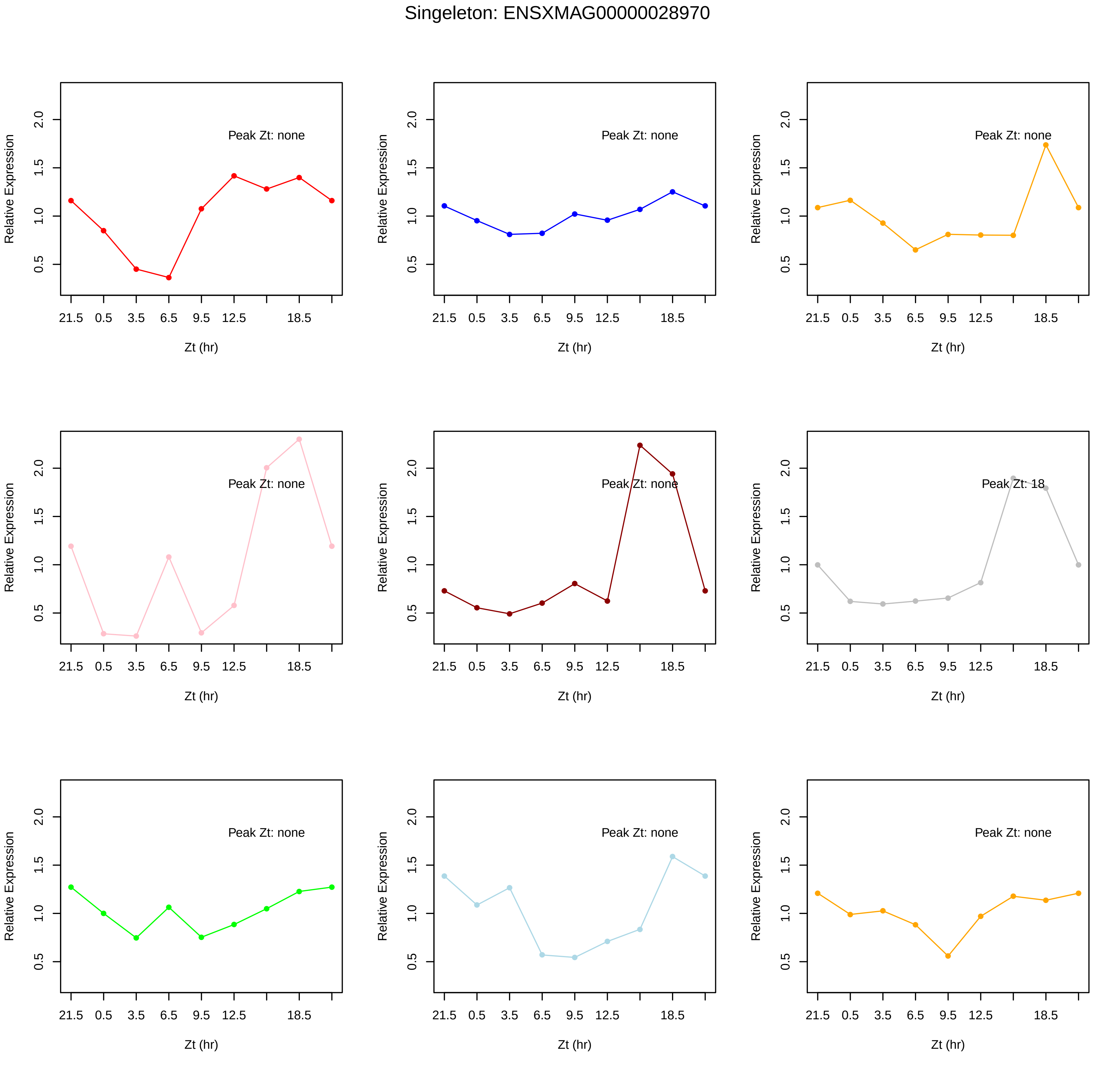
Singeleton: ENSXMAG00000028970
Relative Expression
Zt (hr)
Peak Zt: none
Peak Zt: none
Peak Zt: none
Peak Zt: none
Peak Zt: none
Peak Zt: 18
Peak Zt: none
Peak Zt: none
Peak Zt: none

Singleton: ENSXMAG00000001089 tmcc2

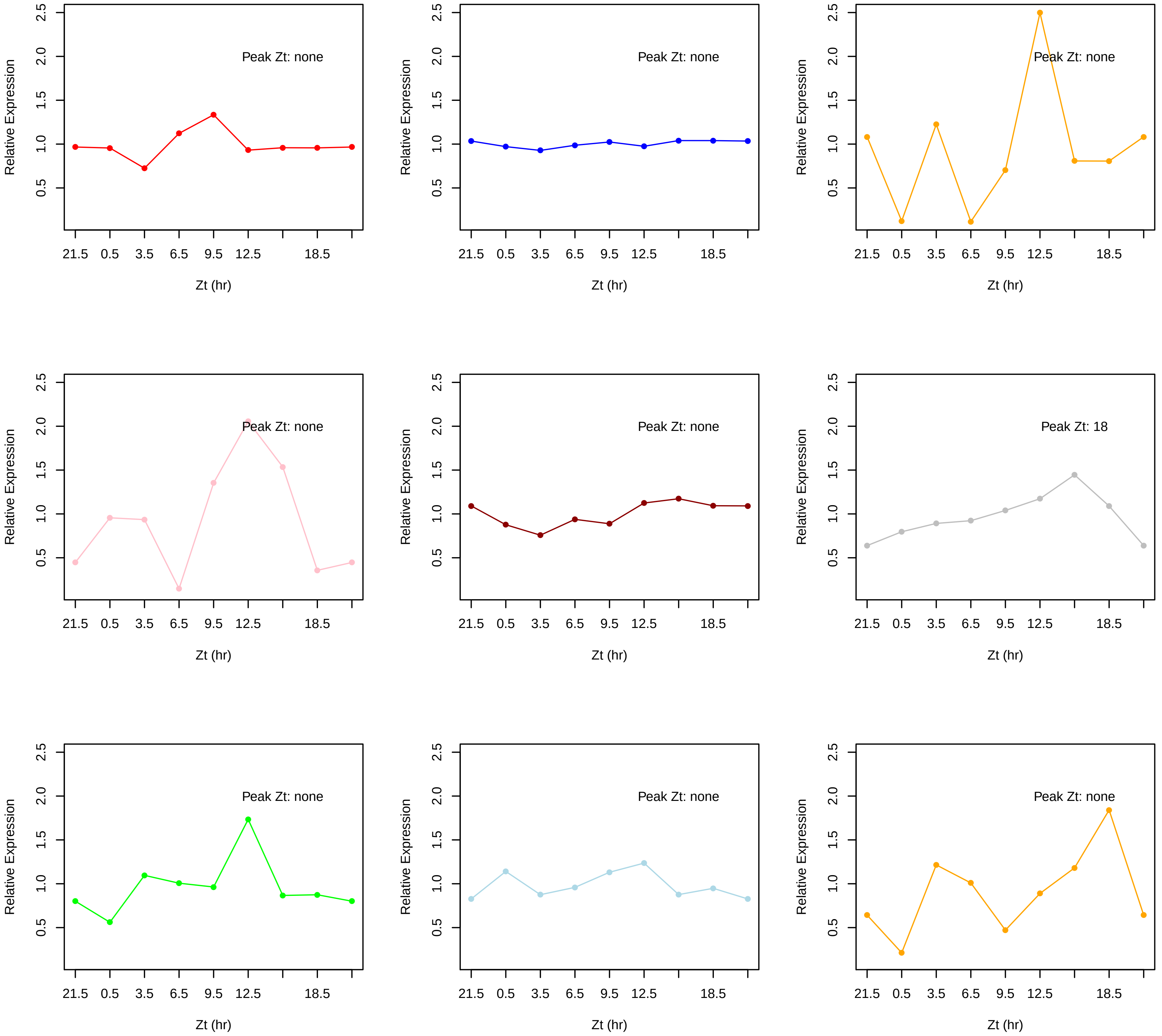

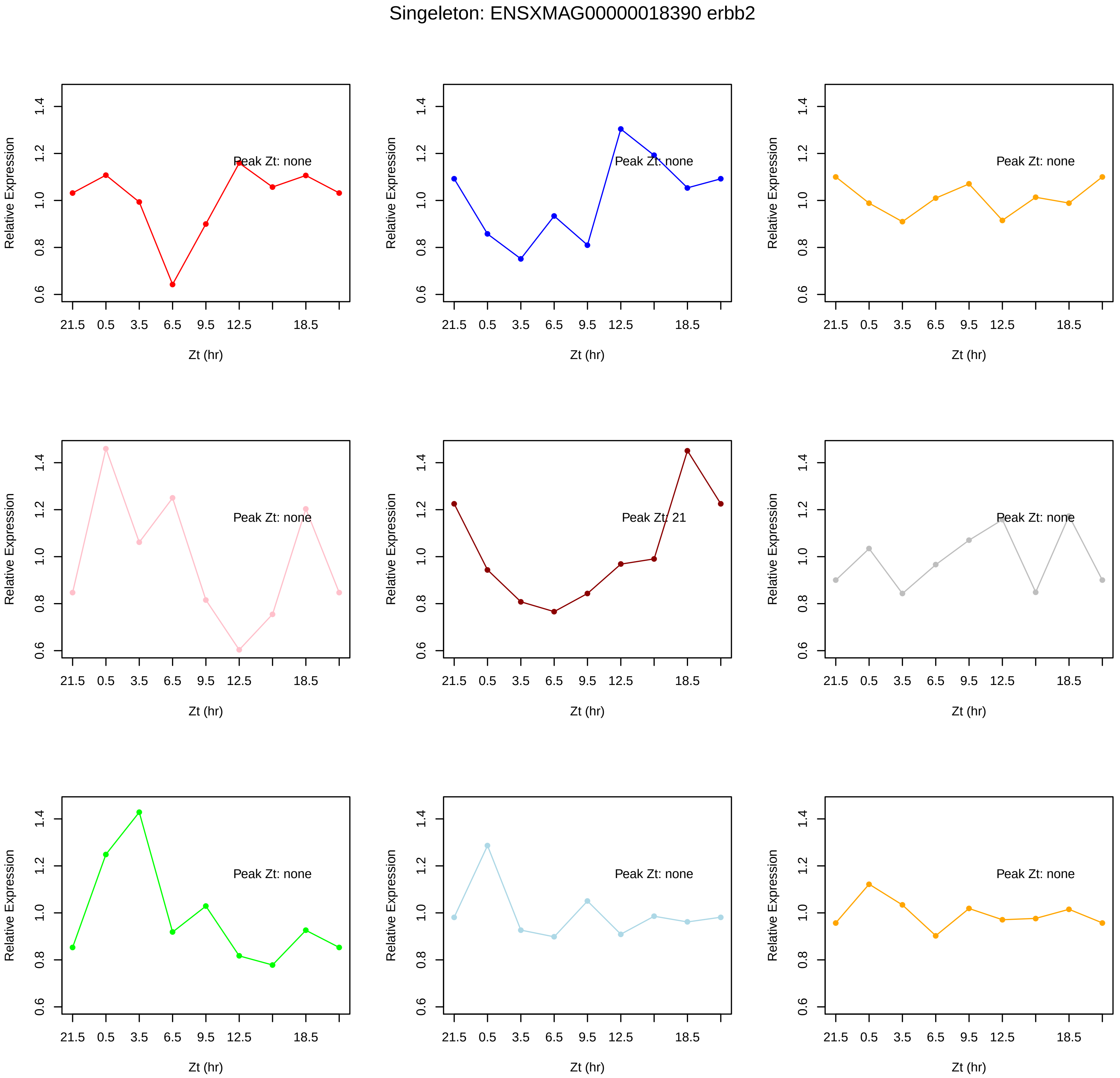

Singleton: ENSXMAG00000018390 erbb2
Relative Expression
Zt (hr)
Peak Zt: none
Peak Zt: none
Peak Zt: none
Peak Zt: none
Peak Zt: 21
Peak Zt: none
Peak Zt: none
Peak Zt: none
Peak Zt: none
21.5 0.5 3.5 6.5 9.5 12.5 18.5
0.6 0.8 1.0 1.2 1.4

Singleton: ENSXMAG00000012401 usp36

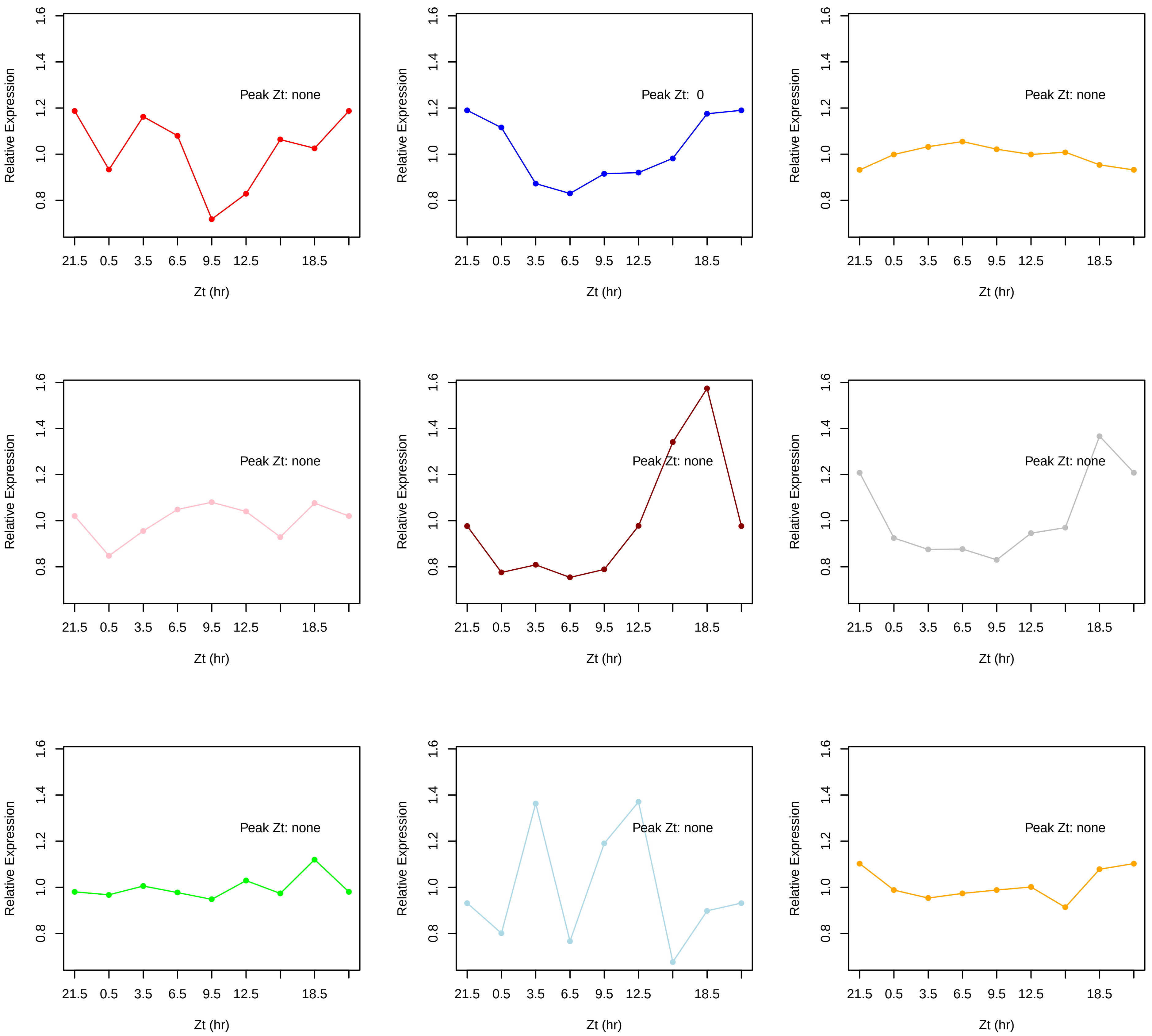

Singeleton: ENSXMAG00000000585 rmdn3

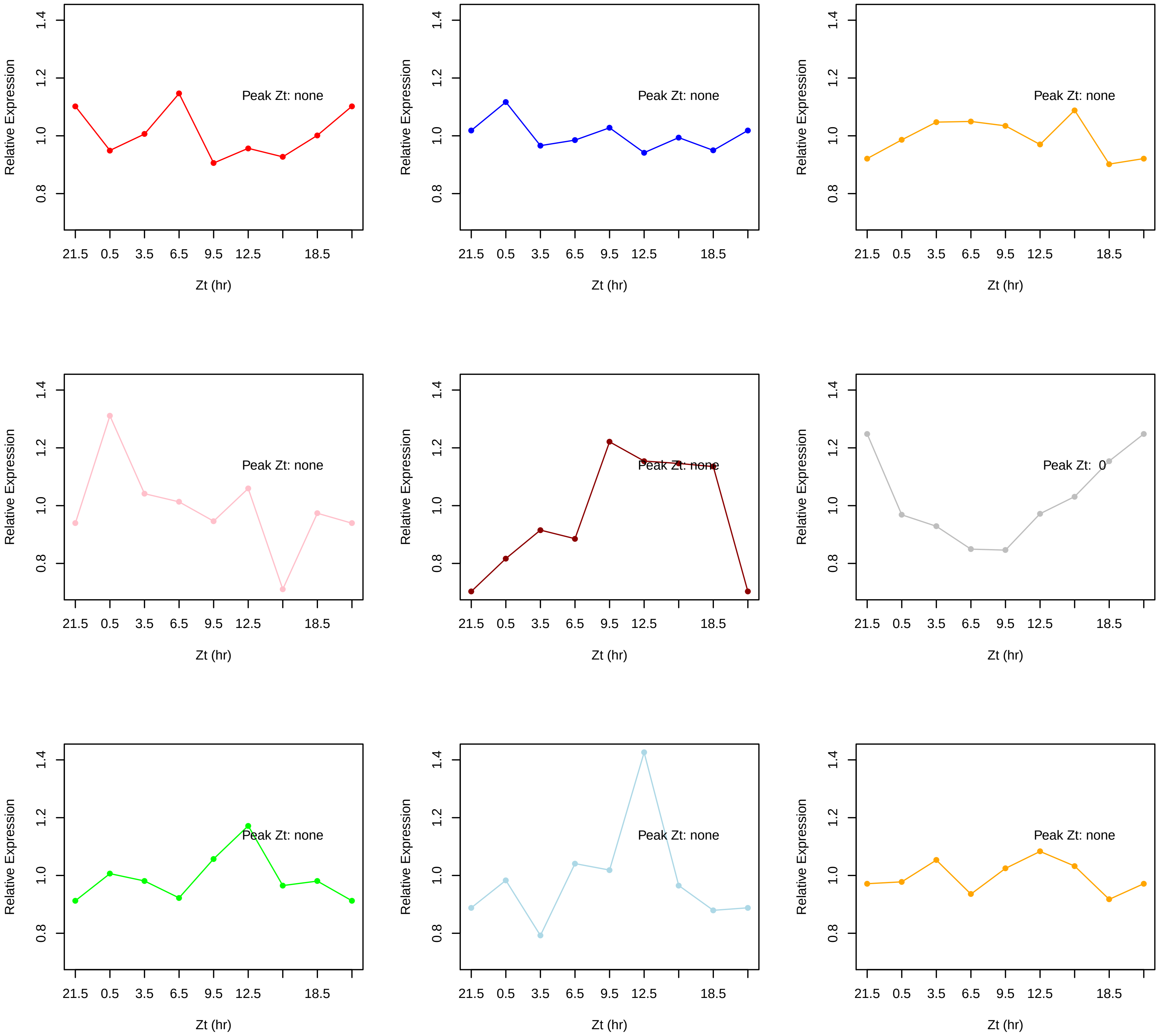

Singeleton: ENSXMAG00000029432 RAP1GDS1

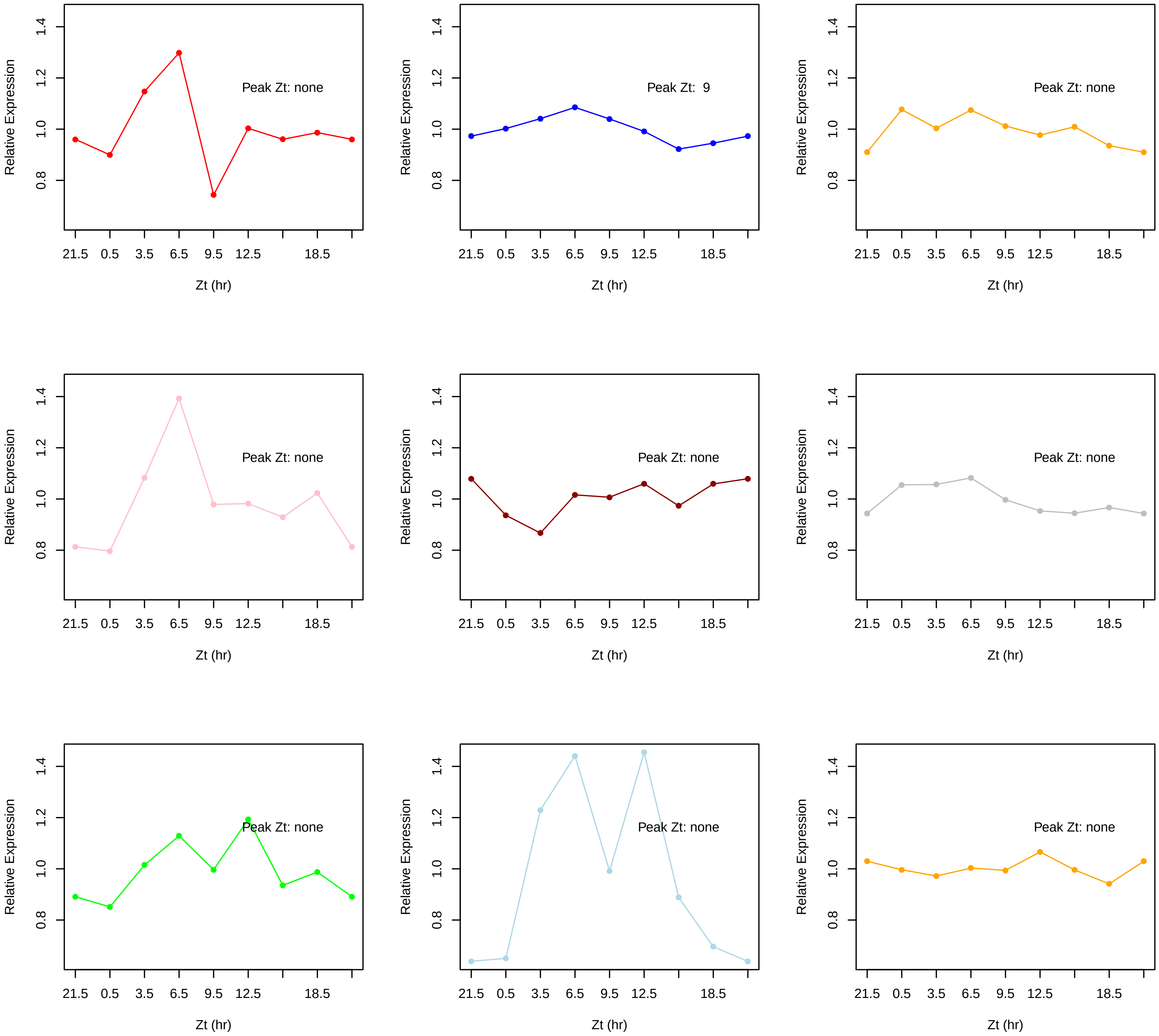

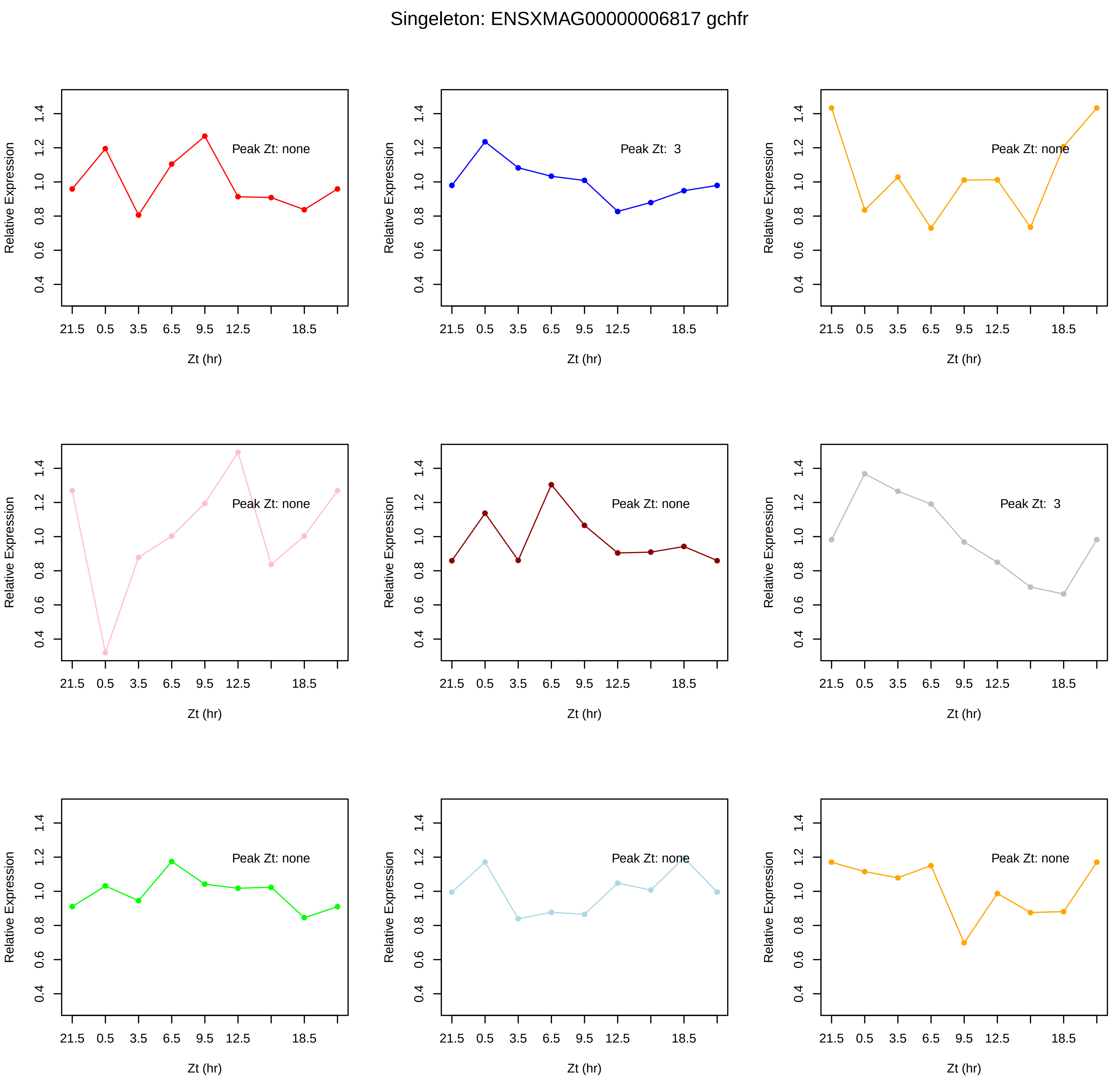

Singeleton: ENSXMAG00000006817 gchfr
Peak Zt: none
Peak Zt: 3
Peak Zt: none
Peak Zt: none
Peak Zt: none
Peak Zt: 3
Peak Zt: none
Peak Zt: none
Peak Zt: none
Relative Expression
Zt (hr)

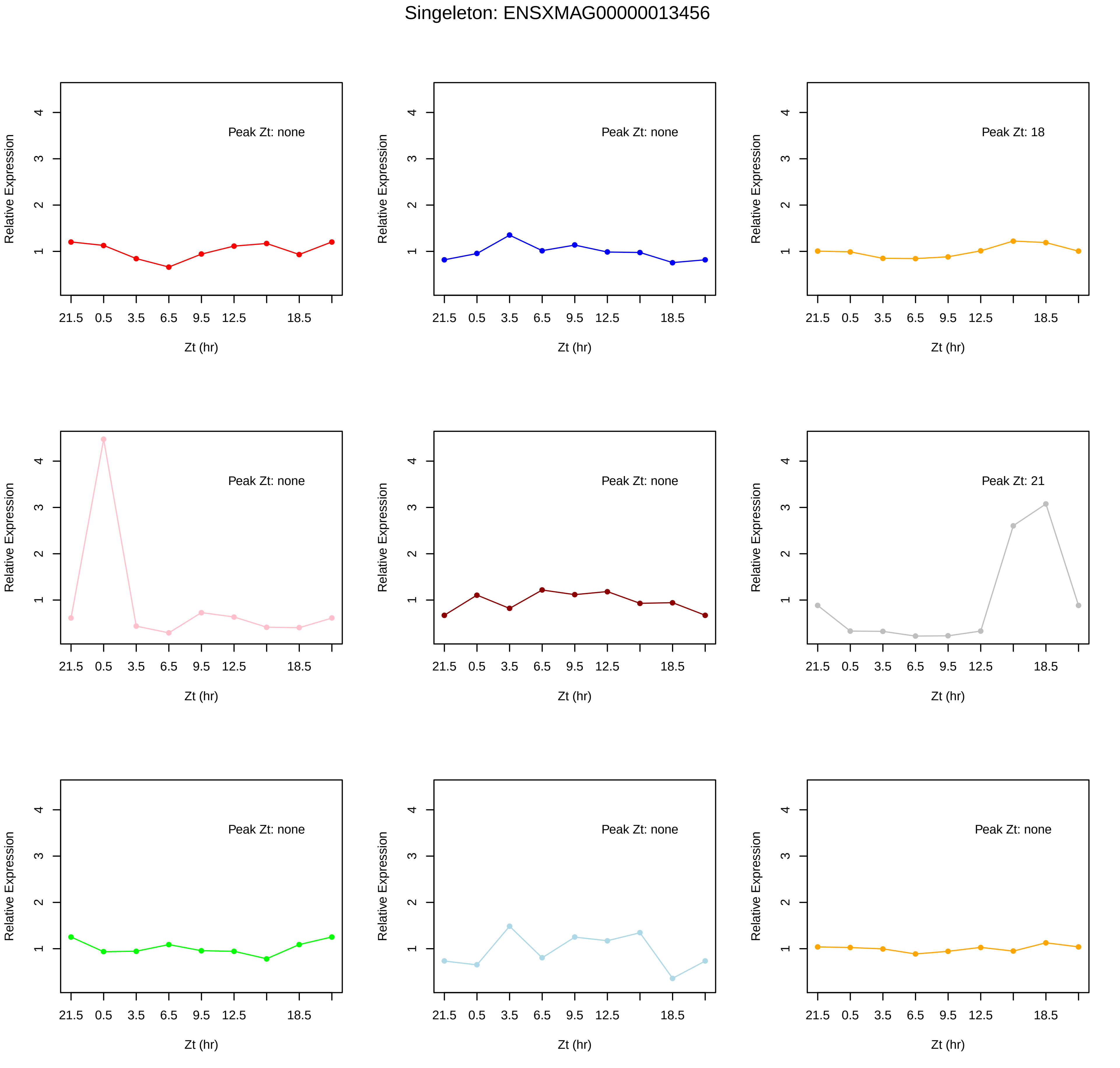
Singeleton: ENSXMAG00000013456
Peak Zt: none
Peak Zt: none
Peak Zt: 18
Peak Zt: none
Peak Zt: none
Peak Zt: 21
Peak Zt: none
Peak Zt: none
Peak Zt: none
Relative Expression
Zt (hr)
21.5 0.5 3.5 6.5 9.5 12.5 18.5

Singeleton: ENSXMAG00000008682 tcap

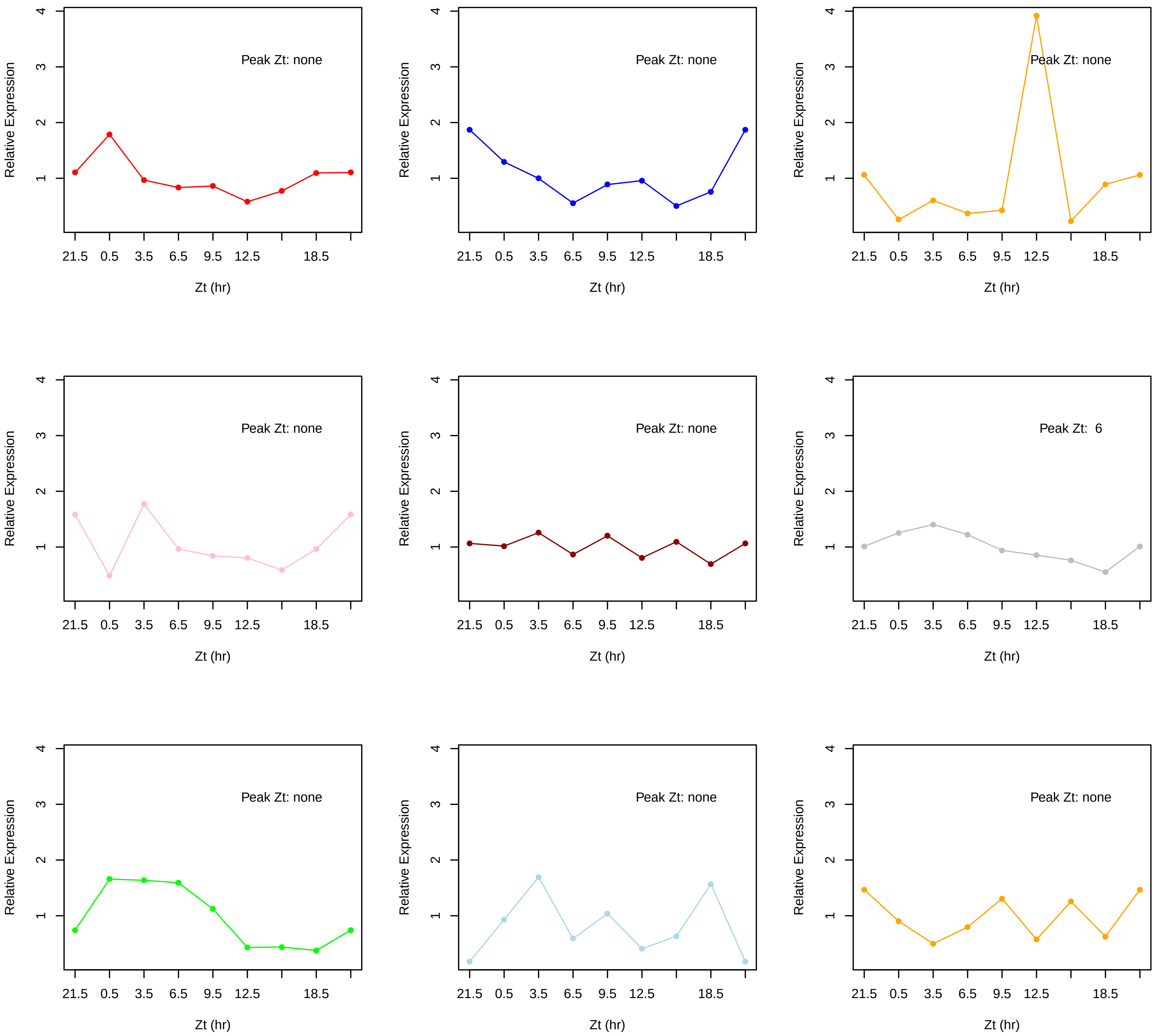

Singeleton: ENSXMAG00000001303 aldh1a3

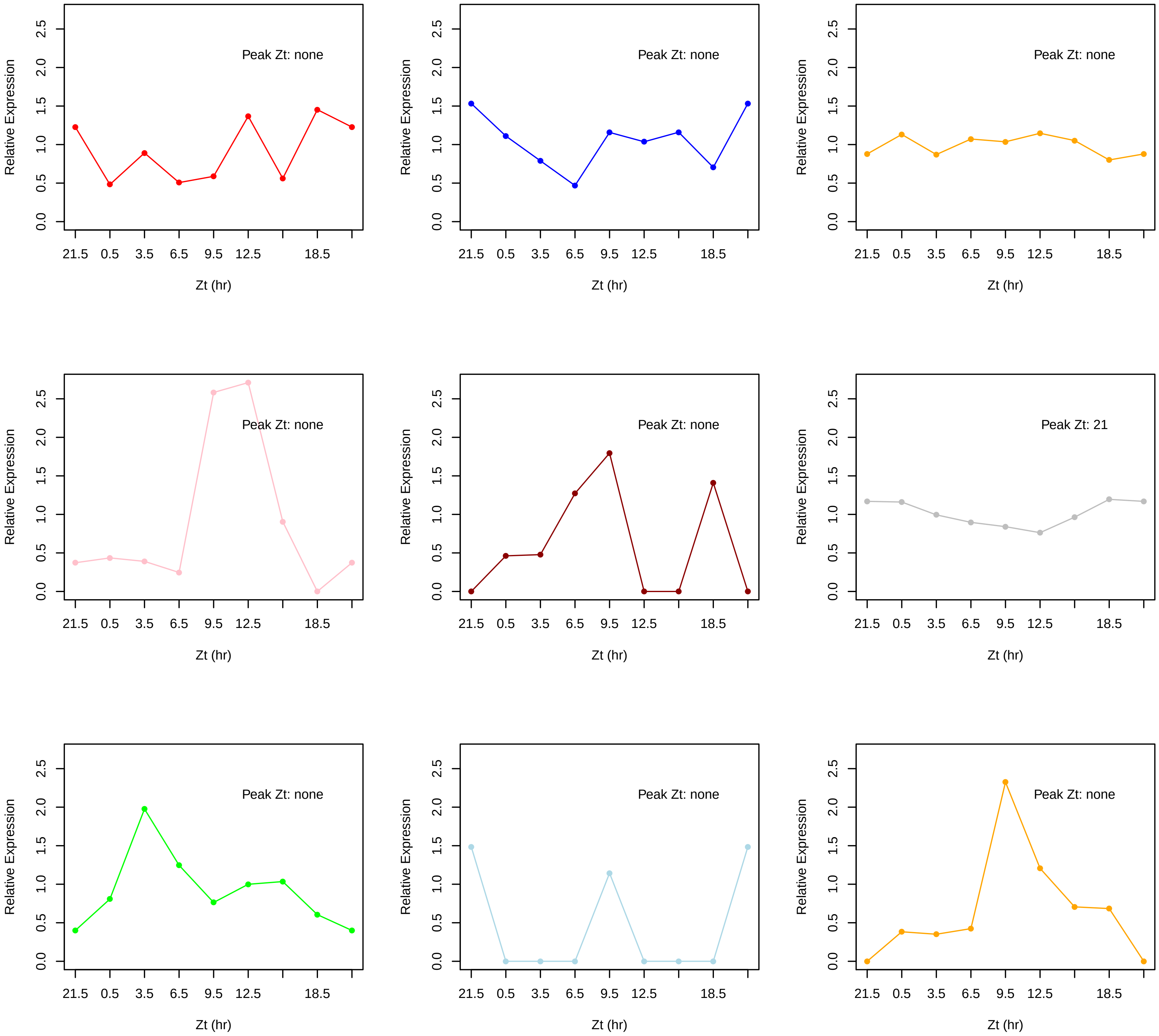

Singeleton: ENSXMAG00000010743 nadk2

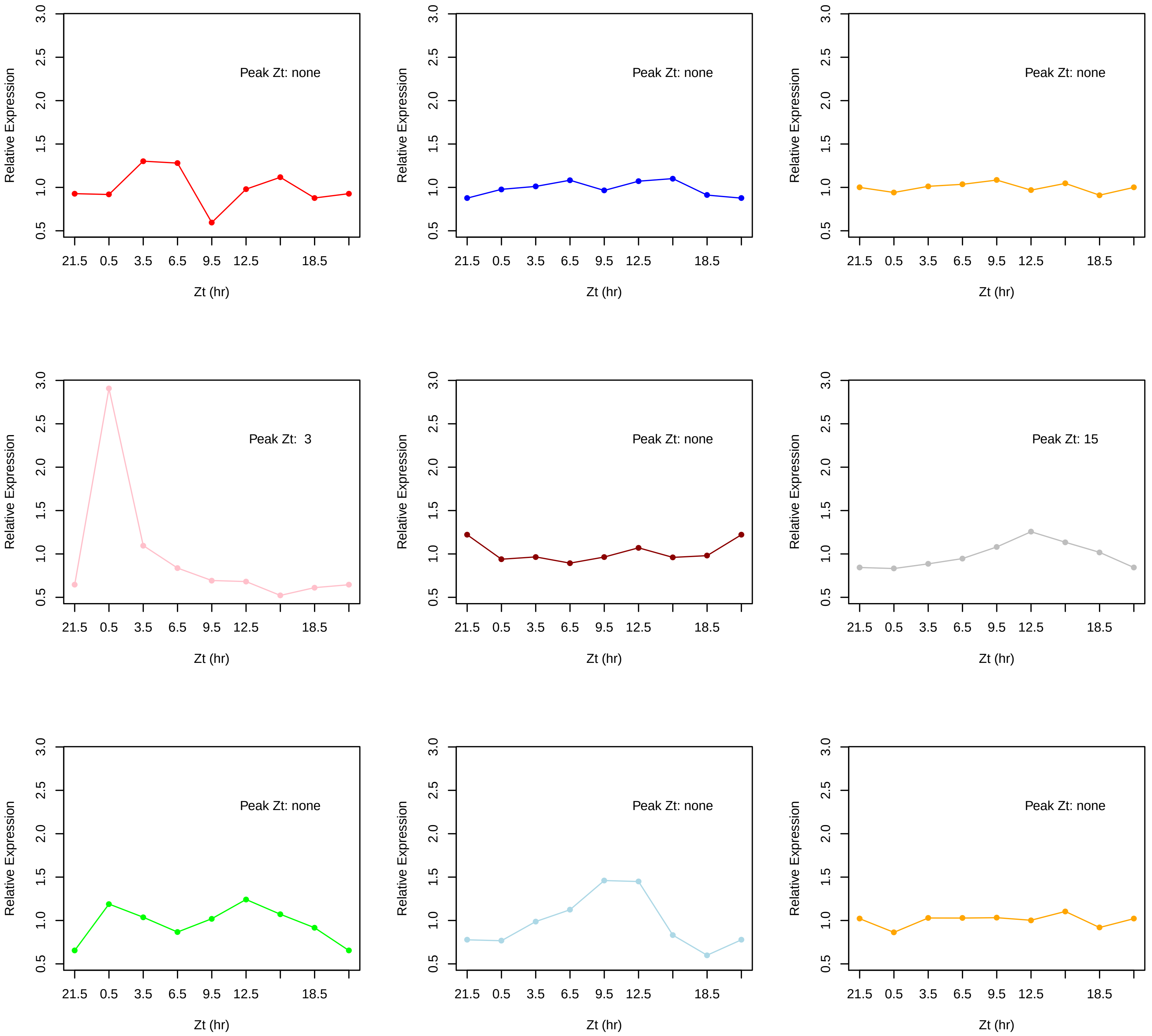

Singeleton: ENSXMAG00000001272 lins1

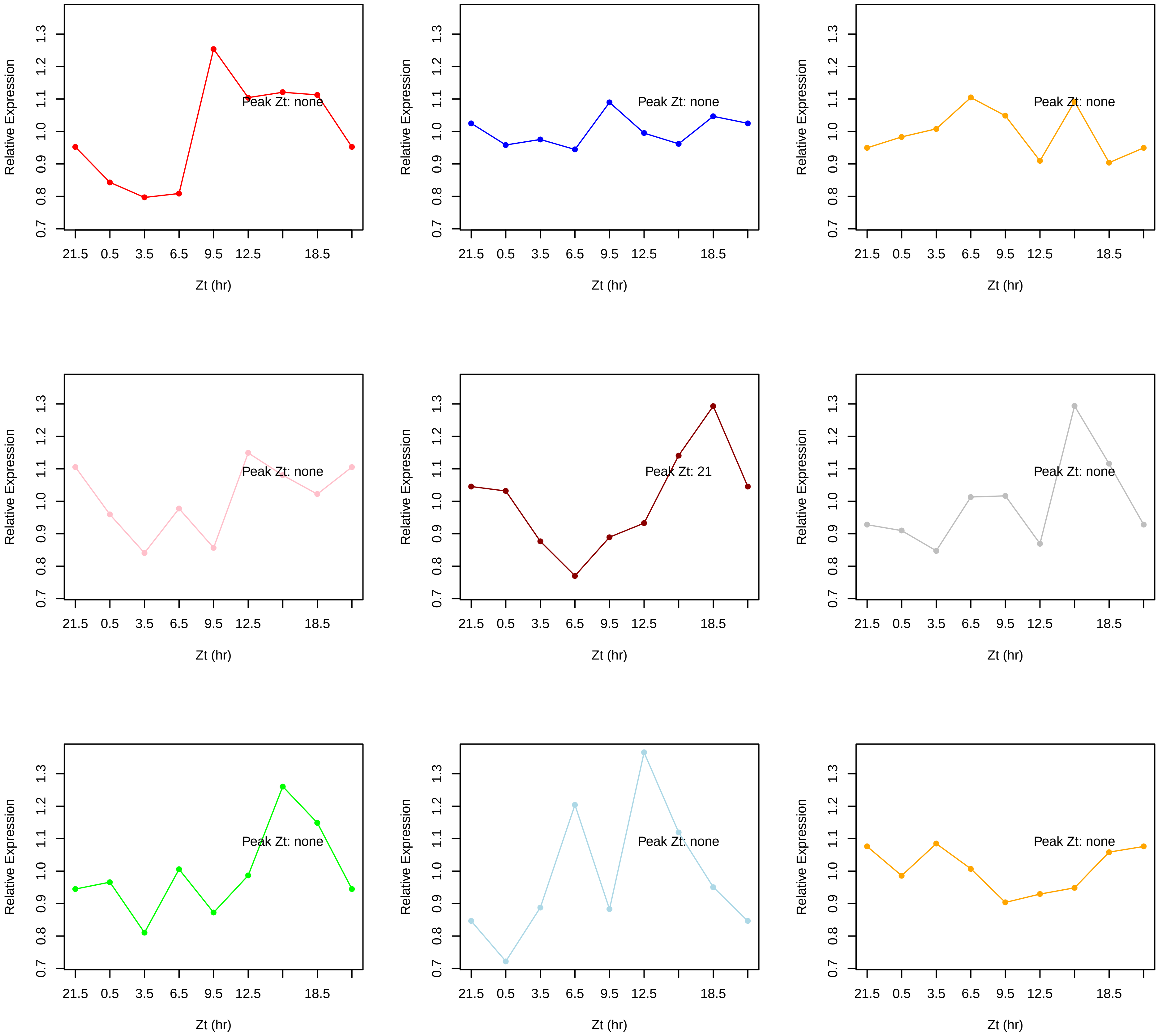

Singleton: ENSXMAG00000013731

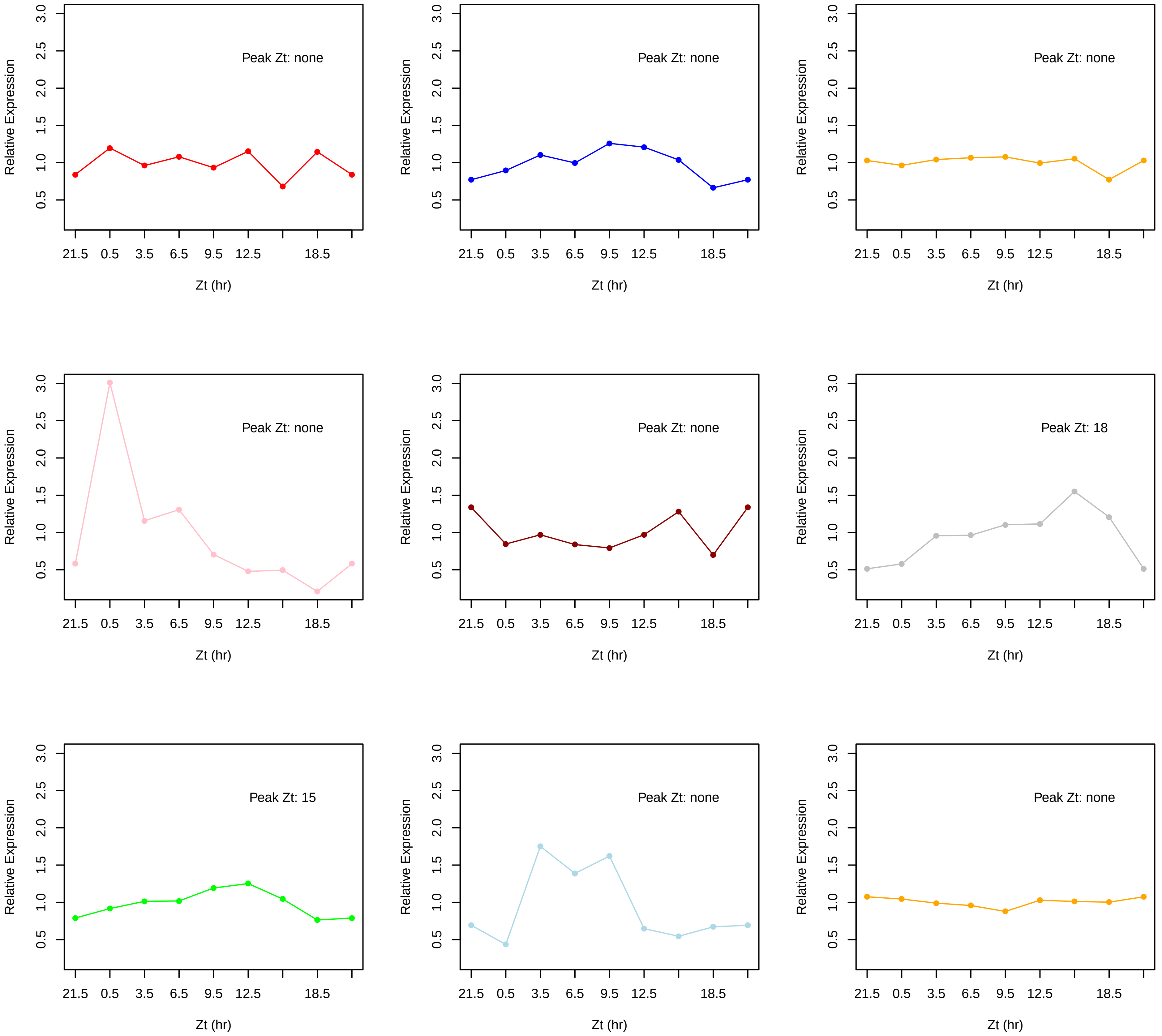

Singleton: ENSXMAG00000001177 adamts17

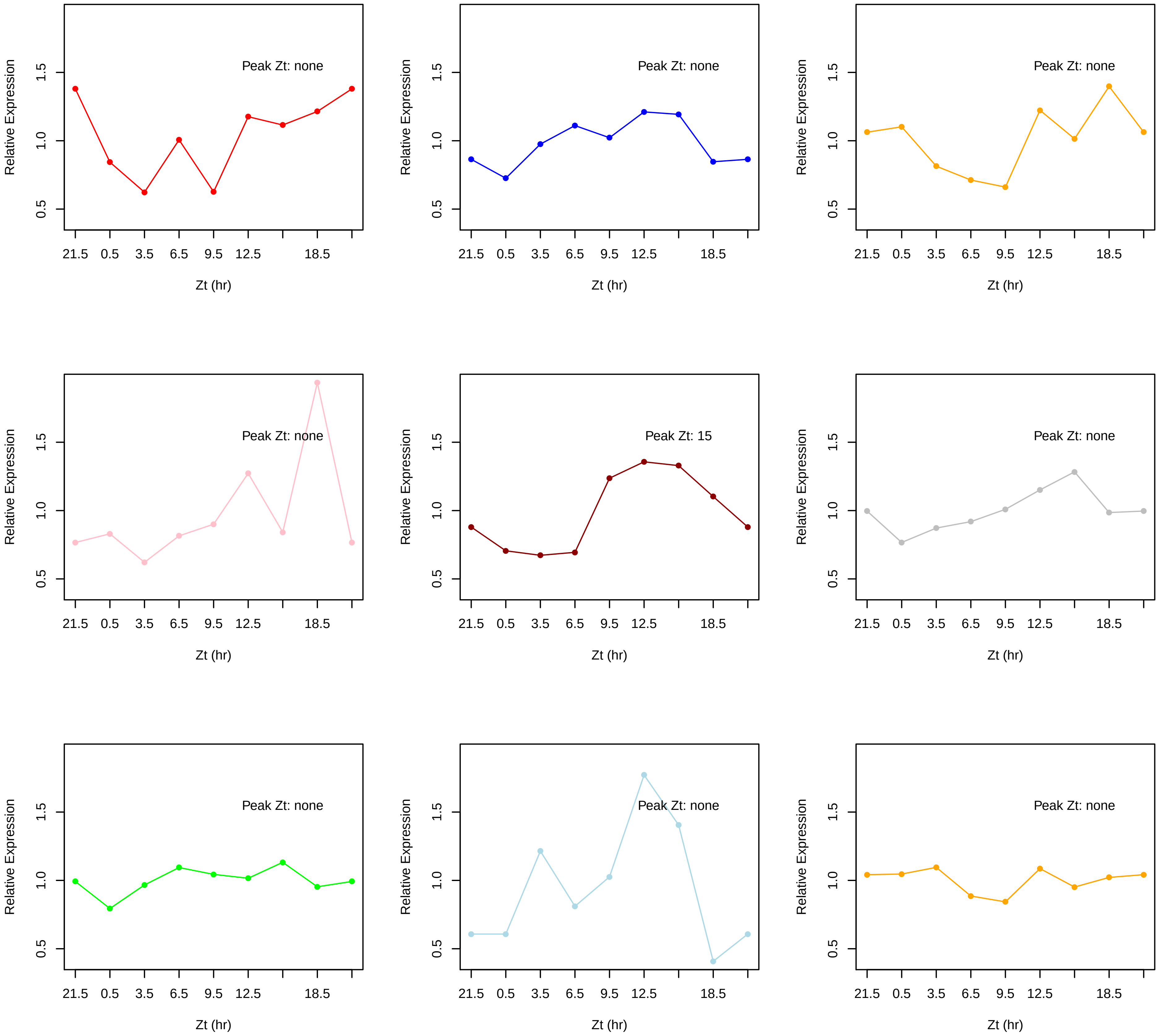

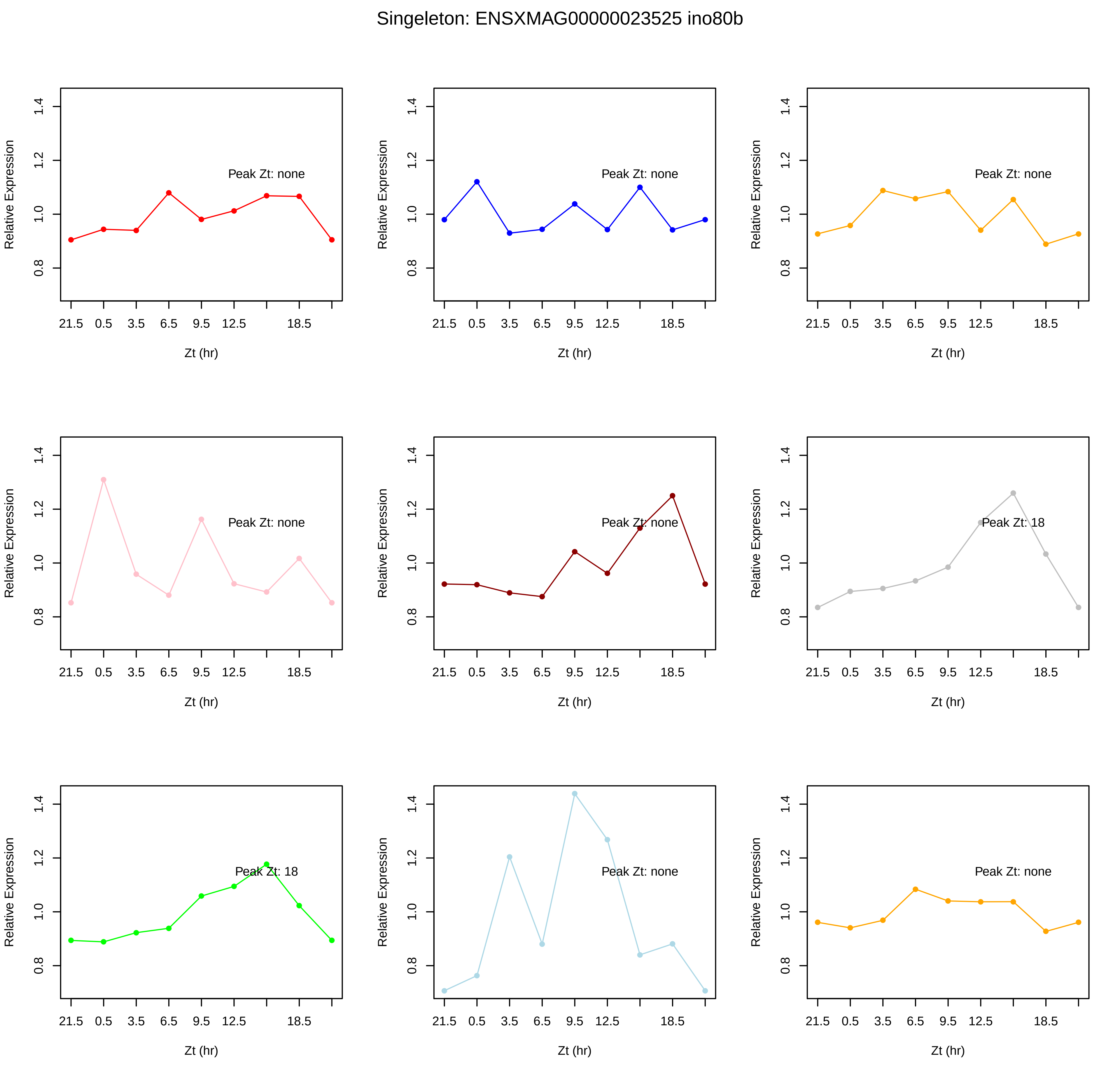

Singeleton: ENSXMAG00000023525 ino80b
Relative Expression
Zt (hr)
21.5
0.5
3.5
6.5
9.5
12.5
18.5
0.8
1.0
1.2
1.4
Peak Zt: none
Peak Zt: 18

Singeleton: ENSXMAG00000006673 akt1

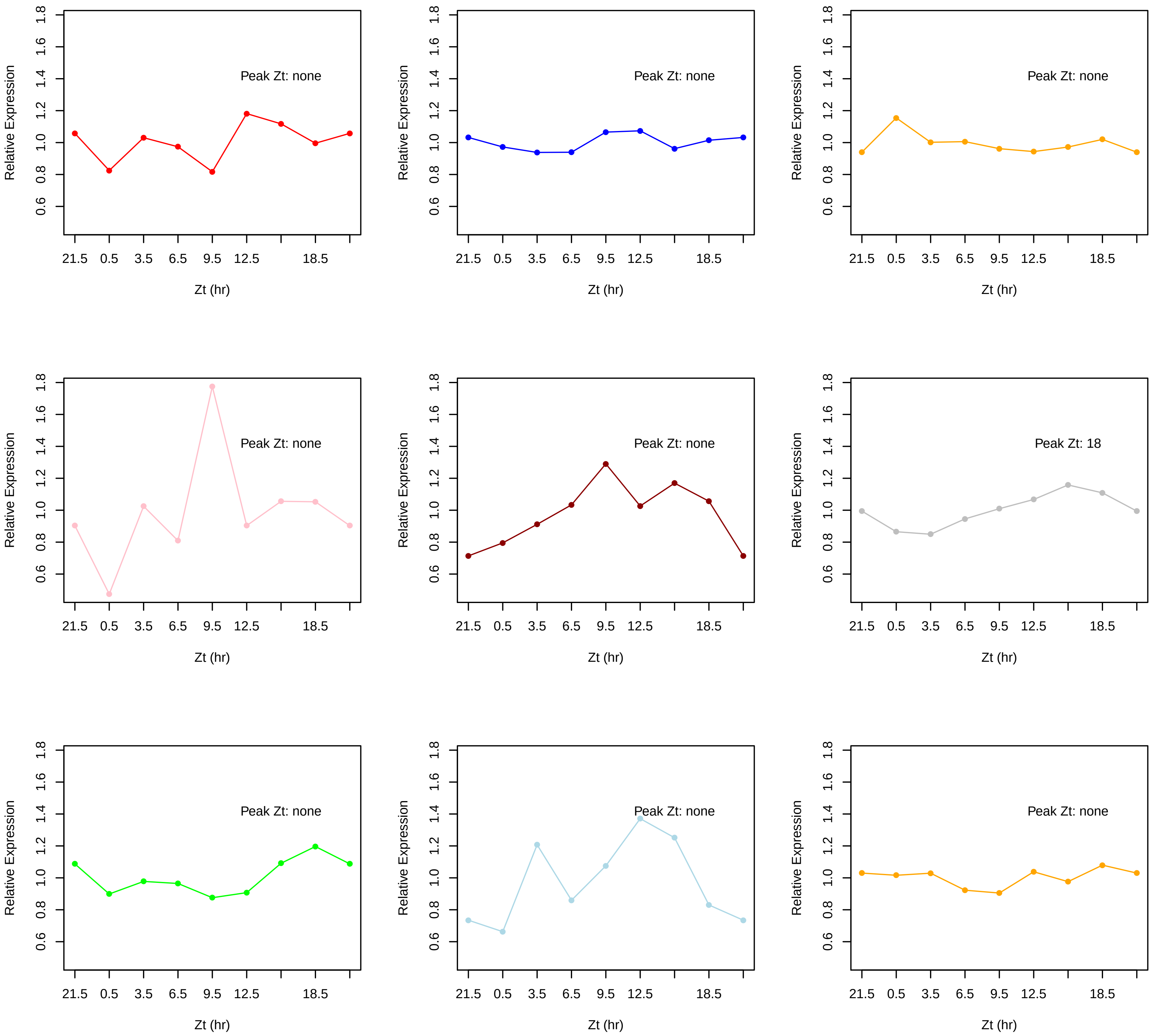

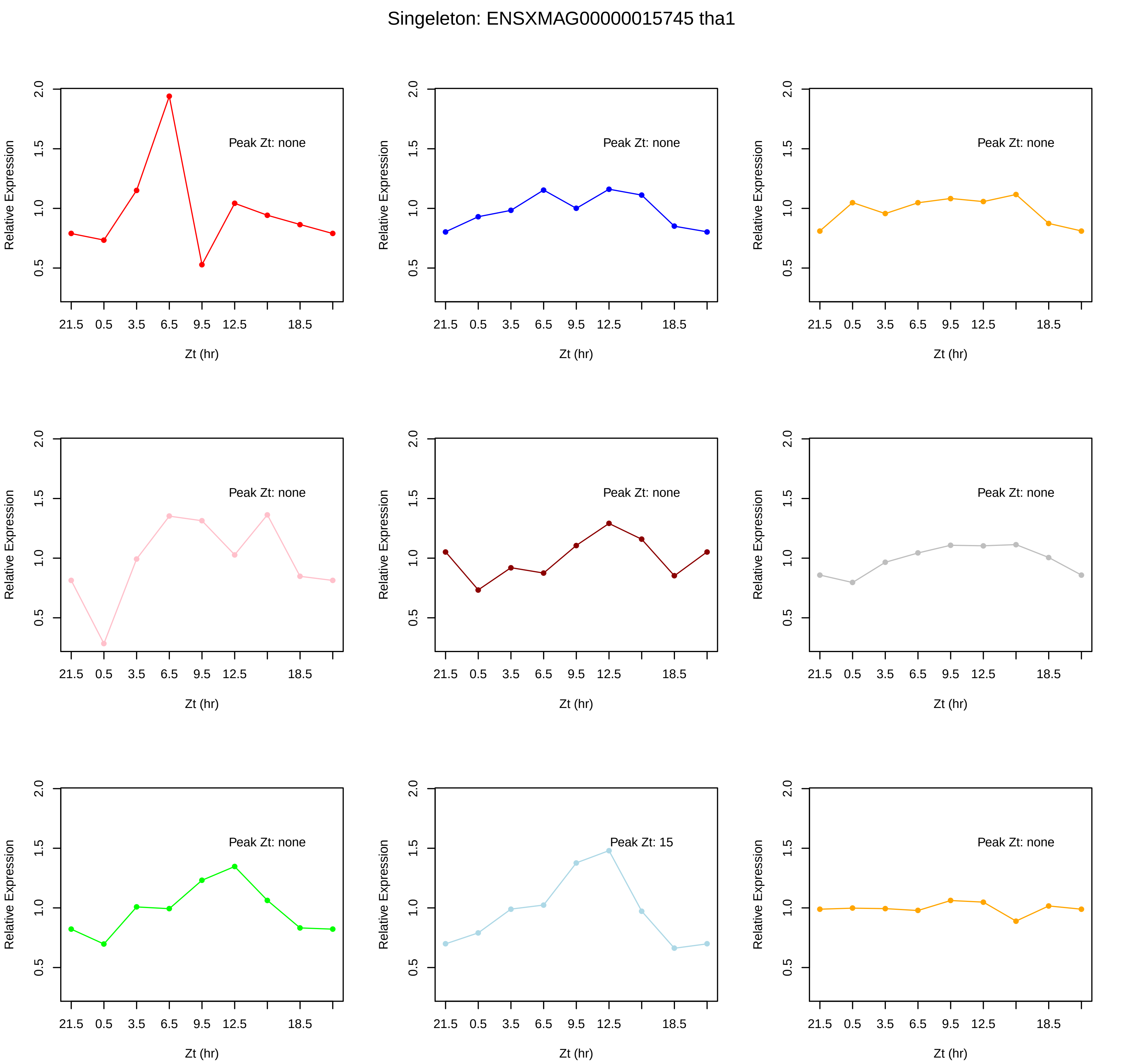
Singeleton: ENSXMAG00000015745 tha1
Peak Zt: none
Peak Zt: 15
Relative Expression
Zt (hr)
21.5 0.5 3.5 6.5 9.5 12.5 18.5
0.5 1.0 1.5 2.0

Singeleton: ENSXMAG00000020009 mthfs

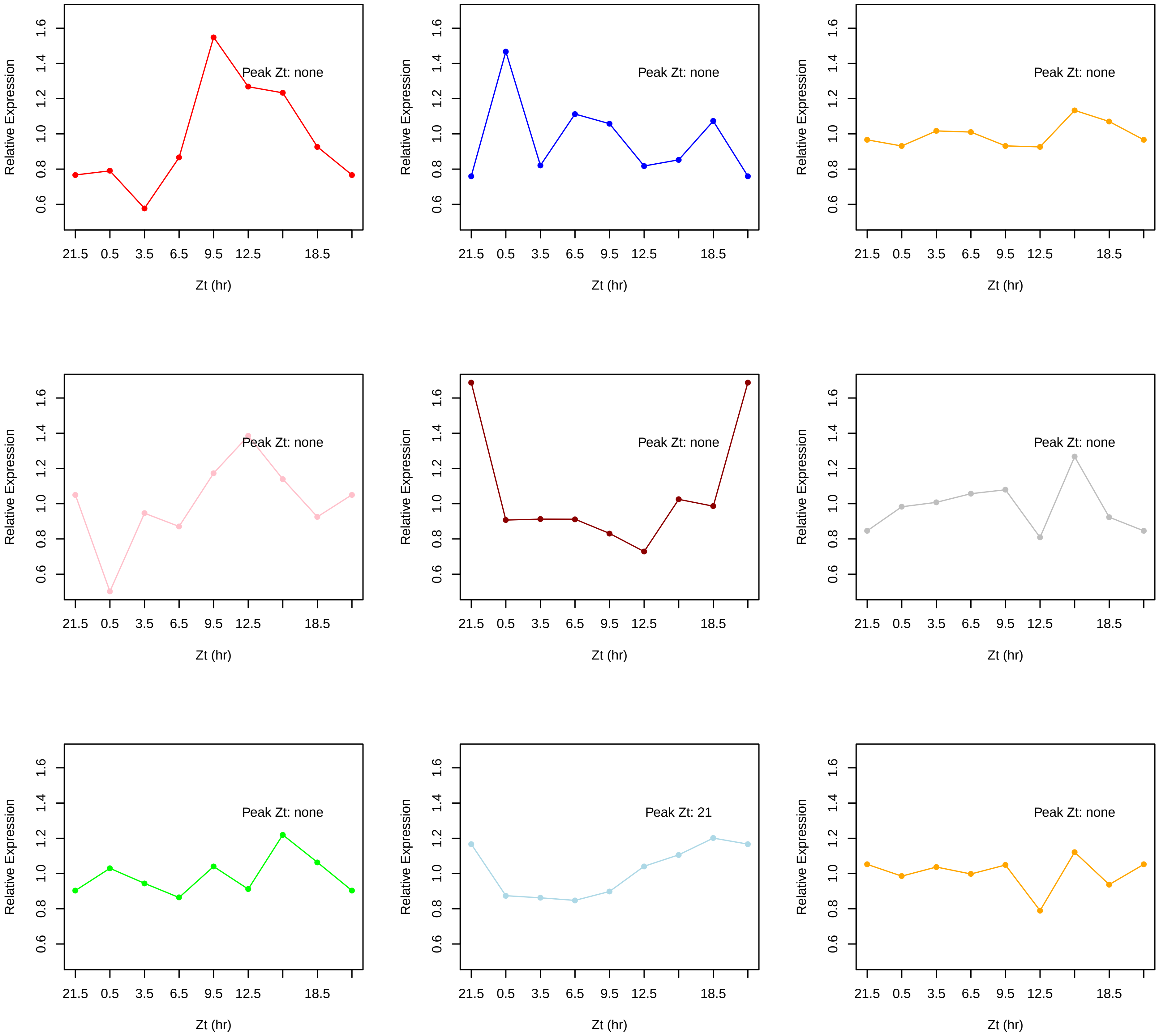

Singleton: ENSXMAG00000013437 CUTC

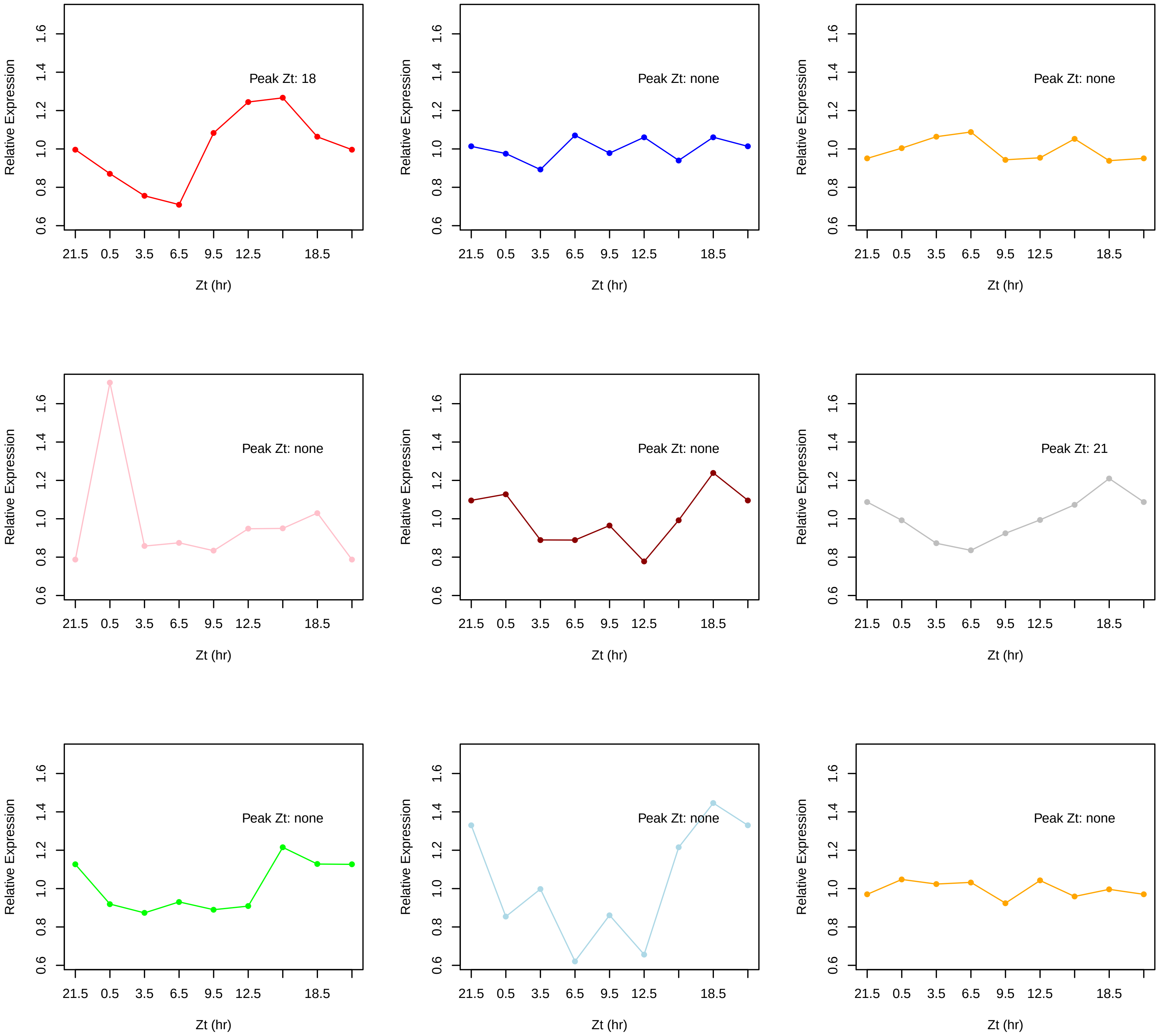

Singeleton: ENSXMAG00000006644 cep170b

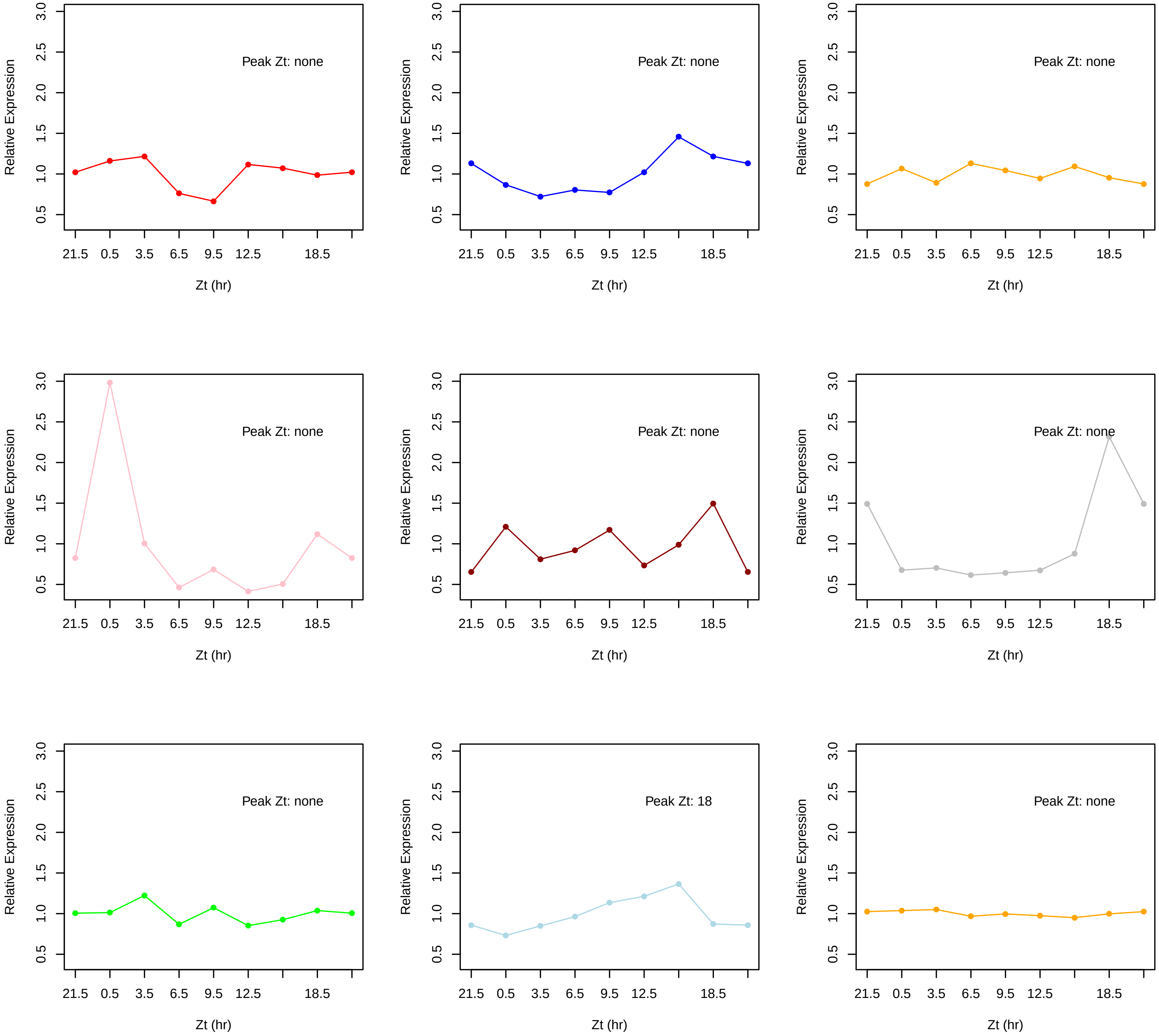

Singleton: ENSXMAG00000029073 fbxl20

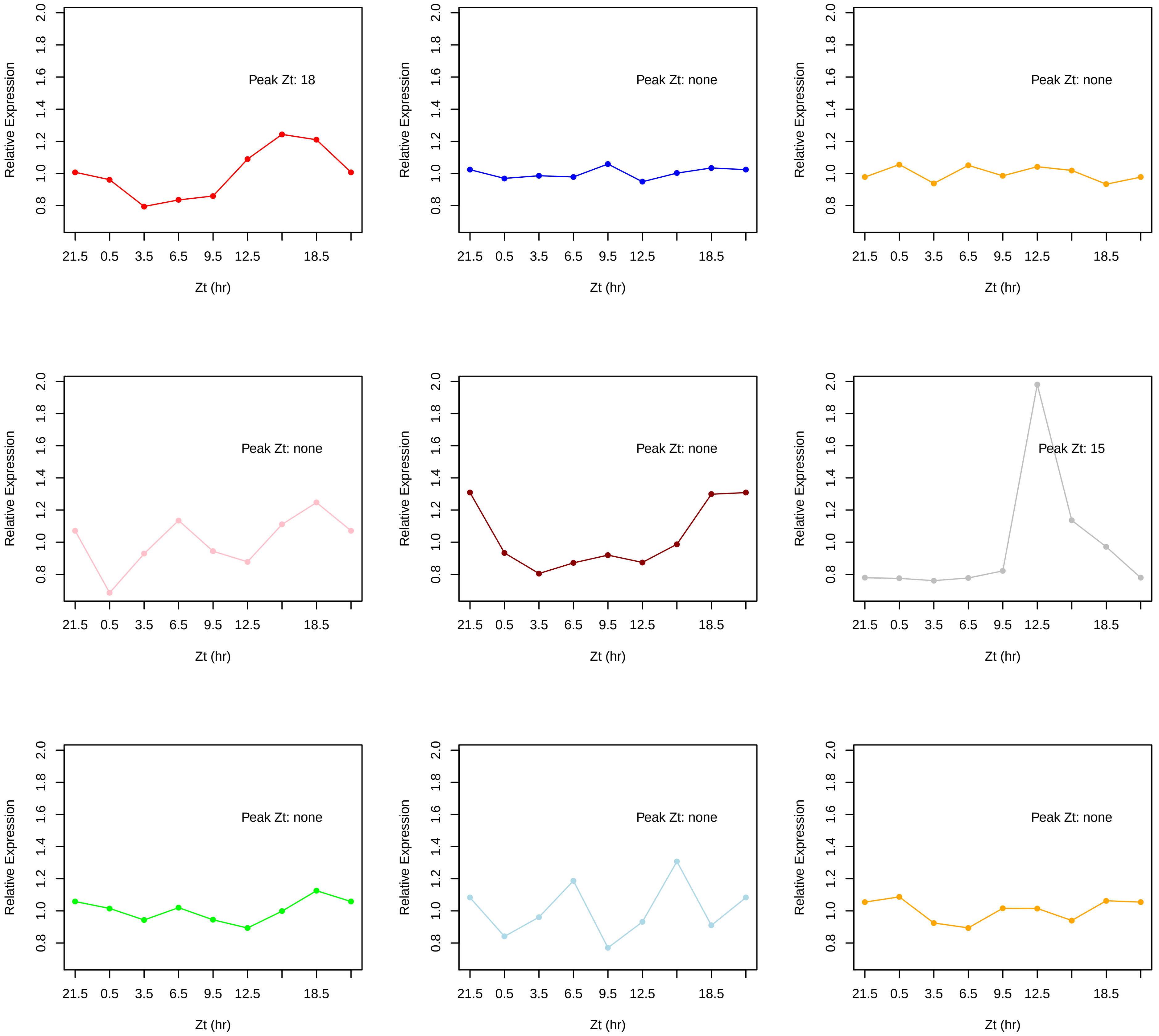

Singeleton: ENSXMAG00000013556 capslb

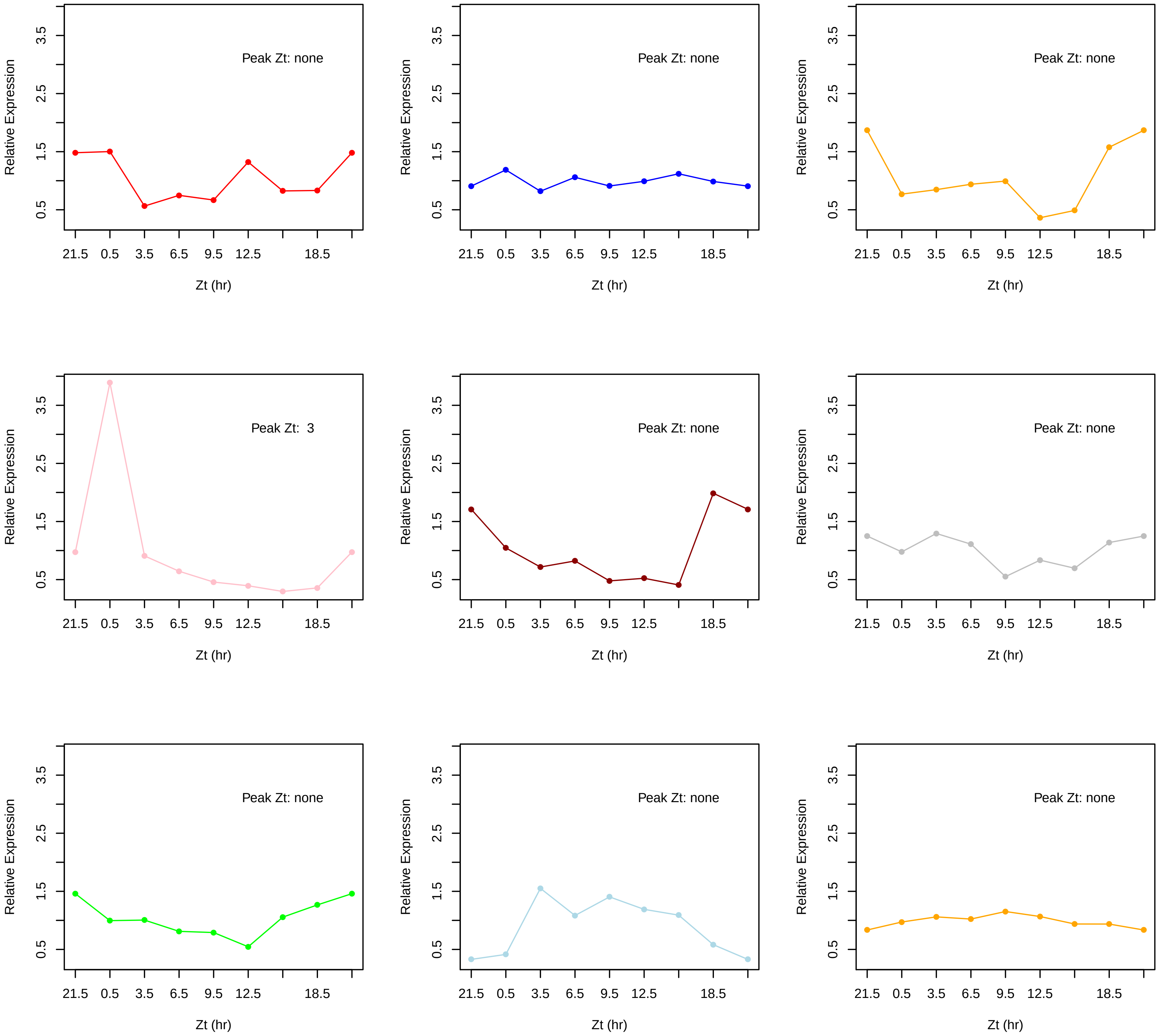

Singeleton: ENSXMAG00000000138 slc39a6

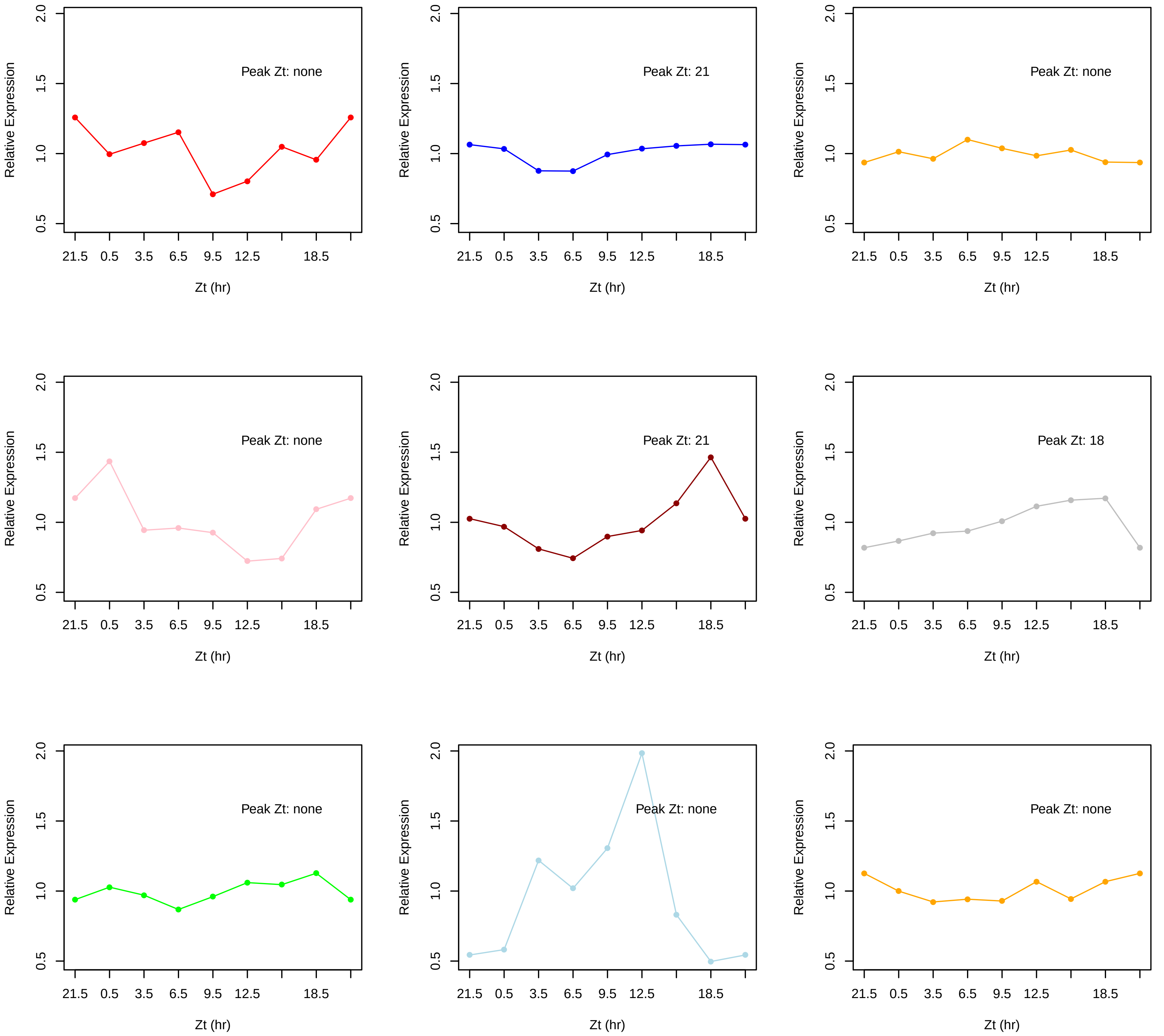

Singeleton: ENSXMAG00000006627

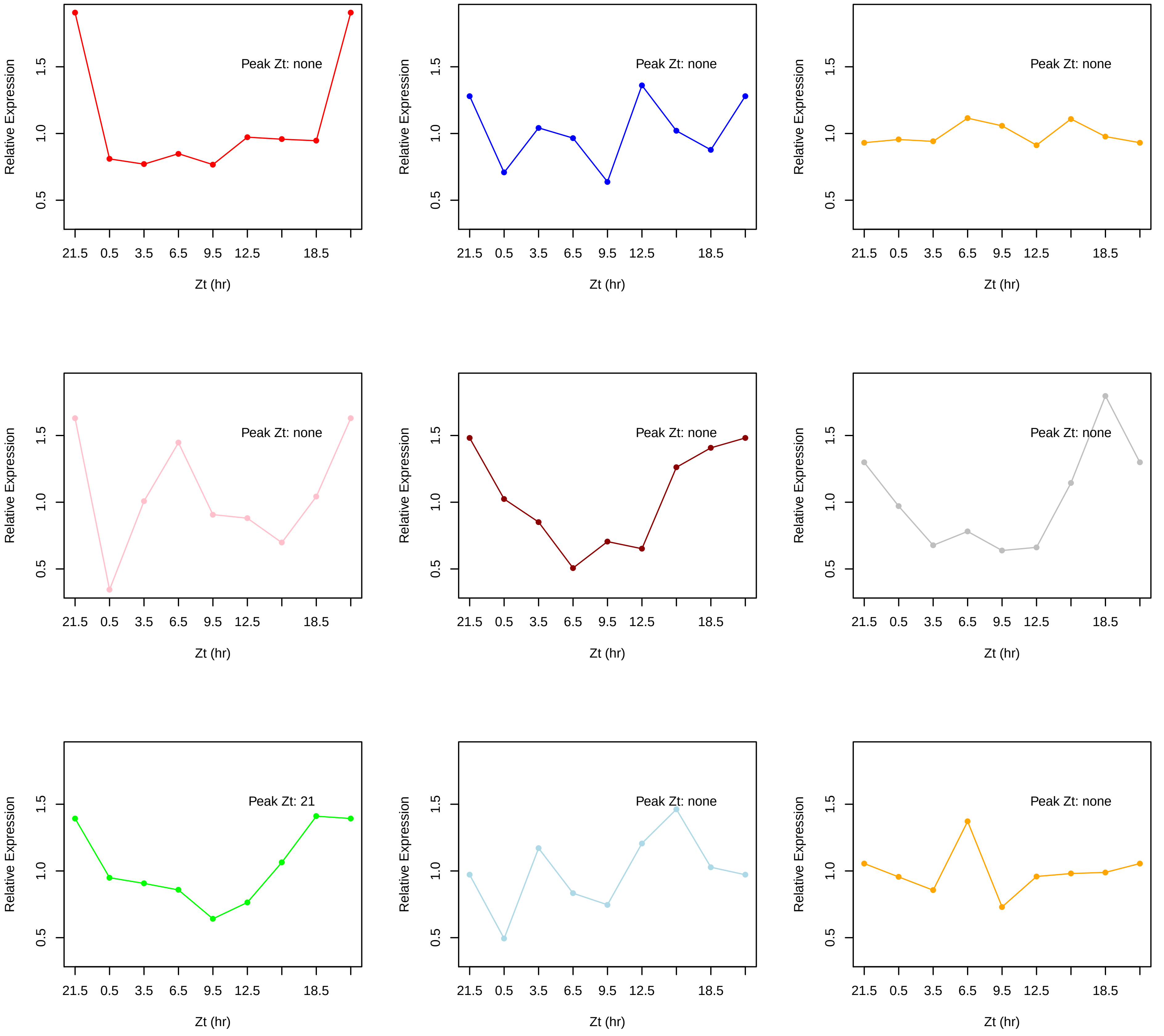

Singeleton: ENSXMAG00000016437 tepsin

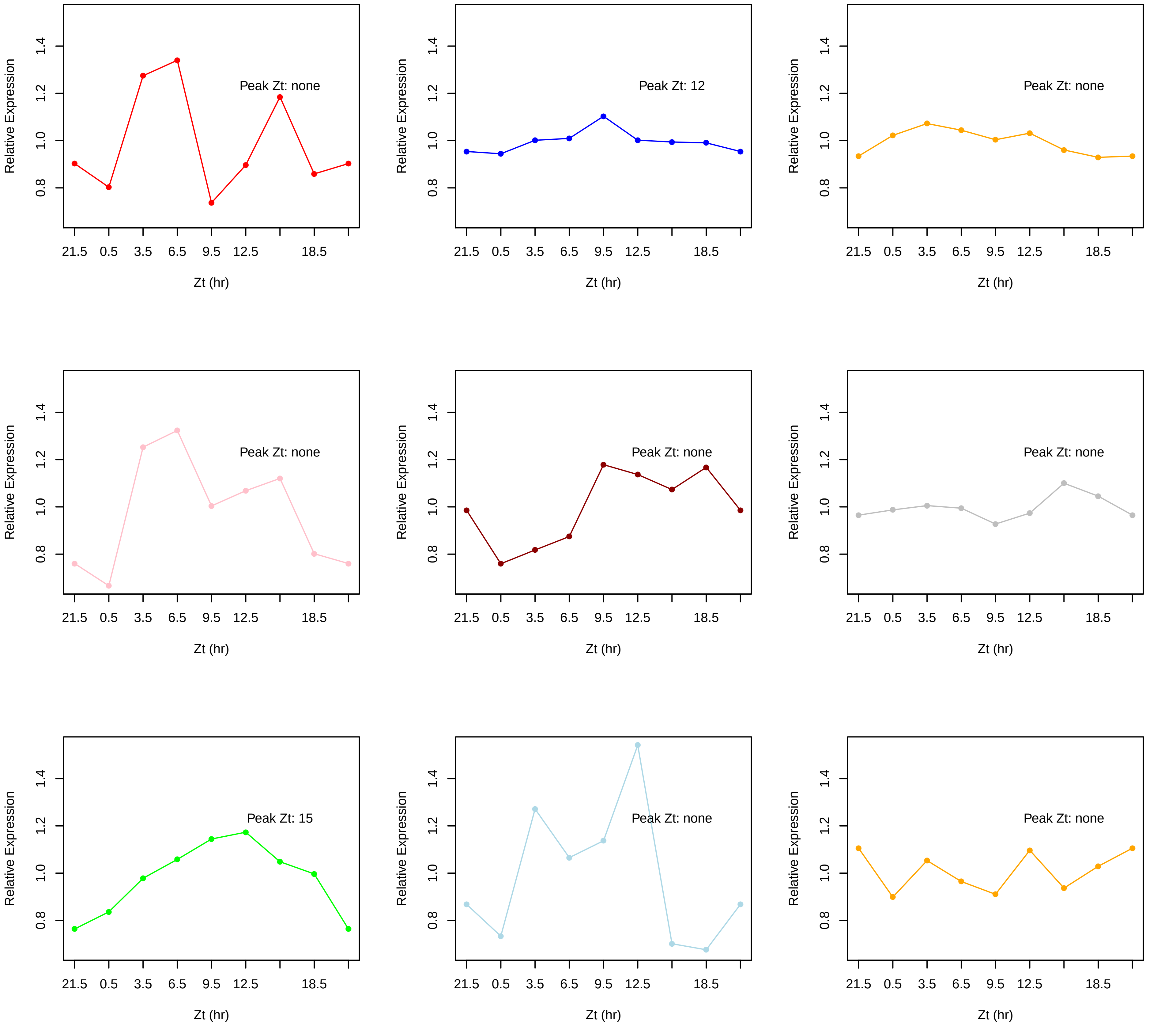

Singeleton: ENSXMAG00000014315 CDH18

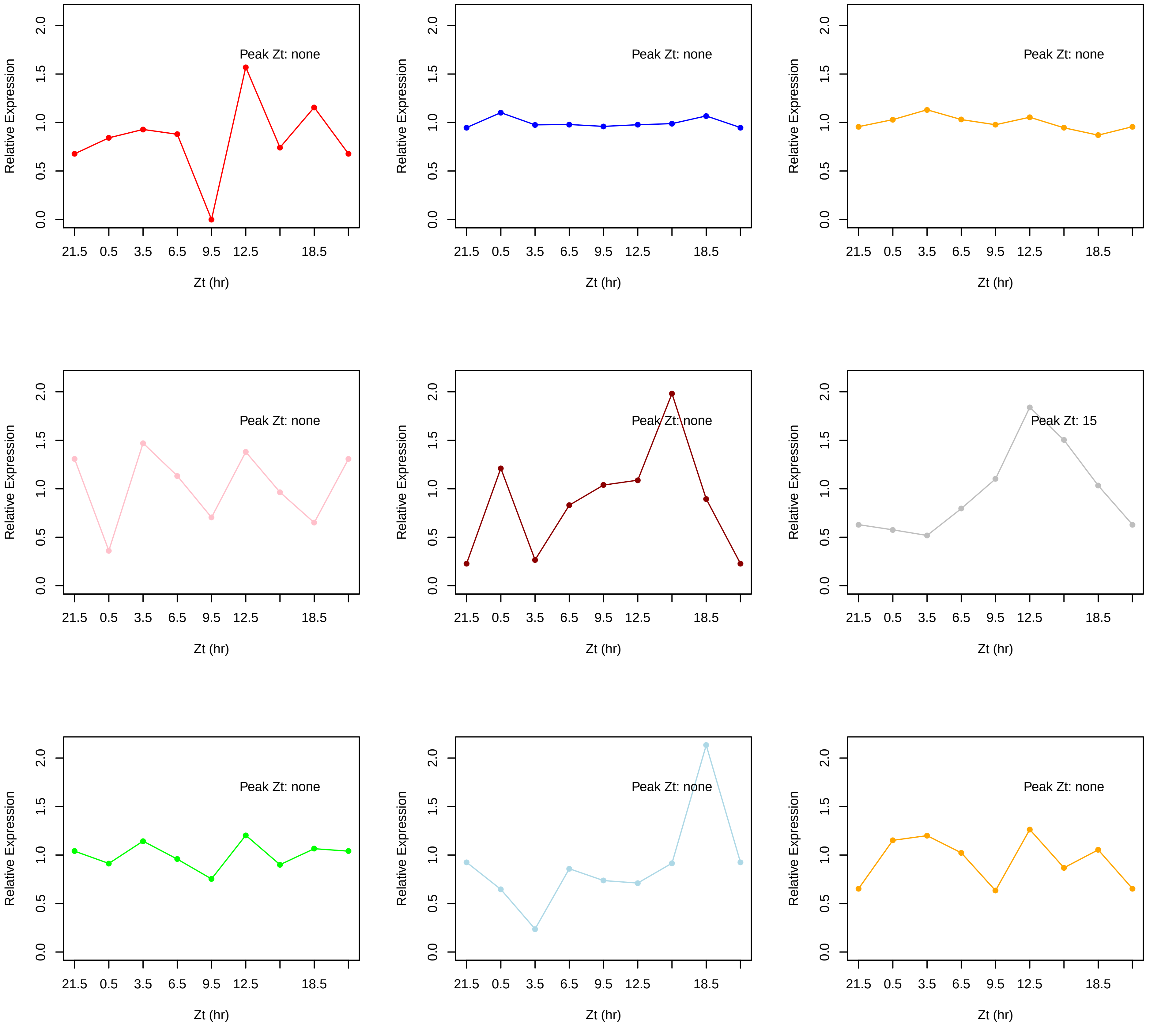

Singeleton: ENSXMAG00000012653 melk

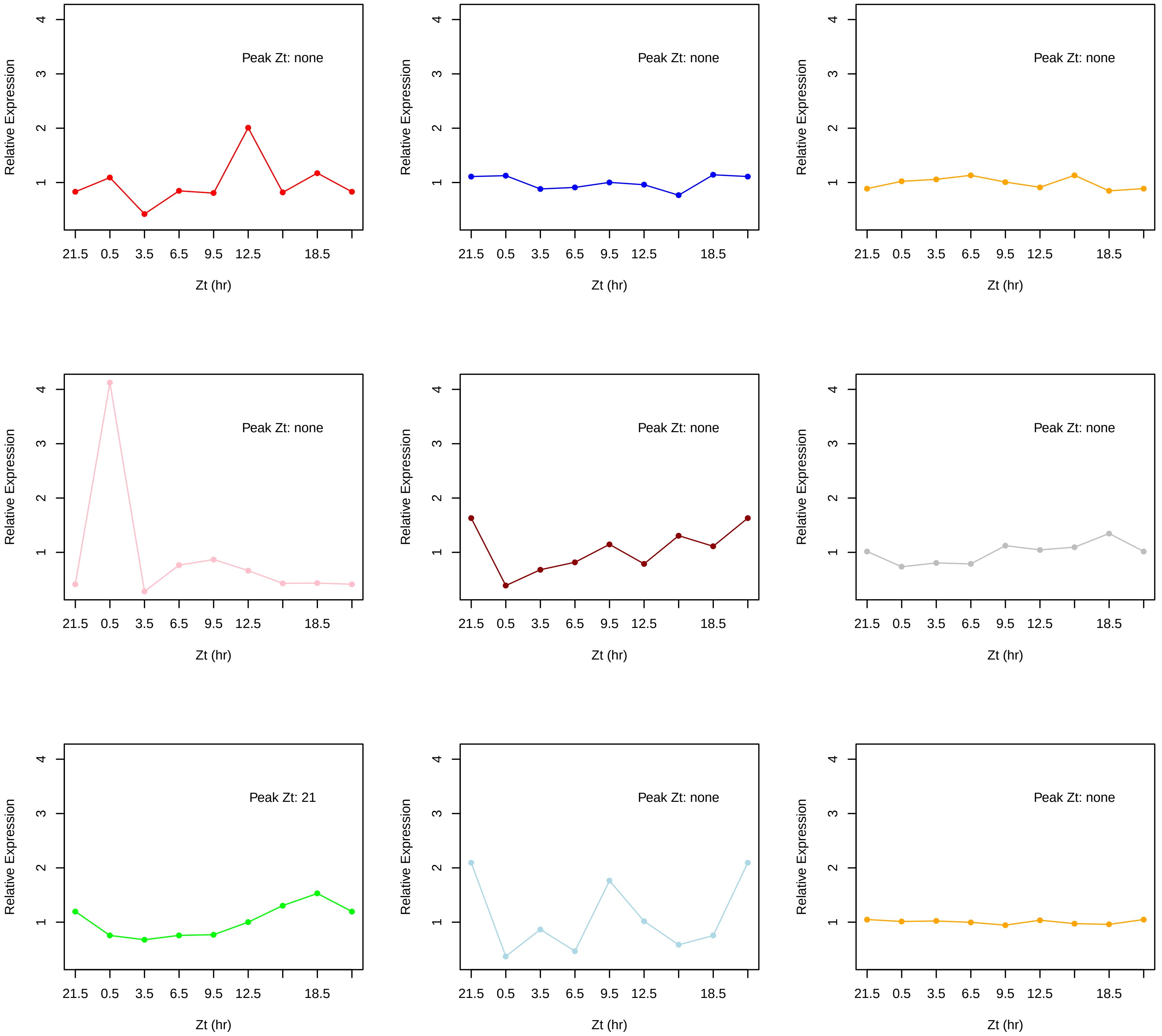

Singeleton: ENSXMAG00000027531 lcor

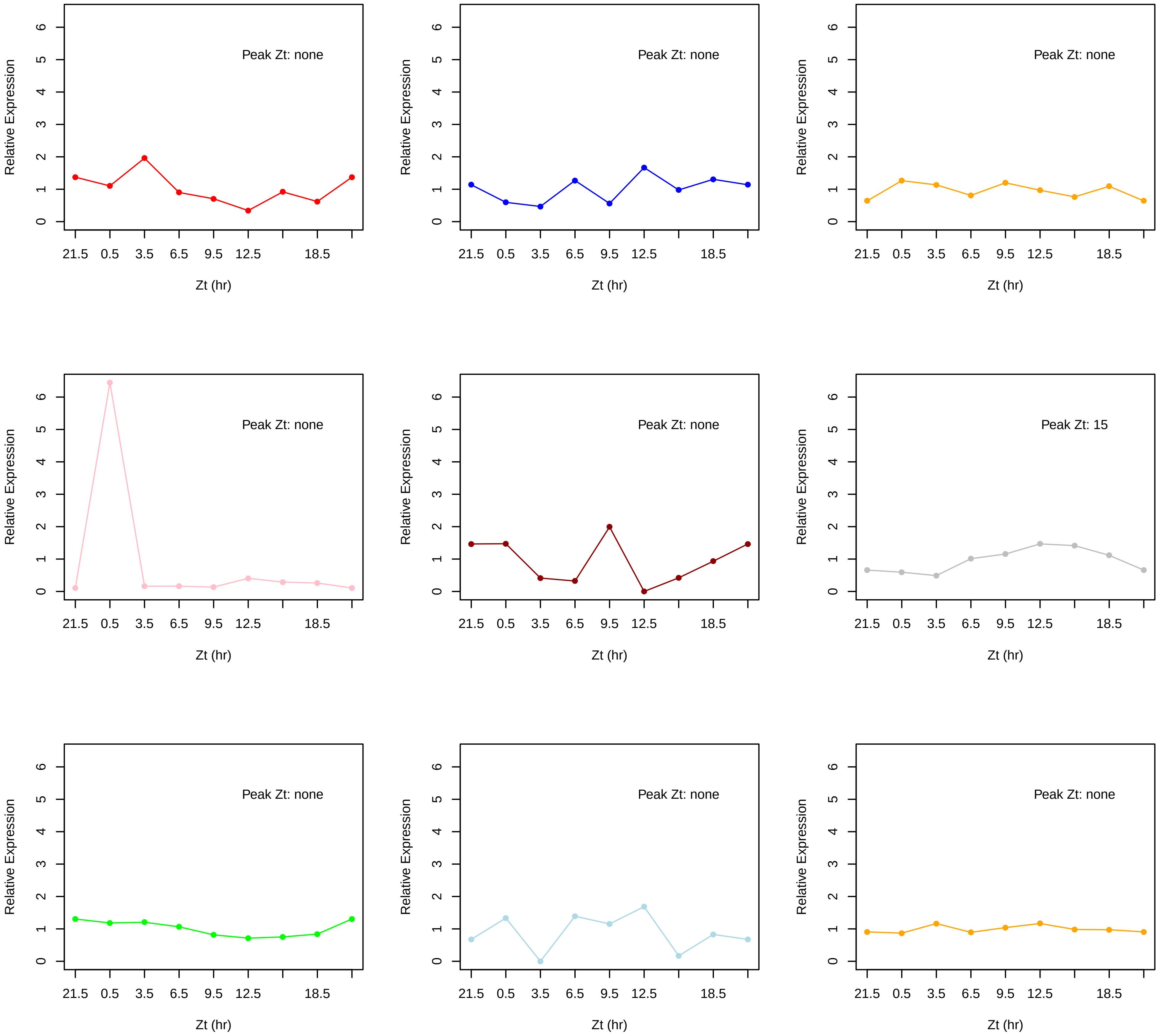

Singleton: ENSXMAG00000016078 mesd

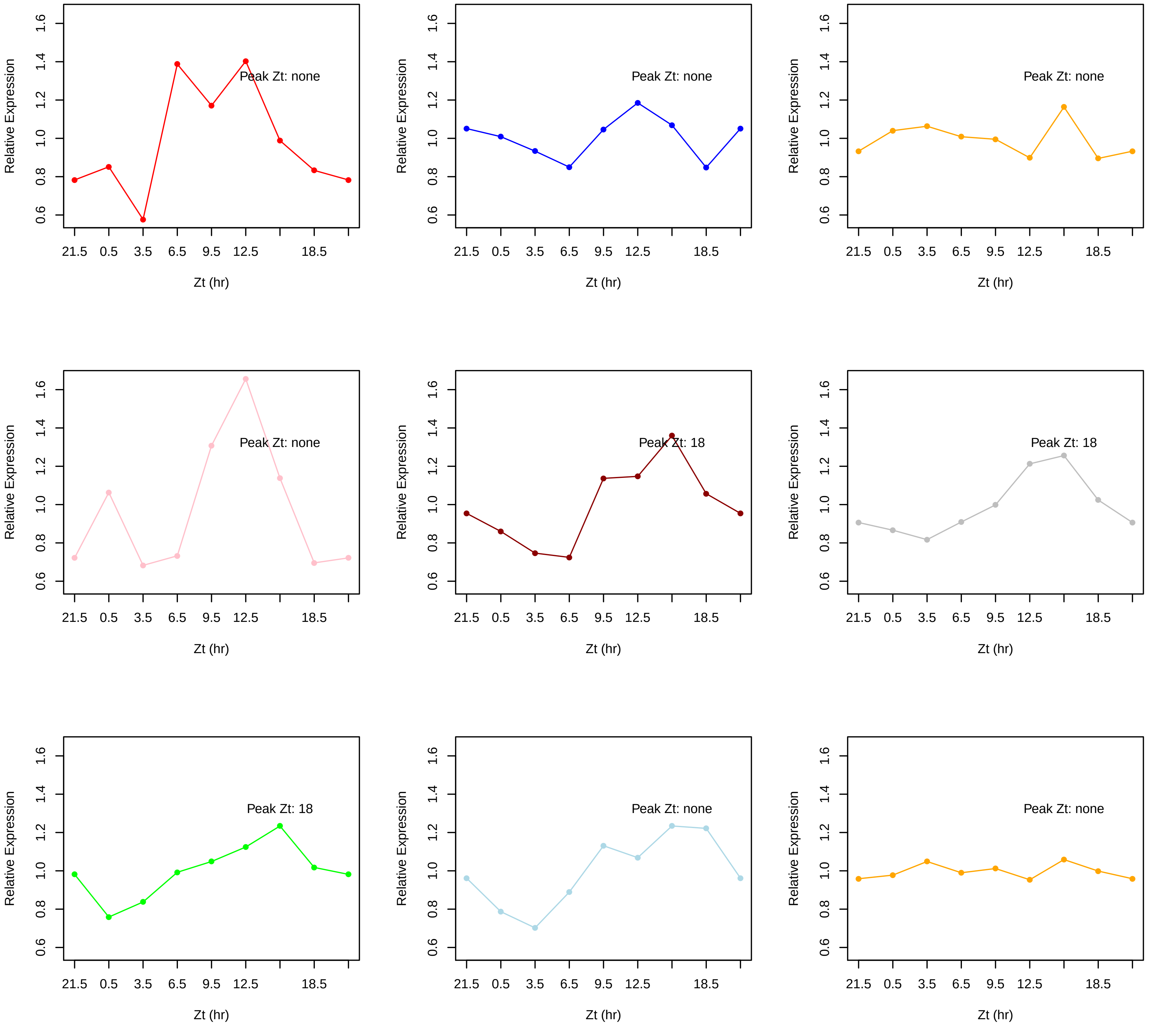

Singeleton: ENSXMAG00000006569 zfyve19

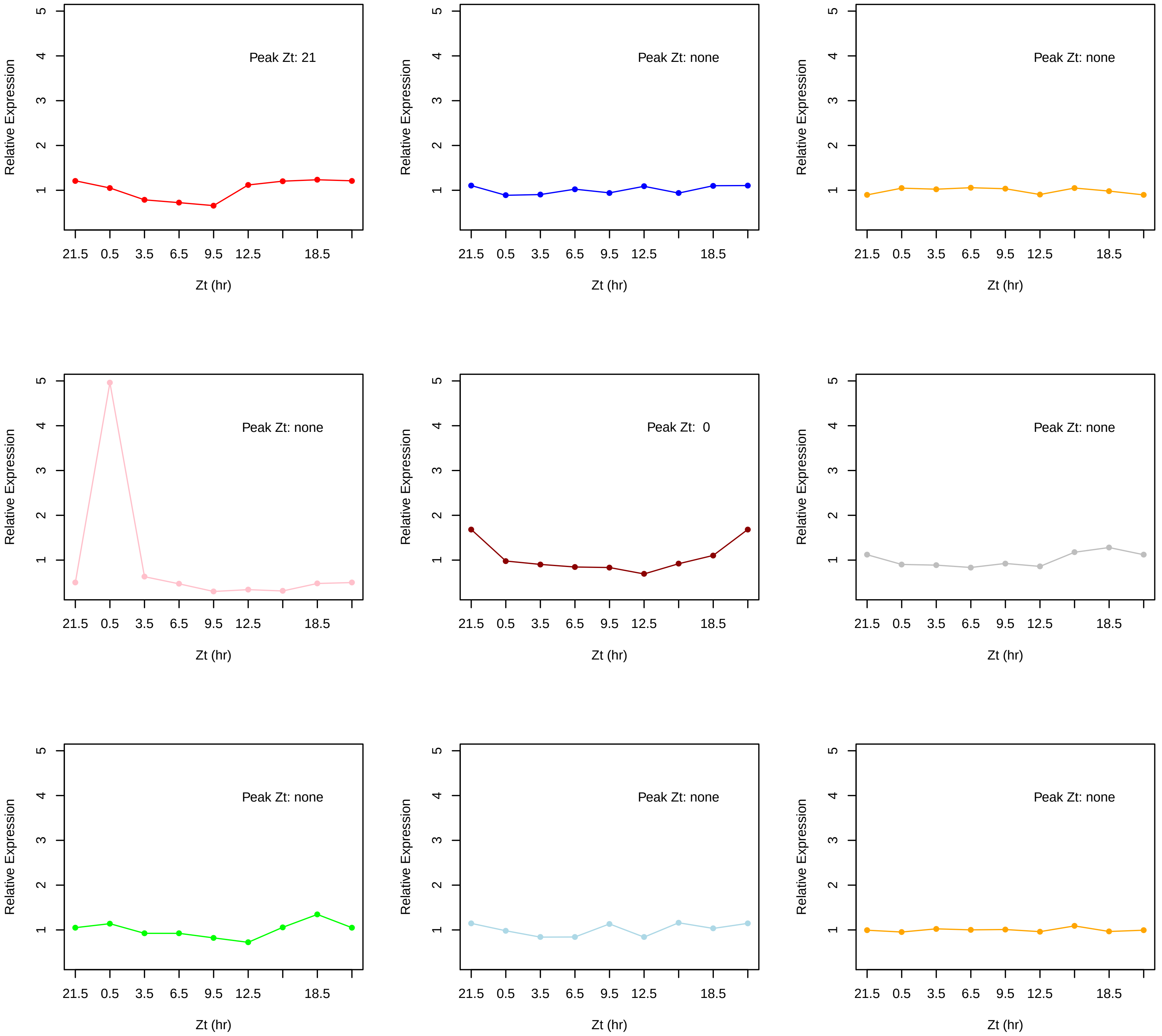

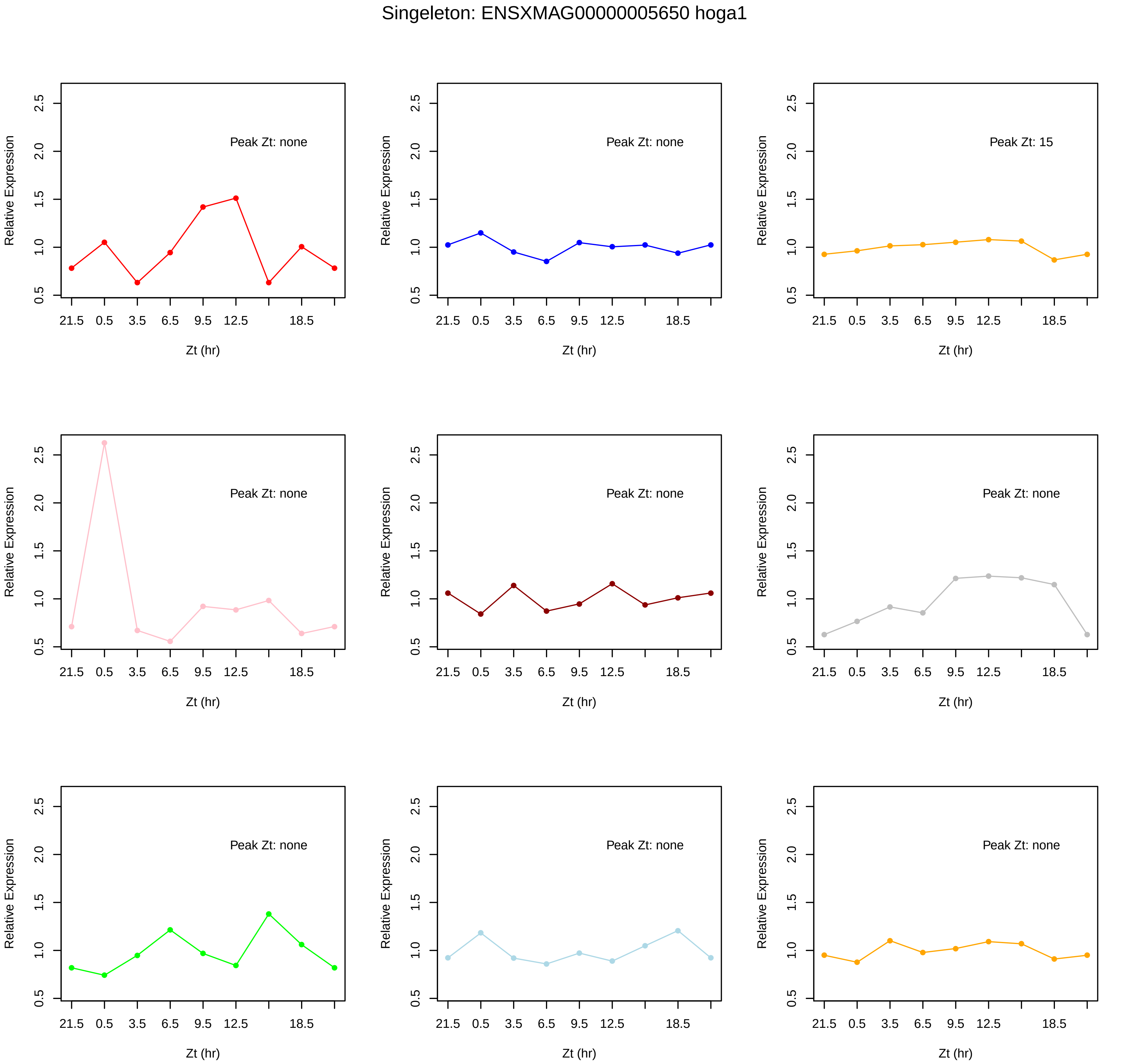

Singeleton: ENSXMAG00000005650 hoga1
Peak Zt: none
Peak Zt: none
Peak Zt: 15
Peak Zt: none
Peak Zt: none
Peak Zt: none
Peak Zt: none
Peak Zt: none
Peak Zt: none
Relative Expression
Zt (hr)
21.5 0.5 3.5 6.5 9.5 12.5 18.5
0.5 1.0 1.5 2.0 2.5

Singeleton: ENSXMAG00000012691 zcchc7

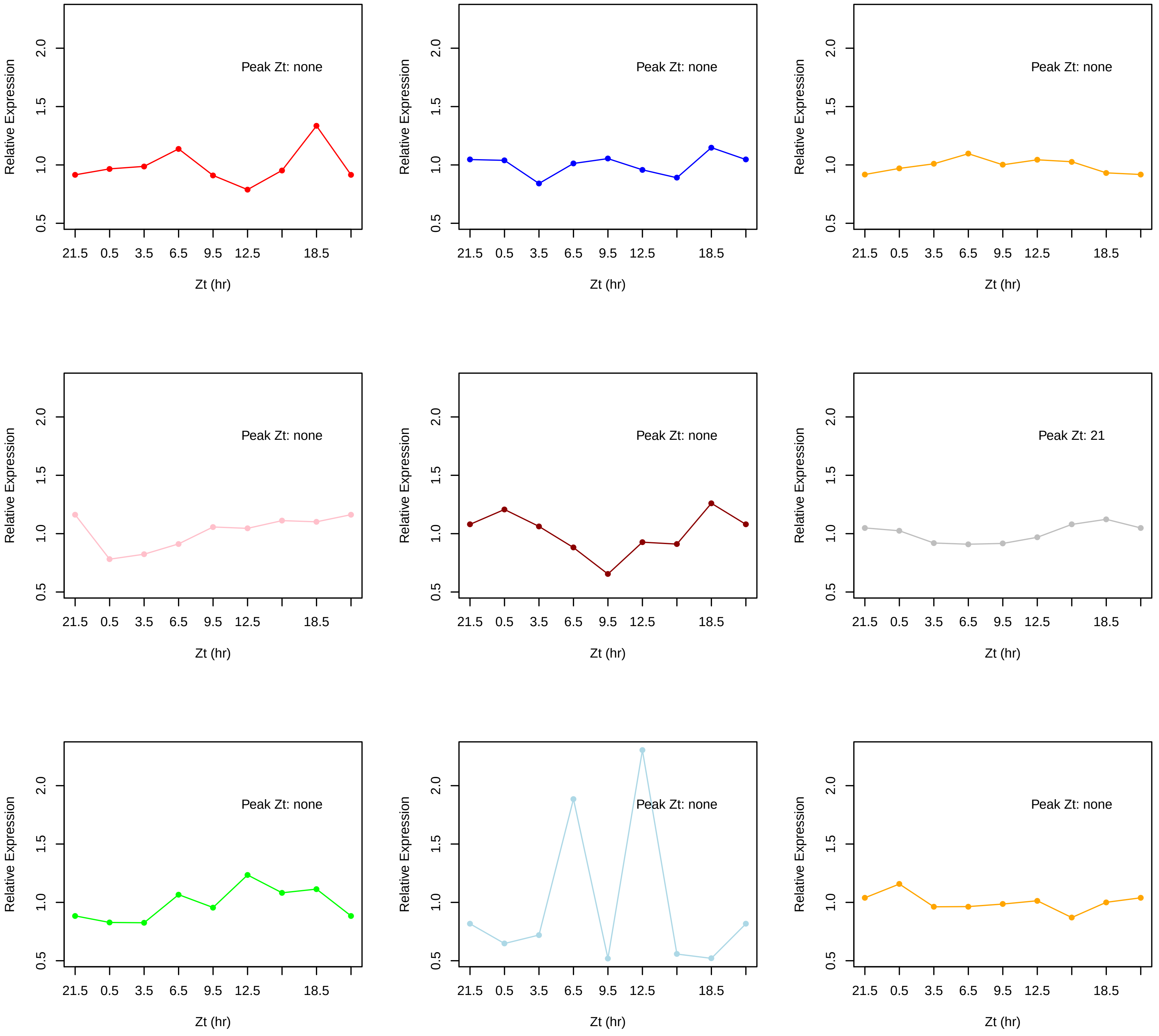

Singeleton: ENSXMAG00000014178 CDH10

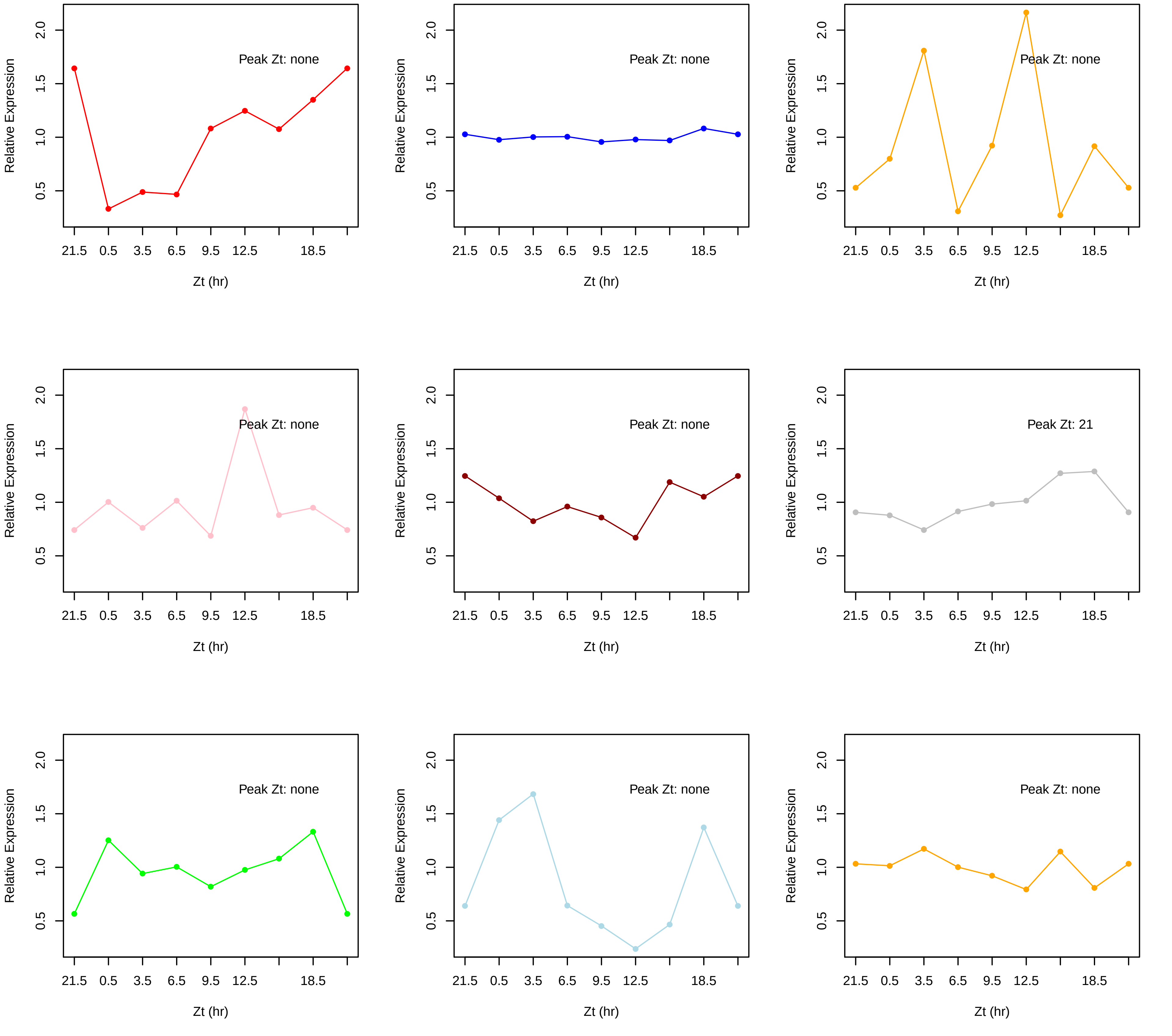

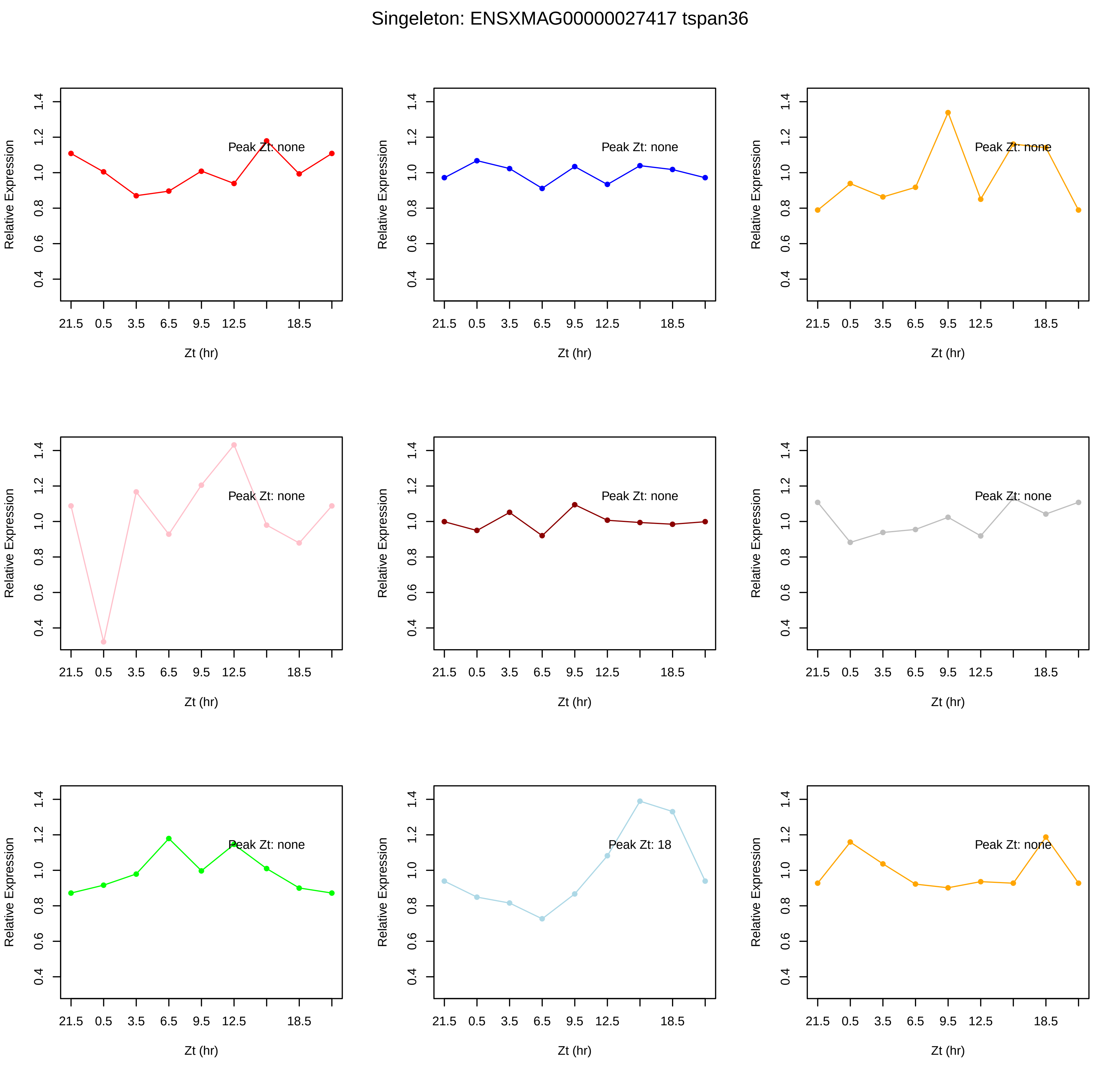
Singeleton: ENSXMAG00000027417 tspan36
Relative Expression
Zt (hr)
Peak Zt: none
Peak Zt: 18
21.5 0.5 3.5 6.5 9.5 12.5 18.5
0.4 0.6 0.8 1.0 1.2 1.4

Singeleton: ENSXMAG00000021985

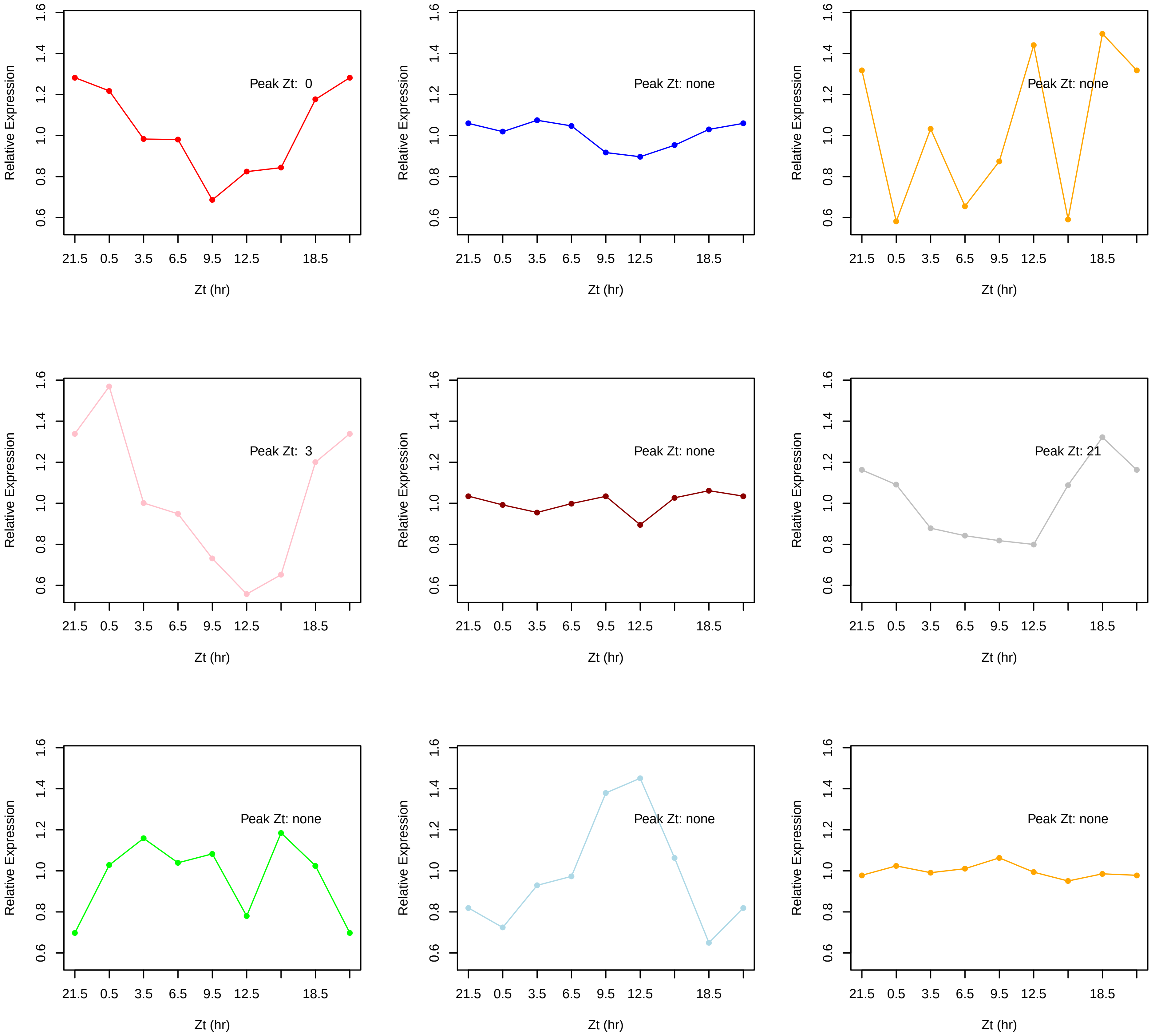

Singleton: ENSXMAG00000026565

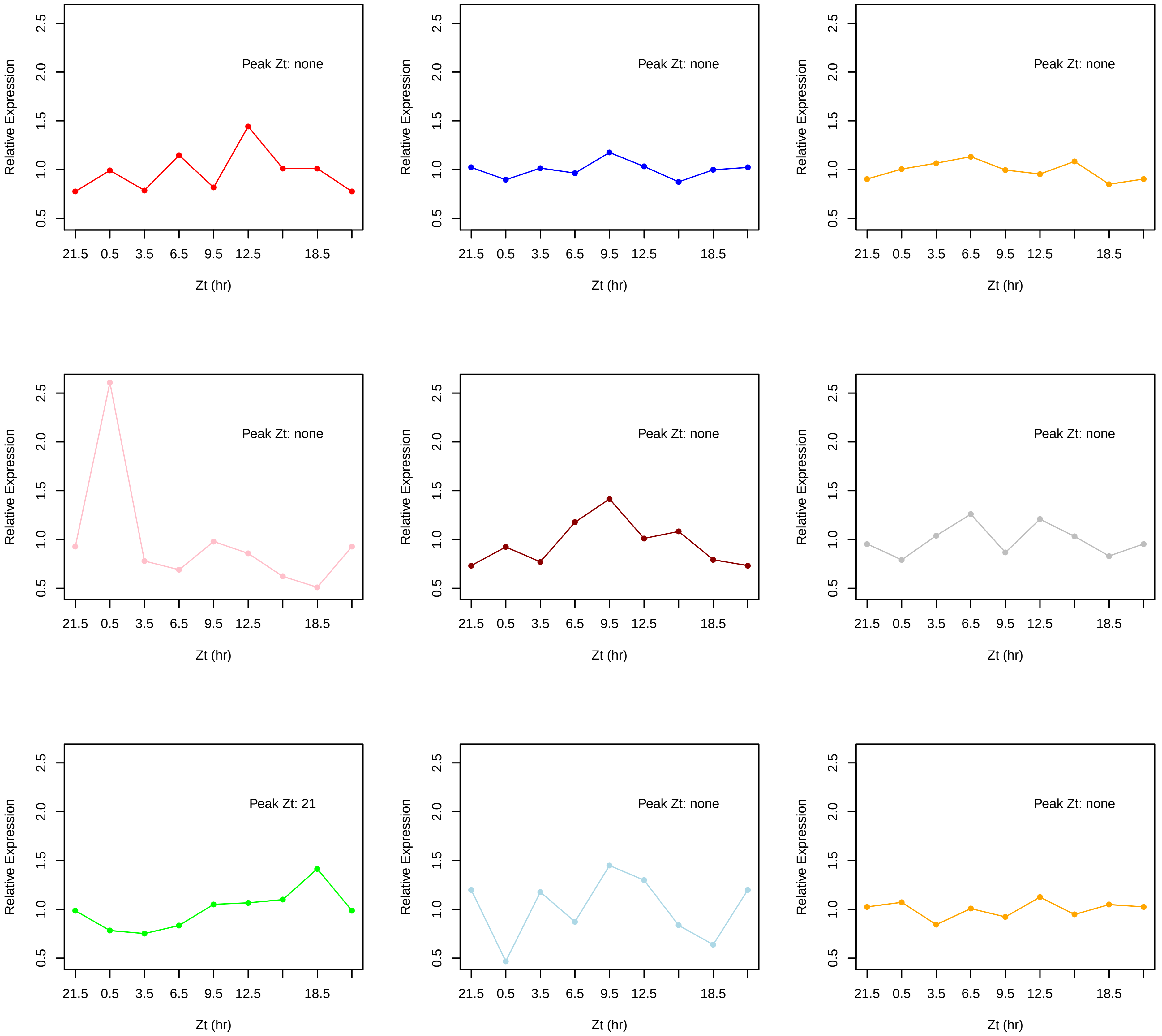

Singeleton: ENSXMAG00000015092 zswim8

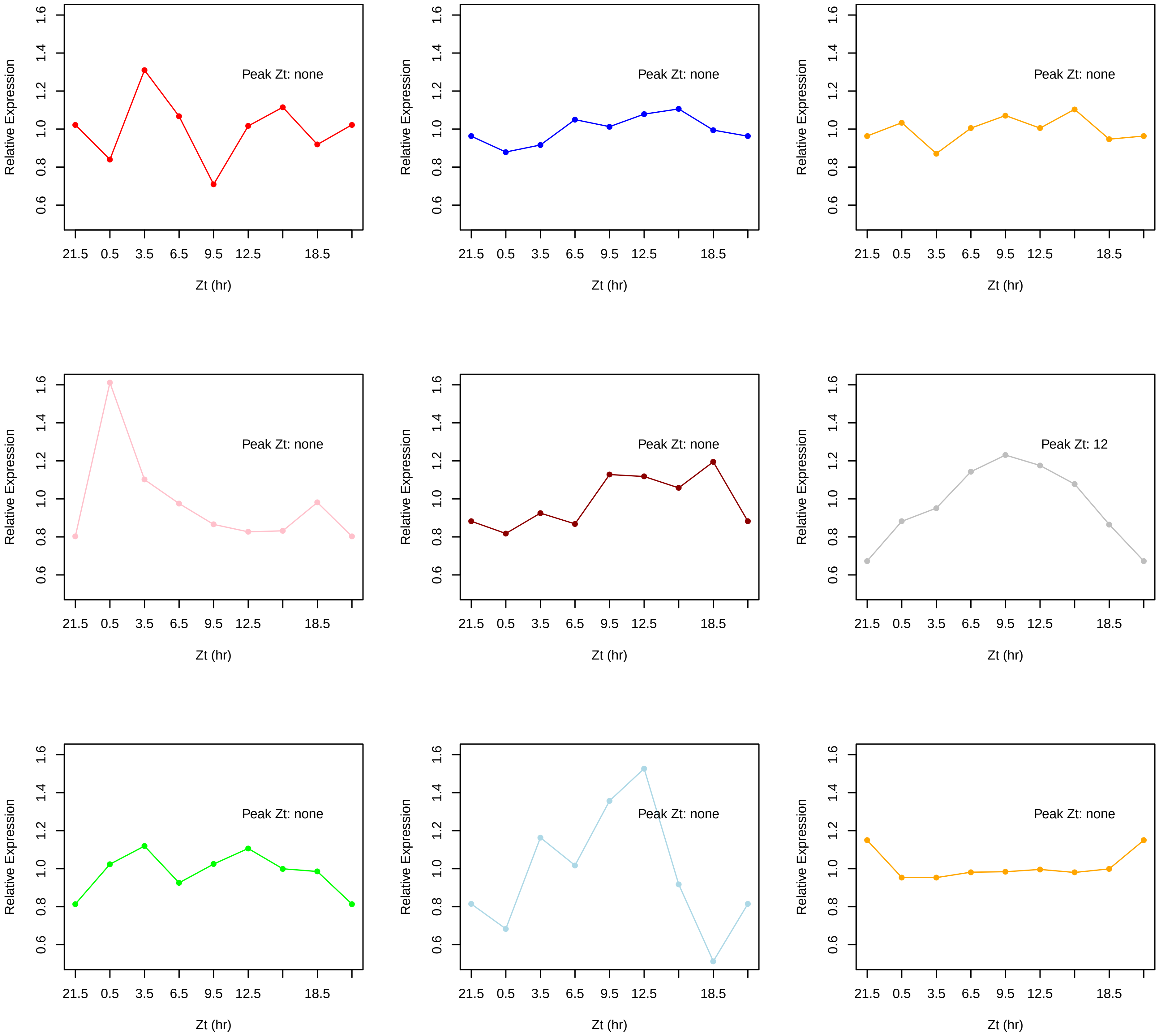

Singeleton: ENSXMAG00000017424 dnai2b

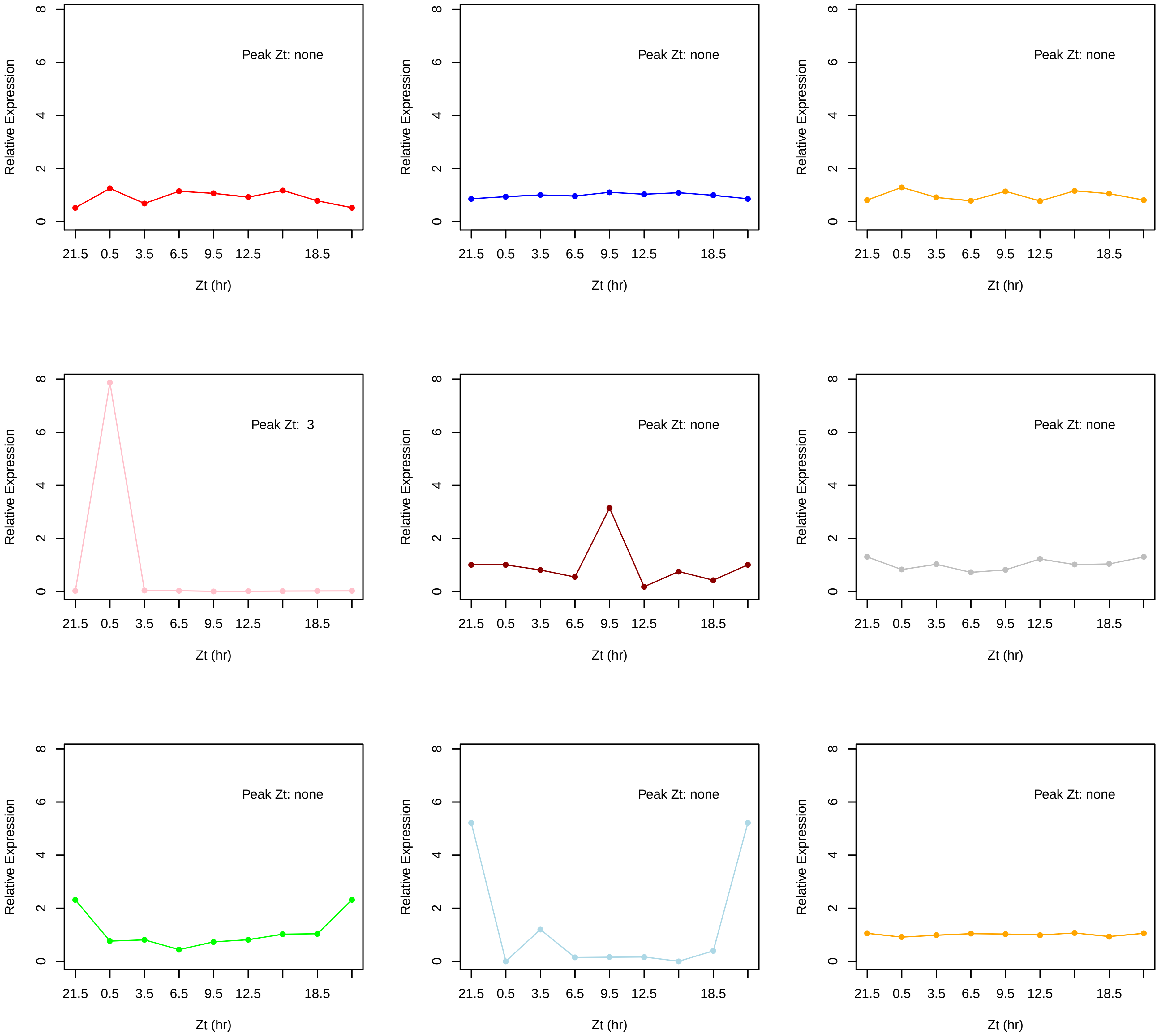

Singleton: ENSXMAG00000015283

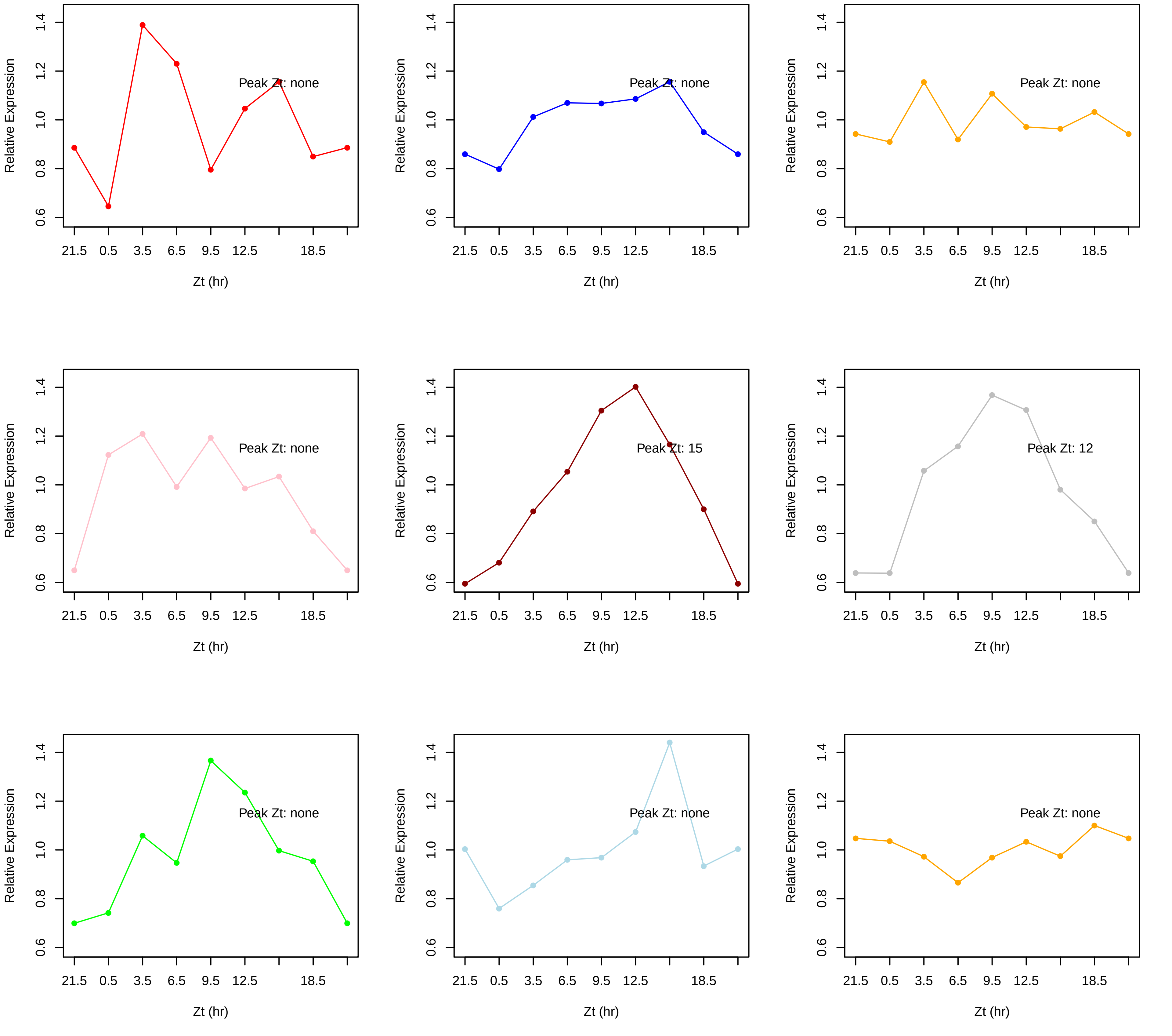

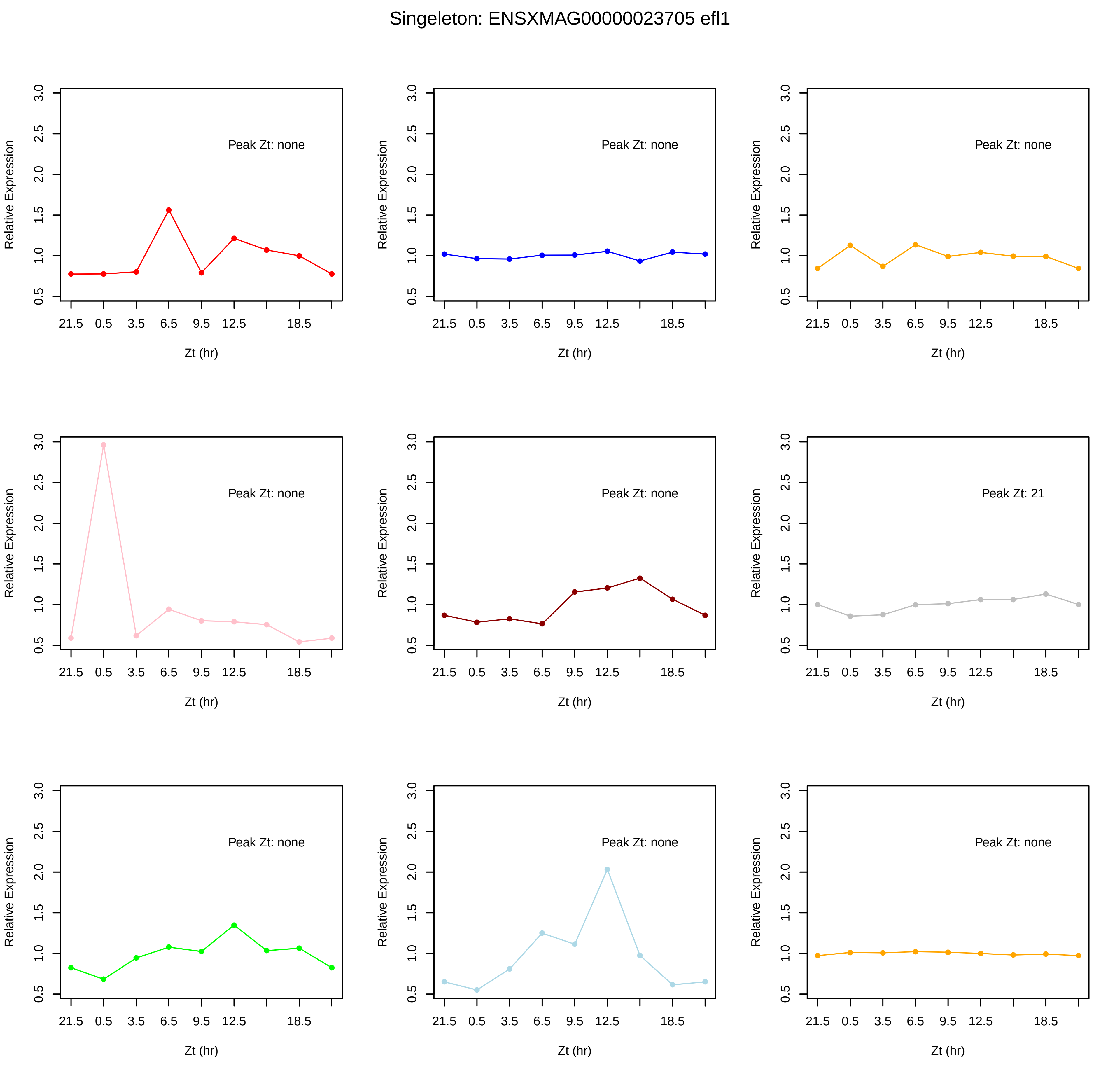
Singeleton: ENSXMAG00000023705 efl1
Peak Zt: none
Peak Zt: none
Peak Zt: none
Peak Zt: none
Peak Zt: none
Peak Zt: 21
Peak Zt: none
Peak Zt: none
Peak Zt: none
Relative Expression
Zt (hr)
21.5
0.5
3.5
6.5
9.5
12.5
18.5

Singeleton: ENSXMAG00000011374 med24

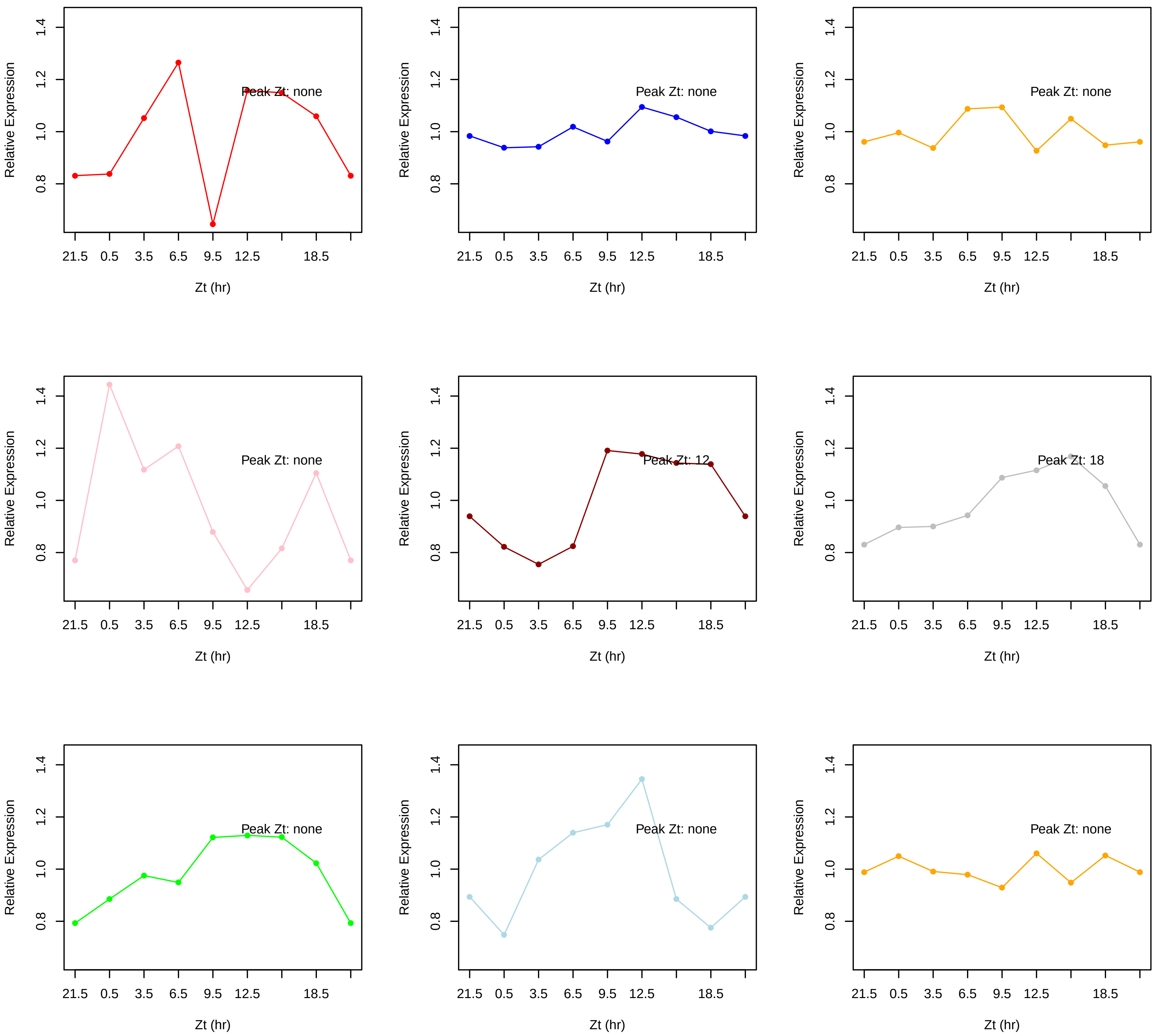

Singeleton: ENSXMAG00000008433 frmpd1b

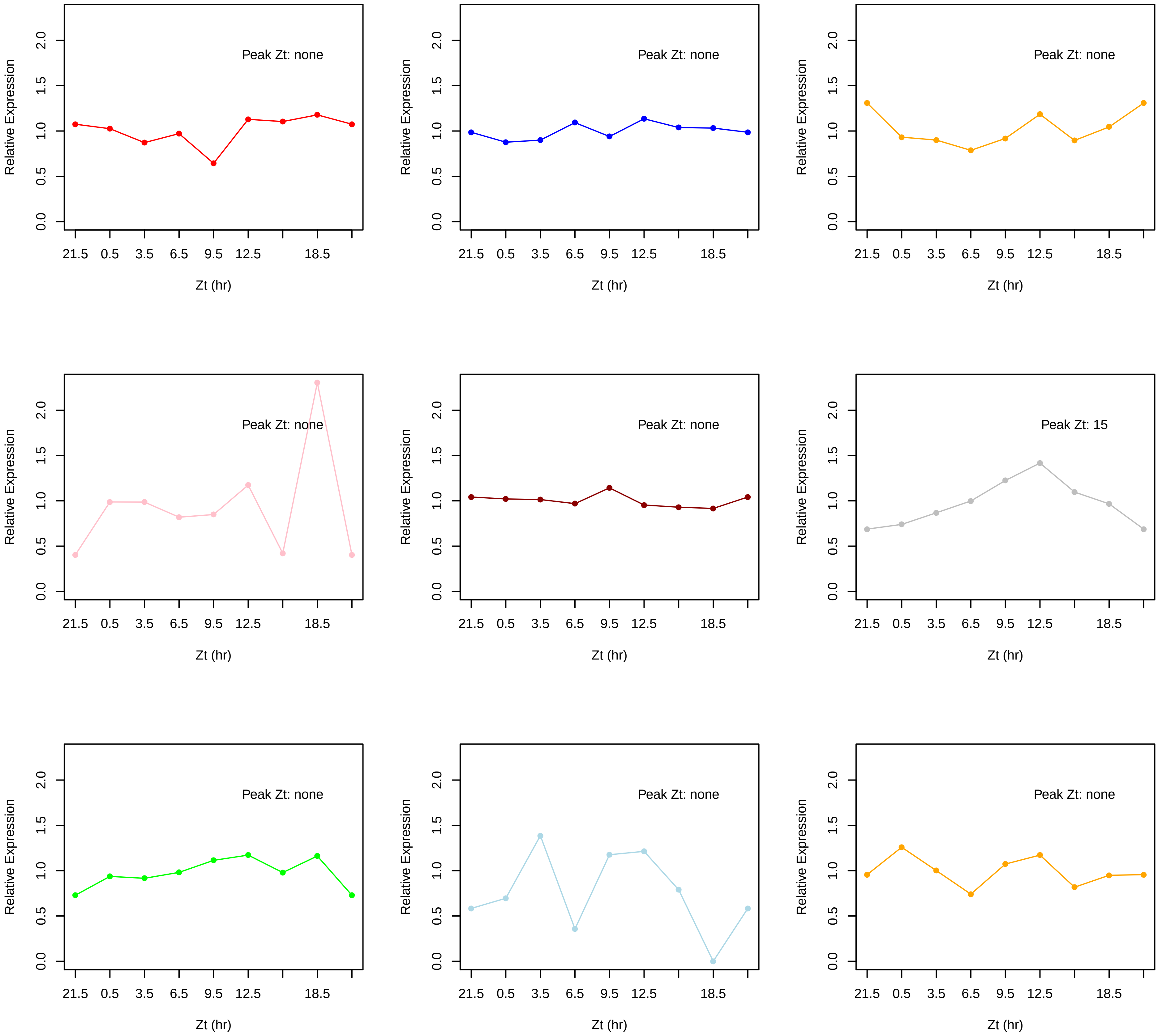

Singleton: ENSXMAG00000008415 ints10

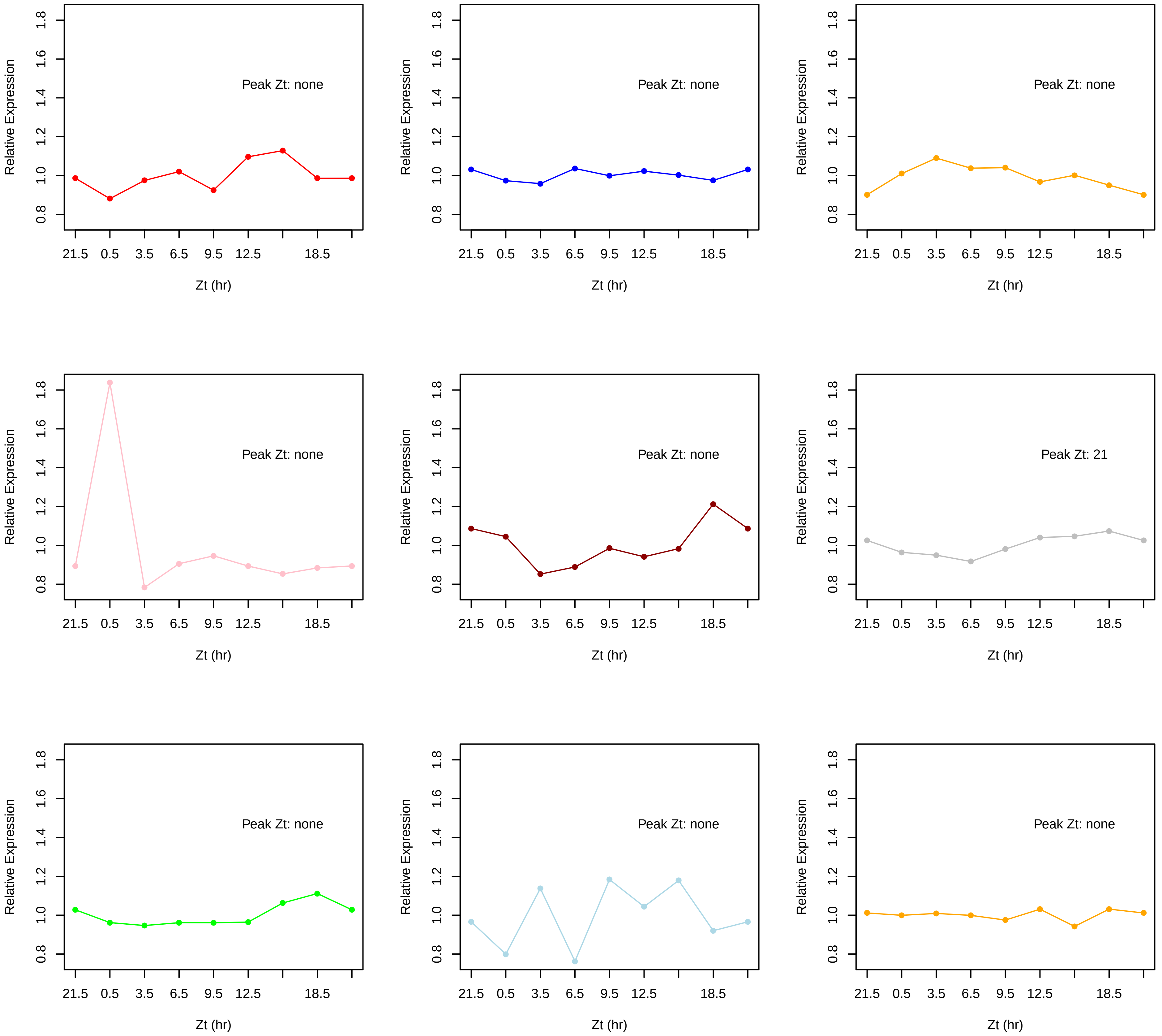

Singeleton: ENSXMAG00000026905 CDH20

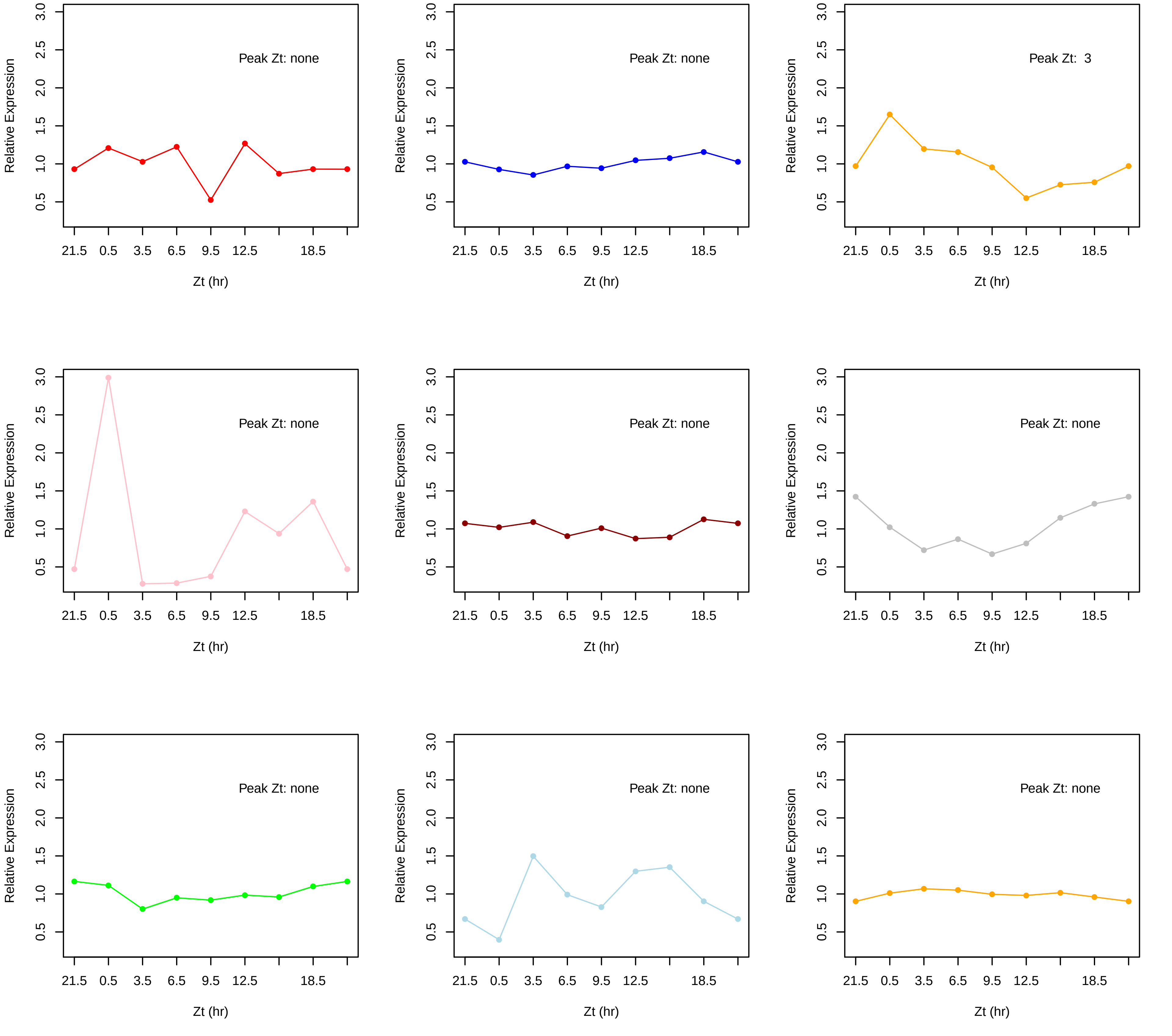

Singleton: ENSXMAG00000014799

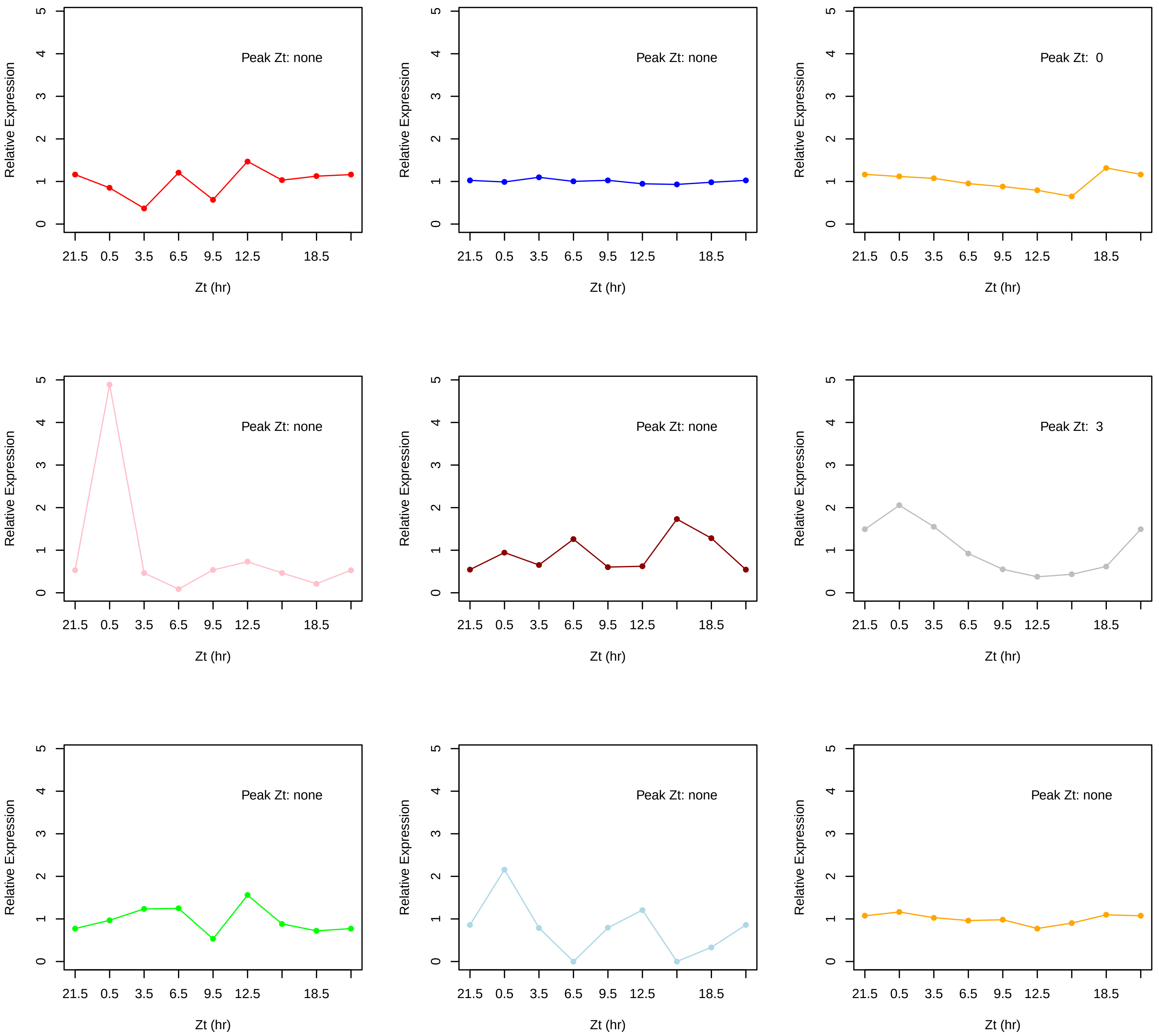

Singeleton: ENSXMAG00000012694 pgm2

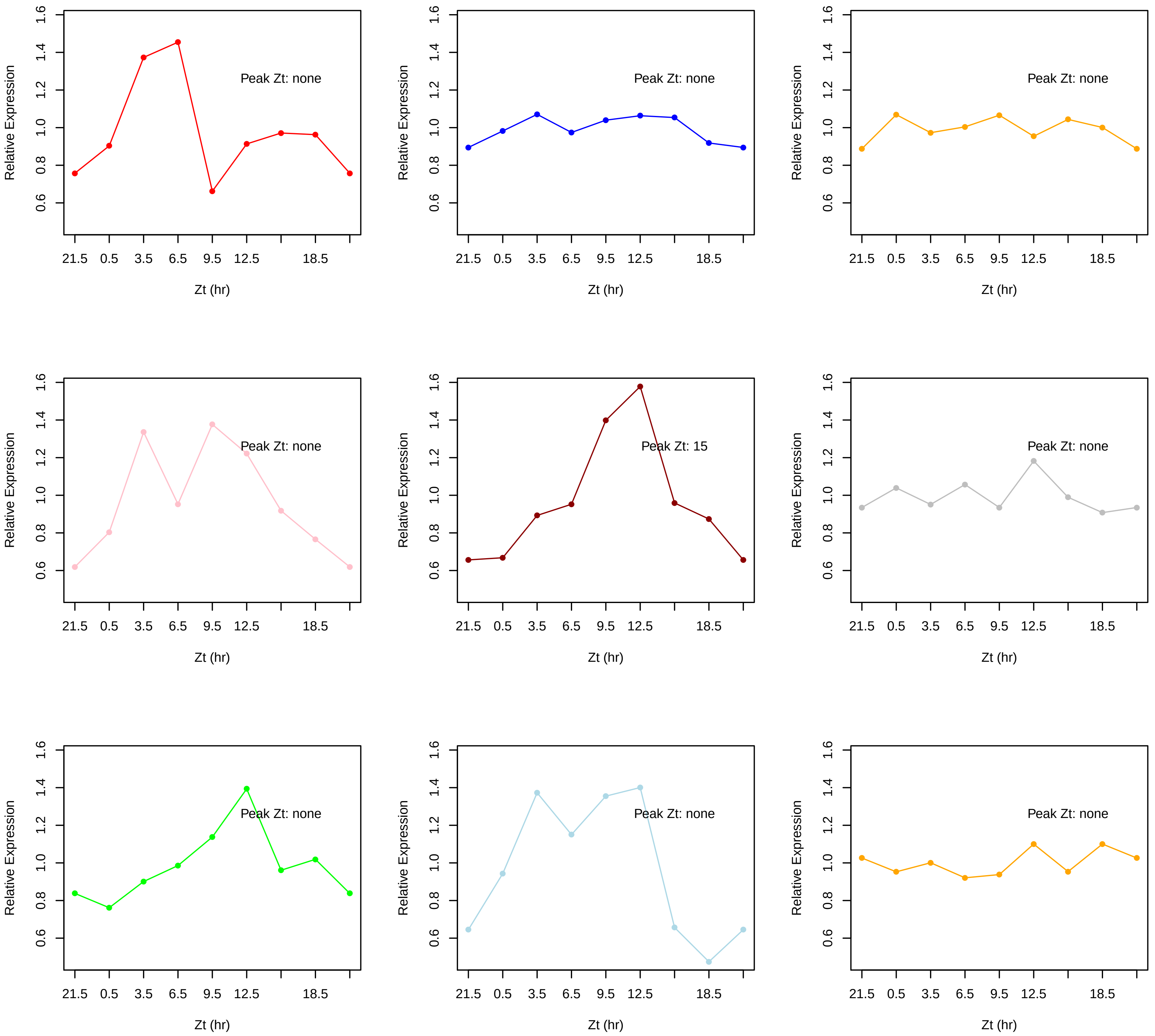

Singeleton: ENSXMAG00000013963 psmd3

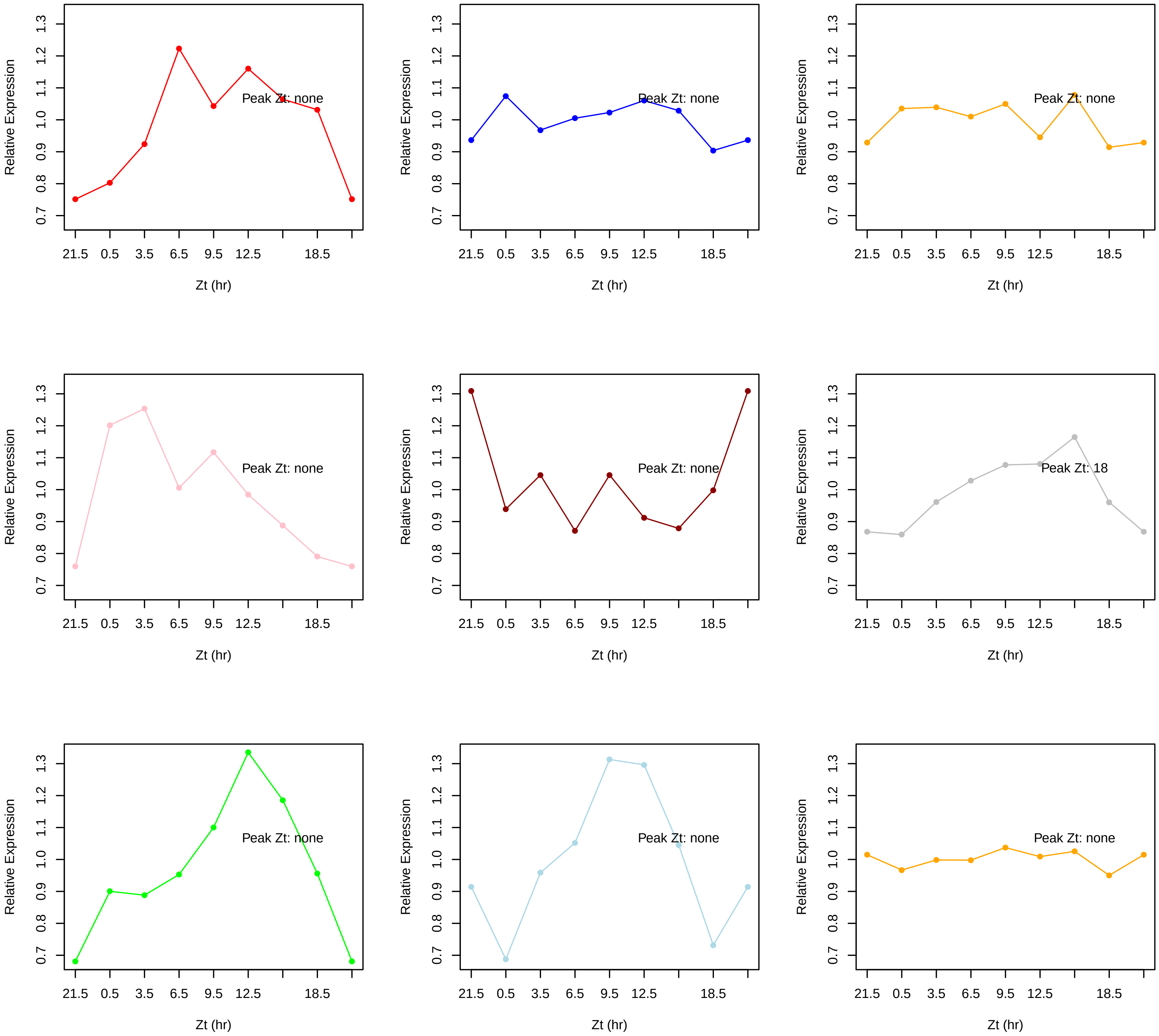

Singeleton: ENSXMAG00000025837 grid2

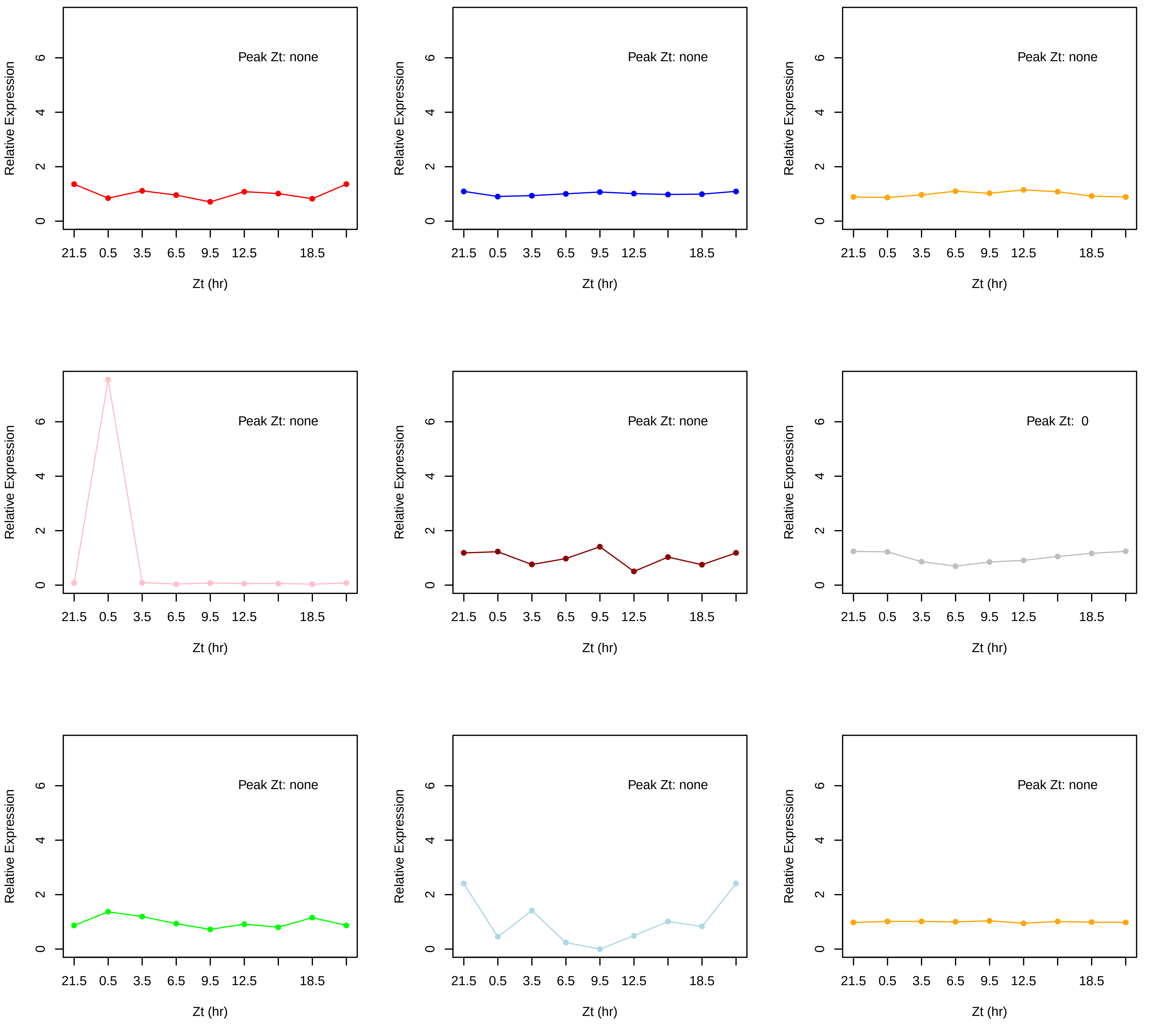

Singeleton: ENSXMAG00000012717 tbc1d1

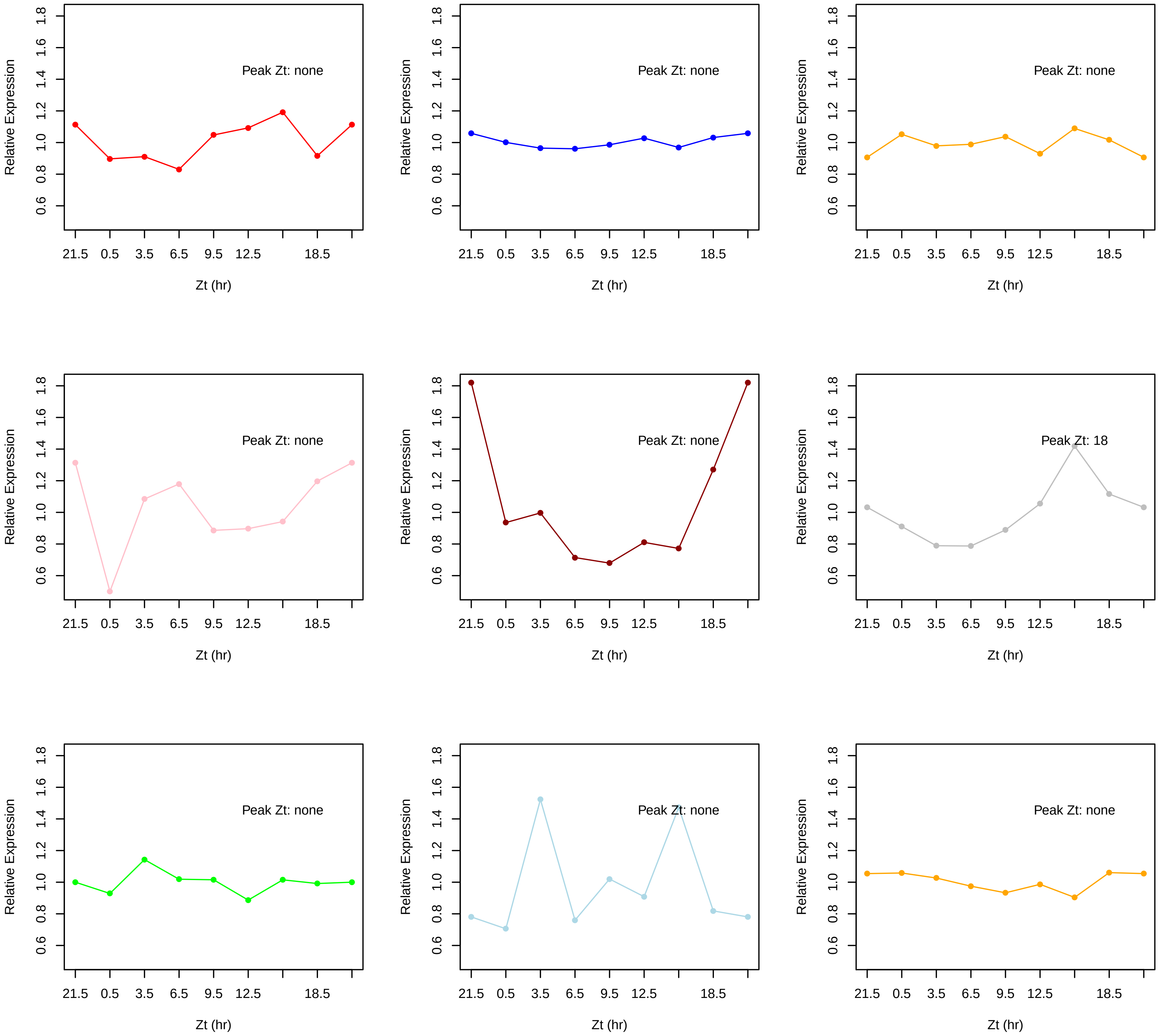

Singleton: ENSXMAG00000011303 top2a

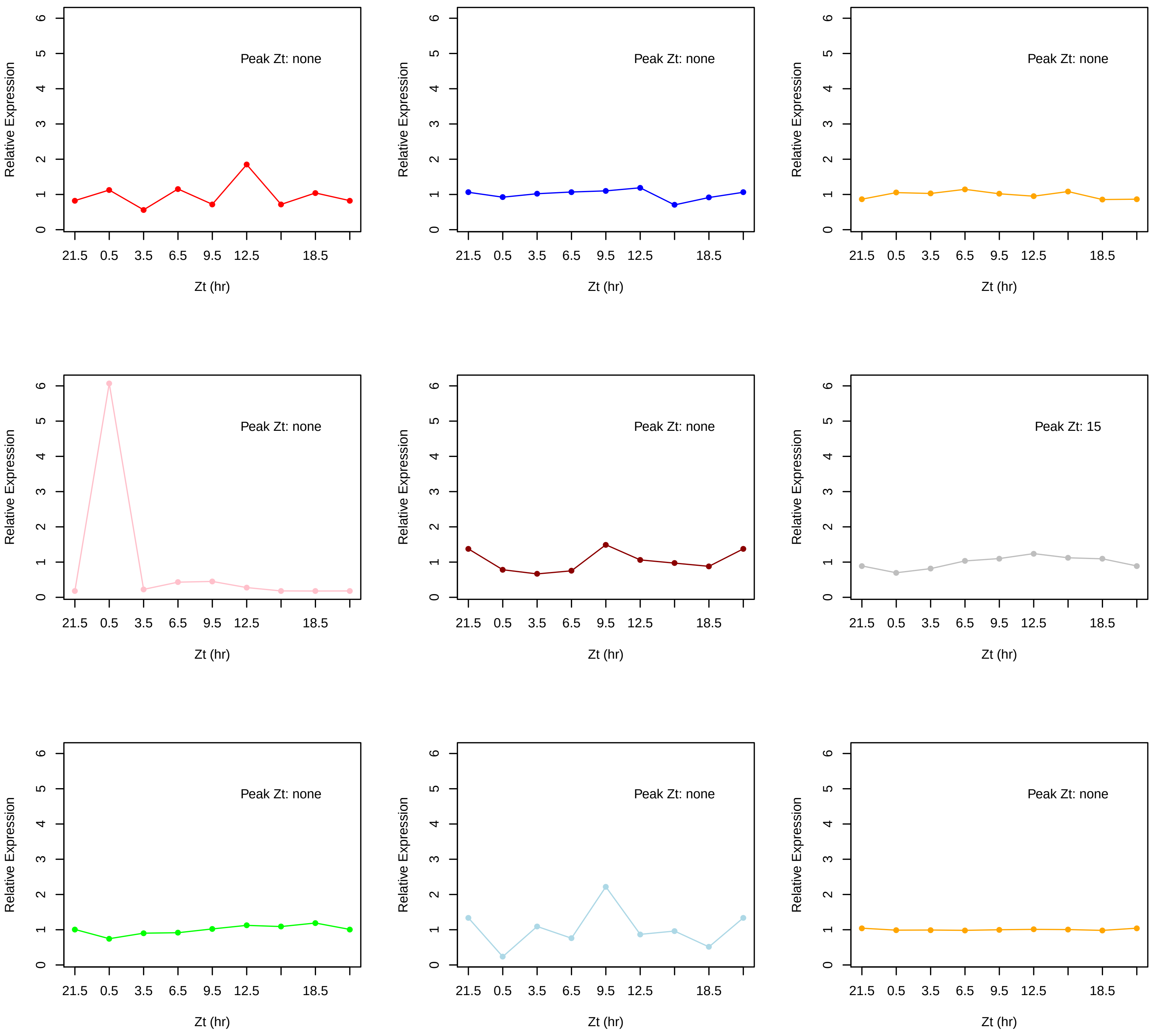

Singeleton: ENSXMAG00000017027 timm23a

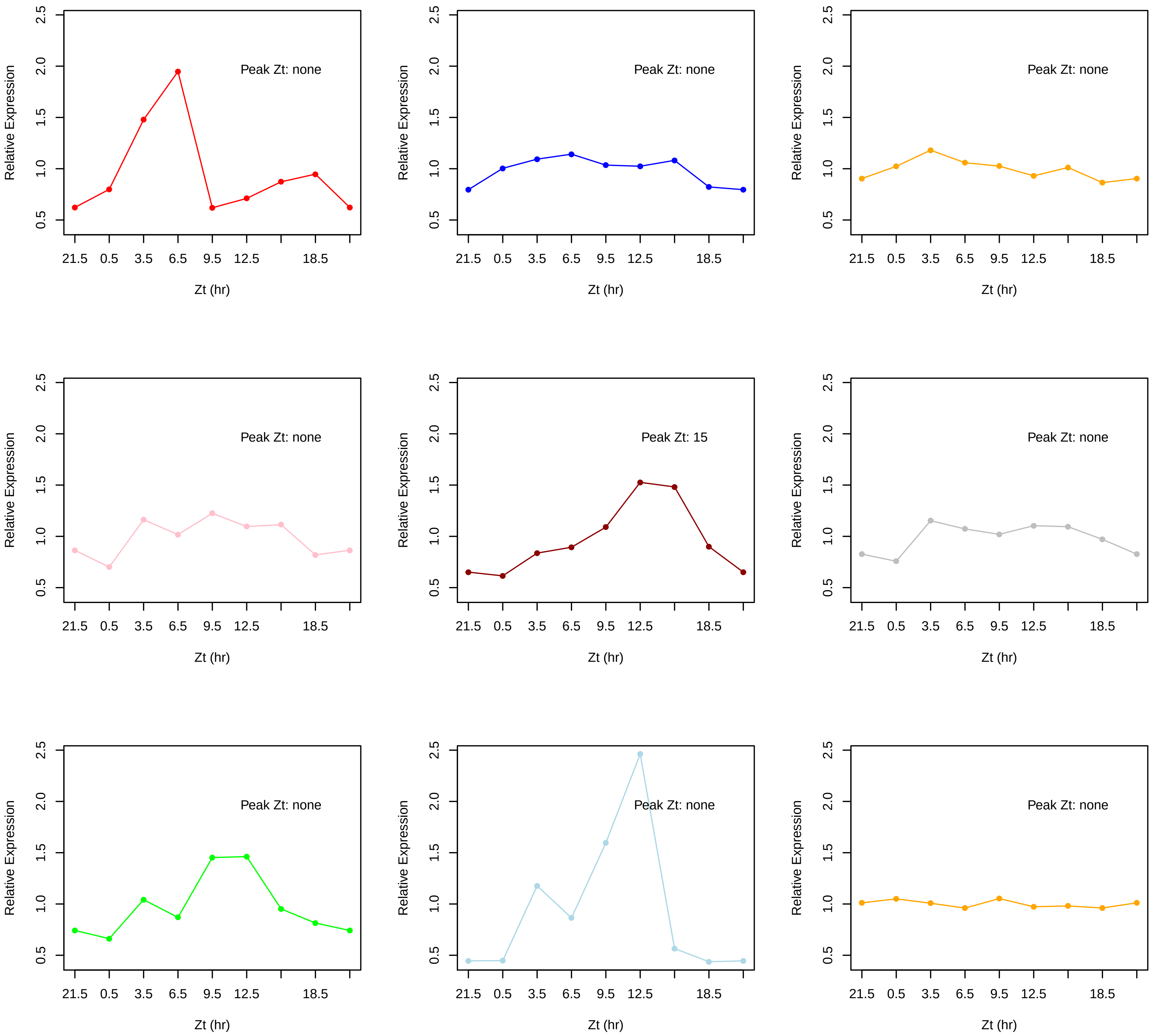

Singeleton: ENSXMAG00000015385 aspg

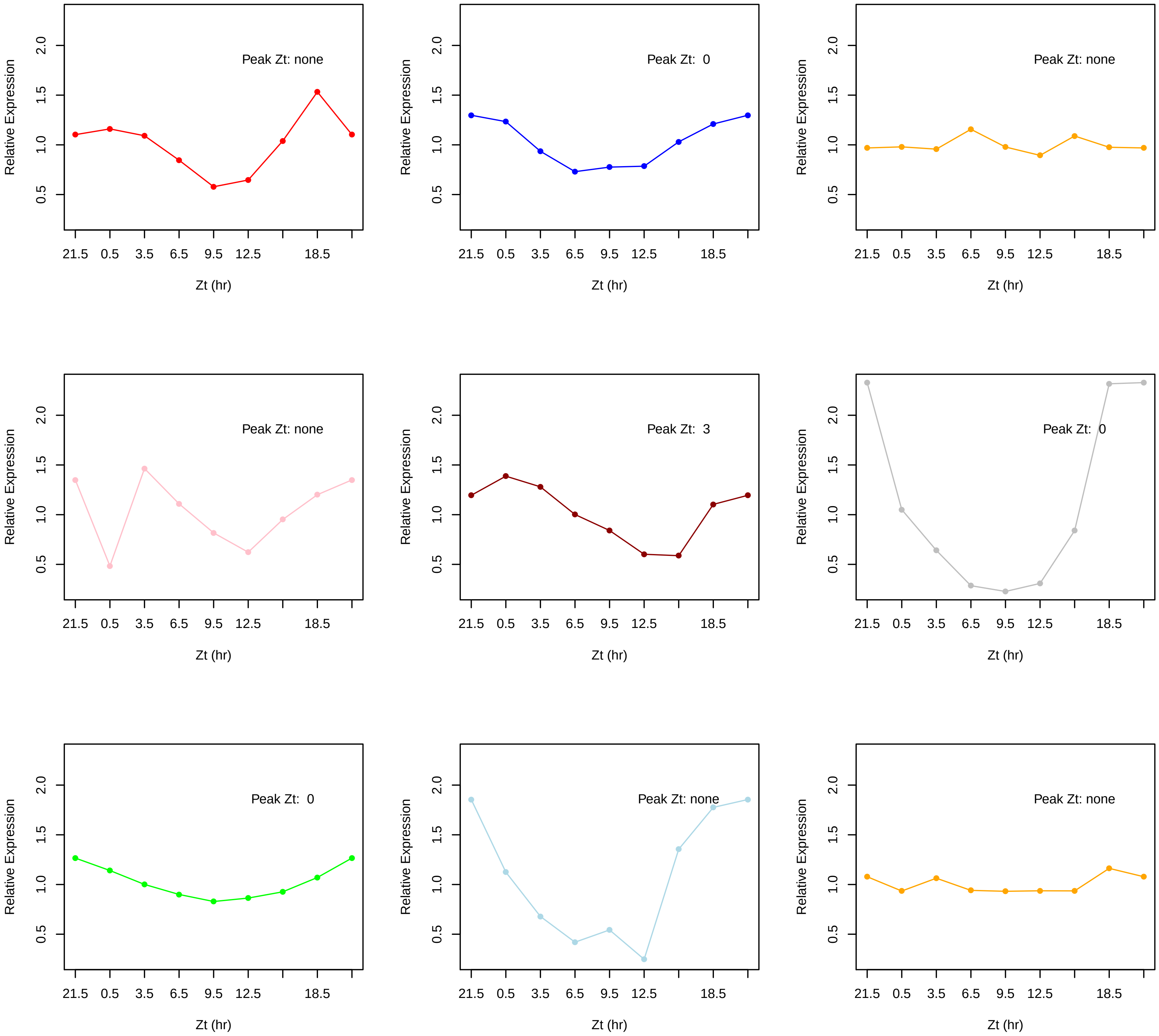

Singeleton: ENSXMAG00000007525

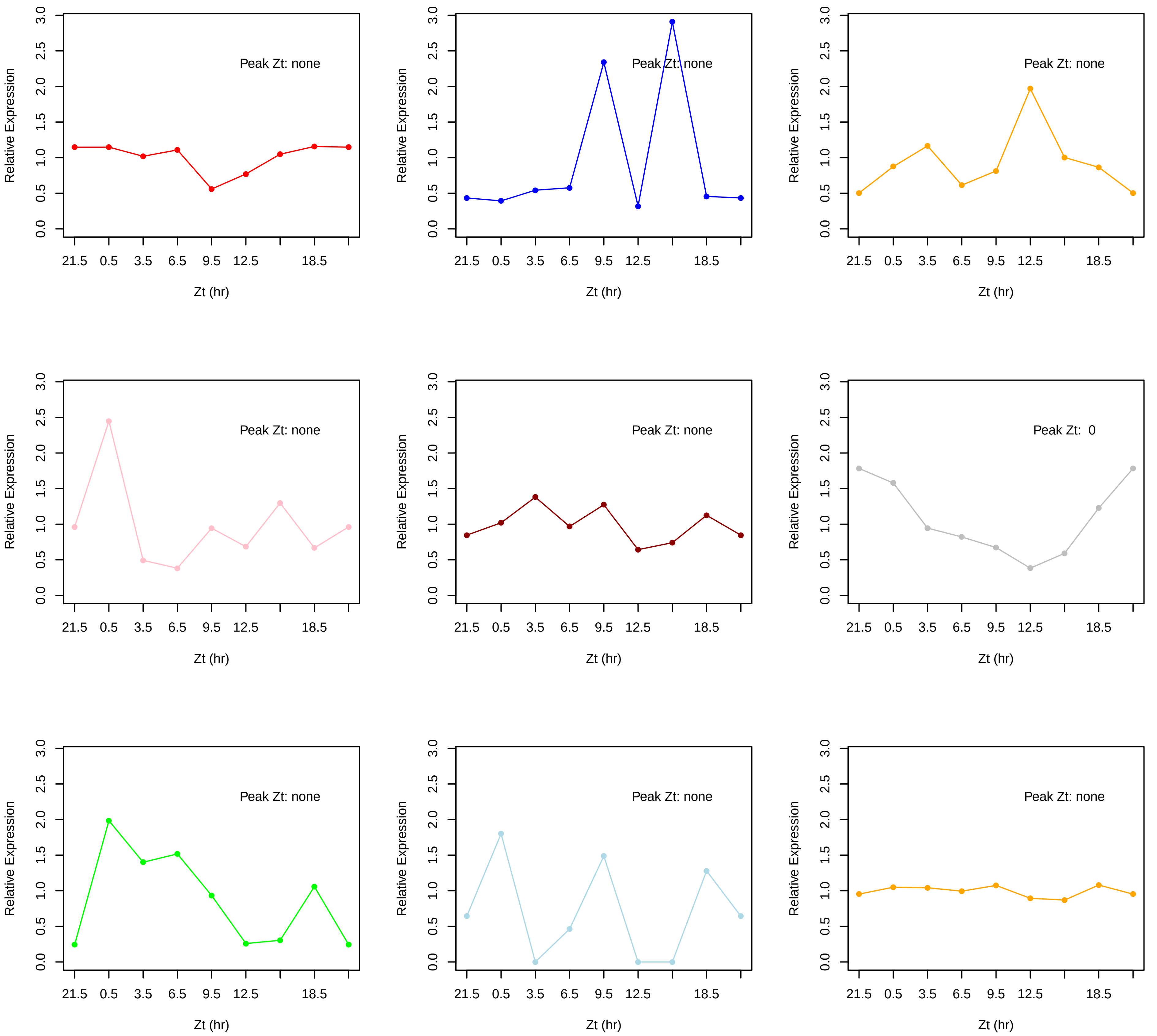

Singeleton: ENSXMAG00000002033 tmem33

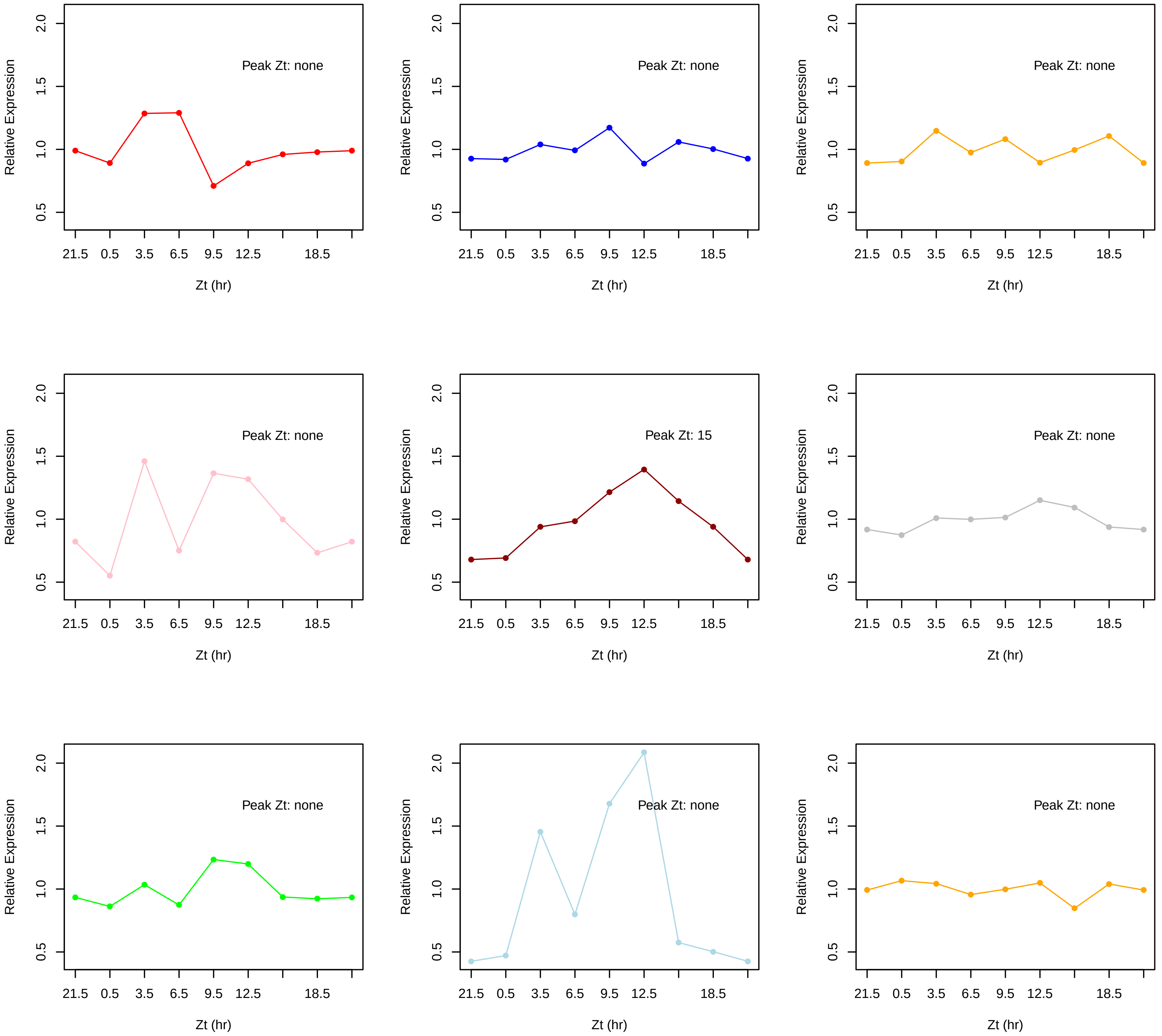

Singeleton: ENSXMAG00000014236 bms1

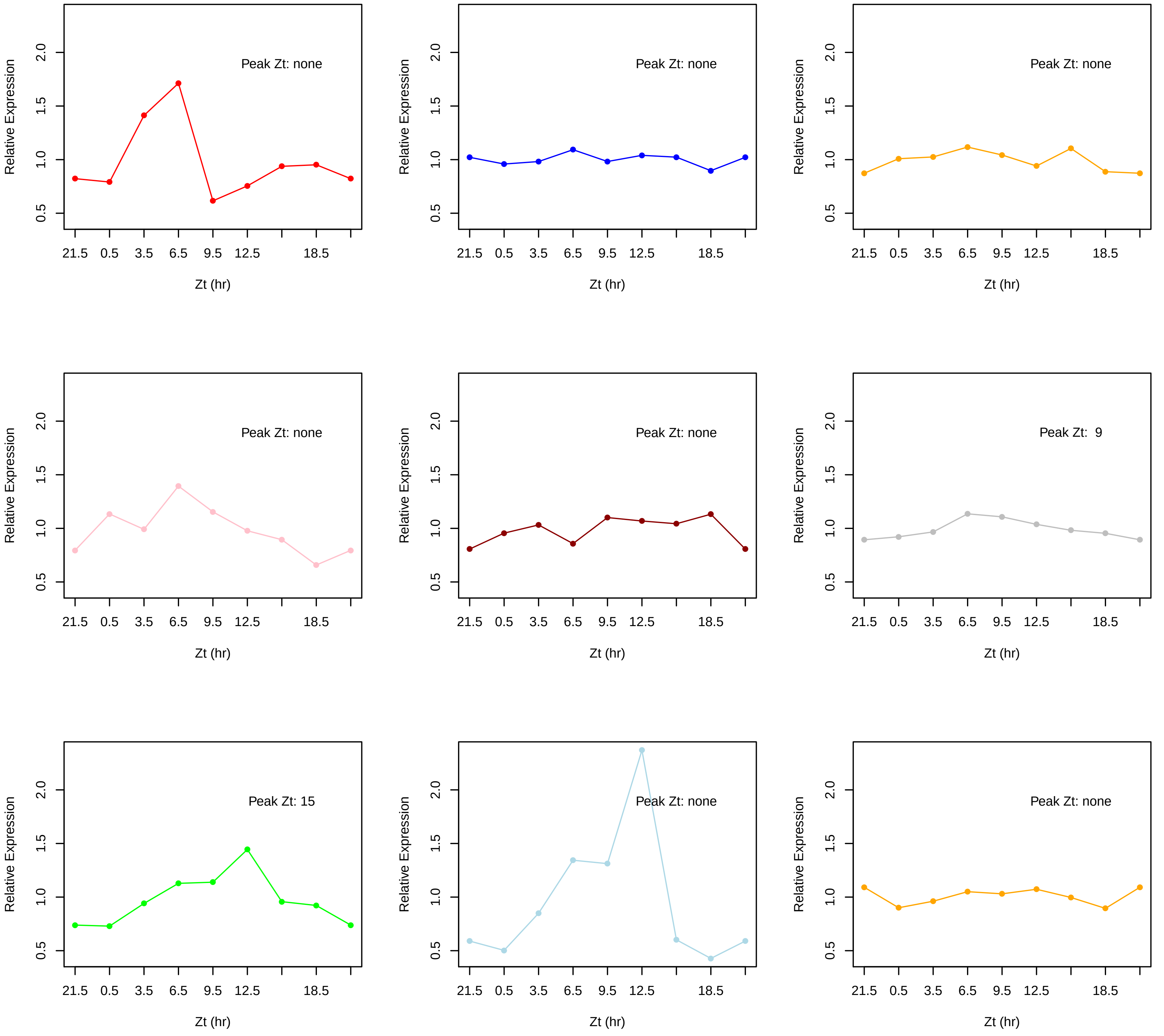

Singeleton: ENSXMAG00000016894 PDE1C

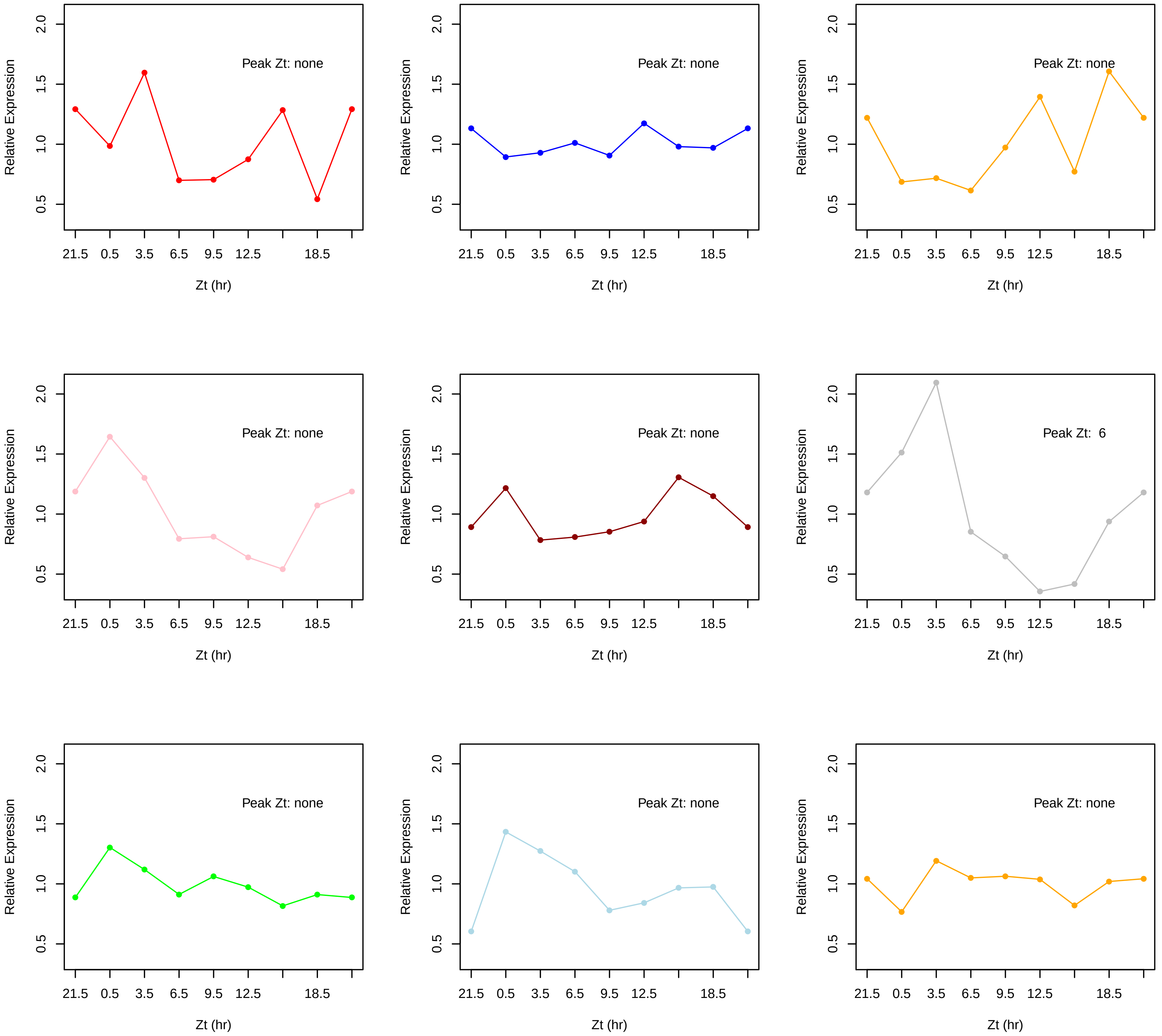

Singleton: ENSXMAG00000026911 TMEM179

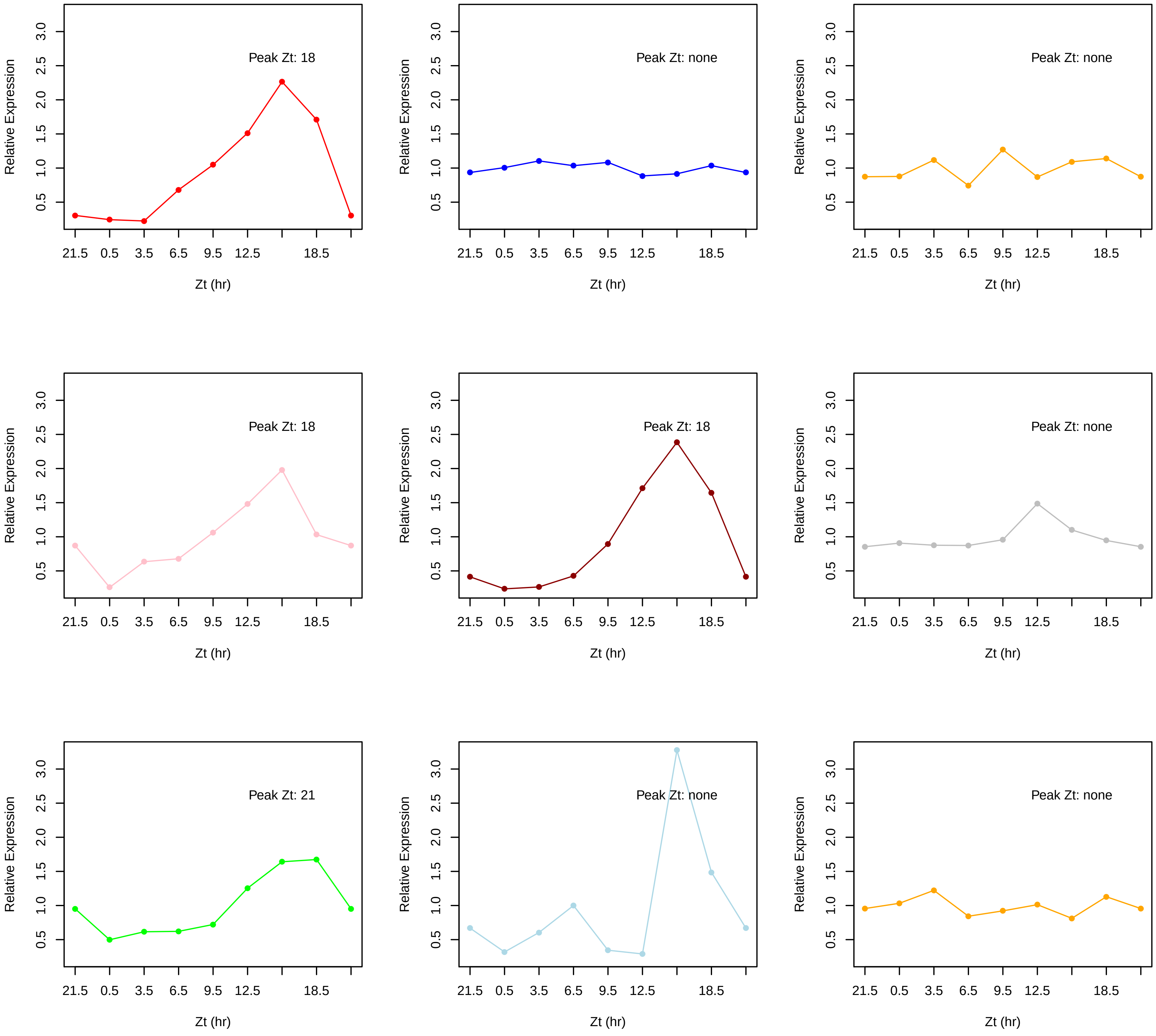

Singleton: ENSXMAG00000008035 uchl1

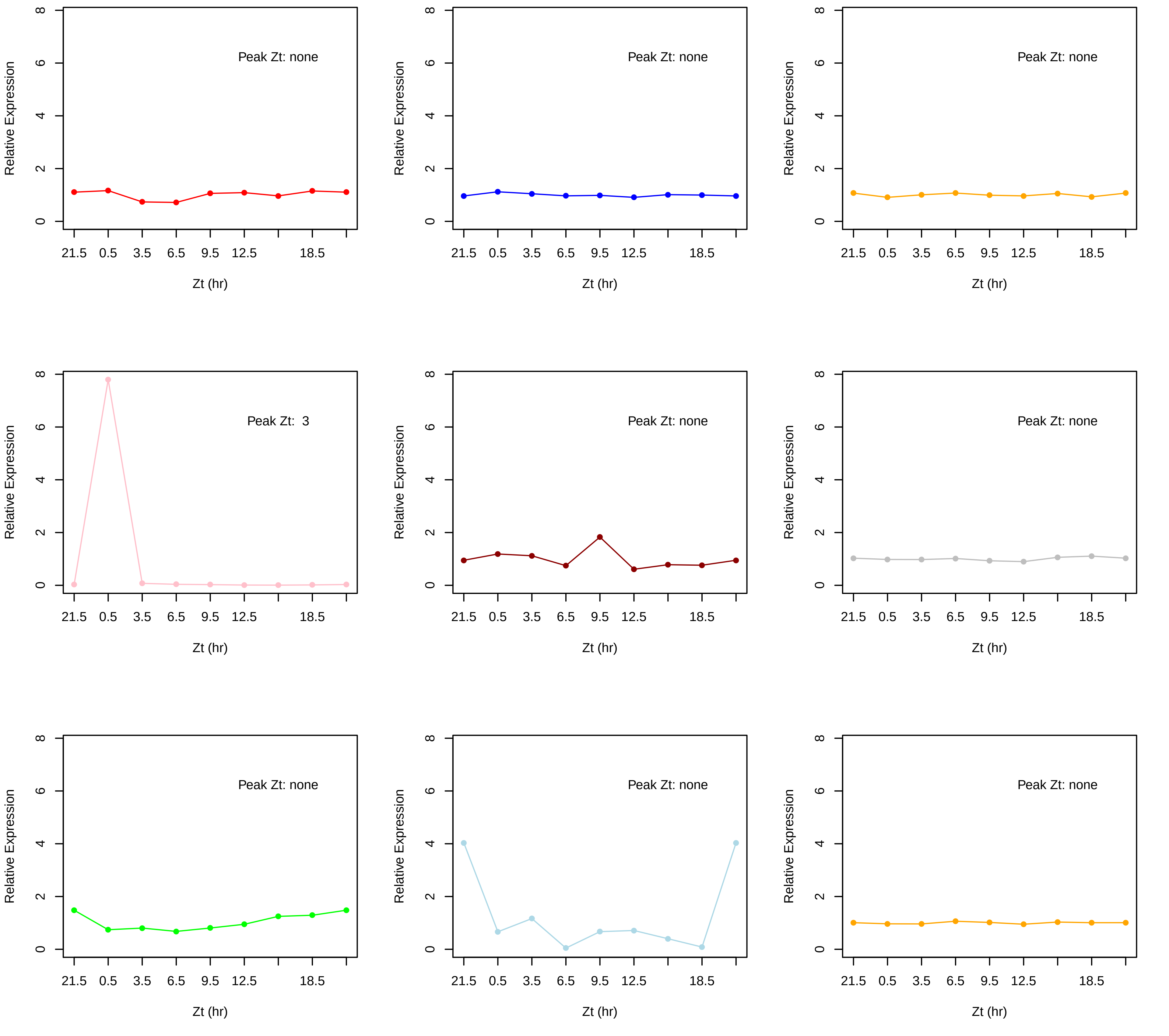

Singeleton: ENSXMAG00000017964

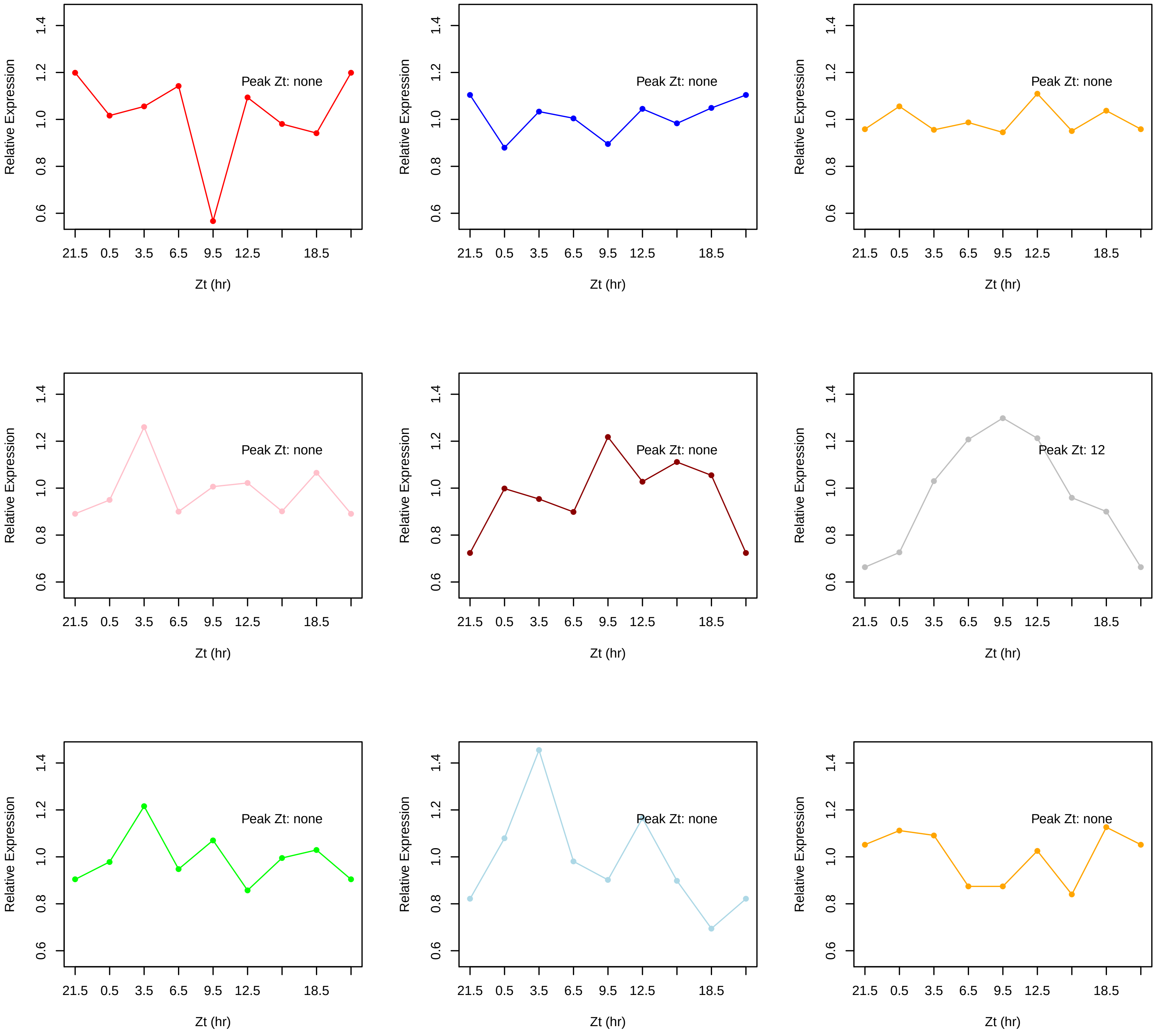

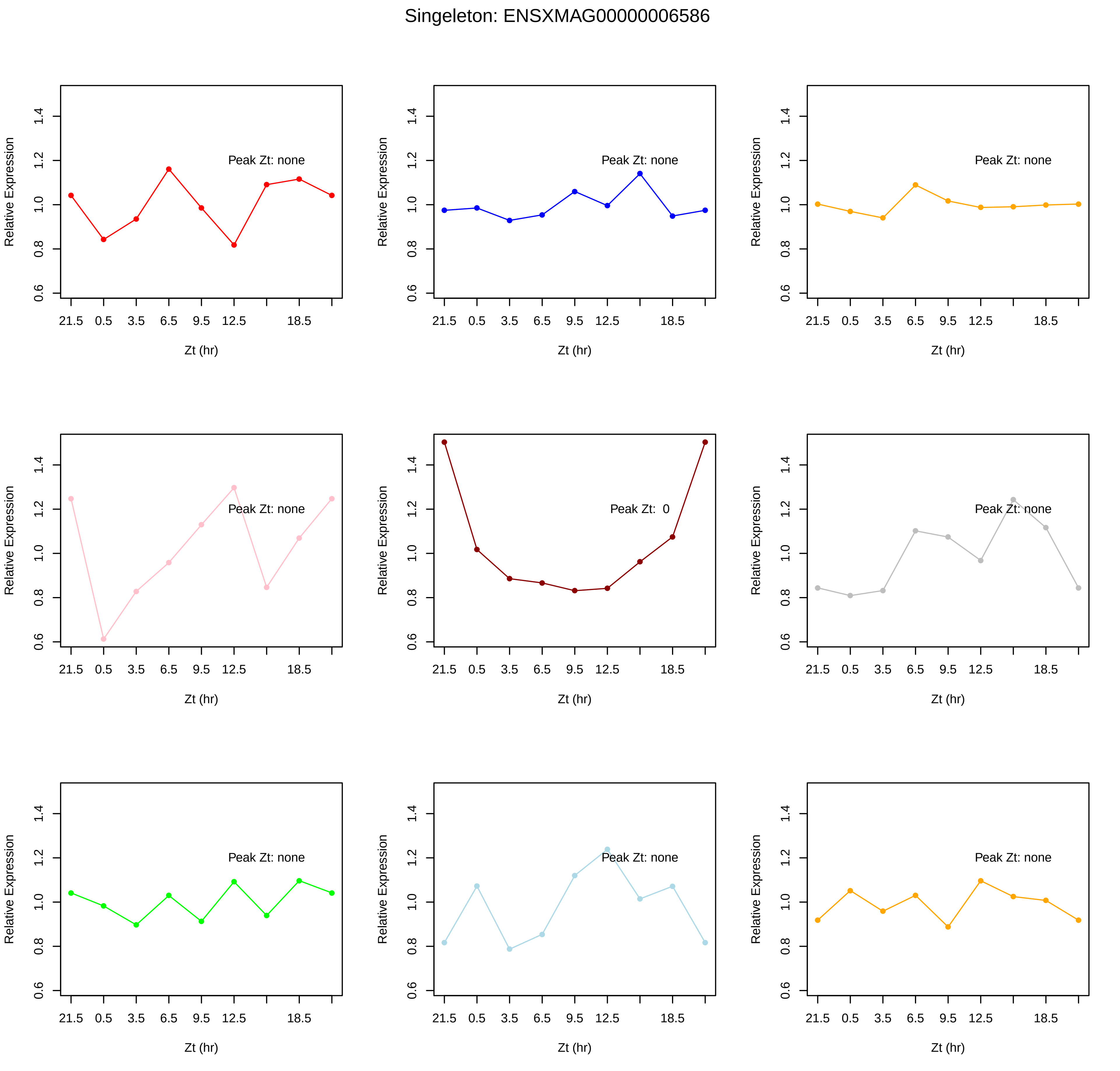
Singeleton: ENSXMAG00000006586
Peak Zt: none
Peak Zt: none
Peak Zt: none
Peak Zt: none
Peak Zt: 0
Peak Zt: none
Peak Zt: none
Peak Zt: none
Peak Zt: none
Relative Expression
Zt (hr)
0.6
0.8
1.0
1.2
1.4
21.5
0.5
3.5
6.5
9.5
12.5
18.5

Singeleton: ENSXMAG00000014501

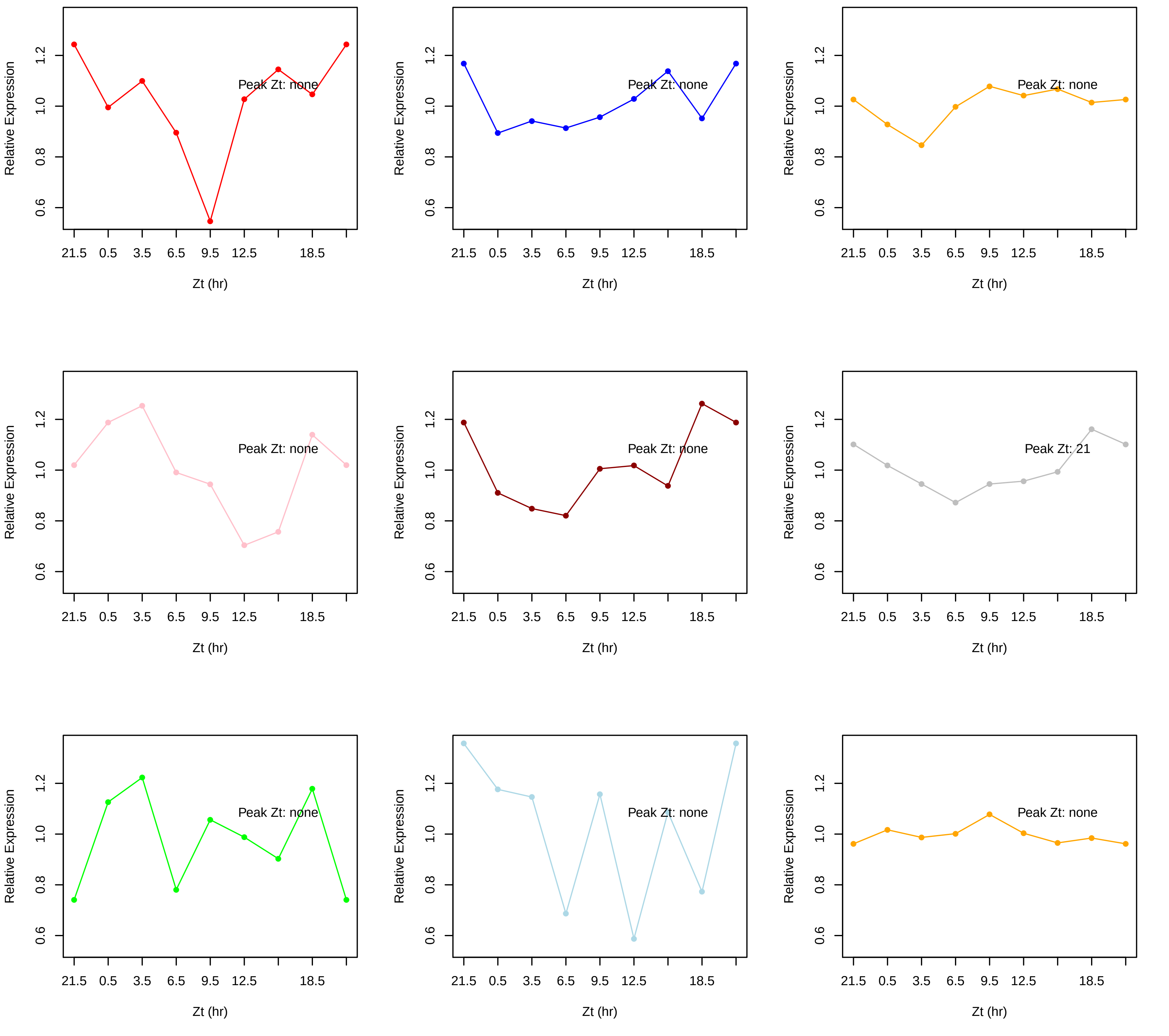

Singeleton: ENSXMAG00000002059 HOMER2

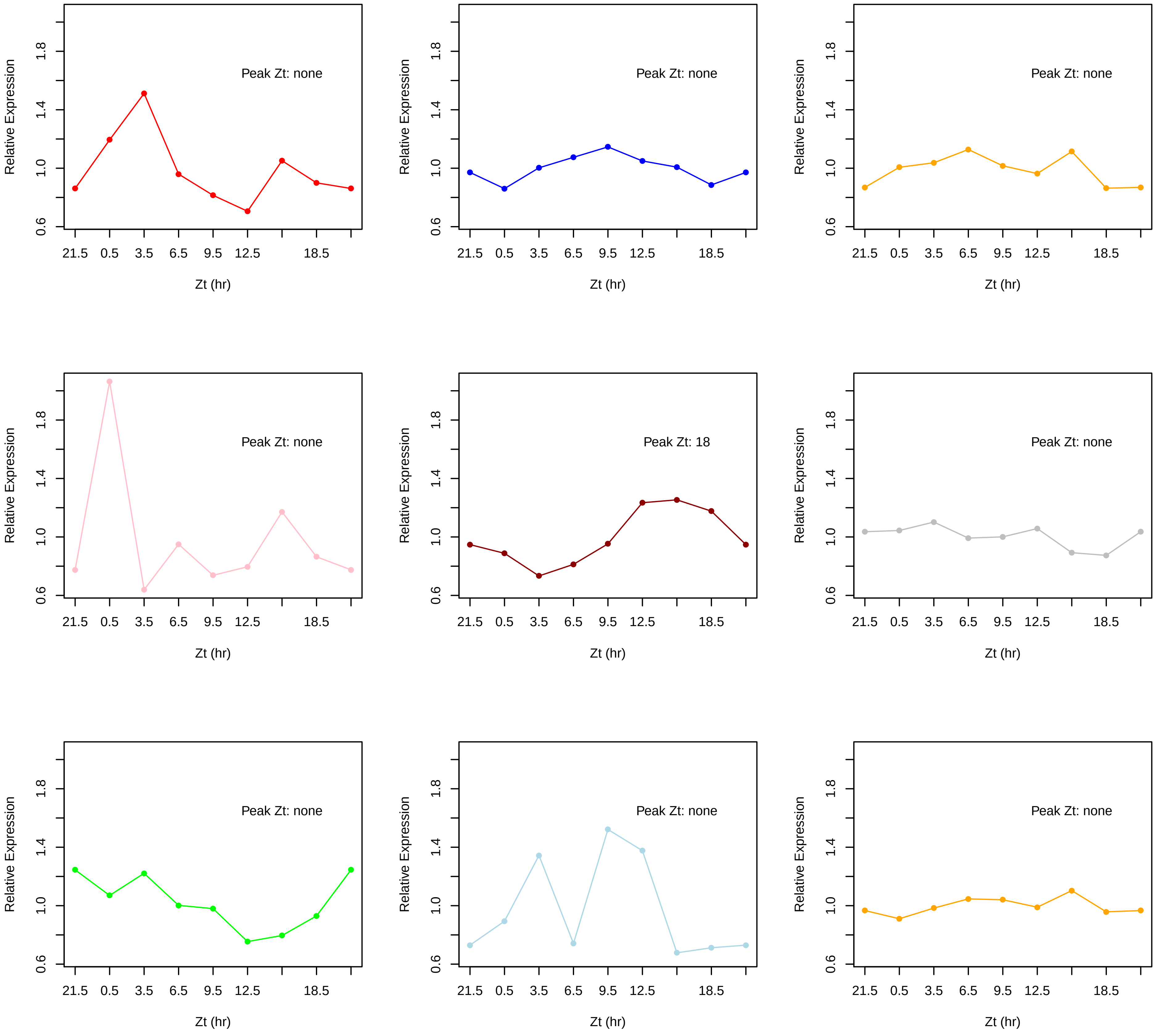

Singeleton: ENSXMAG00000002066 WHAMM

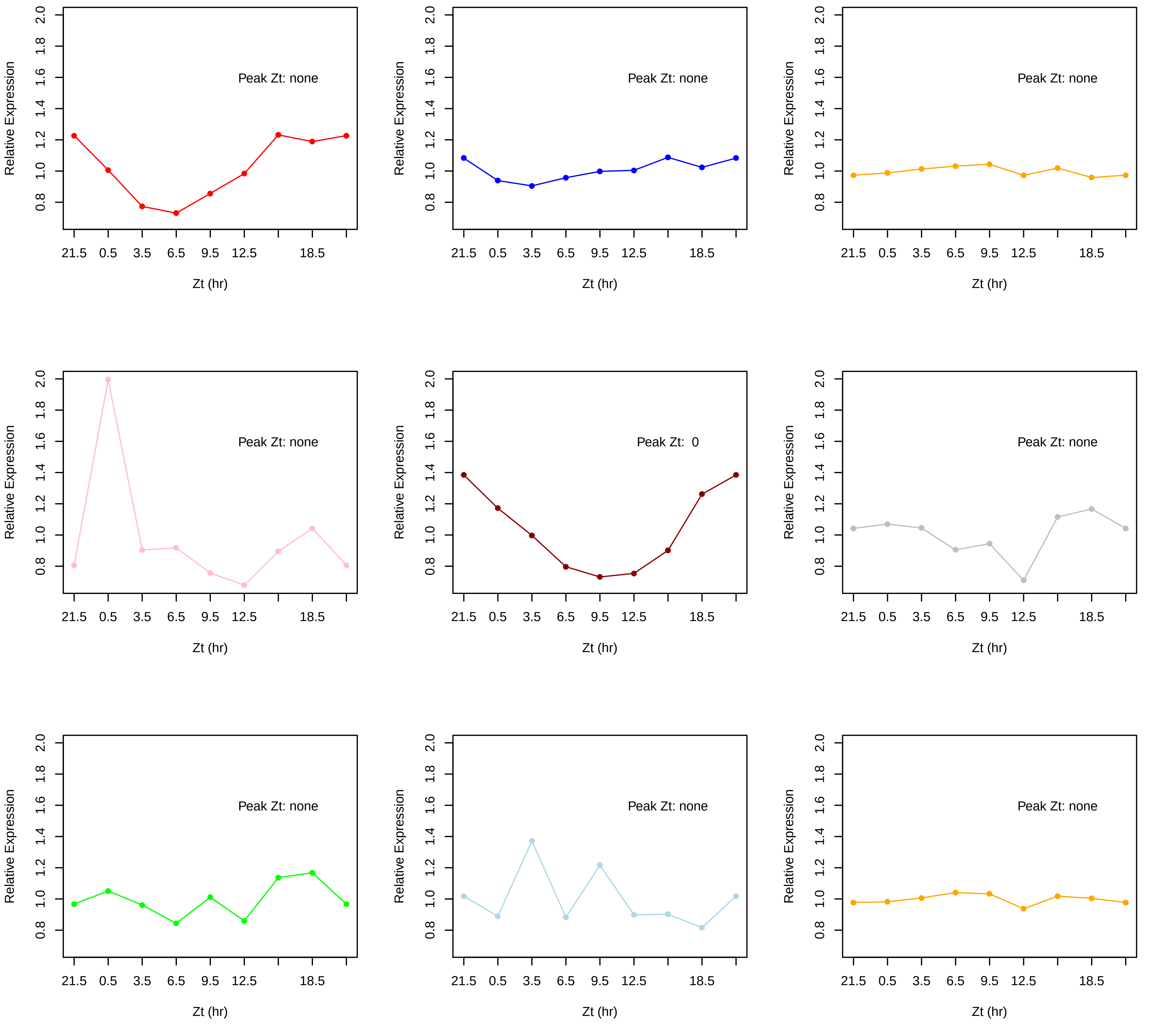

Singeleton: ENSXMAG00000016362 wrnip1

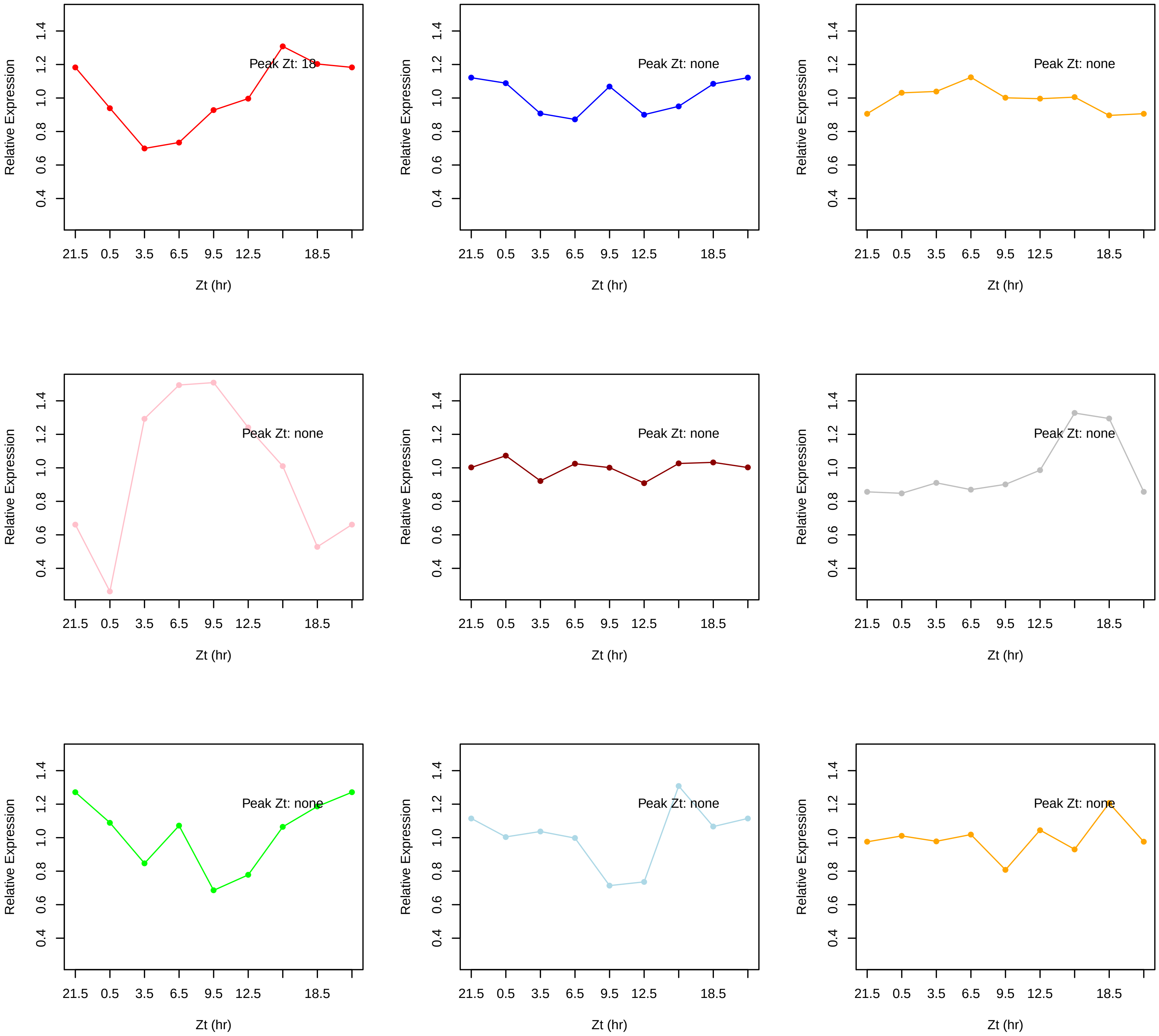

Singleton: ENSXMAG00000002082 pde8a

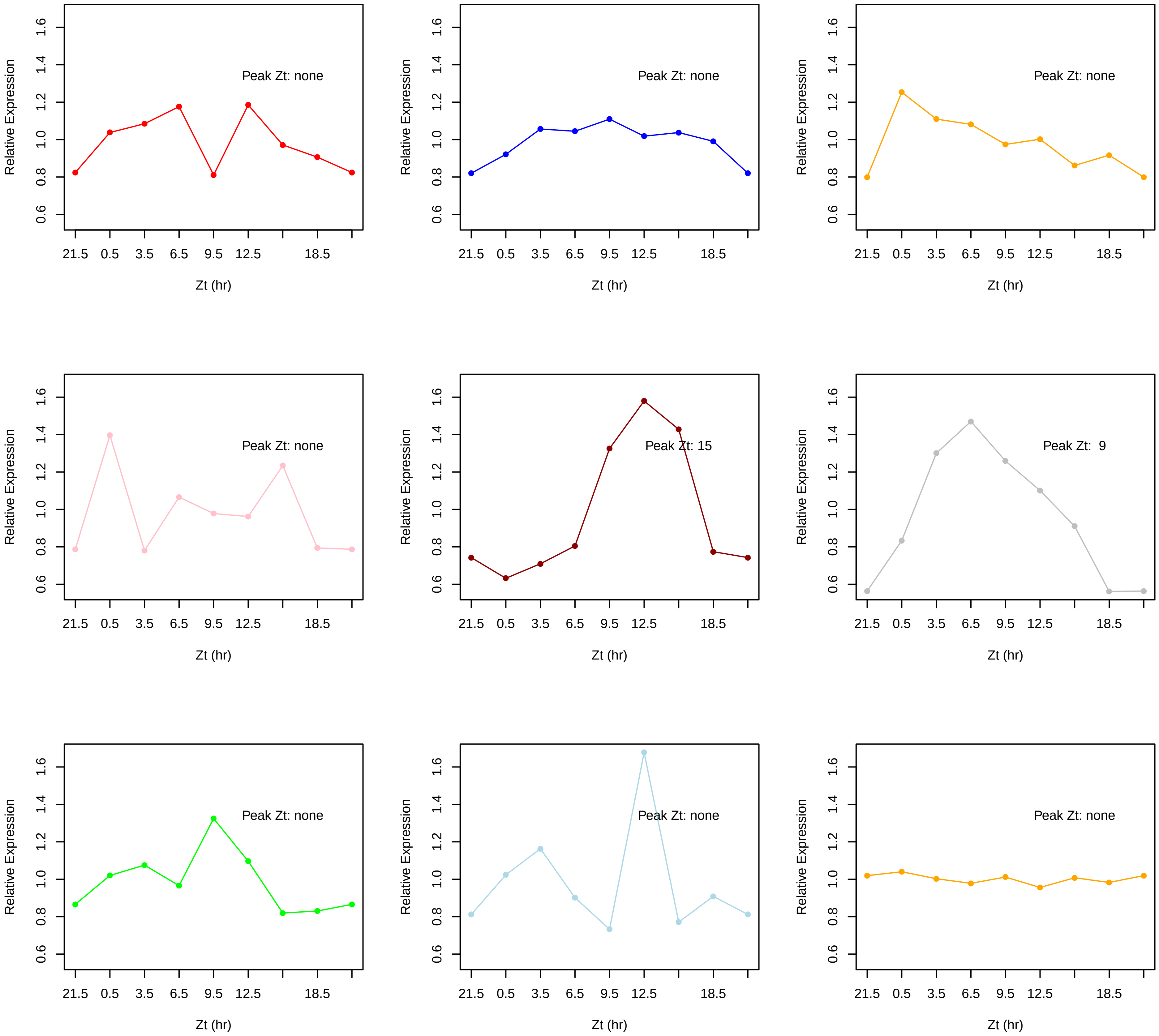

Singleton: ENSXMAG00000008040

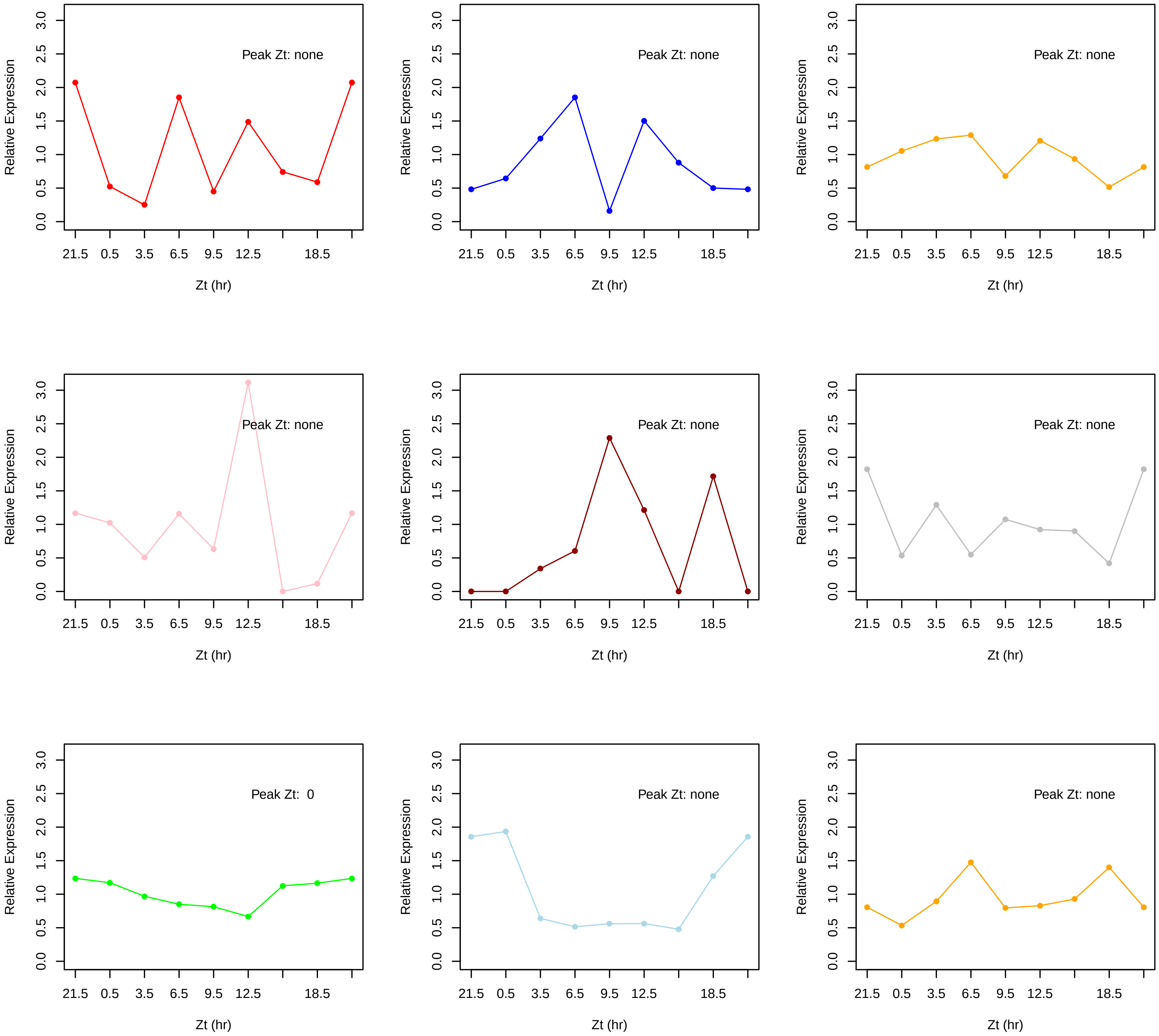

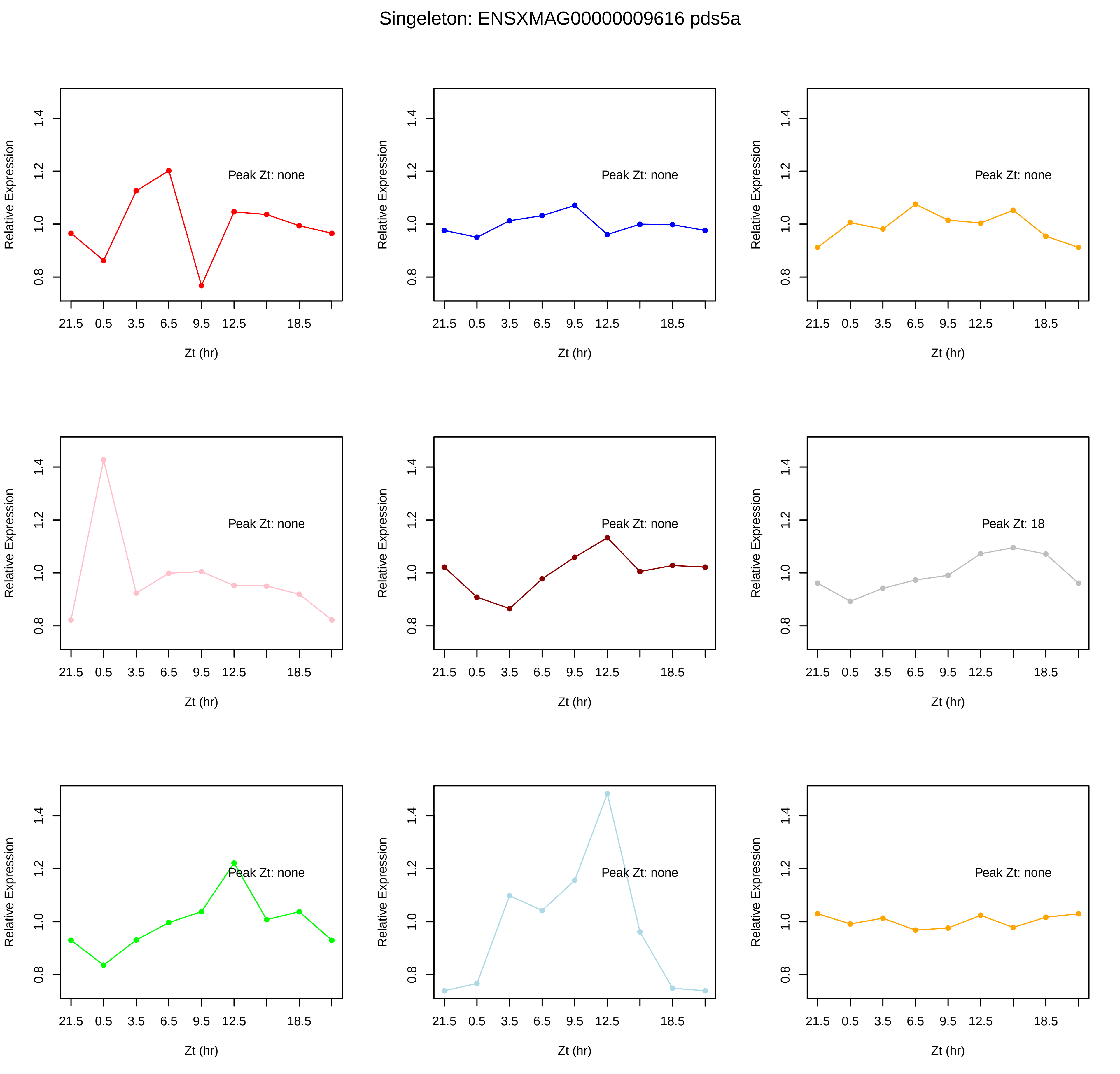
Singleton: ENSXMAG00000009616 pds5a
Peak Zt: none
Peak Zt: none
Peak Zt: none
Peak Zt: none
Peak Zt: none
Peak Zt: 18
Peak Zt: none
Peak Zt: none
Peak Zt: none
Relative Expression
Zt (hr)
21.5
0.5
3.5
6.5
9.5
12.5
18.5
0.8
1.0
1.2
1.4

Singeleton: ENSXMAG00000007555 ppp4r4

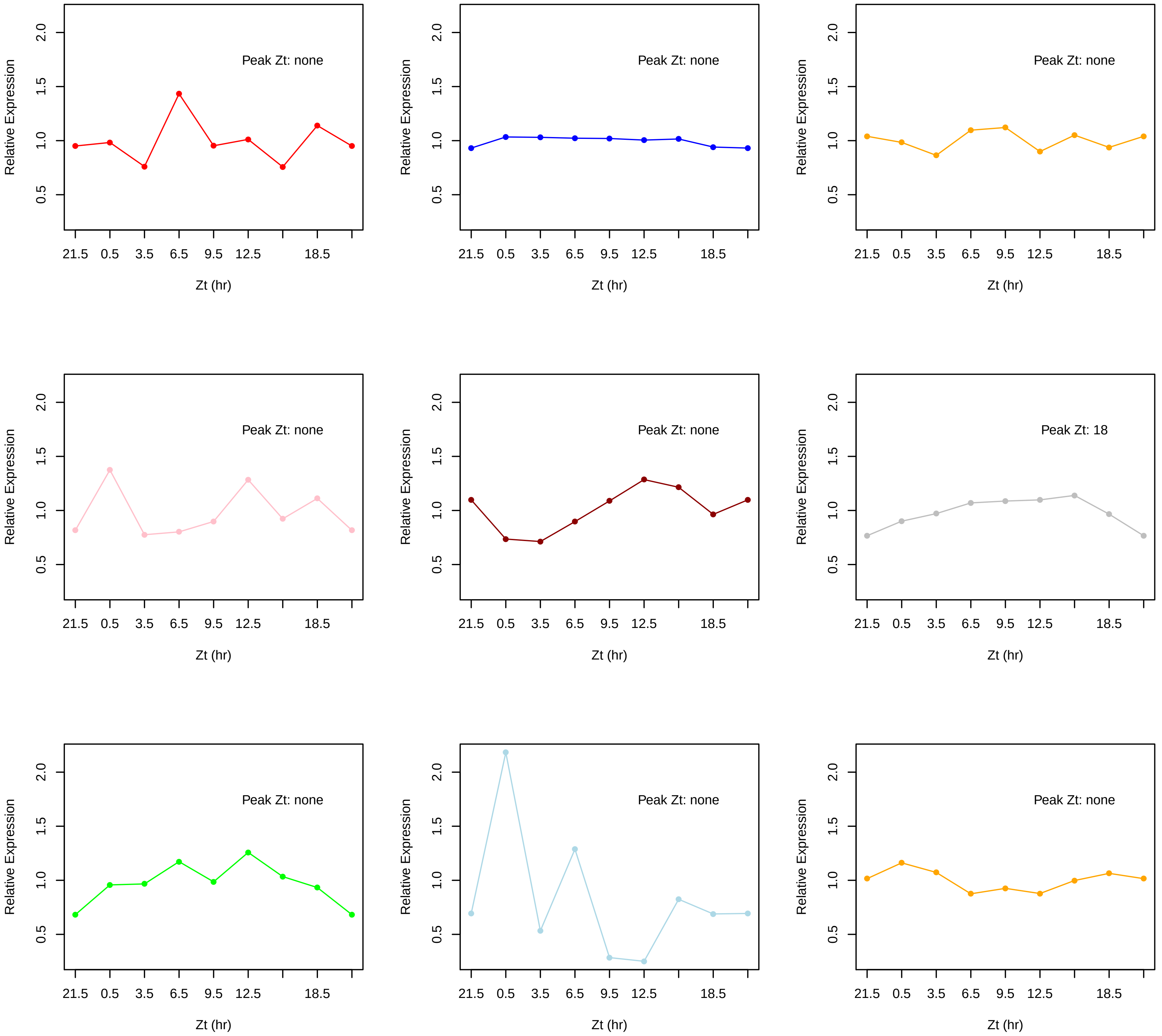

Singeleton: ENSXMAG00000006178 gmds

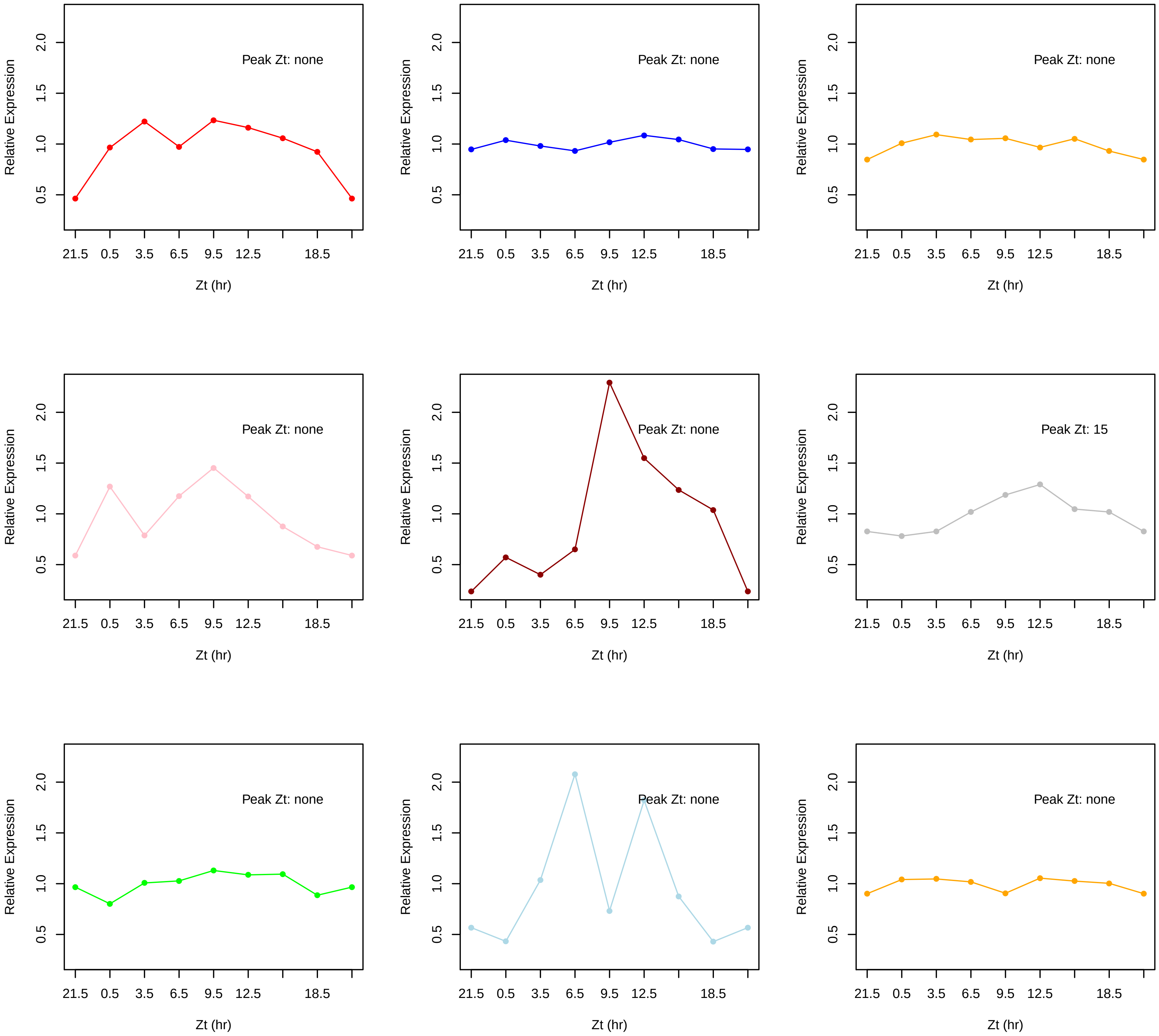

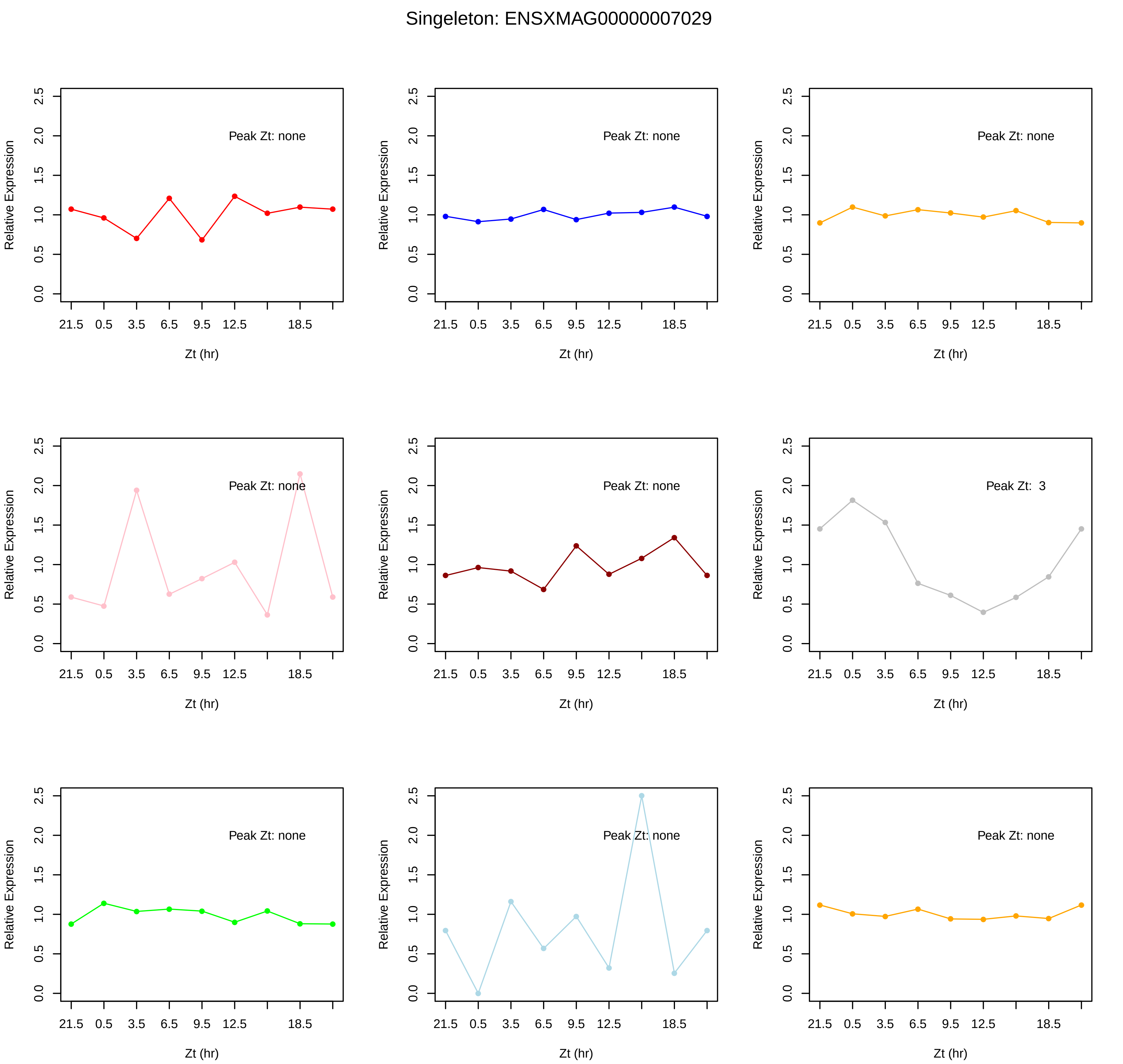

Singeleton: ENSXMAG00000007029
Peak Zt: none
Peak Zt: none
Peak Zt: none
Peak Zt: none
Peak Zt: none
Peak Zt: 3
Peak Zt: none
Peak Zt: none
Peak Zt: none
Relative Expression
Zt (hr)
21.5
0.5
3.5
6.5
9.5
12.5
18.5
0.0
0.5
1.0
1.5
2.0
2.5

Singeleton: ENSXMAG00000009602 ugdh

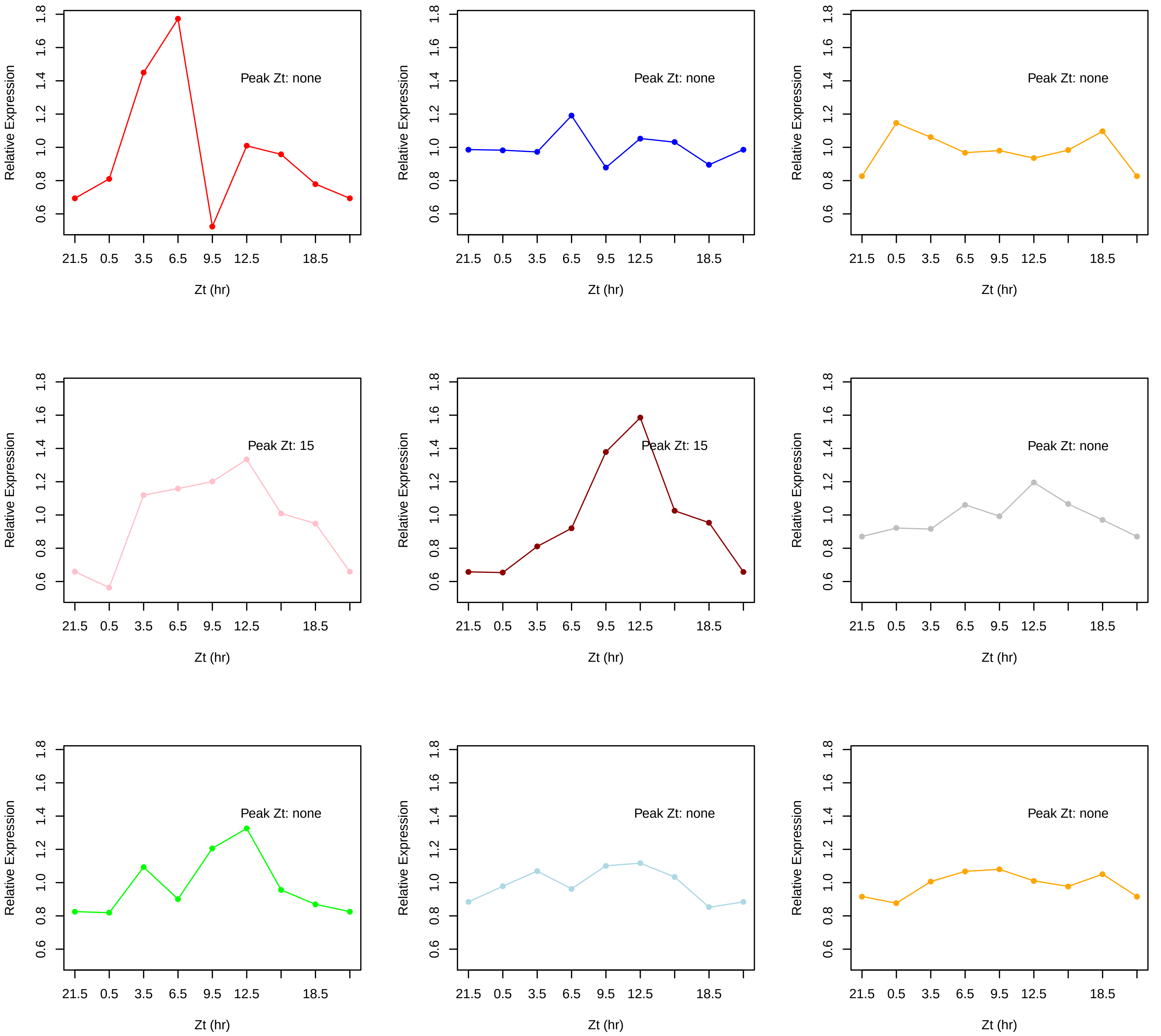

Singleton: ENSXMAG00000013600 ddx24

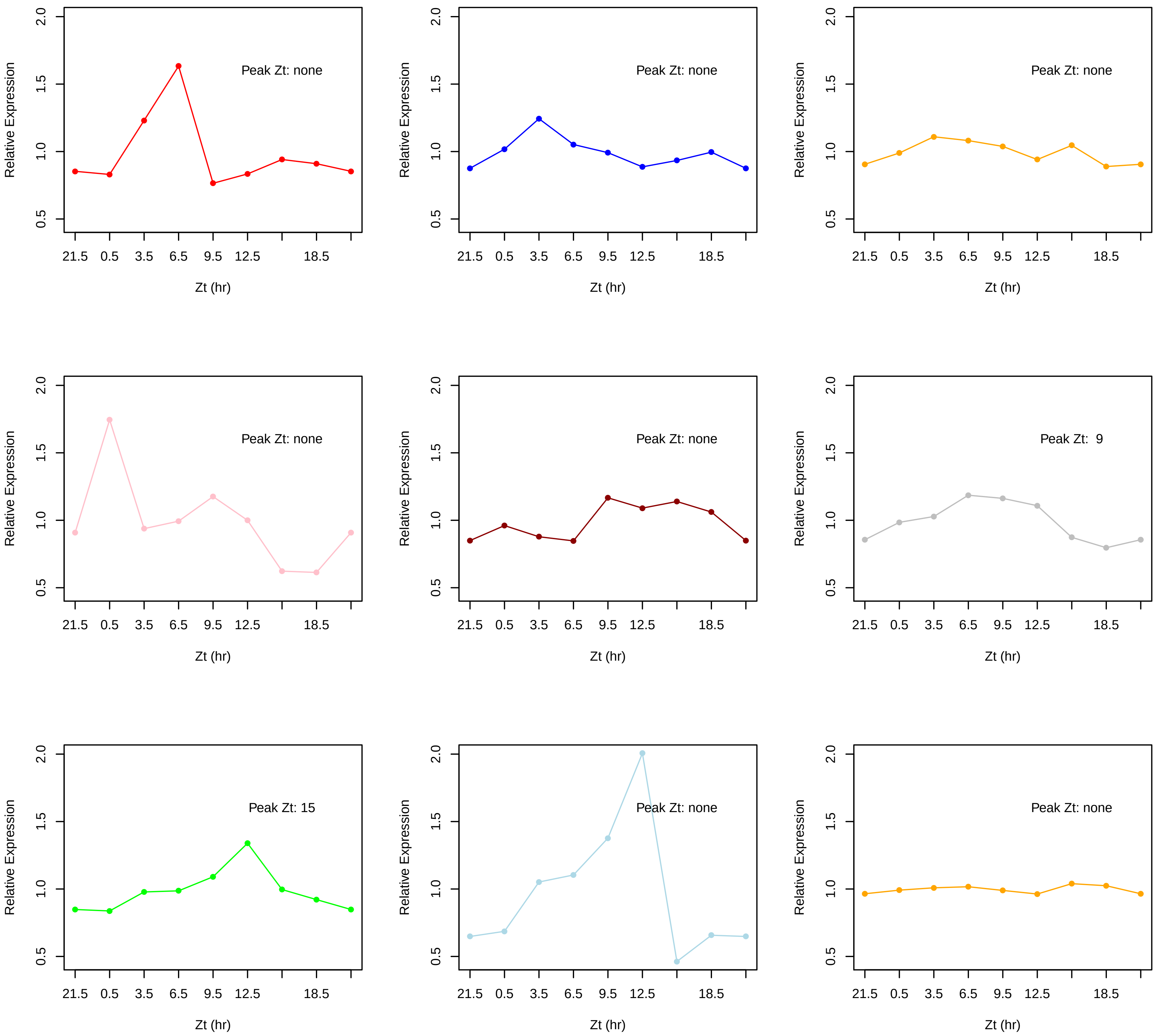

Singeleton: ENSXMAG00000026826 ormdl3

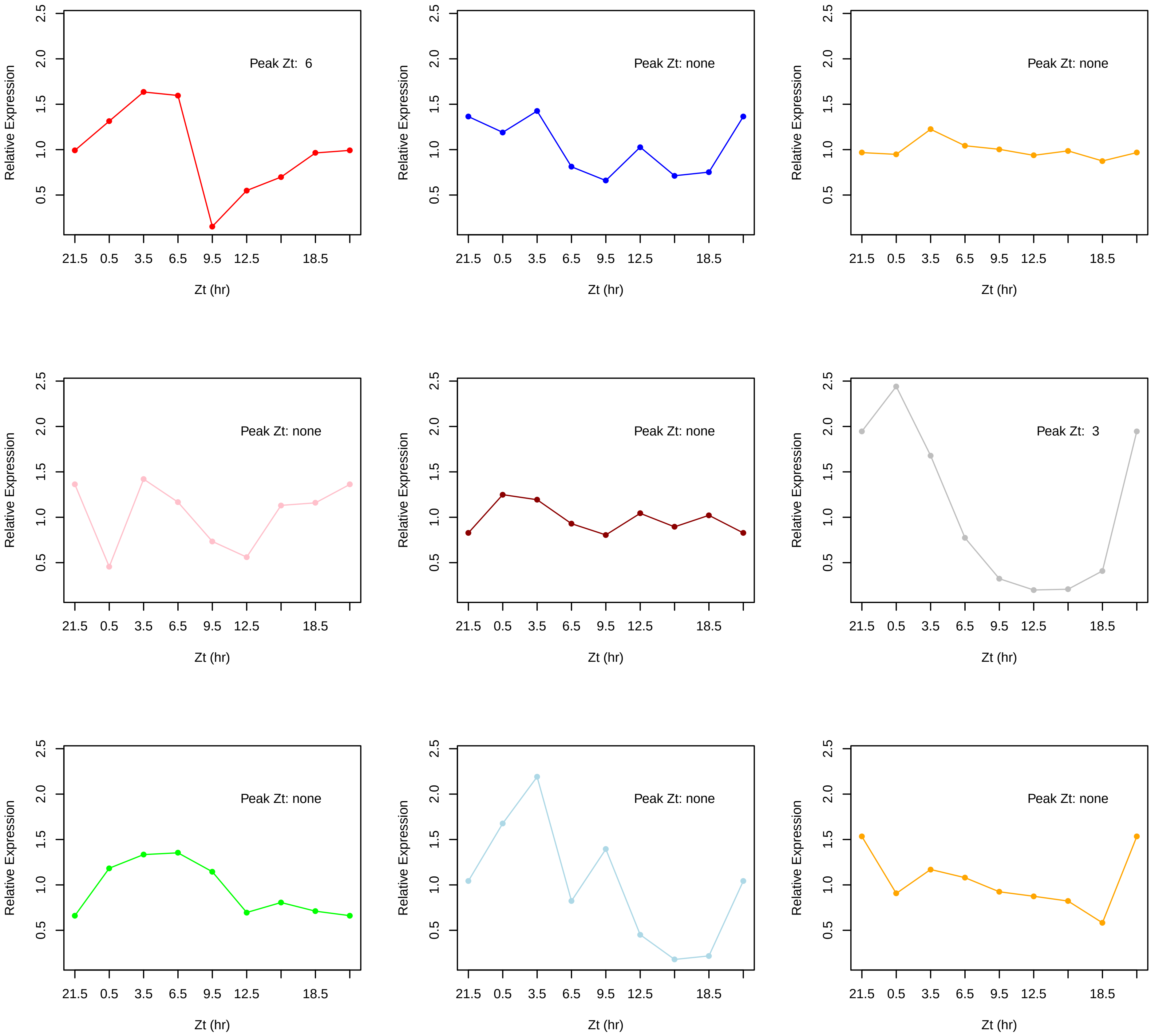

Singeleton: ENSXMAG00000009594 lias

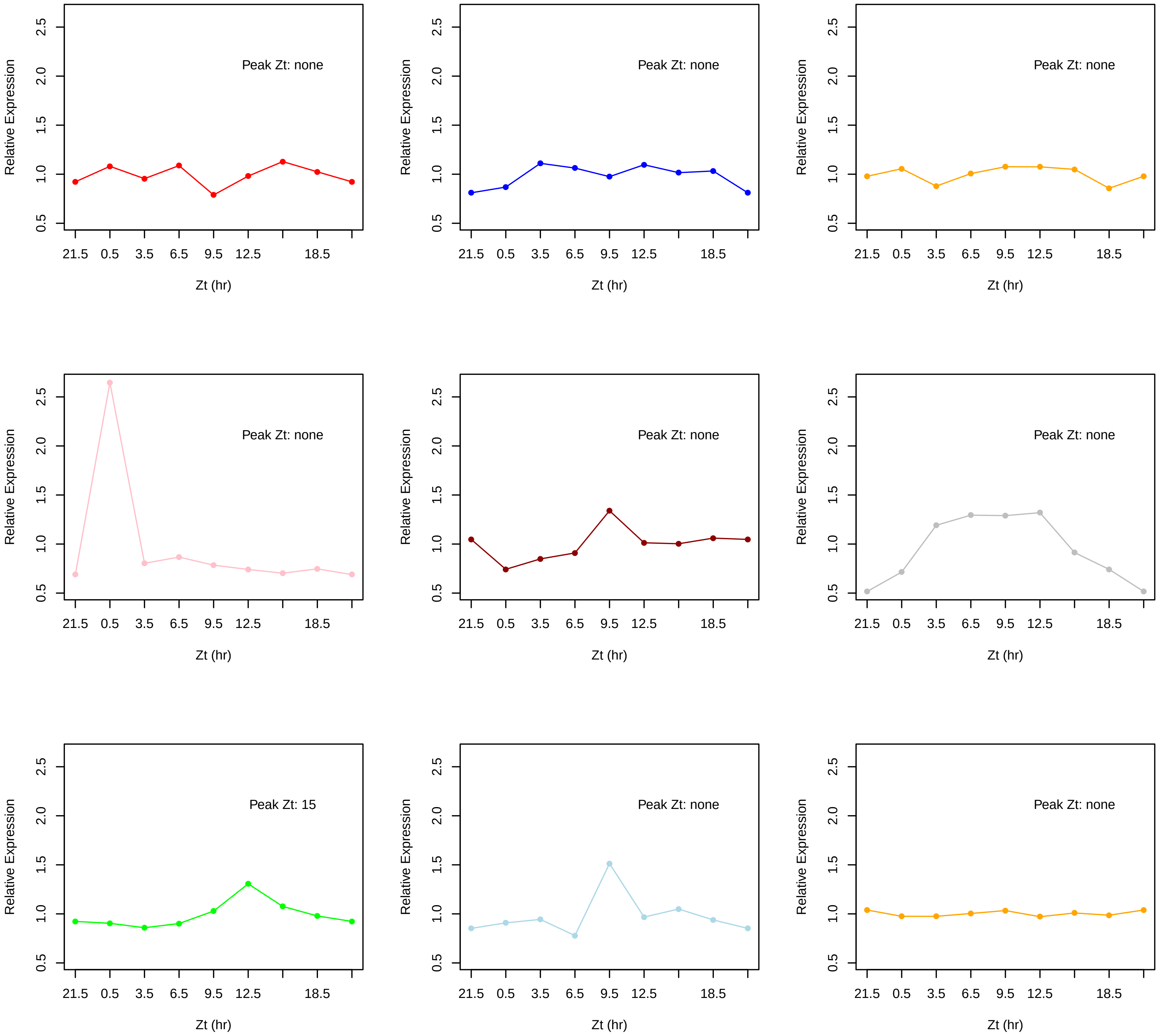

Singeleton: ENSXMAG00000008078 afap1l2

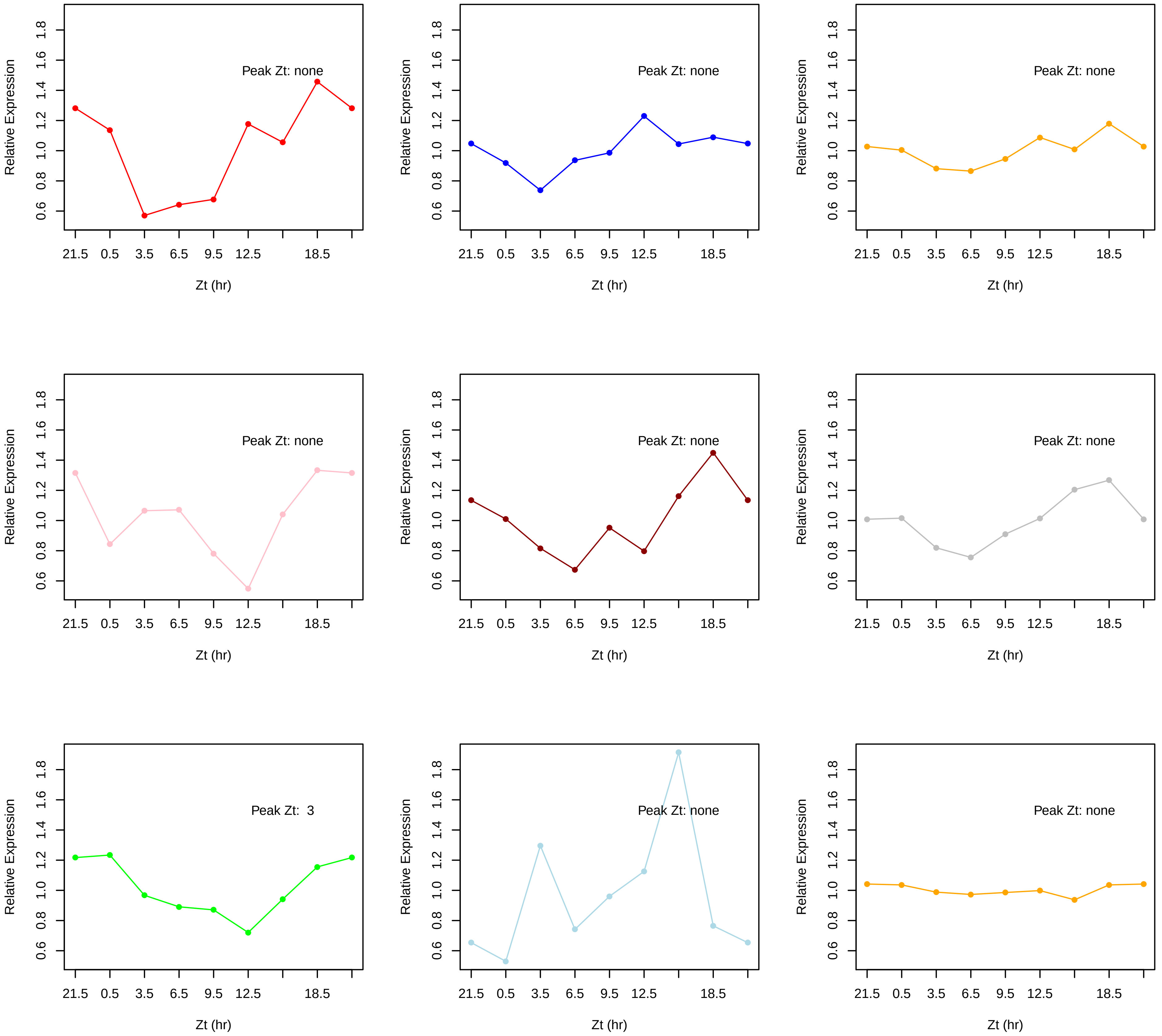

Singeleton: ENSXMAG00000014563 nploc4

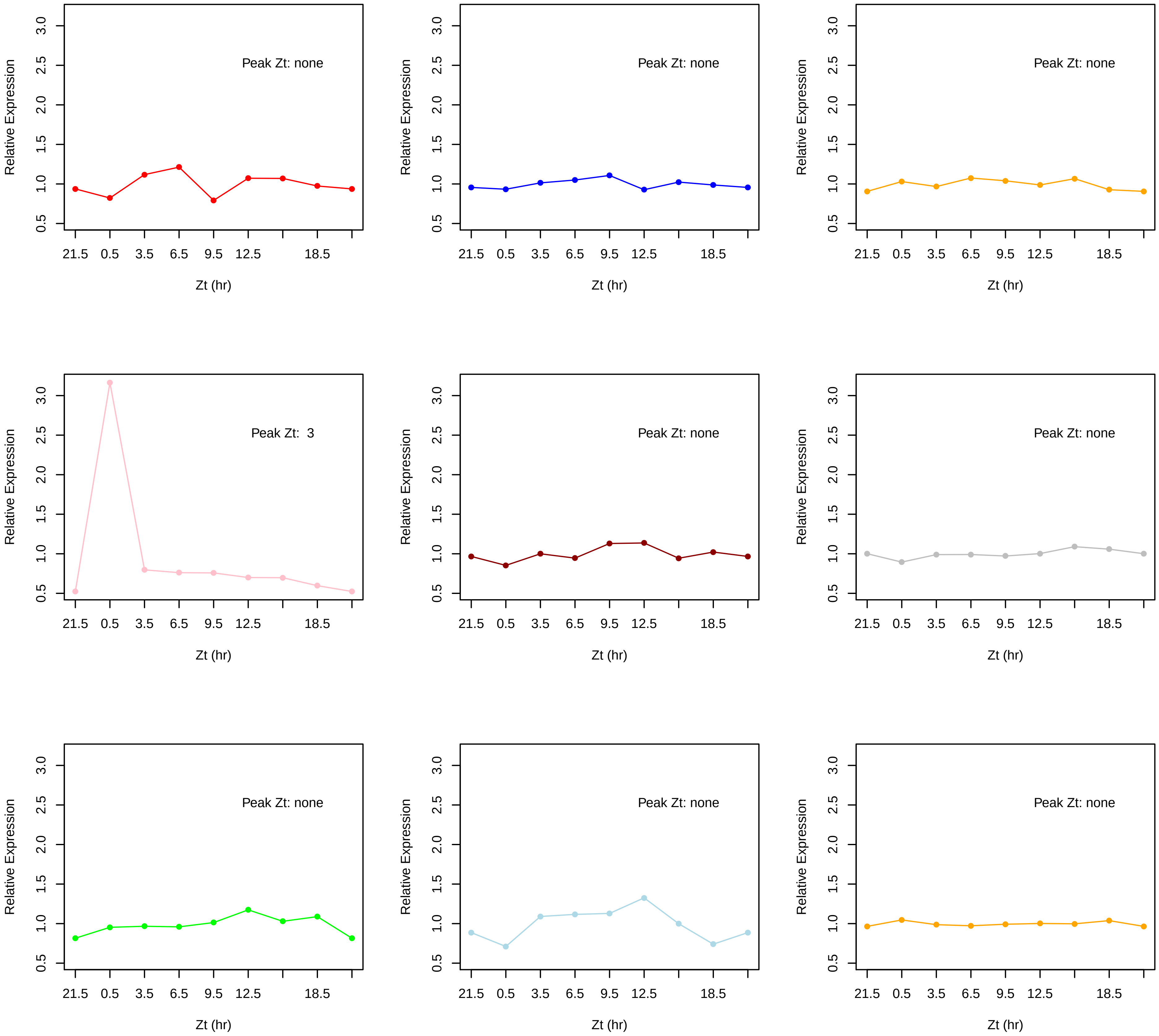

Singeleton: ENSXMAG00000013596 OTUB2

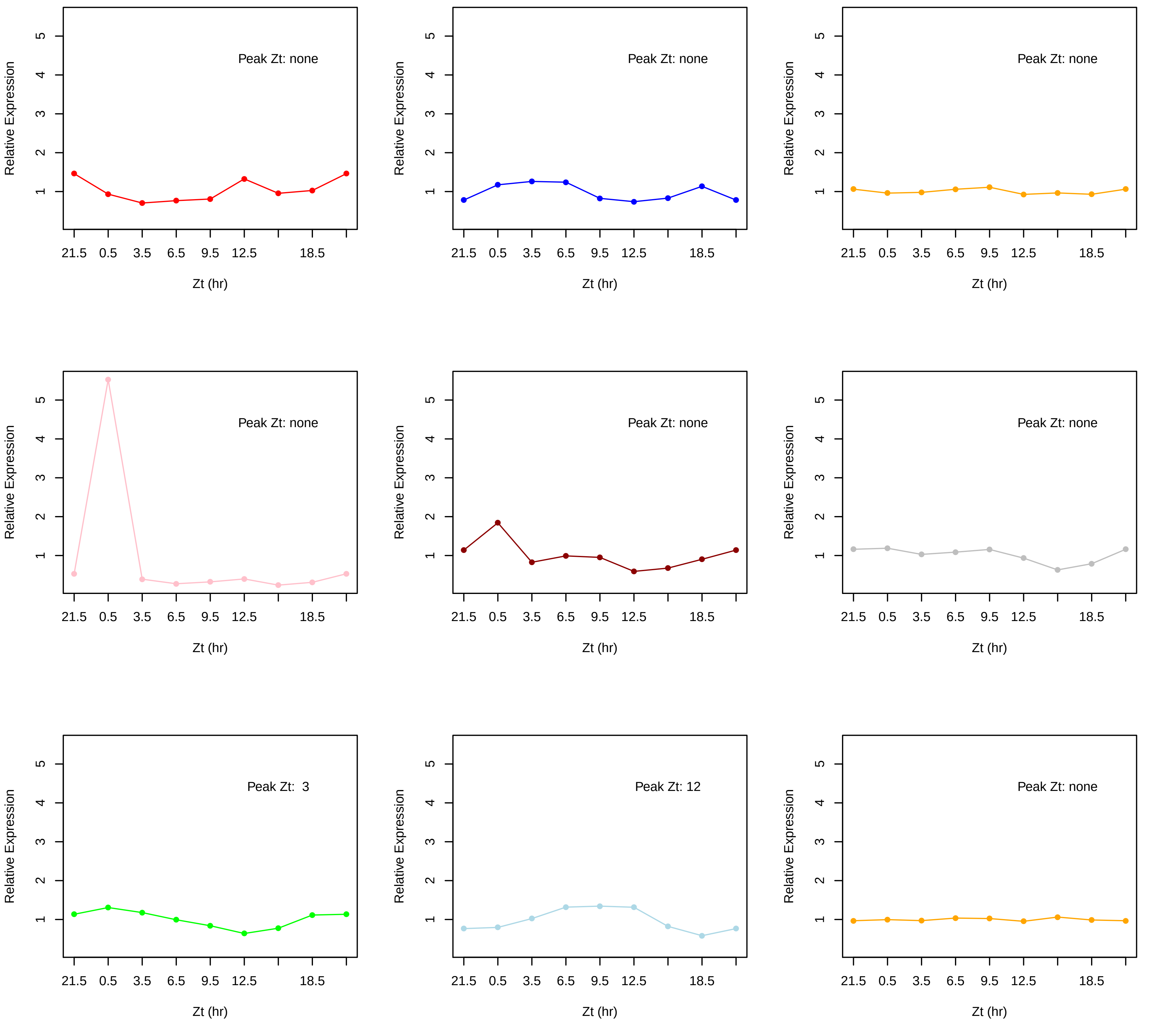

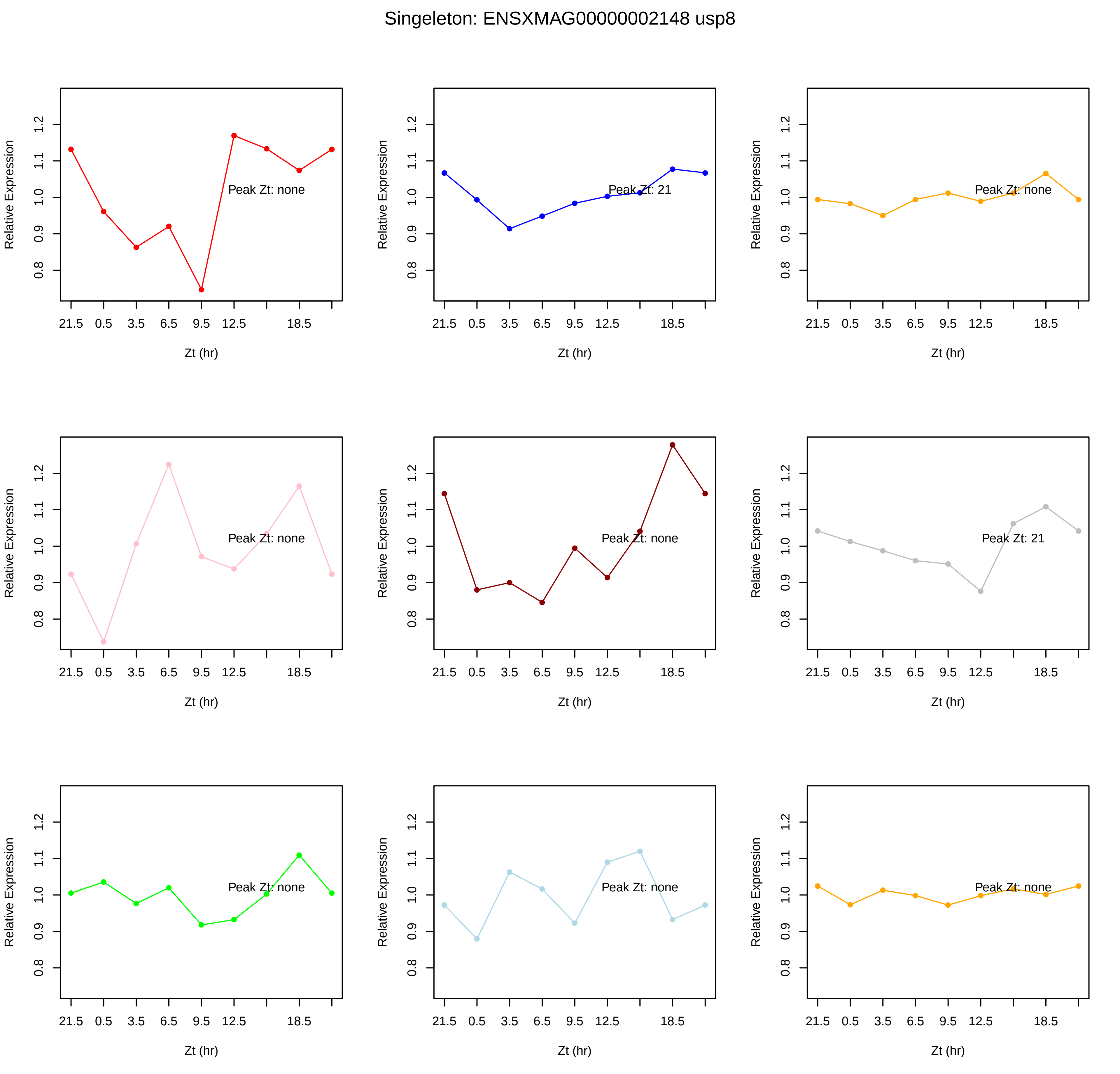
Singeleton: ENSXMAG00000002148 usp8
Relative Expression
Zt (hr)
21.5
0.5
3.5
6.5
9.5
12.5
18.5
0.8
0.9
1.0
1.1
1.2
Peak Zt: none
Peak Zt: 21
Peak Zt: none
Peak Zt: none
Peak Zt: none
Peak Zt: 21
Peak Zt: none
Peak Zt: none
Peak Zt: none

Singeleton: ENSXMAG00000016697

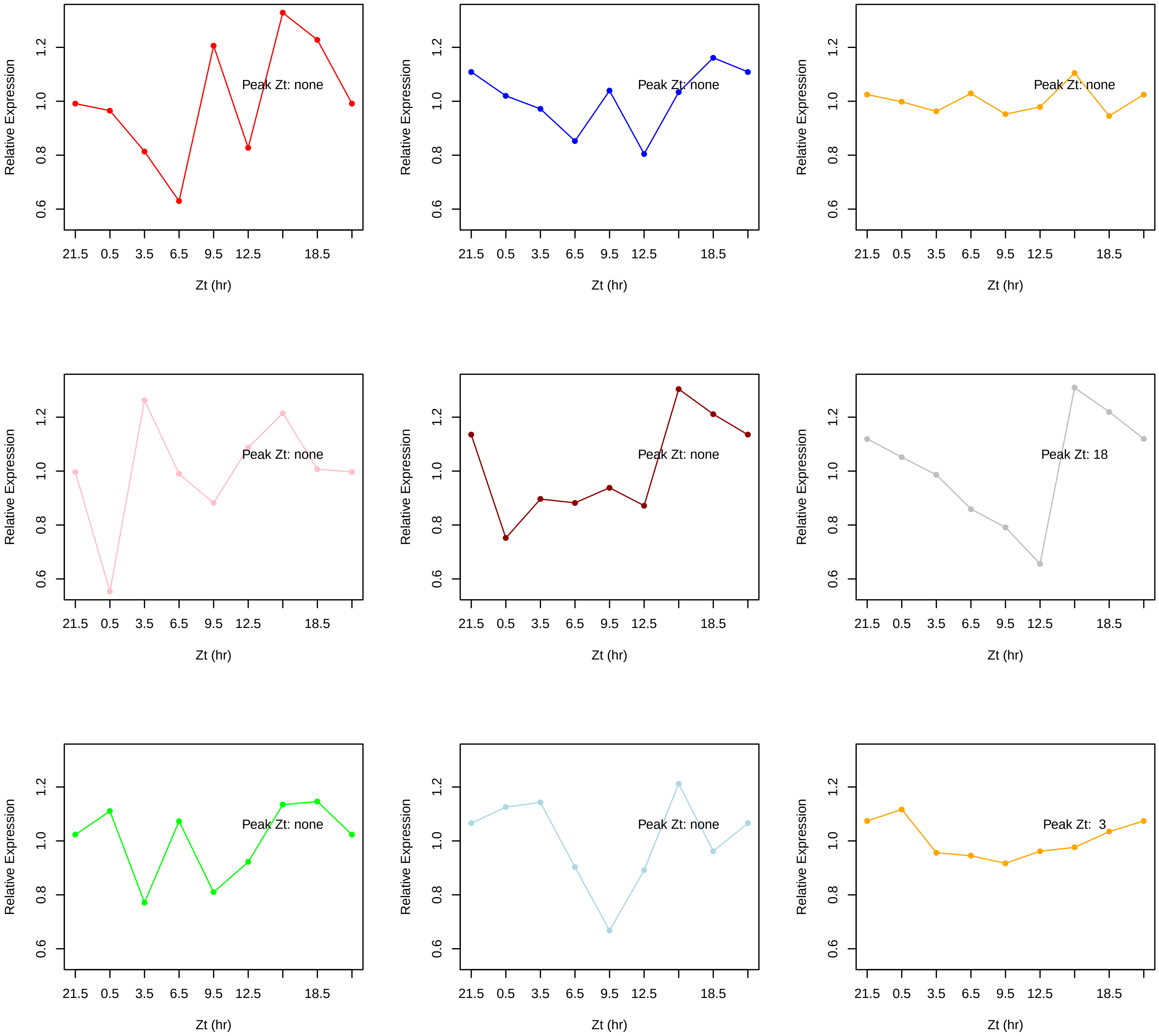

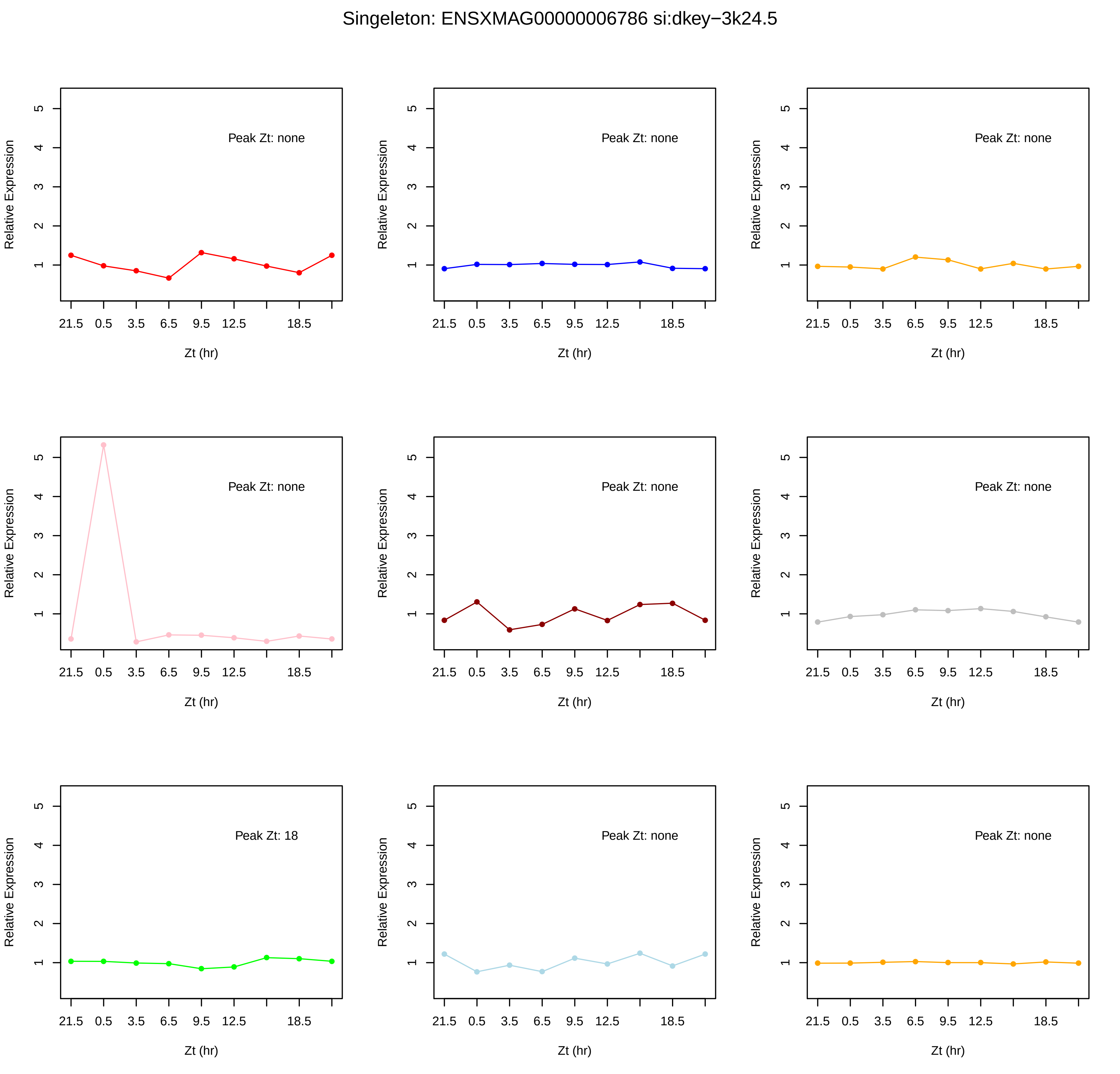

Singeleton: ENSXMAG00000006786 si:dkey−3k24.5
Peak Zt: none
Peak Zt: none
Peak Zt: none
Peak Zt: none
Peak Zt: none
Peak Zt: none
Peak Zt: 18
Peak Zt: none
Peak Zt: none
Relative Expression
Zt (hr)
21.5 0.5 3.5 6.5 9.5 12.5 18.5

Singleton: ENSXMAG00000029585 CCDC137

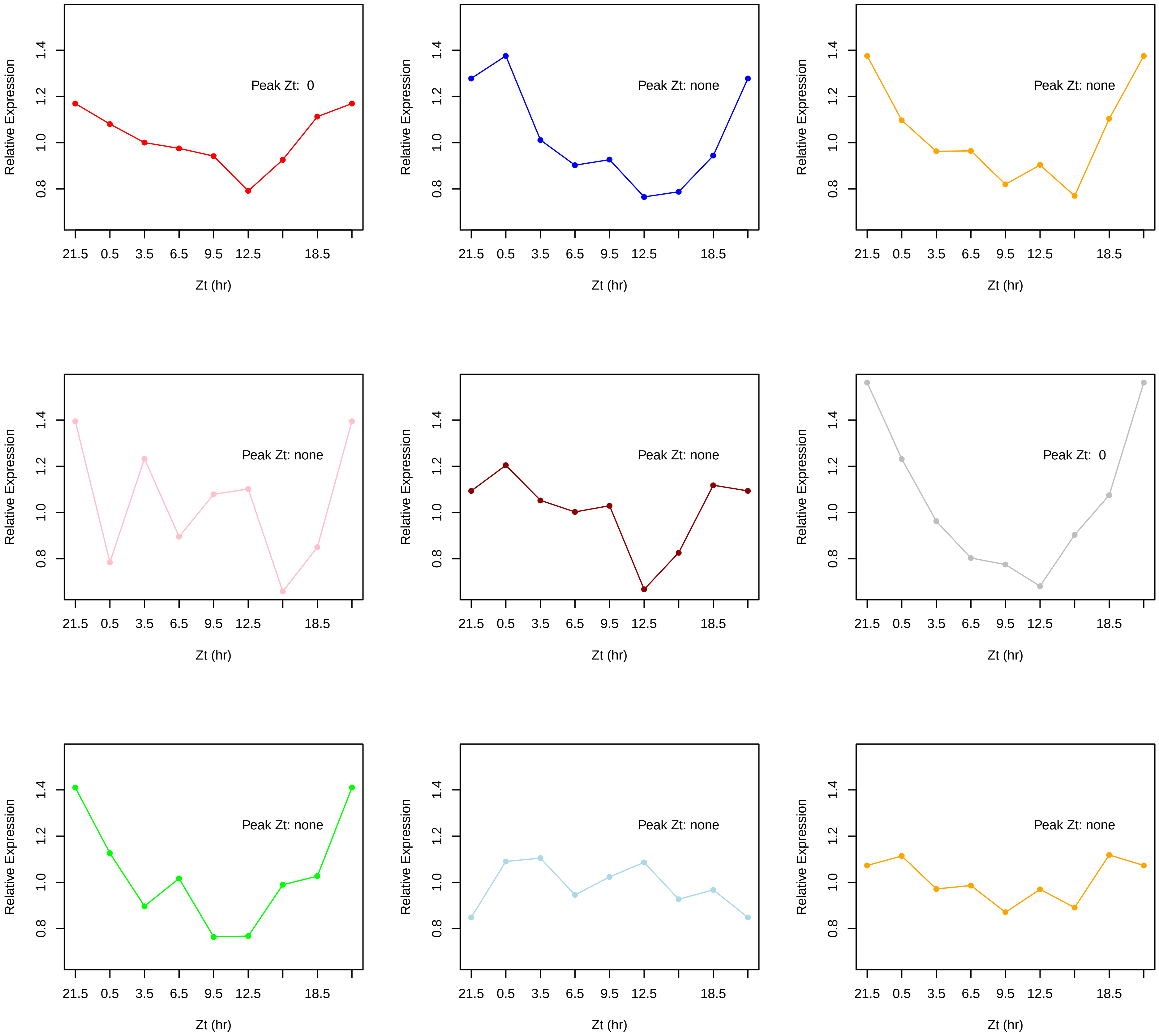

Singleton: ENSXMAG00000014648

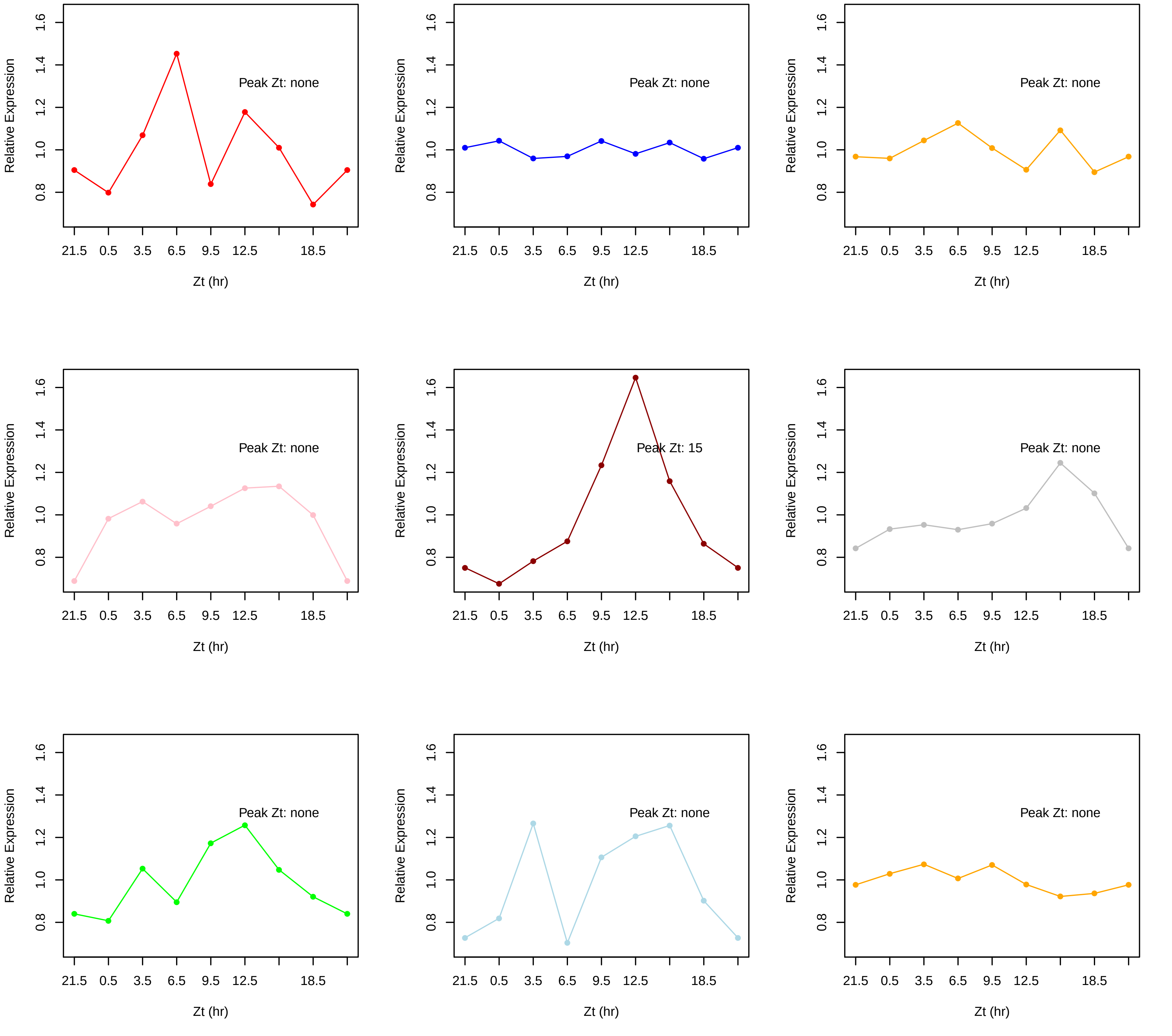

Singleton: ENSXMAG00000008231

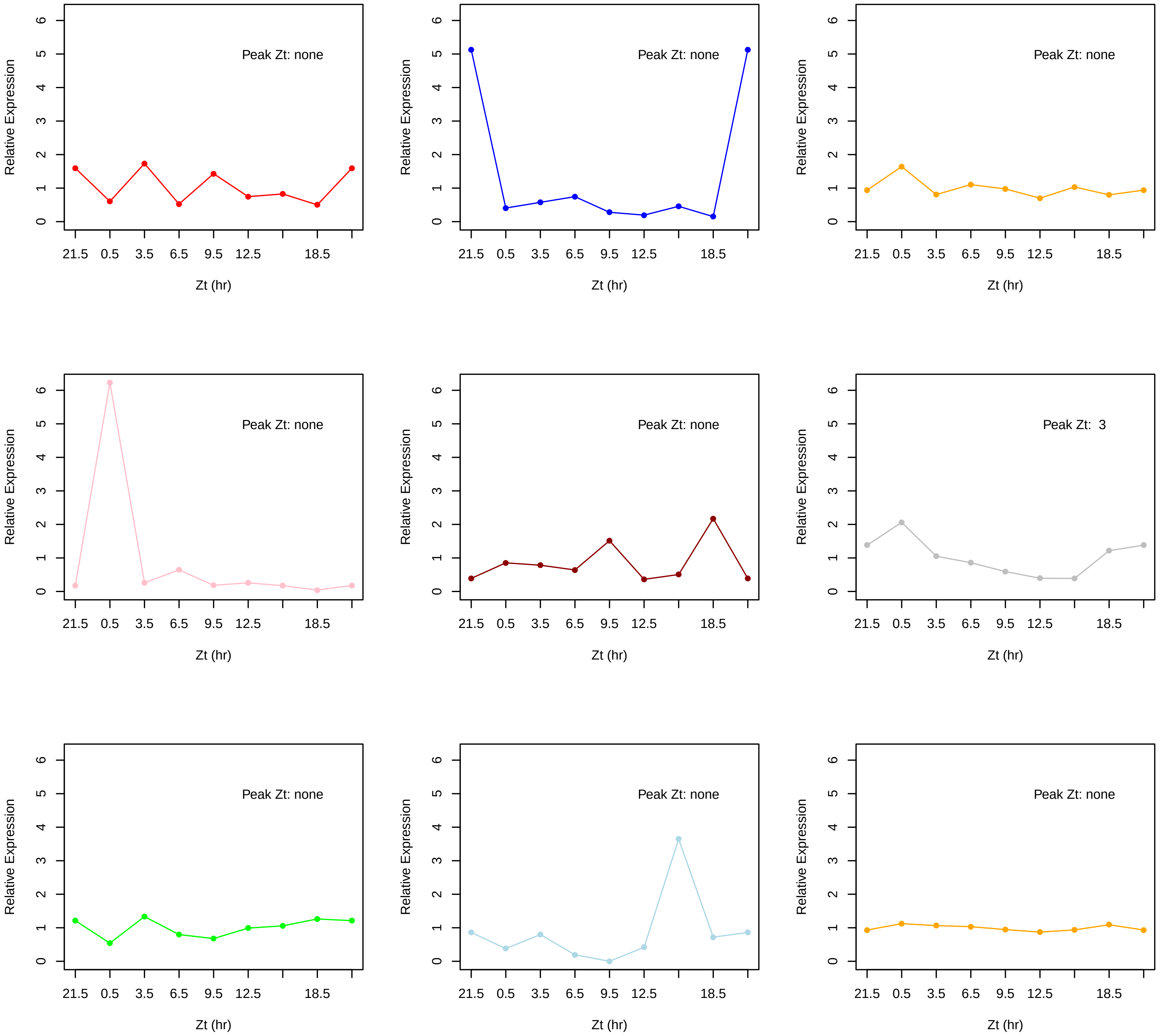

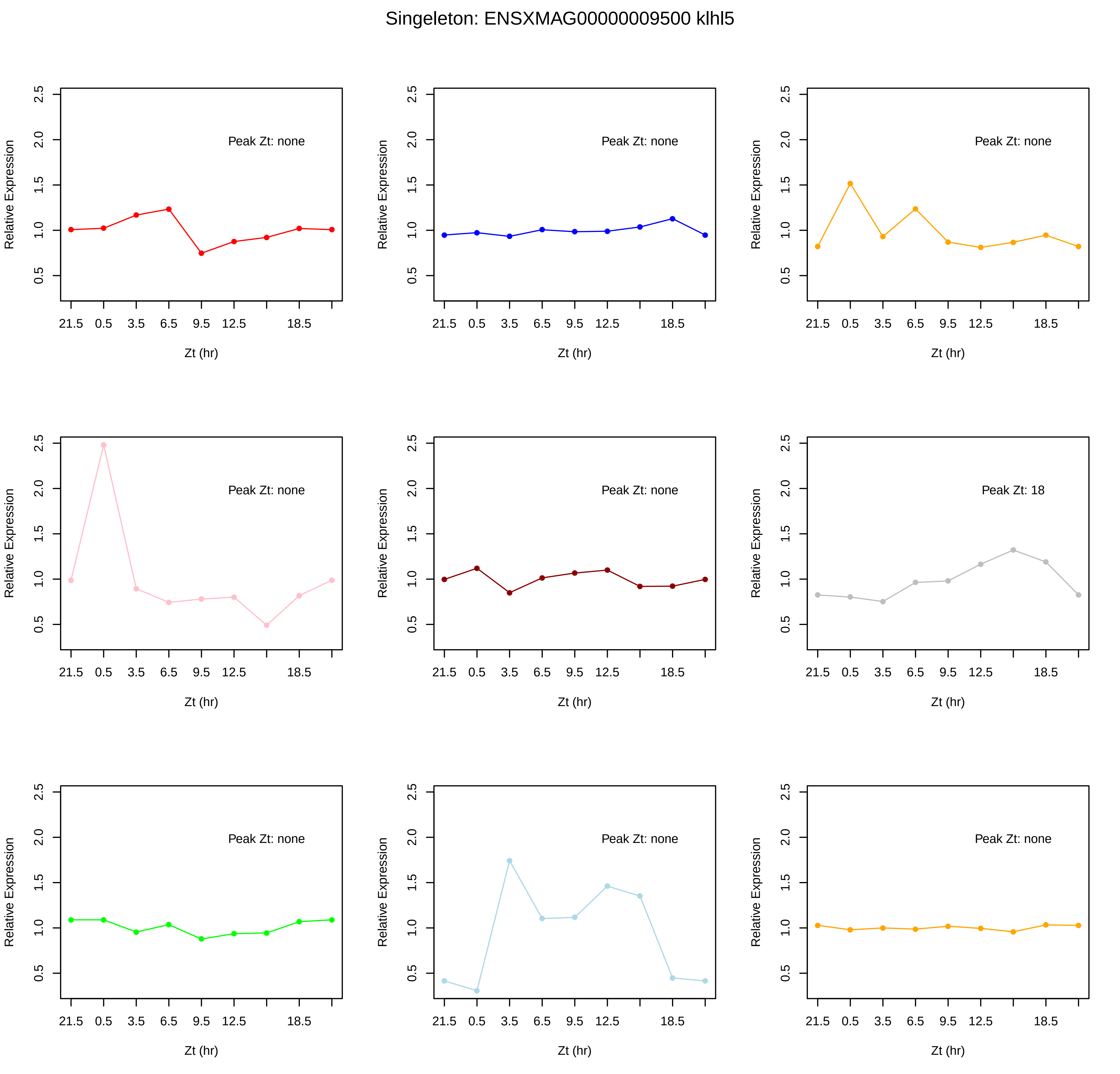
Singeleton: ENSXMAG00000009500 klhl5
Peak Zt: none
Peak Zt: none
Peak Zt: none
Peak Zt: none
Peak Zt: none
Peak Zt: 18
Peak Zt: none
Peak Zt: none
Peak Zt: none
Relative Expression
Zt (hr)
21.5
0.5
3.5
6.5
9.5
12.5
18.5
0.5
1.0
1.5
2.0
2.5

Singleton: ENSXMAG00000017357 IFT122

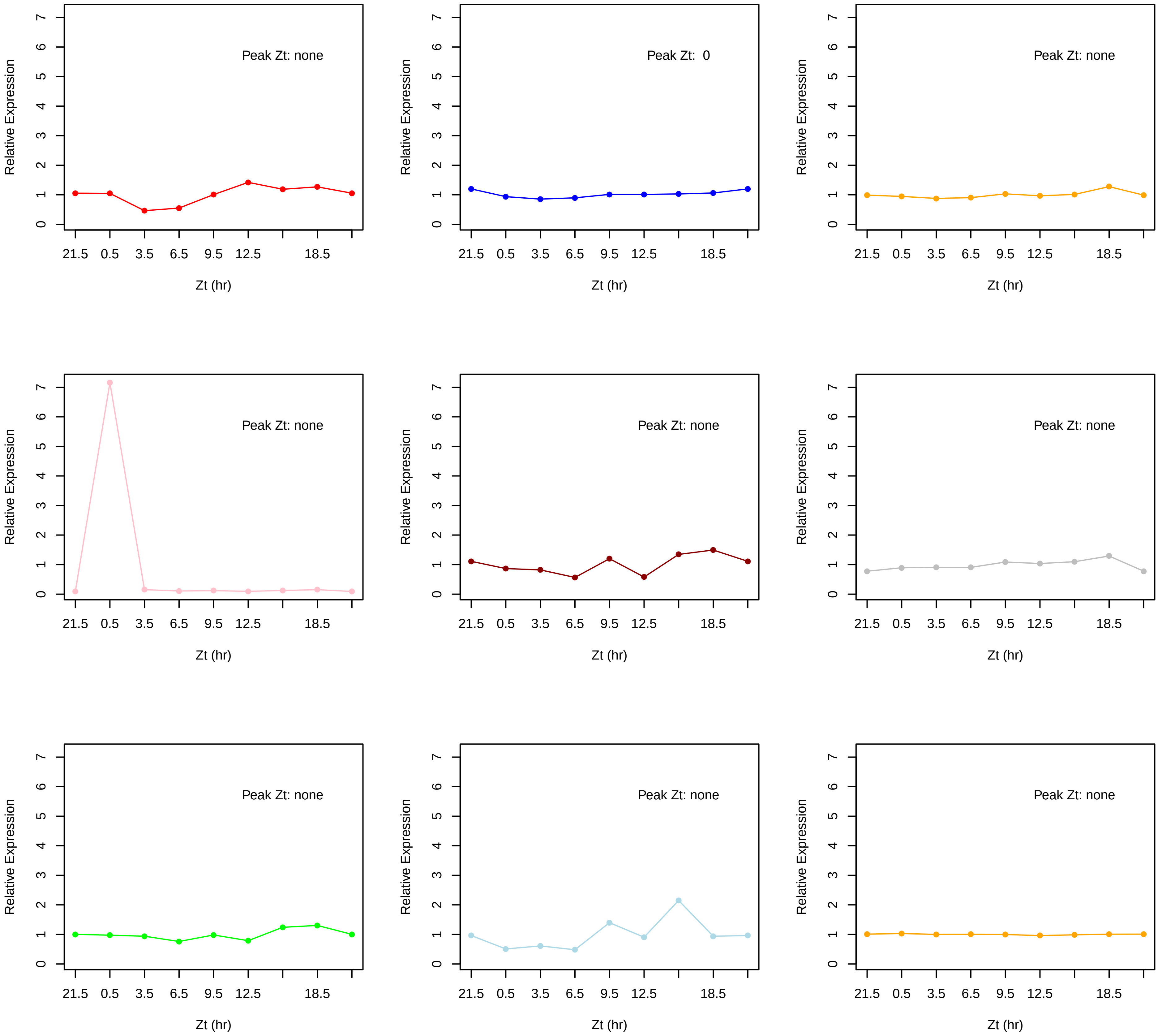

Singeleton: ENSXMAG00000022856 gpr142

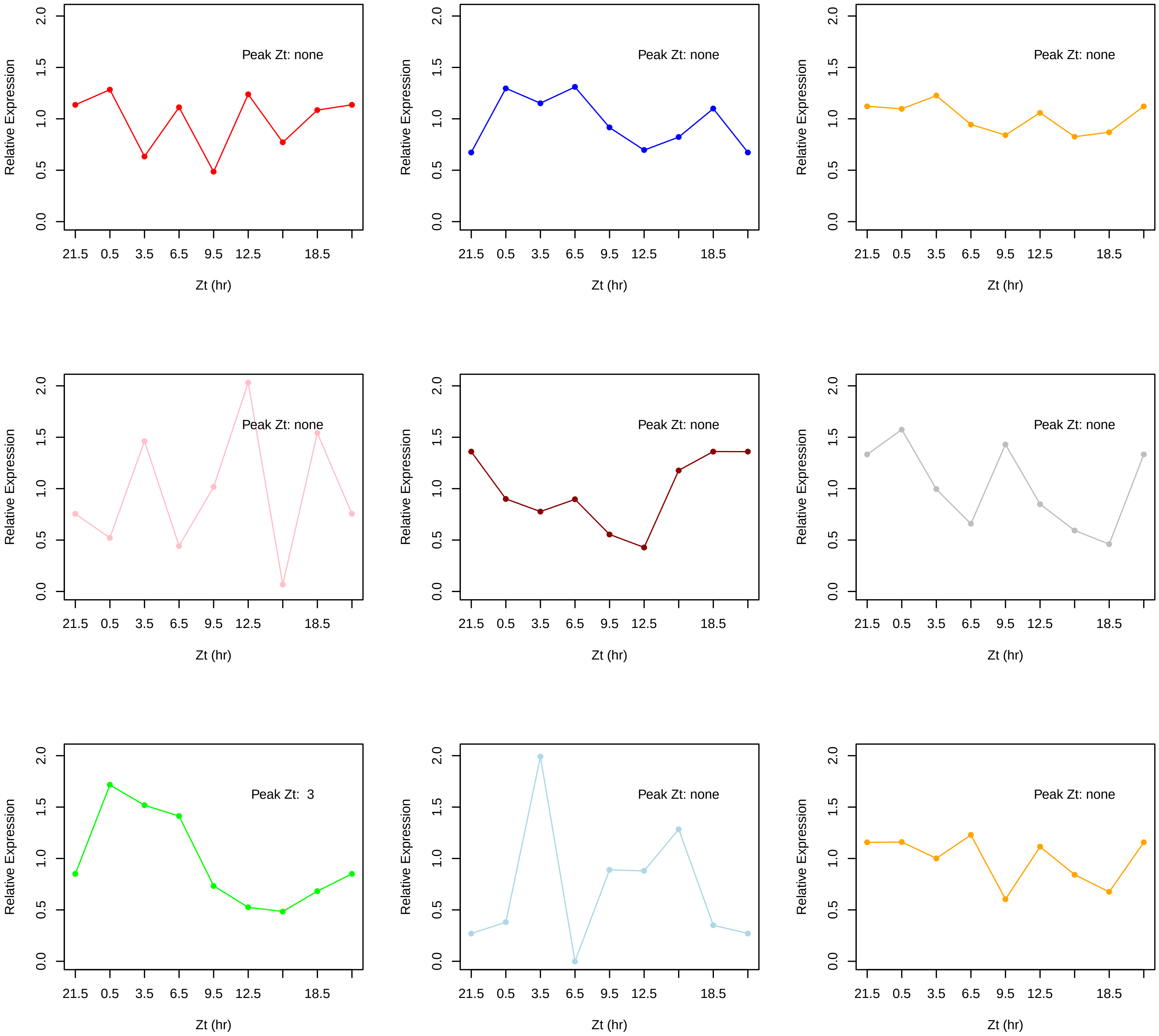

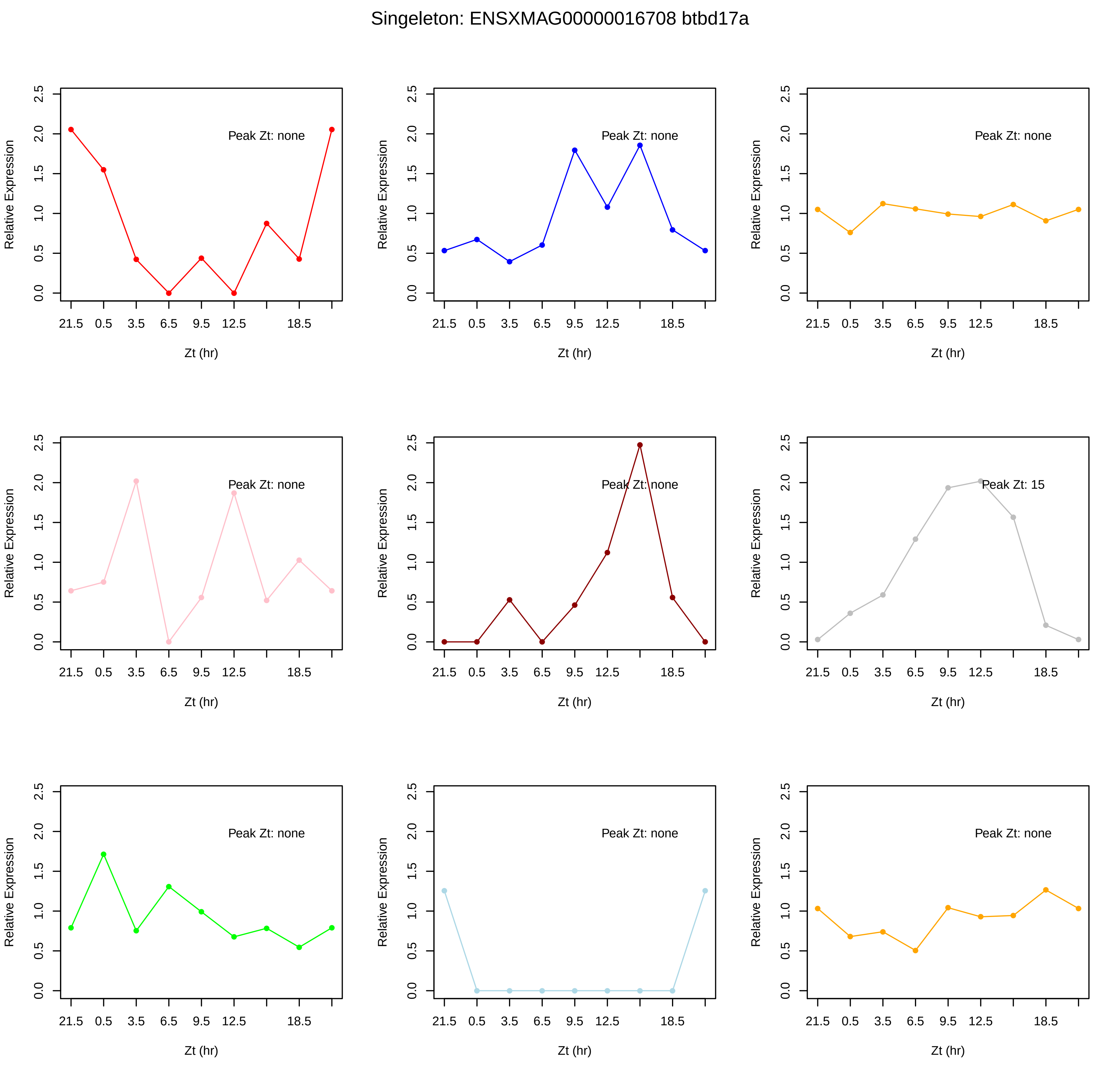

Singeleton: ENSXMAG00000016708 btbd17a
Relative Expression
Zt (hr)
21.5
0.5
3.5
6.5
9.5
12.5
18.5
0.0
0.5
1.0
1.5
2.0
2.5
Peak Zt: none
Peak Zt: none
Peak Zt: none
Peak Zt: none
Peak Zt: none
Peak Zt: 15
Peak Zt: none
Peak Zt: none
Peak Zt: none

Singeleton: ENSXMAG00000021129 mbd4

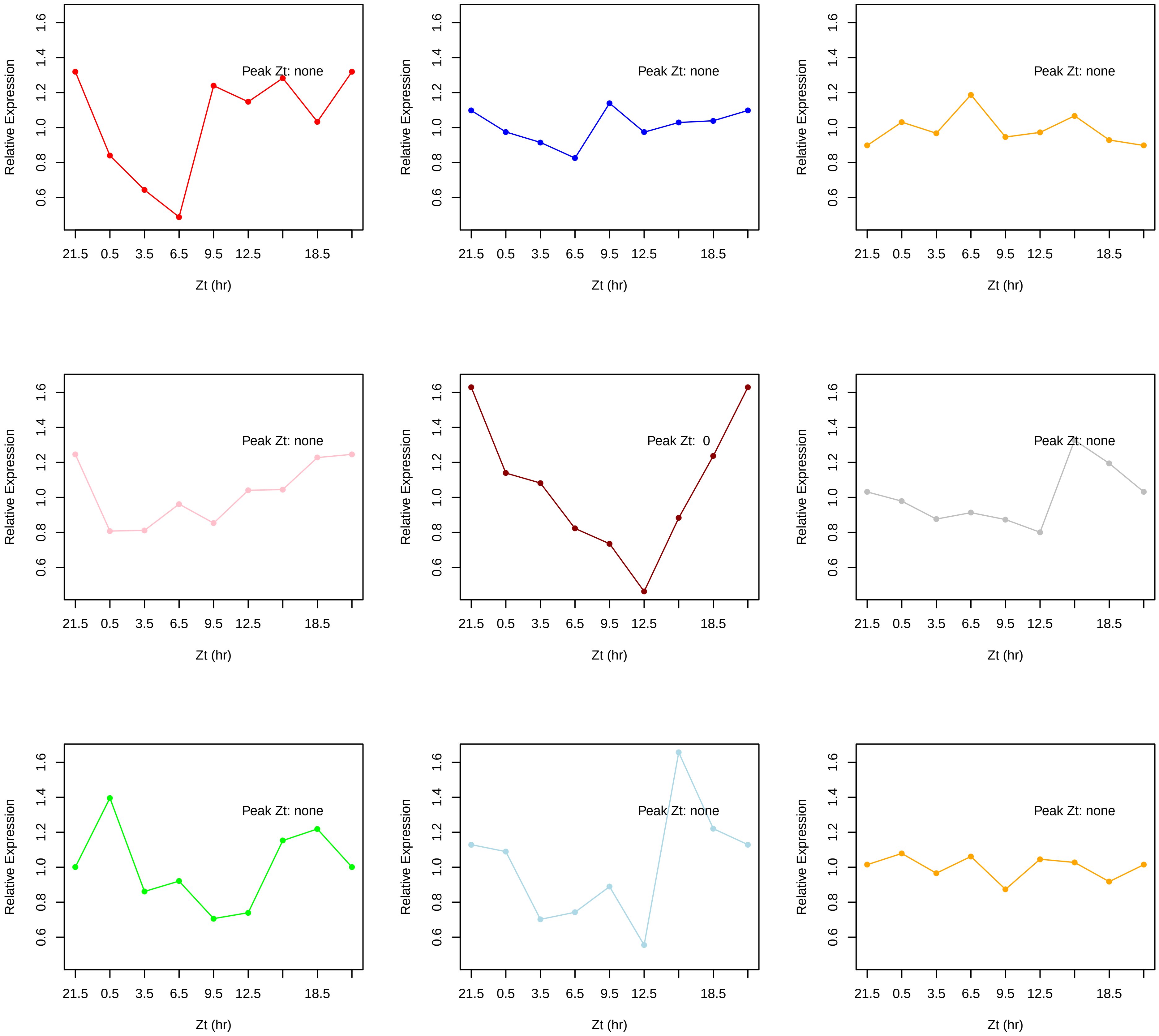

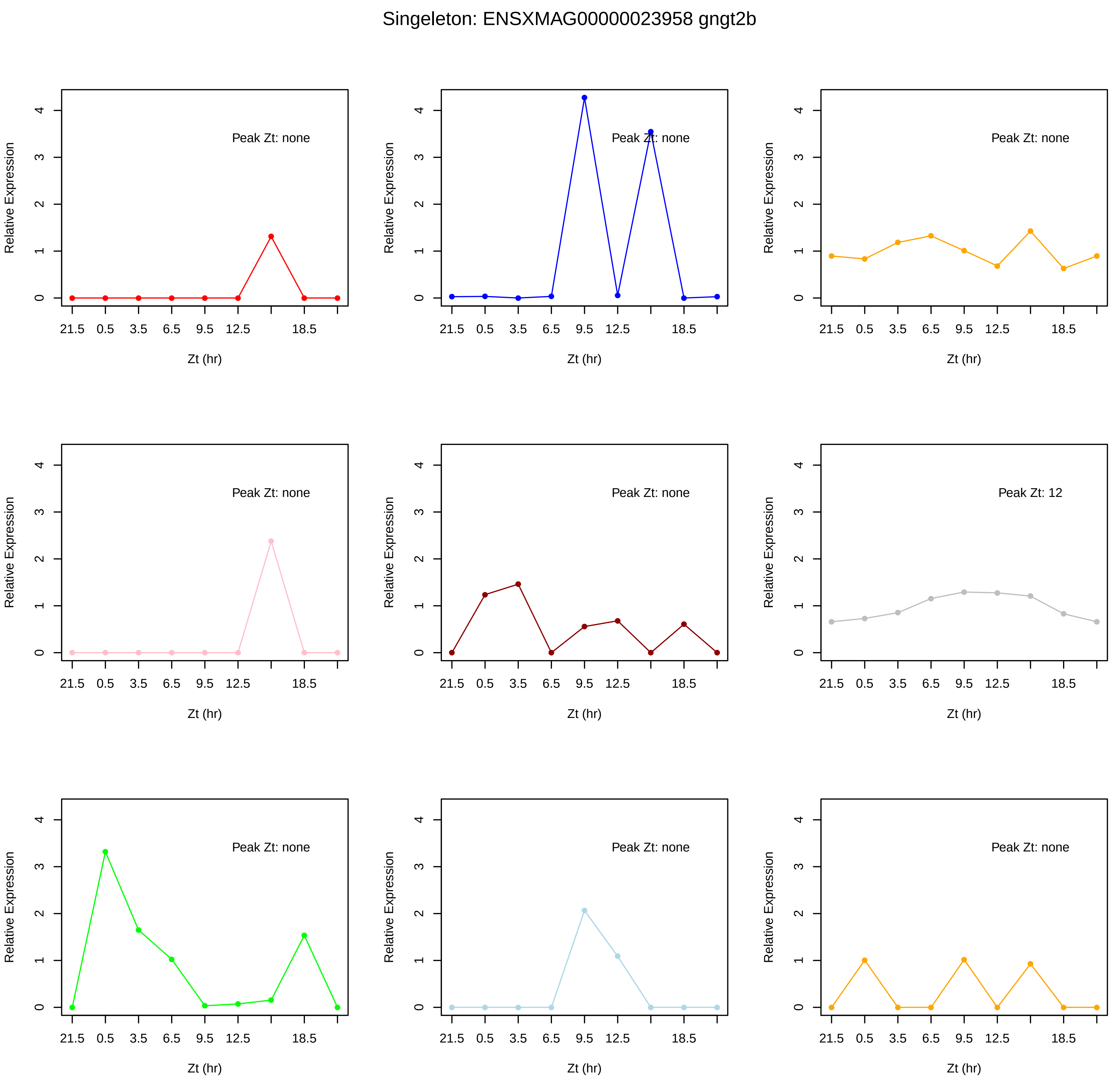
Singeleton: ENSXMAG00000023958 gngt2b
Peak Zt: none
Peak Zt: none
Peak Zt: none
Peak Zt: none
Peak Zt: none
Peak Zt: 12
Peak Zt: none
Peak Zt: none
Peak Zt: none
Relative Expression
Zt (hr)
21.5
0.5
3.5
6.5
9.5
12.5
18.5

Singeleton: ENSXMAG00000019360 xpc

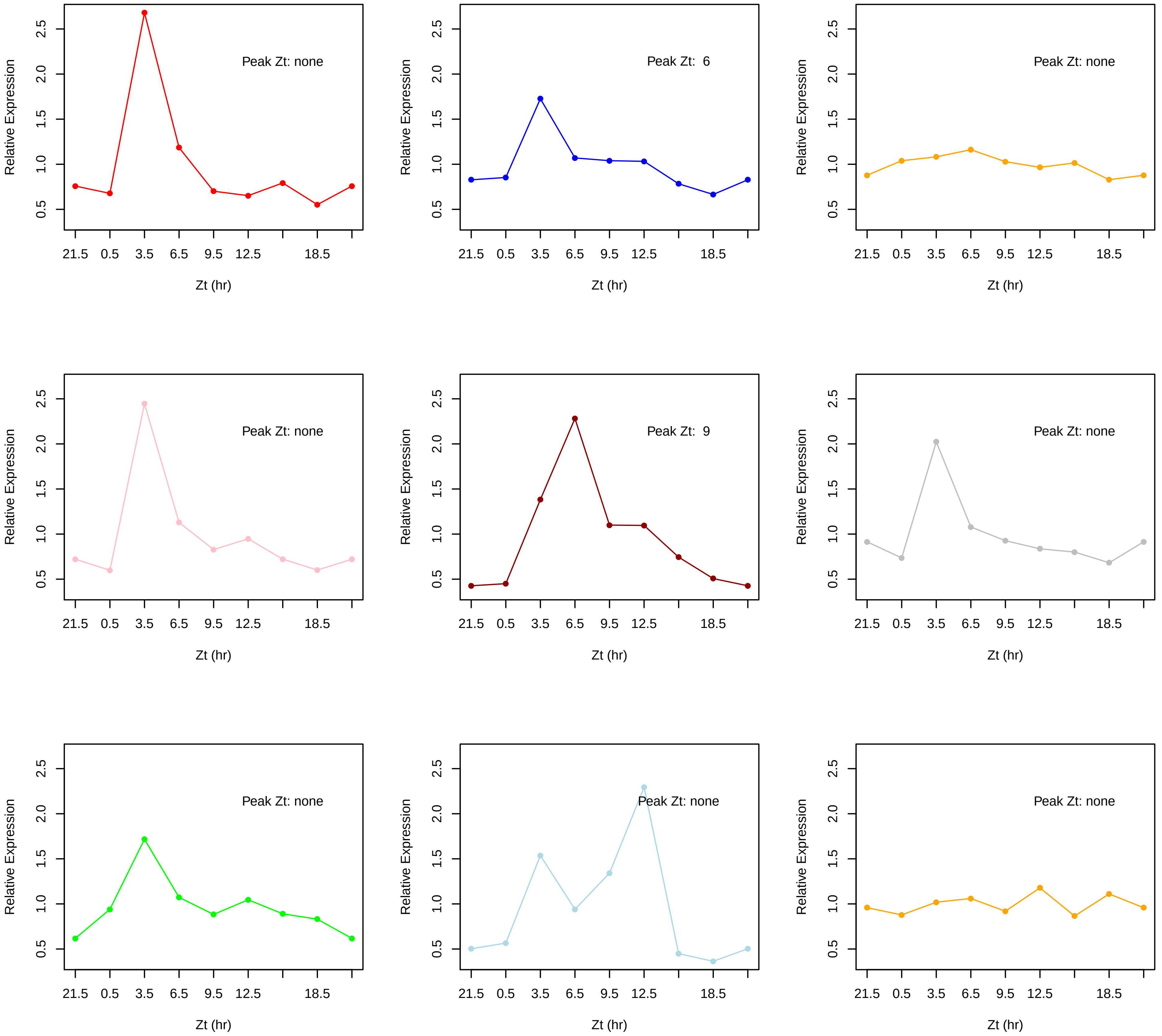

Singeleton: ENSXMAG00000021939 COPS6

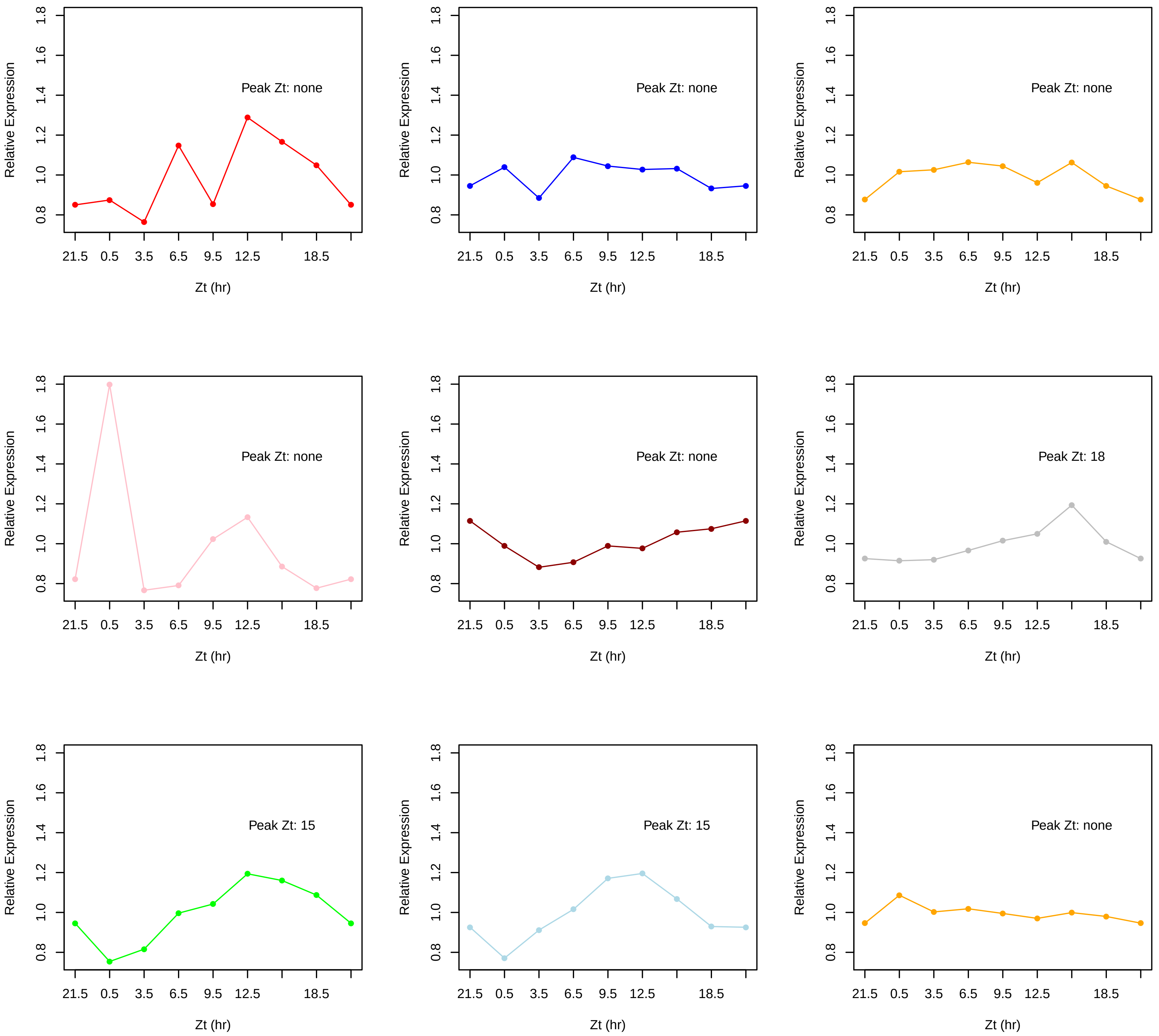

Singeleton: ENSXMAG00000018168

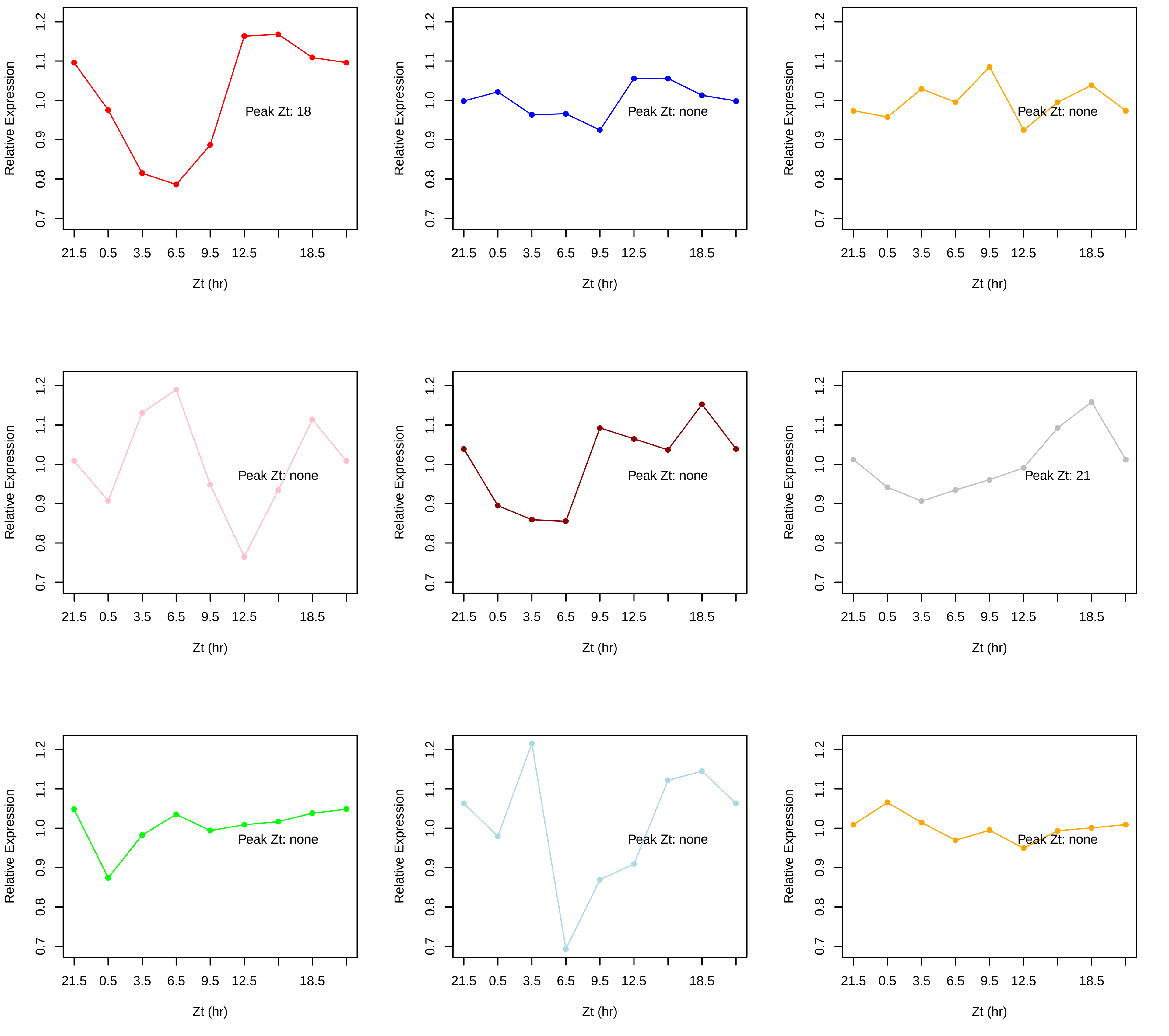

Singeleton: ENSXMAG00000020950 lsm12b

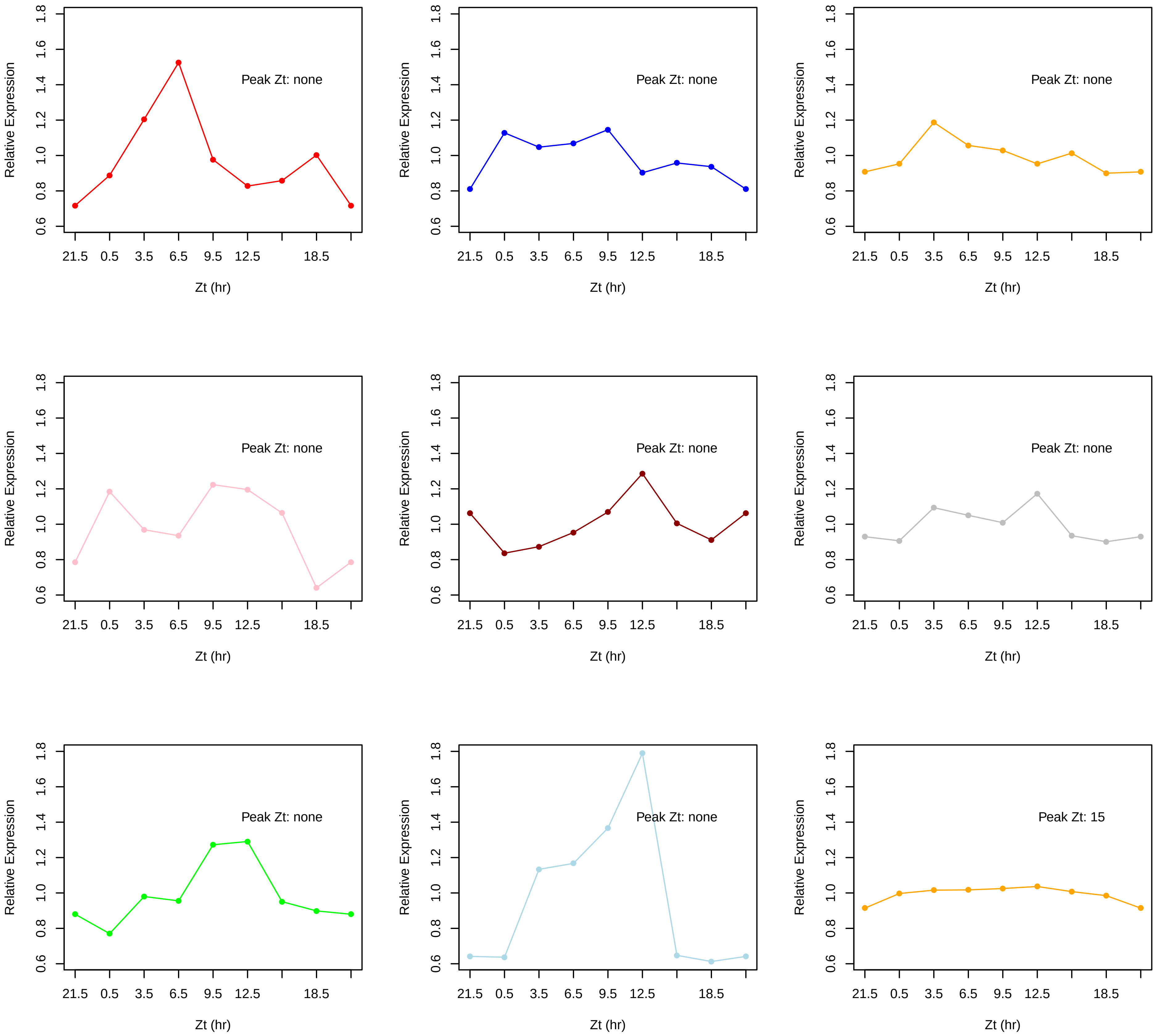

Singeleton: ENSXMAG00000007570 ubr7

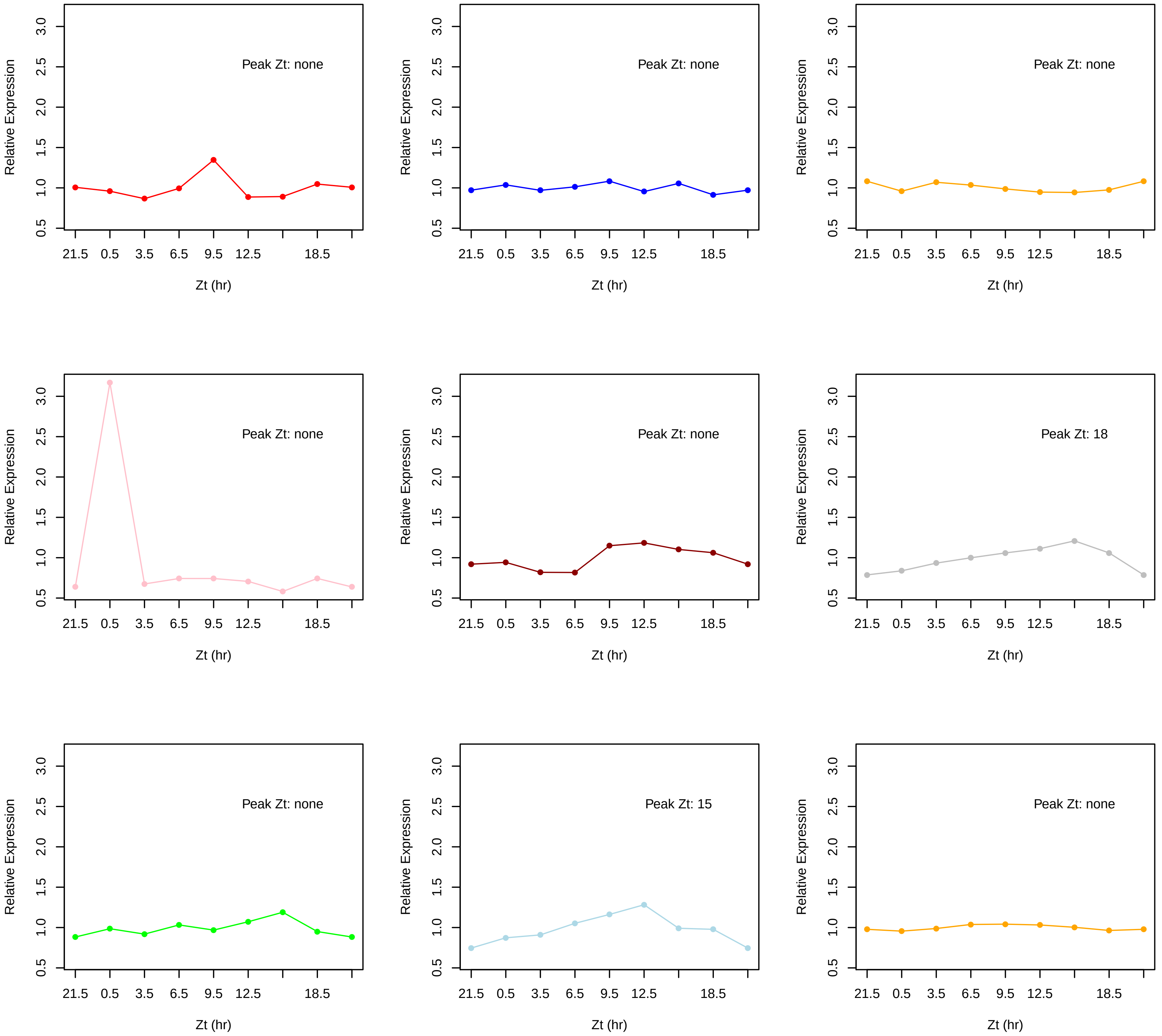

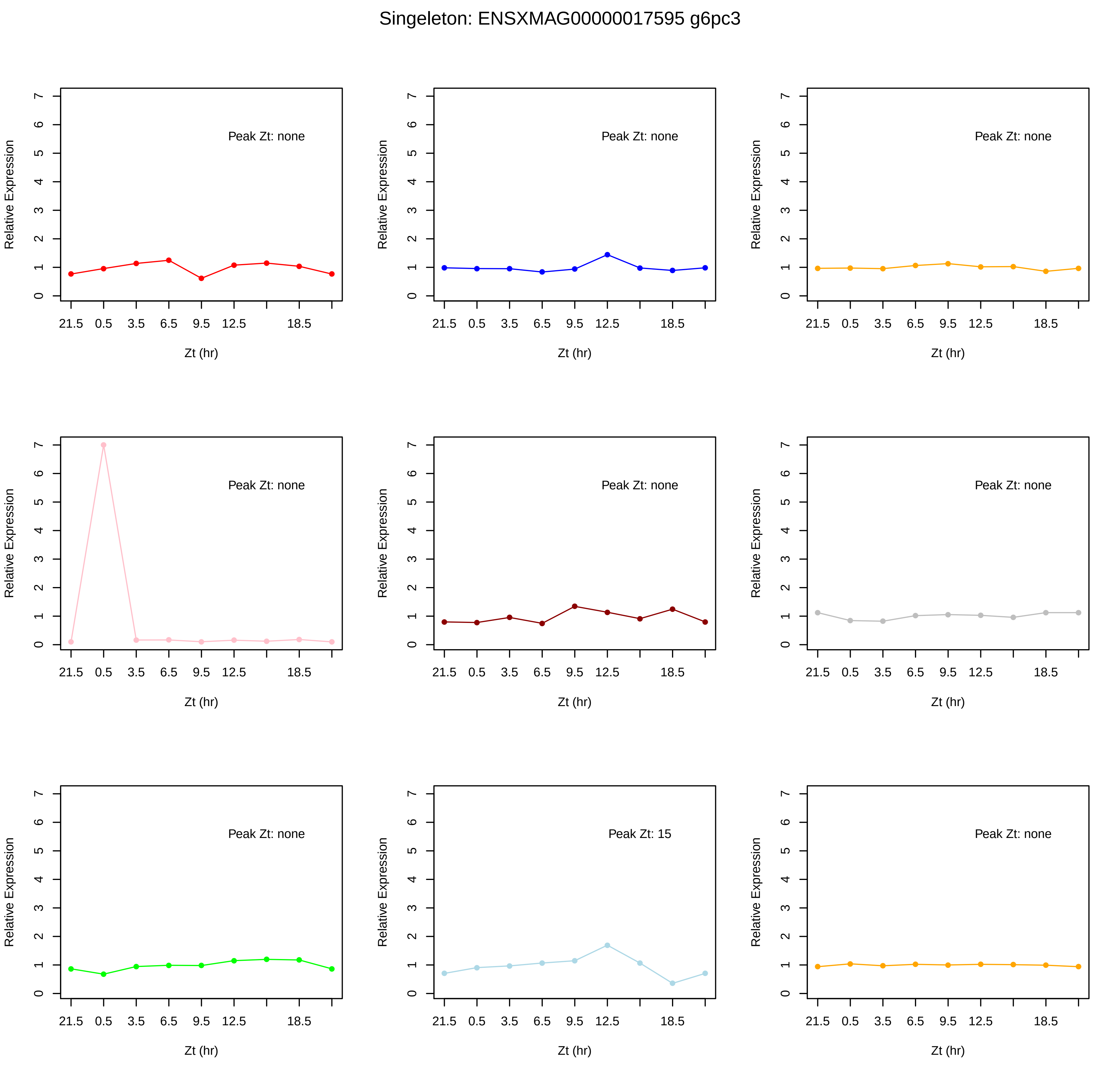
Singleton: ENSXMAG00000017595 g6pc3
Peak Zt: none
Peak Zt: none
Peak Zt: none
Peak Zt: none
Peak Zt: none
Peak Zt: none
Peak Zt: none
Peak Zt: 15
Peak Zt: none
Relative Expression
Zt (hr)
21.5 0.5 3.5 6.5 9.5 12.5 18.5
0 1 2 3 4 5 6 7

Singeleton: ENSXMAG00000006732 ap4m1

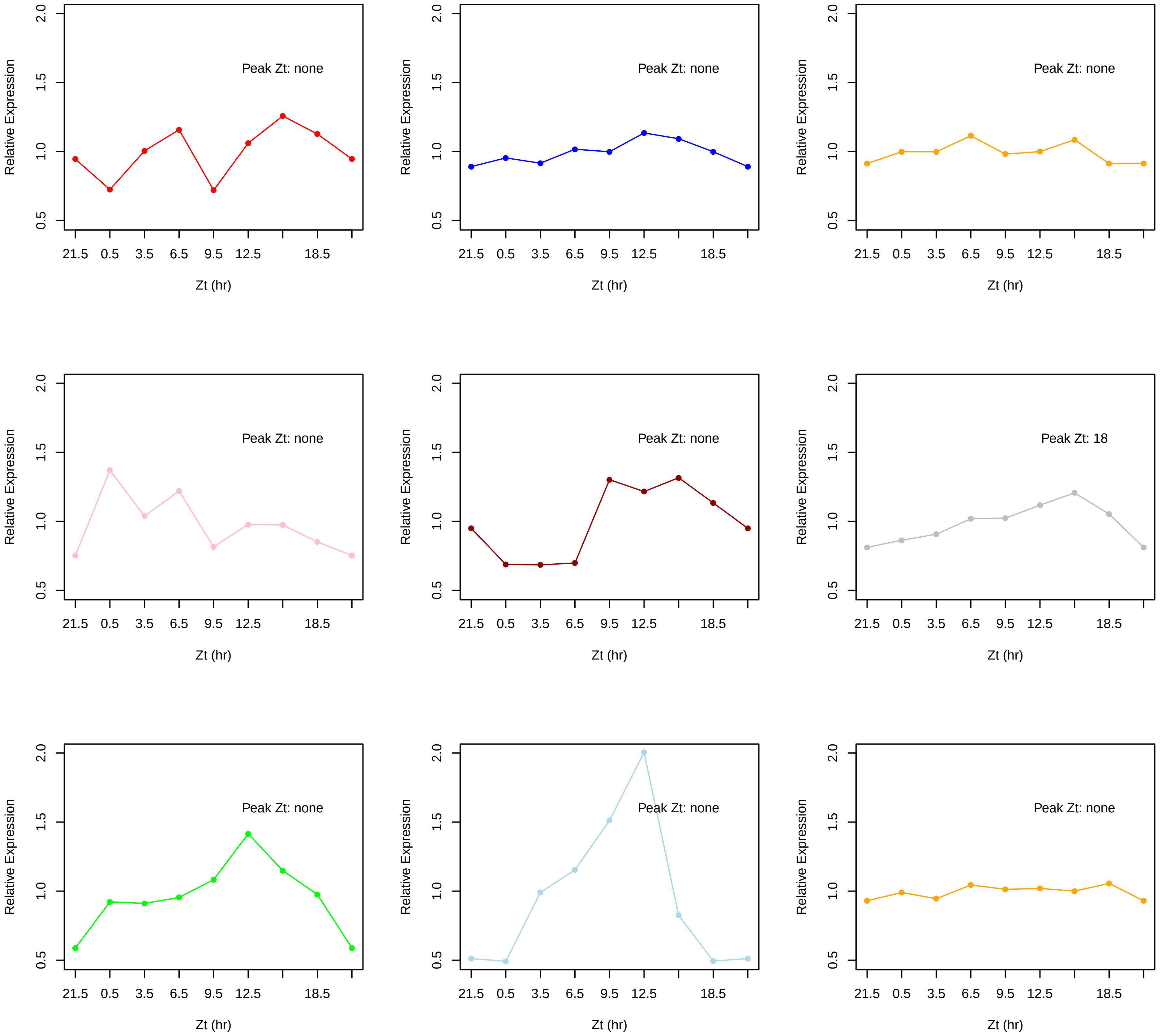

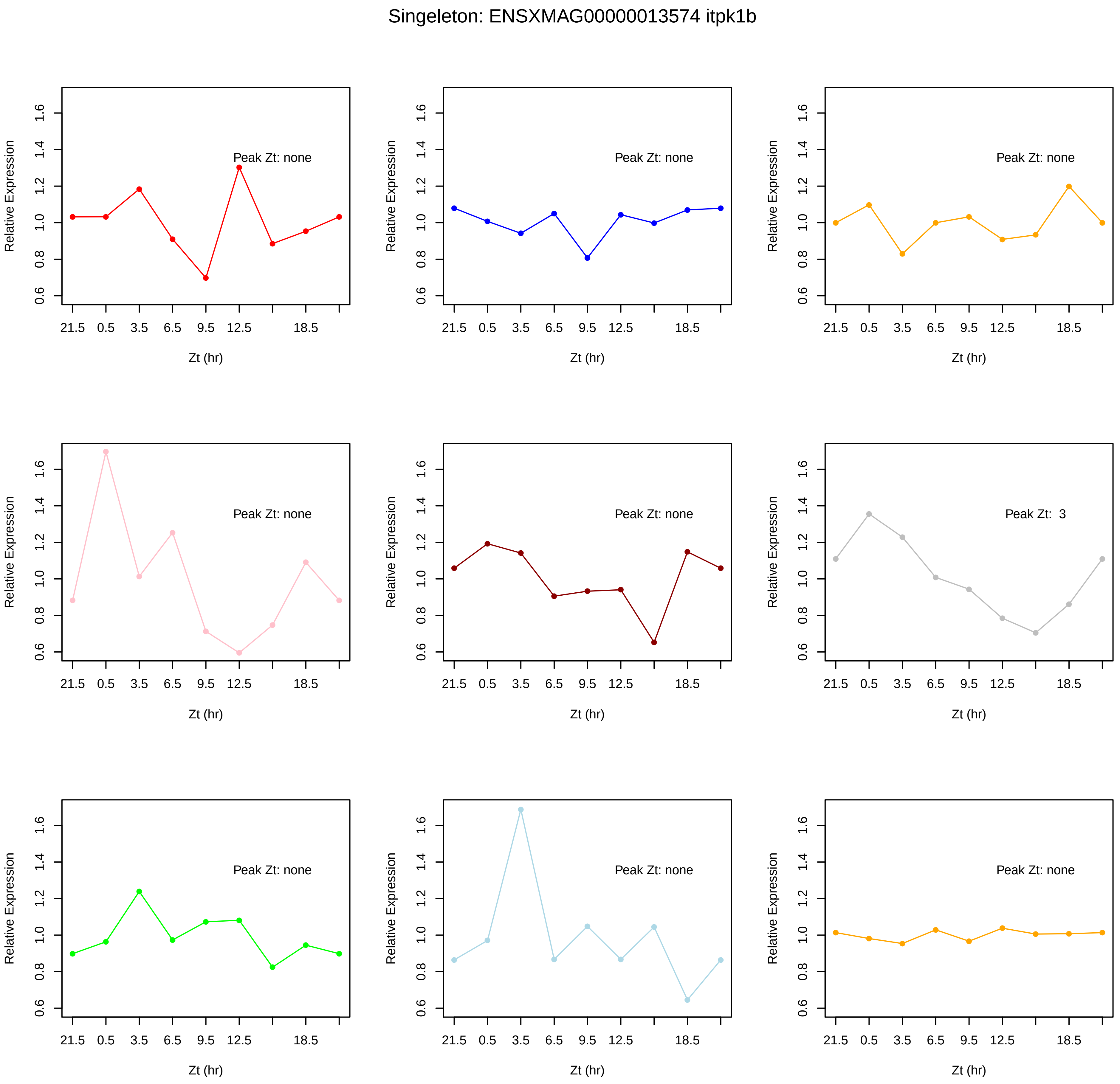
Singleton: ENSXMAG00000013574 itpk1b
Relative Expression
Zt (hr)
21.5
0.5
3.5
6.5
9.5
12.5
18.5
0.6
0.8
1.0
1.2
1.4
1.6
Peak Zt: none
Peak Zt: none
Peak Zt: none
Peak Zt: none
Peak Zt: none
Peak Zt: 3
Peak Zt: none
Peak Zt: none
Peak Zt: none

Singeleton: ENSXMAG00000007483 sqor

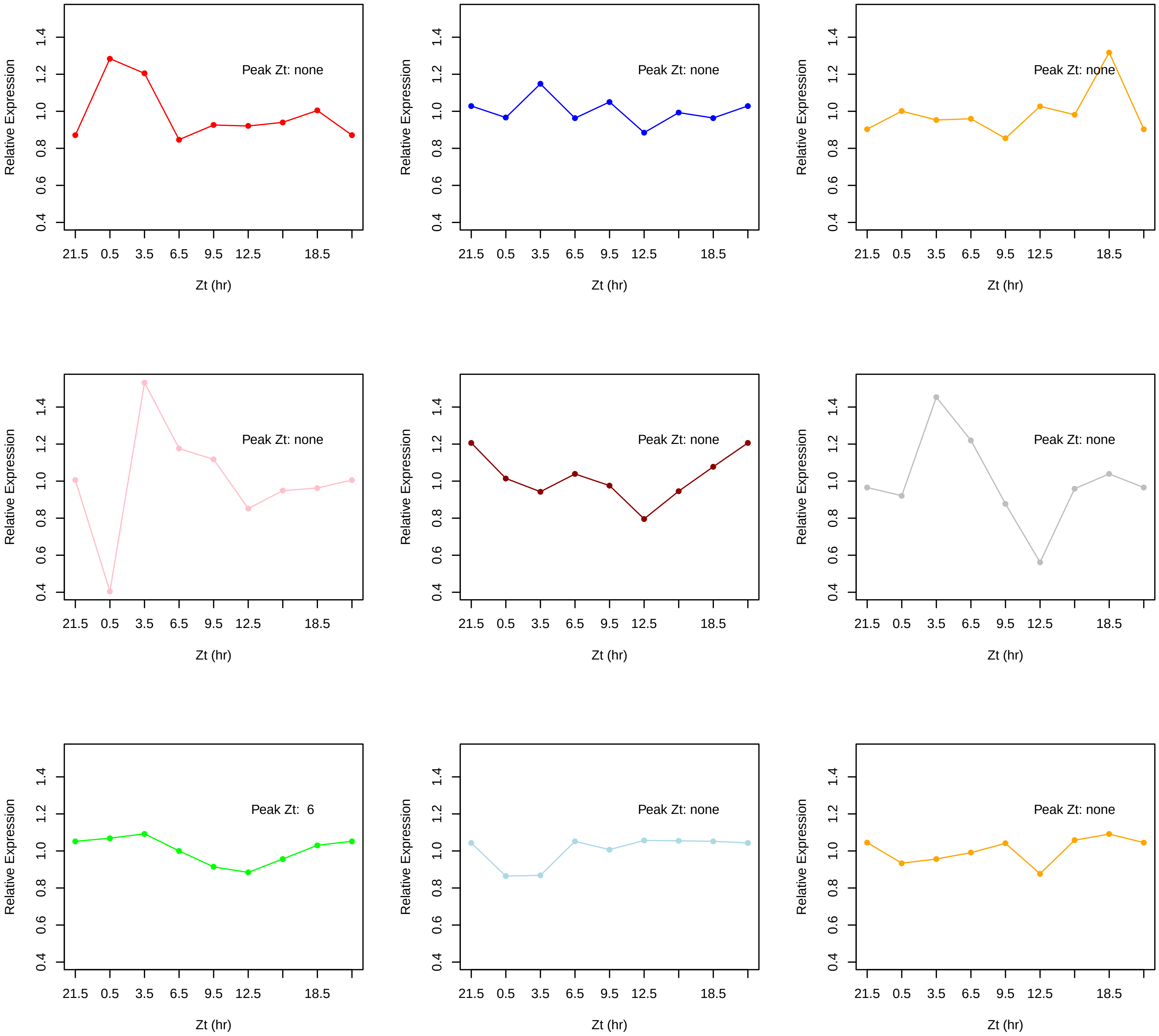

Singleton: ENSXMAG00000007067 ANK2

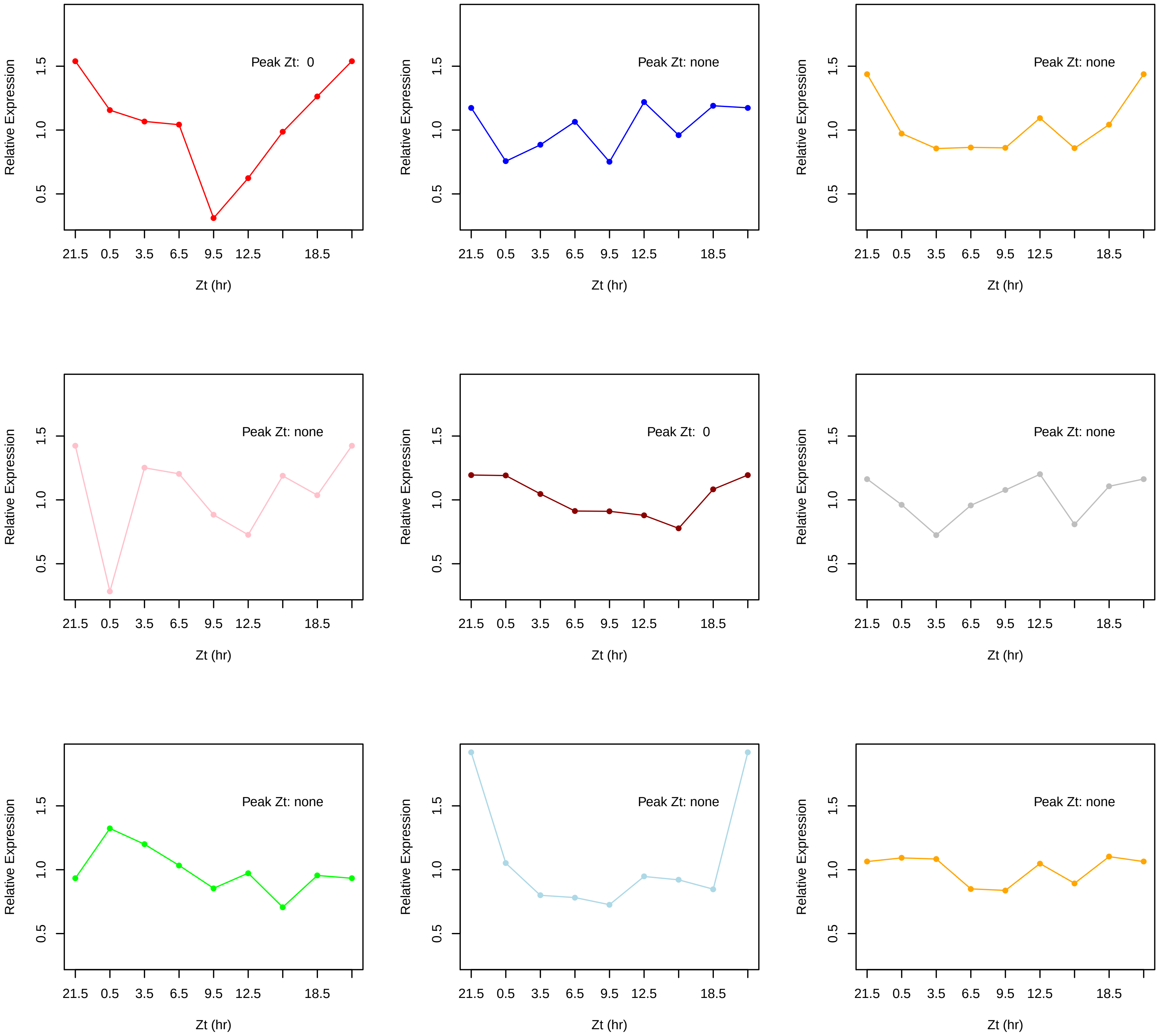

Singeleton: ENSXMAG00000006902 chga

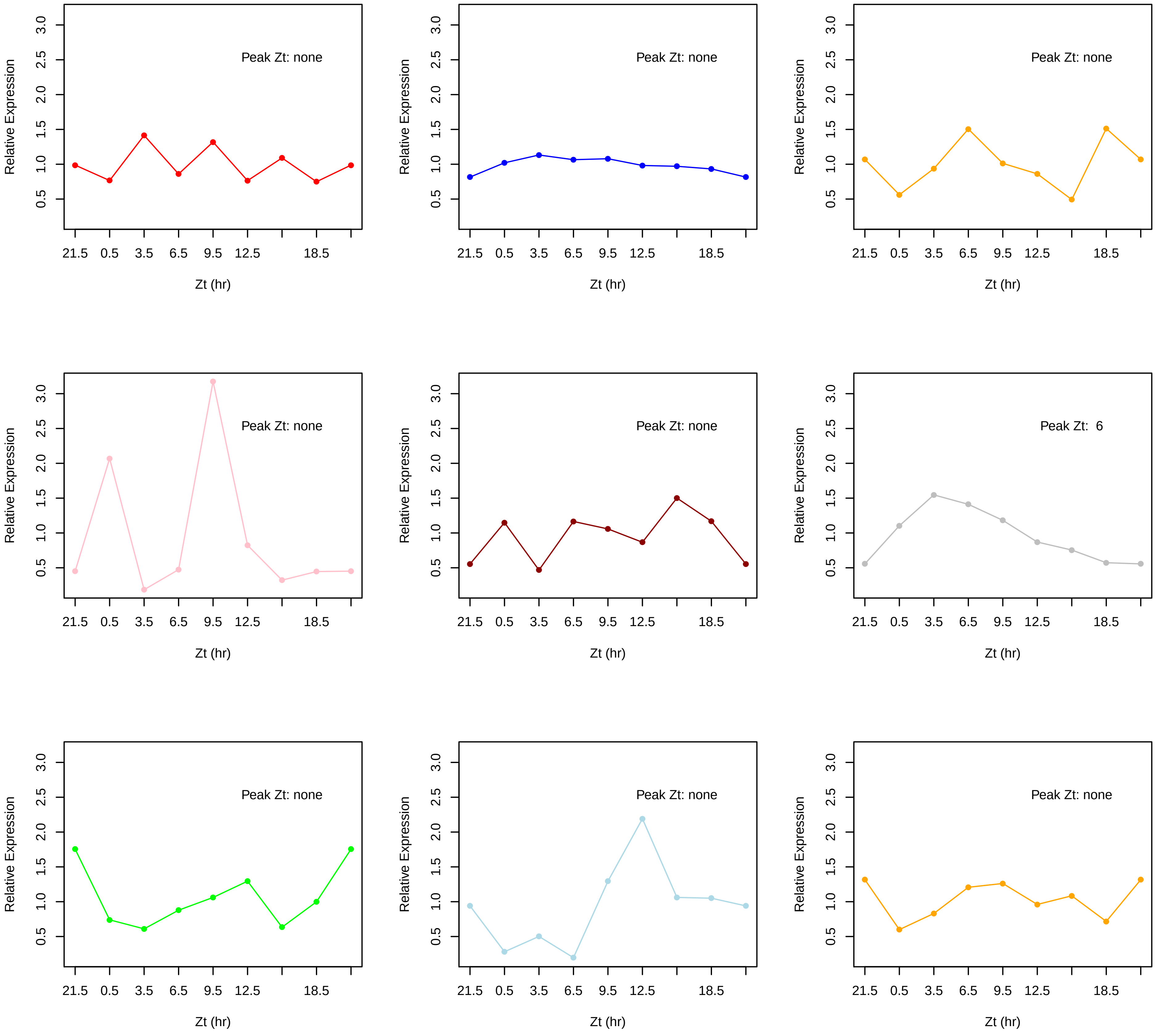

Singeleton: ENSXMAG00000002310 bloc1s6

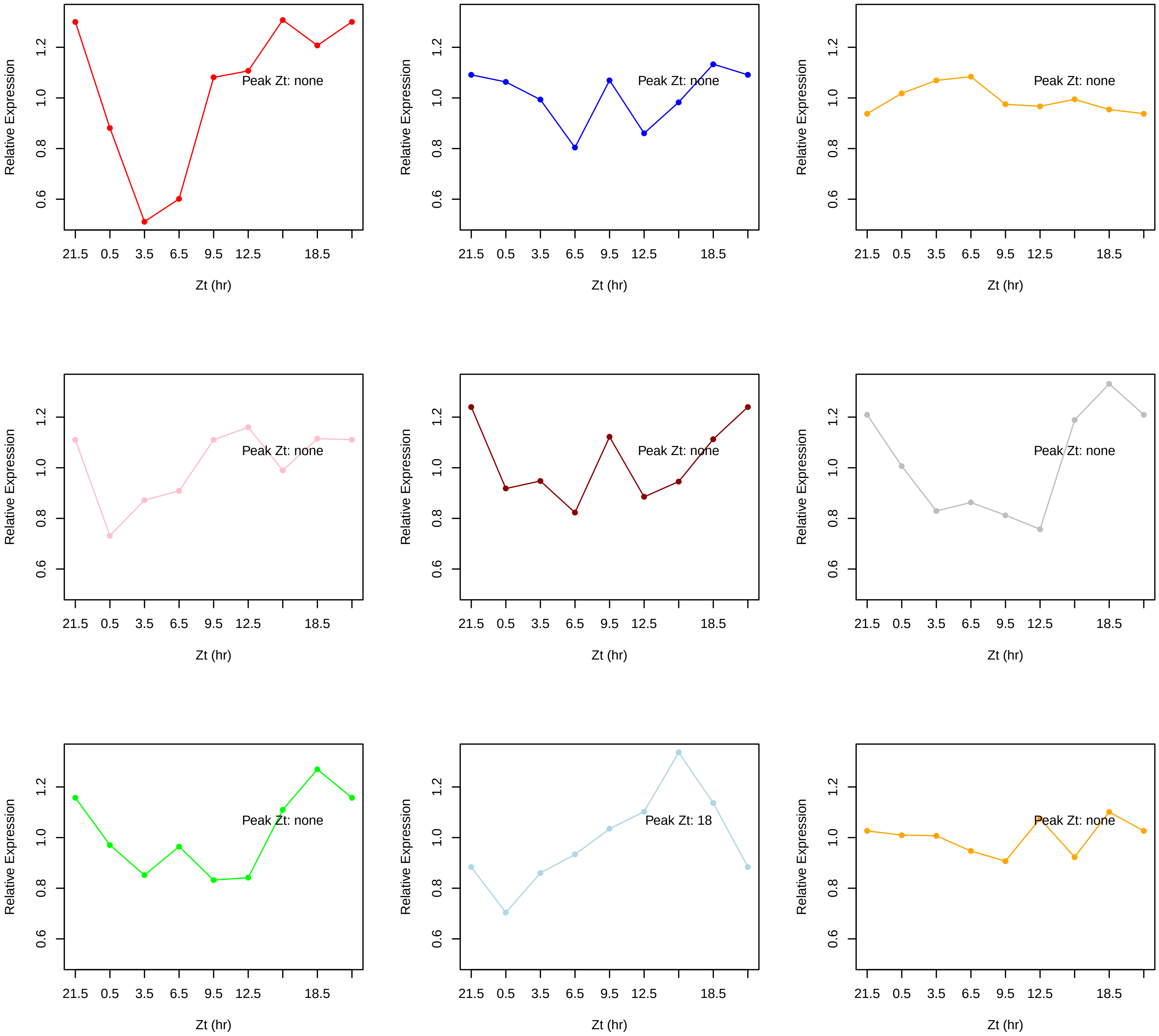

Singeleton: ENSXMAG00000002304 slc30a4

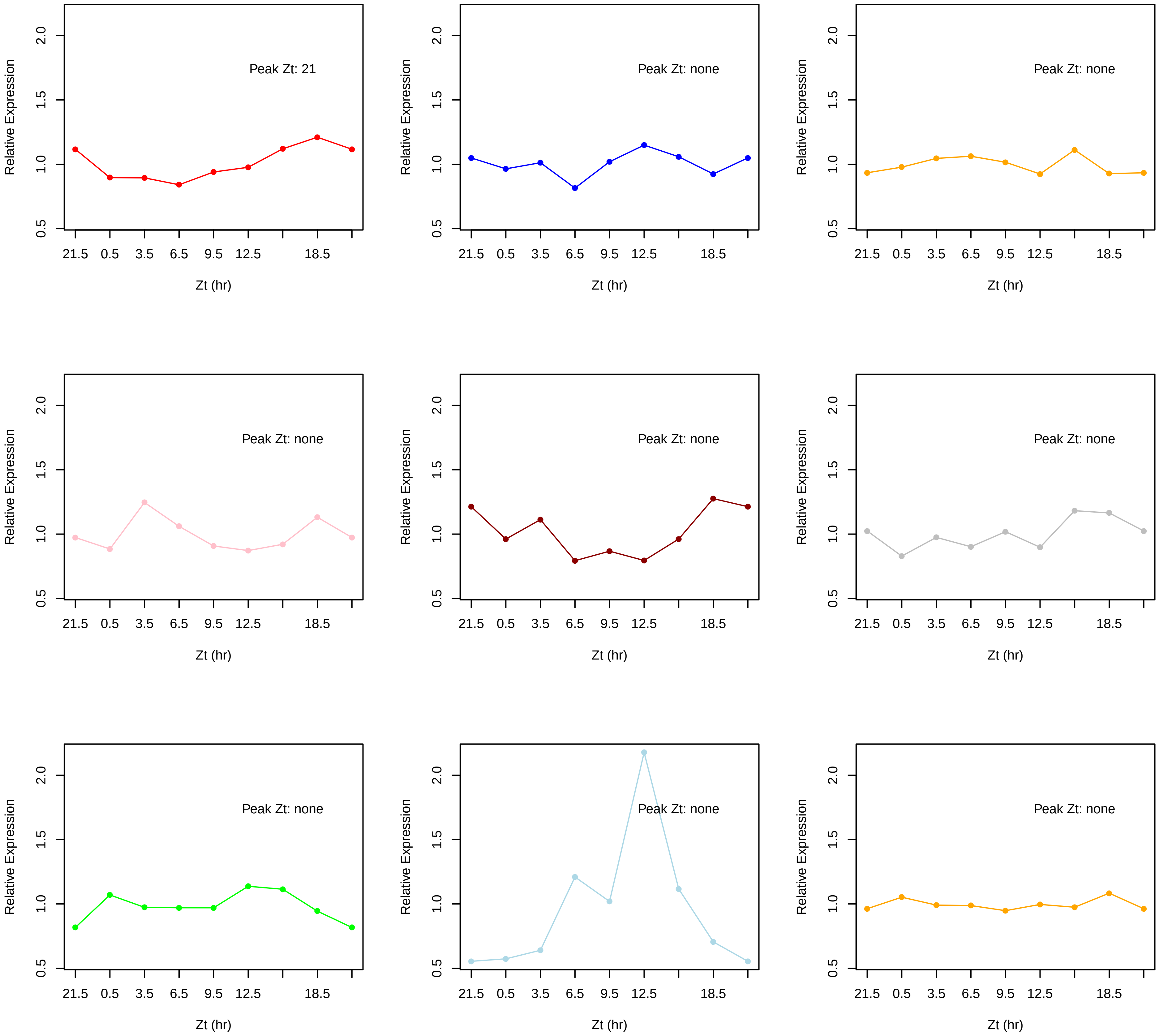

Singeleton: ENSXMAG00000030095 si:dkey−32e6.3

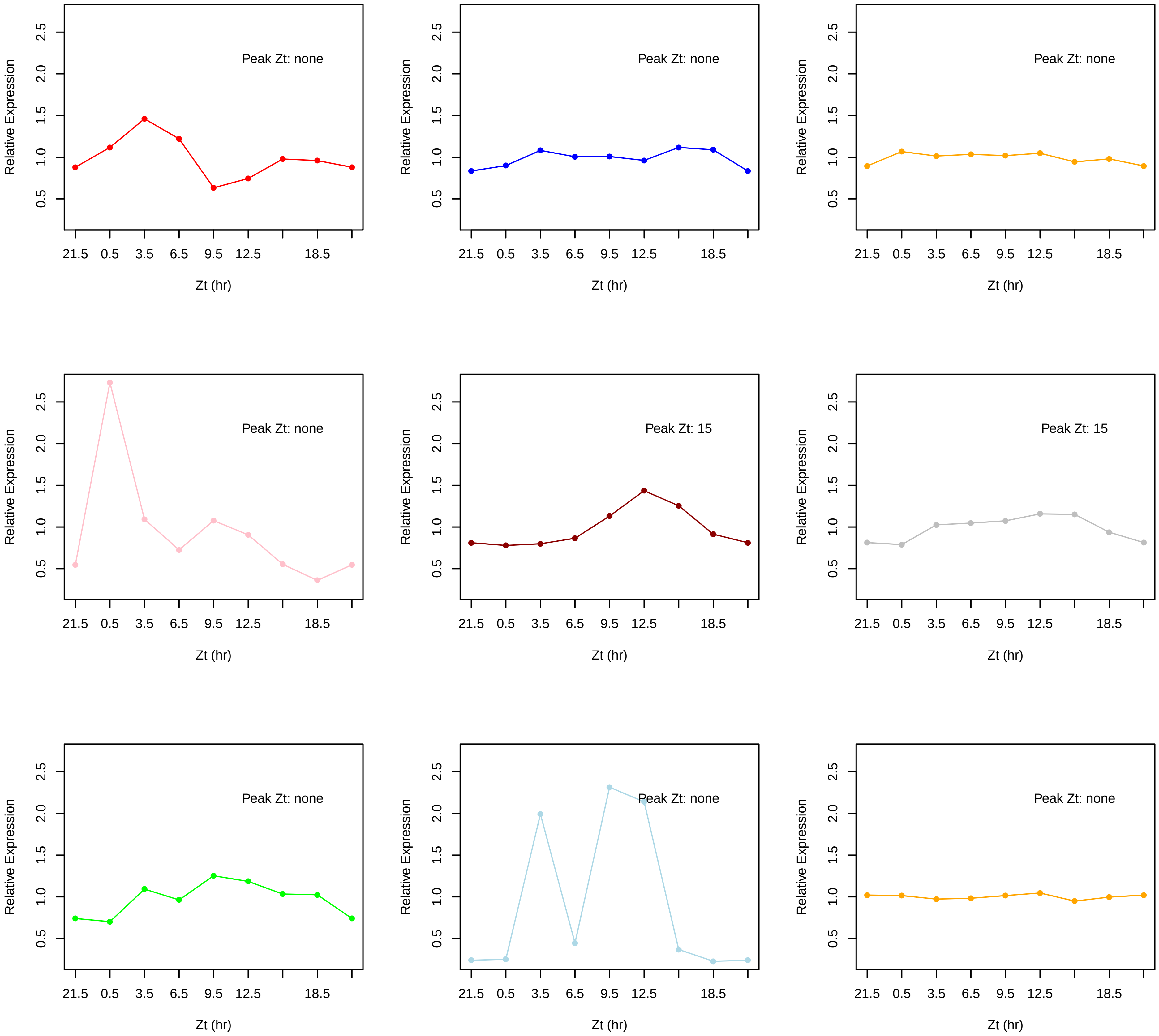

Singeleton: ENSXMAG00000006283 tbc1d7

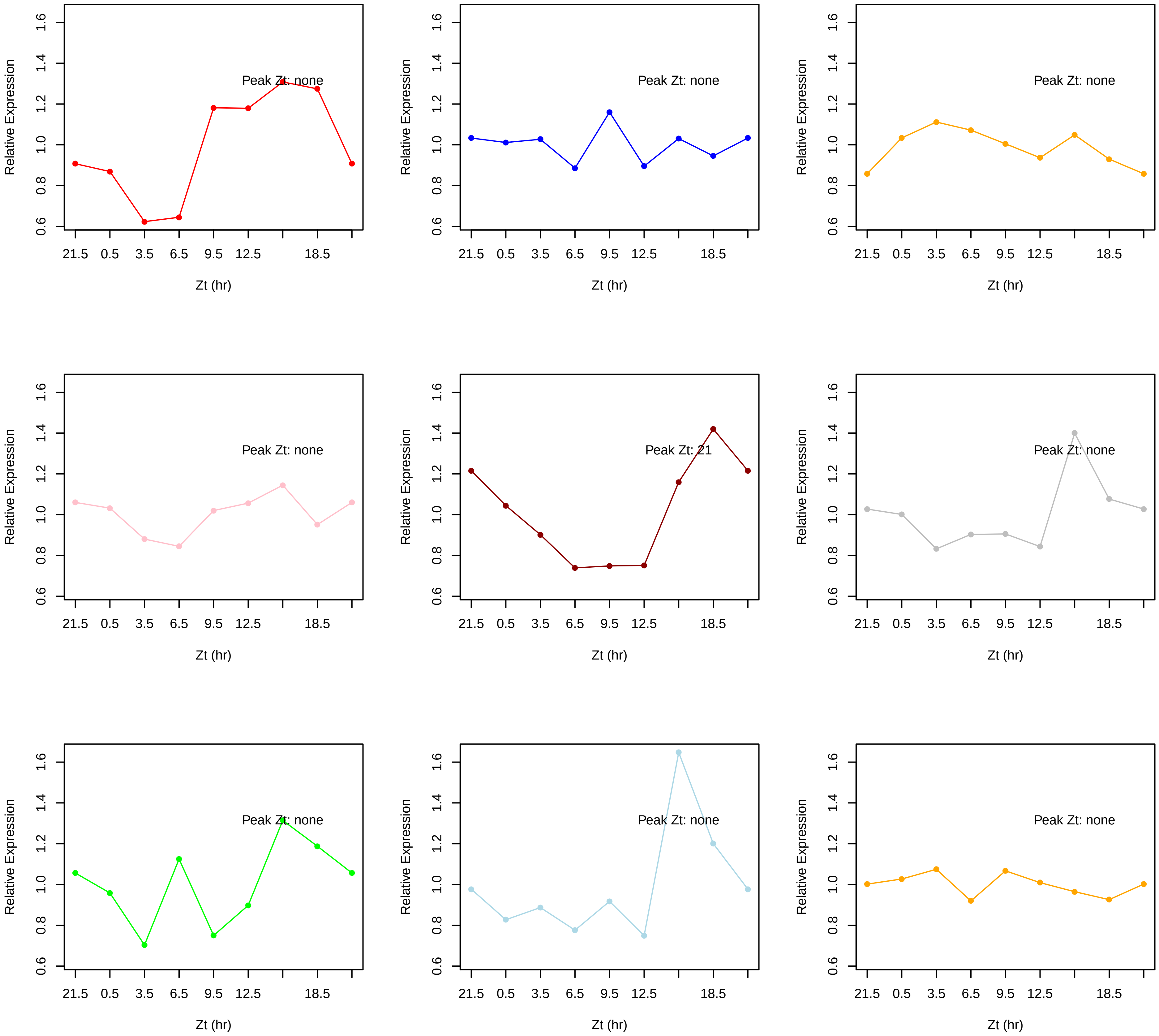

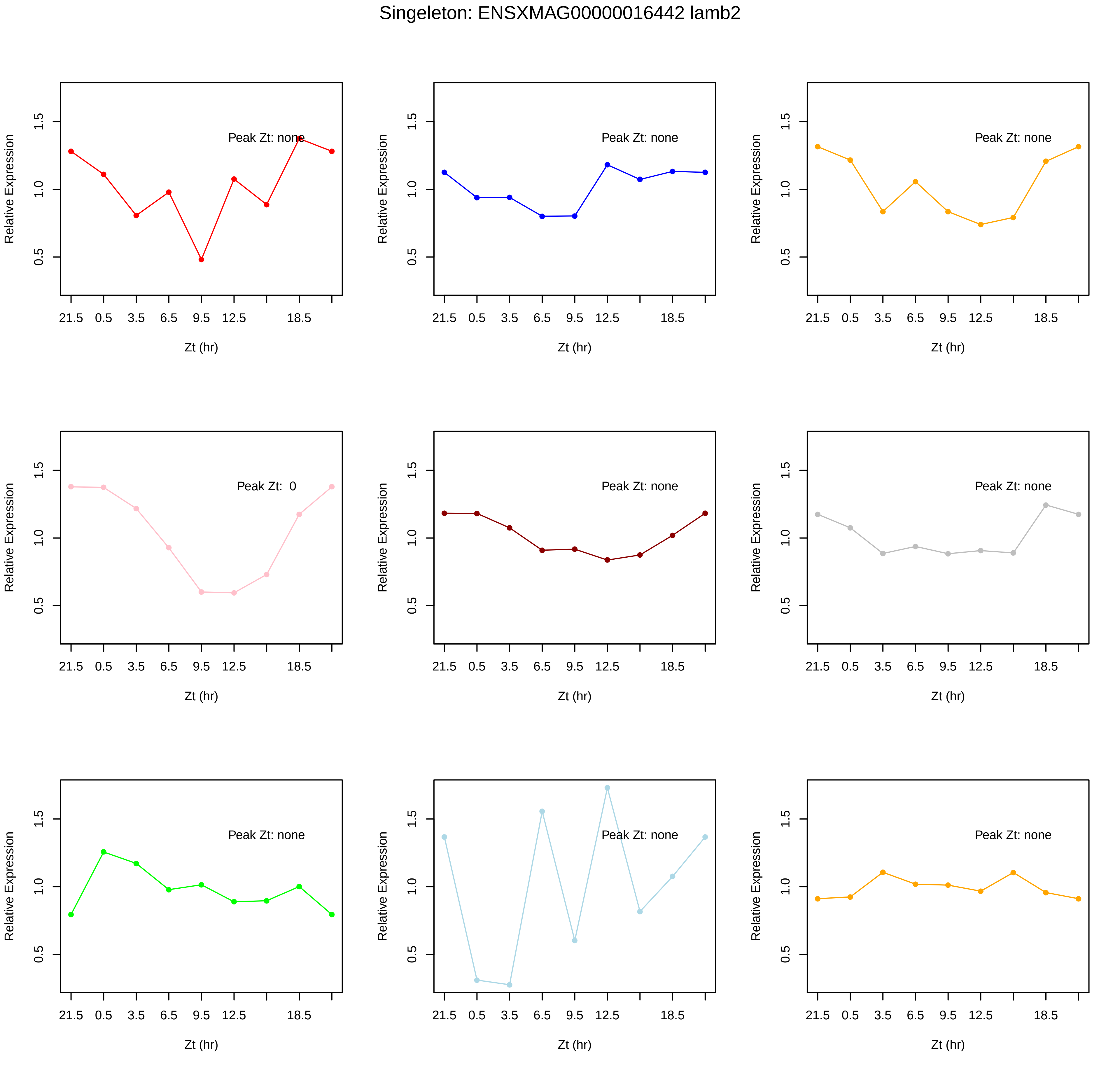
Singleton: ENSXMAG00000016442 lamb2
Relative Expression
Zt (hr)
21.5
0.5
3.5
6.5
9.5
12.5
18.5
0.5
1.0
1.5
Peak Zt: none
Peak Zt: 0

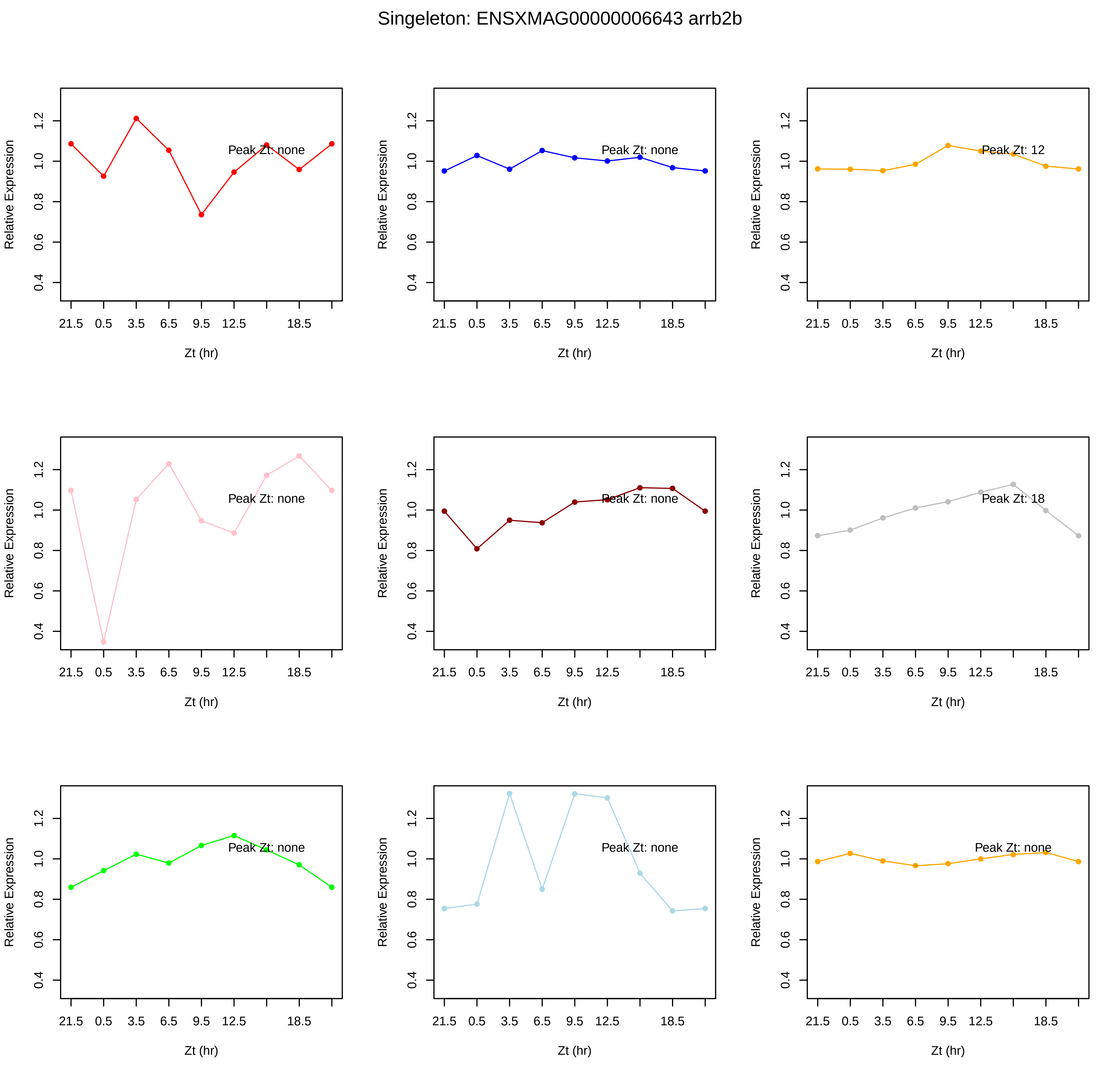

Singeleton: ENSXMAG00000006643 arrb2b
Relative Expression
Zt (hr)
21.5
0.5
3.5
6.5
9.5
12.5
18.5
0.4
0.6
0.8
1.0
1.2
Peak Zt: none
Peak Zt: none
Peak Zt: 12
Peak Zt: none
Peak Zt: none
Peak Zt: 18
Peak Zt: none
Peak Zt: none
Peak Zt: none

Singeleton: ENSXMAG00000012645 abi3a

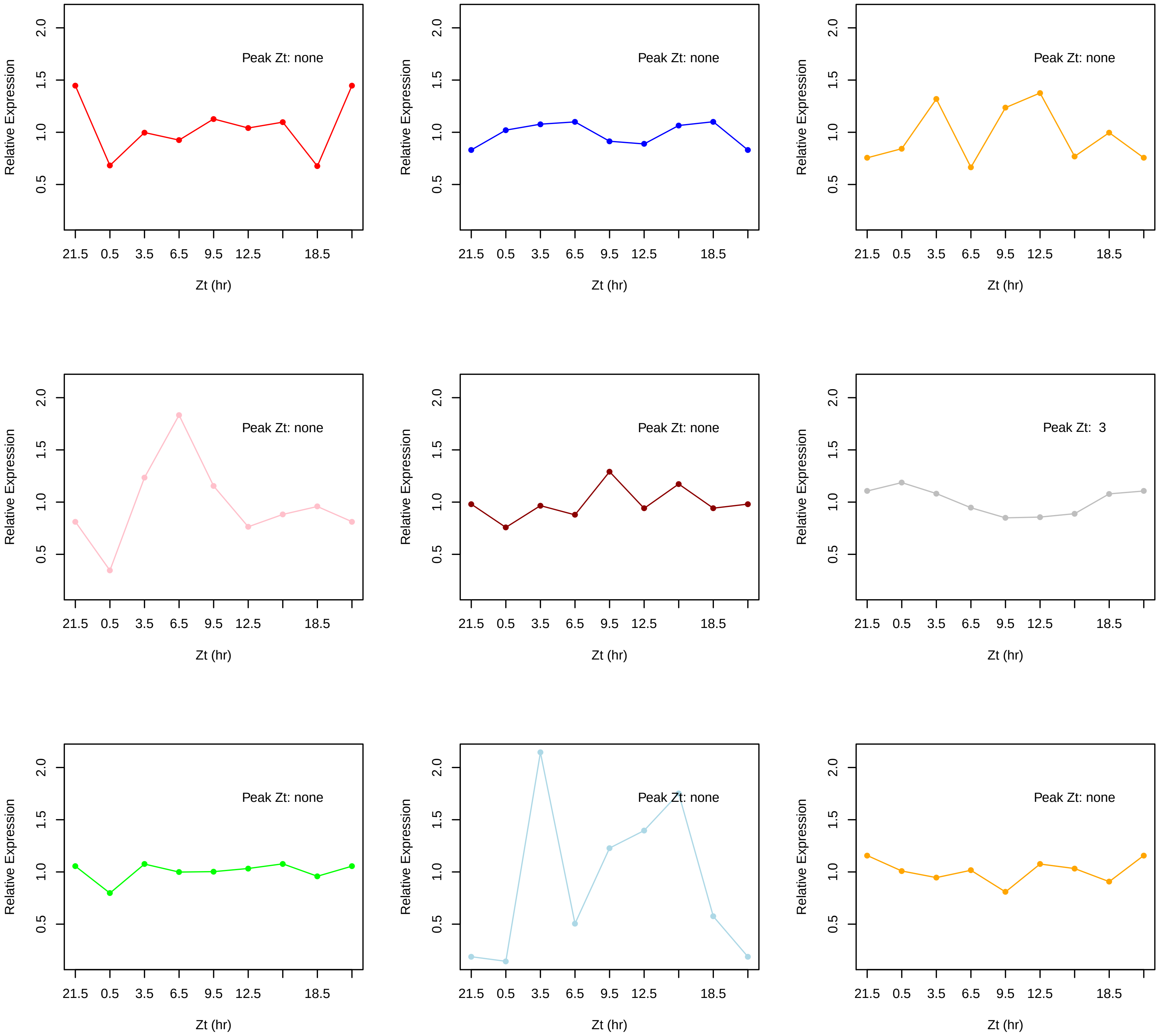

Singeleton: ENSXMAG00000017703 plekhm1

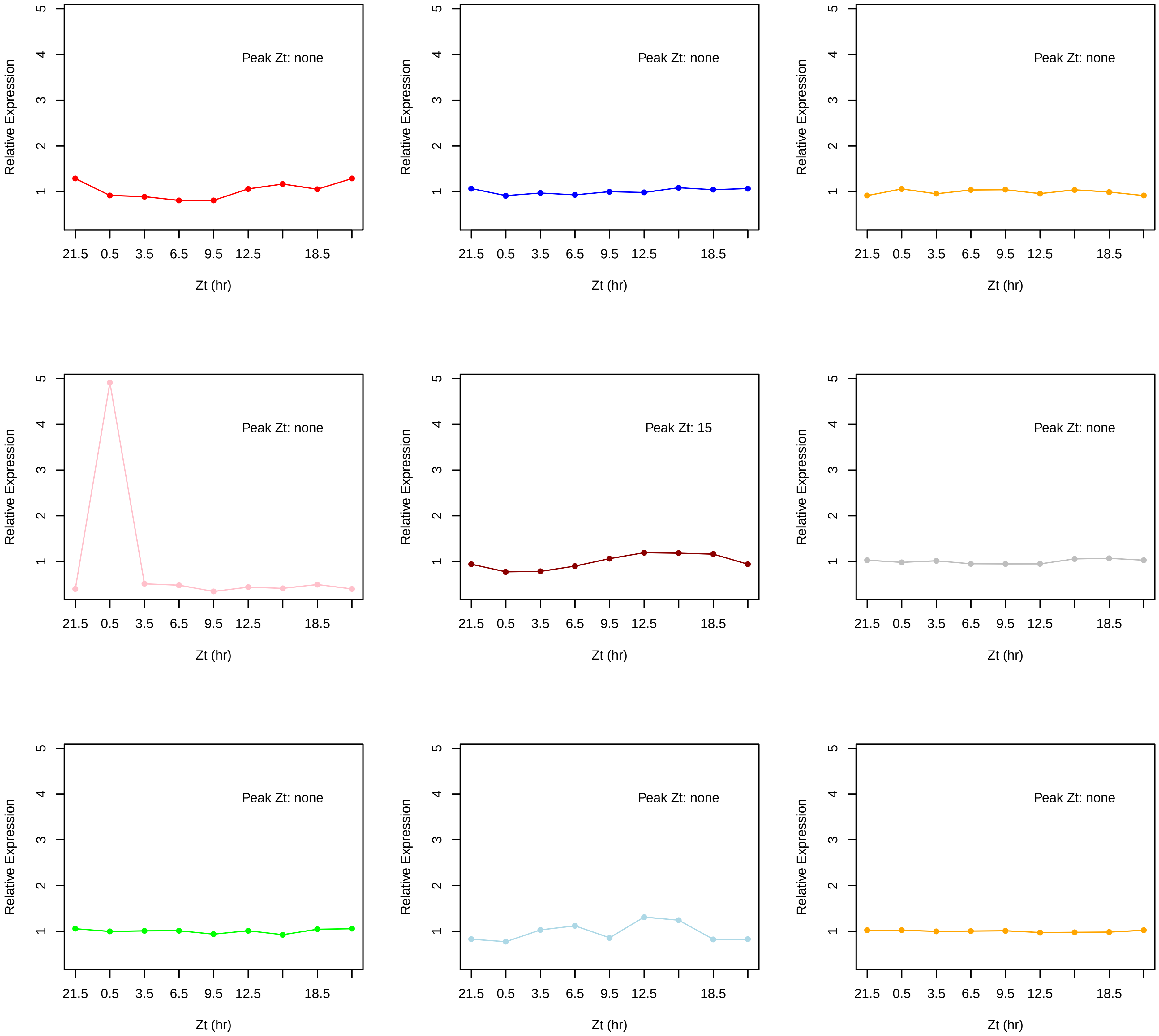

Singeleton: ENSXMAG00000029198 cox10

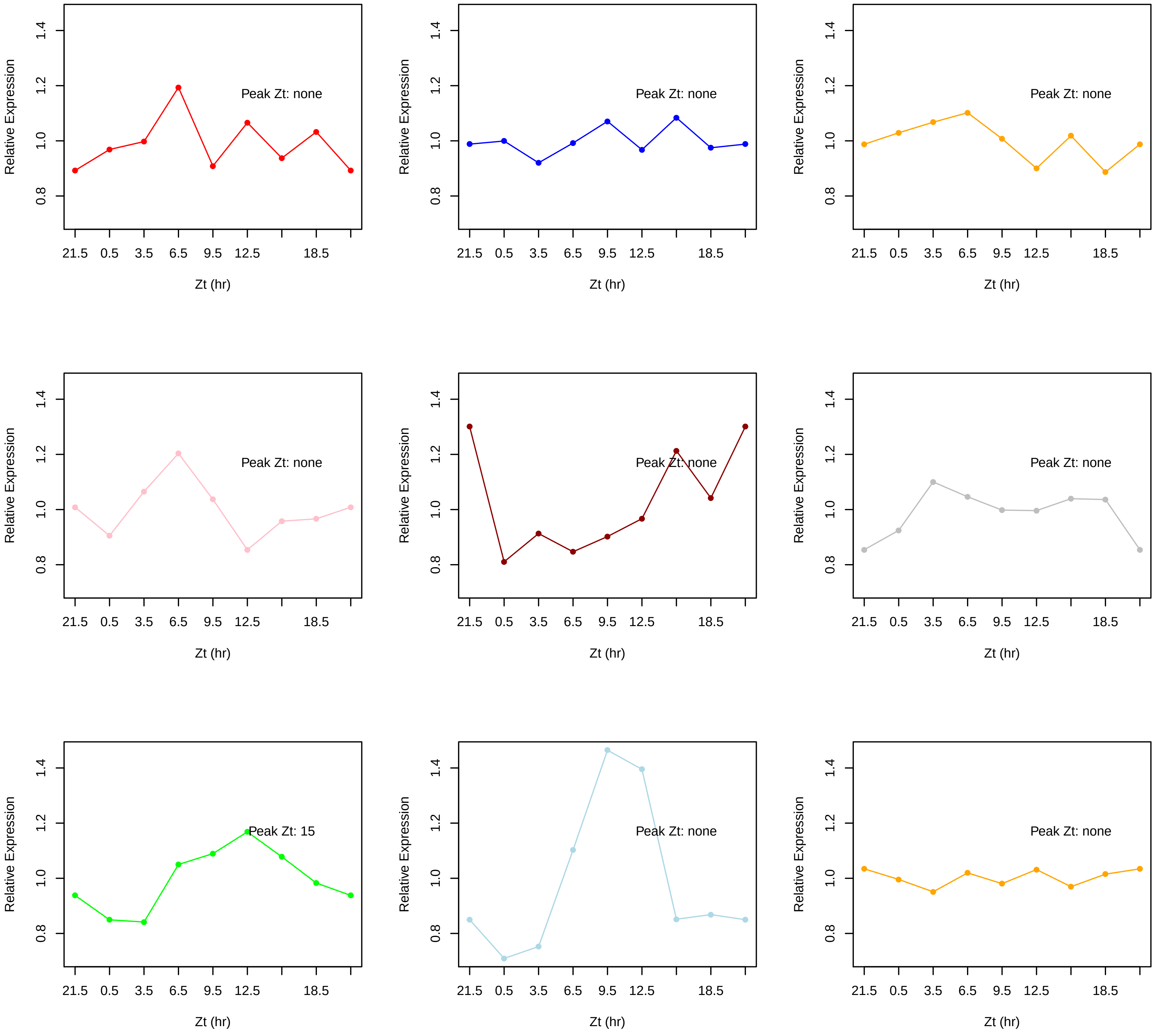

Singeleton: ENSXMAG00000012863 mak

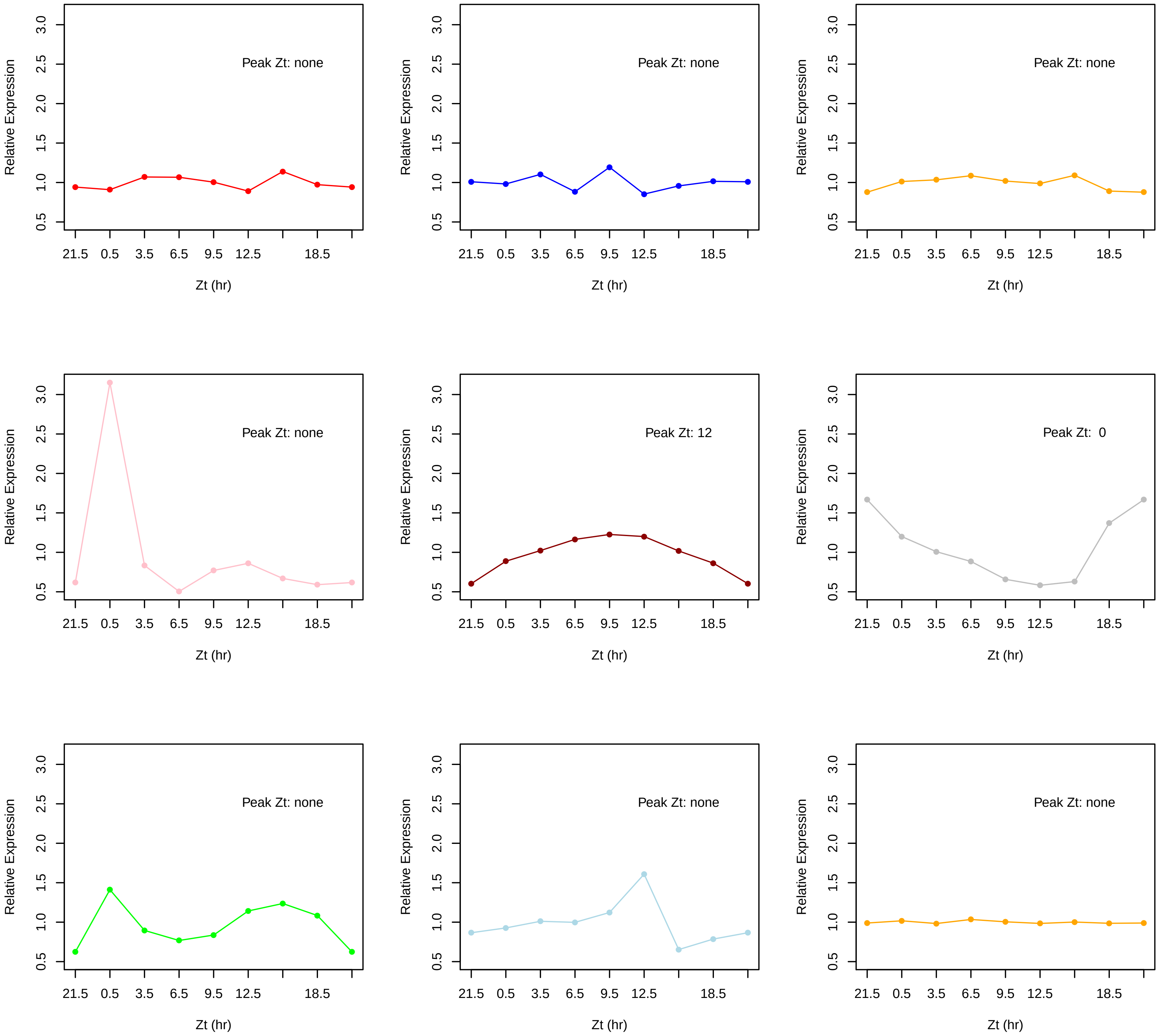

Singeleton: ENSXMAG00000028013

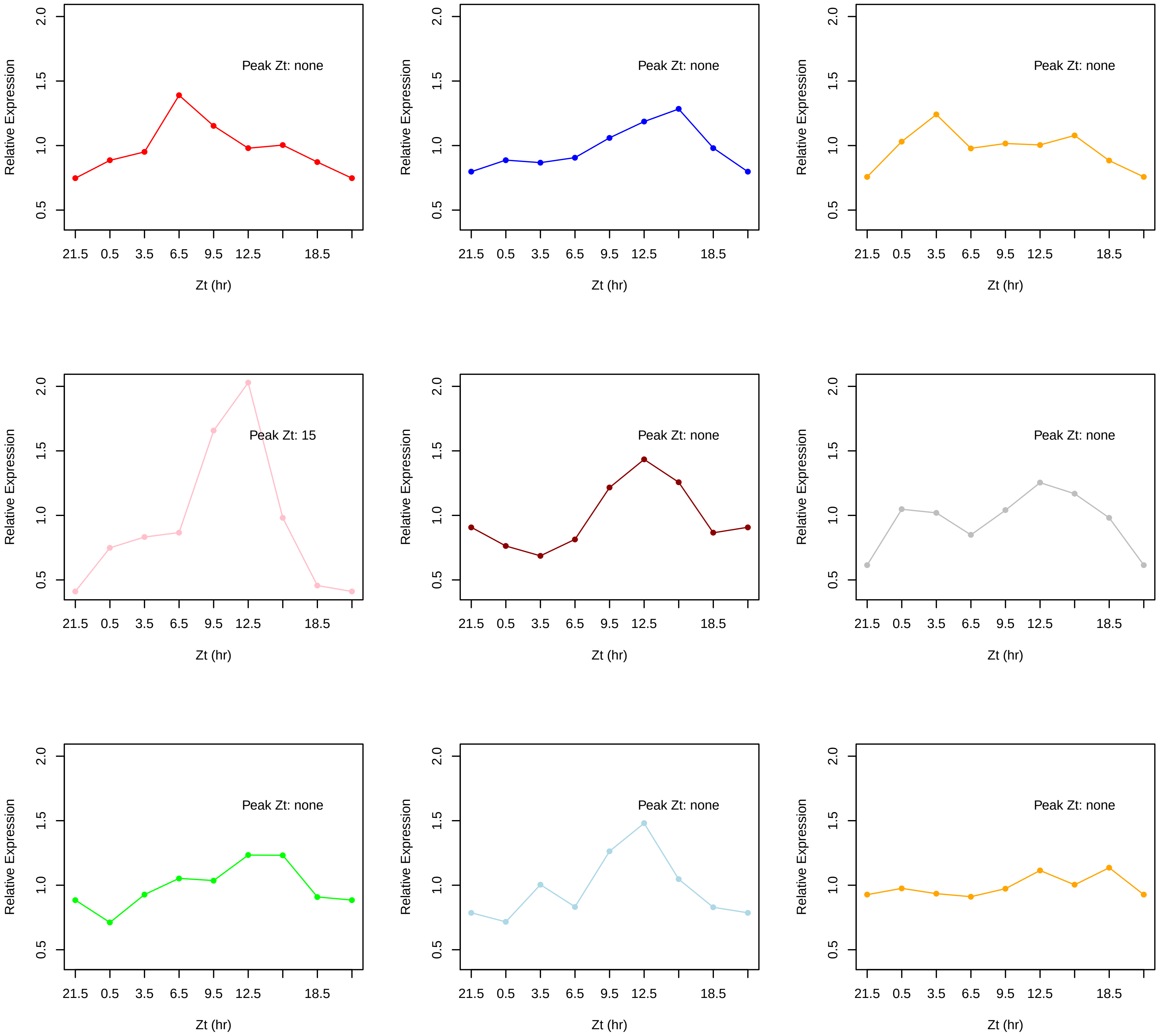

Singeleton: ENSXMAG00000008172 si:dkey−177p2.6

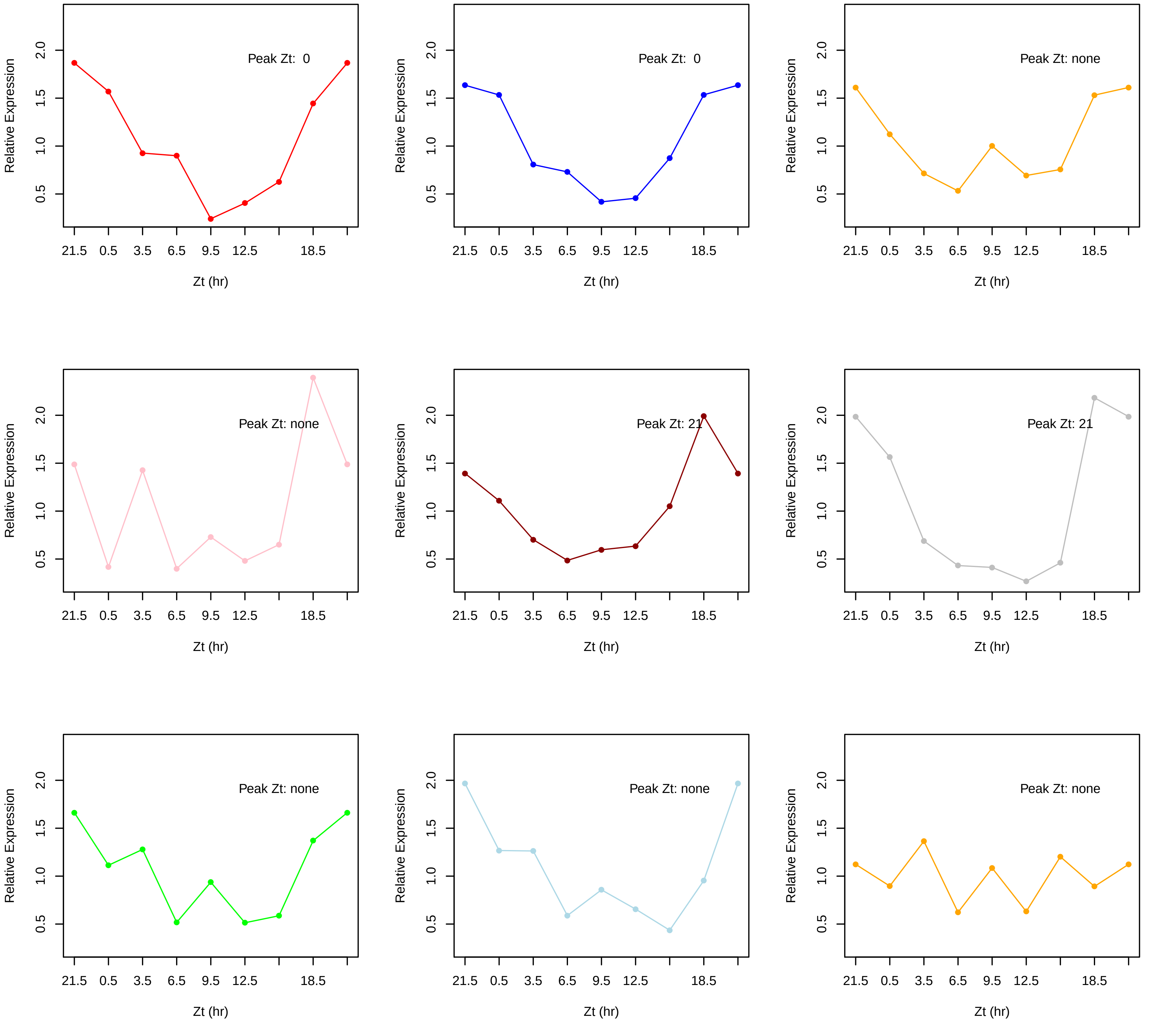

Singeleton: ENSXMAG00000001000 tmem14cb

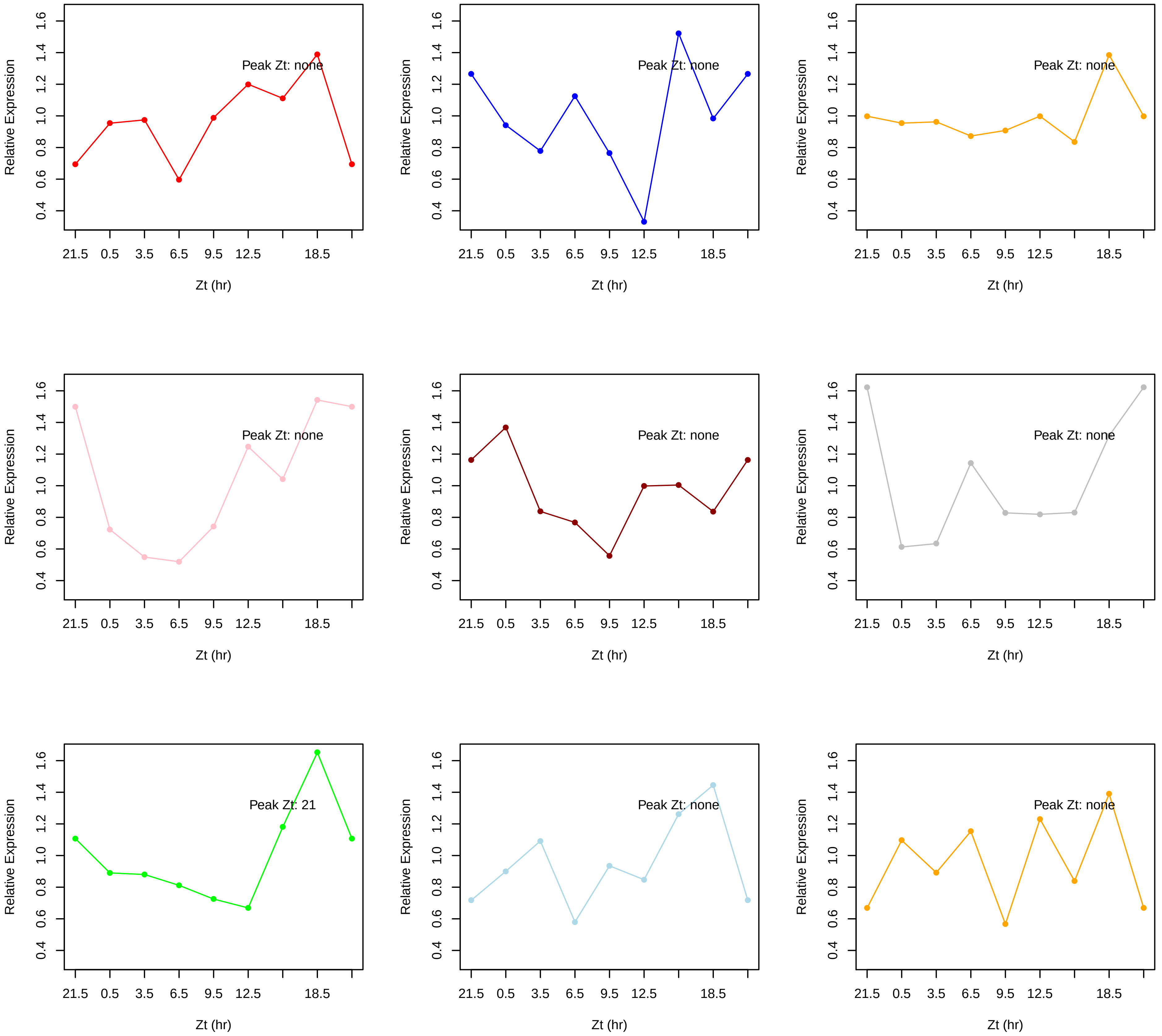

Singeleton: ENSXMAG00000016516 lamb2l

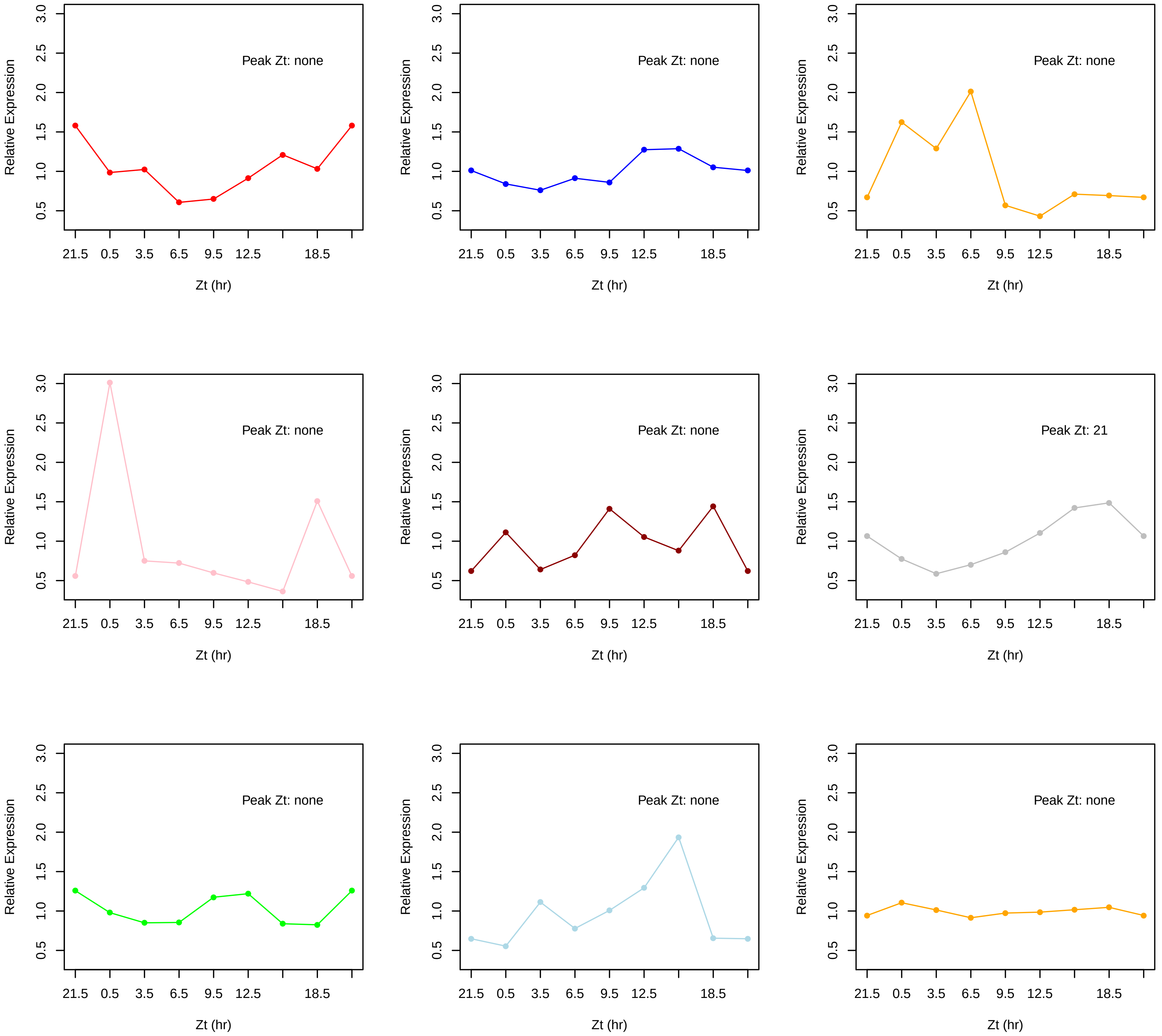

Singeleton: ENSXMAG00000001001 pak1ip1

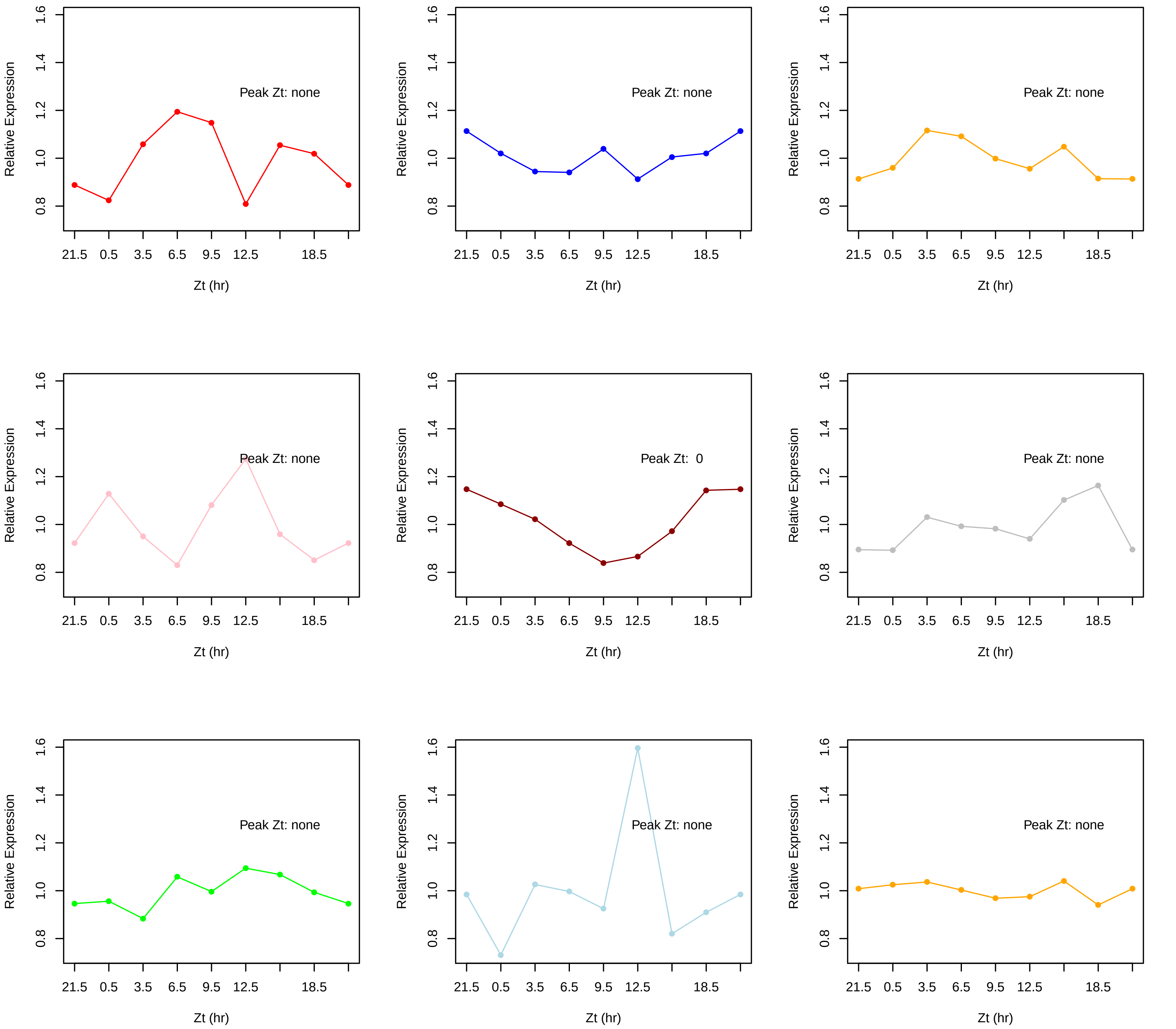

Singeleton: ENSXMAG00000029918 trnau1apb

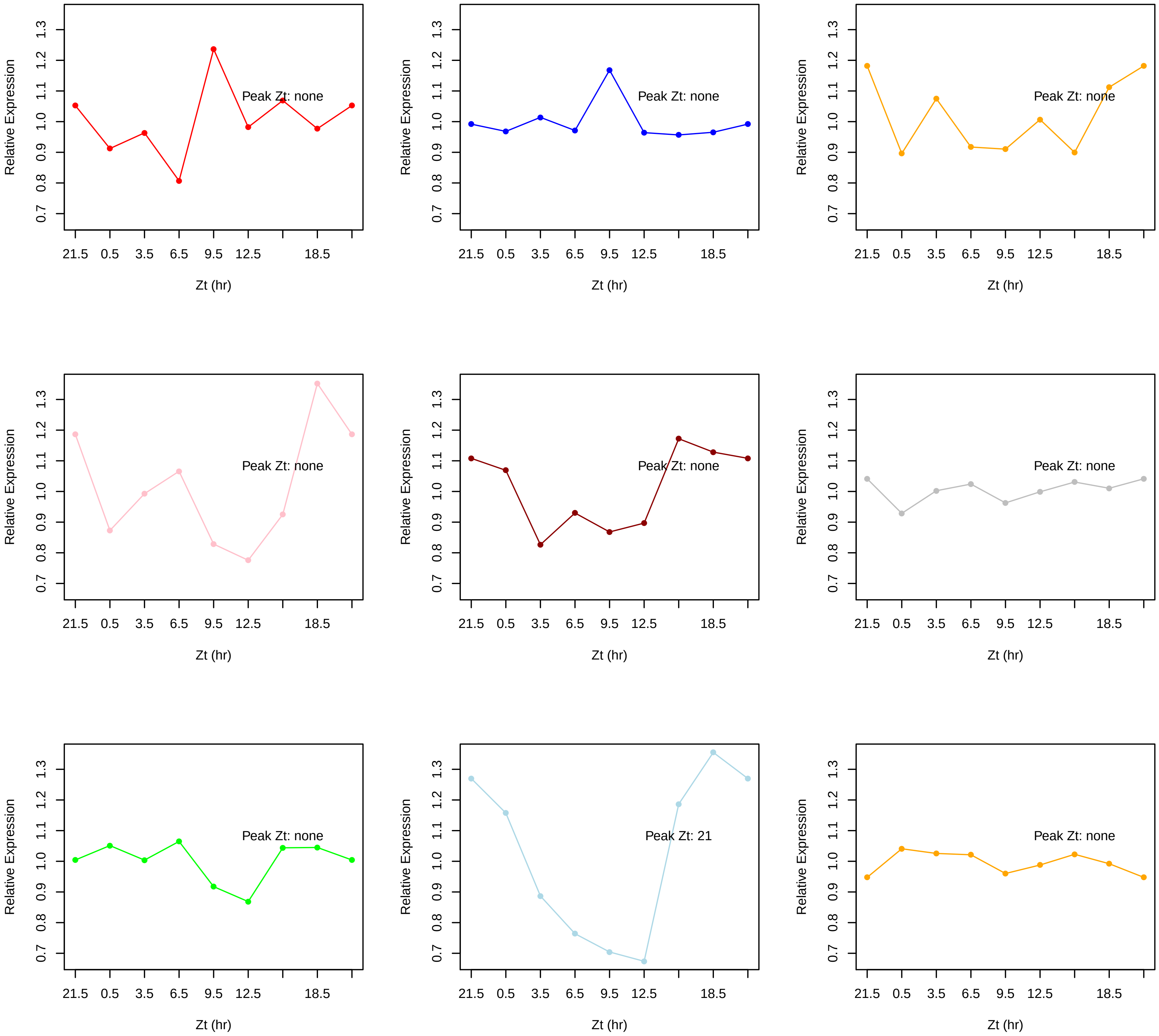

Singeleton: ENSXMAG00000012177 hgsnat

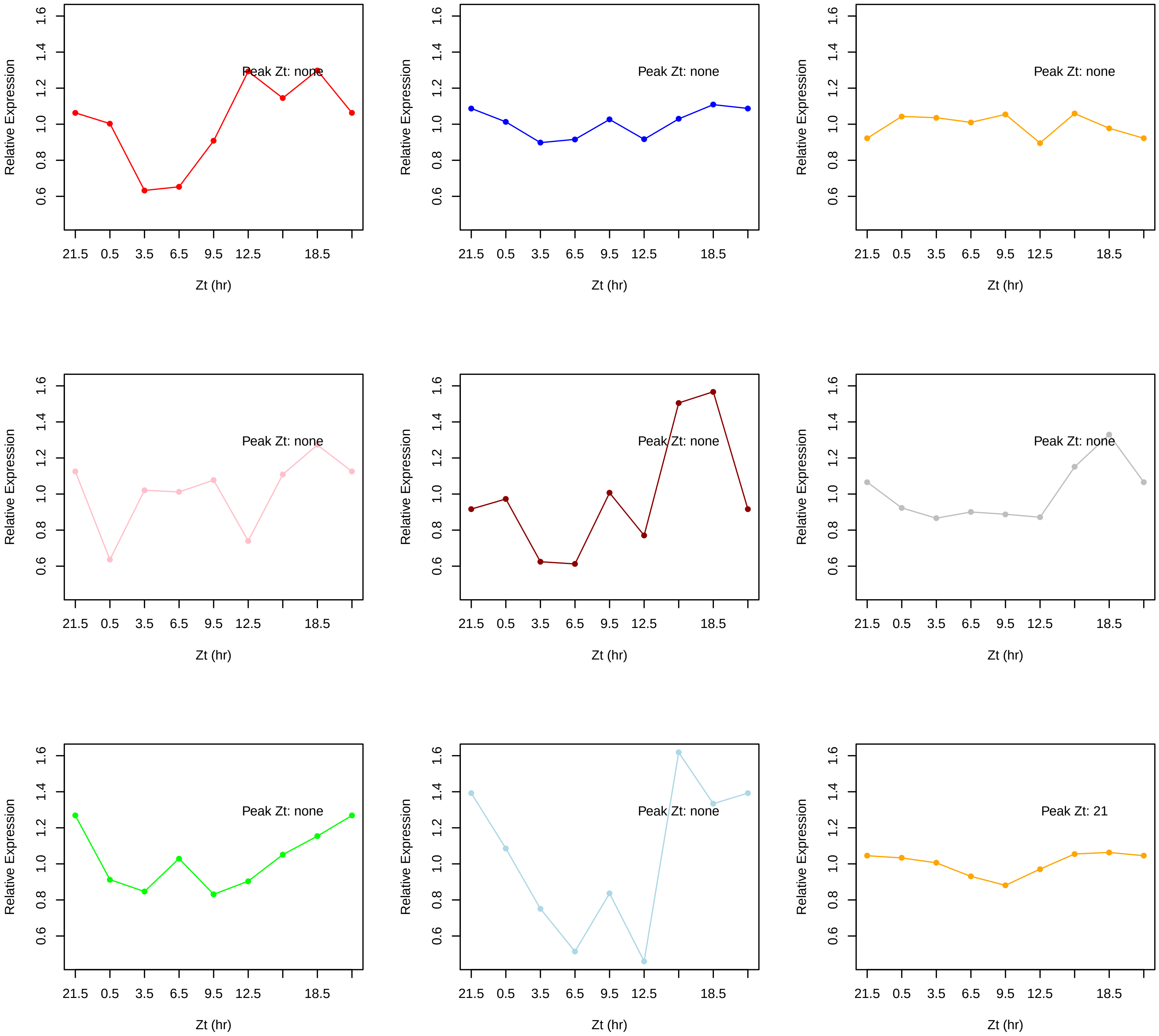

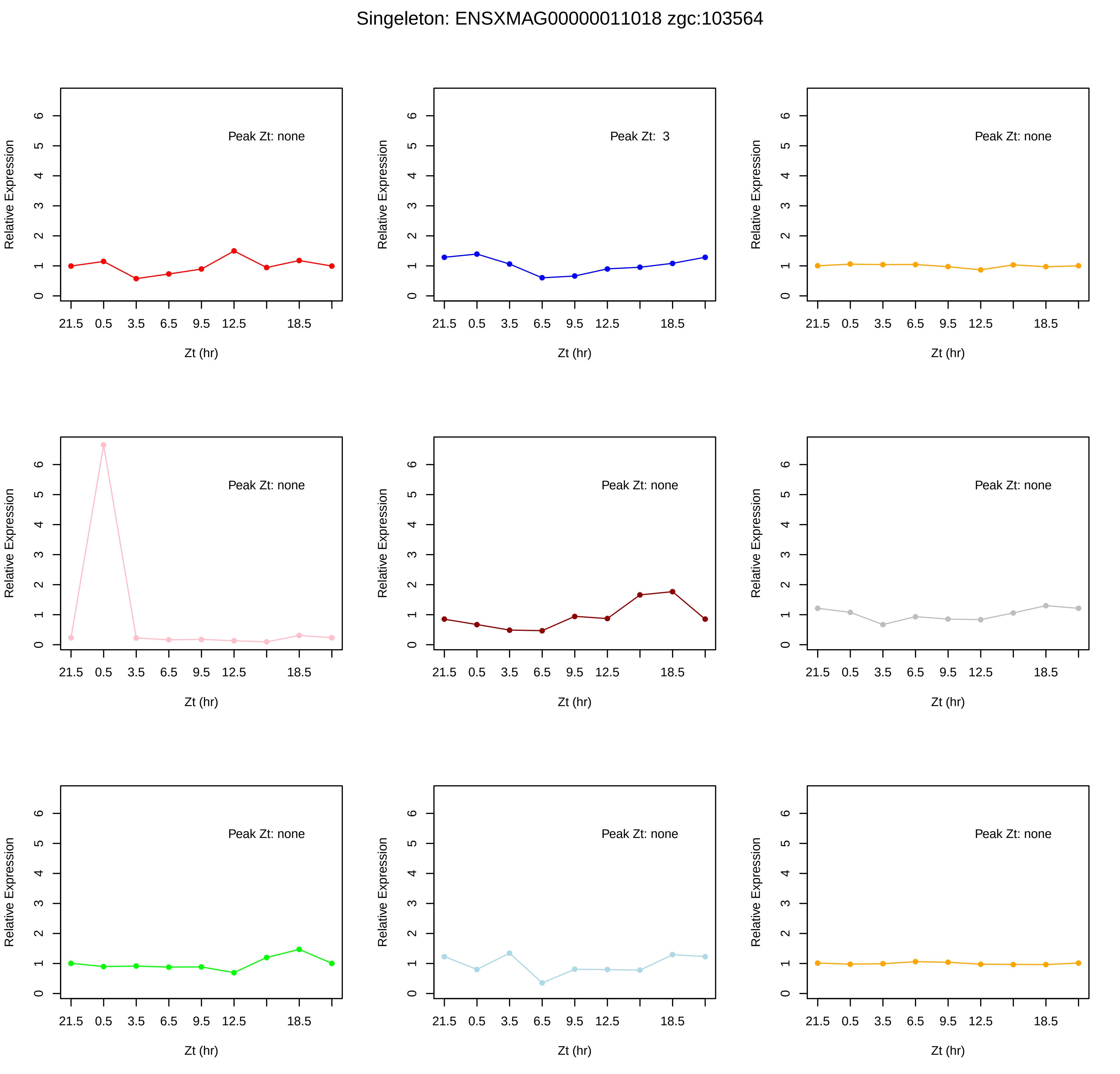

Singeleton: ENSXMAG00000011018 zgc:103564
Peak Zt: none
Peak Zt: 3
Peak Zt: none
Peak Zt: none
Peak Zt: none
Peak Zt: none
Peak Zt: none
Peak Zt: none
Peak Zt: none
Relative Expression
Zt (hr)
21.5
0.5
3.5
6.5
9.5
12.5
18.5

Singeleton: ENSXMAG00000000140 ccdc88c

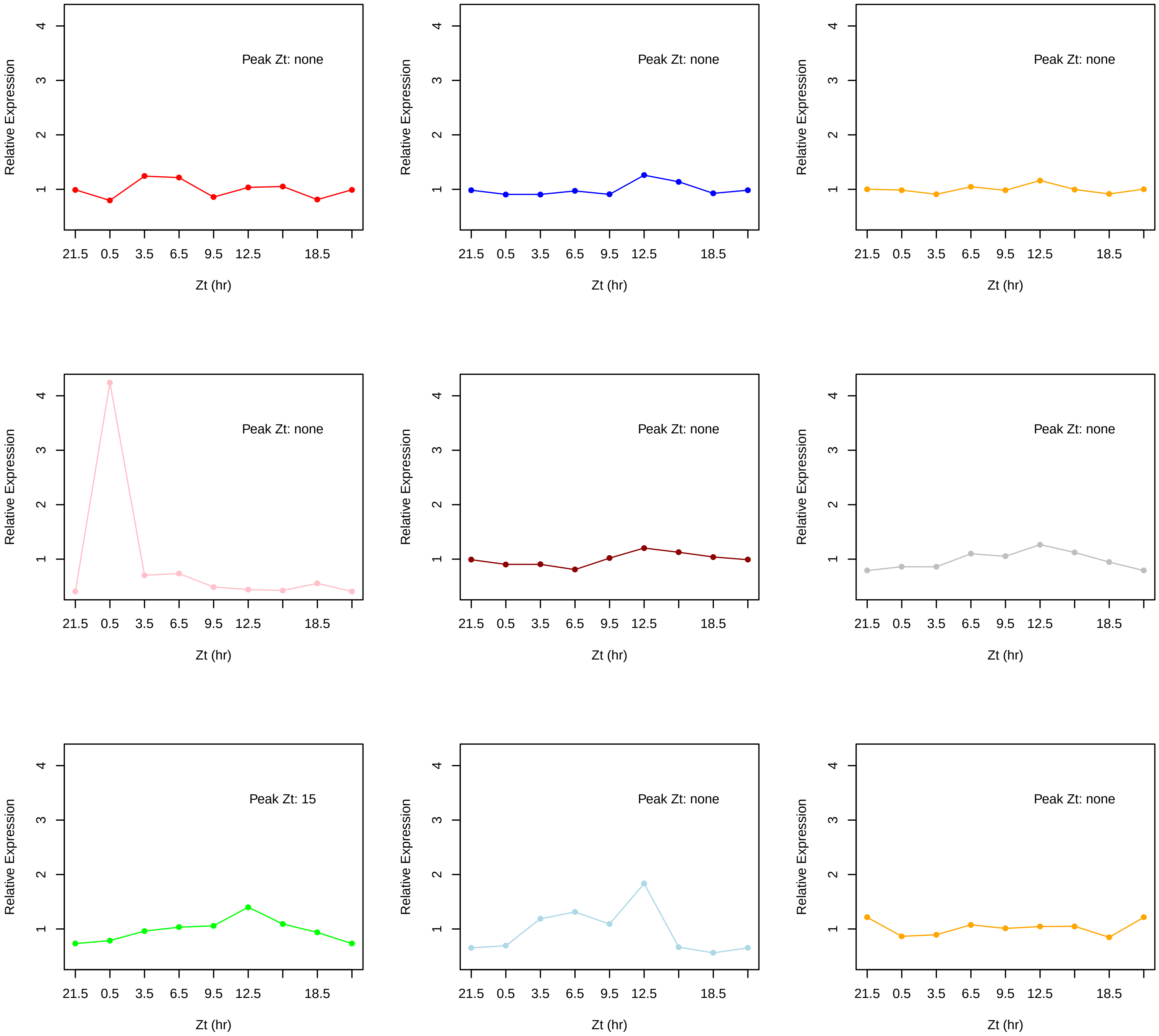

Singeleton: ENSXMAG00000002257 SHC4

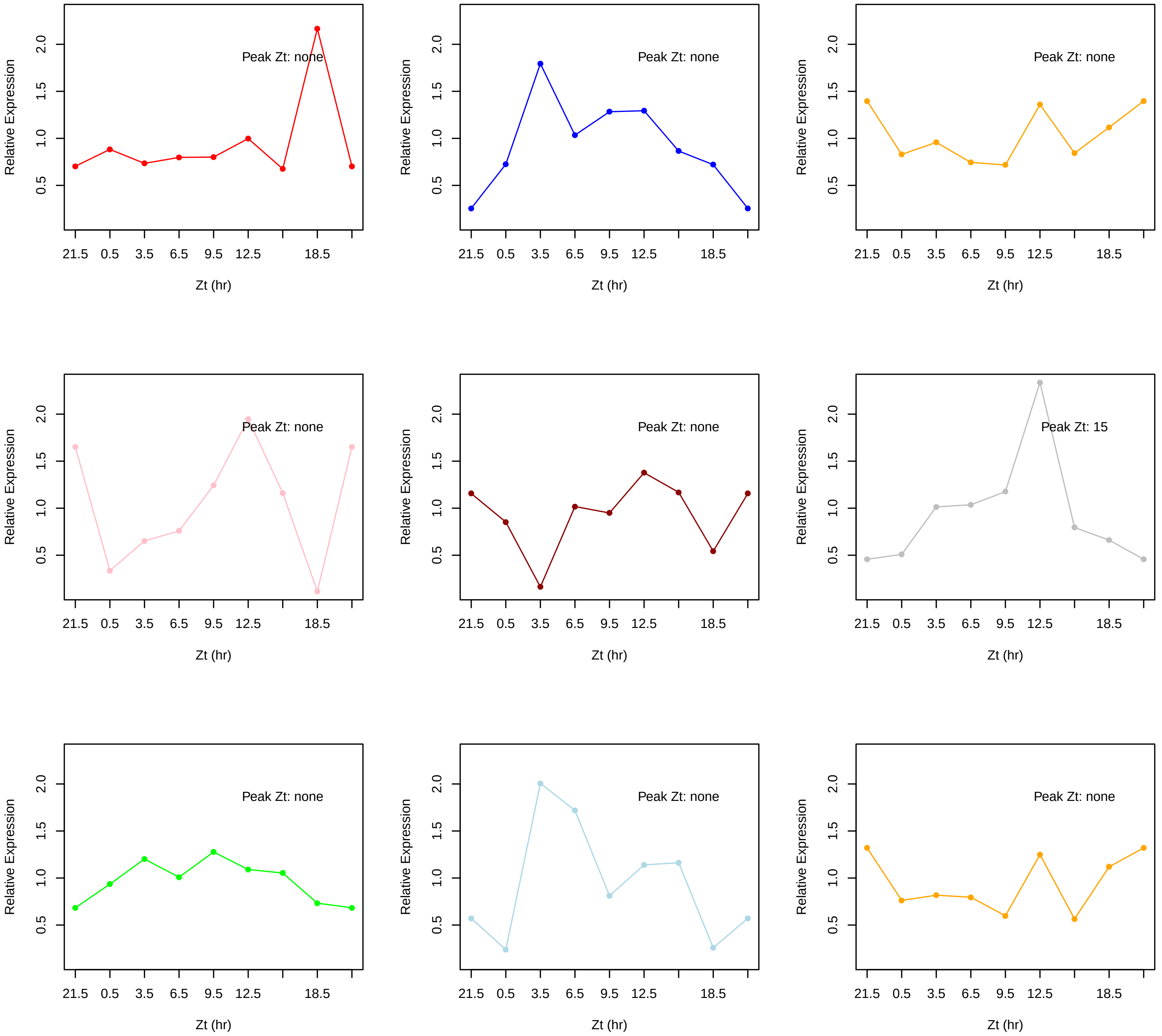

Singleton: ENSXMAG00000027016 tfap2a

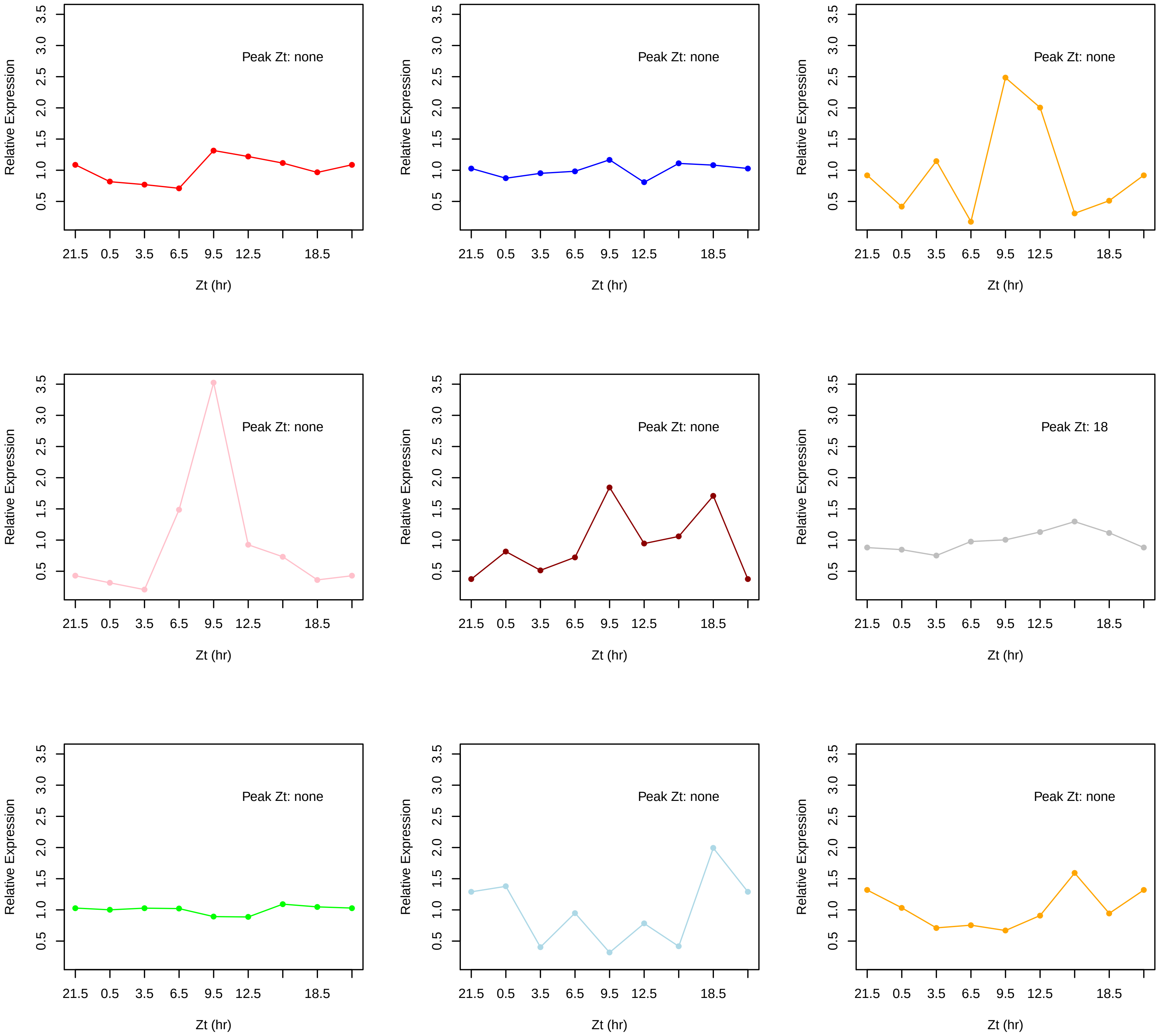

Singeleton: ENSXMAG00000008822 pdhb

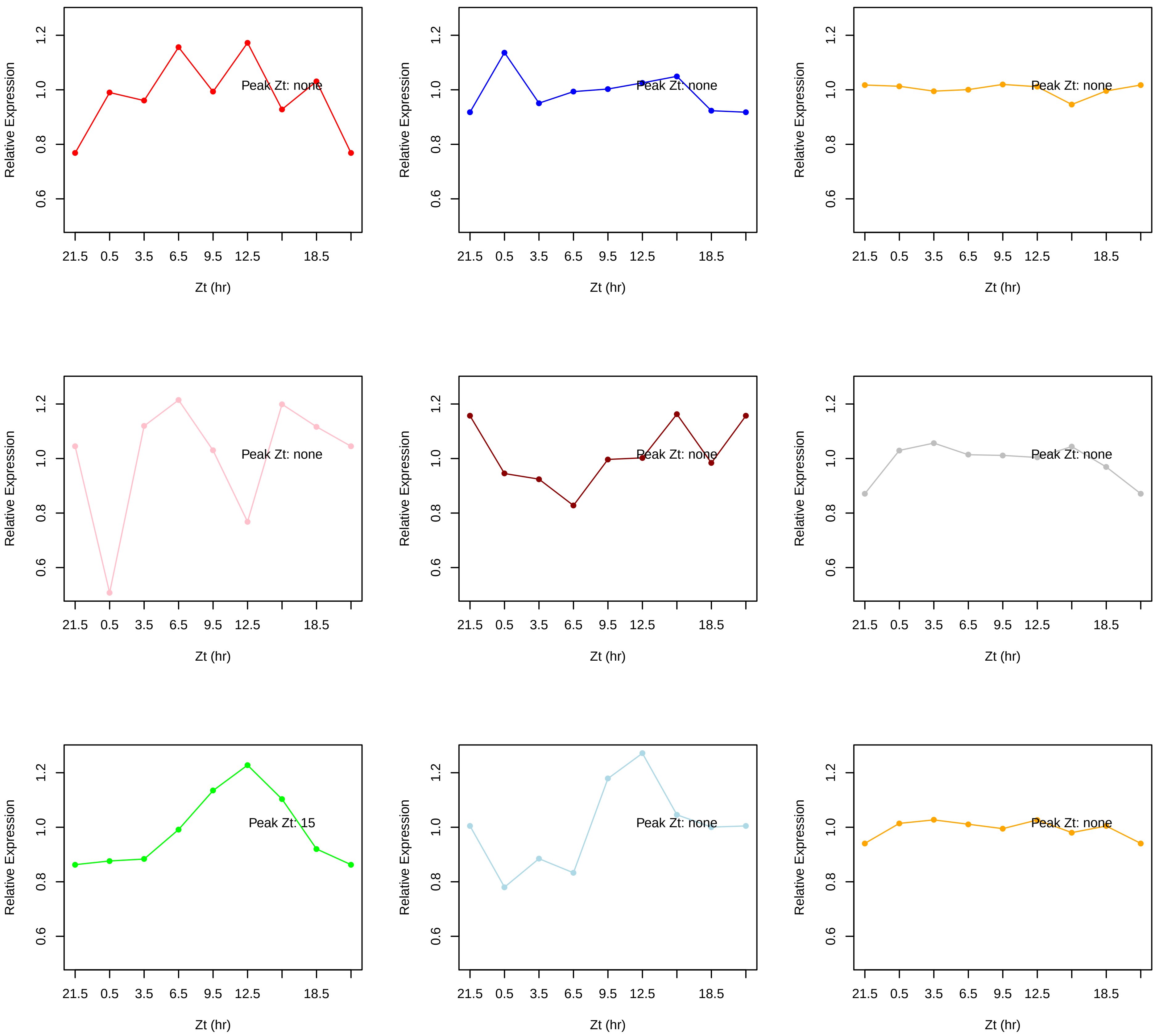

Singeleton: ENSXMAG00000027637 RBPMS

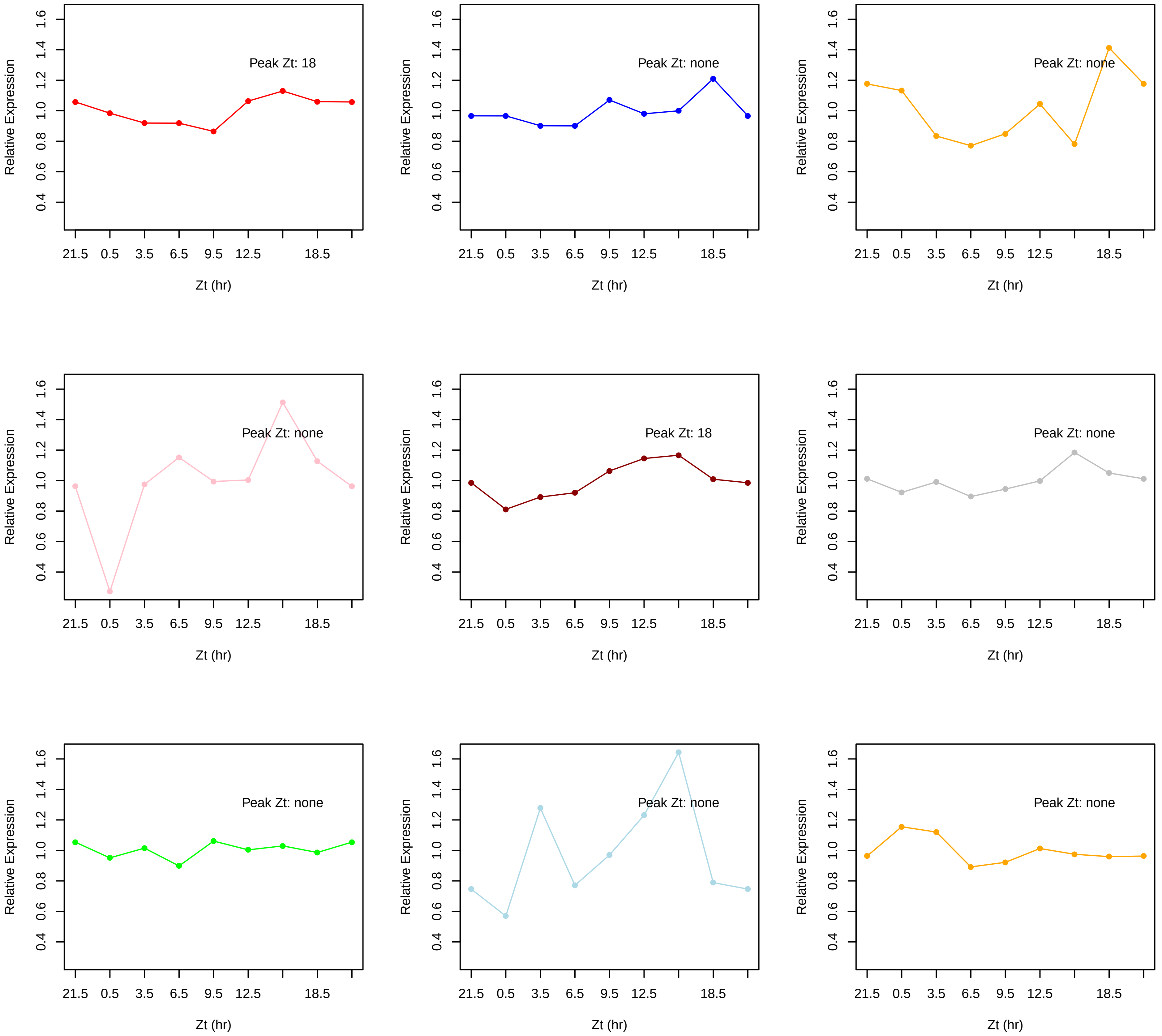

Singeleton: ENSXMAG00000008793 pxk

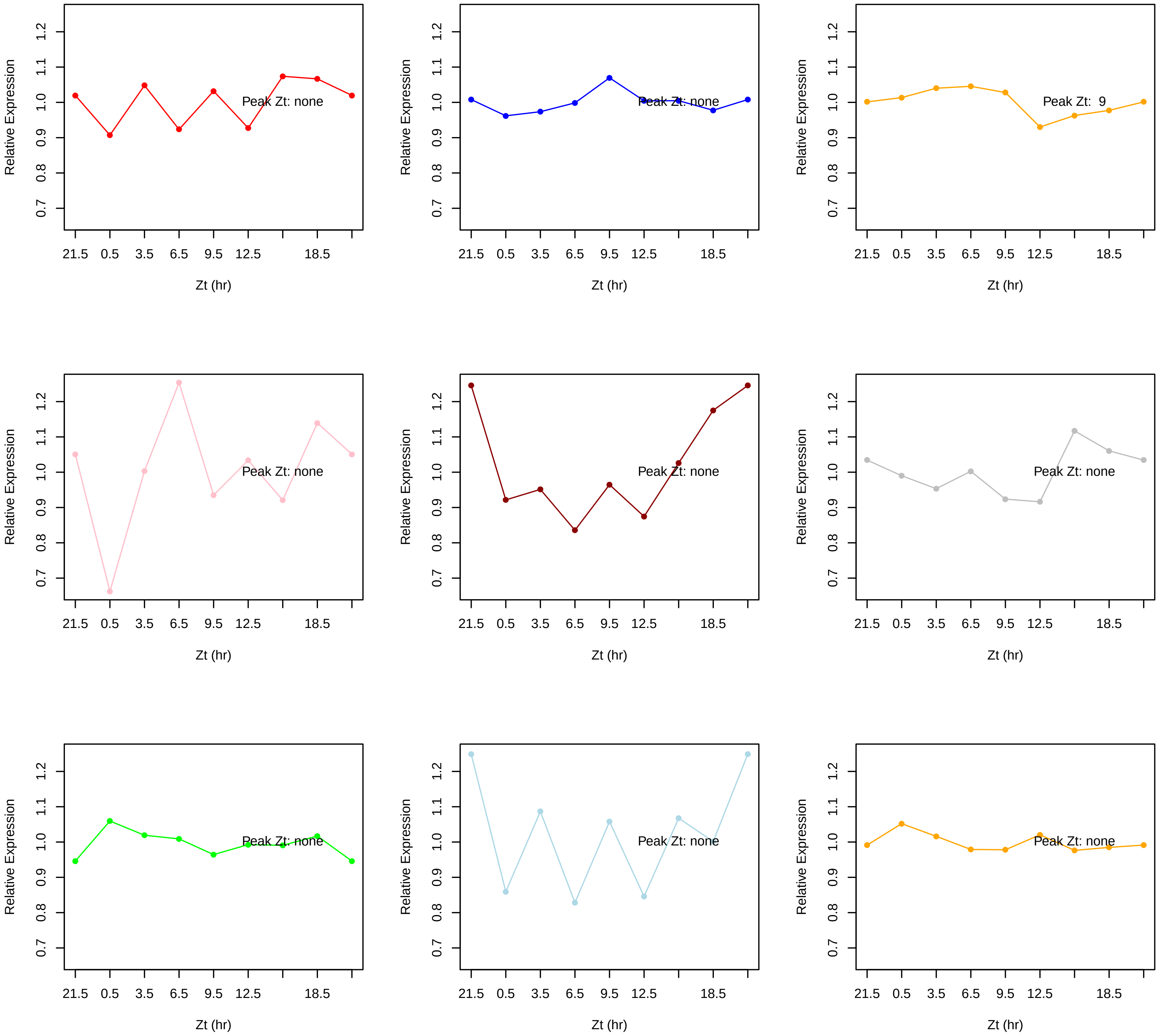

Singeleton: ENSXMAG00000003375 smek1

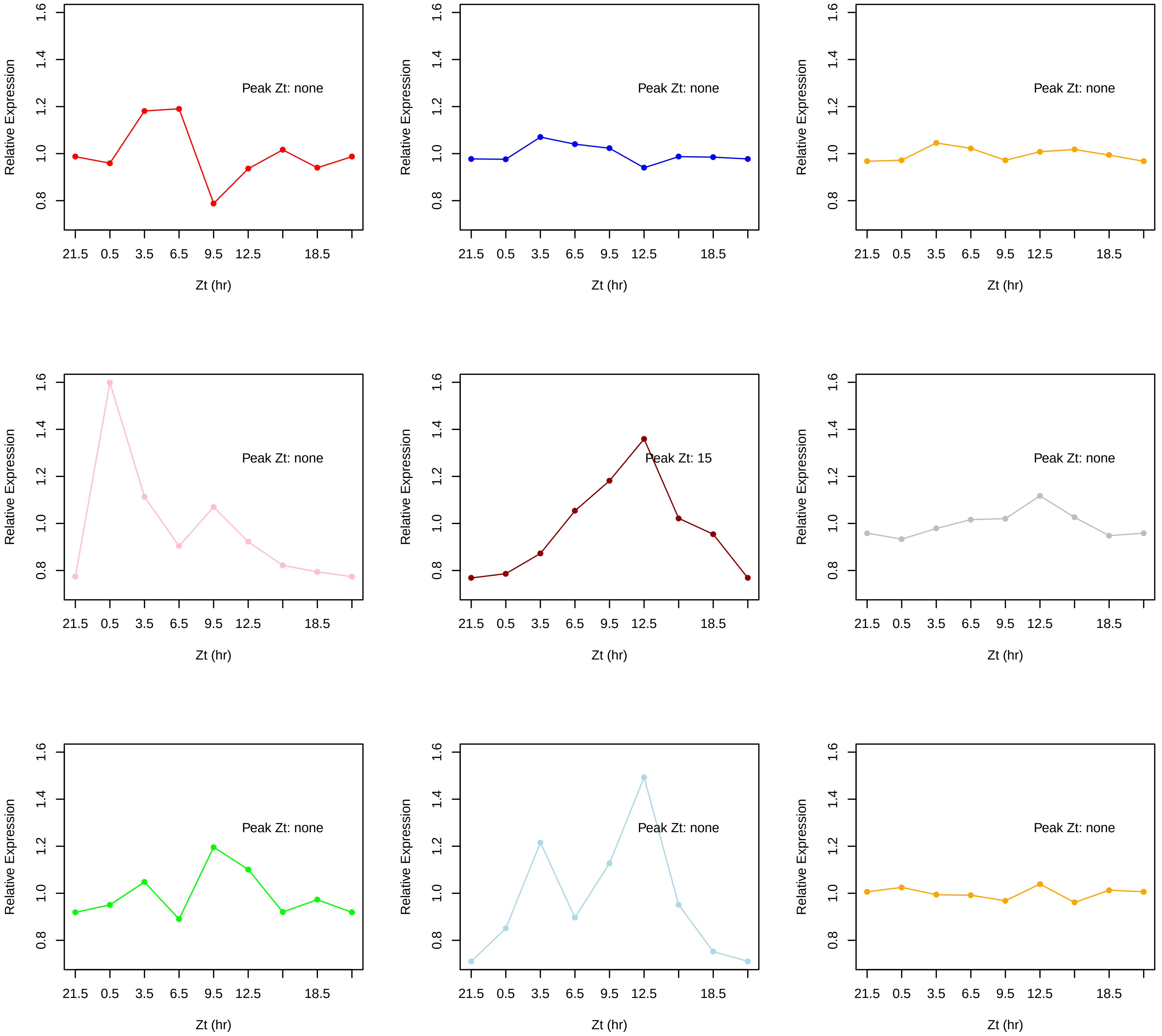

Singleton: ENSXMAG00000020150 HTD2

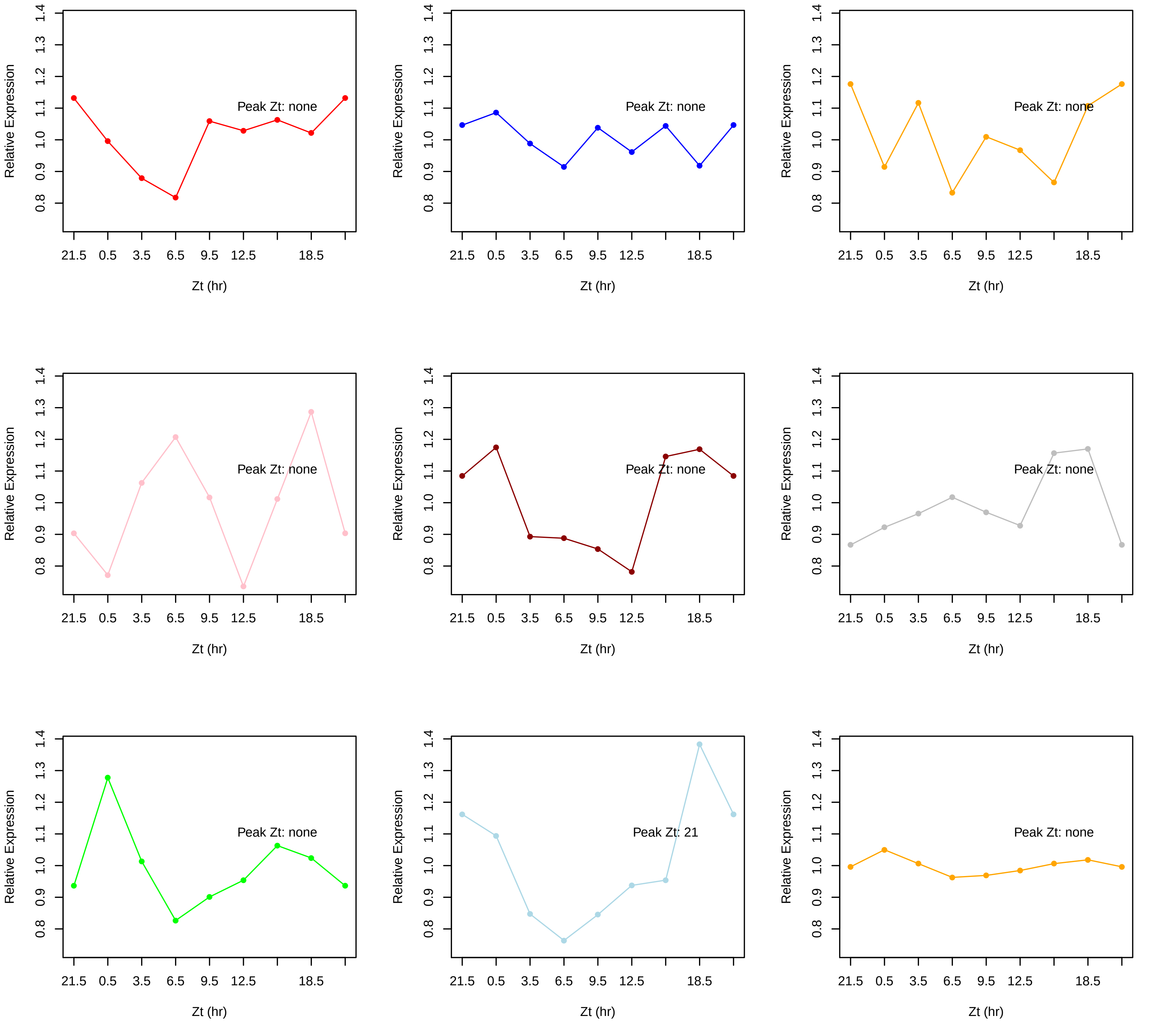

Singeleton: ENSXMAG00000009056 RPP14

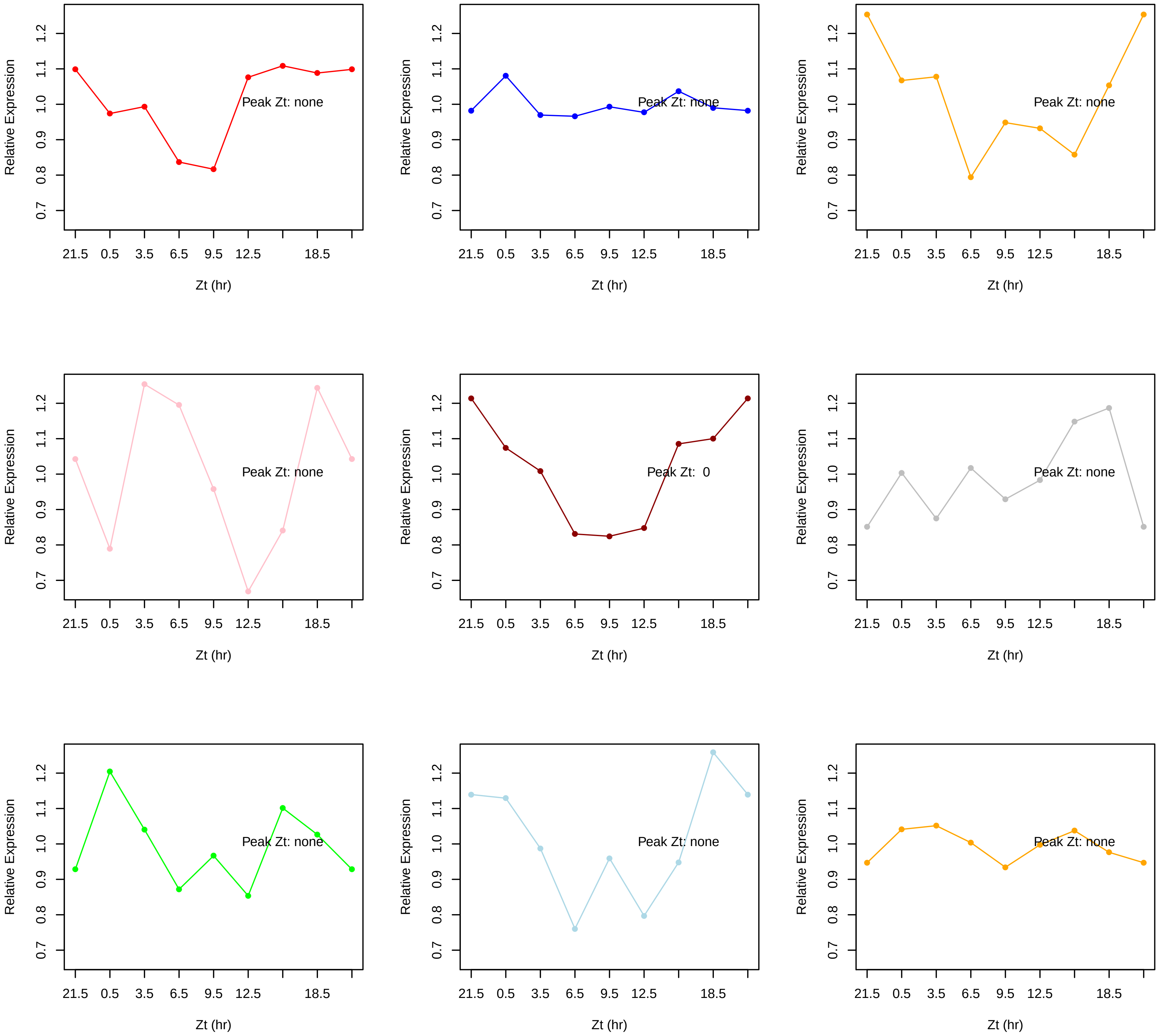

Singeleton: ENSXMAG00000007299 slc35b3

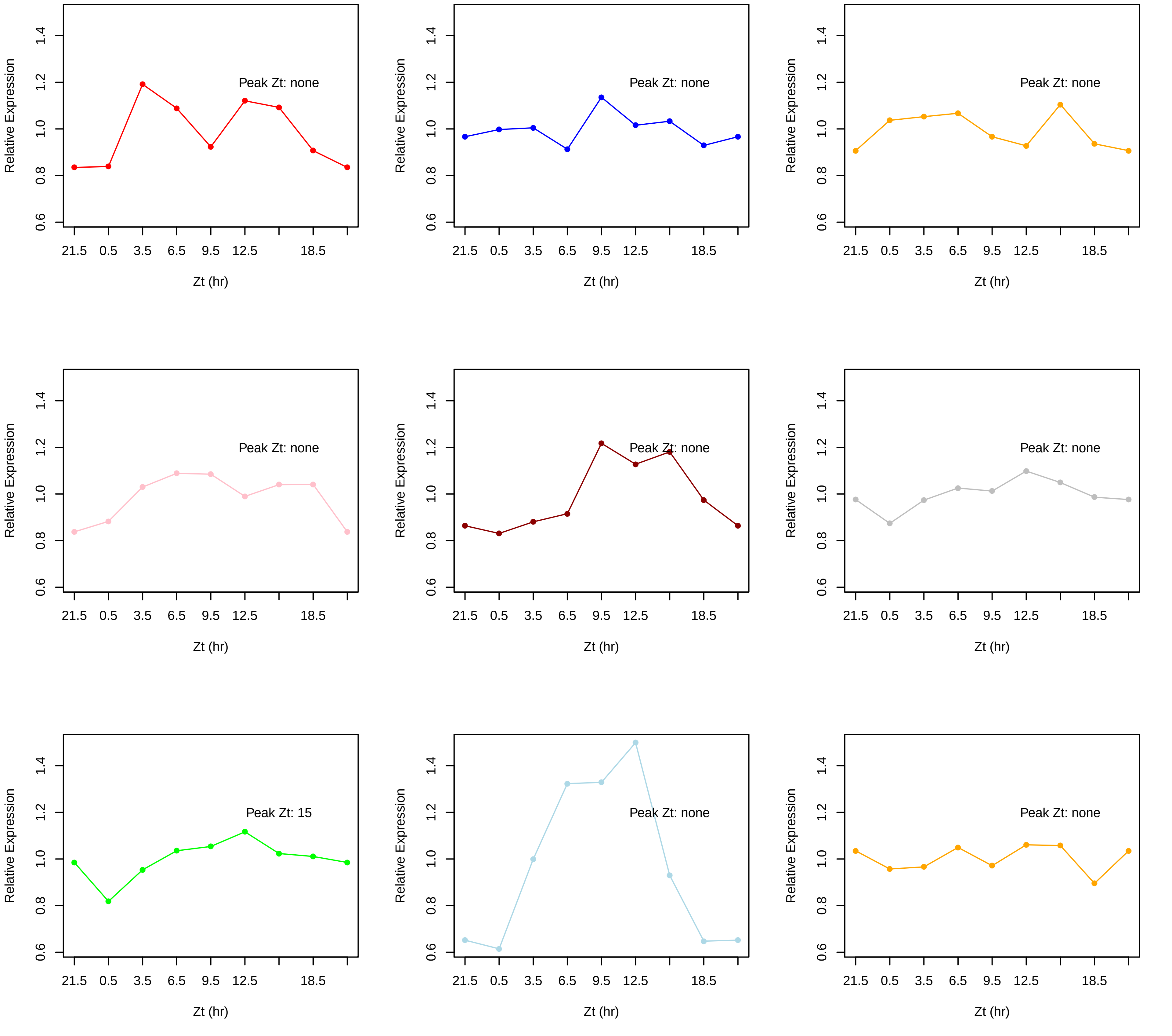

Singleton: ENSXMAG00000005992 eef1e1

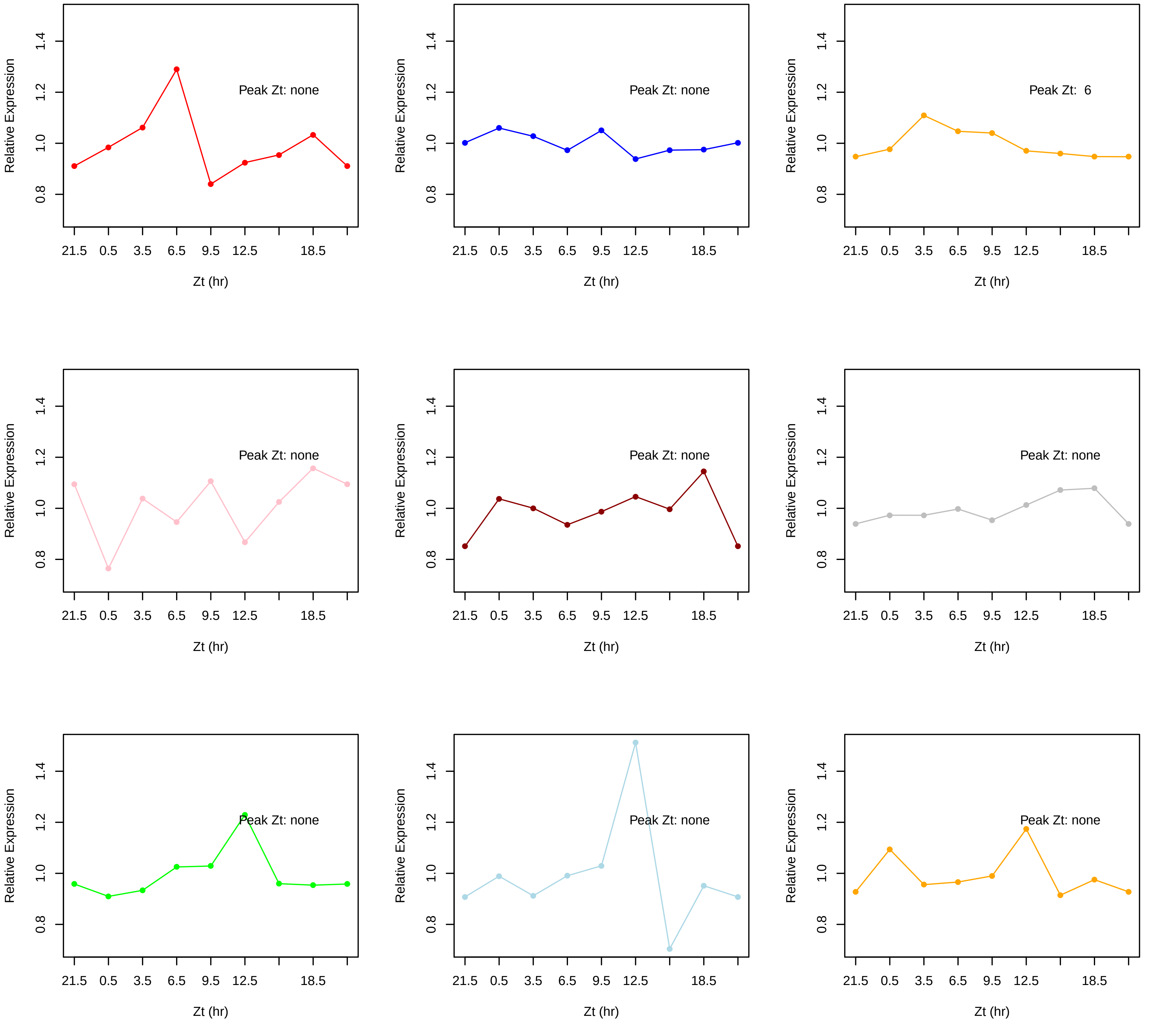

Singeleton: ENSXMAG00000014675 TC2N

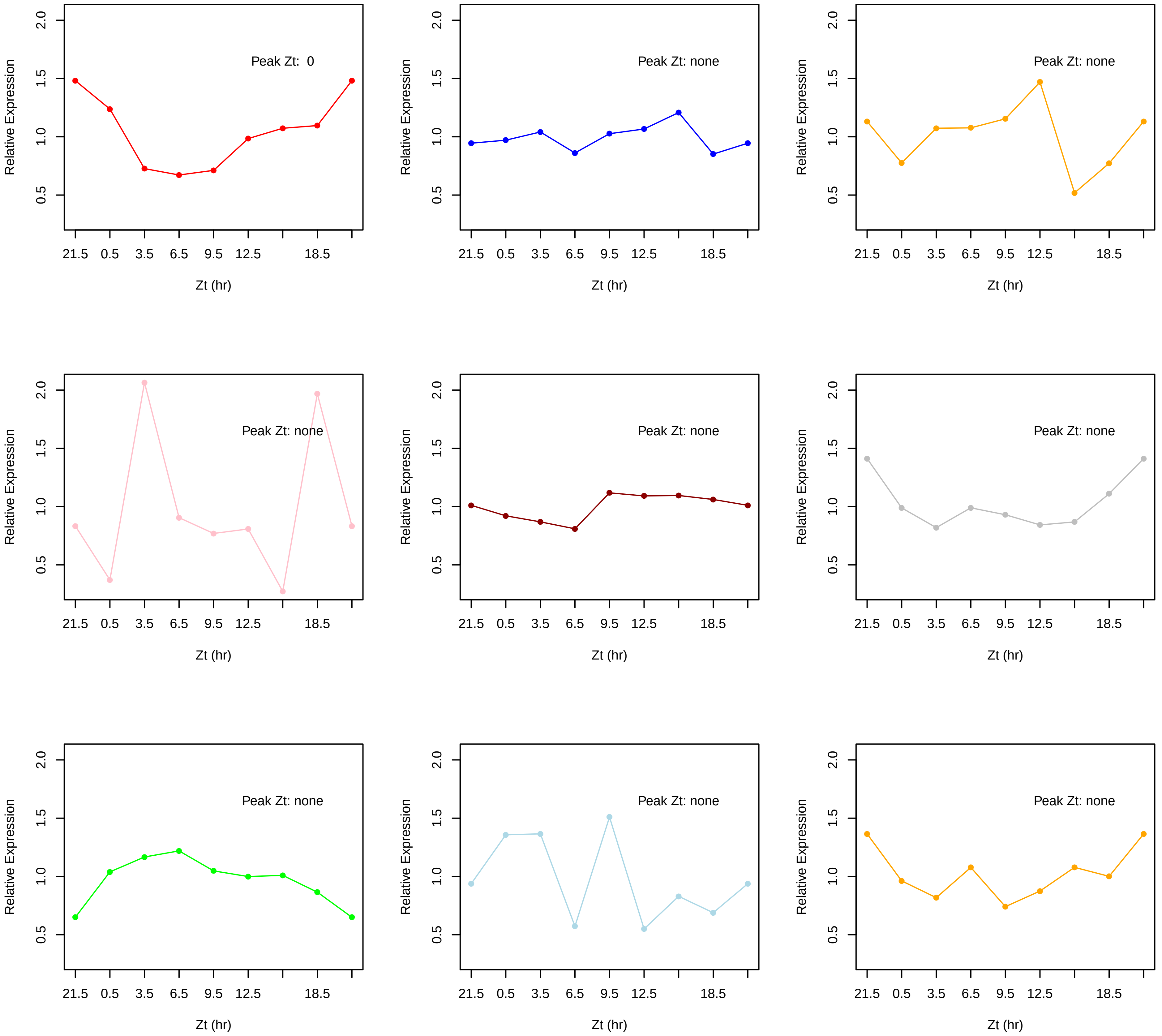

Singeleton: ENSXMAG00000026839 dut

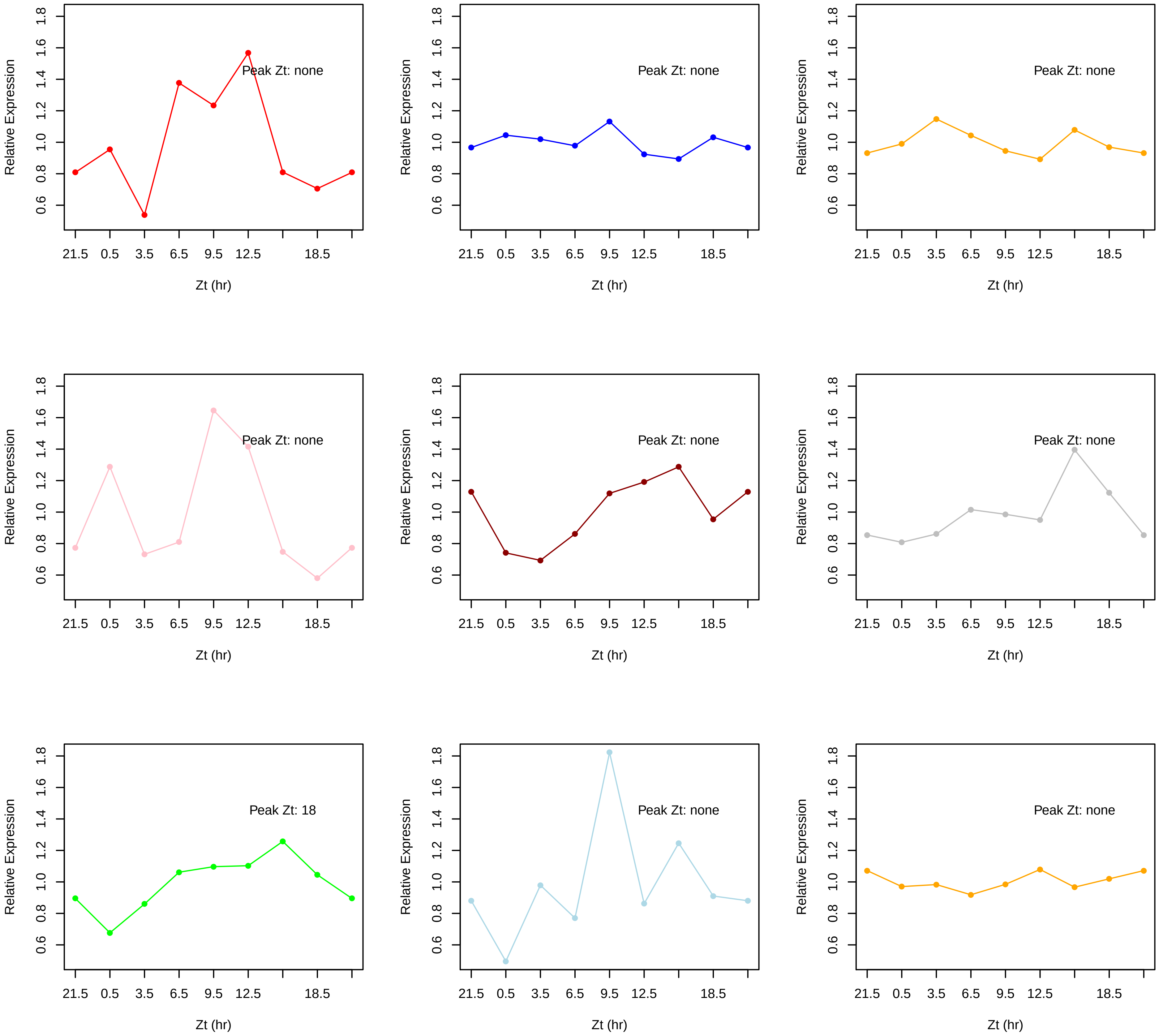

Singleton: ENSXMAG00000012760 cdc27

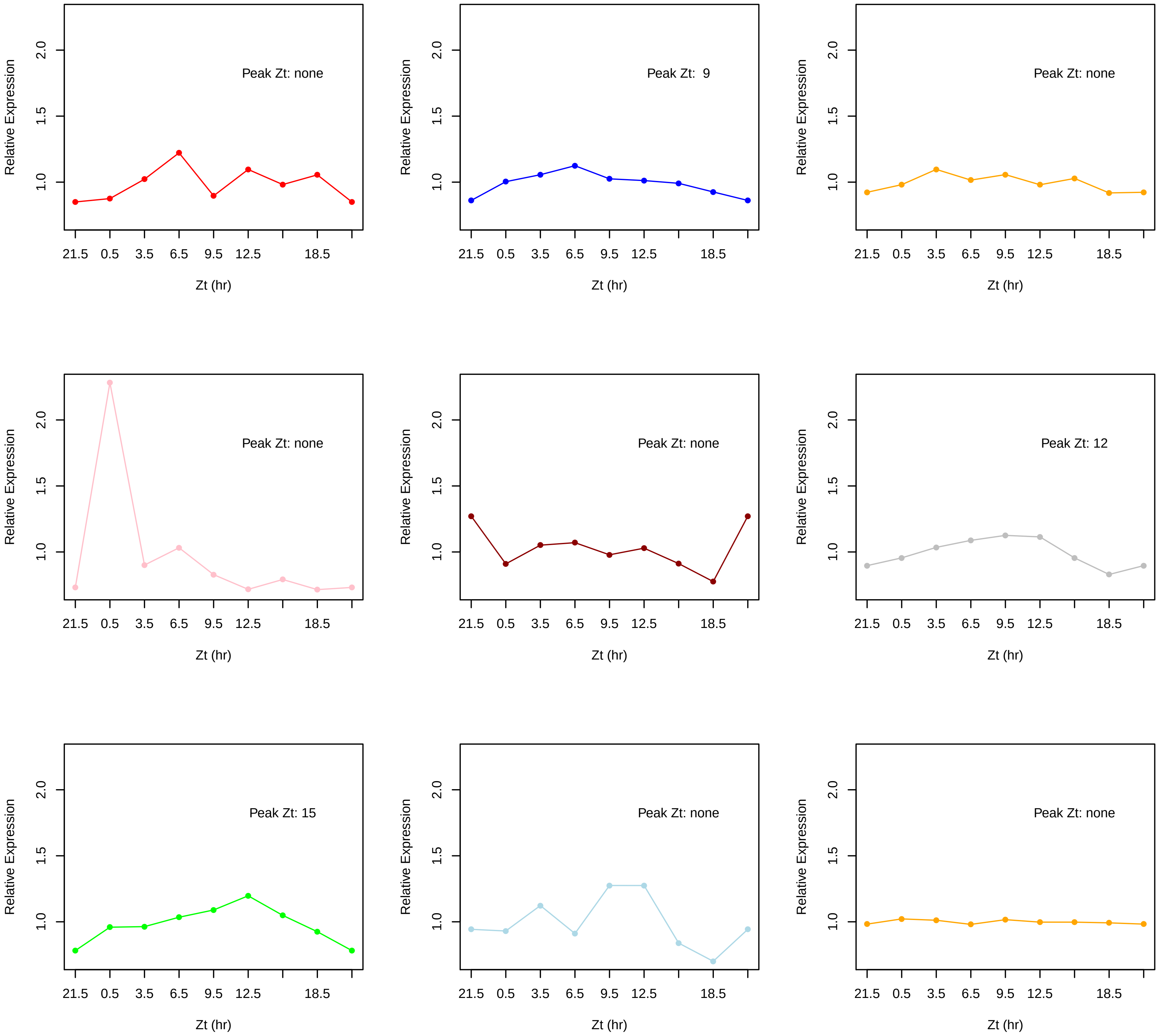

Singeleton: ENSXMAG00000005999 txndc5

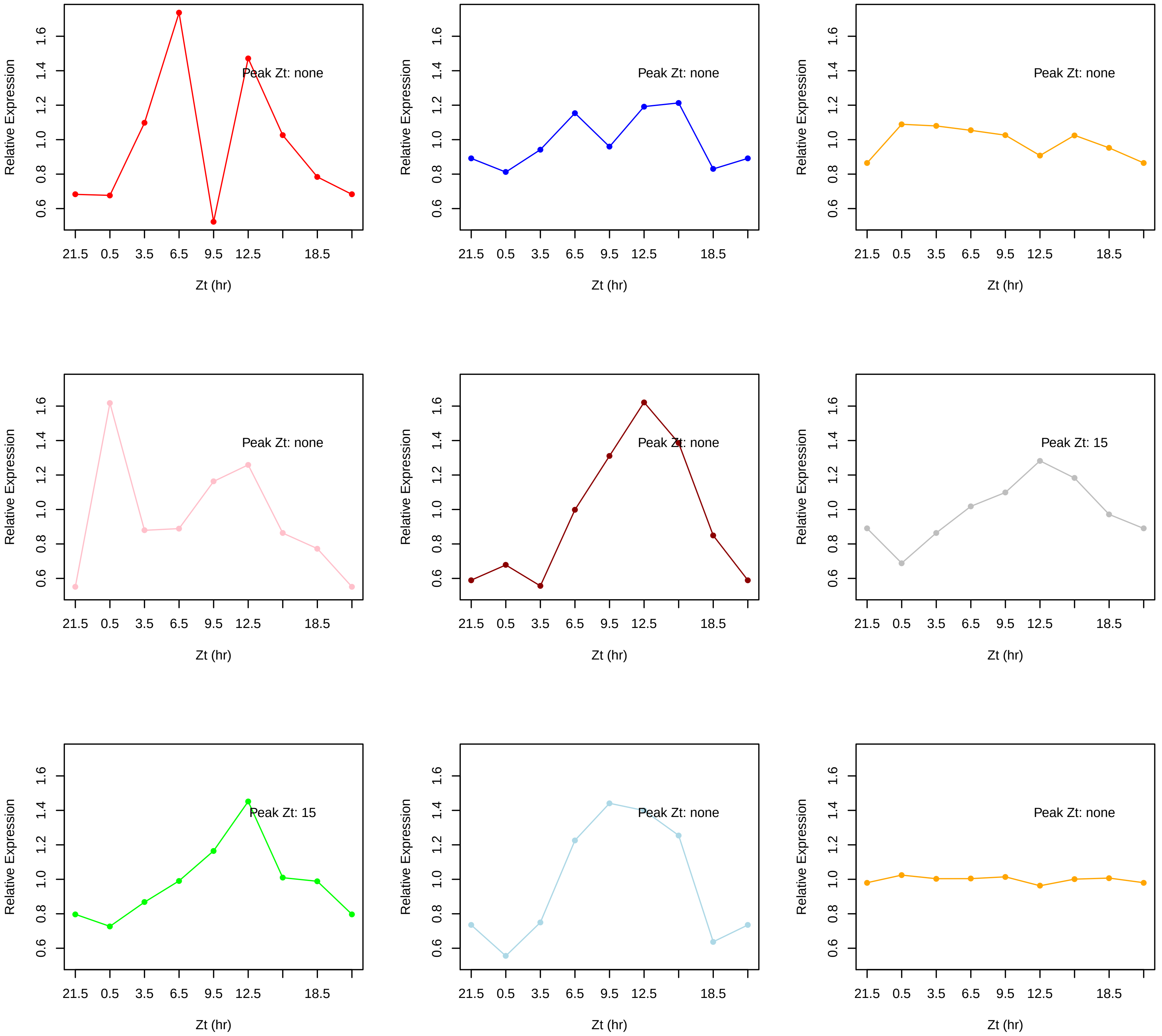

Singeleton: ENSXMAG00000011669 fxr2

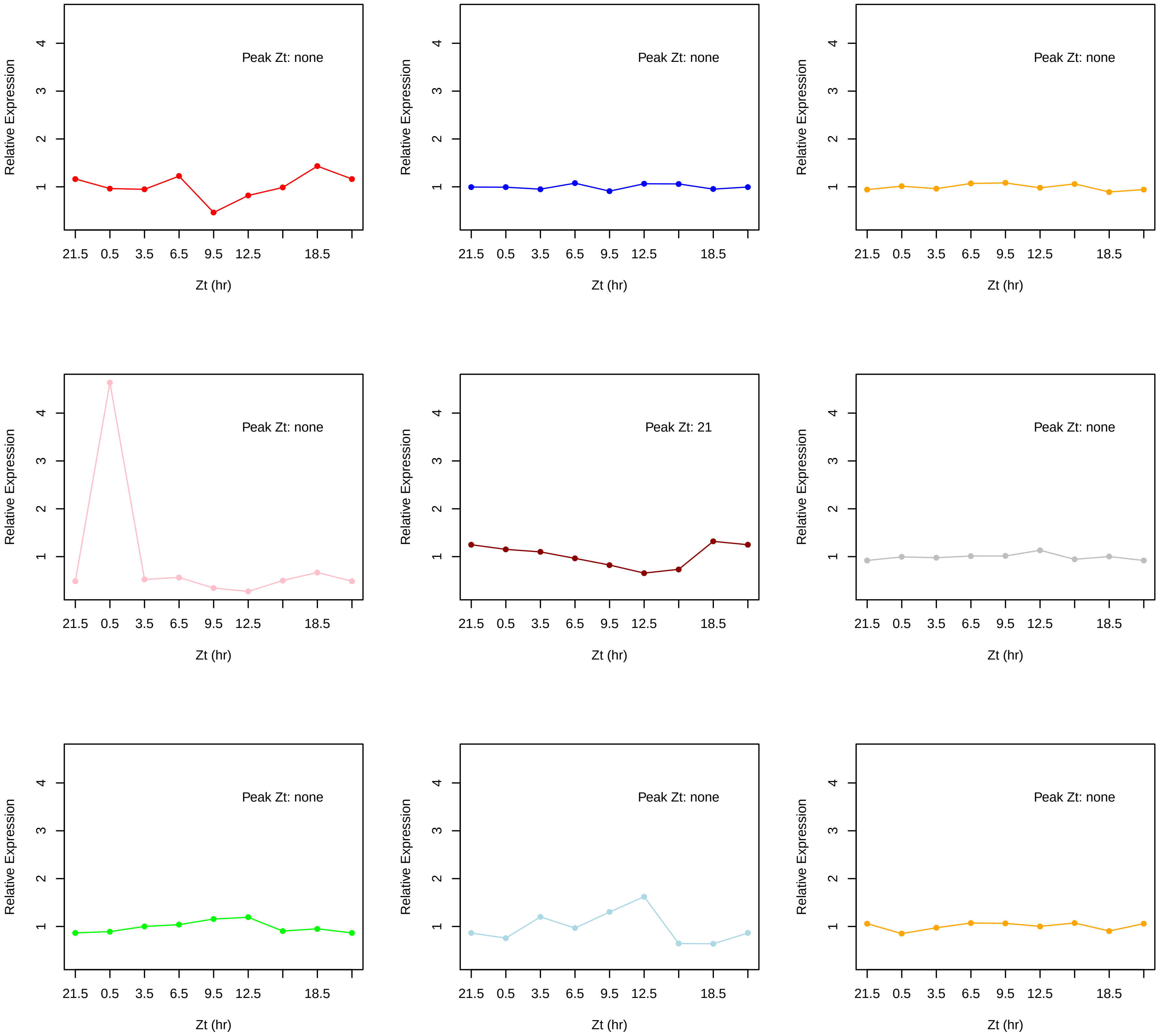

Singleton: ENSXMAG00000016686 myef2

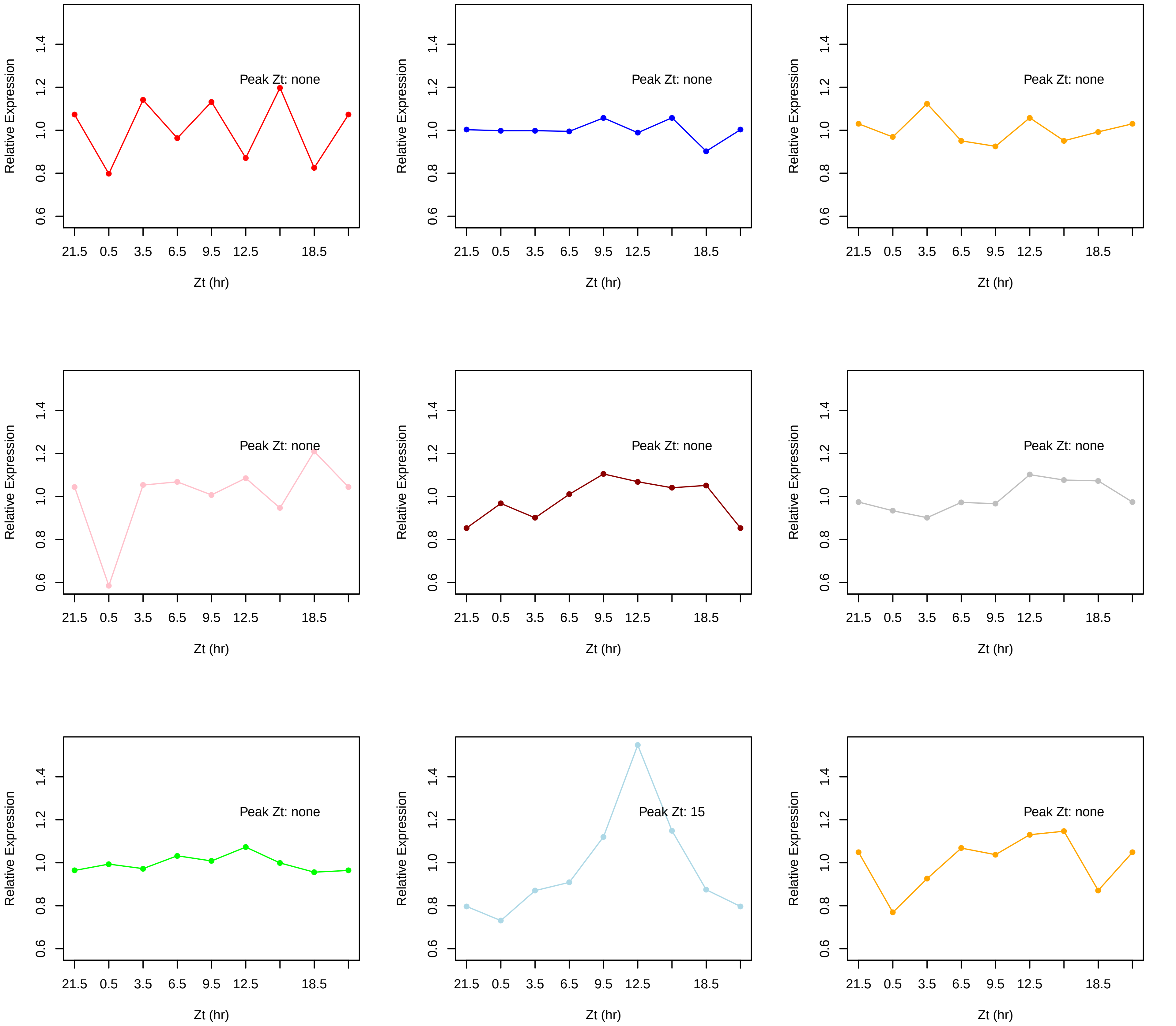

Singeleton: ENSXMAG00000006020 zgc:101569

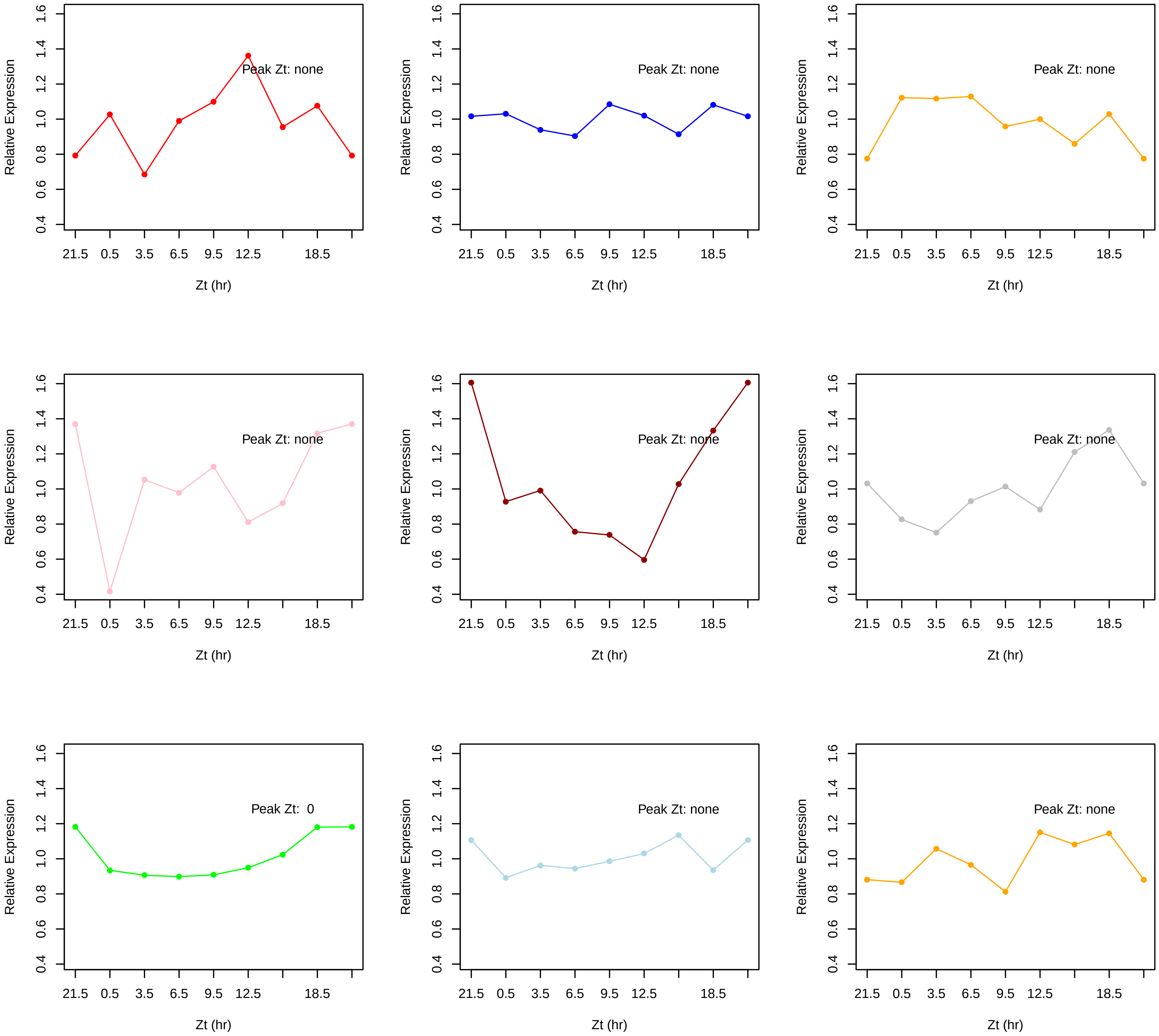

Singeleton: ENSXMAG00000006596 PIK3R6

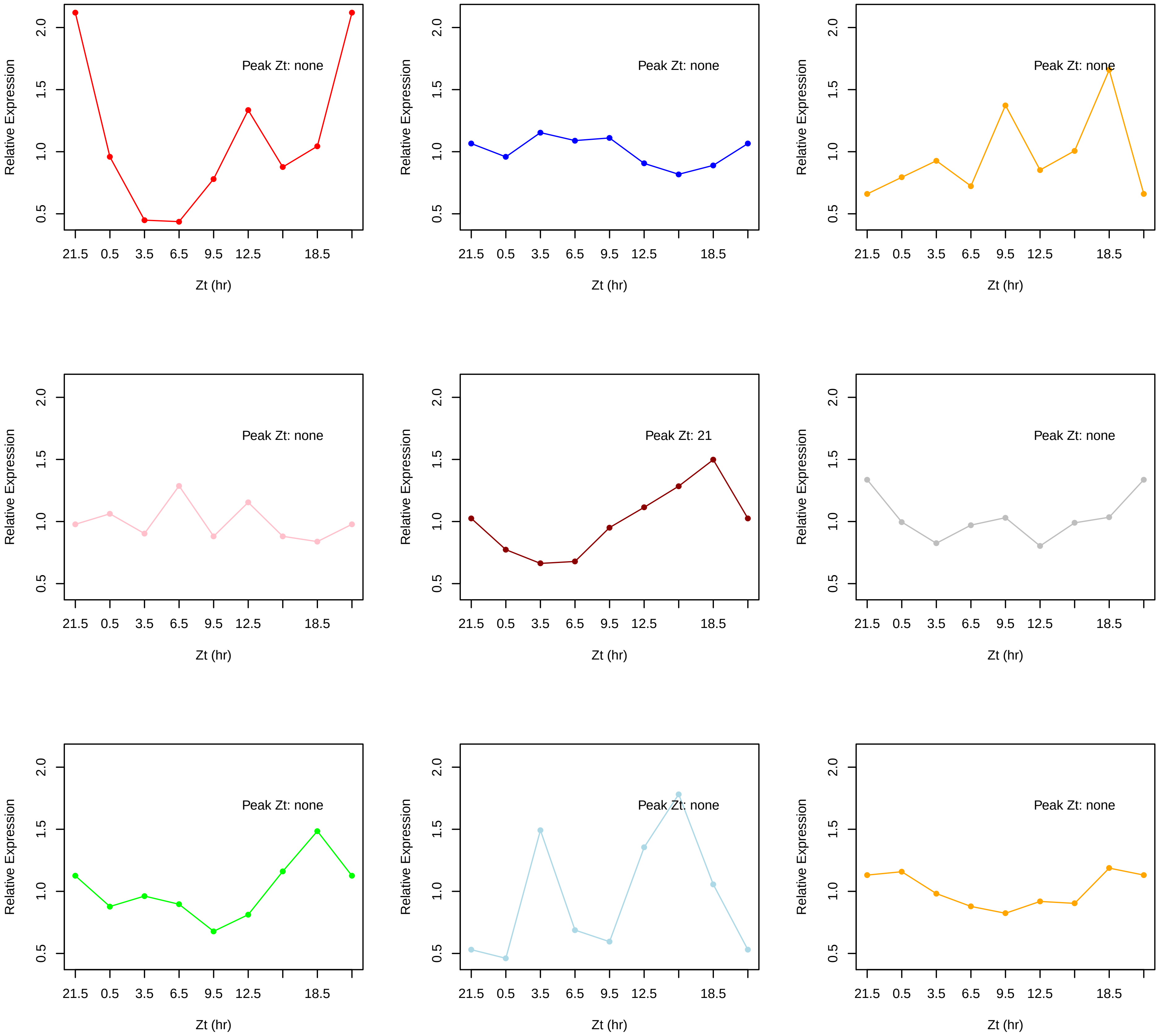

Singleton: ENSXMAG00000025368 pdcd6

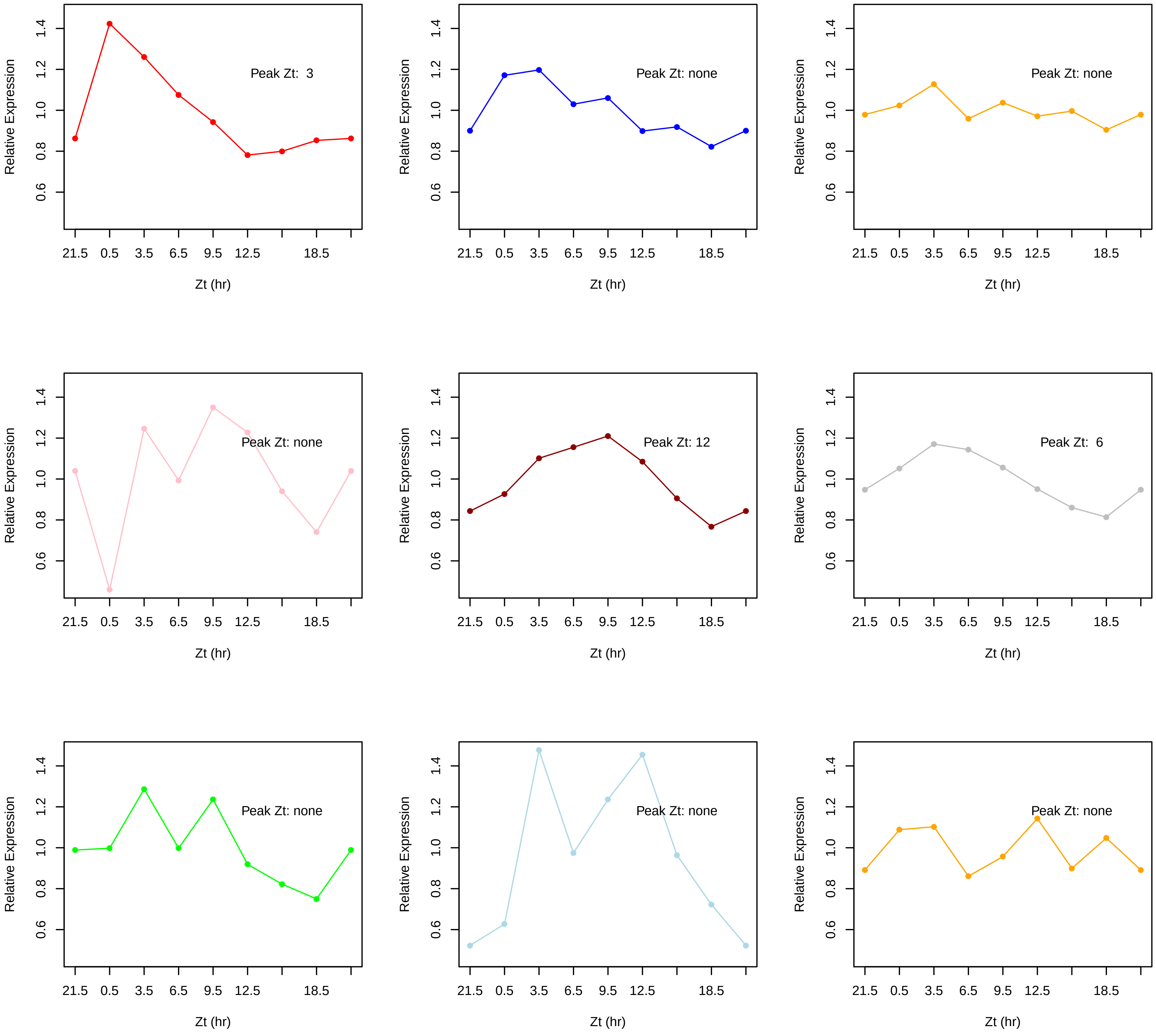

Singeleton: ENSXMAG00000007676 slmapa

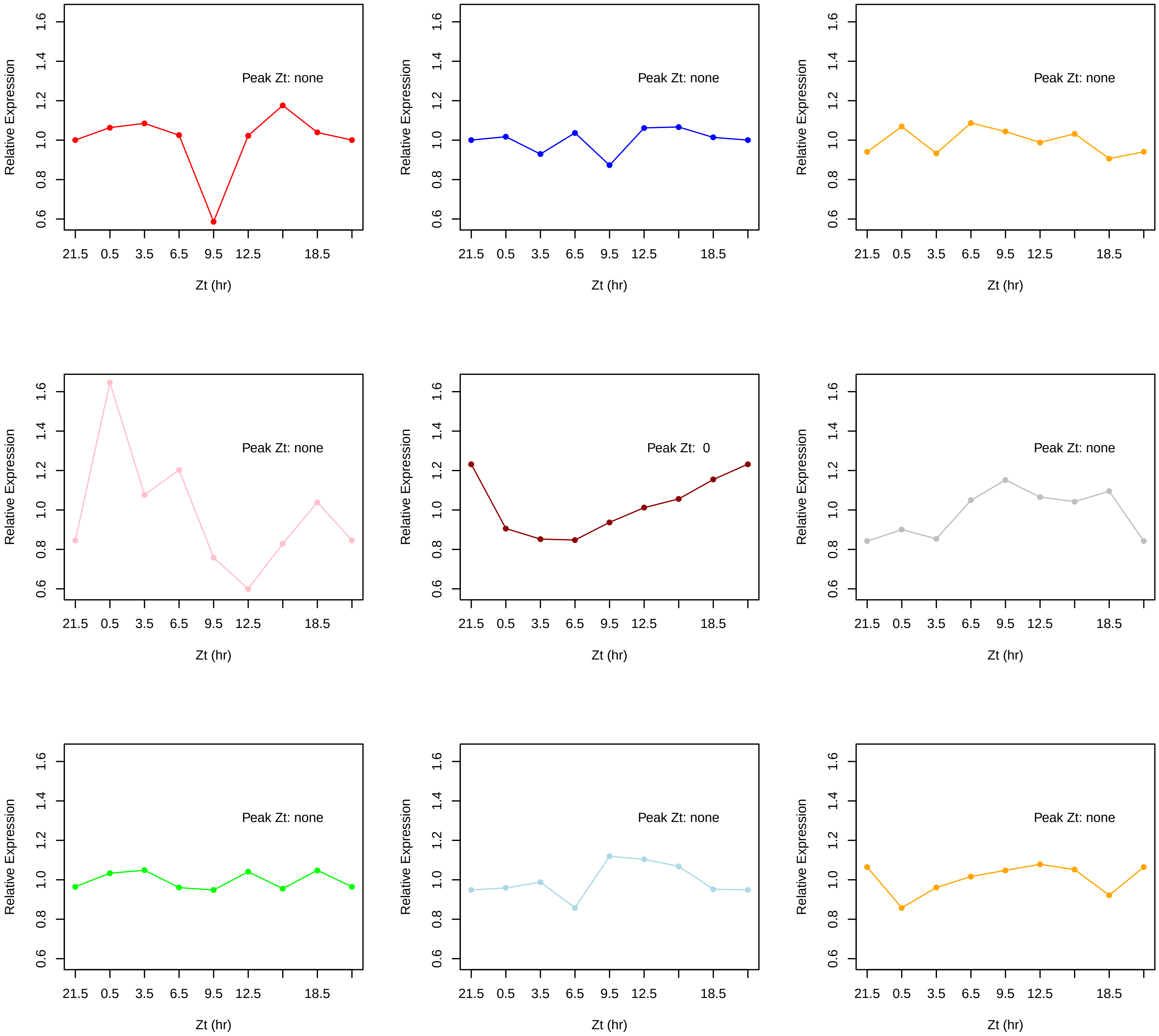

Singleton: ENSXMAG00000004908 LURAP1L

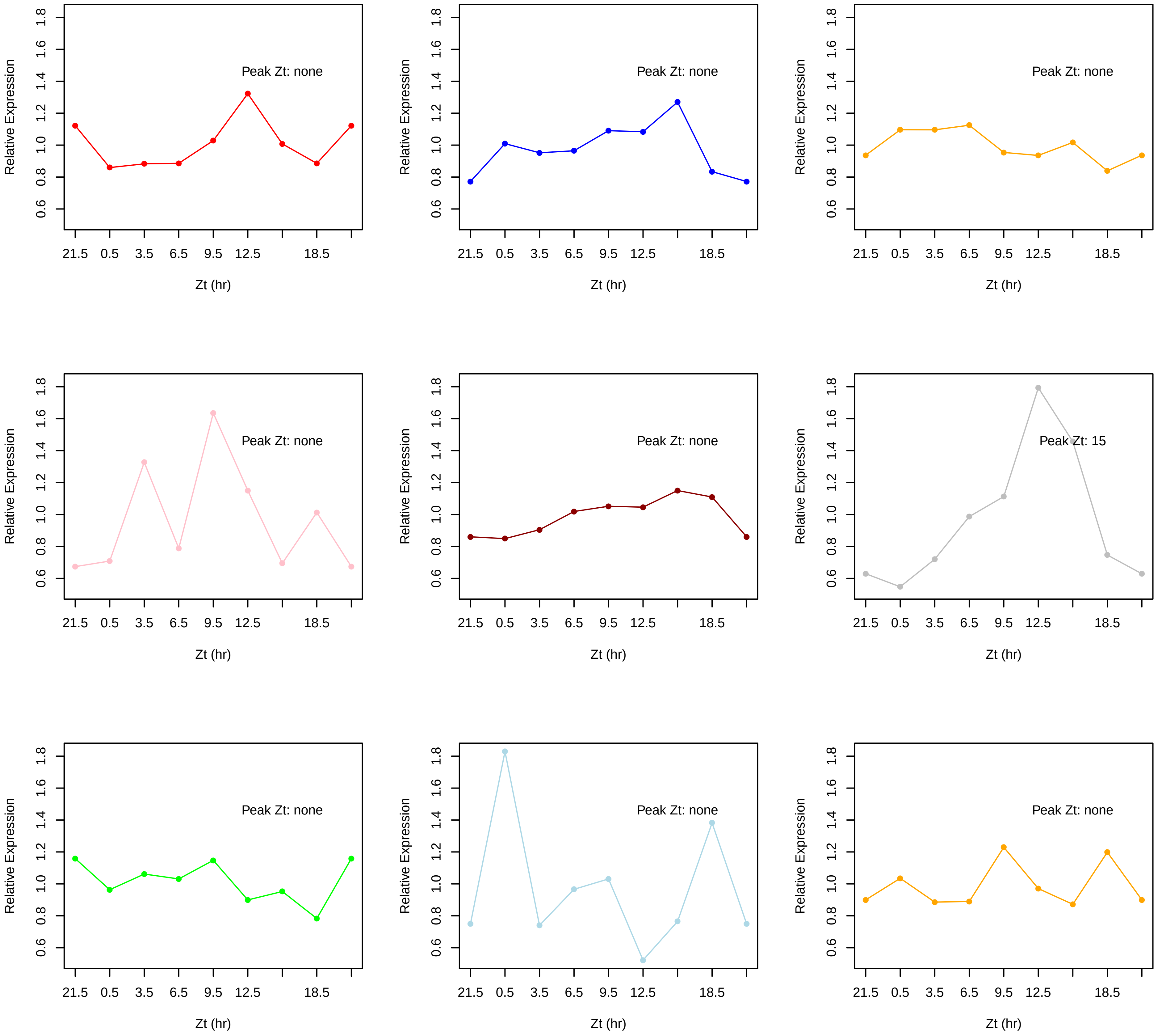

Singleton: ENSXMAG00000002209 dmxl2

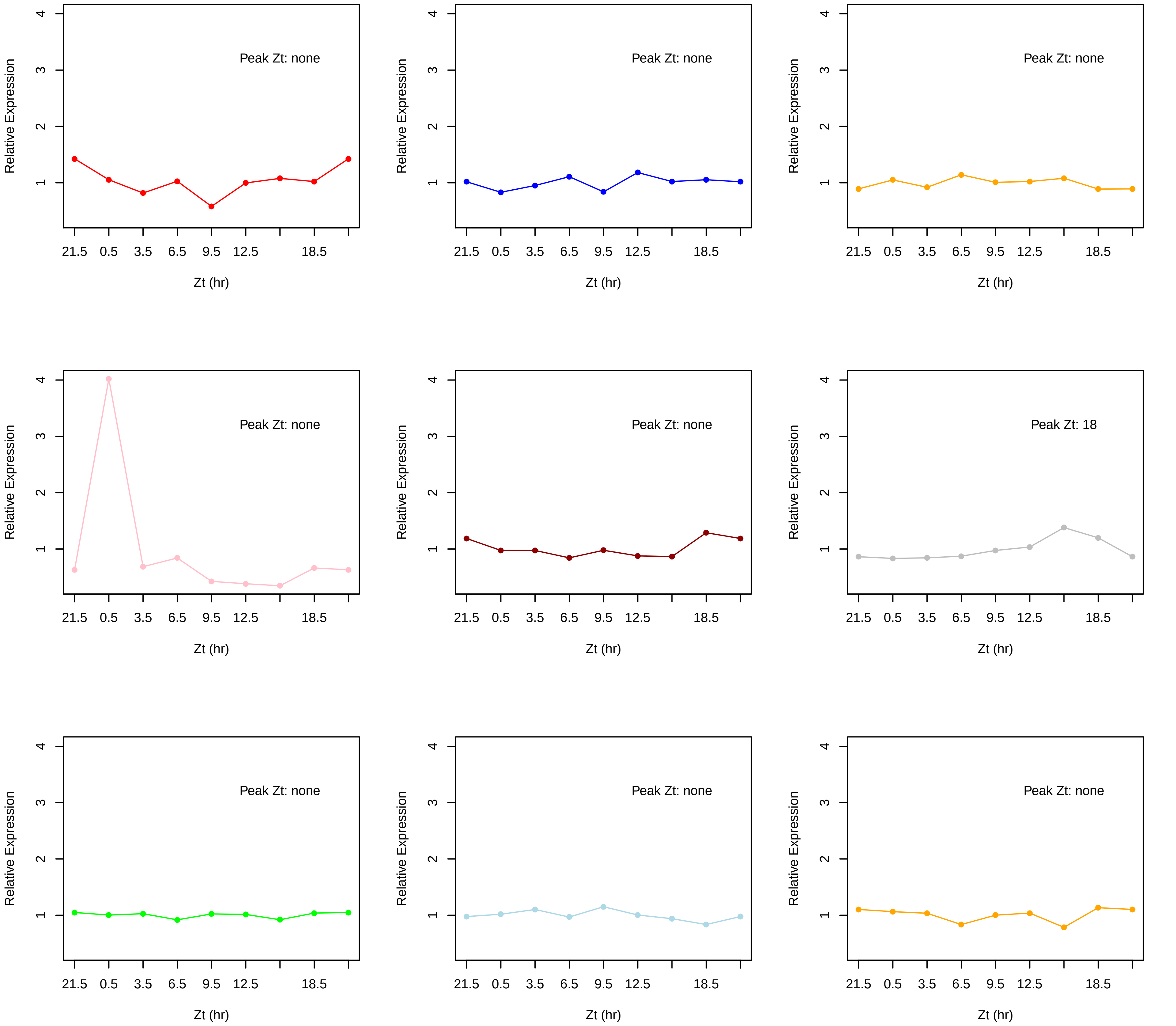

Singeleton: ENSXMAG00000016963 fam171a2b

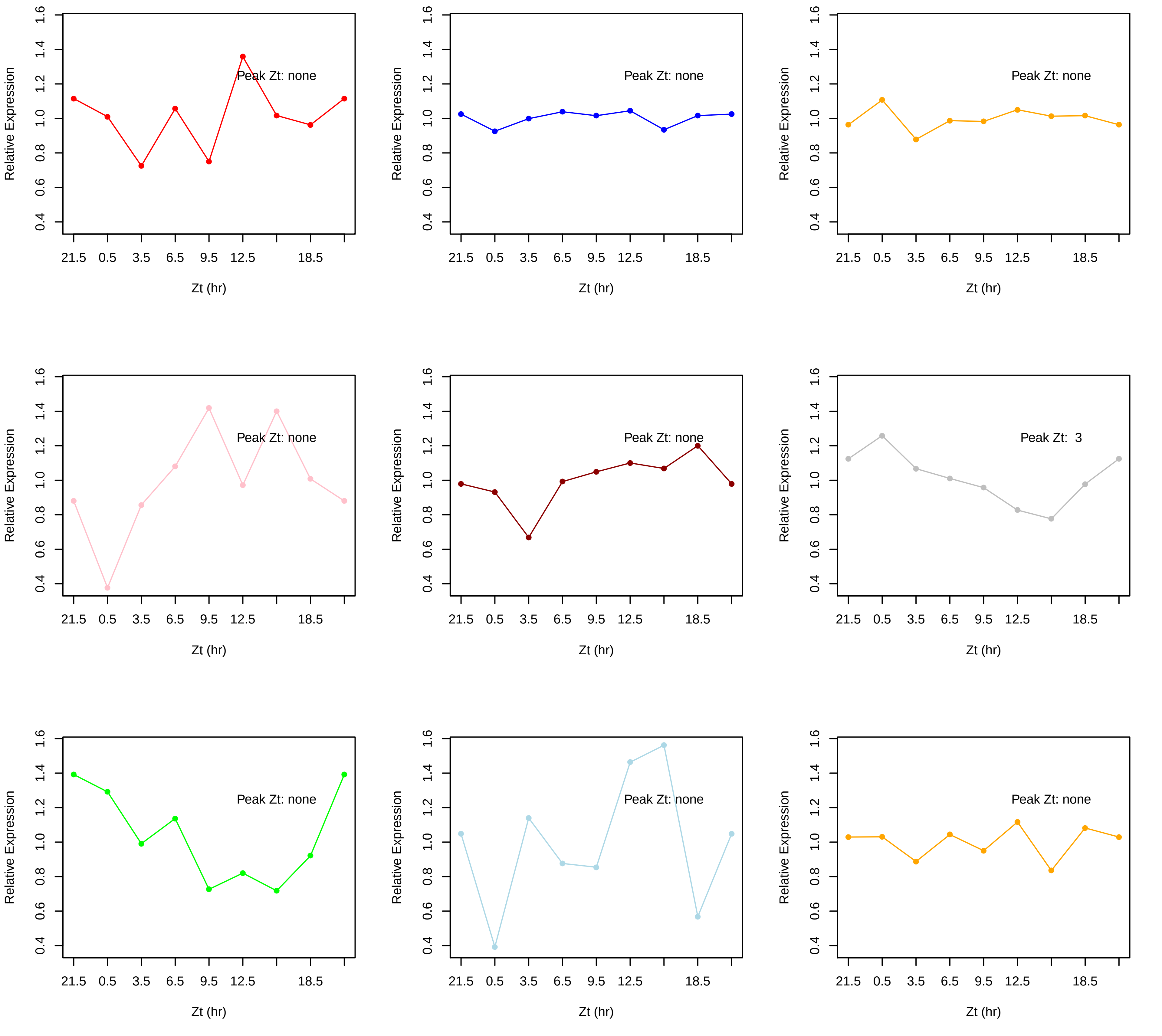

Singeleton: ENSXMAG00000013370 pde12

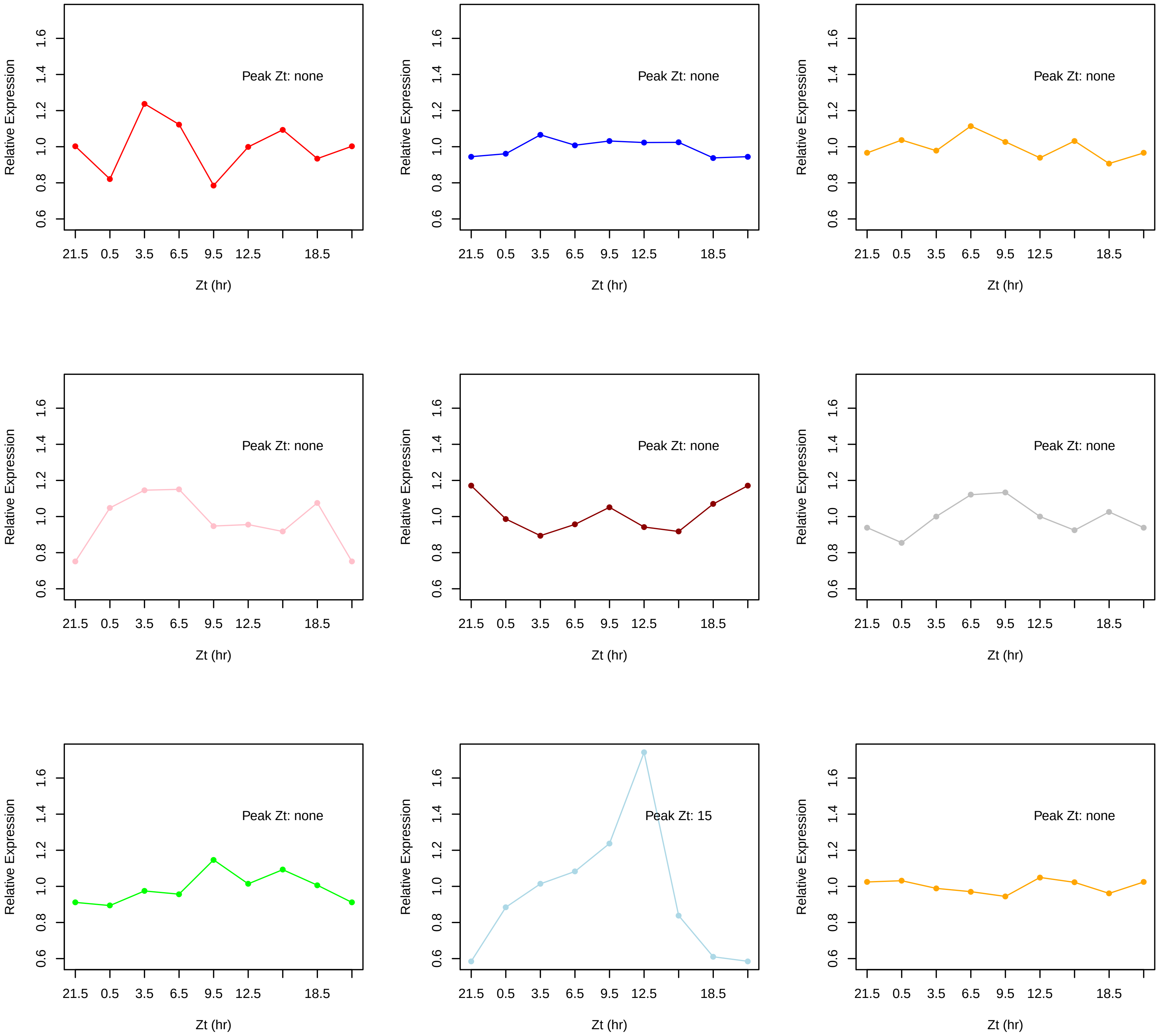

Singeleton: ENSXMAG00000001987 ANKS6

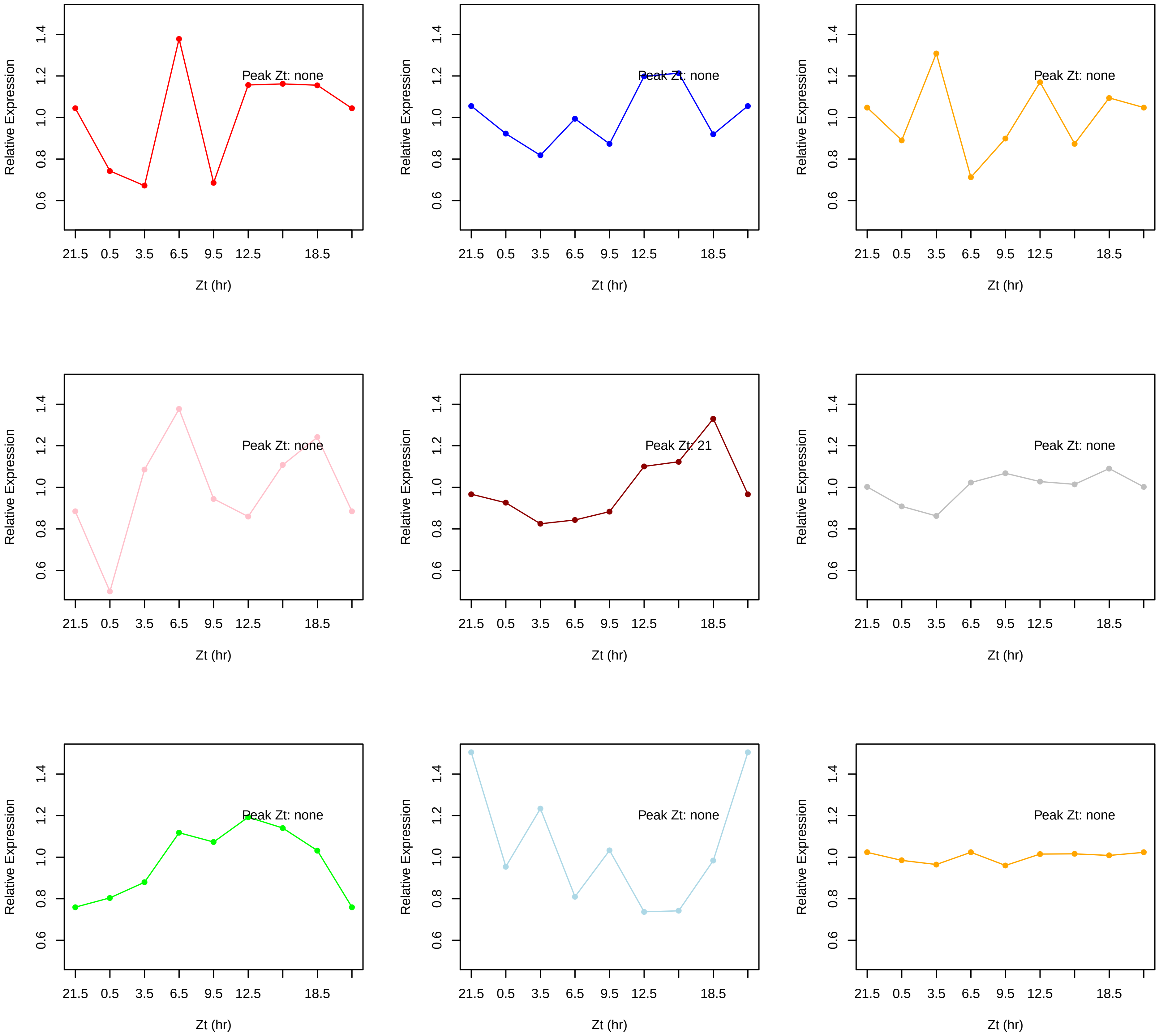

Singeleton: ENSXMAG00000014019 slc25a39

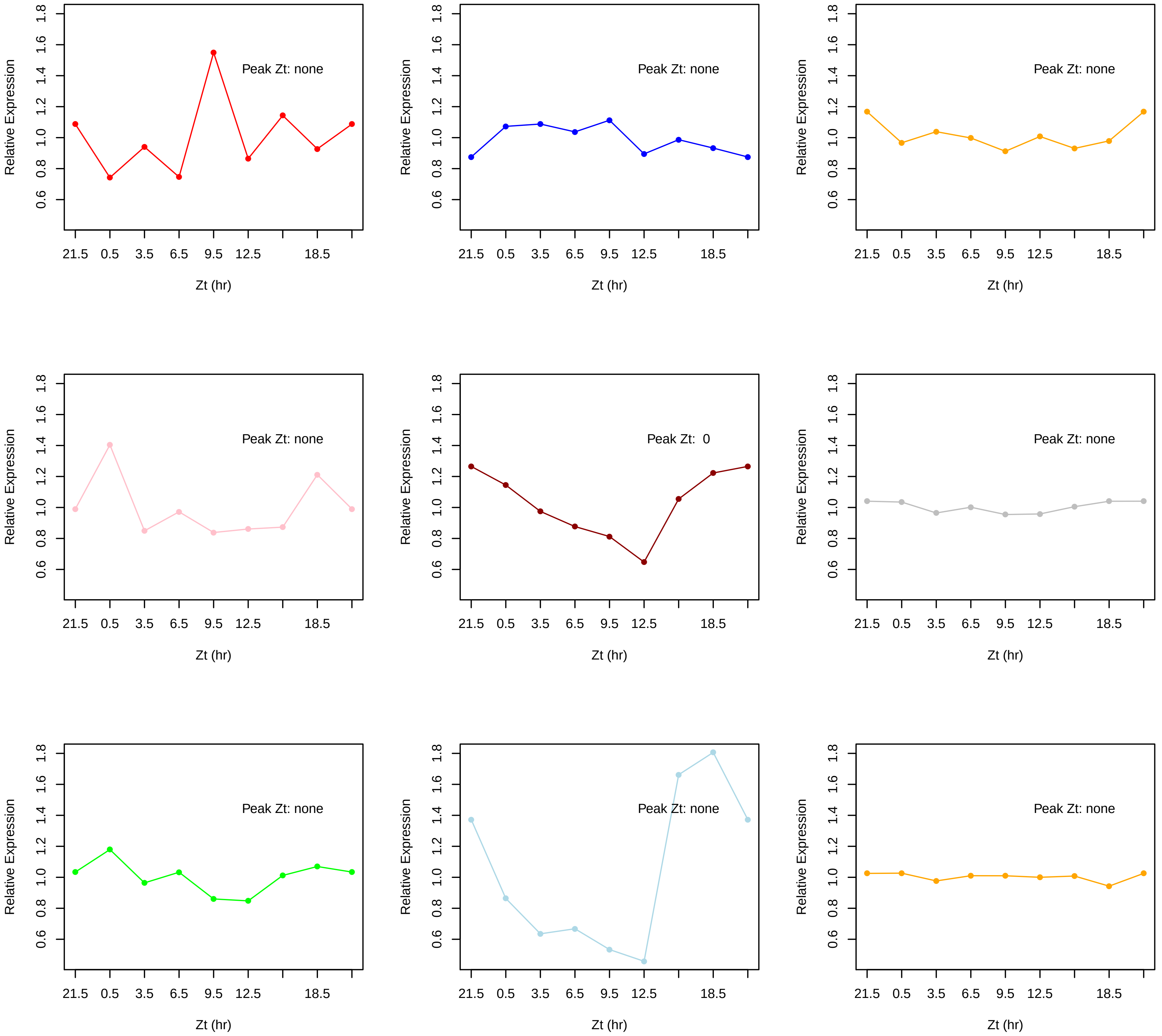

Singeleton: ENSXMAG00000022653

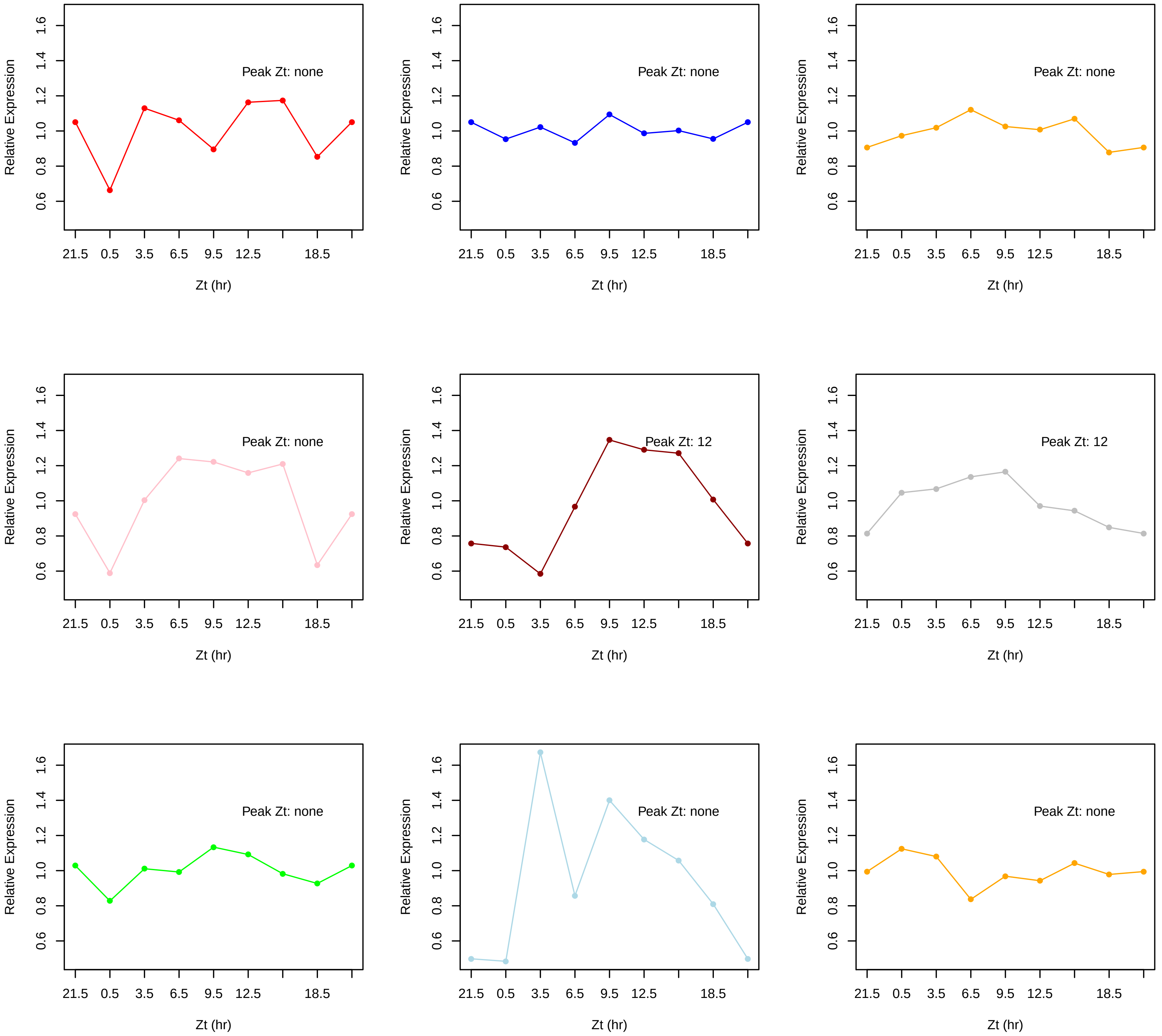

Singeleton: ENSXMAG00000009299 ganc

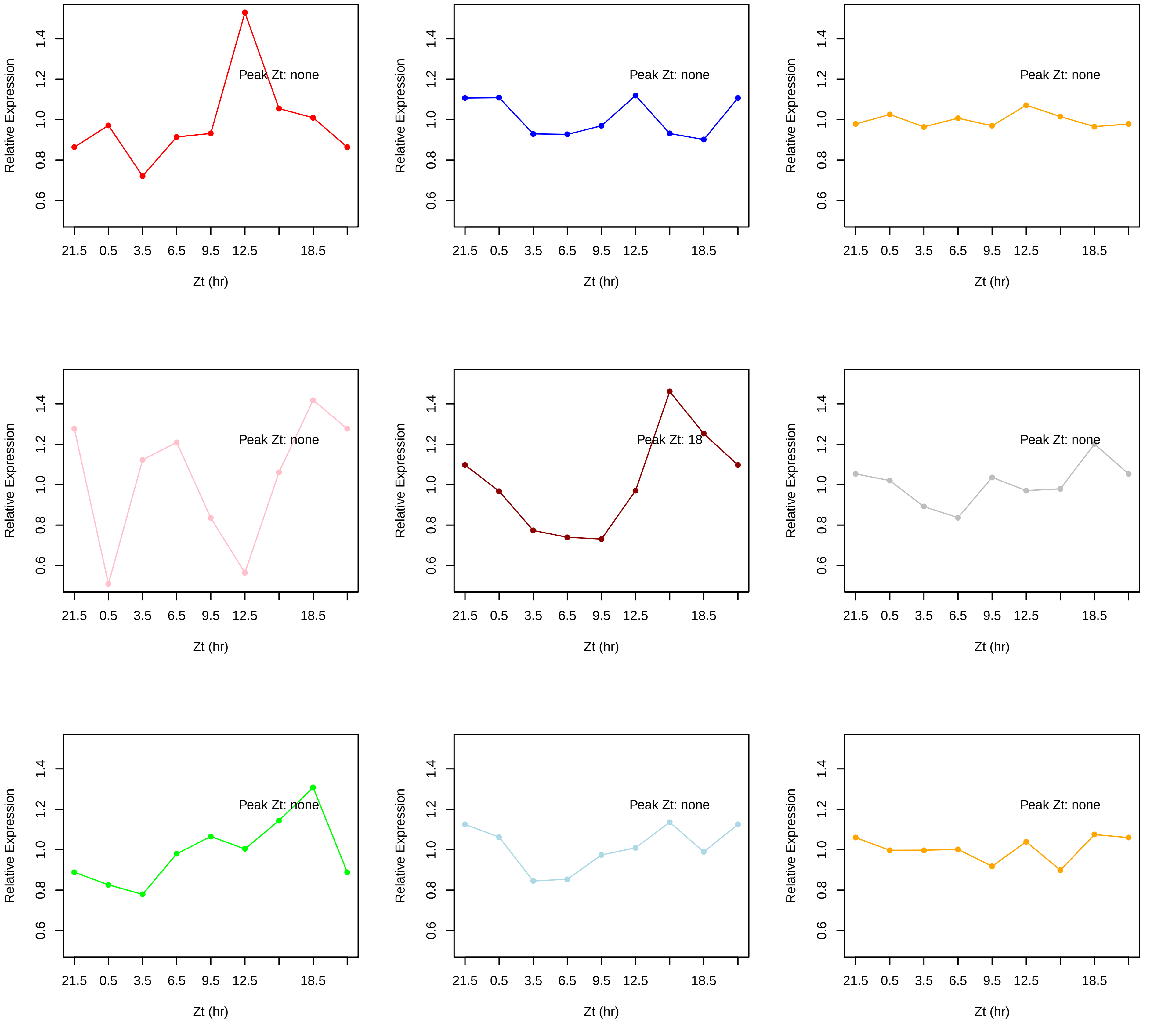

Singeleton: ENSXMAG00000025320 RAMP3

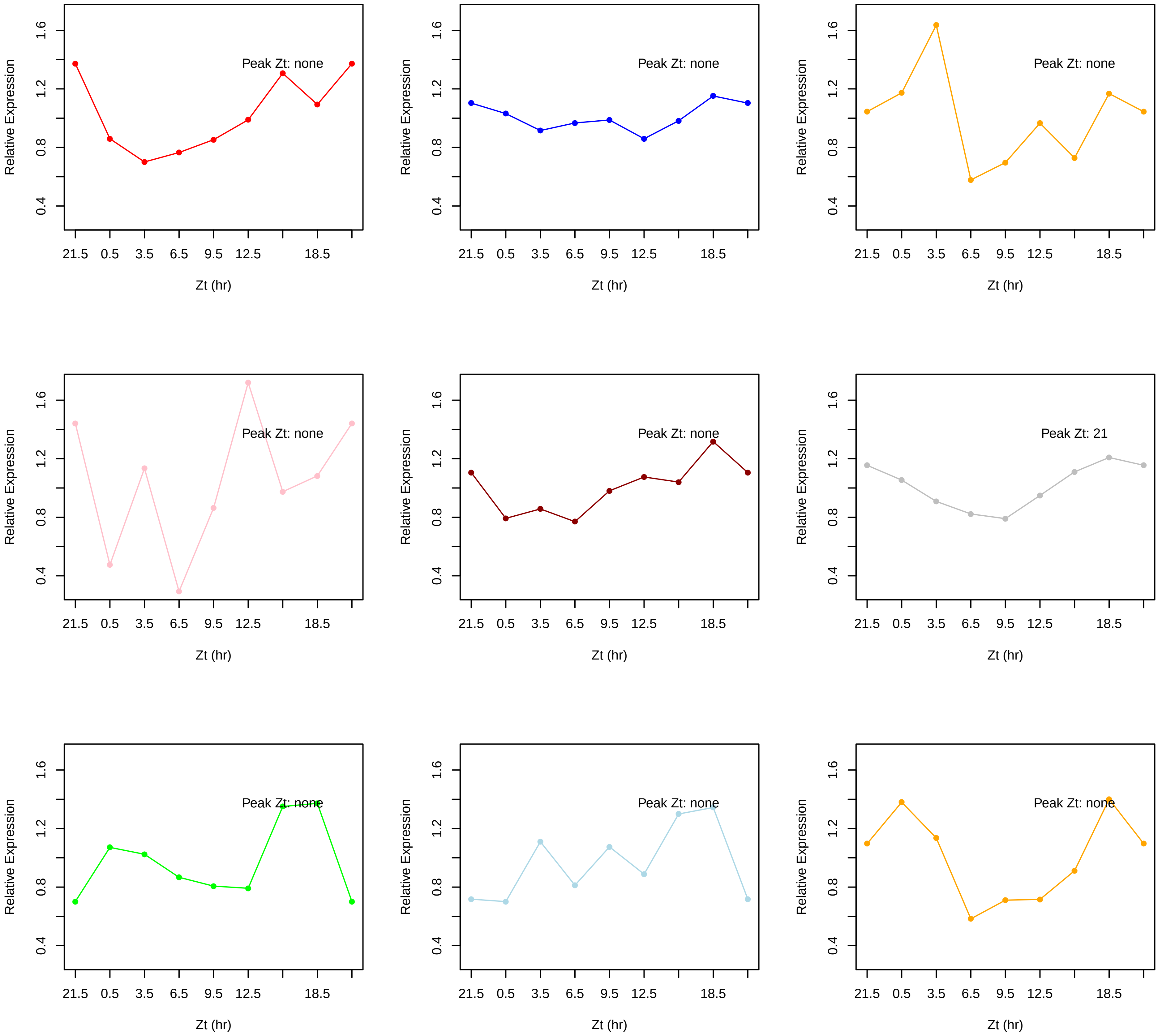

Singleton: ENSXMAG00000015613 bcl6b

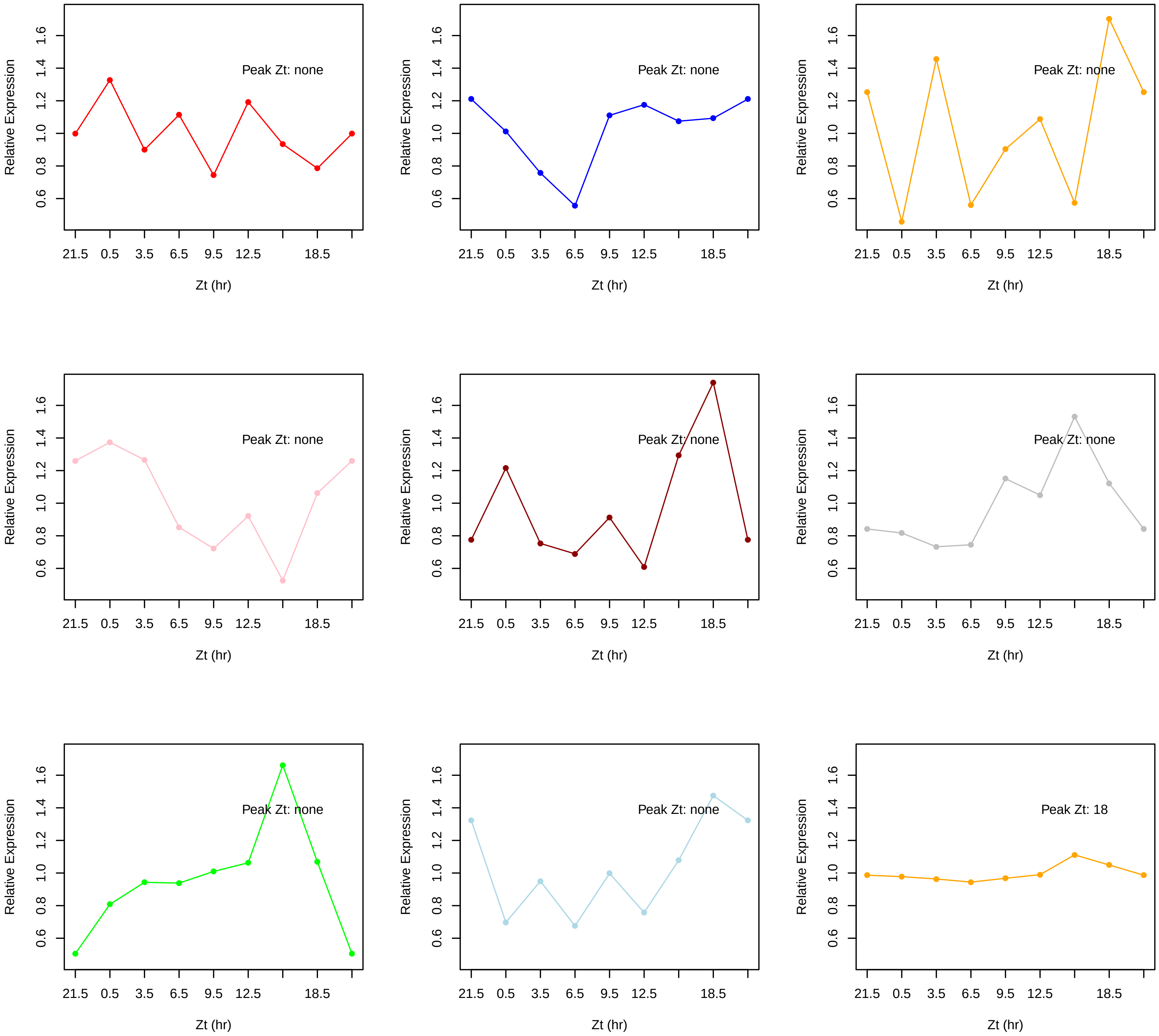

Singeleton: ENSXMAG00000002203 gldn

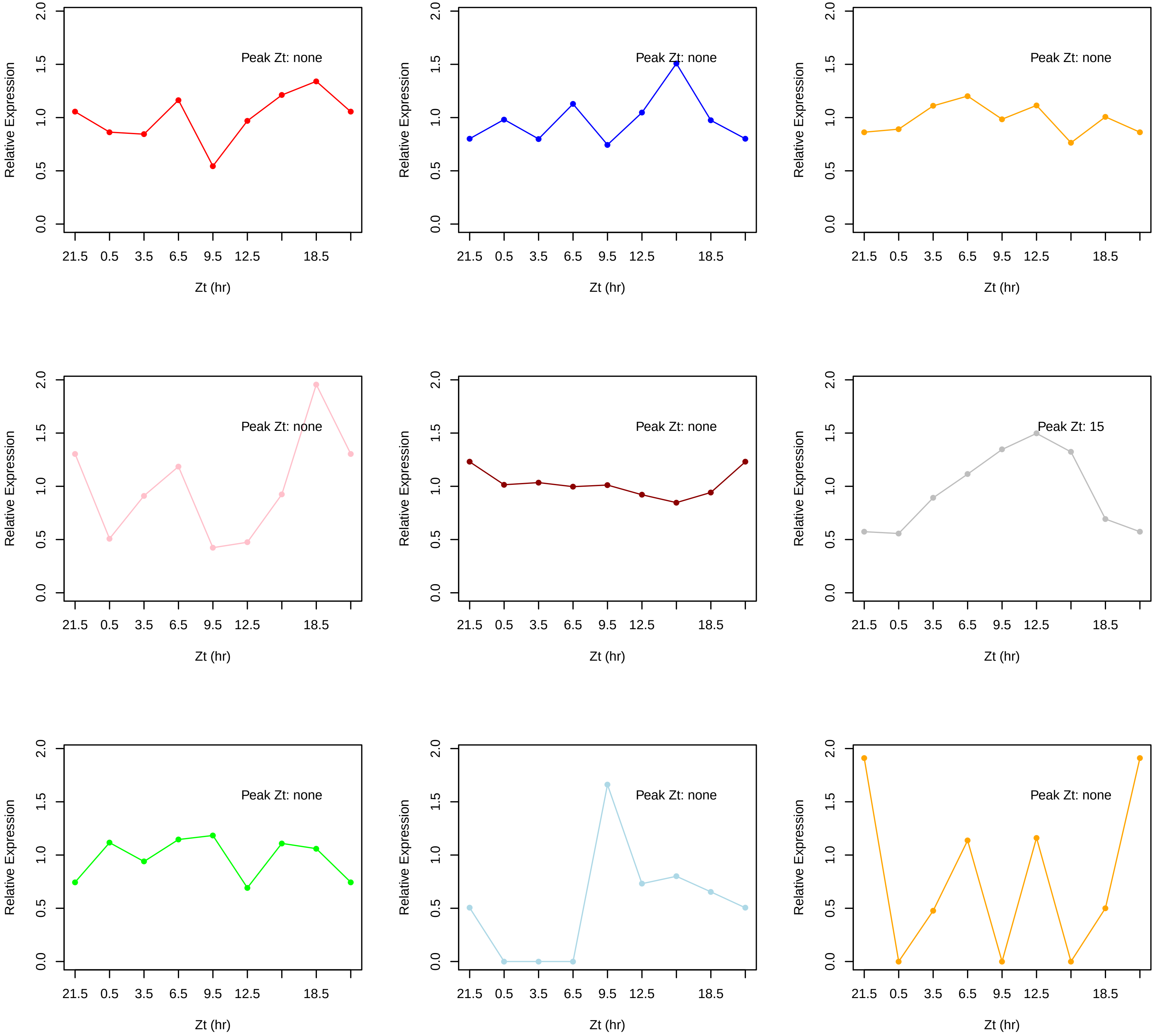

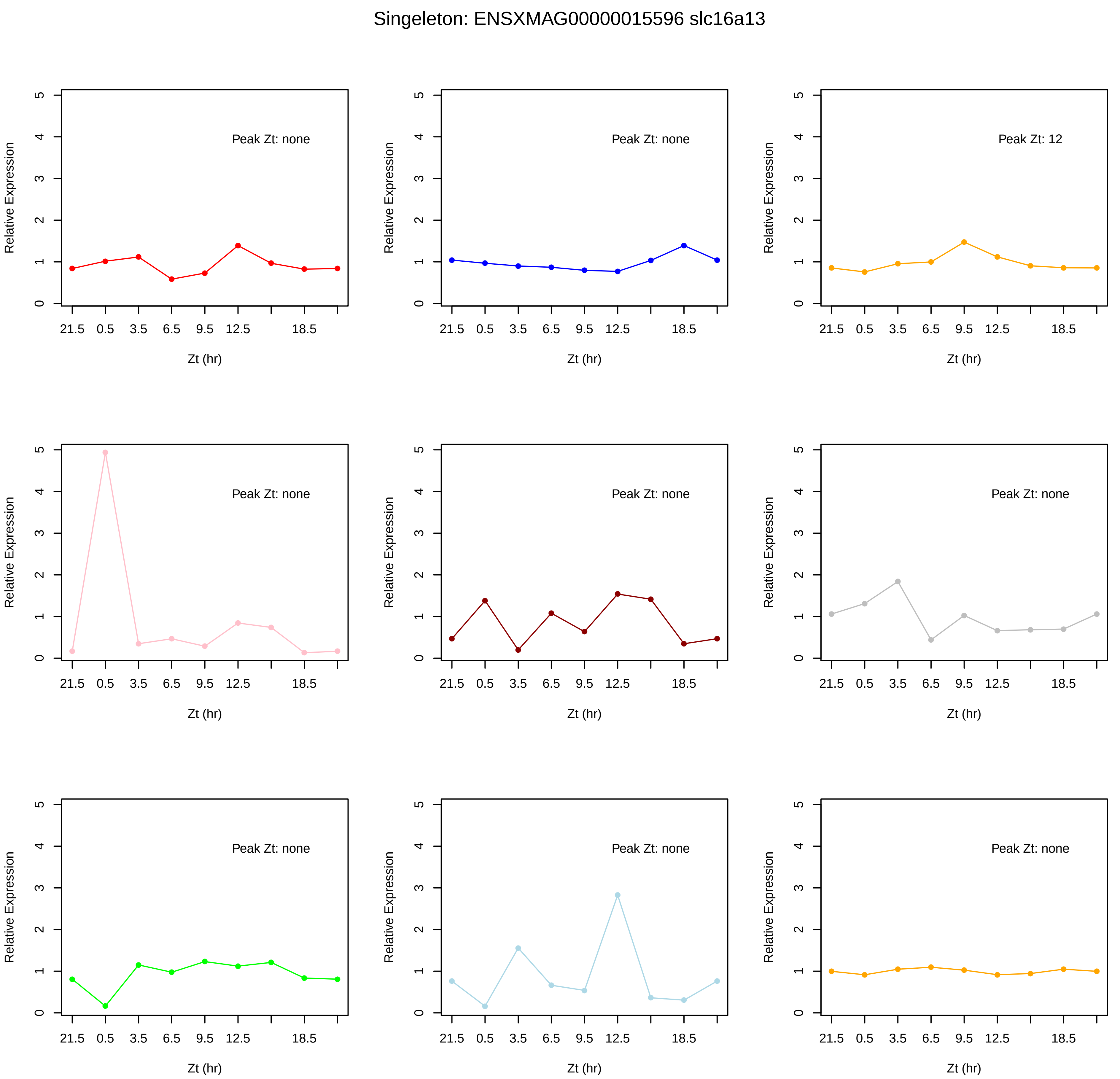
Singeleton: ENSXMAG00000015596 slc16a13
Peak Zt: none
Peak Zt: none
Peak Zt: 12
Peak Zt: none
Peak Zt: none
Peak Zt: none
Peak Zt: none
Peak Zt: none
Peak Zt: none
Relative Expression
Zt (hr)
21.5
0.5
3.5
6.5
9.5
12.5
18.5

Singeleton: ENSXMAG00000023645 kdm4c

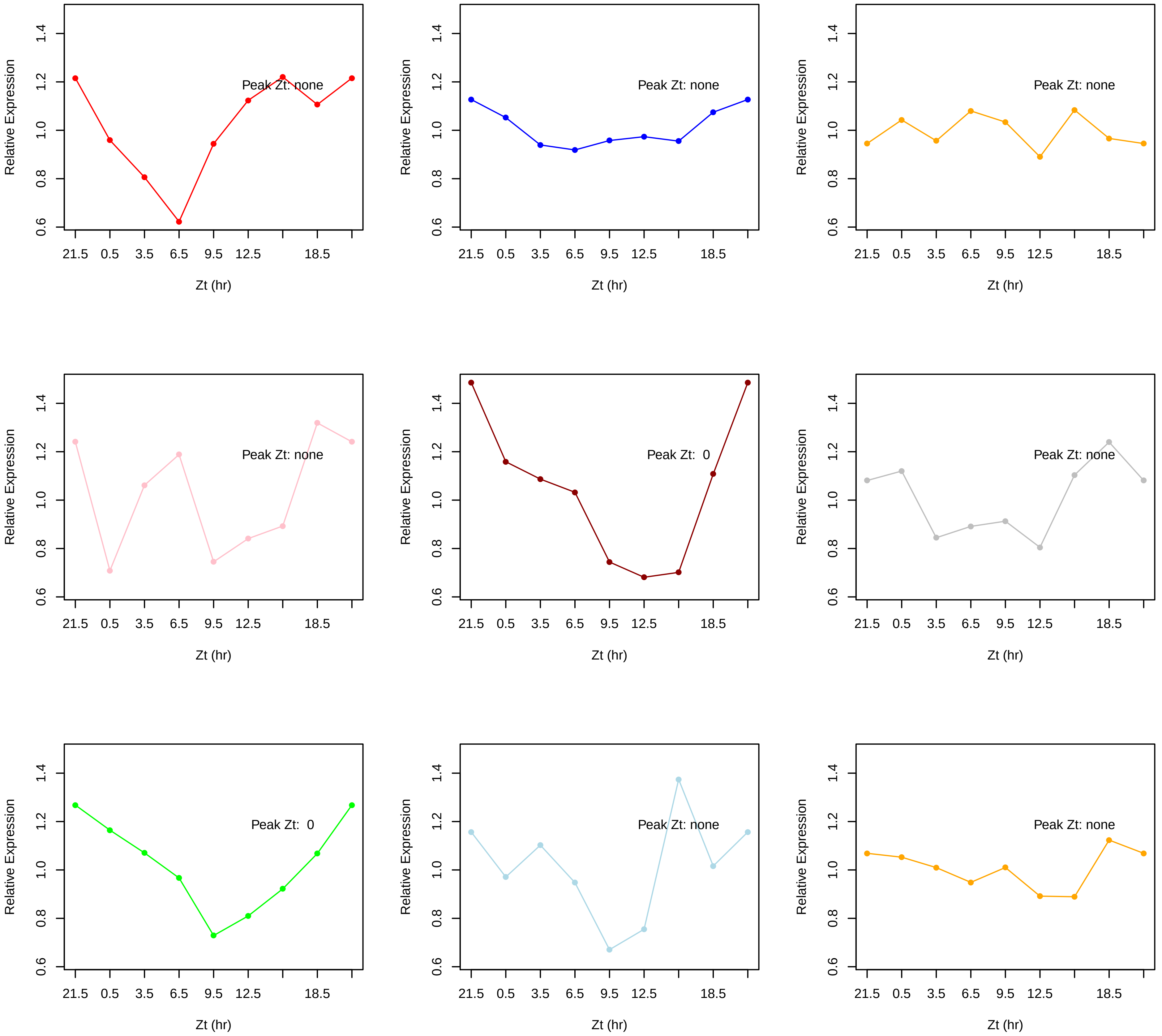

Singleton: ENSXMAG00000015536 acap1

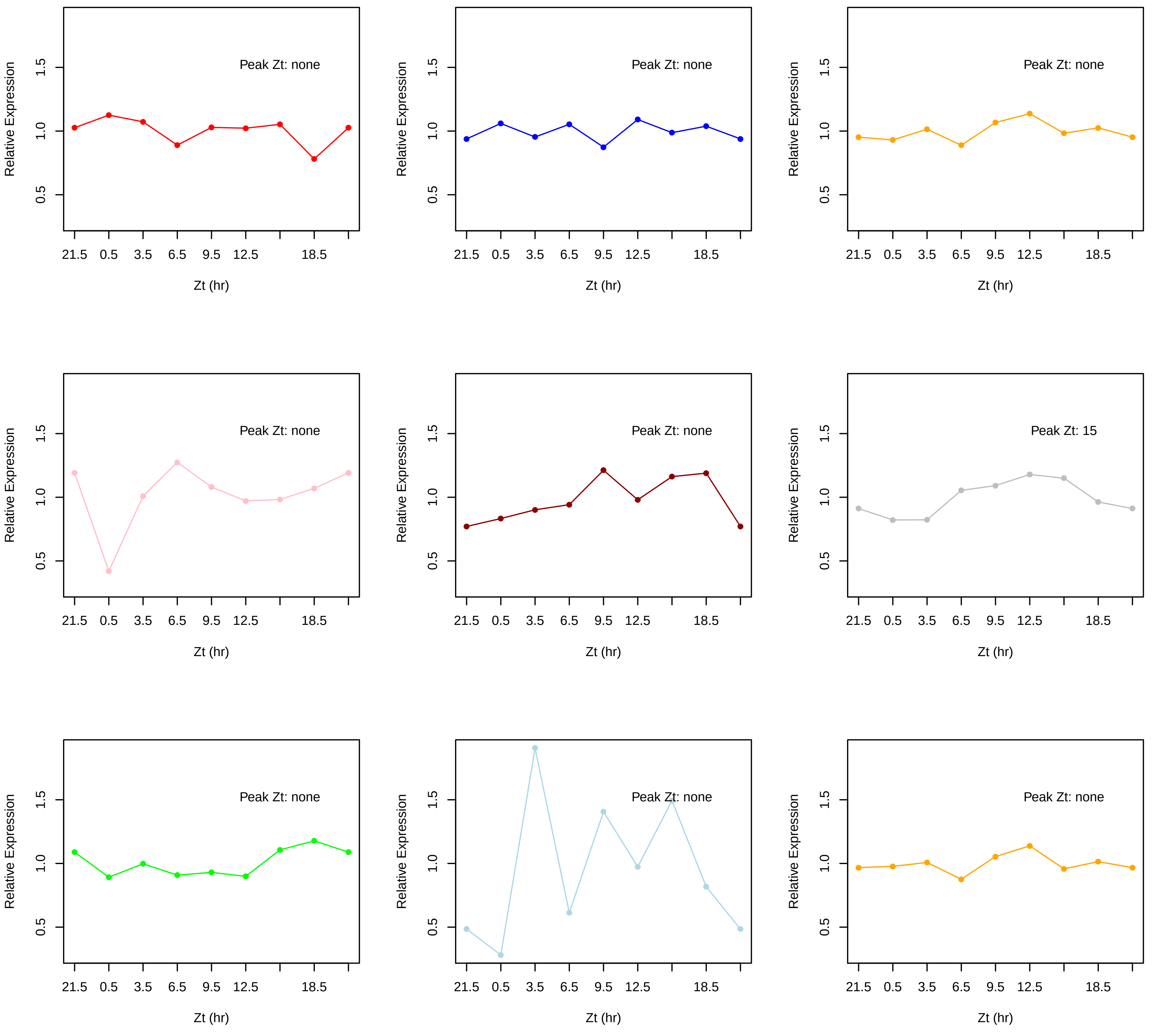

Singeleton: ENSXMAG00000004985 grb2b

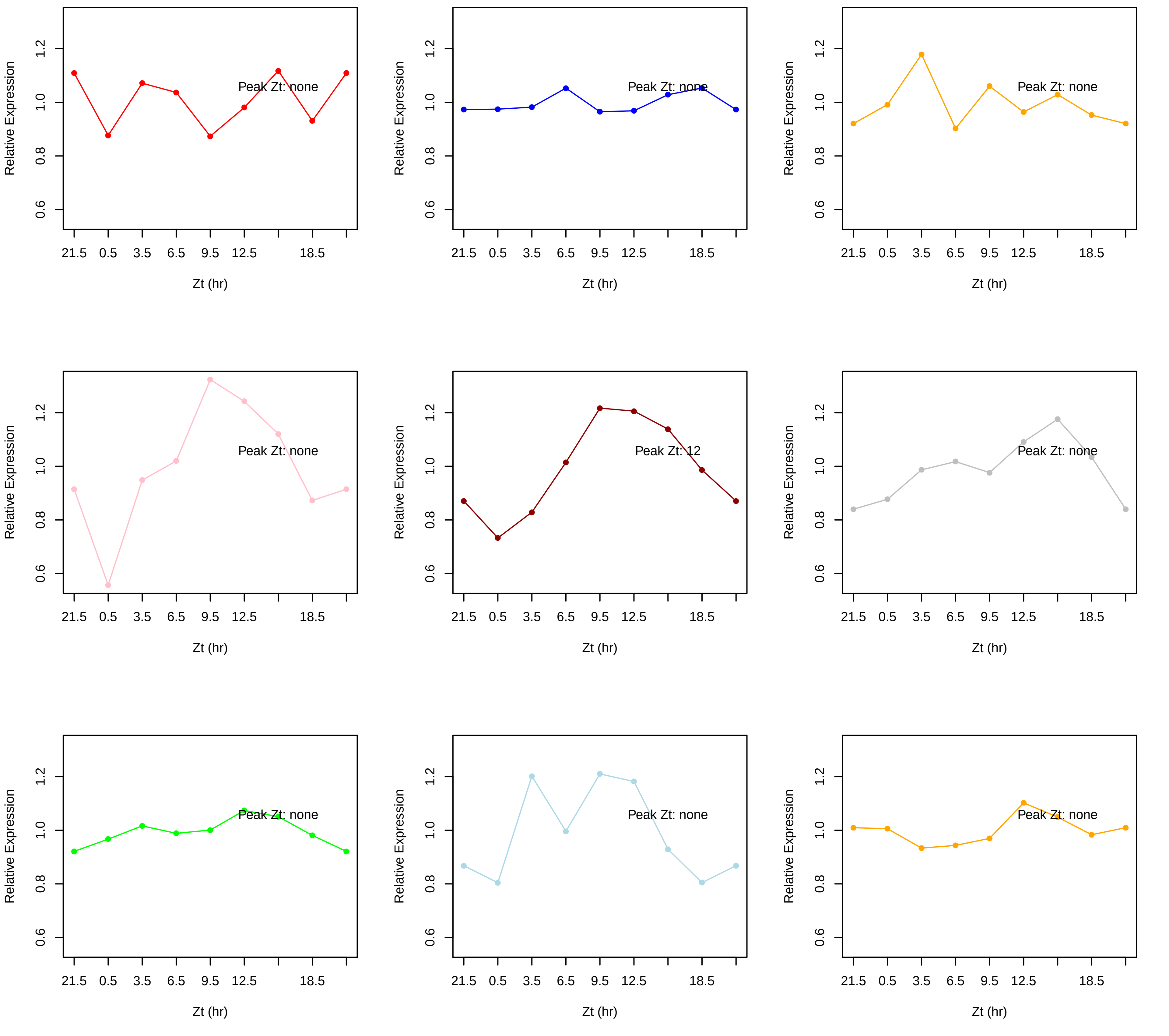

Singeleton: ENSXMAG00000027481 TMEM87A

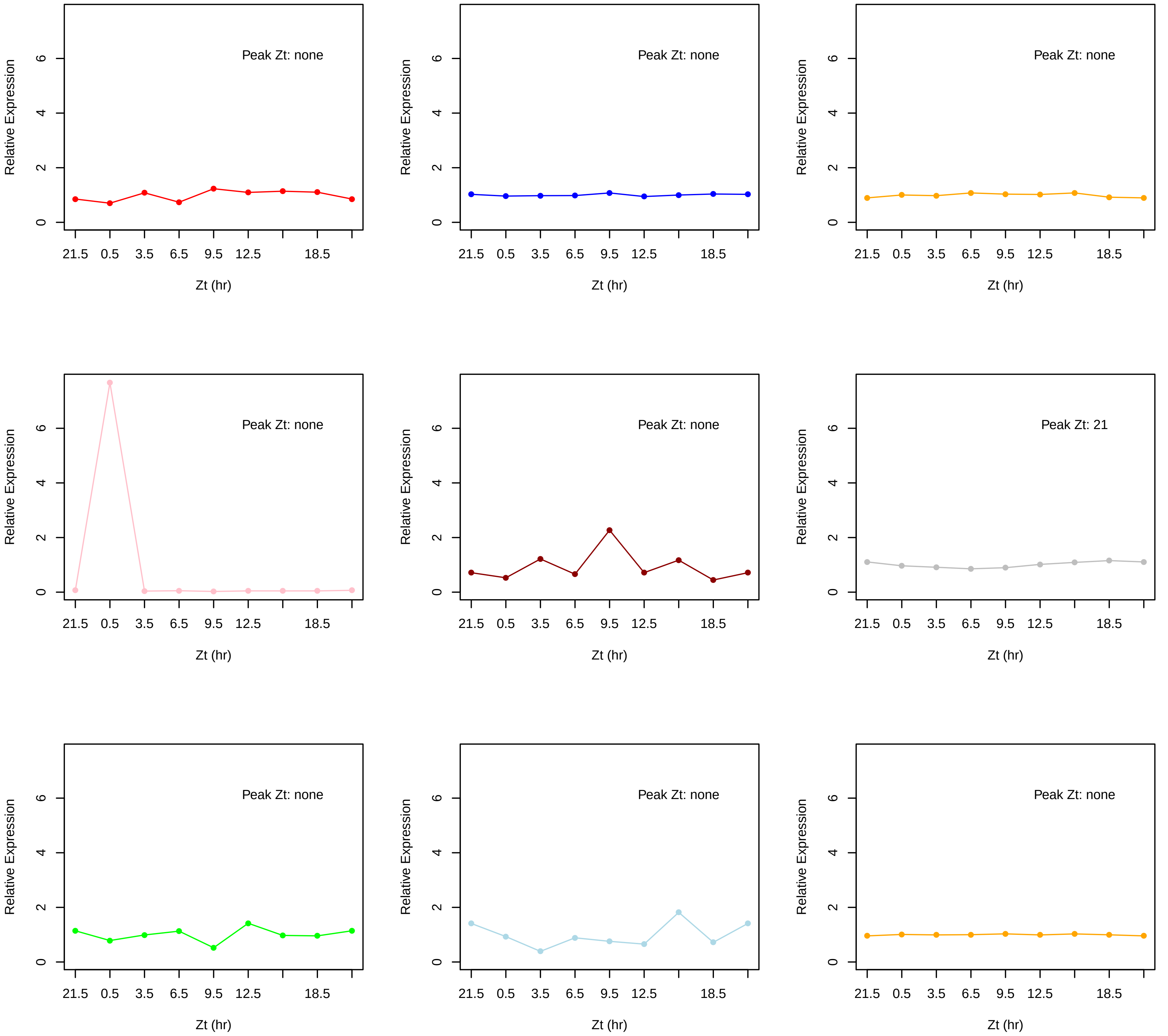

Singeleton: ENSXMAG00000025472 slc25a19

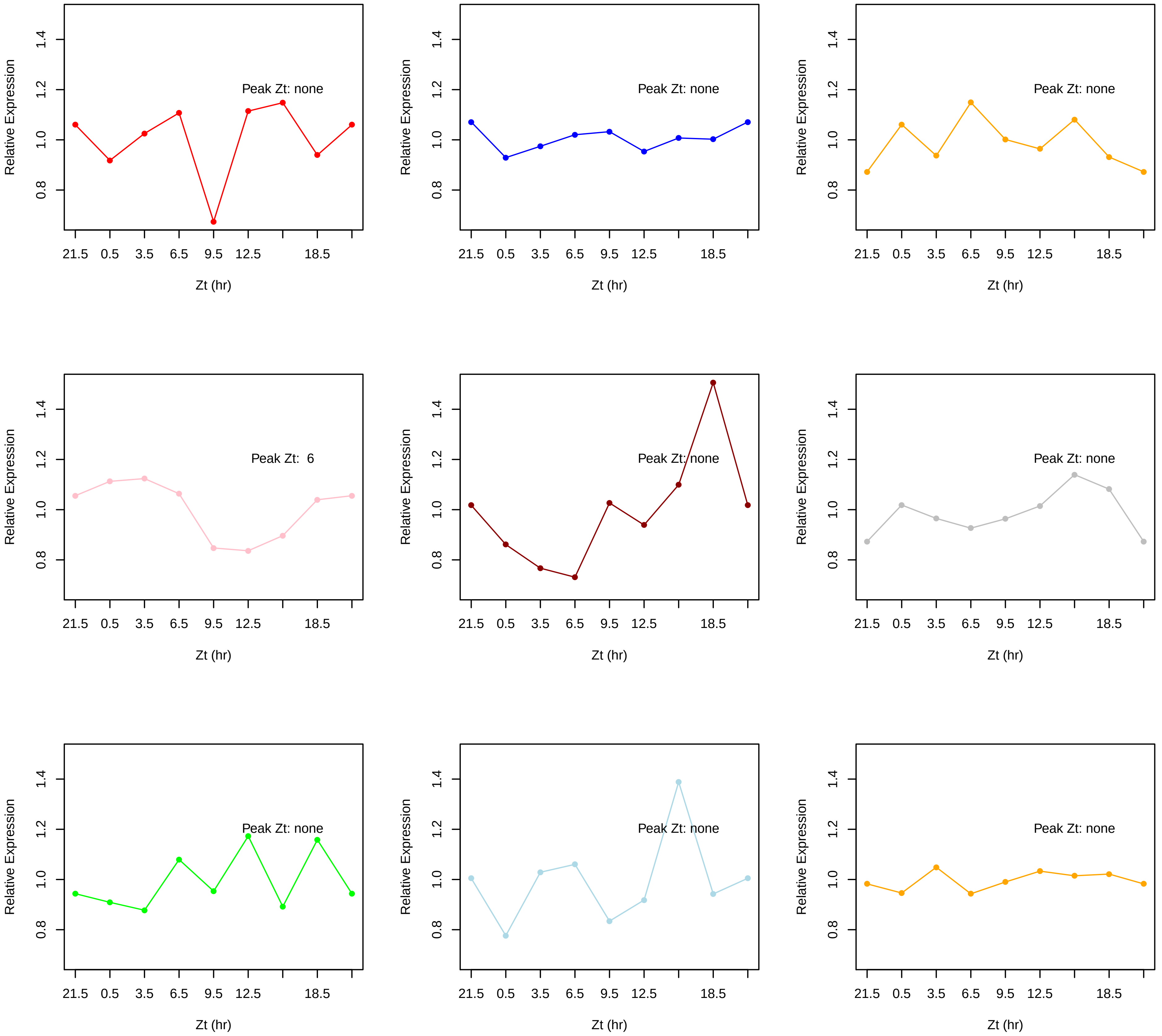

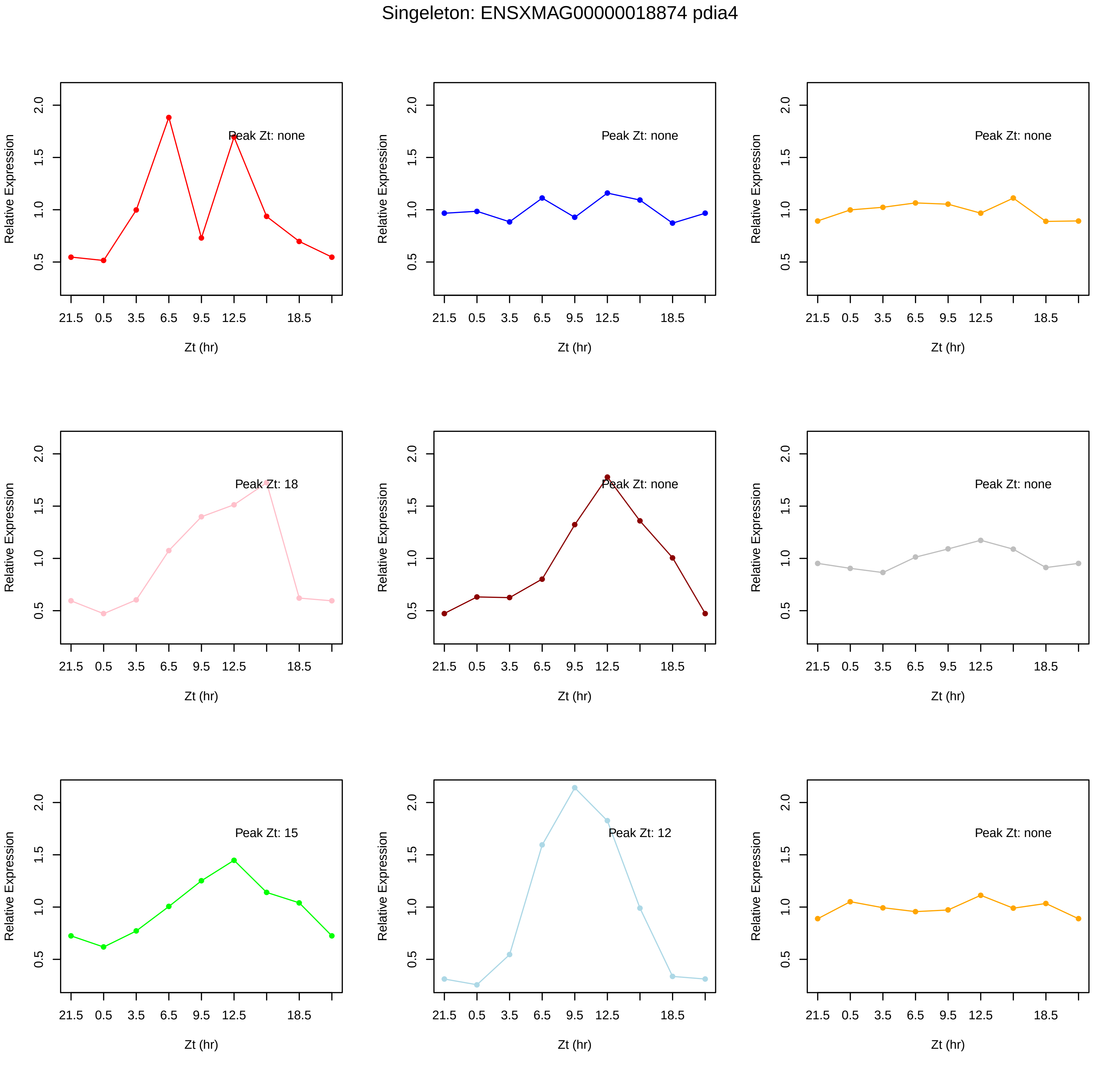
Singeleton: ENSXMAG00000018874 pdia4
Peak Zt: none
Peak Zt: none
Peak Zt: none
Peak Zt: 18
Peak Zt: none
Peak Zt: none
Peak Zt: 15
Peak Zt: 12
Peak Zt: none
Relative Expression
Zt (hr)
21.5
0.5
3.5
6.5
9.5
12.5
18.5

Singleton: ENSXMAG00000009235 vps39

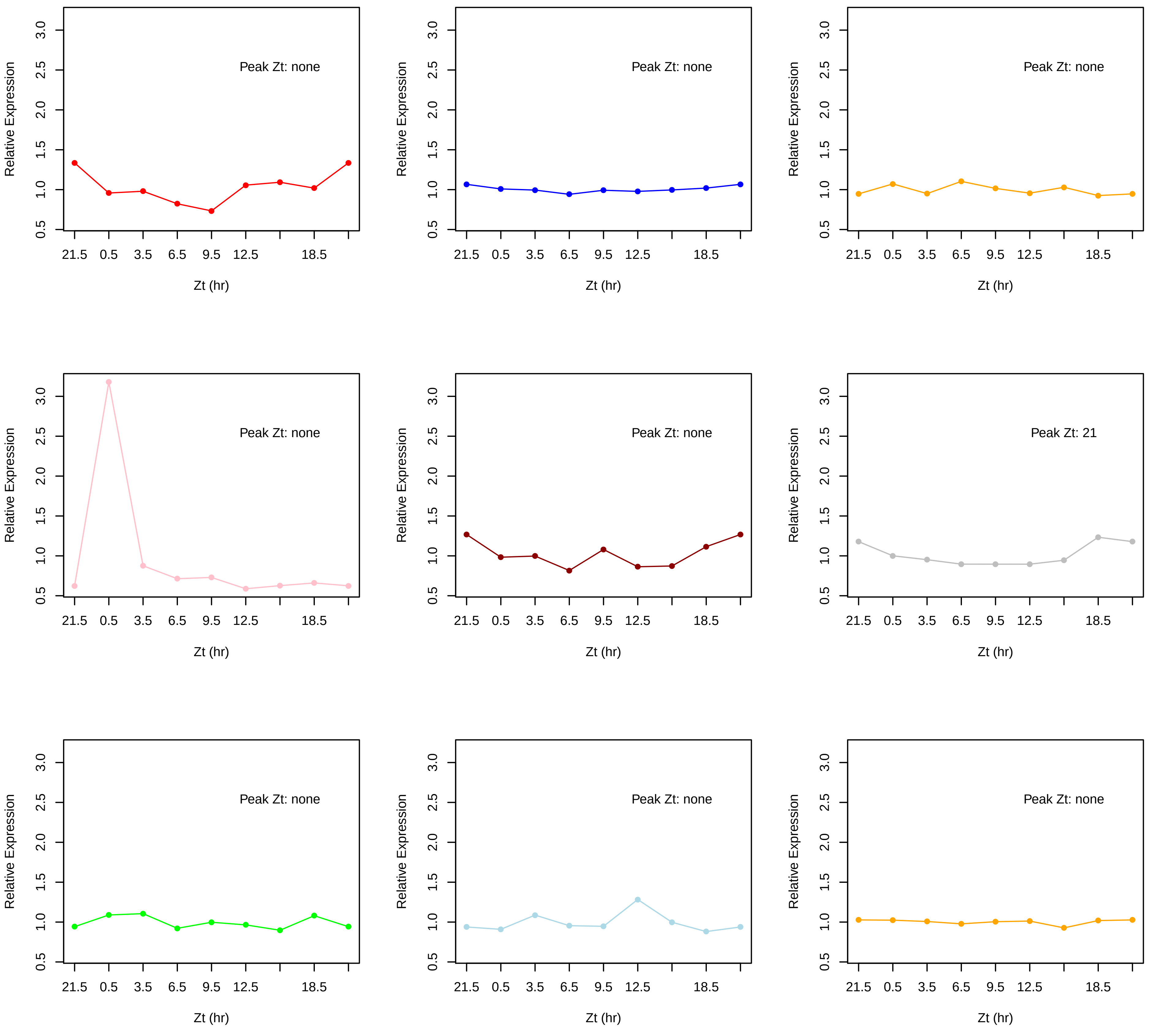

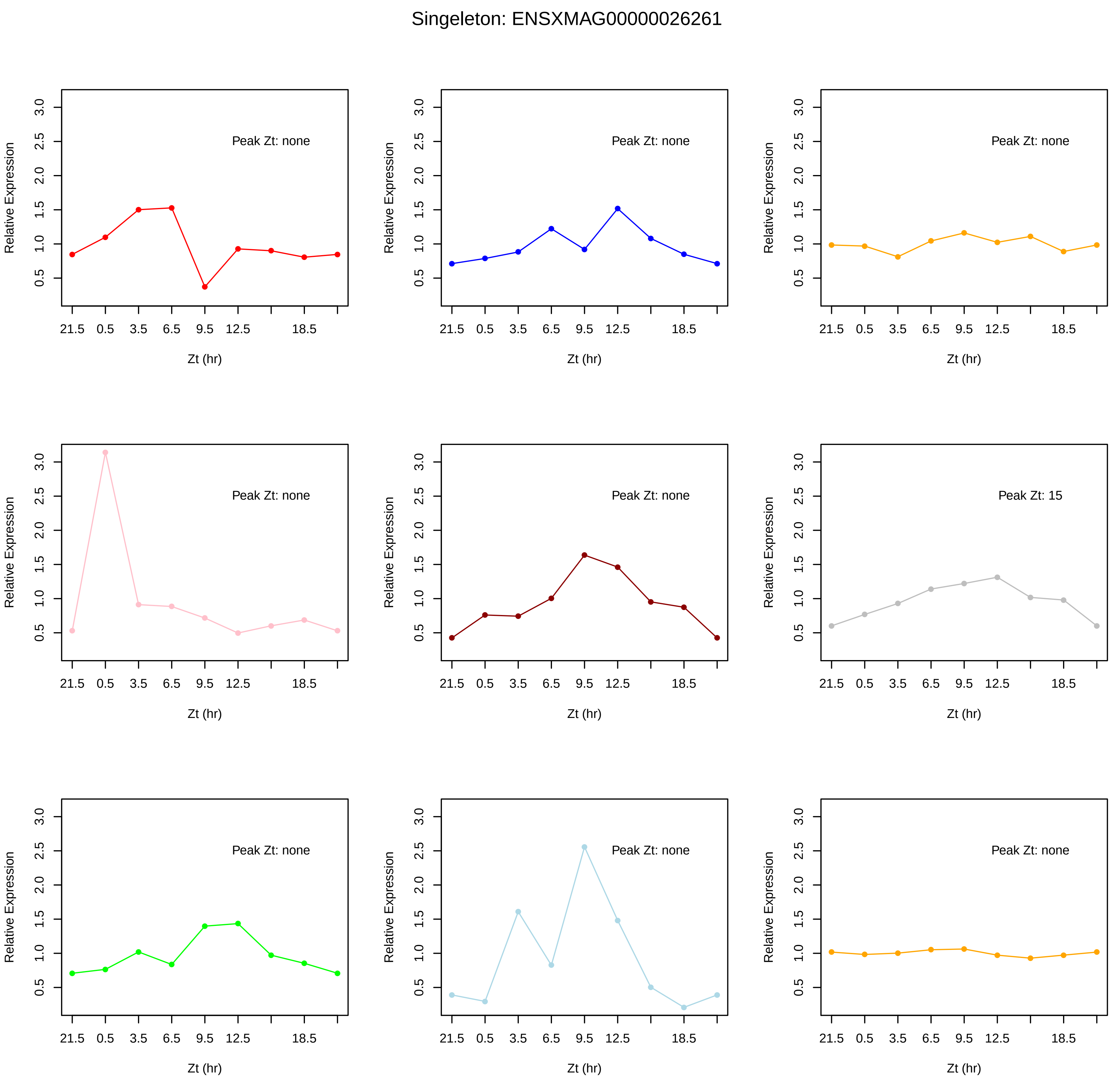

Singeleton: ENSXMAG00000026261
Peak Zt: none
Peak Zt: none
Peak Zt: none
Peak Zt: none
Peak Zt: none
Peak Zt: 15
Peak Zt: none
Peak Zt: none
Peak Zt: none
Relative Expression
Zt (hr)
21.5 0.5 3.5 6.5 9.5 12.5 18.5

Singleton: ENSXMAG00000023411 tbx21

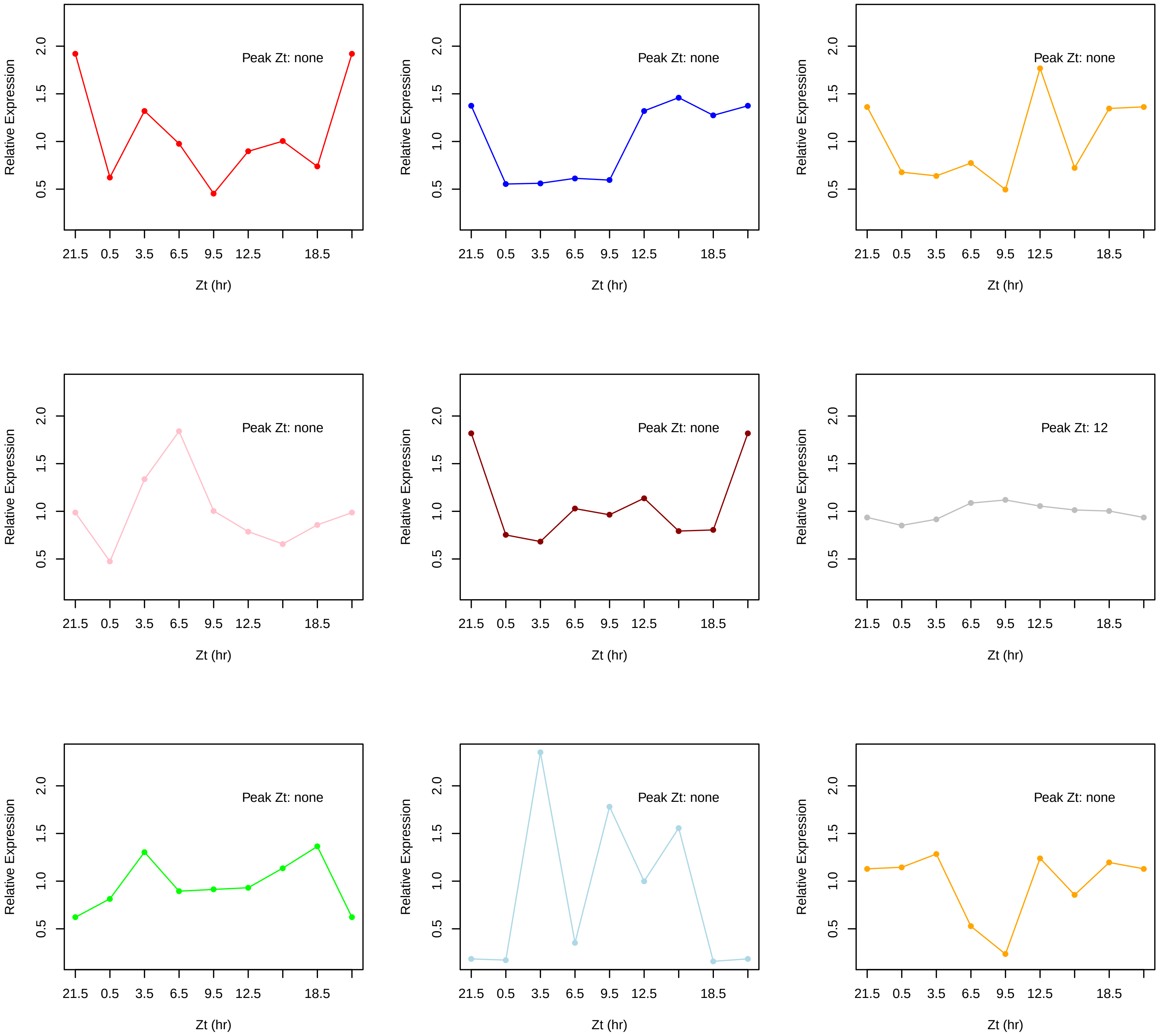

Singleton: ENSXMAG00000004544 smc2

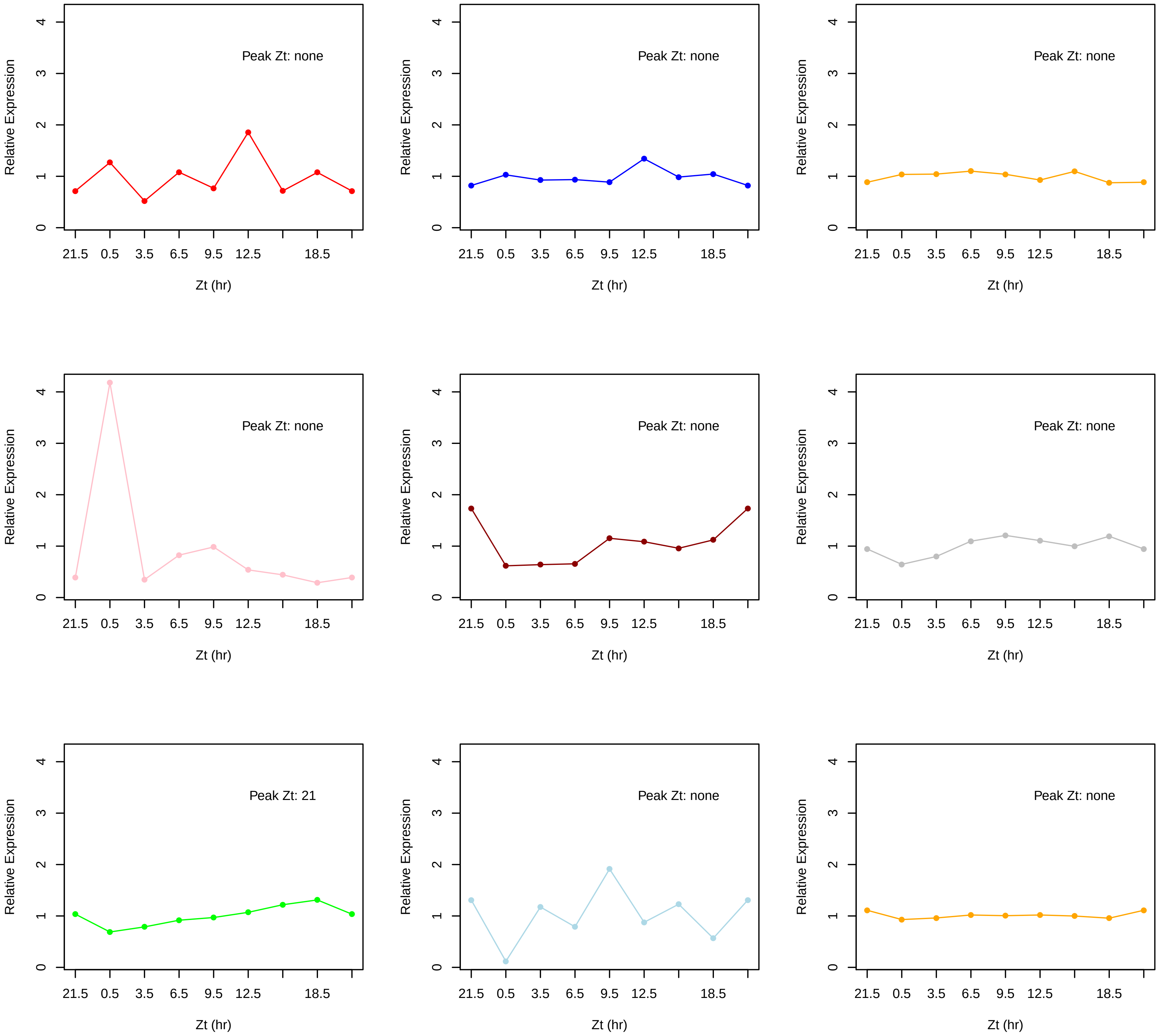

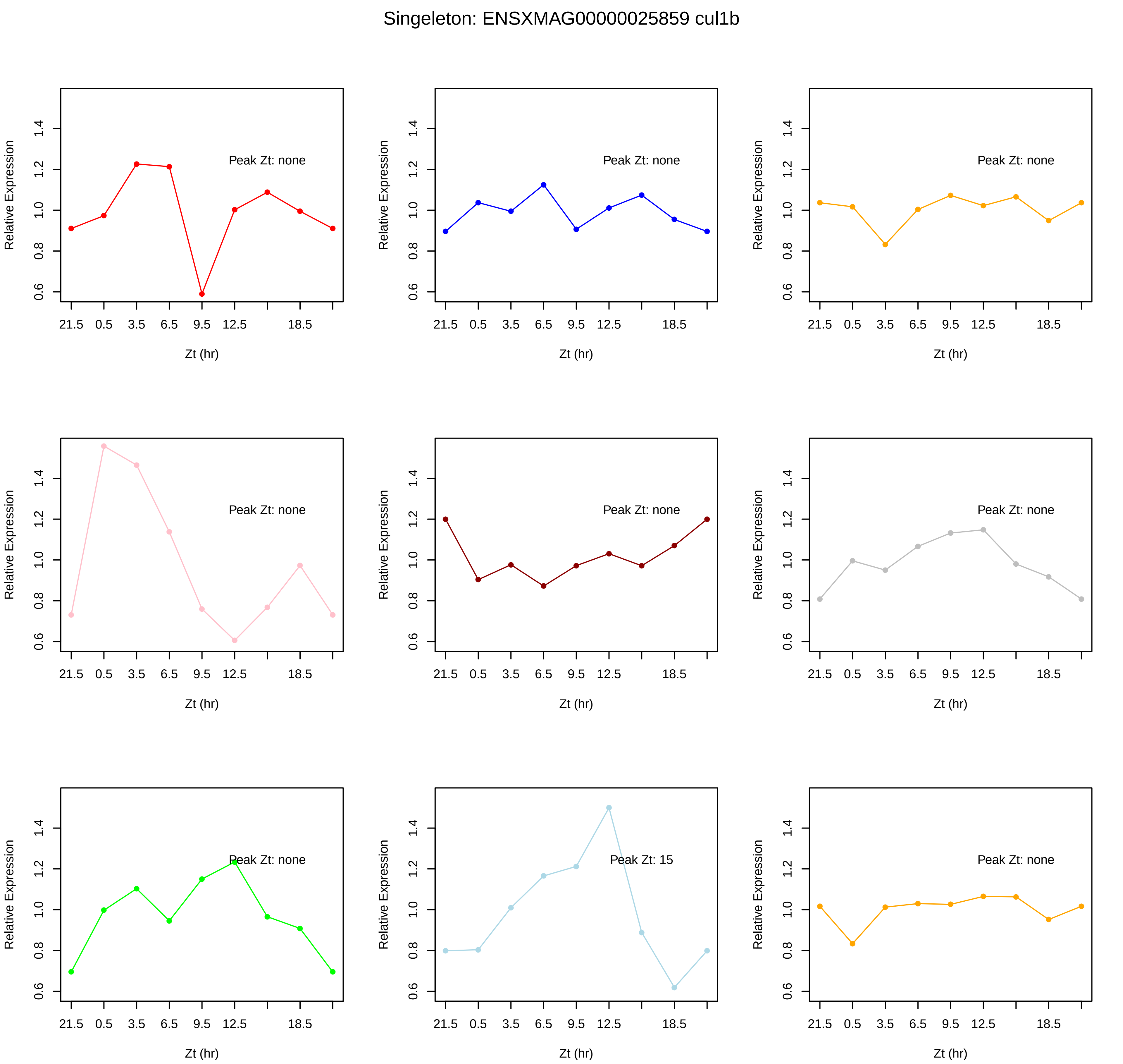
Singleton: ENSXMAG00000025859 cul1b
Relative Expression
Zt (hr)
Peak Zt: none
Peak Zt: 15
21.5
0.5
3.5
6.5
9.5
12.5
18.5
0.6
0.8
1.0
1.2
1.4

Singeleton: ENSXMAG00000024590 numb

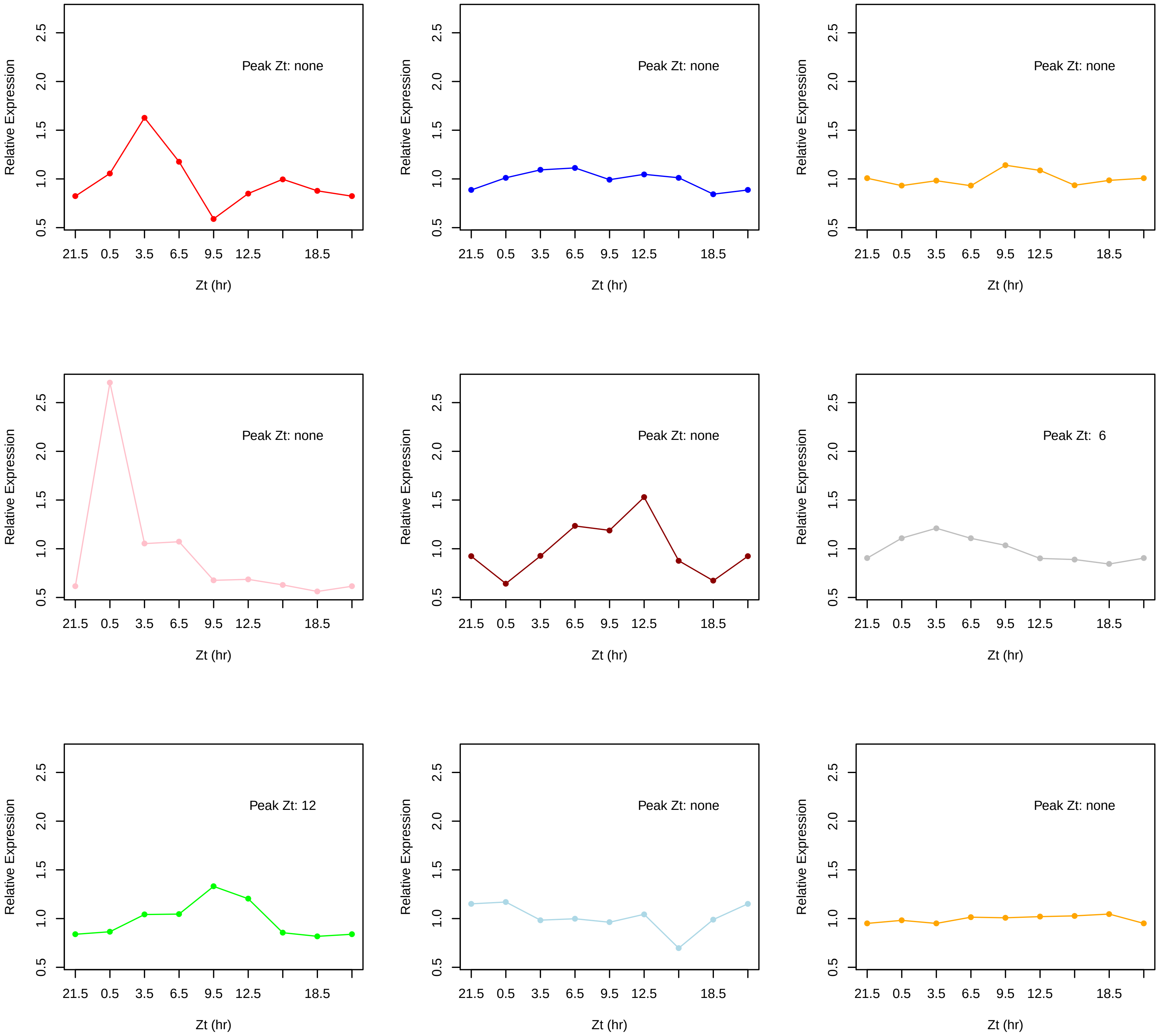

Singleton: ENSXMAG00000015314 per1b

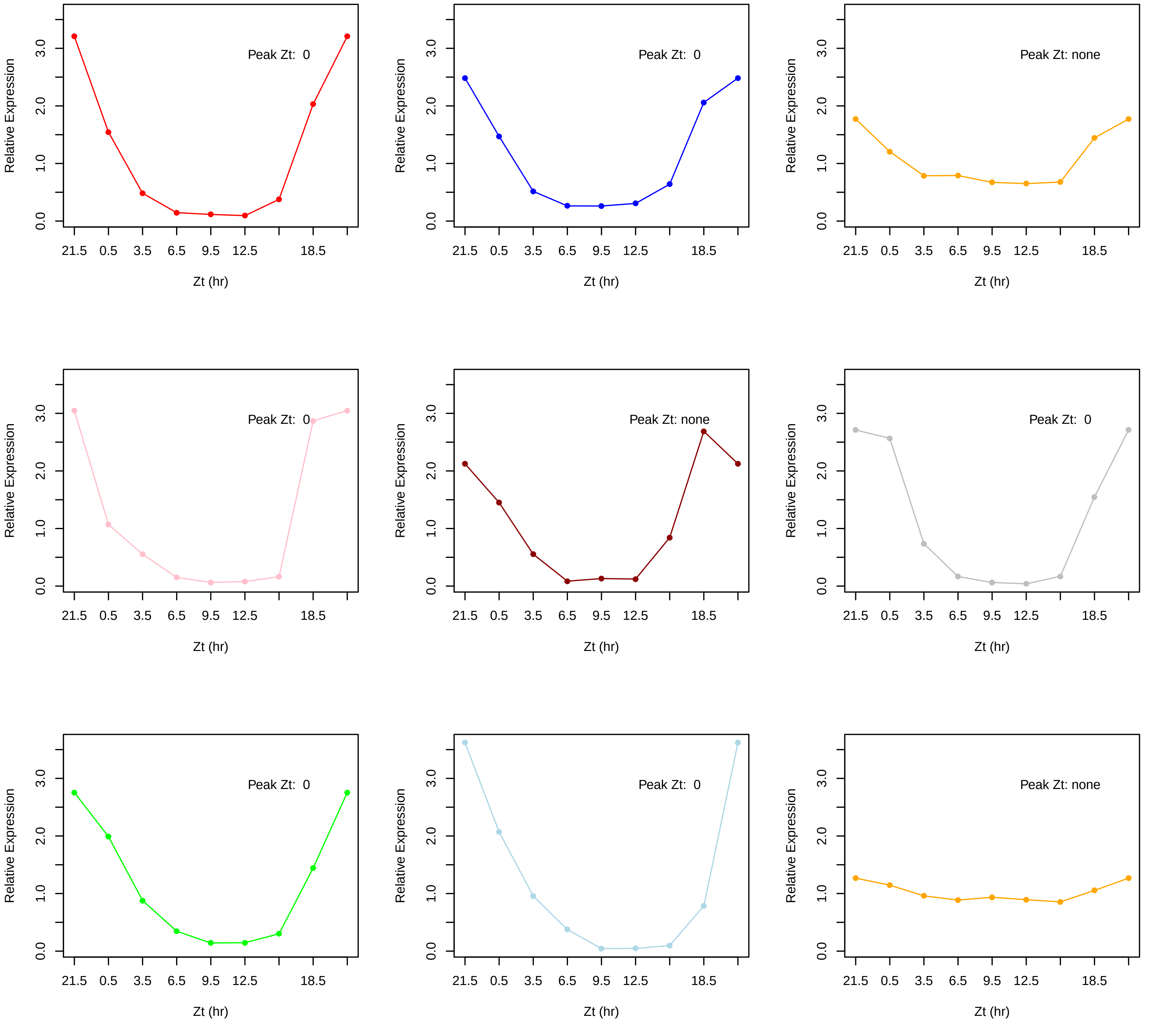

Singleton: ENSXMAG00000000966

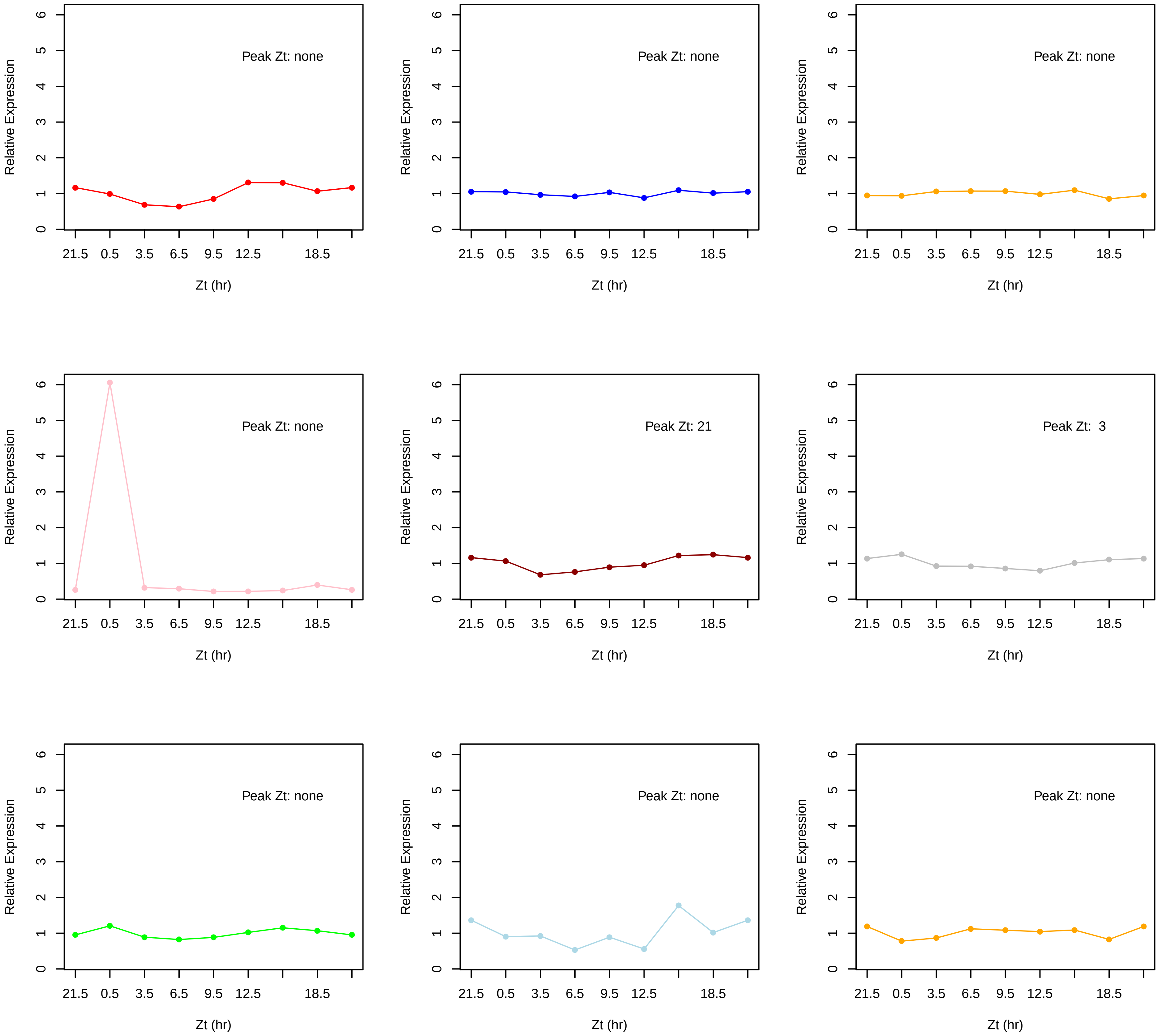

Singleton: ENSXMAG00000014954 pdk2a

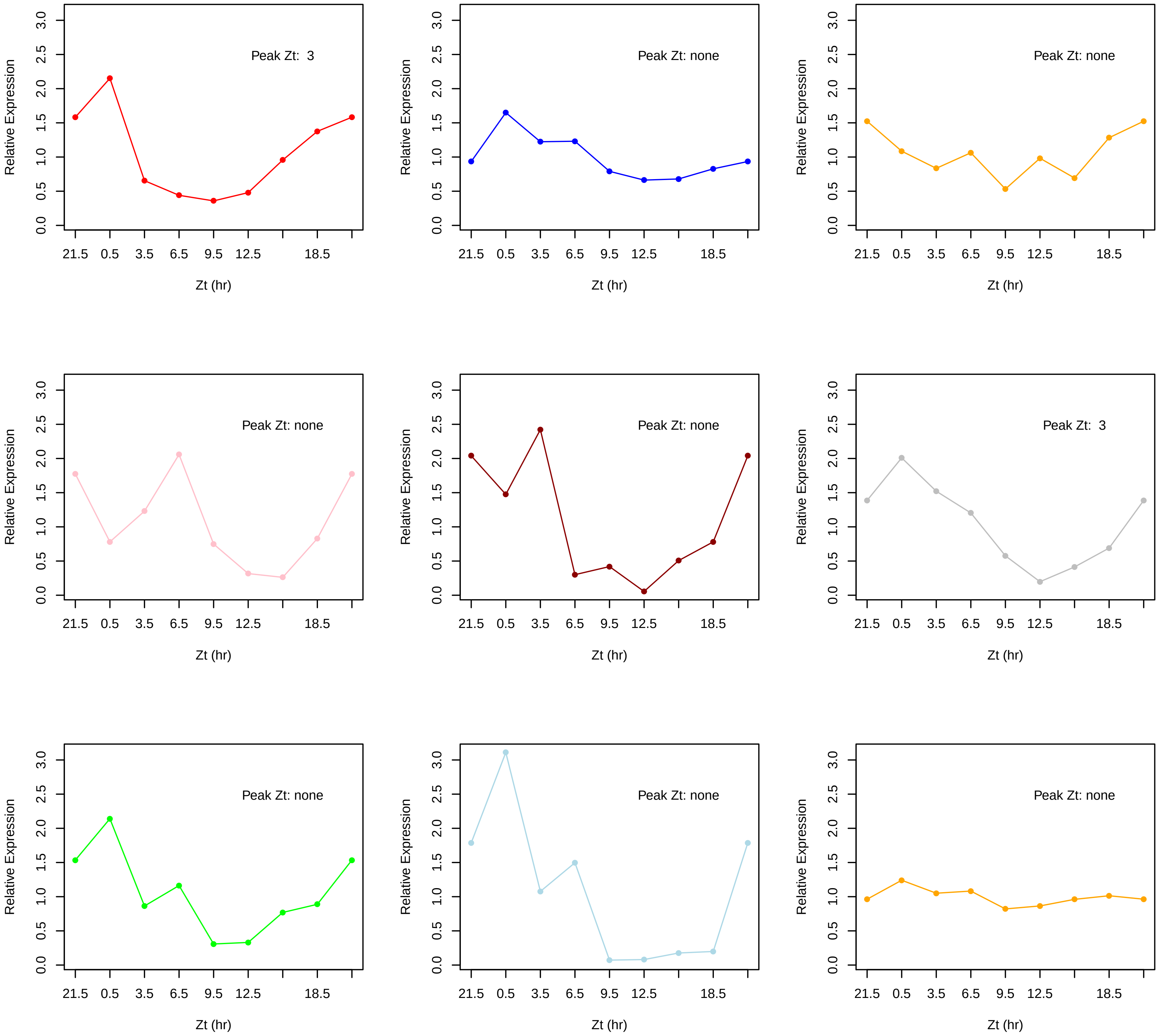

Singeleton: ENSXMAG00000002154 mapk6

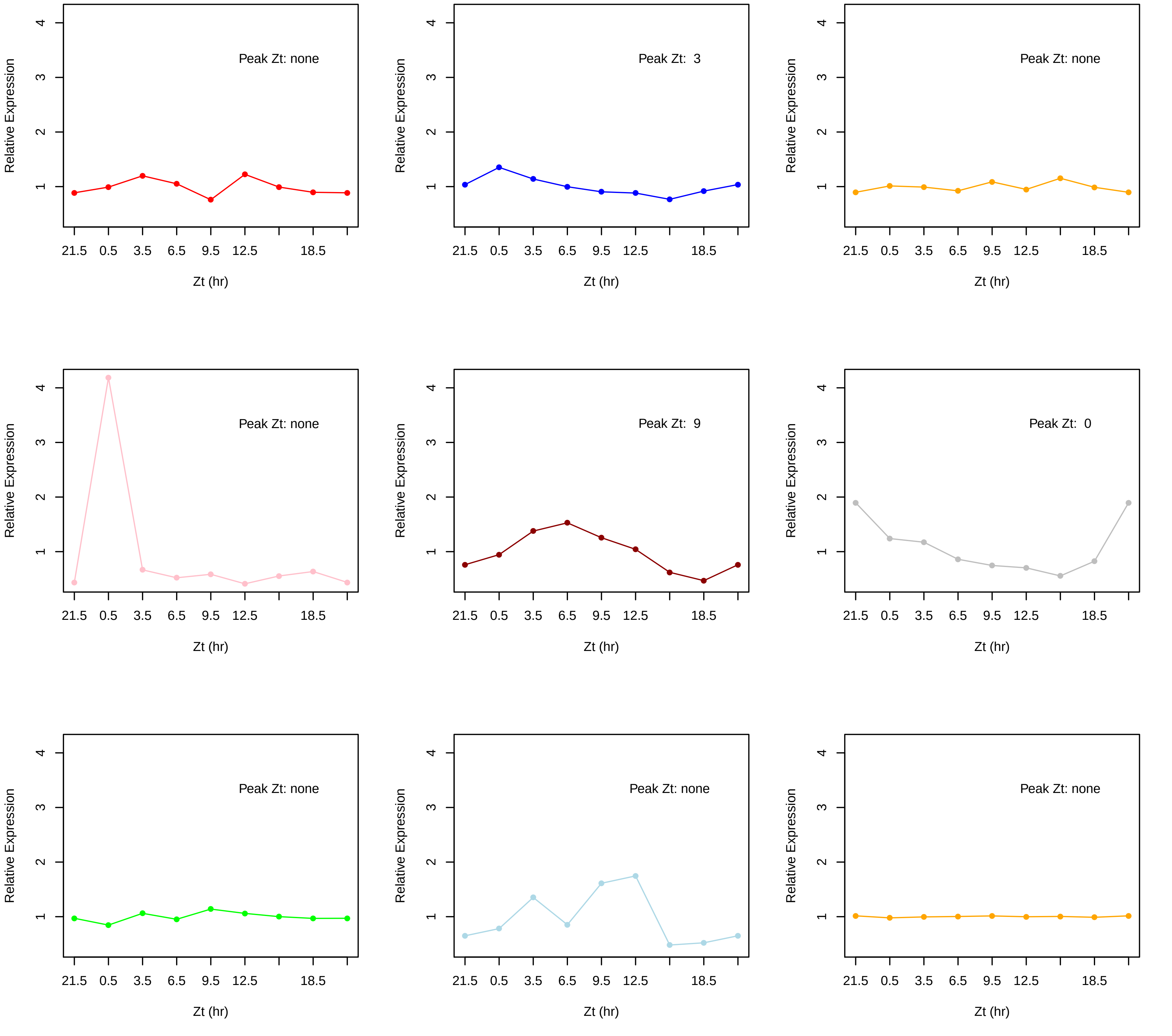

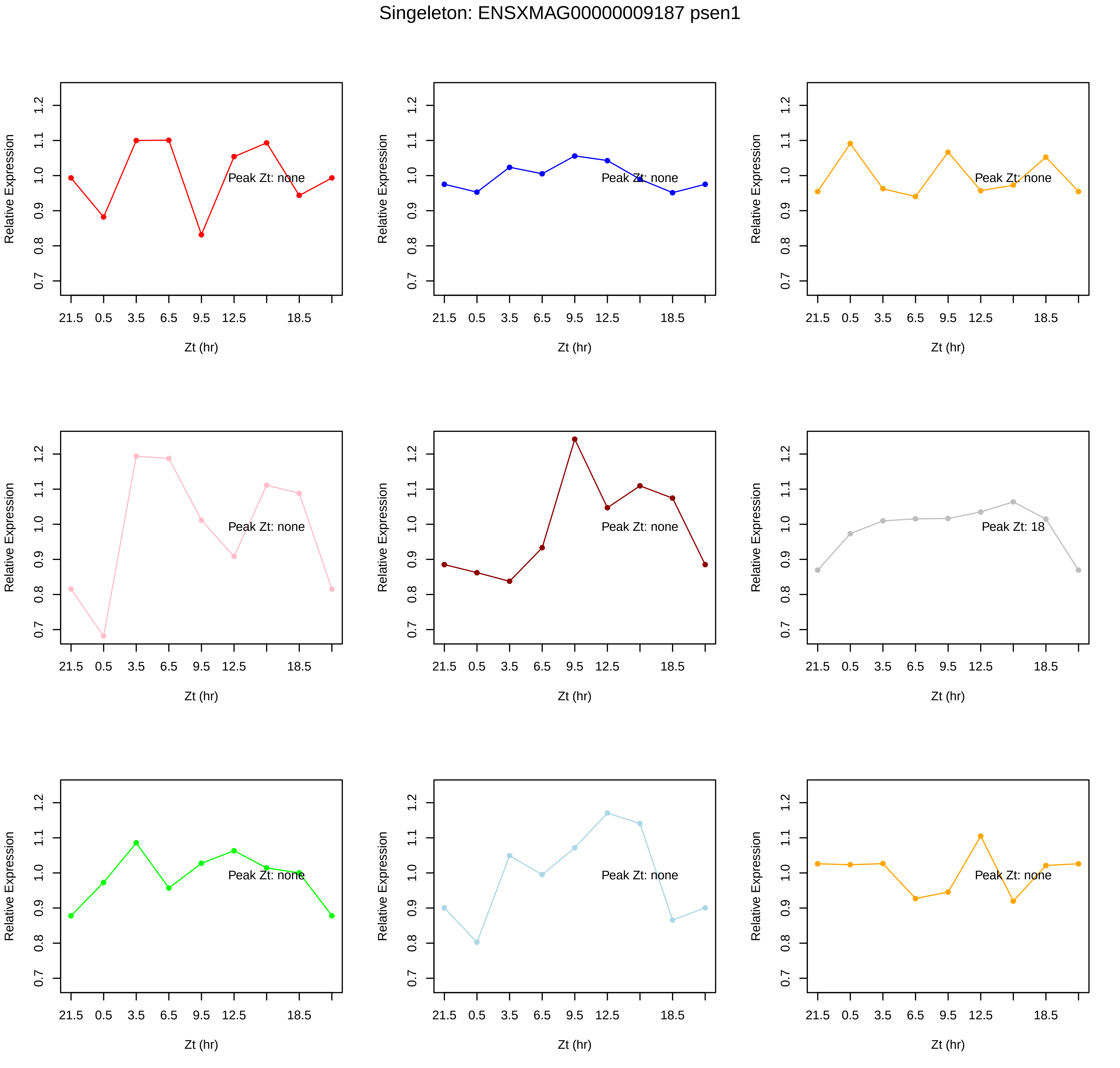
Singleton: ENSXMAG00000009187 psen1
Relative Expression
Zt (hr)
21.5
0.5
3.5
6.5
9.5
12.5
18.5
0.7
0.8
0.9
1.0
1.1
1.2
Peak Zt: none
Peak Zt: 18

Singeleton: ENSXMAG00000013349 gmppb

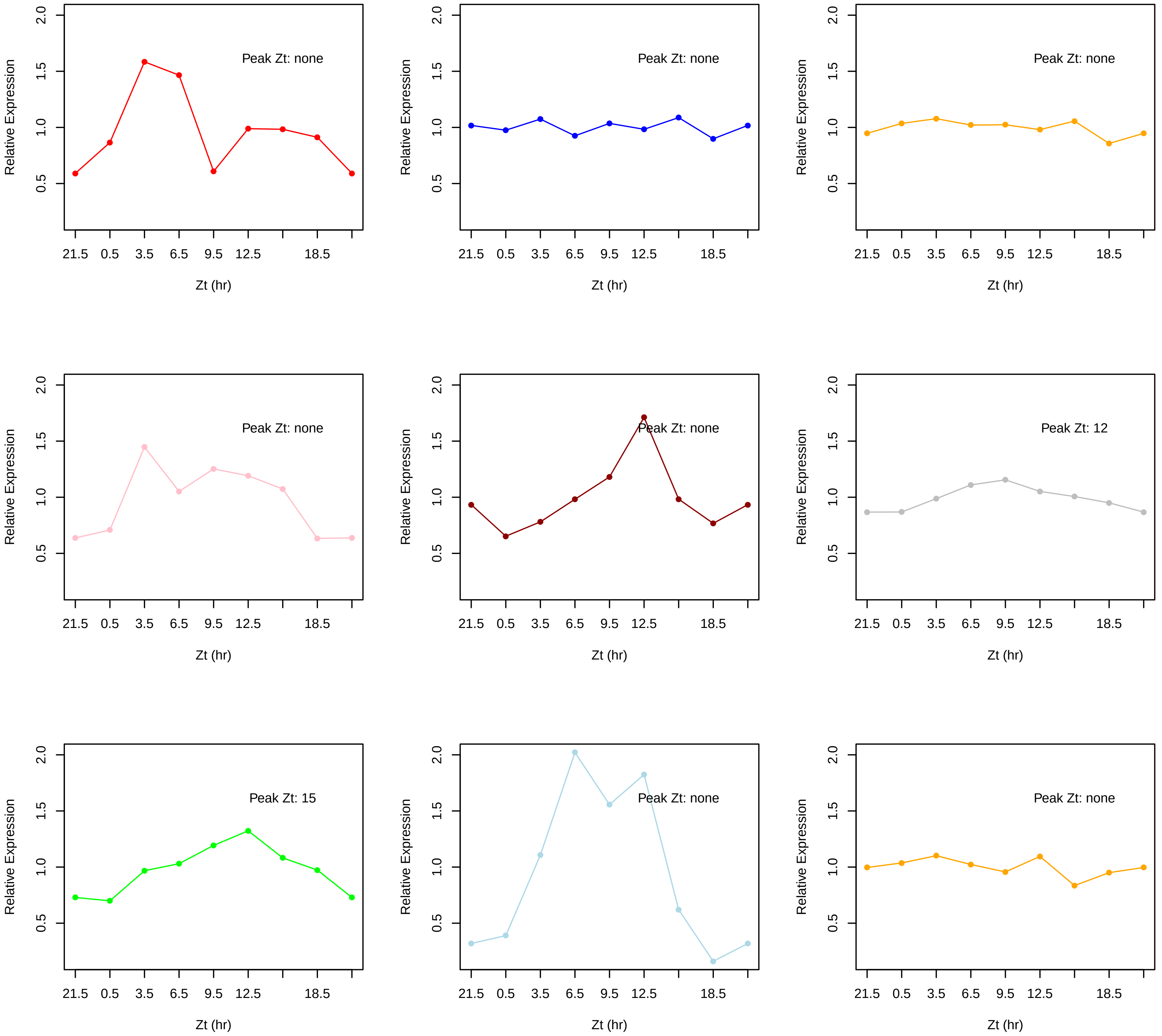

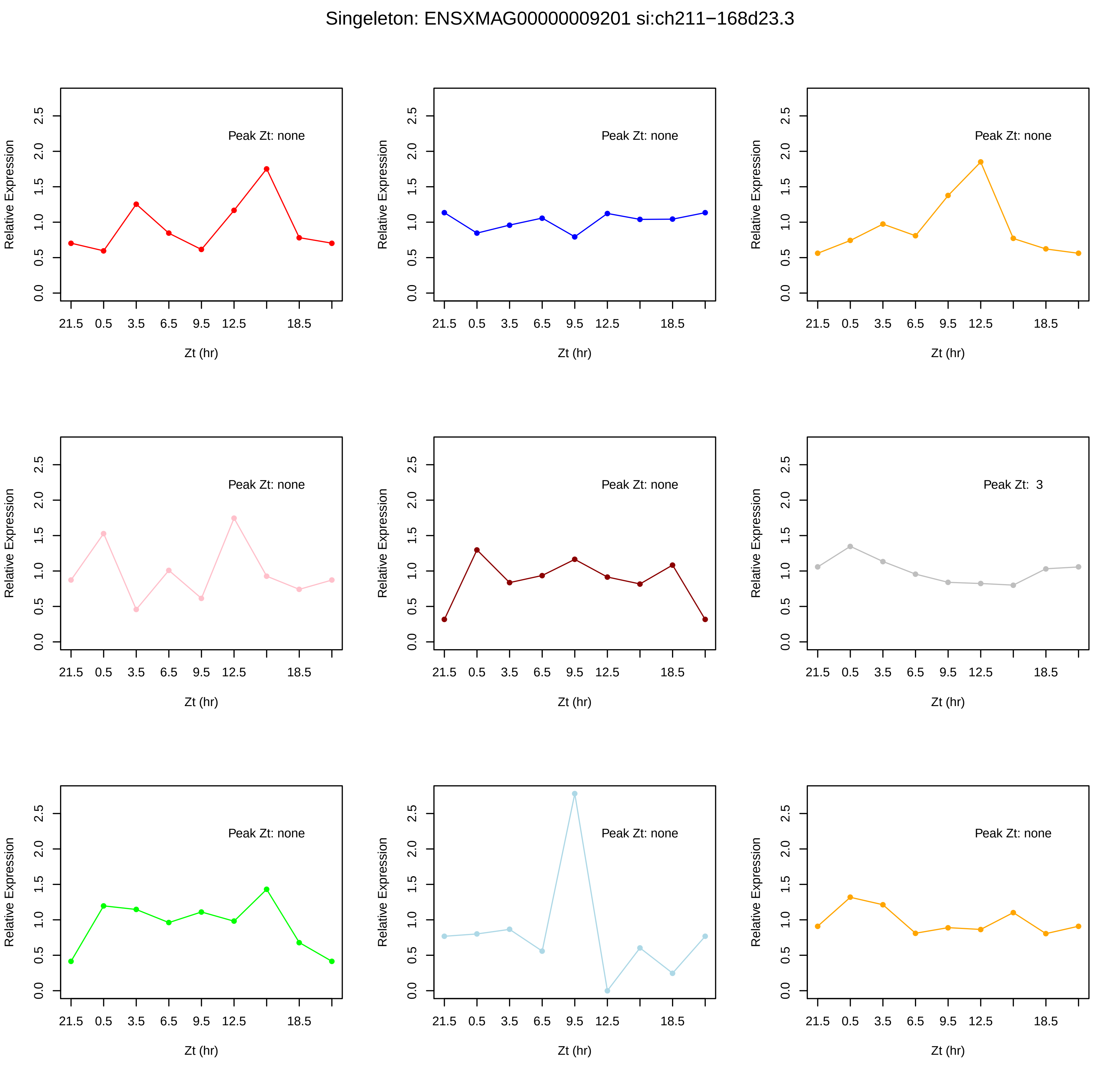

Singleton: ENSXMAG00000009201 si:ch211−168d23.3
Peak Zt: none
Peak Zt: 3
Relative Expression
Zt (hr)
0.0
0.5
1.0
1.5
2.0
2.5
21.5
0.5
3.5
6.5
9.5
12.5
18.5

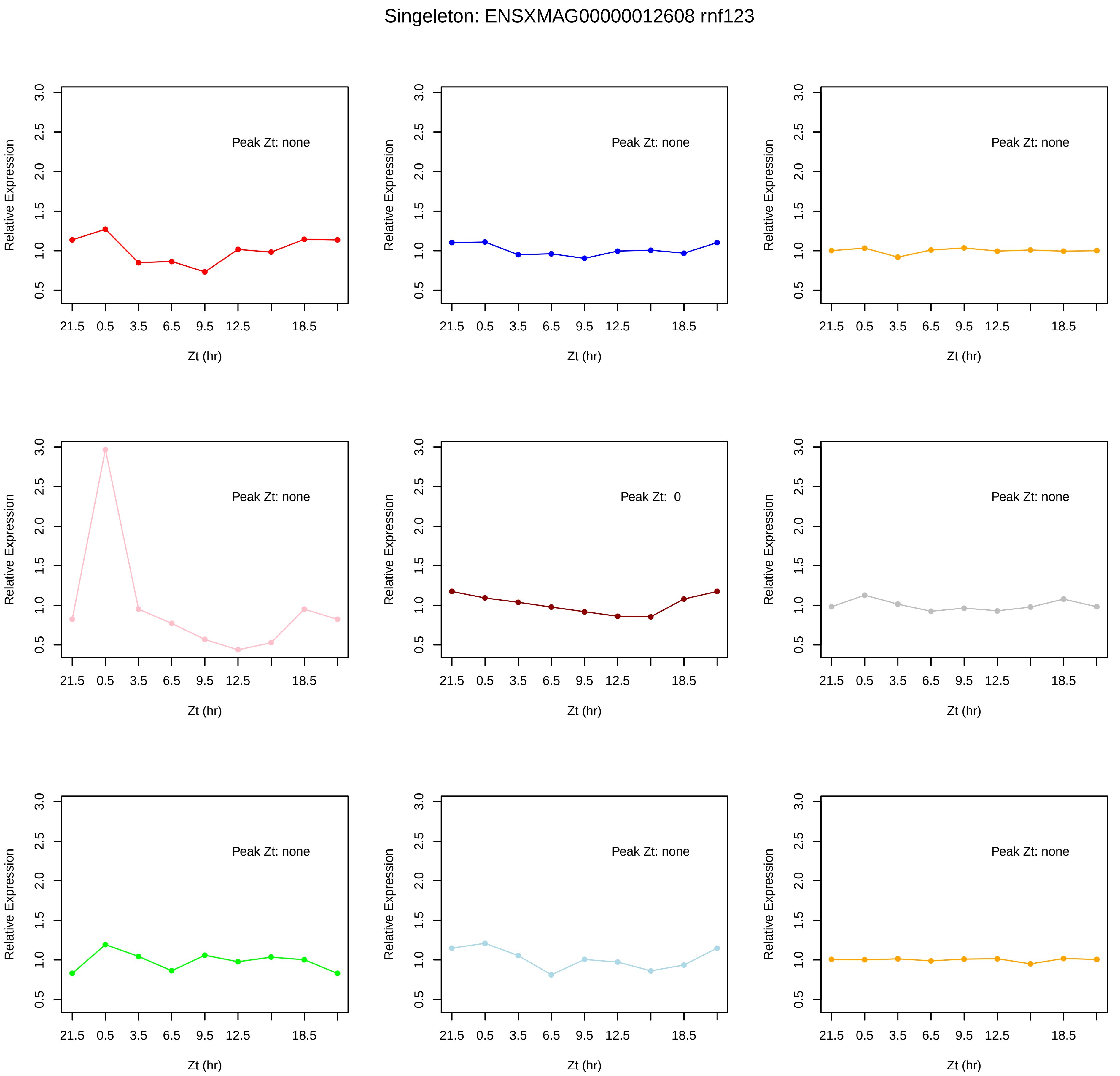

Singeleton: ENSXMAG00000012608 rnf123
Peak Zt: none
Peak Zt: none
Peak Zt: none
Peak Zt: none
Peak Zt: 0
Peak Zt: none
Peak Zt: none
Peak Zt: none
Peak Zt: none
Relative Expression
Zt (hr)
21.5 0.5 3.5 6.5 9.5 12.5 18.5
0.5 1.0 1.5 2.0 2.5 3.0

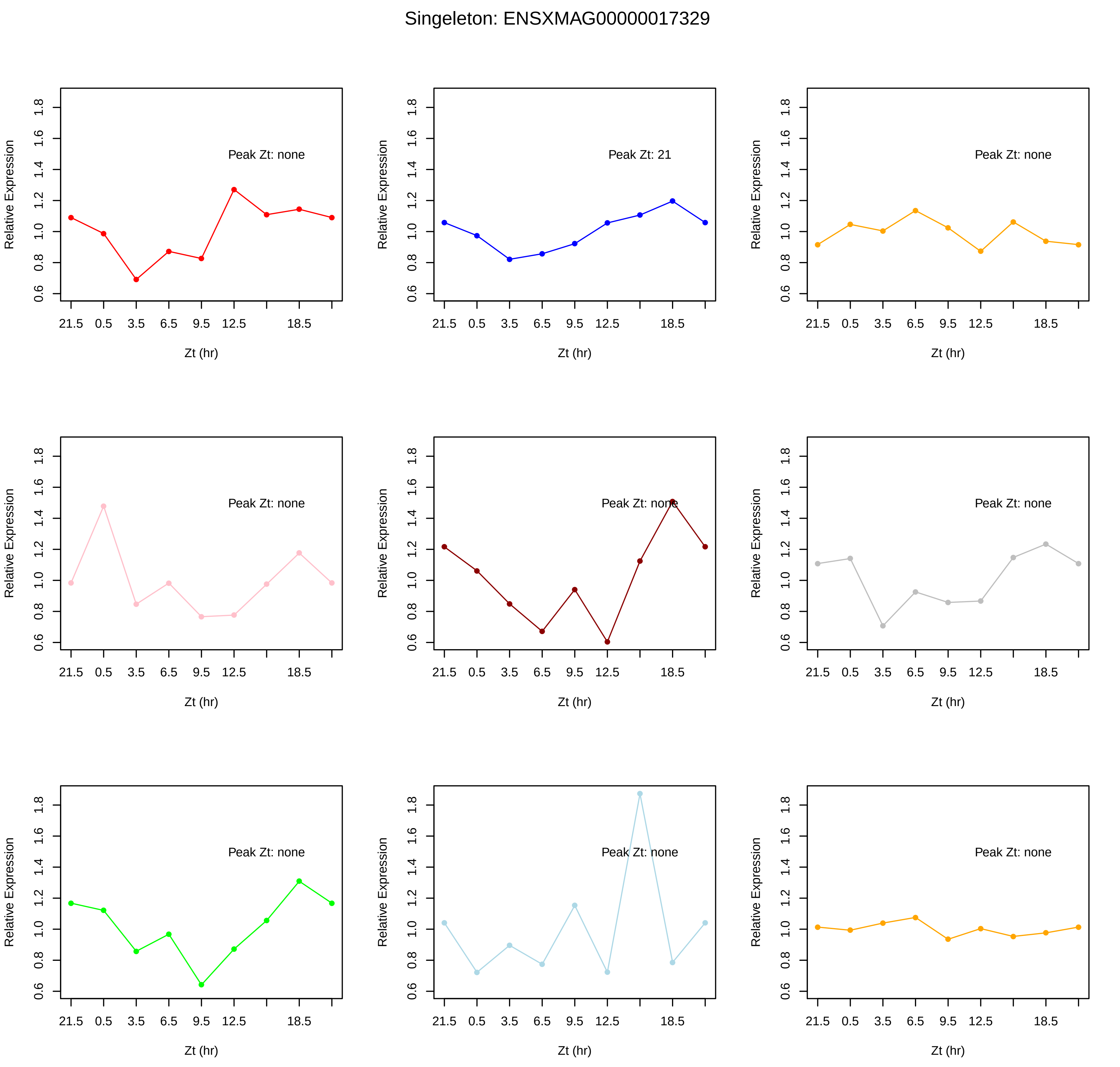
Singeleton: ENSXMAG00000017329
Peak Zt: none
Peak Zt: 21
Peak Zt: none
Peak Zt: none
Peak Zt: none
Peak Zt: none
Peak Zt: none
Peak Zt: none
Peak Zt: none
Relative Expression
Zt (hr)
21.5
0.5
3.5
6.5
9.5
12.5
18.5
0.6
0.8
1.0
1.2
1.4
1.6
1.8

Singeleton: ENSXMAG00000013703 pnpo

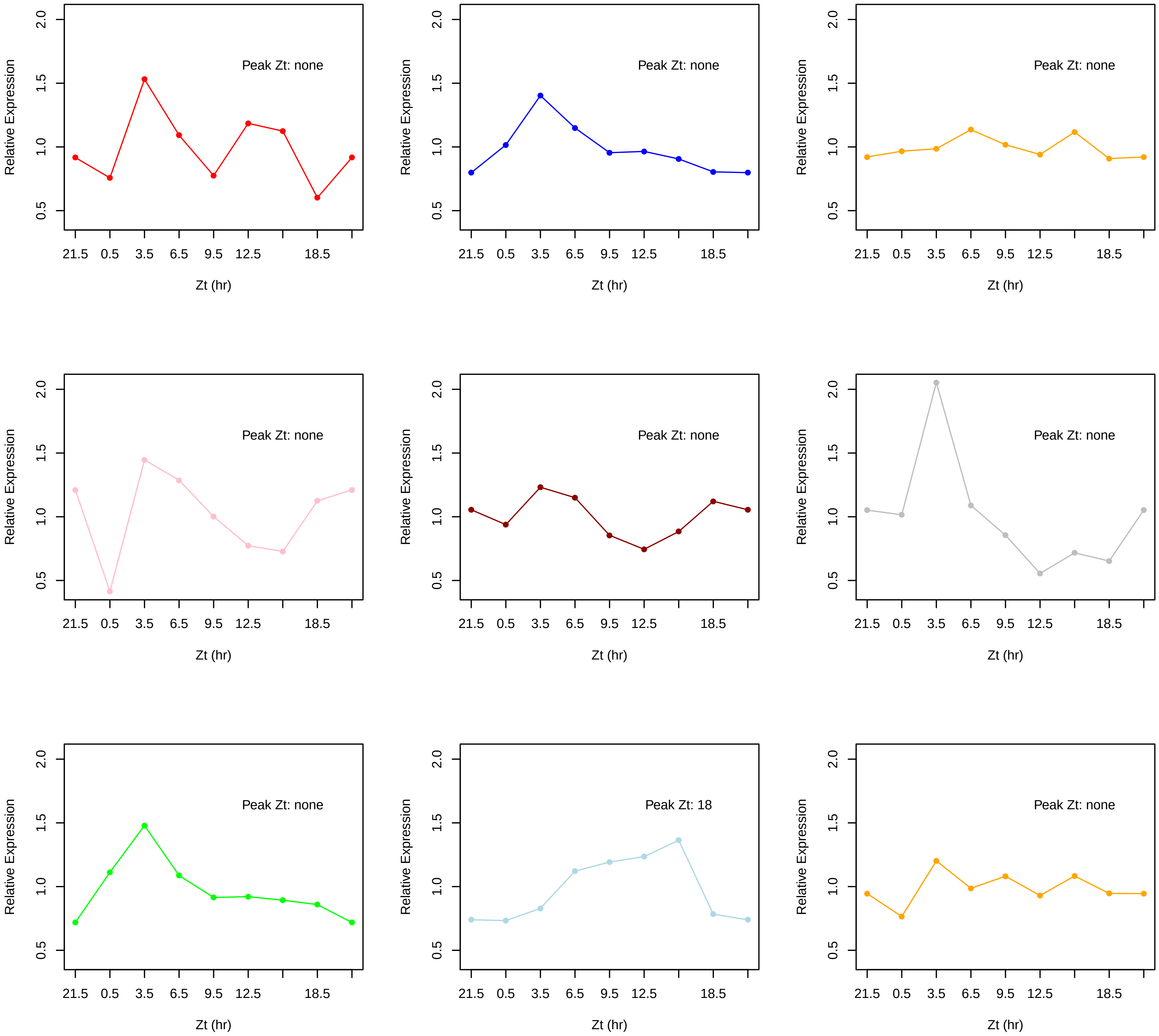

Singeleton: ENSXMAG00000013715 cdk5rap3

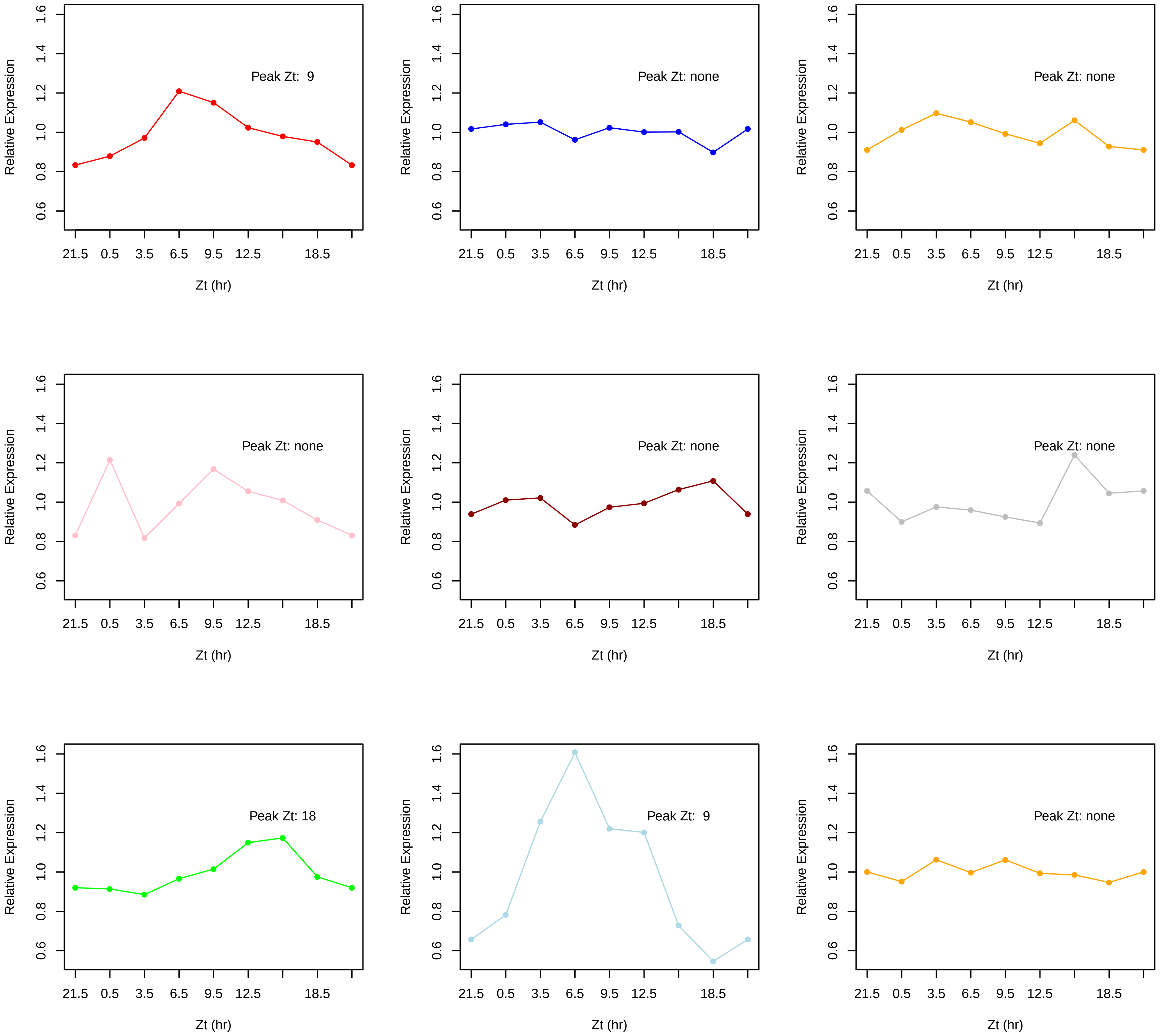

Singleton: ENSXMAG00000024537 nicn1

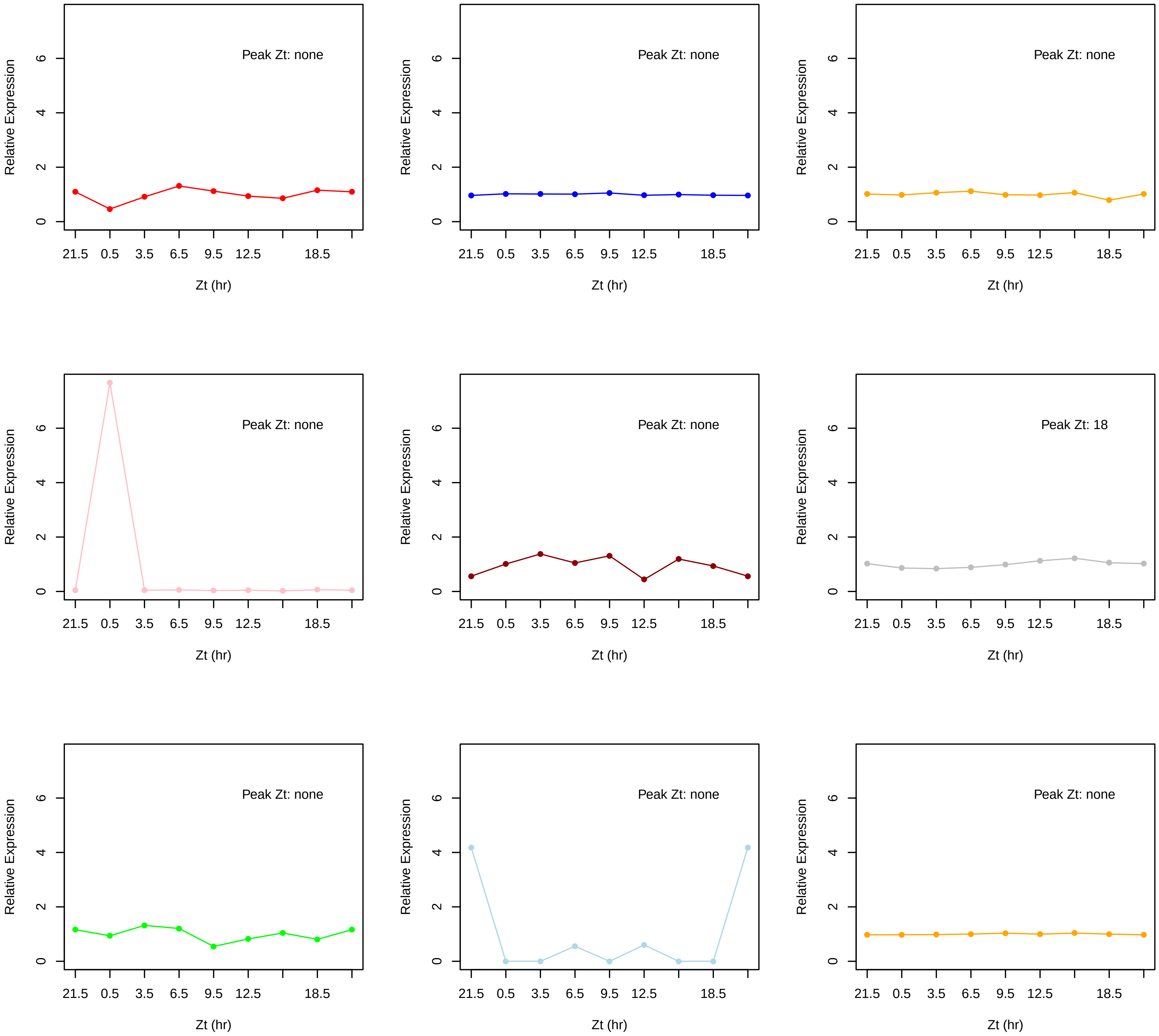

Singeleton: ENSXMAG00000000597 tpk1

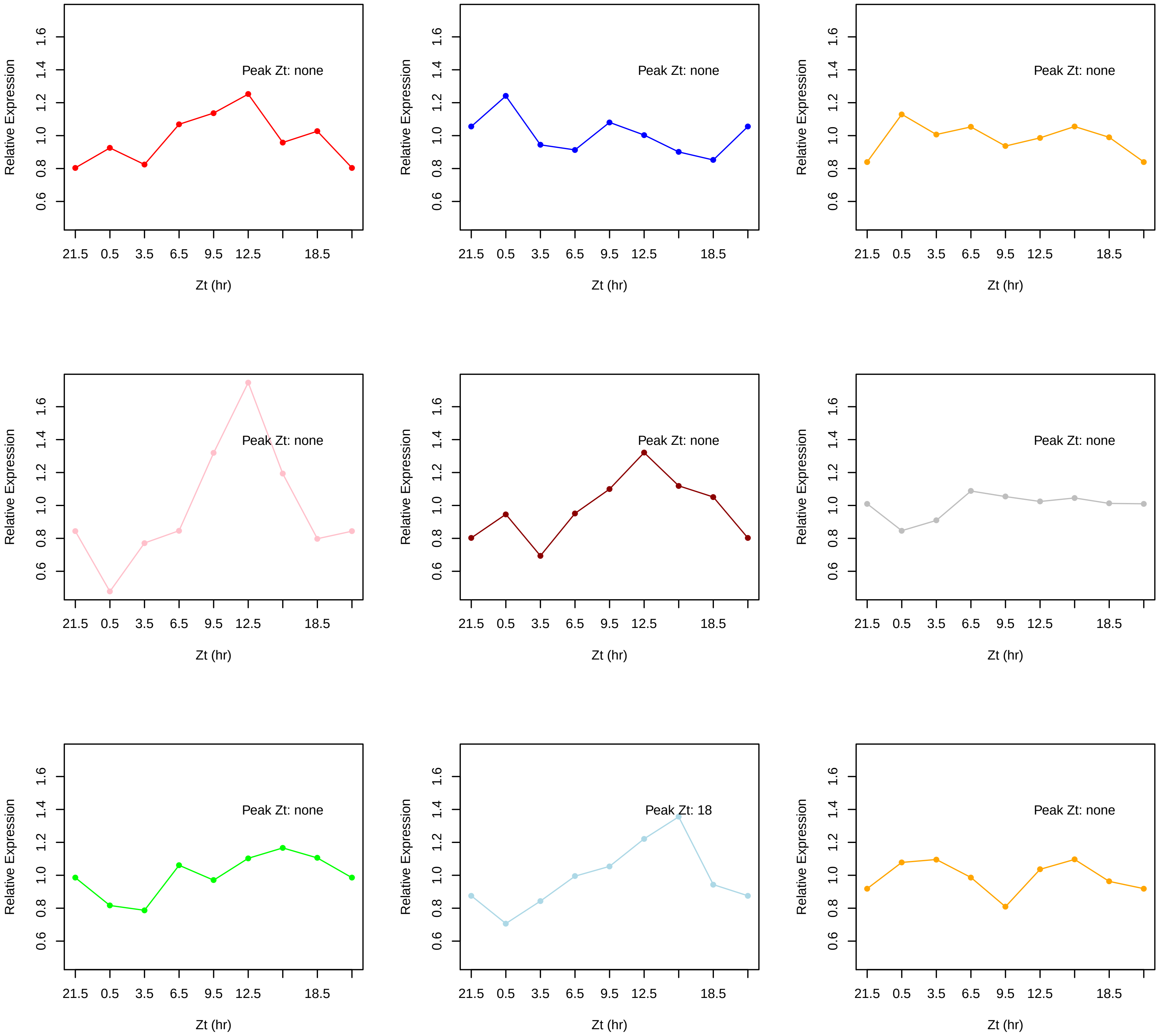

Singeleton: ENSXMAG00000024654 fam214a

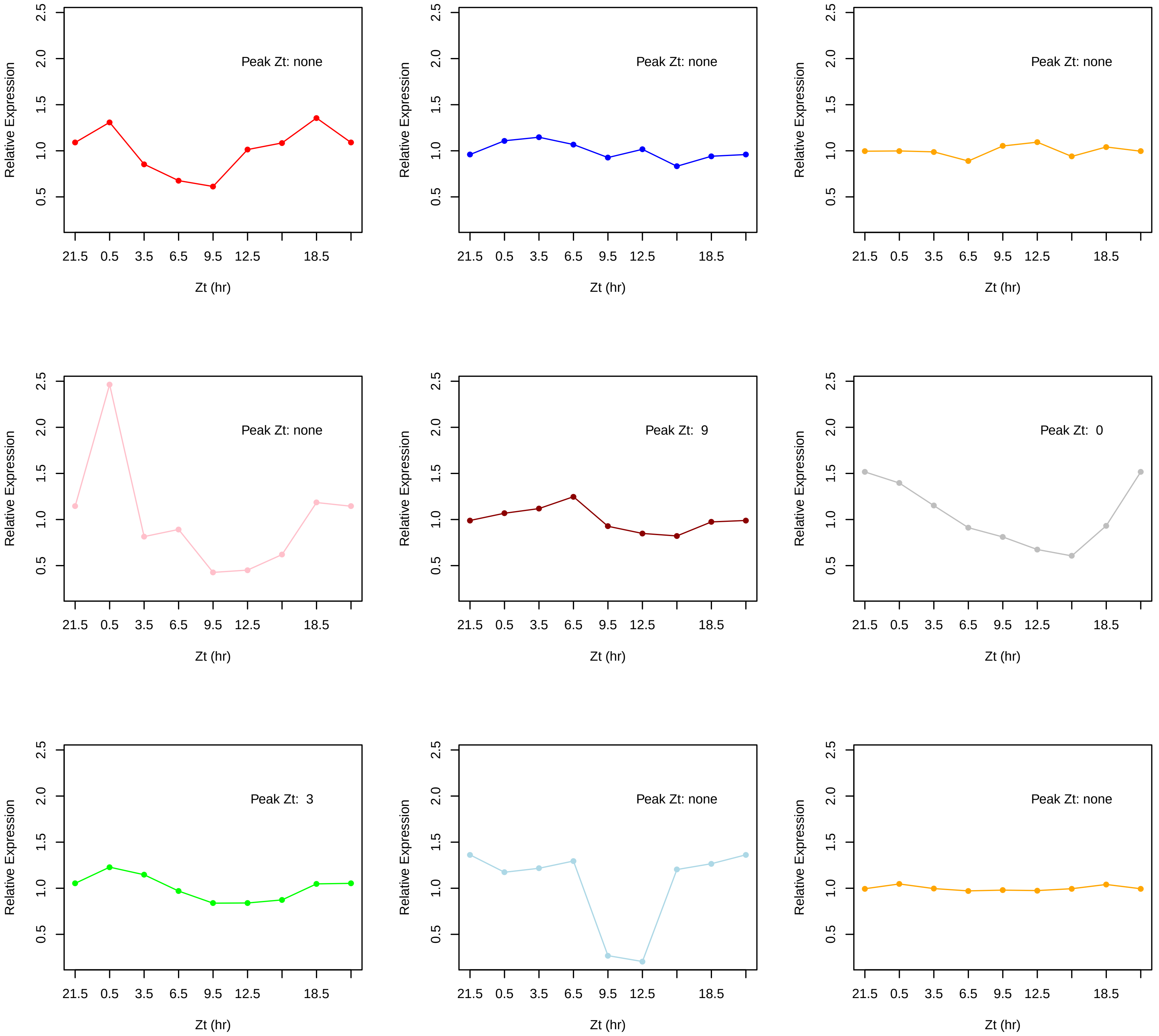

Singeleton: ENSXMAG00000029109 NFE2L1

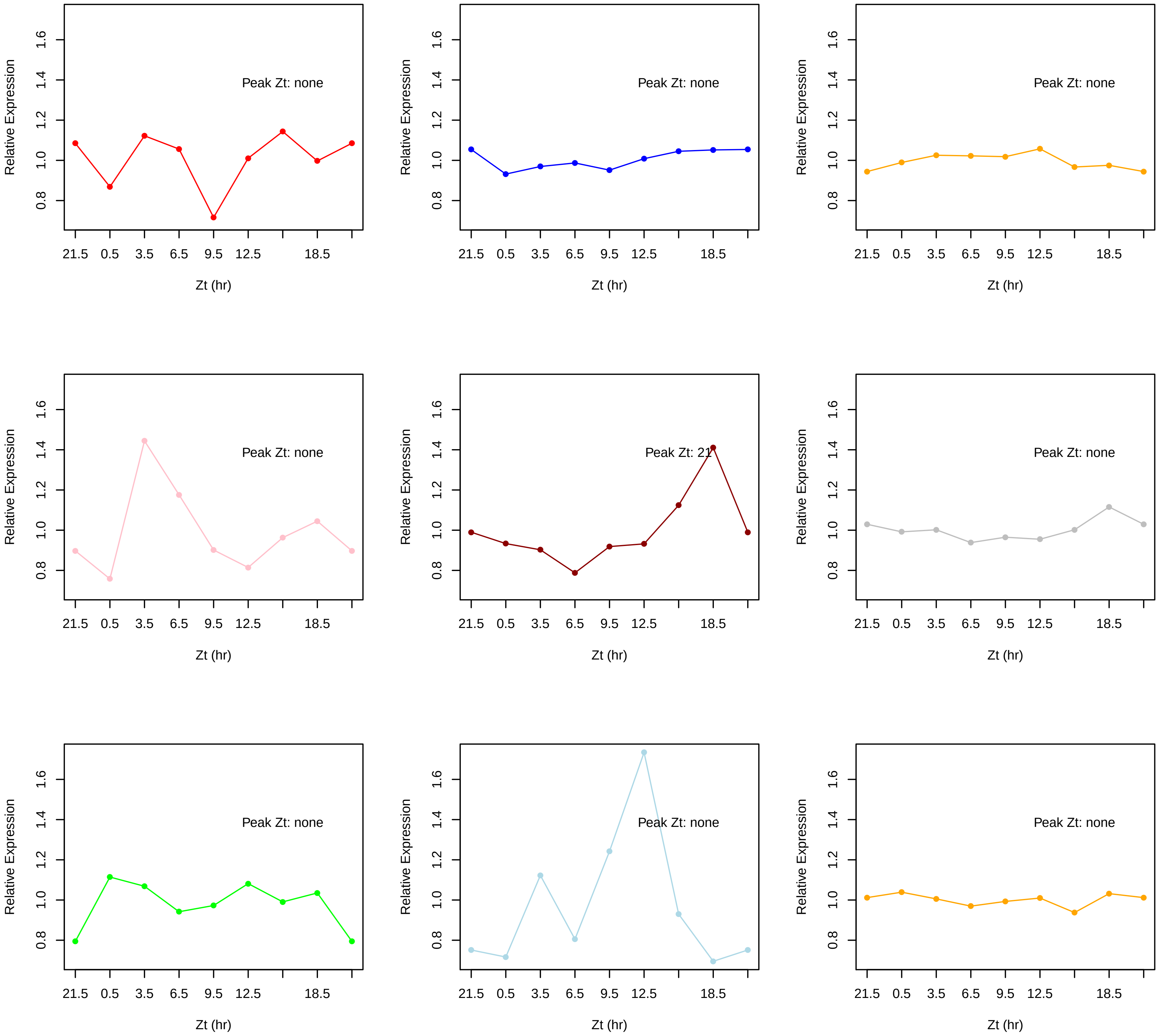

Singeleton: ENSXMAG00000005457 ndufb6

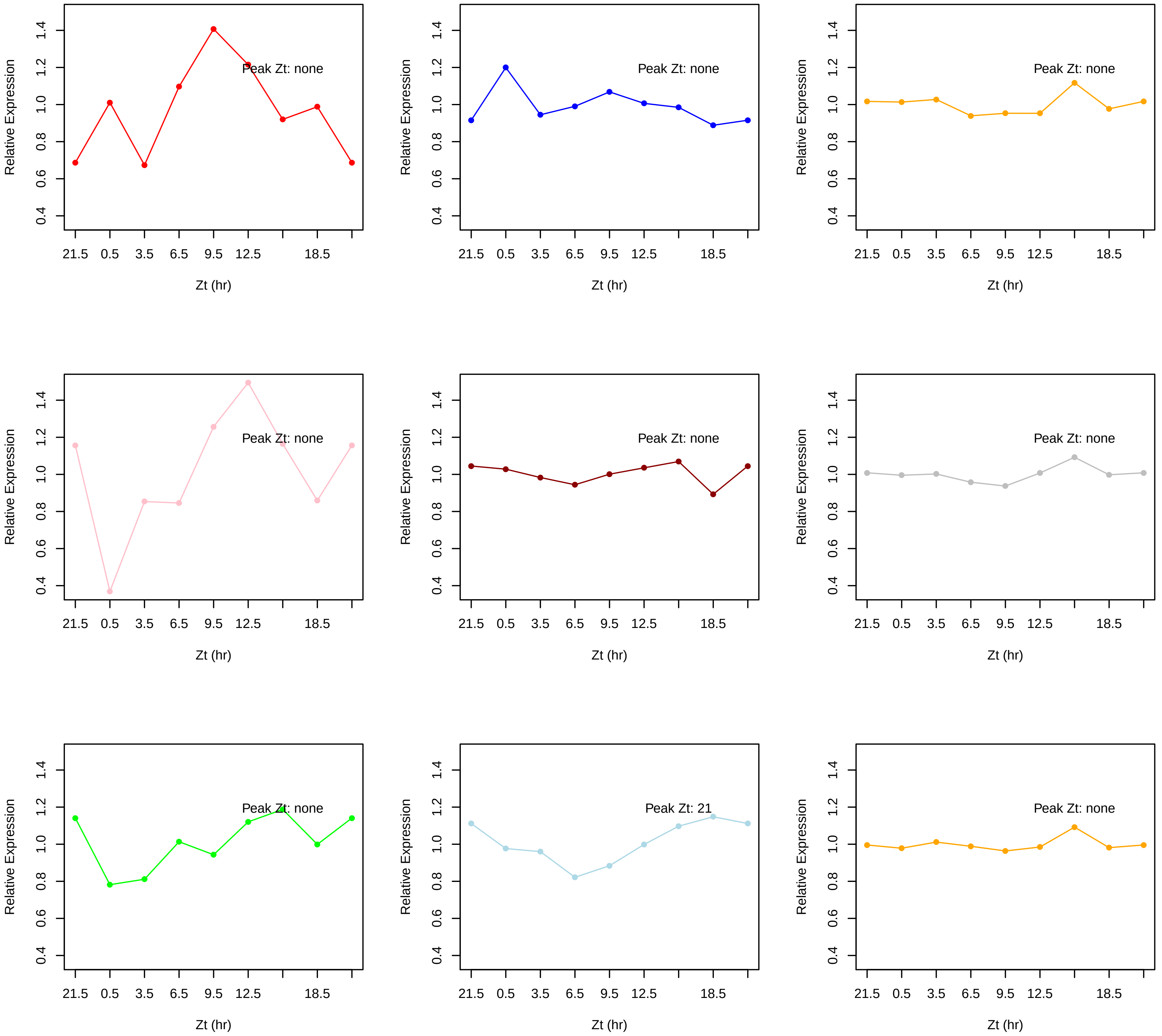

Singeleton: ENSXMAG00000012204 manf

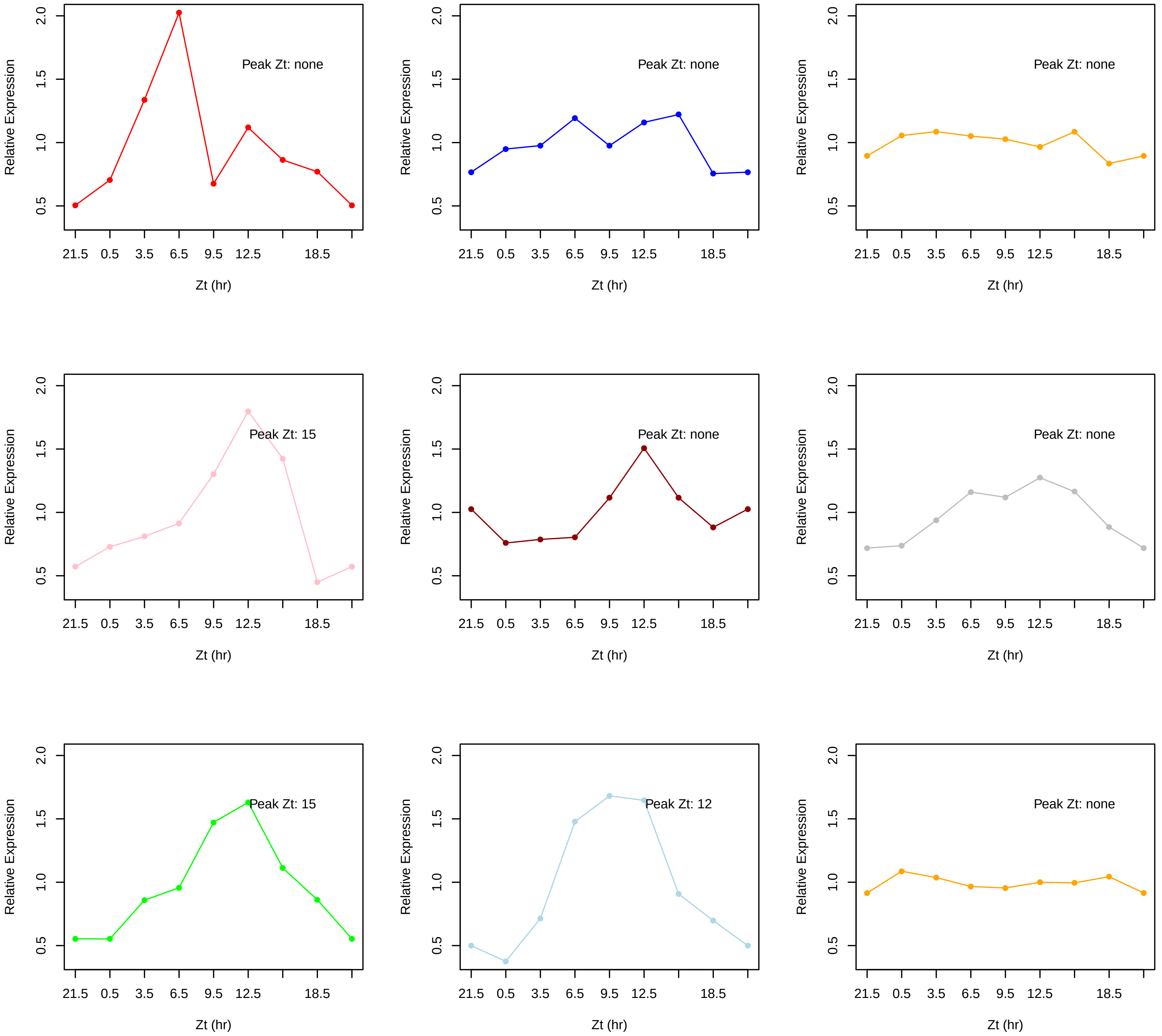

Singleton: ENSXMAG00000025560 cbx1b

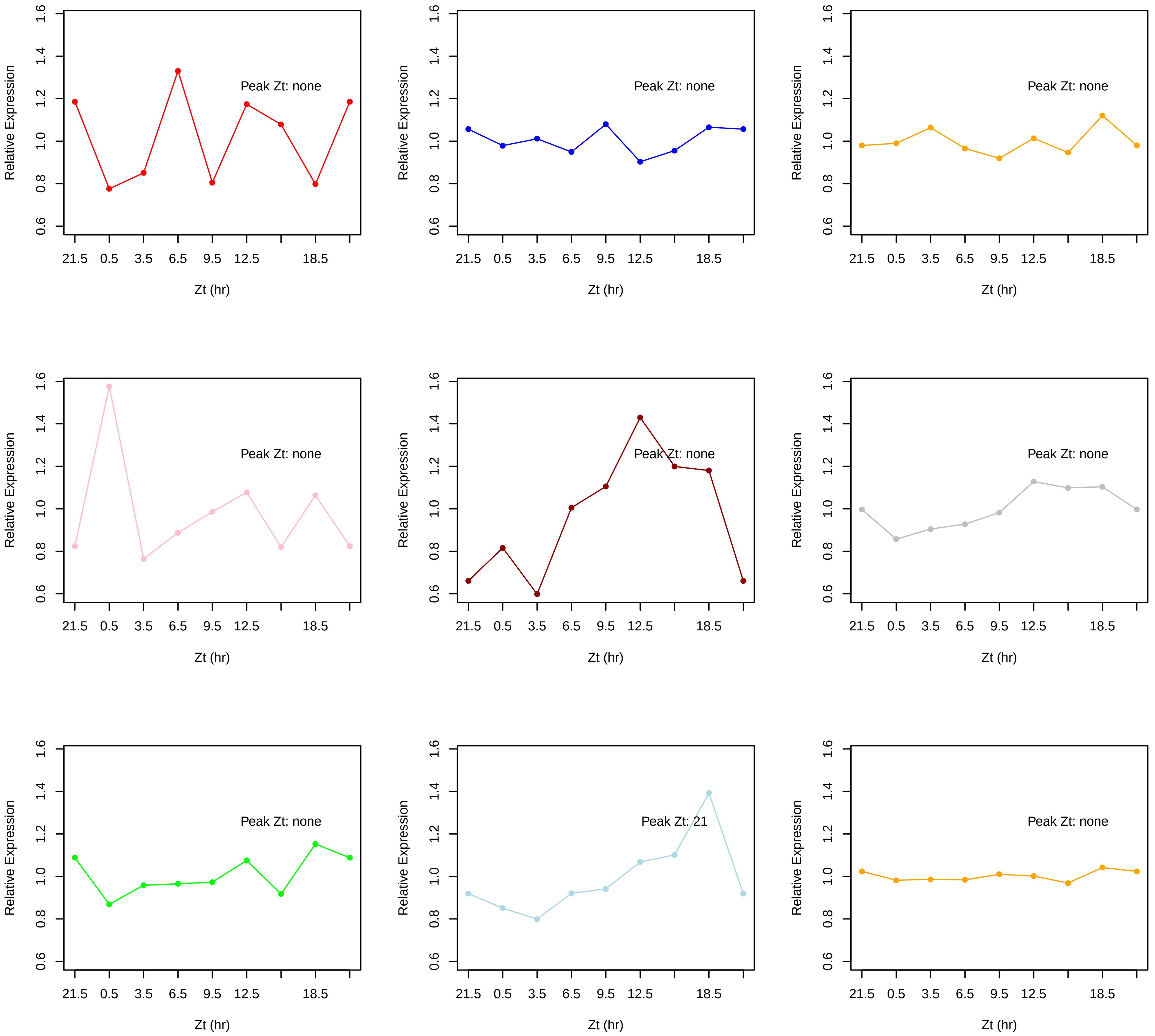

Singeleton: ENSXMAG00000013925 serpinh2

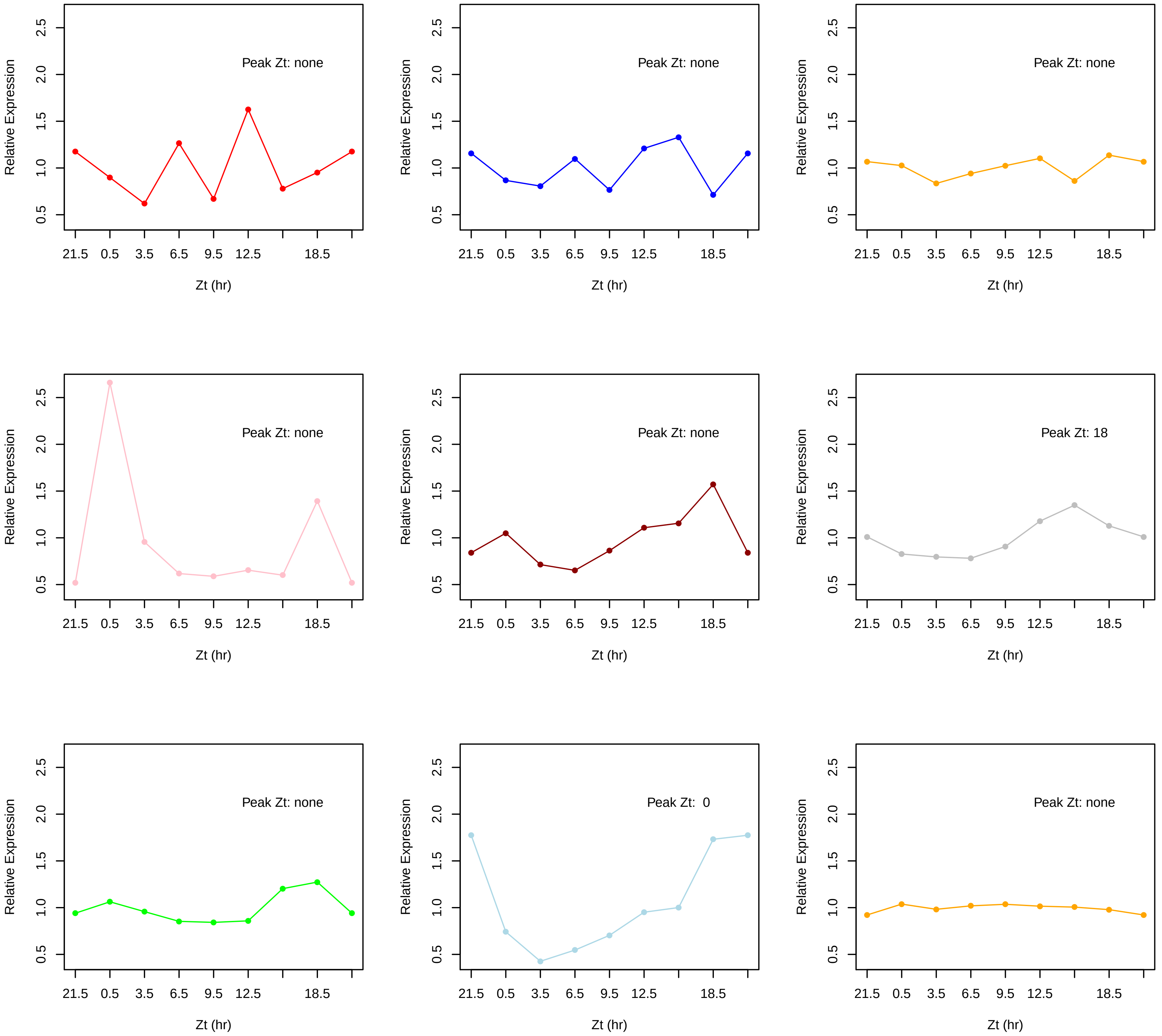

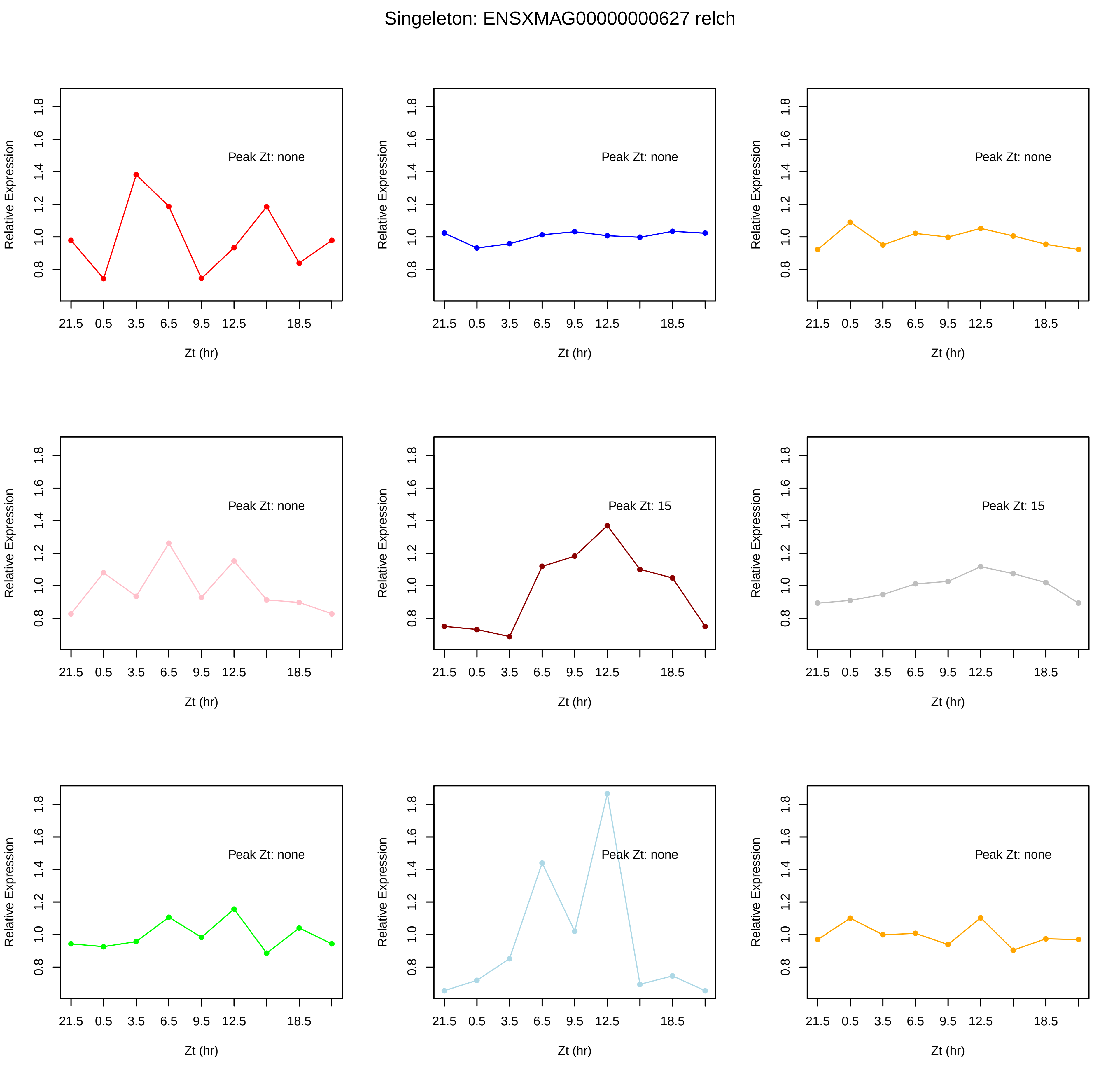

Singeleton: ENSXMAG00000000627 relch
Peak Zt: none
Peak Zt: none
Peak Zt: none
Peak Zt: none
Peak Zt: 15
Peak Zt: 15
Peak Zt: none
Peak Zt: none
Peak Zt: none
Relative Expression
Zt (hr)

Singeleton: ENSXMAG00000018090 UNC13C

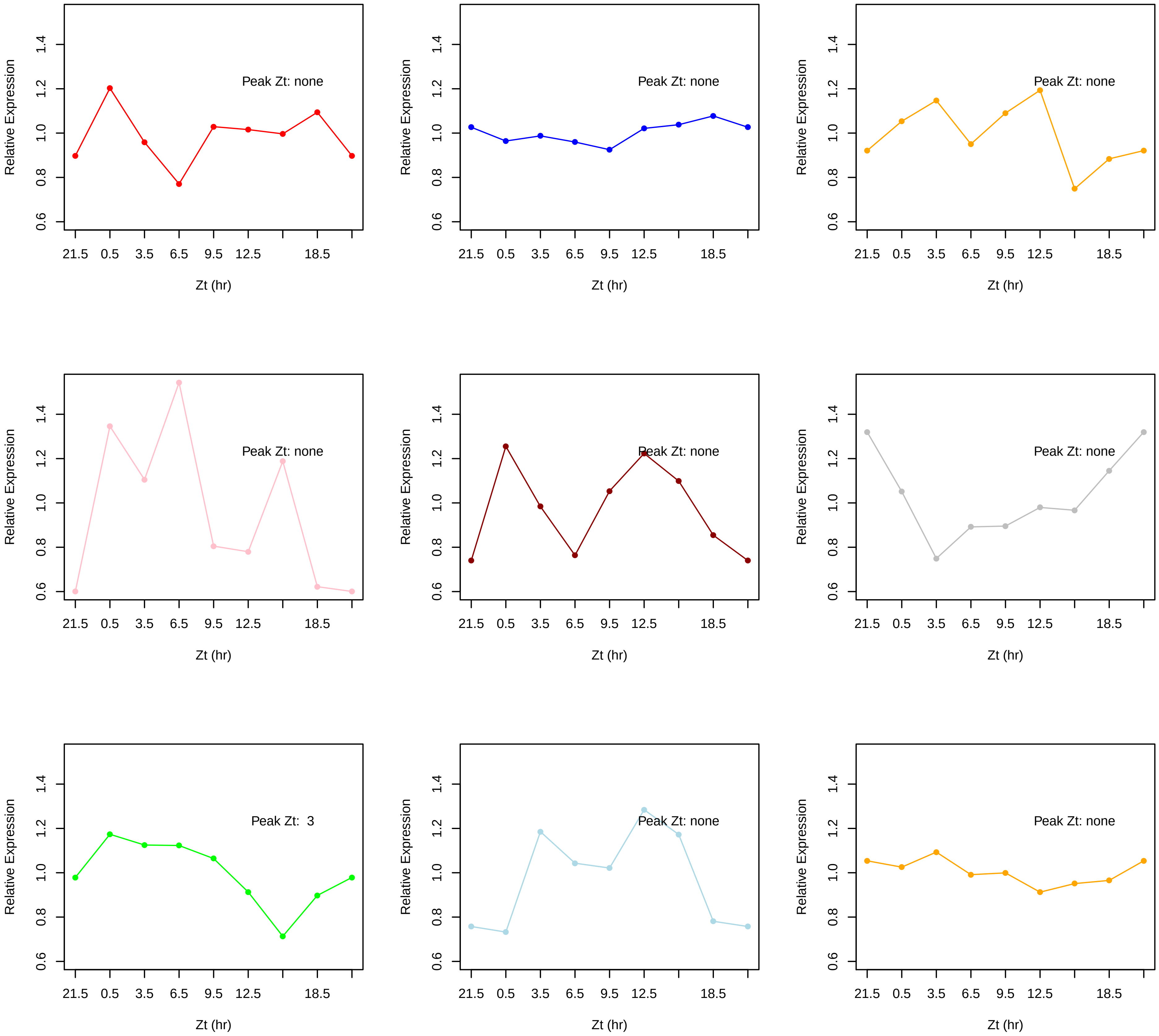

Singeleton: ENSXMAG00000009769

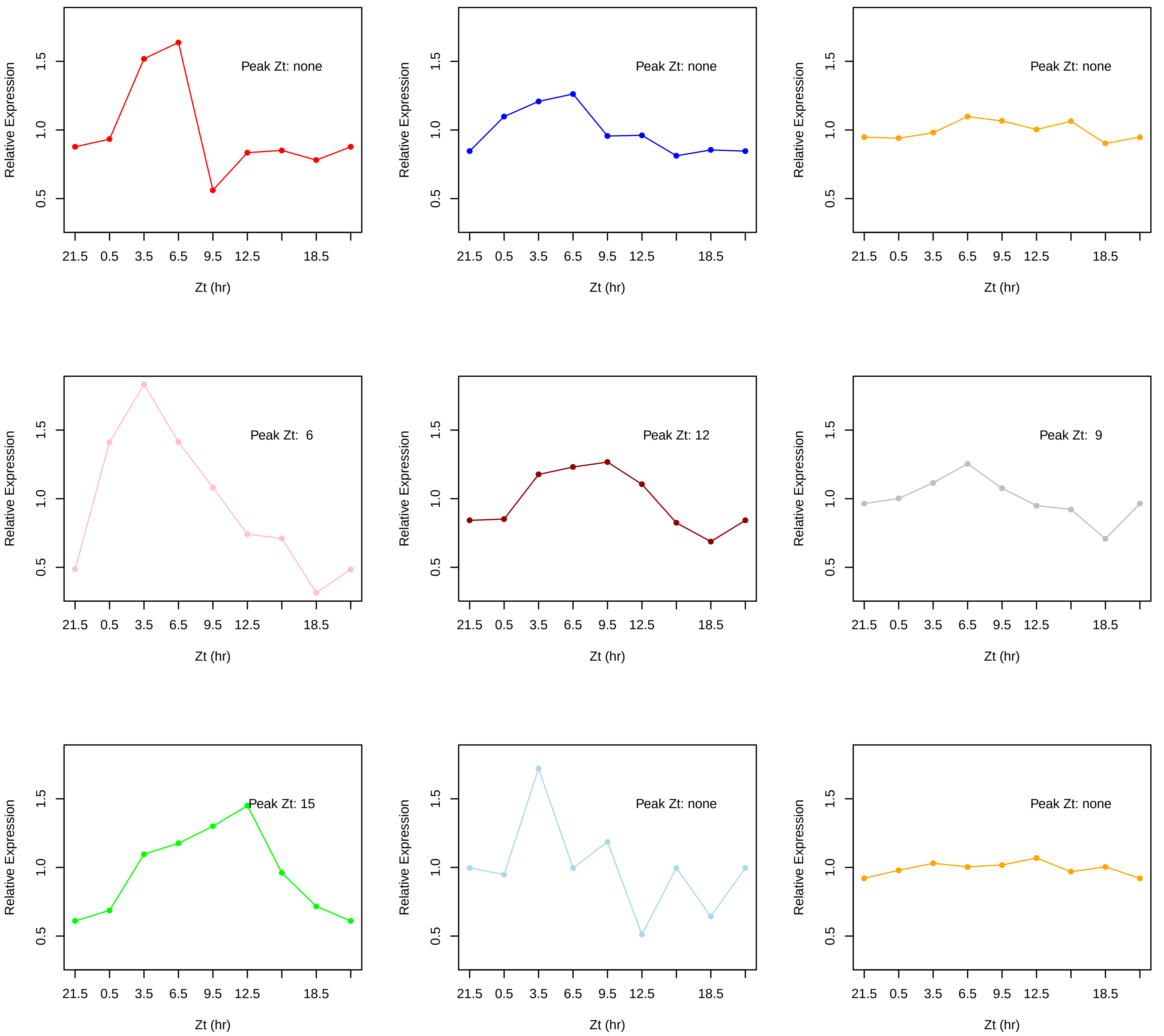

Singeleton: ENSXMAG00000023955 rab27a

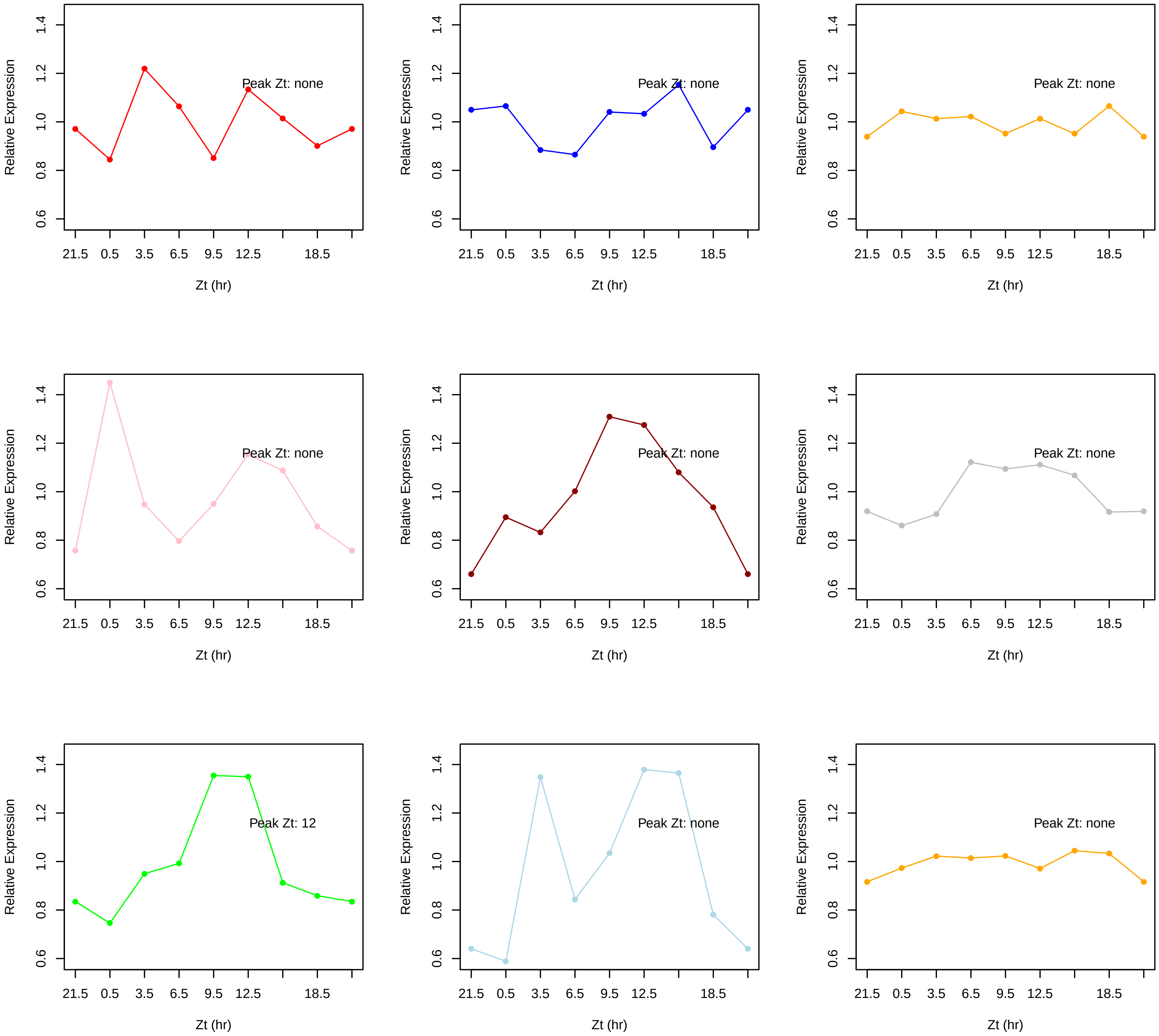

Singeleton: ENSXMAG00000012065 slc12a9

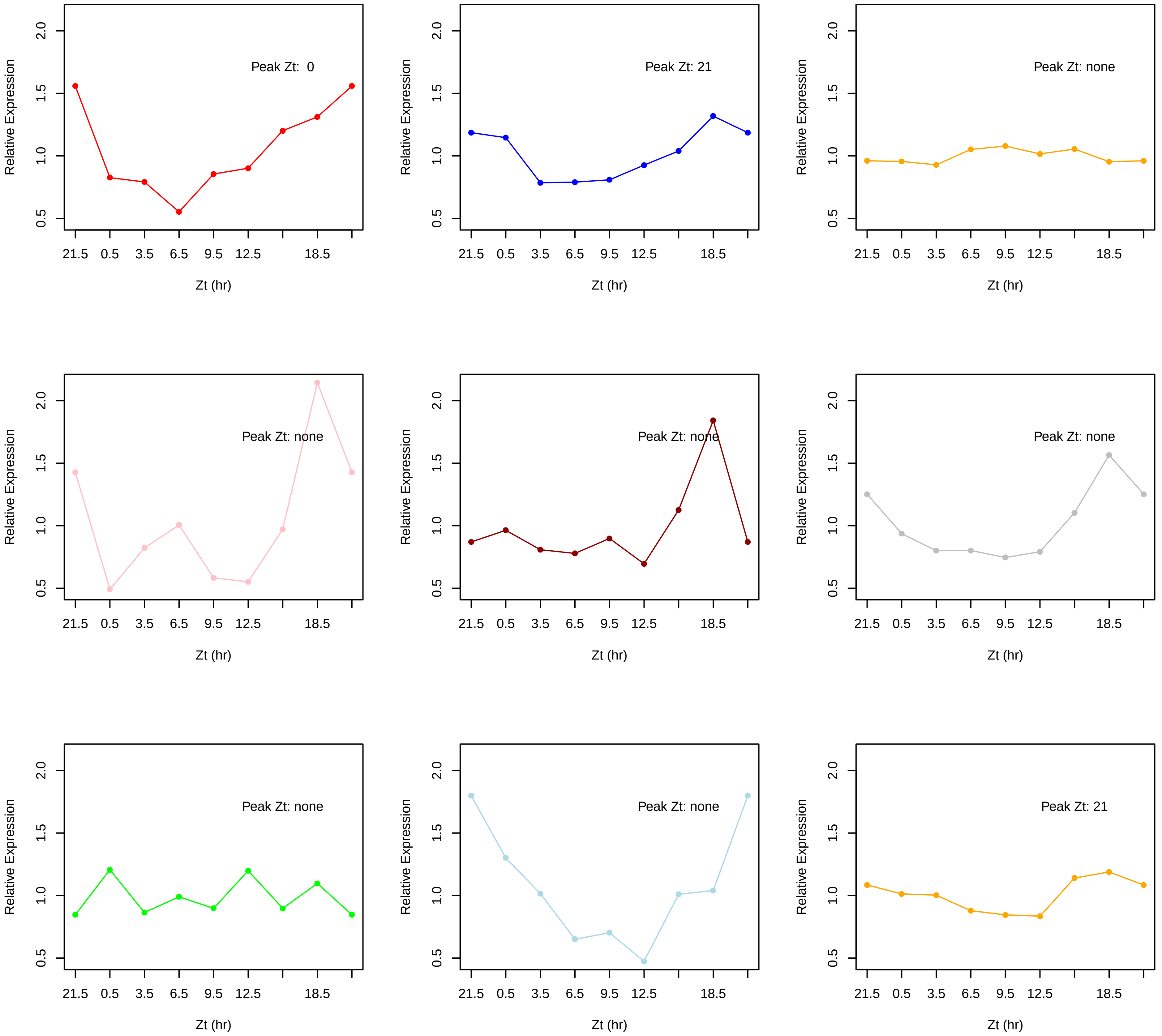

Singleton: ENSXMAG00000014257 PIGB

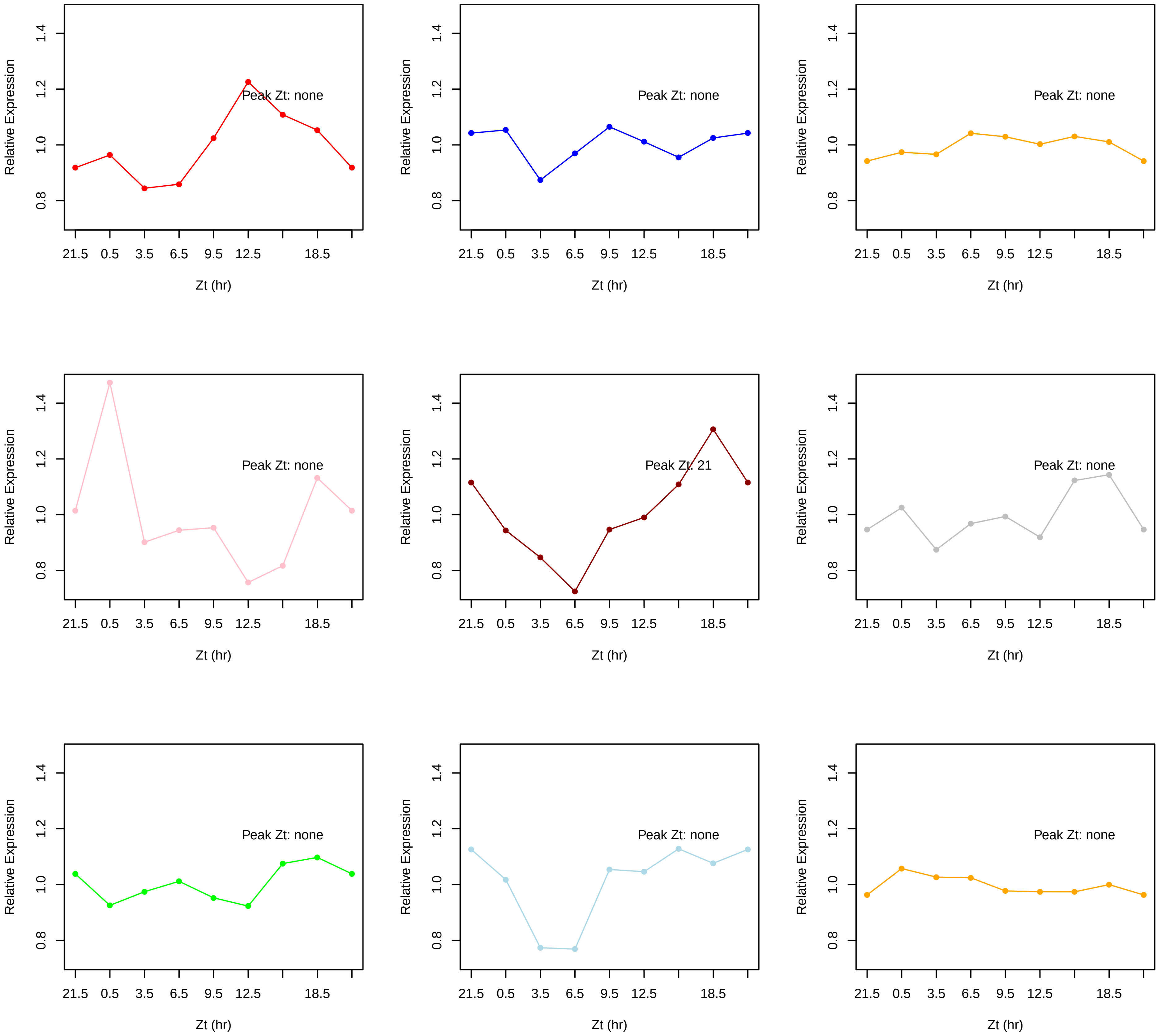

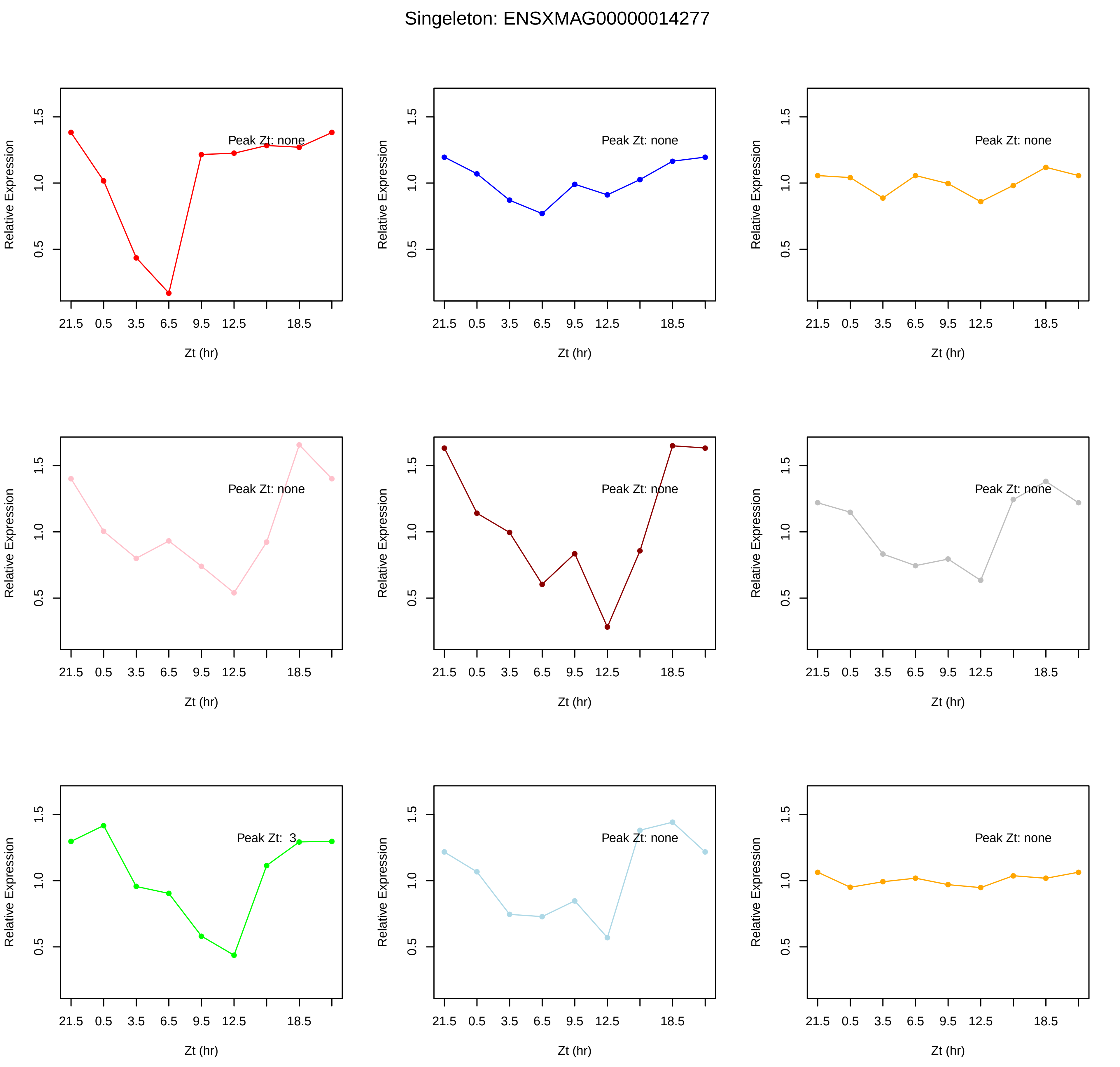
Singeleton: ENSXMAG00000014277
Peak Zt: none
Peak Zt: none
Peak Zt: none
Peak Zt: none
Peak Zt: none
Peak Zt: none
Peak Zt: 3
Peak Zt: none
Peak Zt: none
Relative Expression
Zt (hr)
21.5 0.5 3.5 6.5 9.5 12.5 18.5
0.5 1.0 1.5

Singeleton: ENSXMAG00000010011 g3bp2

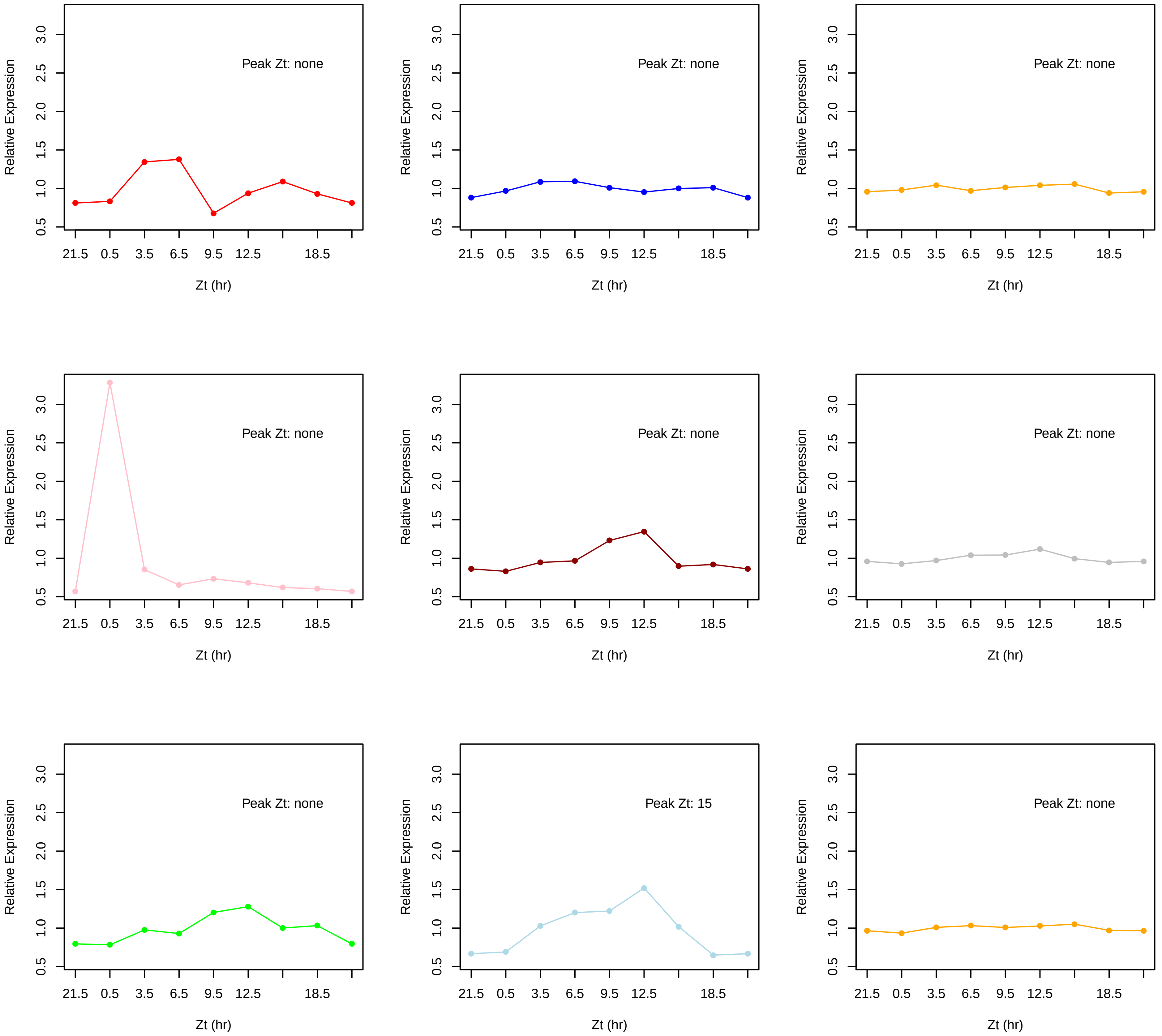

Singeleton: ENSXMAG00000014917

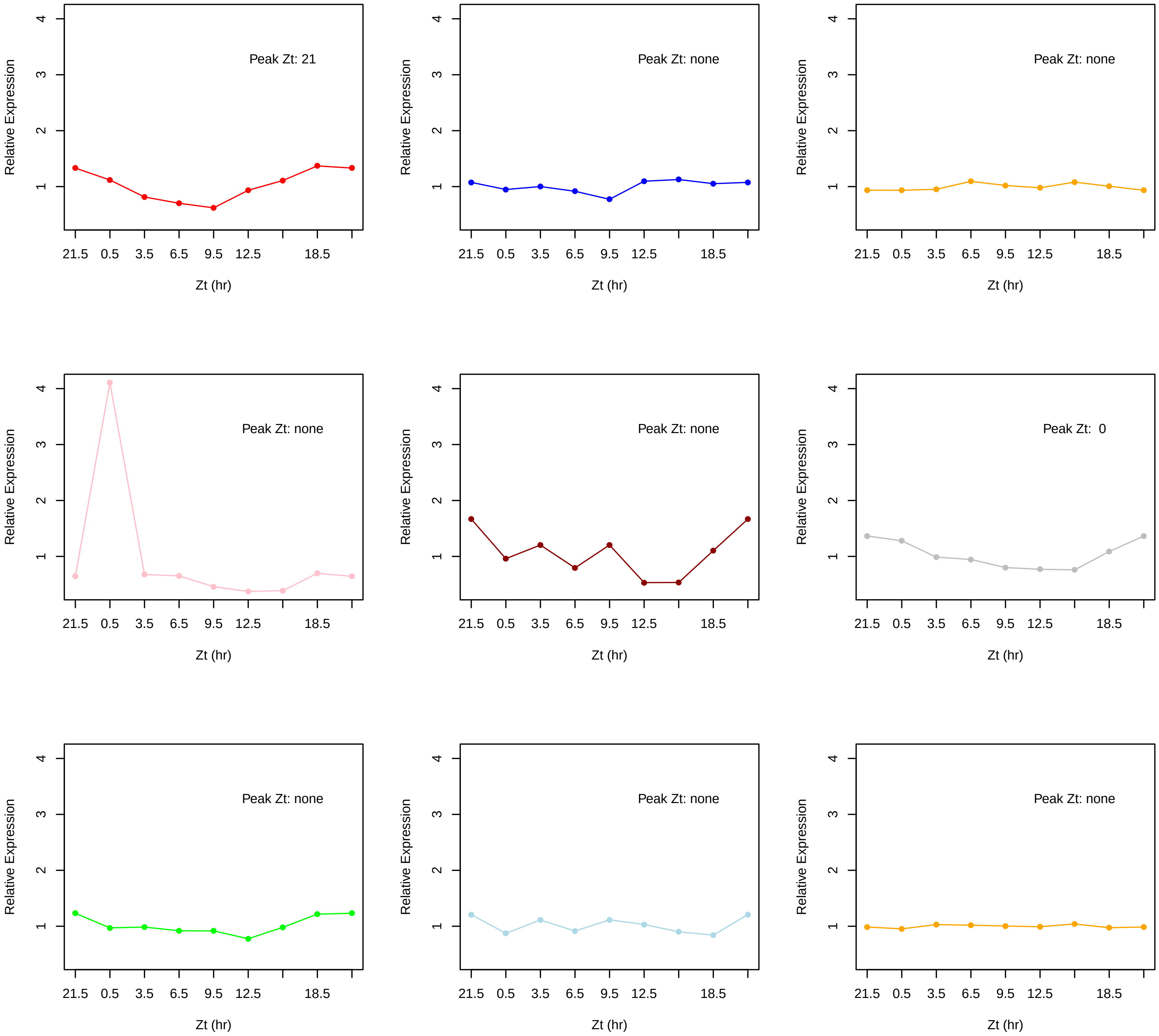

Singeleton: ENSXMAG00000023566 pex2

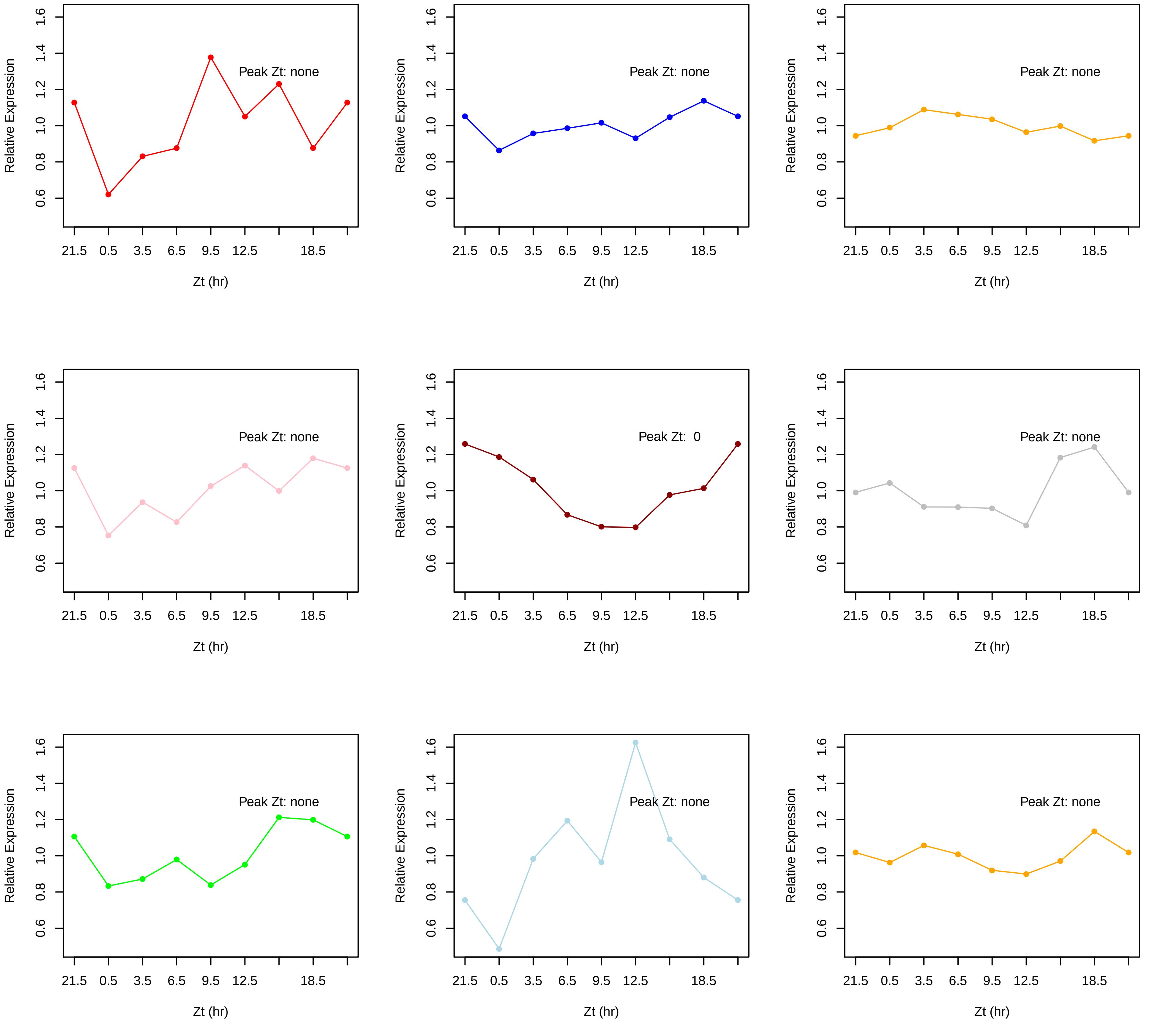

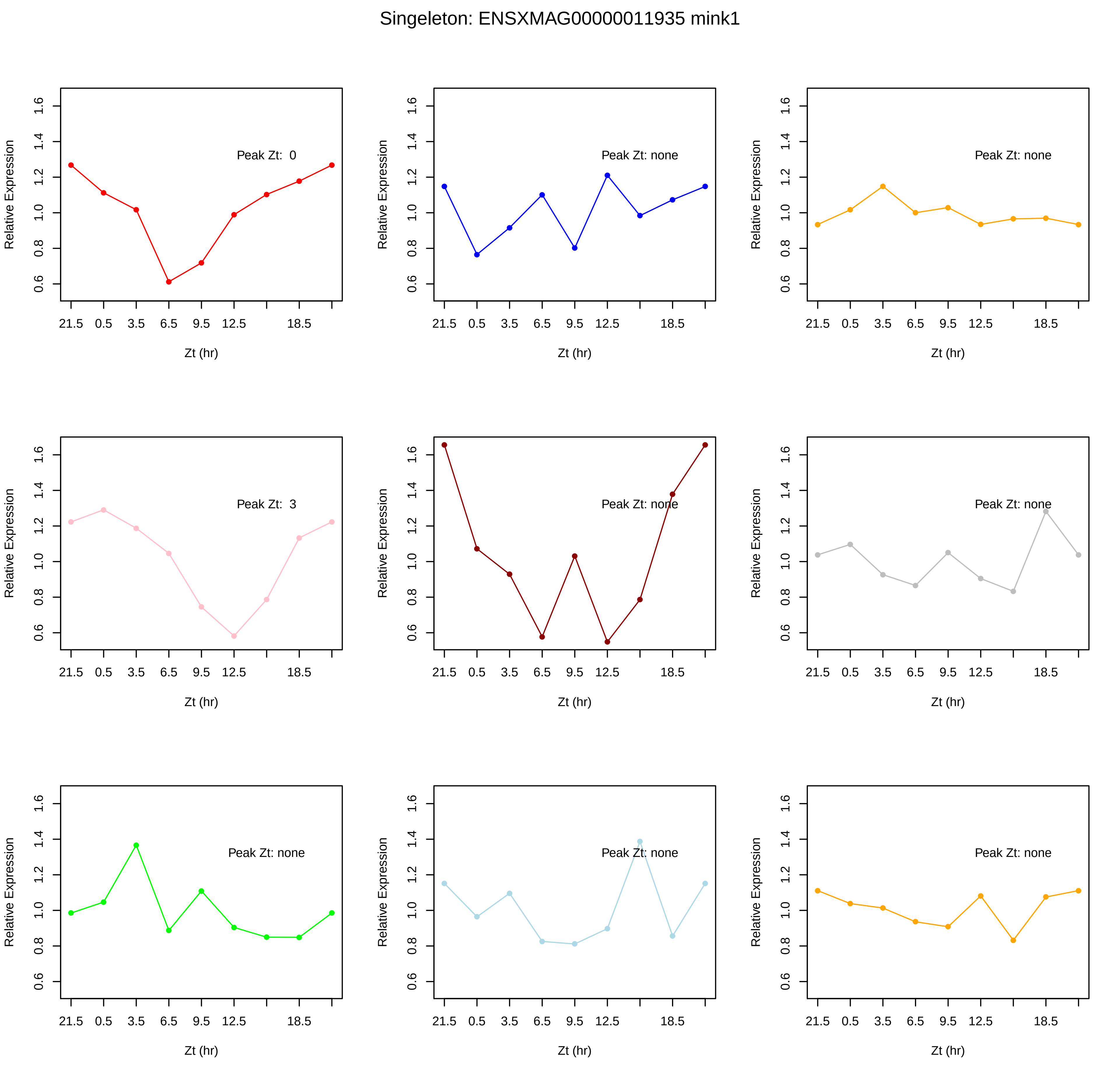
Singleton: ENSXMAG00000011935 mink1
Peak Zt: 0
Peak Zt: none
Peak Zt: none
Peak Zt: 3
Peak Zt: none
Peak Zt: none
Peak Zt: none
Peak Zt: none
Peak Zt: none
Relative Expression
Zt (hr)
21.5 0.5 3.5 6.5 9.5 12.5 18.5
0.6 0.8 1.0 1.2 1.4 1.6

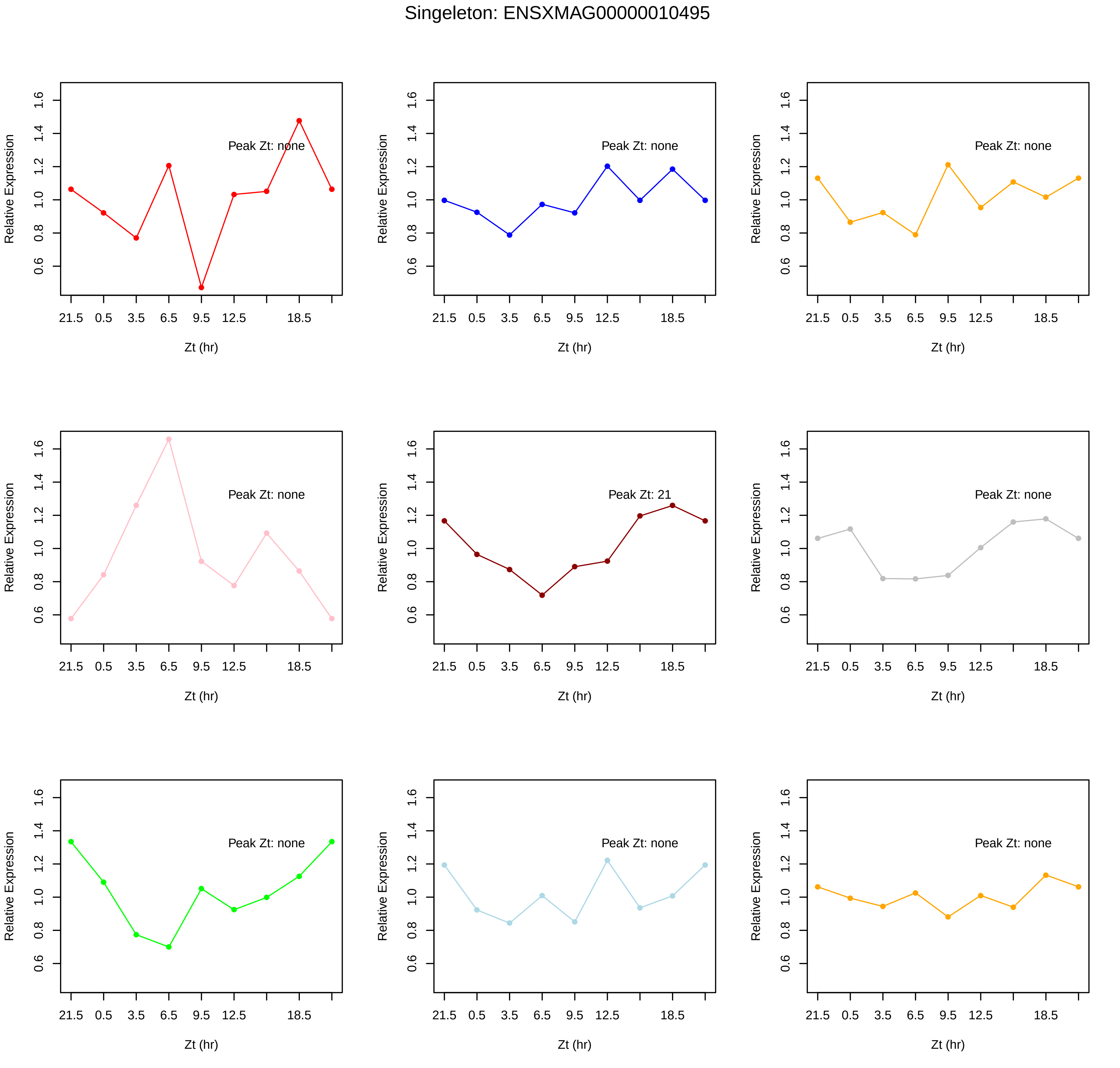
Singleton: ENSXMAG00000010495
Peak Zt: none
Peak Zt: none
Peak Zt: none
Peak Zt: none
Peak Zt: 21
Peak Zt: none
Peak Zt: none
Peak Zt: none
Peak Zt: none
Relative Expression
Zt (hr)
21.5
0.5
3.5
6.5
9.5
12.5
18.5
0.6
0.8
1.0
1.2
1.4
1.6

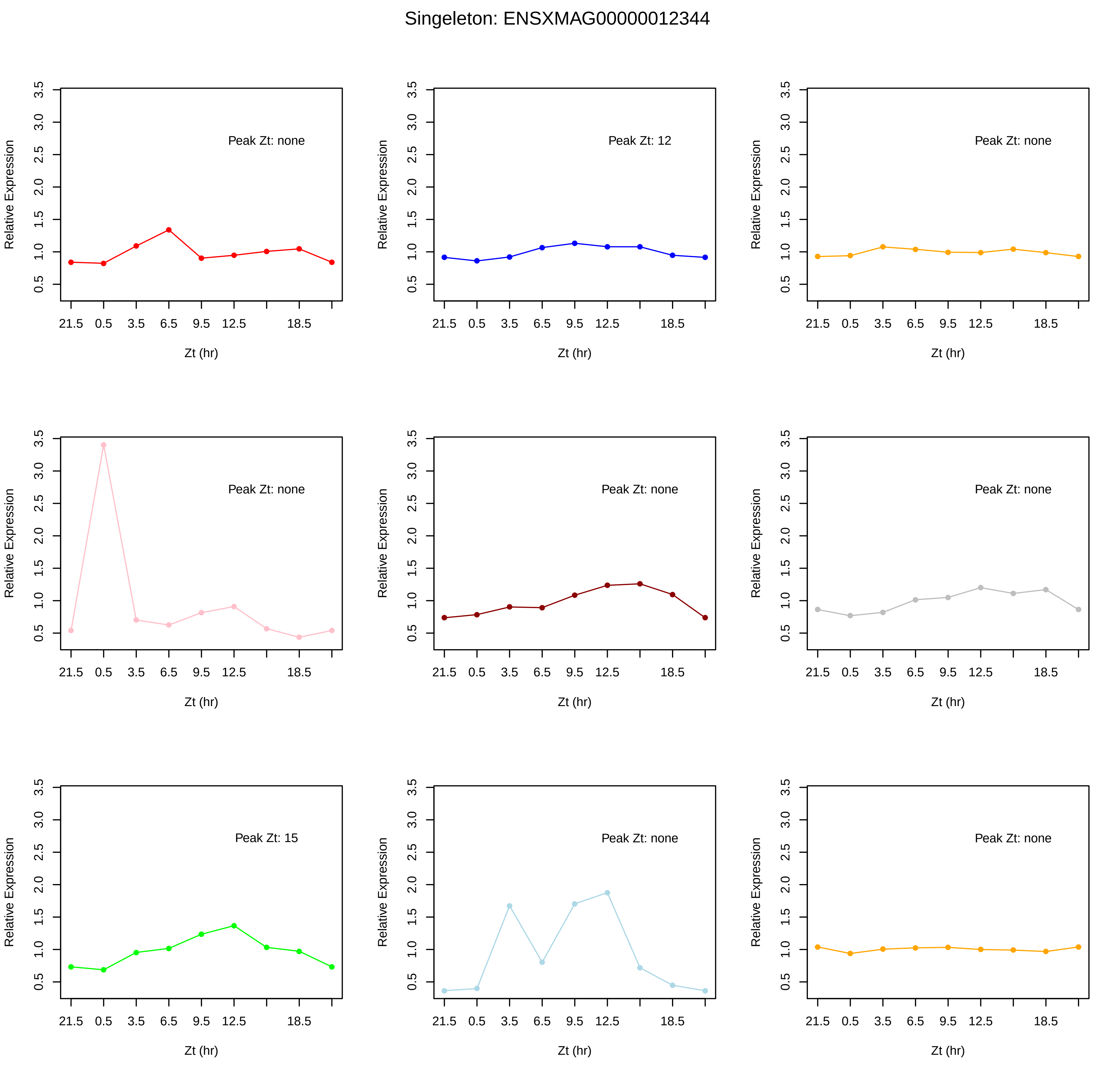
Singeleton: ENSXMAG00000012344
Peak Zt: none
Peak Zt: 12
Peak Zt: none
Peak Zt: none
Peak Zt: none
Peak Zt: none
Peak Zt: 15
Peak Zt: none
Peak Zt: none
Relative Expression
Zt (hr)
21.5
0.5
3.5
6.5
9.5
12.5
18.5
0.5
1.0
1.5
2.0
2.5
3.0
3.5

Singleton: ENSXMAG00000002829 ube2z

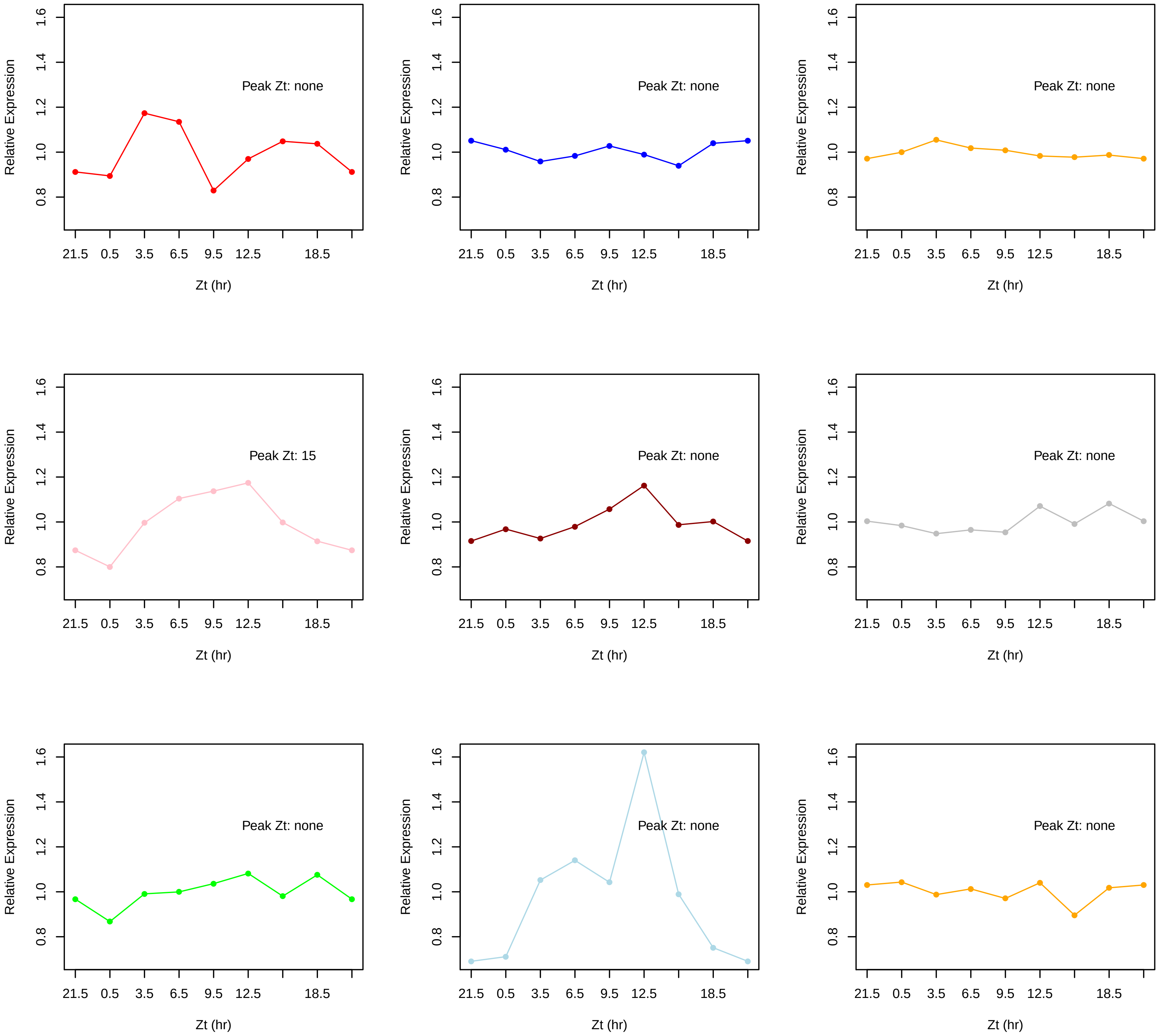

Singeleton: ENSXMAG00000012788 zc2hc1a

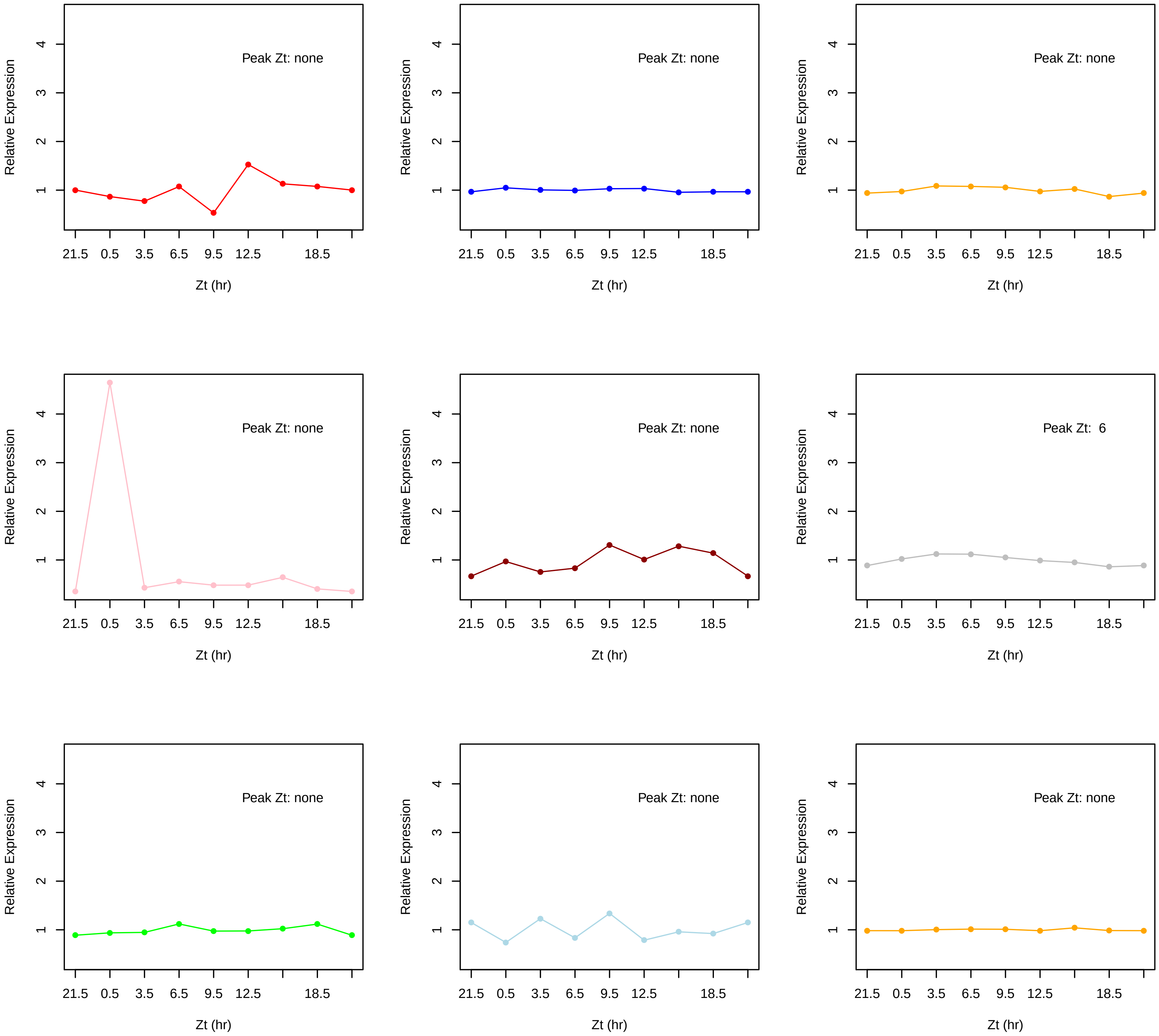

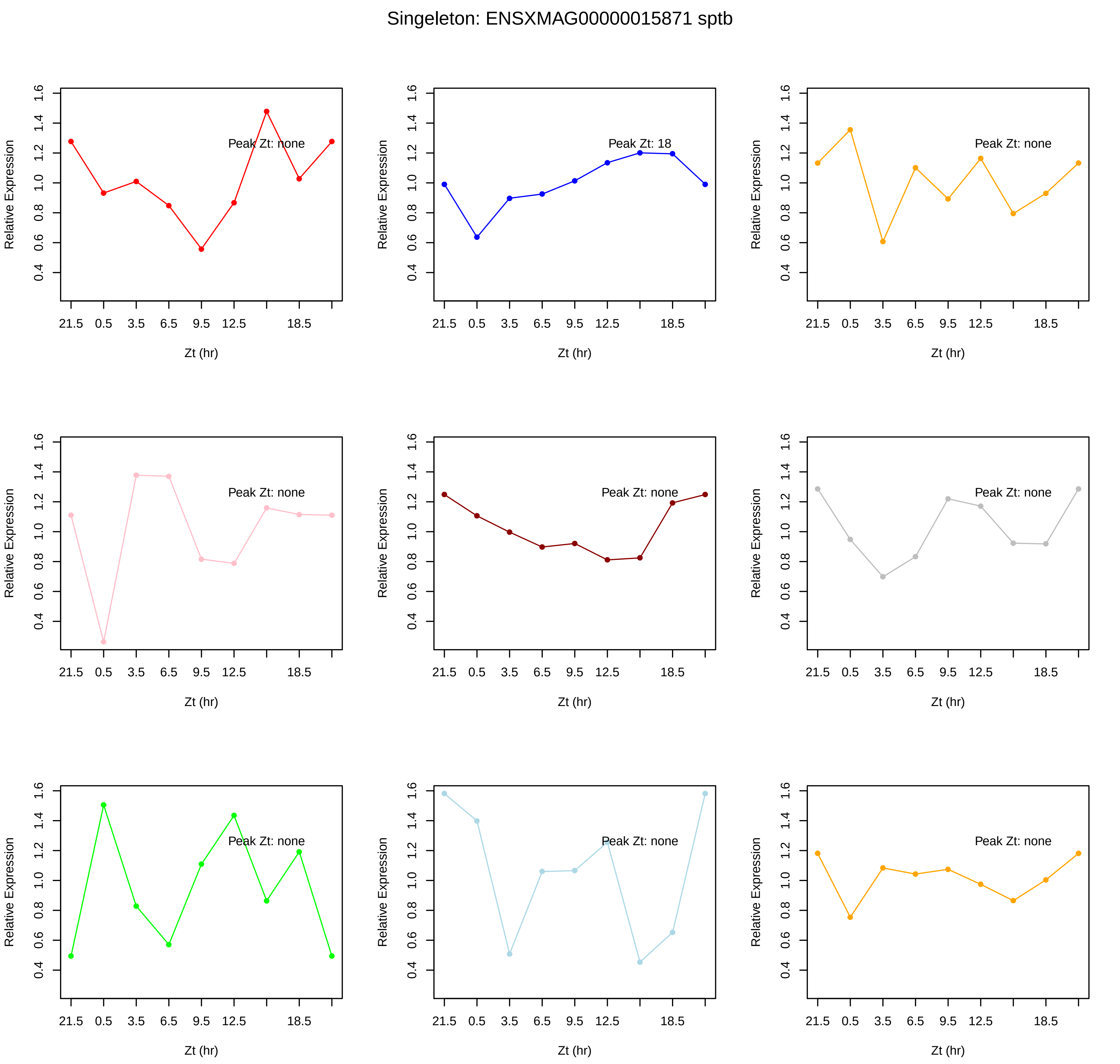
Singeleton: ENSXMAG00000015871 sptb
Peak Zt: none
Peak Zt: 18
Peak Zt: none
Peak Zt: none
Peak Zt: none
Peak Zt: none
Peak Zt: none
Peak Zt: none
Peak Zt: none
Relative Expression
Zt (hr)
21.5
0.5
3.5
6.5
9.5
12.5
18.5
0.4
0.6
0.8
1.0
1.2
1.4
1.6

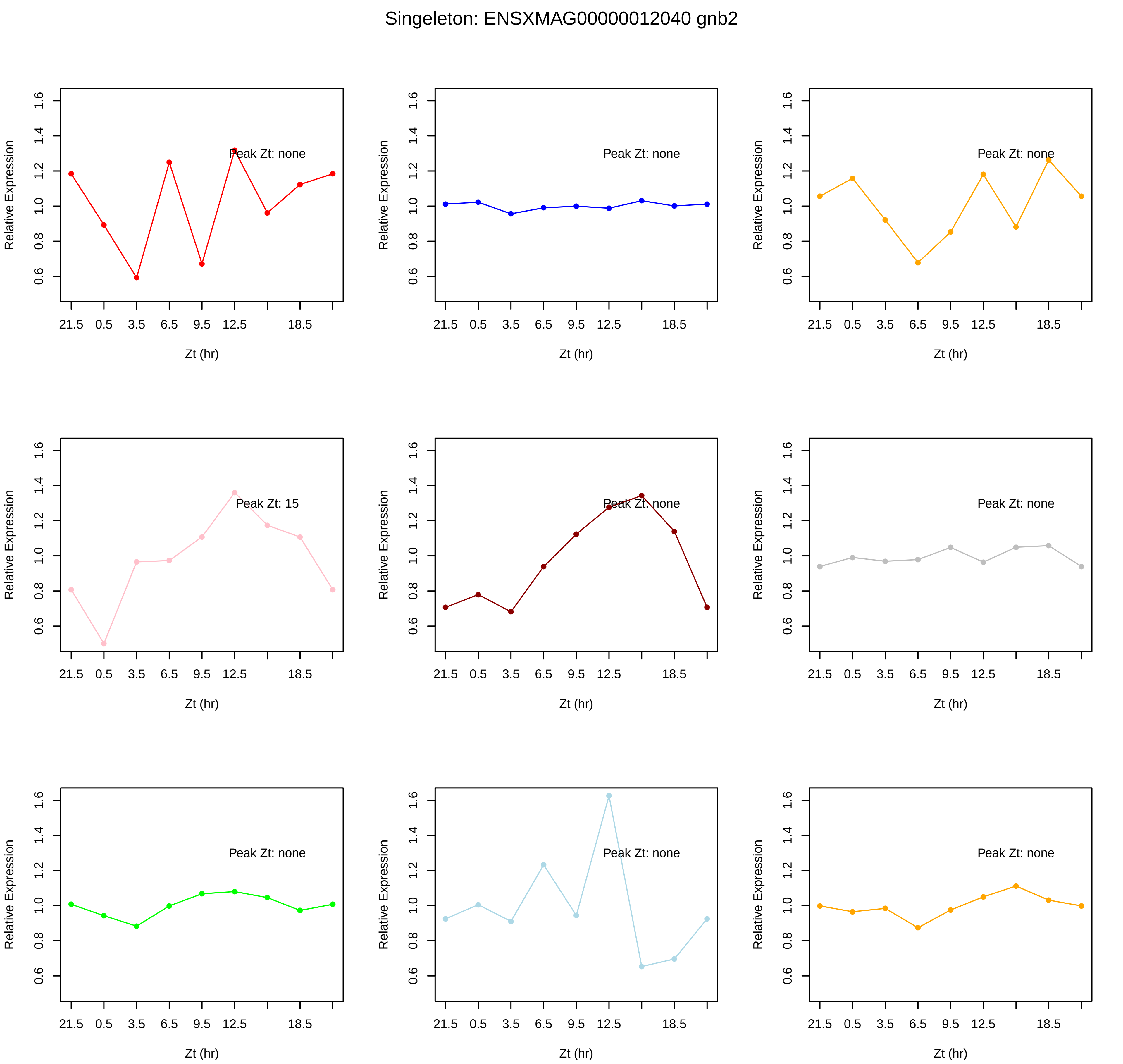
Singeleton: ENSXMAG00000012040 gnb2
Peak Zt: none
Peak Zt: none
Peak Zt: none
Peak Zt: 15
Peak Zt: none
Peak Zt: none
Peak Zt: none
Peak Zt: none
Peak Zt: none
Relative Expression
Zt (hr)

Singleton: ENSXMAG00000014902 msl1b

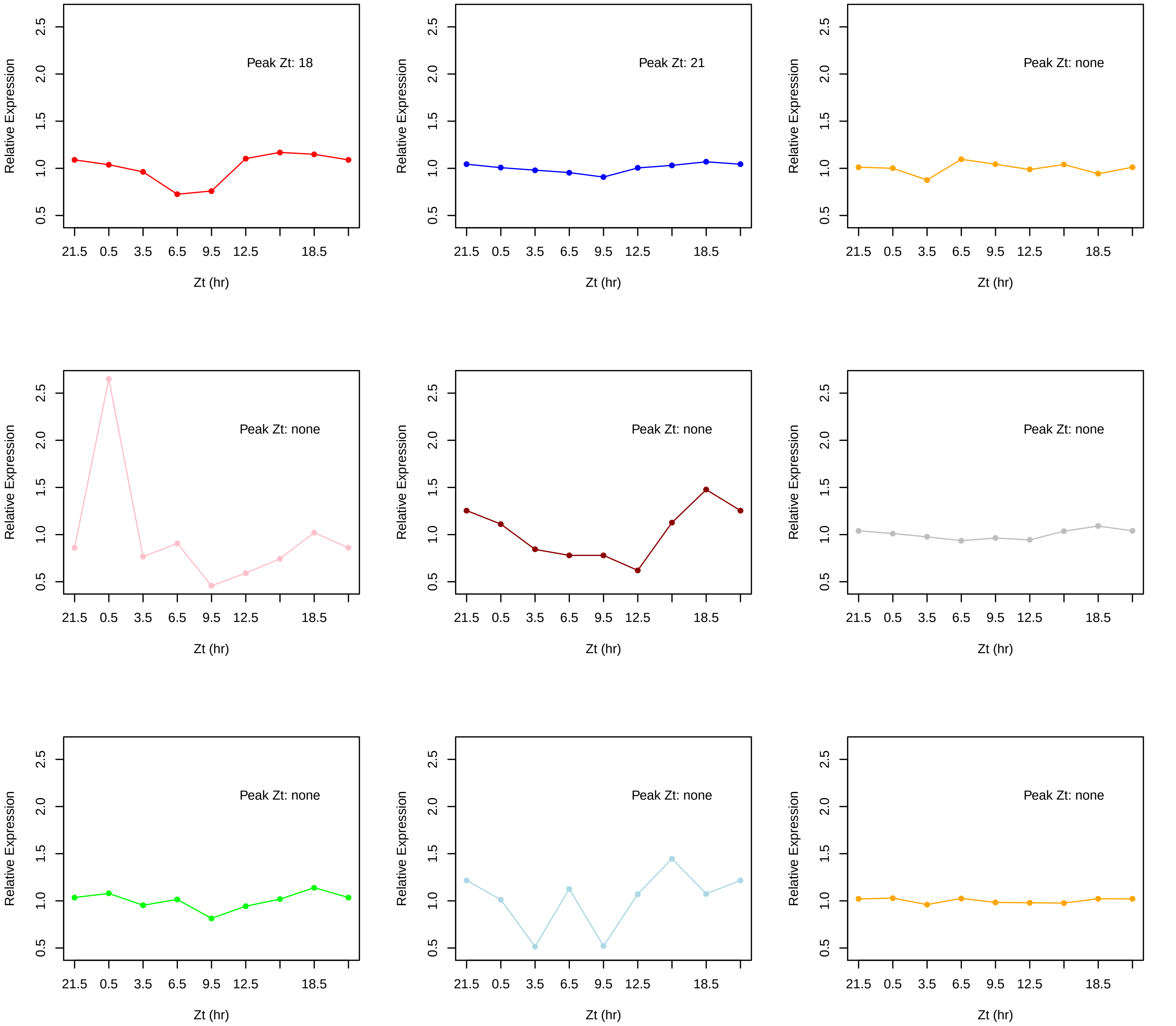

Singleton: ENSXMAG00000006101 srp68

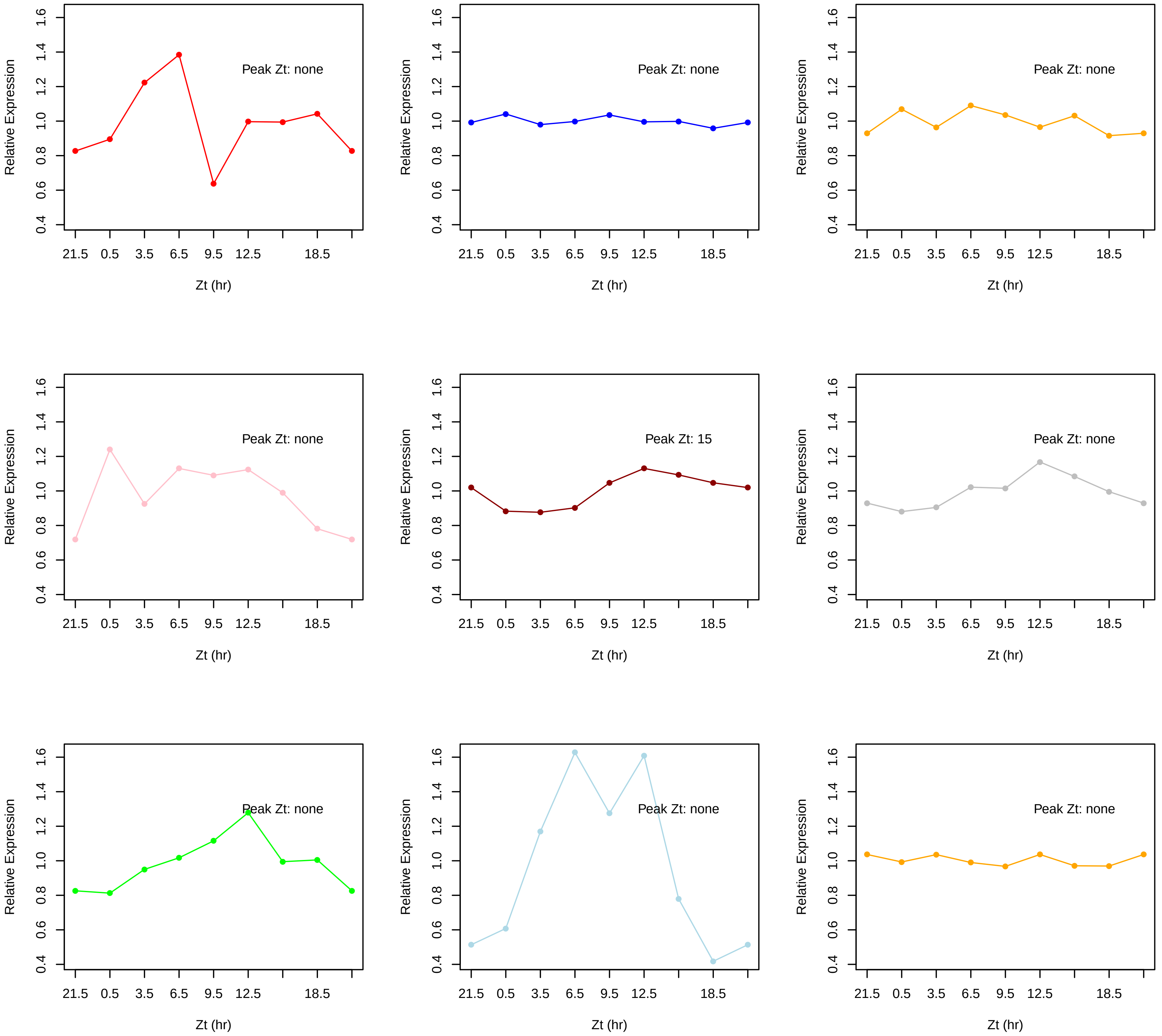

Singeleton: ENSXMAG00000000794 ankrd33ba

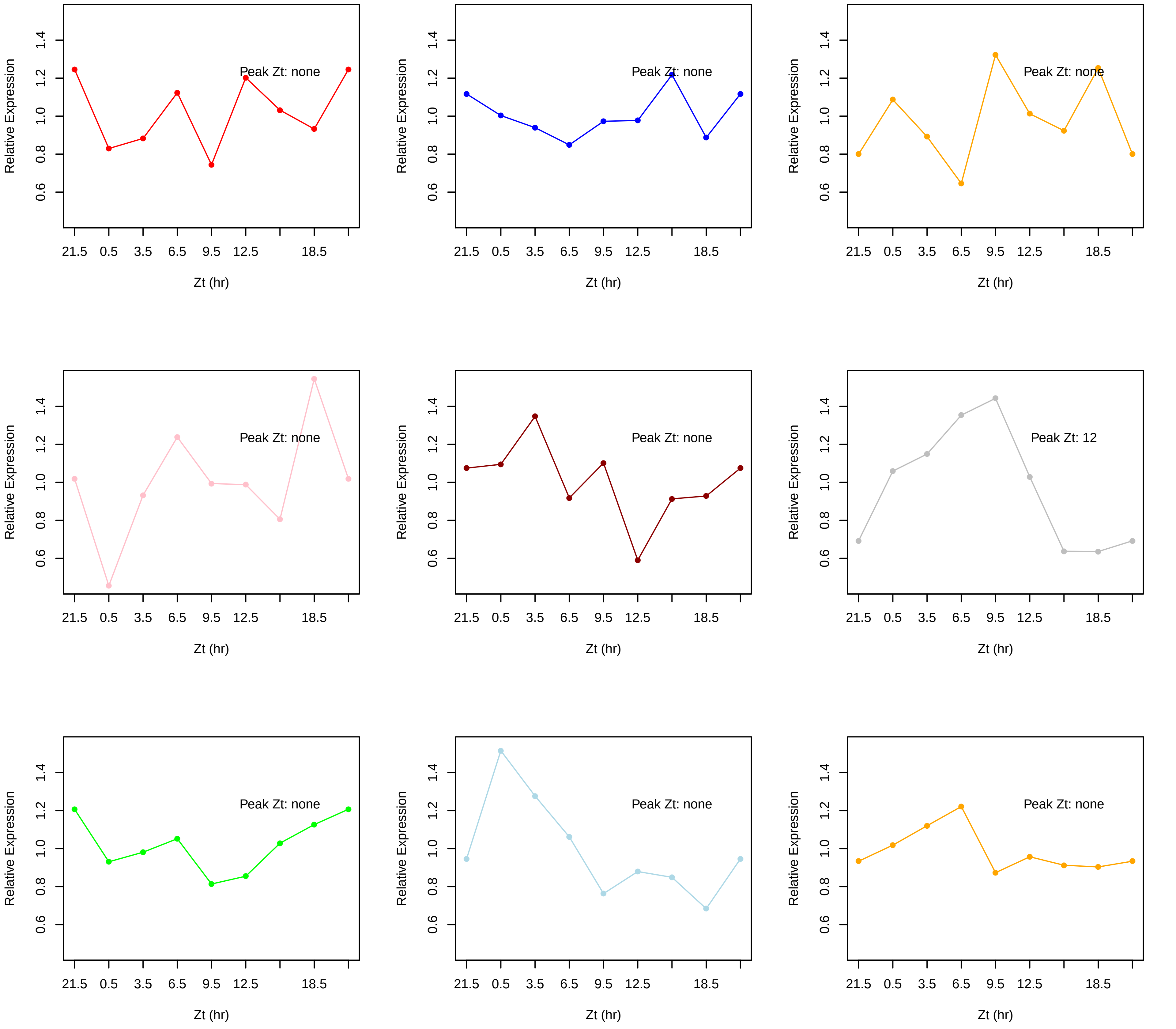

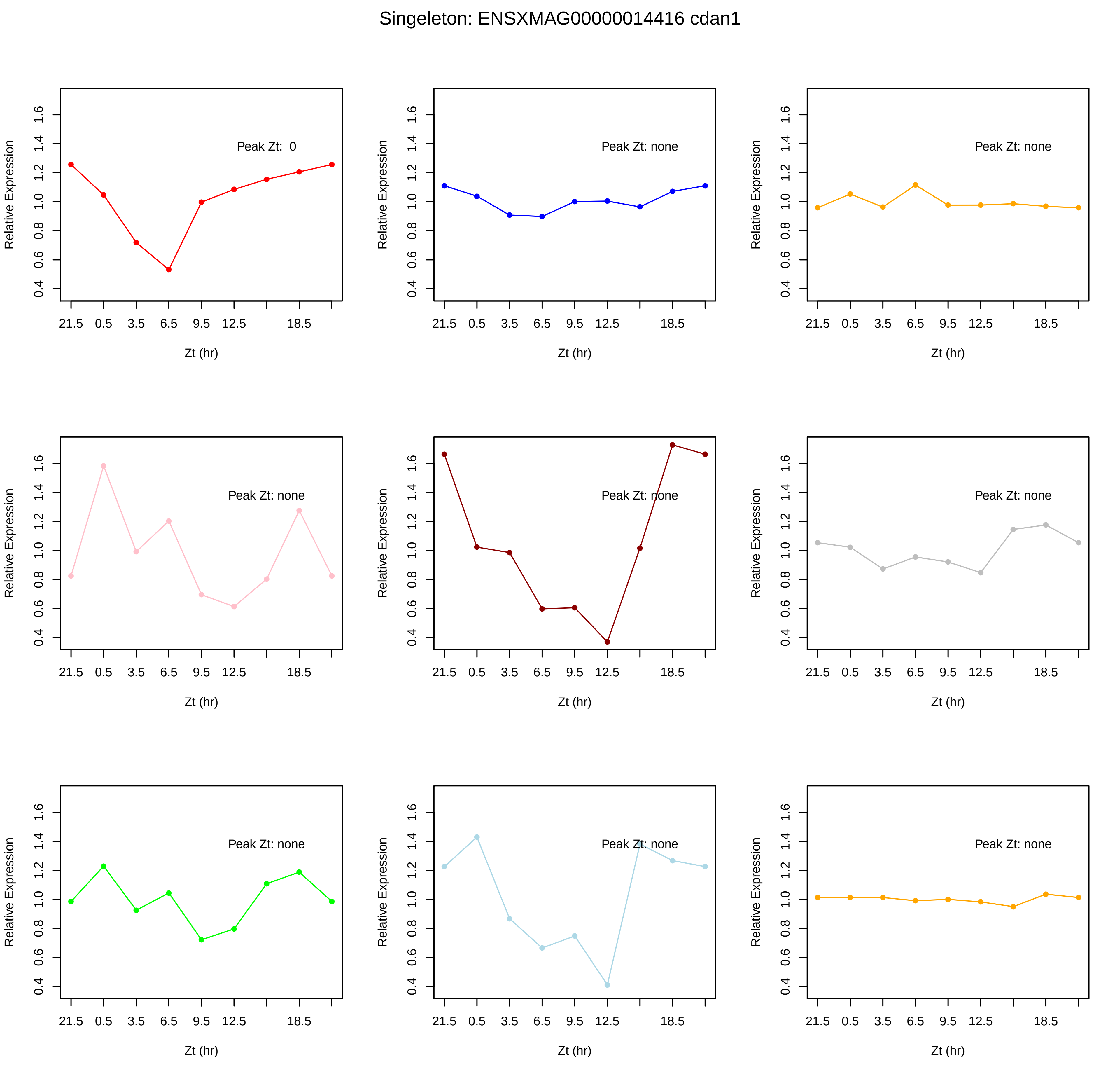

Singleton: ENSXMAG00000014416 cdan1
Peak Zt: 0
Peak Zt: none
Relative Expression
Zt (hr)
21.5
0.5
3.5
6.5
9.5
12.5
18.5
0.4
0.6
0.8
1.0
1.2
1.4
1.6

Singleton: ENSXMAG00000013937 casc3

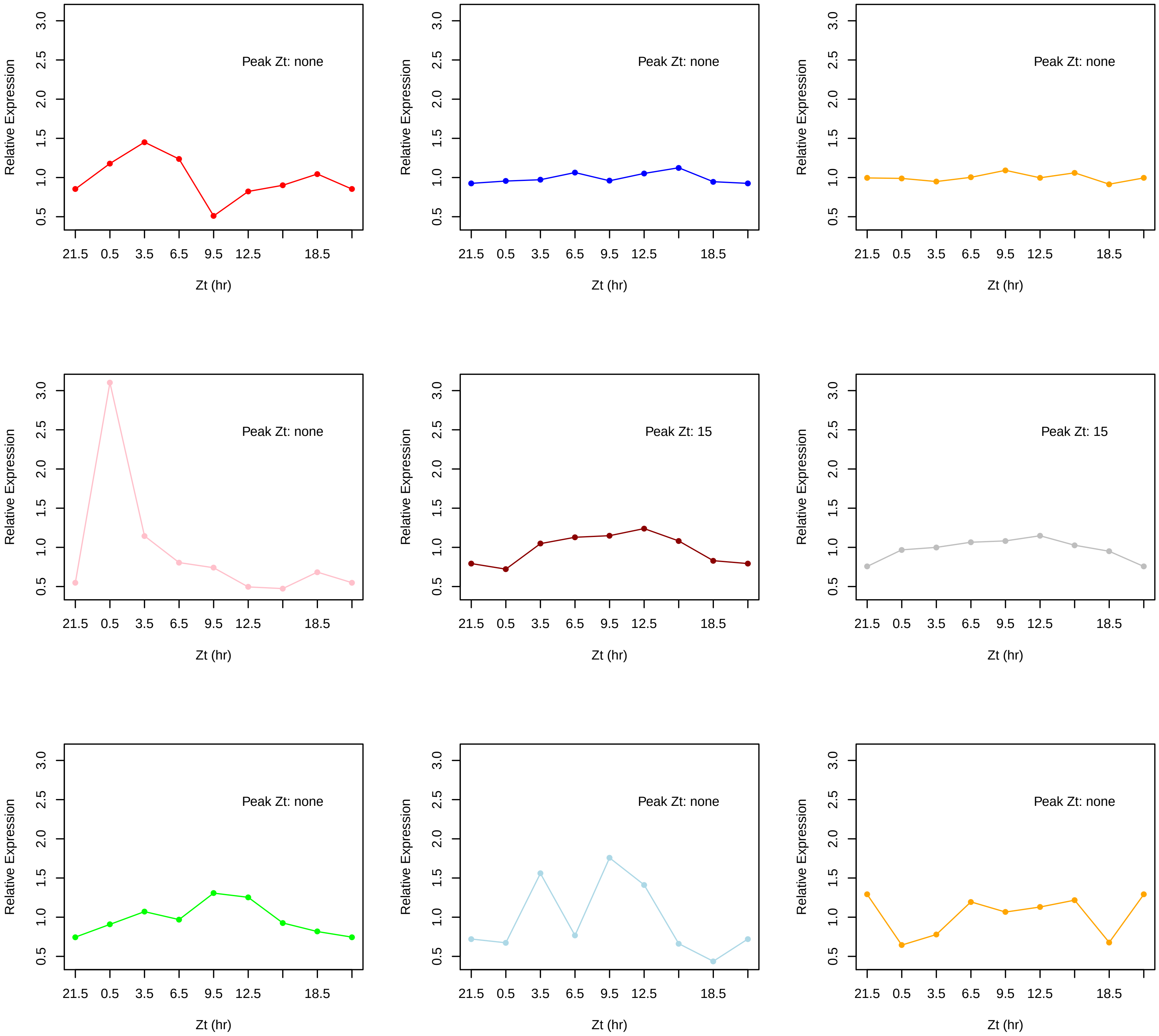

Singeleton: ENSXMAG00000023931 rapgefl1

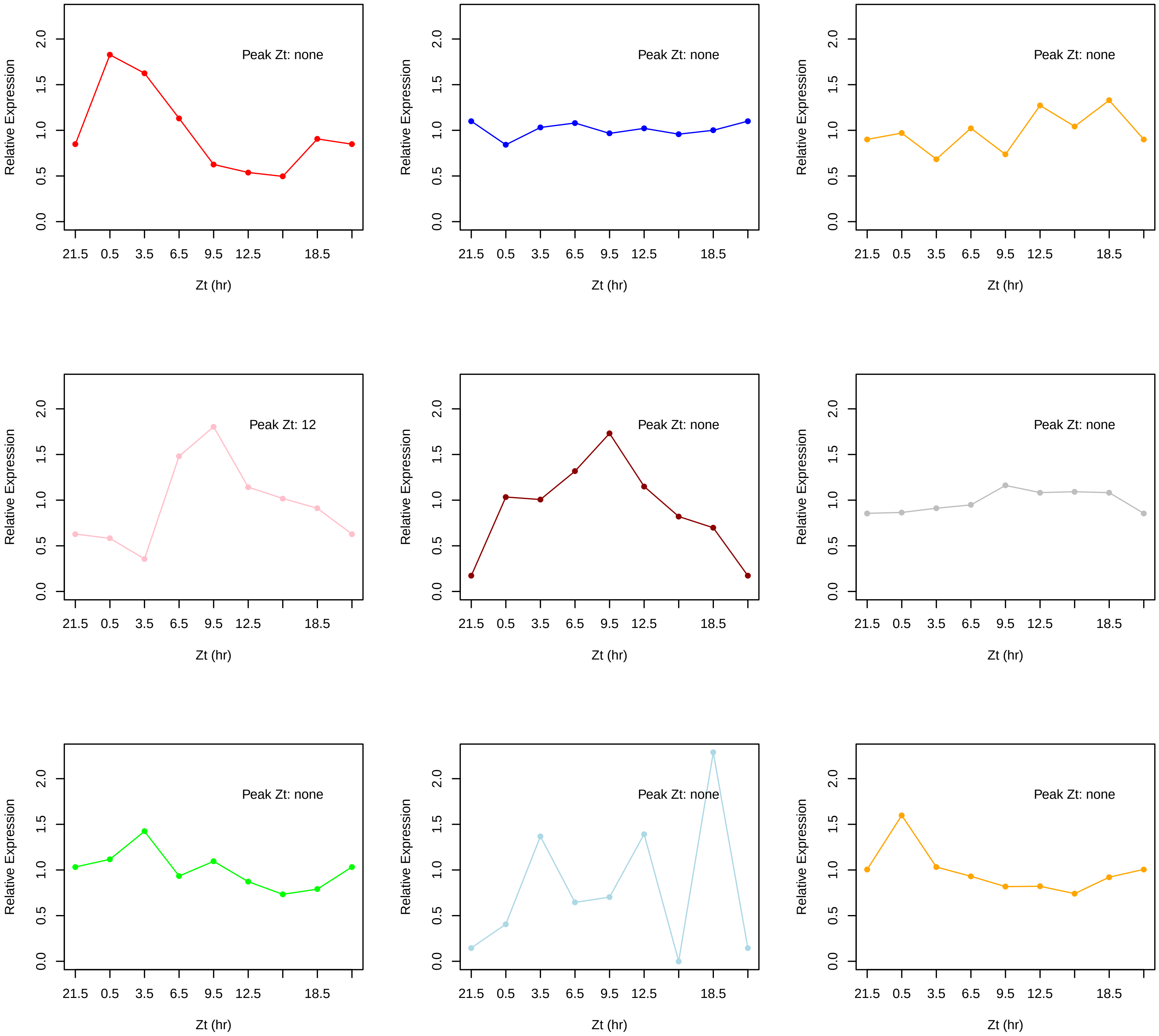

Singeleton: ENSXMAG00000026823 tsc22d2

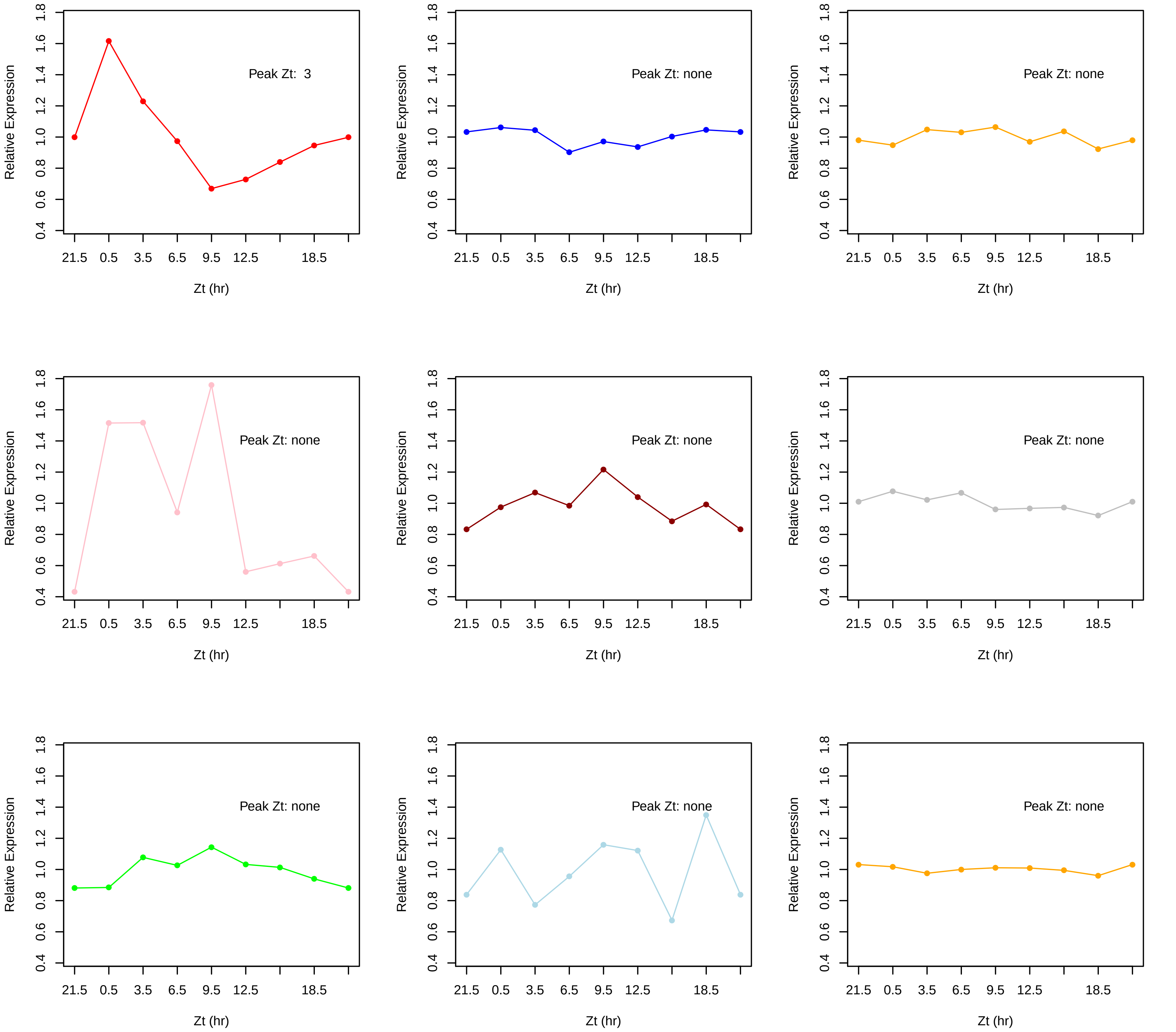

Singeleton: ENSXMAG00000006121 polg2

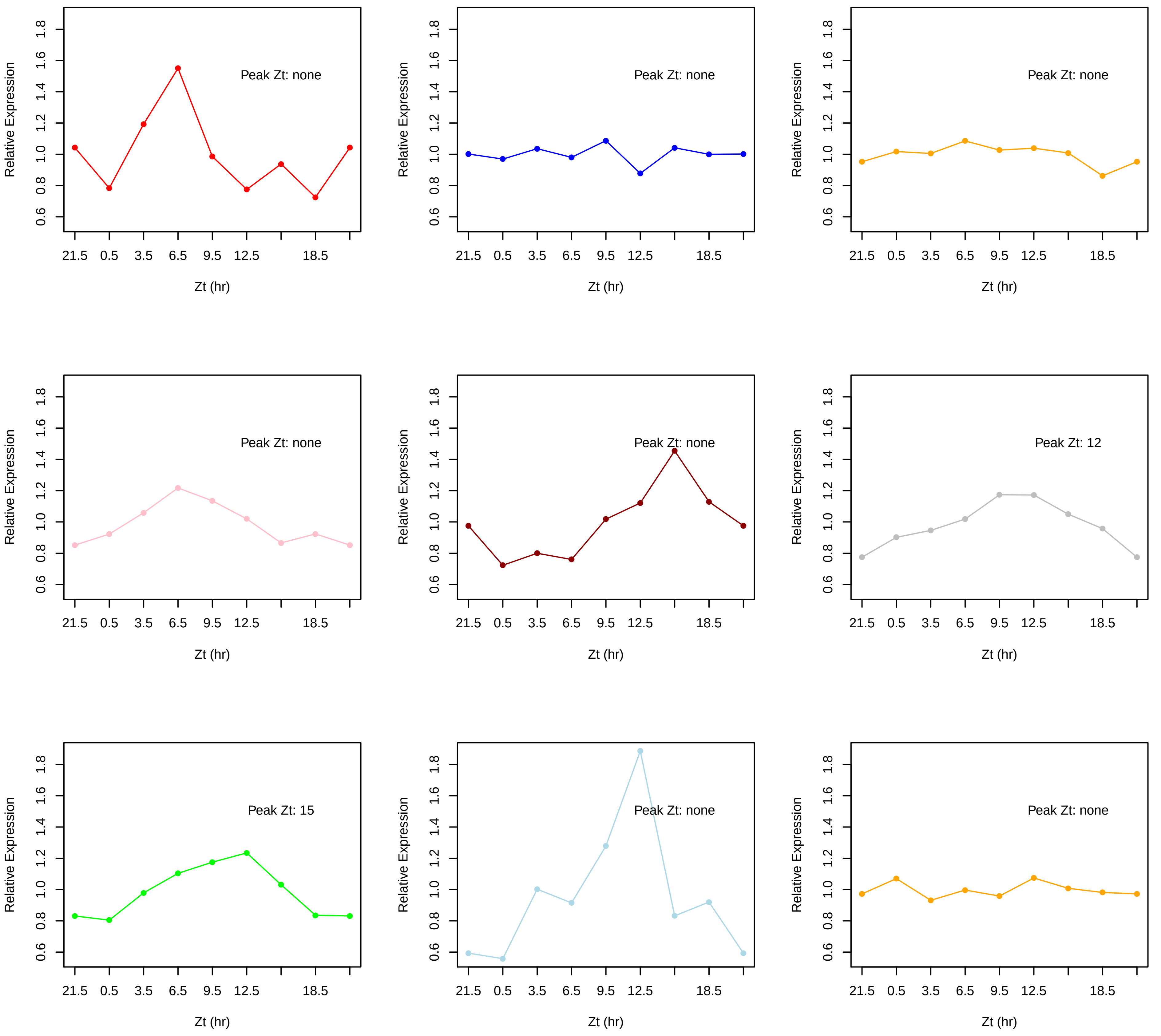

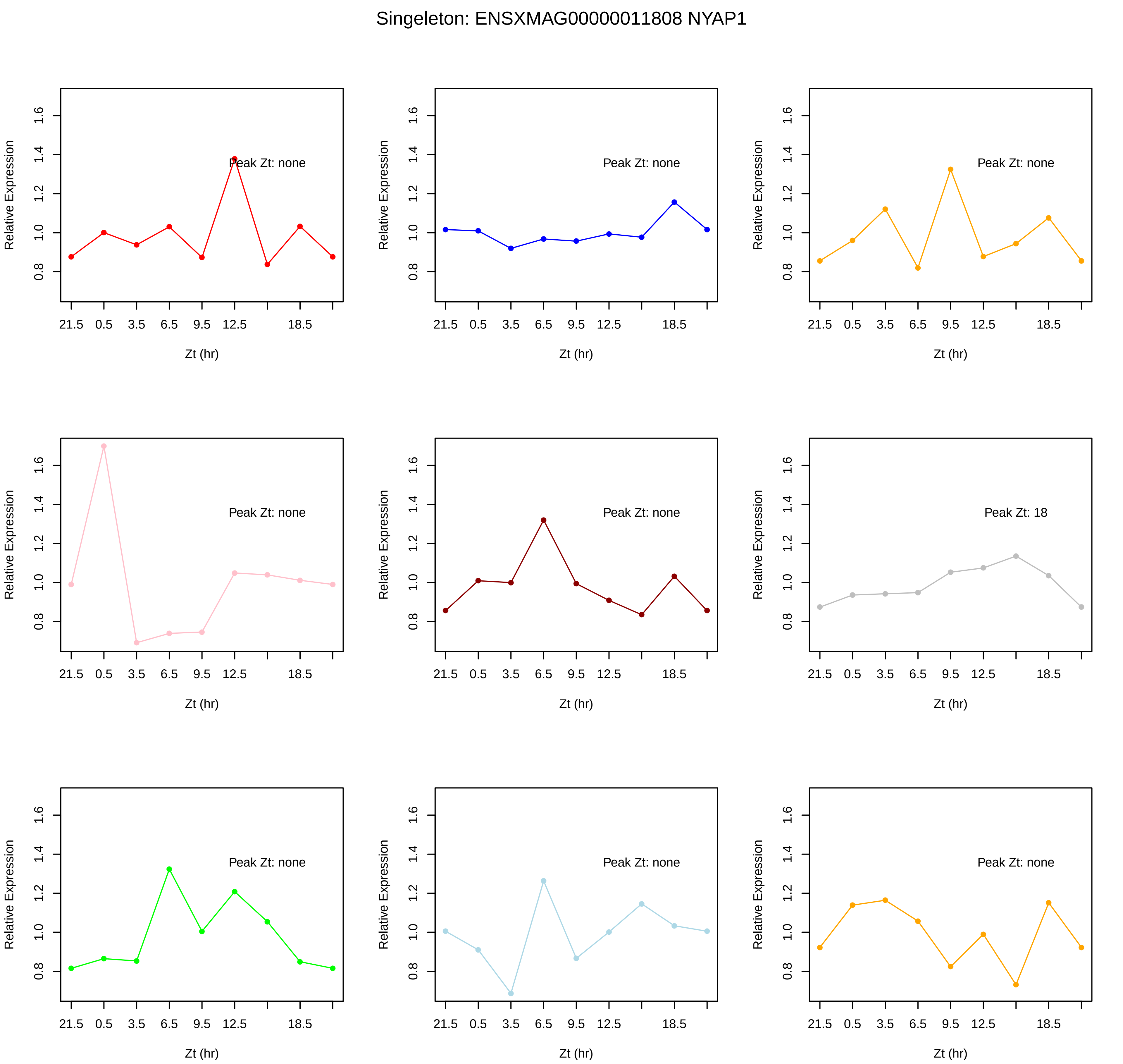

Singeleton: ENSXMAG00000011808 NYAP1
Peak Zt: none
Peak Zt: none
Peak Zt: none
Peak Zt: none
Peak Zt: none
Peak Zt: 18
Peak Zt: none
Peak Zt: none
Peak Zt: none
Relative Expression
Zt (hr)
21.5
0.5
3.5
6.5
9.5
12.5
18.5
0.8
1.0
1.2
1.4
1.6

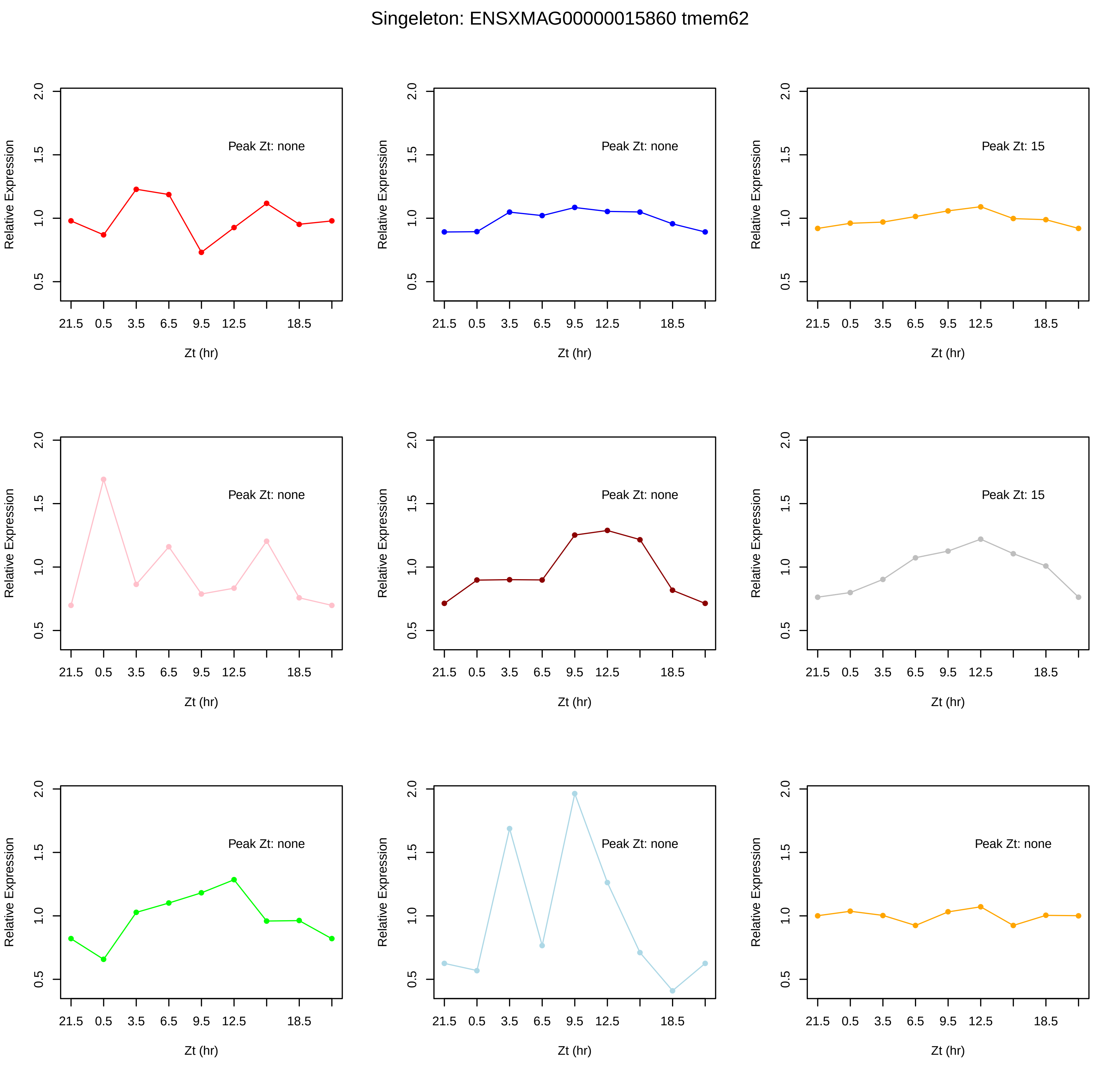
Singeleton: ENSXMAG00000015860 tmem62
Peak Zt: none
Peak Zt: none
Peak Zt: 15
Peak Zt: none
Peak Zt: none
Peak Zt: 15
Peak Zt: none
Peak Zt: none
Peak Zt: none
Relative Expression
Zt (hr)
21.5
0.5
3.5
6.5
9.5
12.5
18.5
0.5
1.0
1.5
2.0

Singeleton: ENSXMAG00000001597

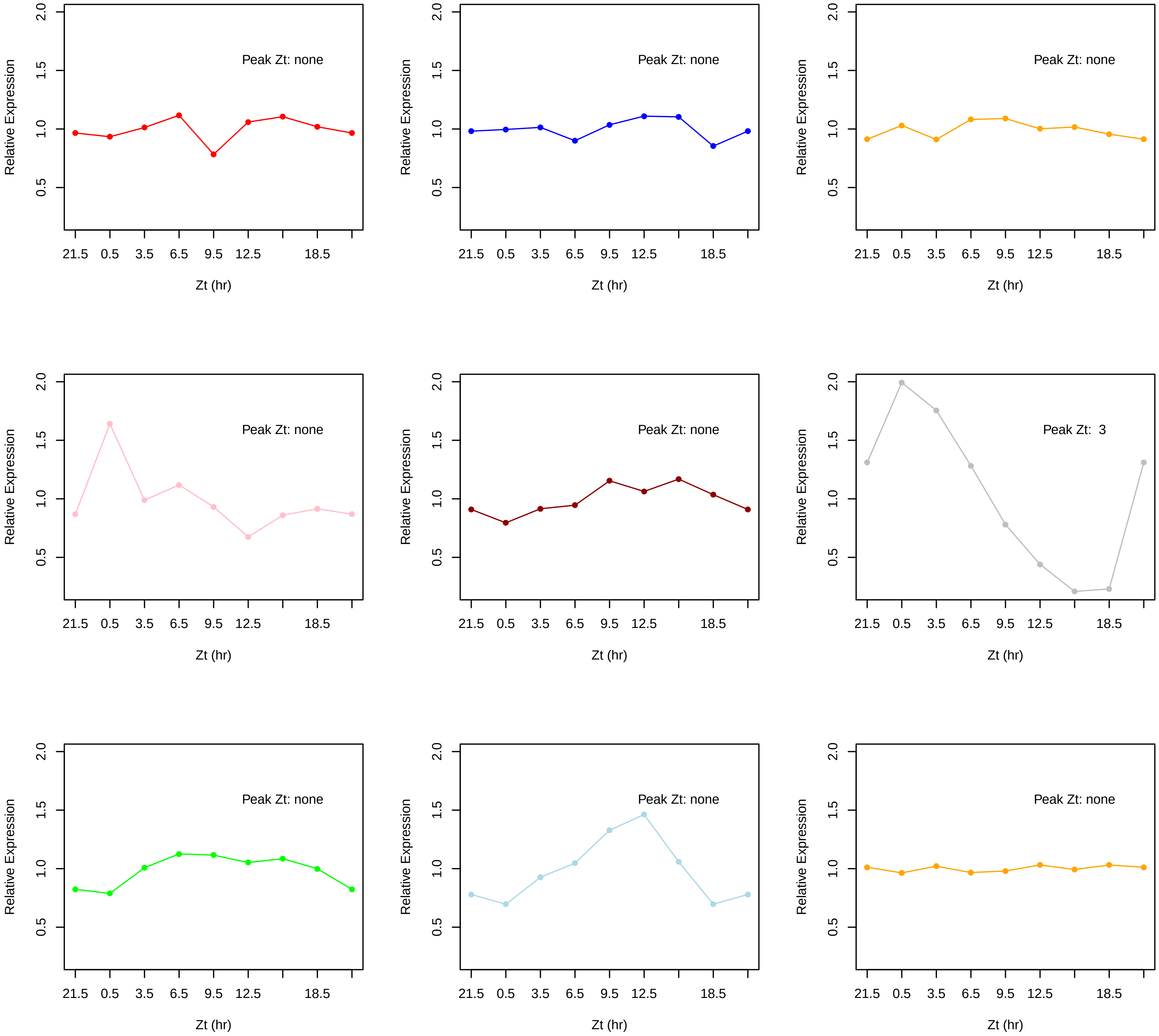

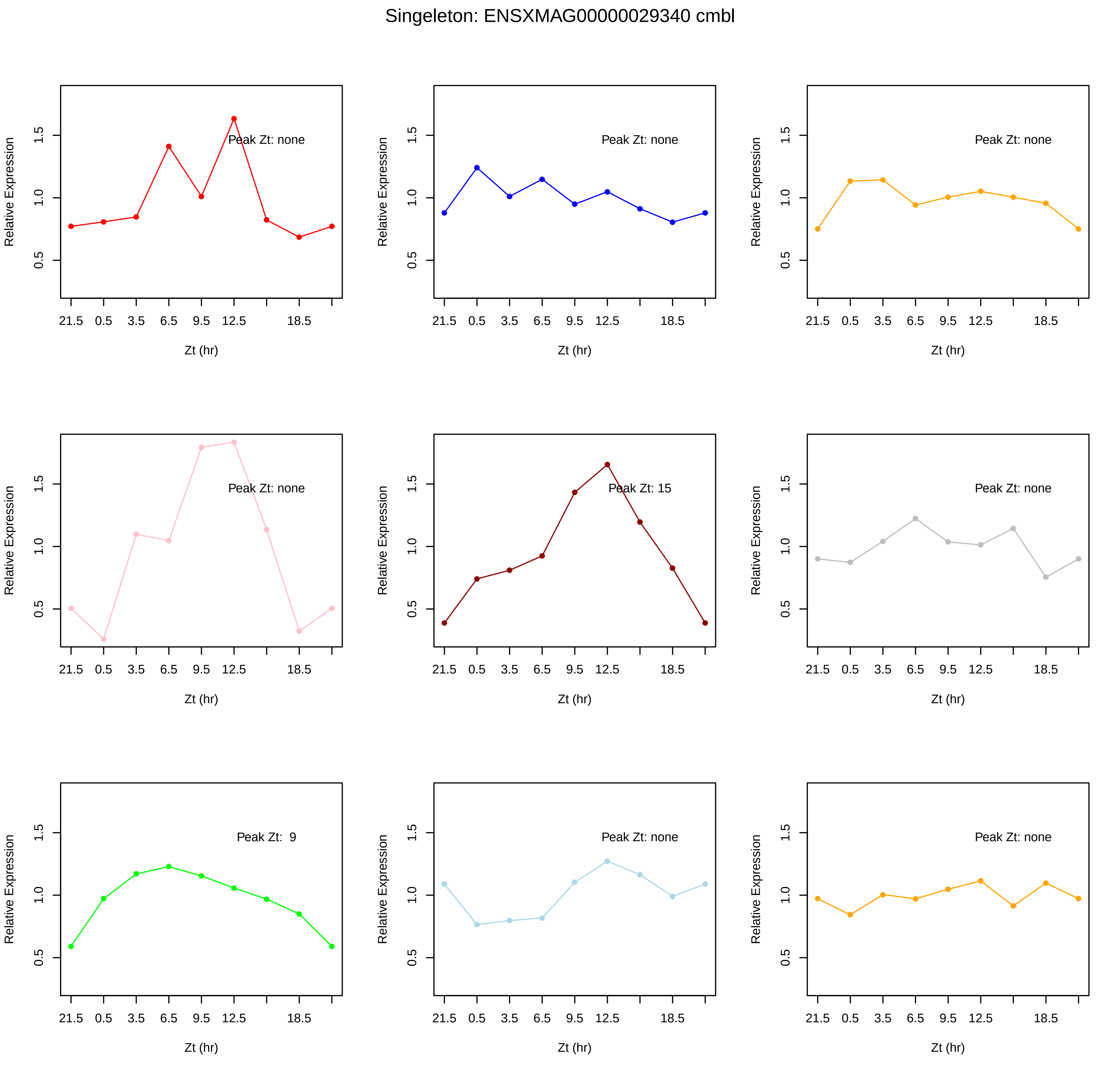
Singeleton: ENSXMAG00000029340 cmbl
Peak Zt: none
Peak Zt: none
Peak Zt: none
Peak Zt: none
Peak Zt: 15
Peak Zt: none
Peak Zt: 9
Peak Zt: none
Peak Zt: none
Relative Expression
Zt (hr)
21.5
0.5
3.5
6.5
9.5
12.5
18.5
0.5
1.0
1.5

Singeleton: ENSXMAG00000027560 tnip2

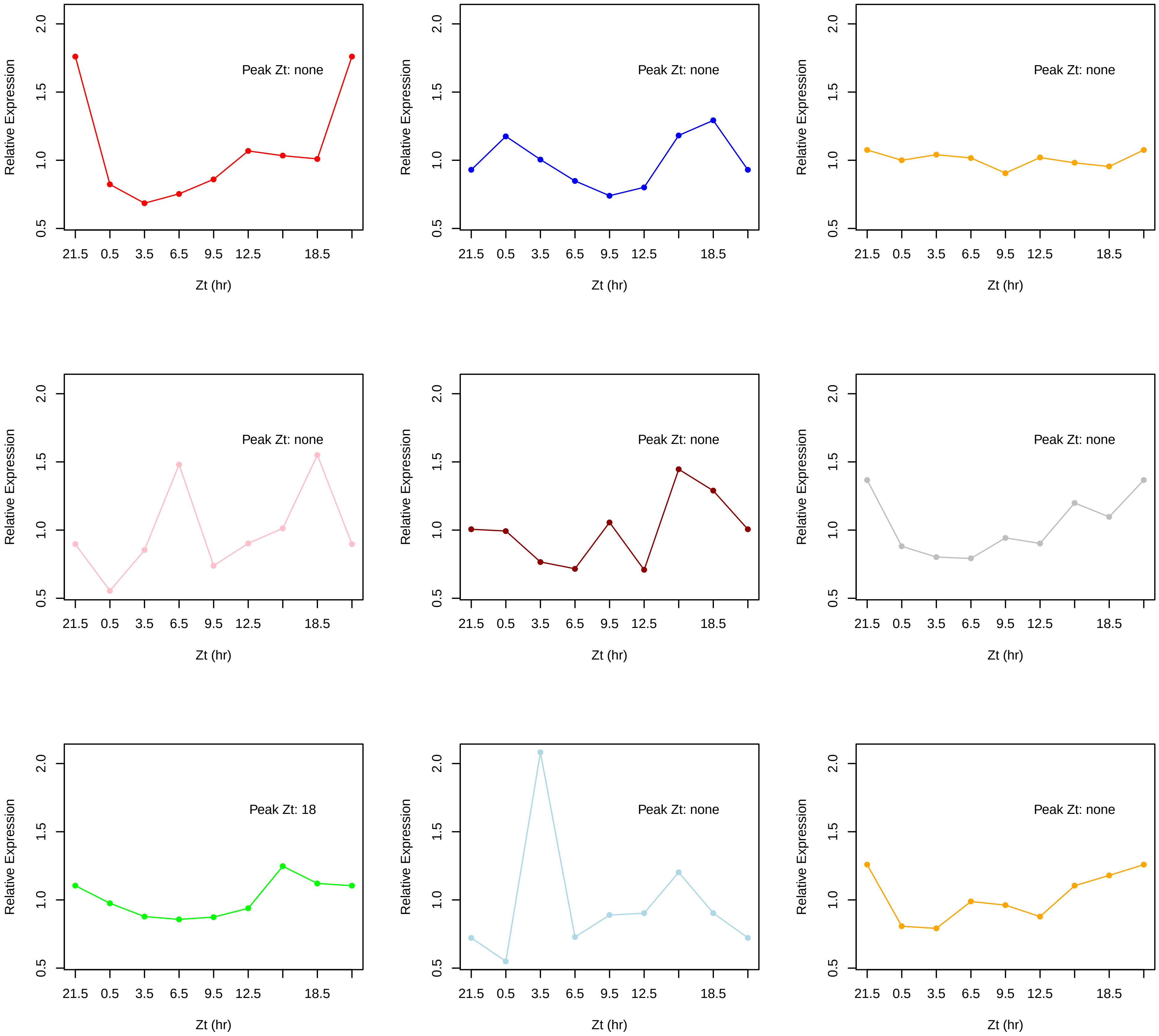

Singleton: ENSXMAG00000011851 chrne

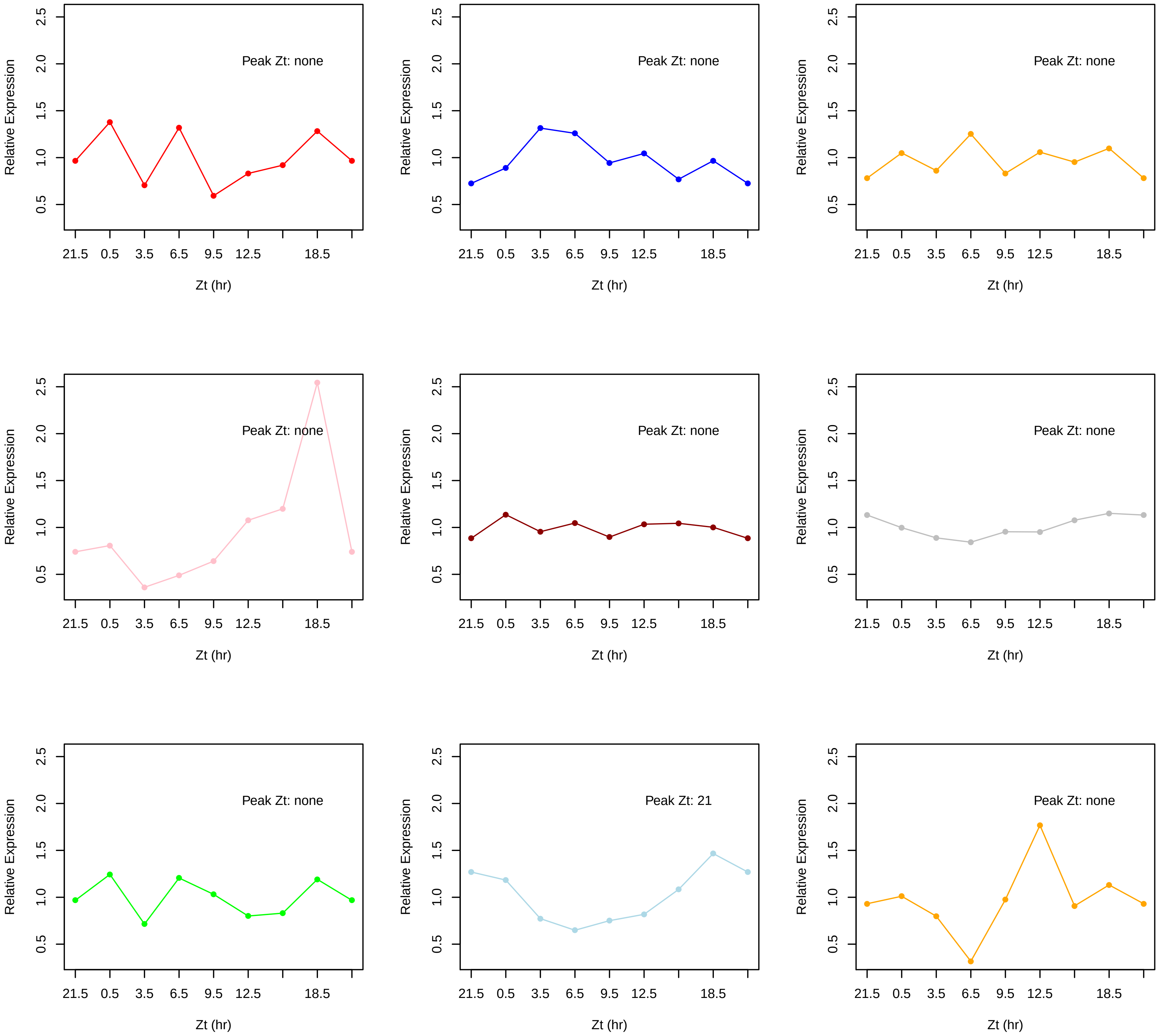

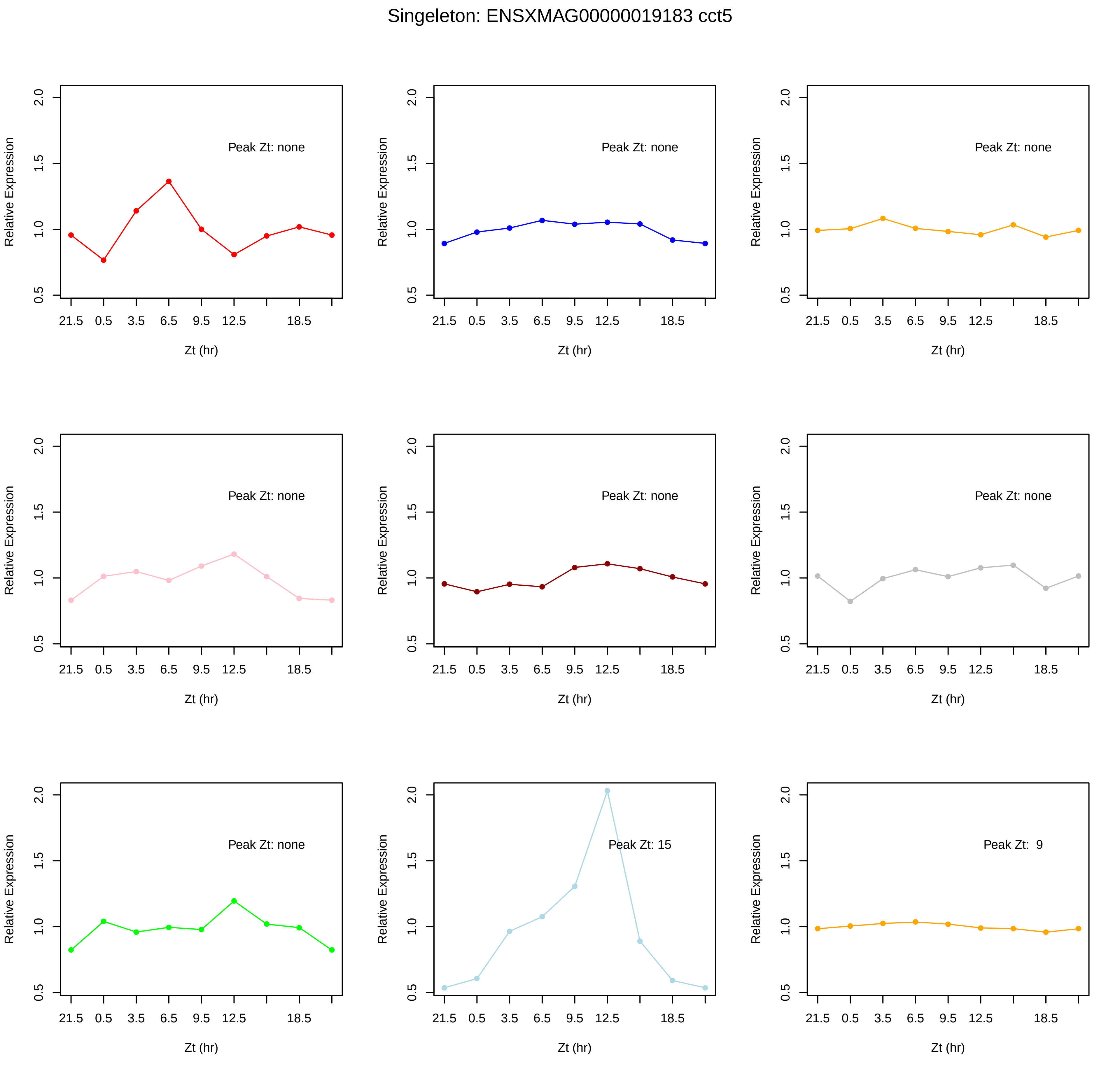
Singeleton: ENSXMAG00000019183 cct5
Peak Zt: none
Peak Zt: none
Peak Zt: none
Peak Zt: none
Peak Zt: none
Peak Zt: none
Peak Zt: none
Peak Zt: 15
Peak Zt: 9
Relative Expression
Zt (hr)
21.5
0.5
3.5
6.5
9.5
12.5
18.5
0.5
1.0
1.5
2.0

Singeleton: ENSXMAG00000009151 lsm3

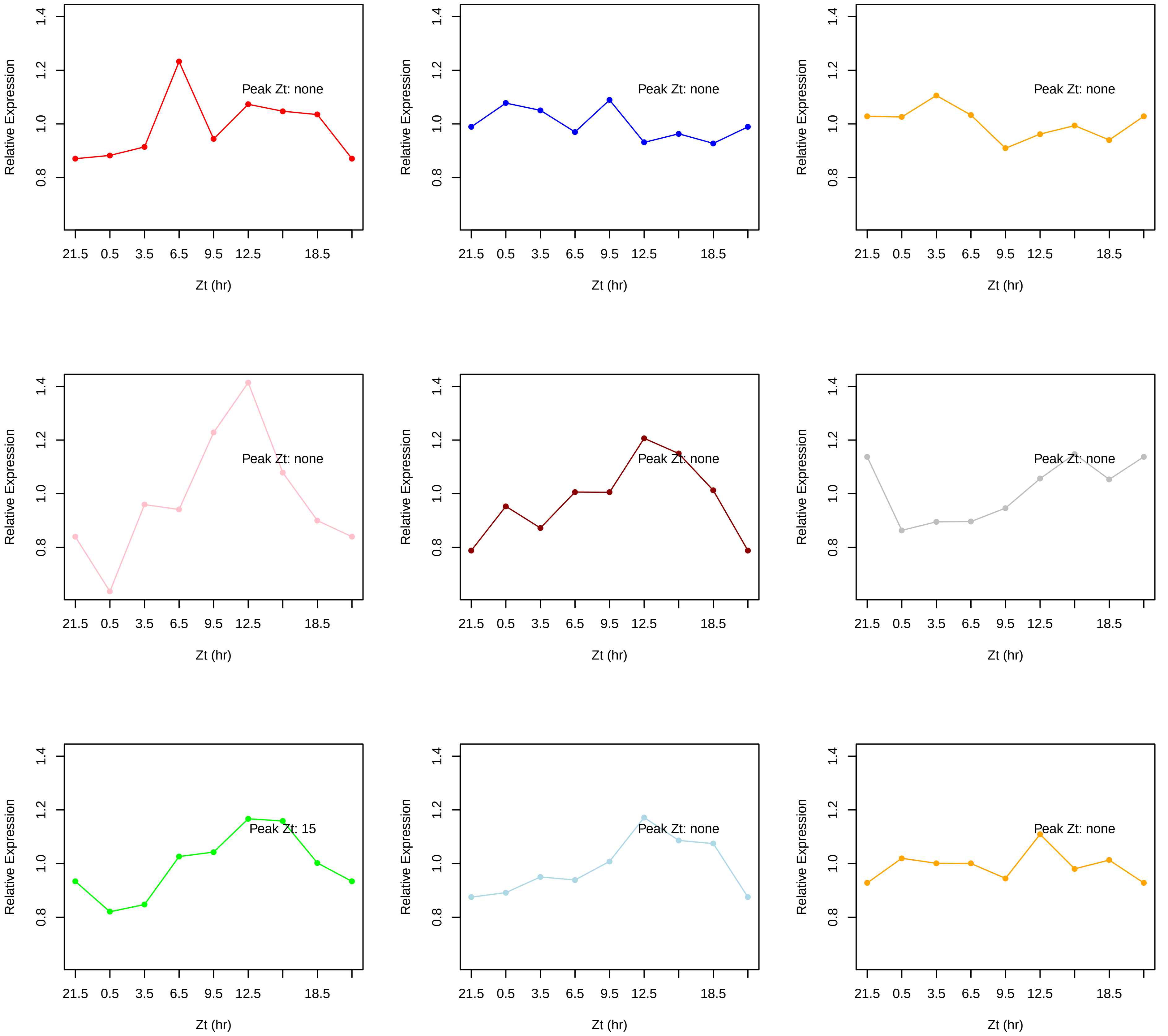

Singeleton: ENSXMAG00000021526

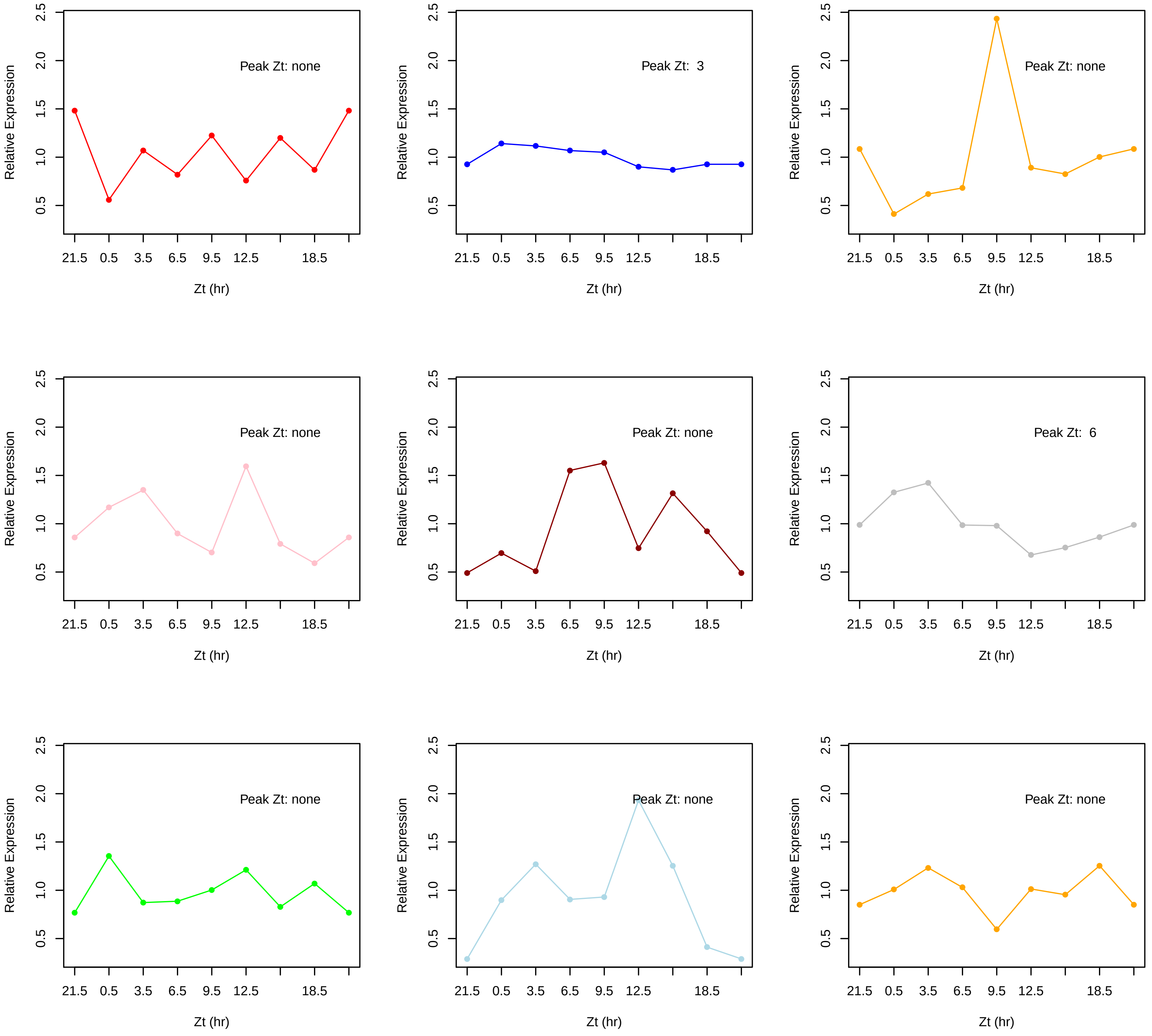

Singeleton: ENSXMAG00000015843 crip1

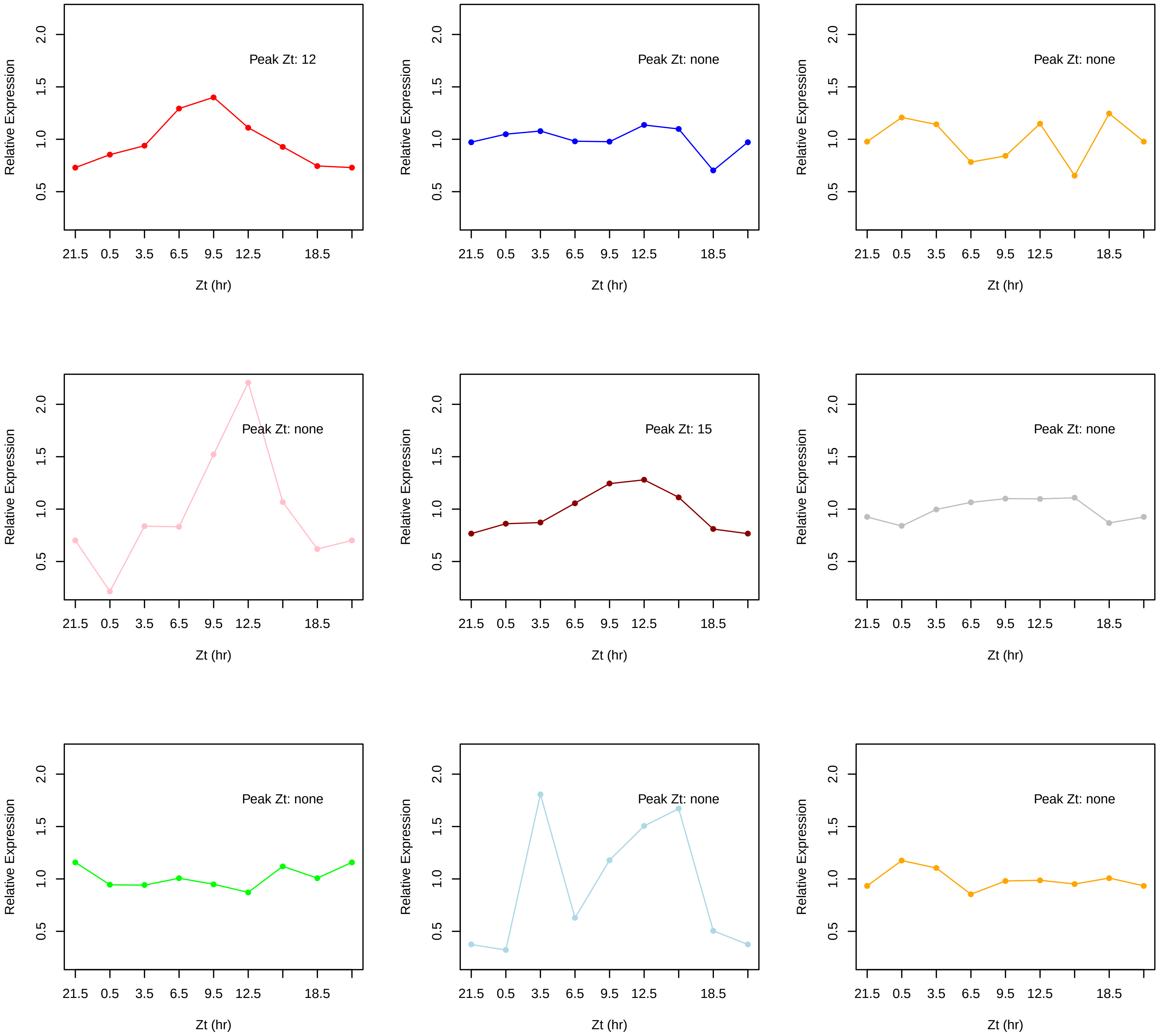

Singeleton: ENSXMAG00000008573 zgc:158659

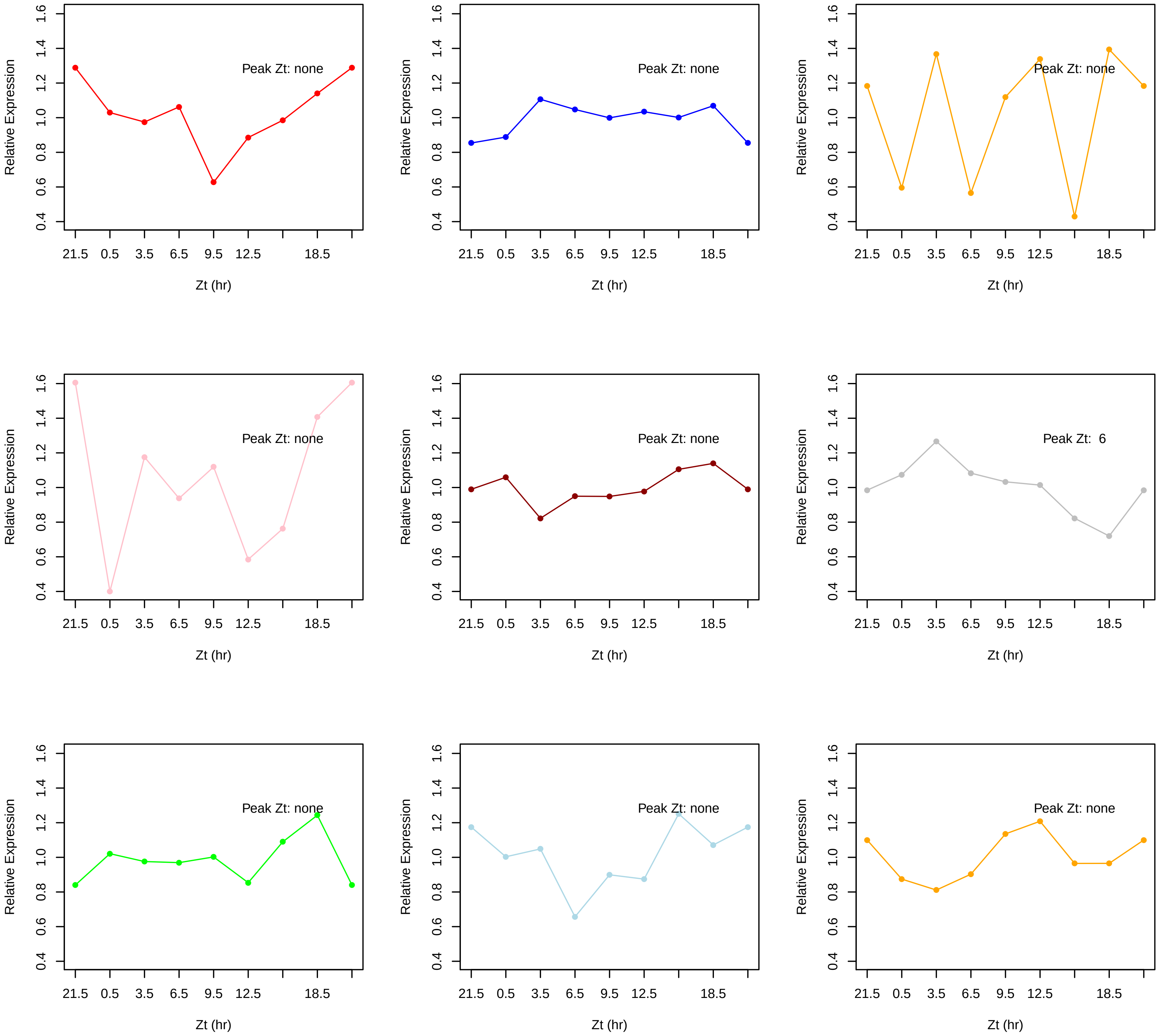

Singeleton: ENSXMAG00000019215 sema5a

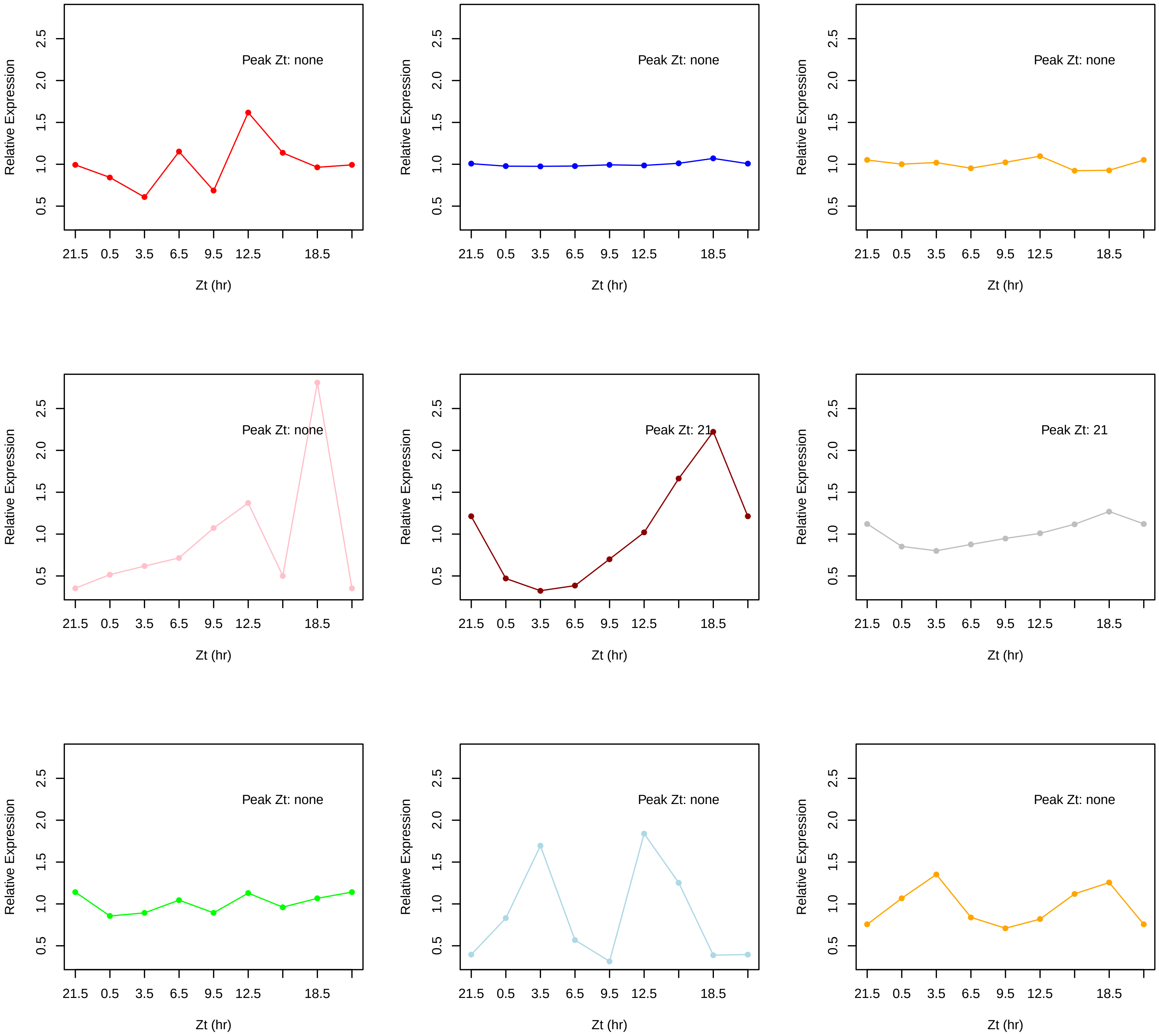

Singleton: ENSXMAG00000023115 TNS4

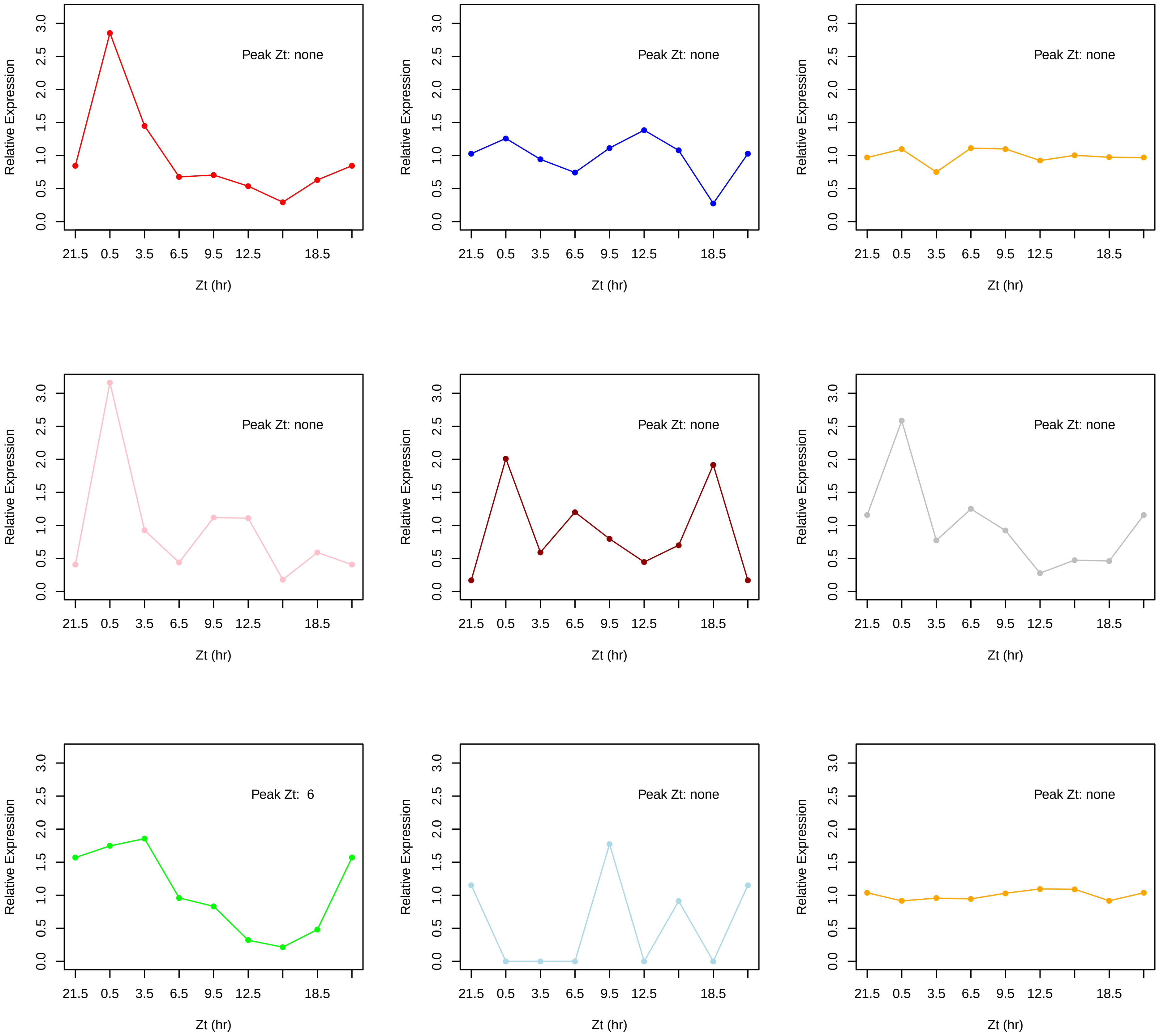

Singeleton: ENSXMAG00000012281 hmgb2a

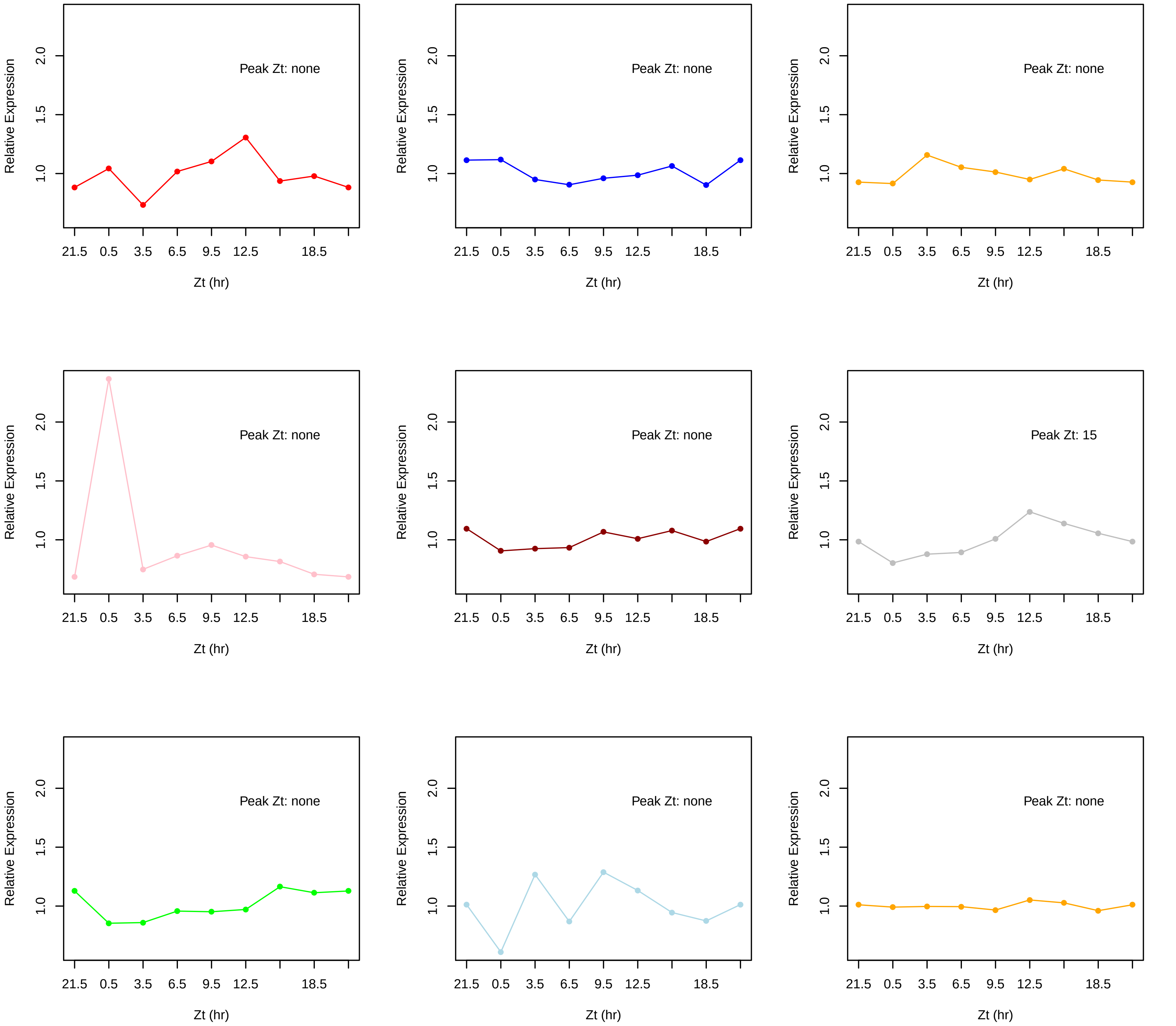

Singleton: ENSXMAG00000021779

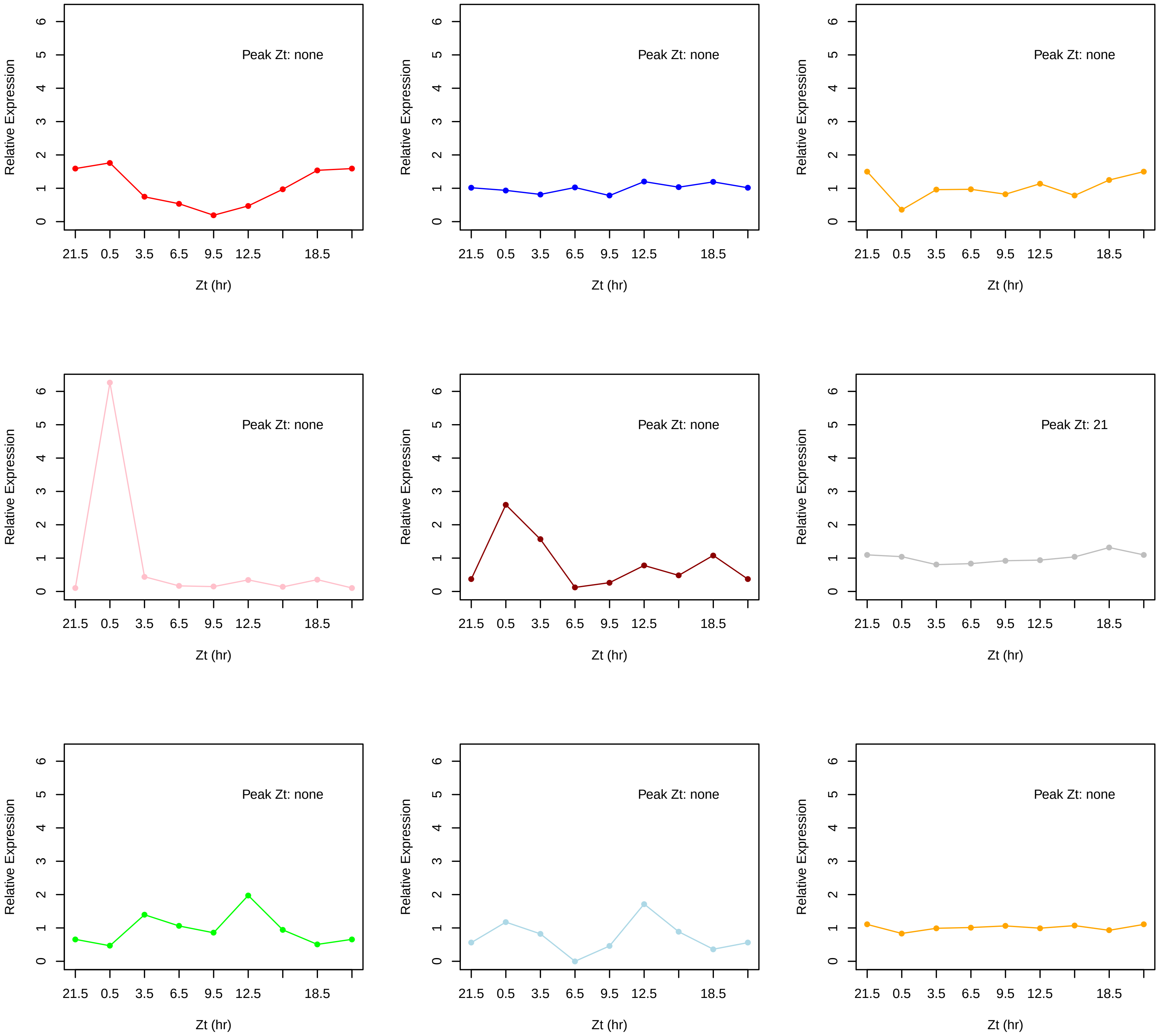

Singeleton: ENSXMAG00000026308 C17orf58

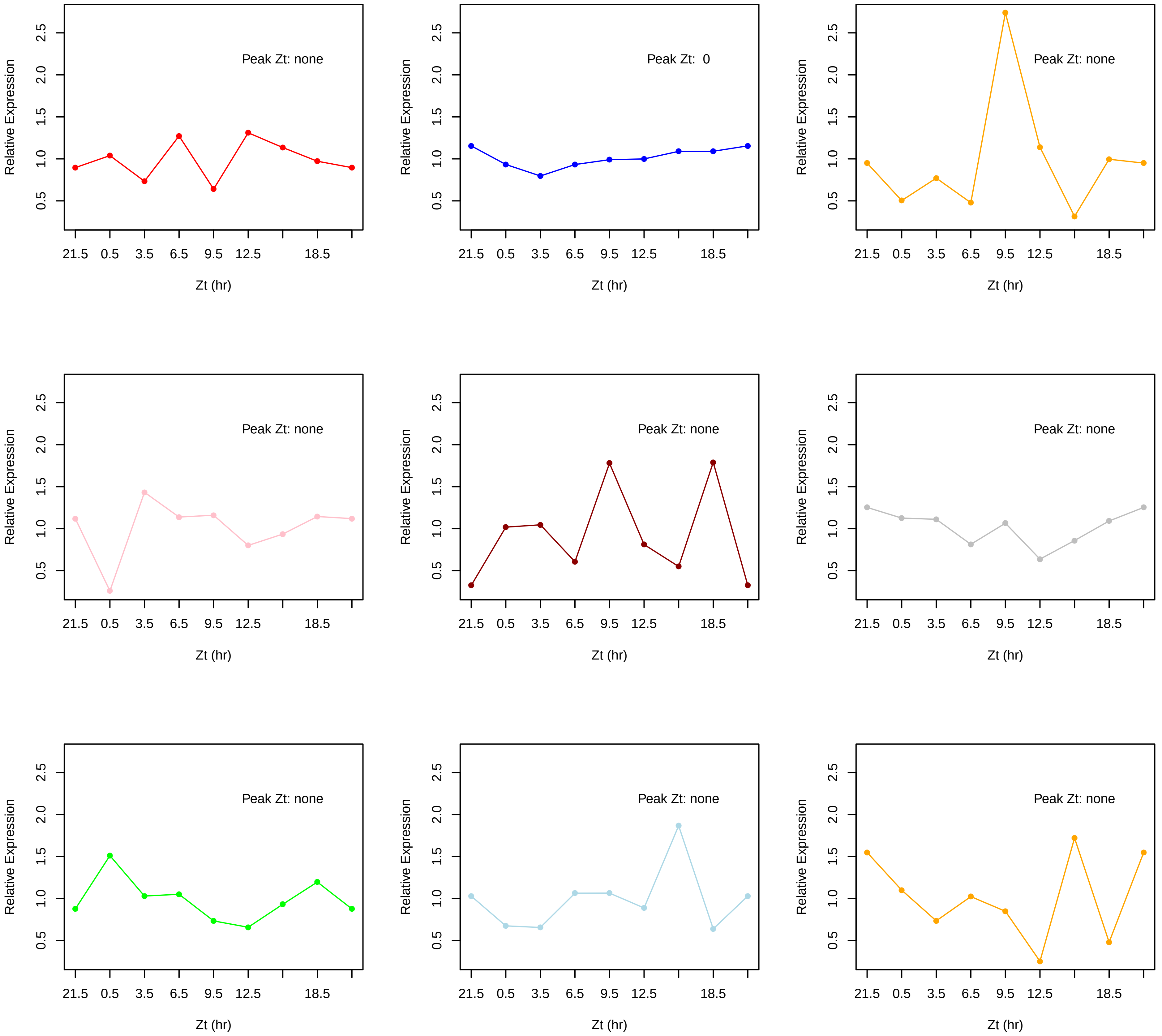

Singeleton: ENSXMAG00000007619 mogat3b

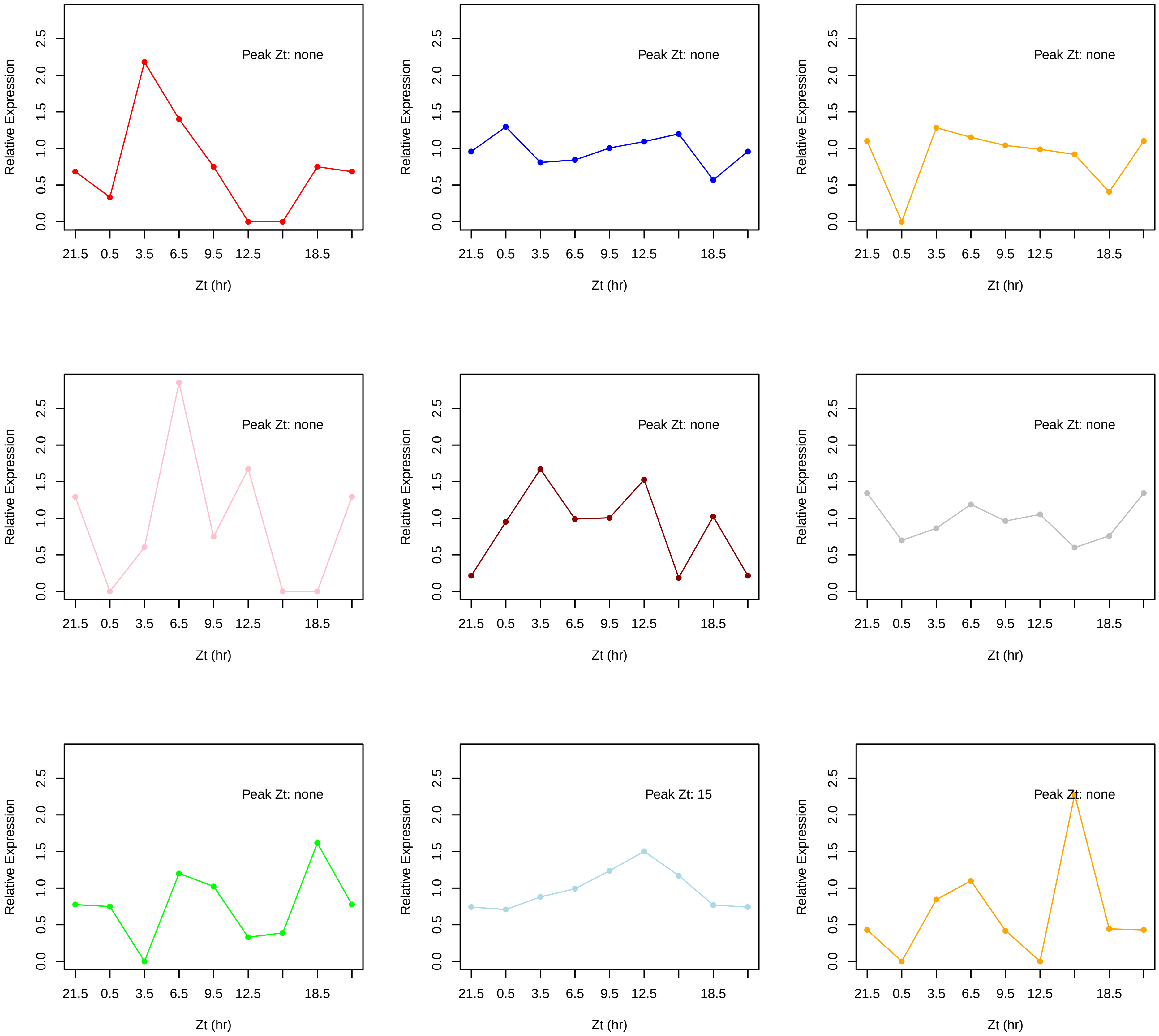

Singleton: ENSXMAG00000006645 nol11

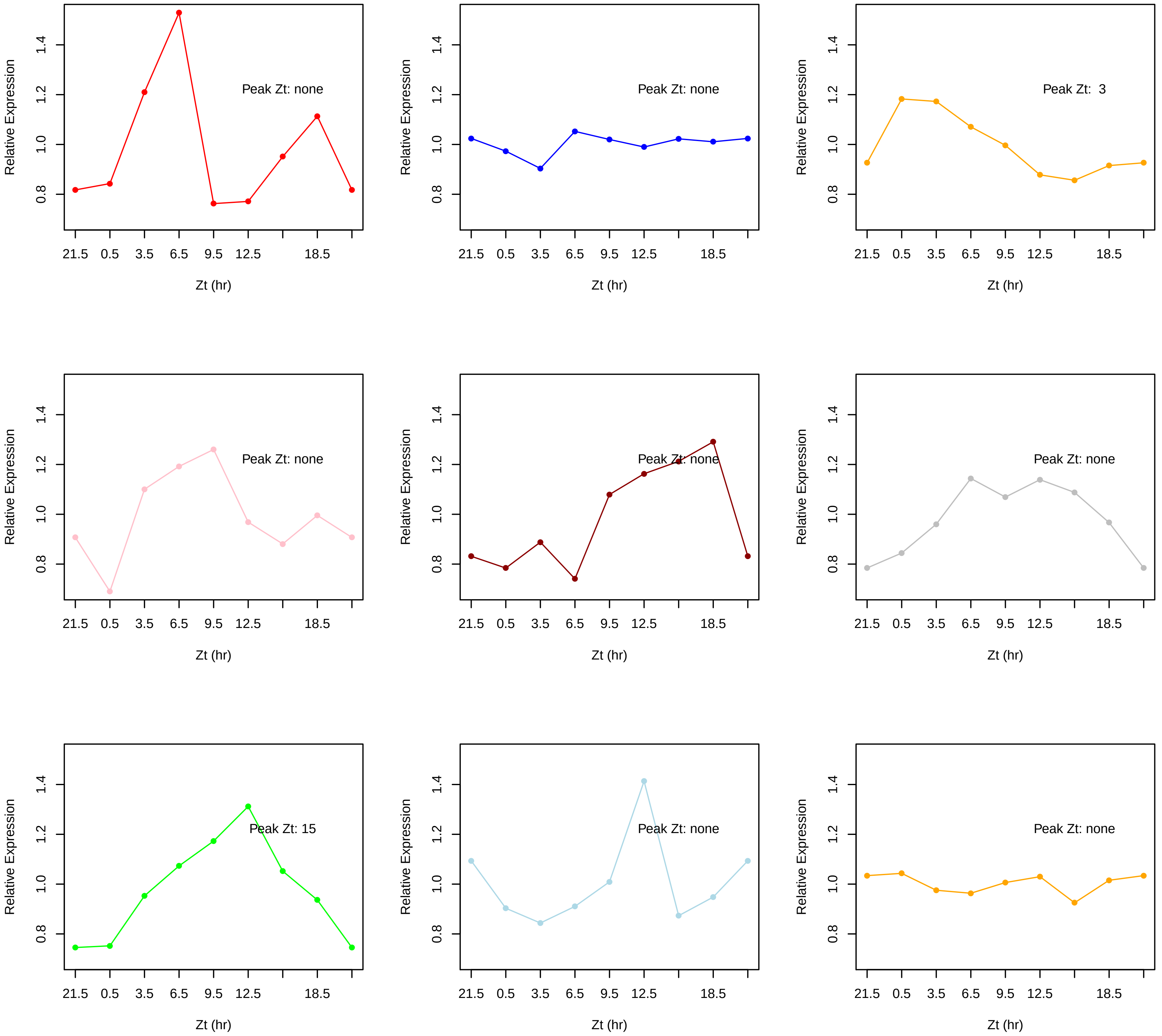

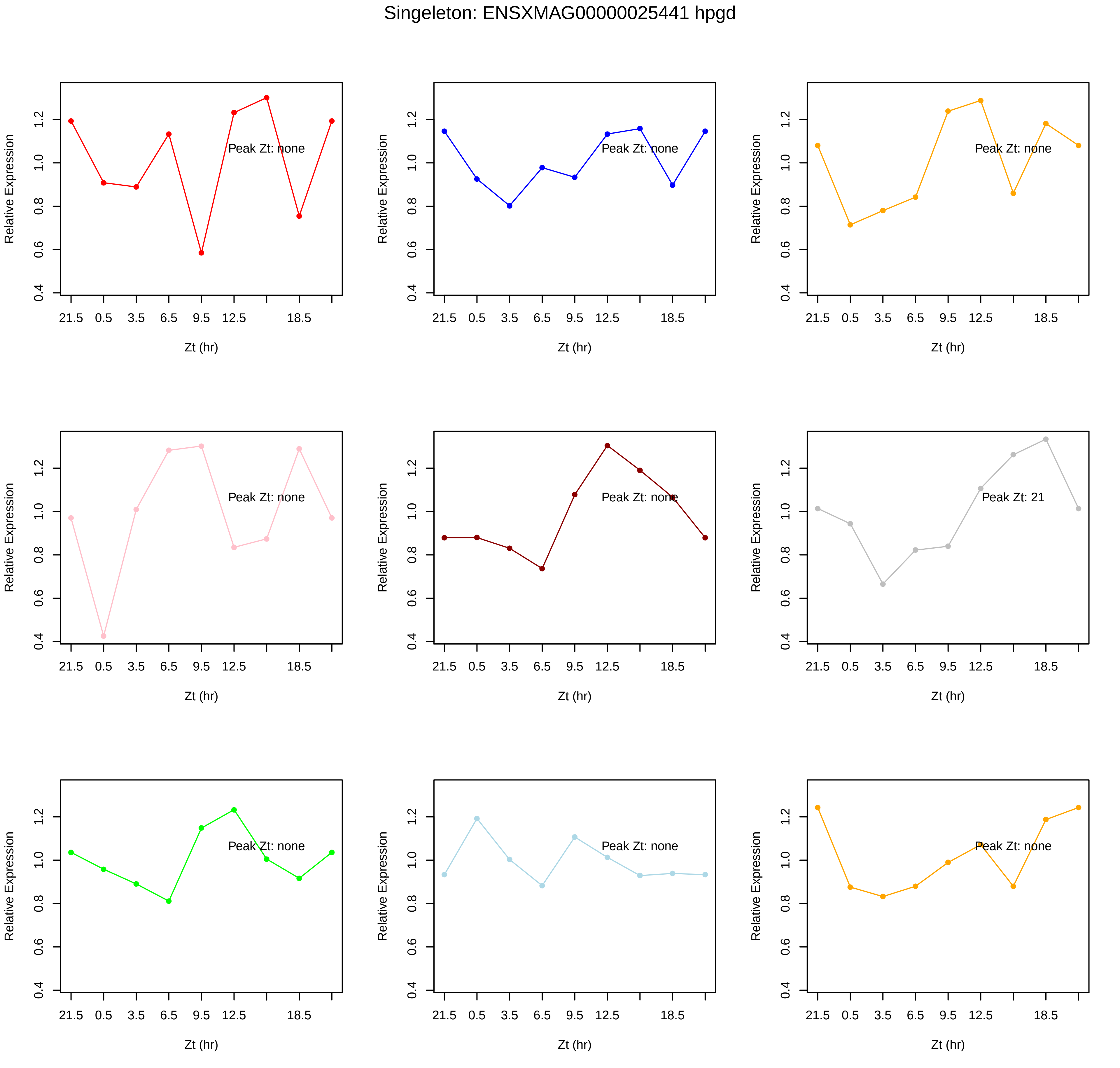

Singeleton: ENSXMAG00000025441 hpgd
Peak Zt: none
Peak Zt: none
Peak Zt: none
Peak Zt: none
Peak Zt: none
Peak Zt: 21
Peak Zt: none
Peak Zt: none
Peak Zt: none
Relative Expression
Zt (hr)
21.5
0.5
3.5
6.5
9.5
12.5
18.5
0.4
0.6
0.8
1.0
1.2

Singeleton: ENSXMAG00000017591 glra3

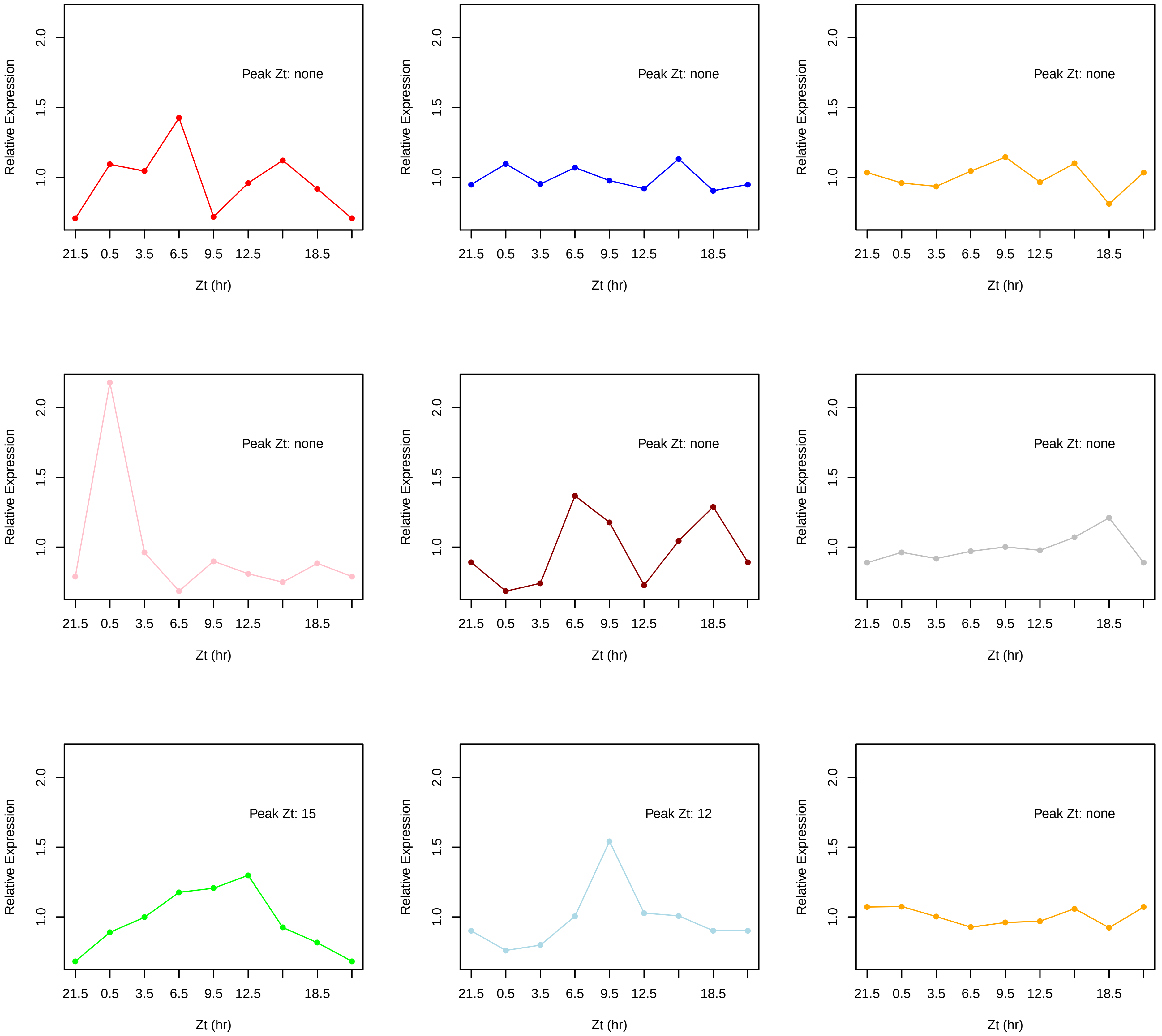

Singeleton: ENSXMAG00000002923

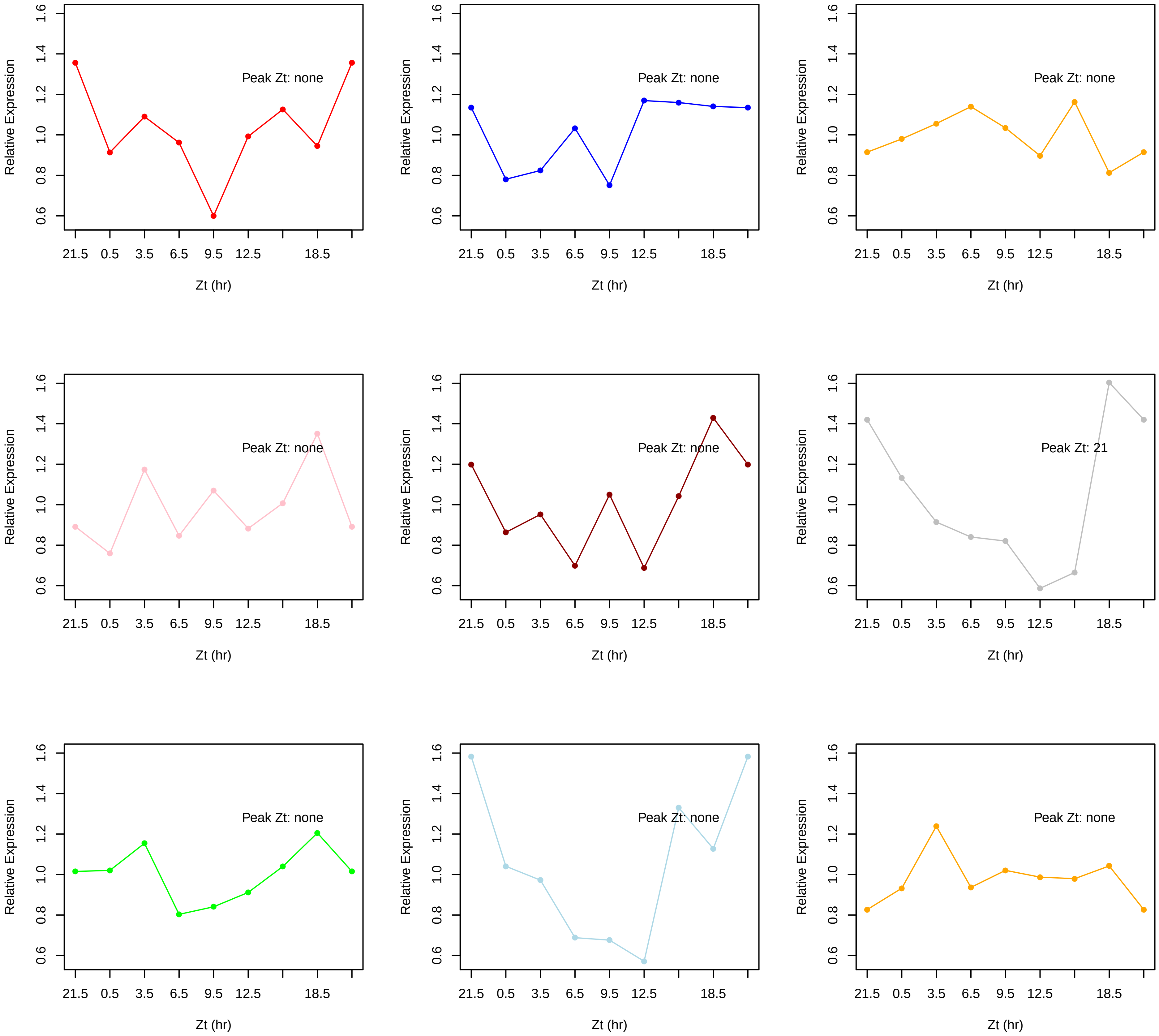

Singeleton: ENSXMAG00000000155 si:ch211−156l18.7

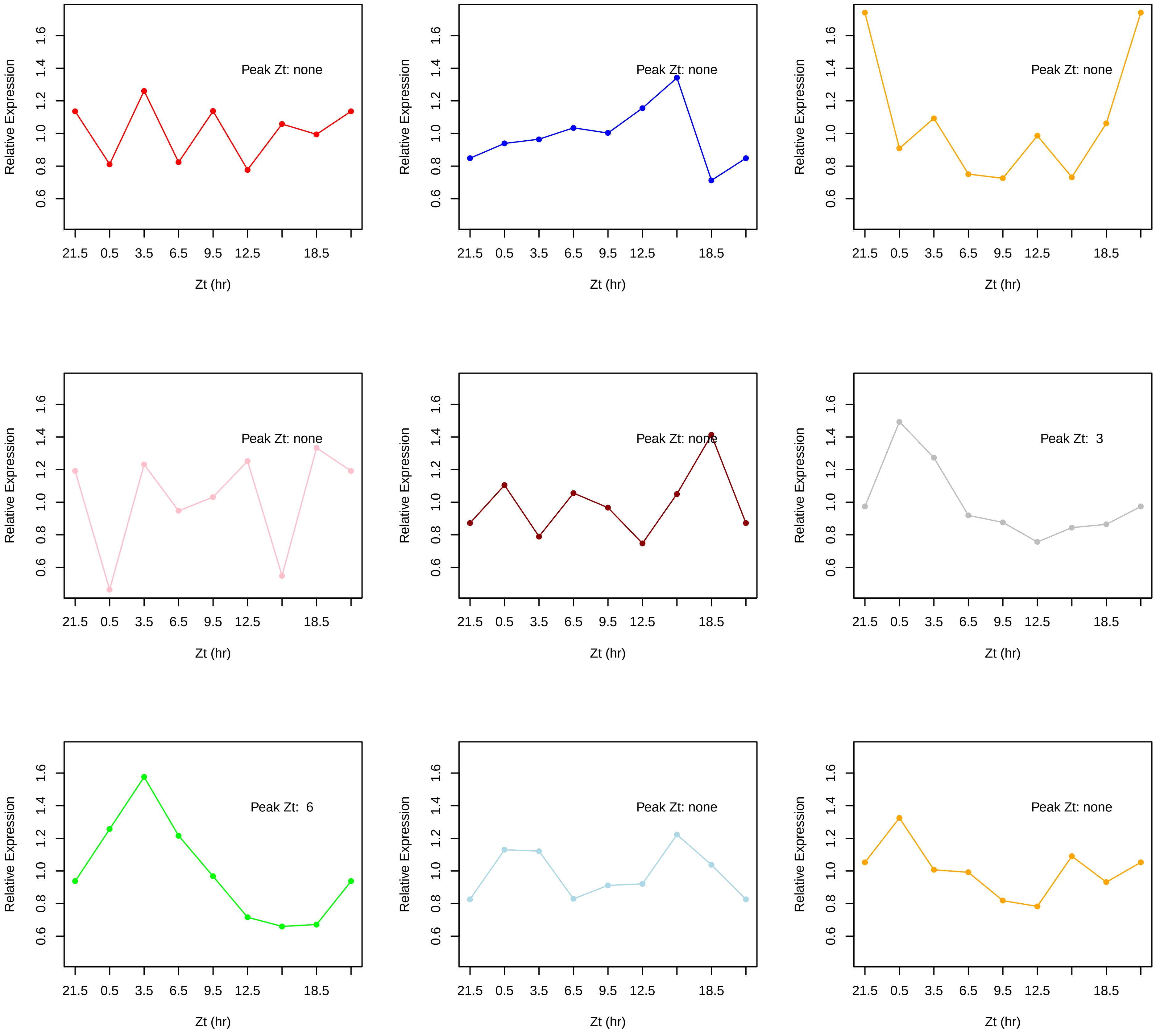

Singeleton: ENSXMAG00000012062 pls3

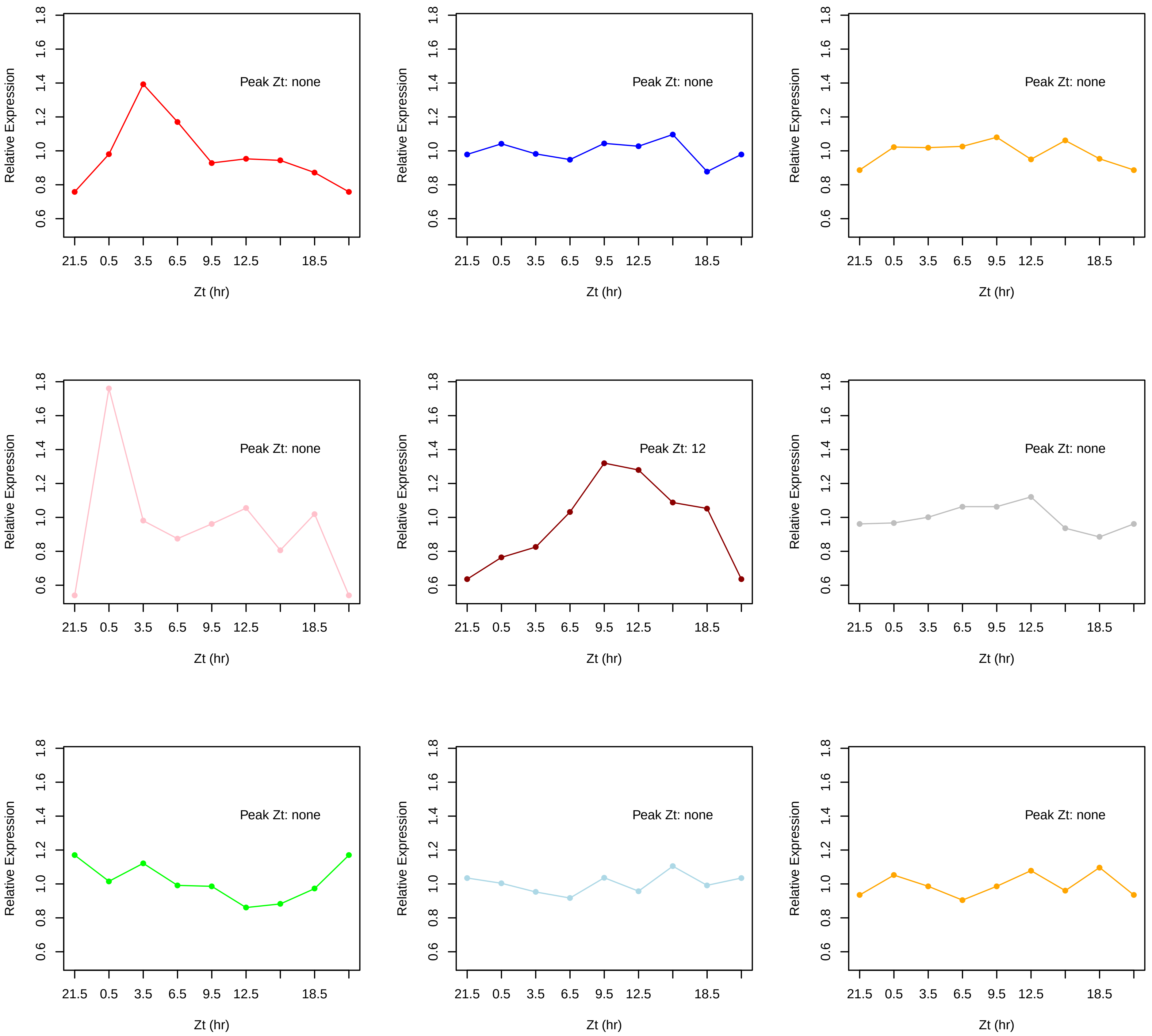

Singeleton: ENSXMAG00000006524 psmd12

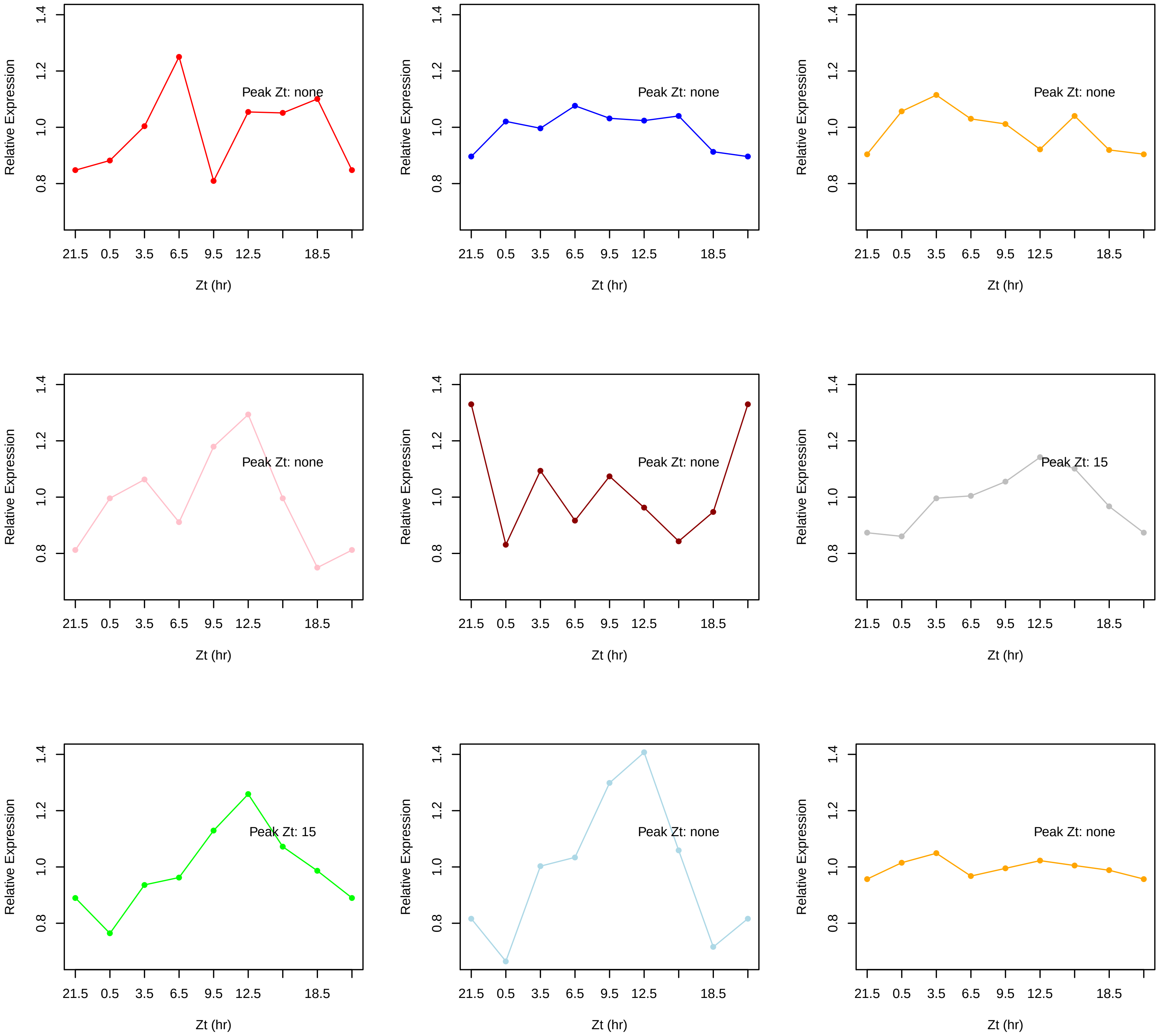

Singeleton: ENSXMAG00000015975

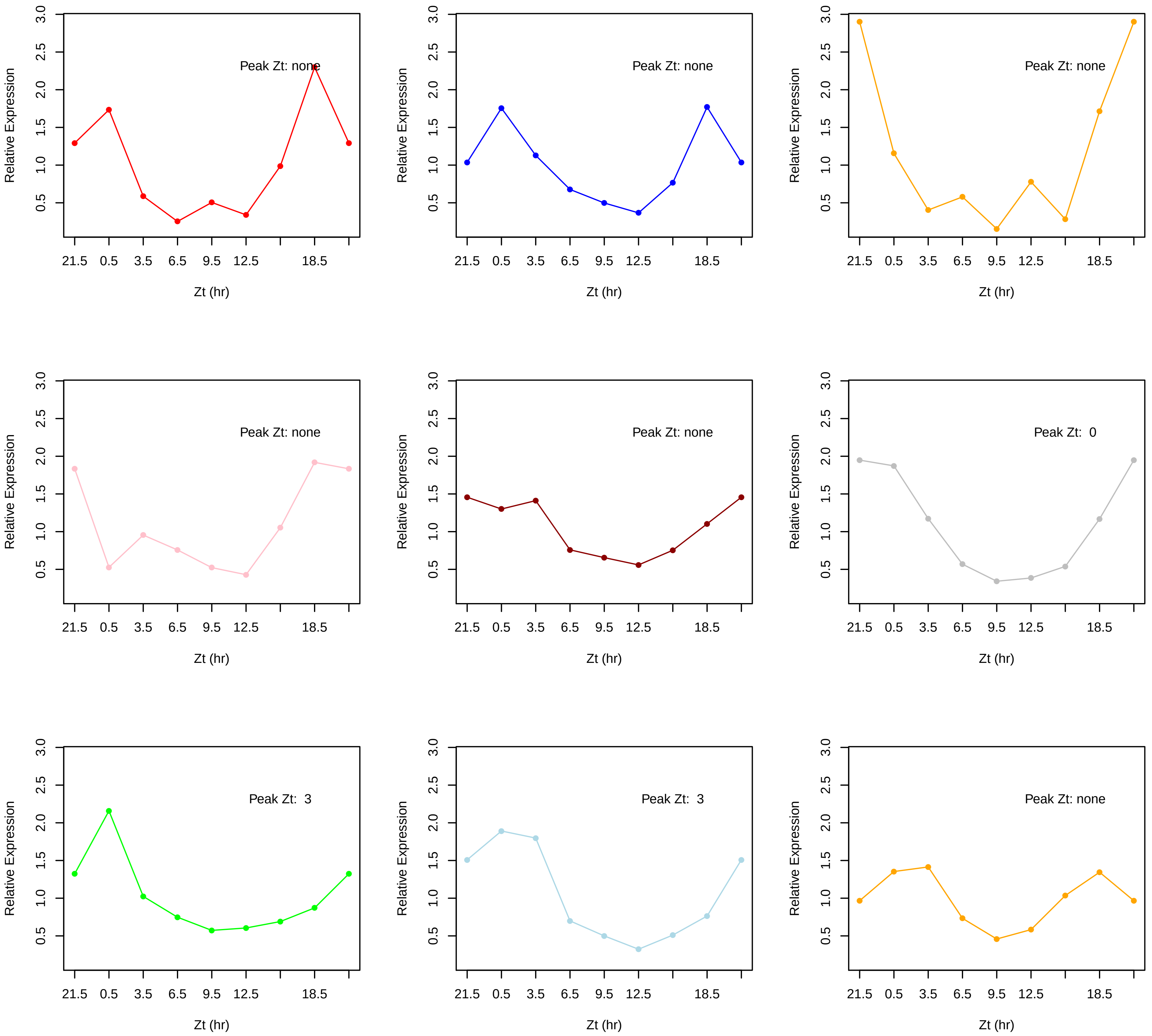

Singeleton: ENSXMAG00000014466 sltm

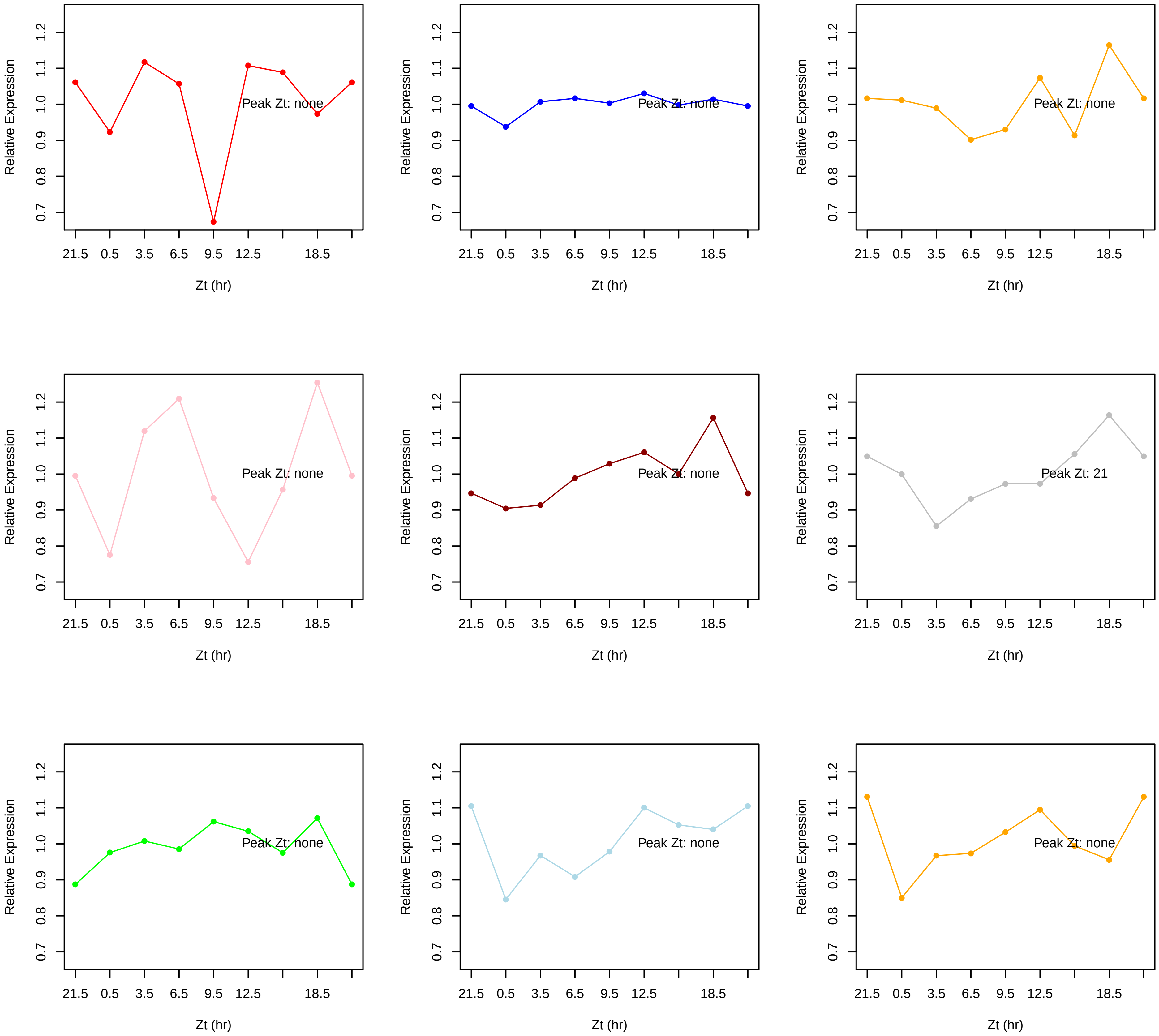

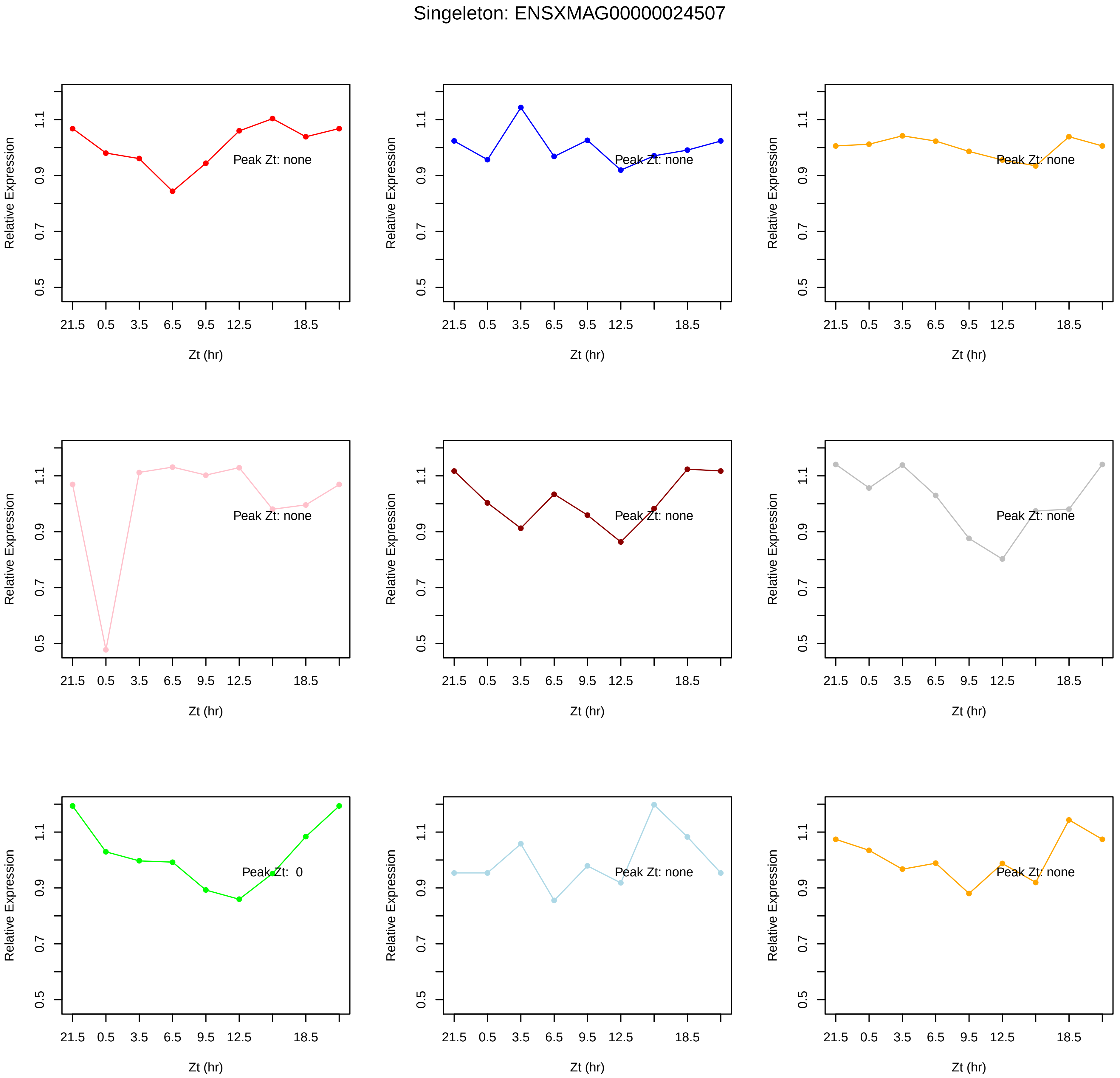
Singeleton: ENSXMAG00000024507
Relative Expression
Zt (hr)
0.5
0.7
0.9
1.1
21.5
0.5
3.5
6.5
9.5
12.5
18.5
Peak Zt: none
Peak Zt: 0

Singleton: ENSXMAG00000015996 spcs3

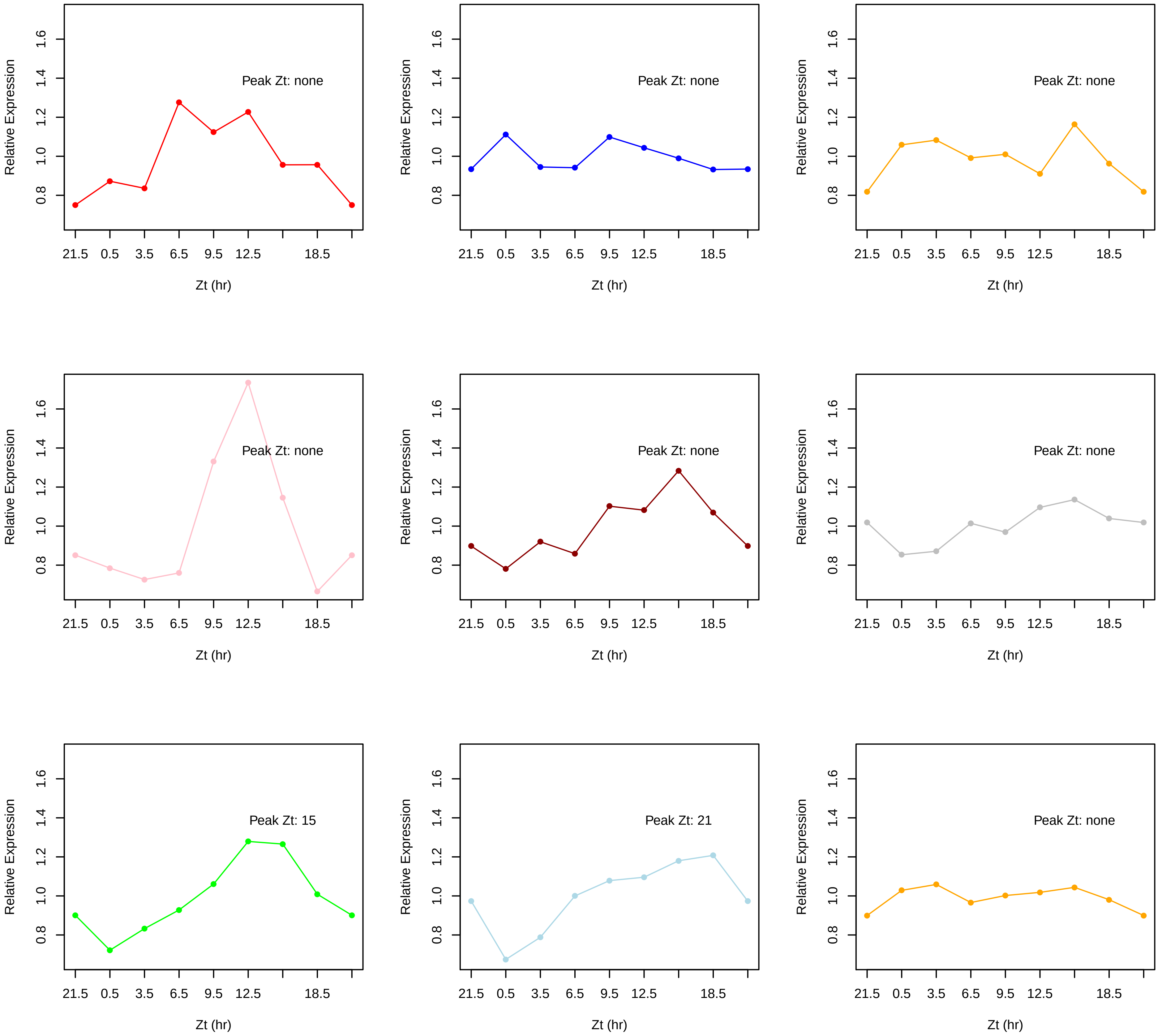

Singeleton: ENSXMAG00000012711 il13ra2

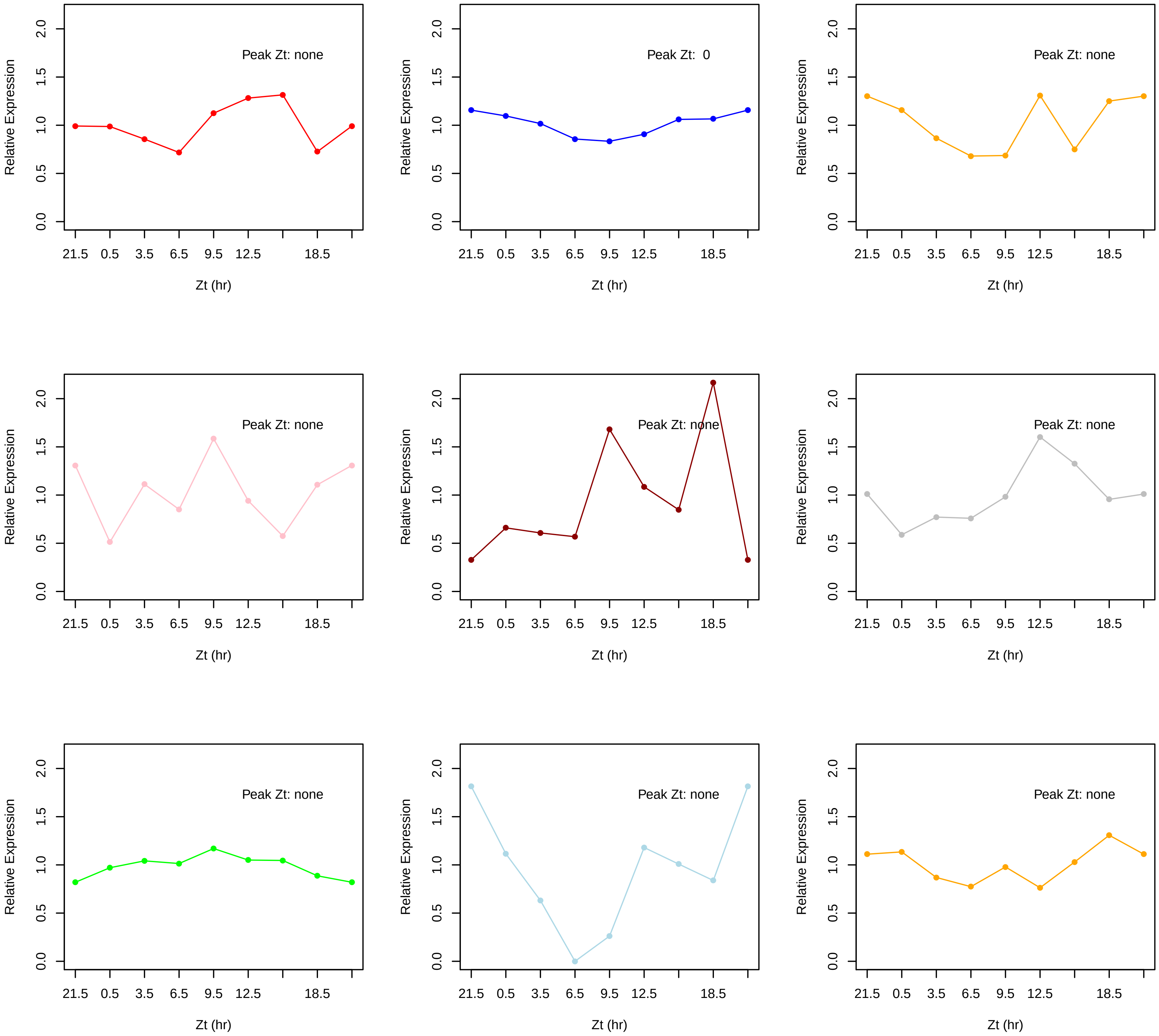

Singleton: ENSXMAG00000011488

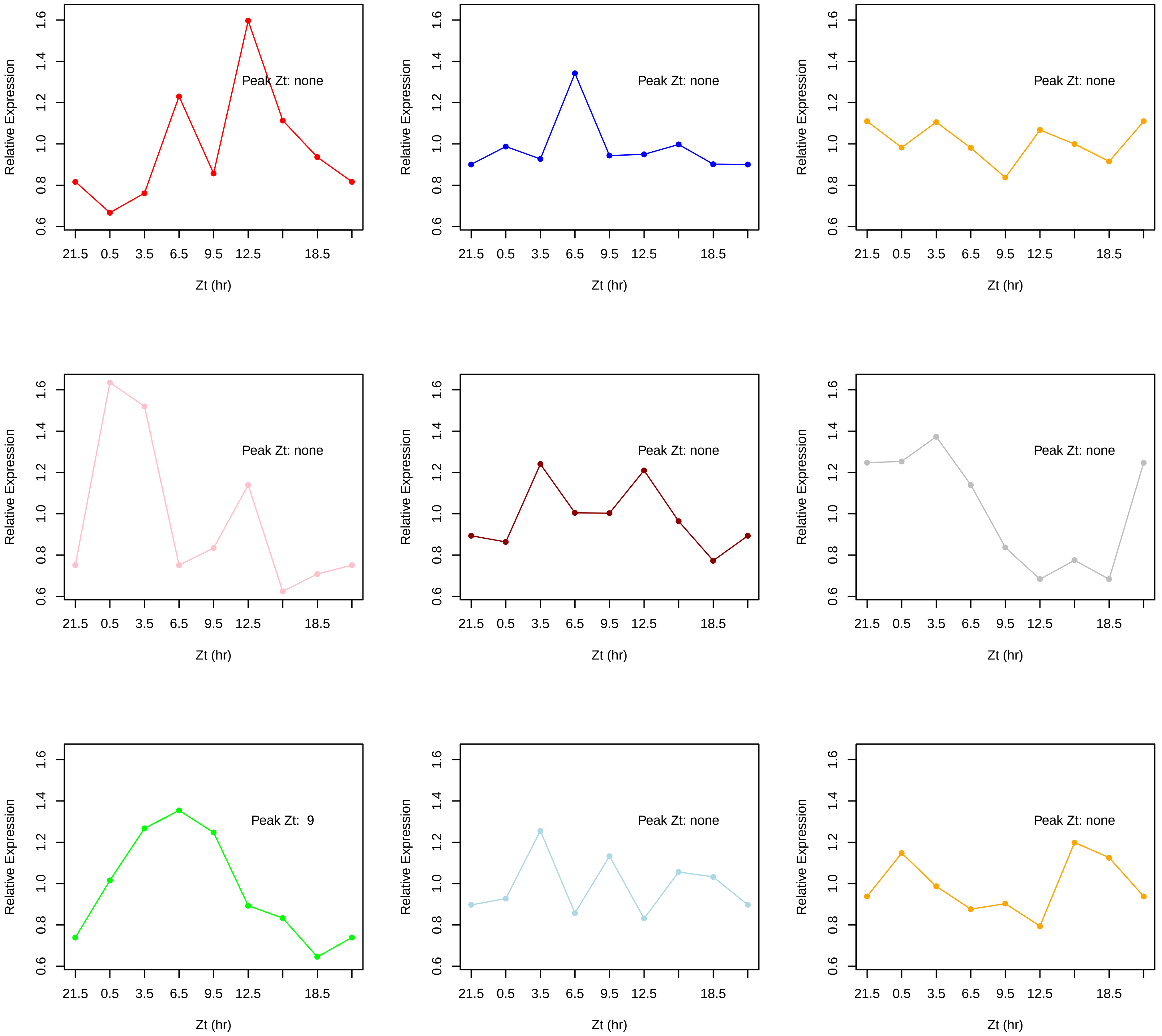

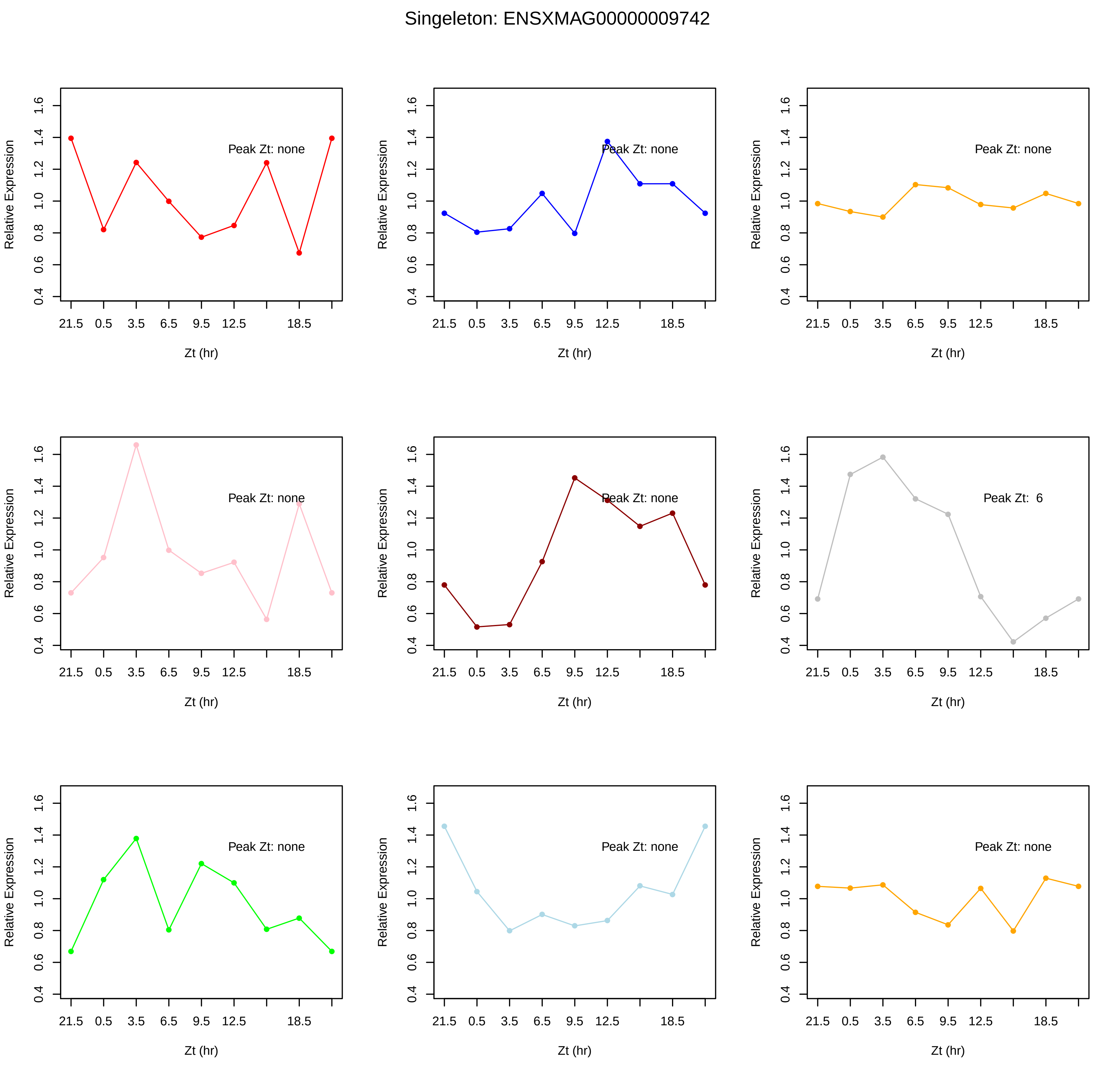

Singeleton: ENSXMAG00000009742
Peak Zt: none
Peak Zt: none
Peak Zt: none
Peak Zt: none
Peak Zt: none
Peak Zt: 6
Peak Zt: none
Peak Zt: none
Peak Zt: none
Relative Expression
Zt (hr)

Singeleton: ENSXMAG00000014476 rnf111

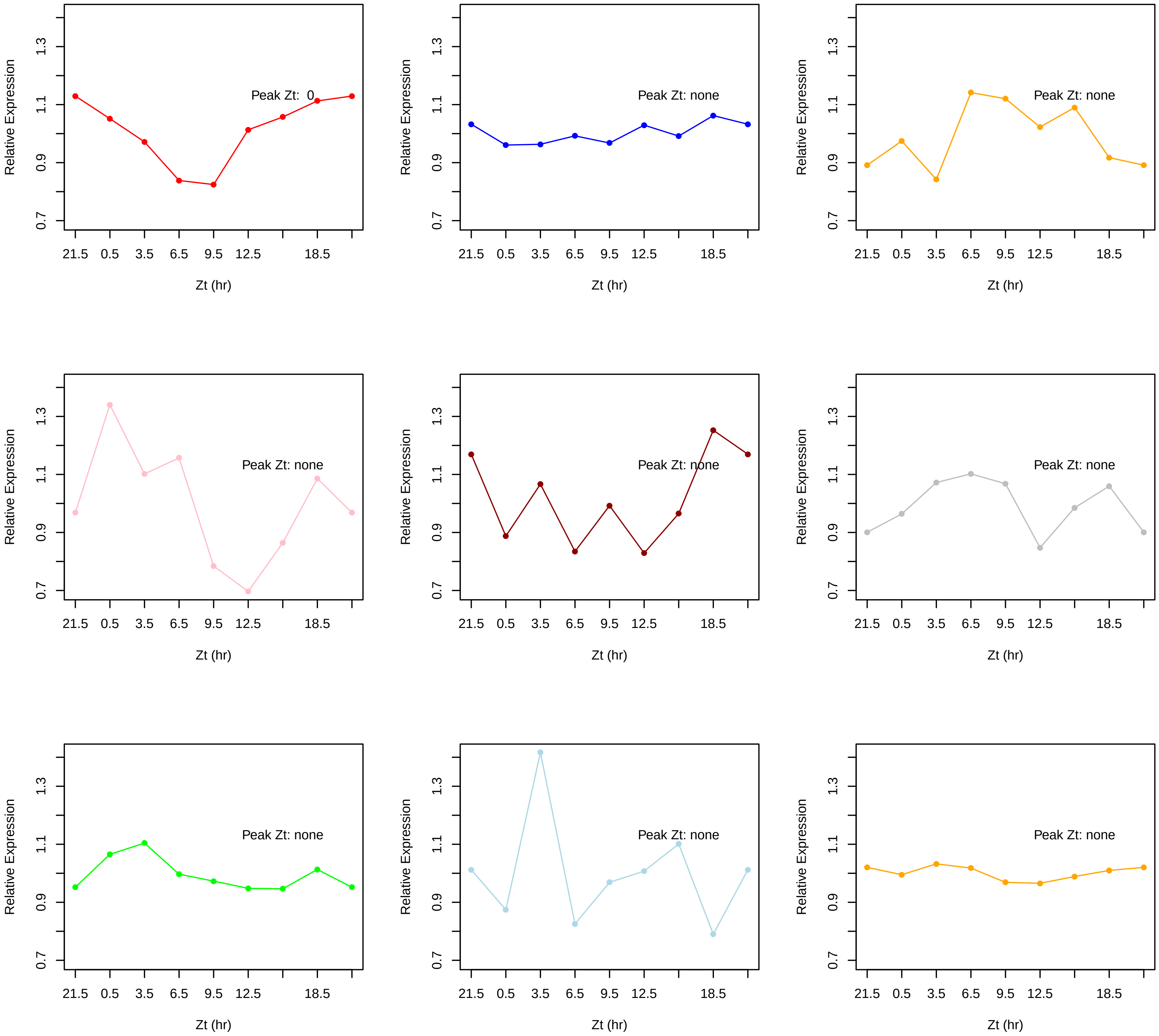

Singeleton: ENSXMAG00000029342 rlim

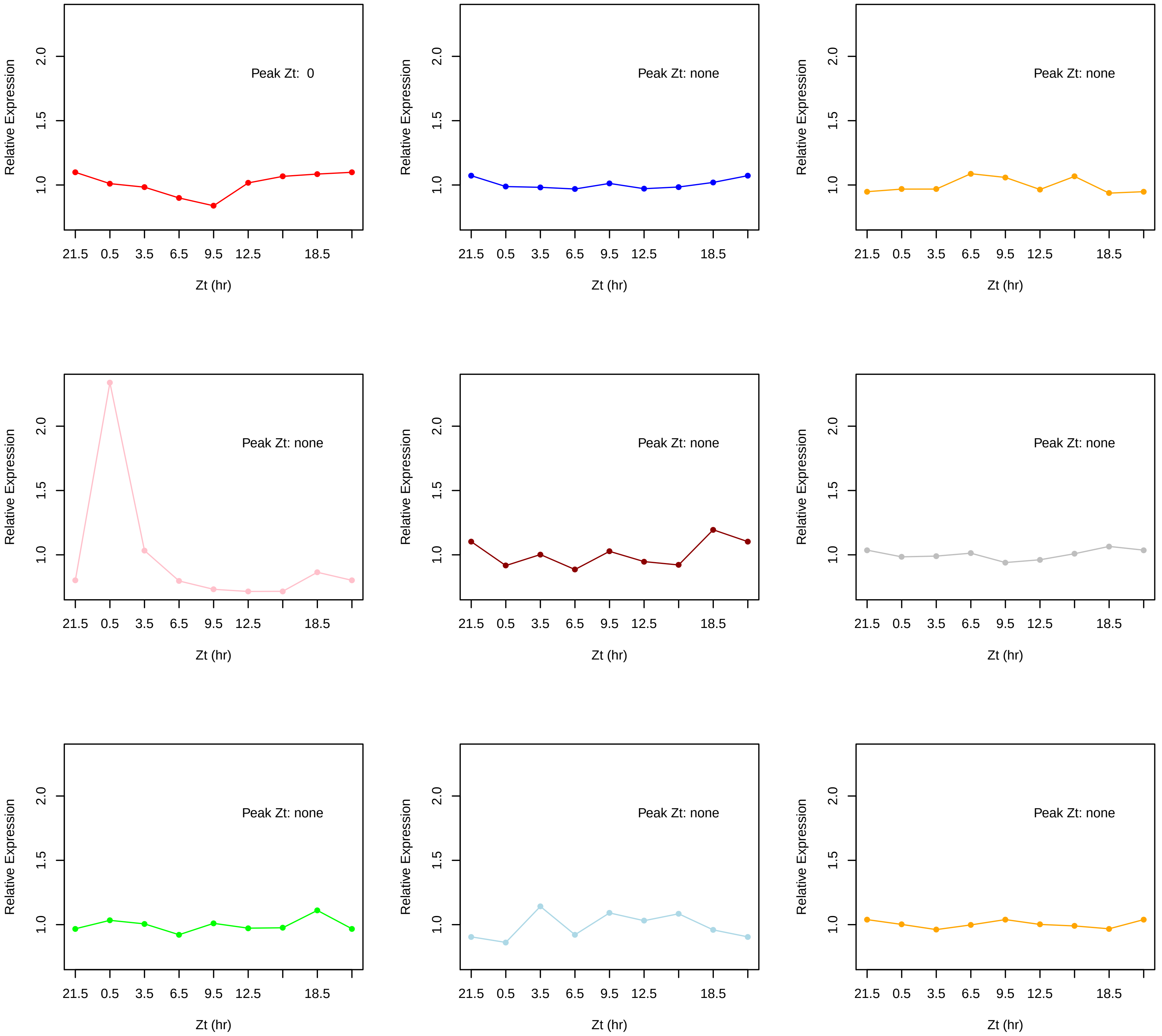

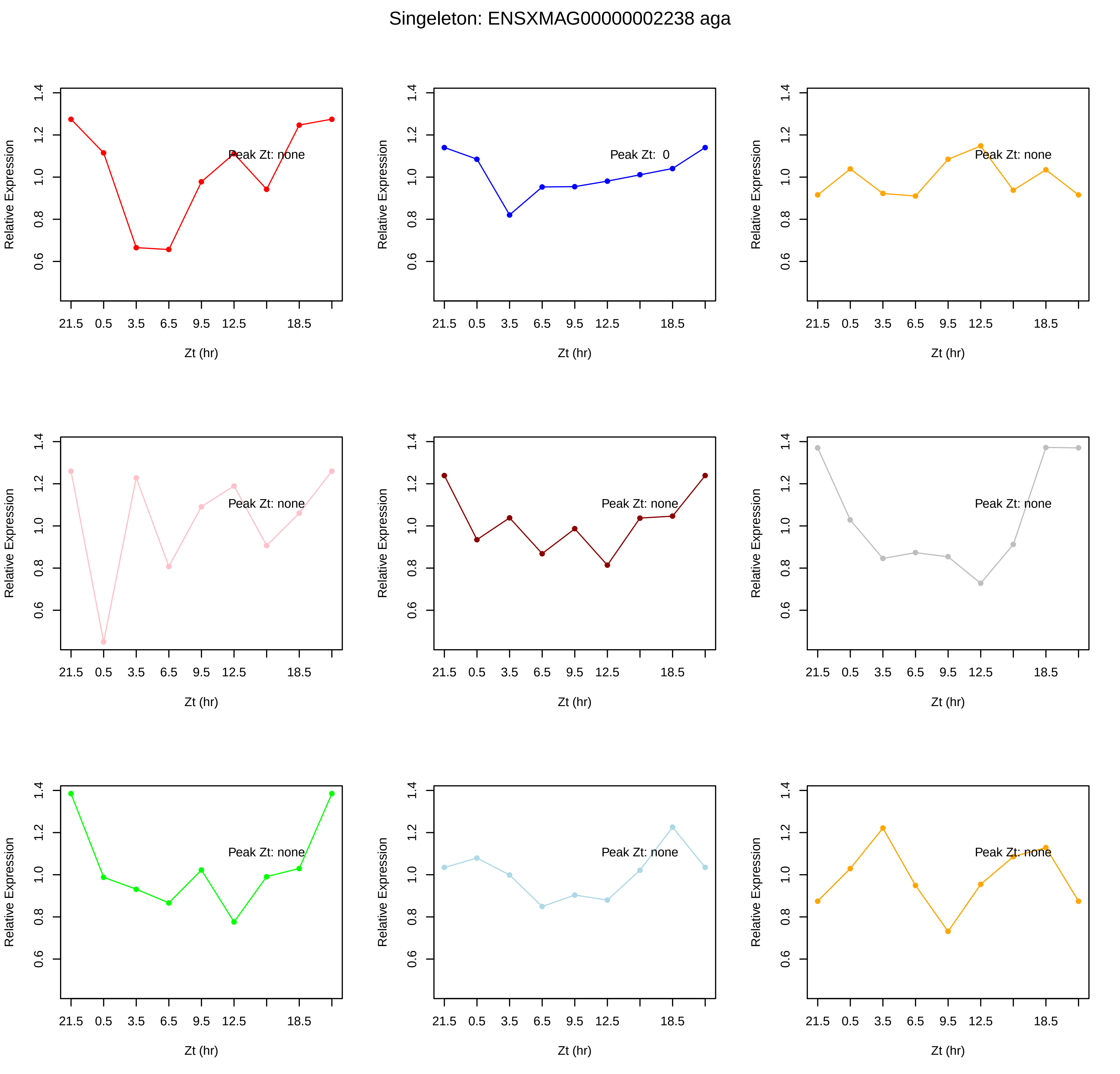
Singeleton: ENSXMAG00000002238 aga
Relative Expression
Zt (hr)
21.5
0.5
3.5
6.5
9.5
12.5
18.5
0.6
0.8
1.0
1.2
1.4
Peak Zt: none
Peak Zt: 0
Peak Zt: none
Peak Zt: none
Peak Zt: none
Peak Zt: none
Peak Zt: none
Peak Zt: none
Peak Zt: none

Singleton: ENSXMAG00000009070 abcb7

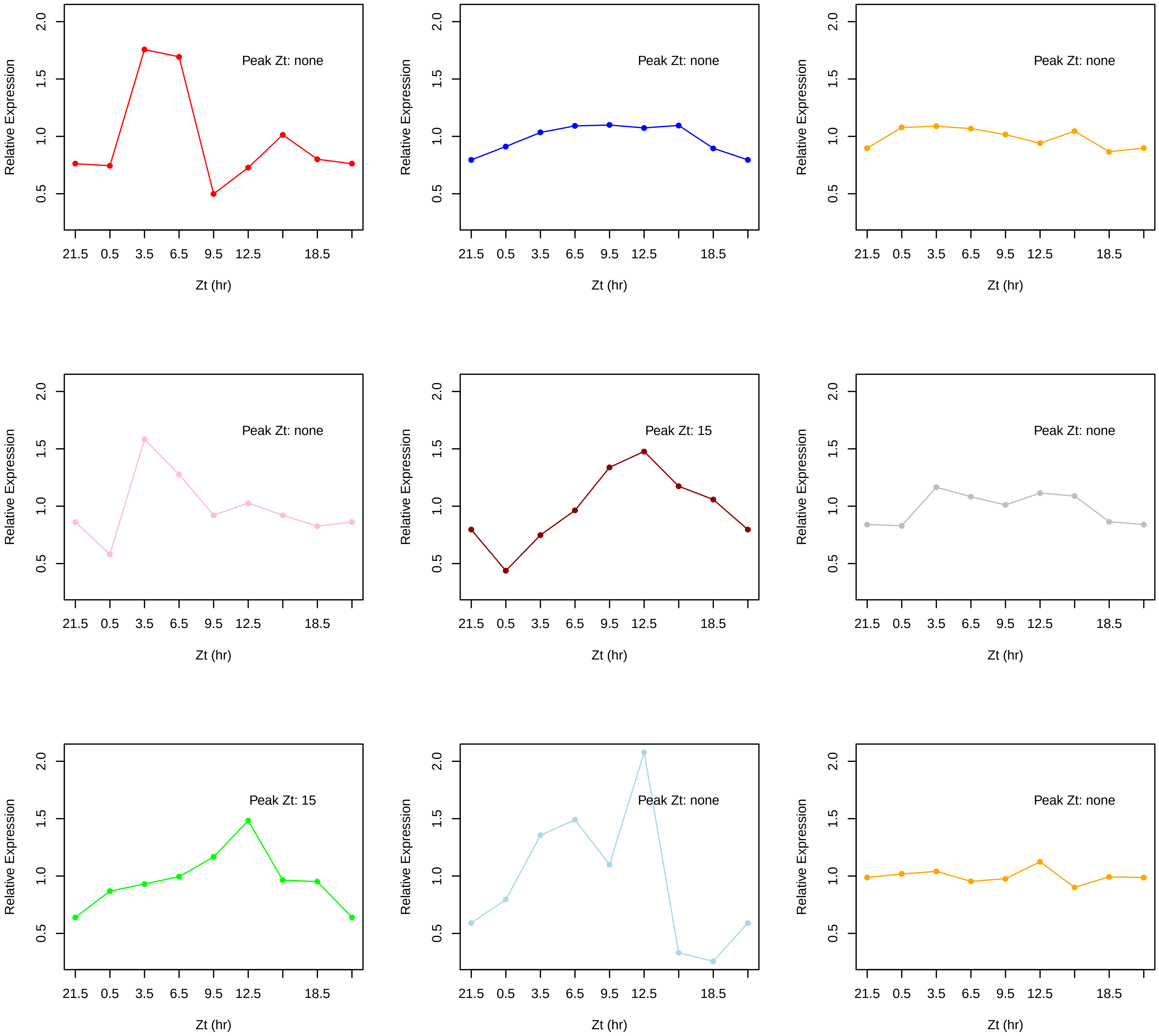

Singeleton: ENSXMAG00000025389 si:dkey−19e4.5

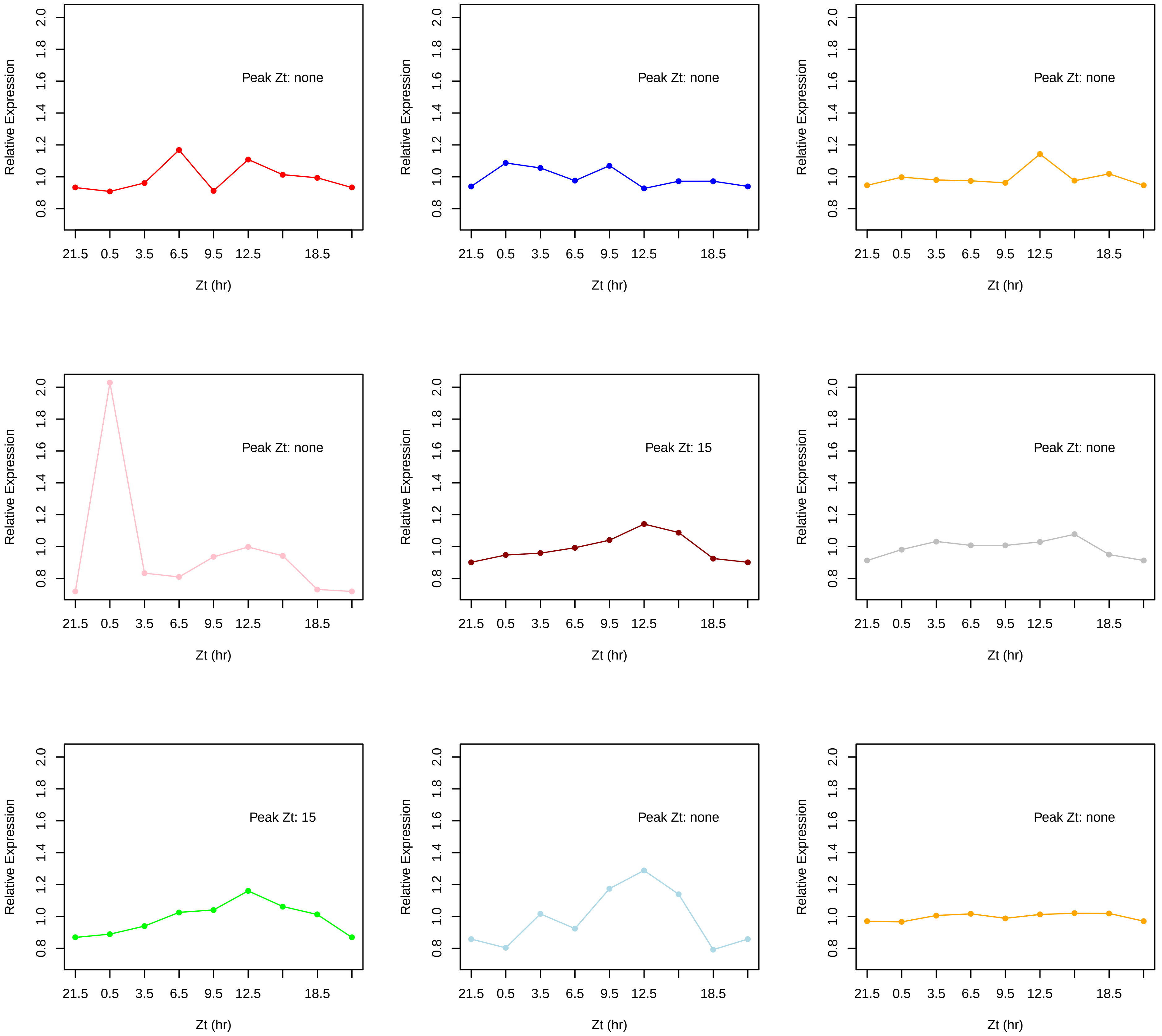

Singeleton: ENSXMAG00000000197 si:dkey−28e7.3

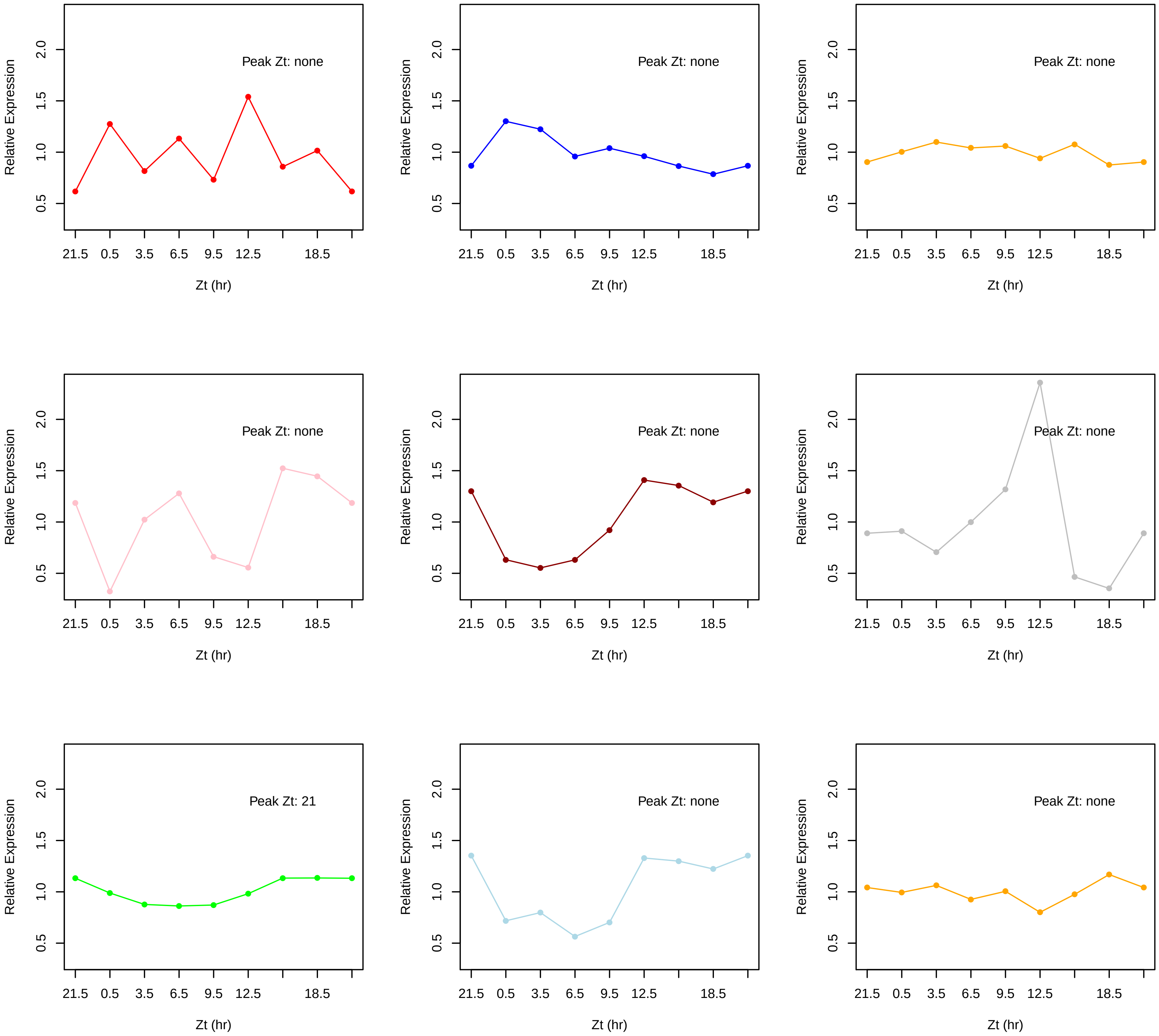

Singeleton: ENSXMAG00000007627 srrt

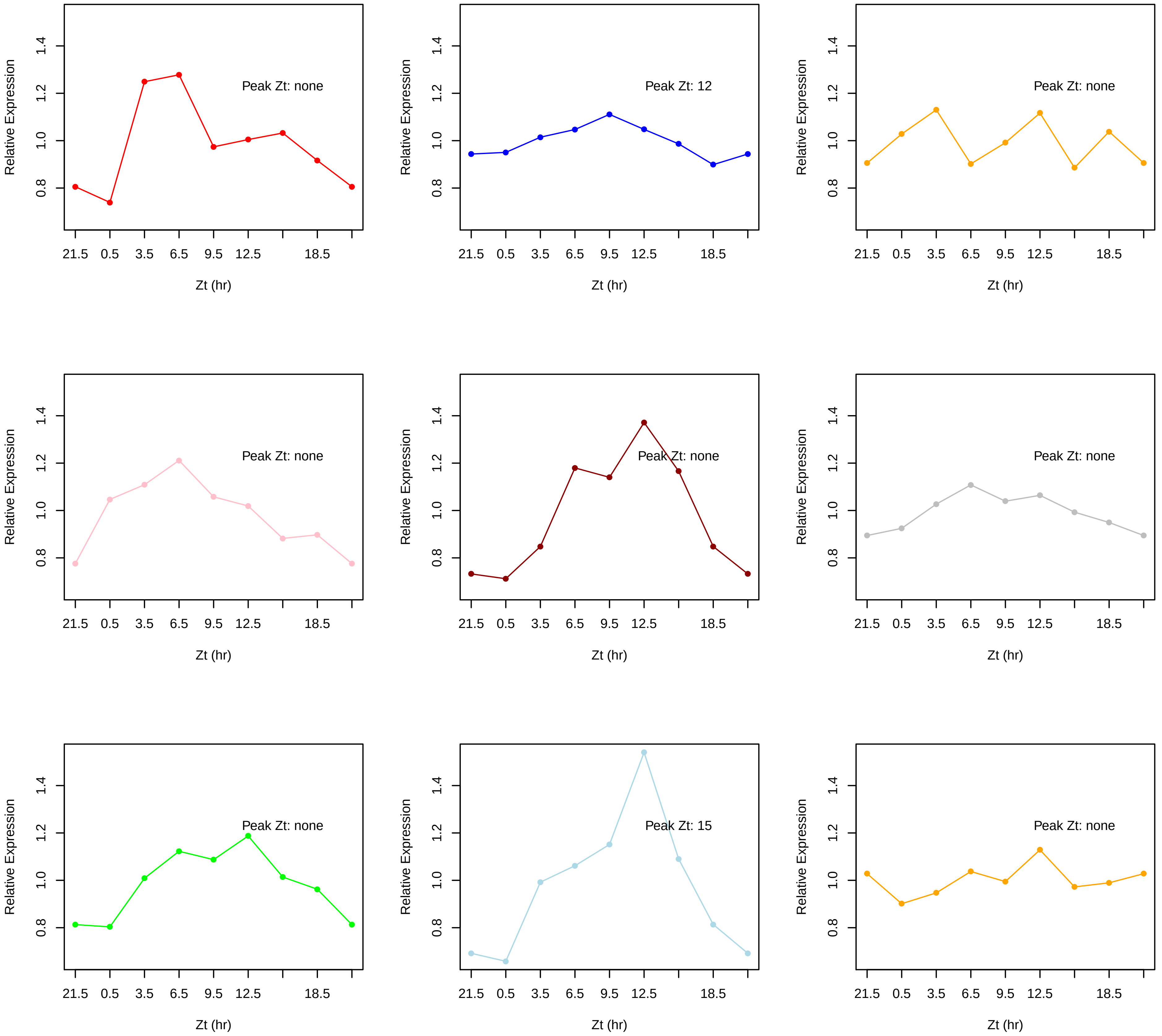

Singeleton: ENSXMAG00000026062 fgf16

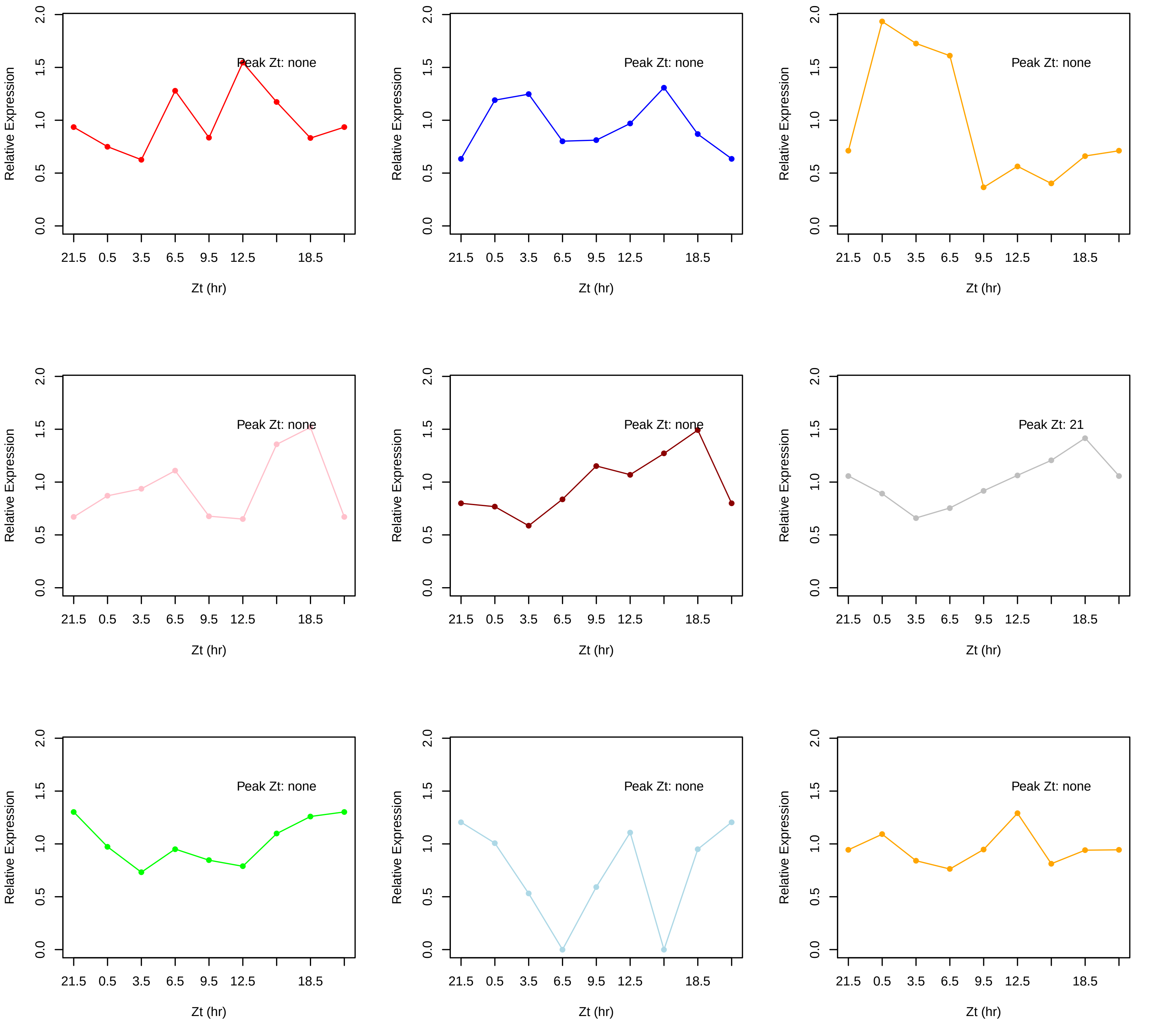

Singeleton: ENSXMAG00000000209 jupa

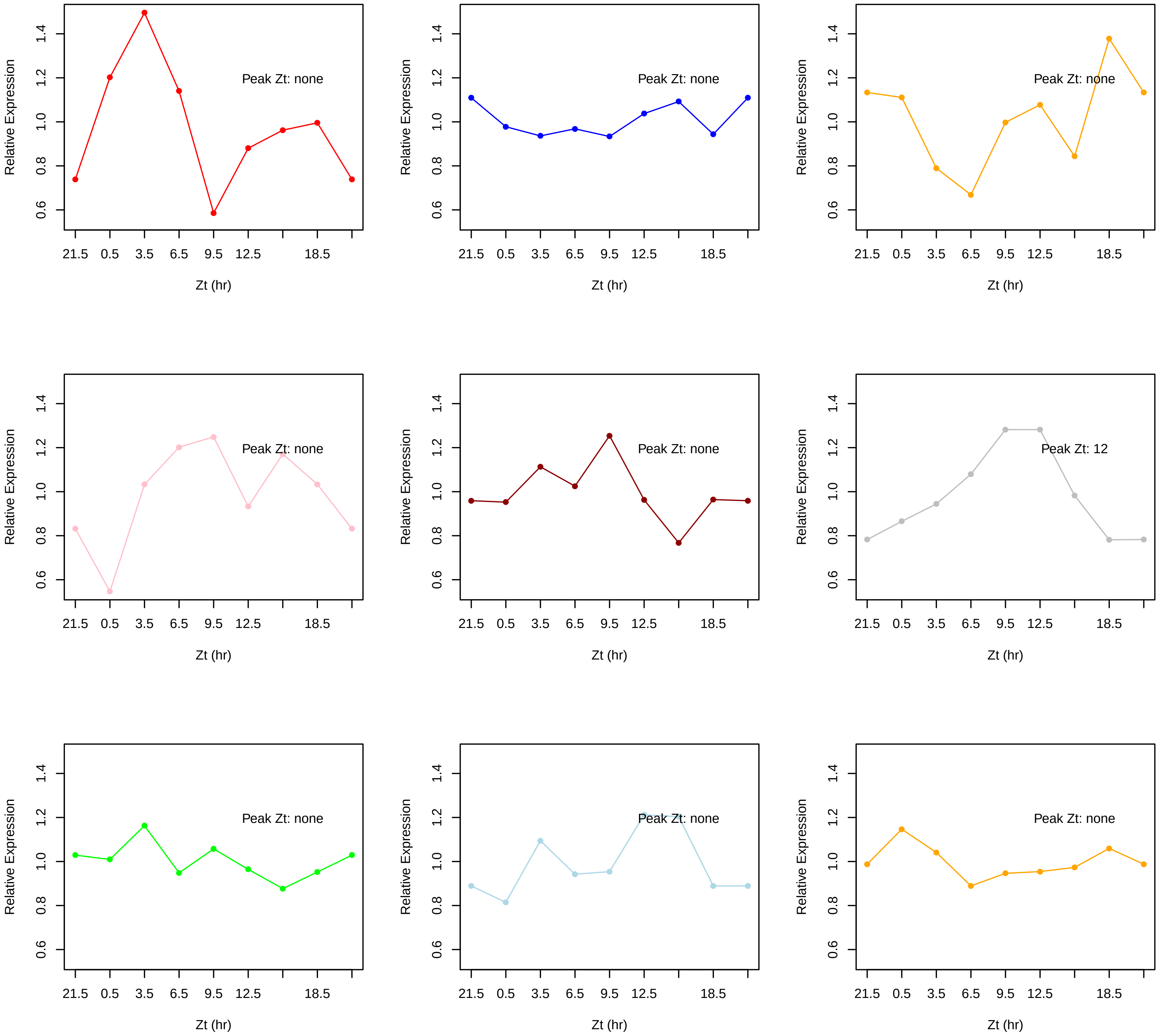

Singeleton: ENSXMAG00000007385 ephb4b

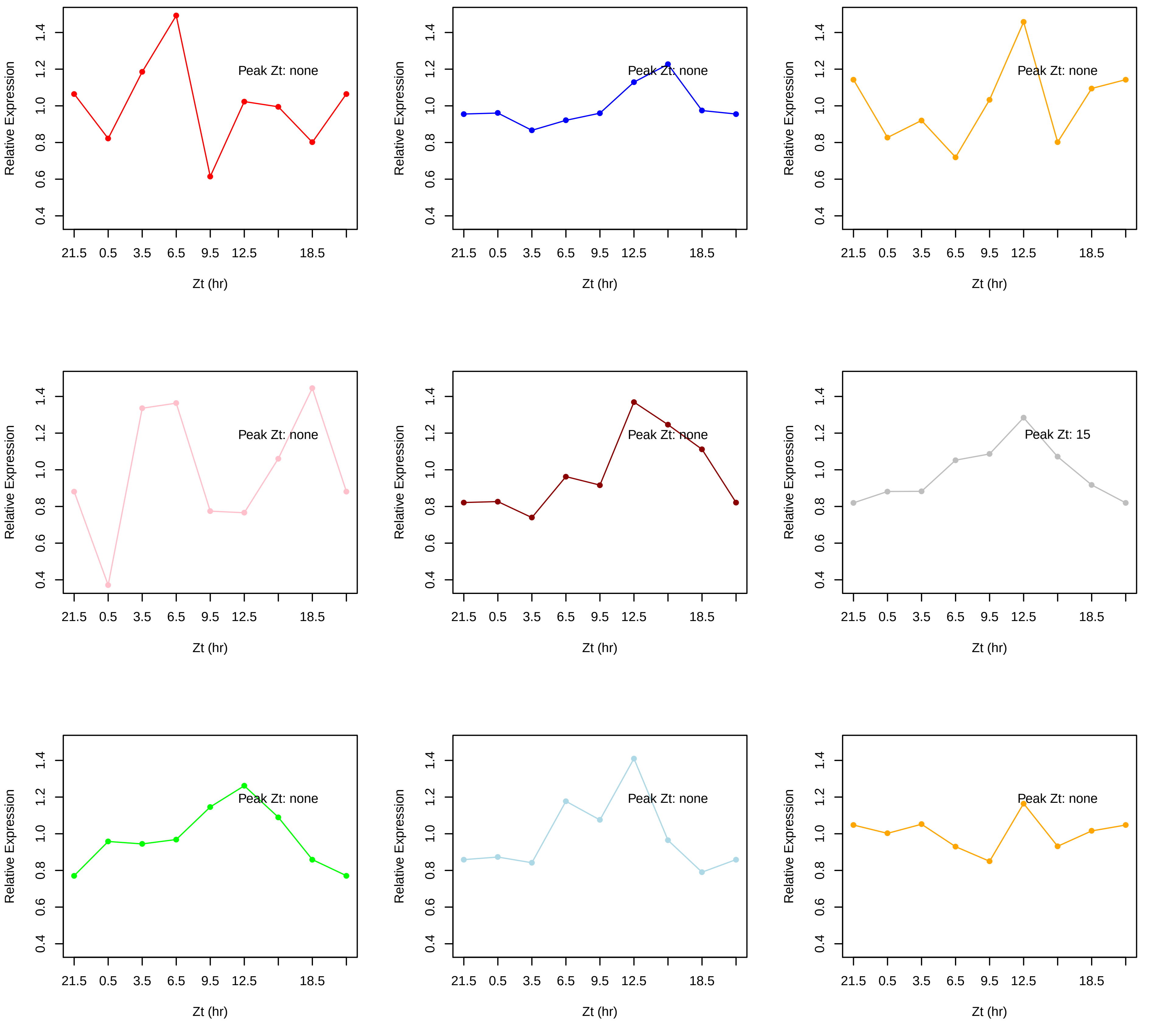

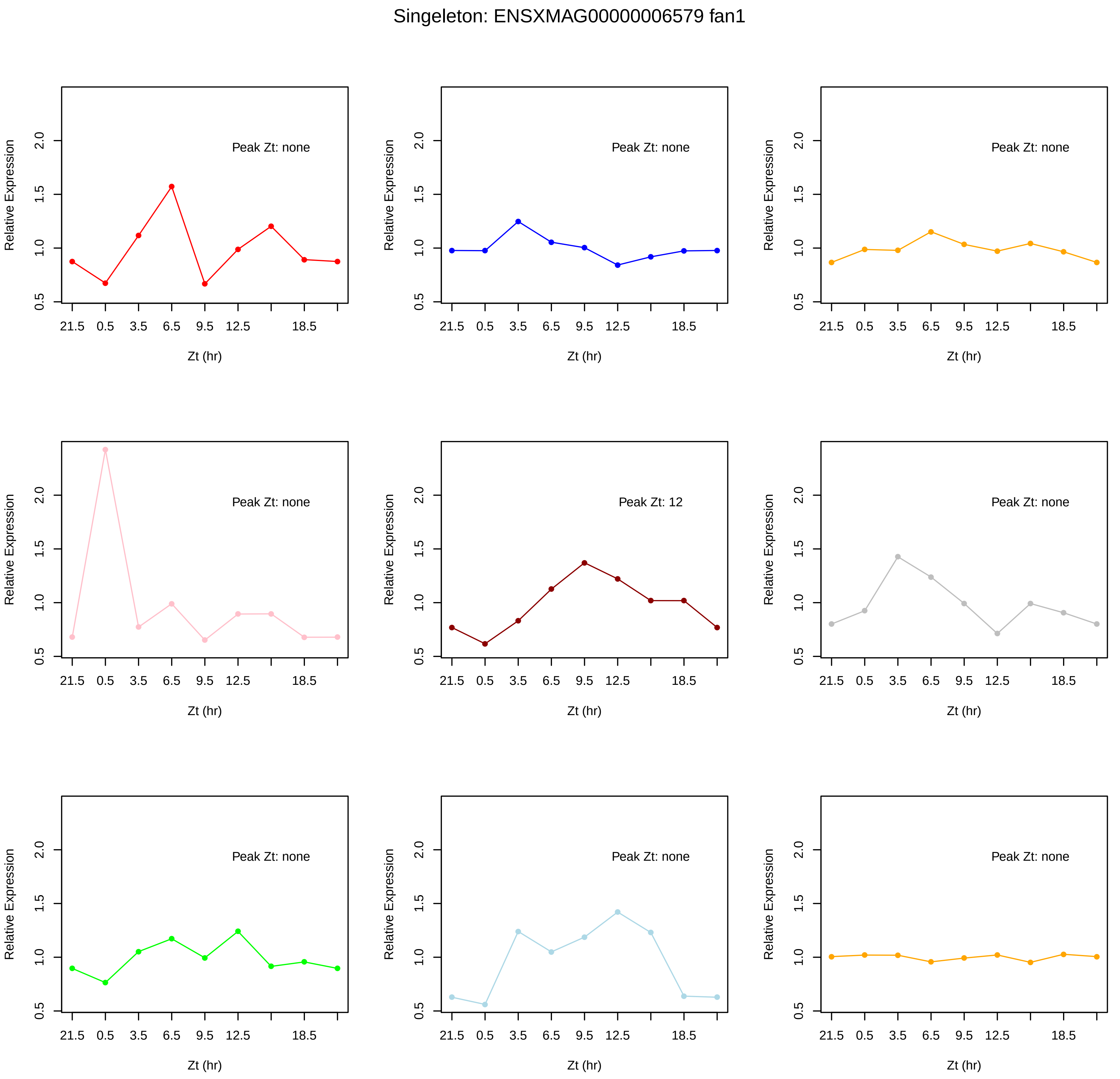
Singeleton: ENSXMAG00000006579 fan1
Peak Zt: none
Peak Zt: none
Peak Zt: none
Peak Zt: none
Peak Zt: 12
Peak Zt: none
Peak Zt: none
Peak Zt: none
Peak Zt: none
Relative Expression
Zt (hr)
0.5
1.0
1.5
2.0
21.5
0.5
3.5
6.5
9.5
12.5
18.5

Singeleton: ENSXMAG00000018298 mphosph10

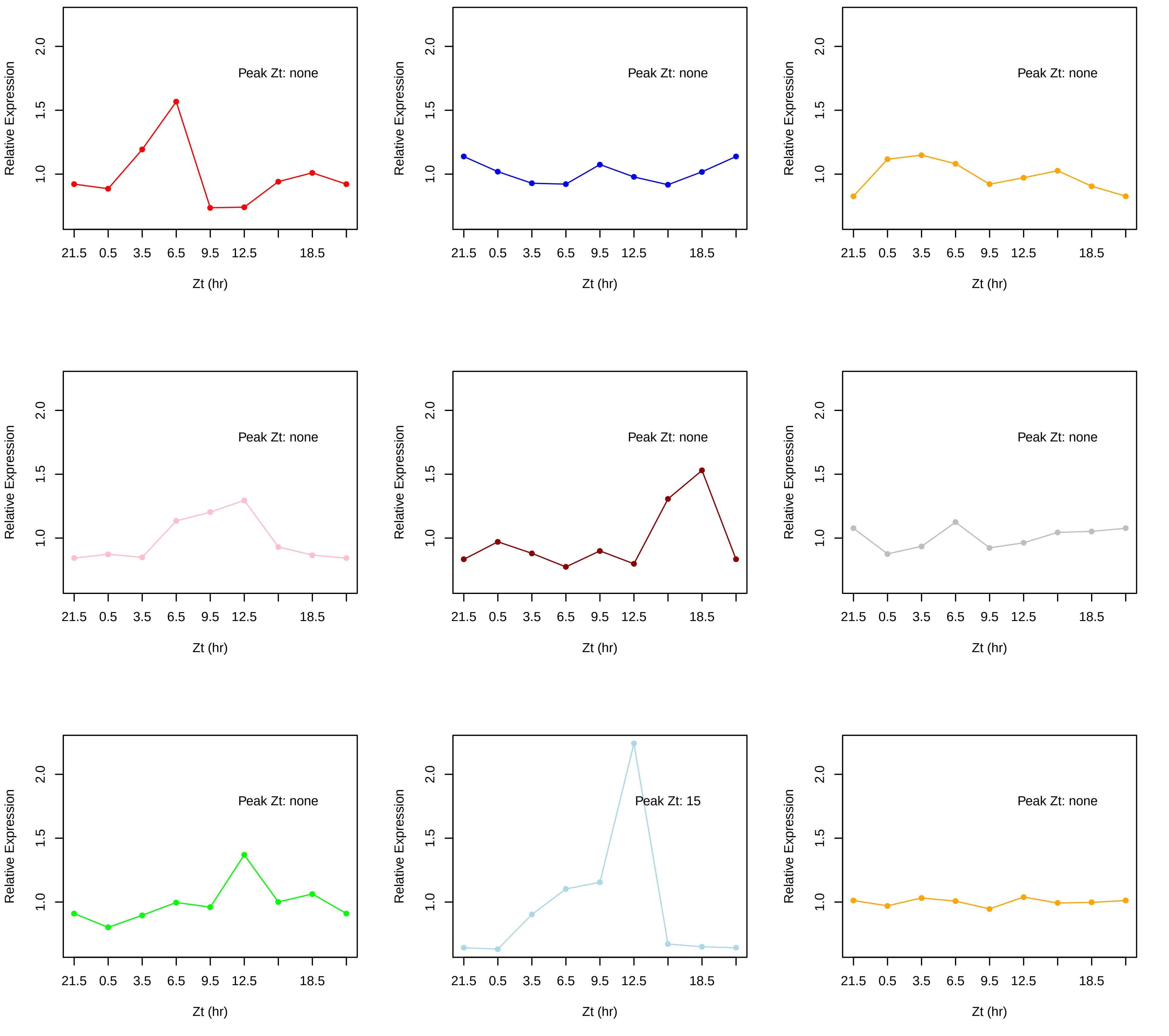

Singeleton: ENSXMAG00000018295 mcee

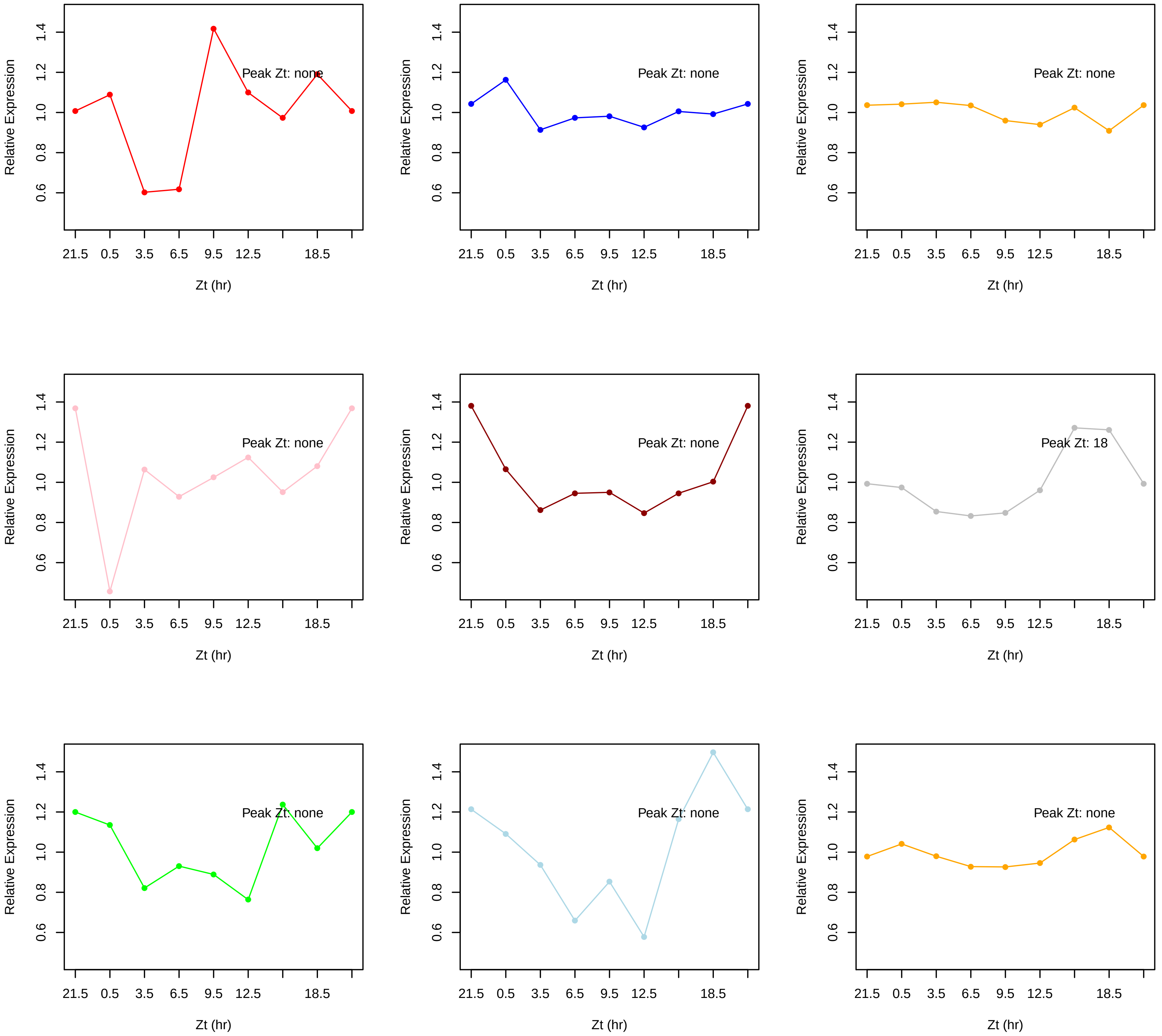

Singeleton: ENSXMAG00000027861

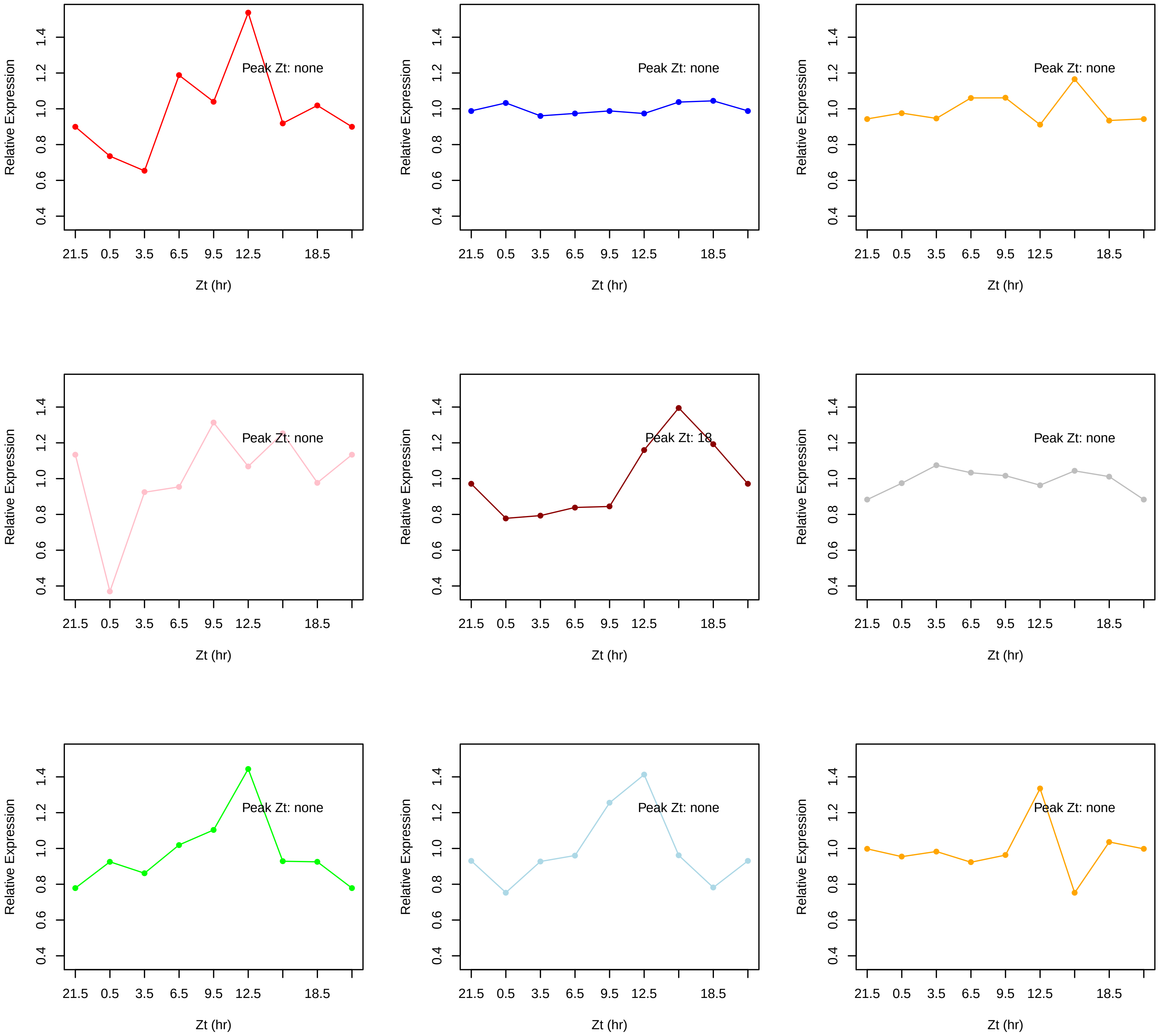

Singeleton: ENSXMAG00000018120 psmb3

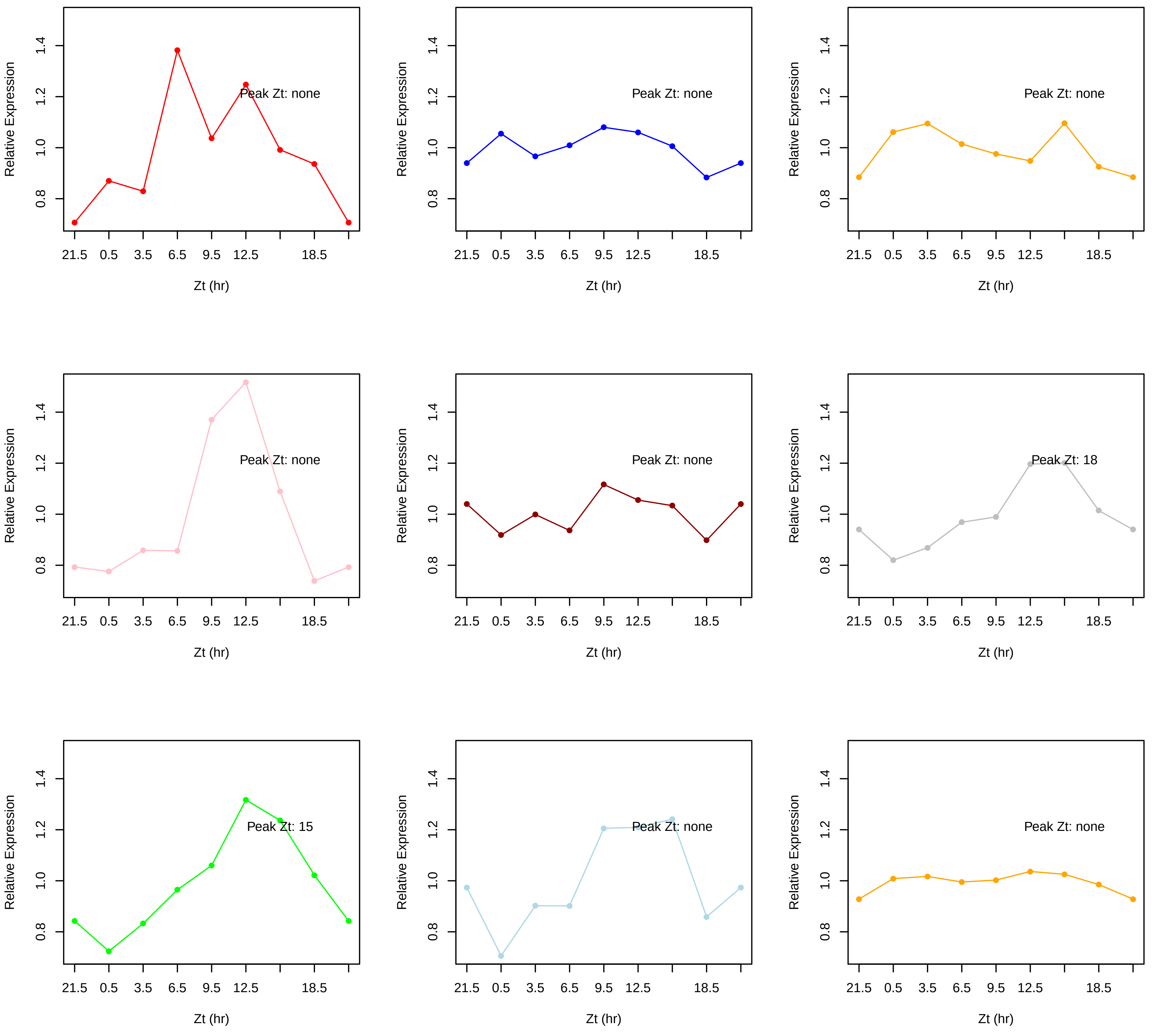

Singeleton: ENSXMAG00000027249 zgc:109889

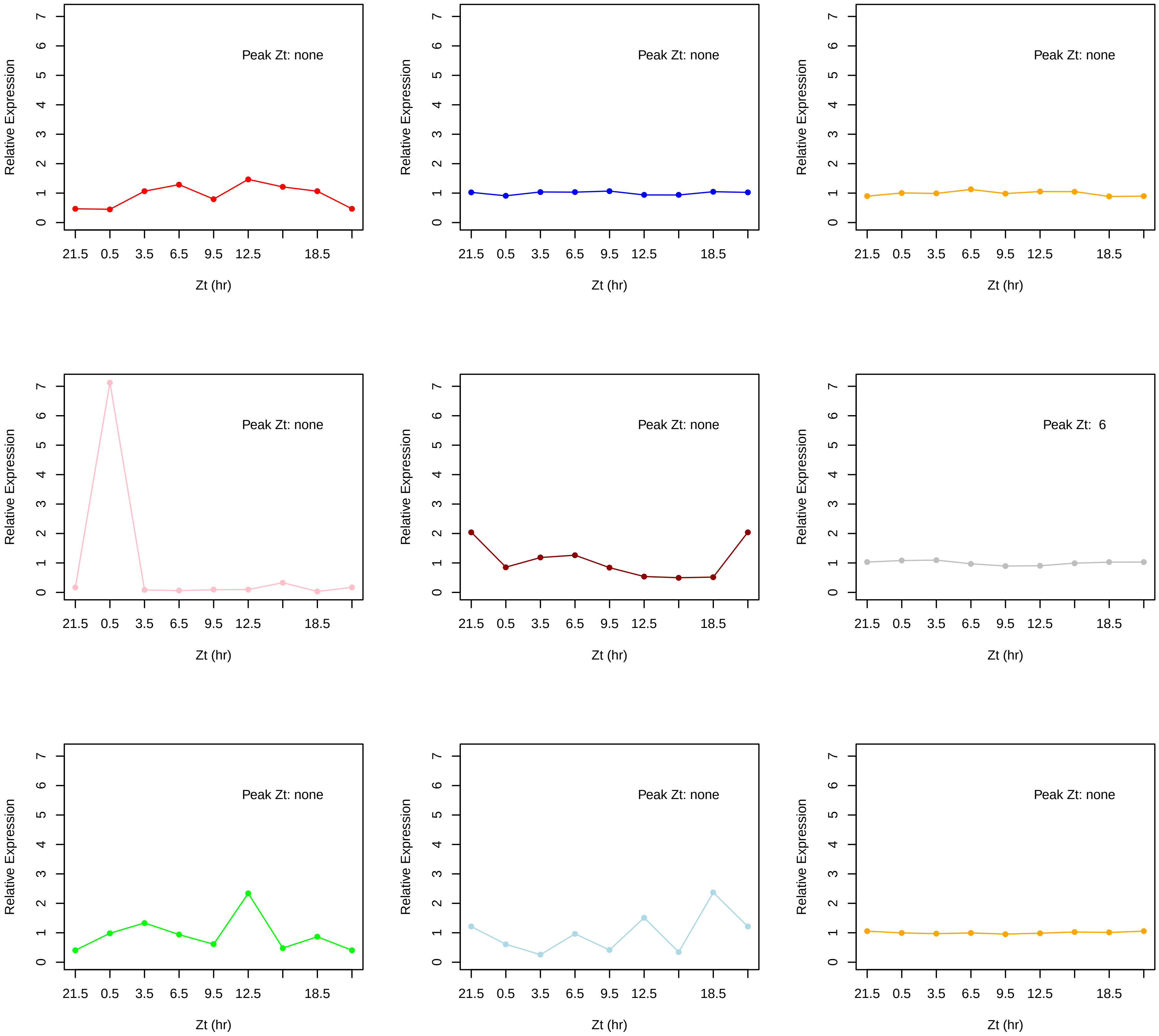

Singeleton: ENSXMAG00000007621

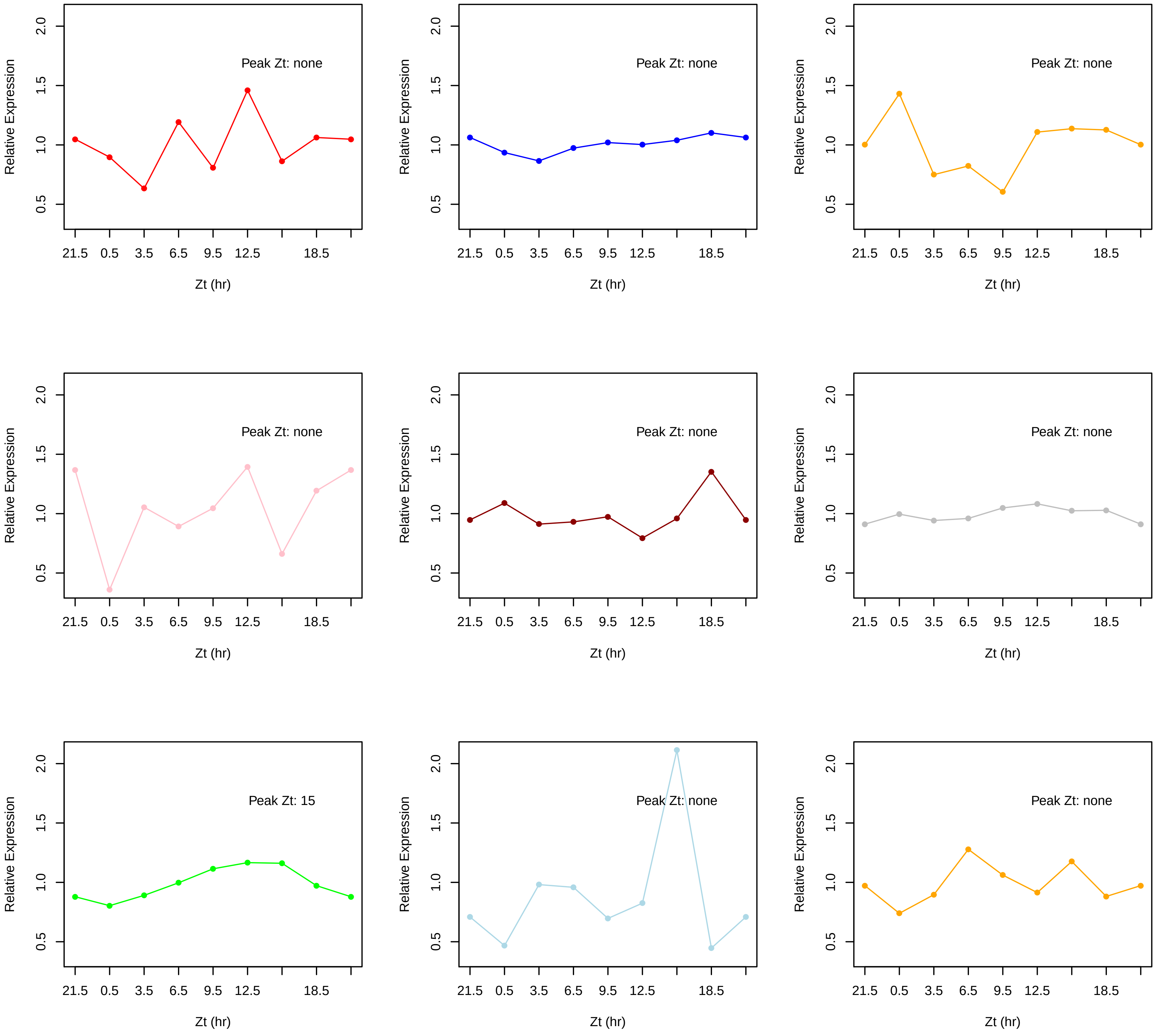

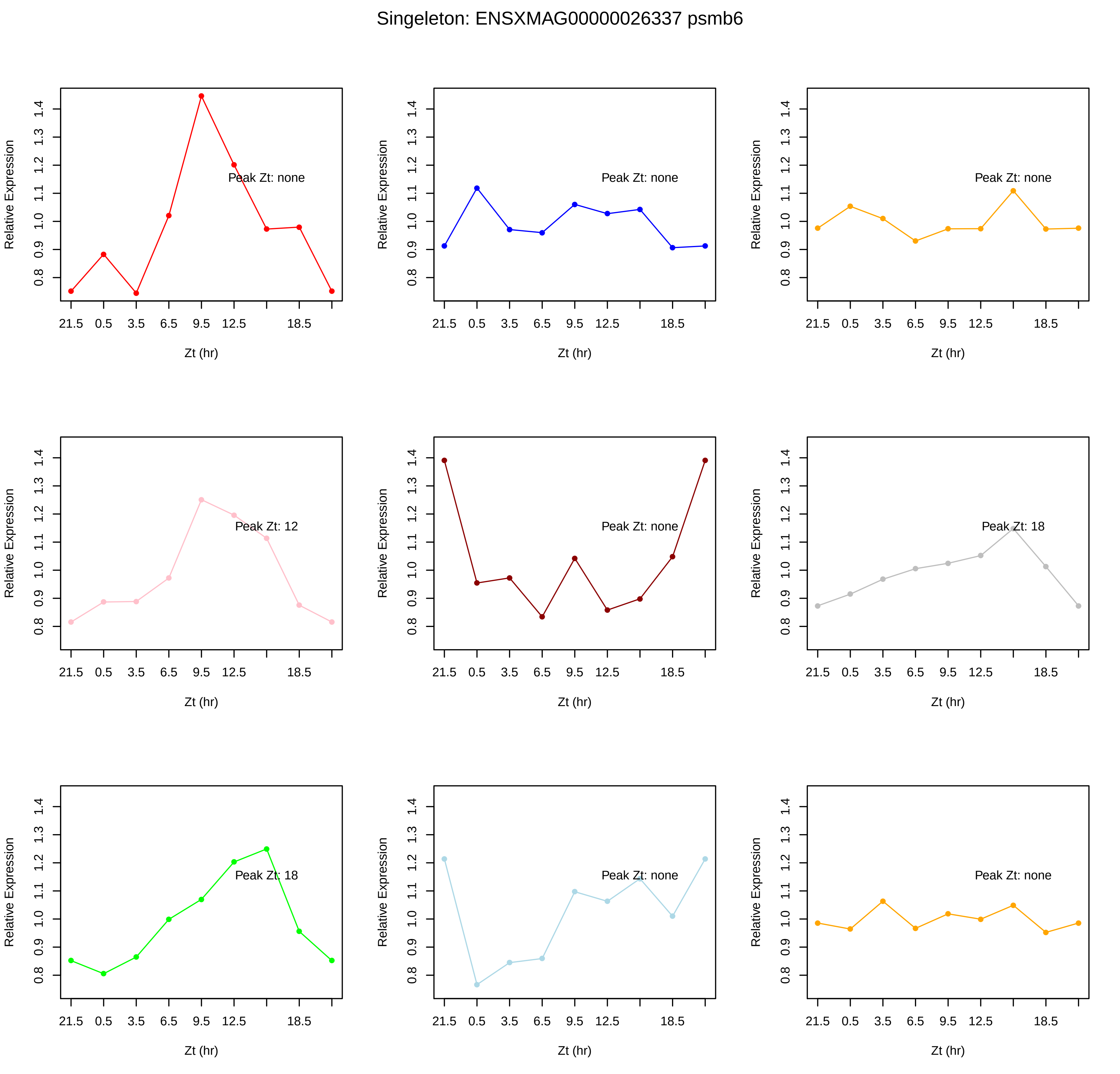

Singeleton: ENSXMAG00000026337 psmb6
Peak Zt: none
Peak Zt: none
Peak Zt: none
Peak Zt: 12
Peak Zt: none
Peak Zt: 18
Peak Zt: 18
Peak Zt: none
Peak Zt: none
Relative Expression
Zt (hr)
21.5
0.5
3.5
6.5
9.5
12.5
18.5
0.8
0.9
1.0
1.1
1.2
1.3
1.4

Singeleton: ENSXMAG00000007705 rwdd

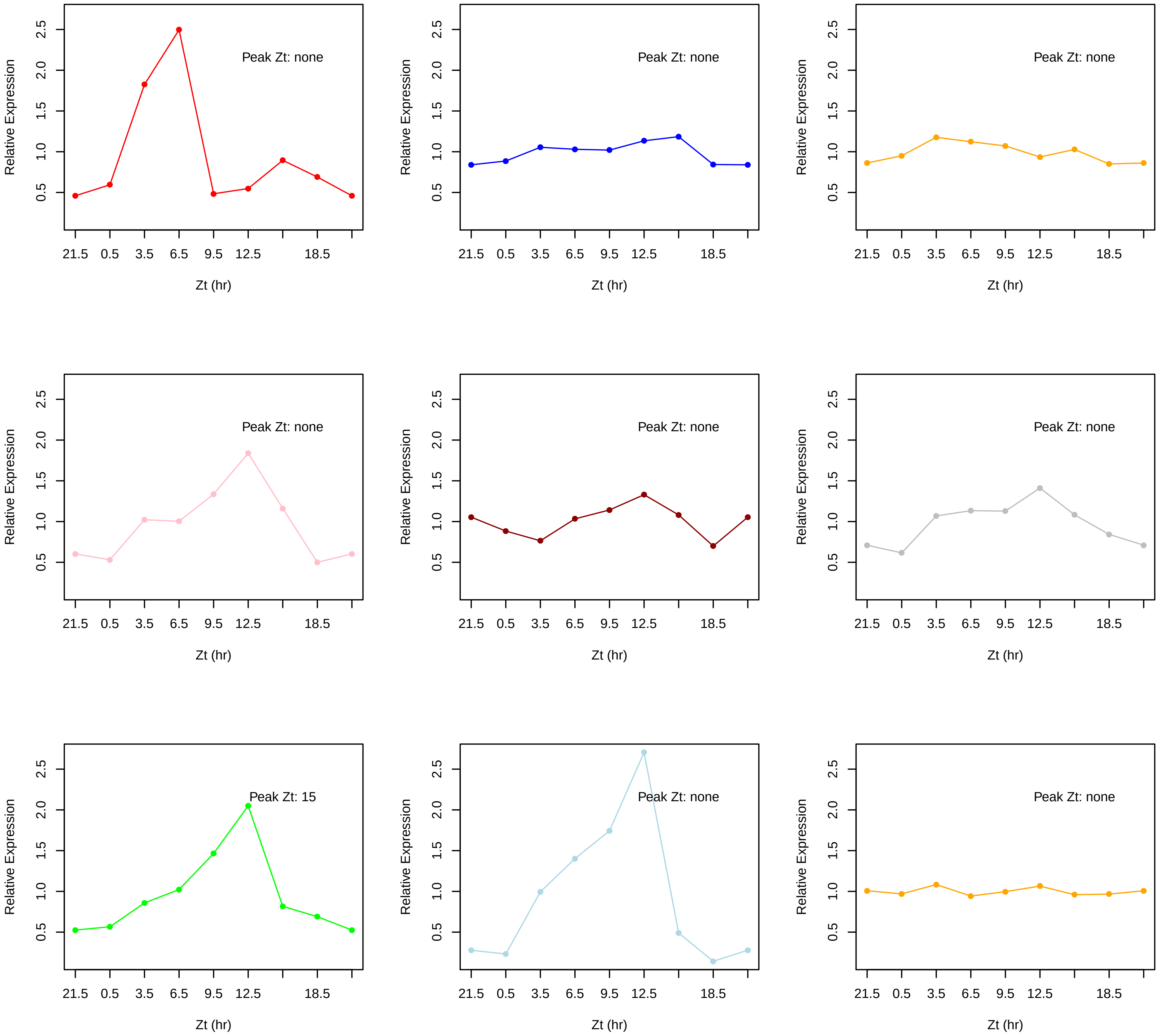

Singeleton: ENSXMAG00000018124 PIP4K2B

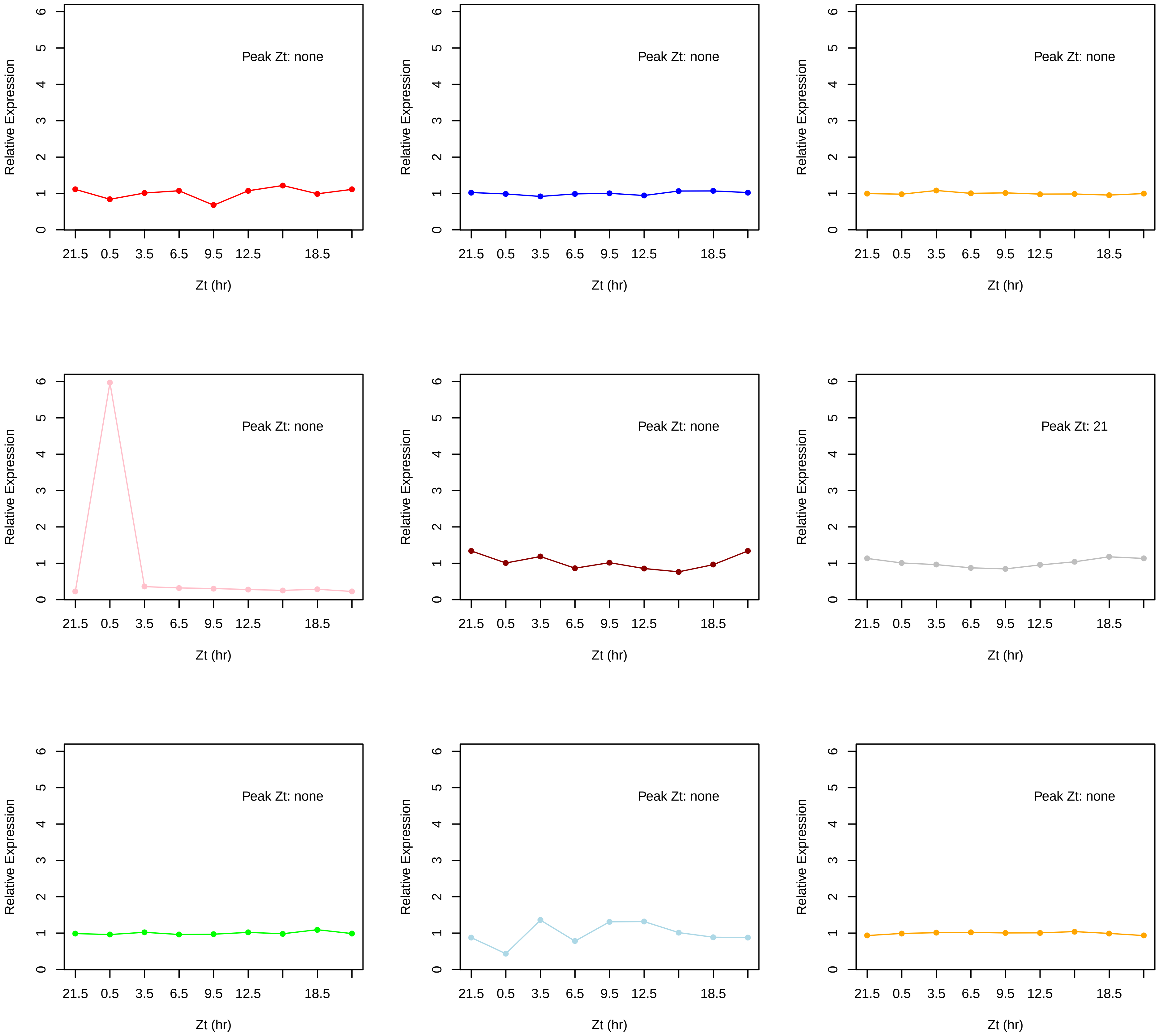

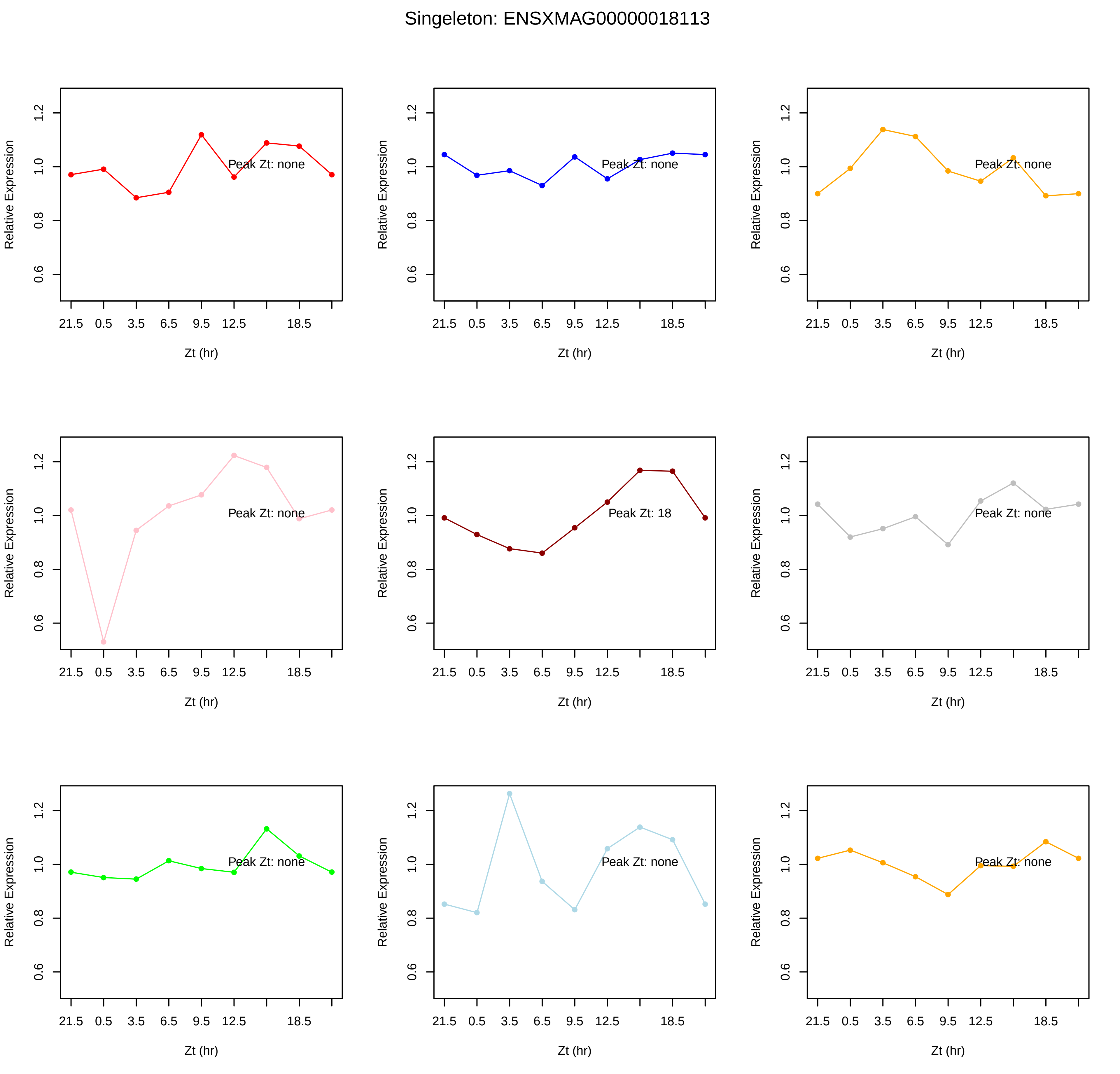
Singeleton: ENSXMAG00000018113
Peak Zt: none
Peak Zt: none
Peak Zt: none
Peak Zt: none
Peak Zt: 18
Peak Zt: none
Peak Zt: none
Peak Zt: none
Peak Zt: none
Relative Expression
Zt (hr)
21.5
0.5
3.5
6.5
9.5
12.5
18.5
0.6
0.8
1.0
1.2

Singeleton: ENSXMAG00000004919 dkc1

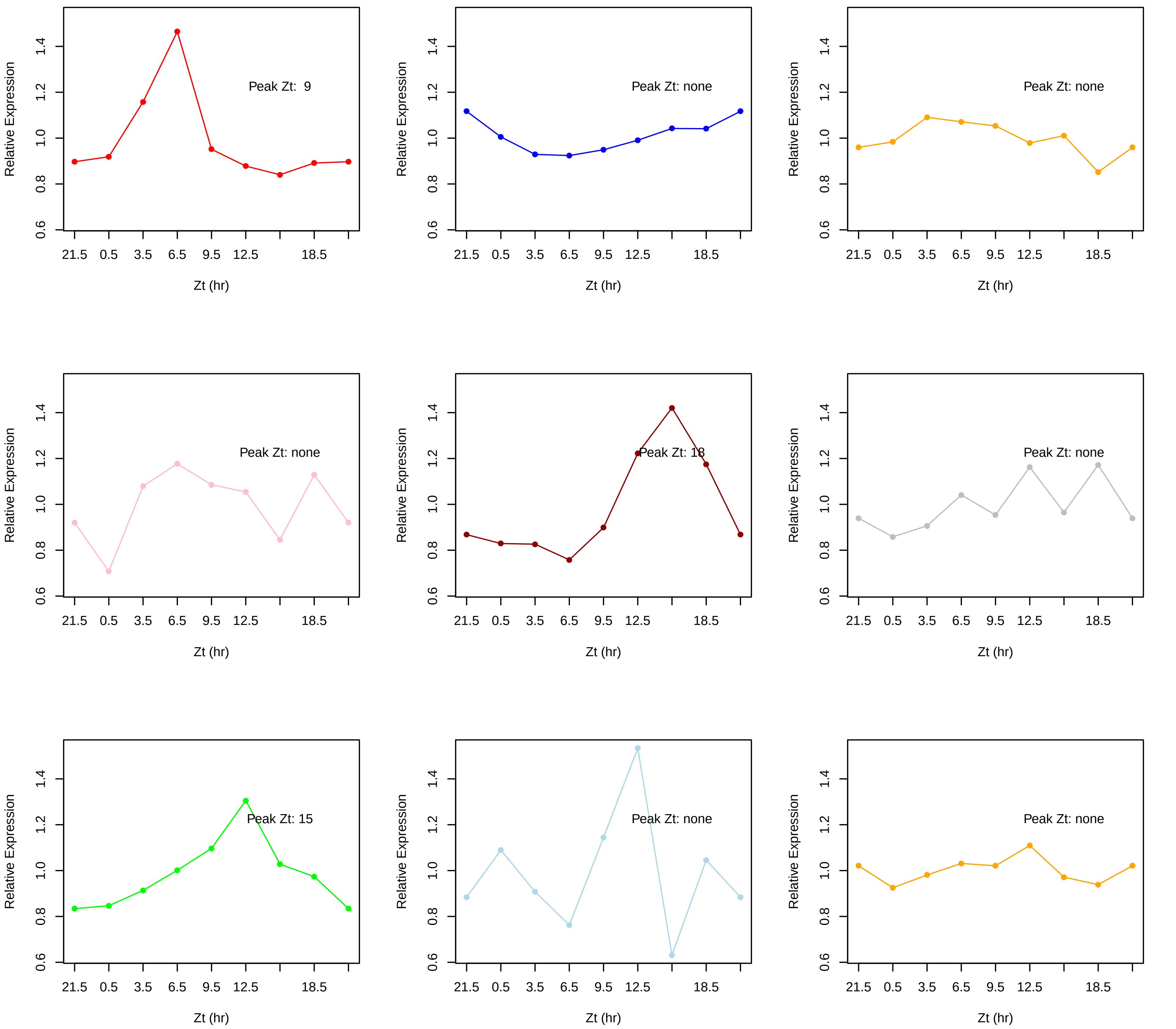

Singleton: ENSXMAG00000018095 scrn2

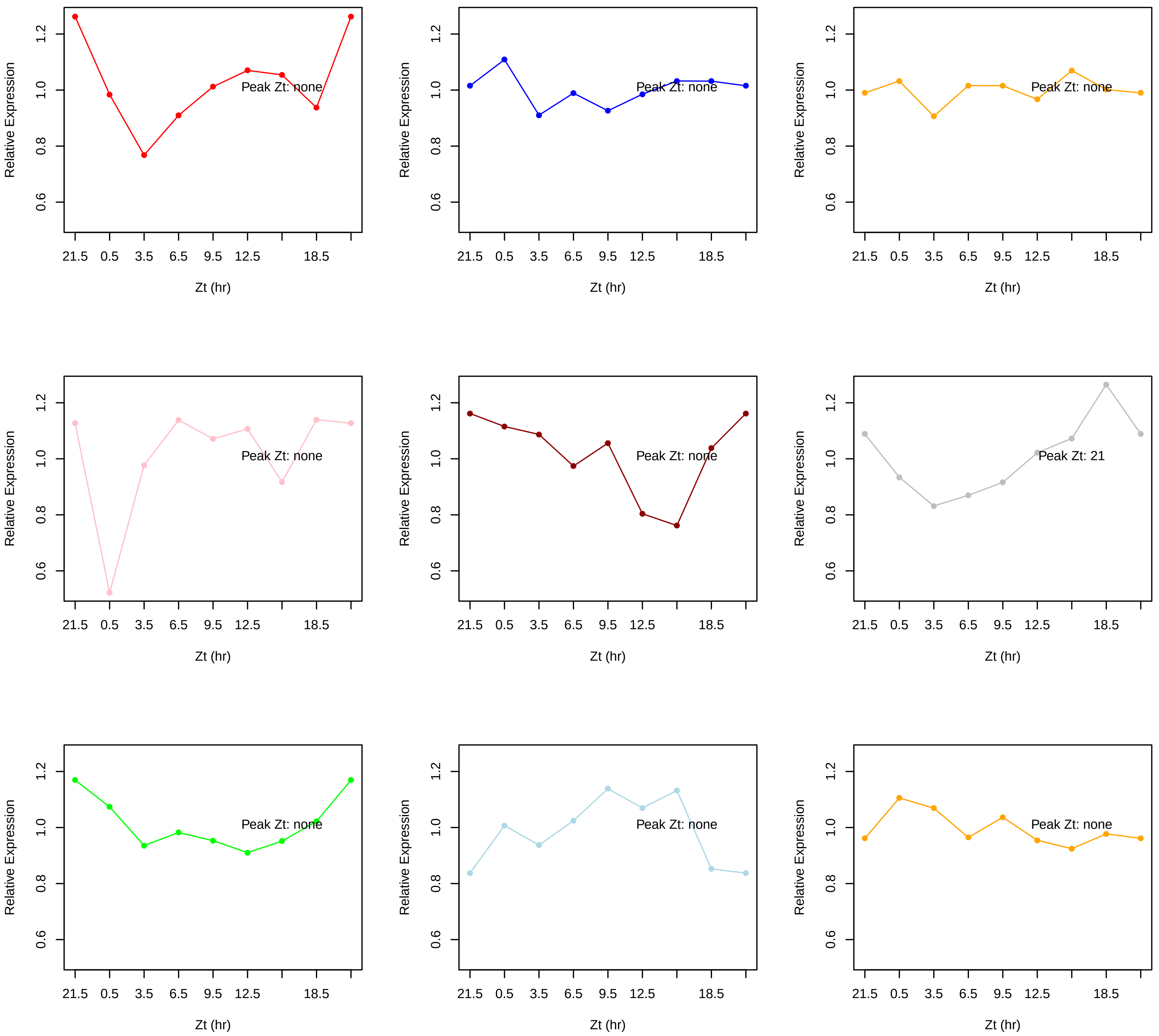

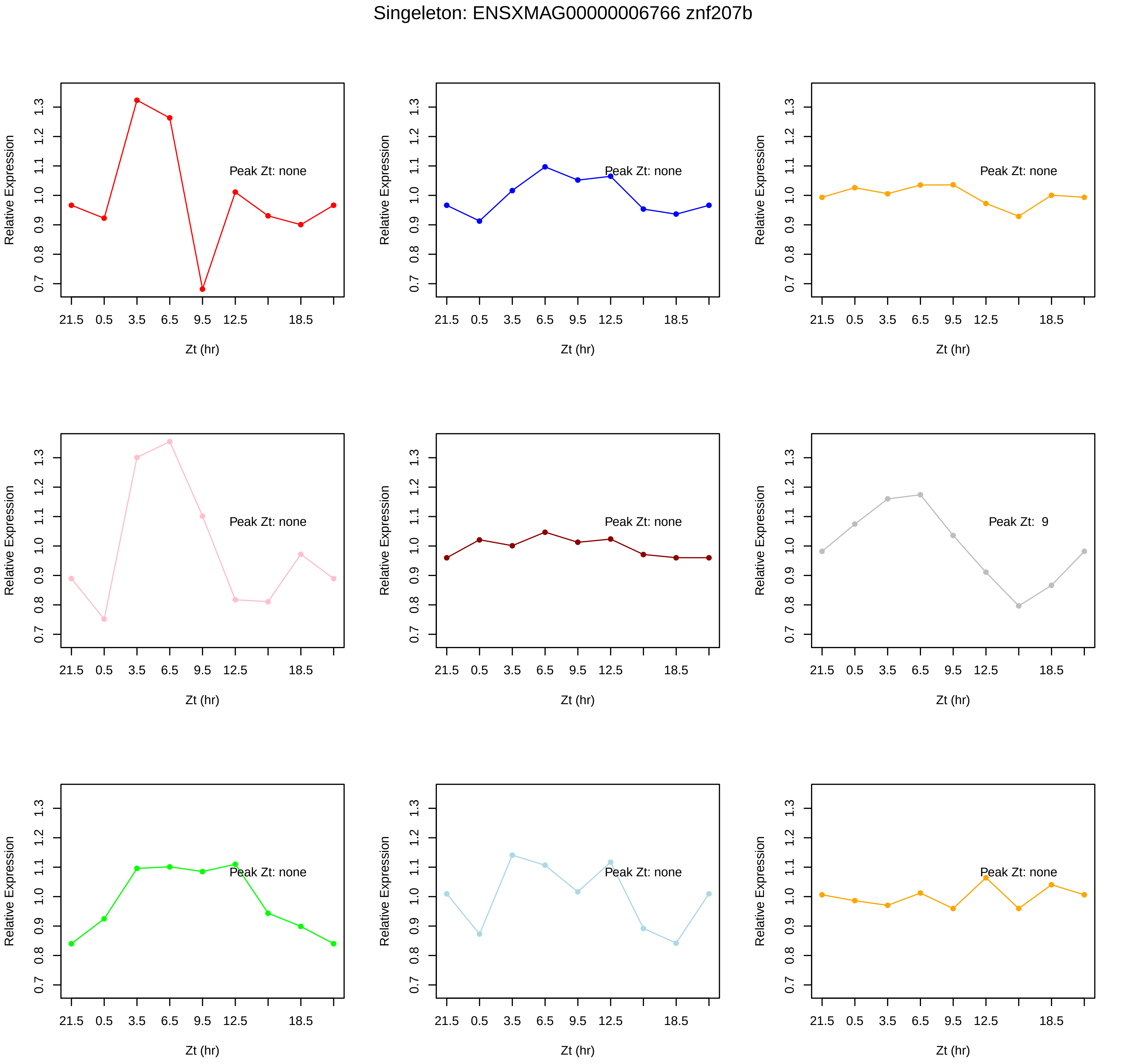
Singeleton: ENSXMAG00000006766 znf207b
Relative Expression
Zt (hr)
21.5 0.5 3.5 6.5 9.5 12.5 18.5
0.7 0.8 0.9 1.0 1.1 1.2 1.3
Peak Zt: none
Peak Zt: none
Peak Zt: none
Peak Zt: none
Peak Zt: none
Peak Zt: 9
Peak Zt: none
Peak Zt: none
Peak Zt: none

Singleton: ENSXMAG00000024624 spag7

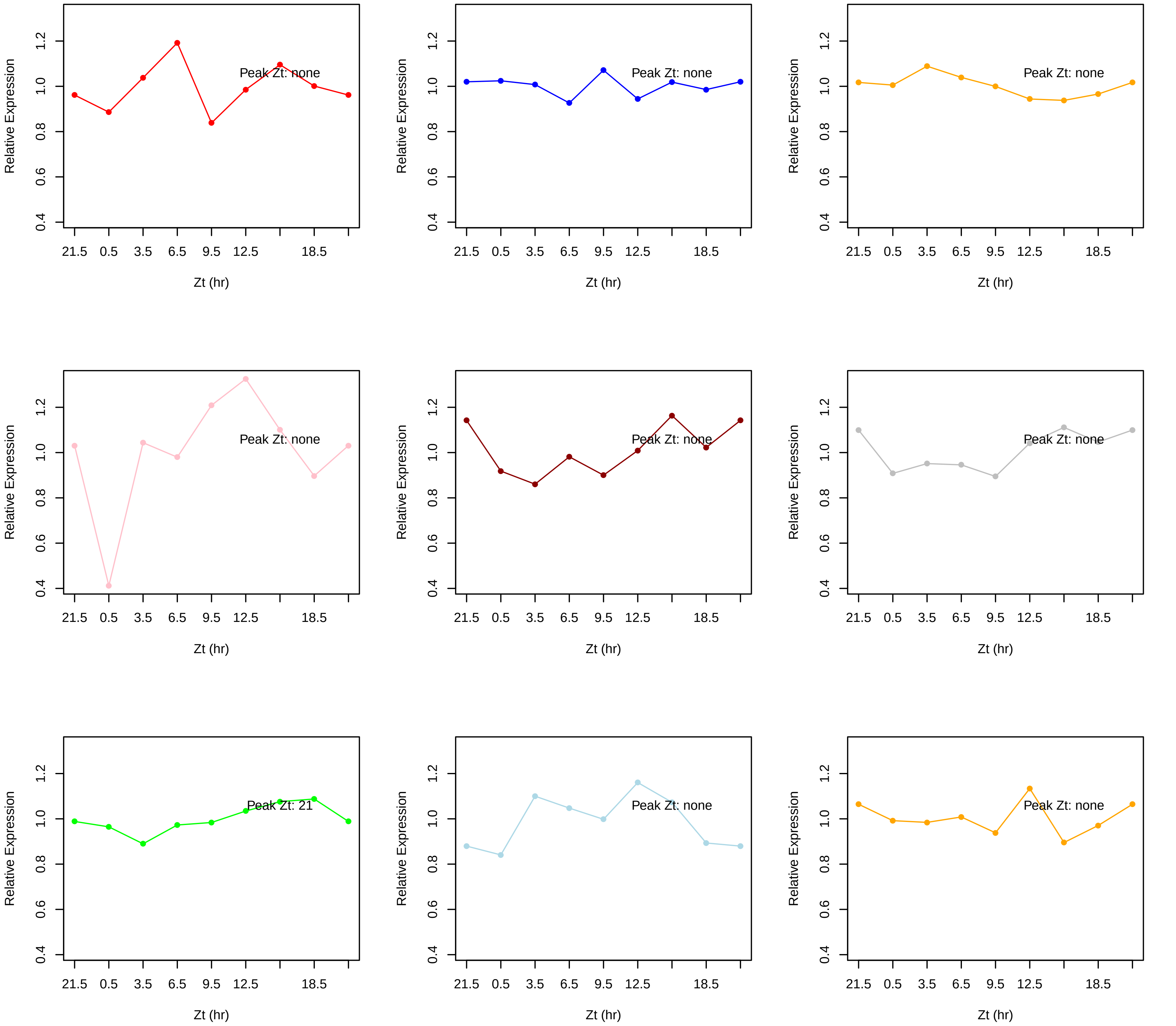

Singleton: ENSXMAG00000014794 enpp6

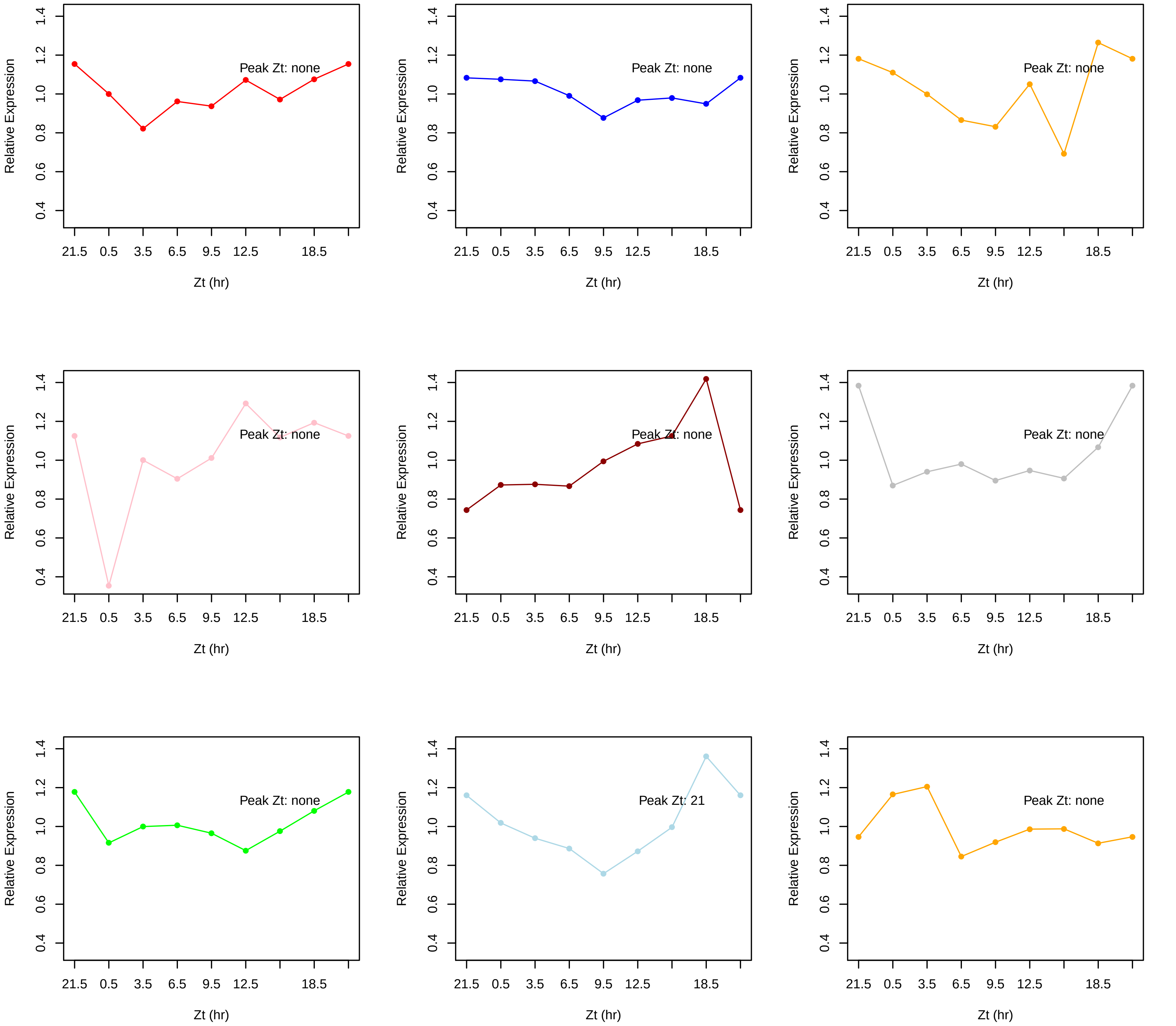

Singleton: ENSXMAG00000001327

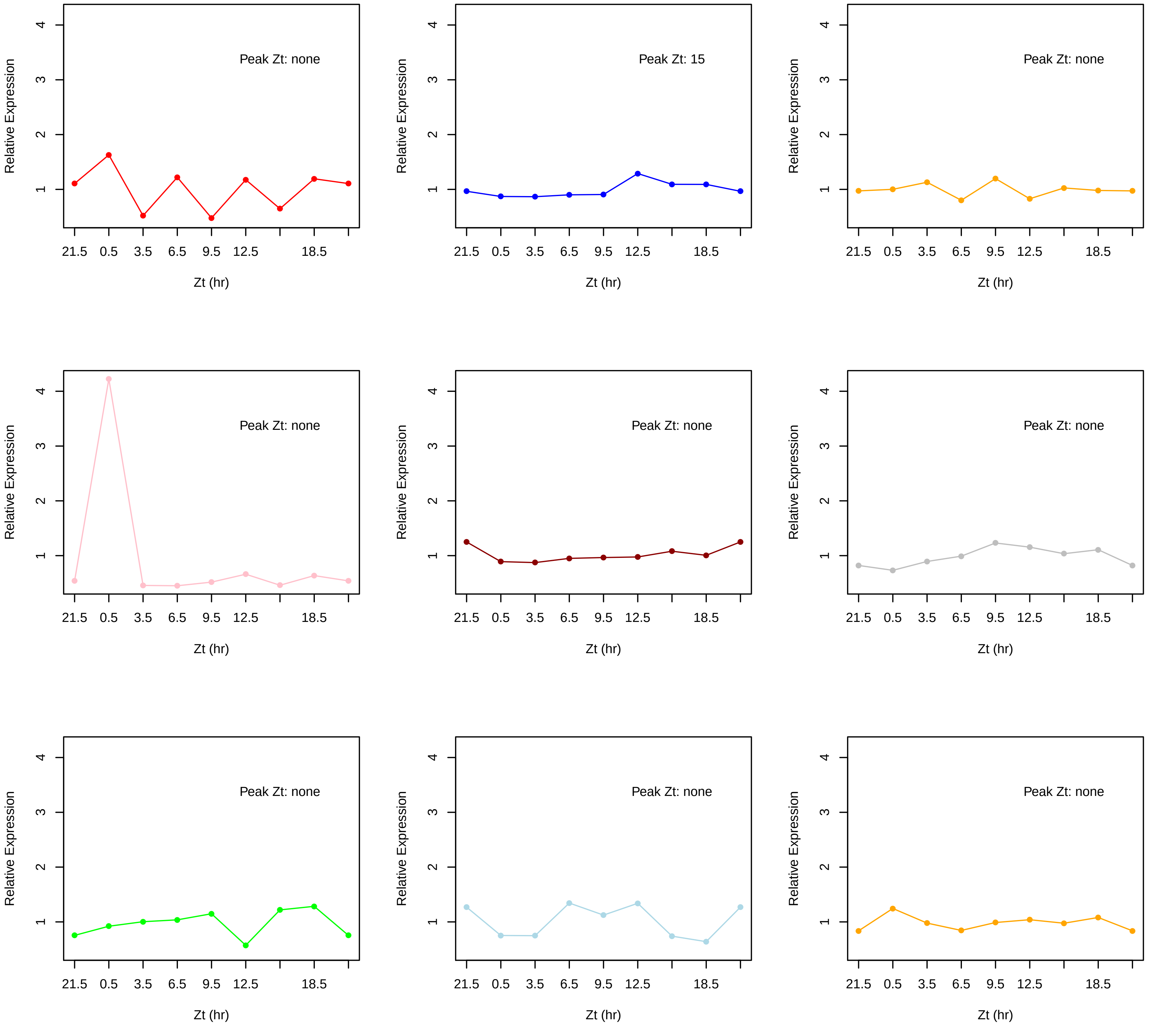

Singleton: ENSXMAG00000004921 mpp1

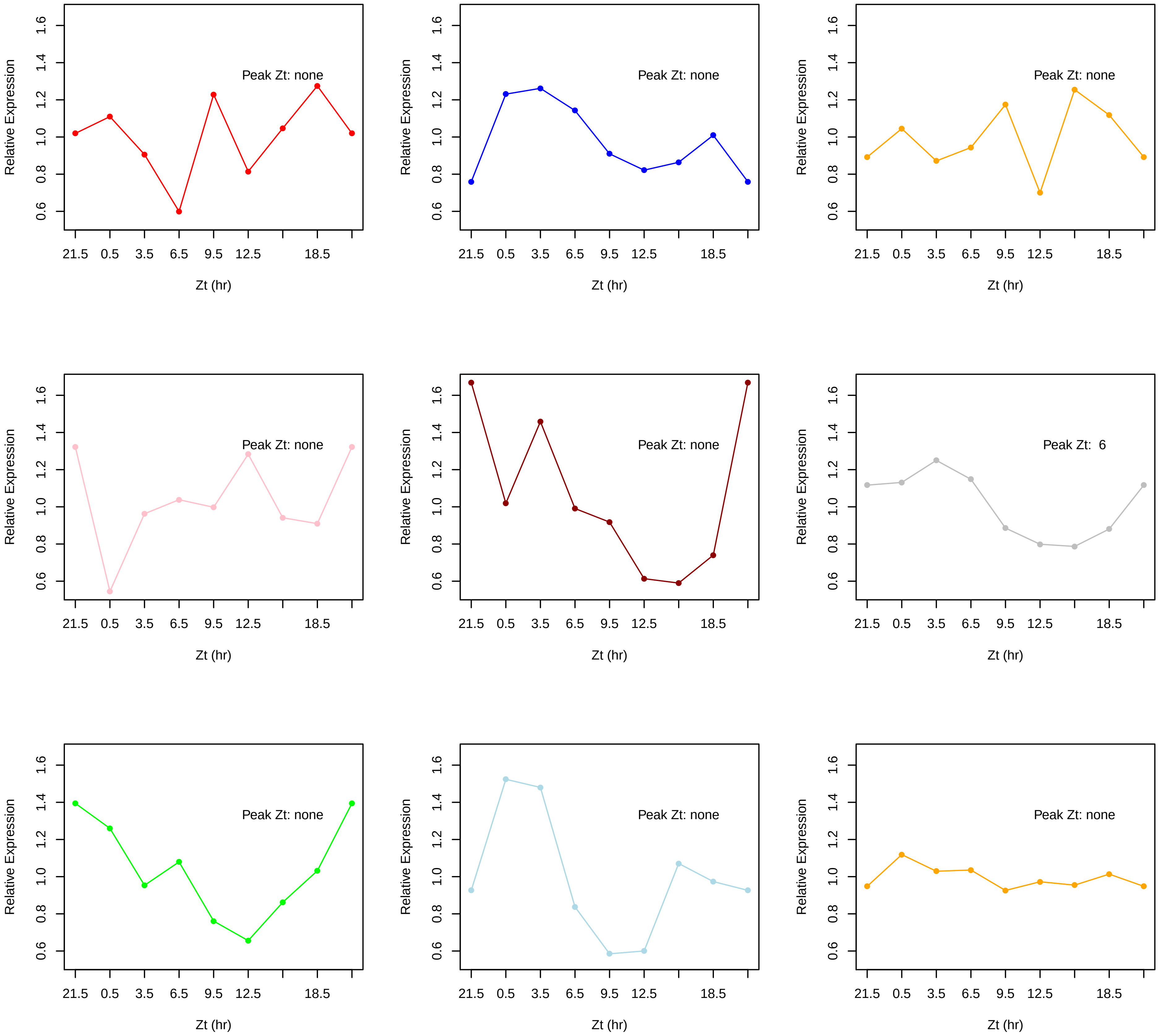

Singleton: ENSXMAG00000009544 txnrd3

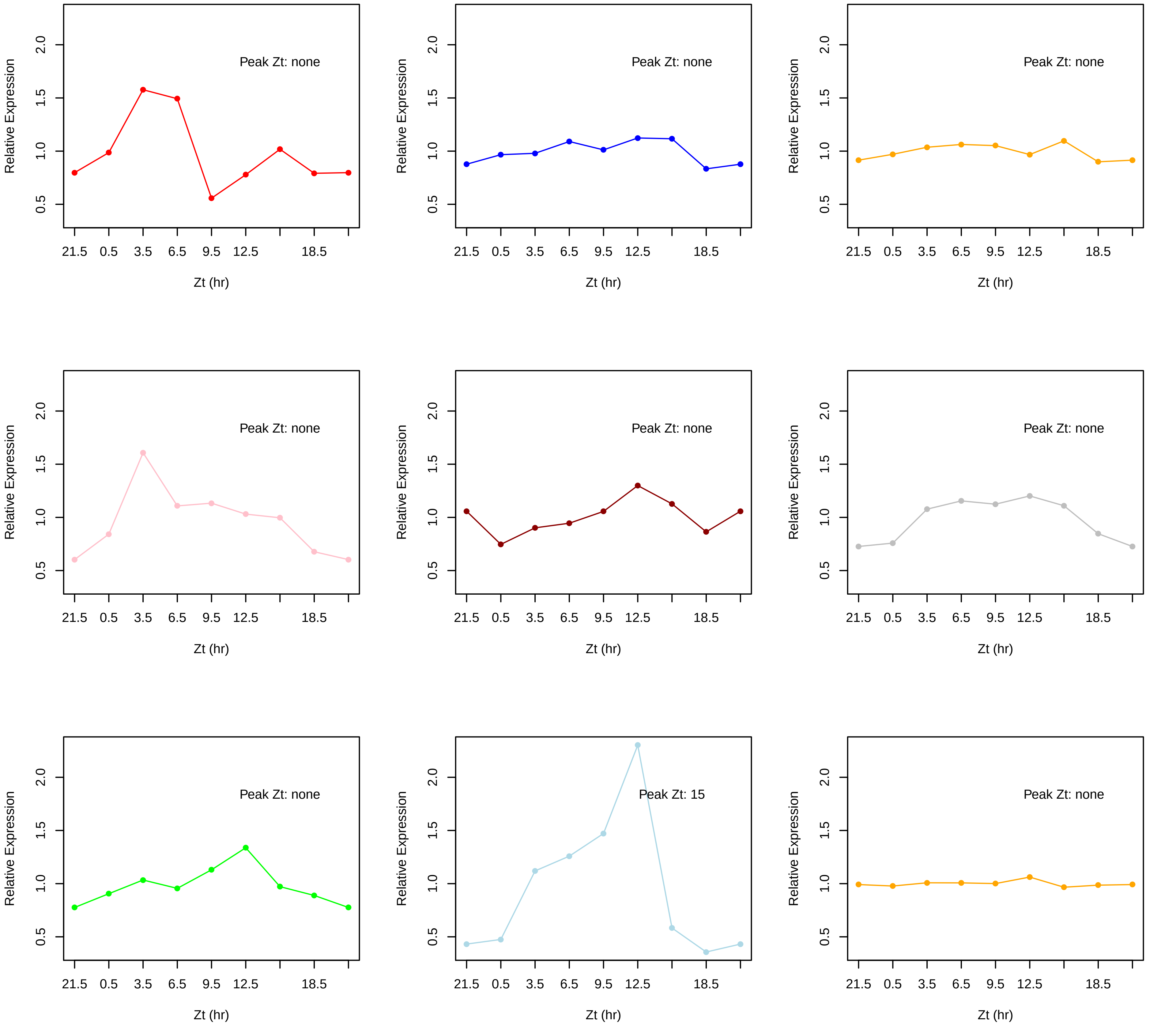

Singeleton: ENSXMAG00000013953

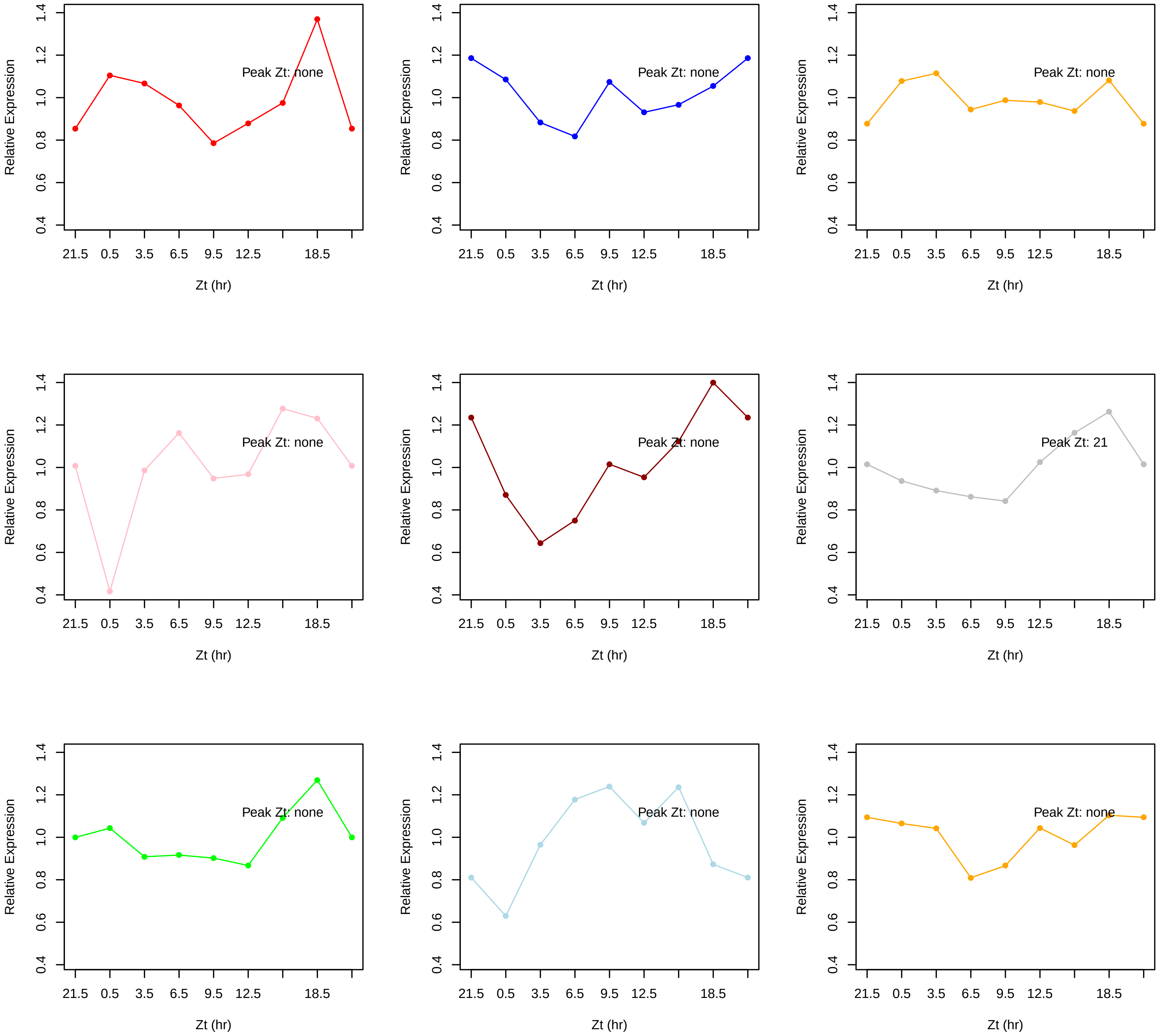

Singeleton: ENSXMAG00000015207 tmem256

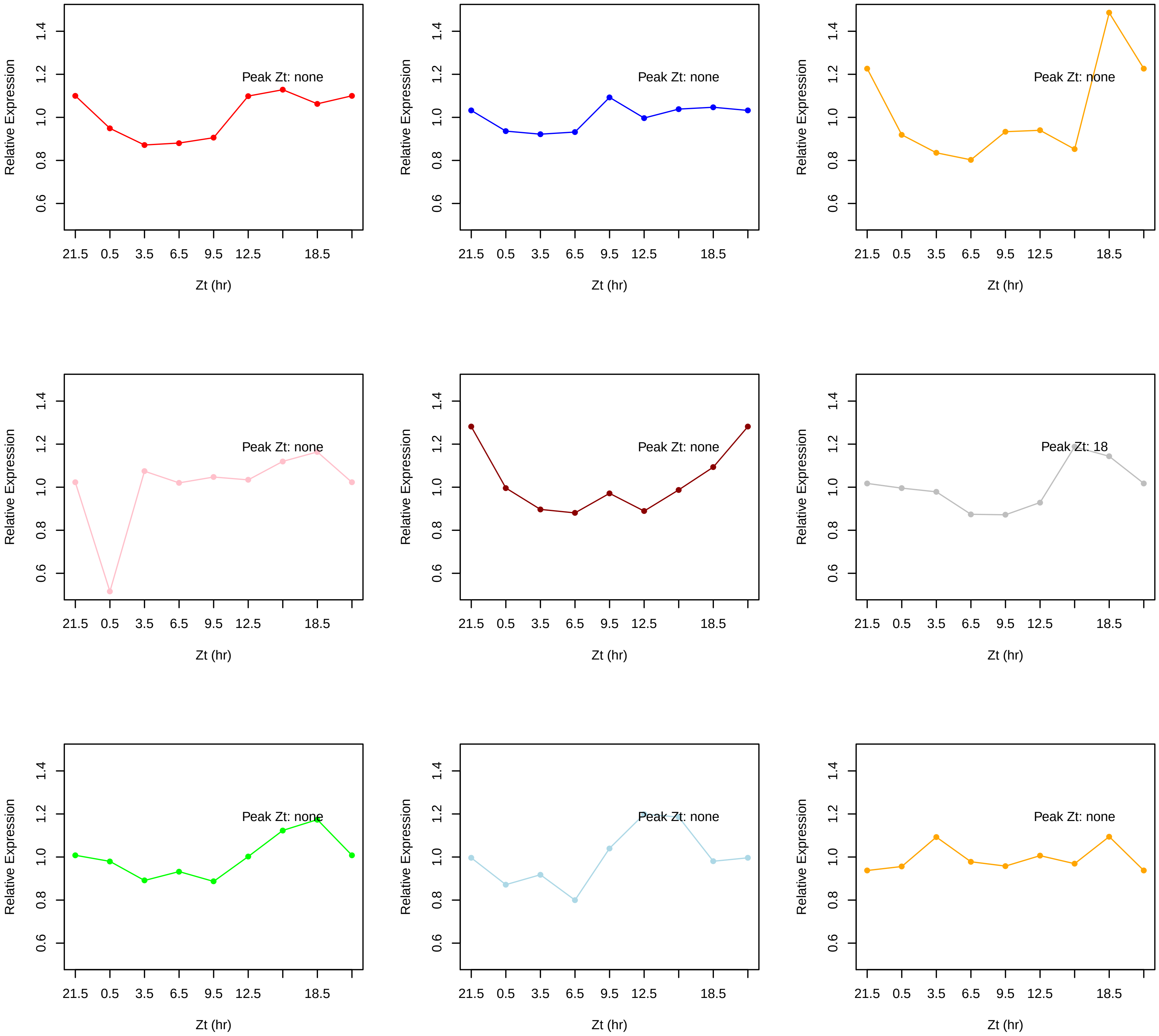

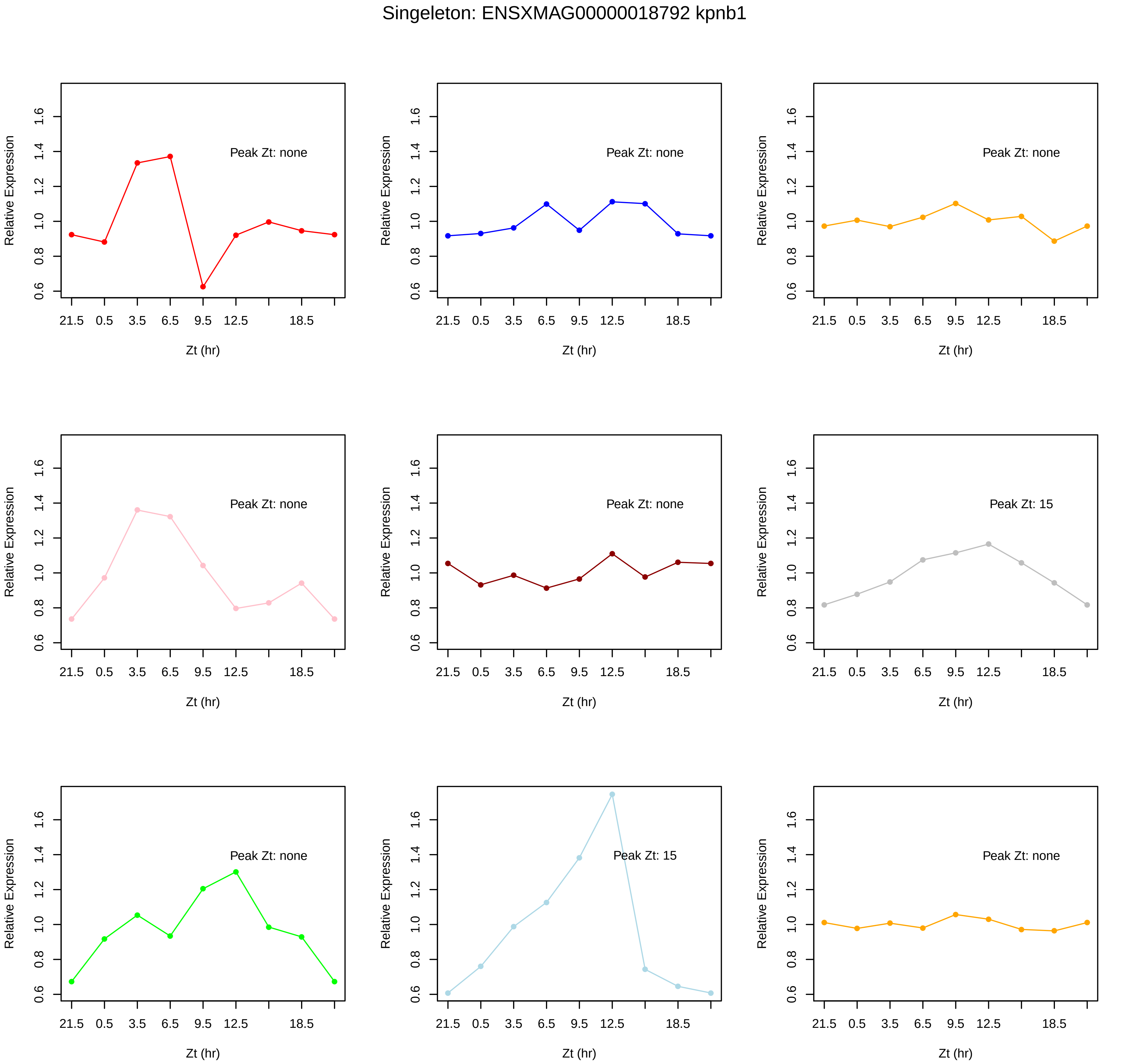
Singleton: ENSXMAG00000018792 kpnb1
Peak Zt: none
Peak Zt: none
Peak Zt: none
Peak Zt: none
Peak Zt: none
Peak Zt: 15
Peak Zt: none
Peak Zt: 15
Peak Zt: none
Relative Expression
Zt (hr)
21.5 0.5 3.5 6.5 9.5 12.5 18.5
0.6 0.8 1.0 1.2 1.4 1.6

Singeleton: ENSXMAG00000005631 helt

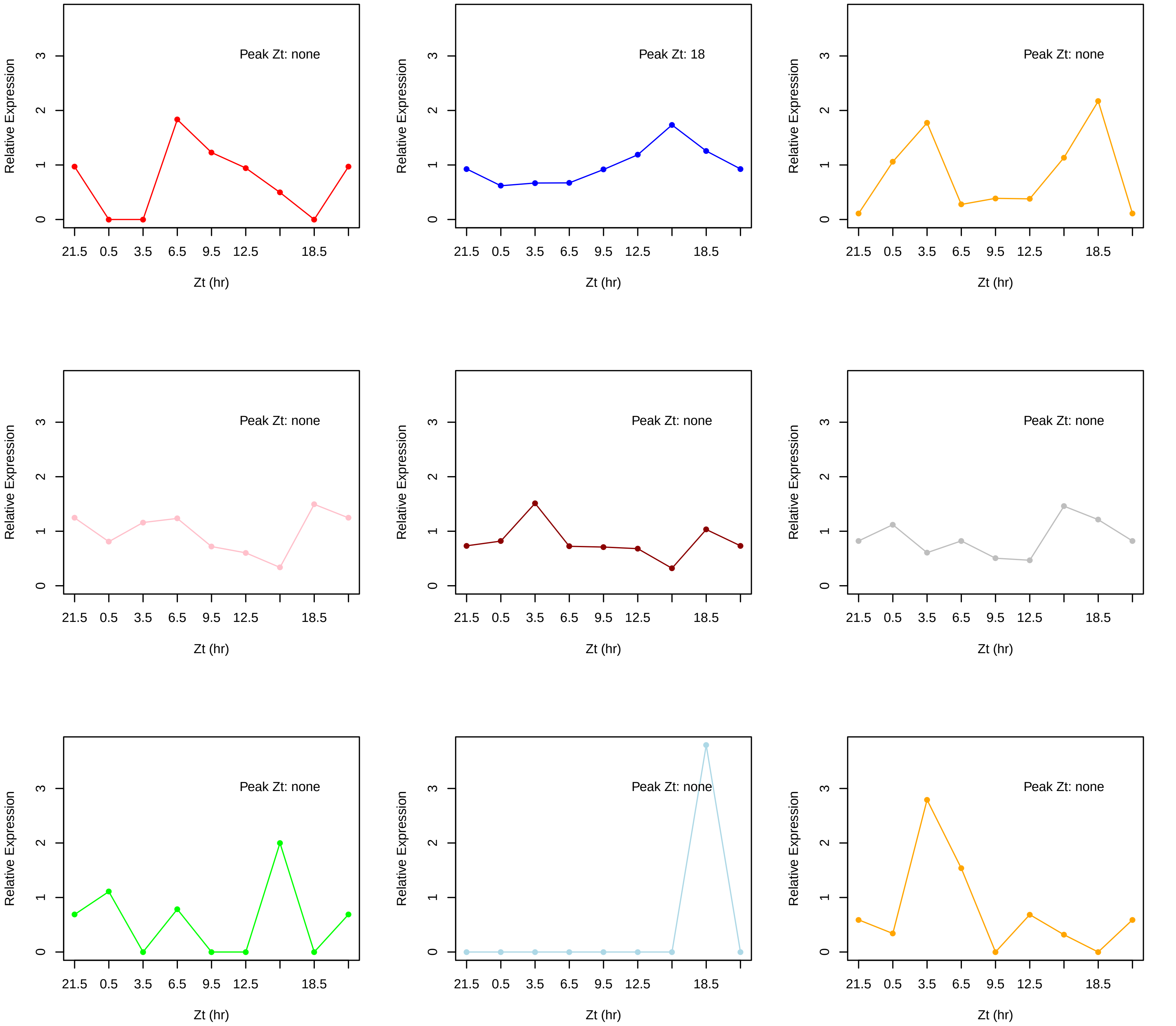

Singeleton: ENSXMAG00000001192 eif3jb

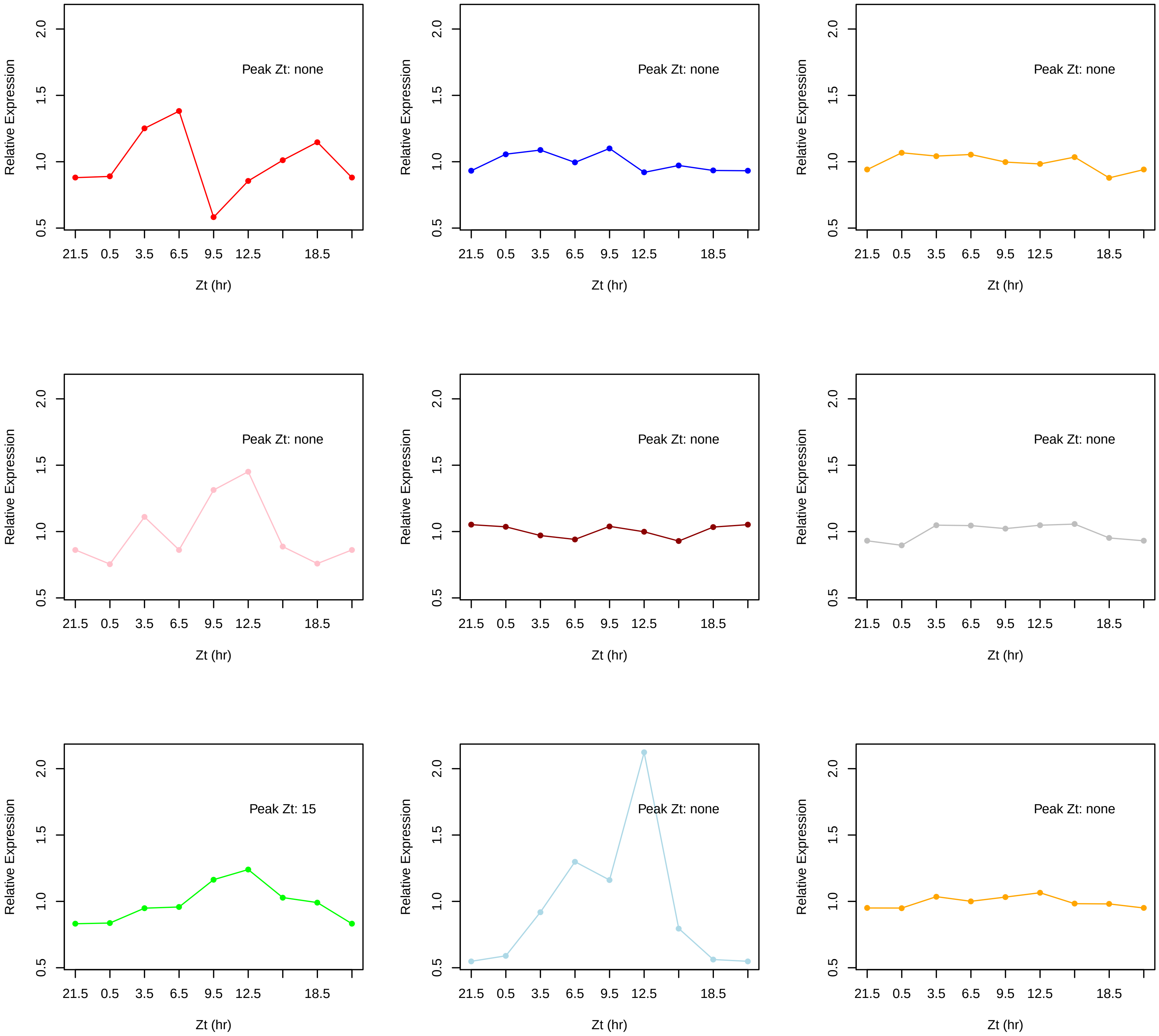

Singeleton: ENSXMAG00000022217 tefm

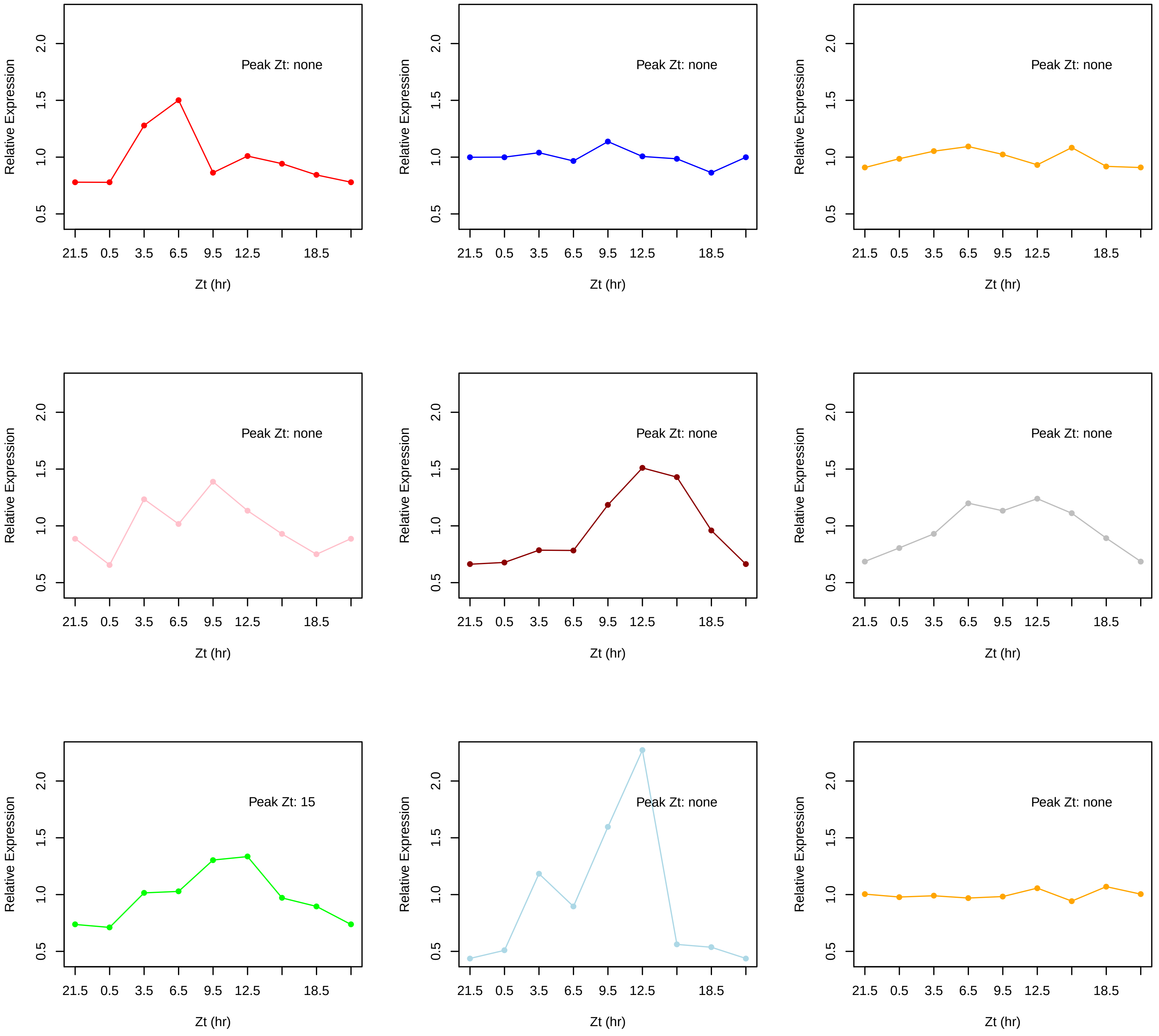

Singleton: ENSXMAG00000020047 brcc3

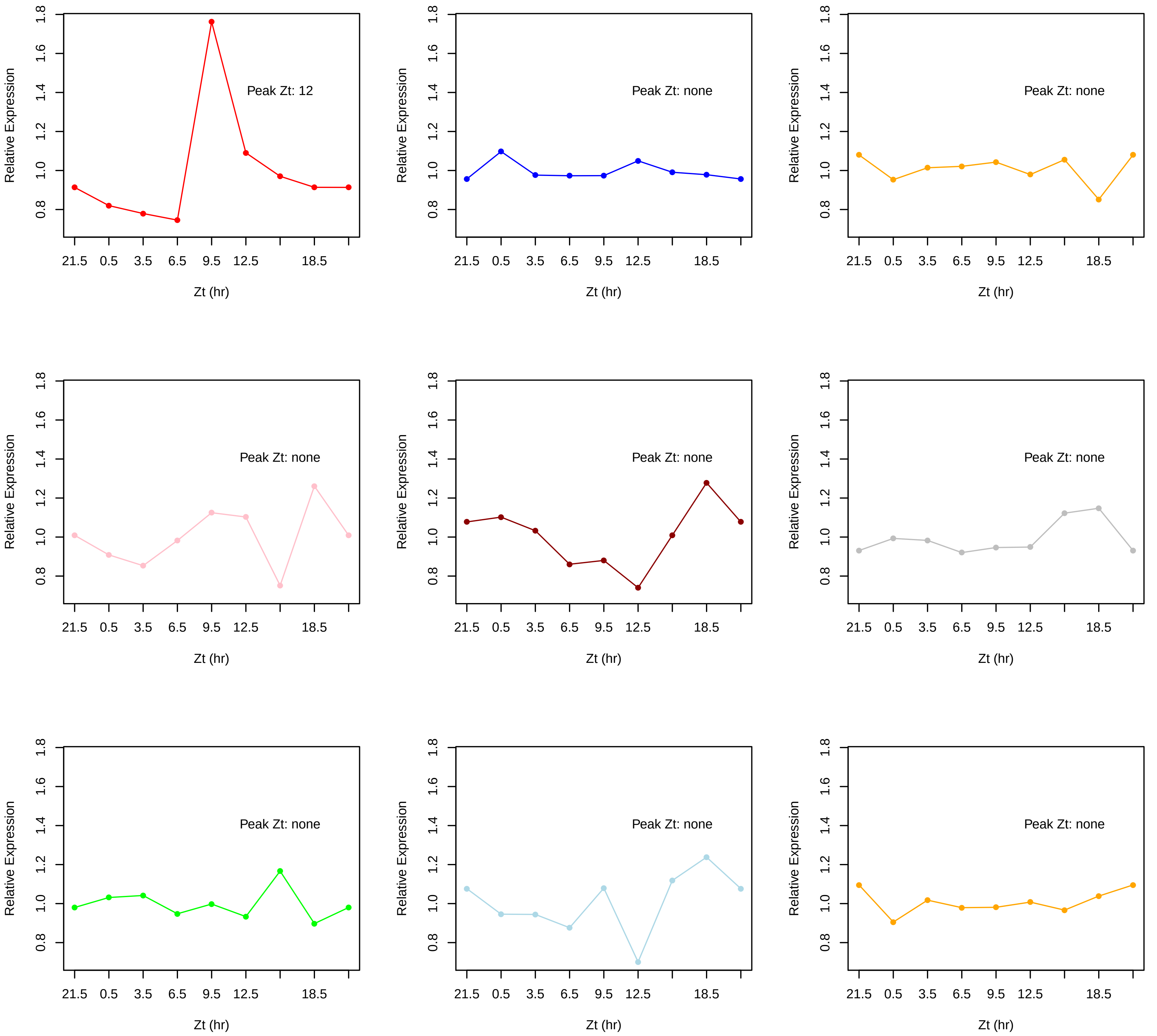

Singeleton: ENSXMAG00000013711 cldn7a

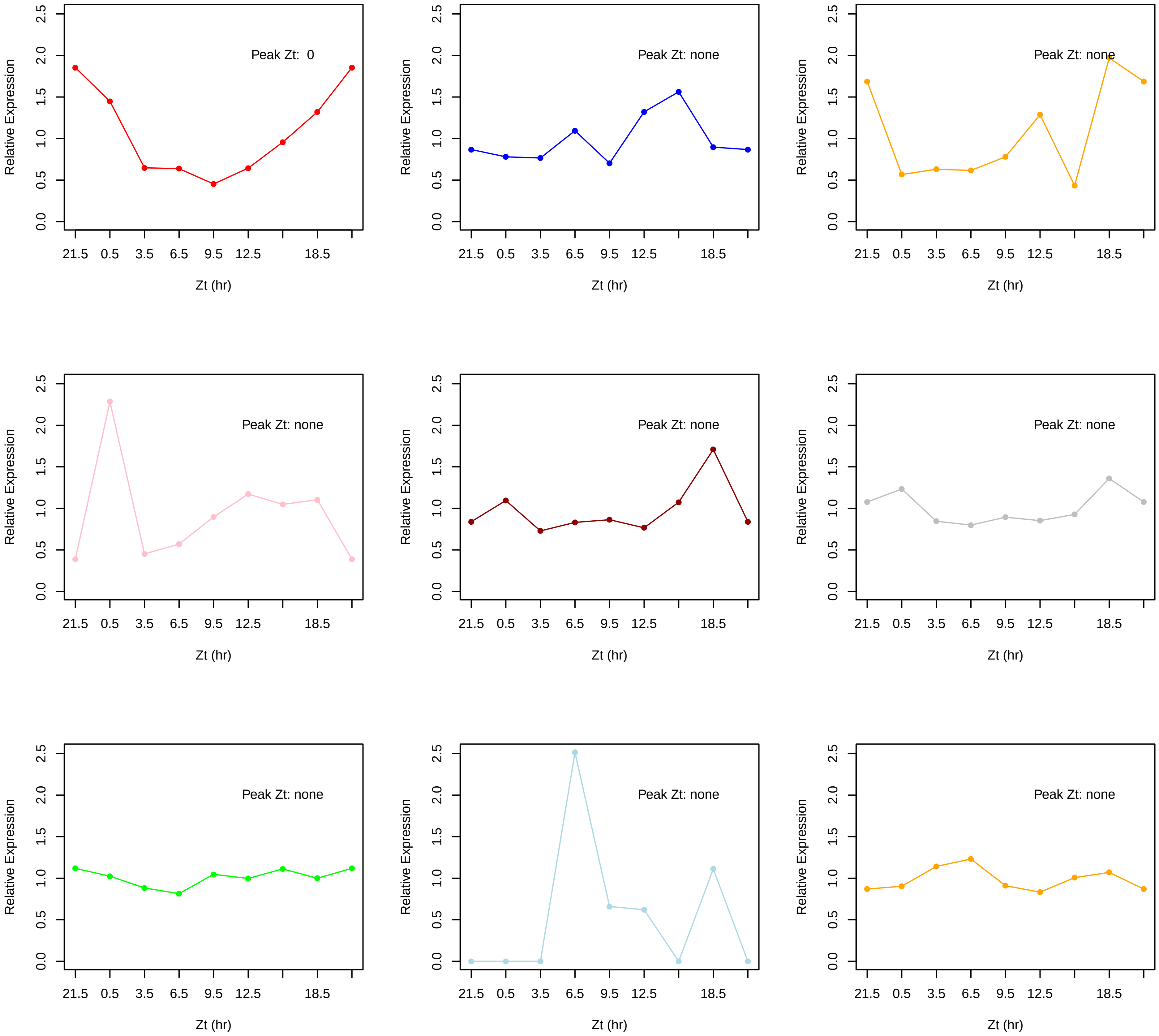

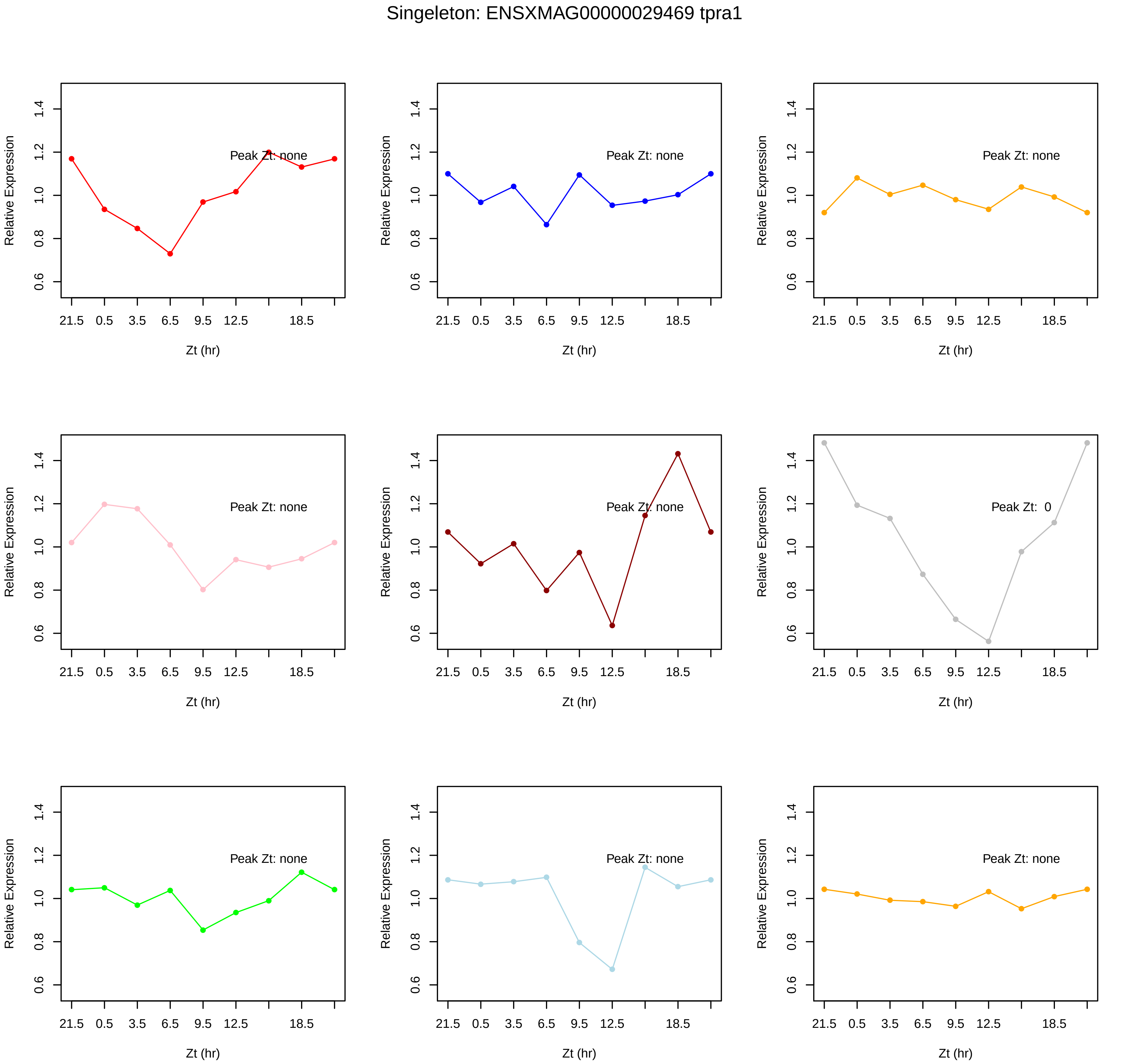
Singeleton: ENSXMAG00000029469 tpra1
Peak Zt: none
Peak Zt: none
Peak Zt: none
Peak Zt: none
Peak Zt: none
Peak Zt: 0
Peak Zt: none
Peak Zt: none
Peak Zt: none
Relative Expression
Zt (hr)
21.5
0.5
3.5
6.5
9.5
12.5
18.5
0.6
0.8
1.0
1.2
1.4

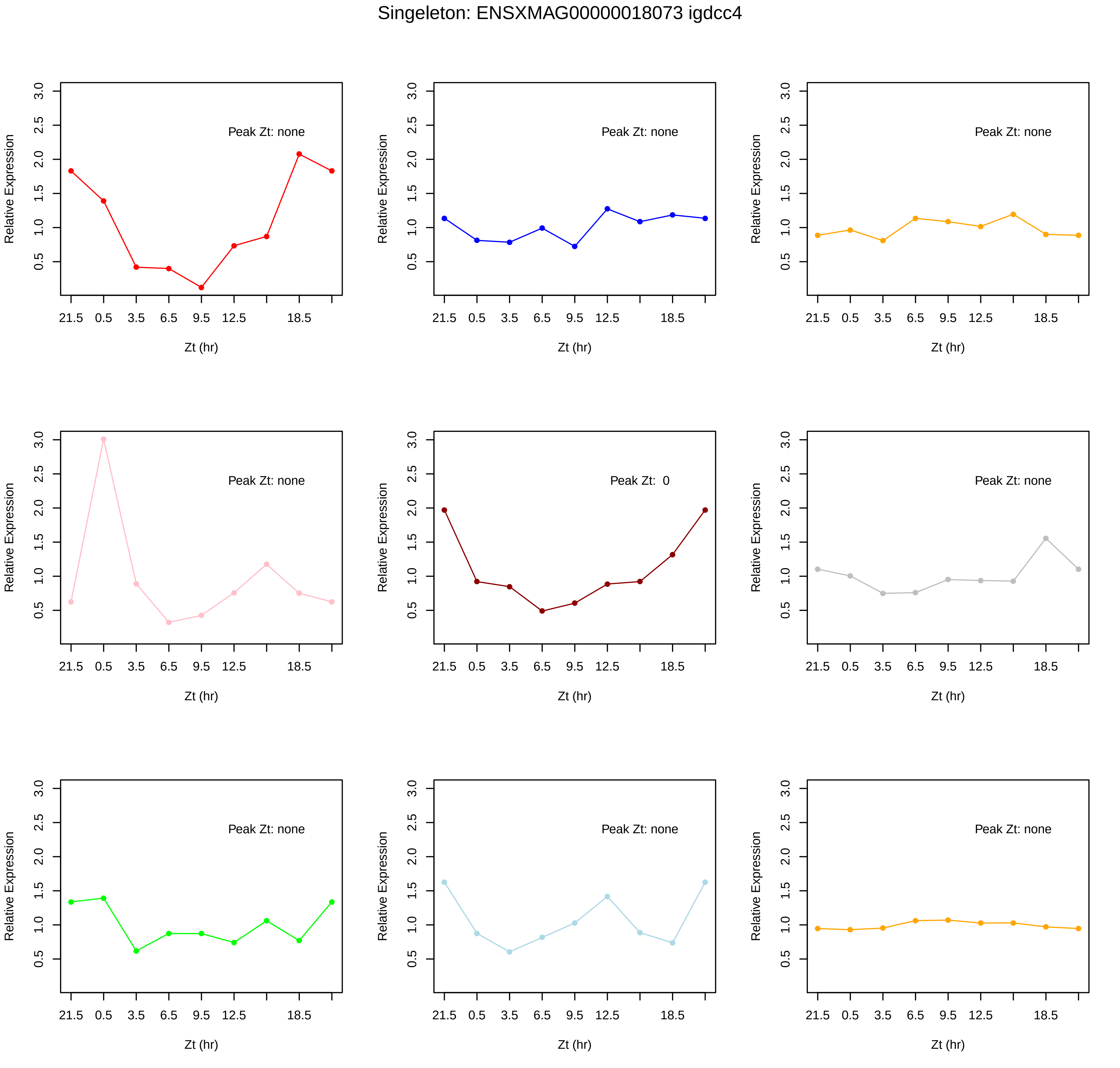
Singleton: ENSXMAG00000018073 igdcc4
Peak Zt: none
Peak Zt: none
Peak Zt: none
Peak Zt: none
Peak Zt: 0
Peak Zt: none
Peak Zt: none
Peak Zt: none
Peak Zt: none
Relative Expression
Zt (hr)
21.5
0.5
3.5
6.5
9.5
12.5
18.5
0.5
1.0
1.5
2.0
2.5
3.0

Singeleton: ENSXMAG00000005647 ufsp2

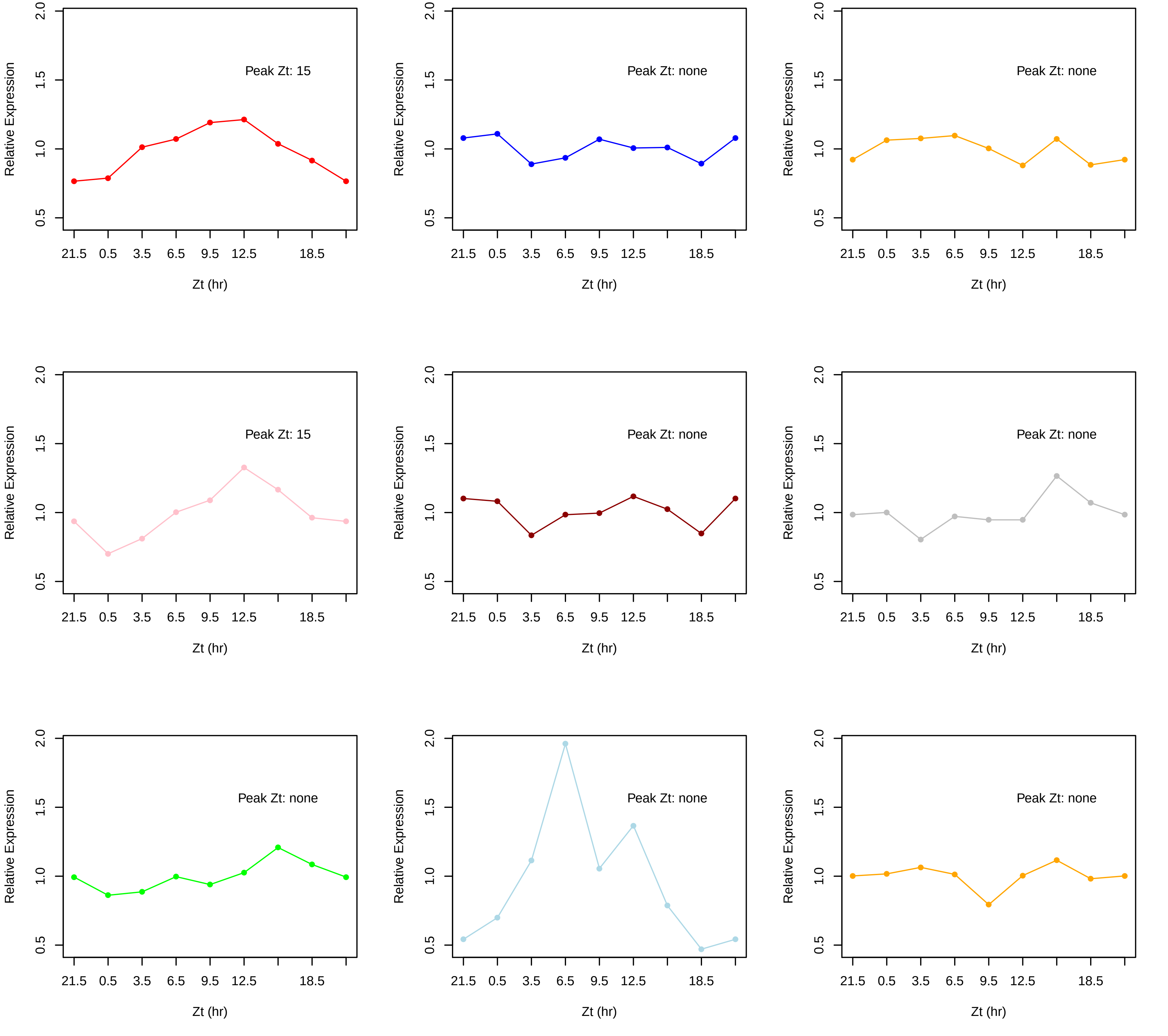

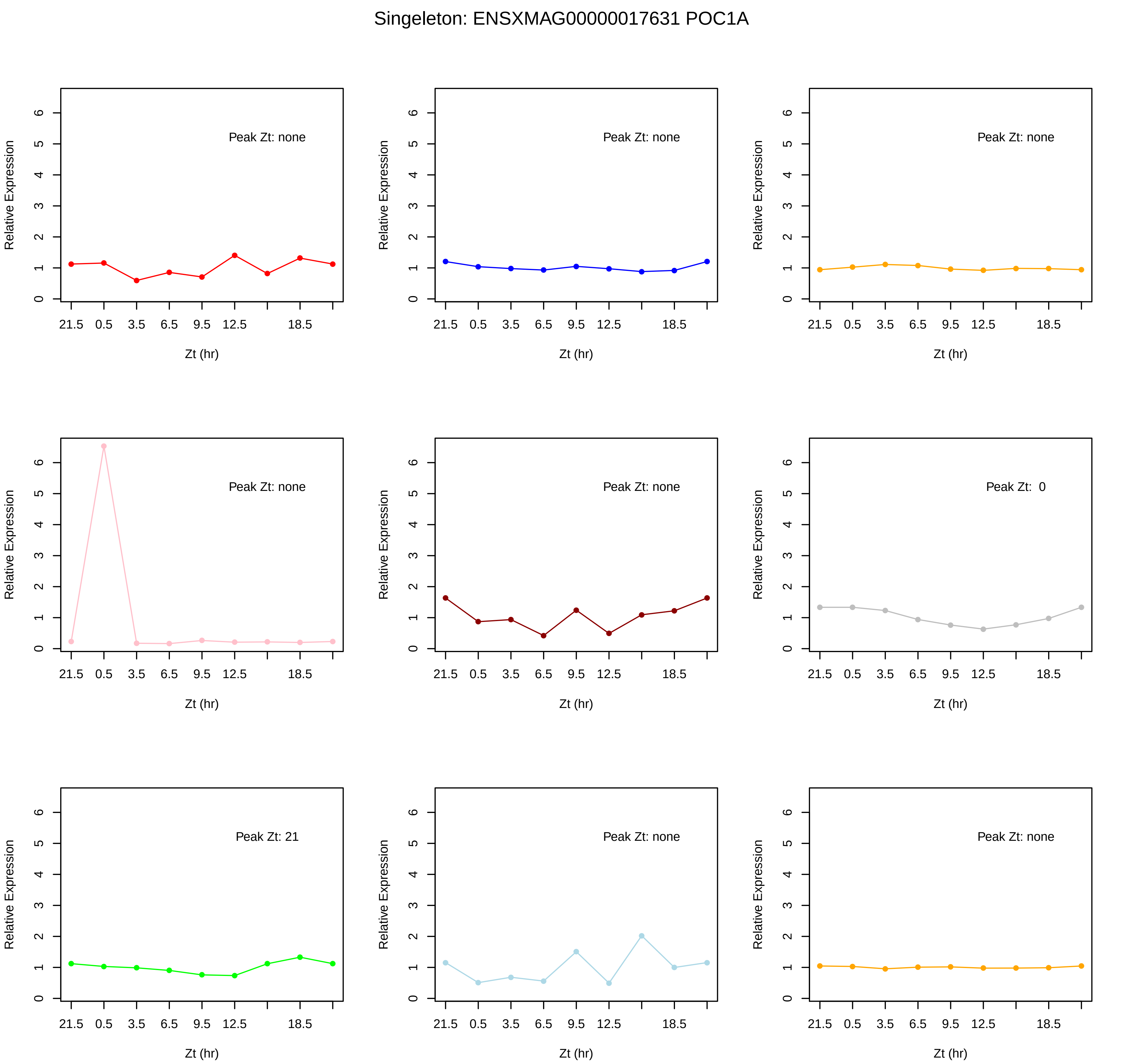
Singeleton: ENSXMAG00000017631 POC1A
Peak Zt: none
Peak Zt: none
Peak Zt: none
Peak Zt: none
Peak Zt: none
Peak Zt: 0
Peak Zt: 21
Peak Zt: none
Peak Zt: none
Relative Expression
Zt (hr)
21.5
0.5
3.5
6.5
9.5
12.5
18.5

Singeleton: ENSXMAG00000018882 sorbs2a

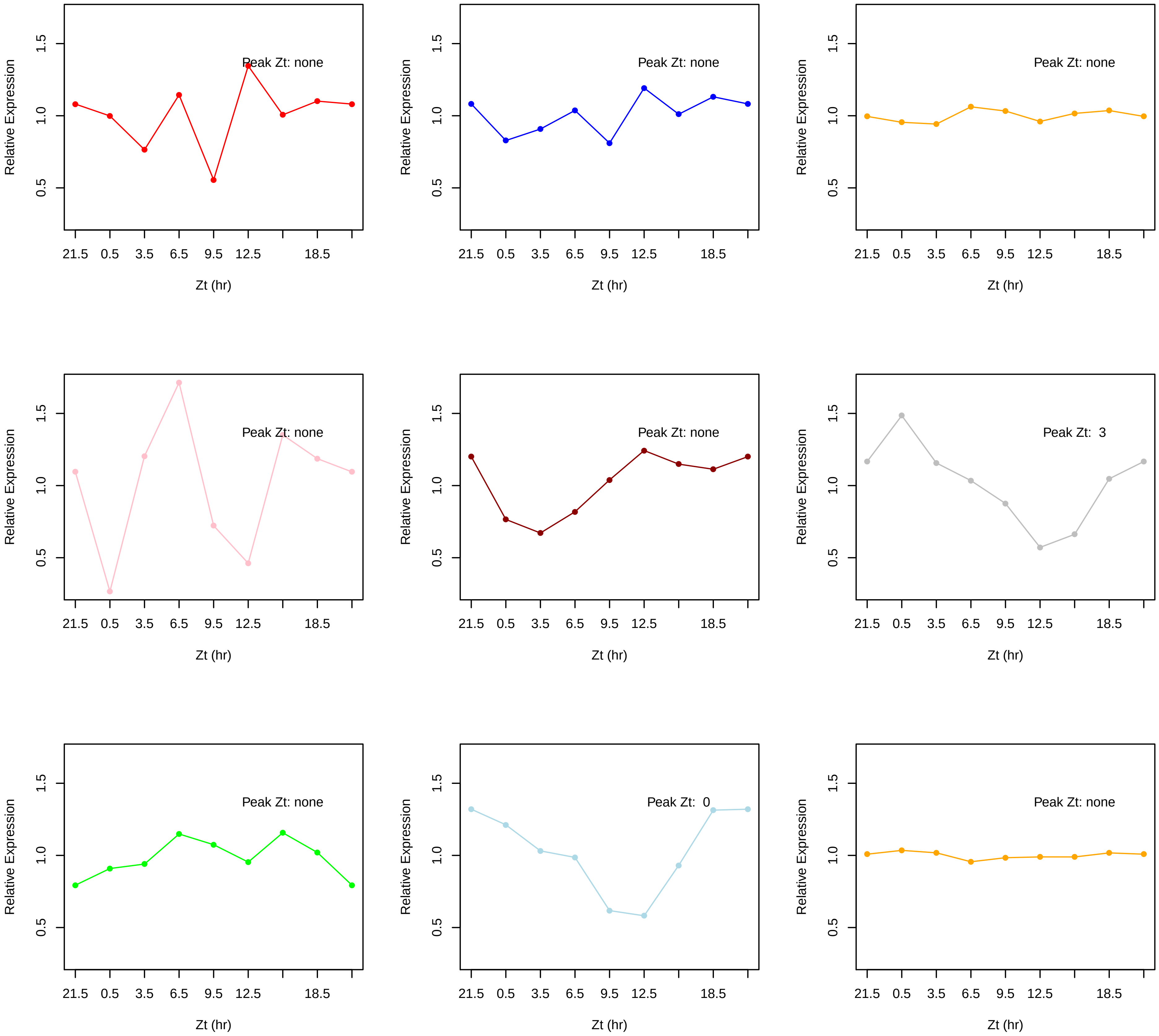

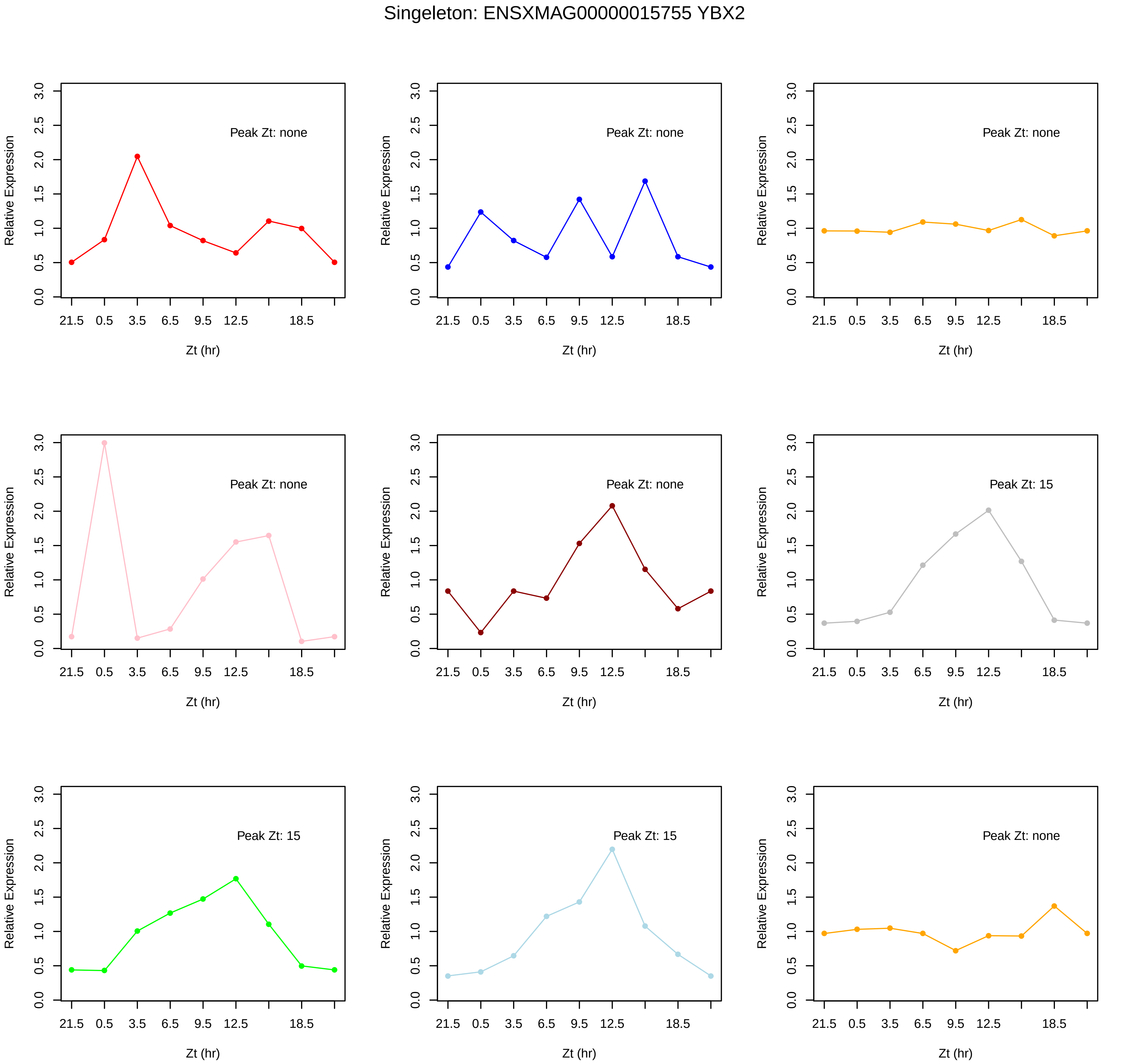
Singleton: ENSXMAG00000015755 YBX2
Peak Zt: none
Peak Zt: none
Peak Zt: none
Peak Zt: none
Peak Zt: none
Peak Zt: 15
Peak Zt: 15
Peak Zt: 15
Peak Zt: none
Relative Expression
Zt (hr)
21.5 0.5 3.5 6.5 9.5 12.5 18.5
0.0 0.5 1.0 1.5 2.0 2.5 3.0

Singeleton: ENSXMAG00000005017 kif4

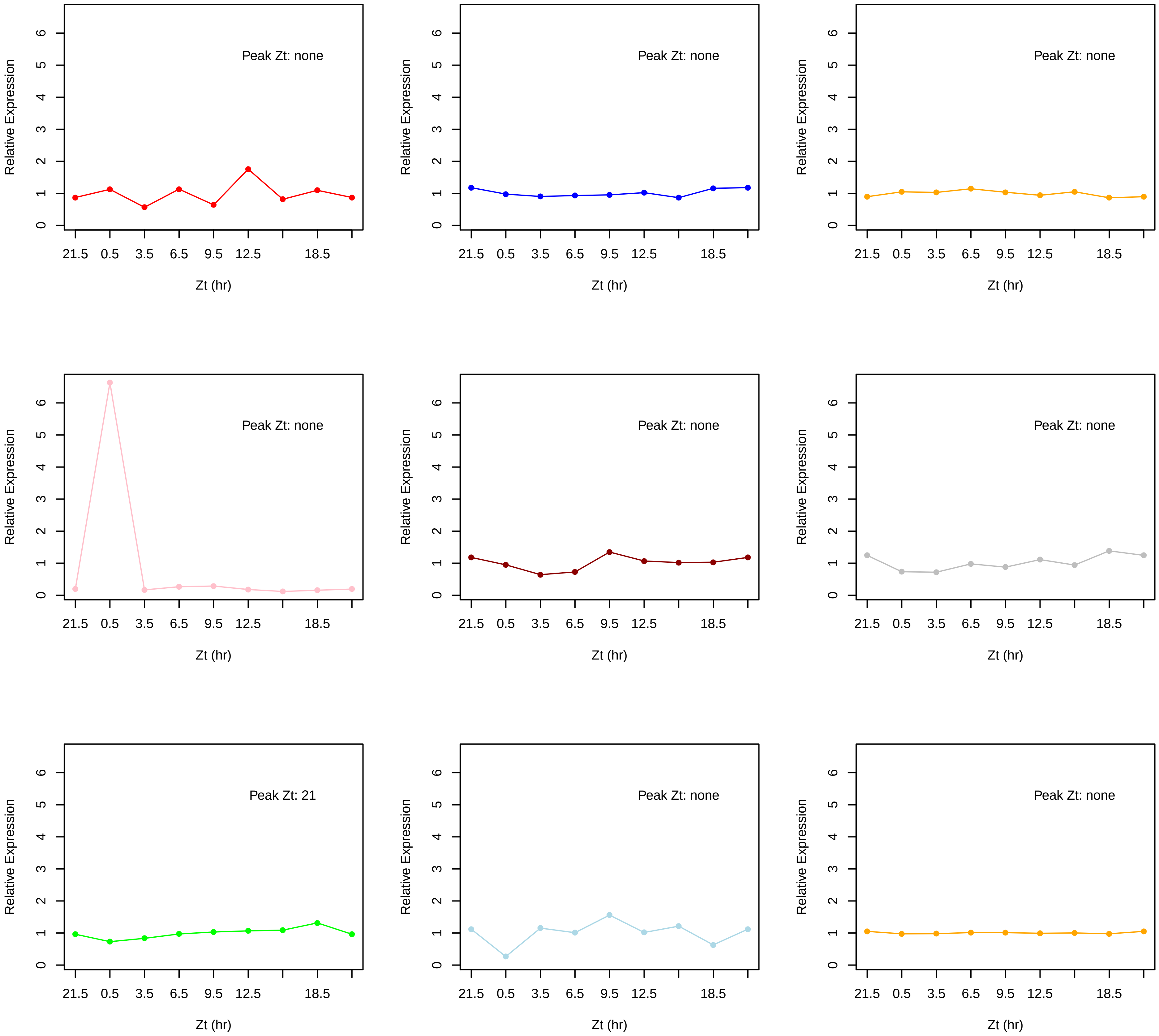

Singeleton: ENSXMAG00000025724 SLC2A4

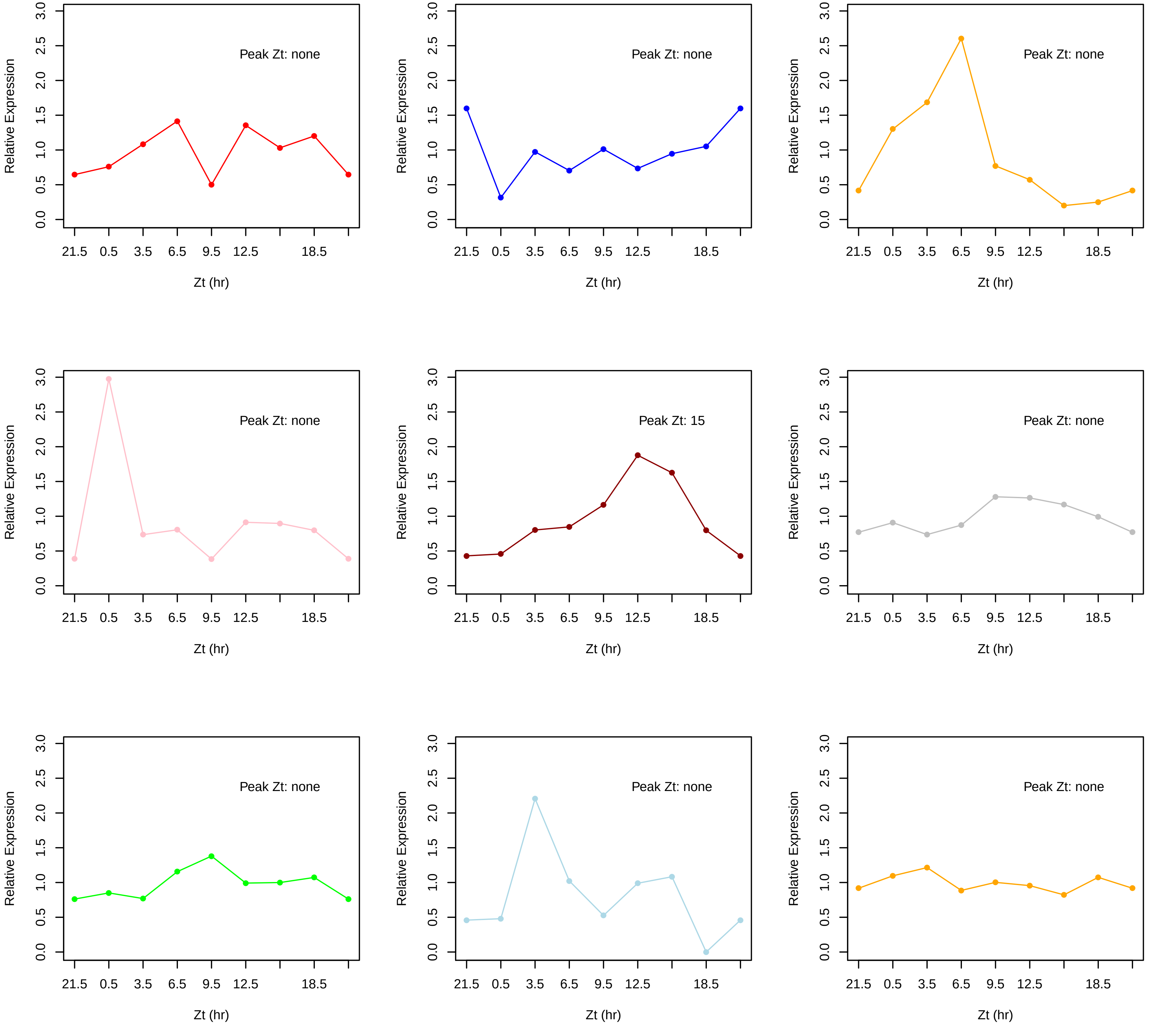

Singeleton: ENSXMAG00000018871

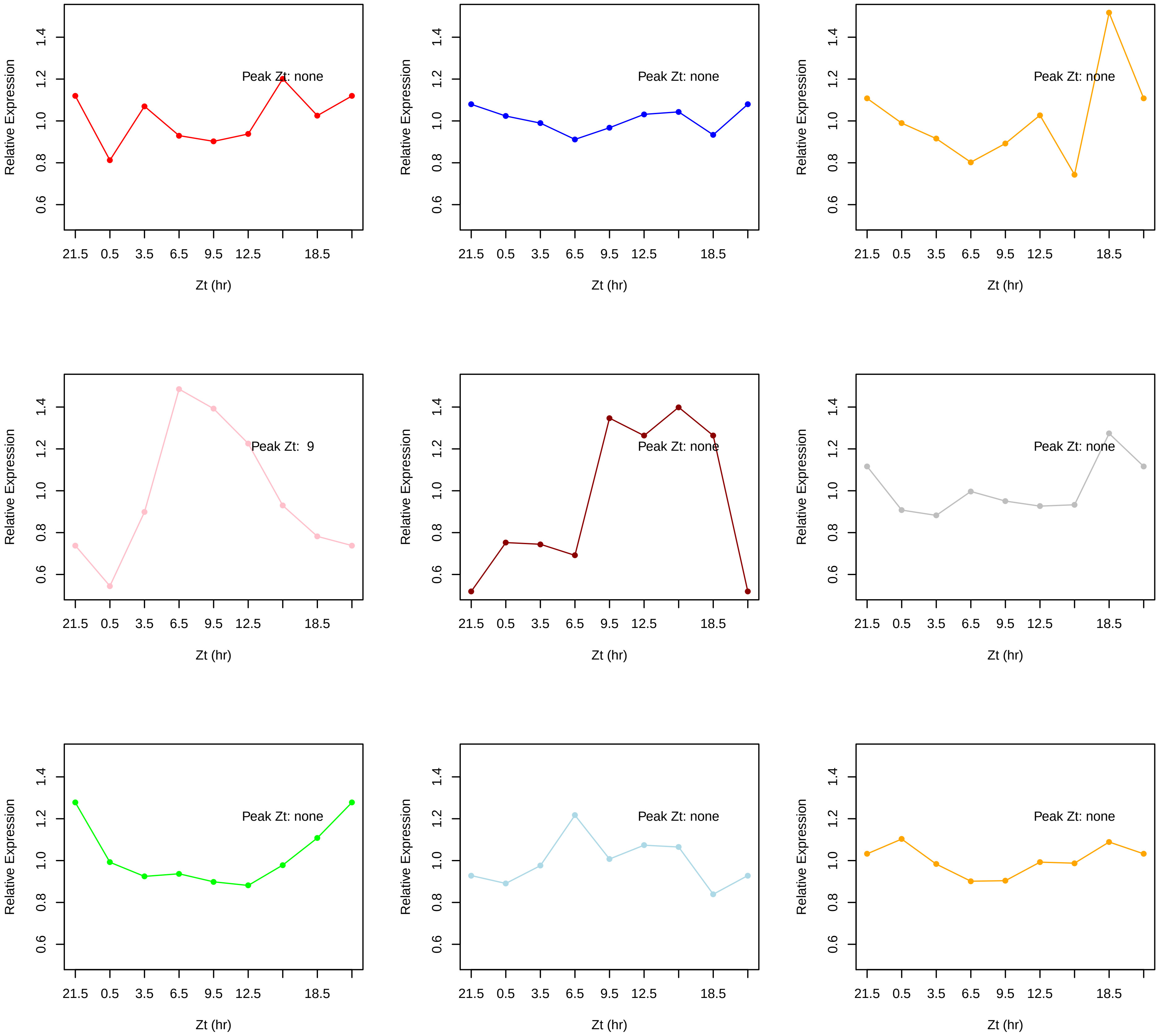

Singleton: ENSXMAG00000012751 PAK3

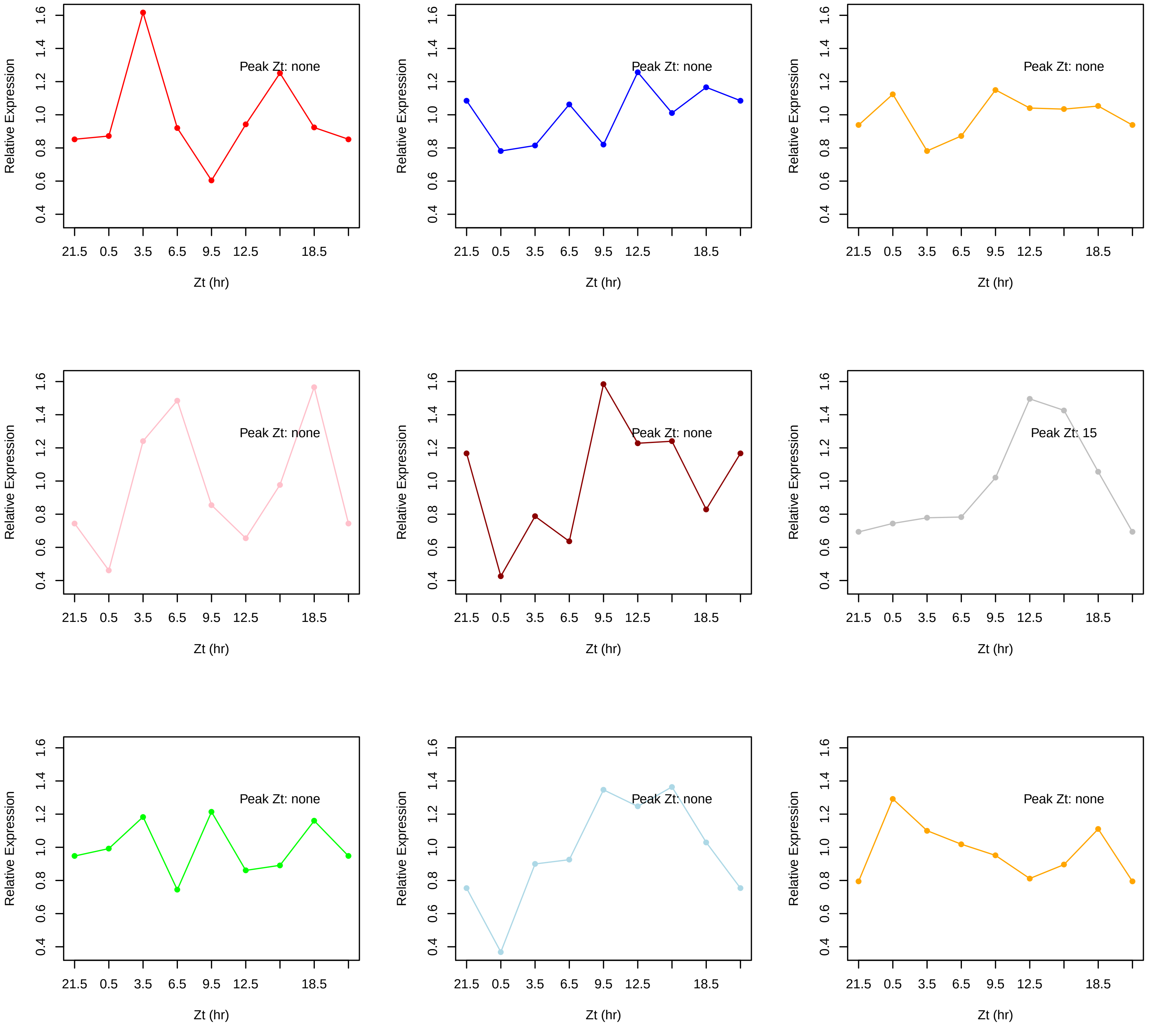

Singeleton: ENSXMAG00000023732 rab11a

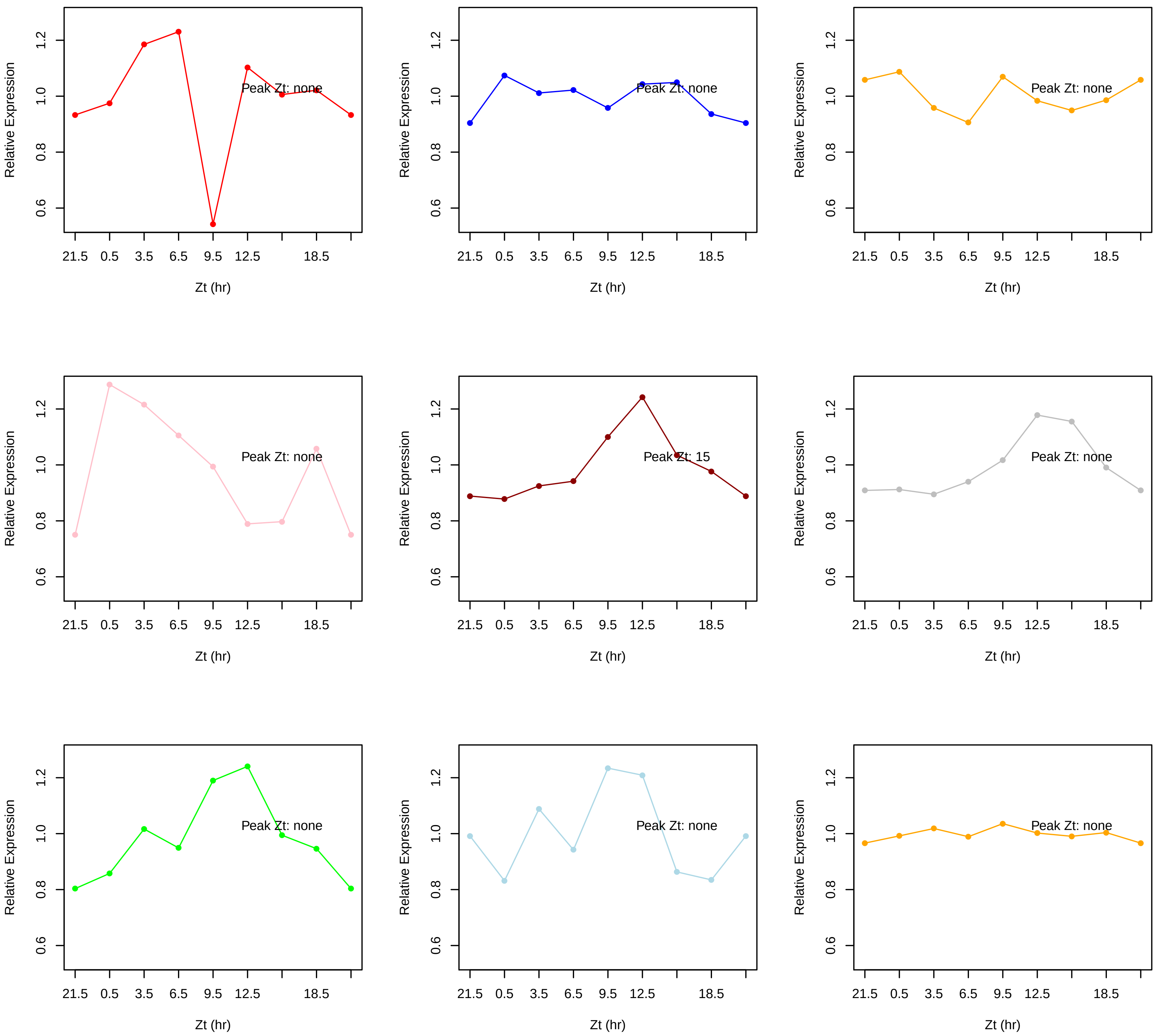

Singleton: ENSXMAG00000010488 ECM2

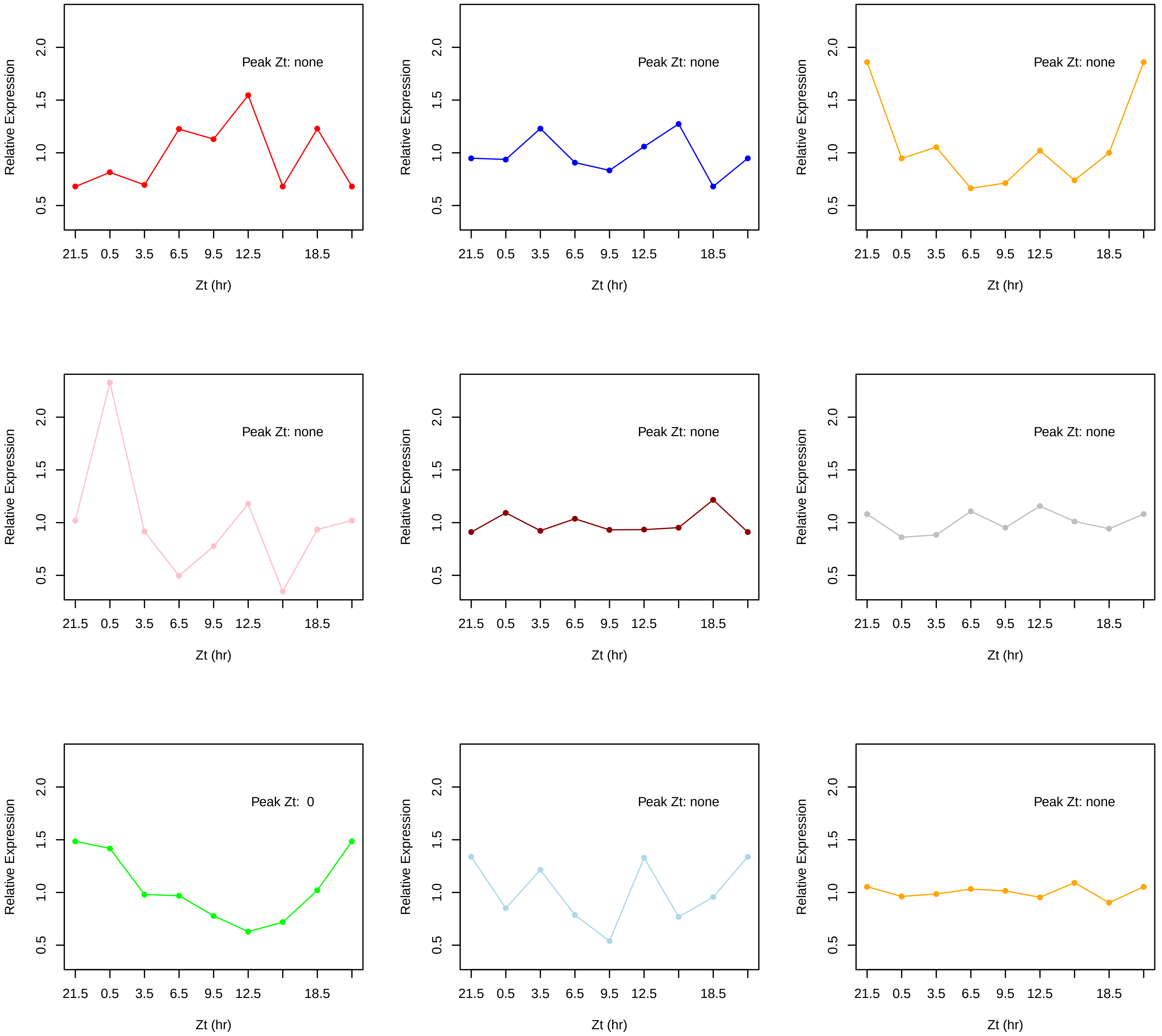

Singeleton: ENSXMAG00000018793 fat1a

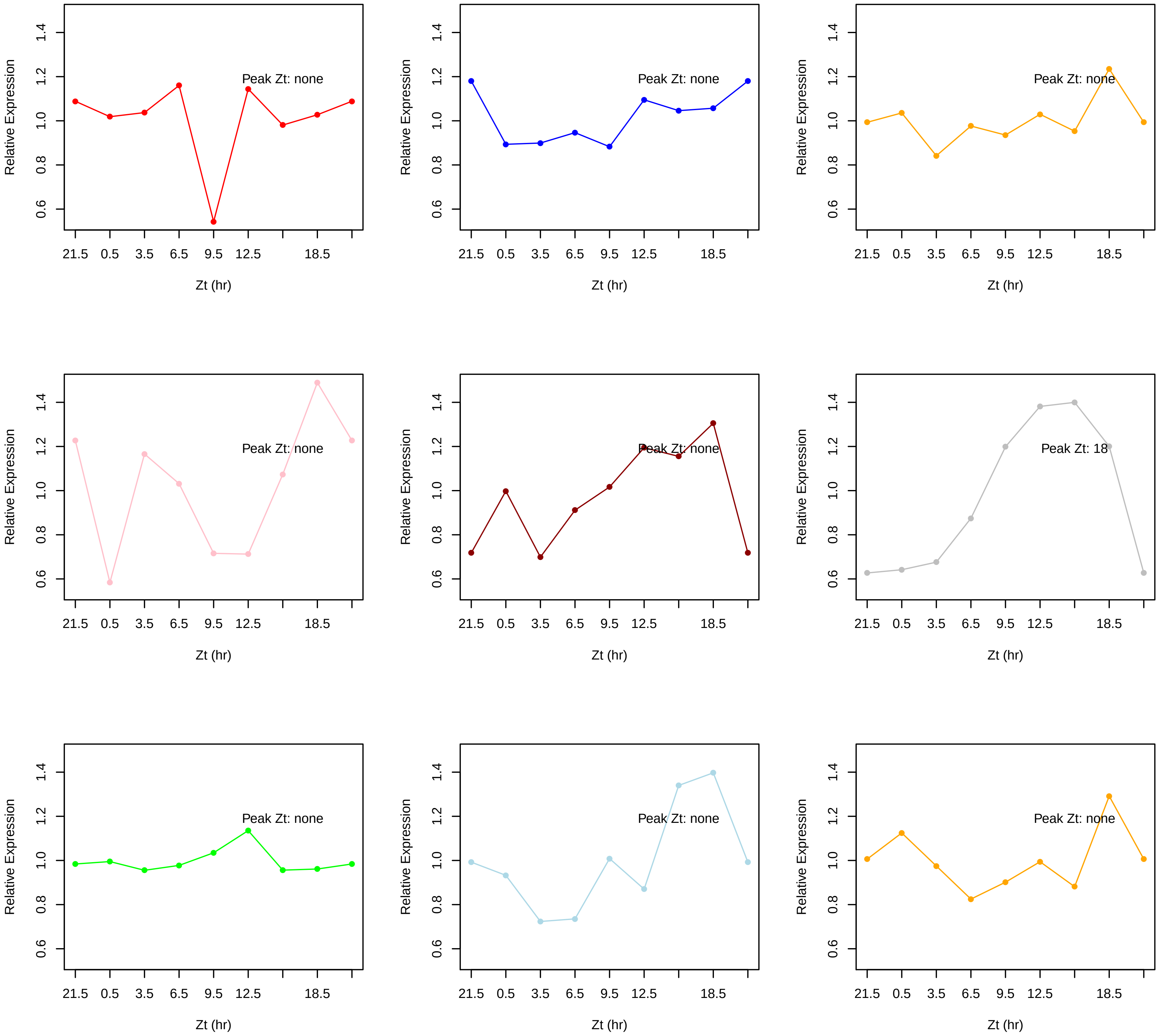

Singeleton: ENSXMAG00000023750 cbx4

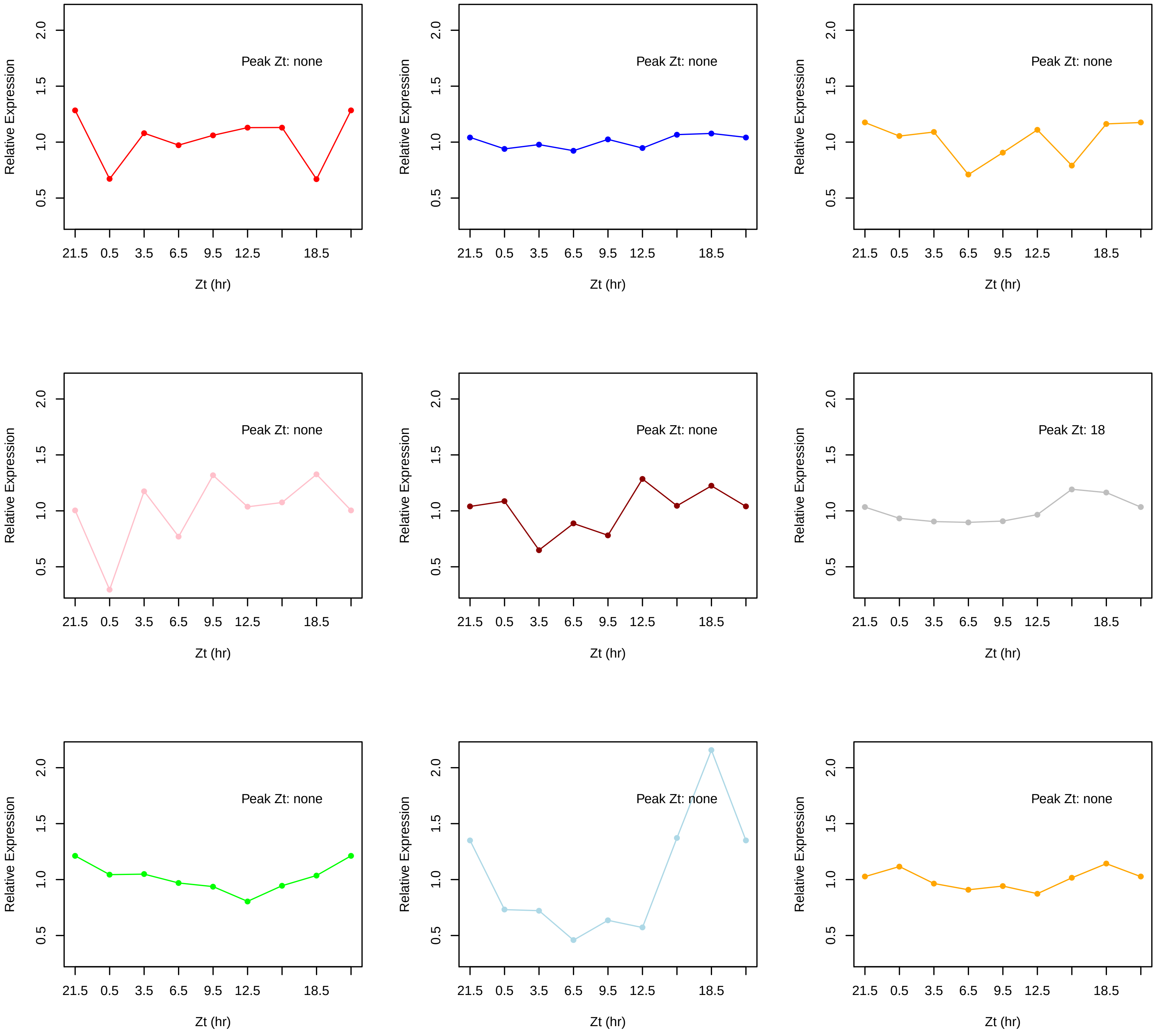

Singeleton: ENSXMAG00000001390 tbc1d16

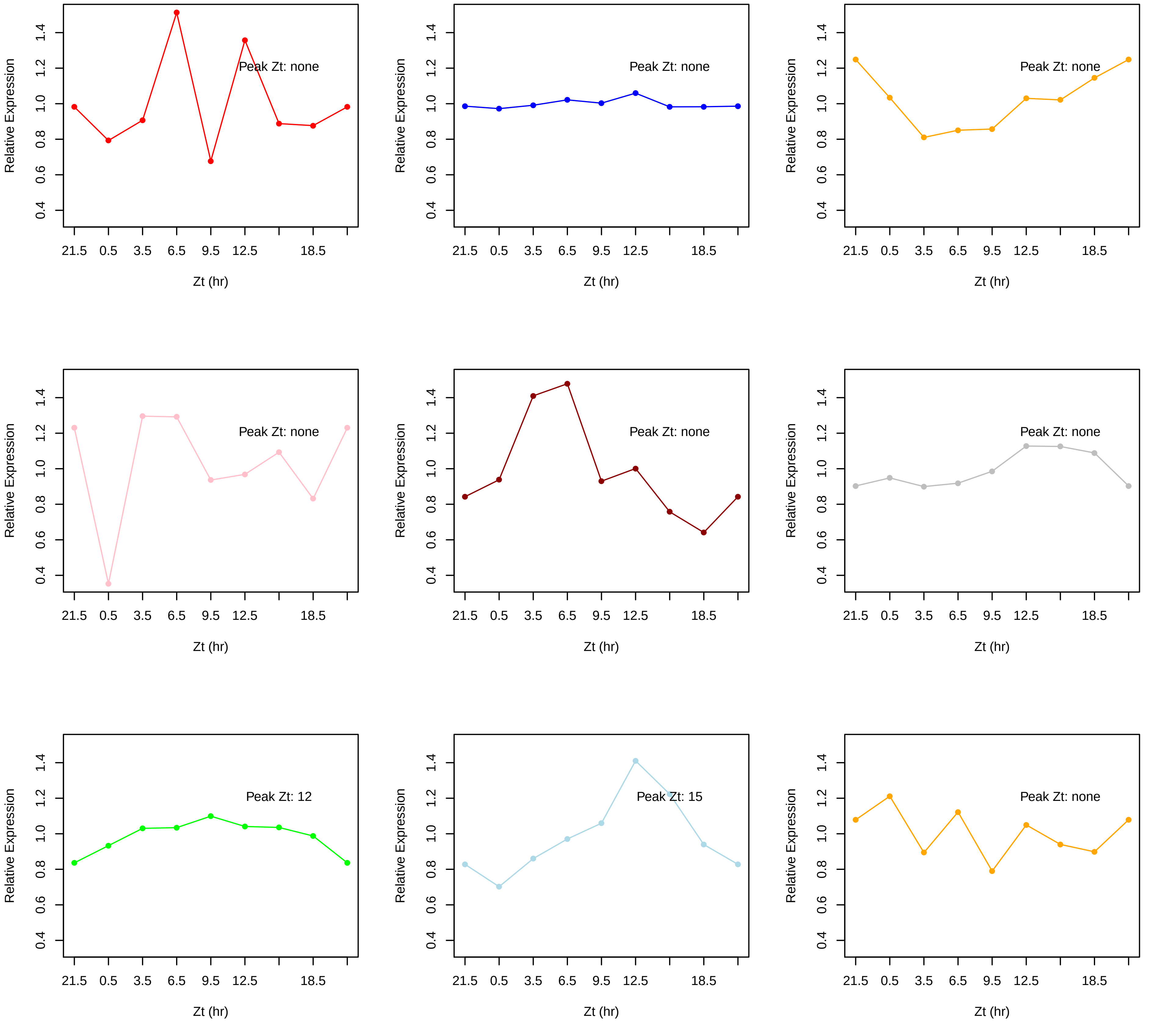

Singeleton: ENSXMAG00000017909 card14

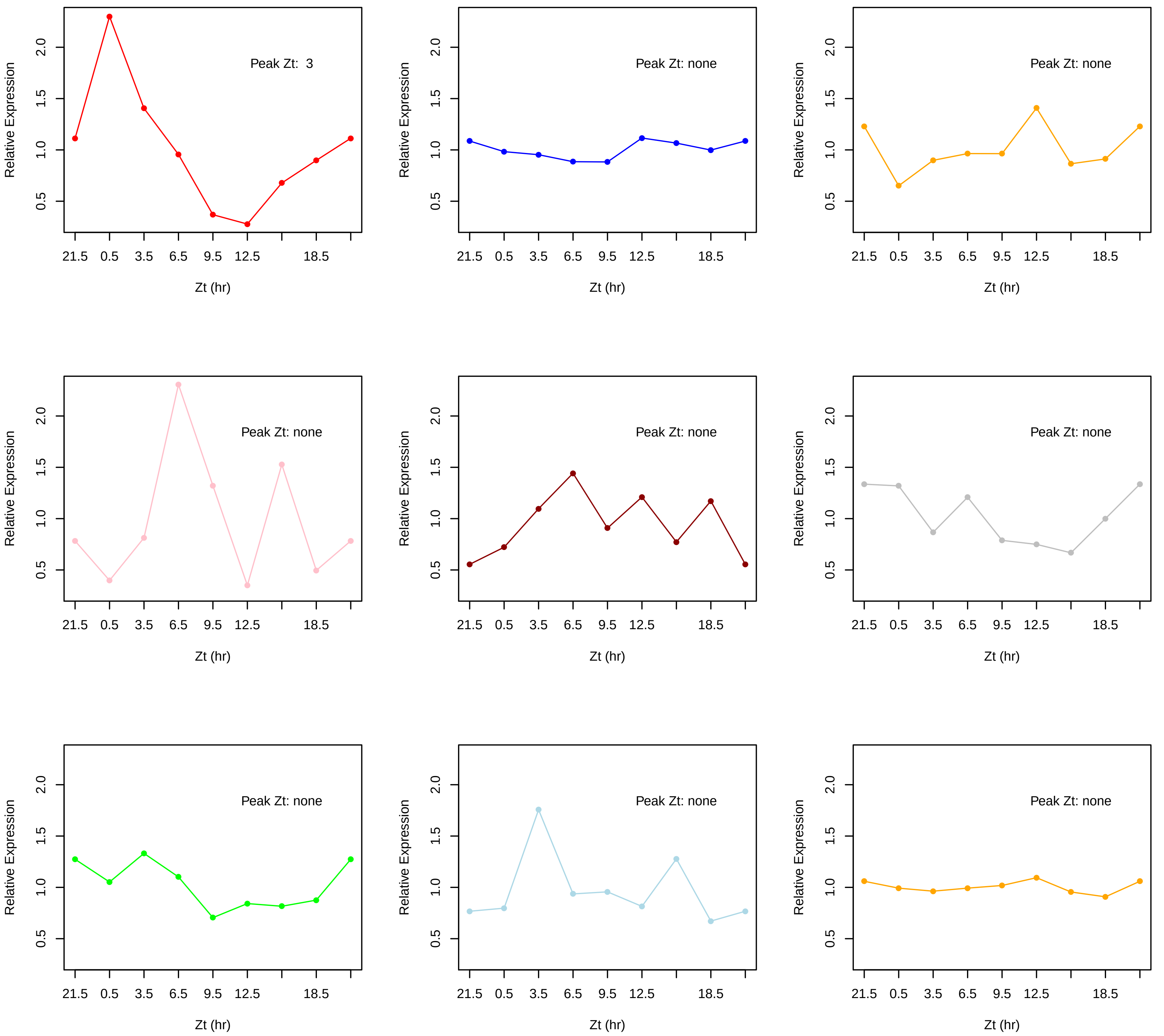

Singeleton: ENSXMAG00000015687 polr2a

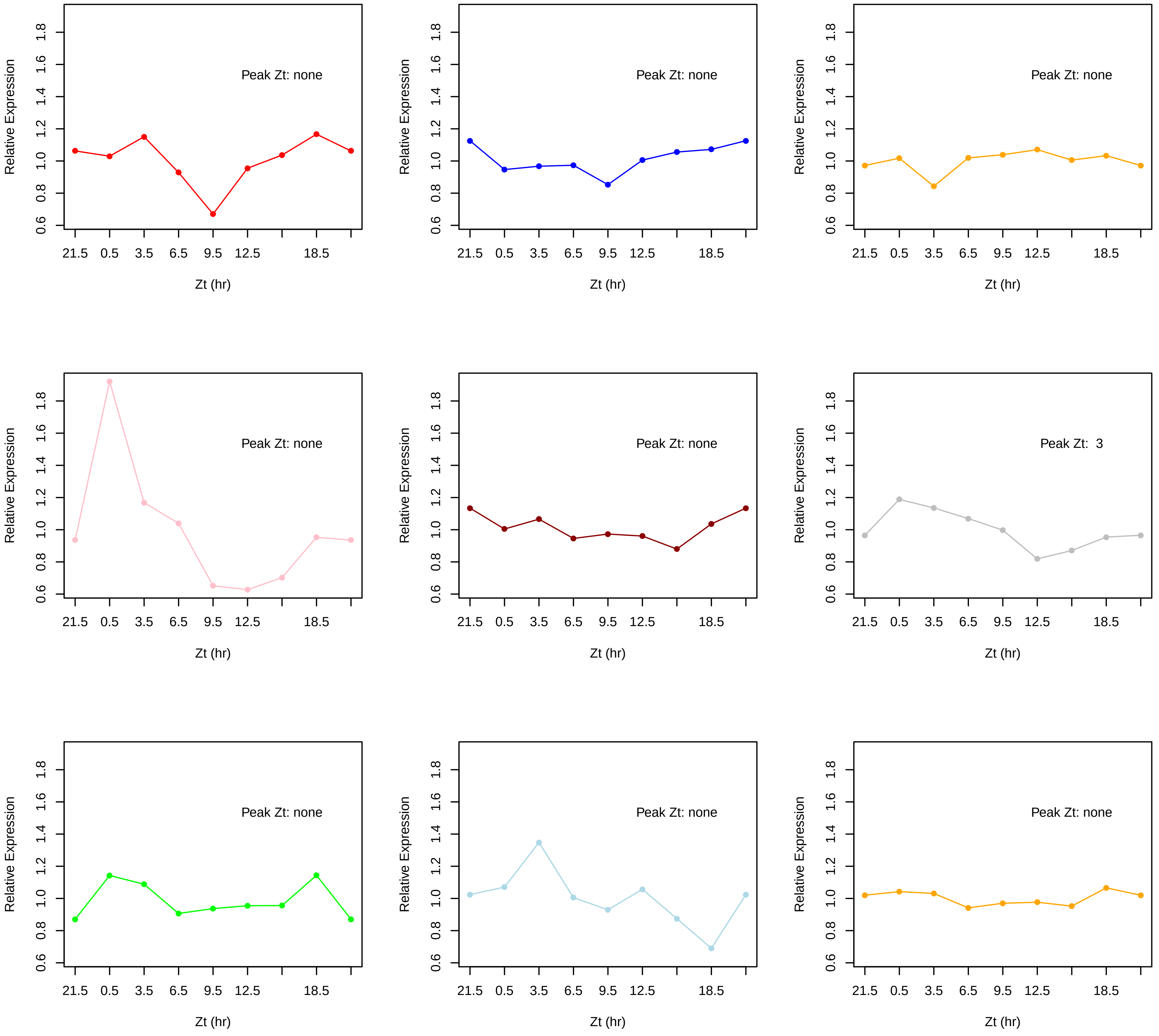

Singeleton: ENSXMAG00000018938 pgk1

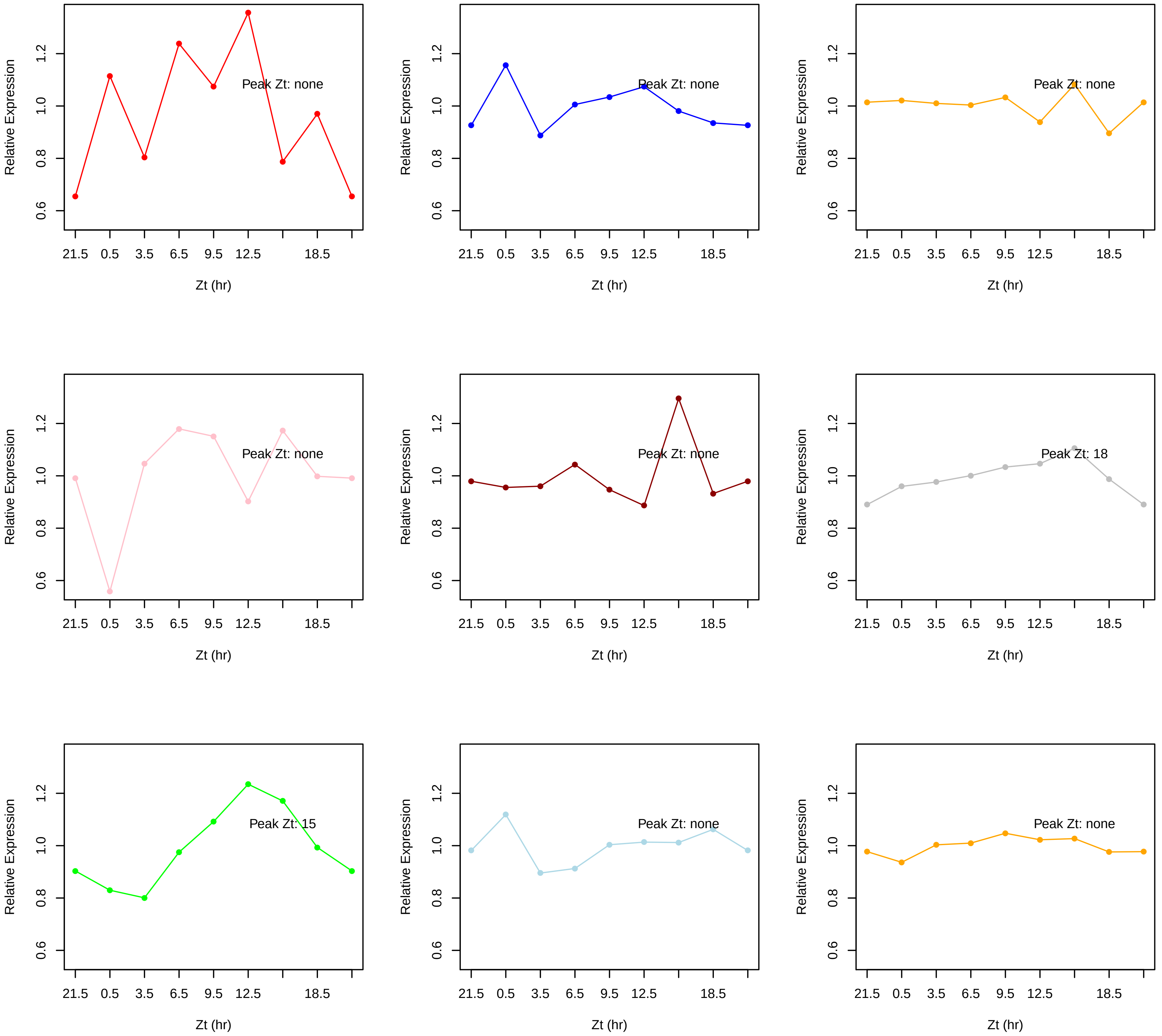

Singeleton: ENSXMAG00000001363 gaa

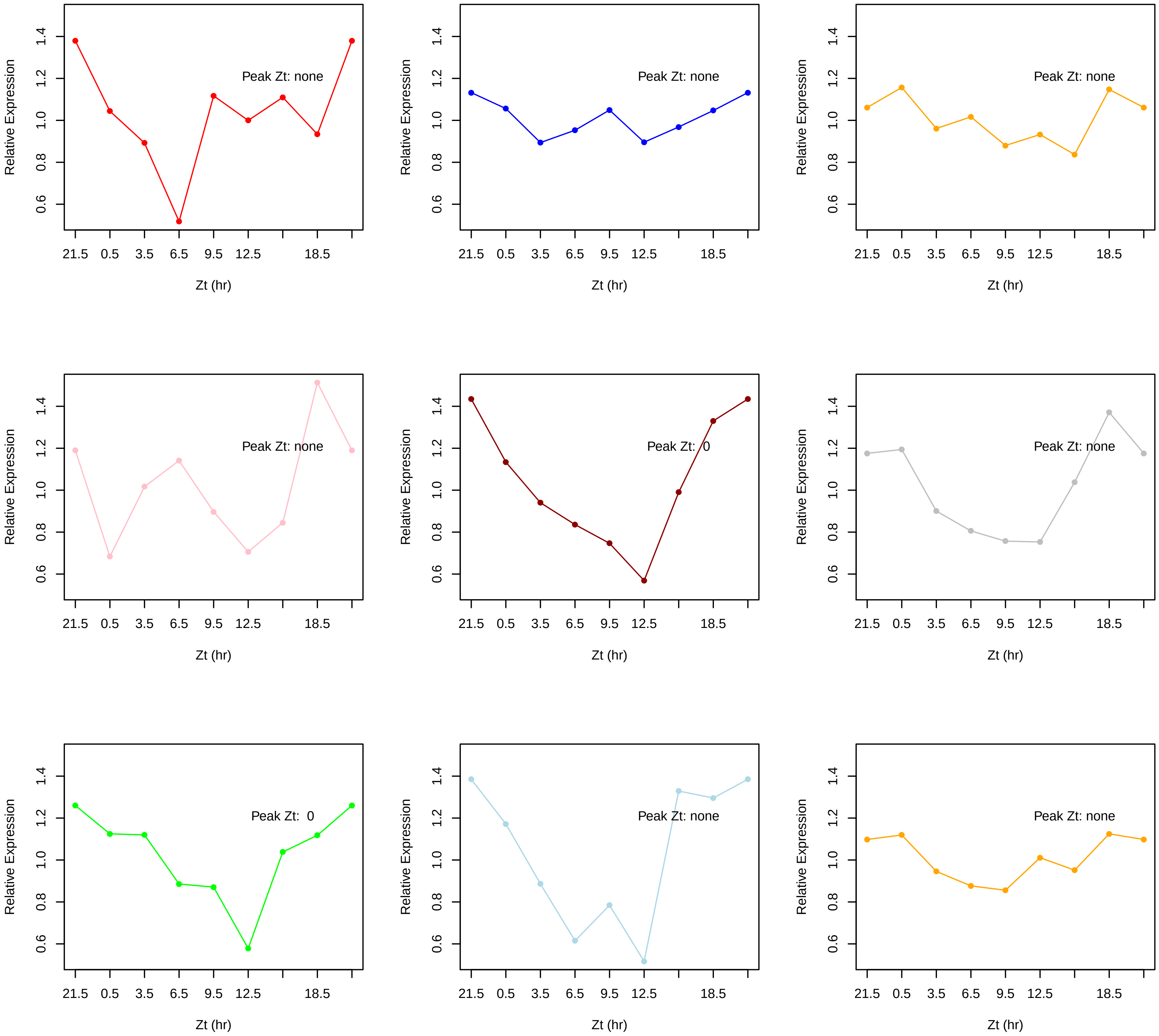

Singleton: ENSXMAG00000017562 iars

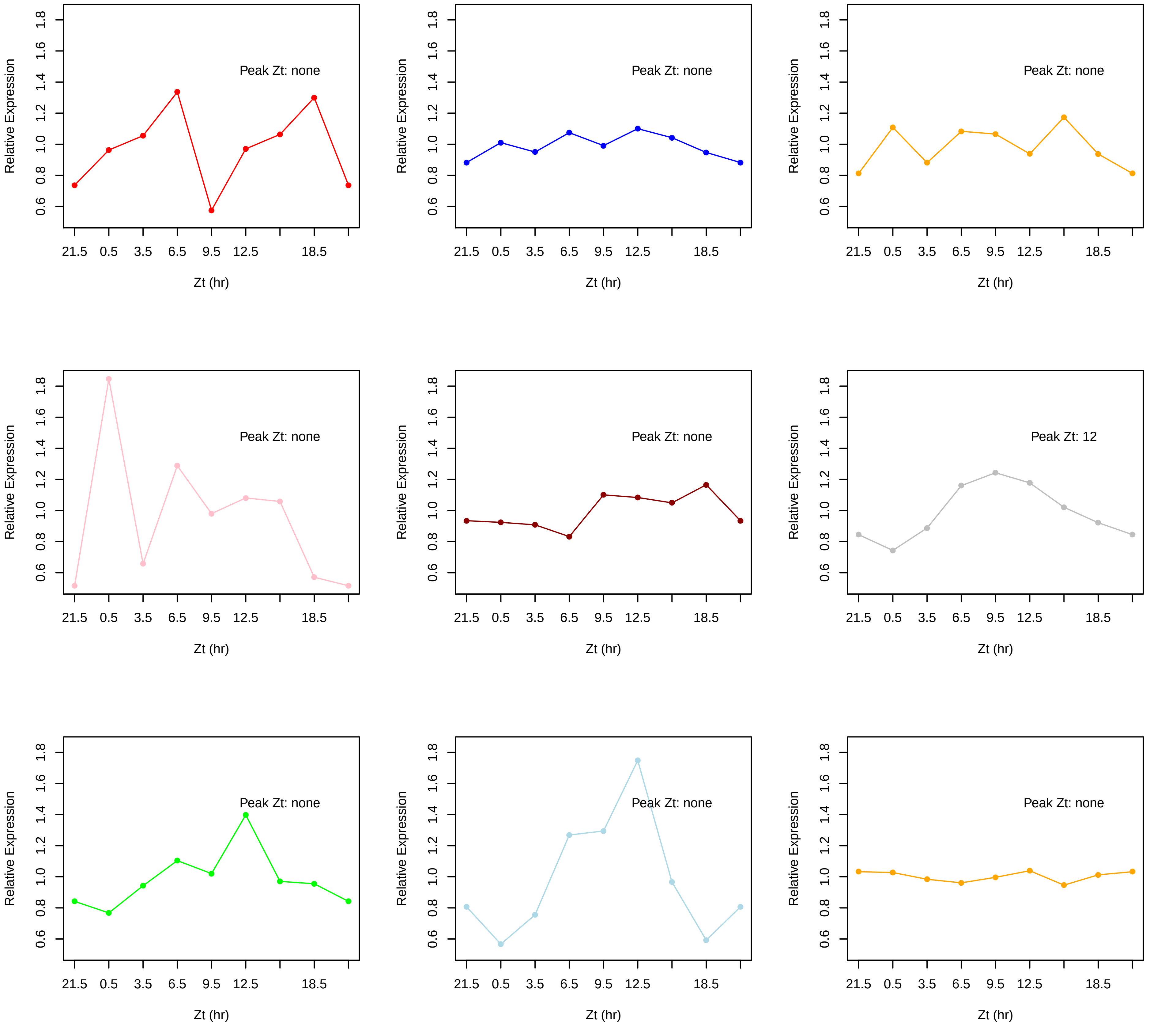

Singleton: ENSXMAG00000023084 asah1b

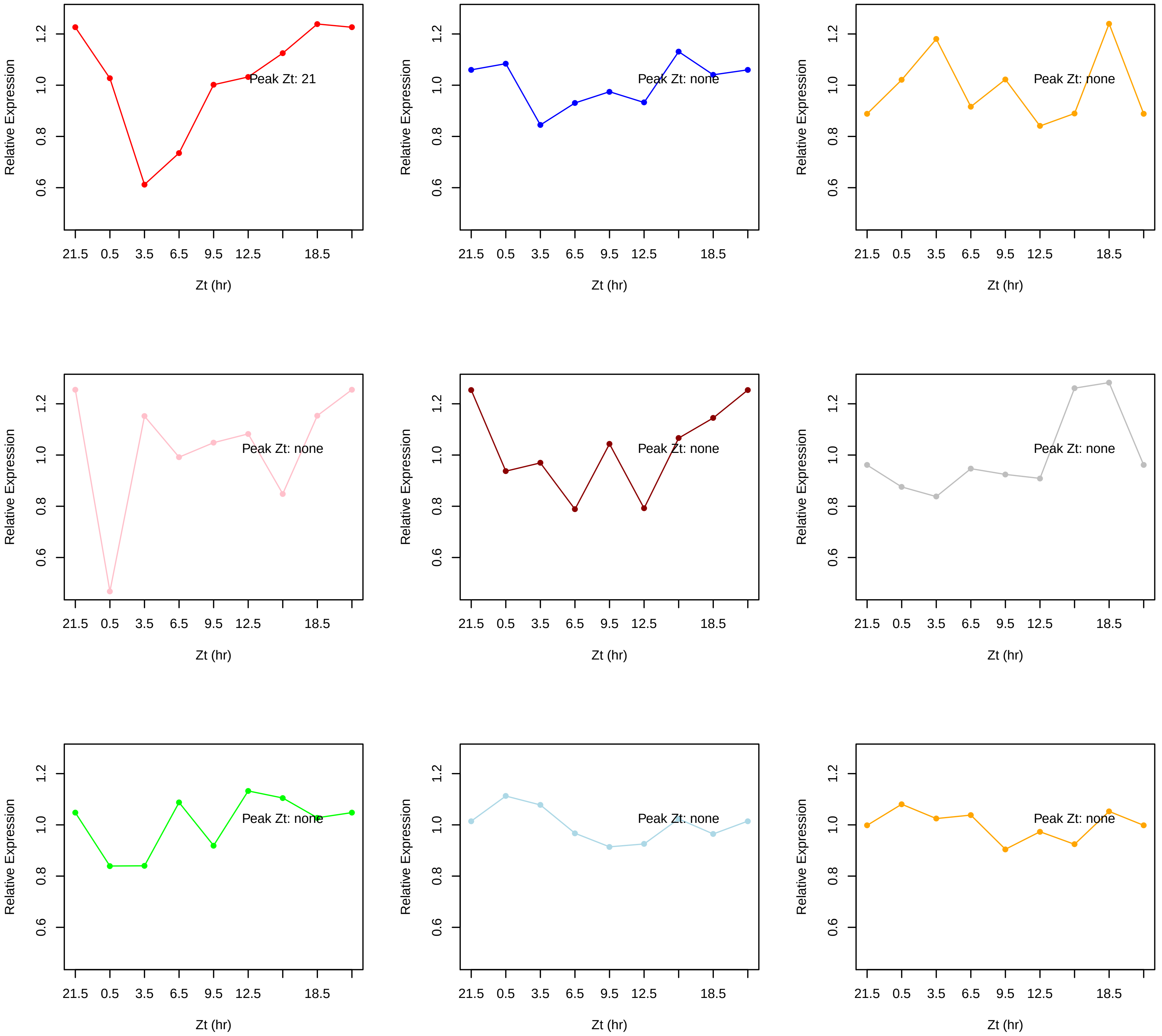

Singeleton: ENSXMAG00000018086

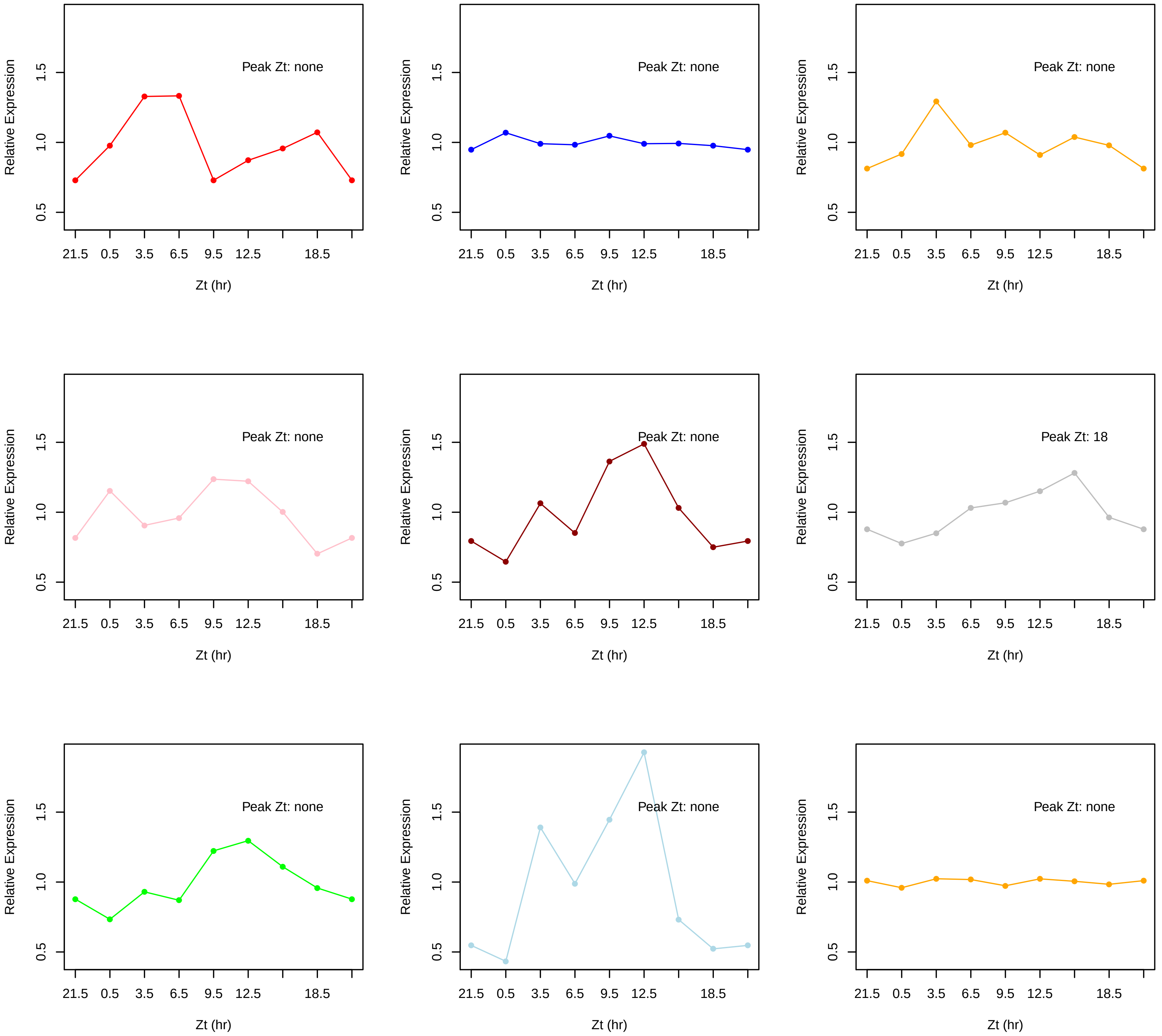

Singeleton: ENSXMAG00000024451

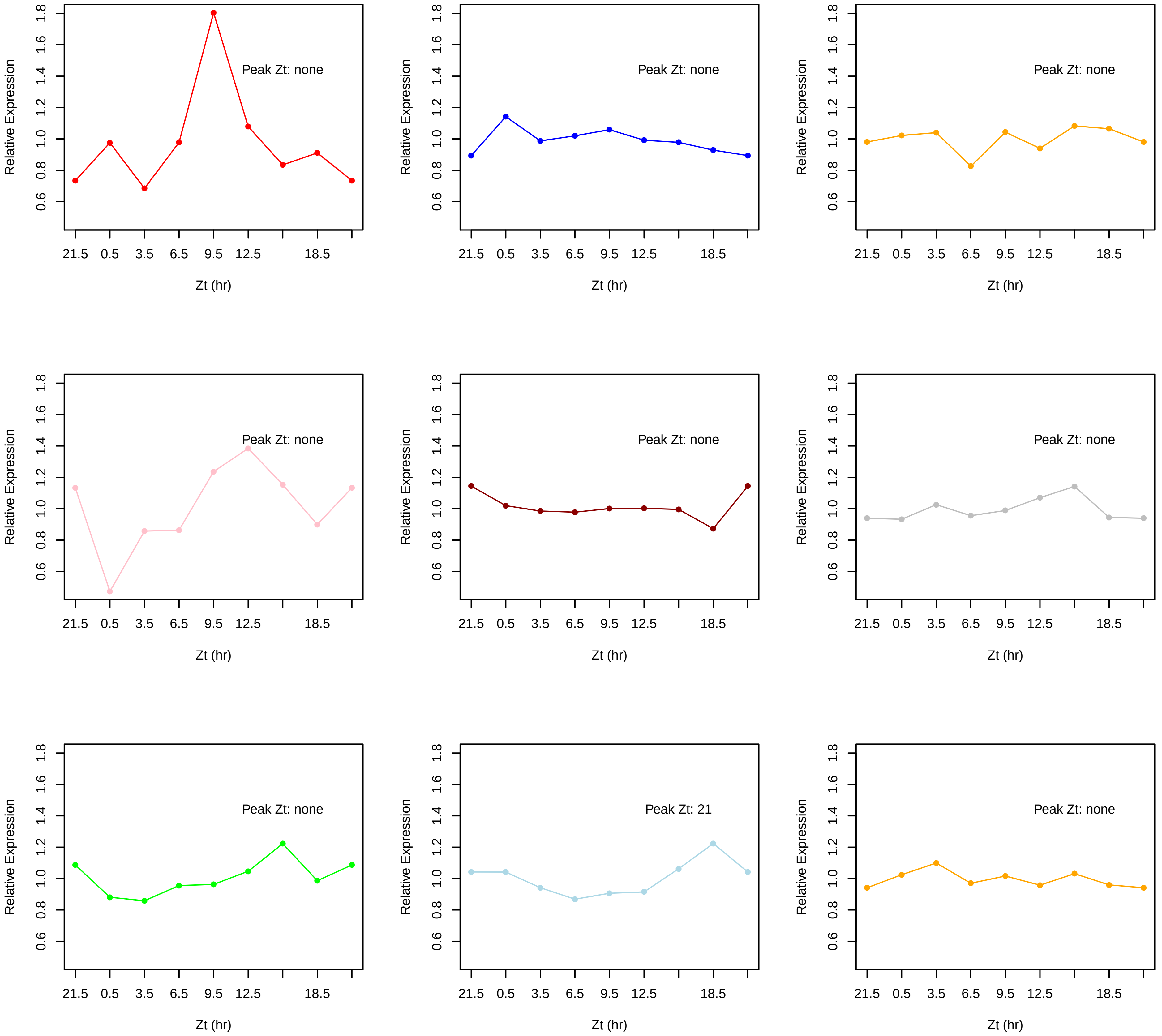

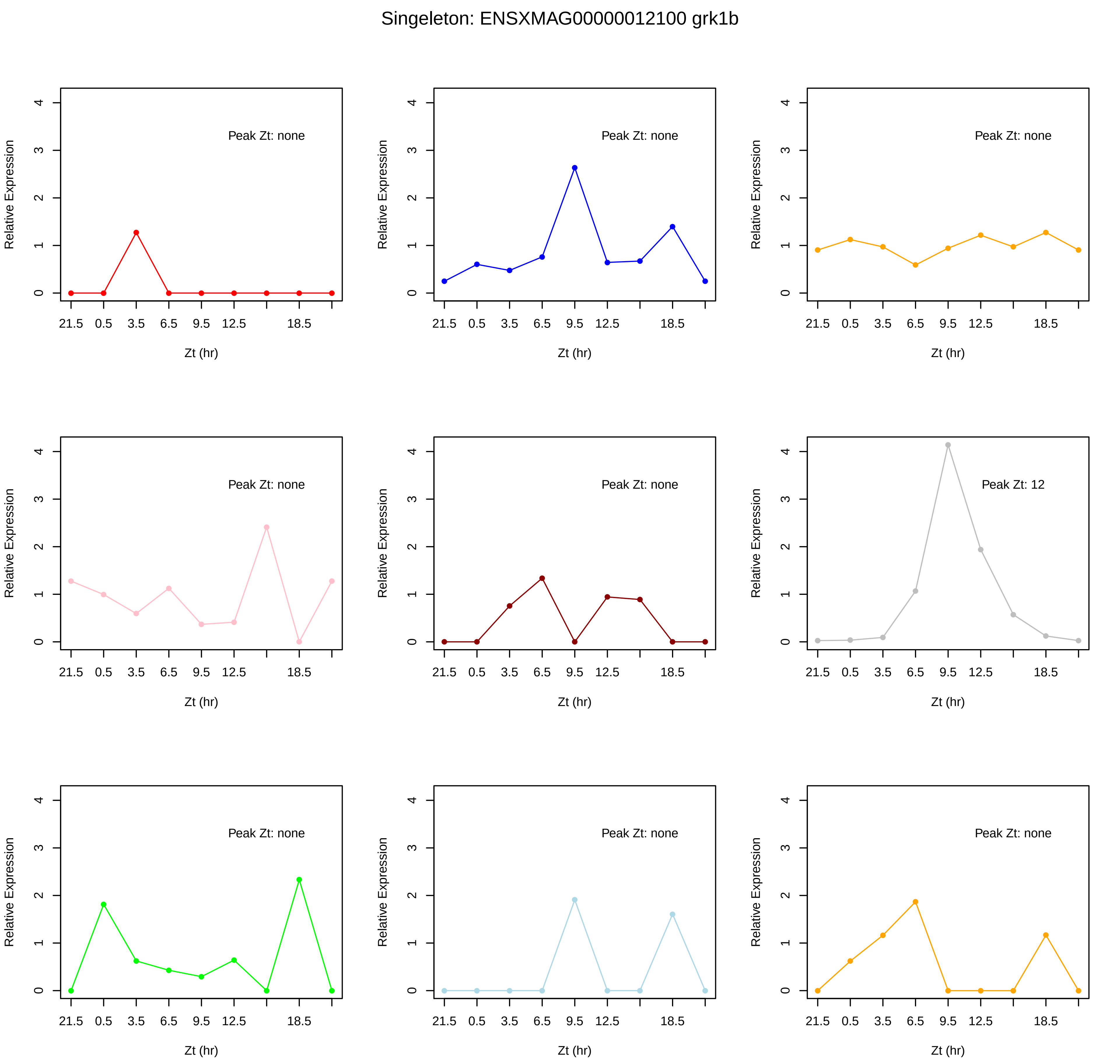
Singeleton: ENSXMAG00000012100 grk1b
Peak Zt: none
Peak Zt: none
Peak Zt: none
Peak Zt: none
Peak Zt: none
Peak Zt: 12
Peak Zt: none
Peak Zt: none
Peak Zt: none
Relative Expression
Zt (hr)
21.5 0.5 3.5 6.5 9.5 12.5 18.5
0 1 2 3 4

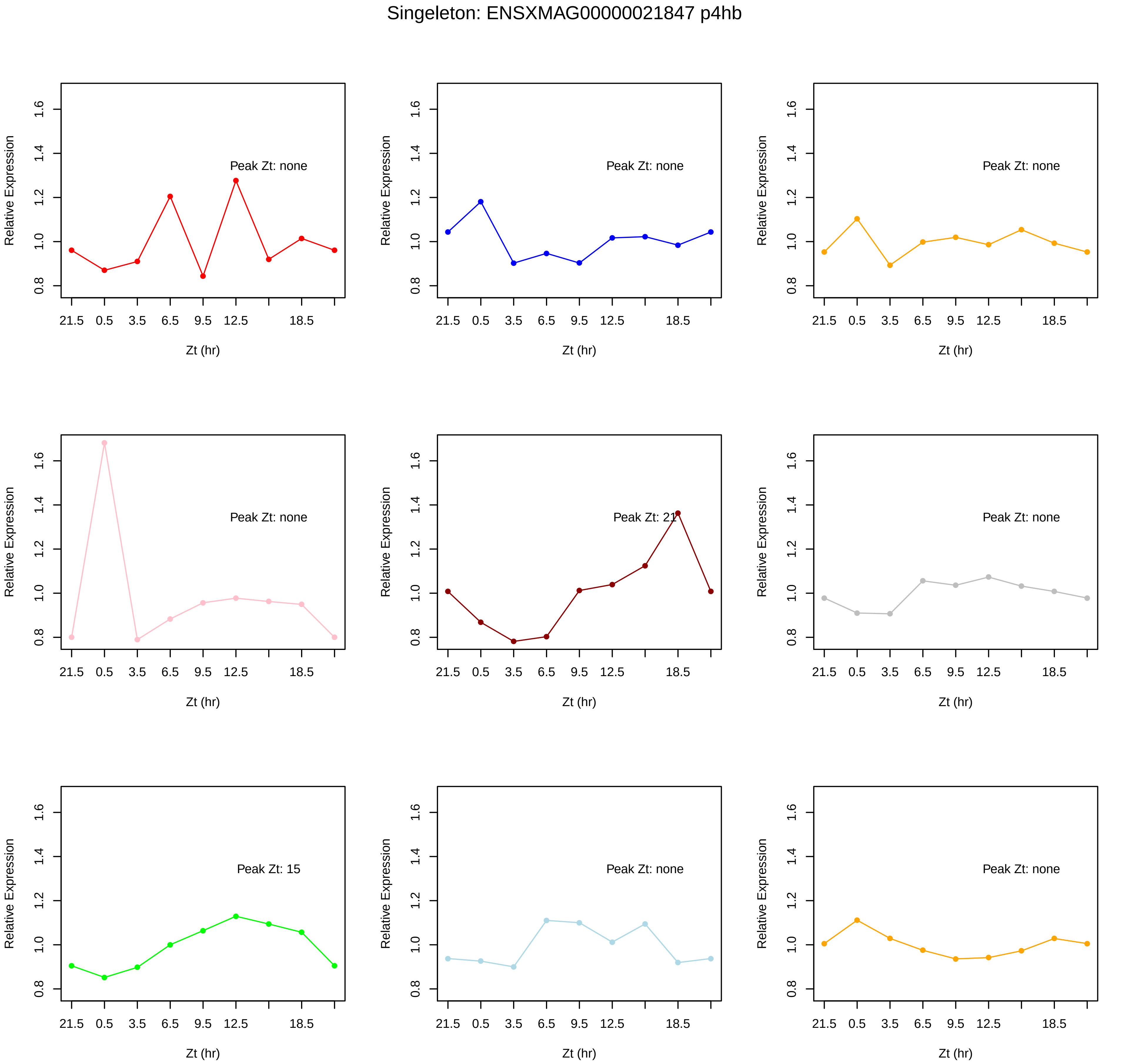
Singeleton: ENSXMAG00000021847 p4hb
Peak Zt: none
Peak Zt: none
Peak Zt: none
Peak Zt: none
Peak Zt: 21
Peak Zt: none
Peak Zt: 15
Peak Zt: none
Peak Zt: none
Relative Expression
Zt (hr)
21.5
0.5
3.5
6.5
9.5
12.5
18.5
0.8
1.0
1.2
1.4
1.6

Singeleton: ENSXMAG00000023616 rangrf

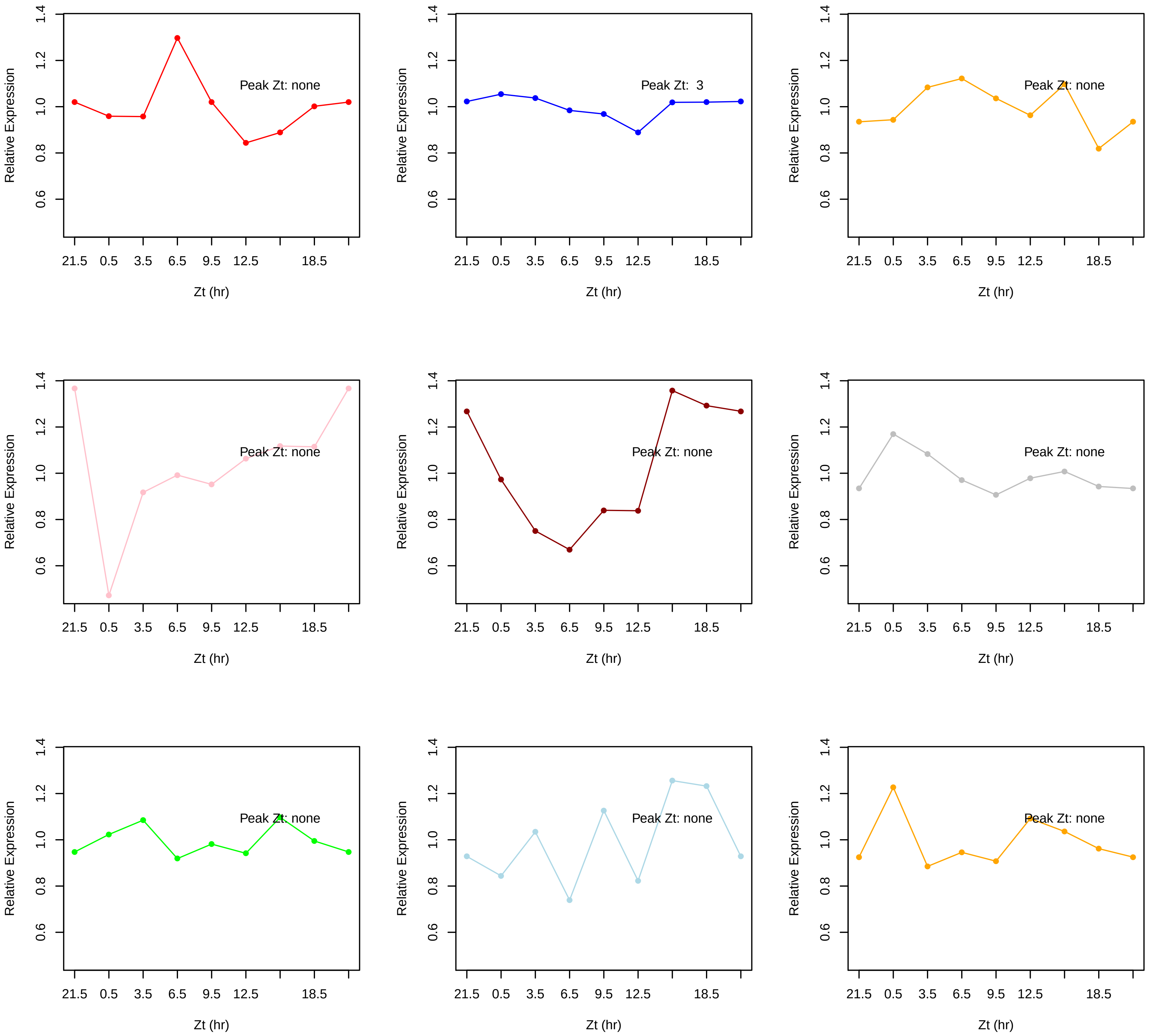

Singeleton: ENSXMAG00000017502 pdgfrl

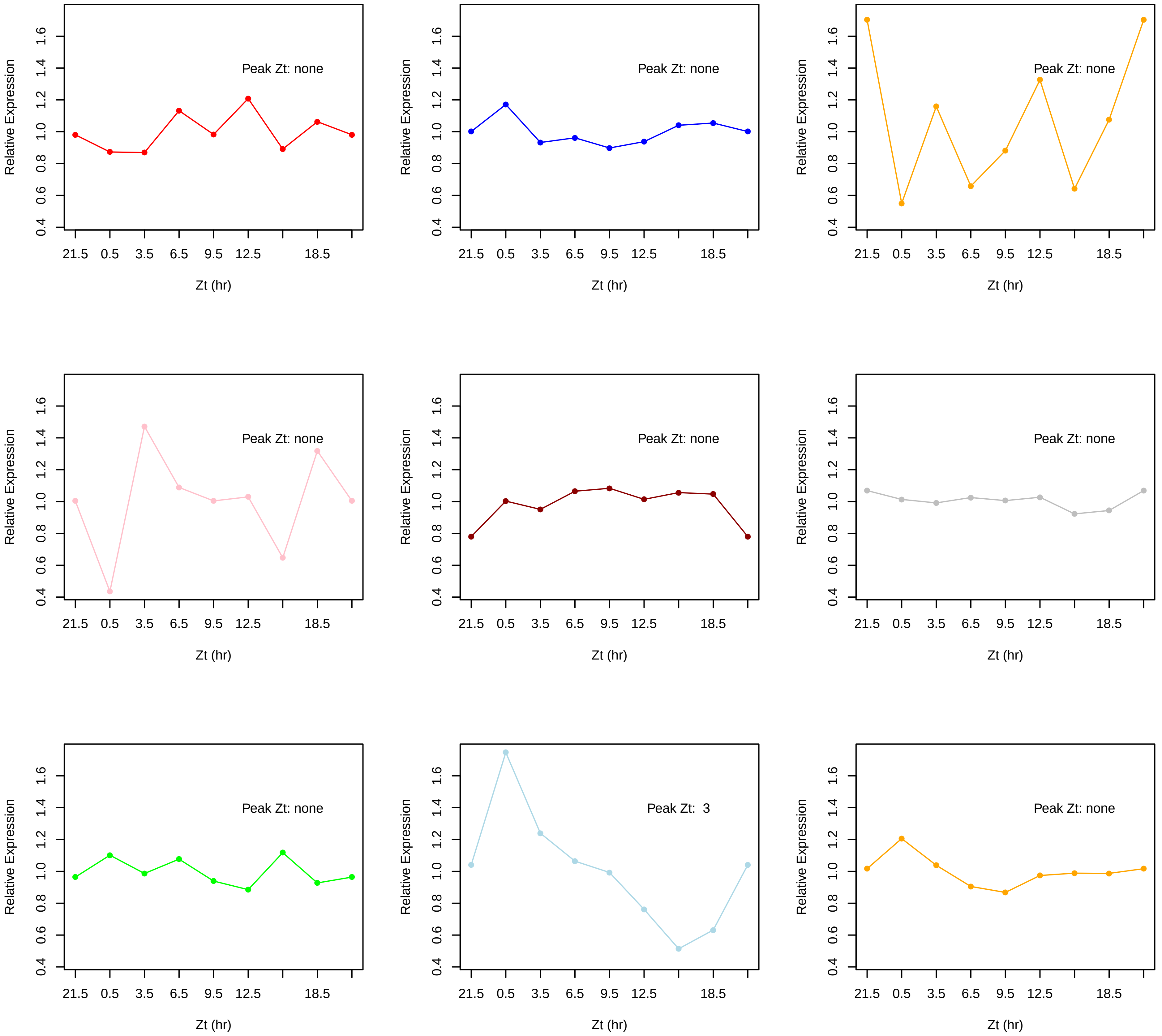

Singeleton: ENSXMAG00000017478 slc6a11b

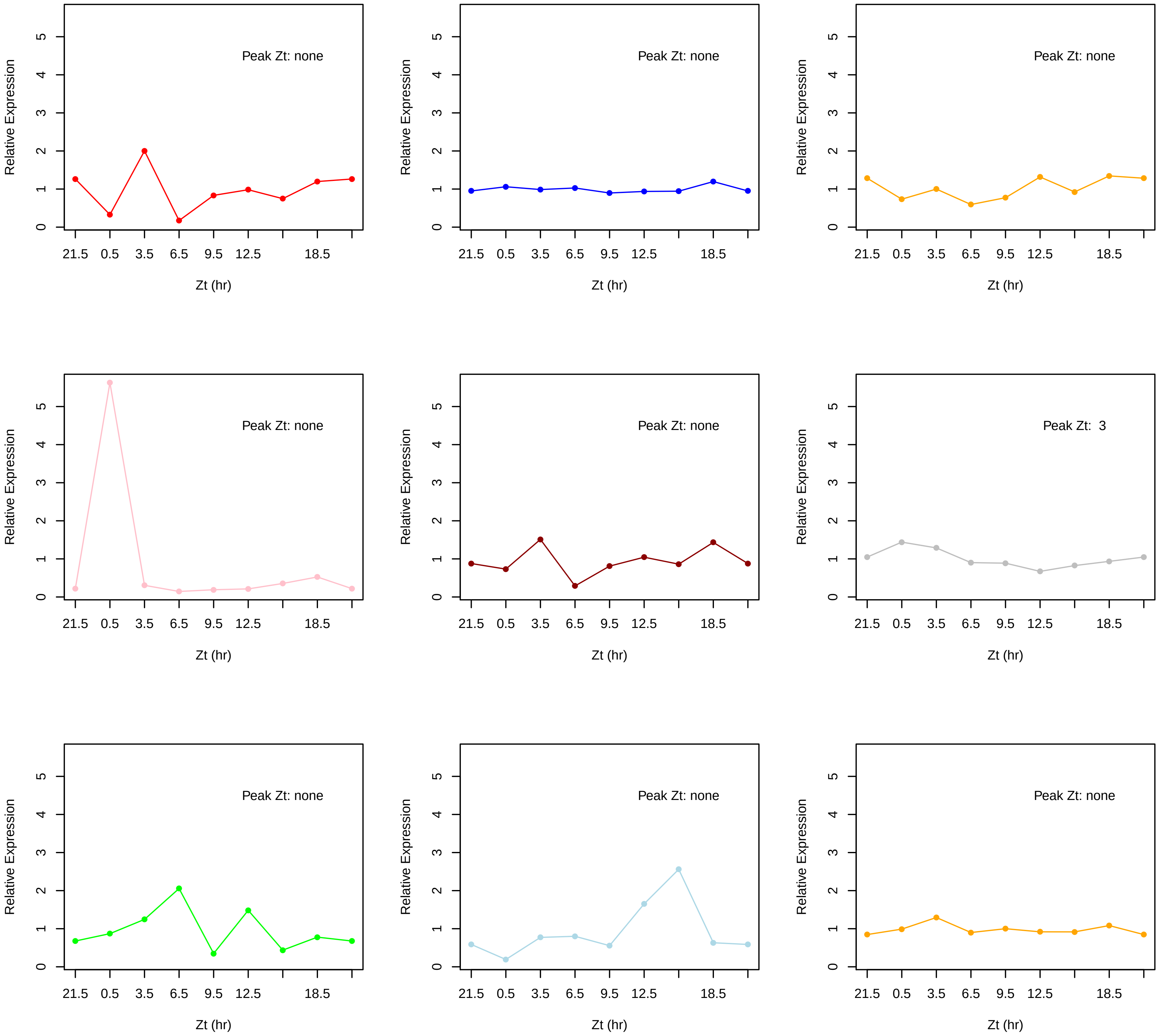

Singleton: ENSXMAG00000003564 aagab

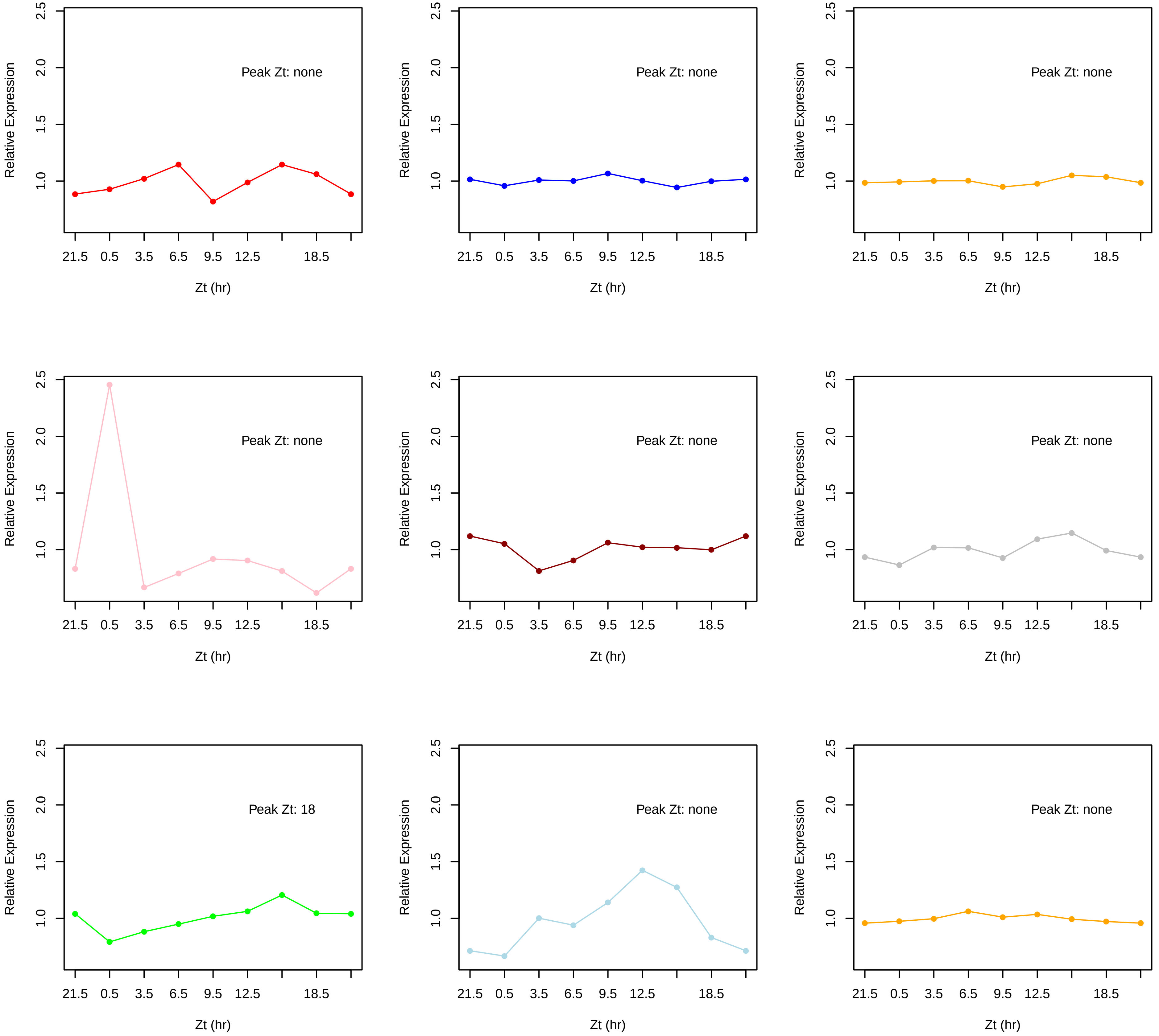

Singleton: ENSXMAG00000024707 si:ch211−220e11.3

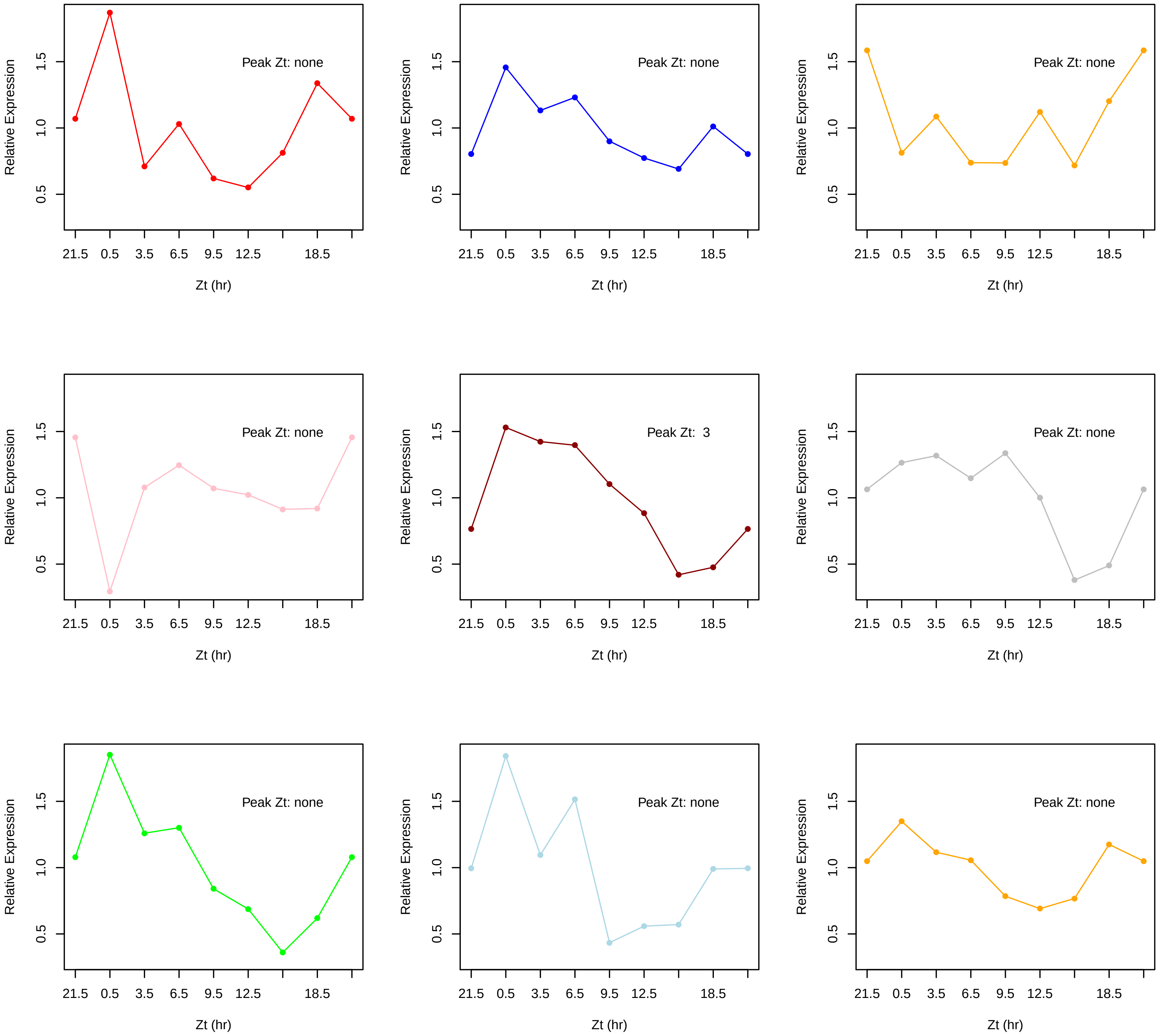

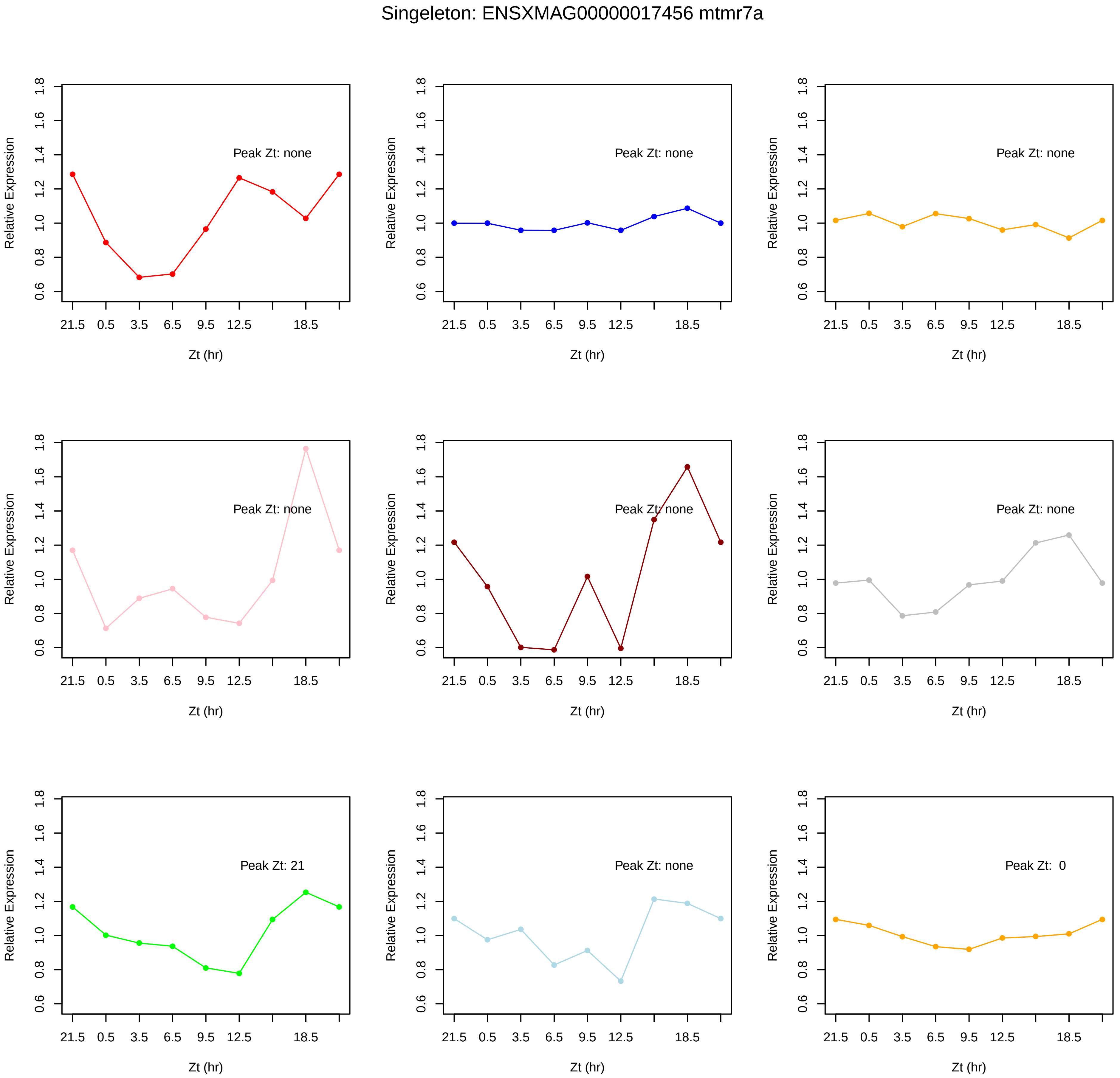
Singeleton: ENSXMAG00000017456 mtmr7a
Peak Zt: none
Peak Zt: none
Peak Zt: none
Peak Zt: none
Peak Zt: none
Peak Zt: none
Peak Zt: 21
Peak Zt: none
Peak Zt: 0
Relative Expression
Zt (hr)
21.5 0.5 3.5 6.5 9.5 12.5 18.5
0.6 0.8 1.0 1.2 1.4 1.6 1.8

Singeleton: ENSXMAG00000022123 zgc:162898

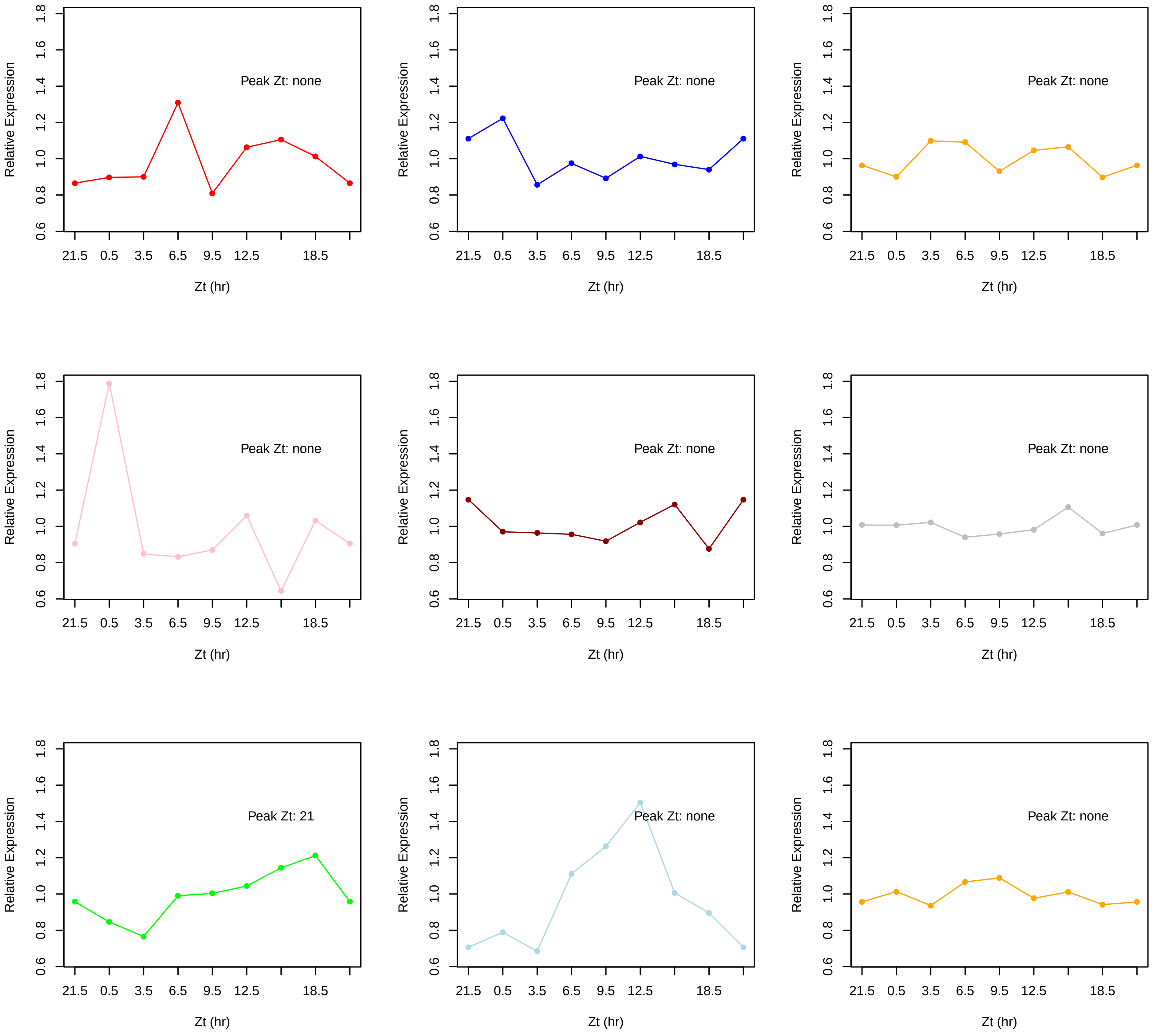

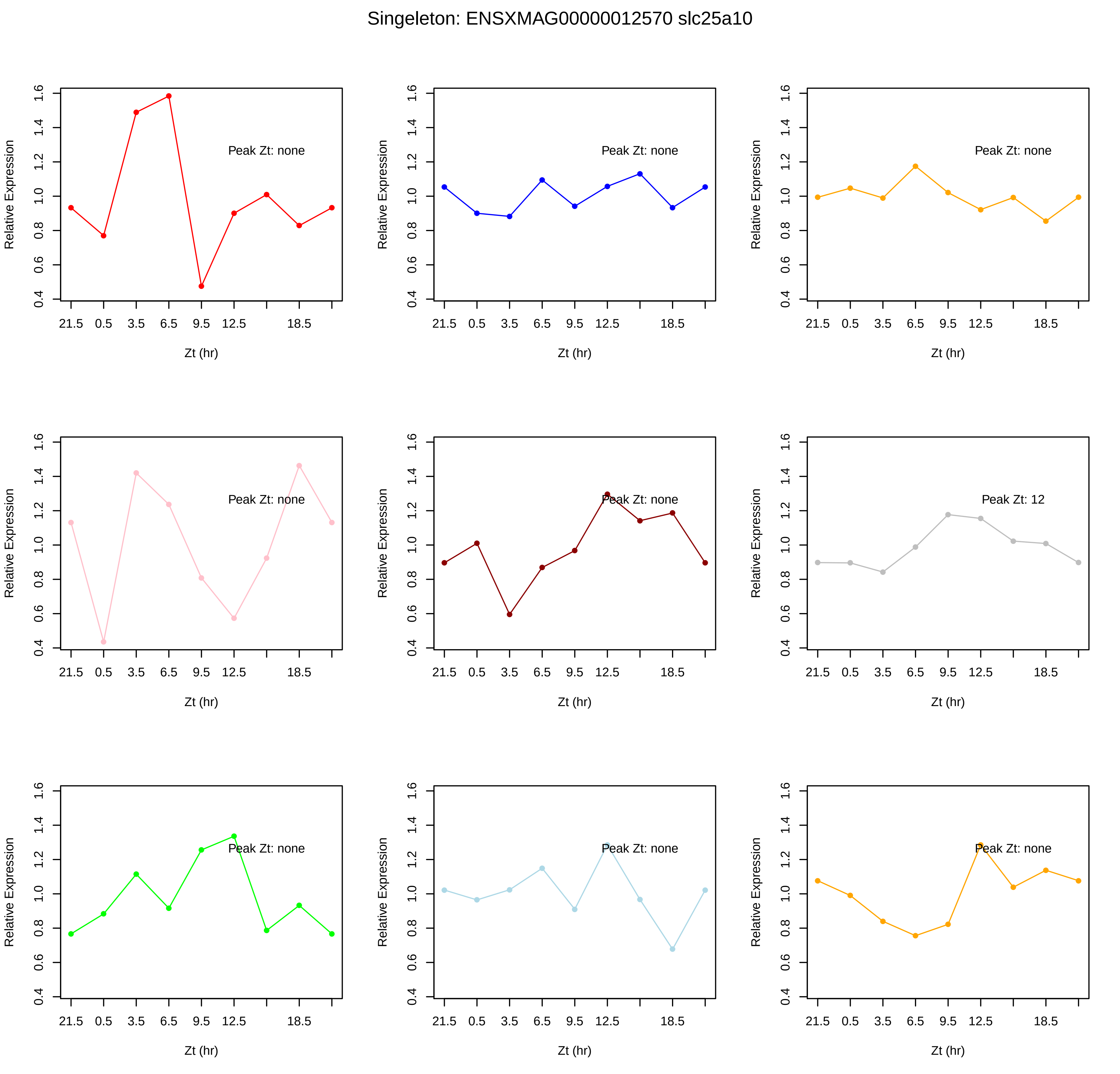
Singeleton: ENSXMAG00000012570 slc25a10
Peak Zt: none
Peak Zt: none
Peak Zt: none
Peak Zt: none
Peak Zt: none
Peak Zt: 12
Peak Zt: none
Peak Zt: none
Peak Zt: none
Relative Expression
Zt (hr)
21.5
0.5
3.5
6.5
9.5
12.5
18.5

Singeleton: ENSXMAG00000017429 atg7

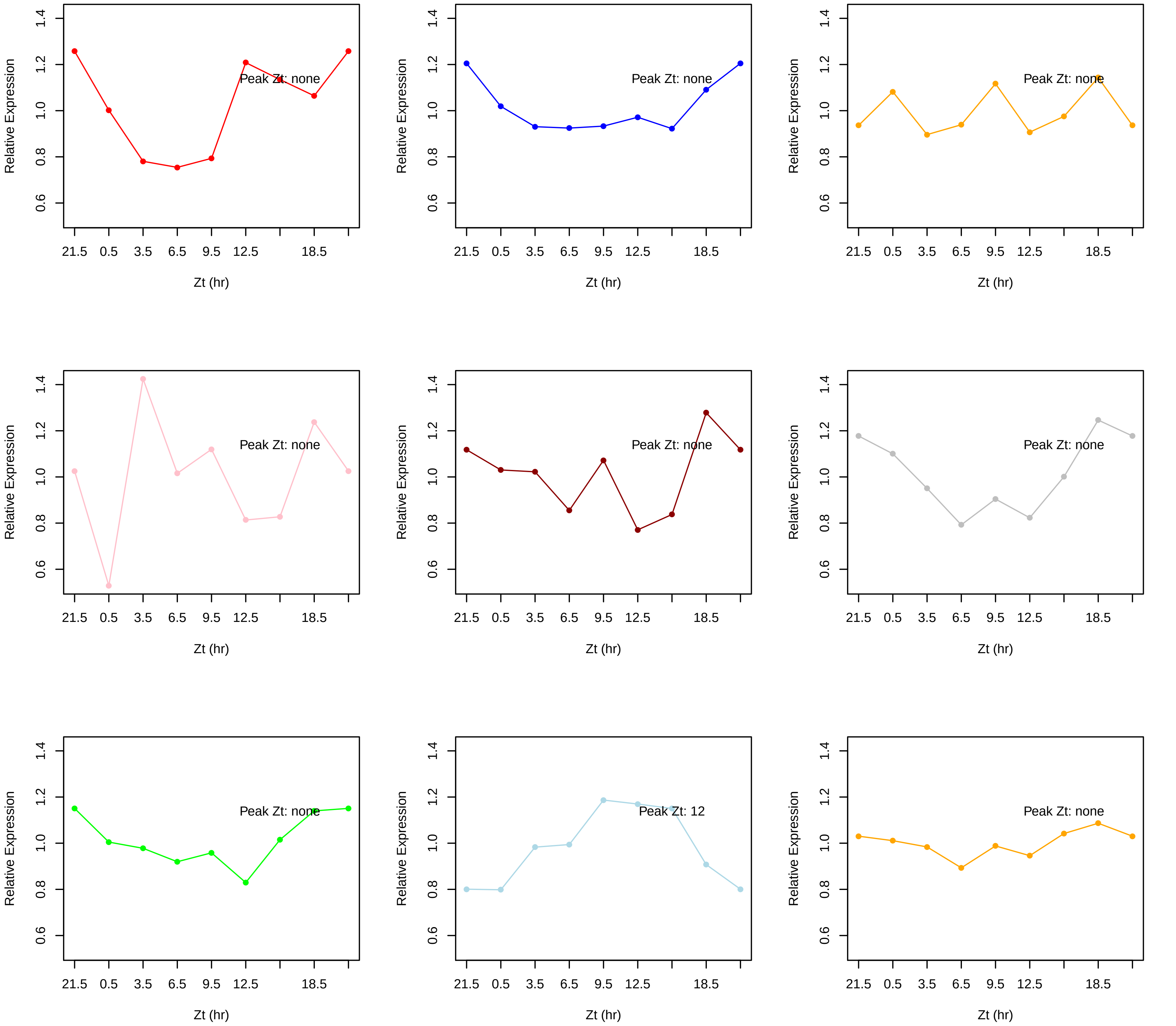

Singeleton: ENSXMAG00000015629 si:dkey−38p12.3

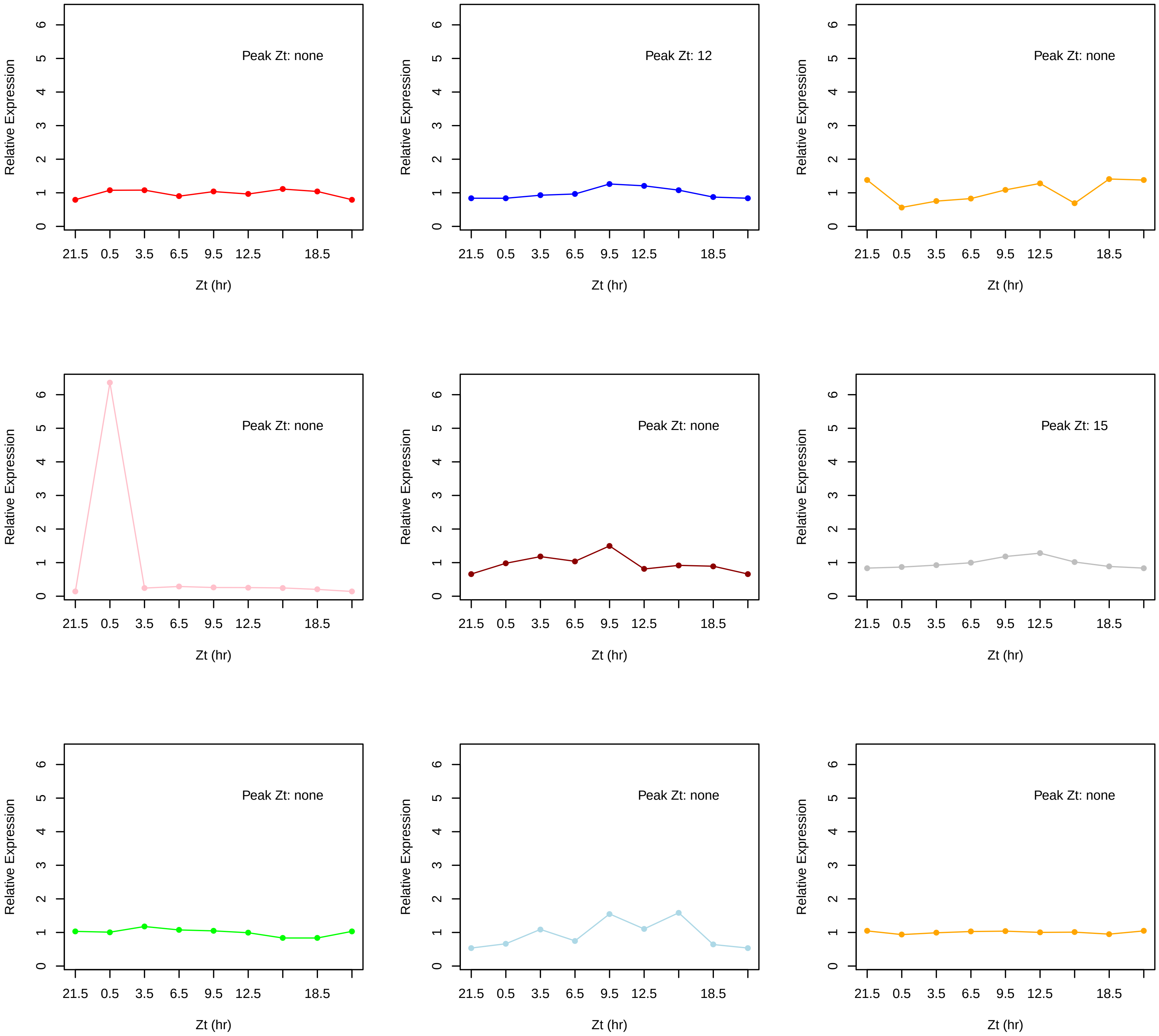

Singeleton: ENSXMAG00000017407 tamm41

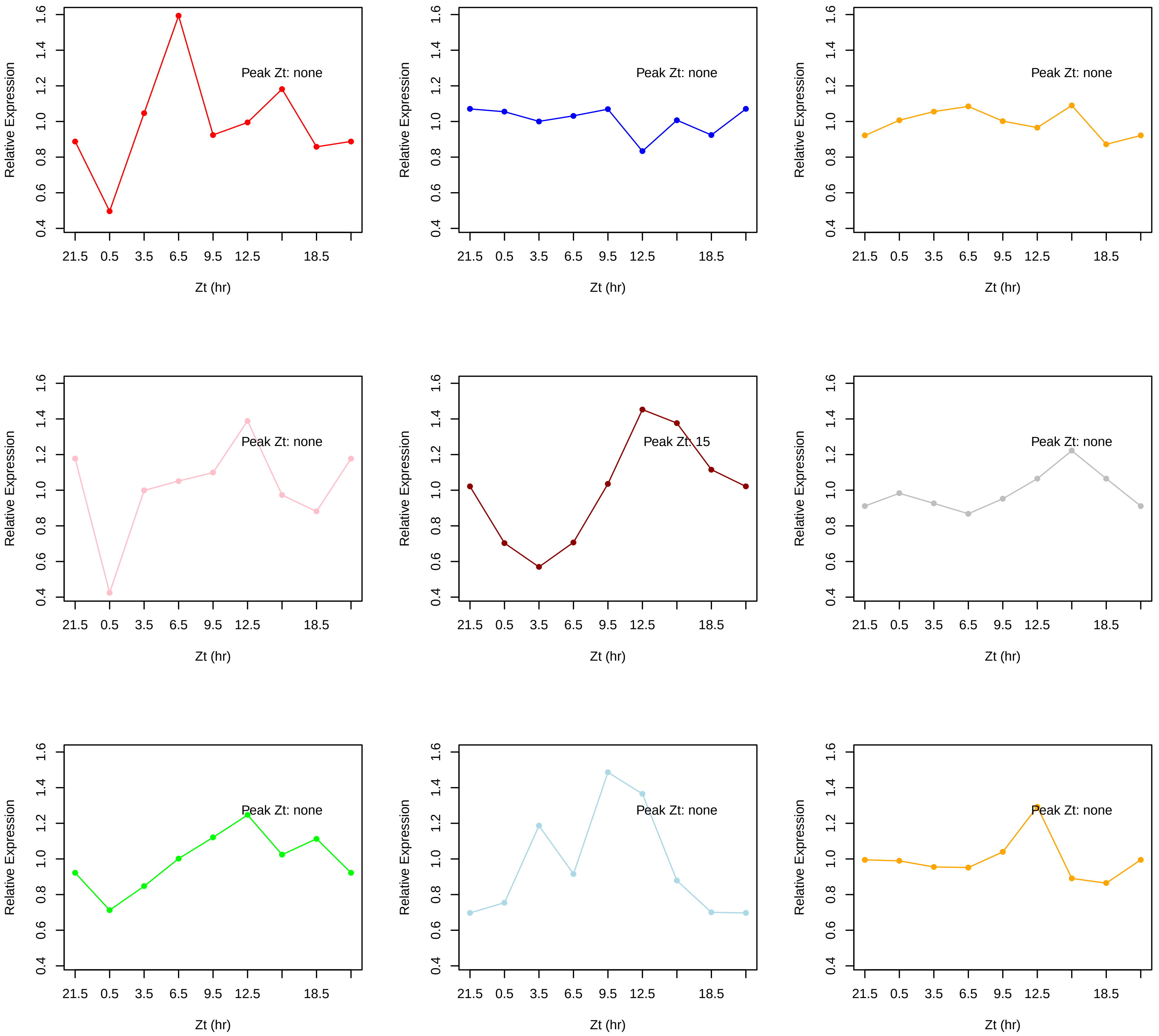

Singeleton: ENSXMAG00000017447 cnot7

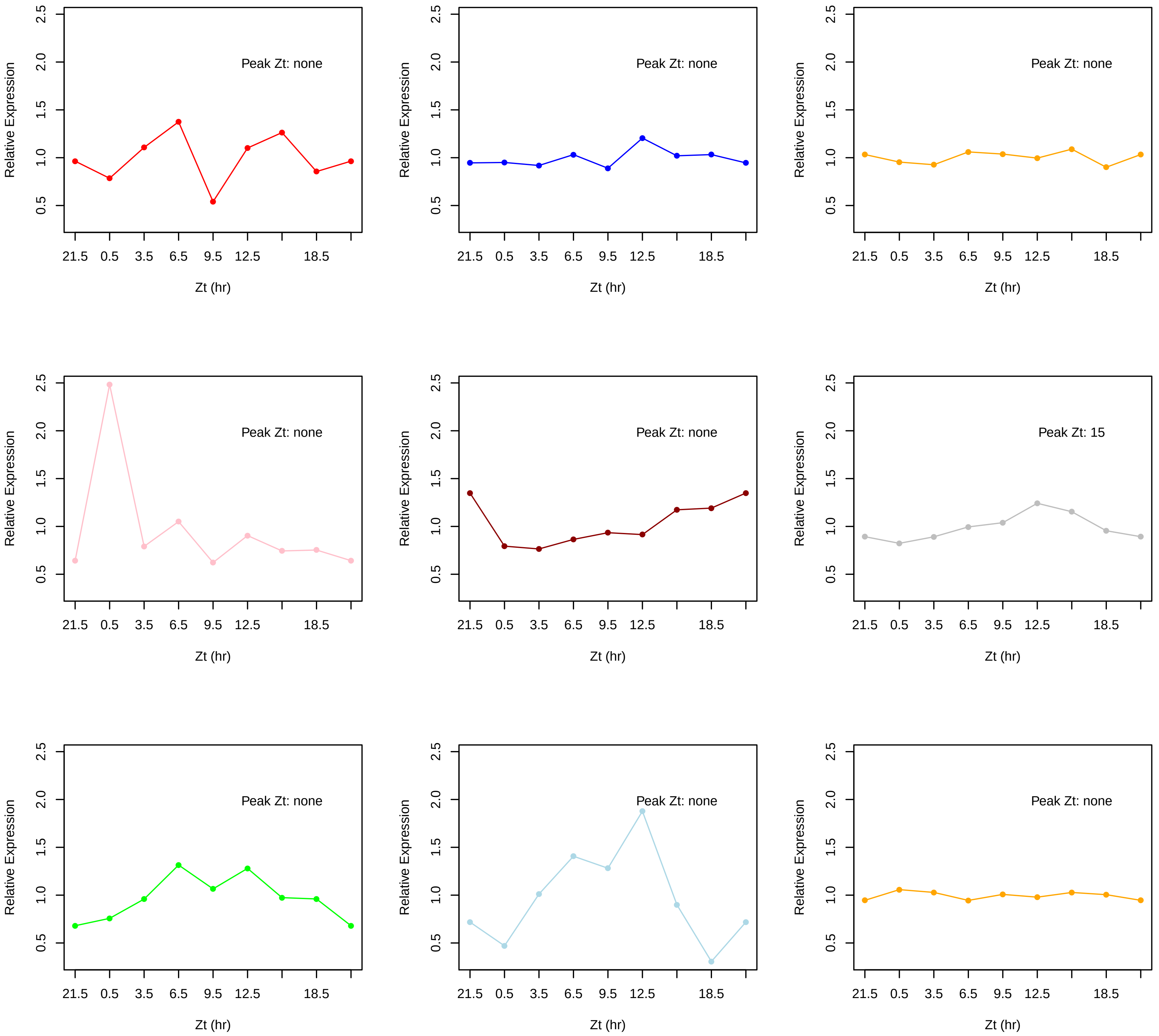

Singleton: ENSXMAG00000017725 syn2a

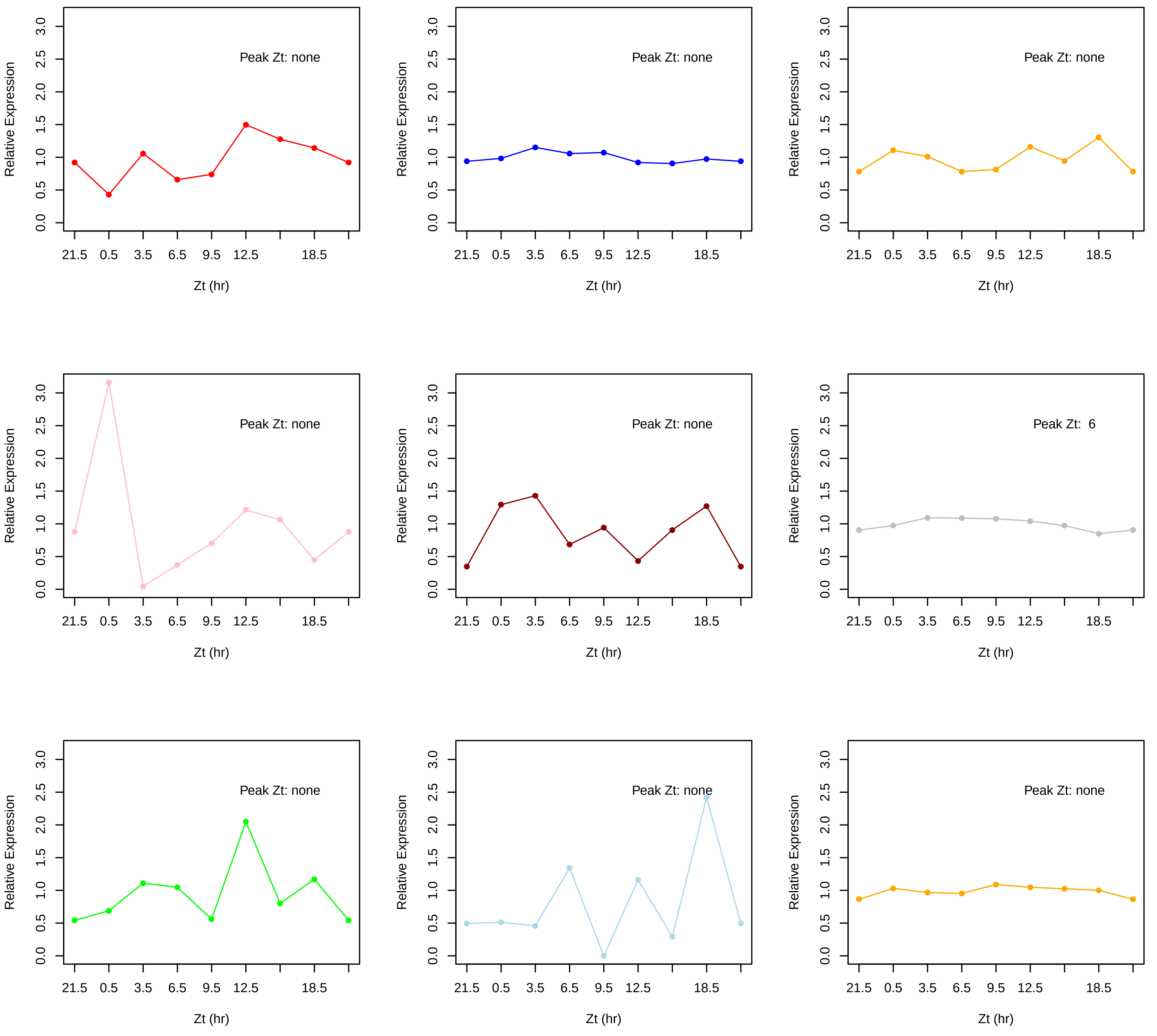

Singeleton: ENSXMAG00000000510

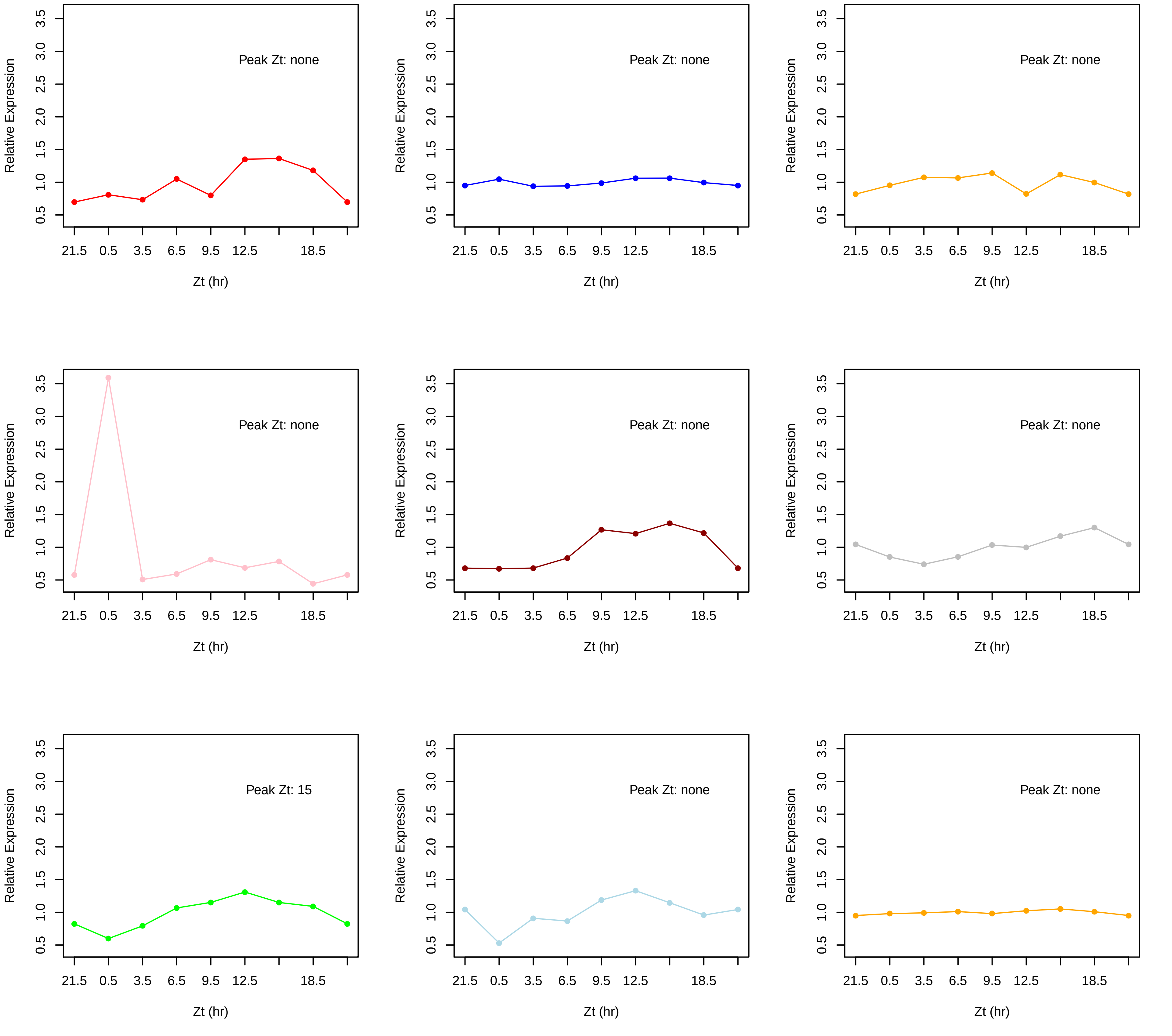

Singleton: ENSXMAG00000009578 fem1b

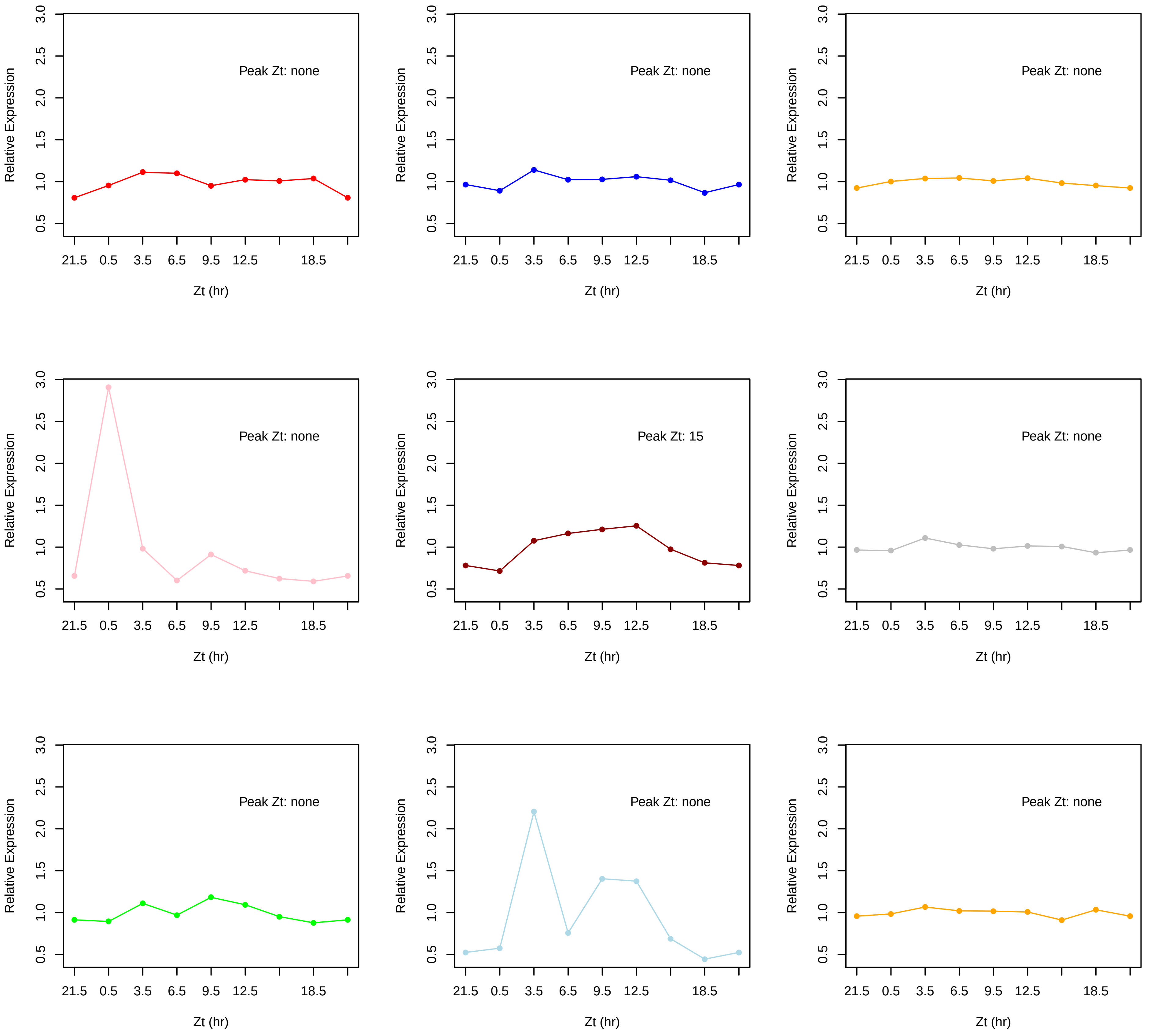

Singeleton: ENSXMAG00000028986 timp4.3

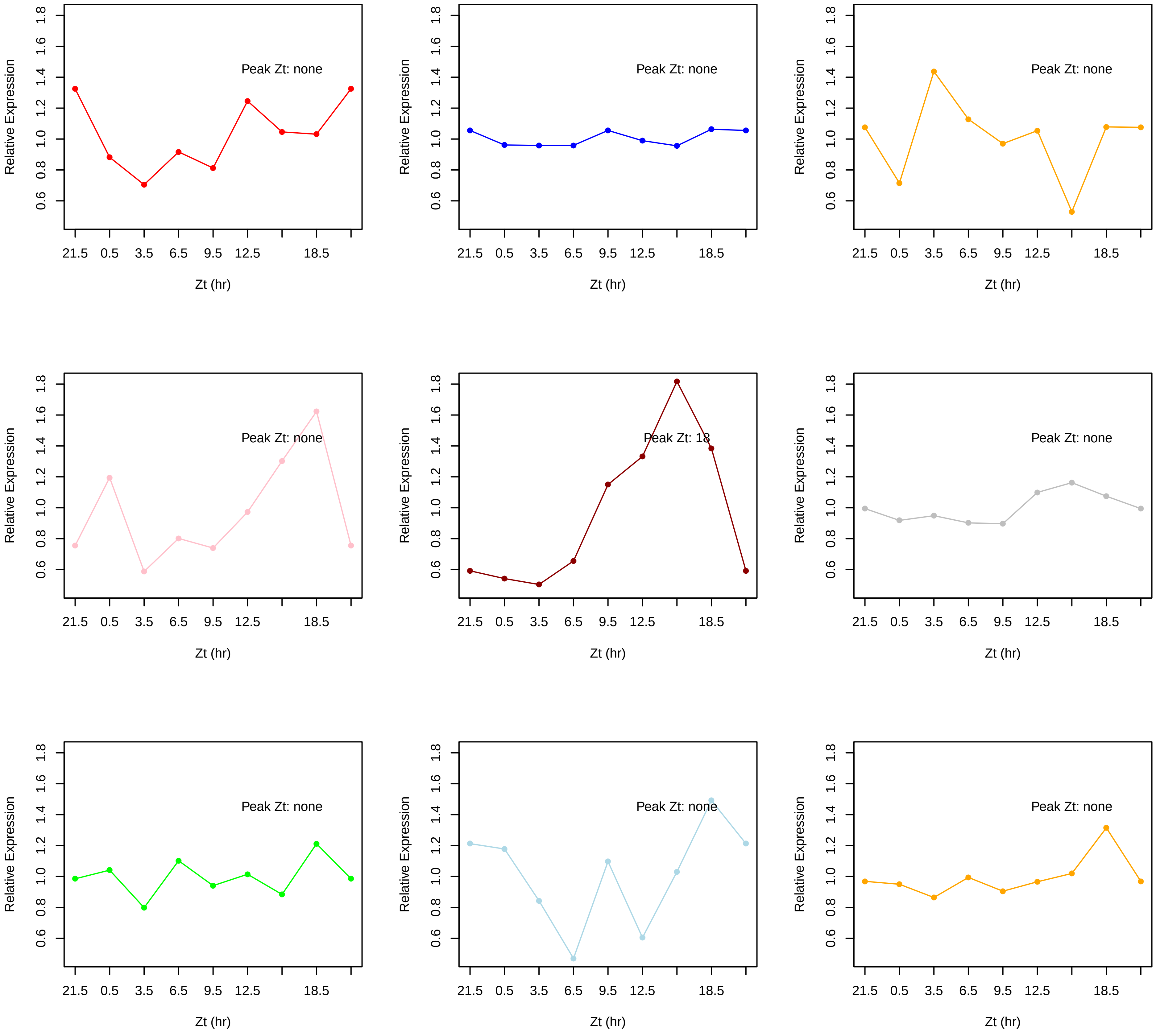

Singeleton: ENSXMAG00000017330 PPARG

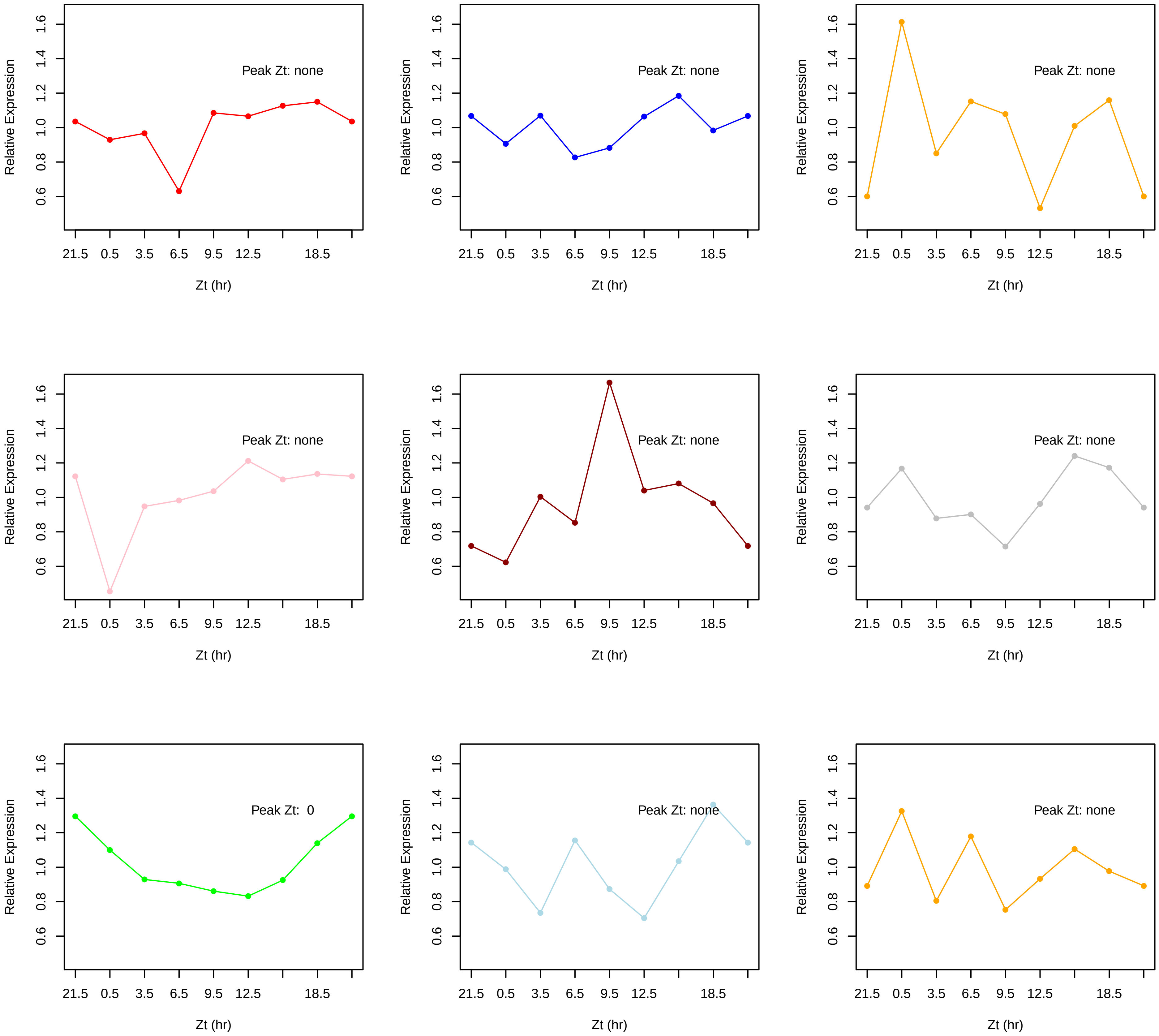

Singeleton: ENSXMAG00000012479

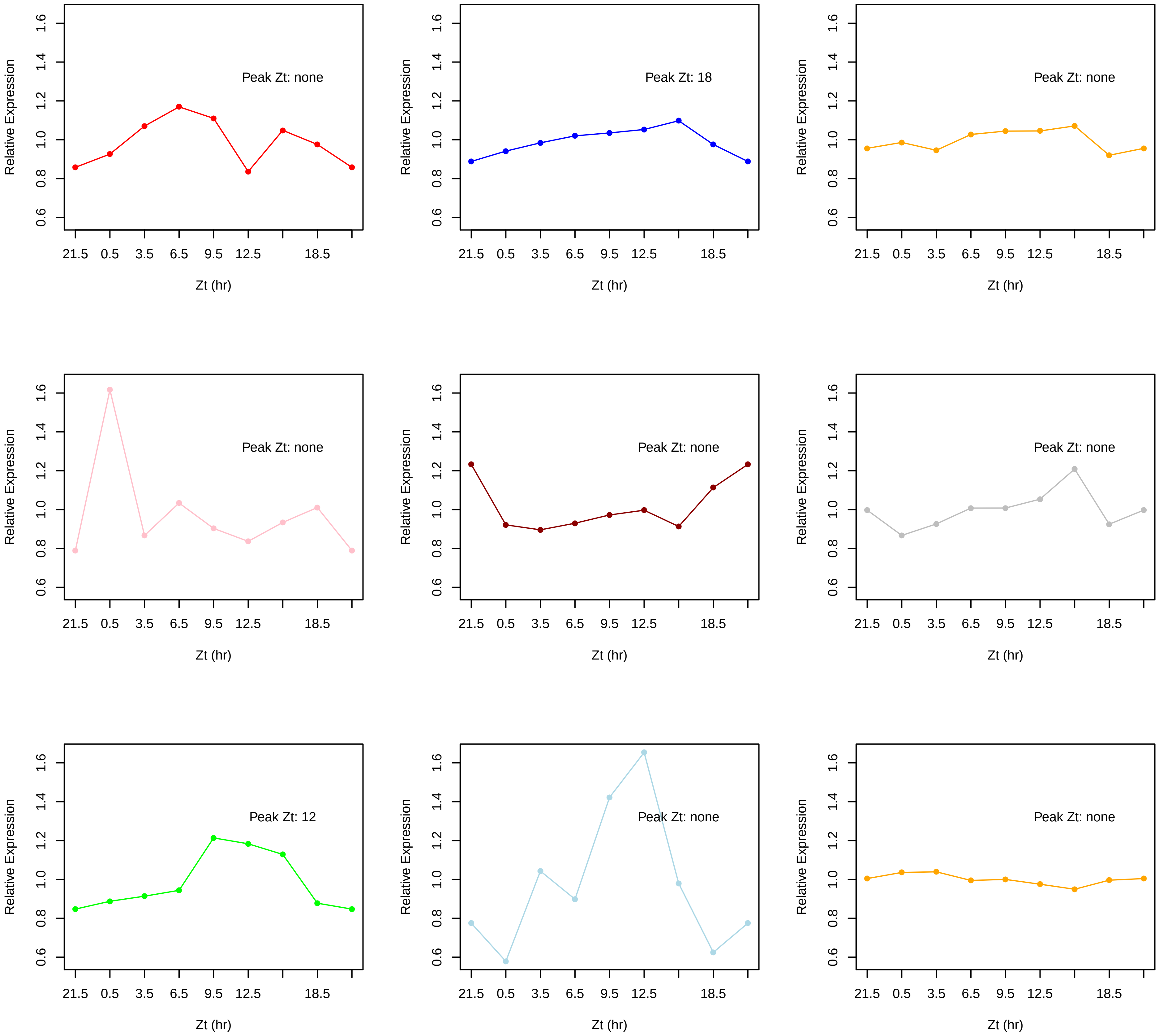

Singeleton: ENSXMAG00000025997 si:dkey−19b23.7

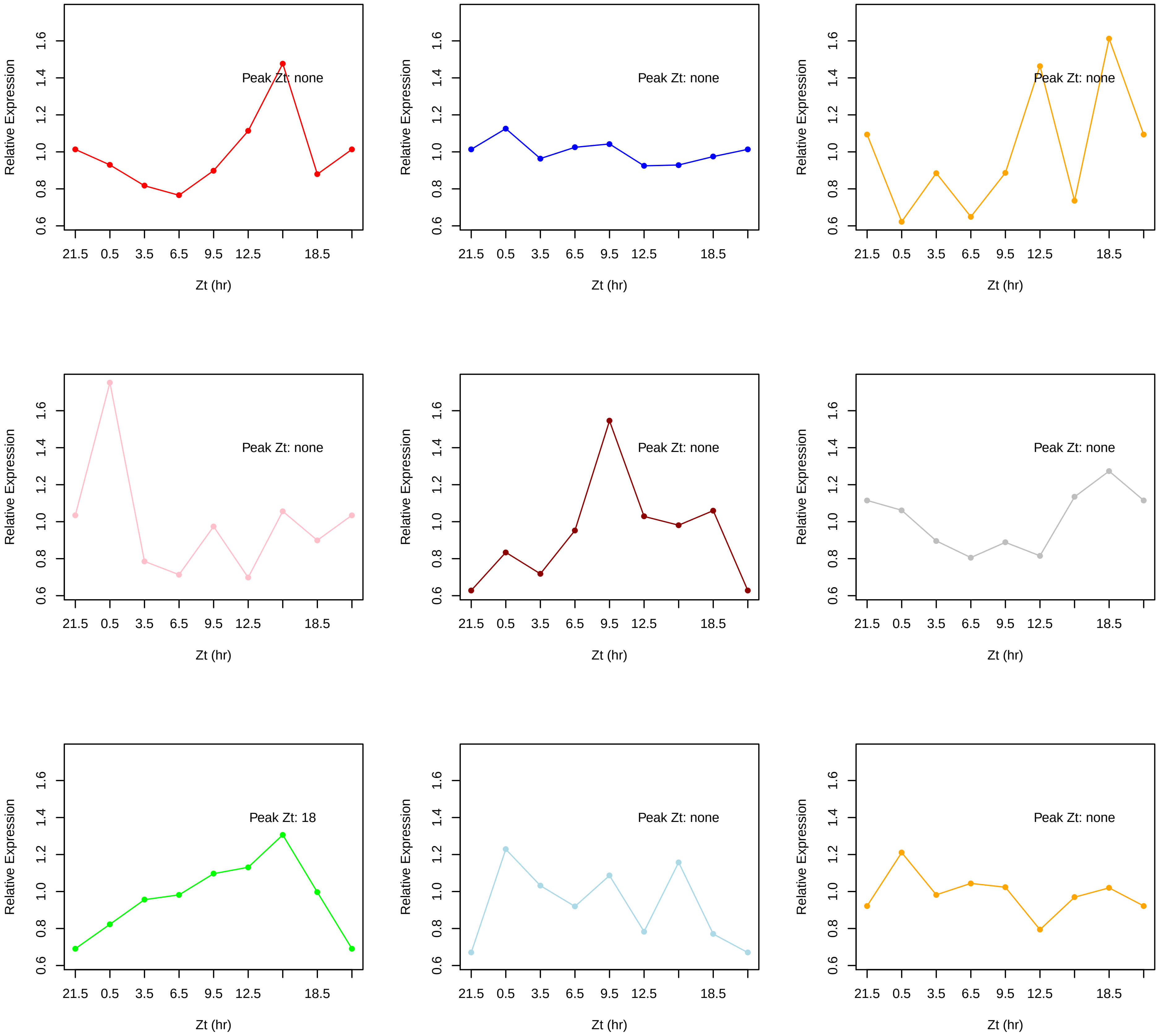

Singeleton: ENSXMAG00000015779 plekhh1

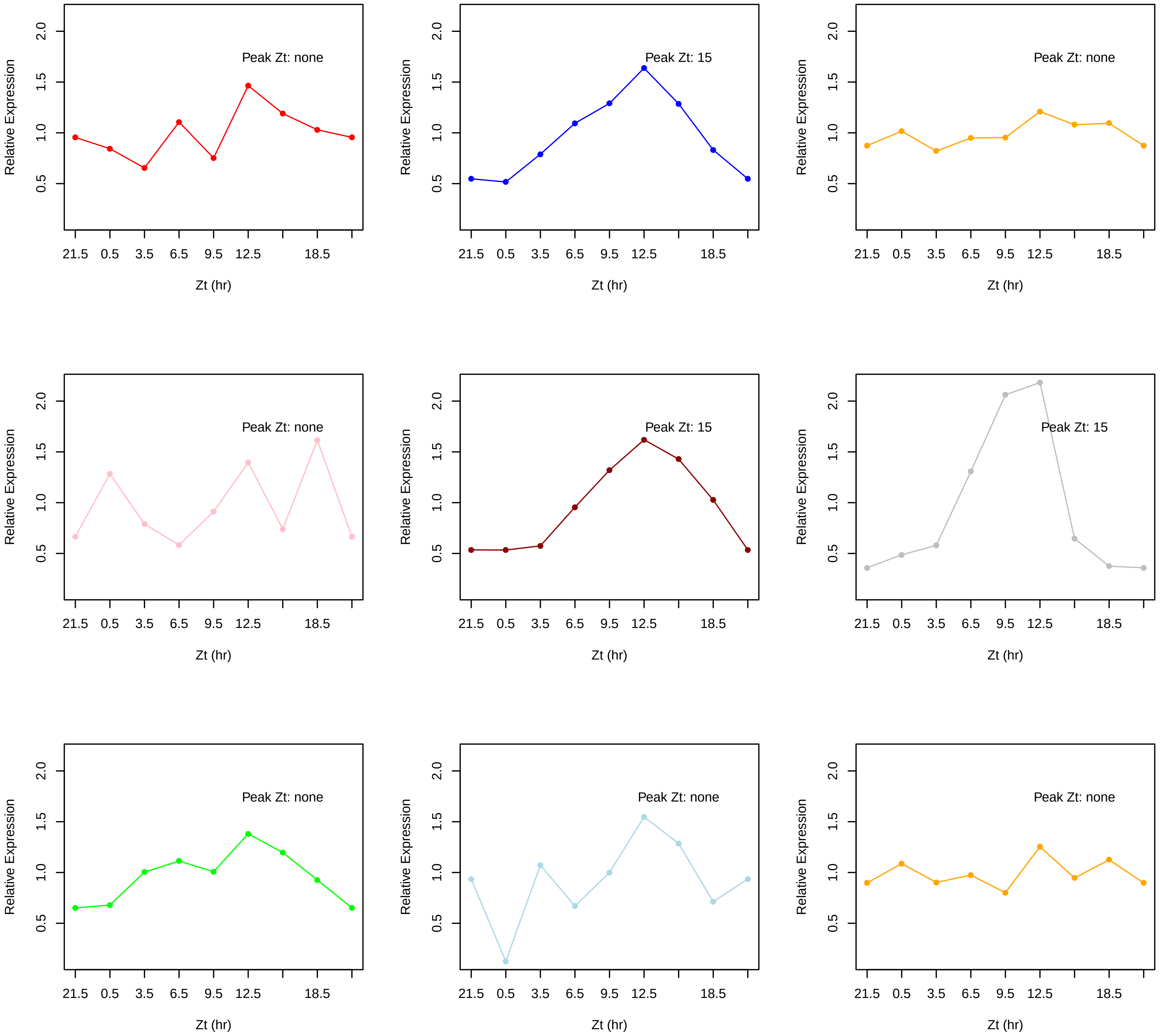

Singeleton: ENSXMAG00000016035 chd3

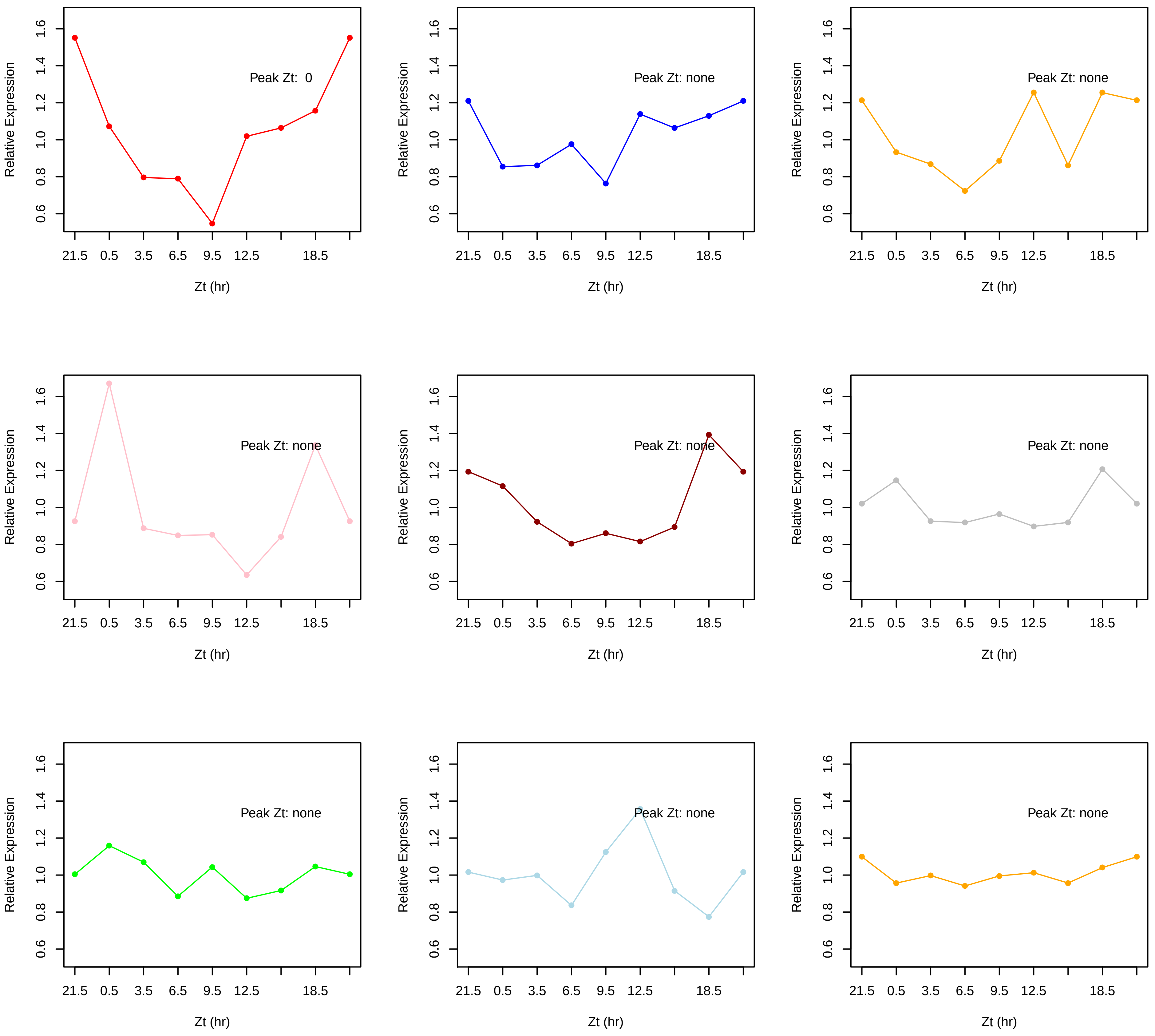

Singeleton: ENSXMAG00000000471 coro2ba

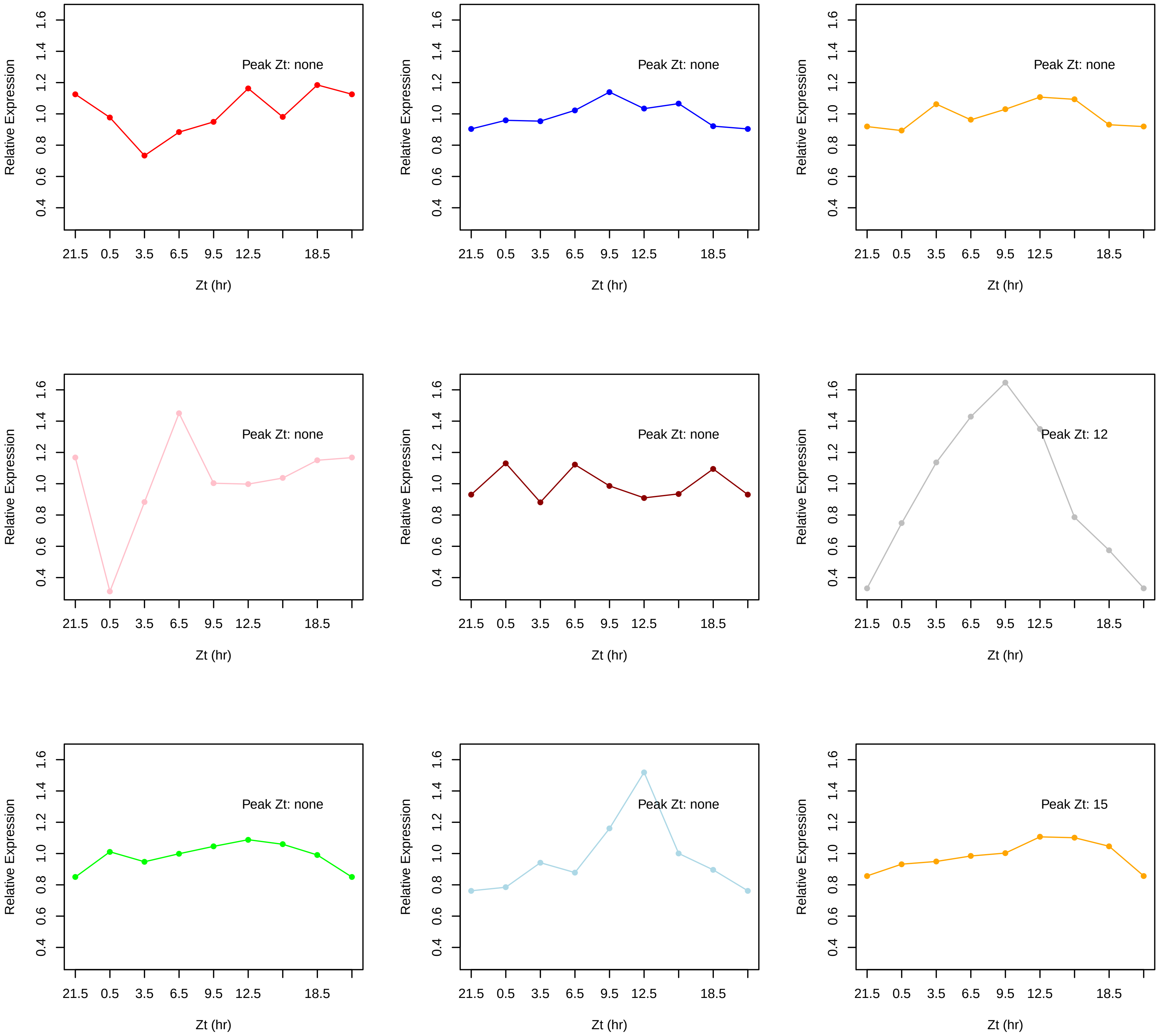

Singleton: ENSXMAG00000023035 tusc3

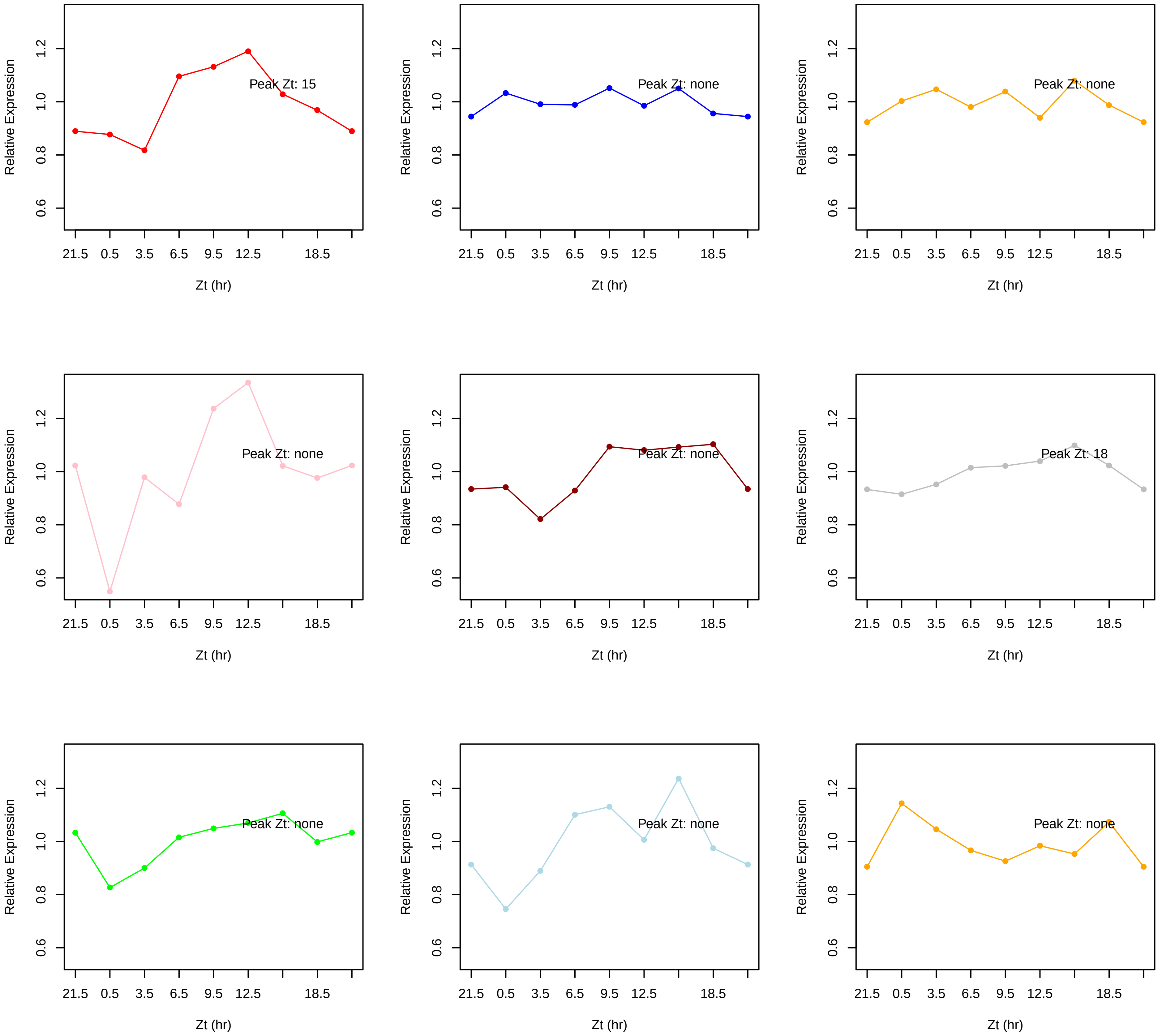

Singeleton: ENSXMAG00000000465 anp32a

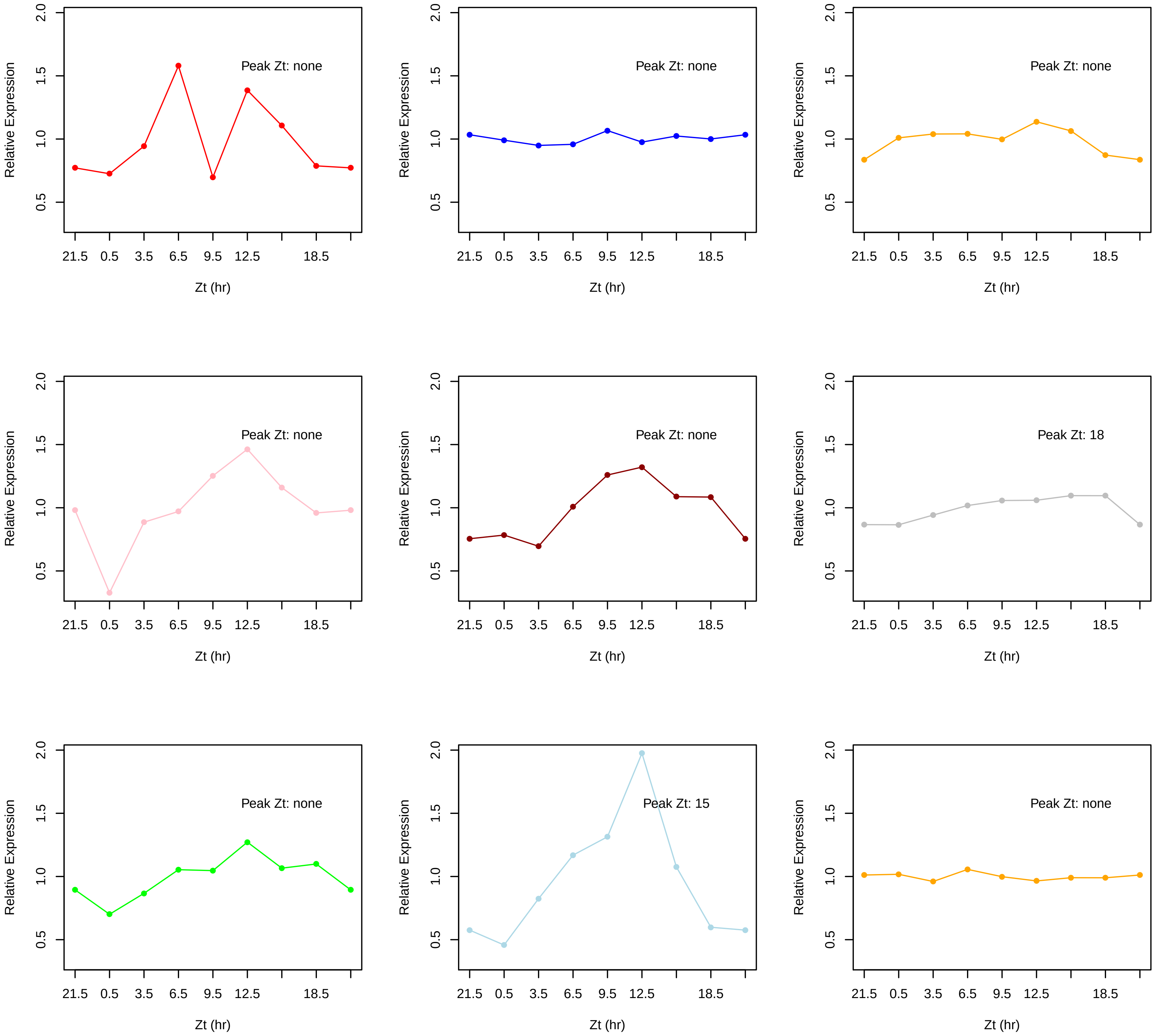

Singeleton: ENSXMAG00000006758 nox5

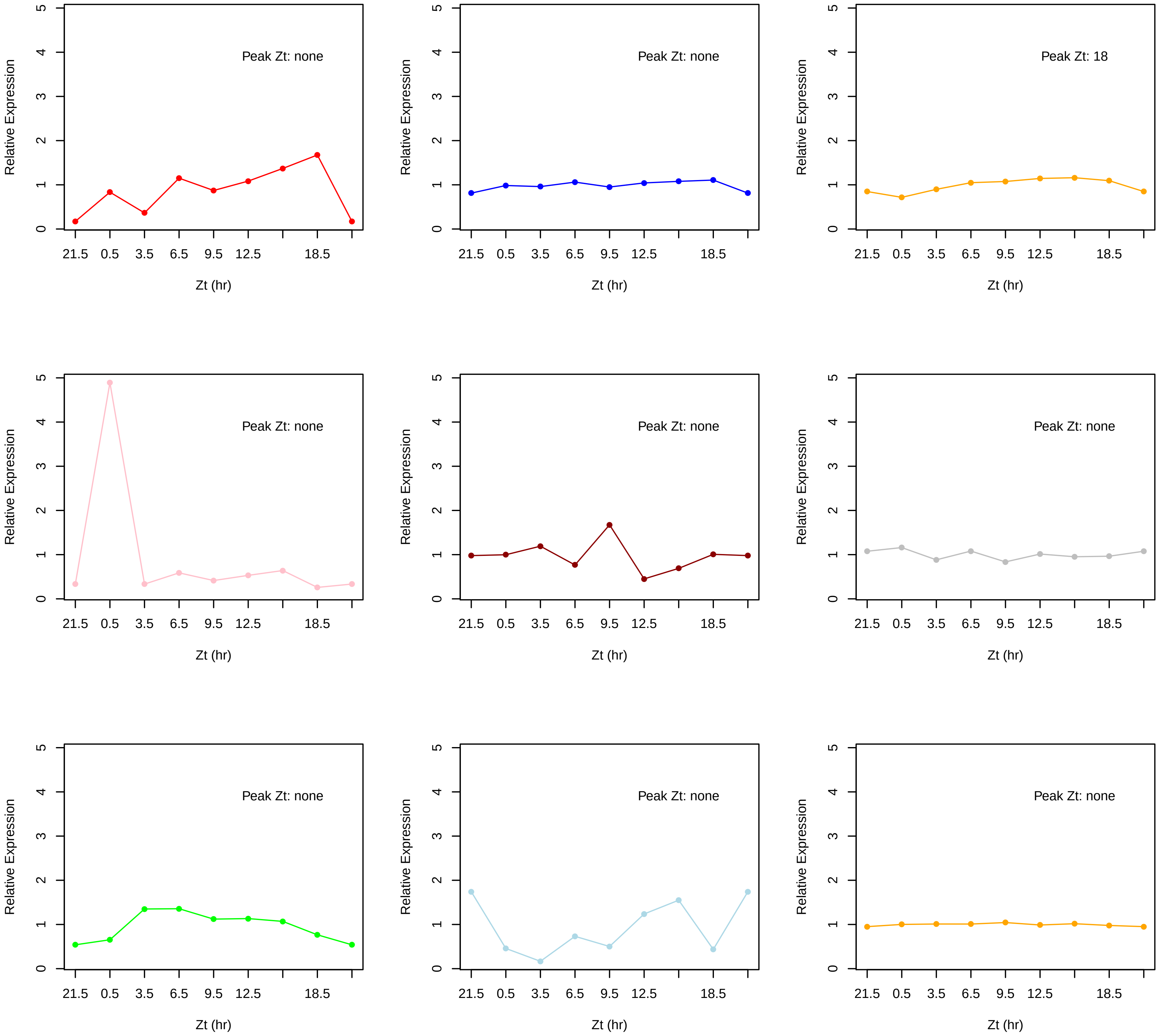

Singeleton: ENSXMAG00000012607 b3gntl1

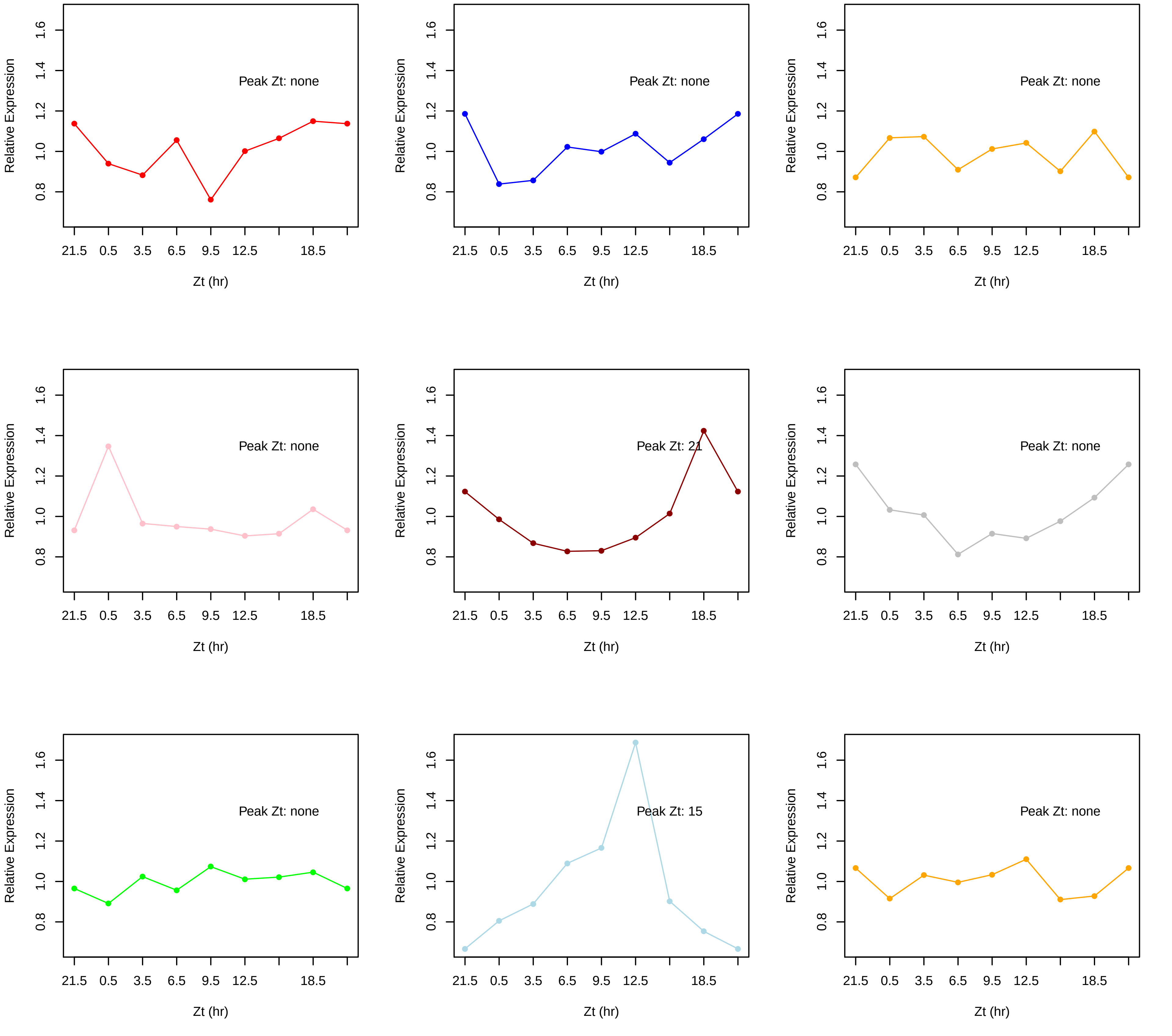

Singeleton: ENSXMAG00000012874 tbcd

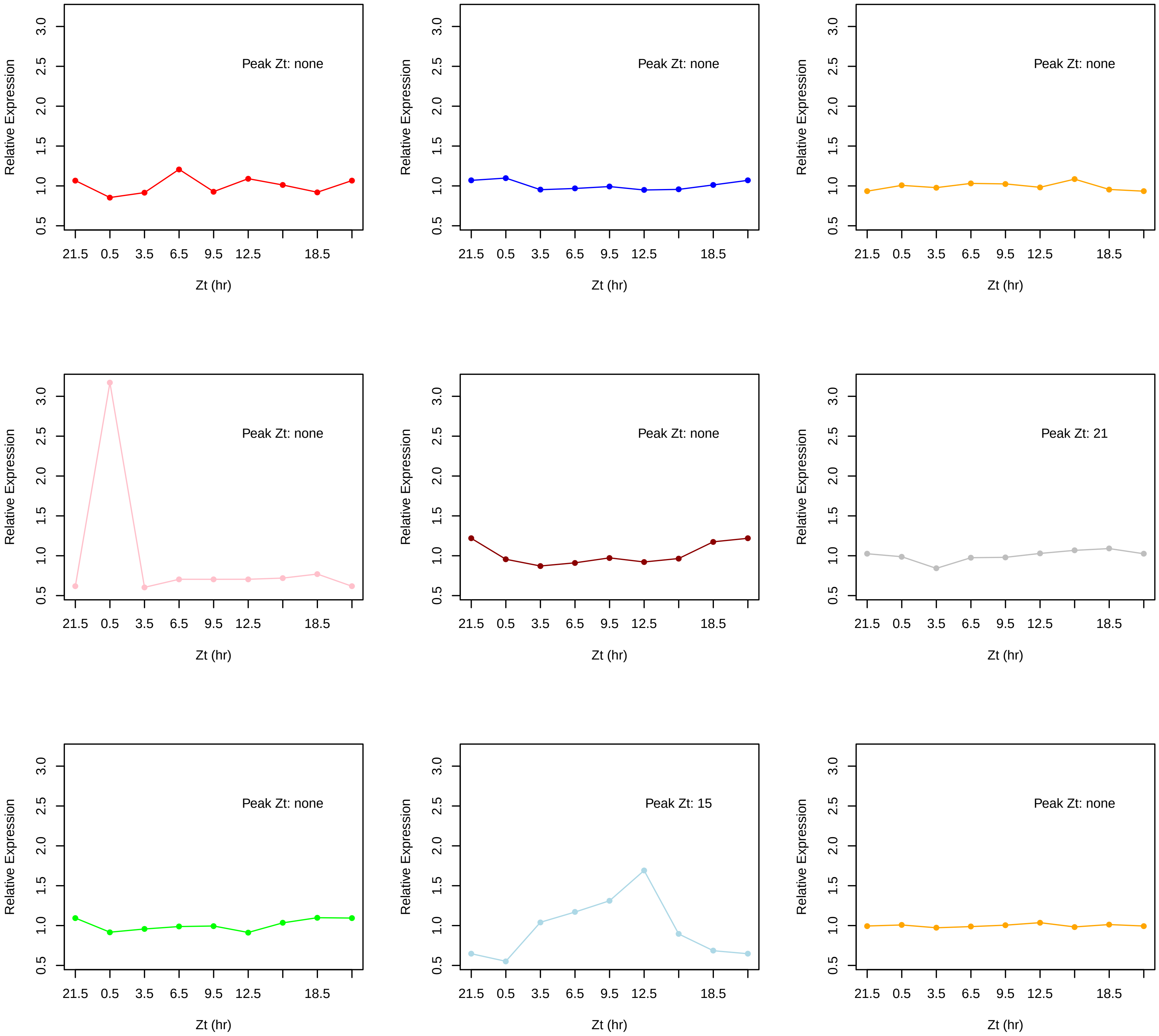

Singeleton: ENSXMAG00000010671 dnajc3b

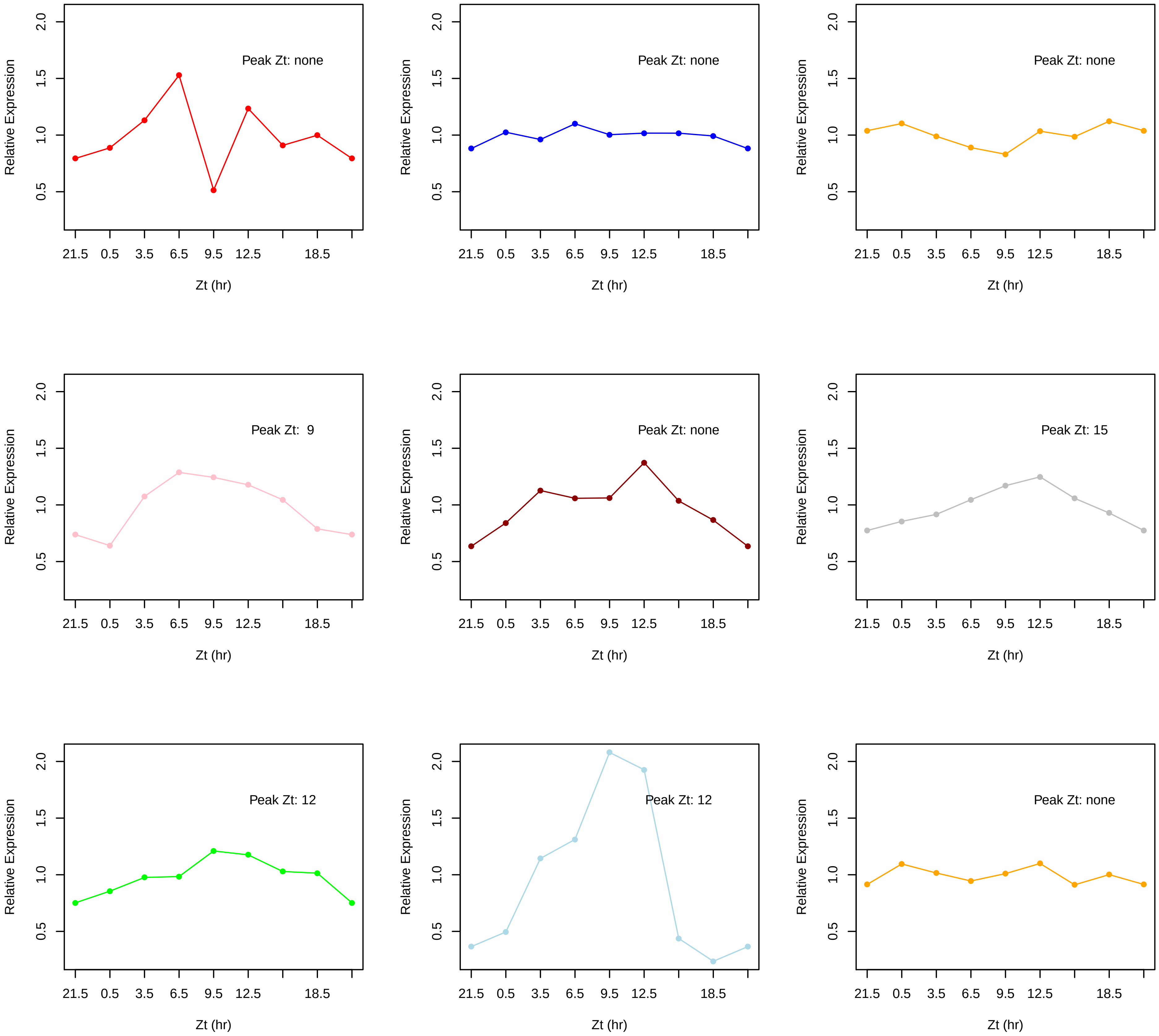

Singleton: ENSXMAG00000024938 cnbpa

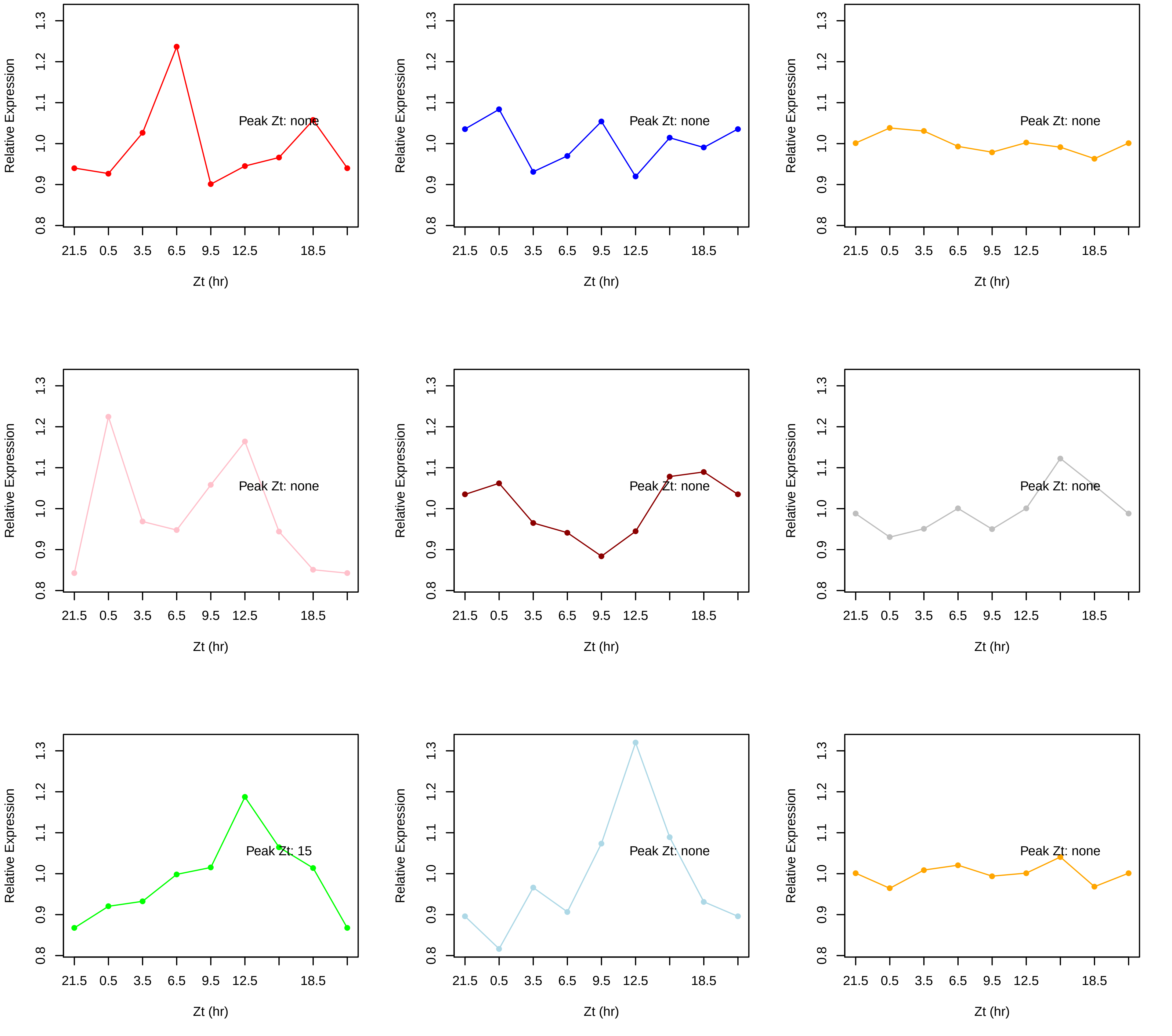

Singeleton: ENSXMAG00000015763 slc25a21

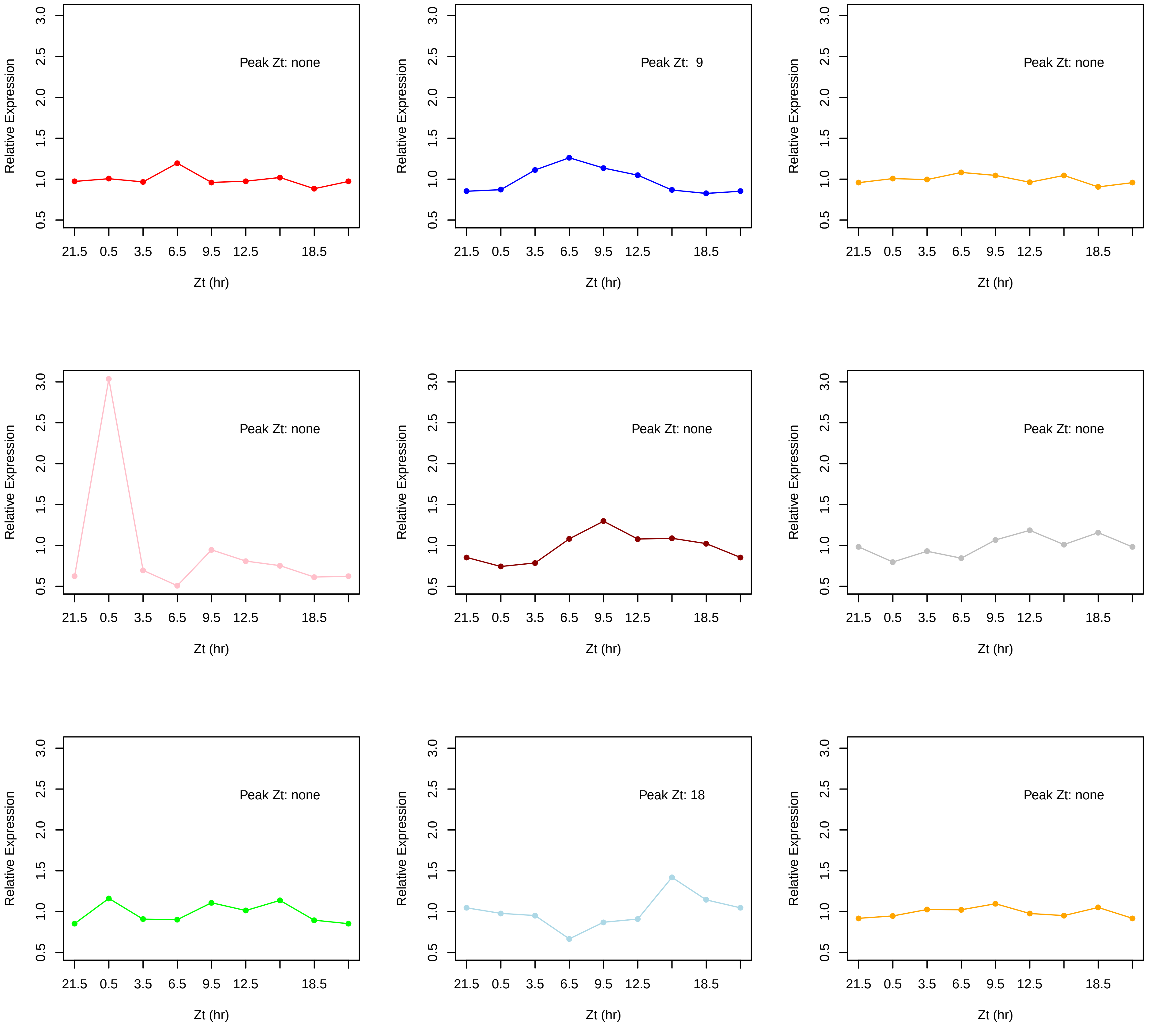

Singleton: ENSXMAG00000025024 paqr5a

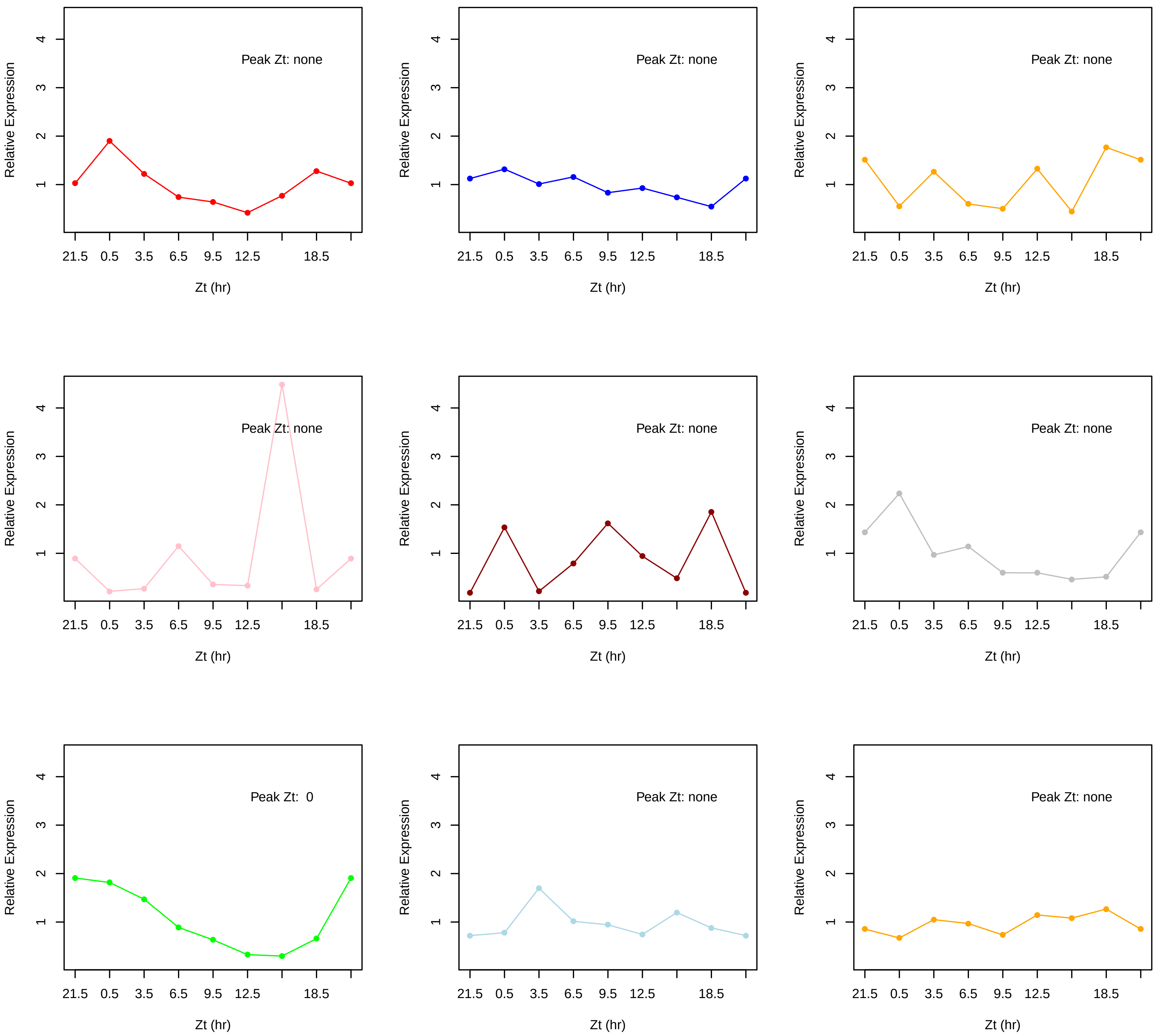

Singleton: ENSXMAG00000009974 lonrf1l

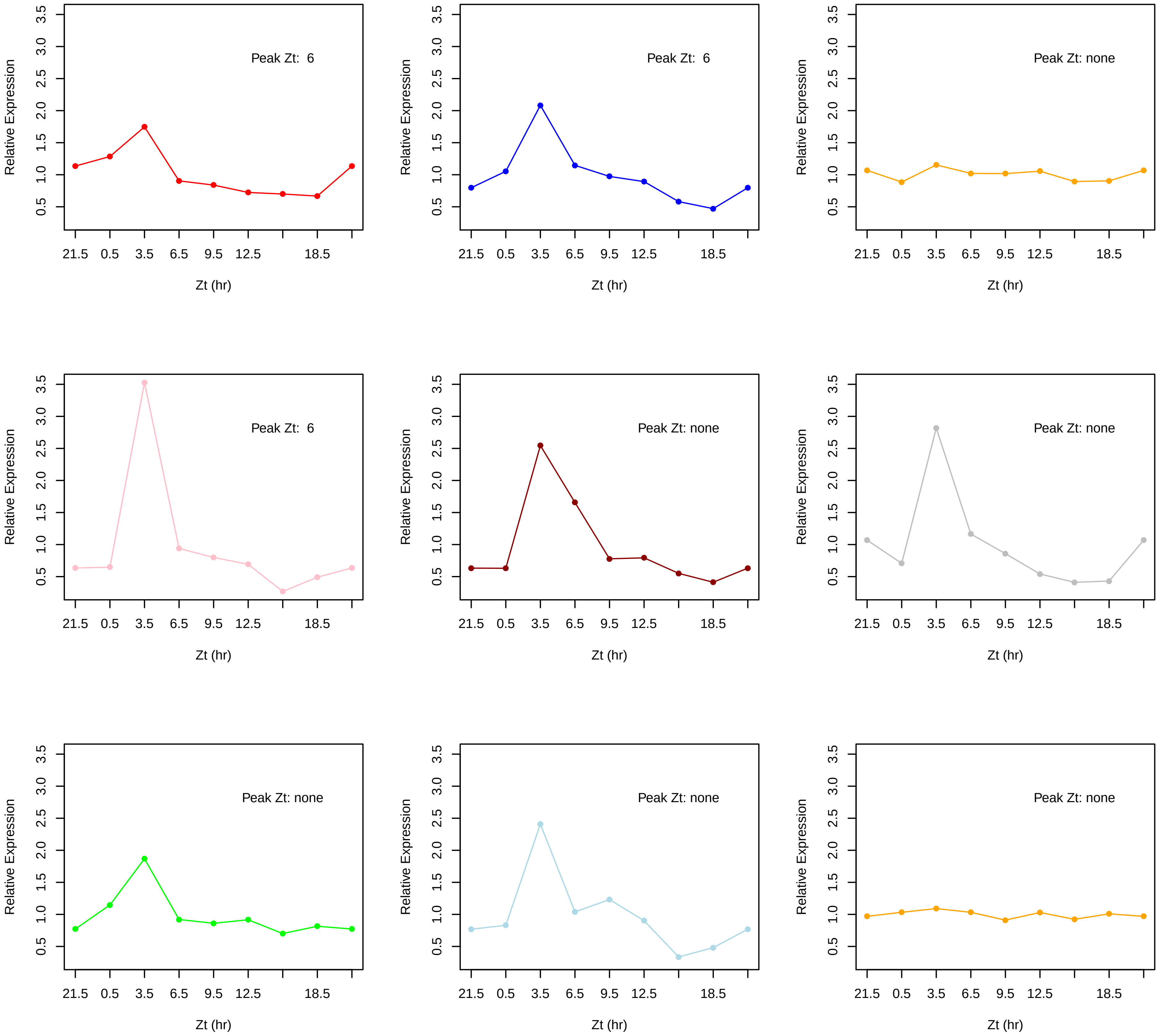

Singeleton: ENSXMAG00000016822 paics

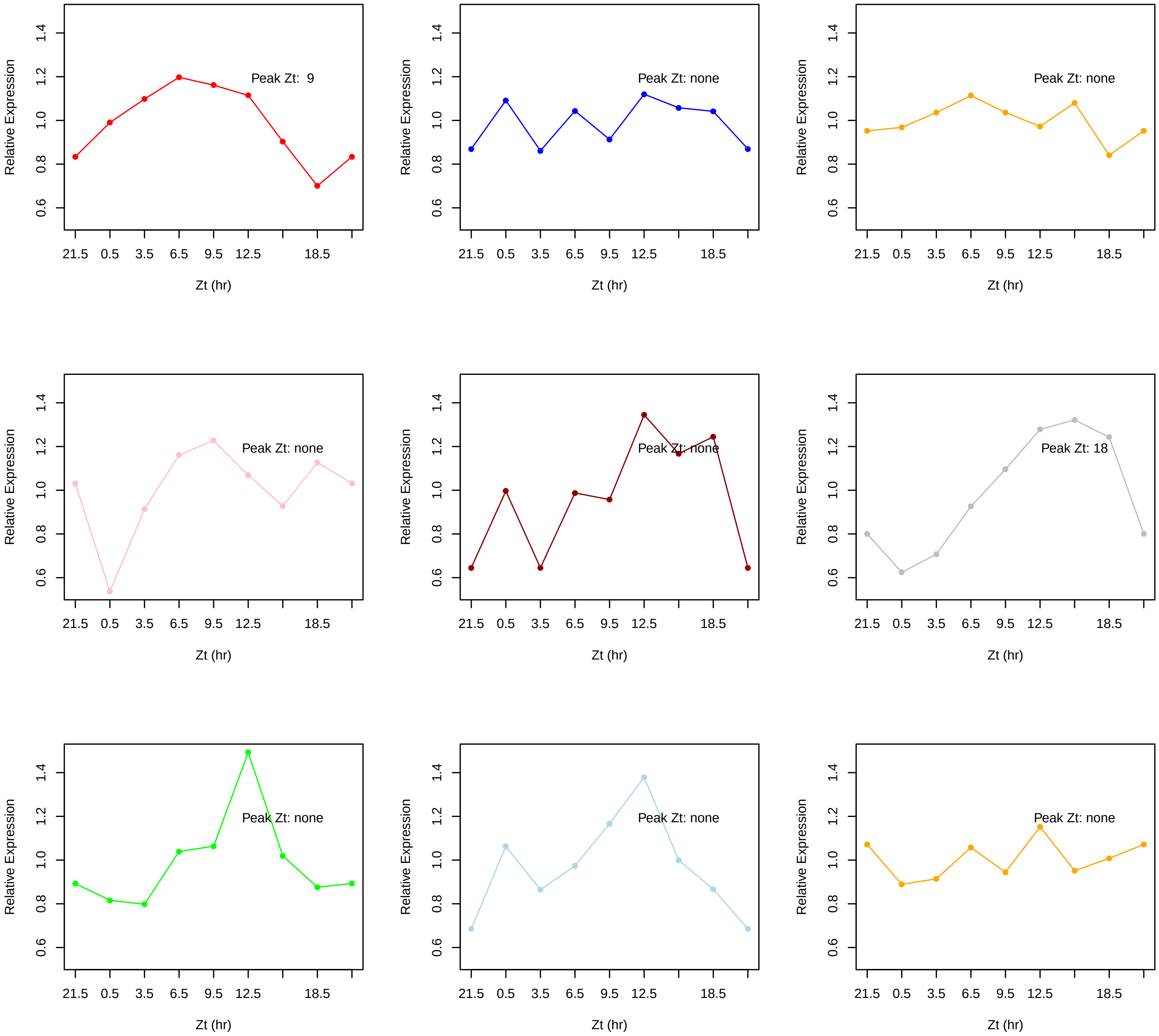

Singleton: ENSXMAG00000029129 sat2a

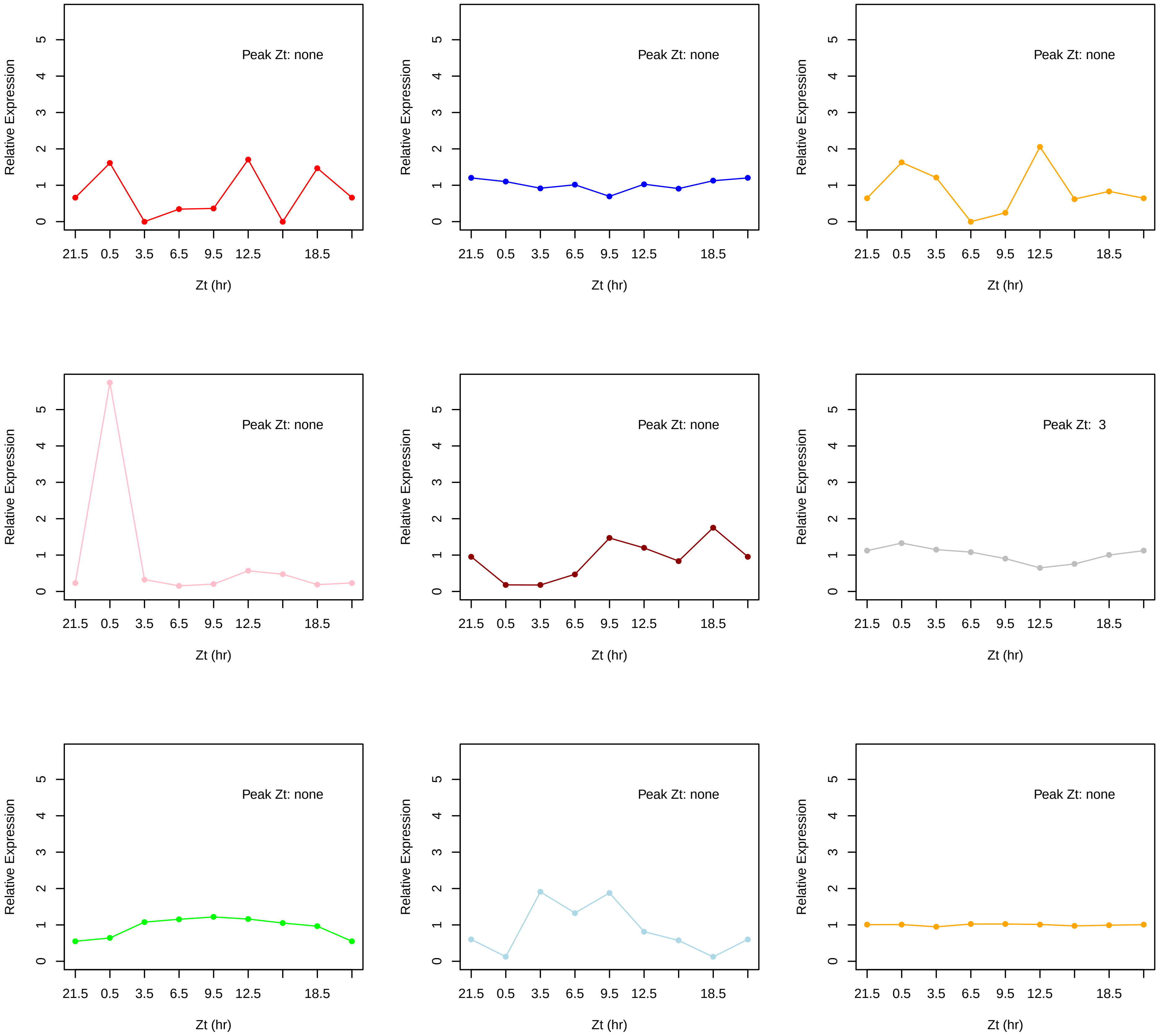

Singeleton: ENSXMAG00000027208 ppat

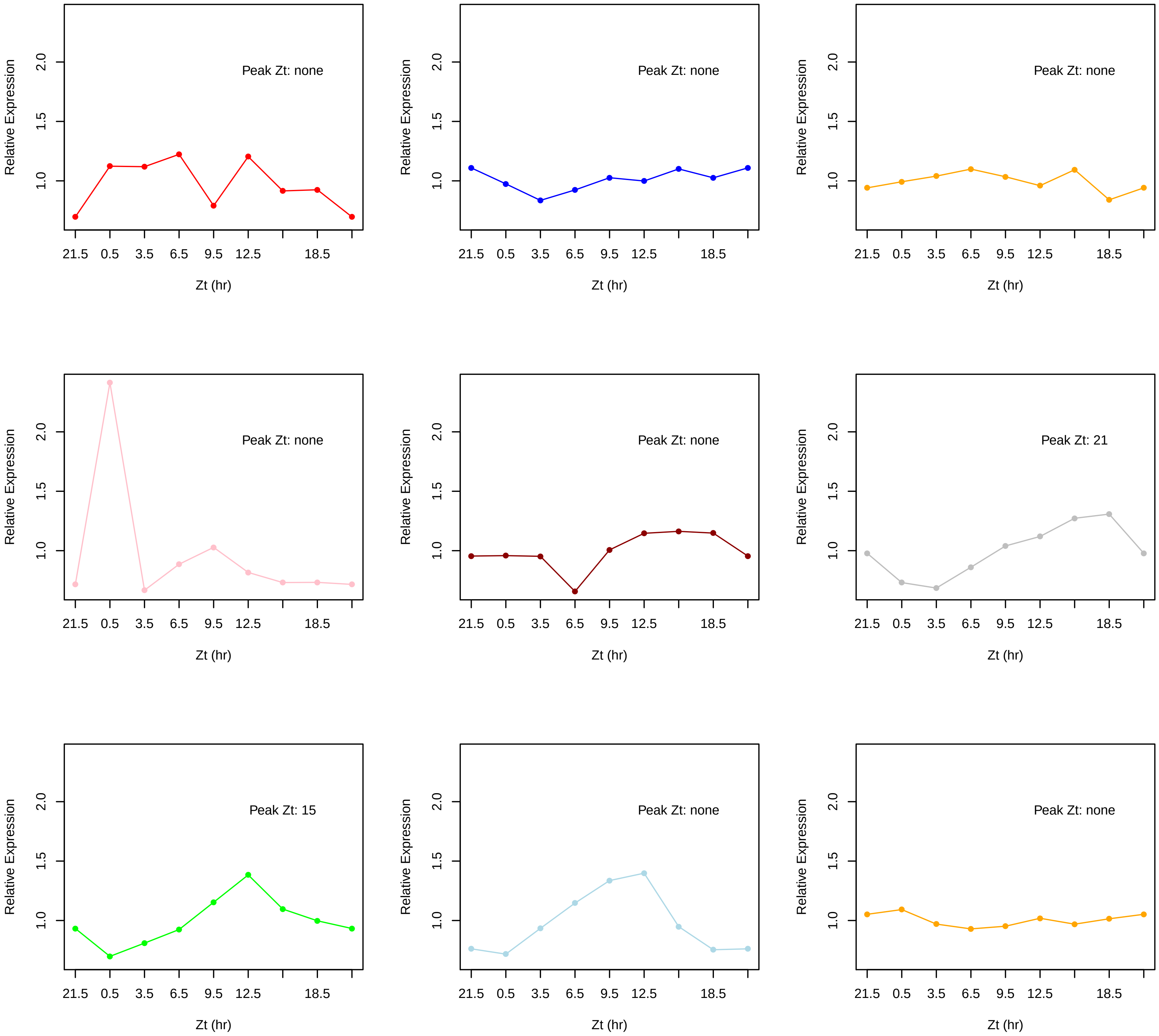

Singeleton: ENSXMAG00000015712 rps6ka5

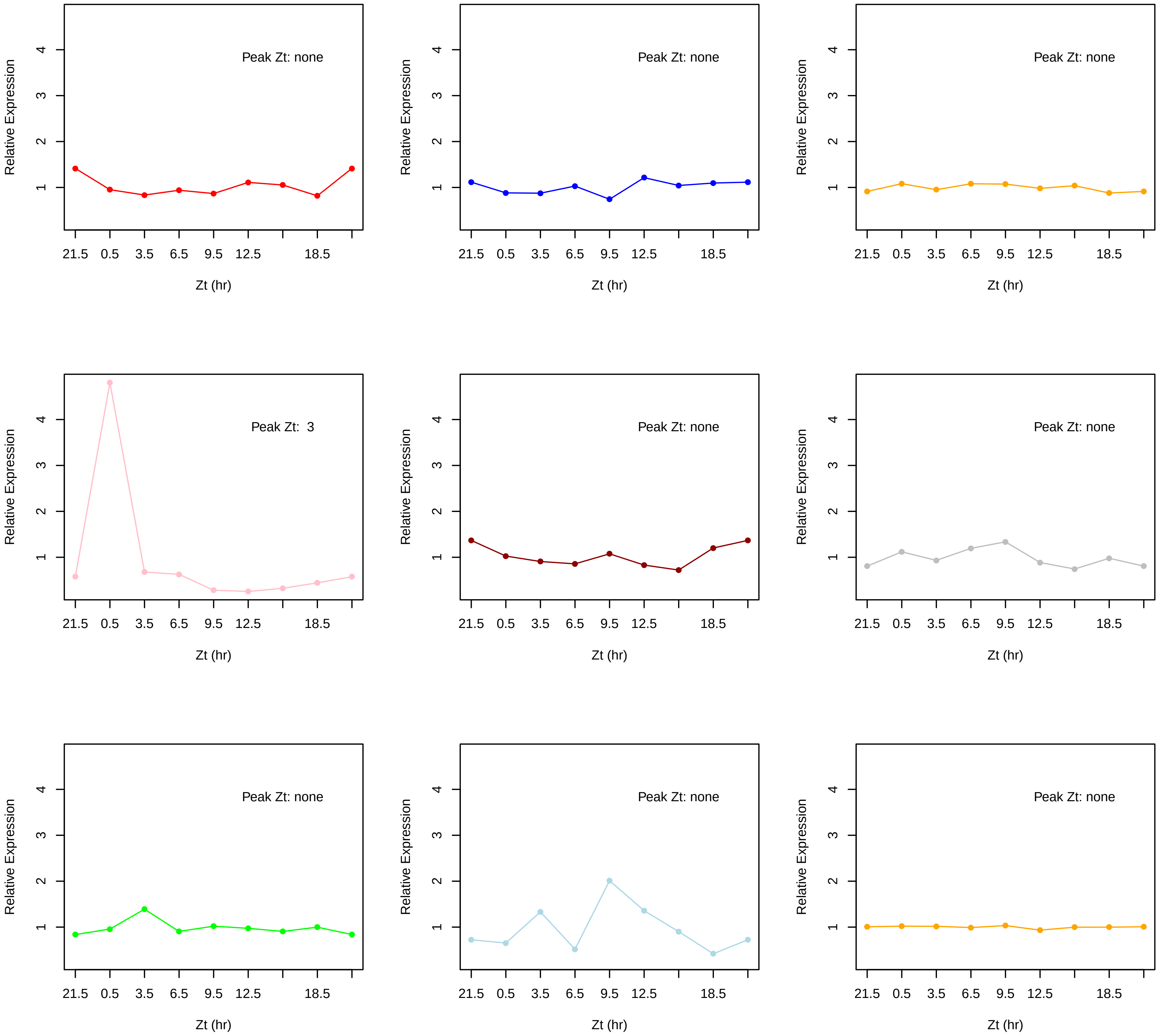

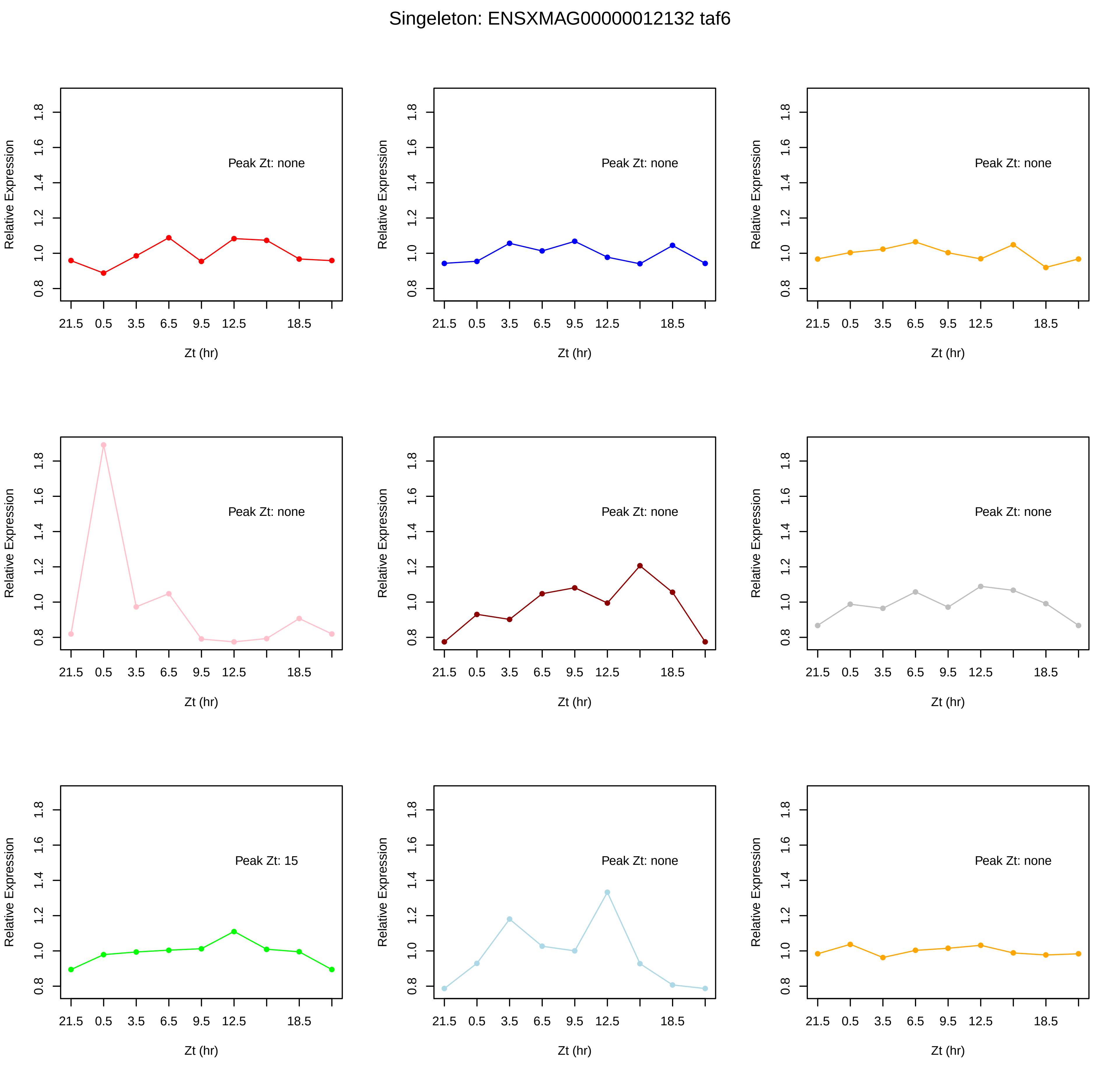
Singeleton: ENSXMAG00000012132 taf6
Peak Zt: none
Peak Zt: none
Peak Zt: none
Peak Zt: none
Peak Zt: none
Peak Zt: none
Peak Zt: 15
Peak Zt: none
Peak Zt: none
Relative Expression
Zt (hr)
21.5 0.5 3.5 6.5 9.5 12.5 18.5
0.8 1.0 1.2 1.4 1.6 1.8

Singleton: ENSXMAG00000000731 si:dkeyp−117h8.2

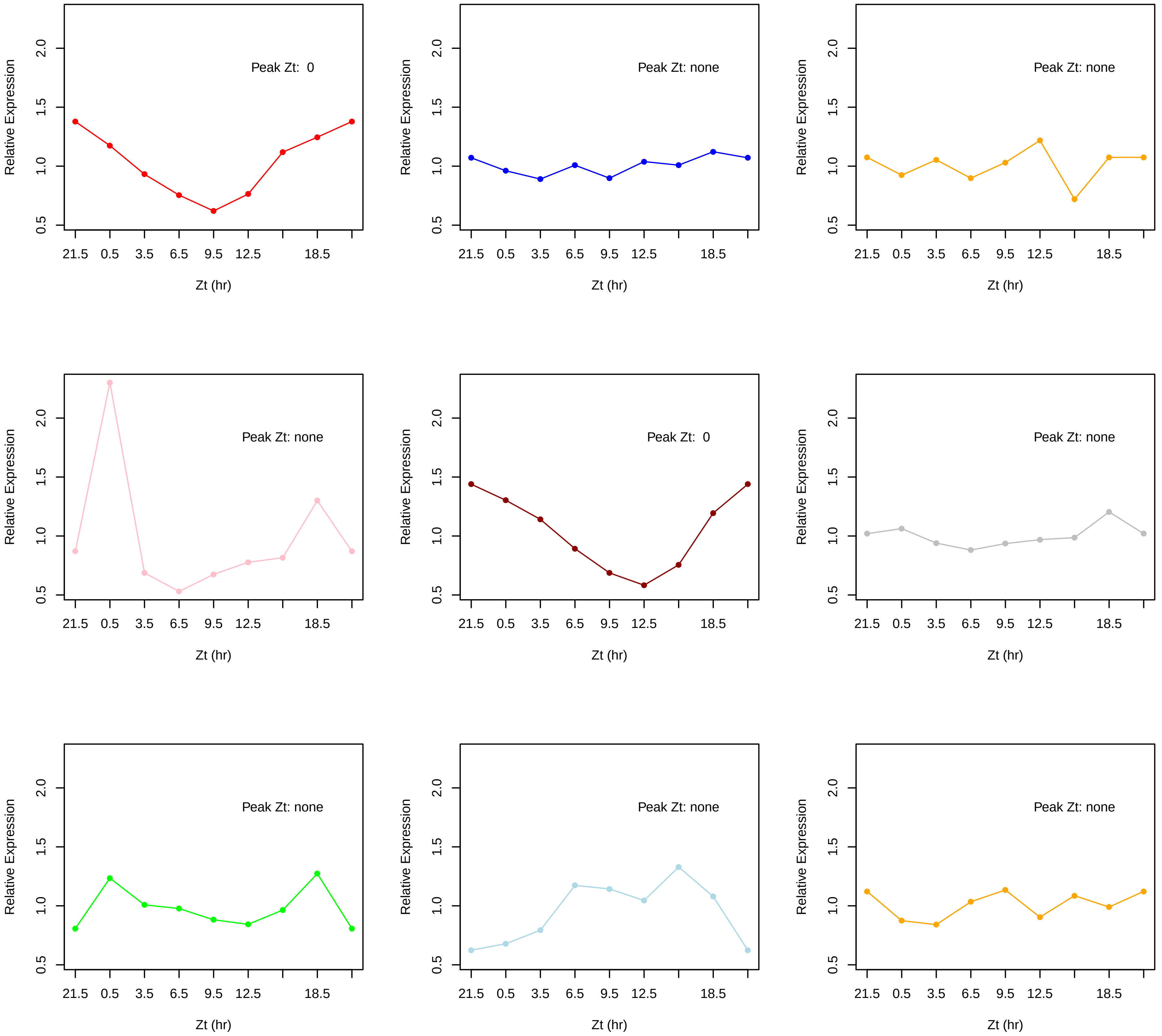

Singeleton: ENSXMAG00000030086 chst14

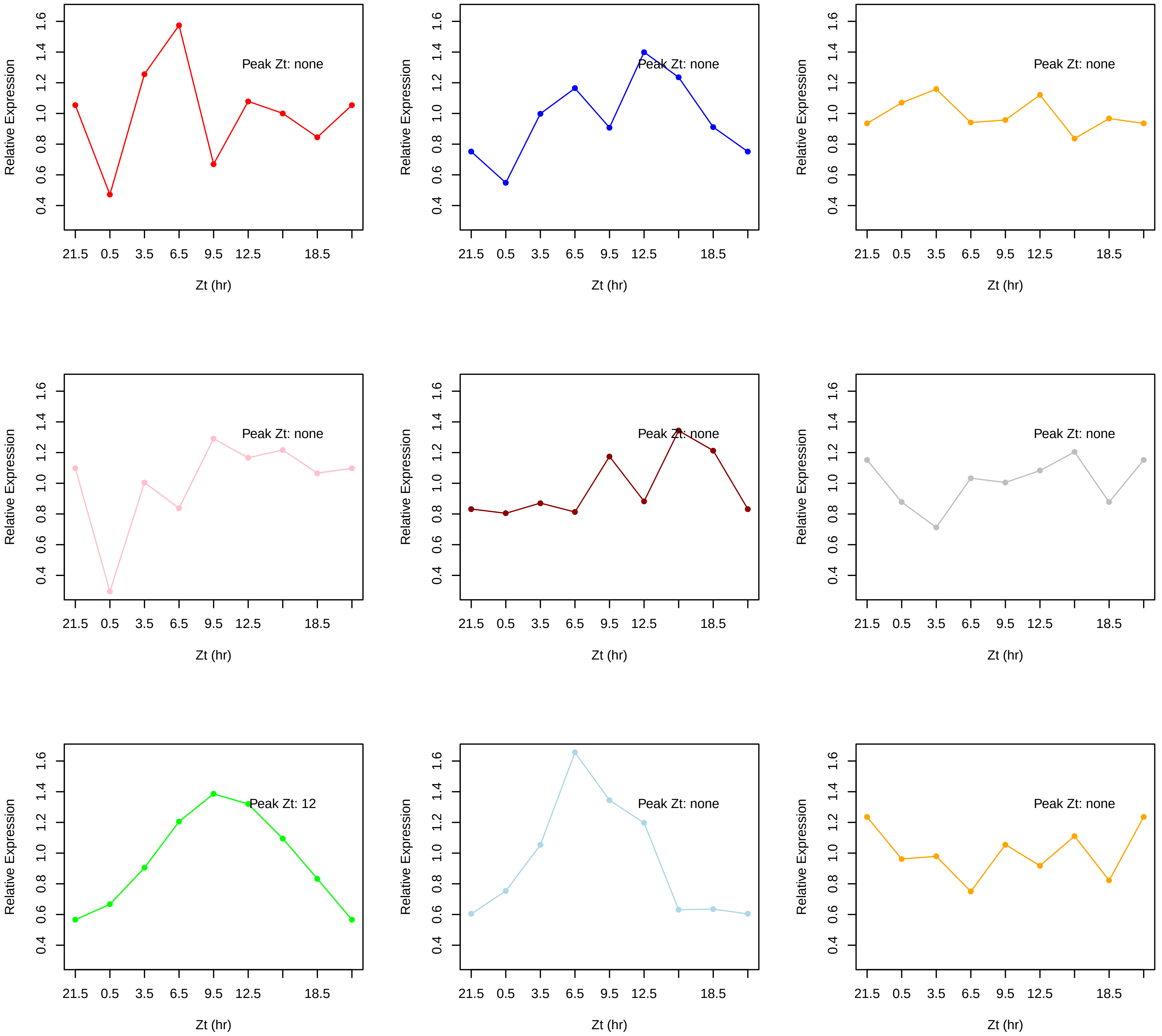

Singeleton: ENSXMAG00000015373 cnpy4

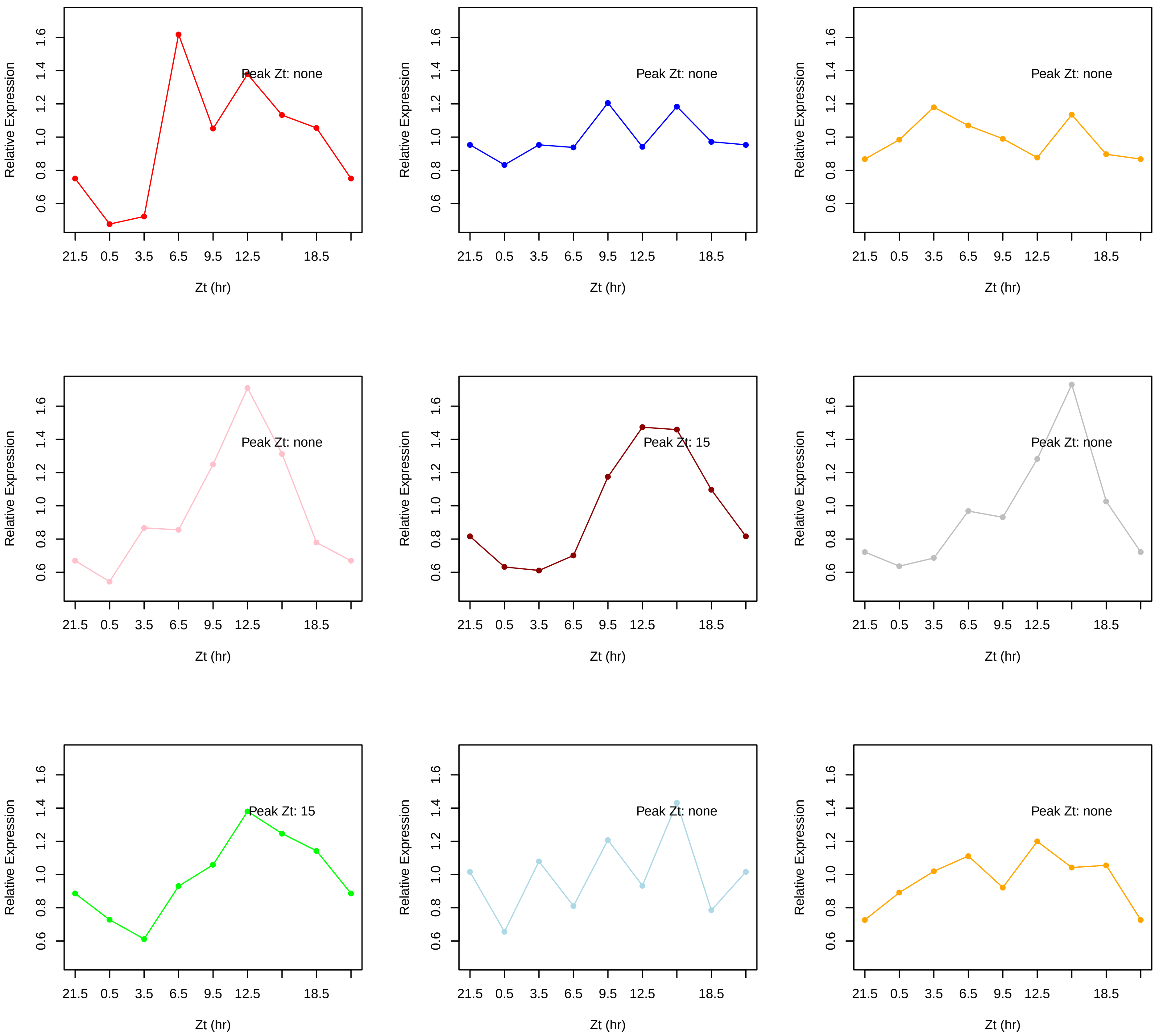

Singeleton: ENSXMAG00000008215 mblac1

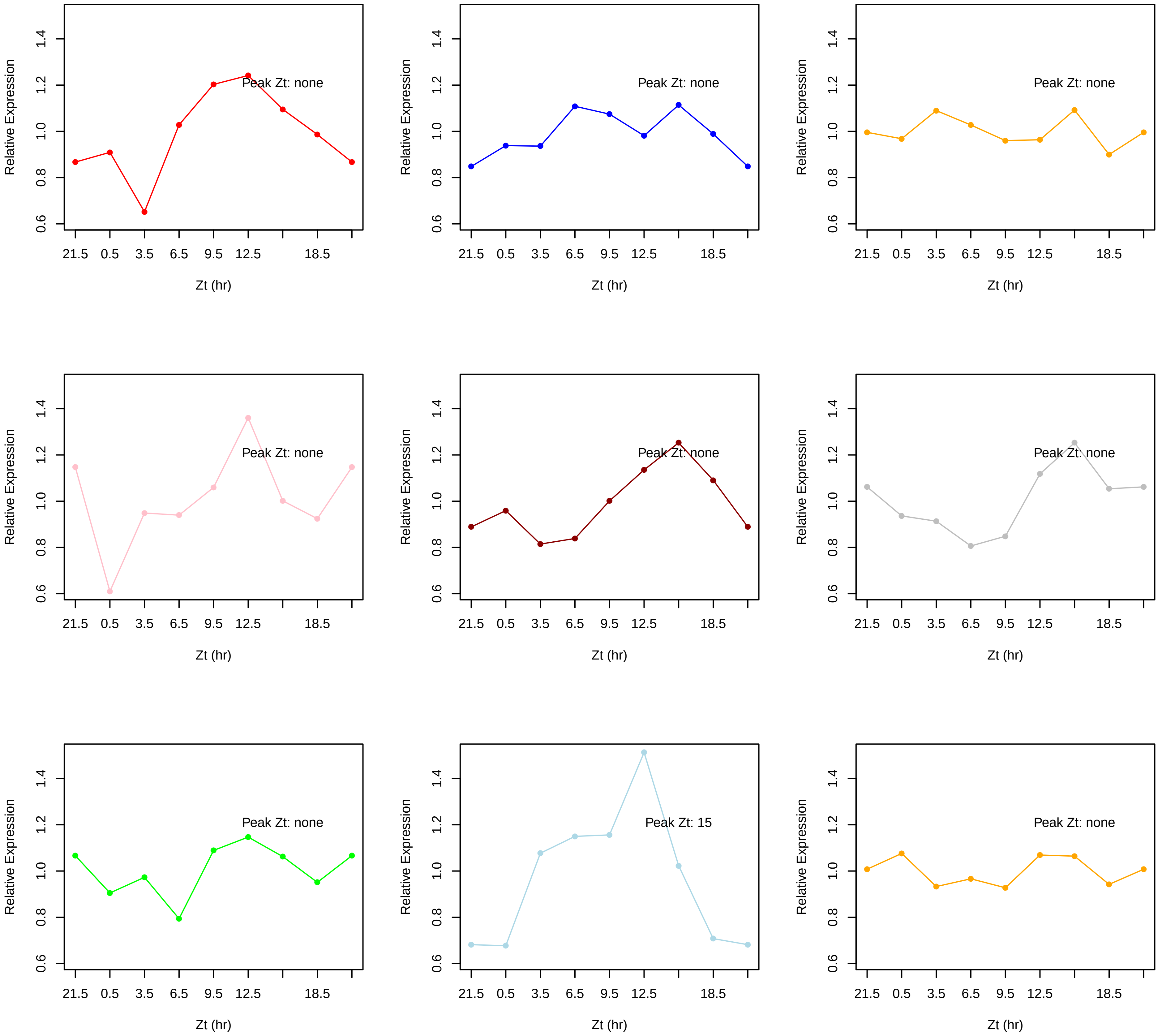

Singleton: ENSXMAG00000013341 pcnx1

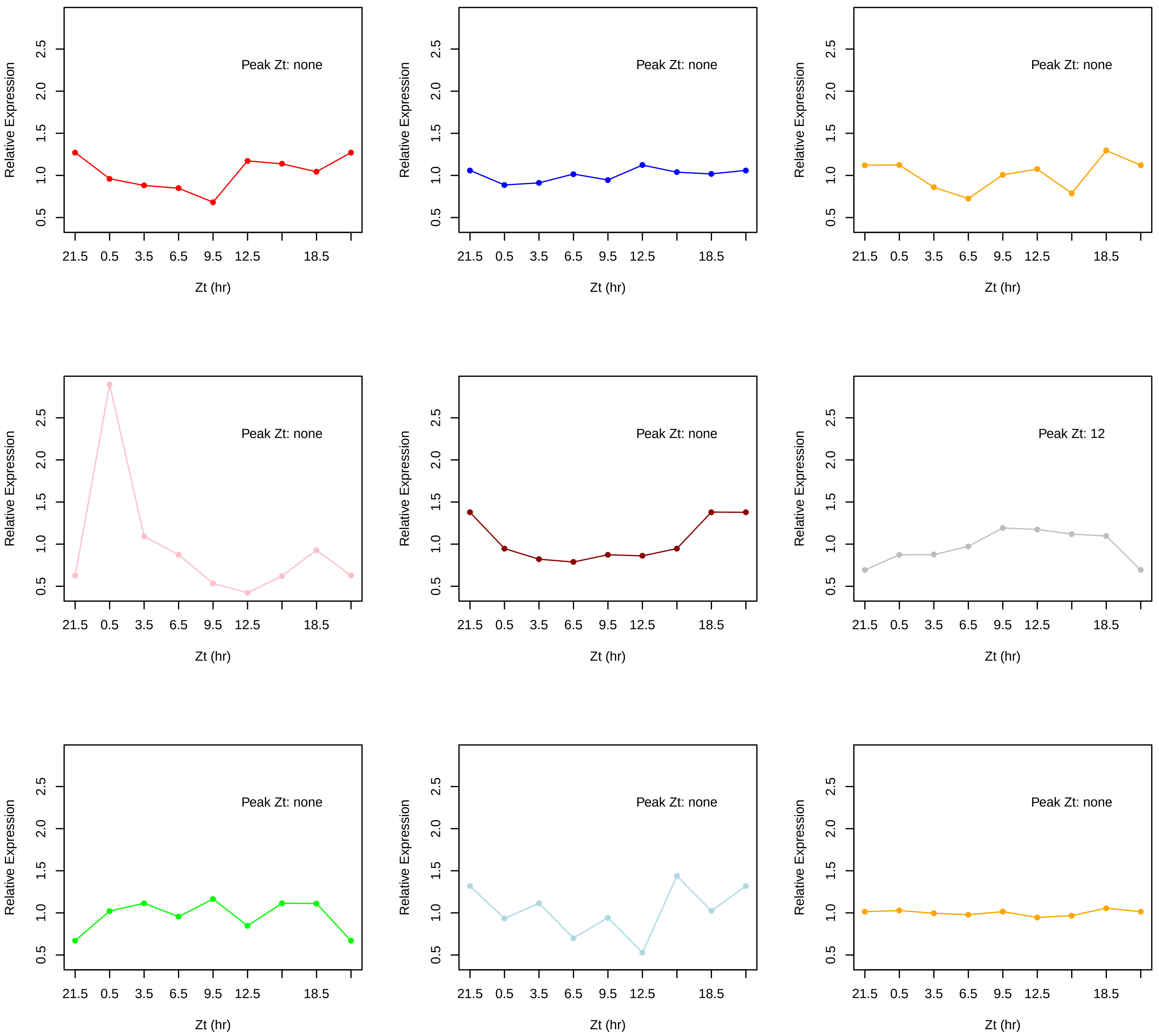

Singleton: ENSXMAG00000006984

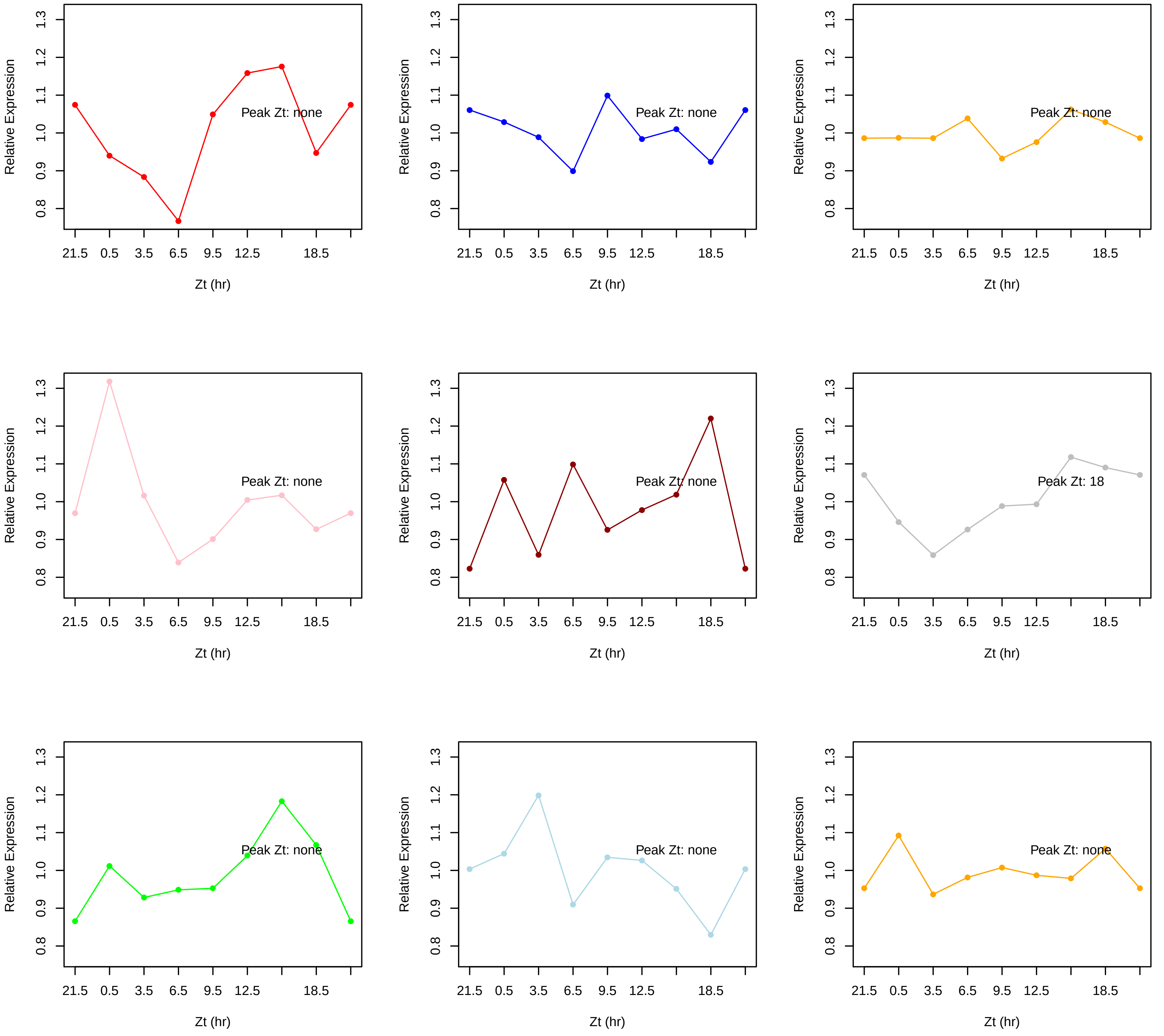

Singeleton: ENSXMAG00000007491 hdac11

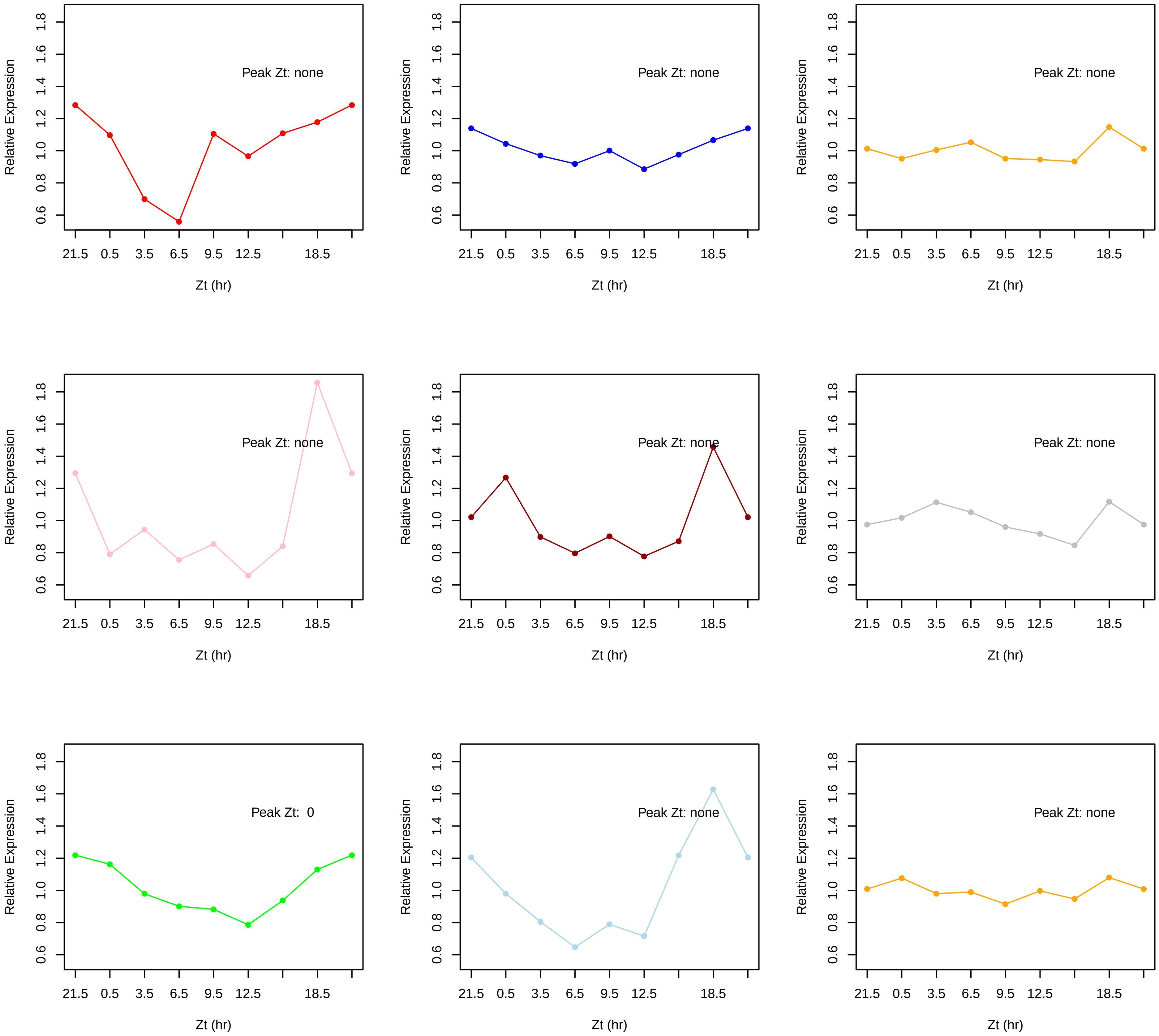

Singeleton: ENSXMAG00000014520 map3k9

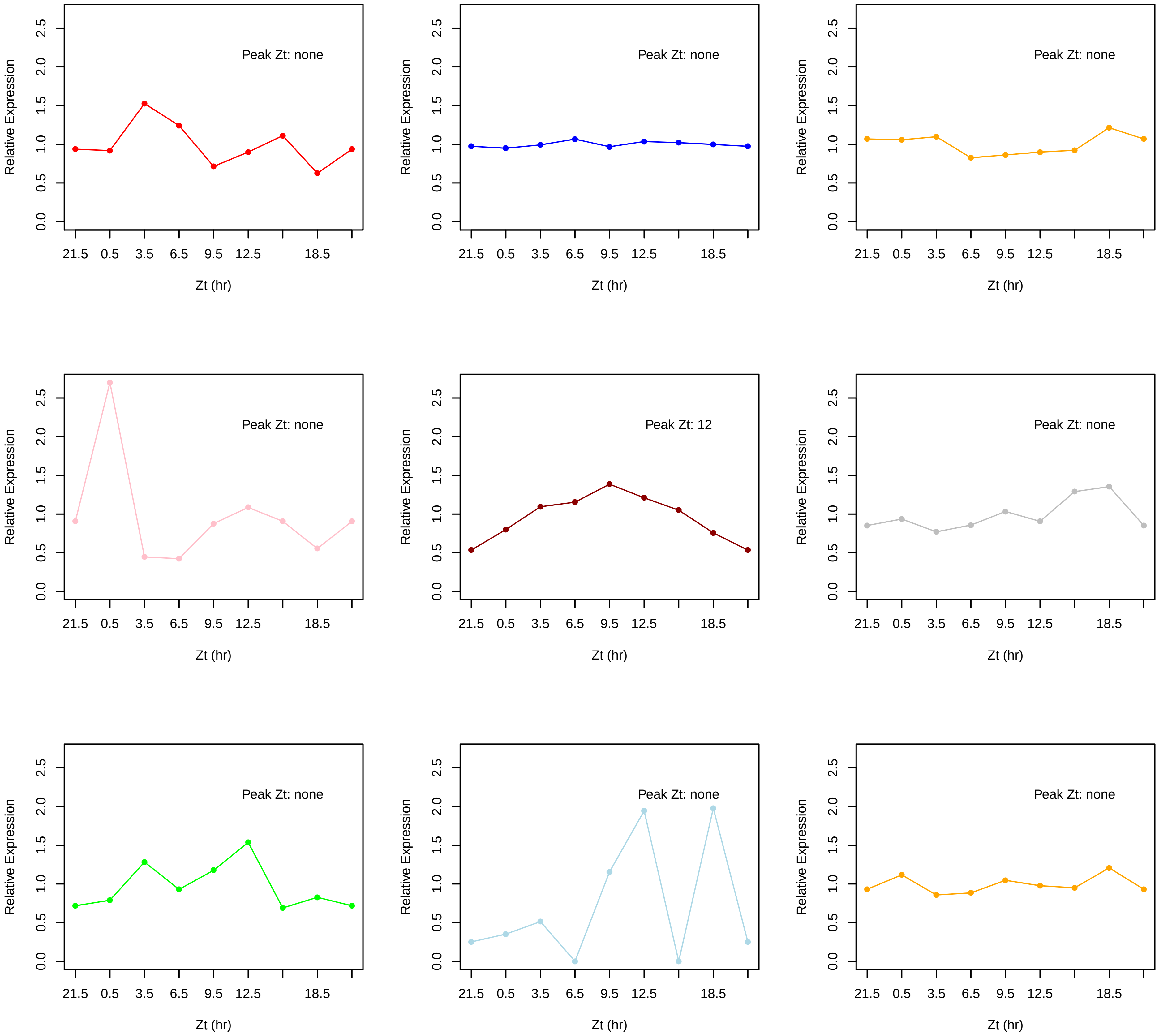

Singeleton: ENSXMAG00000011952 serpine1

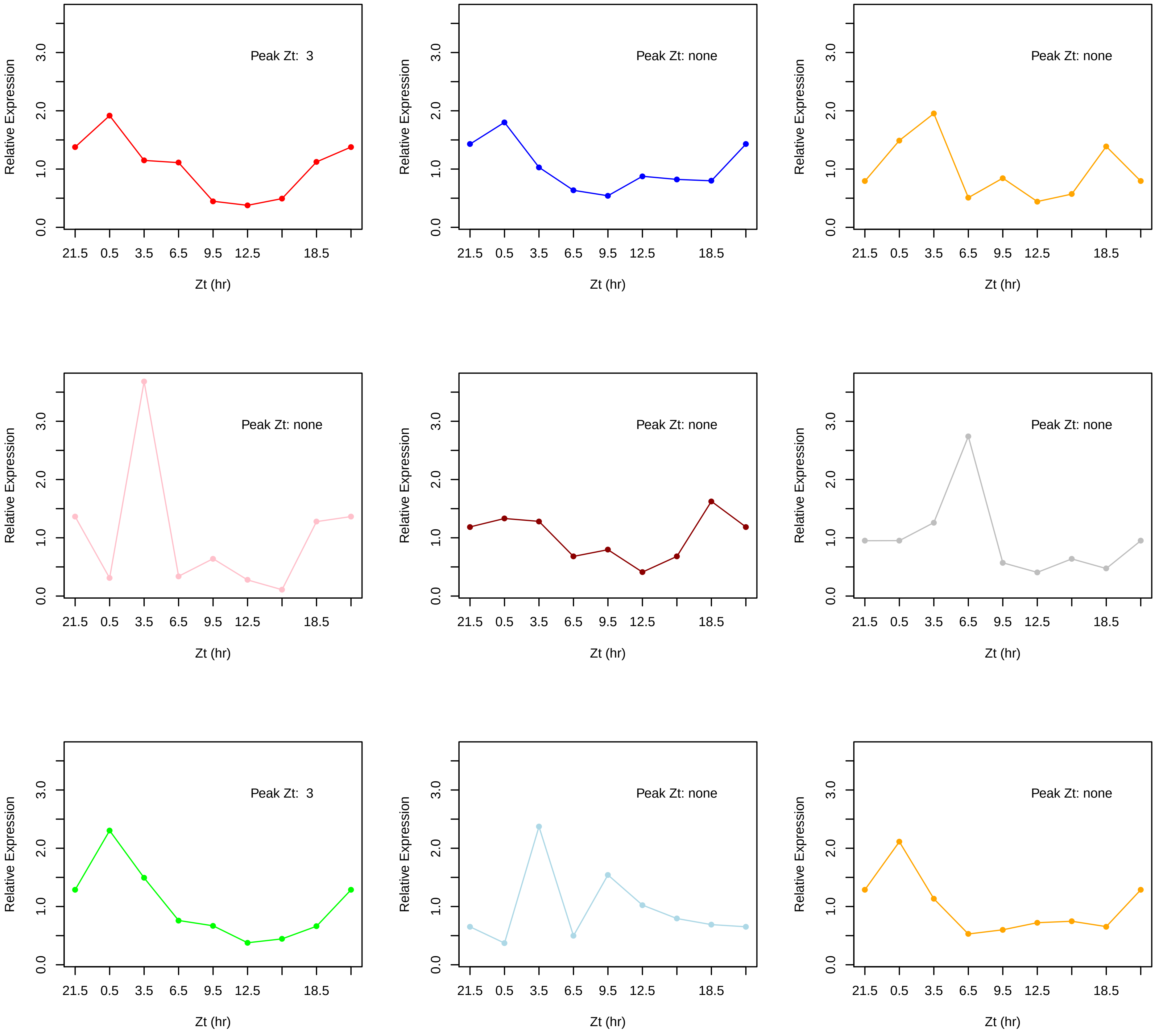

Singeleton: ENSXMAG00000016958 tmem165

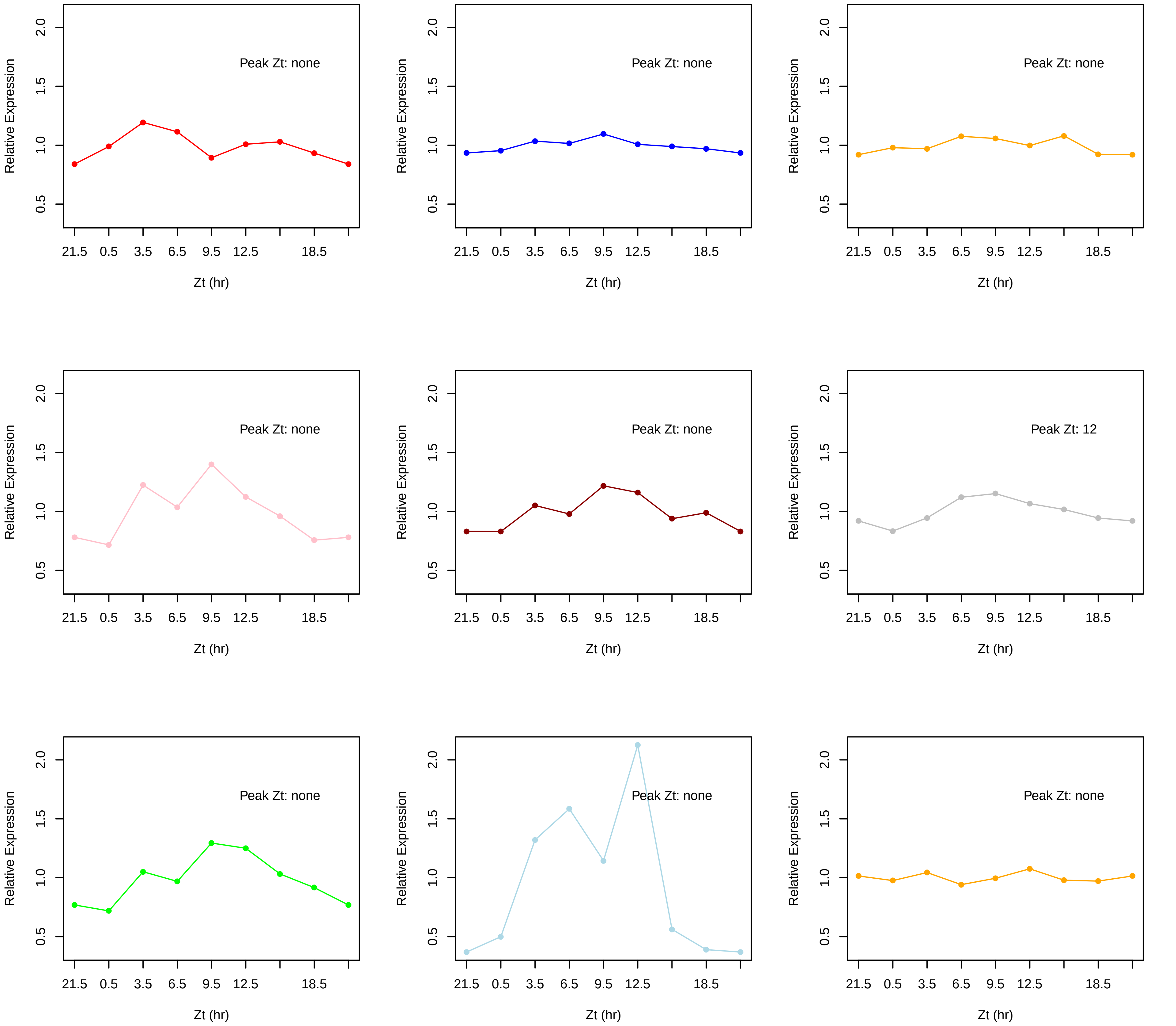

Singleton: ENSXMAG00000014534 slc8a3

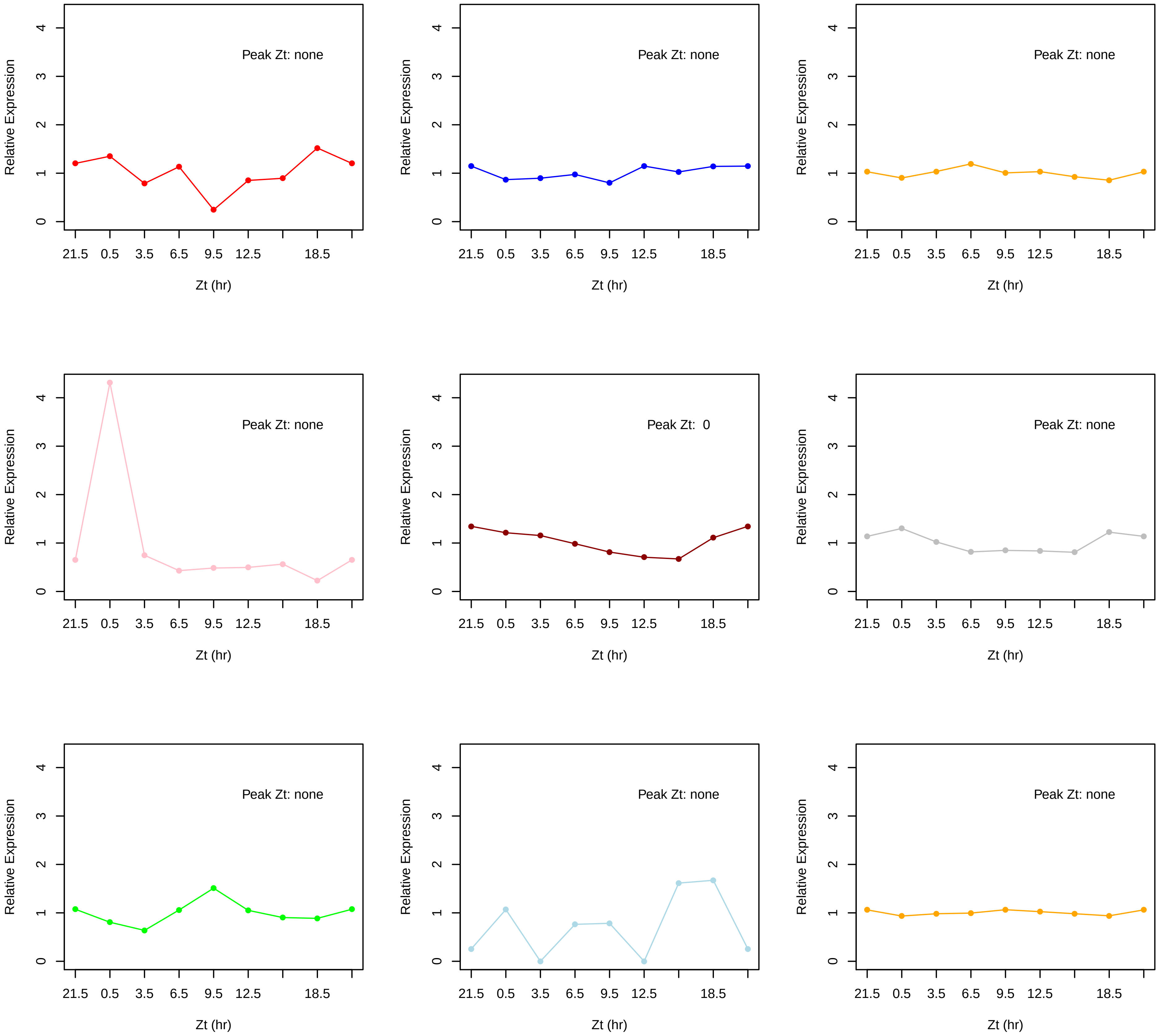

Singleton: ENSXMAG00000017604 si:ch73−142c19.1

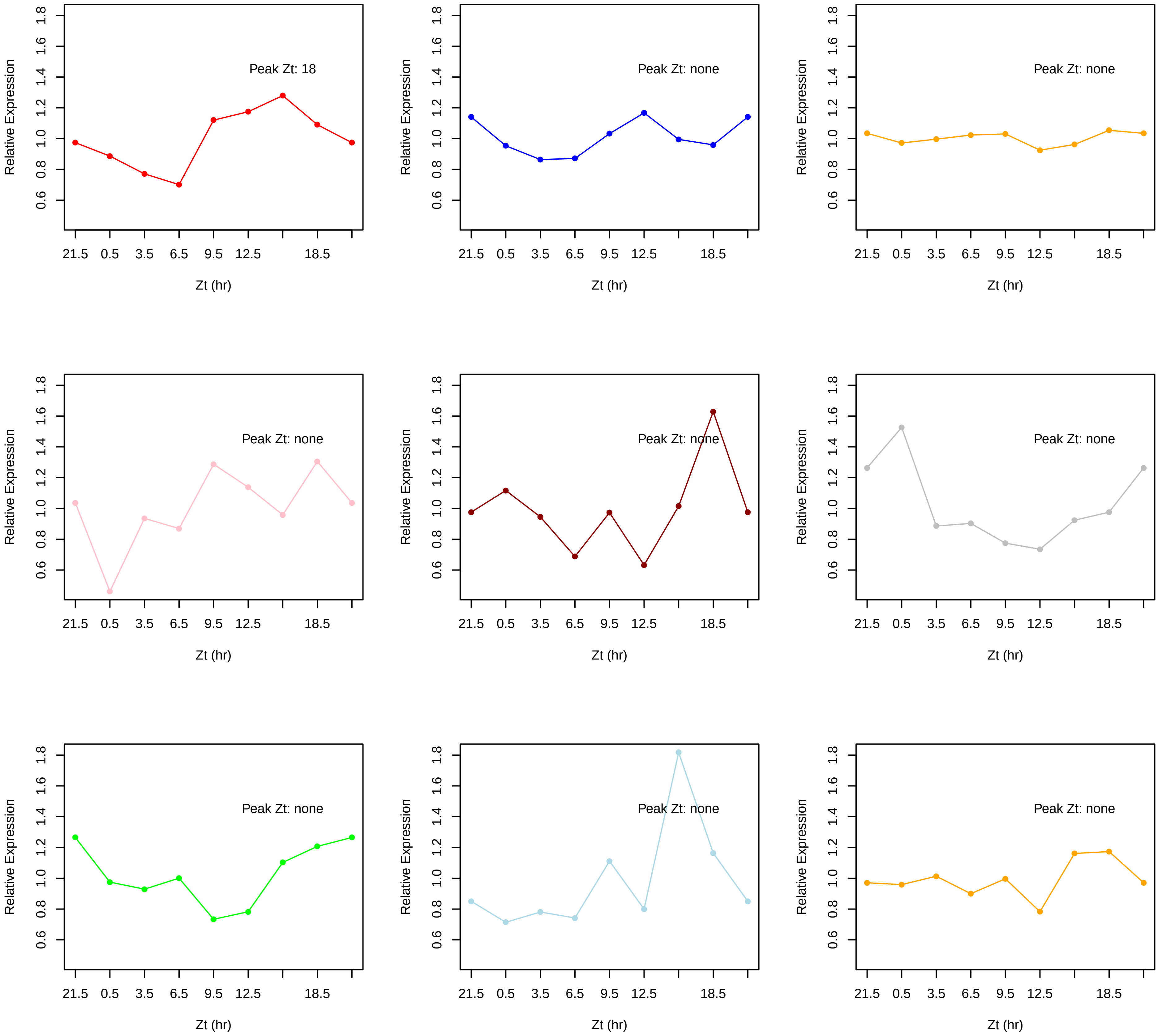

Singeleton: ENSXMAG00000011744 anapc4

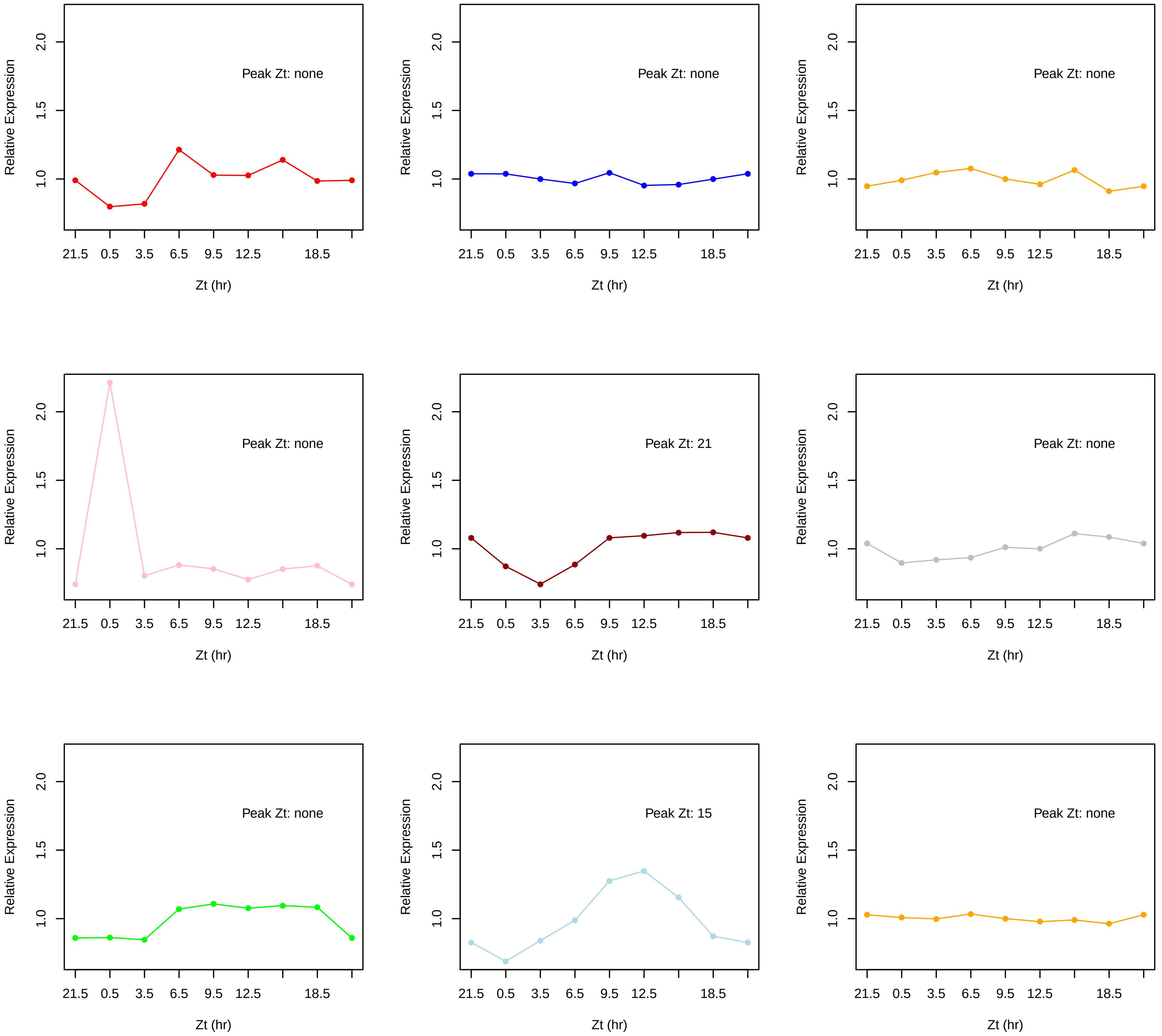

Singleton: ENSXMAG00000011931 ap1s1

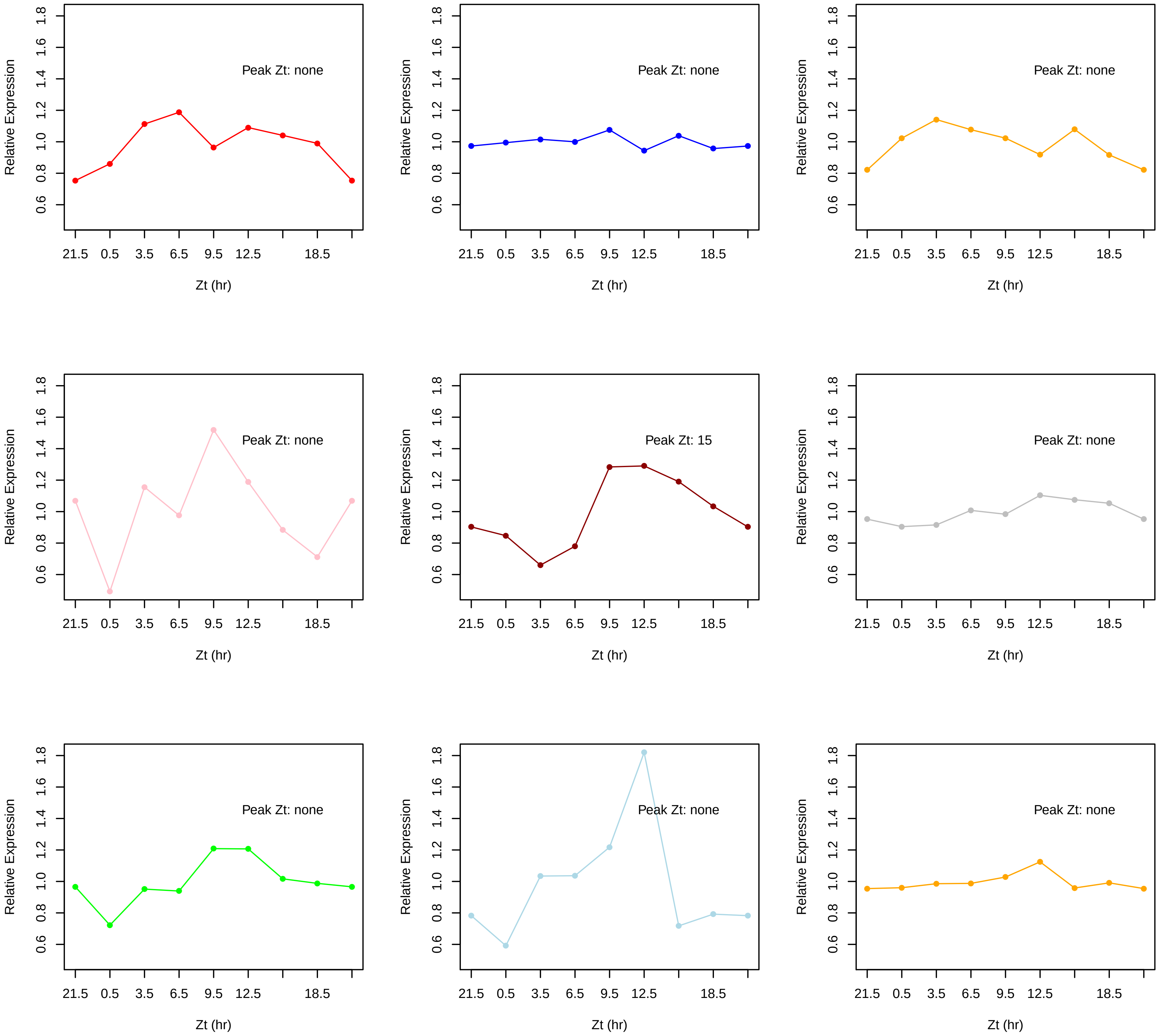

Singeleton: ENSXMAG00000014545 actn1

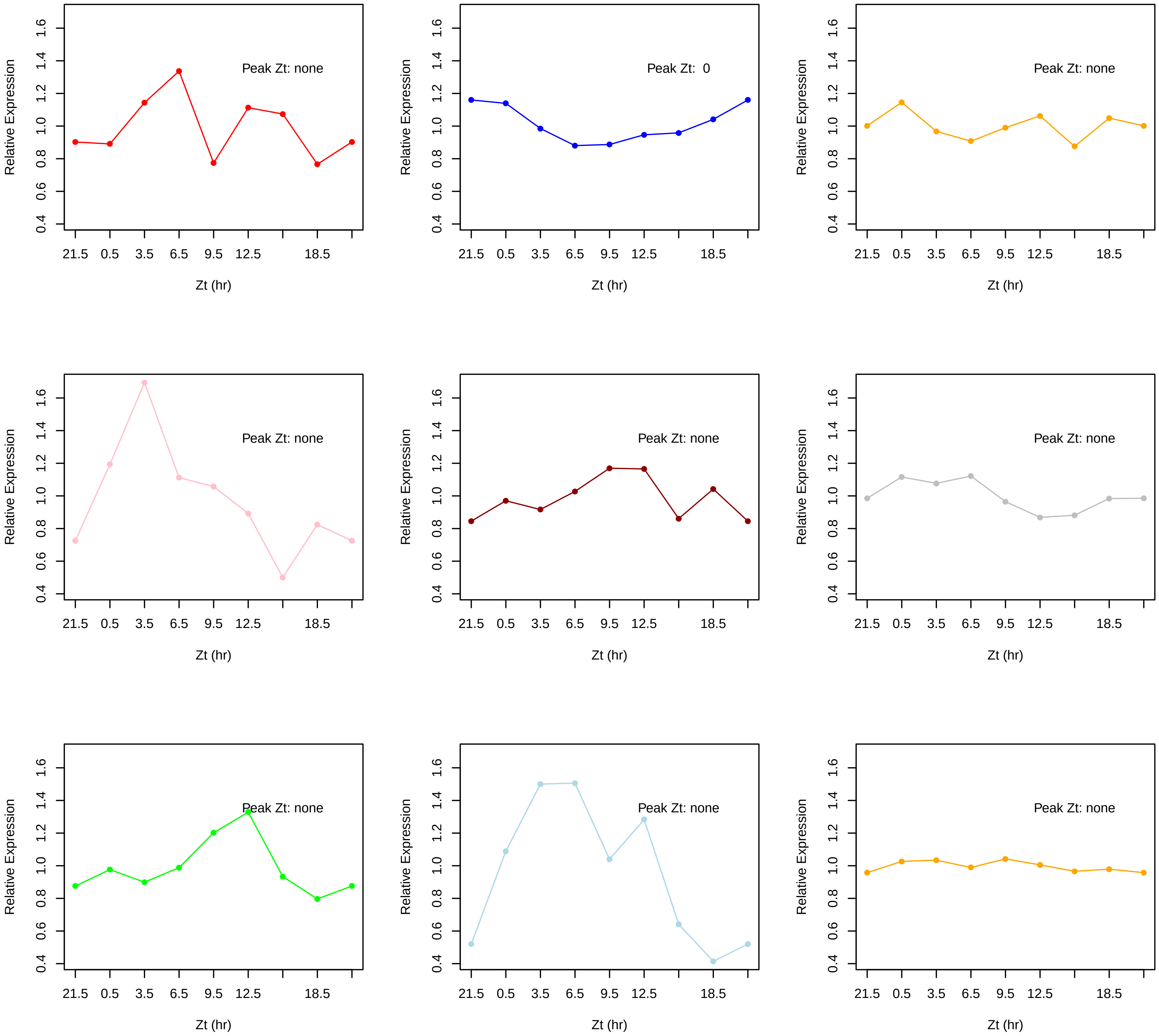

Singleton: ENSXMAG00000011841 nat16

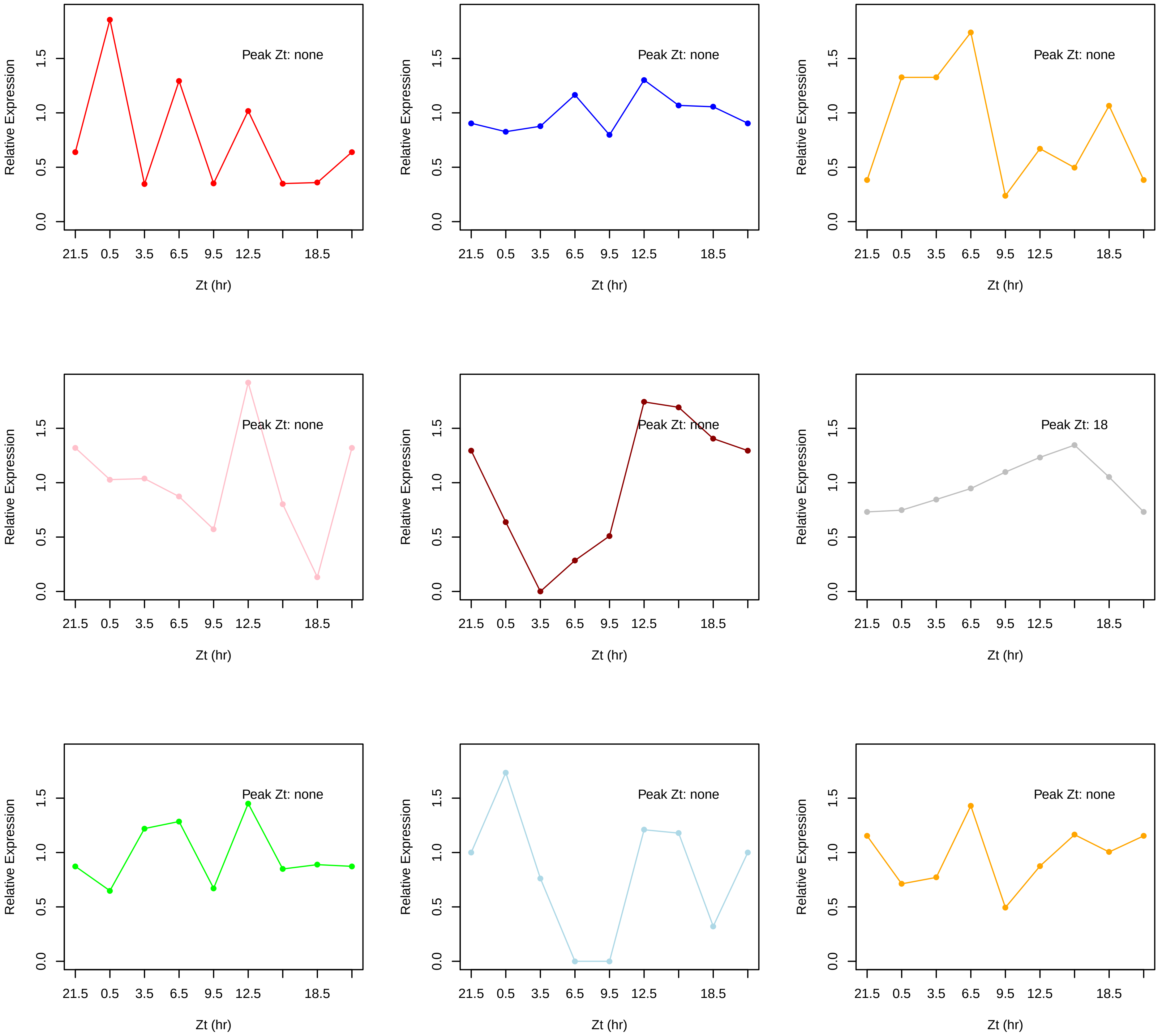

Singeleton: ENSXMAG00000027683

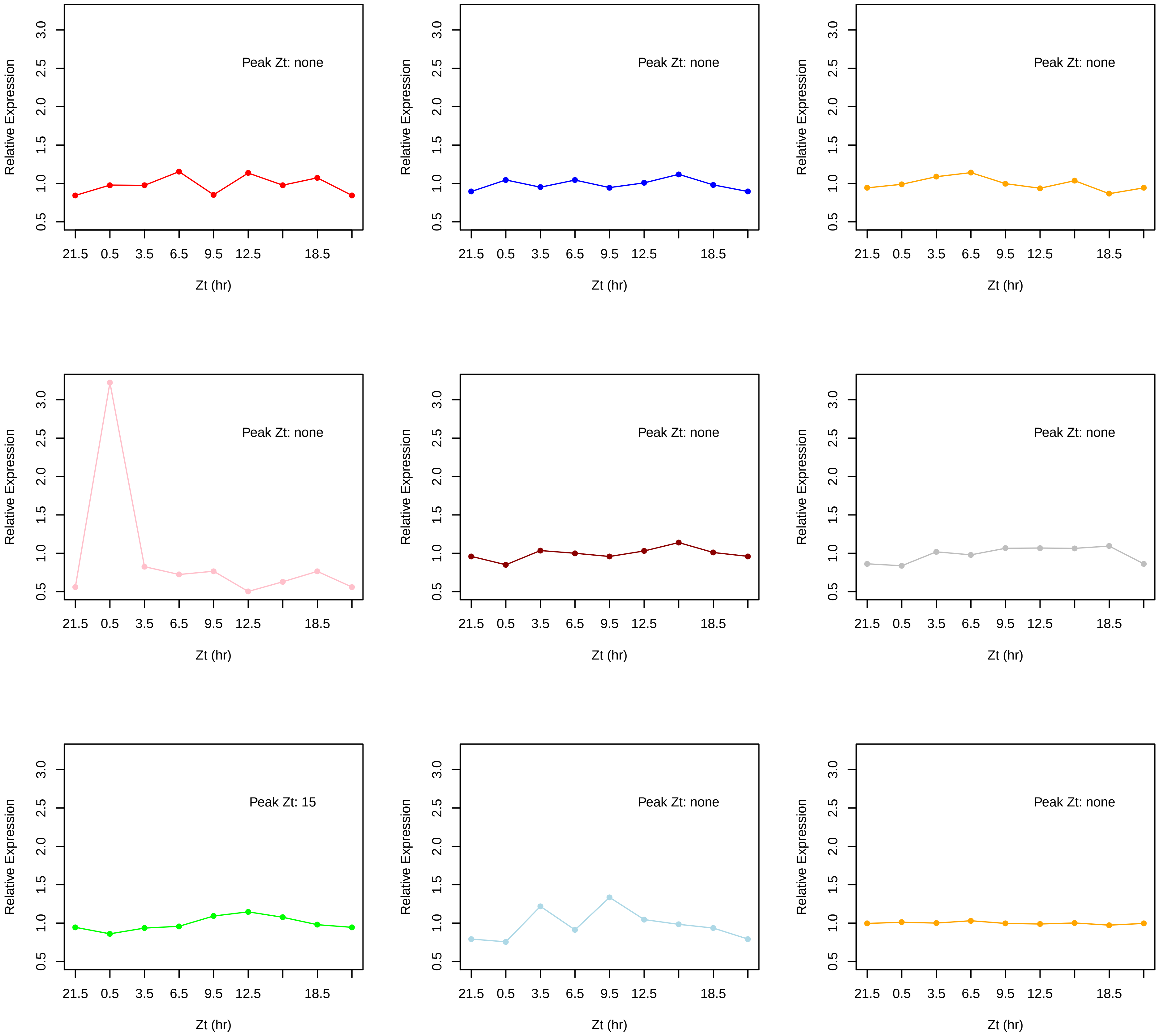

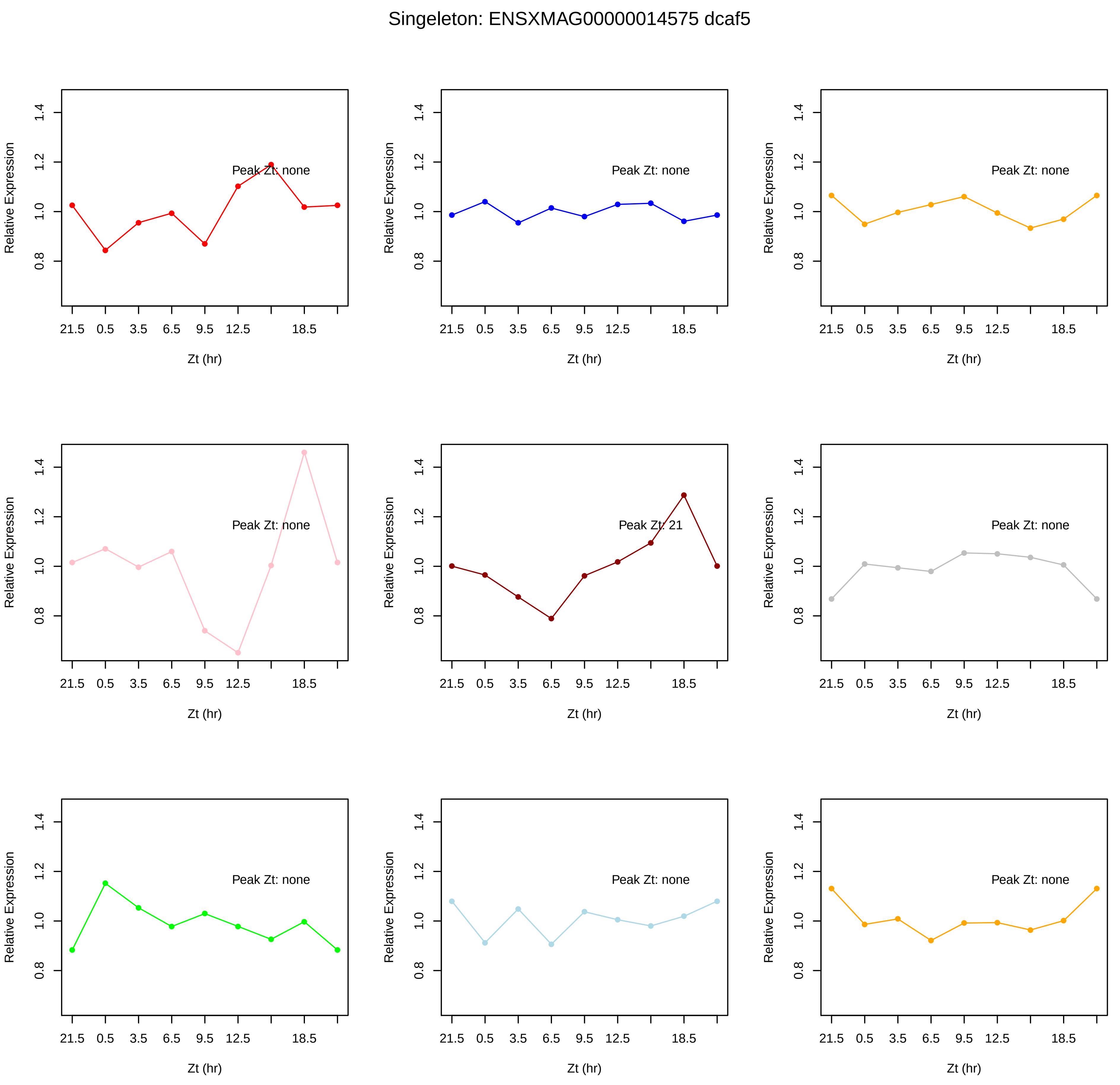
Singleton: ENSXMAG00000014575 dcaf5
Relative Expression
Zt (hr)
21.5
0.5
3.5
6.5
9.5
12.5
18.5
0.8
1.0
1.2
1.4
Peak Zt: none
Peak Zt: none
Peak Zt: none
Peak Zt: none
Peak Zt: 21
Peak Zt: none
Peak Zt: none
Peak Zt: none
Peak Zt: none

Singleton: ENSXMAG00000011799 slain2

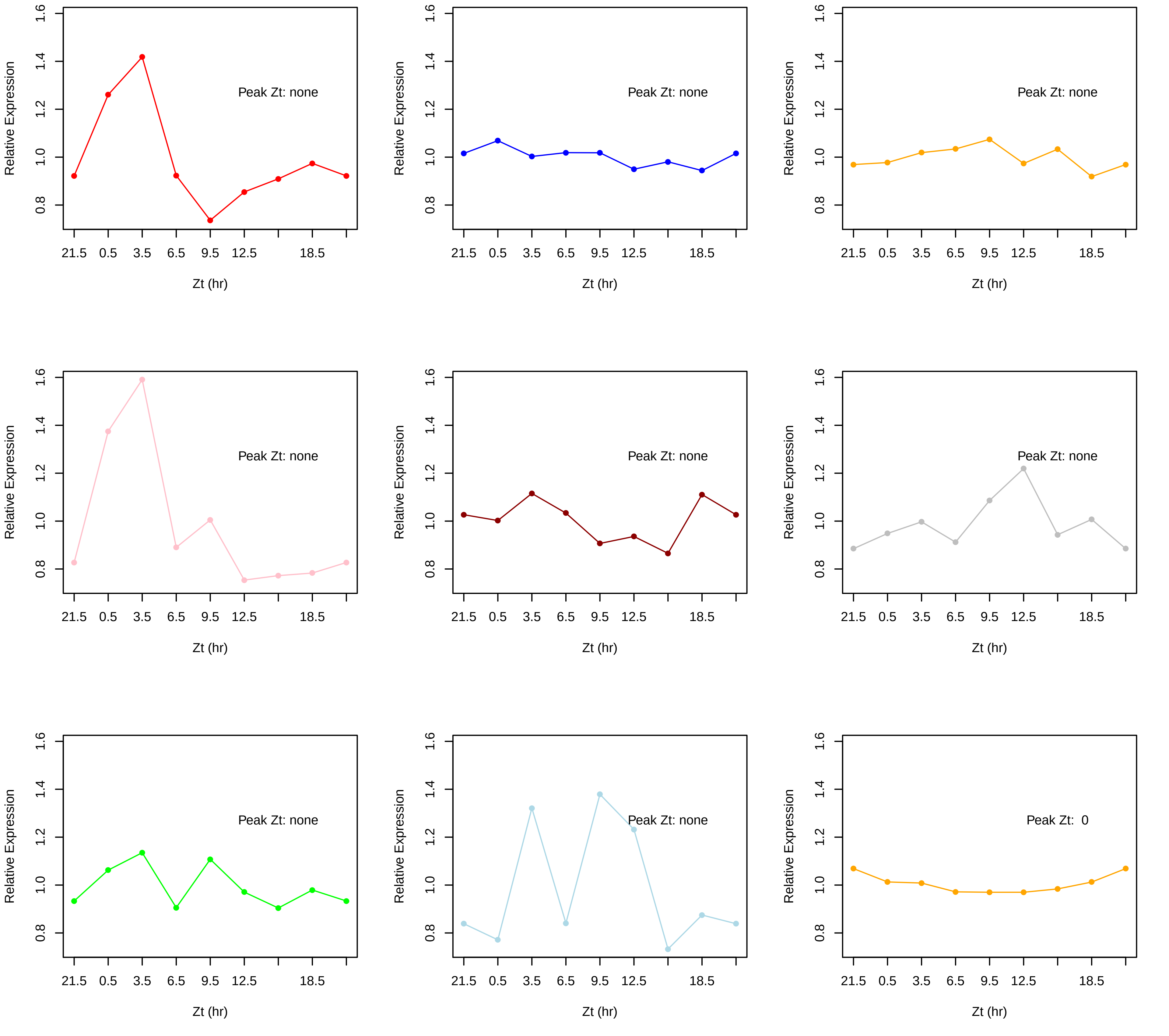

Singleton: ENSXMAG00000012823 srsf5a

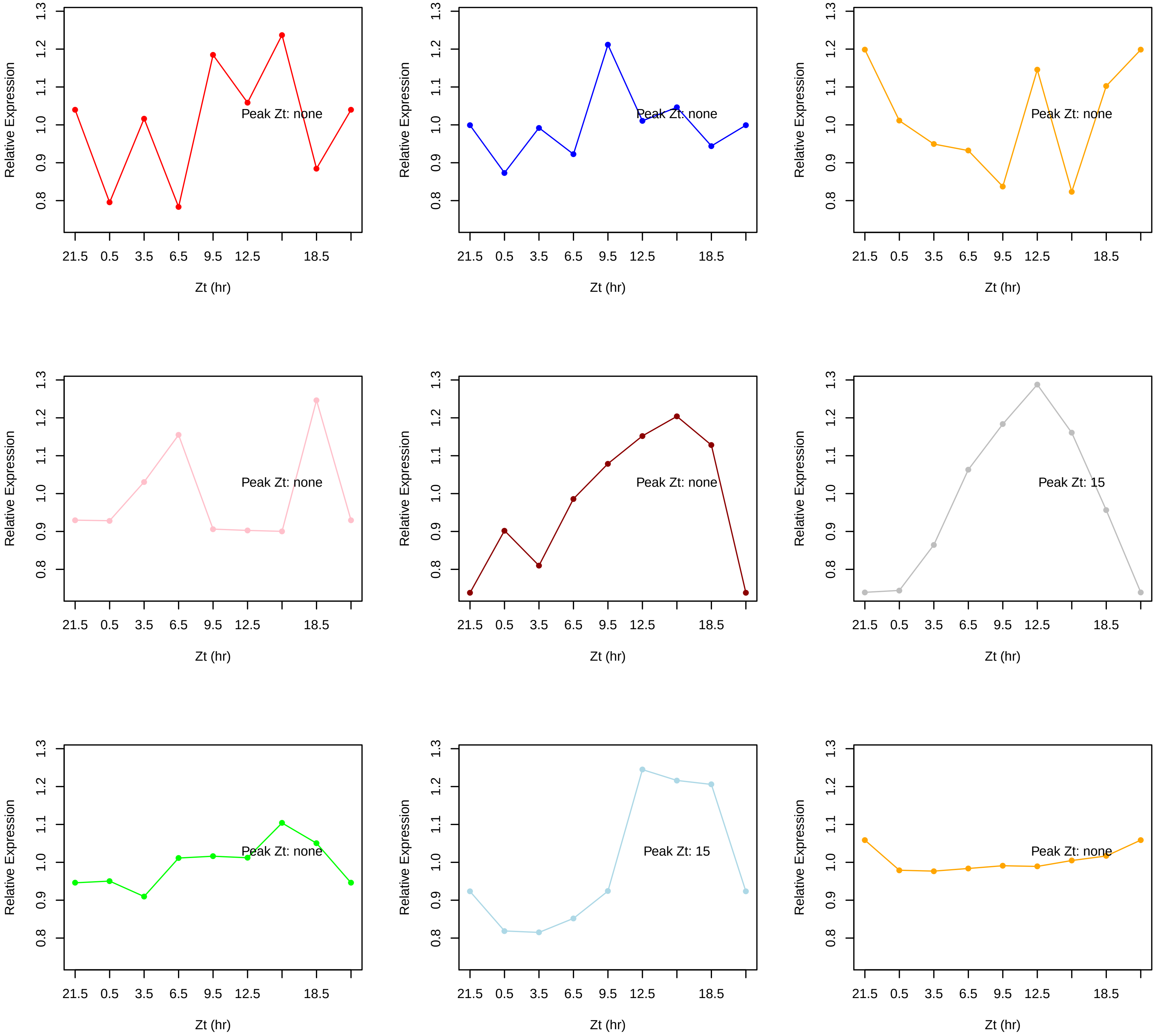

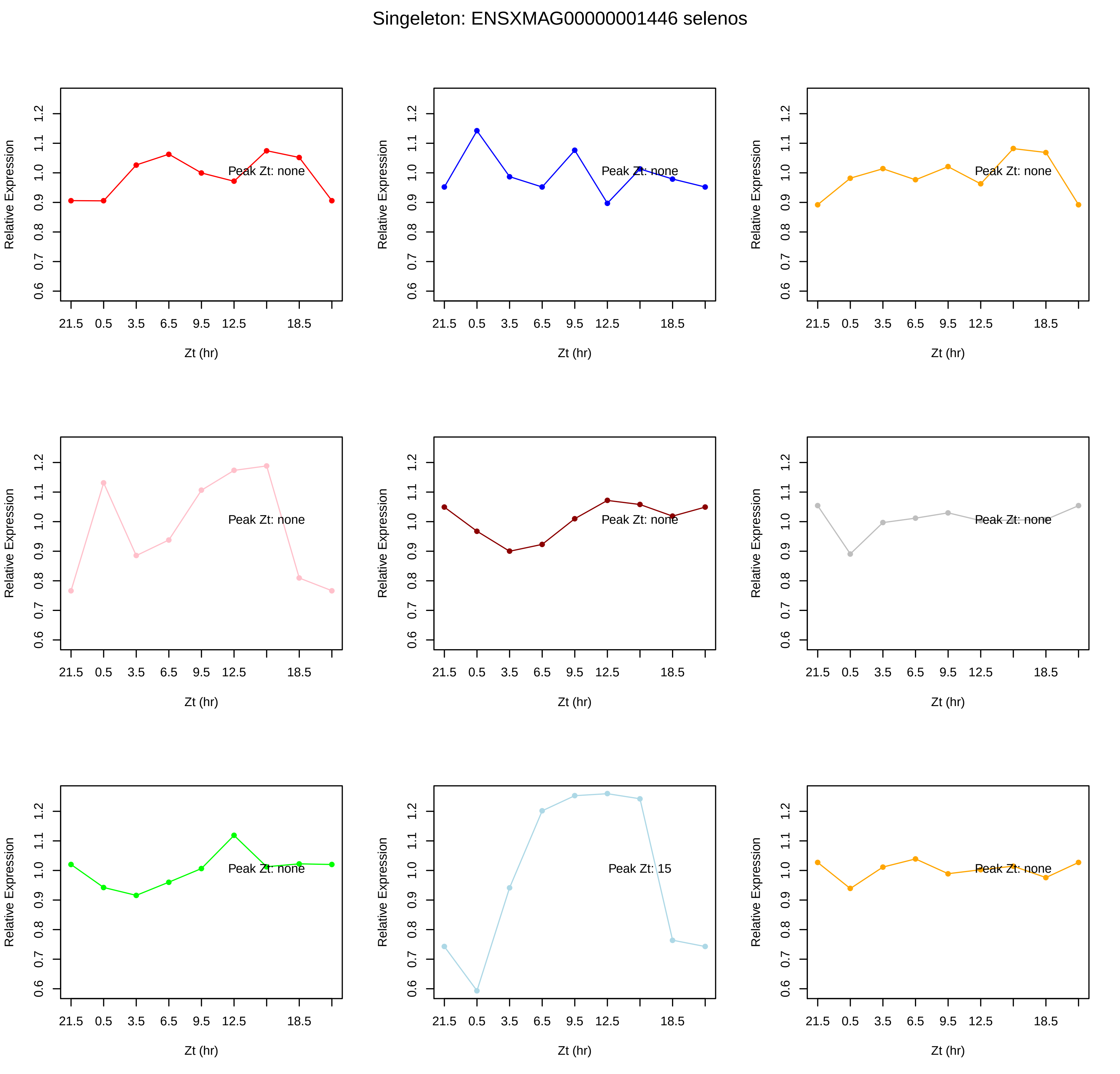
Singeleton: ENSXMAG00000001446 selenos
Relative Expression
Zt (hr)
21.5 0.5 3.5 6.5 9.5 12.5 18.5
0.6 0.7 0.8 0.9 1.0 1.1 1.2
Peak Zt: none
Peak Zt: none
Peak Zt: none
Peak Zt: none
Peak Zt: none
Peak Zt: none
Peak Zt: none
Peak Zt: 15
Peak Zt: none

Singleton: ENSXMAG00000014578 pacs2

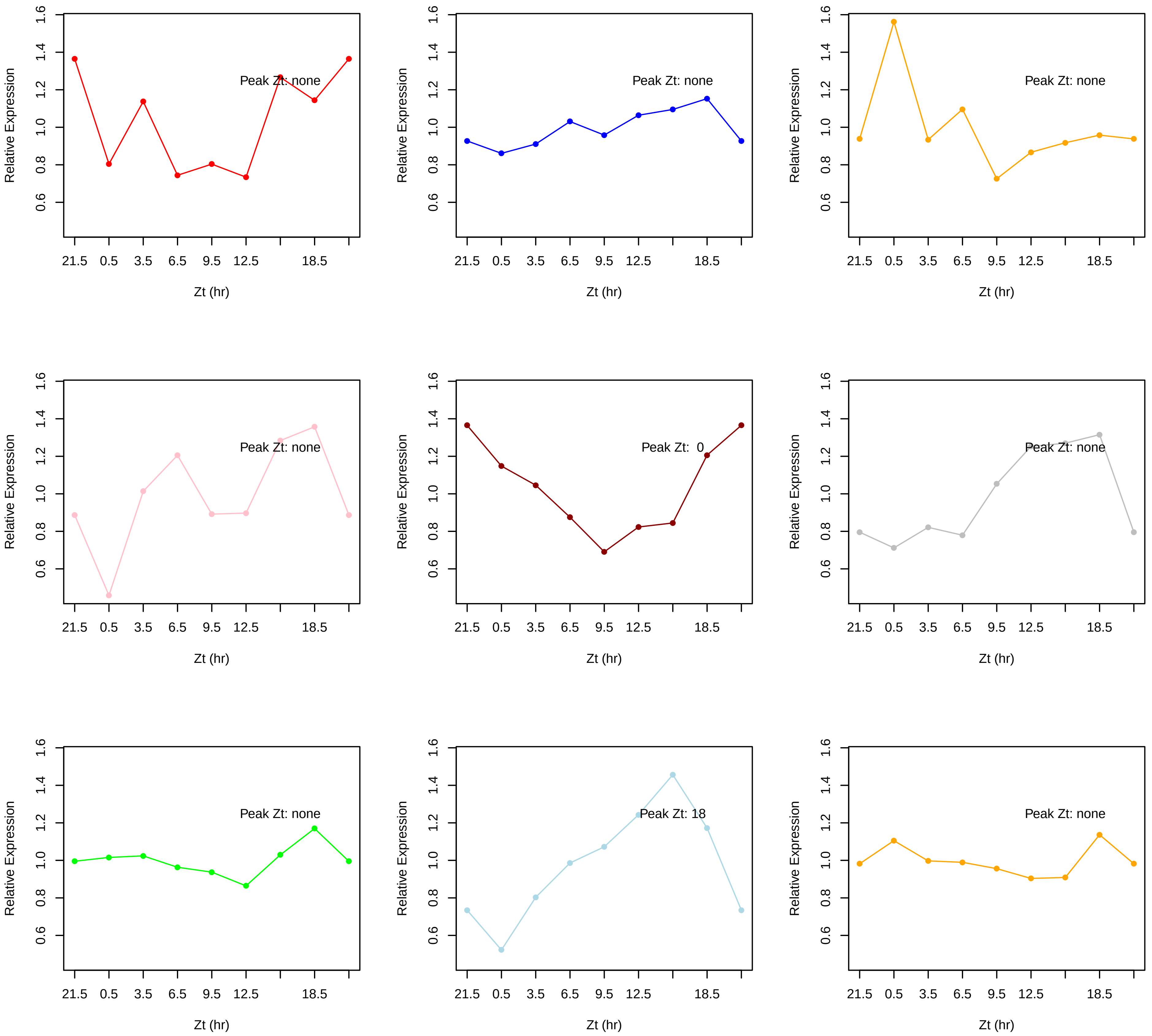

Singeleton: ENSXMAG00000001455 snrpa1

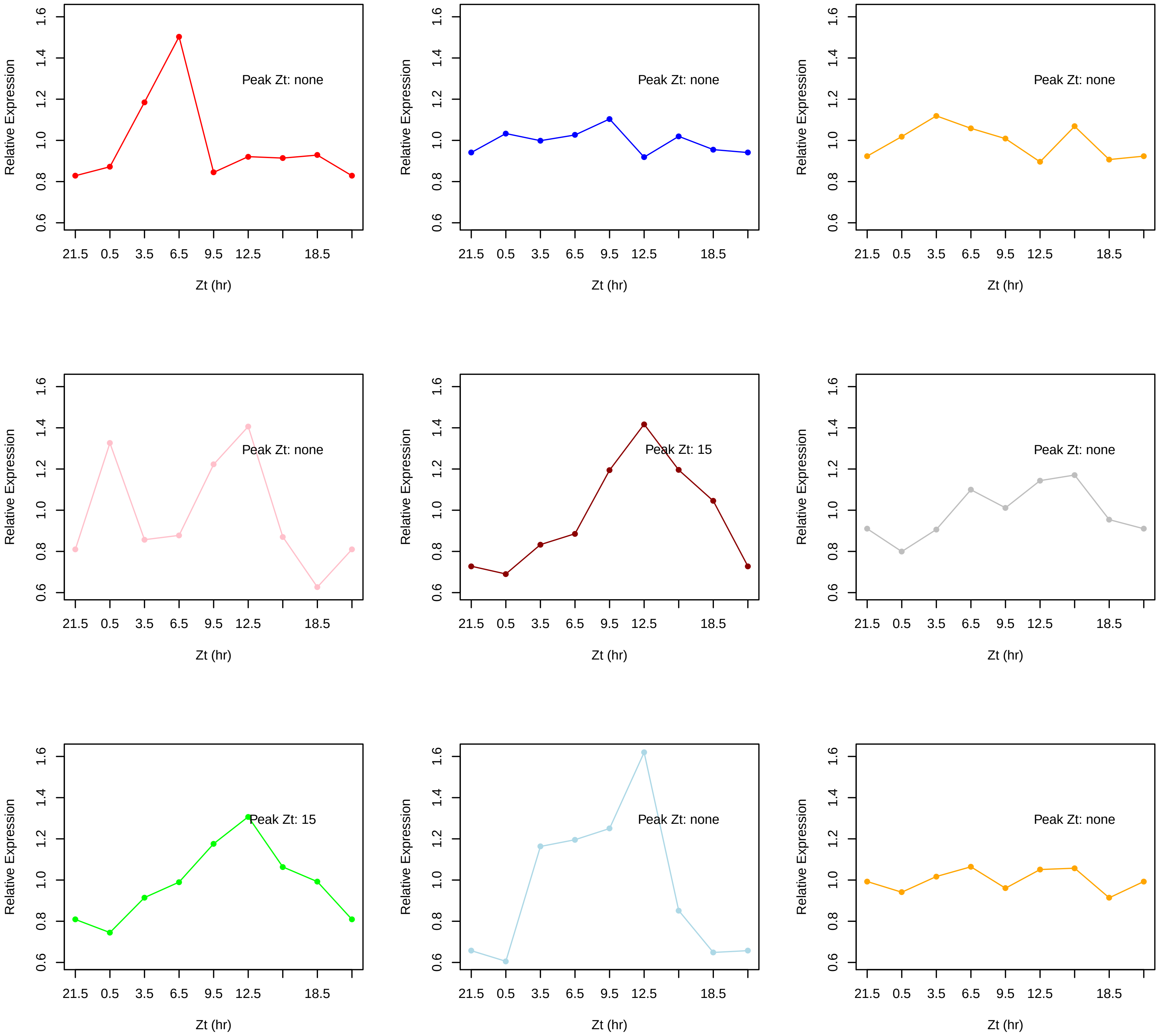

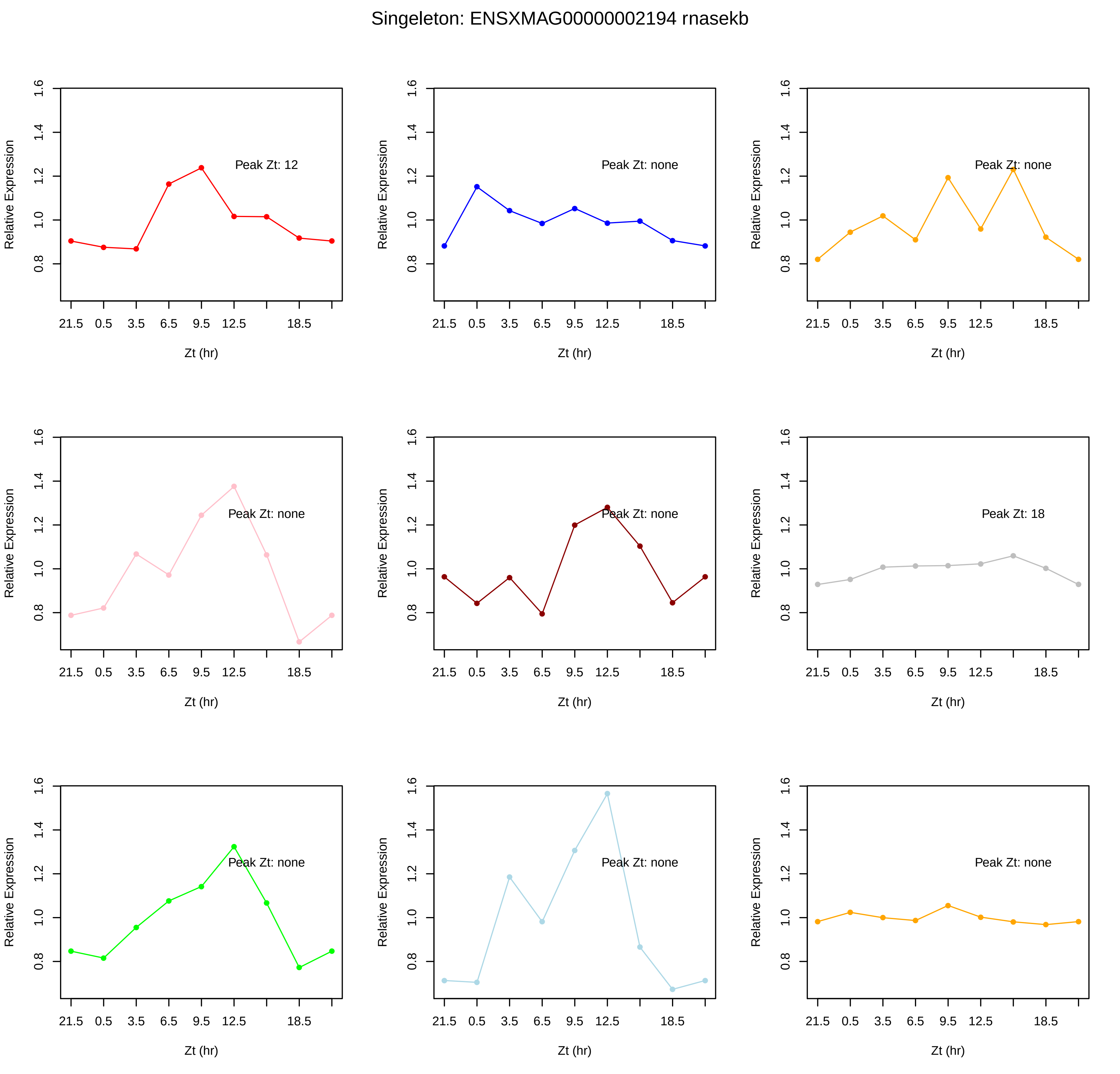
Singeleton: ENSXMAG00000002194 rnasekb
Peak Zt: 12
Peak Zt: none
Peak Zt: none
Peak Zt: none
Peak Zt: none
Peak Zt: 18
Peak Zt: none
Peak Zt: none
Peak Zt: none
Relative Expression
Zt (hr)
0.8
1.0
1.2
1.4
1.6
21.5
0.5
3.5
6.5
9.5
12.5
18.5

Singleton: ENSXMAG00000017525 FGD3

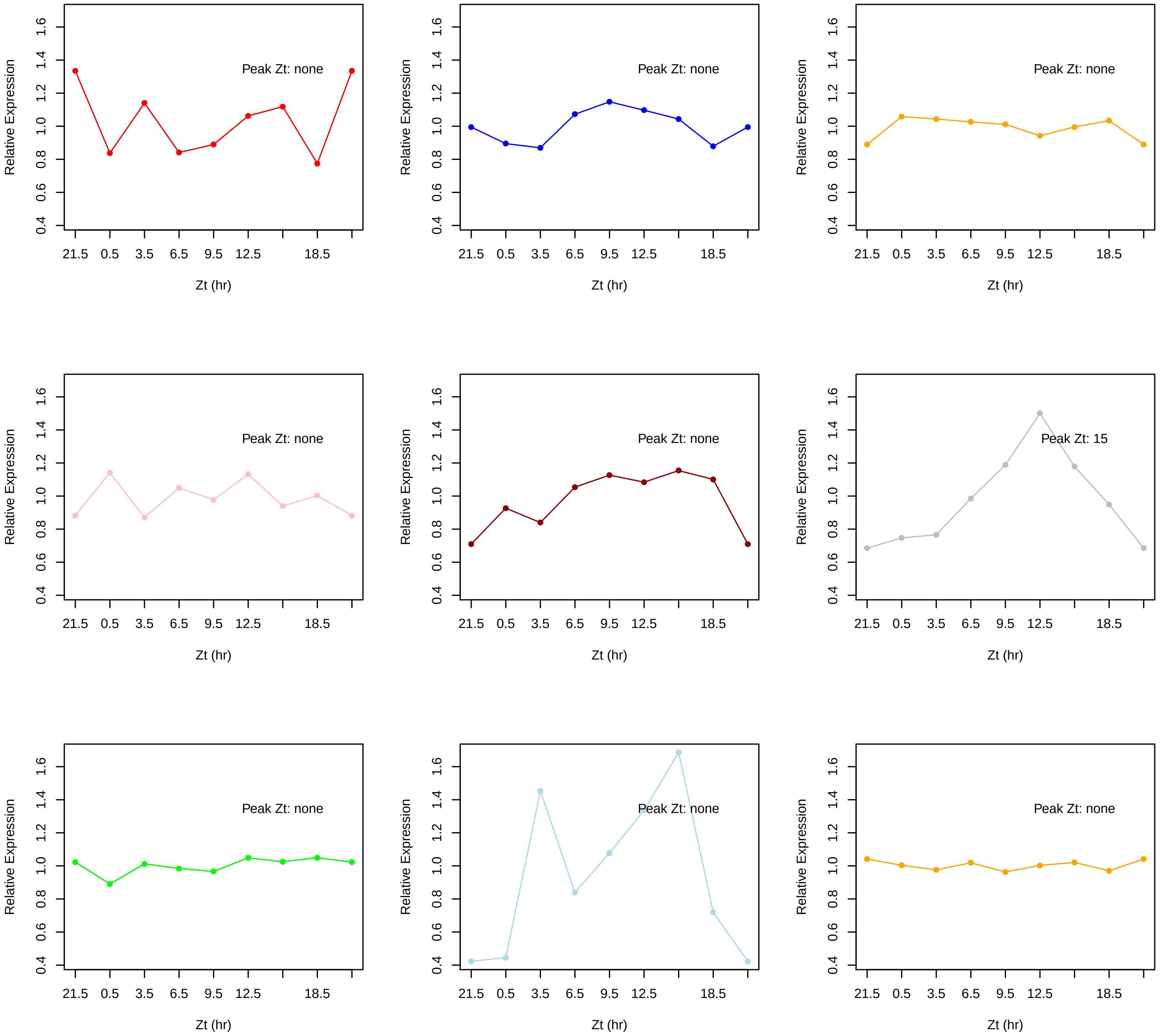

Singeleton: ENSXMAG00000008158 pelp1

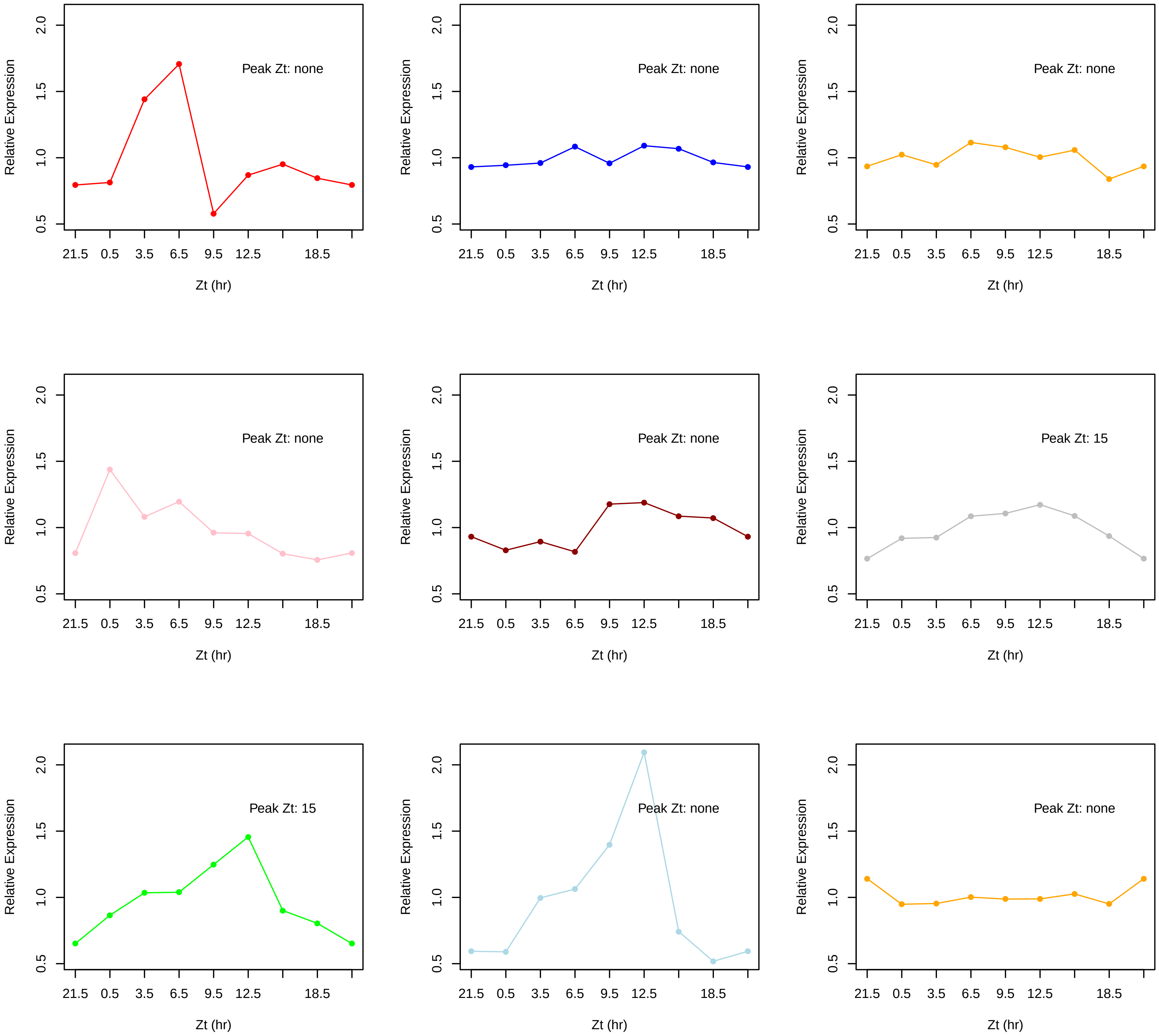

Singleton: ENSXMAG00000000831

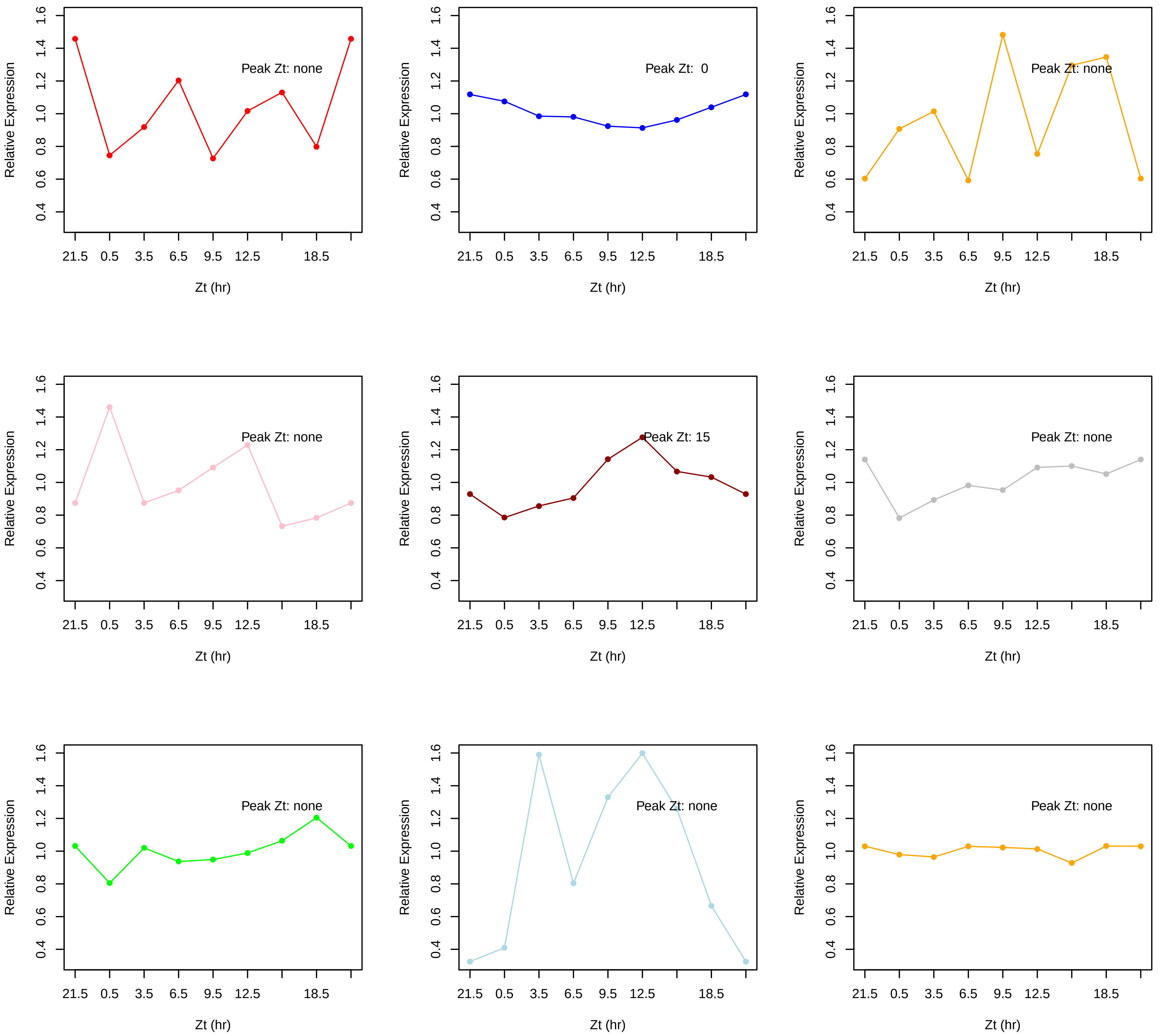

Singeleton: ENSXMAG00000017048 cpsf2

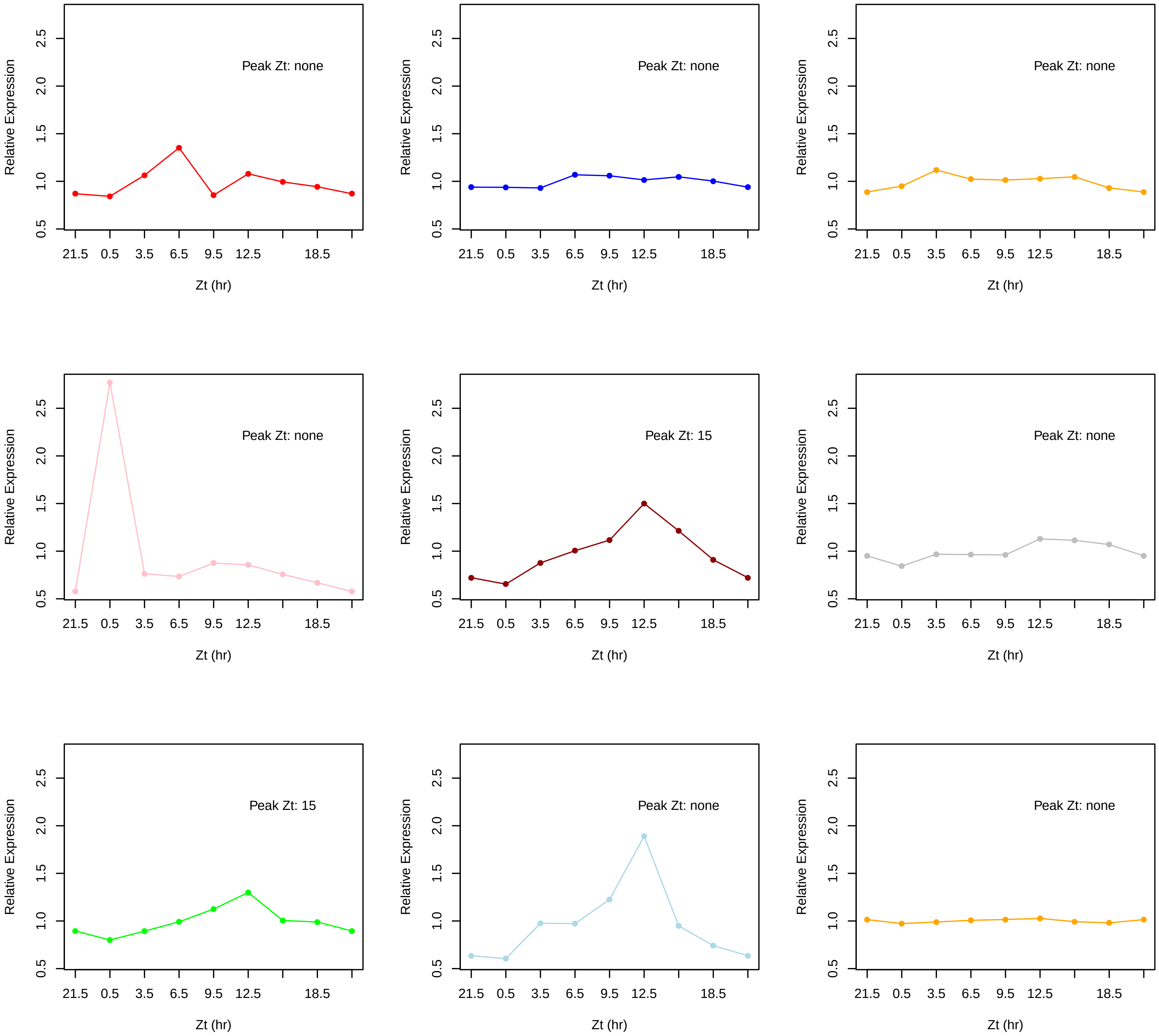

Singleton: ENSXMAG00000011516 spg21

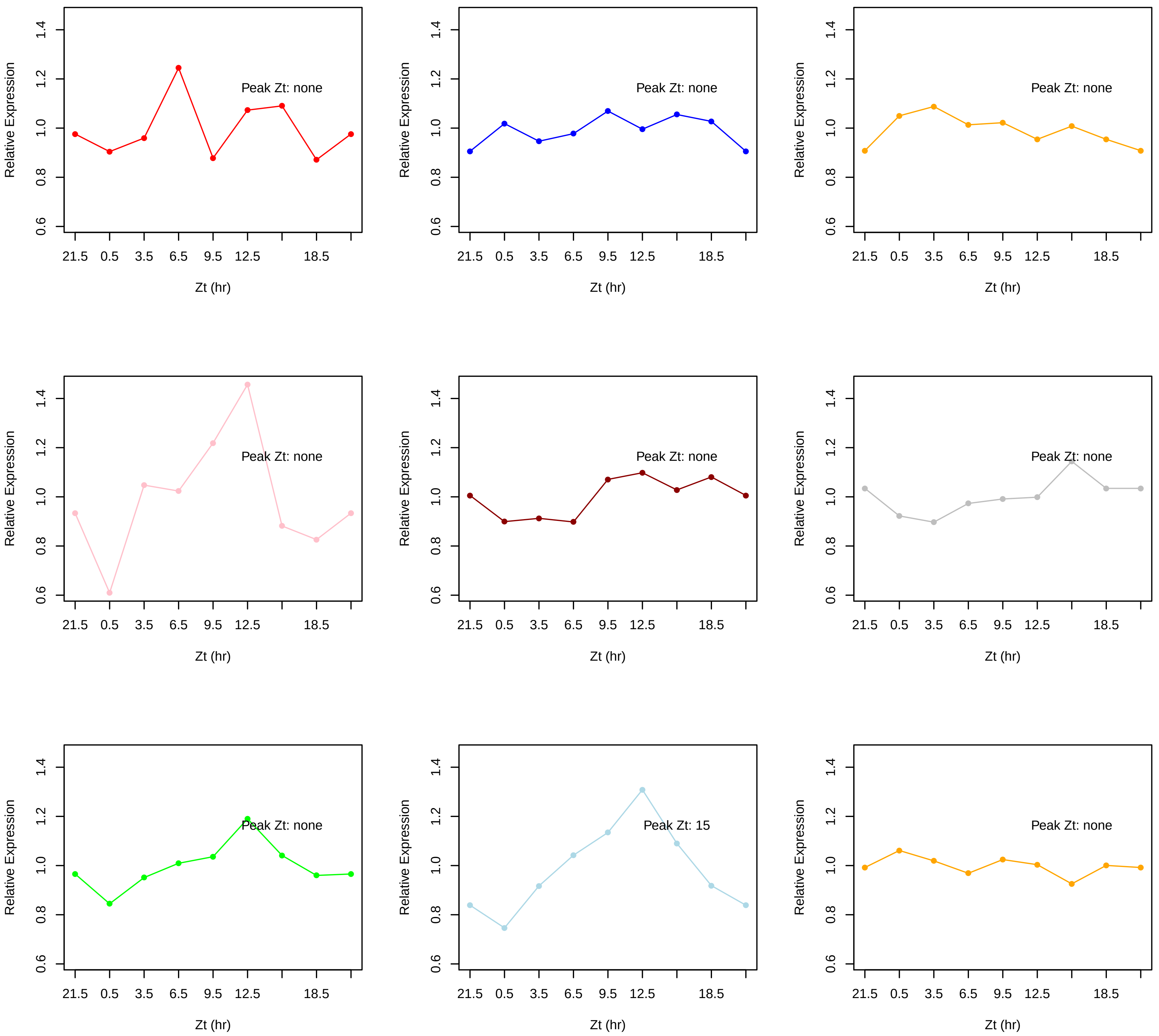

Singeleton: ENSXMAG00000009790

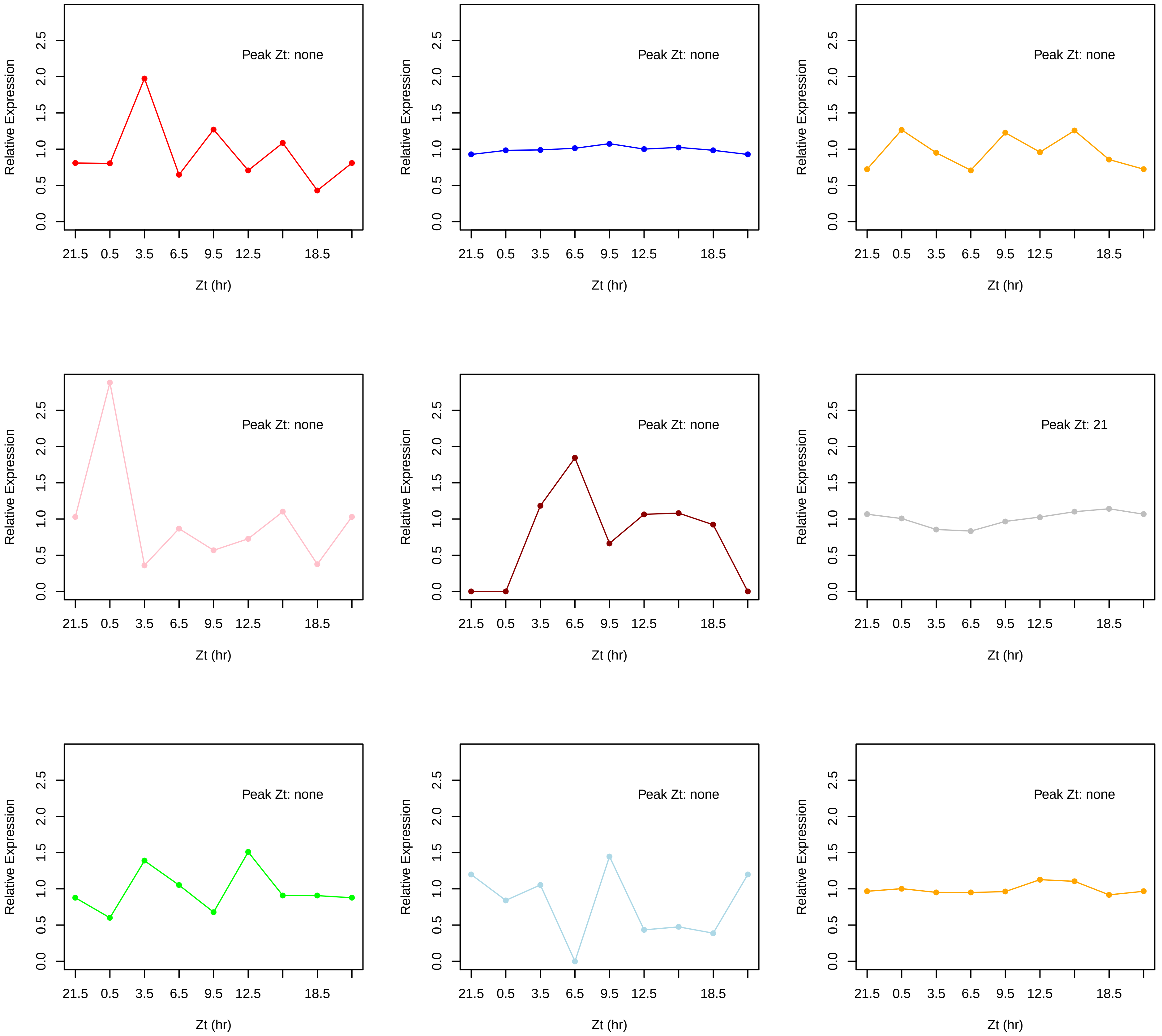

Singeleton: ENSXMAG00000014298 spata18

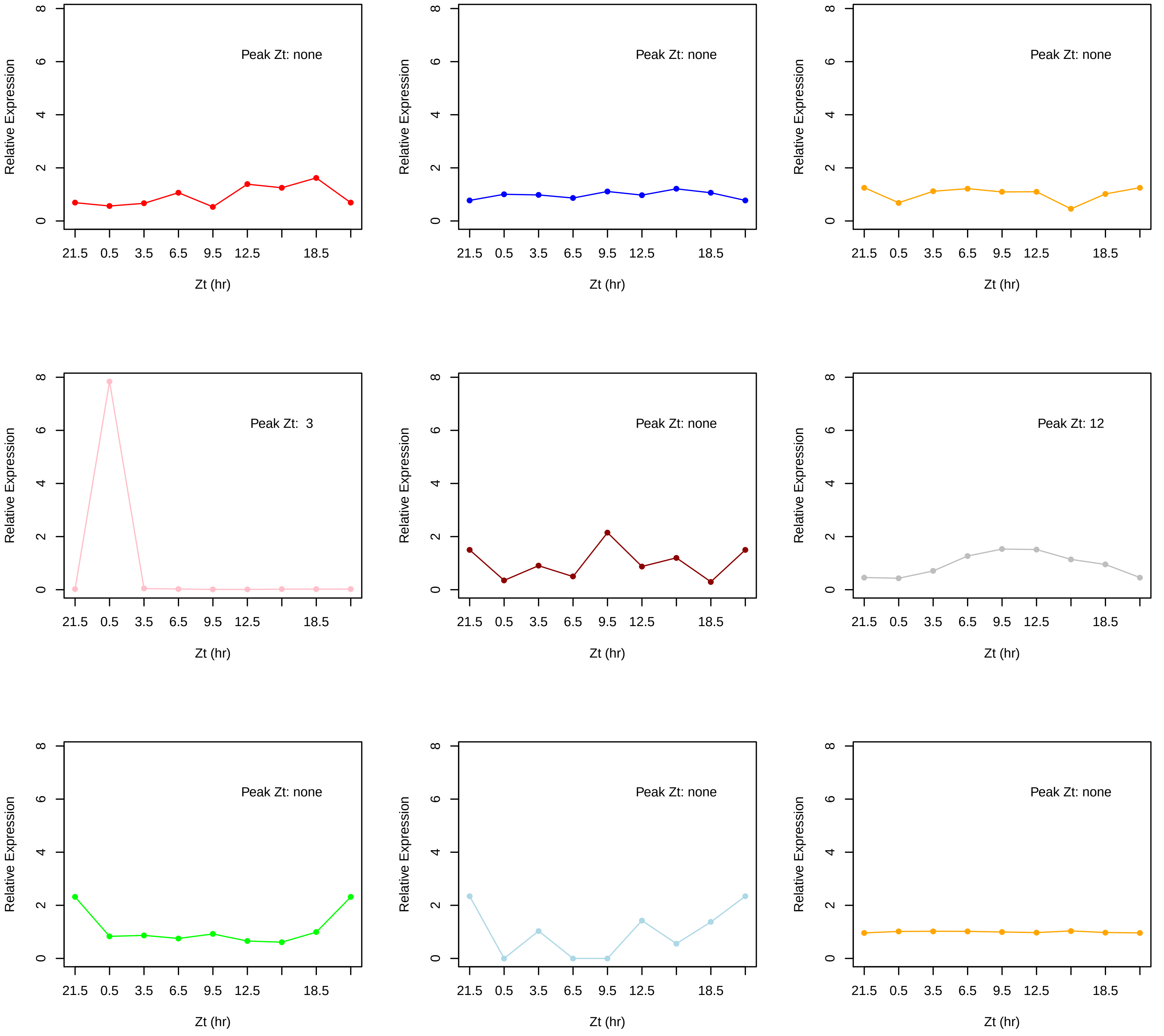

Singeleton: ENSXMAG00000006841

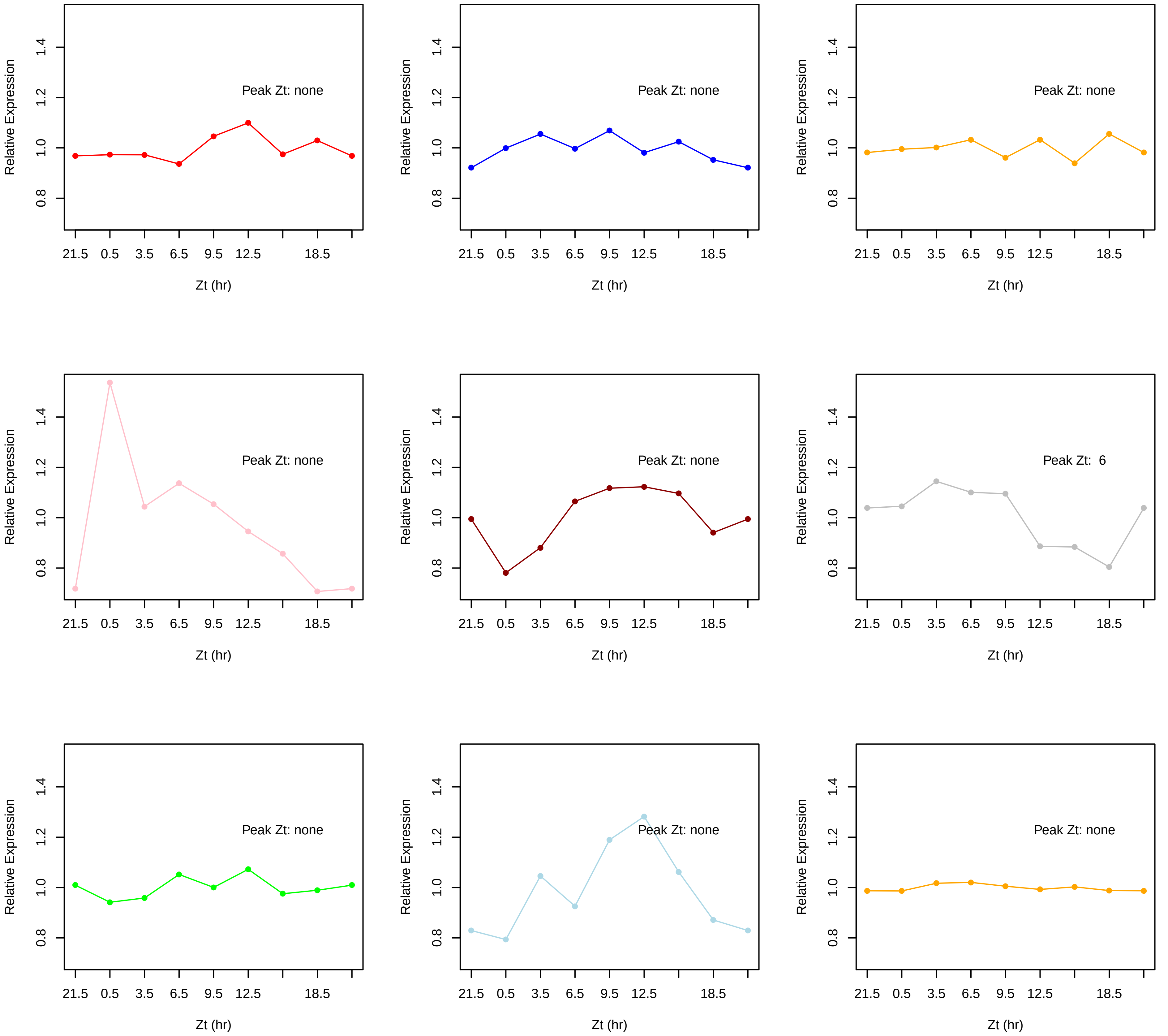

Singleton: ENSXMAG00000021952 pdcd7

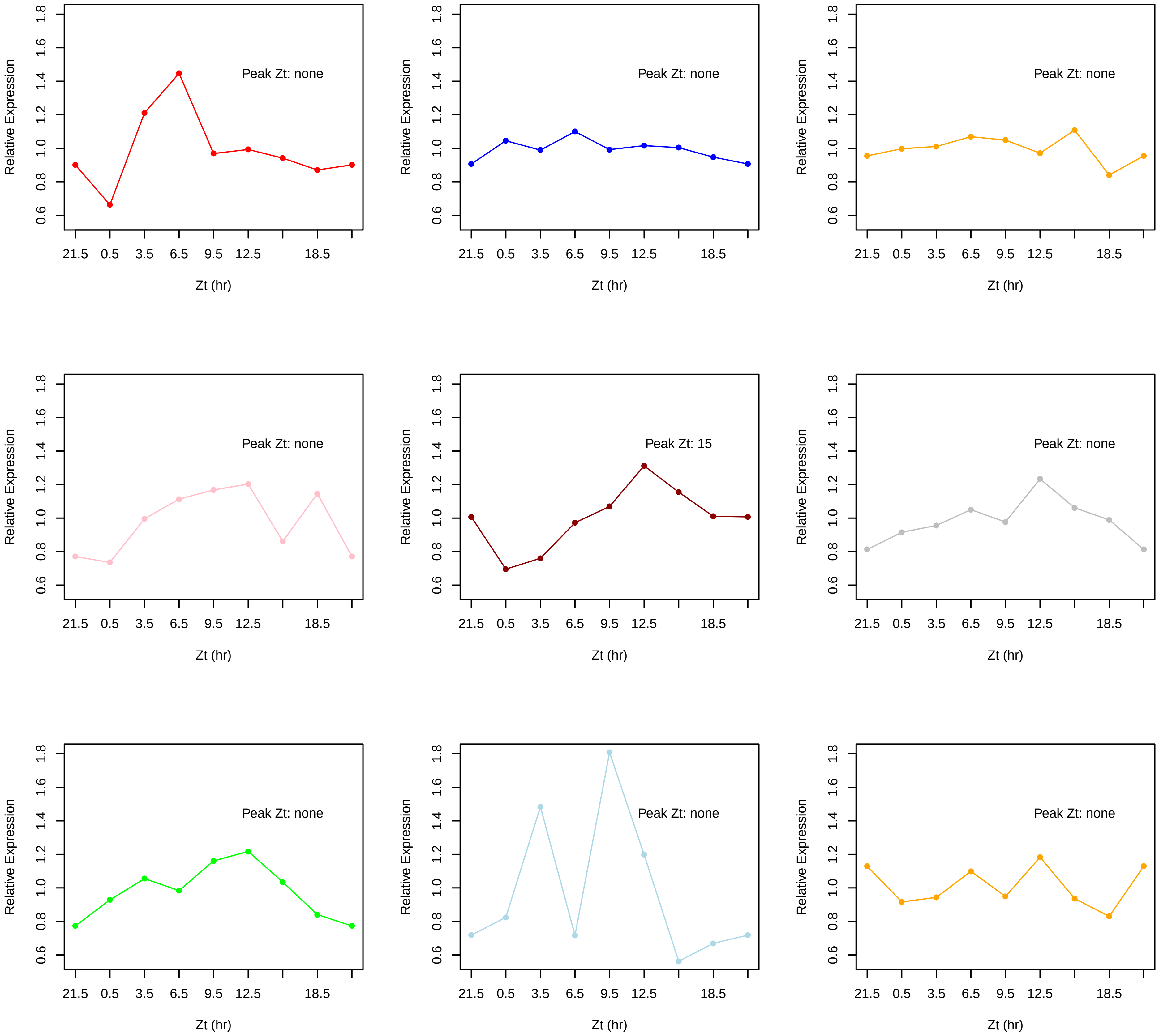

Singleton: ENSXMAG00000014314 usp46

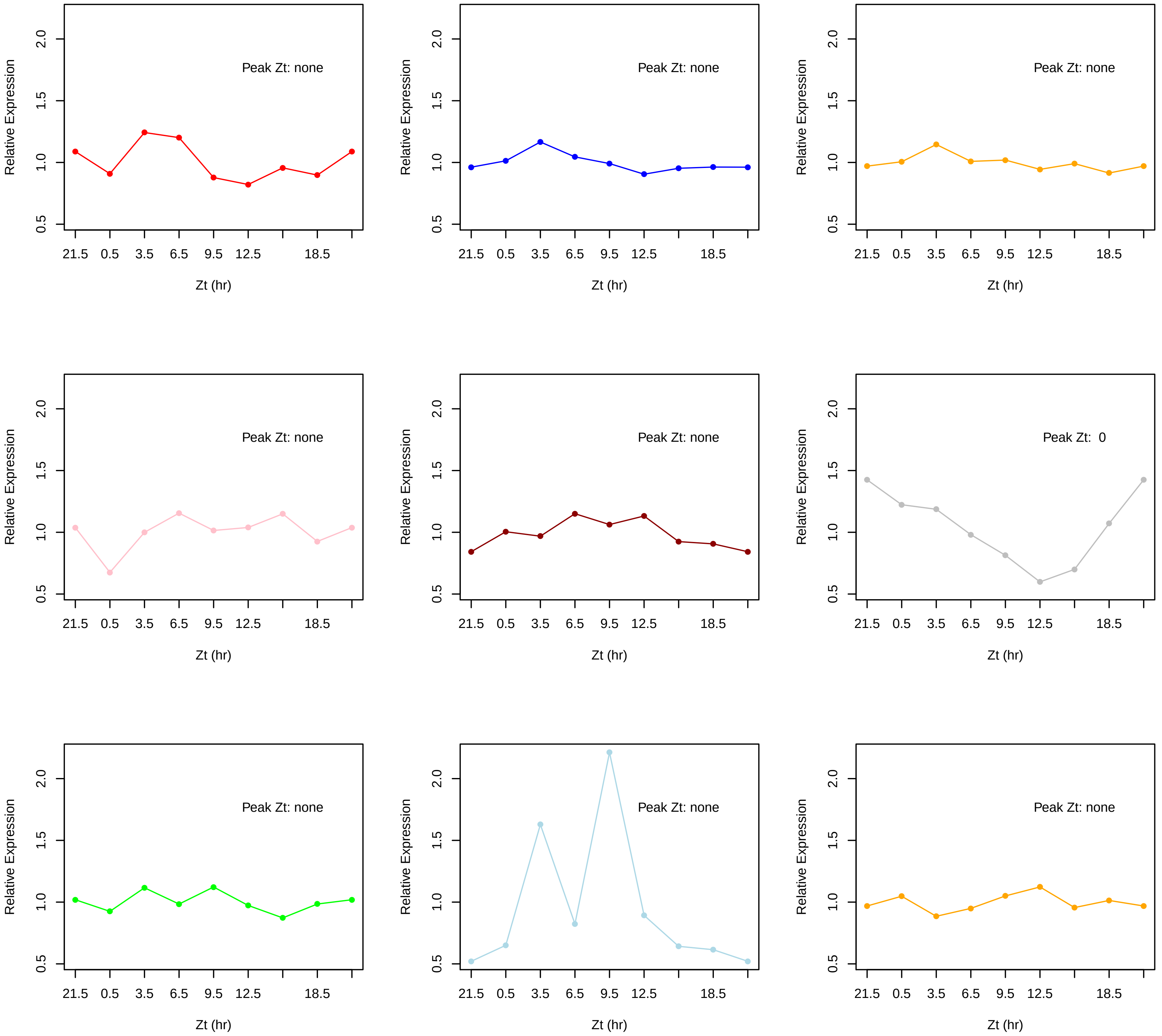

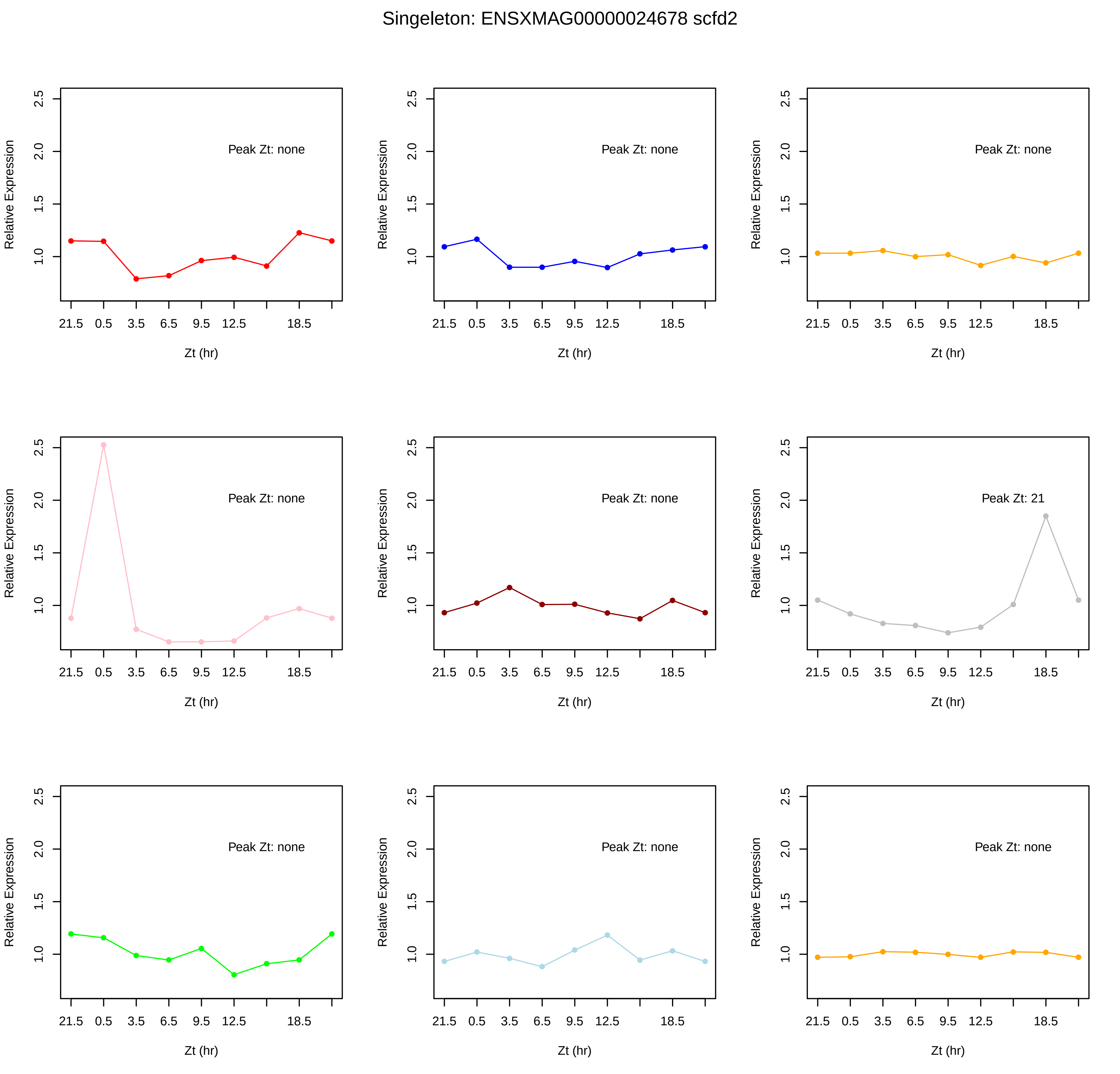
Singeleton: ENSXMAG00000024678 scfd2
Peak Zt: none
Peak Zt: none
Peak Zt: none
Peak Zt: none
Peak Zt: none
Peak Zt: 21
Peak Zt: none
Peak Zt: none
Peak Zt: none
Relative Expression
Zt (hr)
21.5 0.5 3.5 6.5 9.5 12.5 18.5
1.0 1.5 2.0 2.5

Singeleton: ENSXMAG00000003975 MTFMT

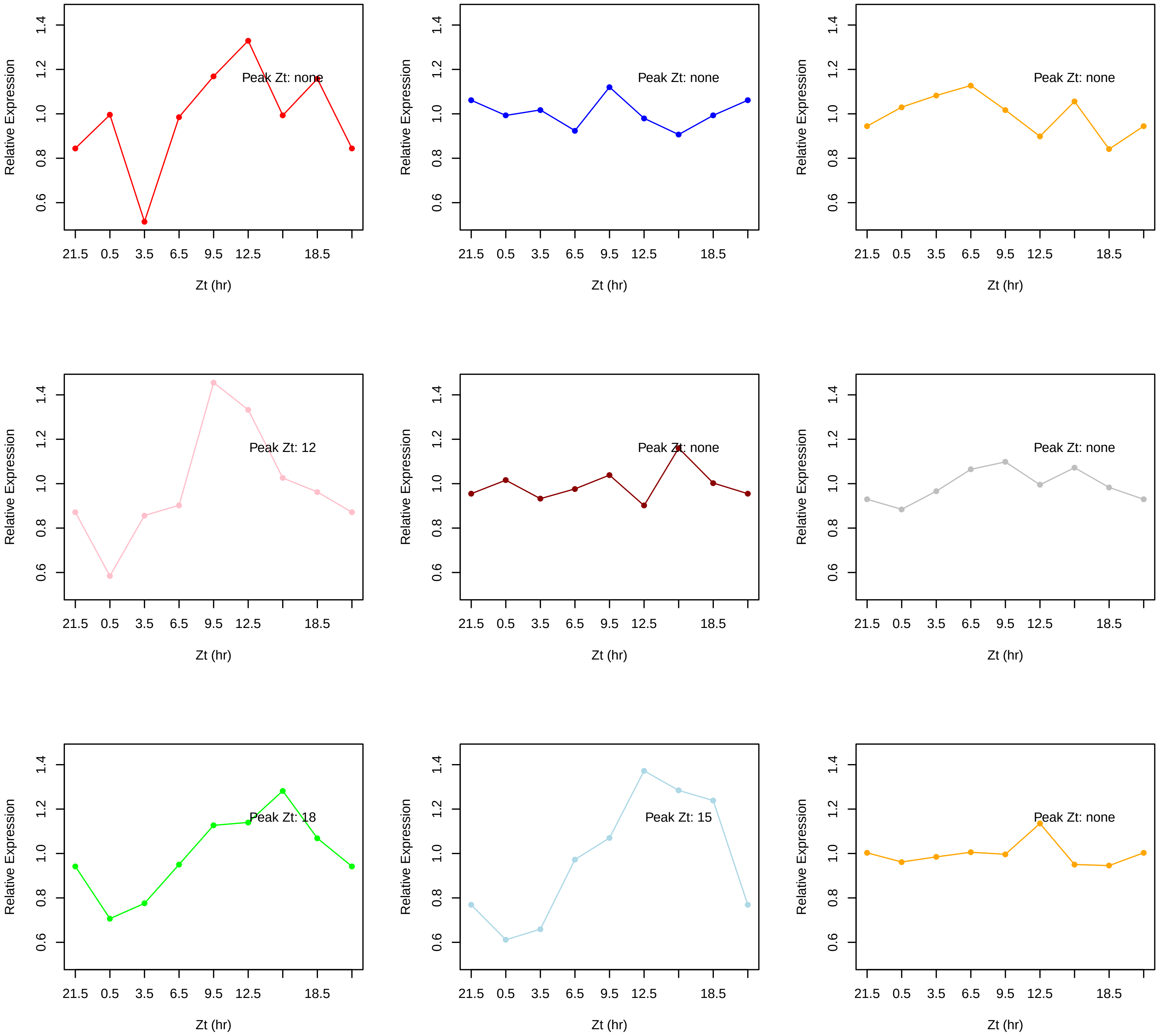

Singeleton: ENSXMAG00000017148 fut8b

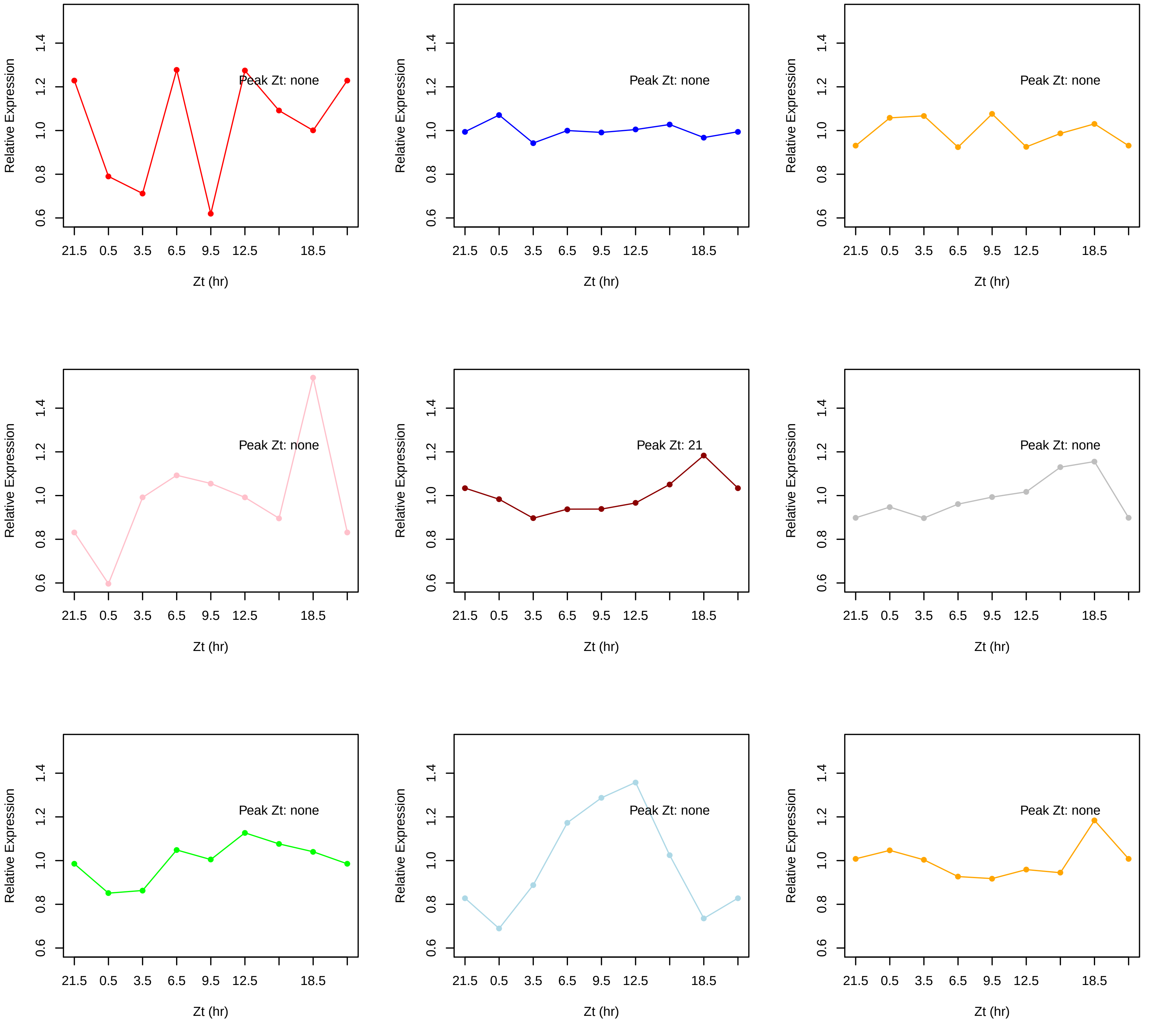

Singleton: ENSXMAG00000017179 gphnb

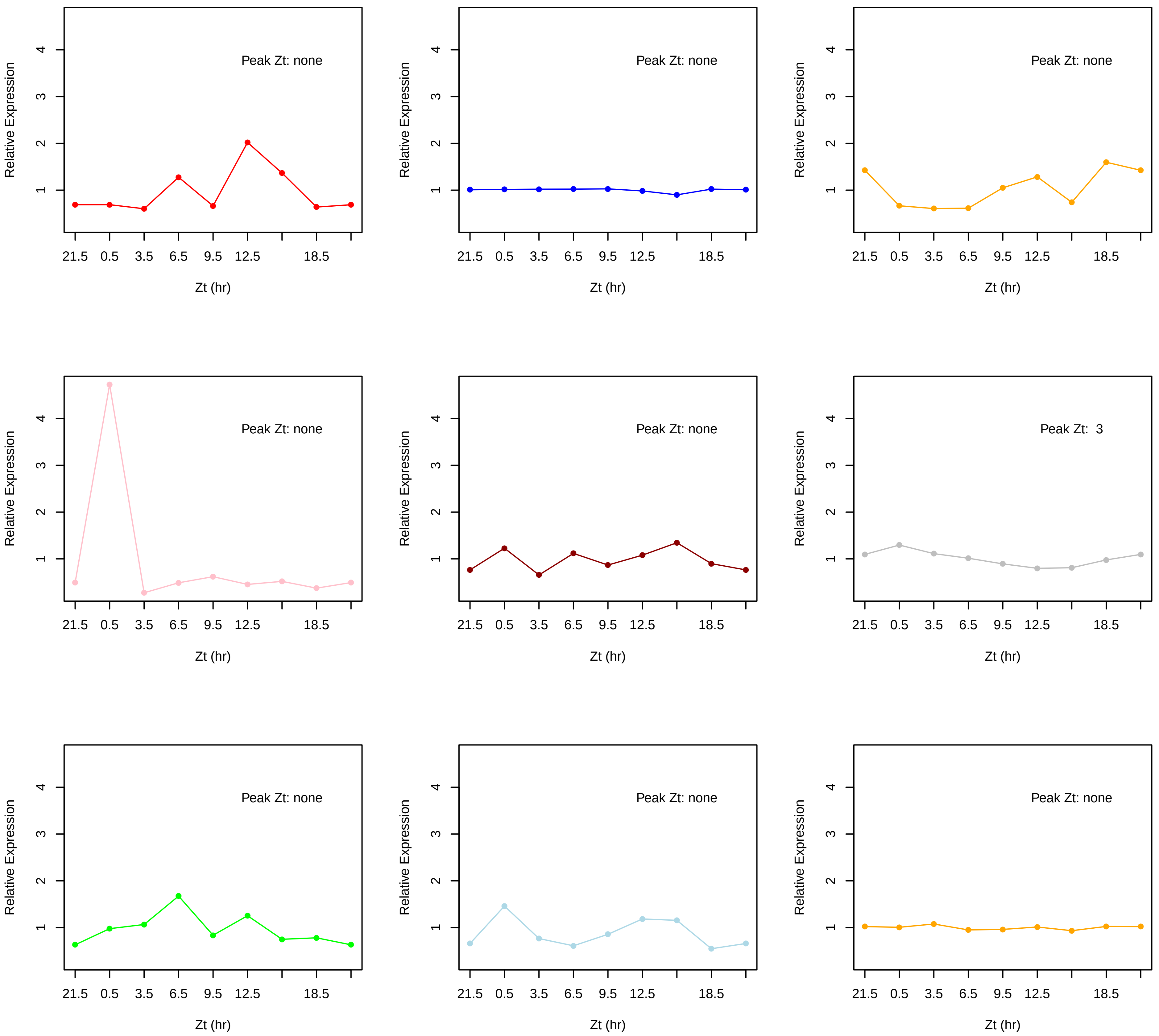

Singeleton: ENSXMAG00000018080 vps33b

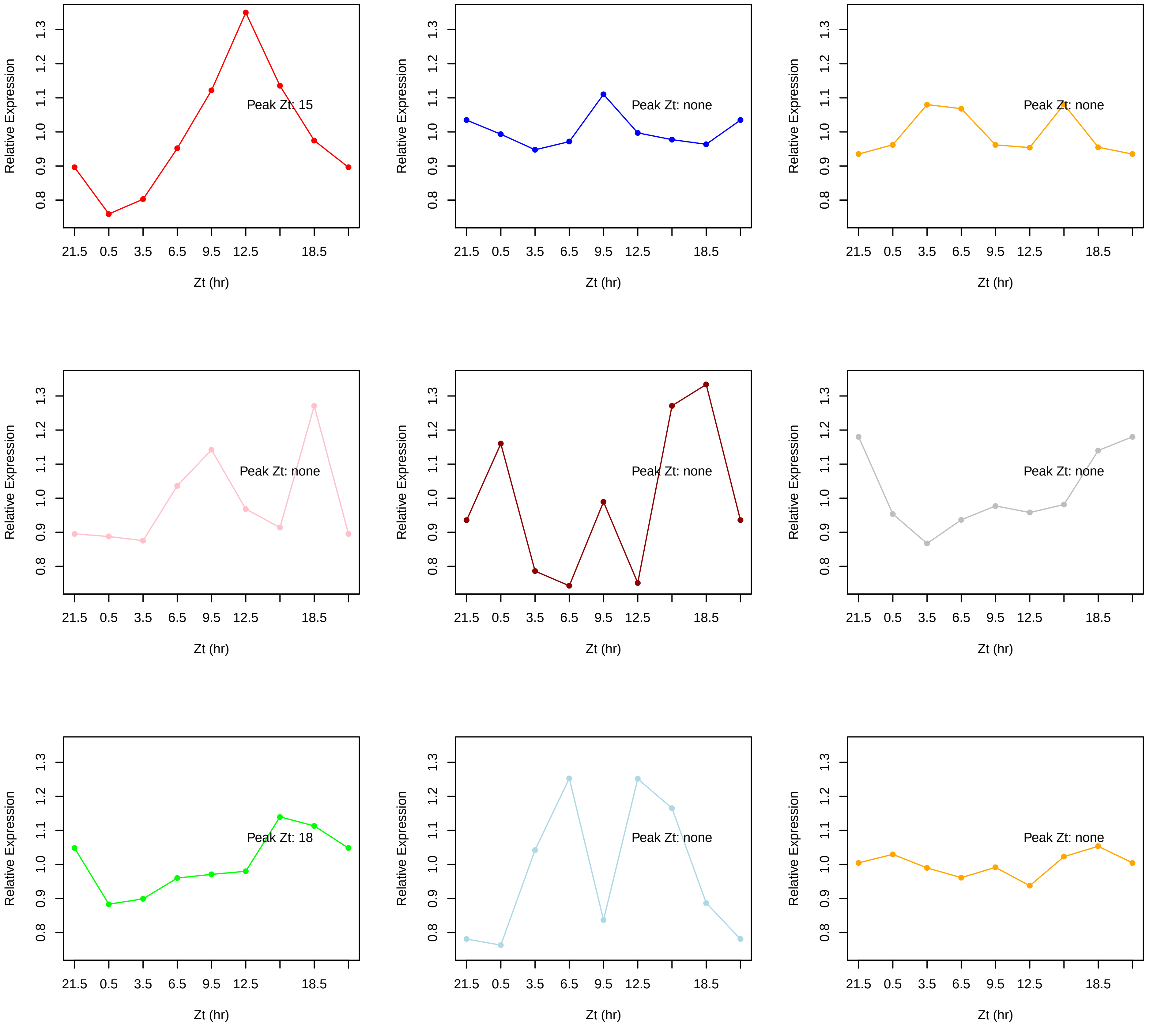

Singeleton: ENSXMAG00000014362 gsx2

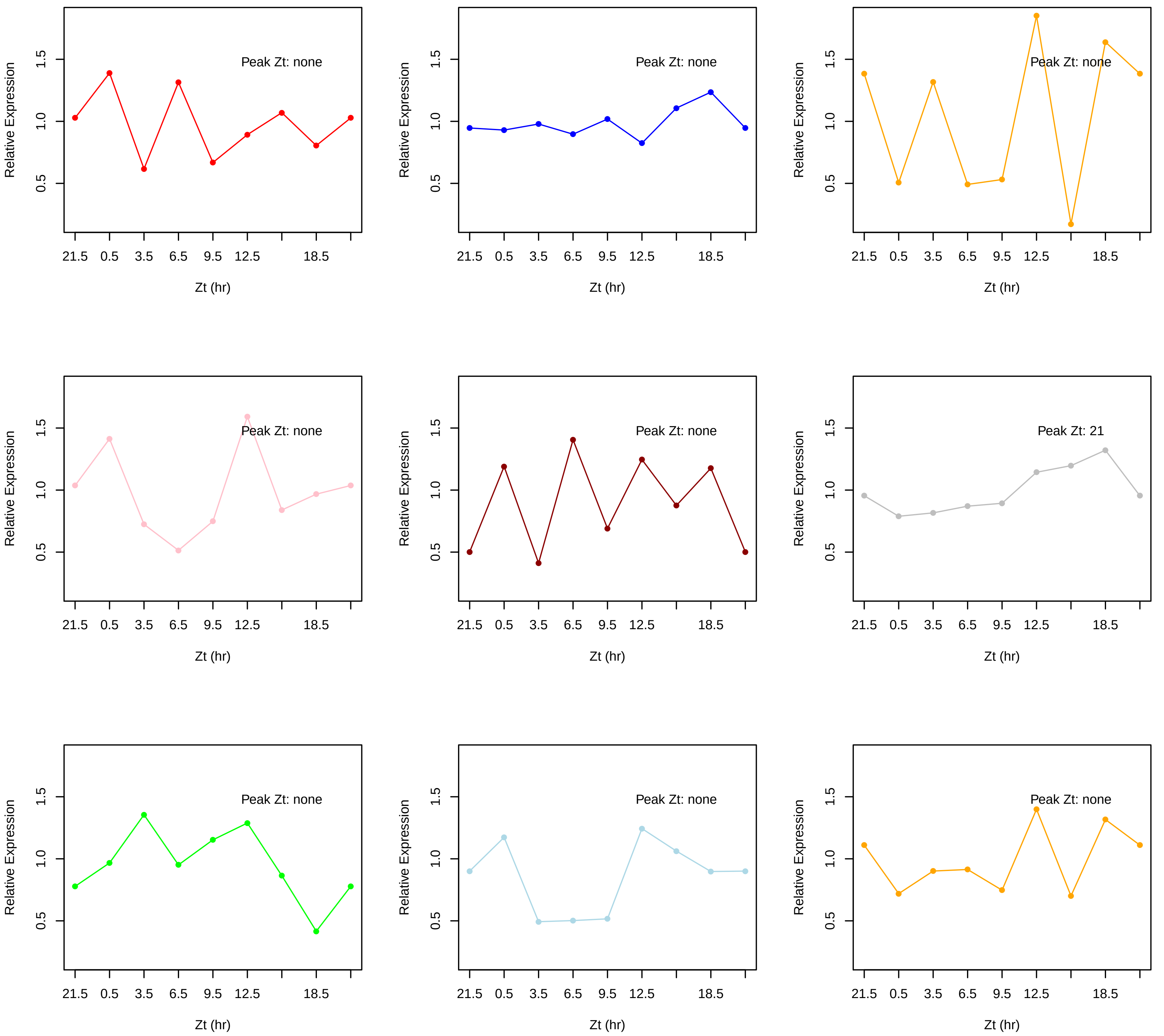

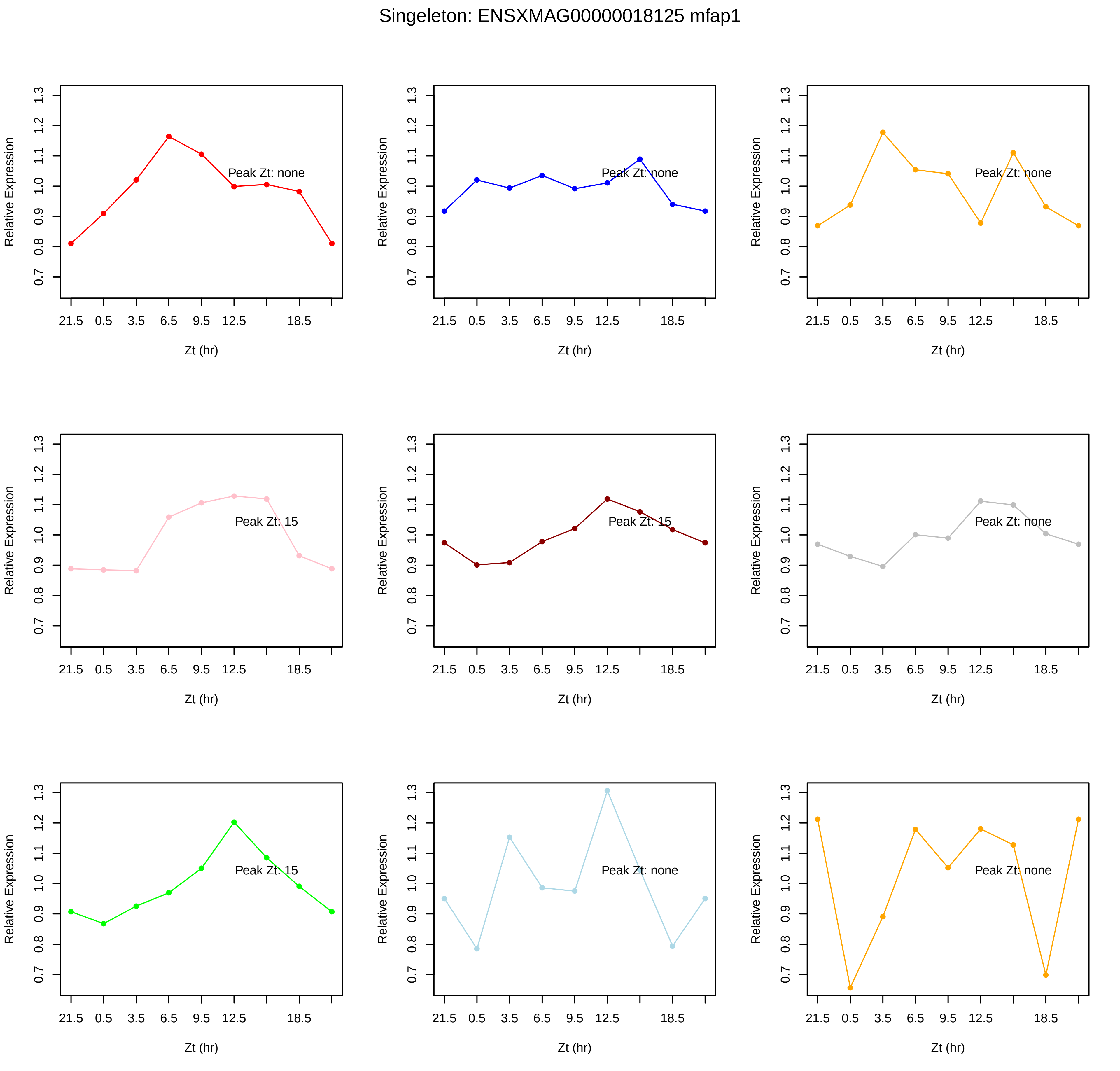

Singleton: ENSXMAG00000018125 mfap1
Relative Expression
Zt (hr)
21.5
0.5
3.5
6.5
9.5
12.5
18.5
0.7
0.8
0.9
1.0
1.1
1.2
1.3
Peak Zt: none
Peak Zt: none
Peak Zt: none
Peak Zt: 15
Peak Zt: 15
Peak Zt: none
Peak Zt: 15
Peak Zt: none
Peak Zt: none

Singeleton: ENSXMAG00000019281 chdh

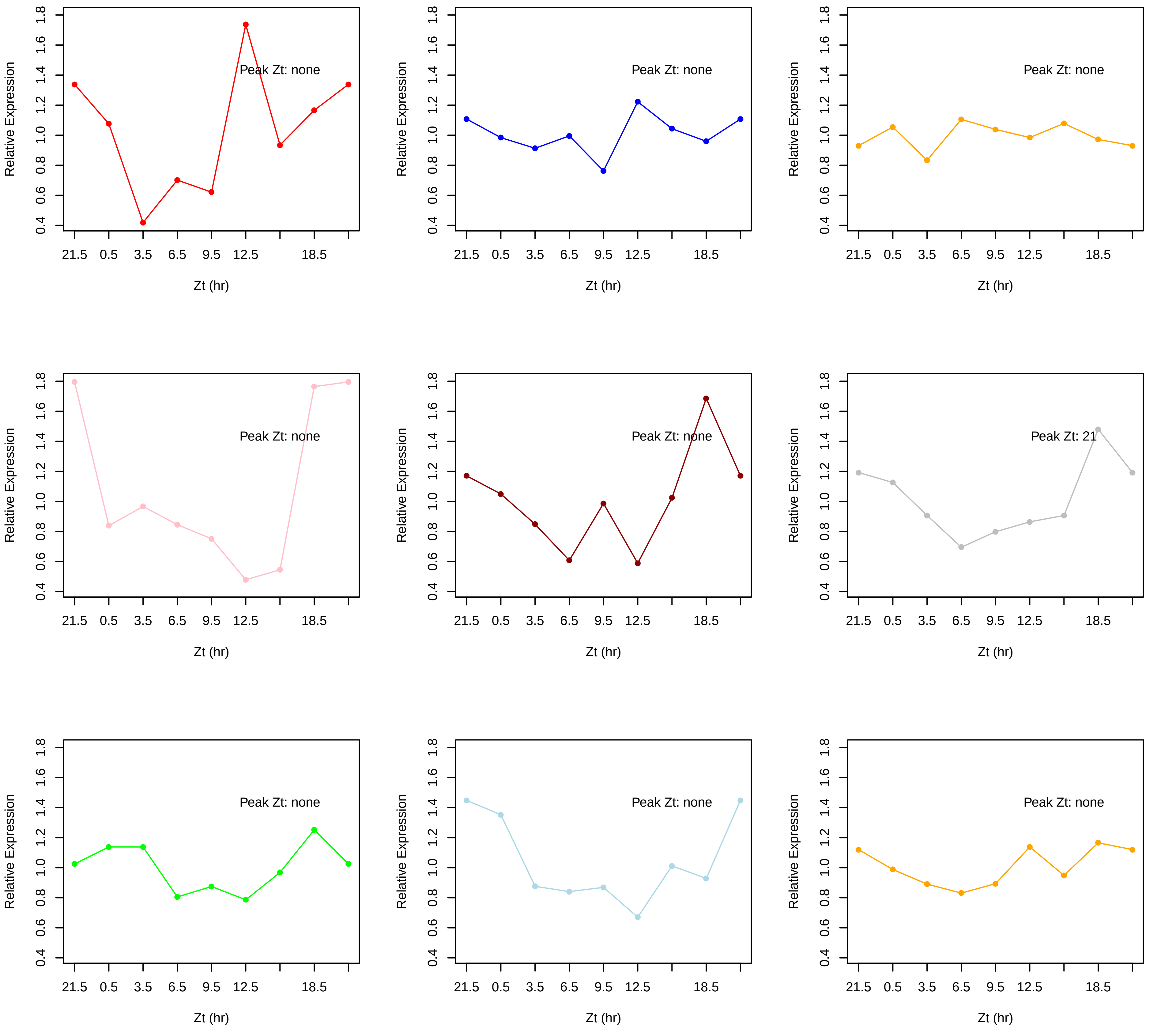

Singleton: ENSXMAG00000001149 tgfb1a

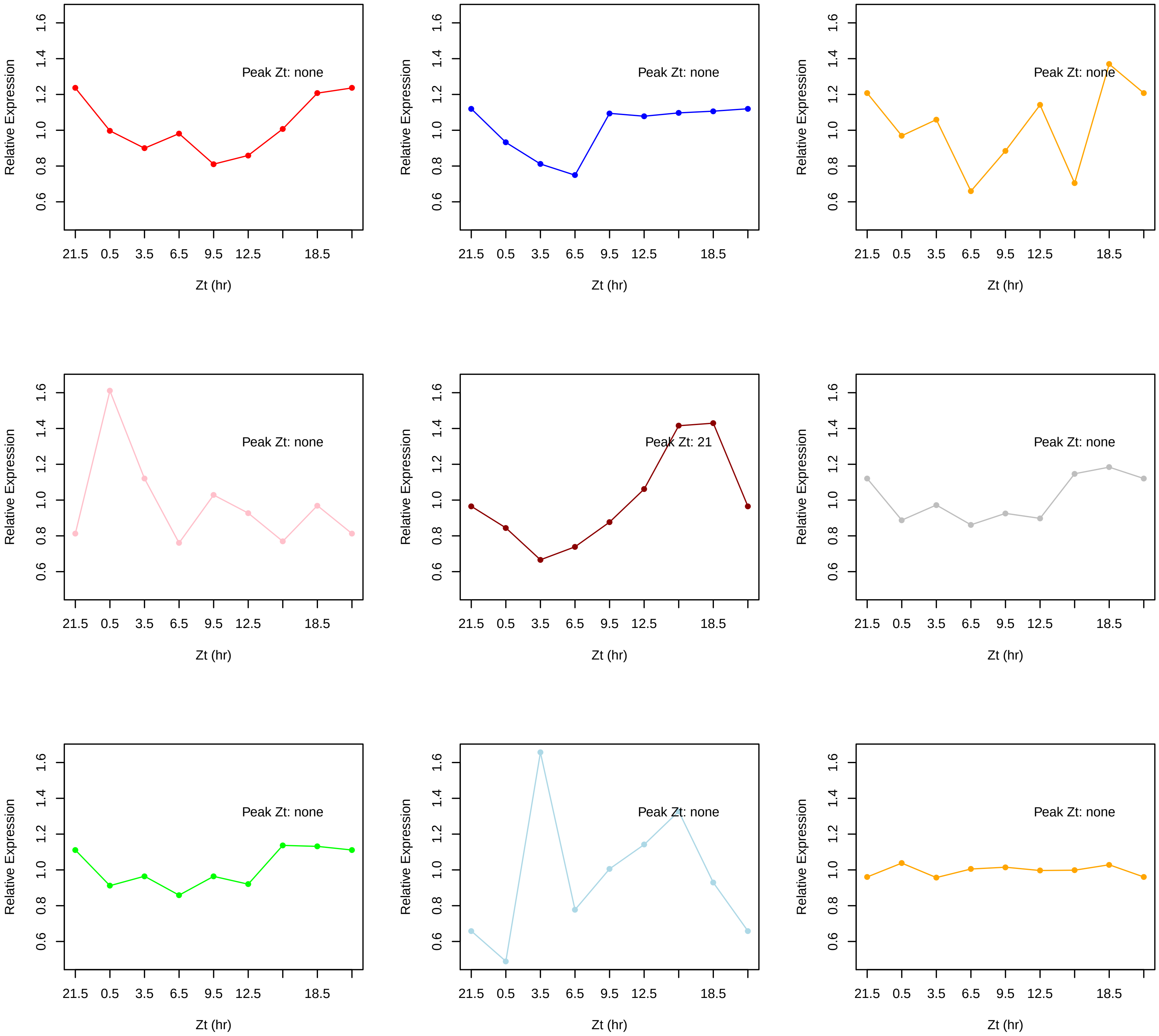

Singeleton: ENSXMAG00000019284 actr8

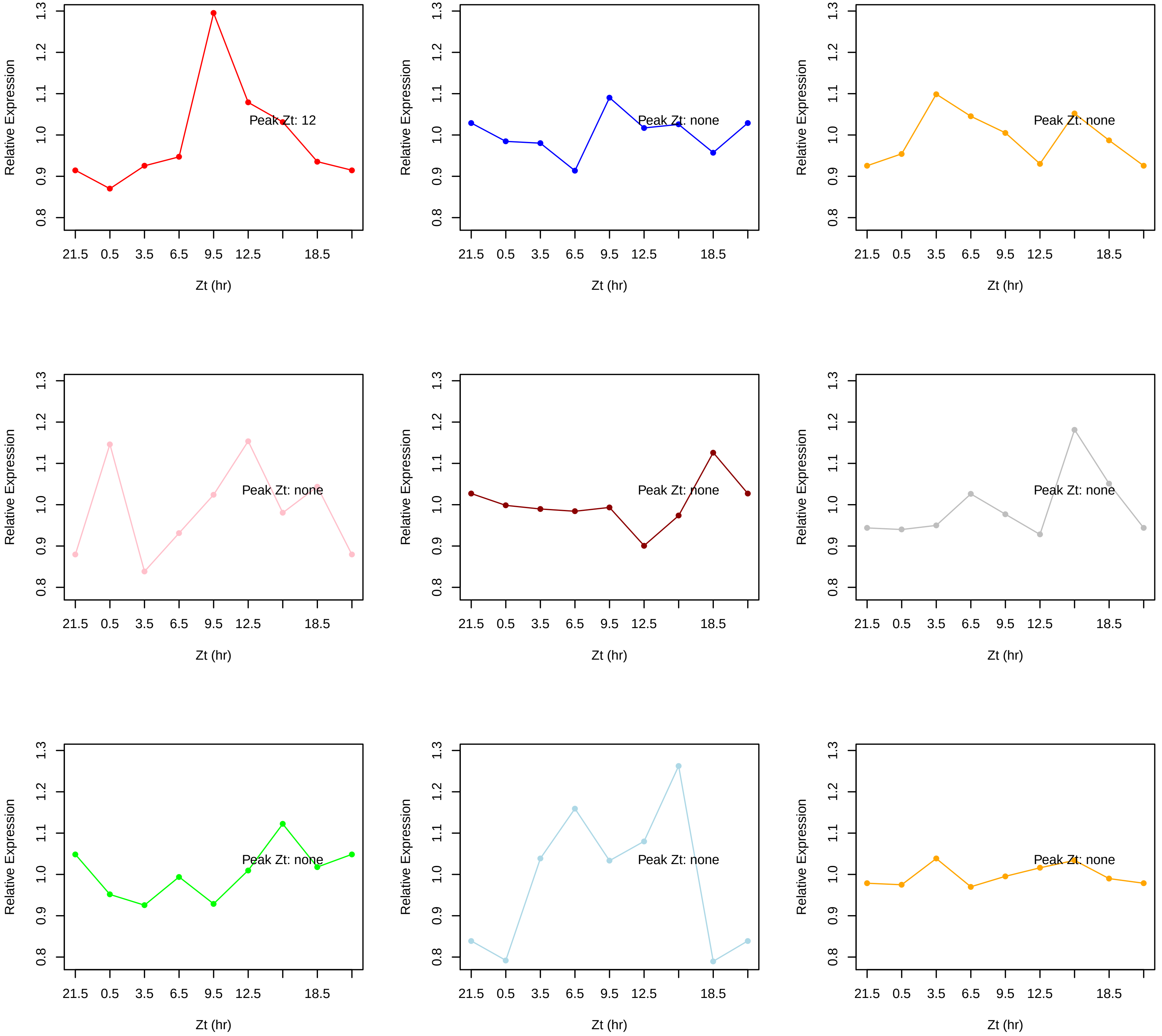

Singeleton: ENSXMAG00000014723 zfyve26

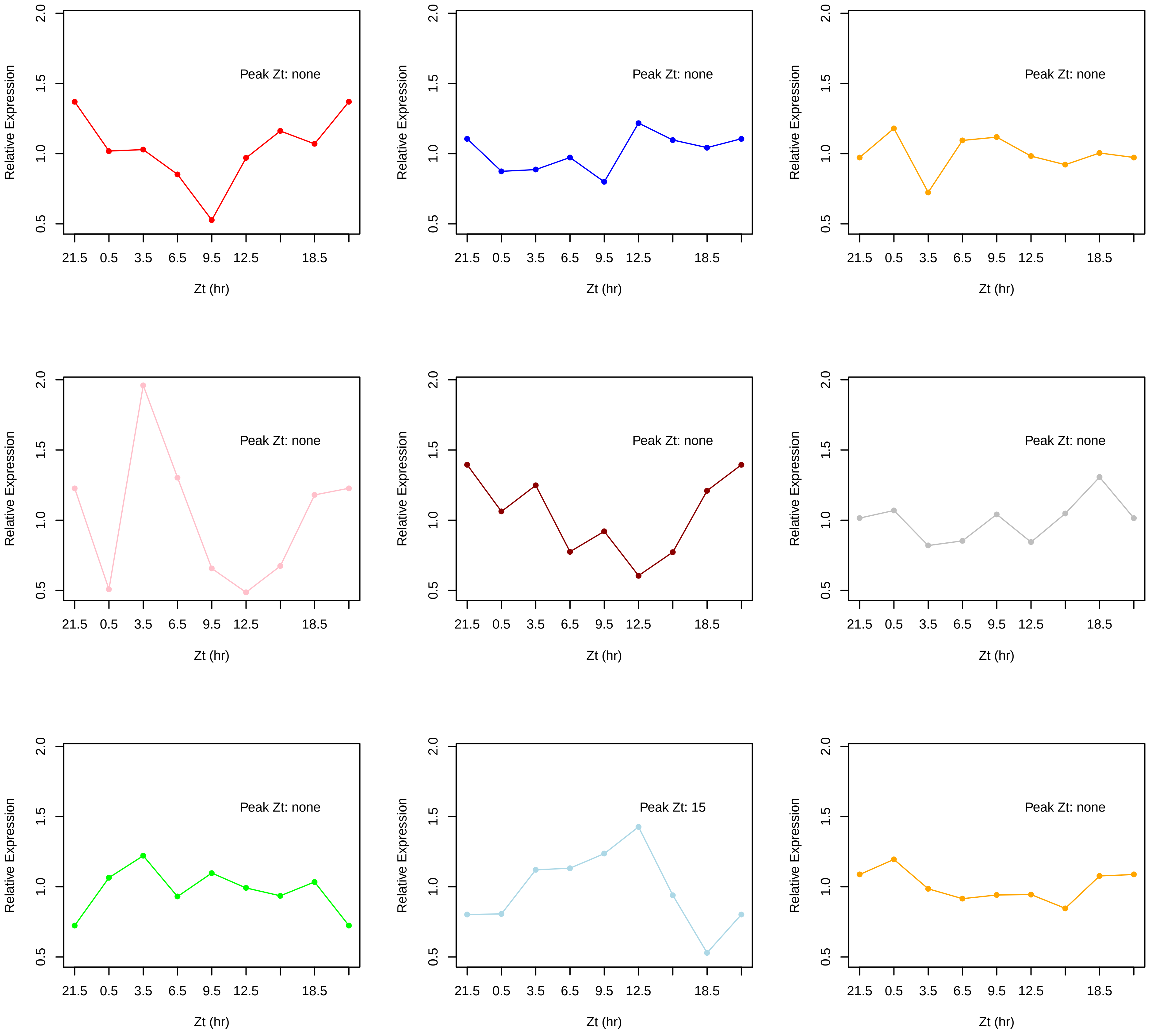

Singeleton: ENSXMAG00000013541 kdr

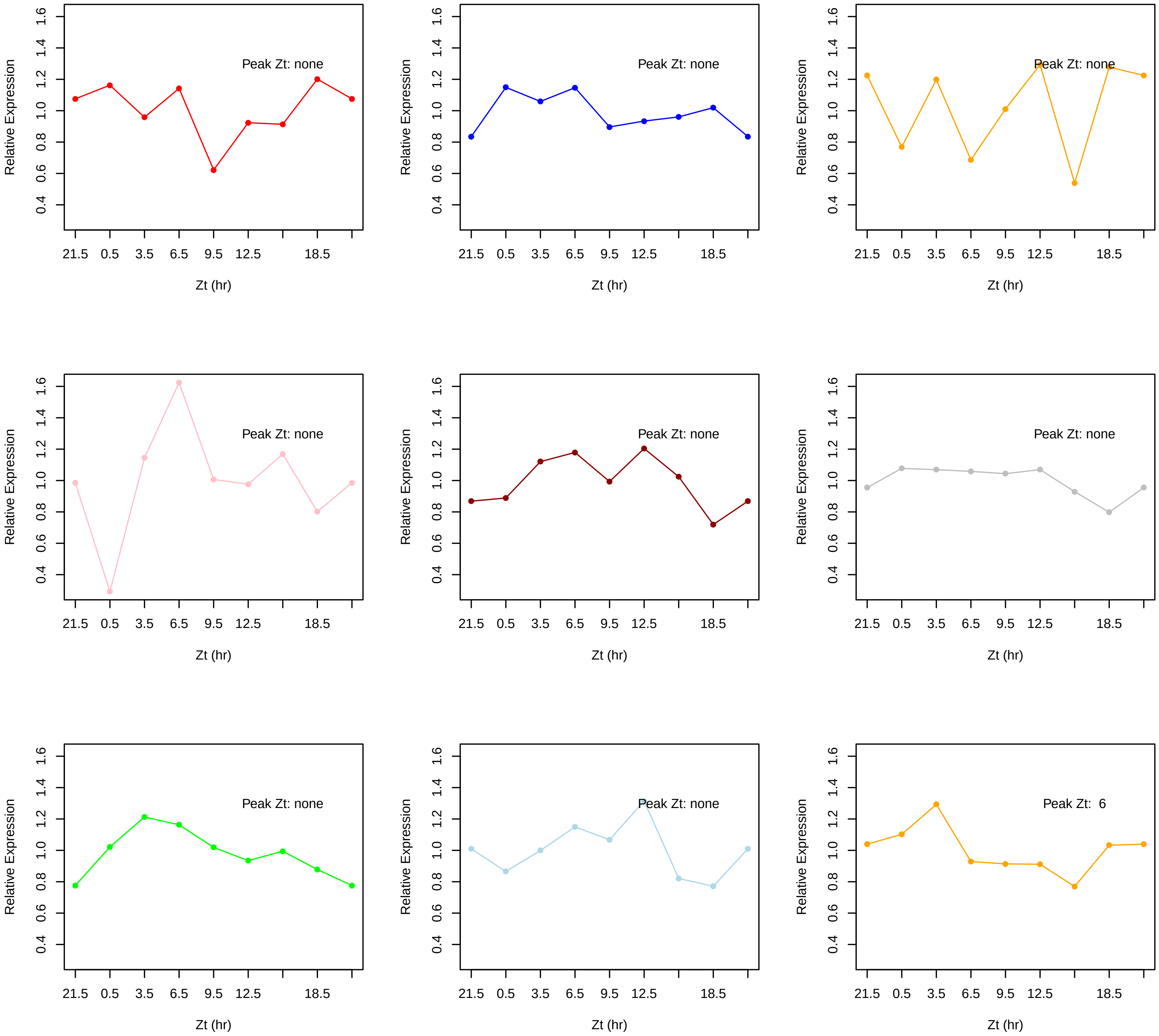

Singeleton: ENSXMAG00000018240 tubgcp4

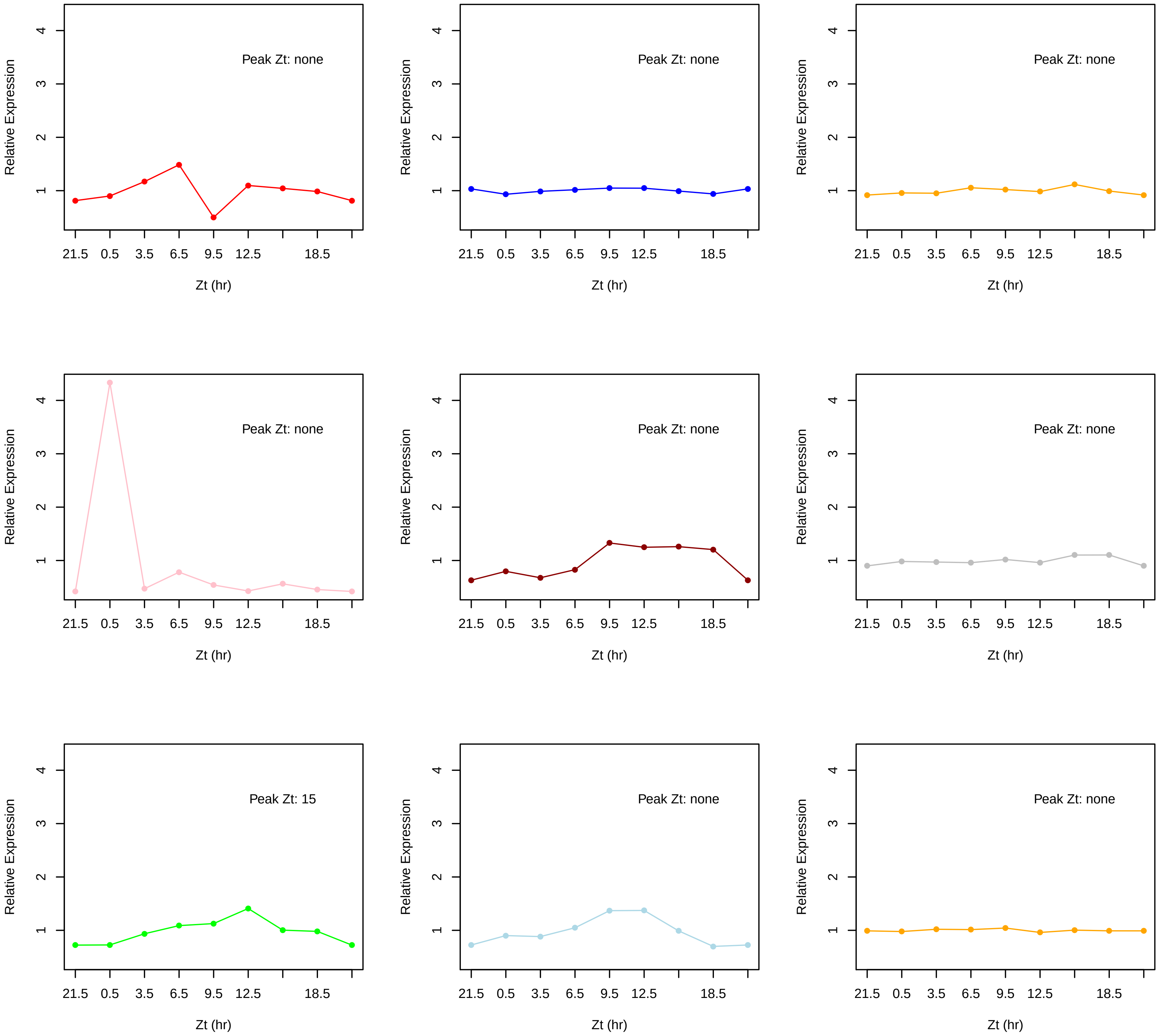

Singleton: ENSXMAG00000020818 rdh12

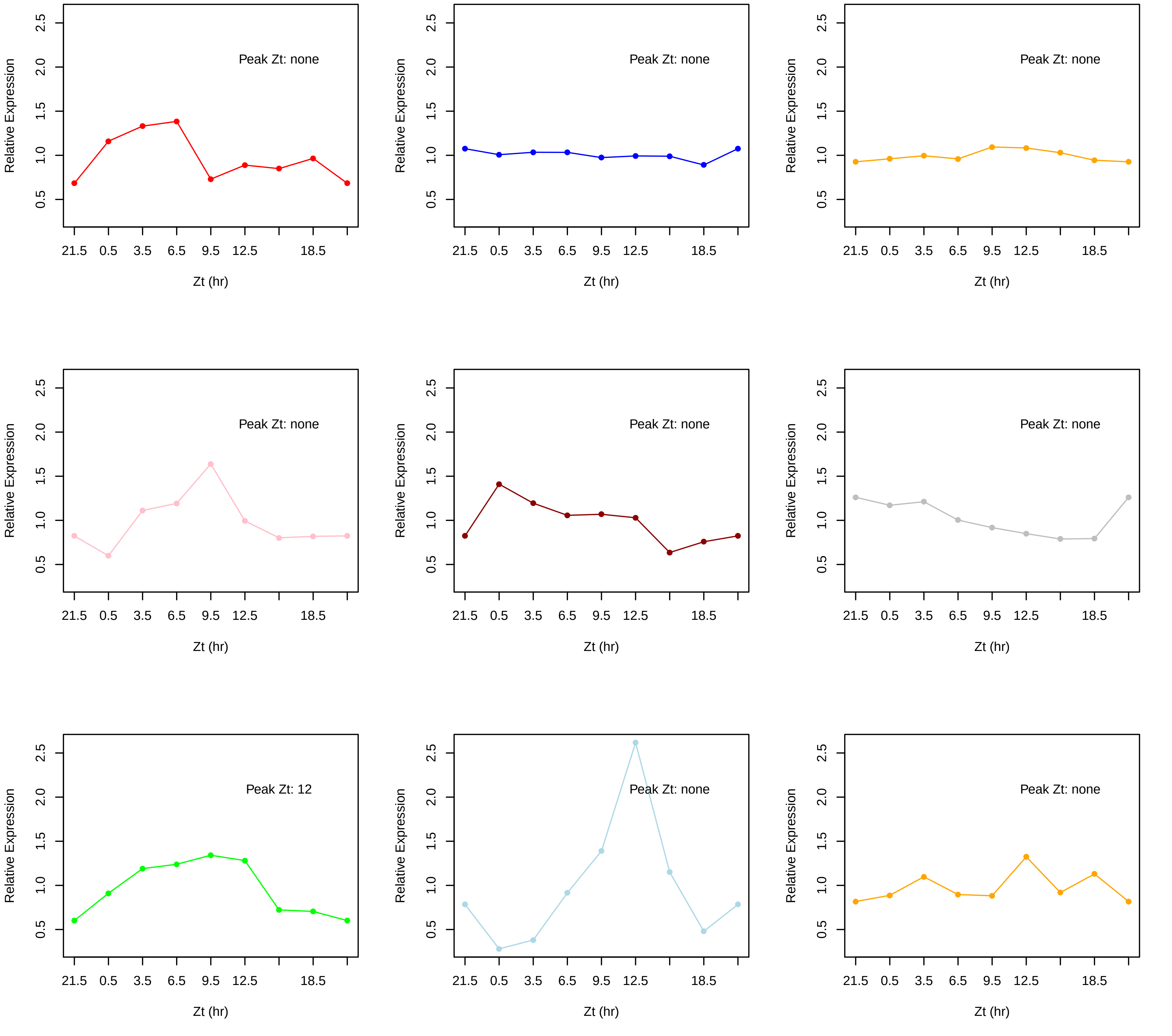

Singeleton: ENSXMAG00000002605

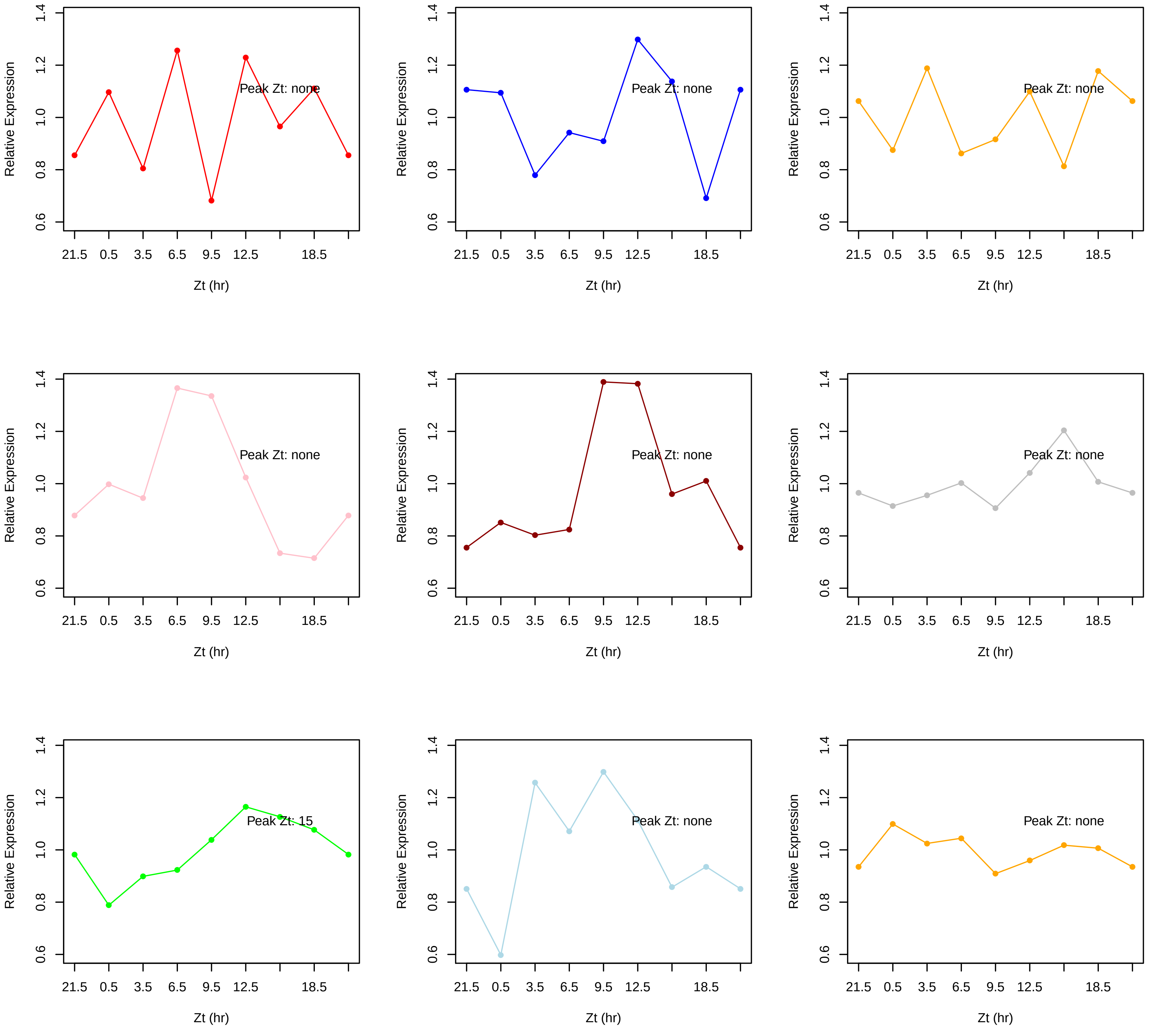

Singeleton: ENSXMAG00000014792 arg2

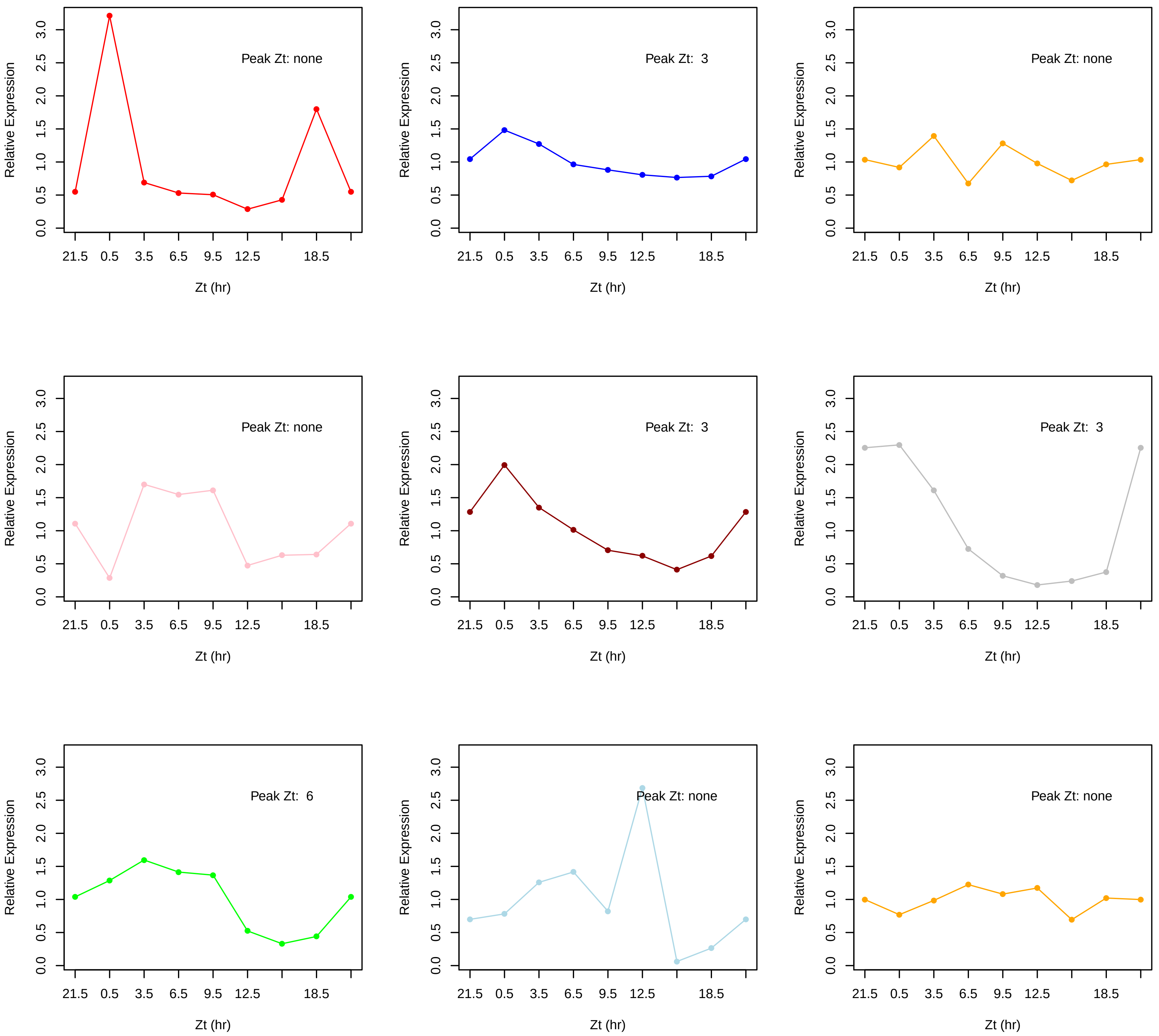

Singleton: ENSXMAG00000029157 lrtm1

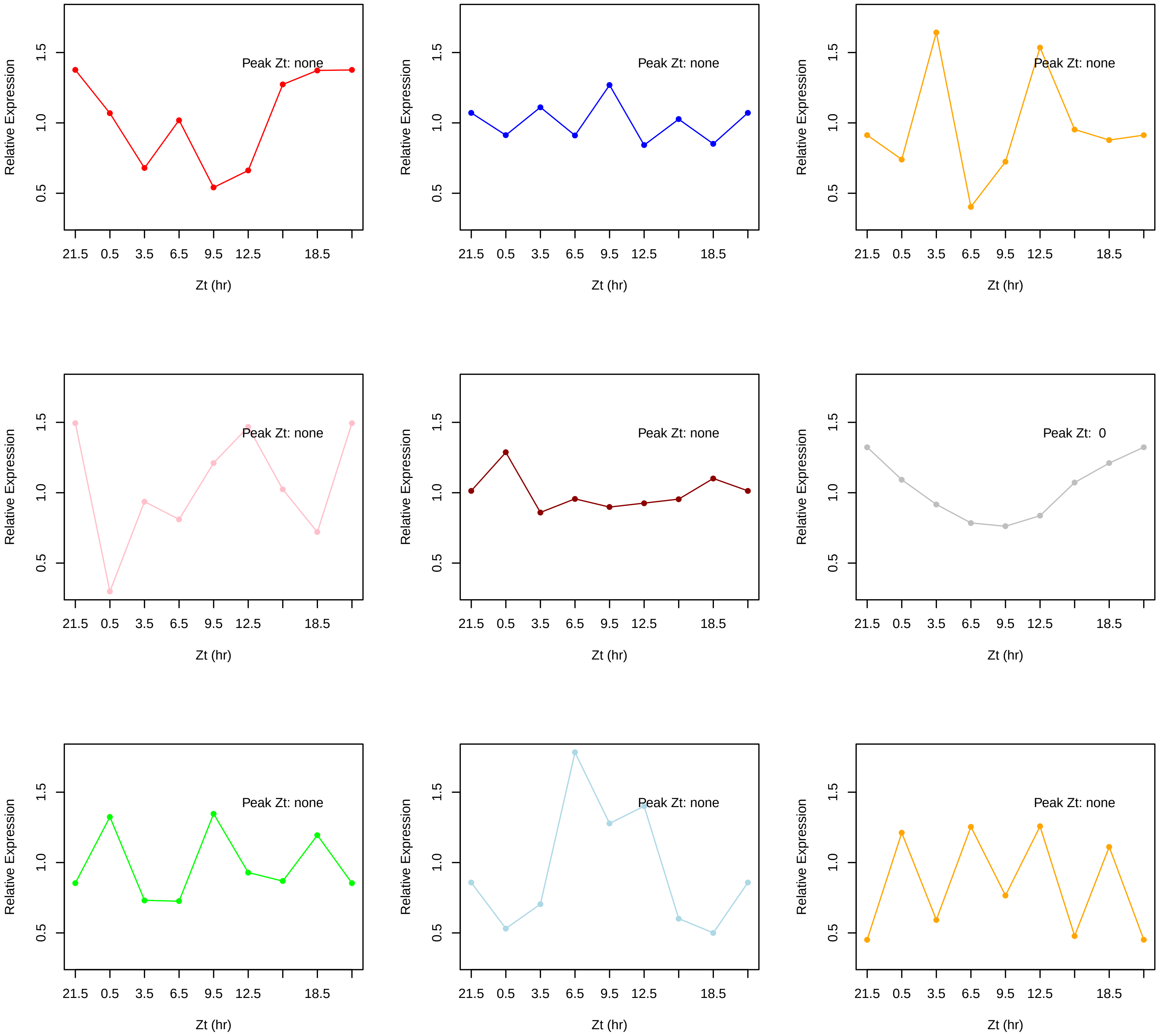

Singleton: ENSXMAG00000002613 sars2

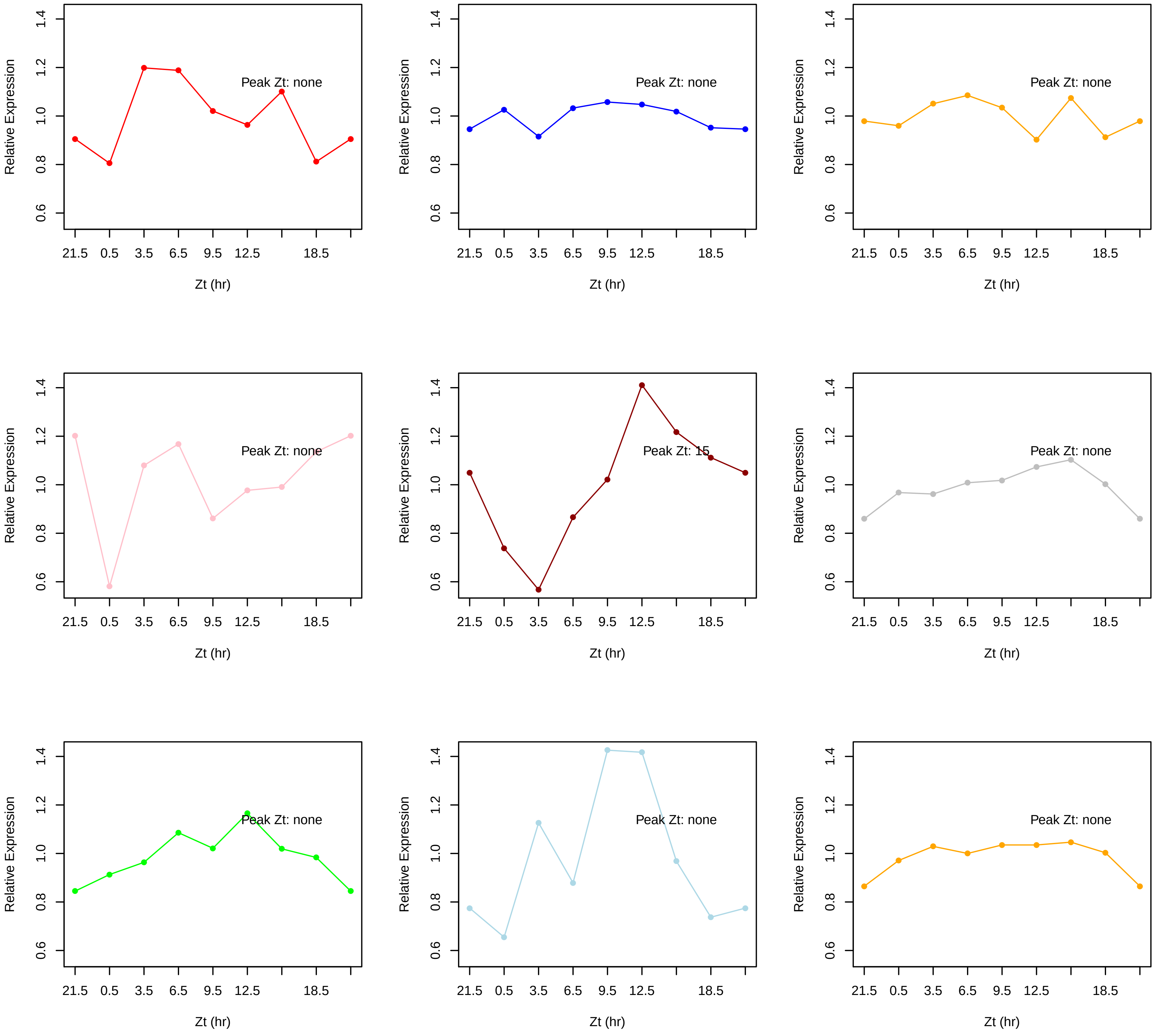

Singeleton: ENSXMAG00000018260 tp53bp1

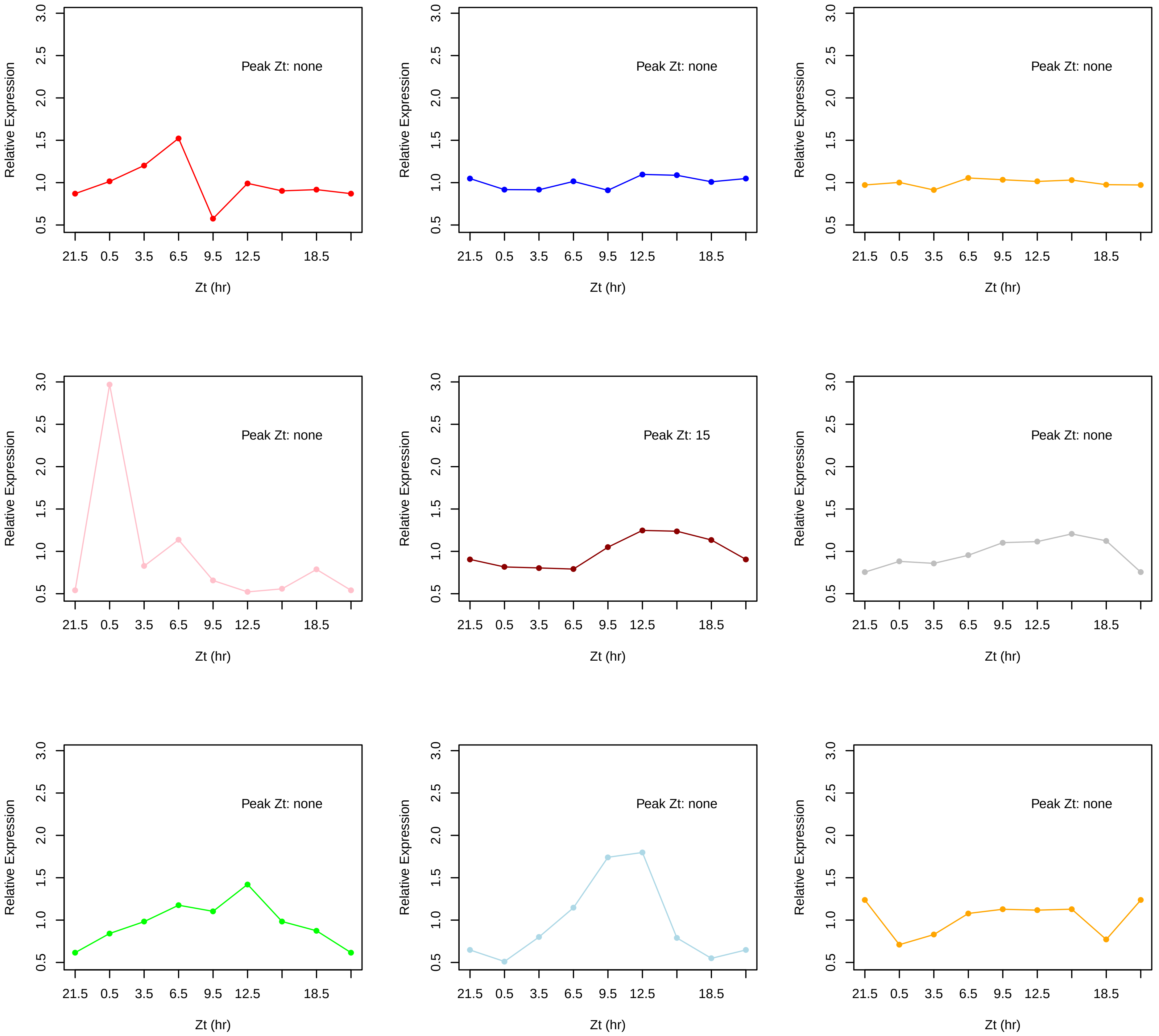

Singeleton: ENSXMAG00000007685 psmd8

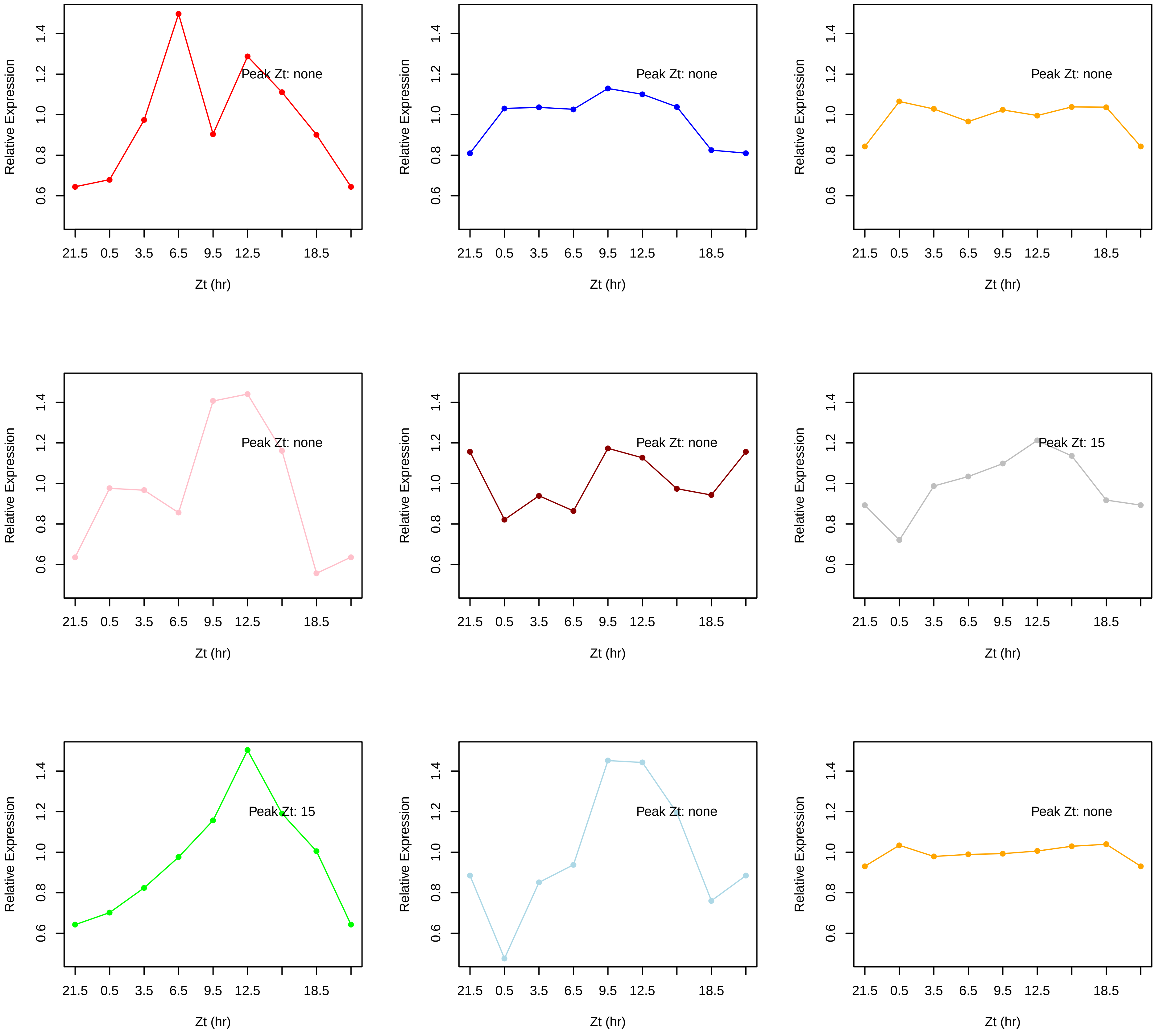

Singeleton: ENSXMAG00000022774 slc39a9

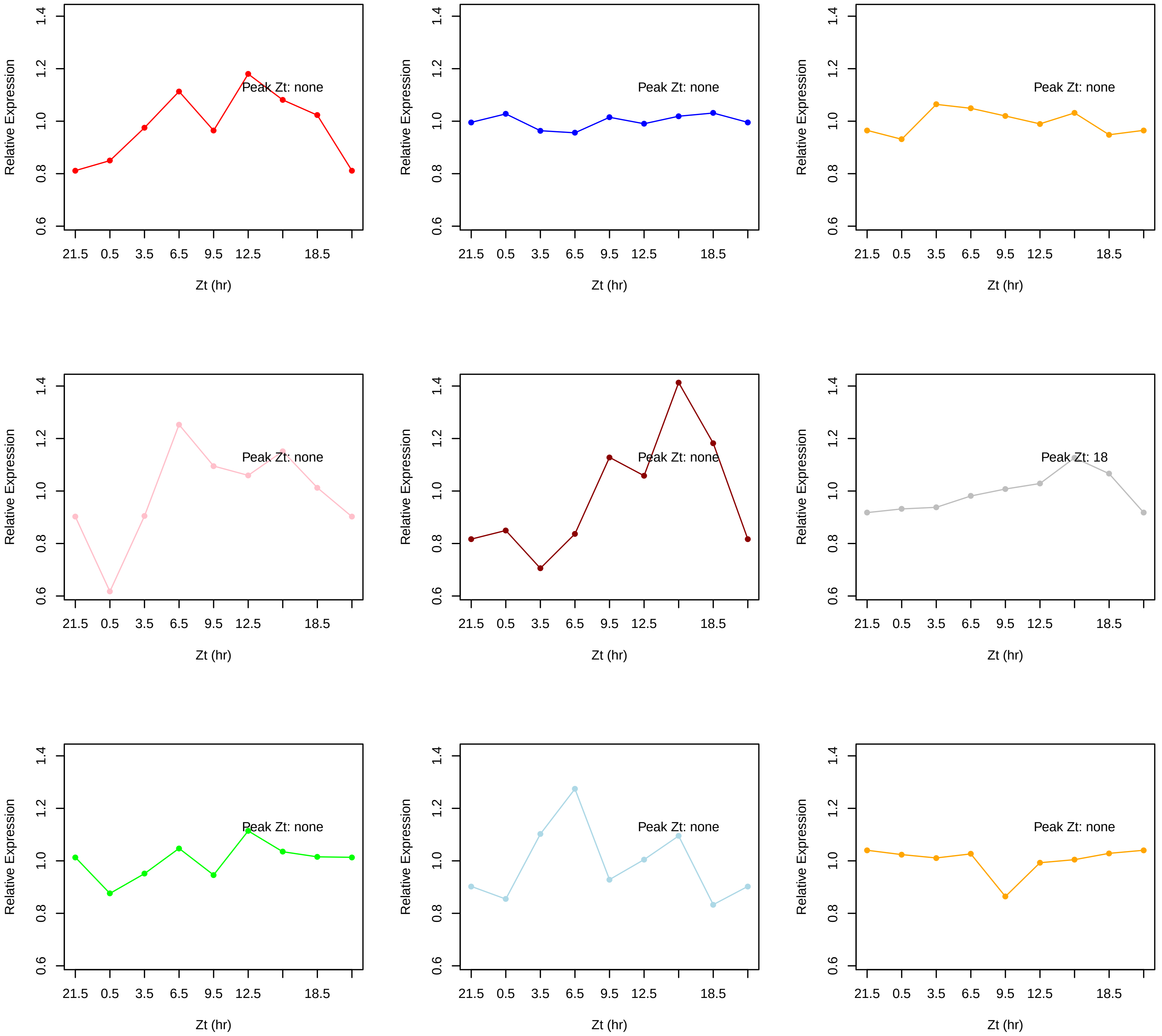

Singeleton: ENSXMAG00000009118 plekhd1

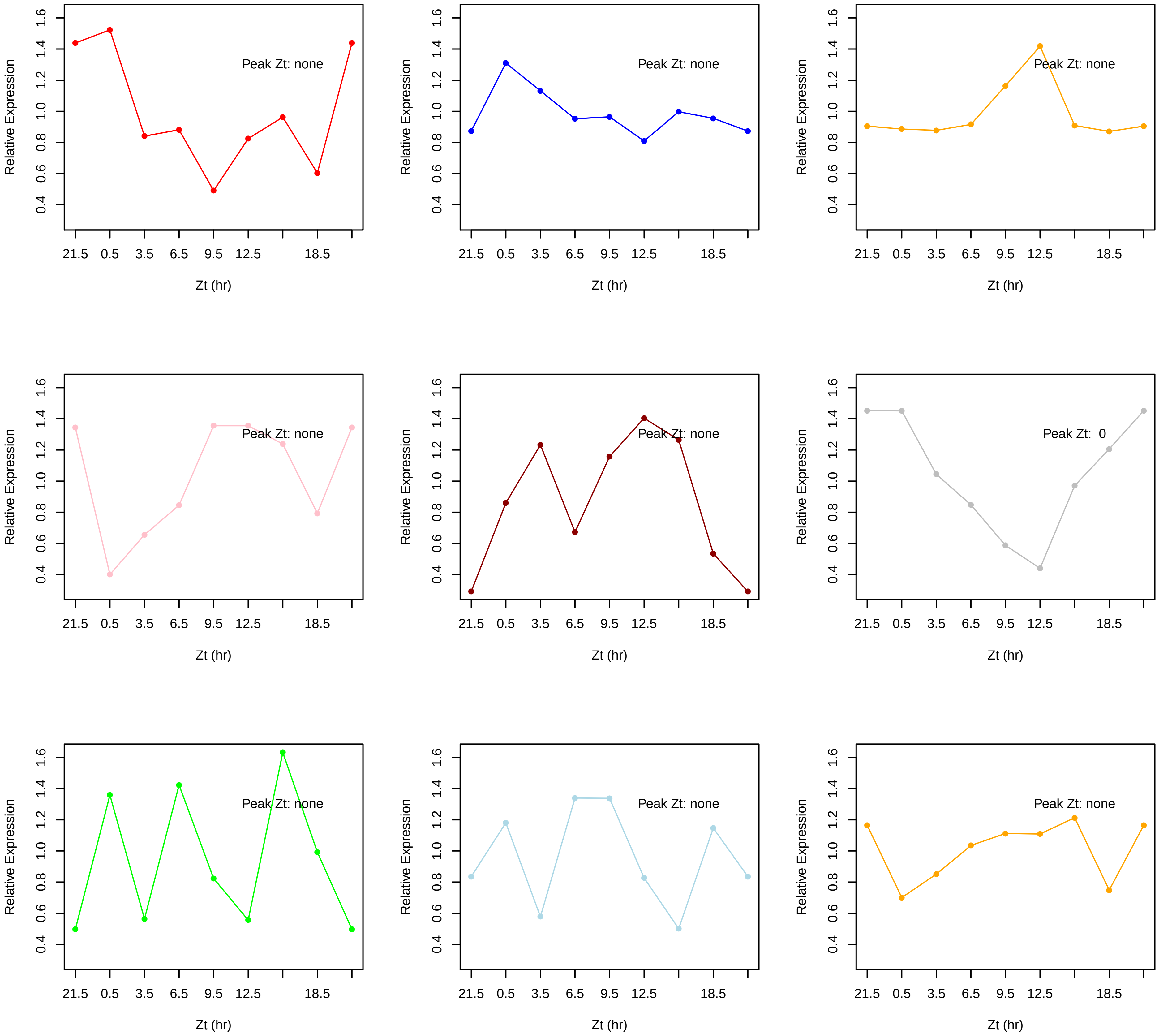

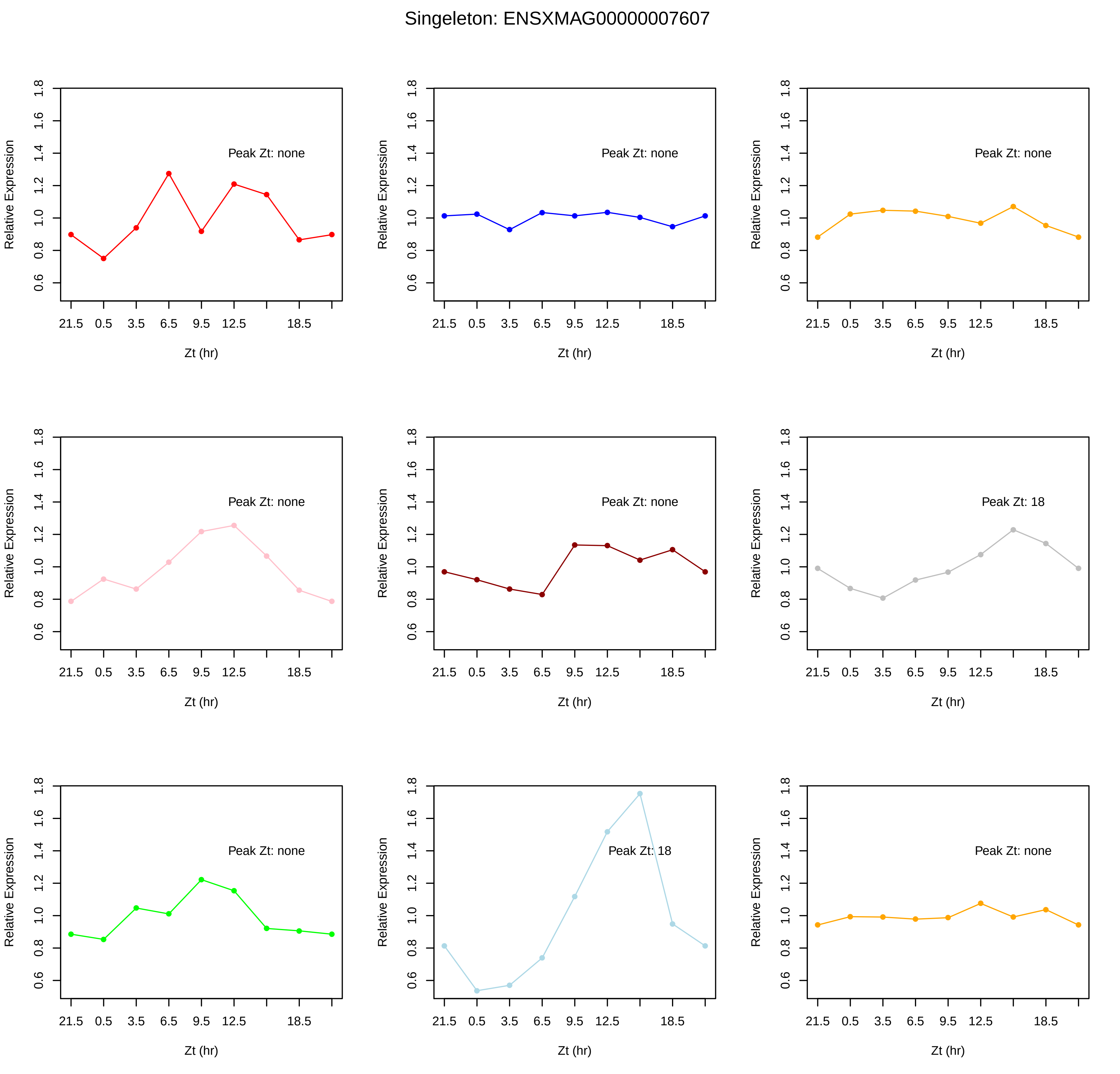

Singleton: ENSXMAG00000007607
Peak Zt: none
Peak Zt: none
Peak Zt: none
Peak Zt: none
Peak Zt: none
Peak Zt: 18
Peak Zt: none
Peak Zt: 18
Peak Zt: none
Relative Expression
Zt (hr)
21.5
0.5
3.5
6.5
9.5
12.5
18.5
0.6
0.8
1.0
1.2
1.4
1.6
1.8

Singleton: ENSXMAG00000007662 napab

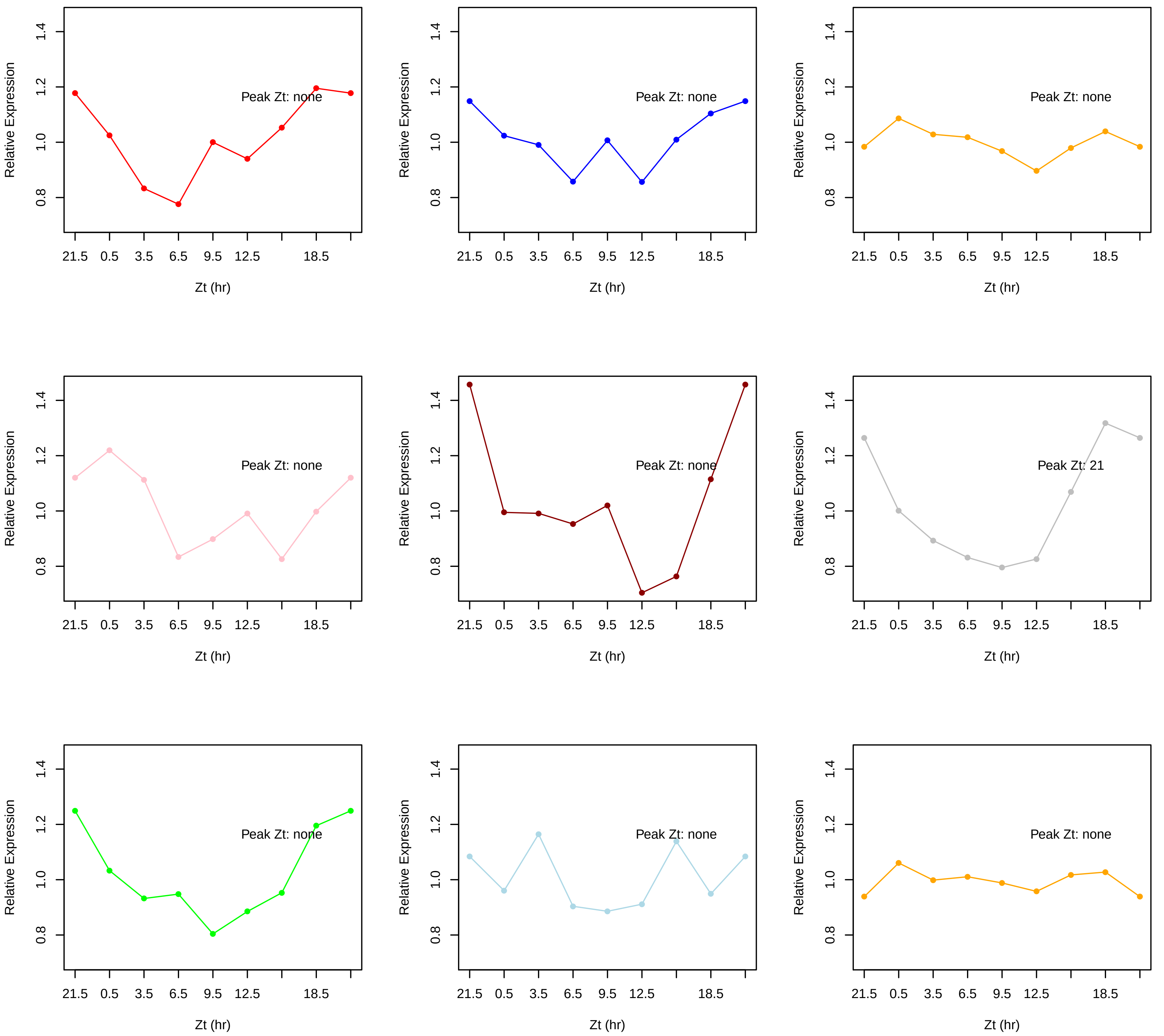

Singleton: ENSXMAG00000009131 smoc1

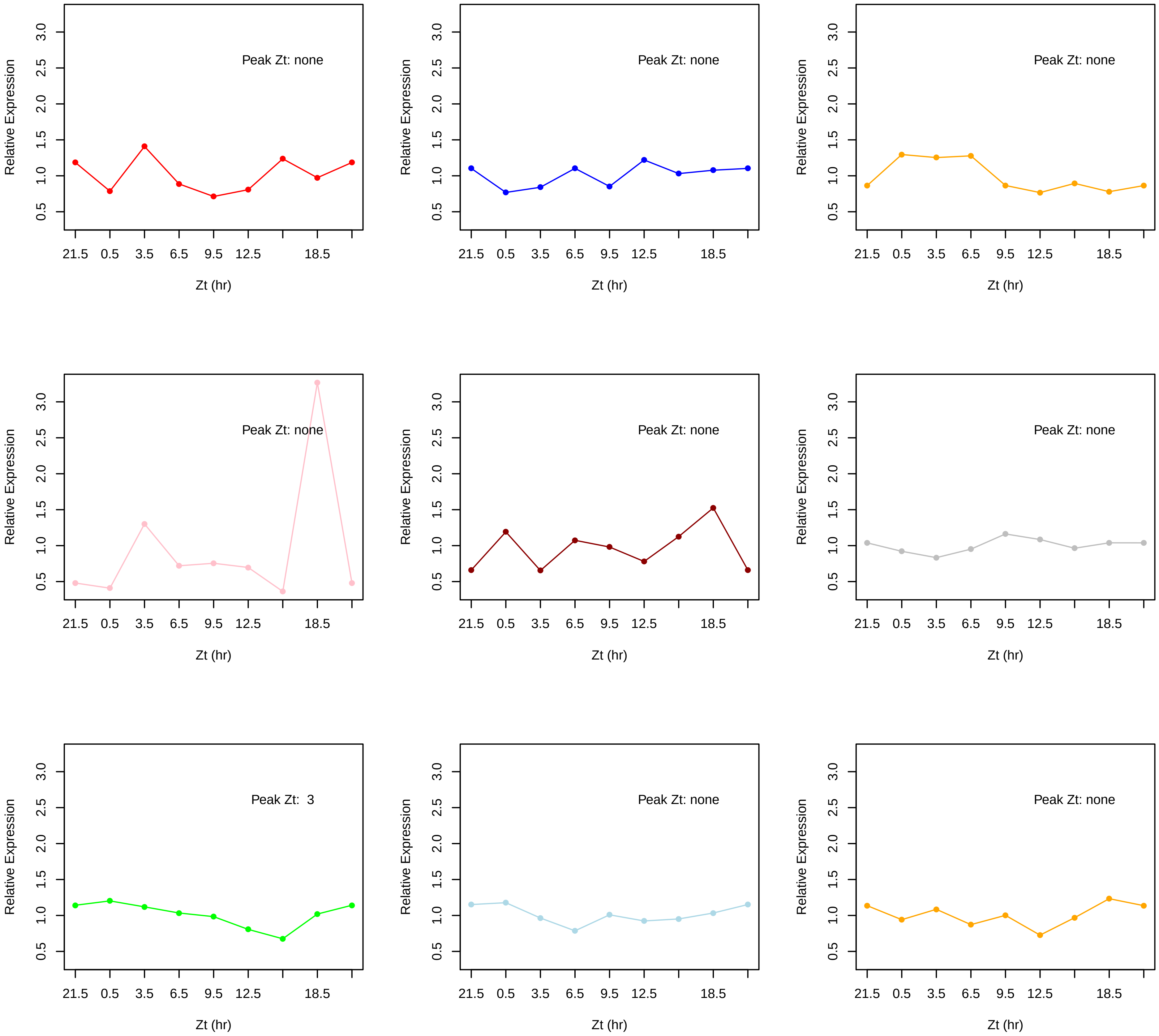

Singeleton: ENSXMAG00000007649 bckdha

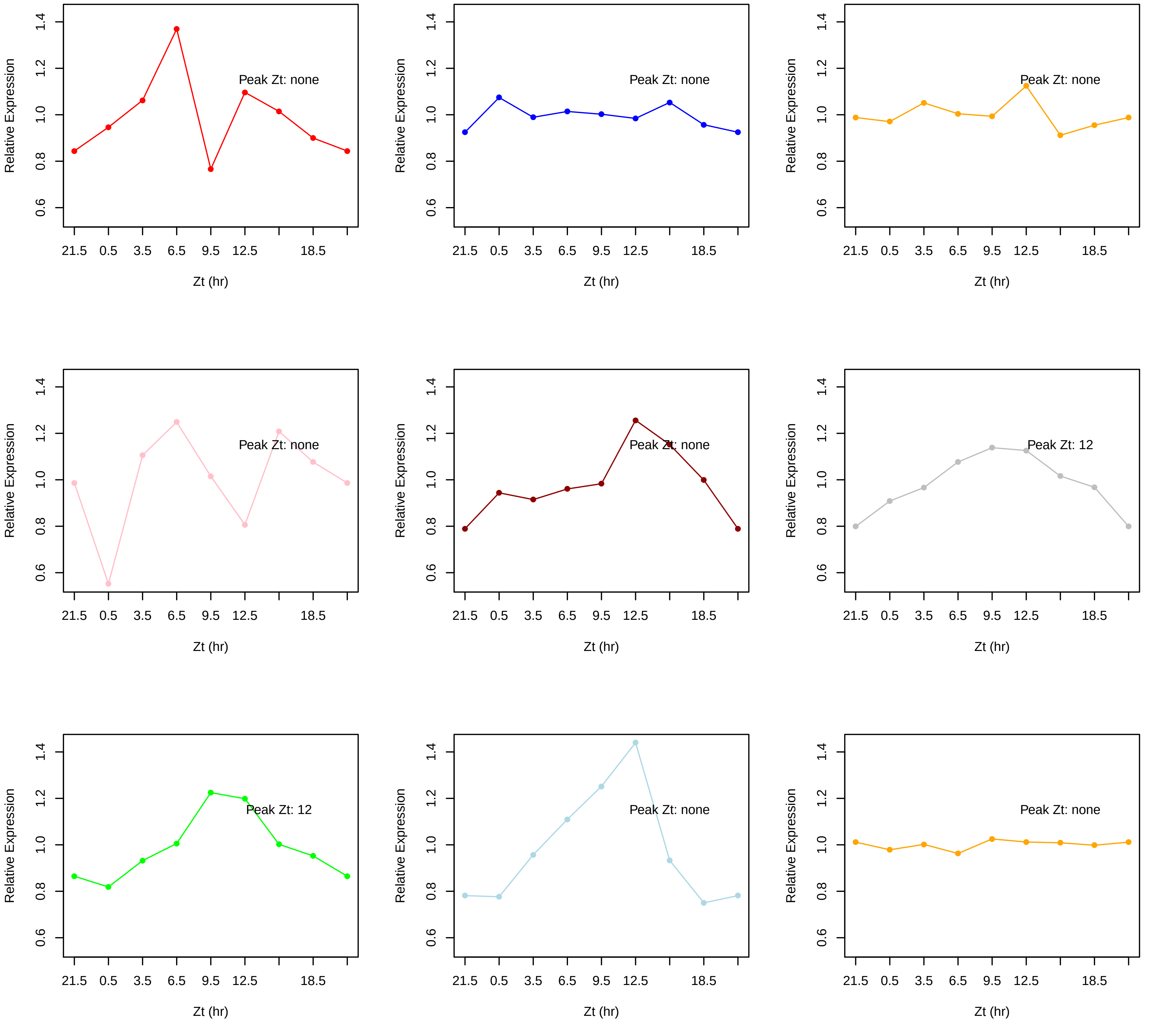

Singeleton: ENSXMAG00000004786 exosc5

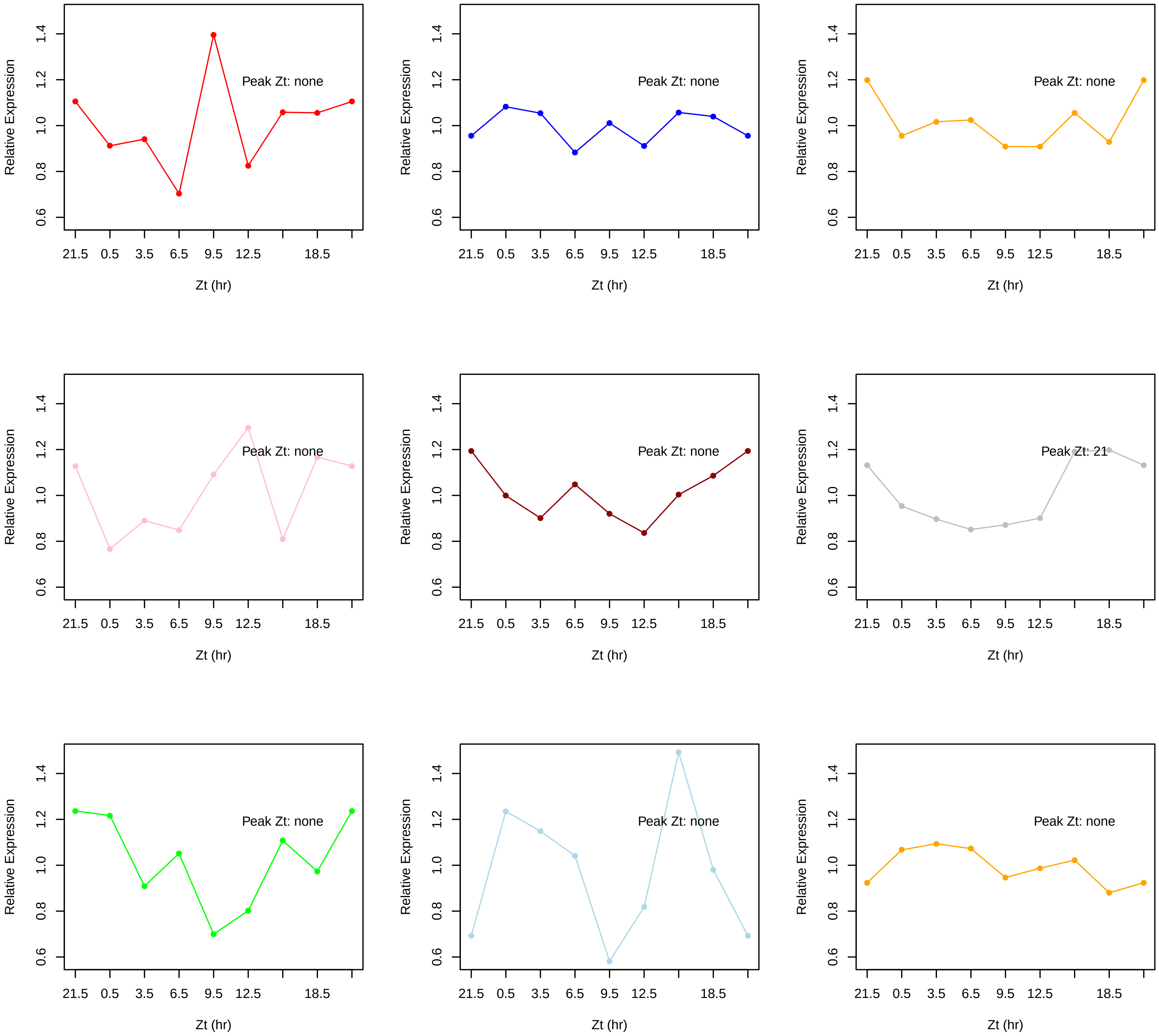

Singeleton: ENSXMAG00000005737 sympk

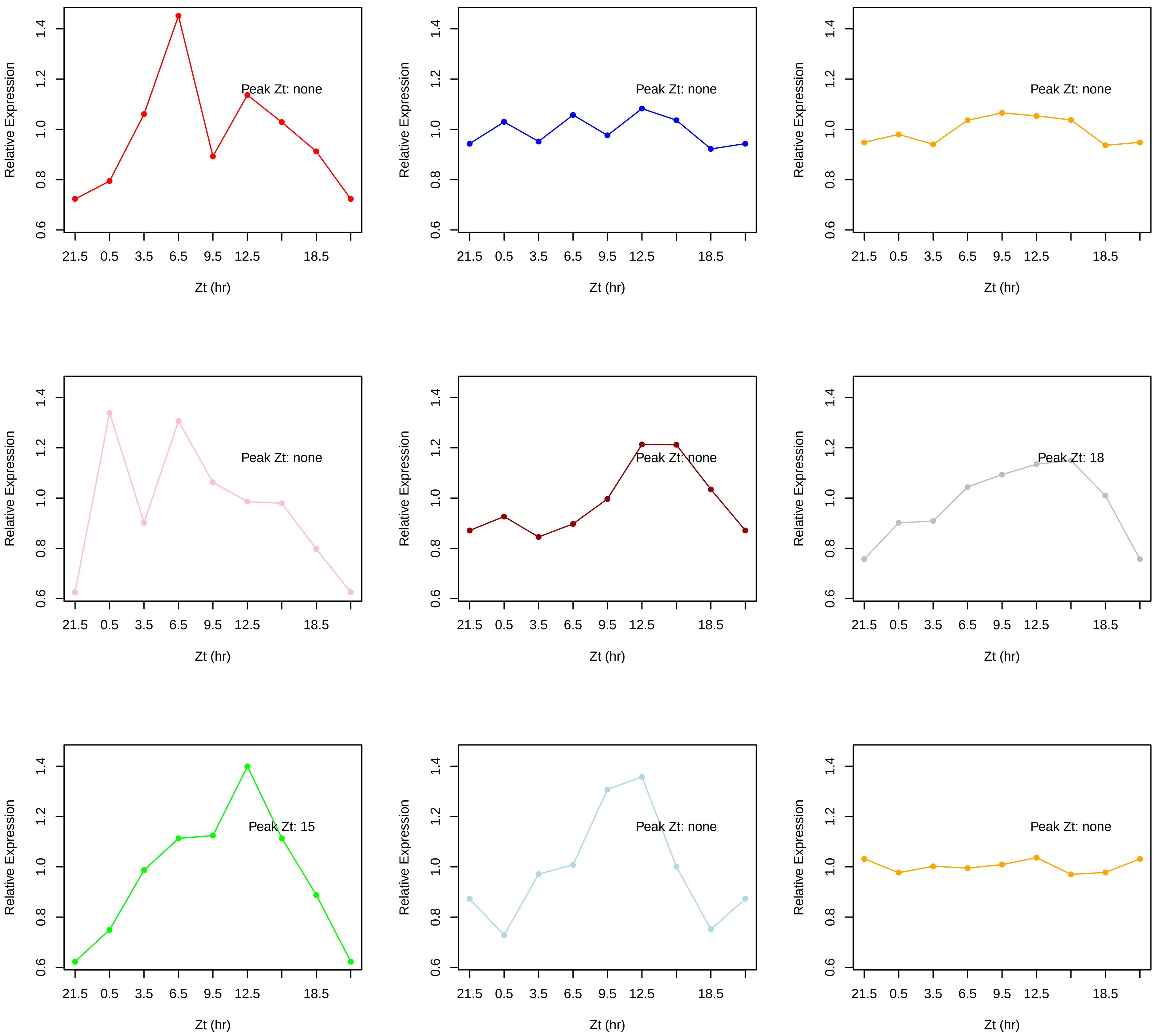

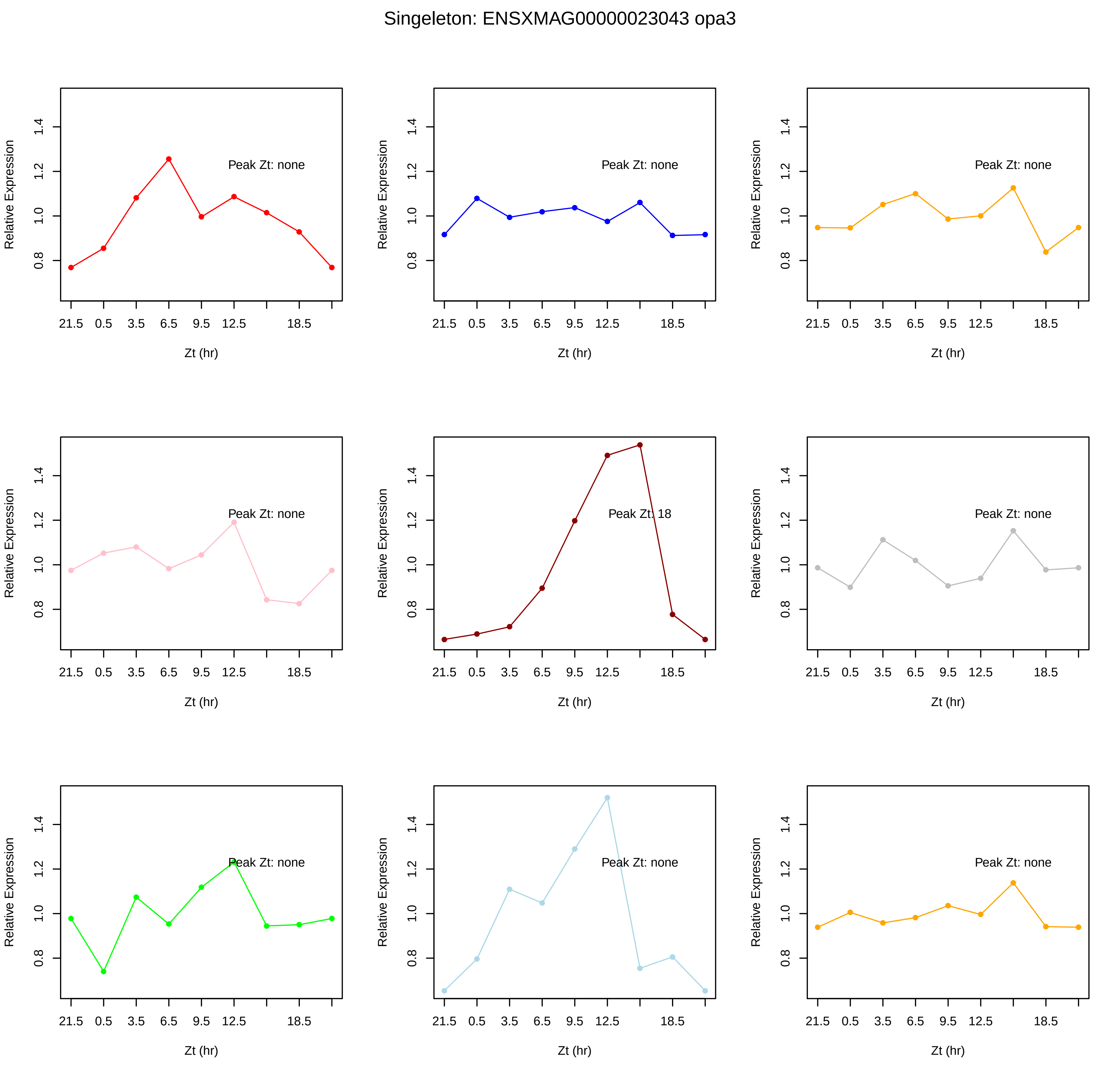
Singeleton: ENSXMAG00000023043 opa3
Peak Zt: none
Peak Zt: none
Peak Zt: none
Peak Zt: none
Peak Zt: 18
Peak Zt: none
Peak Zt: none
Peak Zt: none
Peak Zt: none
Relative Expression
Zt (hr)
21.5
0.5
3.5
6.5
9.5
12.5
18.5
0.8
1.0
1.2
1.4

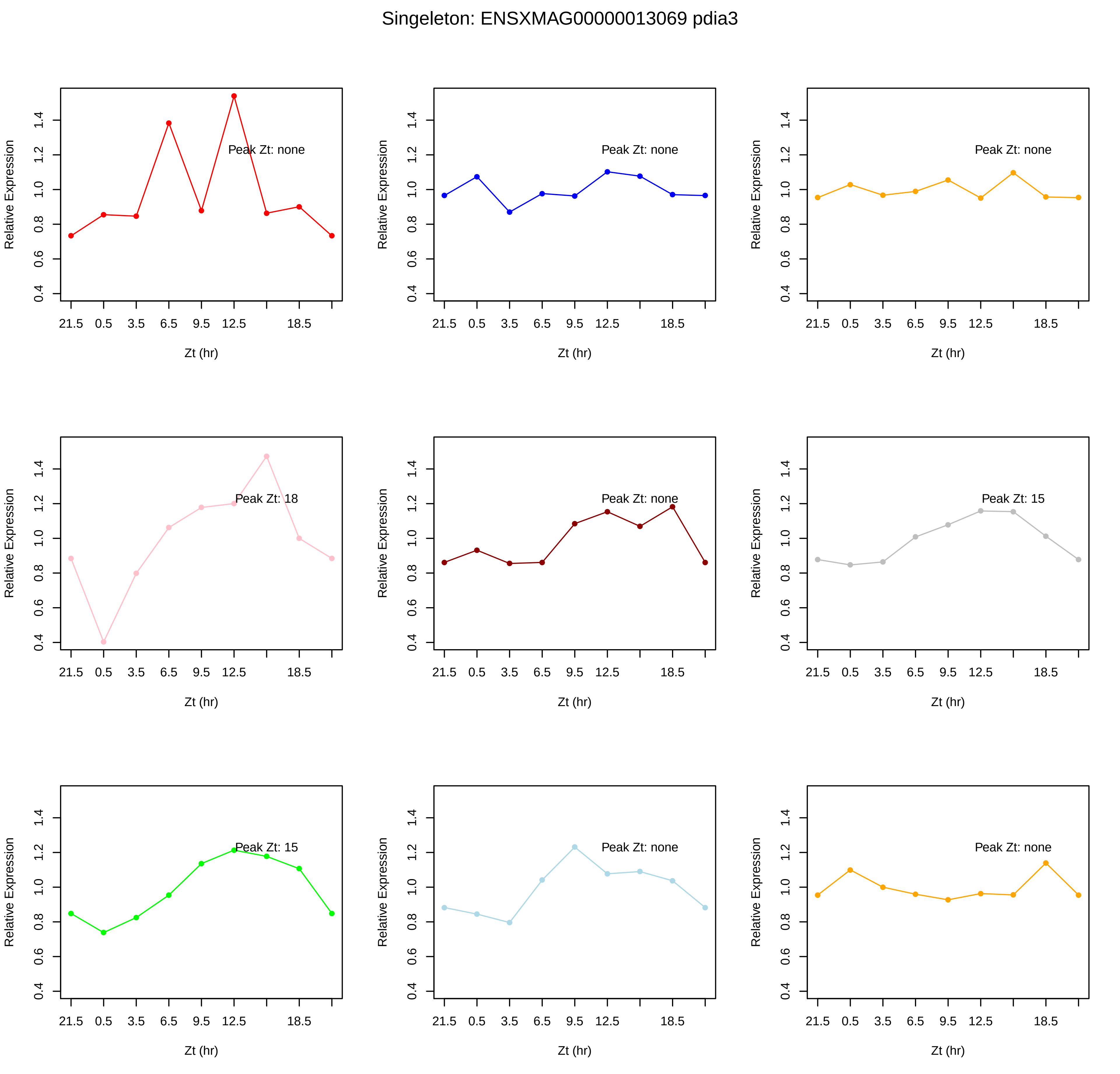

Singleton: ENSXMAG00000013069 pdia3
Peak Zt: none
Peak Zt: none
Peak Zt: none
Peak Zt: 18
Peak Zt: none
Peak Zt: 15
Peak Zt: 15
Peak Zt: none
Peak Zt: none
Relative Expression
Zt (hr)

Singeleton: ENSXMAG00000017241 acy1

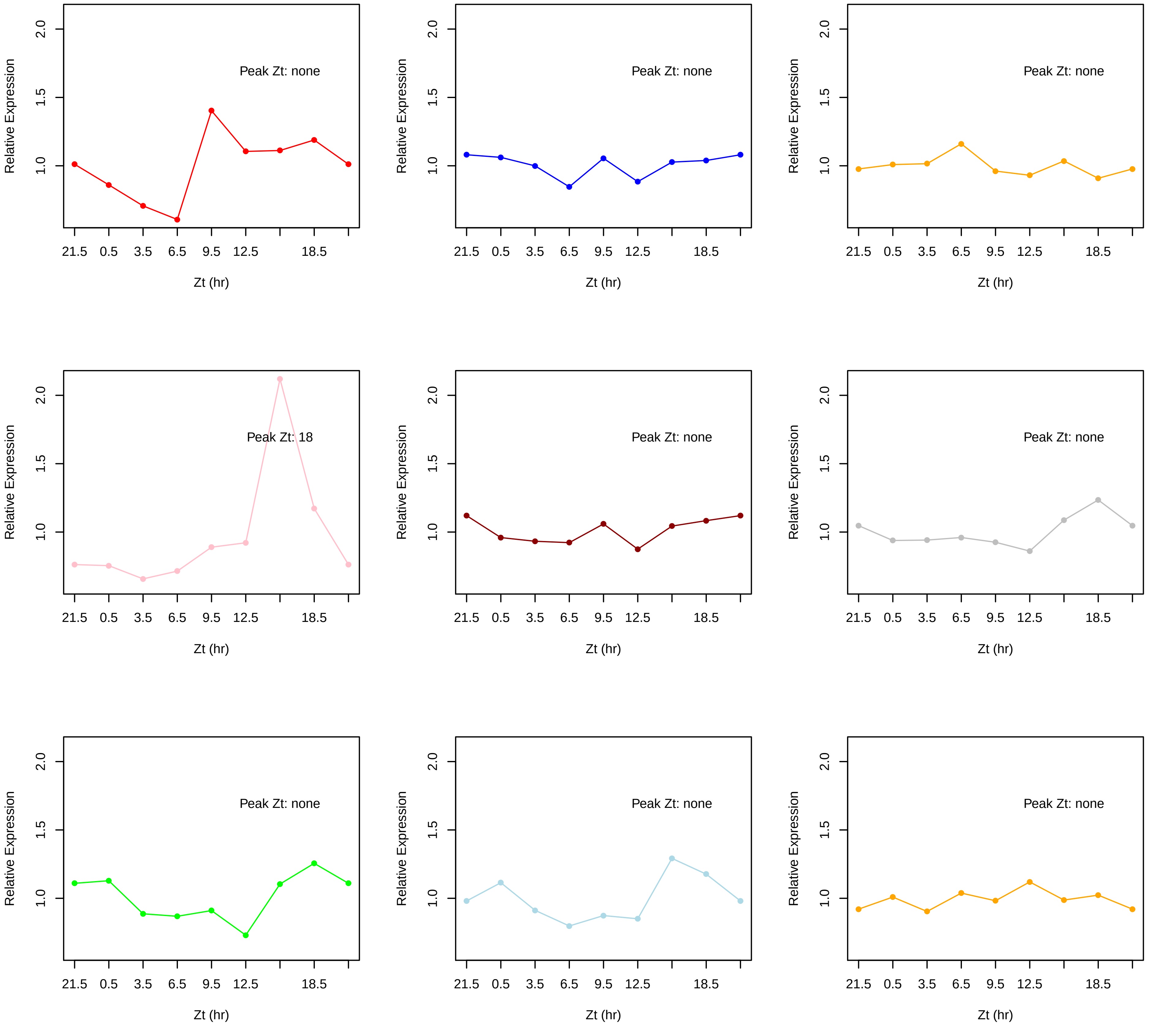

Singleton: ENSXMAG00000007297 snx33

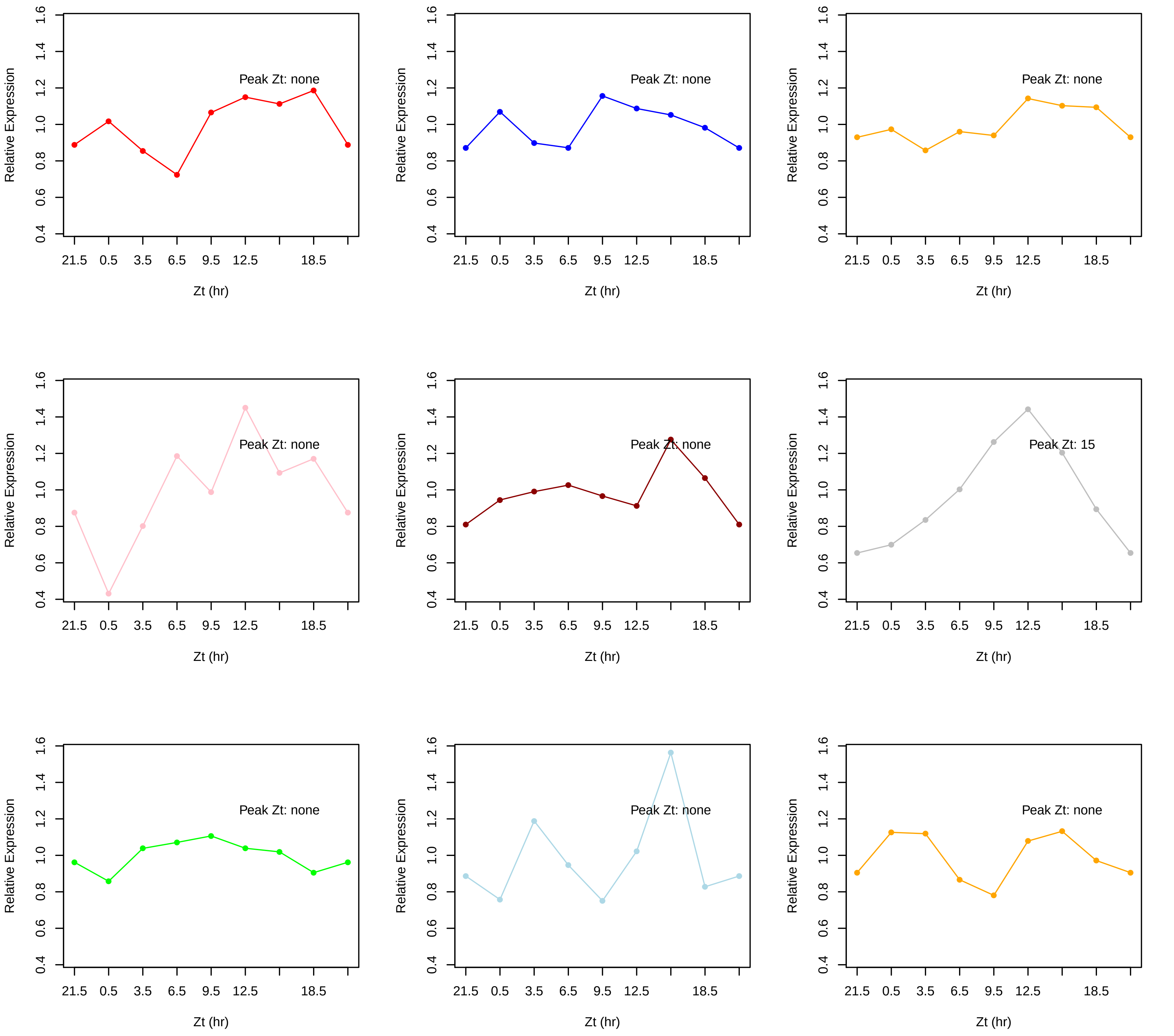

Singleton: ENSXMAG00000029983

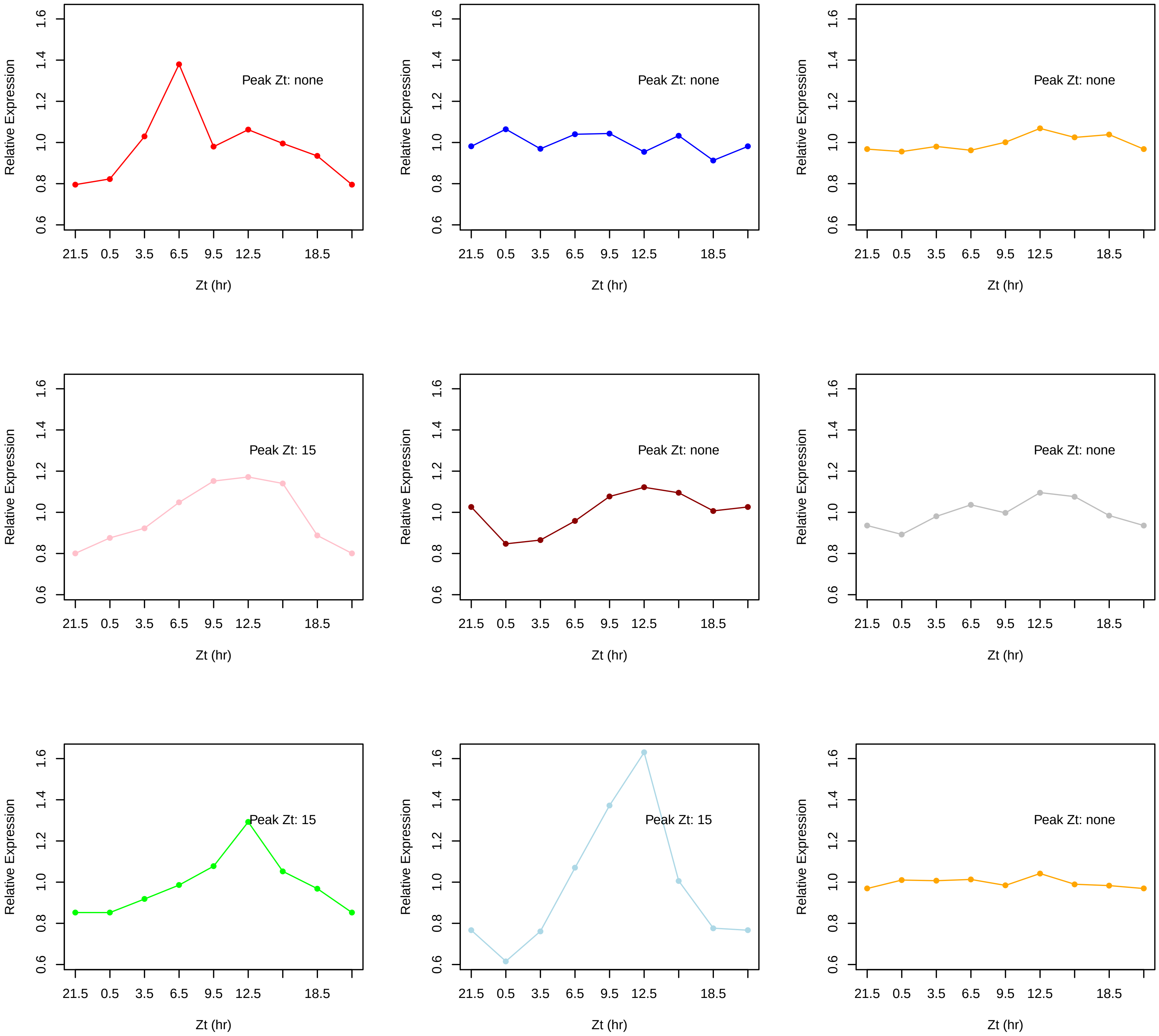

Singleton: ENSXMAG00000025736 peak1

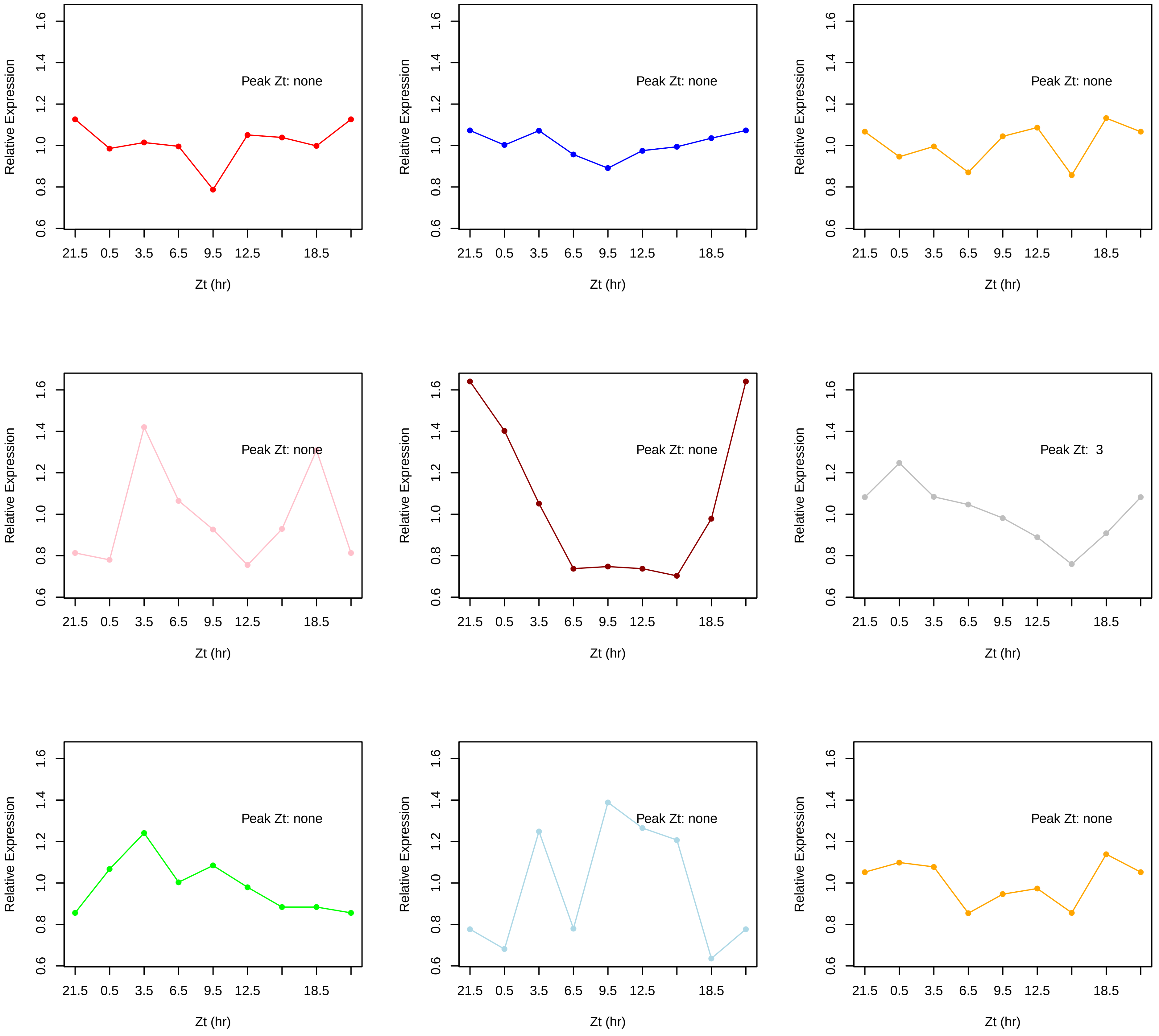

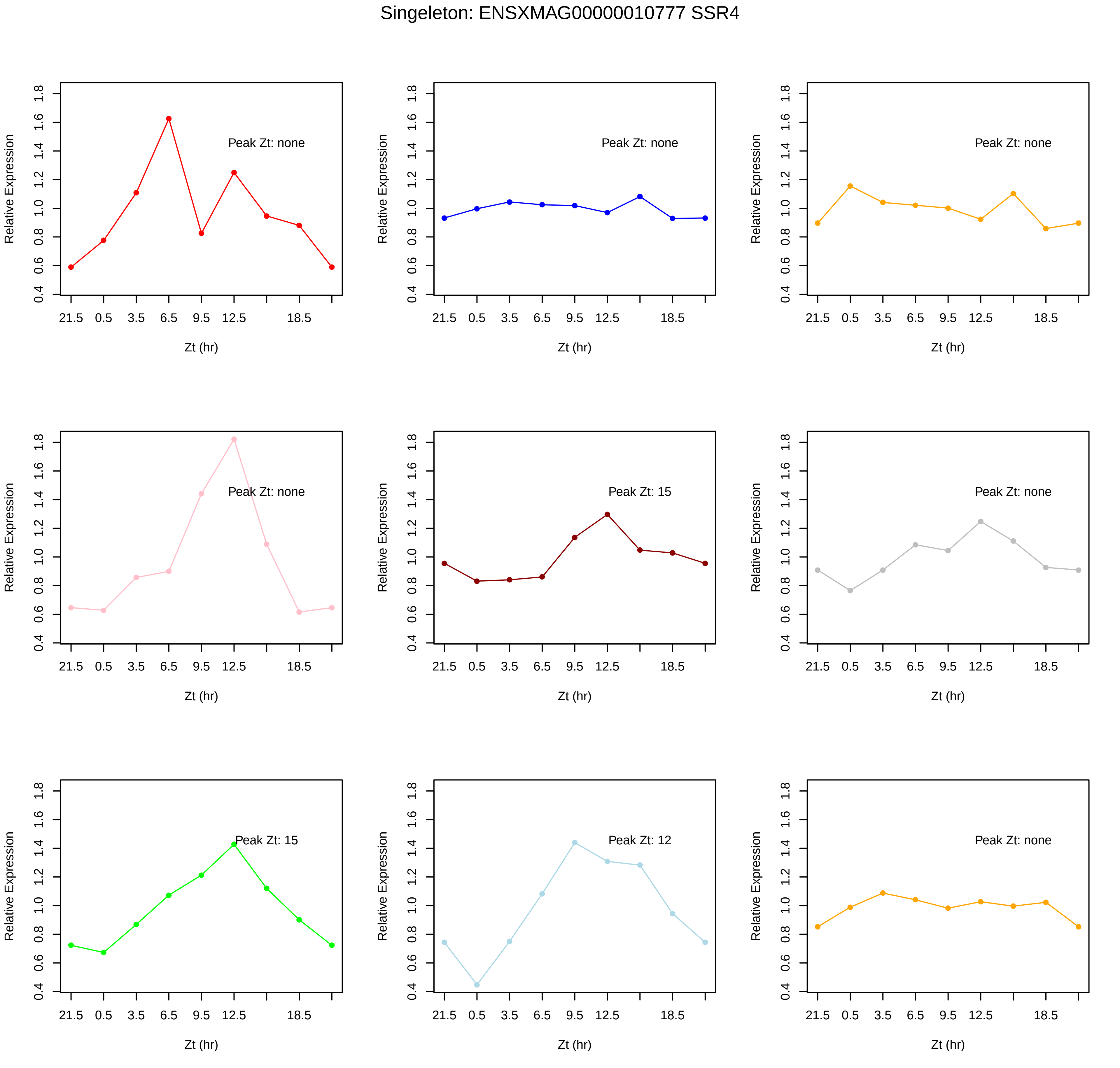
Singeleton: ENSXMAG00000010777 SSR4
Peak Zt: none
Peak Zt: none
Peak Zt: none
Peak Zt: none
Peak Zt: 15
Peak Zt: none
Peak Zt: 15
Peak Zt: 12
Peak Zt: none
Relative Expression
Zt (hr)

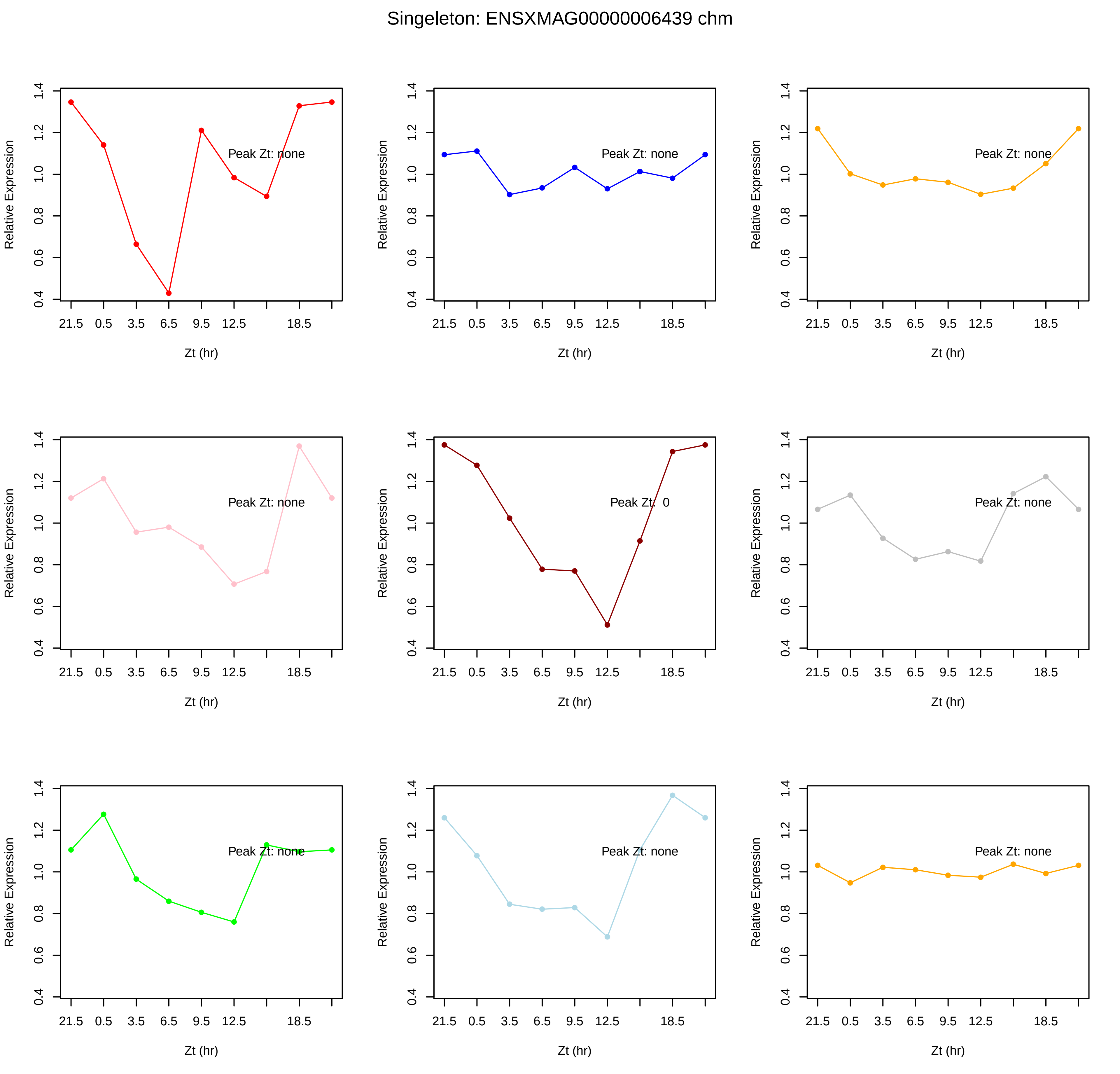
Singeleton: ENSXMAG00000006439 chm
Relative Expression
Zt (hr)
Peak Zt: none
Peak Zt: 0
21.5
0.5
3.5
6.5
9.5
12.5
18.5
0.4
0.6
0.8
1.0
1.2
1.4

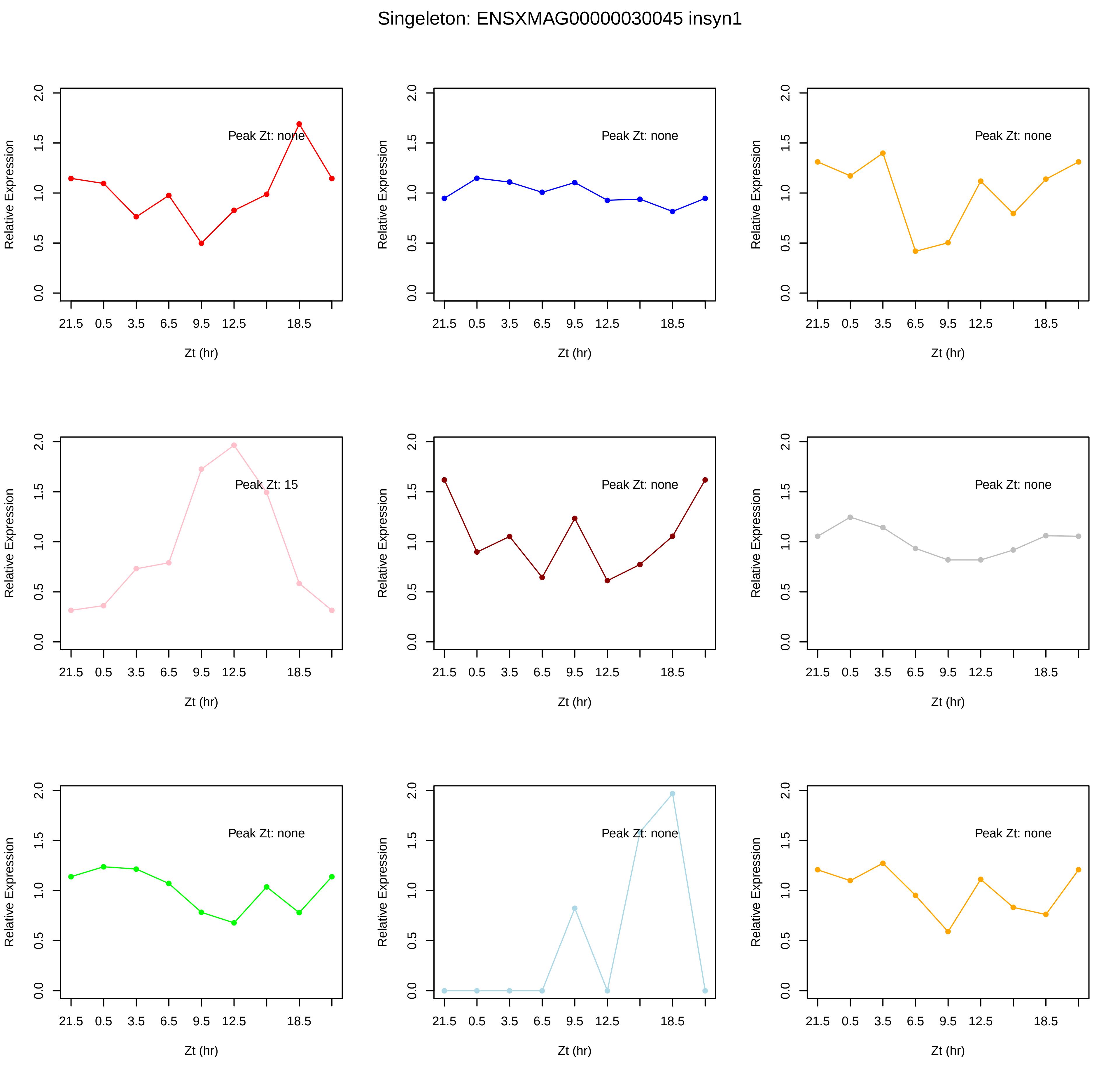
Singleton: ENSXMAG00000030045 insyn1
Peak Zt: none
Peak Zt: none
Peak Zt: none
Peak Zt: 15
Peak Zt: none
Peak Zt: none
Peak Zt: none
Peak Zt: none
Peak Zt: none
Relative Expression
Zt (hr)
21.5
0.5
3.5
6.5
9.5
12.5
18.5
0.0
0.5
1.0
1.5
2.0

Singleton: ENSXMAG00000022292 cd276

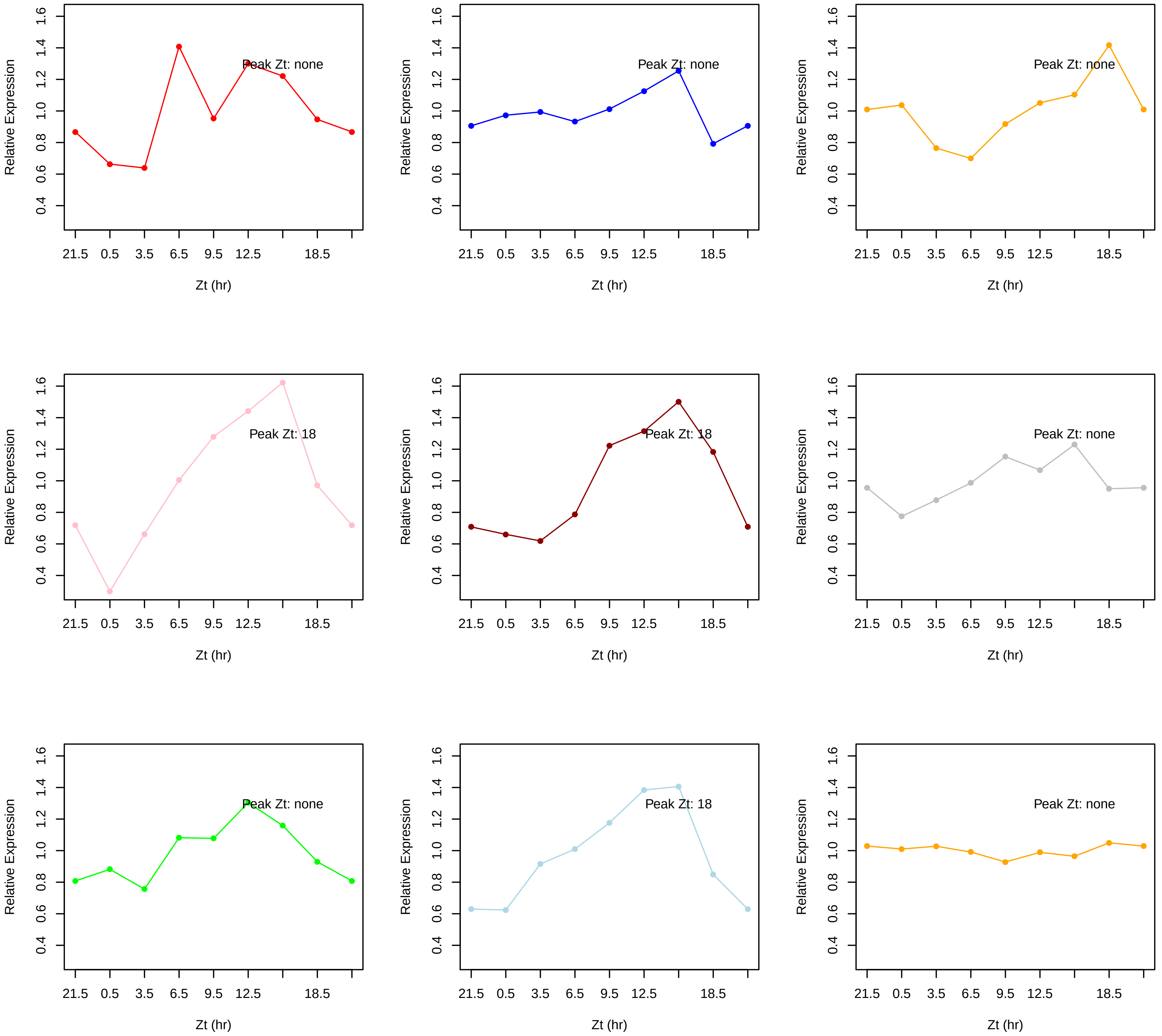

Singeleton: ENSXMAG00000016739

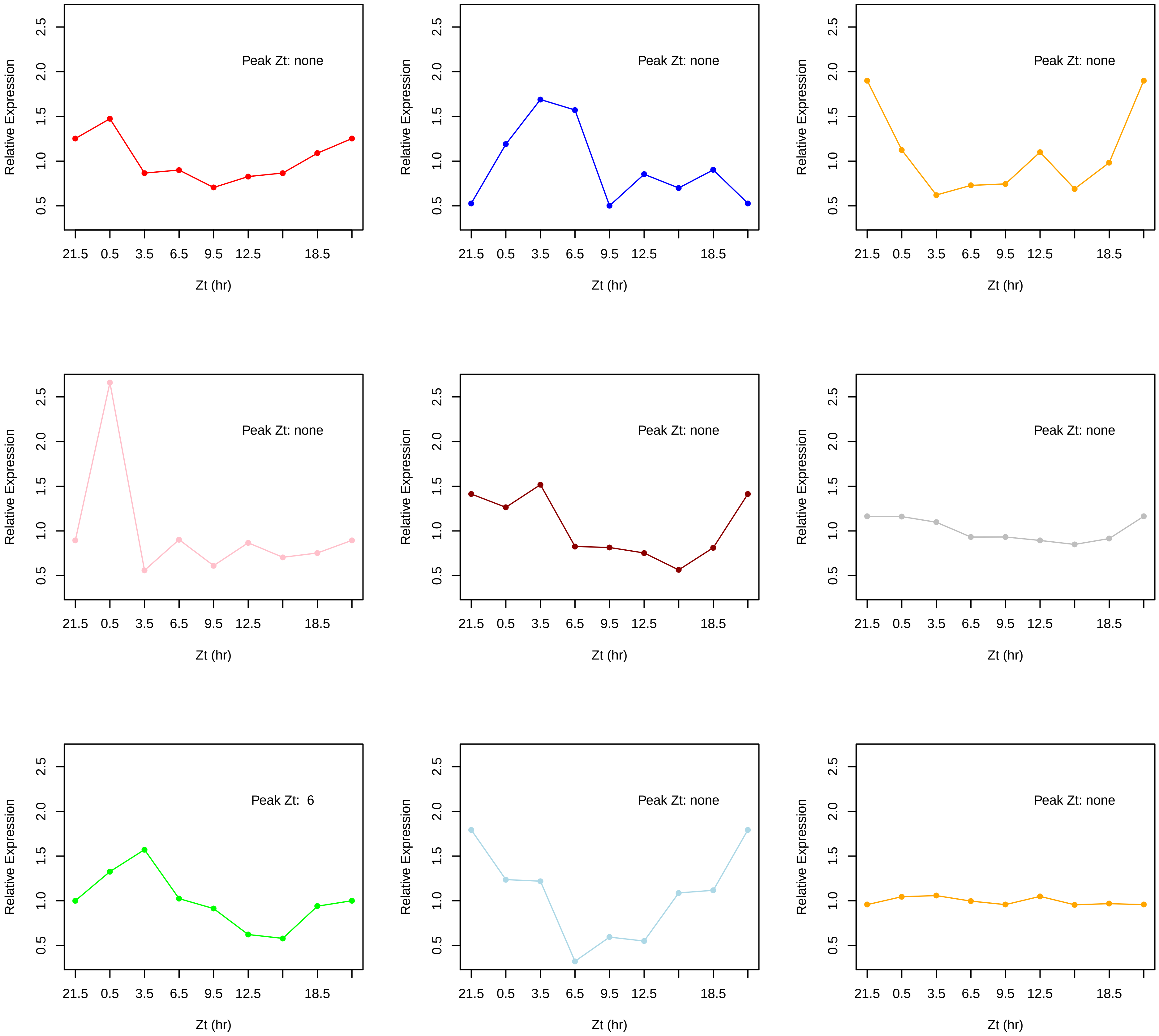

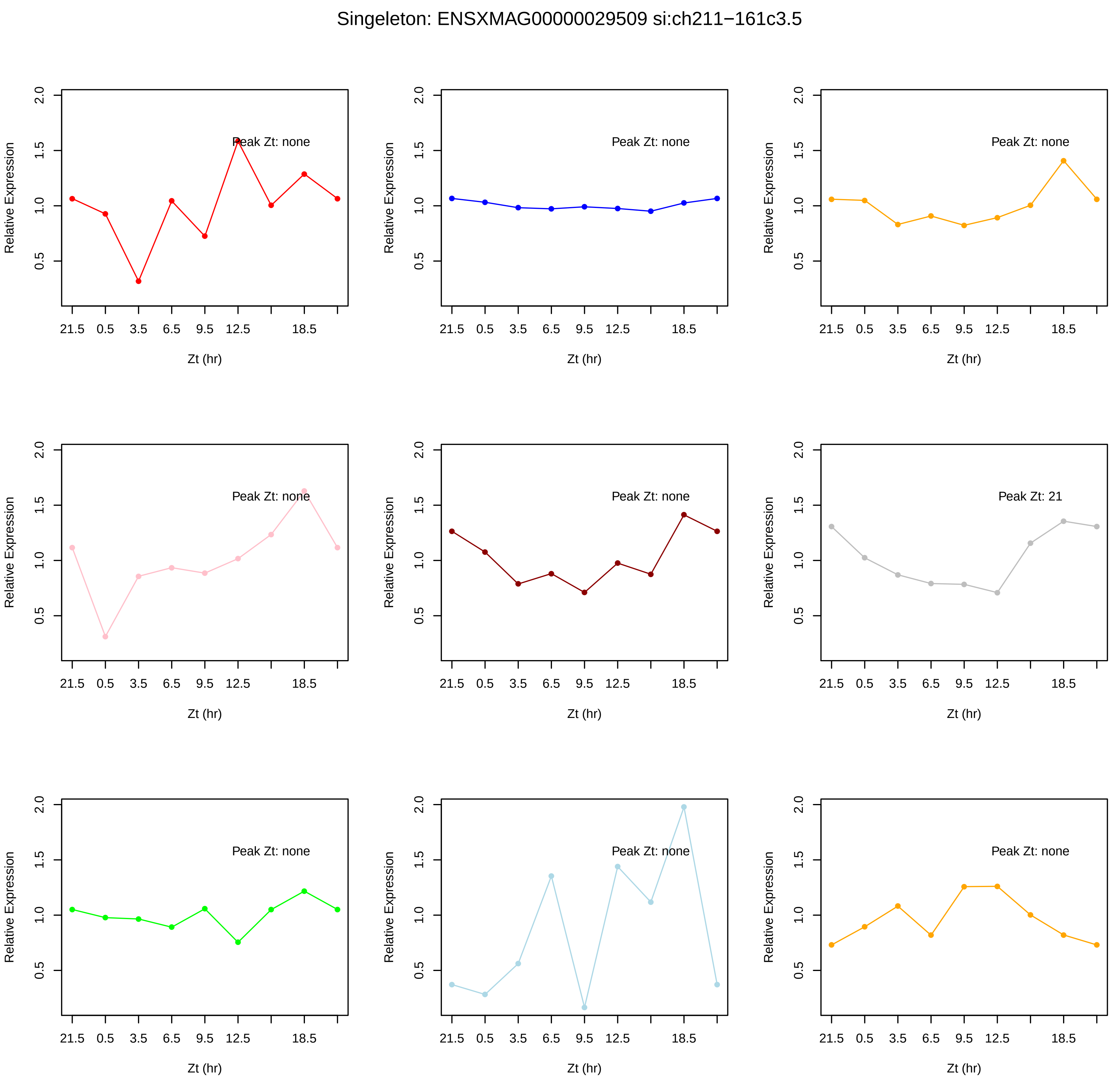
Singleton: ENSXMAG00000029509 si:ch211−161c3.5
Peak Zt: none
Peak Zt: none
Peak Zt: none
Peak Zt: none
Peak Zt: none
Peak Zt: 21
Peak Zt: none
Peak Zt: none
Peak Zt: none
Relative Expression
Zt (hr)
21.5
0.5
3.5
6.5
9.5
12.5
18.5
0.5
1.0
1.5
2.0

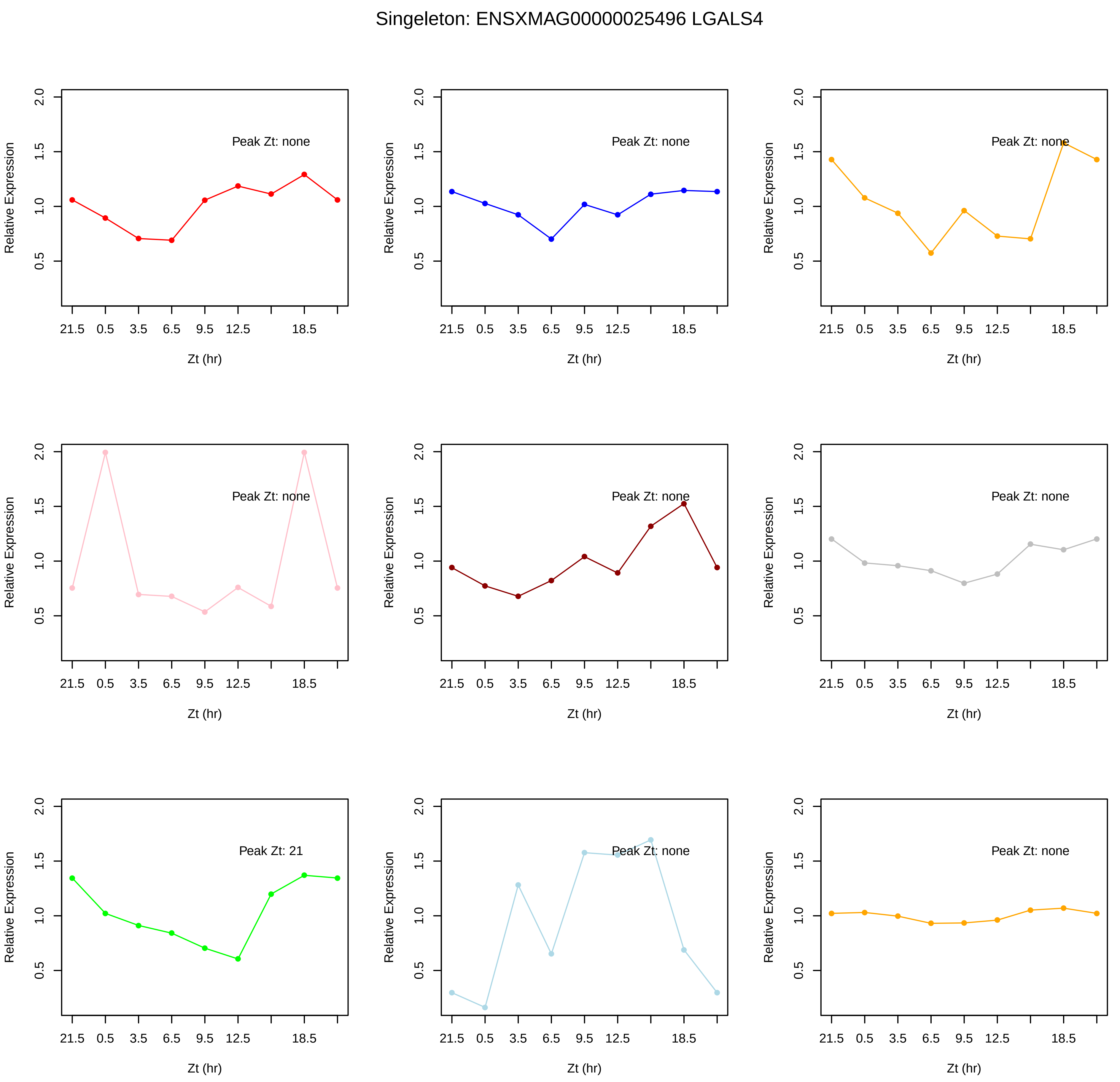
Singeleton: ENSXMAG00000025496 LGALS4
Peak Zt: none
Peak Zt: none
Peak Zt: none
Peak Zt: none
Peak Zt: none
Peak Zt: none
Peak Zt: 21
Peak Zt: none
Peak Zt: none
Relative Expression
Zt (hr)
21.5 0.5 3.5 6.5 9.5 12.5 18.5

Singeleton: ENSXMAG00000000028

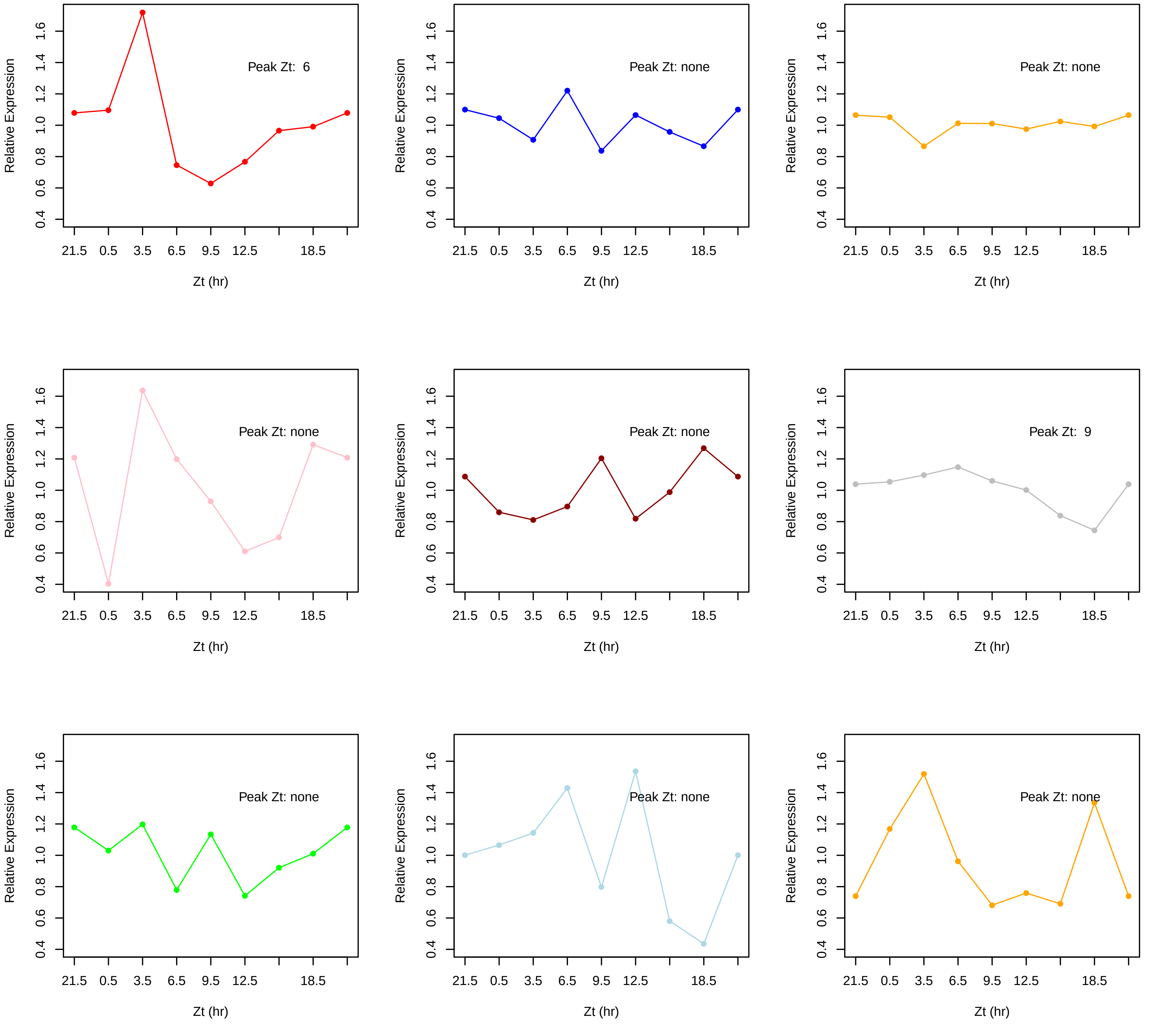

Singleton: ENSXMAG00000011013 cnpy3

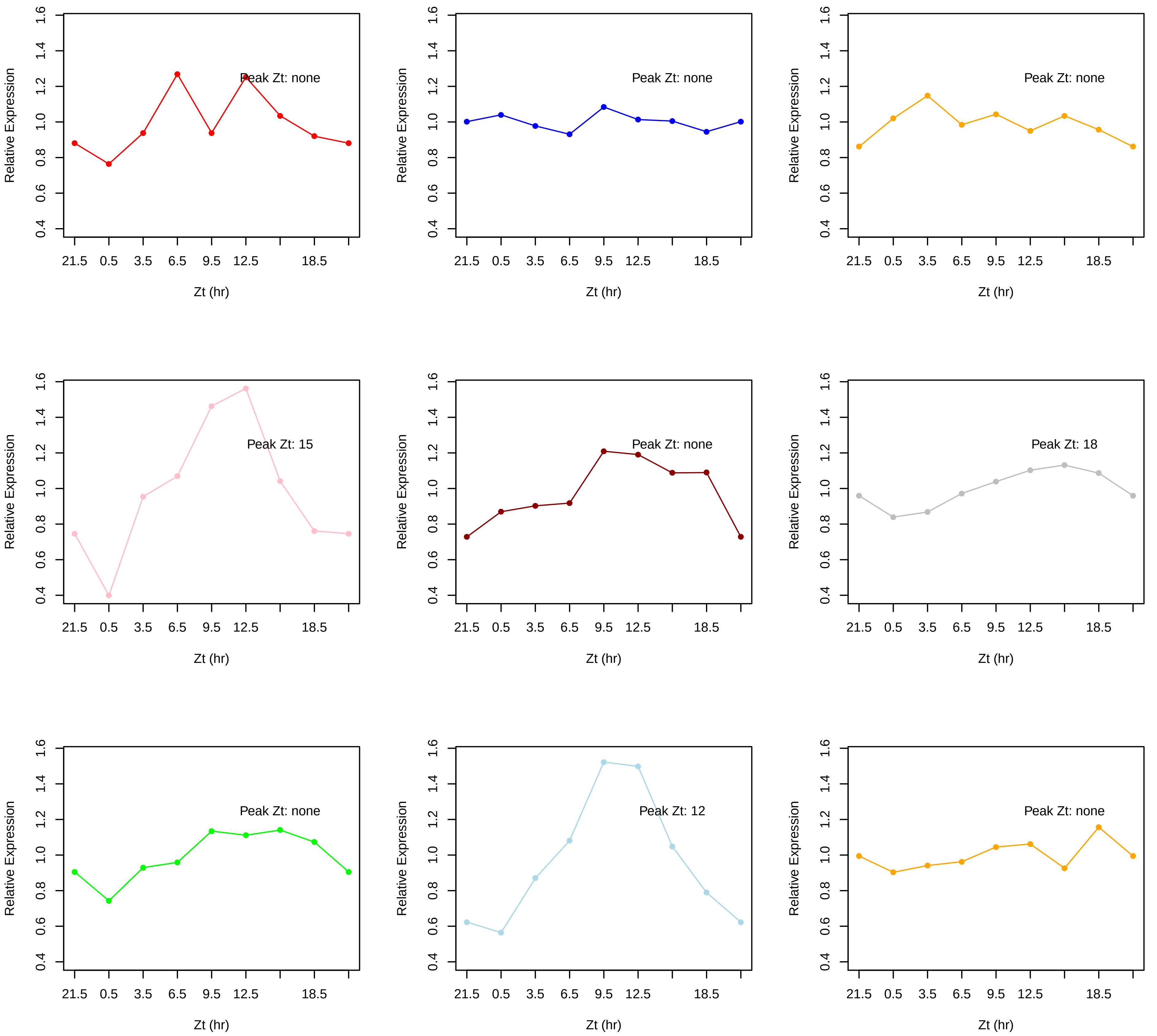

Singeleton: ENSXMAG00000017878 kptn

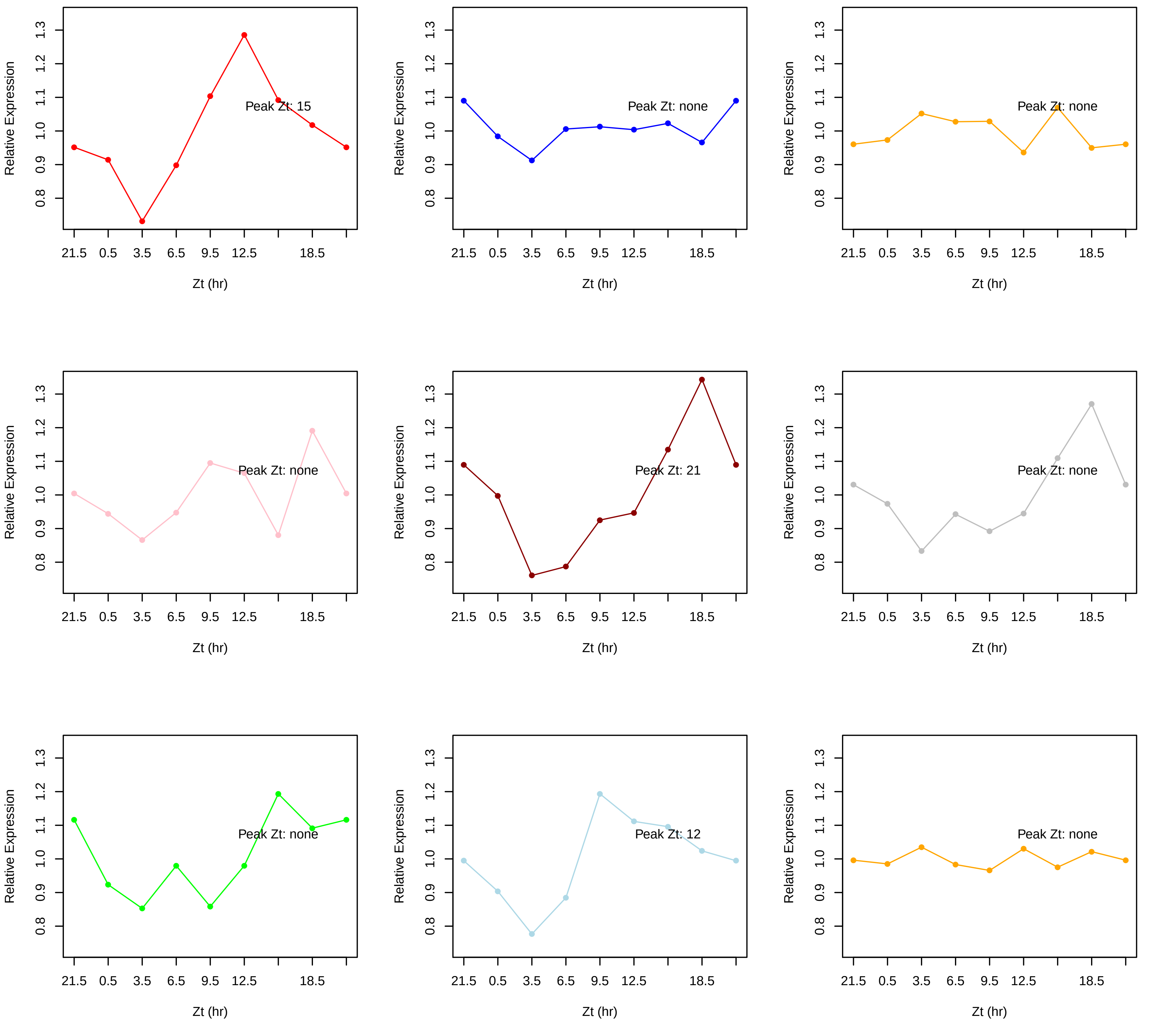

Singeleton: ENSXMAG00000019318 ifrd2

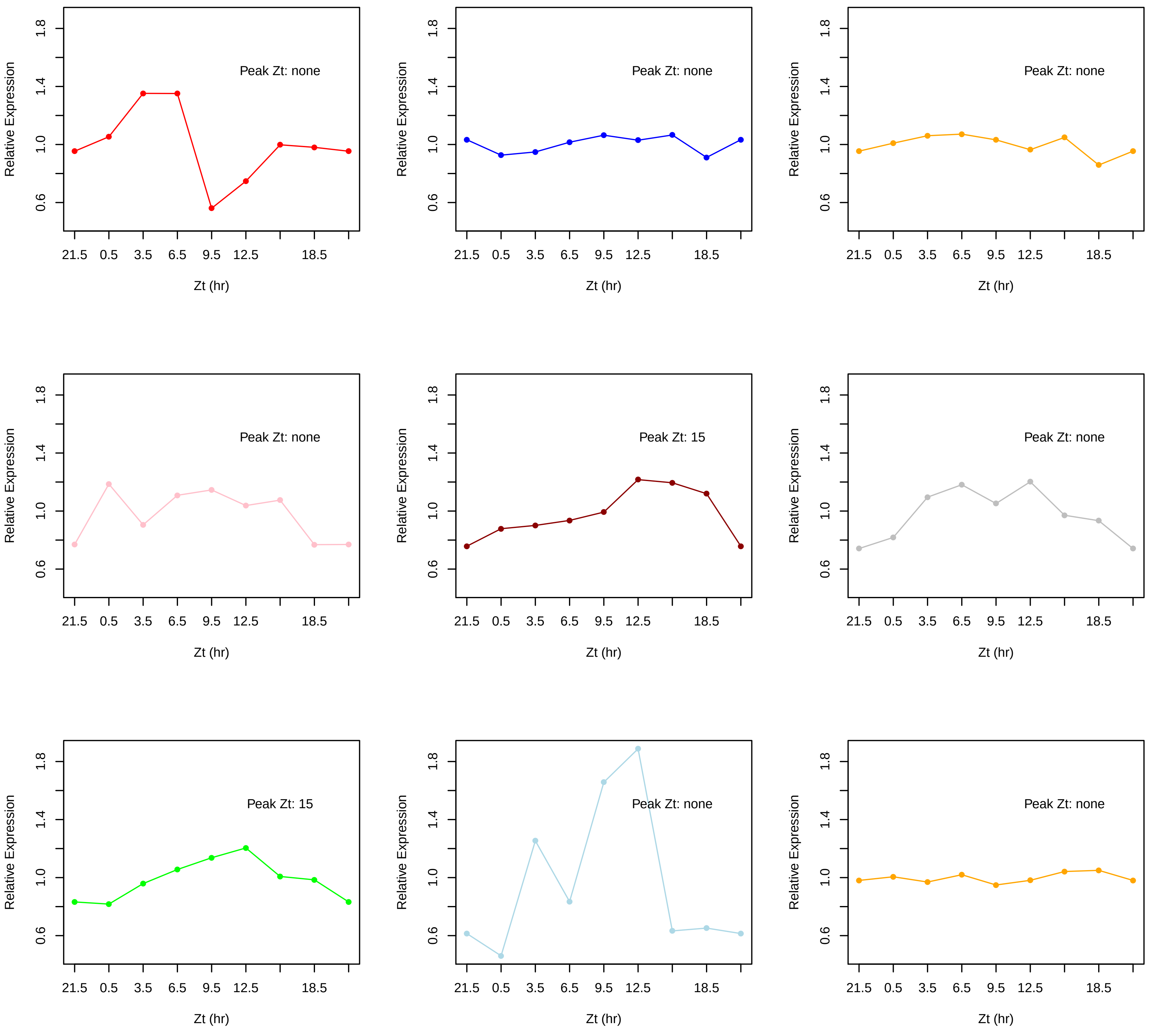

Singleton: ENSXMAG00000008307 usp21

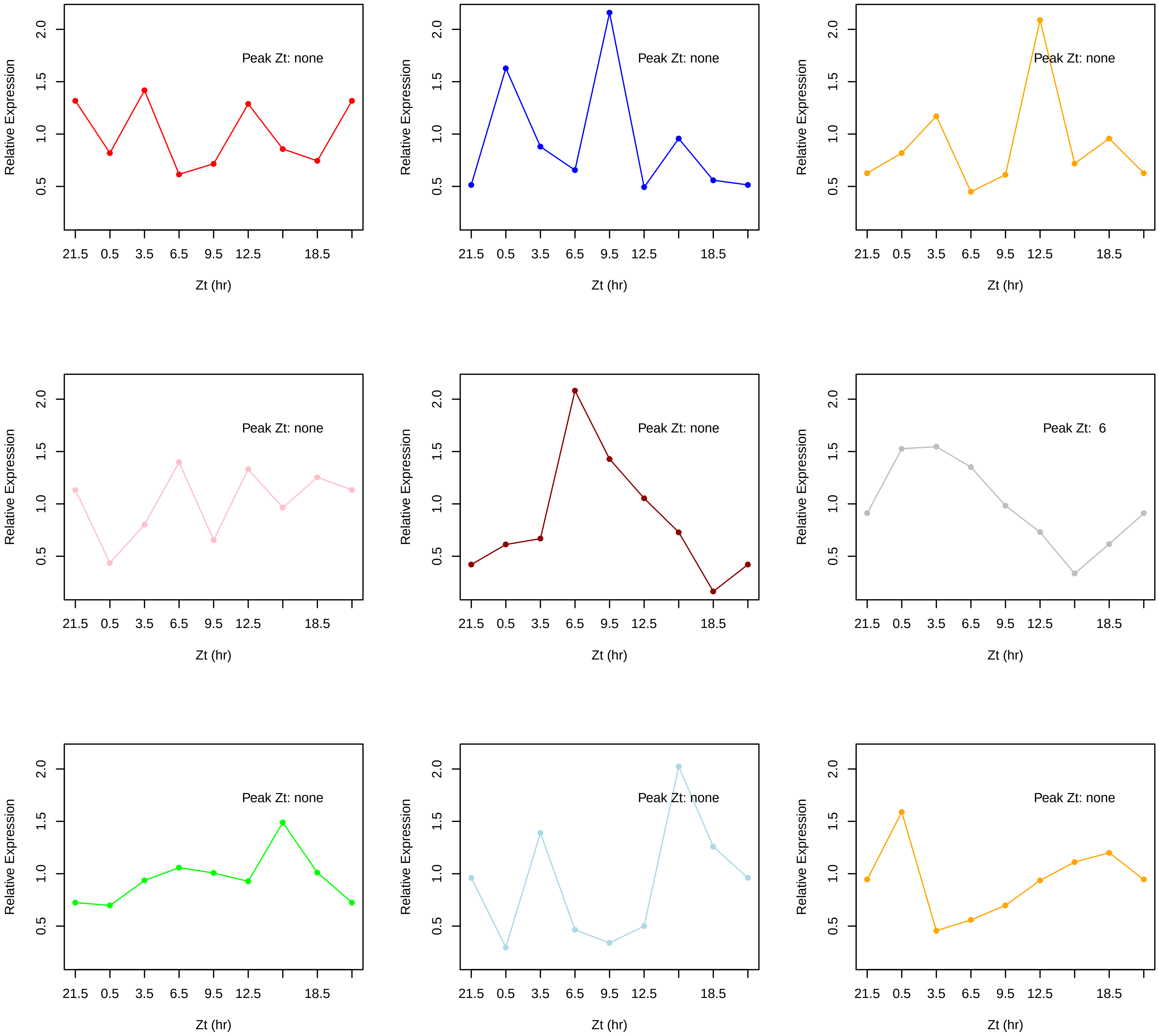

Singeleton: ENSXMAG00000019315 zgc:136971

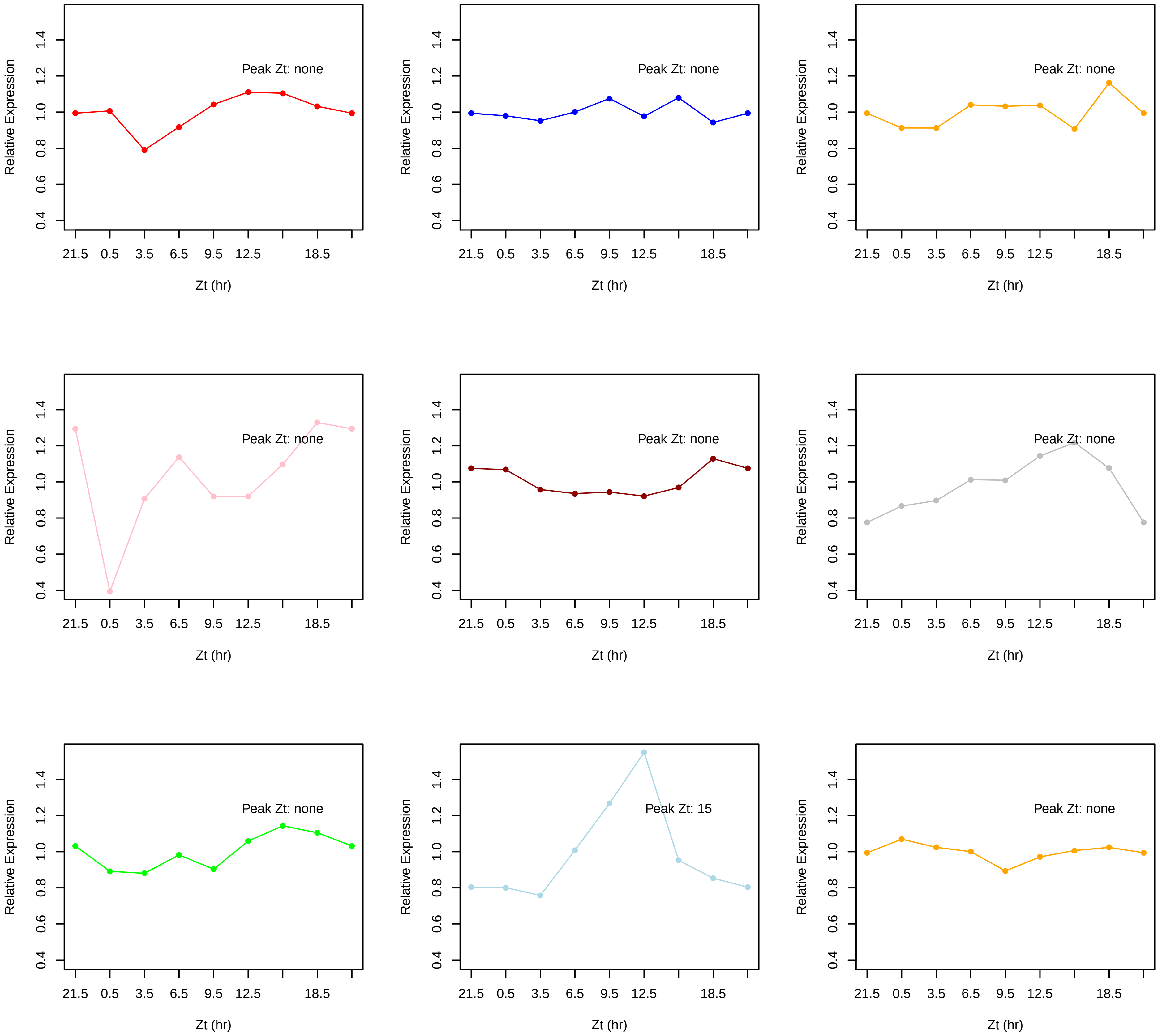

Singeleton: ENSXMAG00000012836

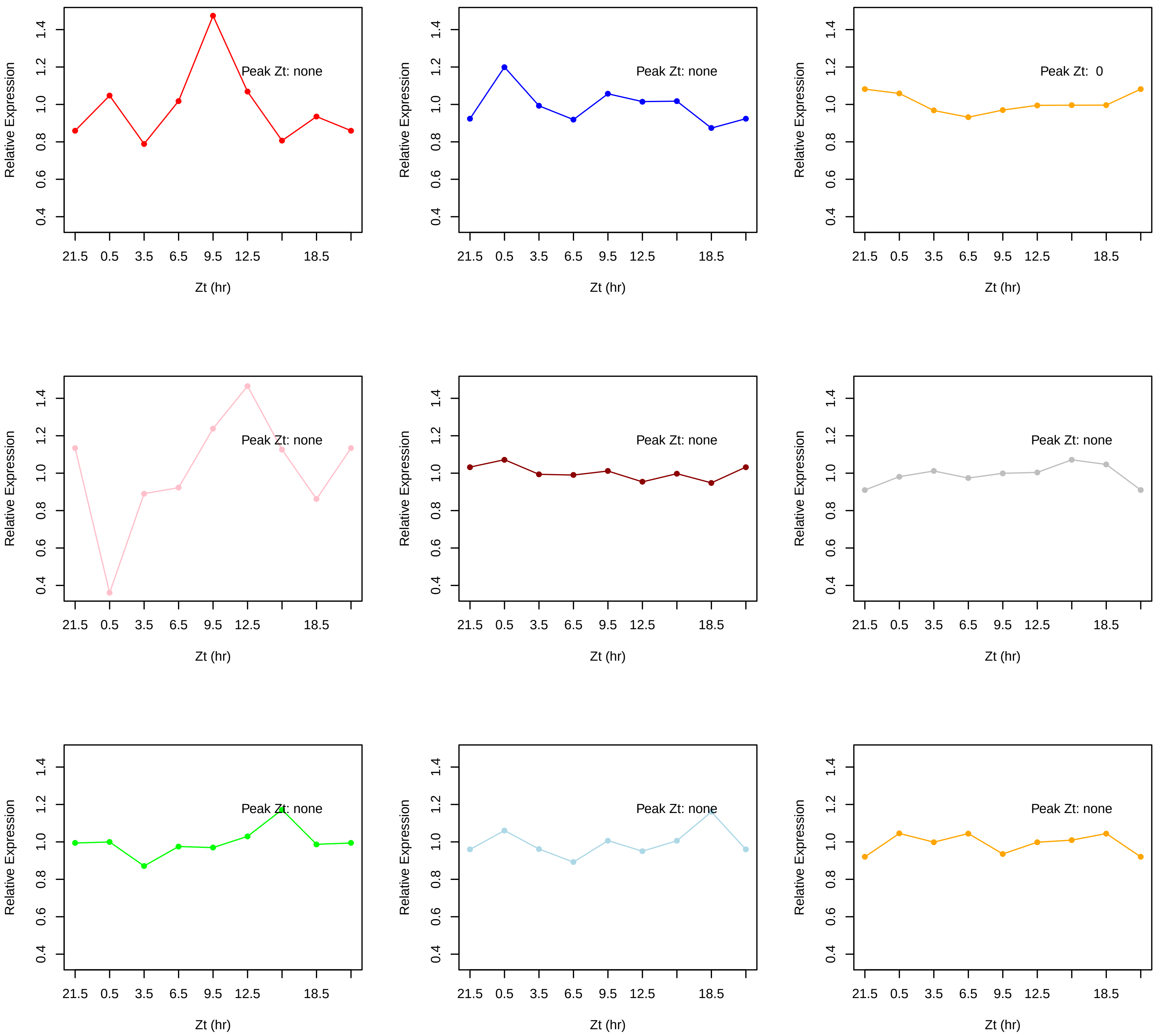

Singleton: ENSXMAG00000002549 meis3

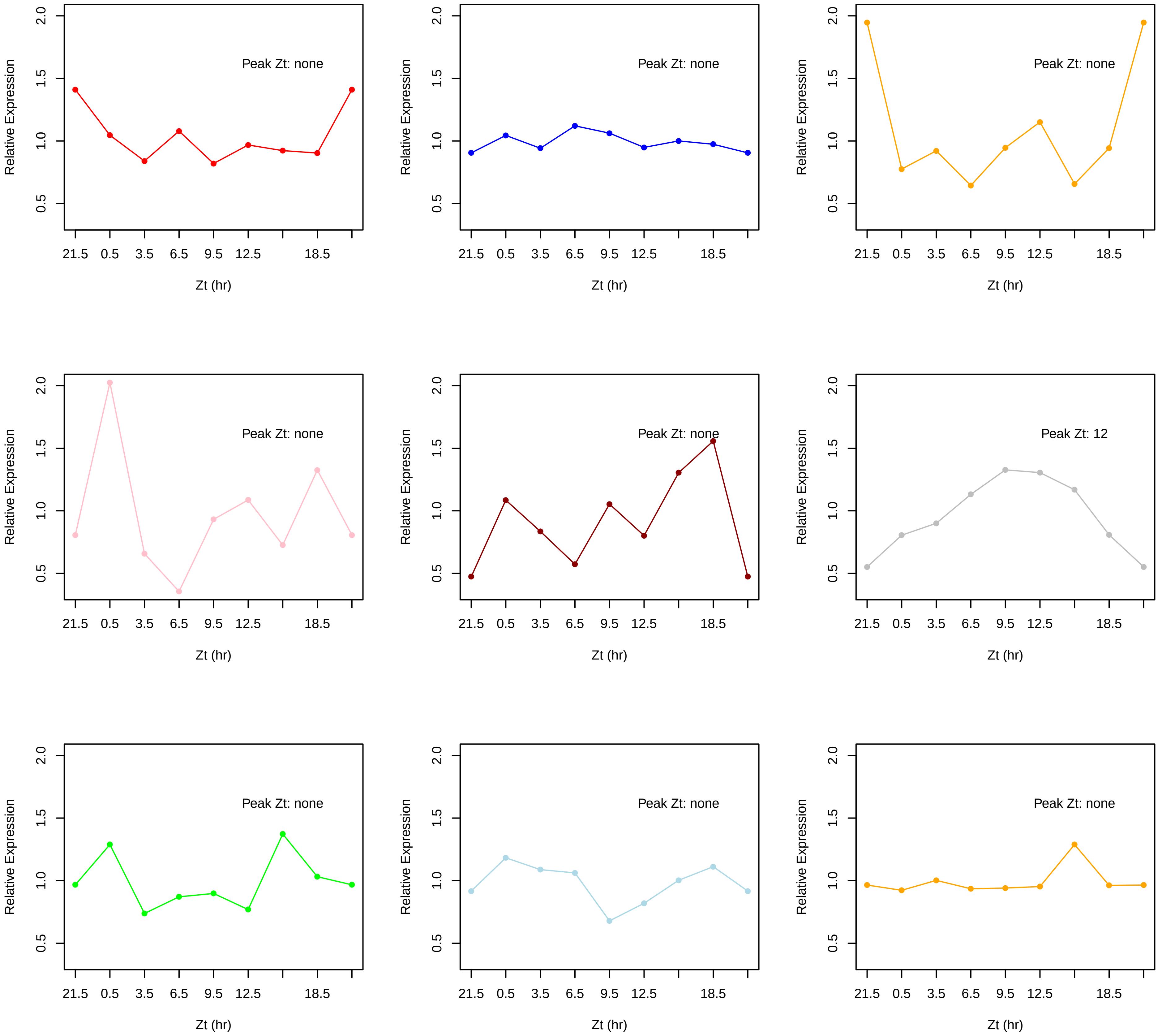

Singeleton: ENSXMAG00000019294 sema3bl

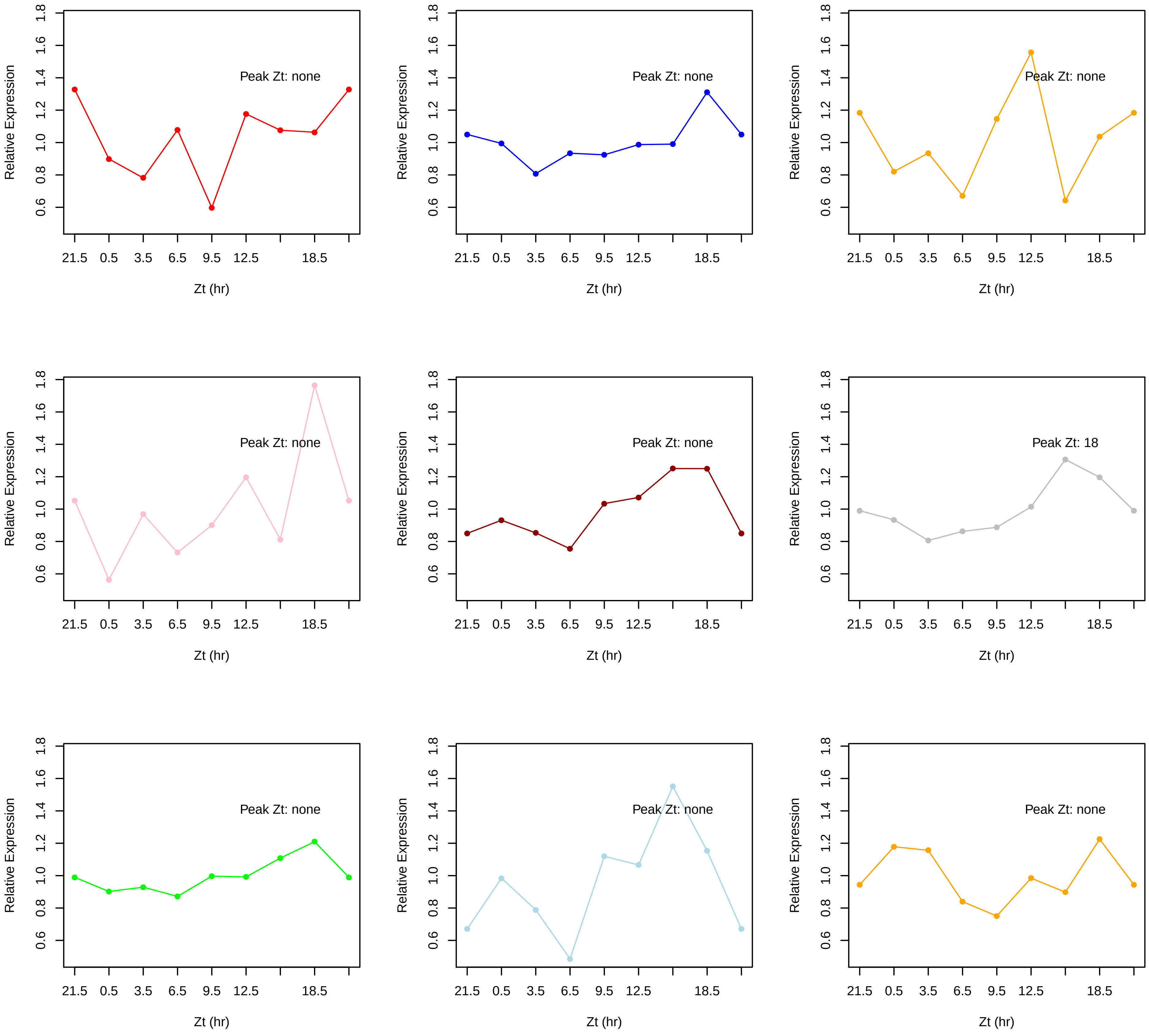

Singleton: ENSXMAG00000003050 srsf7a

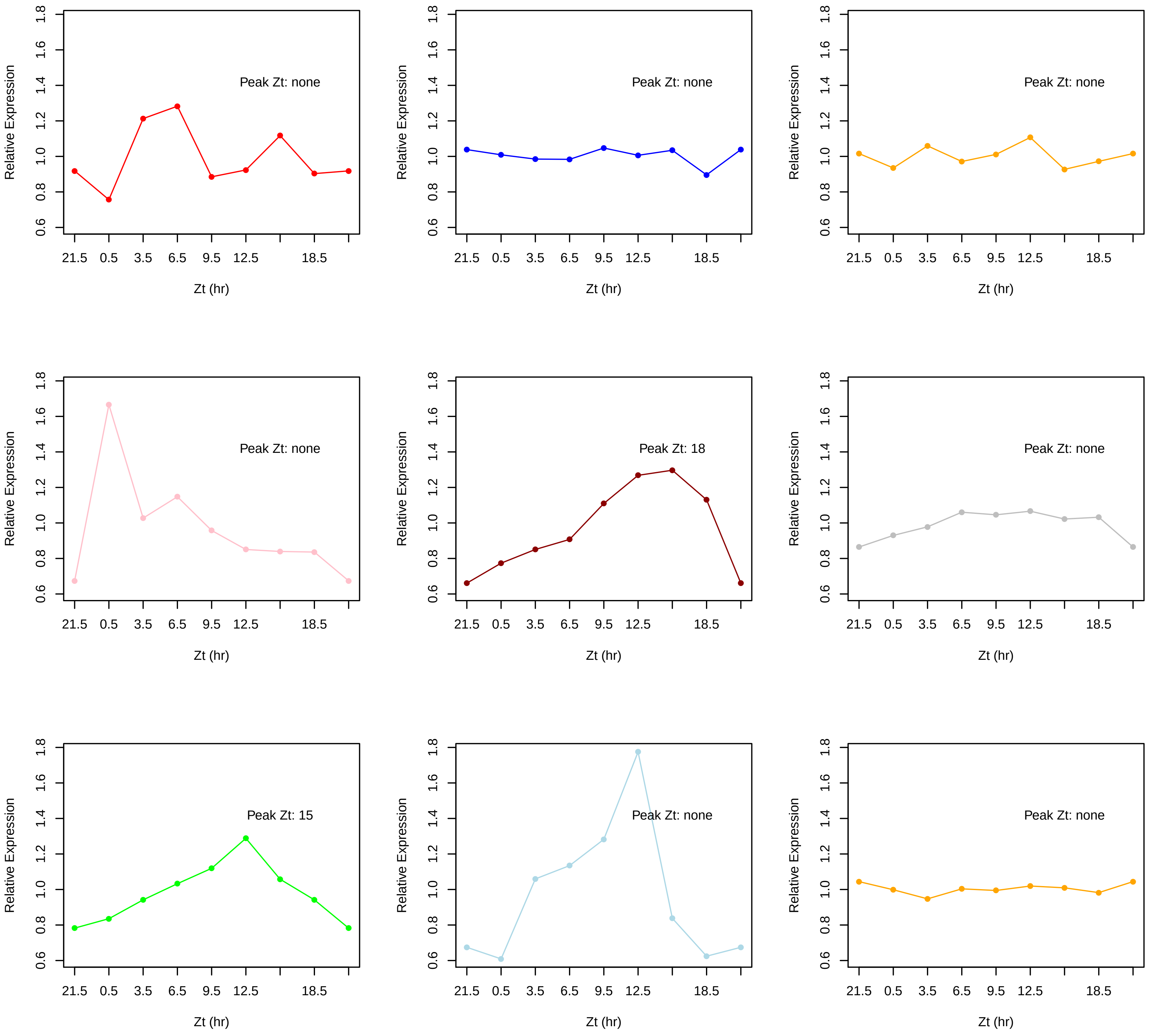

Singeleton: ENSXMAG00000008297 arhgef3l

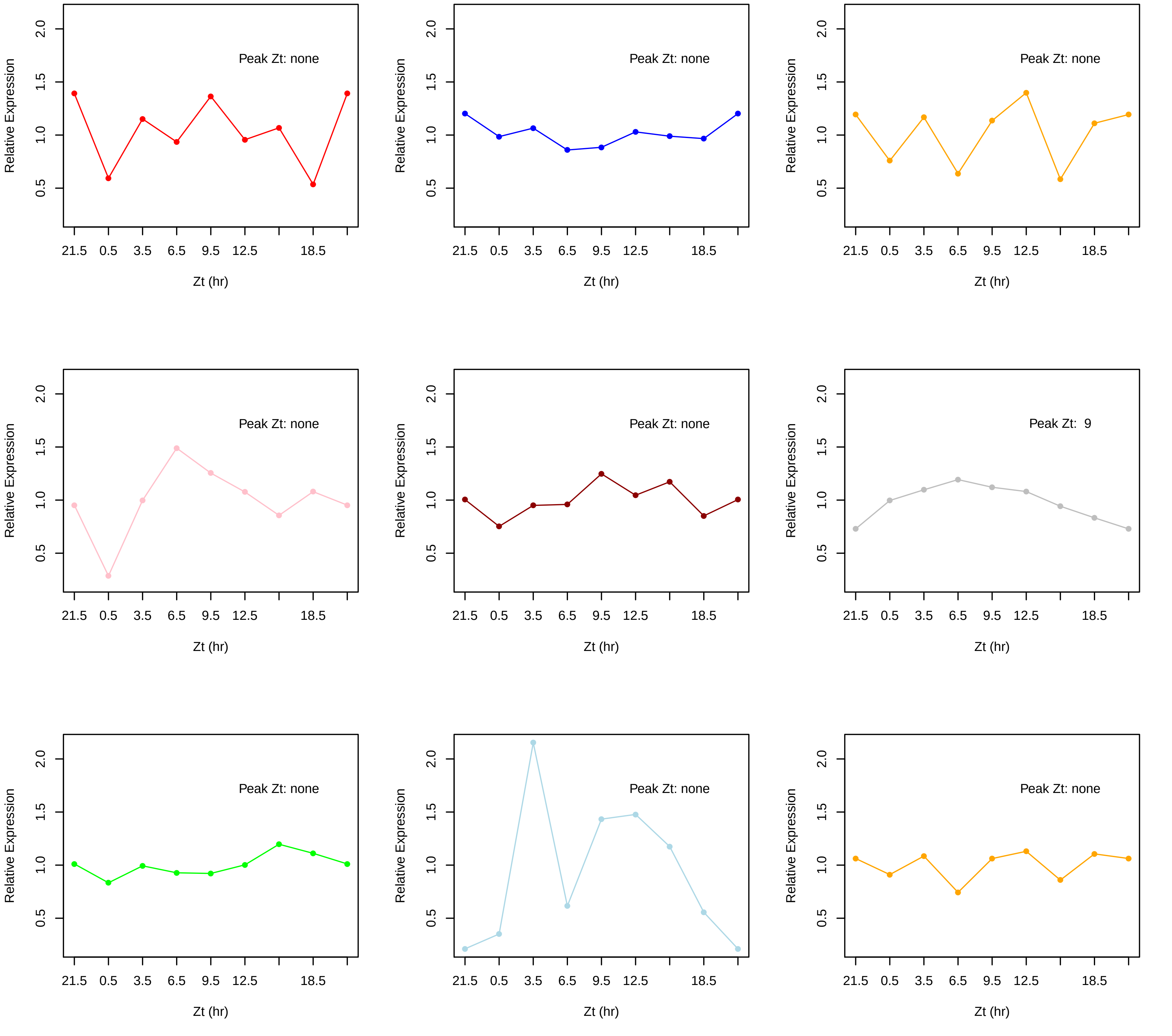

Singeleton: ENSXMAG00000016983 tmed3

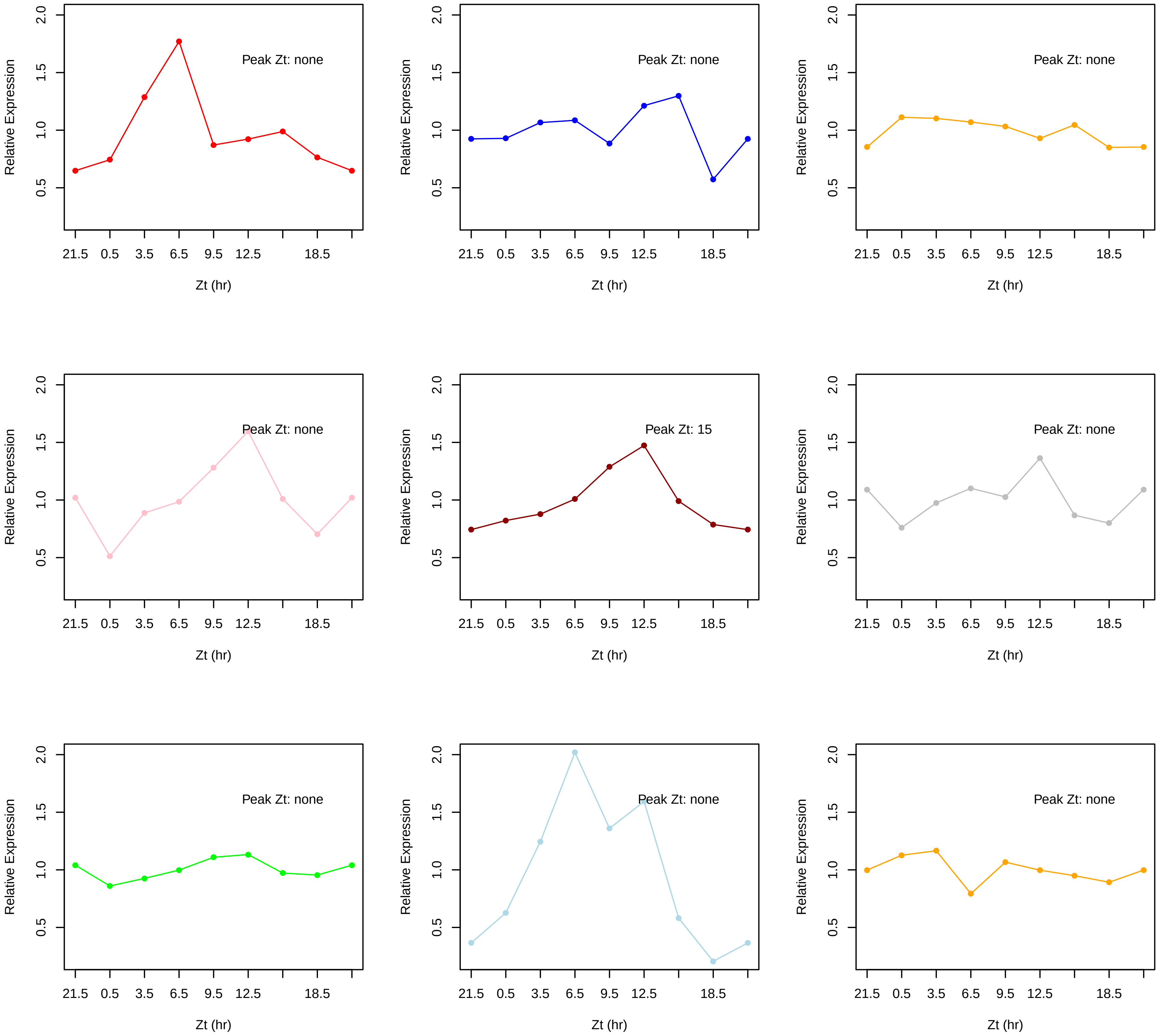

Singeleton: ENSXMAG00000019303

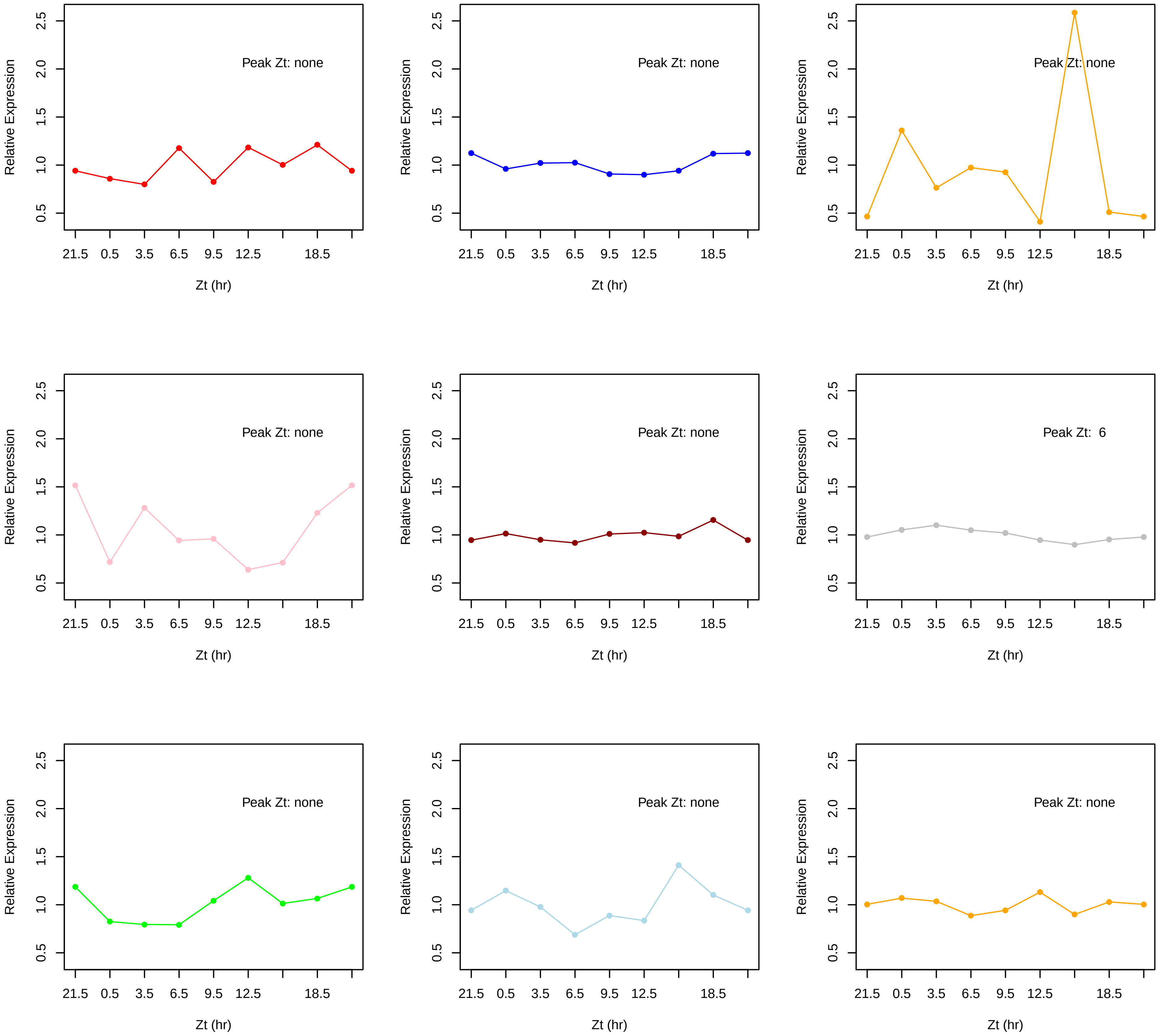

Singeleton: ENSXMAG00000003111

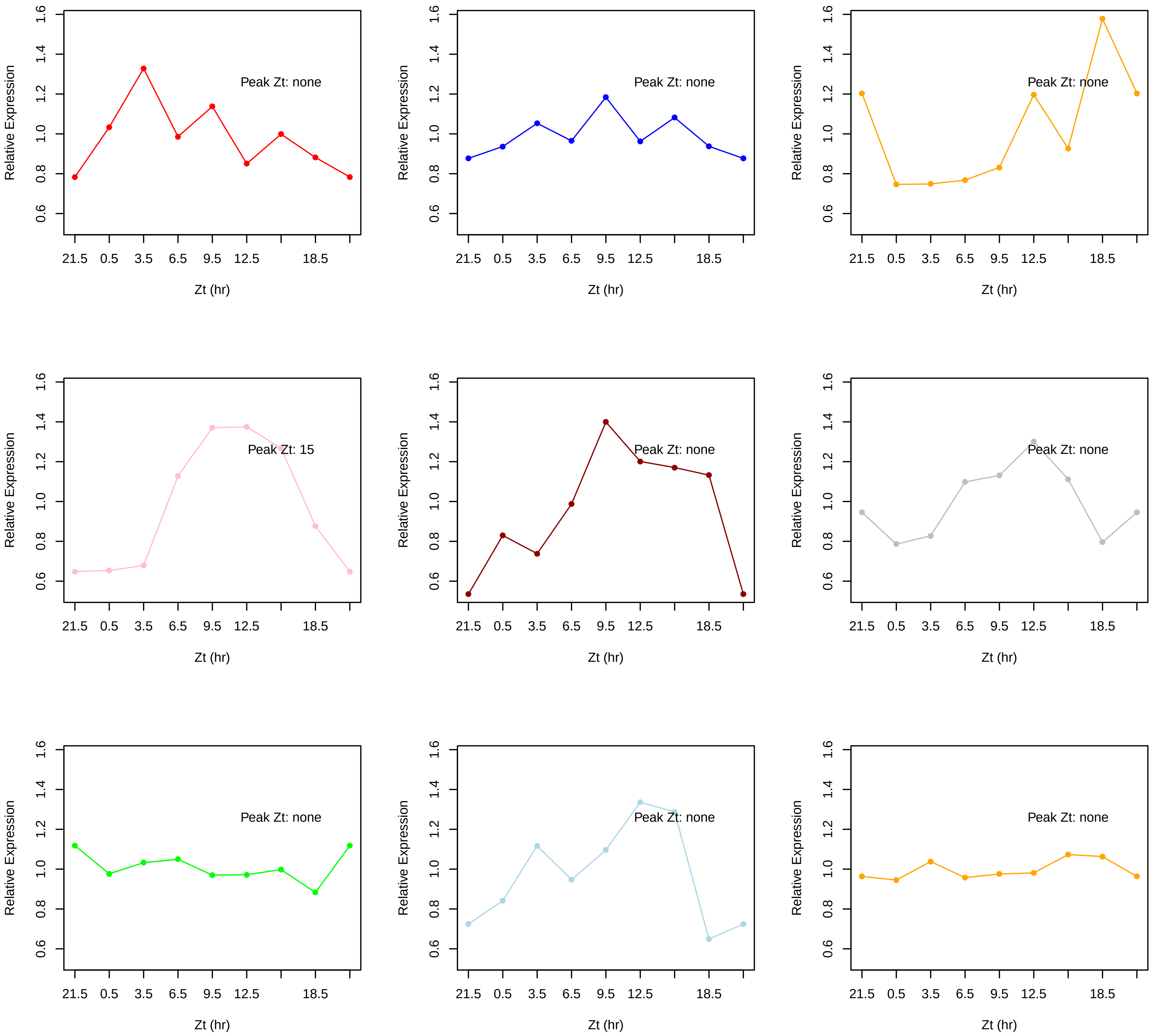

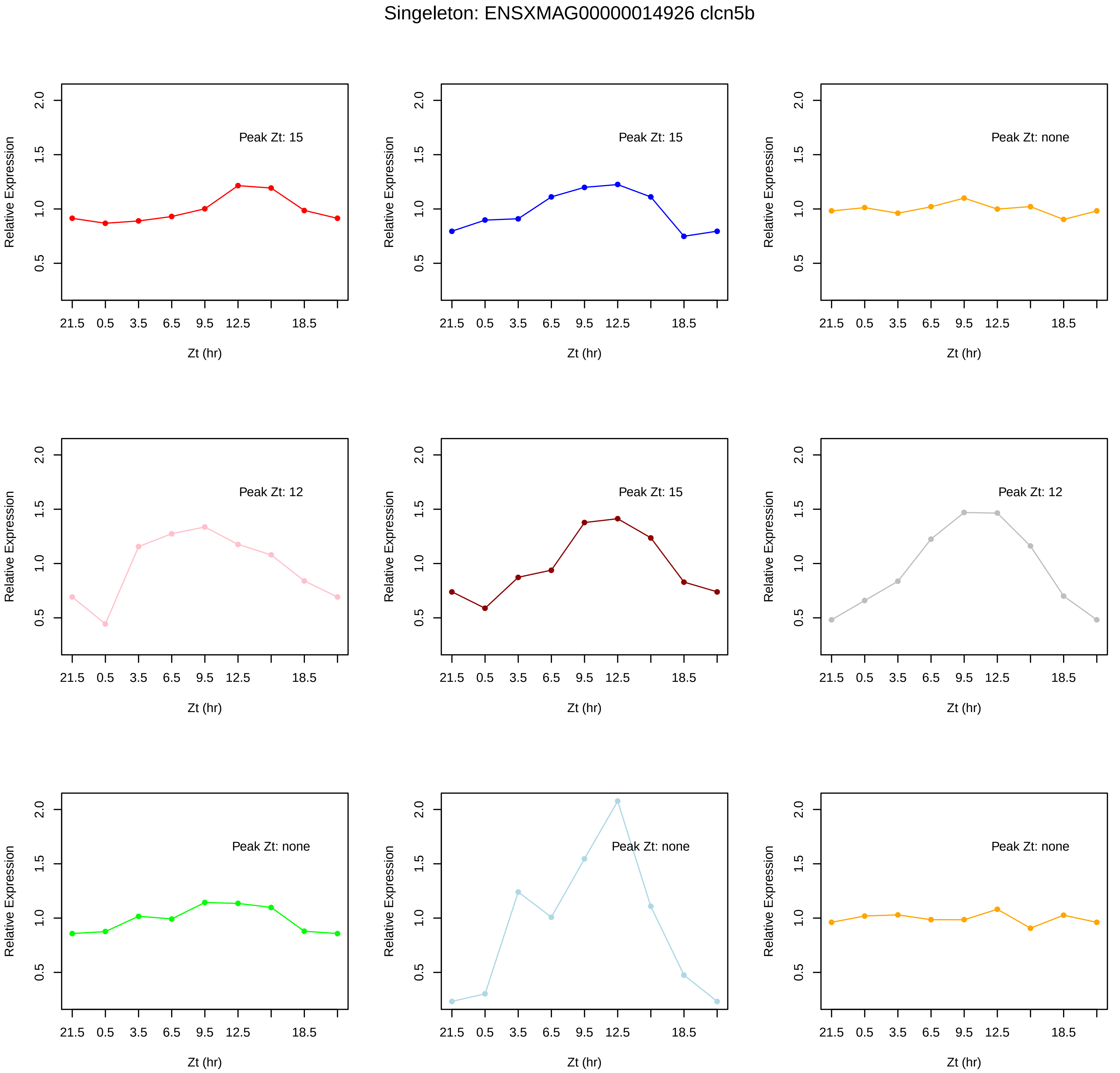
Singeleton: ENSXMAG00000014926 clcn5b
Peak Zt: 15
Peak Zt: 15
Peak Zt: none
Peak Zt: 12
Peak Zt: 15
Peak Zt: 12
Peak Zt: none
Peak Zt: none
Peak Zt: none
Relative Expression
Zt (hr)
21.5
0.5
3.5
6.5
9.5
12.5
18.5
0.5
1.0
1.5
2.0

Singeleton: ENSXMAG00000008274 uba1

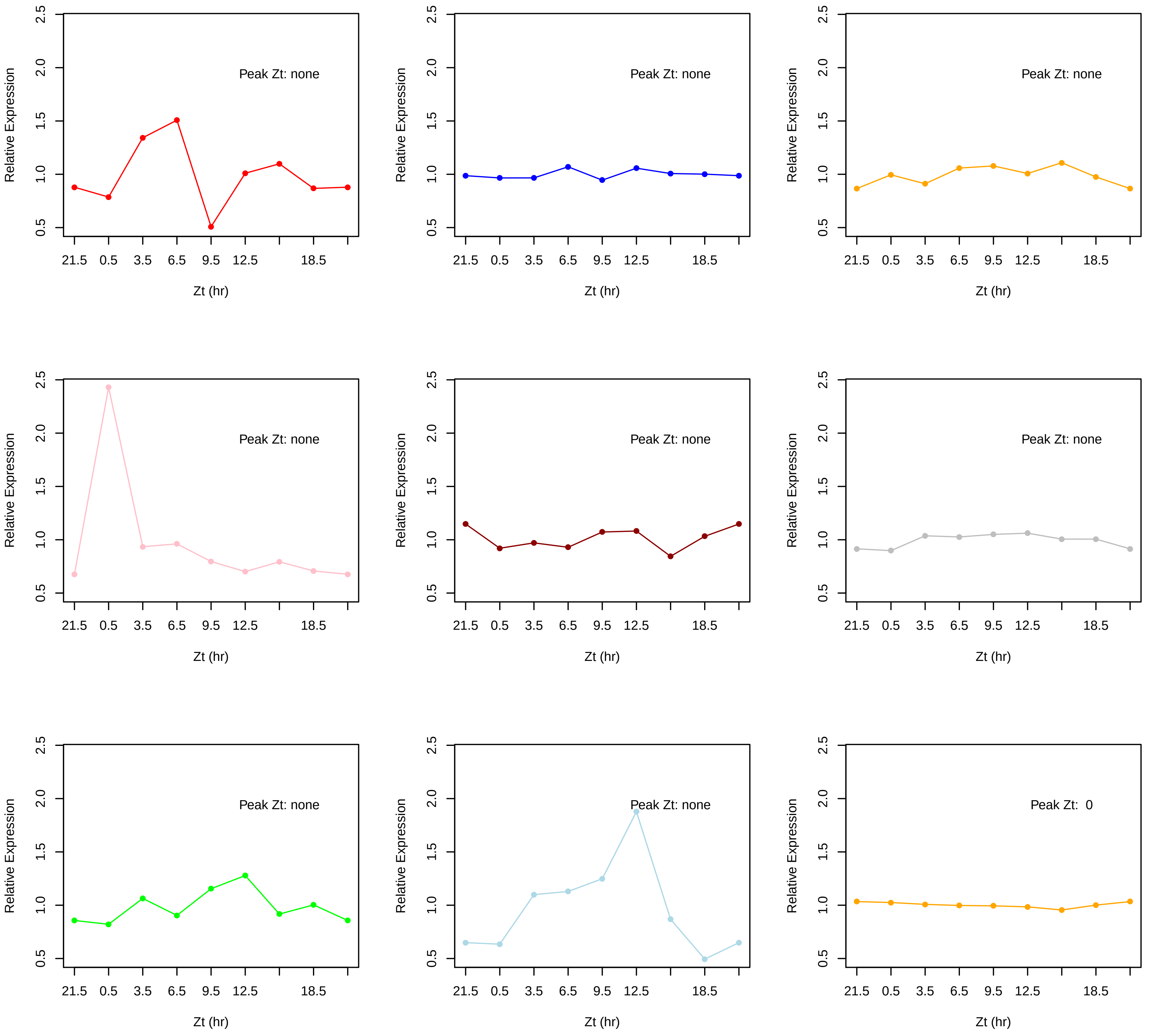

Singeleton: ENSXMAG00000008129 fkbpl

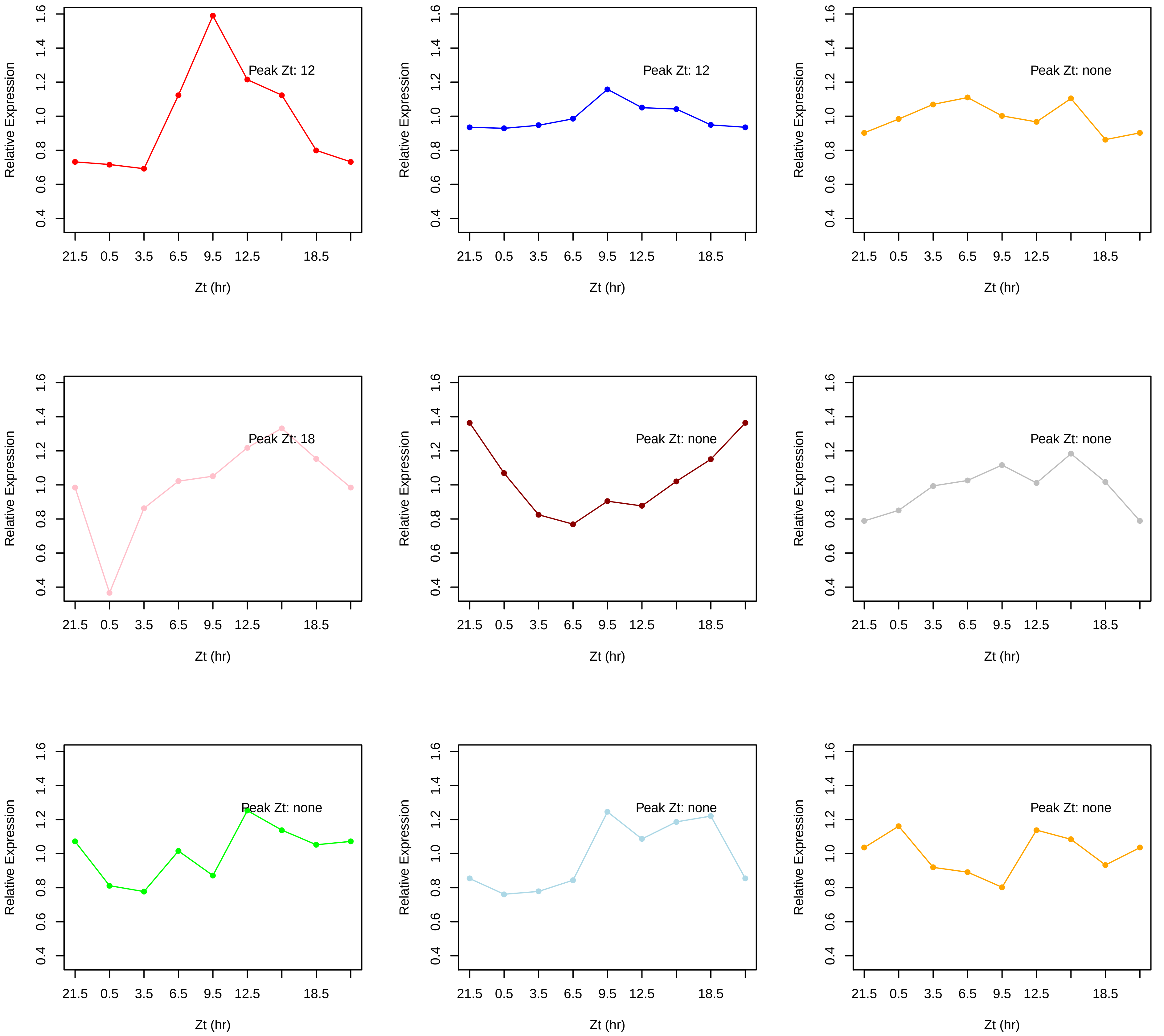

Singeleton: ENSXMAG00000008130 ebp

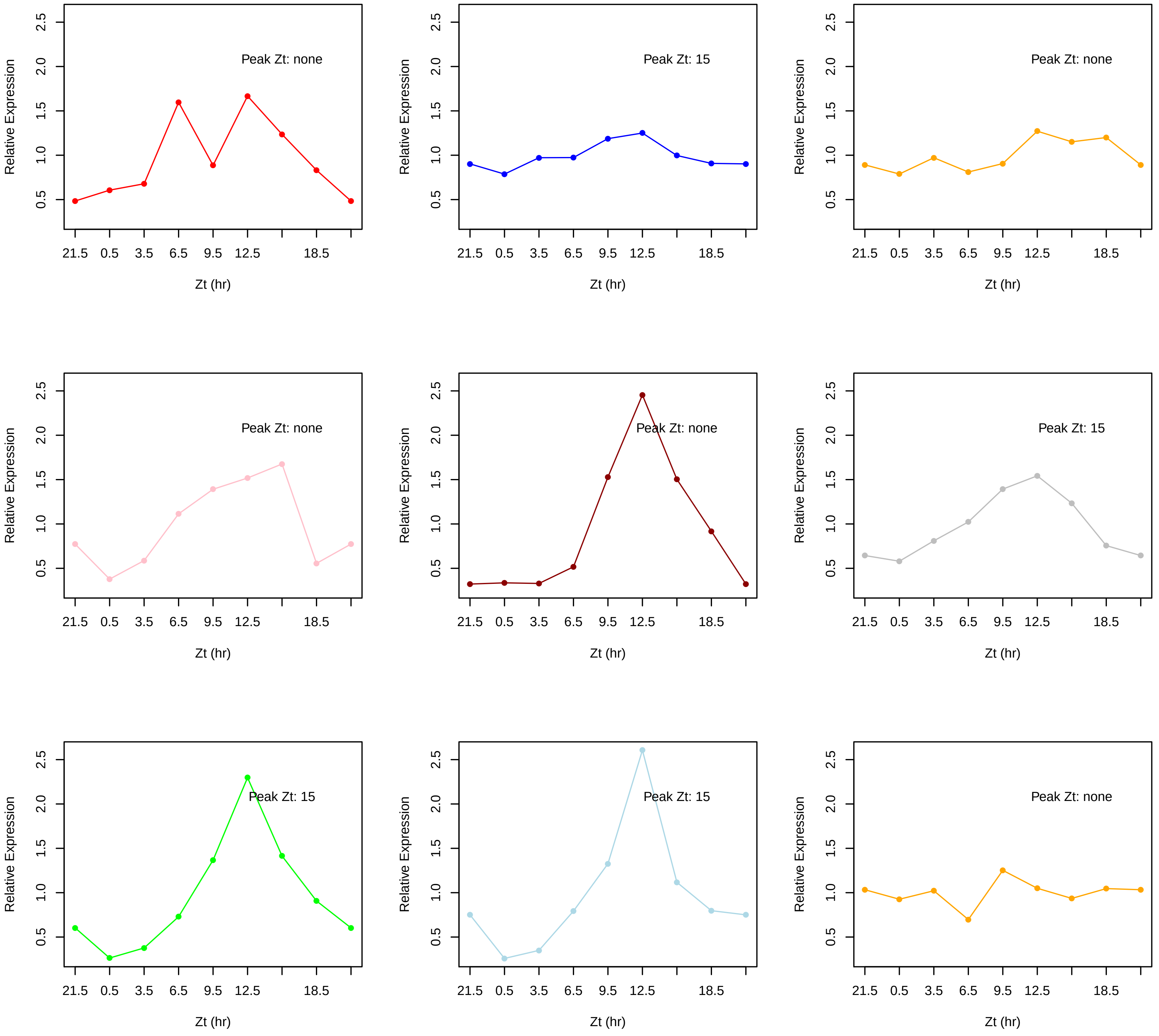

Singeleton: ENSXMAG00000018319 PORCN

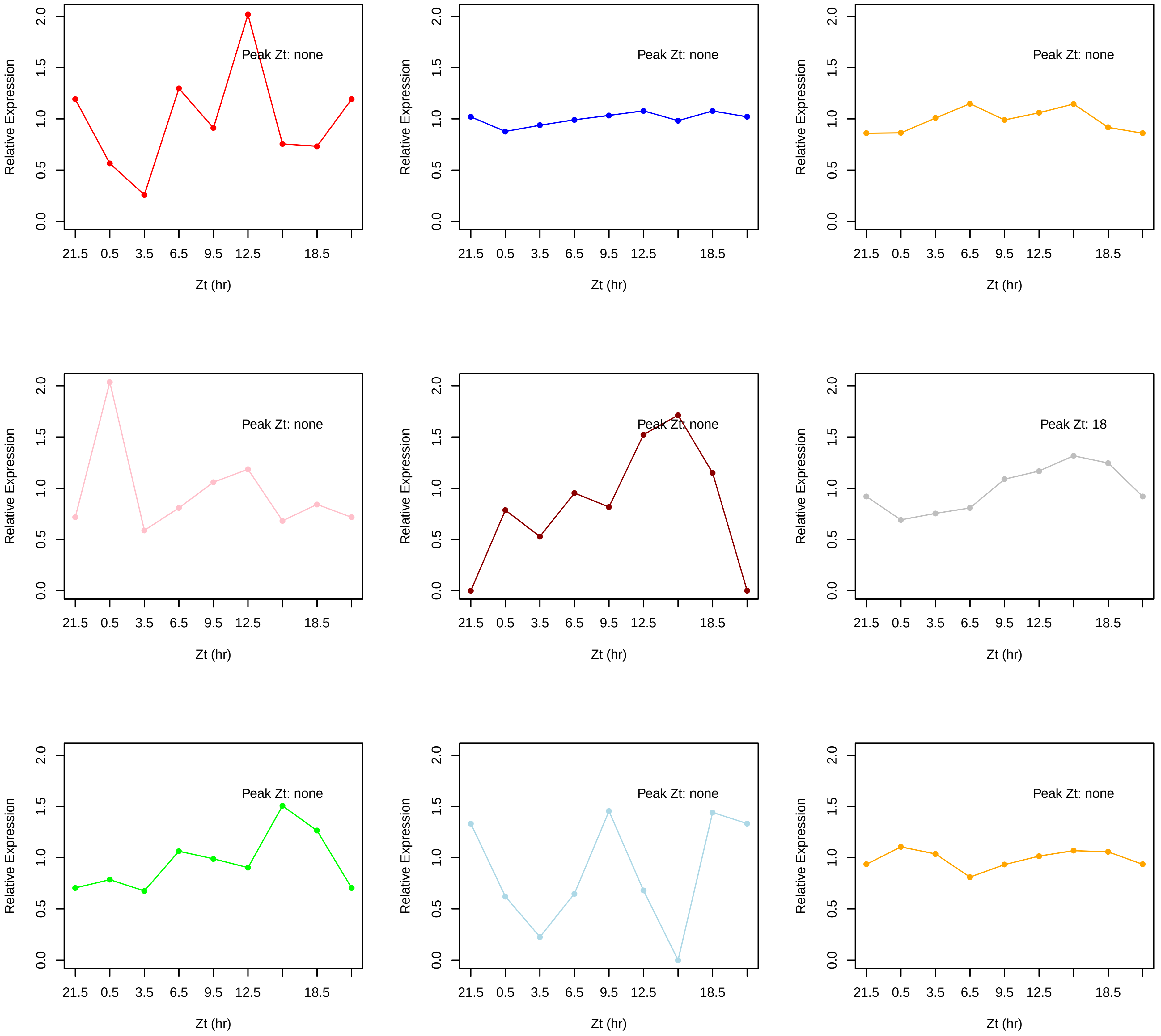

Singeleton: ENSXMAG00000022361 si:ch211−39k3.2

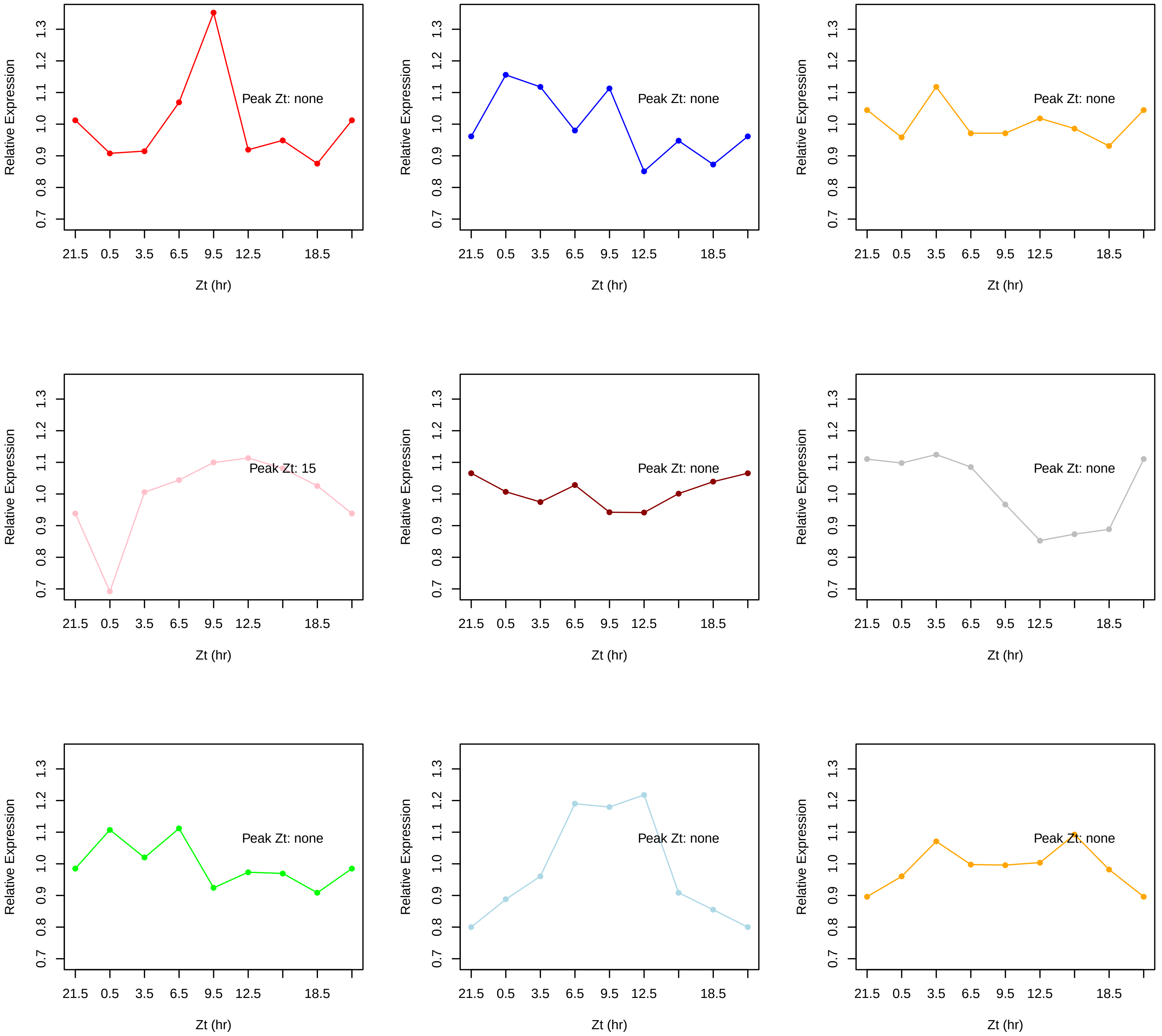

Singeleton: ENSXMAG00000007523

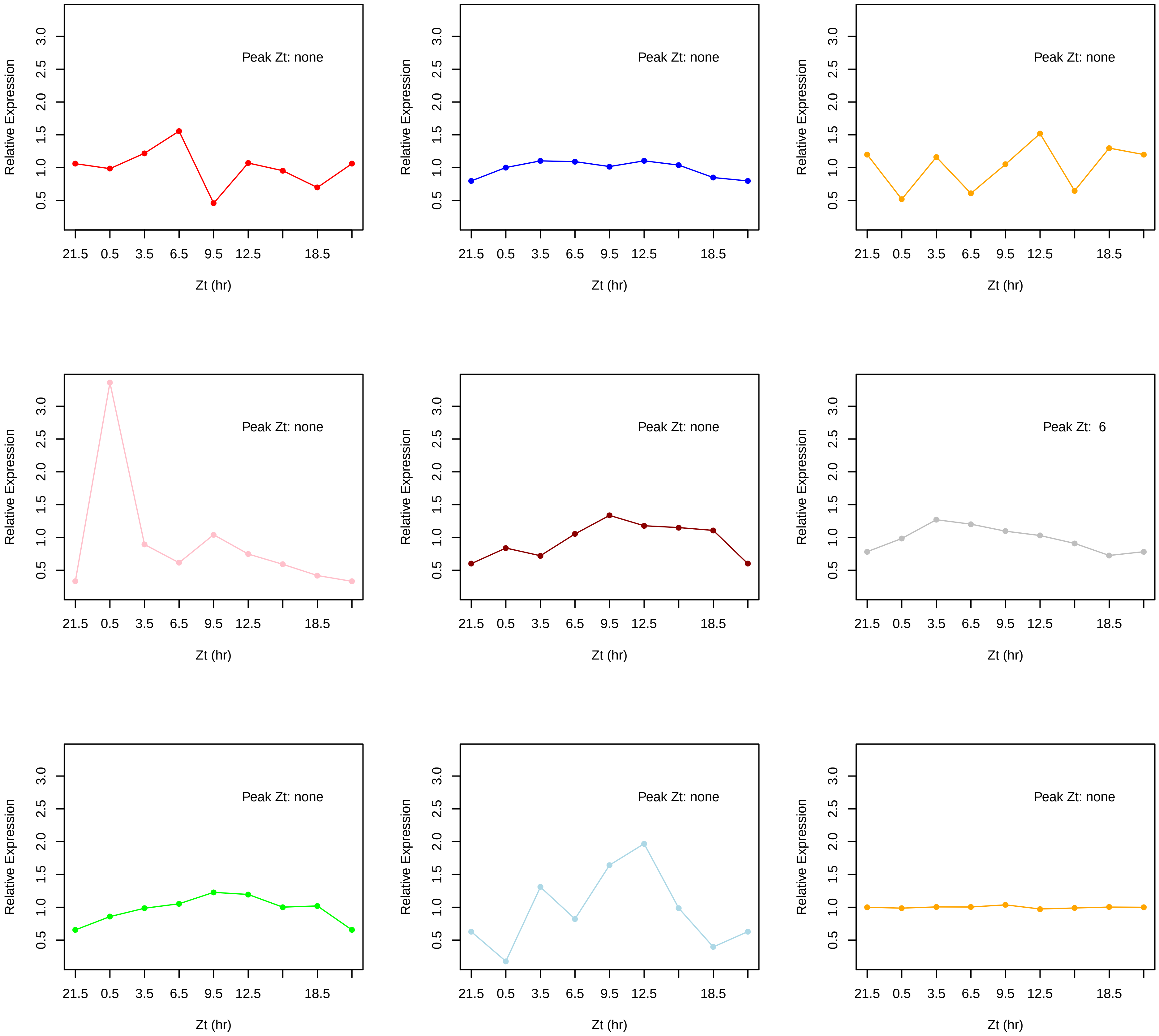

Singeleton: ENSXMAG00000003186 hif1al

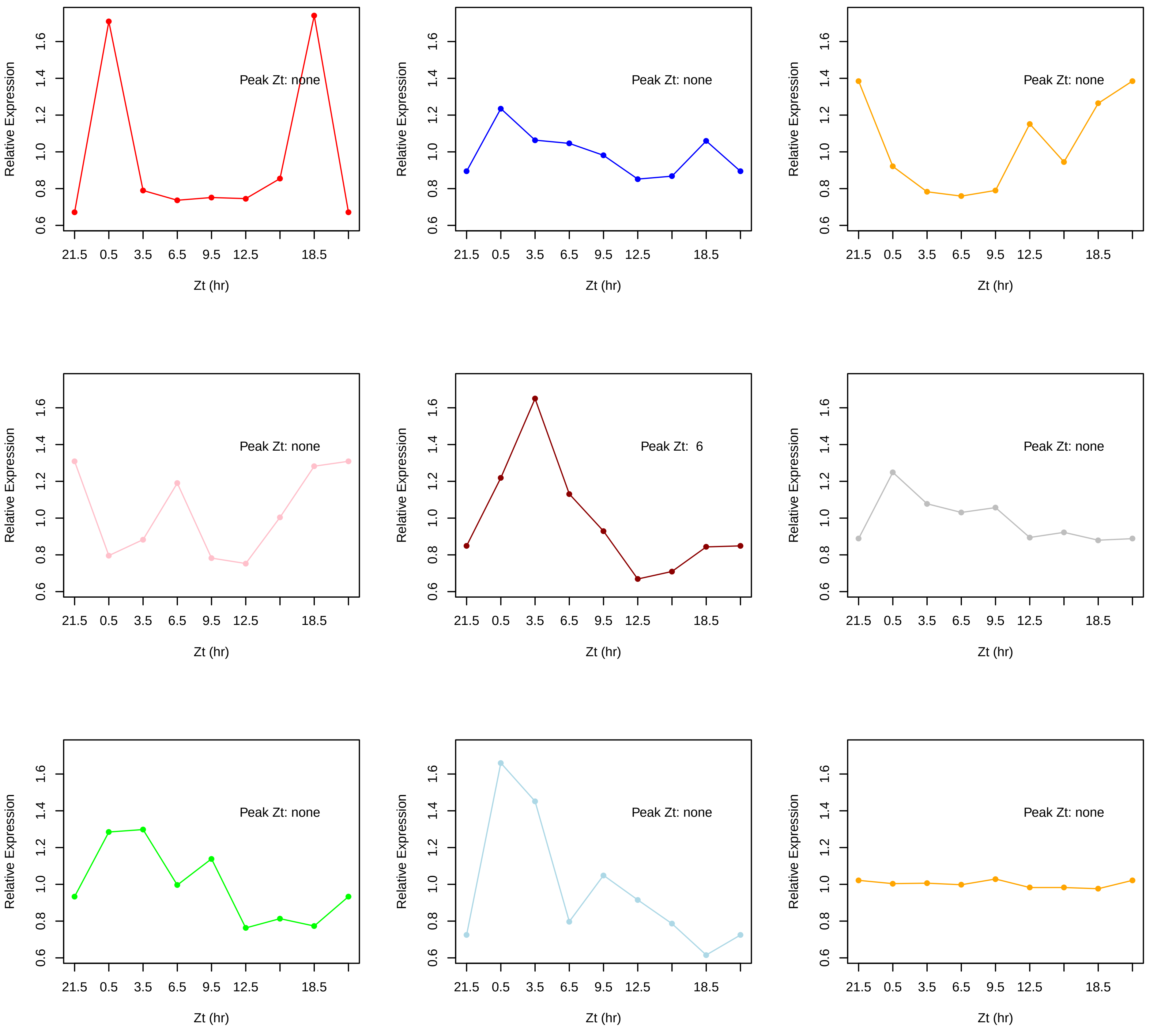

Singeleton: ENSXMAG00000003193 zgc:152863

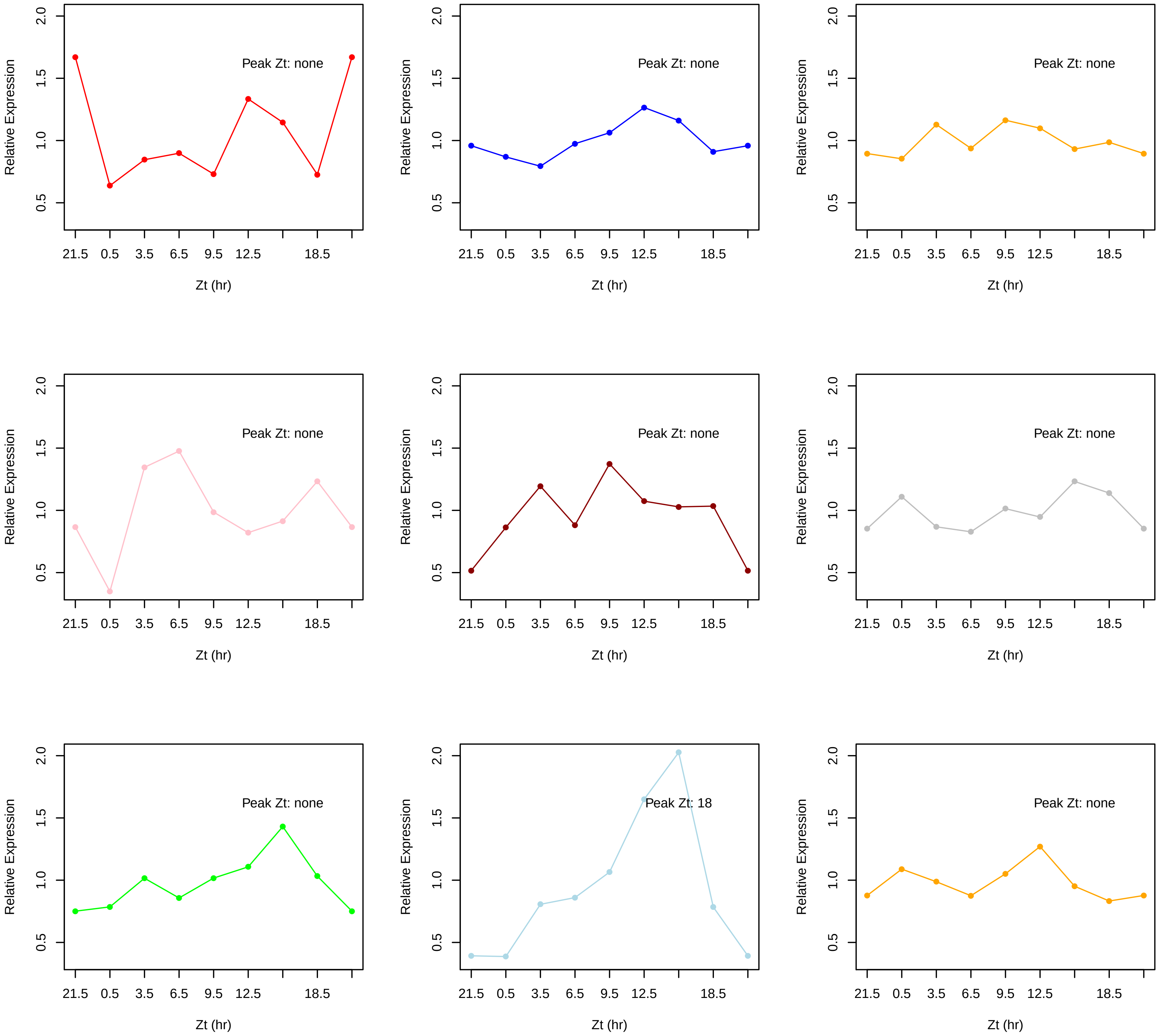

Singeleton: ENSXMAG00000014667 COL4A5

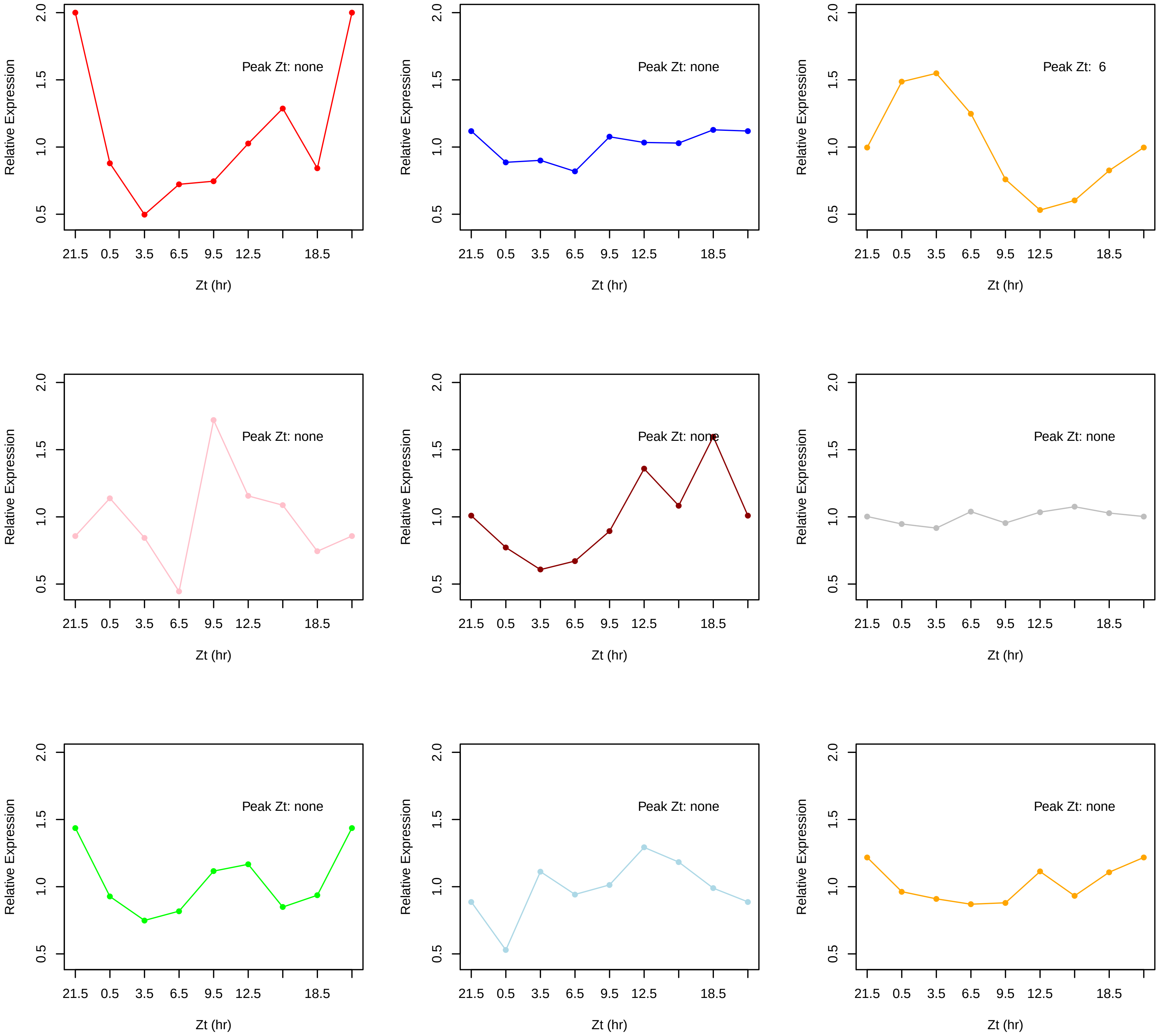

Singeleton: ENSXMAG00000003960 sae1

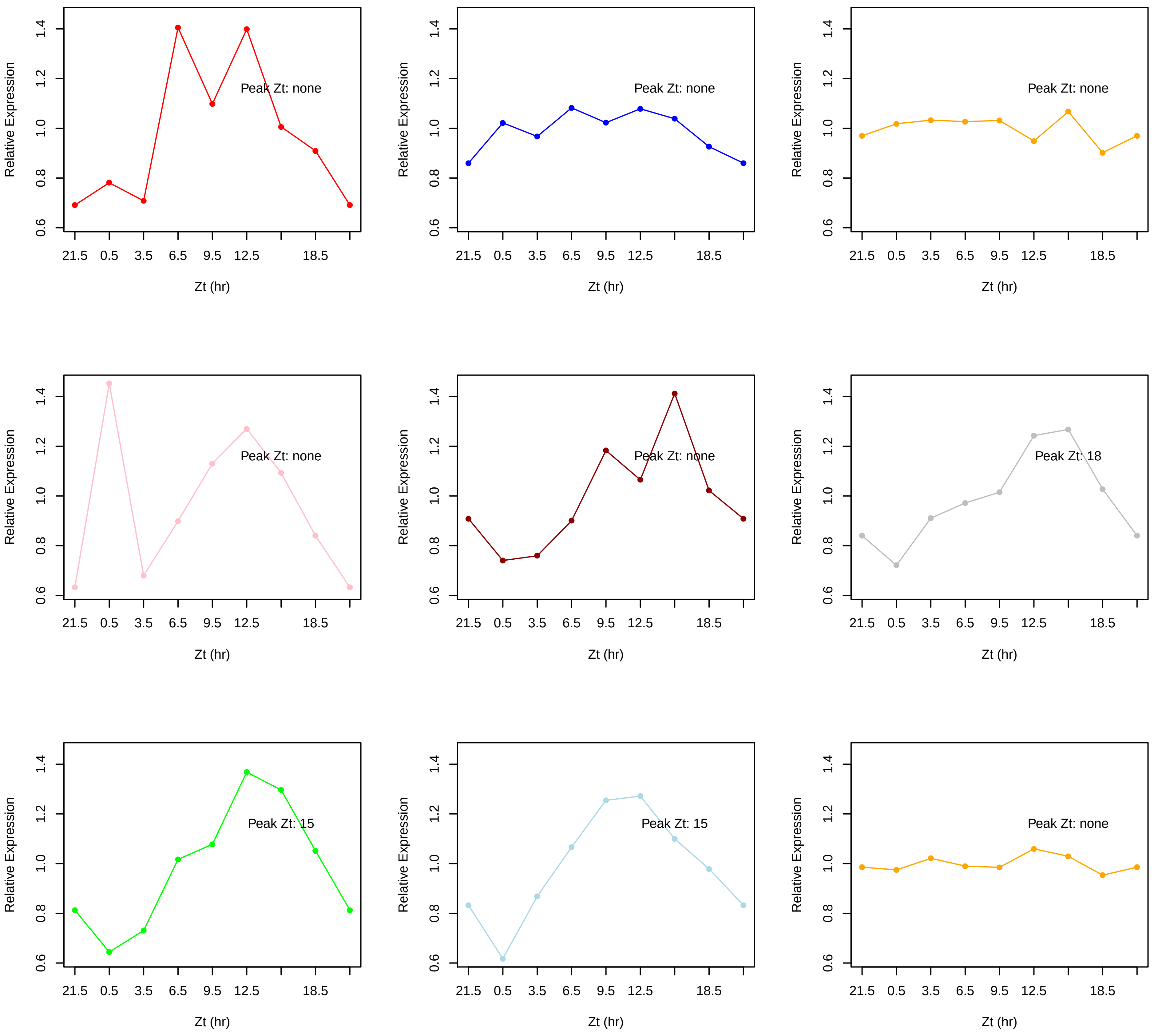

Singeleton: ENSXMAG00000029856 si:ch211−207e14.4

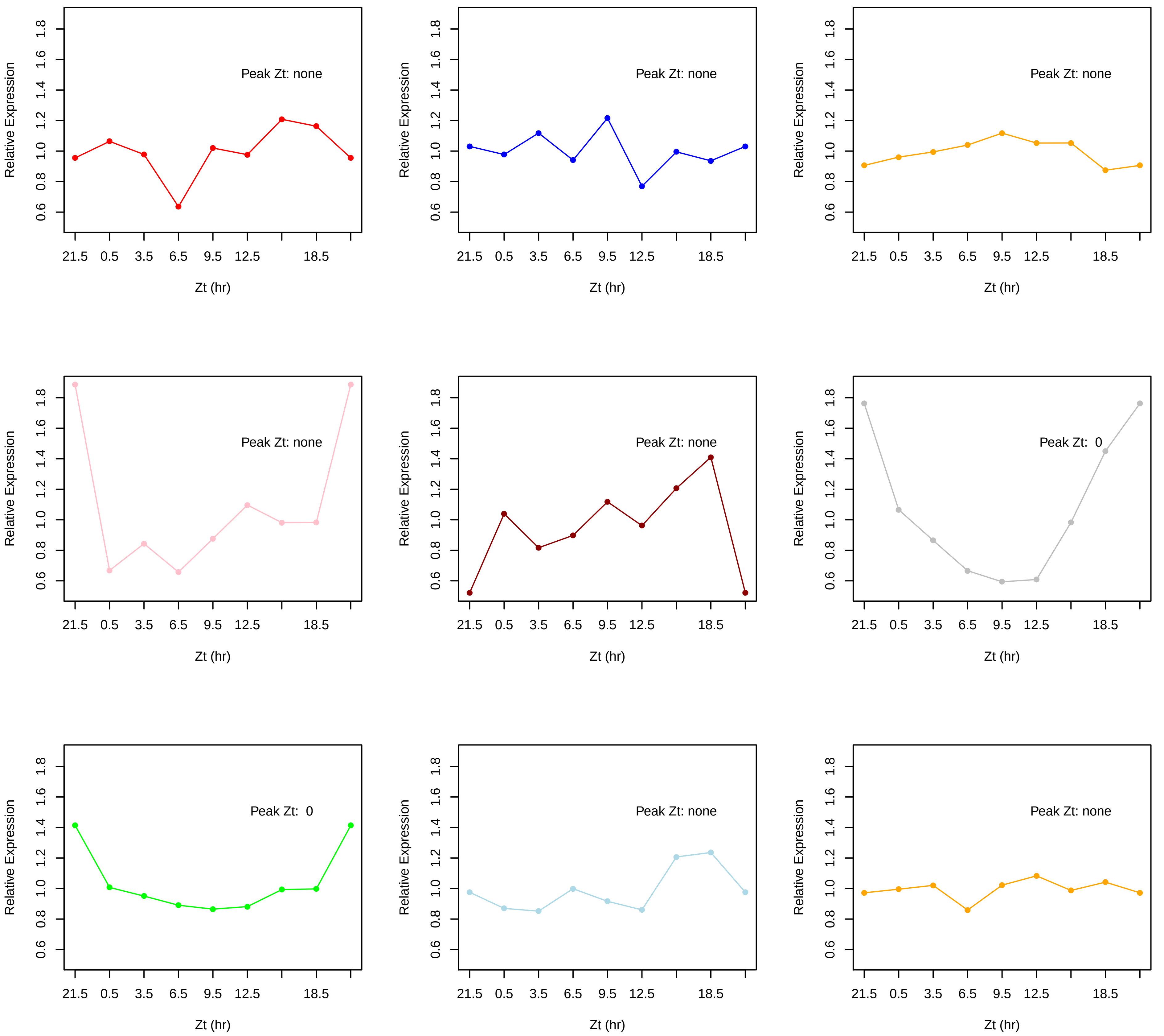

Singeleton: ENSXMAG00000016806 nckipsd

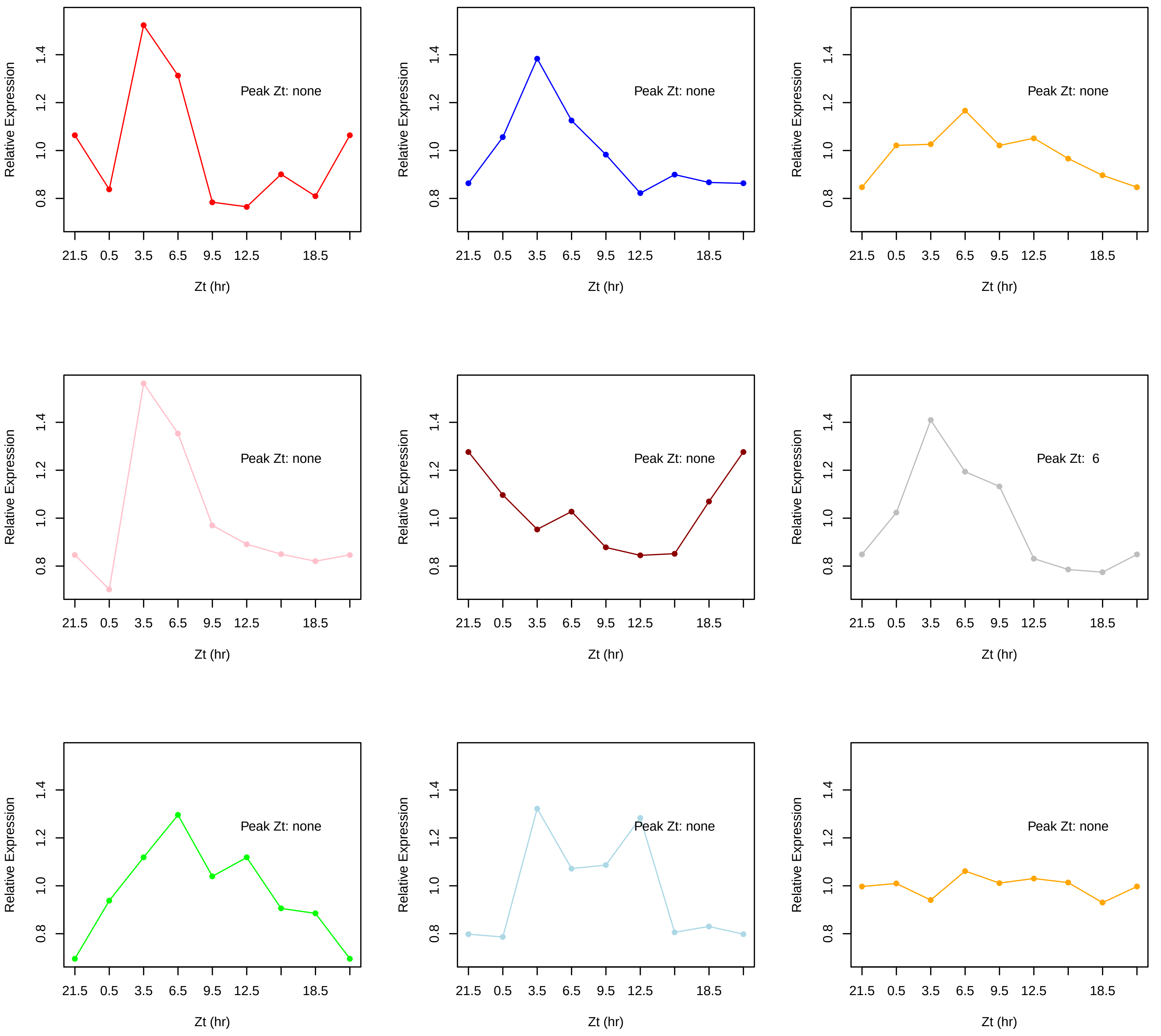

Singeleton: ENSXMAG00000003922 ercc2

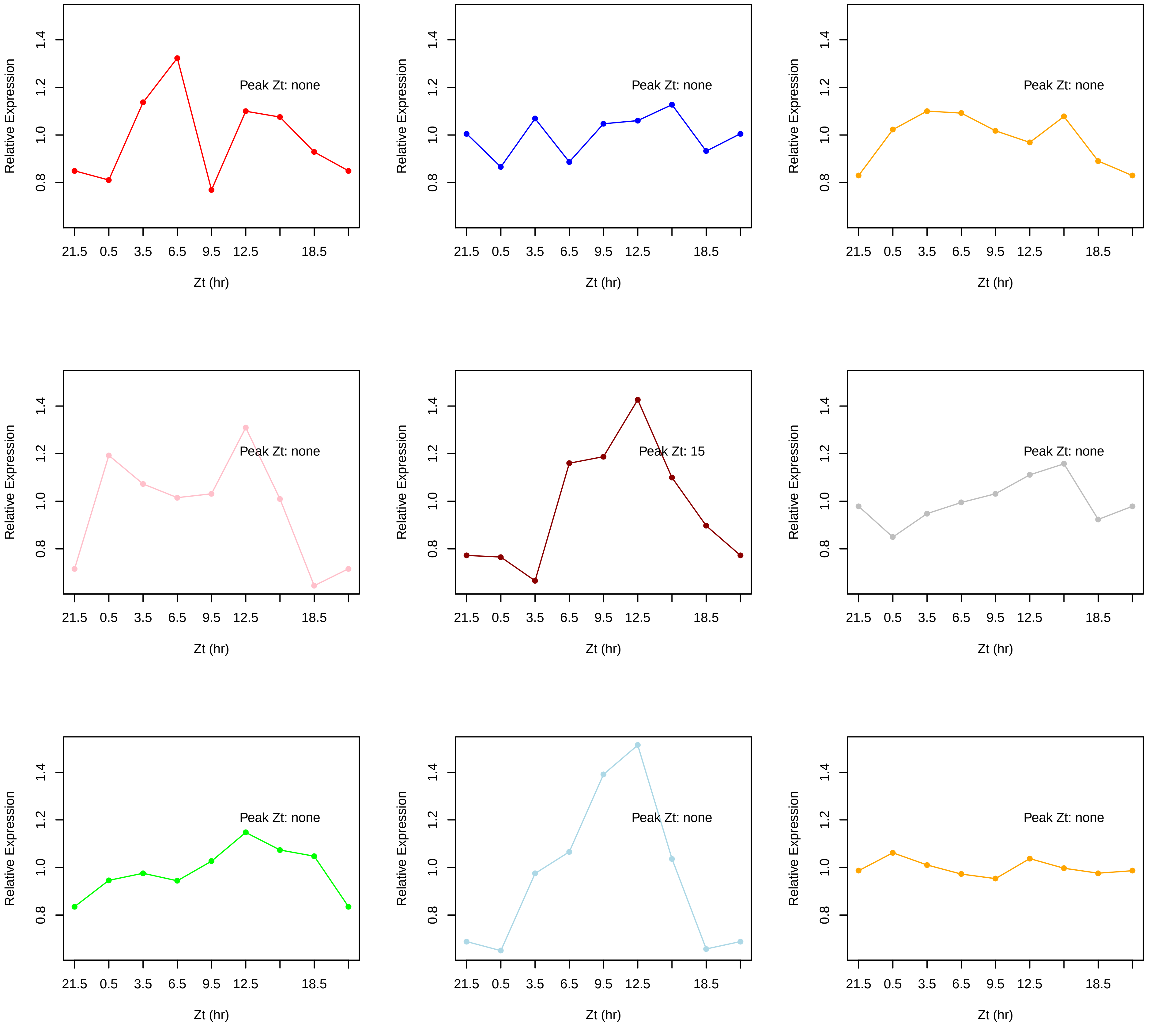

Singeleton: ENSXMAG00000002707 chd2

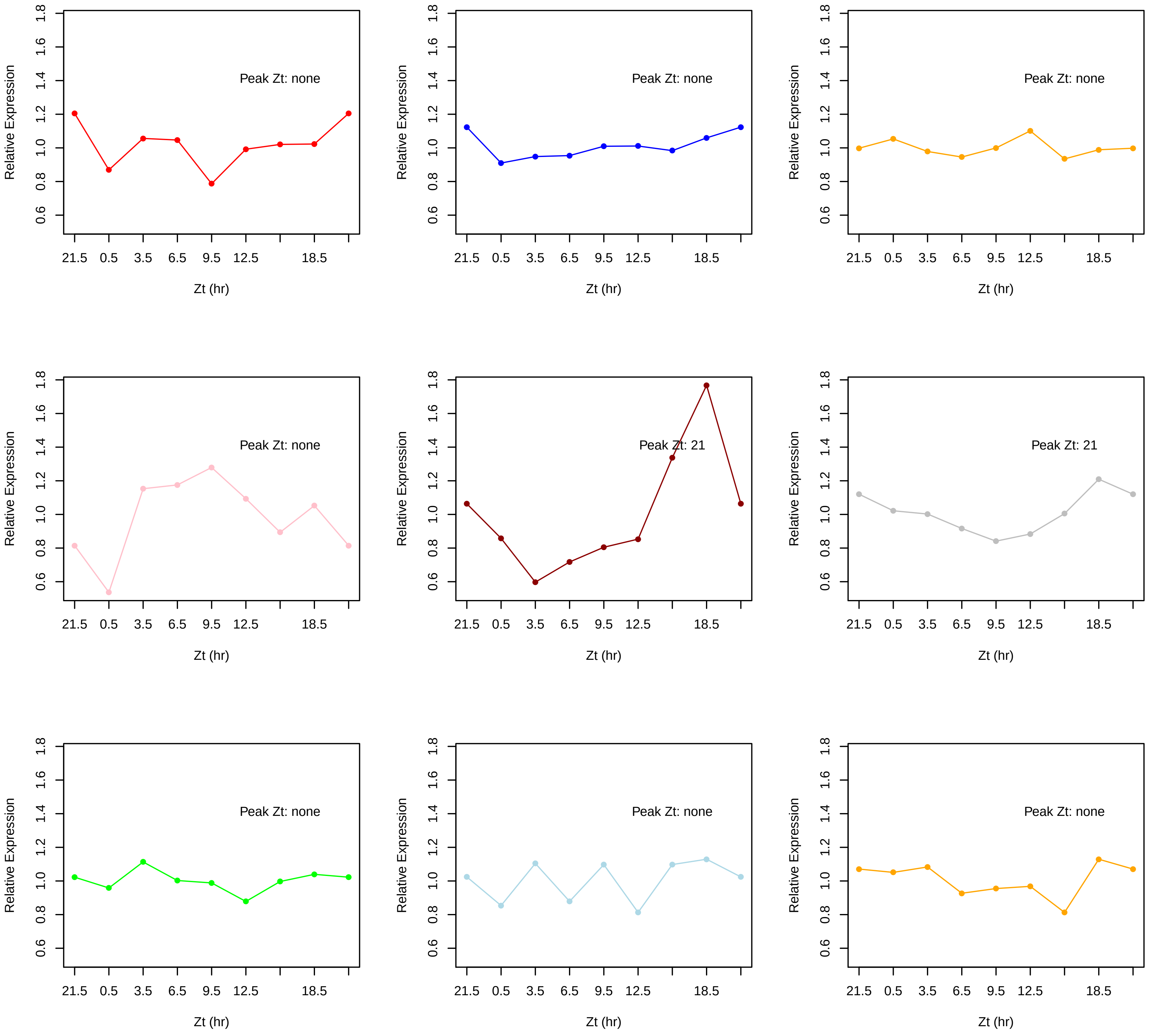

Singeleton: ENSXMAG00000002710 dpf1

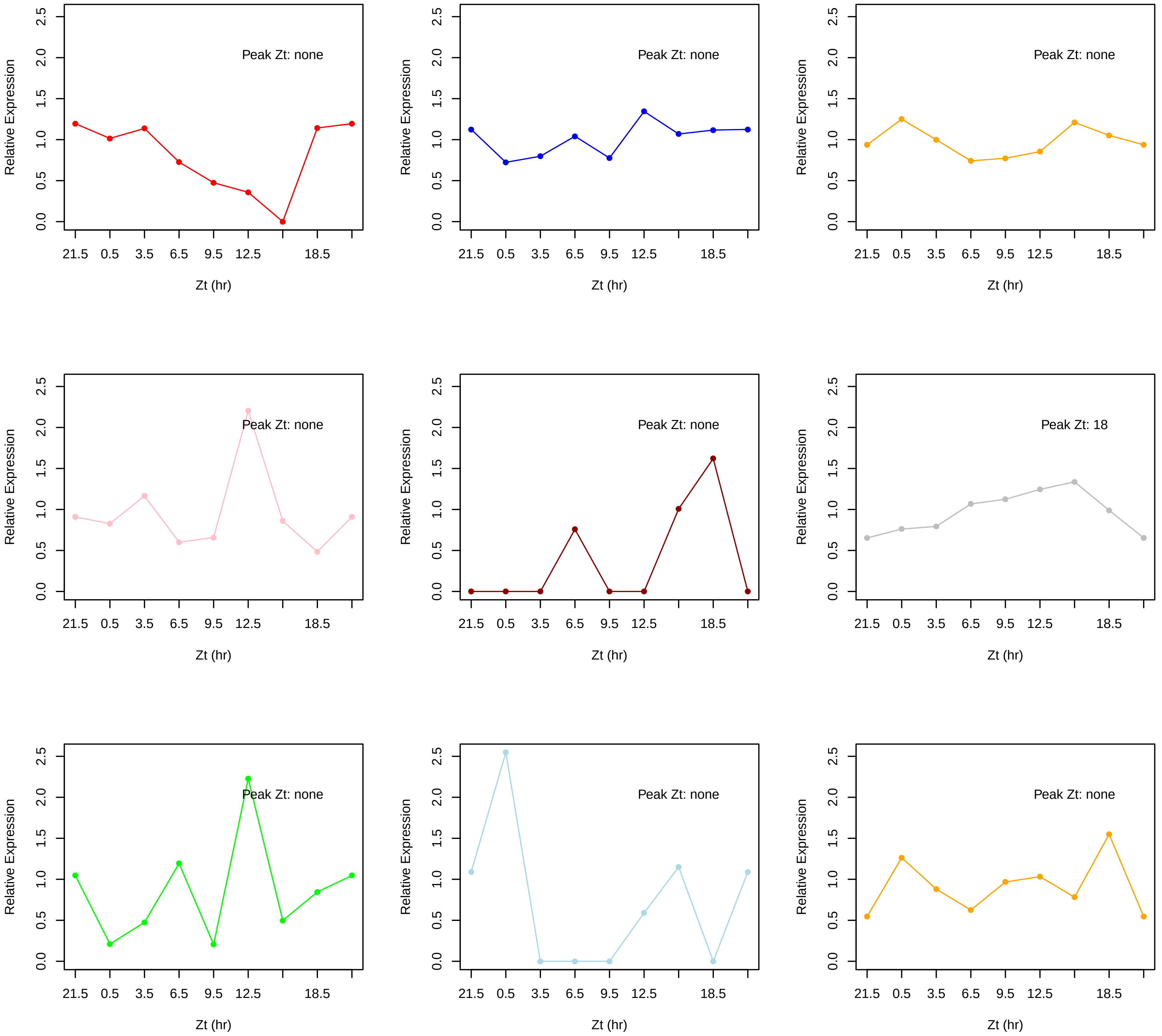

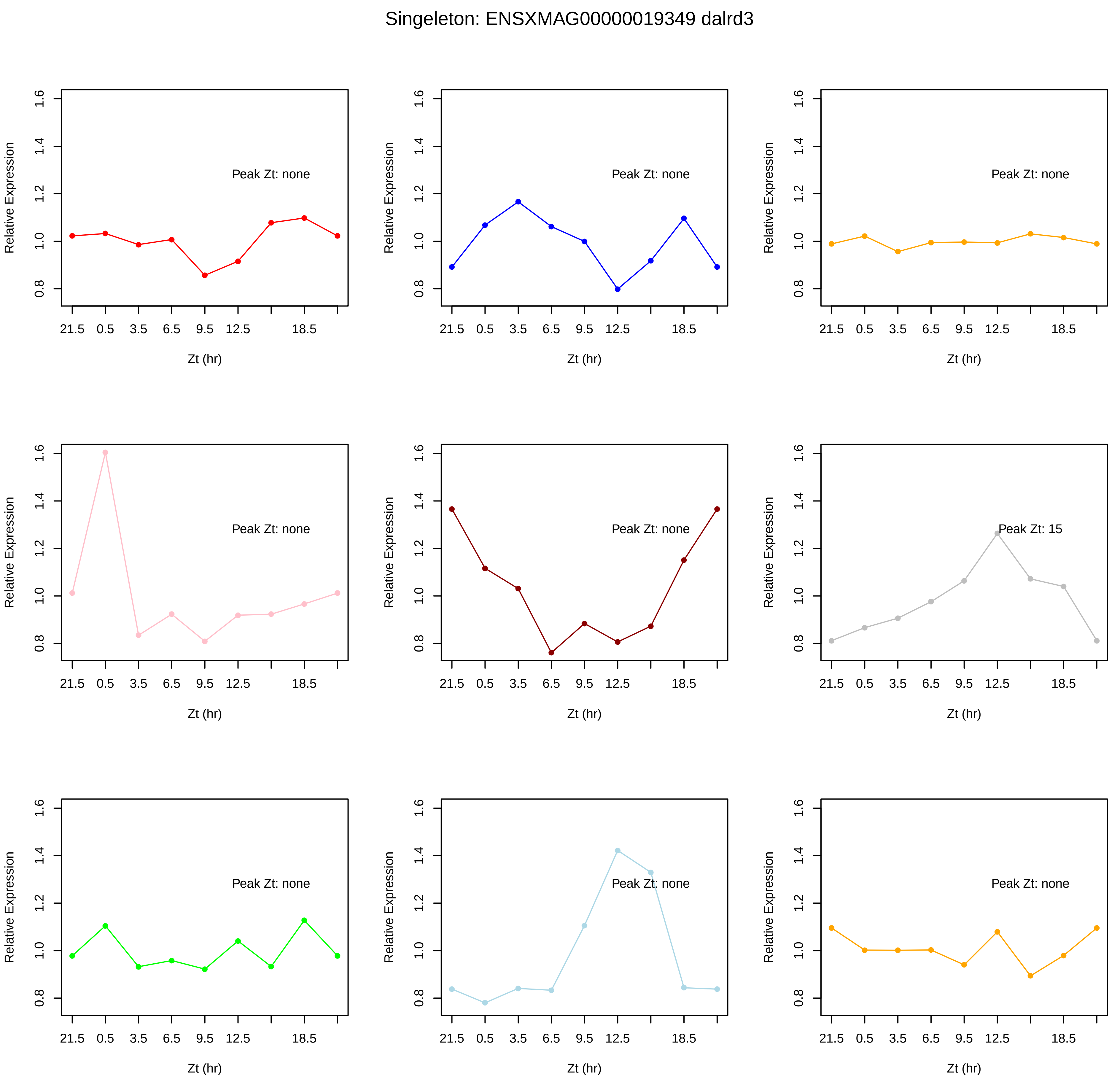
Singleton: ENSXMAG00000019349 dalrd3
Relative Expression
Zt (hr)
21.5
0.5
3.5
6.5
9.5
12.5
18.5
0.8
1.0
1.2
1.4
1.6
Peak Zt: none
Peak Zt: none
Peak Zt: none
Peak Zt: none
Peak Zt: none
Peak Zt: 15
Peak Zt: none
Peak Zt: none
Peak Zt: none

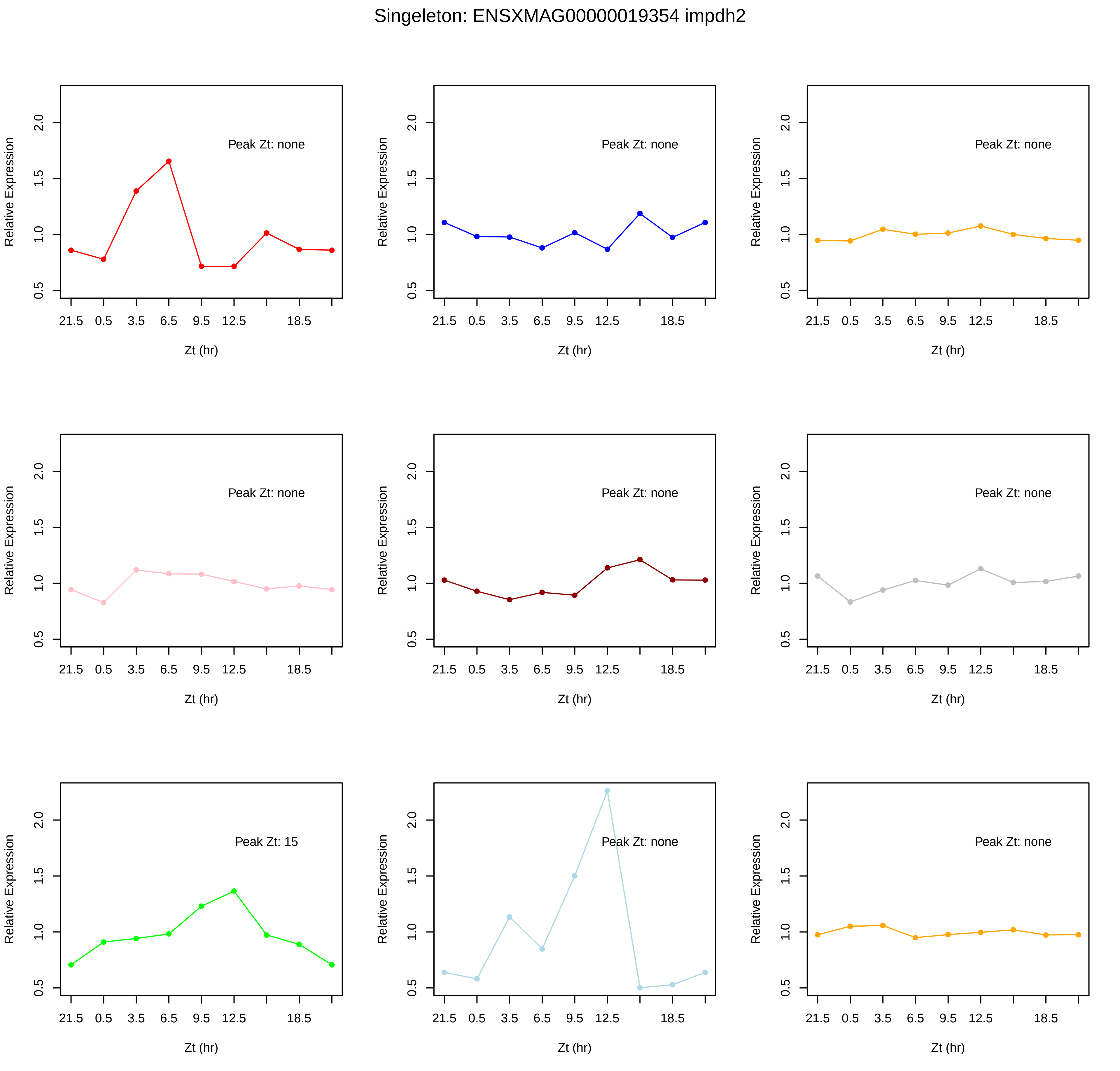
Singeleton: ENSXMAG00000019354 impdh2
Peak Zt: none
Peak Zt: none
Peak Zt: none
Peak Zt: none
Peak Zt: none
Peak Zt: none
Peak Zt: 15
Peak Zt: none
Peak Zt: none
Relative Expression
Zt (hr)
21.5
0.5
3.5
6.5
9.5
12.5
18.5
0.5
1.0
1.5
2.0

Singeleton: ENSXMAG00000002704 rgma

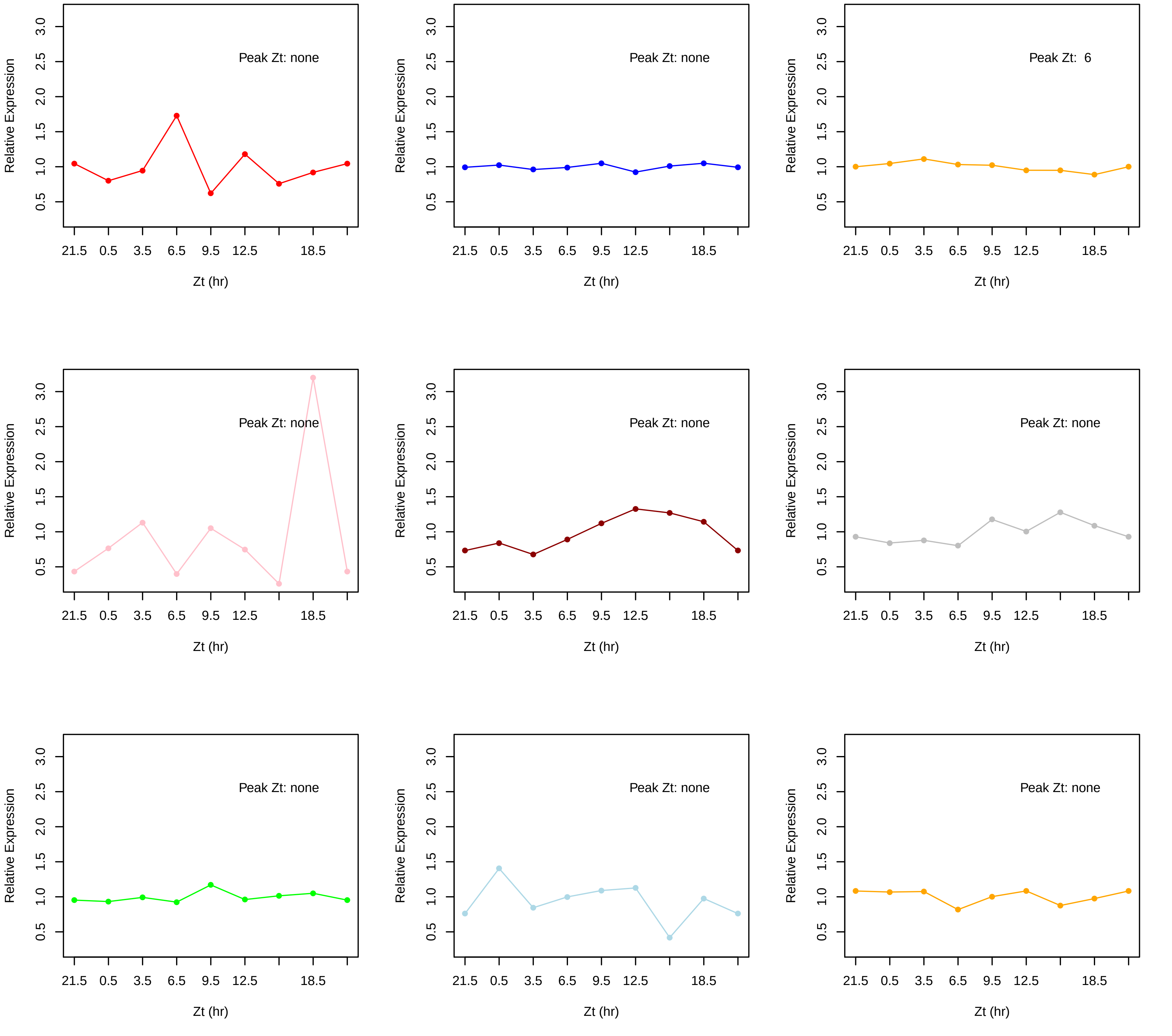

Singeleton: ENSXMAG00000004915 imp4

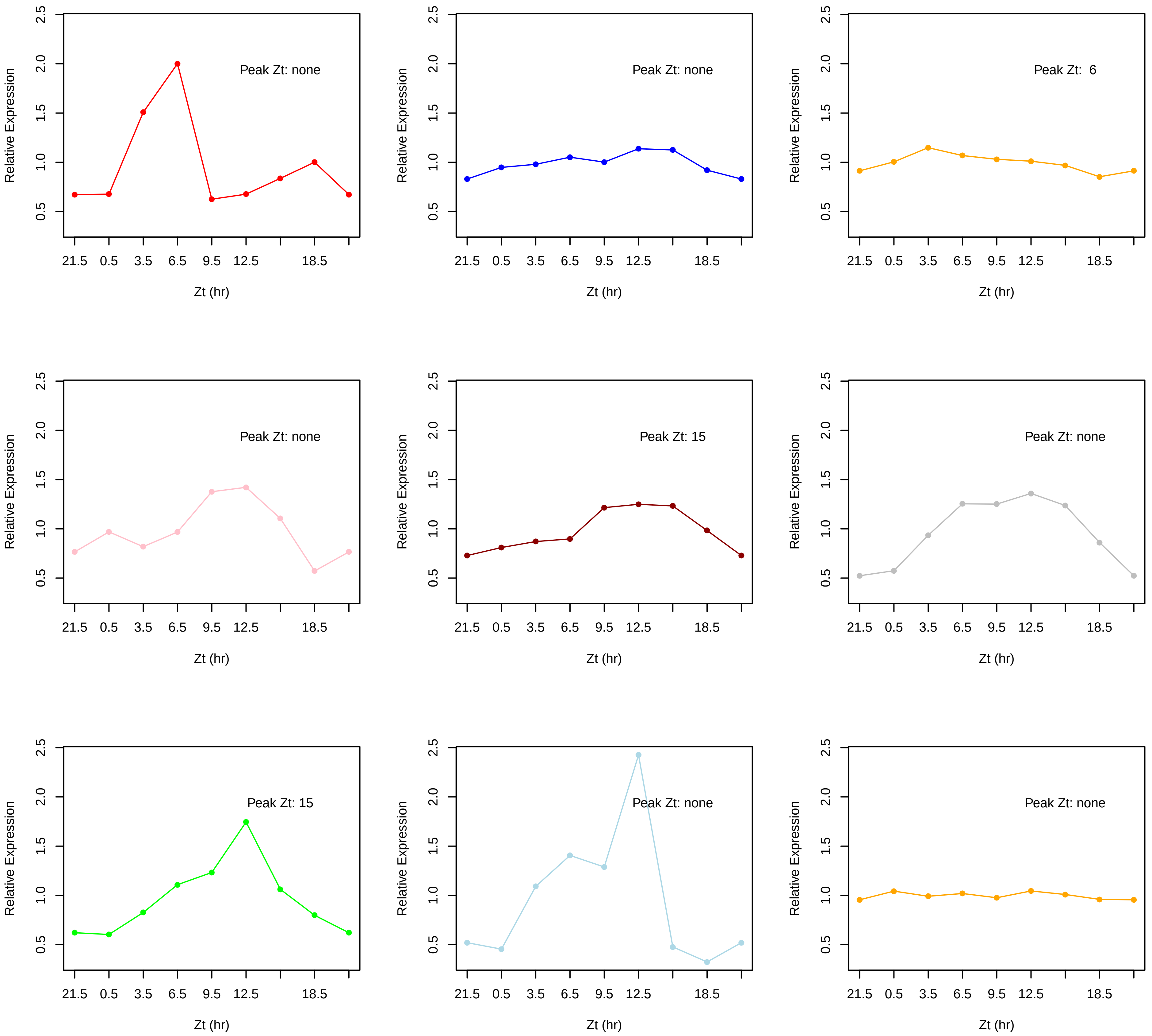

Singleton: ENSXMAG00000019355 arih2

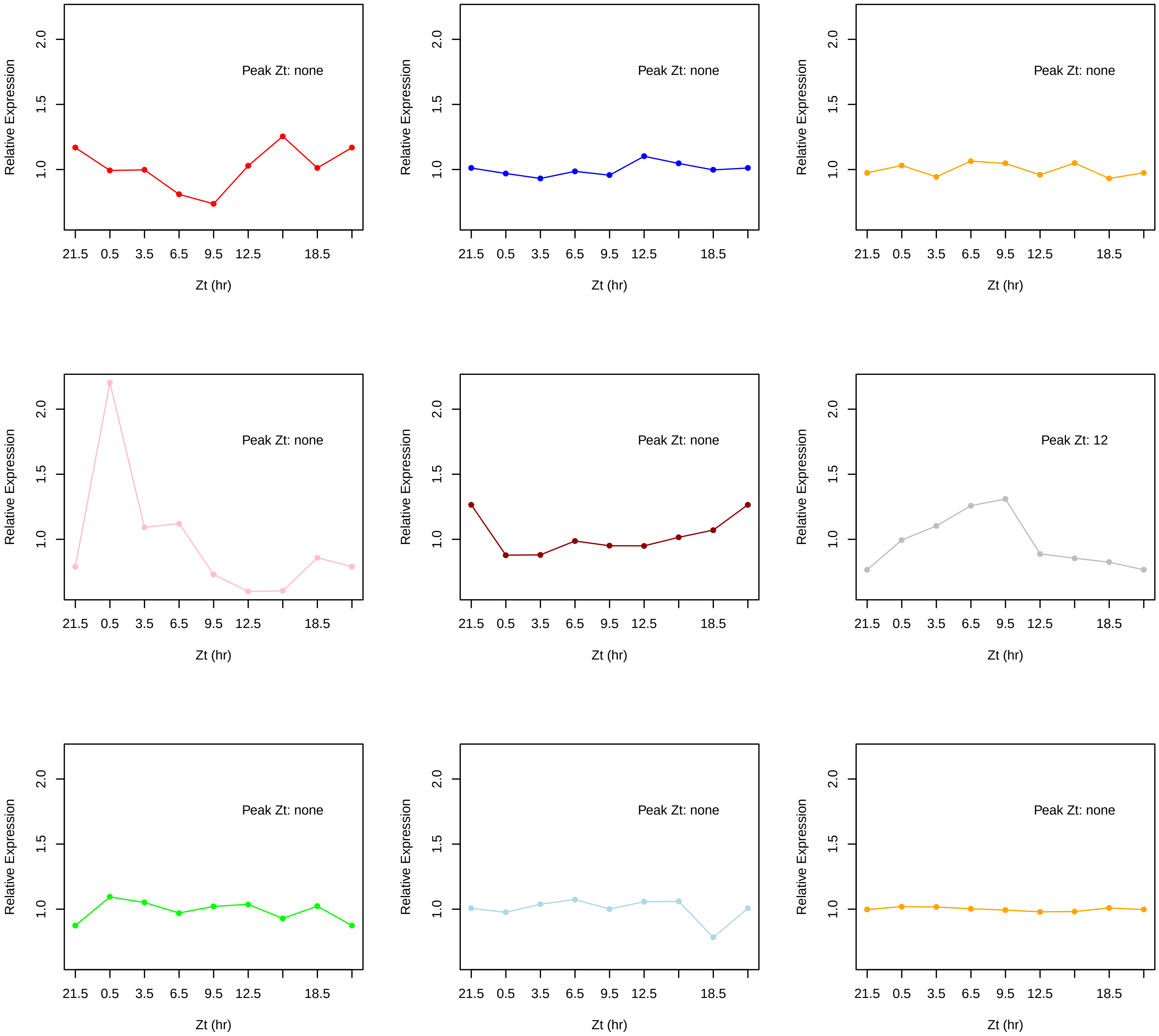

Singeleton: ENSXMAG00000015071 syn1

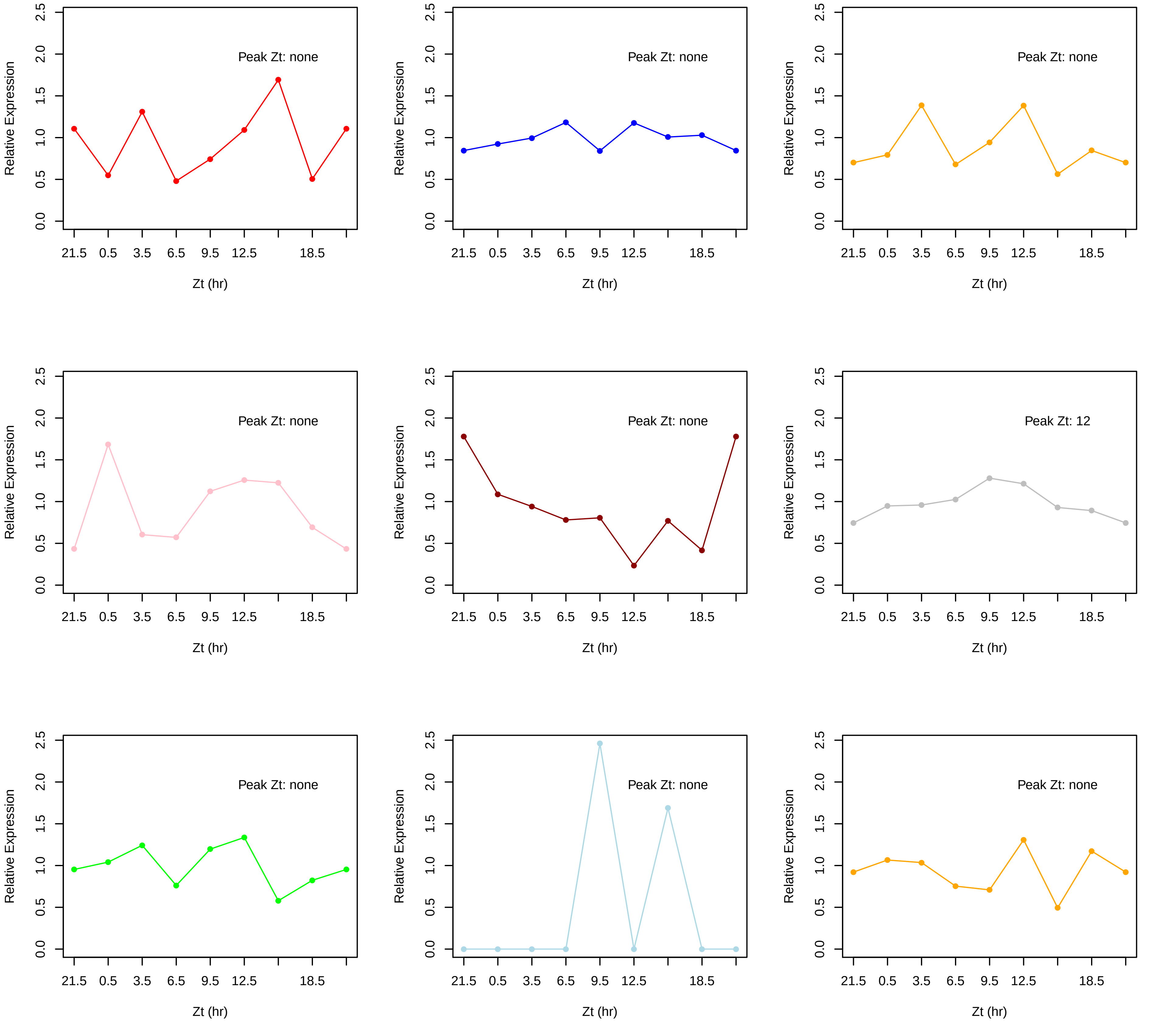

Singeleton: ENSXMAG00000014729 gc2

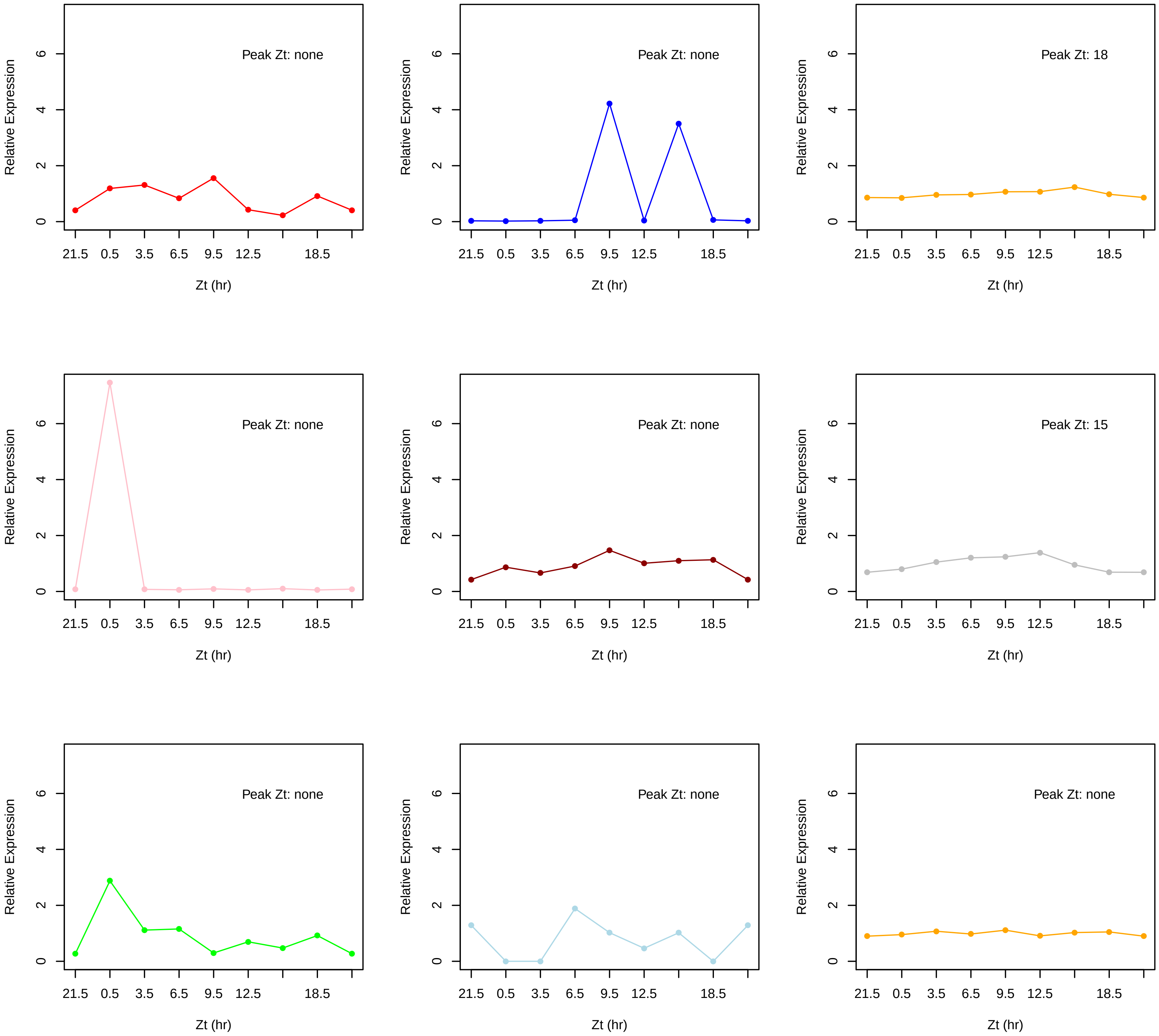

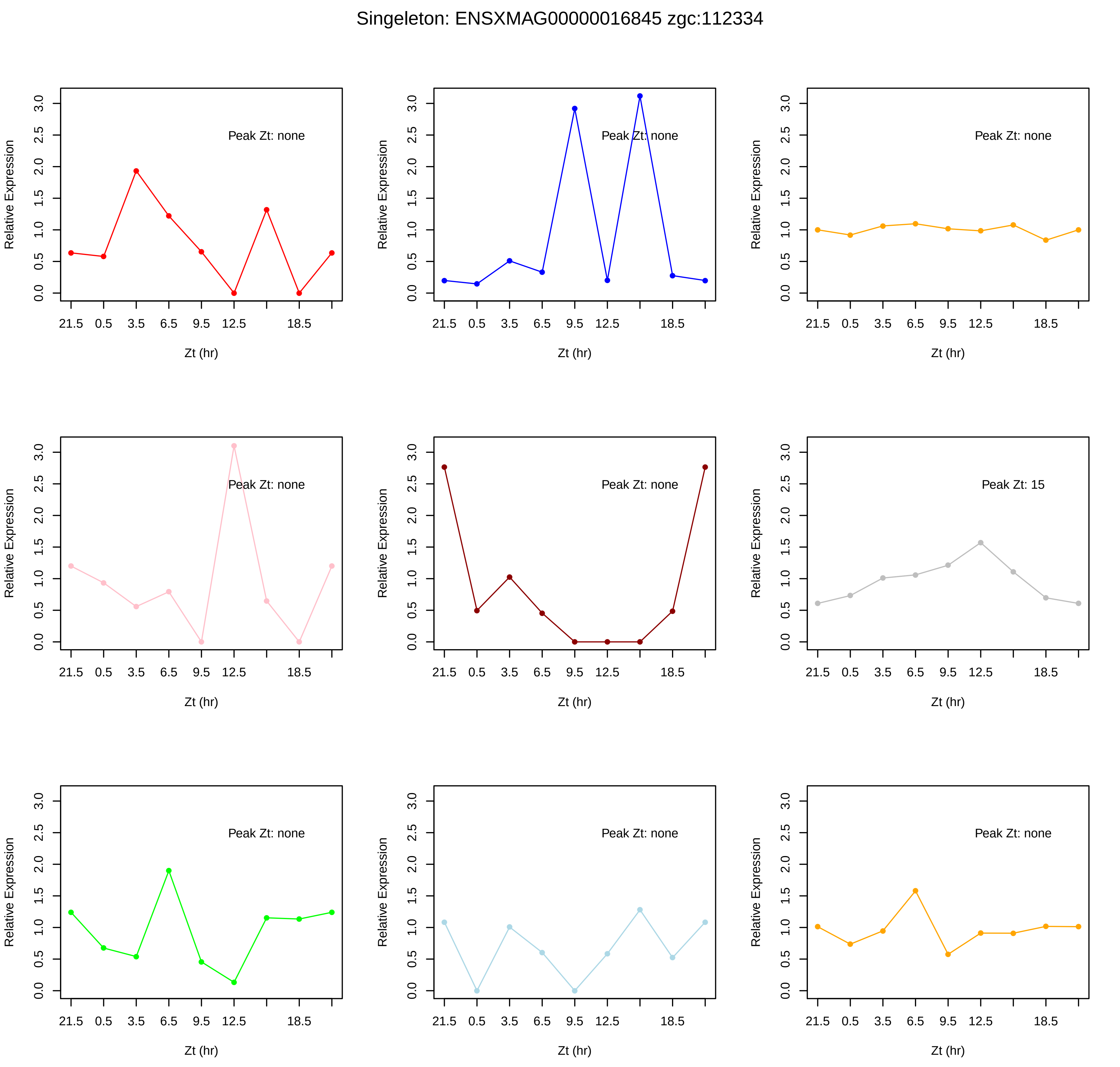
Singeleton: ENSXMAG00000016845 zgc:112334
Peak Zt: none
Peak Zt: none
Peak Zt: none
Peak Zt: none
Peak Zt: none
Peak Zt: 15
Peak Zt: none
Peak Zt: none
Peak Zt: none
Relative Expression
Zt (hr)
21.5
0.5
3.5
6.5
9.5
12.5
18.5

Singeleton: ENSXMAG00000008131 ccdc115

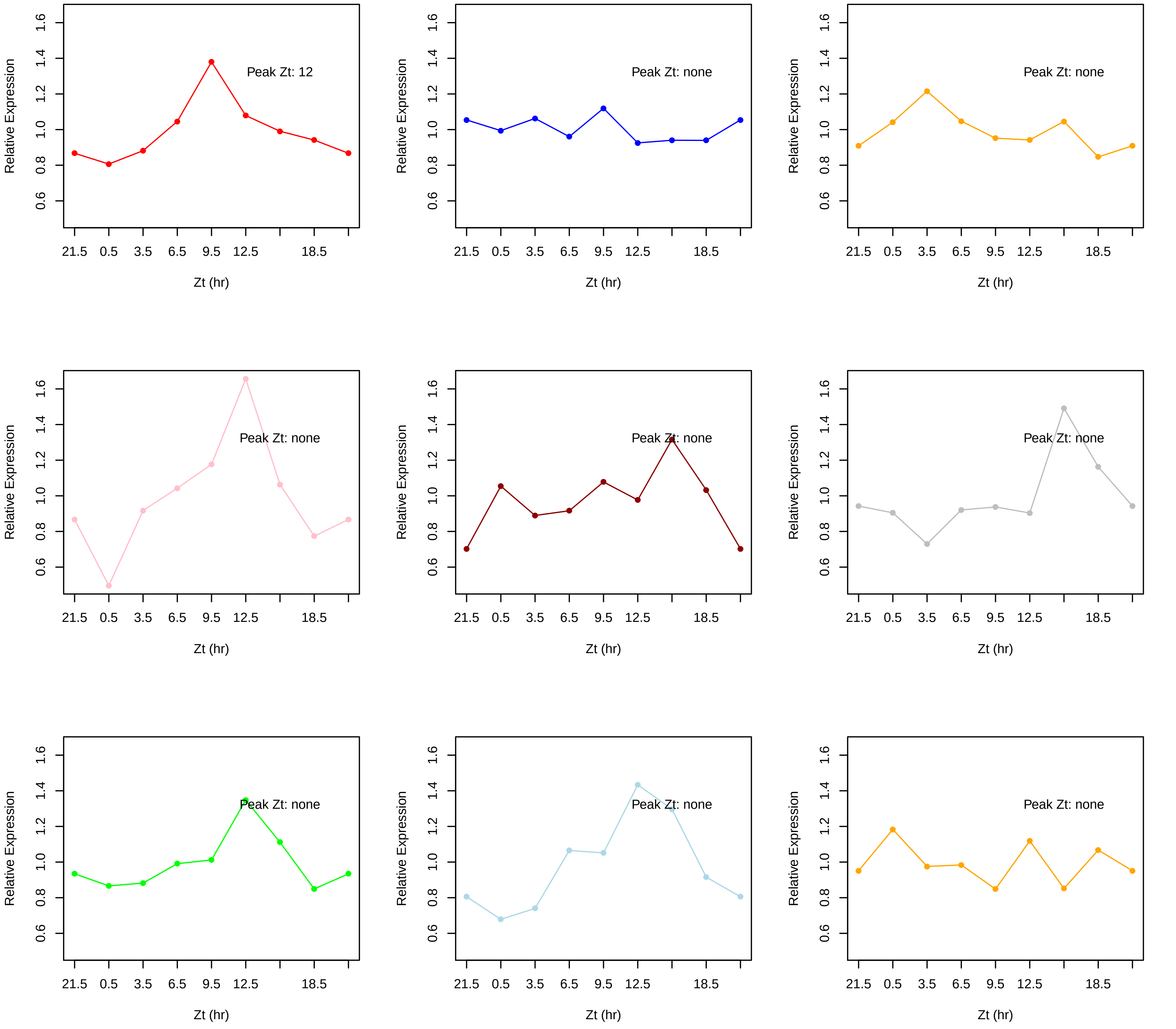

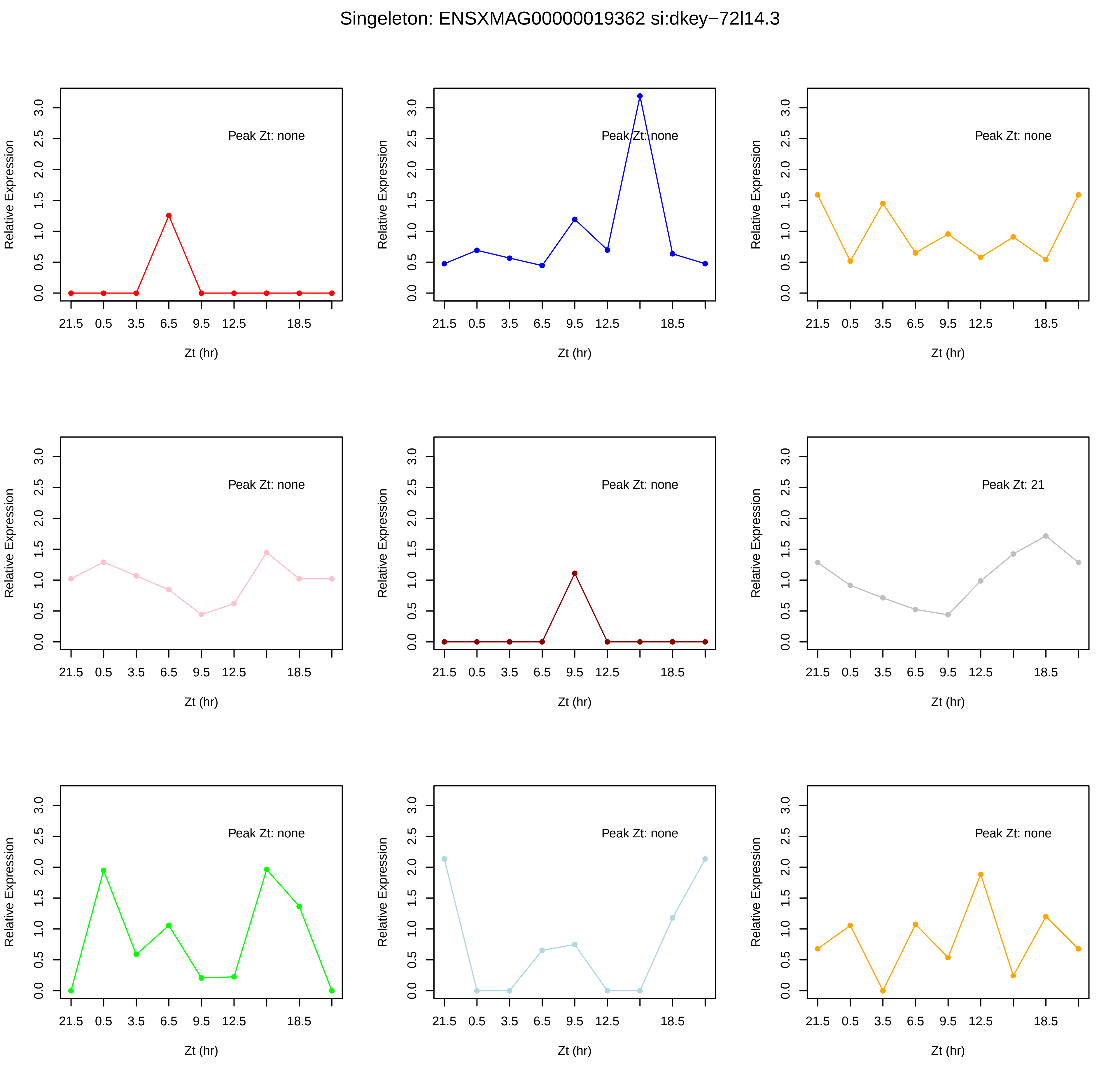
Singeleton: ENSXMAG00000019362 si:dkey−72l14.3
Peak Zt: none
Peak Zt: none
Peak Zt: none
Peak Zt: none
Peak Zt: none
Peak Zt: 21
Peak Zt: none
Peak Zt: none
Peak Zt: none
Relative Expression
Zt (hr)
21.5
0.5
3.5
6.5
9.5
12.5
18.5

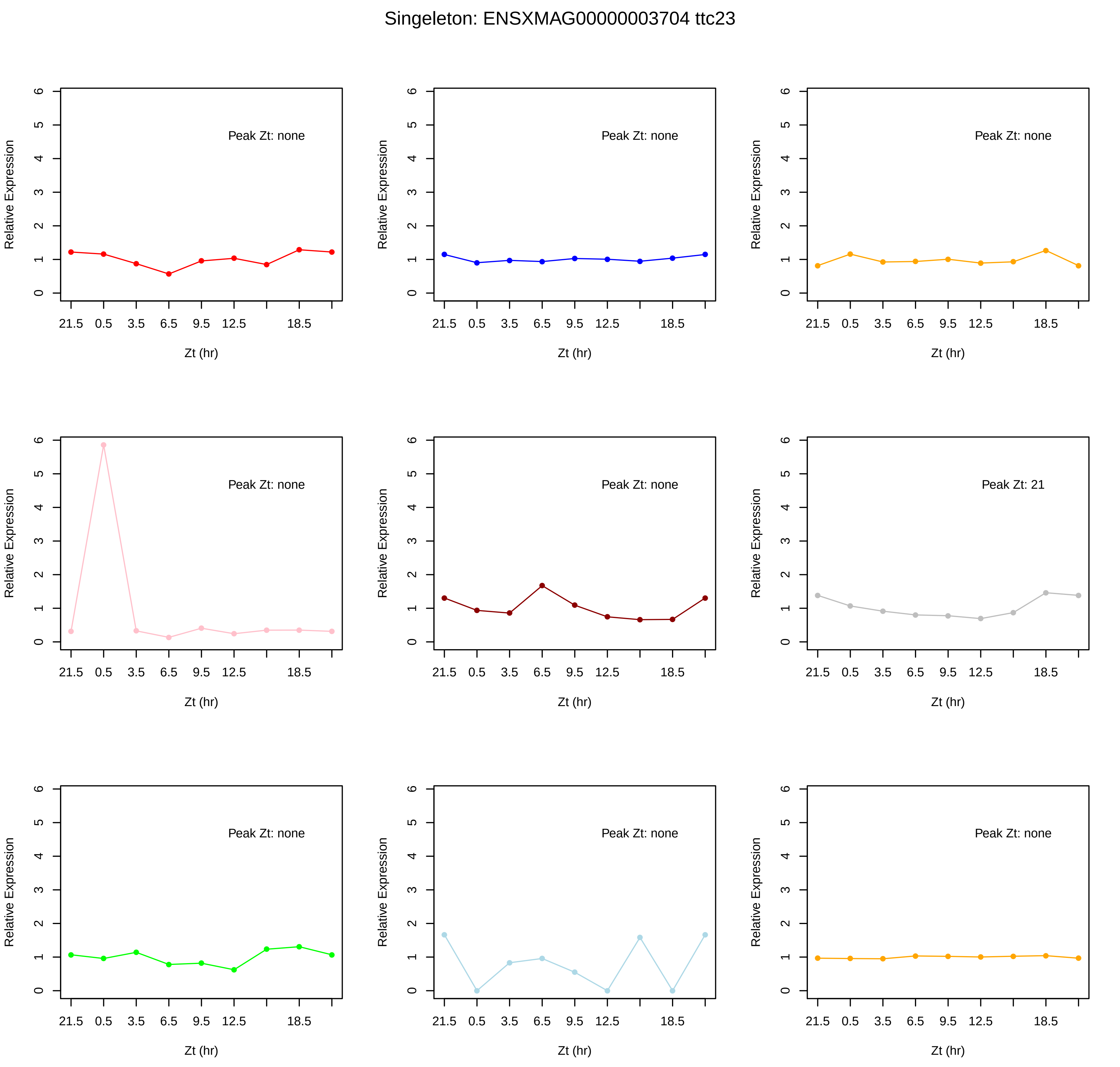
Singeleton: ENSXMAG00000003704 ttc23
Peak Zt: none
Peak Zt: none
Peak Zt: none
Peak Zt: none
Peak Zt: none
Peak Zt: 21
Peak Zt: none
Peak Zt: none
Peak Zt: none
Relative Expression
Zt (hr)
21.5
0.5
3.5
6.5
9.5
12.5
18.5

Singeleton: ENSXMAG00000023032 rasl11a

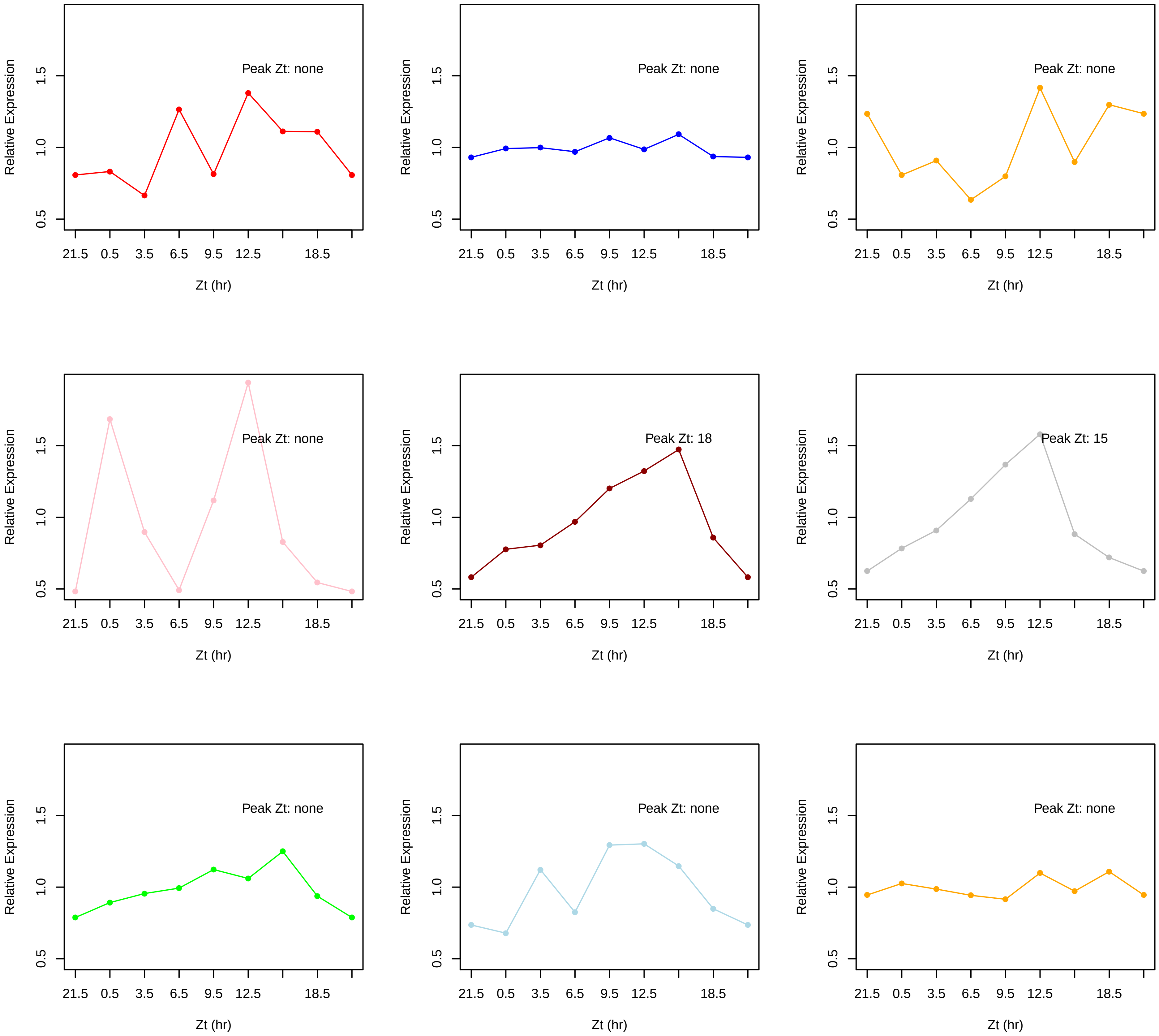

Singeleton: ENSXMAG00000003710 synm

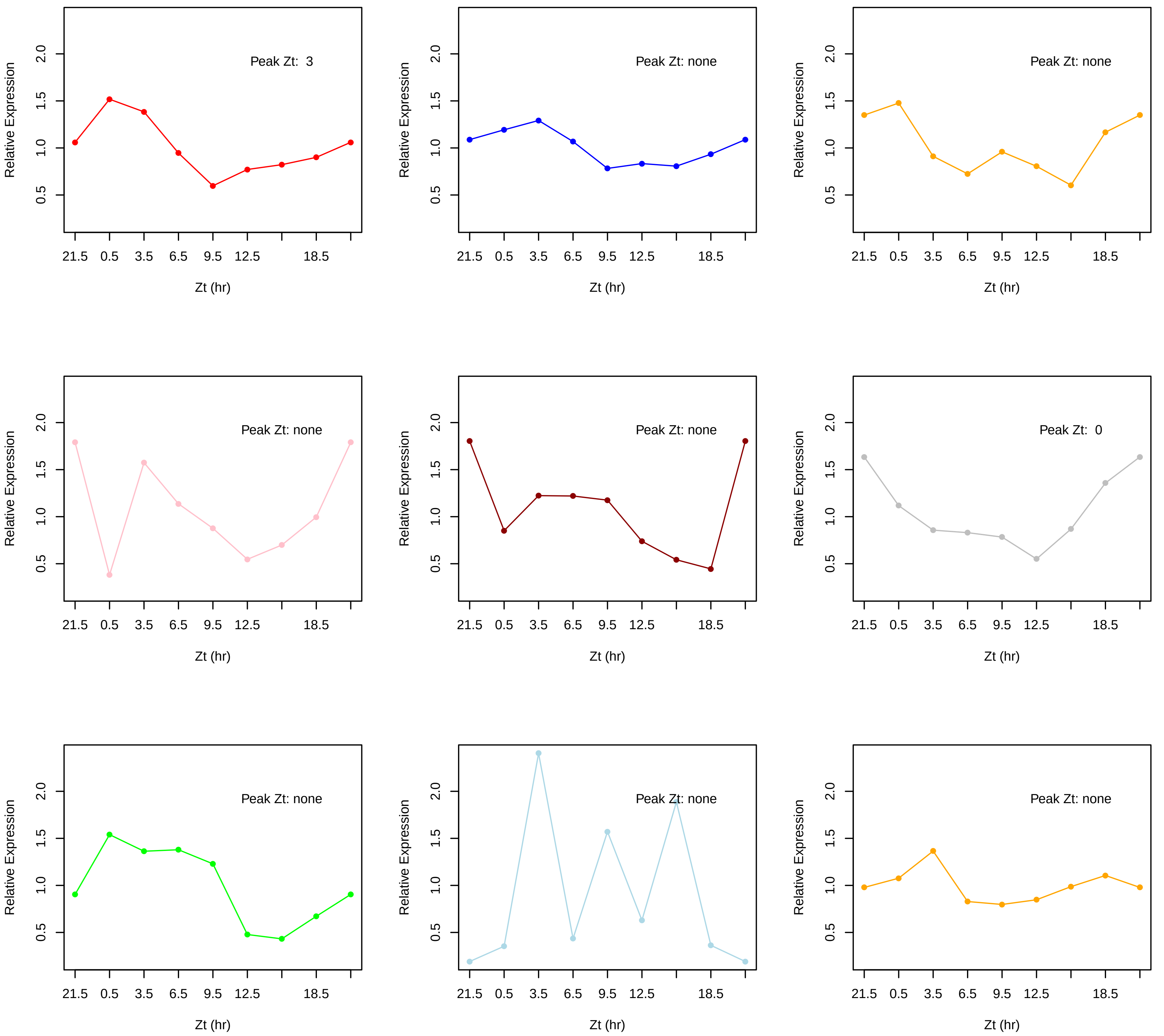

Singeleton: ENSXMAG00000025552 pgpep1l

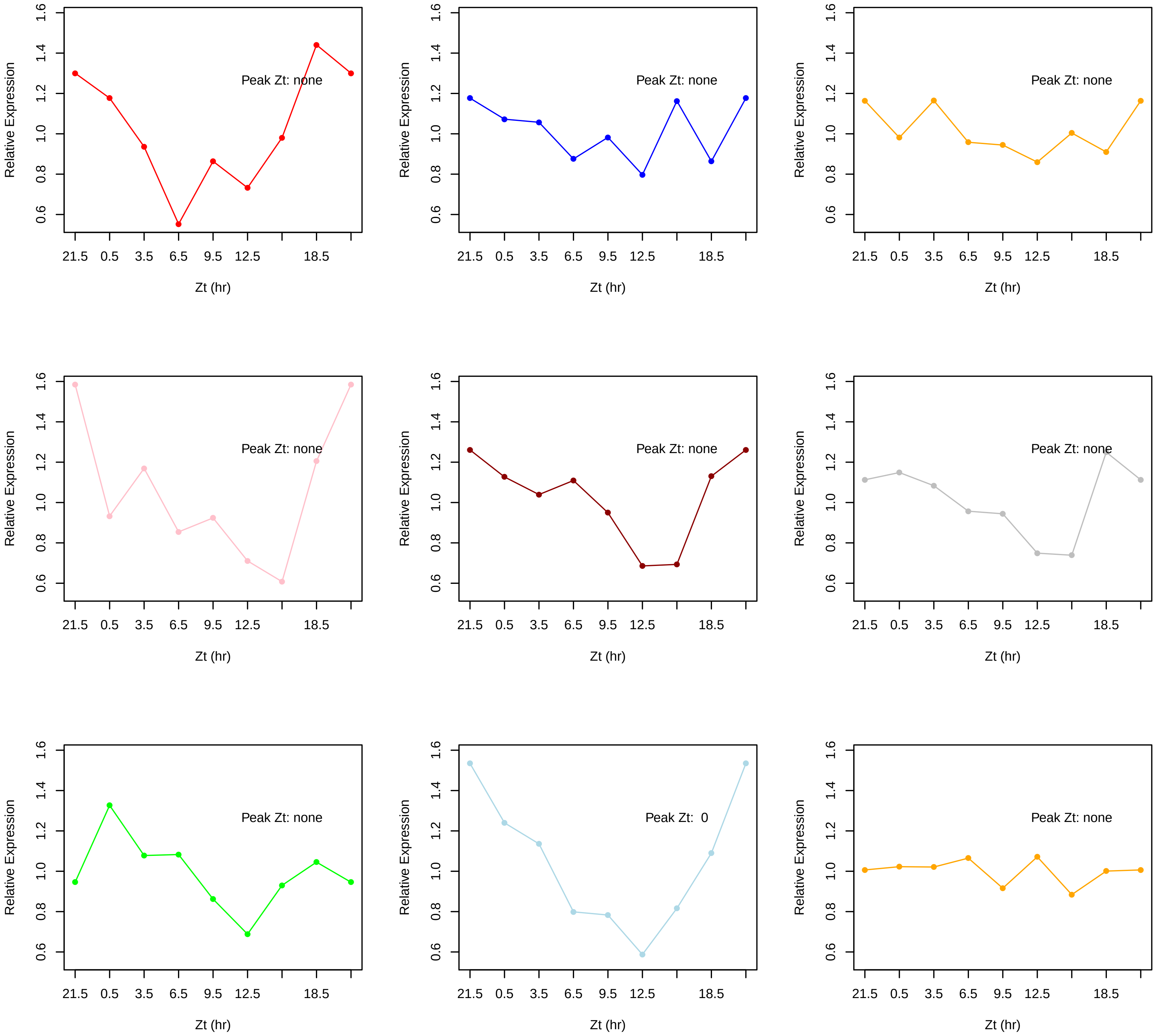

Singeleton: ENSXMAG00000019366 sema3h

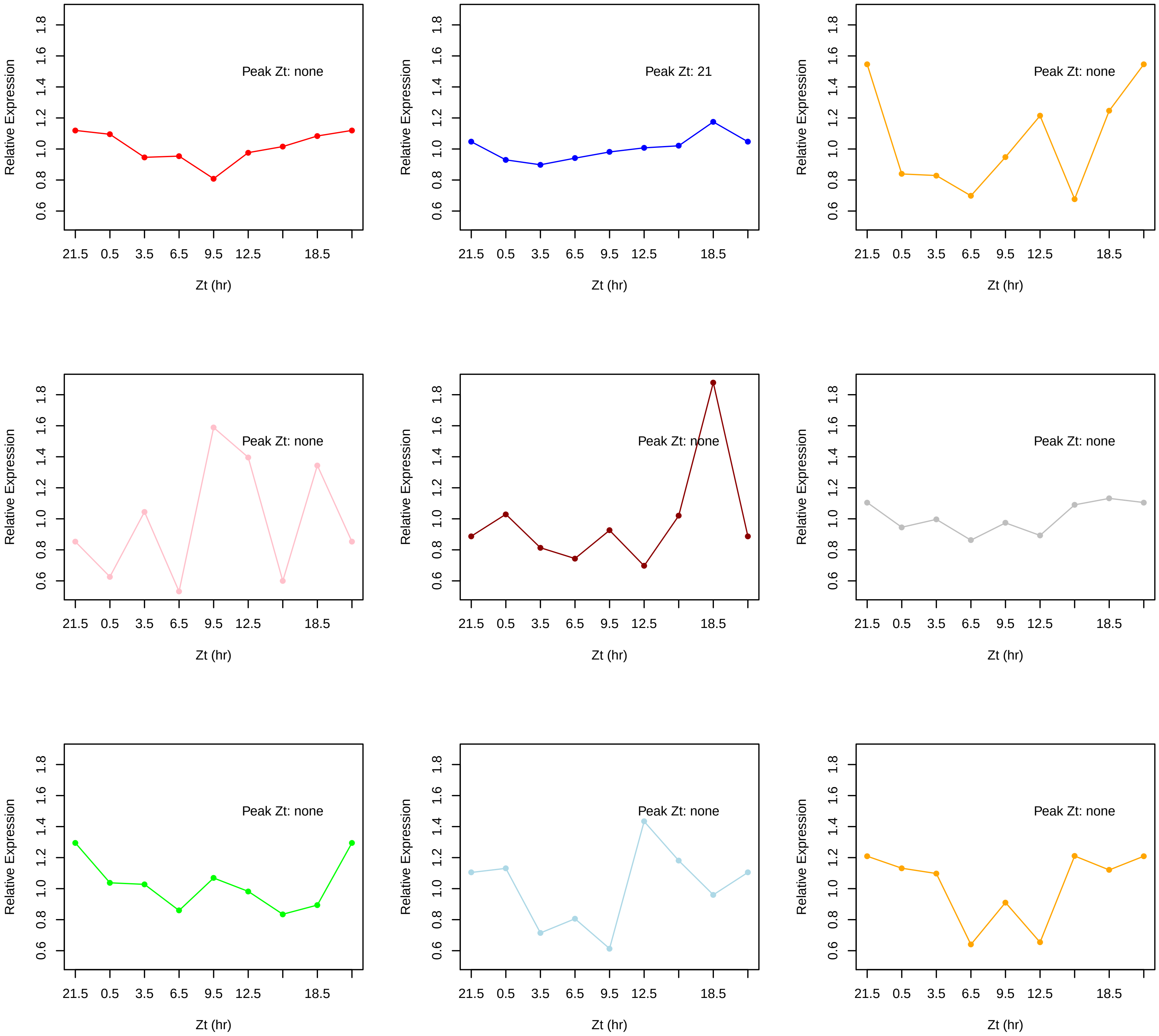

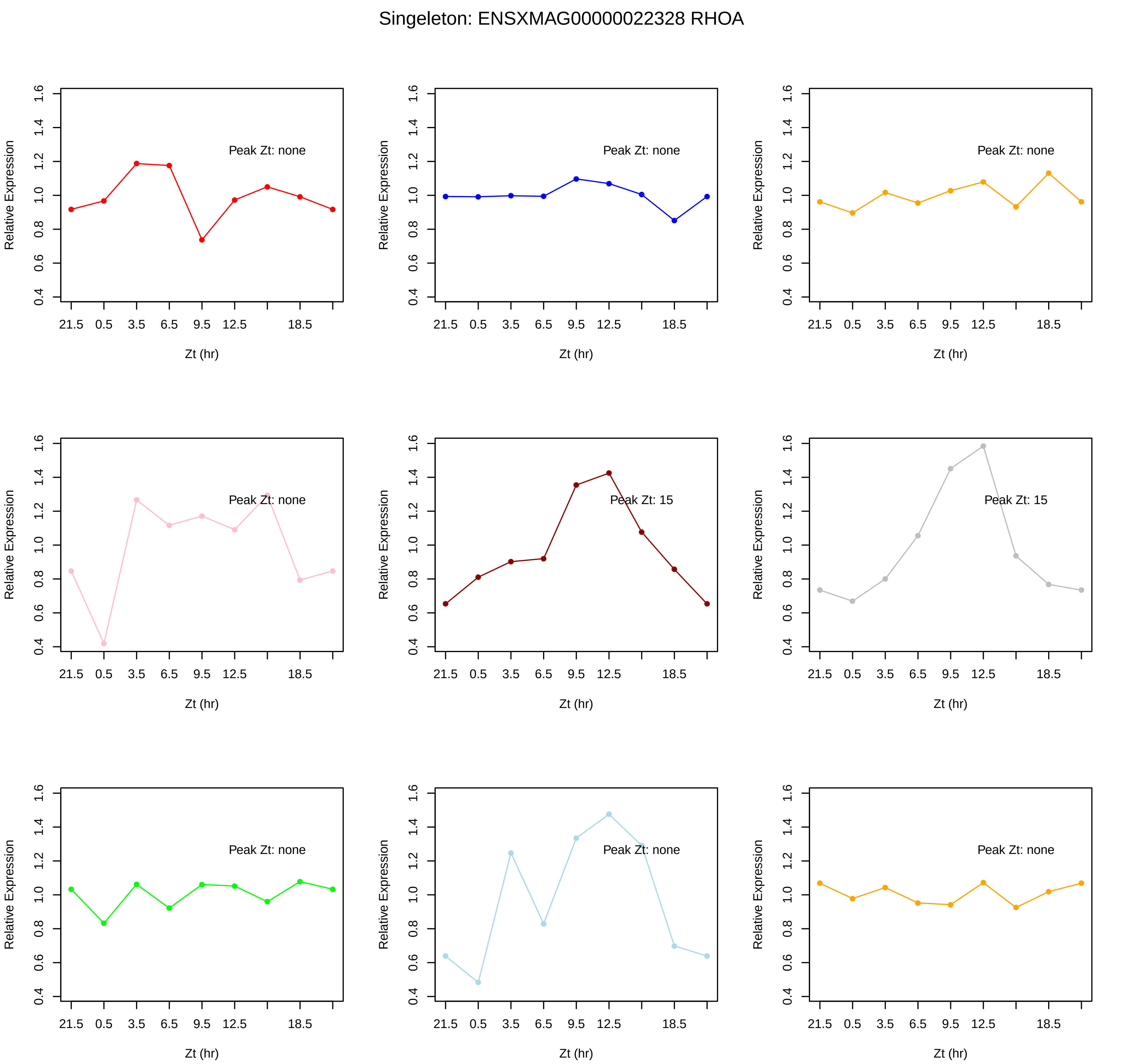
Singeleton: ENSXMAG00000022328 RHOA
Peak Zt: none
Peak Zt: none
Peak Zt: none
Peak Zt: none
Peak Zt: 15
Peak Zt: 15
Peak Zt: none
Peak Zt: none
Peak Zt: none
Relative Expression
Zt (hr)
21.5
0.5
3.5
6.5
9.5
12.5
18.5
0.4
0.6
0.8
1.0
1.2
1.4
1.6

Singeleton: ENSXMAG00000018350 tspy

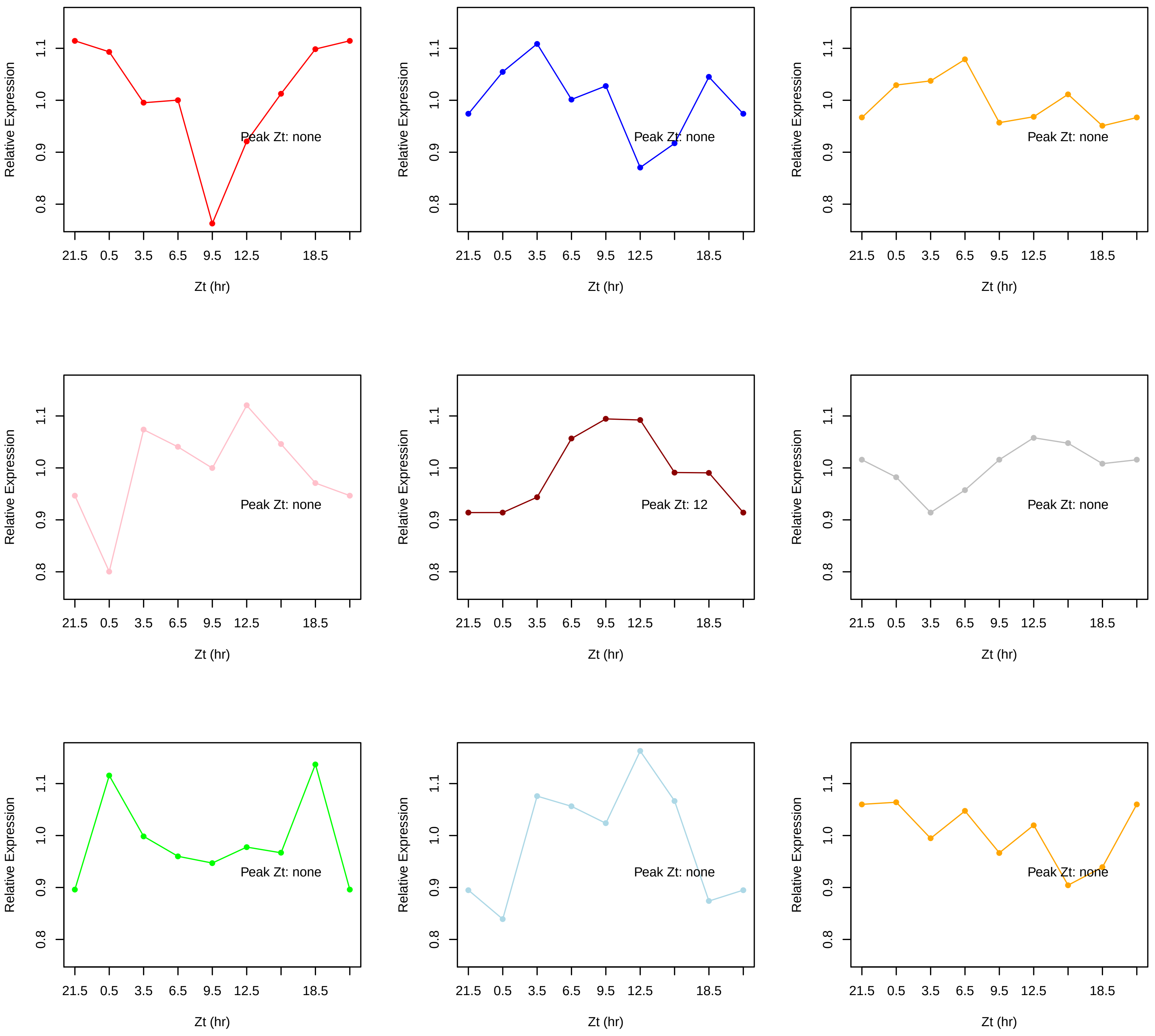

Singeleton: ENSXMAG00000026540 hemk1

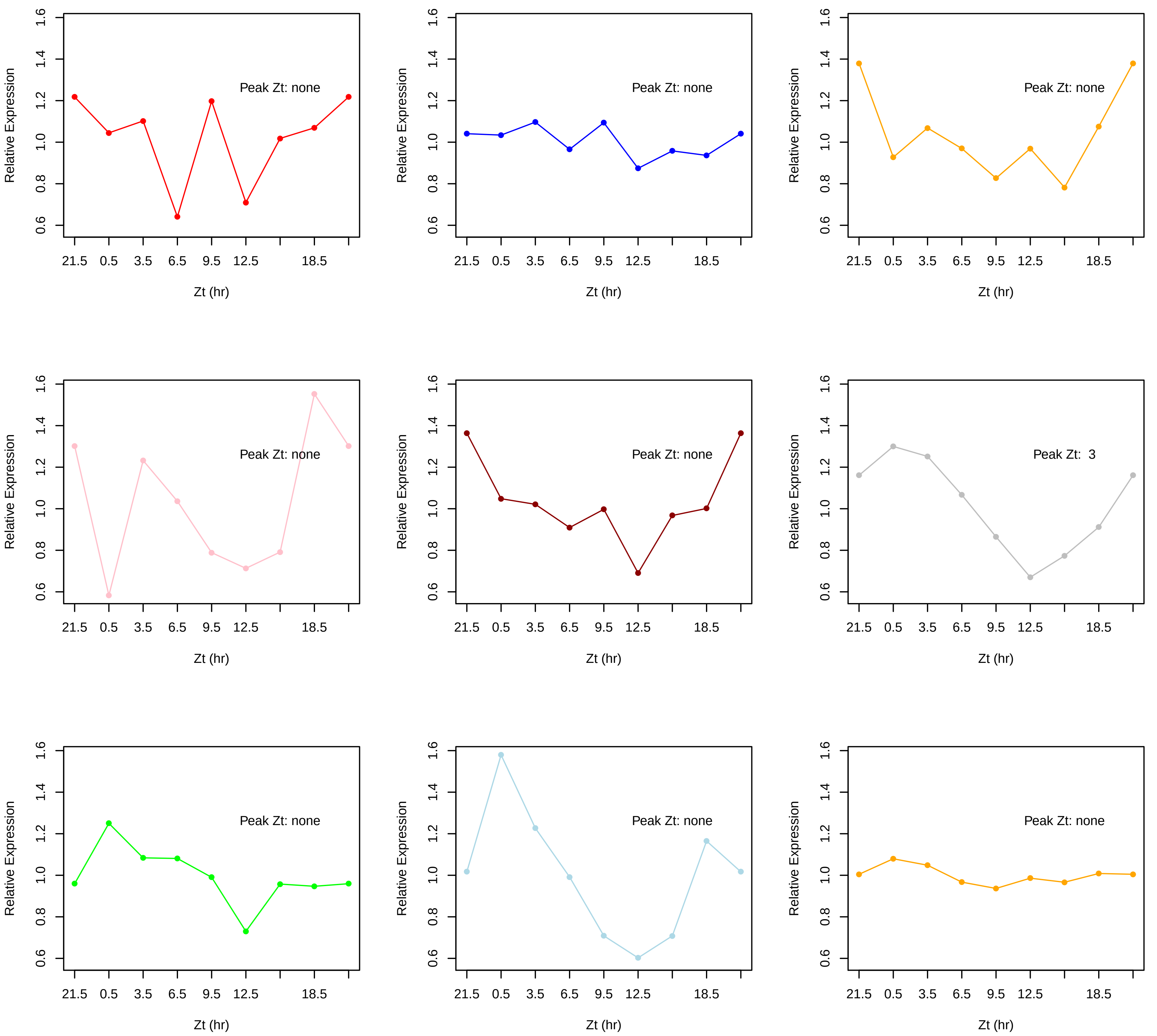

Singeleton: ENSXMAG00000026871 ntn5

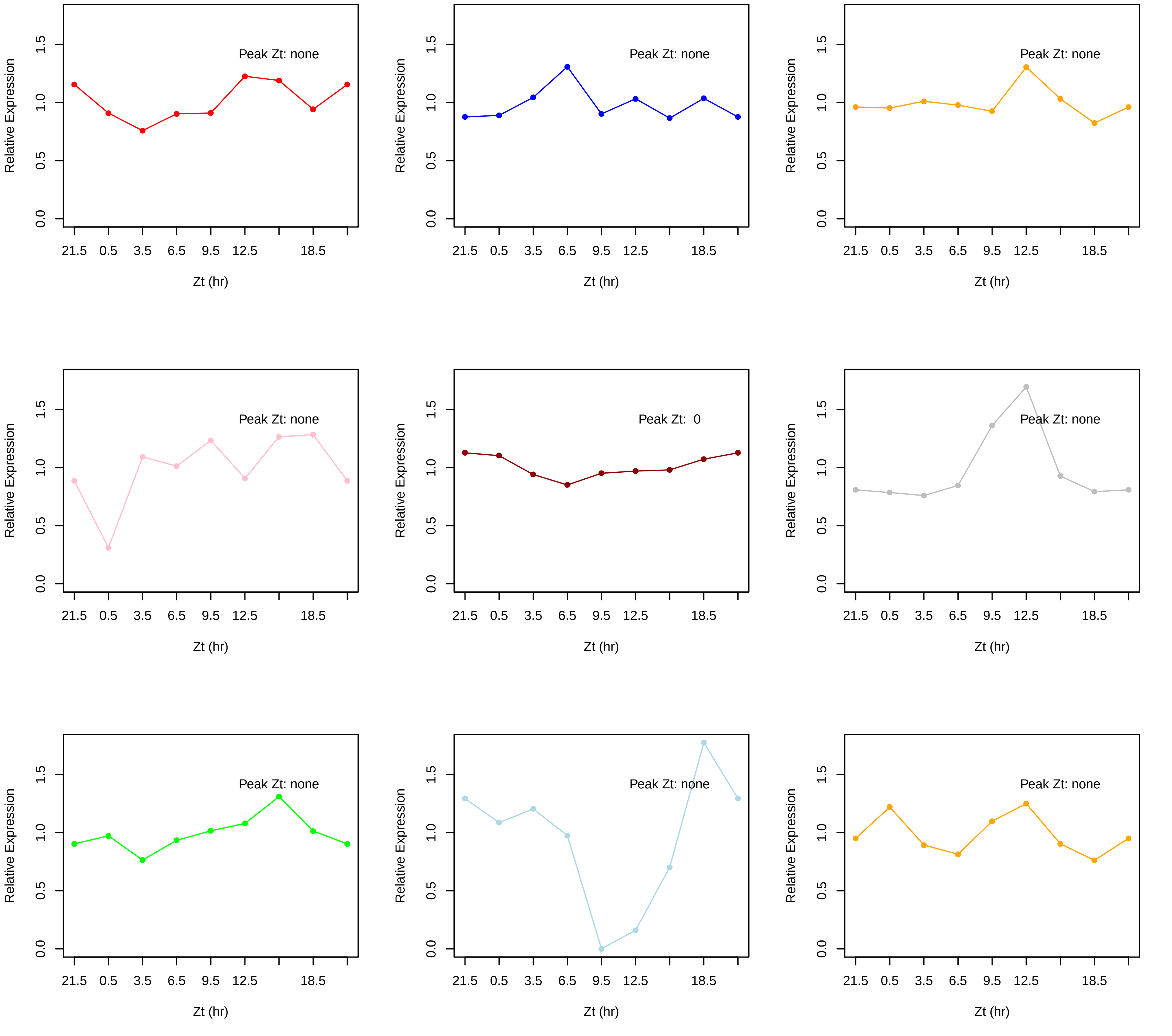

Singeleton: ENSXMAG00000024271 mpdu1b

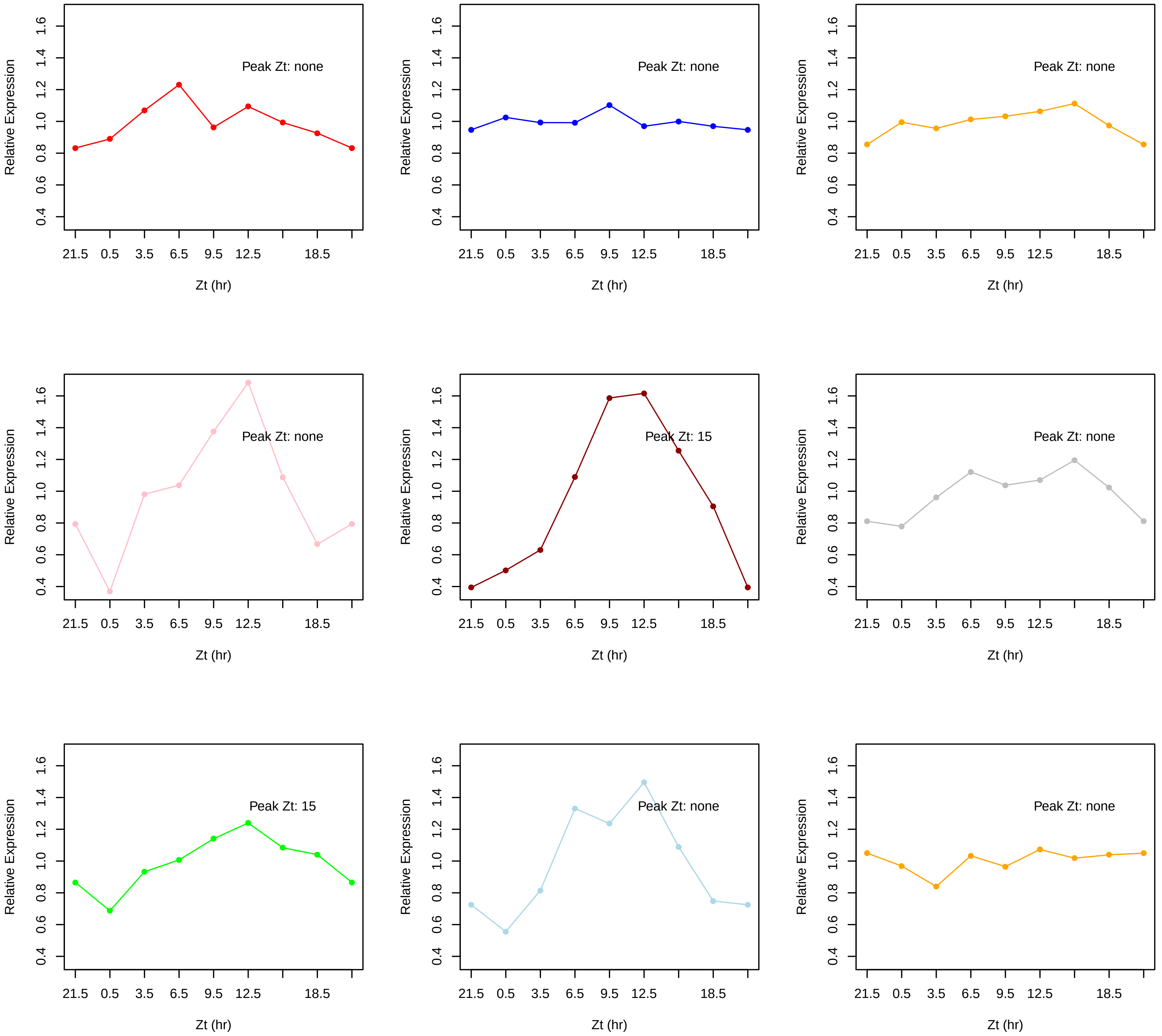

Singleton: ENSXMAG00000008210 sox19b

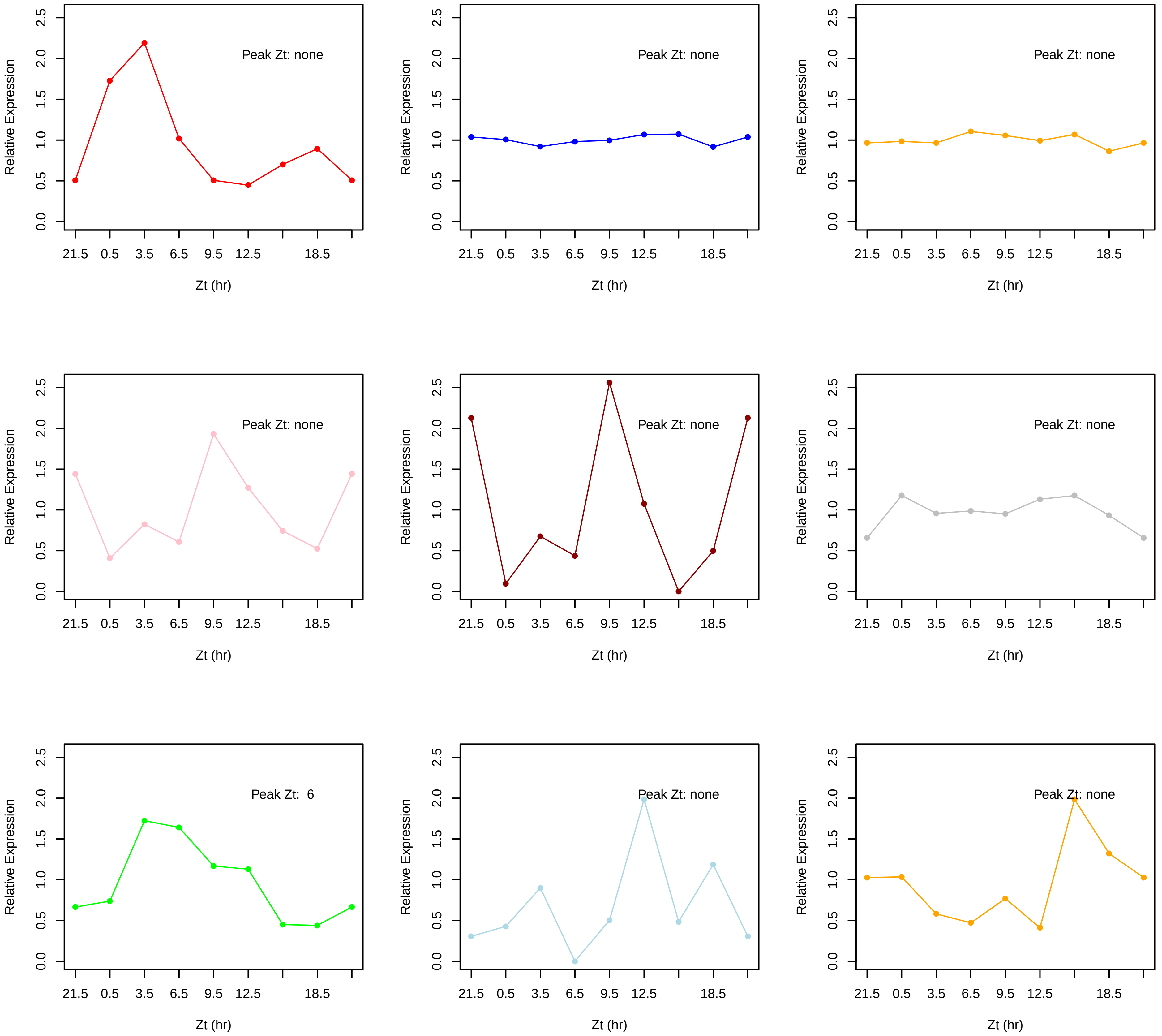

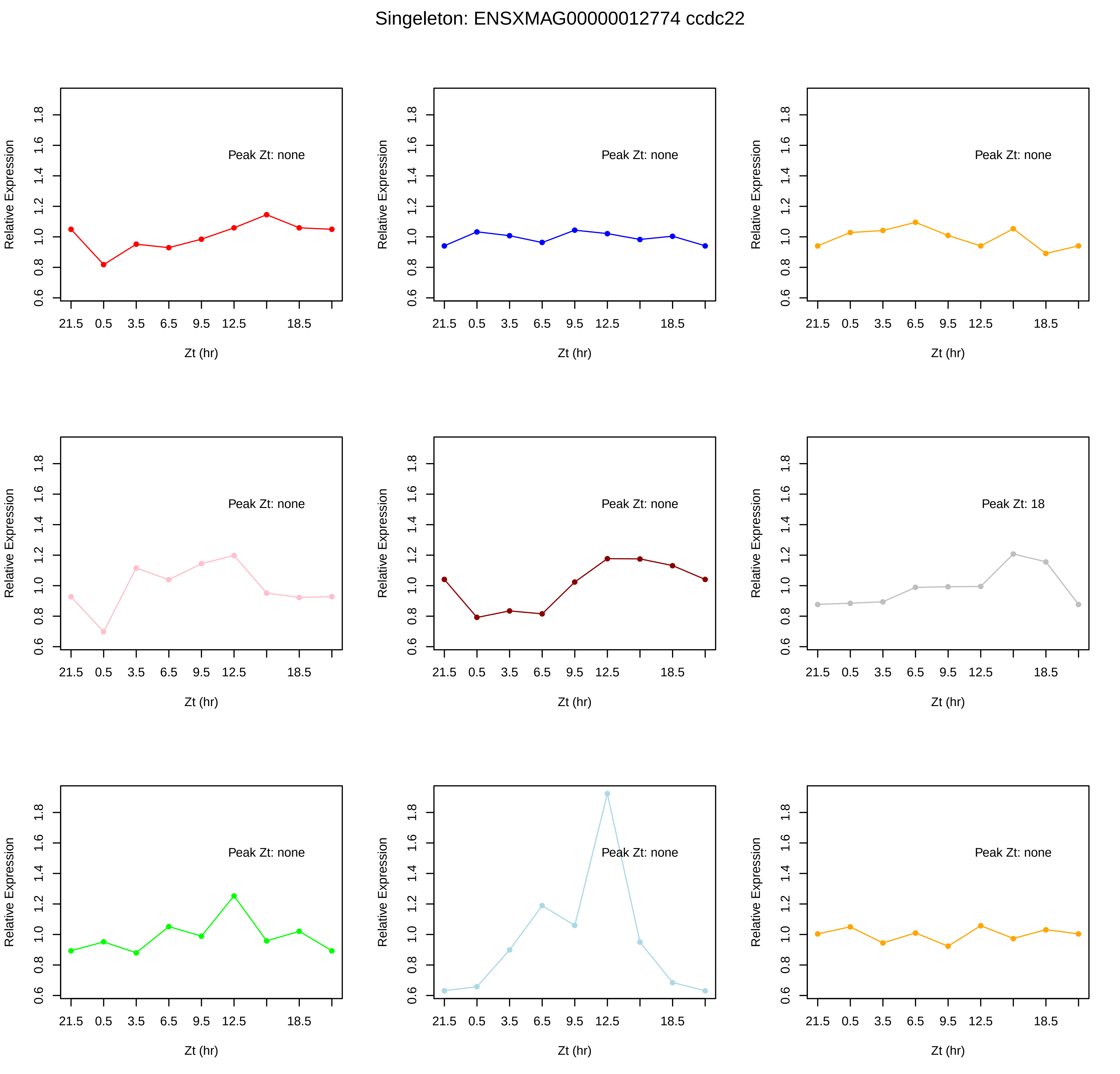
Singeleton: ENSXMAG00000012774 ccdc22
Relative Expression
Zt (hr)
21.5 0.5 3.5 6.5 9.5 12.5 18.5
0.6 0.8 1.0 1.2 1.4 1.6 1.8
Peak Zt: none
Peak Zt: none
Peak Zt: none
Peak Zt: none
Peak Zt: none
Peak Zt: 18
Peak Zt: none
Peak Zt: none
Peak Zt: none

Singeleton: ENSXMAG00000001947 ankrd46b

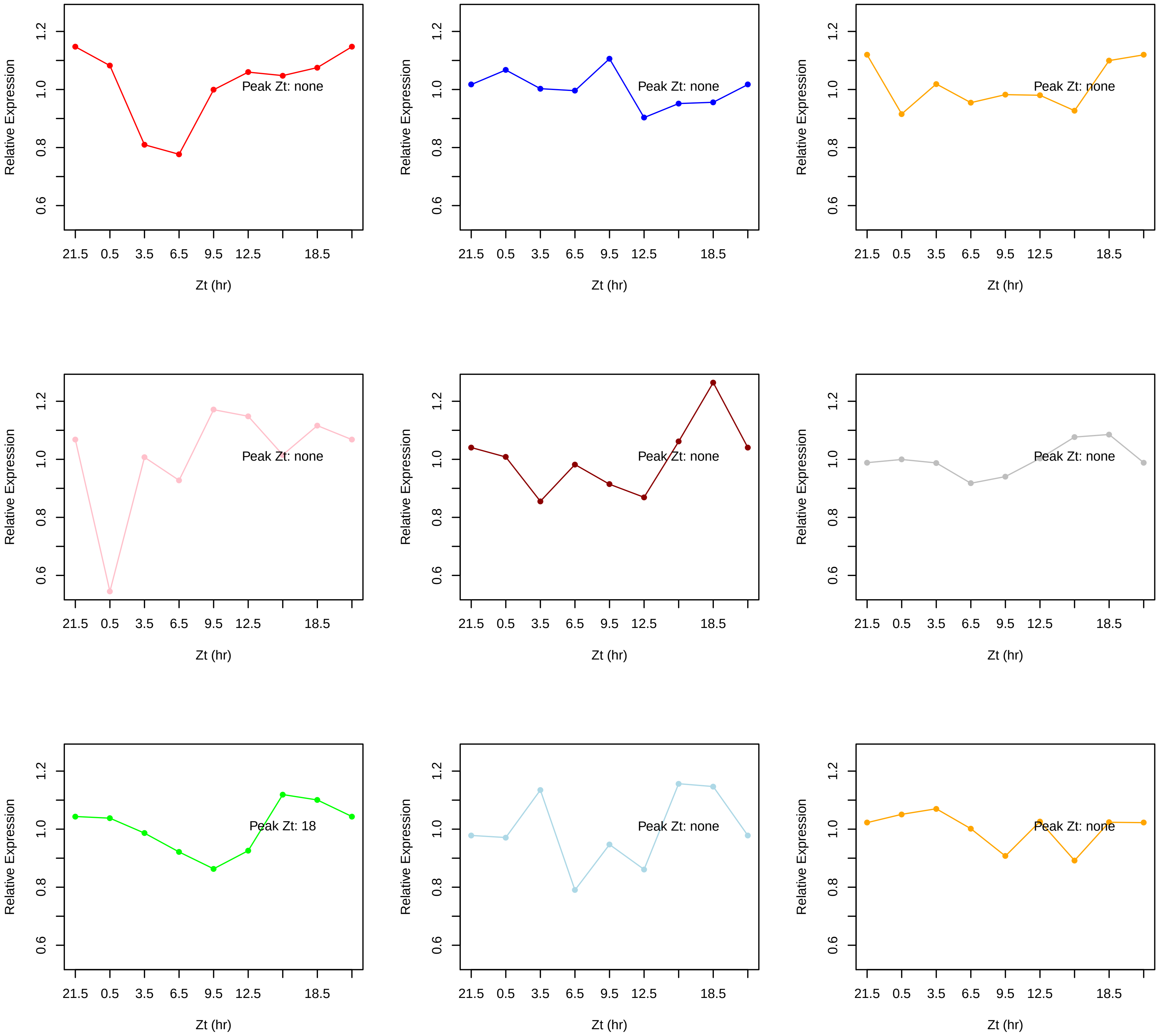

Singeleton: ENSXMAG00000001855 rft1

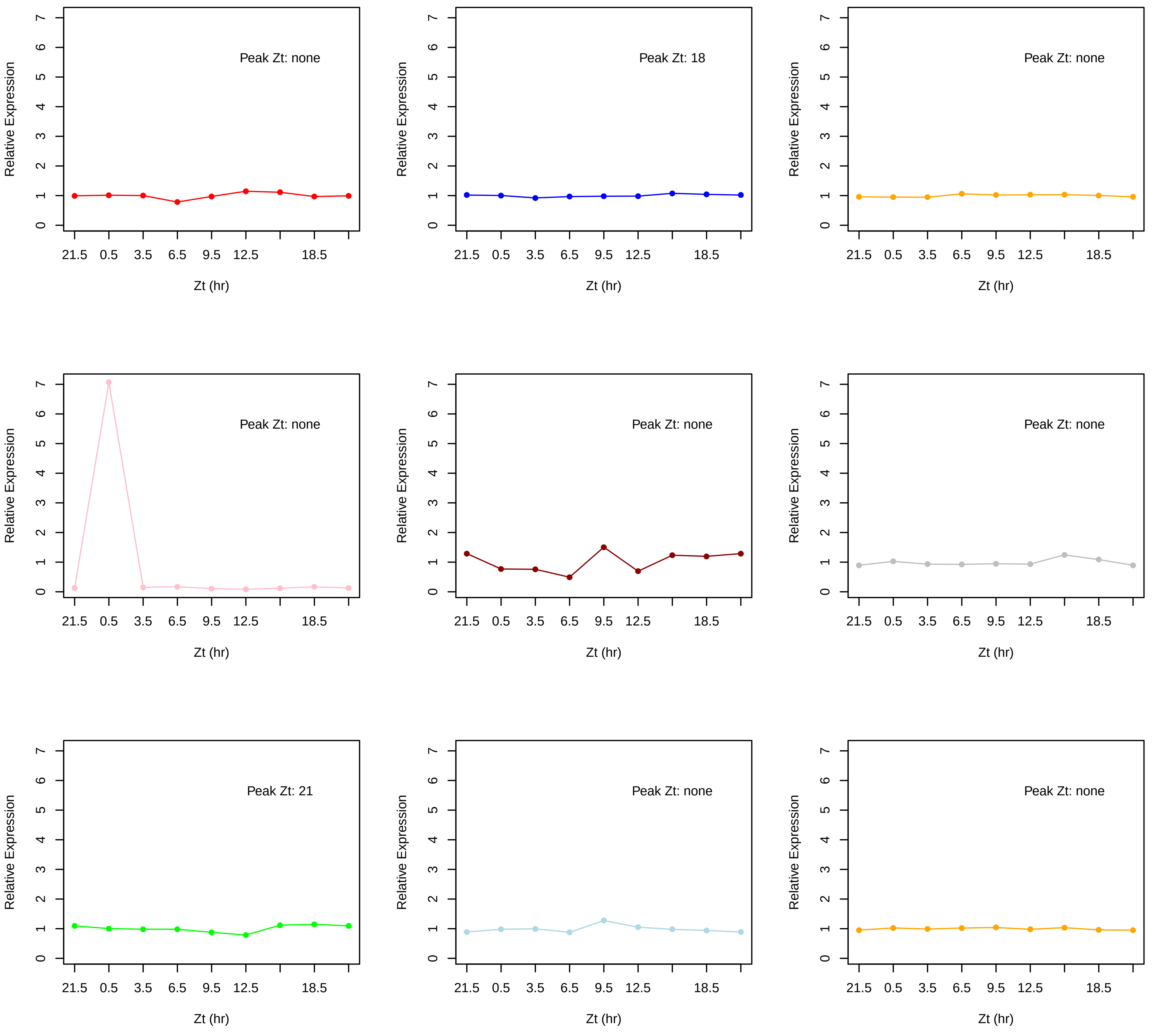

Singeleton: ENSXMAG00000016109 nt5dc2

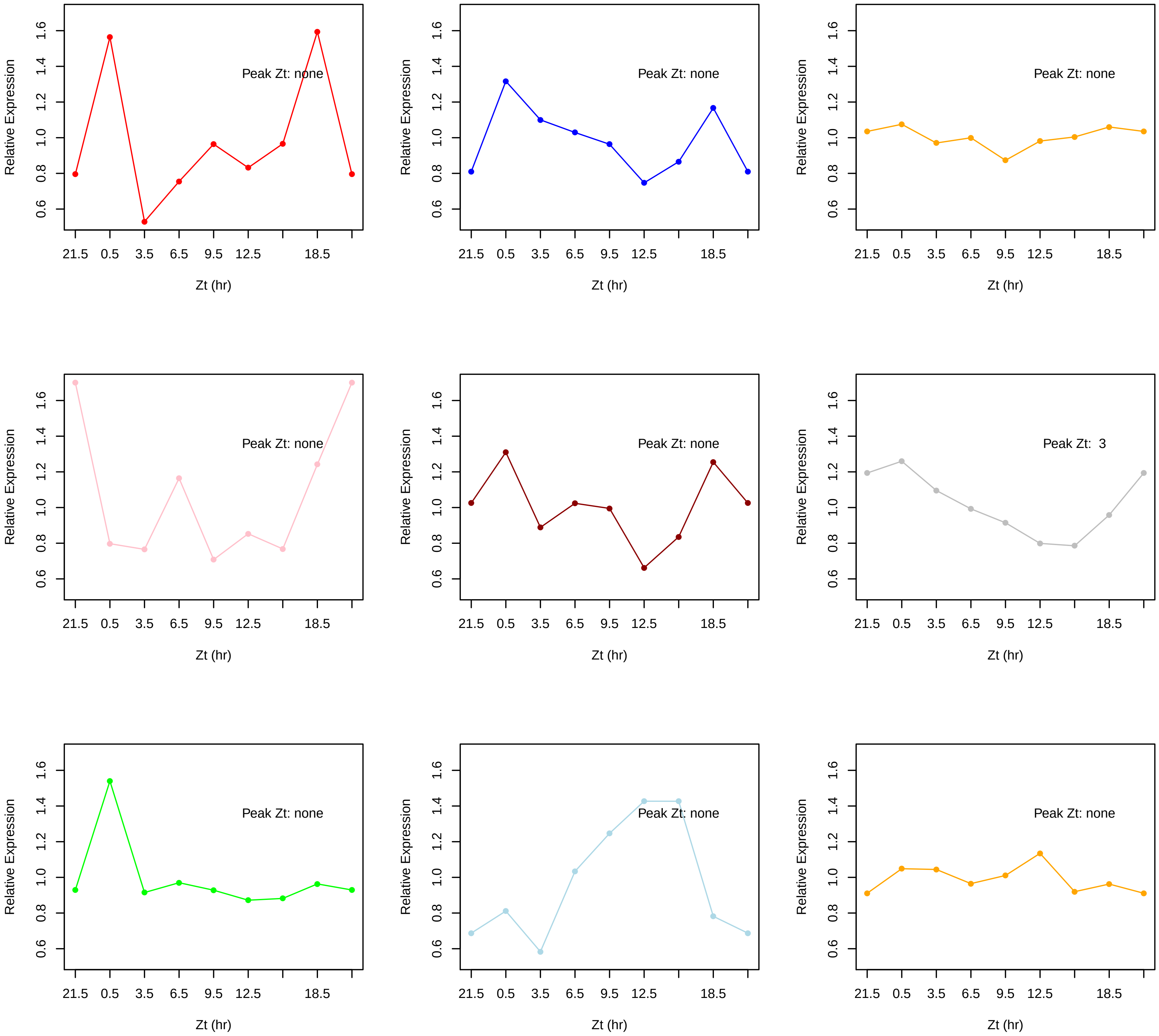

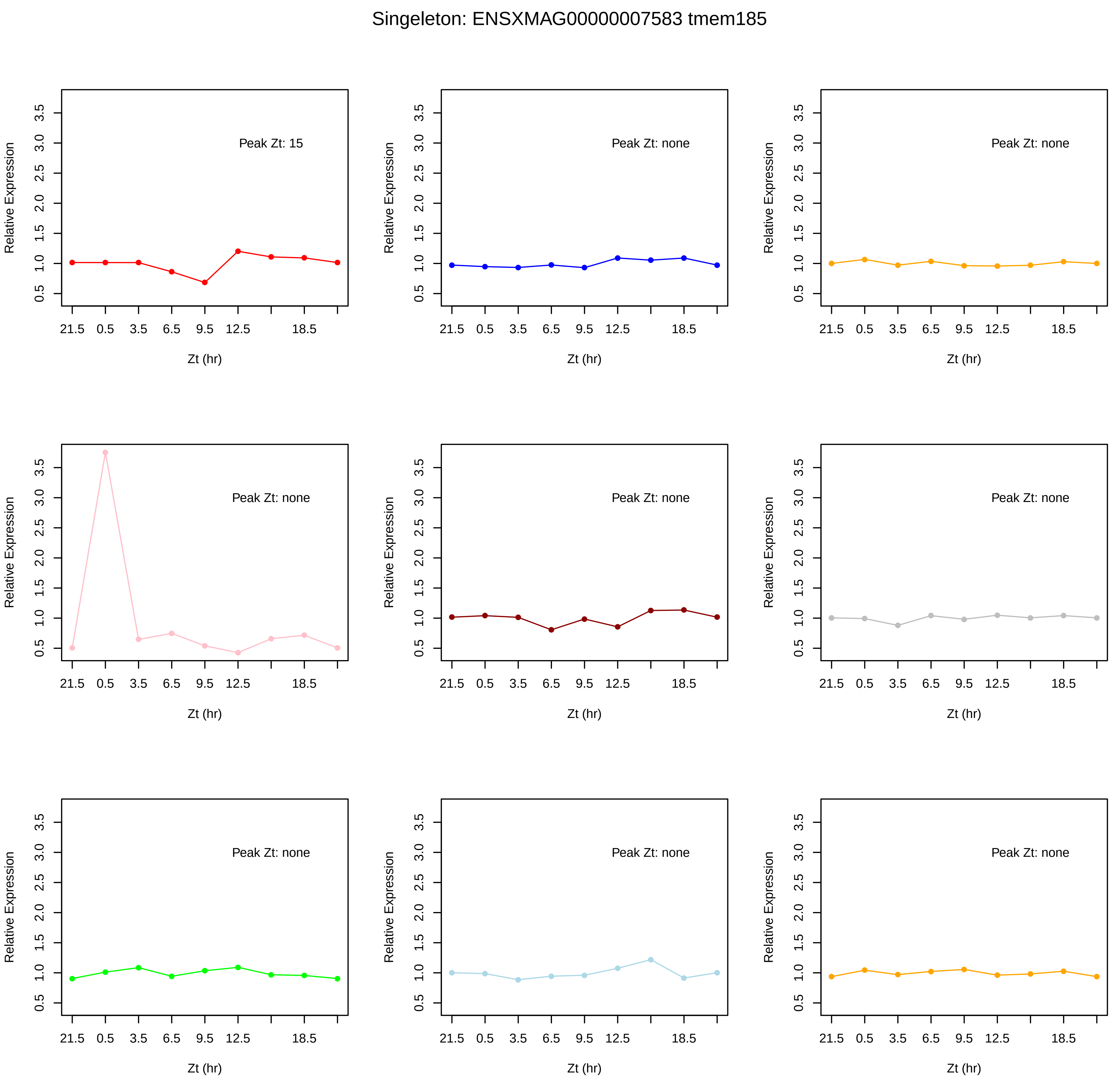
Singeleton: ENSXMAG00000007583 tmem185
Peak Zt: 15
Peak Zt: none
Peak Zt: none
Peak Zt: none
Peak Zt: none
Peak Zt: none
Peak Zt: none
Peak Zt: none
Peak Zt: none
Relative Expression
Zt (hr)
21.5
0.5
3.5
6.5
9.5
12.5
18.5
0.5
1.0
1.5
2.0
2.5
3.0
3.5

Singeleton: ENSXMAG00000015788 ctdnep1b

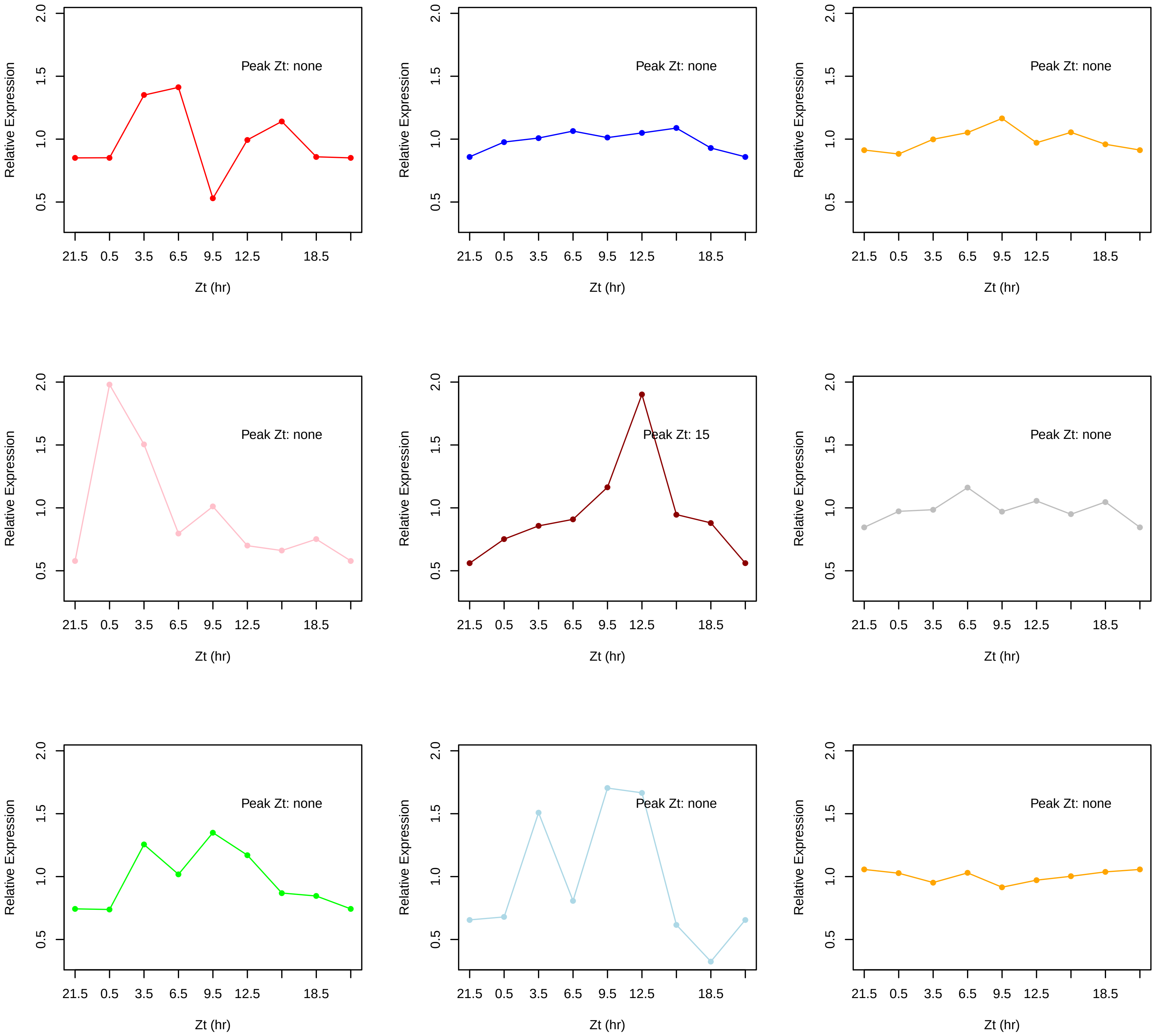

Singeleton: ENSXMAG00000022890 gabarapb

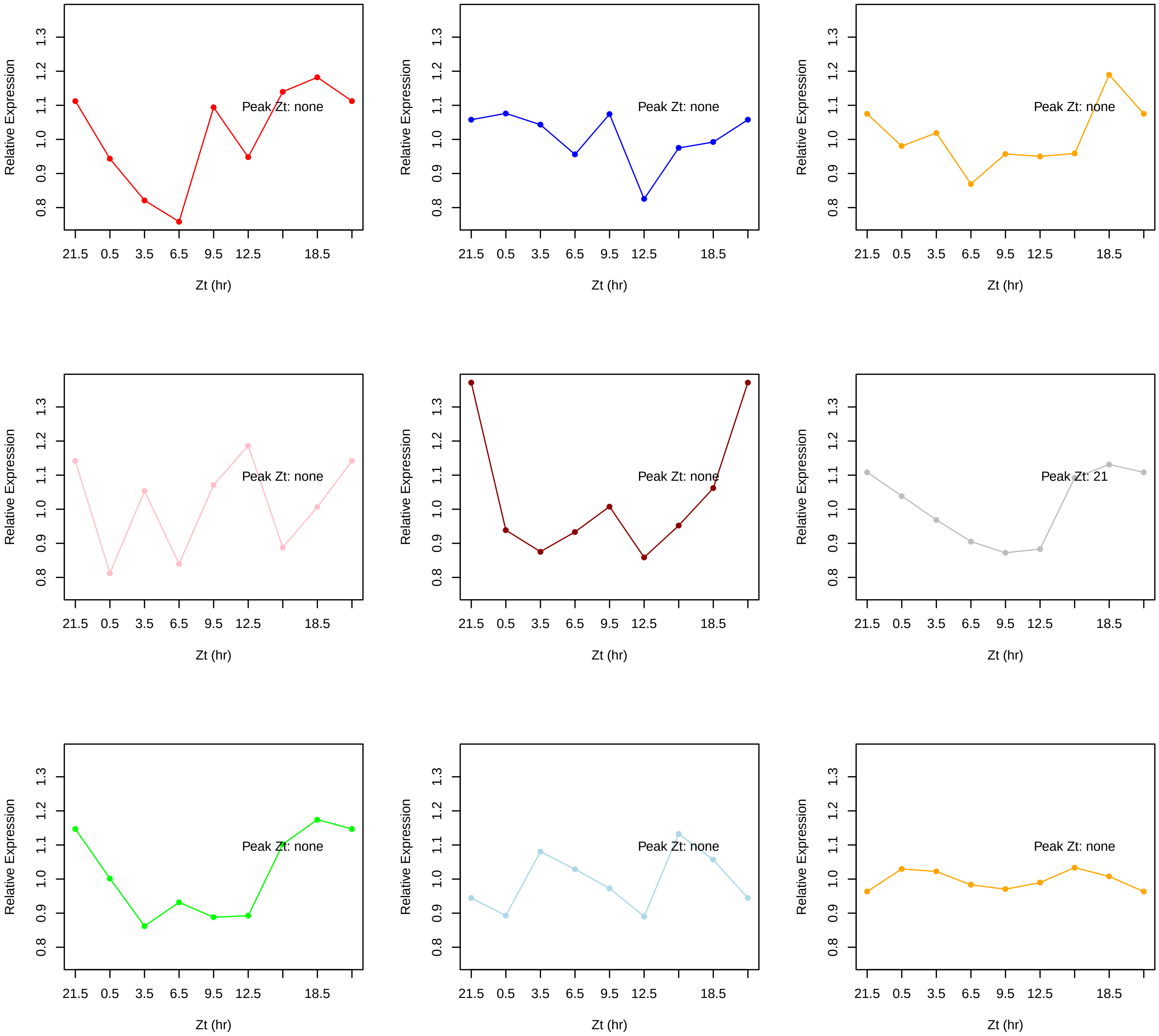

Singeleton: ENSXMAG00000016974 mon1bb

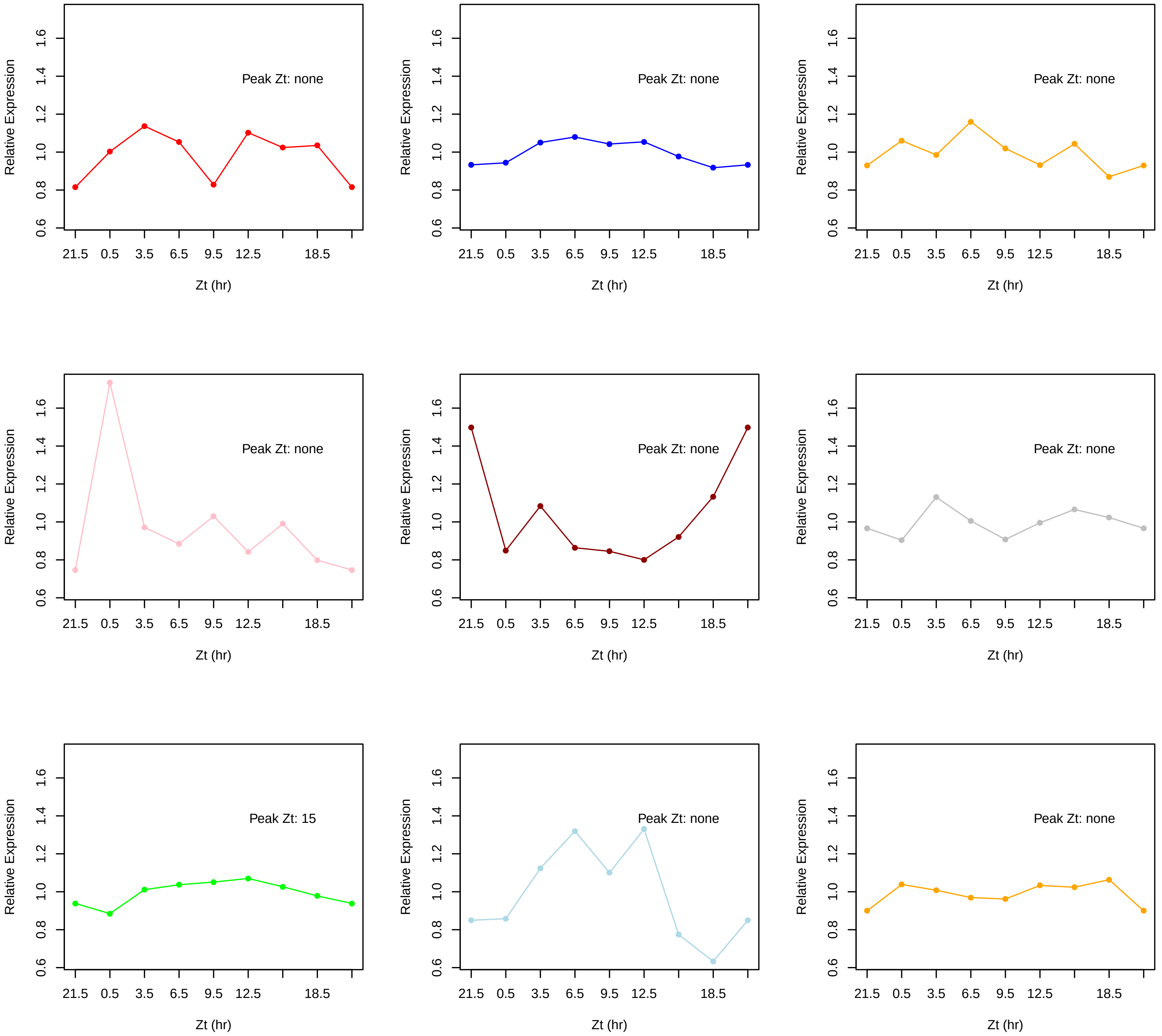

Singeleton: ENSXMAG00000011106

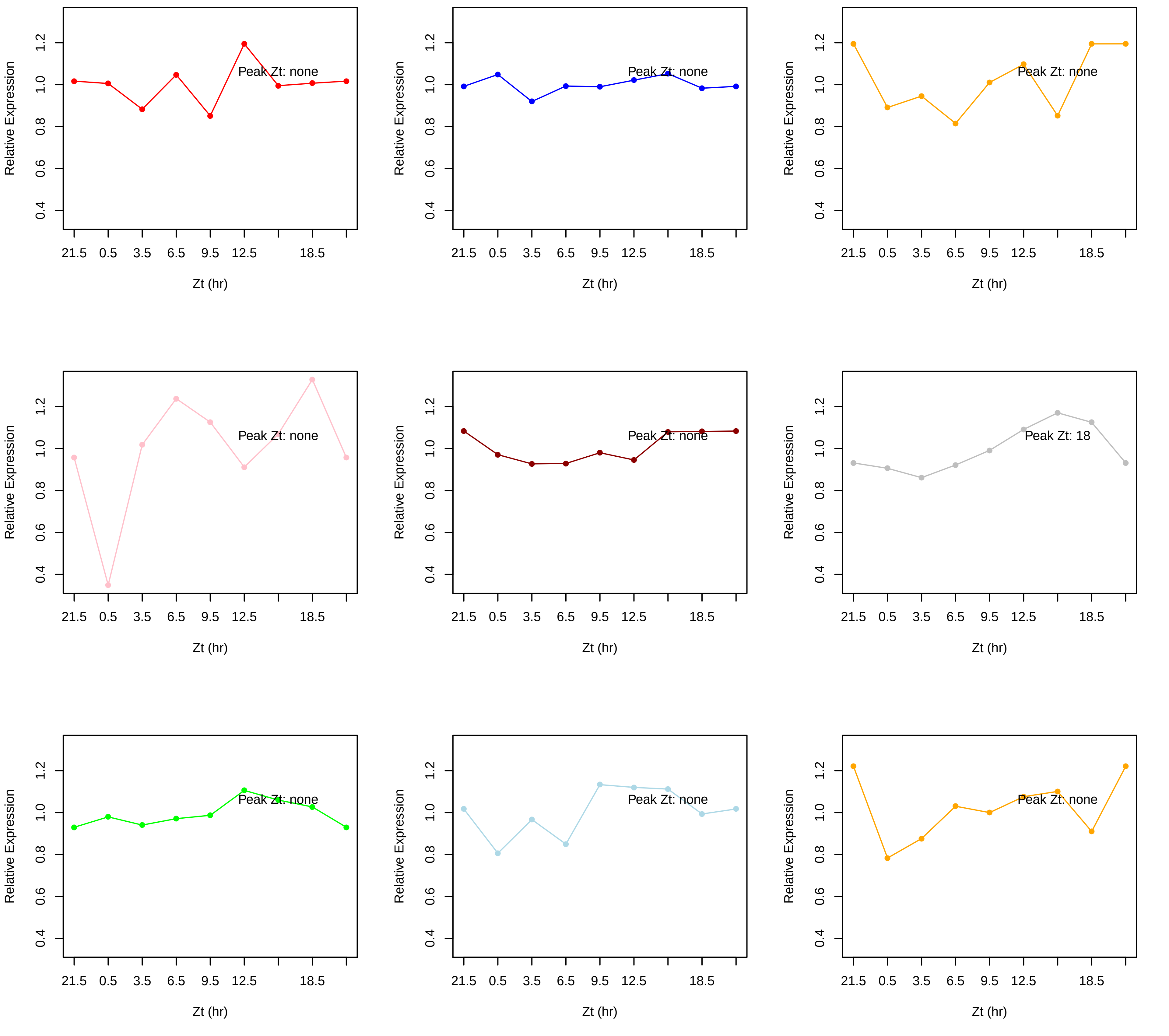

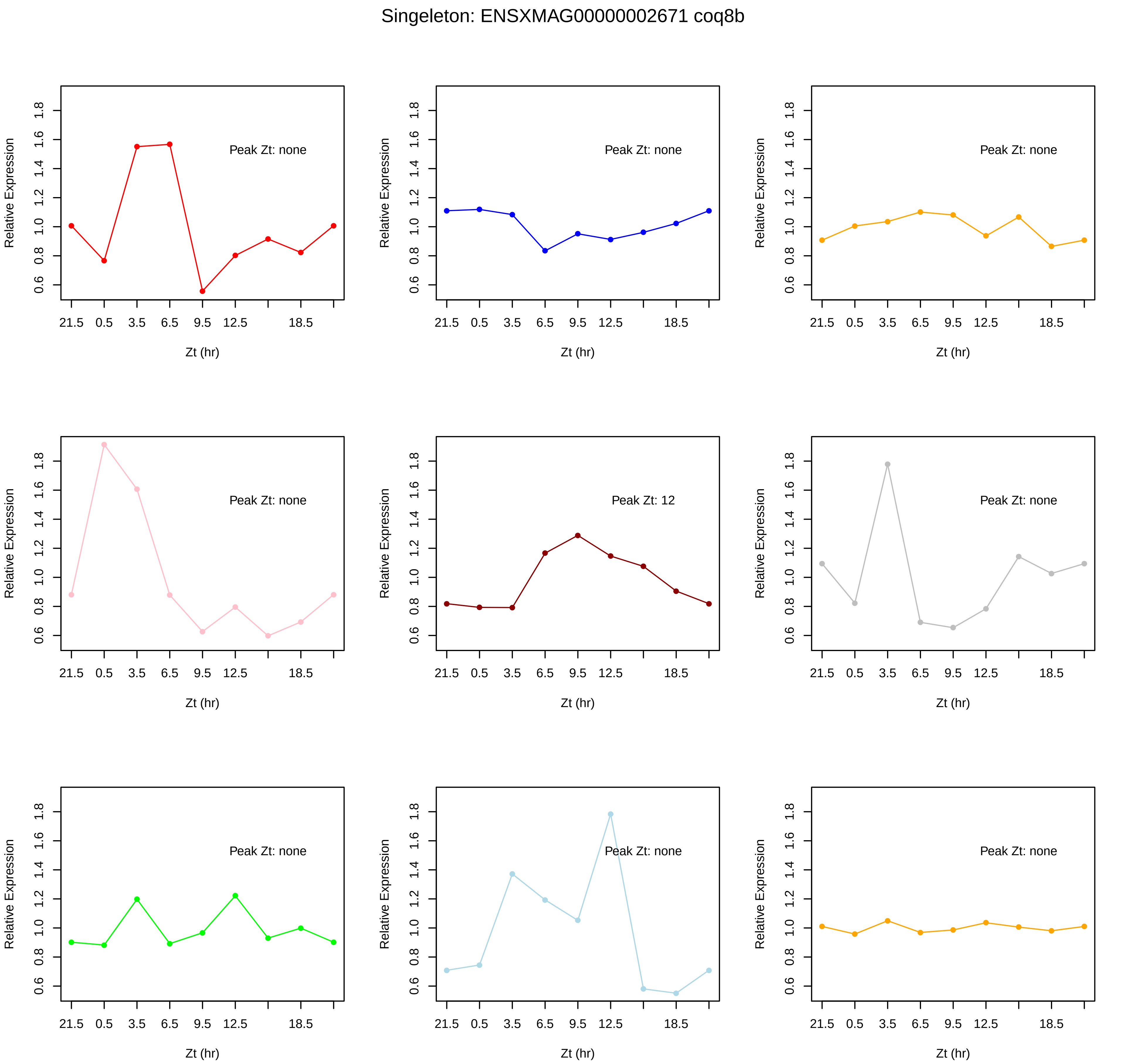
Singleton: ENSXMAG00000002671 coq8b
Relative Expression
Zt (hr)
21.5
0.5
3.5
6.5
9.5
12.5
18.5
0.6
0.8
1.0
1.2
1.4
1.6
1.8
Peak Zt: none
Peak Zt: none
Peak Zt: none
Peak Zt: none
Peak Zt: 12
Peak Zt: none
Peak Zt: none
Peak Zt: none
Peak Zt: none

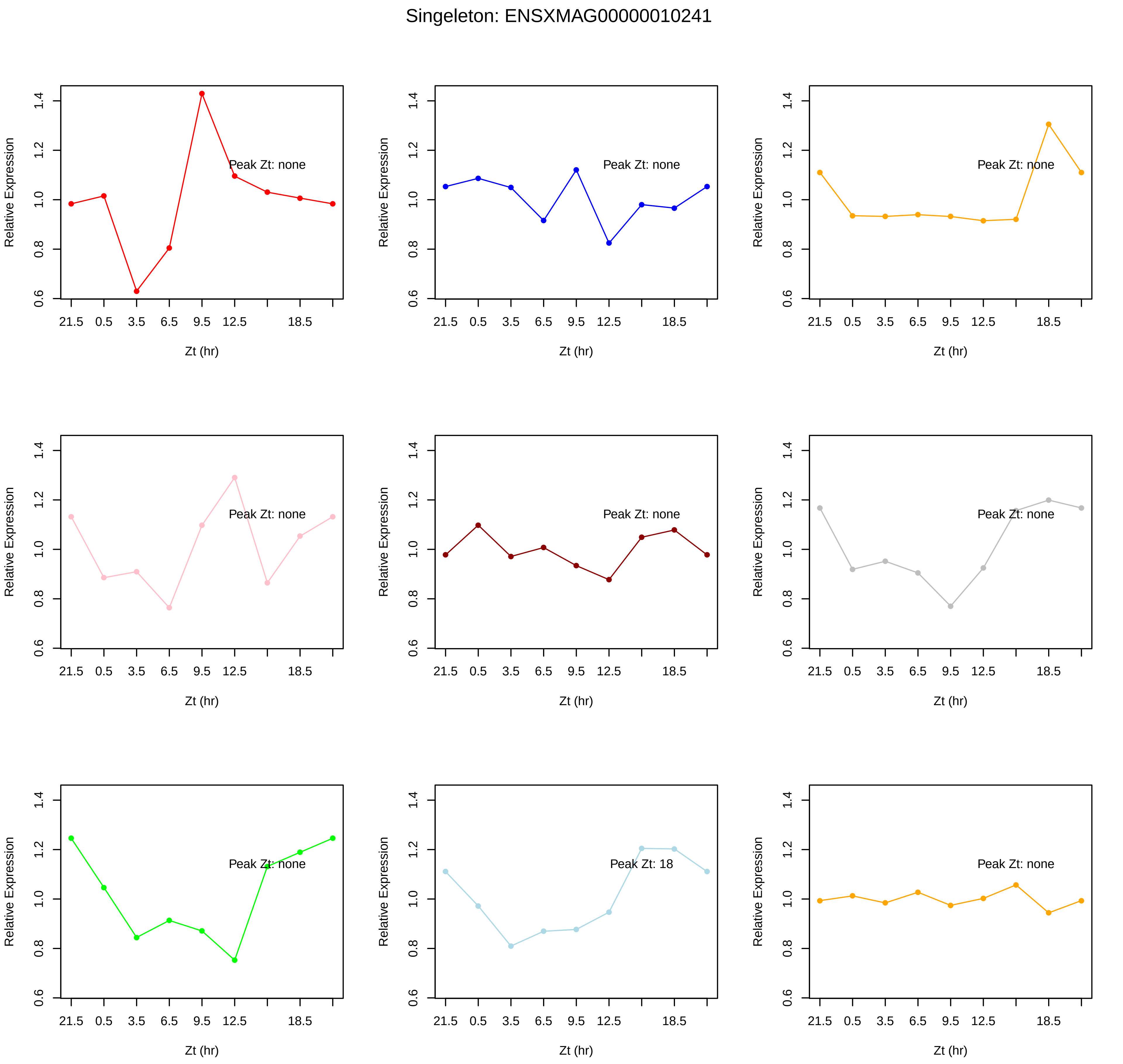

Singleton: ENSXMAG00000010241
Peak Zt: none
Peak Zt: none
Peak Zt: none
Peak Zt: none
Peak Zt: none
Peak Zt: none
Peak Zt: none
Peak Zt: 18
Peak Zt: none
Relative Expression
Zt (hr)
21.5
0.5
3.5
6.5
9.5
12.5
18.5
0.6
0.8
1.0
1.2
1.4

Singleton: ENSXMAG00000006825 acsl4a

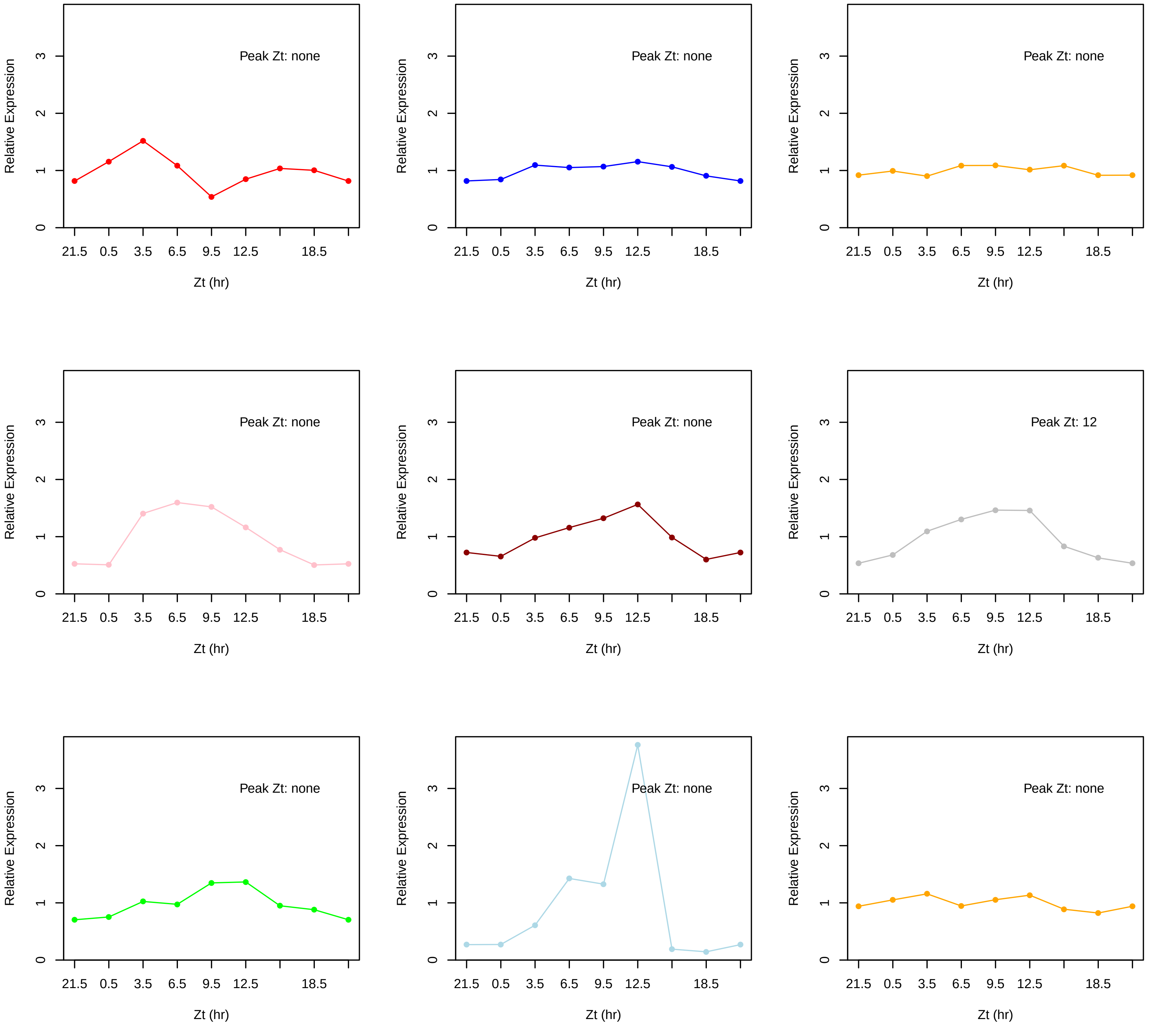

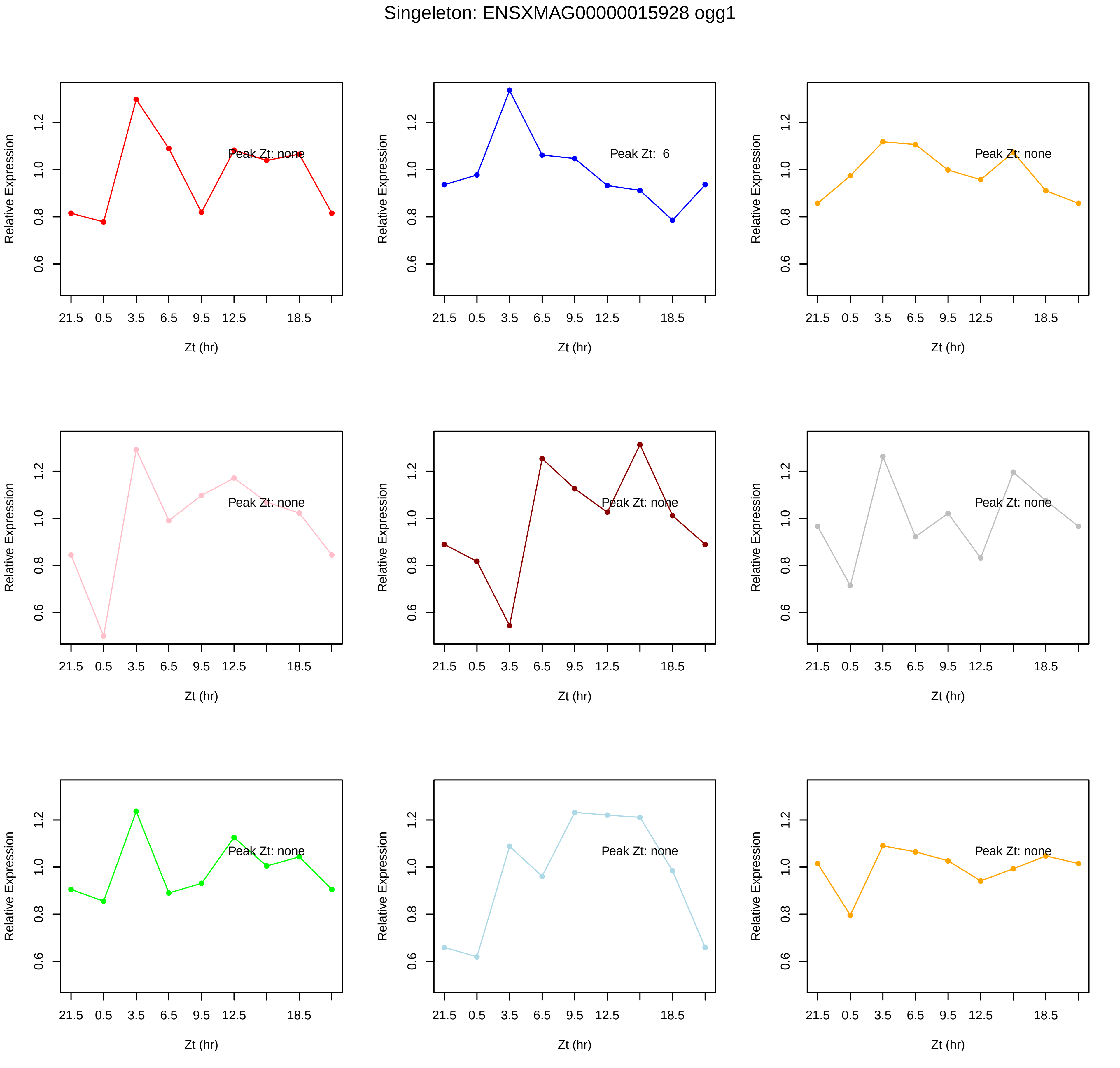
Singleton: ENSXMAG00000015928 ogg1
Peak Zt: none
Peak Zt: 6
Peak Zt: none
Peak Zt: none
Peak Zt: none
Peak Zt: none
Peak Zt: none
Peak Zt: none
Peak Zt: none
Relative Expression
Zt (hr)
21.5
0.5
3.5
6.5
9.5
12.5
18.5
0.6
0.8
1.0
1.2

Singeleton: ENSXMAG00000022277 nxt2

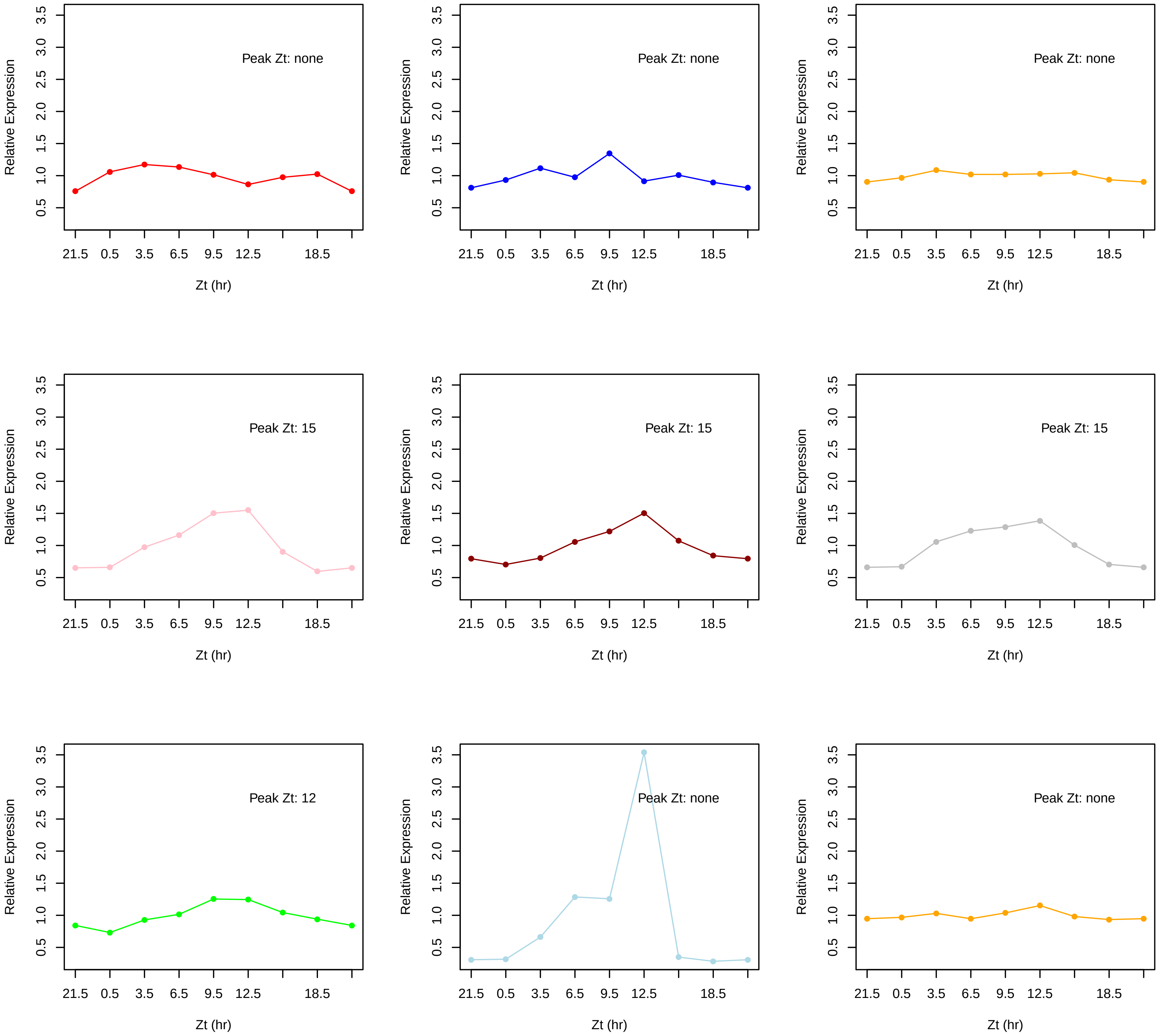

Singeleton: ENSXMAG00000028393

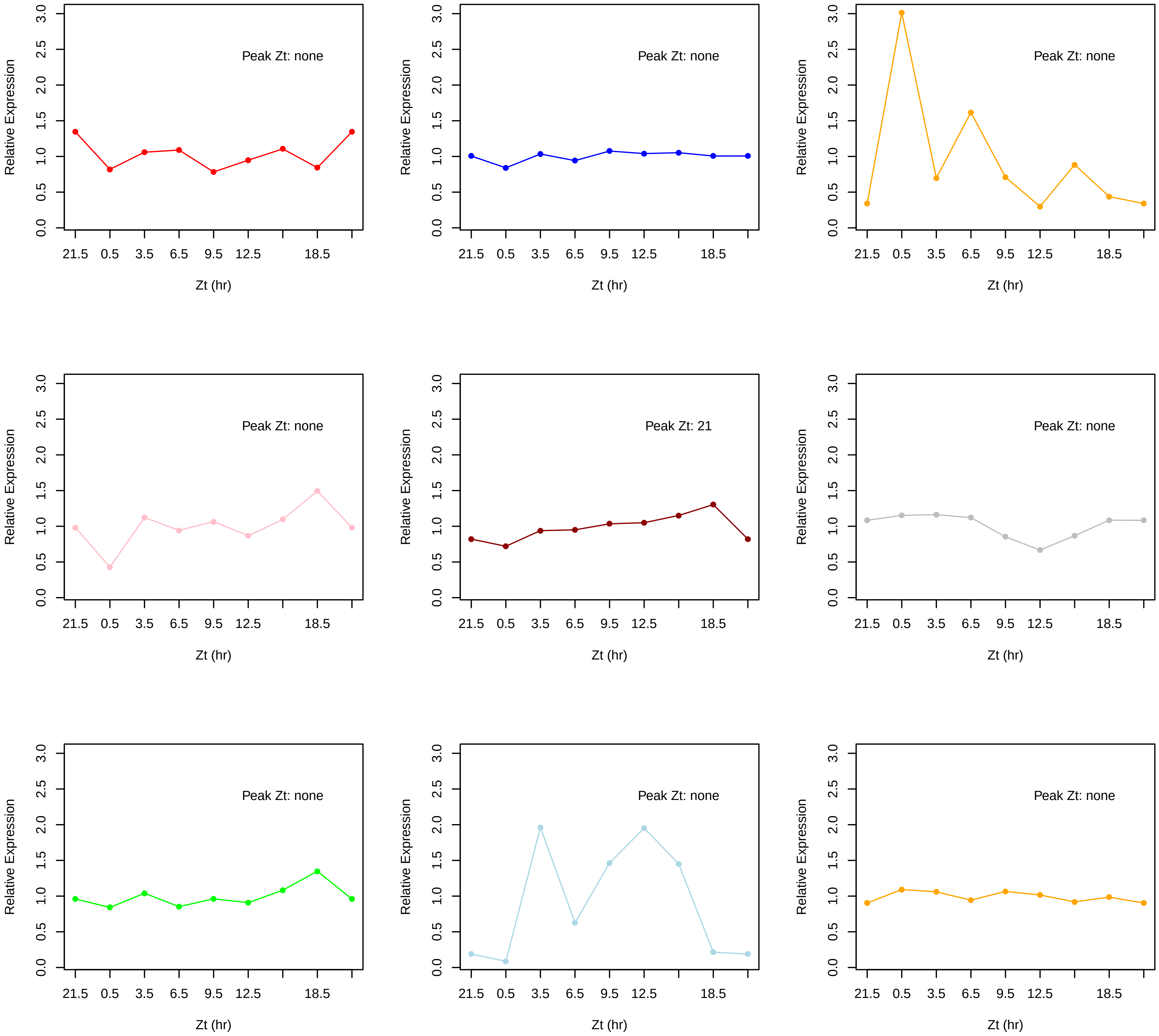

Singeleton: ENSXMAG00000028475

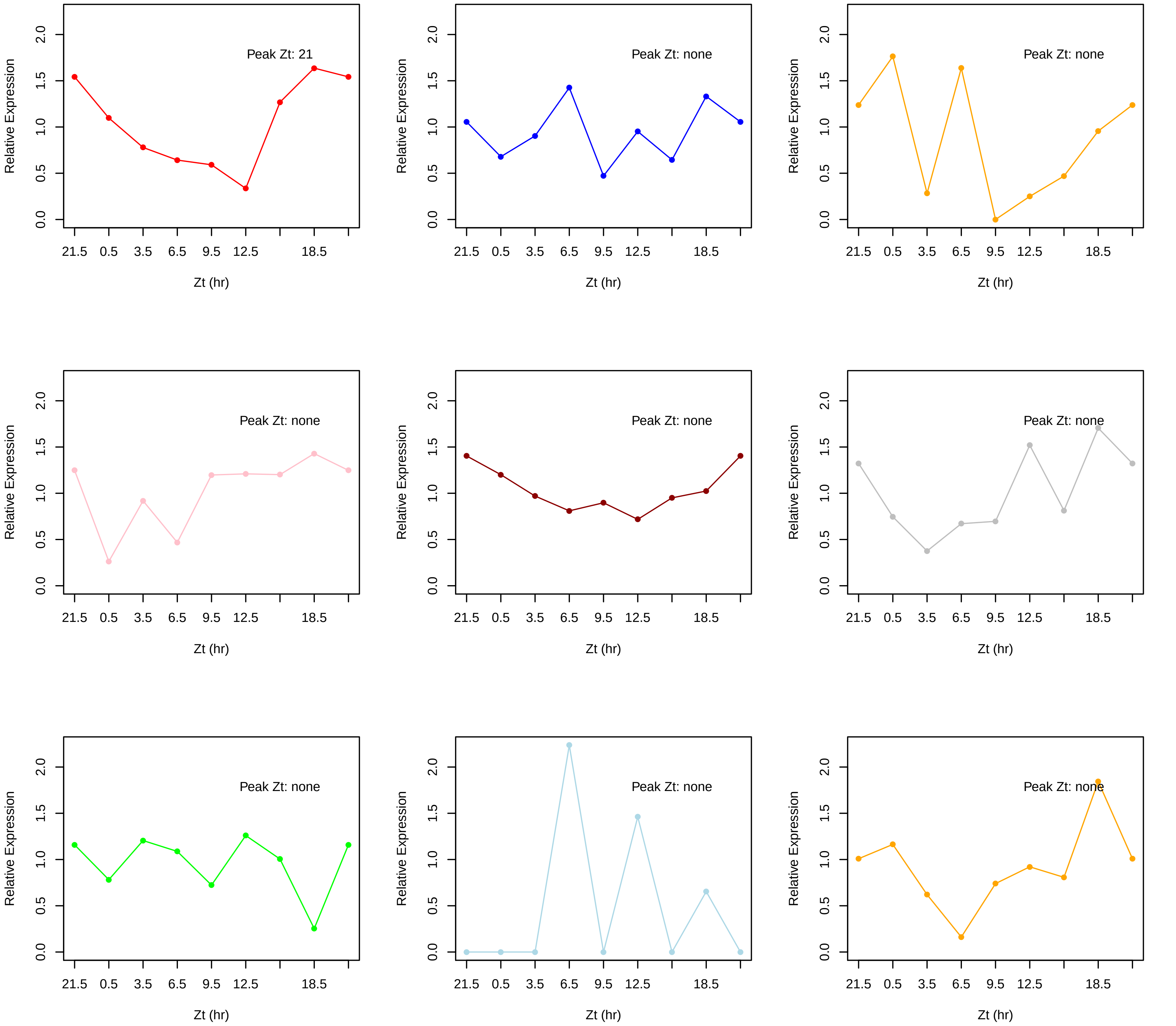

Singleton: ENSXMAG00000018093 mrpl46

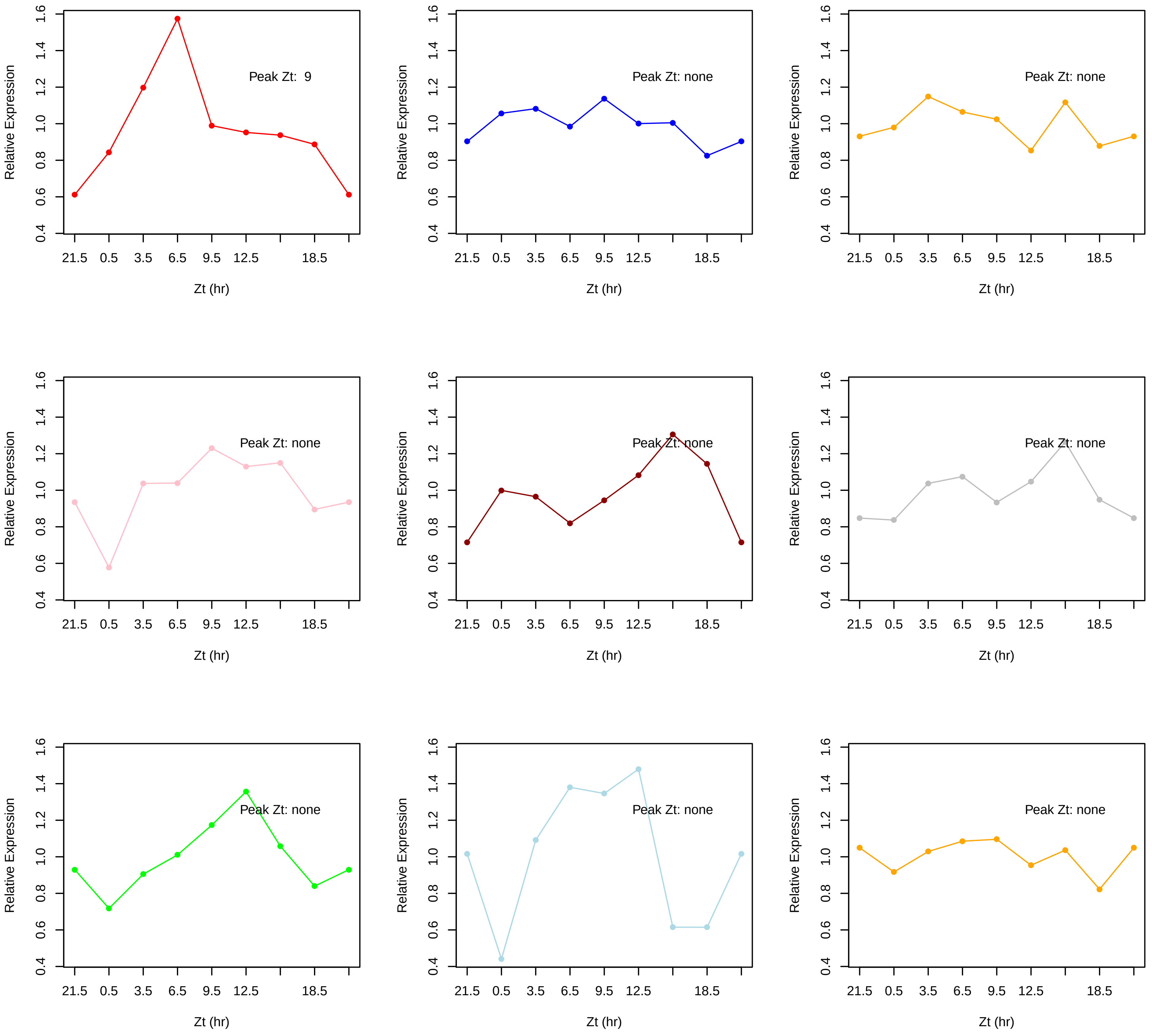

Singeleton: ENSXMAG00000011607 mrps11

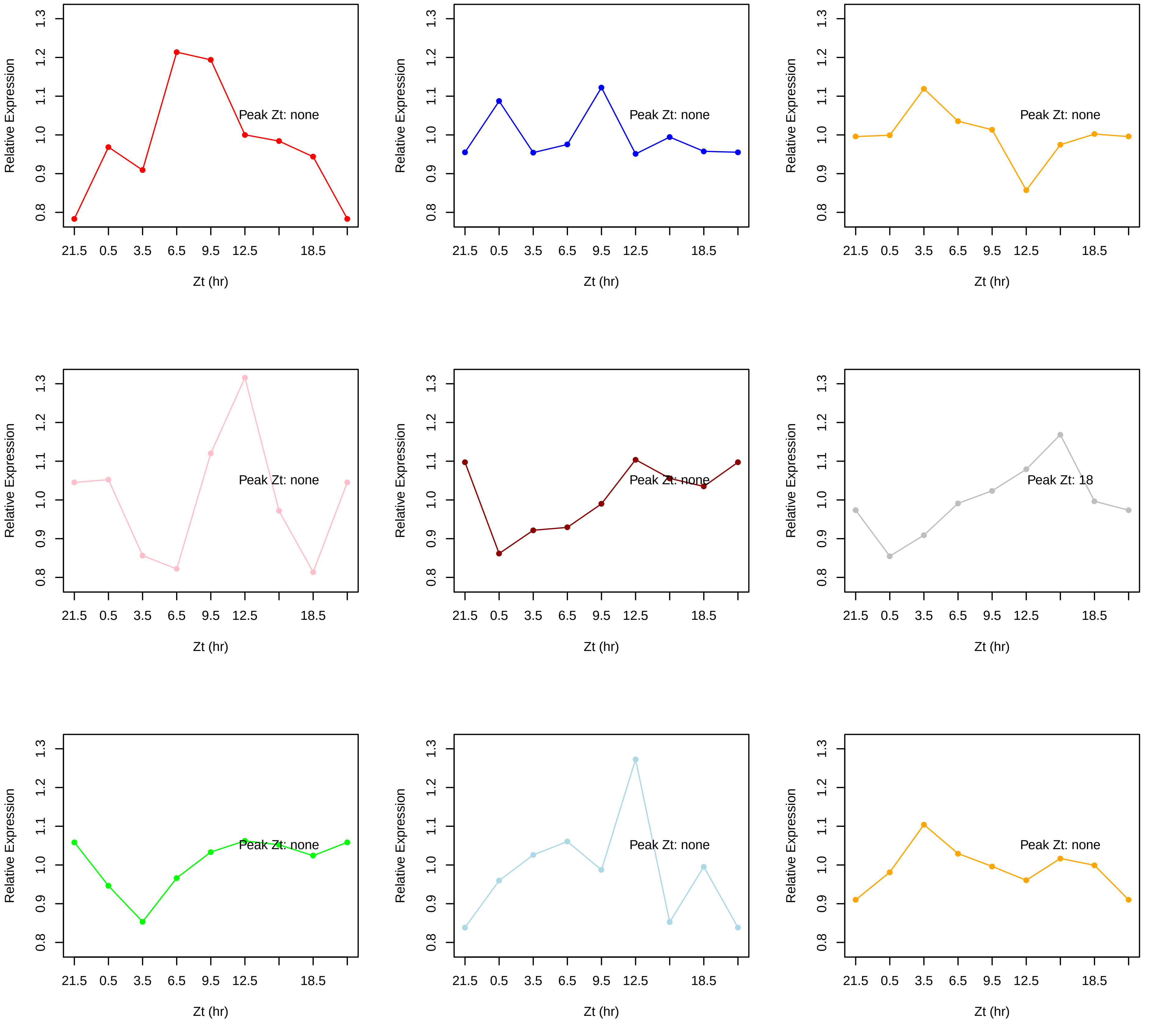

Singleton: ENSXMAG00000001847 ruvbl1

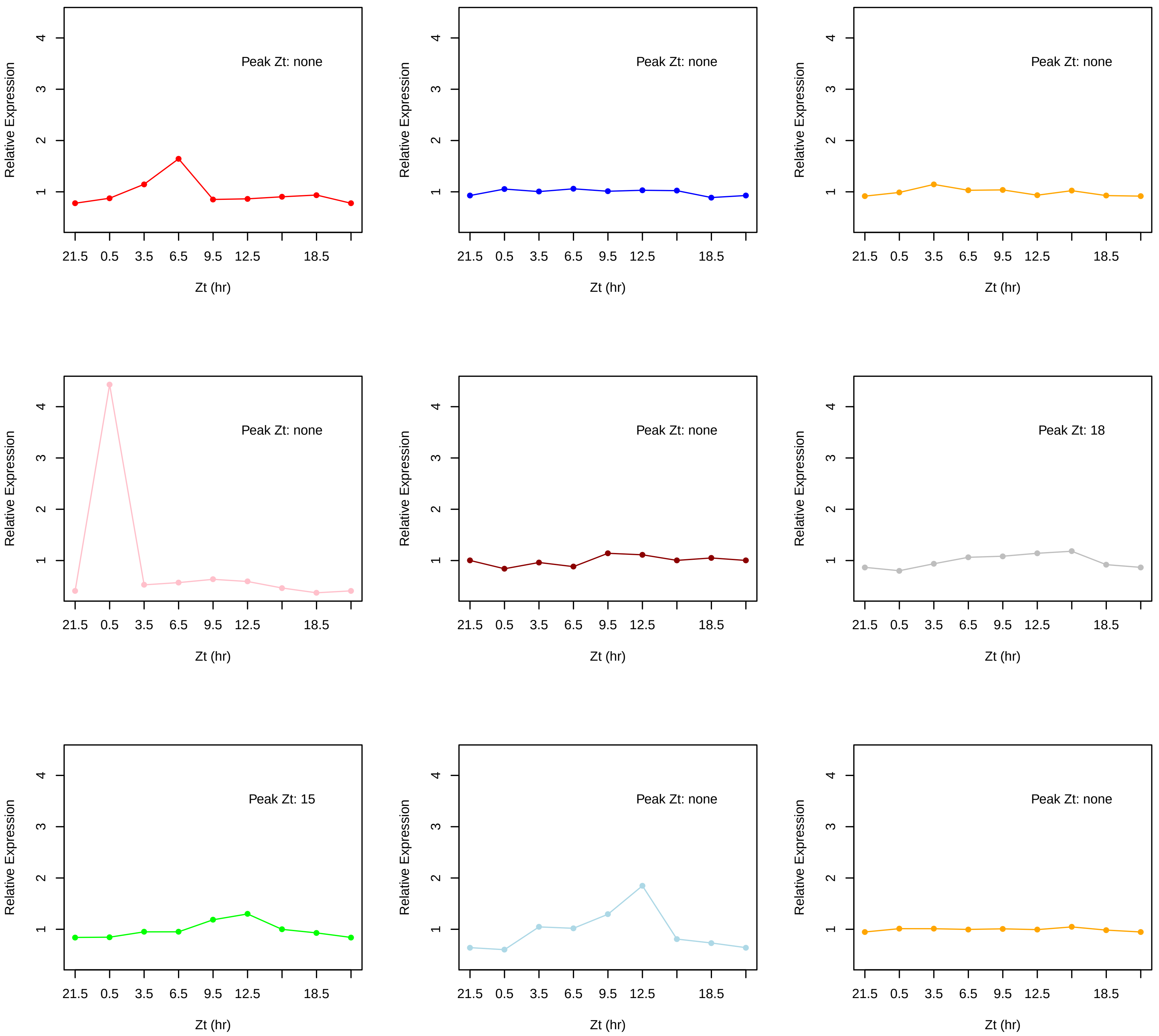

Singeleton: ENSXMAG00000001839 eefsec

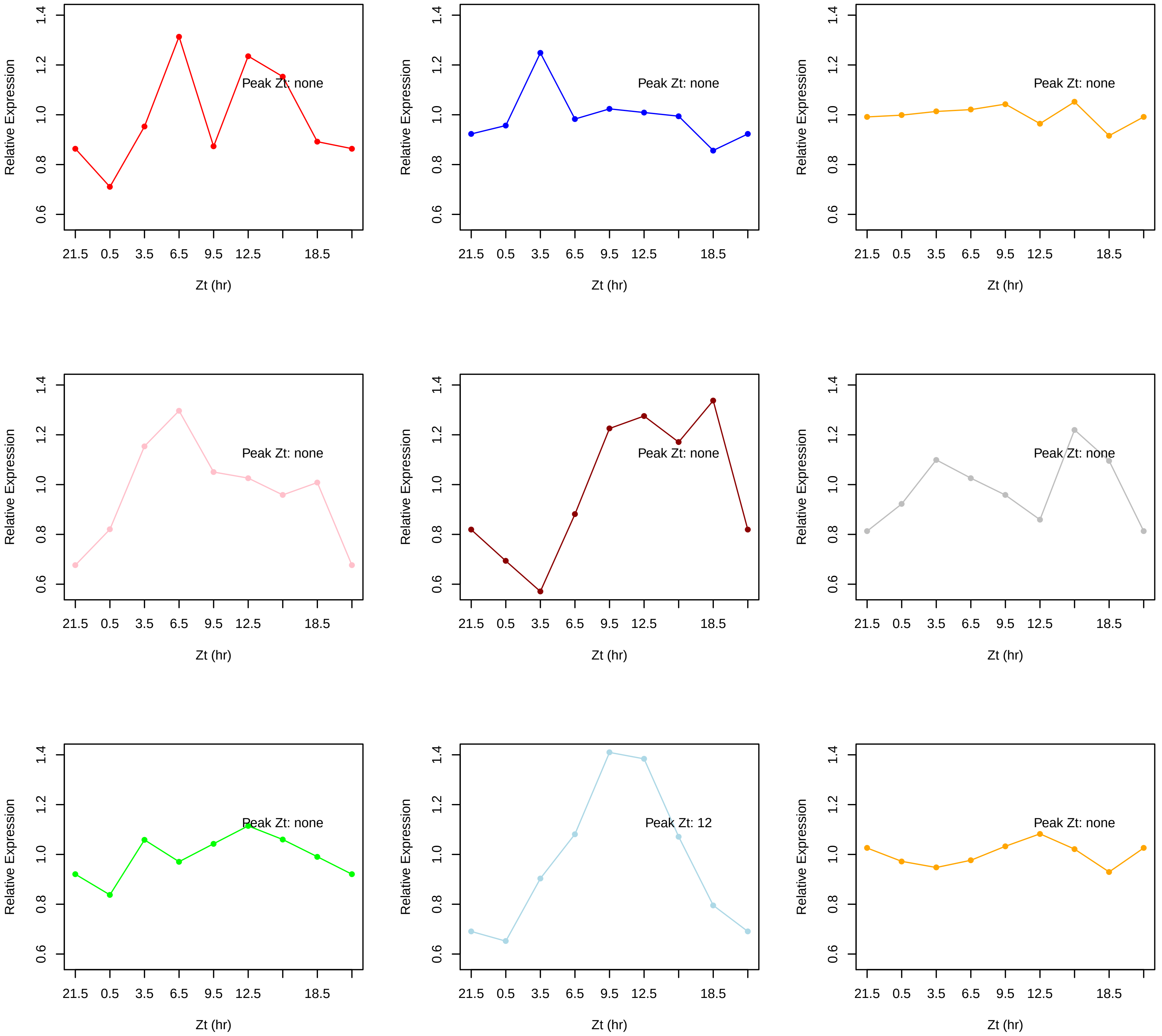

Singeleton: ENSXMAG00000025836 si:dkey−8e10.3

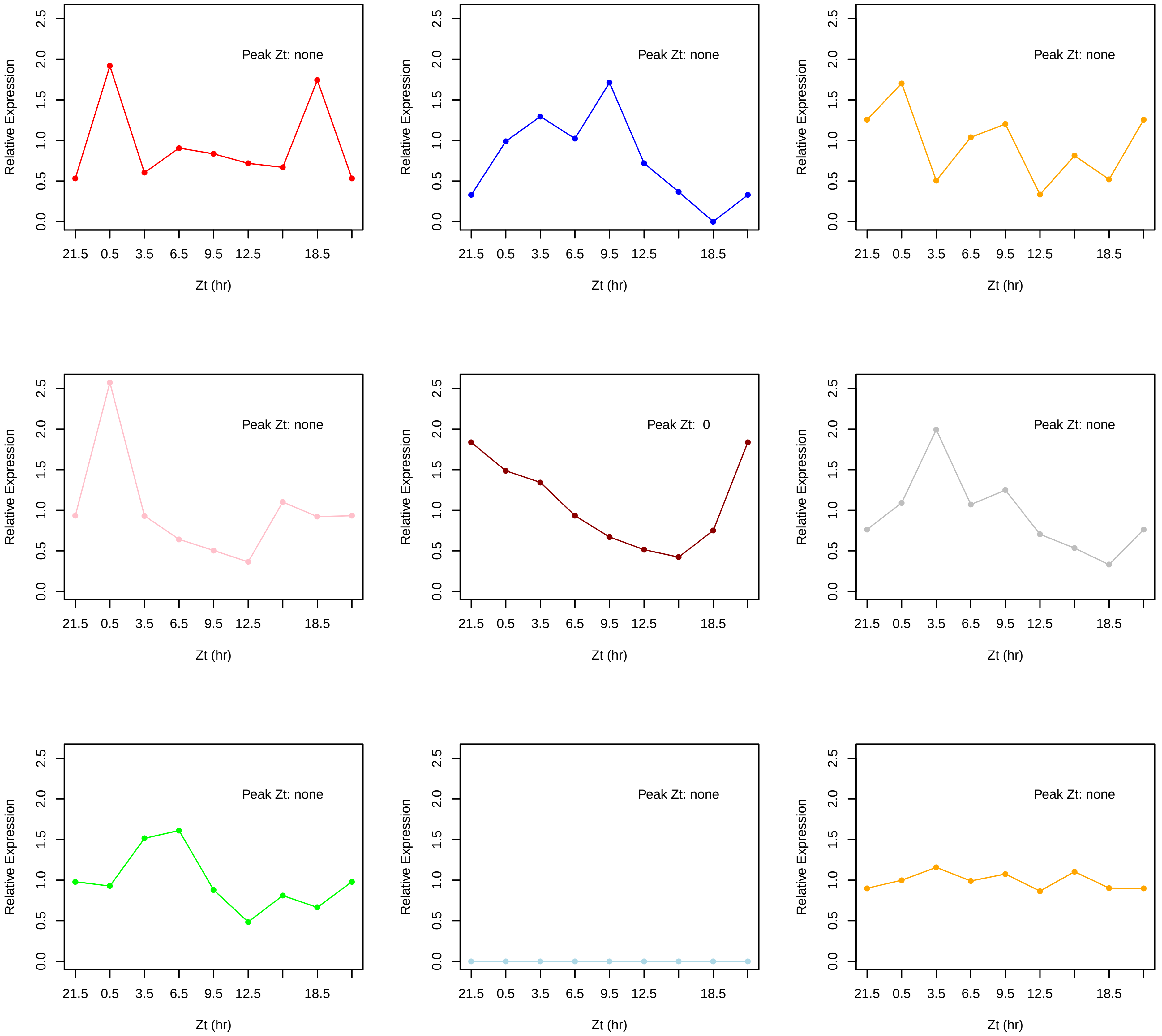

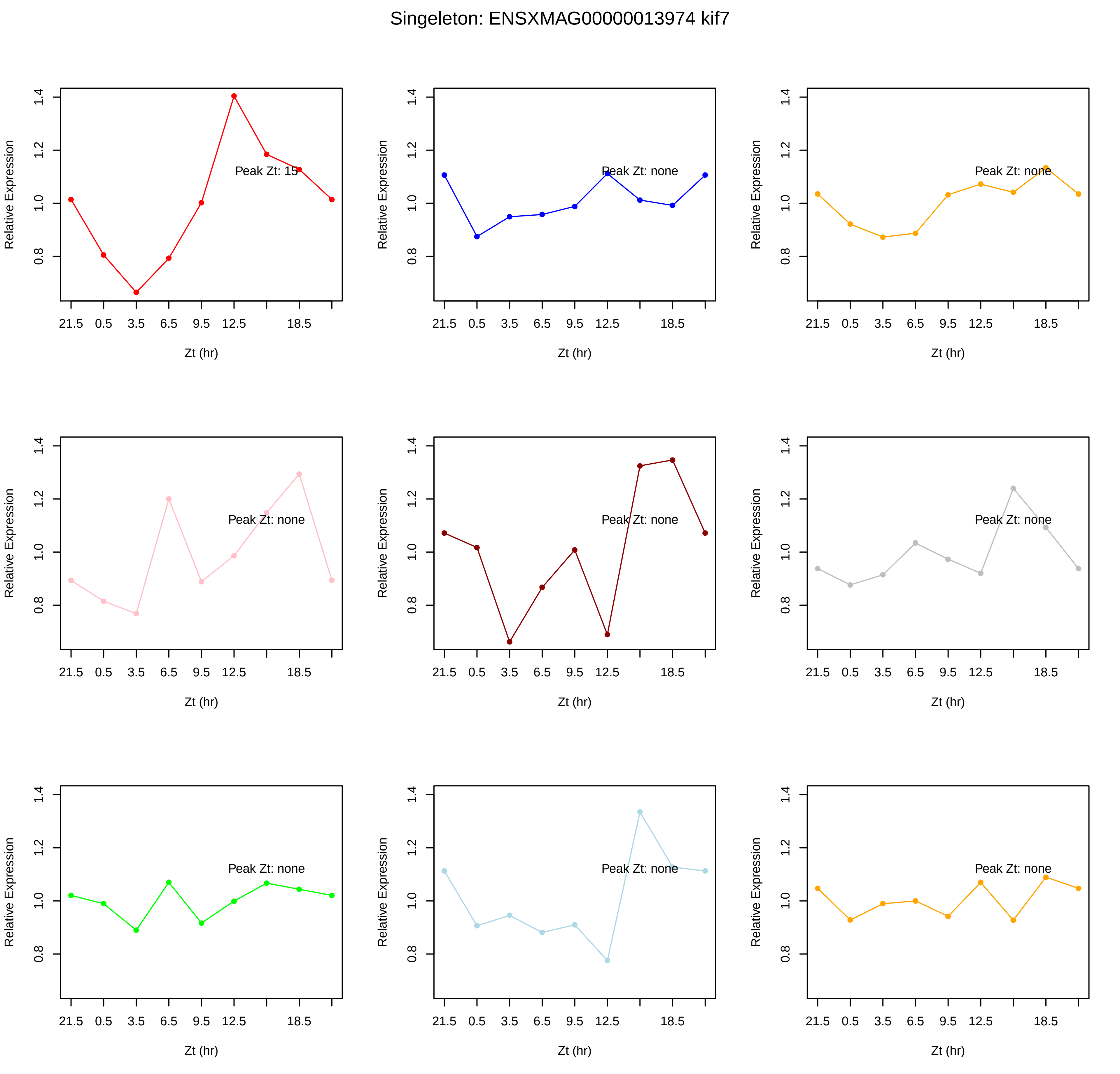
Singeleton: ENSXMAG00000013974 kif7
Relative Expression
Zt (hr)
21.5
0.5
3.5
6.5
9.5
12.5
18.5
0.8
1.0
1.2
1.4
Peak Zt: 15
Peak Zt: none

Singleton: ENSXMAG00000016690

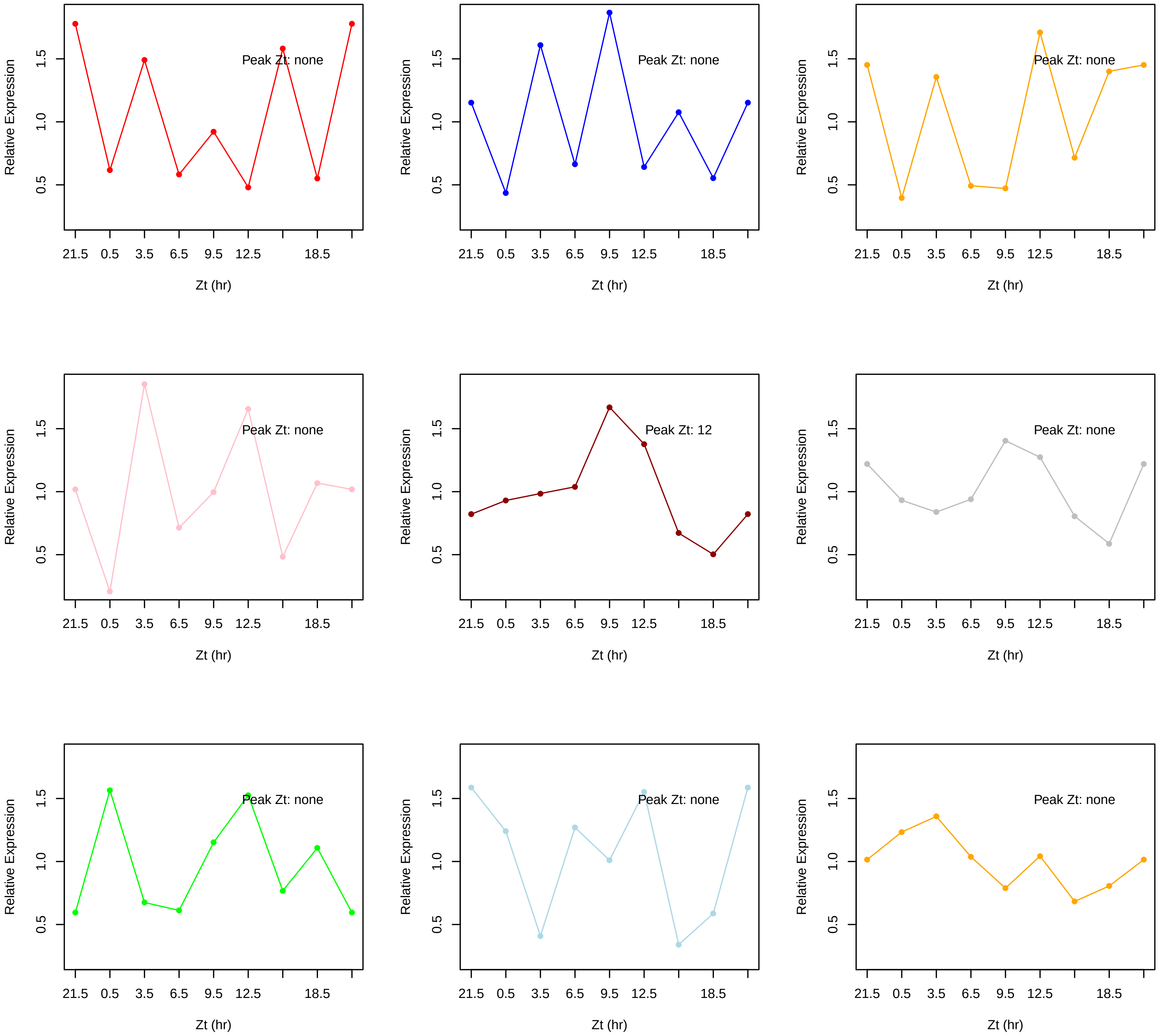

Singeleton: ENSXMAG00000018896 rpn1

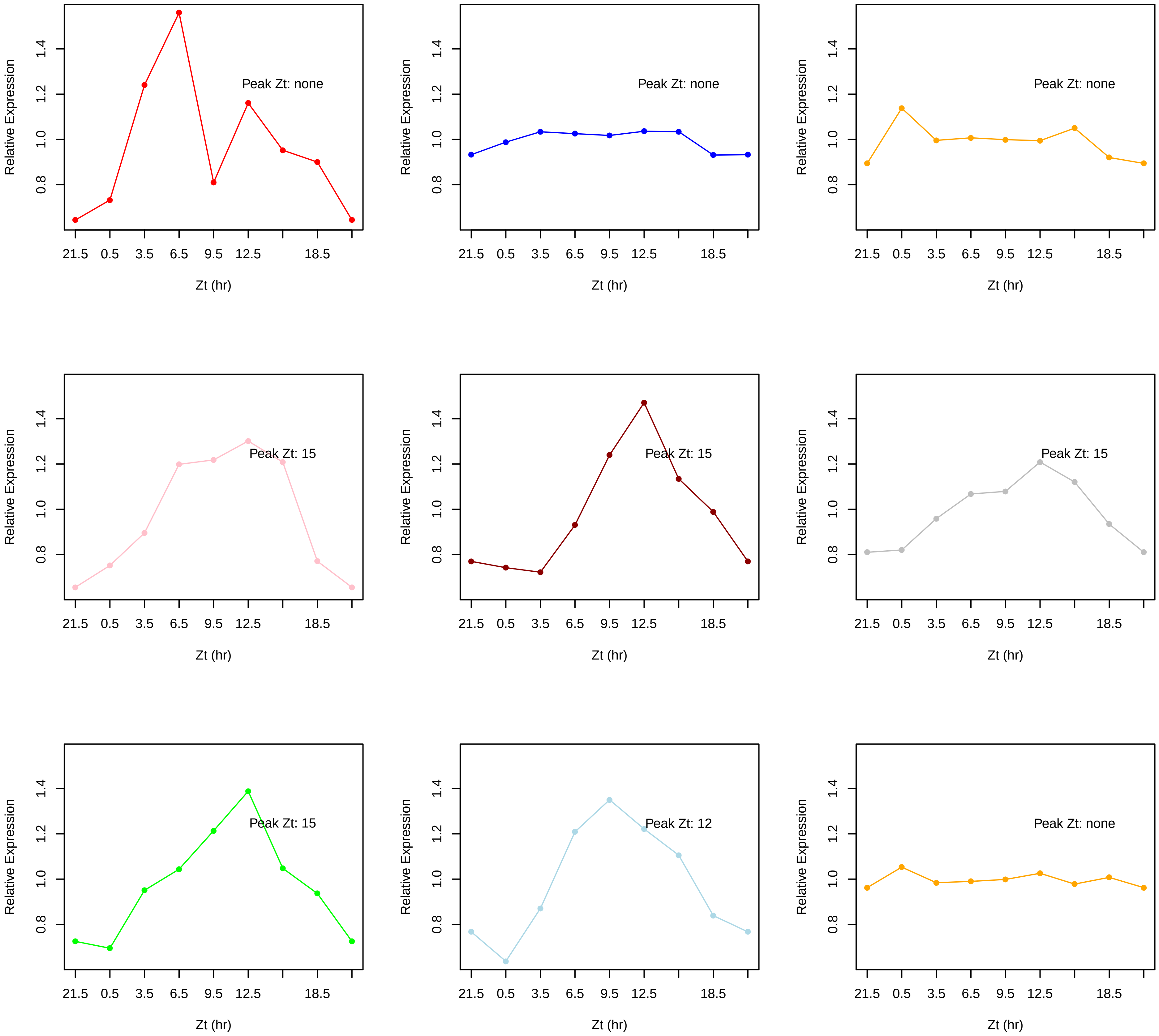

Singeleton: ENSXMAG00000014900

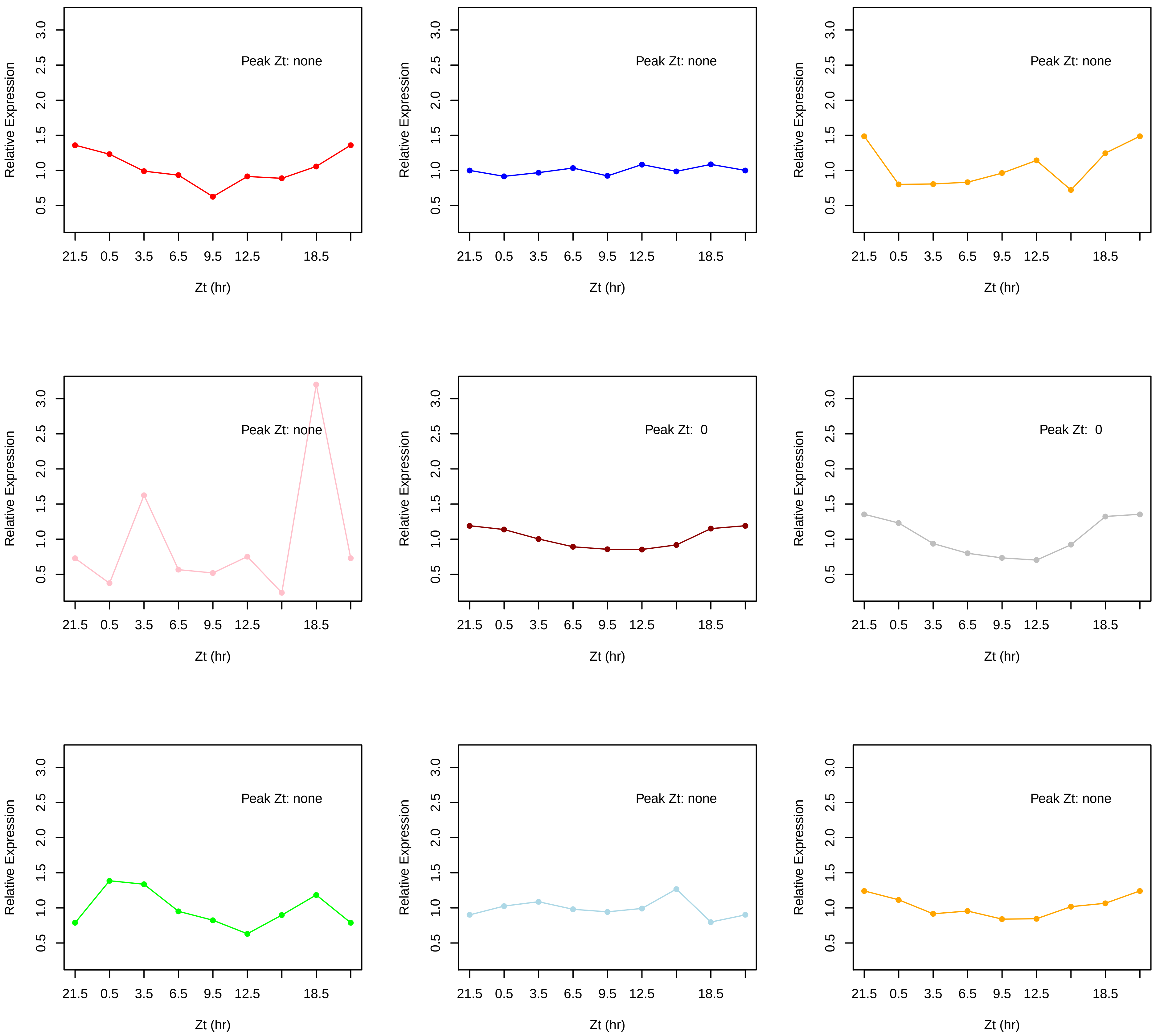

Singleton: ENSXMAG00000011604 THOC2

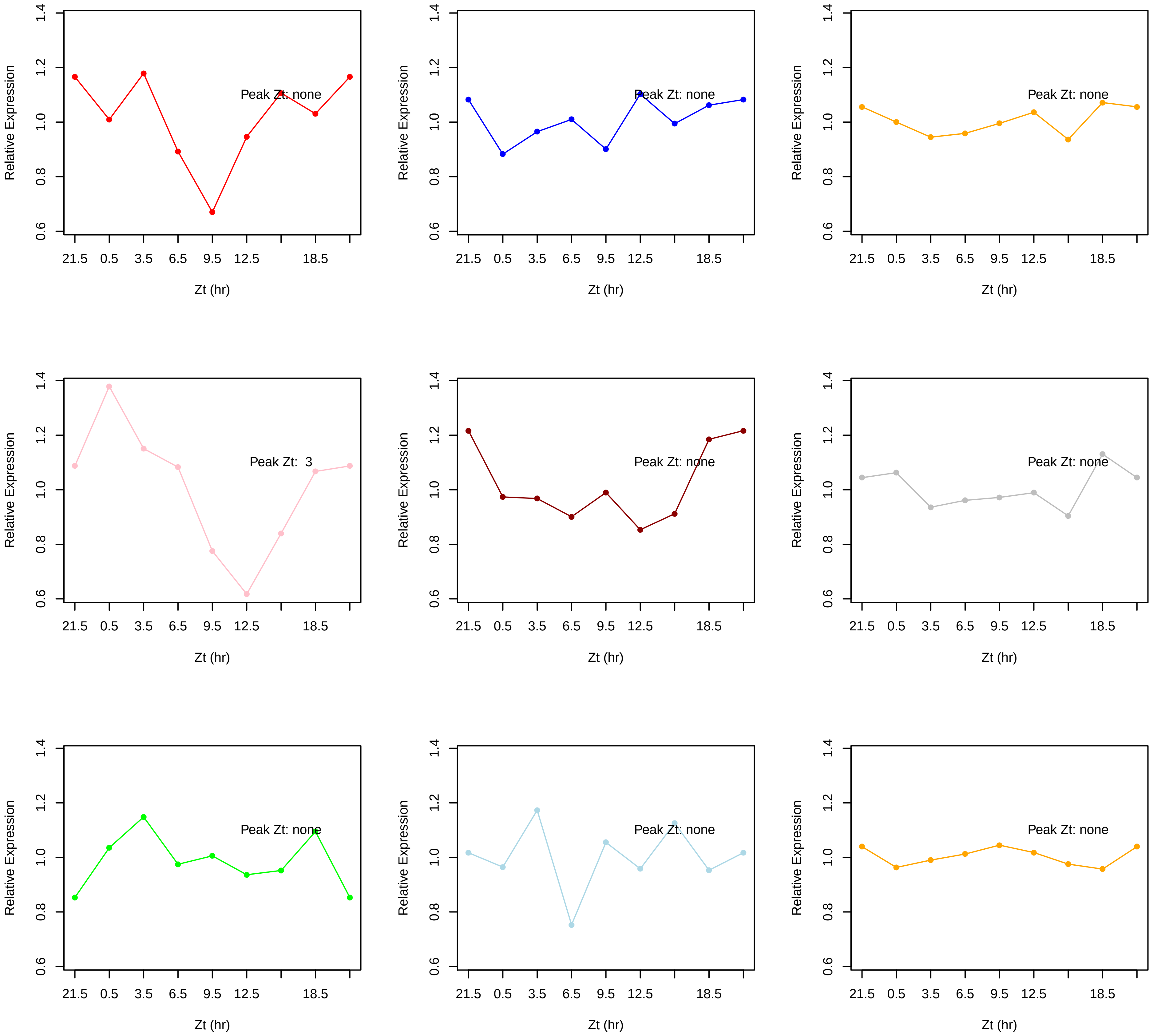

Singeleton: ENSXMAG00000023736 fam50a

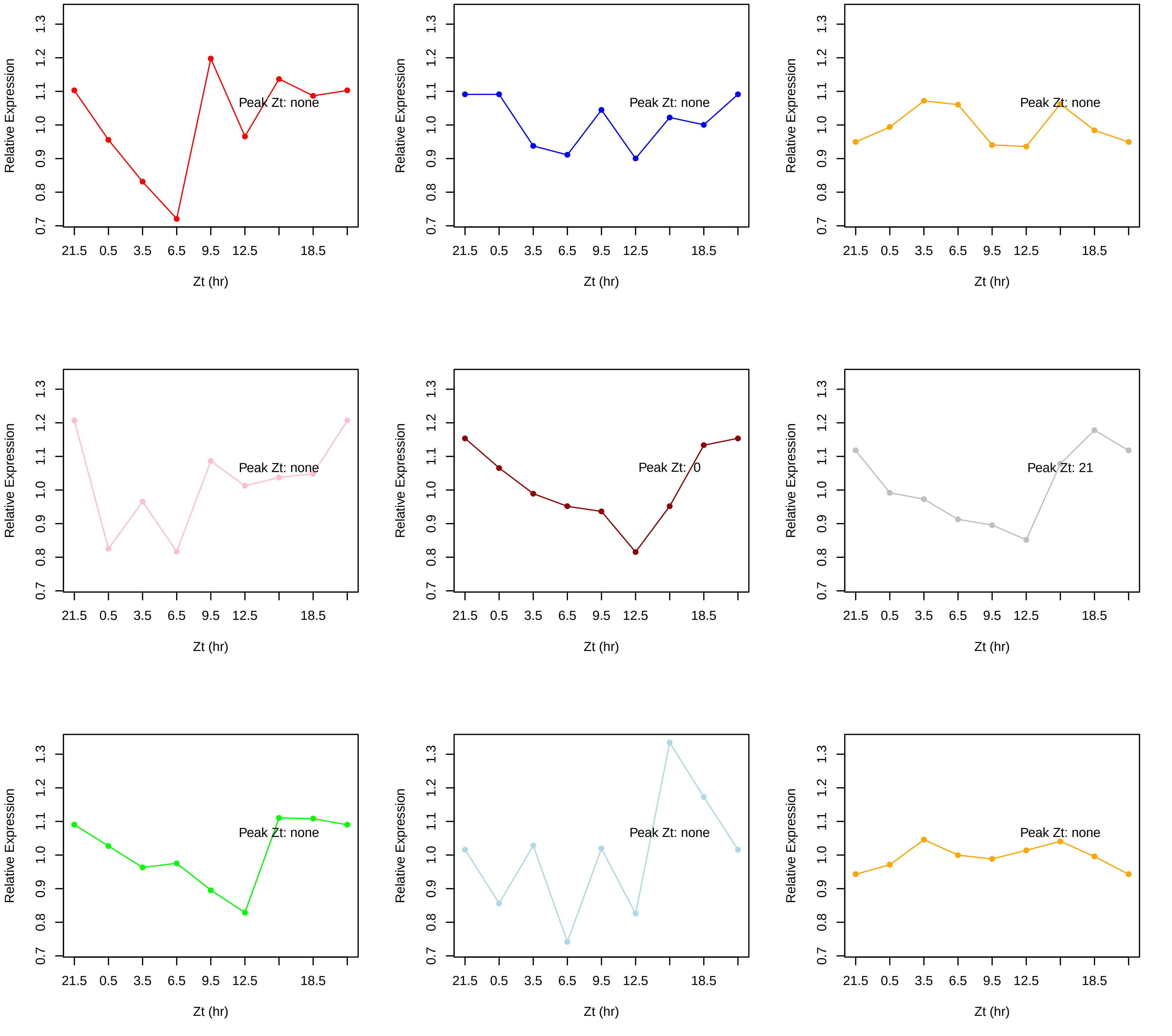

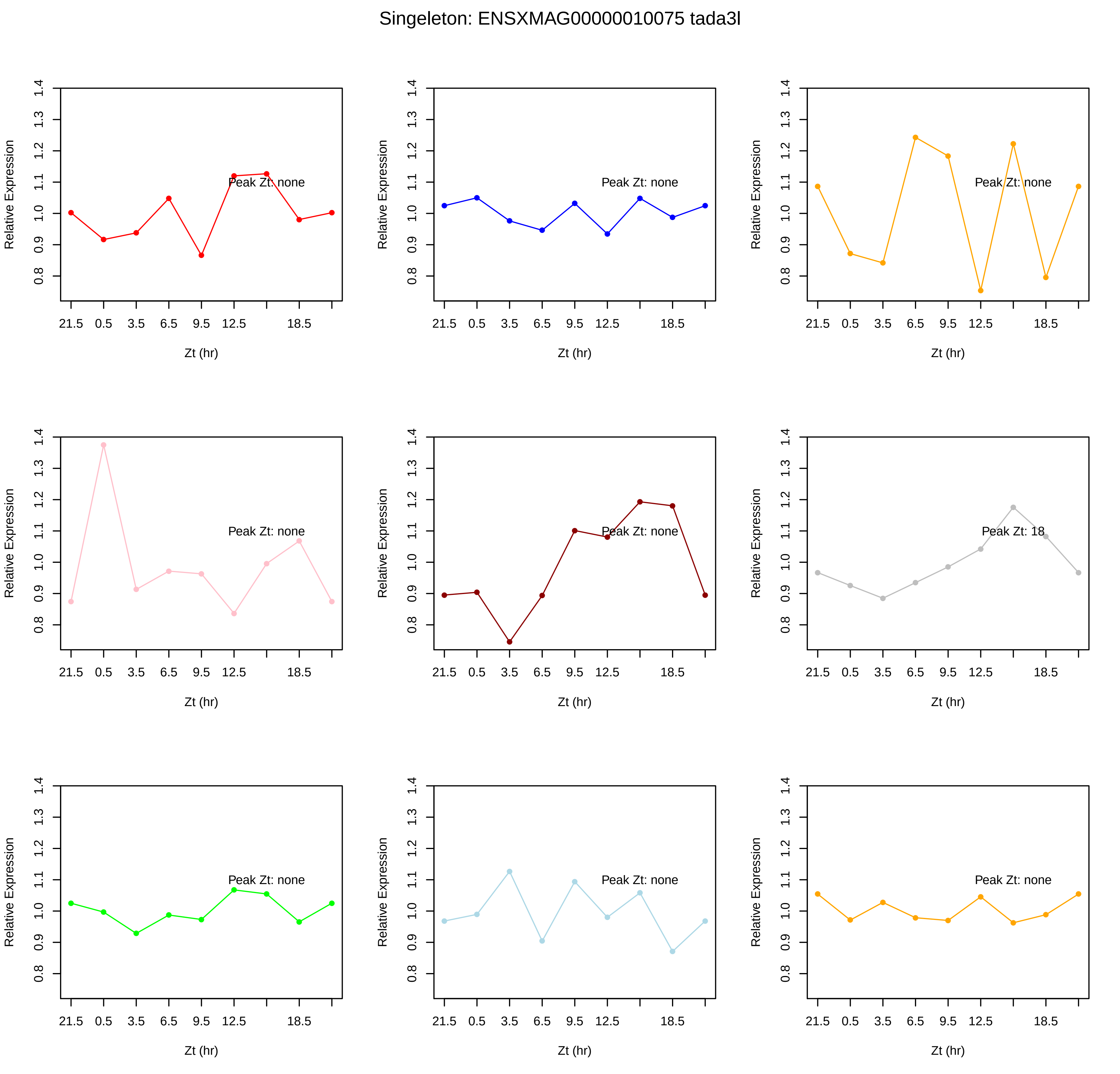
Singleton: ENSXMAG00000010075 tada3l
Relative Expression
Zt (hr)
21.5
0.5
3.5
6.5
9.5
12.5
18.5
0.8
0.9
1.0
1.1
1.2
1.3
1.4
Peak Zt: none
Peak Zt: 18

Singeleton: ENSXMAG00000023124 esrrd

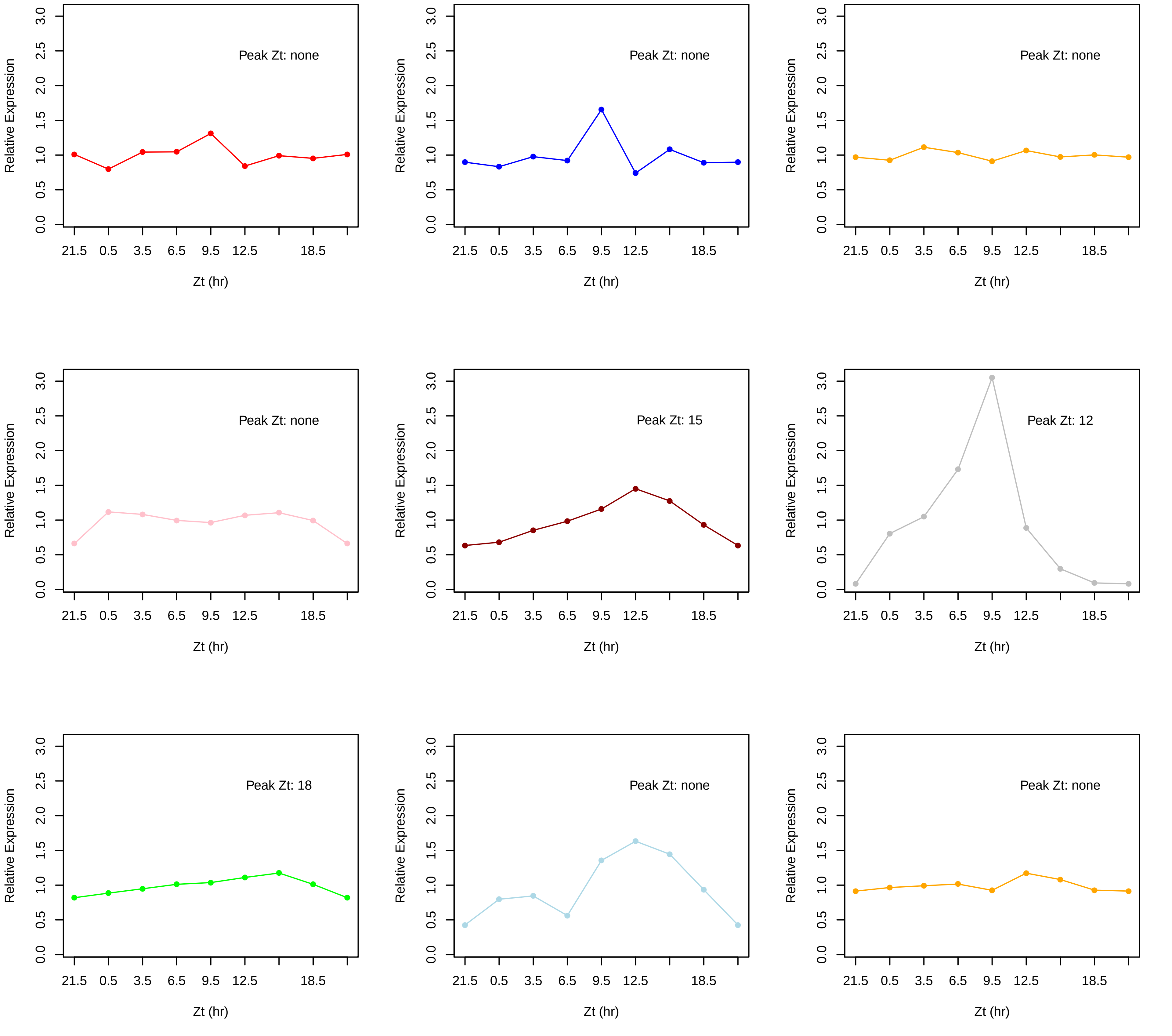

Singleton: ENSXMAG00000002300

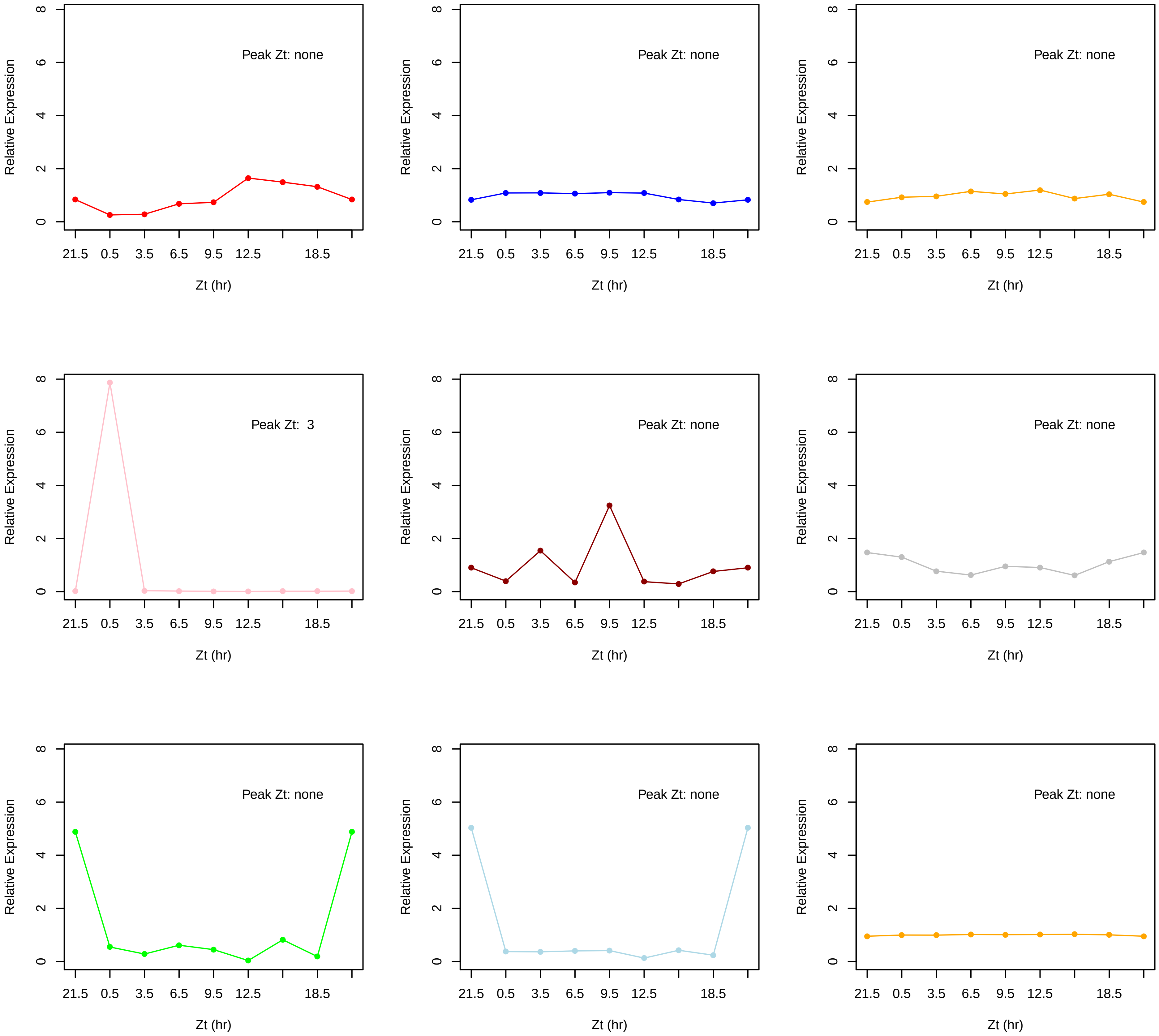

Singeleton: ENSXMAG00000005592 comtb

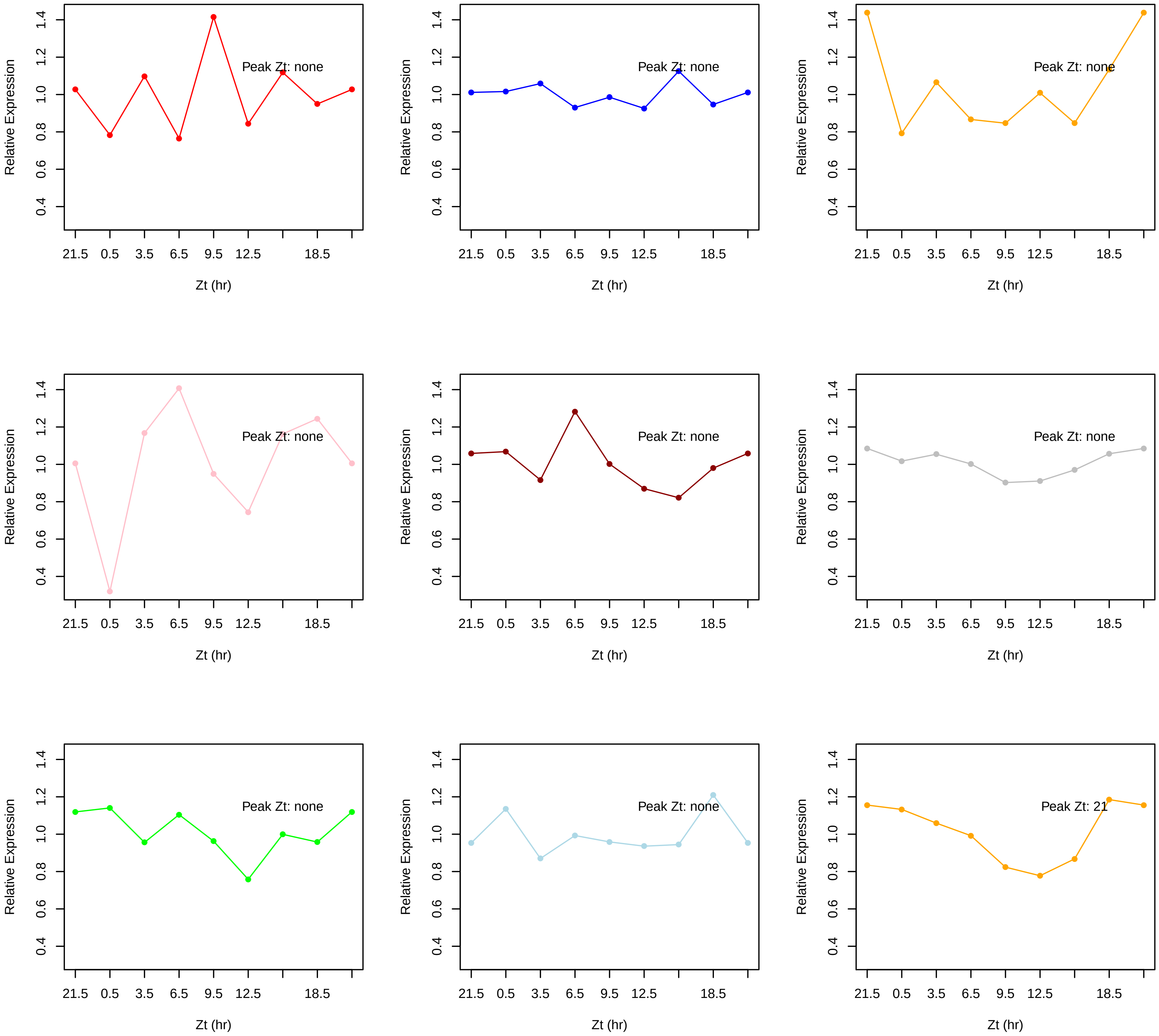

Singeleton: ENSXMAG00000002290

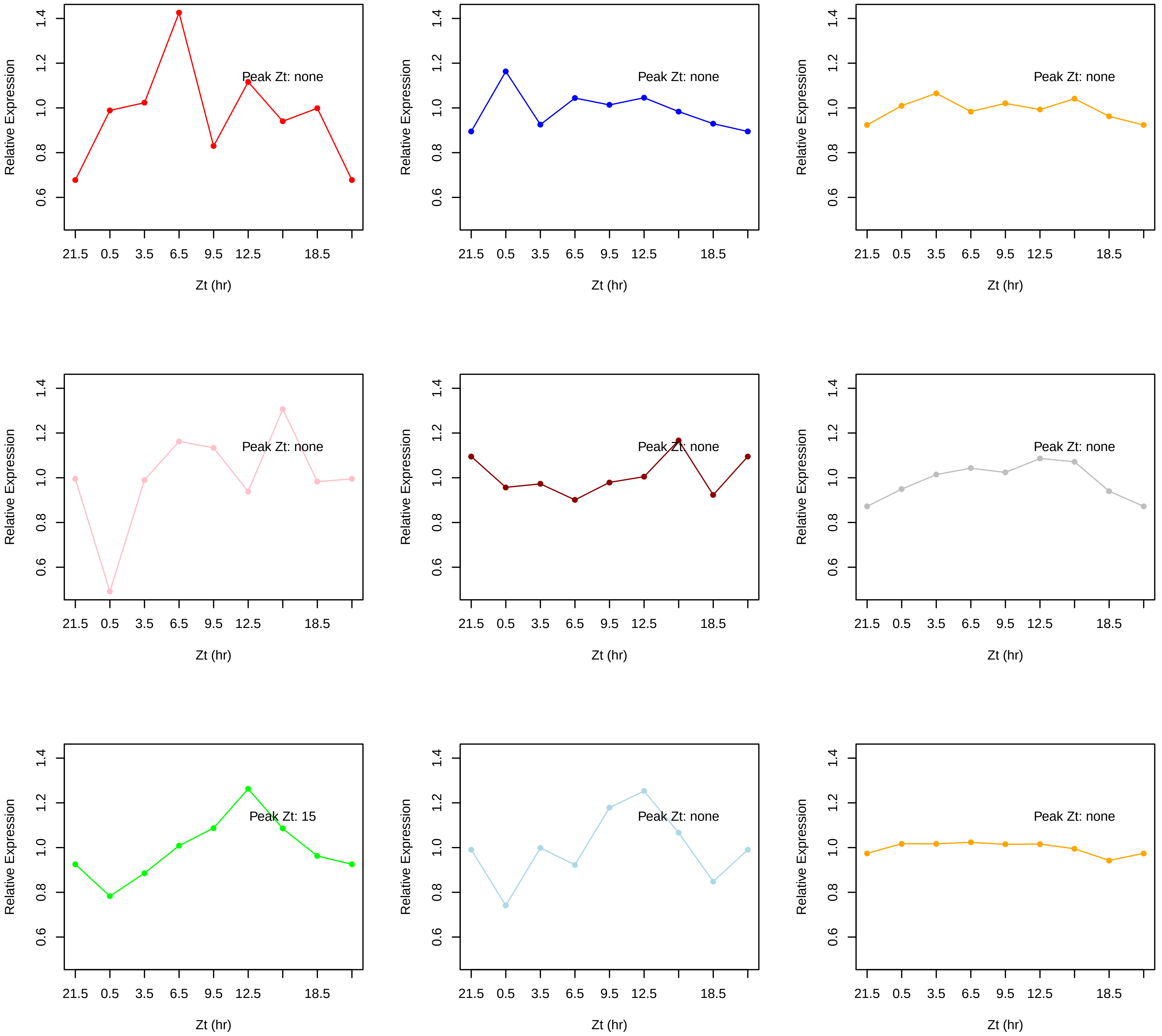

Singeleton: ENSXMAG00000006723 stag2b

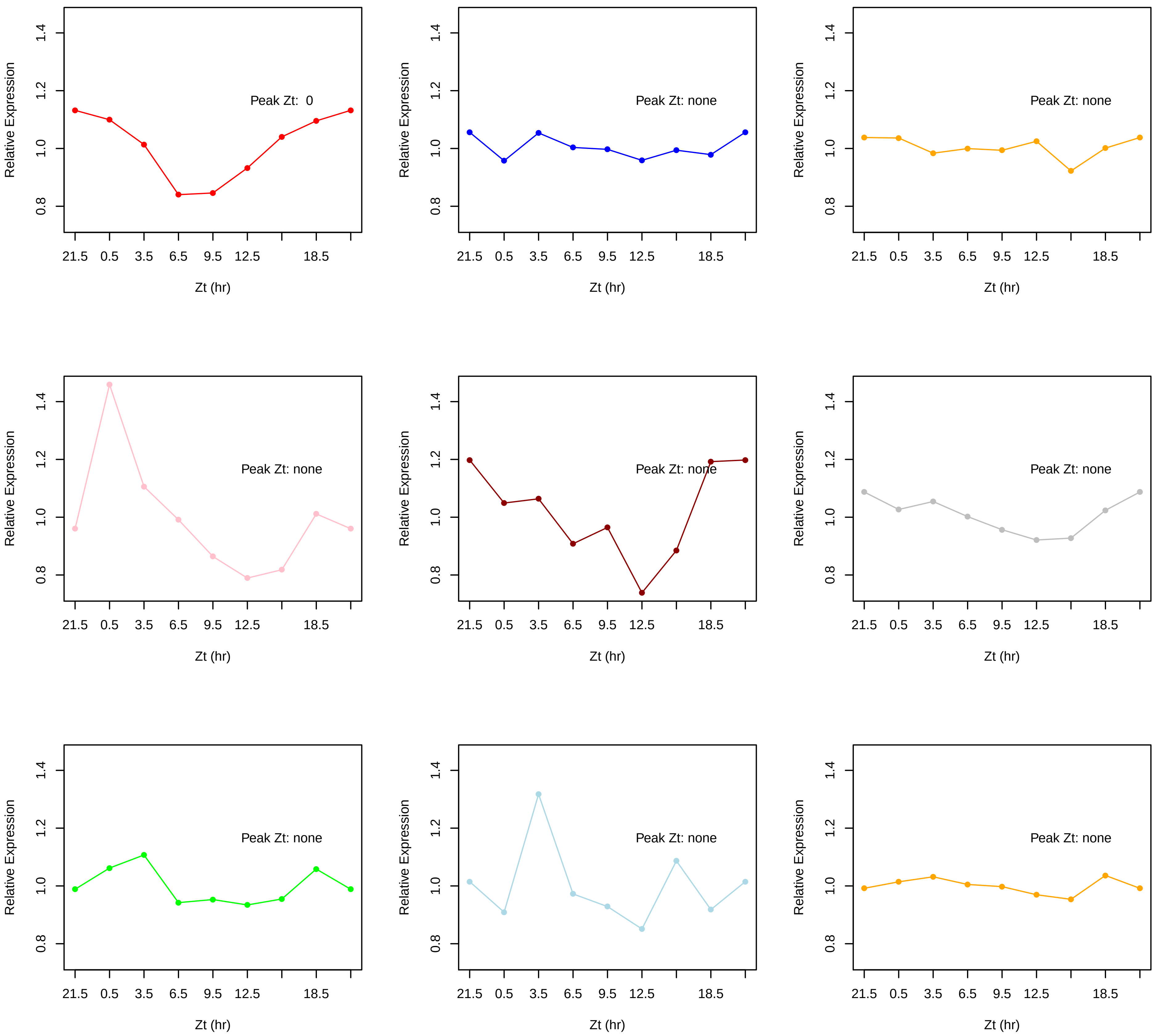

Singeleton: ENSXMAG00000001372

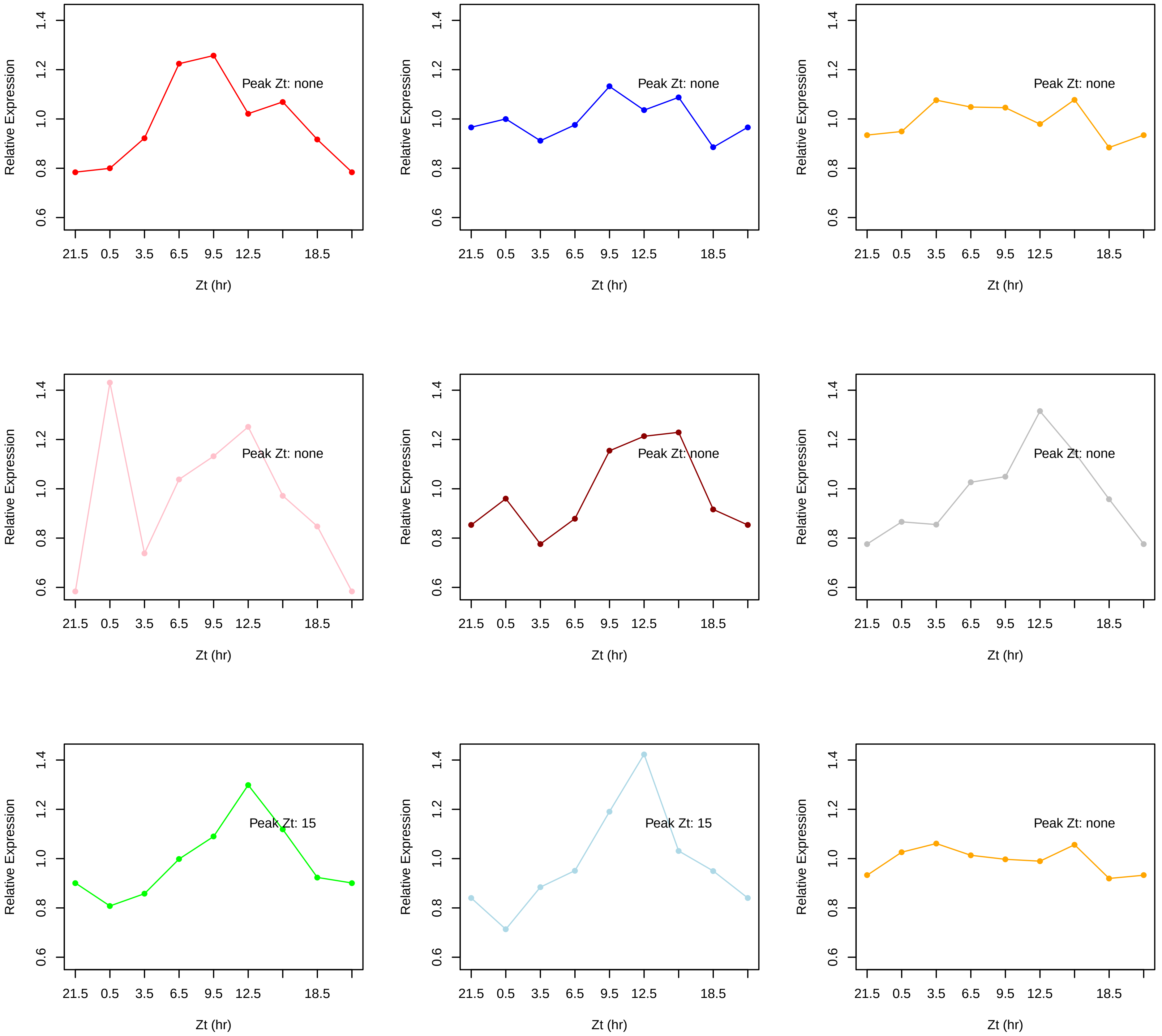

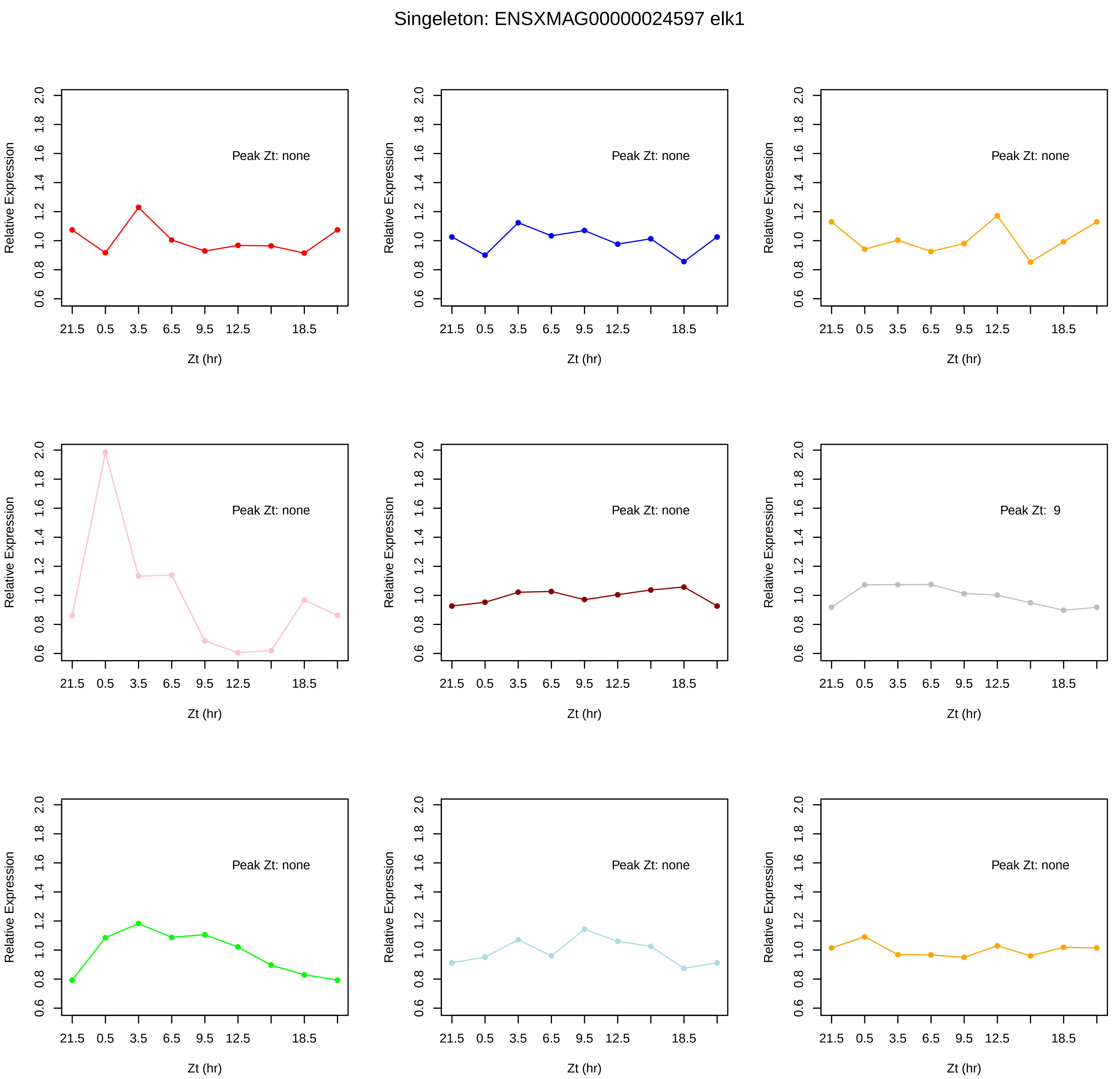
Singeleton: ENSXMAG00000024597 elk1
Peak Zt: none
Peak Zt: none
Peak Zt: none
Peak Zt: none
Peak Zt: none
Peak Zt: 9
Peak Zt: none
Peak Zt: none
Peak Zt: none
Relative Expression
Zt (hr)
21.5
0.5
3.5
6.5
9.5
12.5
18.5

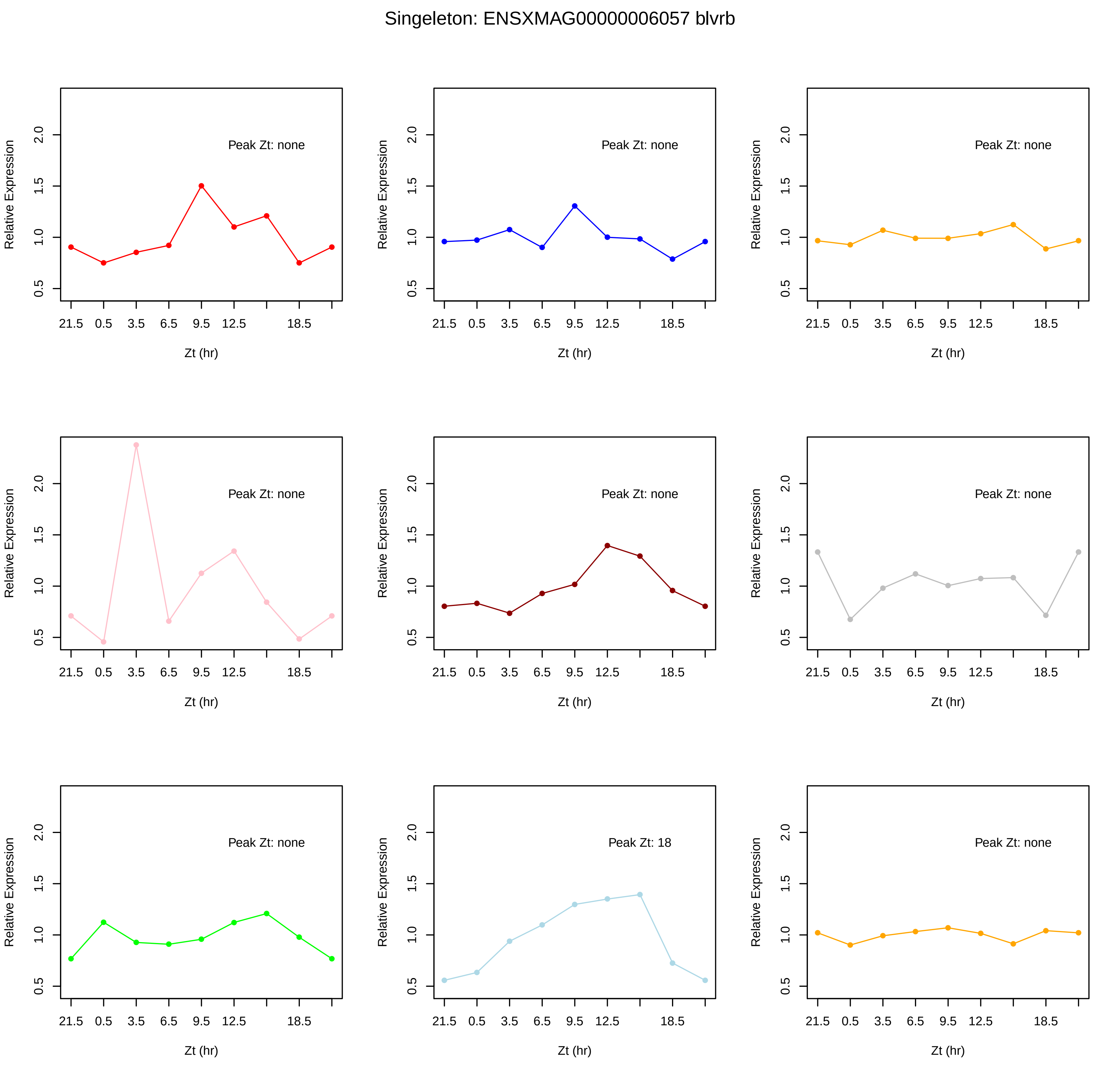
Singeleton: ENSXMAG00000006057 blvrb
Peak Zt: none
Peak Zt: none
Peak Zt: none
Peak Zt: none
Peak Zt: none
Peak Zt: none
Peak Zt: none
Peak Zt: 18
Peak Zt: none
Relative Expression
Zt (hr)
21.5 0.5 3.5 6.5 9.5 12.5 18.5
0.5 1.0 1.5 2.0

Singeleton: ENSXMAG00000013220 pim2

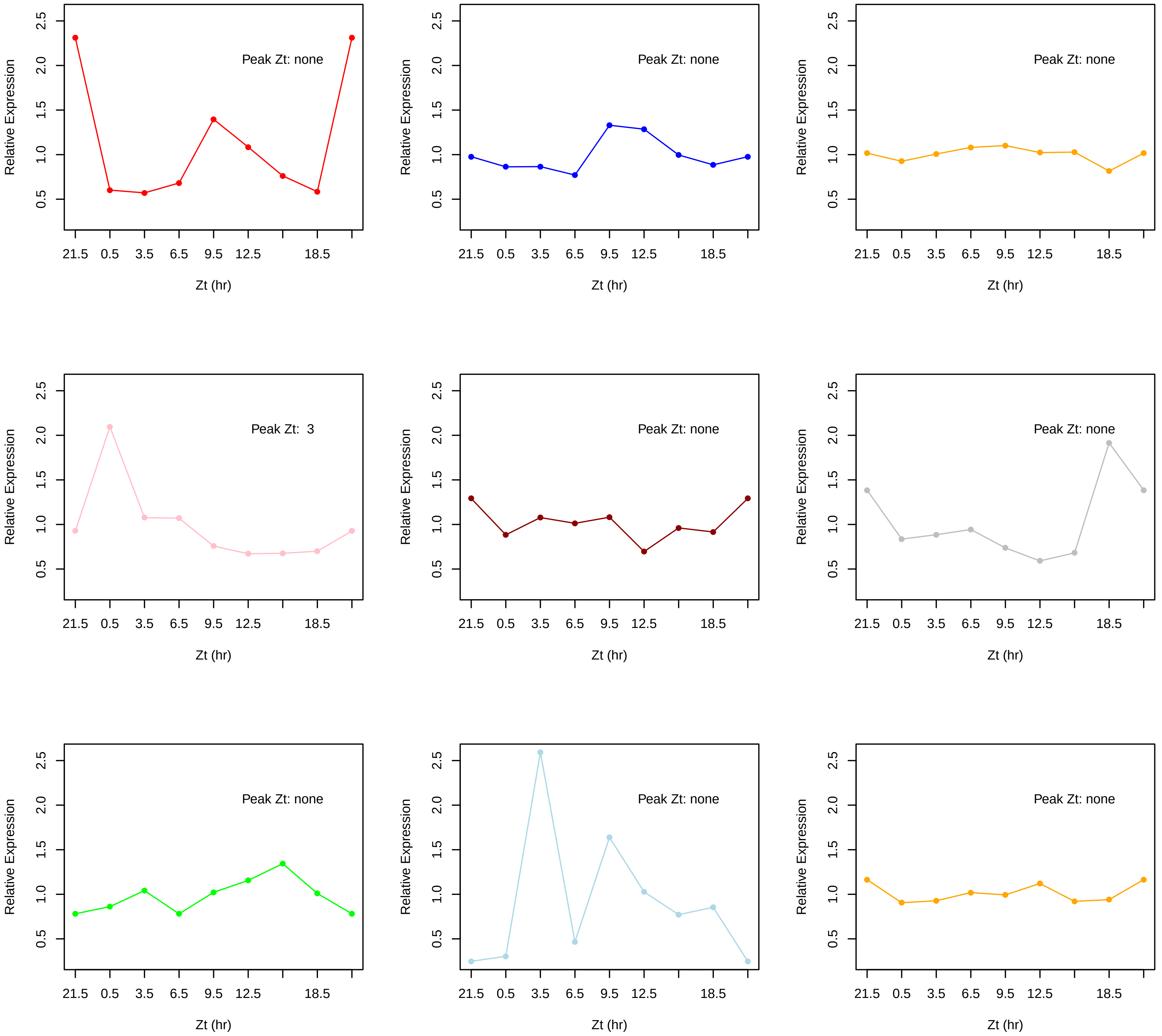

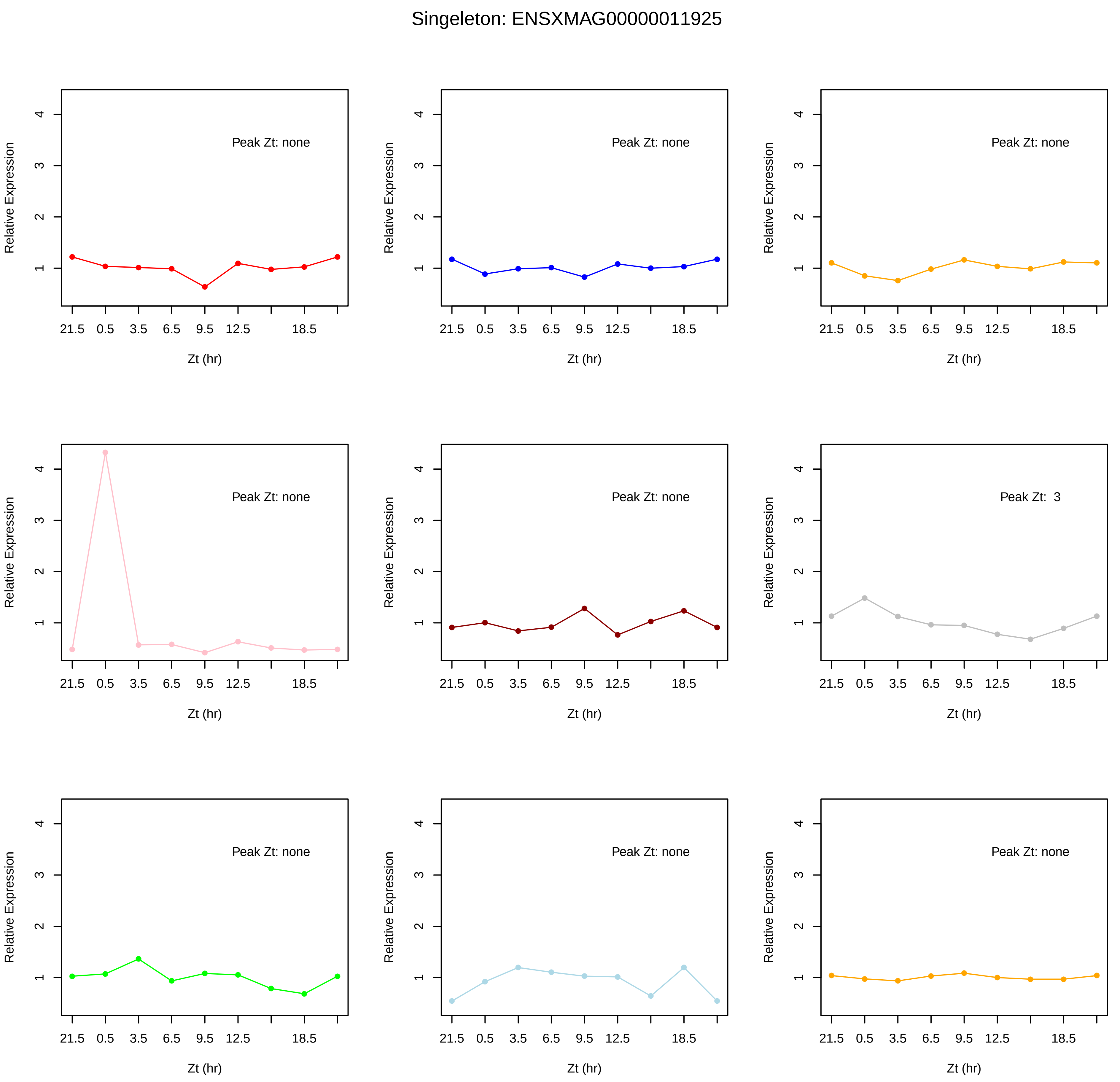
Singeleton: ENSXMAG00000011925
Peak Zt: none
Peak Zt: none
Peak Zt: none
Peak Zt: none
Peak Zt: none
Peak Zt: 3
Peak Zt: none
Peak Zt: none
Peak Zt: none
Relative Expression
Zt (hr)
21.5 0.5 3.5 6.5 9.5 12.5 18.5
1 2 3 4

Singeleton: ENSXMAG00000016602 LRRC49

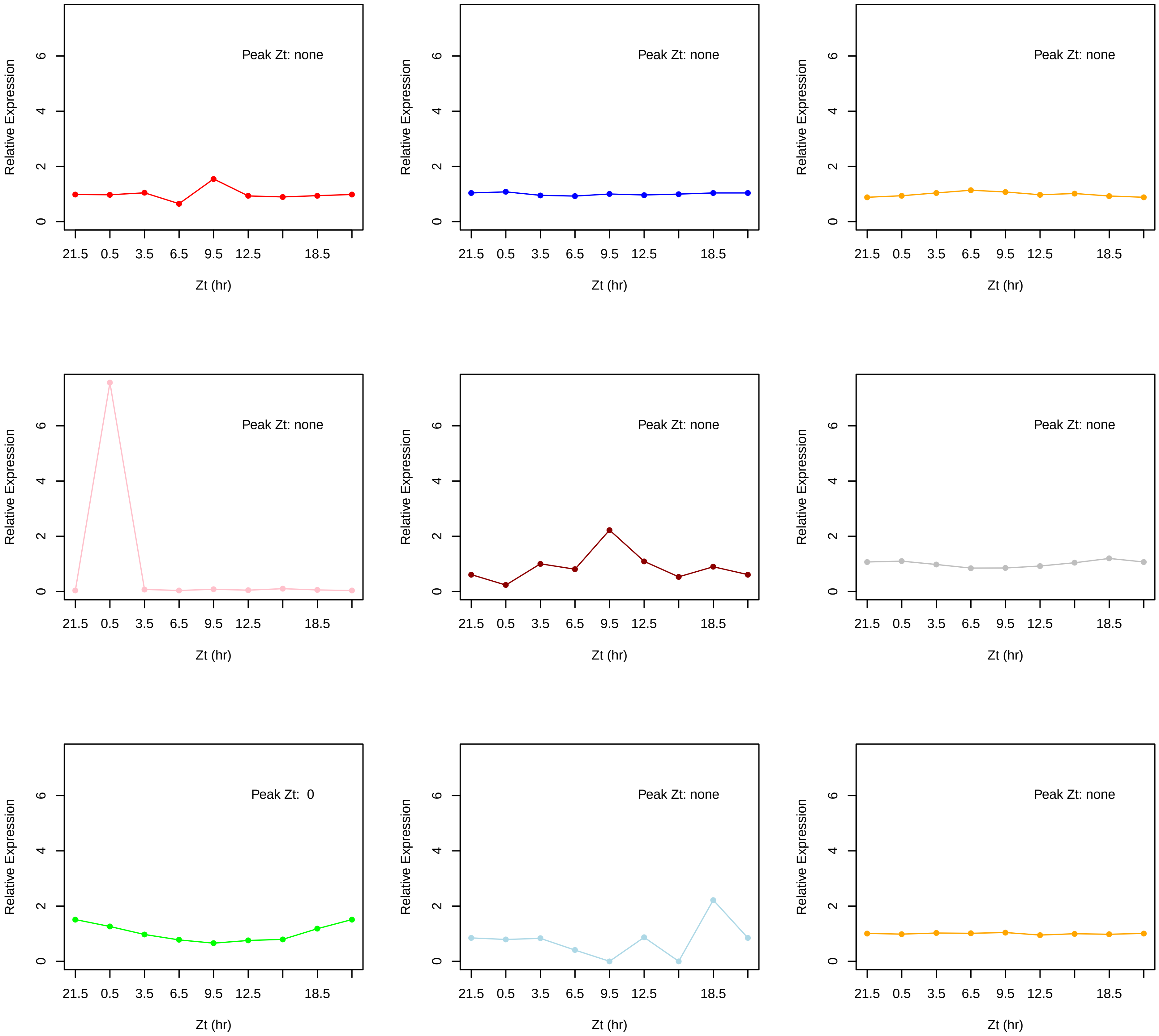

Singeleton: ENSXMAG00000027837 bcap31

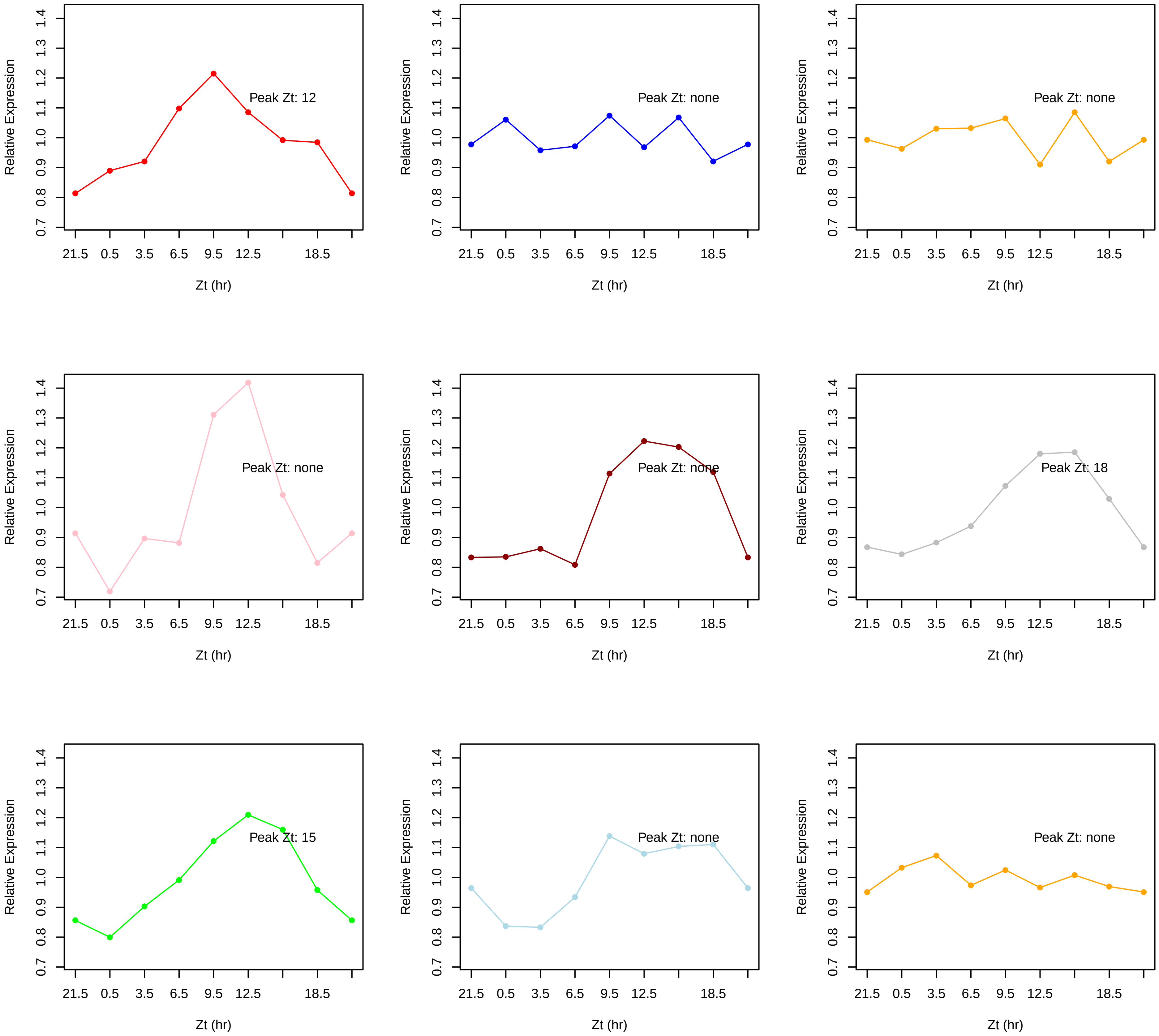

Singeleton: ENSXMAG00000004681 sb:cb81

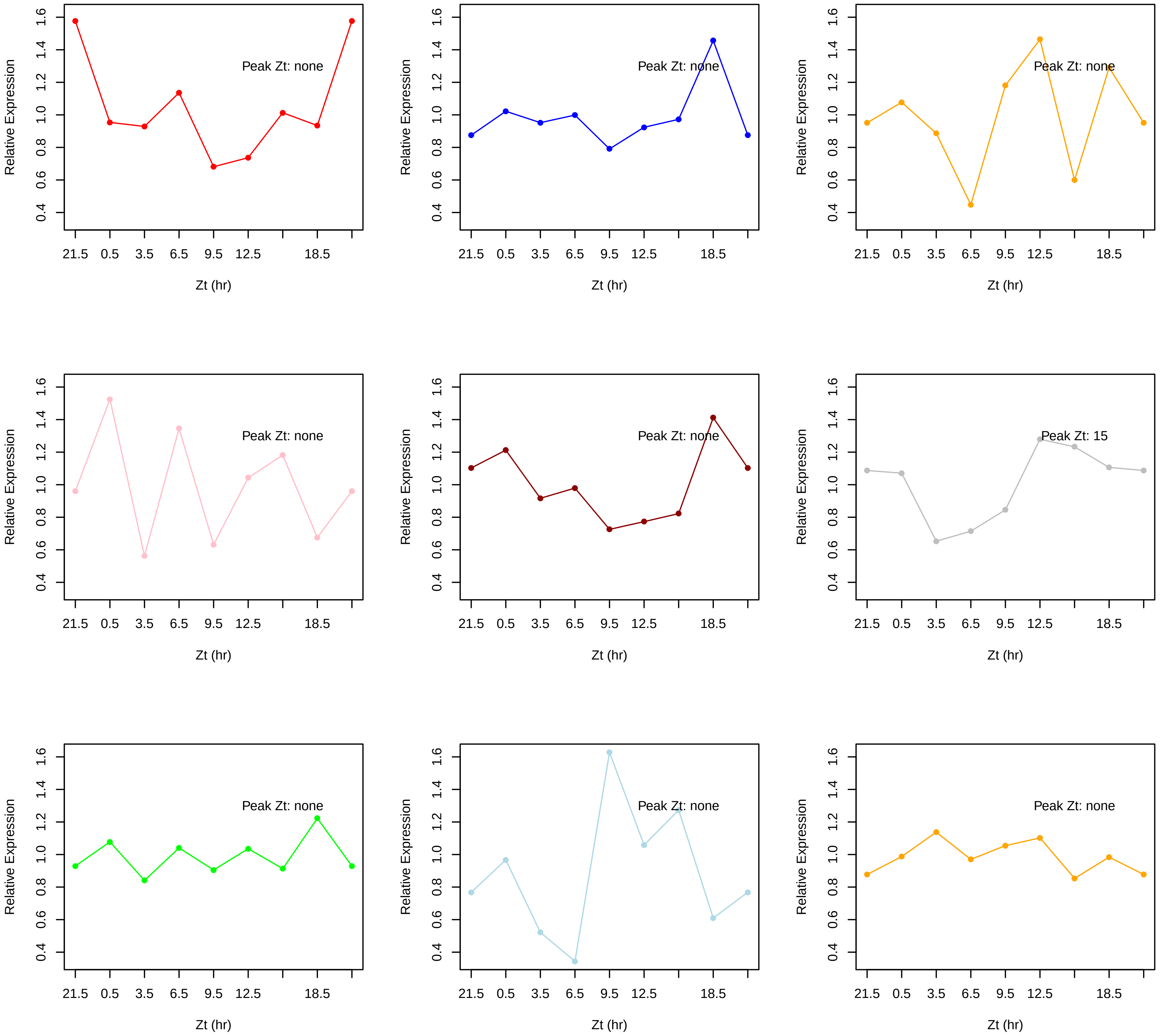

Singleton: ENSXMAG00000009678 SMARCA1

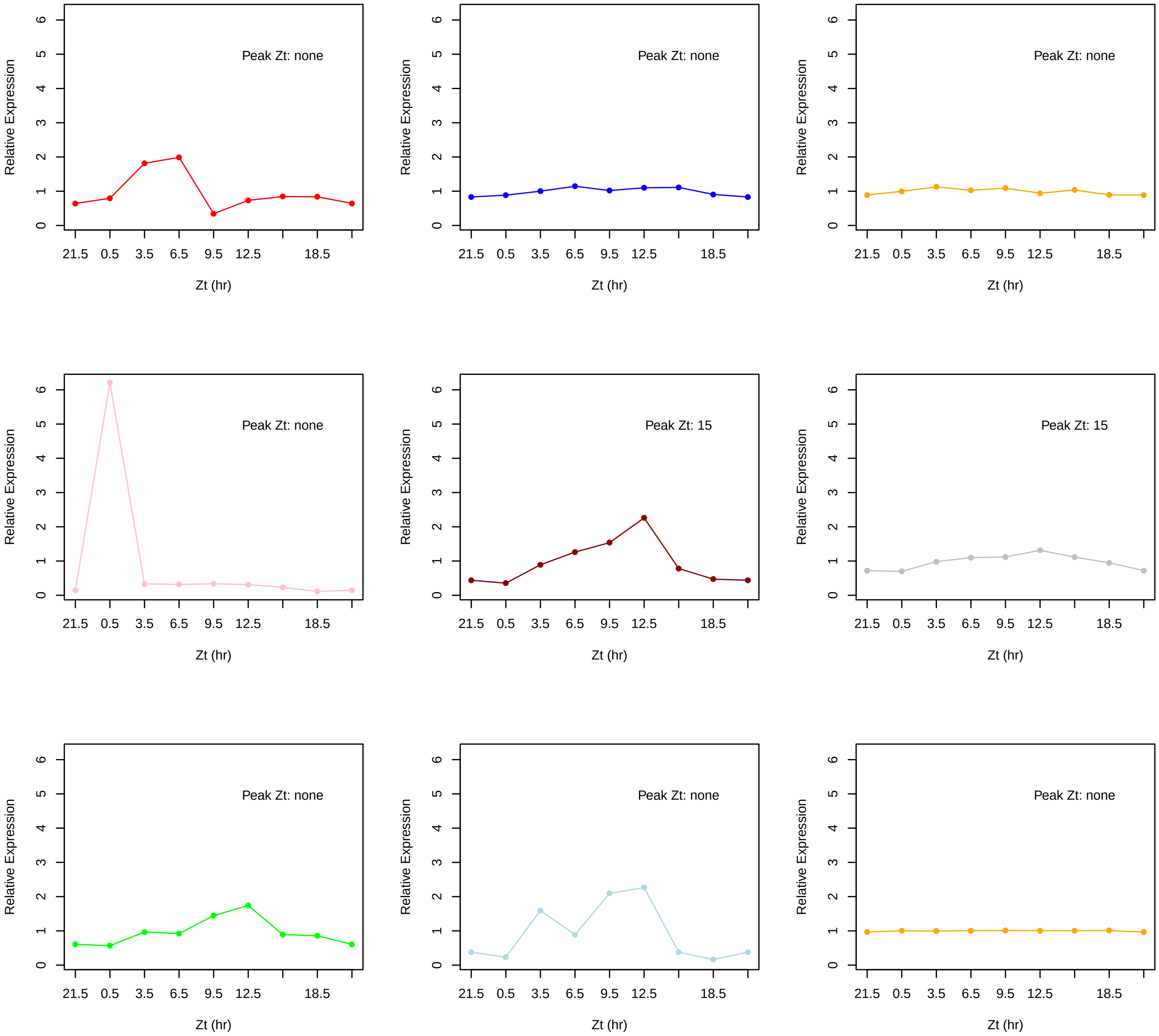

Singeleton: ENSXMAG00000018203 trappc6bl

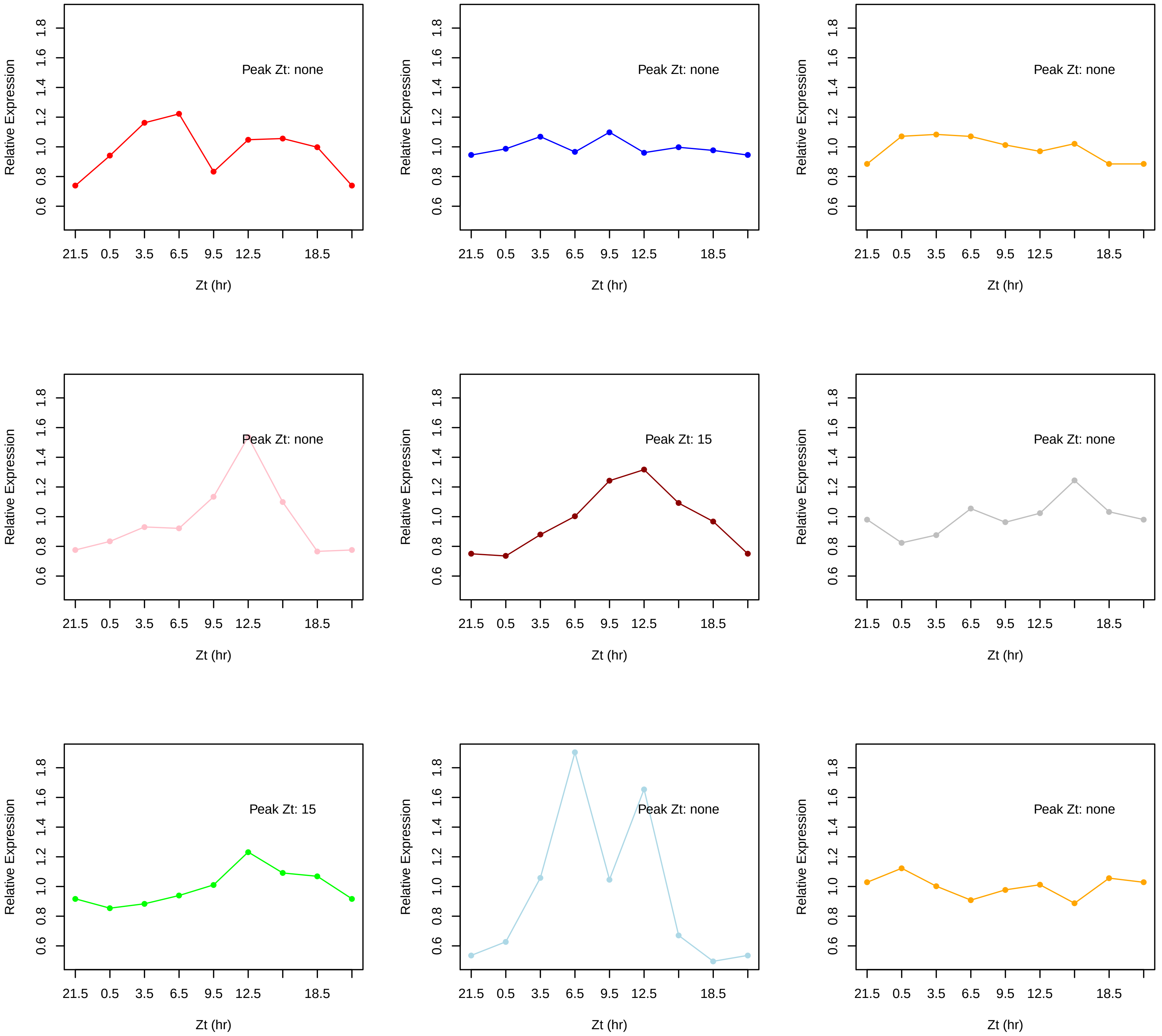

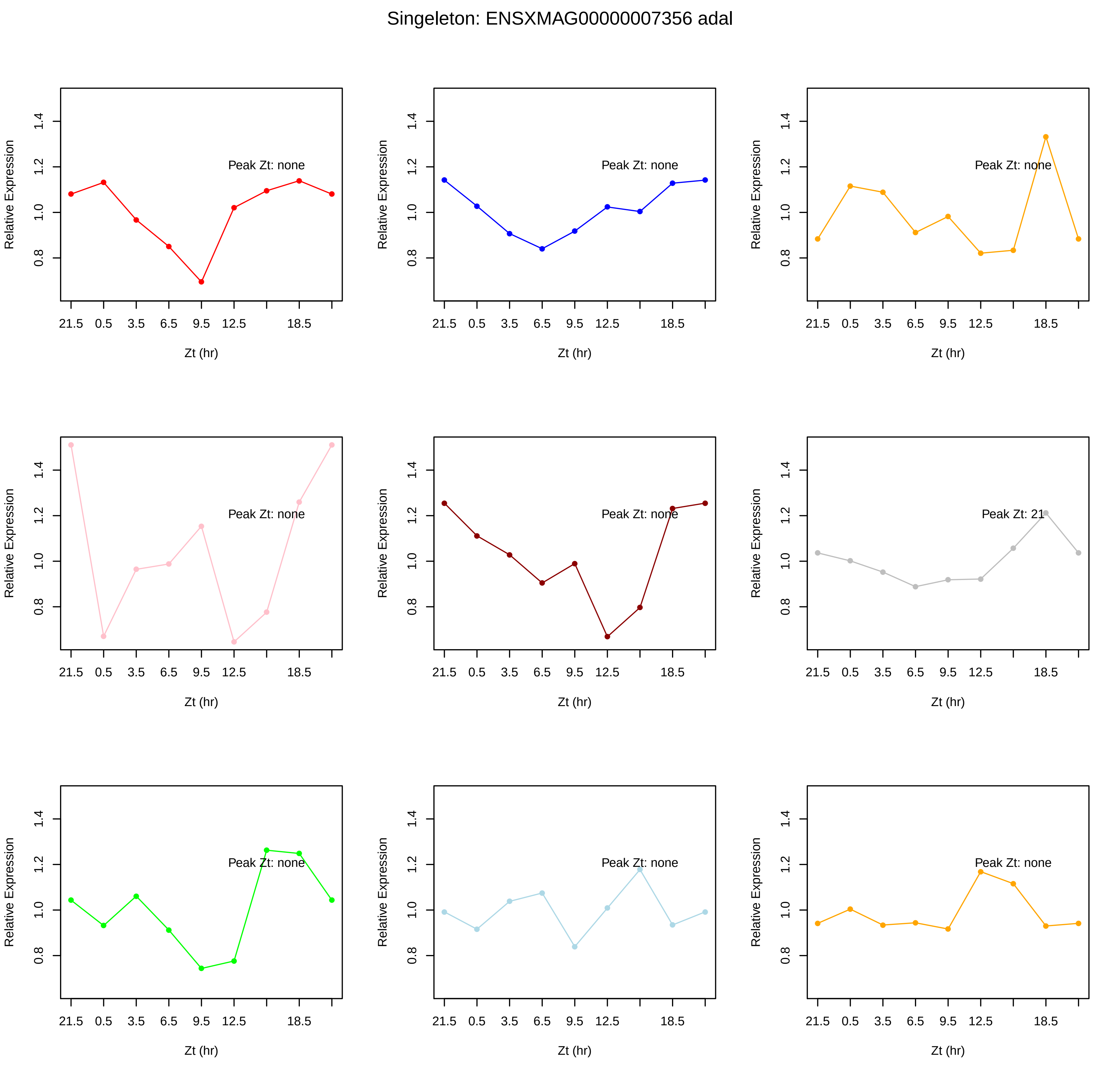

Singeleton: ENSXMAG00000007356 adal
Relative Expression
Zt (hr)
21.5 0.5 3.5 6.5 9.5 12.5 18.5
0.8 1.0 1.2 1.4
Peak Zt: none
Peak Zt: 21

Singleton: ENSXMAG00000022710 tm2d3

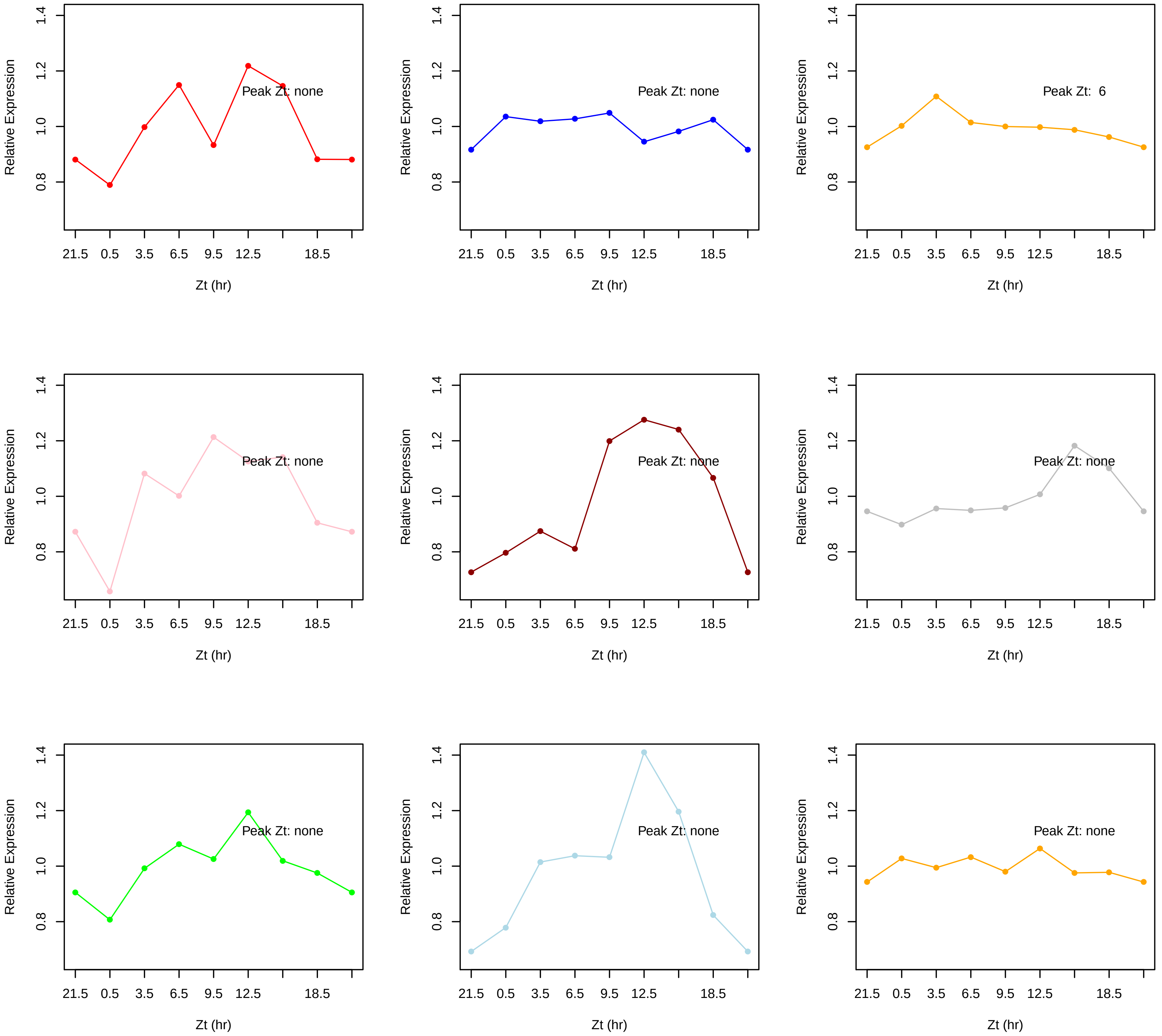

Singeleton: ENSXMAG00000004192

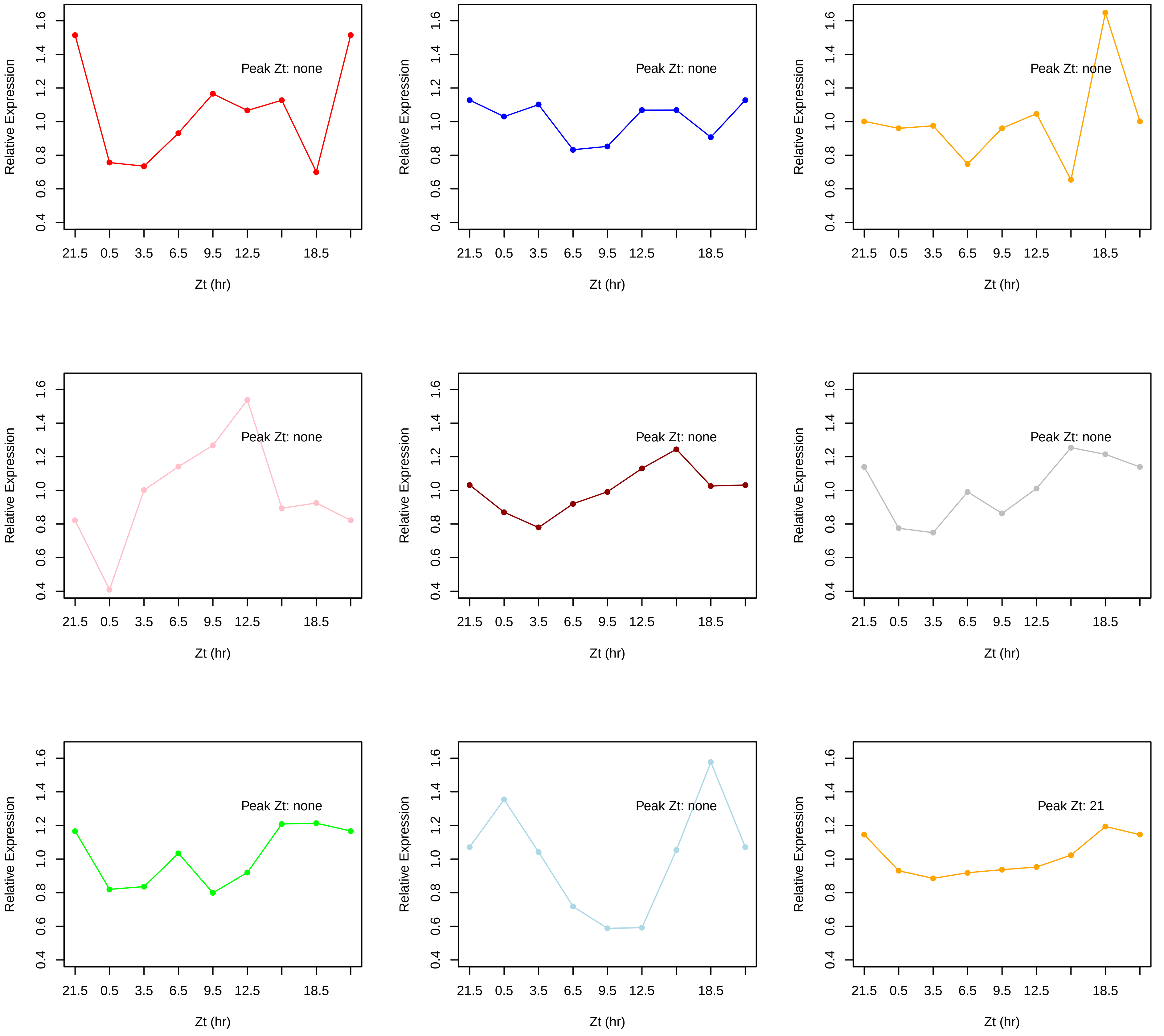

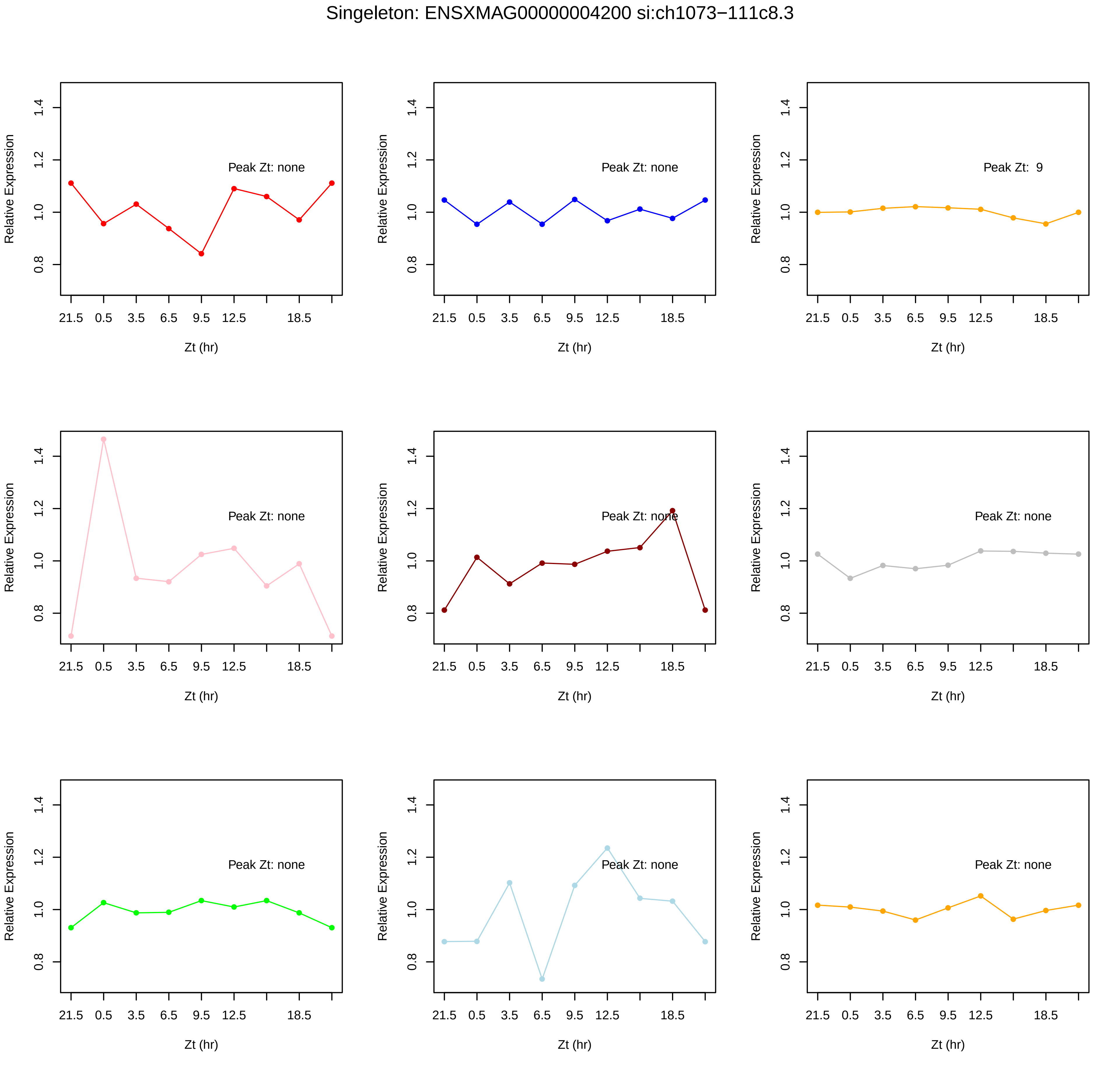
Singeleton: ENSXMAG00000004200 si:ch1073−111c8.3
Relative Expression
Zt (hr)
21.5
0.5
3.5
6.5
9.5
12.5
18.5
0.8
1.0
1.2
1.4
Peak Zt: none
Peak Zt: none
Peak Zt: 9
Peak Zt: none
Peak Zt: none
Peak Zt: none
Peak Zt: none
Peak Zt: none
Peak Zt: none

Singeleton: ENSXMAG00000006663 arhgef9b

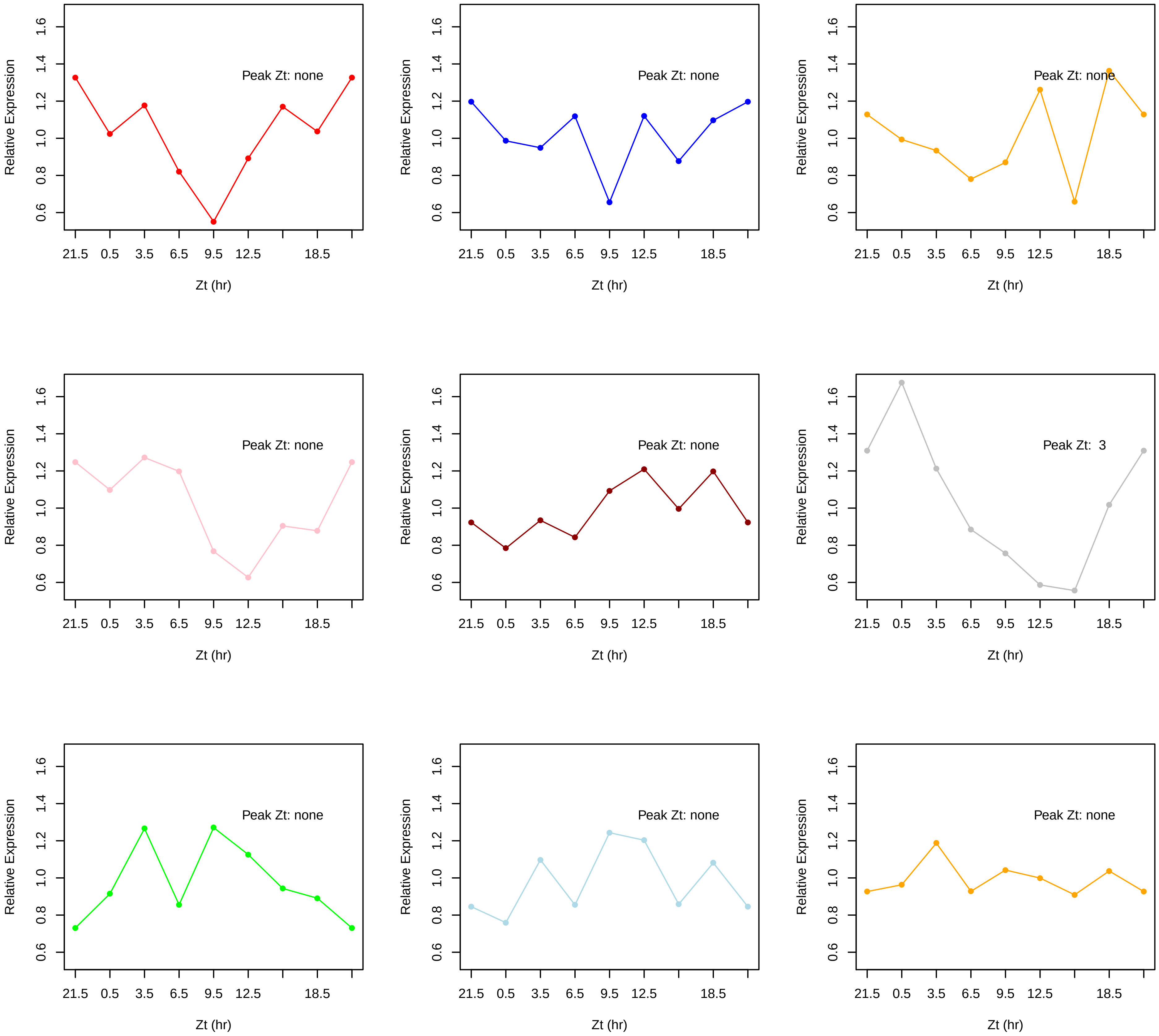

Singleton: ENSXMAG00000019579 socs9

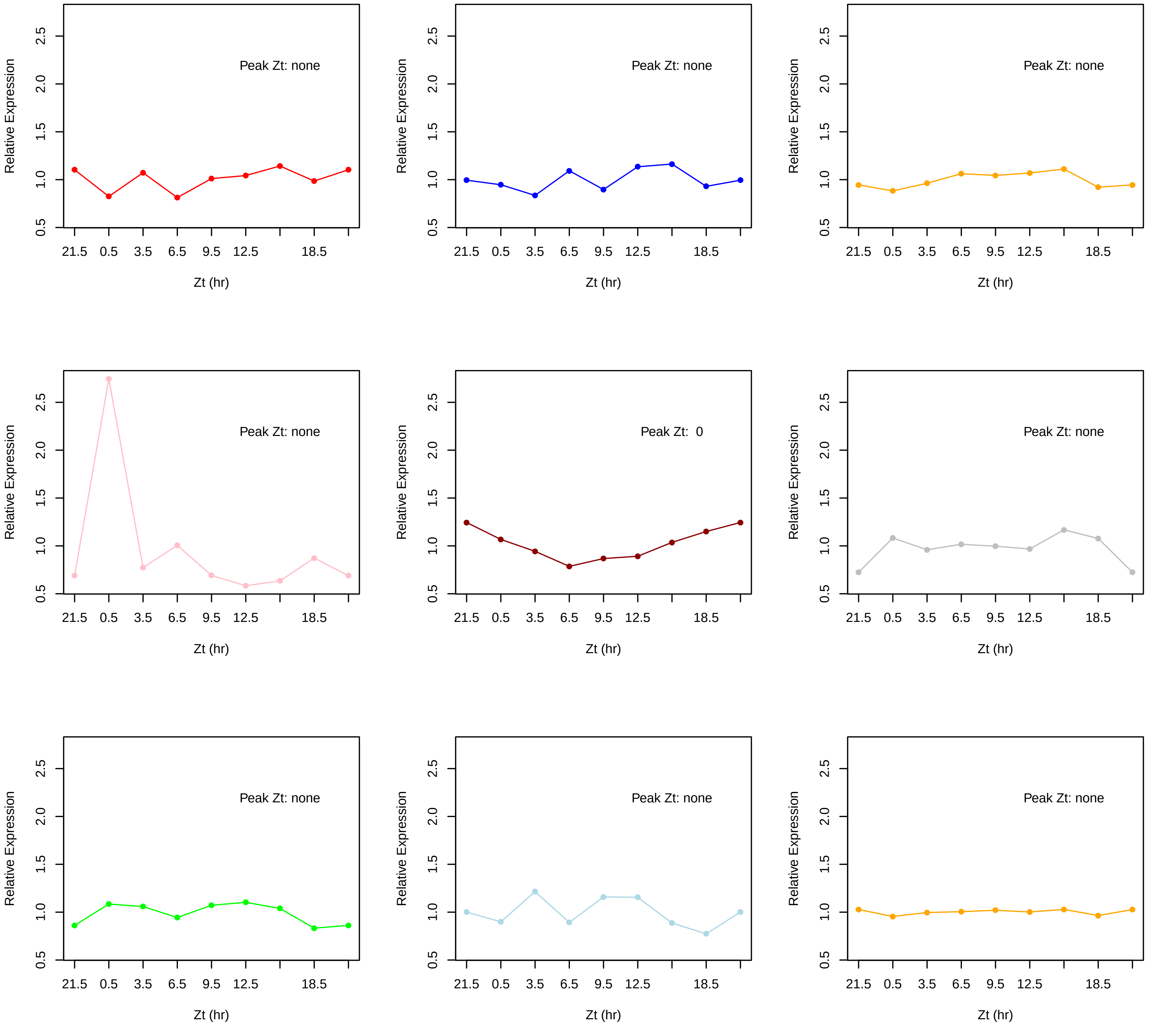

Singeleton: ENSXMAG00000021118 sipa1l3

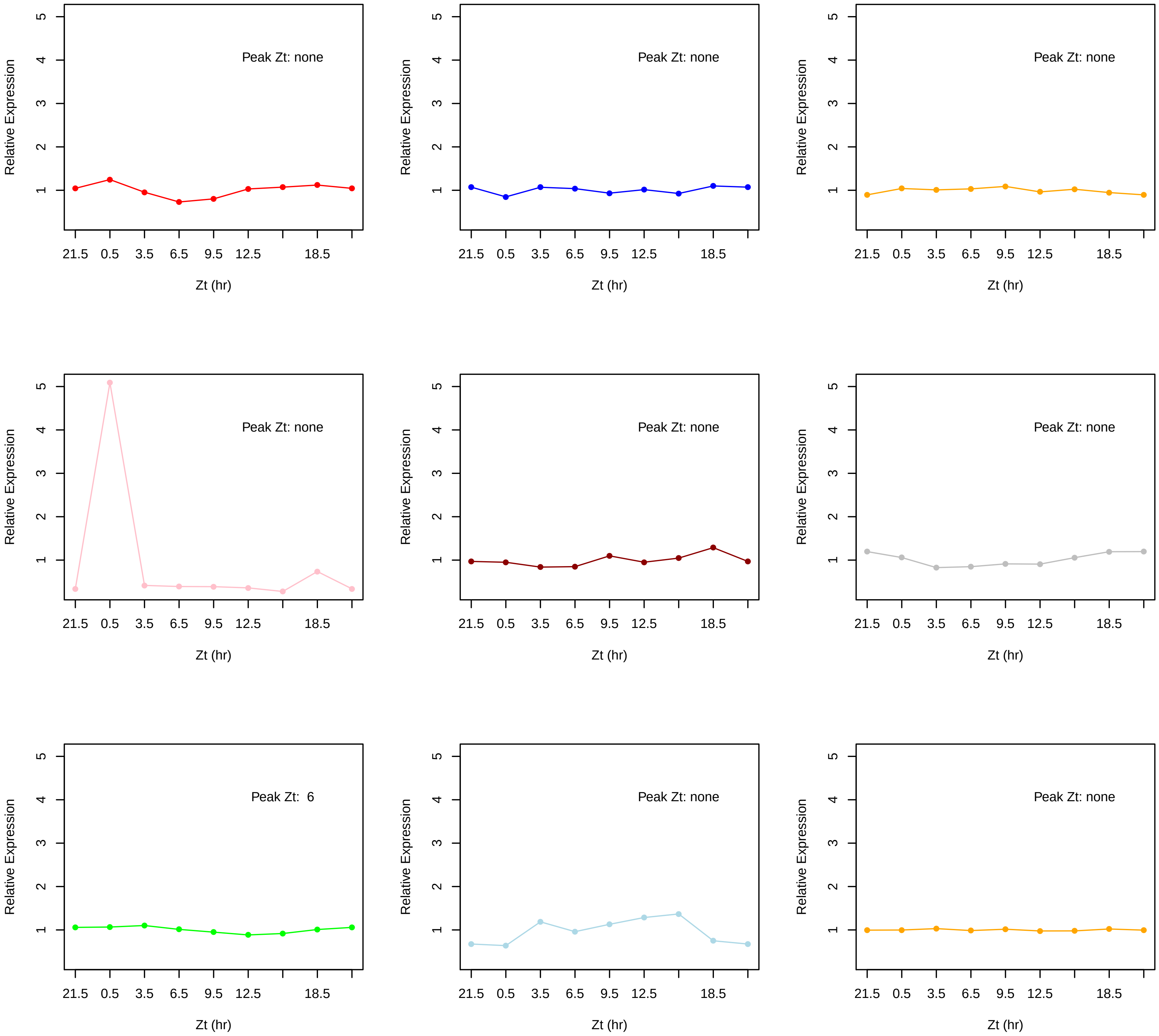

Singeleton: ENSXMAG00000026598 cplx3a

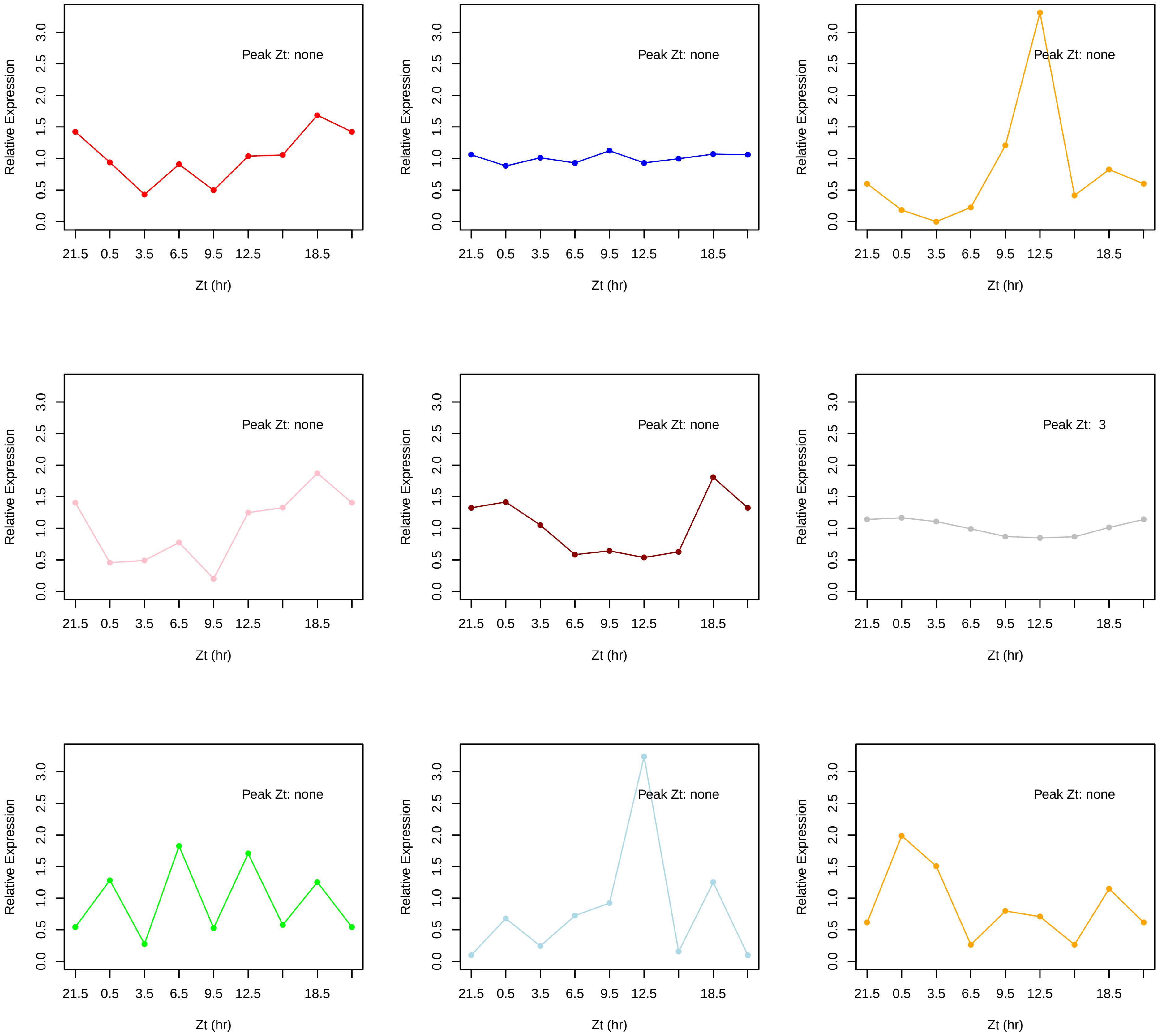

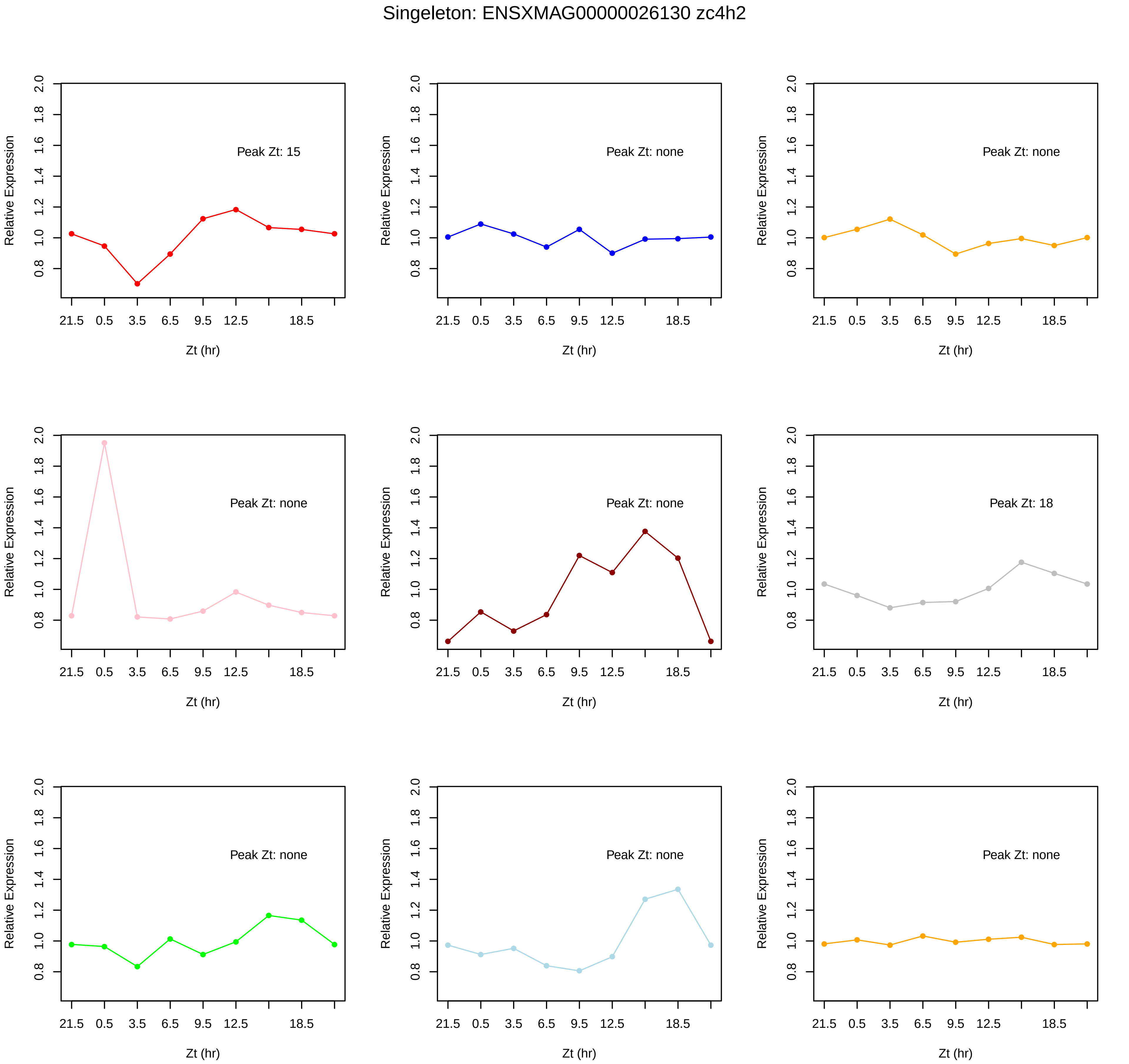
Singleton: ENSXMAG00000026130 zc4h2
Peak Zt: 15
Peak Zt: none
Peak Zt: none
Peak Zt: none
Peak Zt: none
Peak Zt: 18
Peak Zt: none
Peak Zt: none
Peak Zt: none
Relative Expression
Zt (hr)
21.5
0.5
3.5
6.5
9.5
12.5
18.5
0.8
1.0
1.2
1.4
1.6
1.8
2.0

Singleton: ENSXMAG00000016761 cyp1a

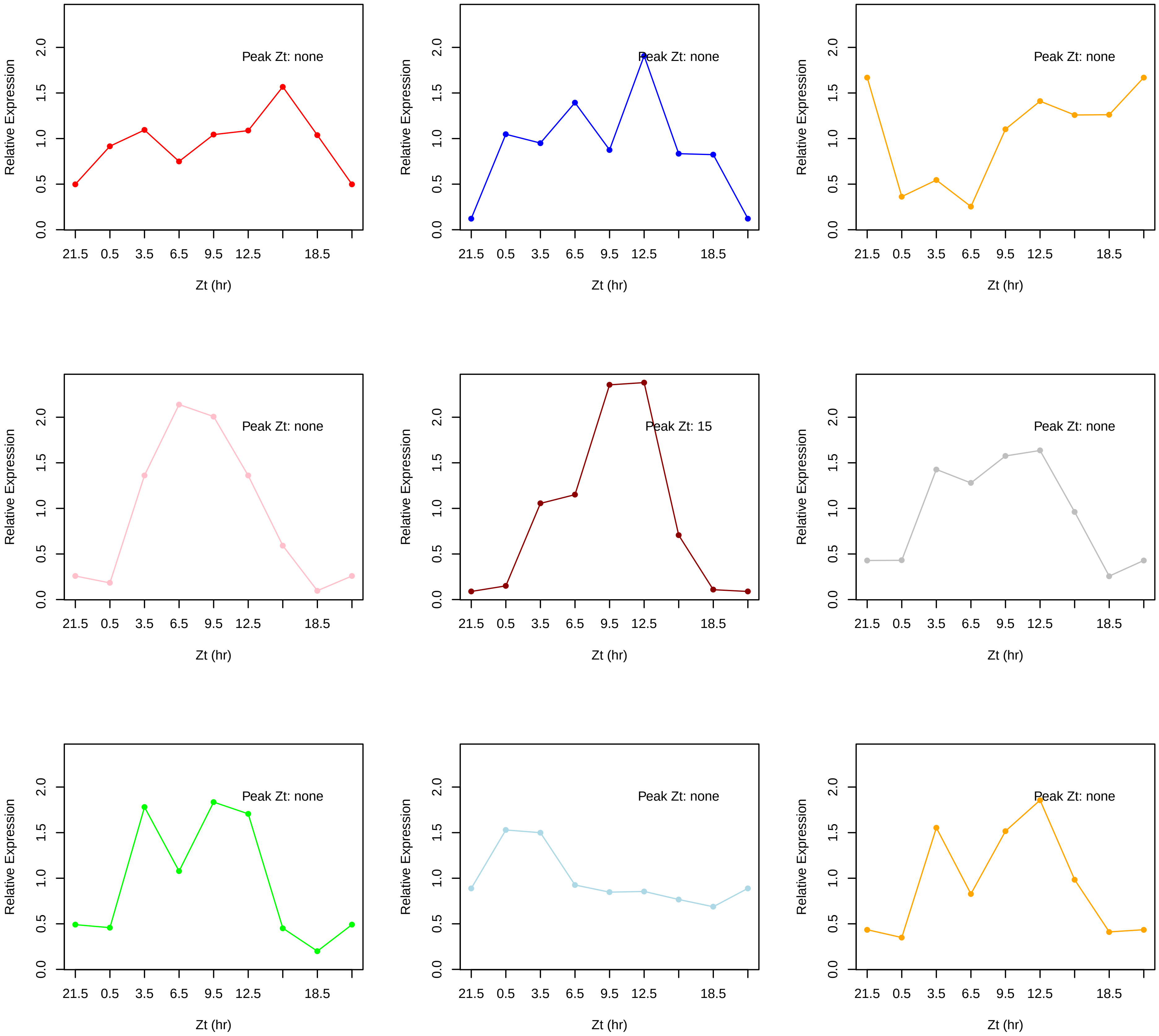

Singeleton: ENSXMAG00000018670 si:ch211−160d20.3

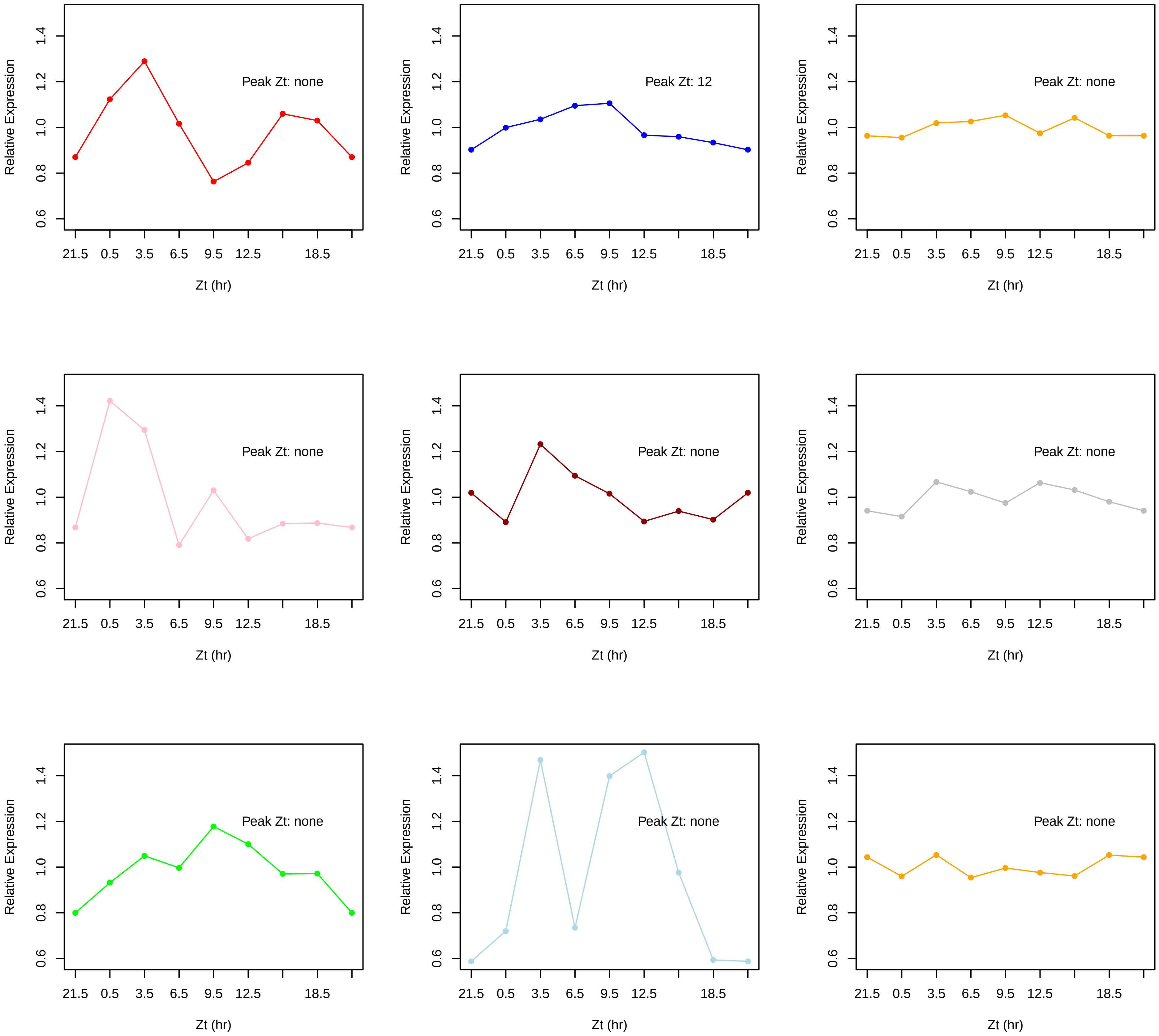

Singeleton: ENSXMAG00000018623 capn12

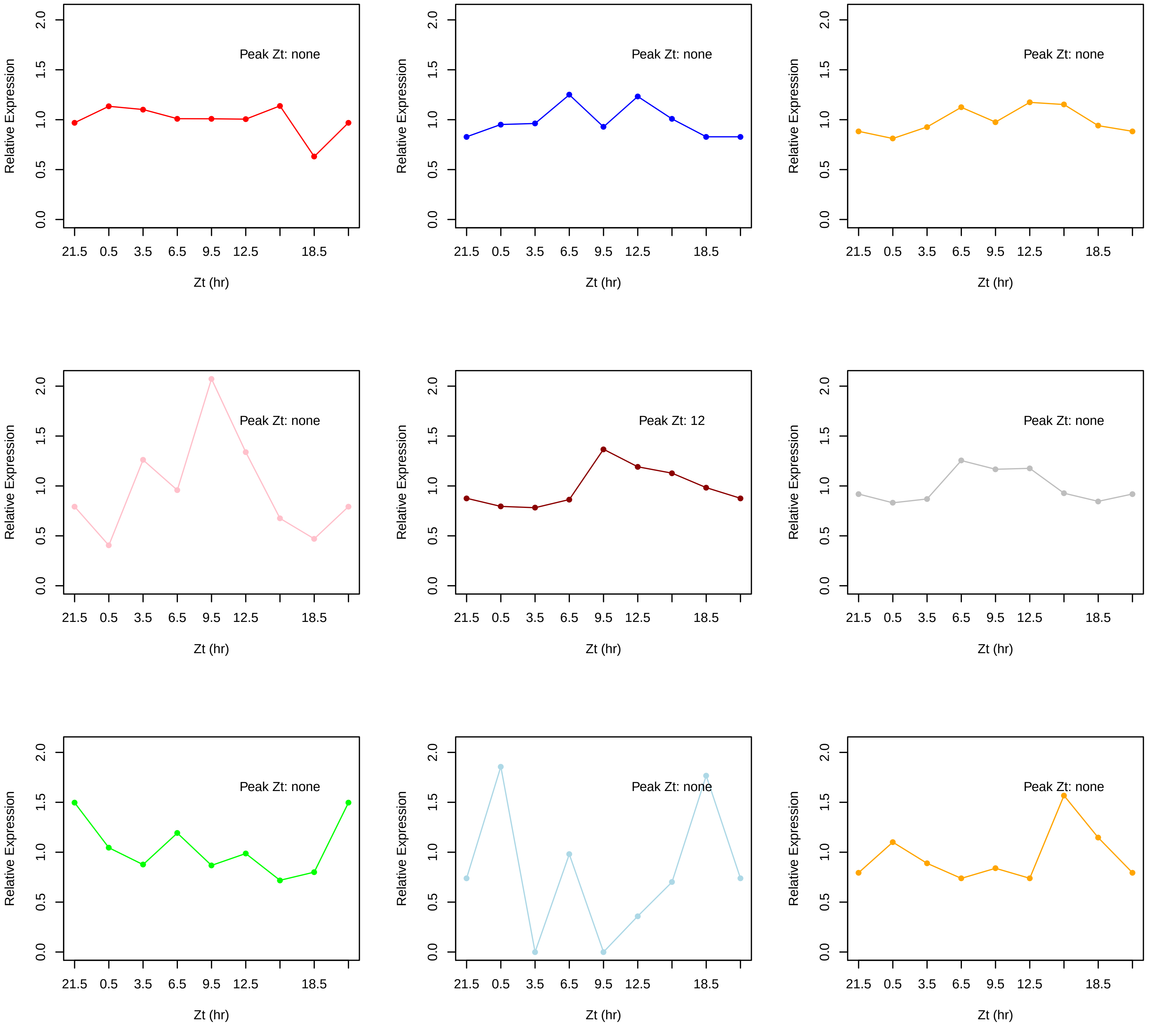

Singeleton: ENSXMAG00000023987

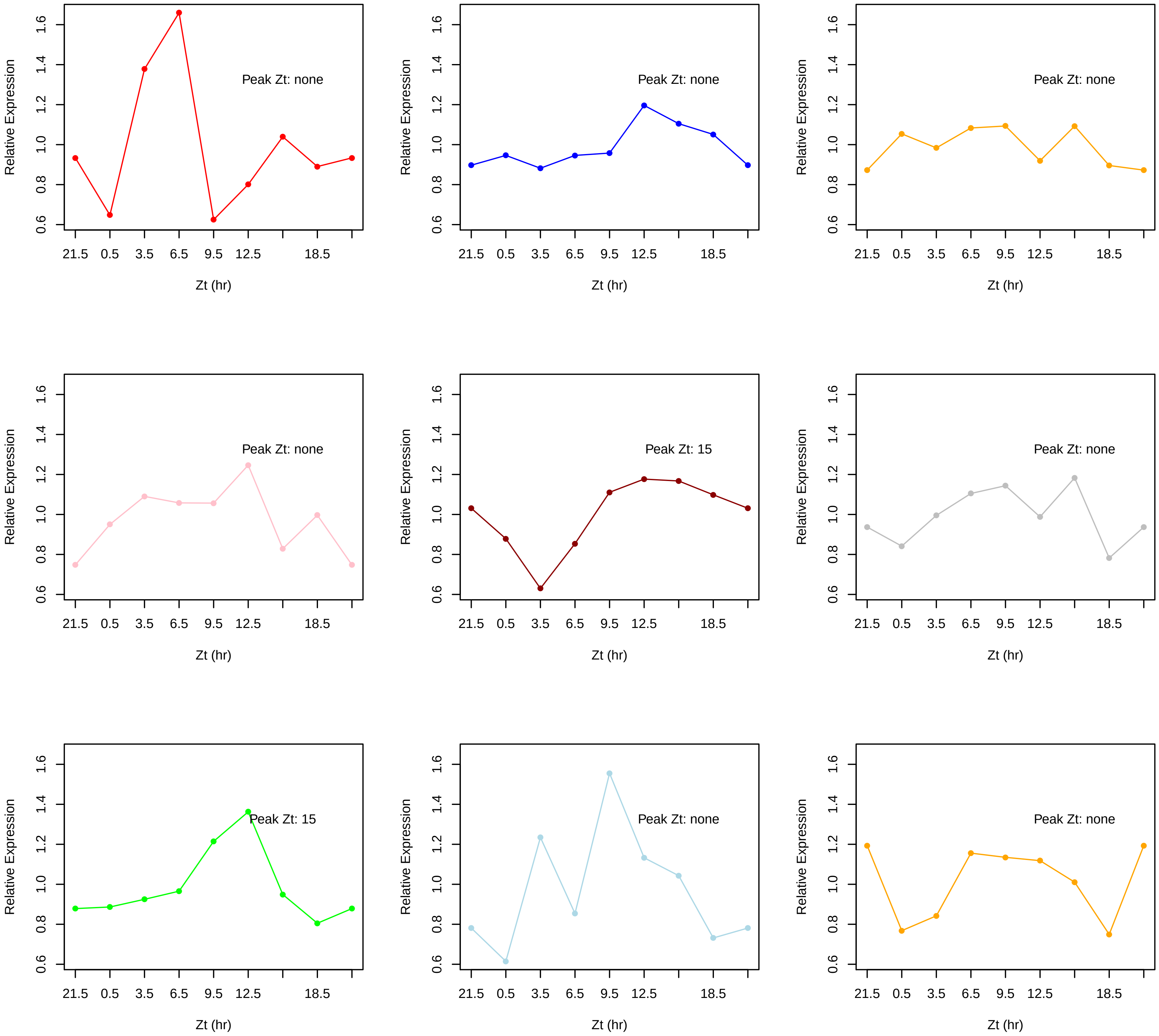

Singleton: ENSXMAG00000017055 wdr76

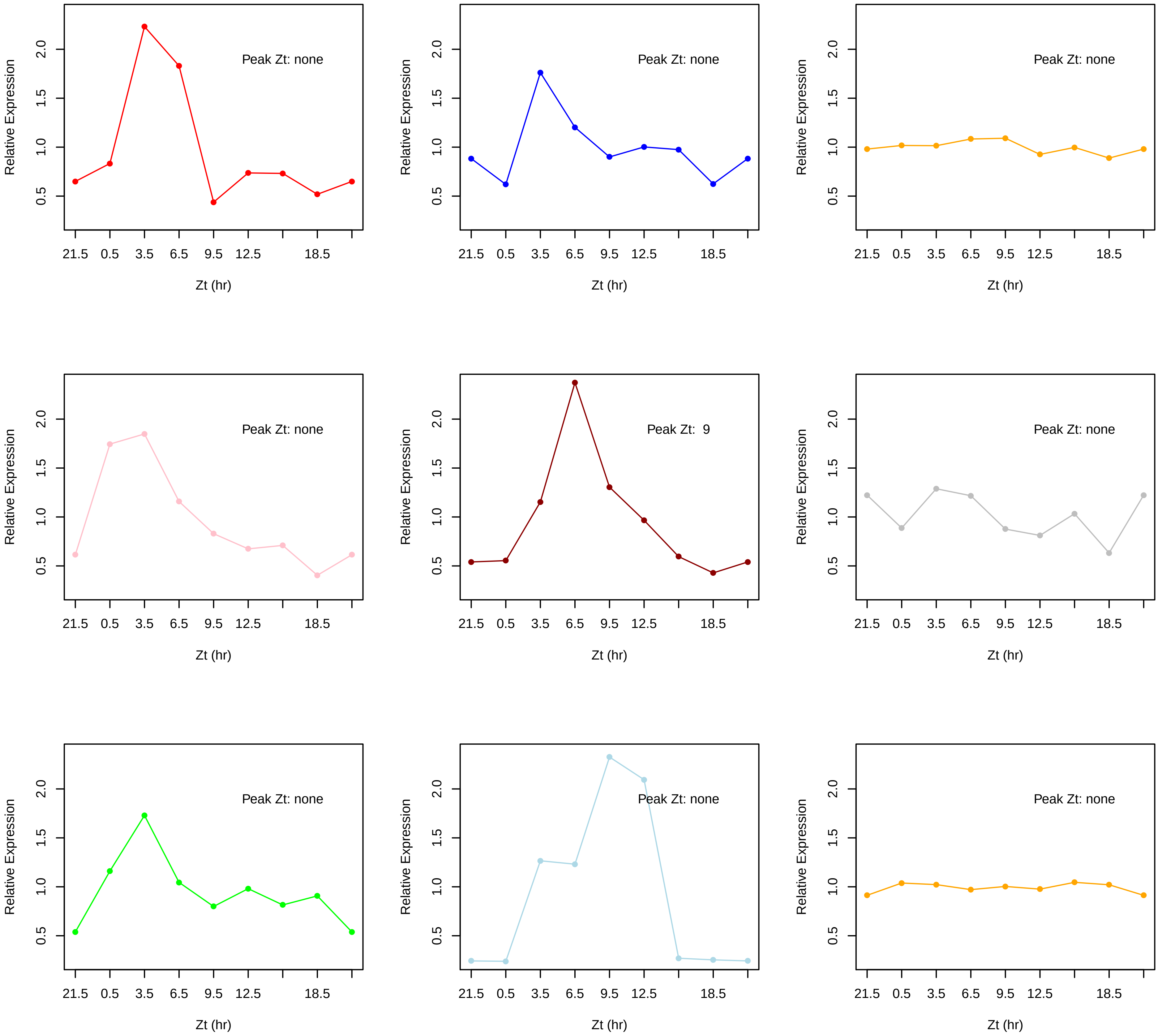

Singeleton: ENSXMAG00000018525 MSN

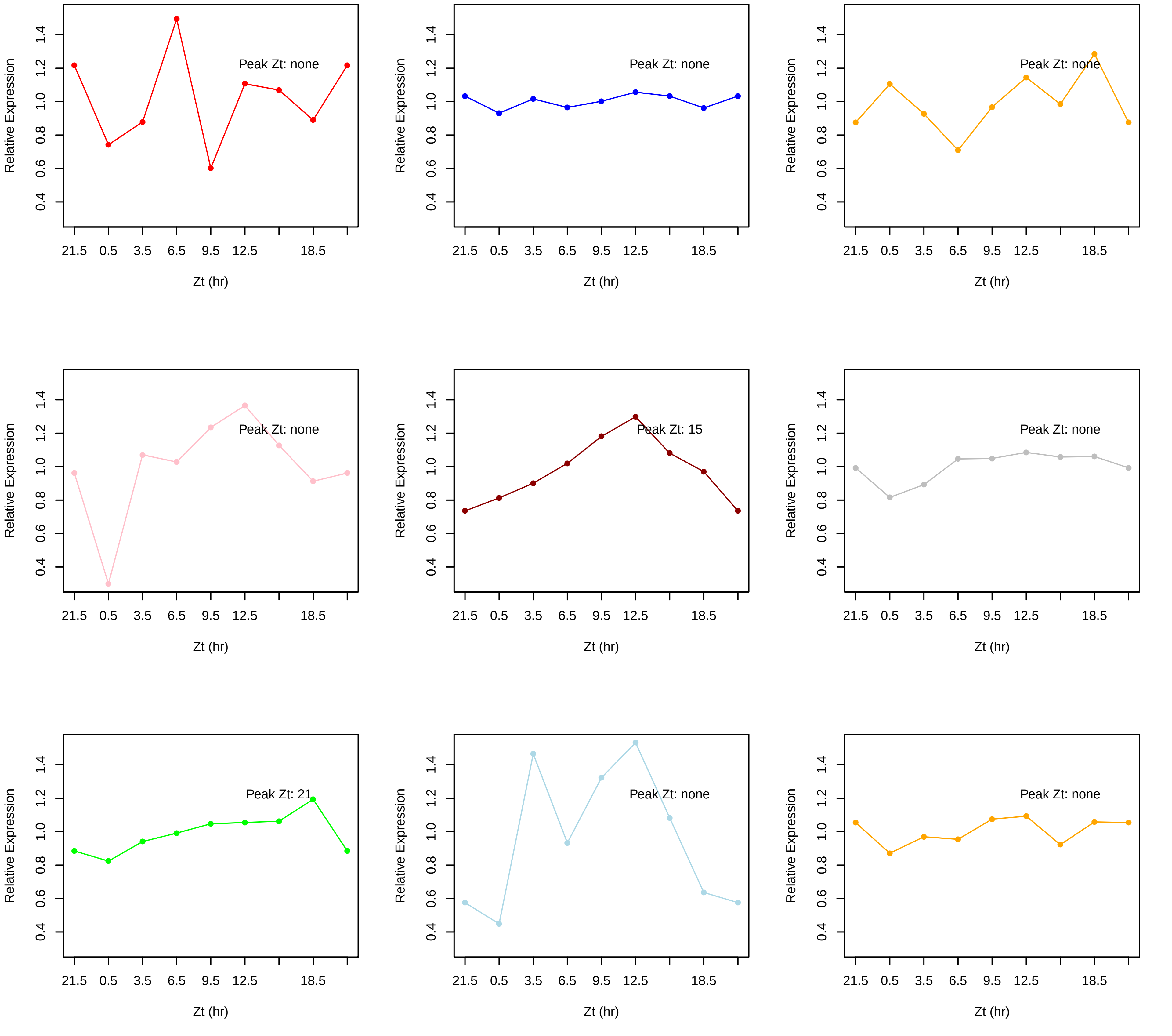

Singeleton: ENSXMAG00000007242

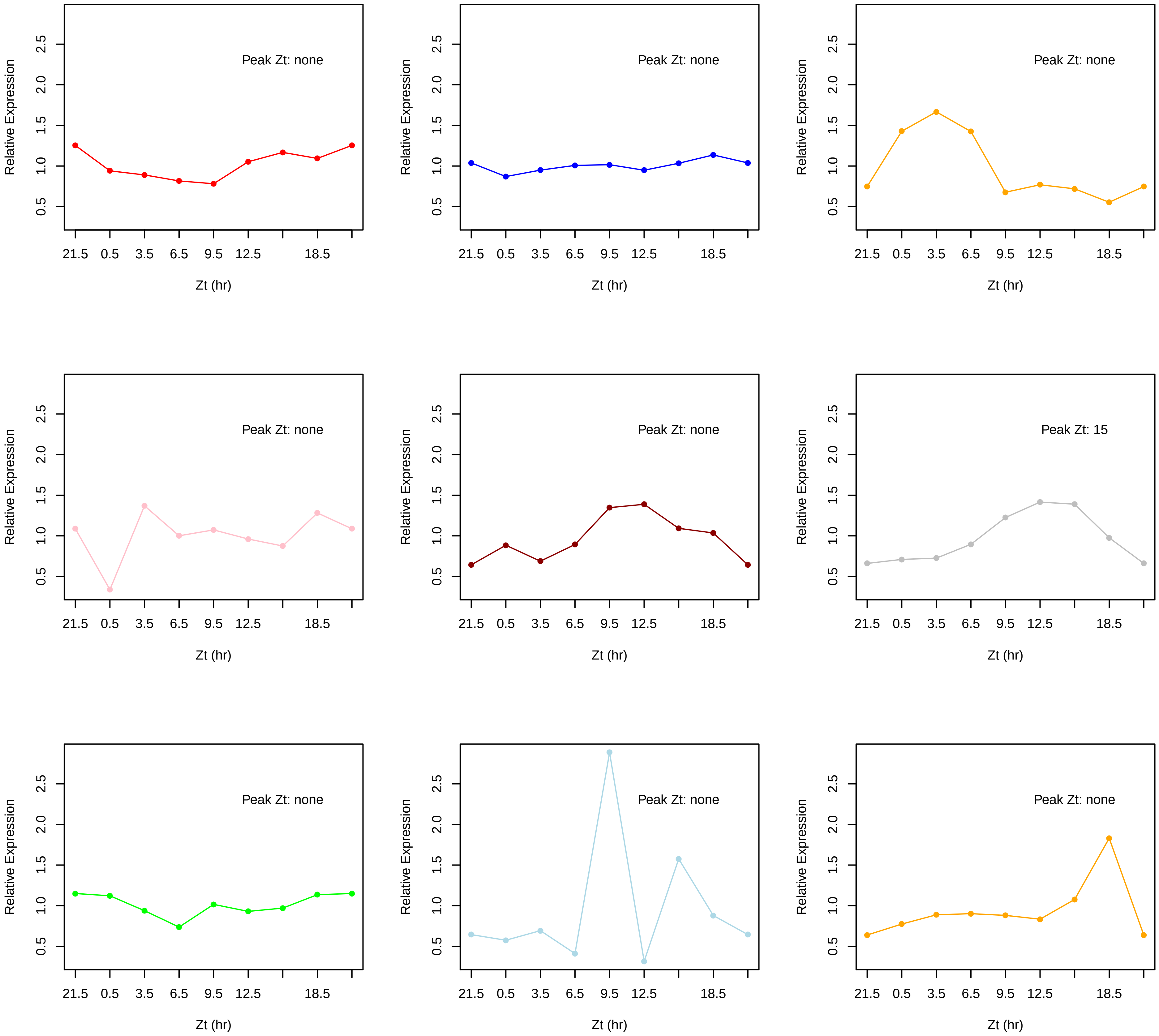

Singeleton: ENSXMAG00000002975 malt3

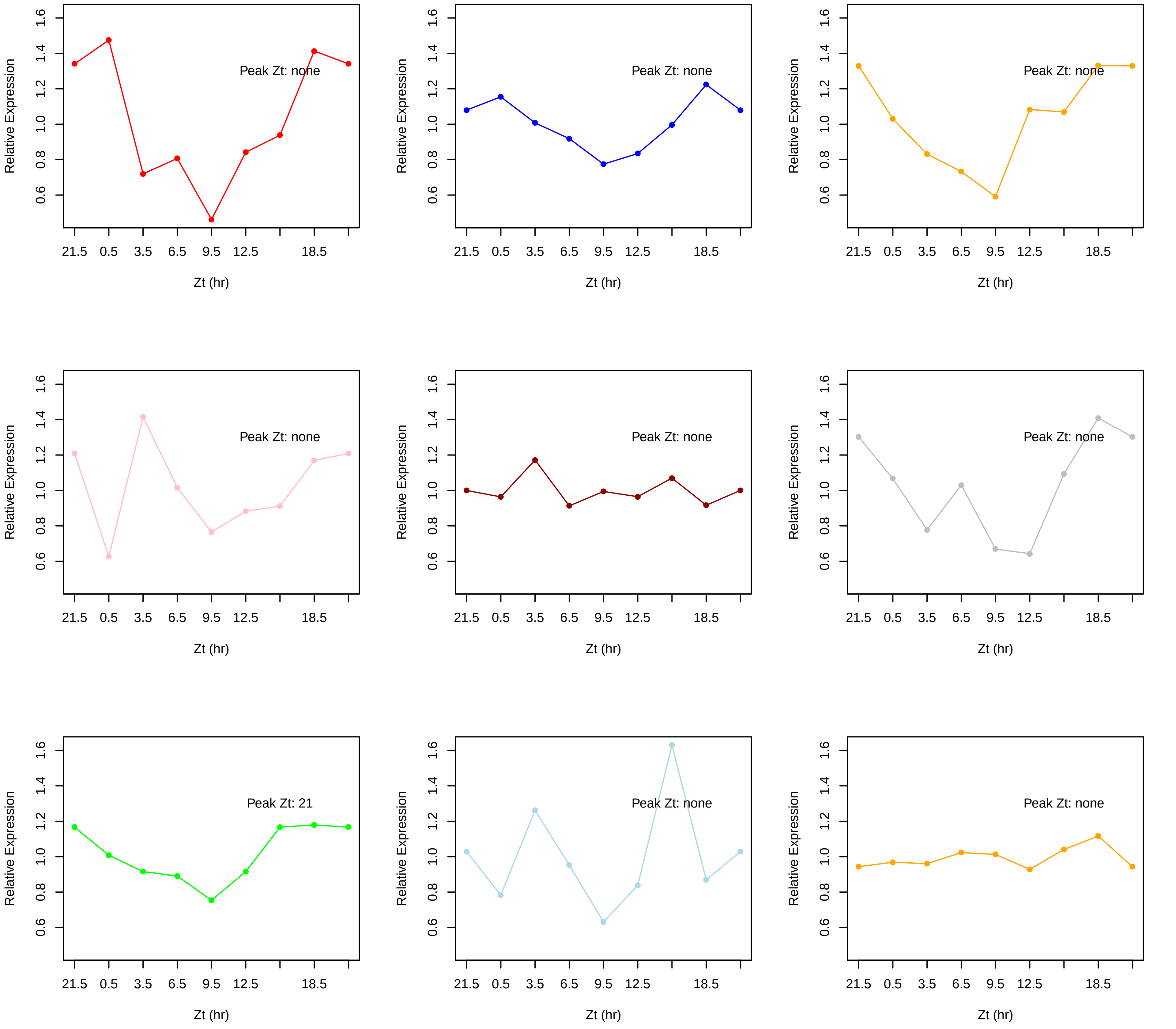

Singeleton: ENSXMAG00000001466 slc35c1

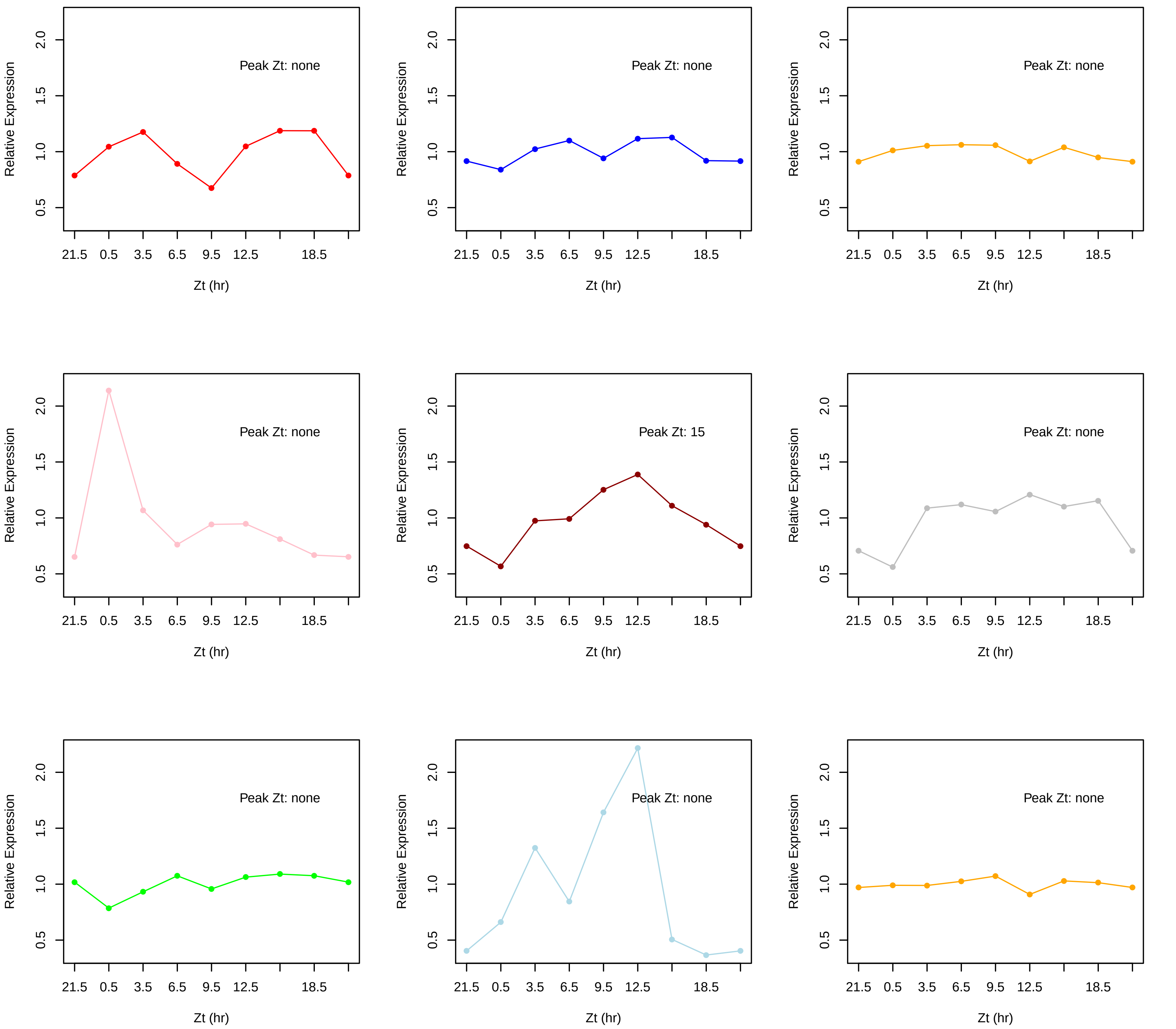

Singeleton: ENSXMAG00000006417 cry2

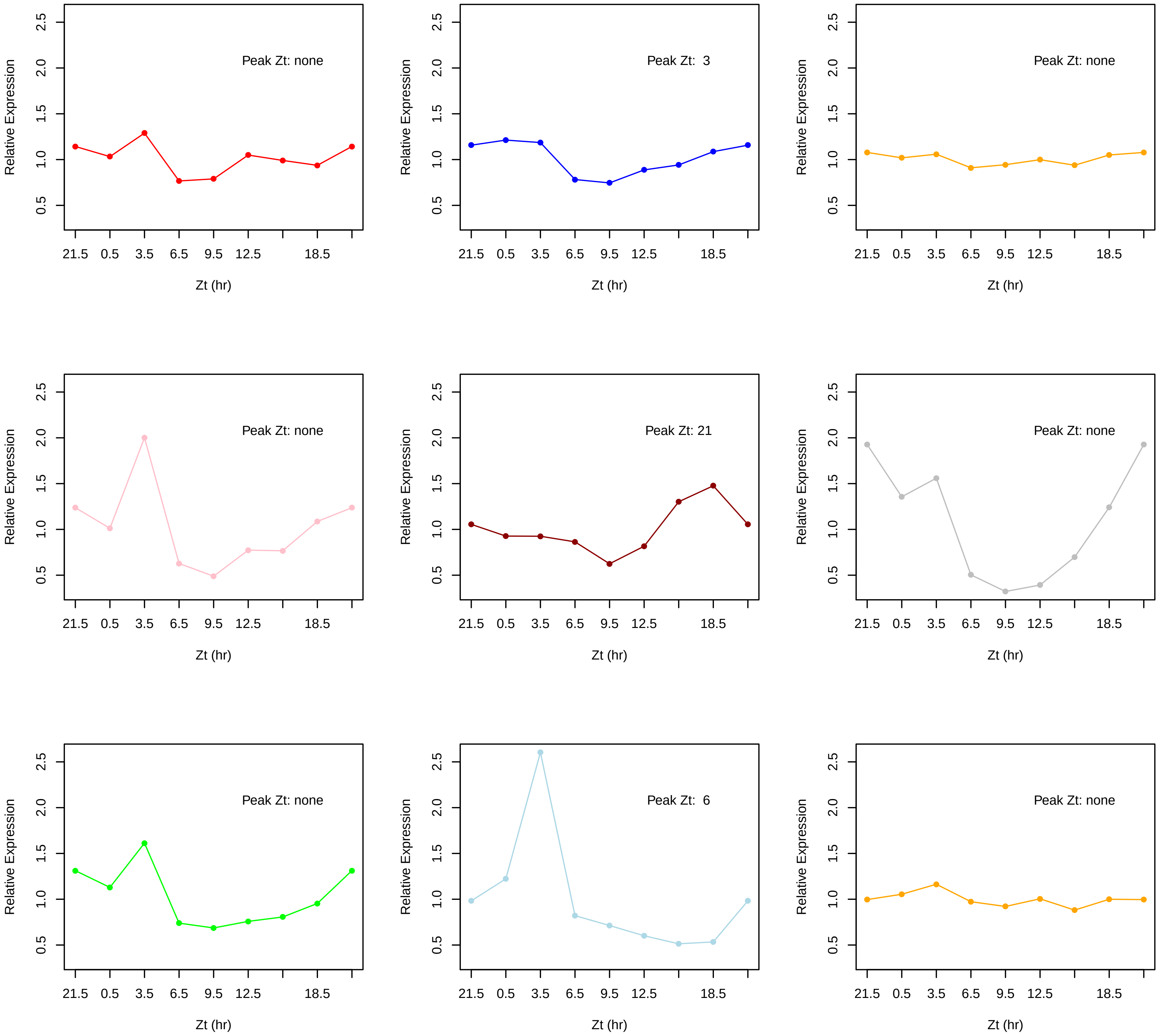

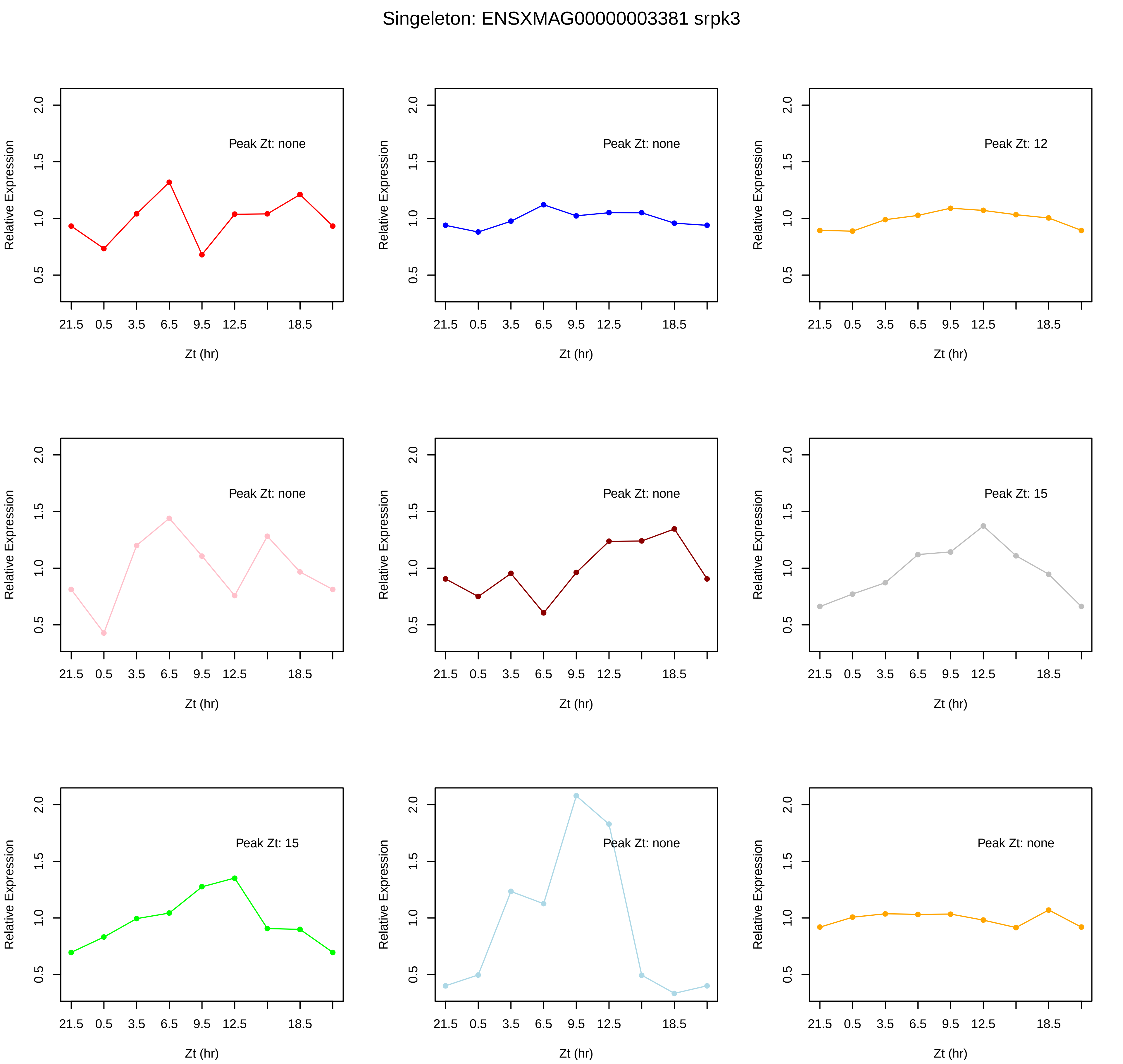
Singeleton: ENSXMAG00000003381 srpk3
Peak Zt: none
Peak Zt: none
Peak Zt: 12
Peak Zt: none
Peak Zt: none
Peak Zt: 15
Peak Zt: 15
Peak Zt: none
Peak Zt: none
Relative Expression
Zt (hr)
21.5
0.5
3.5
6.5
9.5
12.5
18.5
0.5
1.0
1.5
2.0

Singeleton: ENSXMAG00000003393 plxnb3

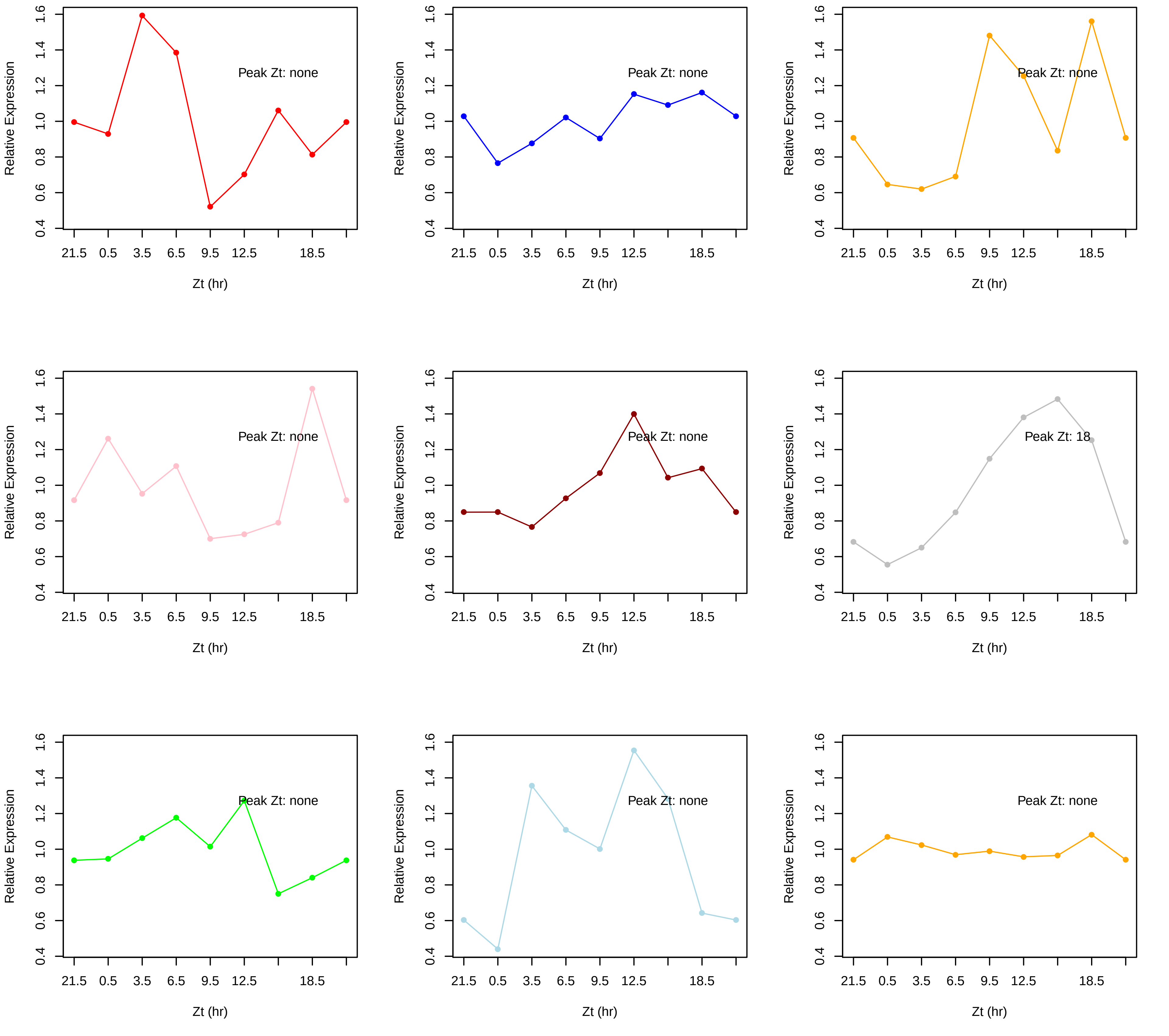

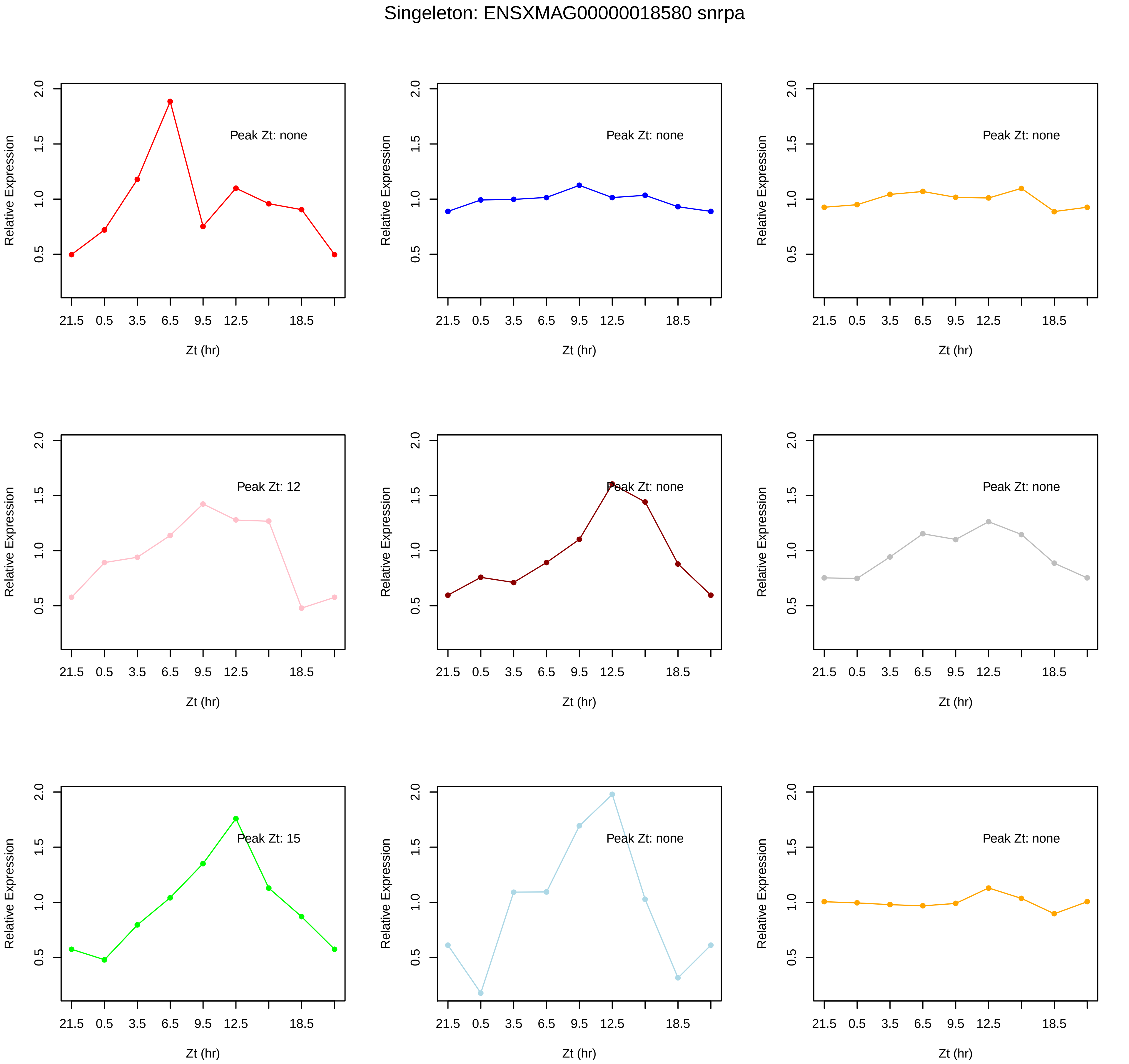

Singleton: ENSXMAG00000018580 snrpa
Relative Expression
Zt (hr)
21.5 0.5 3.5 6.5 9.5 12.5 18.5
Peak Zt: none
Peak Zt: none
Peak Zt: none
Peak Zt: 12
Peak Zt: none
Peak Zt: none
Peak Zt: 15
Peak Zt: none
Peak Zt: none

Singeleton: ENSXMAG00000018577 zgc:153119

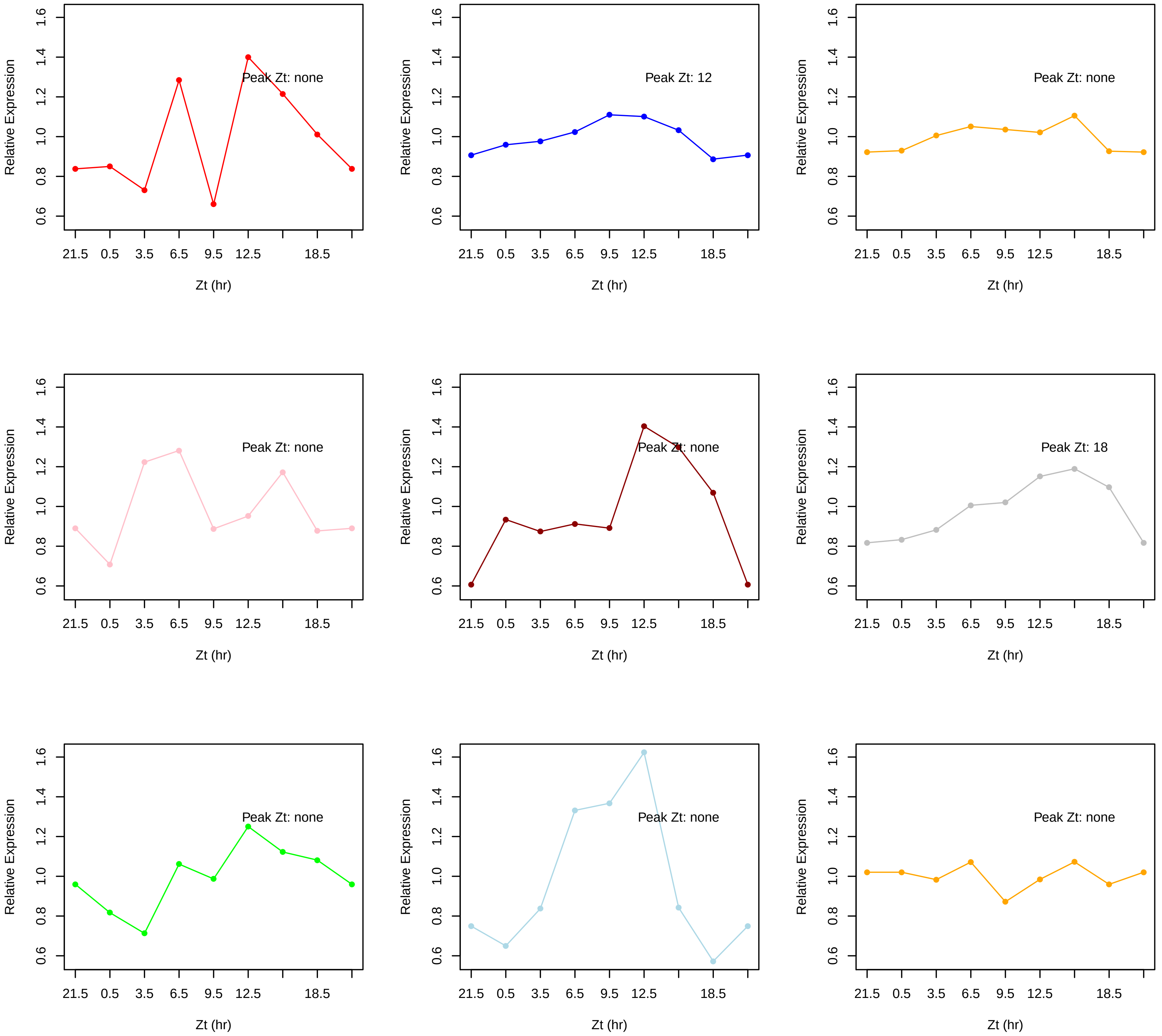

Singleton: ENSXMAG00000018575 itpkcb

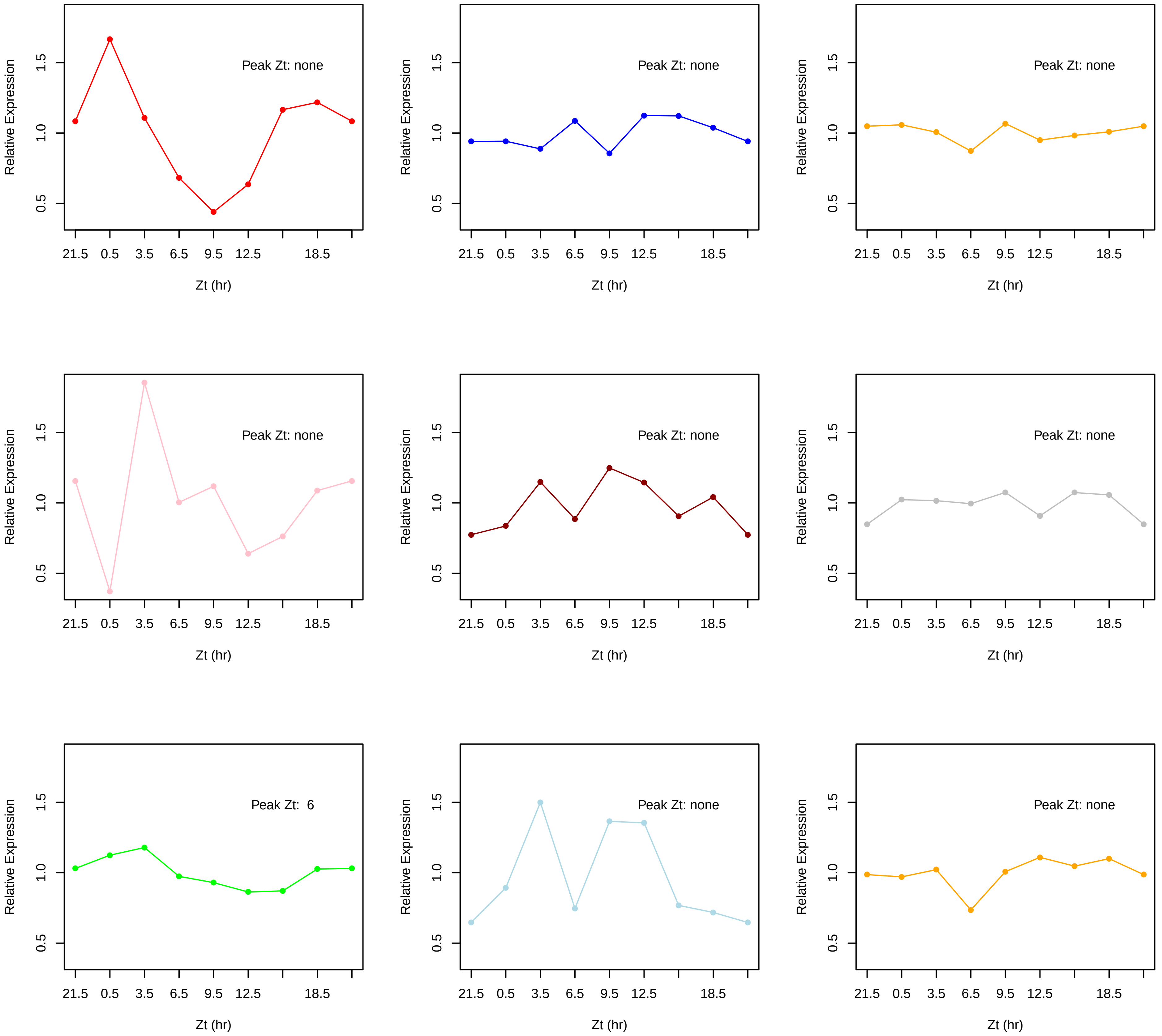

Singeleton: ENSXMAG00000006426 zgc:92873

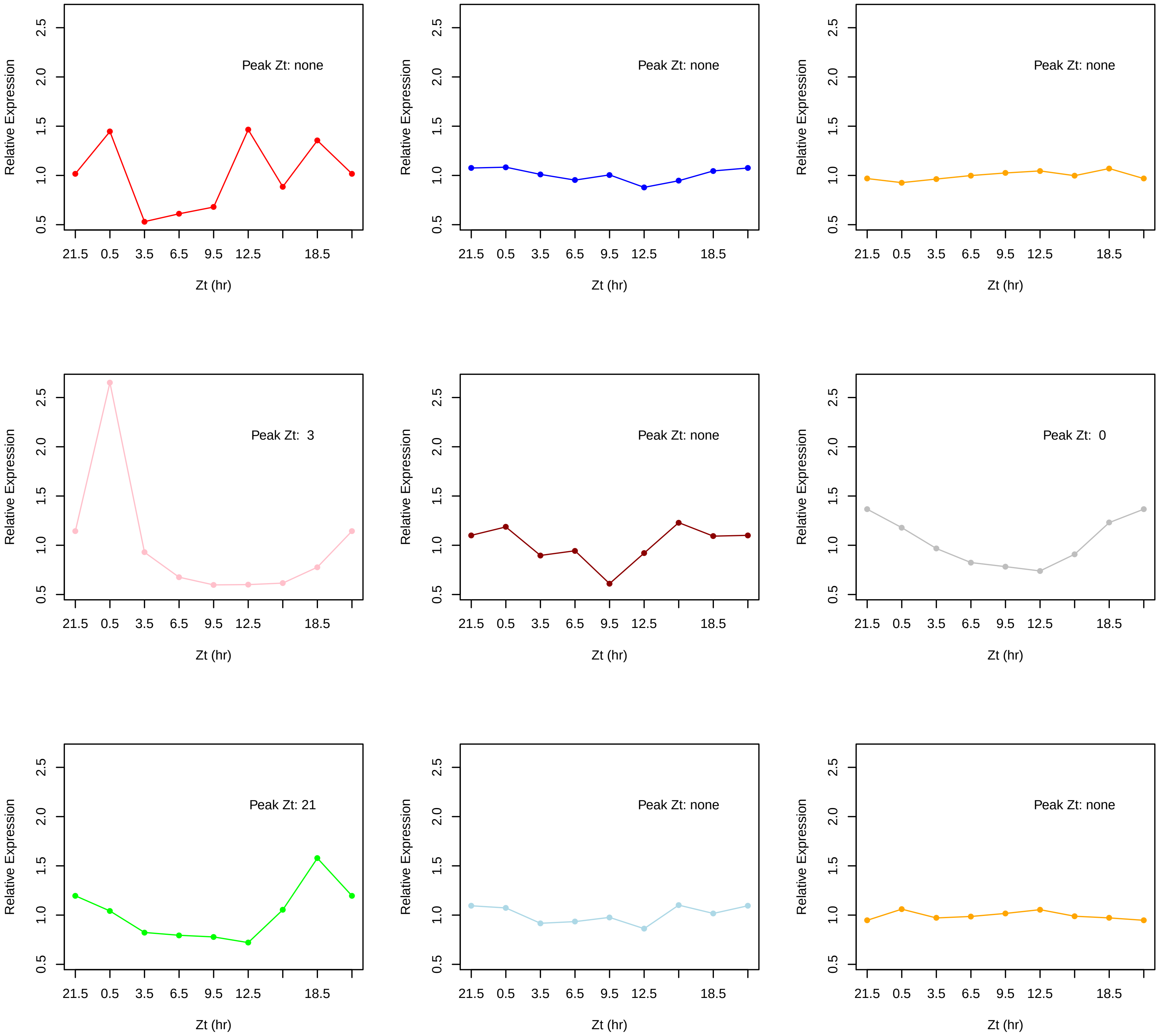

Singeleton: ENSXMAG00000017133 sec11a

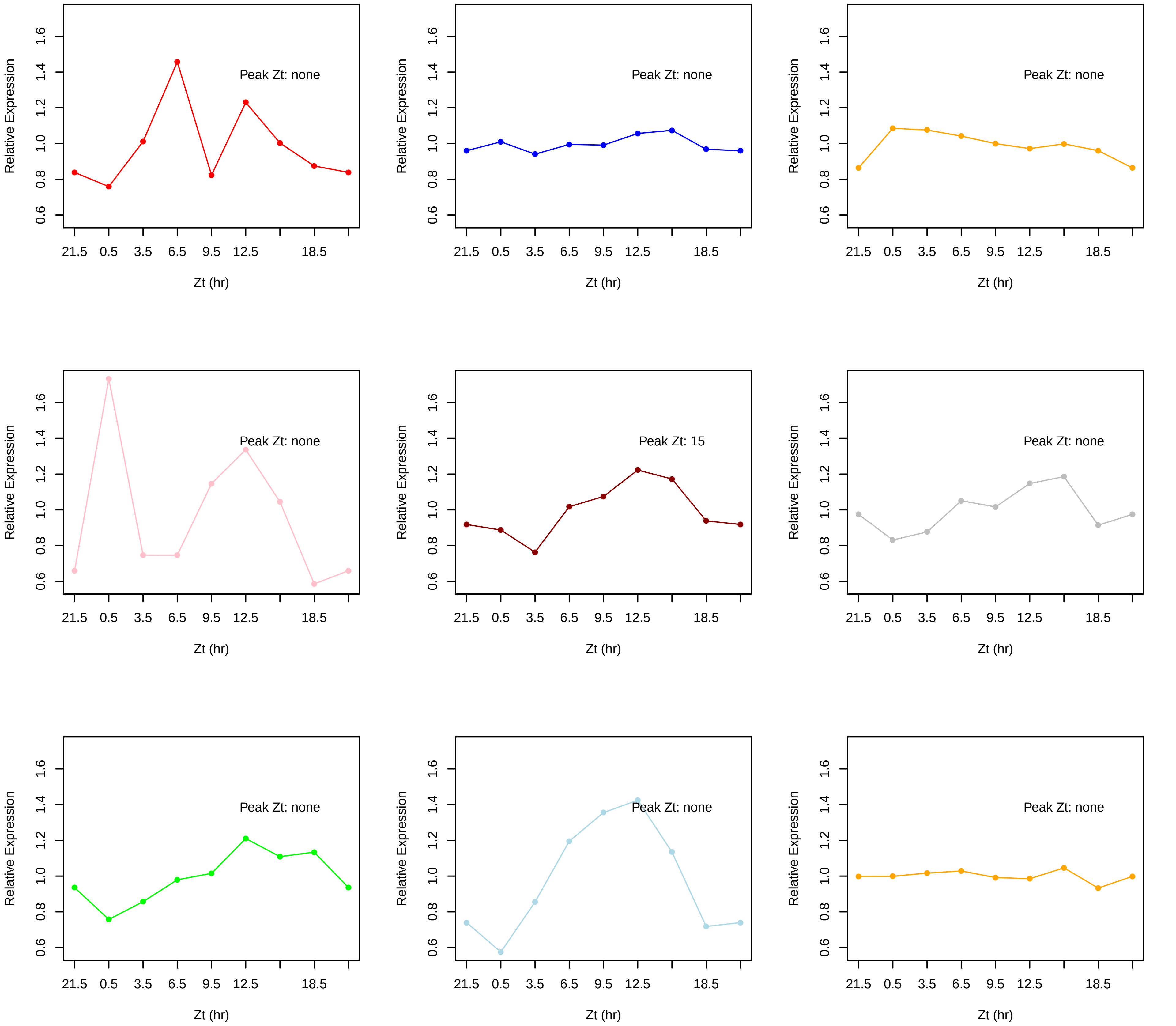

Singleton: ENSXMAG00000001380 LRP4

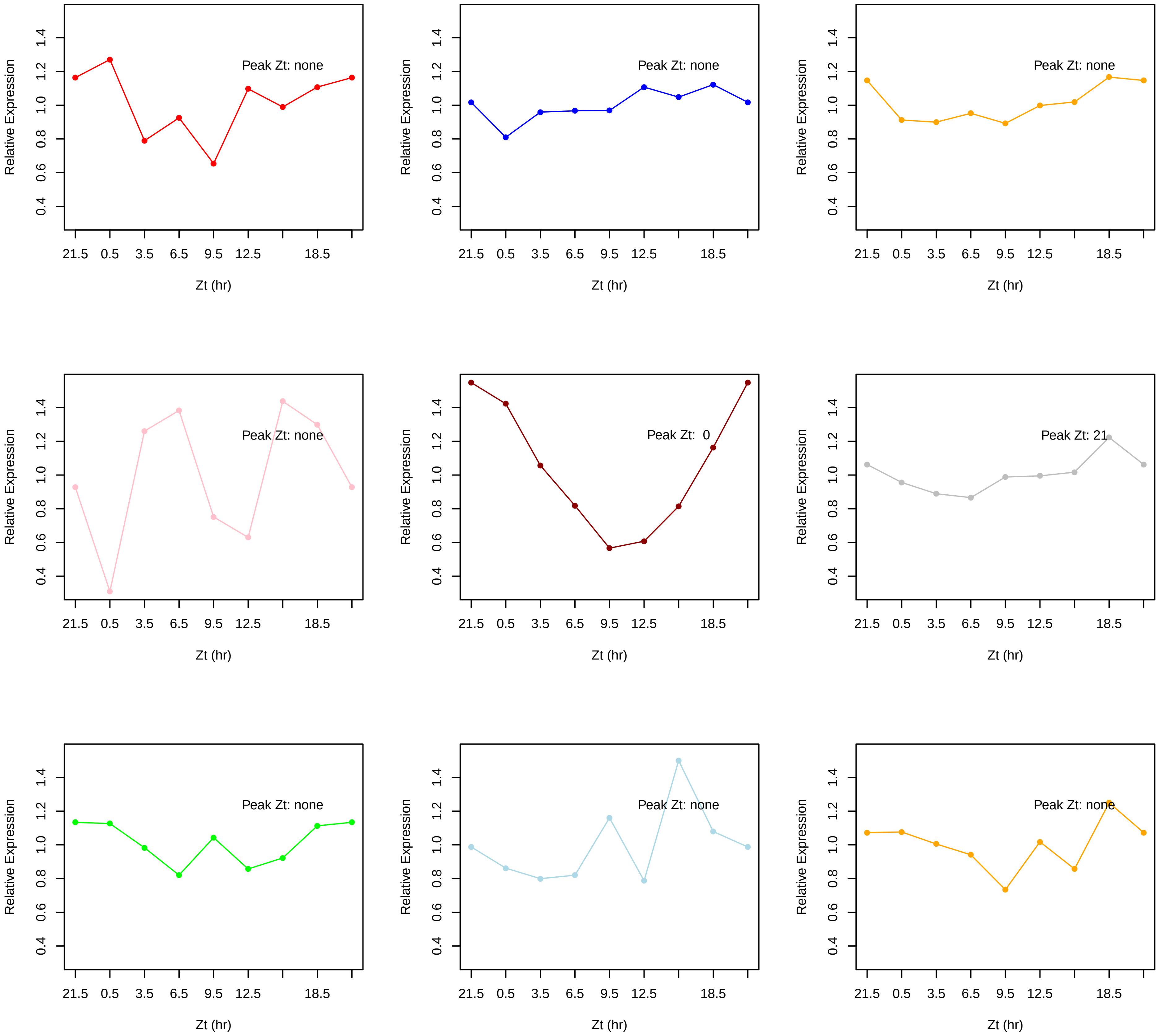

Singeleton: ENSXMAG00000021685 ophn1

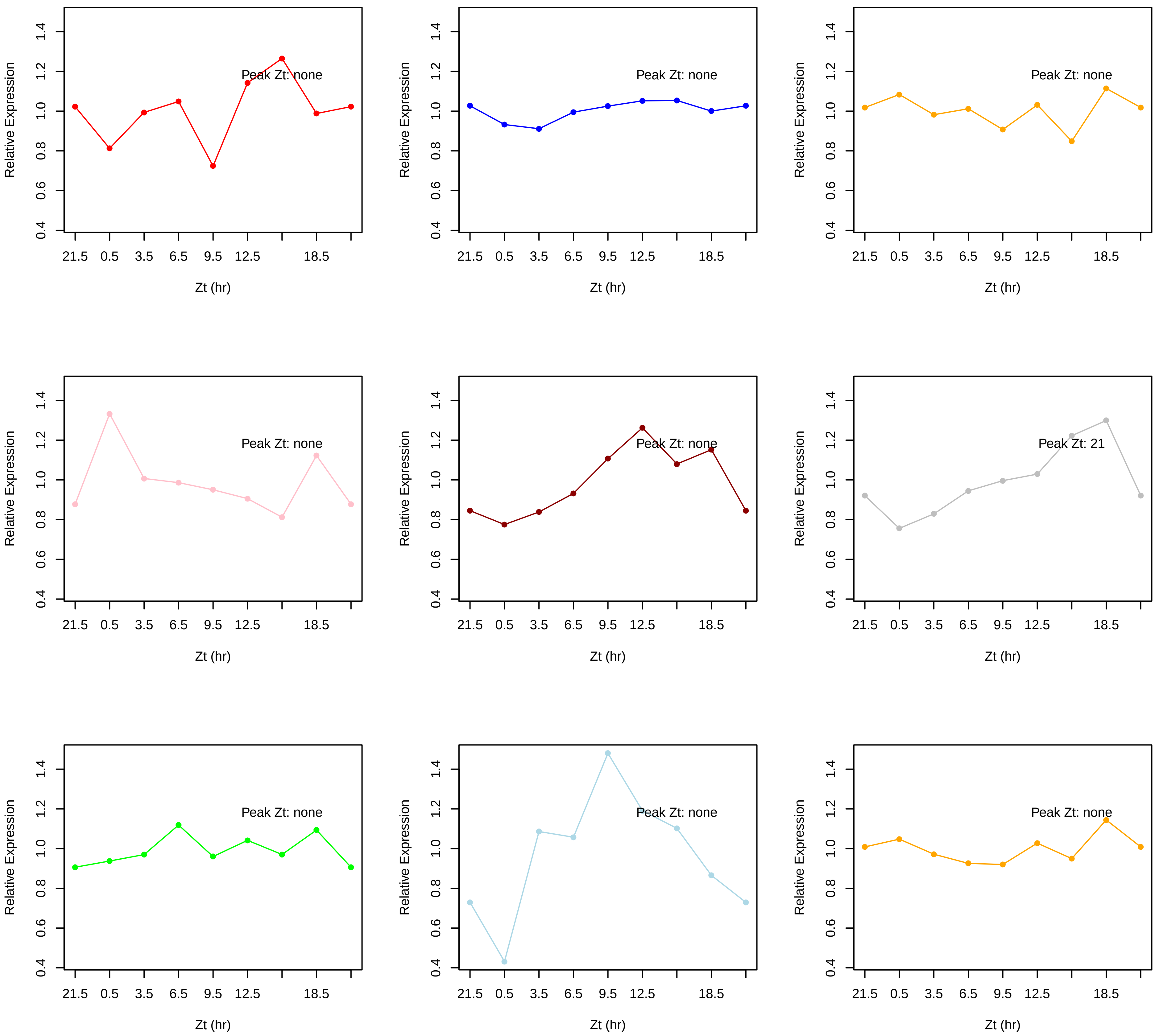

Singleton: ENSXMAG00000003466 abcd1

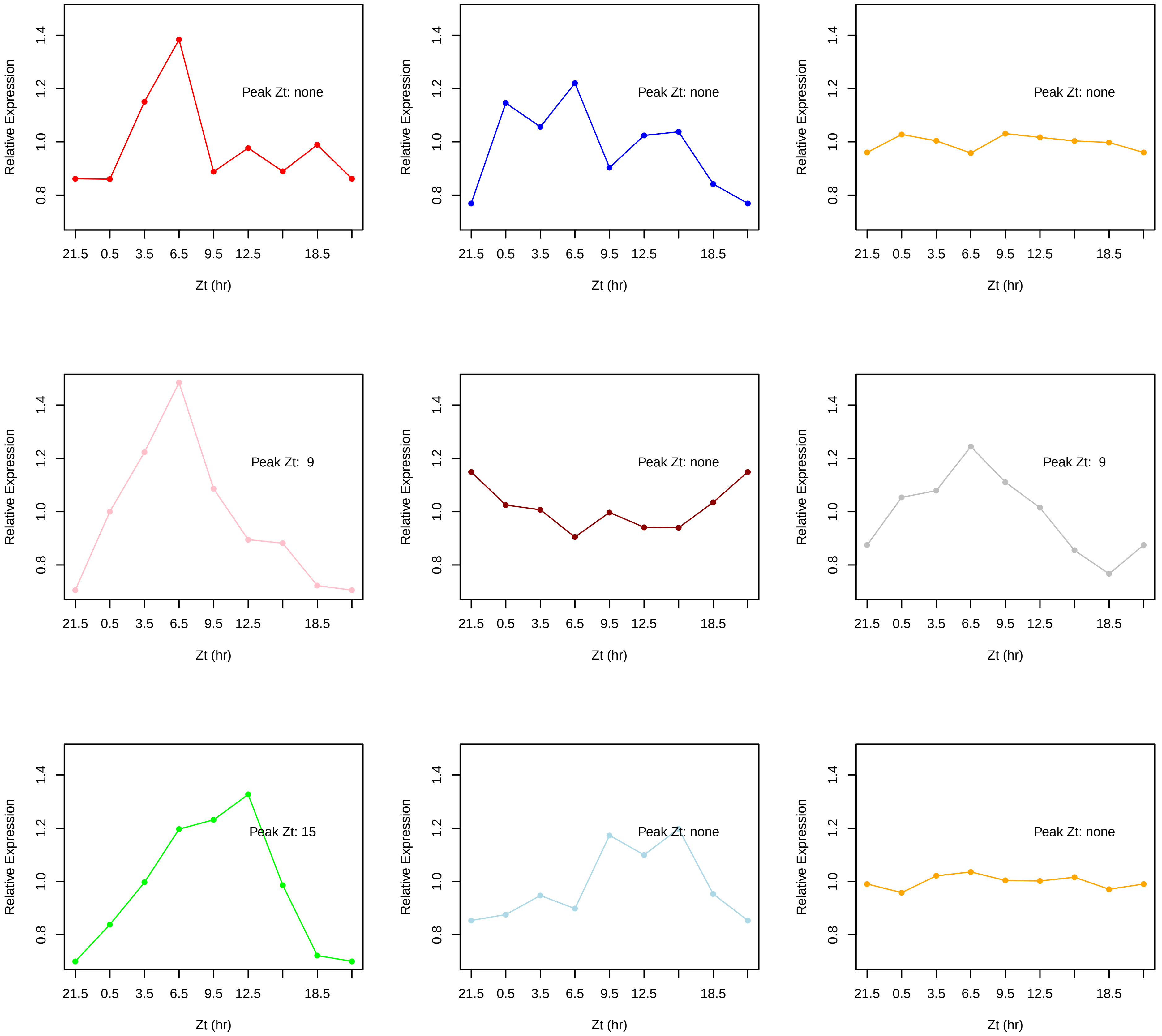

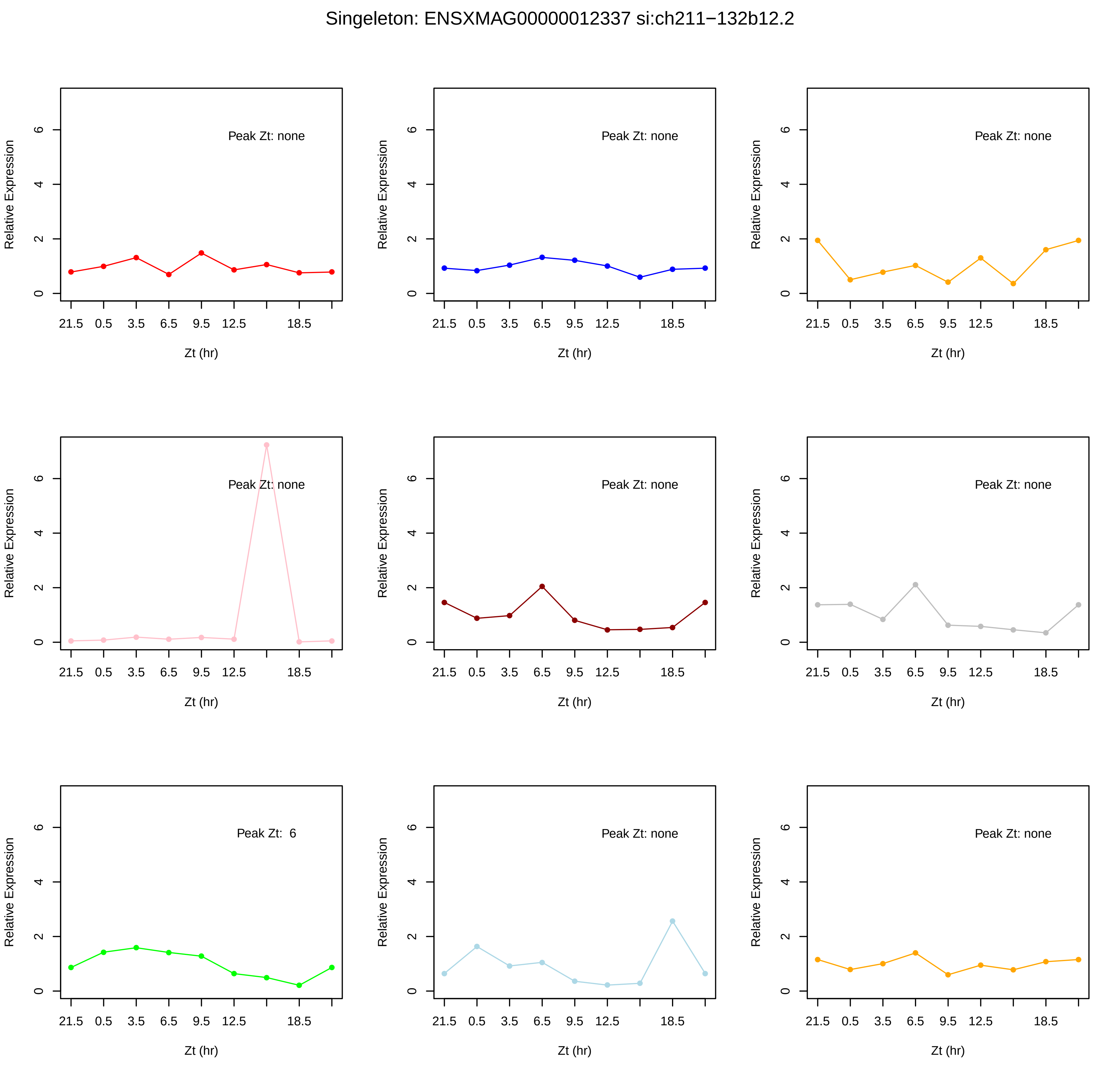
Singeleton: ENSXMAG00000012337 si:ch211−132b12.2
Peak Zt: none
Peak Zt: none
Peak Zt: none
Peak Zt: none
Peak Zt: none
Peak Zt: none
Peak Zt: 6
Peak Zt: none
Peak Zt: none
Relative Expression
Zt (hr)
21.5 0.5 3.5 6.5 9.5 12.5 18.5
0 2 4 6

Singeleton: ENSXMAG00000028556 yipf6

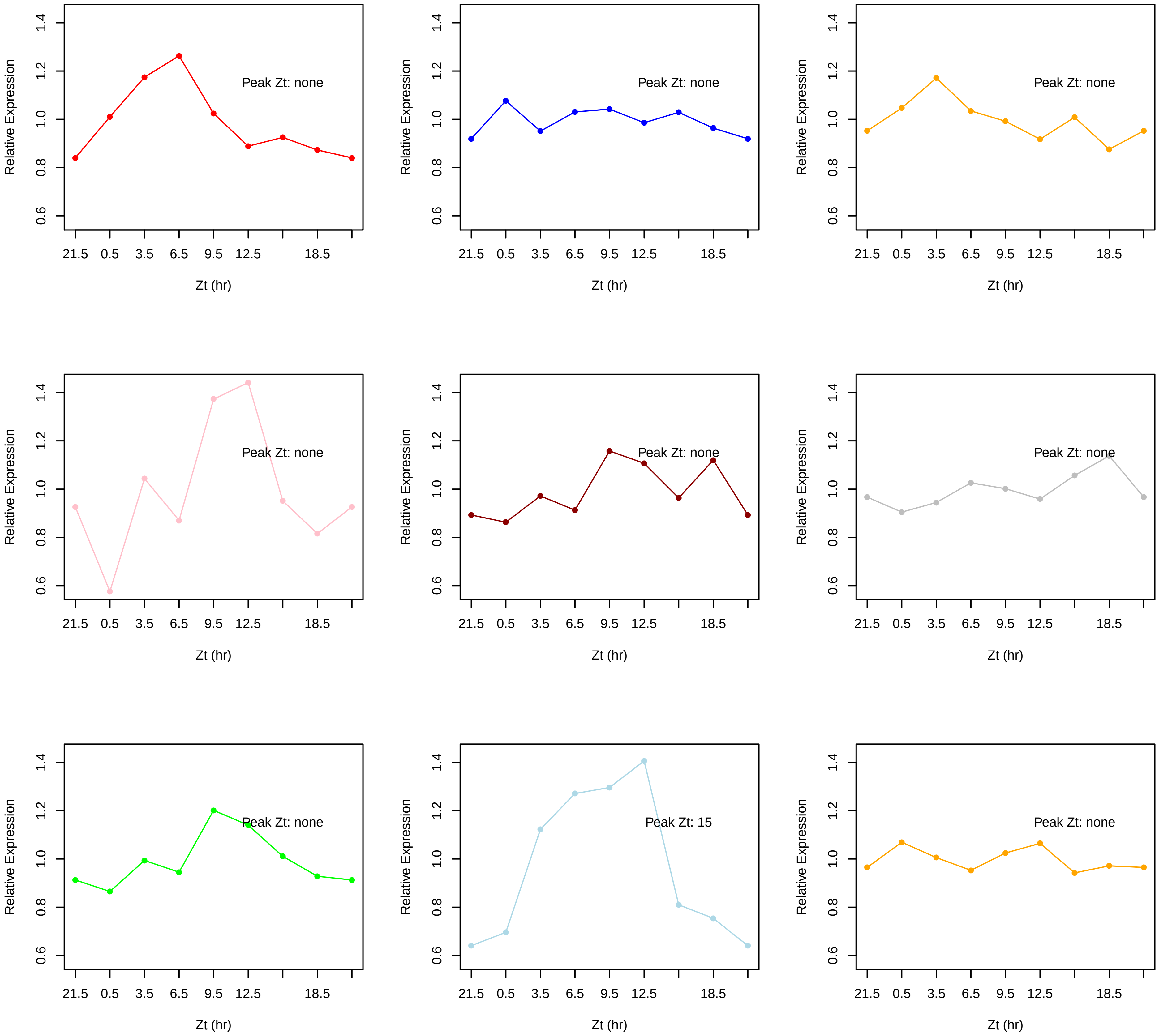

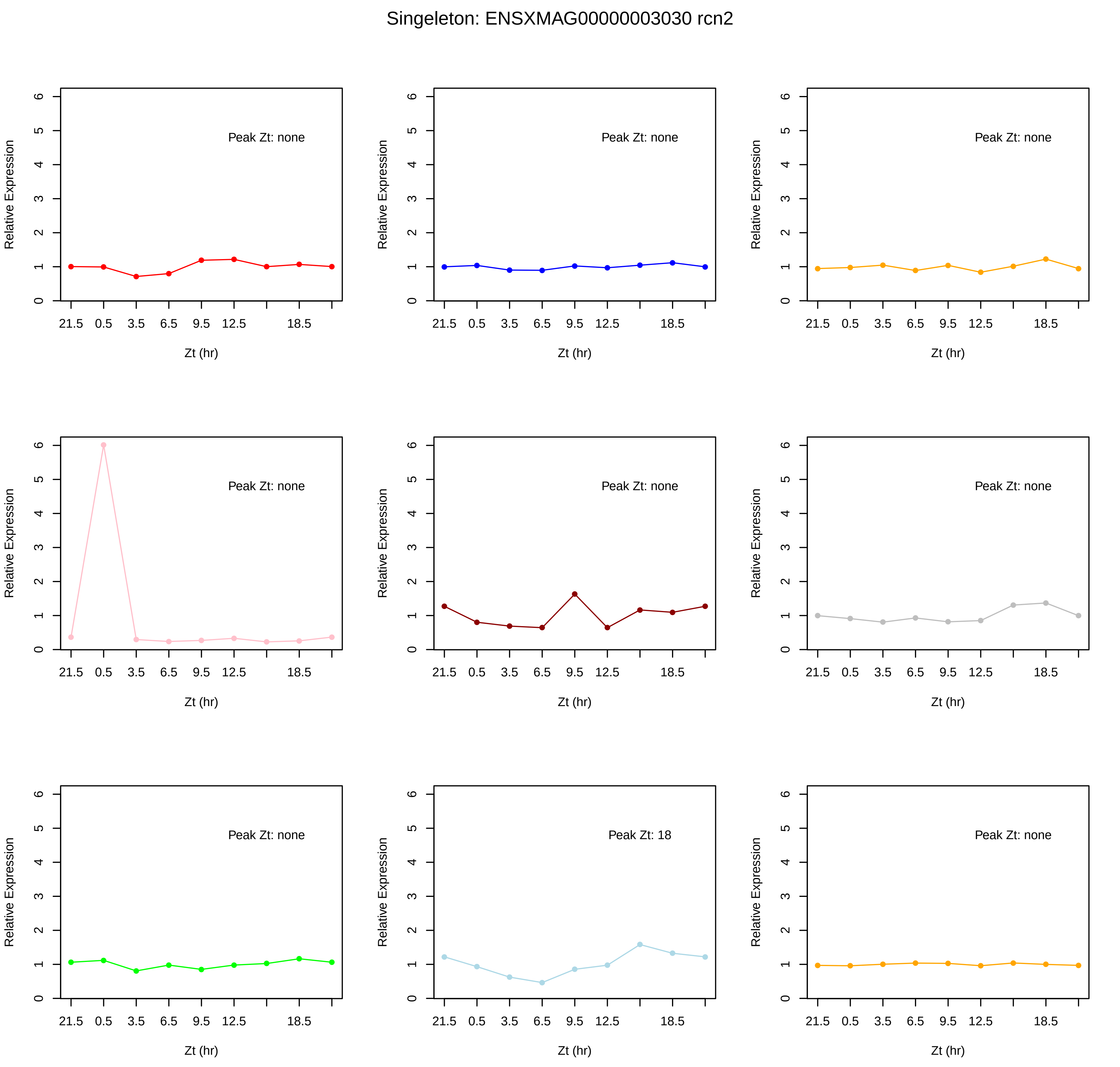
Singeleton: ENSXMAG00000003030 rcn2
Relative Expression
Zt (hr)
21.5 0.5 3.5 6.5 9.5 12.5 18.5
Peak Zt: none
Peak Zt: none
Peak Zt: none
Peak Zt: none
Peak Zt: none
Peak Zt: none
Peak Zt: none
Peak Zt: 18
Peak Zt: none

Singeleton: ENSXMAG00000003057 scaper

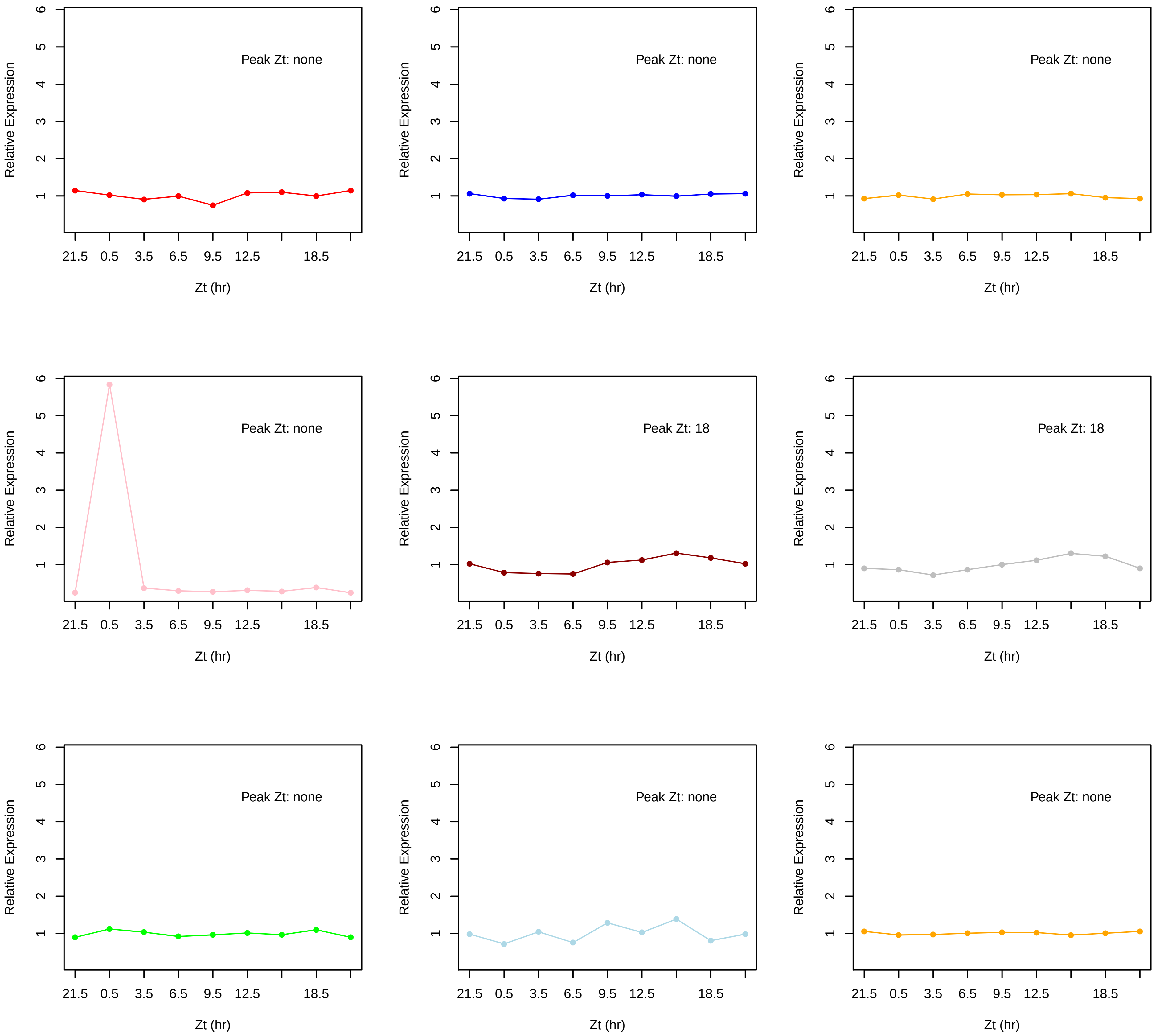

Singeleton: ENSXMAG00000003528 fgd1

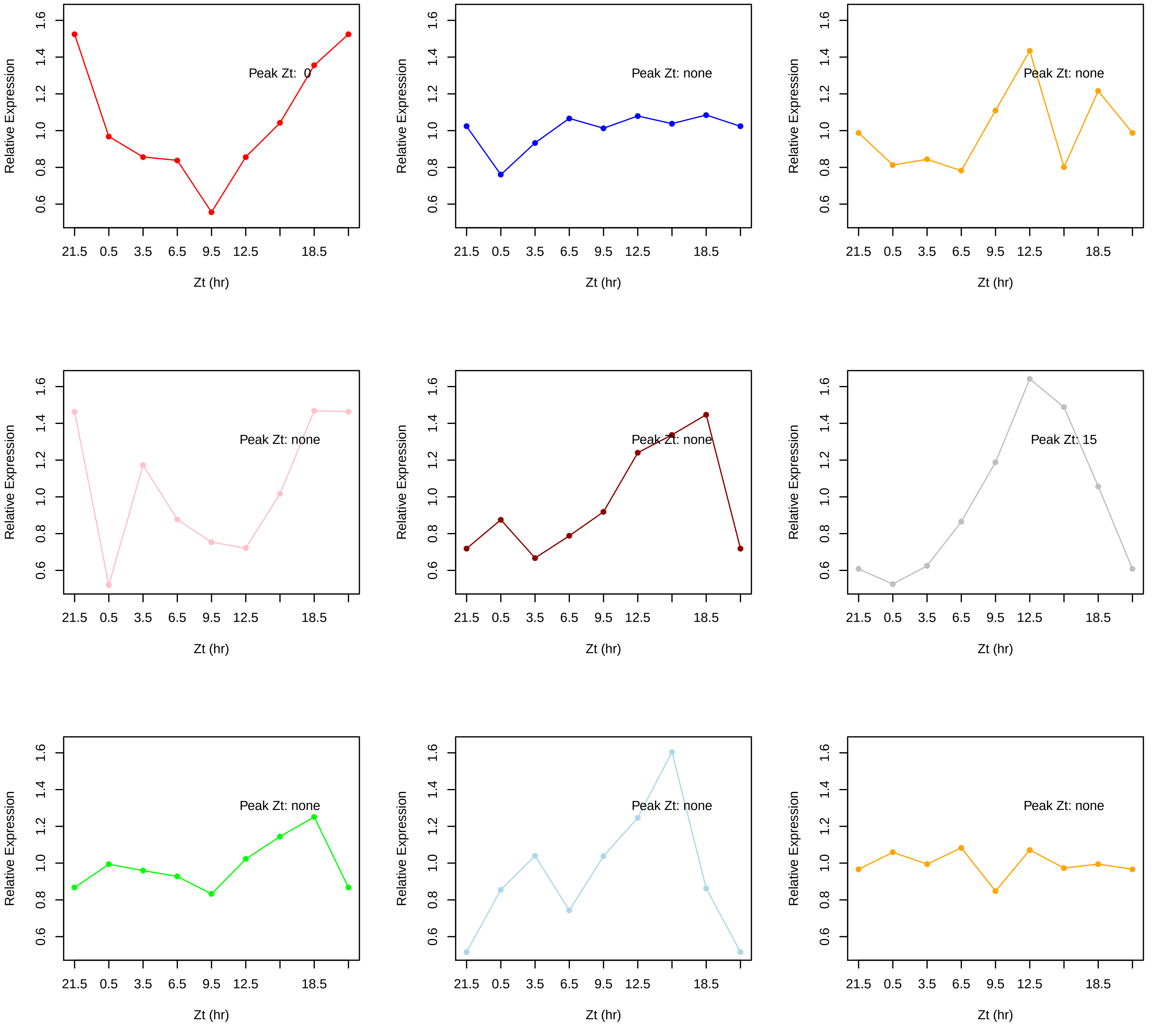

Singeleton: ENSXMAG00000000505 timm50

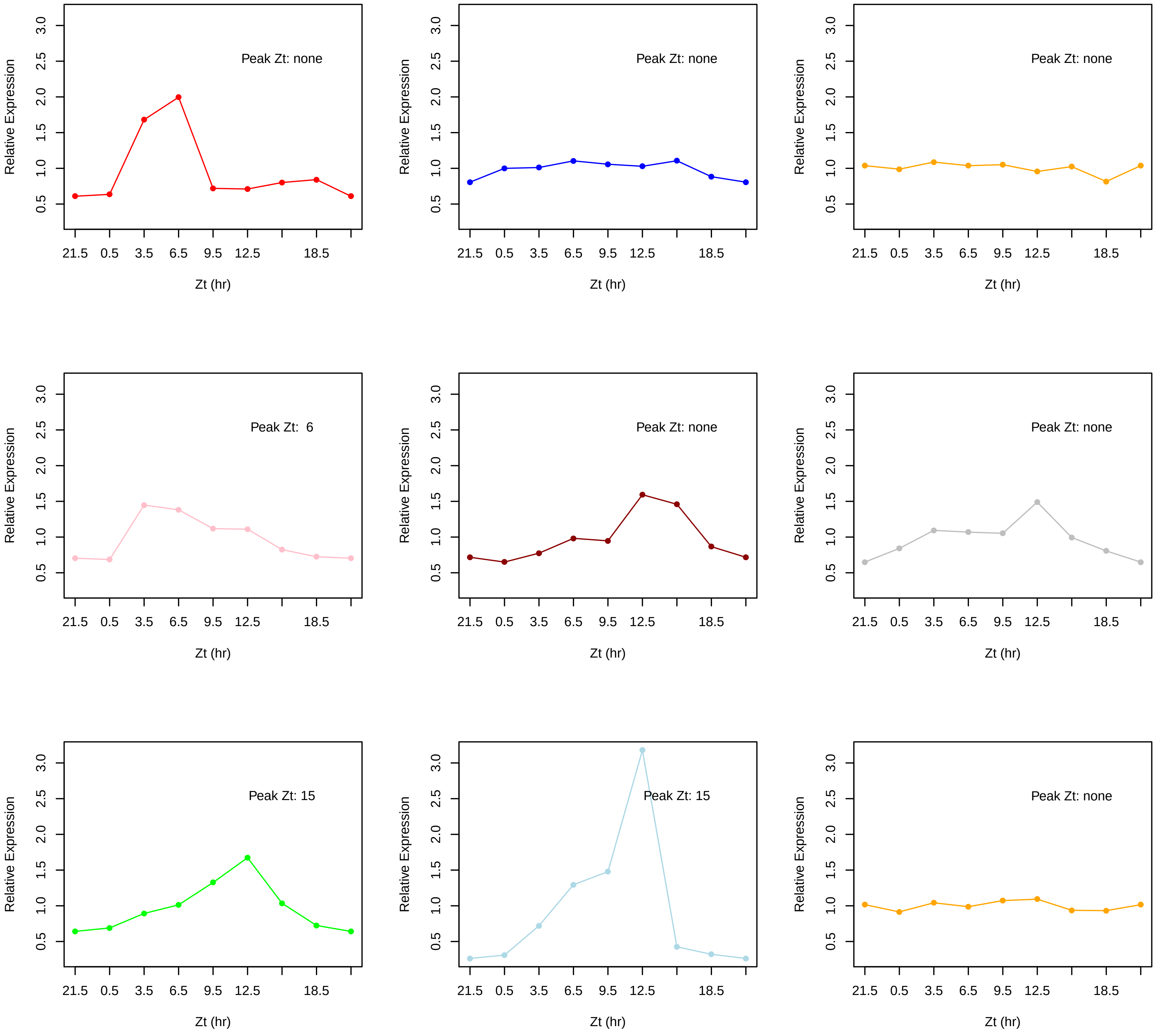

Singeleton: ENSXMAG00000006108 si:dkey−13p1.4

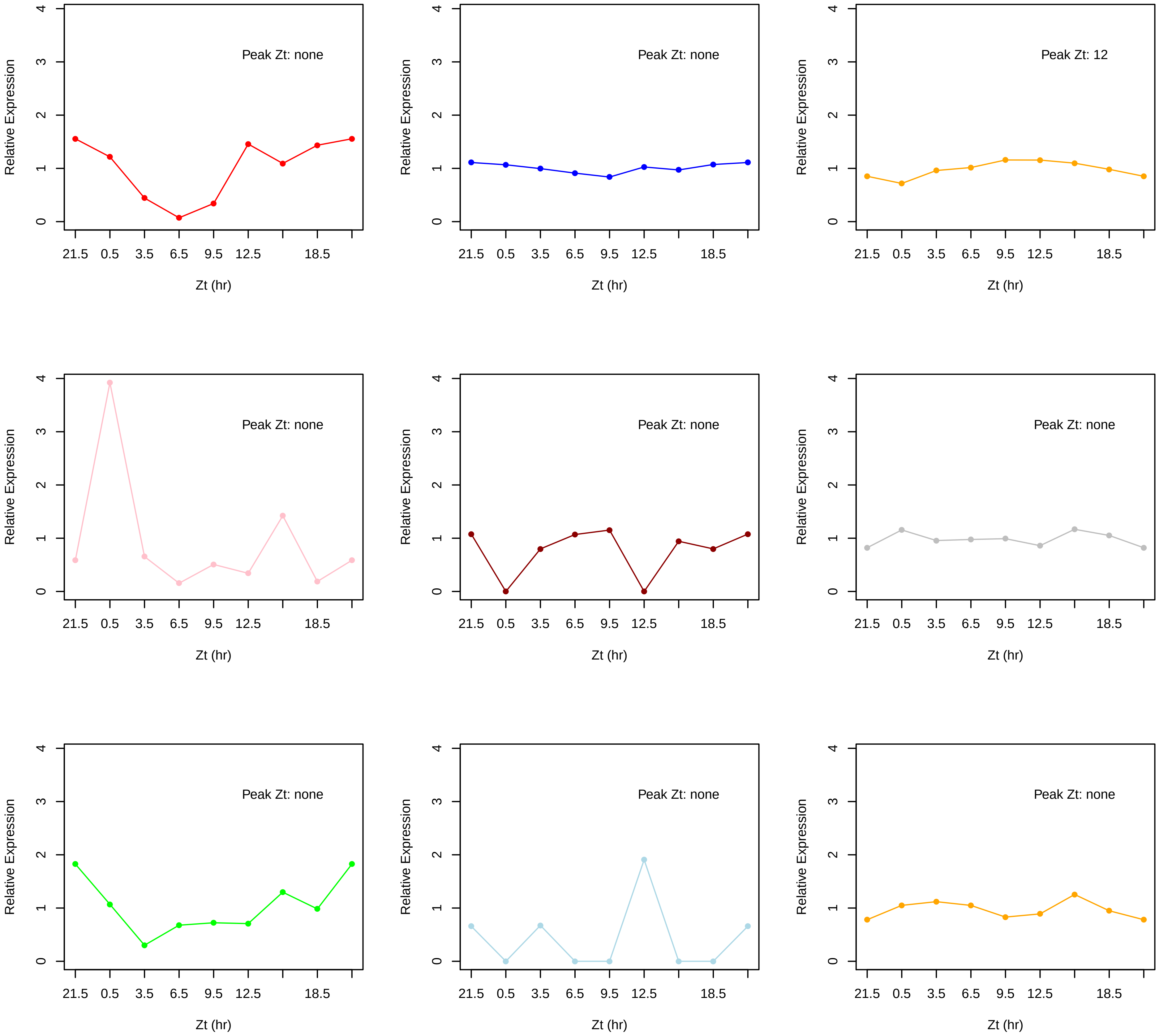

Singeleton: ENSXMAG00000019476 zgc:103697

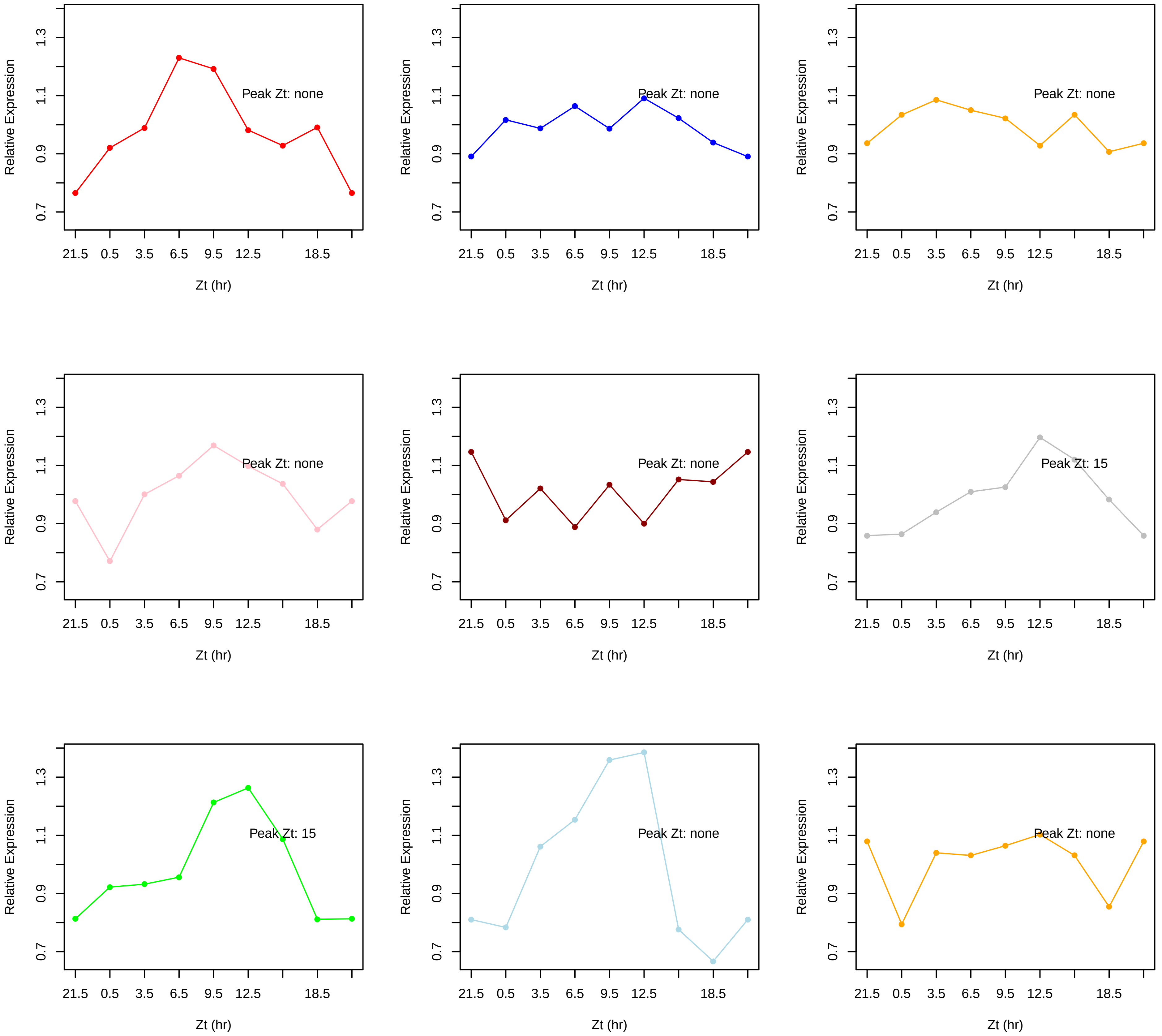

Singleton: ENSXMAG00000028371 efnb1

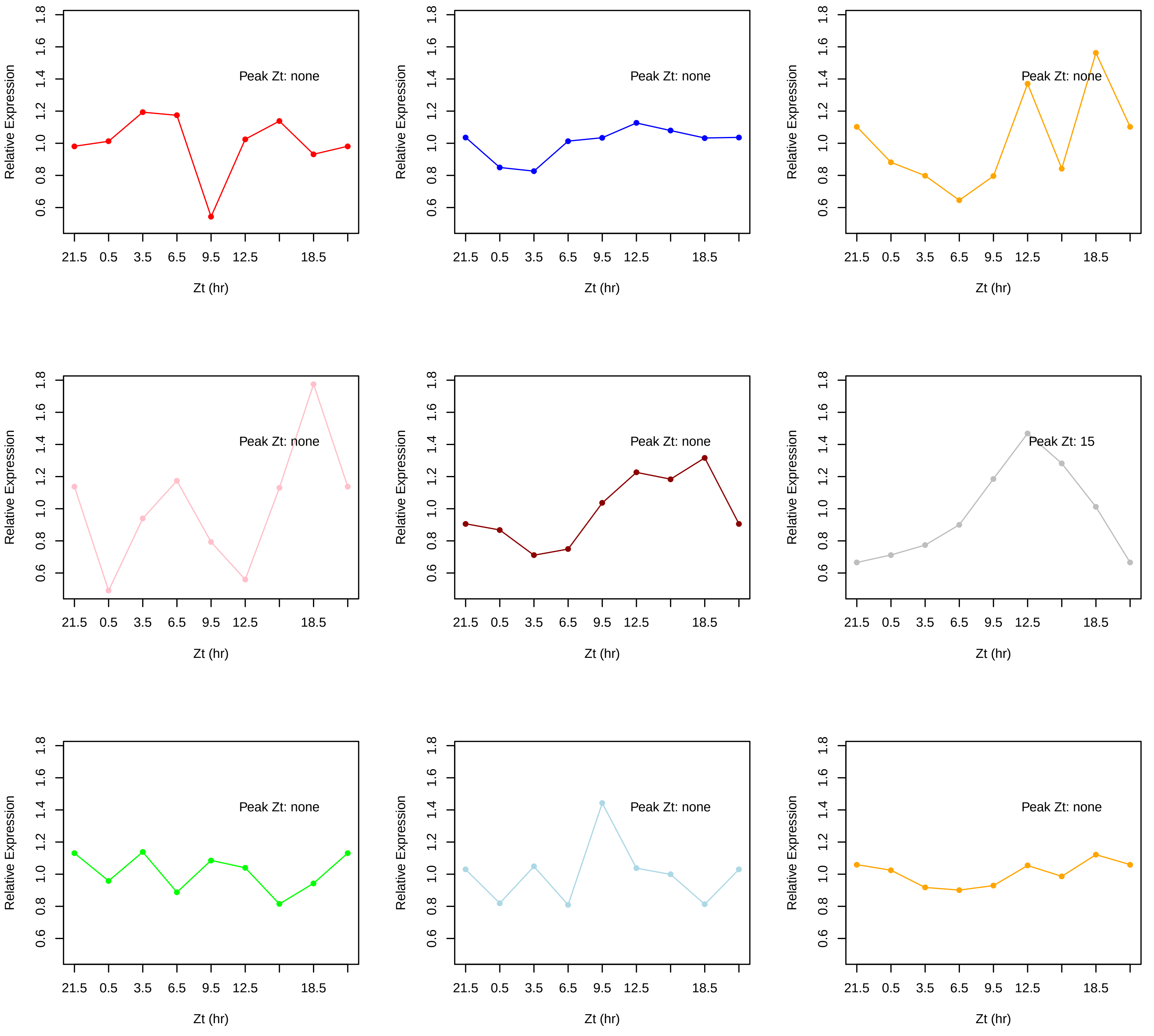

Singeleton: ENSXMAG00000003102 etfa

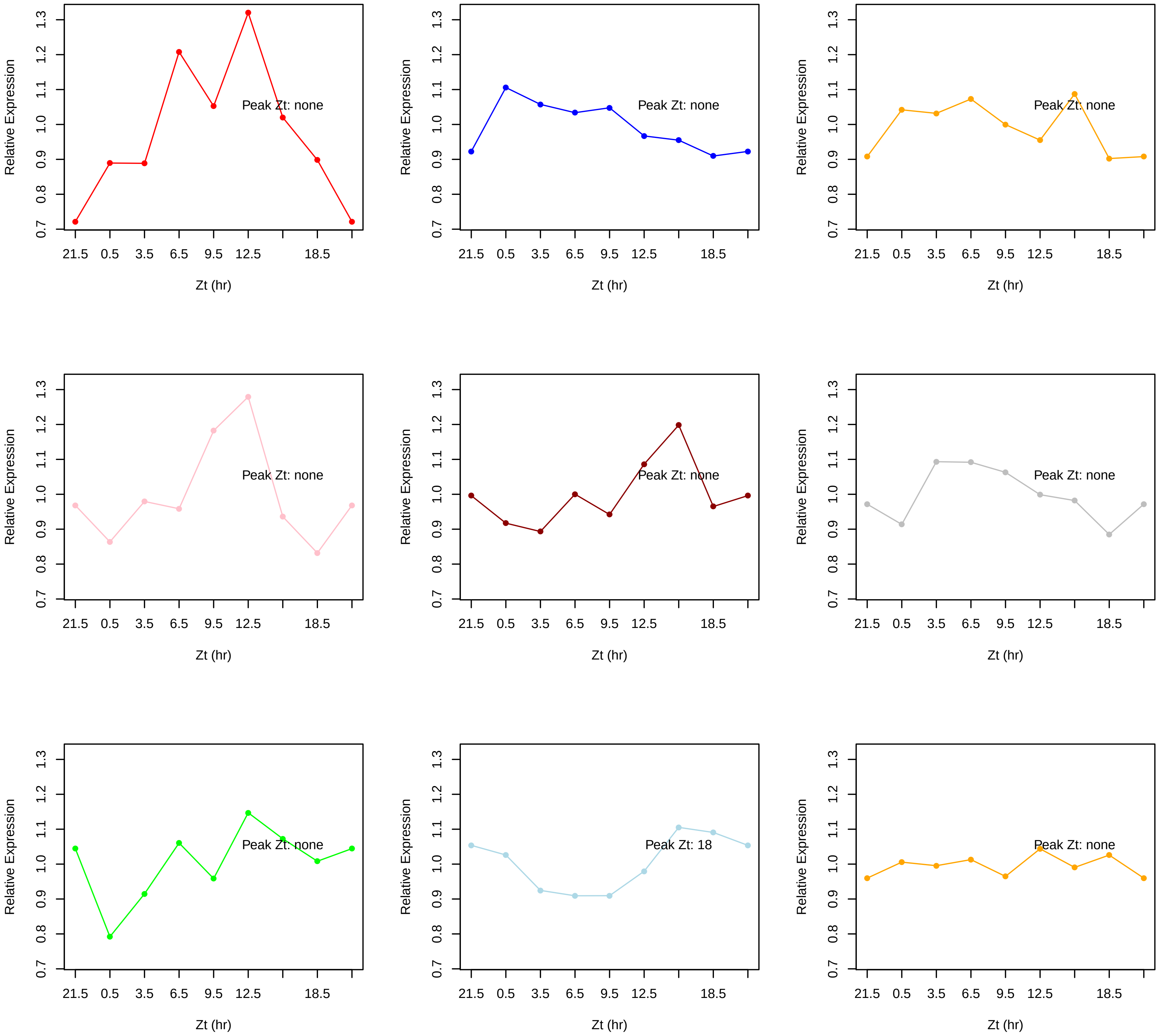

Singeleton: ENSXMAG00000015037

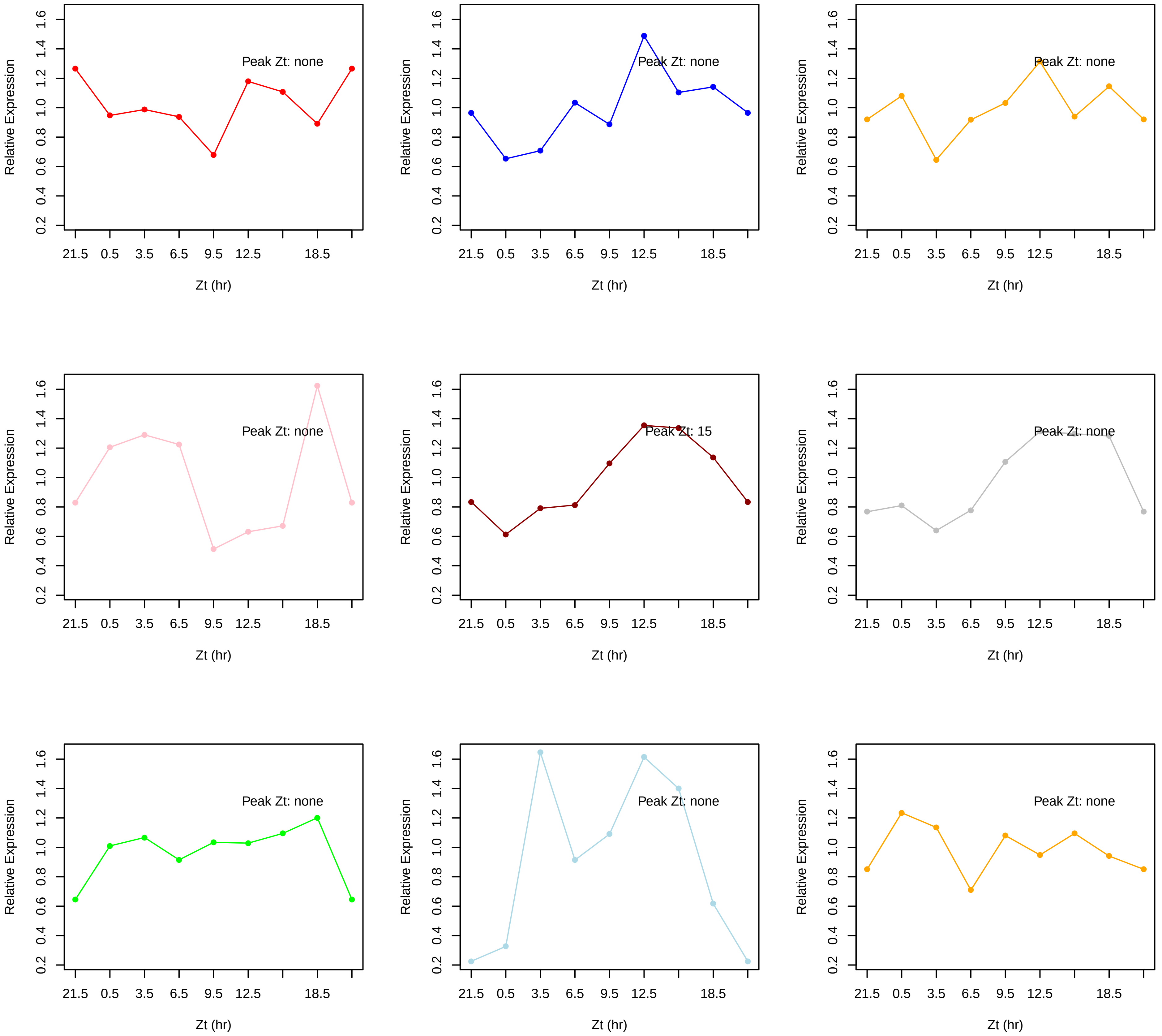

Singeleton: ENSXMAG00000009549 opn1sw2

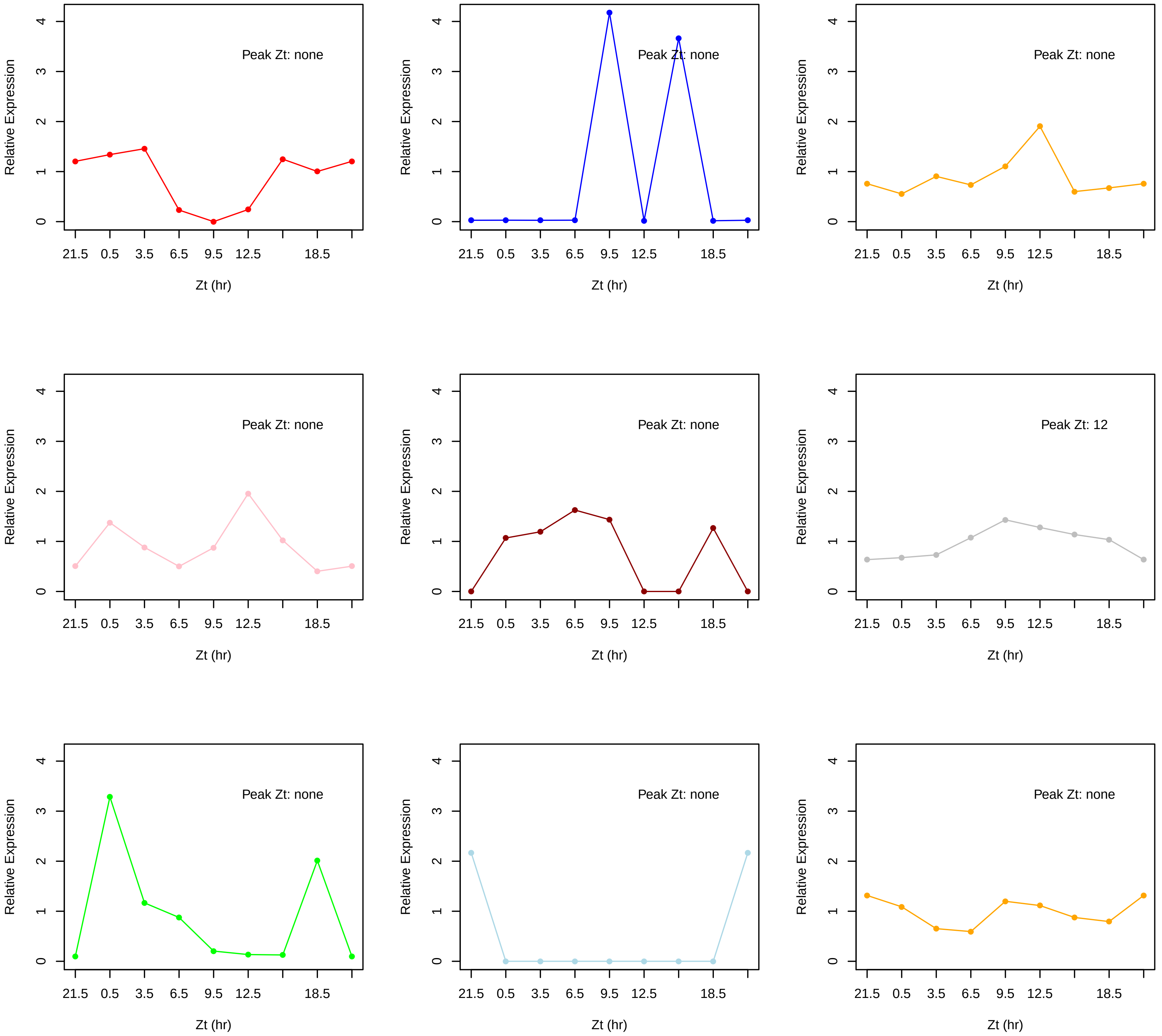

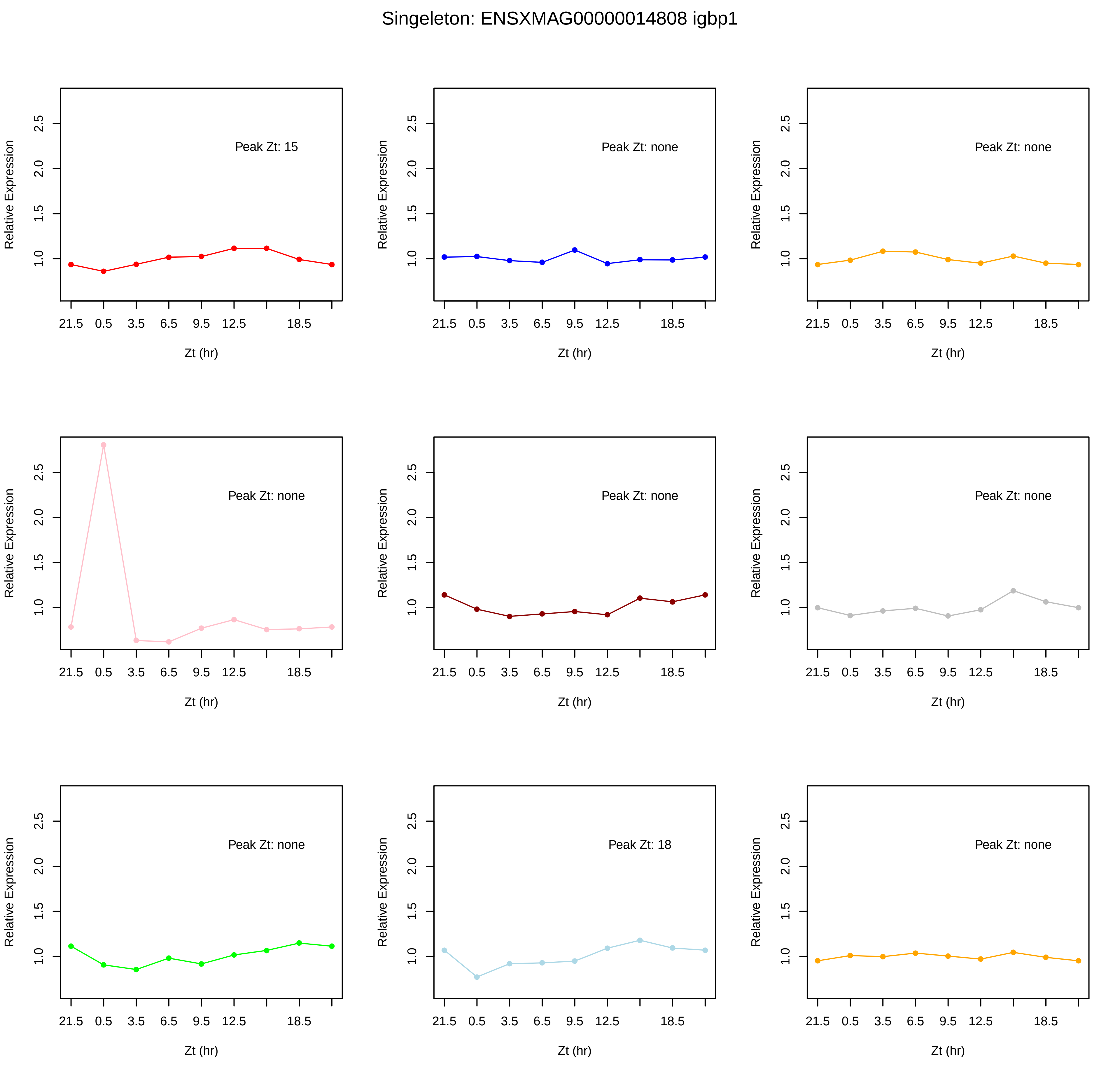
Singeleton: ENSXMAG00000014808 igbp1
Peak Zt: 15
Peak Zt: none
Peak Zt: none
Peak Zt: none
Peak Zt: none
Peak Zt: none
Peak Zt: none
Peak Zt: 18
Peak Zt: none
Relative Expression
Zt (hr)
21.5
0.5
3.5
6.5
9.5
12.5
18.5
1.0
1.5
2.0
2.5

Singeleton: ENSXMAG00000003138 lactb

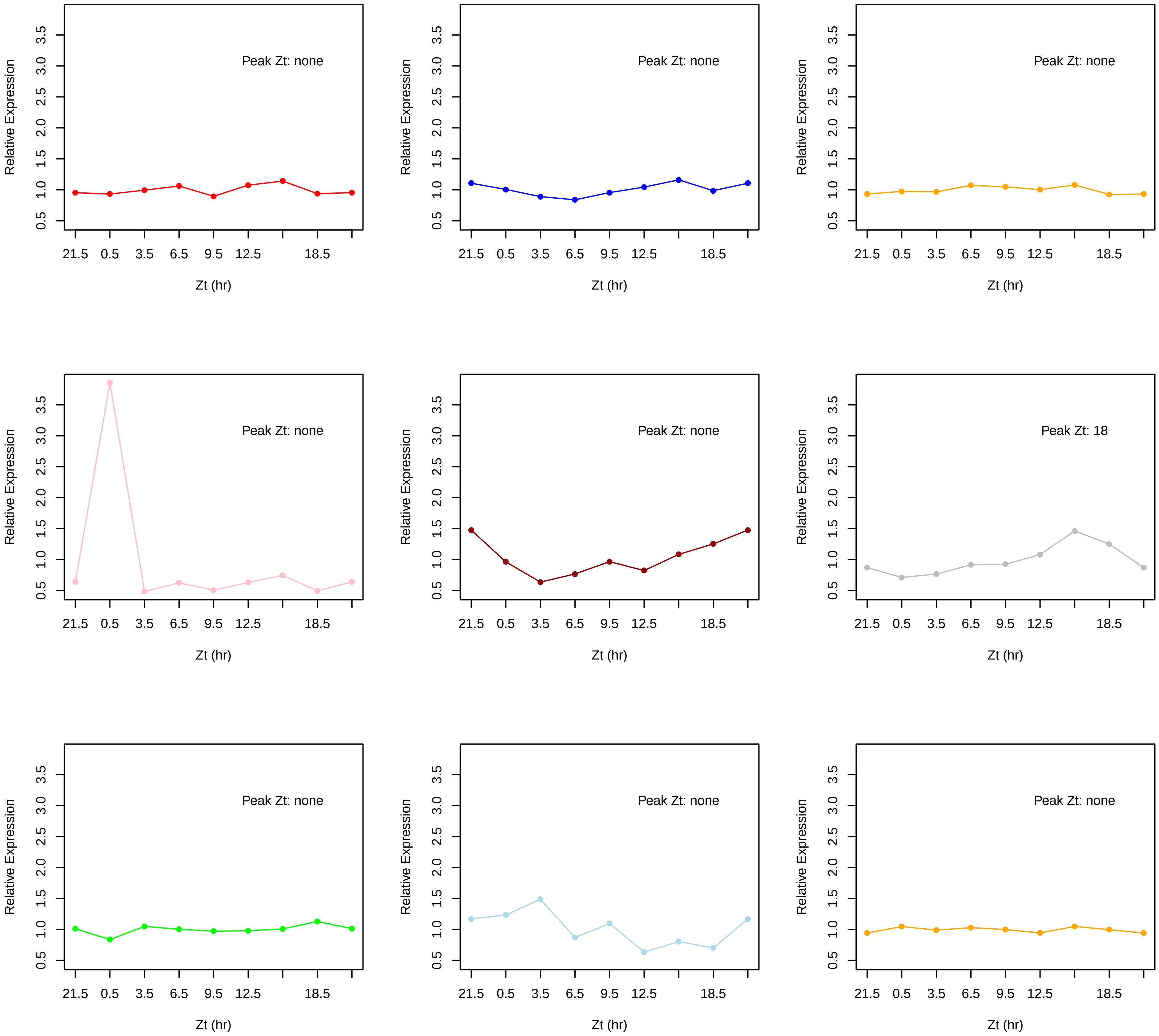

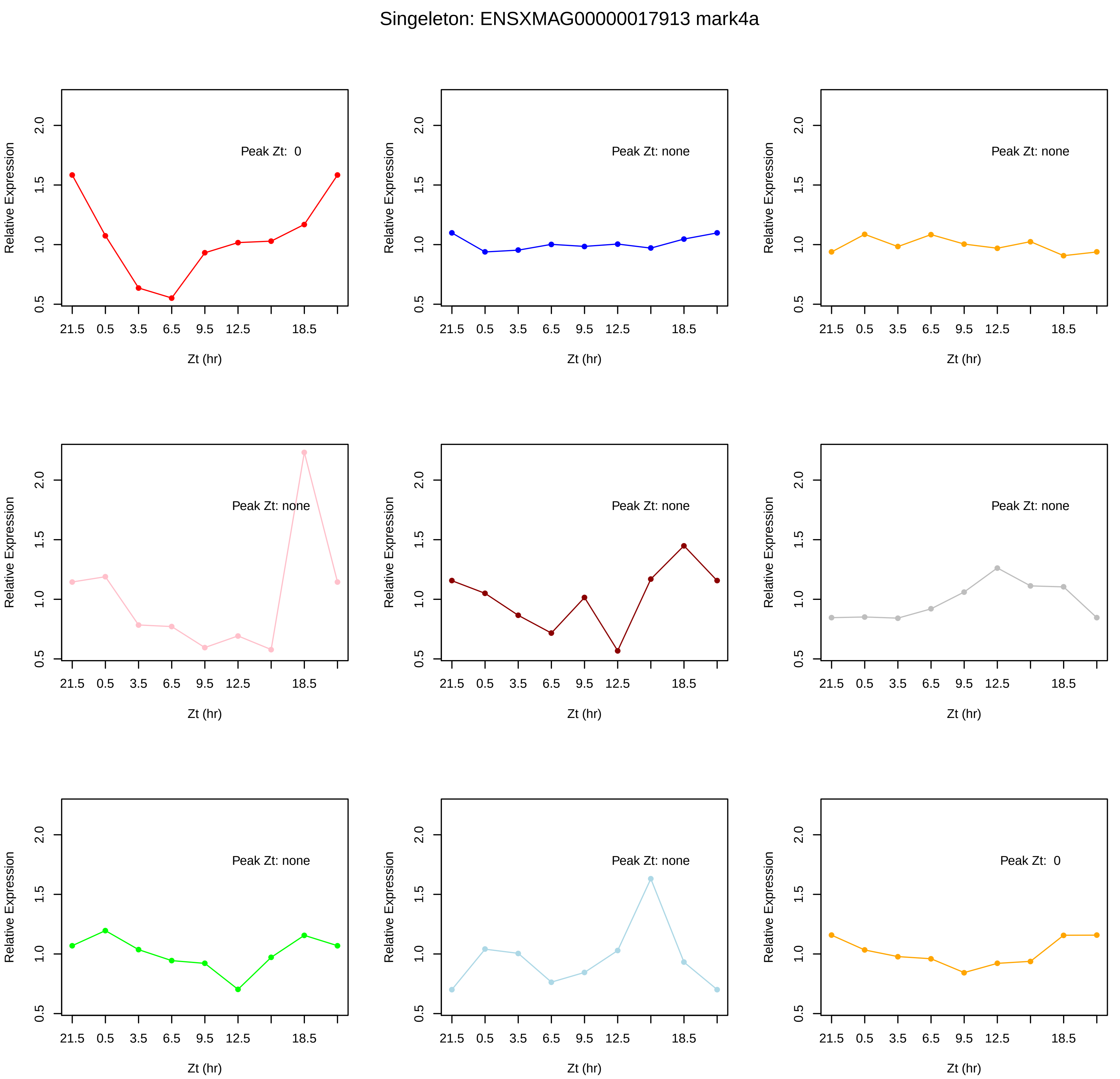
Singeleton: ENSXMAG00000017913 mark4a
Peak Zt: 0
Peak Zt: none
Peak Zt: none
Peak Zt: none
Peak Zt: none
Peak Zt: none
Peak Zt: none
Peak Zt: none
Peak Zt: 0
Relative Expression
Zt (hr)
21.5
0.5
3.5
6.5
9.5
12.5
18.5
0.5
1.0
1.5
2.0

Singeleton: ENSXMAG00000019883 nono

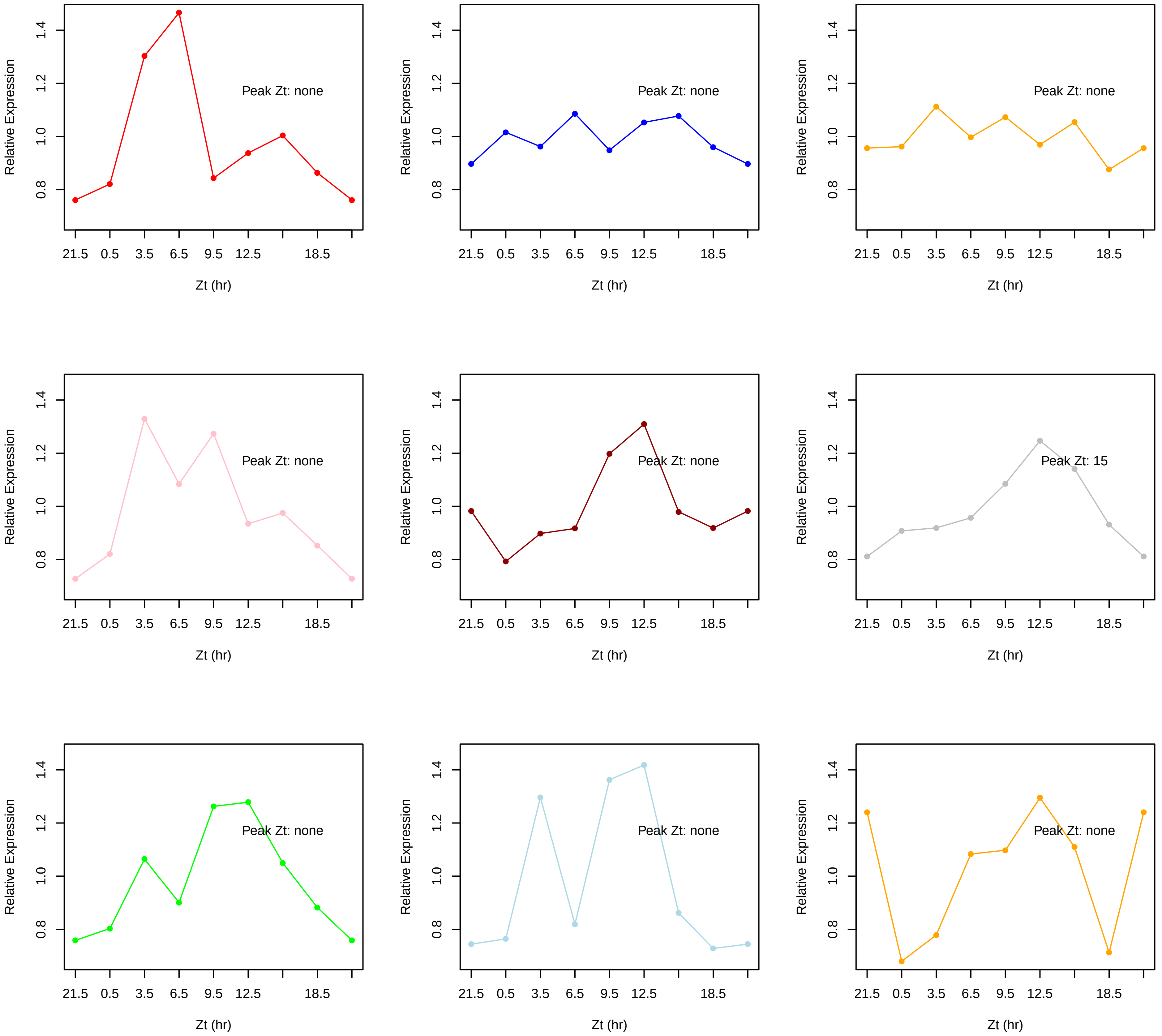

Singeleton: ENSXMAG00000021653 mtnr1c

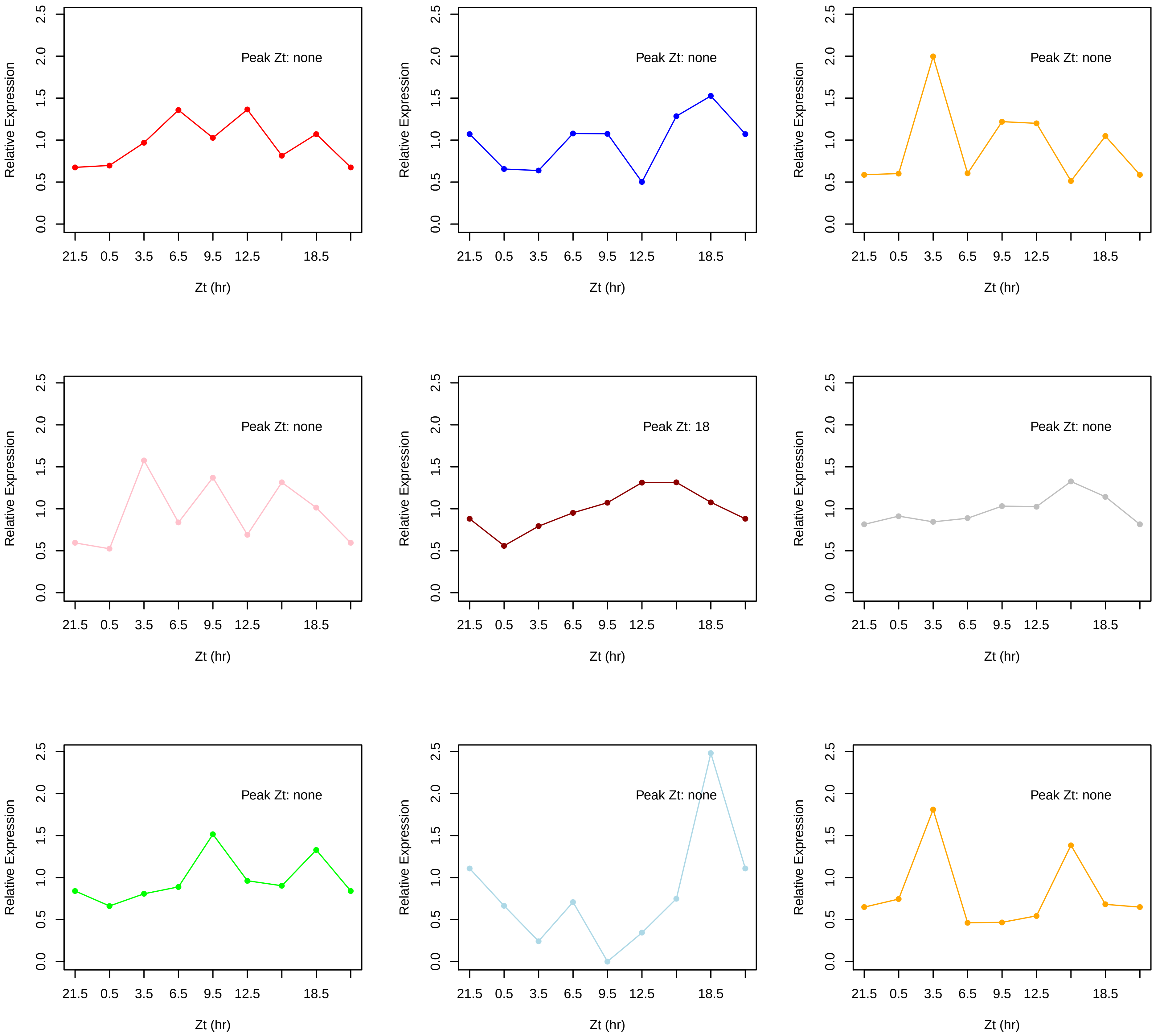

Singeleton: ENSXMAG00000001420 klc3

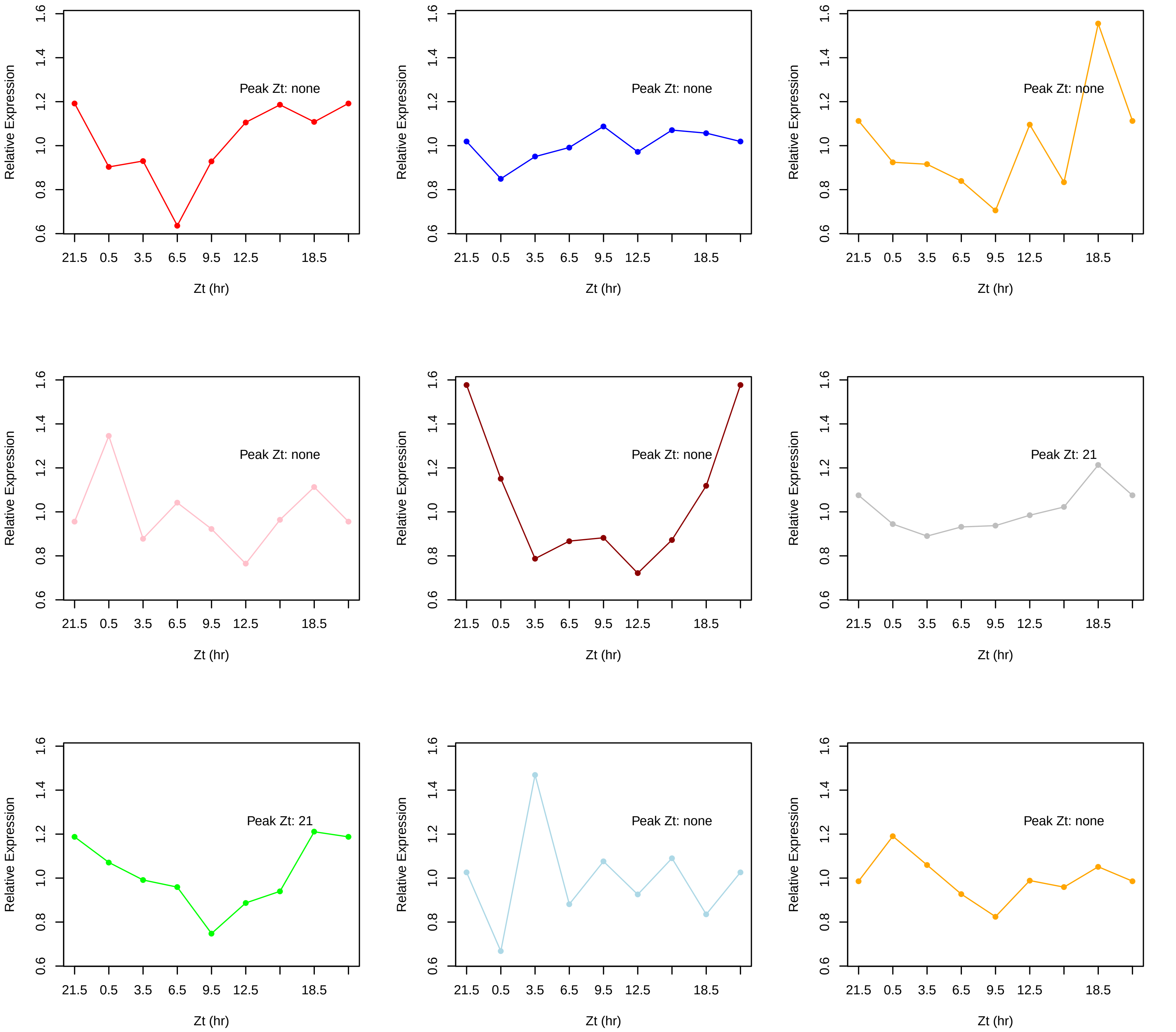

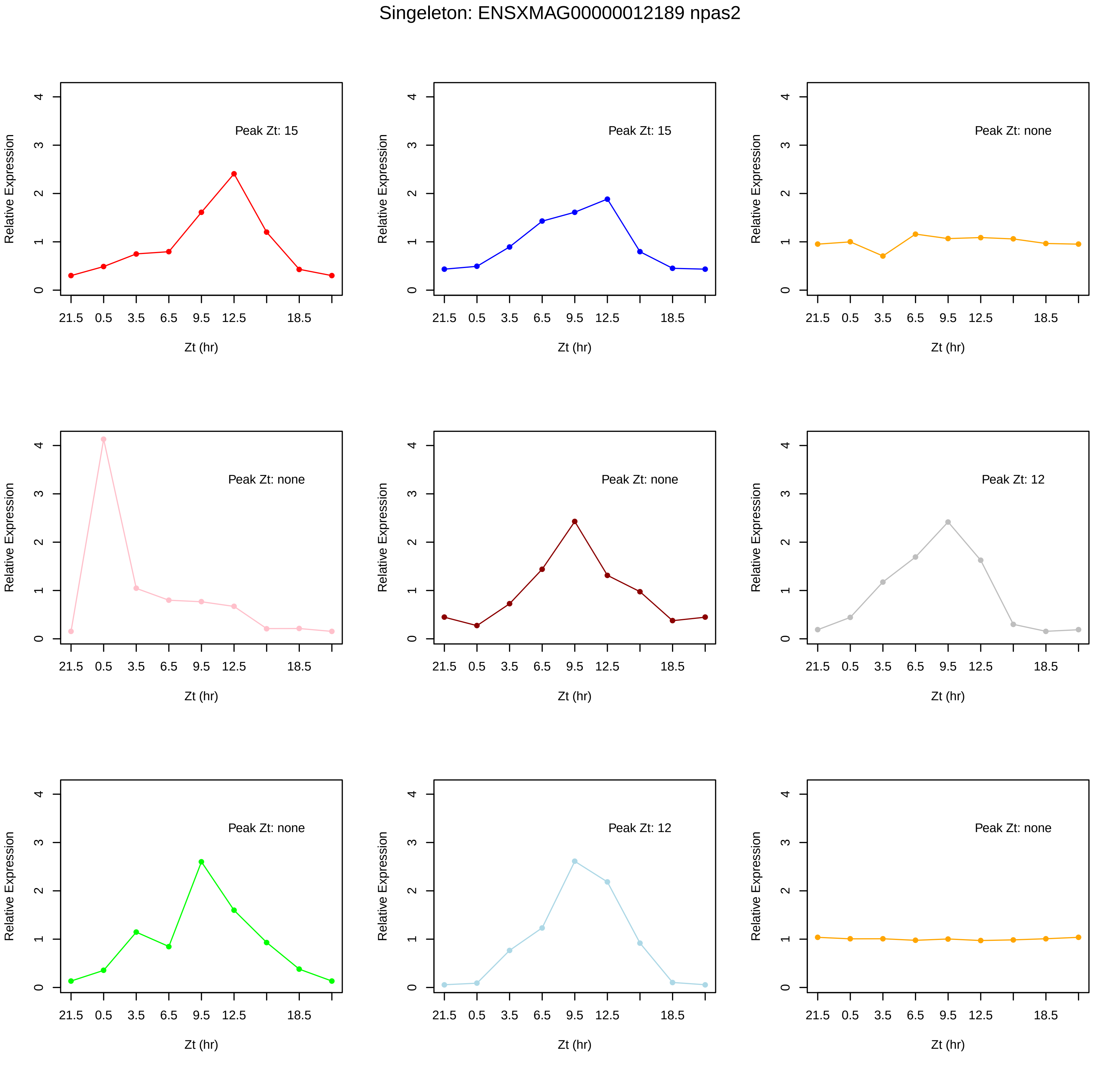
Singeleton: ENSXMAG00000012189 npas2
Peak Zt: 15
Peak Zt: 15
Peak Zt: none
Peak Zt: none
Peak Zt: none
Peak Zt: 12
Peak Zt: none
Peak Zt: 12
Peak Zt: none
Relative Expression
Zt (hr)
21.5
0.5
3.5
6.5
9.5
12.5
18.5
0
1
2
3
4

Singeleton: ENSXMAG00000013327 pcmtl

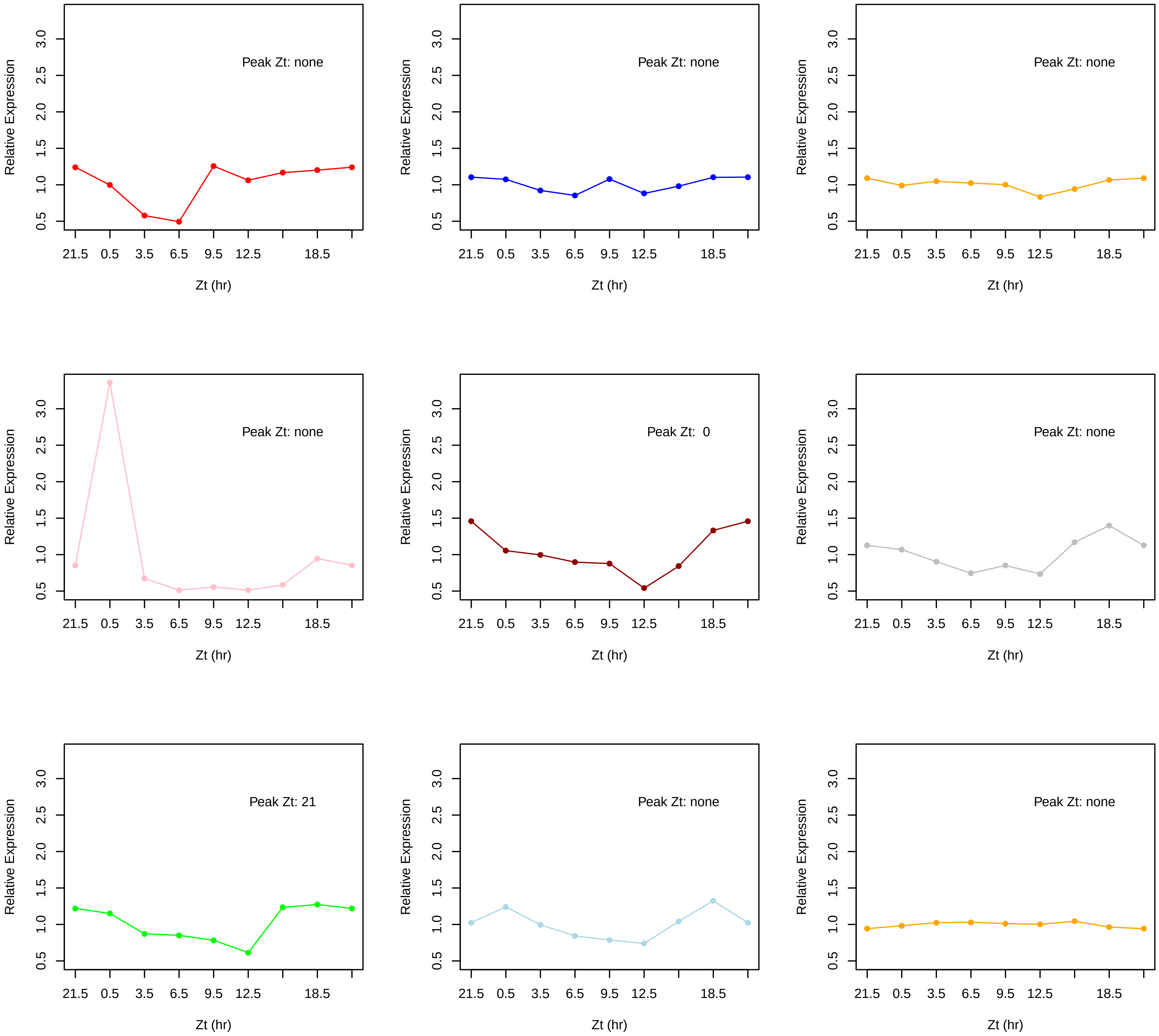

Singeleton: ENSXMAG00000024533 gata1a

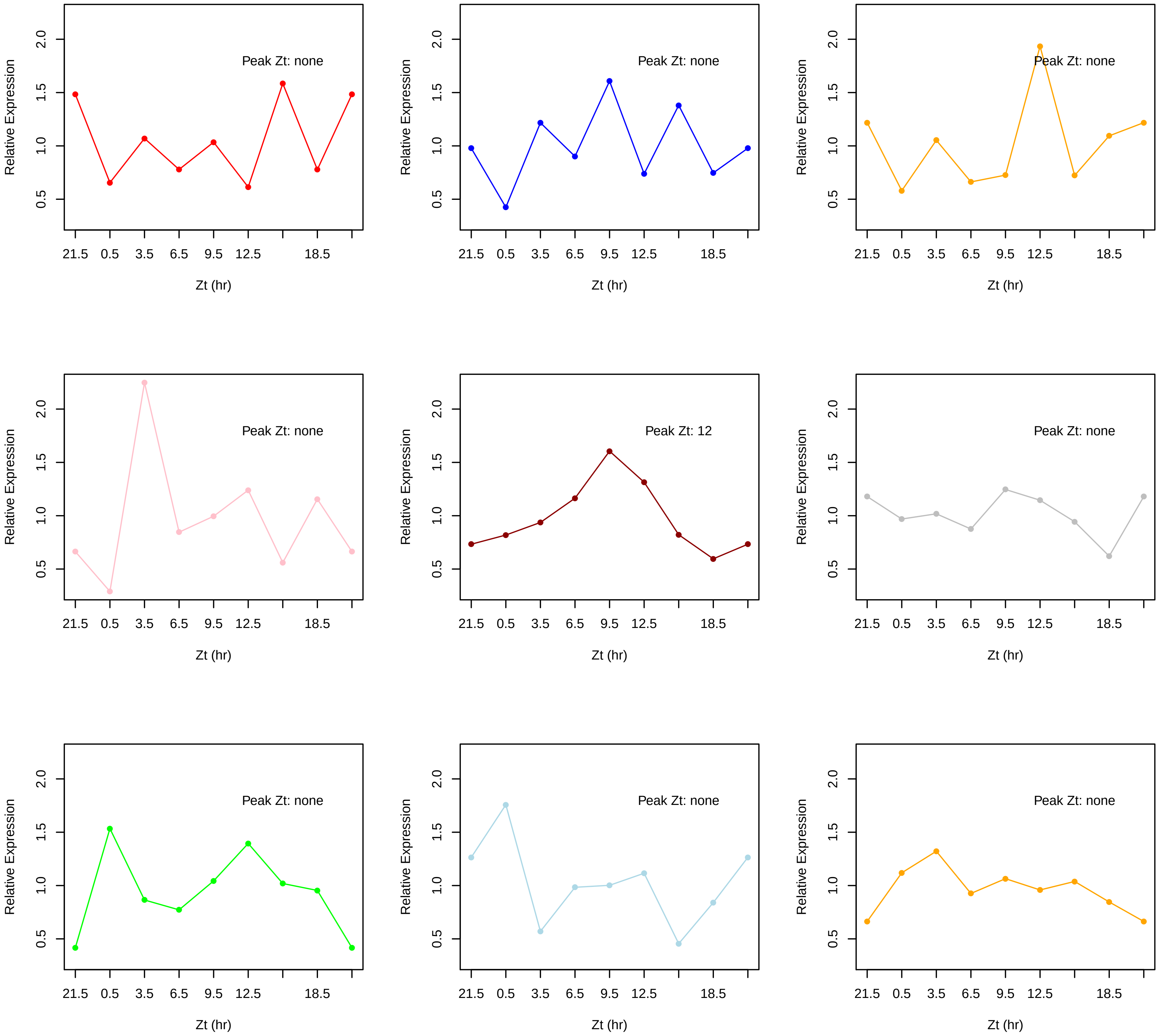

Singeleton: ENSXMAG00000017746 pkdccb

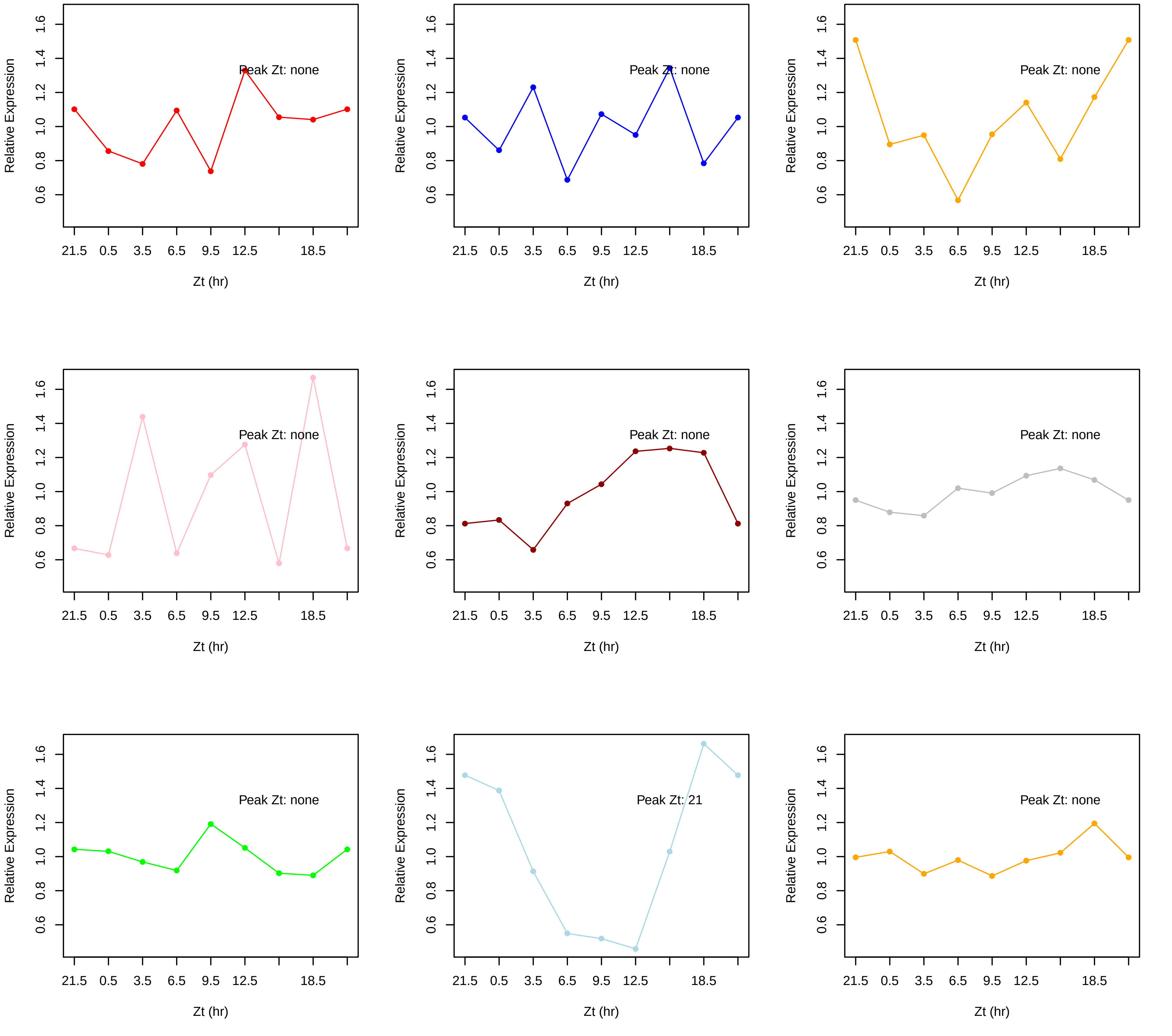

Singleton: ENSXMAG00000013014 hdac6

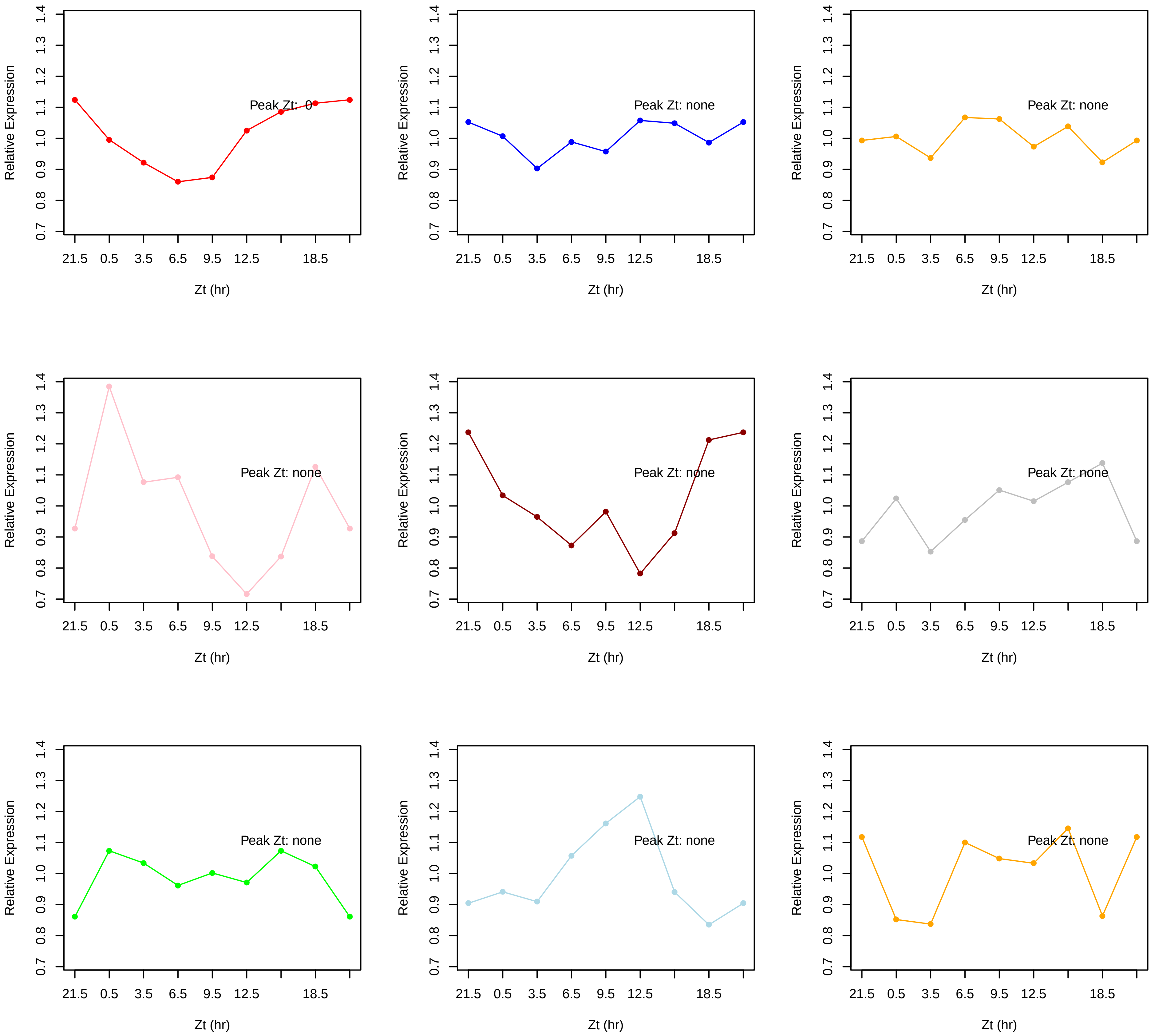

Singeleton: ENSXMAG00000013348 eml1

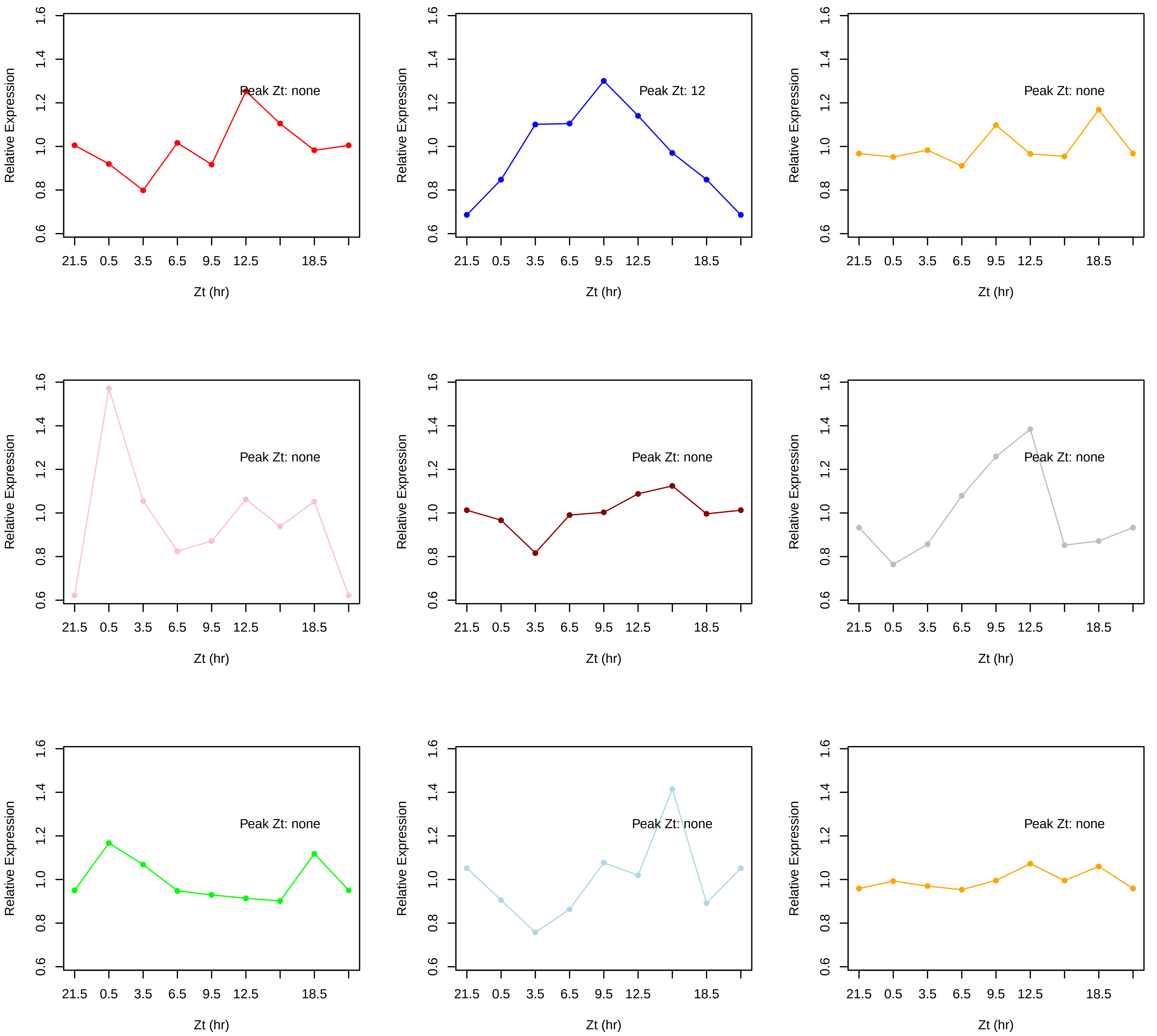

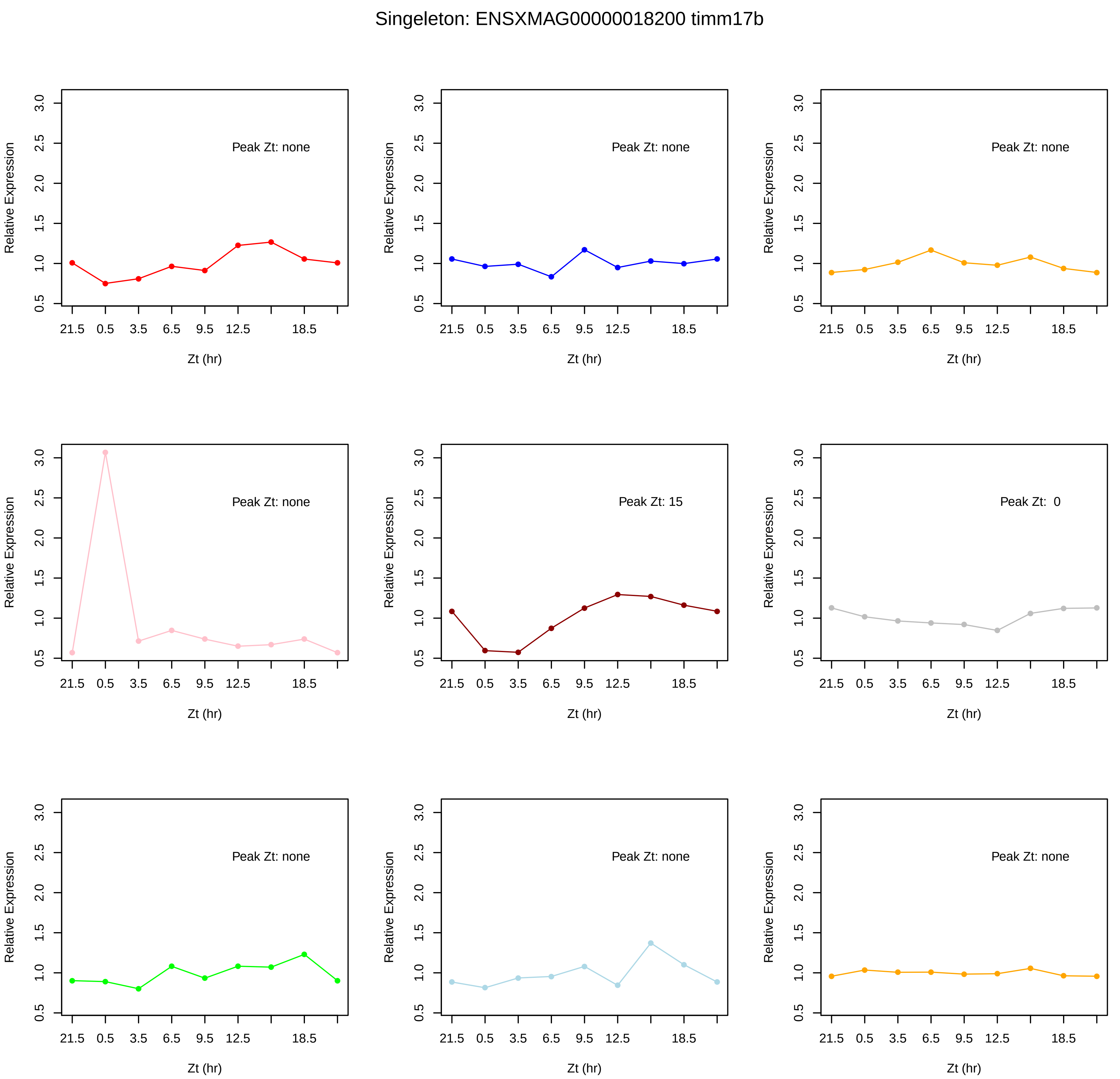
Singeleton: ENSXMAG00000018200 timm17b
Peak Zt: none
Peak Zt: none
Peak Zt: none
Peak Zt: none
Peak Zt: 15
Peak Zt: 0
Peak Zt: none
Peak Zt: none
Peak Zt: none
Relative Expression
Zt (hr)

Singeleton: ENSXMAG00000020048

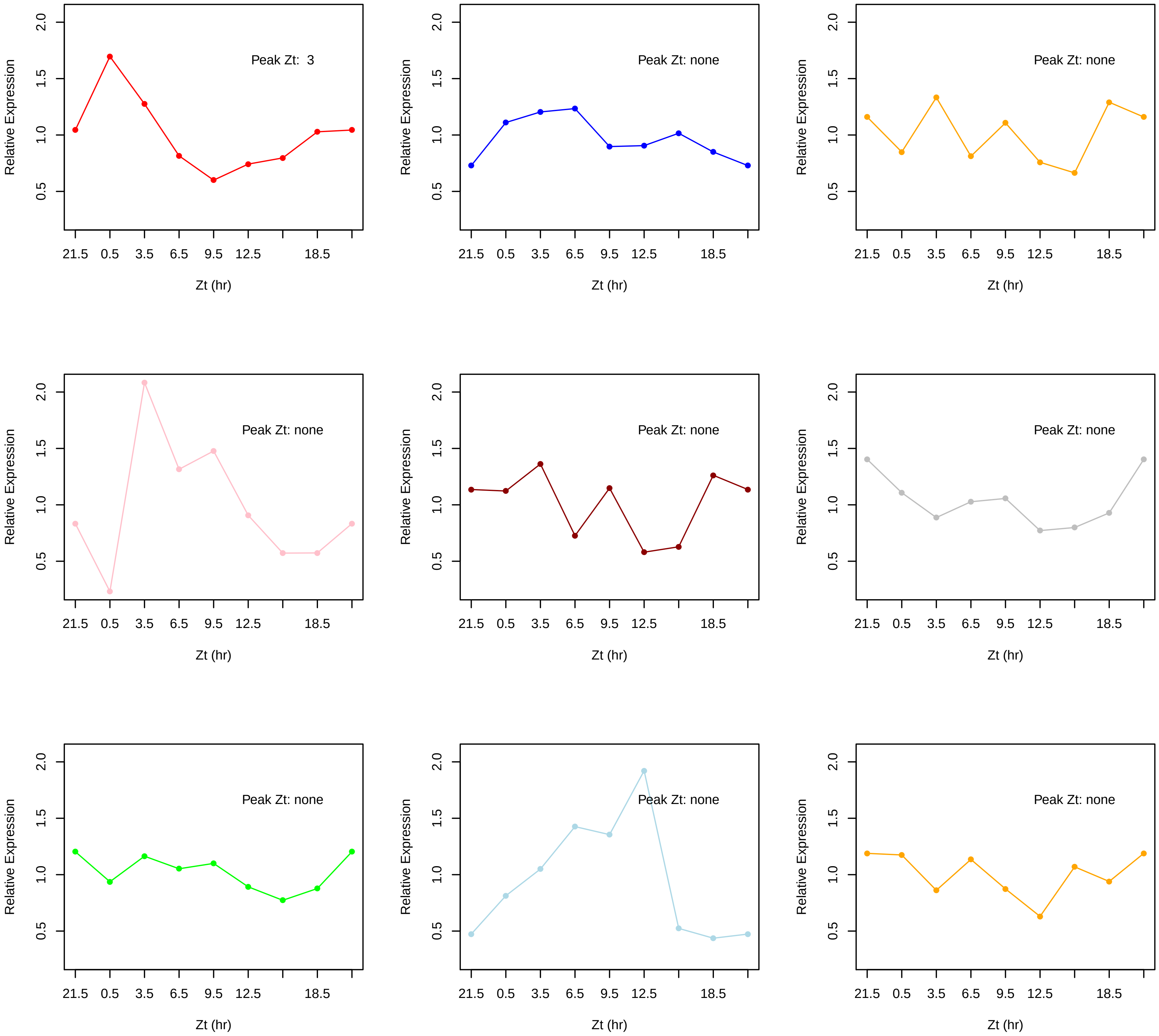

Singeleton: ENSXMAG00000012287

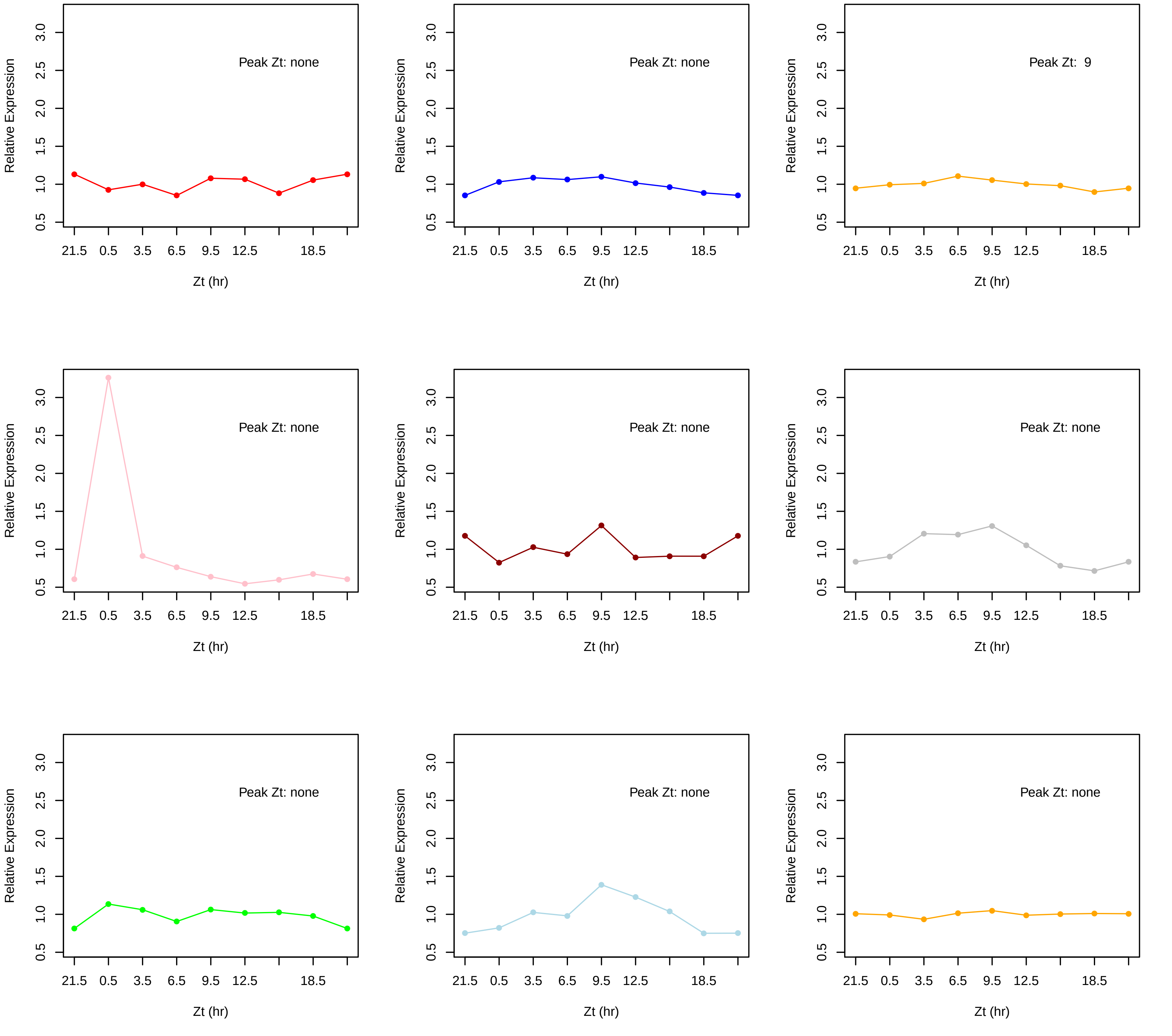

Singleton: ENSXMAG00000003861

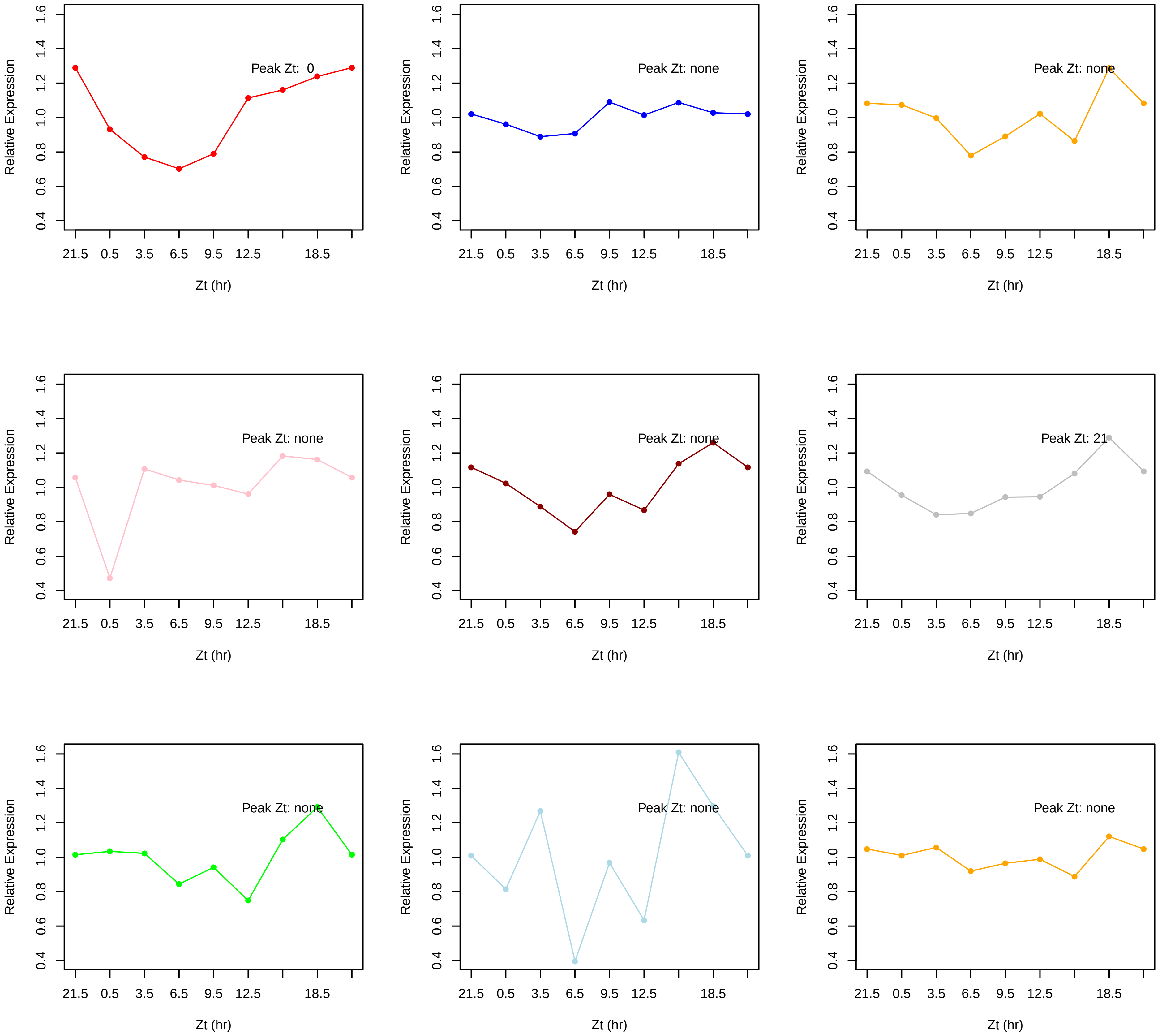

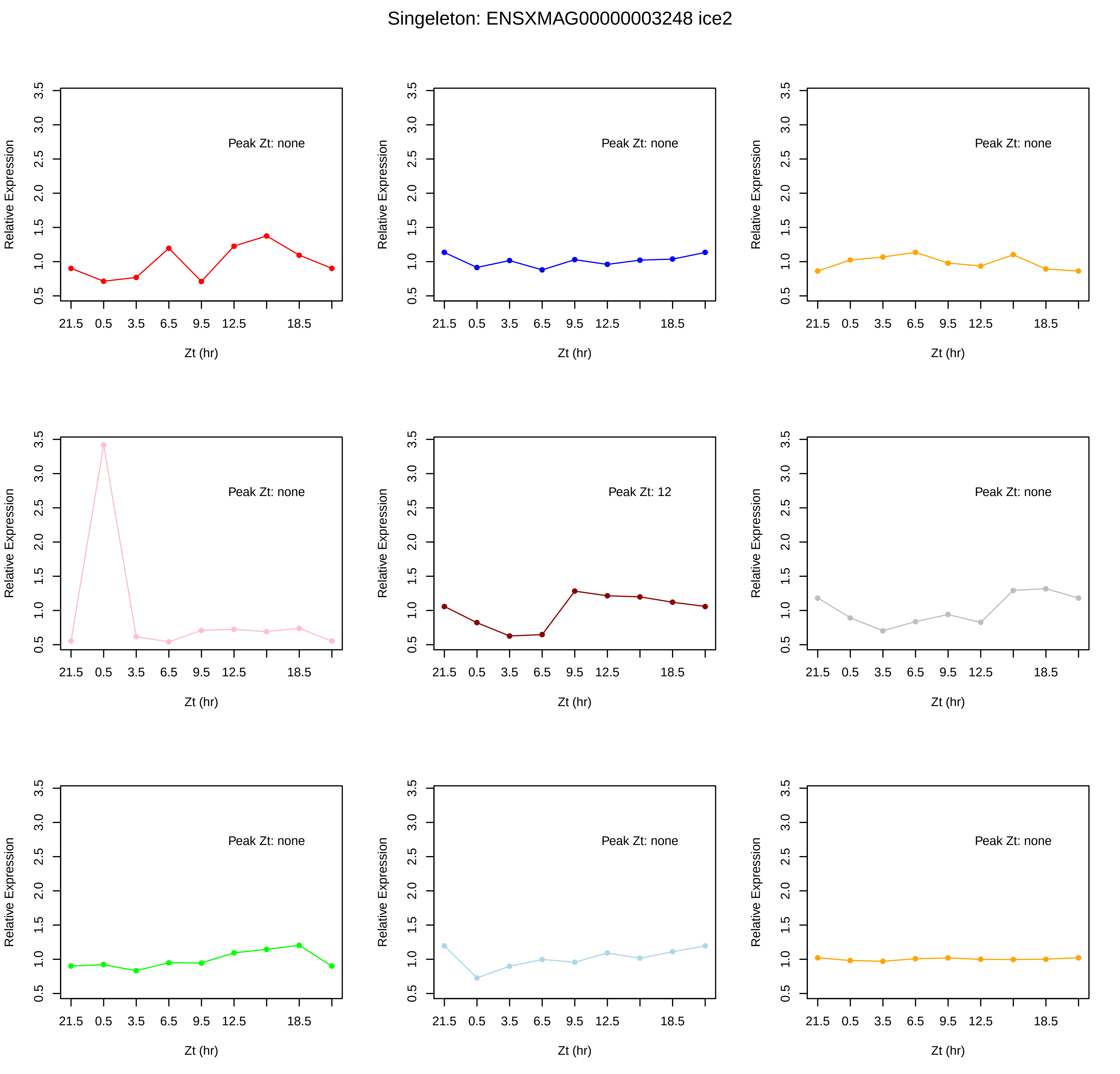
Singeleton: ENSXMAG00000003248 ice2
Relative Expression
Zt (hr)
21.5
0.5
3.5
6.5
9.5
12.5
18.5
0.5
1.0
1.5
2.0
2.5
3.0
3.5
Peak Zt: none
Peak Zt: 12

Singleton: ENSXMAG00000021004

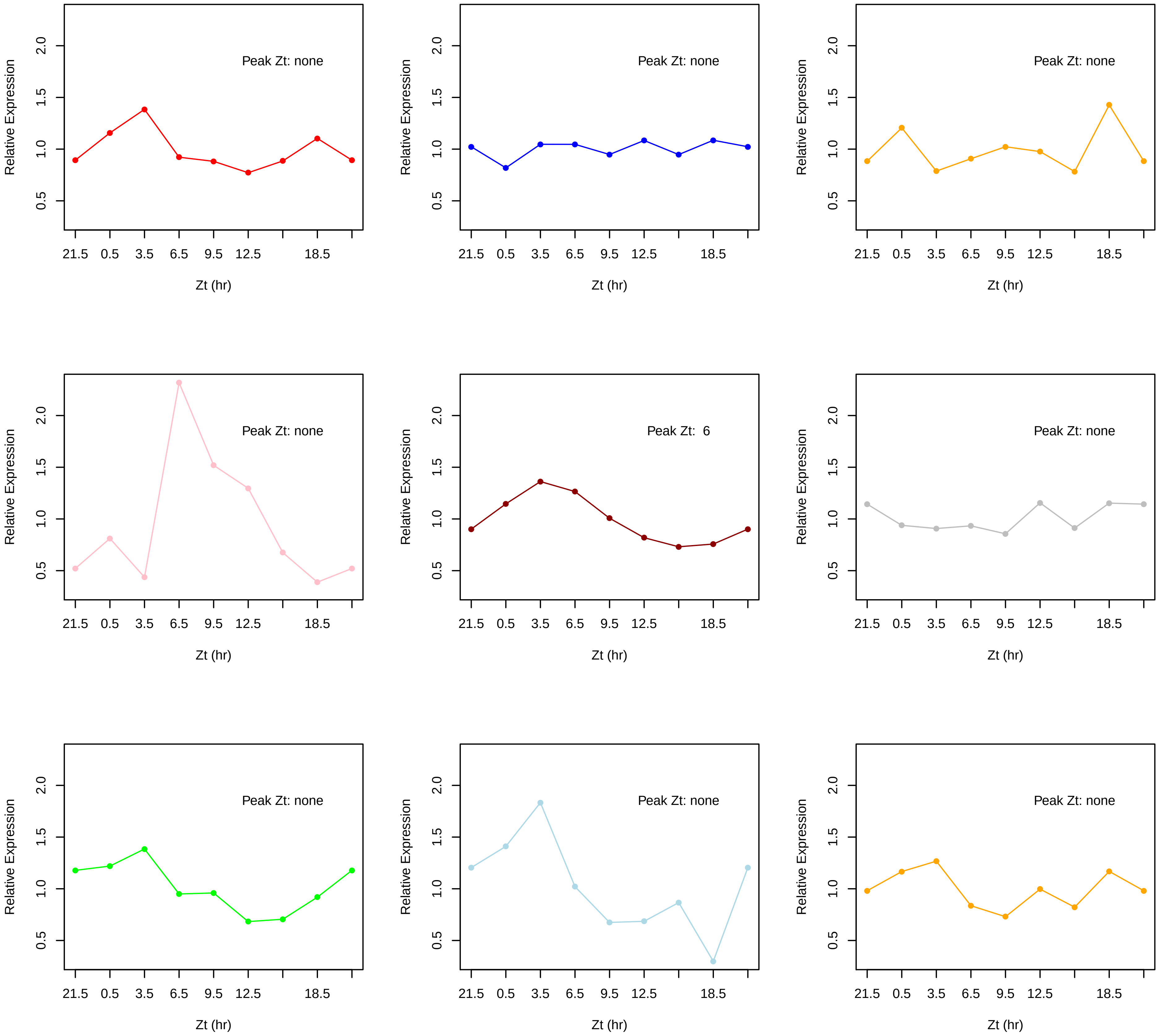

Singeleton: ENSXMAG00000003898

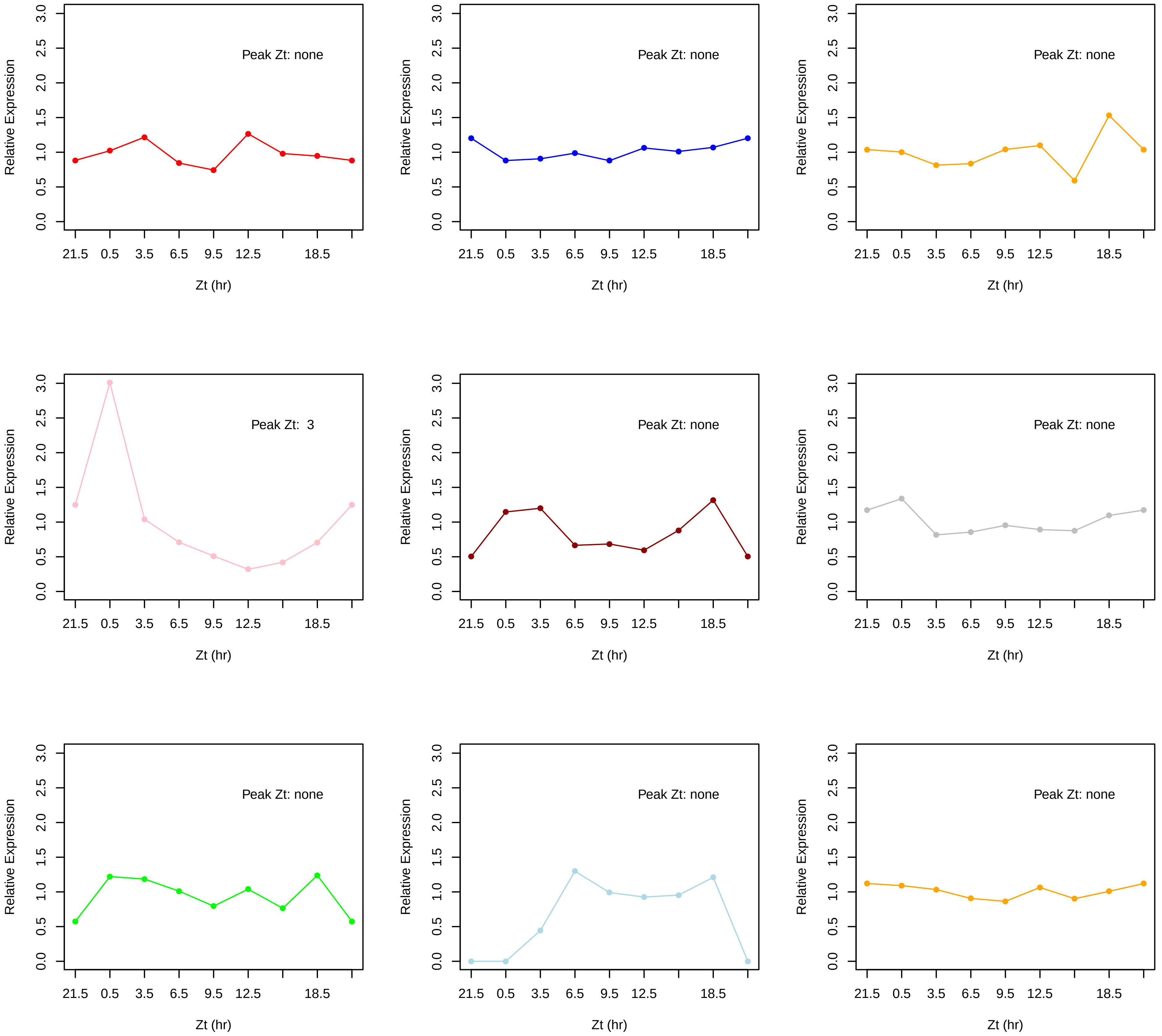

Singeleton: ENSXMAG00000027547 gtf2a2

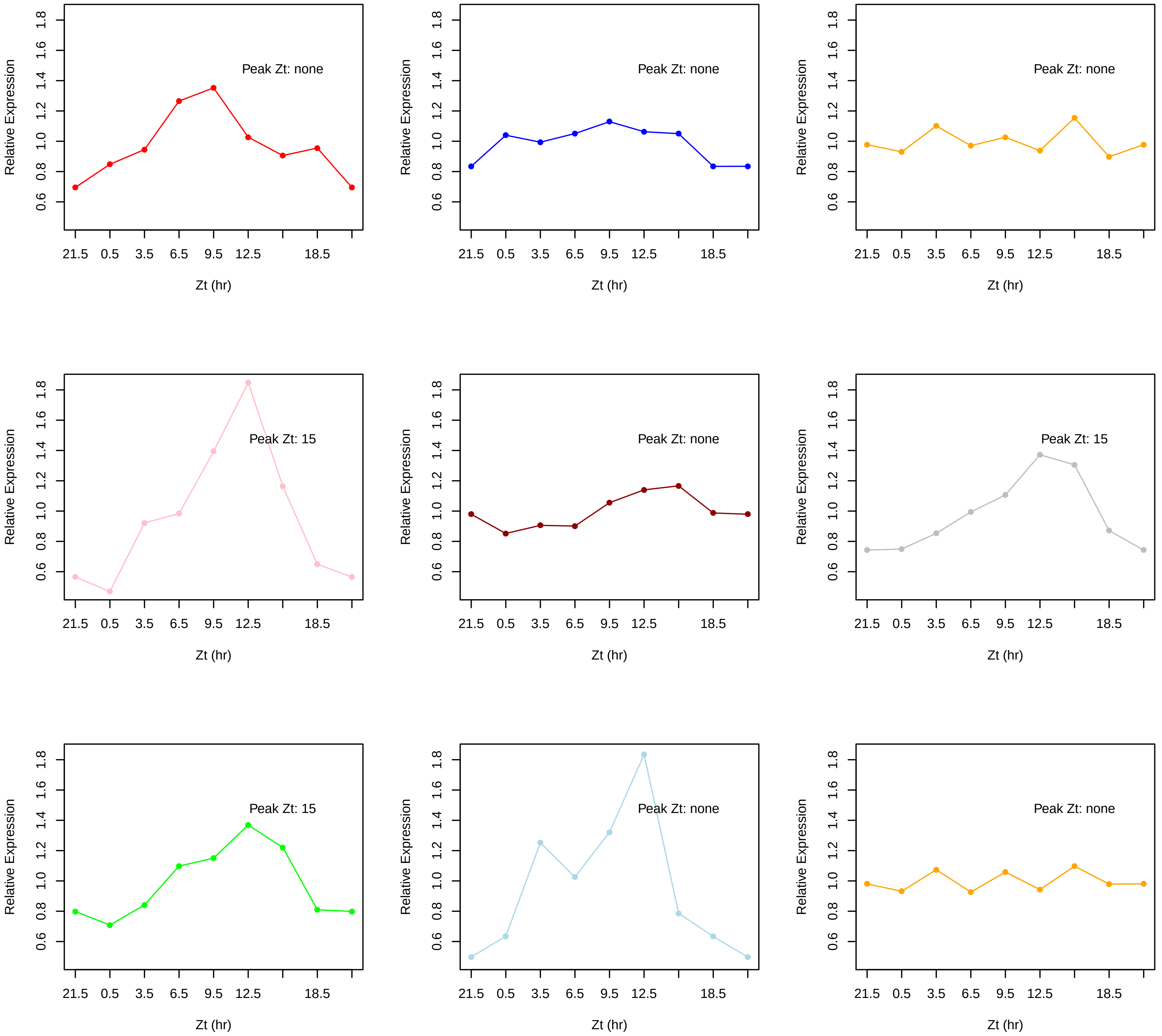

Singeleton: ENSXMAG00000010828 huwe1

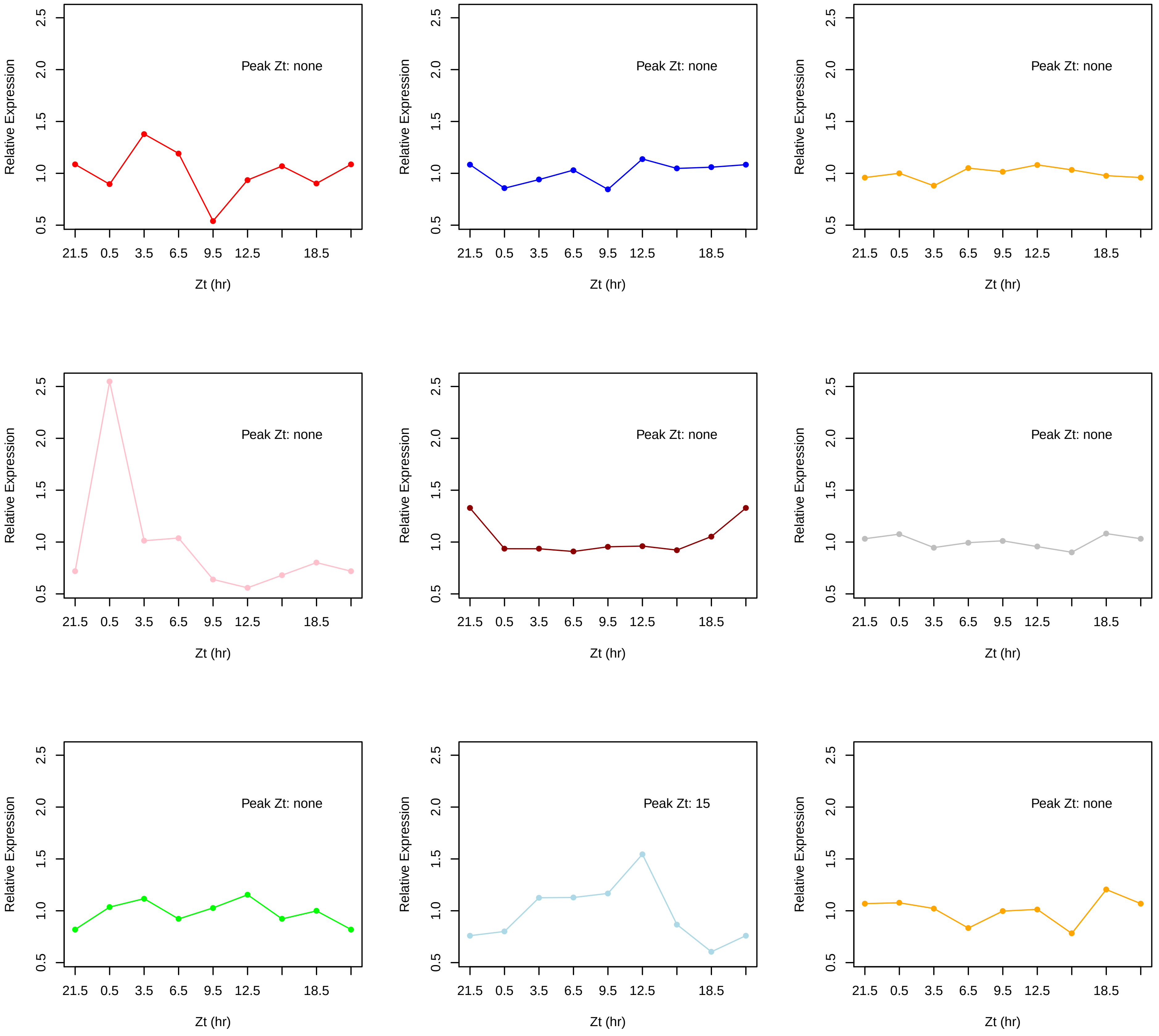

Singeleton: ENSXMAG00000015785 LONRF3

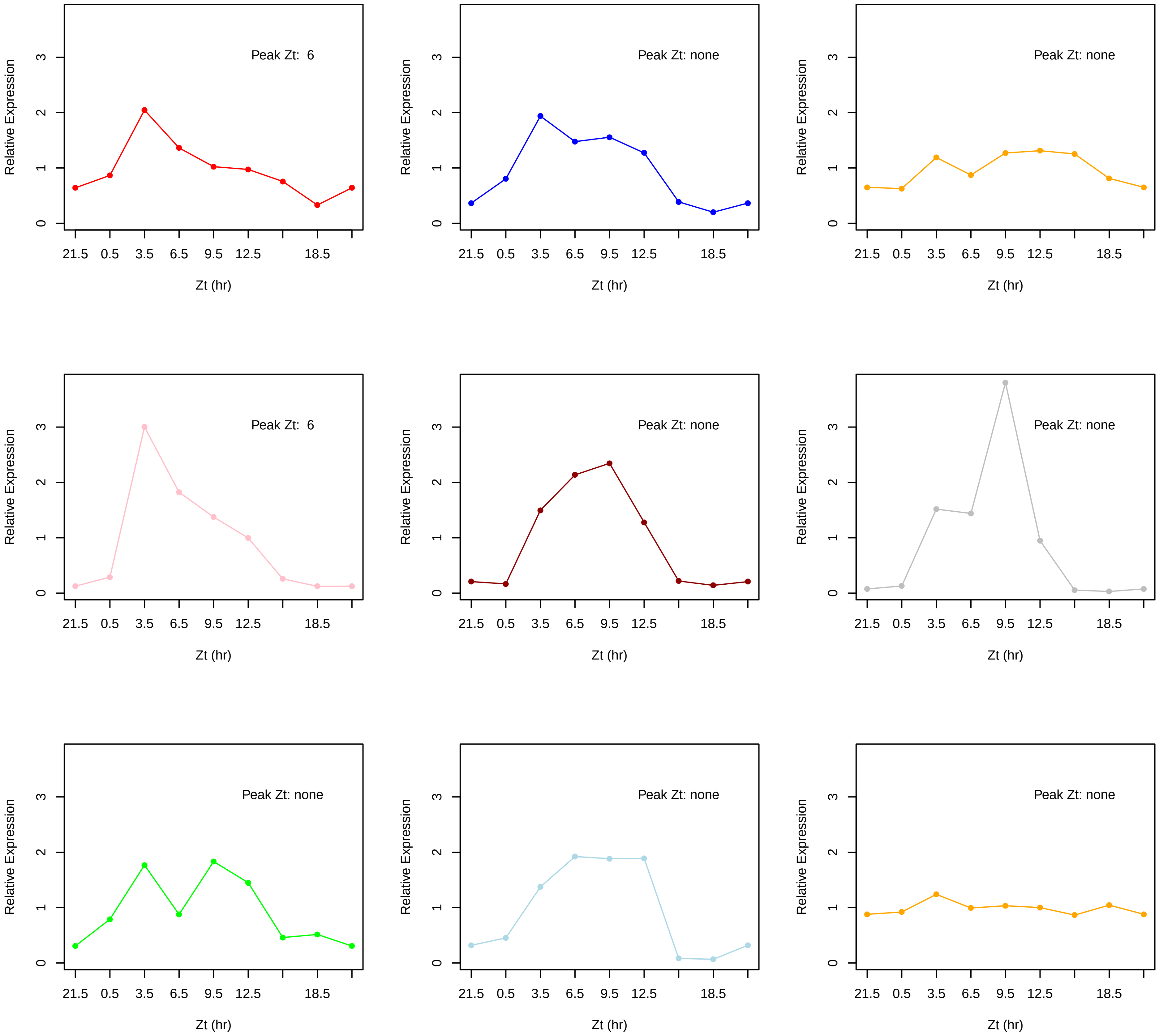

Singleton: ENSXMAG00000009563 nlgn3a

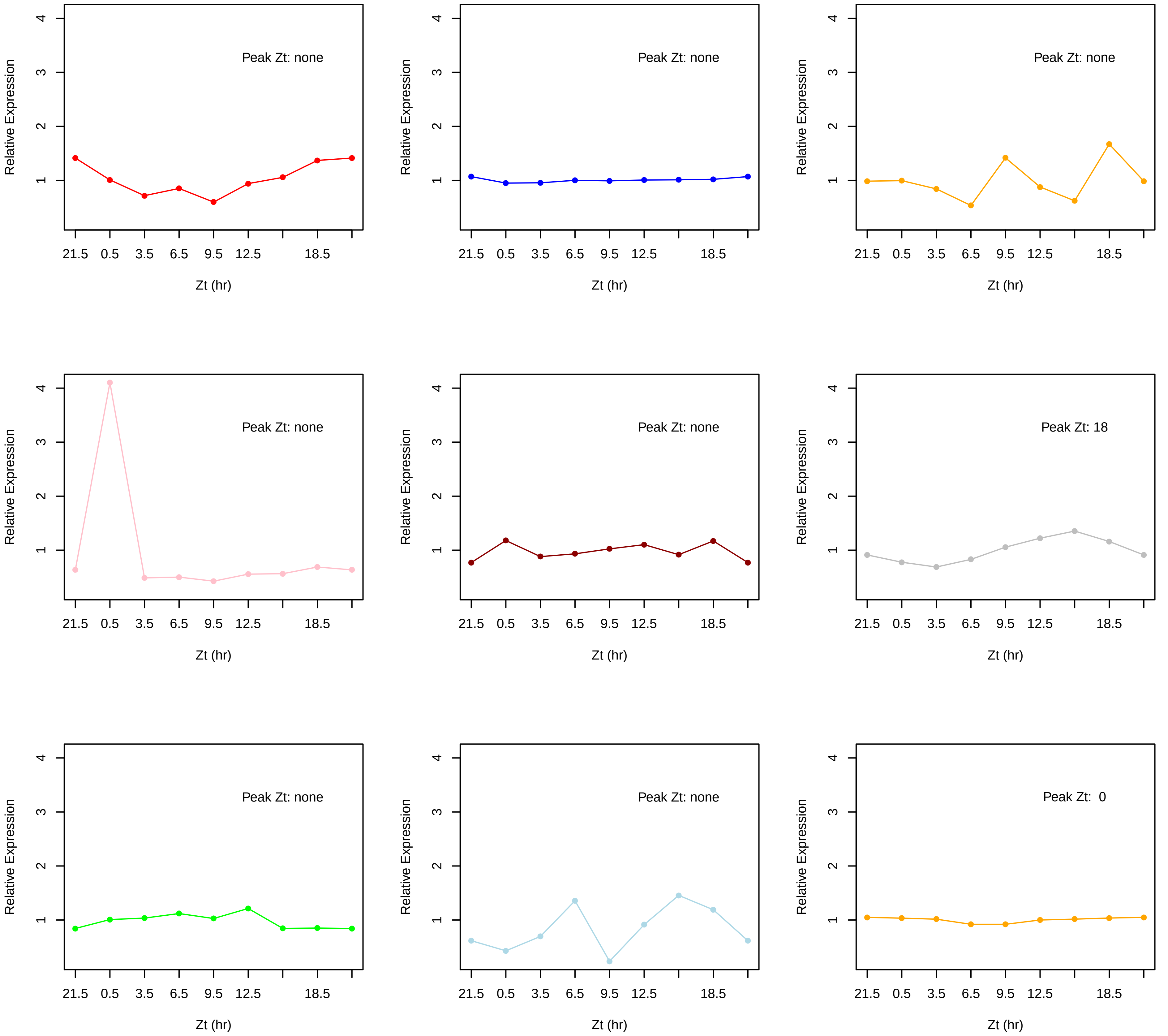

Singeleton: ENSXMAG00000013059 pld3

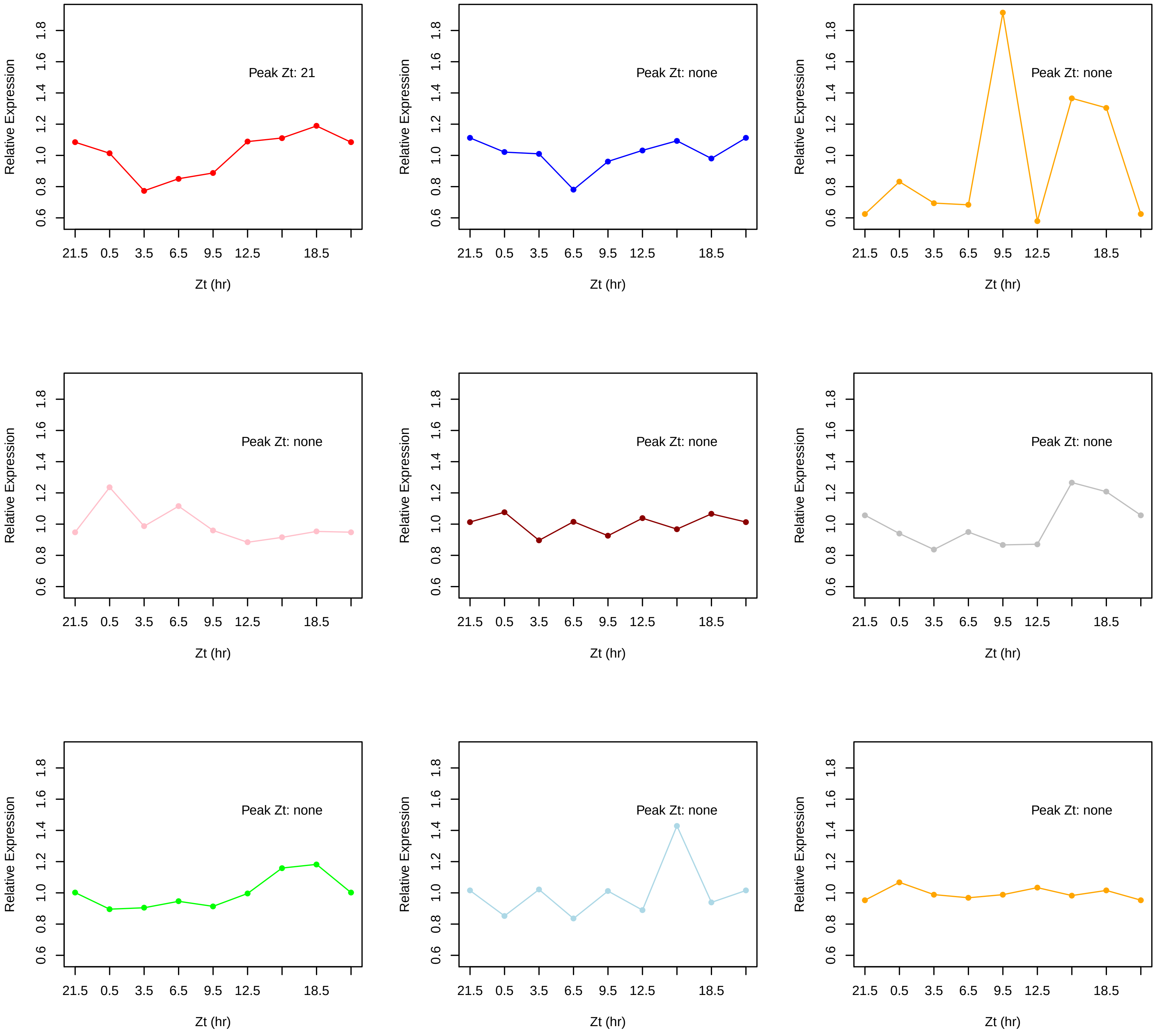

Singeleton: ENSXMAG00000003323 DNAJA4

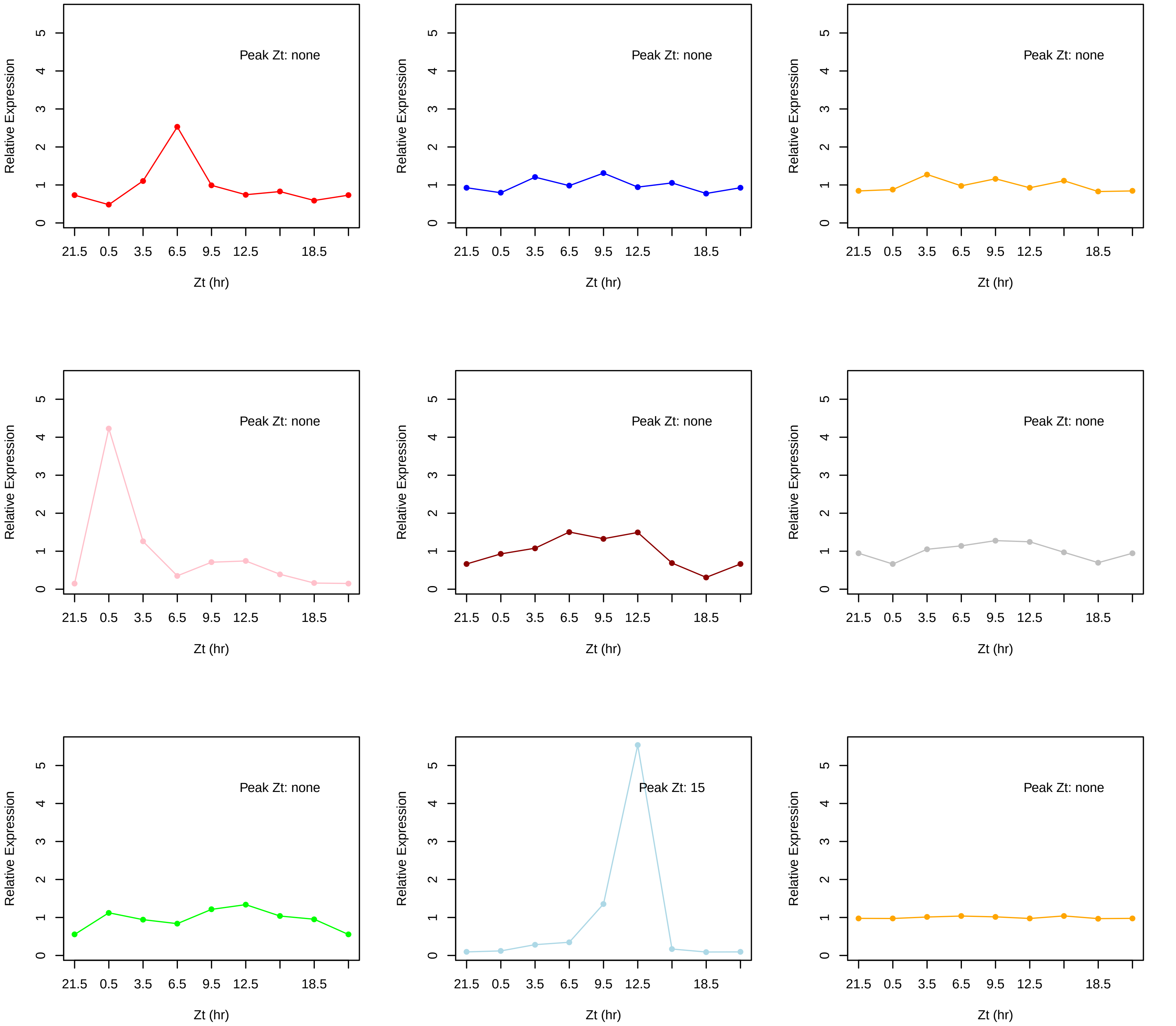

Singeleton: ENSXMAG00000021278 polr2i

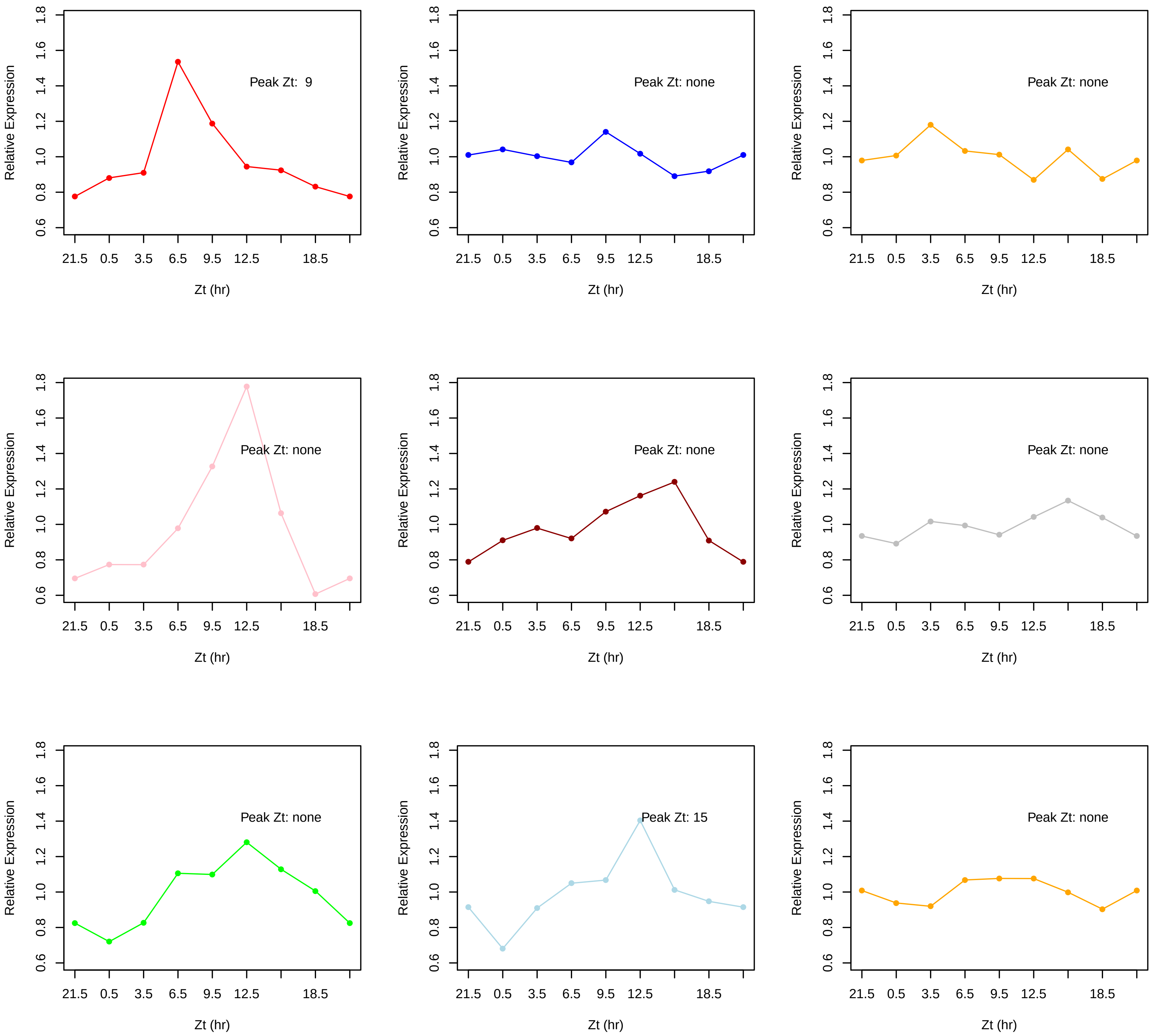

Singeleton: ENSXMAG00000001018 snrpd2

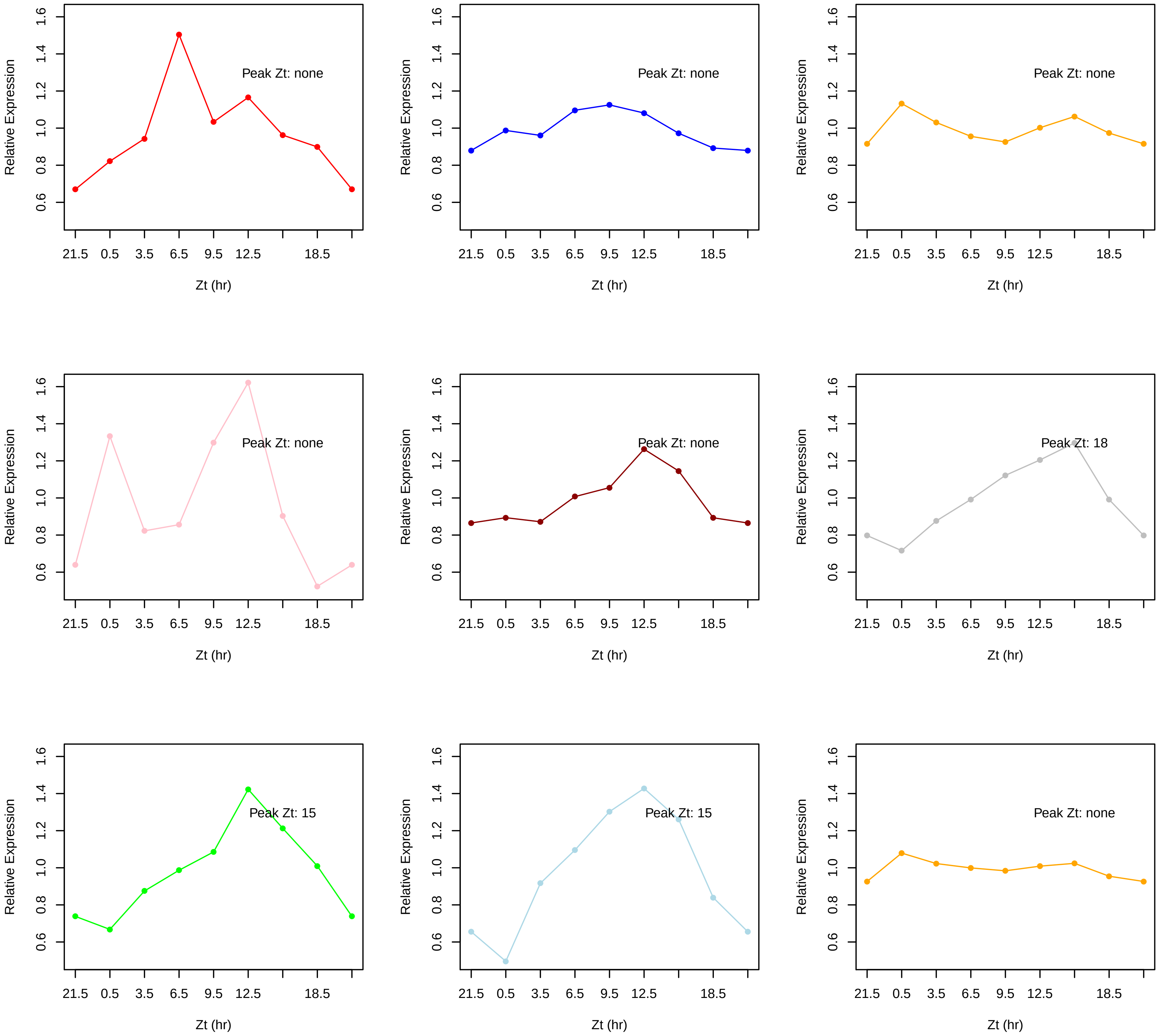

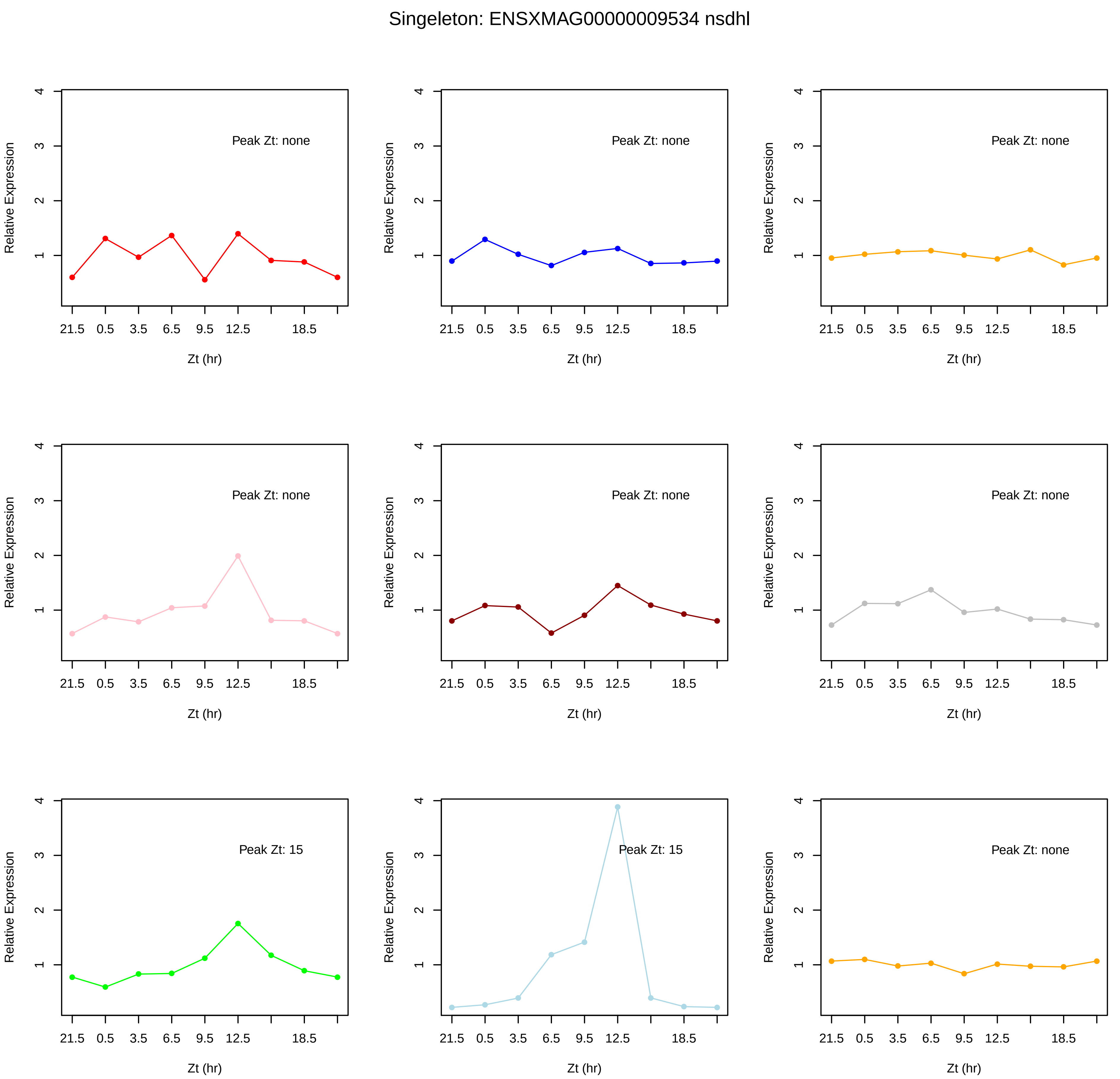
Singeleton: ENSXMAG00000009534 nsdhl
Peak Zt: none
Peak Zt: none
Peak Zt: none
Peak Zt: none
Peak Zt: none
Peak Zt: none
Peak Zt: 15
Peak Zt: 15
Peak Zt: none
Relative Expression
Zt (hr)
21.5 0.5 3.5 6.5 9.5 12.5 18.5

Singleton: ENSXMAG00000017403

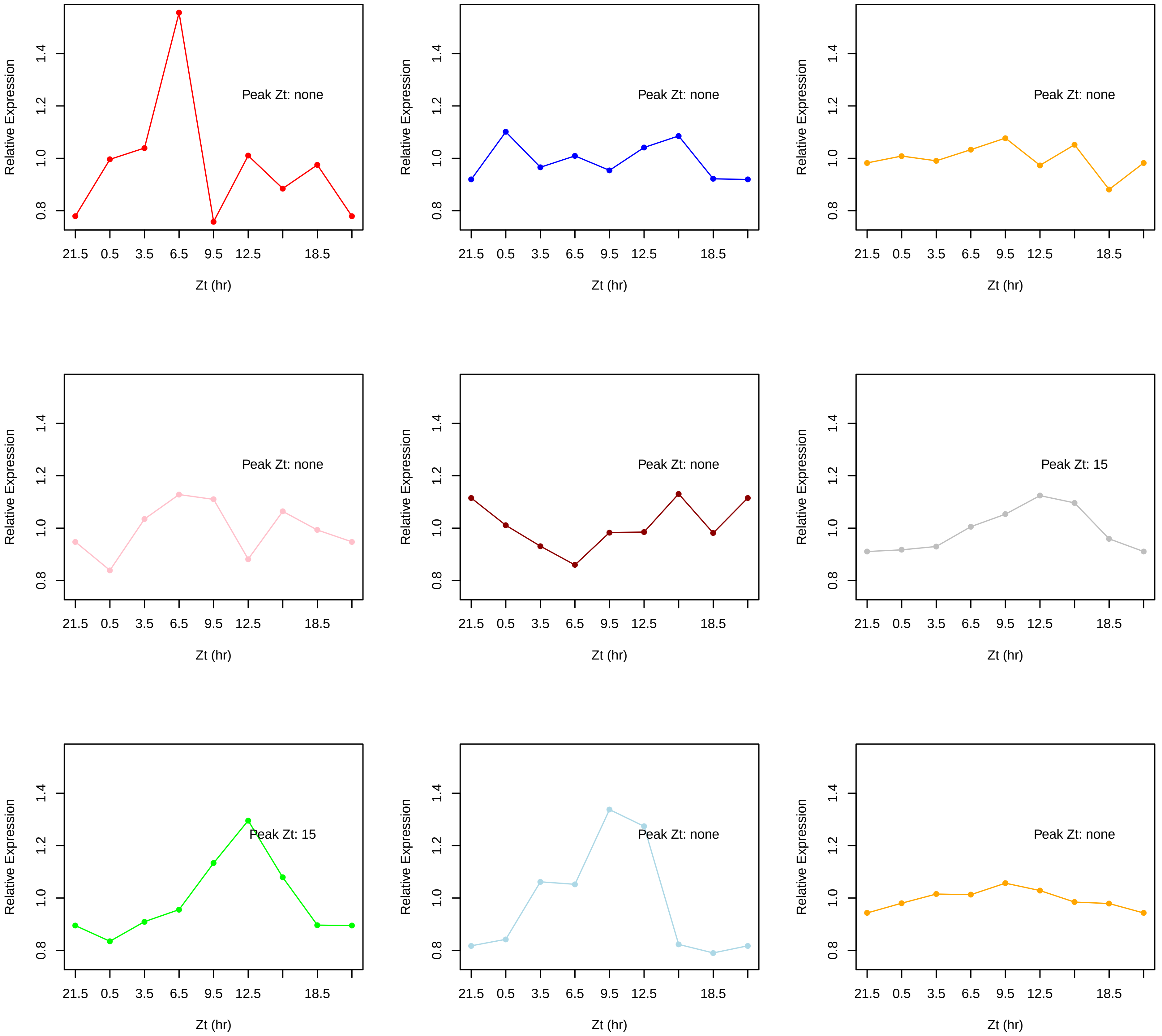

Singleton: ENSXMAG00000014318 wdr62

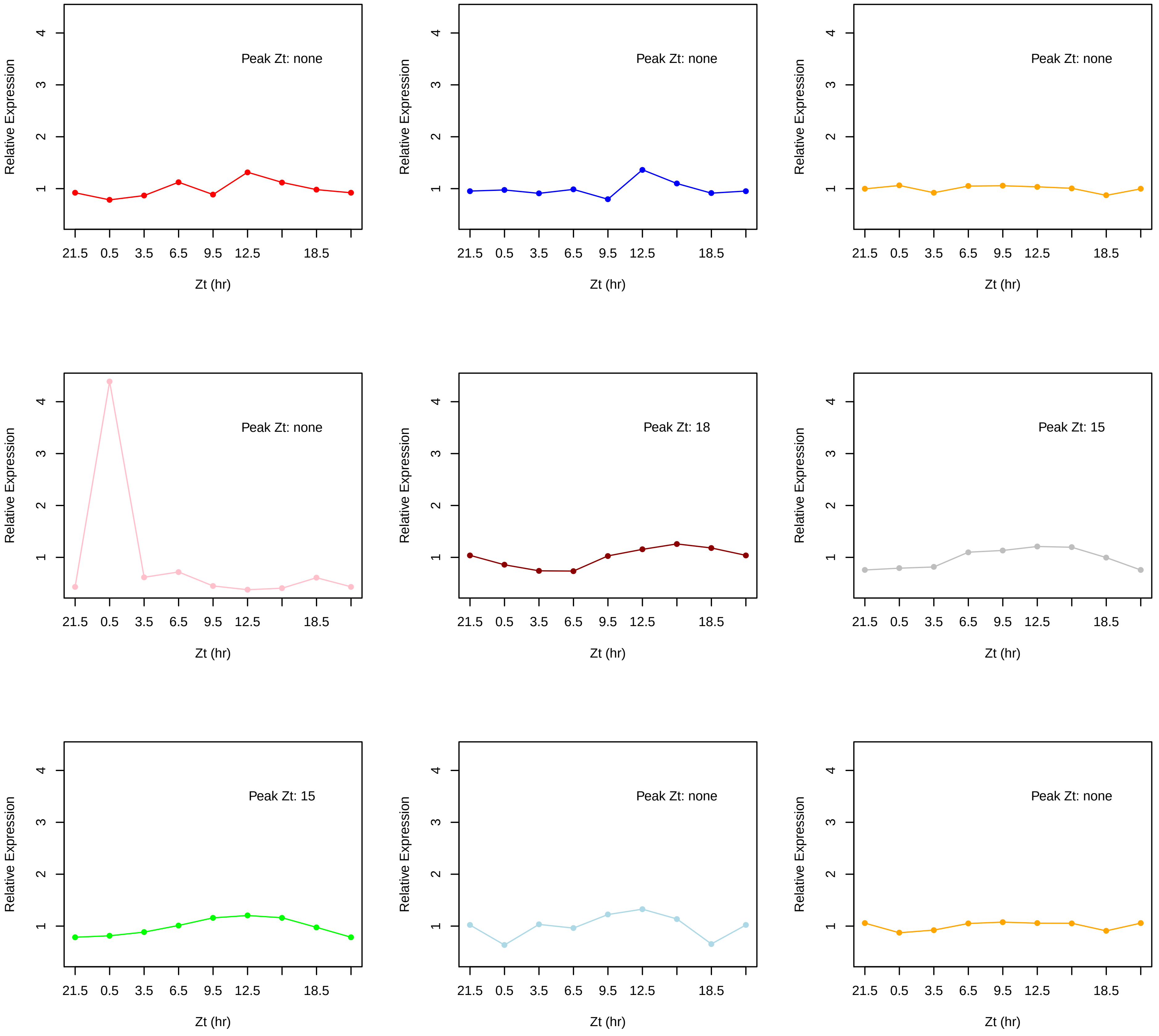

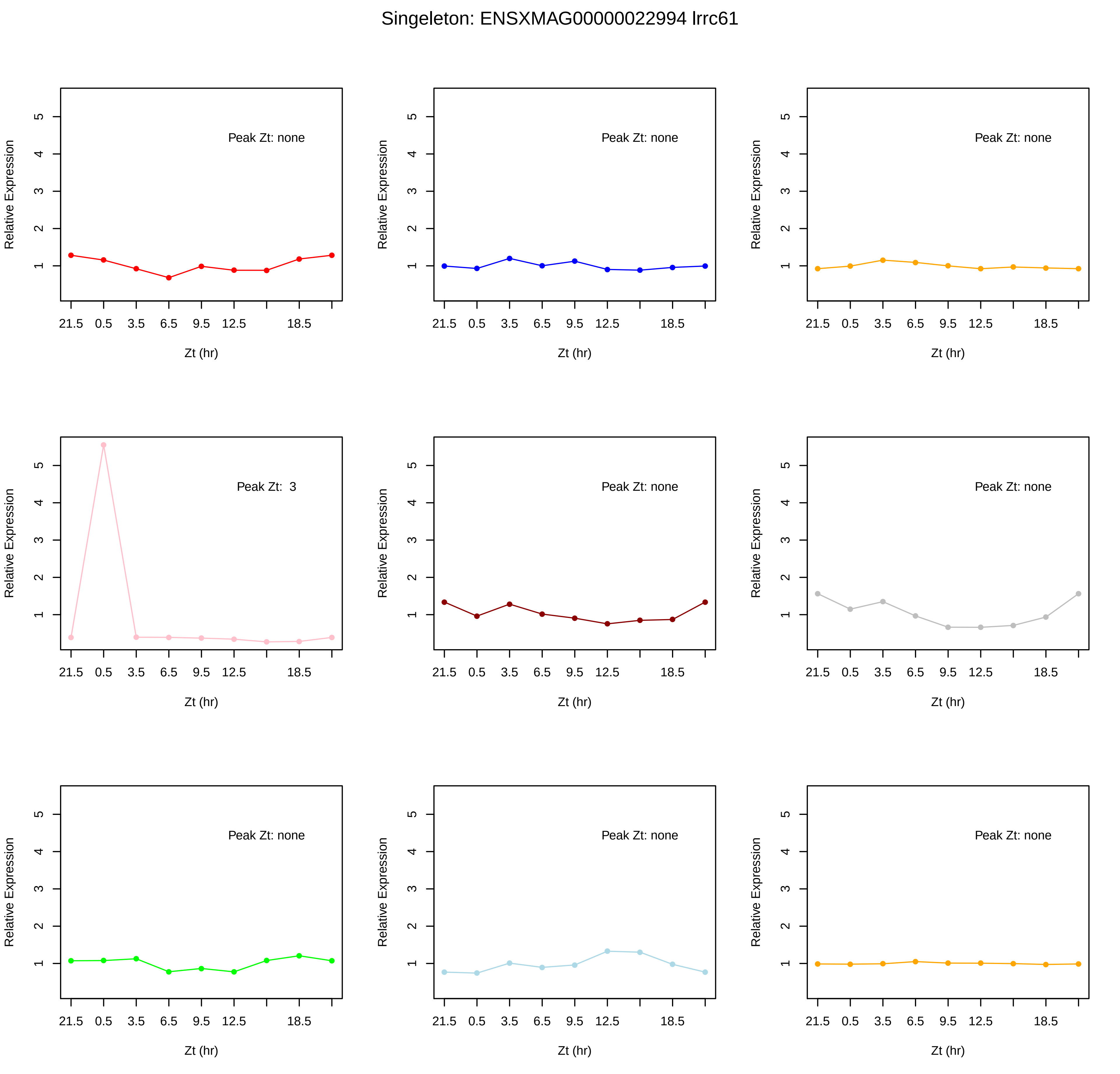
Singleton: ENSXMAG00000022994 lrrc61
Peak Zt: none
Peak Zt: none
Peak Zt: none
Peak Zt: 3
Peak Zt: none
Peak Zt: none
Peak Zt: none
Peak Zt: none
Peak Zt: none
Relative Expression
Zt (hr)
21.5
0.5
3.5
6.5
9.5
12.5
18.5

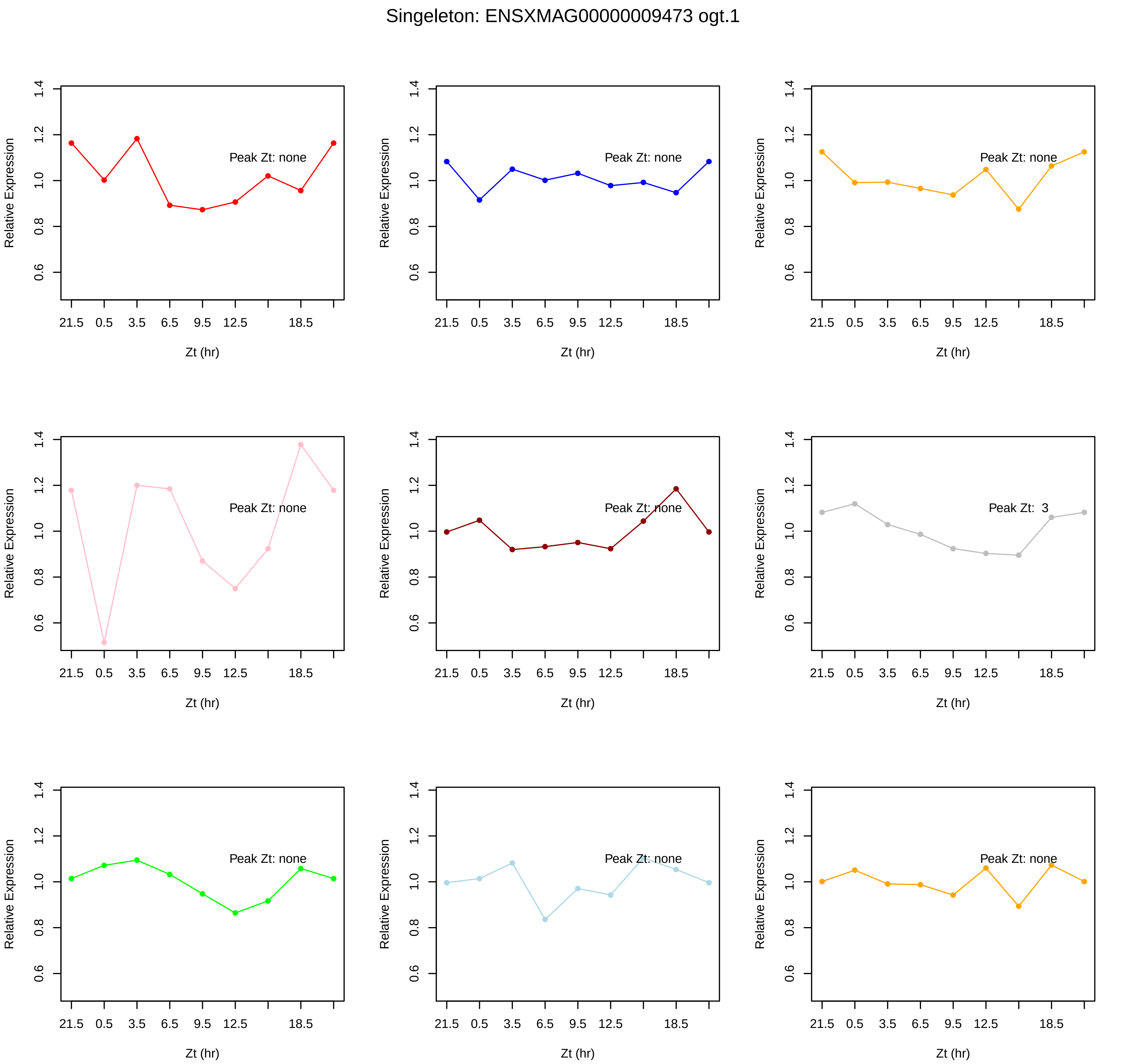
Singeleton: ENSXMAG00000009473 ogt.1
Peak Zt: none
Peak Zt: none
Peak Zt: none
Peak Zt: none
Peak Zt: none
Peak Zt: 3
Peak Zt: none
Peak Zt: none
Peak Zt: none
Relative Expression
Zt (hr)
21.5
0.5
3.5
6.5
9.5
12.5
18.5
0.6
0.8
1.0
1.2
1.4

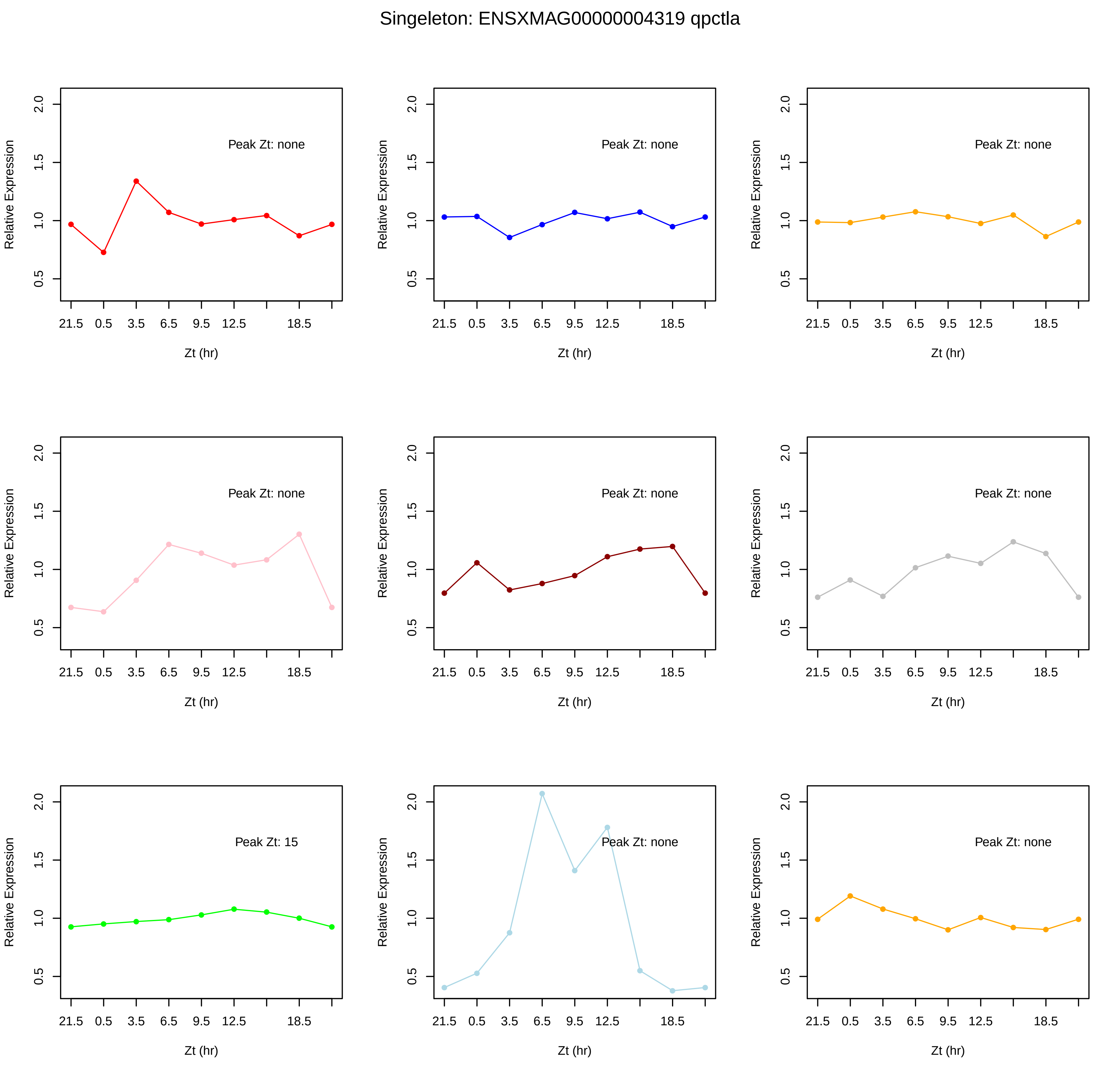
Singleton: ENSXMAG00000004319 qpctla
Peak Zt: none
Peak Zt: none
Peak Zt: none
Peak Zt: none
Peak Zt: none
Peak Zt: none
Peak Zt: 15
Peak Zt: none
Peak Zt: none
Relative Expression
Zt (hr)
21.5
0.5
3.5
6.5
9.5
12.5
18.5
0.5
1.0
1.5
2.0

Singeleton: ENSXMAG00000004309 tbcb

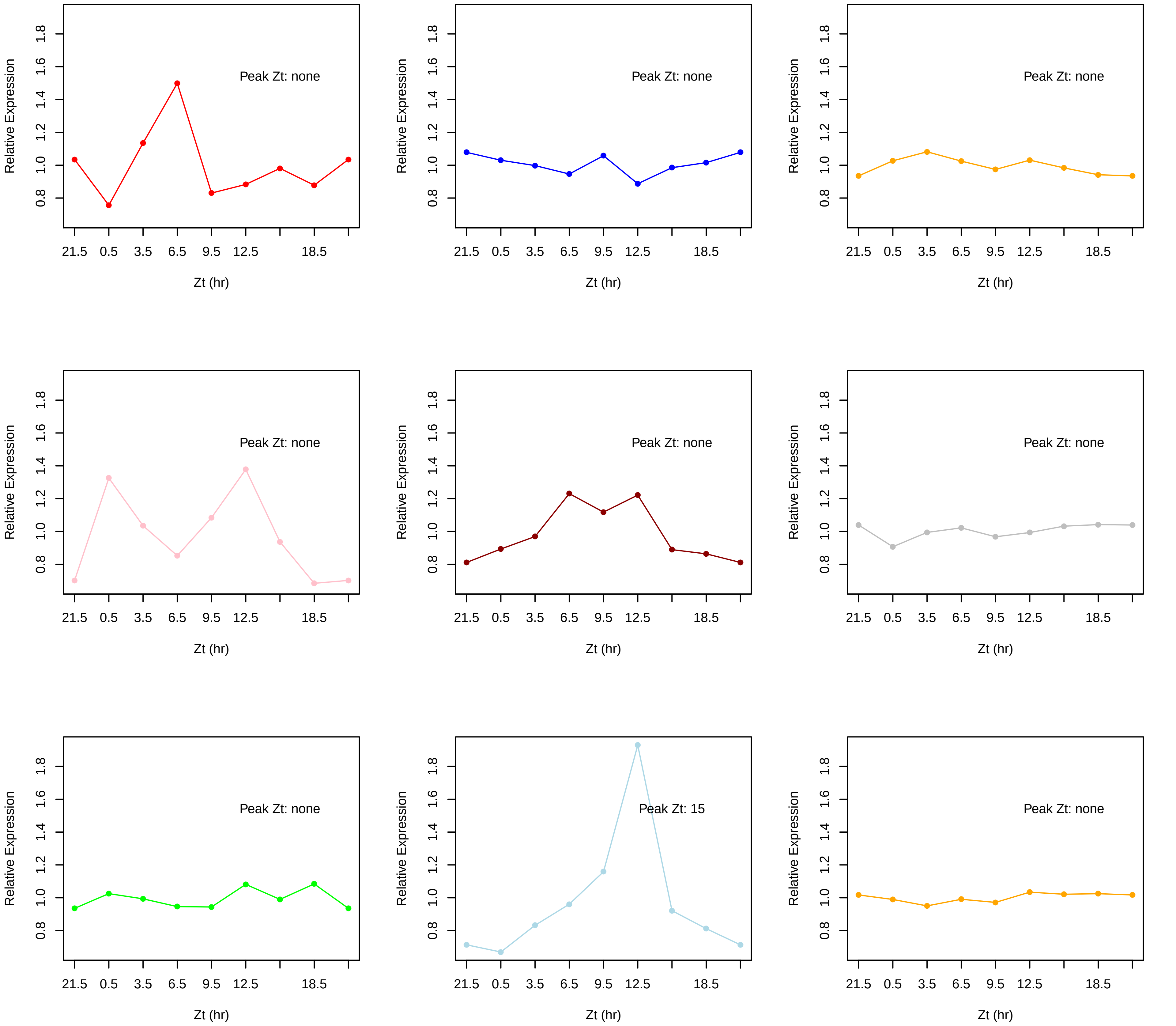

Singeleton: ENSXMAG00000004298 clptm1

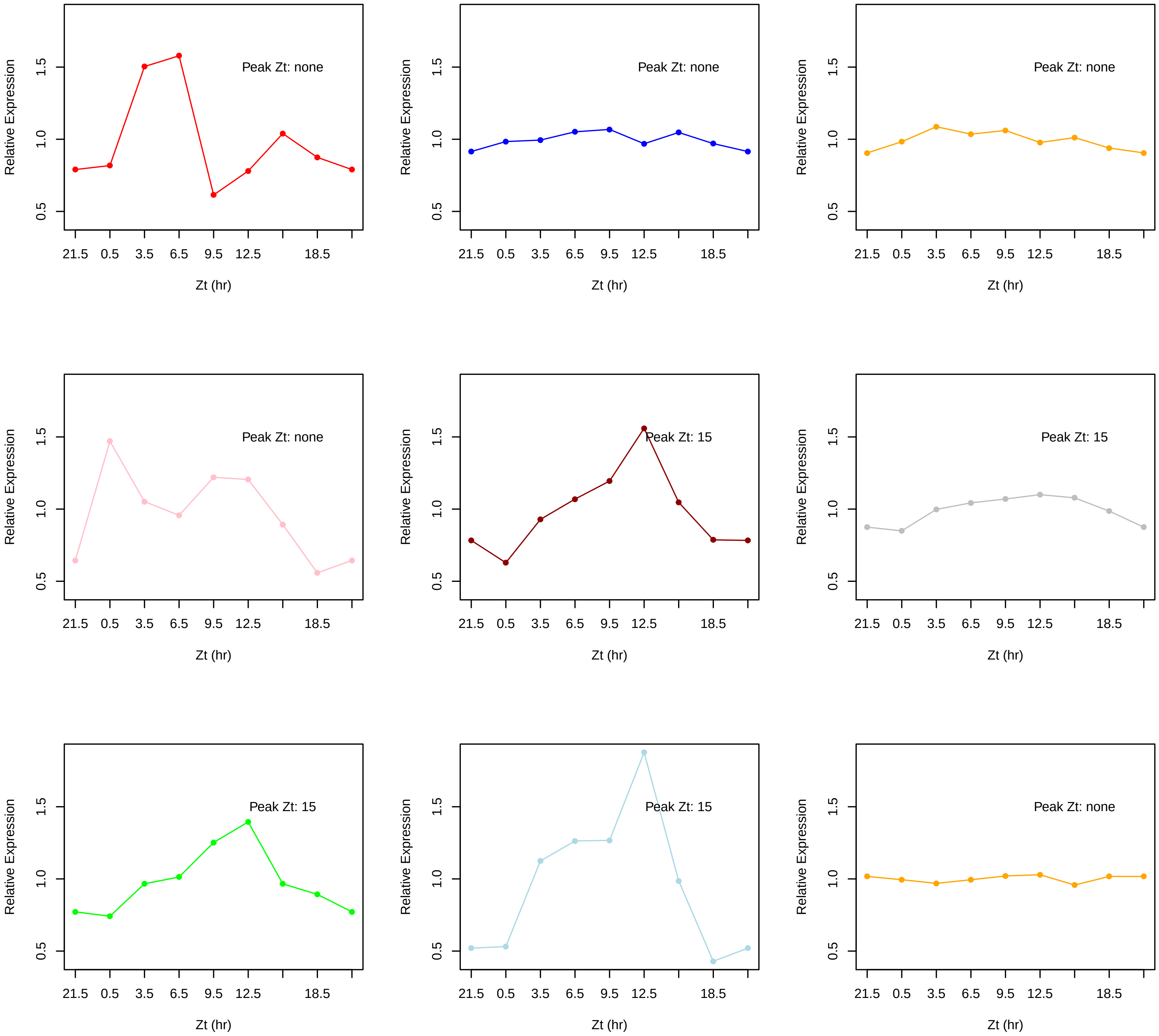

Singeleton: ENSXMAG00000011406 taf1

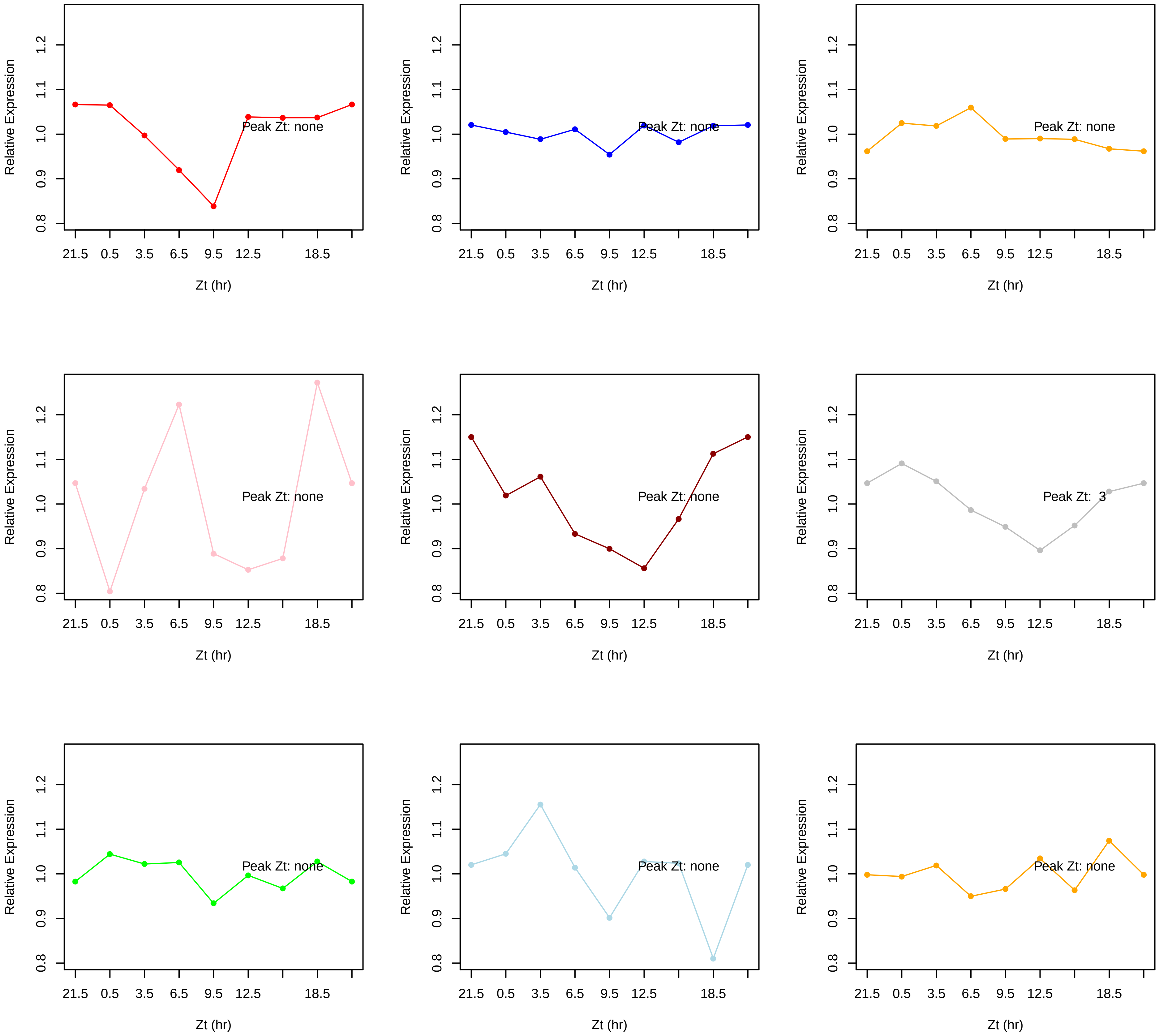

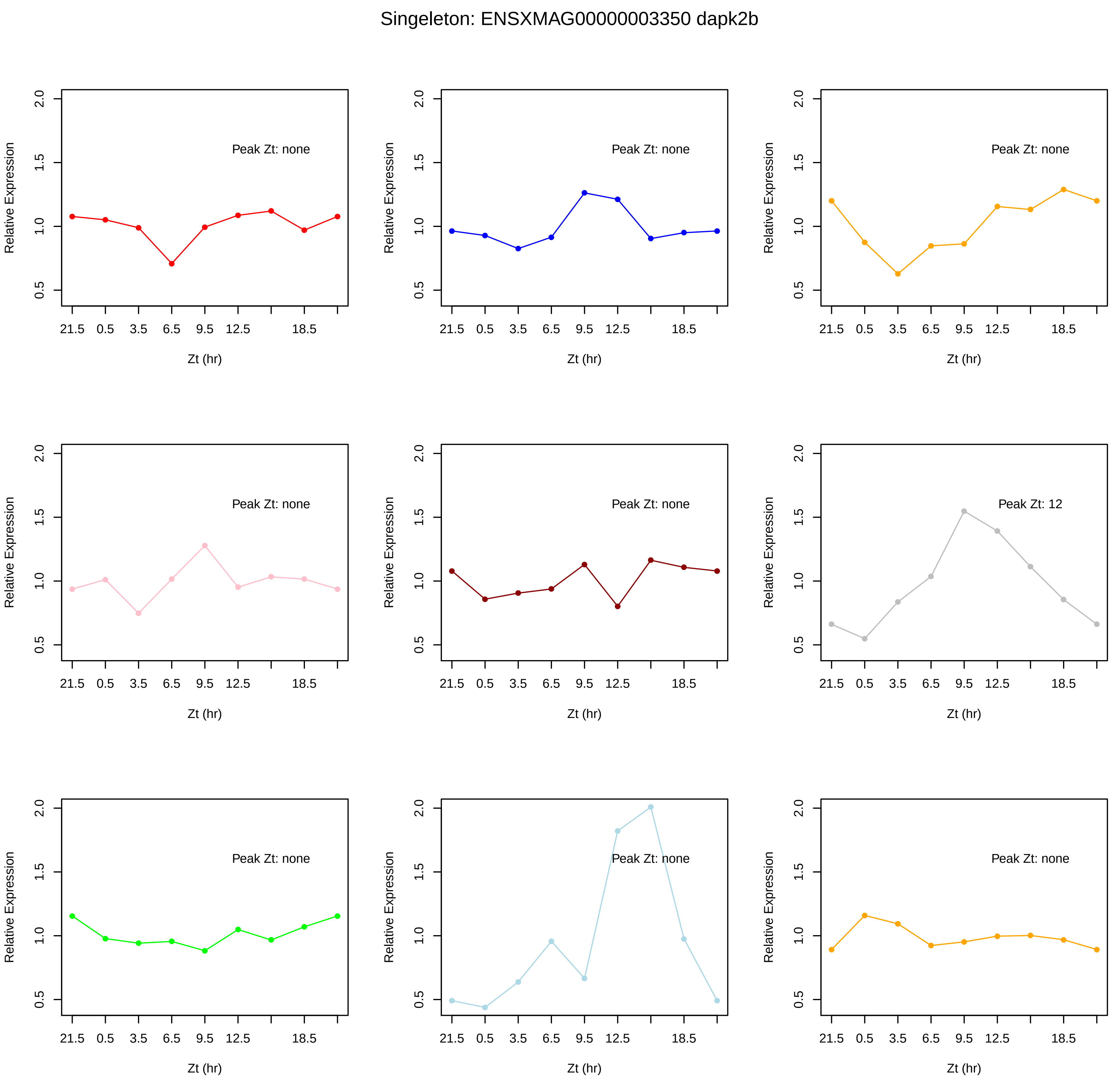
Singeleton: ENSXMAG00000003350 dapk2b
Peak Zt: none
Peak Zt: none
Peak Zt: none
Peak Zt: none
Peak Zt: none
Peak Zt: 12
Peak Zt: none
Peak Zt: none
Peak Zt: none
Relative Expression
Zt (hr)
21.5
0.5
3.5
6.5
9.5
12.5
18.5
0.5
1.0
1.5
2.0

Singeleton: ENSXMAG00000004297 relb

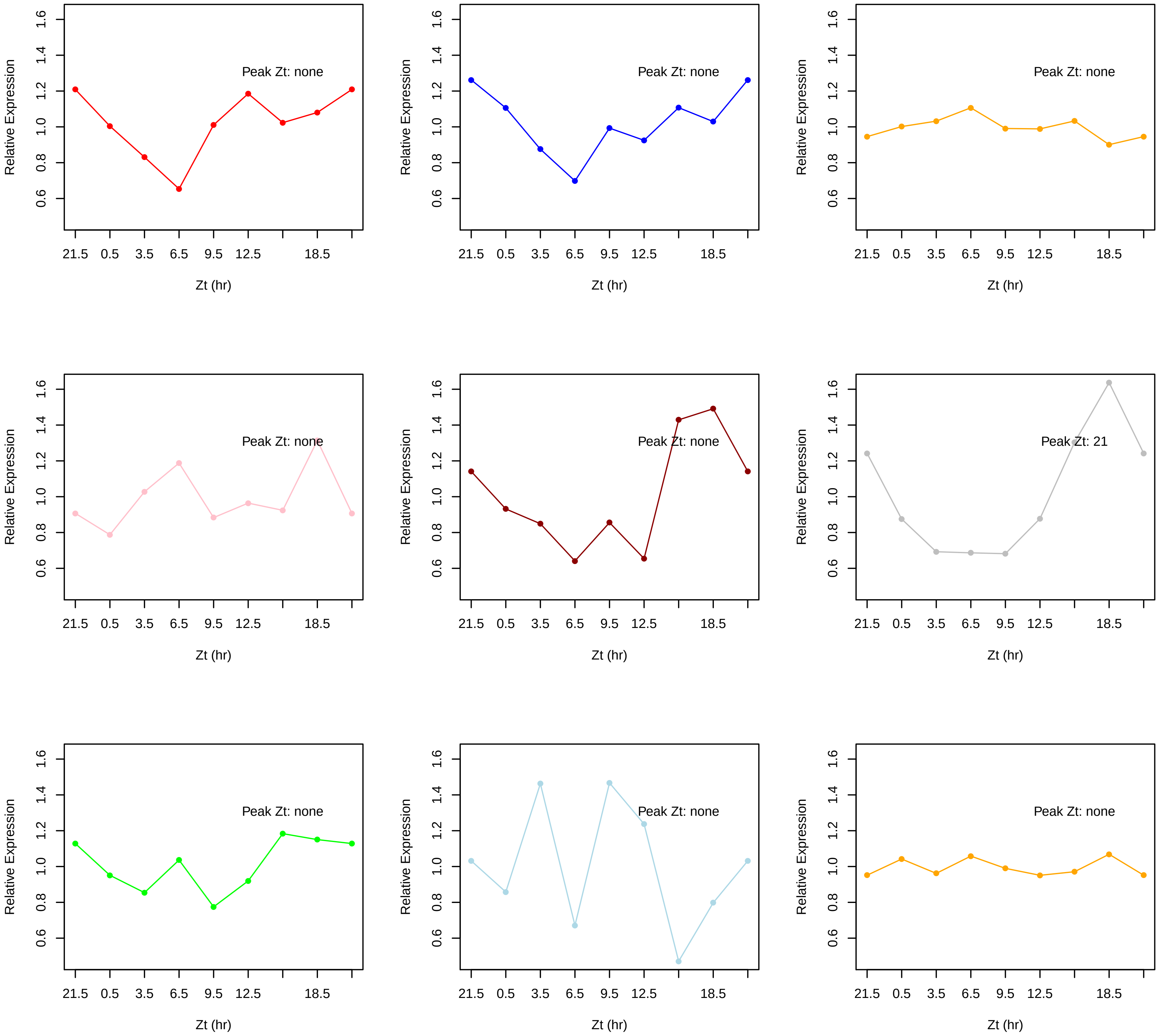

Singeleton: ENSXMAG00000000652

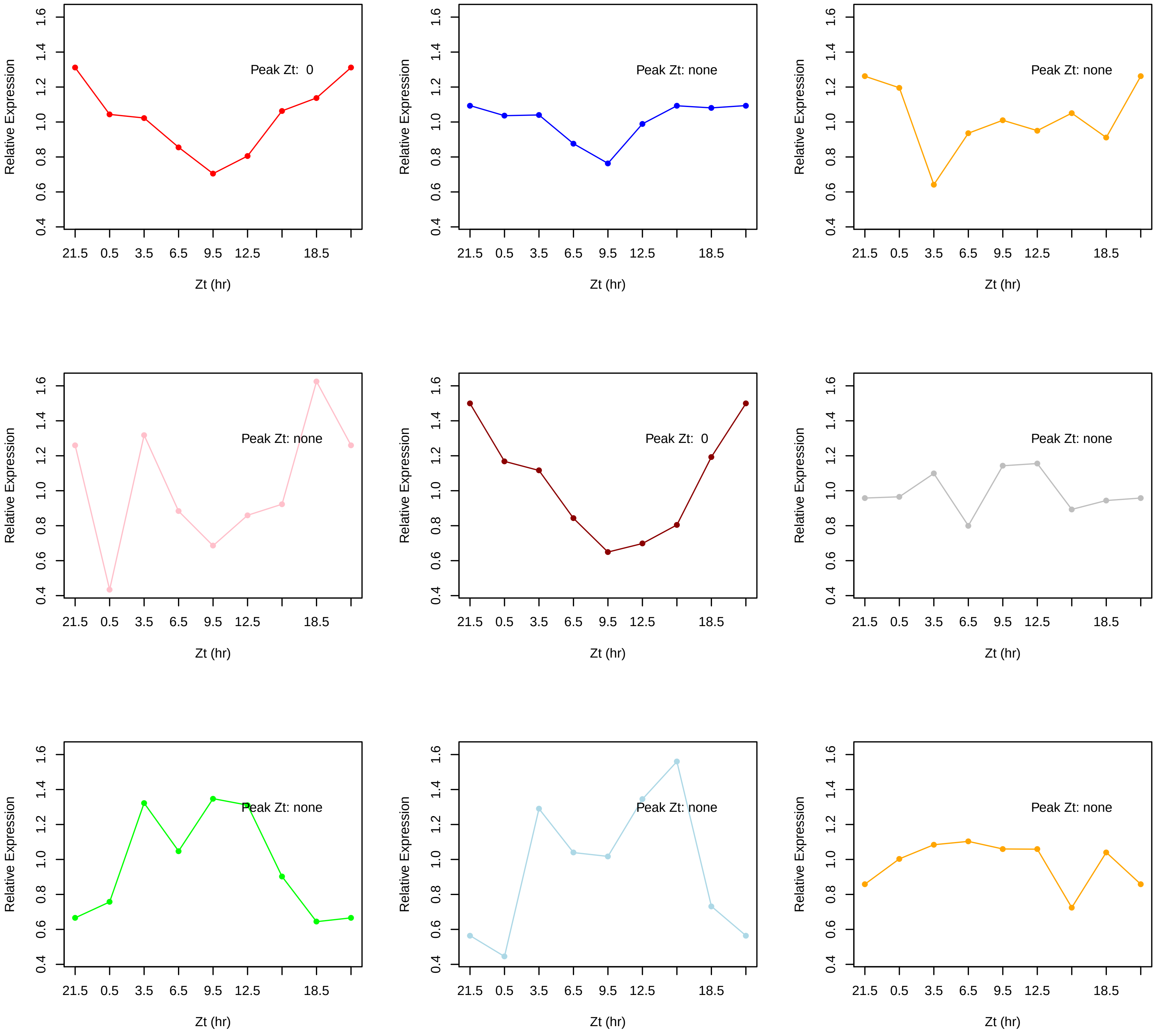

Singleton: ENSXMAG00000026233

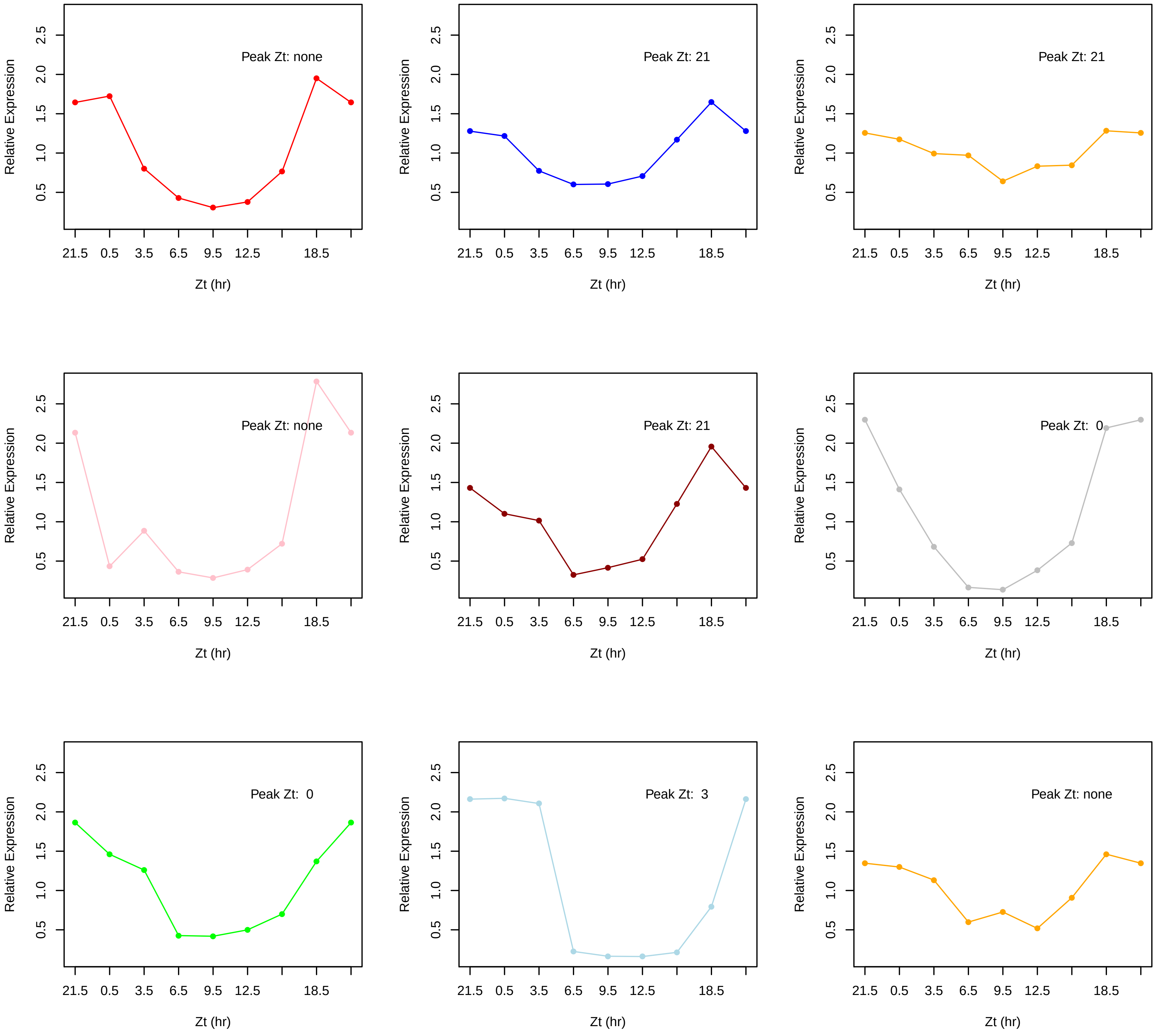

Singleton: ENSXMAG00000003361 fbxl22

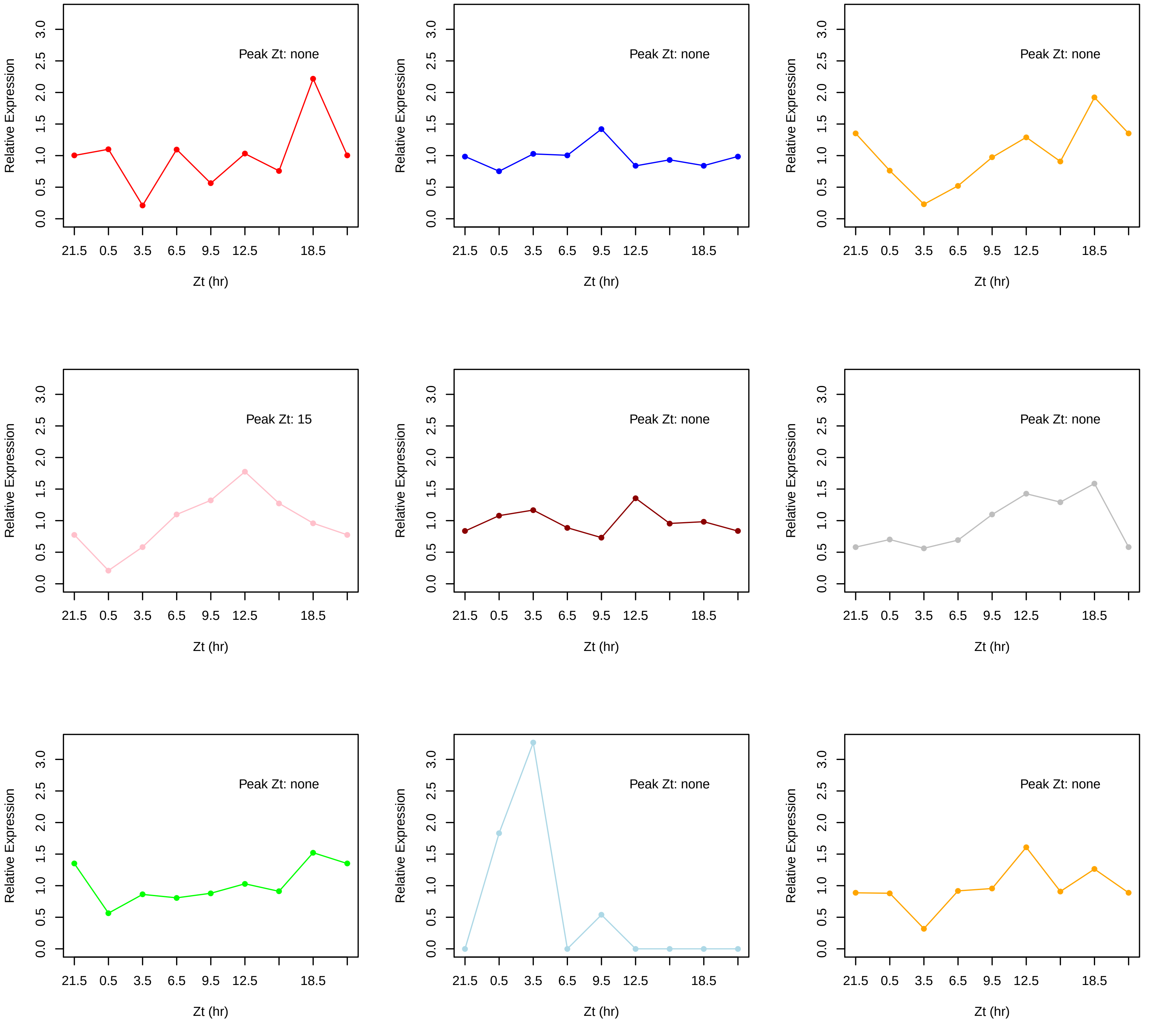

Singeleton: ENSXMAG00000003362 usp3

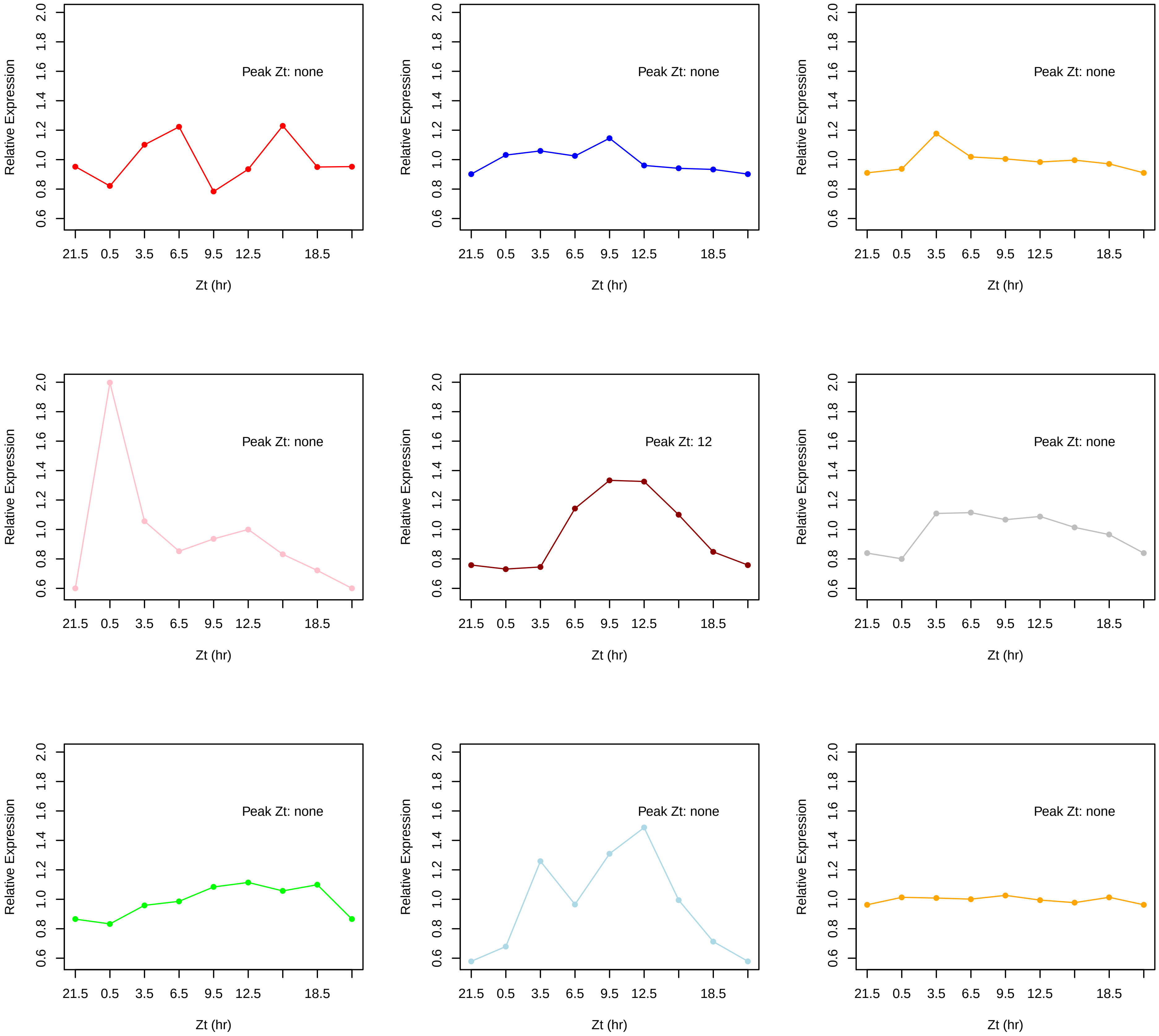

Singeleton: ENSXMAG00000009419 frmpd3

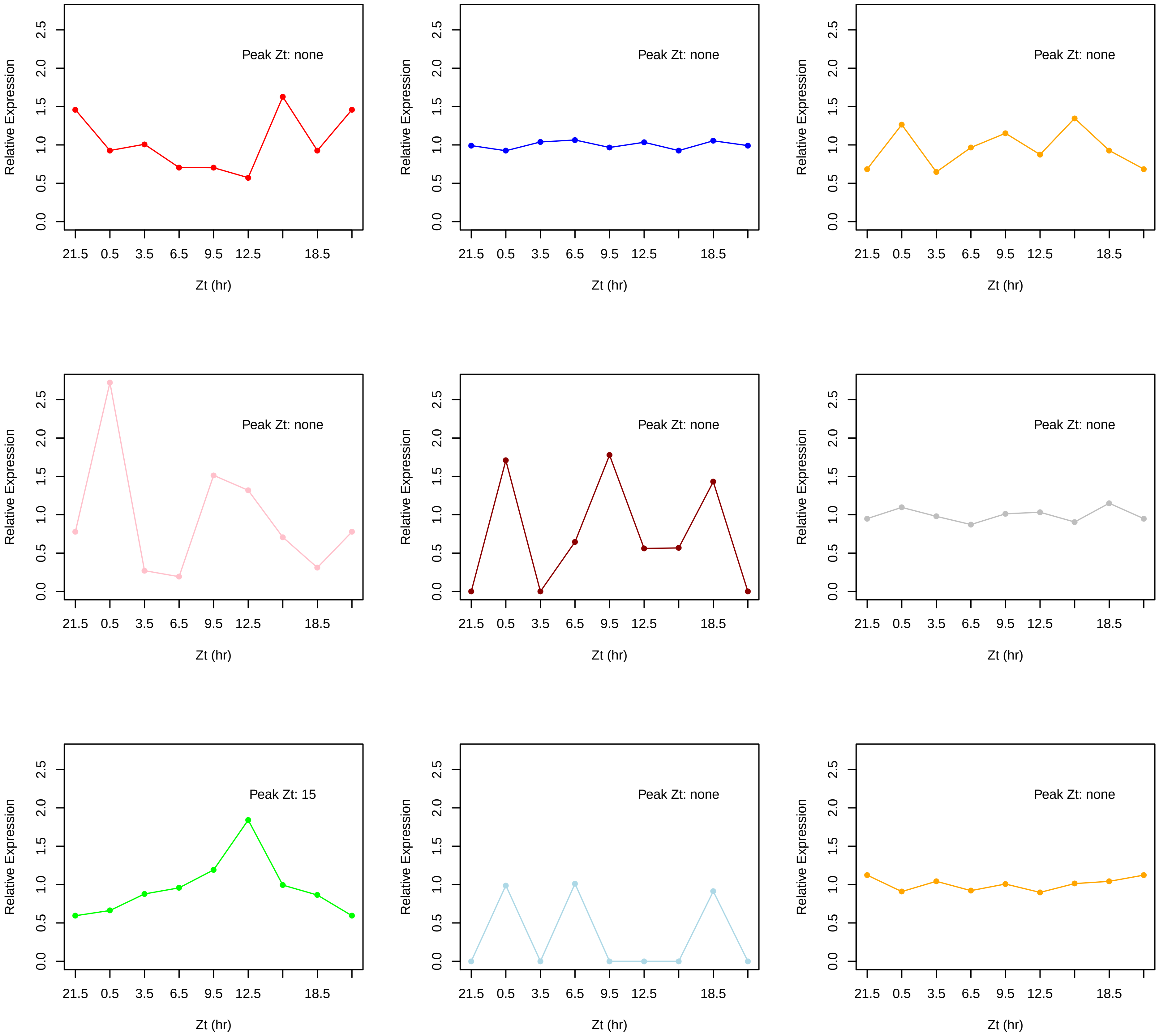

Singleton: ENSXMAG00000022375 rhoub

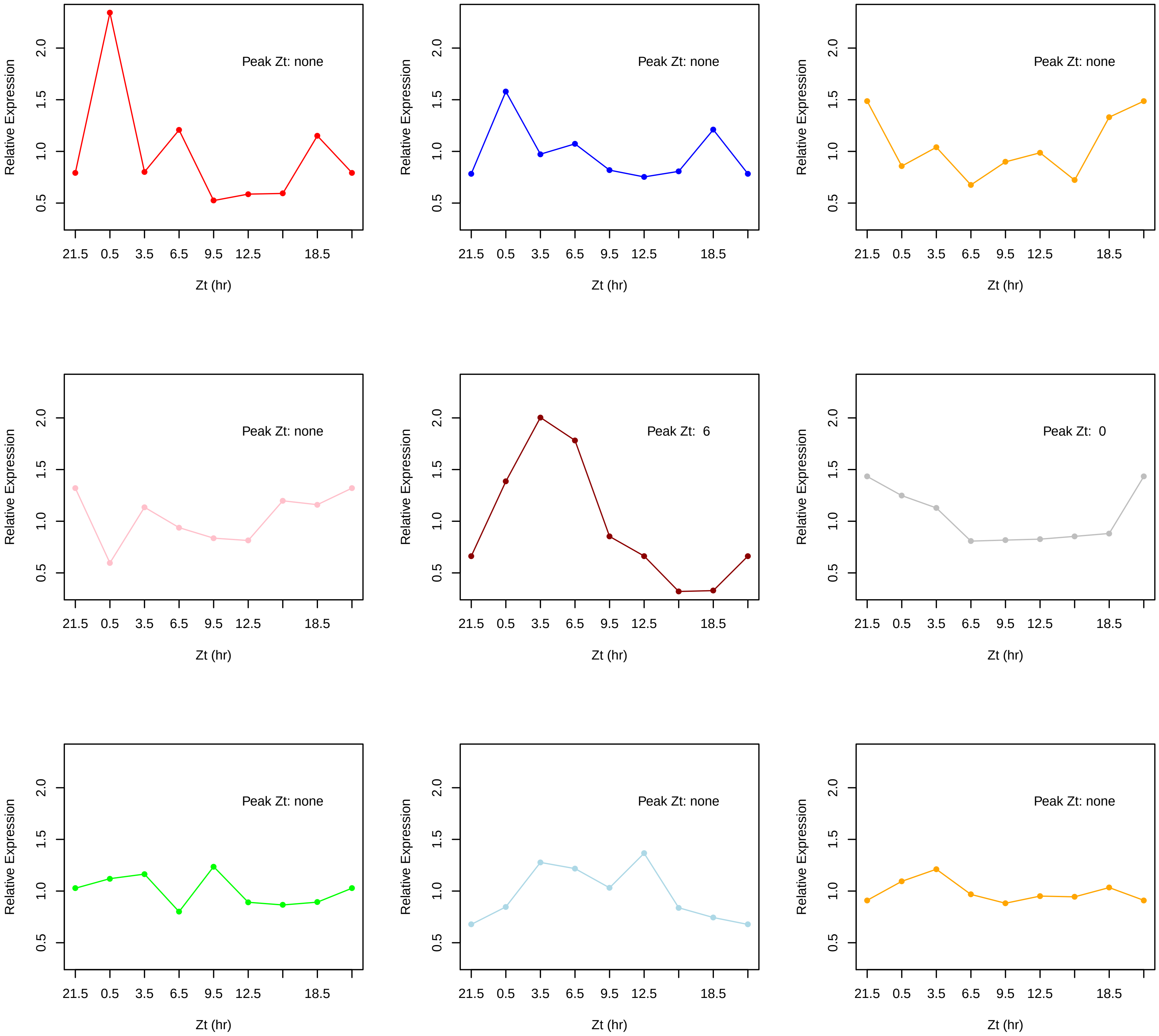

Singeleton: ENSXMAG00000009431 zgc:66447

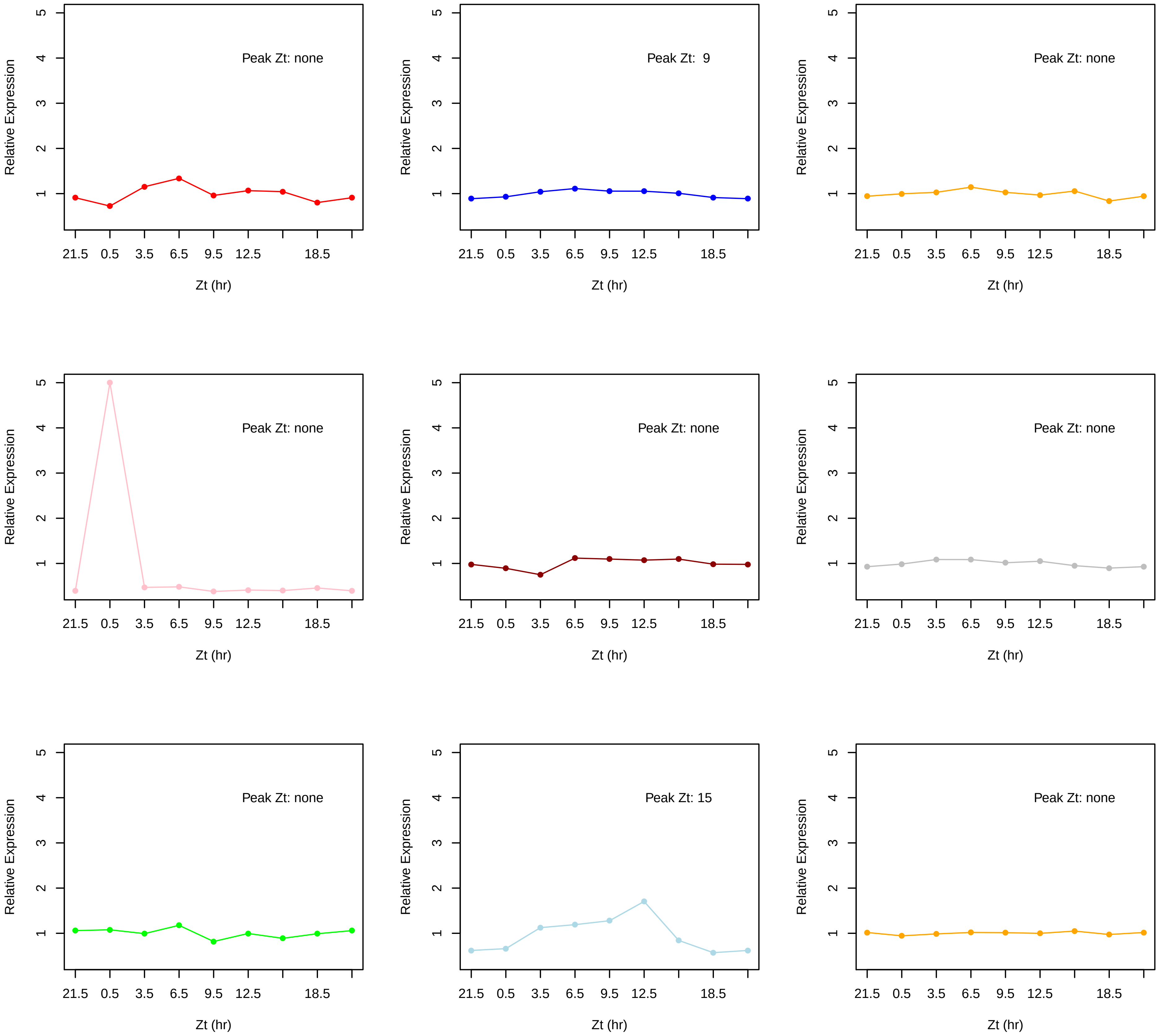

Singleton: ENSXMAG00000009804 aph1b

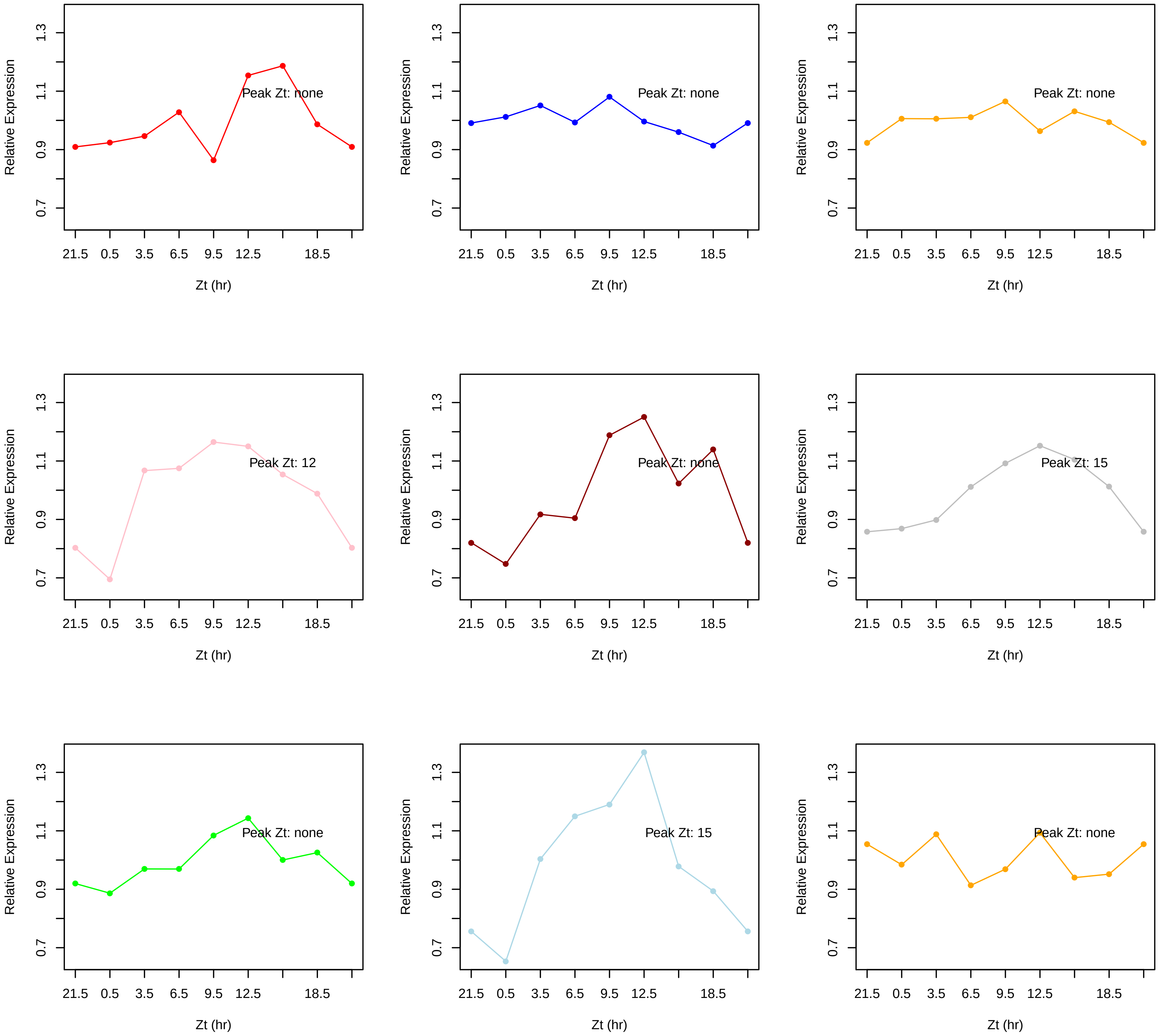

Singleton: ENSXMAG00000004235 ap2s1

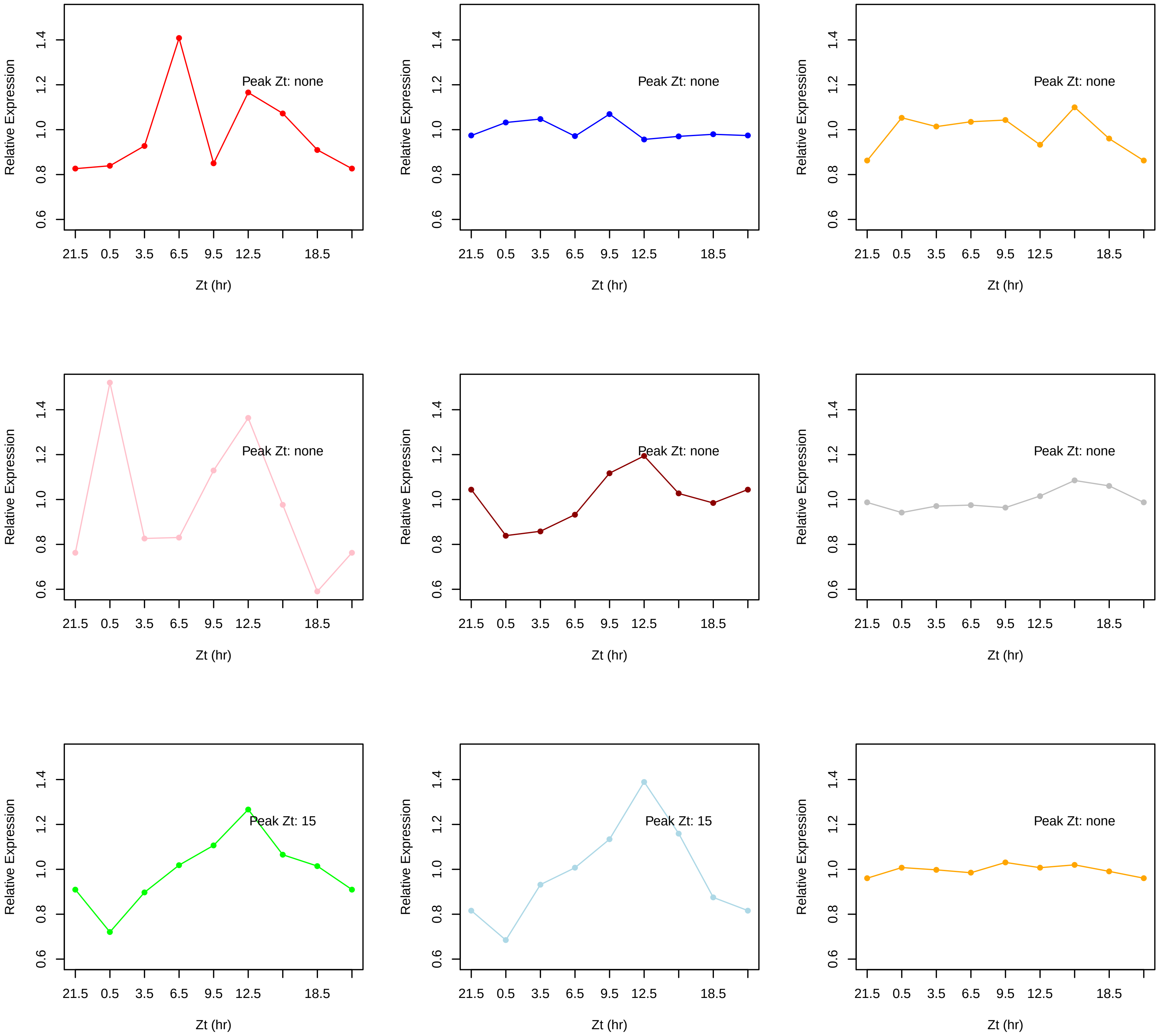

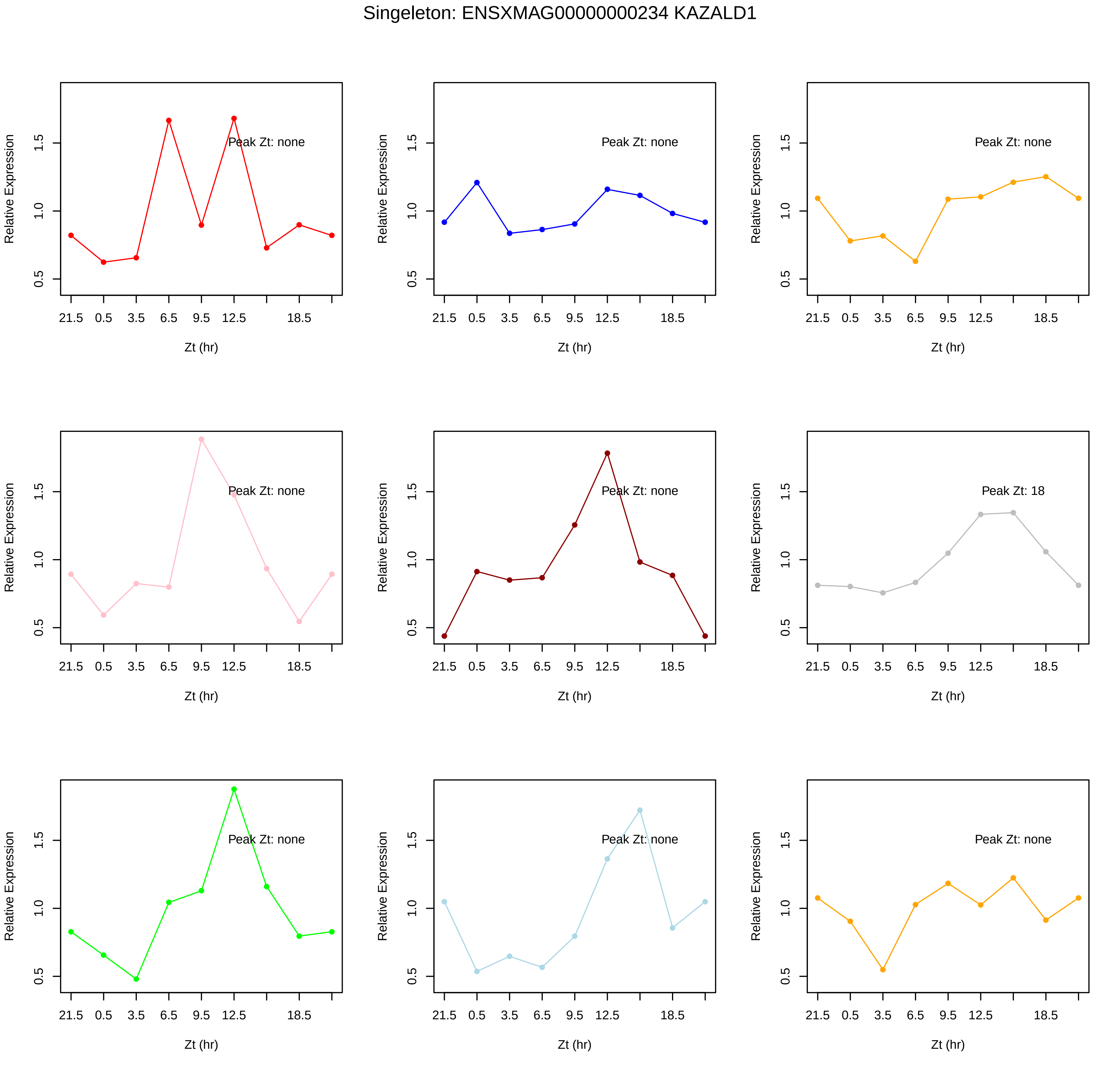
Singeleton: ENSXMAG00000000234 KAZALD1
Peak Zt: none
Peak Zt: none
Peak Zt: none
Peak Zt: none
Peak Zt: none
Peak Zt: 18
Peak Zt: none
Peak Zt: none
Peak Zt: none
Relative Expression
Zt (hr)
21.5
0.5
3.5
6.5
9.5
12.5
18.5
0.5
1.0
1.5

Singleton: ENSXMAG00000028521 npas1

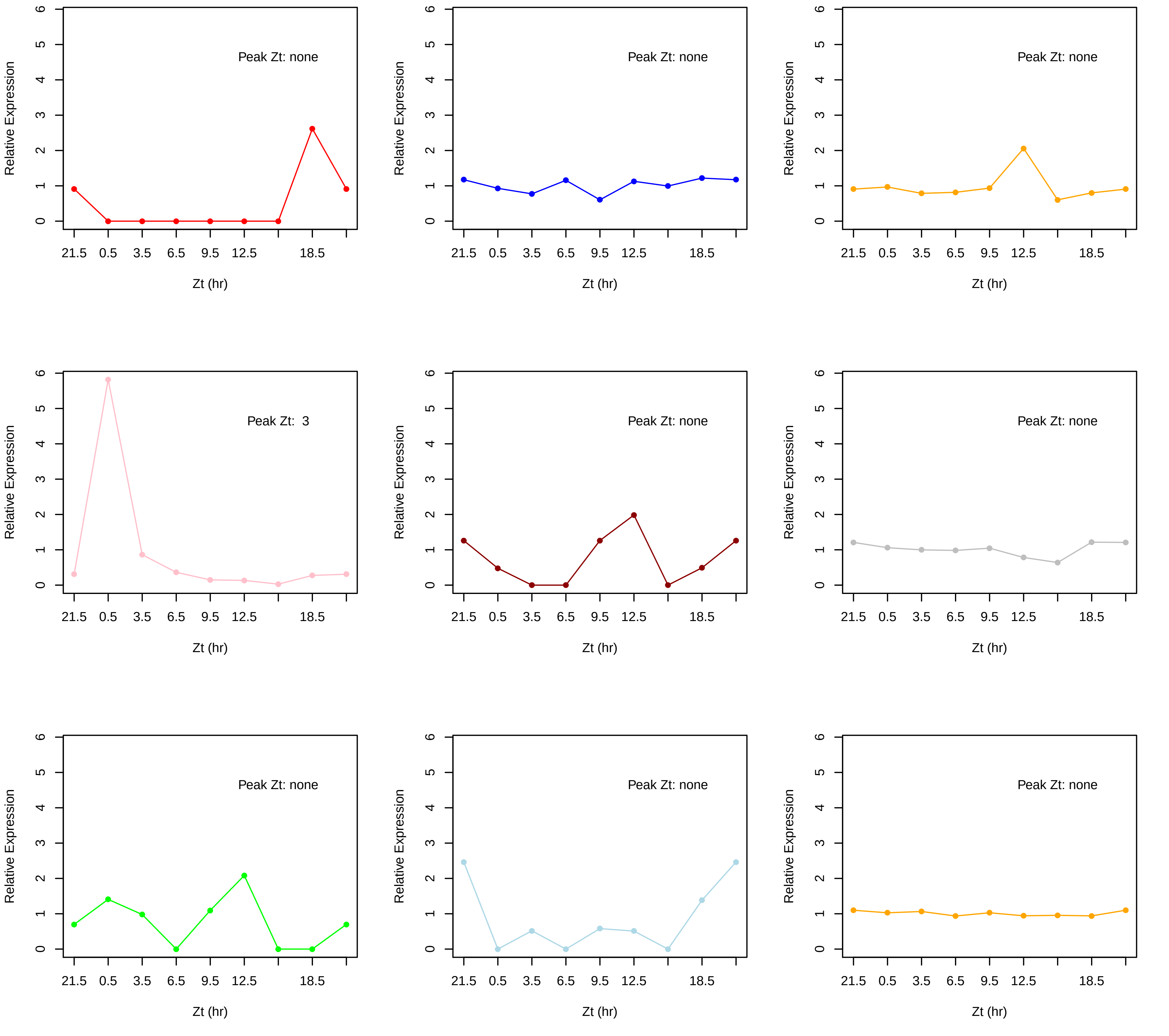

Singeleton: ENSXMAG00000004210 tmem160

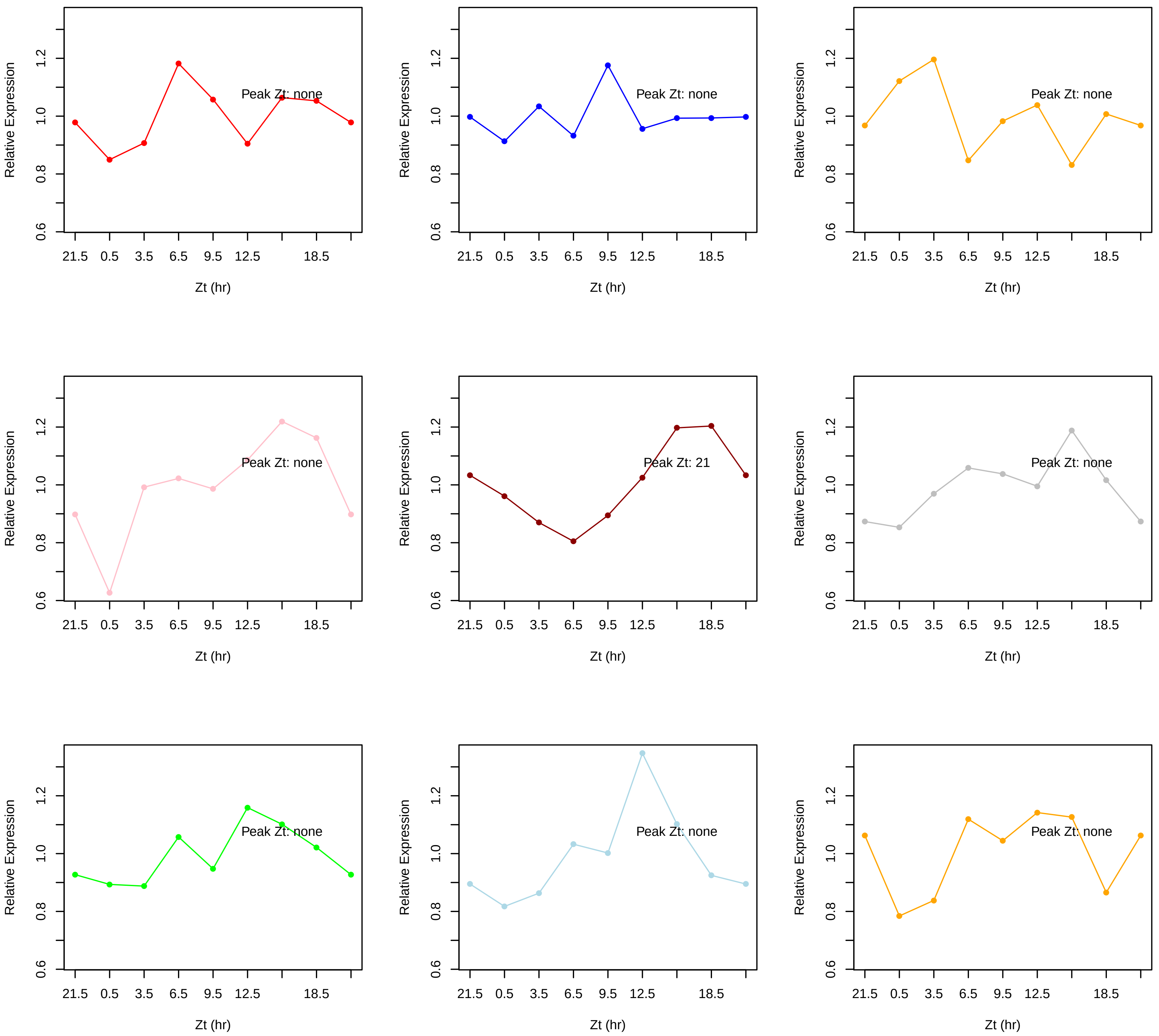

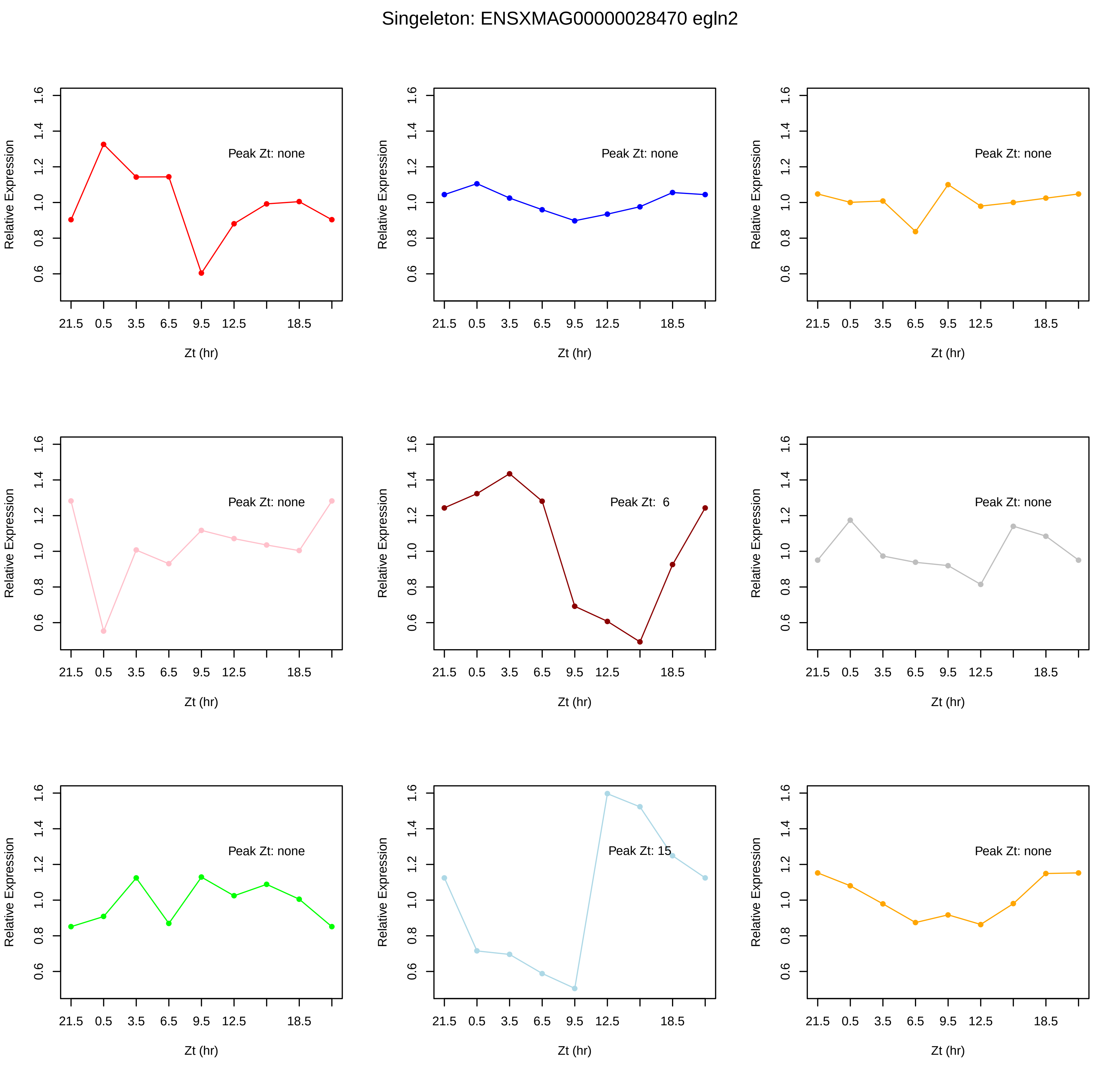
Singeleton: ENSXMAG00000028470 egln2
Peak Zt: none
Peak Zt: none
Peak Zt: none
Peak Zt: none
Peak Zt: 6
Peak Zt: none
Peak Zt: none
Peak Zt: 15
Peak Zt: none
Relative Expression
Zt (hr)
21.5
0.5
3.5
6.5
9.5
12.5
18.5
0.6
0.8
1.0
1.2
1.4
1.6

Singeleton: ENSXMAG00000004179 slc1a5

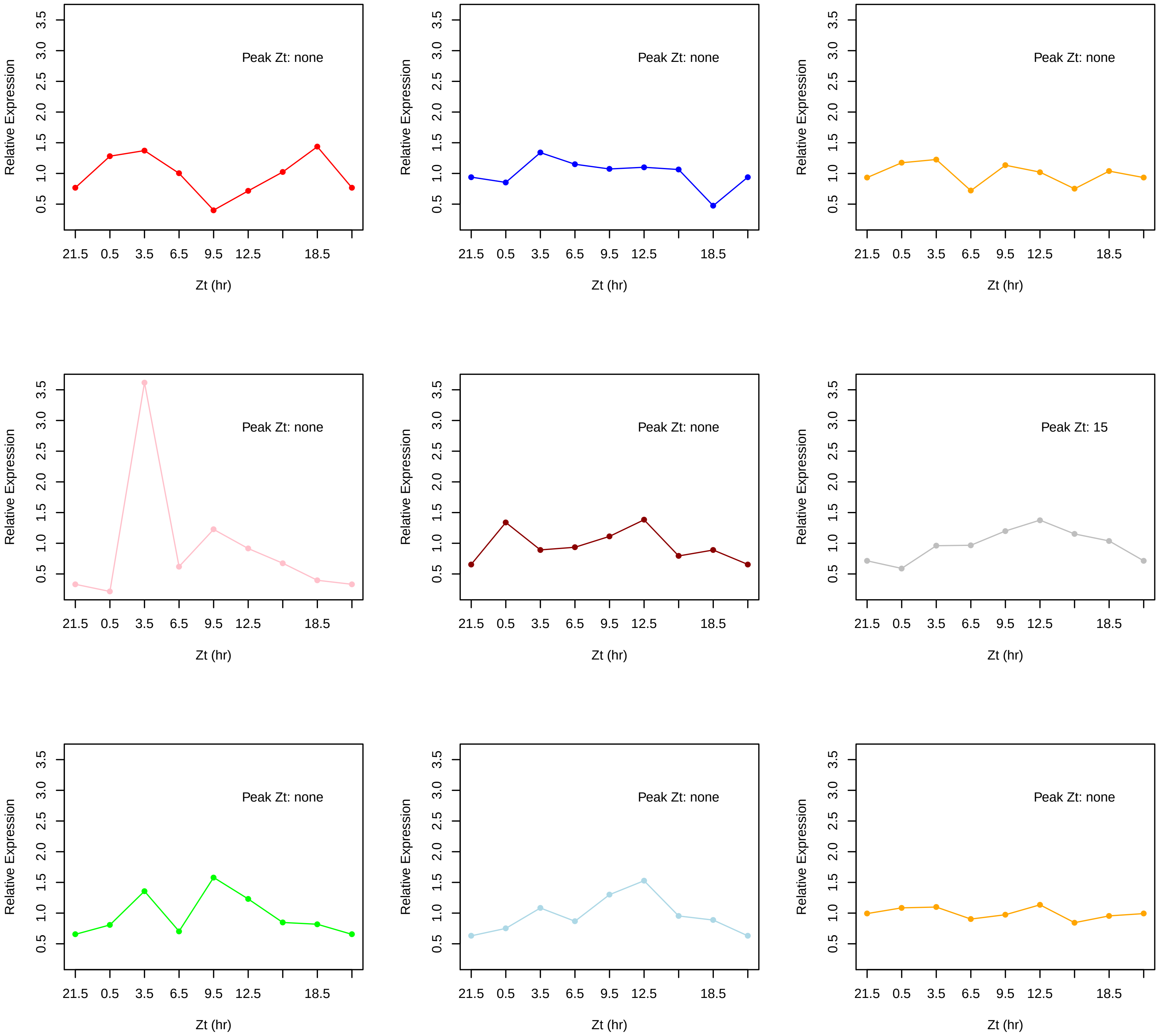

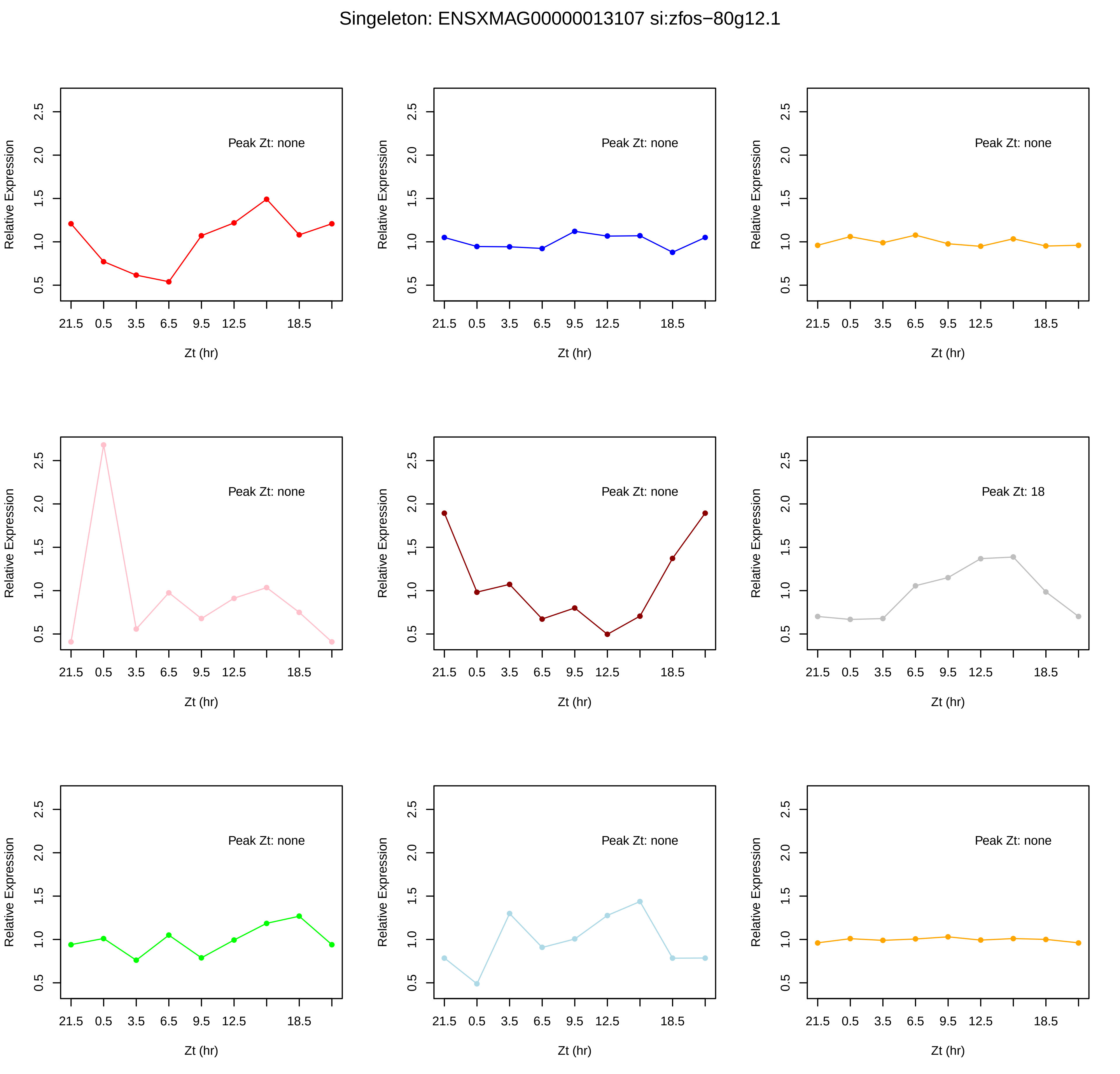

Singeleton: ENSXMAG00000013107 si:zfos−80g12.1
Peak Zt: none
Peak Zt: none
Peak Zt: none
Peak Zt: none
Peak Zt: none
Peak Zt: 18
Peak Zt: none
Peak Zt: none
Peak Zt: none
Relative Expression
Zt (hr)
21.5
0.5
3.5
6.5
9.5
12.5
18.5
0.5
1.0
1.5
2.0
2.5

Singleton: ENSXMAG00000013124 foxo4

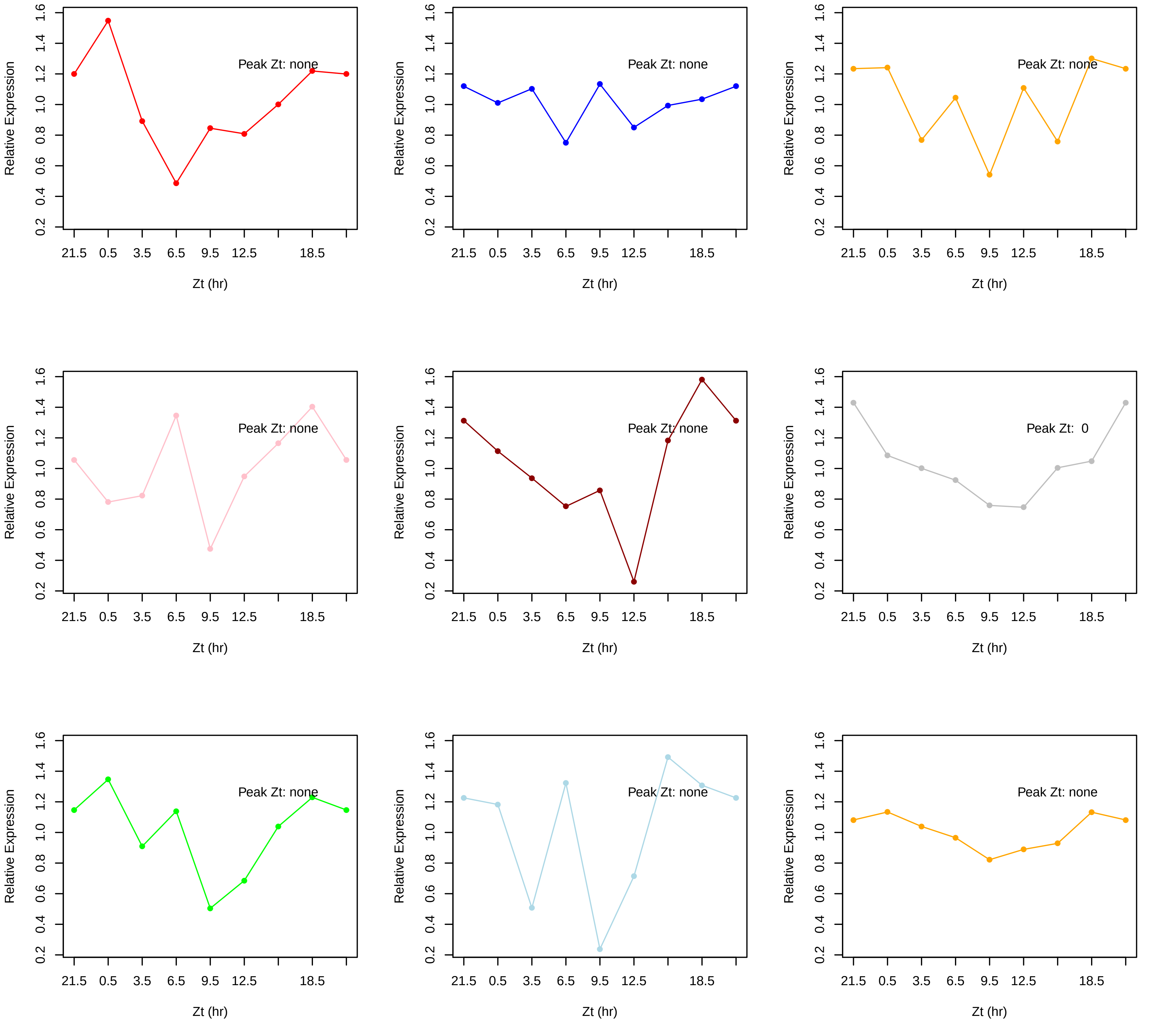

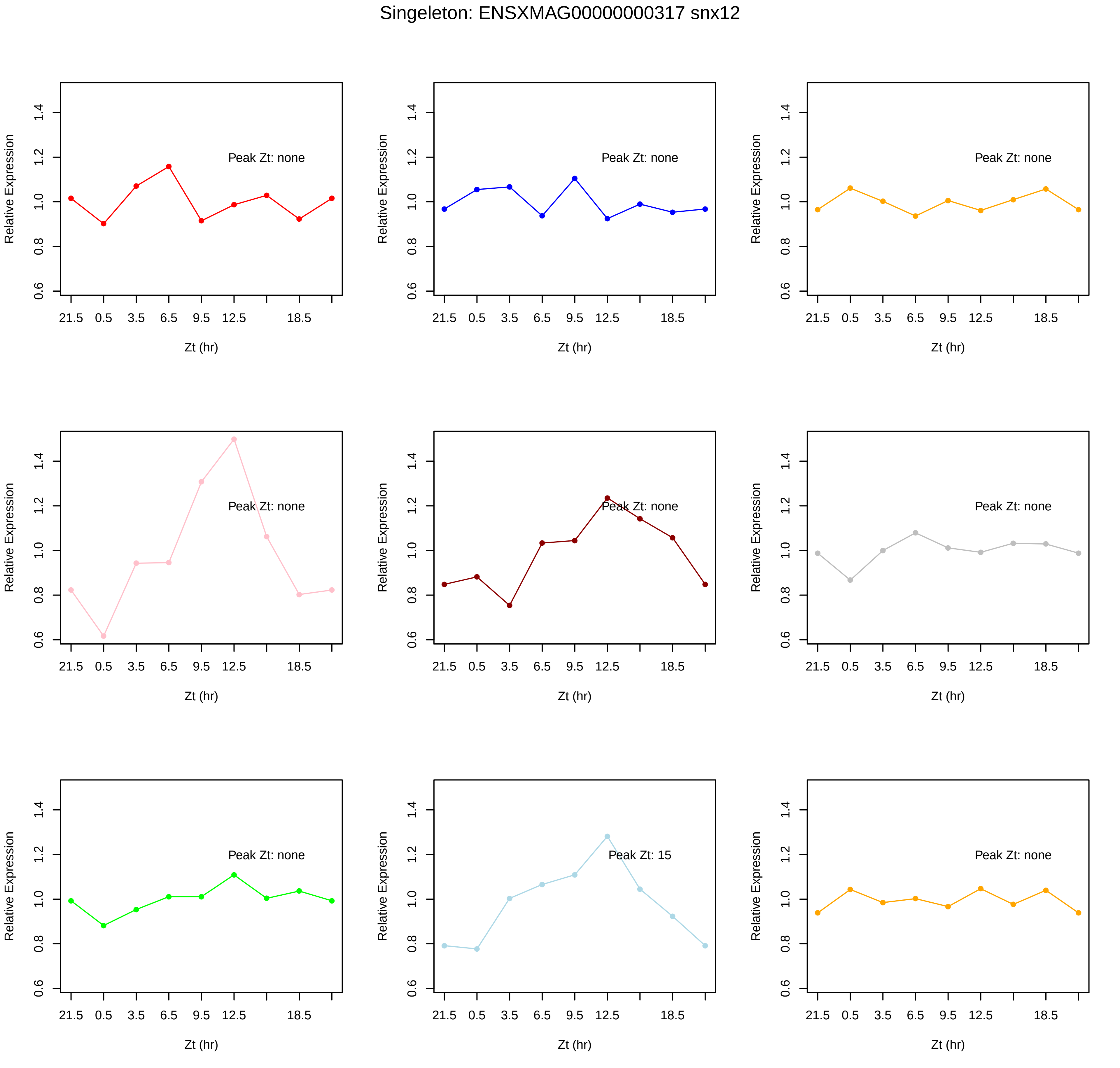
Singleton: ENSXMAG00000000317 snx12
Relative Expression
Zt (hr)
21.5 0.5 3.5 6.5 9.5 12.5 18.5
0.6 0.8 1.0 1.2 1.4
Peak Zt: none
Peak Zt: 15

Singeleton: ENSXMAG00000000319 slc7a3a

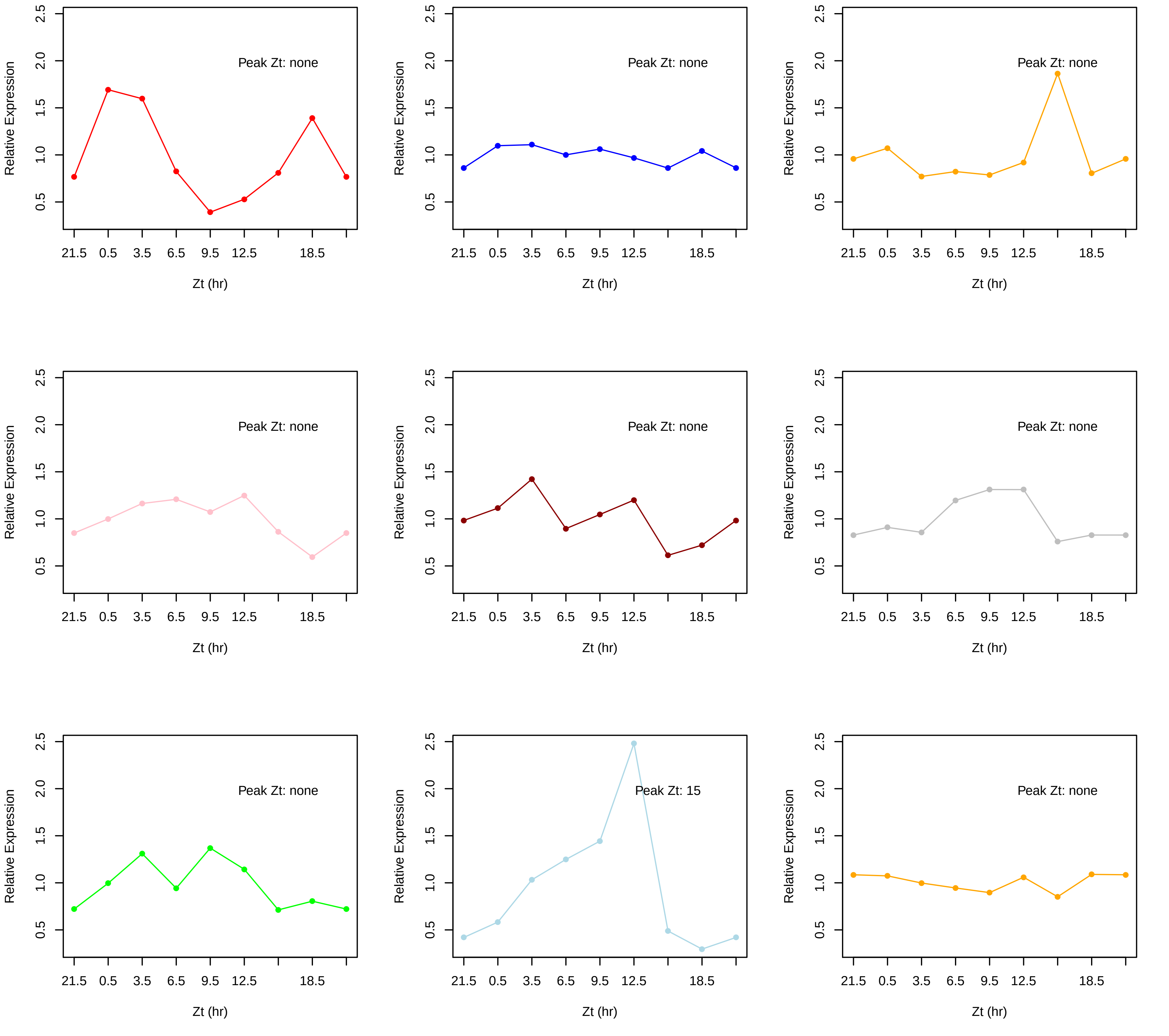

Singeleton: ENSXMAG00000015850 stoml1

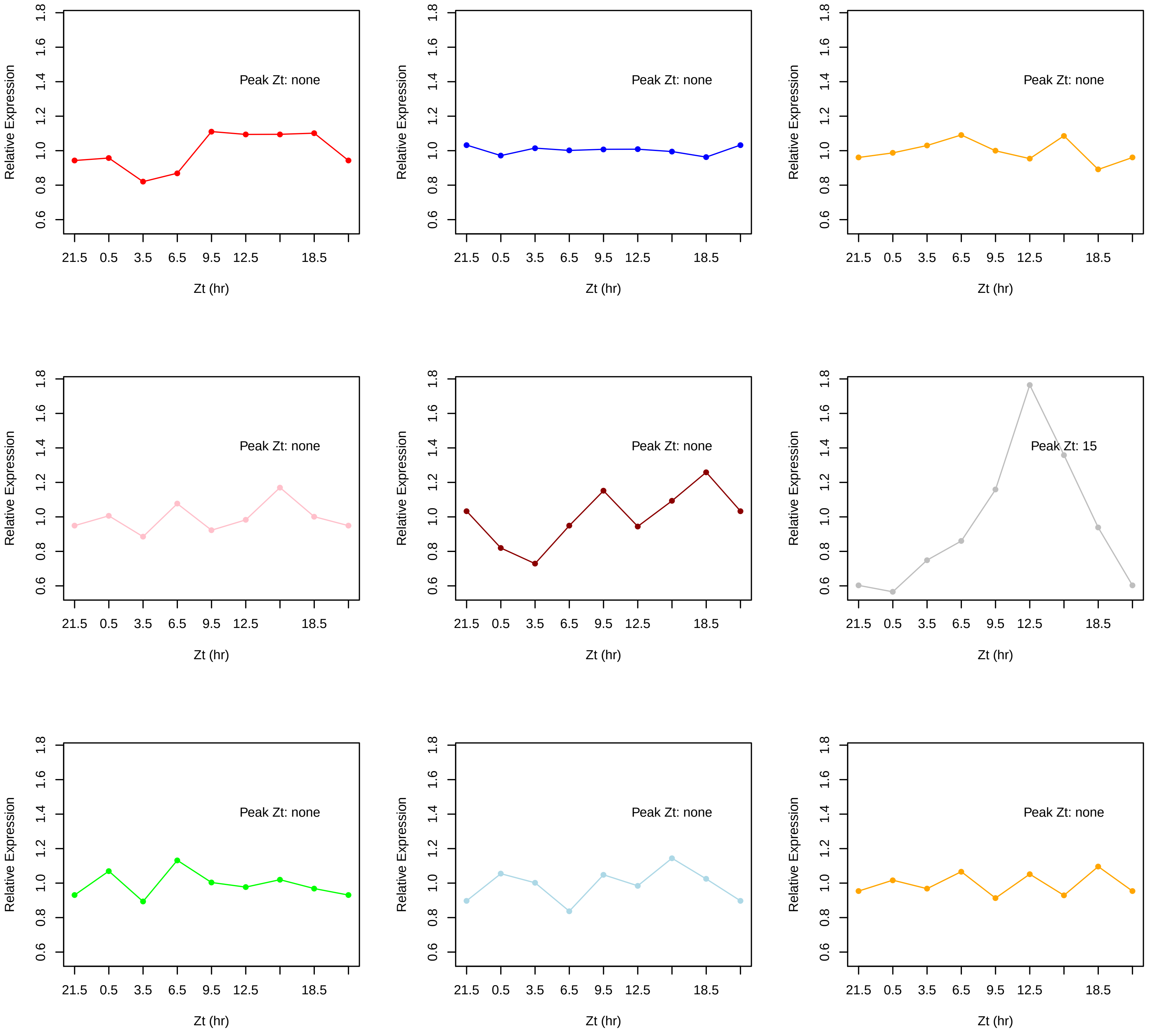

Singeleton: ENSXMAG00000026528 BIN3

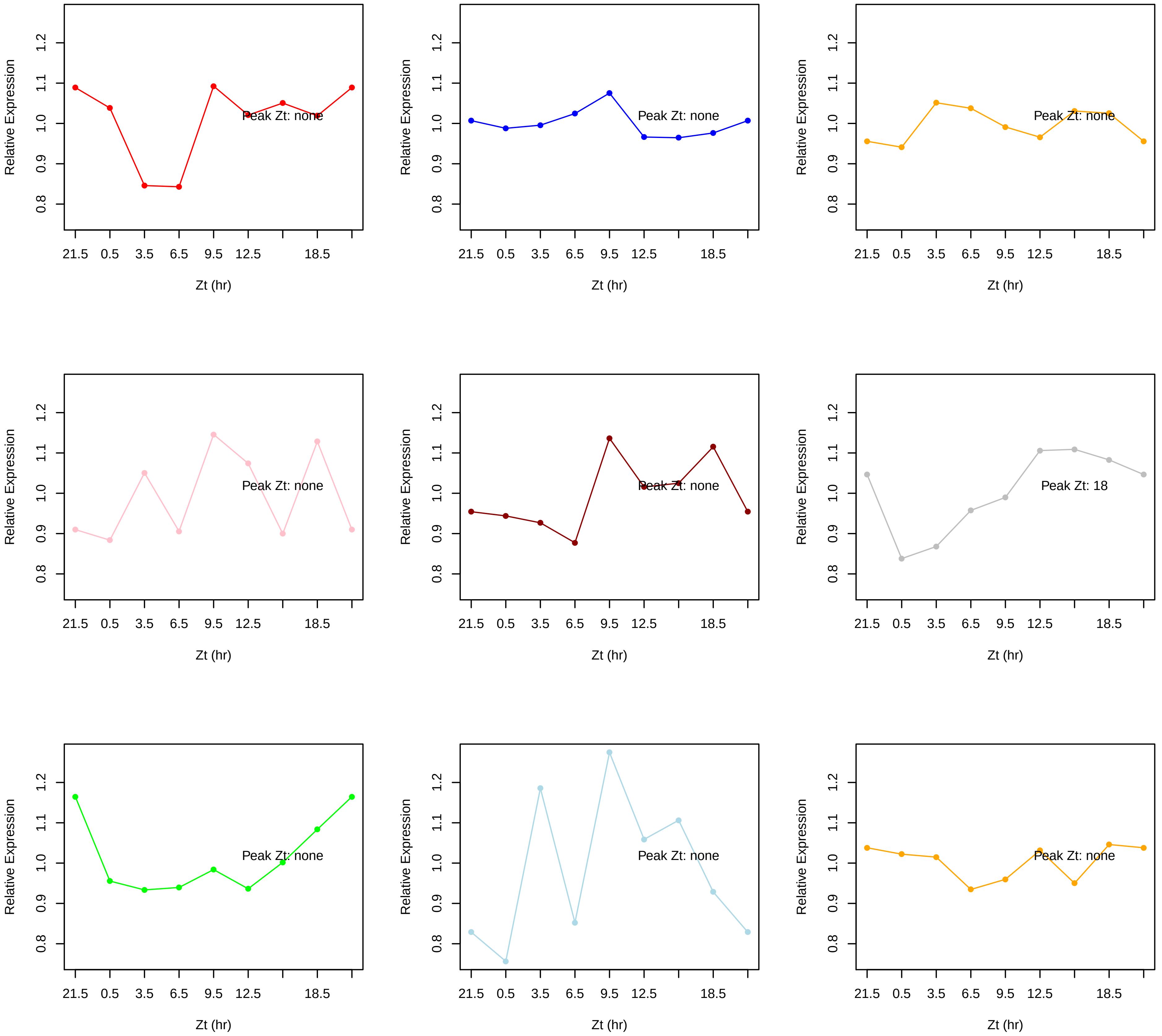

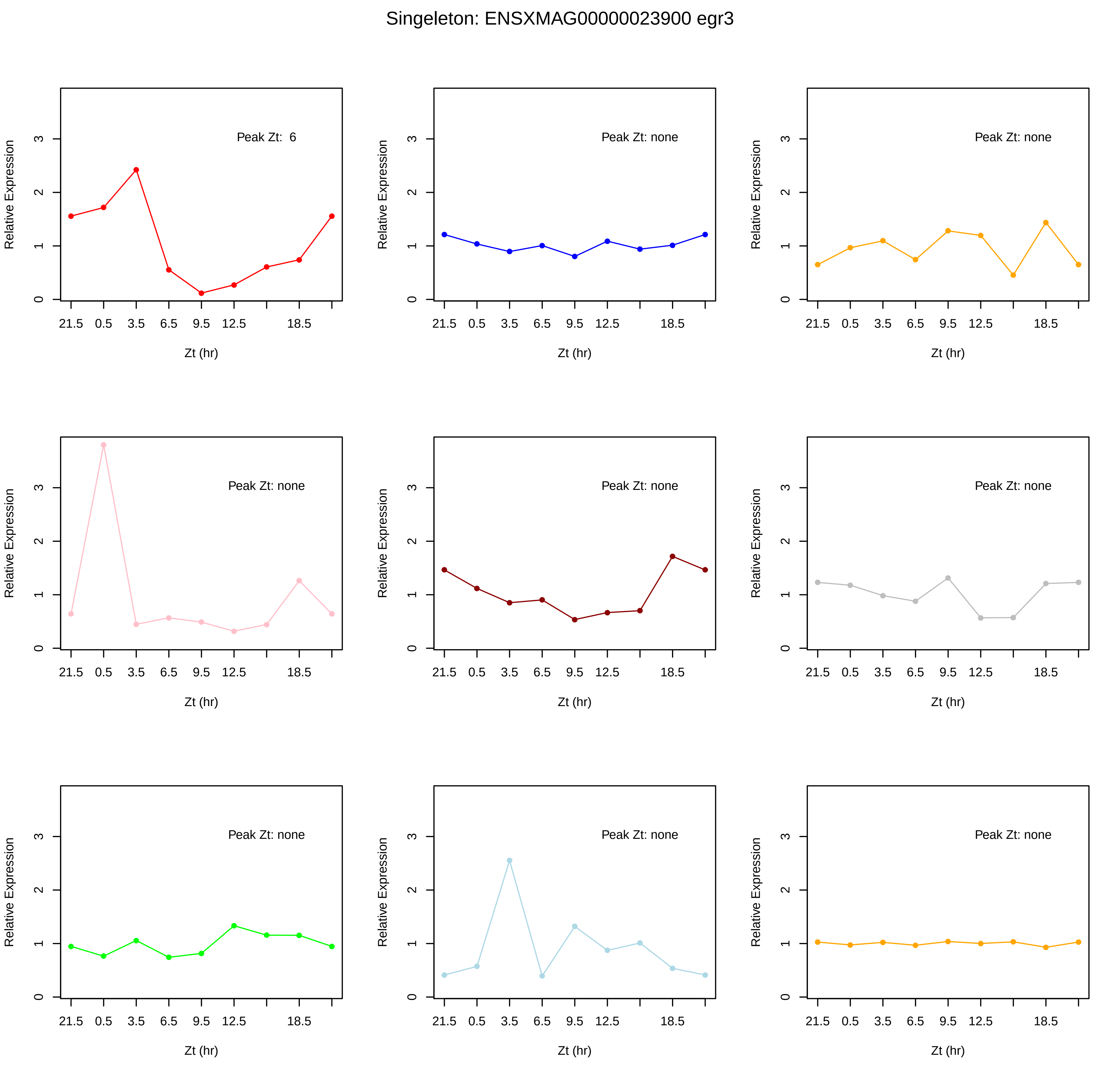
Singeleton: ENSXMAG00000023900 egr3
Peak Zt: 6
Peak Zt: none
Peak Zt: none
Peak Zt: none
Peak Zt: none
Peak Zt: none
Peak Zt: none
Peak Zt: none
Peak Zt: none
Relative Expression
Zt (hr)
21.5 0.5 3.5 6.5 9.5 12.5 18.5
0 1 2 3

Singeleton: ENSXMAG00000001153 RHOBTB2

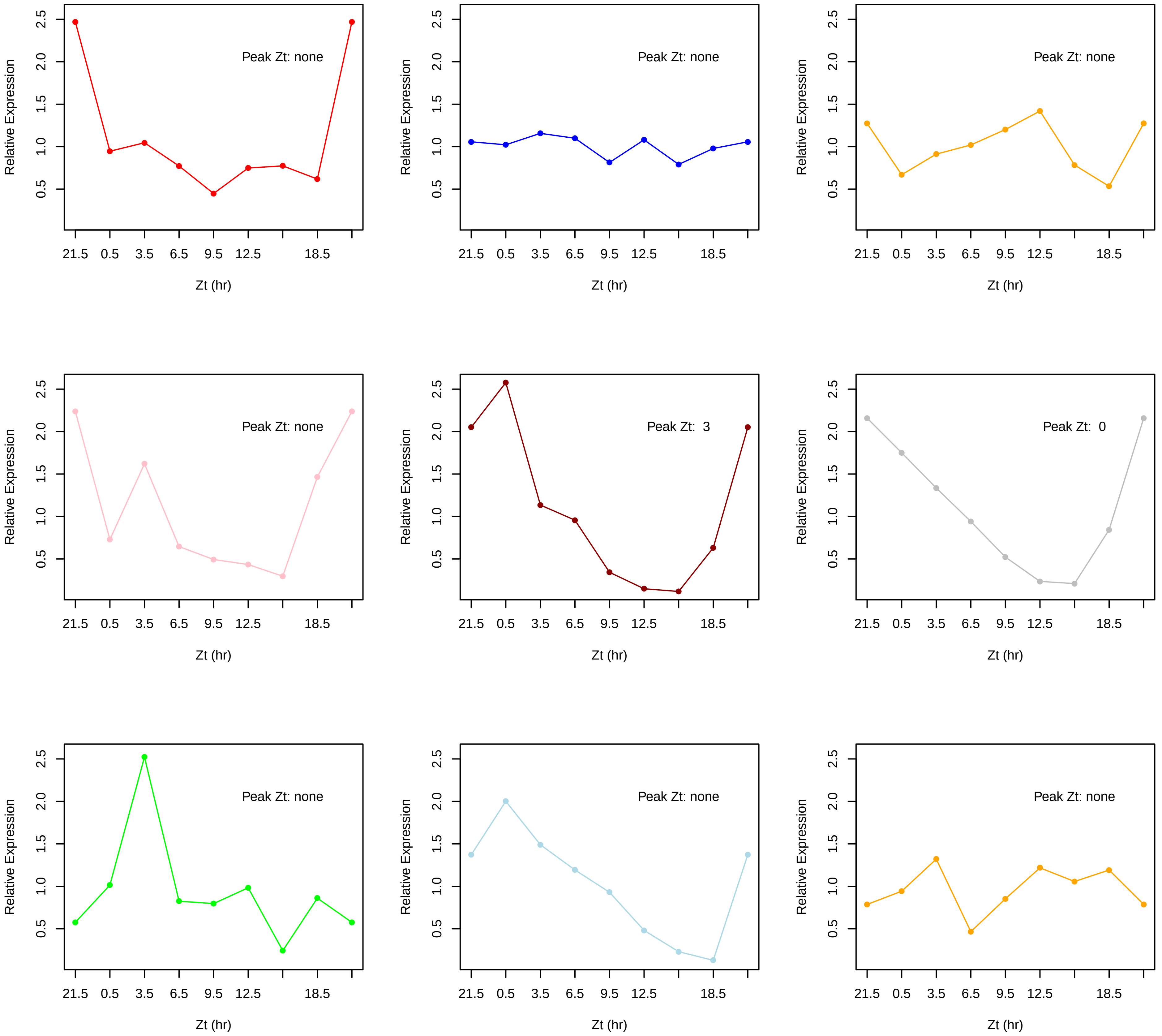

Singleton: ENSXMAG00000012946 mpi

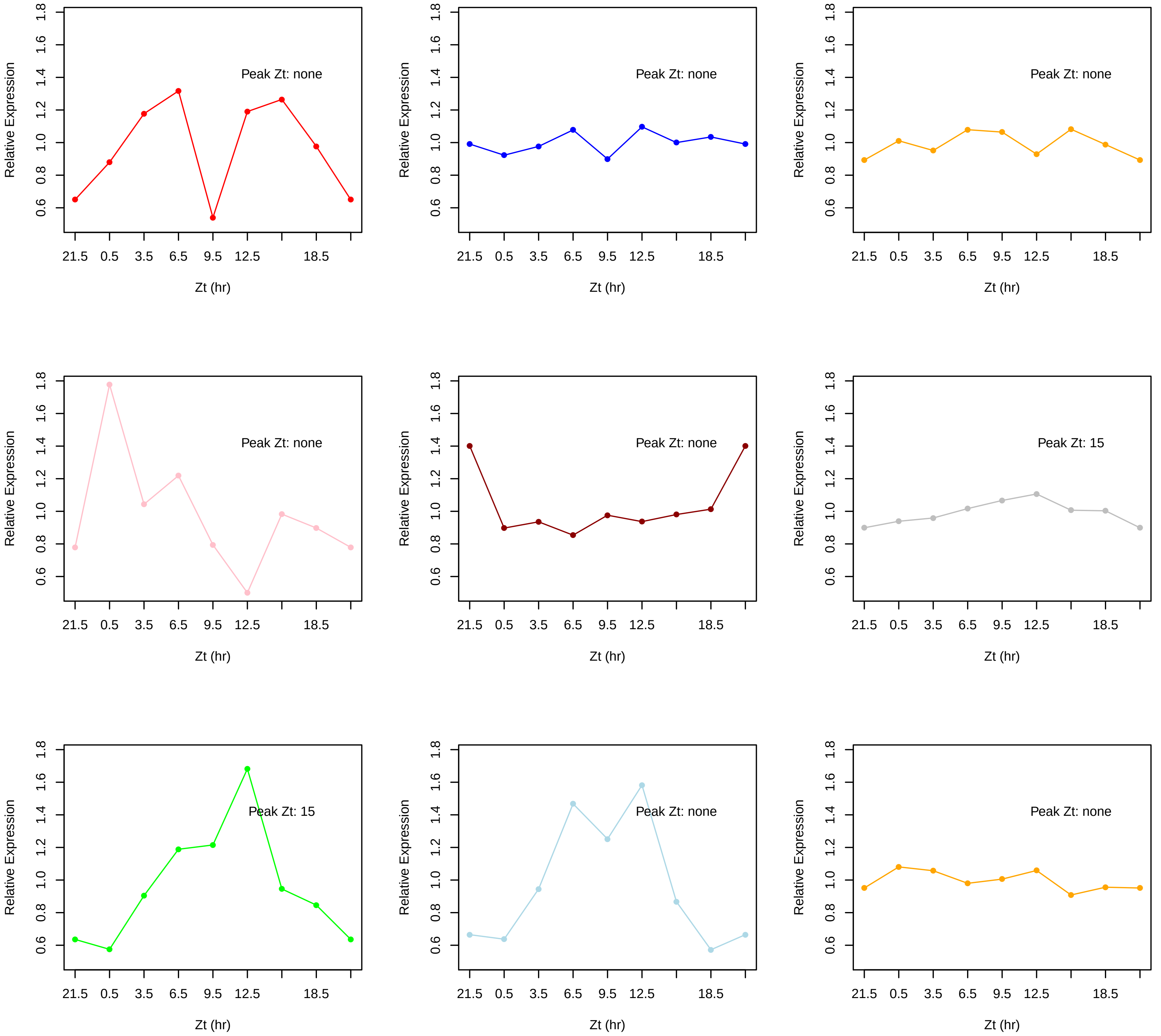

Singeleton: ENSXMAG00000001865 sorbs3

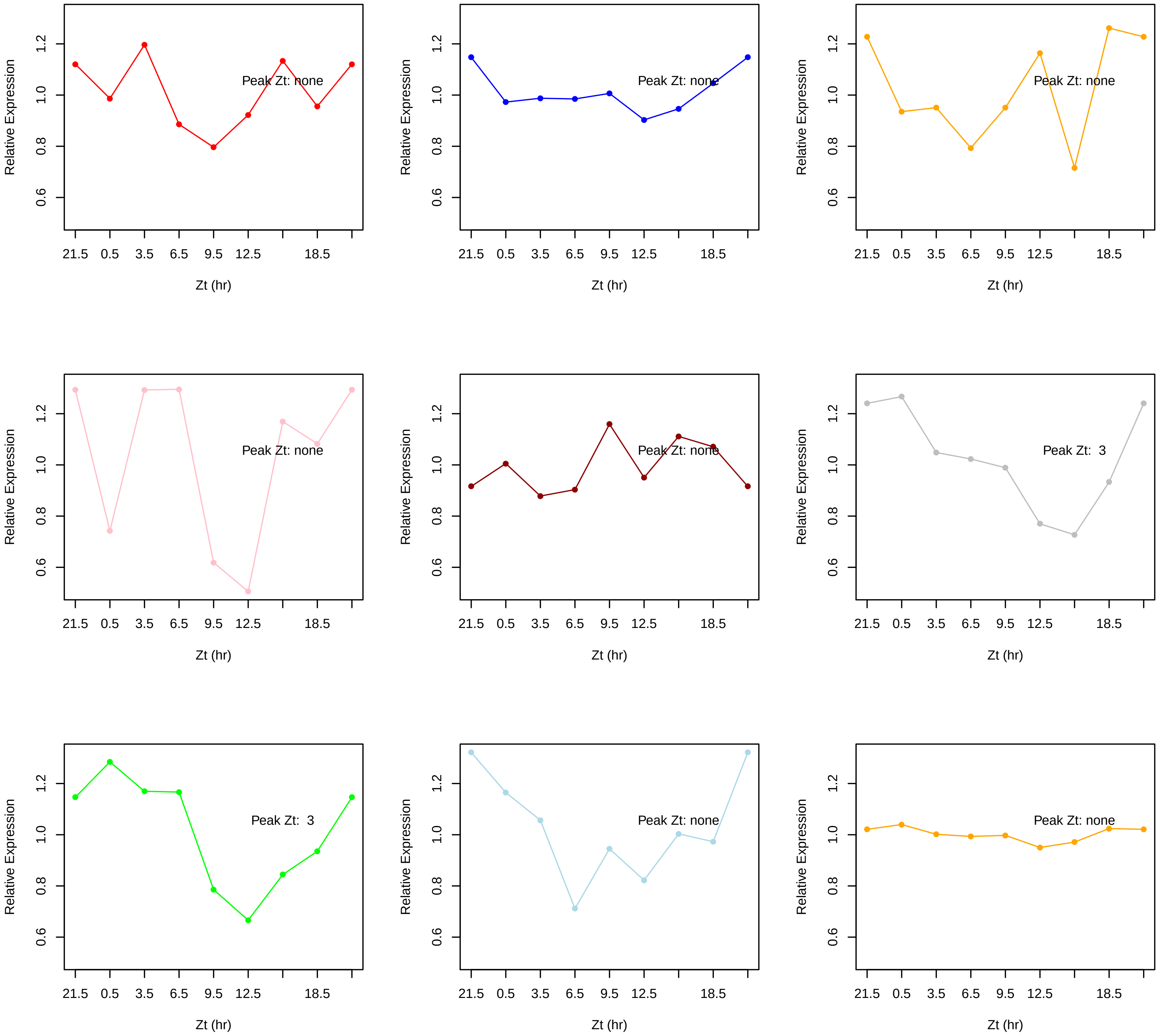

Singleton: ENSXMAG00000022895 siah2l

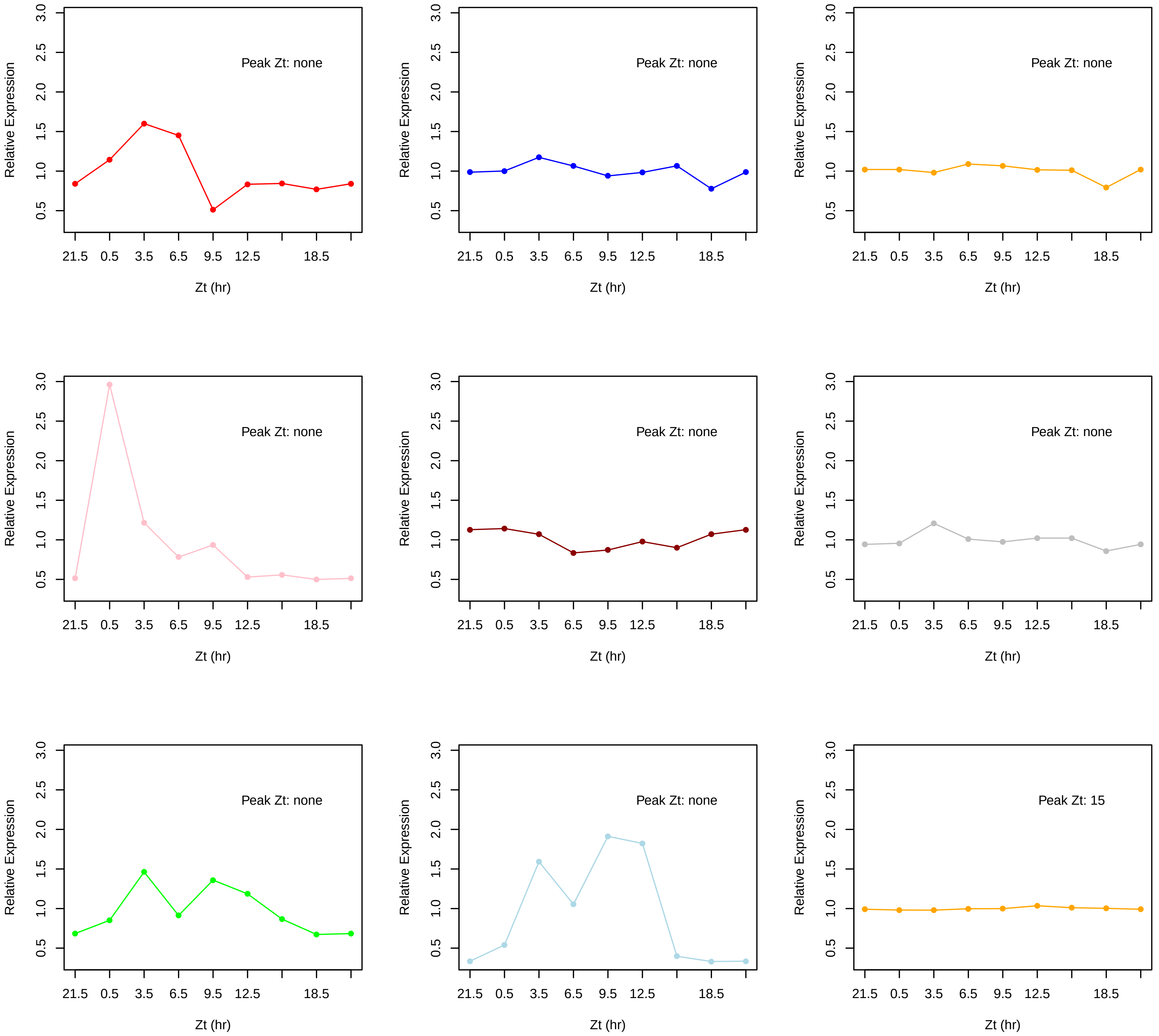

Singeleton: ENSXMAG00000010383 bbs4

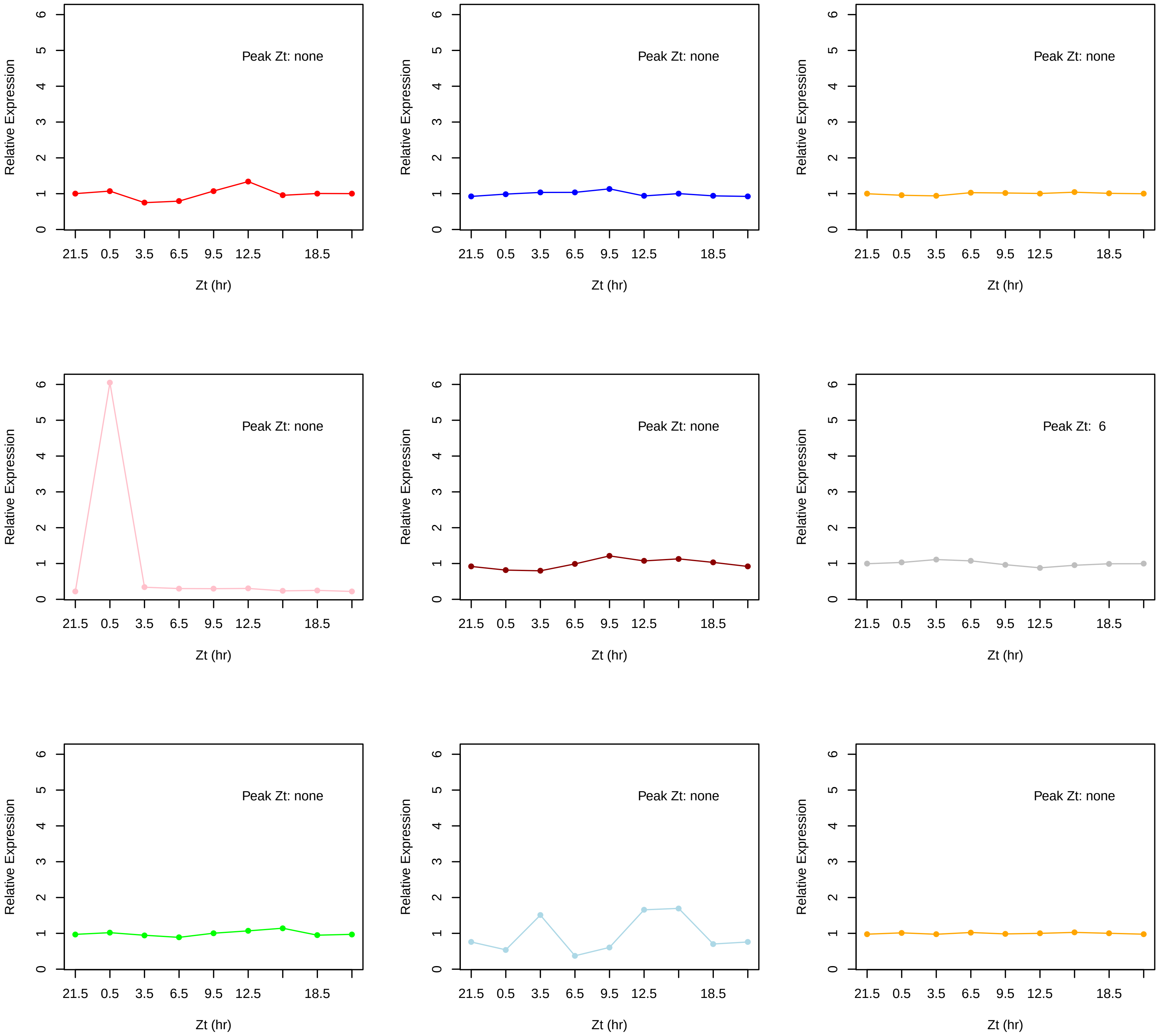

Singeleton: ENSXMAG00000021313 pdlim2

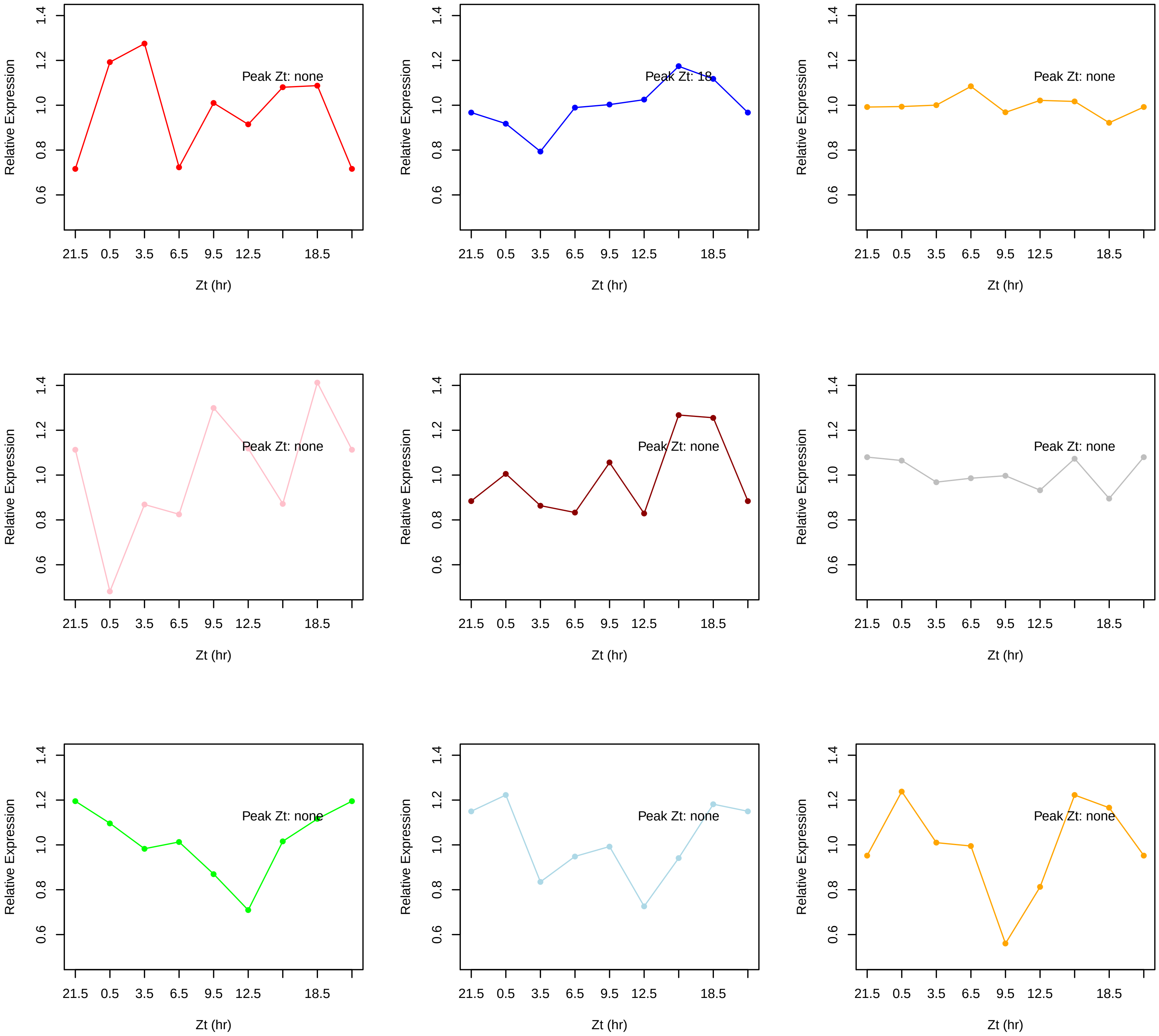

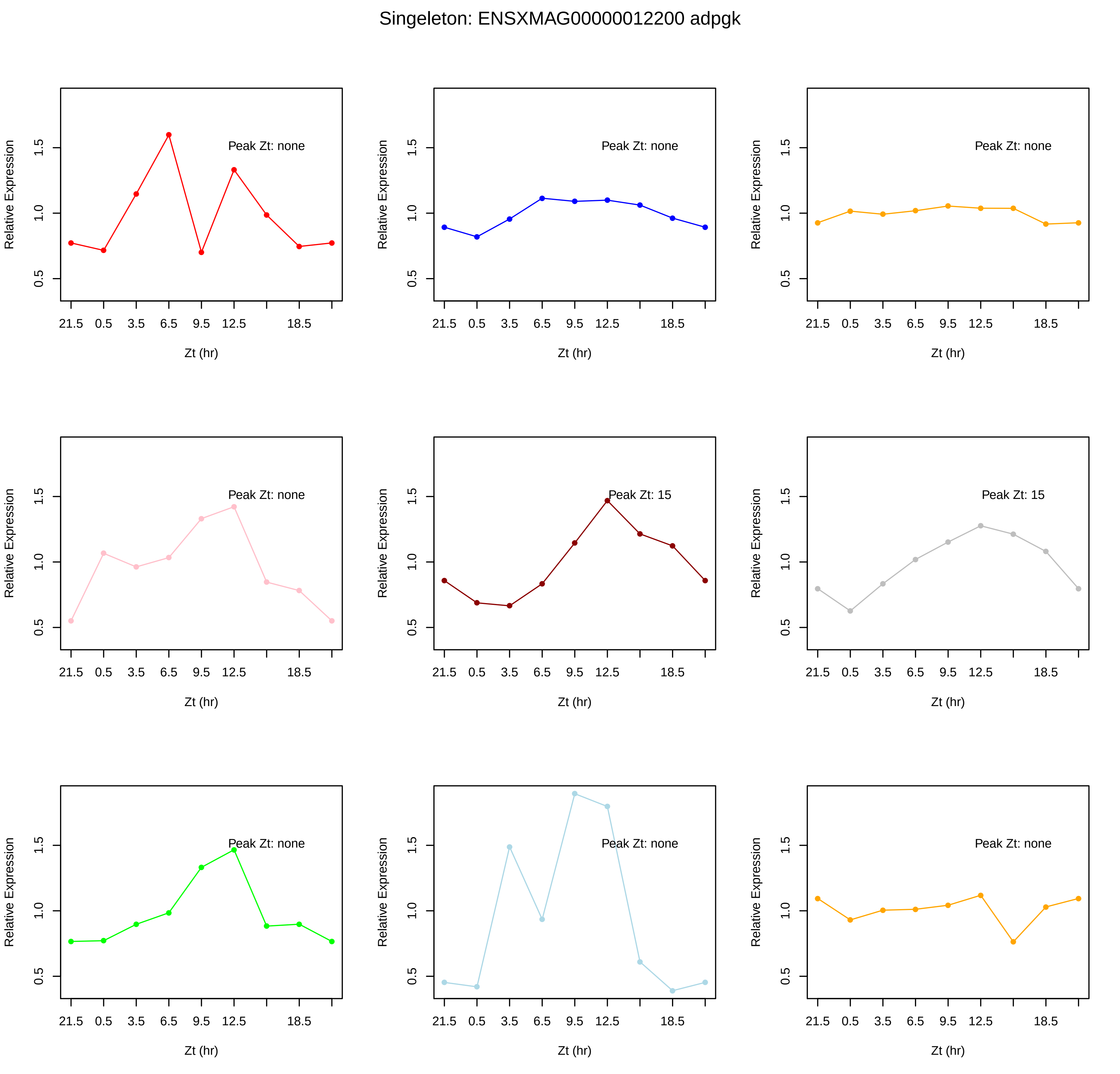
Singleton: ENSXMAG00000012200 adpgk
Peak Zt: none
Peak Zt: none
Peak Zt: none
Peak Zt: none
Peak Zt: 15
Peak Zt: 15
Peak Zt: none
Peak Zt: none
Peak Zt: none
Relative Expression
Zt (hr)
21.5
0.5
3.5
6.5
9.5
12.5
18.5
0.5
1.0
1.5

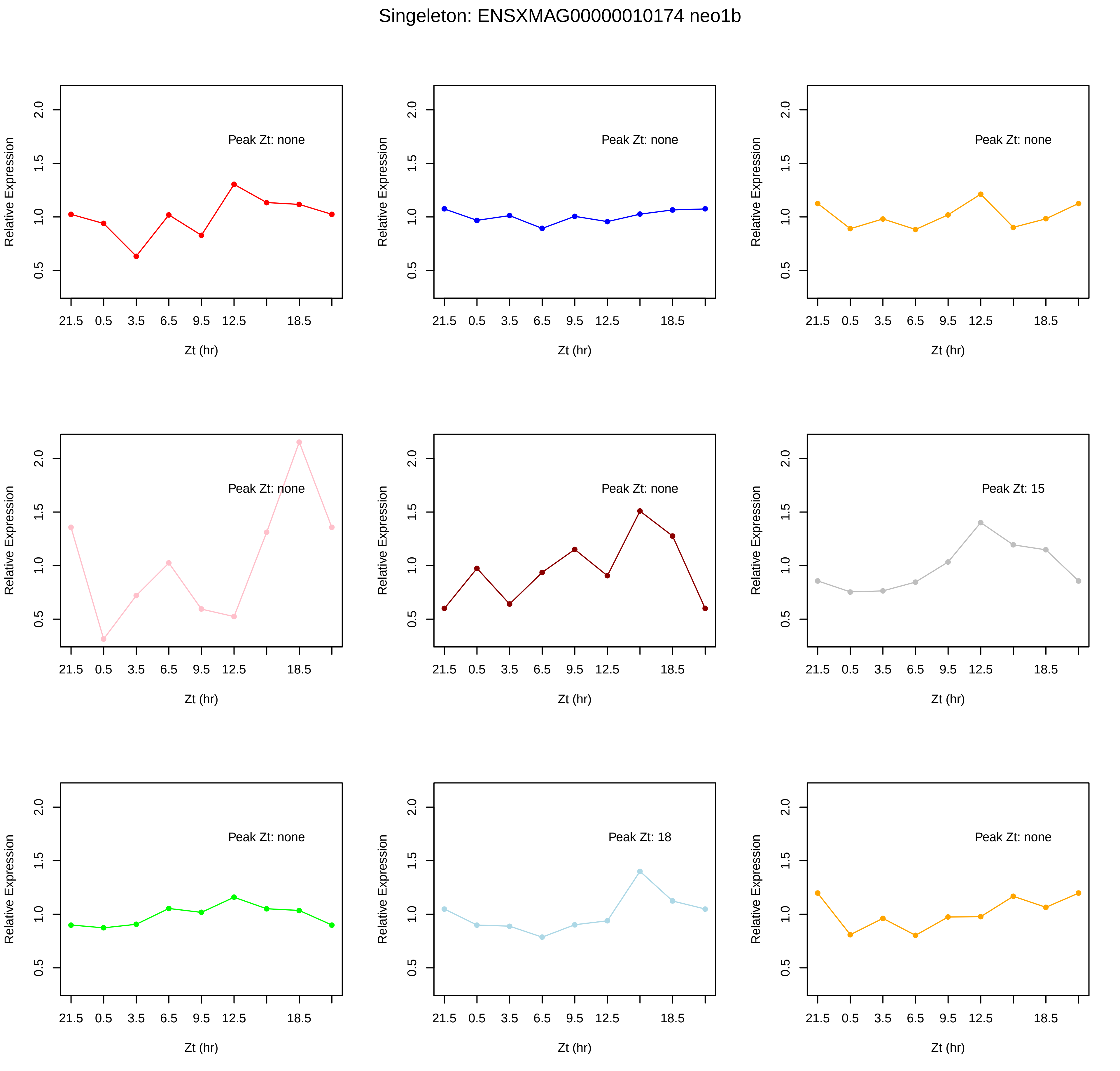
Singleton: ENSXMAG00000010174 neo1b
Peak Zt: none
Peak Zt: none
Peak Zt: none
Peak Zt: none
Peak Zt: none
Peak Zt: 15
Peak Zt: none
Peak Zt: 18
Peak Zt: none
Relative Expression
Zt (hr)
21.5
0.5
3.5
6.5
9.5
12.5
18.5
0.5
1.0
1.5
2.0

Singeleton: ENSXMAG00000007445 klf8

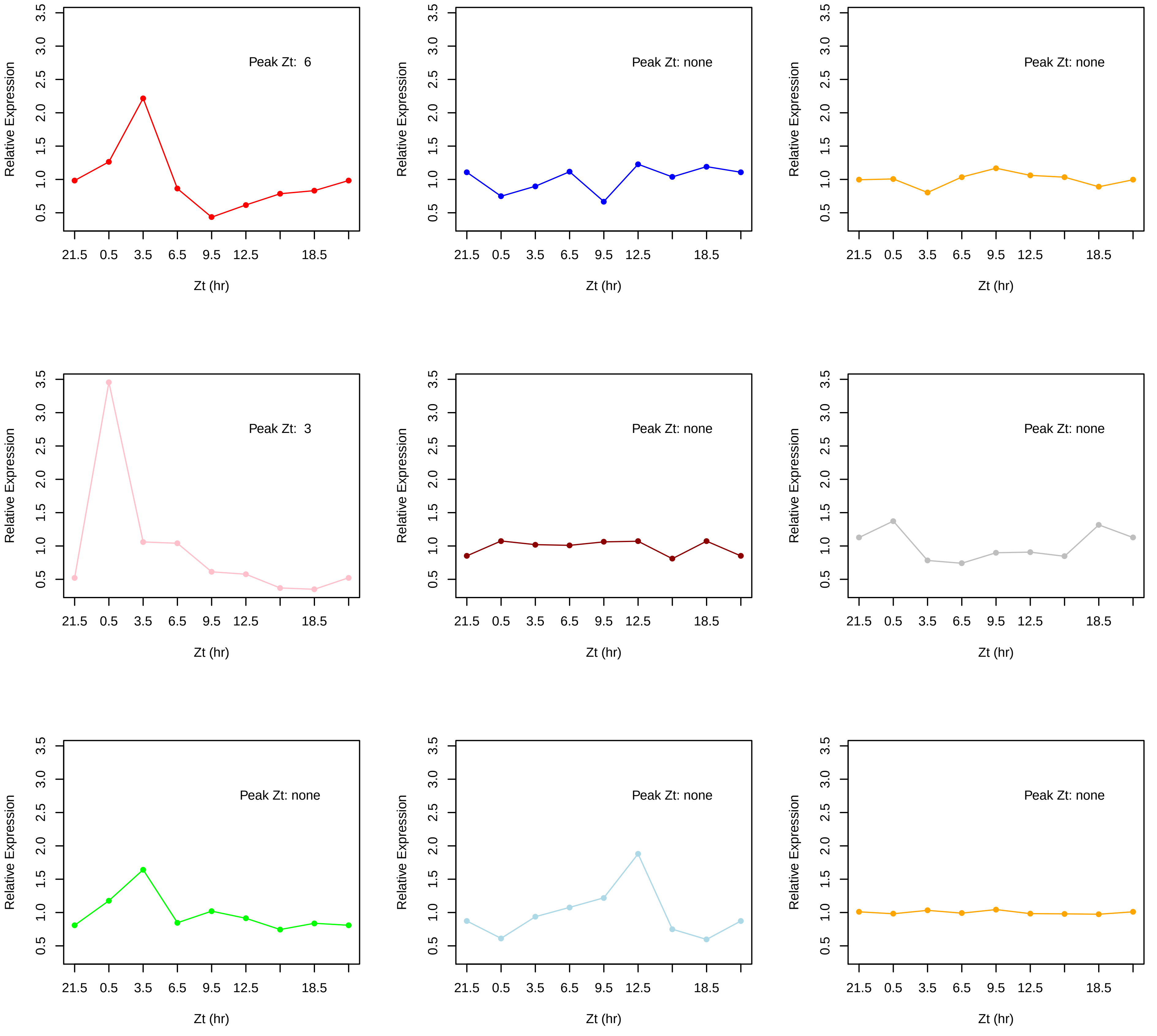

Singeleton: ENSXMAG00000015819 arih1

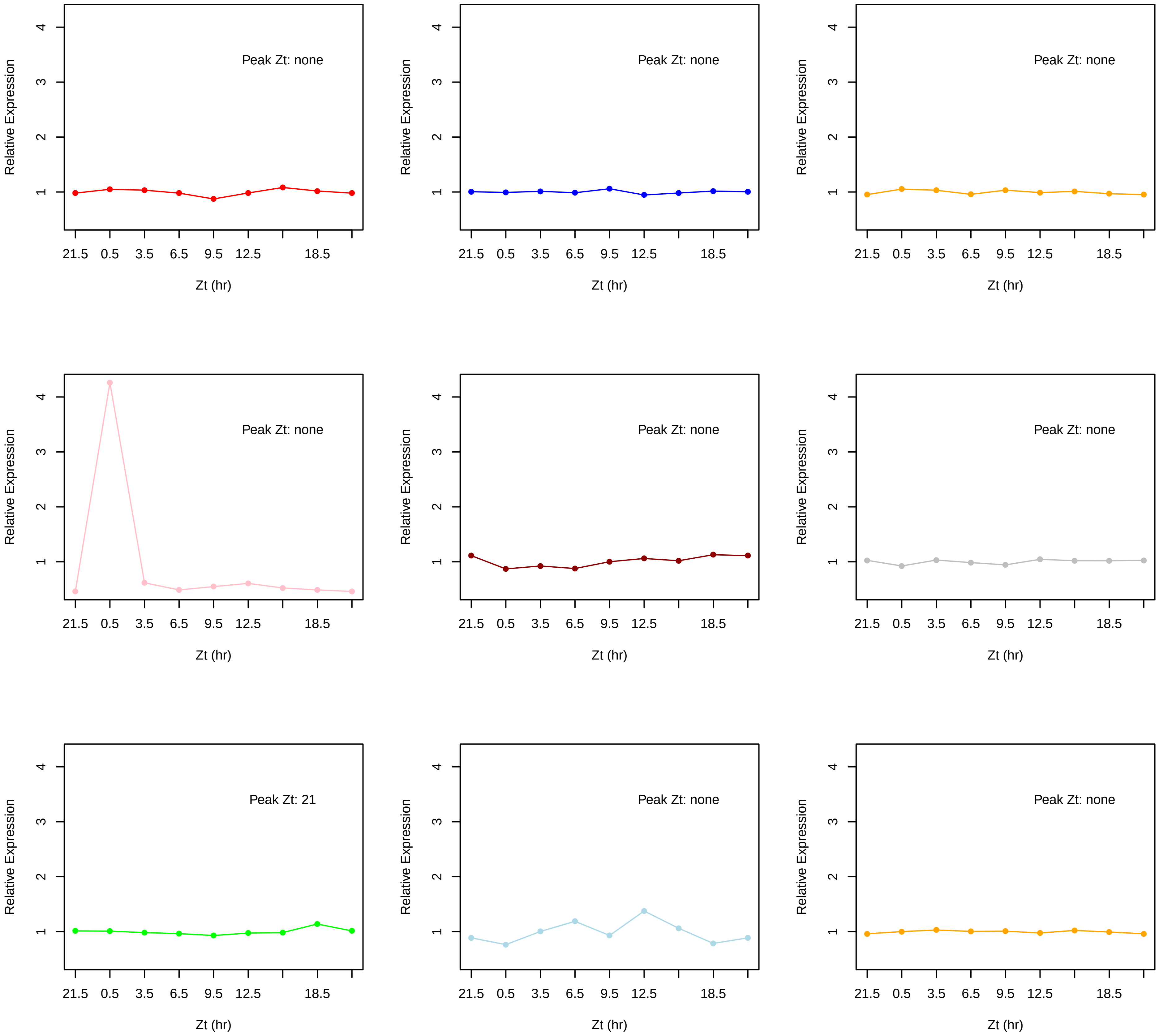

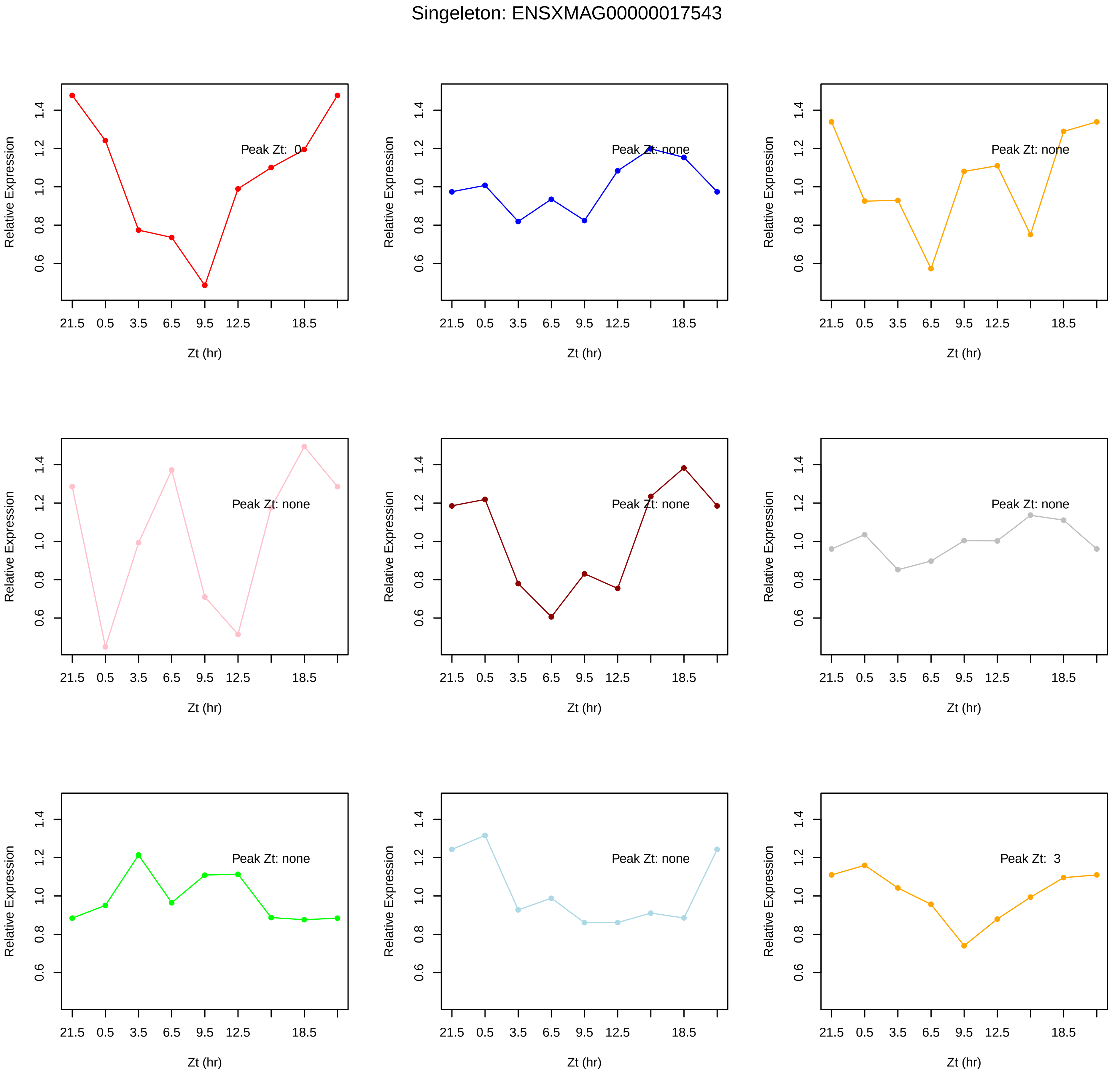
Singeleton: ENSXMAG00000017543
Peak Zt: 0
Peak Zt: none
Peak Zt: none
Peak Zt: none
Peak Zt: none
Peak Zt: none
Peak Zt: none
Peak Zt: none
Peak Zt: 3
Relative Expression
Zt (hr)
21.5
0.5
3.5
6.5
9.5
12.5
18.5

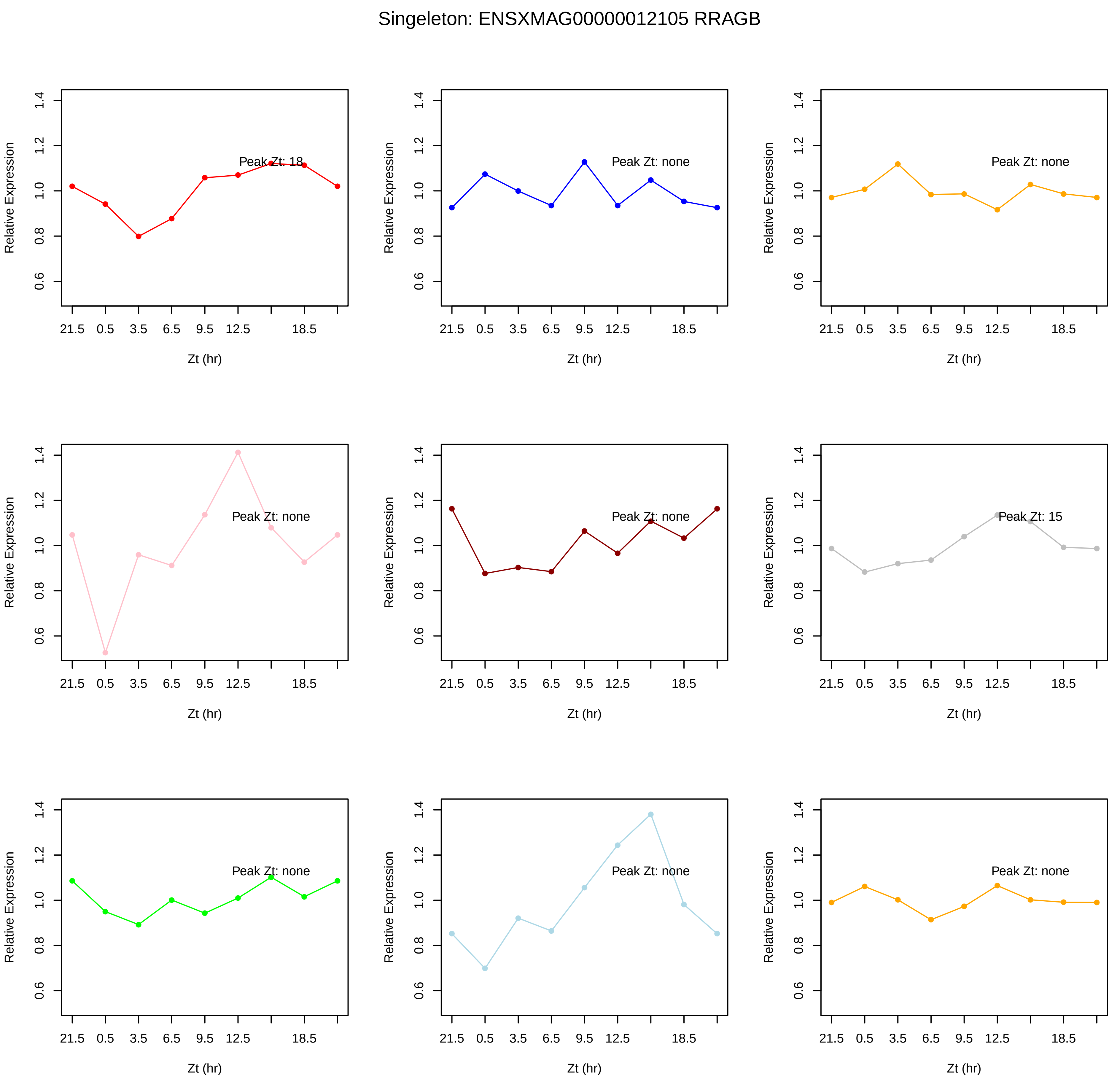
Singeleton: ENSXMAG00000012105 RRAGB
Relative Expression
Zt (hr)
Peak Zt: 18
Peak Zt: none
Peak Zt: none
Peak Zt: none
Peak Zt: none
Peak Zt: 15
Peak Zt: none
Peak Zt: none
Peak Zt: none
21.5
0.5
3.5
6.5
9.5
12.5
18.5
0.6
0.8
1.0
1.2
1.4

Singeleton: ENSXMAG00000000355 actr1

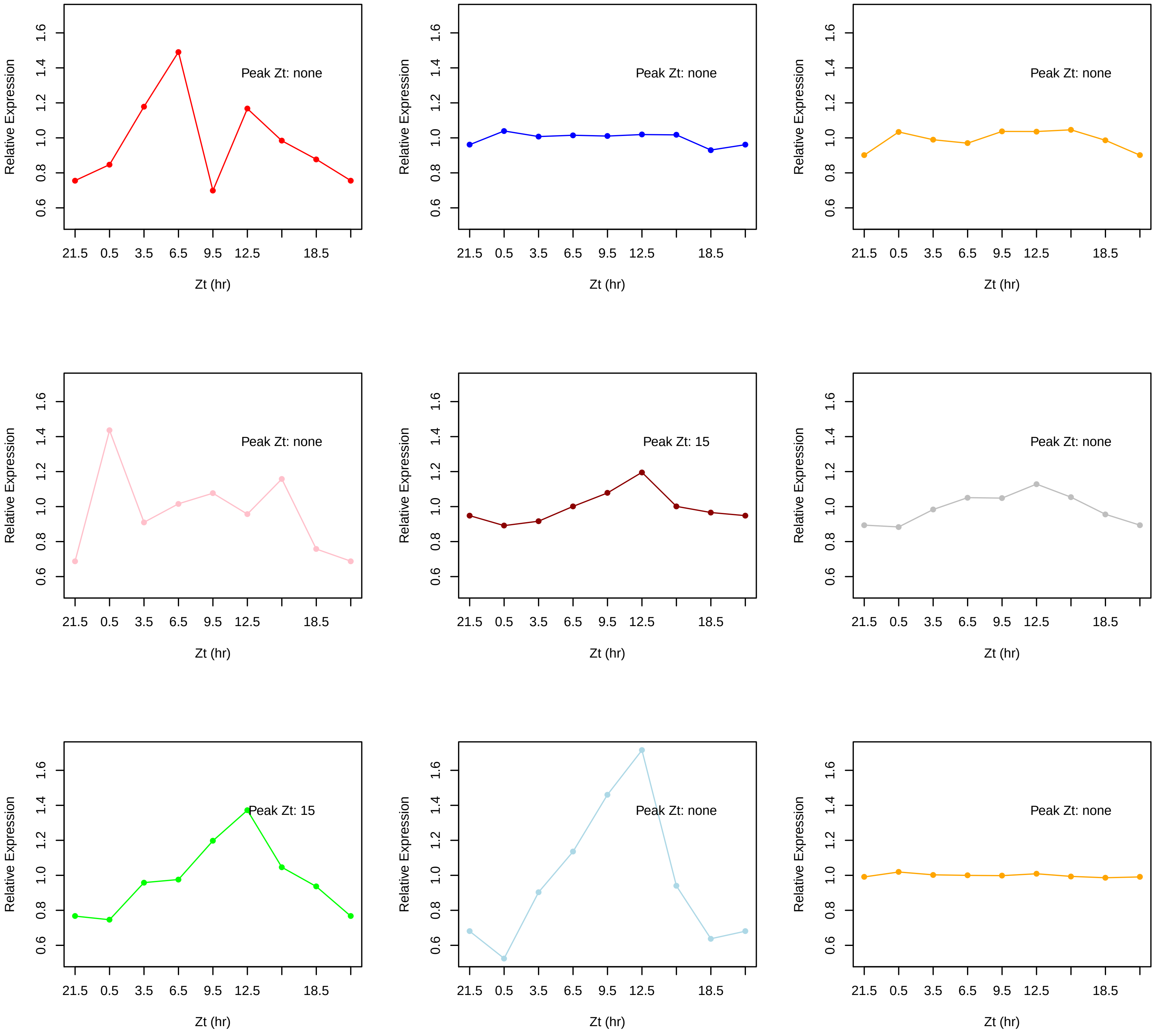

Singeleton: ENSXMAG00000022314 kctd9a

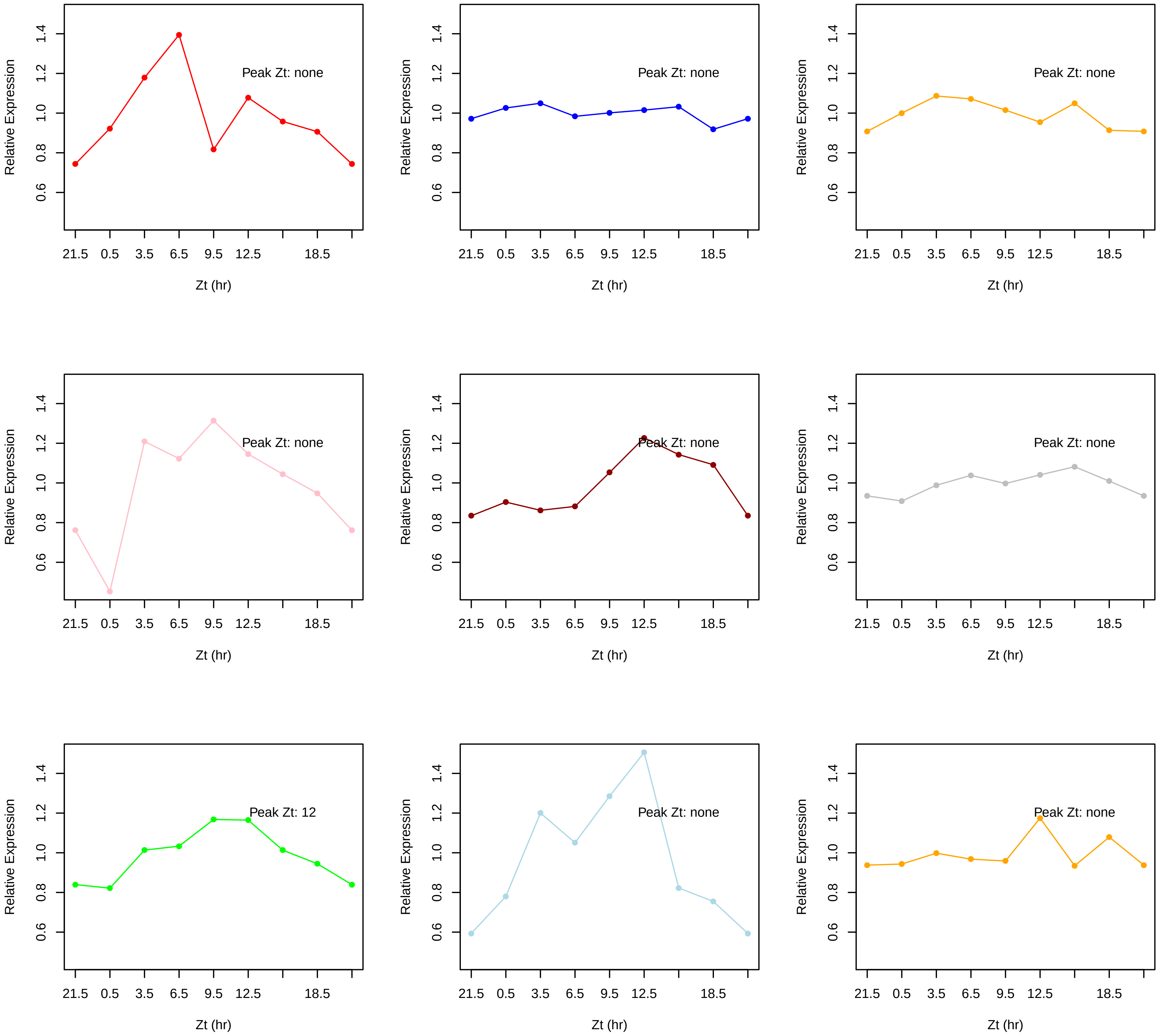

Singleton: ENSXMAG00000021086 gramd2aa

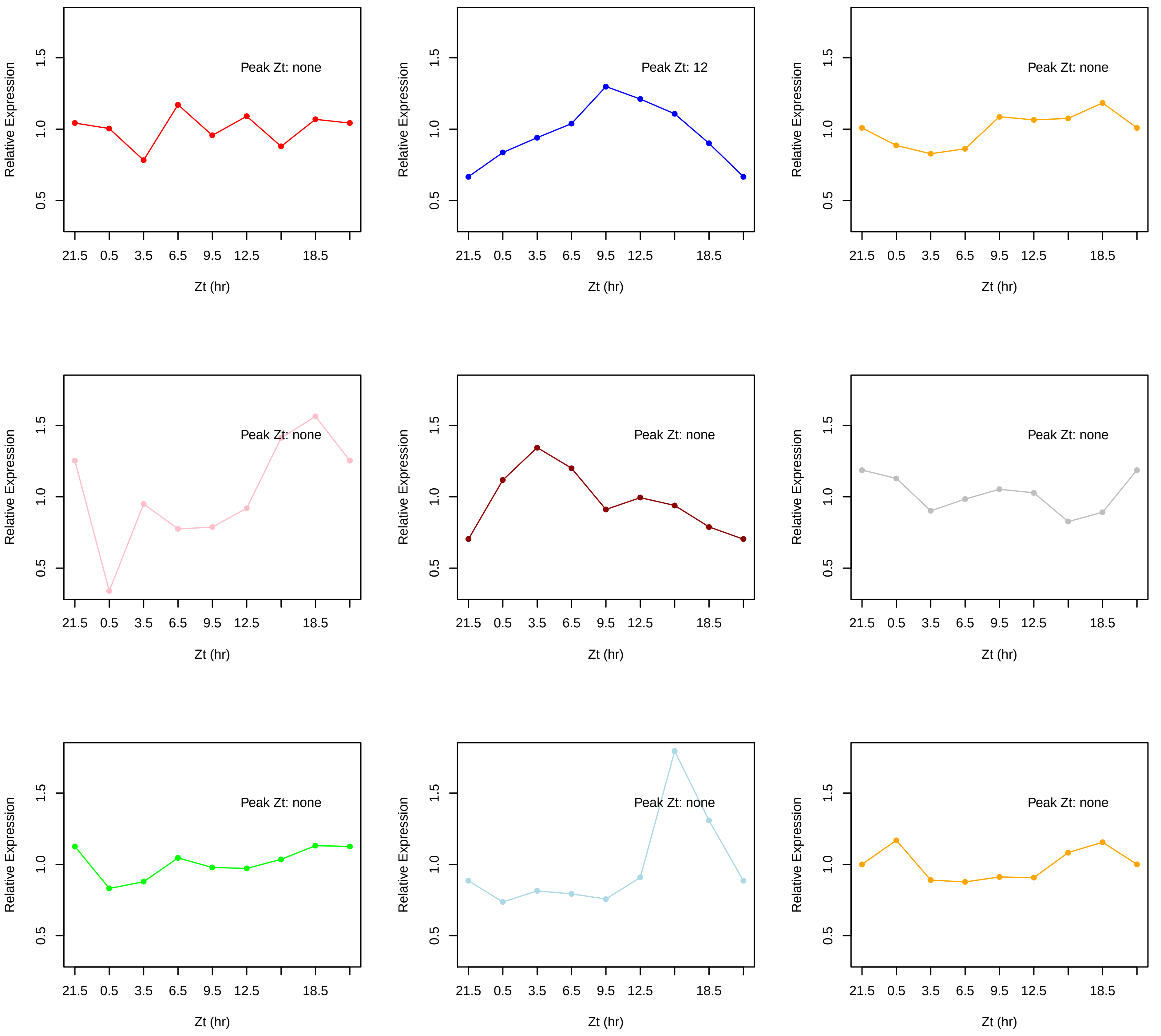

Singleton: ENSXMAG00000012127 brwd3

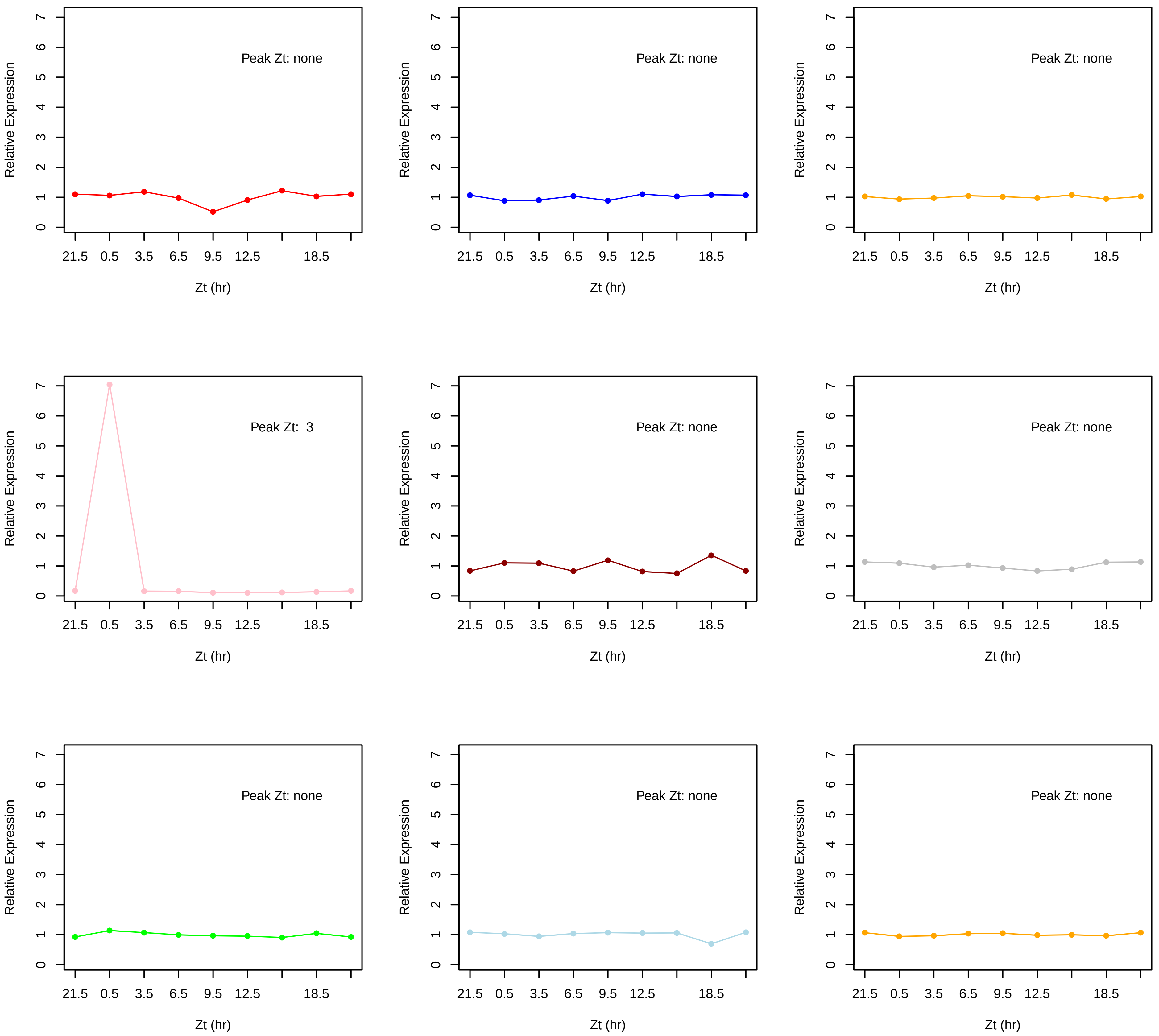

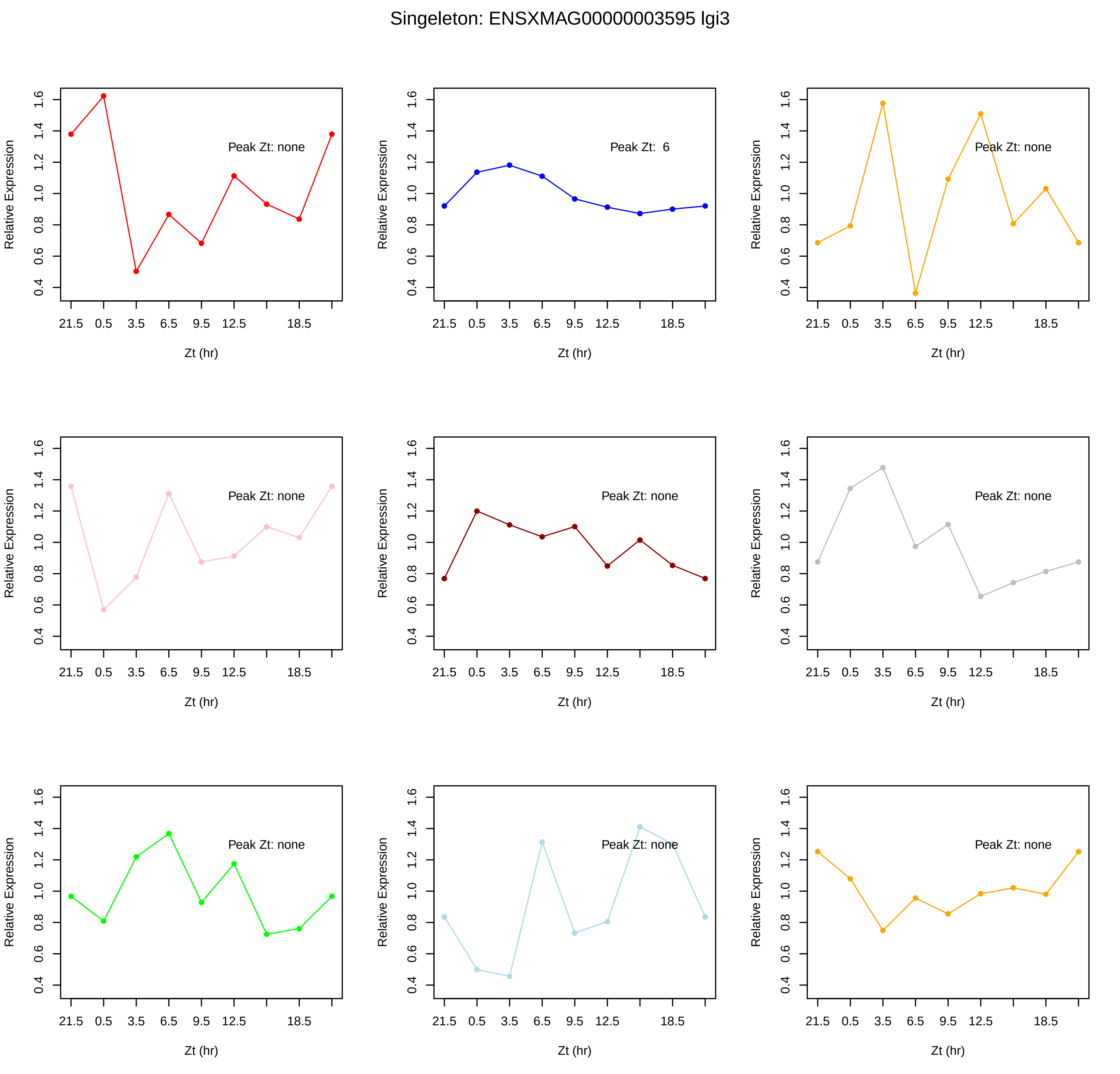
Singeleton: ENSXMAG00000003595 lgi3
Peak Zt: none
Peak Zt: 6
Peak Zt: none
Peak Zt: none
Peak Zt: none
Peak Zt: none
Peak Zt: none
Peak Zt: none
Peak Zt: none
Relative Expression
Zt (hr)
21.5
0.5
3.5
6.5
9.5
12.5
18.5
0.4
0.6
0.8
1.0
1.2
1.4
1.6

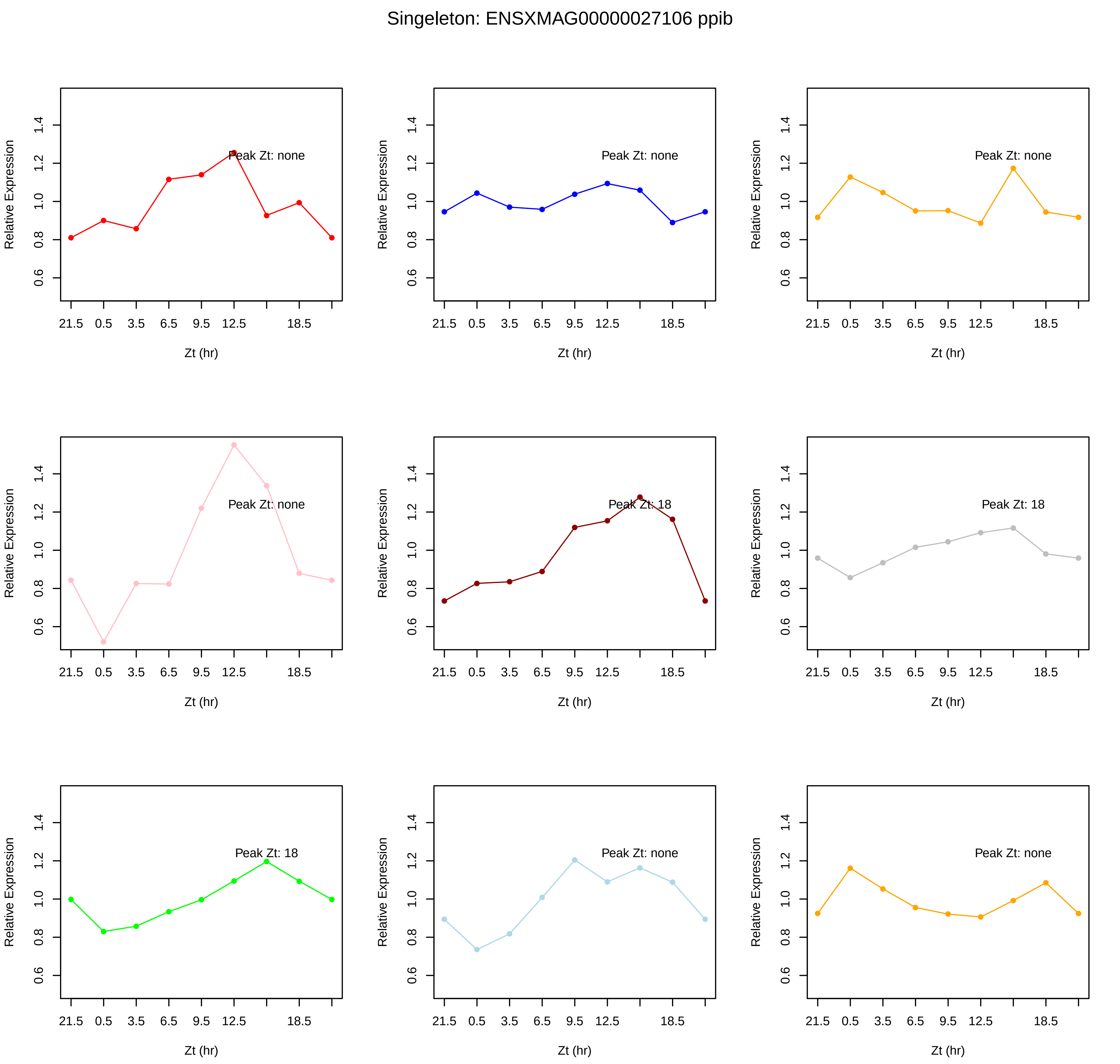

Singeleton: ENSXMAG00000027106 ppib
Peak Zt: none
Peak Zt: none
Peak Zt: none
Peak Zt: none
Peak Zt: 18
Peak Zt: 18
Peak Zt: 18
Peak Zt: none
Peak Zt: none
Relative Expression
Zt (hr)
21.5
0.5
3.5
6.5
9.5
12.5
18.5
0.6
0.8
1.0
1.2
1.4

Singleton: ENSXMAG00000022428

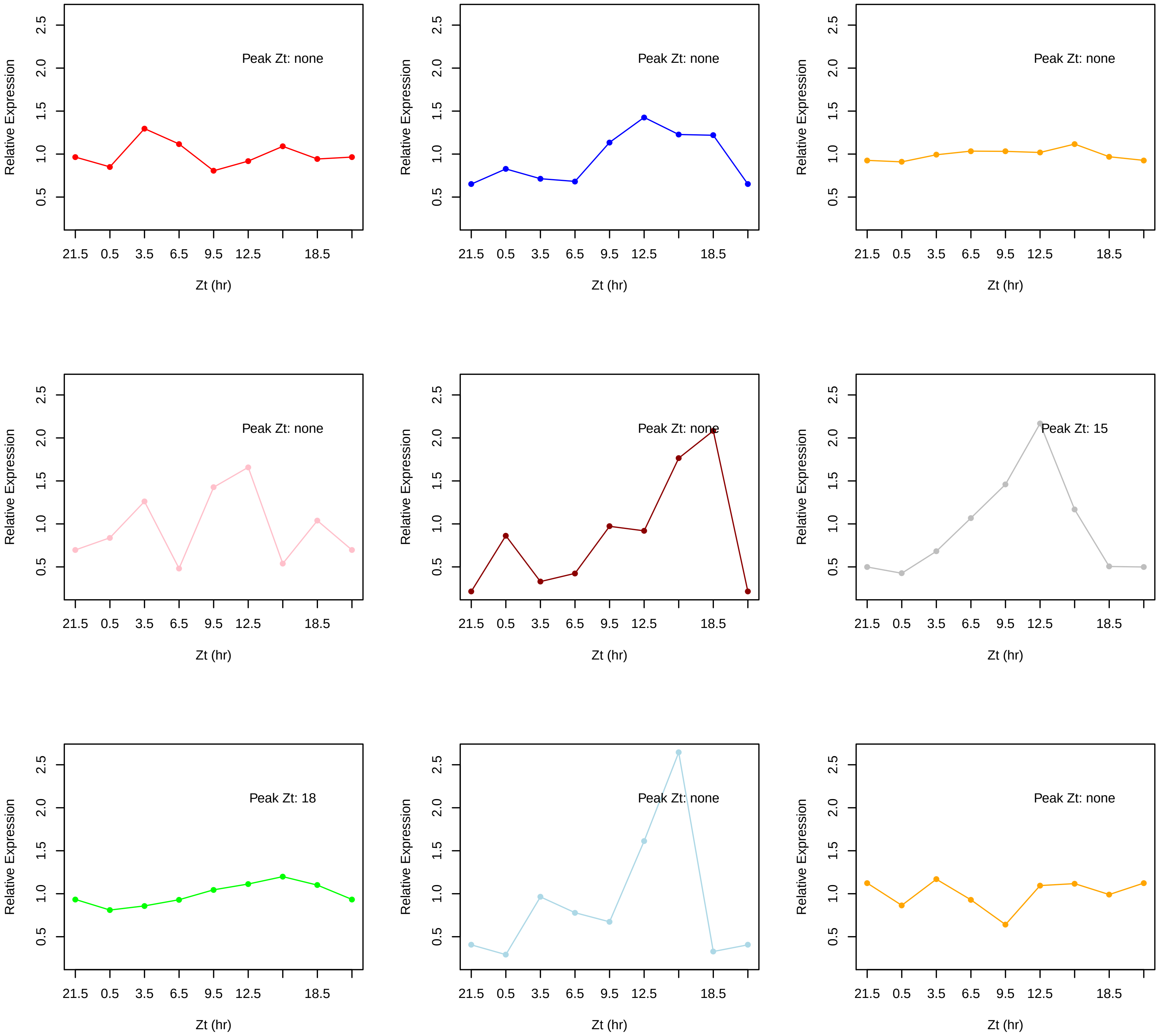

Singeleton: ENSXMAG00000016490 nt5dc3

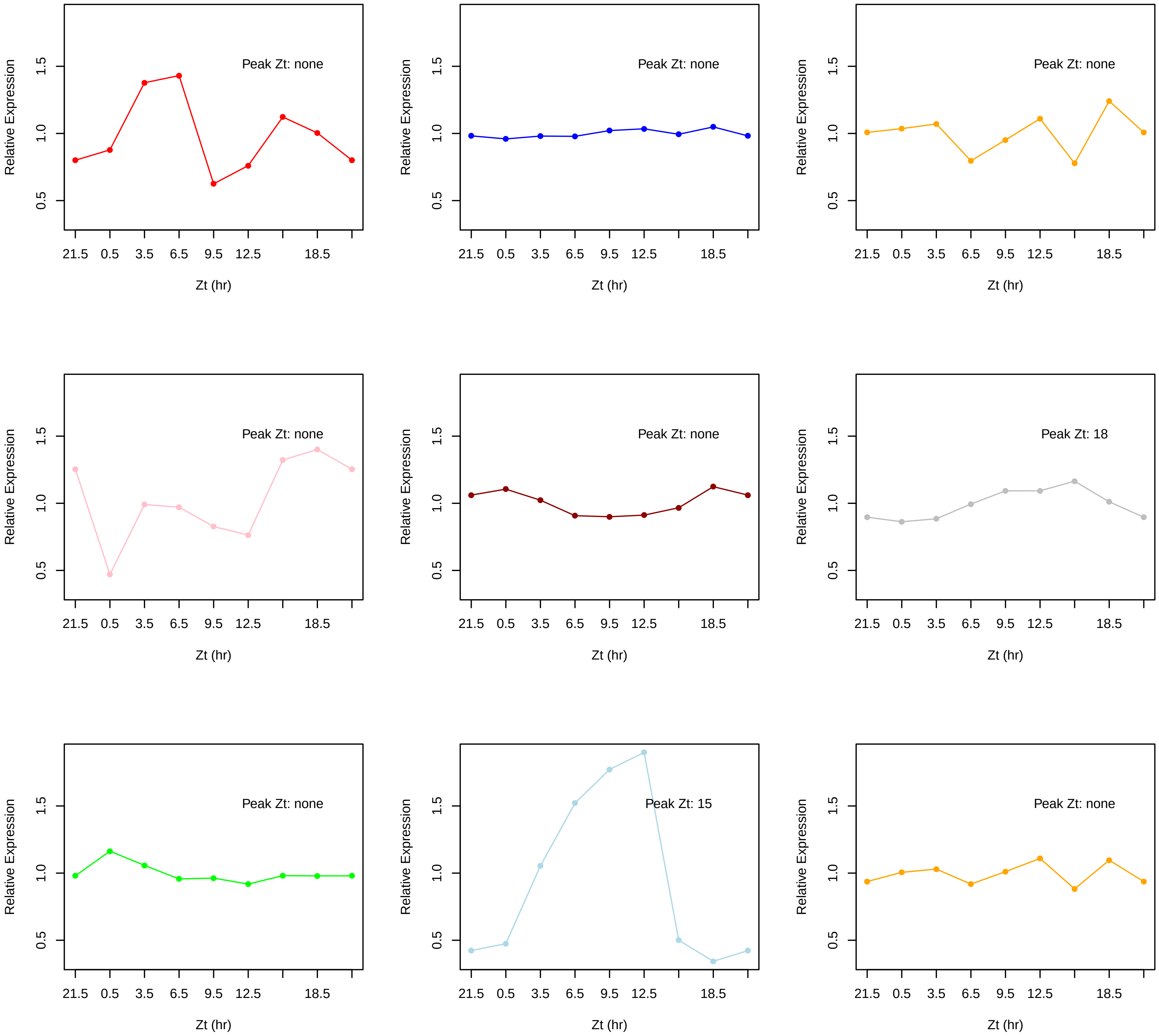

Singeleton: ENSXMAG00000001222 entpd4

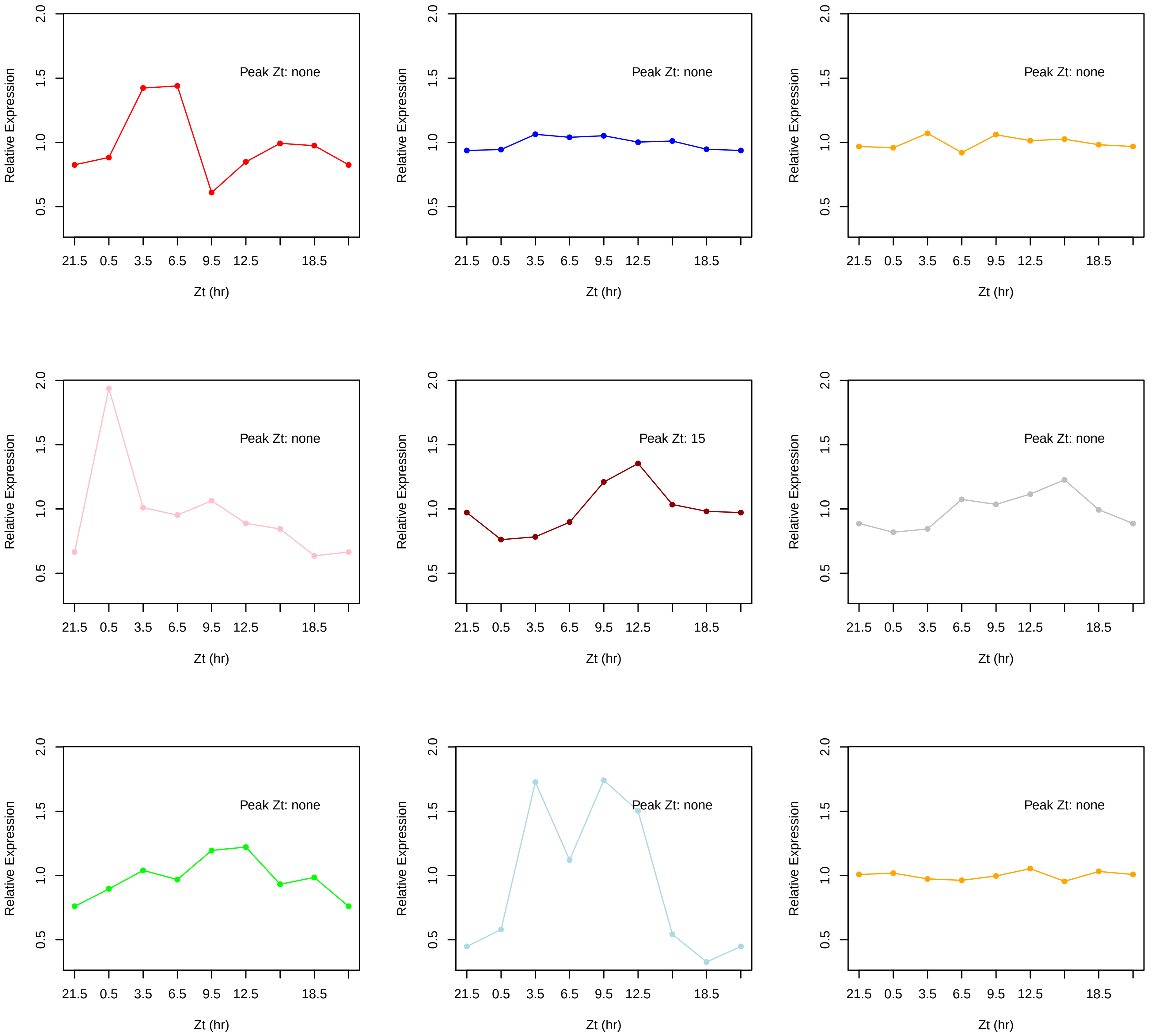

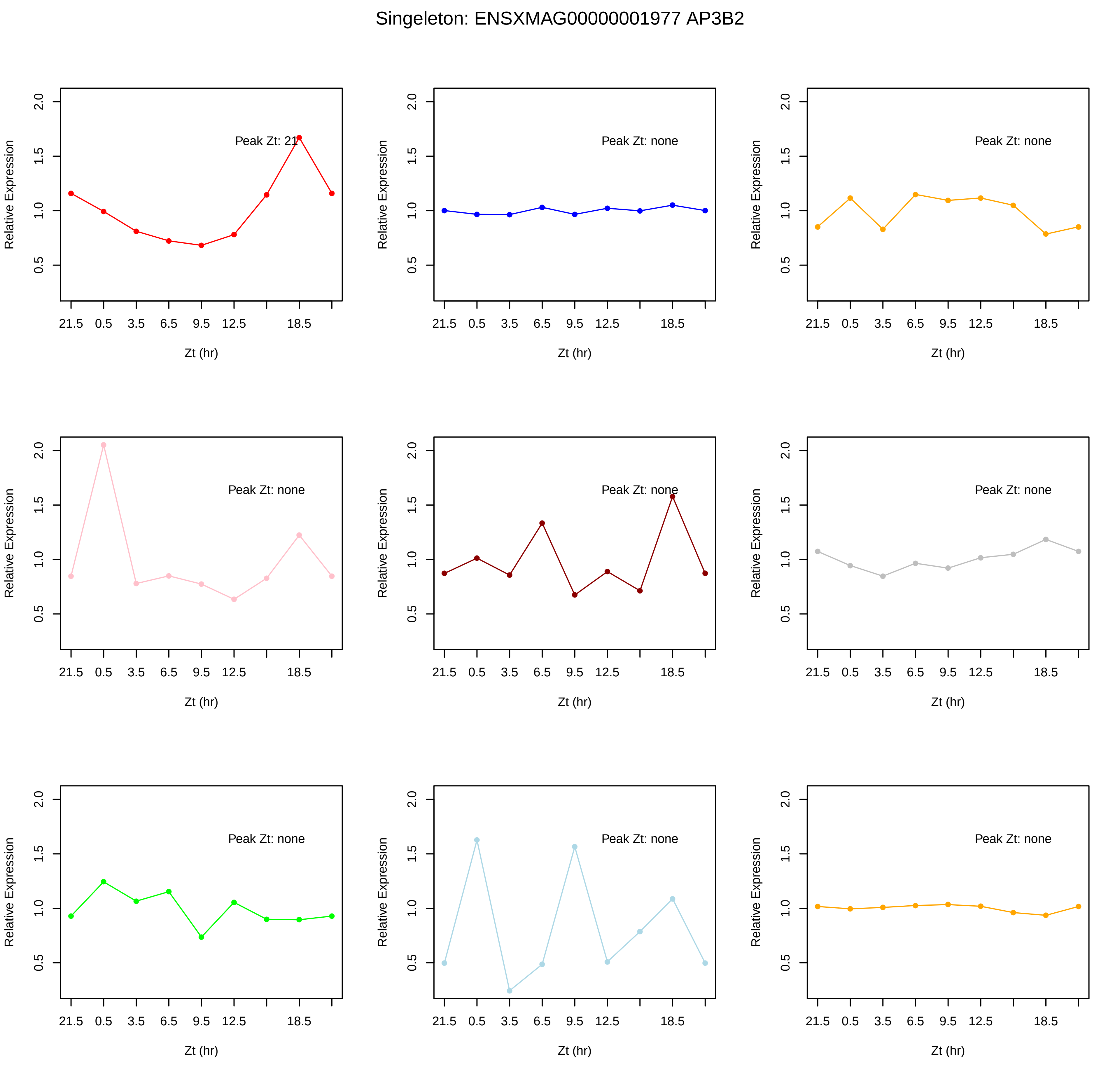
Singeleton: ENSXMAG00000001977 AP3B2
Peak Zt: 21
Peak Zt: none
Peak Zt: none
Peak Zt: none
Peak Zt: none
Peak Zt: none
Peak Zt: none
Peak Zt: none
Peak Zt: none
Relative Expression
Zt (hr)
21.5
0.5
3.5
6.5
9.5
12.5
18.5
0.5
1.0
1.5
2.0

Singeleton: ENSXMAG00000016460 hsp90b1

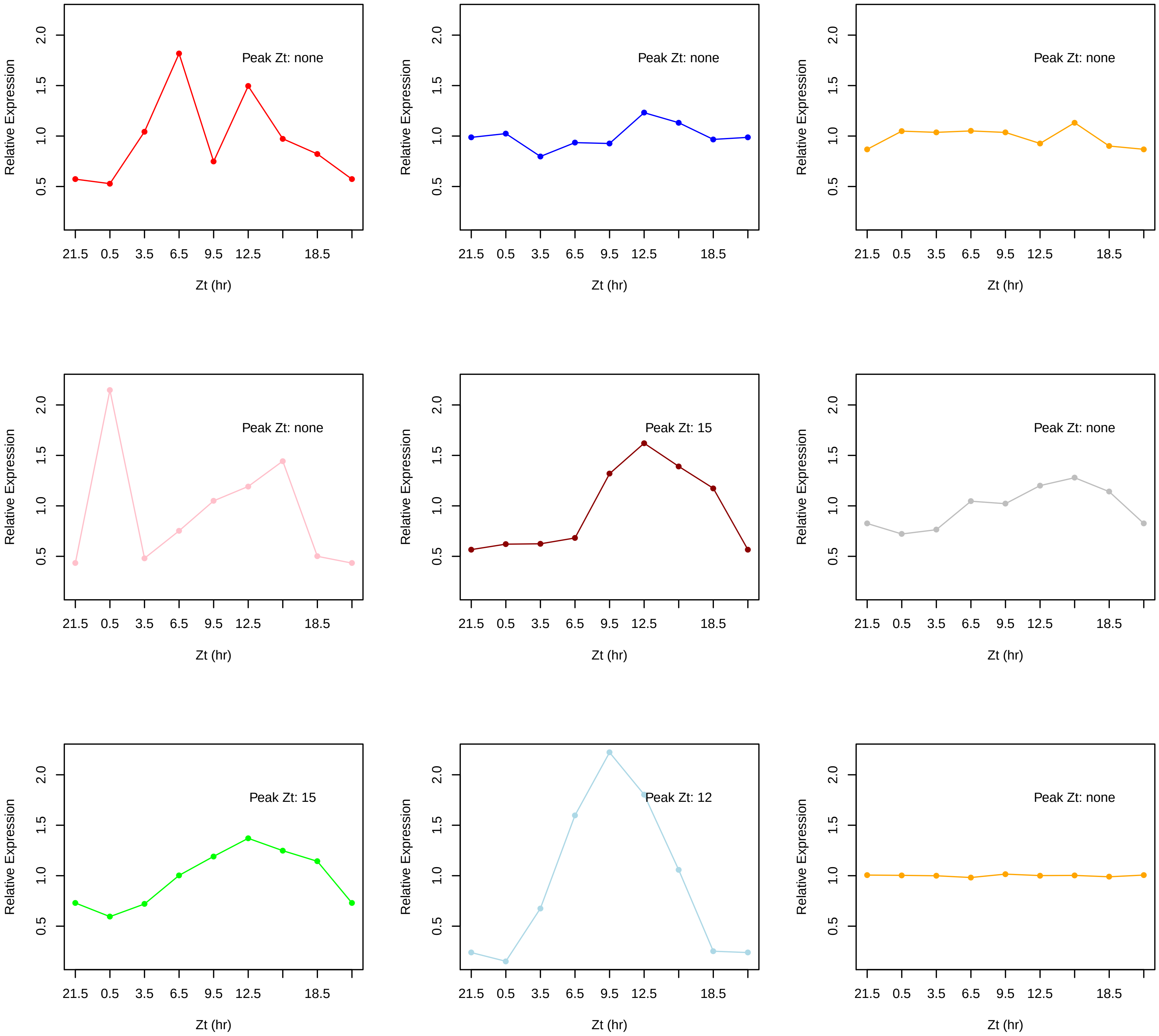

Singeleton: ENSXMAG00000011817 SH3BGRL

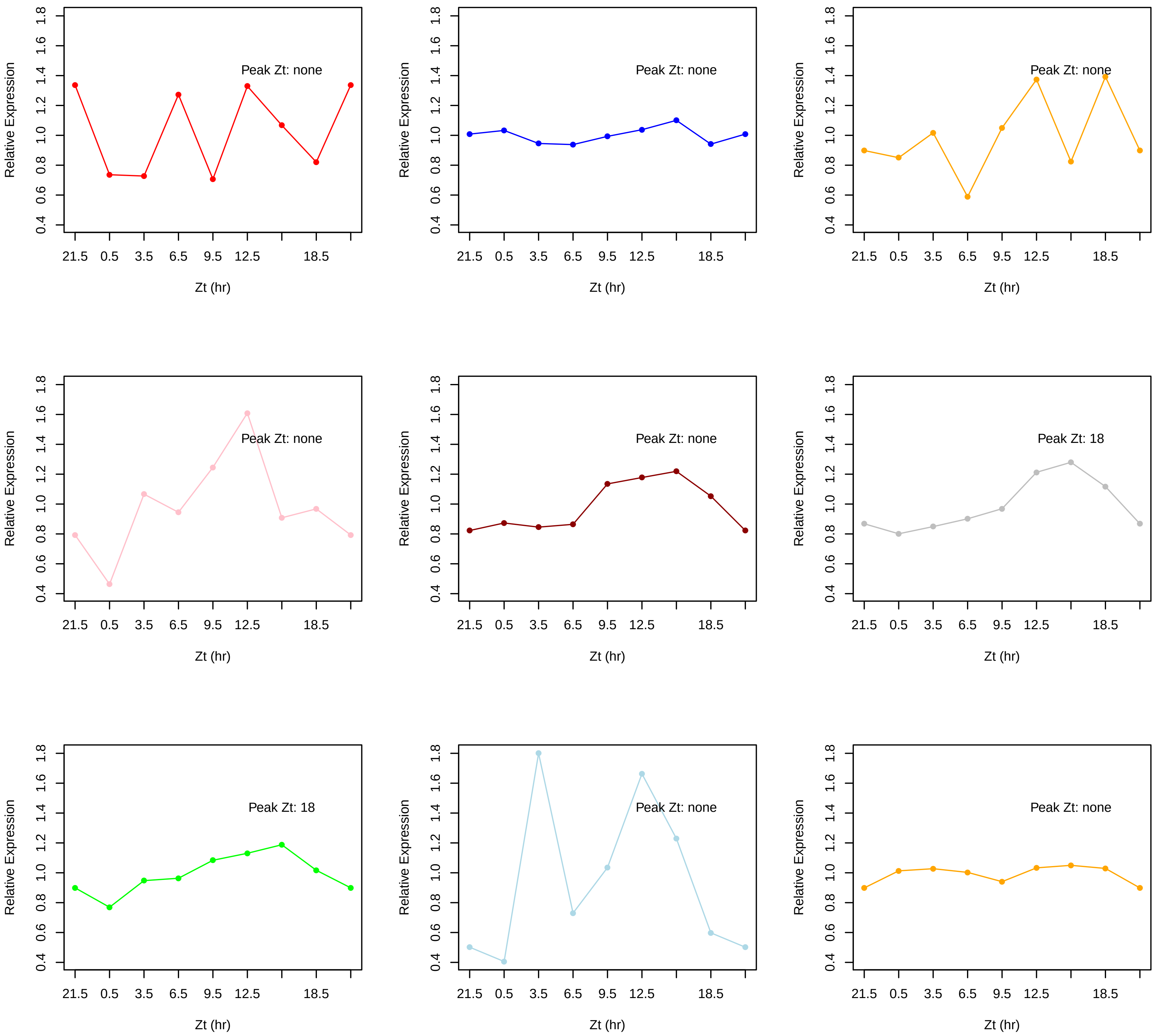

Singleton: ENSXMAG00000022470 oaz2a

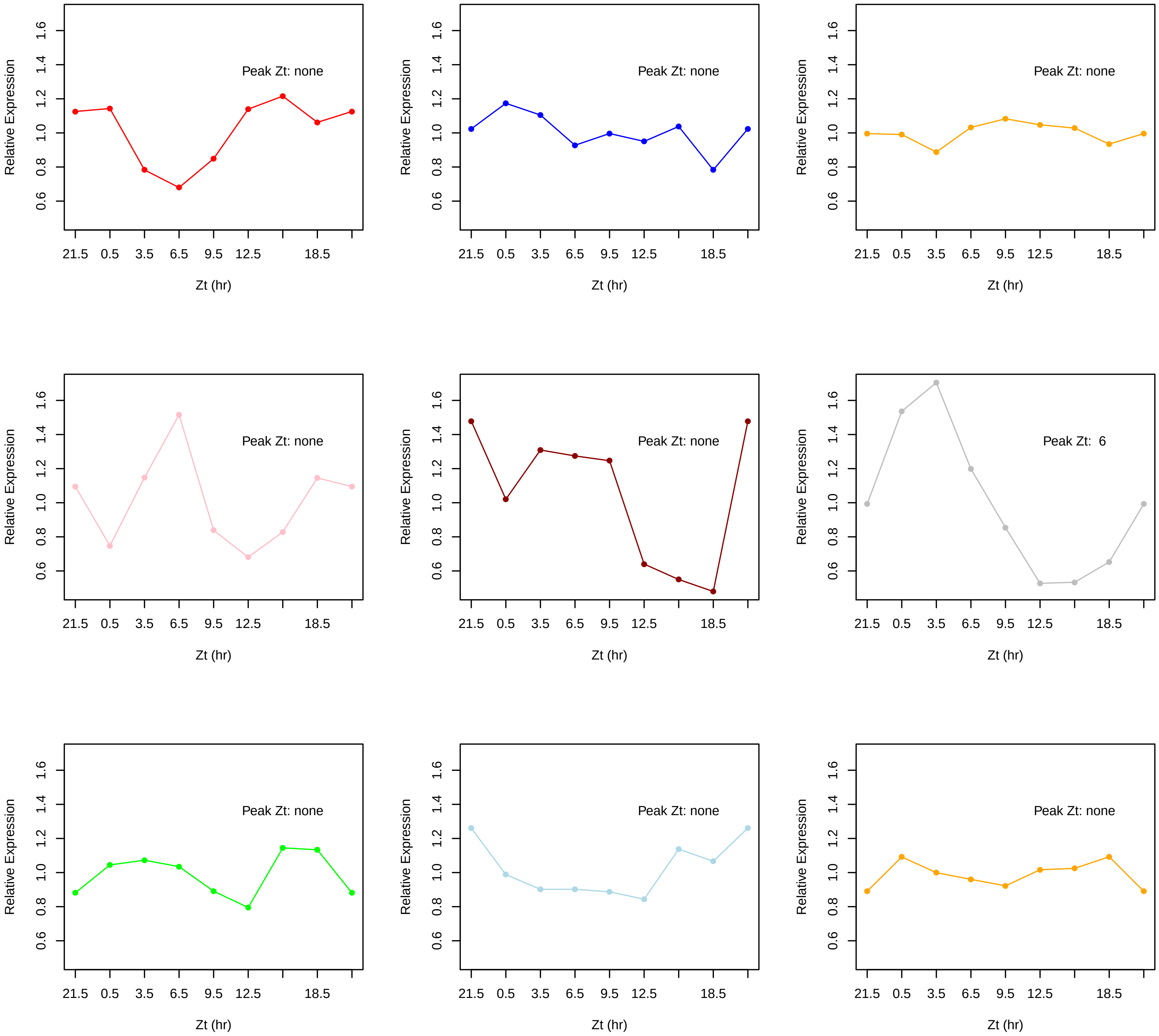

Singeleton: ENSXMAG00000011091 PSMA4

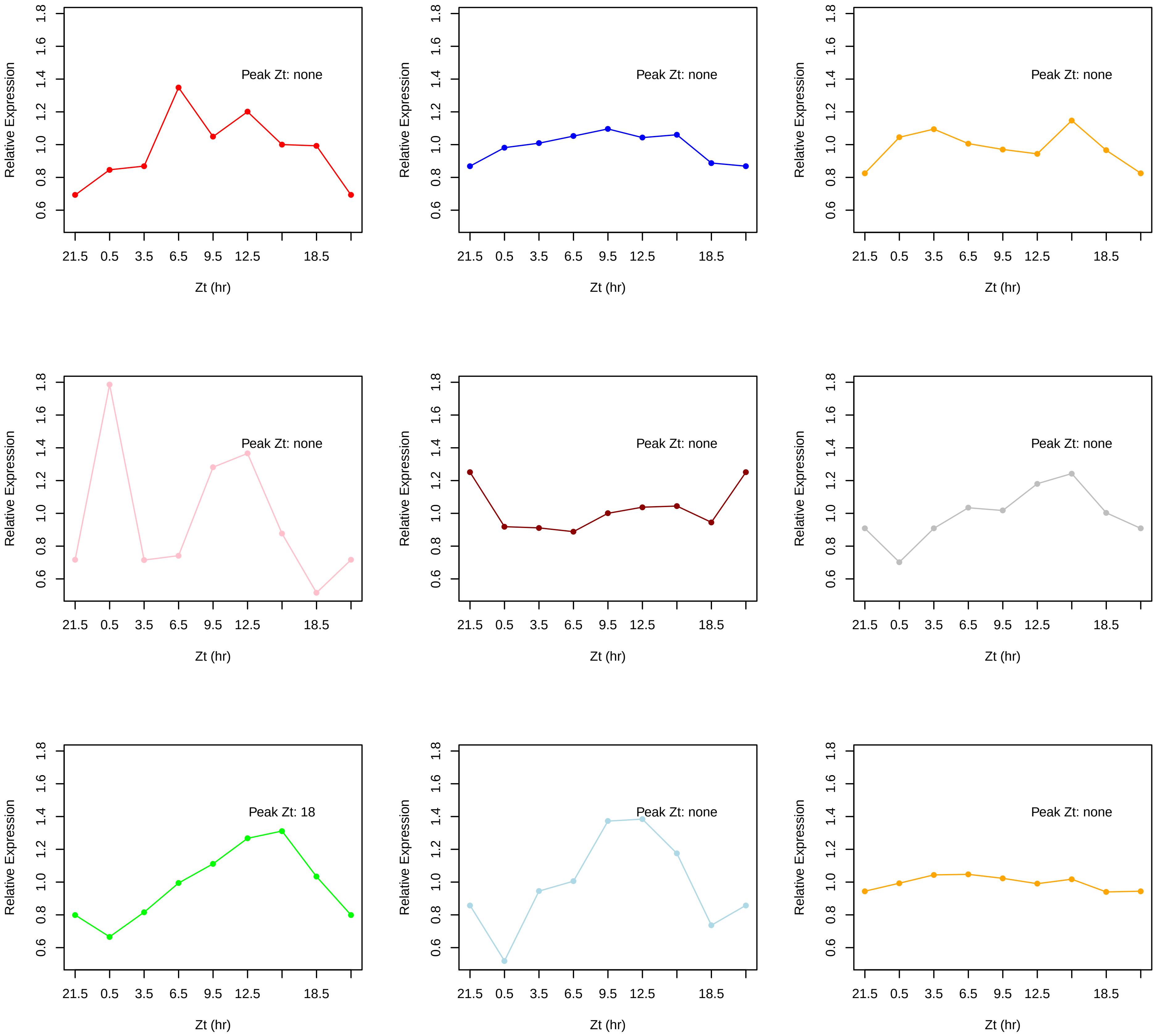

Singeleton: ENSXMAG00000002195 ireb2

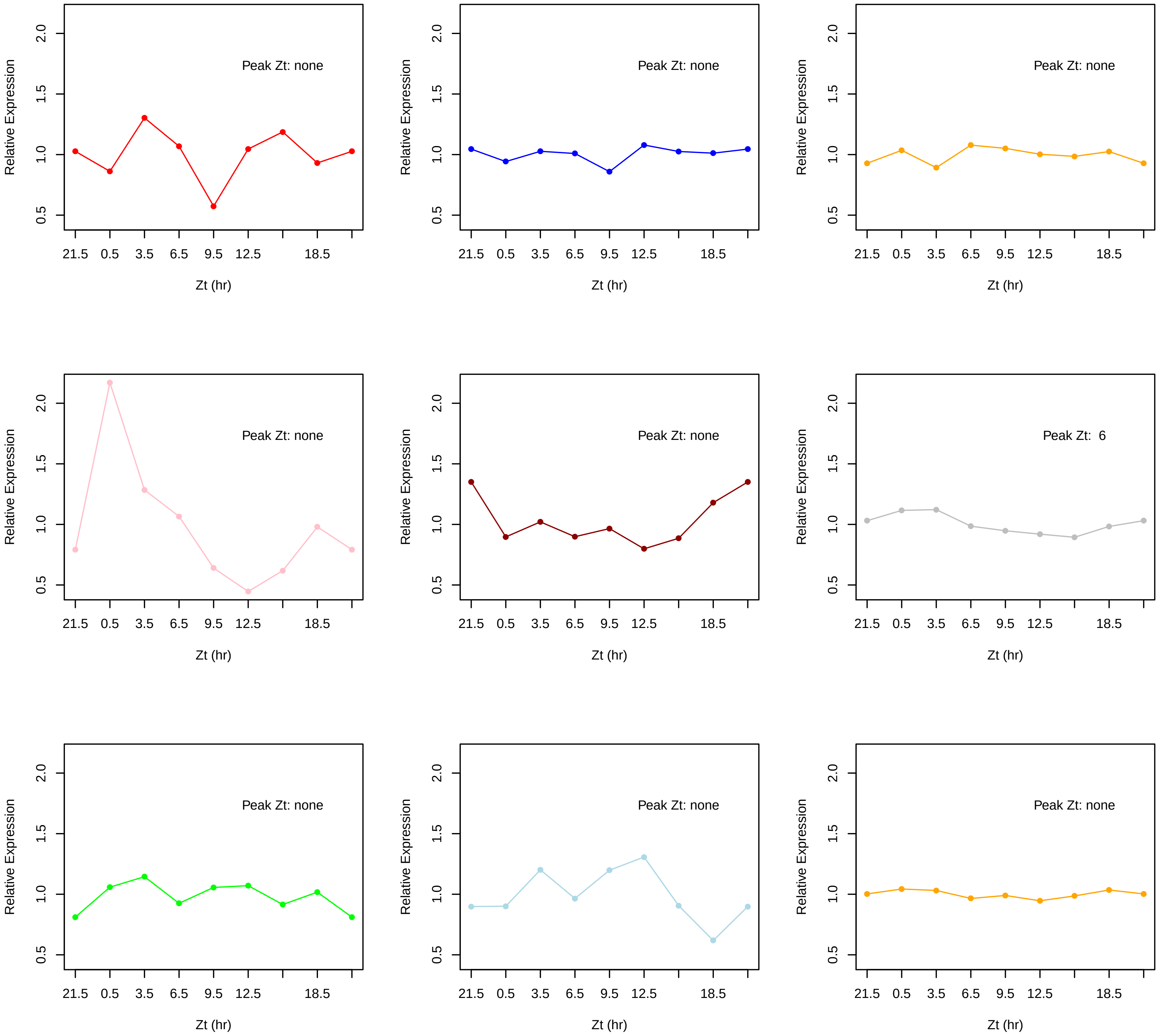

Singeleton: ENSXMAG00000011838 rps6kal

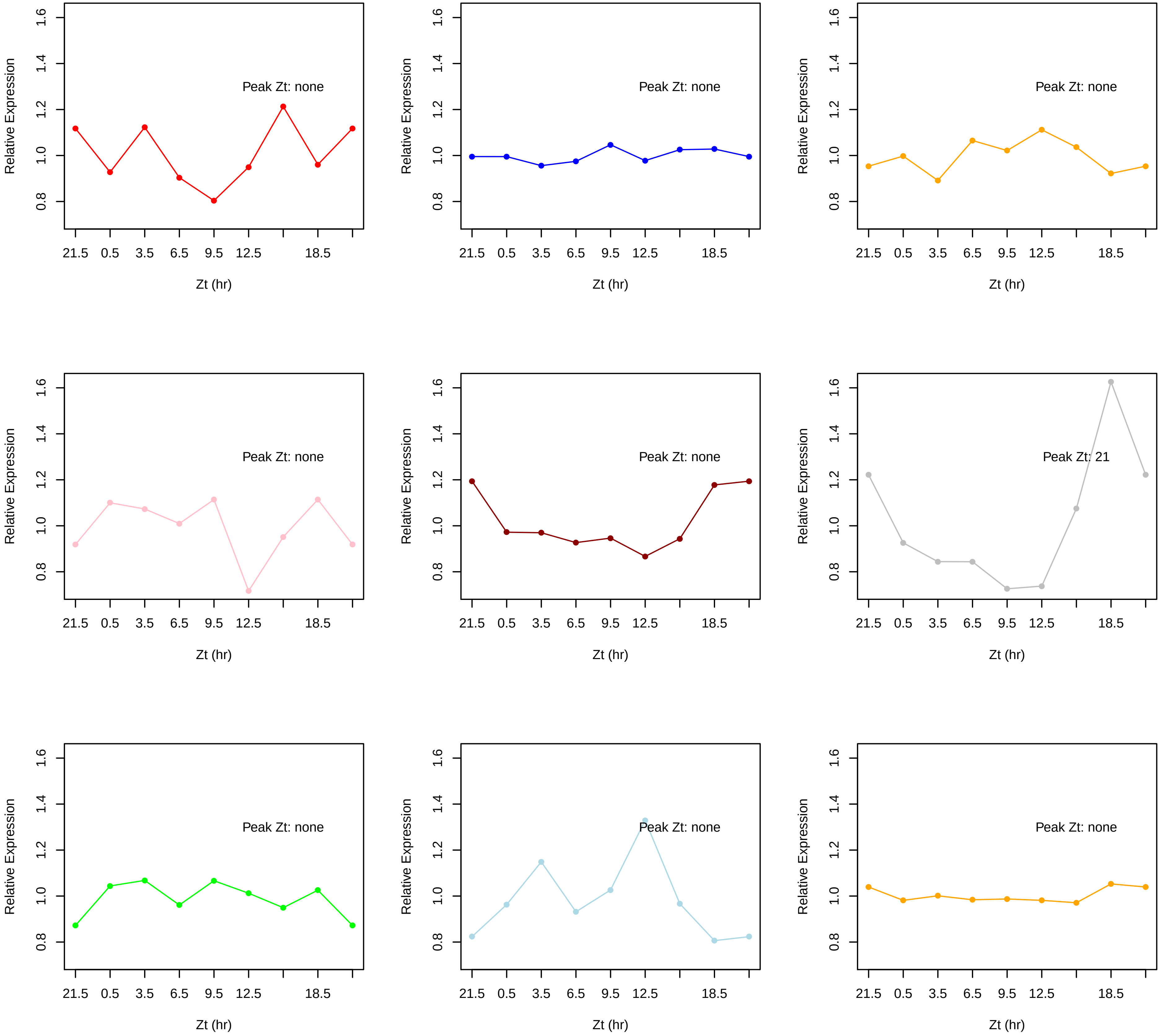

Singeleton: ENSXMAG00000005234 nfybb

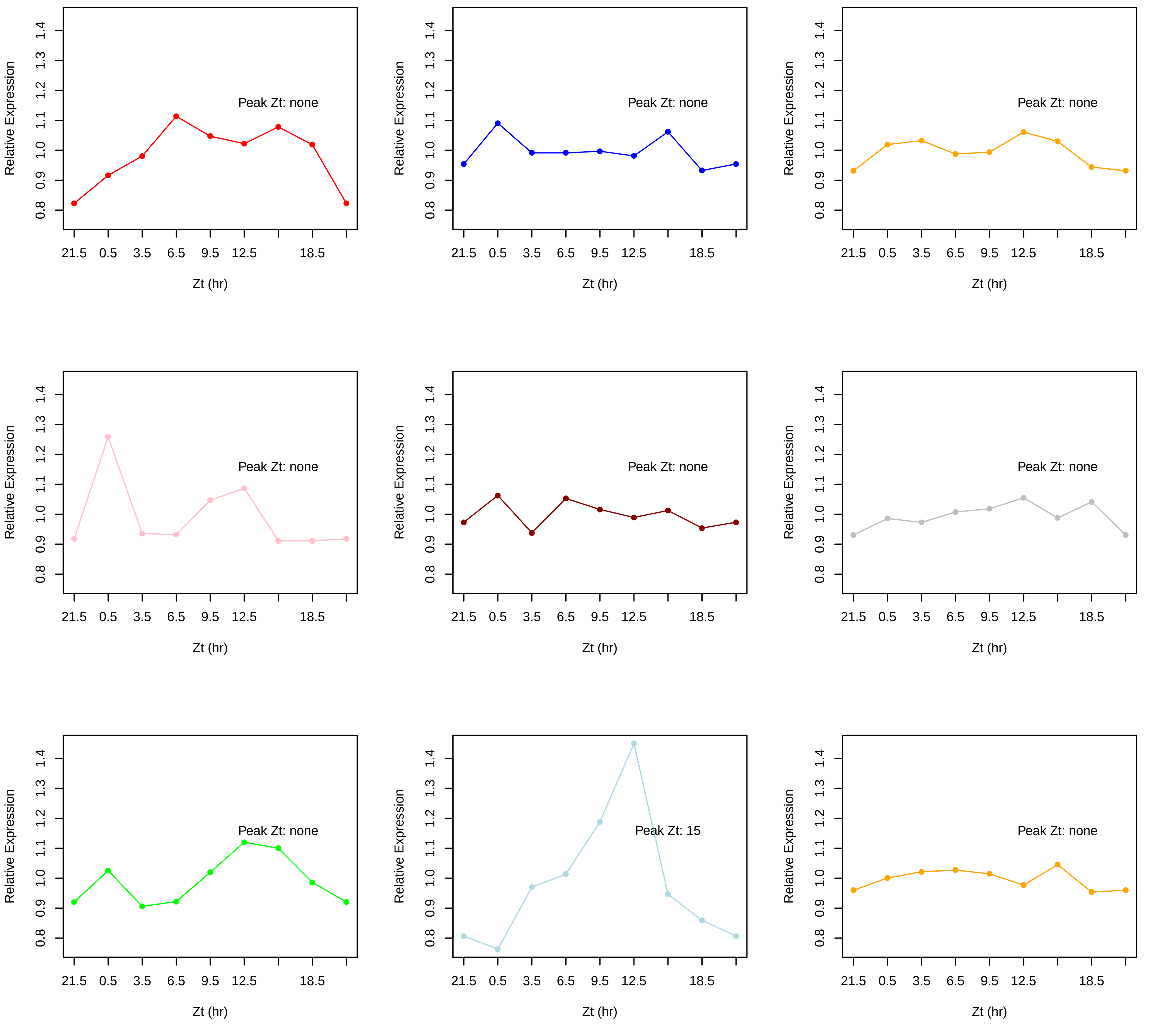

Singeleton: ENSXMAG00000005250 slc41a2b

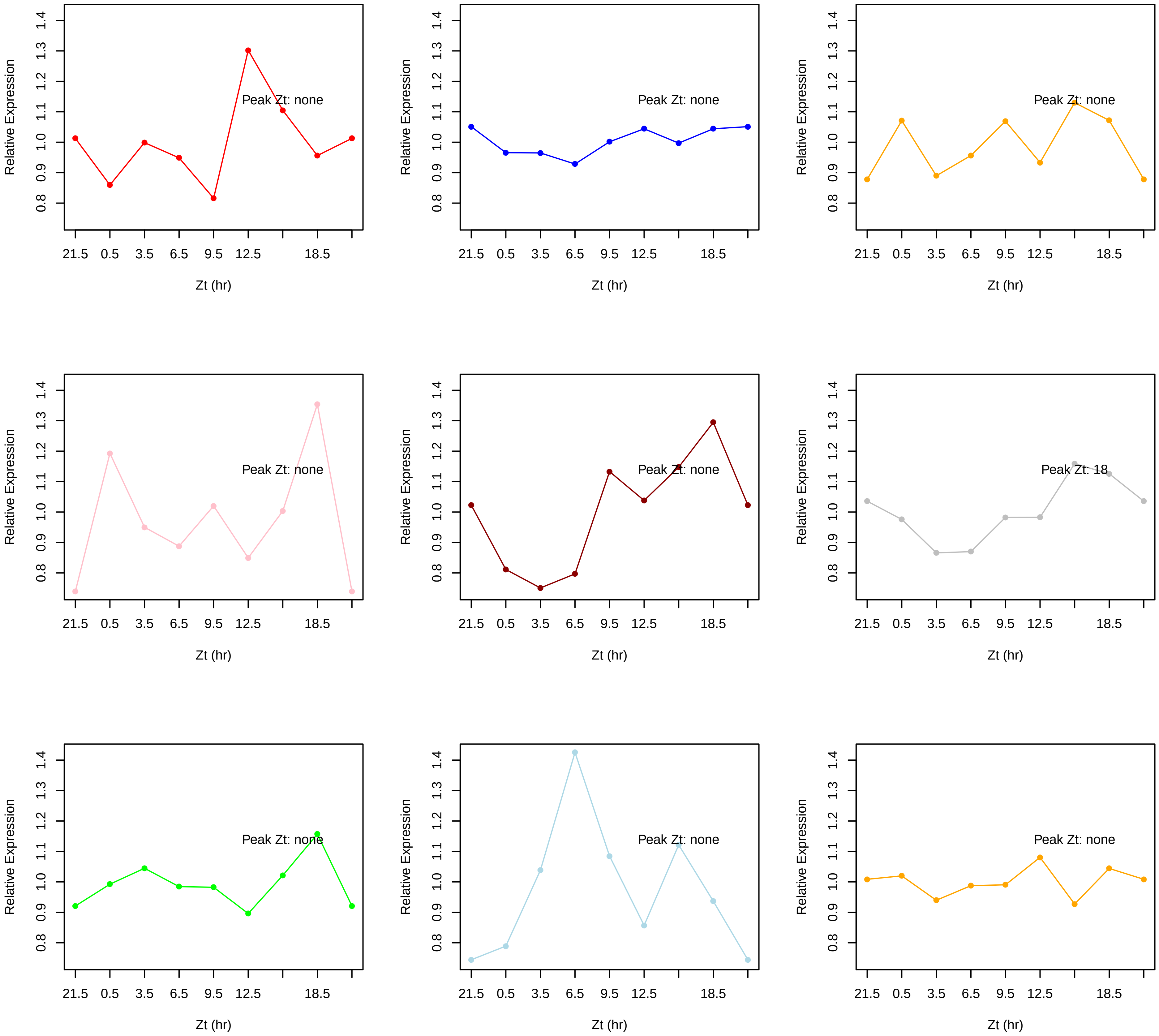

Singeleton: ENSXMAG00000011901 hdx

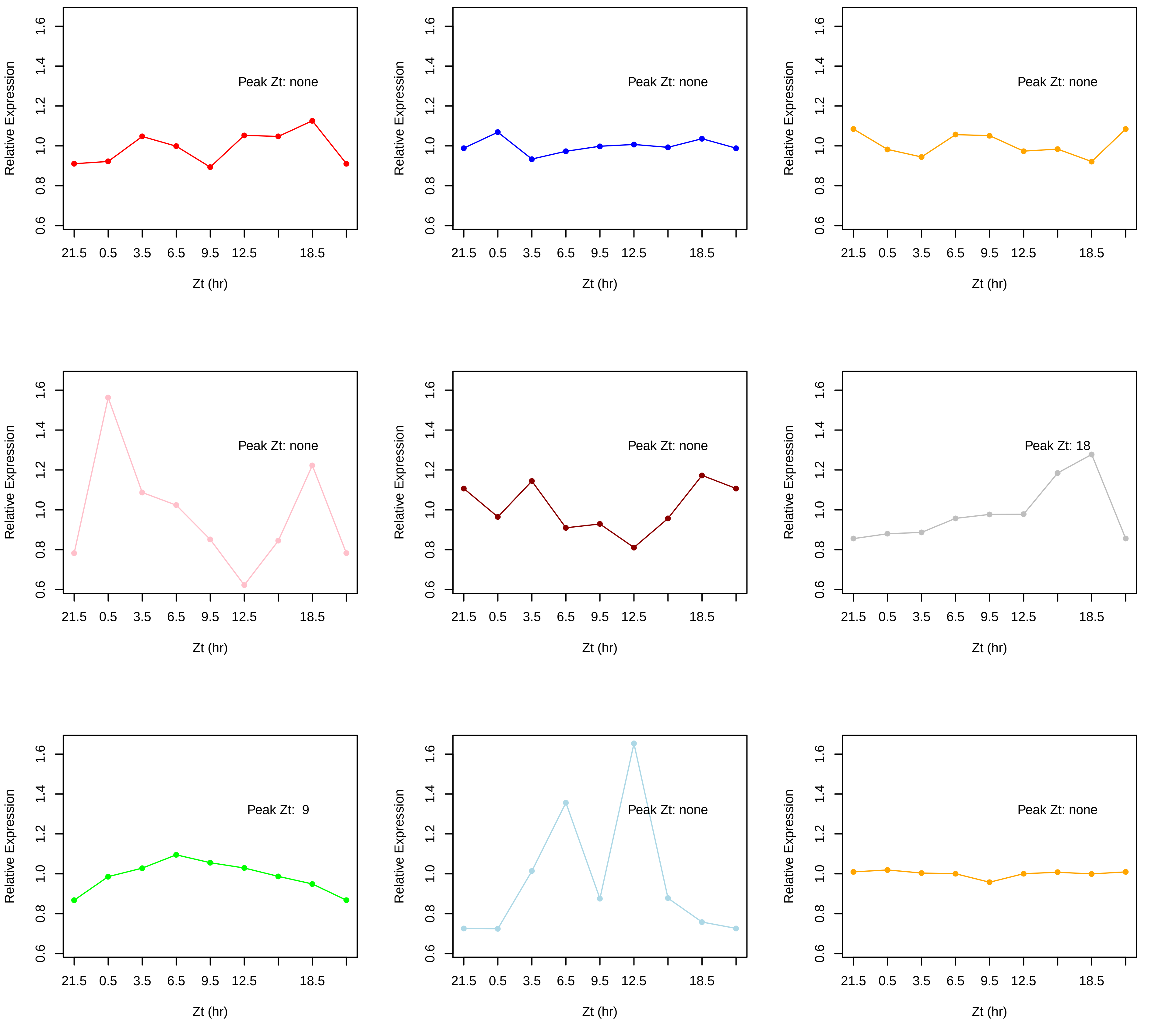

Singeleton: ENSXMAG00000002207 wdr61

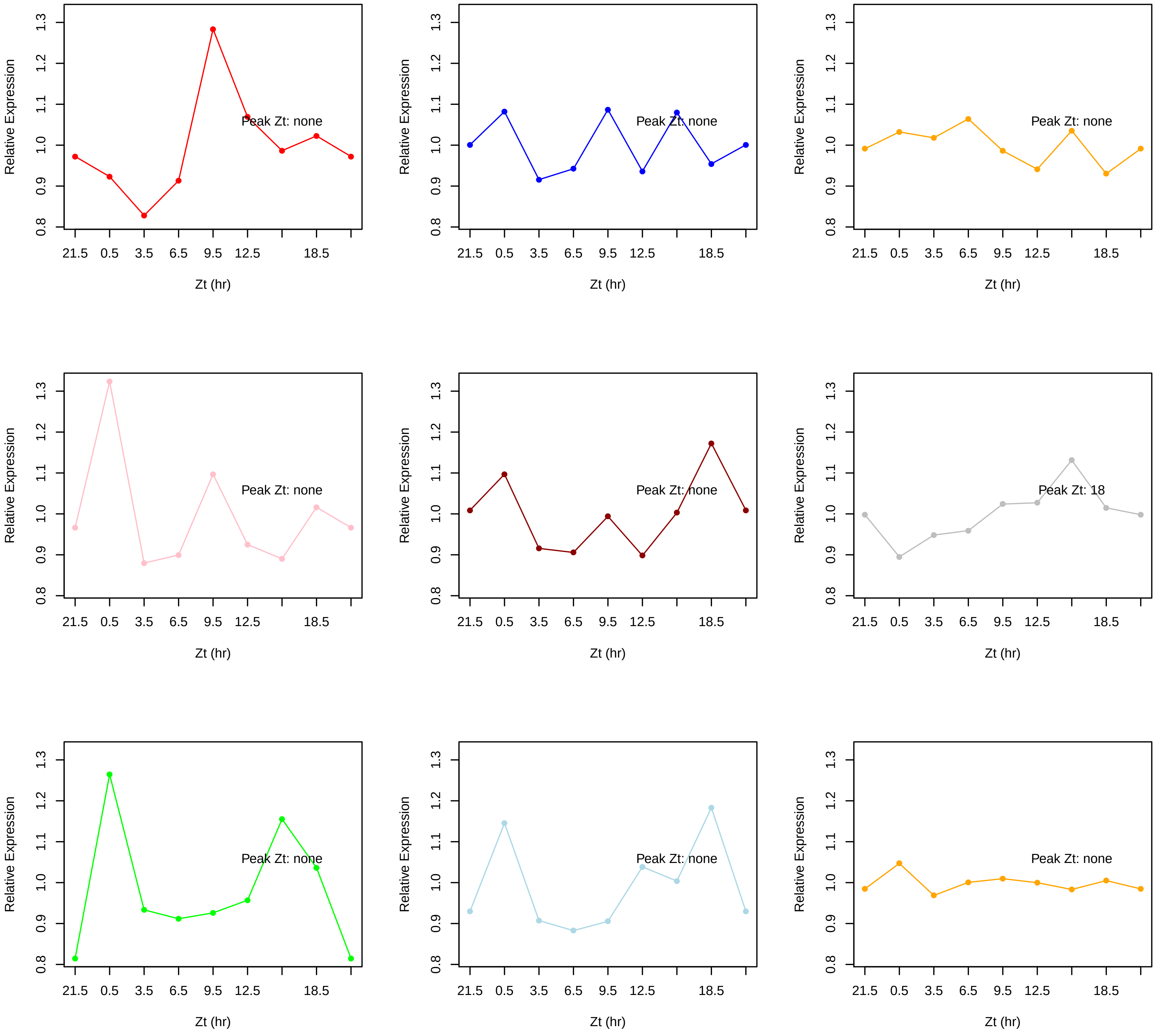

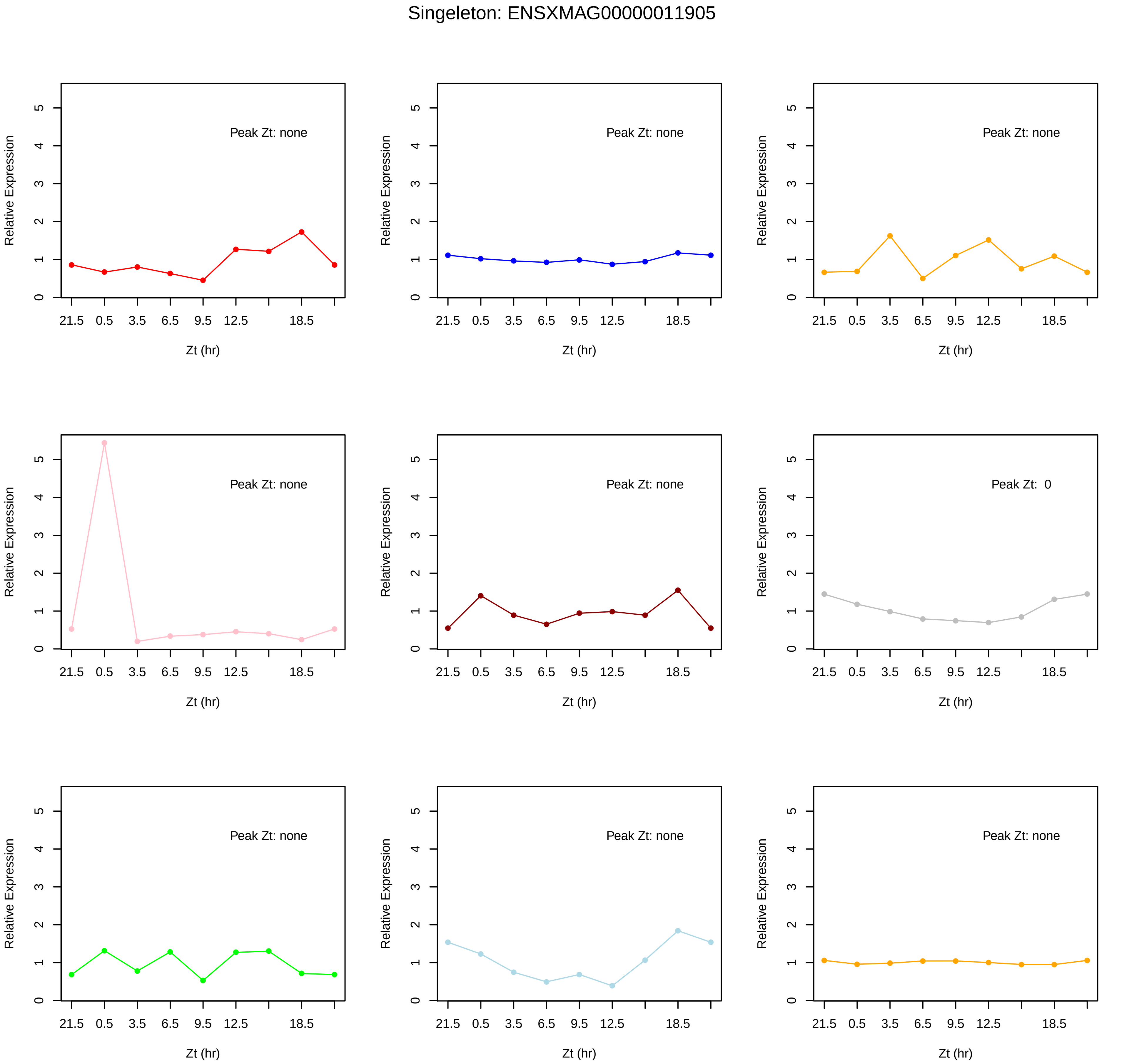
Singleton: ENSXMAG00000011905
Peak Zt: none
Peak Zt: none
Peak Zt: none
Peak Zt: none
Peak Zt: none
Peak Zt: 0
Peak Zt: none
Peak Zt: none
Peak Zt: none
Relative Expression
Zt (hr)
21.5
0.5
3.5
6.5
9.5
12.5
18.5
0
1
2
3
4
5

Singleton: ENSXMAG00000005276 aldh1l2

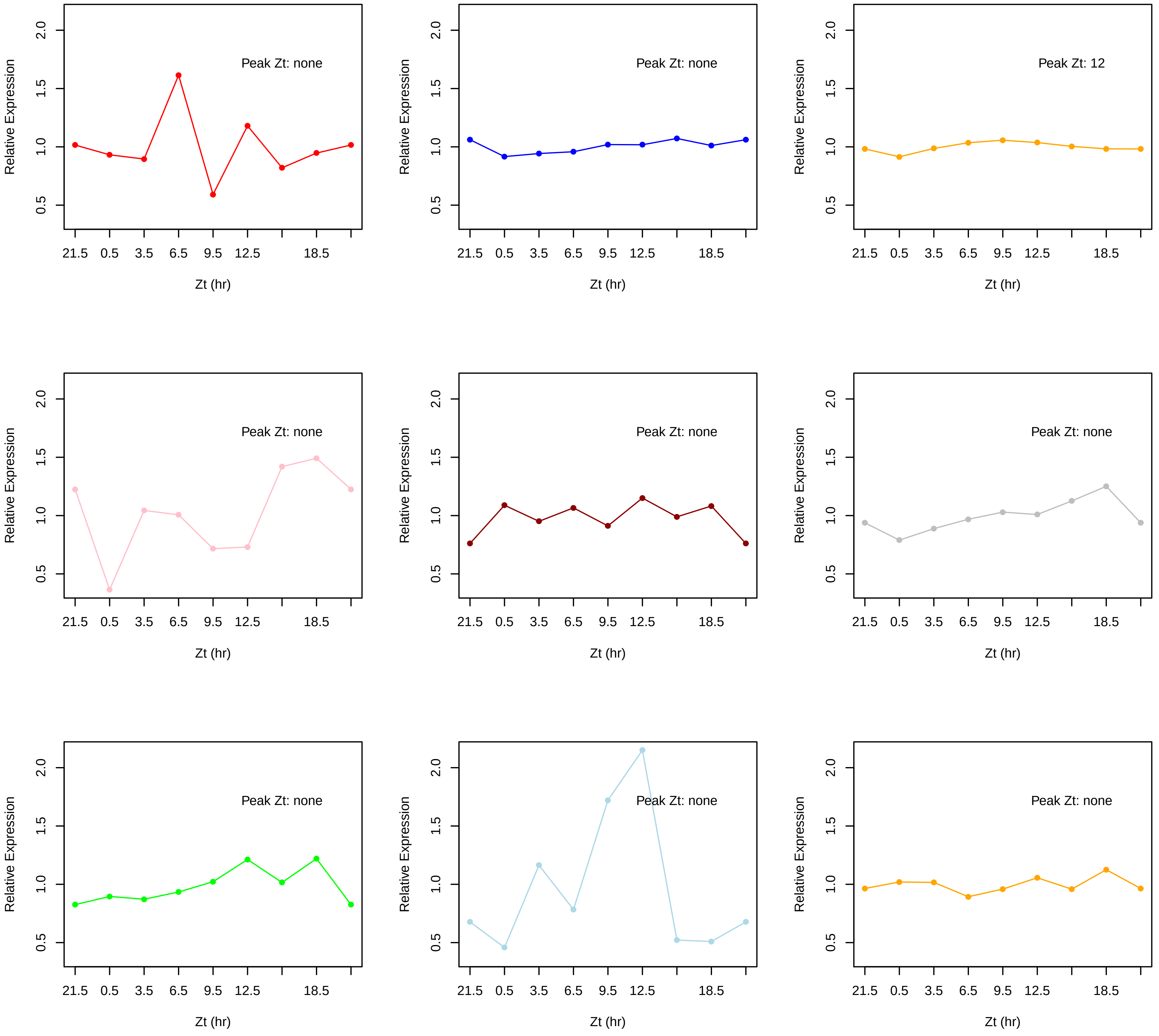

Singeleton: ENSXMAG00000000374

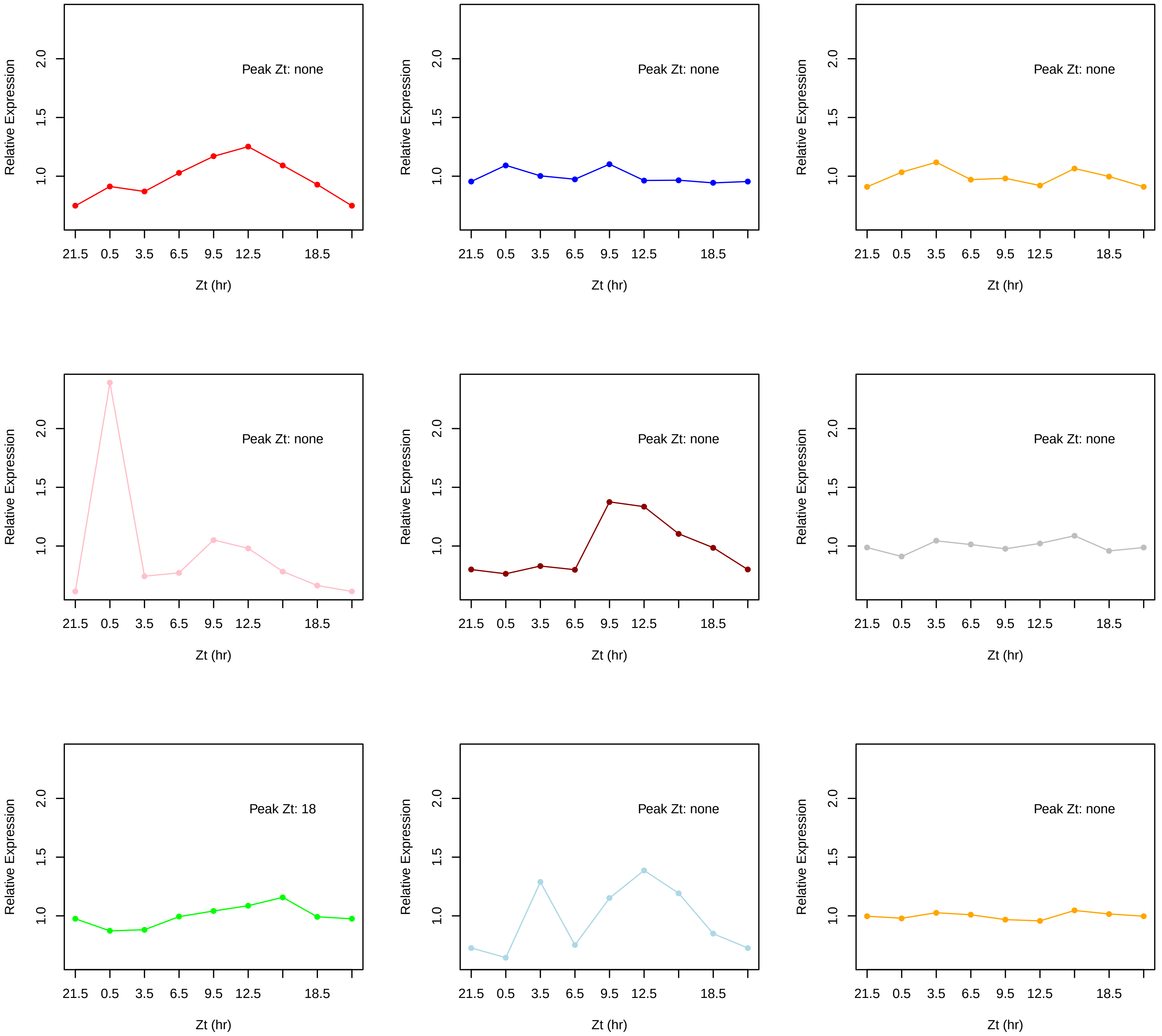

Singeleton: ENSXMAG00000019275 washc4

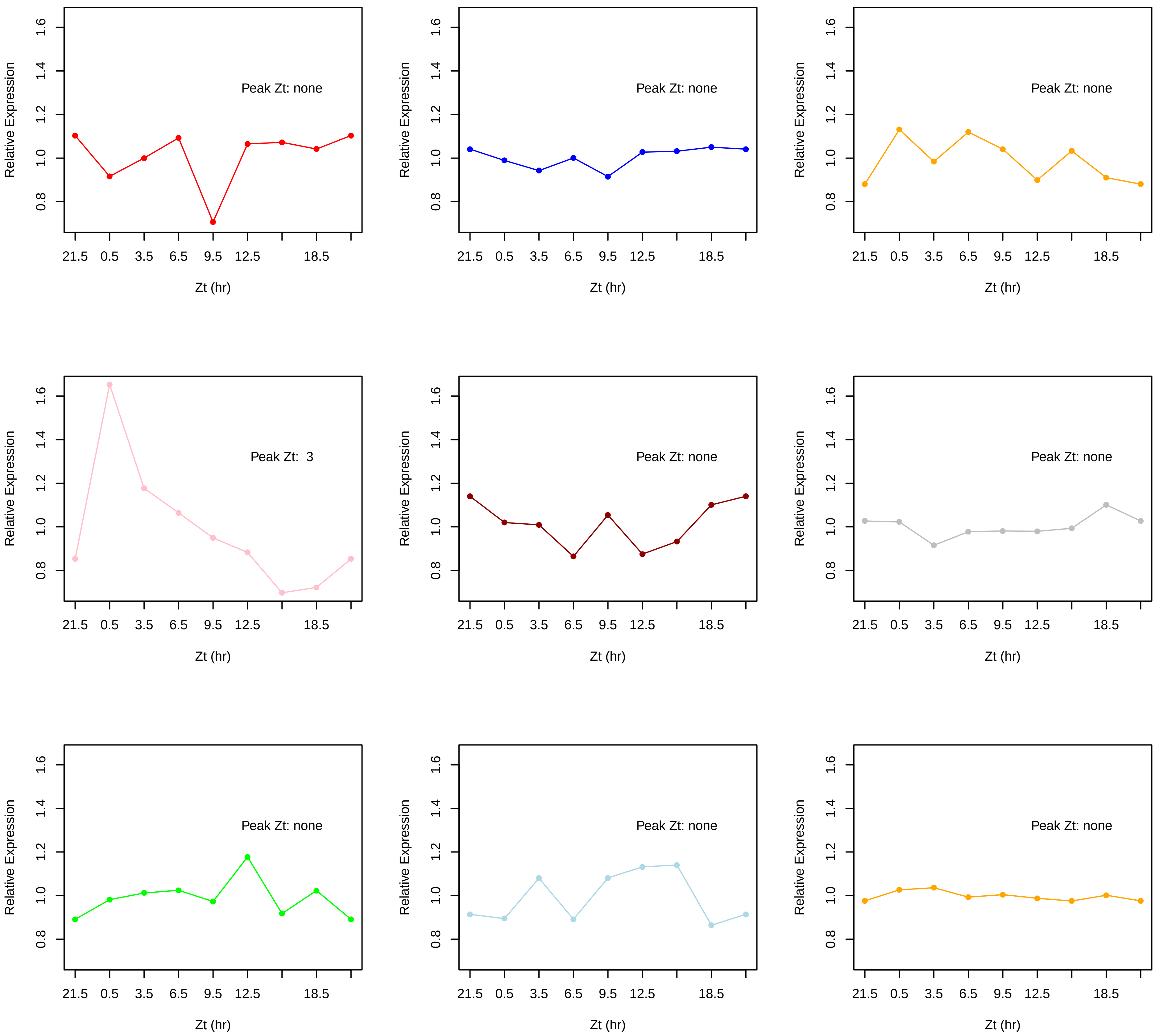

Singleton: ENSXMAG00000001885 dbnlb

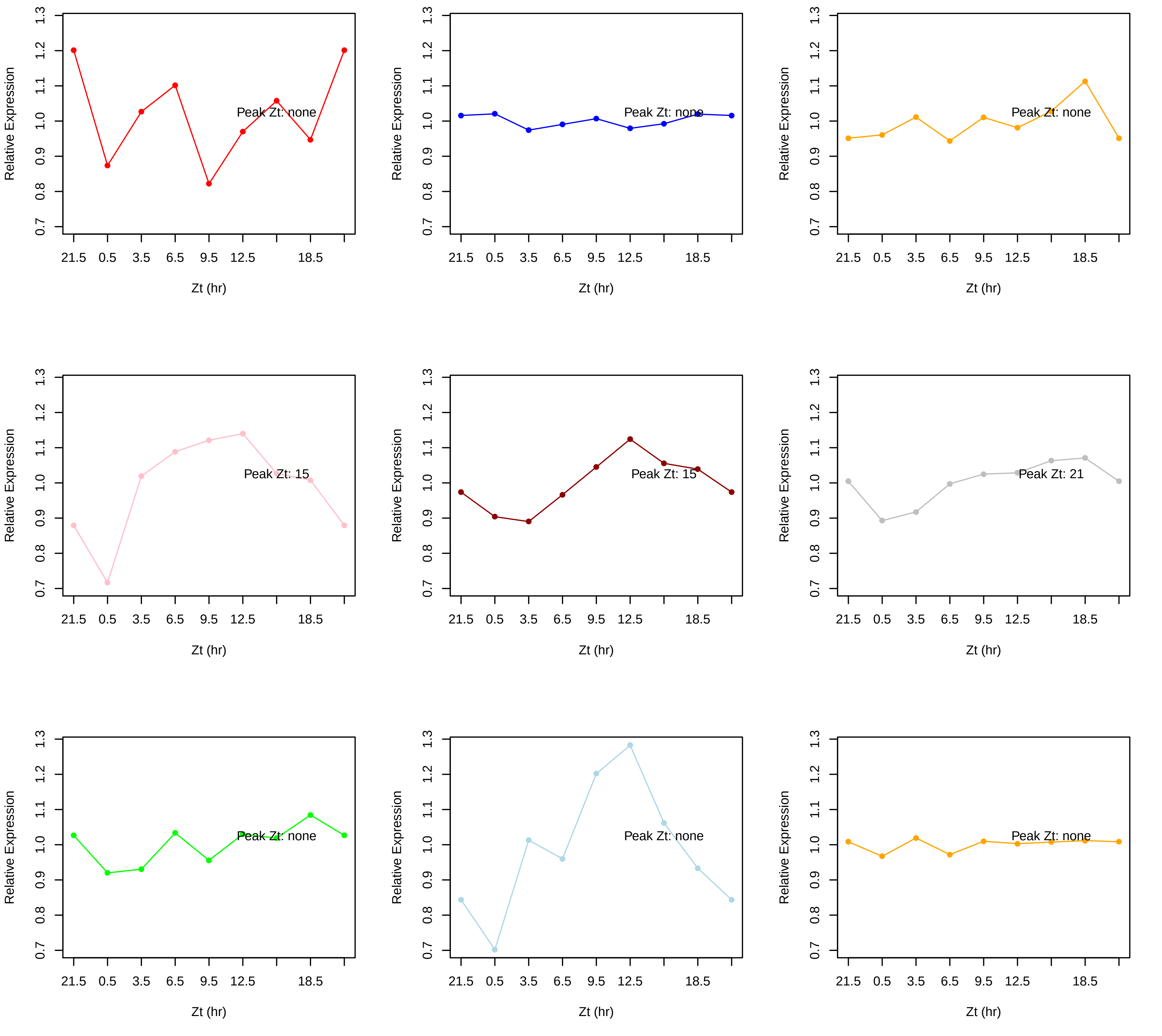

Singleton: ENSXMAG00000022360 rap2c

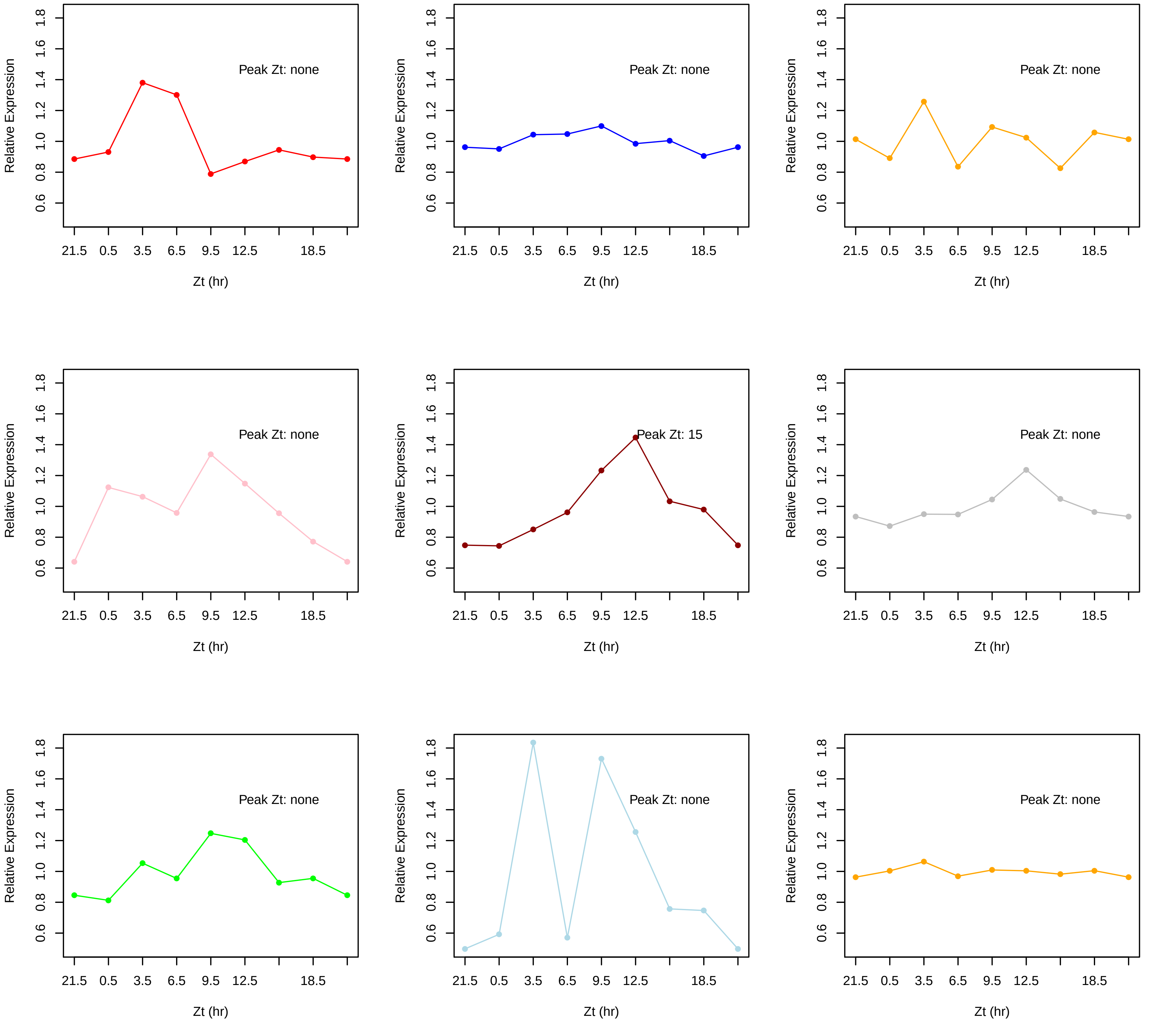

Singleton: ENSXMAG00000006634 snupn

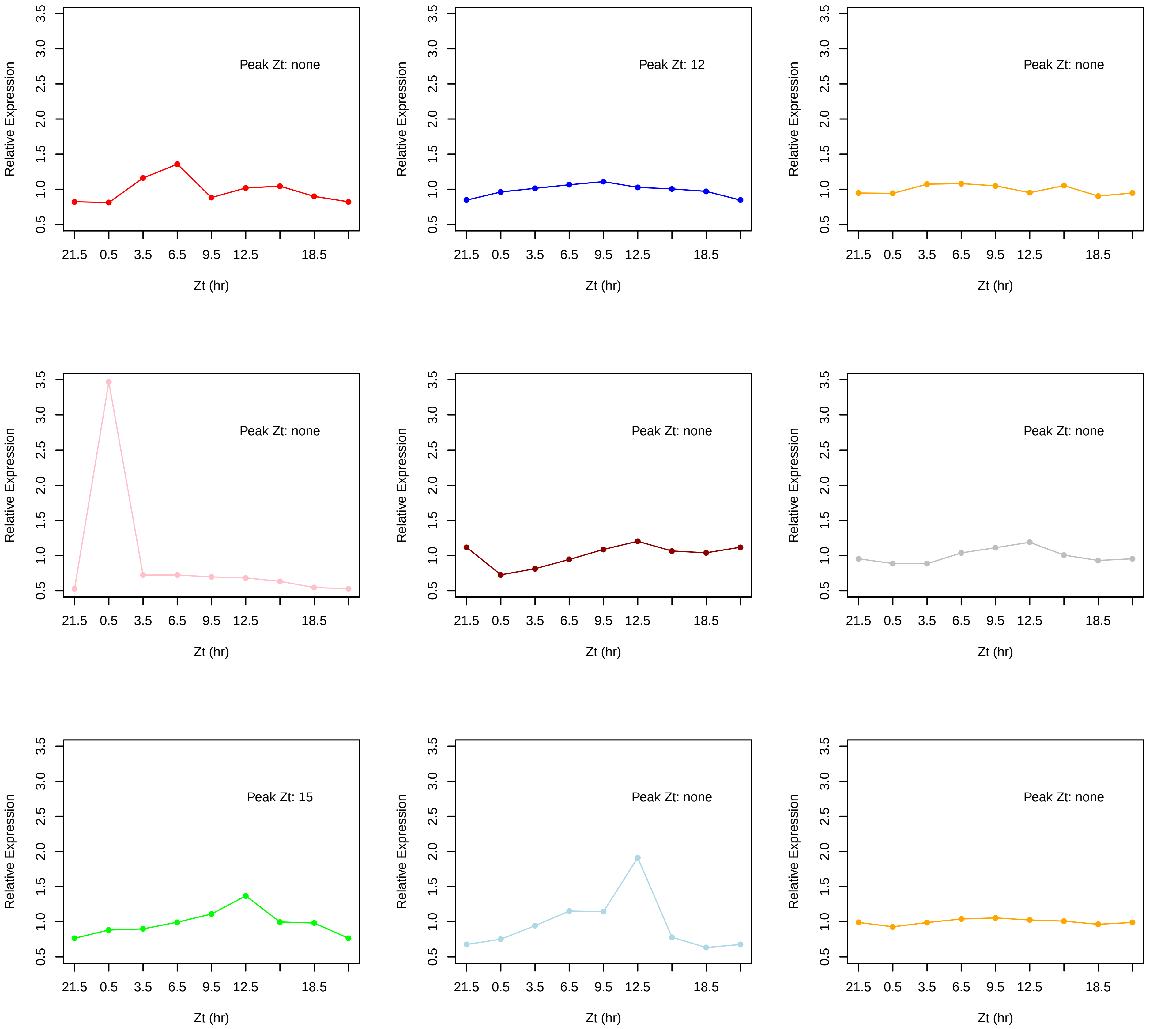

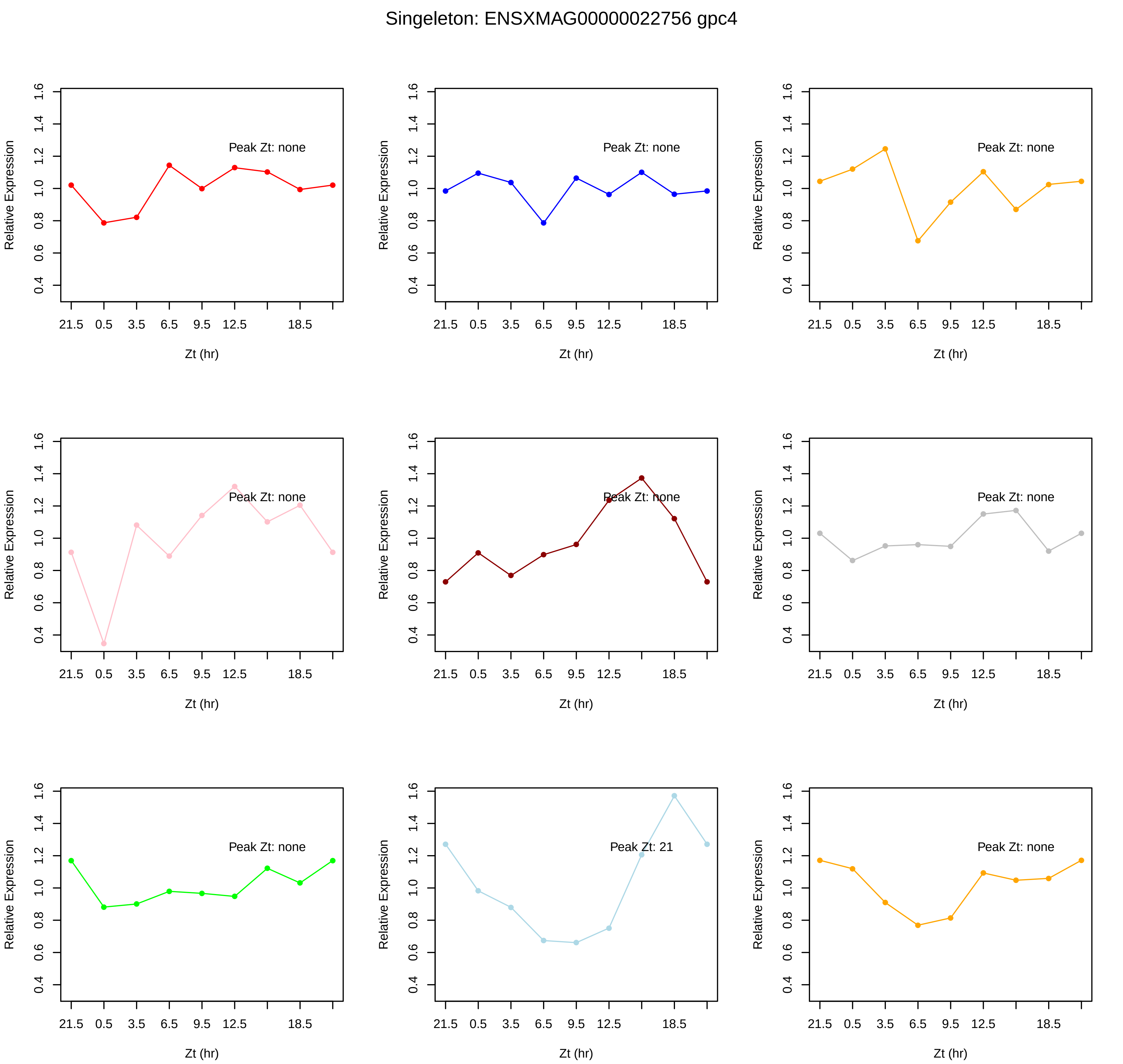
Singeleton: ENSXMAG00000022756 gpc4
Relative Expression
Zt (hr)
21.5 0.5 3.5 6.5 9.5 12.5 18.5
0.4 0.6 0.8 1.0 1.2 1.4 1.6
Peak Zt: none
Peak Zt: none
Peak Zt: none
Peak Zt: none
Peak Zt: none
Peak Zt: none
Peak Zt: none
Peak Zt: 21
Peak Zt: none

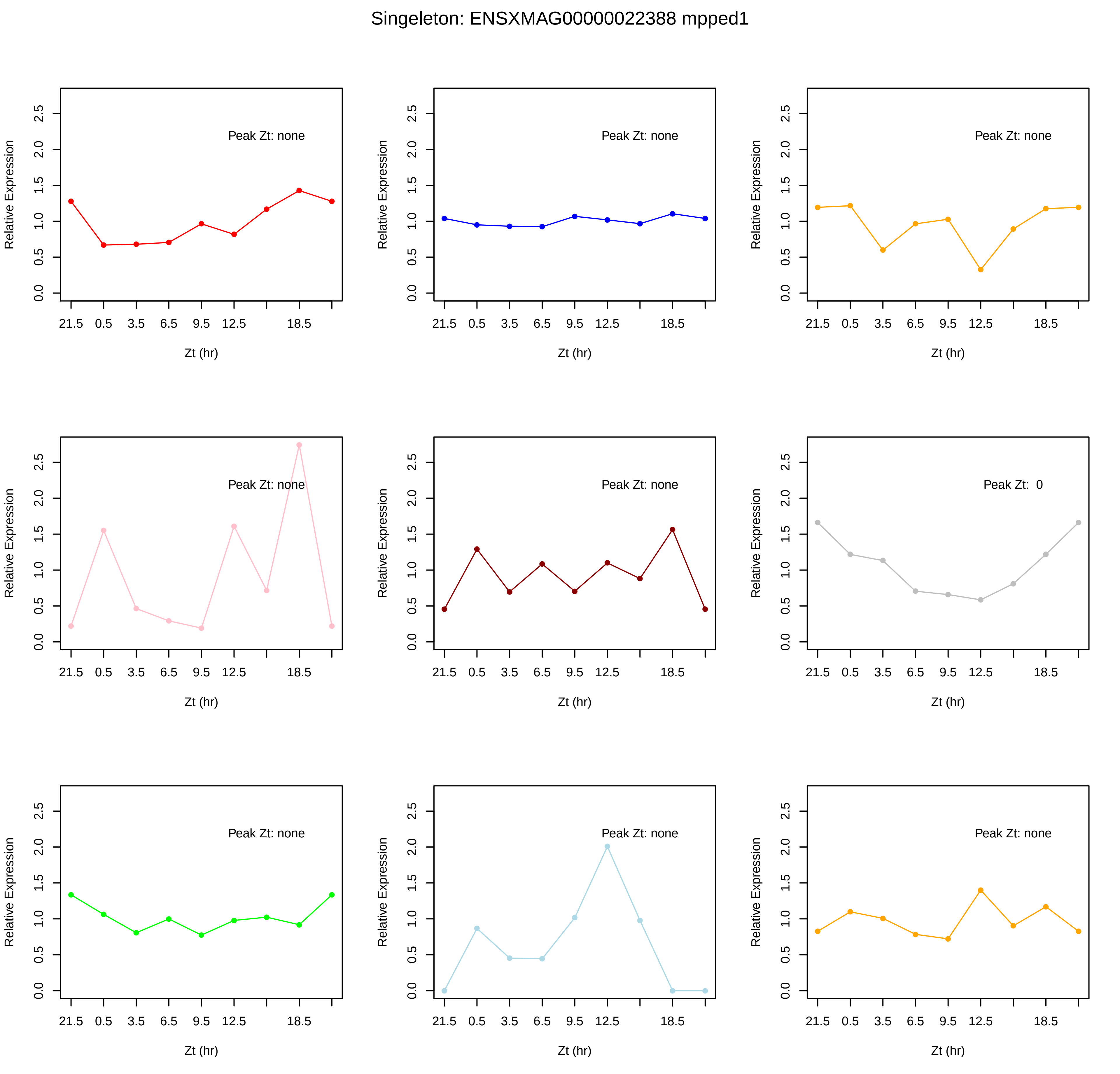

Singeleton: ENSXMAG00000022388 mpped1
Peak Zt: none
Peak Zt: none
Peak Zt: none
Peak Zt: none
Peak Zt: none
Peak Zt: 0
Peak Zt: none
Peak Zt: none
Peak Zt: none
Relative Expression
Zt (hr)
21.5 0.5 3.5 6.5 9.5 12.5 18.5
0.0 0.5 1.0 1.5 2.0 2.5

Singeleton: ENSXMAG00000023611

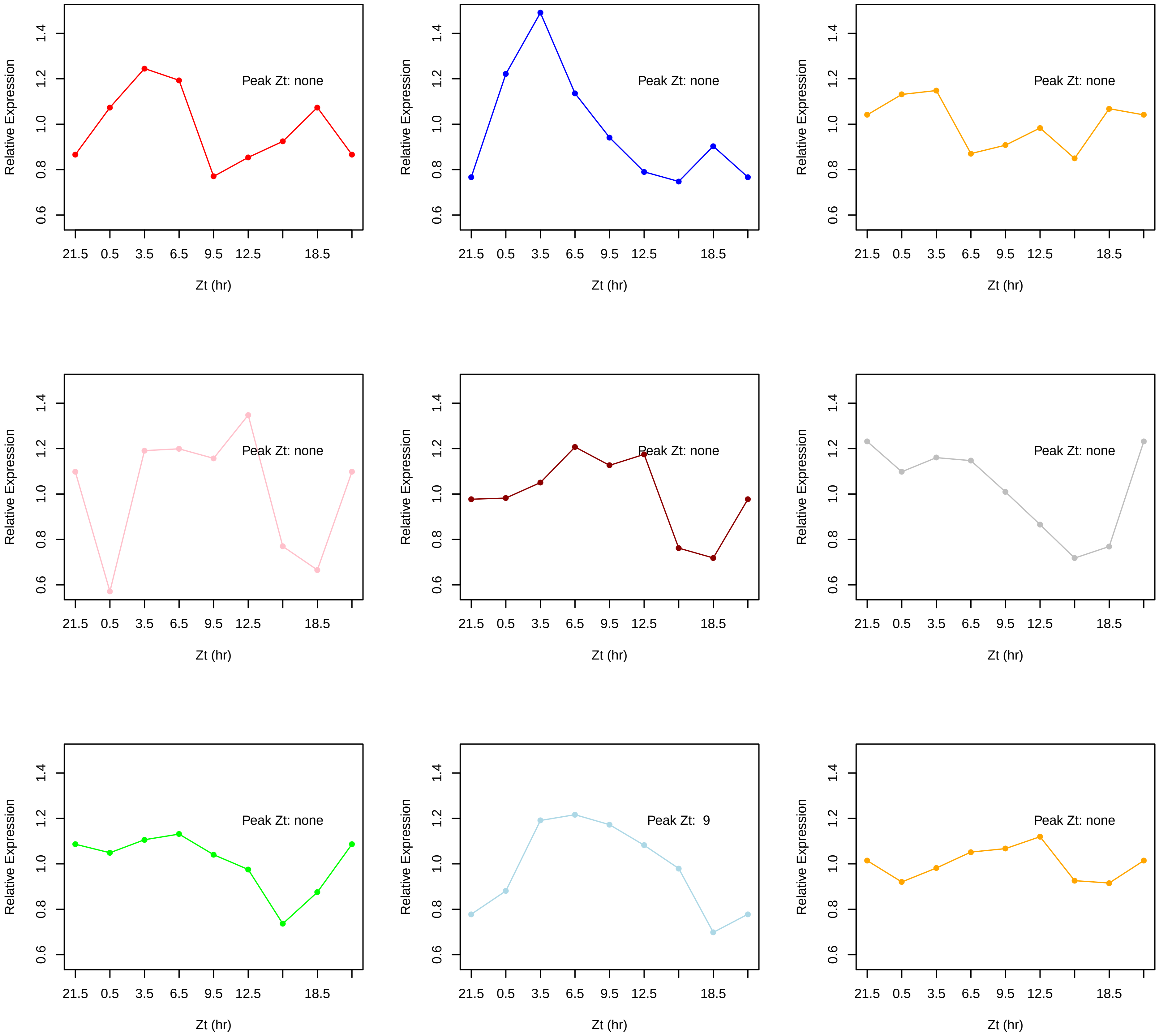

Singeleton: ENSXMAG00000017347 phf6

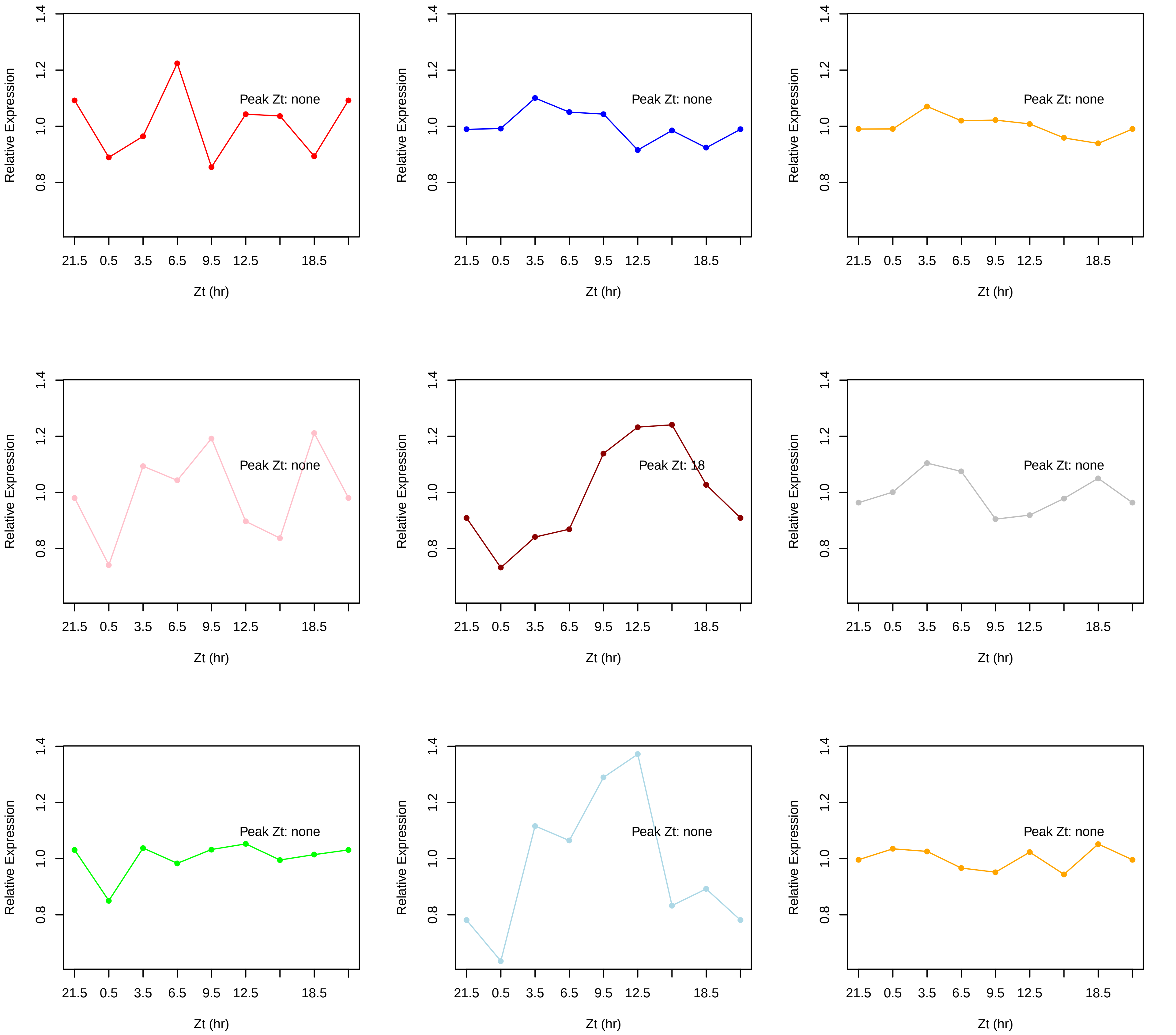

Singeleton: ENSXMAG00000021509 sult4a1

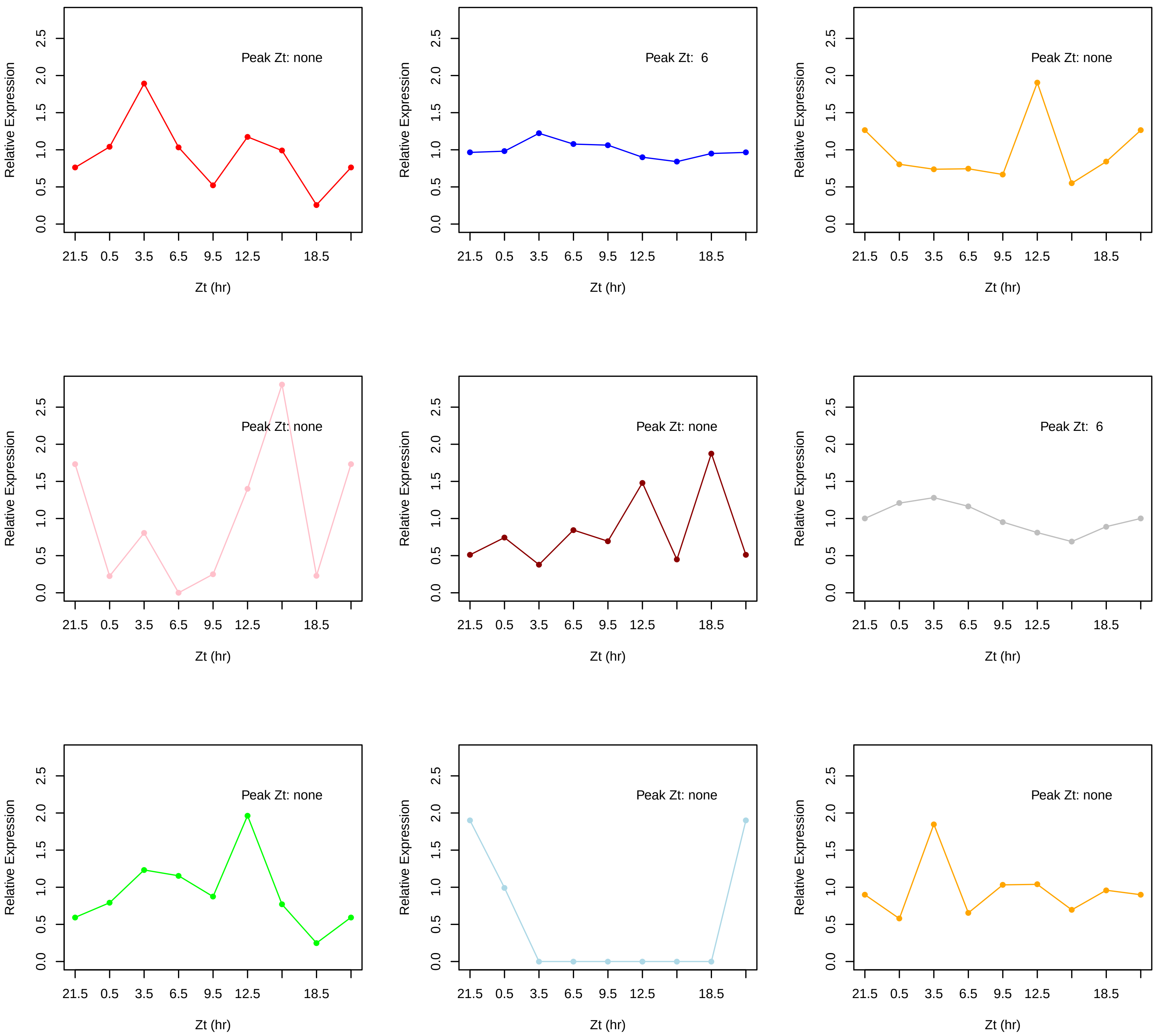

Singeleton: ENSXMAG00000025803 hprt1

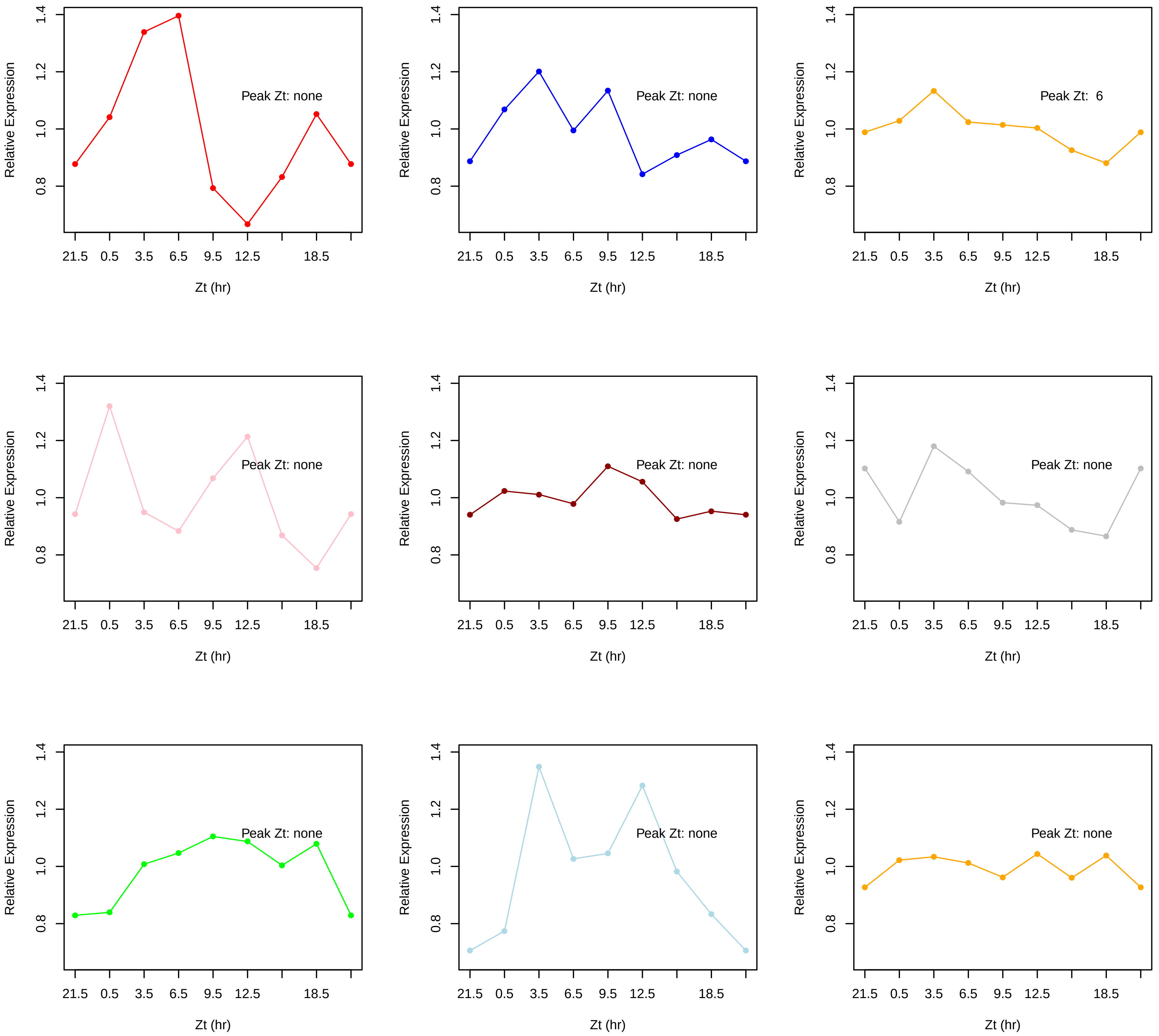

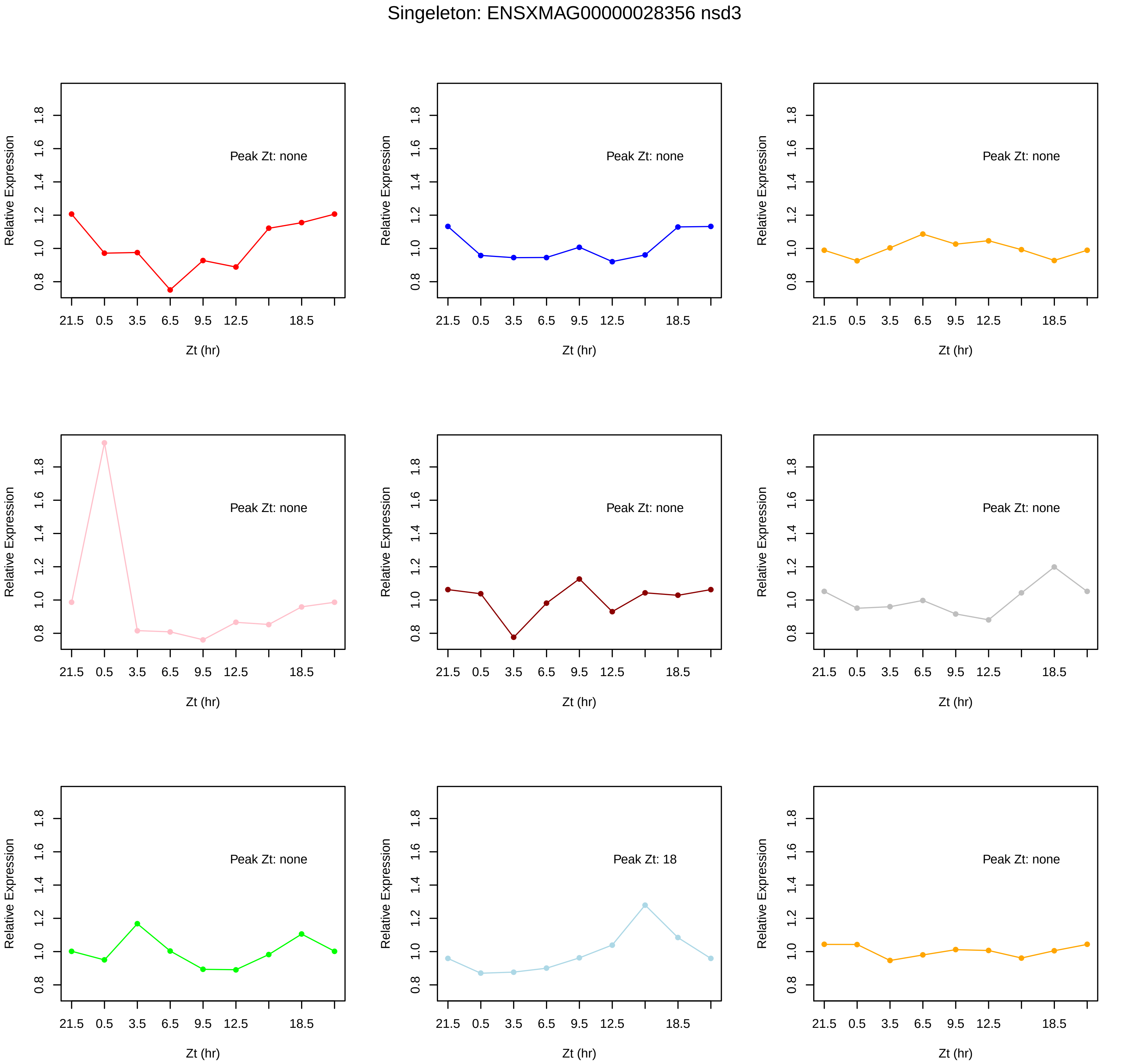

Singeleton: ENSXMAG00000028356 nsd3
Peak Zt: none
Peak Zt: none
Peak Zt: none
Peak Zt: none
Peak Zt: none
Peak Zt: none
Peak Zt: none
Peak Zt: 18
Peak Zt: none
Relative Expression
Zt (hr)
21.5
0.5
3.5
6.5
9.5
12.5
18.5
0.8
1.0
1.2
1.4
1.6
1.8

Singeleton: ENSXMAG00000021654 ttll12

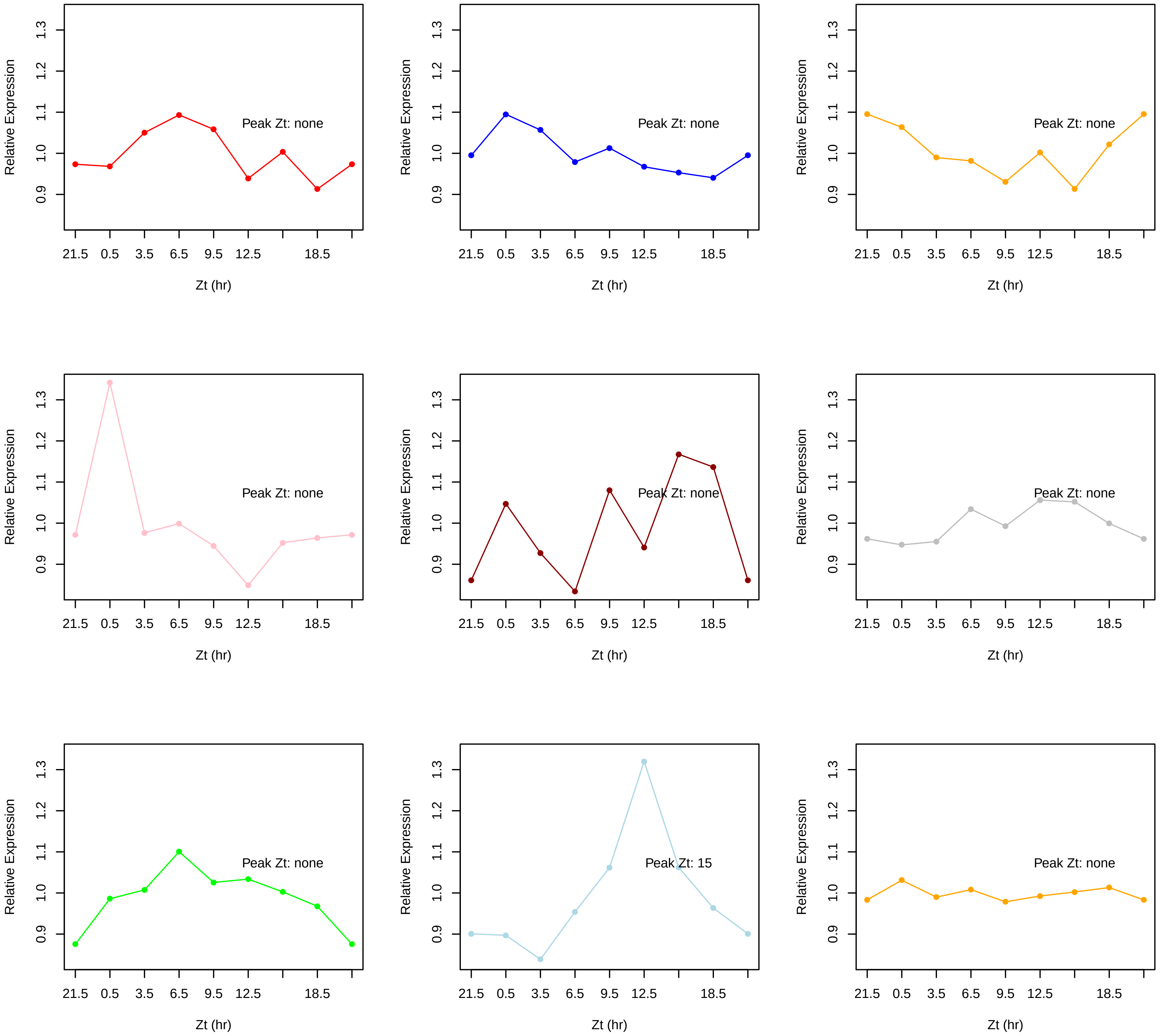

Singeleton: ENSXMAG00000017365 fam122b

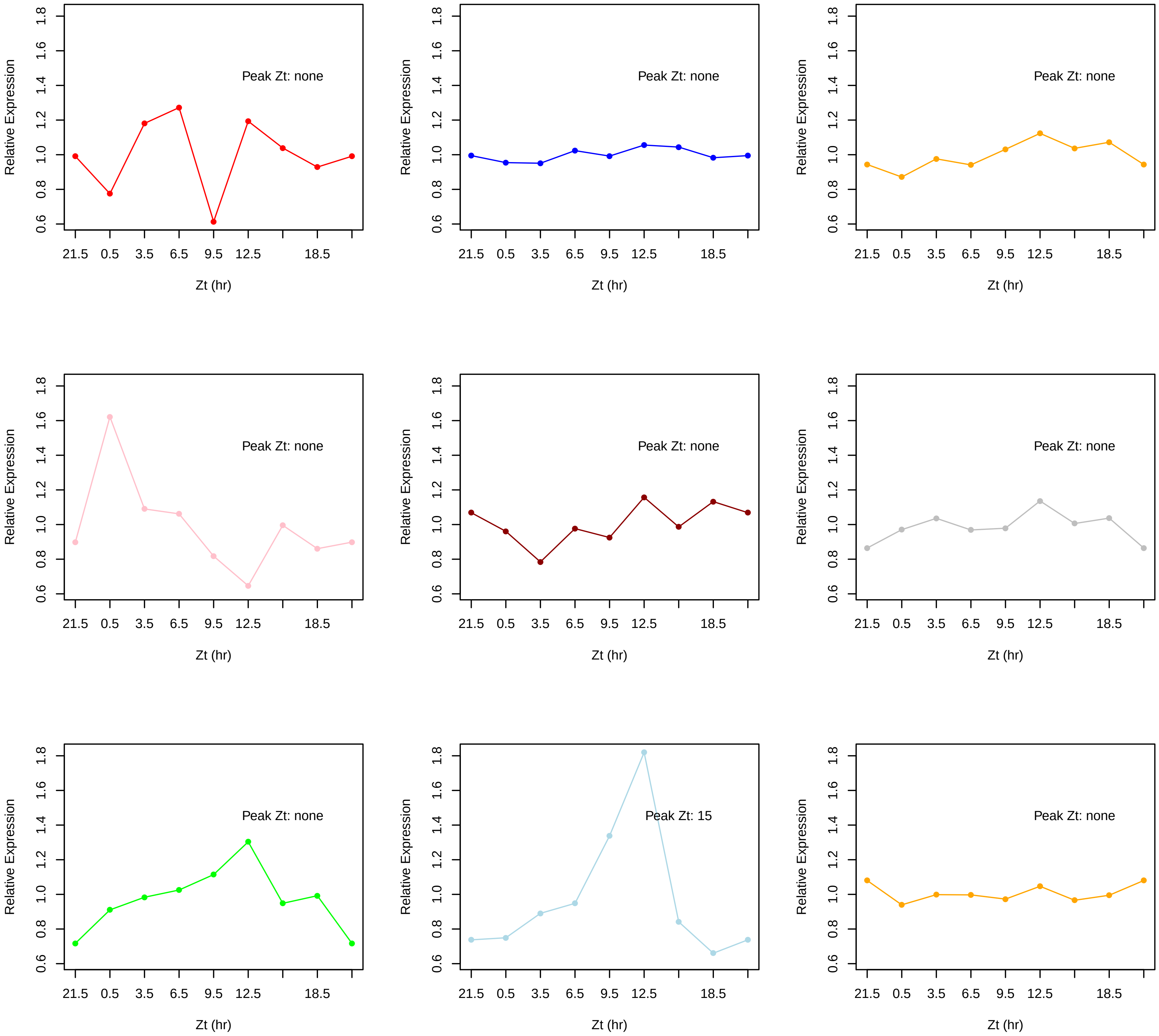

Singeleton: ENSXMAG00000017374 ints6l

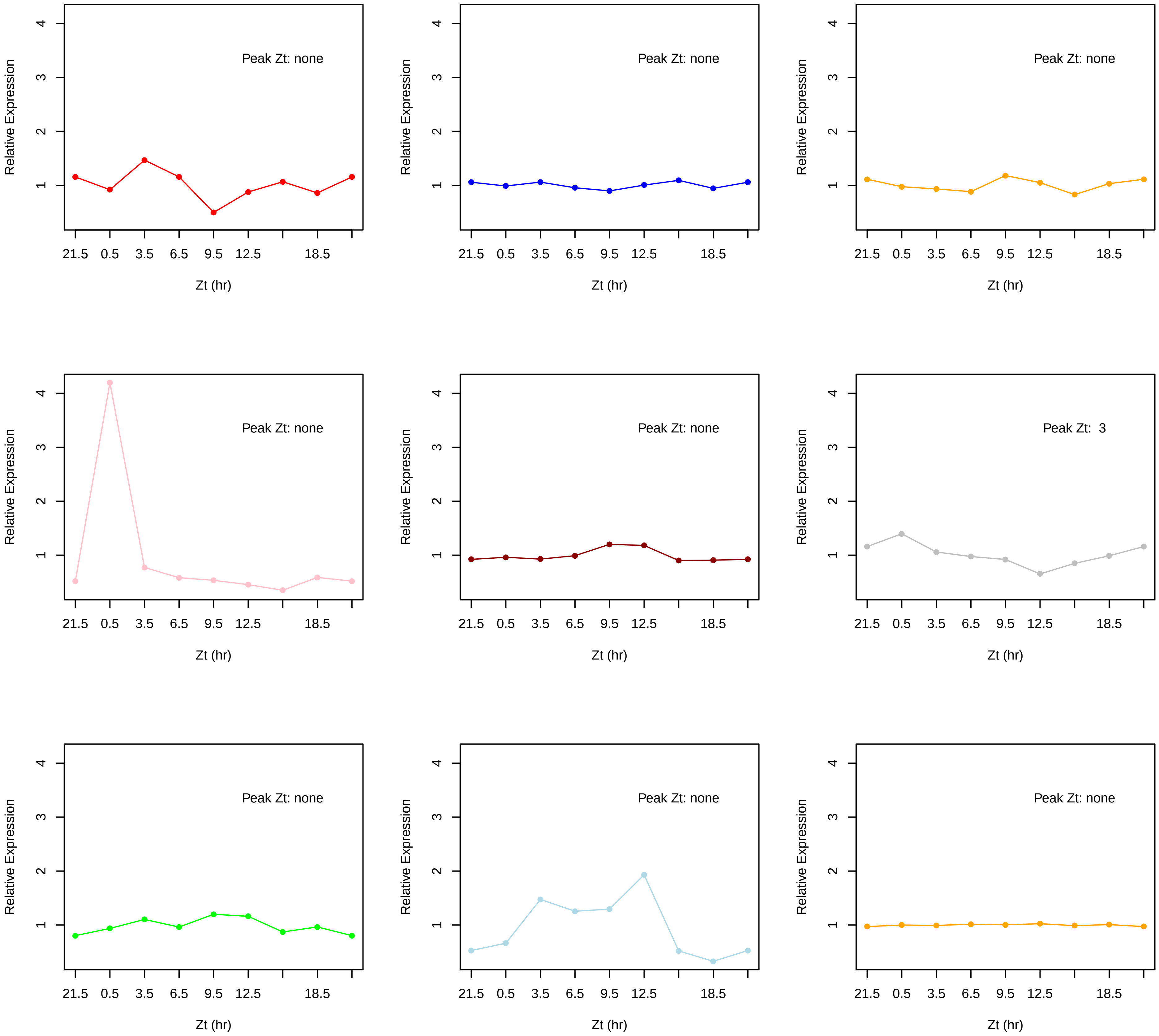

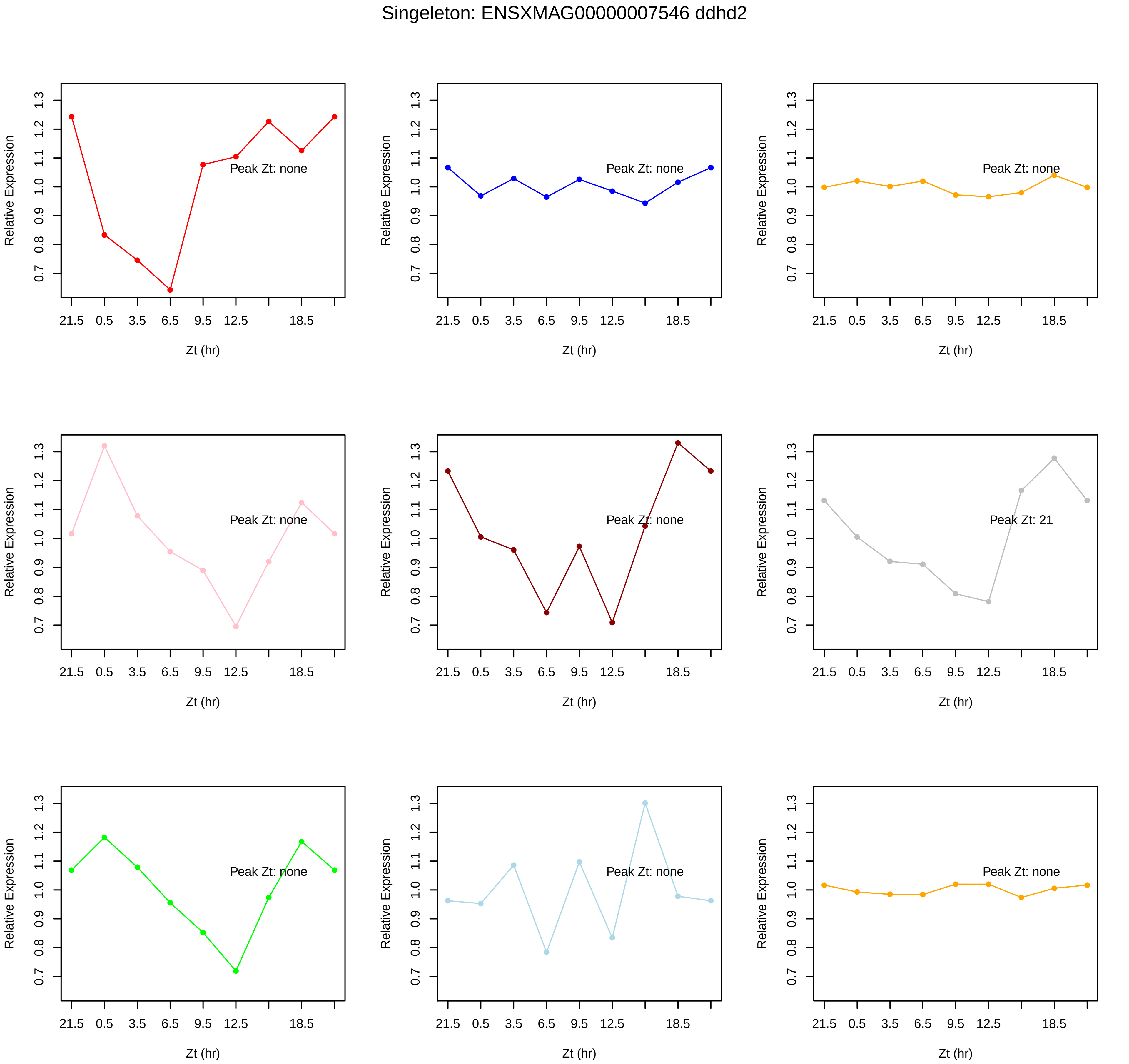
Singleton: ENSXMAG00000007546 ddhd2
Relative Expression
Zt (hr)
21.5 0.5 3.5 6.5 9.5 12.5 18.5
0.7 0.8 0.9 1.0 1.1 1.2 1.3
Peak Zt: none
Peak Zt: none
Peak Zt: none
Peak Zt: none
Peak Zt: none
Peak Zt: 21
Peak Zt: none
Peak Zt: none
Peak Zt: none

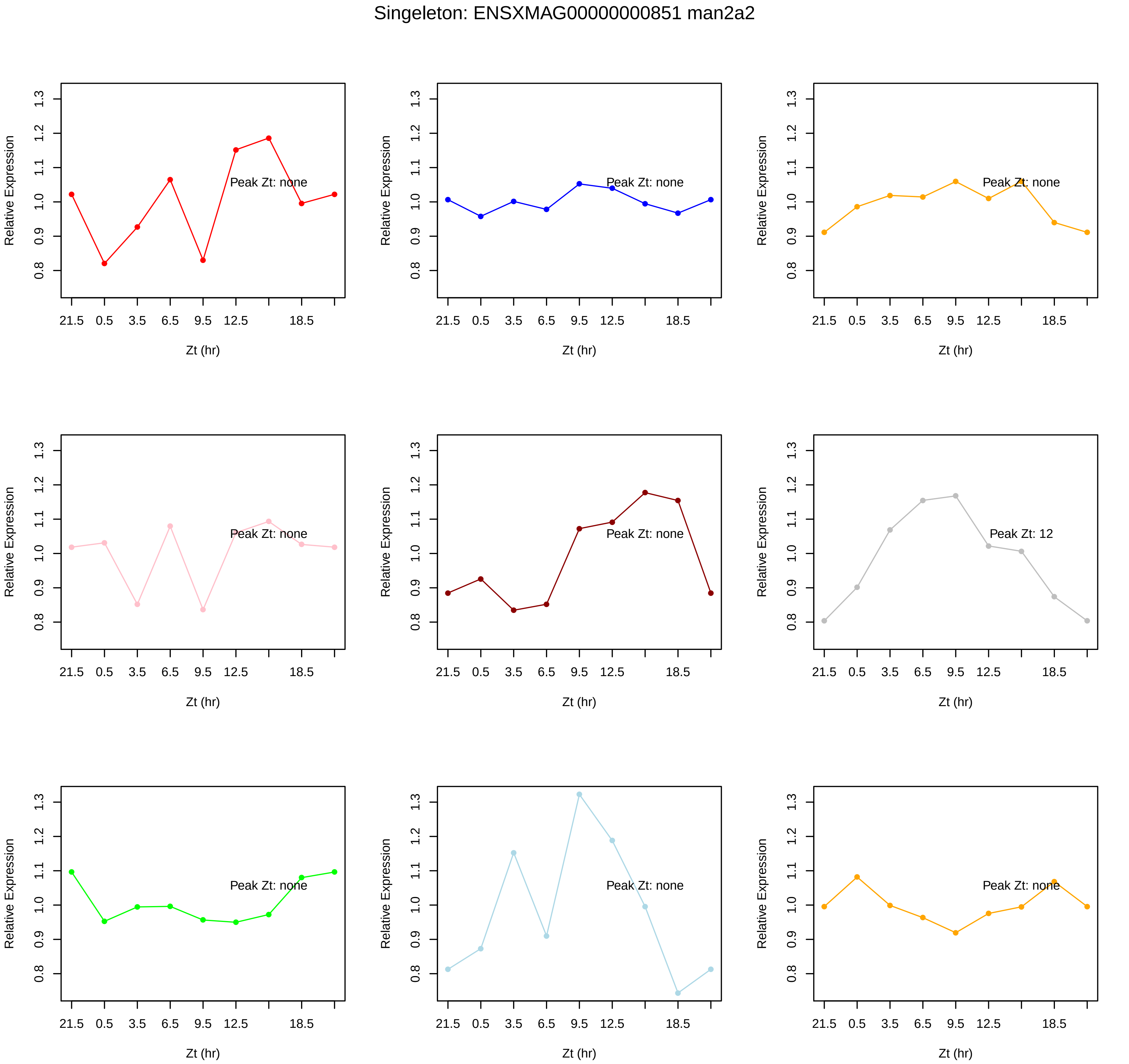
Singeleton: ENSXMAG00000000851 man2a2
Relative Expression
Zt (hr)
Peak Zt: none
Peak Zt: 12
21.5 0.5 3.5 6.5 9.5 12.5 18.5
0.8 0.9 1.0 1.1 1.2 1.3

Singleton: ENSXMAG00000022106 si:ch211−204d2.4

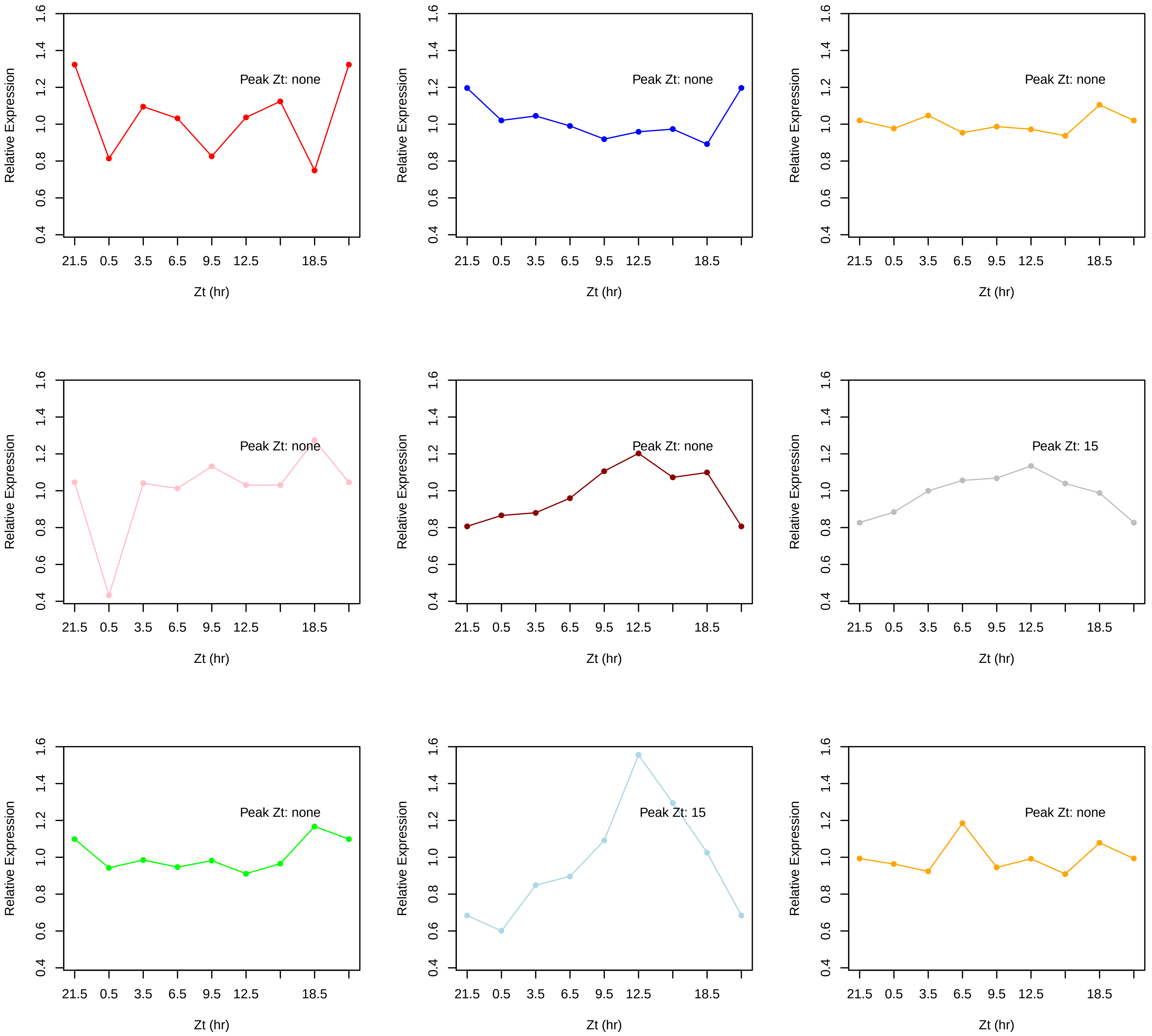

Singeleton: ENSXMAG00000008581 ipo8

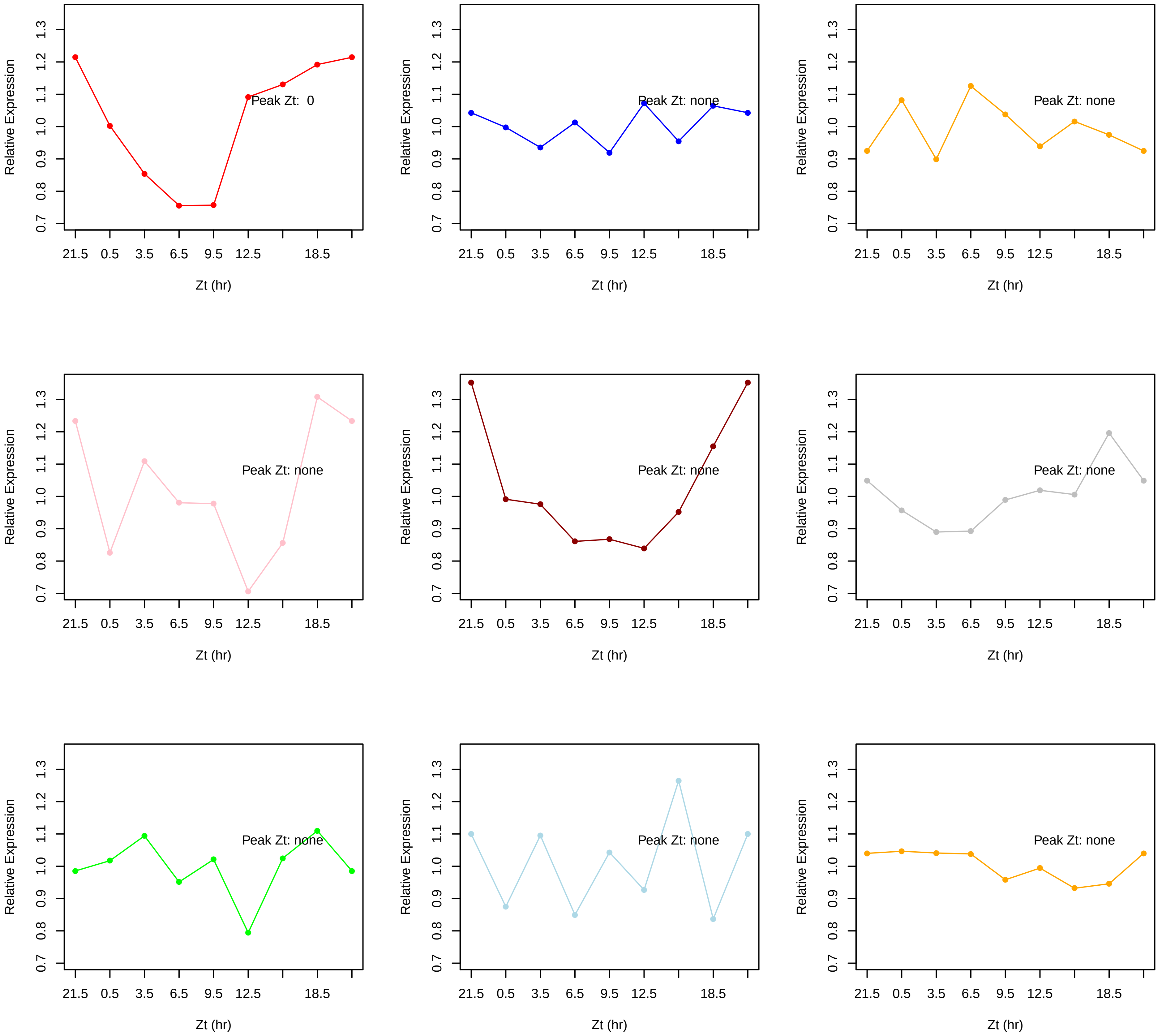

Singeleton: ENSXMAG00000000874 unc45a

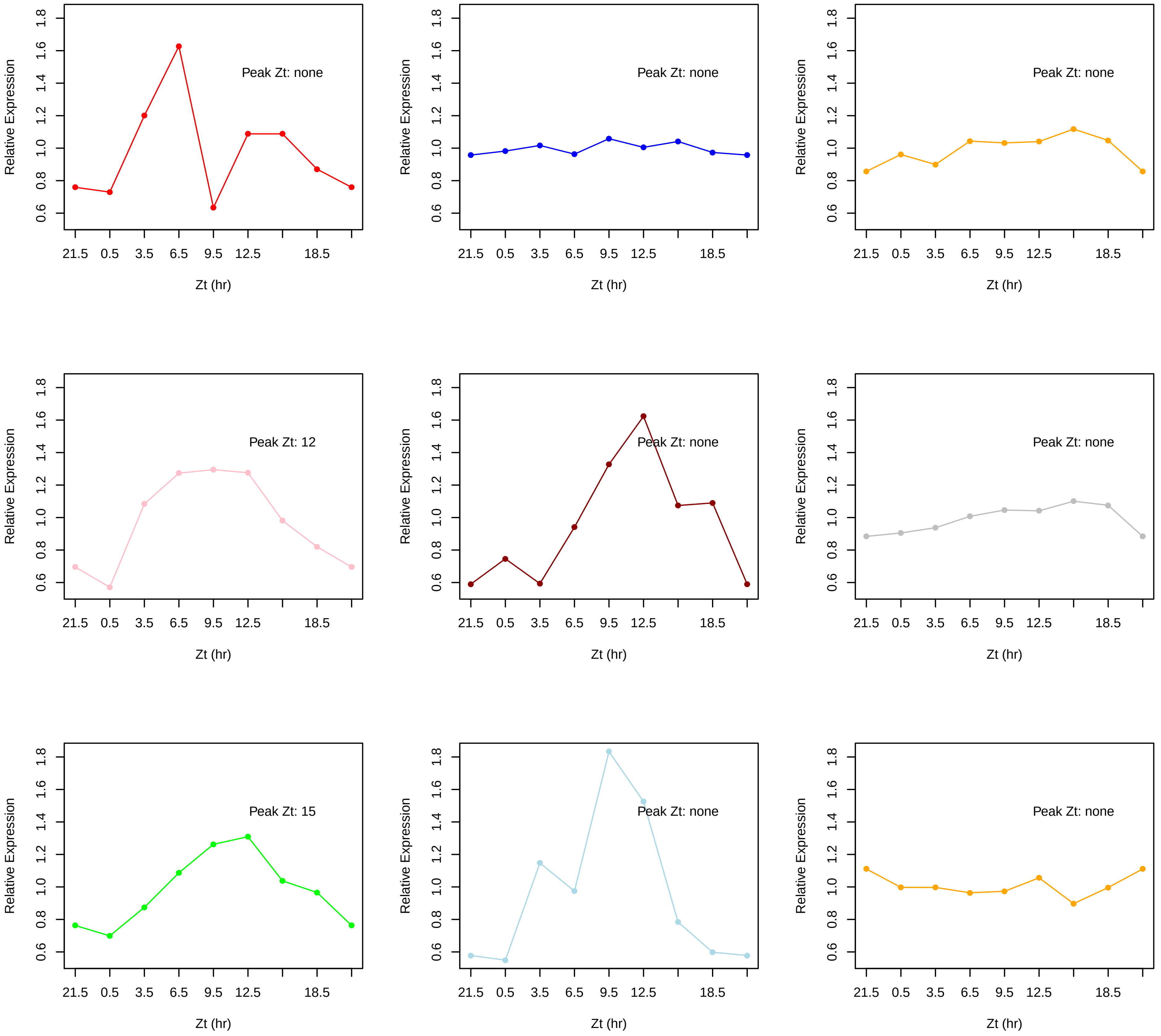

Singleton: ENSXMAG00000027970 TMTC1

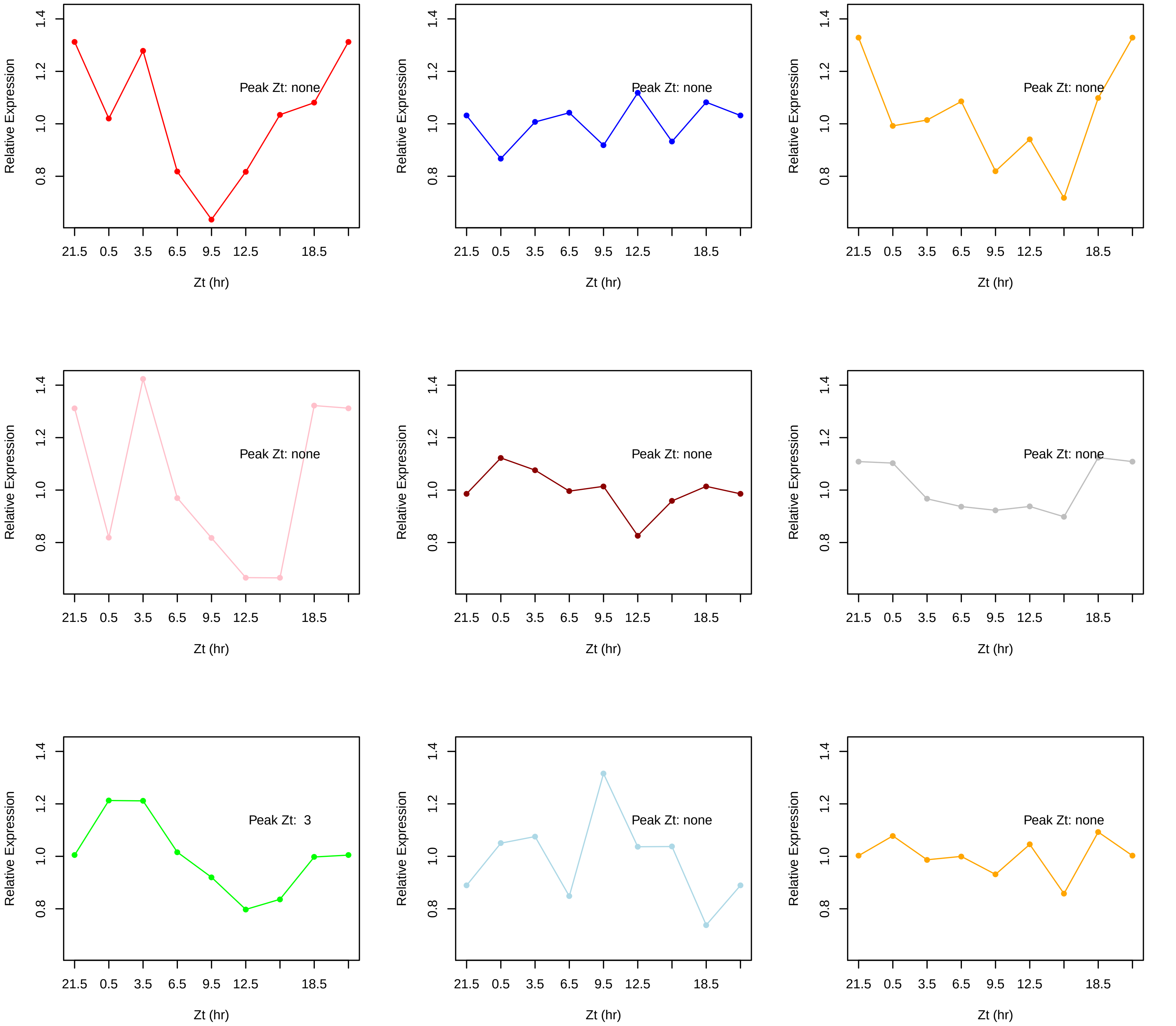

Singeleton: ENSXMAG00000013428 map7d3

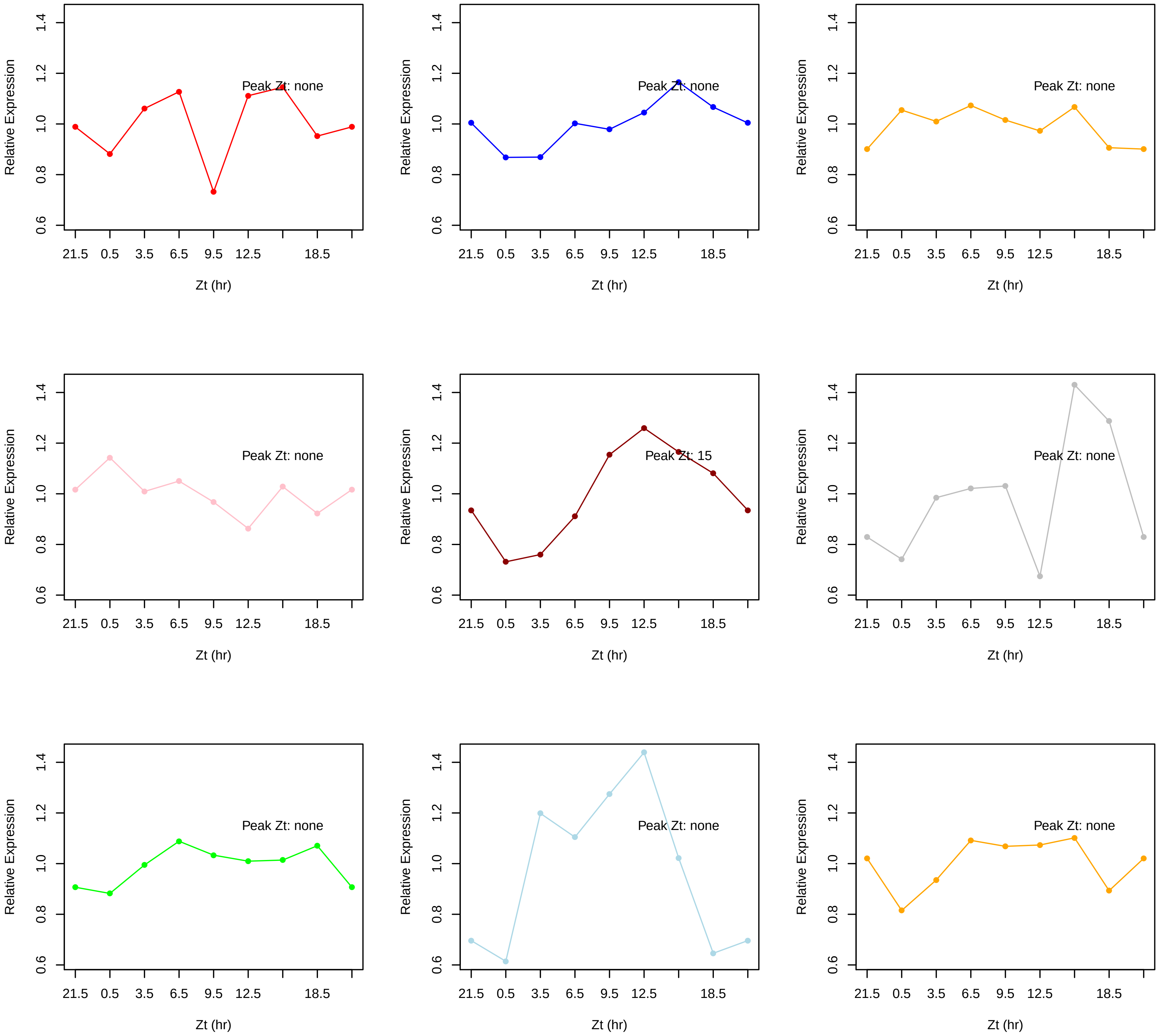

Singeleton: ENSXMAG00000000888 CIB1

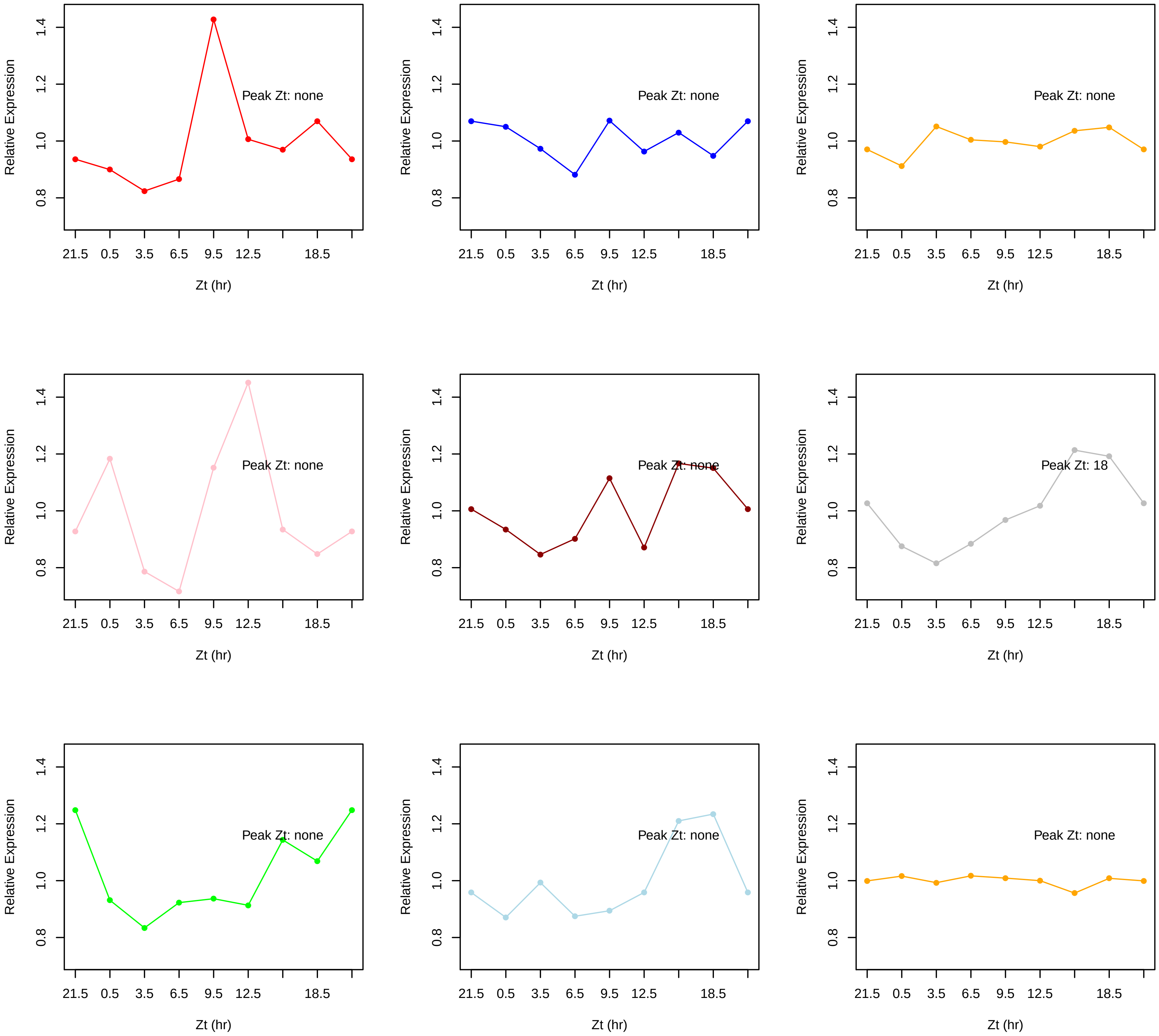

Singeleton: ENSXMAG00000004652

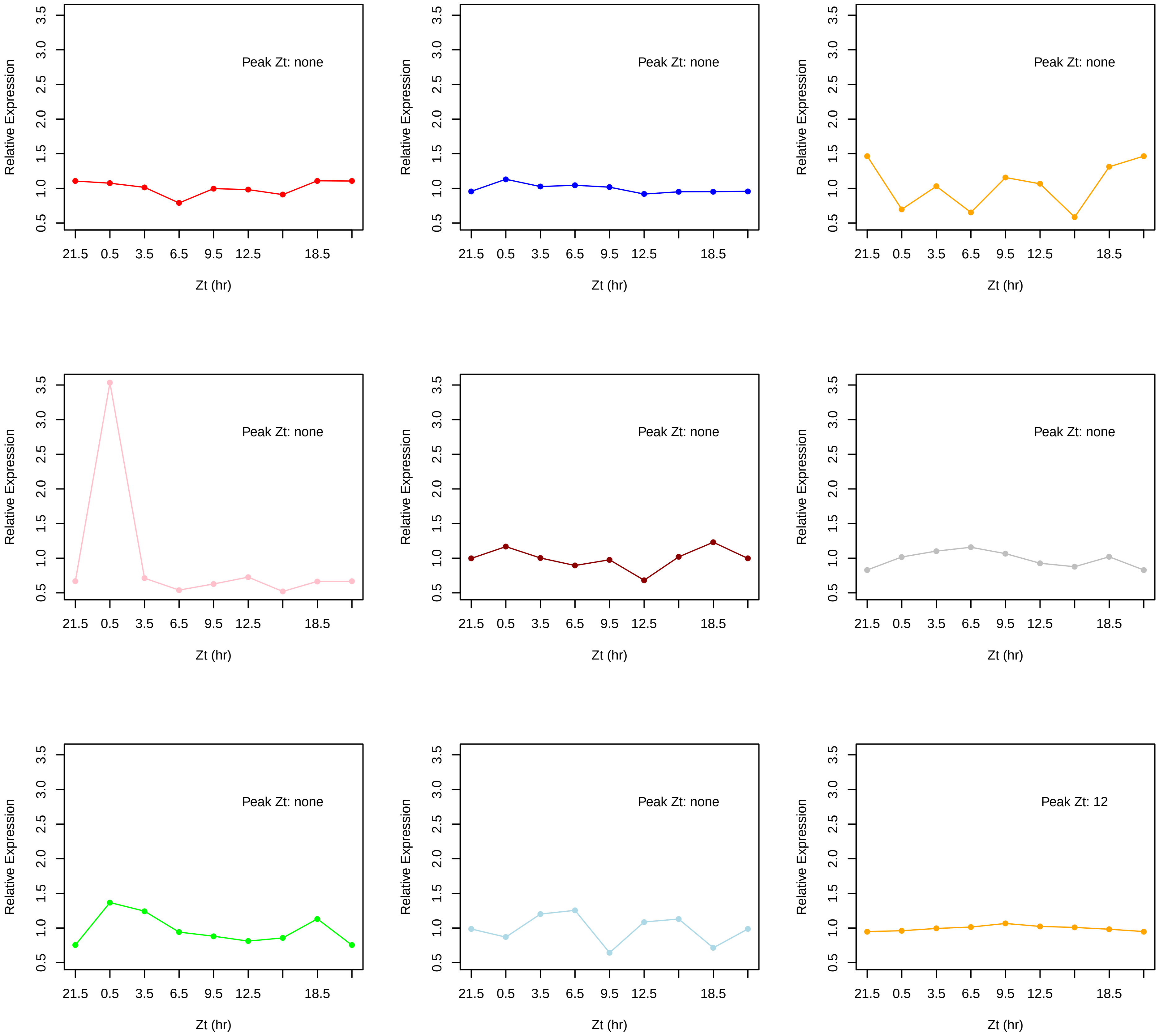

Singeleton: ENSXMAG00000000490 htatsf1

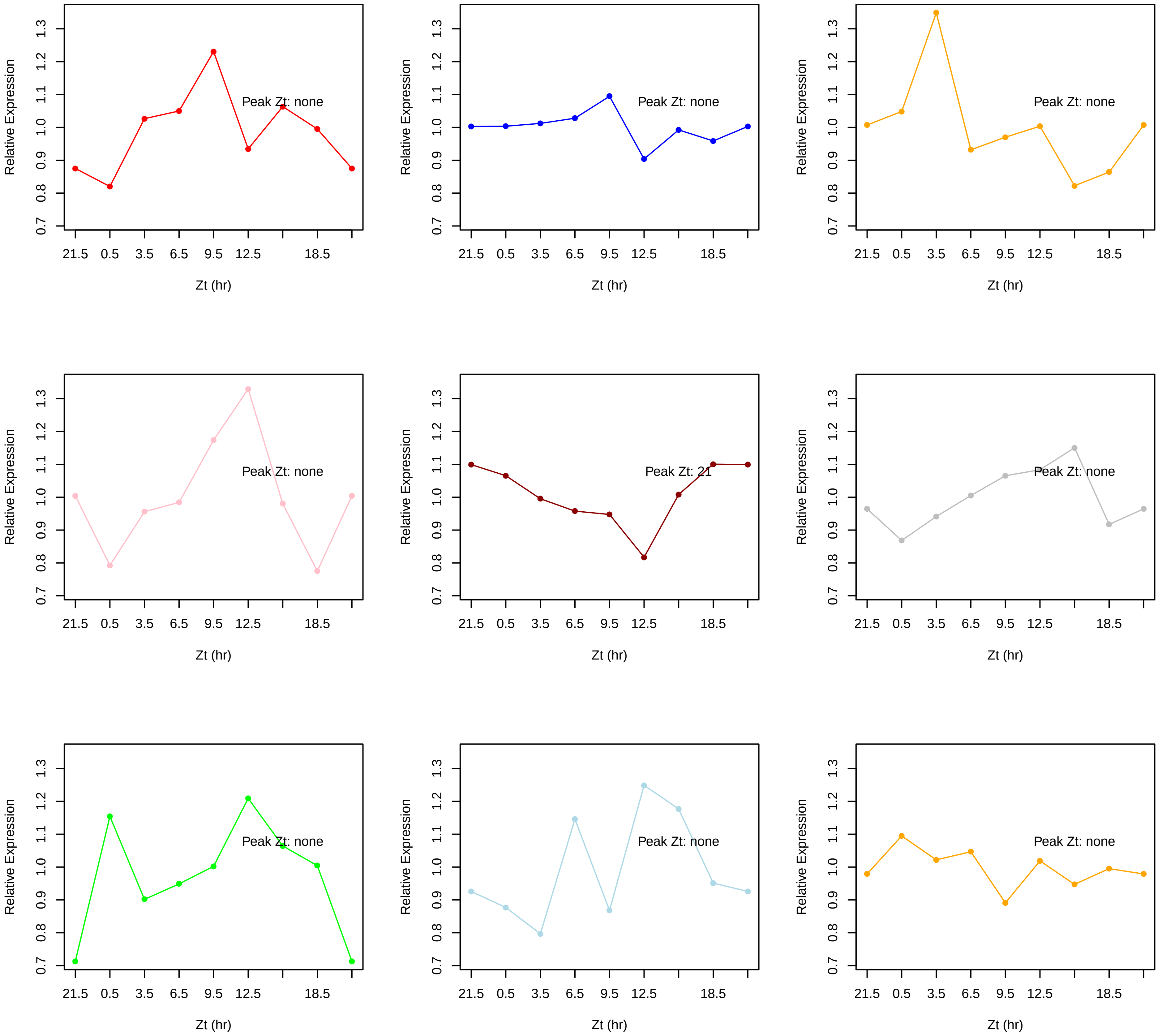

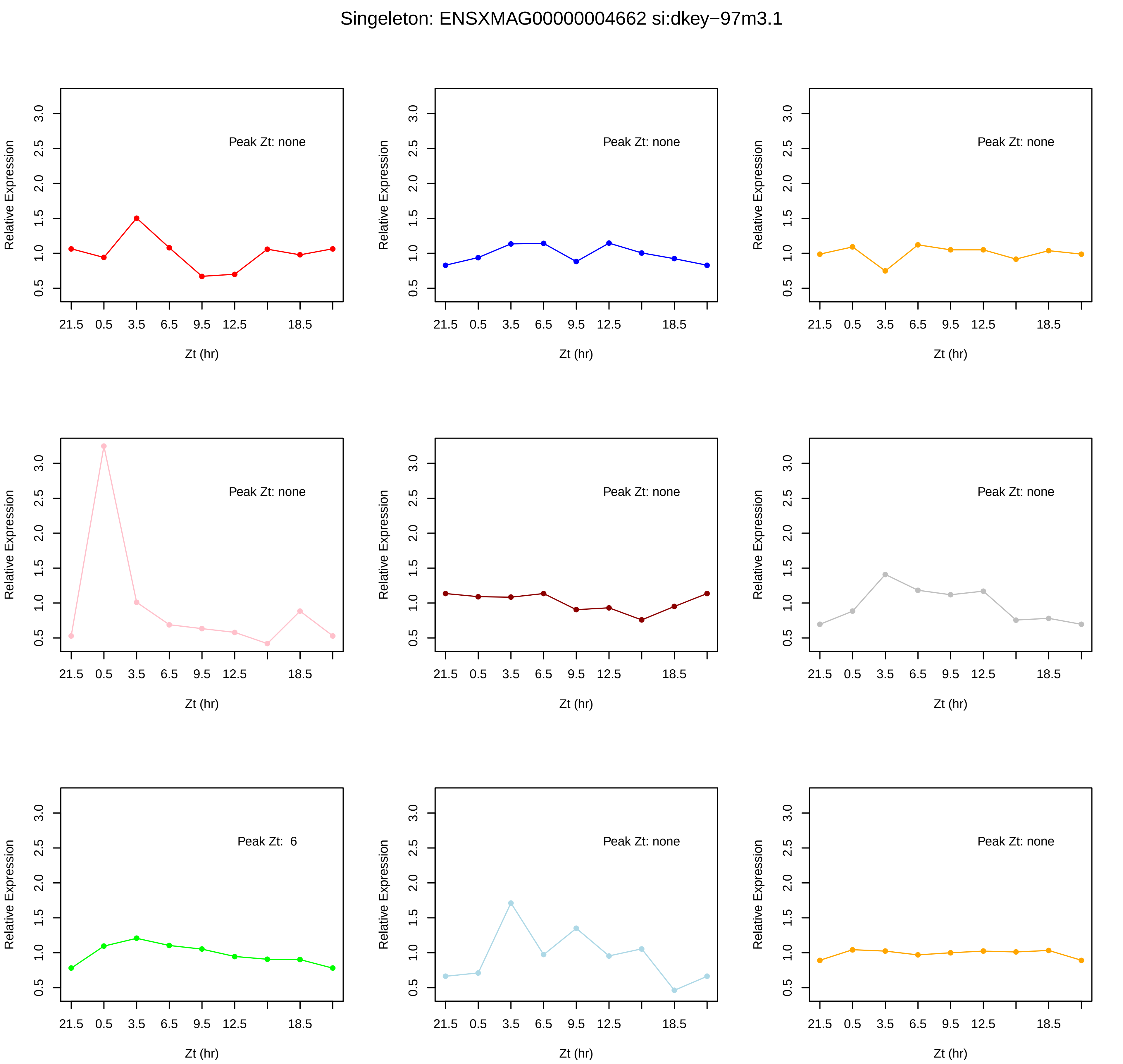
Singeleton: ENSXMAG00000004662 si:dkey−97m3.1
Peak Zt: none
Peak Zt: none
Peak Zt: none
Peak Zt: none
Peak Zt: none
Peak Zt: none
Peak Zt: 6
Peak Zt: none
Peak Zt: none
Relative Expression
Zt (hr)

Singeleton: ENSXMAG00000001421 def6c

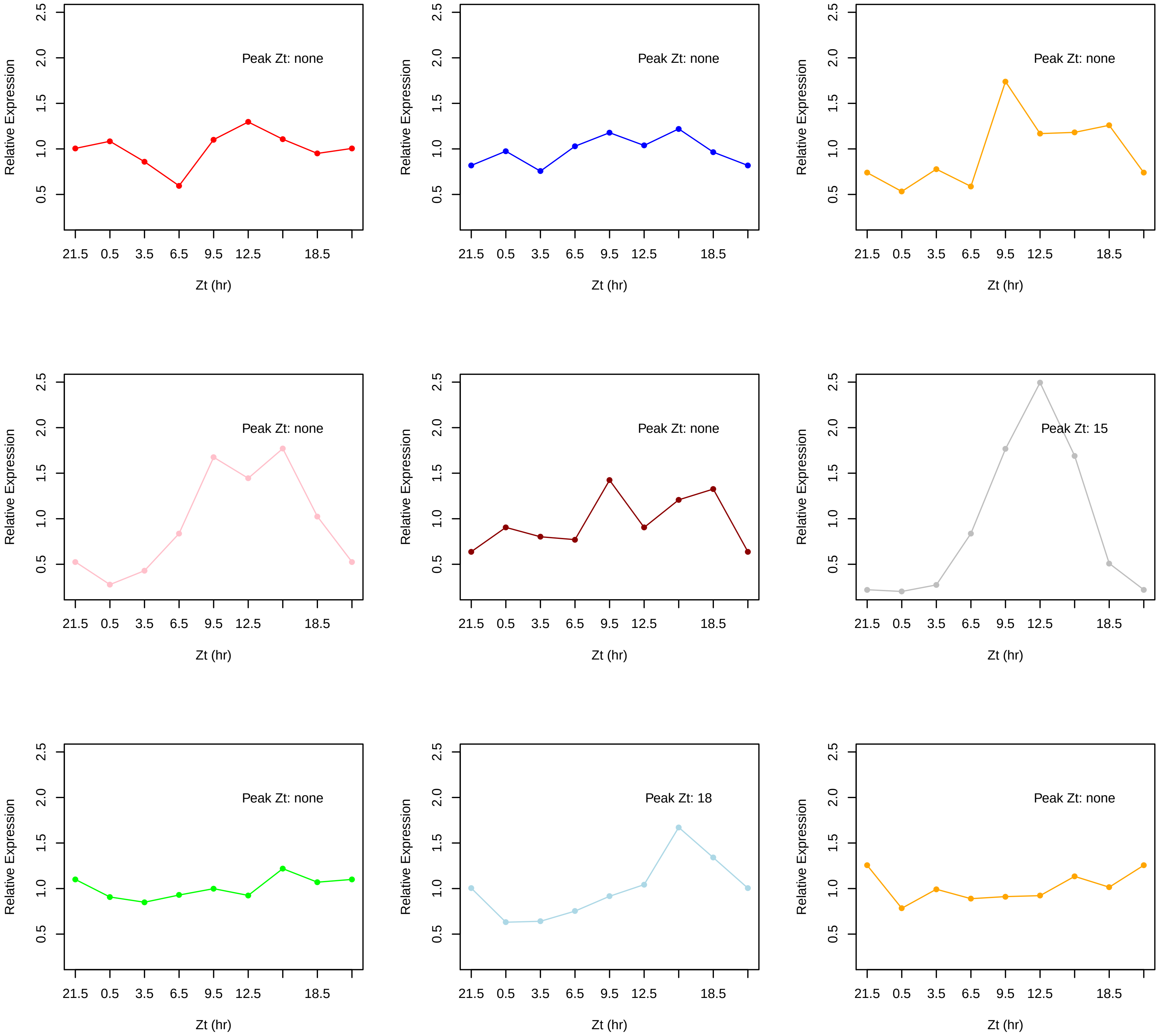

Singeleton: ENSXMAG00000021275 mcat

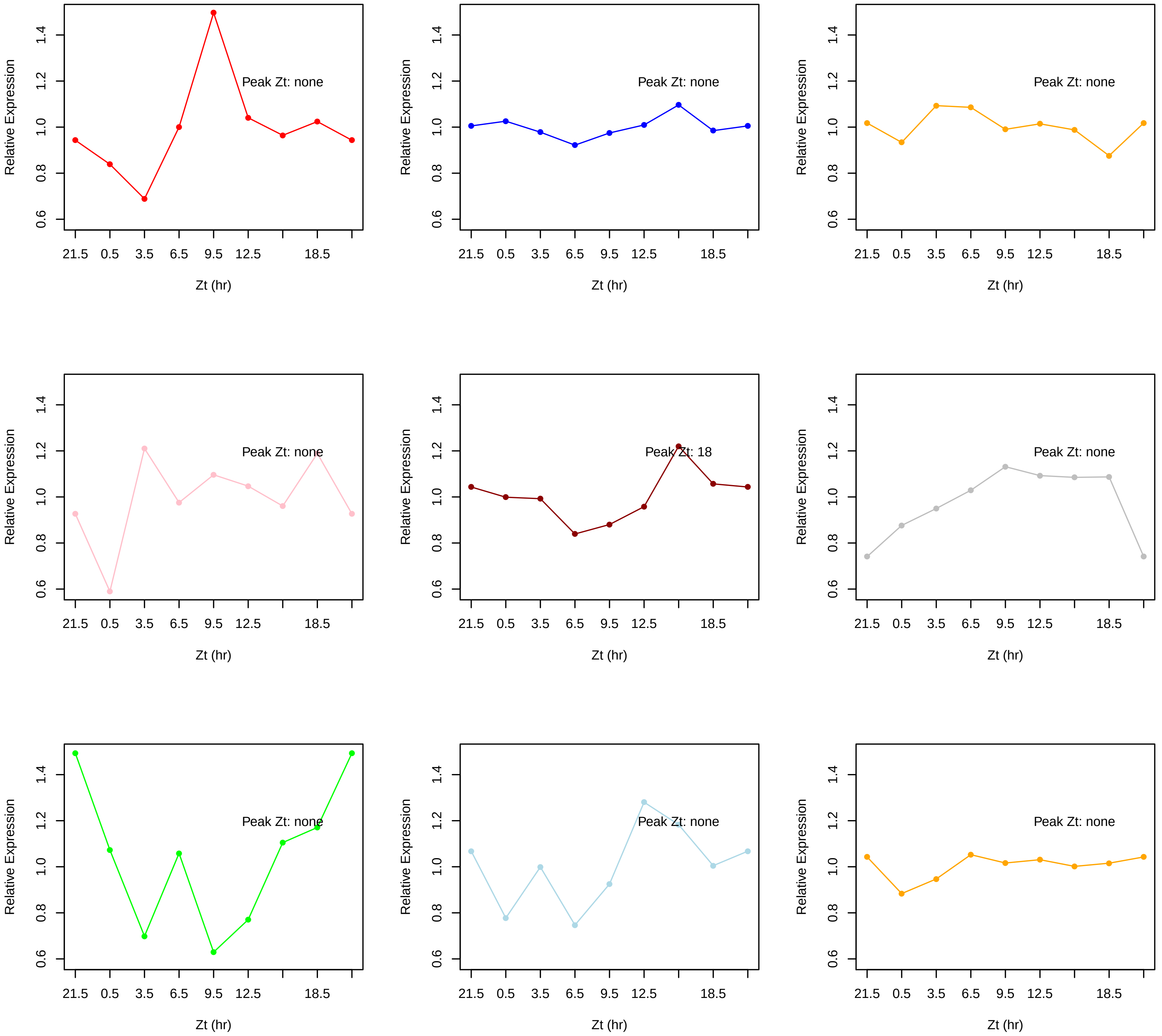

Singeleton: ENSXMAG00000001367 pacsin2

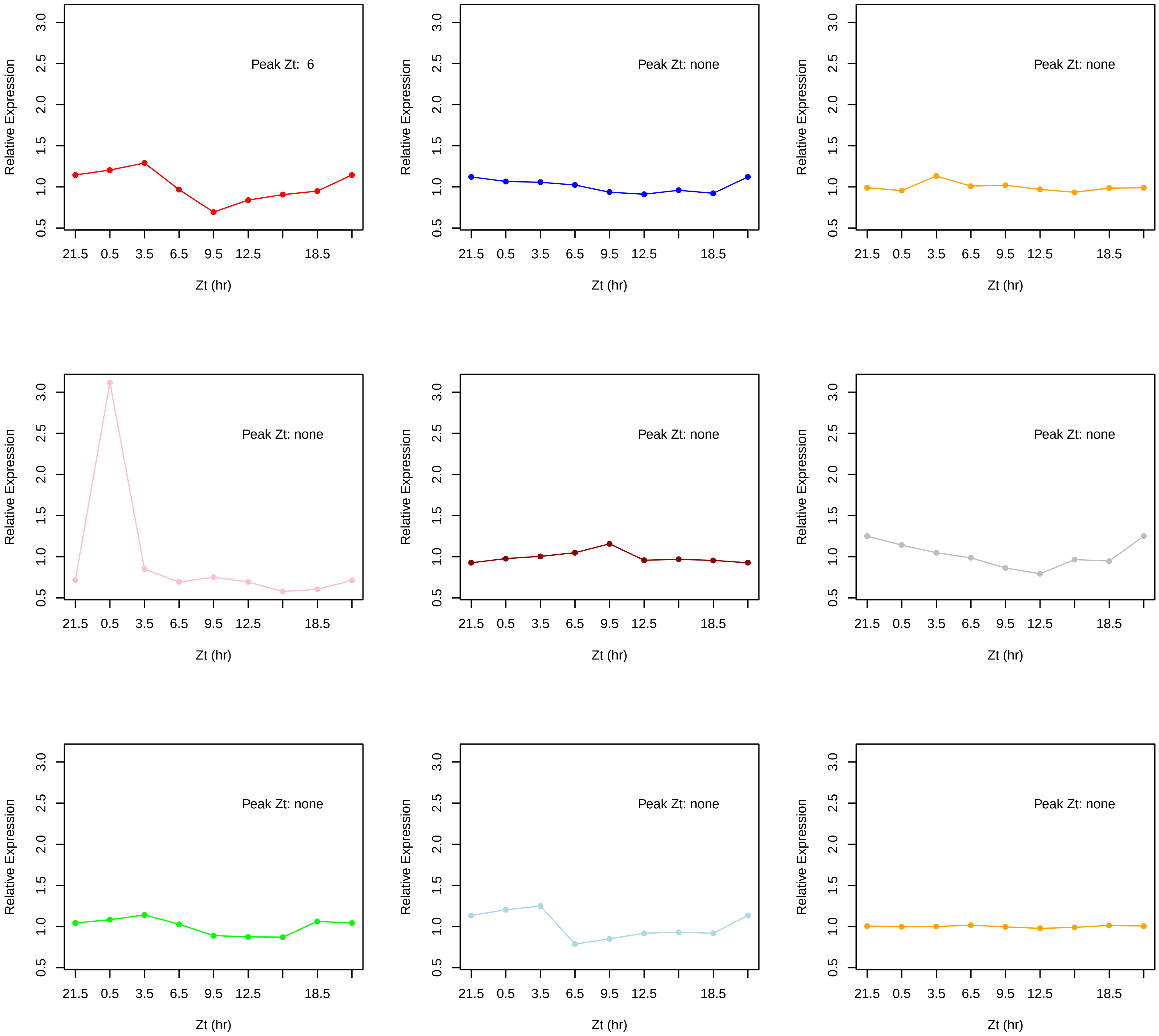

Singeleton: ENSXMAG00000016507

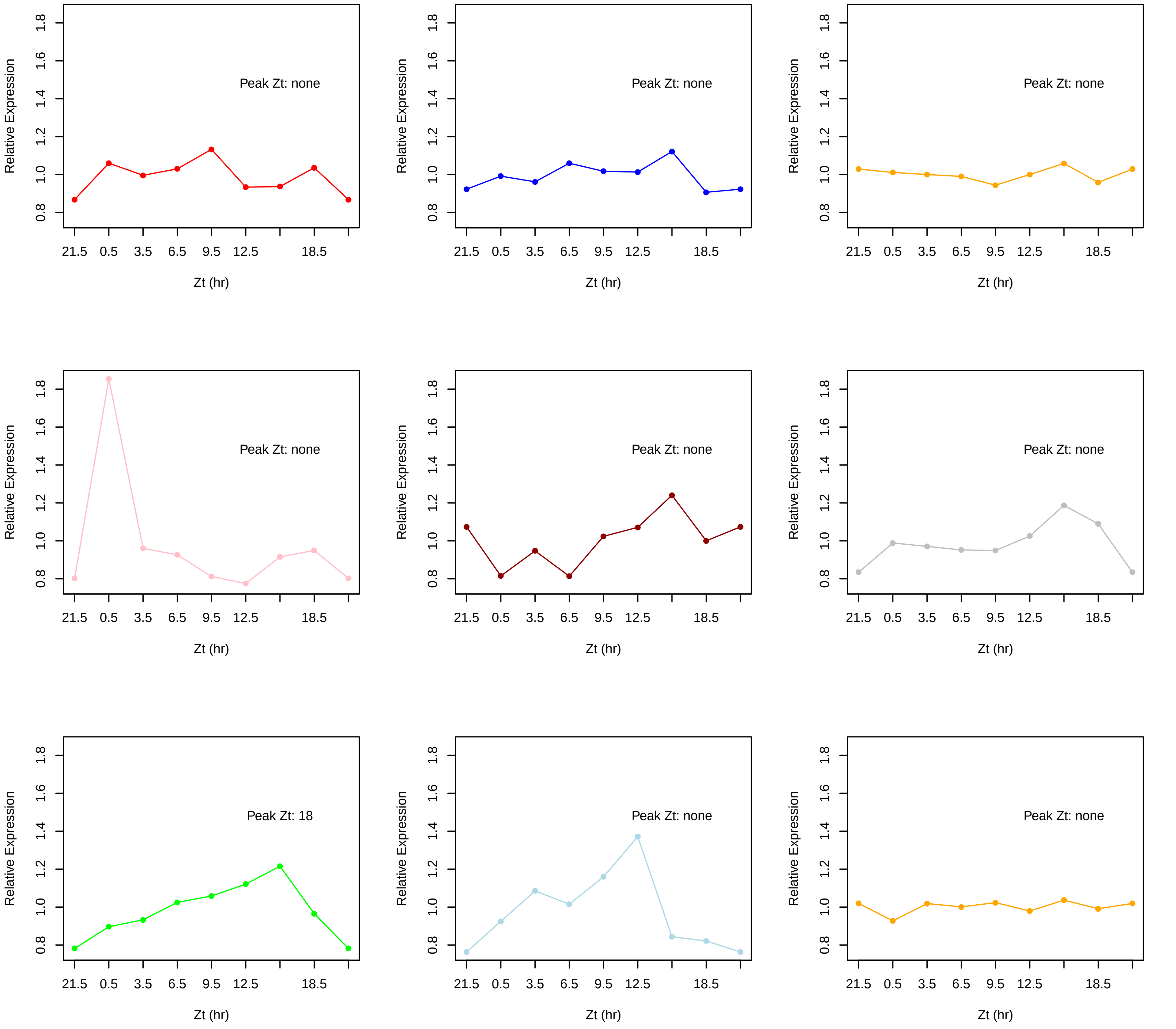

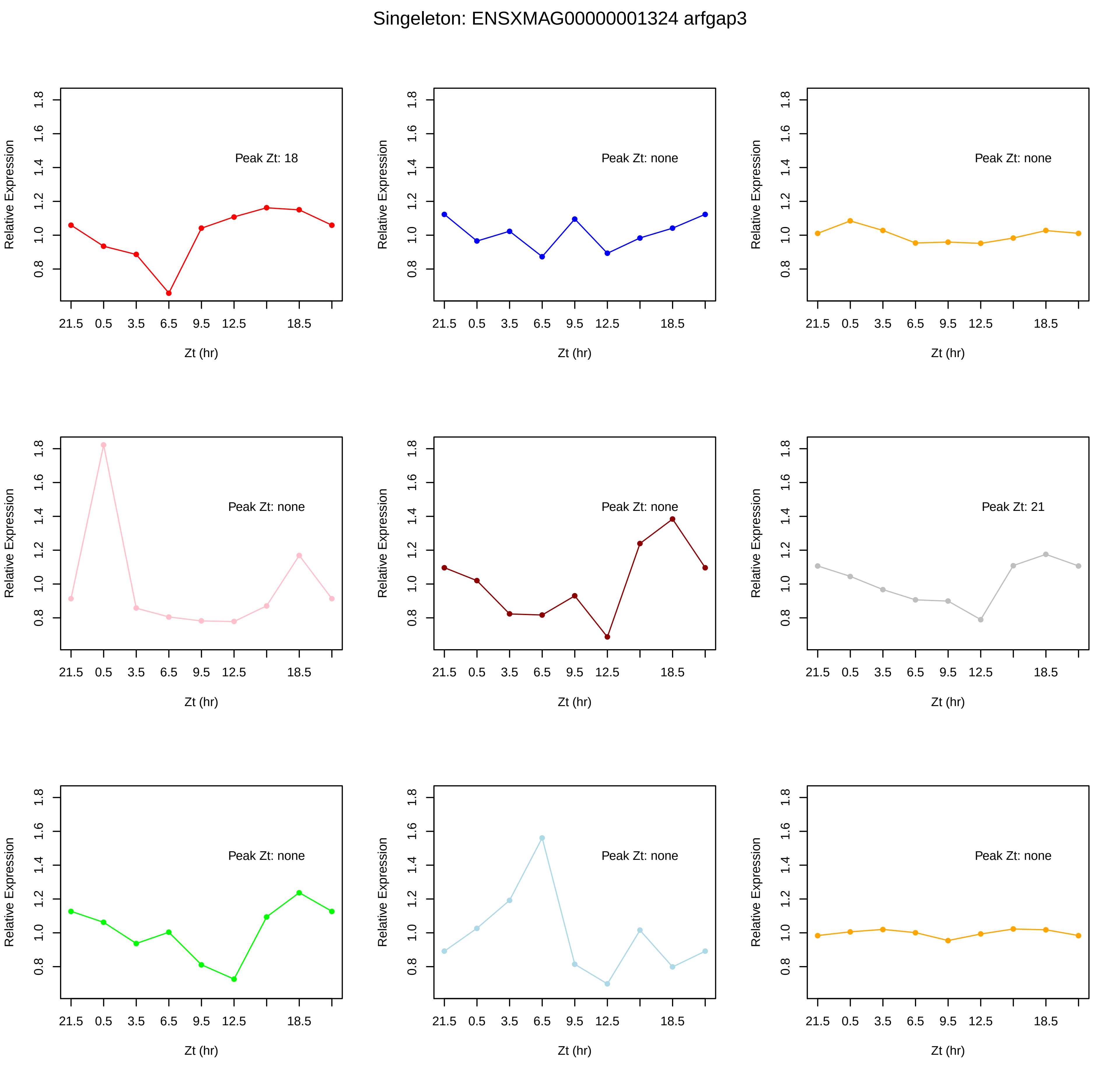

Singeleton: ENSXMAG00000001324 arfgap3
Peak Zt: 18
Peak Zt: none
Peak Zt: none
Peak Zt: none
Peak Zt: none
Peak Zt: 21
Peak Zt: none
Peak Zt: none
Peak Zt: none
Relative Expression
Zt (hr)

Singeleton: ENSXMAG00000029335 mak16

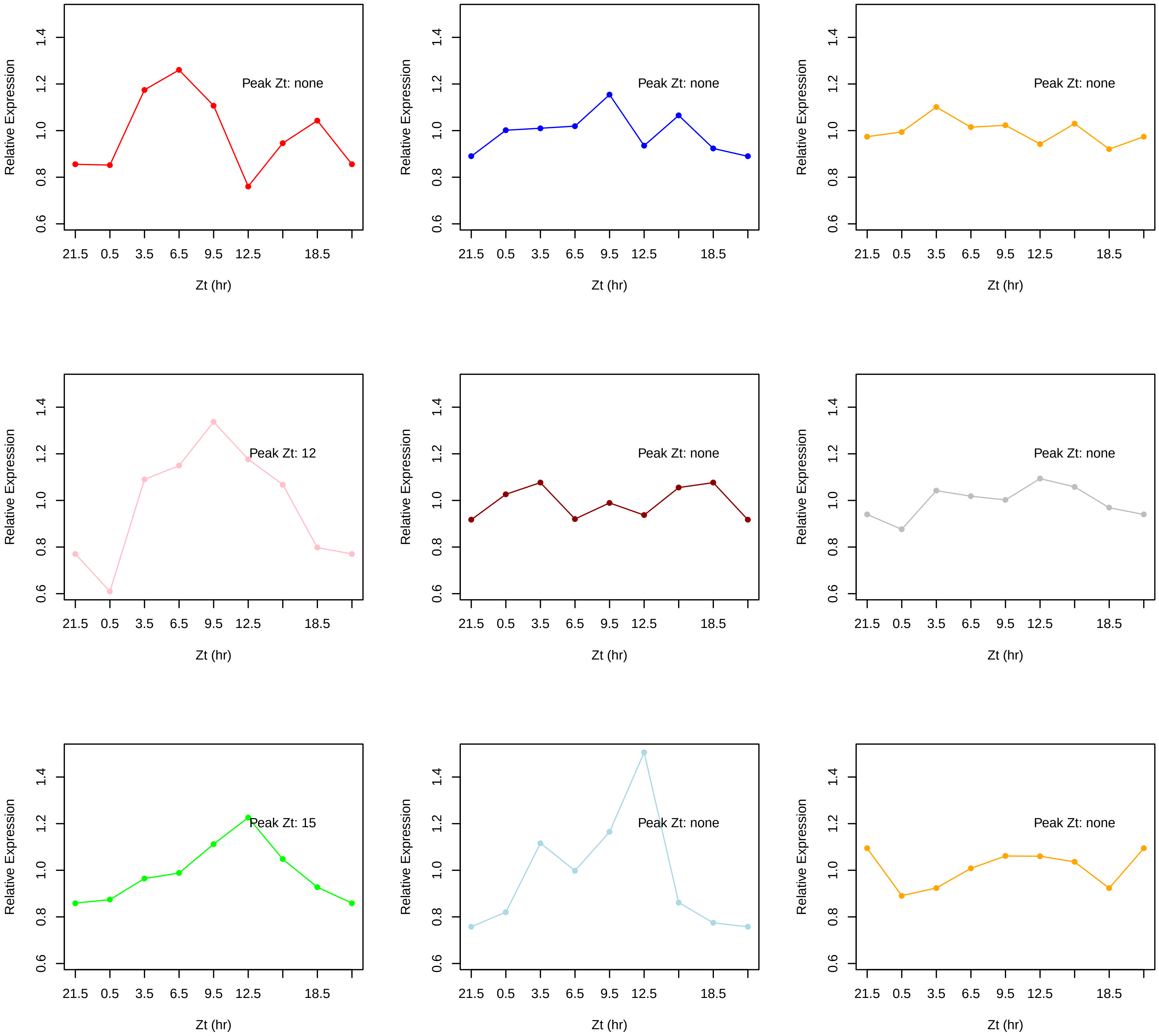

Singeleton: ENSXMAG00000001321

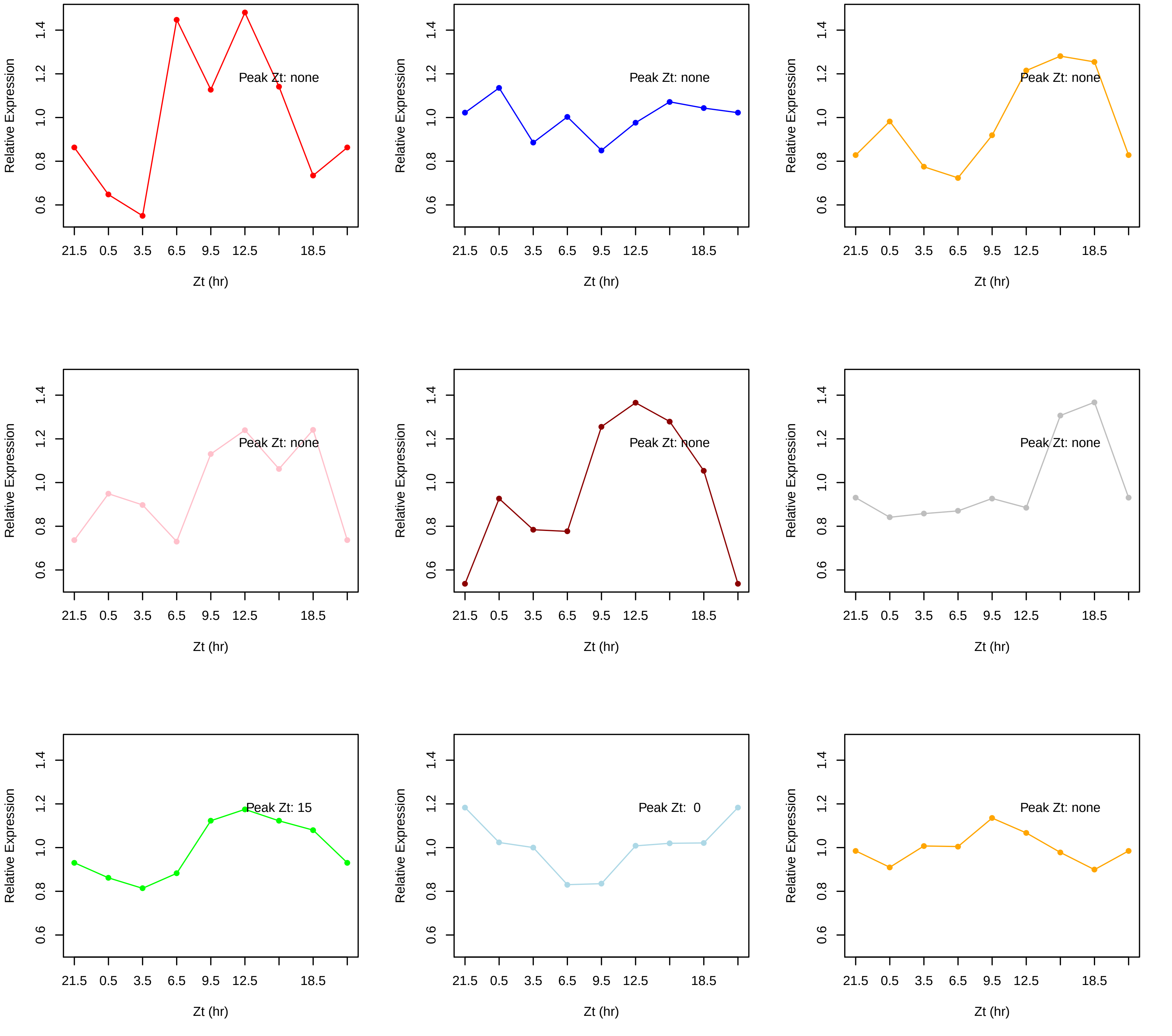

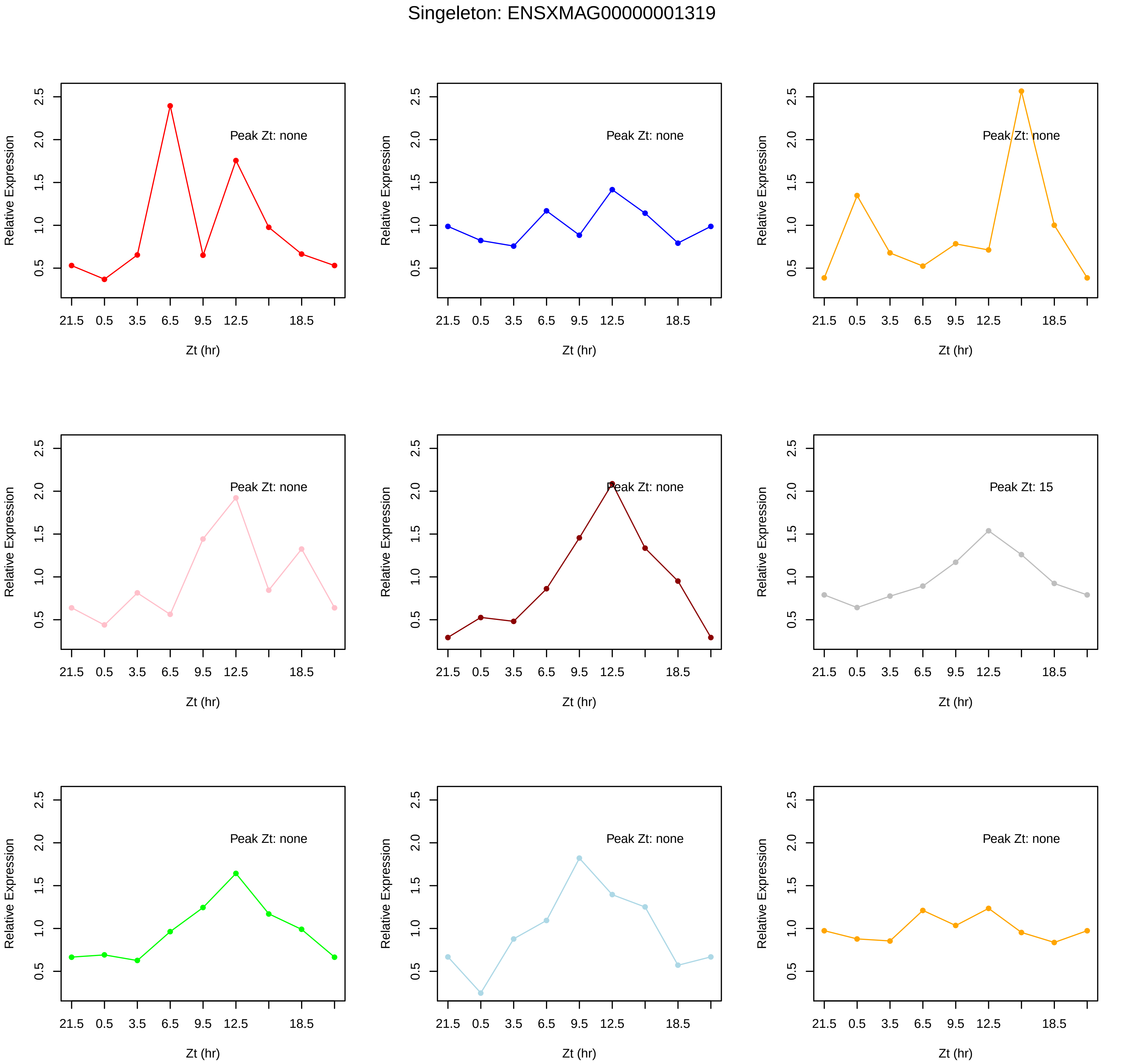
Singeleton: ENSXMAG00000001319
Peak Zt: none
Peak Zt: none
Peak Zt: none
Peak Zt: none
Peak Zt: none
Peak Zt: 15
Peak Zt: none
Peak Zt: none
Peak Zt: none
Relative Expression
Zt (hr)
21.5
0.5
3.5
6.5
9.5
12.5
18.5
0.5
1.0
1.5
2.0
2.5

Singeleton: ENSXMAG00000016203 si:ch1073−392o20.1

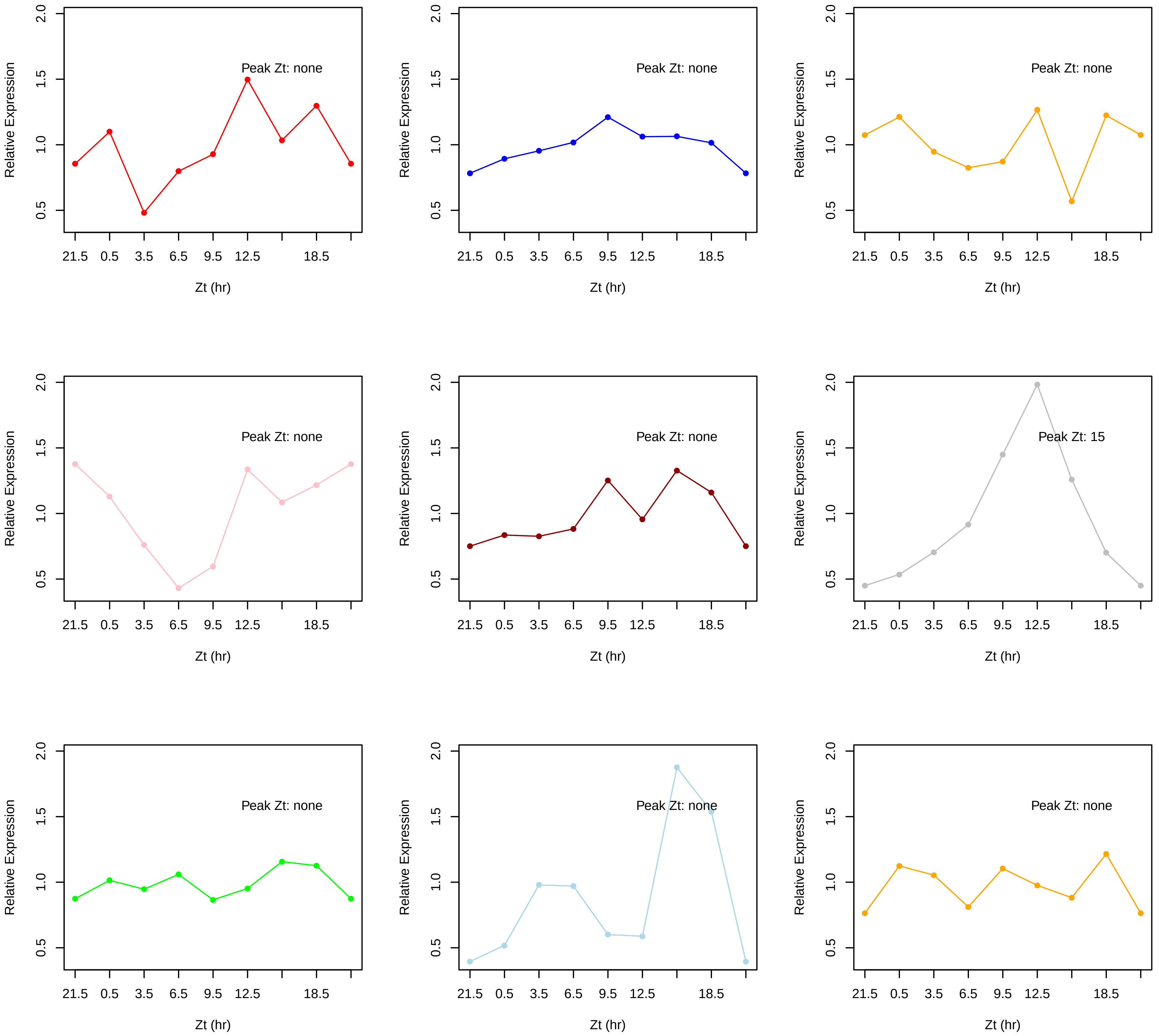

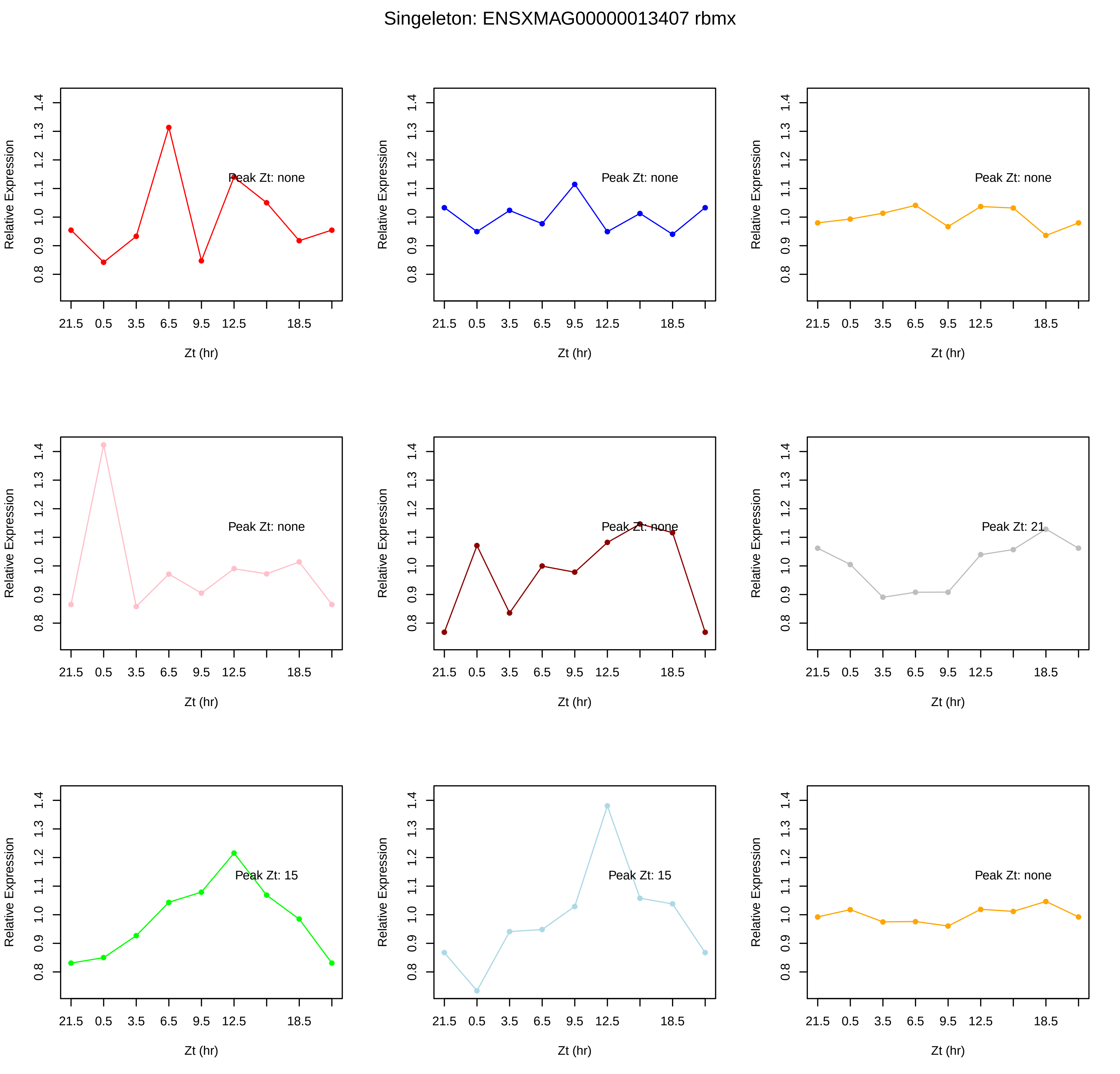
Singeleton: ENSXMAG00000013407 rbmx
Relative Expression
Zt (hr)
21.5
0.5
3.5
6.5
9.5
12.5
18.5
0.8
0.9
1.0
1.1
1.2
1.3
1.4
Peak Zt: none
Peak Zt: none
Peak Zt: none
Peak Zt: none
Peak Zt: none
Peak Zt: 21
Peak Zt: 15
Peak Zt: 15
Peak Zt: none

Singleton: ENSXMAG00000001252 anks1b

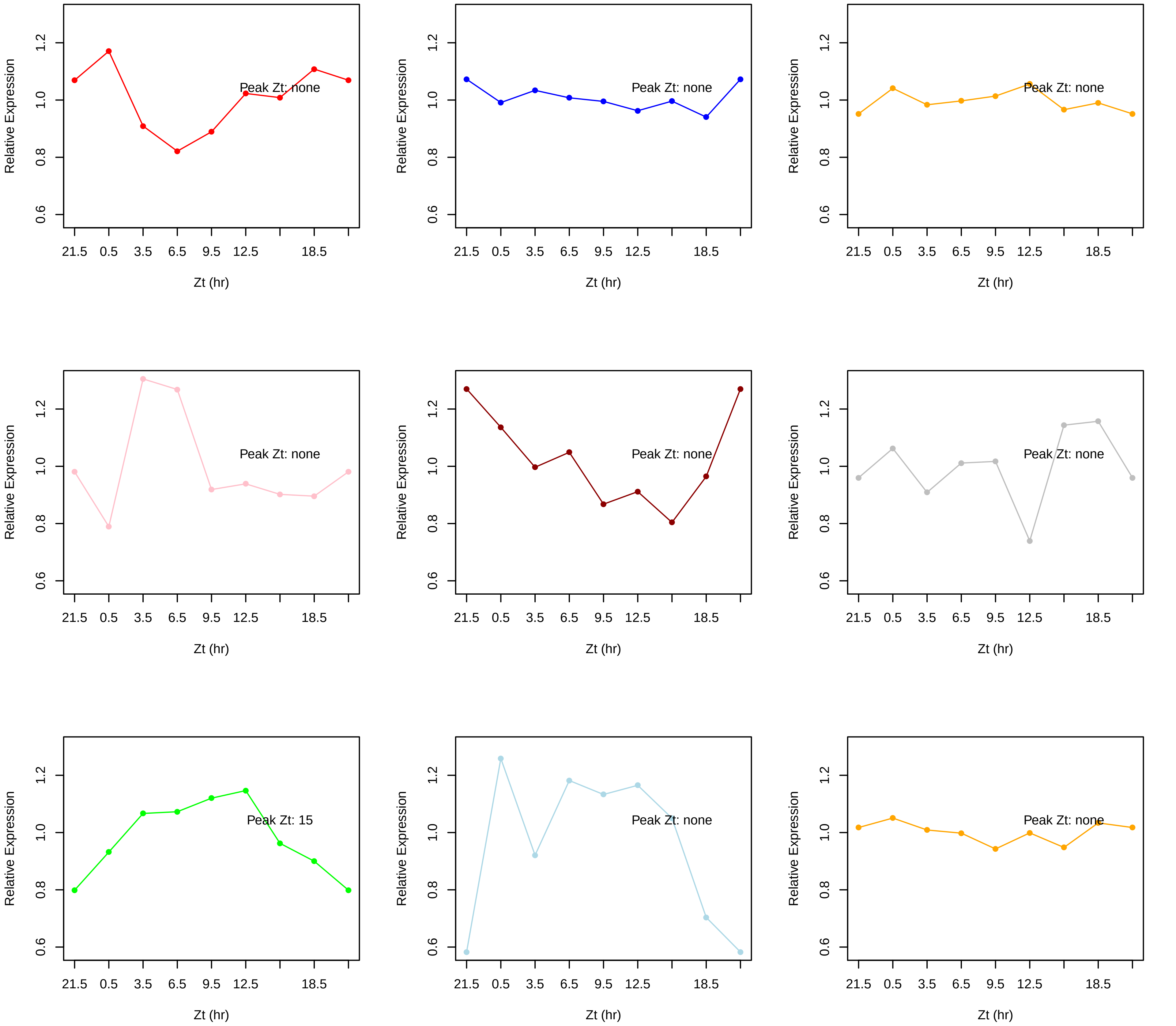

Singleton: ENSXMAG00000015473 bag4

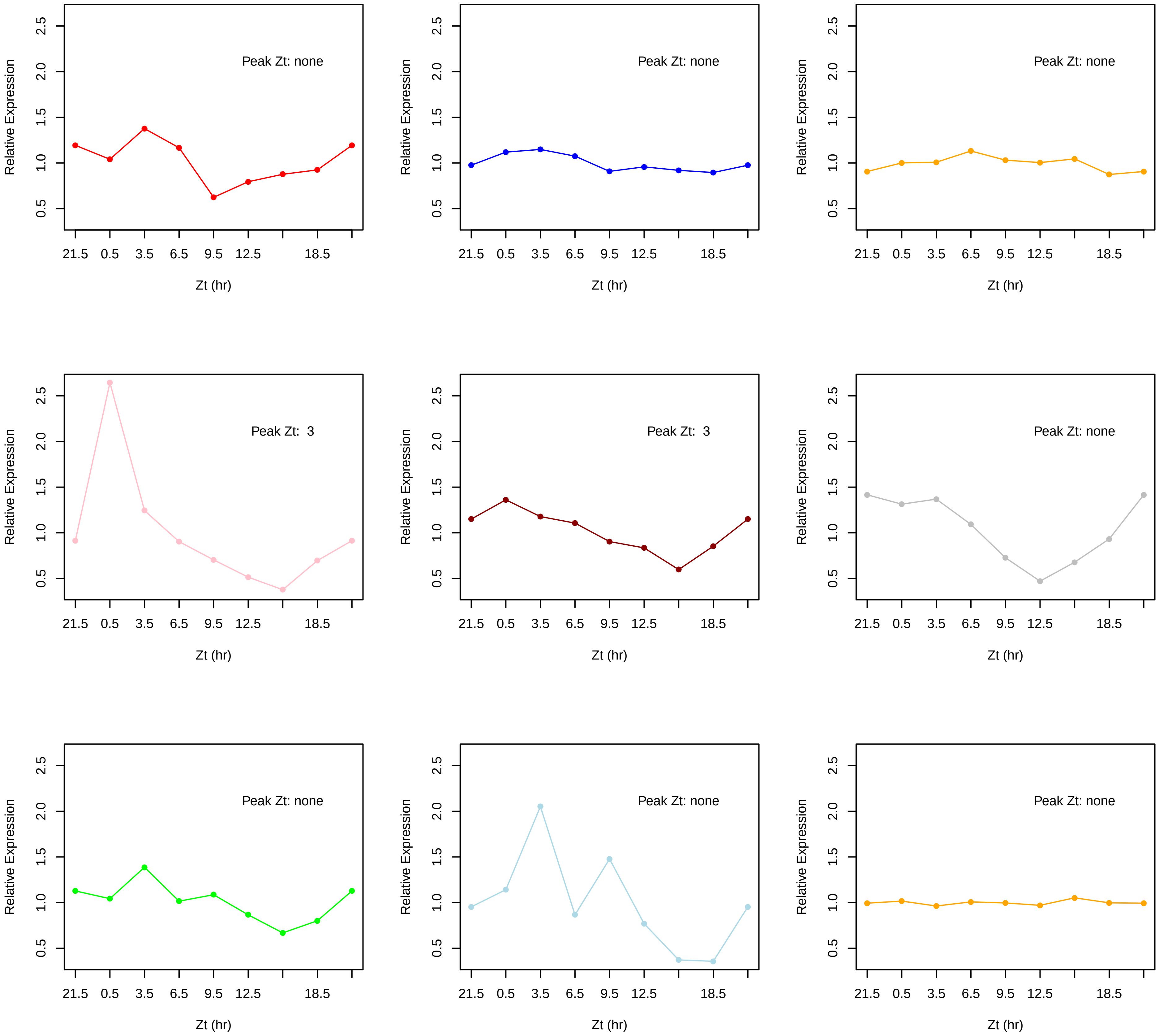

Singeleton: ENSXMAG00000026637 lsm1

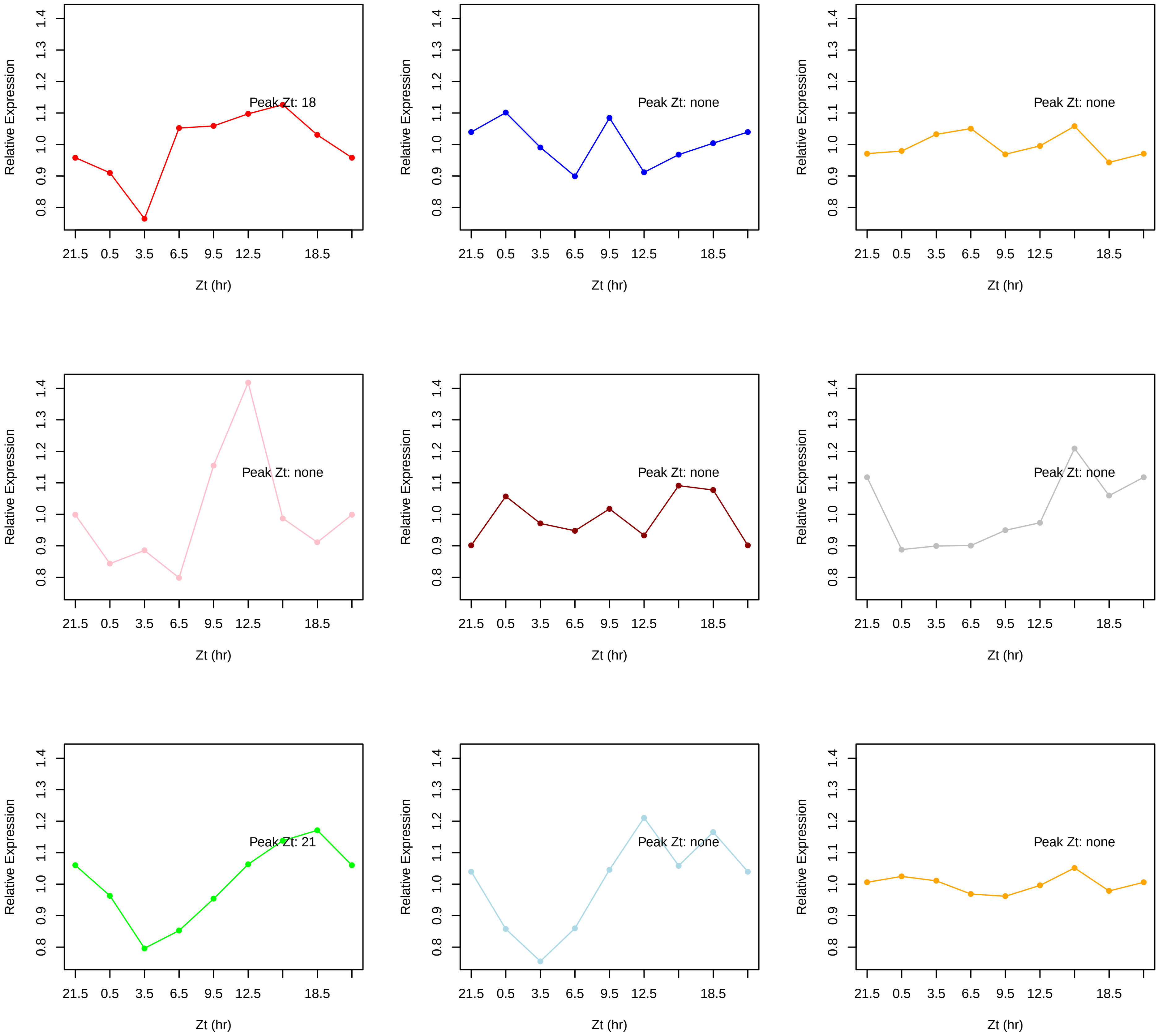

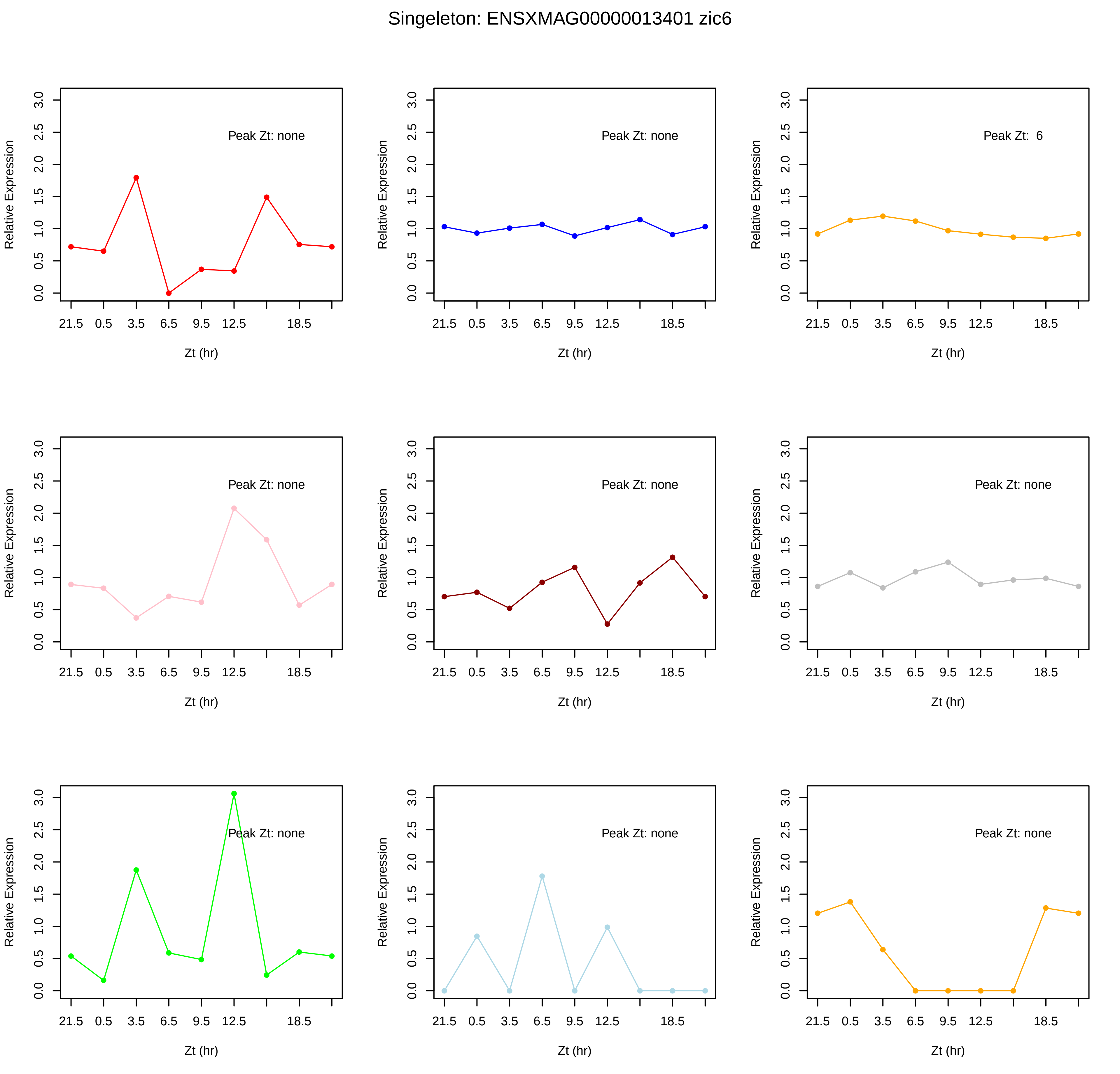

Singeleton: ENSXMAG00000013401 zic6
Peak Zt: none
Peak Zt: none
Peak Zt: 6
Peak Zt: none
Peak Zt: none
Peak Zt: none
Peak Zt: none
Peak Zt: none
Peak Zt: none
Relative Expression
Zt (hr)

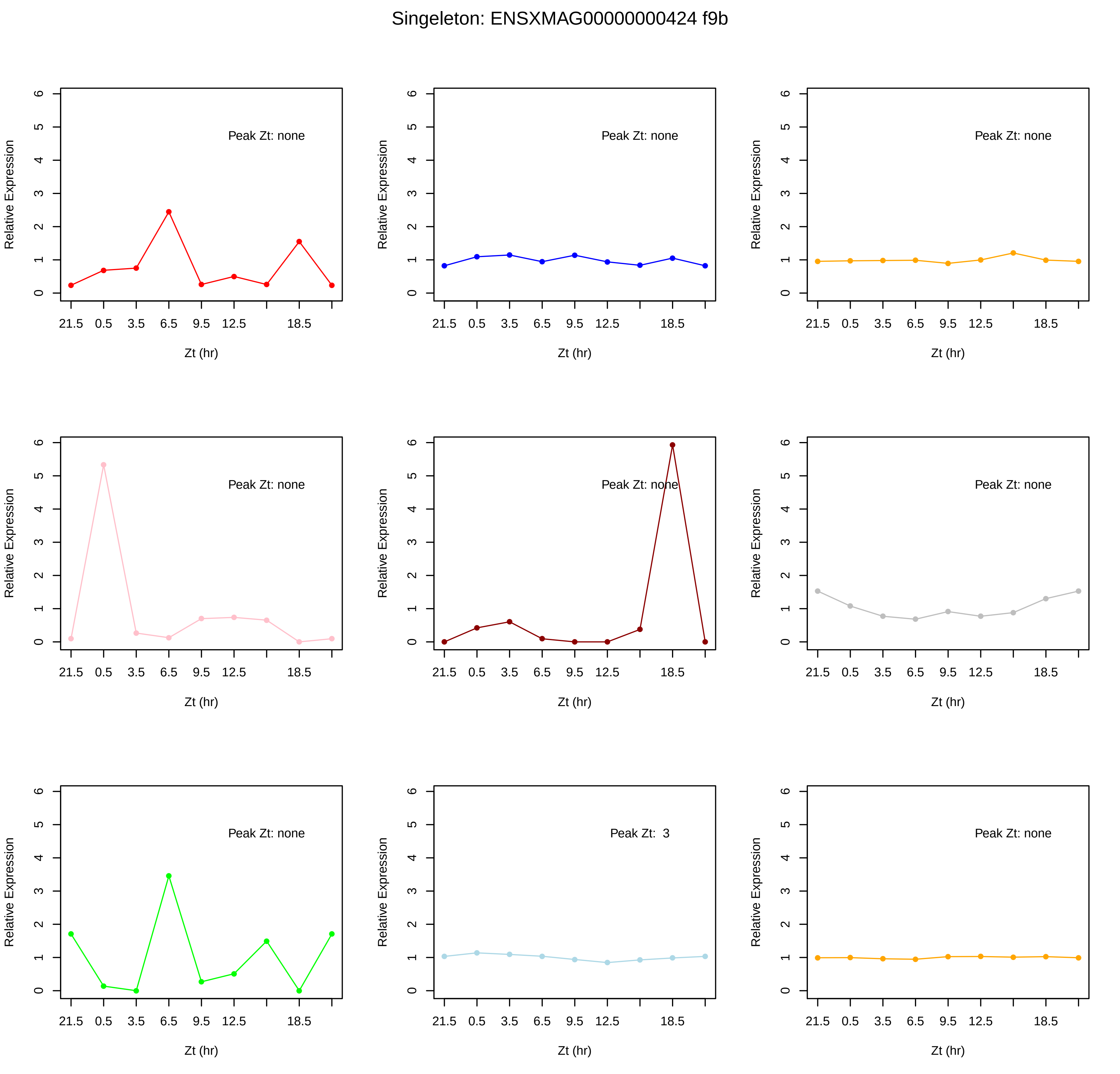
Singeleton: ENSXMAG00000000424 f9b
Peak Zt: none
Peak Zt: none
Peak Zt: none
Peak Zt: none
Peak Zt: none
Peak Zt: none
Peak Zt: none
Peak Zt: 3
Peak Zt: none
Relative Expression
Zt (hr)
21.5
0.5
3.5
6.5
9.5
12.5
18.5

Singeleton: ENSXMAG00000018543 tcp11l2

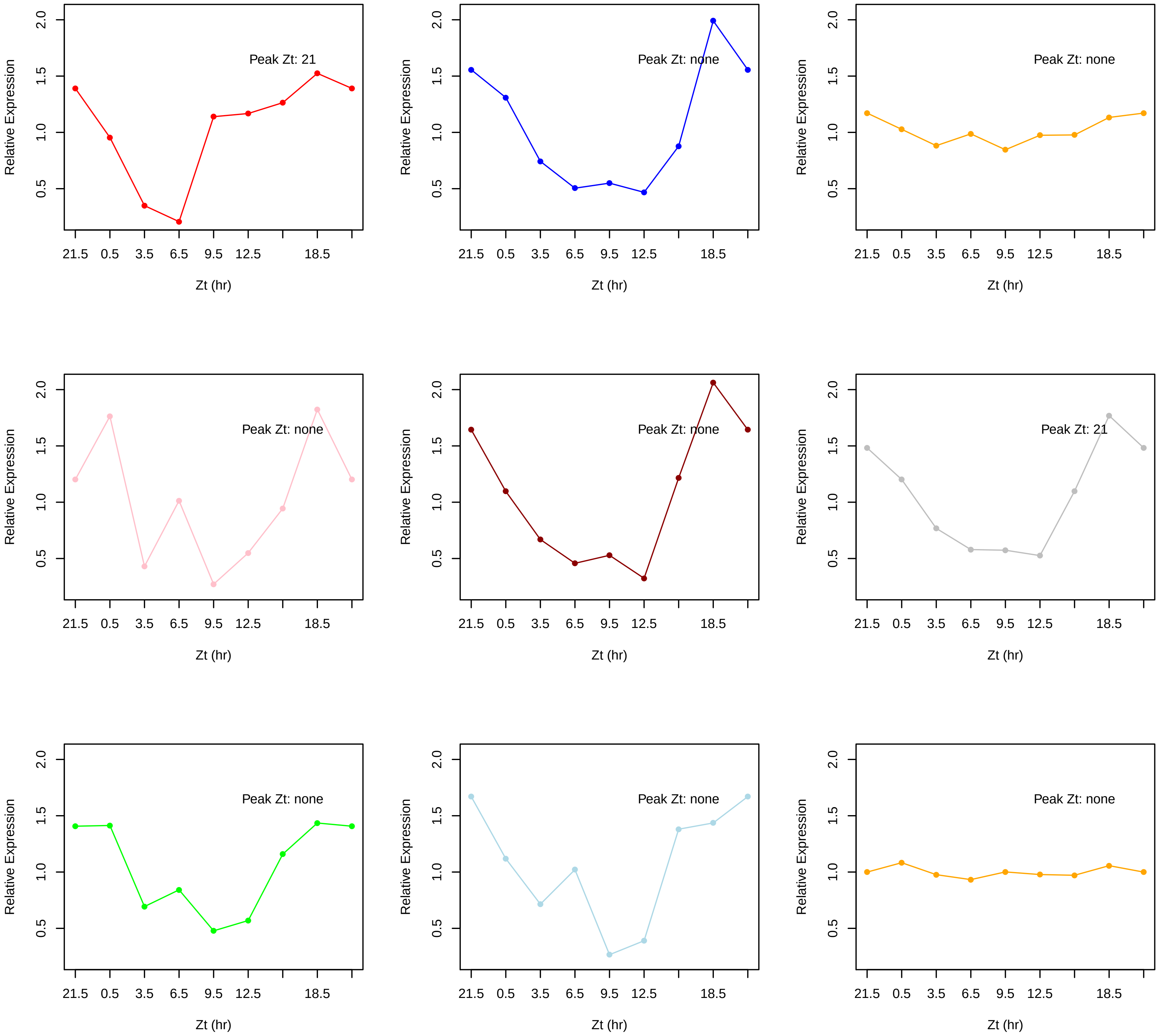

Singeleton: ENSXMAG00000028565 elmod3

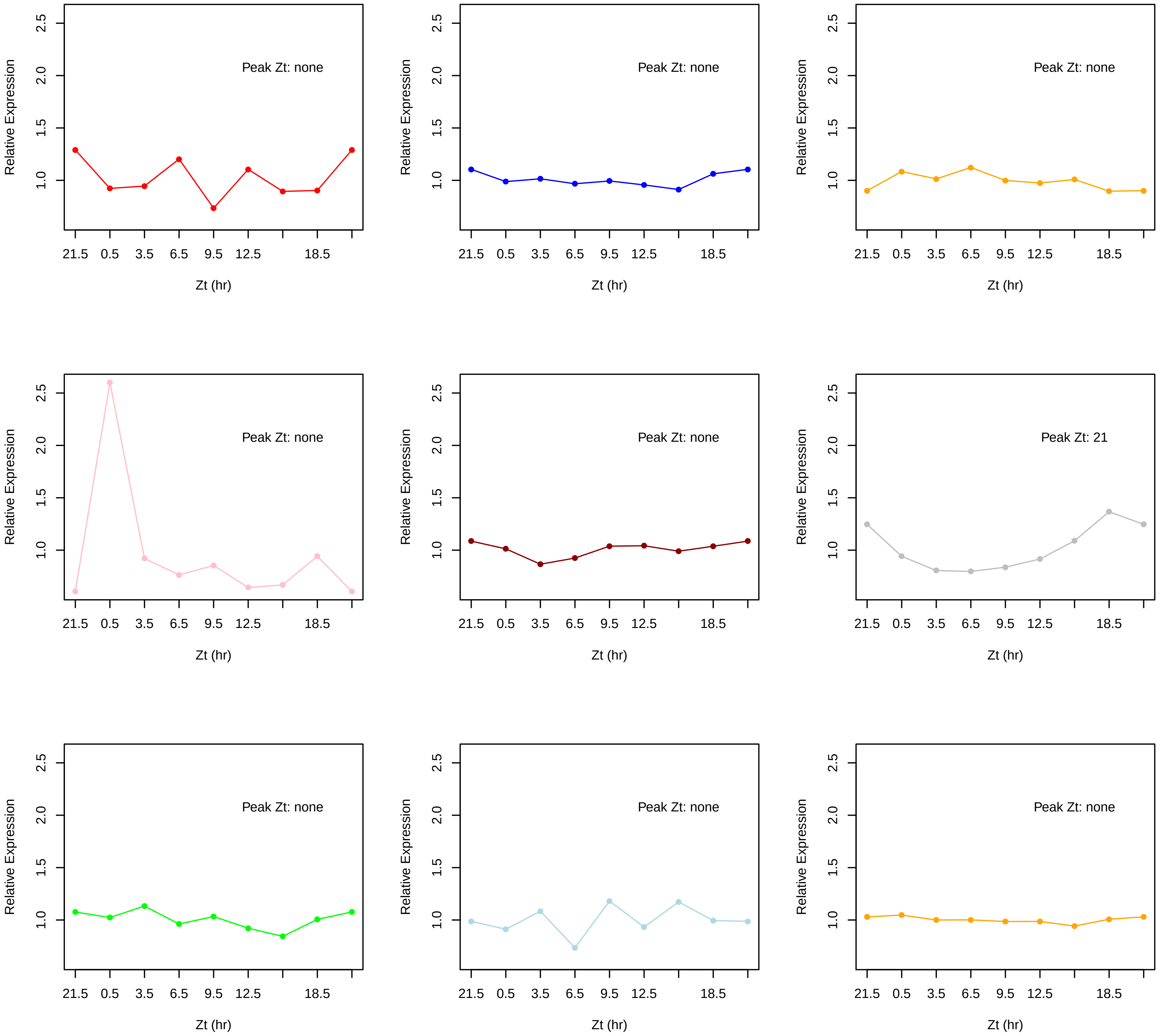

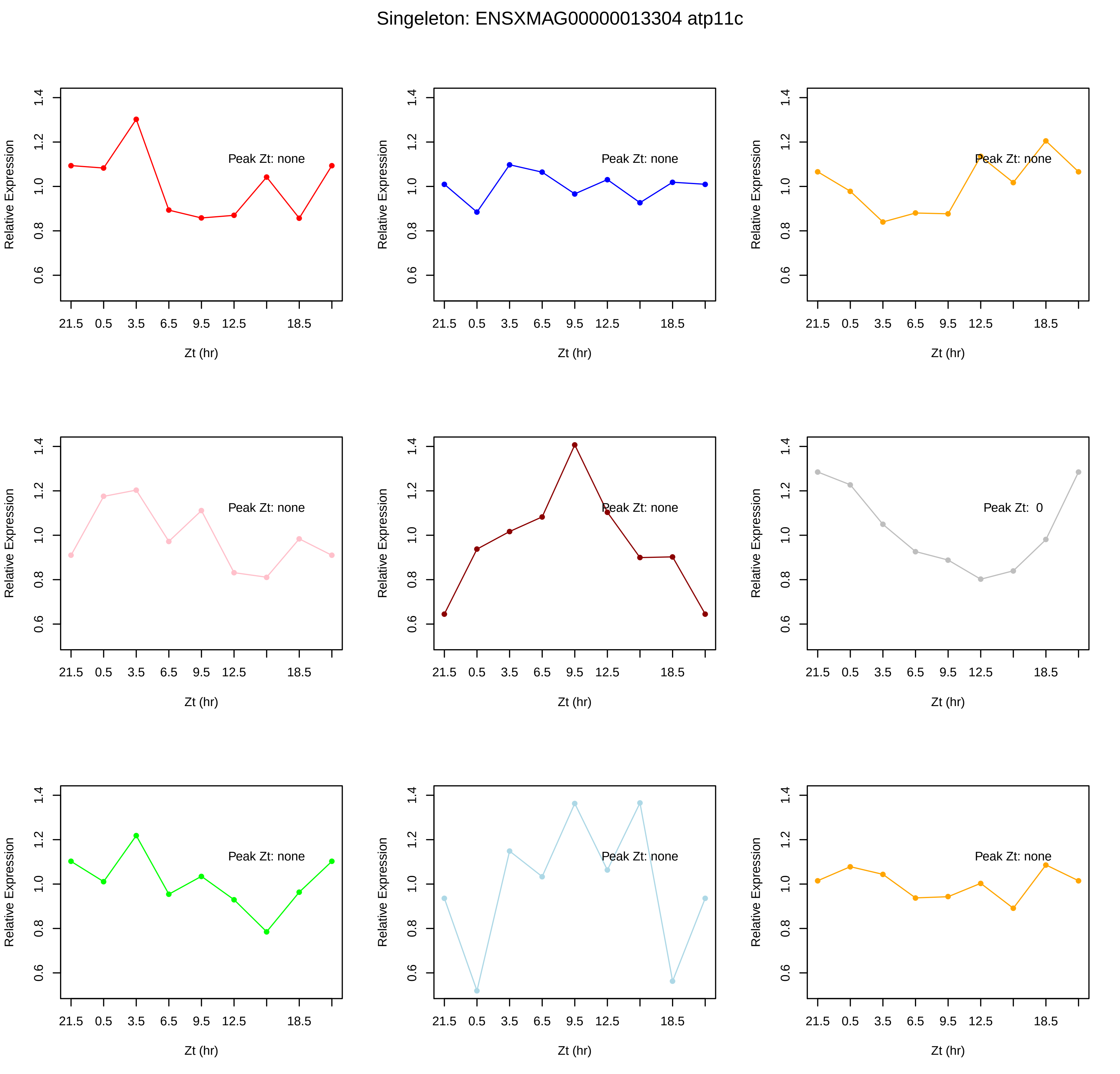

Singeleton: ENSXMAG00000013304 atp11c
Relative Expression
Zt (hr)
21.5
0.5
3.5
6.5
9.5
12.5
18.5
0.6
0.8
1.0
1.2
1.4
Peak Zt: none
Peak Zt: 0

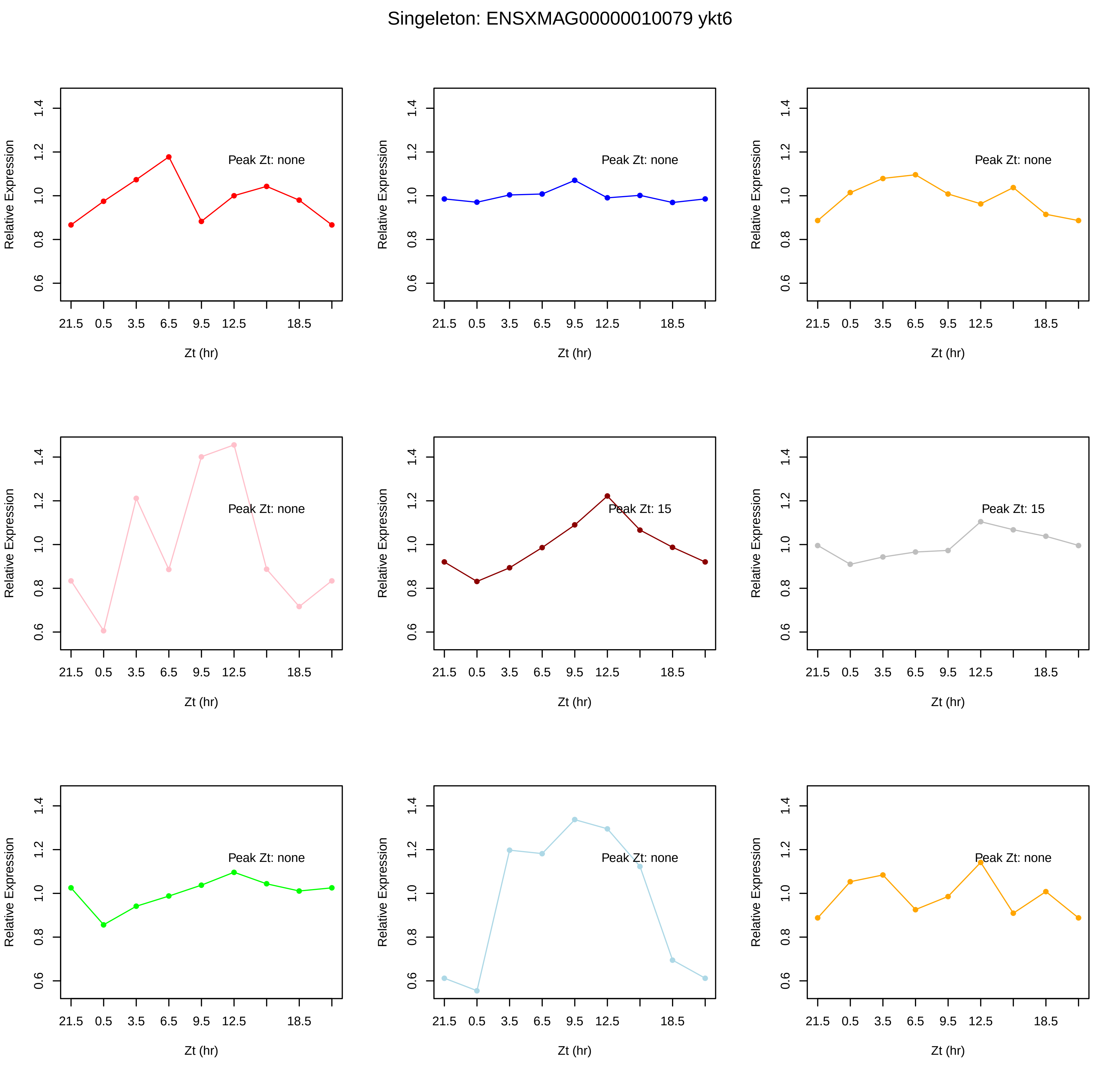
Singeleton: ENSXMAG00000010079 ykt6
Peak Zt: none
Peak Zt: none
Peak Zt: none
Peak Zt: none
Peak Zt: 15
Peak Zt: 15
Peak Zt: none
Peak Zt: none
Peak Zt: none
Relative Expression
Zt (hr)
21.5
0.5
3.5
6.5
9.5
12.5
18.5
0.6
0.8
1.0
1.2
1.4

Singeleton: ENSXMAG00000001470 gnptab

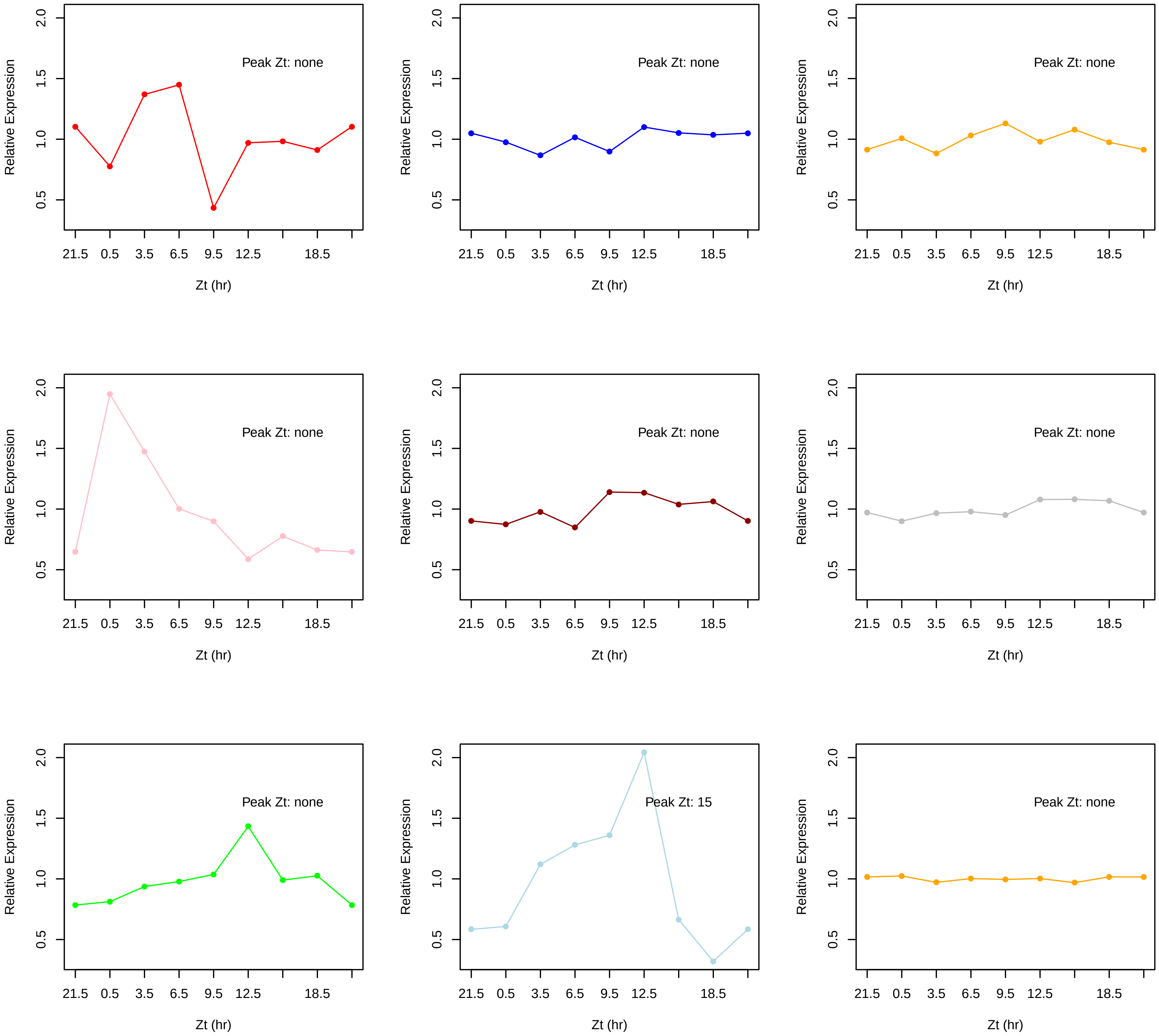

Singeleton: ENSXMAG00000001481 chpt1

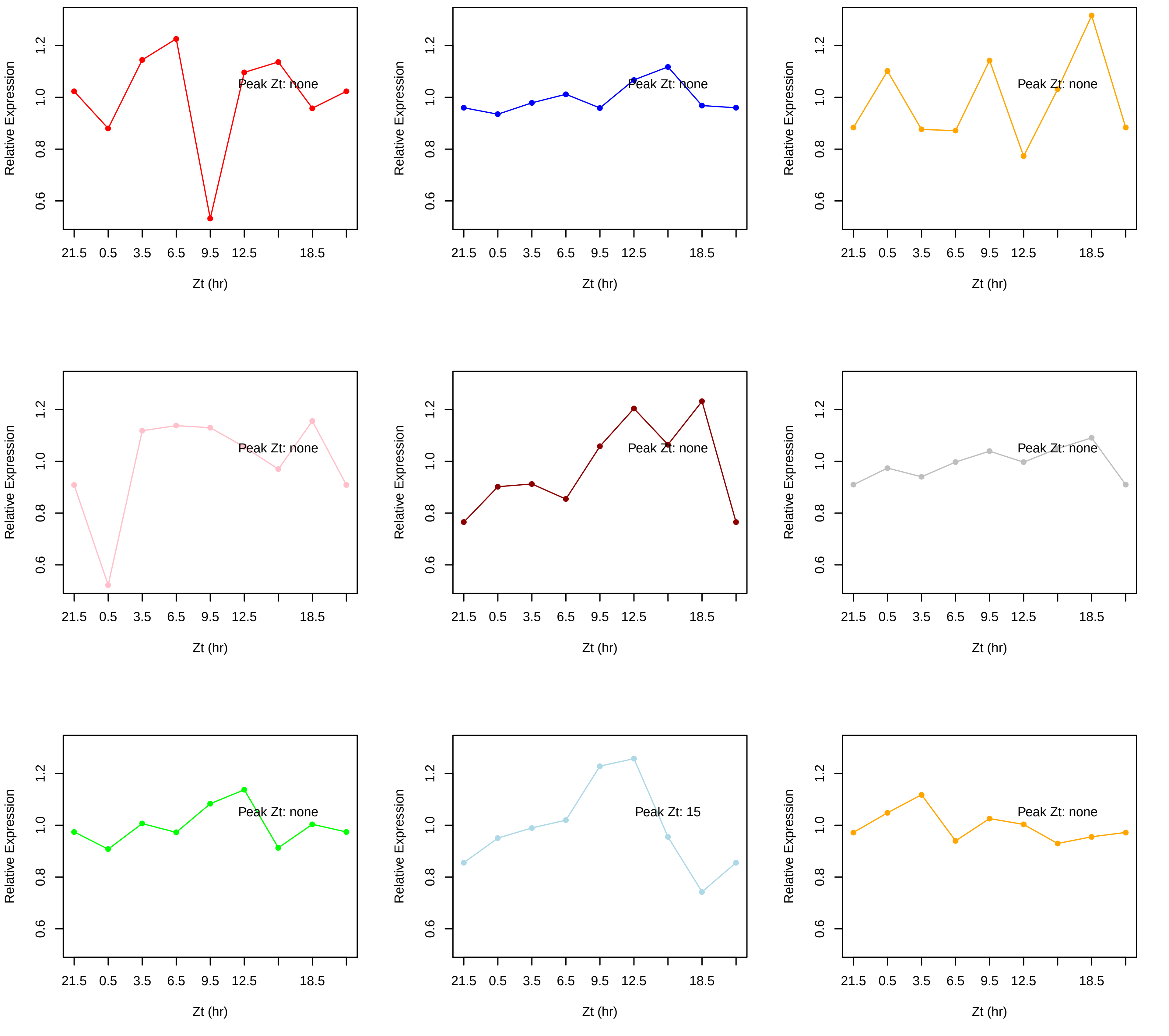

Singeleton: ENSXMAG00000007569 nt5c2l1

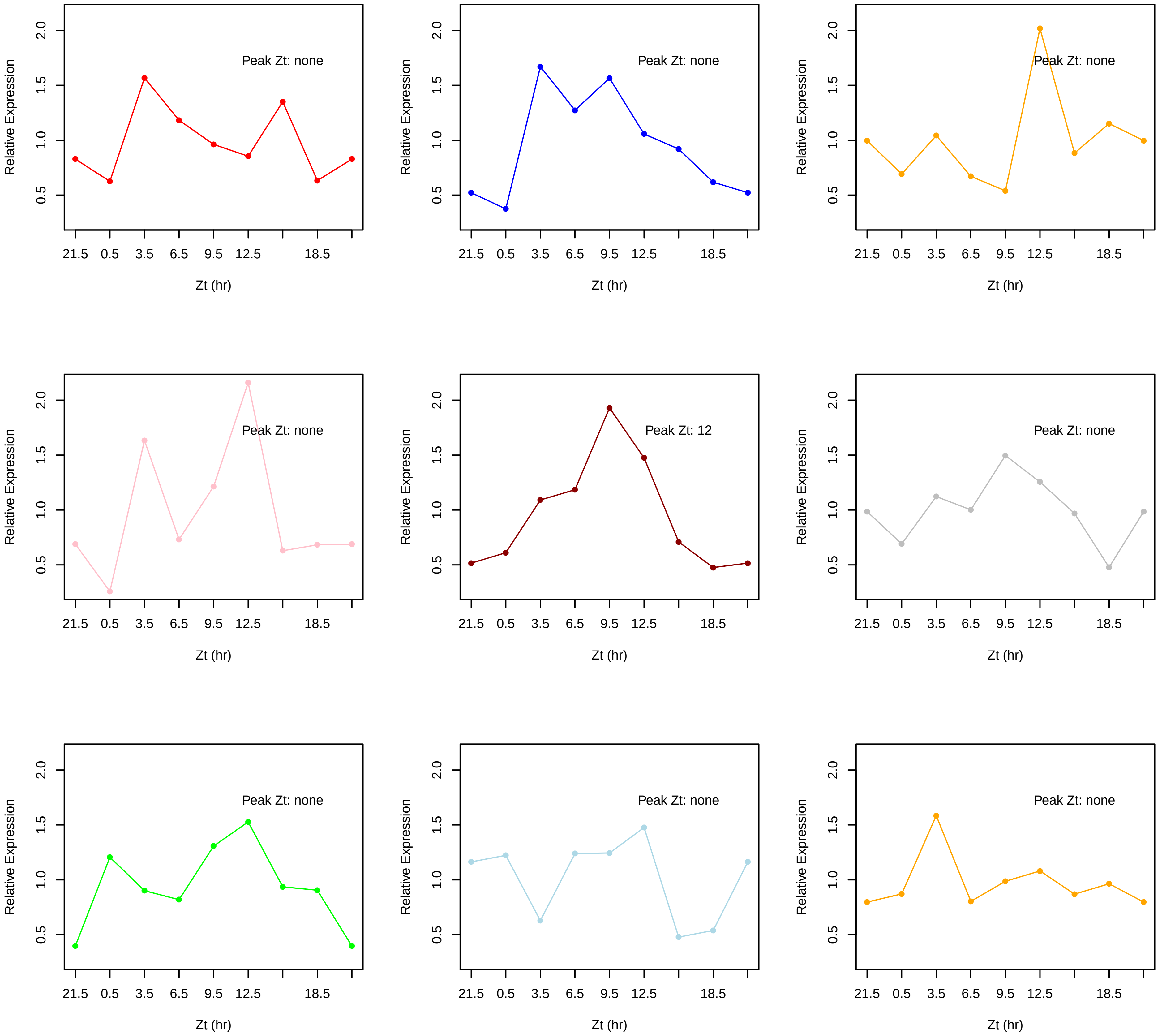

Singeleton: ENSXMAG00000028338 arl1

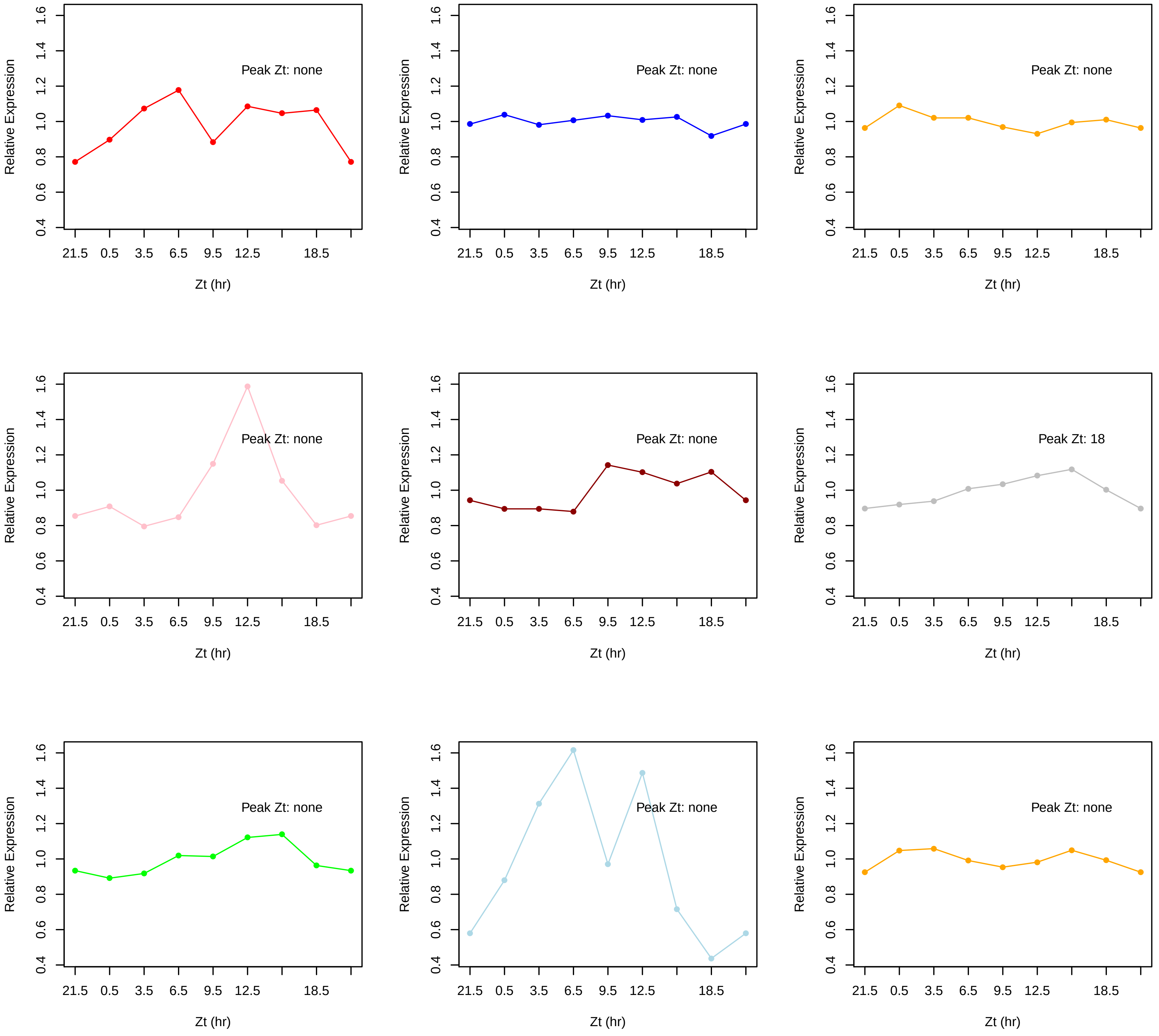

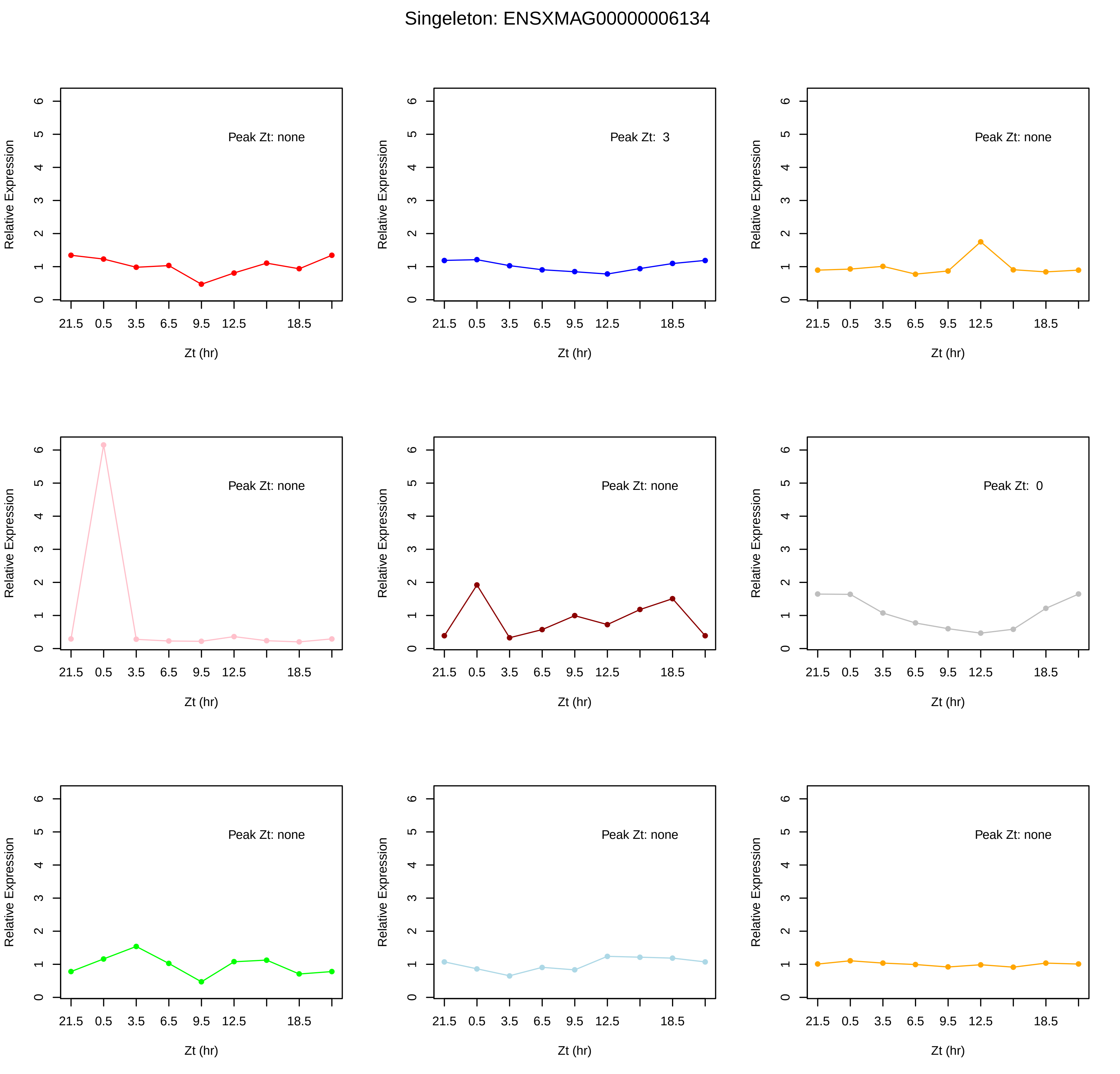

Singeleton: ENSXMAG00000006134
Peak Zt: none
Peak Zt: 3
Peak Zt: none
Peak Zt: none
Peak Zt: none
Peak Zt: 0
Peak Zt: none
Peak Zt: none
Peak Zt: none
Relative Expression
Zt (hr)
21.5
0.5
3.5
6.5
9.5
12.5
18.5

Singeleton: ENSXMAG00000008963 gas2l3

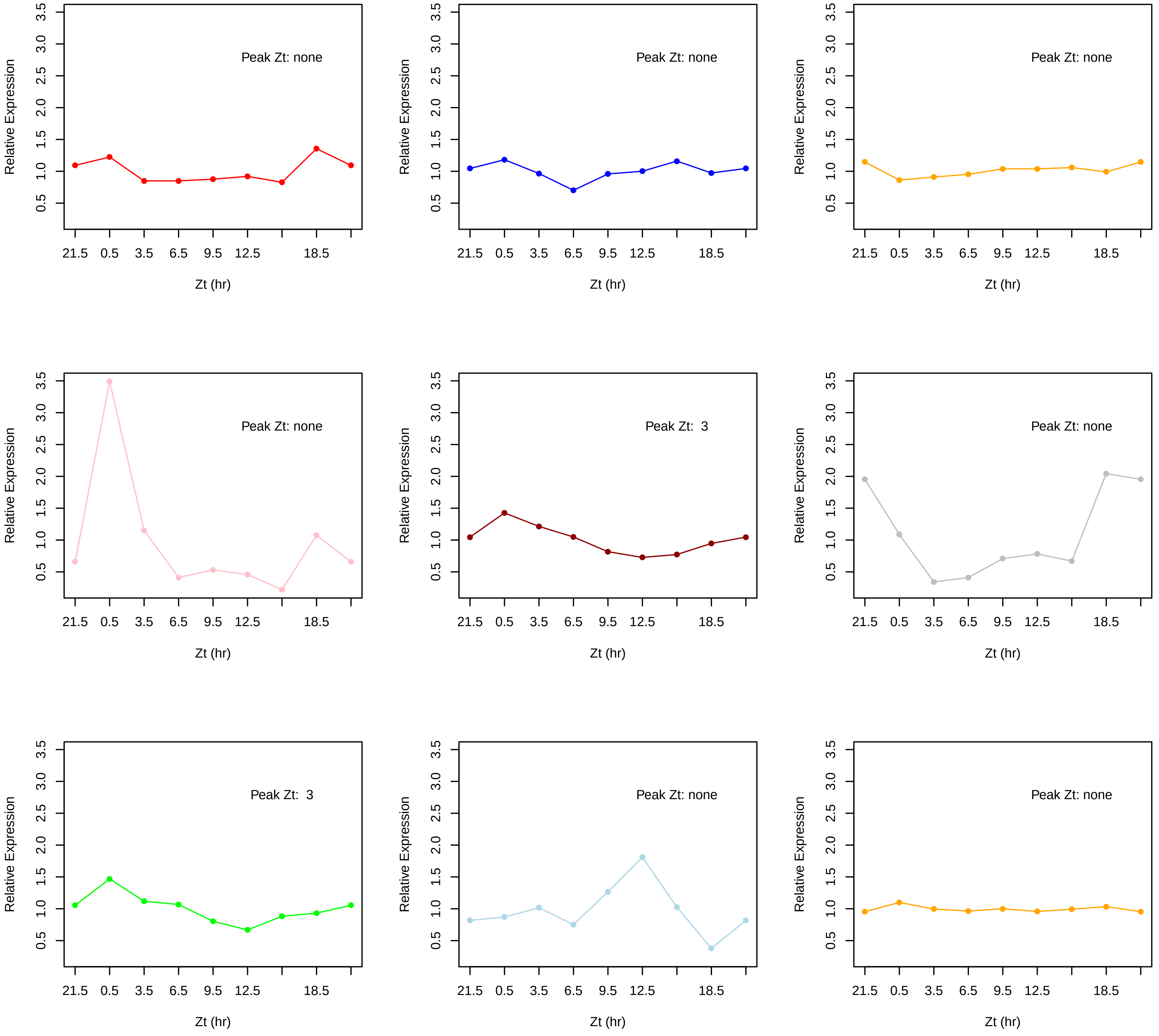

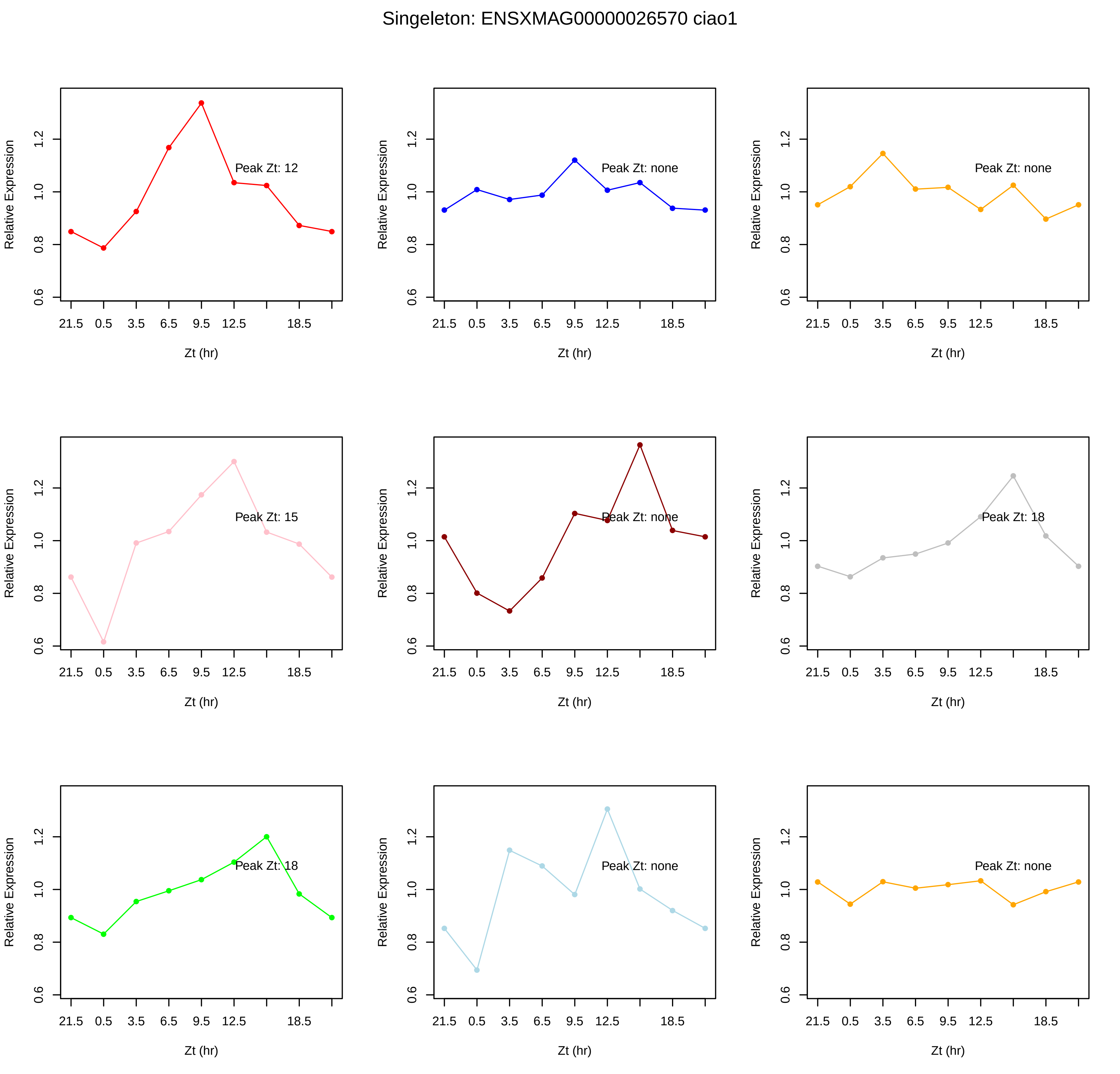
Singeleton: ENSXMAG00000026570 ciao1
Peak Zt: 12
Peak Zt: none
Peak Zt: none
Peak Zt: 15
Peak Zt: none
Peak Zt: 18
Peak Zt: 18
Peak Zt: none
Peak Zt: none
Relative Expression
Zt (hr)
21.5
0.5
3.5
6.5
9.5
12.5
18.5
0.6
0.8
1.0
1.2

Singeleton: ENSXMAG00000003033 ggcx

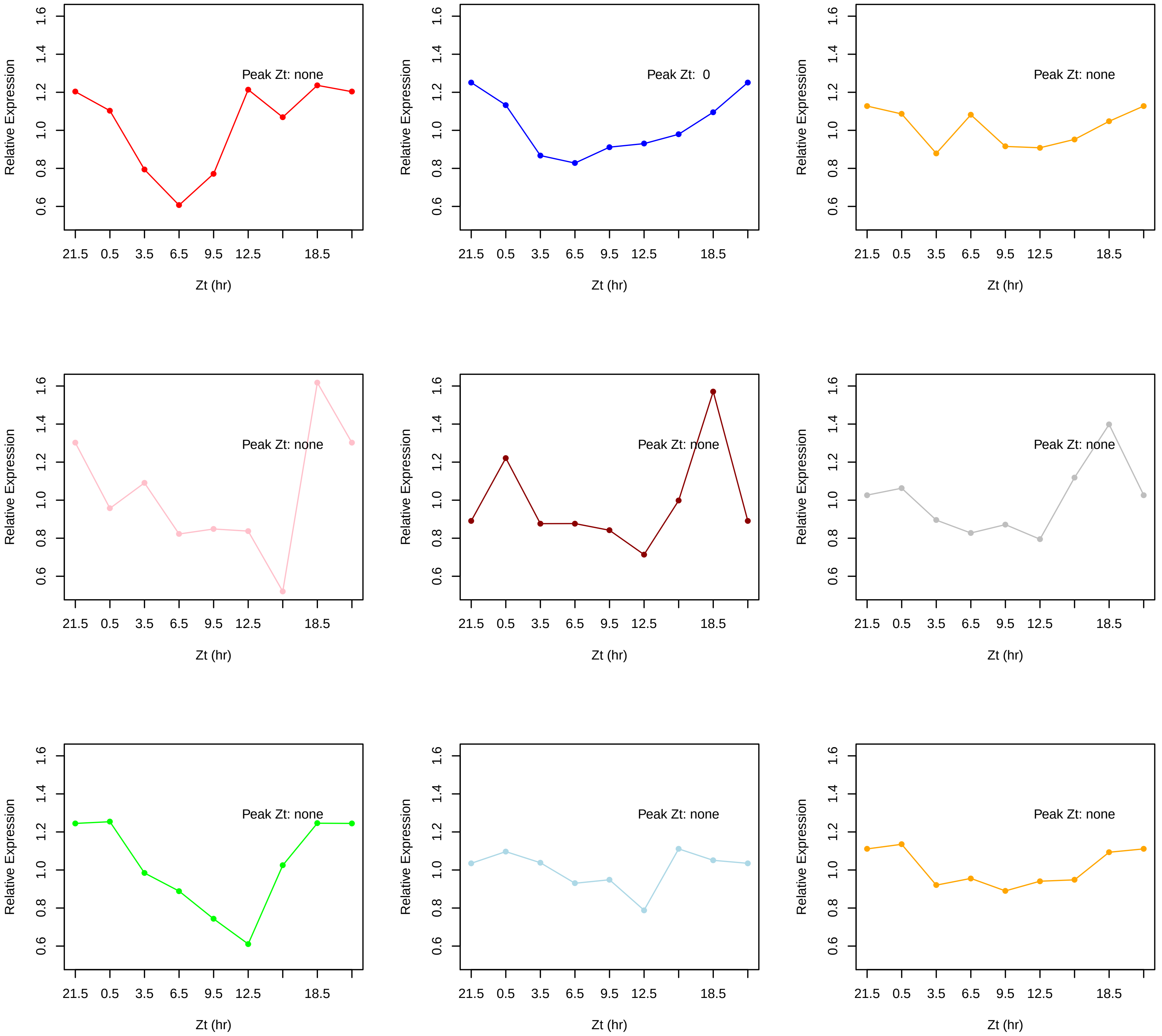

Singeleton: ENSXMAG00000001463 slc17a8

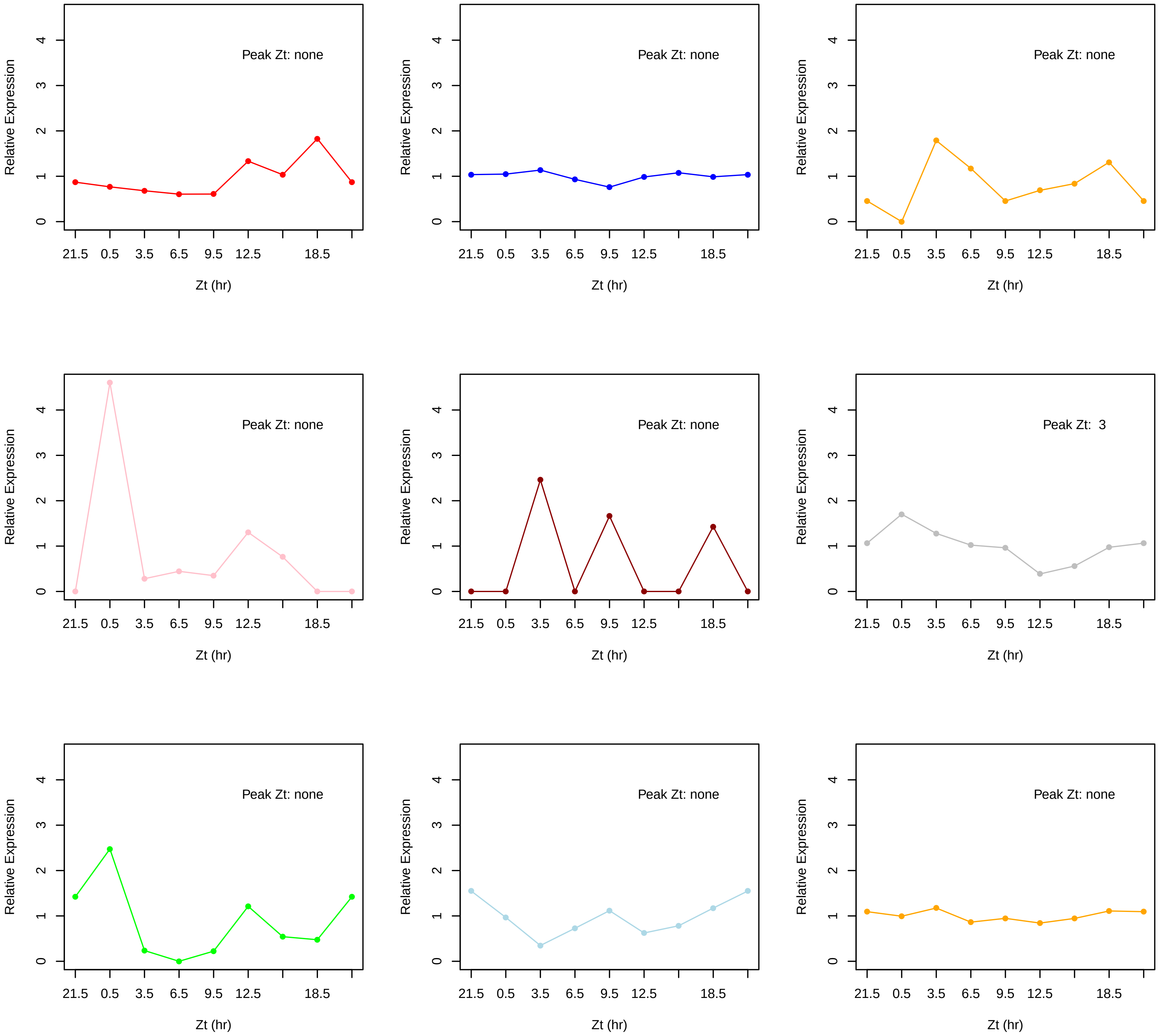

Singleton: ENSXMAG00000017793 usp39

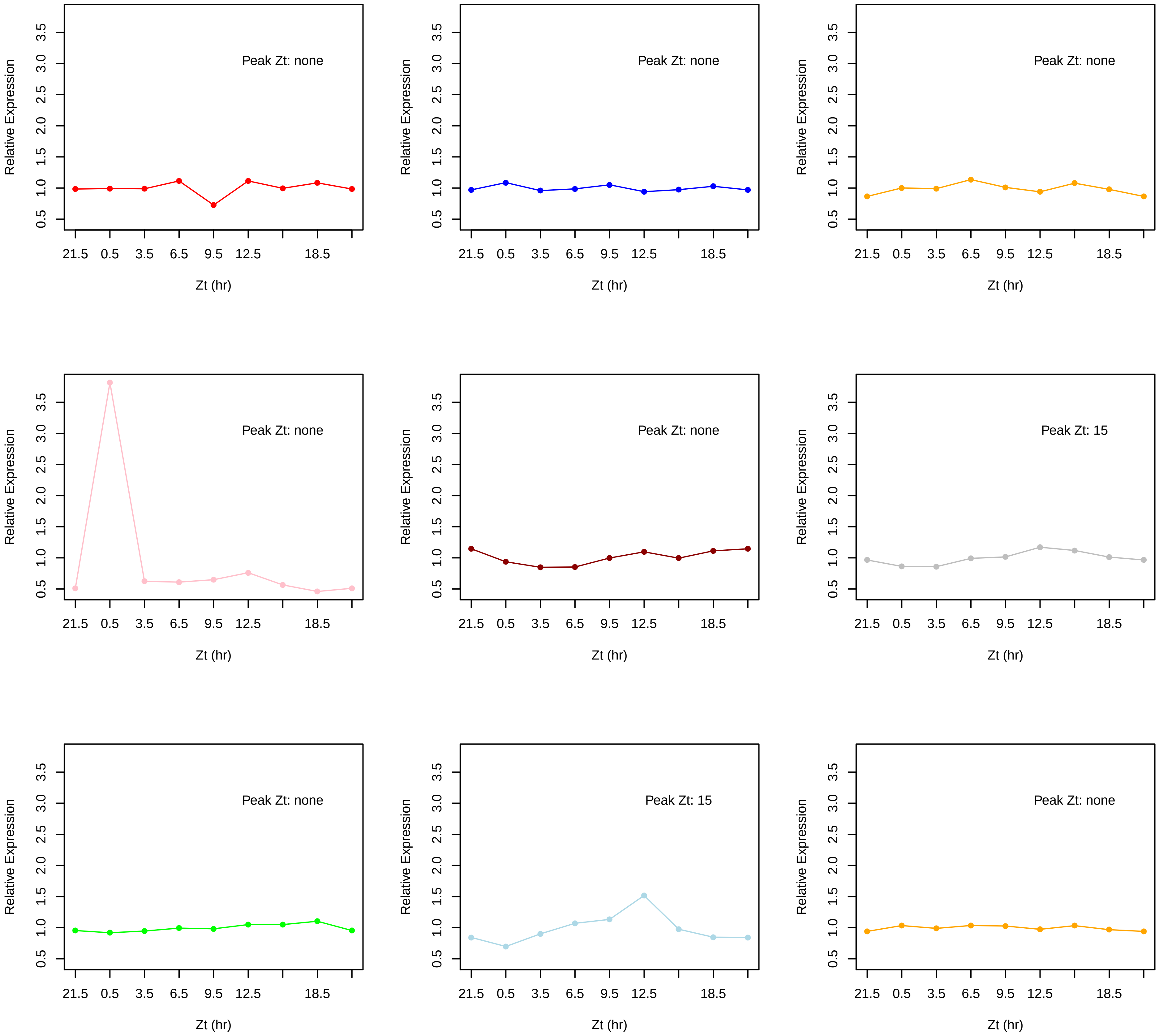

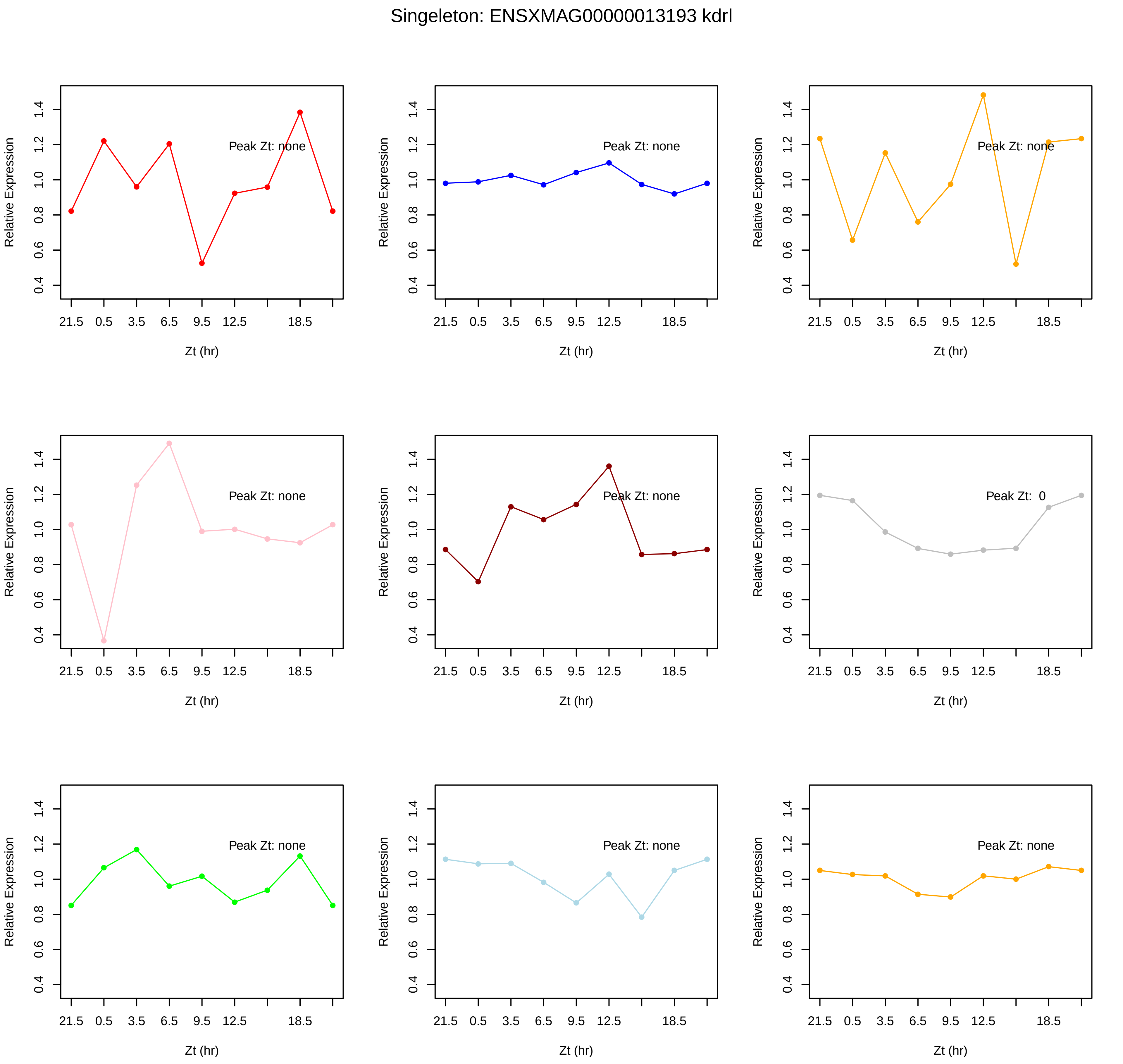
Singeleton: ENSXMAG00000013193 kdrl
Relative Expression
Zt (hr)
21.5
0.5
3.5
6.5
9.5
12.5
18.5
0.4
0.6
0.8
1.0
1.2
1.4
Peak Zt: none
Peak Zt: 0

Singeleton: ENSXMAG00000017859 ebf2

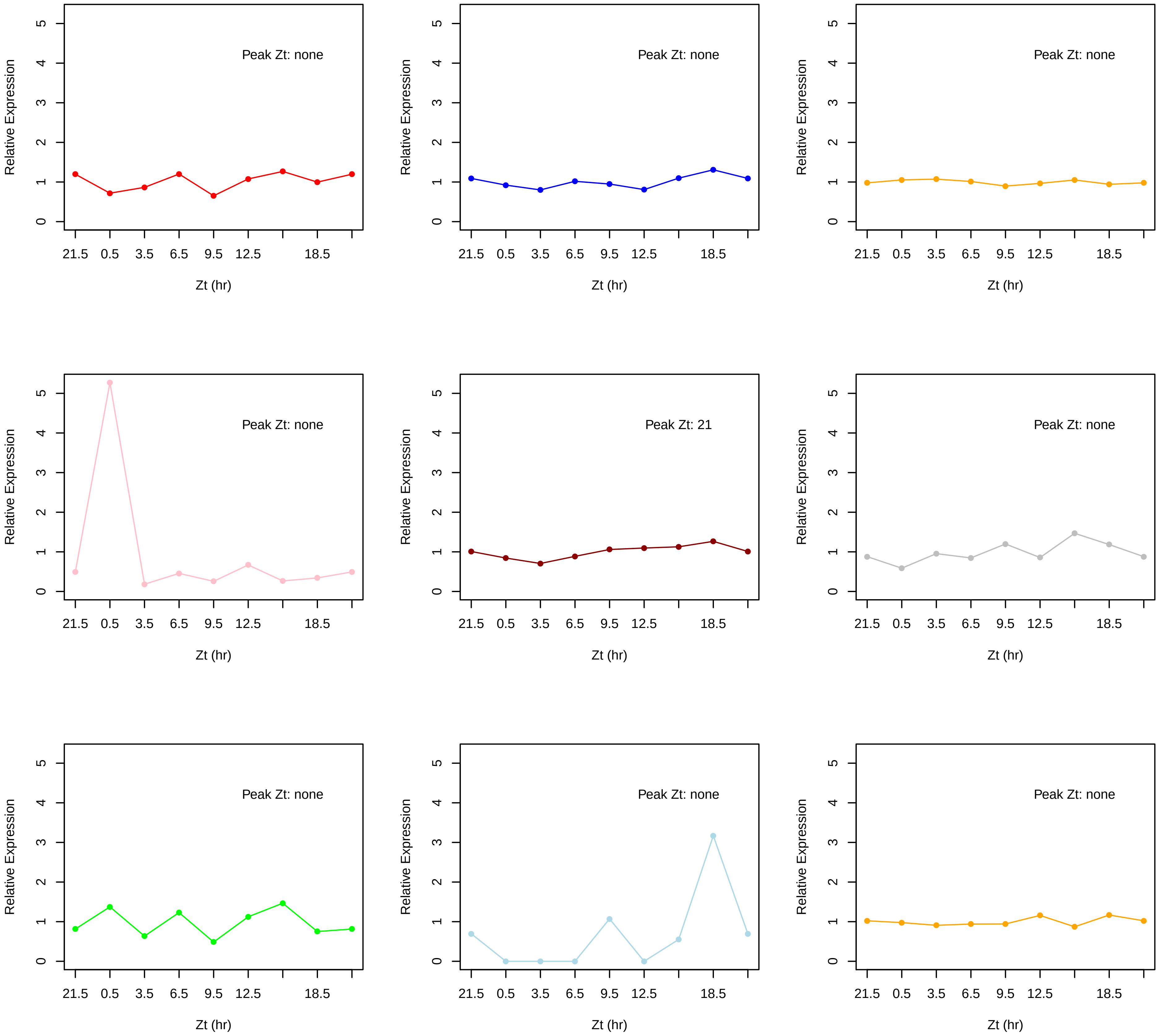

Singleton: ENSXMAG00000013699 sept6

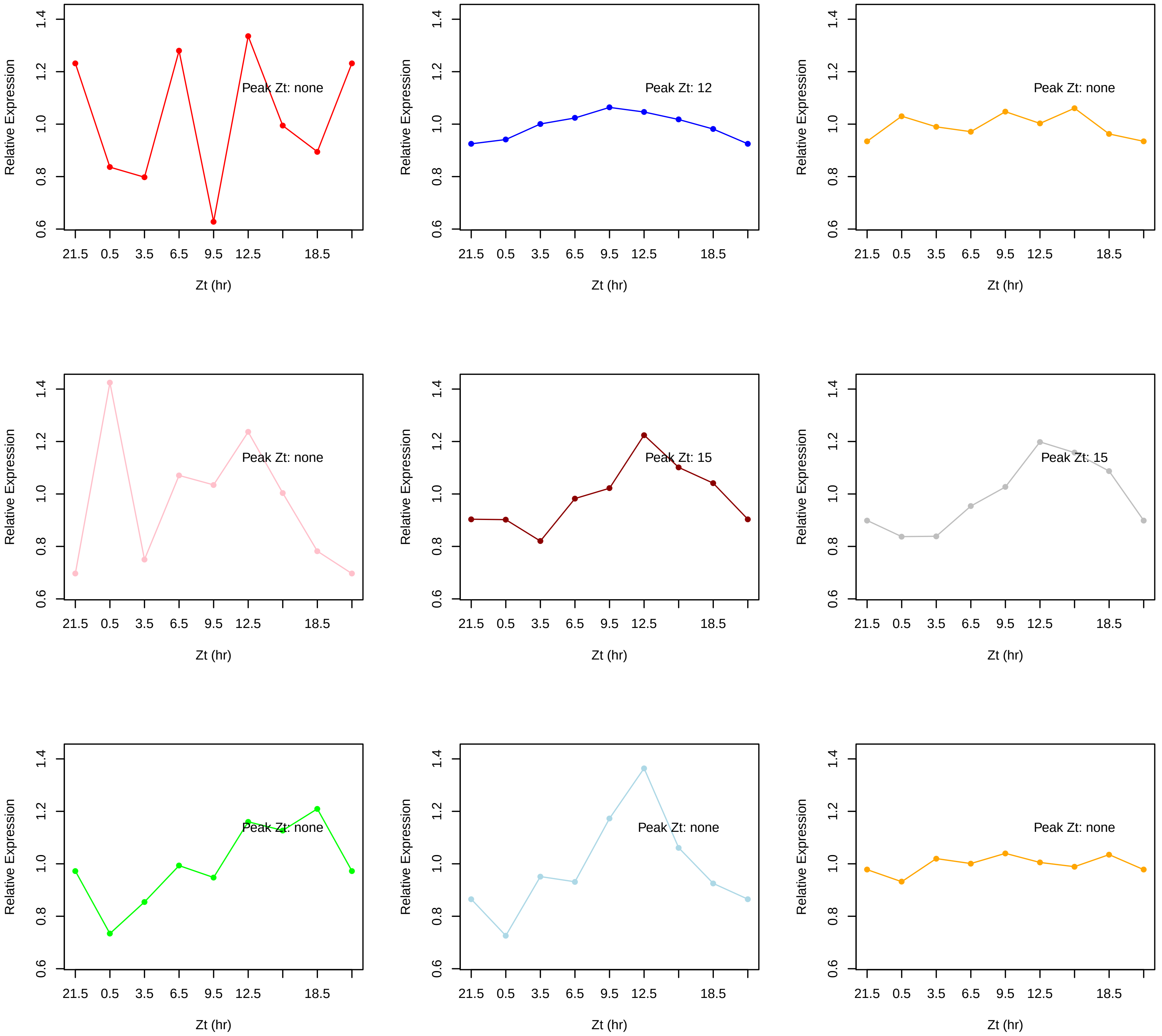

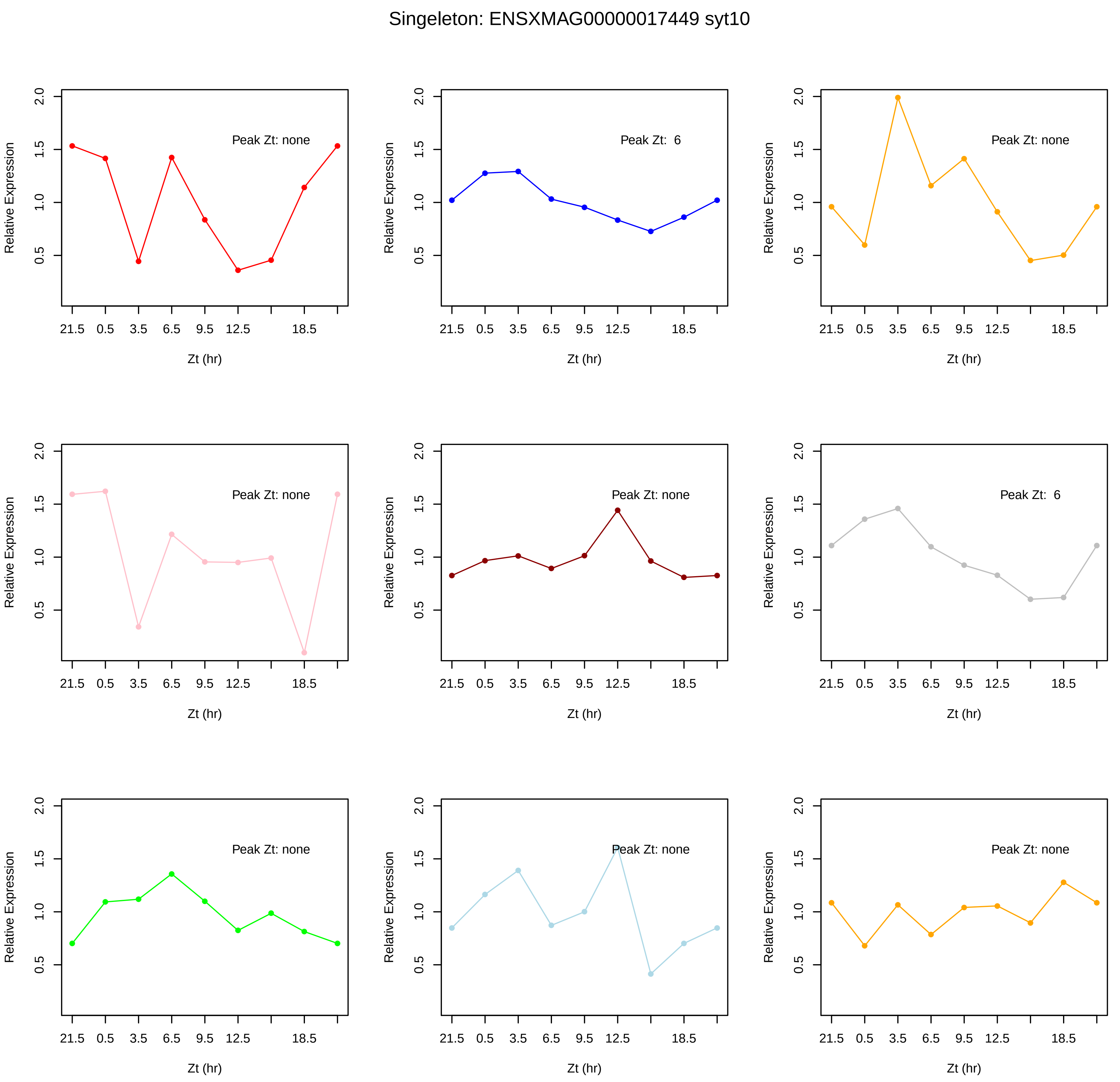
Singeleton: ENSXMAG00000017449 syt10
Peak Zt: none
Peak Zt: 6
Peak Zt: none
Peak Zt: none
Peak Zt: none
Peak Zt: 6
Peak Zt: none
Peak Zt: none
Peak Zt: none
Relative Expression
Zt (hr)
21.5
0.5
3.5
6.5
9.5
12.5
18.5
0.5
1.0
1.5
2.0

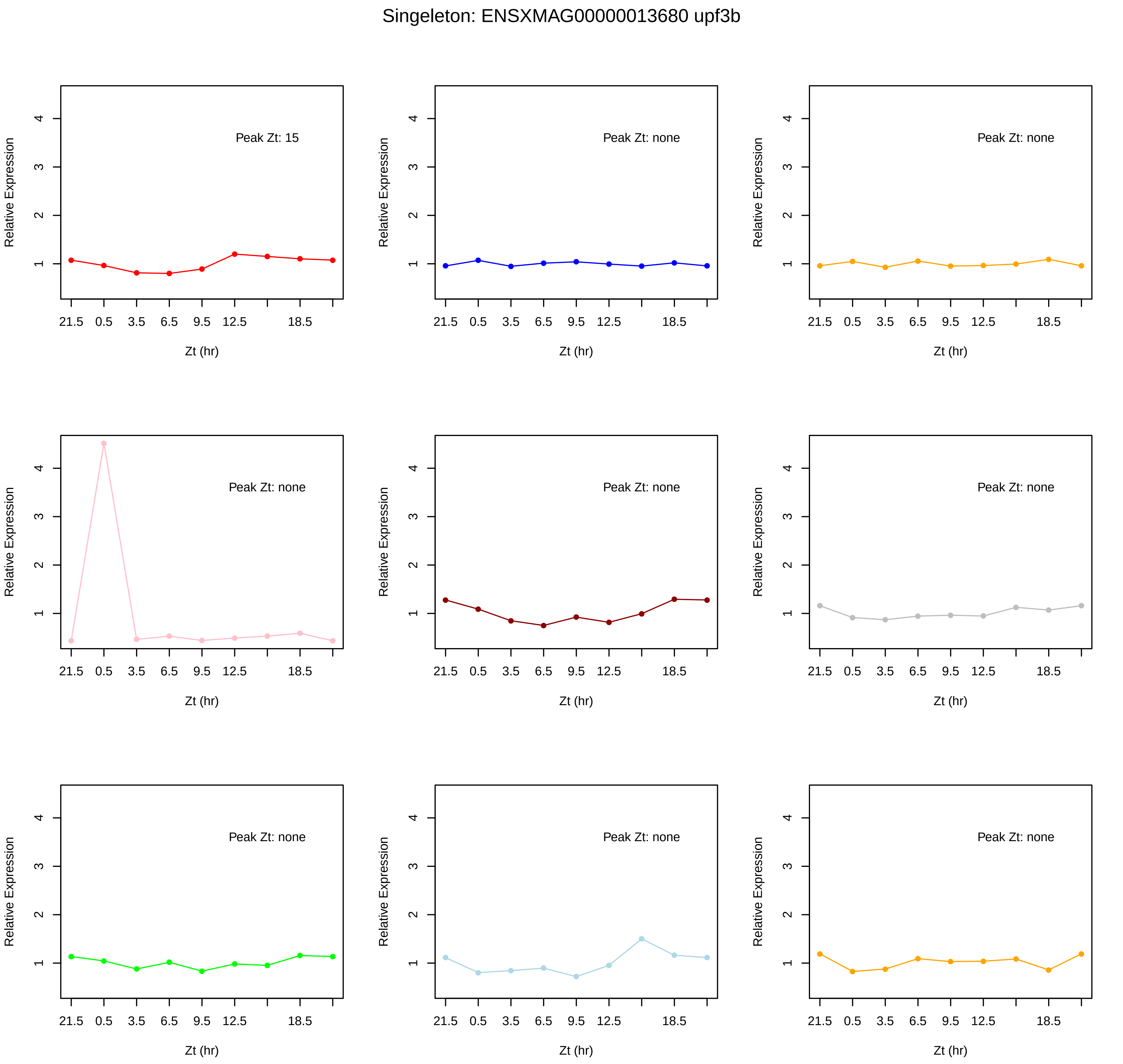
Singleton: ENSXMAG00000013680 upf3b
Peak Zt: 15
Peak Zt: none
Peak Zt: none
Peak Zt: none
Peak Zt: none
Peak Zt: none
Peak Zt: none
Peak Zt: none
Peak Zt: none
Relative Expression
Zt (hr)
21.5 0.5 3.5 6.5 9.5 12.5 18.5
1 2 3 4

Singeleton: ENSXMAG00000017087

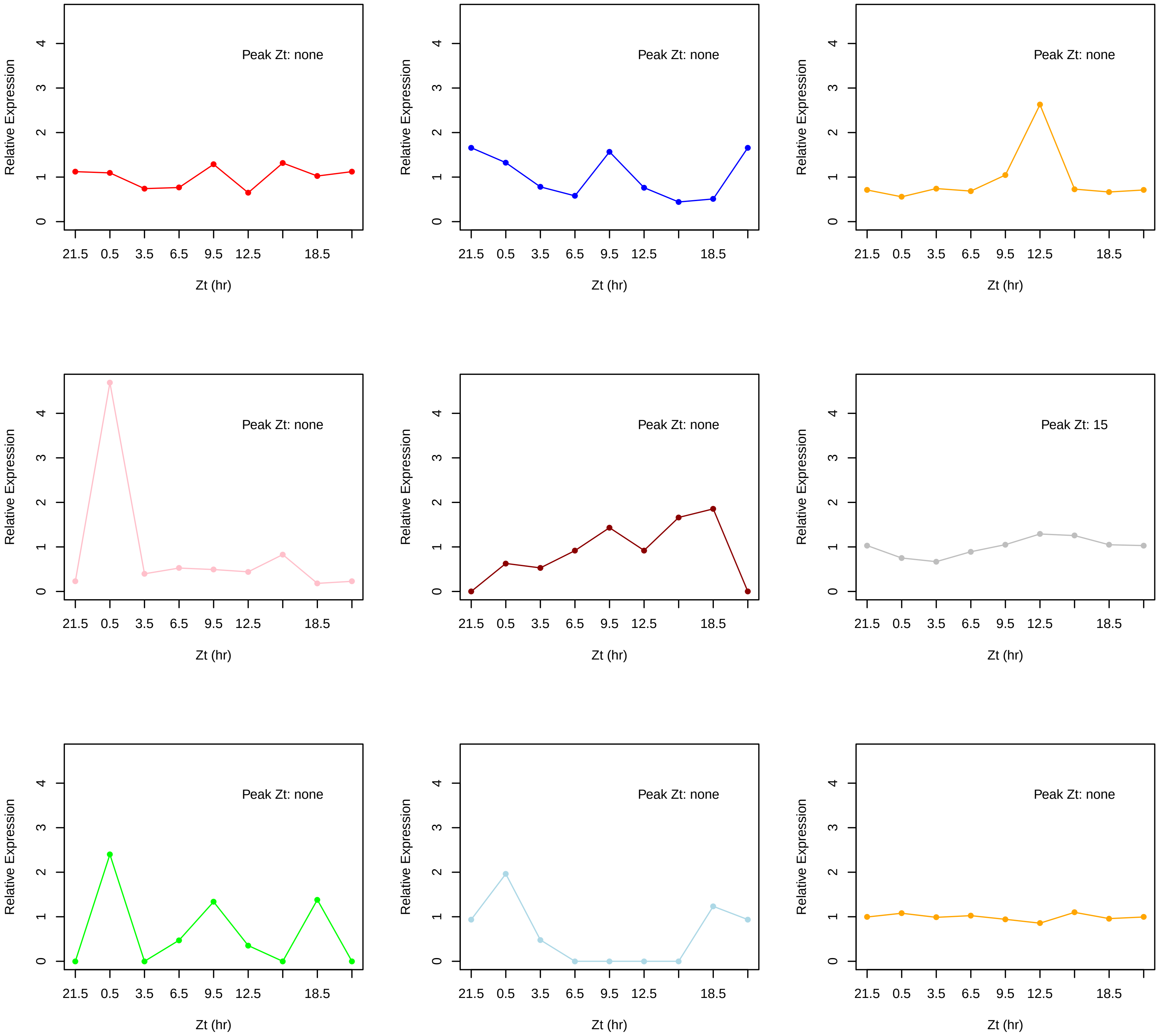

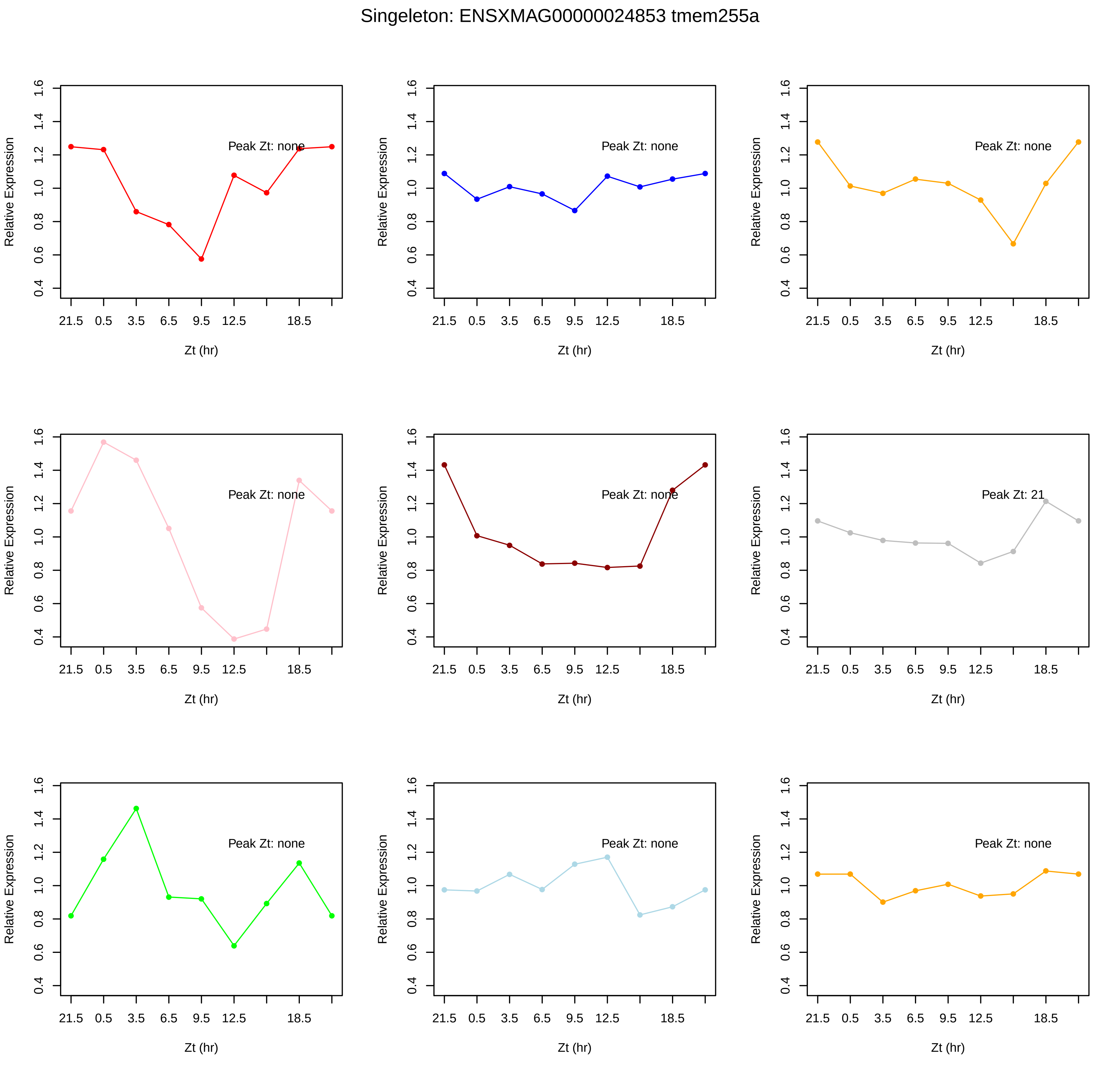
Singeleton: ENSXMAG00000024853 tmem255a
Relative Expression
Zt (hr)
21.5
0.5
3.5
6.5
9.5
12.5
18.5
Peak Zt: none
Peak Zt: 21

Singeleton: ENSXMAG00000017411 cyb5r3

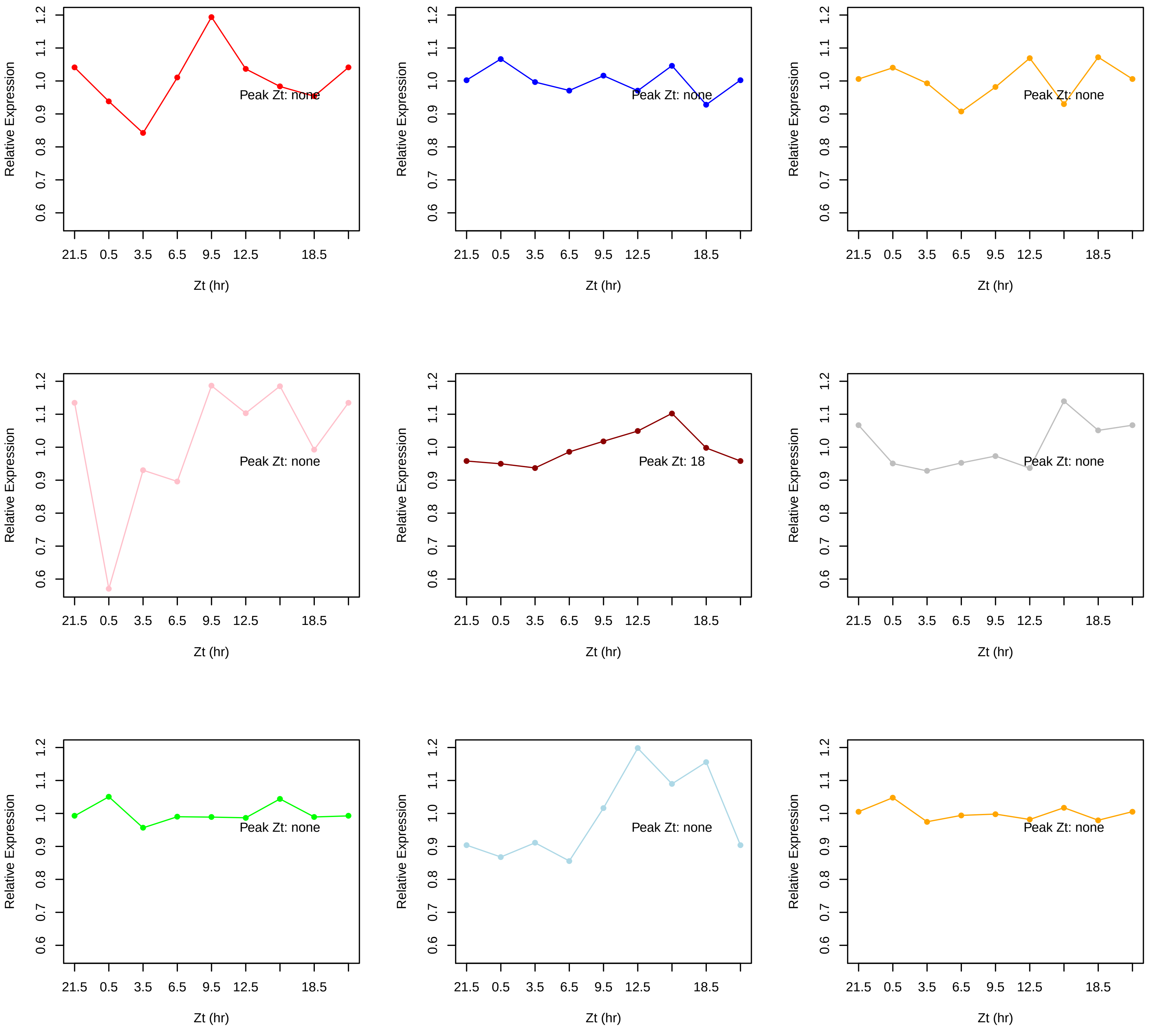

Singeleton: ENSXMAG00000001568 polr3b

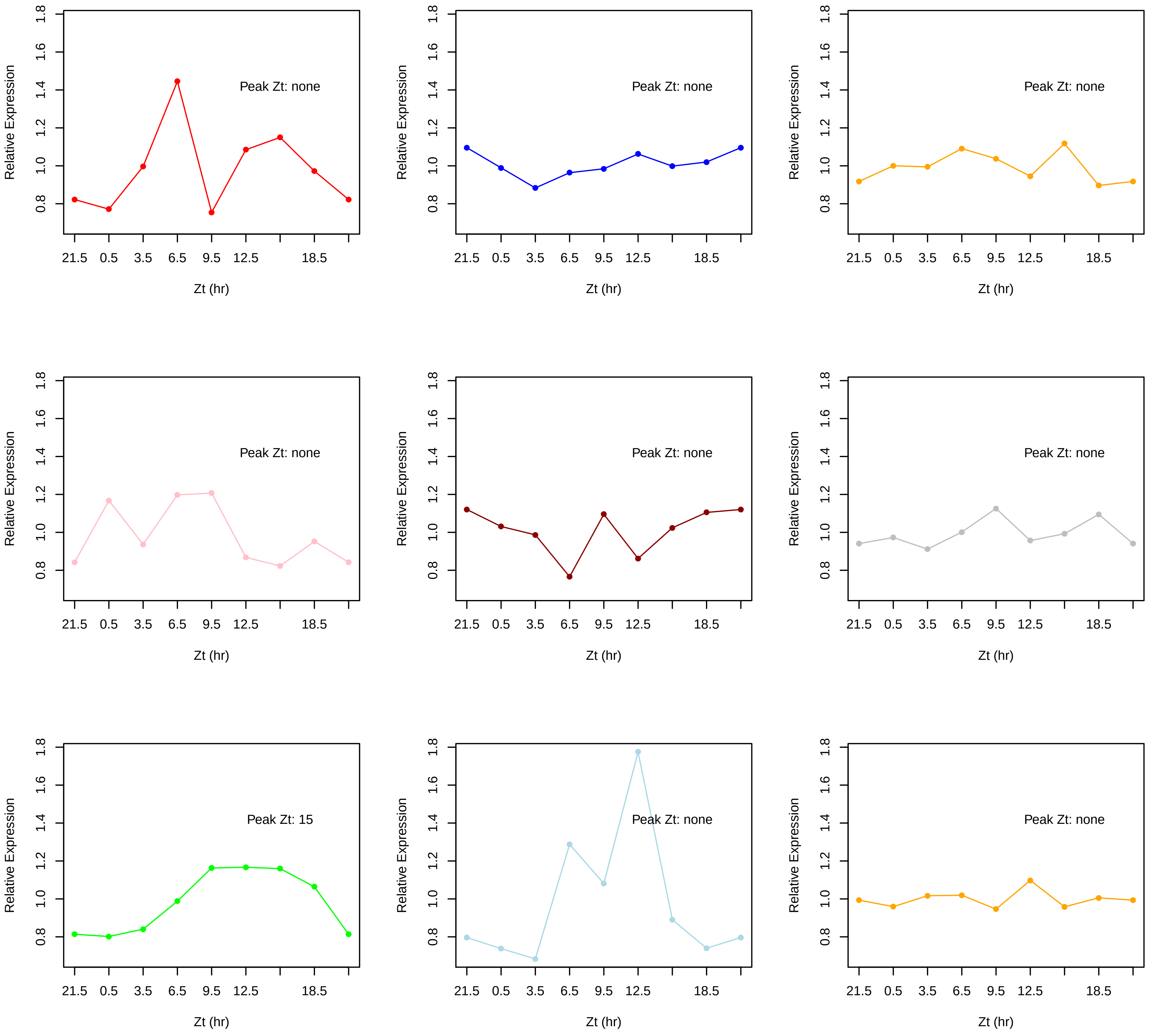

Singleton: ENSXMAG00000013631 lamp2

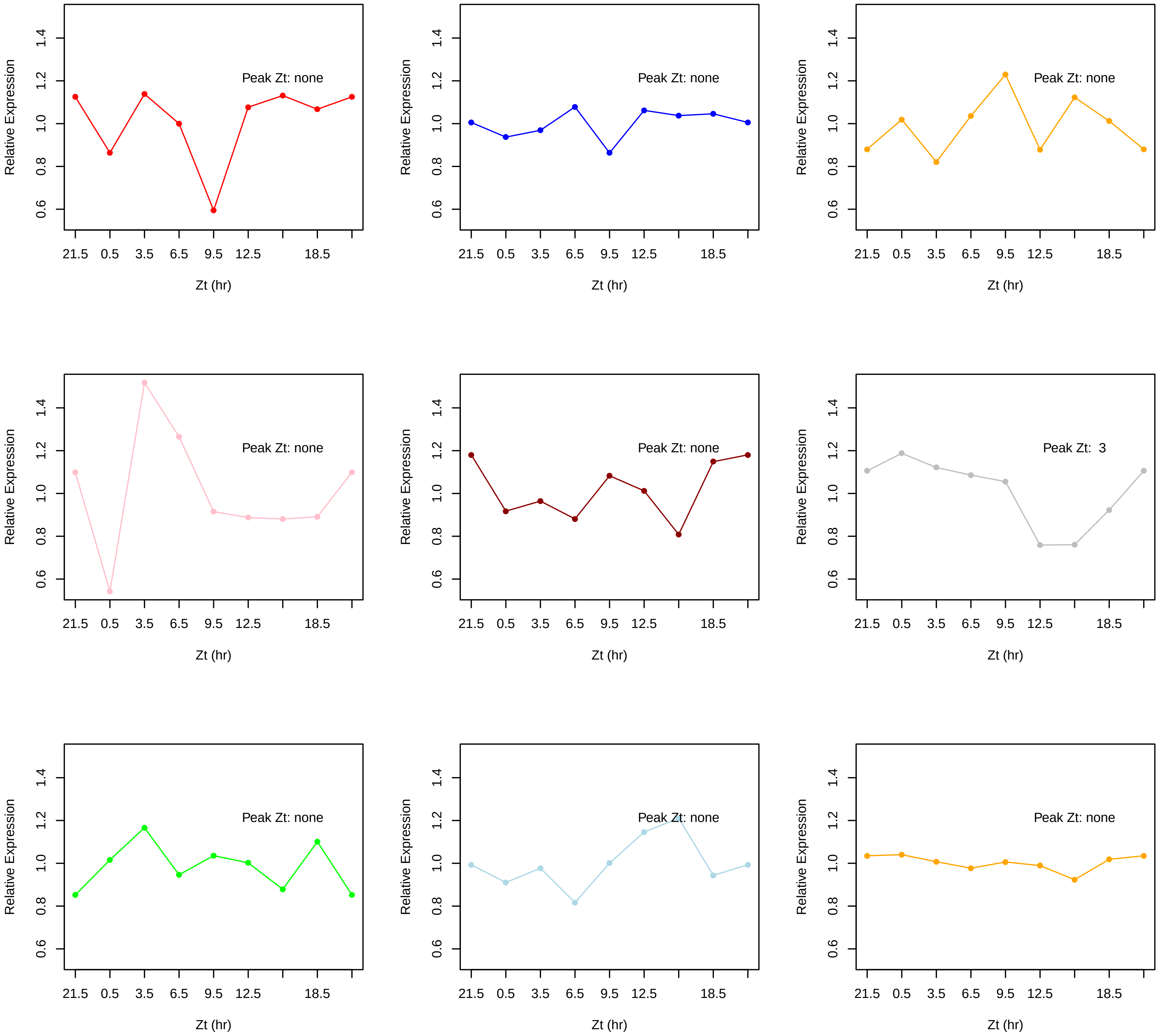

Singleton: ENSXMAG00000013613 cul4b

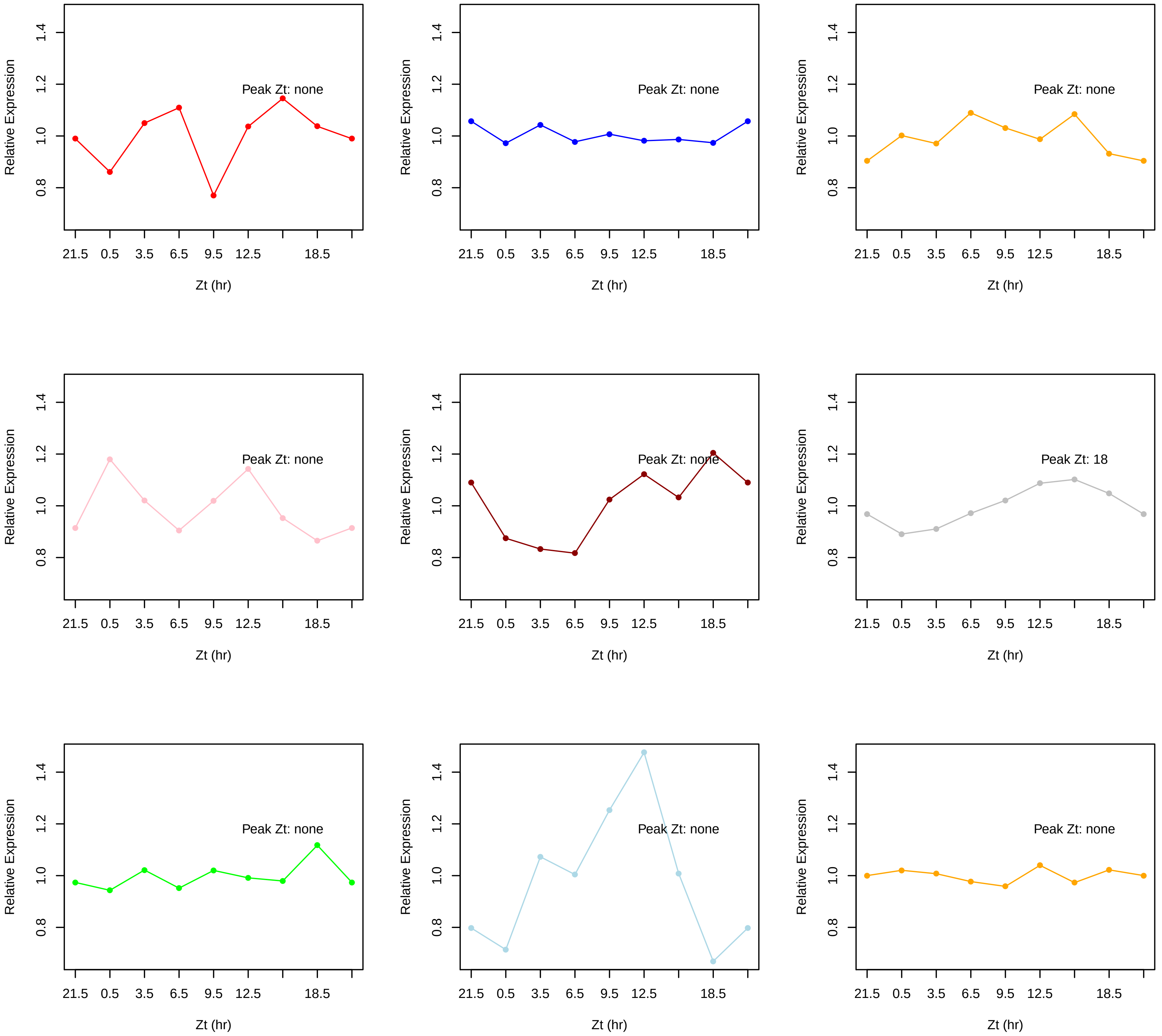

Singleton: ENSXMAG00000005449 wbp1

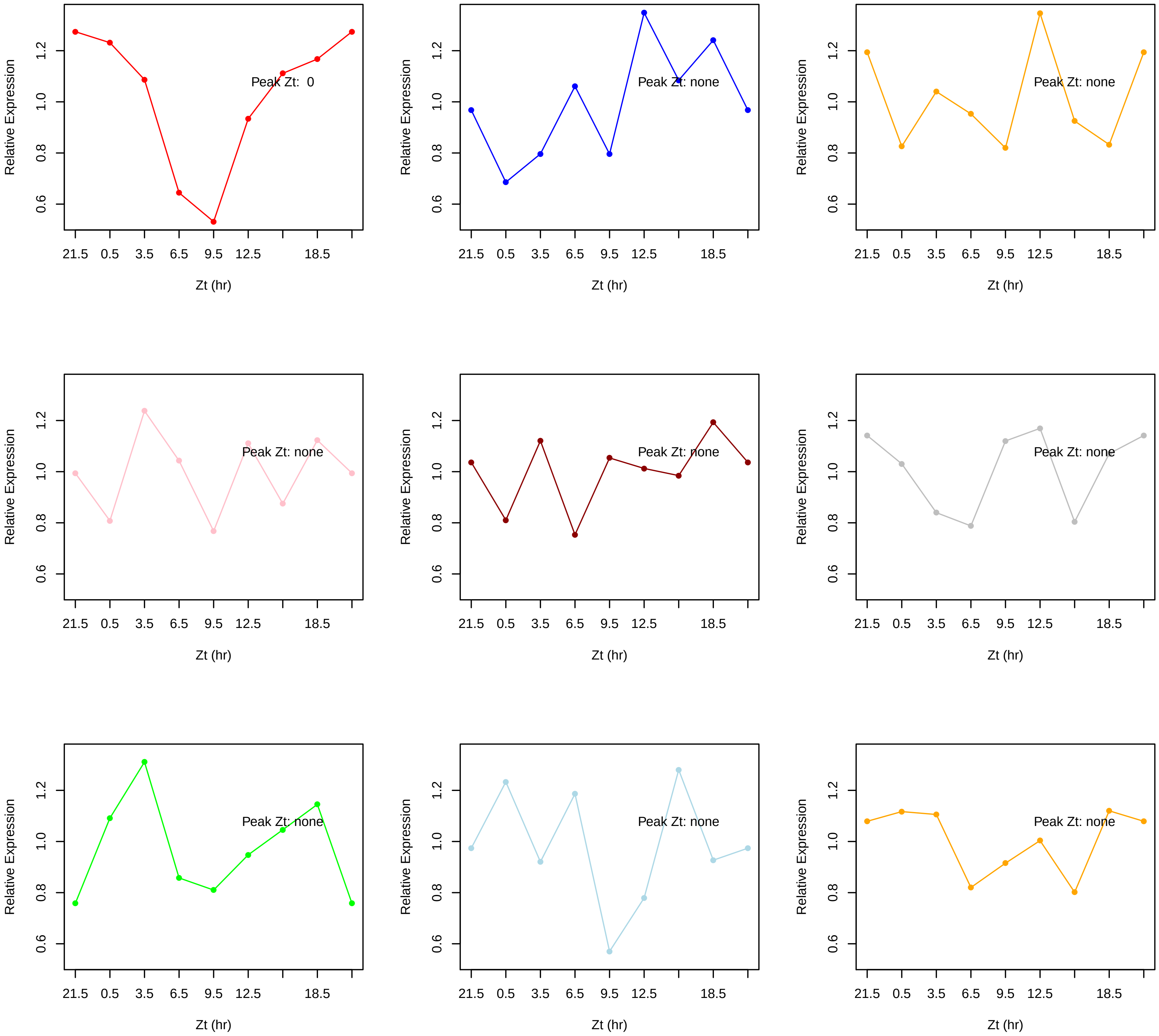

Singleton: ENSXMAG00000023339 mcts1

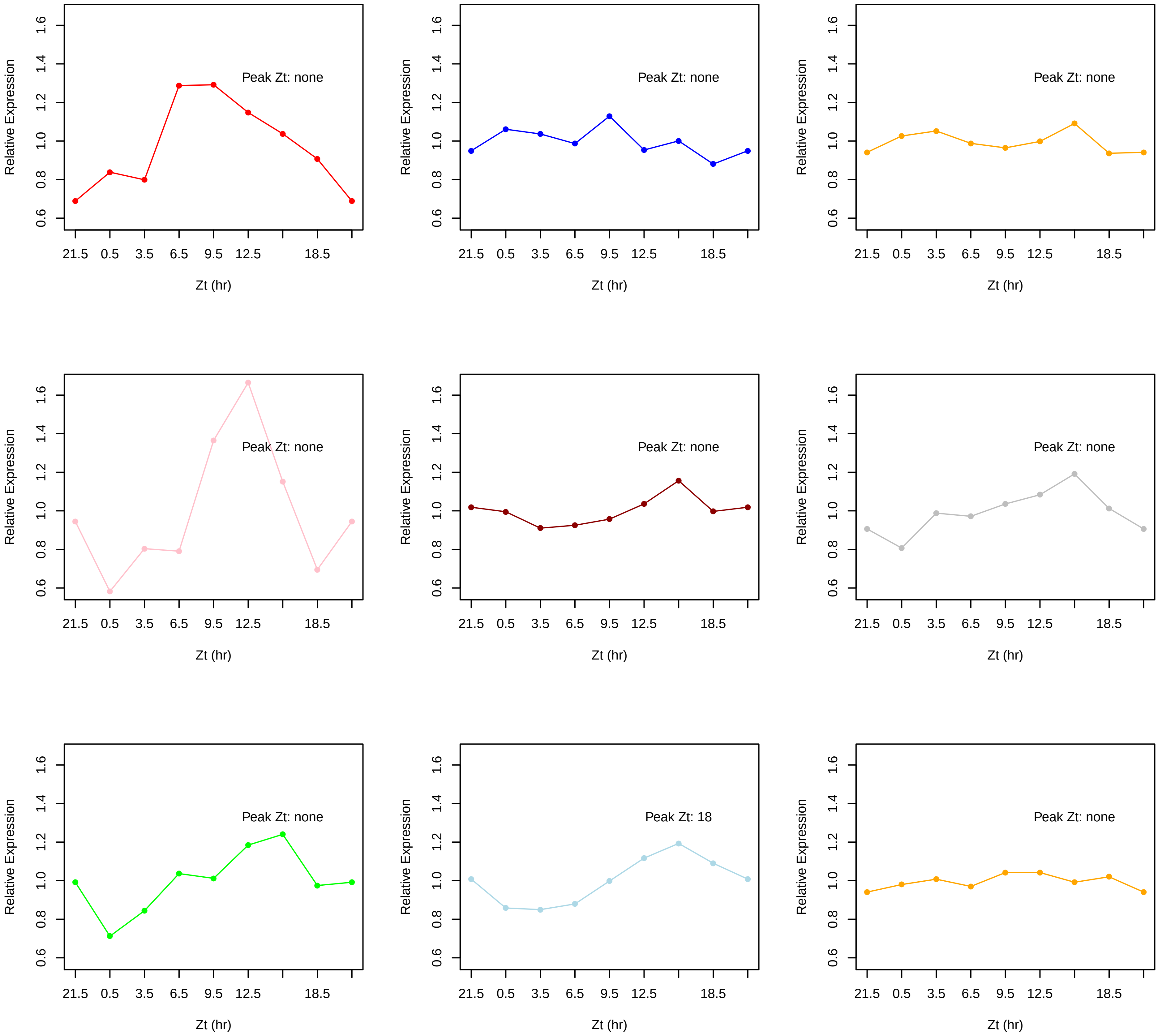

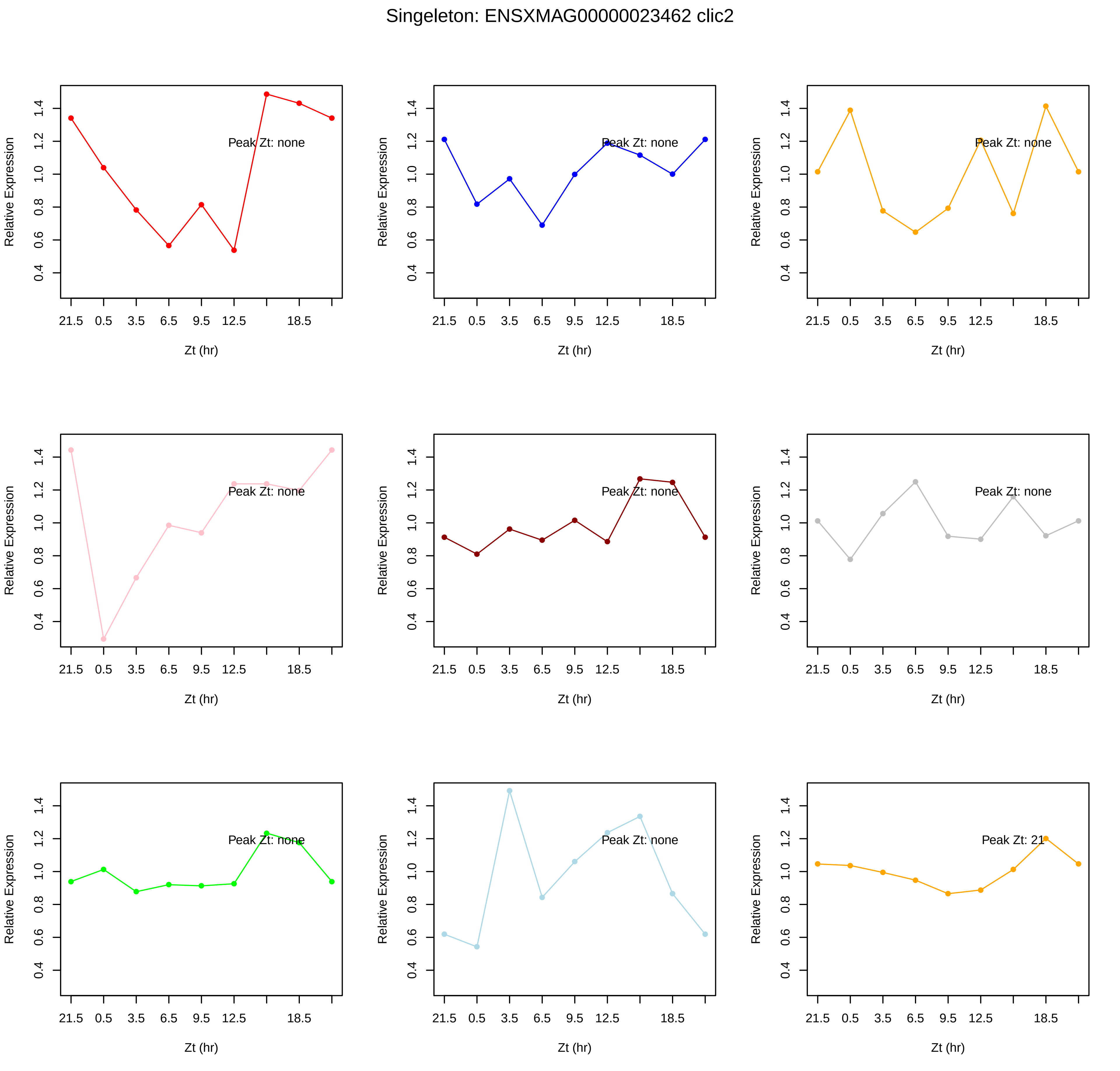
Singeleton: ENSXMAG00000023462 clic2
Relative Expression
0.4
0.6
0.8
1.0
1.2
1.4
21.5
0.5
3.5
6.5
9.5
12.5
18.5
Zt (hr)
Peak Zt: none
Peak Zt: 21

Singeleton: ENSXMAG00000005479

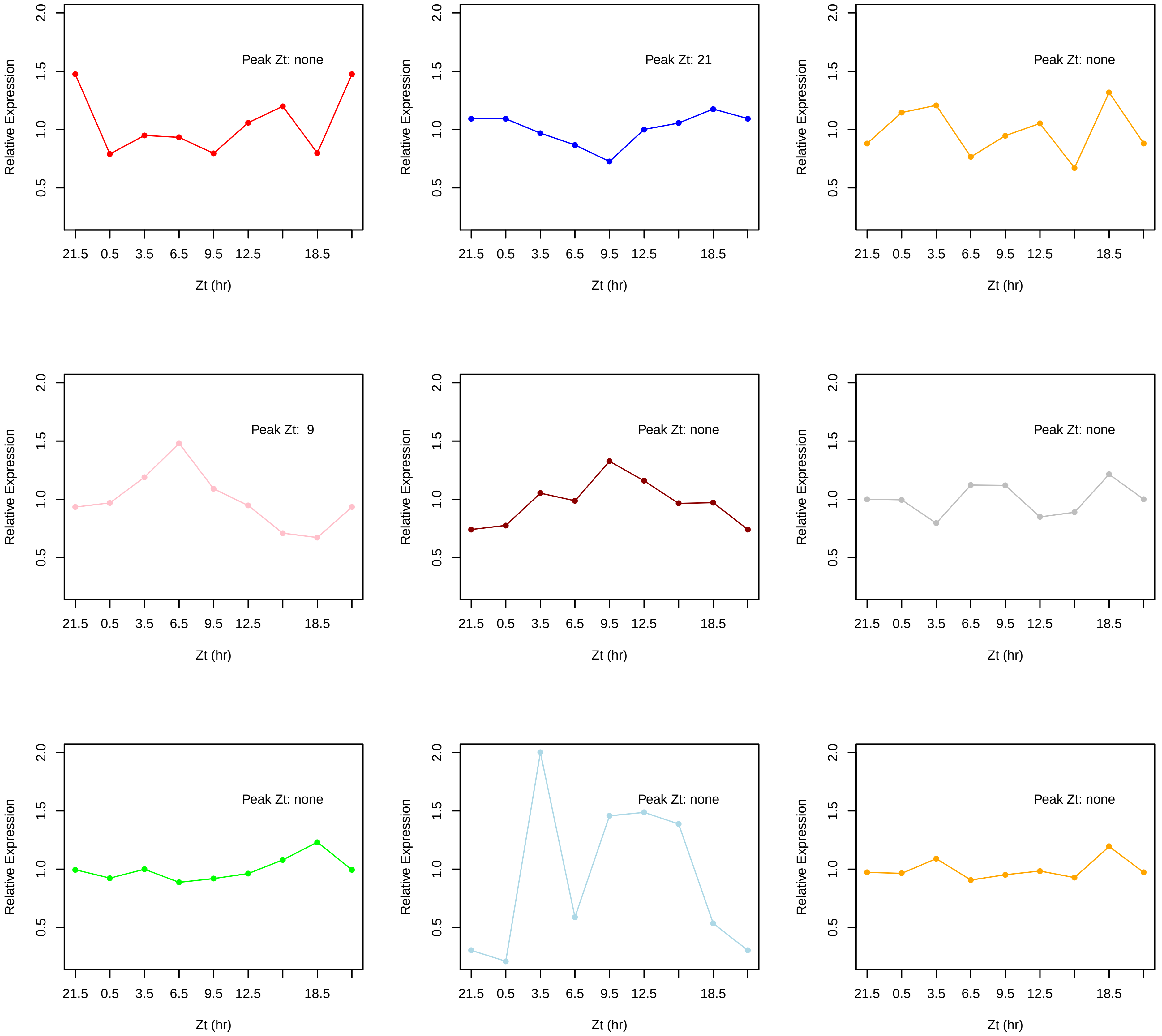

Singeleton: ENSXMAG00000001138 polm

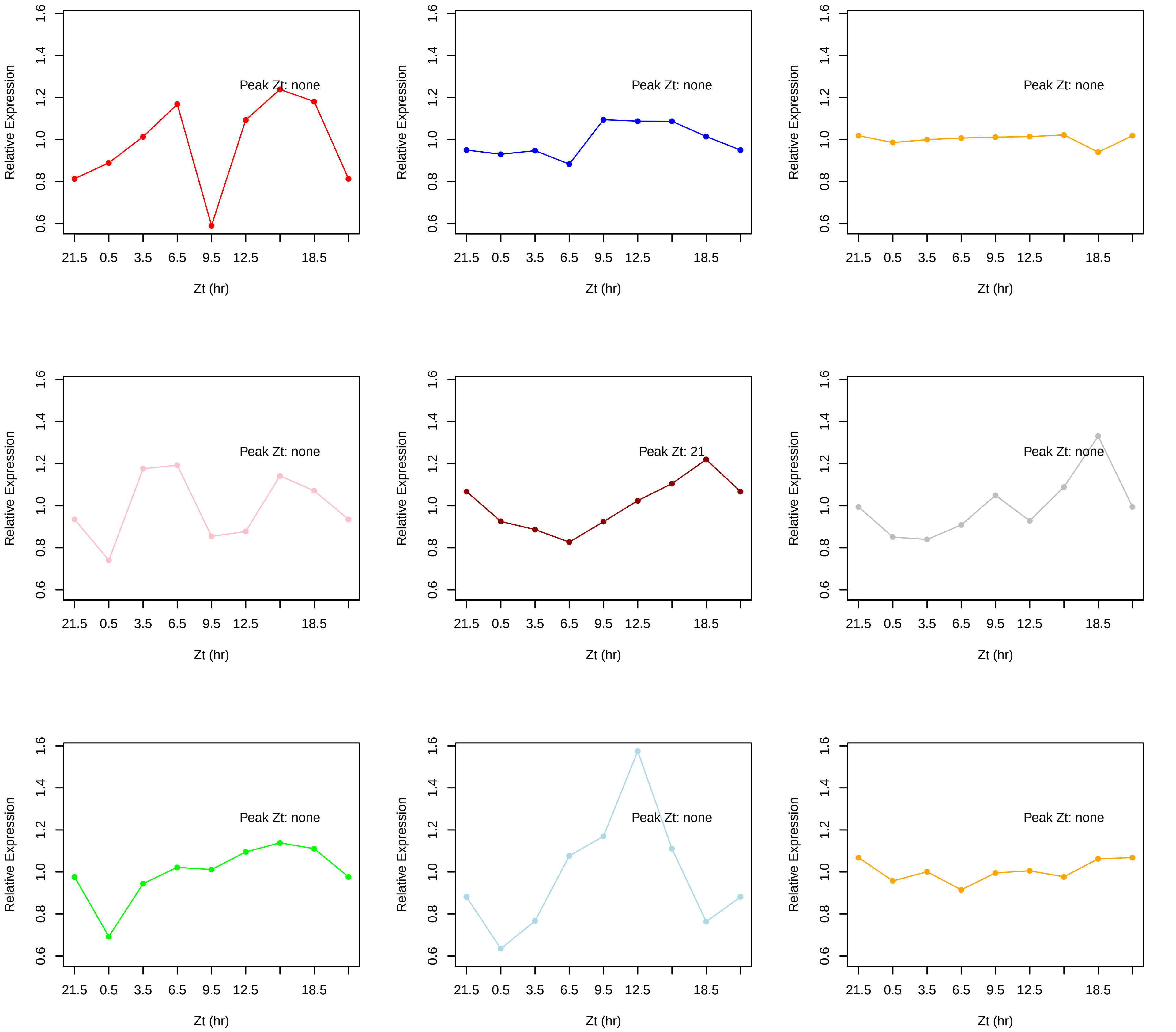

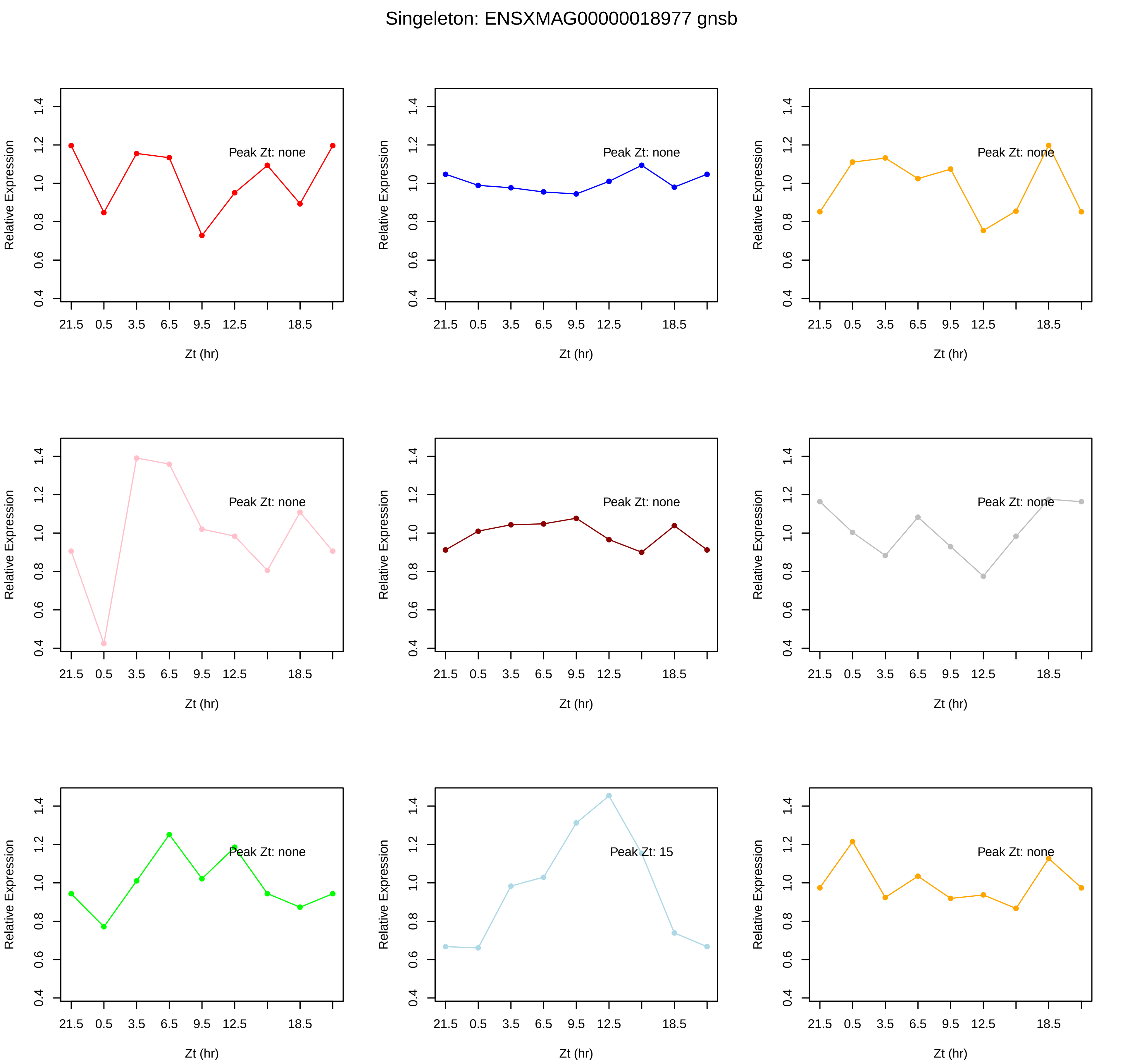
Singeleton: ENSXMAG00000018977 gnsb
Peak Zt: none
Peak Zt: none
Peak Zt: none
Peak Zt: none
Peak Zt: none
Peak Zt: none
Peak Zt: none
Peak Zt: 15
Peak Zt: none
Relative Expression
Zt (hr)
21.5
0.5
3.5
6.5
9.5
12.5
18.5
0.4
0.6
0.8
1.0
1.2
1.4

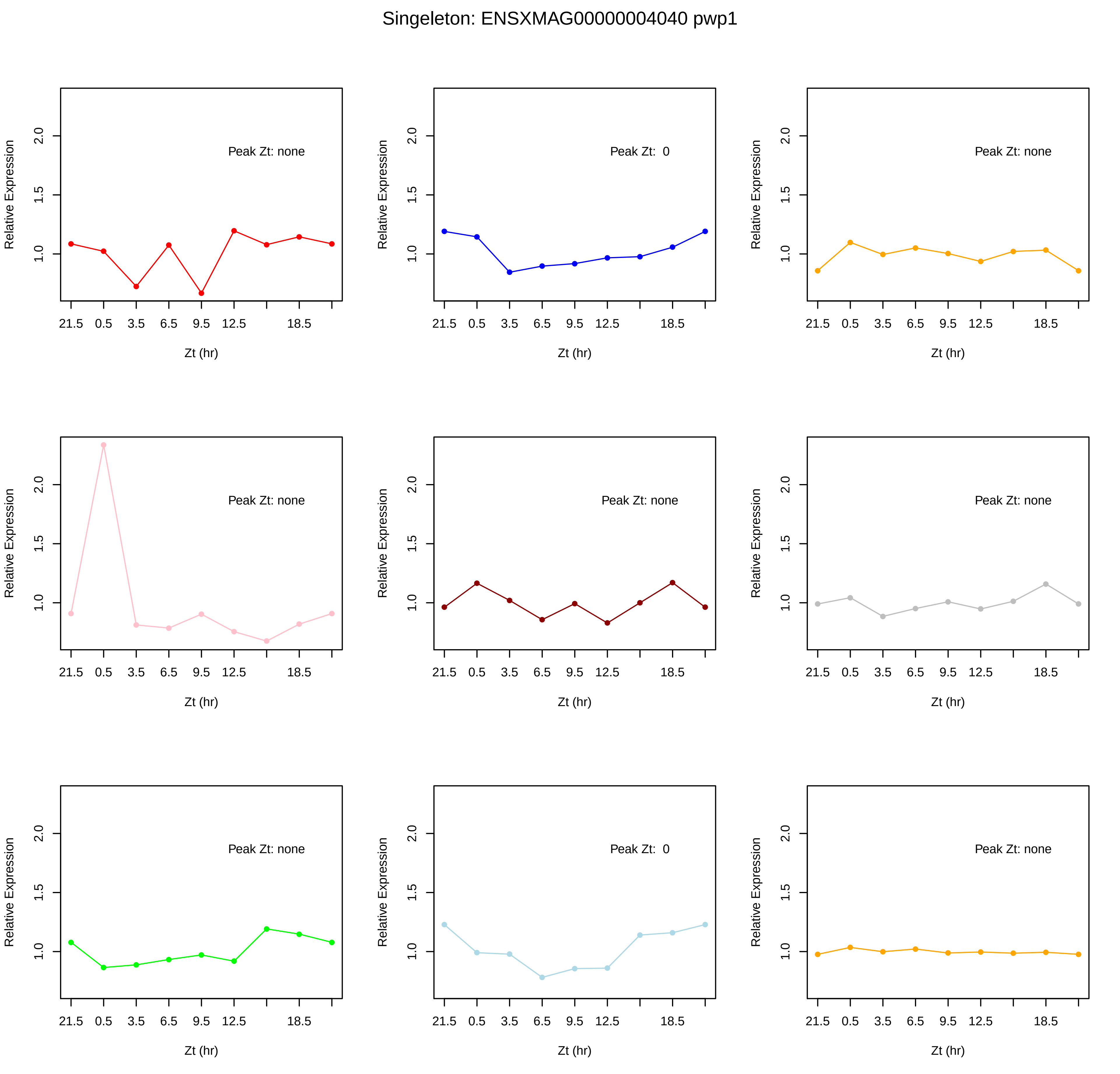
Singeleton: ENSXMAG00000004040 pwp1
Peak Zt: none
Peak Zt: 0
Peak Zt: none
Peak Zt: none
Peak Zt: none
Peak Zt: none
Peak Zt: none
Peak Zt: 0
Peak Zt: none
Relative Expression
Zt (hr)
21.5 0.5 3.5 6.5 9.5 12.5 18.5
1.0 1.5 2.0

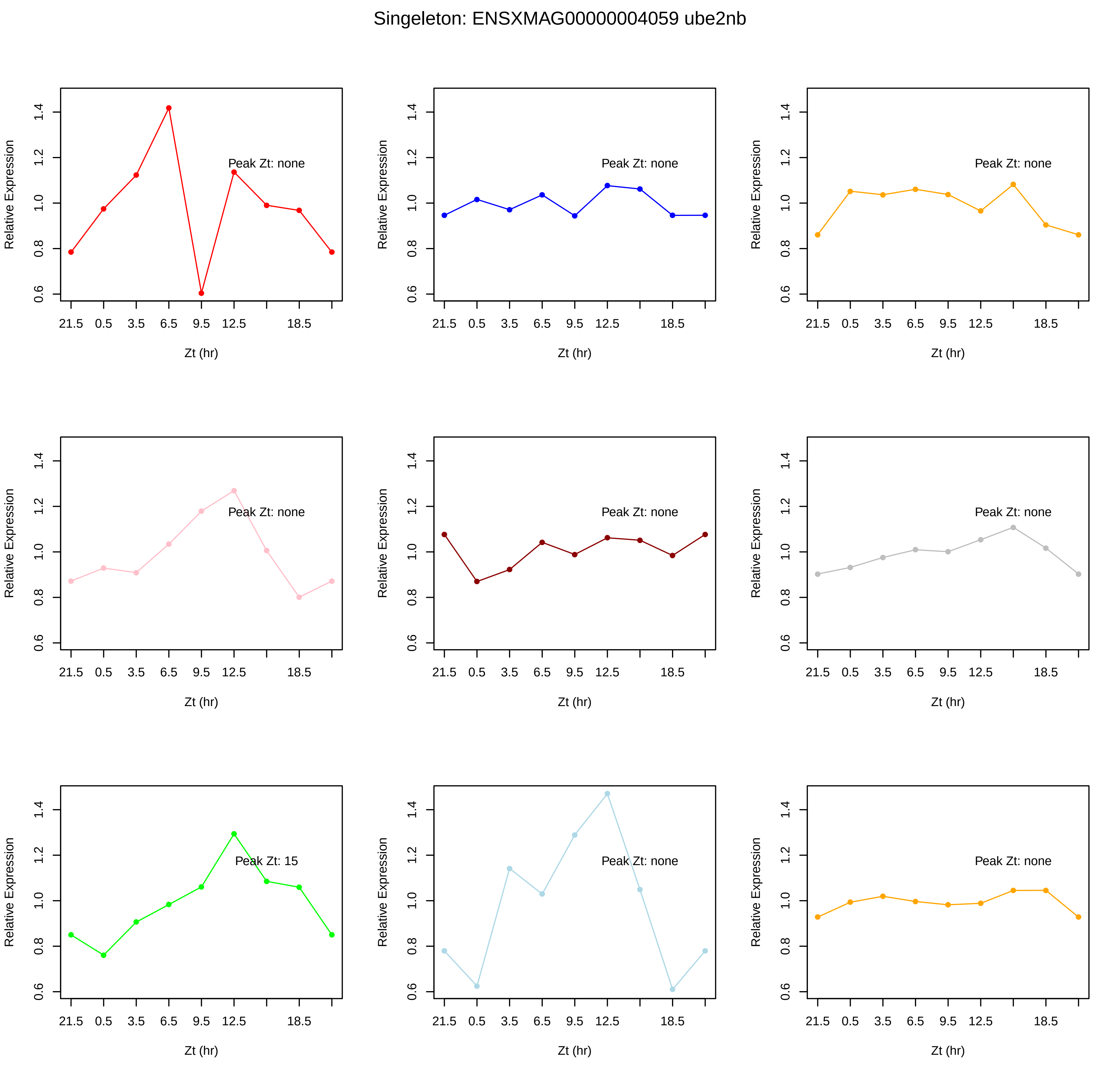

Singleton: ENSXMAG00000004059 ube2nb
Relative Expression
Zt (hr)
21.5 0.5 3.5 6.5 9.5 12.5 18.5
0.6 0.8 1.0 1.2 1.4
Peak Zt: none
Peak Zt: none
Peak Zt: none
Peak Zt: none
Peak Zt: none
Peak Zt: none
Peak Zt: 15
Peak Zt: none
Peak Zt: none

Singeleton: ENSXMAG00000013554 zdhhc9

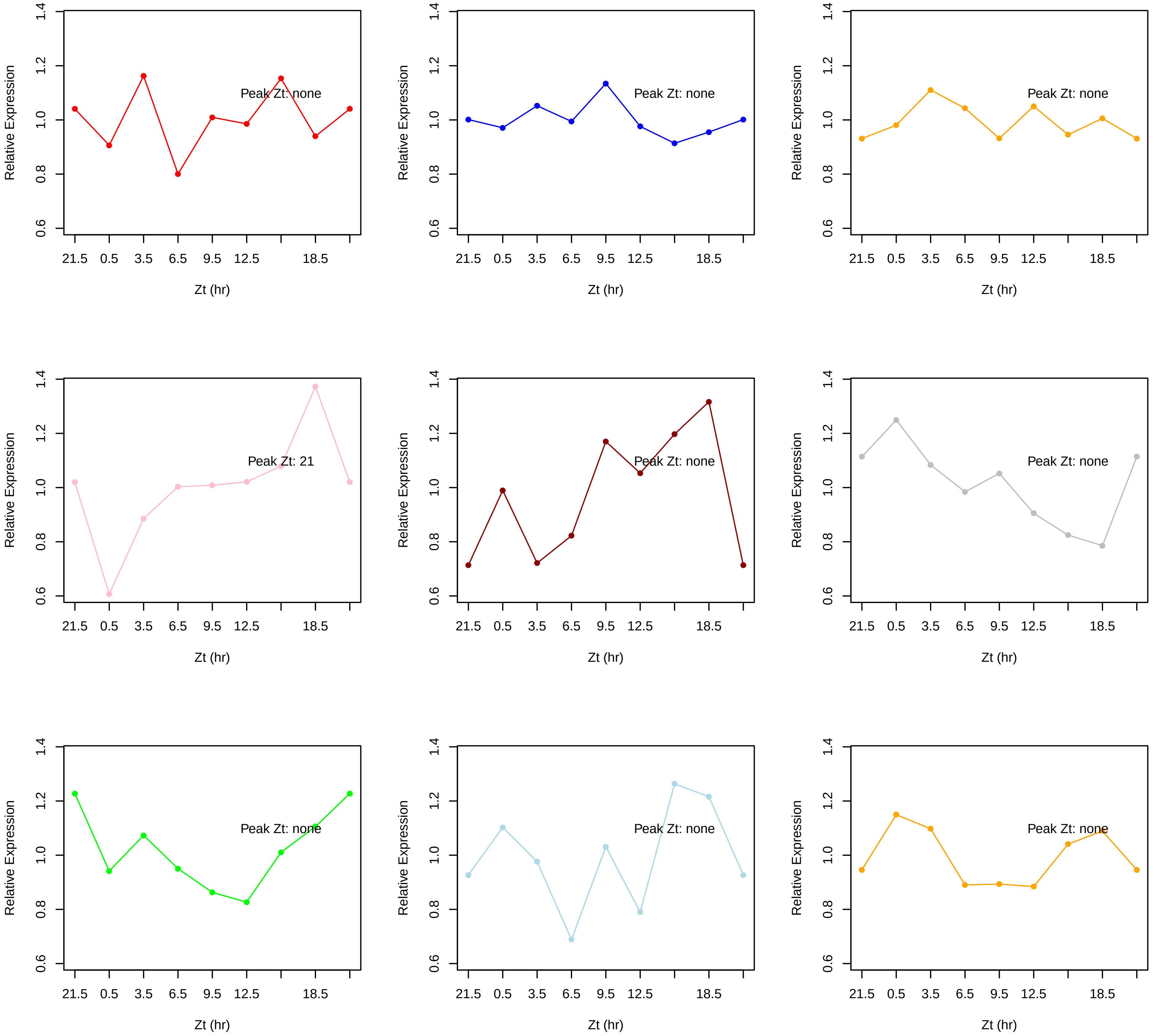

Singeleton: ENSXMAG00000018813 pargl

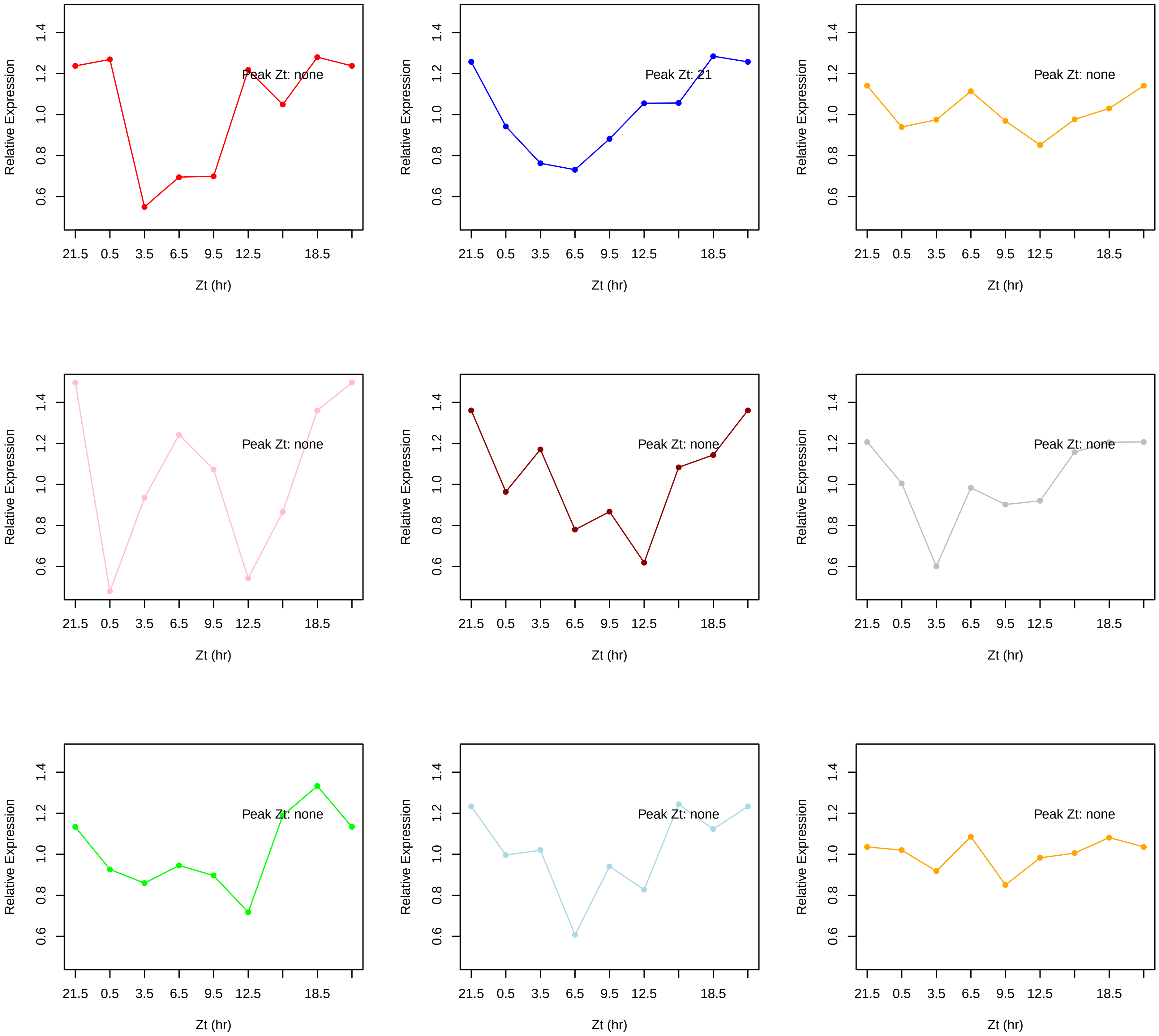

Singeleton: ENSXMAG00000025318 trmt12

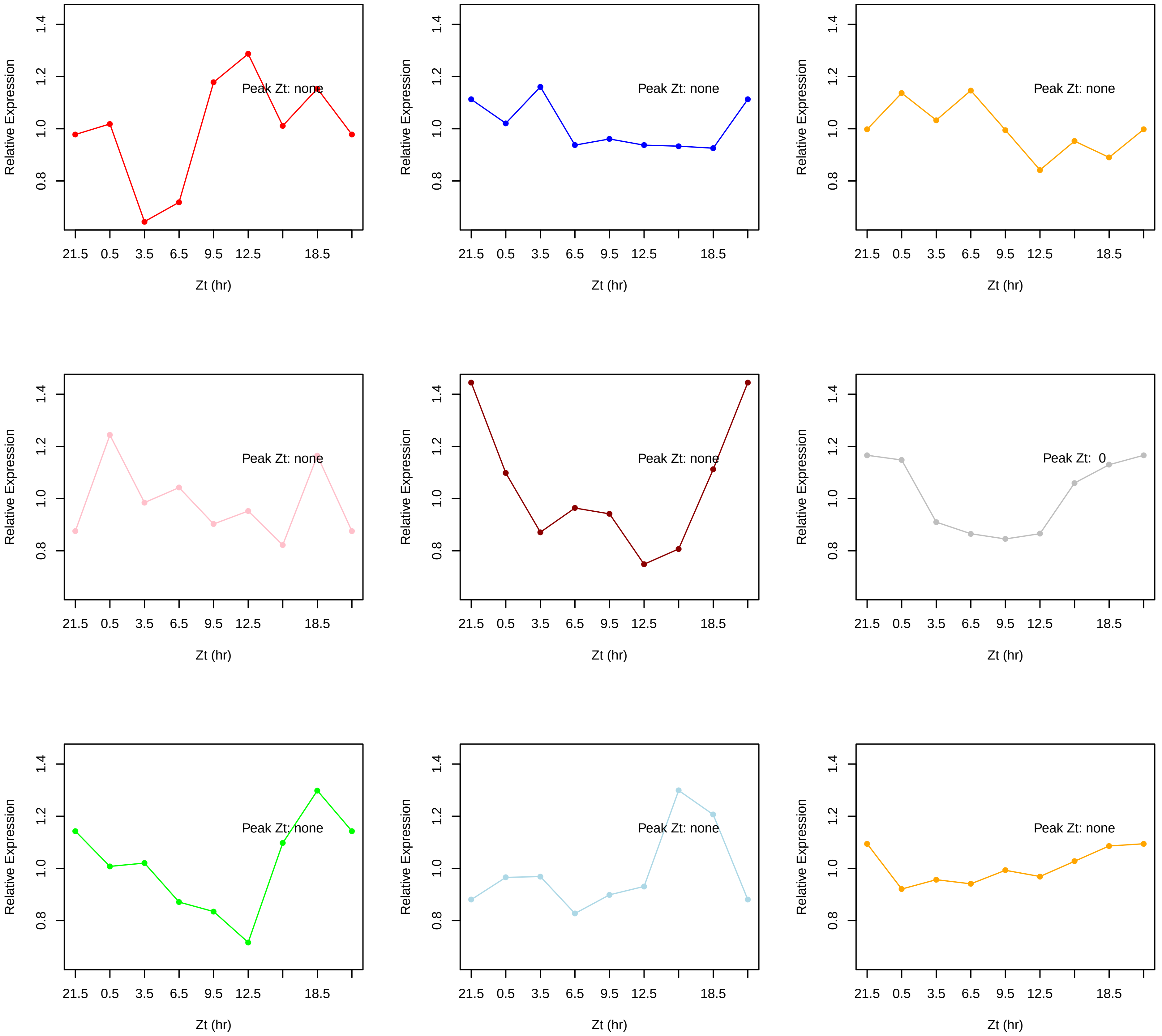

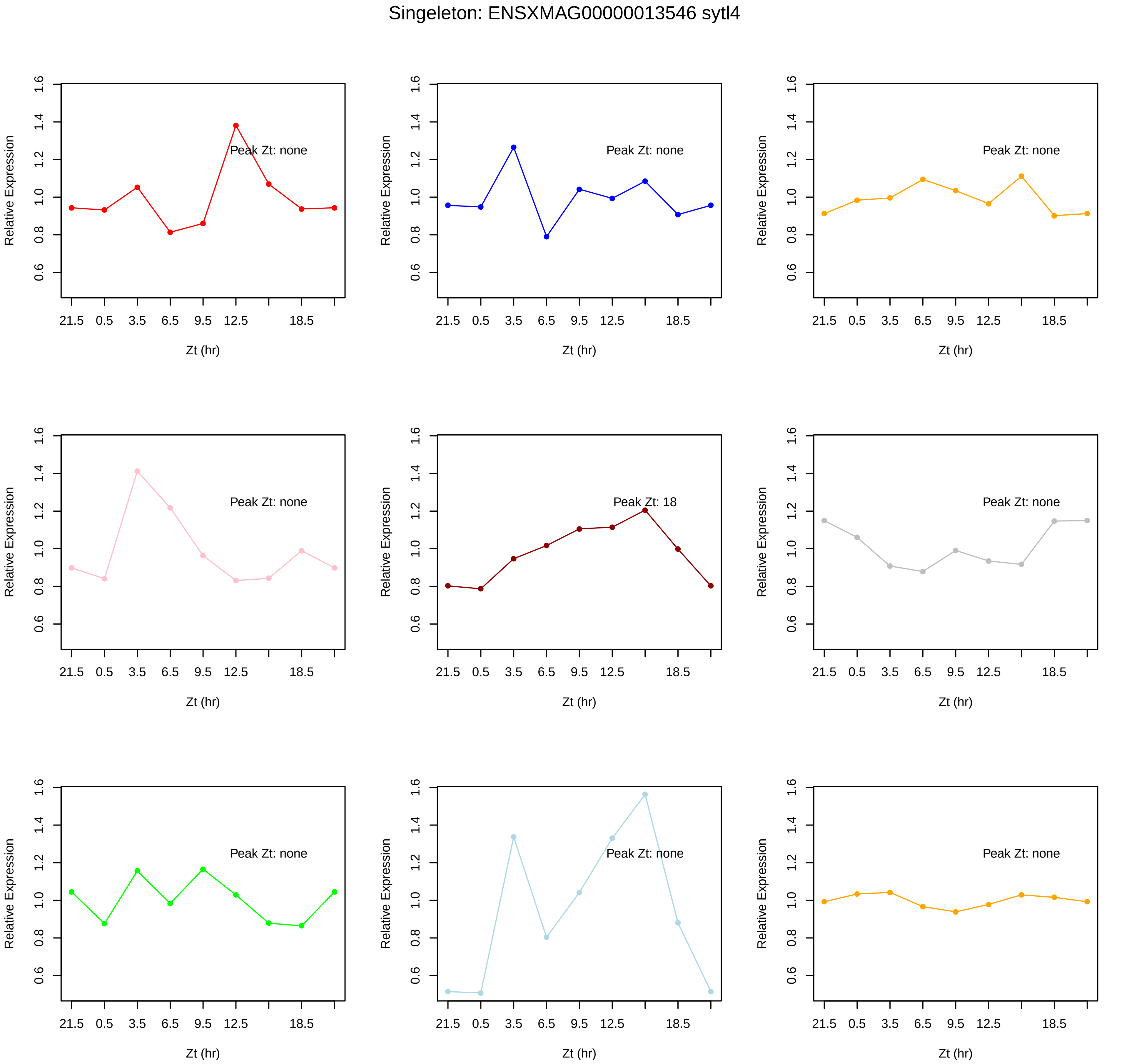
Singeleton: ENSXMAG00000013546 sytl4
Peak Zt: none
Peak Zt: none
Peak Zt: none
Peak Zt: none
Peak Zt: 18
Peak Zt: none
Peak Zt: none
Peak Zt: none
Peak Zt: none
Relative Expression
Zt (hr)
21.5
0.5
3.5
6.5
9.5
12.5
18.5
0.6
0.8
1.0
1.2
1.4
1.6

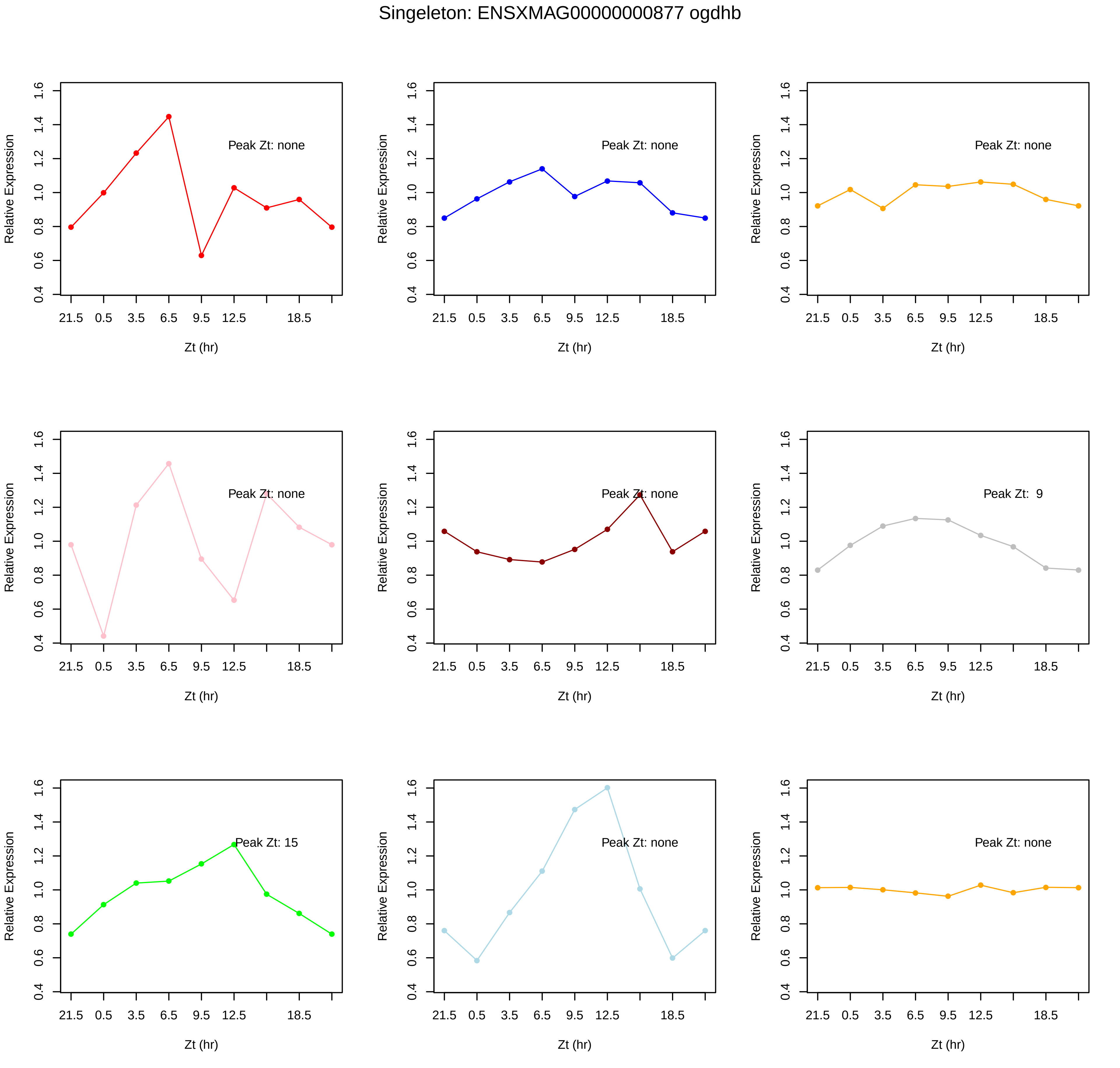
Singeleton: ENSXMAG00000000877 ogdhb
Peak Zt: none
Peak Zt: none
Peak Zt: none
Peak Zt: none
Peak Zt: none
Peak Zt: 9
Peak Zt: 15
Peak Zt: none
Peak Zt: none
Relative Expression
Zt (hr)
21.5
0.5
3.5
6.5
9.5
12.5
18.5
0.4
0.6
0.8
1.0
1.2
1.4
1.6

Singeleton: ENSXMAG00000001345 plekhg7

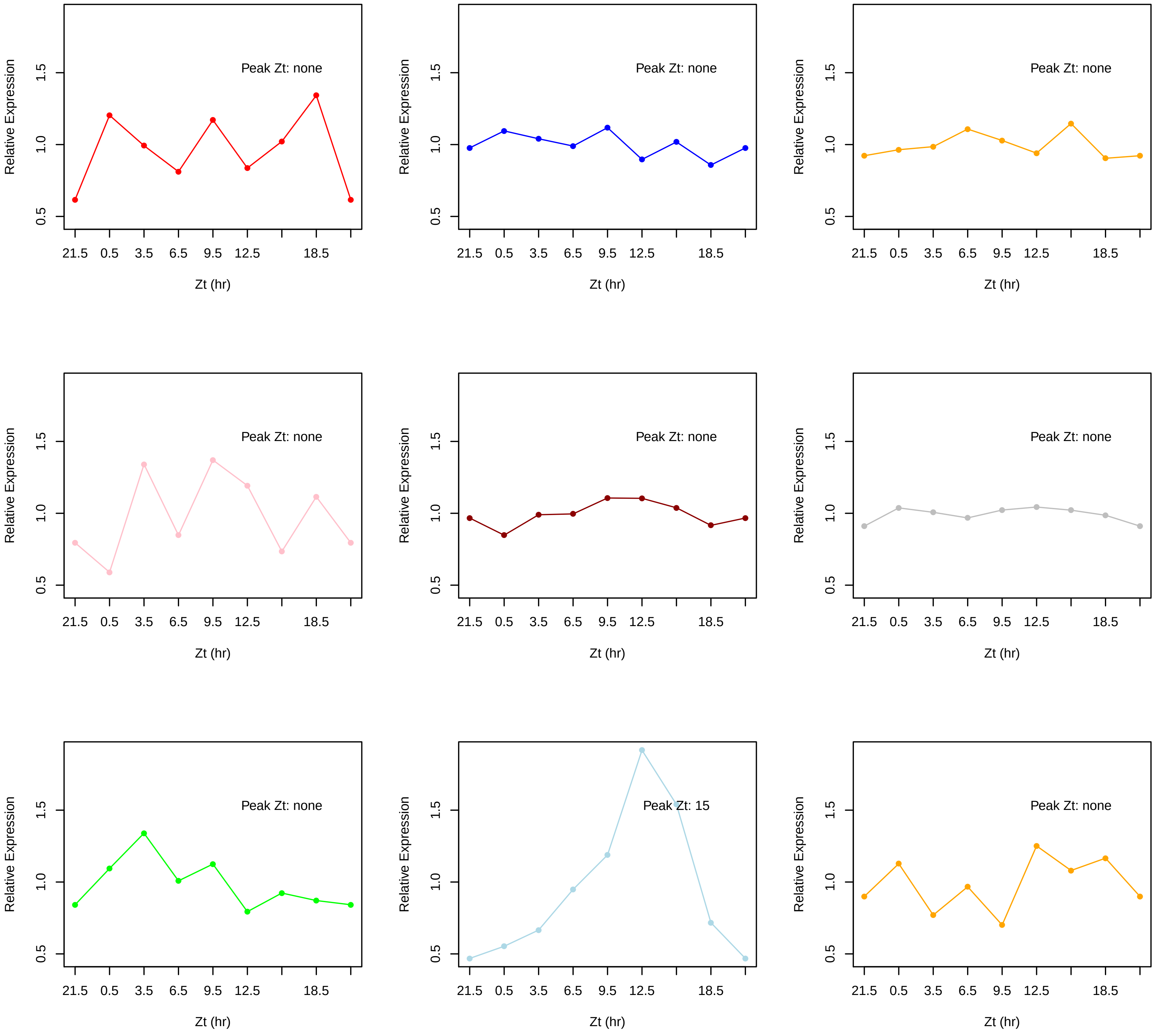

Singeleton: ENSXMAG00000013531

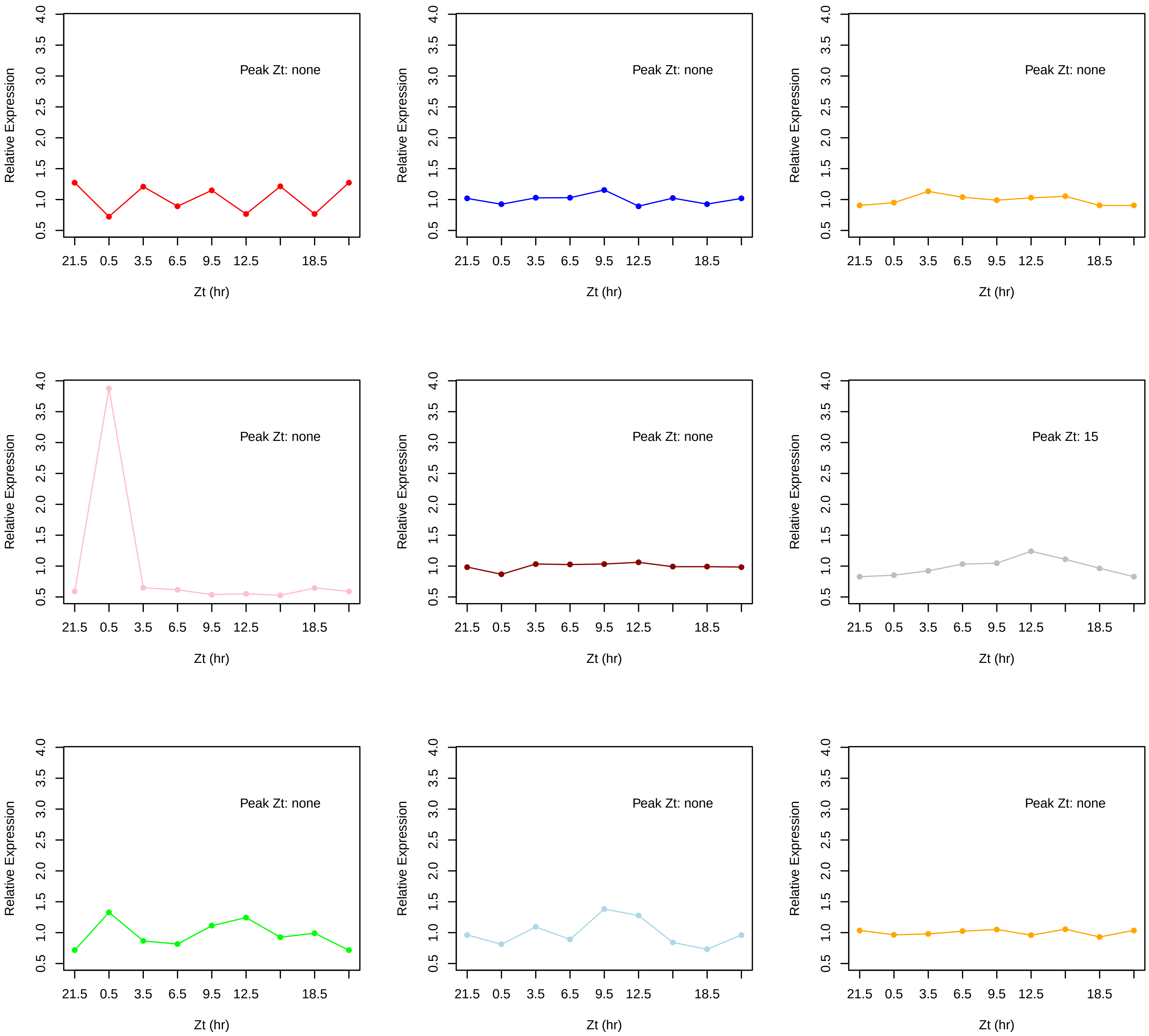

Singeleton: ENSXMAG00000018966 nudcd3

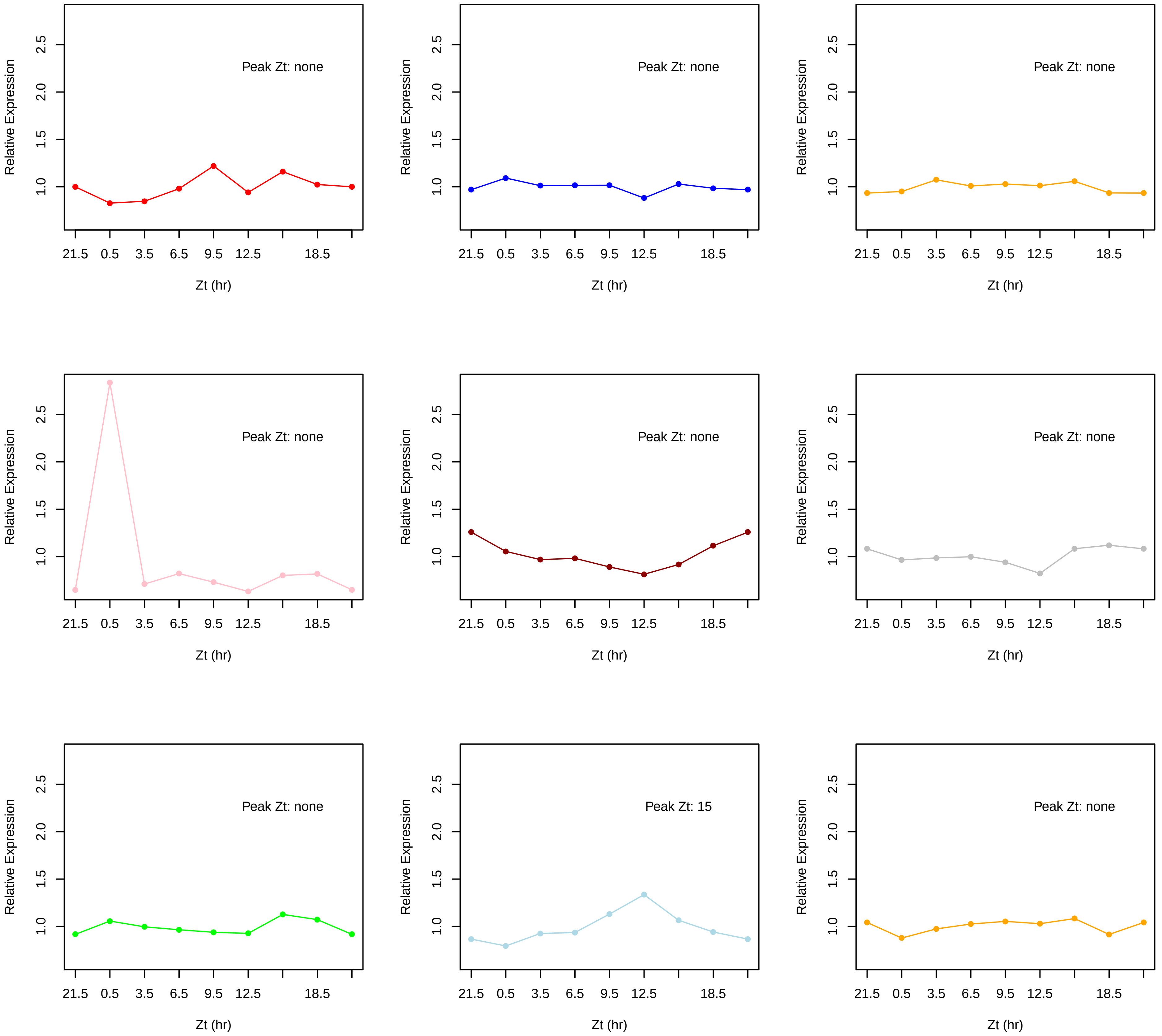

Singeleton: ENSXMAG00000018973 mrps24

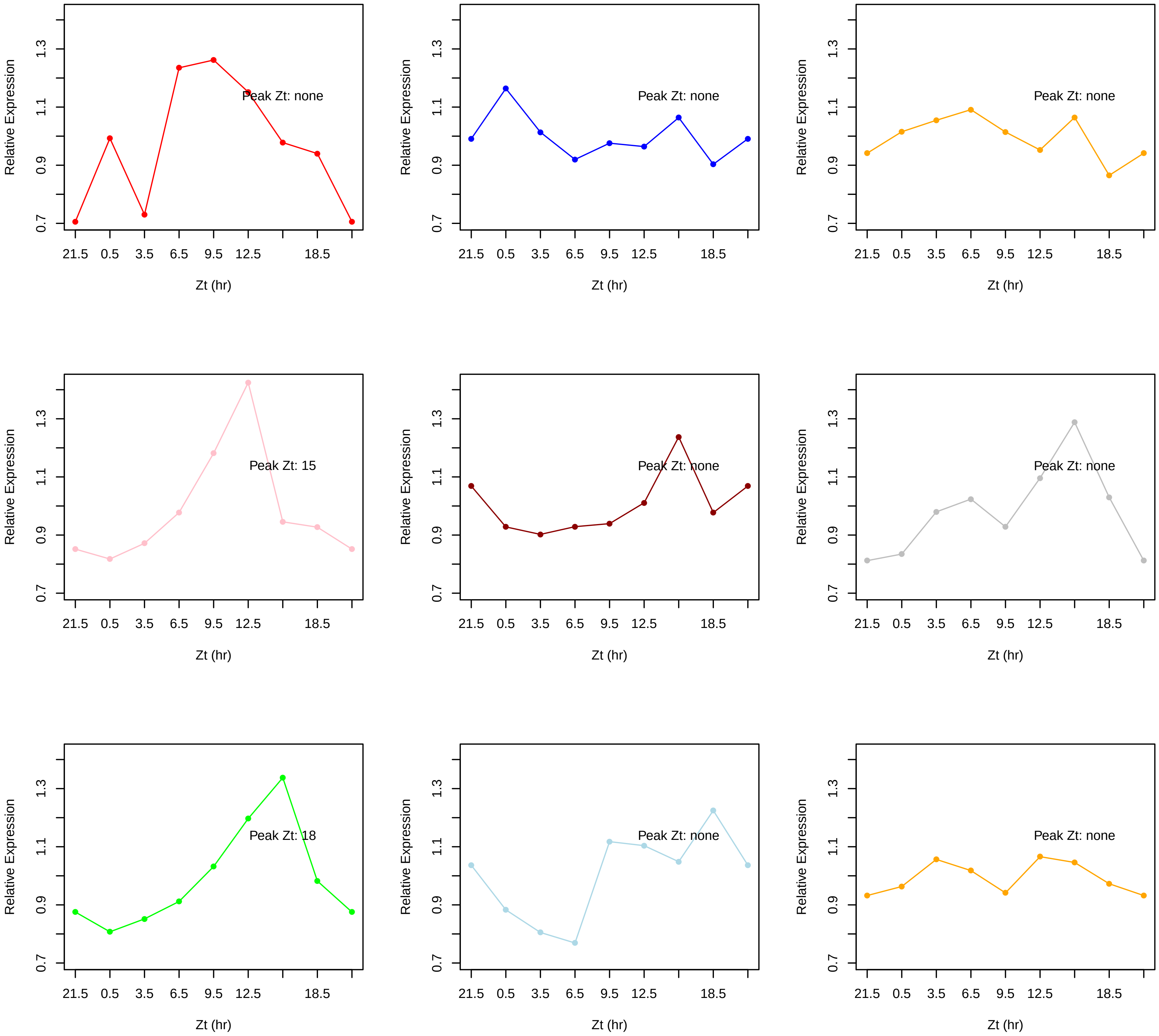

Singeleton: ENSXMAG00000013483 tmem35

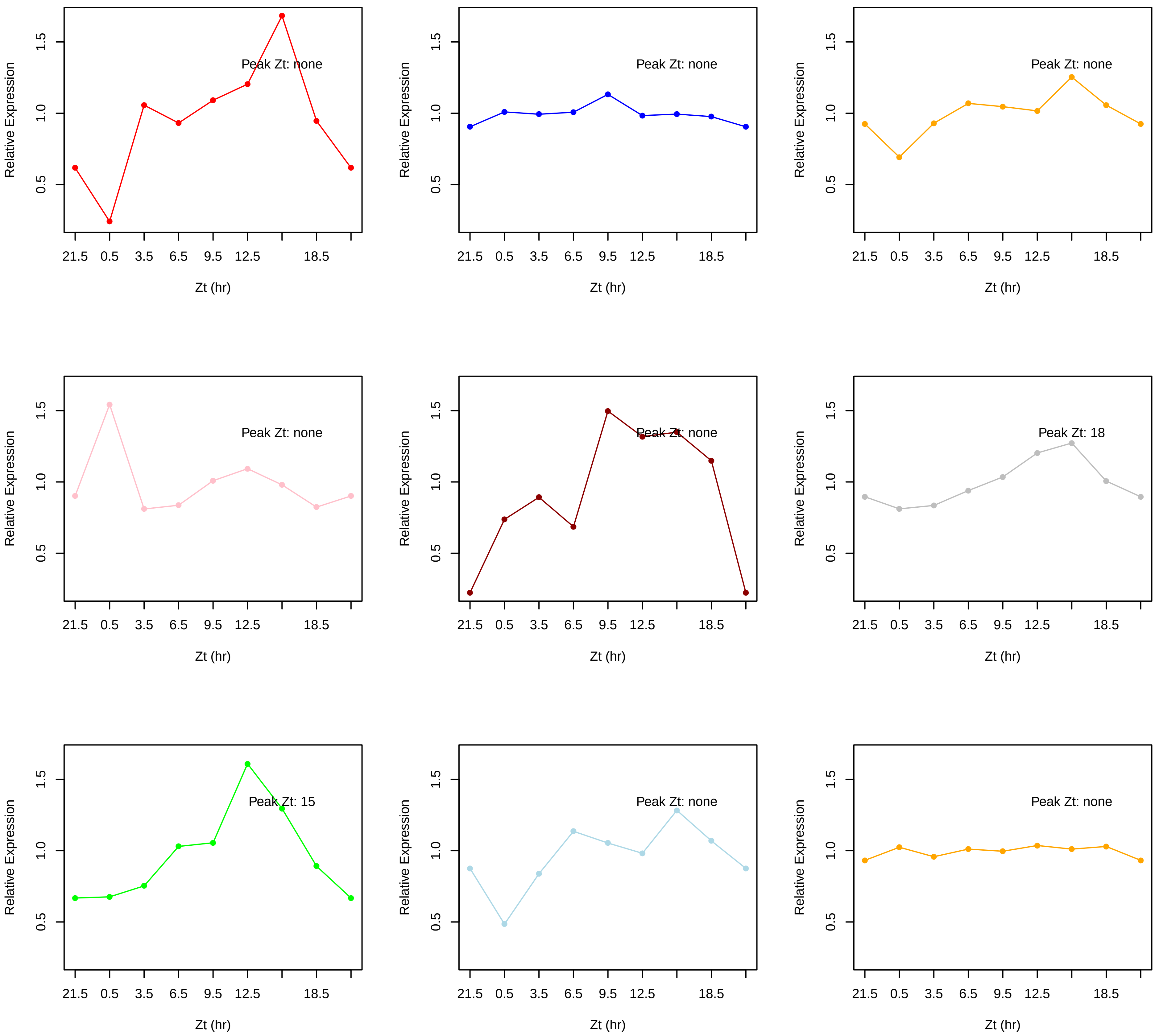

Singeleton: ENSXMAG00000004680 hk2

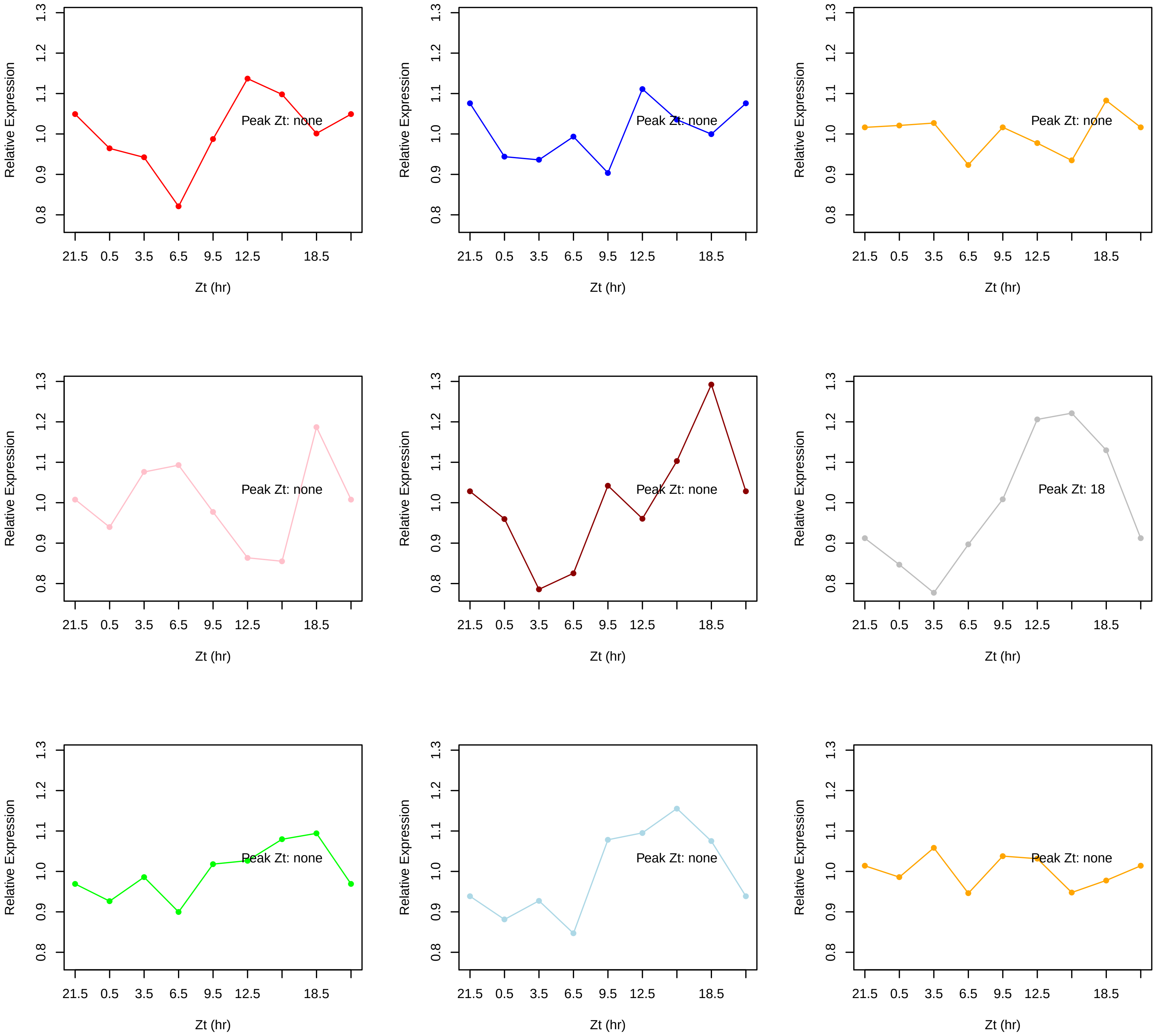

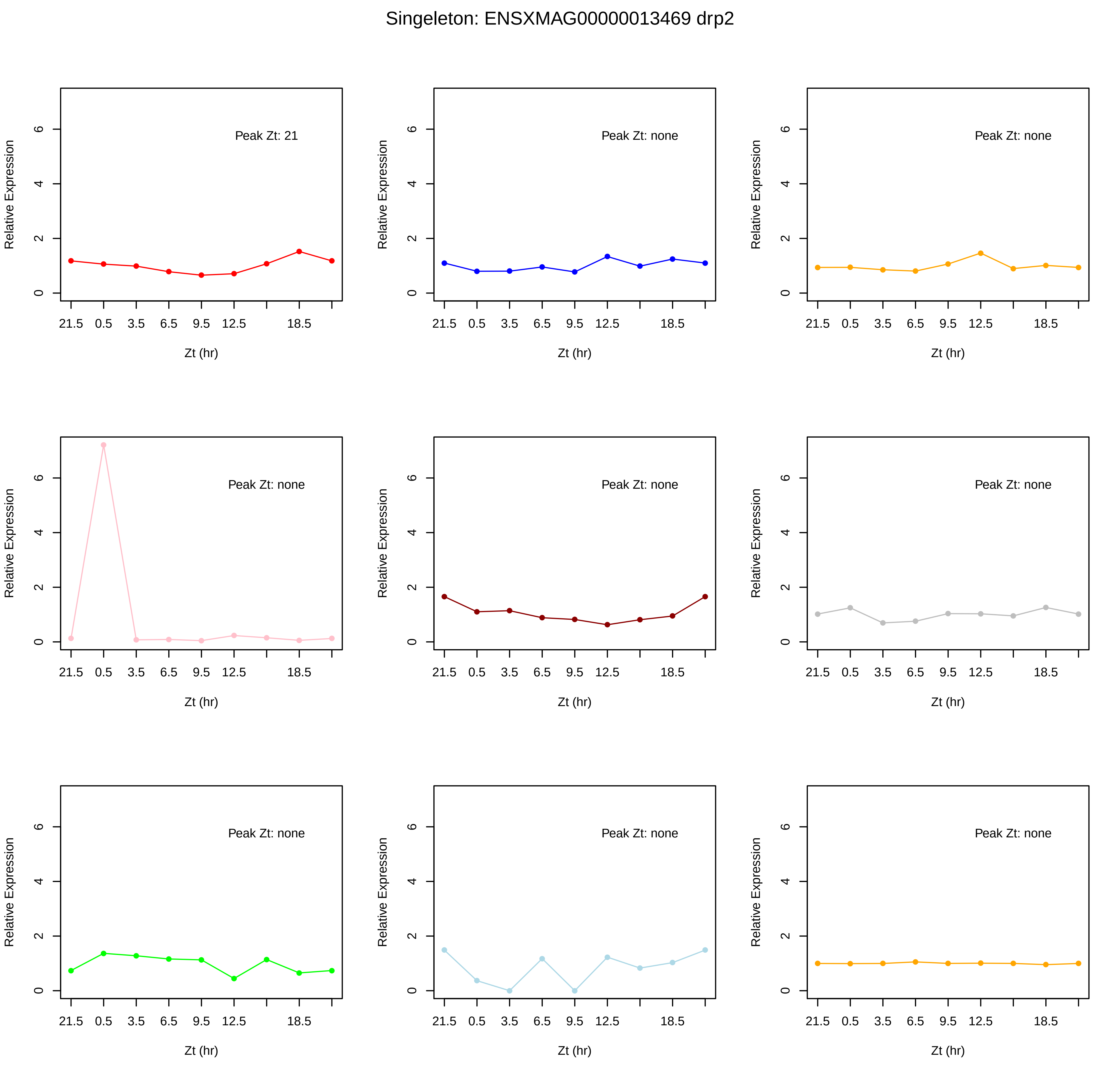
Singeleton: ENSXMAG00000013469 drp2
Peak Zt: 21
Peak Zt: none
Peak Zt: none
Peak Zt: none
Peak Zt: none
Peak Zt: none
Peak Zt: none
Peak Zt: none
Peak Zt: none
Relative Expression
Zt (hr)
21.5
0.5
3.5
6.5
9.5
12.5
18.5
0
2
4
6

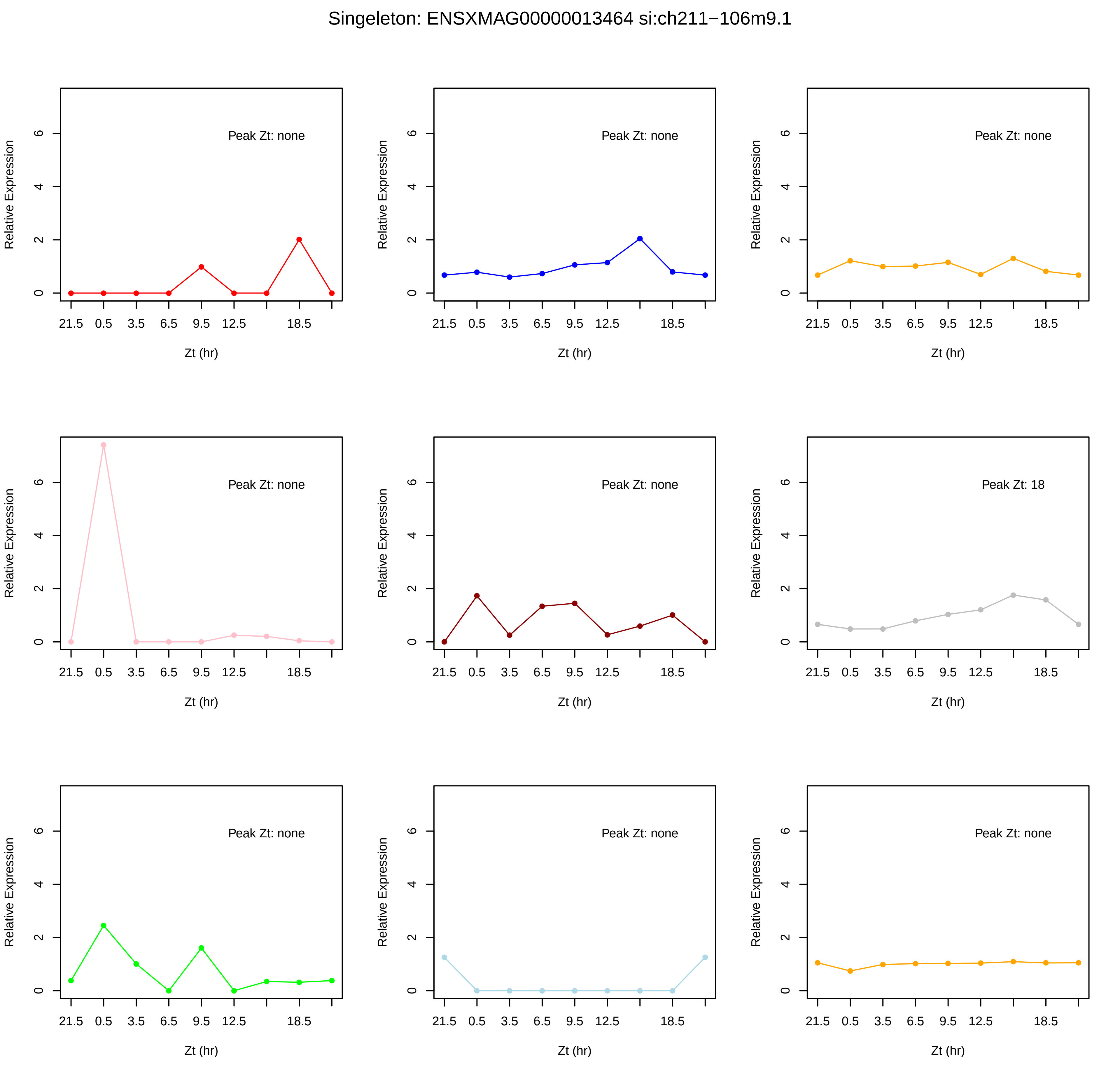
Singeleton: ENSXMAG00000013464 si:ch211−106m9.1
Peak Zt: none
Peak Zt: none
Peak Zt: none
Peak Zt: none
Peak Zt: none
Peak Zt: 18
Peak Zt: none
Peak Zt: none
Peak Zt: none
Relative Expression
Zt (hr)
21.5
0.5
3.5
6.5
9.5
12.5
18.5
0
2
4
6

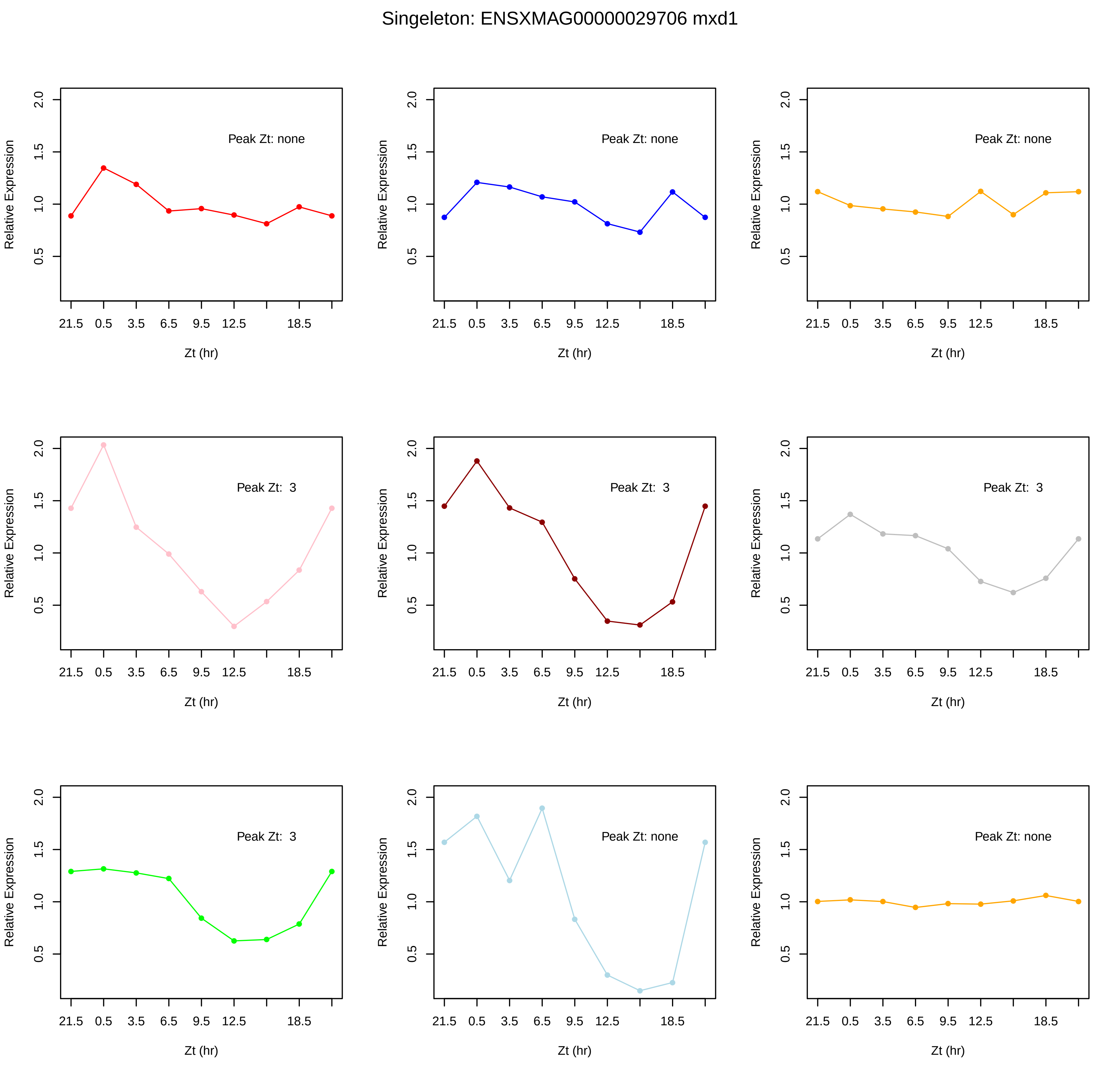
Singleton: ENSXMAG00000029706 mxd1
Peak Zt: none
Peak Zt: none
Peak Zt: none
Peak Zt: 3
Peak Zt: 3
Peak Zt: 3
Peak Zt: 3
Peak Zt: none
Peak Zt: none
Relative Expression
Zt (hr)
21.5
0.5
3.5
6.5
9.5
12.5
18.5
0.5
1.0
1.5
2.0

Singeleton: ENSXMAG00000022168 timm8a

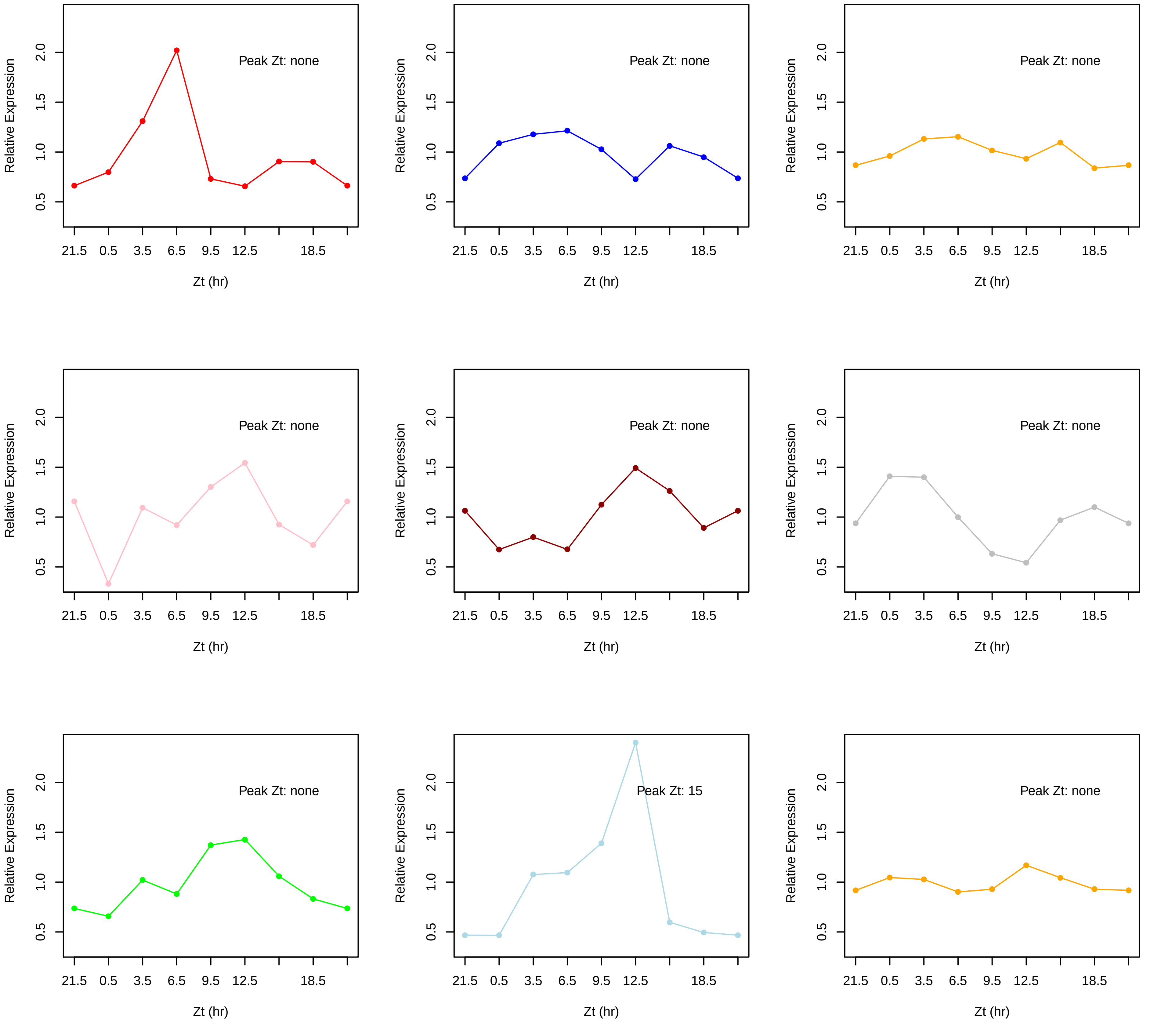

Singeleton: ENSXMAG00000021943 sspn

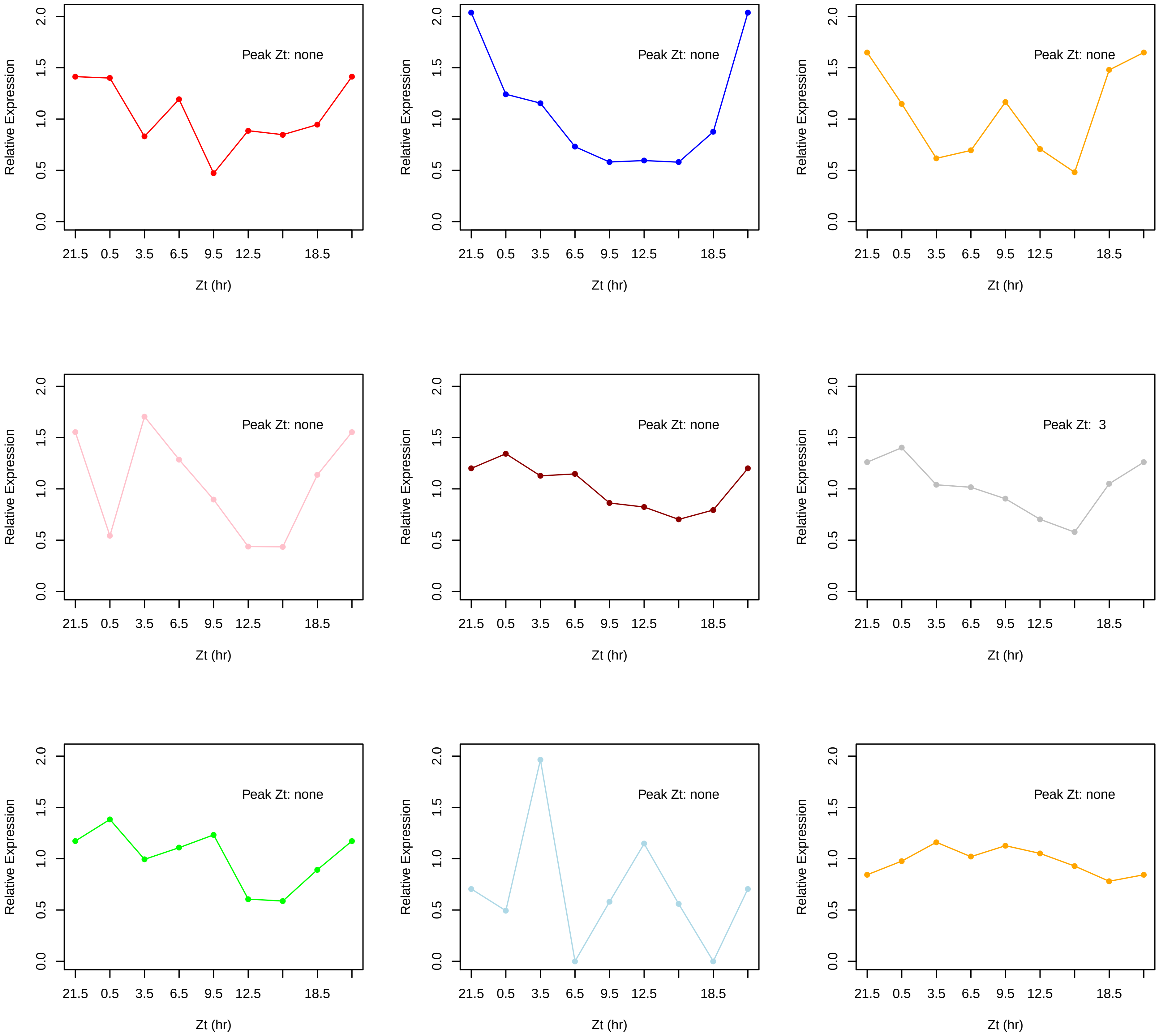

Singeleton: ENSXMAG00000001113 bhlhe41

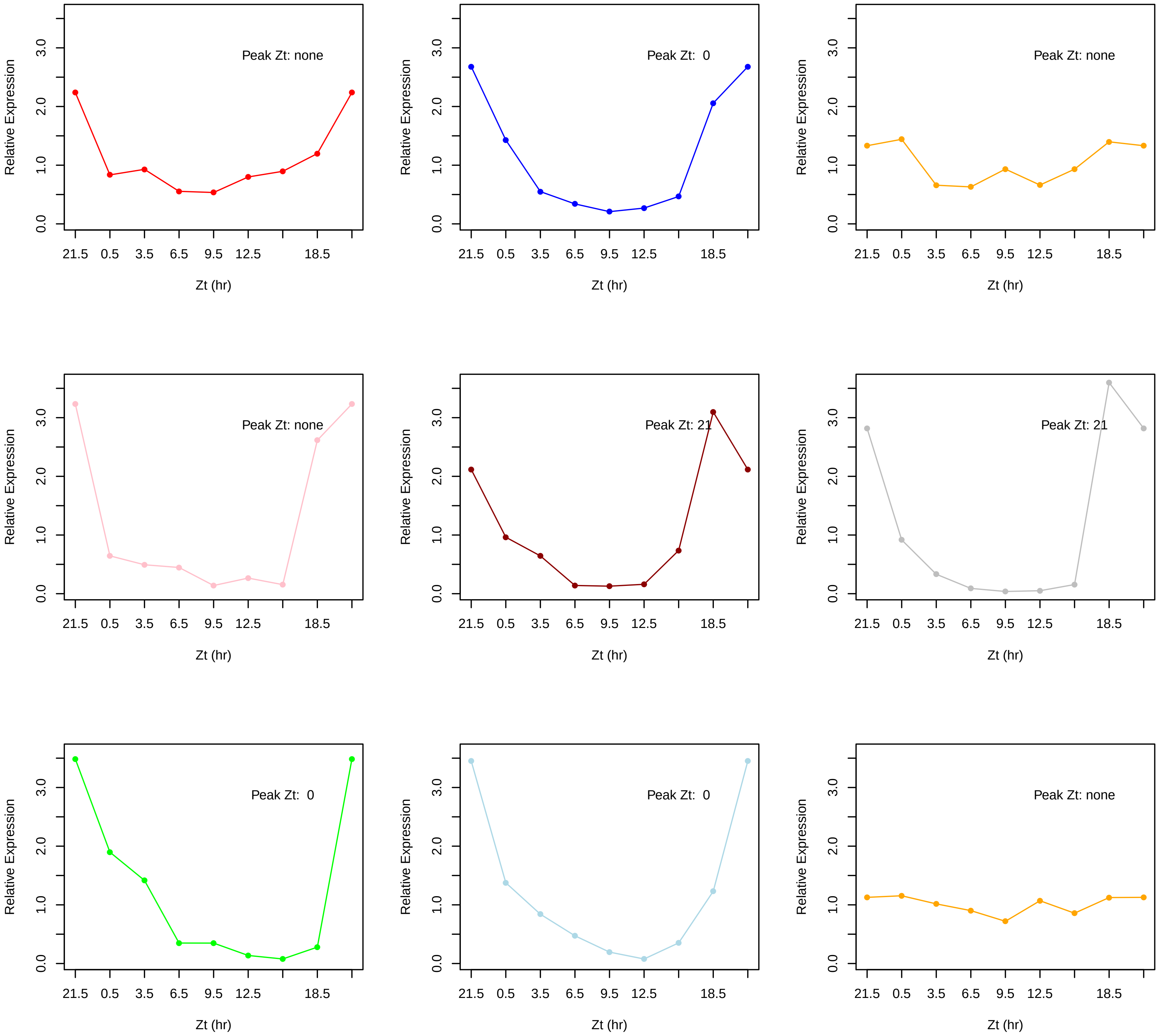

Singeleton: ENSXMAG00000025790 kras

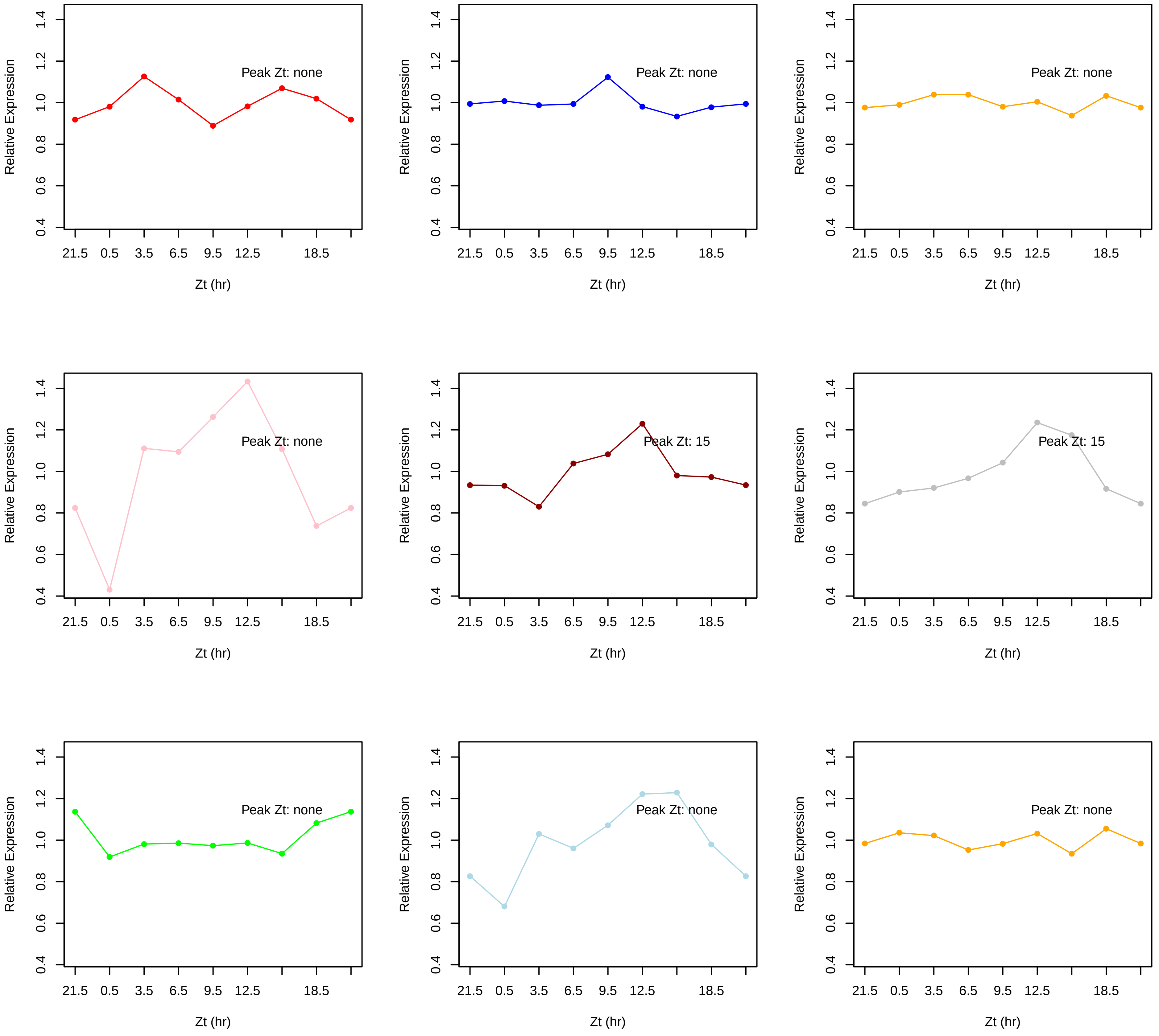

Singeleton: ENSXMAG00000006670 si:ch211−59o9.10

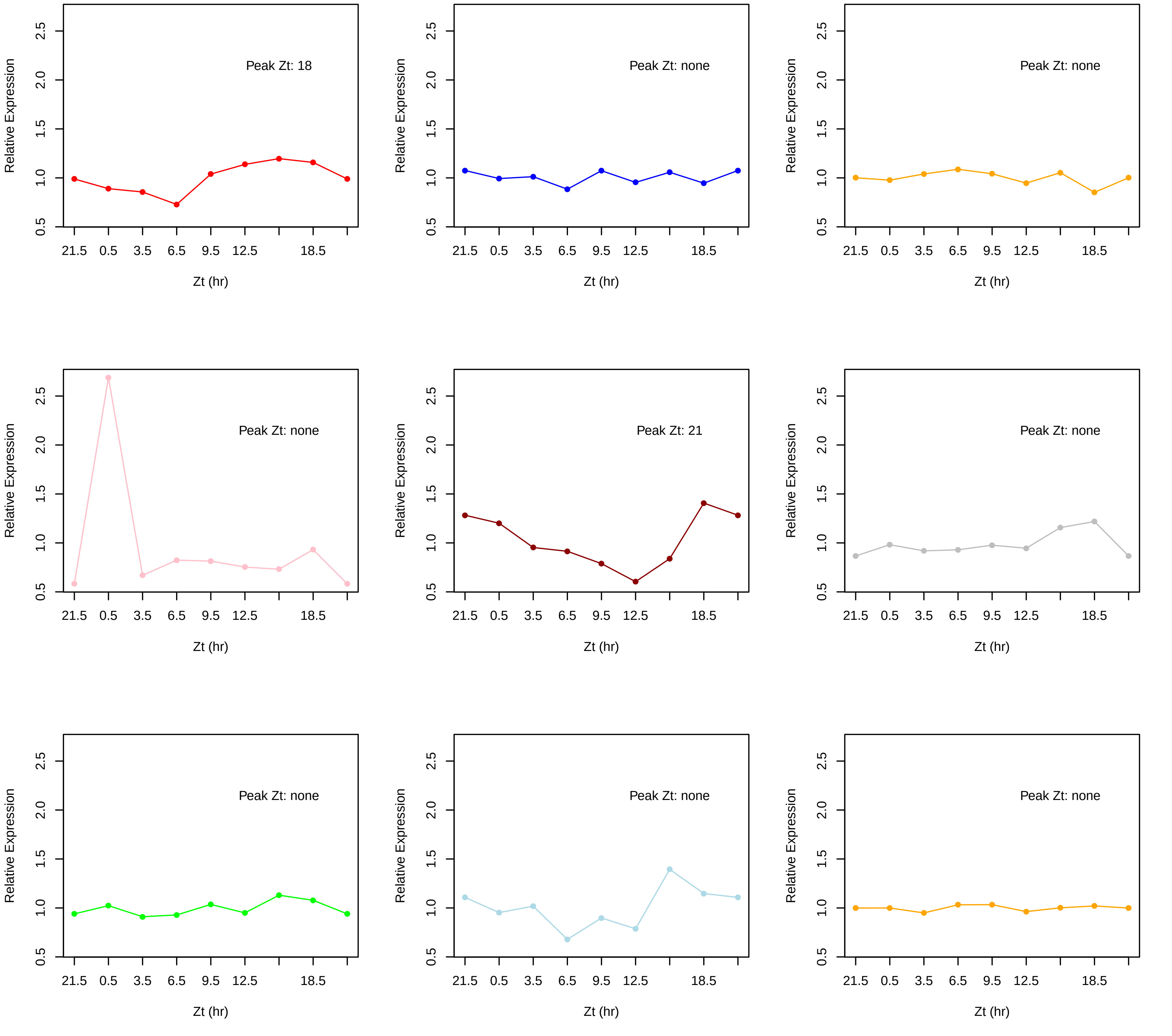

Singeleton: ENSXMAG00000008690 casc1

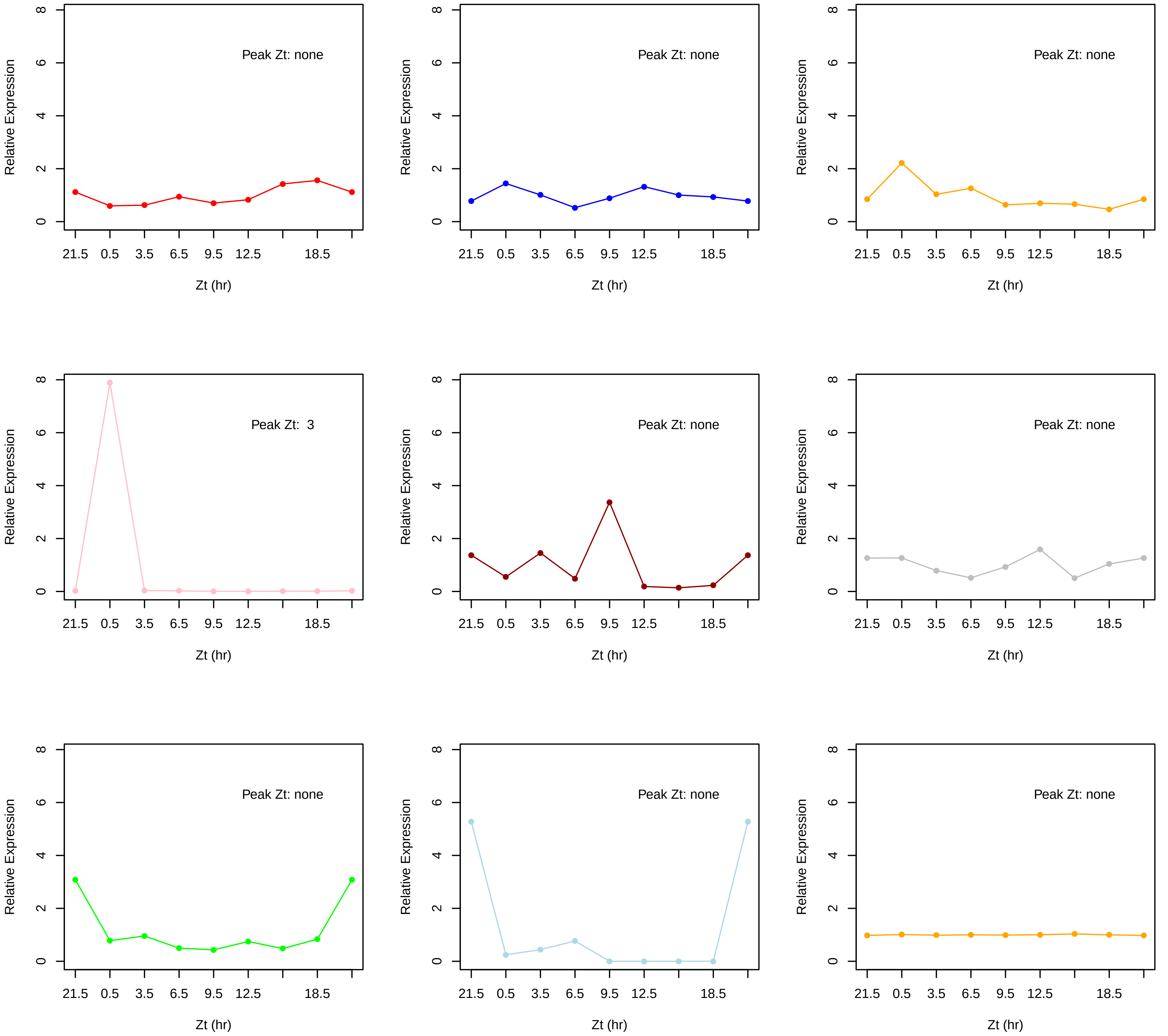

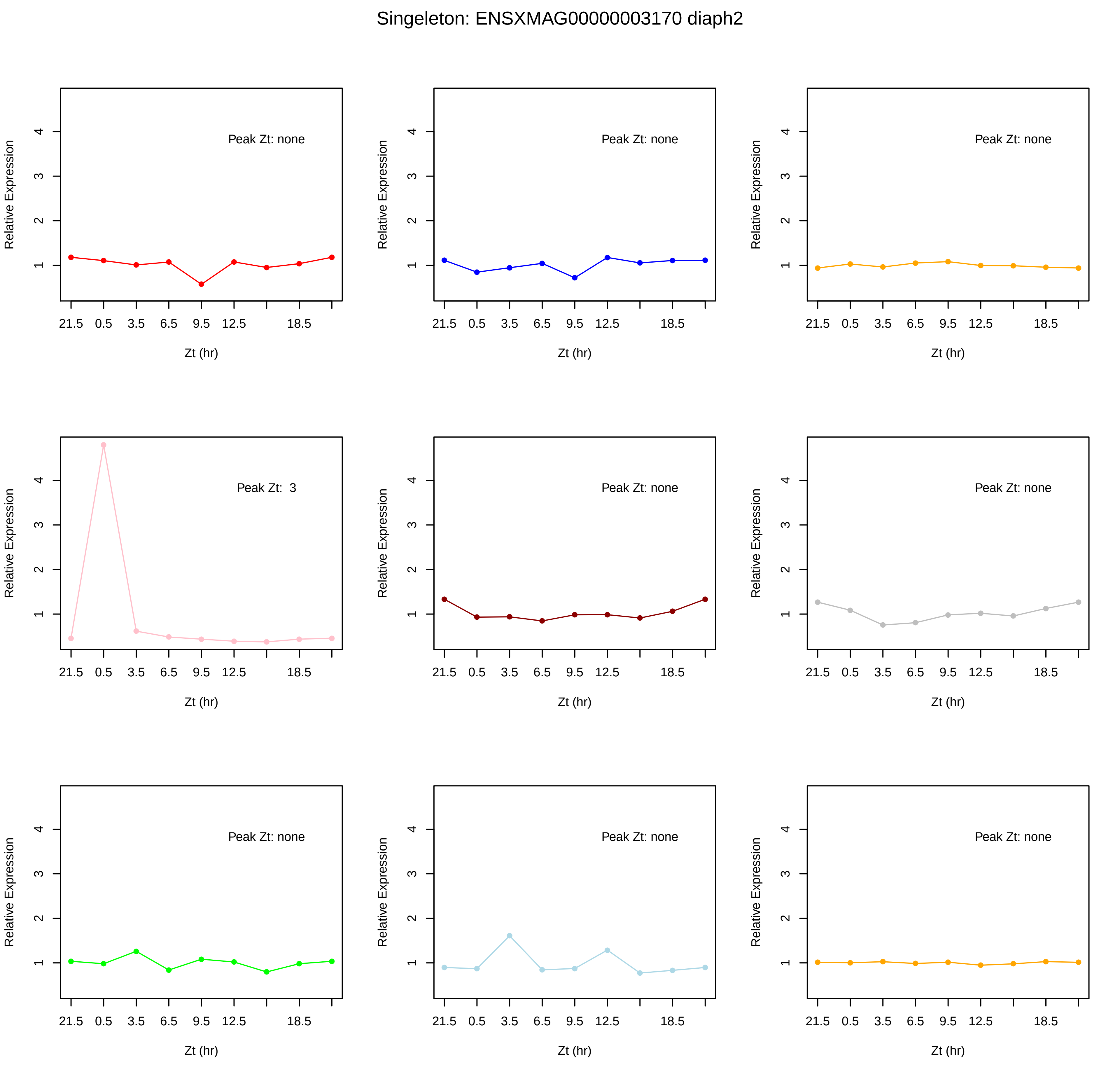
Singeleton: ENSXMAG00000003170 diaph2
Peak Zt: none
Peak Zt: none
Peak Zt: none
Peak Zt: 3
Peak Zt: none
Peak Zt: none
Peak Zt: none
Peak Zt: none
Peak Zt: none
Relative Expression
Zt (hr)
21.5
0.5
3.5
6.5
9.5
12.5
18.5
1
2
3
4

Singeleton: ENSXMAG00000006532 lrmp

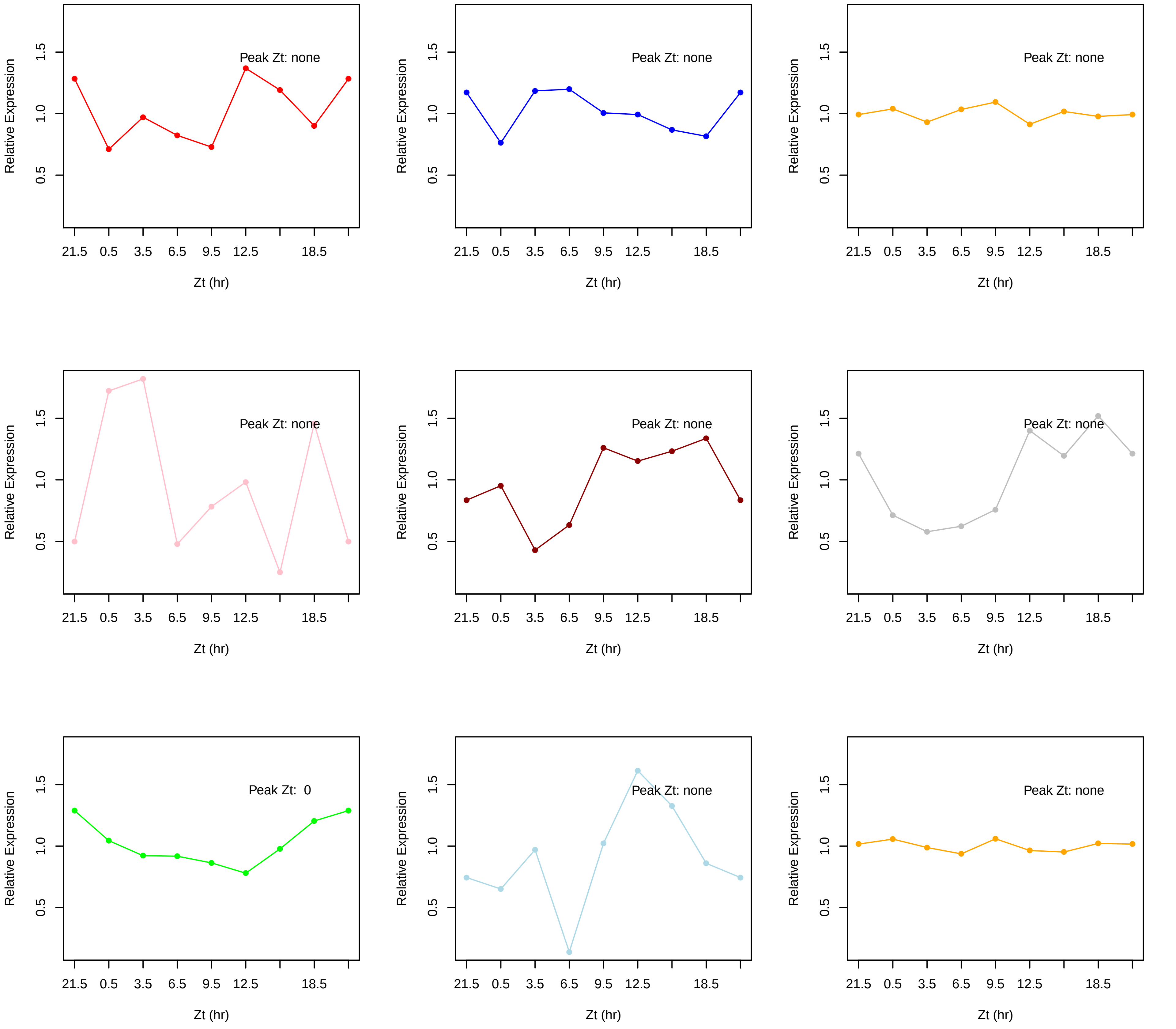

Singeleton: ENSXMAG00000004613 arhgap25

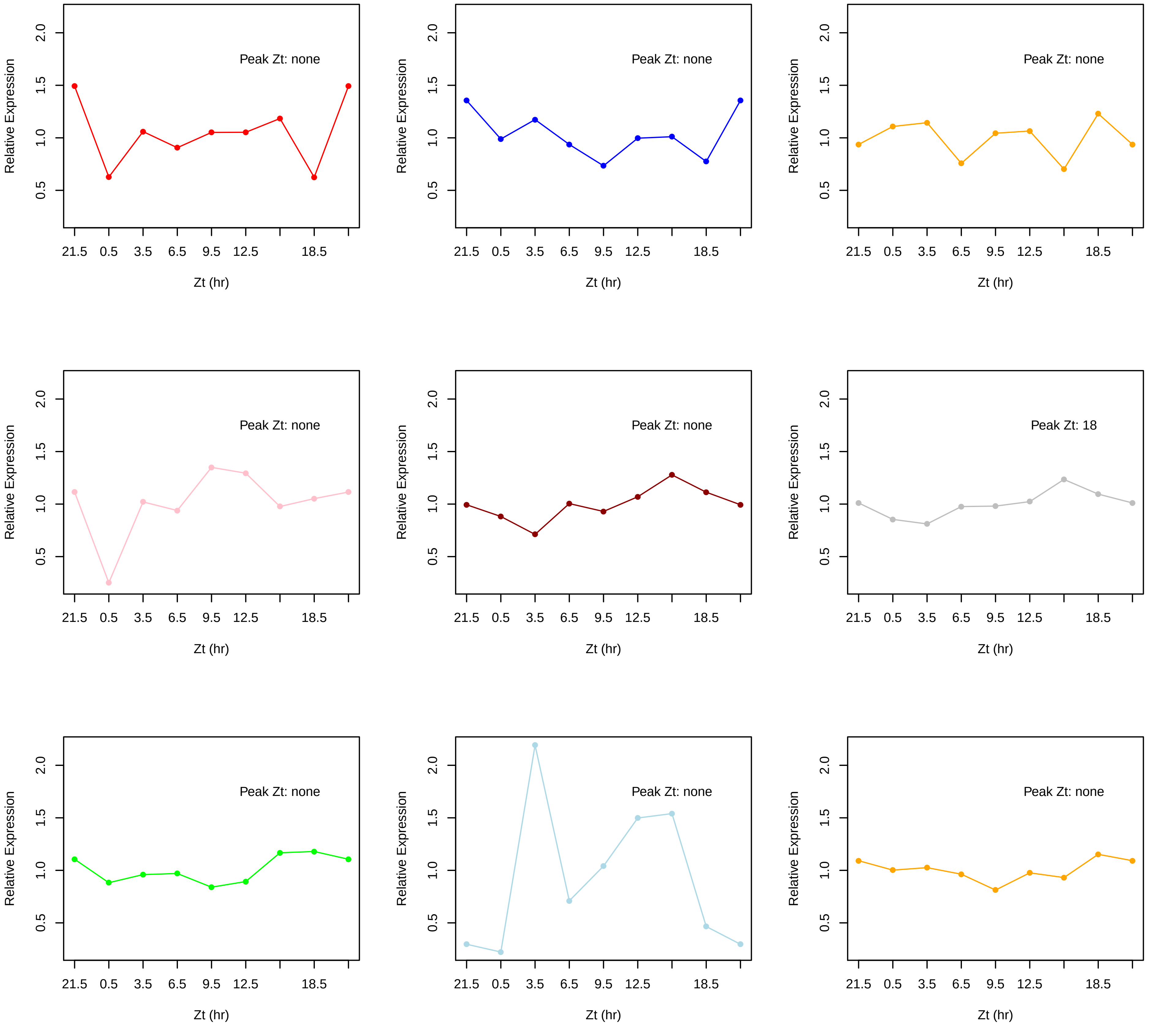

Singeleton: ENSXMAG00000029016 strap

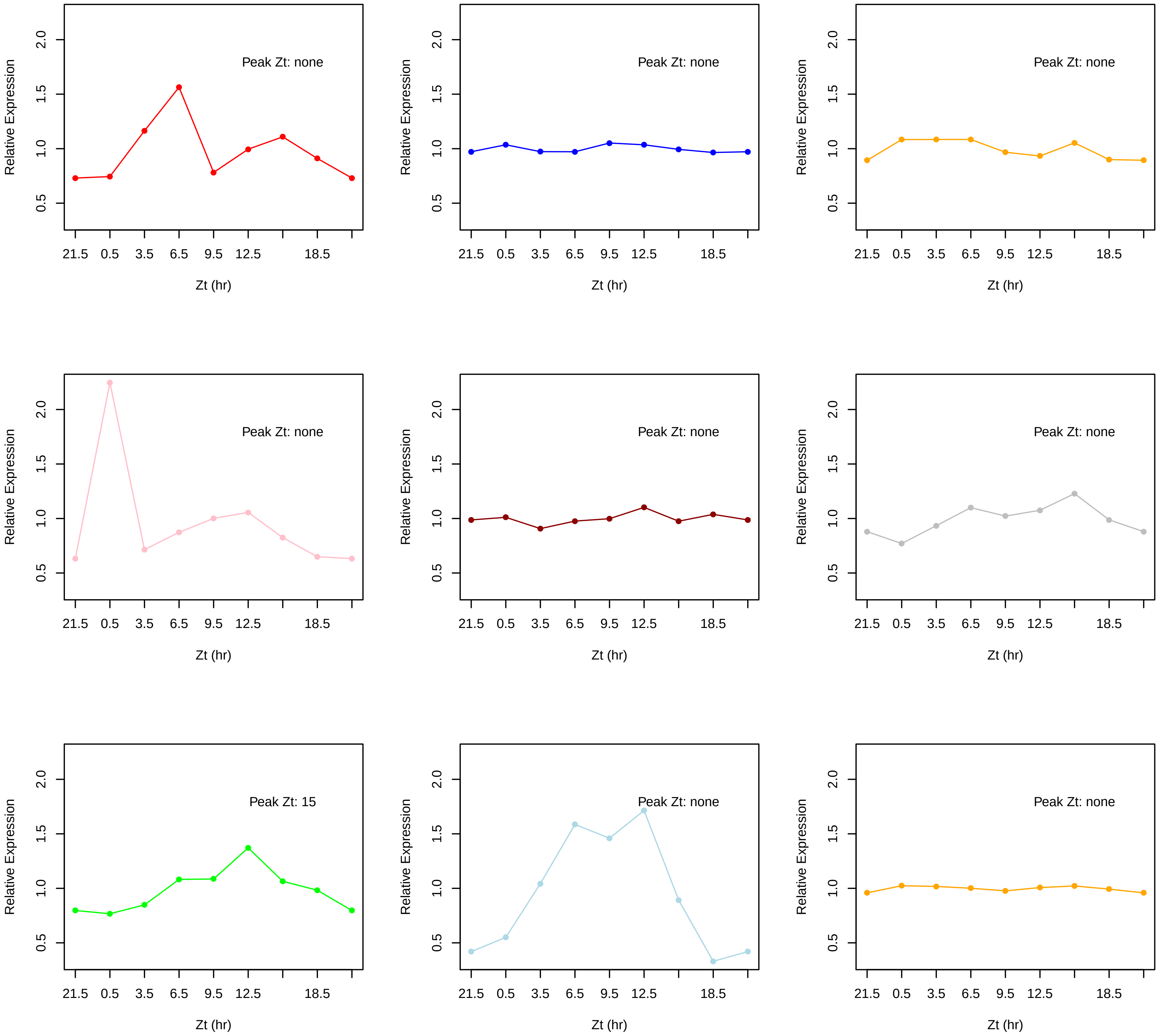

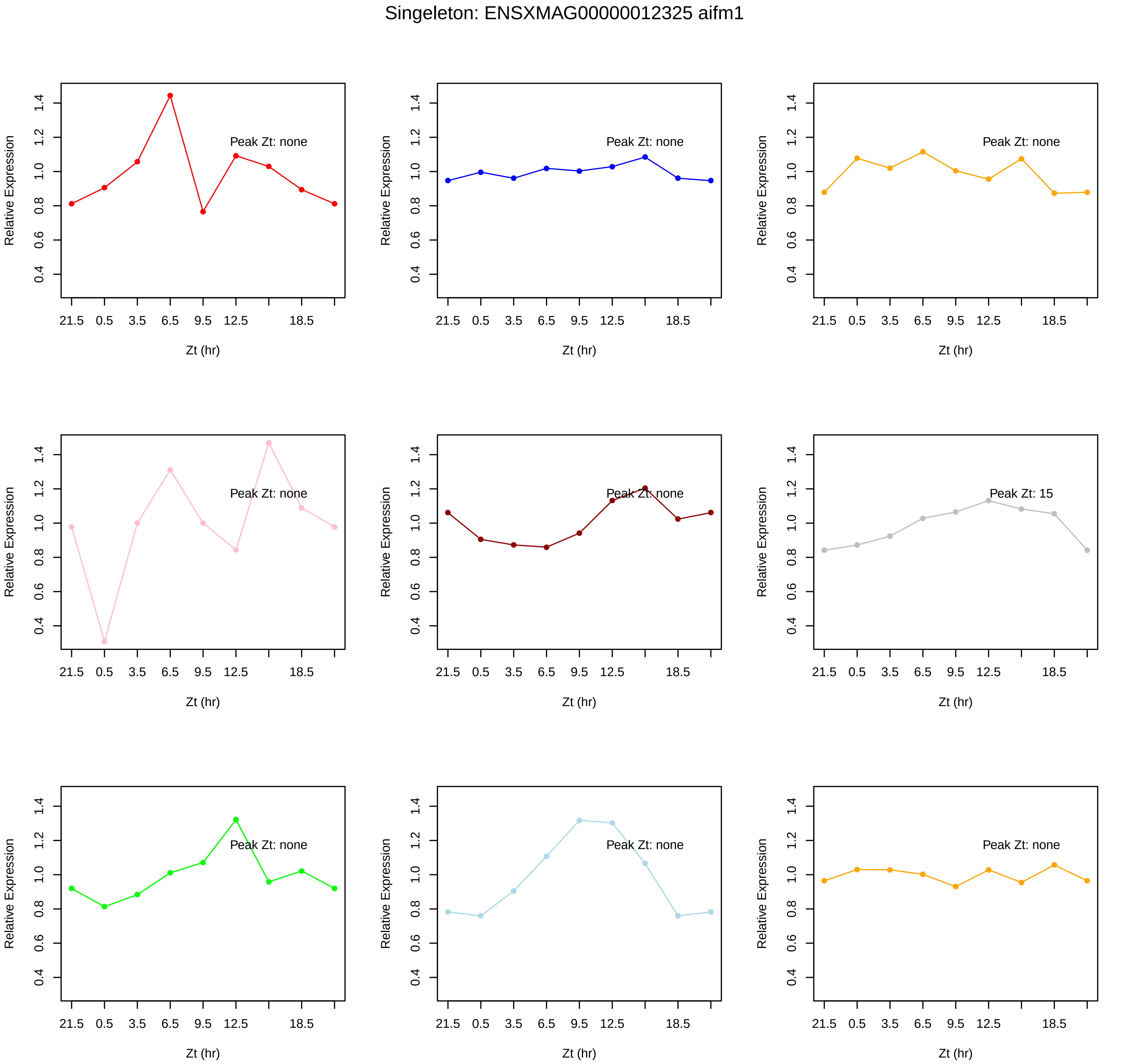
Singleton: ENSXMAG00000012325 aifm1
Relative Expression
Zt (hr)
Peak Zt: none
Peak Zt: 15
21.5
0.5
3.5
6.5
9.5
12.5
18.5
0.4
0.6
0.8
1.0
1.2
1.4

Singleton: ENSXMAG00000006674 nedd1

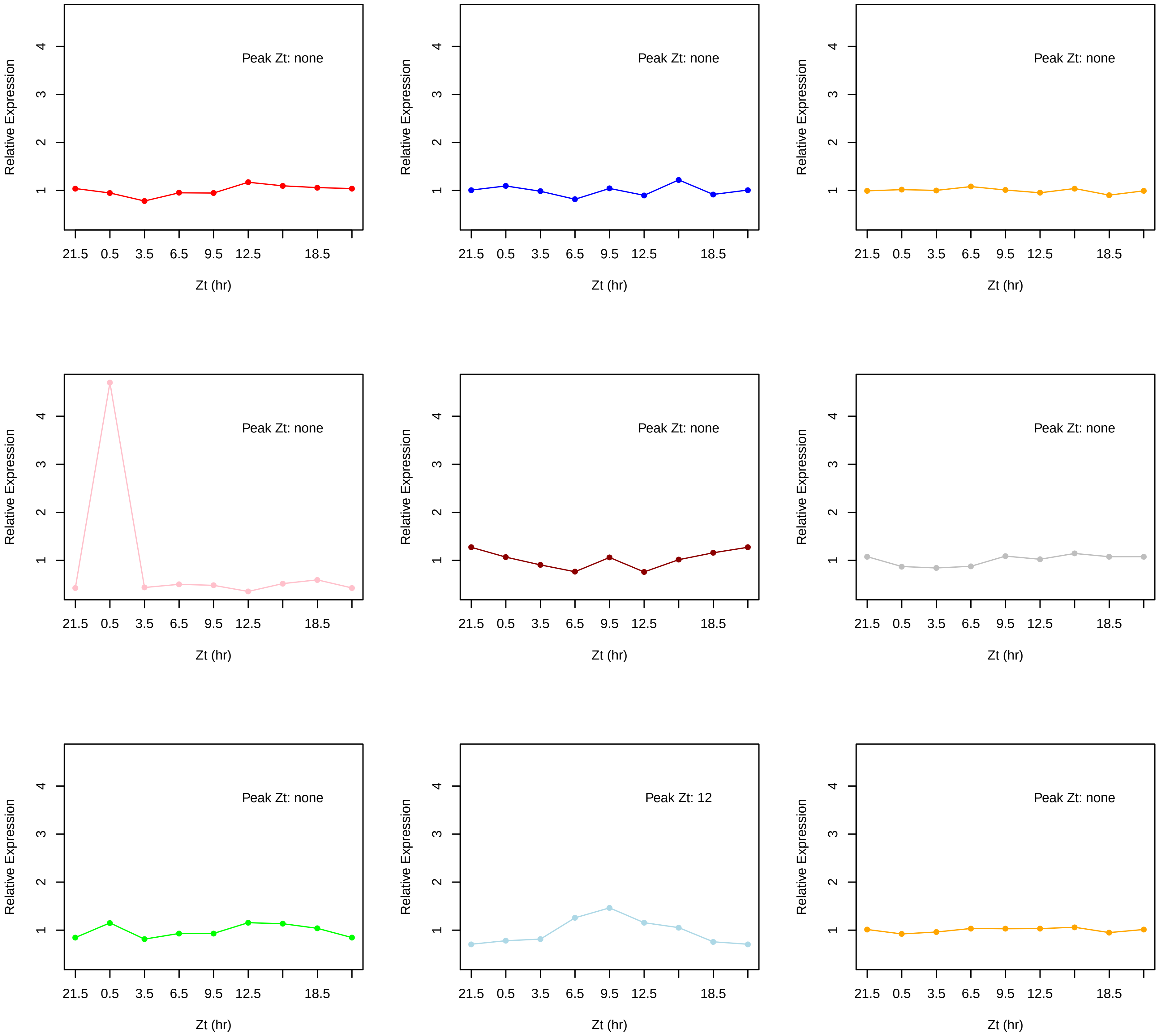

Singleton: ENSXMAG00000000296 mars2

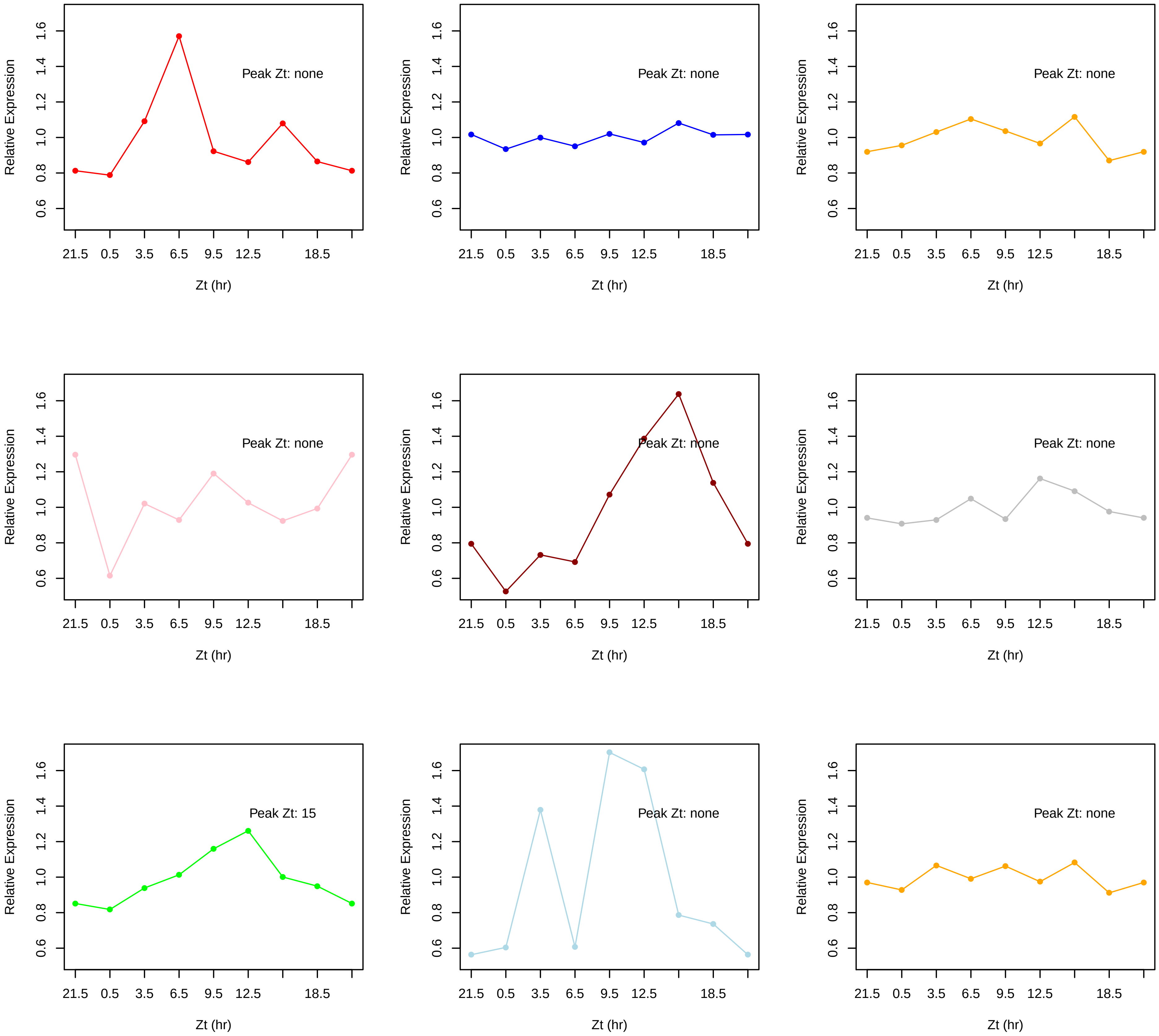

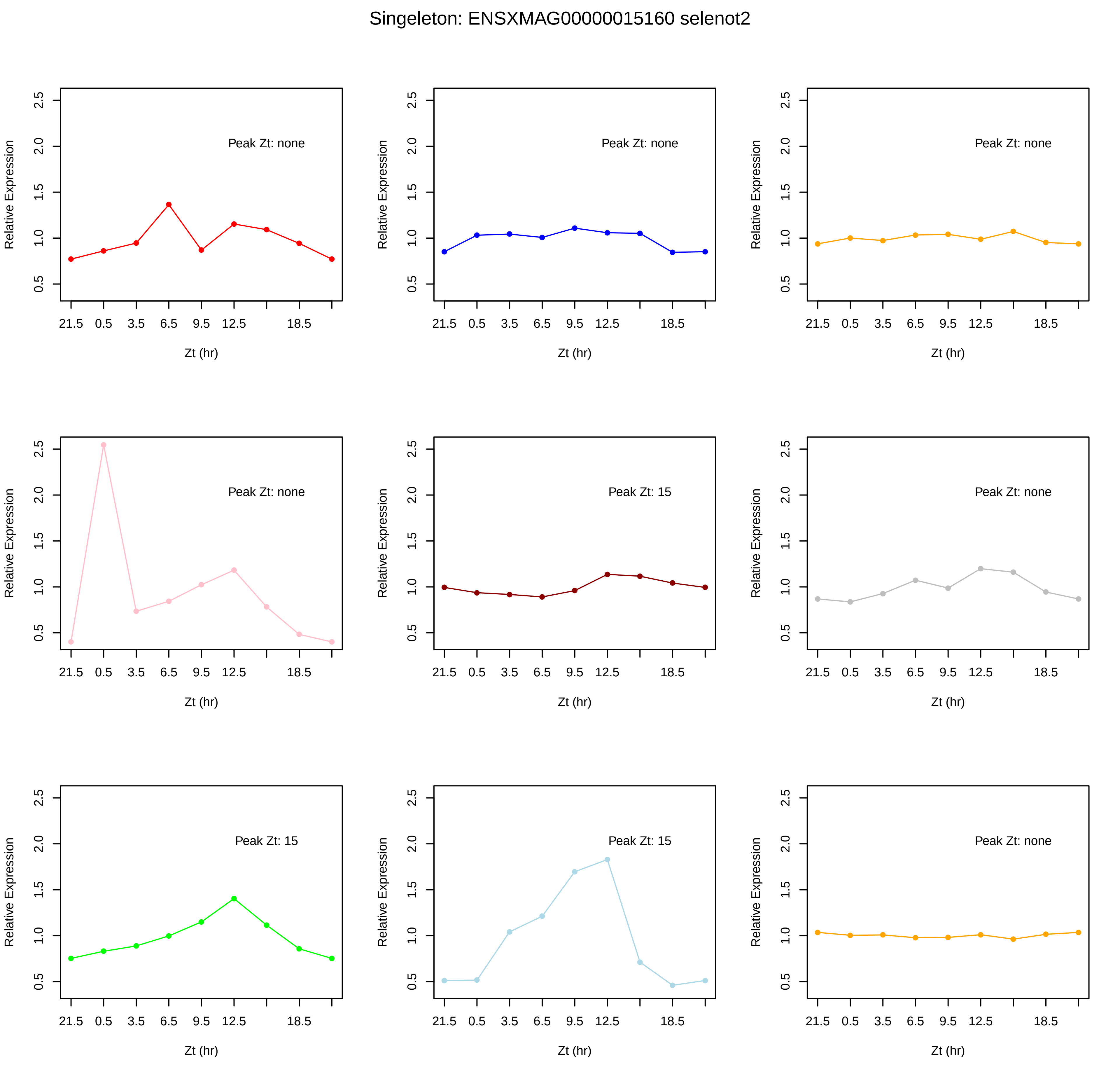
Singeleton: ENSXMAG00000015160 selenot2
Peak Zt: none
Peak Zt: none
Peak Zt: none
Peak Zt: none
Peak Zt: 15
Peak Zt: none
Peak Zt: 15
Peak Zt: 15
Peak Zt: none
Relative Expression
Zt (hr)
21.5
0.5
3.5
6.5
9.5
12.5
18.5

Singeleton: ENSXMAG00000000252 si:ch211−221f10.2

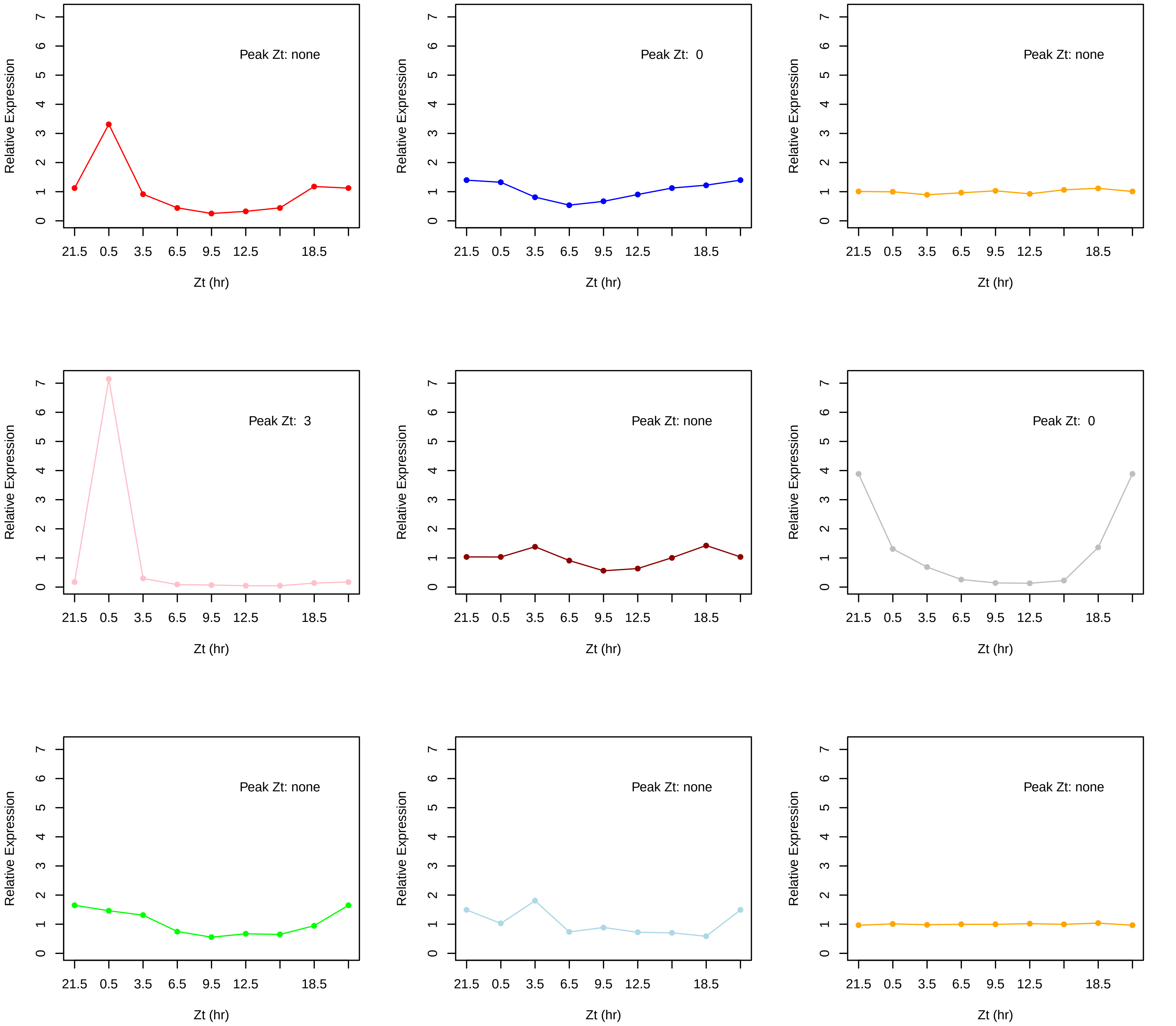

Singeleton: ENSXMAG00000020954 parvg

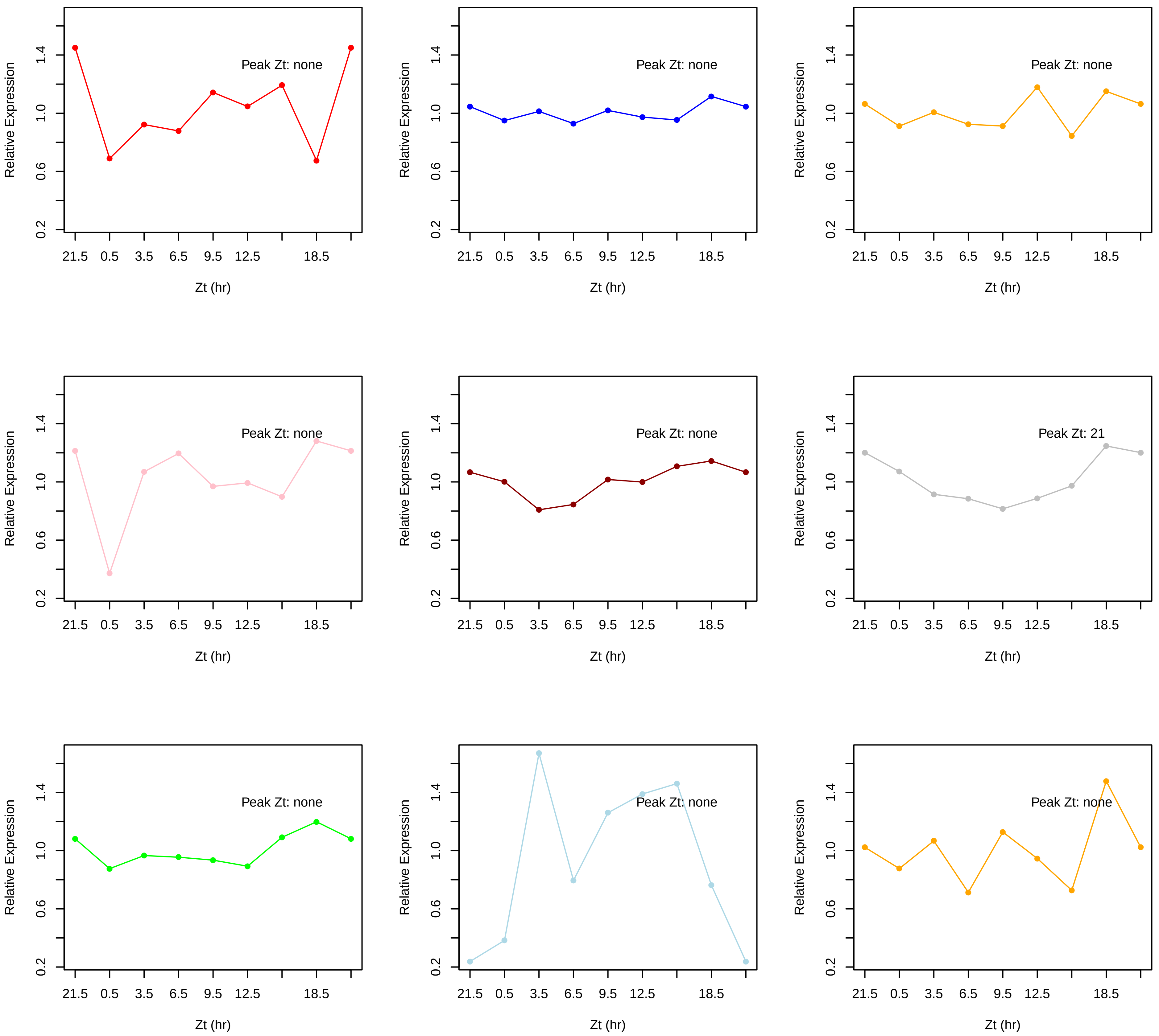

Singeleton: ENSXMAG00000010226 zgc:110329

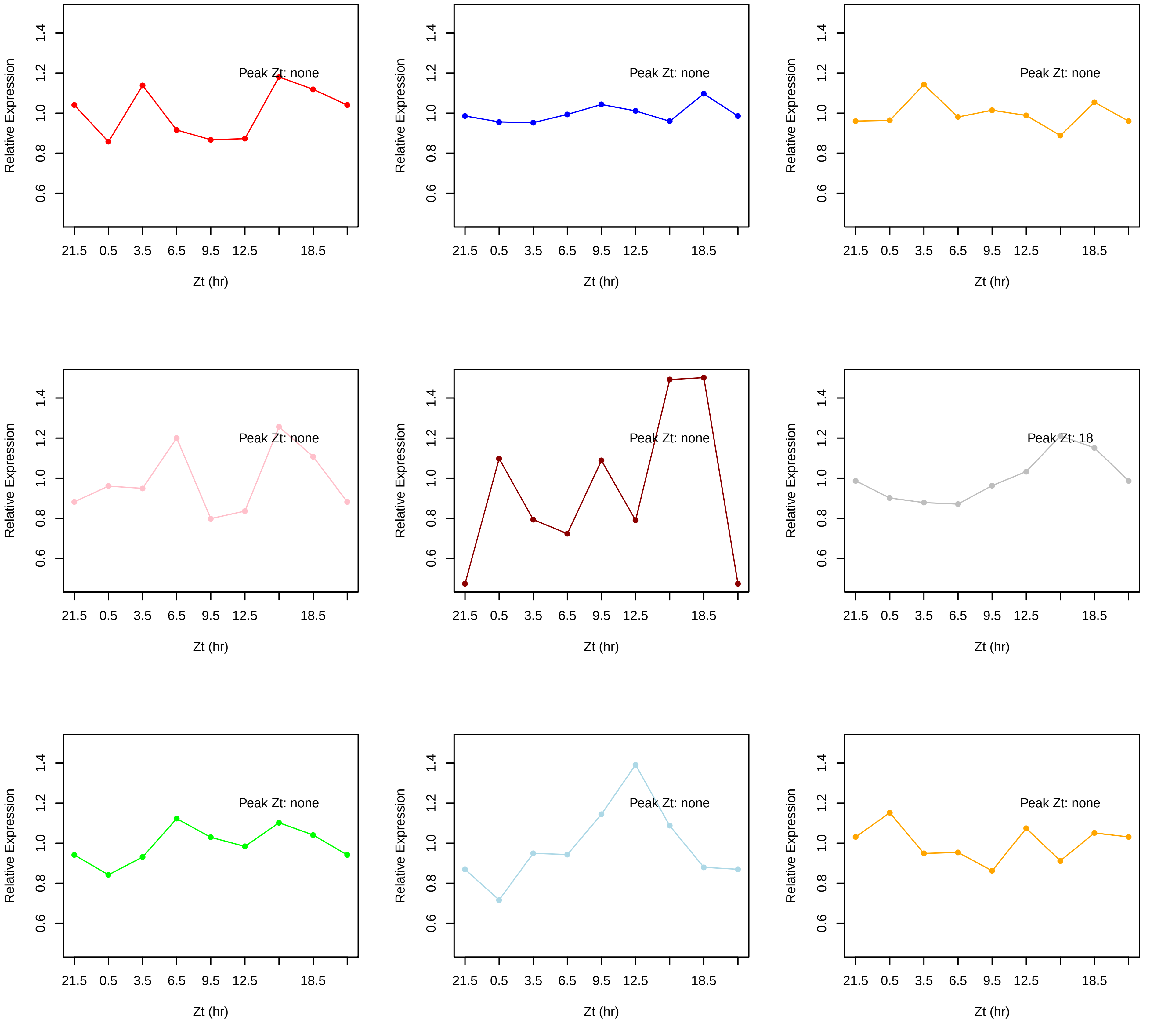

Singleton: ENSXMAG00000002888

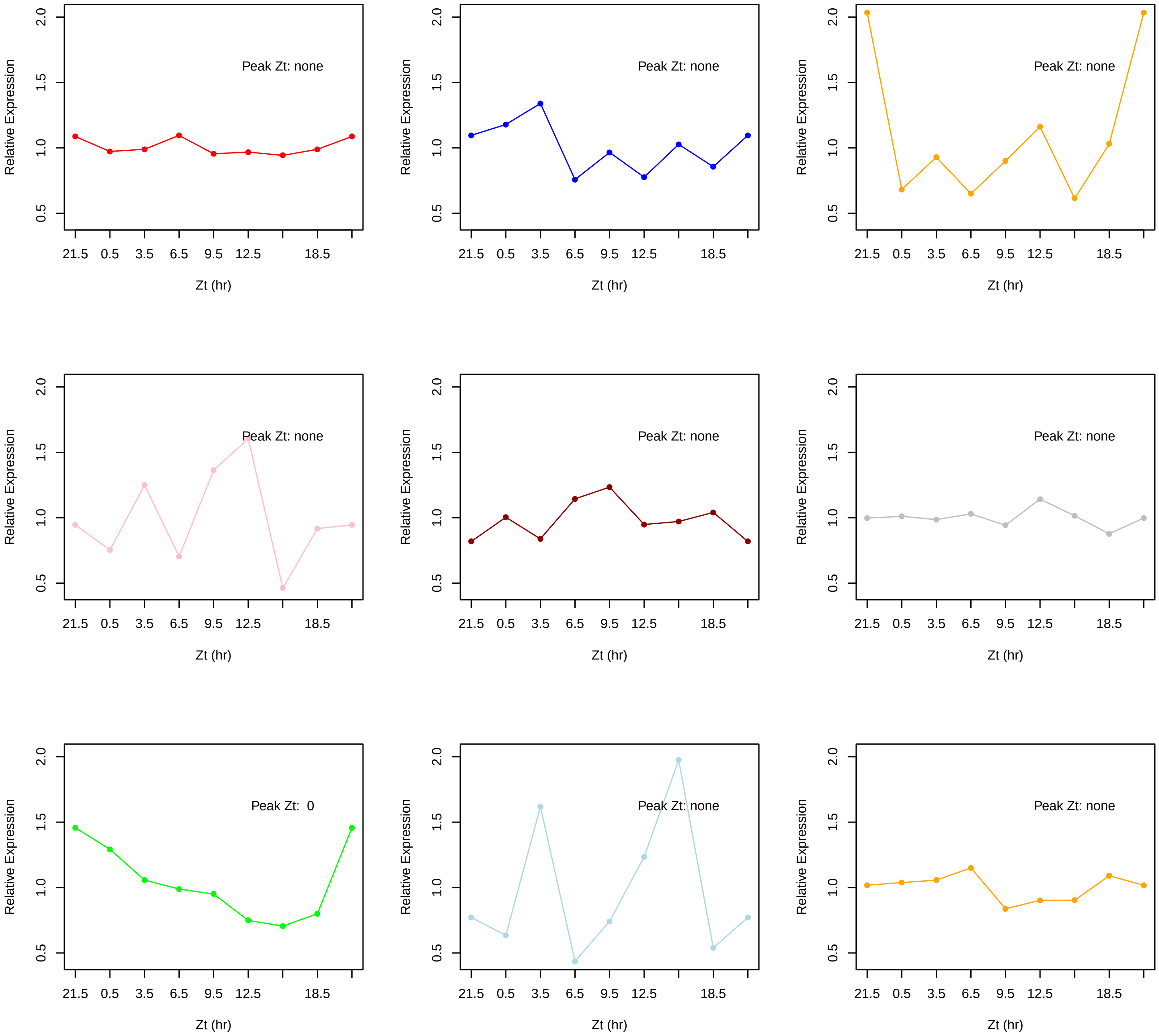

Singeleton: ENSXMAG00000016907 PNPT1

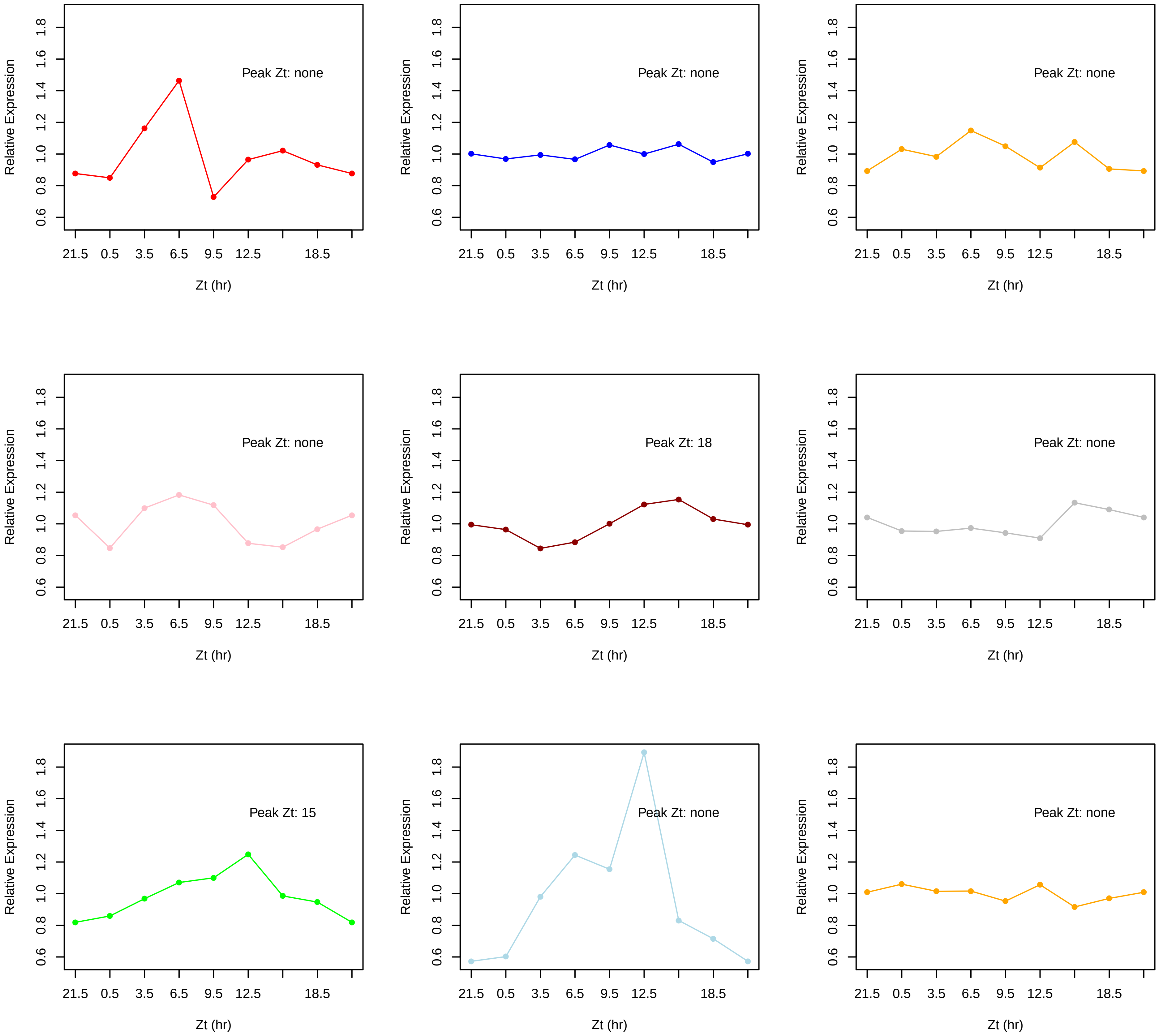

Singleton: ENSXMAG00000016873 cfap36

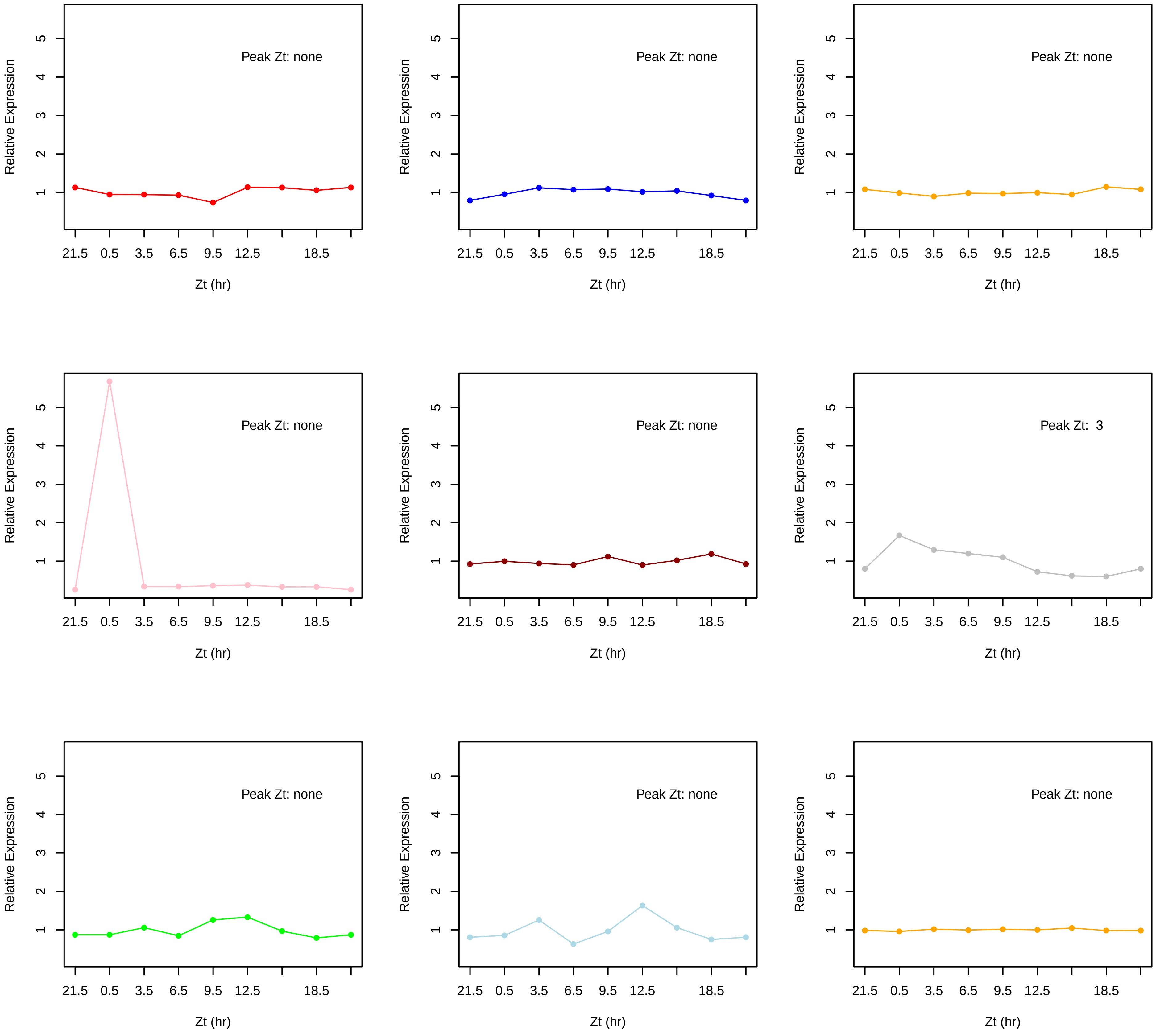

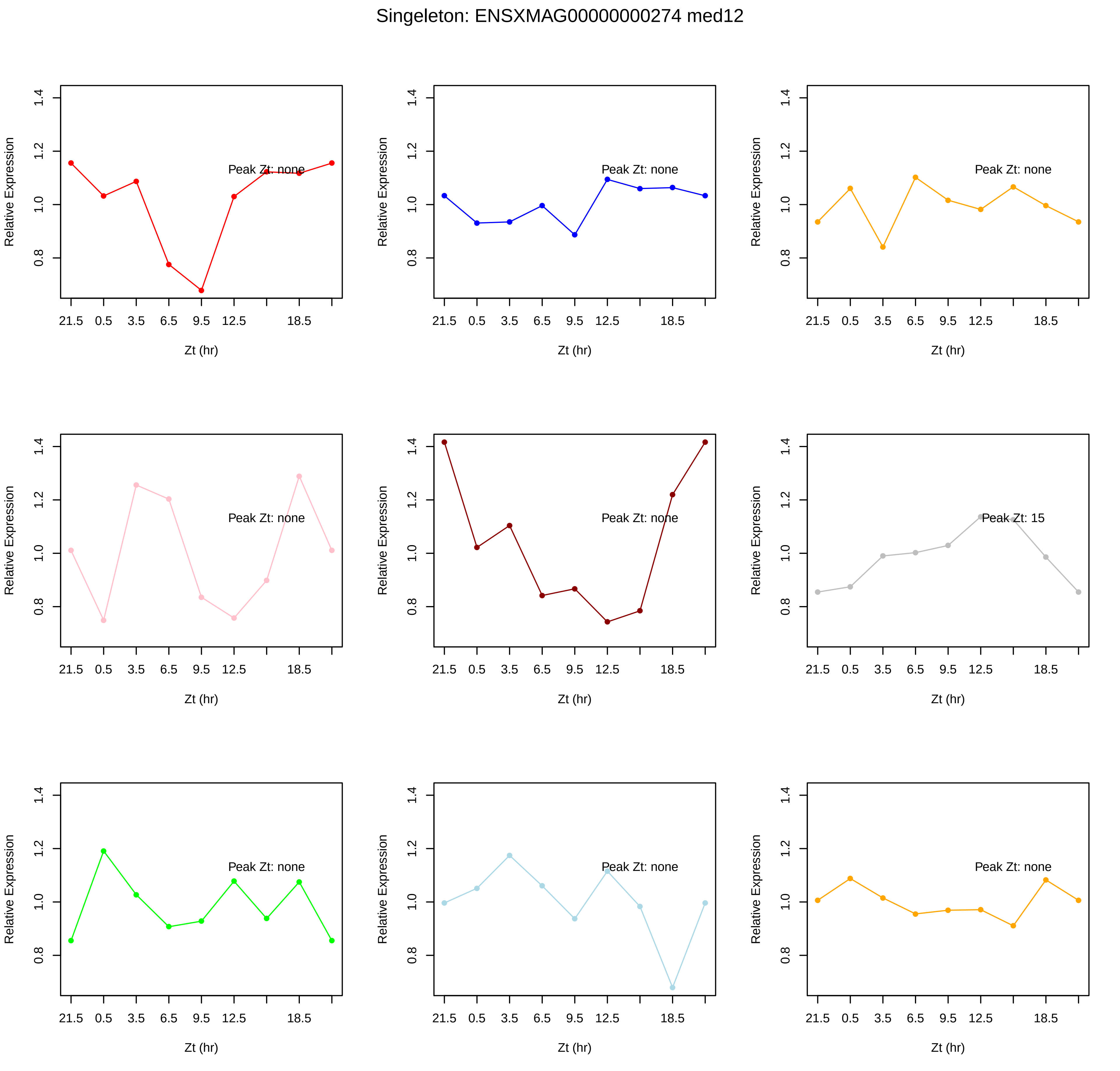
Singeleton: ENSXMAG00000000274 med12
Peak Zt: none
Peak Zt: none
Peak Zt: none
Peak Zt: none
Peak Zt: none
Peak Zt: 15
Peak Zt: none
Peak Zt: none
Peak Zt: none
Relative Expression
Zt (hr)
21.5
0.5
3.5
6.5
9.5
12.5
18.5
0.8
1.0
1.2
1.4

Singleton: ENSXMAG00000018174 cep170aa

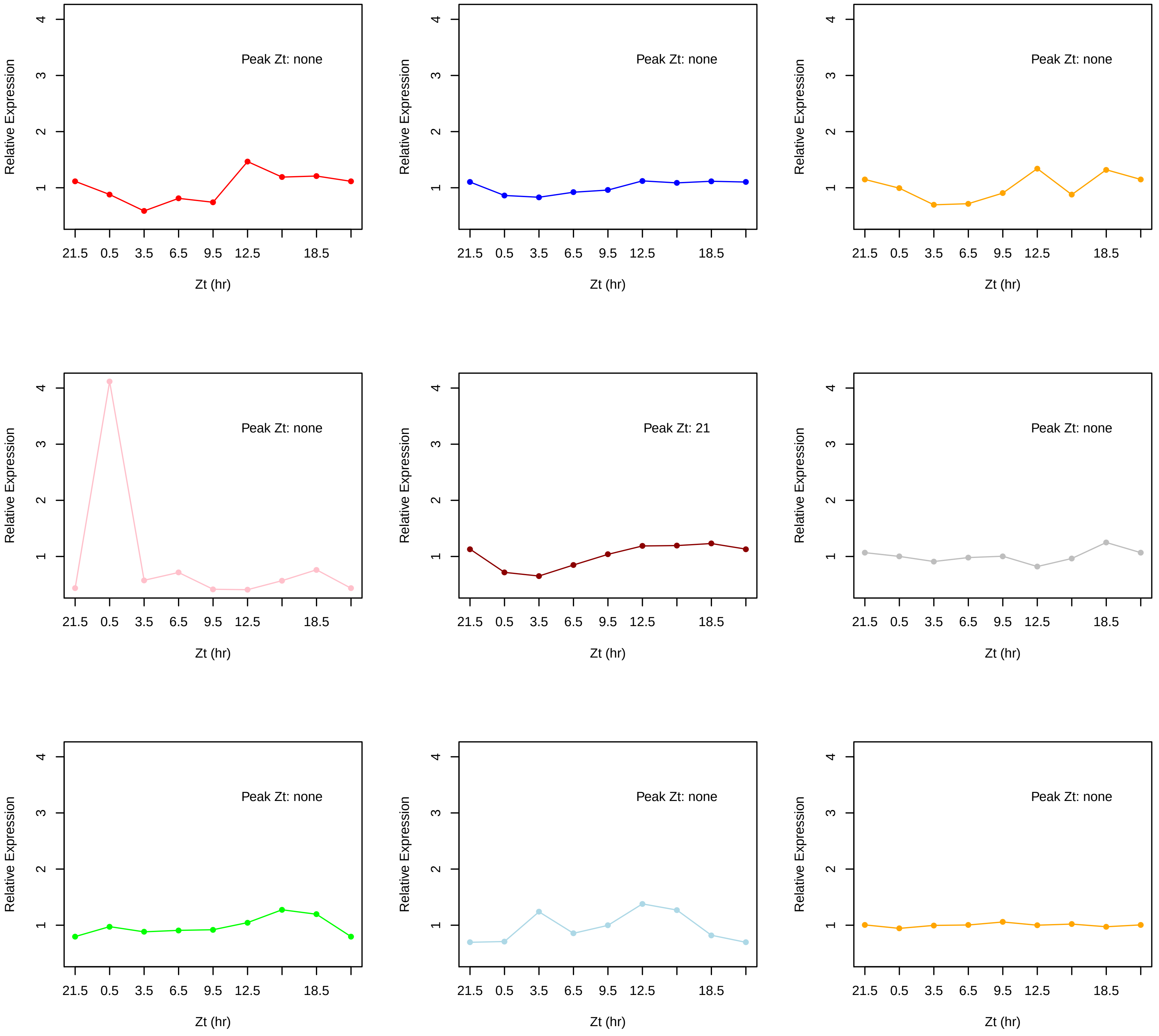

Singeleton: ENSXMAG00000024334 gys2

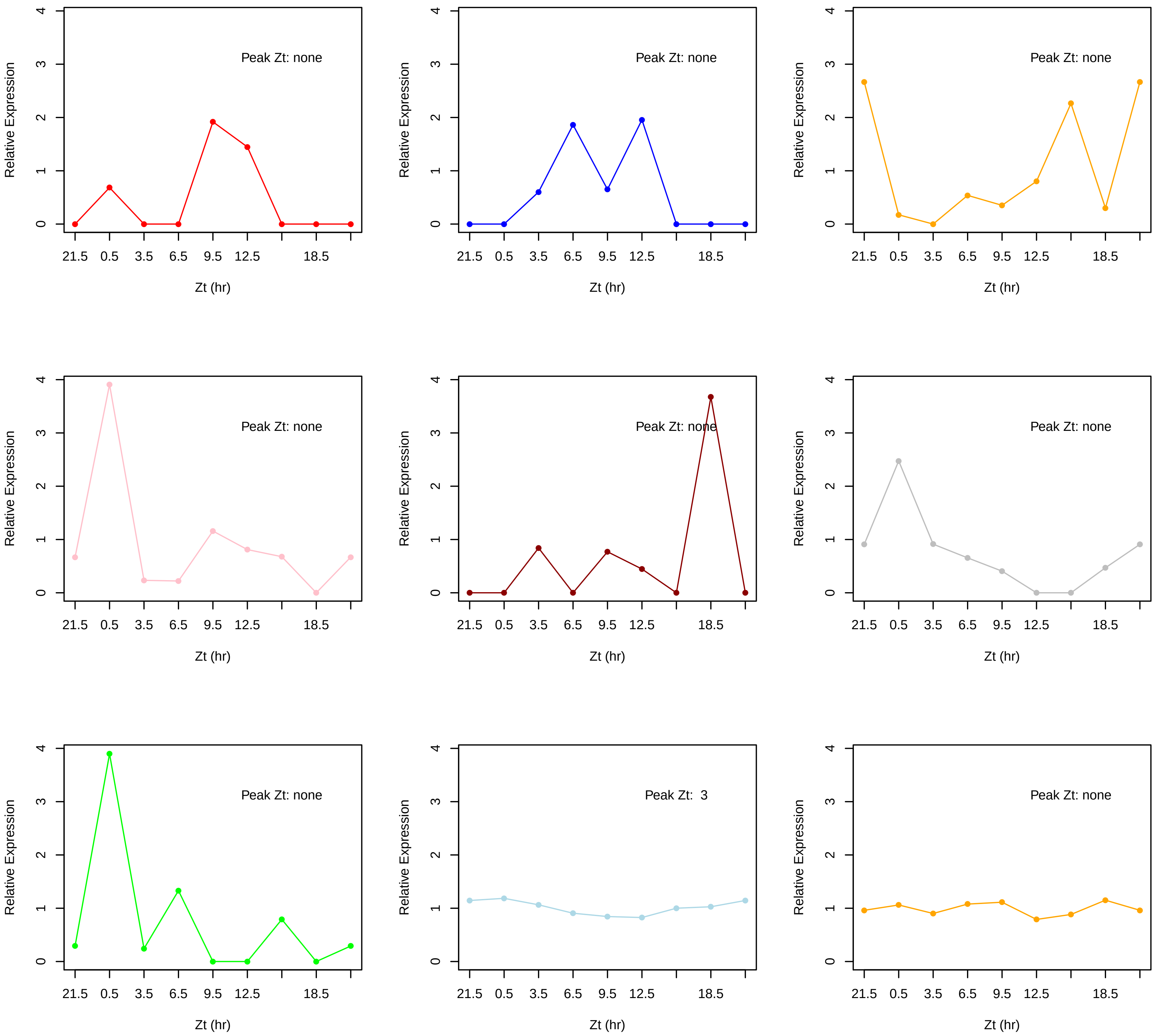

Singeleton: ENSXMAG00000015000 nudt18

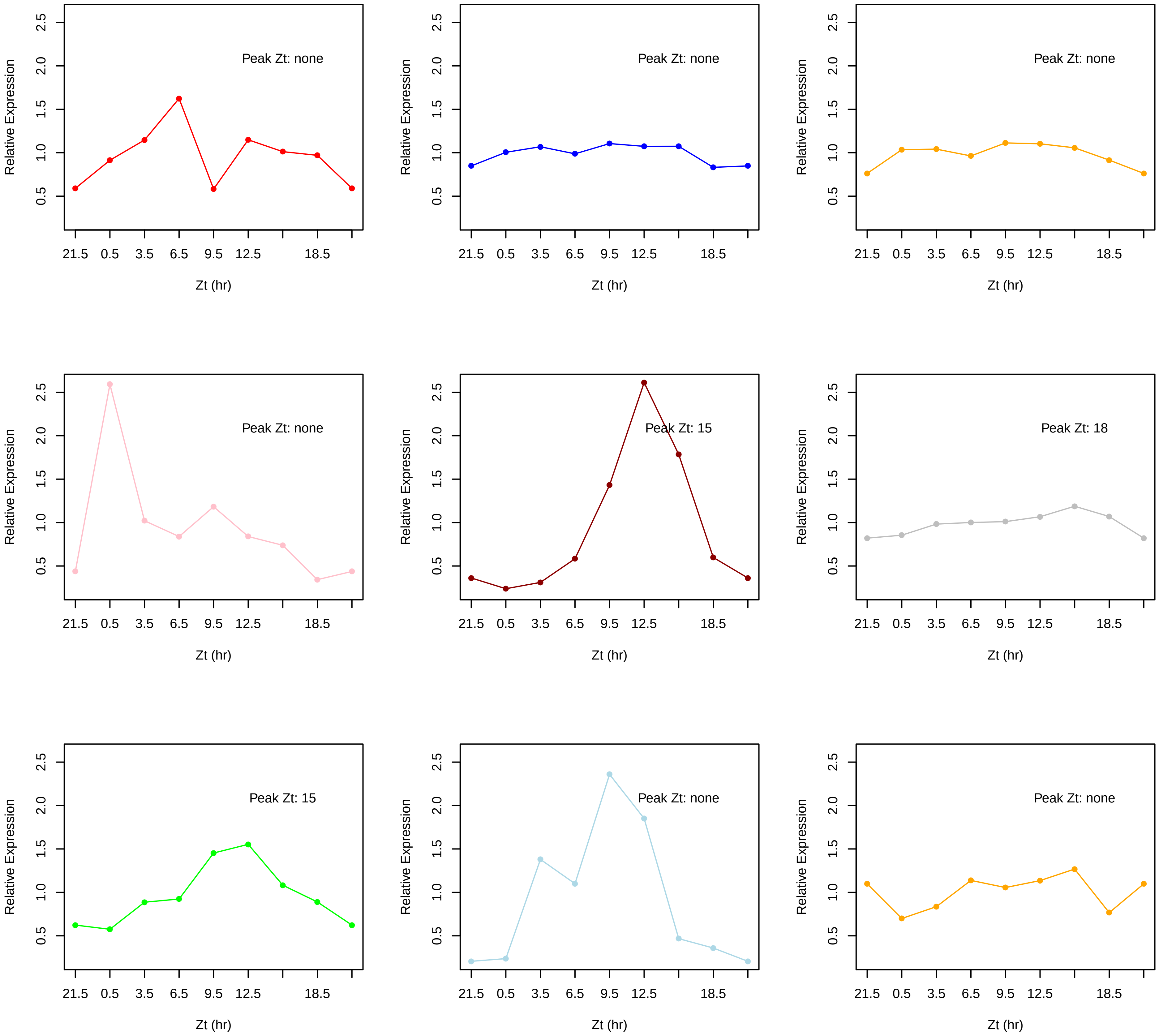

Singeleton: ENSXMAG00000000901 adam9

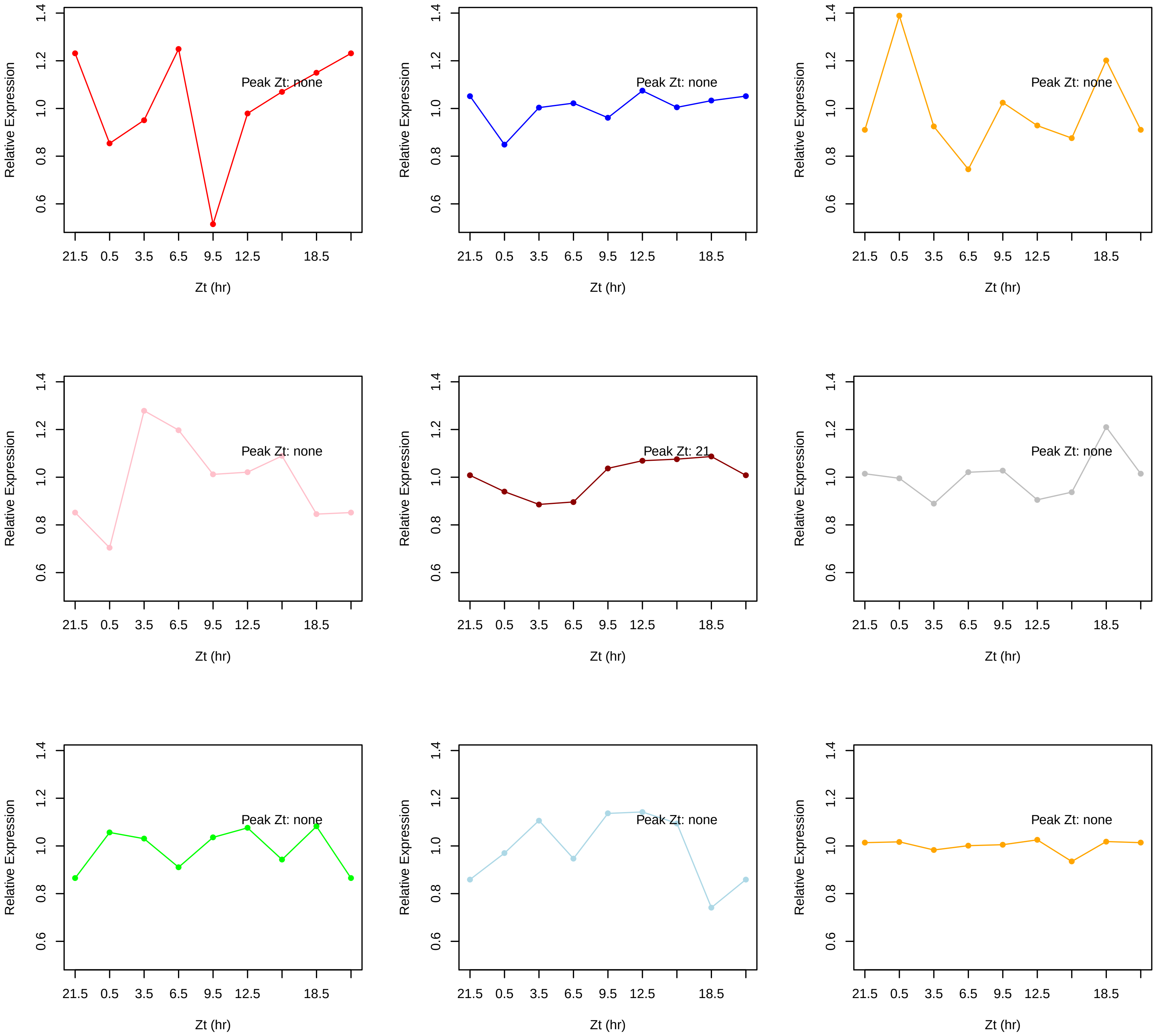

Singeleton: ENSXMAG00000018224 sdccag8

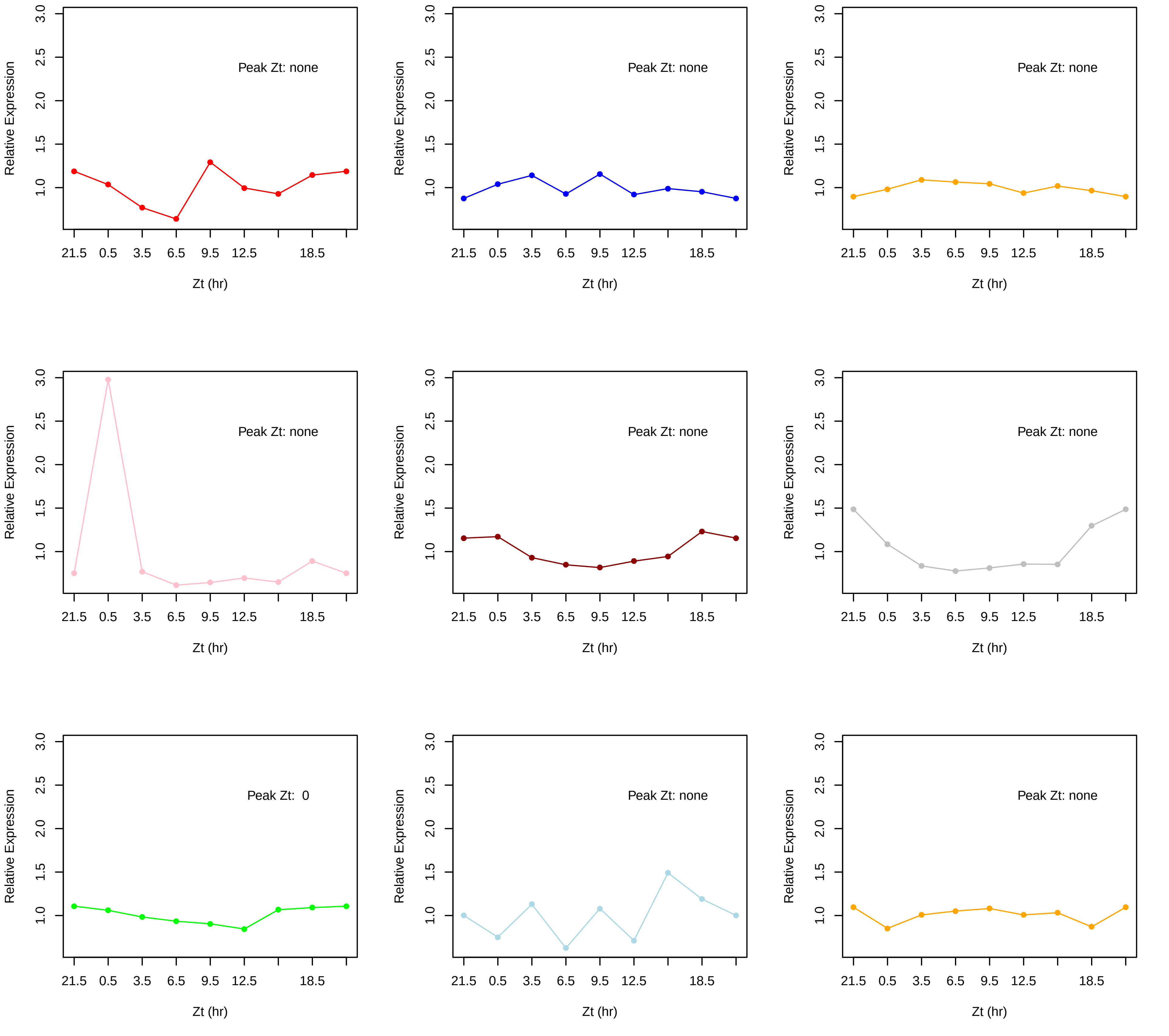

Singeleton: ENSXMAG00000029316 recql

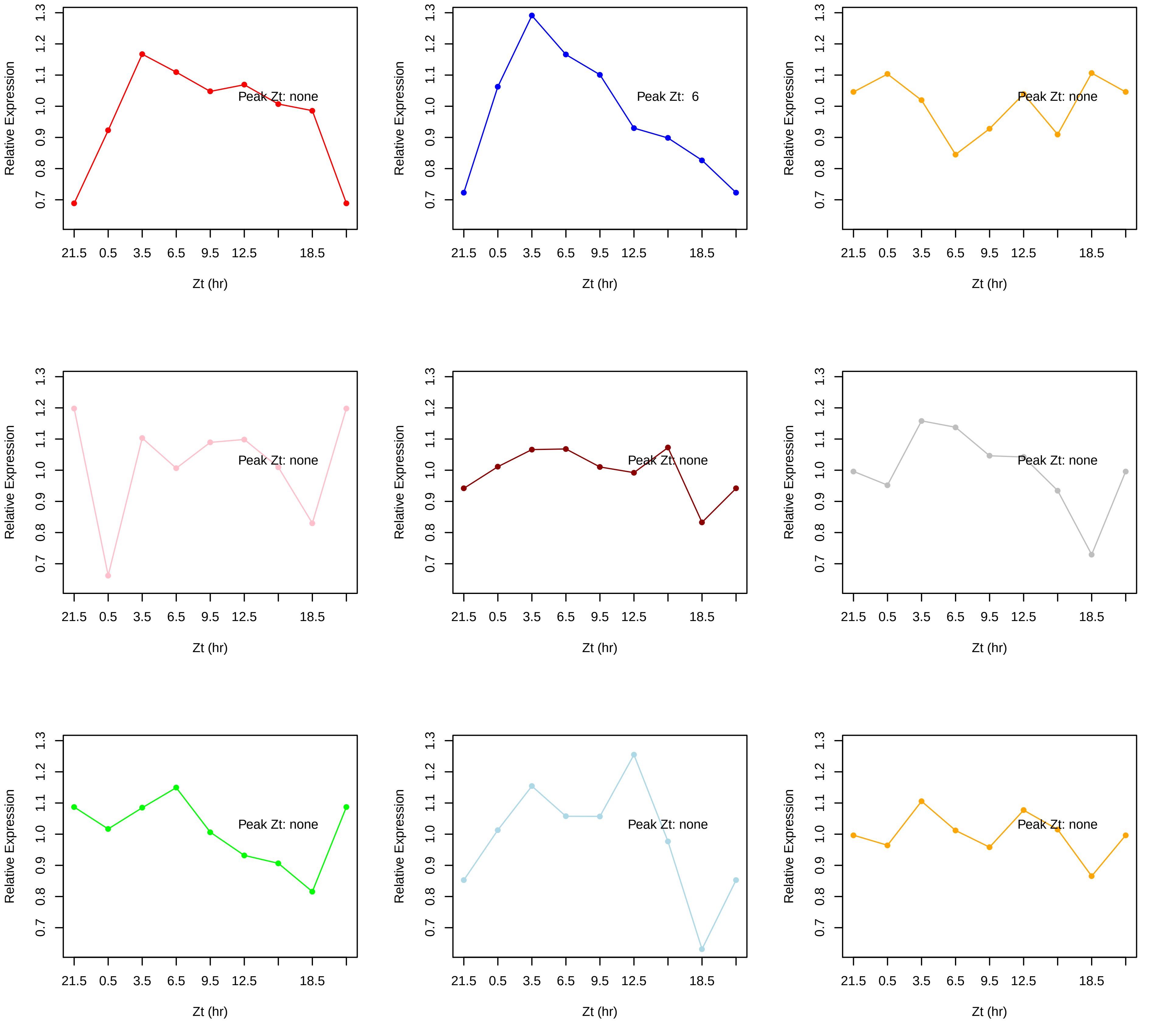

Singeleton: ENSXMAG00000006587 pyroxd1

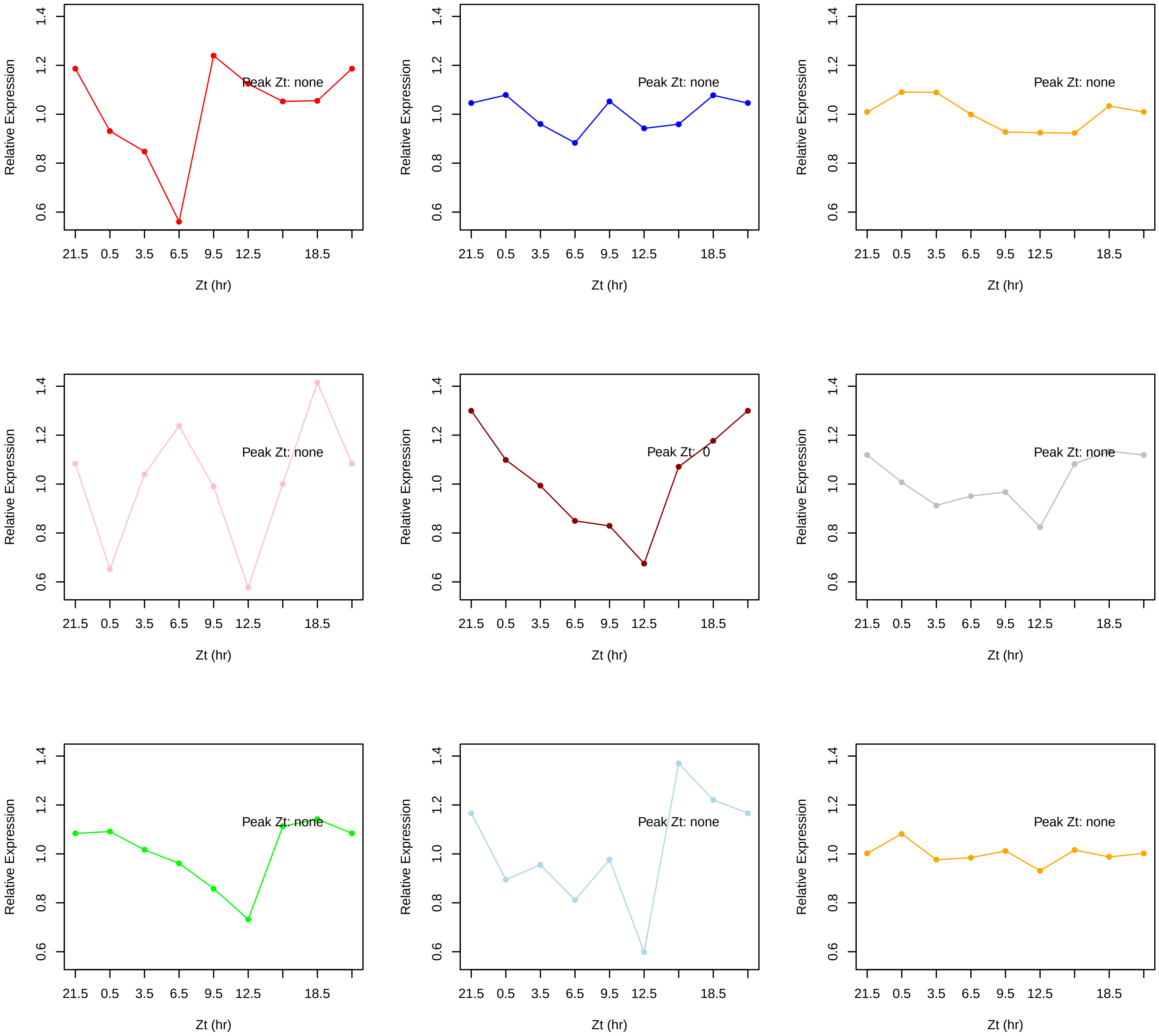

Singleton: ENSXMAG00000013812 akt3a

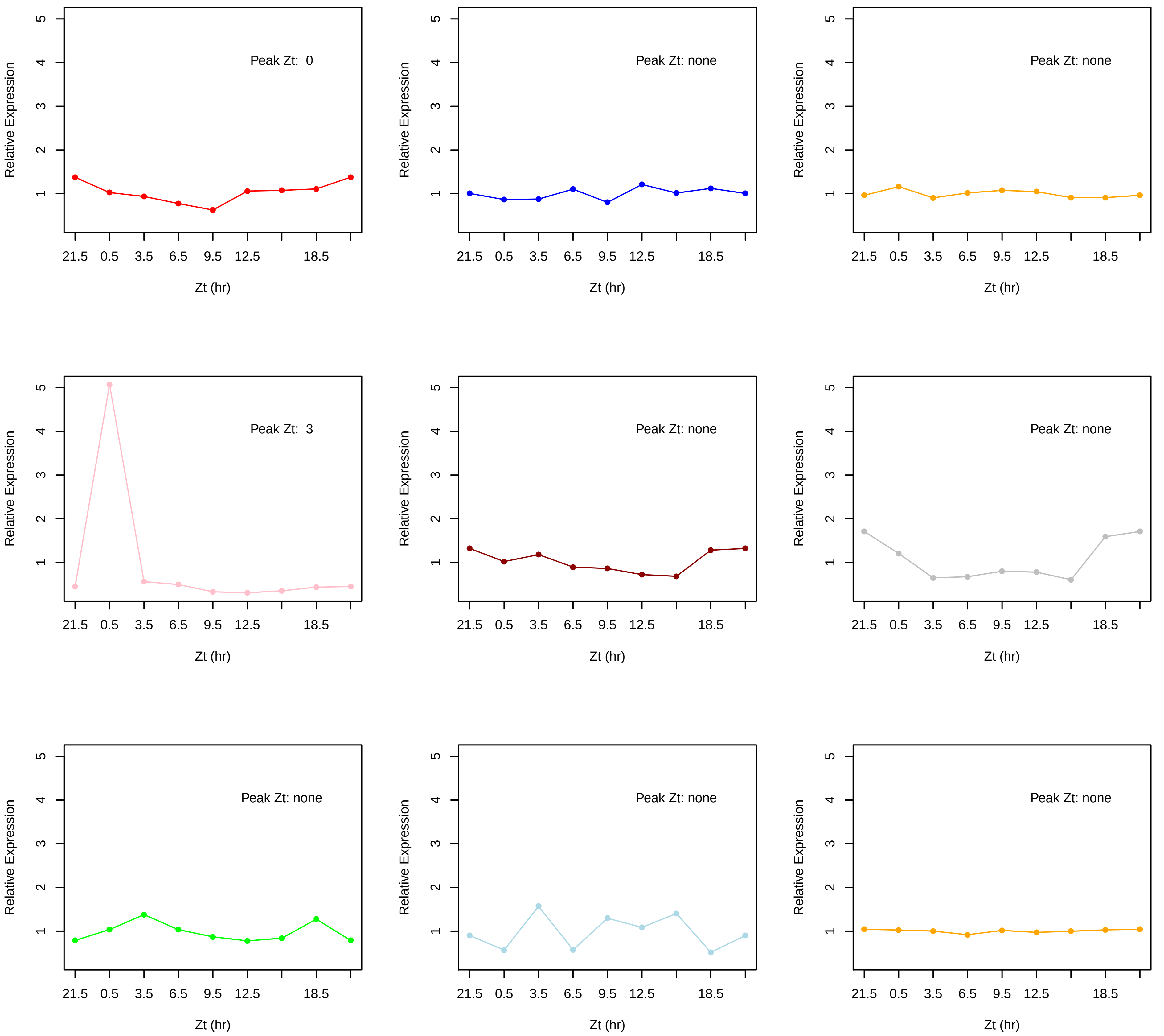

Singleton: ENSXMAG00000006603 slco1e1

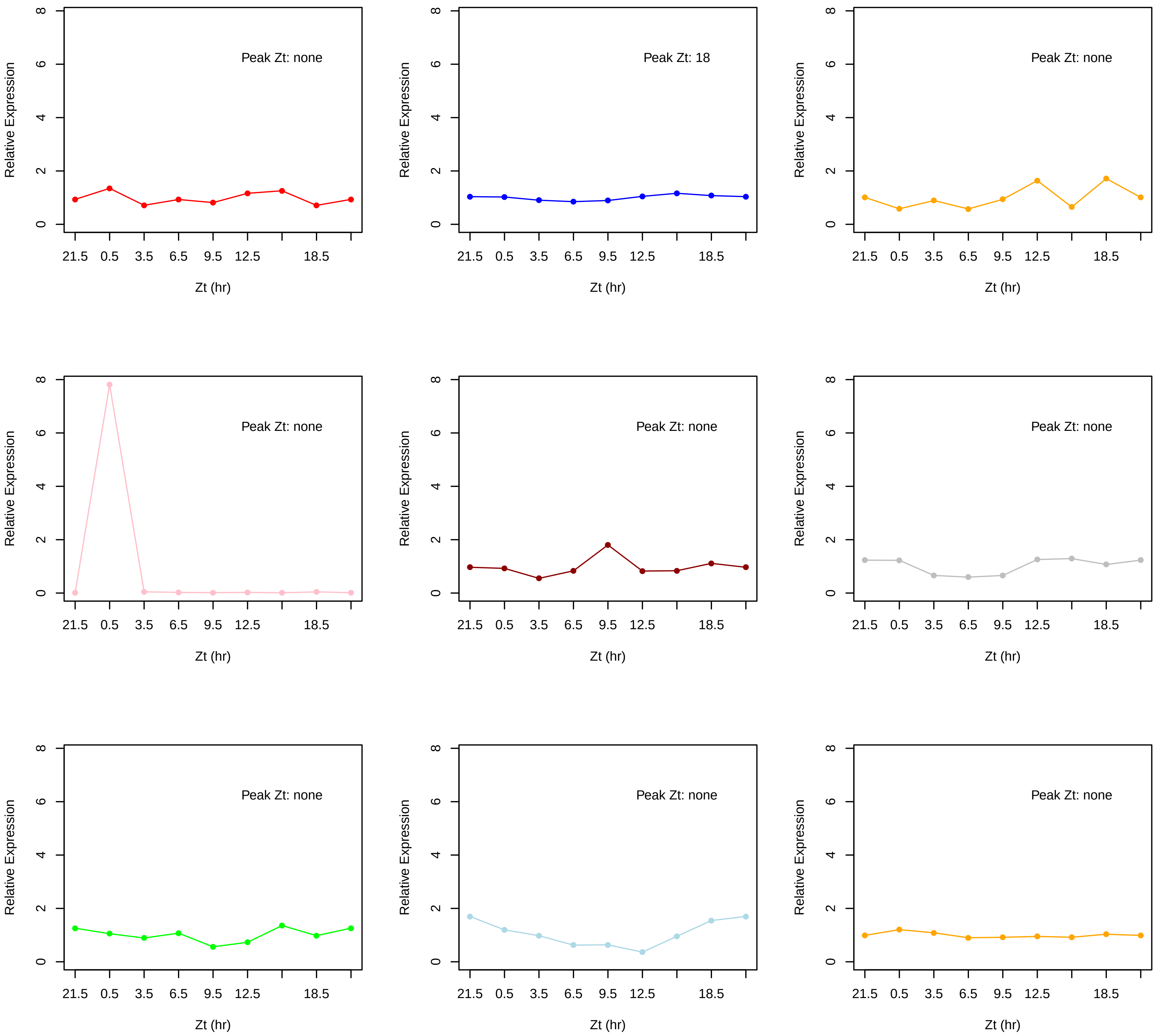

Singeleton: ENSXMAG00000015826 aff2

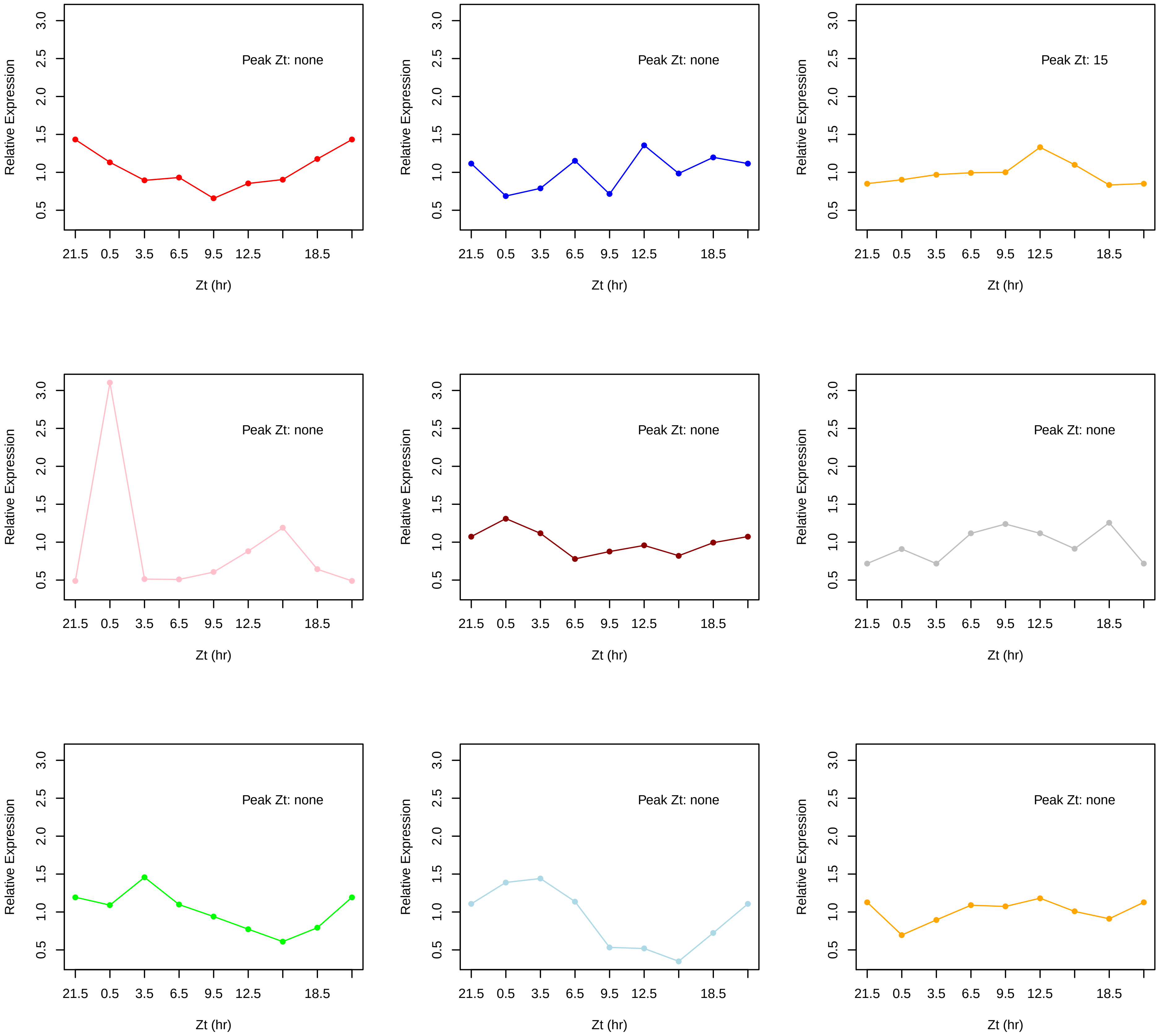

Singeleton: ENSXMAG00000017961 atad1a

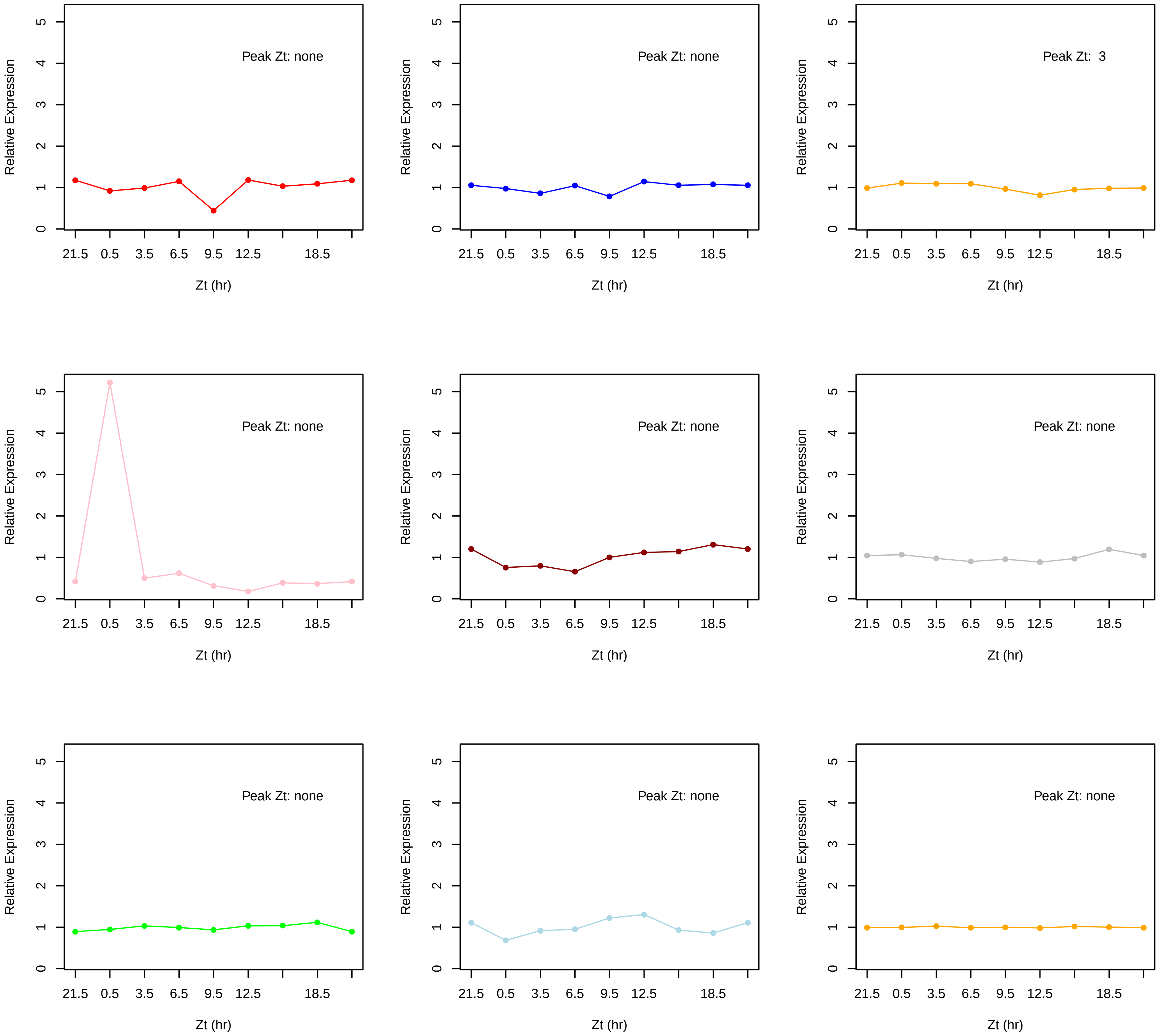

Singleton: ENSXMAG00000024233

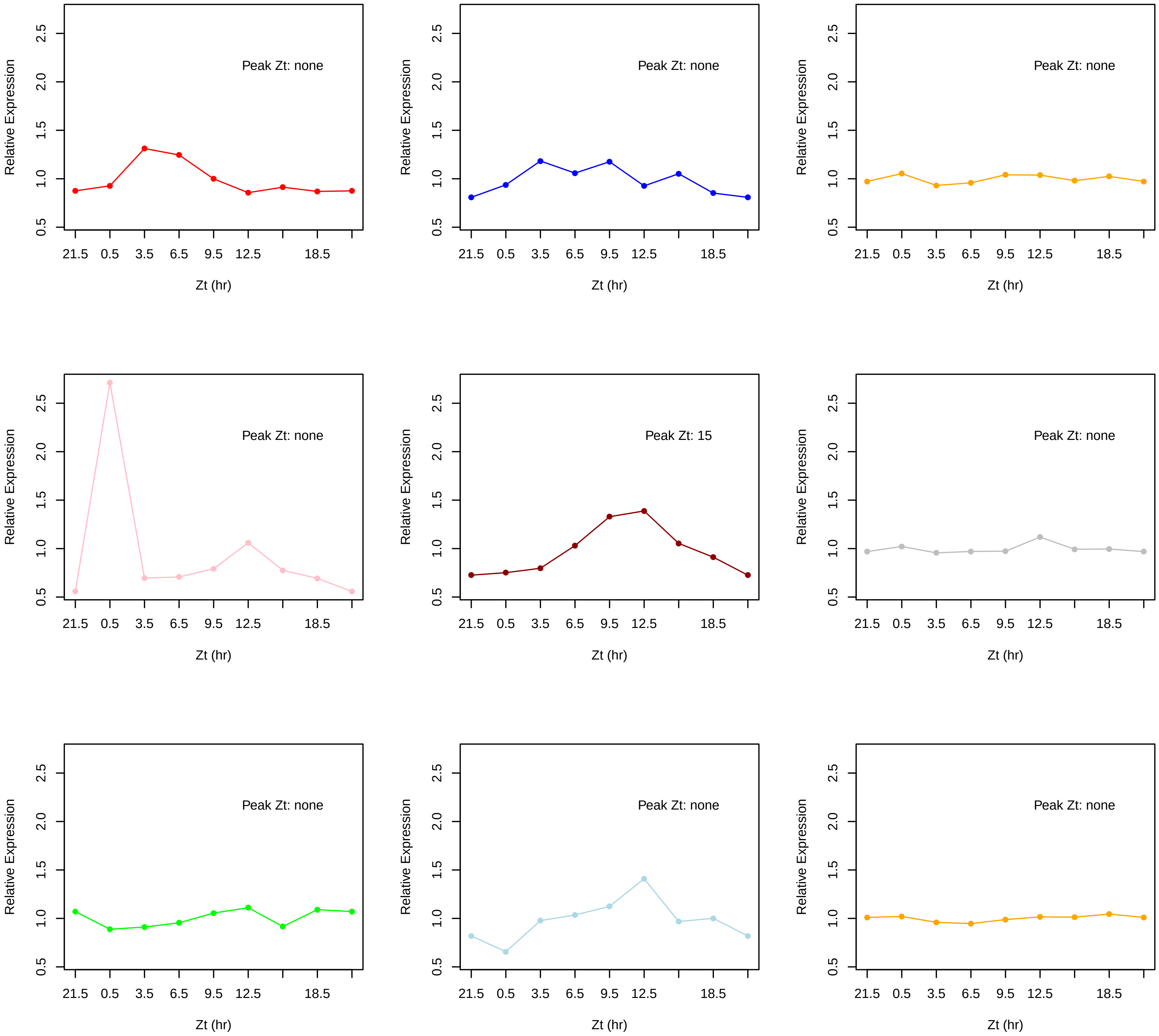

Singleton: ENSXMAG00000012602 klhl13

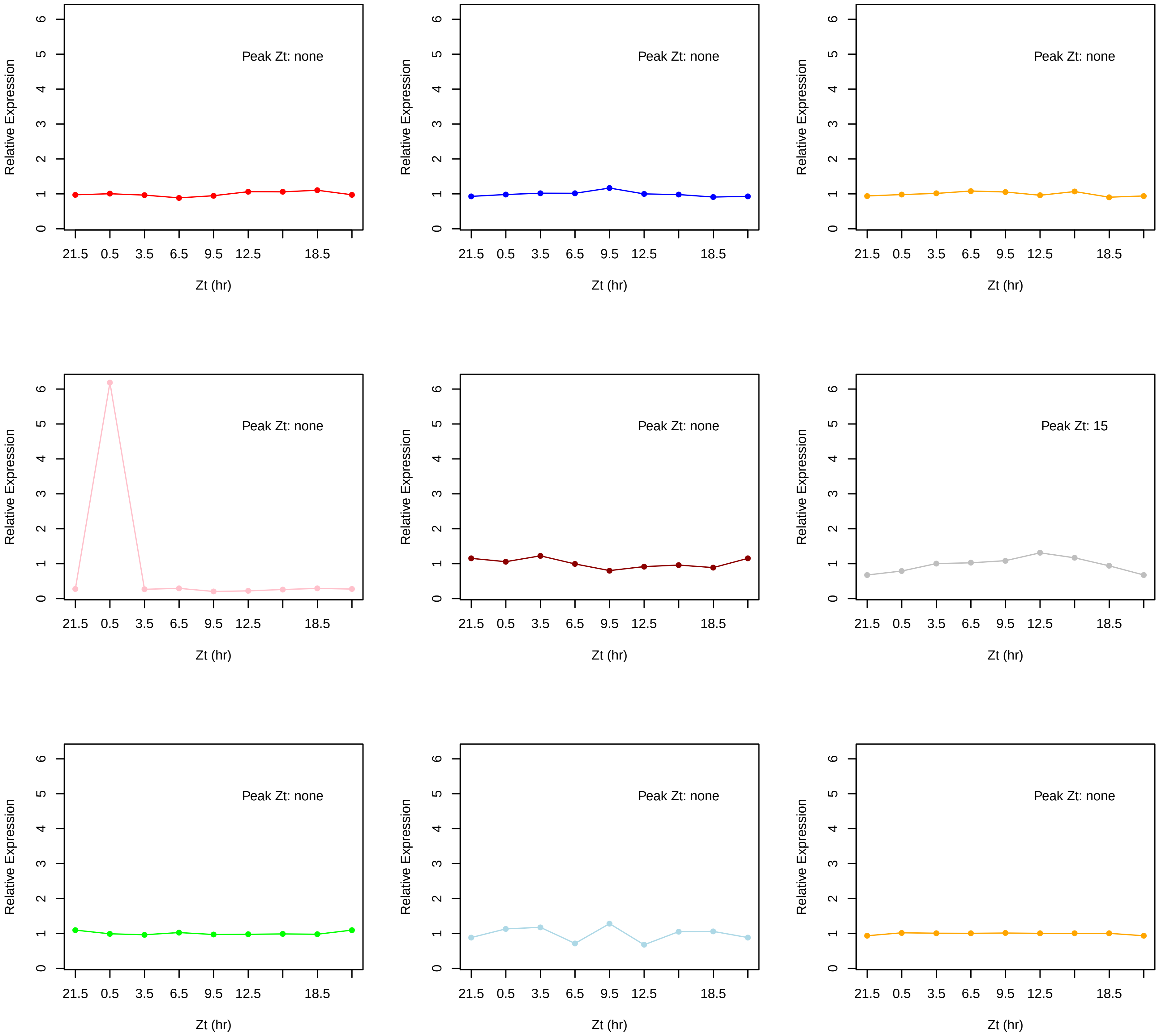

Singeleton: ENSXMAG00000000406 ppp3ccb

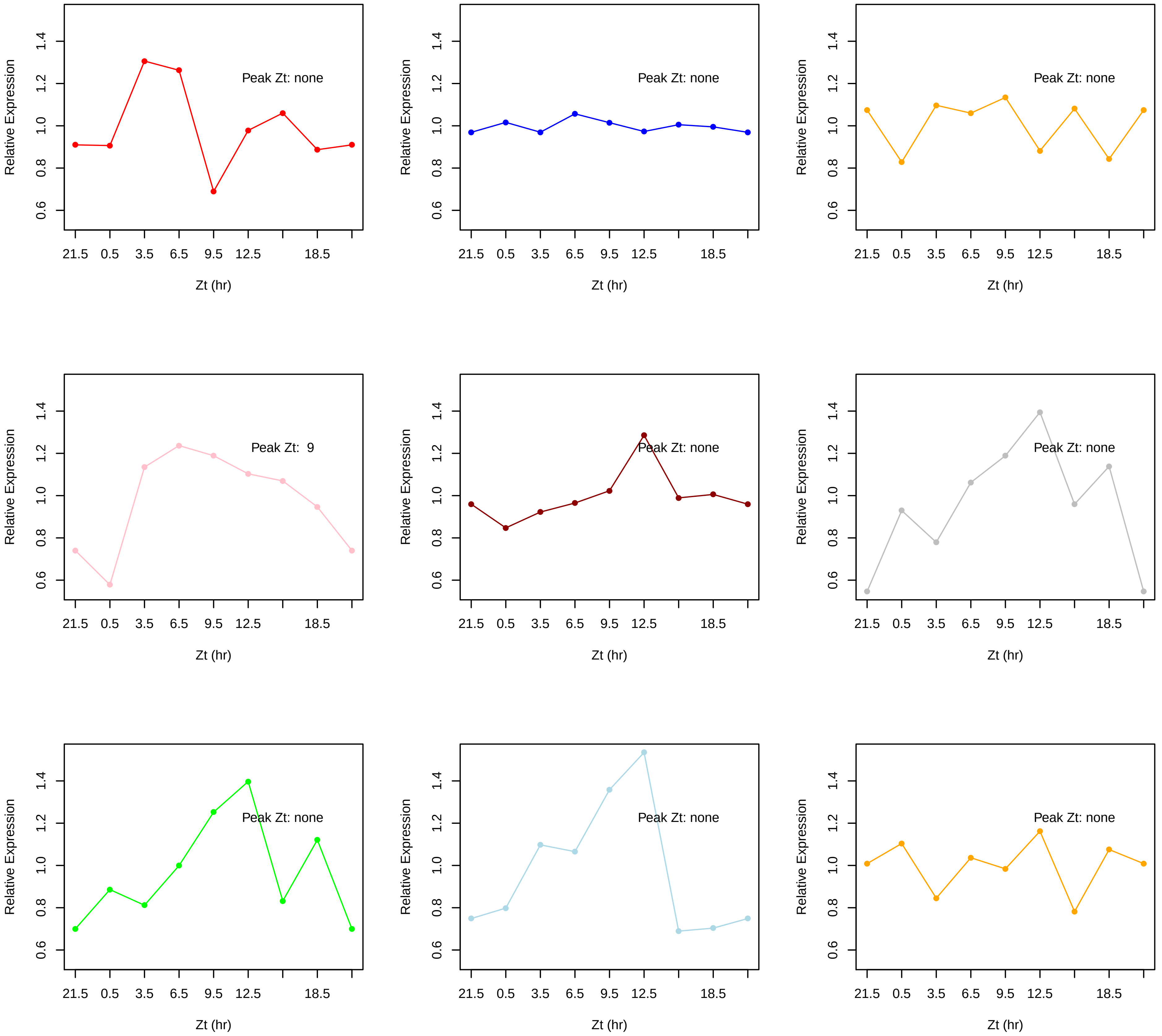

Singeleton: ENSXMAG00000026936 tnfaip3

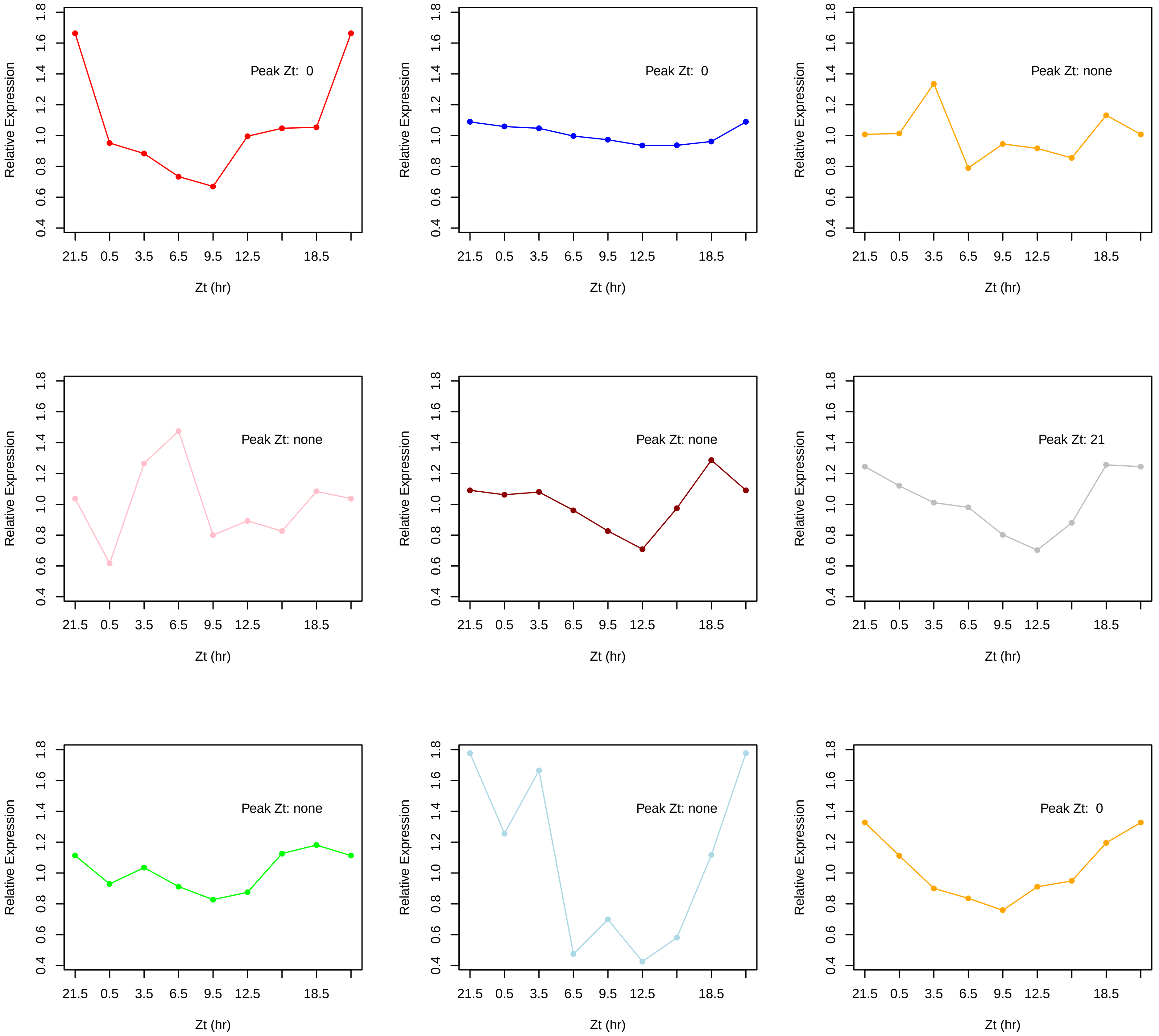

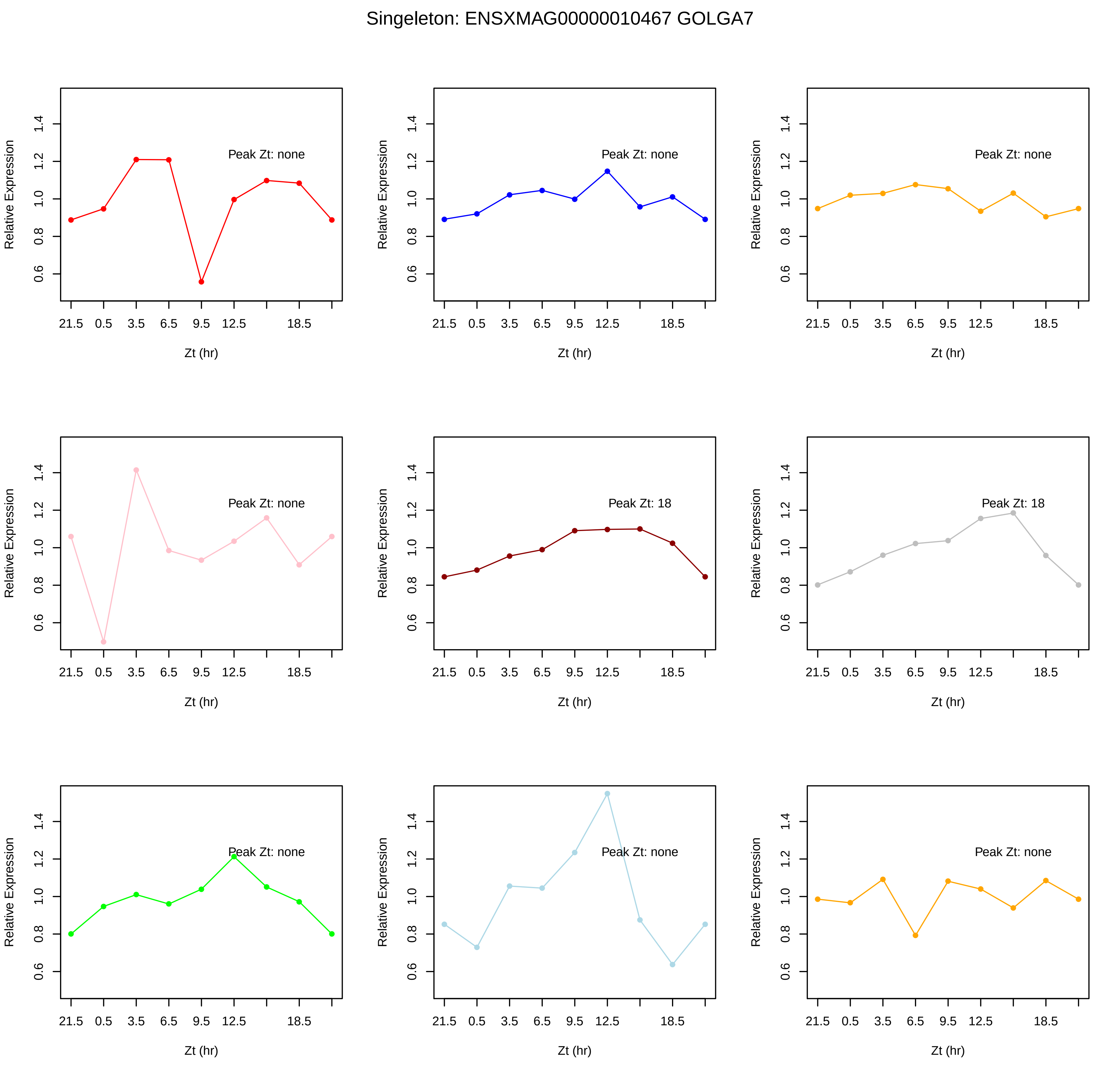

Singleton: ENSXMAG00000010467 GOLGA7
Relative Expression
Zt (hr)
Peak Zt: none
Peak Zt: none
Peak Zt: none
Peak Zt: none
Peak Zt: 18
Peak Zt: 18
Peak Zt: none
Peak Zt: none
Peak Zt: none
21.5
0.5
3.5
6.5
9.5
12.5
18.5
0.6
0.8
1.0
1.2
1.4

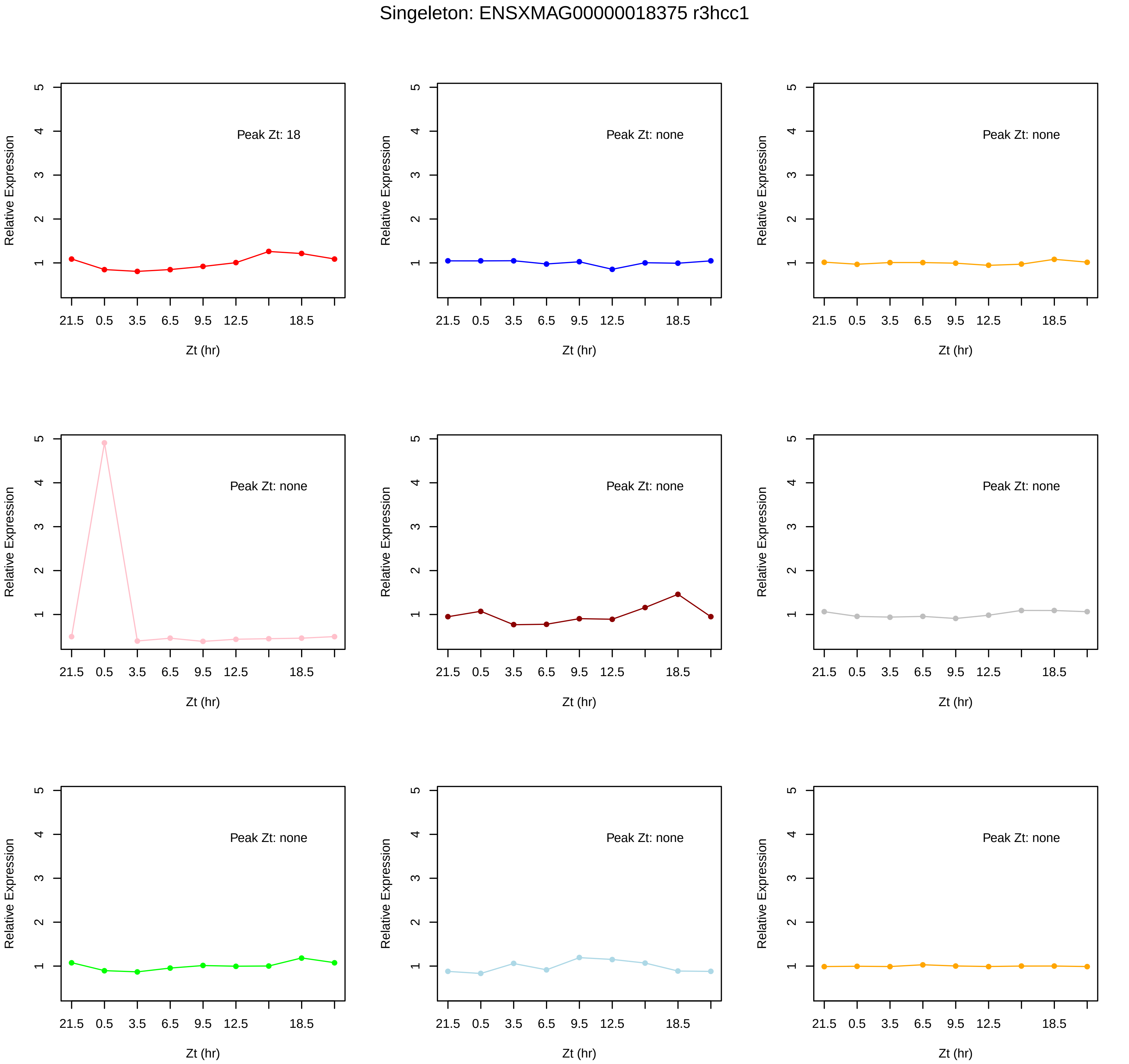
Singeleton: ENSXMAG00000018375 r3hcc1
Peak Zt: 18
Peak Zt: none
Peak Zt: none
Peak Zt: none
Peak Zt: none
Peak Zt: none
Peak Zt: none
Peak Zt: none
Peak Zt: none
Relative Expression
Zt (hr)
21.5
0.5
3.5
6.5
9.5
12.5
18.5
1
2
3
4
5

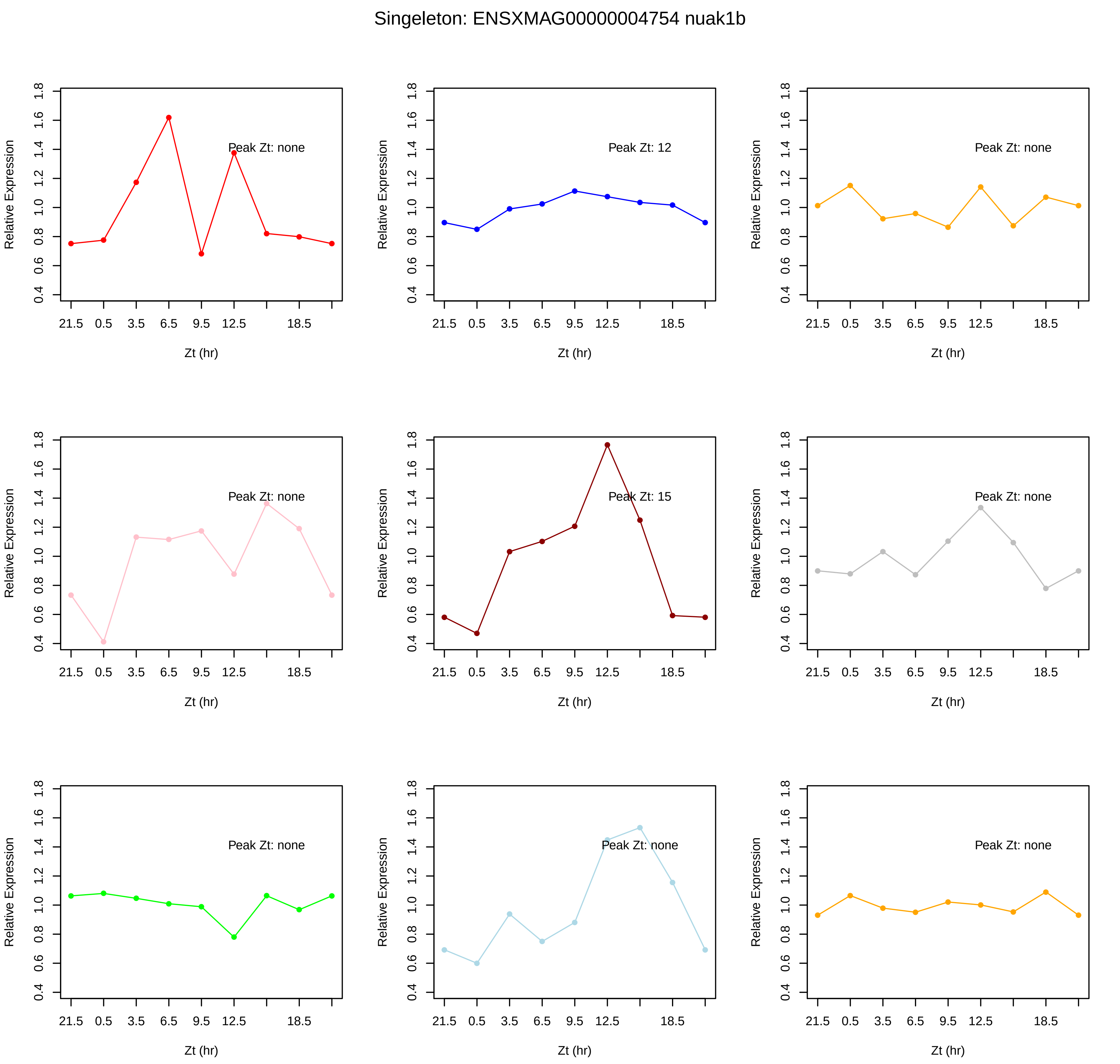
Singeleton: ENSXMAG00000004754 nuak1b
Peak Zt: none
Peak Zt: 12
Peak Zt: none
Peak Zt: none
Peak Zt: 15
Peak Zt: none
Peak Zt: none
Peak Zt: none
Peak Zt: none
Relative Expression
Zt (hr)
21.5
0.5
3.5
6.5
9.5
12.5
18.5
0.4
0.6
0.8
1.0
1.2
1.4
1.6
1.8

Singeleton: ENSXMAG00000022102 pgbd5

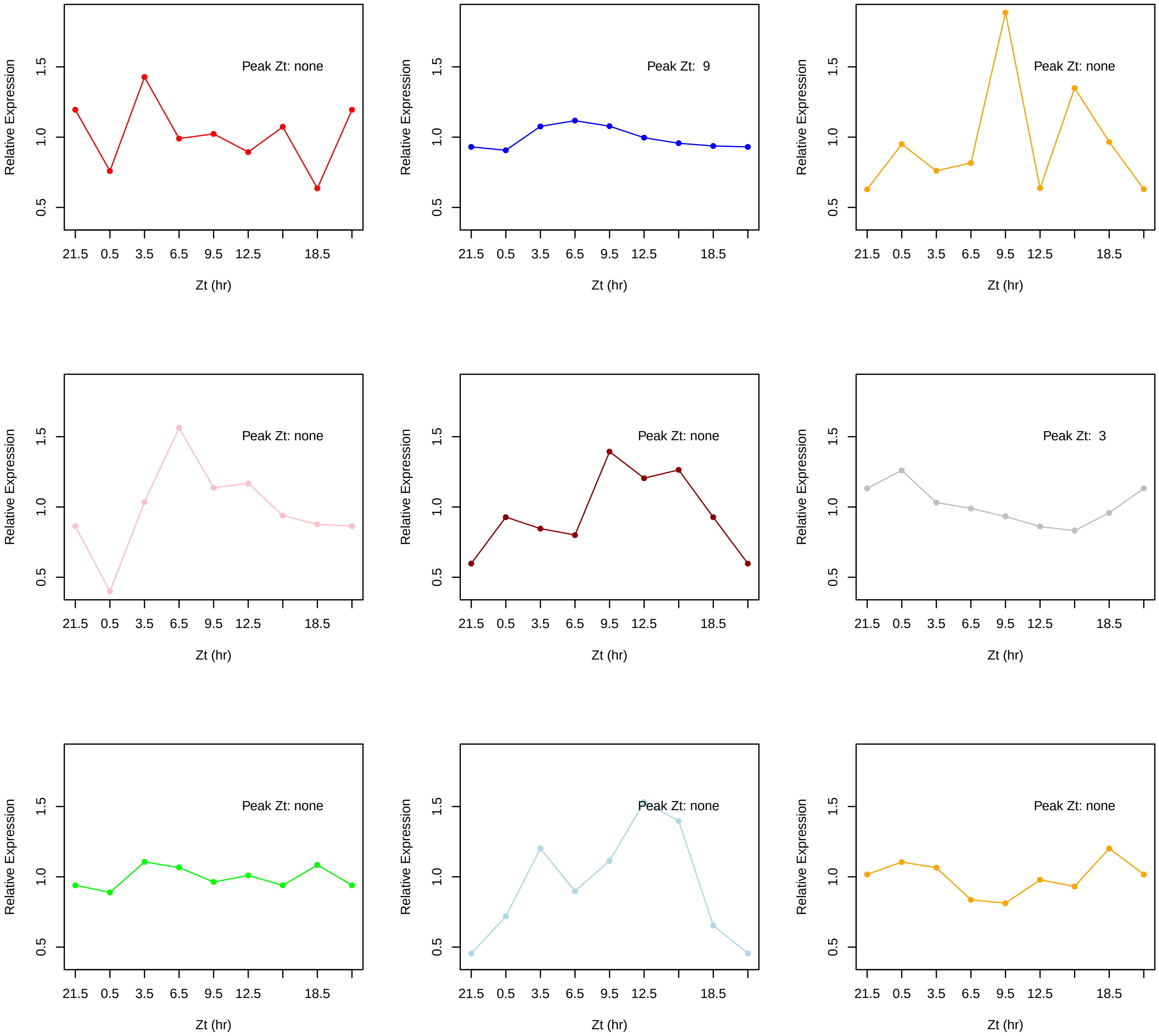

Singleton: ENSXMAG00000015073 galnt2

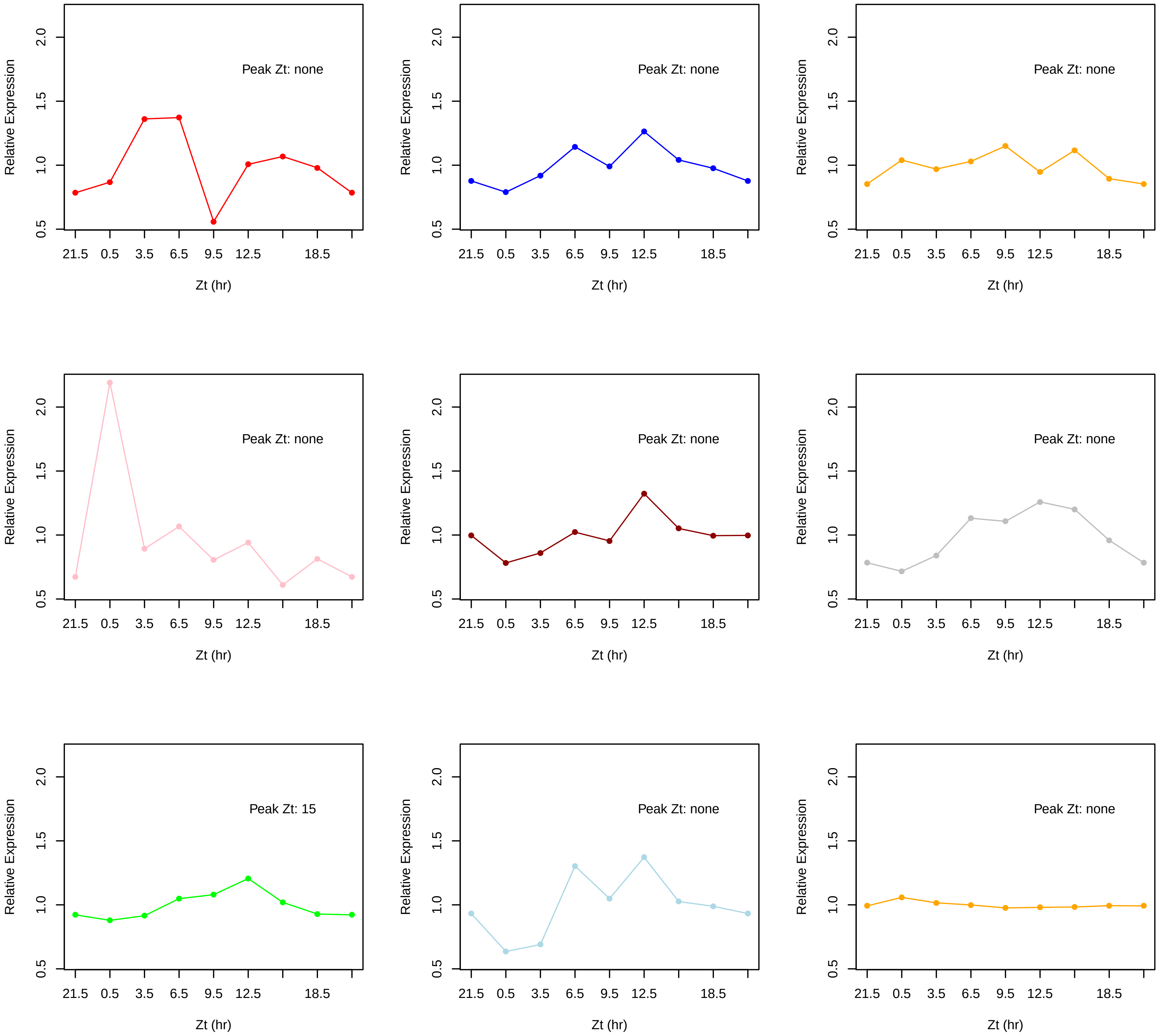

Singeleton: ENSXMAG00000023303 taf5l

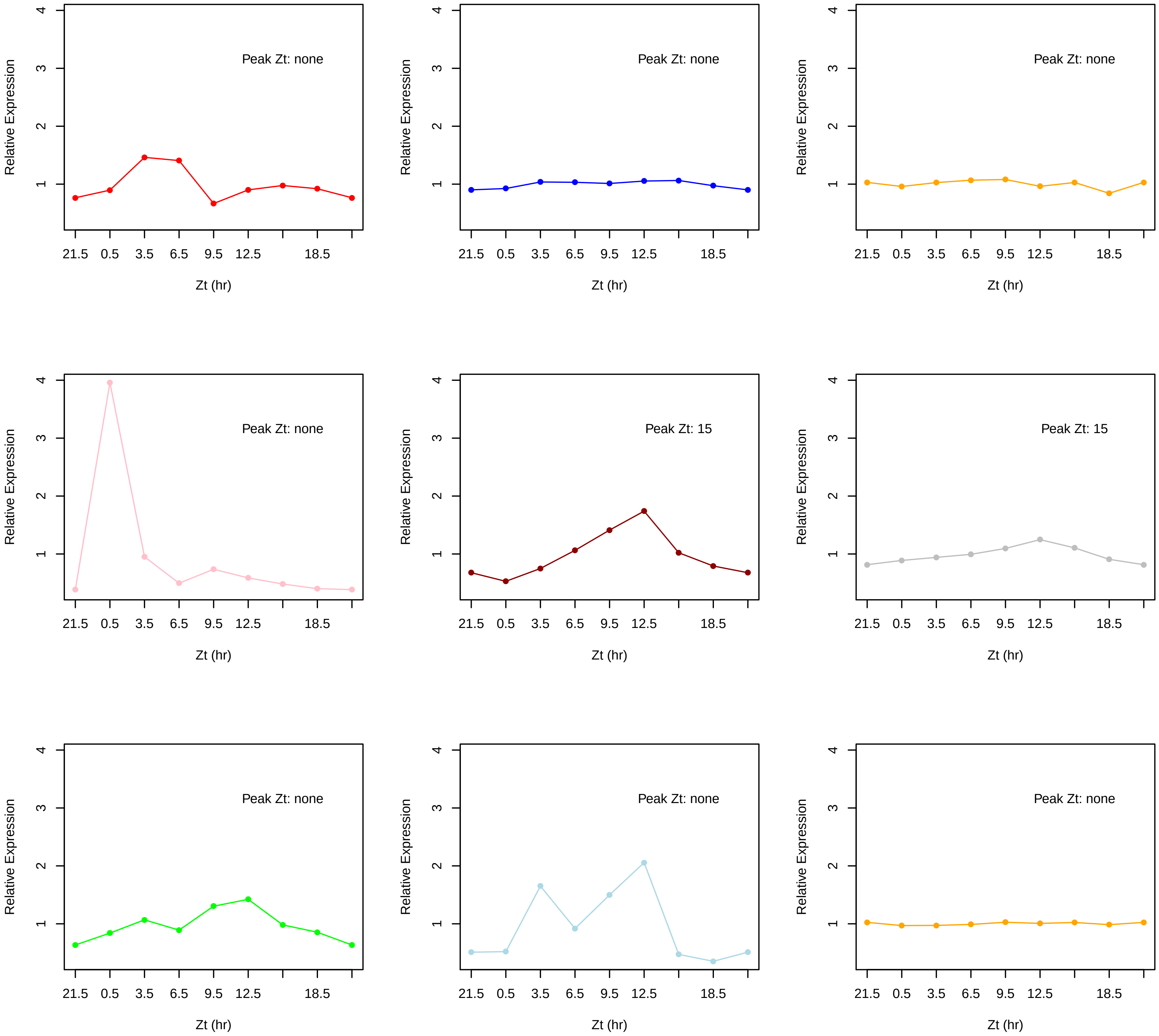

Singeleton: ENSXMAG00000000254

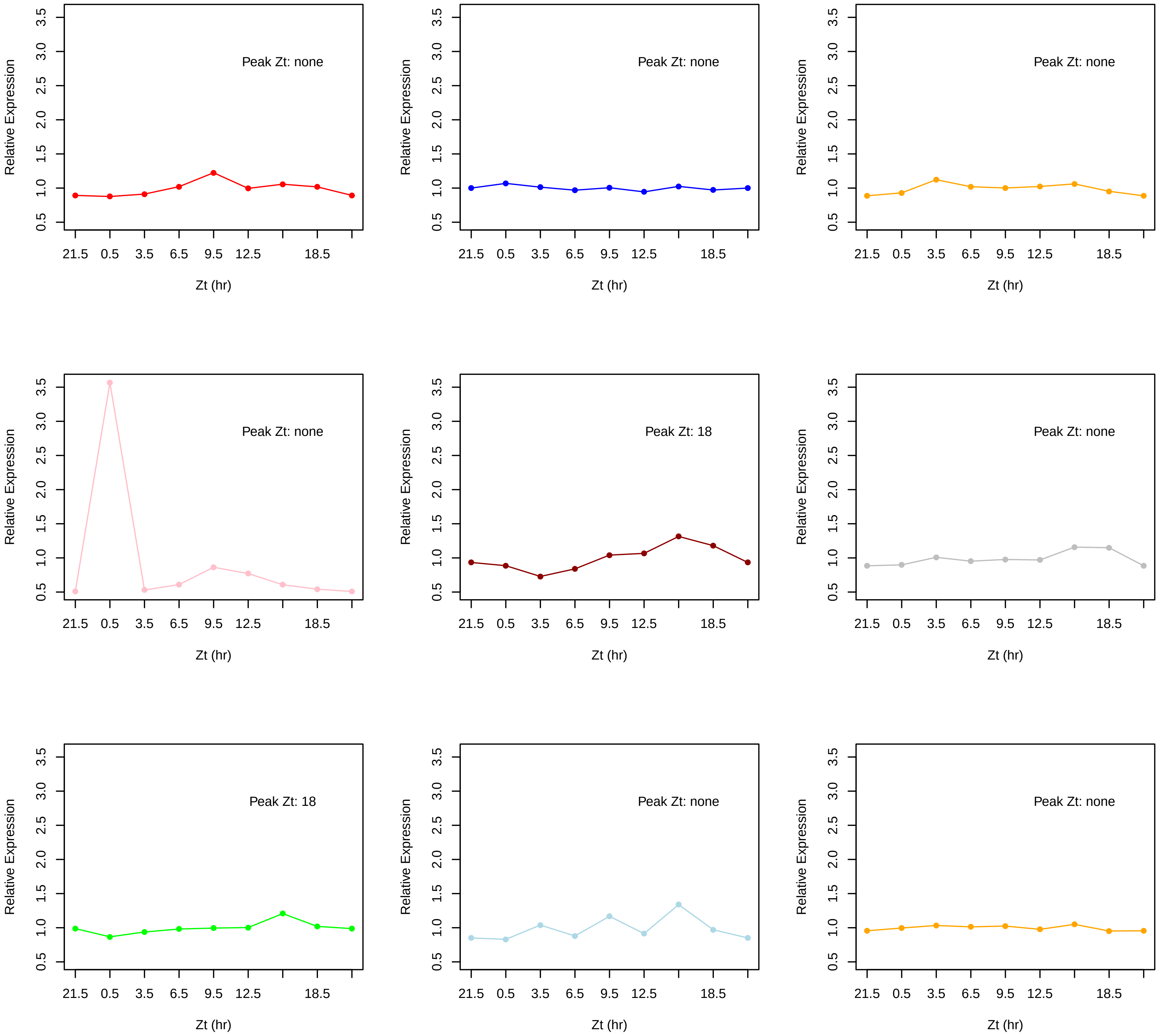

Singeleton: ENSXMAG00000014130 nup133

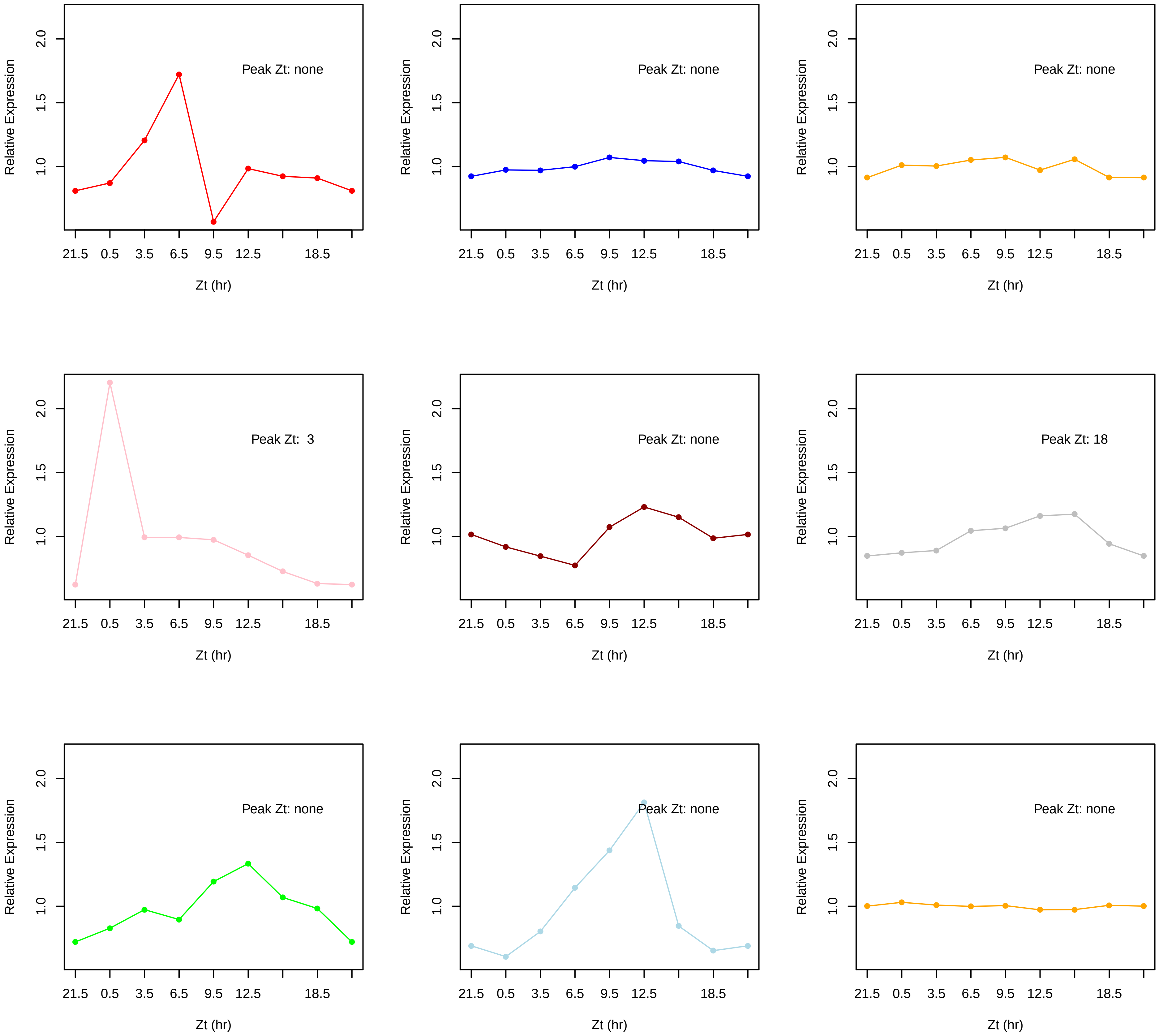

Singleton: ENSXMAG00000028378 guca1a

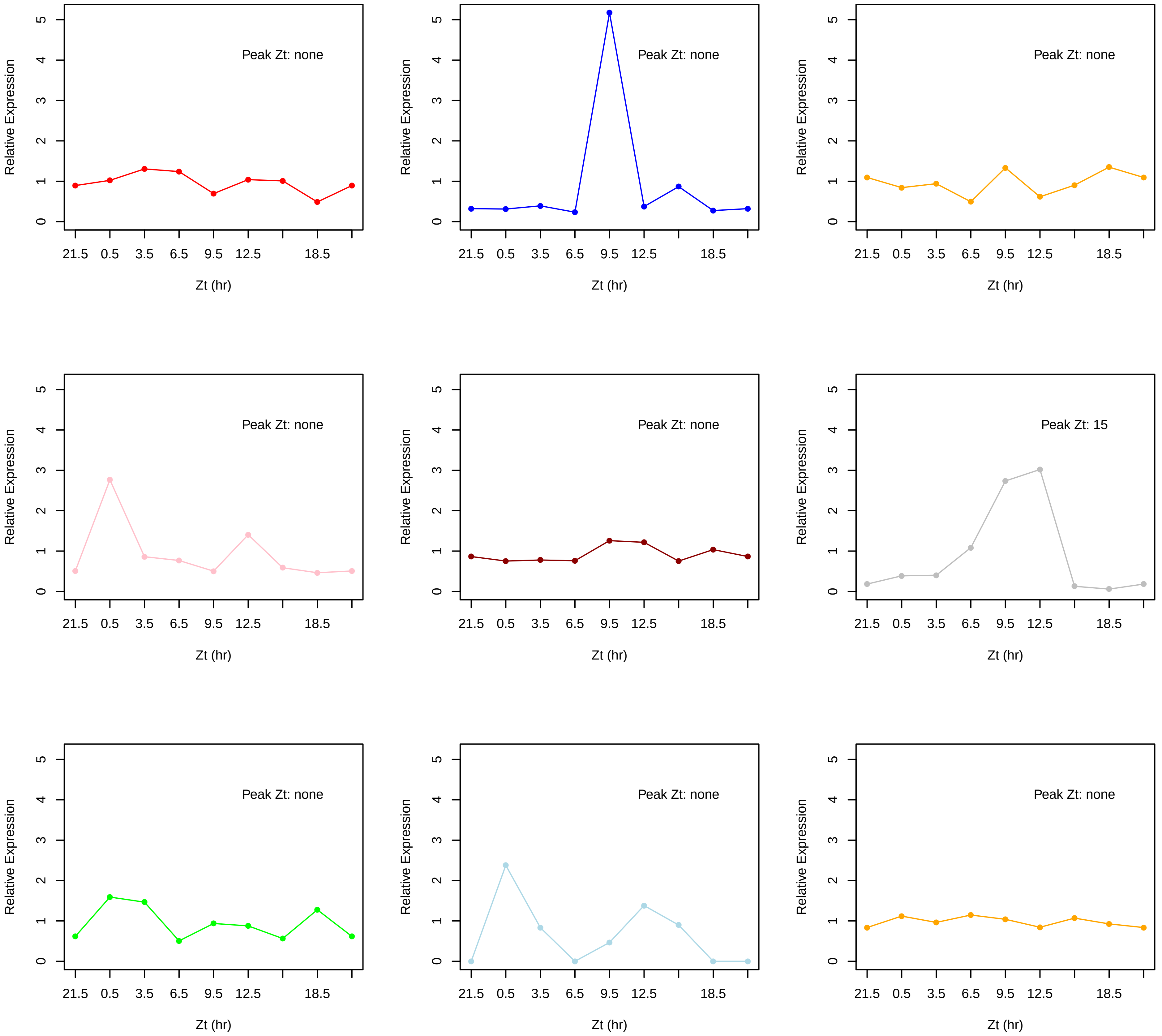

Singeleton: ENSXMAG00000030012

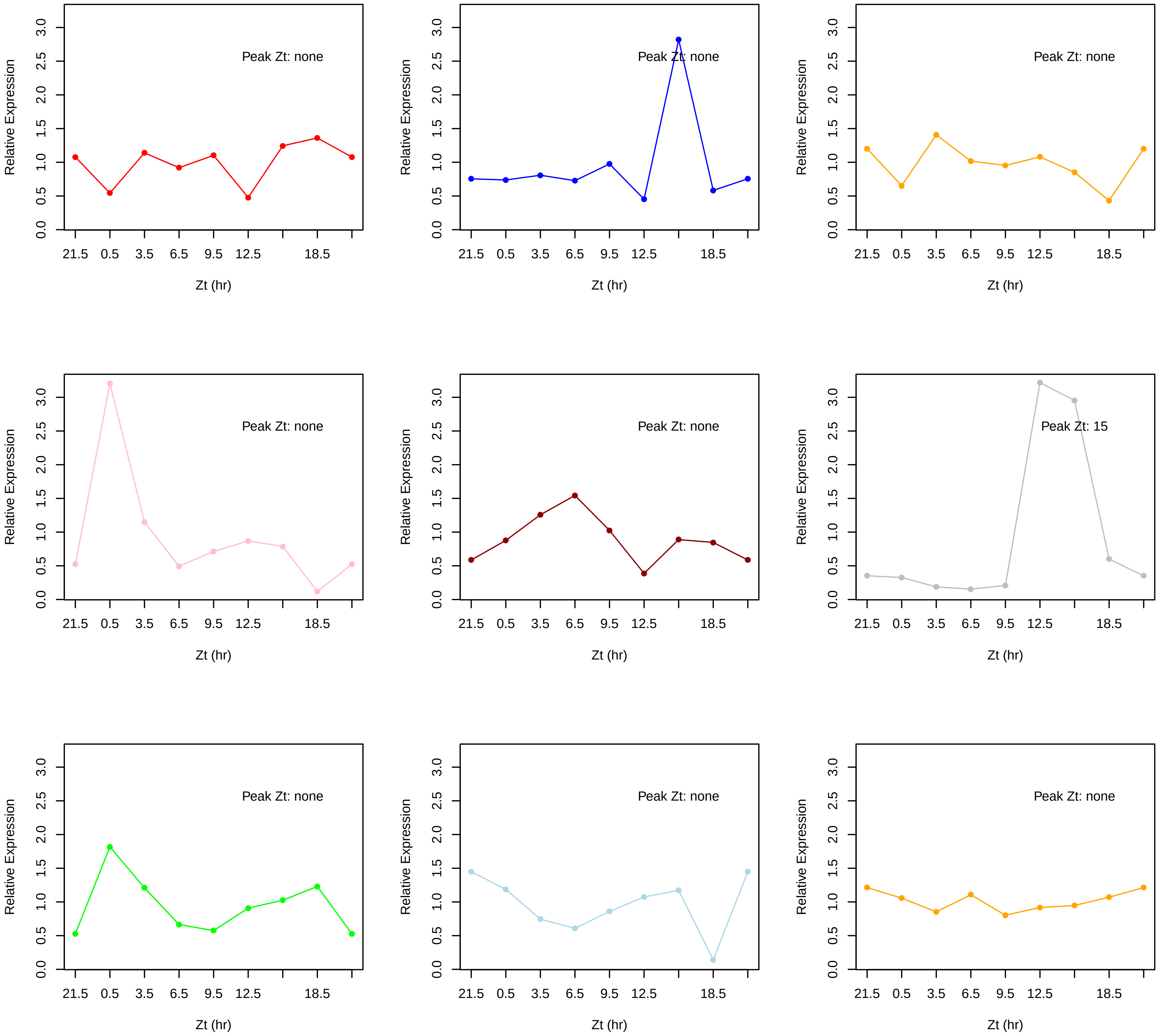

Singleton: ENSXMAG00000014929 pdcd2

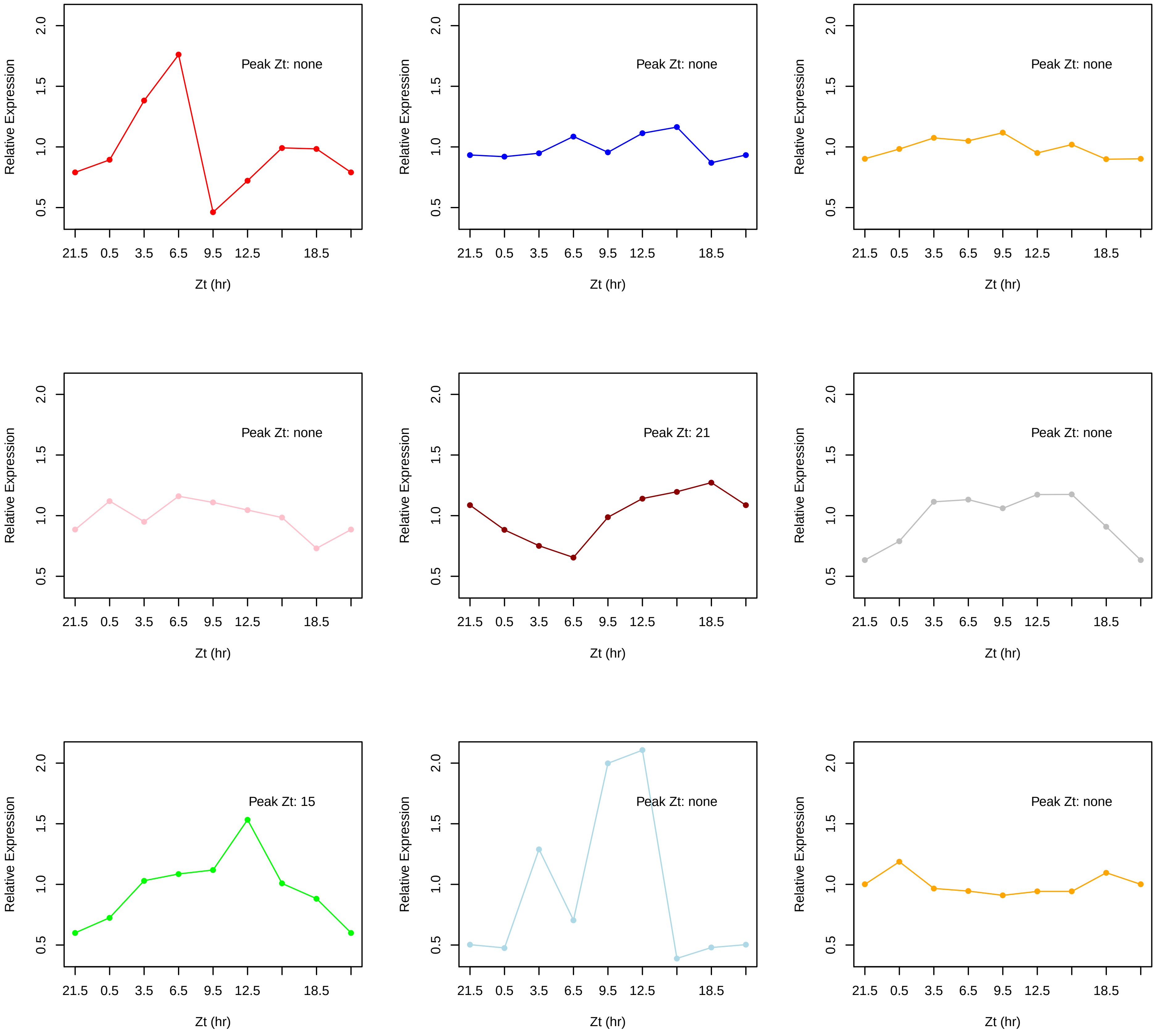

Singeleton: ENSXMAG00000000265 taz

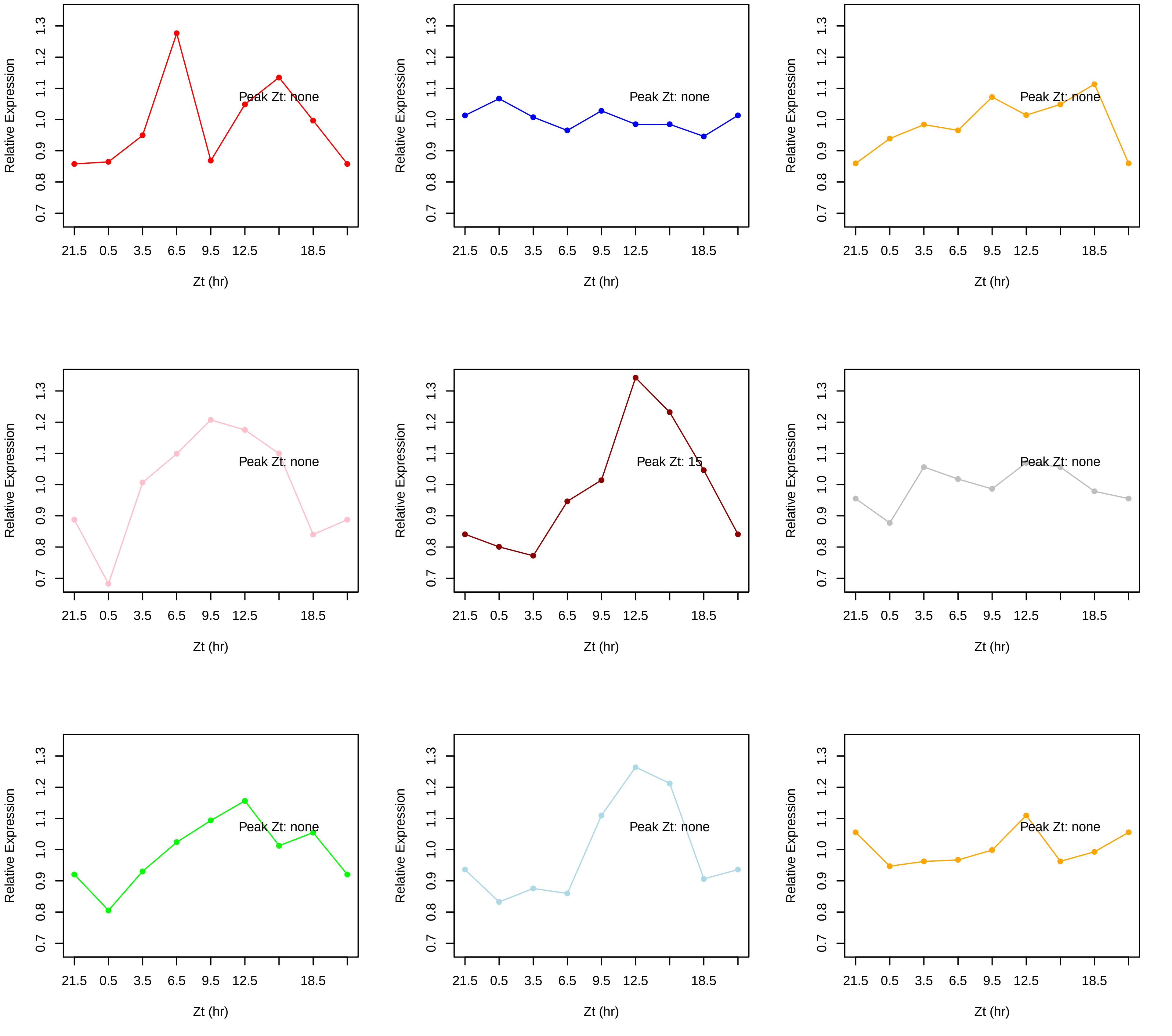

Singeleton: ENSXMAG00000012620 SRPK2

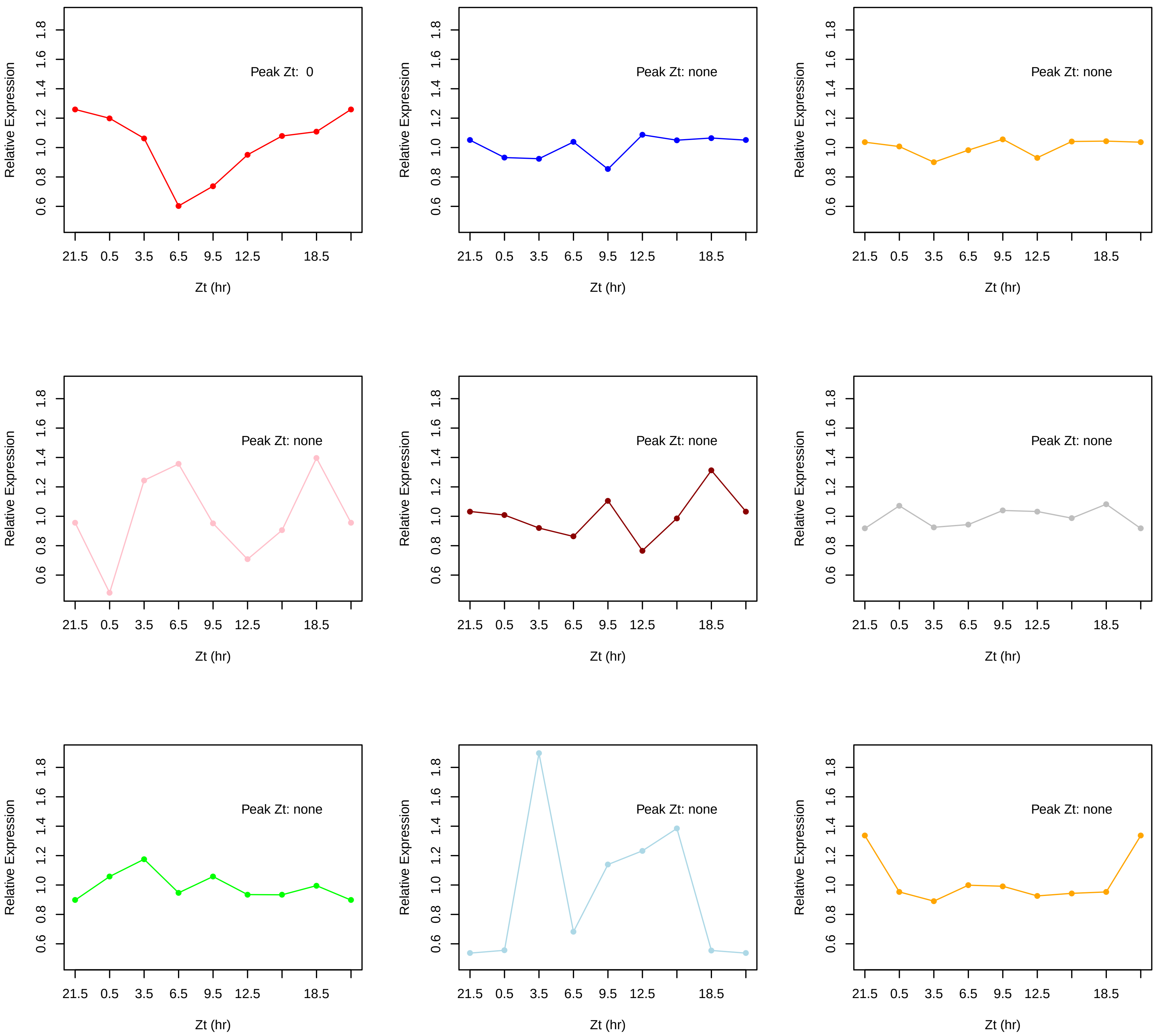

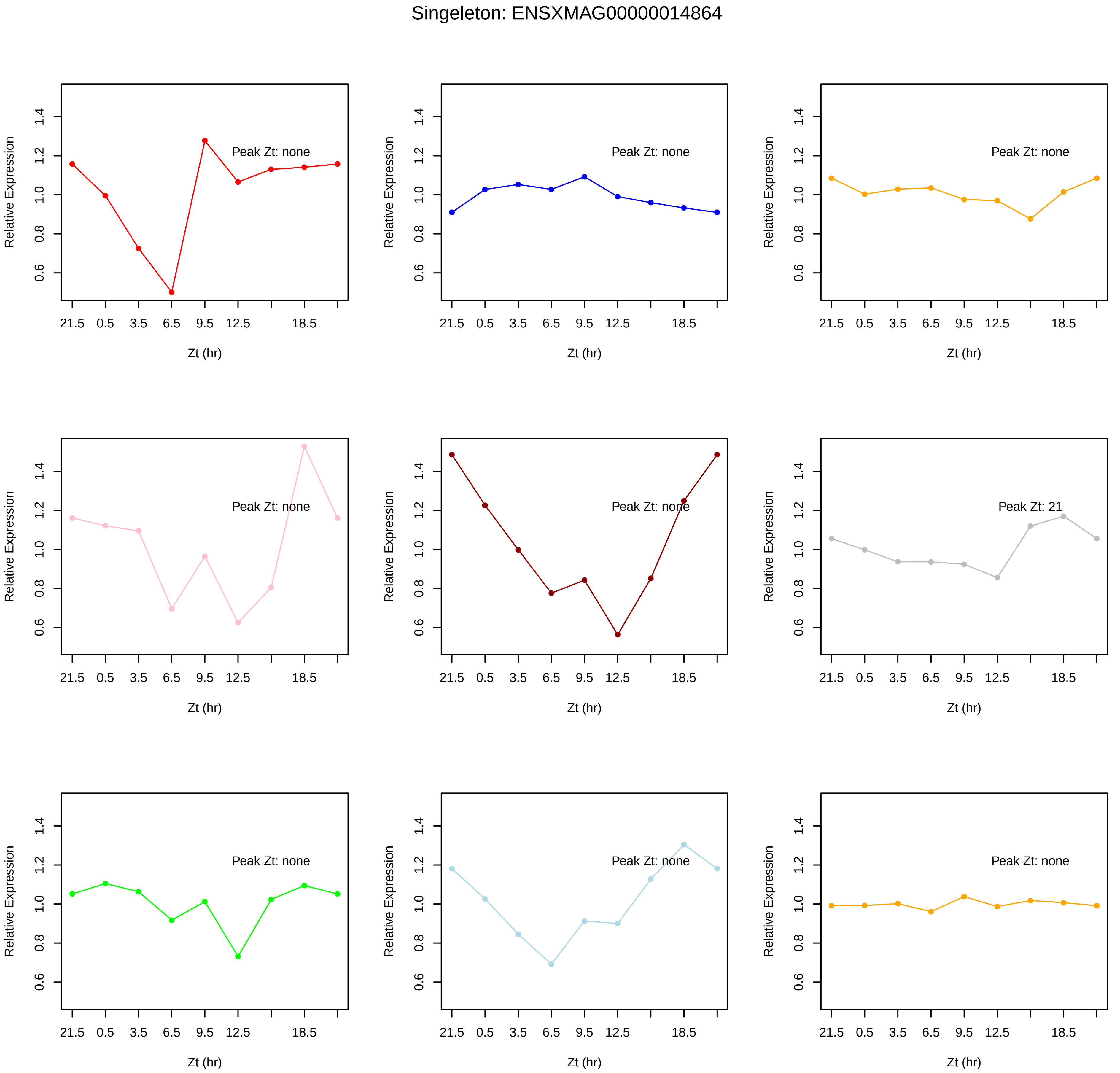

Singeleton: ENSXMAG00000014864
Relative Expression
Zt (hr)
21.5
0.5
3.5
6.5
9.5
12.5
18.5
0.6
0.8
1.0
1.2
1.4
Peak Zt: none
Peak Zt: 21

Singleton: ENSXMAG00000005014 RINT1

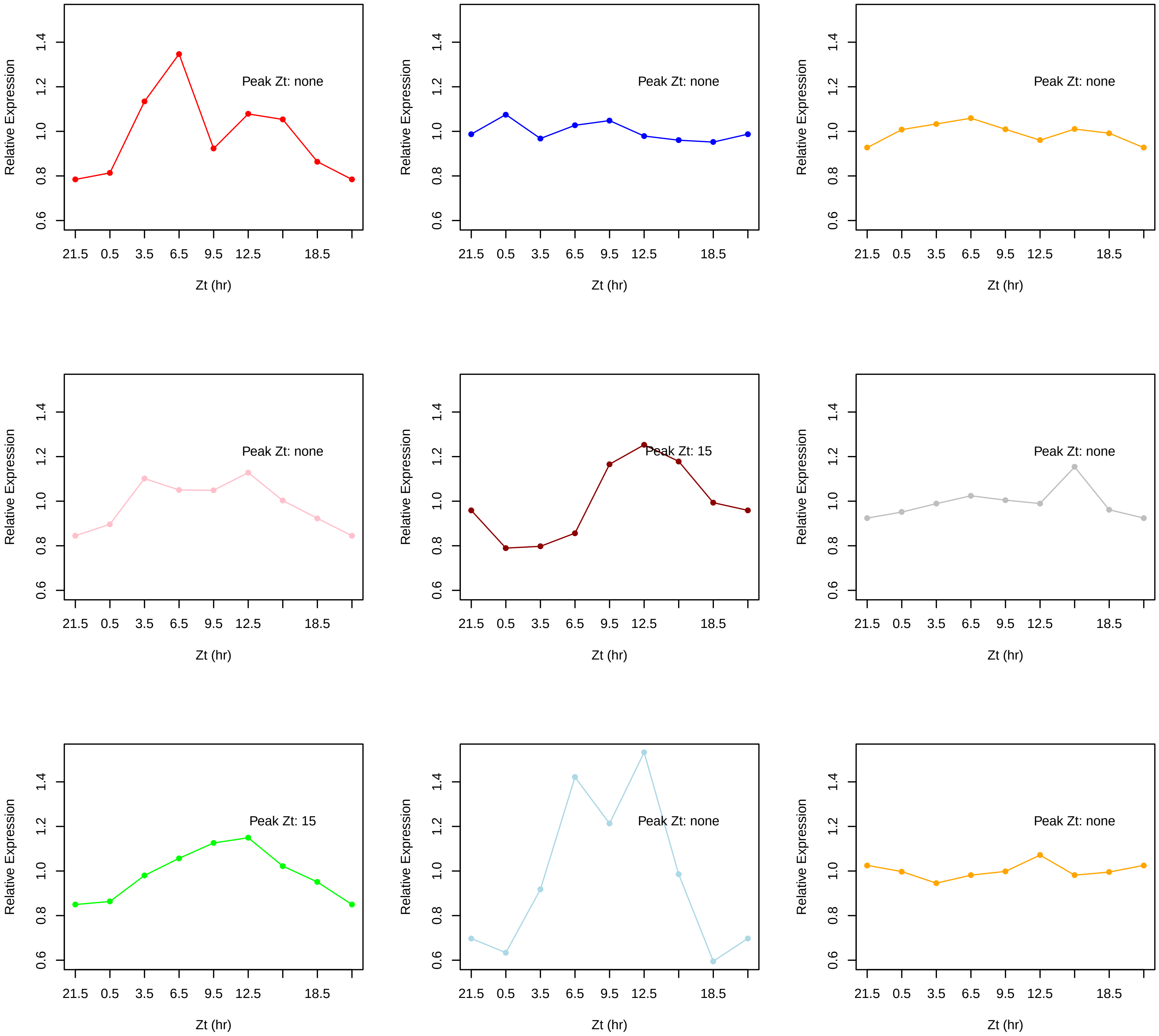

Singeleton: ENSXMAG00000014897 ppm1g

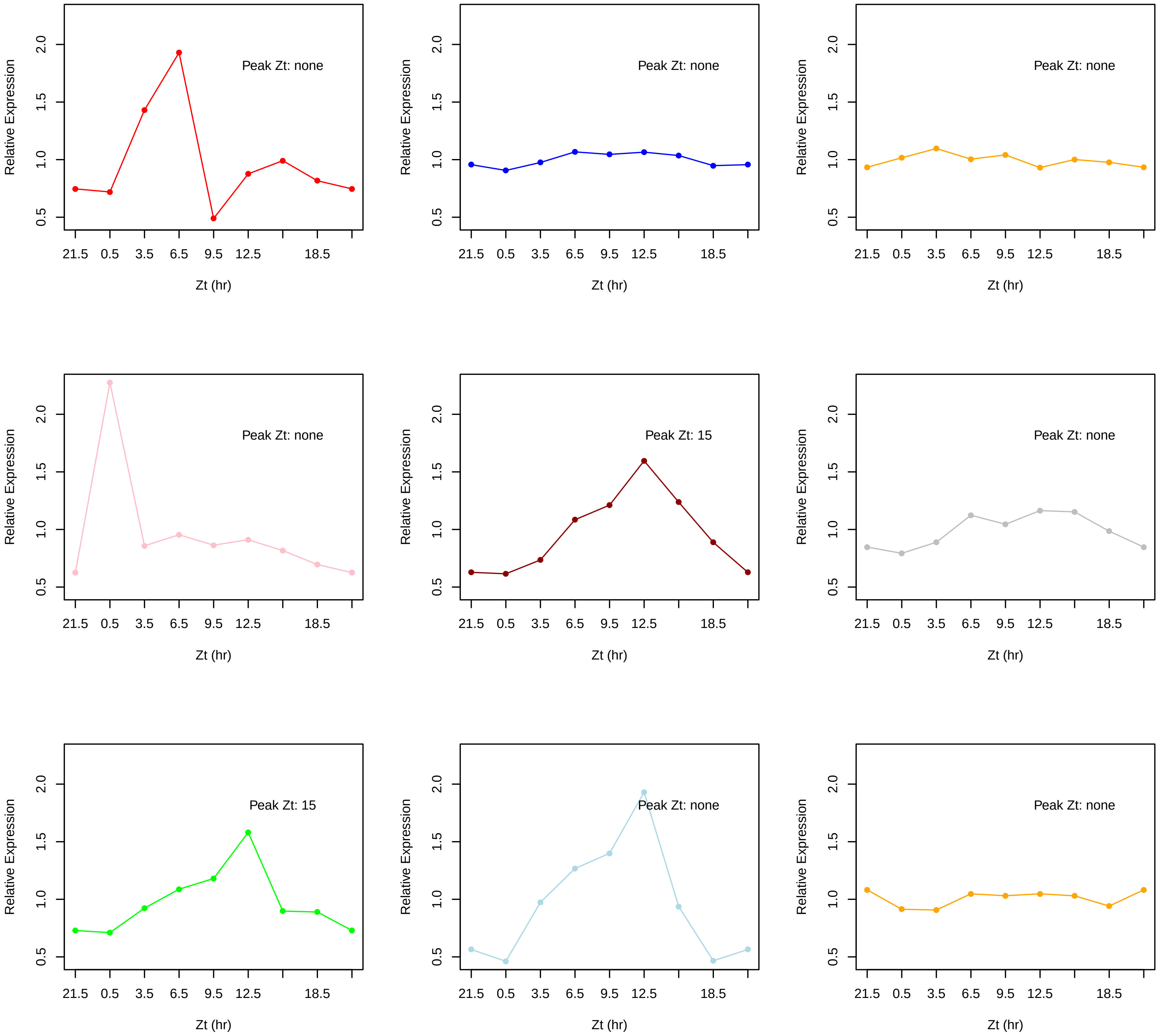

Singeleton: ENSXMAG00000004788 rpl10

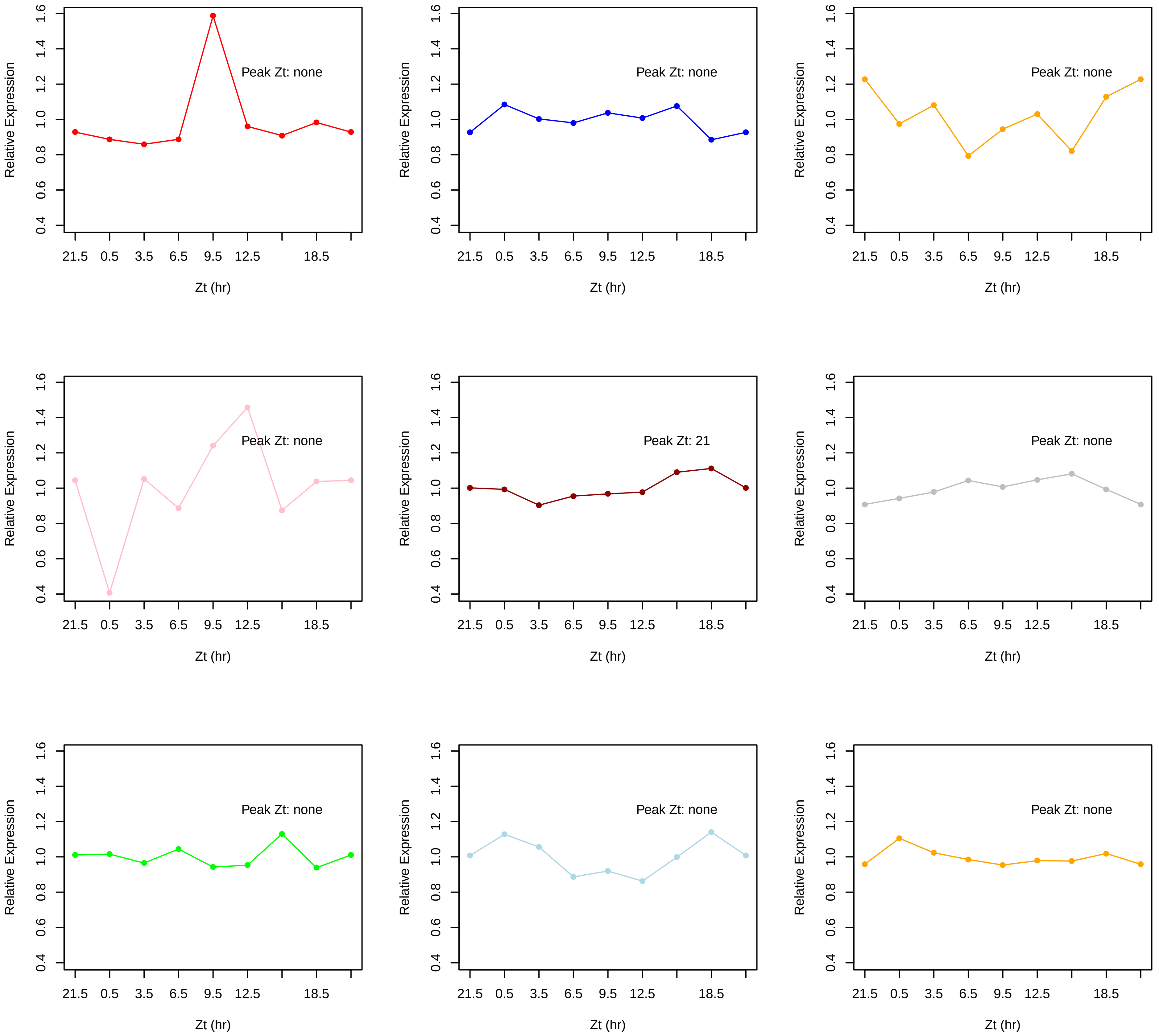

Singeleton: ENSXMAG00000017158

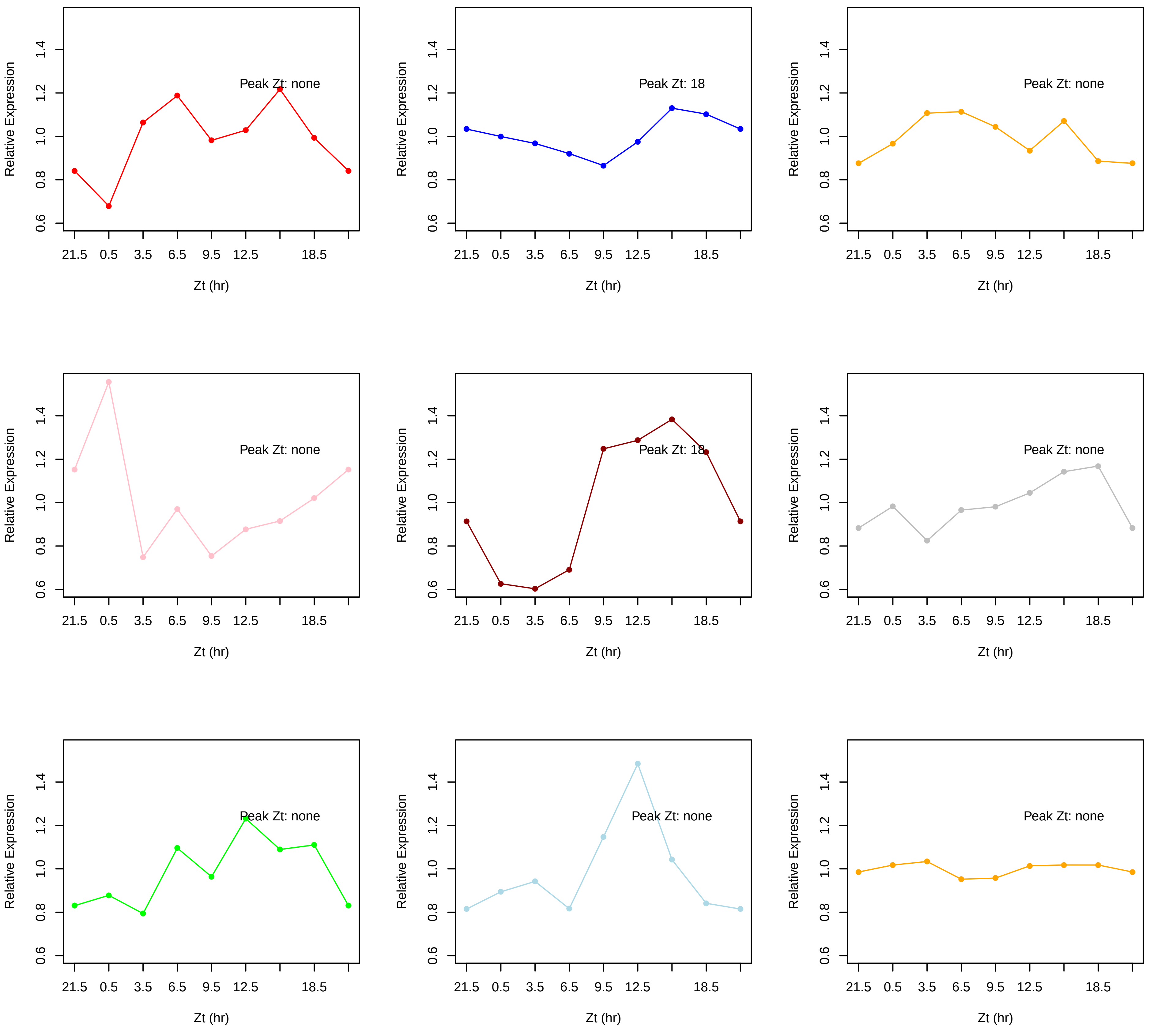

Singleton: ENSXMAG00000019204 panx2

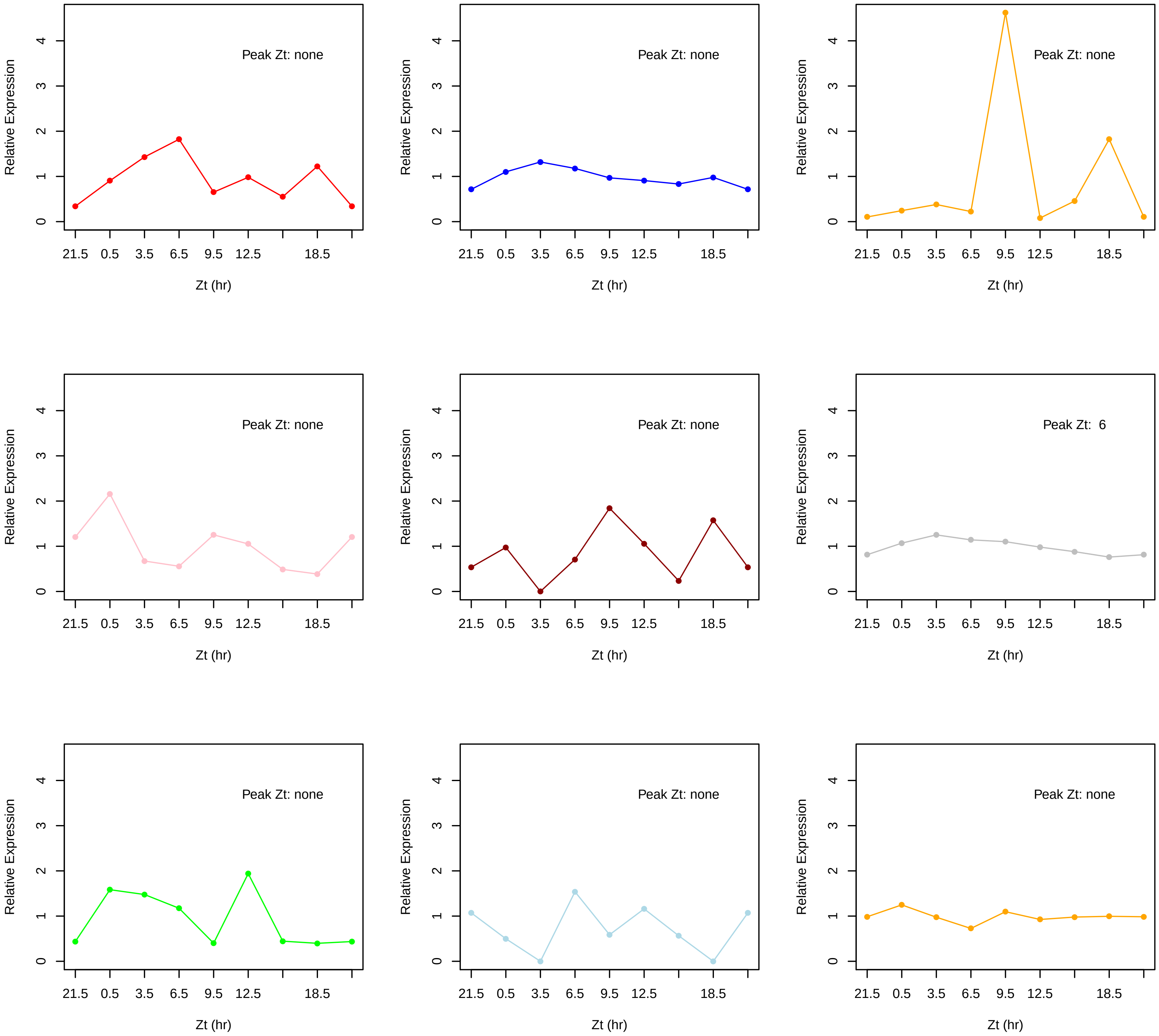

Singeleton: ENSXMAG00000017180 vip

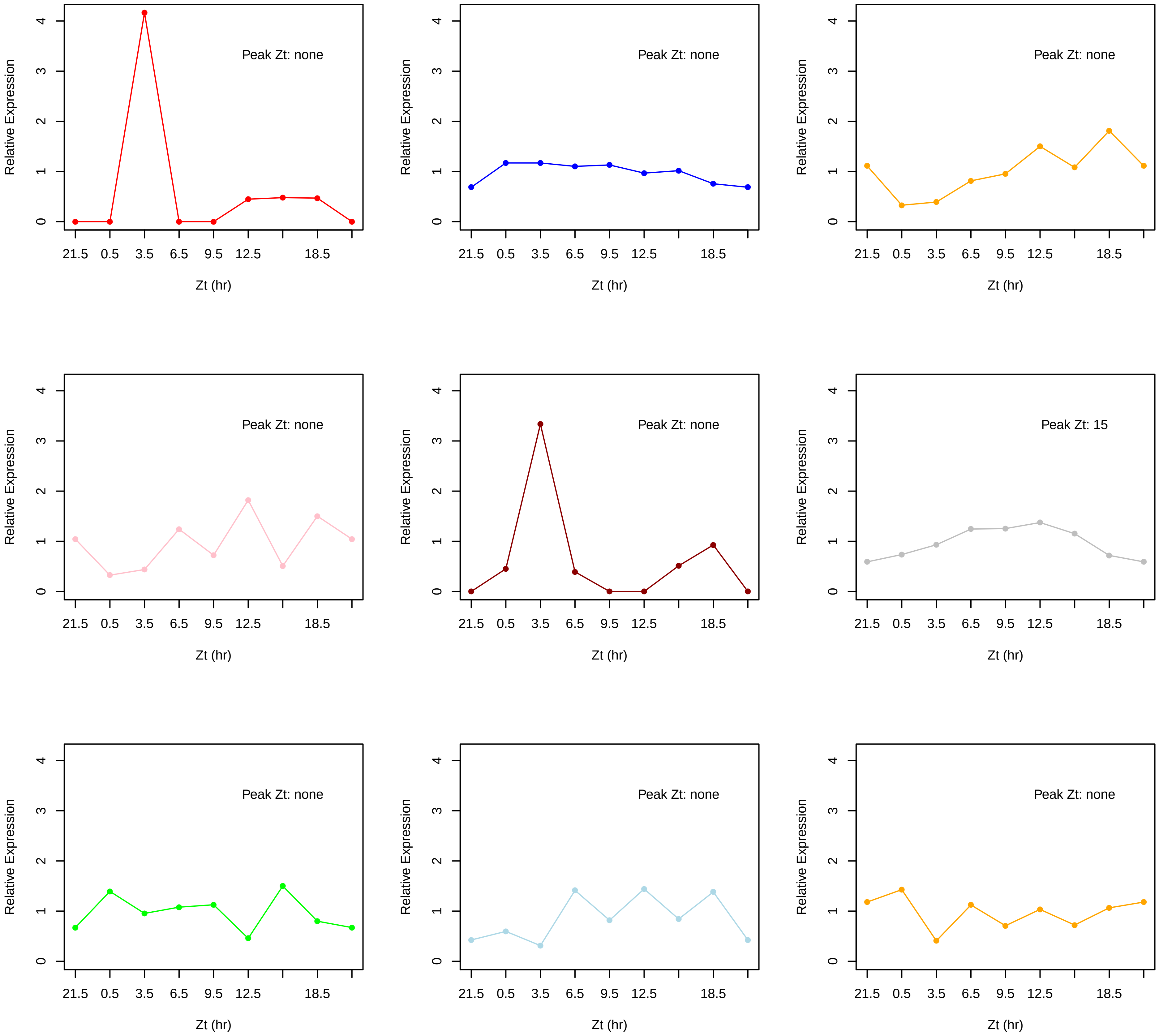

Singeleton: ENSXMAG00000019211 mlc1

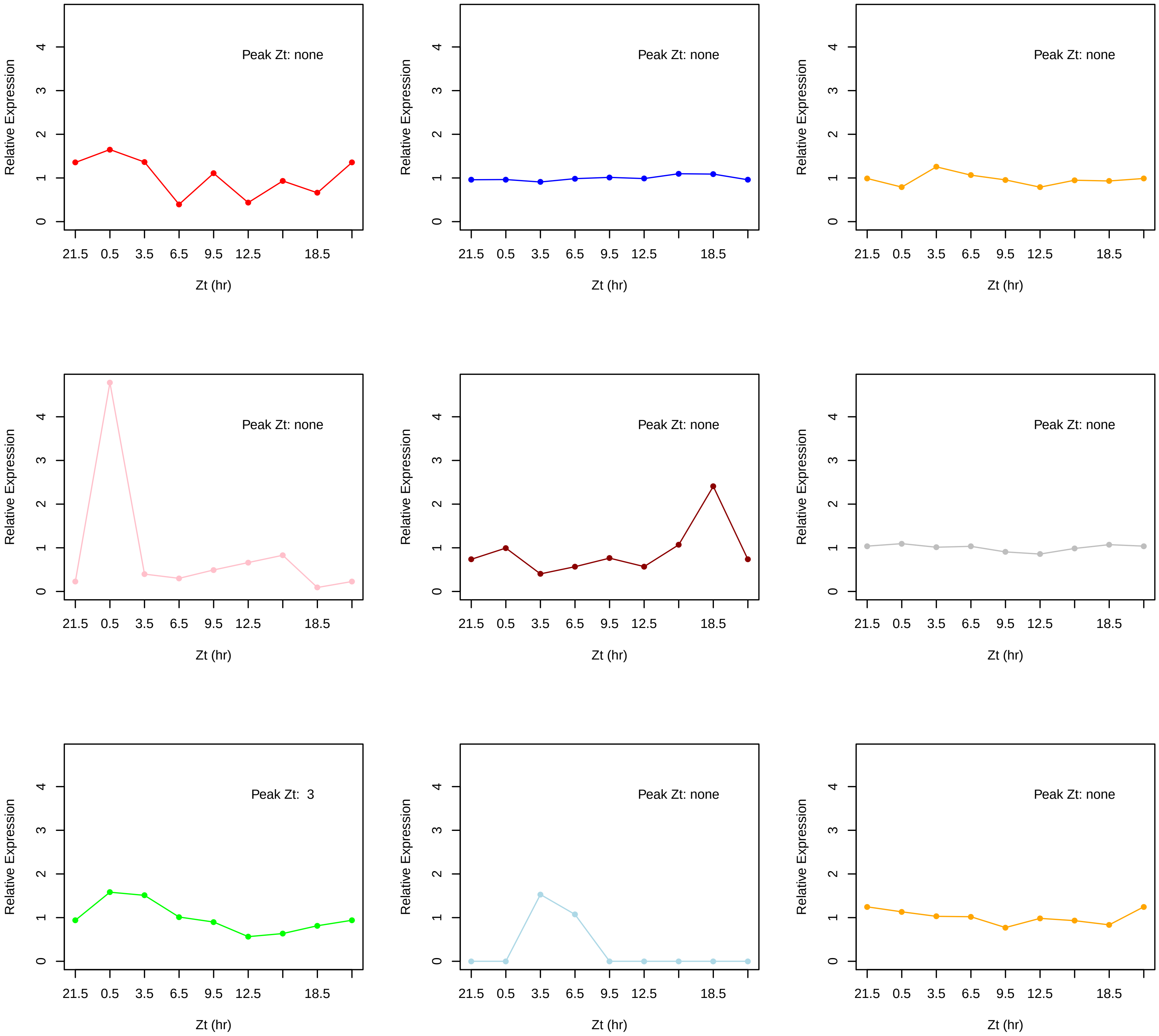

Singleton: ENSXMAG00000006199 osgep

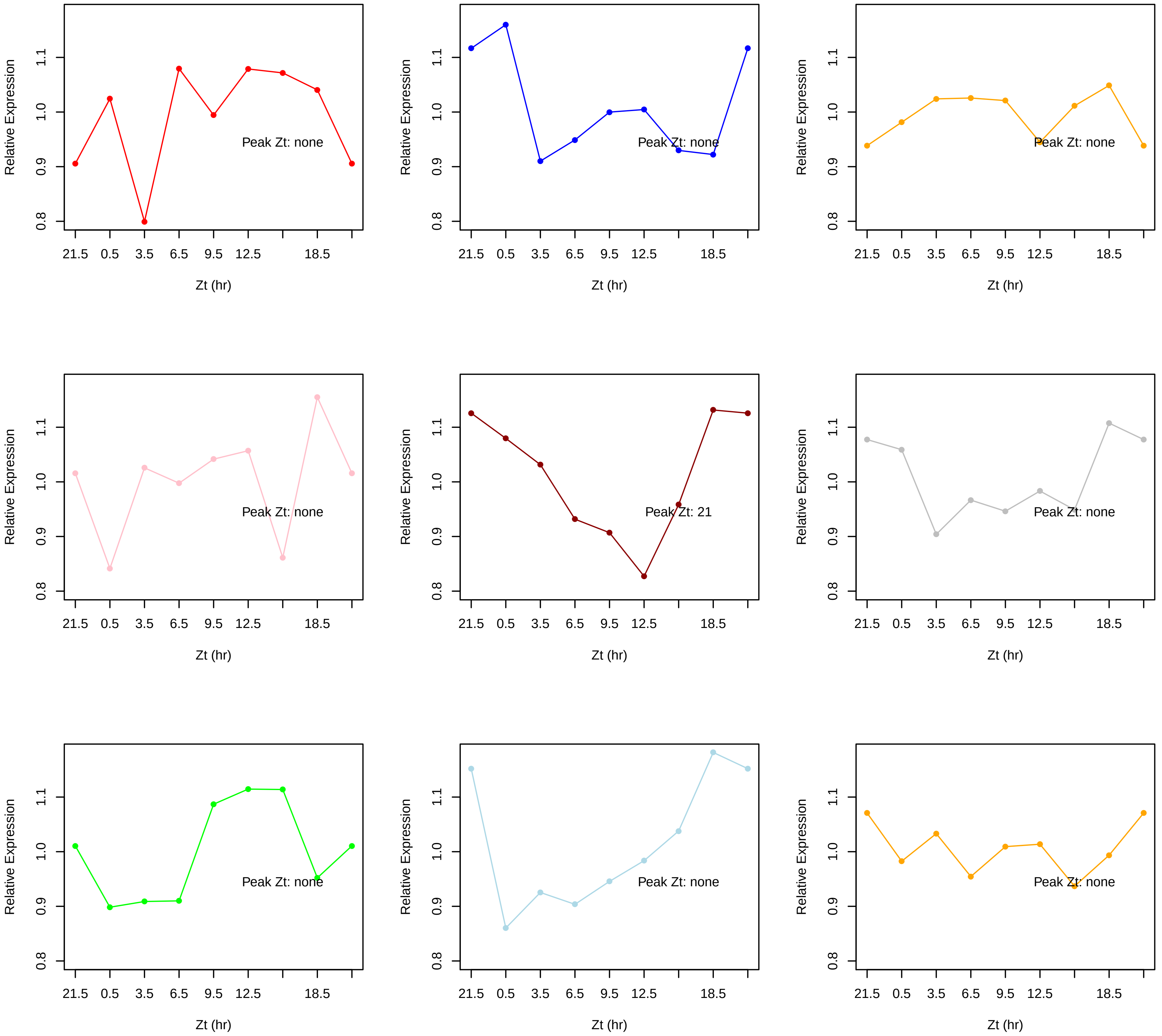

Singleton: ENSXMAG00000023884 si:ch211−150o23.2

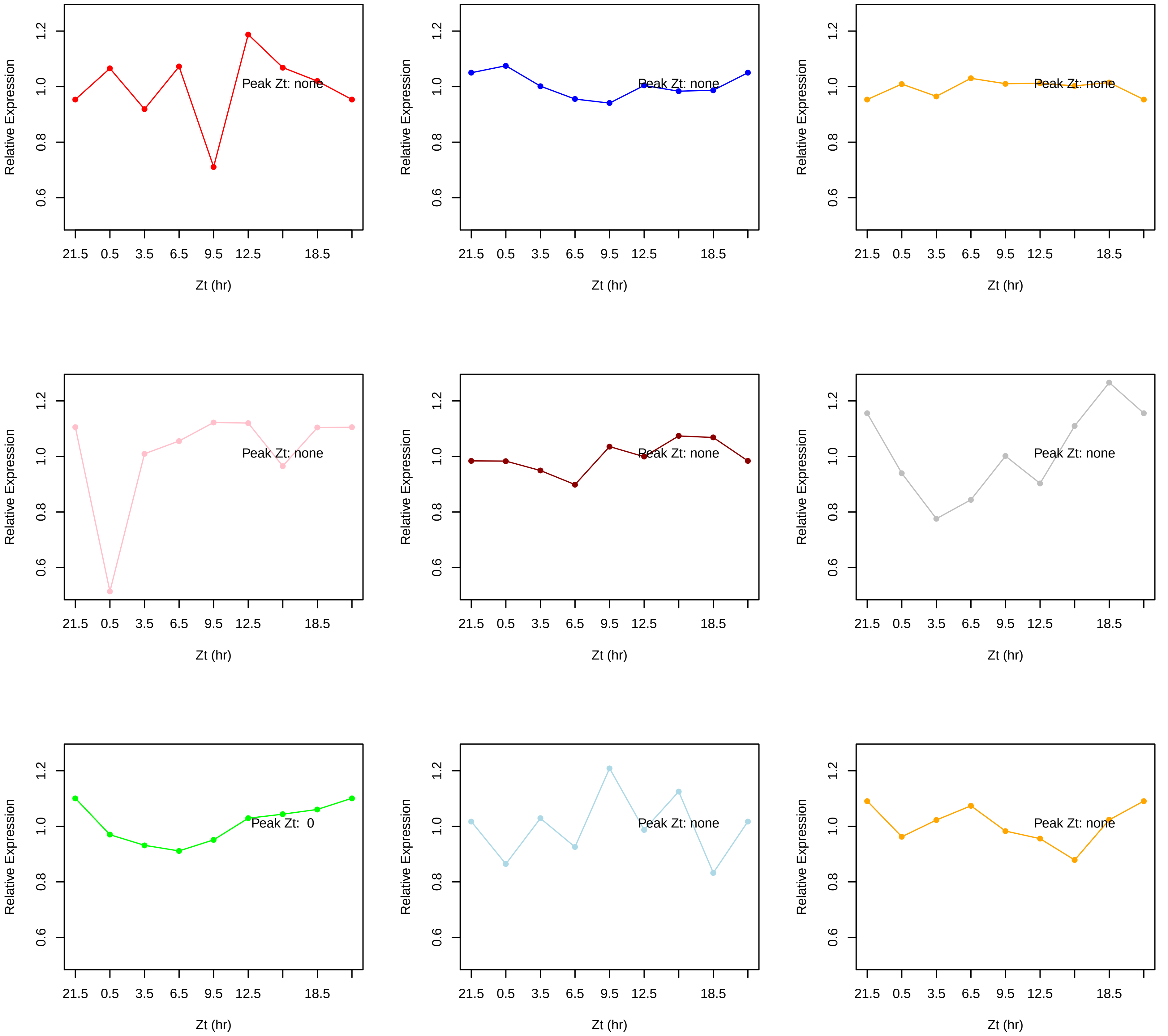

Singeleton: ENSXMAG00000021734 pclob

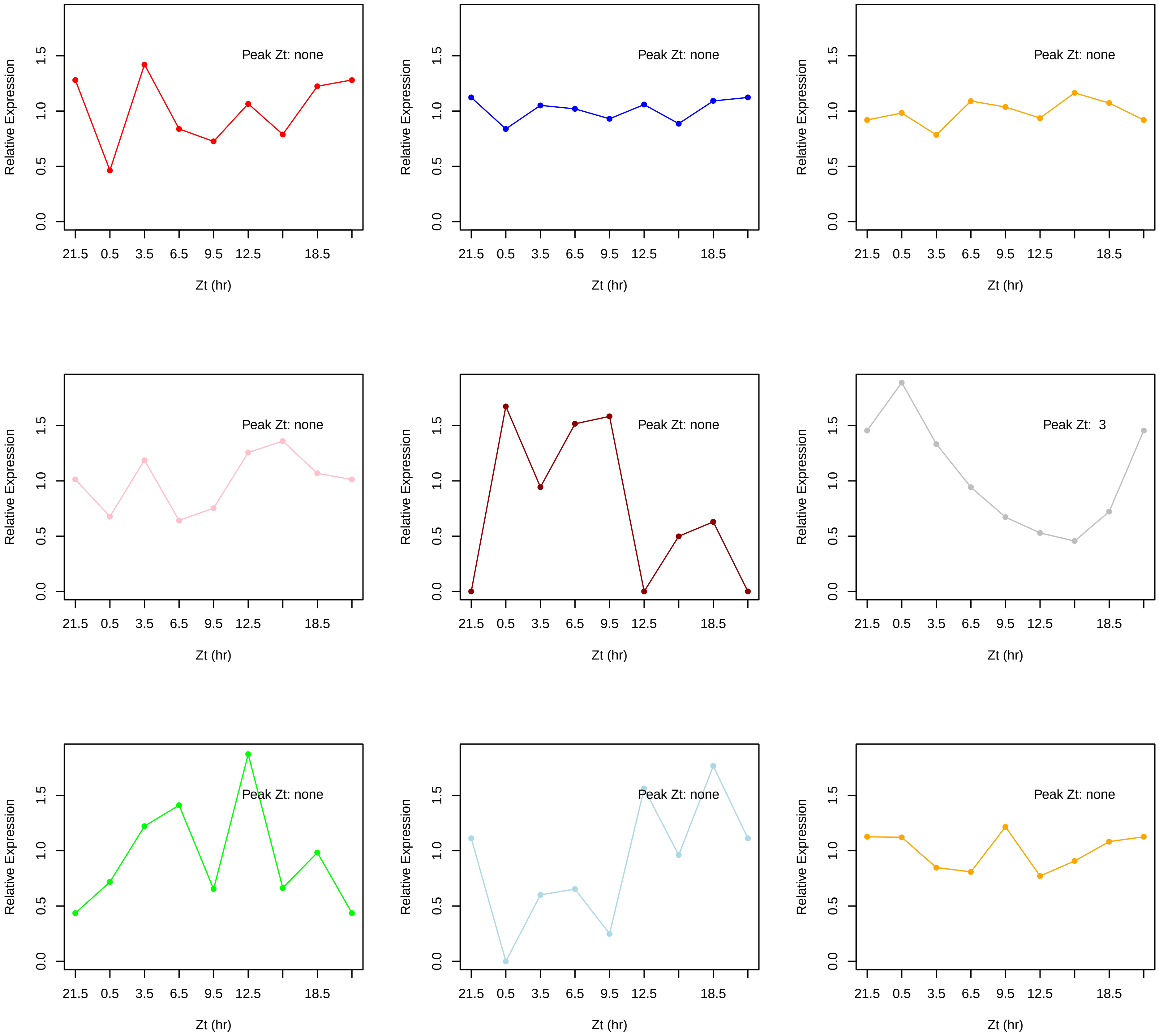

Singeleton: ENSXMAG00000008350 bgnb

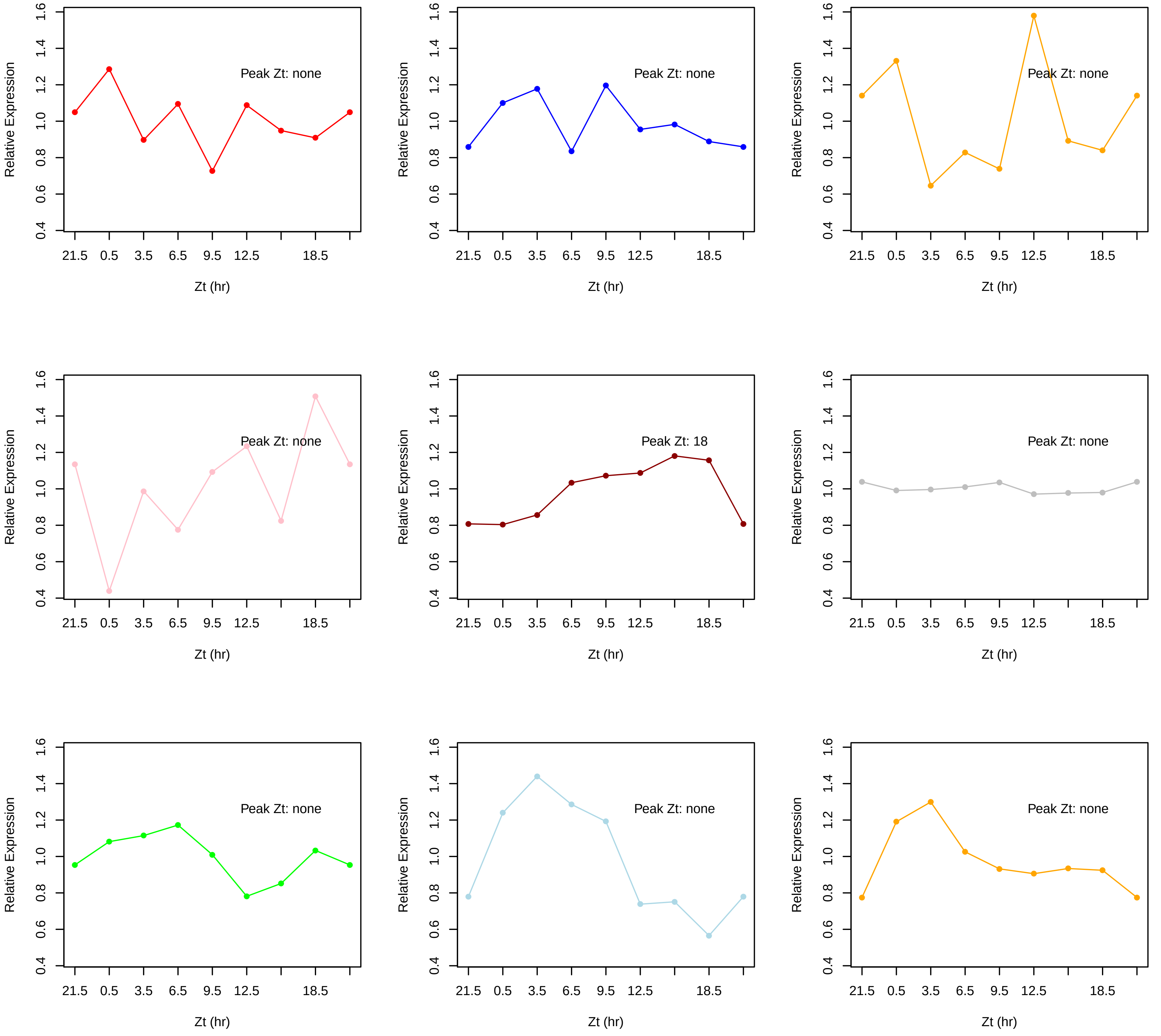

Singeleton: ENSXMAG00000013701

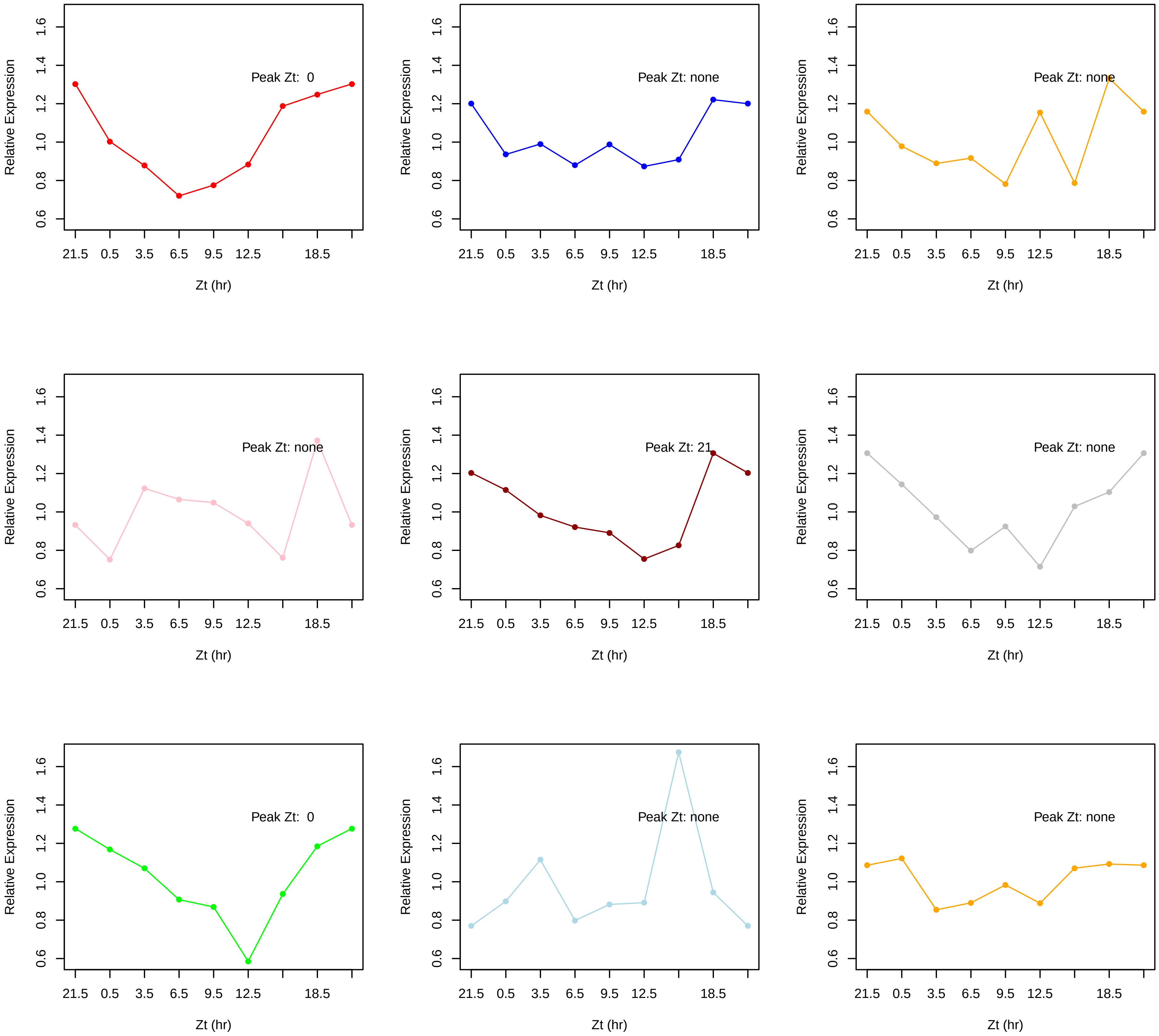

Singleton: ENSXMAG00000004468 EML6

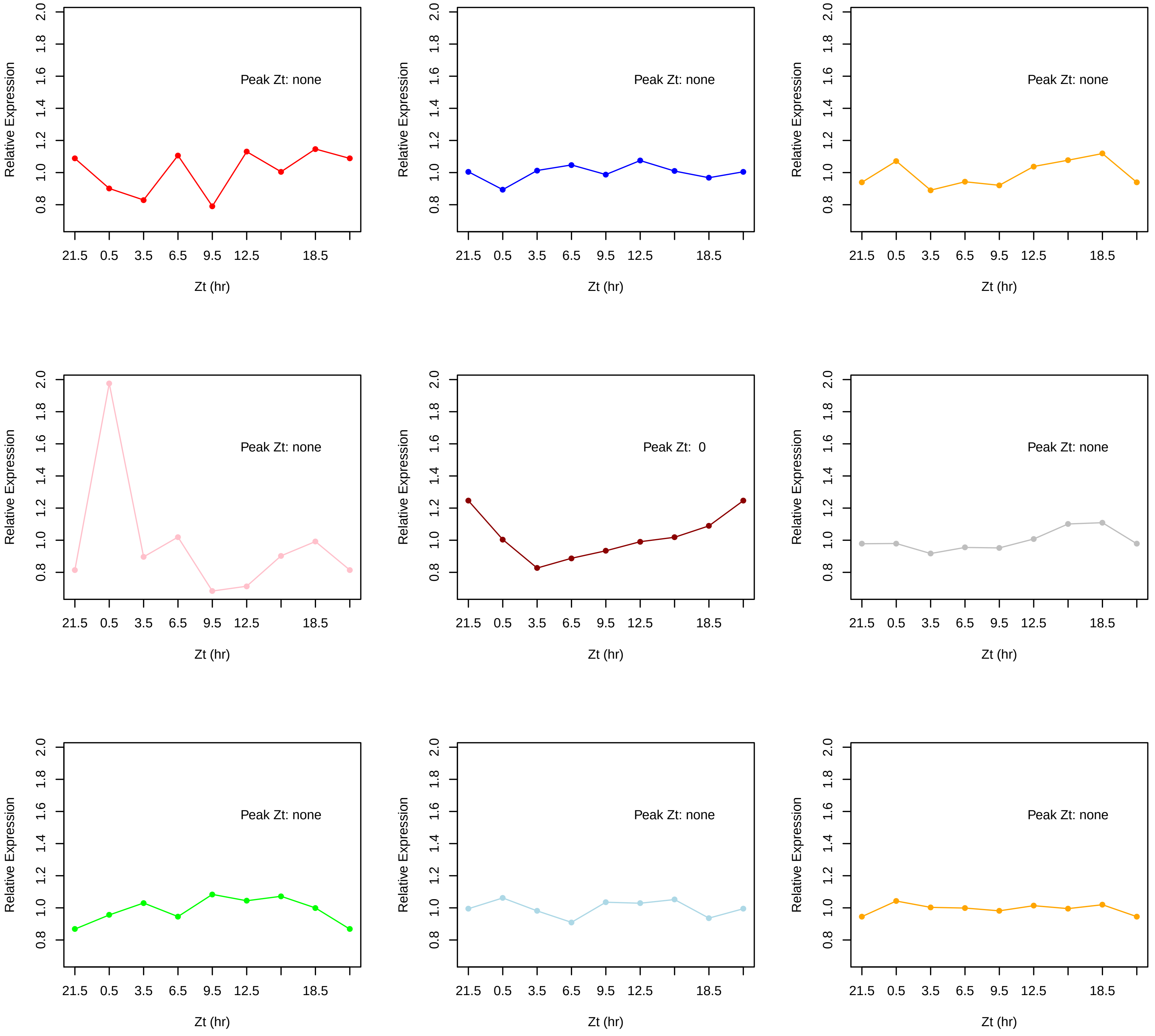

Singleton: ENSXMAG00000000174

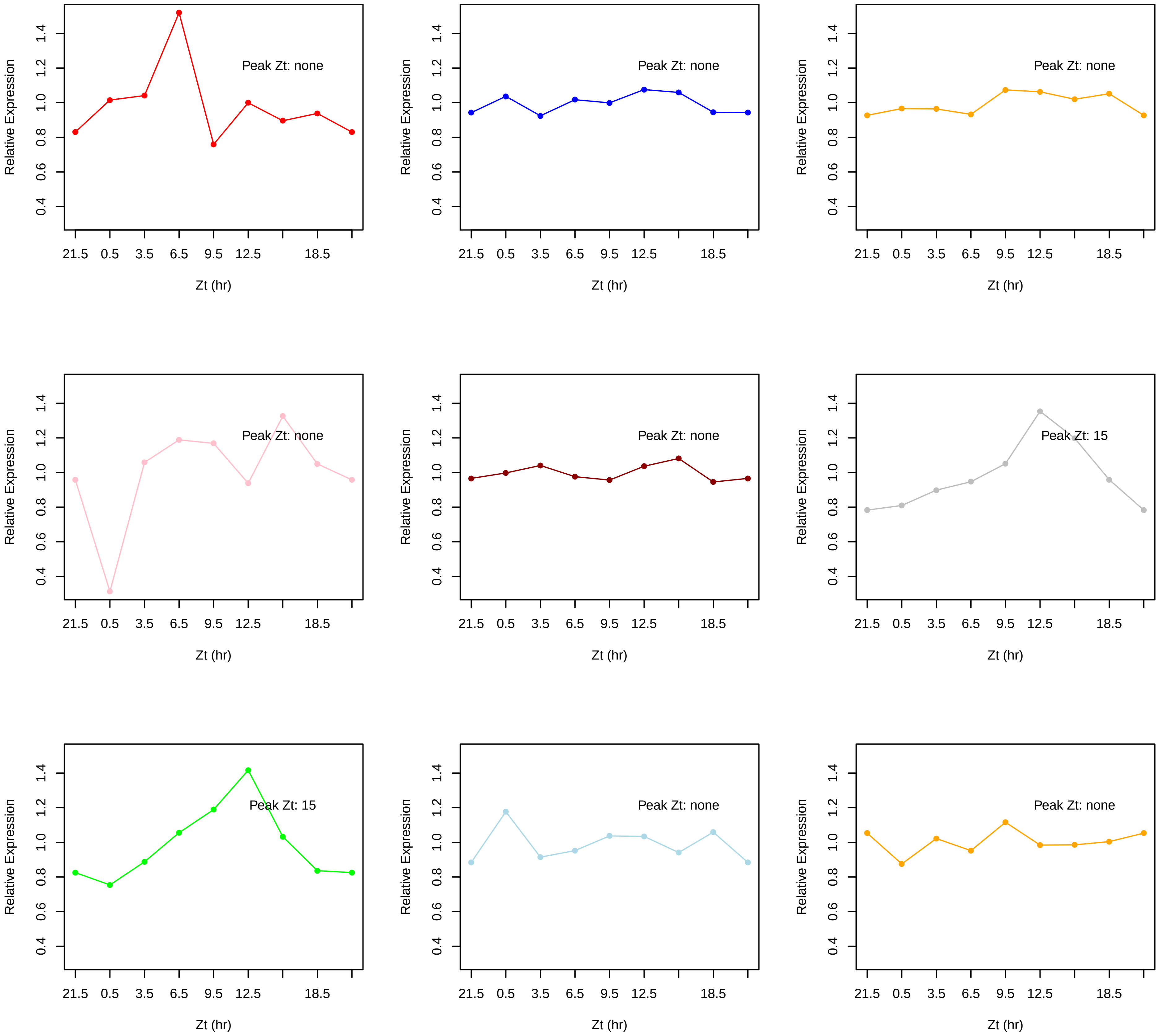

Singleton: ENSXMAG00000022781

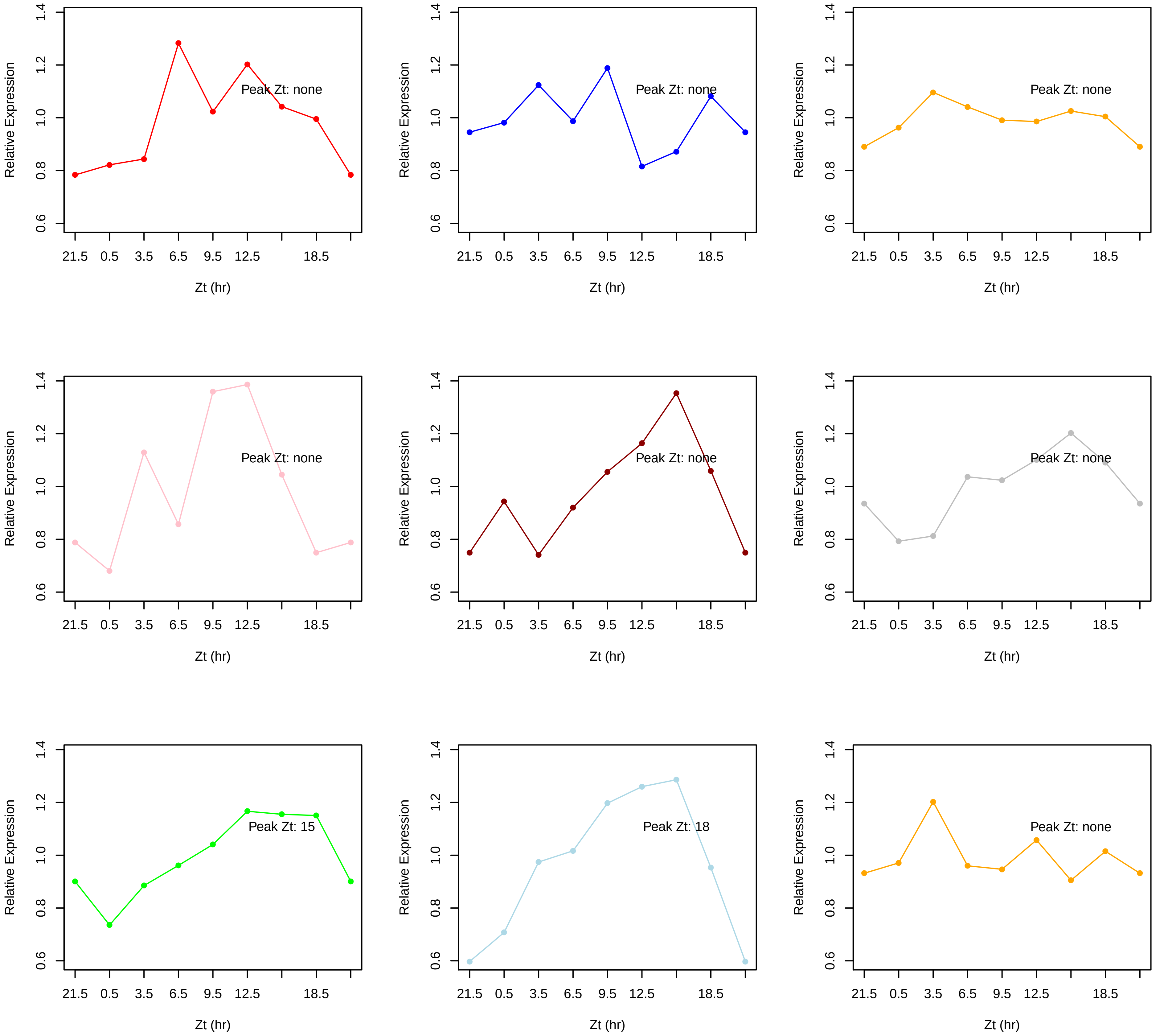

Singeleton: ENSXMAG00000004661

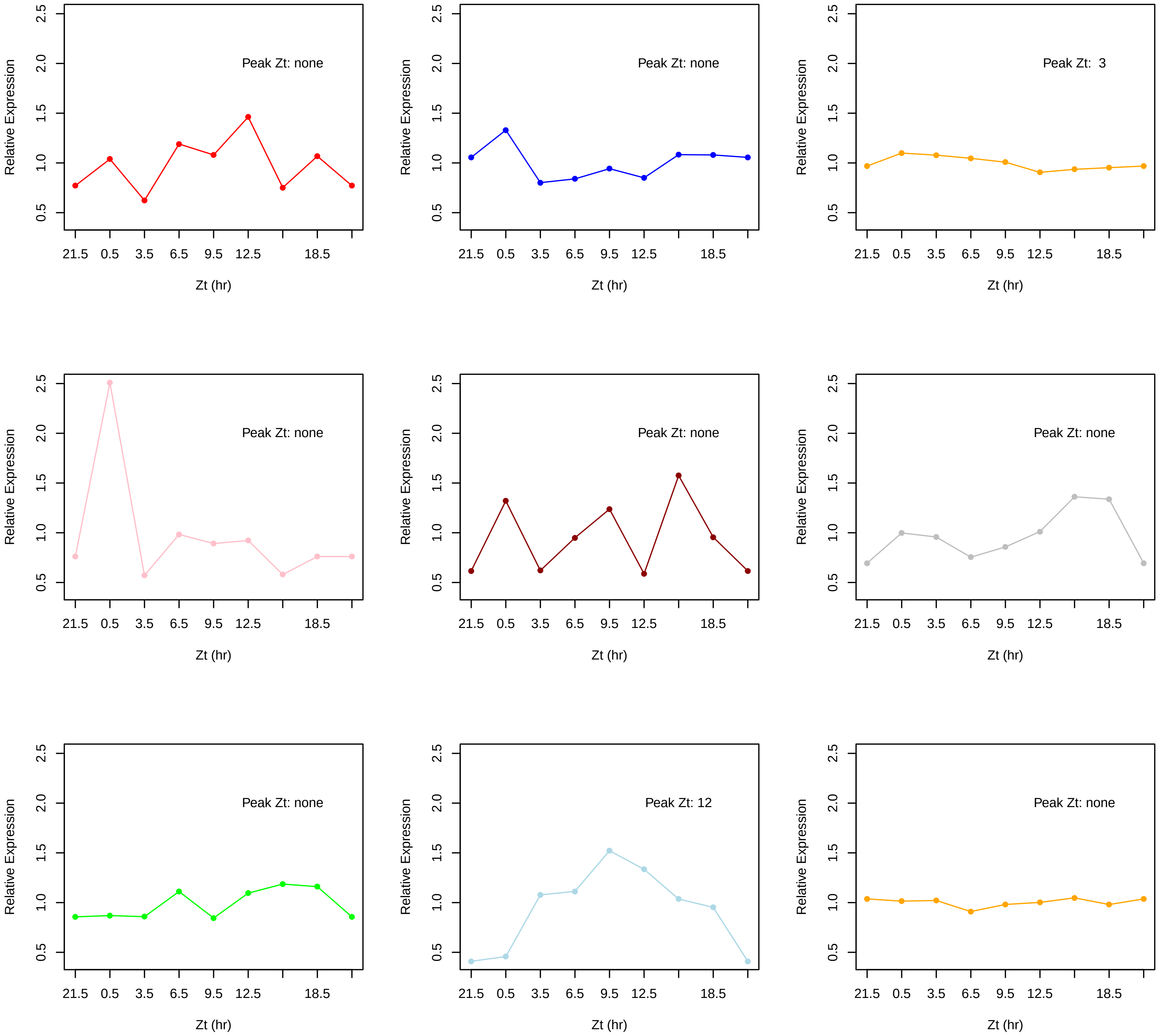

Singleton: ENSXMAG00000013062 tnpo3

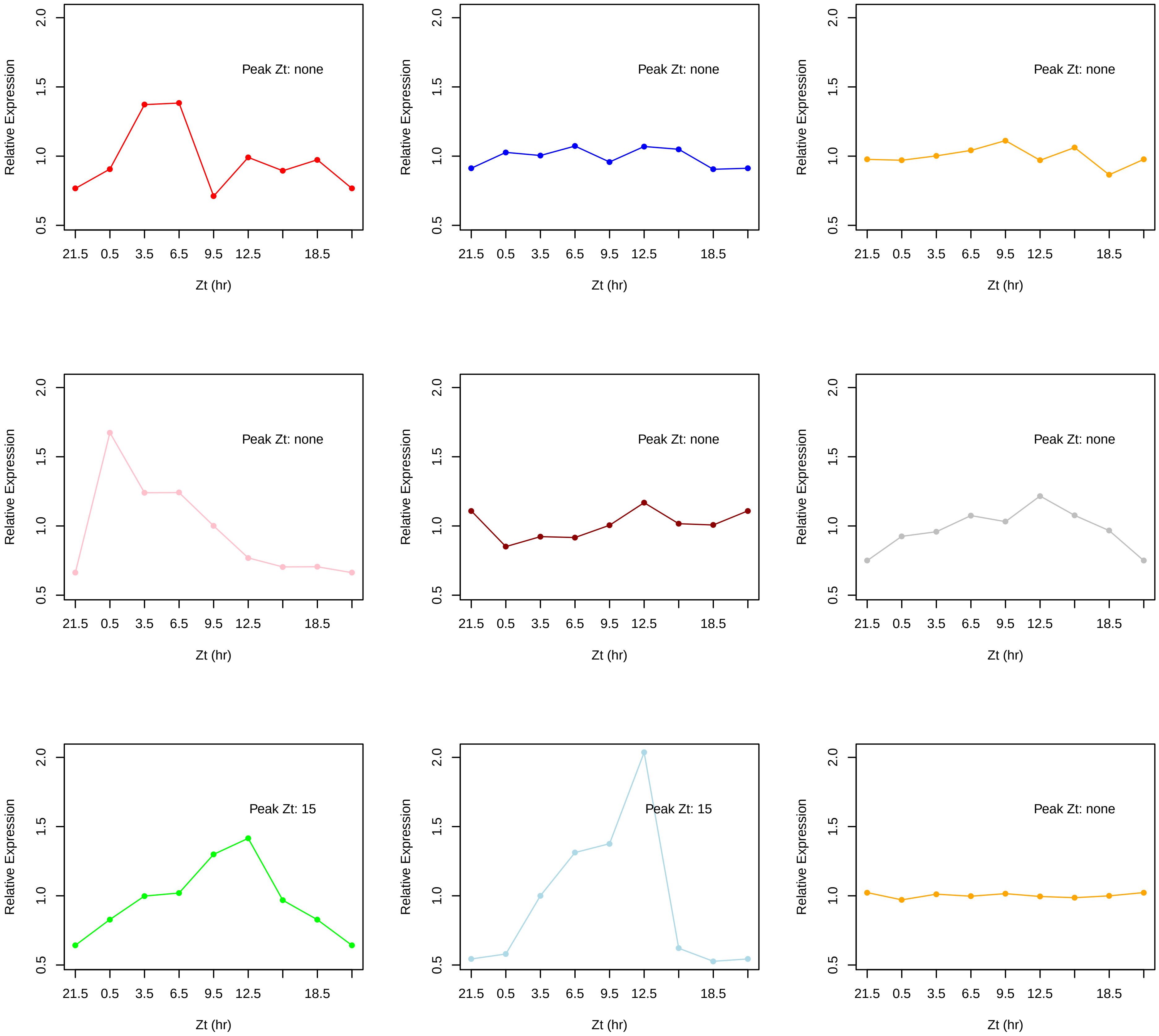

Singeleton: ENSXMAG00000028479 zgc:64201

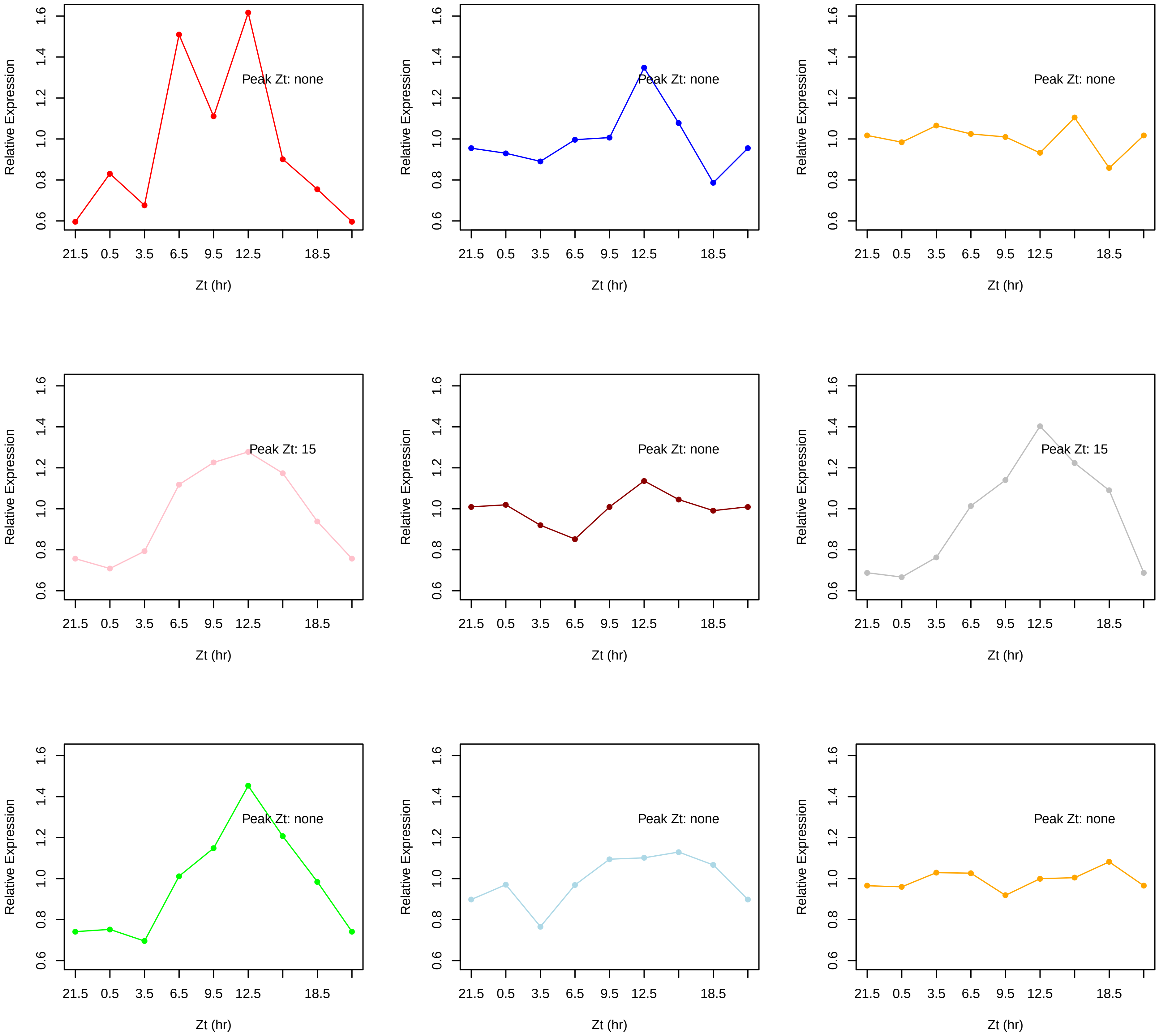

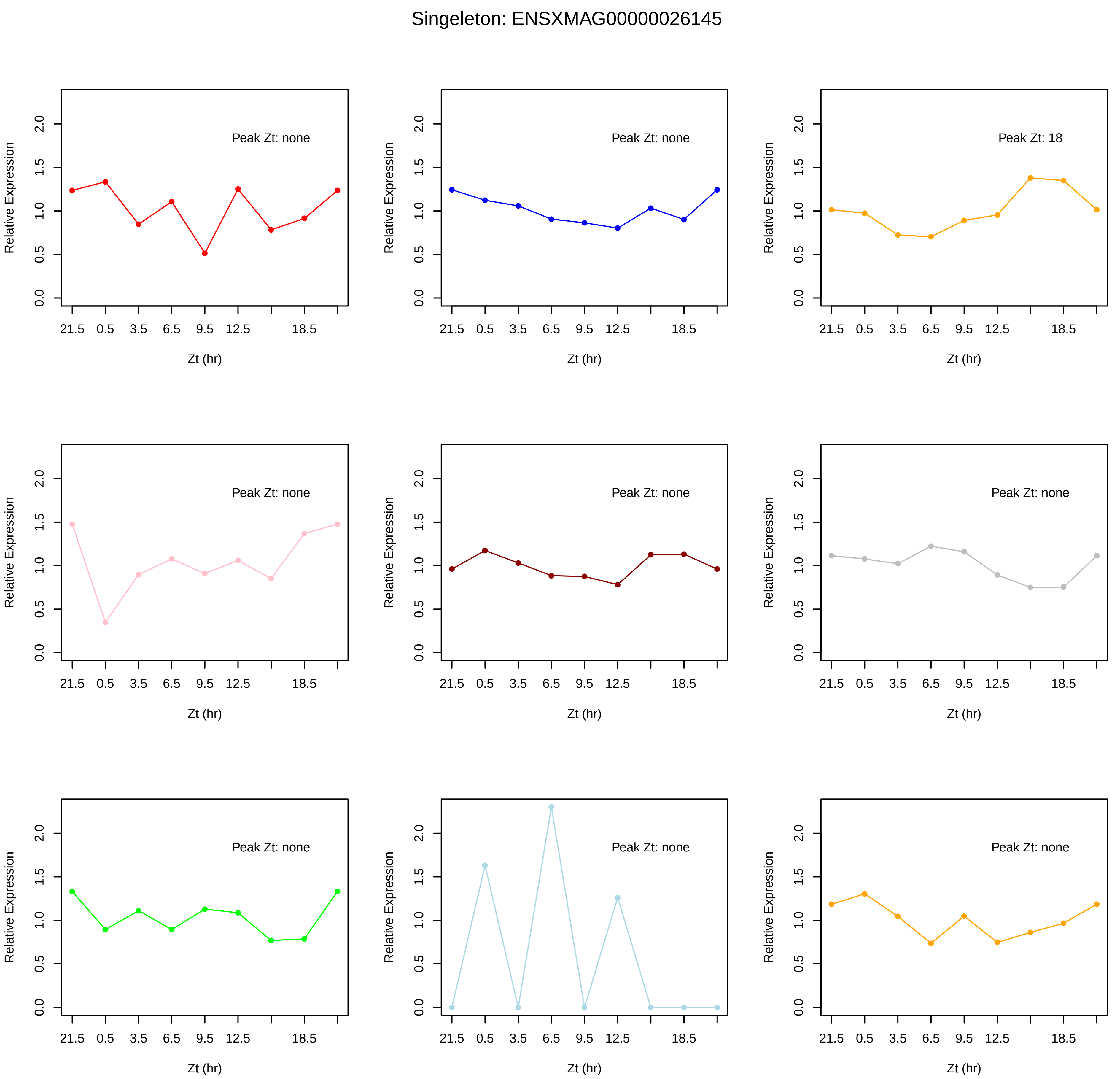
Singeleton: ENSXMAG00000026145
Peak Zt: none
Peak Zt: none
Peak Zt: 18
Peak Zt: none
Peak Zt: none
Peak Zt: none
Peak Zt: none
Peak Zt: none
Peak Zt: none
Relative Expression
Zt (hr)
21.5
0.5
3.5
6.5
9.5
12.5
18.5

Singleton: ENSXMAG00000013048

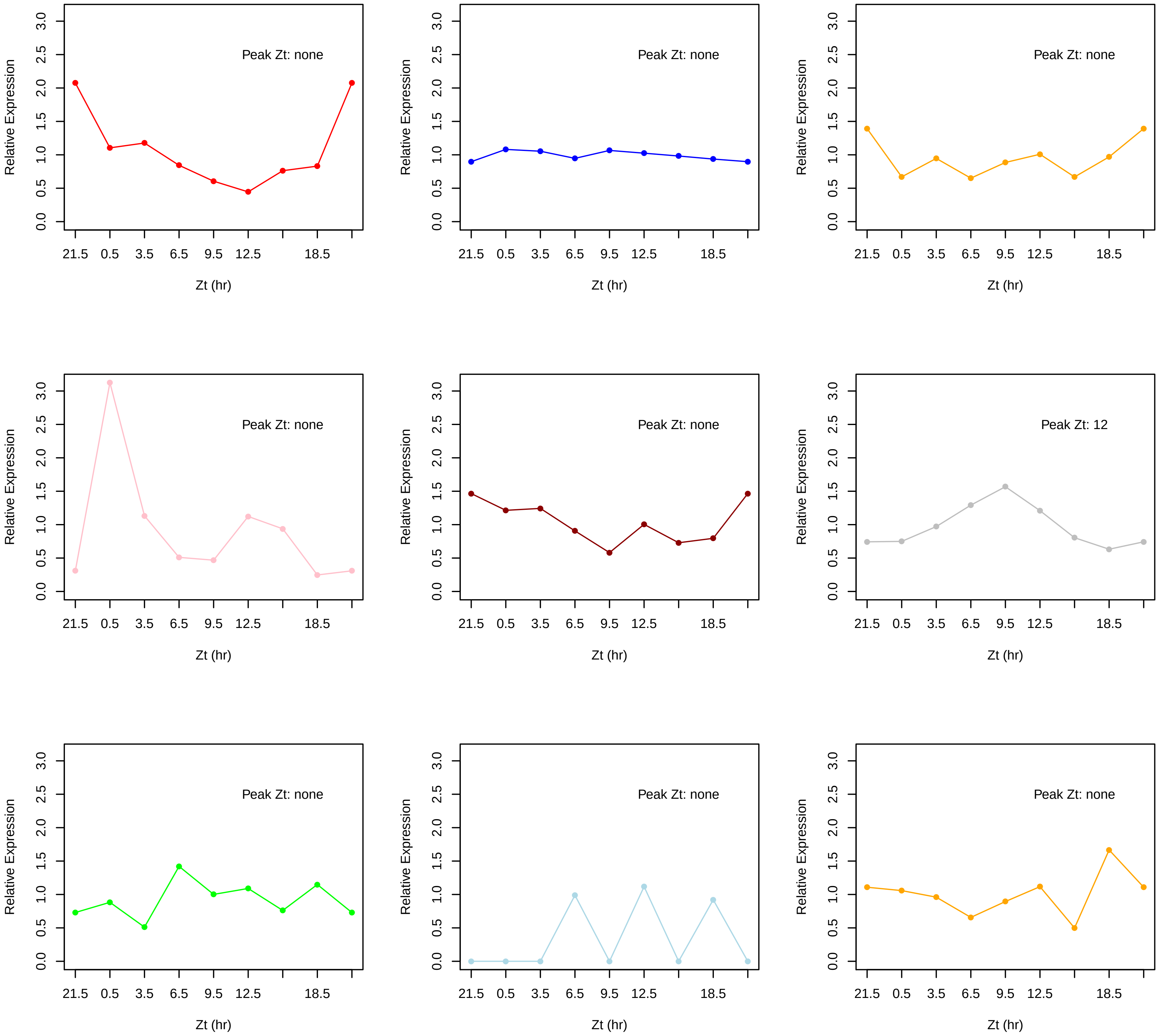

Singleton: ENSXMAG00000024959 si:ch211−272n13.3

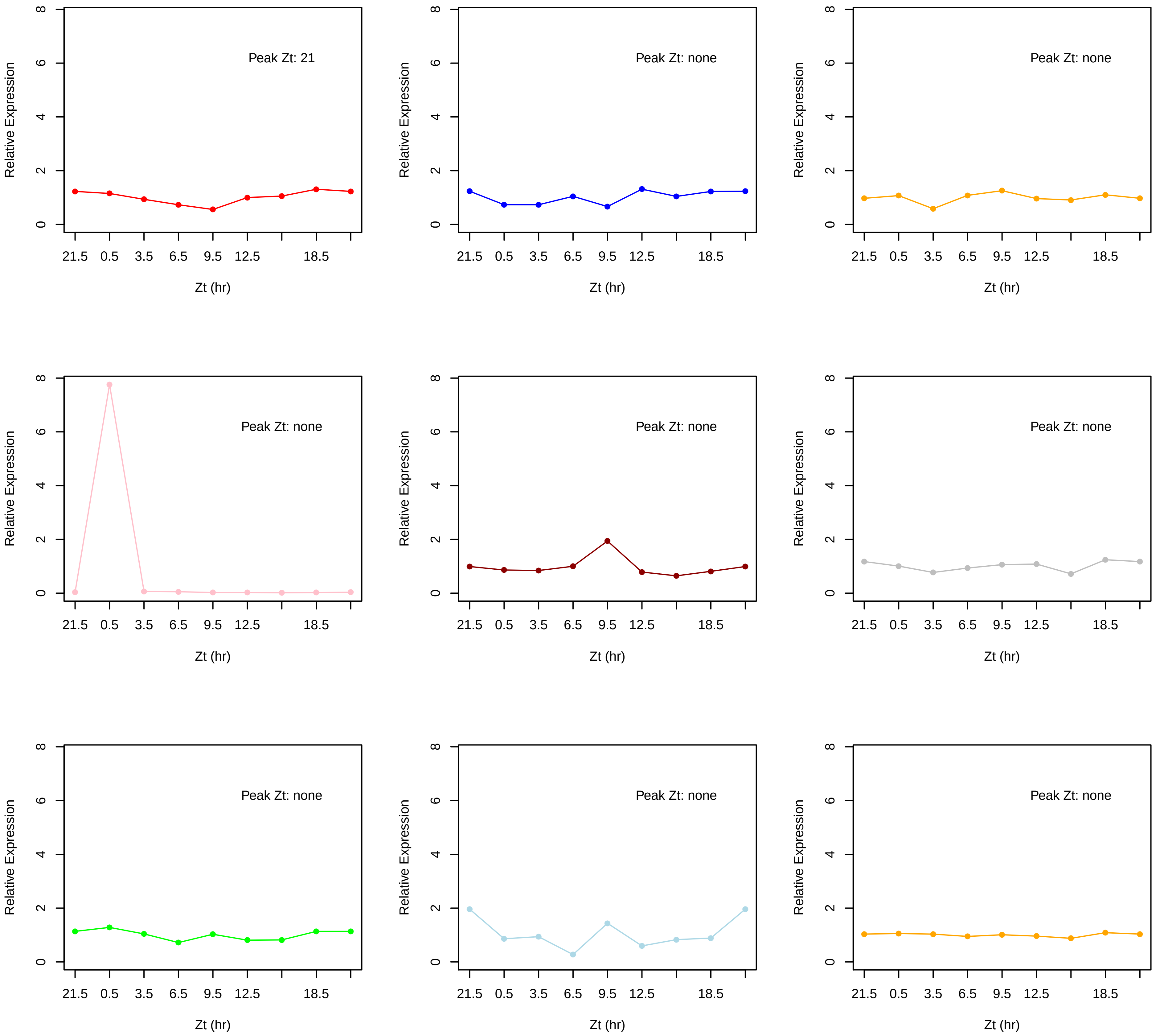

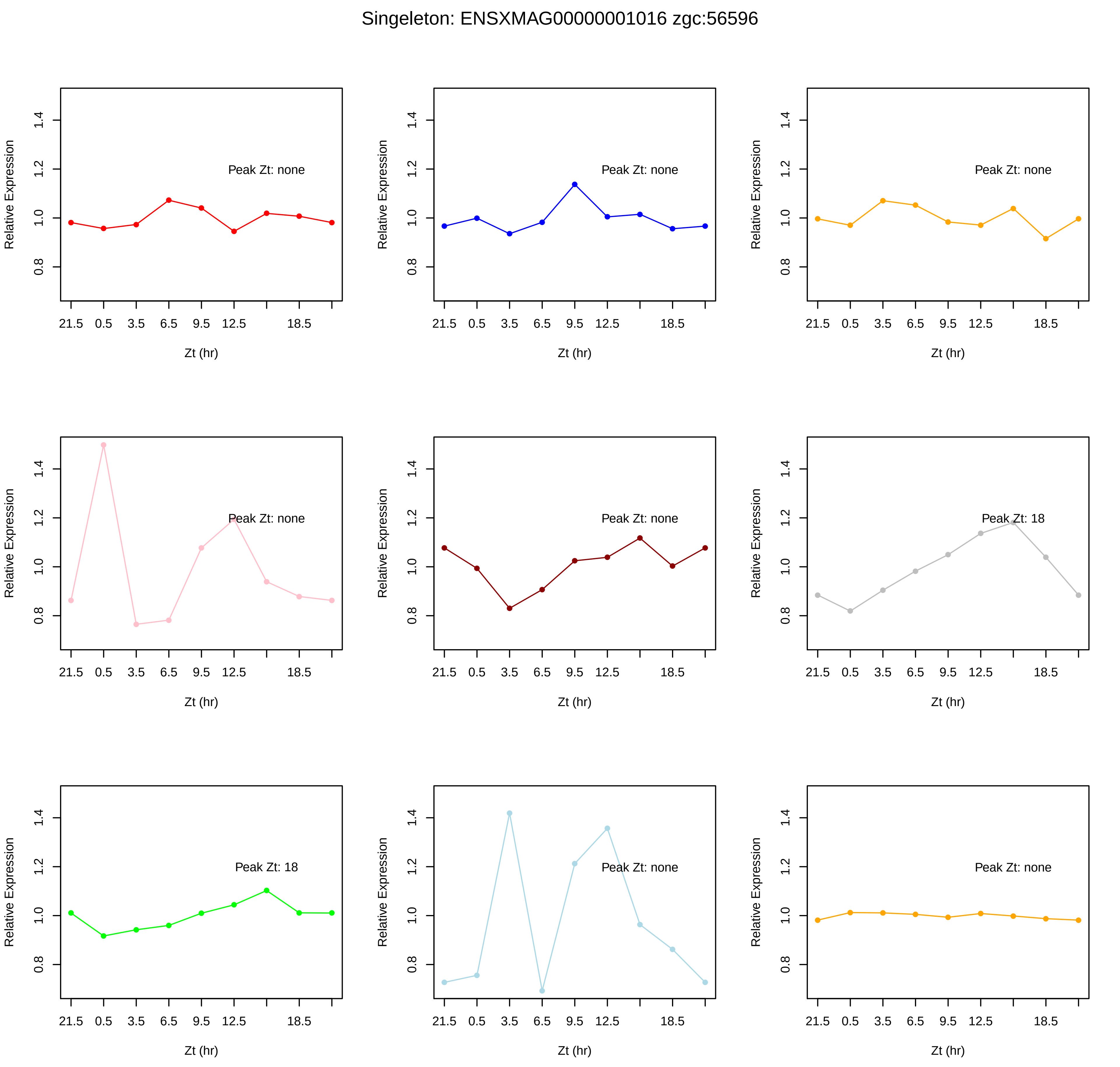

Singeleton: ENSXMAG00000001016 zgc:56596
Relative Expression
Zt (hr)
Peak Zt: none
Peak Zt: 18
1.4
1.2
1.0
0.8
21.5
0.5
3.5
6.5
9.5
12.5
18.5

Singeleton: ENSXMAG00000016389

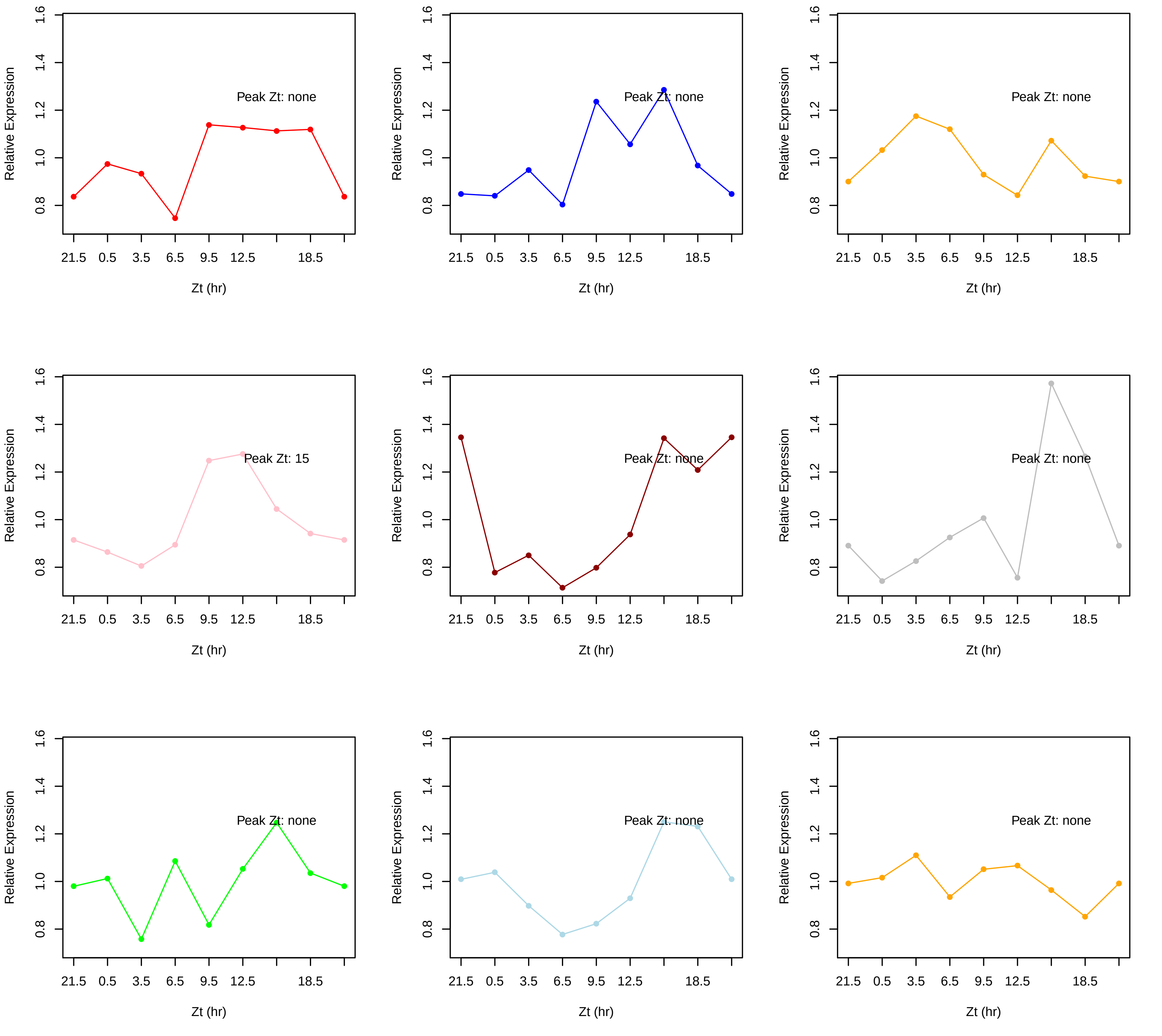

Singeleton: ENSXMAG00000011487 cttnbp2

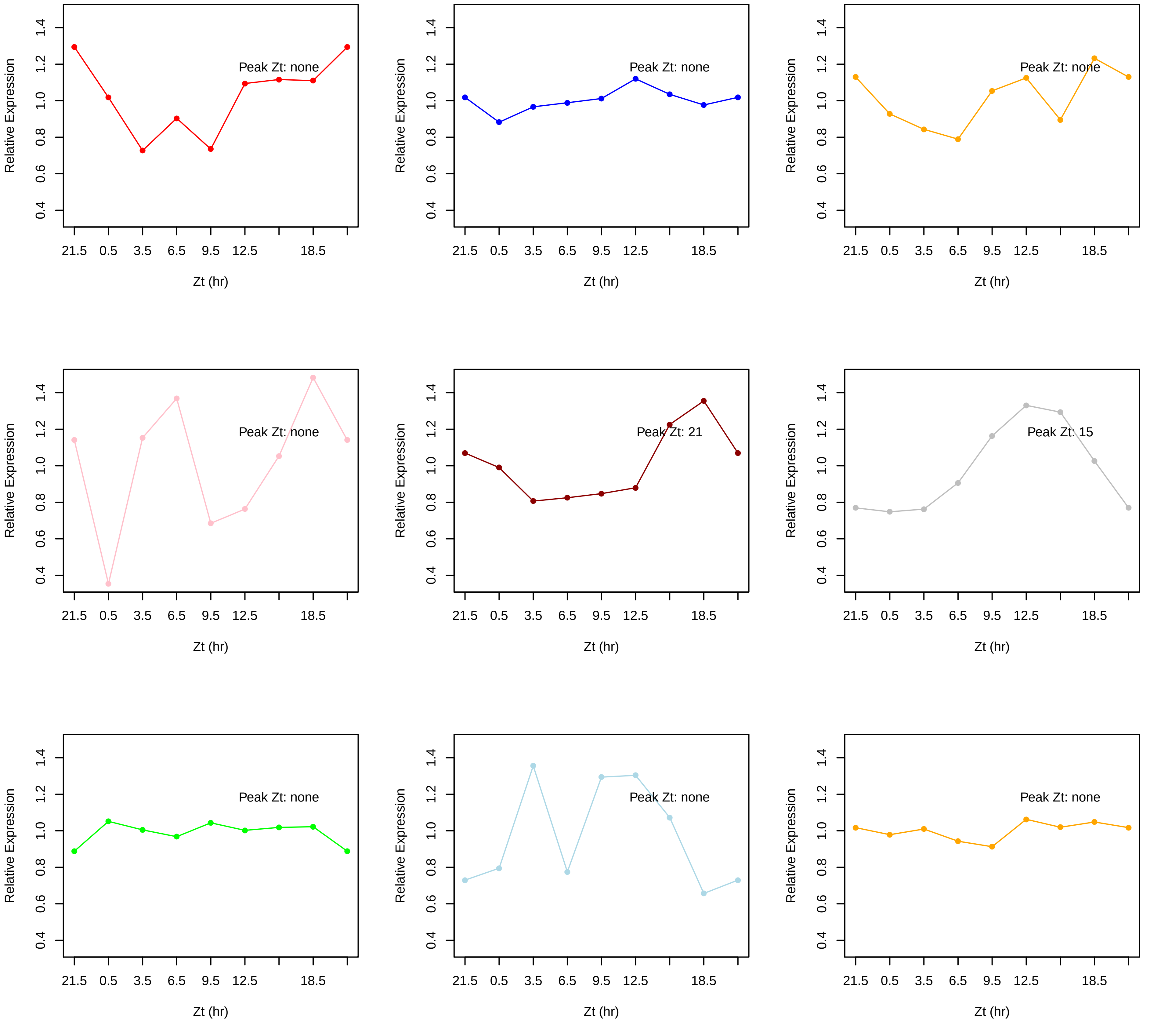

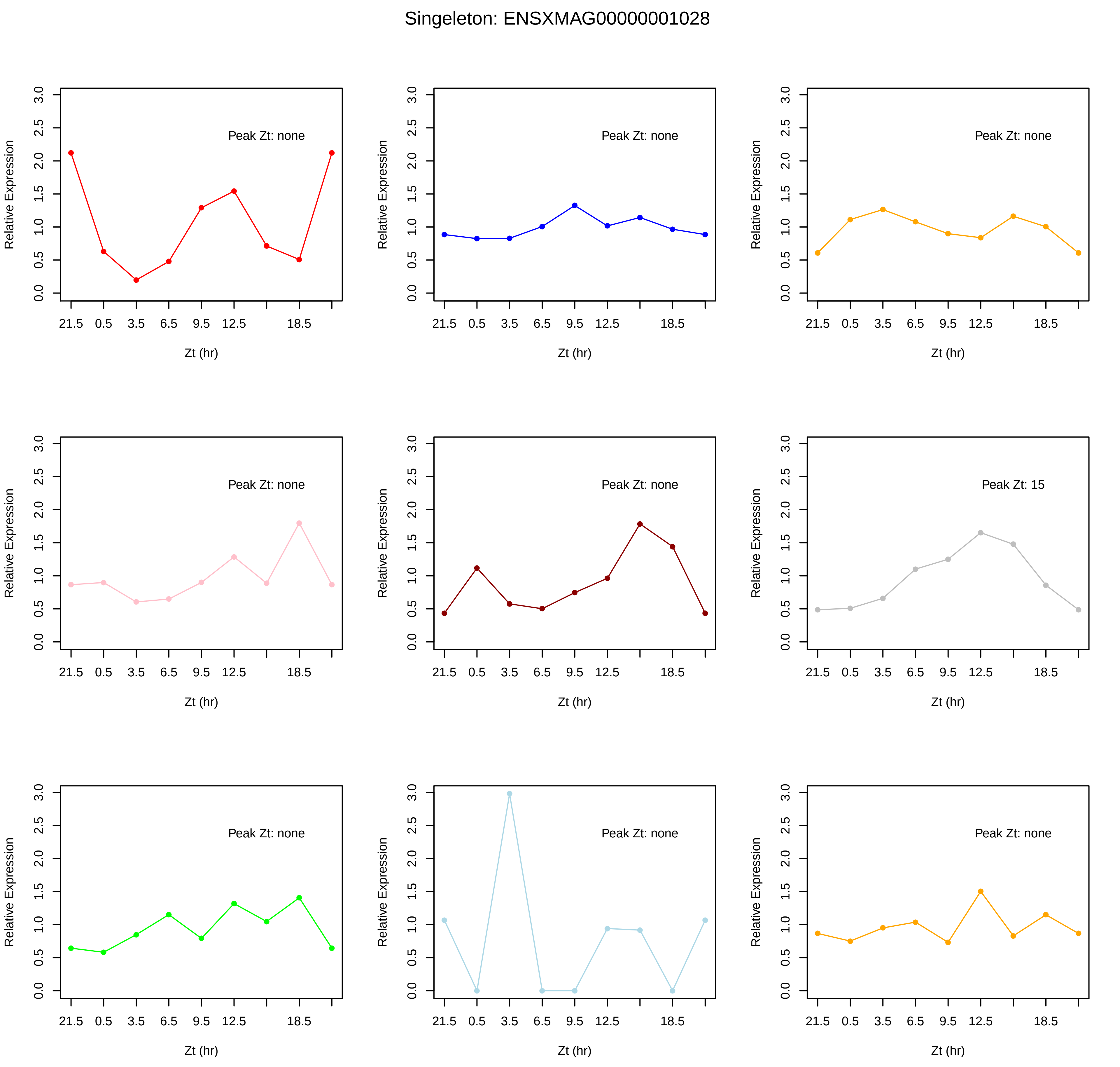
Singeleton: ENSXMAG00000001028
Peak Zt: none
Peak Zt: none
Peak Zt: none
Peak Zt: none
Peak Zt: none
Peak Zt: 15
Peak Zt: none
Peak Zt: none
Peak Zt: none
Relative Expression
Zt (hr)
21.5
0.5
3.5
6.5
9.5
12.5
18.5

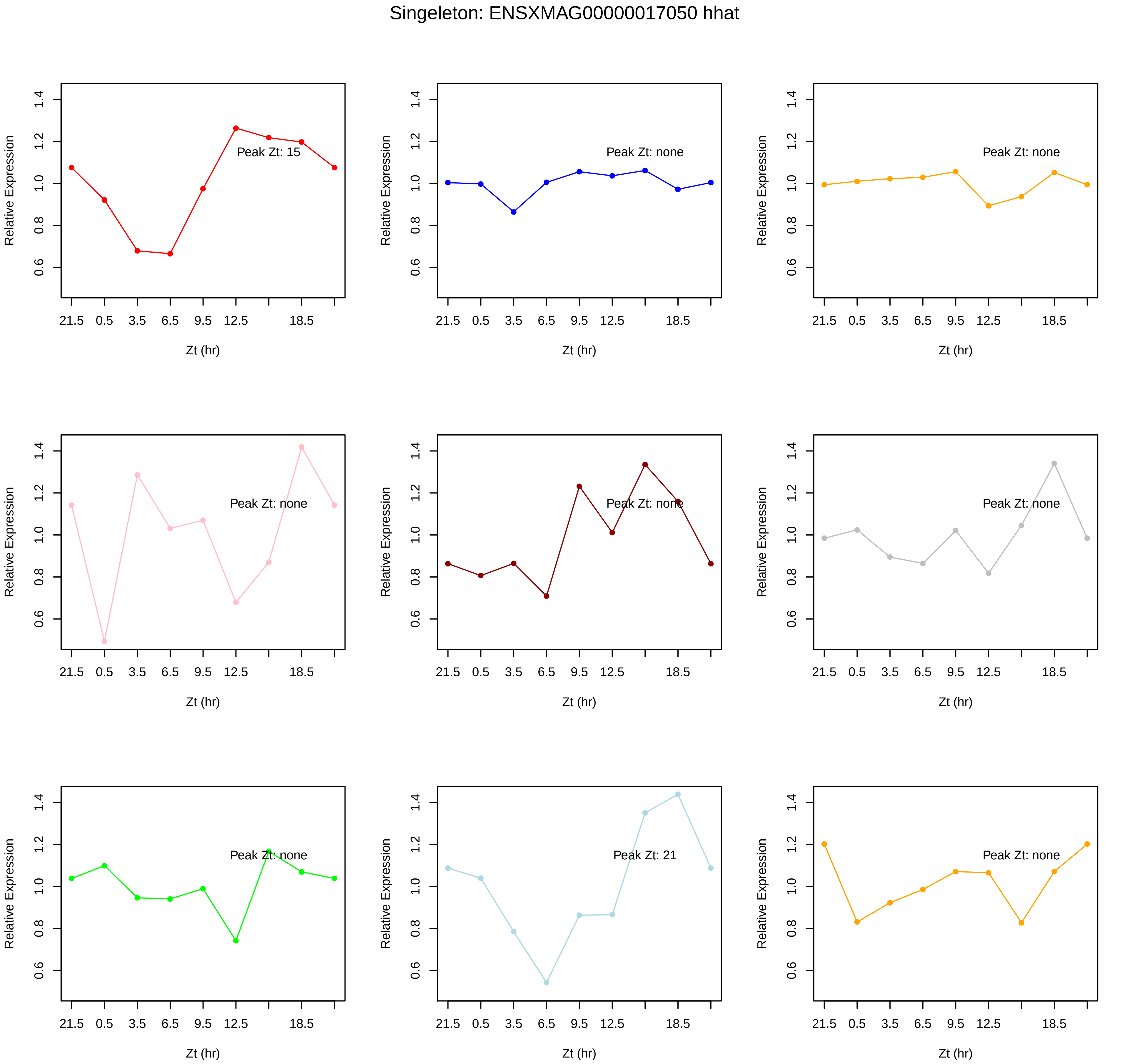
Singeleton: ENSXMAG00000017050 hhat
Peak Zt: 15
Peak Zt: none
Peak Zt: none
Peak Zt: none
Peak Zt: none
Peak Zt: none
Peak Zt: none
Peak Zt: 21
Peak Zt: none
Relative Expression
Zt (hr)
21.5
0.5
3.5
6.5
9.5
12.5
18.5
0.6
0.8
1.0
1.2
1.4

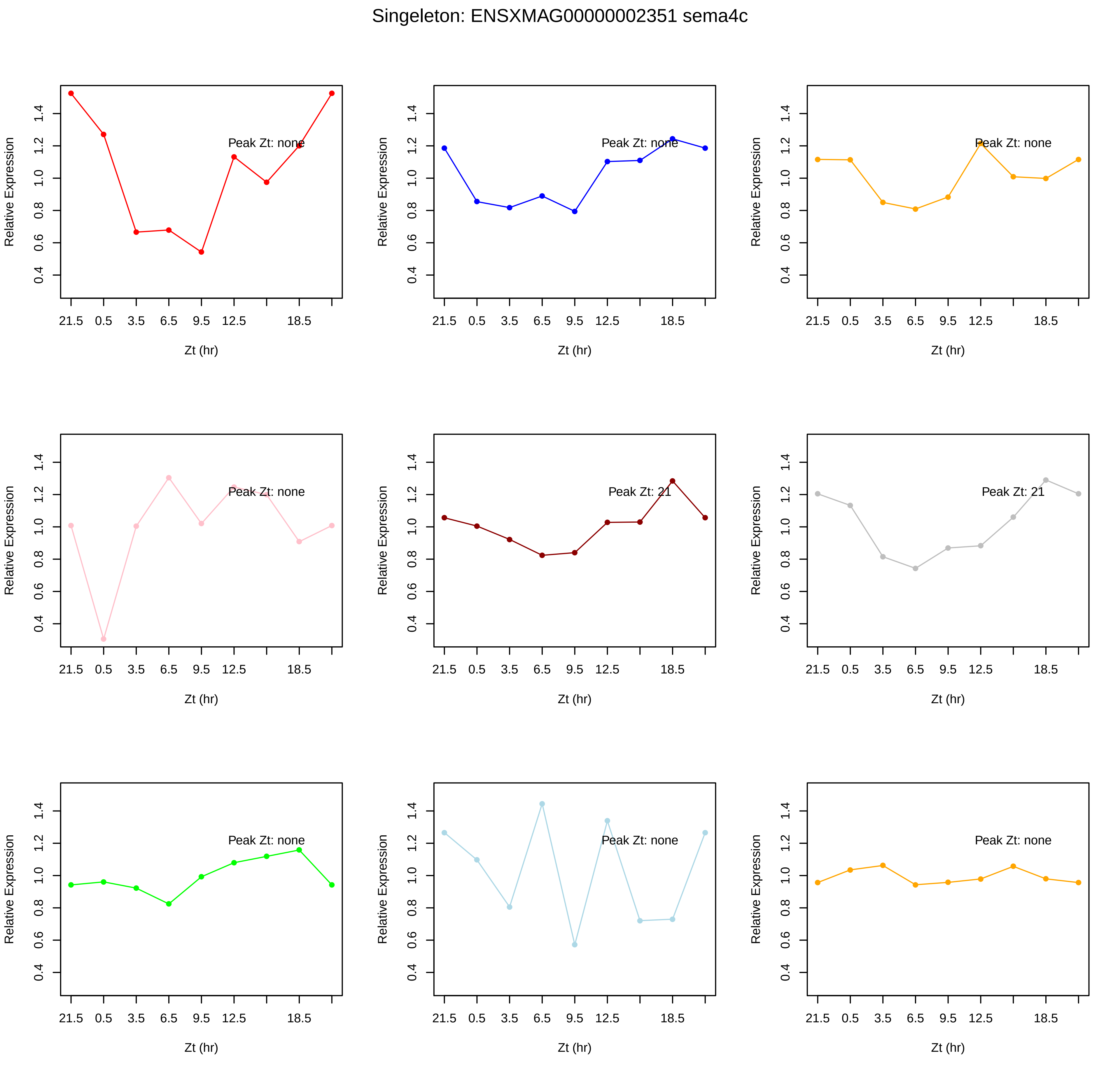

Singeleton: ENSXMAG00000002351 sema4c
Peak Zt: none
Peak Zt: none
Peak Zt: none
Peak Zt: none
Peak Zt: 21
Peak Zt: 21
Peak Zt: none
Peak Zt: none
Peak Zt: none
Relative Expression
Zt (hr)
21.5
0.5
3.5
6.5
9.5
12.5
18.5
0.4
0.6
0.8
1.0
1.2
1.4

Singeleton: ENSXMAG00000014457 met

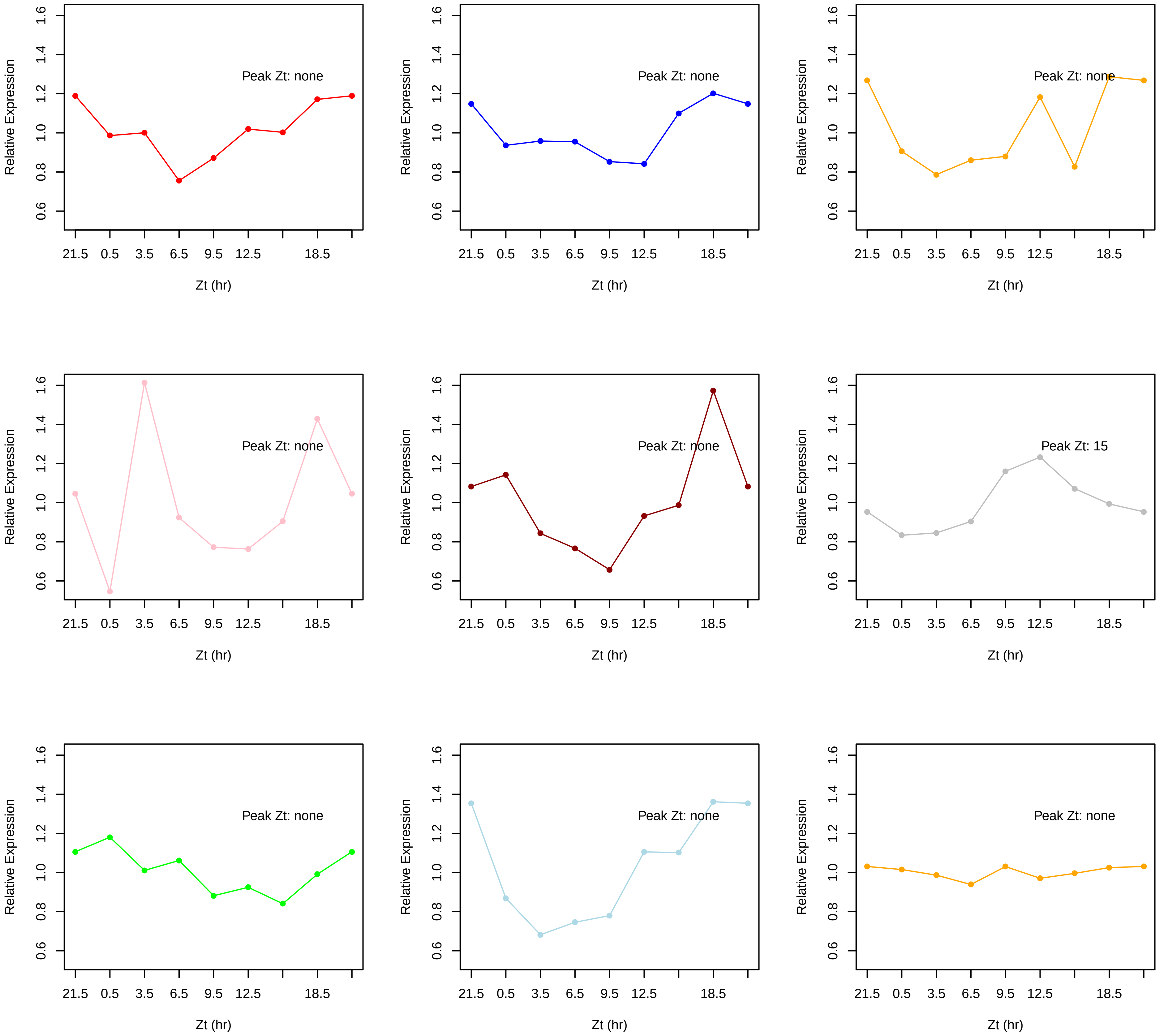

Singeleton: ENSXMAG00000023118

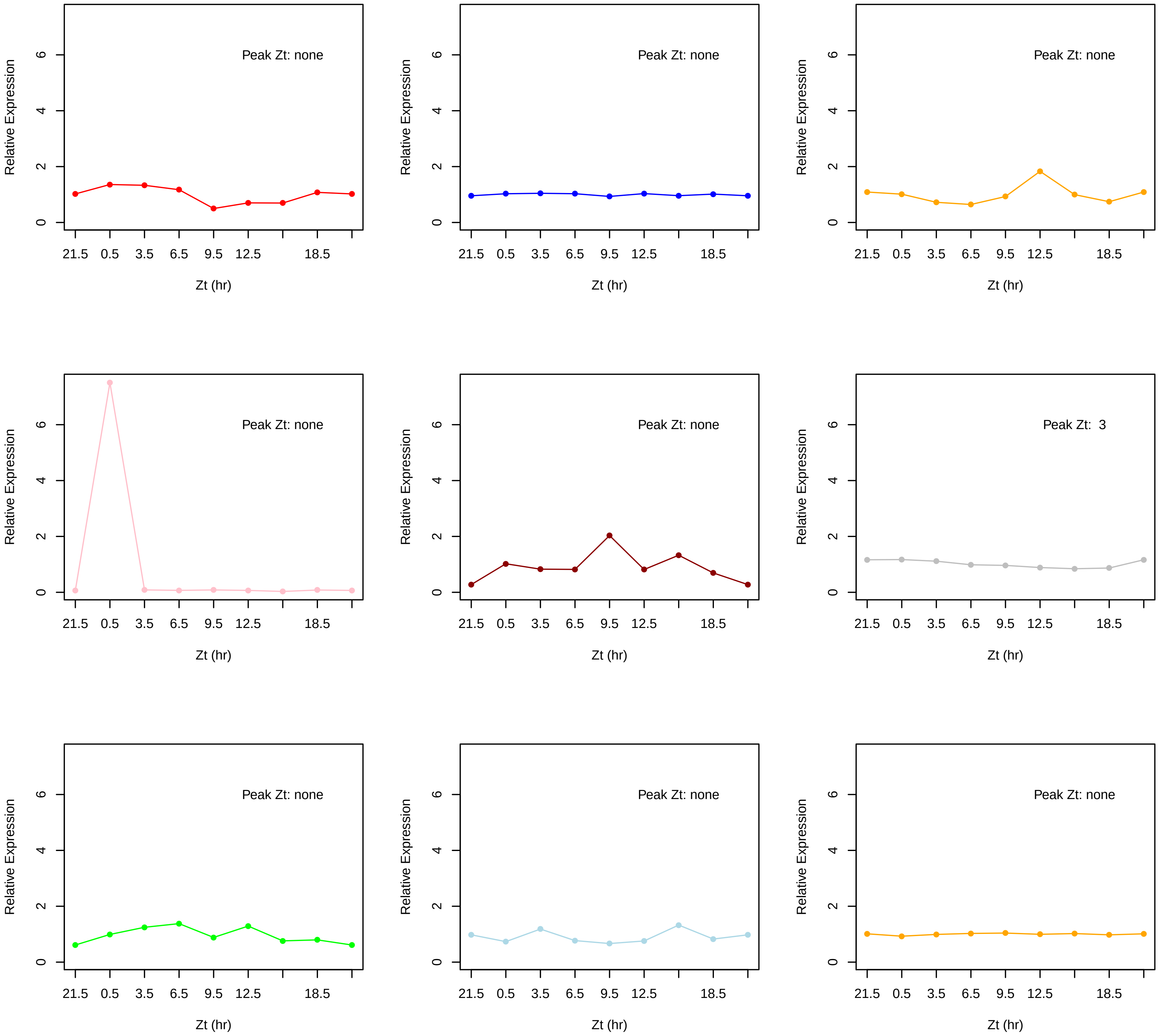

Singeleton: ENSXMAG00000014447 tes

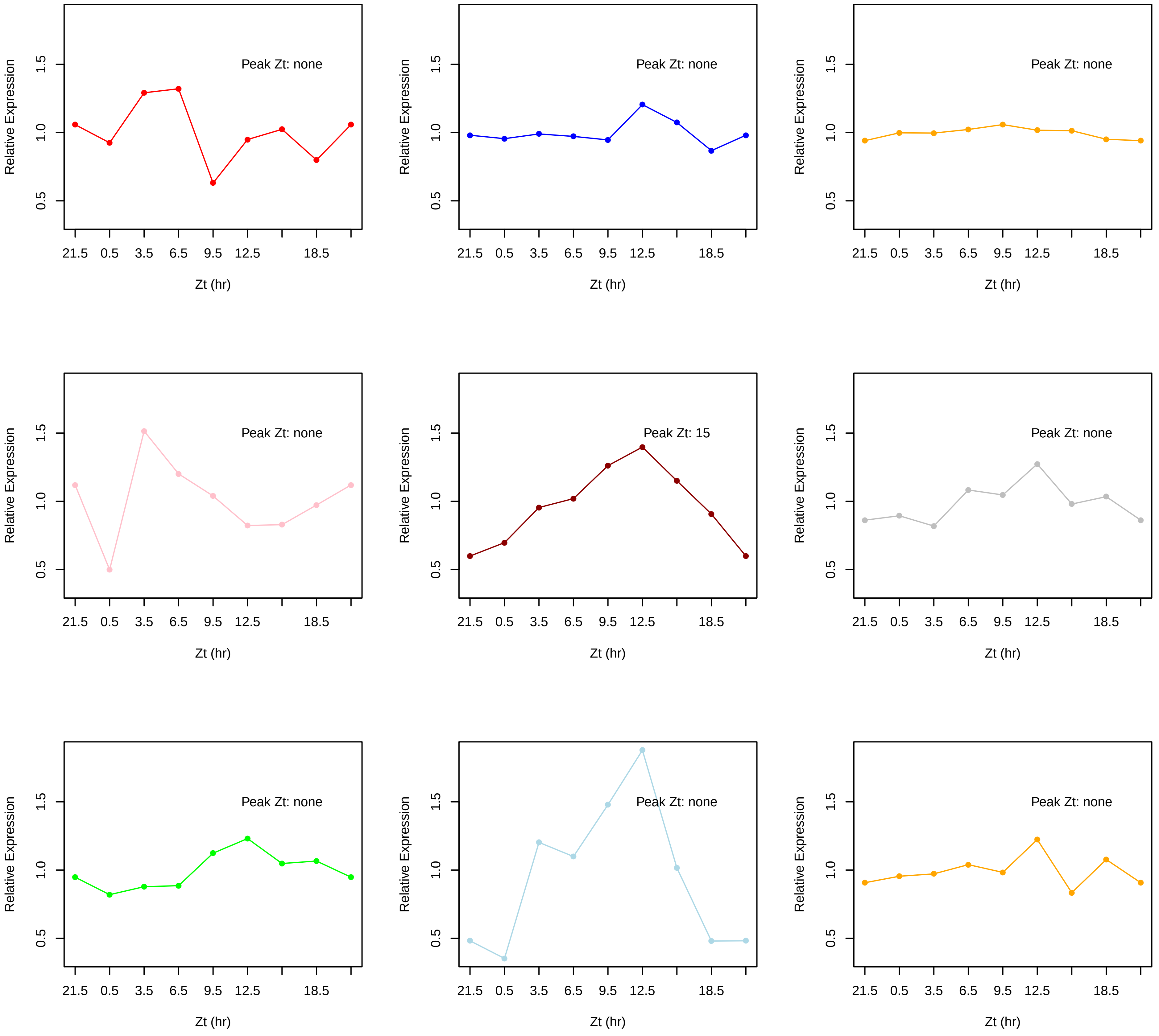

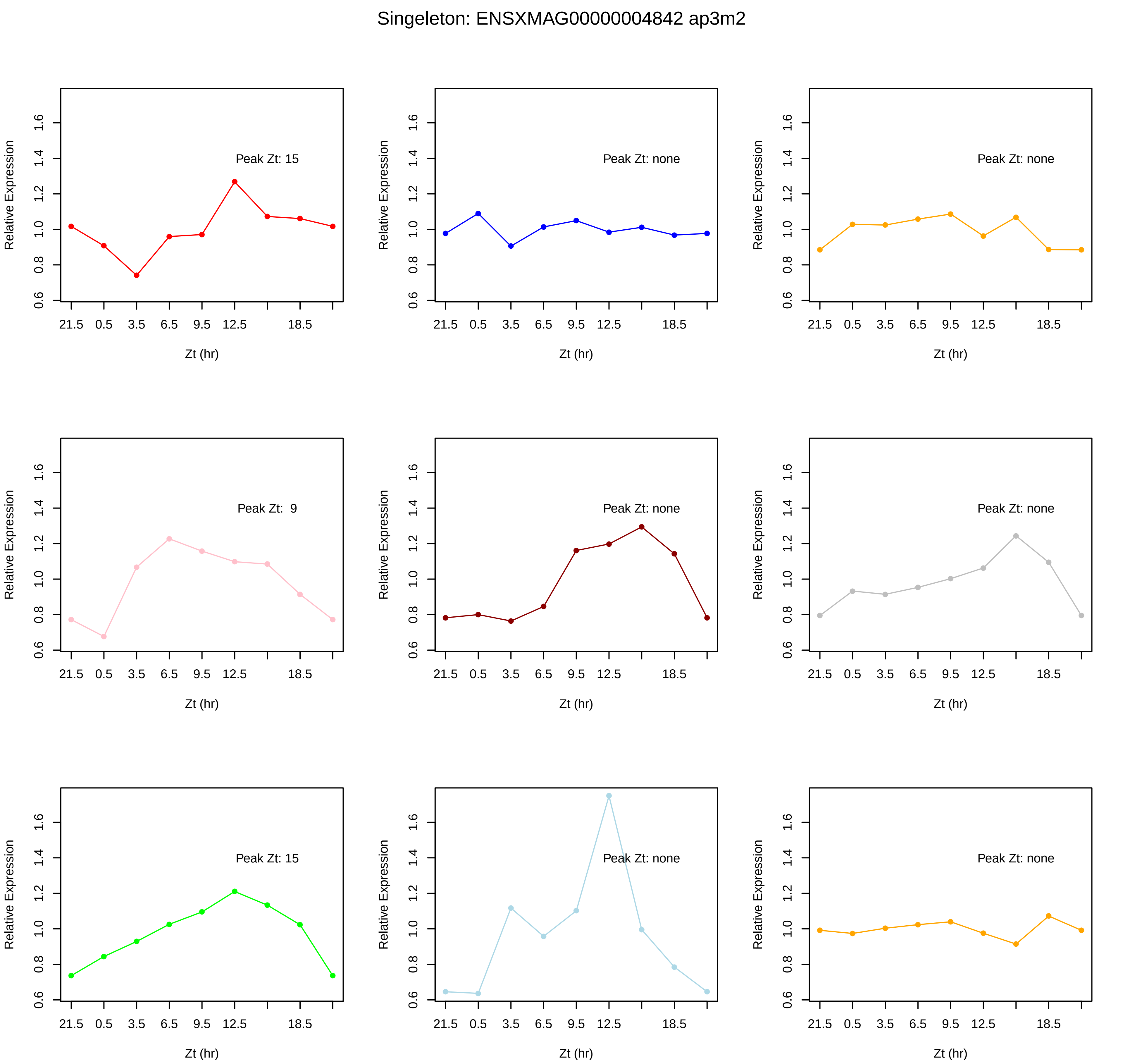

Singeleton: ENSXMAG00000004842 ap3m2
Peak Zt: 15
Peak Zt: none
Peak Zt: none
Peak Zt: 9
Peak Zt: none
Peak Zt: none
Peak Zt: 15
Peak Zt: none
Peak Zt: none
Relative Expression
Zt (hr)
0.6
0.8
1.0
1.2
1.4
1.6
21.5
0.5
3.5
6.5
9.5
12.5
18.5

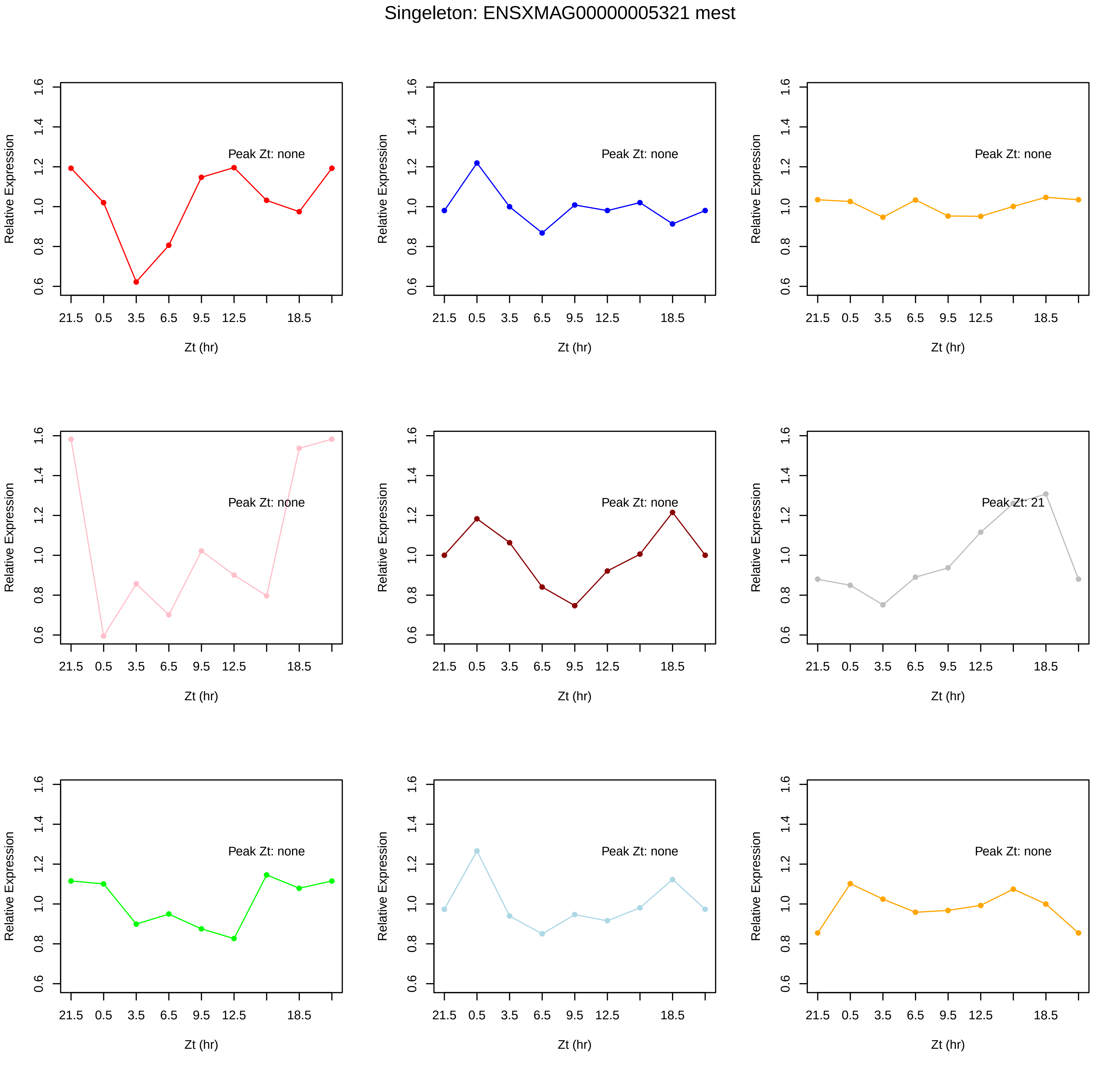
Singeleton: ENSXMAG00000005321 mest
Peak Zt: none
Peak Zt: none
Peak Zt: none
Peak Zt: none
Peak Zt: none
Peak Zt: 21
Peak Zt: none
Peak Zt: none
Peak Zt: none
Relative Expression
Zt (hr)
21.5 0.5 3.5 6.5 9.5 12.5 18.5
0.6 0.8 1.0 1.2 1.4 1.6

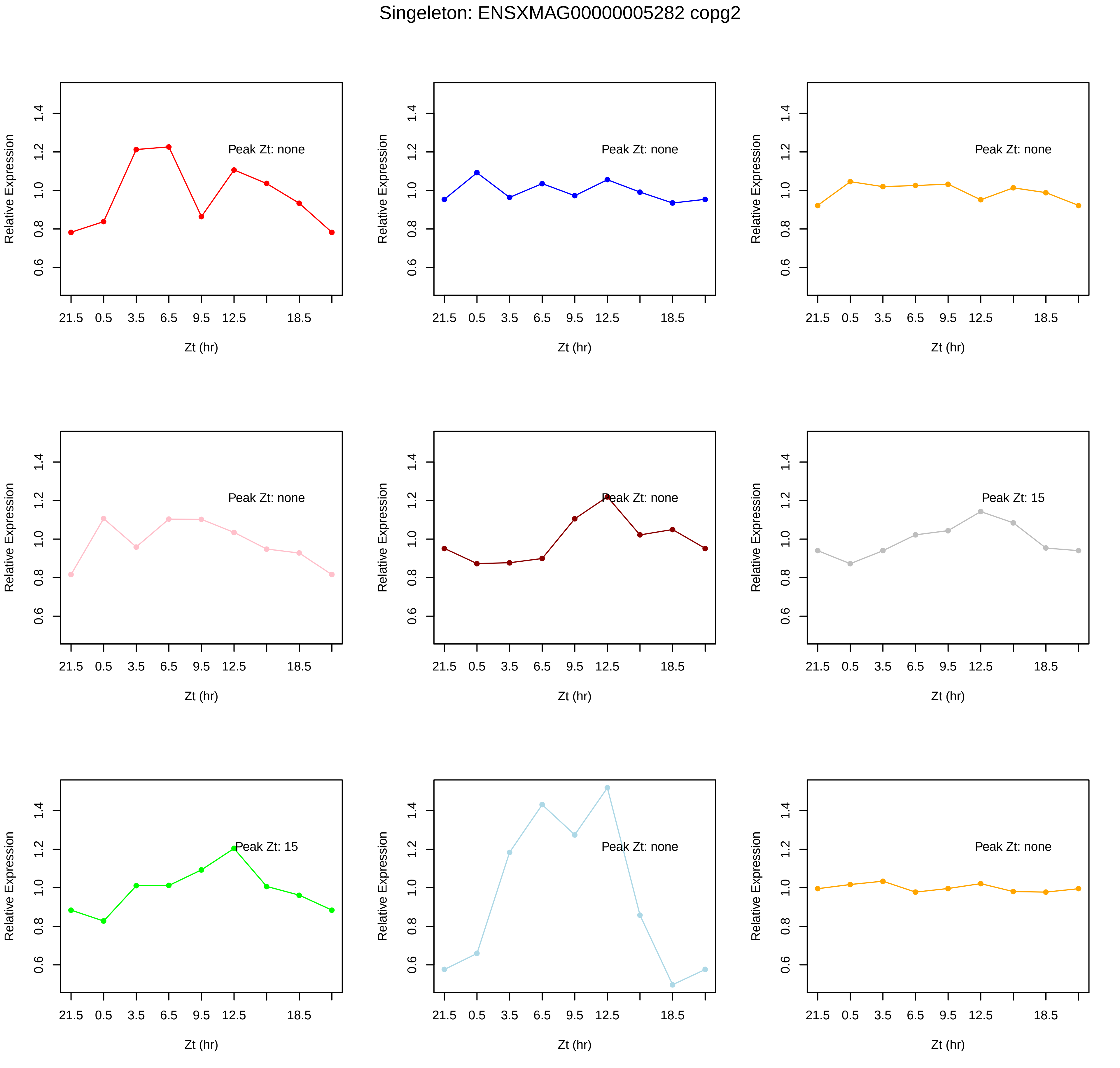
Singleton: ENSXMAG00000005282 copg2
Relative Expression
Zt (hr)
Peak Zt: none
Peak Zt: none
Peak Zt: none
Peak Zt: none
Peak Zt: none
Peak Zt: 15
Peak Zt: 15
Peak Zt: none
Peak Zt: none
0.6
0.8
1.0
1.2
1.4
21.5
0.5
3.5
6.5
9.5
12.5
18.5

Singeleton: ENSXMAG00000021855

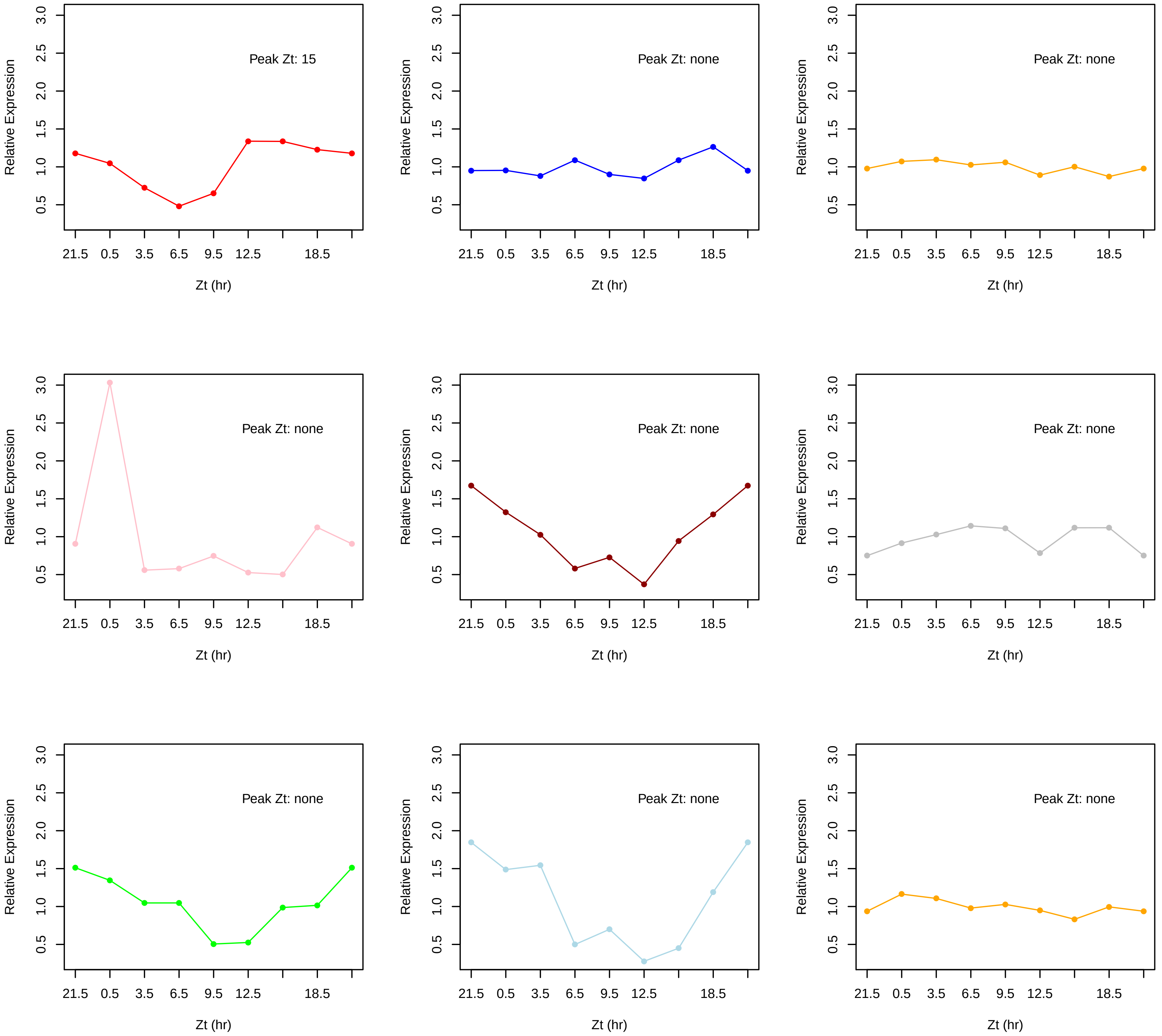

Singeleton: ENSXMAG00000016942 slc35b4

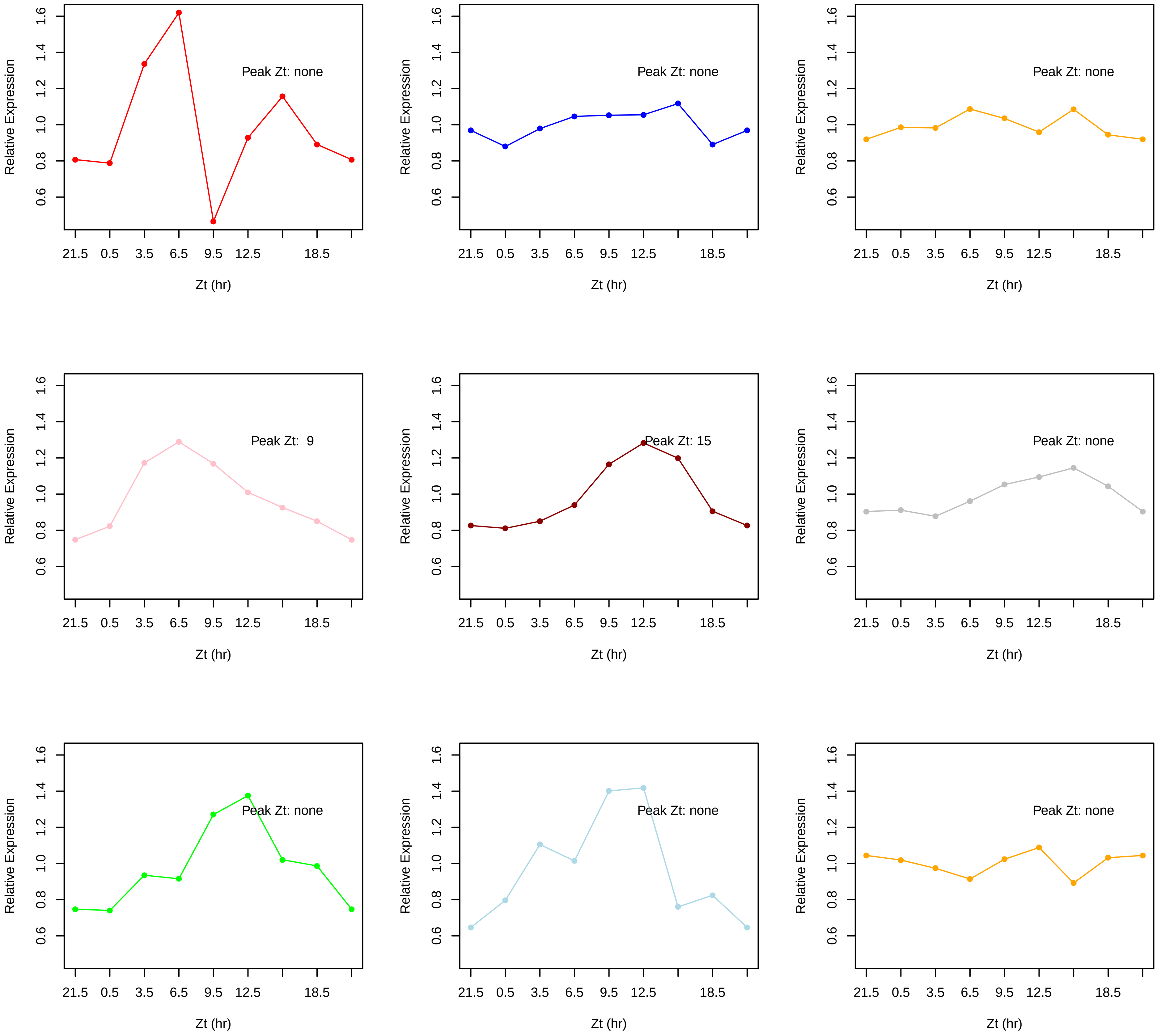

Singeleton: ENSXMAG00000014844 rassf2a

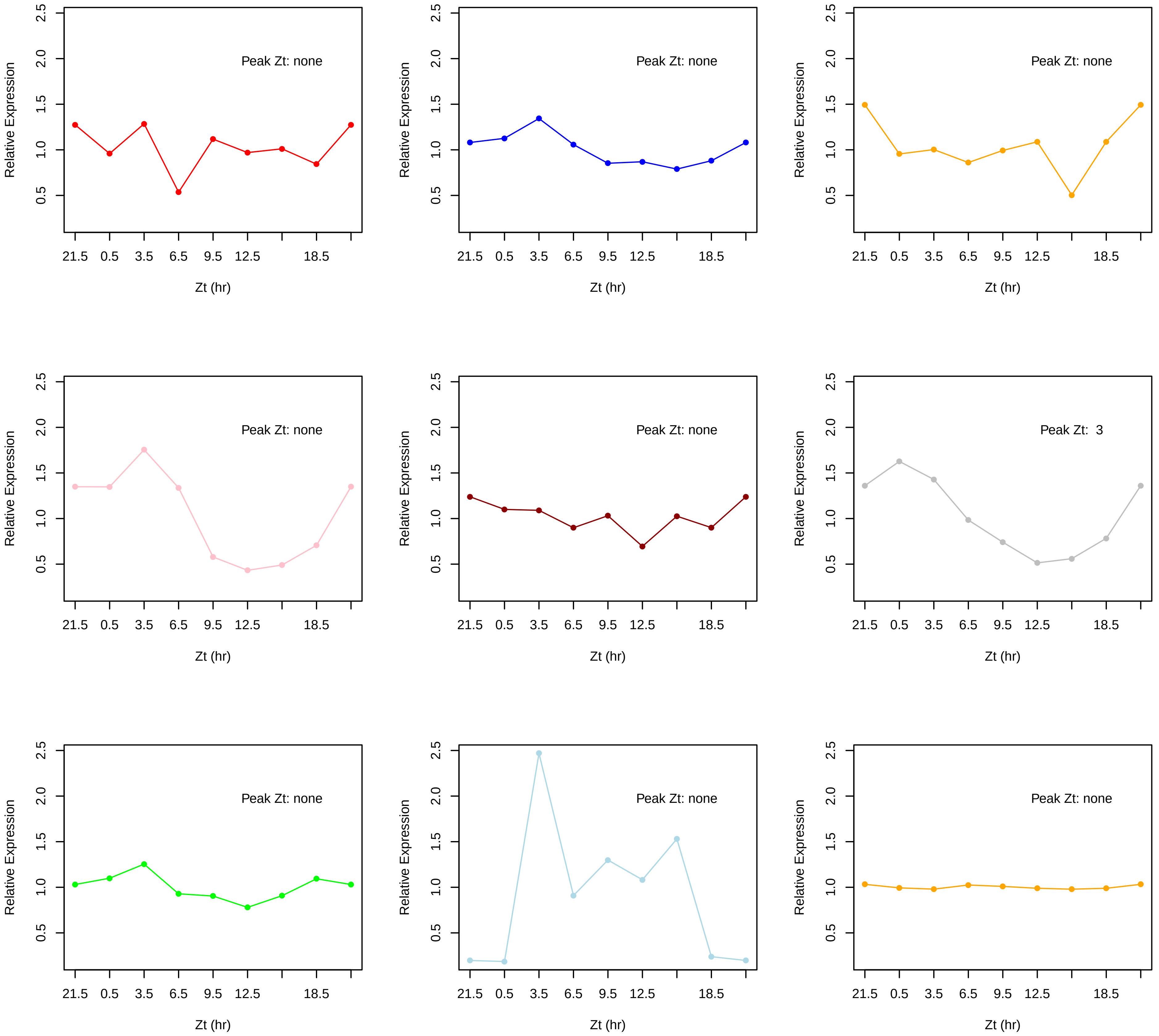

Singeleton: ENSXMAG00000014856 SLC23A2

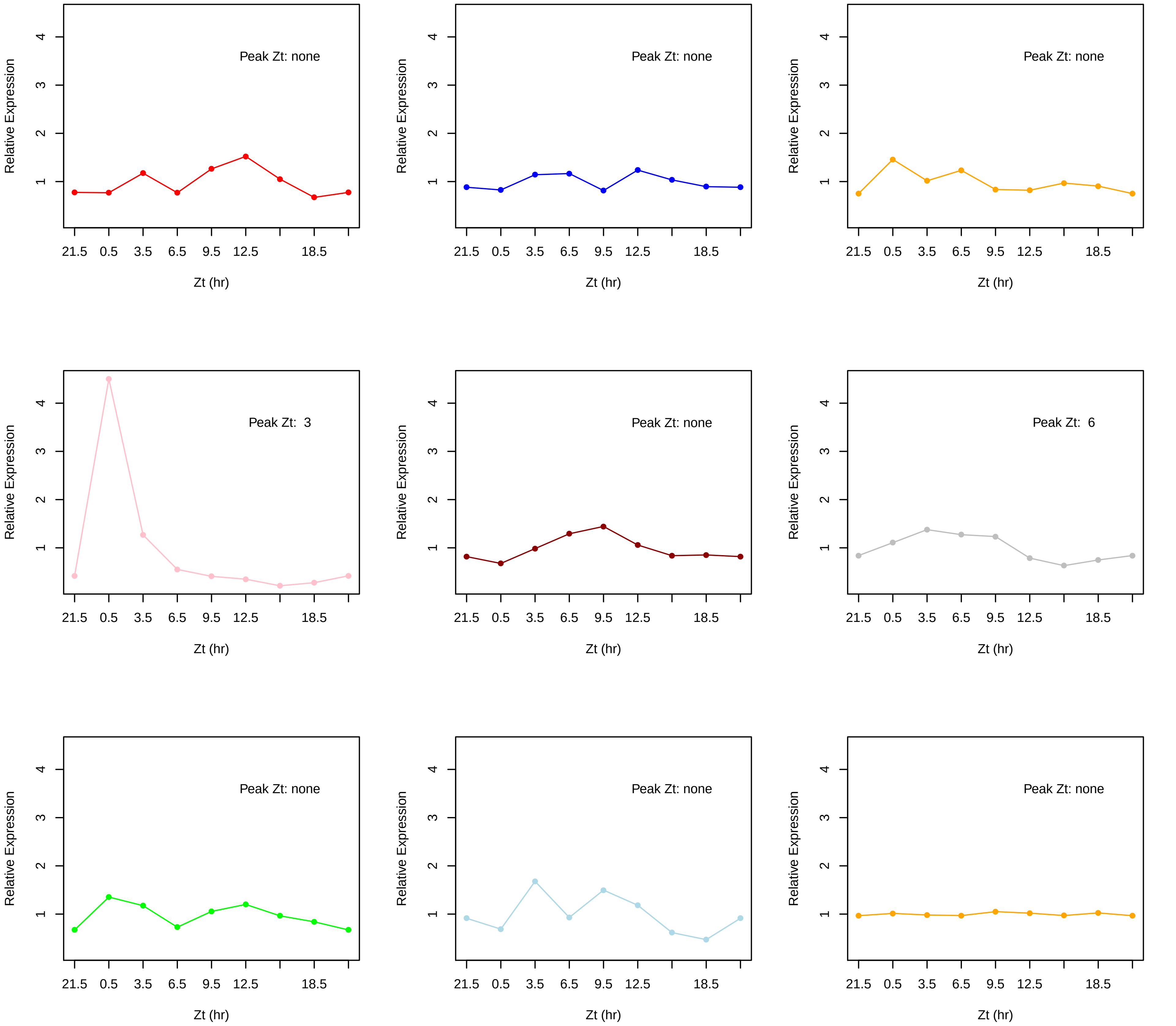

Singeleton: ENSXMAG00000024229 MRPL14

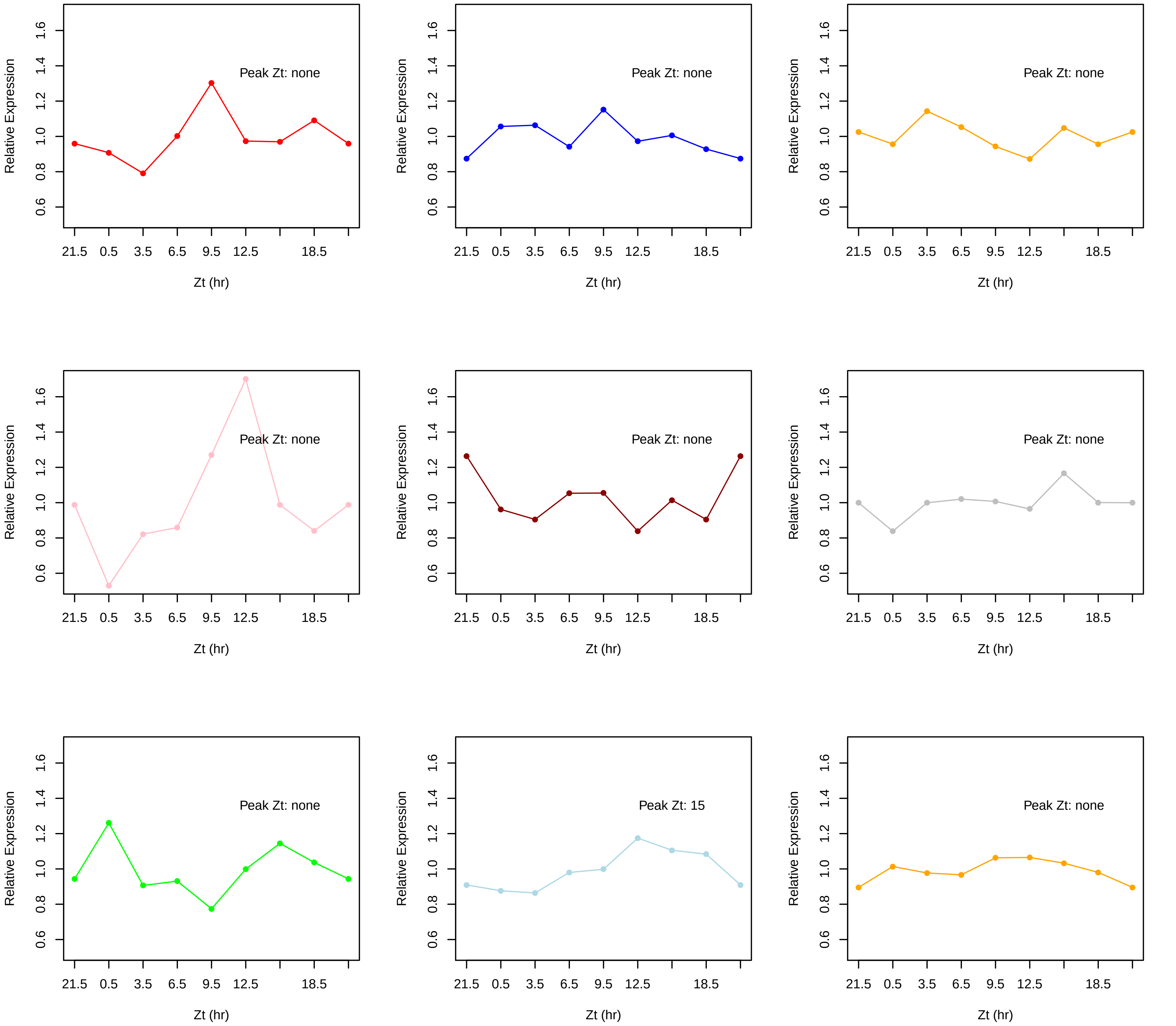

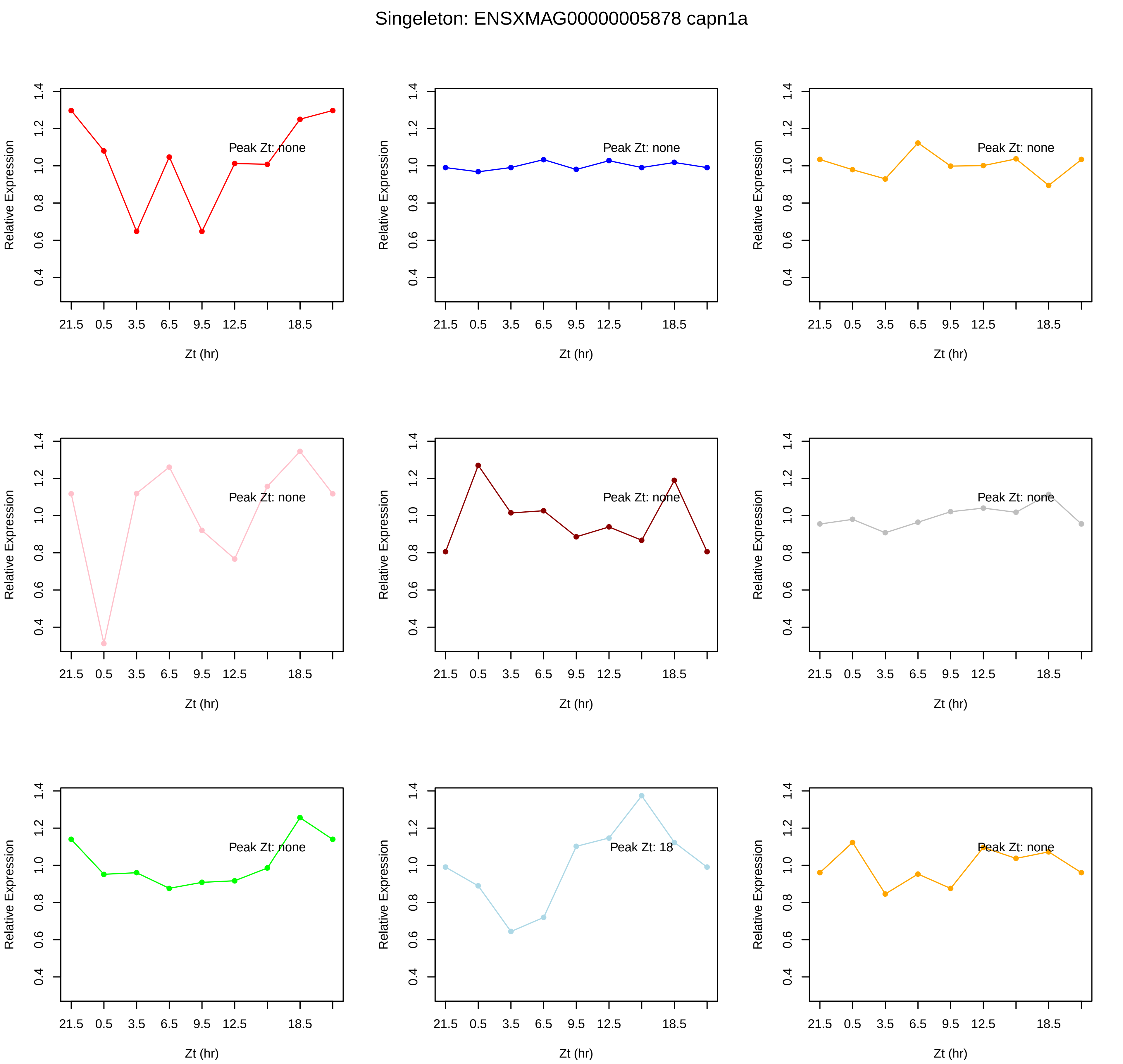
Singeleton: ENSXMAG00000005878 capn1a
Peak Zt: none
Peak Zt: none
Peak Zt: none
Peak Zt: none
Peak Zt: none
Peak Zt: none
Peak Zt: none
Peak Zt: 18
Peak Zt: none
Relative Expression
Zt (hr)
21.5
0.5
3.5
6.5
9.5
12.5
18.5
0.4
0.6
0.8
1.0
1.2
1.4

Singeleton: ENSXMAG00000014891 pcna

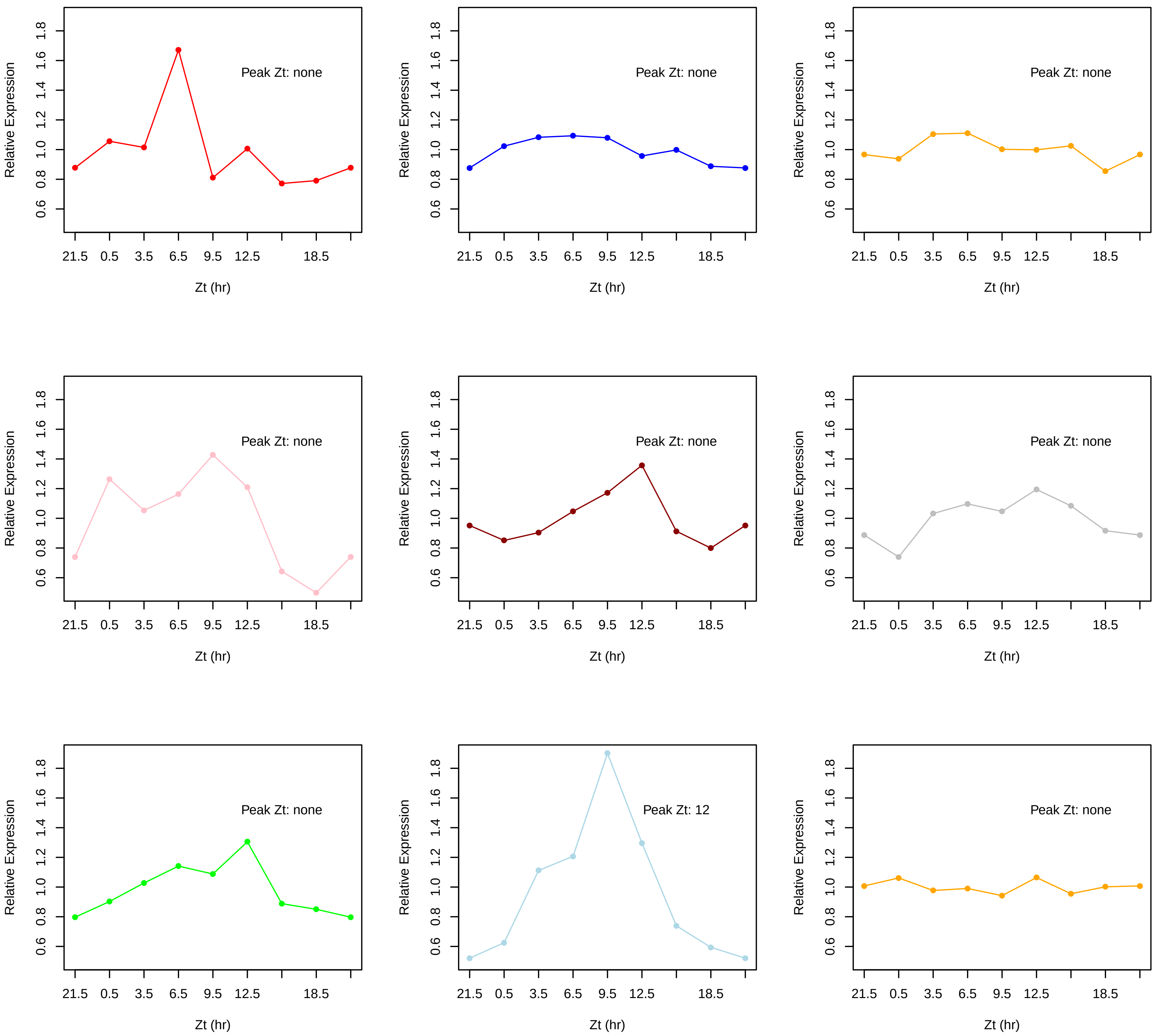

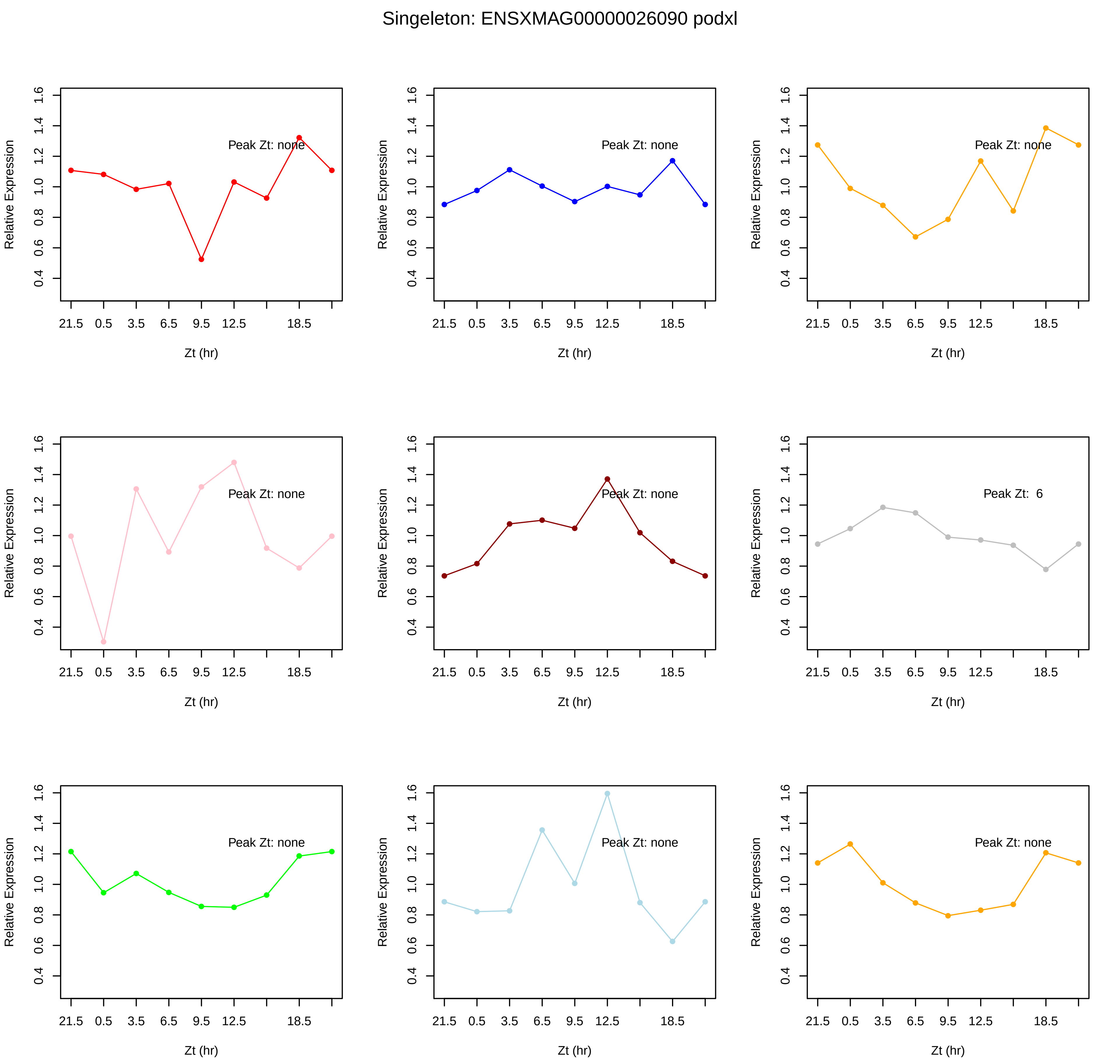
Singleton: ENSXMAG00000026090 podxl
Relative Expression
Zt (hr)
21.5
0.5
3.5
6.5
9.5
12.5
18.5
0.4
0.6
0.8
1.0
1.2
1.4
1.6
Peak Zt: none
Peak Zt: 6

Singeleton: ENSXMAG00000020317 adra2b

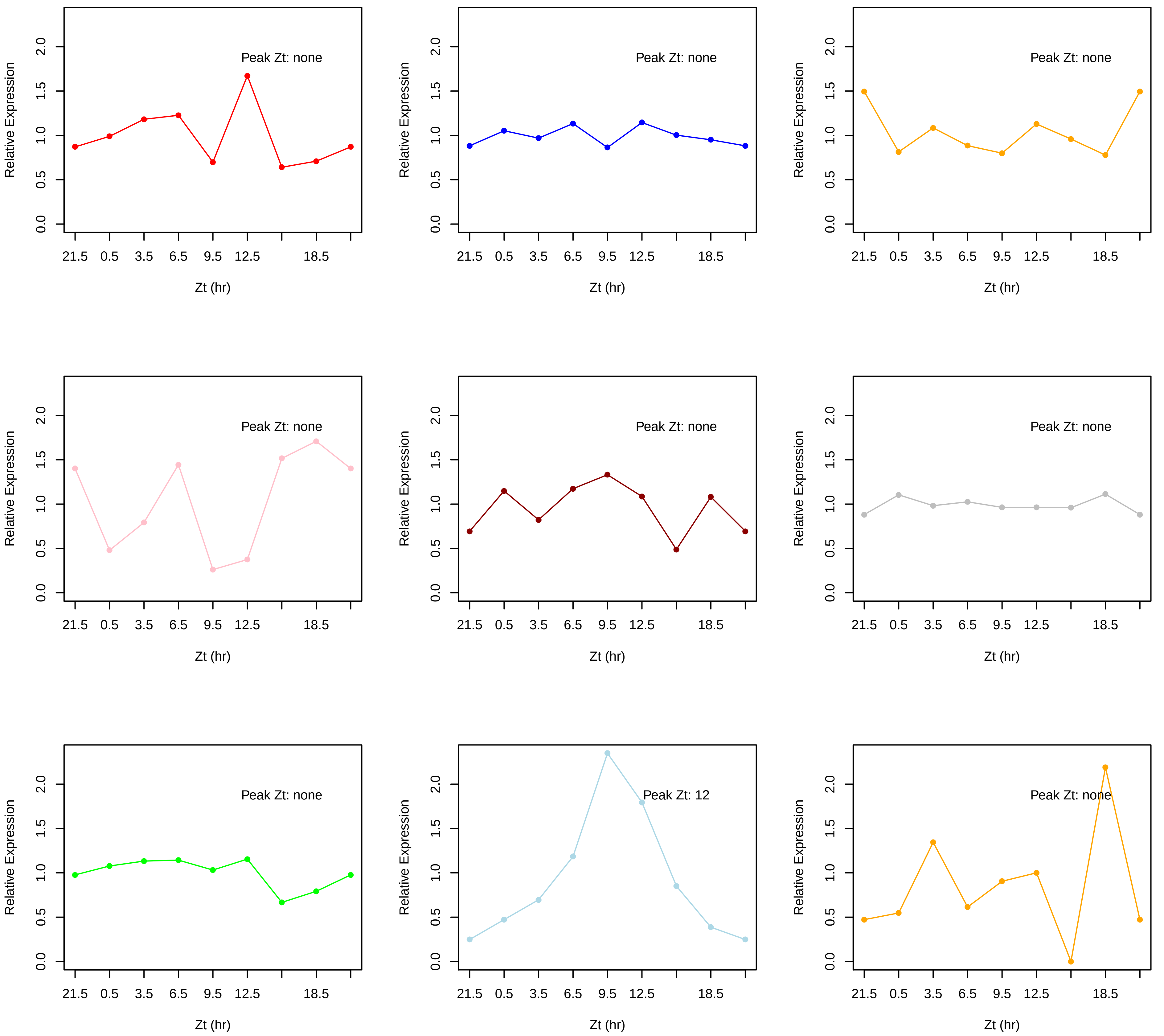

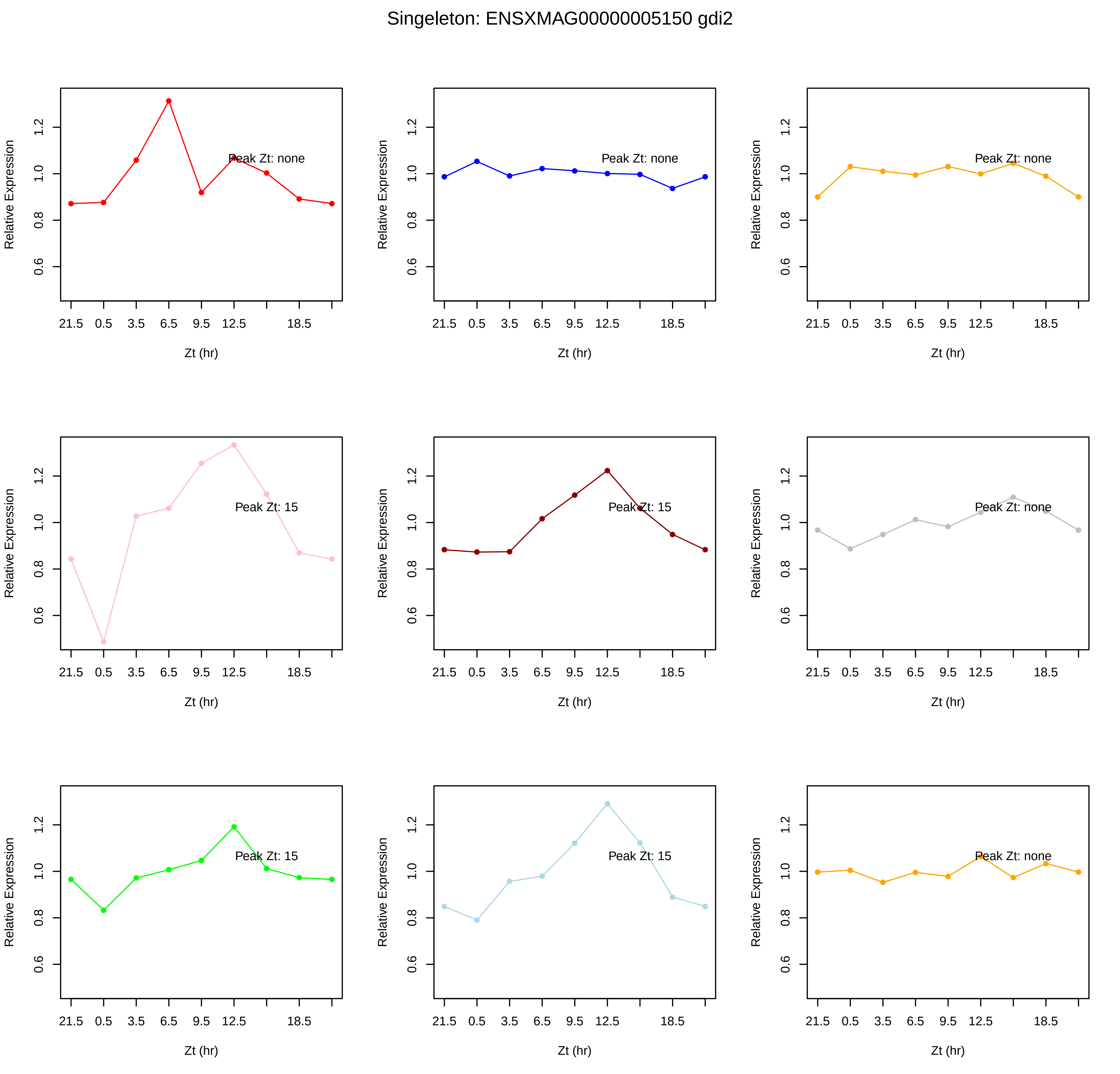
Singeleton: ENSXMAG00000005150 gdi2
Peak Zt: none
Peak Zt: none
Peak Zt: none
Peak Zt: 15
Peak Zt: 15
Peak Zt: none
Peak Zt: 15
Peak Zt: 15
Peak Zt: none
Relative Expression
Zt (hr)
21.5
0.5
3.5
6.5
9.5
12.5
18.5
0.6
0.8
1.0
1.2

Singleton: ENSXMAG00000014933 dusp2

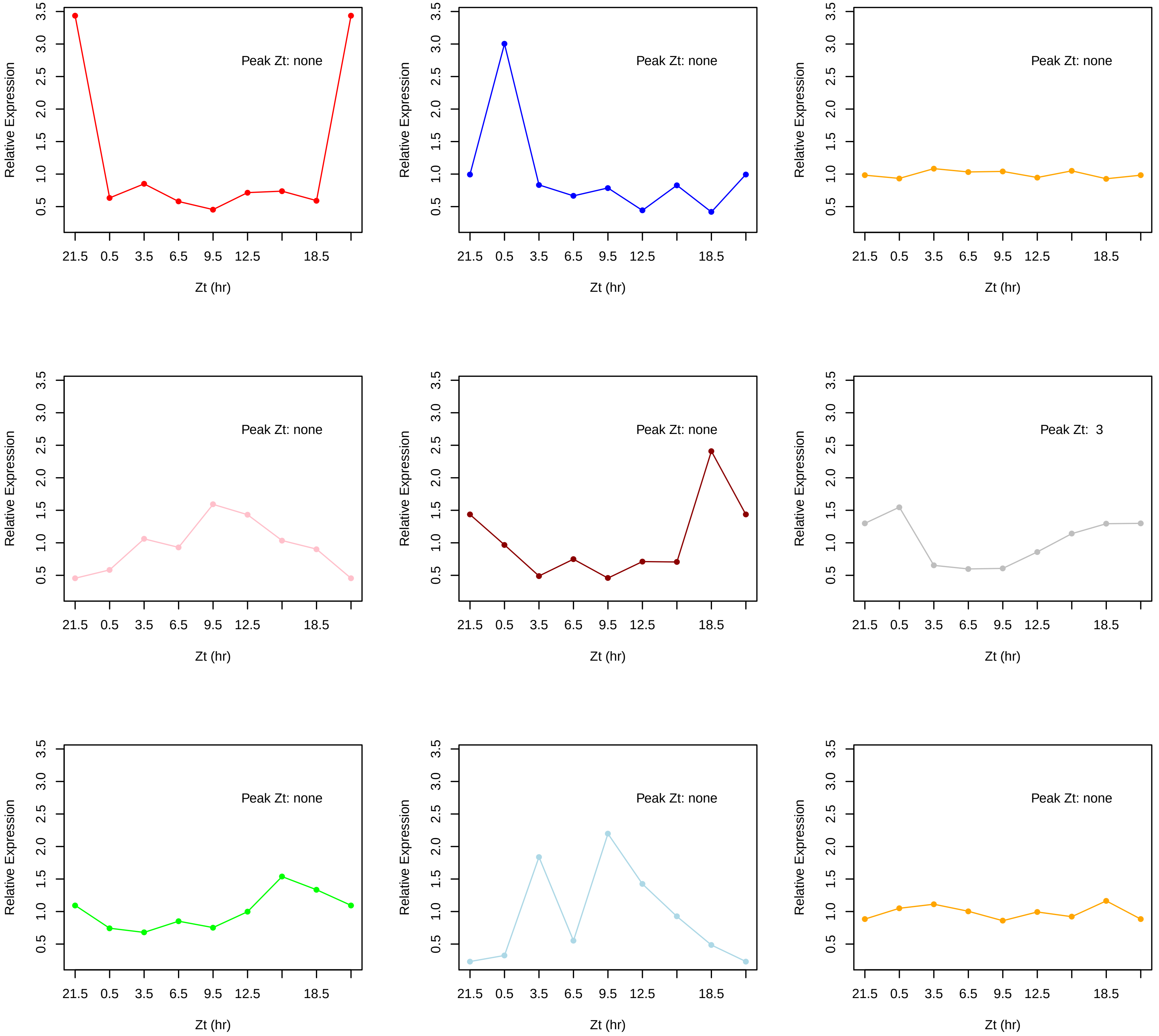

Singeleton: ENSXMAG00000019200 fbxo18

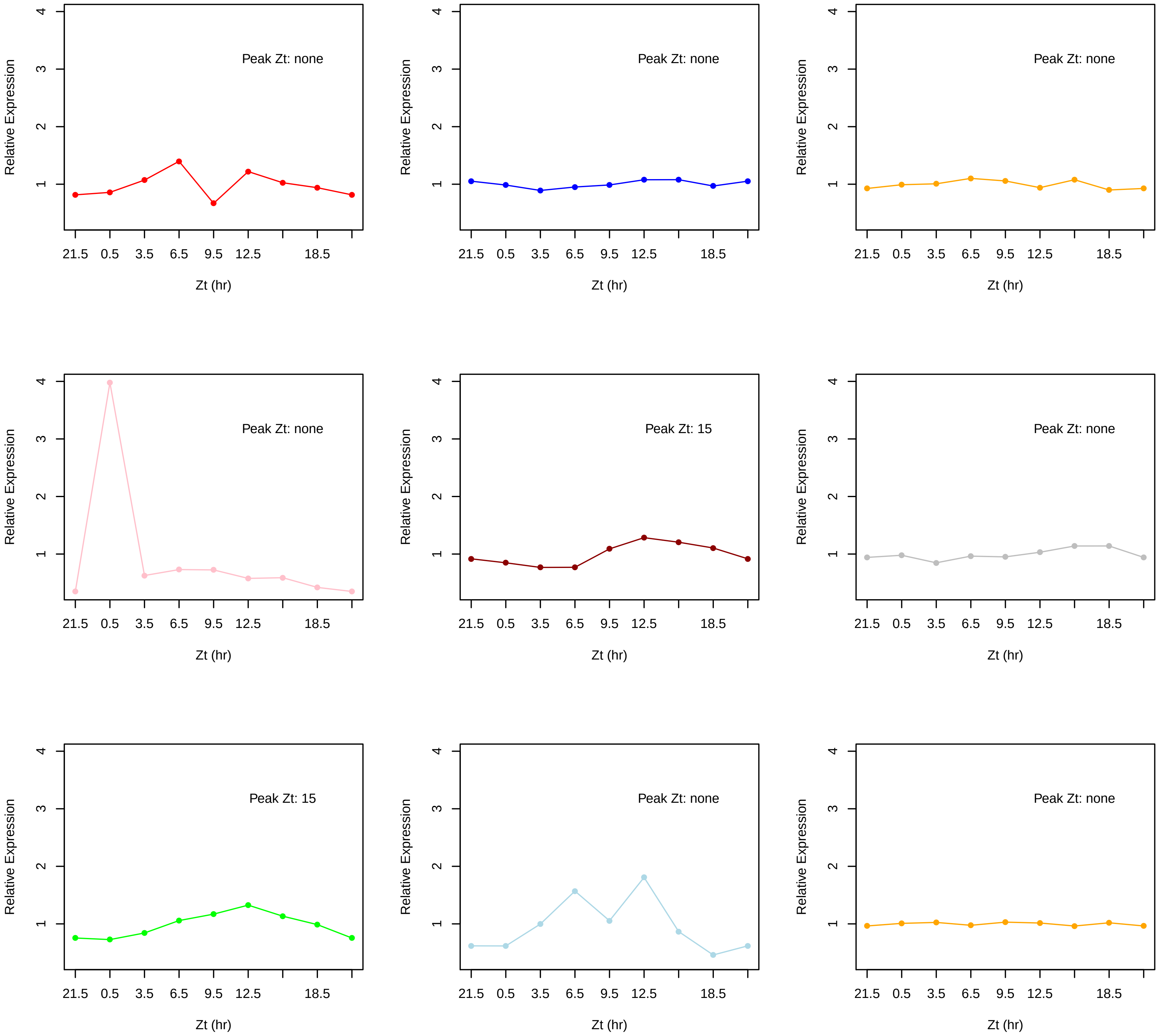

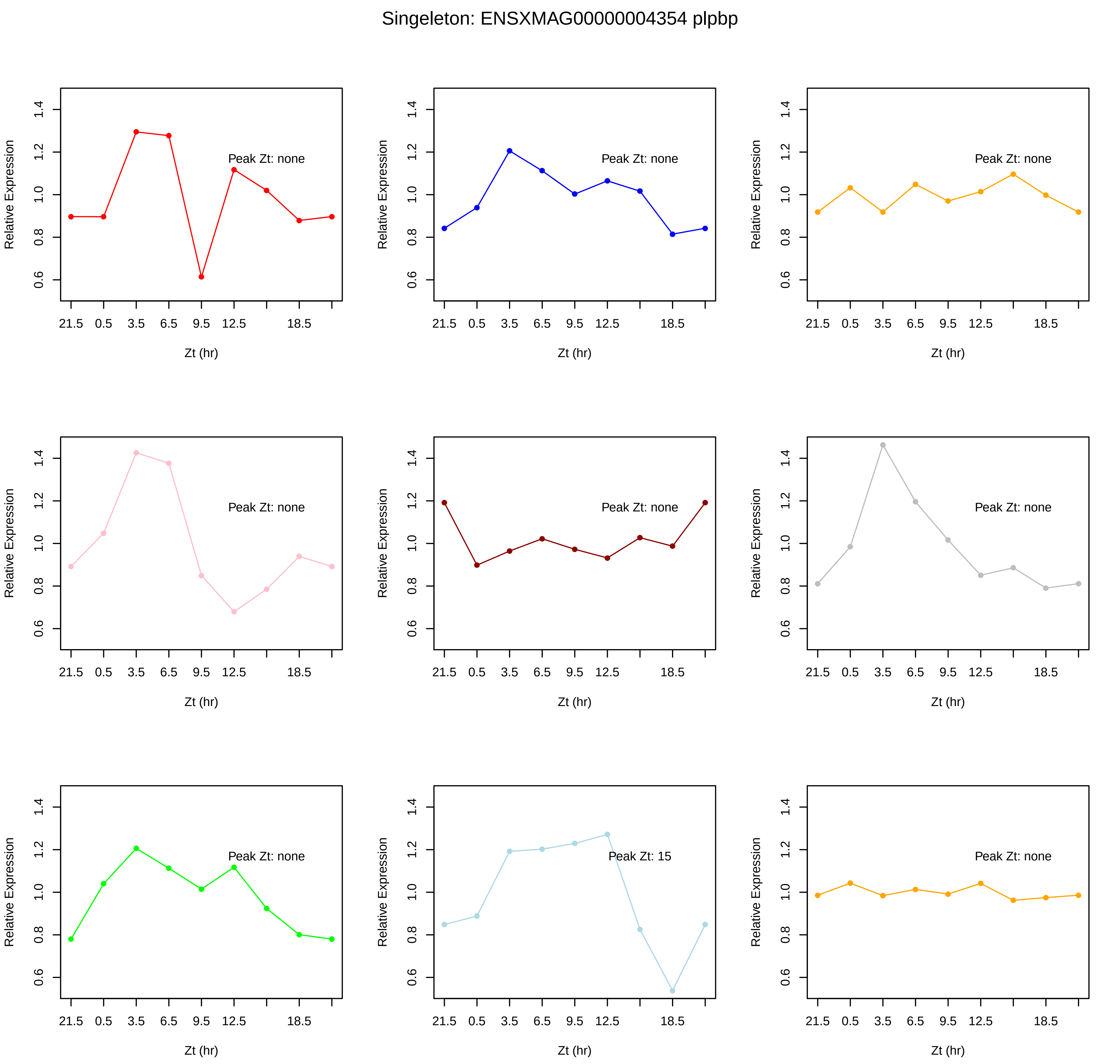
Singleton: ENSXMAG00000004354 plpbp
Relative Expression
Zt (hr)
21.5 0.5 3.5 6.5 9.5 12.5 18.5
0.6 0.8 1.0 1.2 1.4
Peak Zt: none
Peak Zt: 15

Singleton: ENSXMAG00000004254 pcyox1

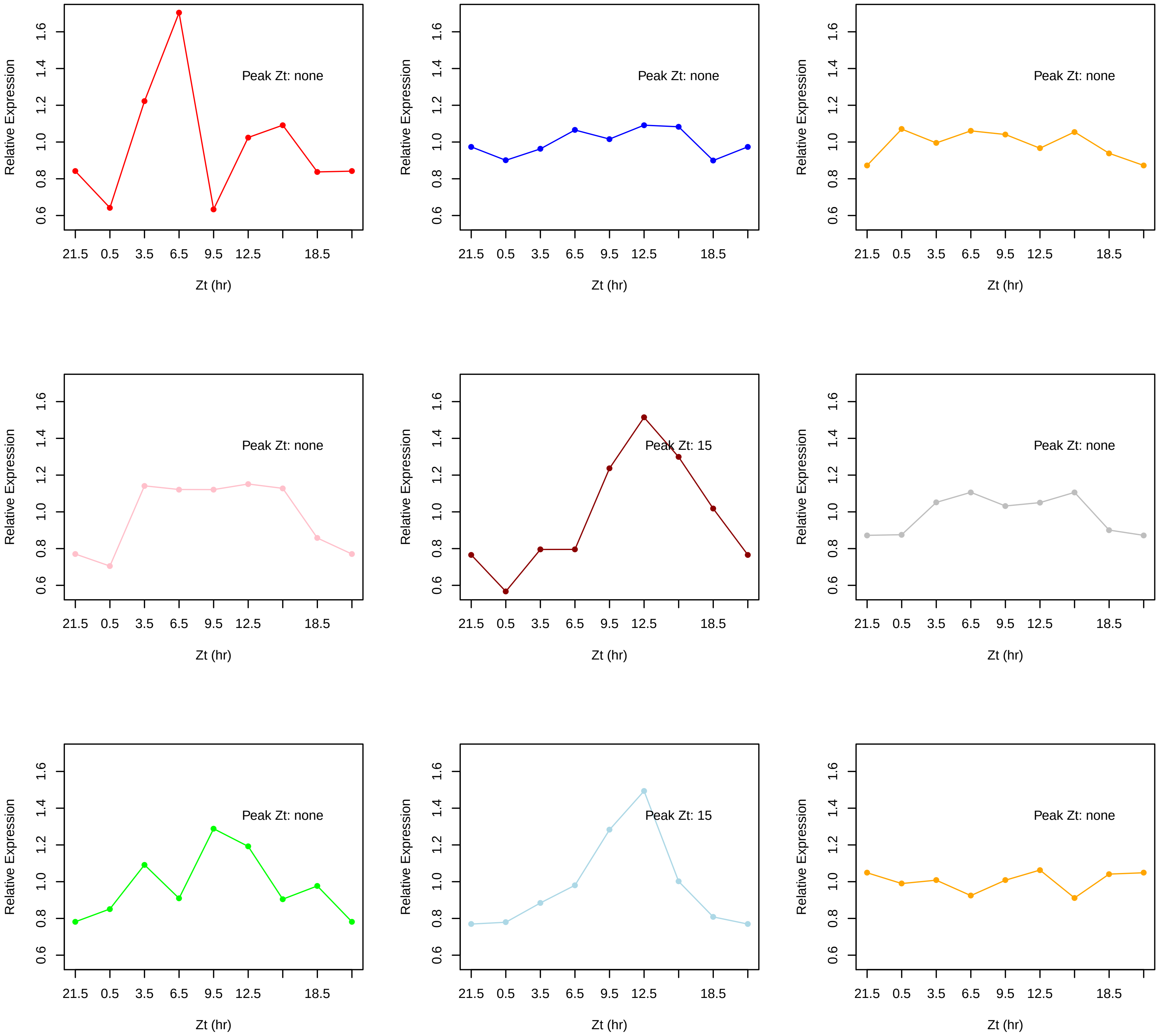

Singleton: ENSXMAG00000008689 pfkfb3

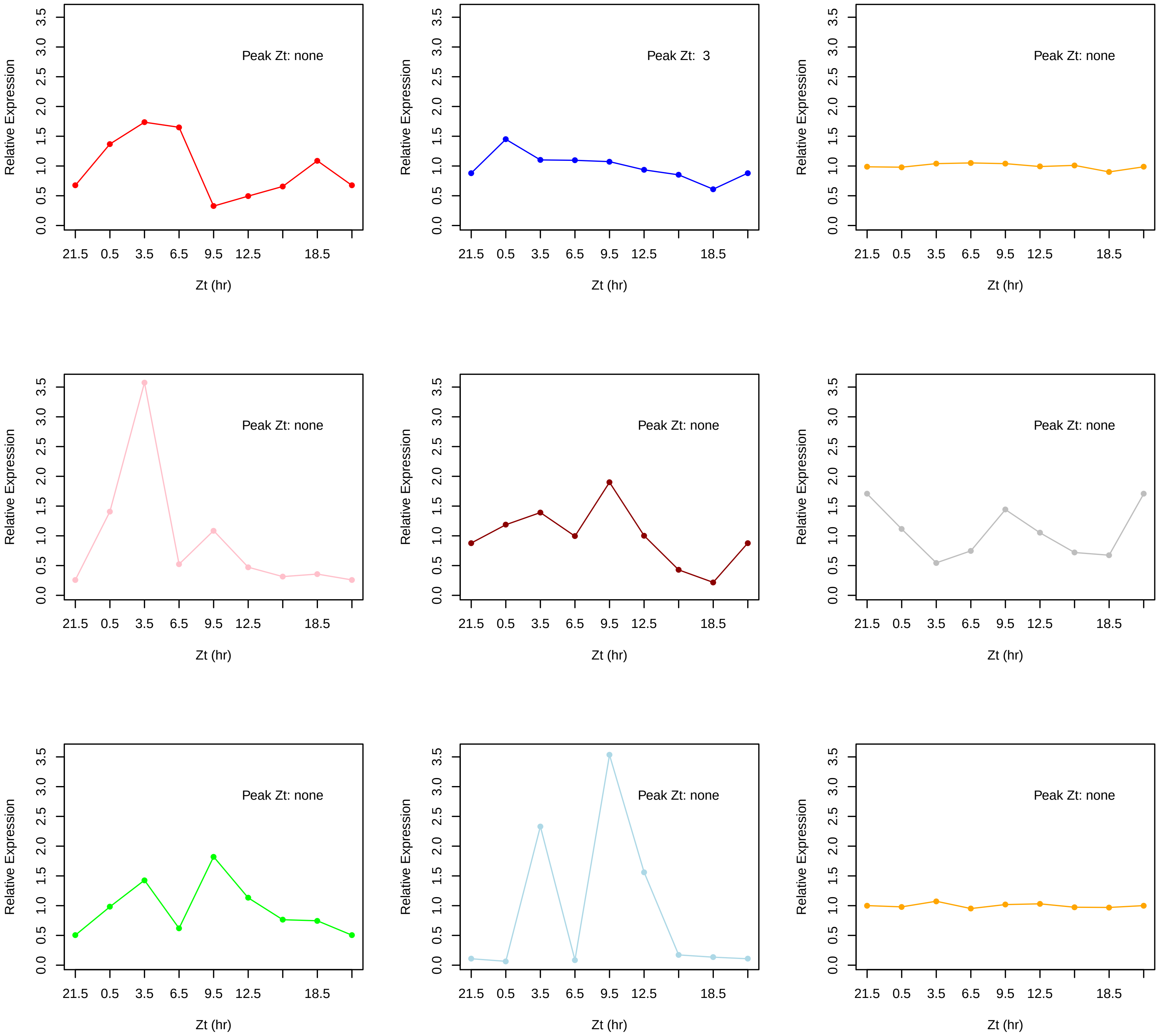

Singeleton: ENSXMAG00000004248 fam136a

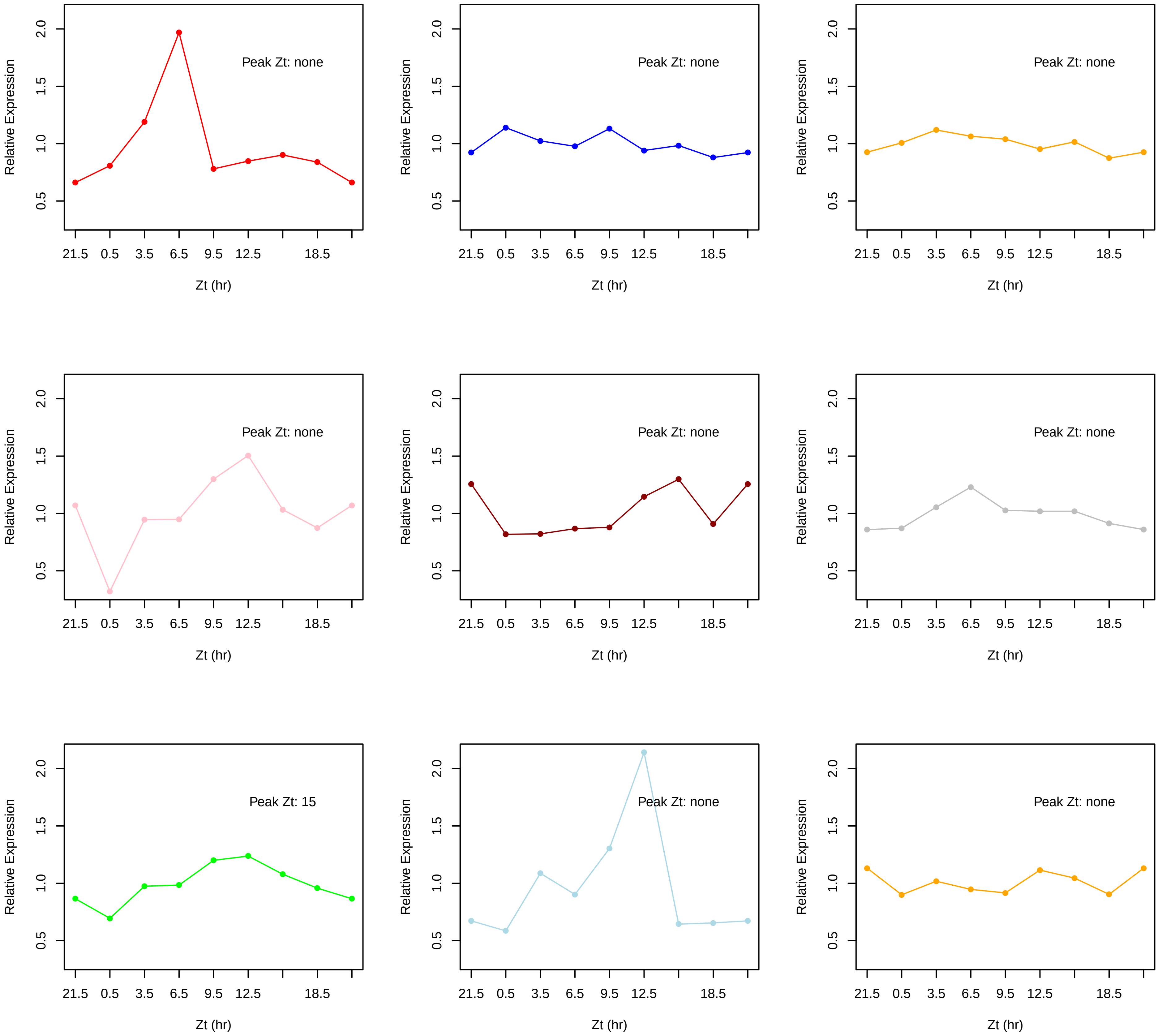

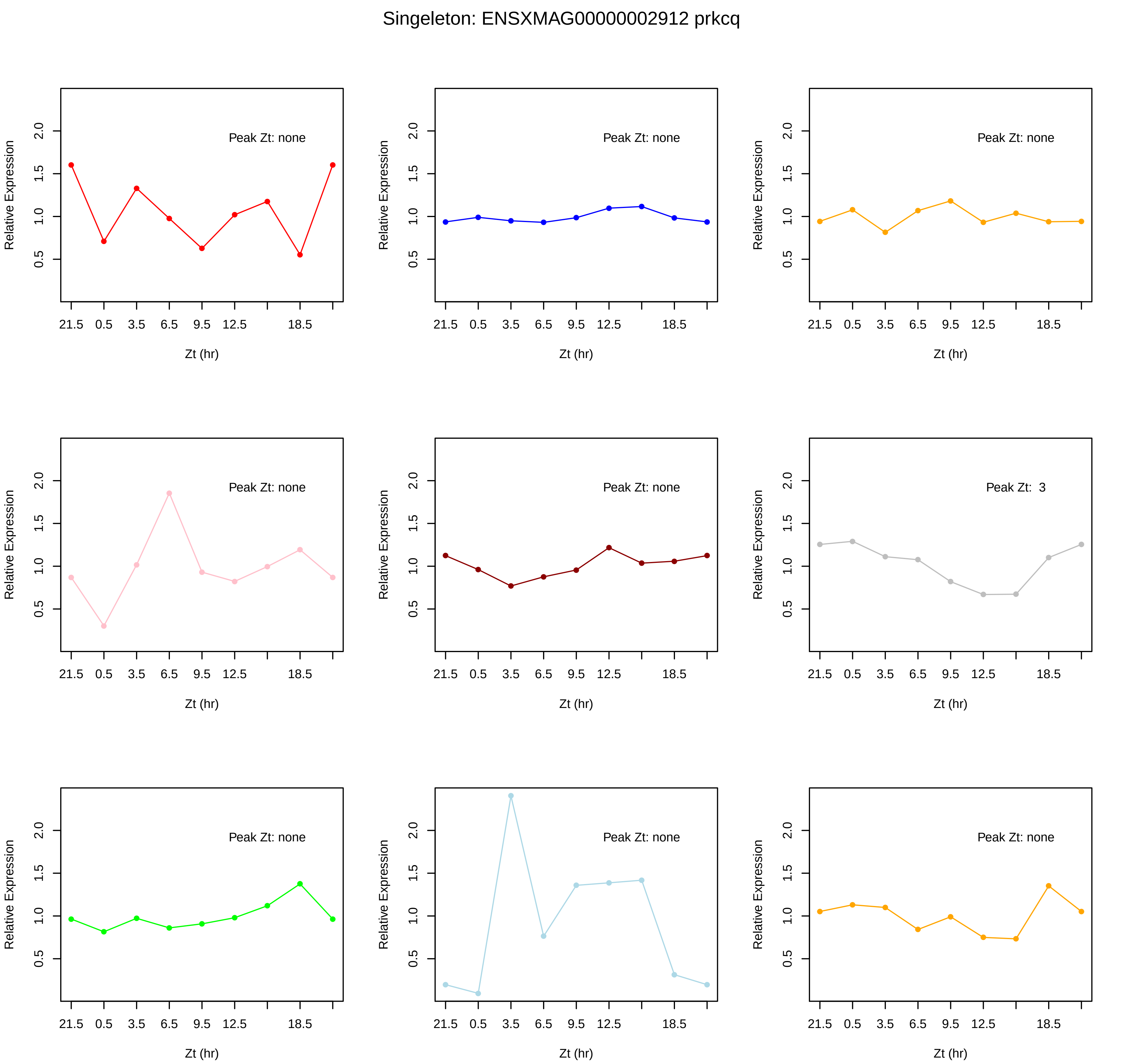
Singeleton: ENSXMAG00000002912 prkcq
Peak Zt: none
Peak Zt: none
Peak Zt: none
Peak Zt: none
Peak Zt: none
Peak Zt: 3
Peak Zt: none
Peak Zt: none
Peak Zt: none
Relative Expression
Zt (hr)
21.5
0.5
3.5
6.5
9.5
12.5
18.5
0.5
1.0
1.5
2.0

Singeleton: ENSXMAG00000002967 sfmbt2

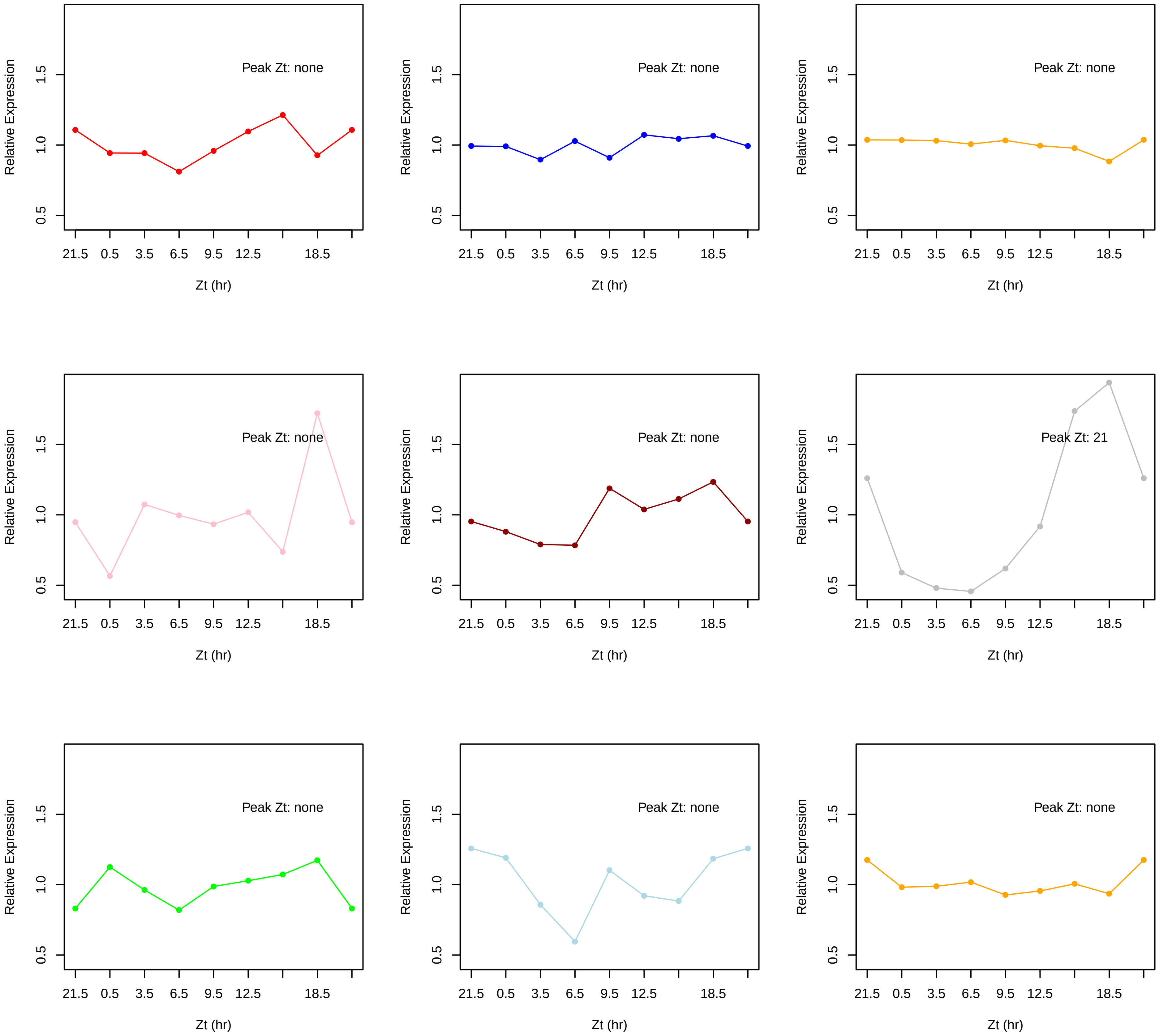

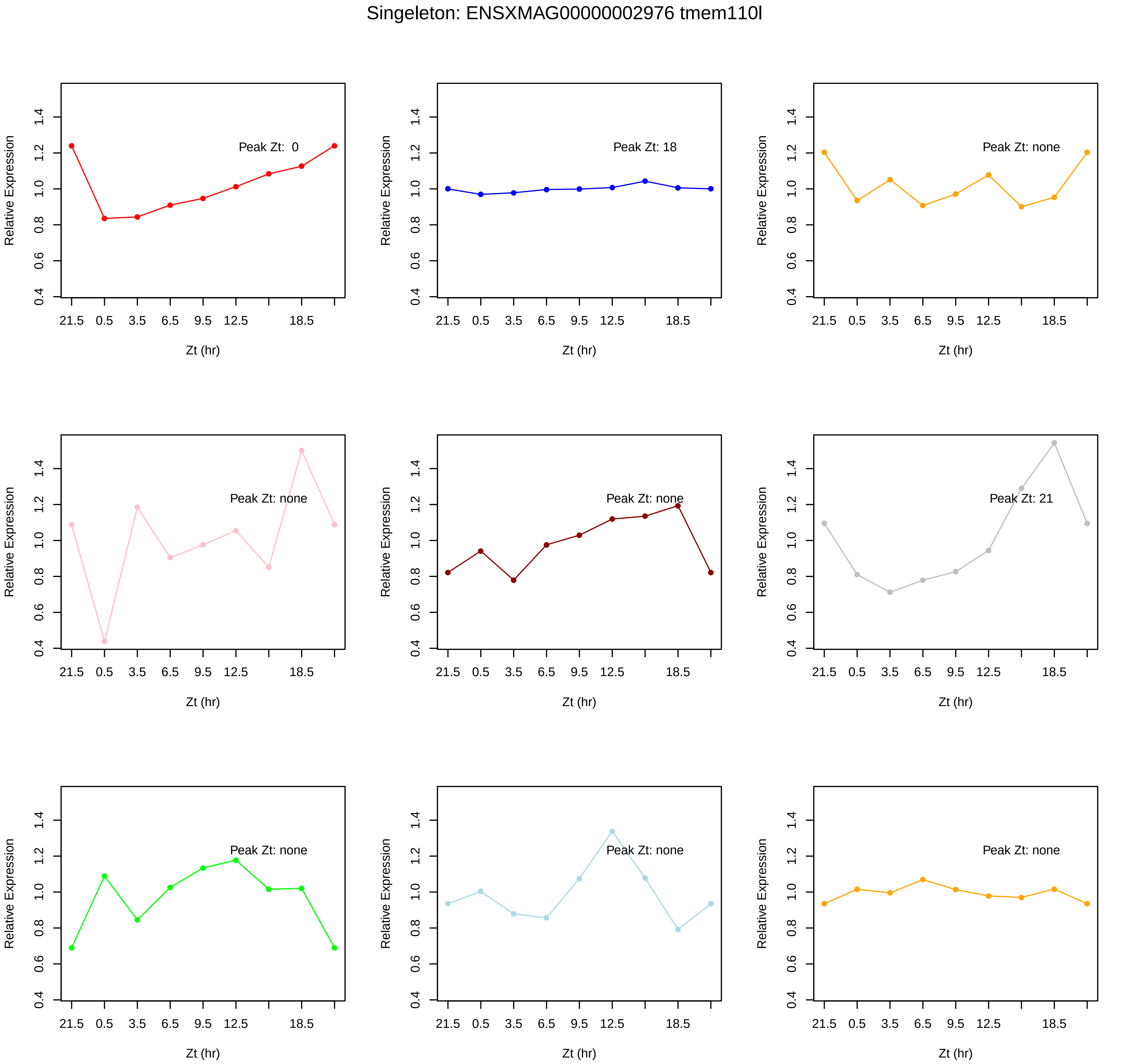

Singleton: ENSXMAG00000002976 tmem110l
Peak Zt: 0
Peak Zt: 18
Peak Zt: none
Peak Zt: none
Peak Zt: none
Peak Zt: 21
Peak Zt: none
Peak Zt: none
Peak Zt: none
Relative Expression
Zt (hr)
0.4
0.6
0.8
1.0
1.2
1.4
21.5
0.5
3.5
6.5
9.5
12.5
18.5

Singleton: ENSXMAG00000008742 aars2

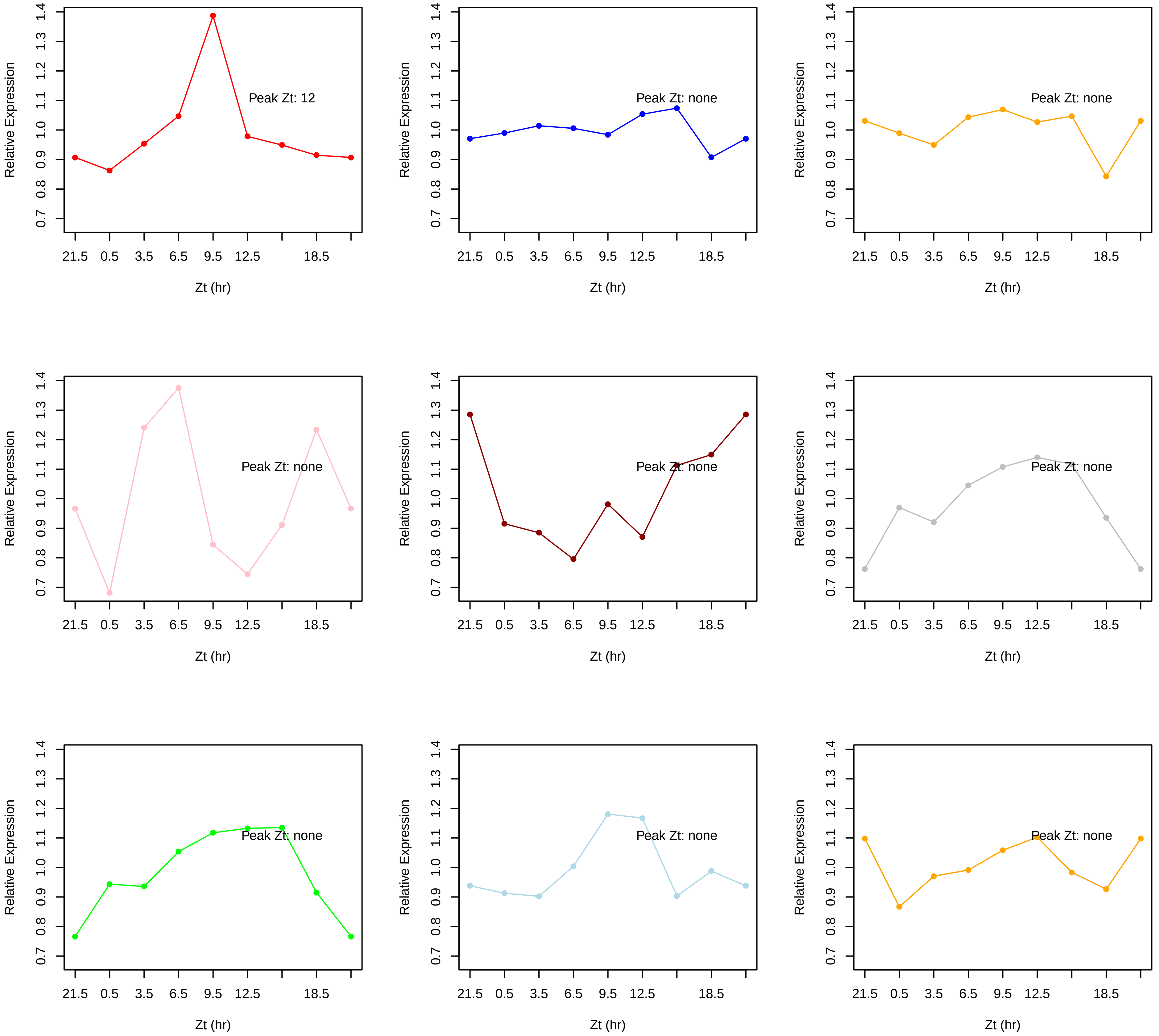

Singeleton: ENSXMAG00000006142 rrbp1a

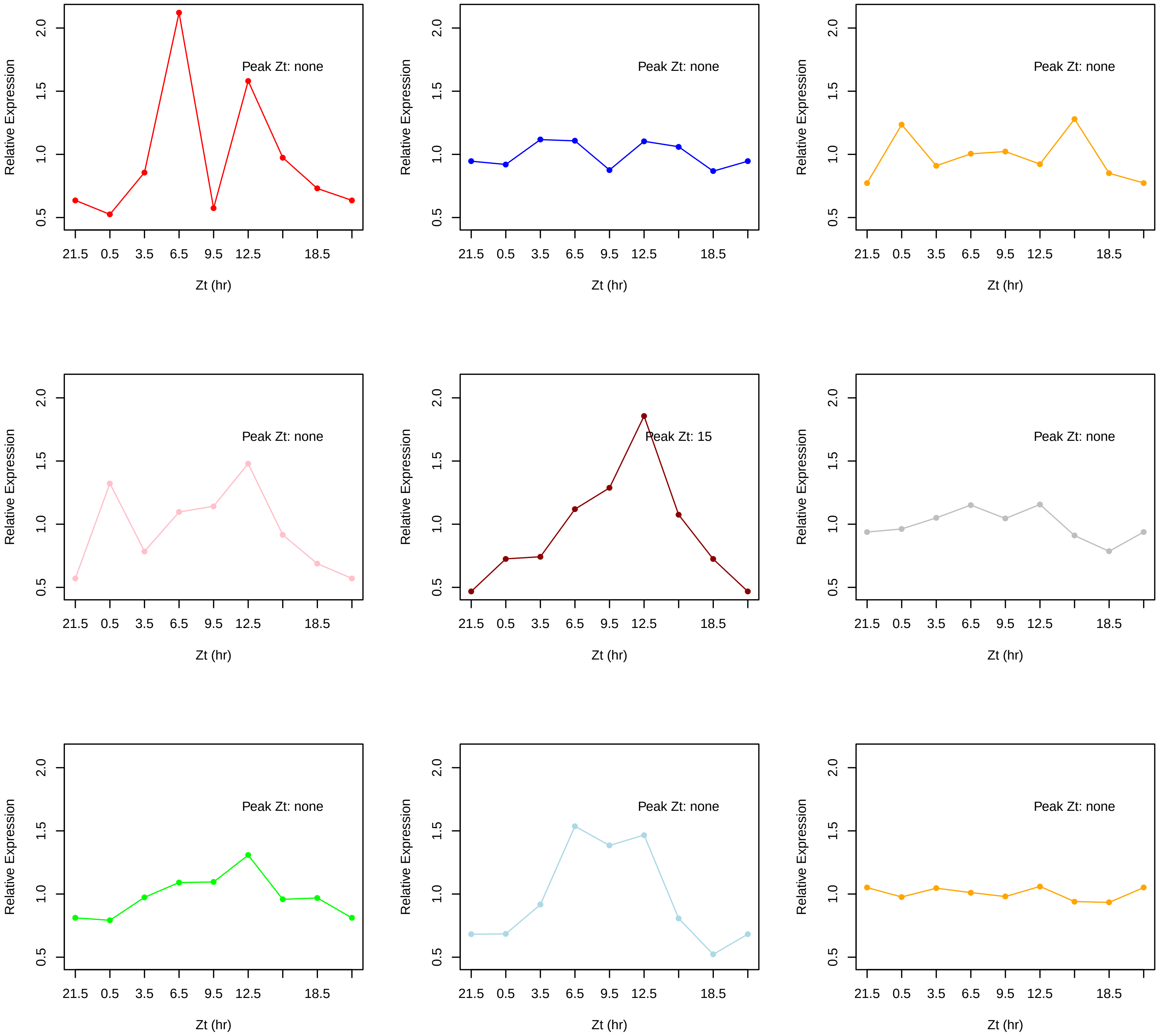

Singeleton: ENSXMAG00000006160 snx5

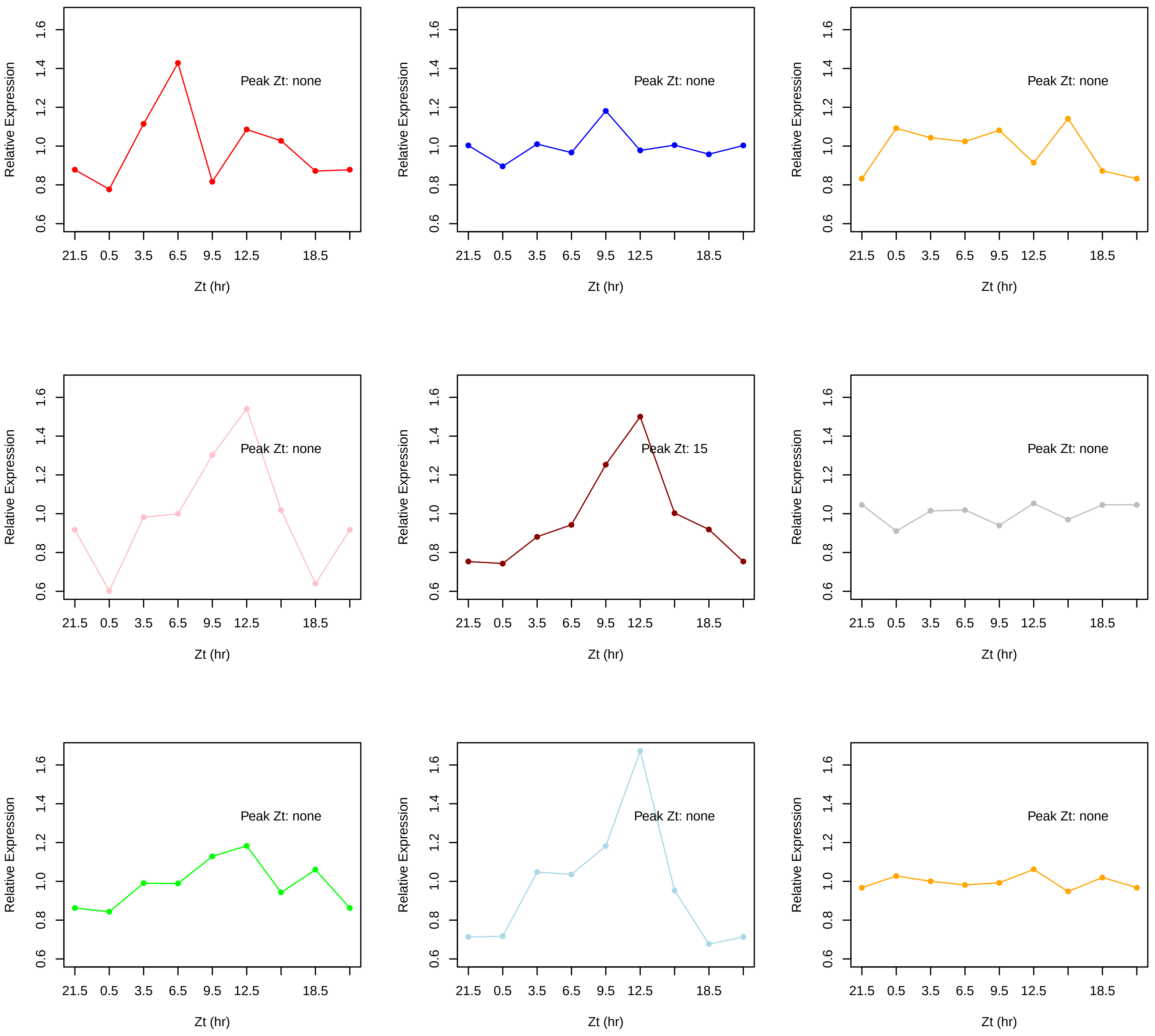

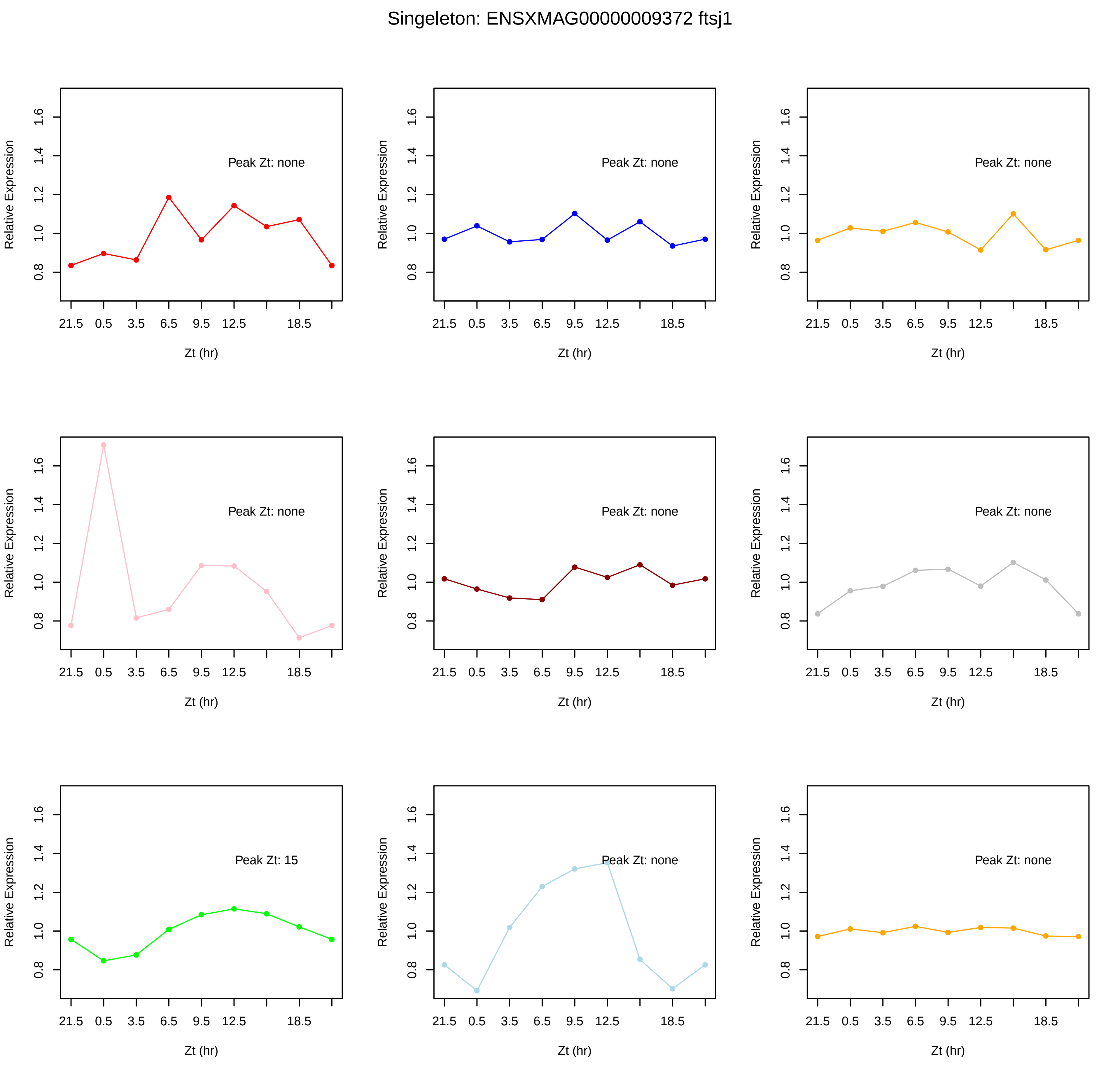
Singeleton: ENSXMAG00000009372 ftsj1
Relative Expression
Zt (hr)
Peak Zt: none
Peak Zt: none
Peak Zt: none
Peak Zt: none
Peak Zt: none
Peak Zt: none
Peak Zt: 15
Peak Zt: none
Peak Zt: none
21.5 0.5 3.5 6.5 9.5 12.5 18.5
0.8 1.0 1.2 1.4 1.6

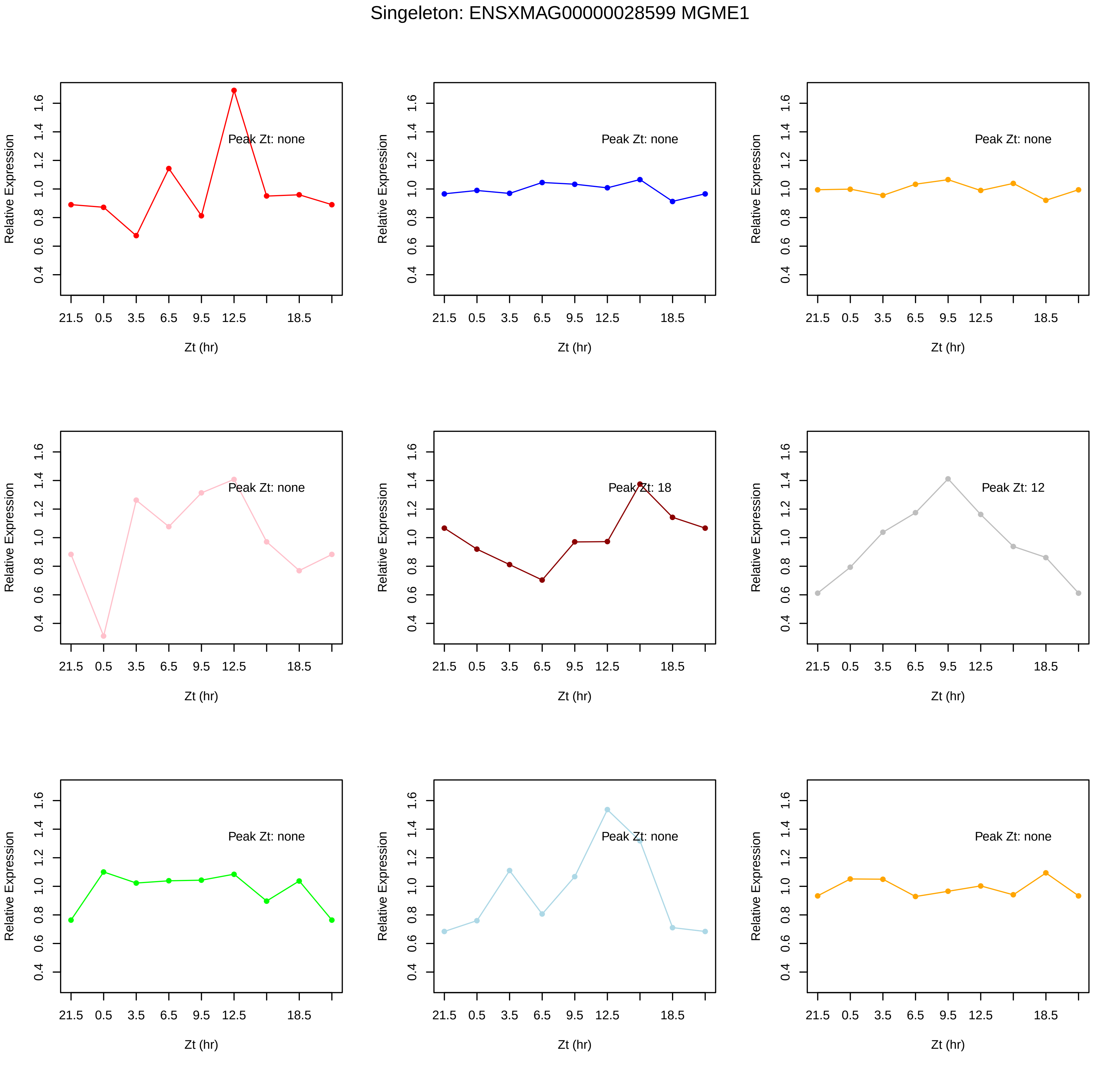

Singeleton: ENSXMAG00000028599 MGME1
Relative Expression
Zt (hr)
Peak Zt: none
Peak Zt: none
Peak Zt: none
Peak Zt: none
Peak Zt: 18
Peak Zt: 12
Peak Zt: none
Peak Zt: none
Peak Zt: none
21.5
0.5
3.5
6.5
9.5
12.5
18.5

Singeleton: ENSXMAG00000009773 PET117

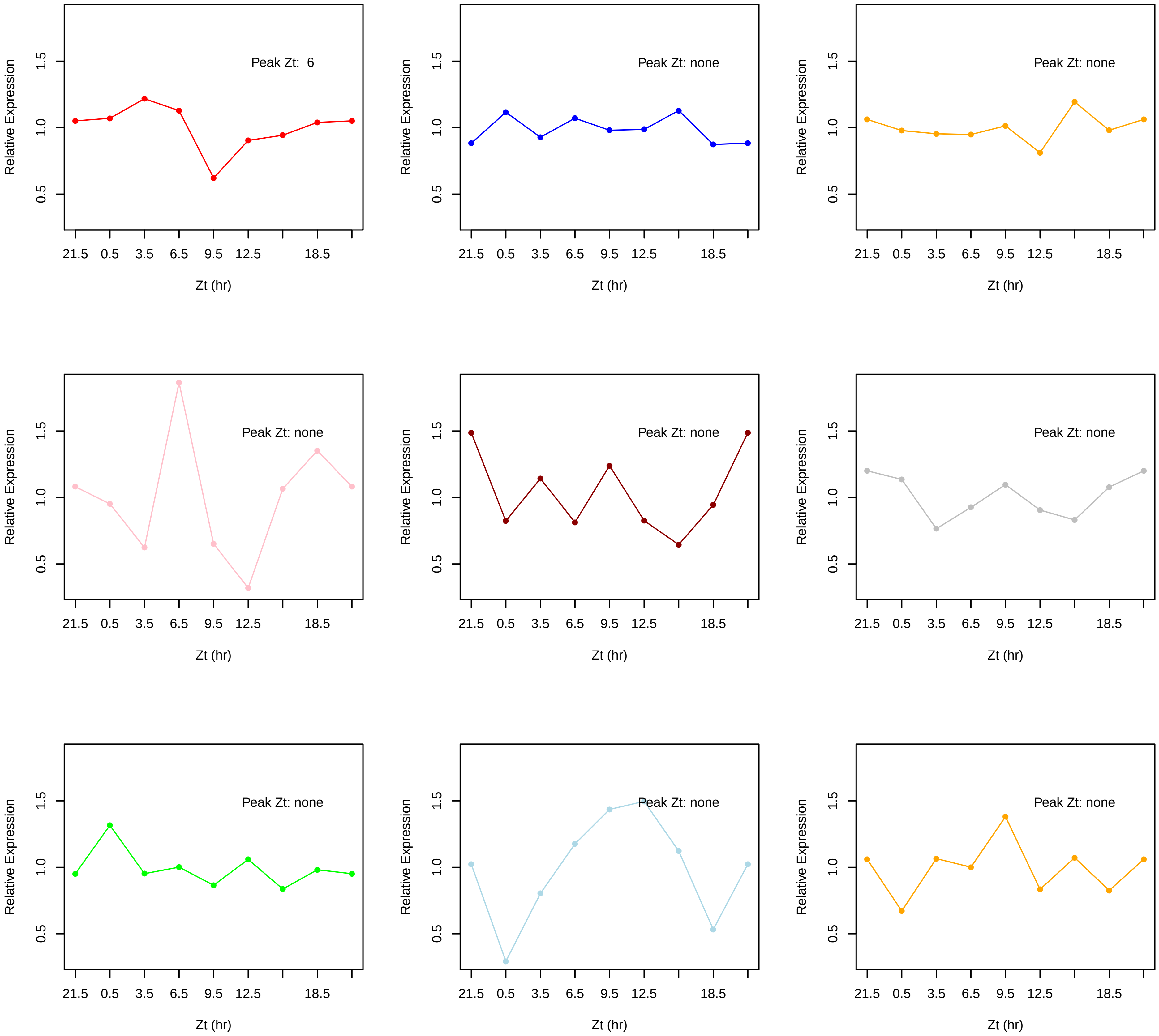

Singeleton: ENSXMAG00000017026 riox1

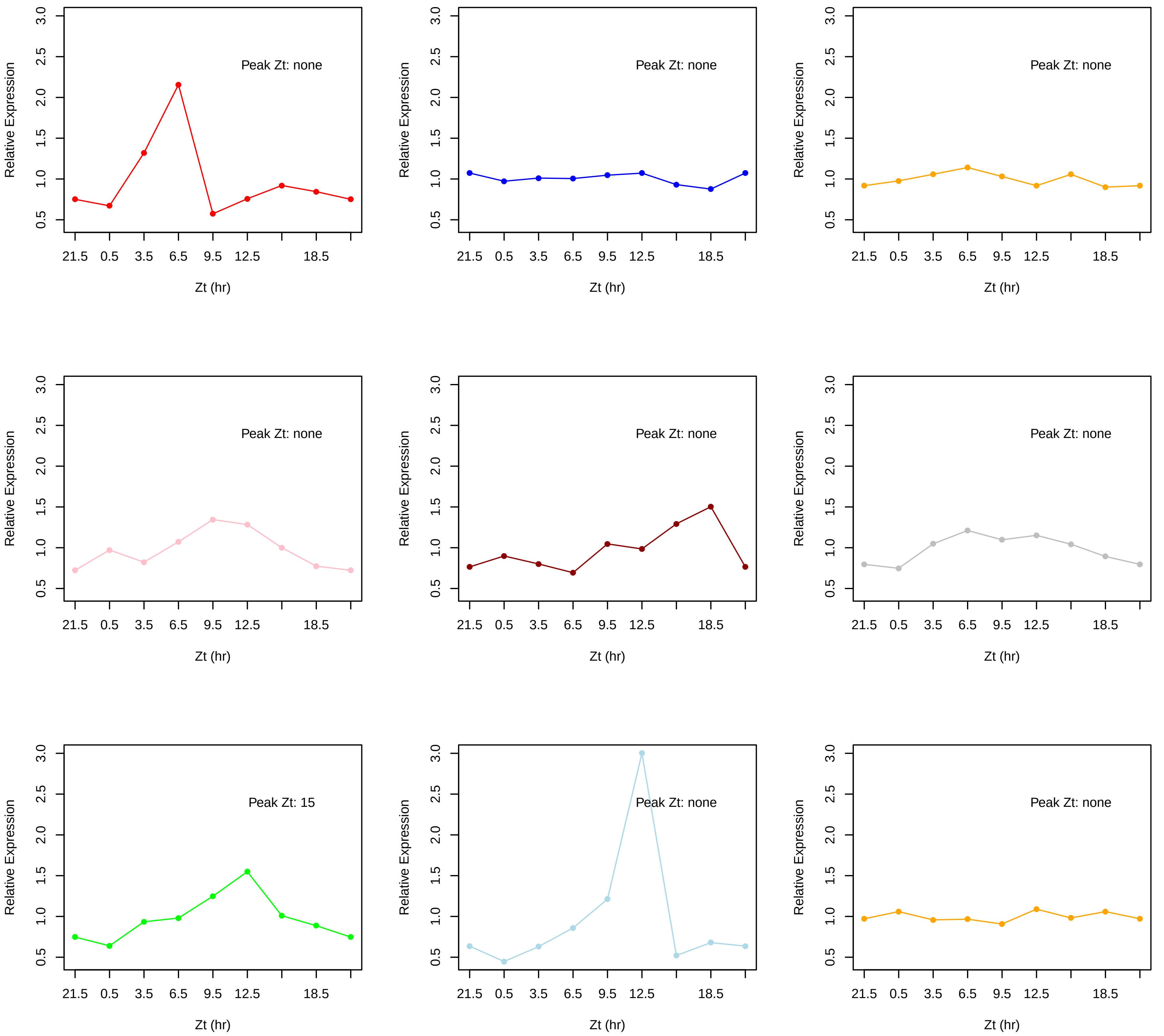

Singleton: ENSXMAG00000004604 polr3f

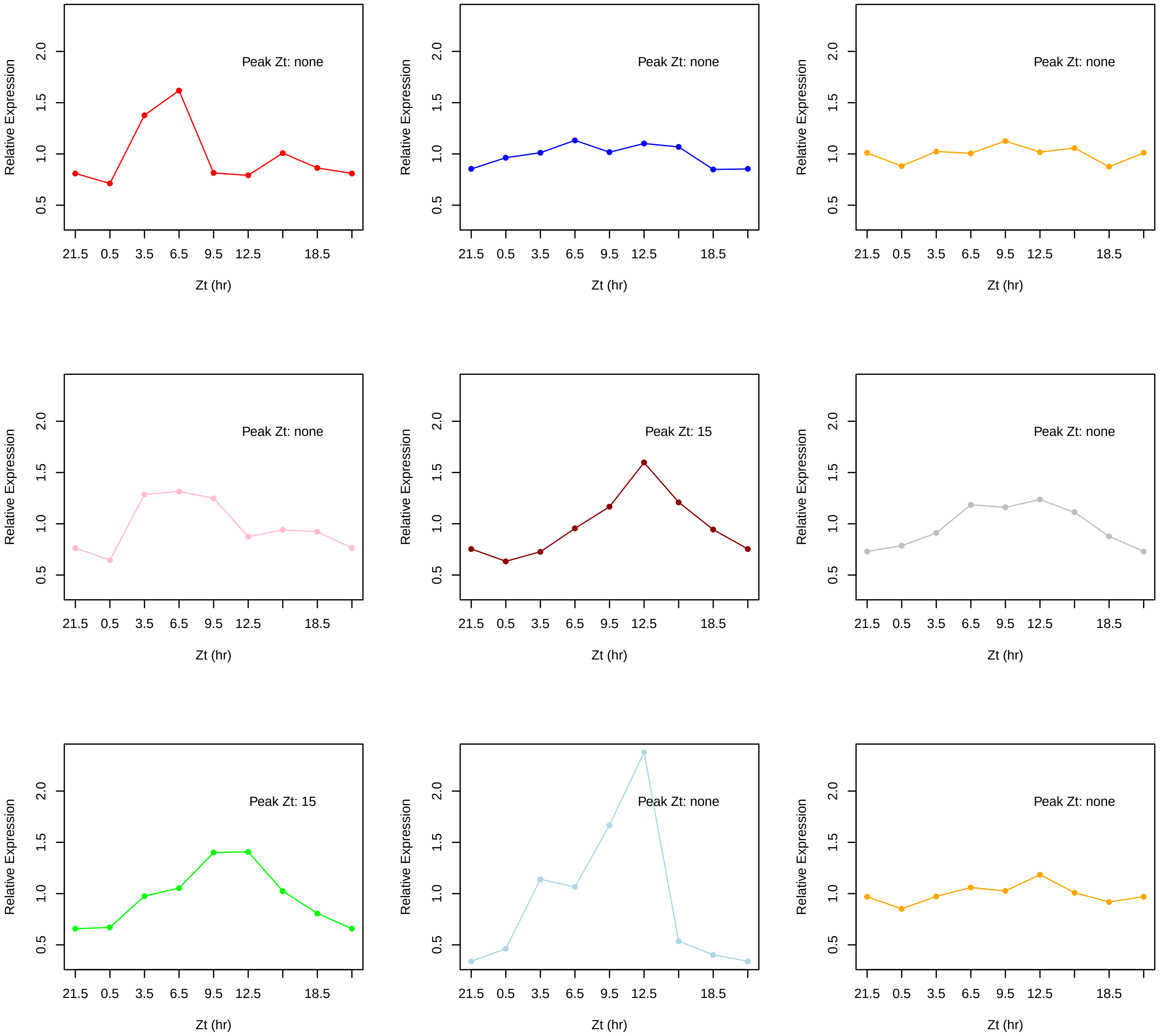

Singeleton: ENSXMAG00000023808 clec11a

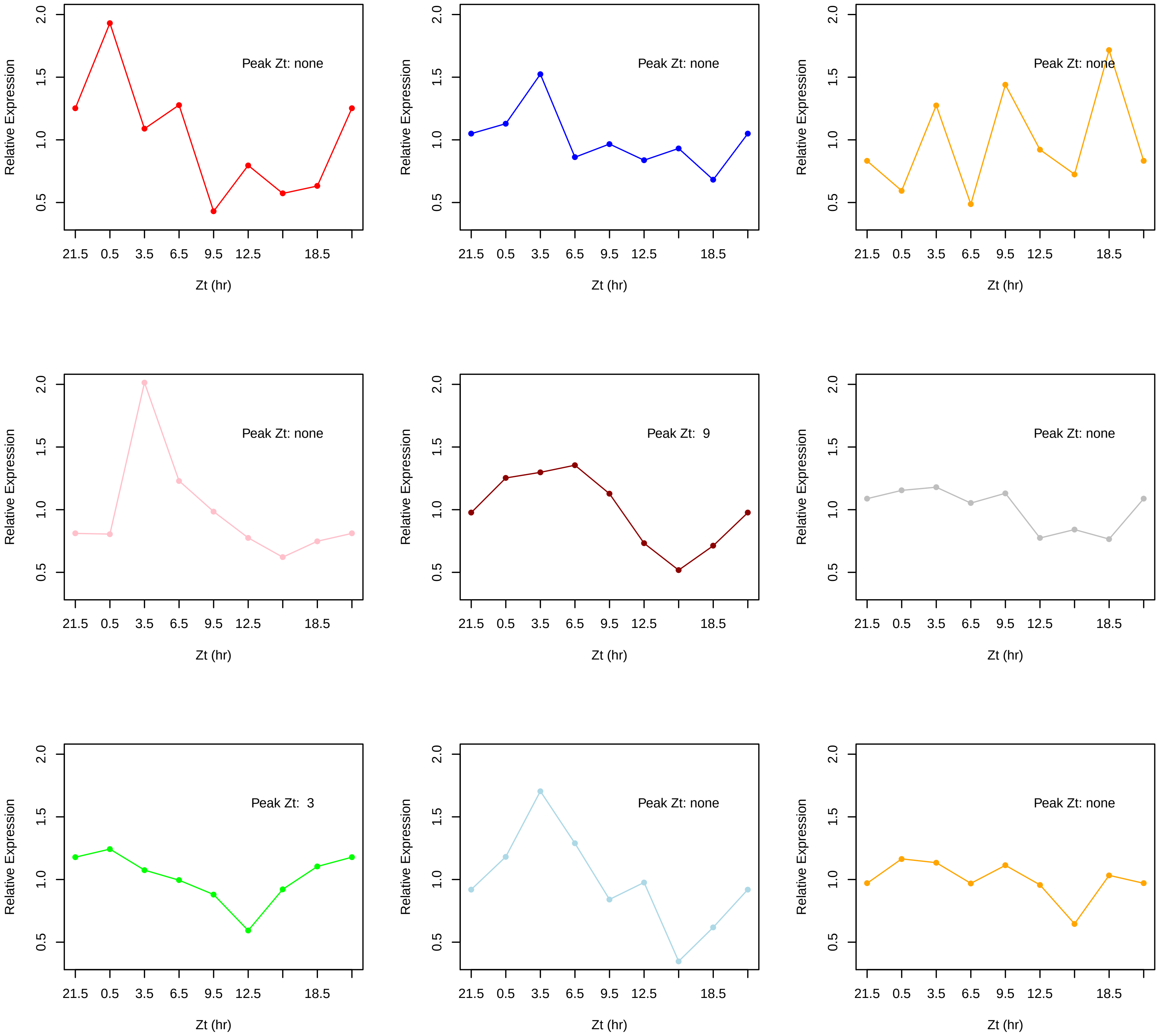

Singleton: ENSXMAG00000006434 si:ch73−111k22.2

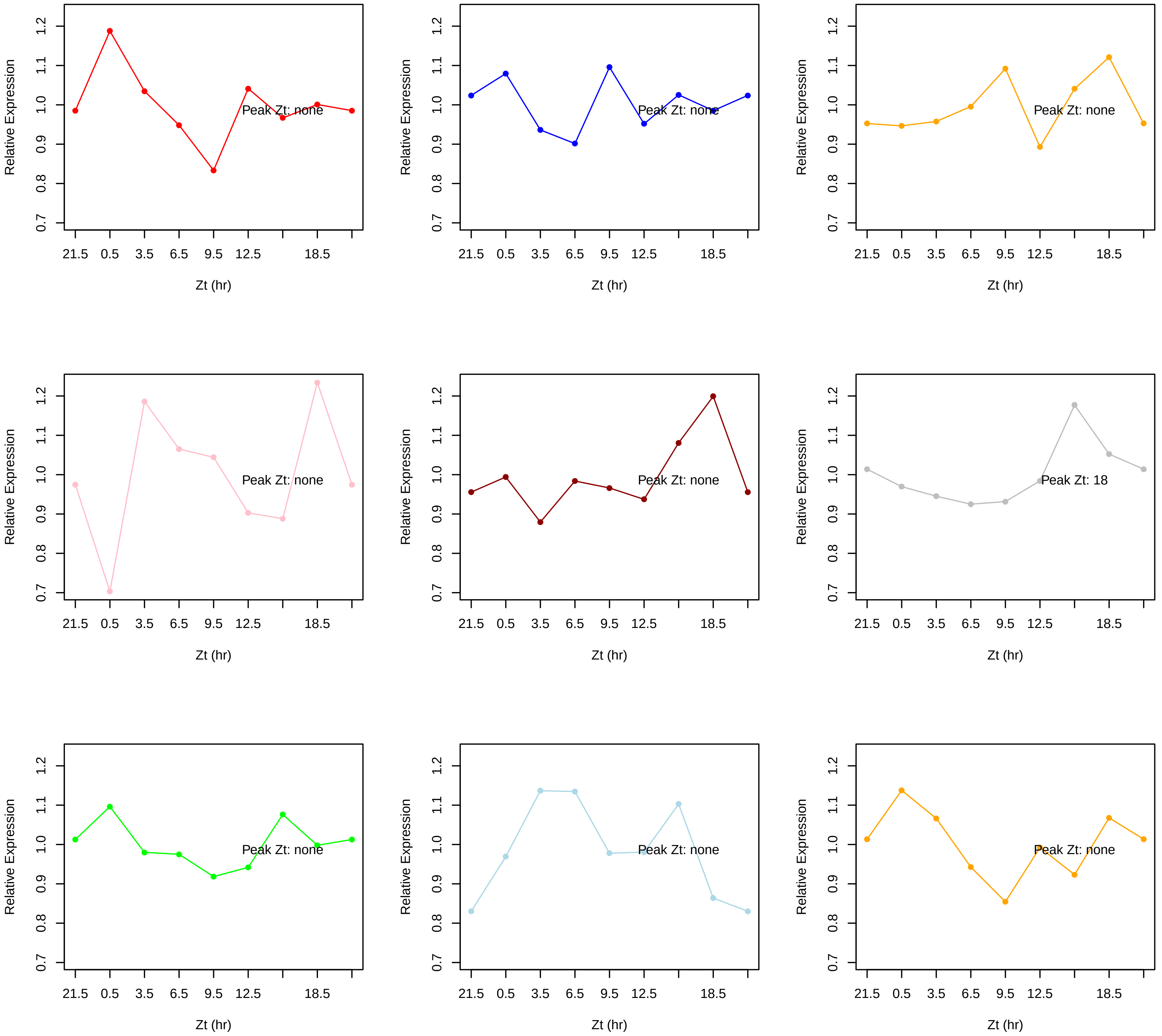

Singeleton: ENSXMAG00000005516 igf2r

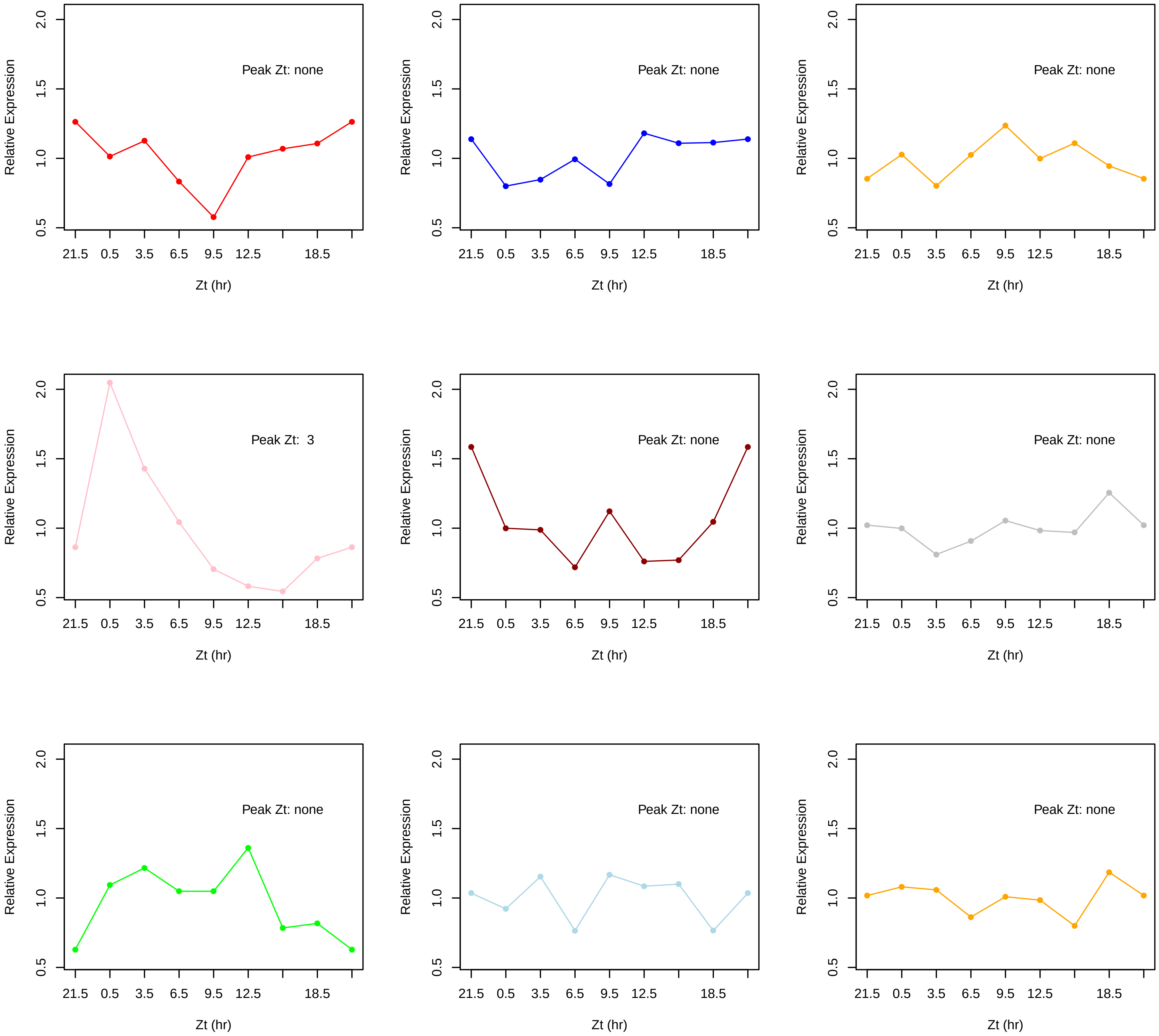

Singeleton: ENSXMAG00000006208 si:dkey−76k16.5

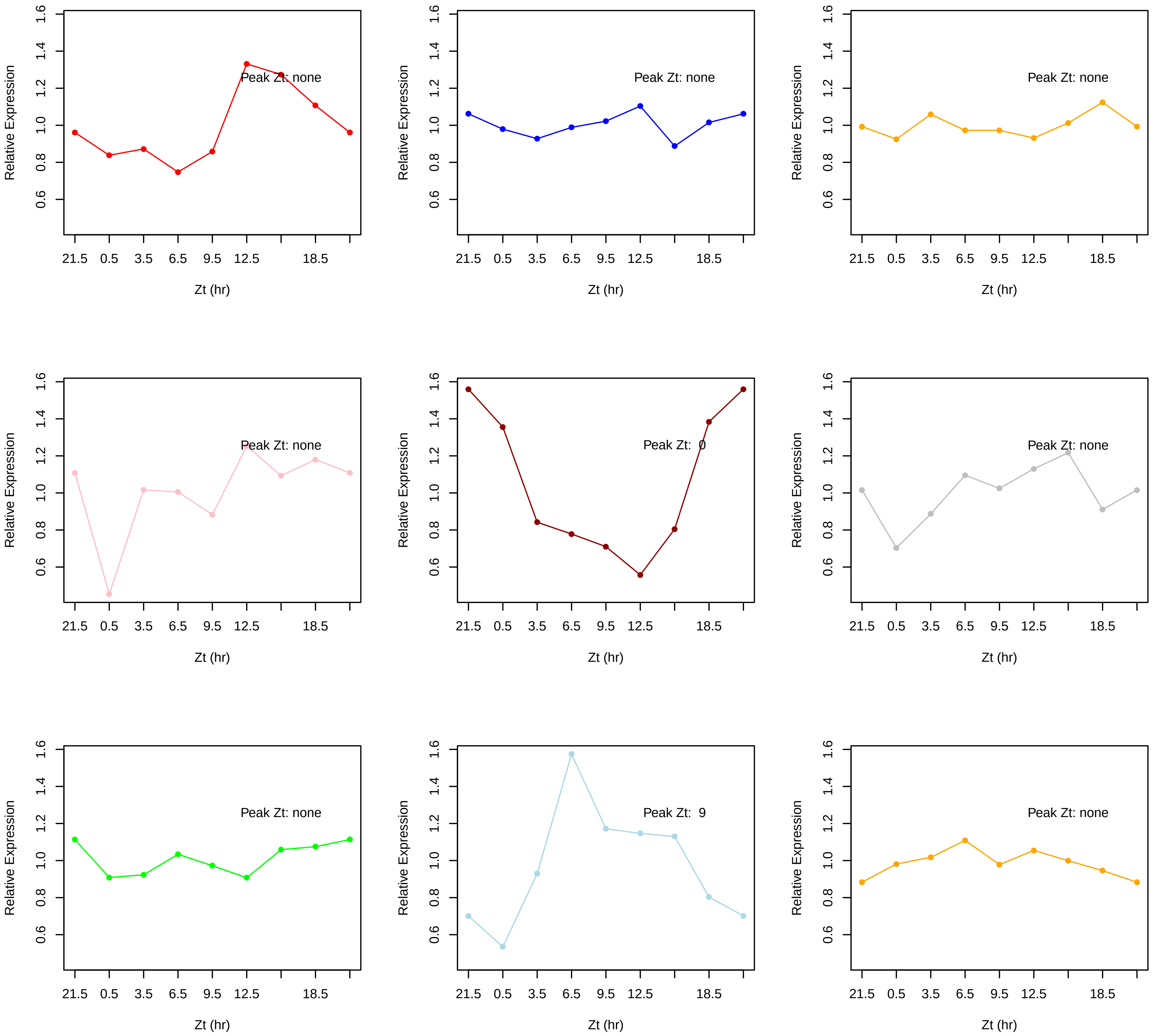

Singeleton: ENSXMAG00000029127 nudt5

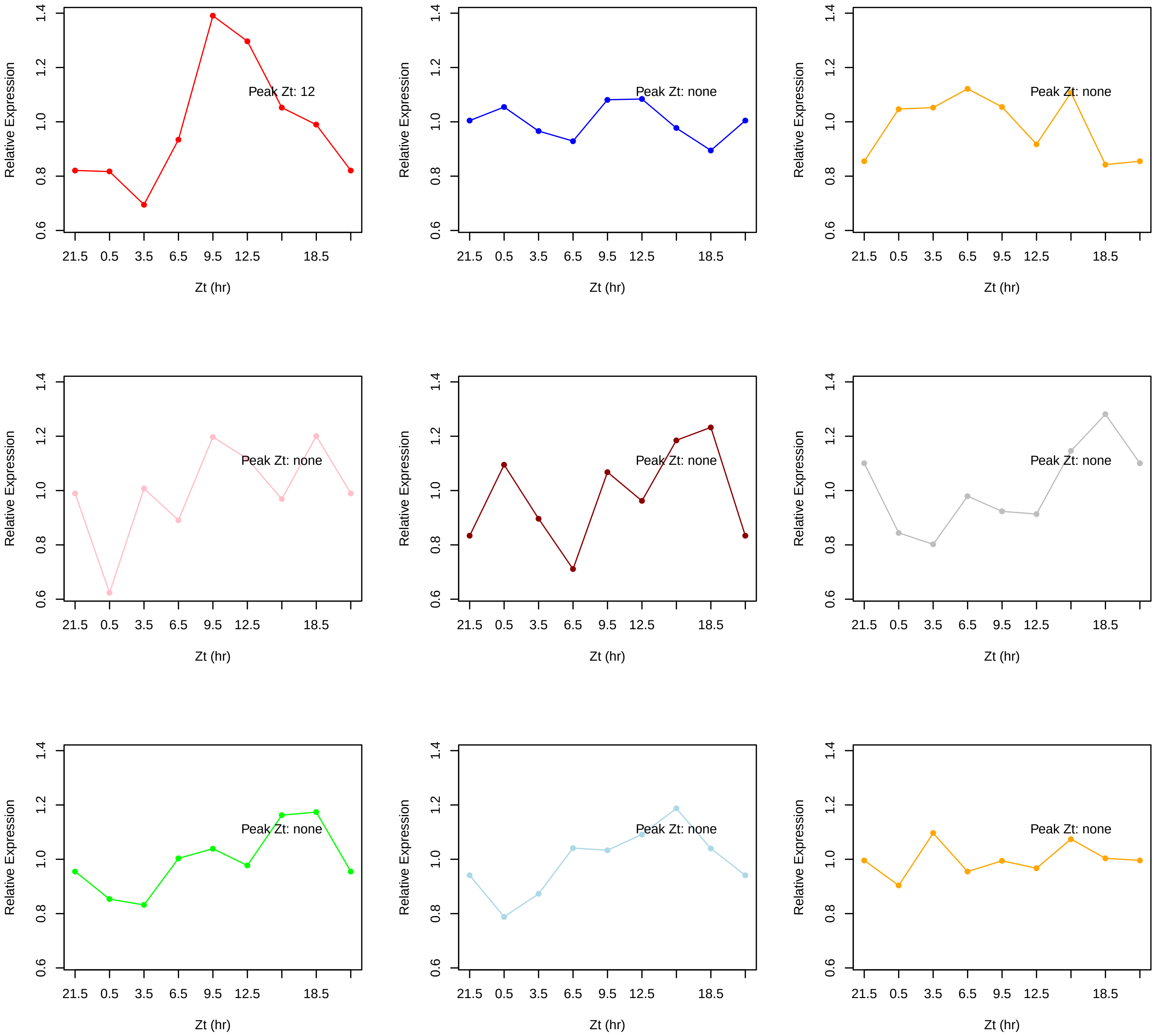

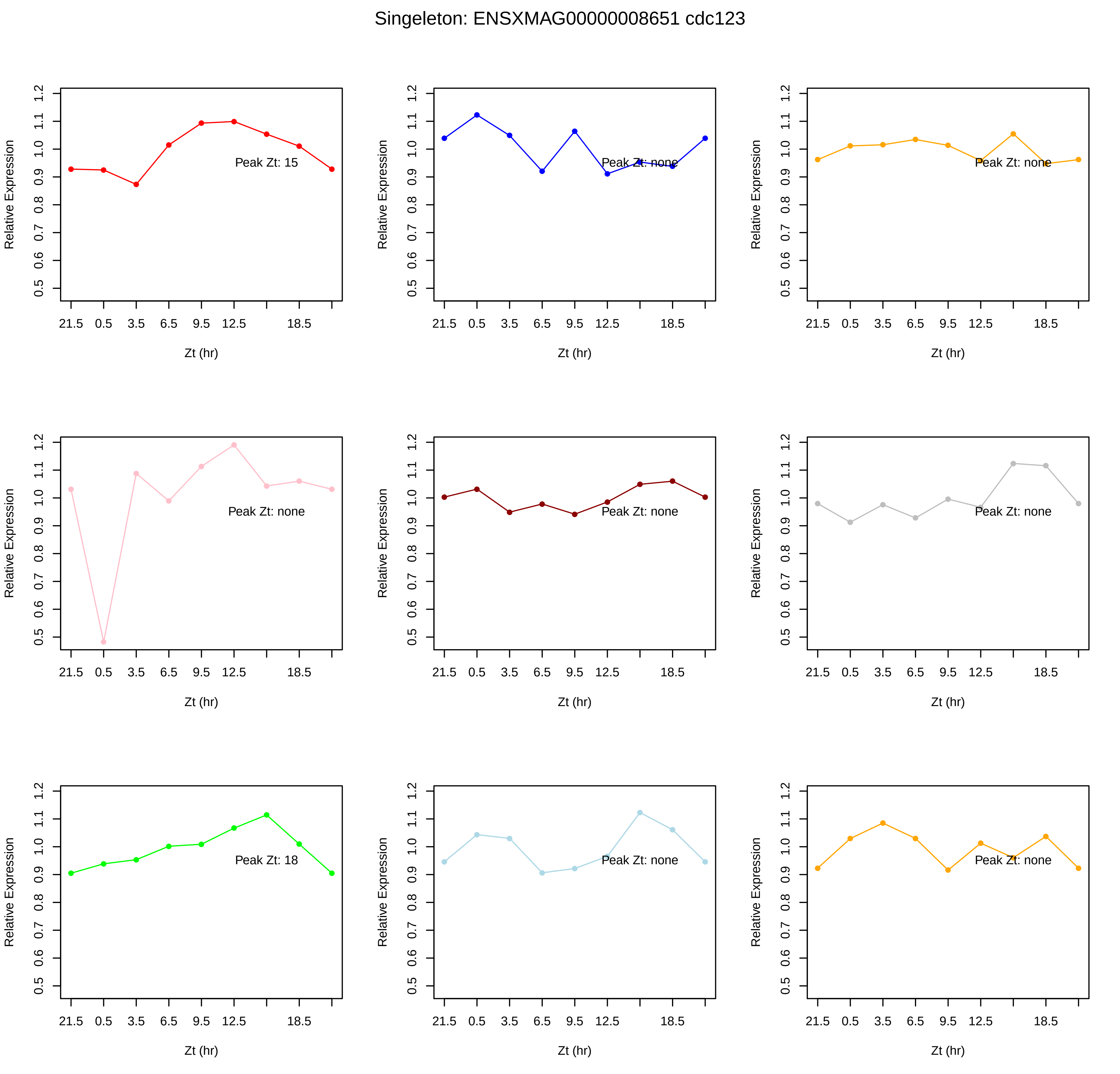
Singeleton: ENSXMAG00000008651 cdc123
Relative Expression
Zt (hr)
21.5 0.5 3.5 6.5 9.5 12.5 18.5
0.5 0.6 0.7 0.8 0.9 1.0 1.1 1.2
Peak Zt: 15
Peak Zt: none
Peak Zt: none
Peak Zt: none
Peak Zt: none
Peak Zt: none
Peak Zt: 18
Peak Zt: none
Peak Zt: none

Singeleton: ENSXMAG00000027219 ccdc3a

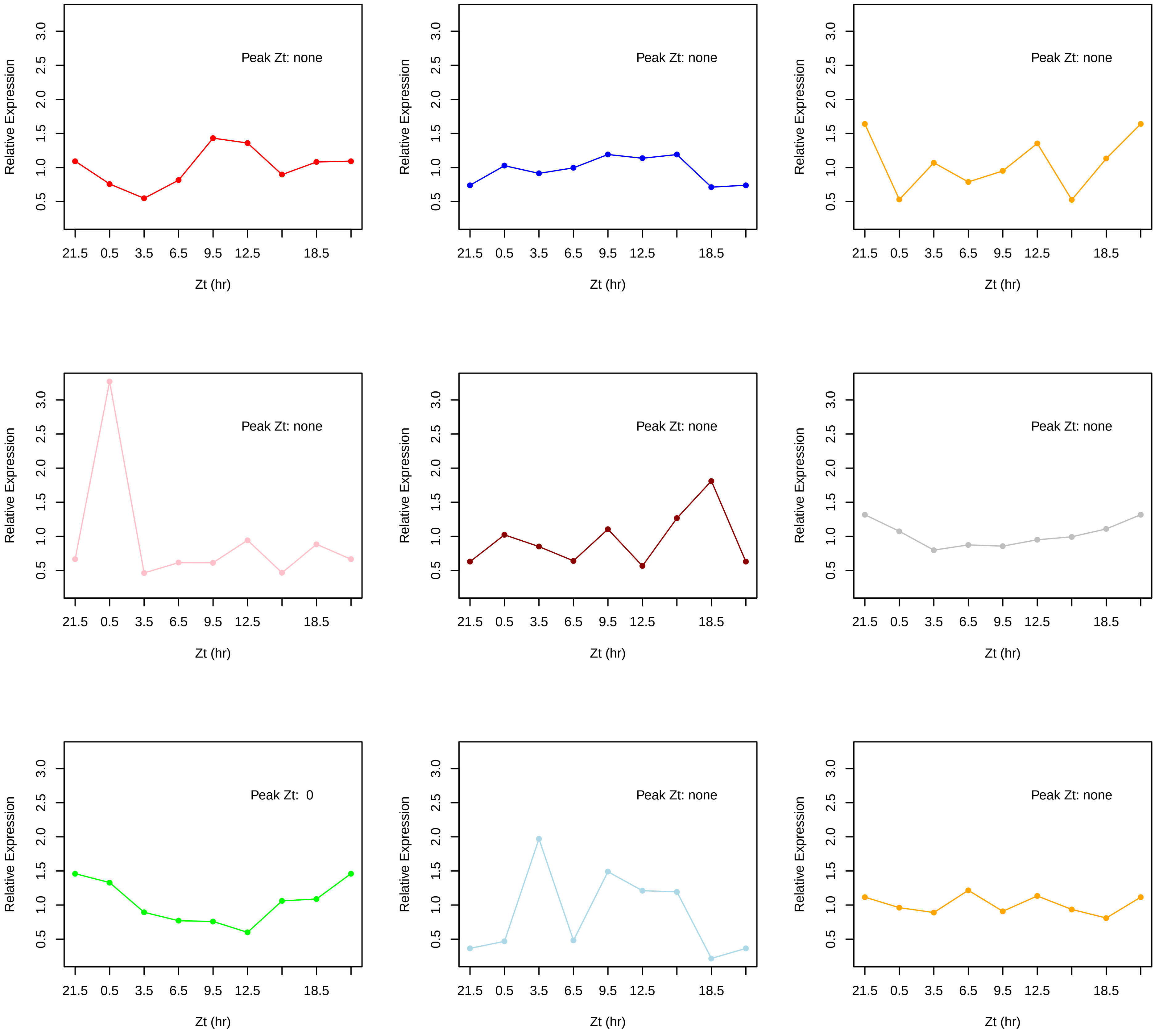

Singeleton: ENSXMAG00000016767 ELK3

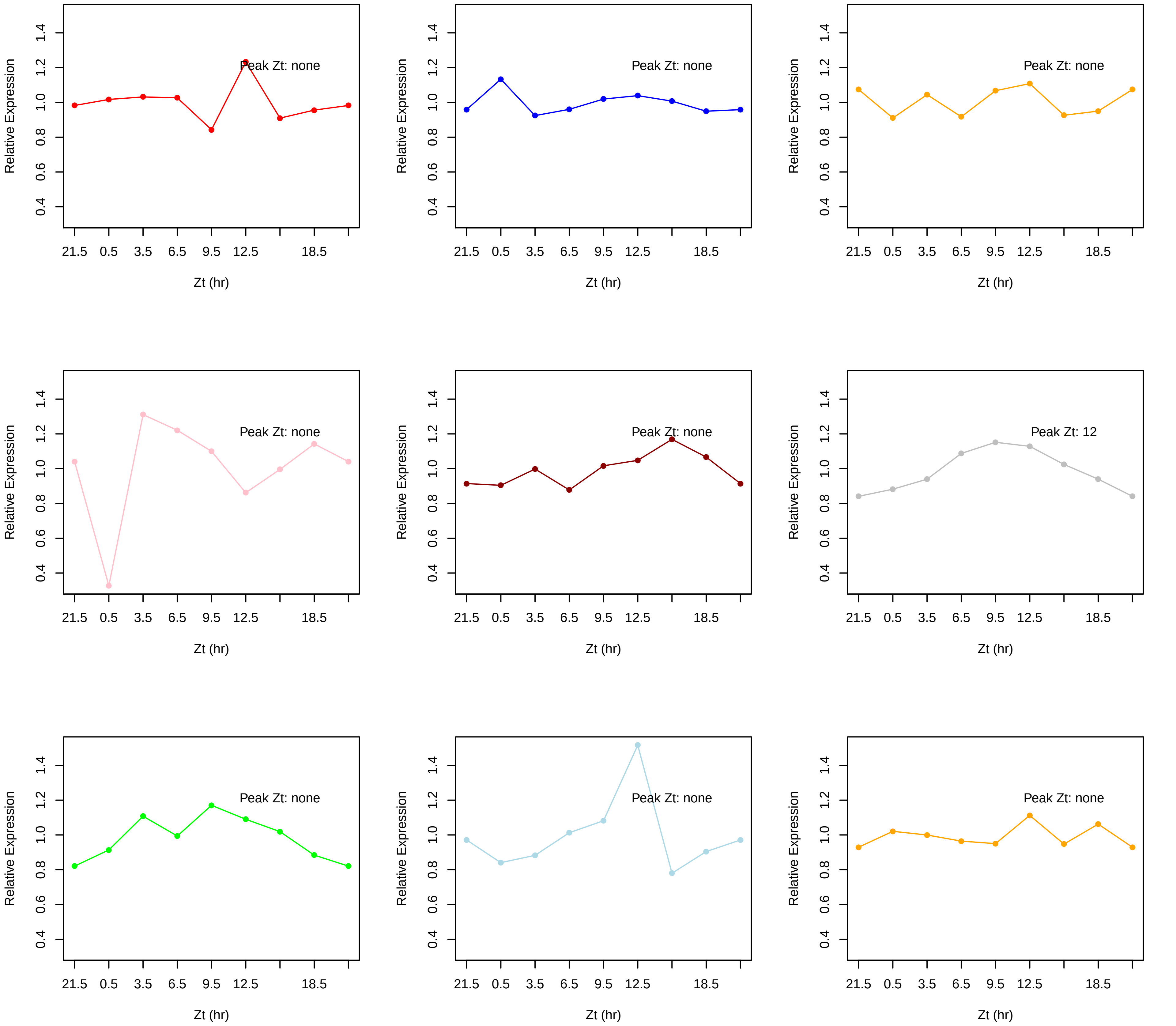

Singeleton: ENSXMAG00000015455 AMDHD1

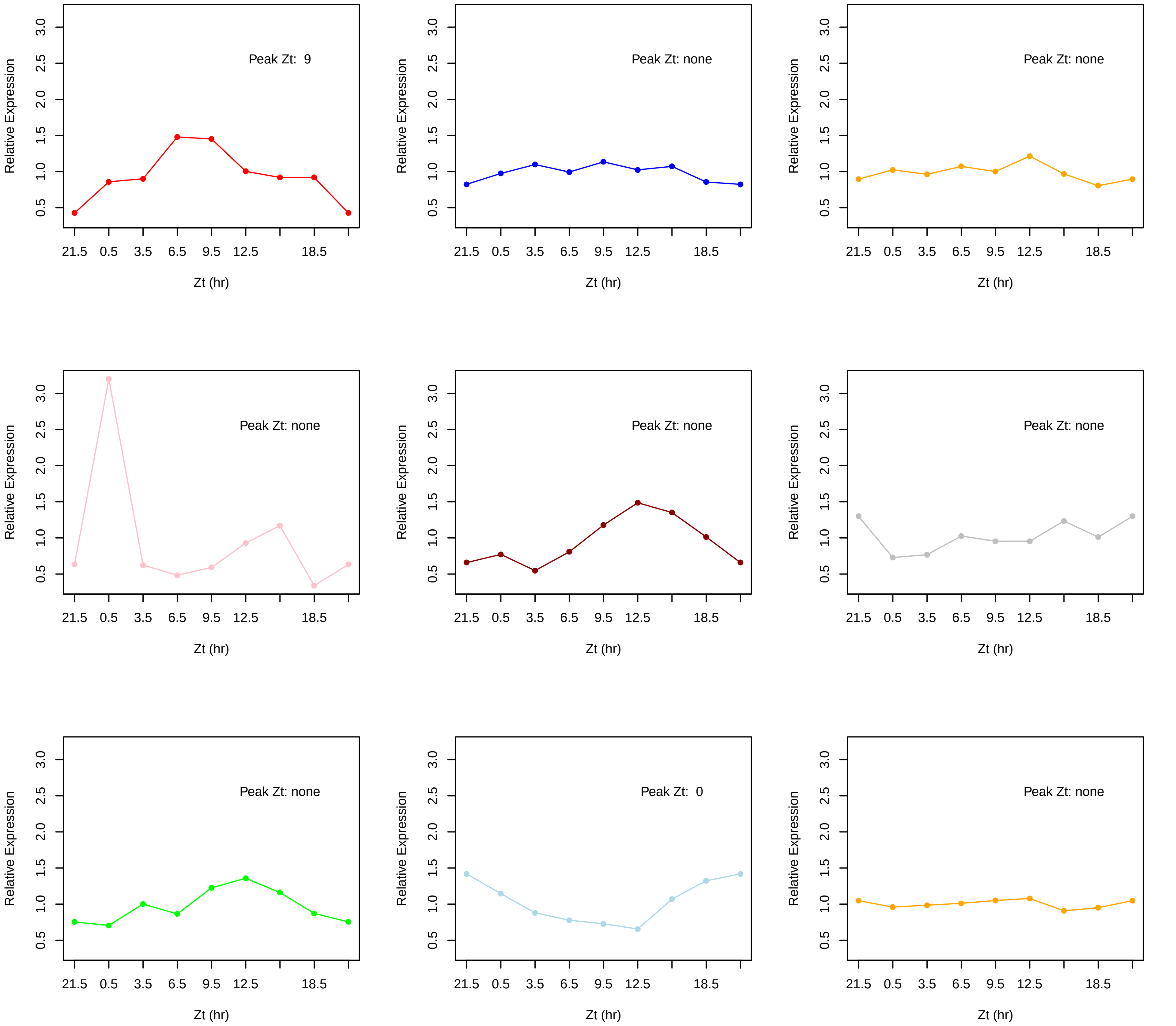

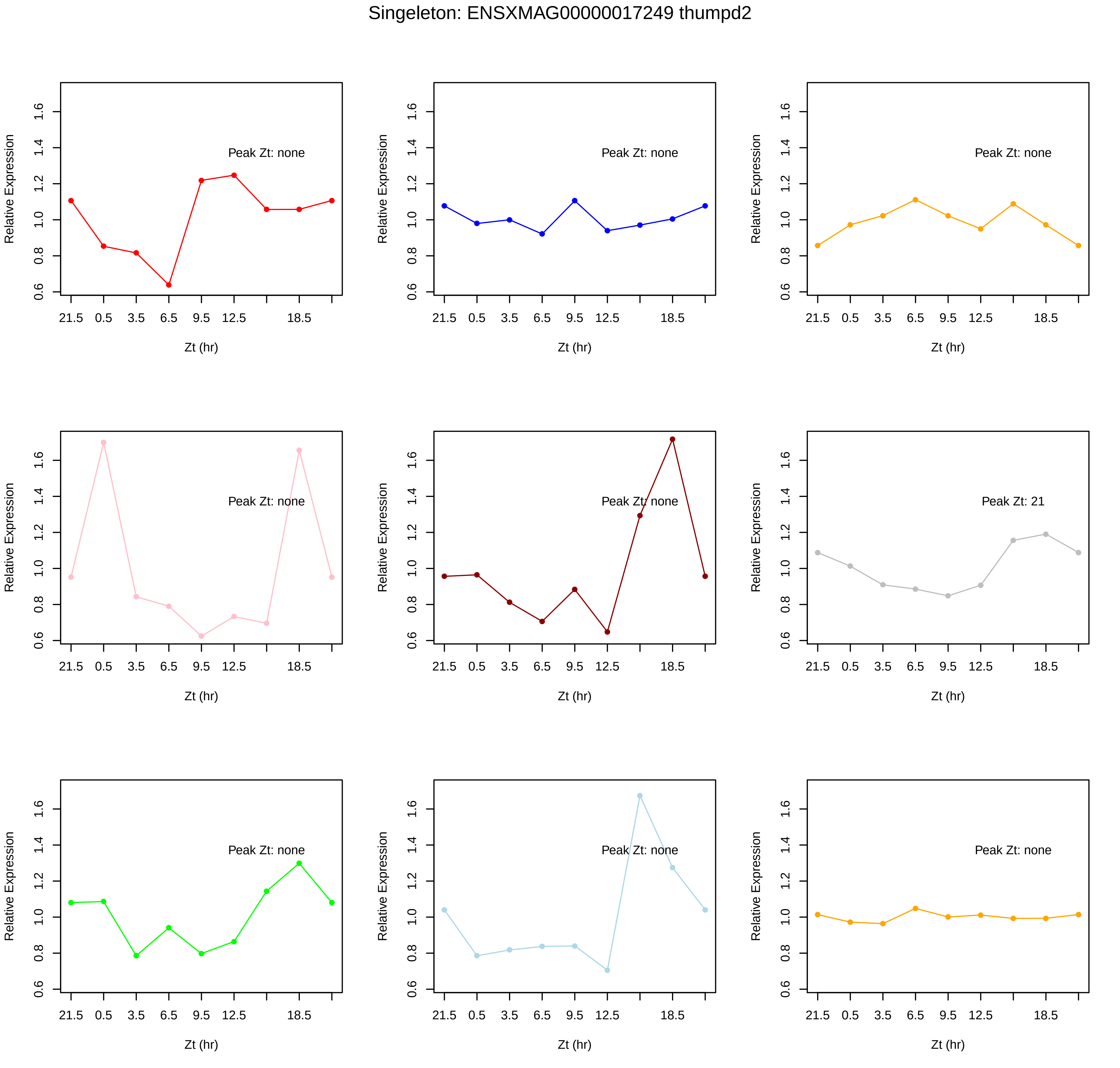

Singeleton: ENSXMAG00000017249 thumpd2
Peak Zt: none
Peak Zt: none
Peak Zt: none
Peak Zt: none
Peak Zt: none
Peak Zt: 21
Peak Zt: none
Peak Zt: none
Peak Zt: none
Relative Expression
Zt (hr)
0.6
0.8
1.0
1.2
1.4
1.6
21.5
0.5
3.5
6.5
9.5
12.5
18.5

Singeleton: ENSXMAG00000008904 vezt

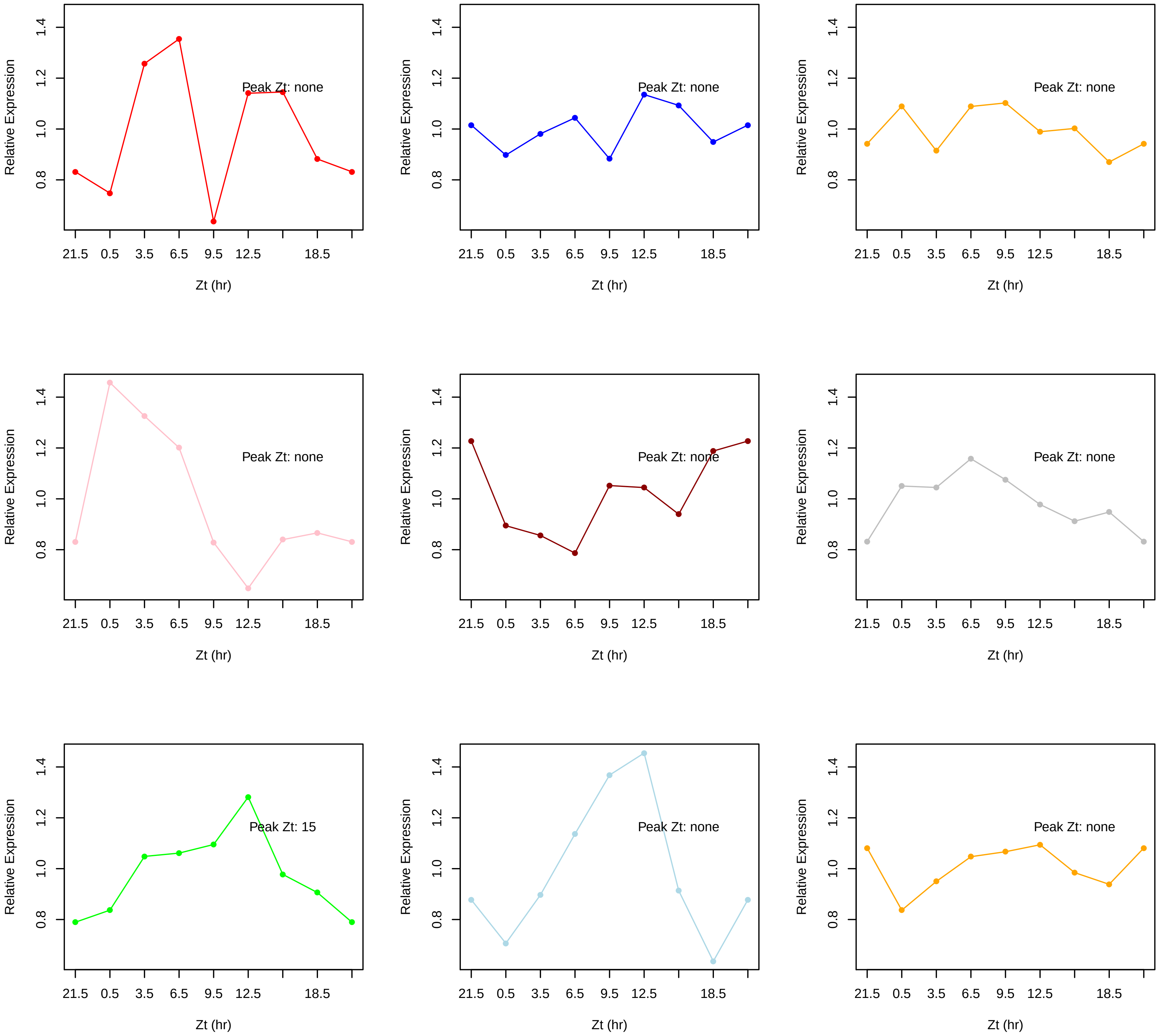

Singeleton: ENSXMAG00000016651 NR2C1

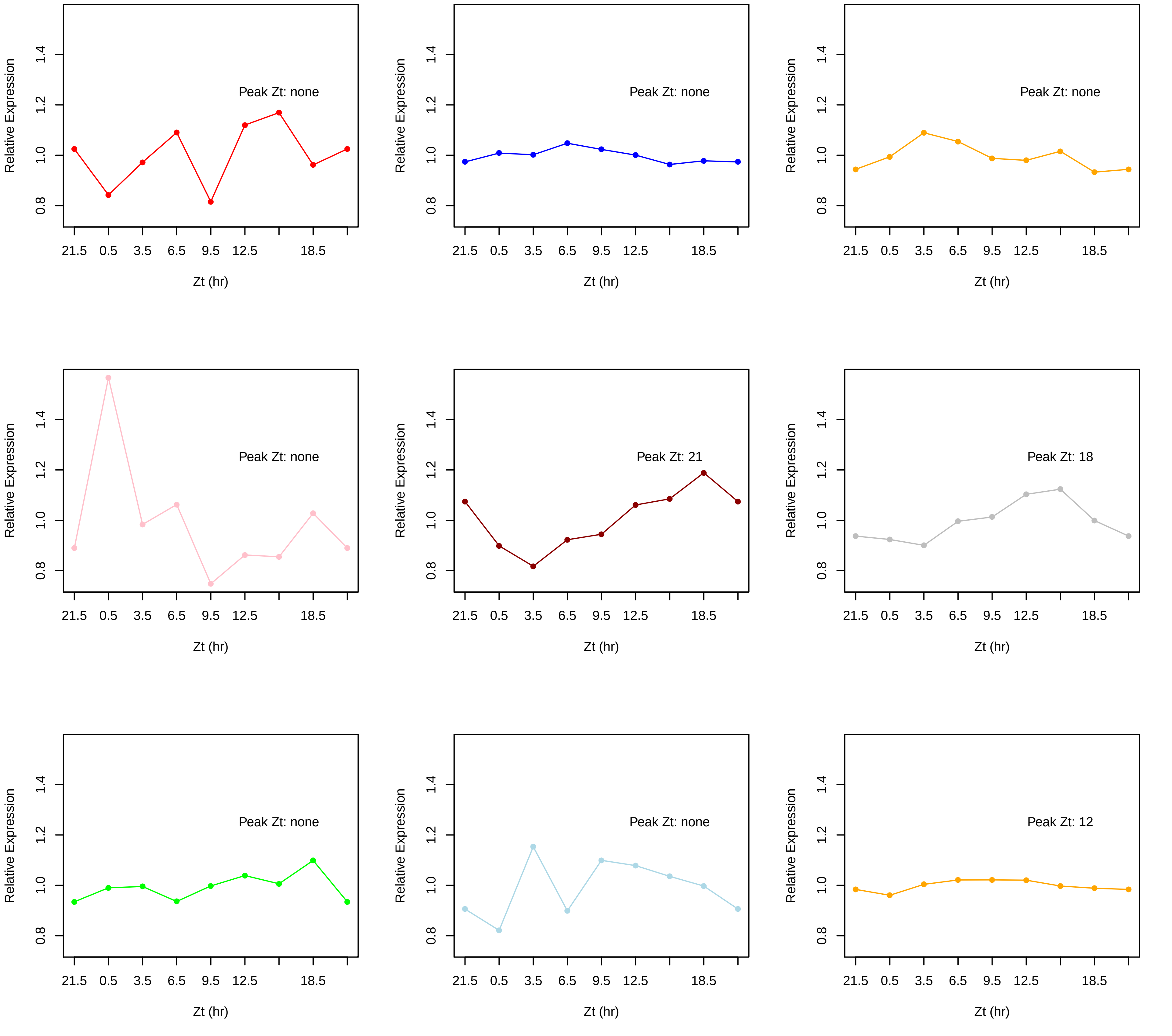

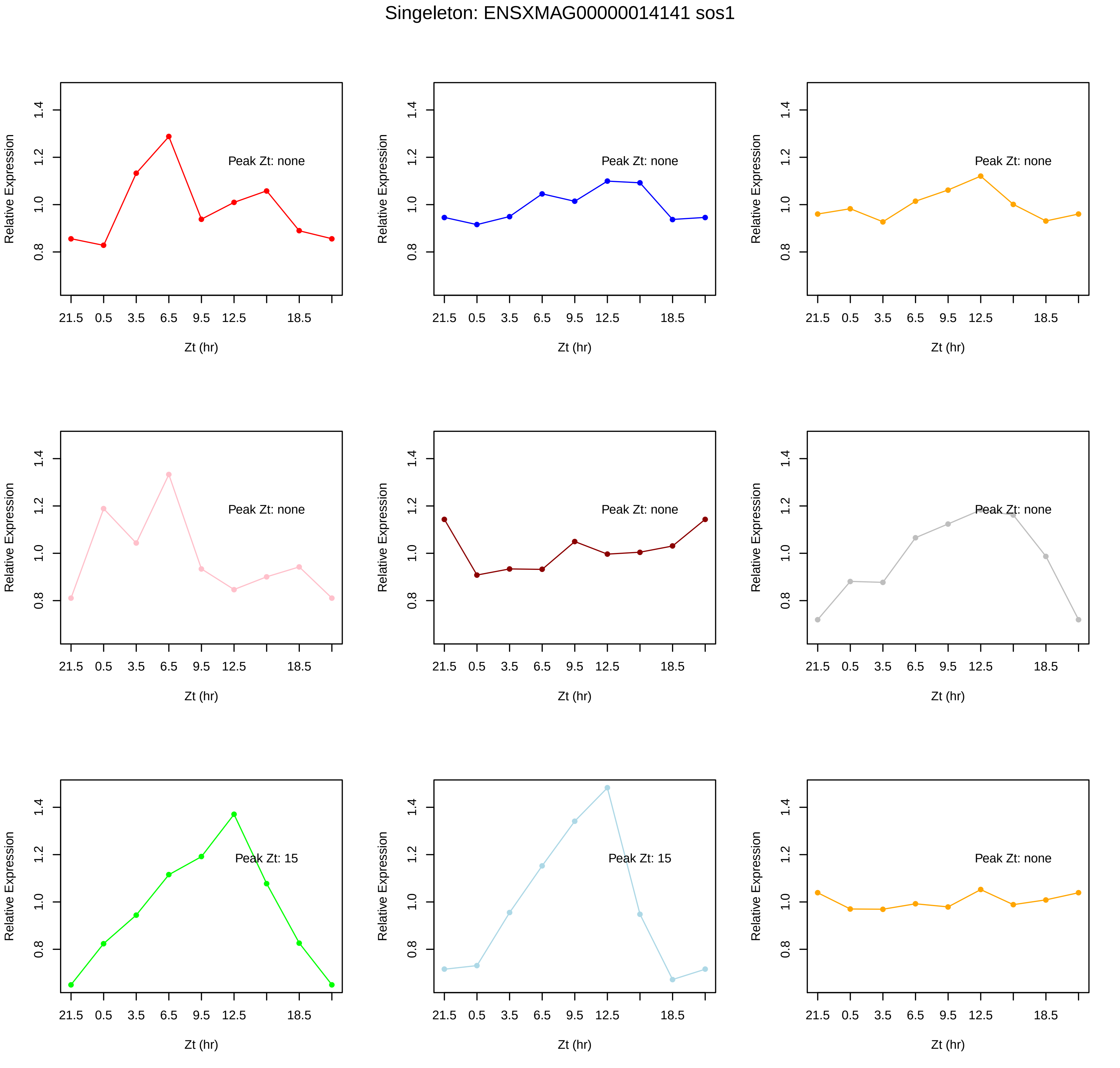
Singeleton: ENSXMAG00000014141 sos1
Relative Expression
Zt (hr)
Peak Zt: none
Peak Zt: none
Peak Zt: none
Peak Zt: none
Peak Zt: none
Peak Zt: none
Peak Zt: 15
Peak Zt: 15
Peak Zt: none

Singeleton: ENSXMAG00000006391 sprtn

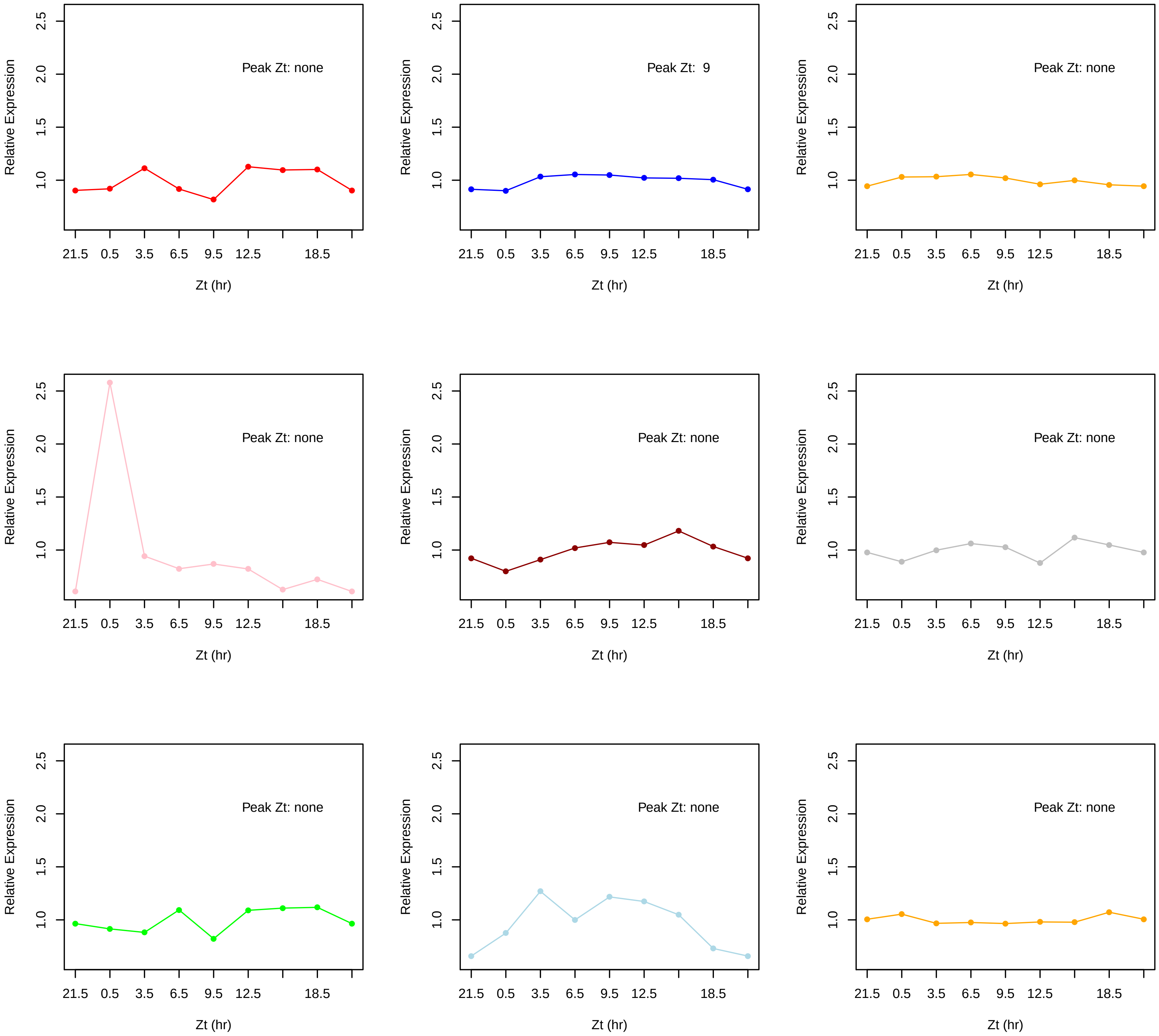

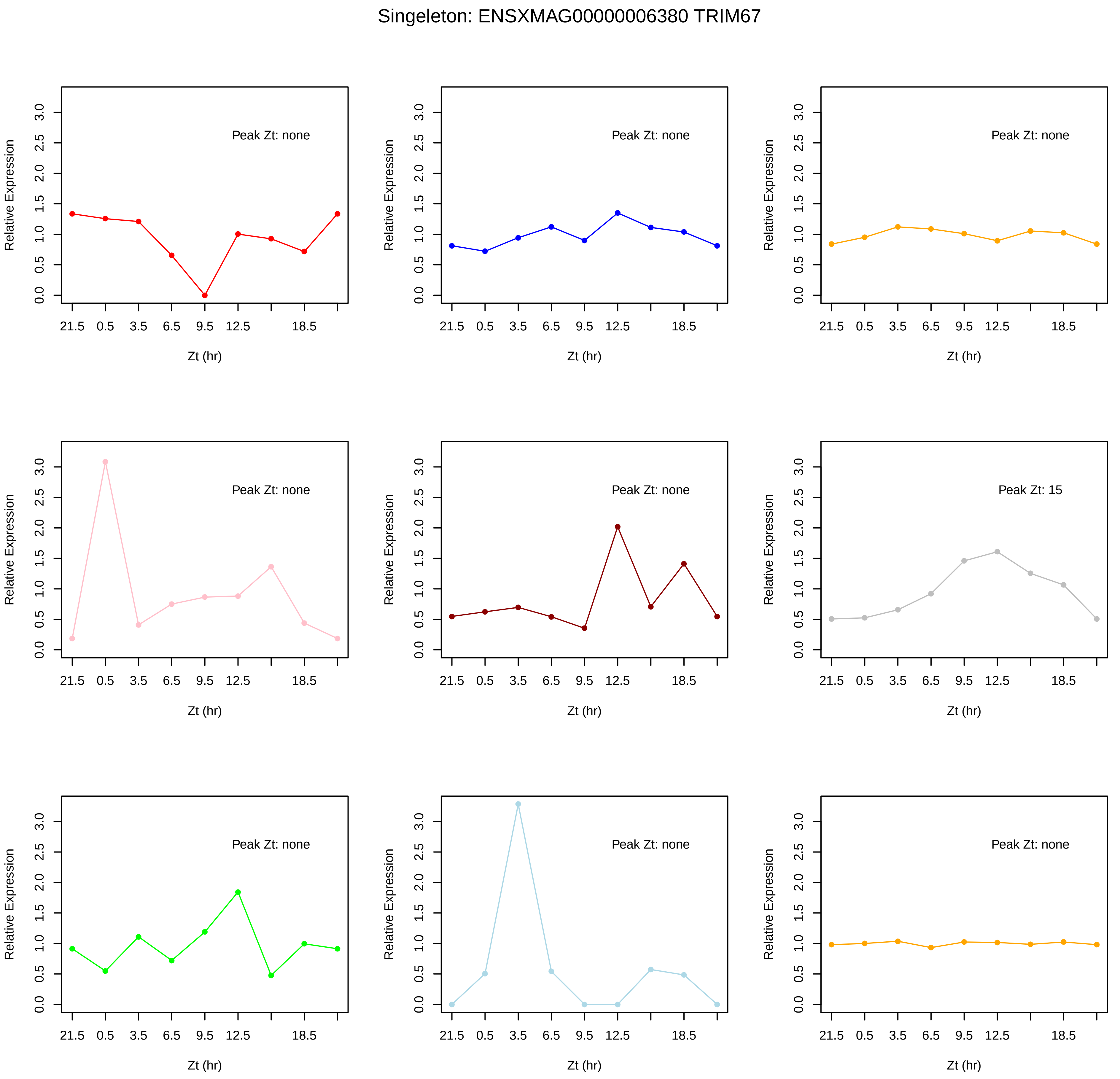
Singeleton: ENSXMAG00000006380 TRIM67
Peak Zt: none
Peak Zt: none
Peak Zt: none
Peak Zt: none
Peak Zt: none
Peak Zt: 15
Peak Zt: none
Peak Zt: none
Peak Zt: none
Relative Expression
Zt (hr)
21.5 0.5 3.5 6.5 9.5 12.5 18.5
0.0 0.5 1.0 1.5 2.0 2.5 3.0

Singeleton: ENSXMAG00000006356 gnpat

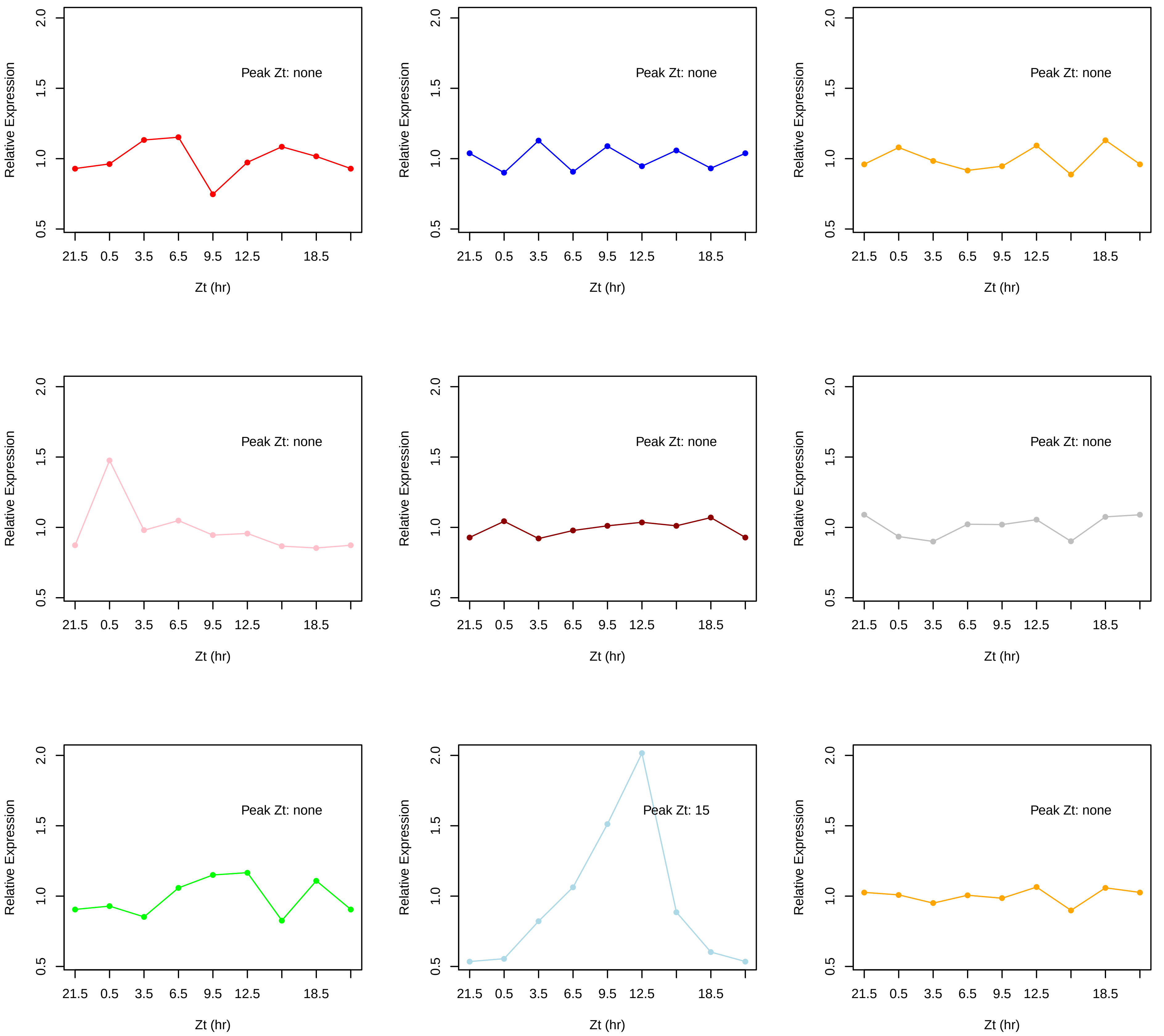

Singeleton: ENSXMAG00000009329 cradd

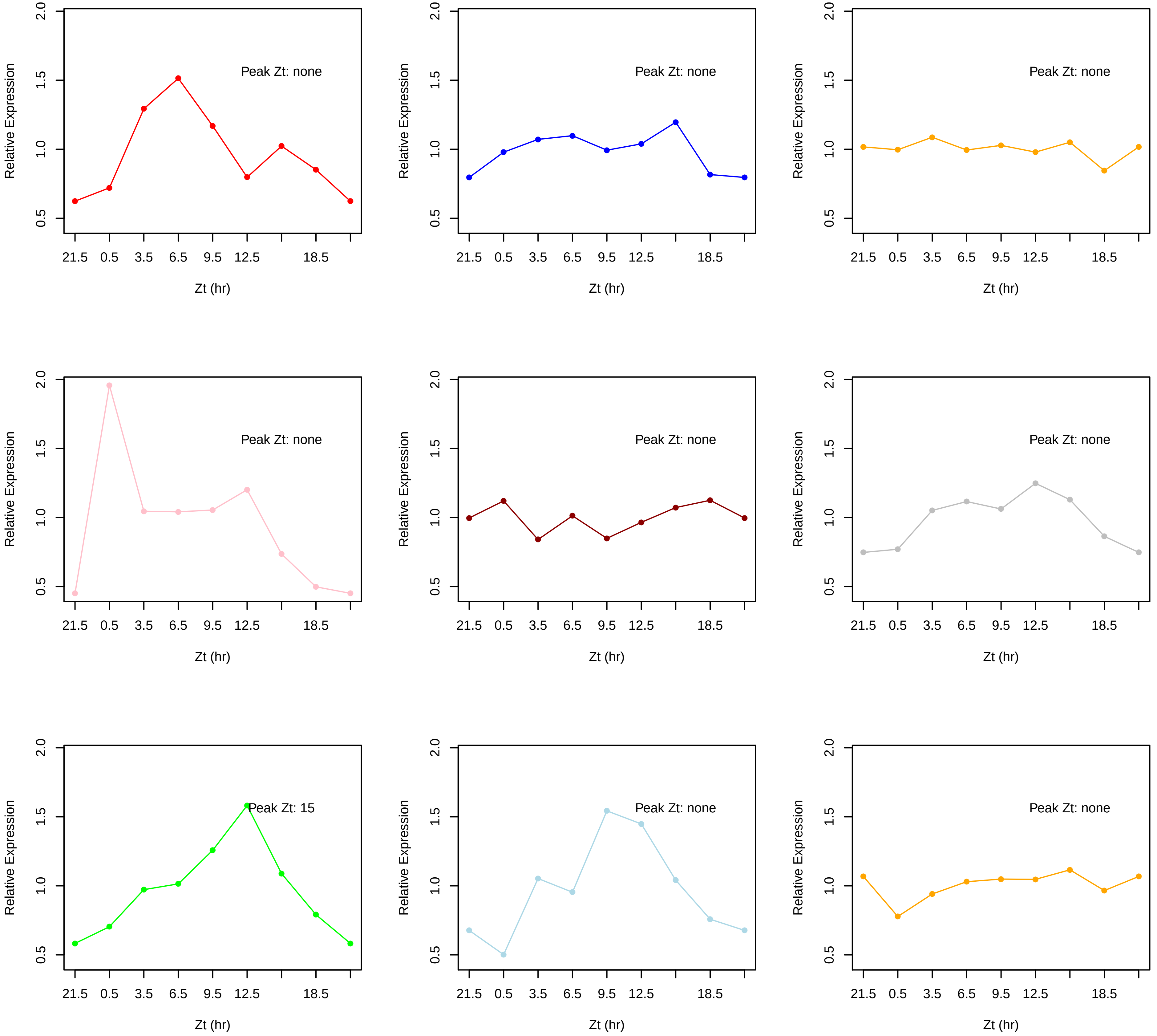

Singleton: ENSXMAG00000004136 dhx57

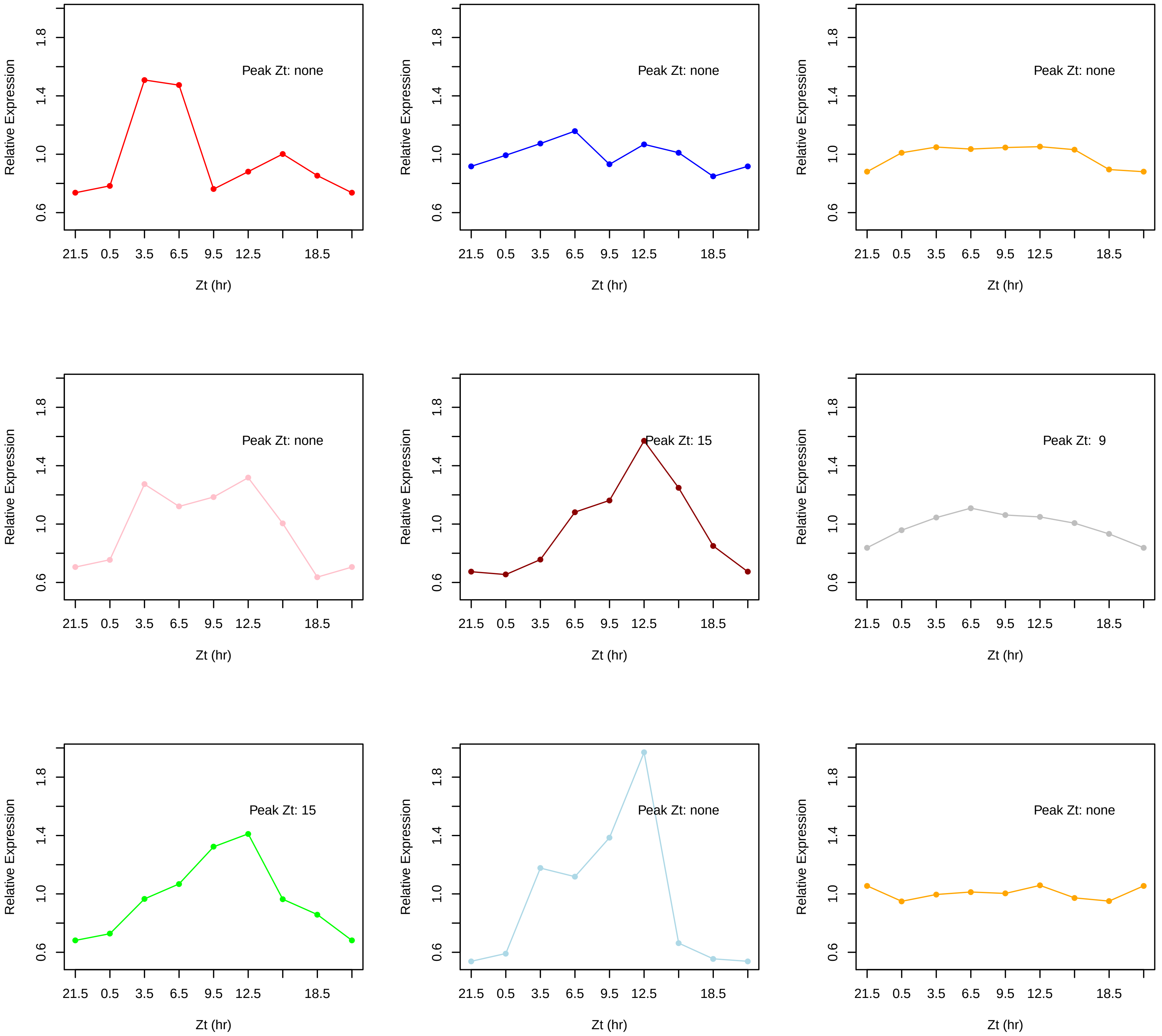

Singeleton: ENSXMAG00000014191 gemin6

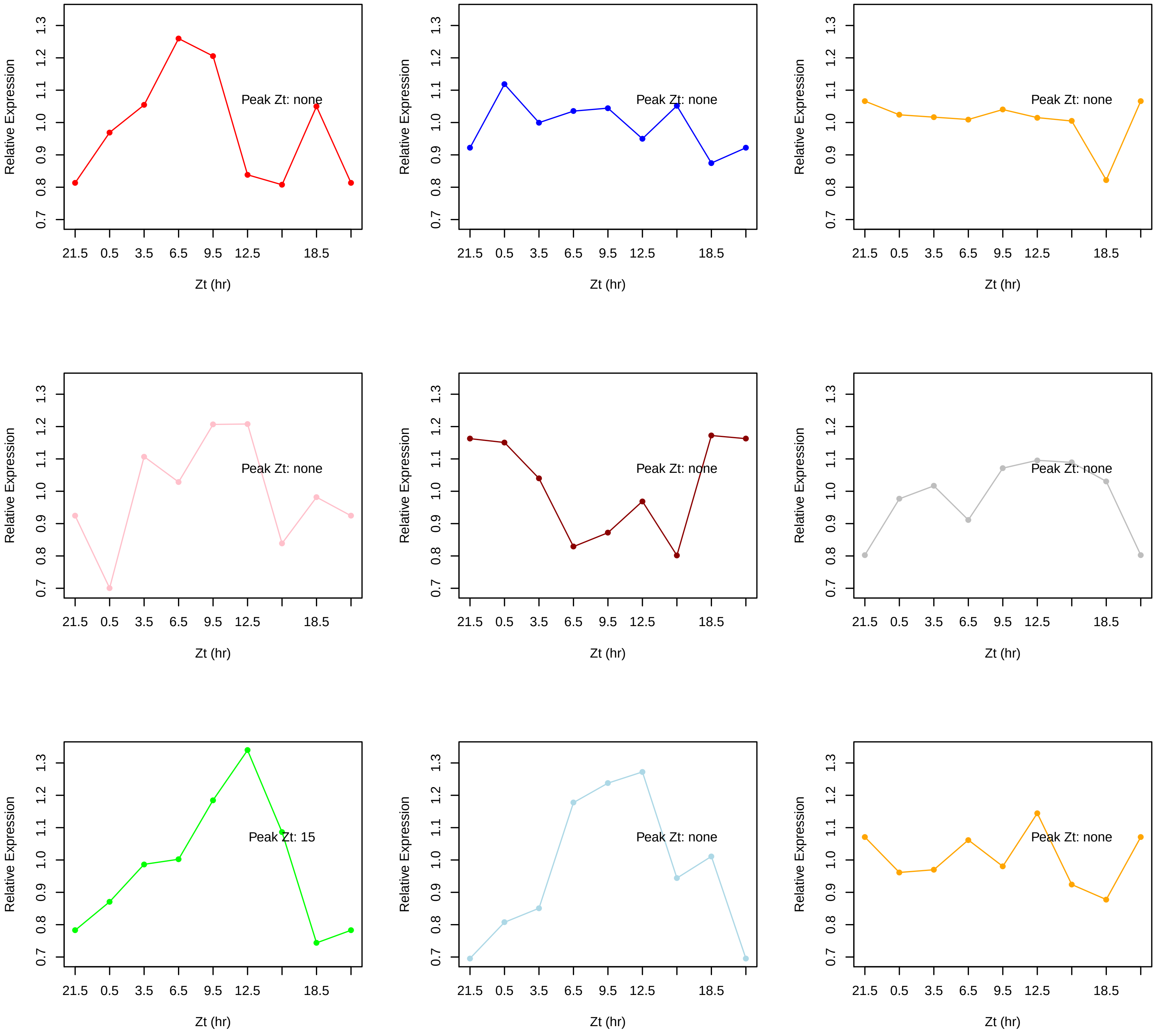

Singeleton: ENSXMAG00000014782

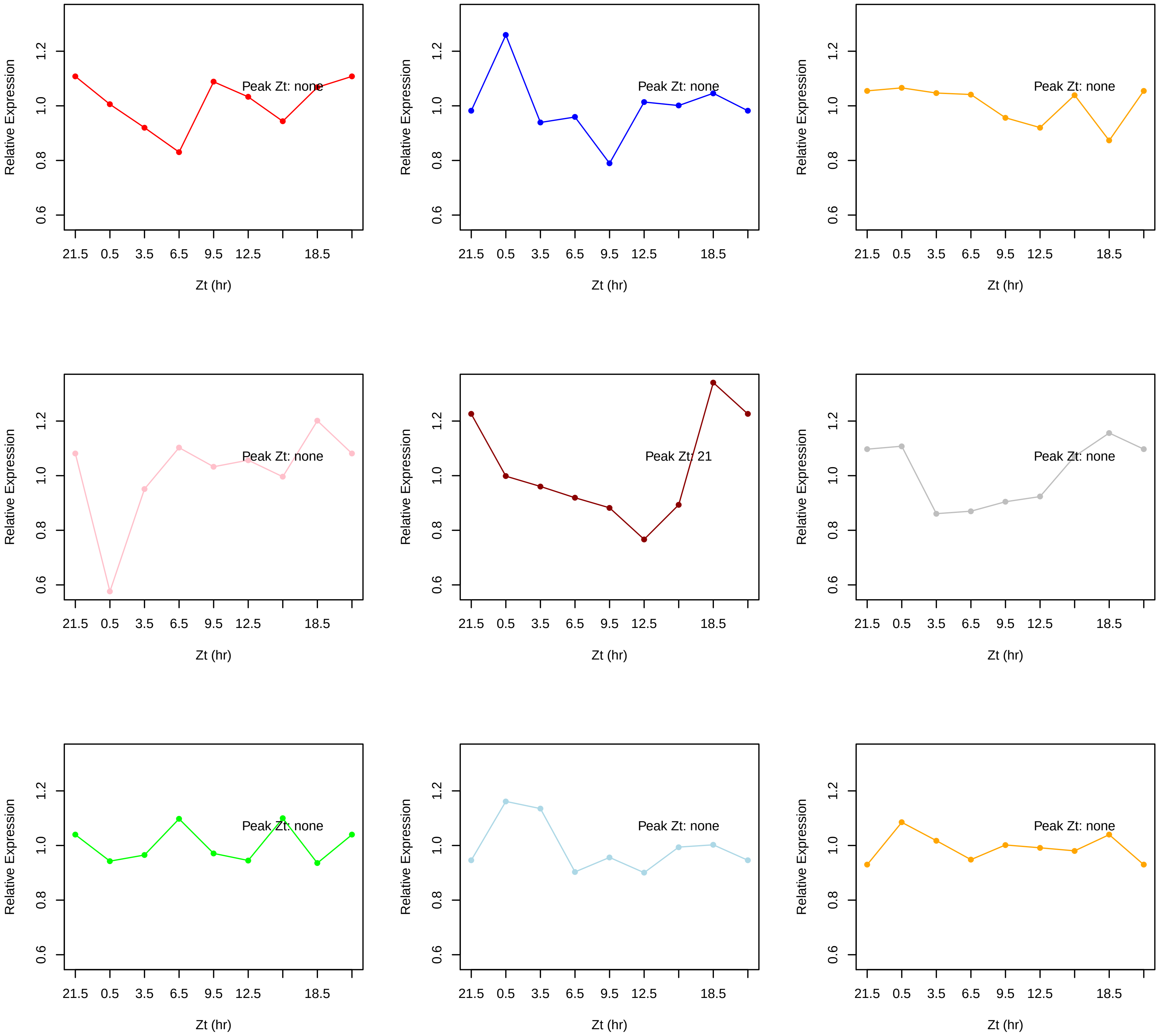

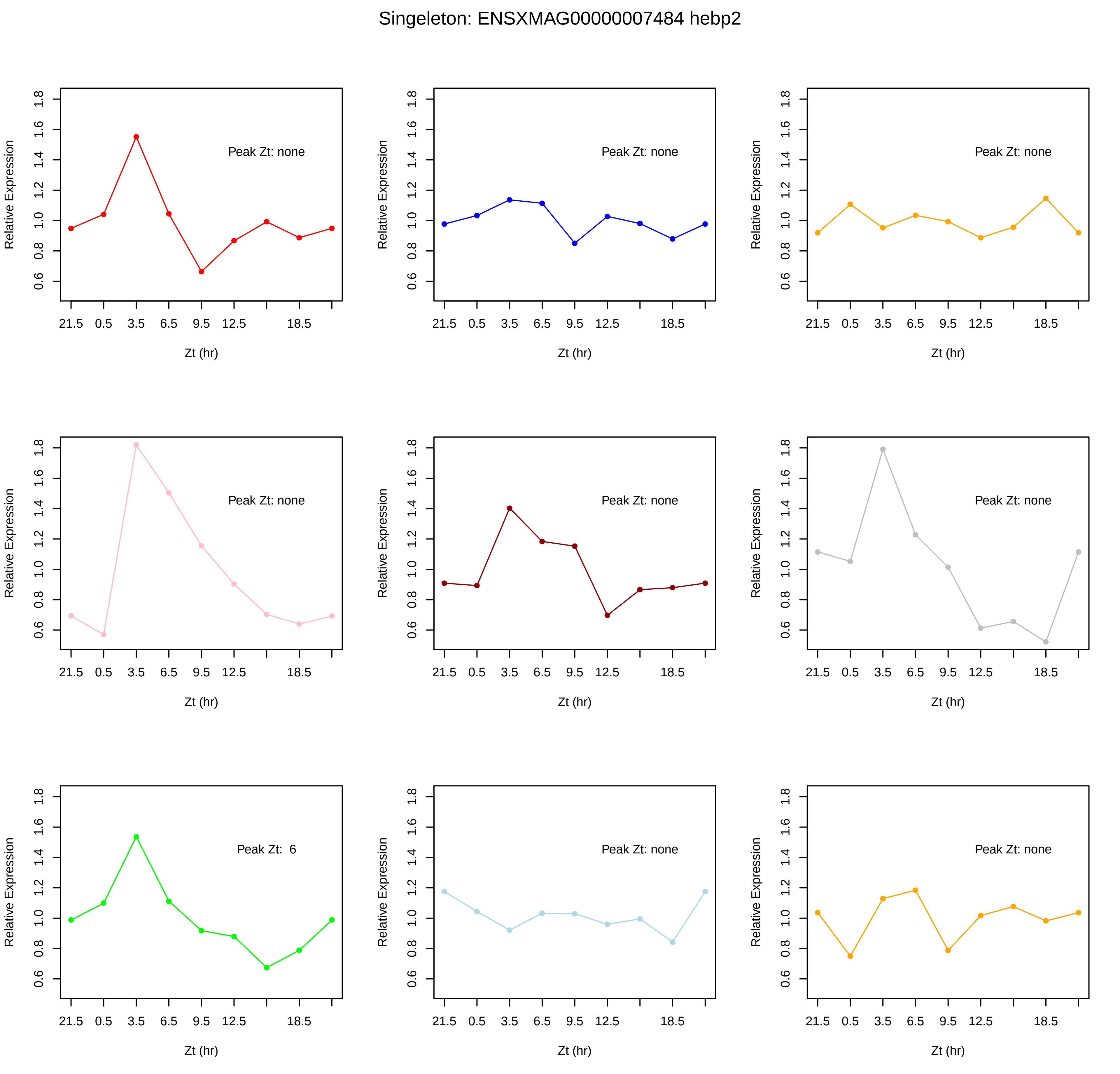
Singleton: ENSXMAG00000007484 hebp2
Relative Expression
Zt (hr)
21.5
0.5
3.5
6.5
9.5
12.5
18.5
0.6
0.8
1.0
1.2
1.4
1.6
1.8
Peak Zt: none
Peak Zt: 6

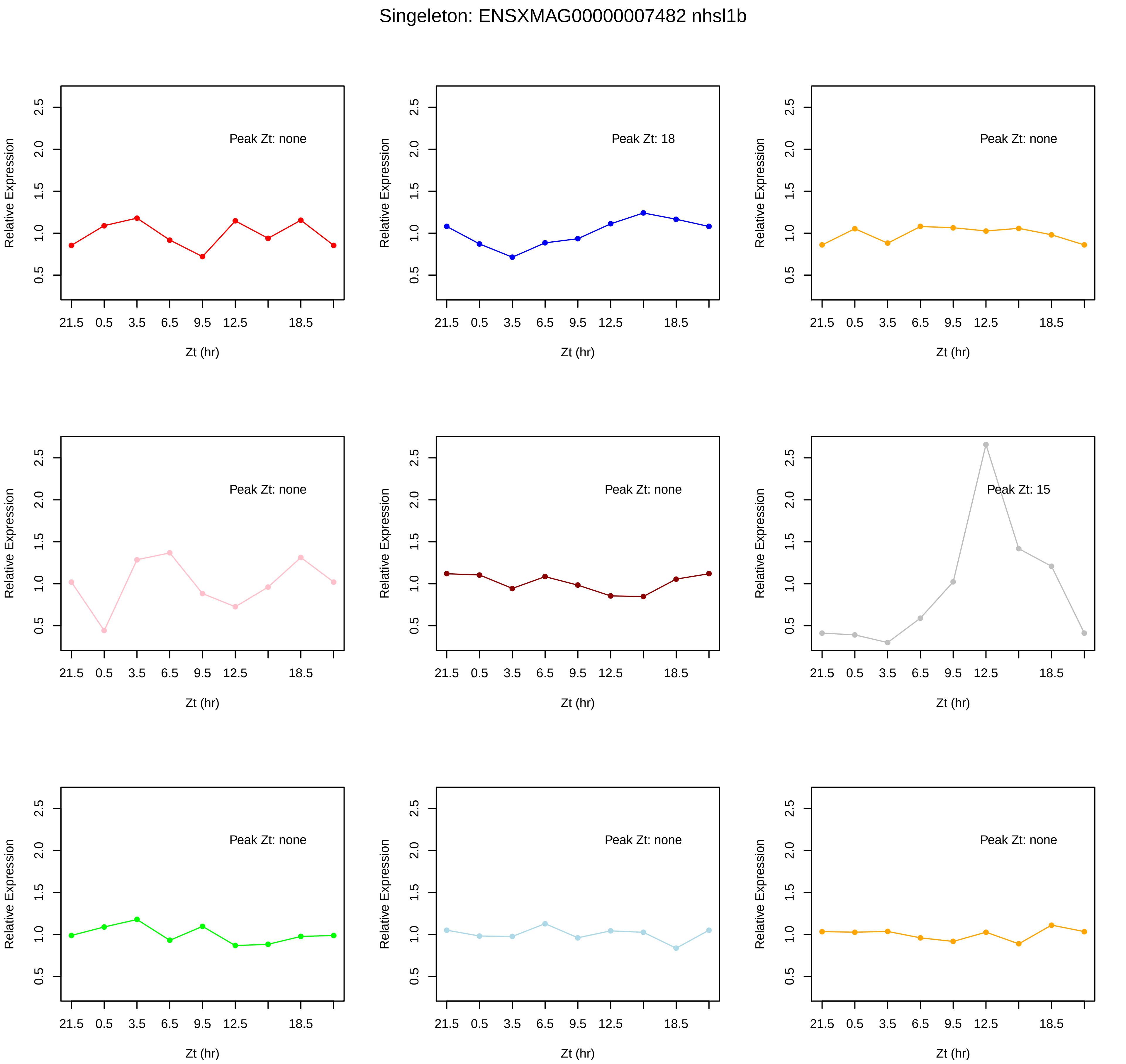
Singeleton: ENSXMAG00000007482 nhsl1b
Peak Zt: none
Peak Zt: 18
Peak Zt: none
Peak Zt: none
Peak Zt: none
Peak Zt: 15
Peak Zt: none
Peak Zt: none
Peak Zt: none
Relative Expression
Zt (hr)
21.5
0.5
3.5
6.5
9.5
12.5
18.5
0.5
1.0
1.5
2.0
2.5

Singleton: ENSXMAG00000014111 eif4a3

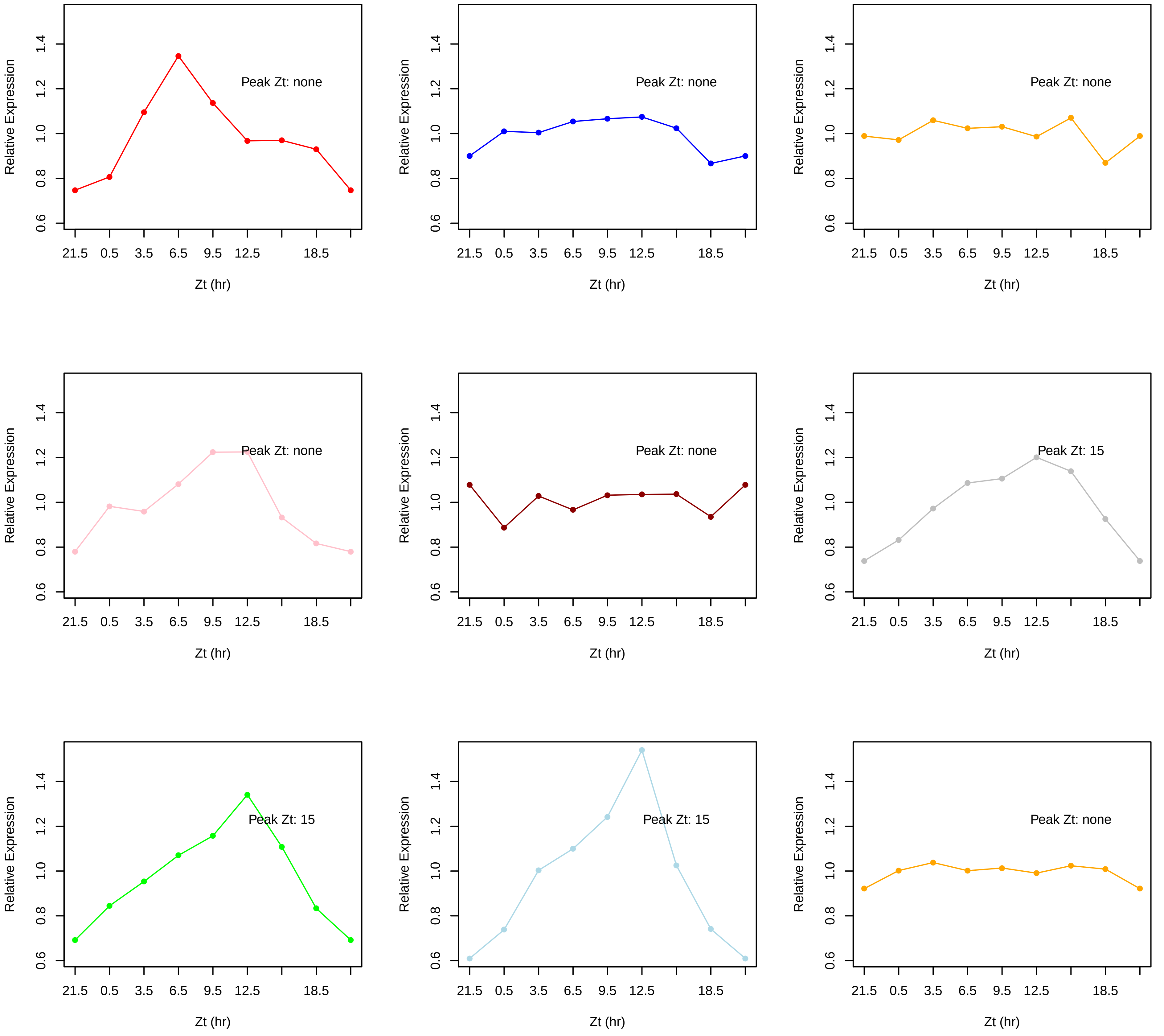

Singeleton: ENSXMAG00000021110 cbll1

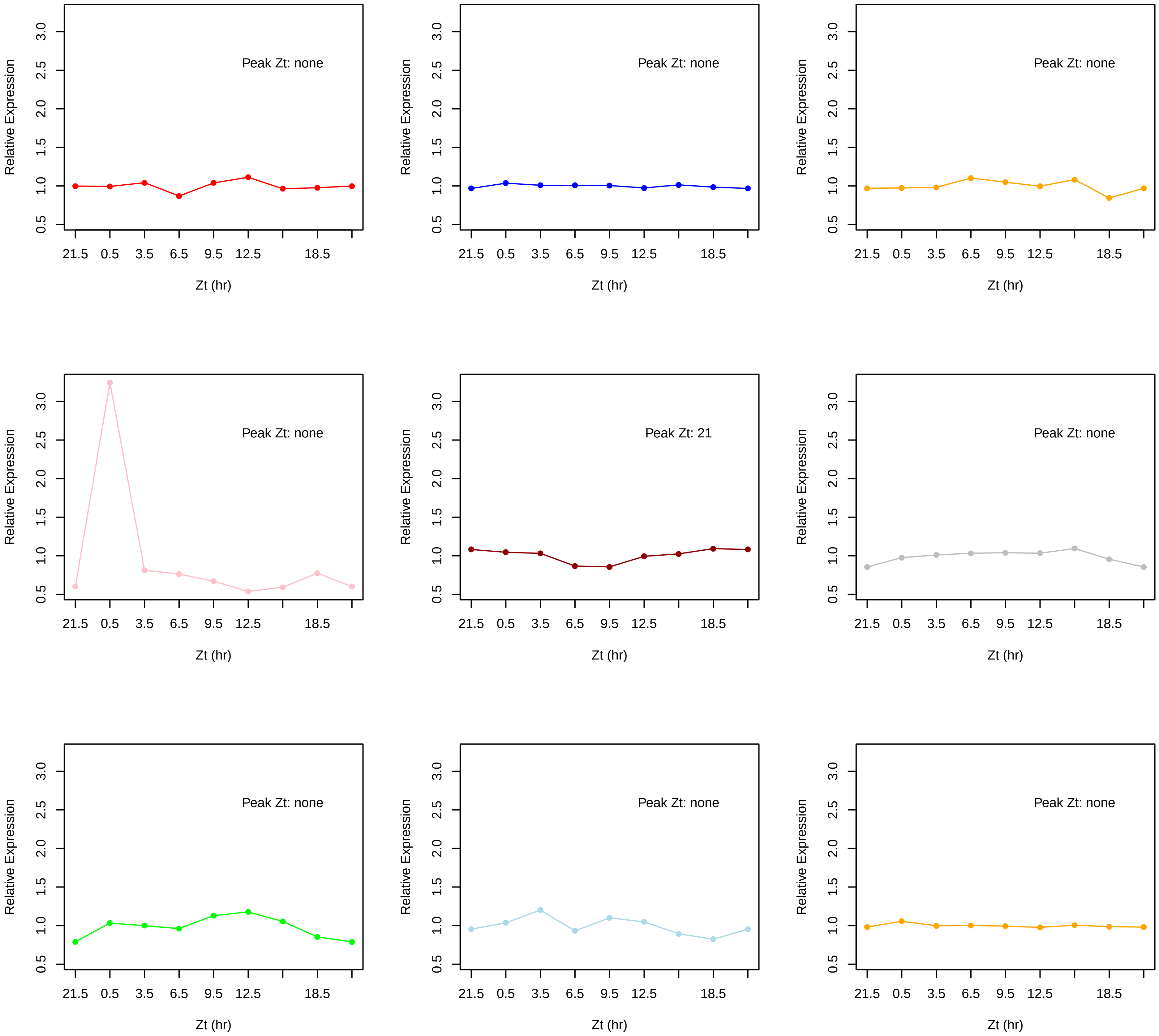

Singeleton: ENSXMAG00000026338

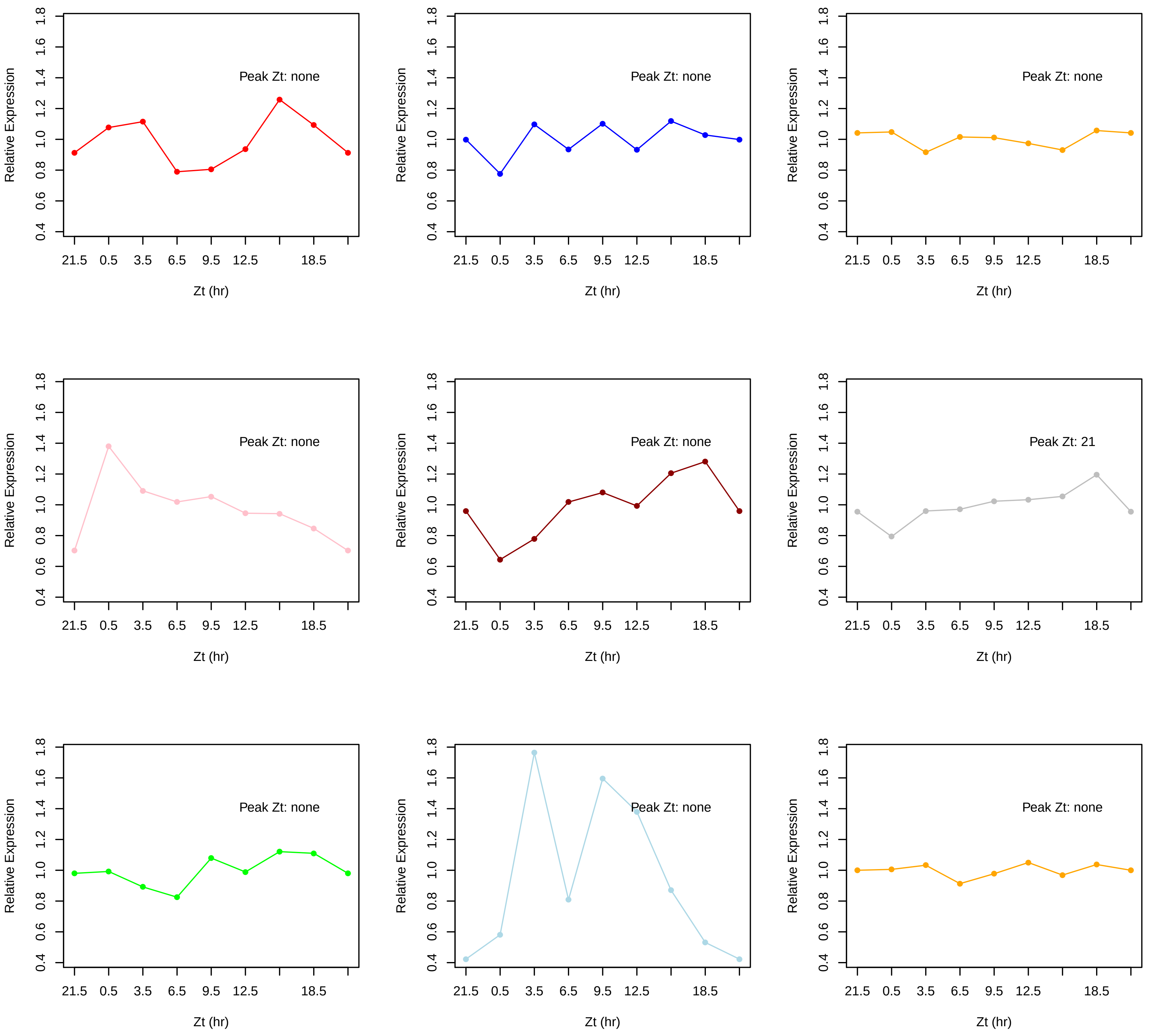

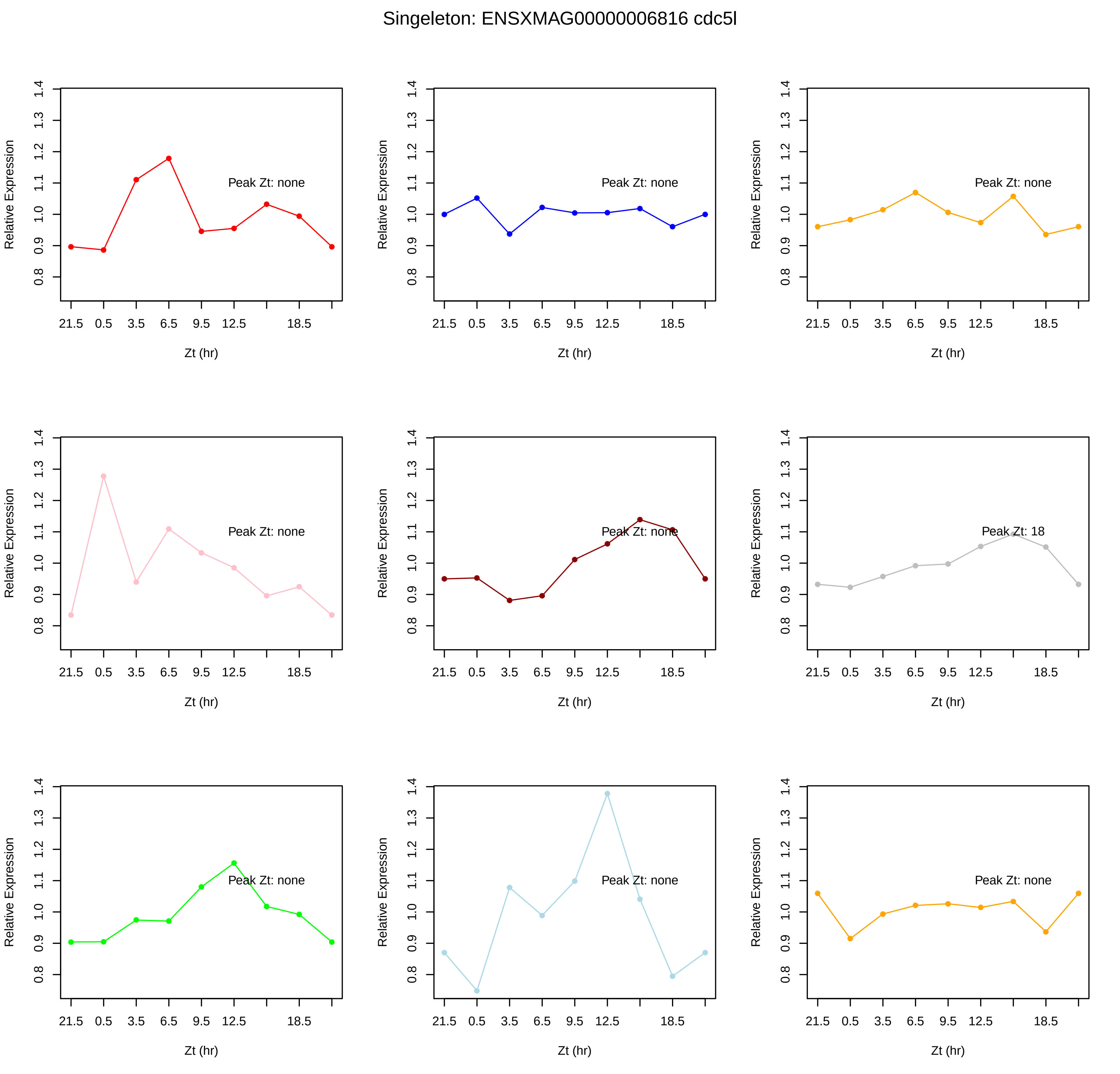
Singeleton: ENSXMAG00000006816 cdc5l
Relative Expression
Zt (hr)
Peak Zt: none
Peak Zt: 18
21.5
0.5
3.5
6.5
9.5
12.5
18.5
0.8
0.9
1.0
1.1
1.2
1.3
1.4

Singleton: ENSXMAG00000006720

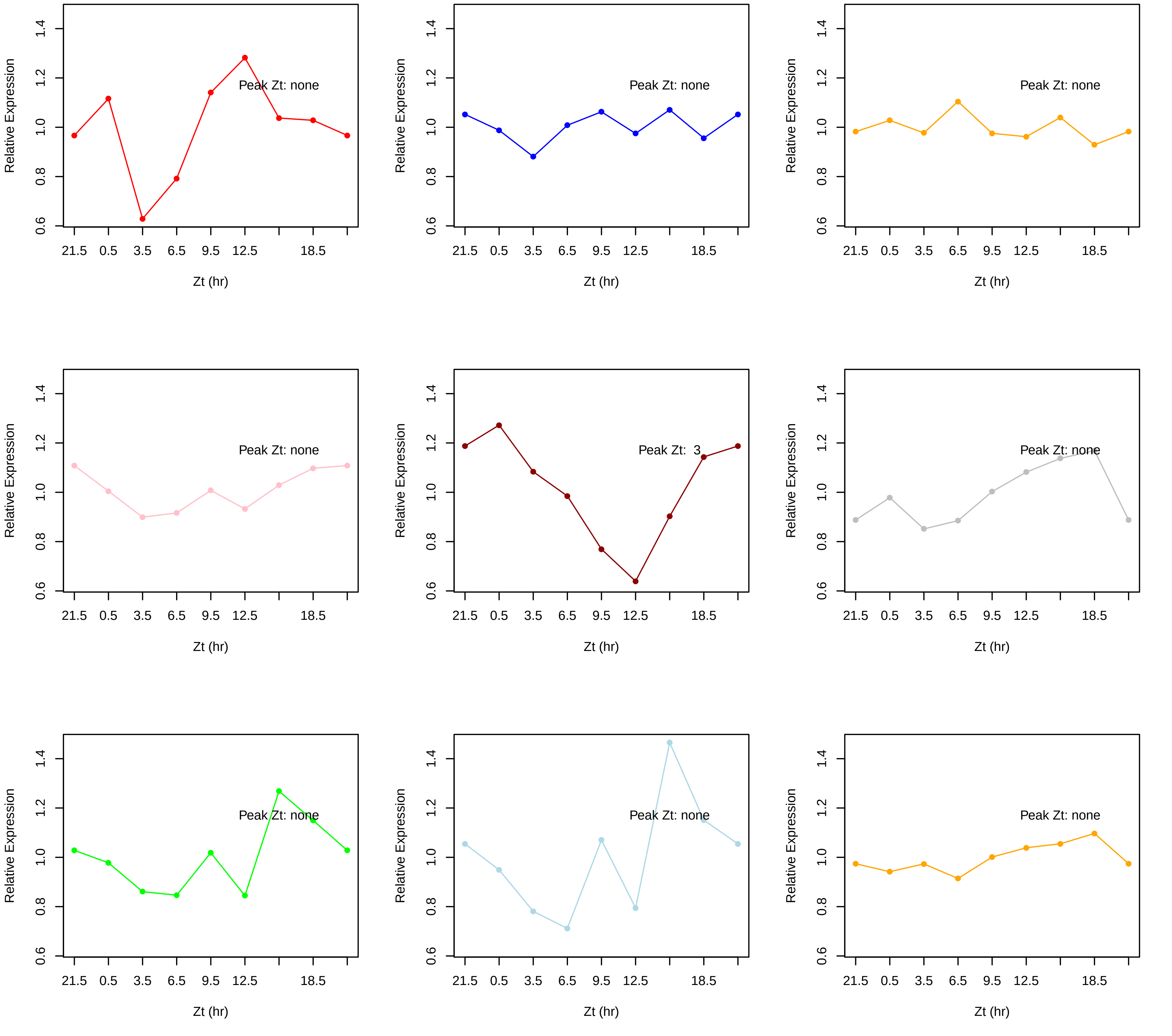

Singeleton: ENSXMAG00000008454 dus4l

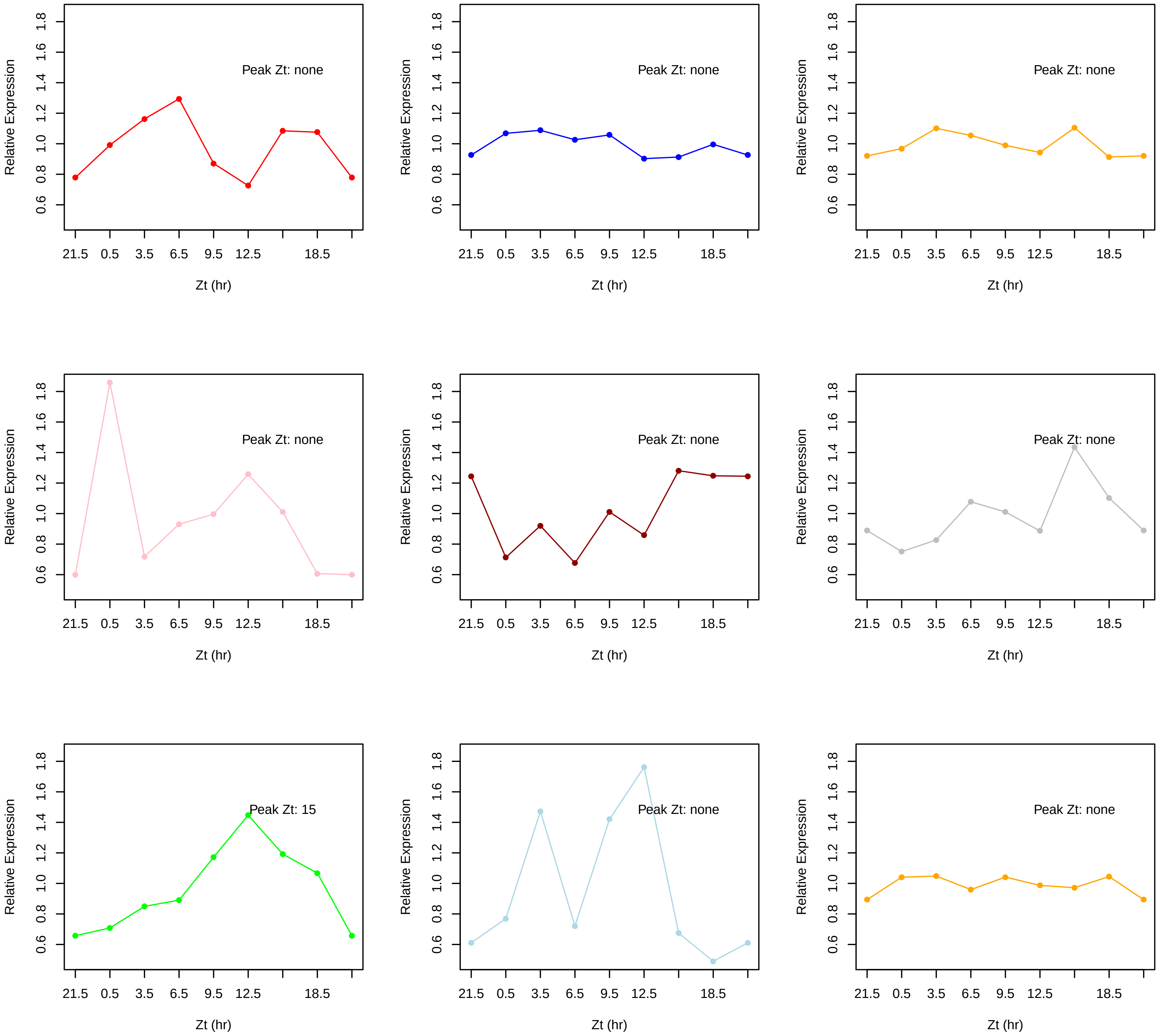

Singleton: ENSXMAG00000007831 enpp4

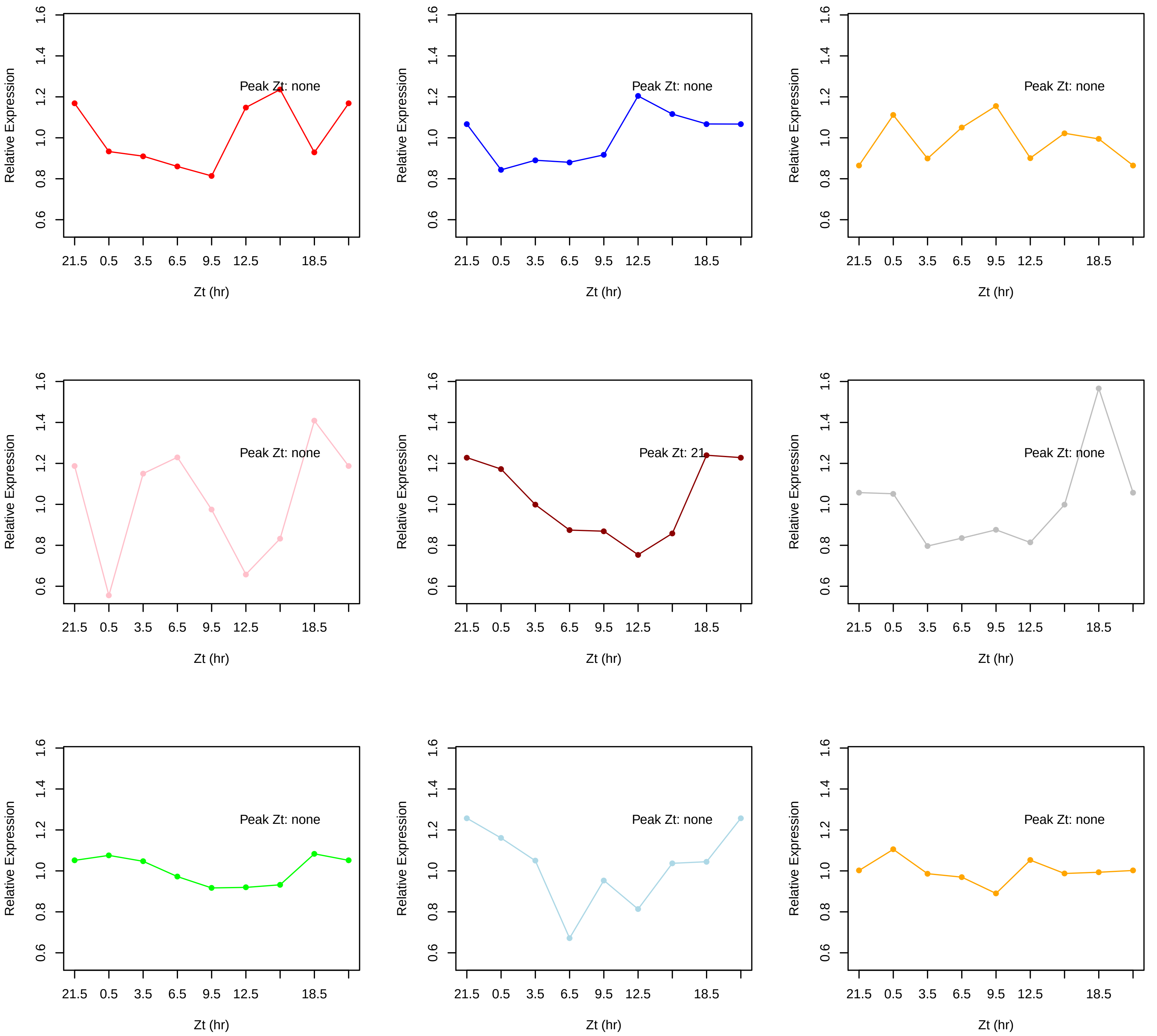

Singleton: ENSXMAG00000004278 fam167ab

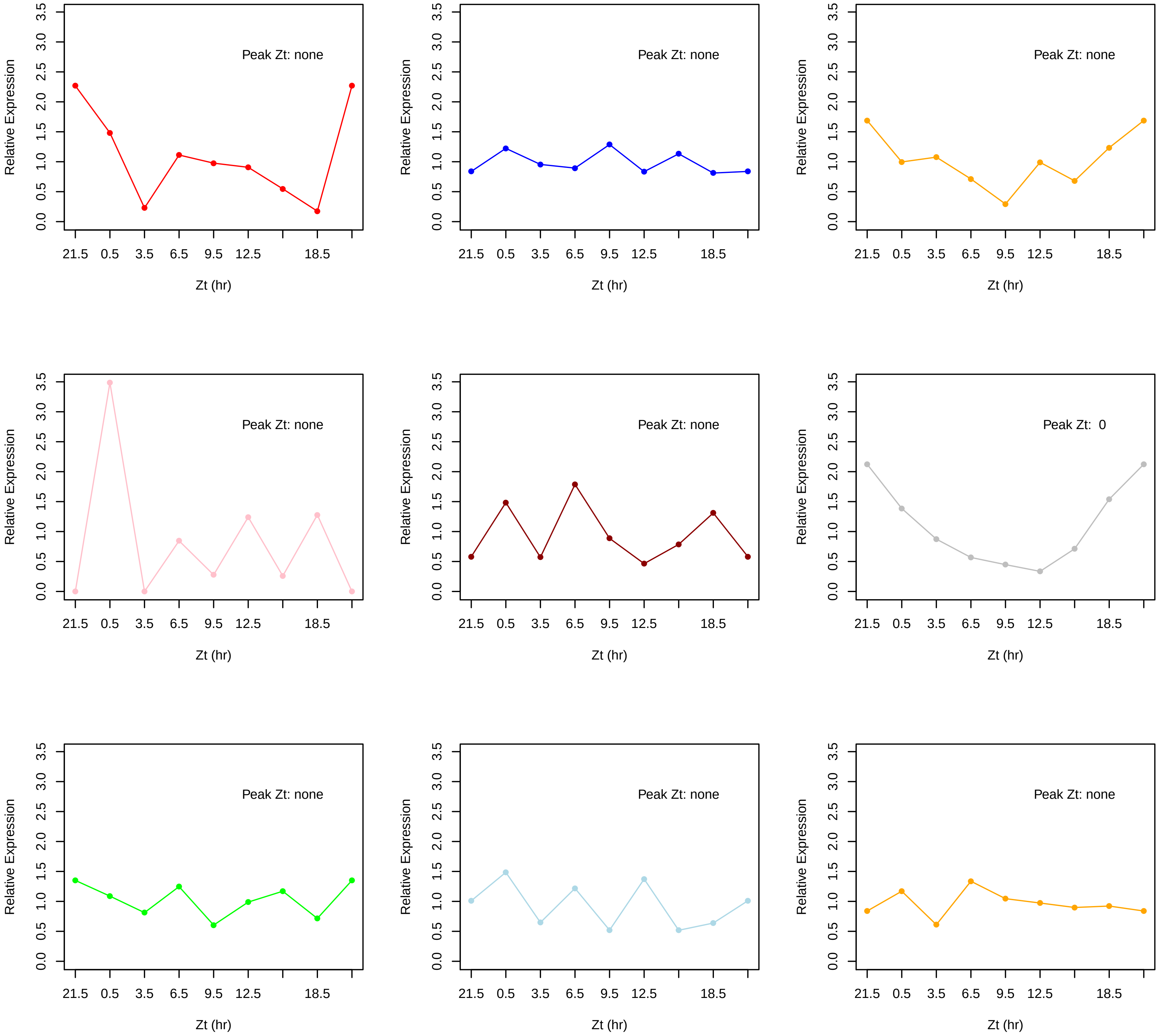

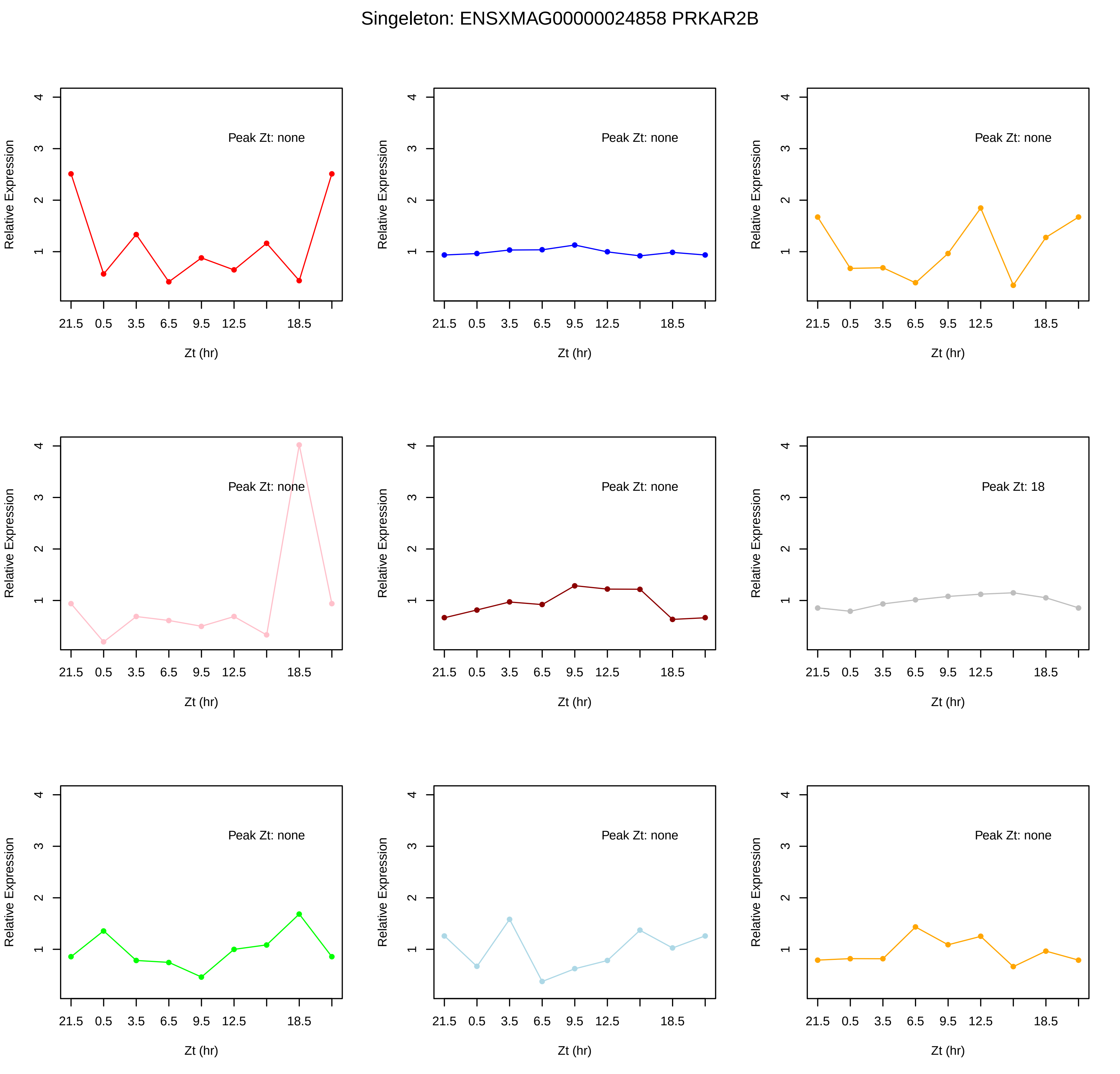
Singeleton: ENSXMAG00000024858 PRKAR2B
Peak Zt: none
Peak Zt: none
Peak Zt: none
Peak Zt: none
Peak Zt: none
Peak Zt: 18
Peak Zt: none
Peak Zt: none
Peak Zt: none
Relative Expression
Zt (hr)
21.5 0.5 3.5 6.5 9.5 12.5 18.5
1 2 3 4

Singeleton: ENSXMAG00000006729 egln1a

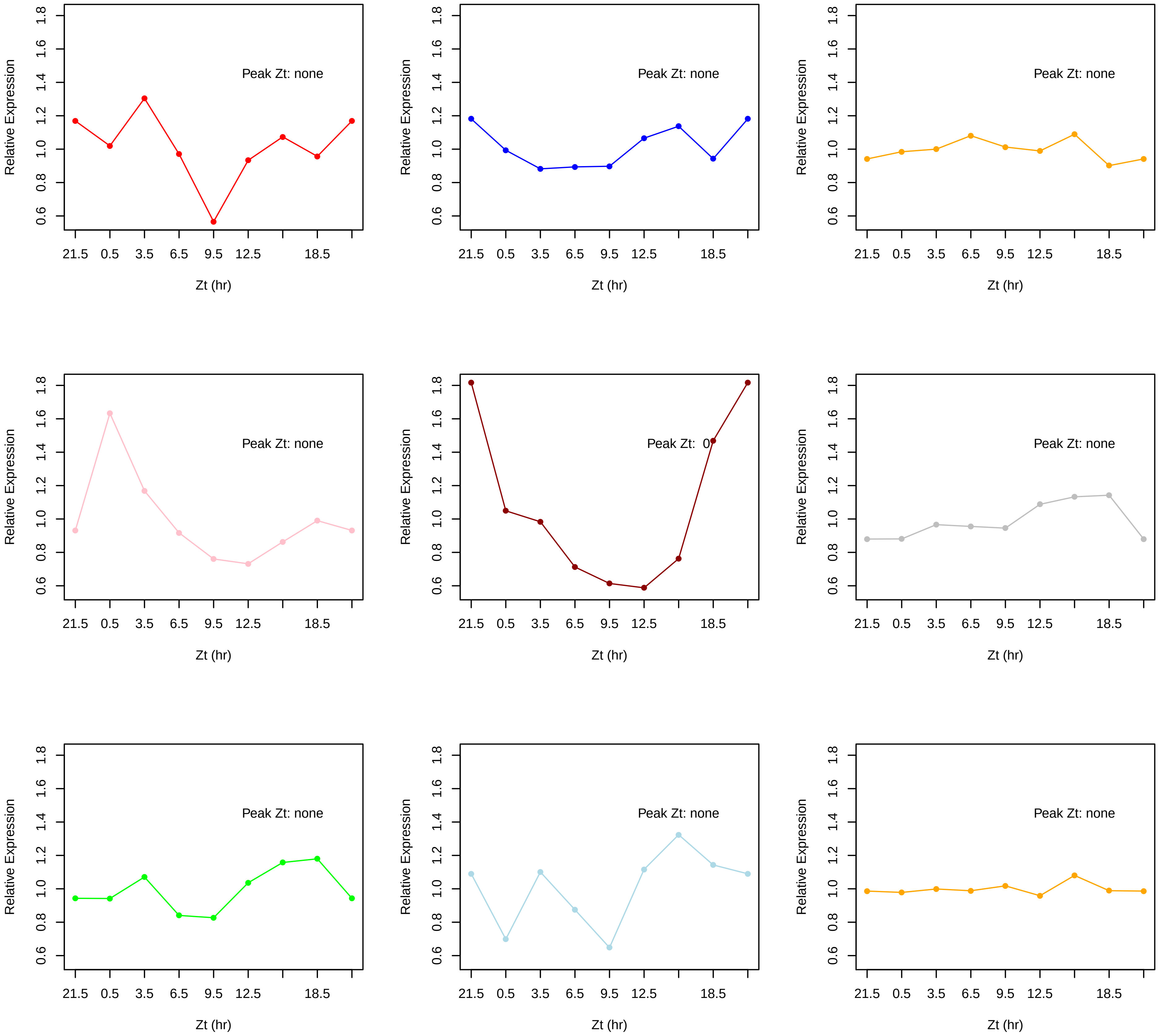

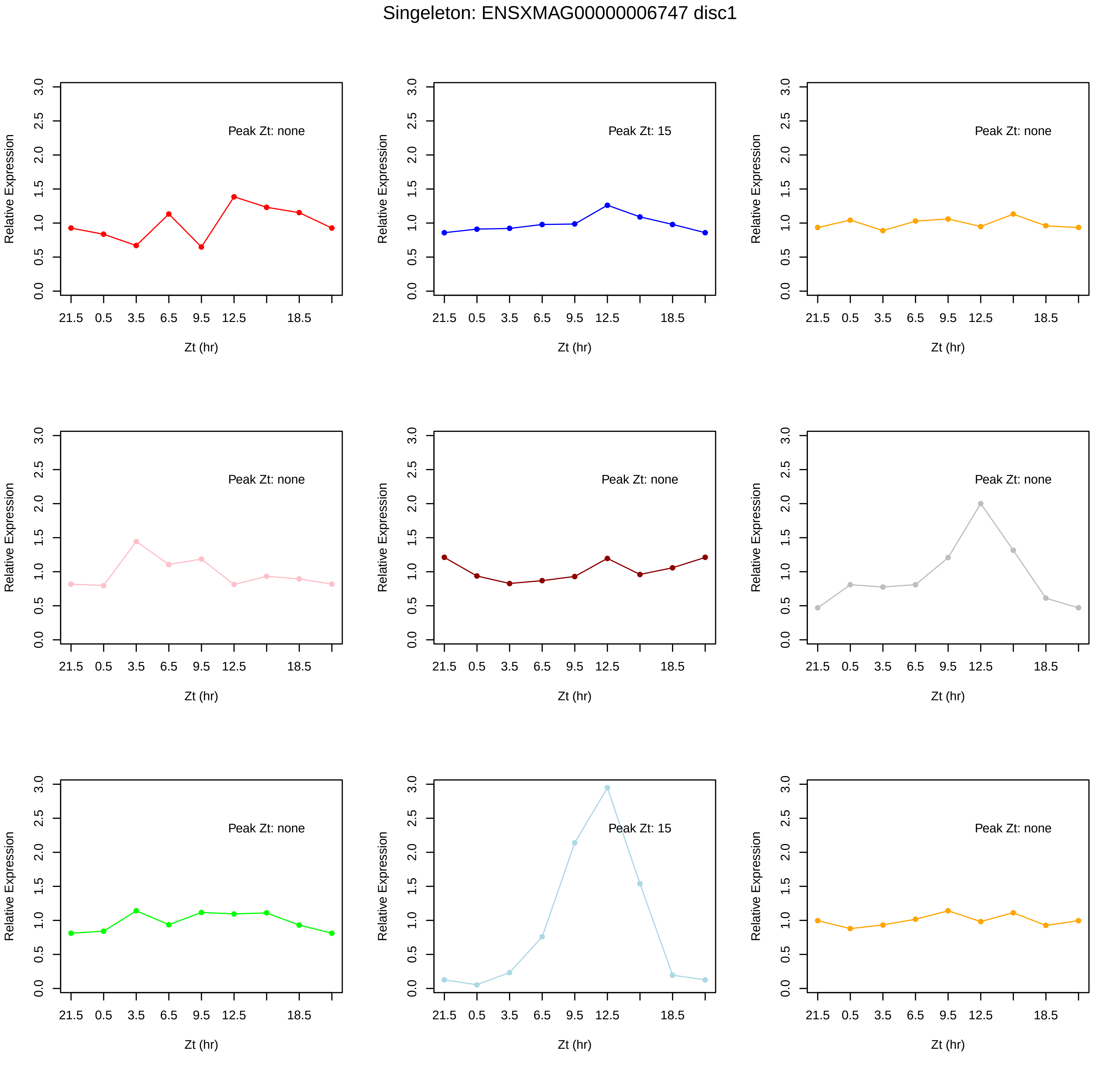
Singeleton: ENSXMAG00000006747 disc1
Peak Zt: none
Peak Zt: 15
Peak Zt: none
Peak Zt: none
Peak Zt: none
Peak Zt: none
Peak Zt: none
Peak Zt: 15
Peak Zt: none
Relative Expression
Zt (hr)
21.5
0.5
3.5
6.5
9.5
12.5
18.5
0.0
0.5
1.0
1.5
2.0
2.5
3.0

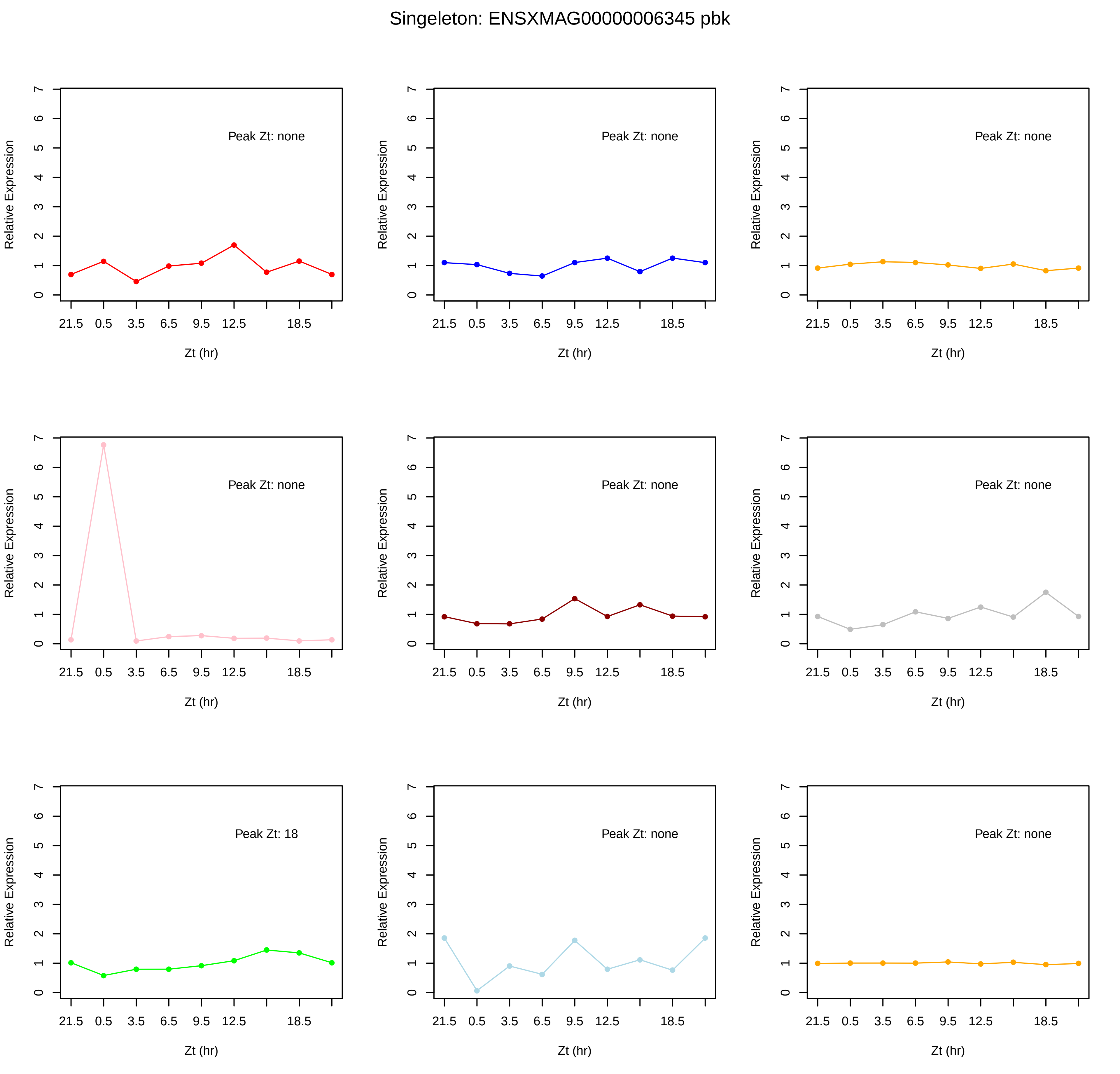

Singeleton: ENSXMAG00000006345 pbk
Peak Zt: none
Peak Zt: none
Peak Zt: none
Peak Zt: none
Peak Zt: none
Peak Zt: none
Peak Zt: 18
Peak Zt: none
Peak Zt: none
Relative Expression
Zt (hr)
21.5
0.5
3.5
6.5
9.5
12.5
18.5

Singeleton: ENSXMAG00000009482 grm8a

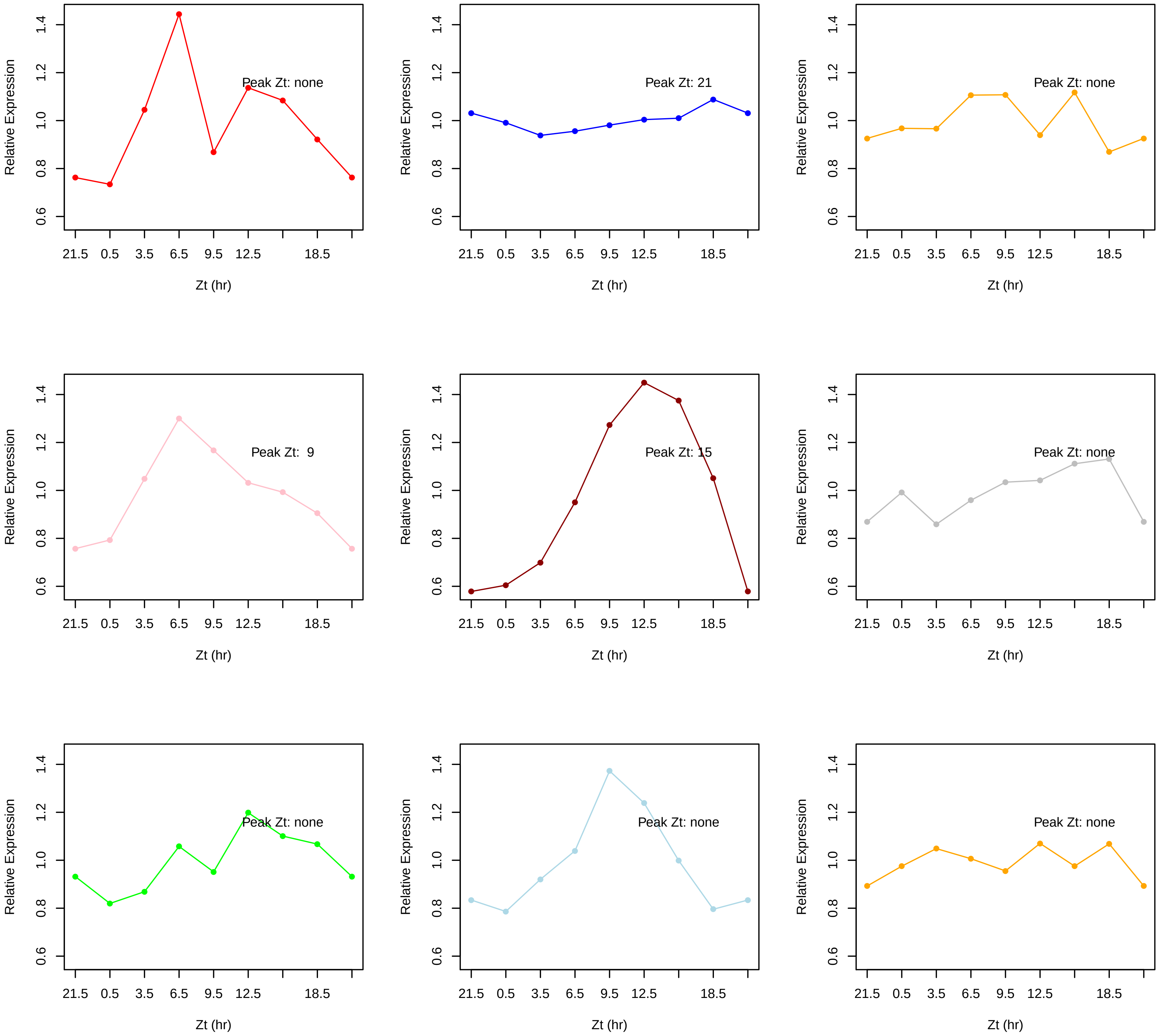

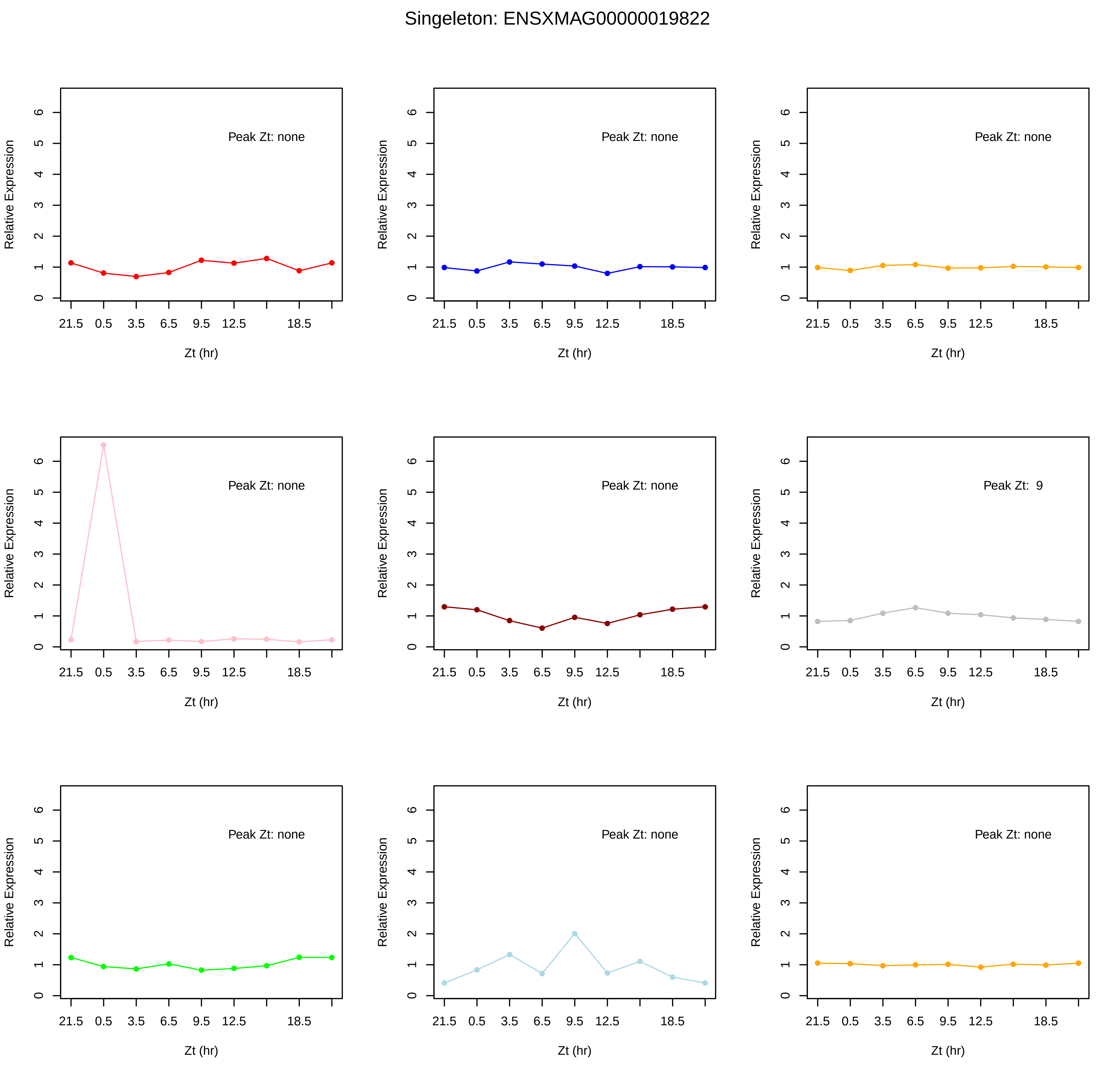
Singeleton: ENSXMAG00000019822
Peak Zt: none
Peak Zt: none
Peak Zt: none
Peak Zt: none
Peak Zt: none
Peak Zt: 9
Peak Zt: none
Peak Zt: none
Peak Zt: none
Relative Expression
Zt (hr)
21.5
0.5
3.5
6.5
9.5
12.5
18.5
0
1
2
3
4
5
6

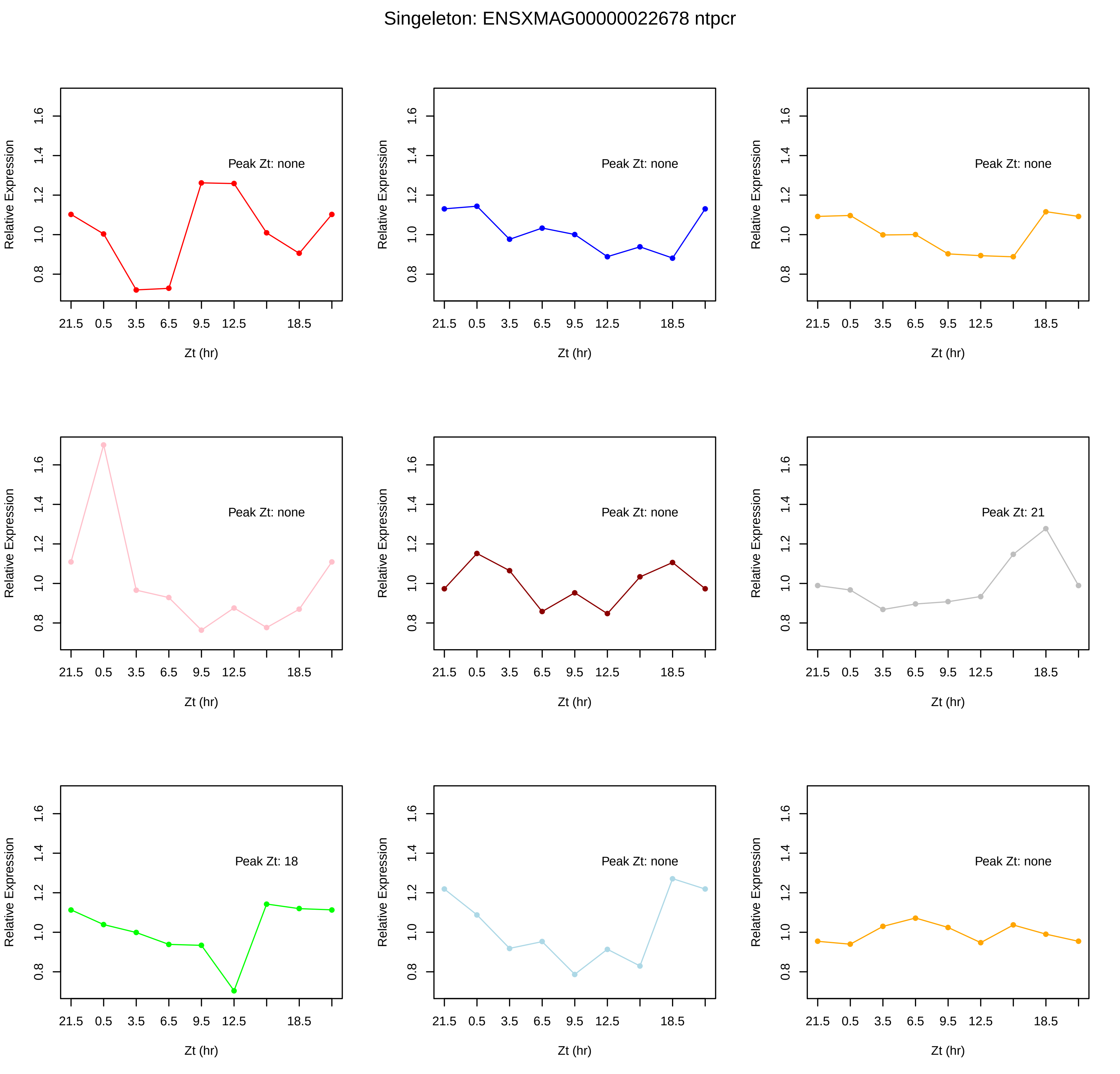
Singeleton: ENSXMAG00000022678 ntpcr
Peak Zt: none
Peak Zt: none
Peak Zt: none
Peak Zt: none
Peak Zt: none
Peak Zt: 21
Peak Zt: 18
Peak Zt: none
Peak Zt: none
Relative Expression
Zt (hr)
21.5
0.5
3.5
6.5
9.5
12.5
18.5
0.8
1.0
1.2
1.4
1.6

Singeleton: ENSXMAG00000023274 klhdc3

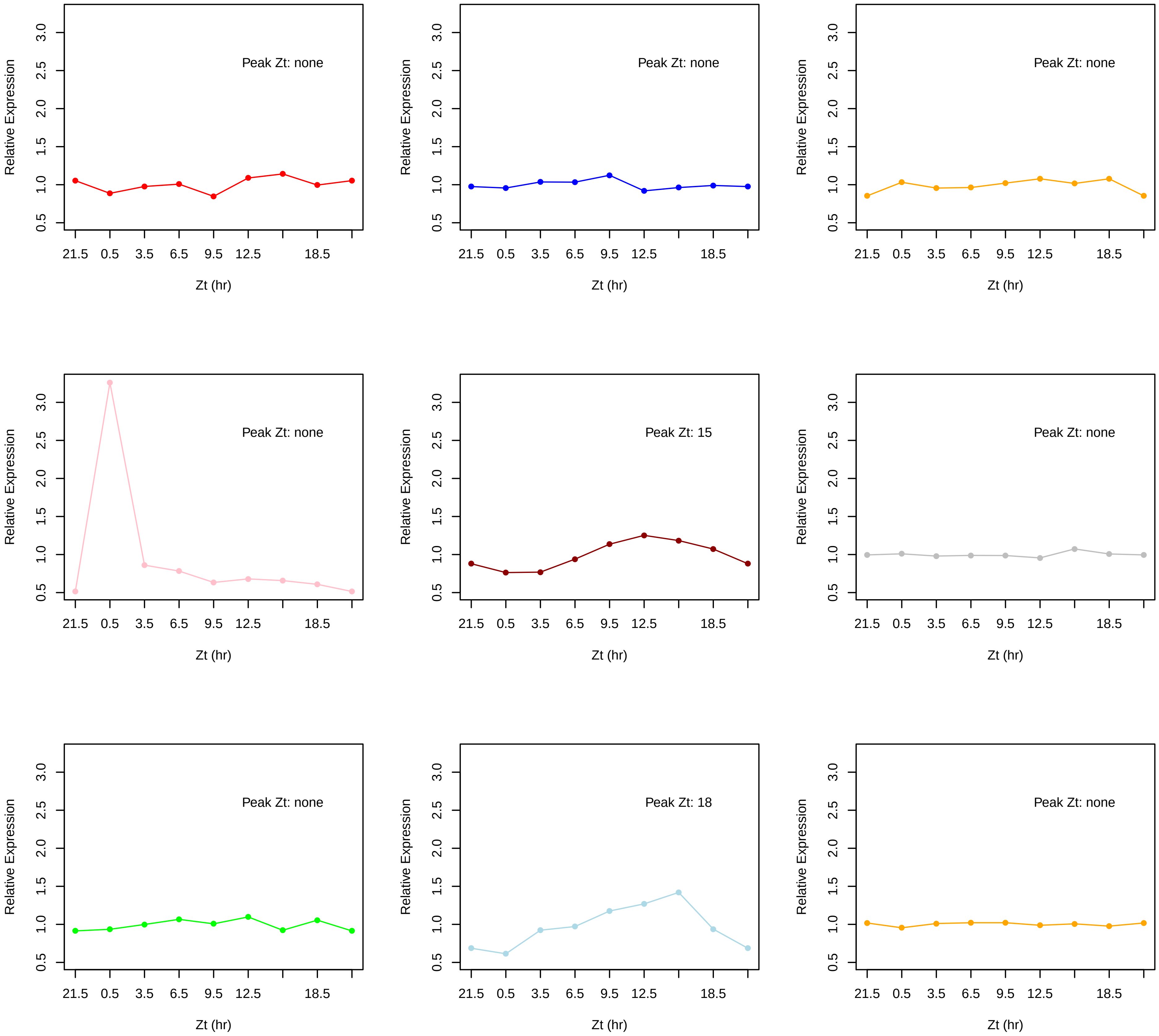

Singeleton: ENSXMAG00000014419 spam1

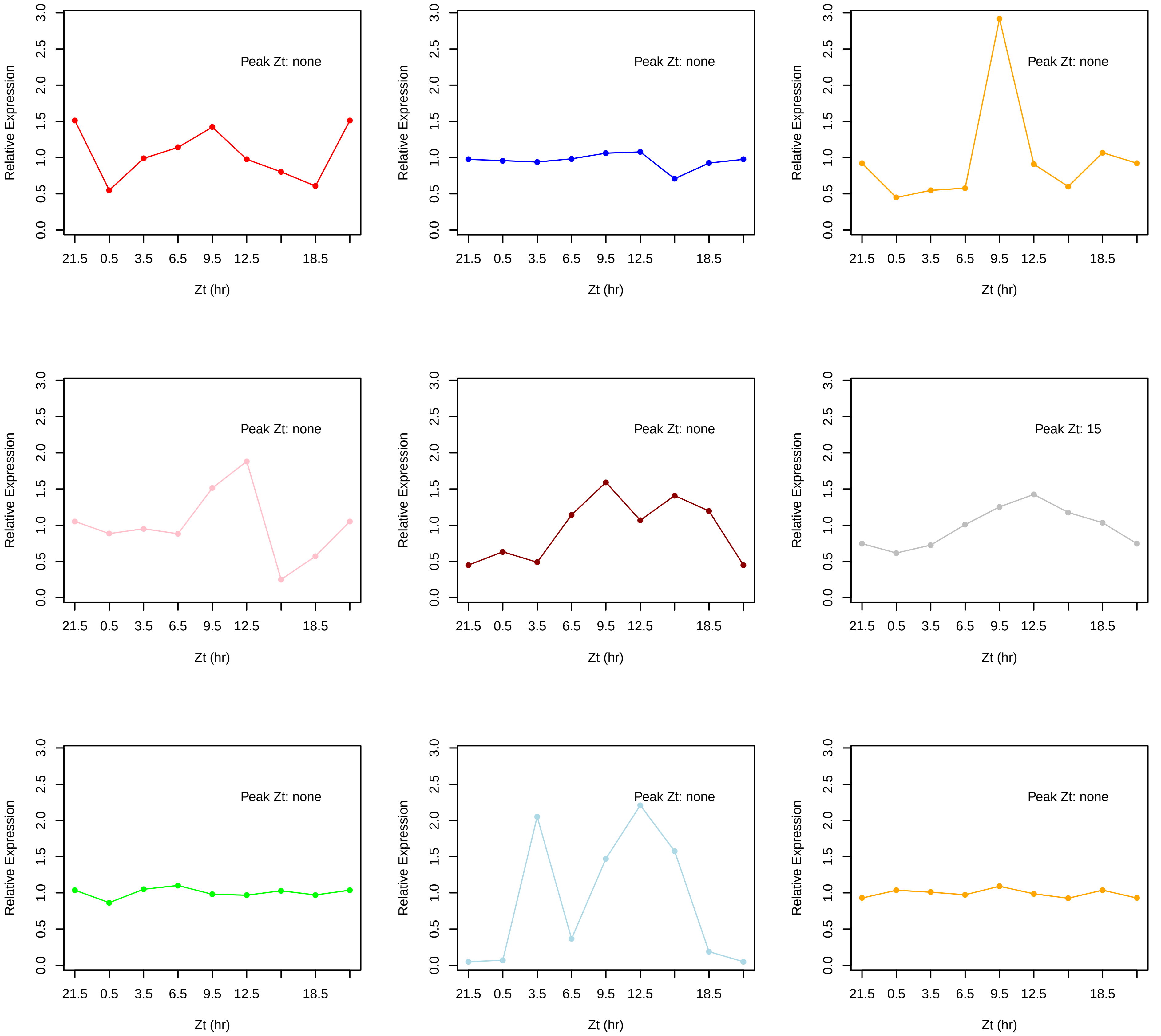

Singeleton: ENSXMAG00000014417 hyal6

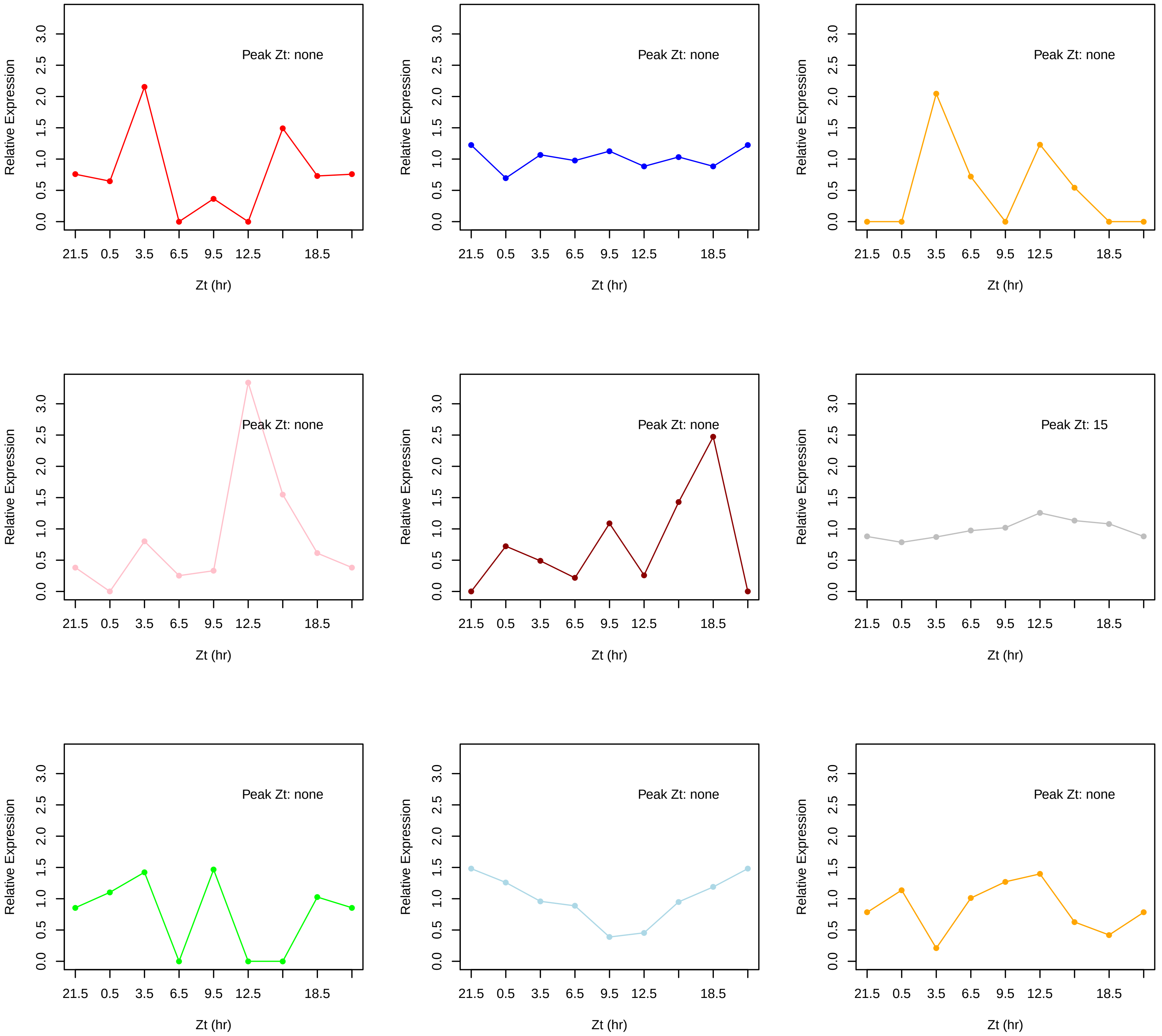

Singeleton: ENSXMAG00000013804 rrp36

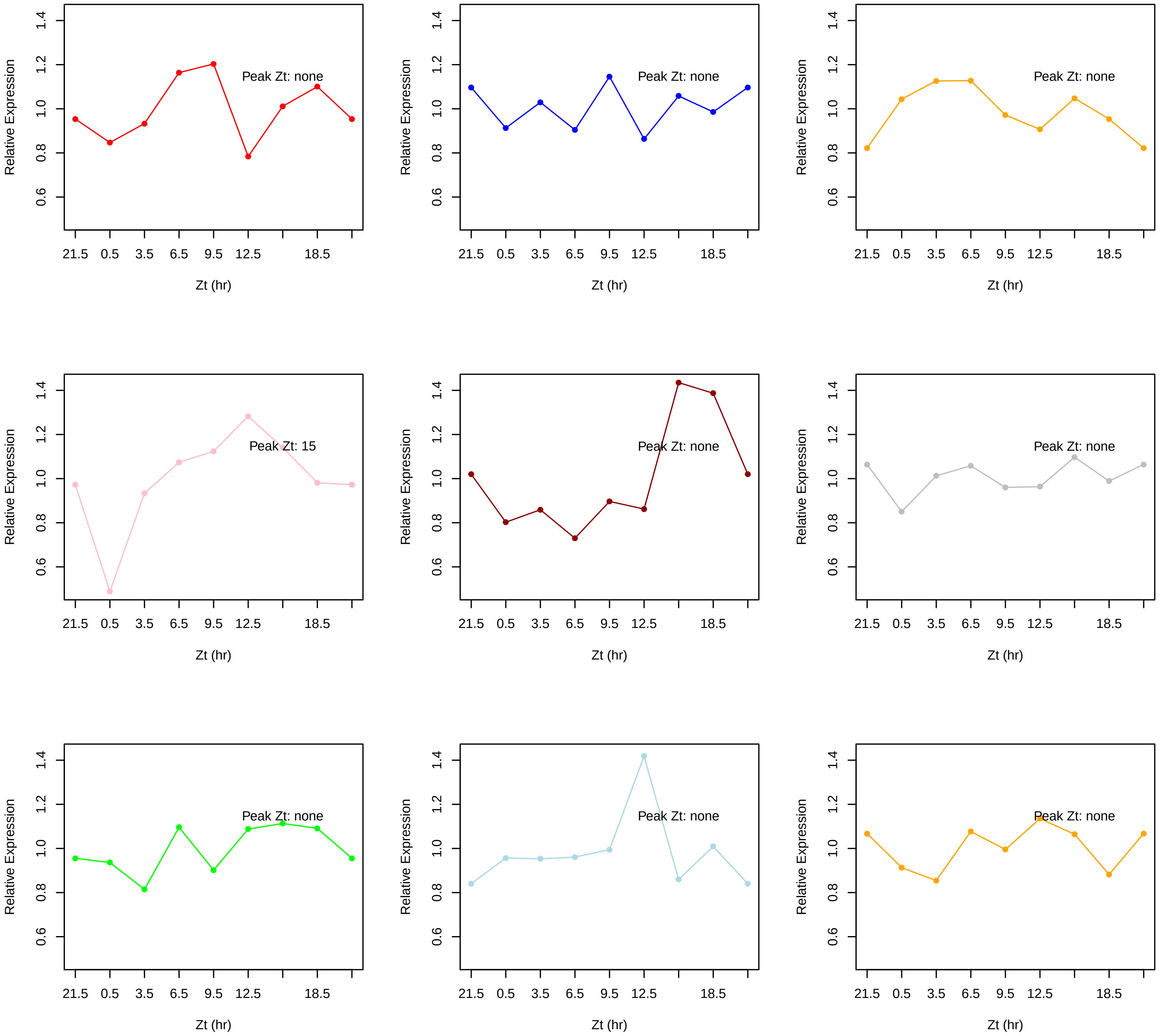

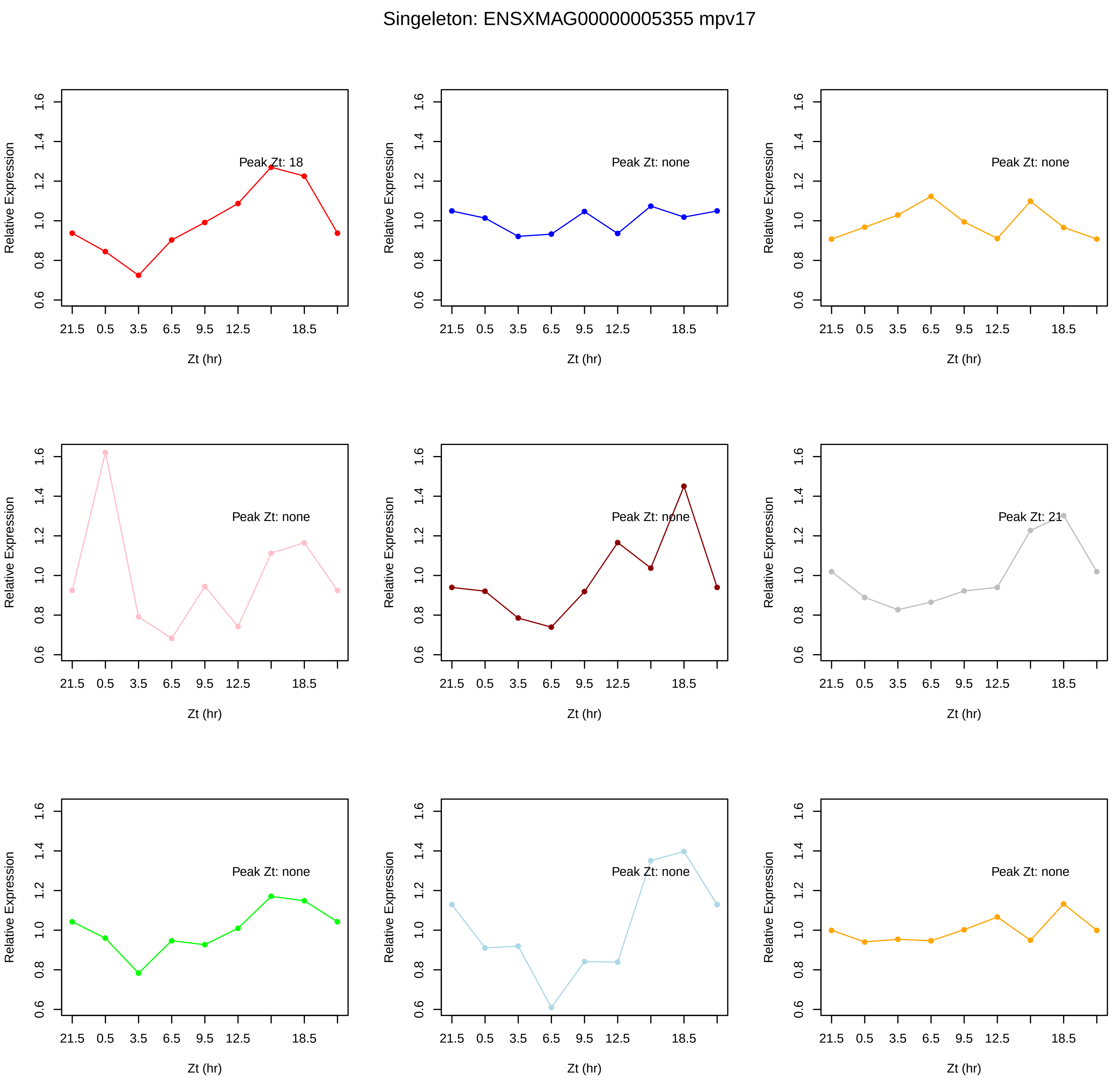
Singeleton: ENSXMAG00000005355 mpv17
Peak Zt: 18
Peak Zt: none
Peak Zt: none
Peak Zt: none
Peak Zt: none
Peak Zt: 21
Peak Zt: none
Peak Zt: none
Peak Zt: none
Relative Expression
Zt (hr)

Singeleton: ENSXMAG00000000116 asb15b

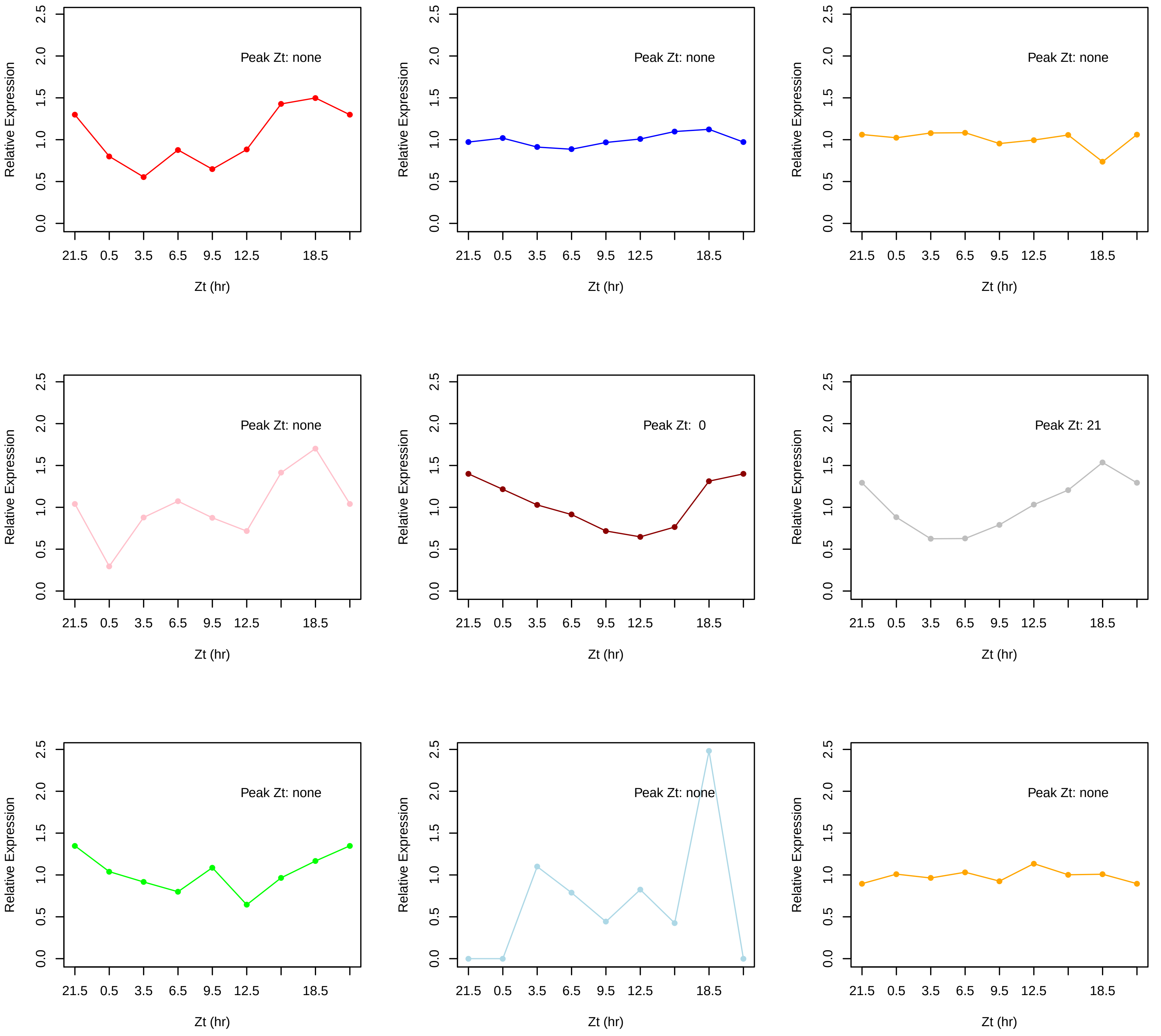

Singeleton: ENSXMAG00000006842

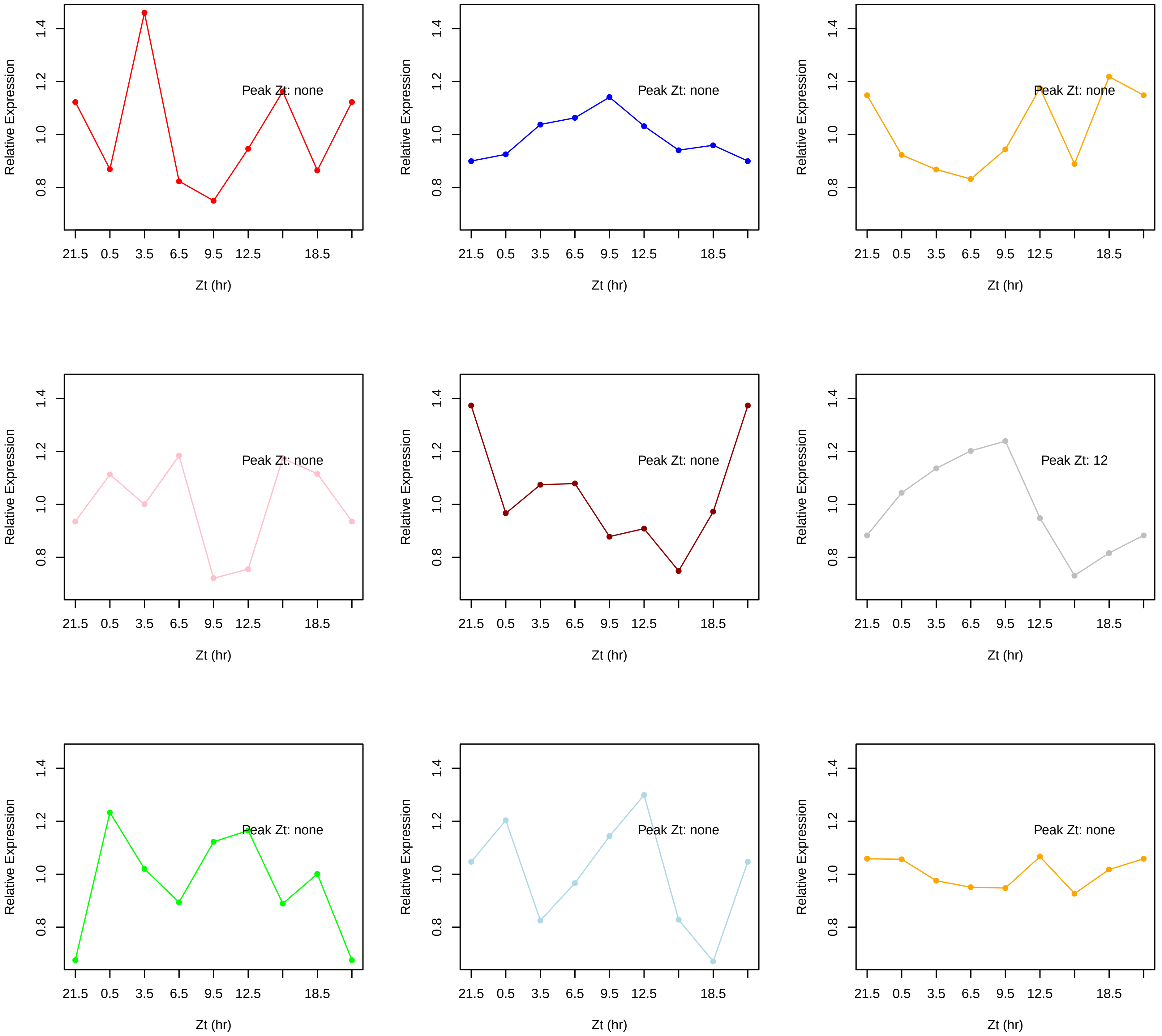

Singeleton: ENSXMAG00000005460 dnajc5ga

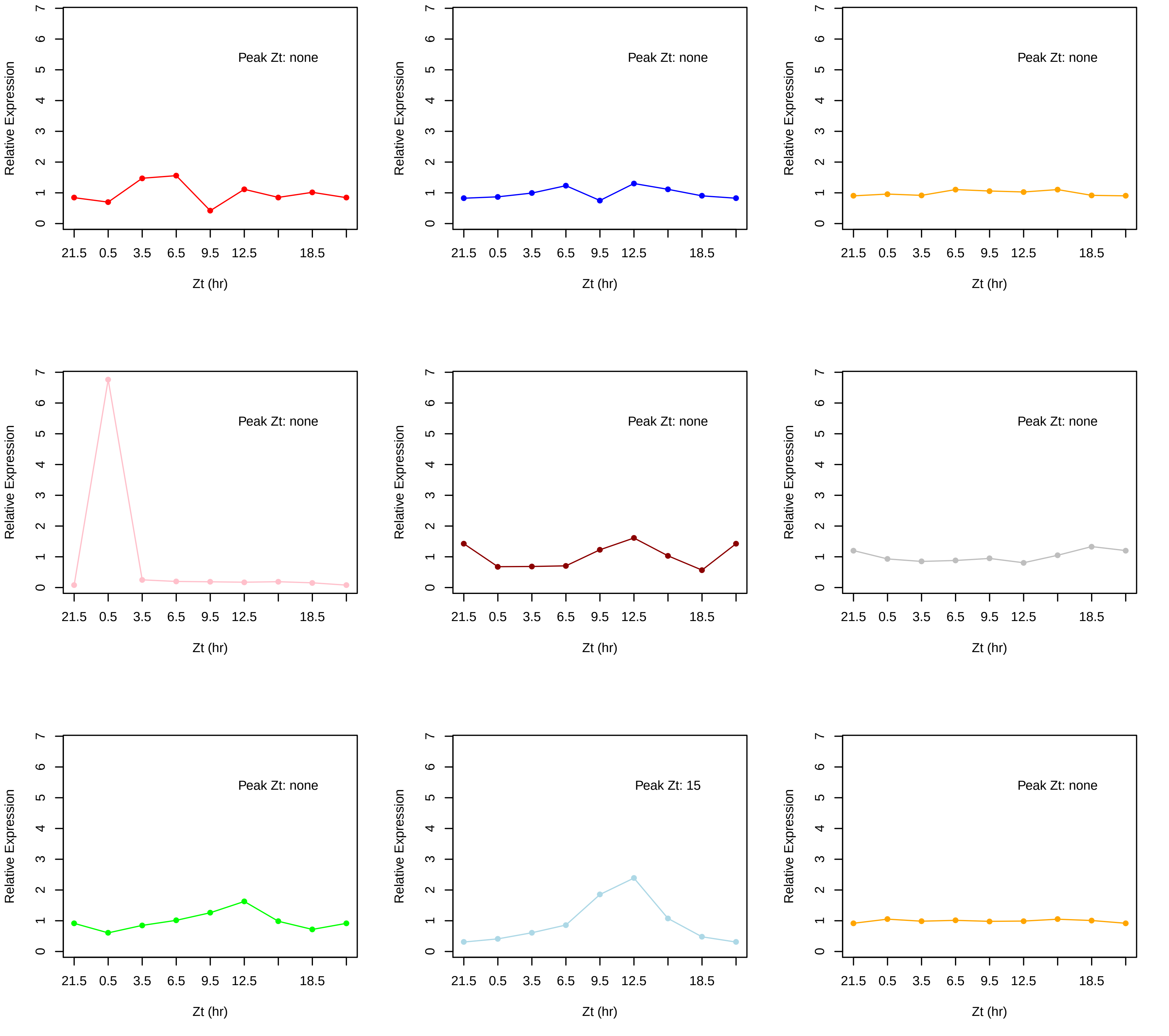

Singeleton: ENSXMAG00000010676

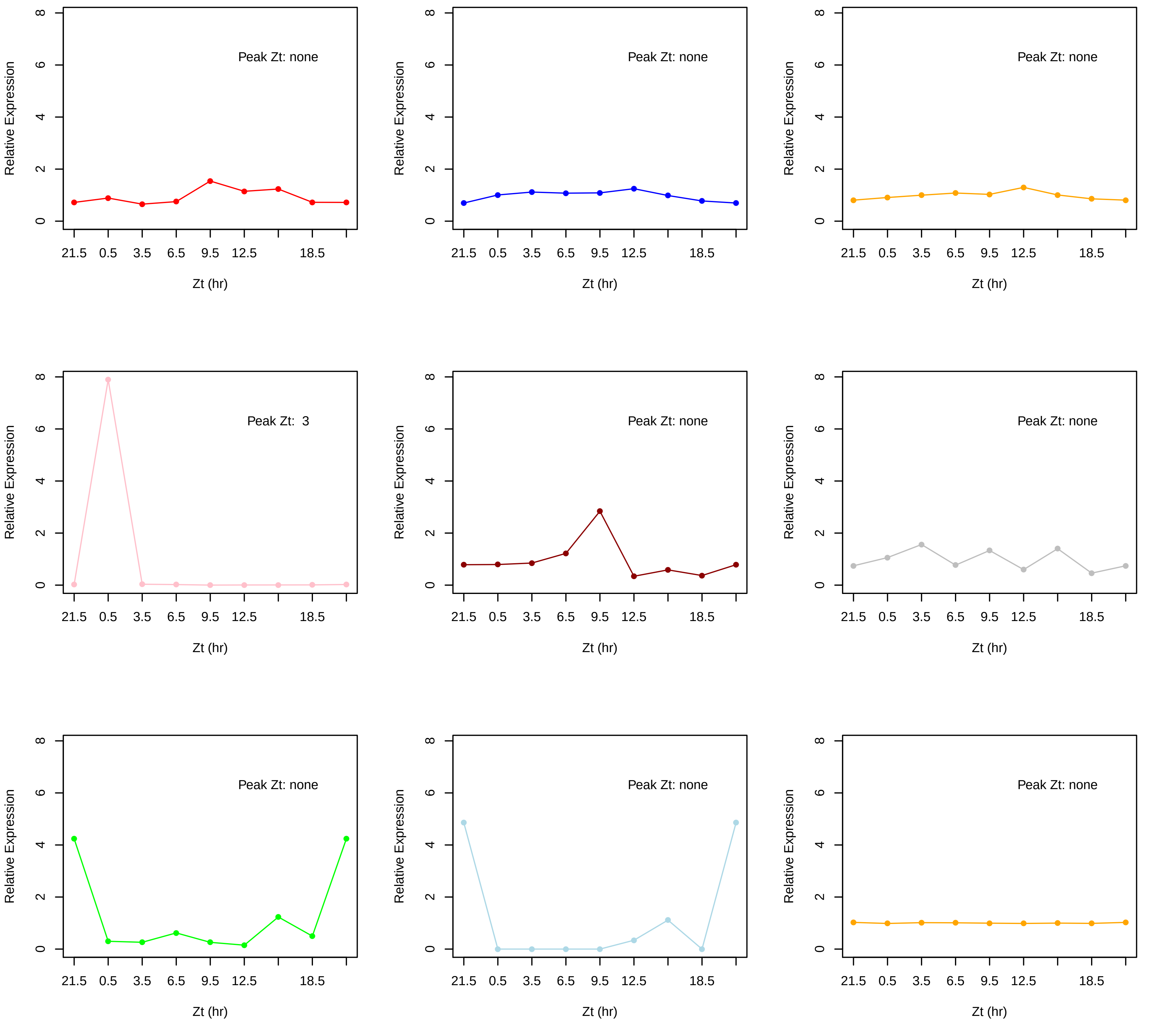

Singleton: ENSXMAG00000006879 mrpl2

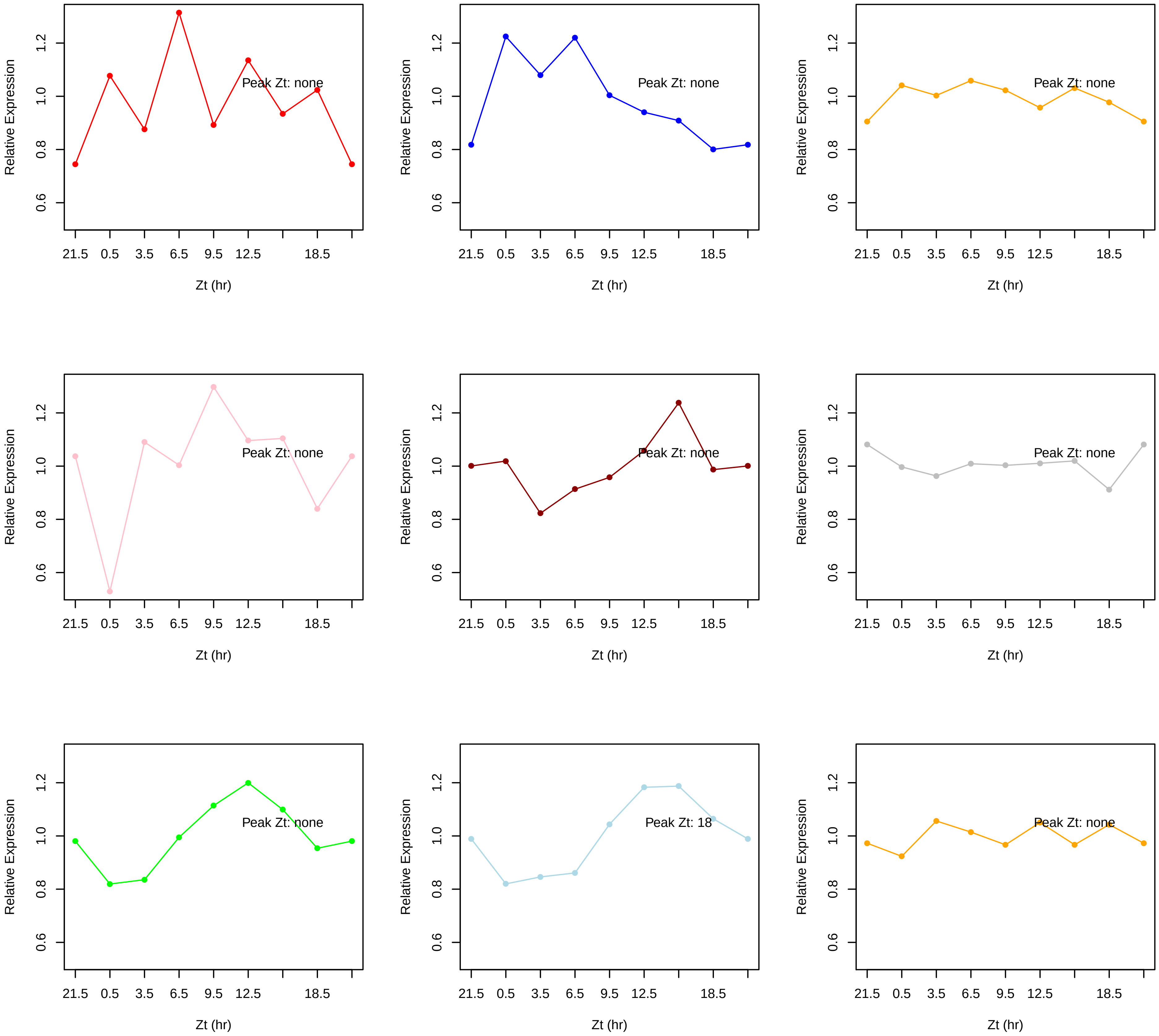

Singleton: ENSXMAG00000006889

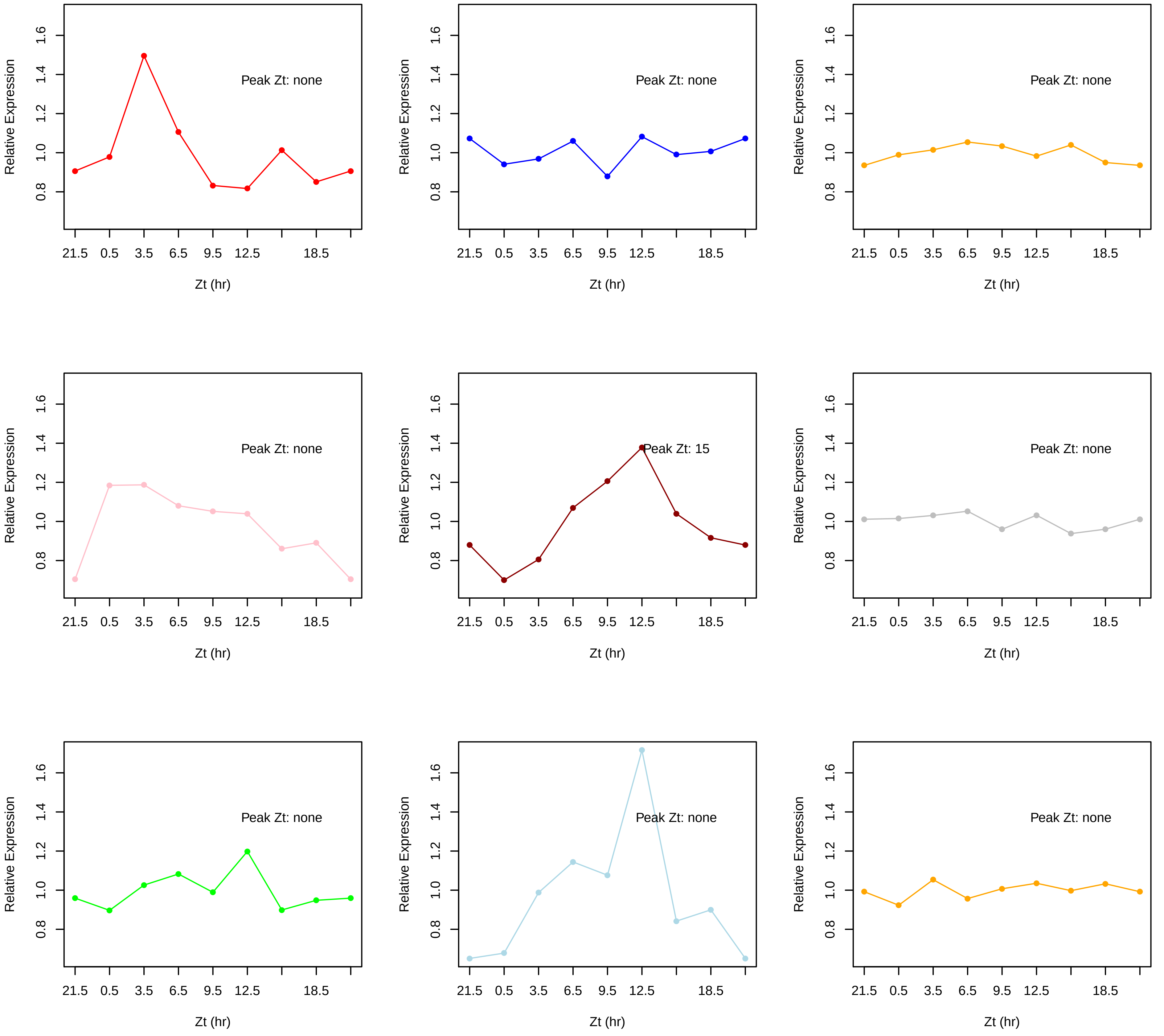

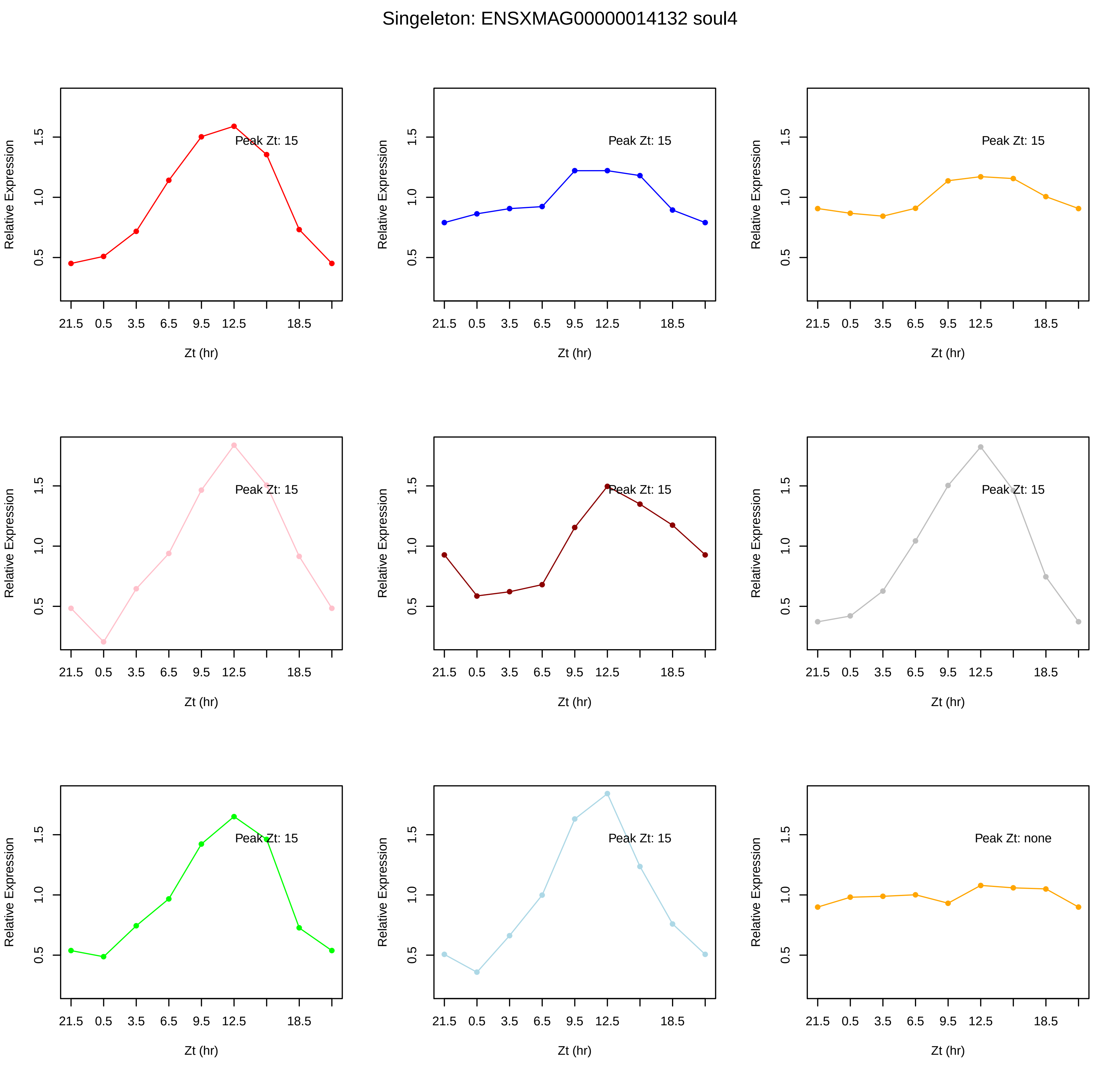

Singeleton: ENSXMAG00000014132 soul4
Peak Zt: 15
Peak Zt: 15
Peak Zt: 15
Peak Zt: 15
Peak Zt: 15
Peak Zt: 15
Peak Zt: 15
Peak Zt: 15
Peak Zt: none
Relative Expression
Zt (hr)
21.5
0.5
3.5
6.5
9.5
12.5
18.5
0.5
1.0
1.5

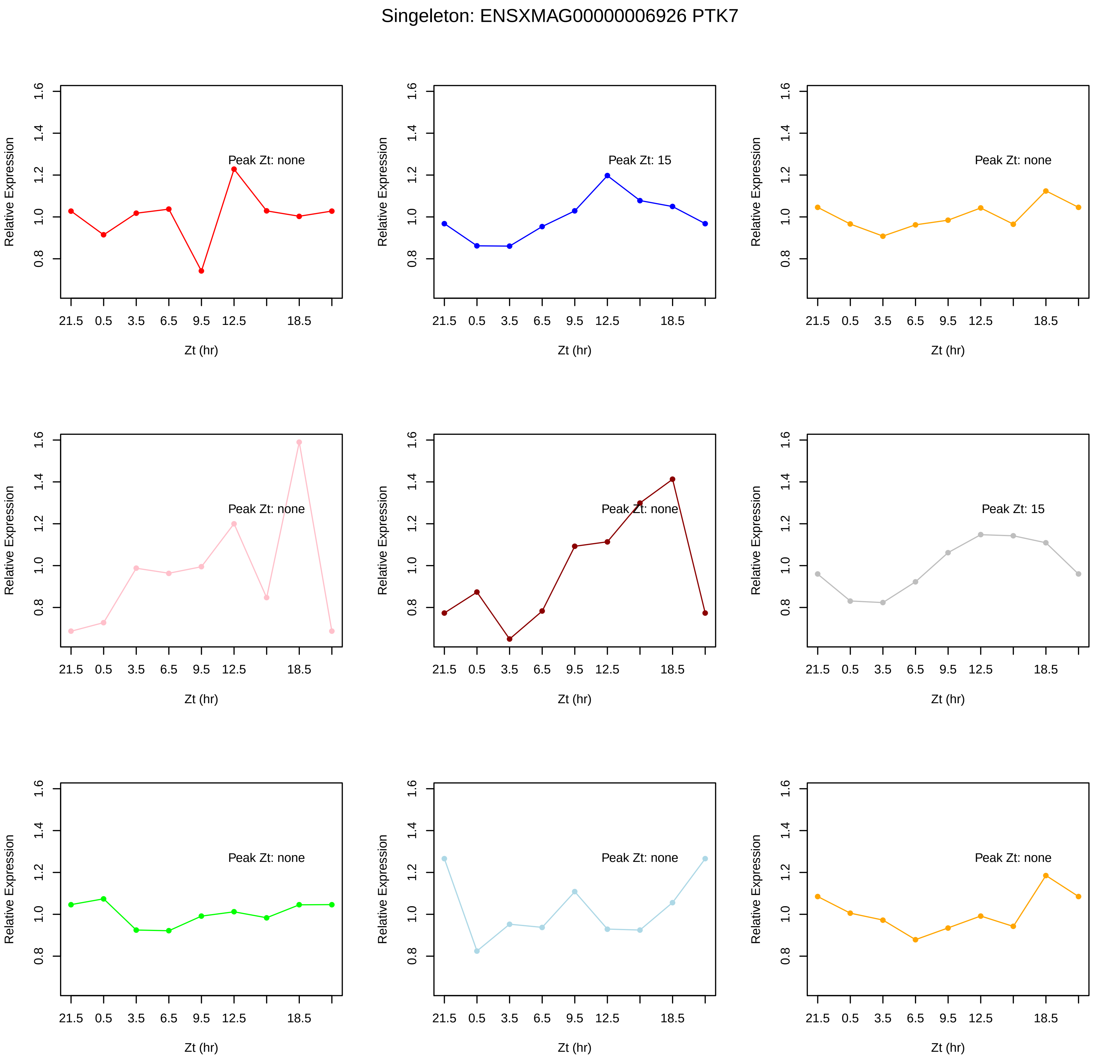
Singleton: ENSXMAG00000006926 PTK7
Peak Zt: none
Peak Zt: 15
Peak Zt: none
Peak Zt: none
Peak Zt: none
Peak Zt: 15
Peak Zt: none
Peak Zt: none
Peak Zt: none
Relative Expression
Zt (hr)
21.5
0.5
3.5
6.5
9.5
12.5
18.5
0.8
1.0
1.2
1.4
1.6

Singeleton: ENSXMAG00000015128

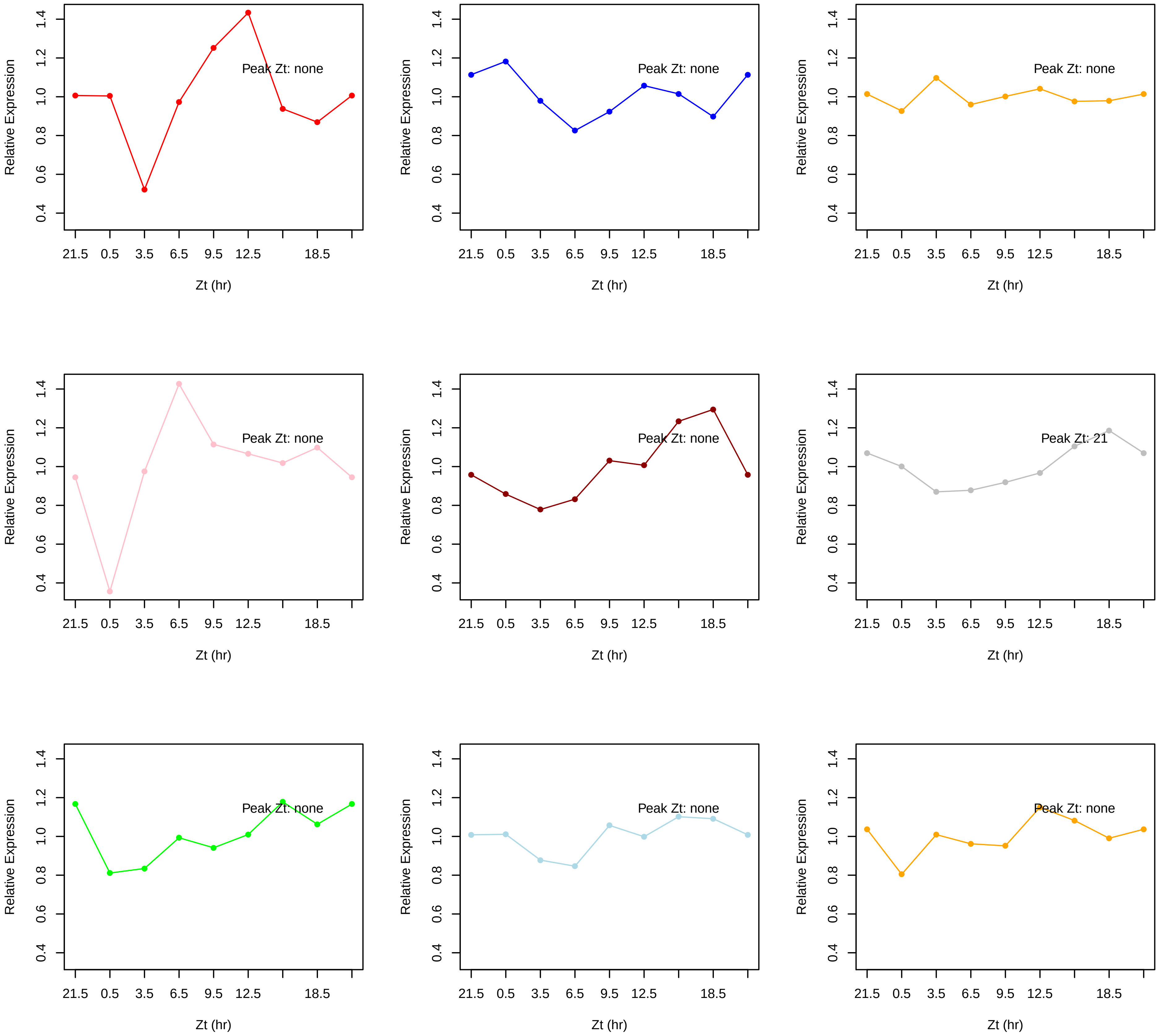

Singleton: ENSXMAG00000015146 cenpo

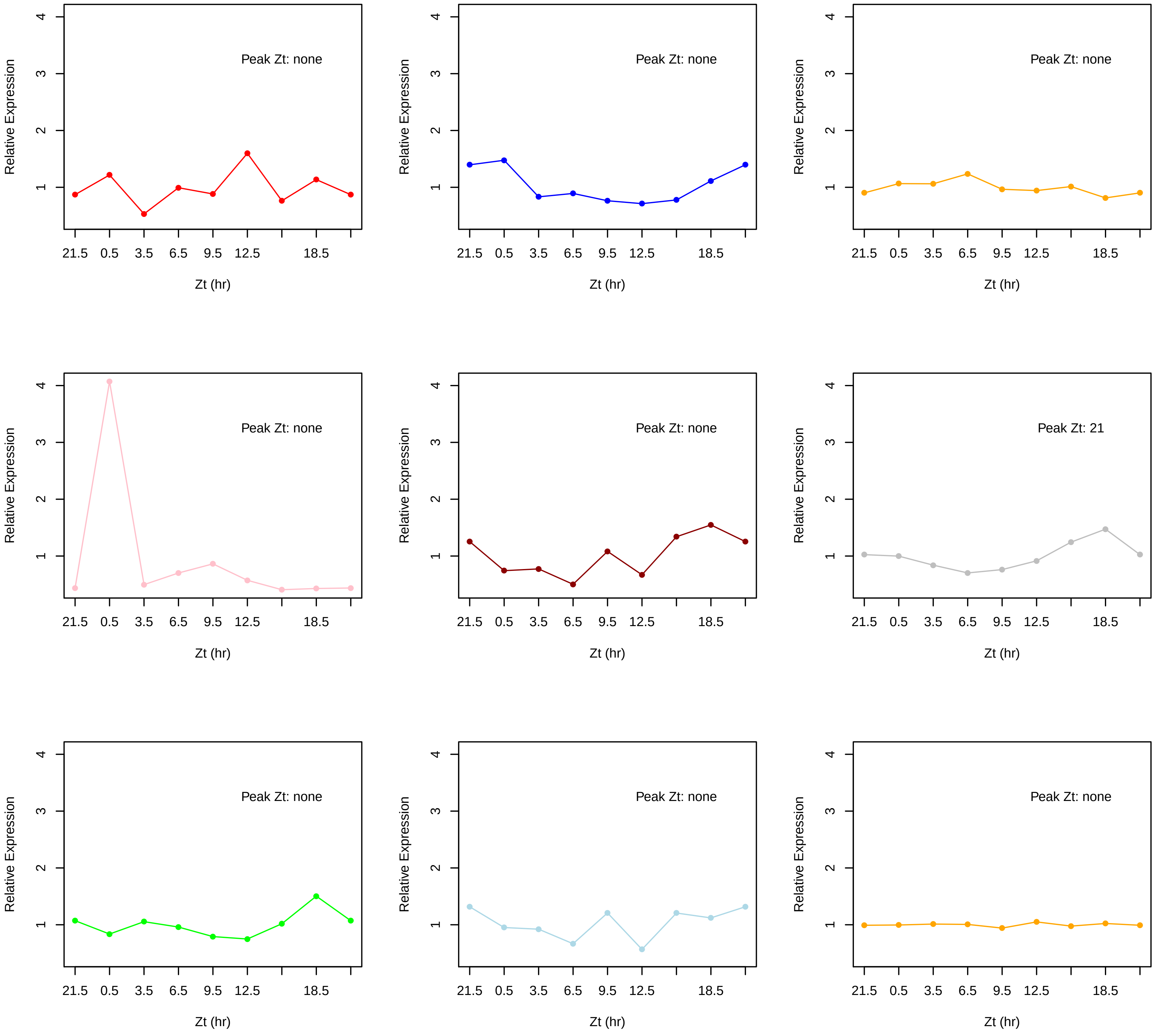

Singeleton: ENSXMAG00000024458 ptrhd1

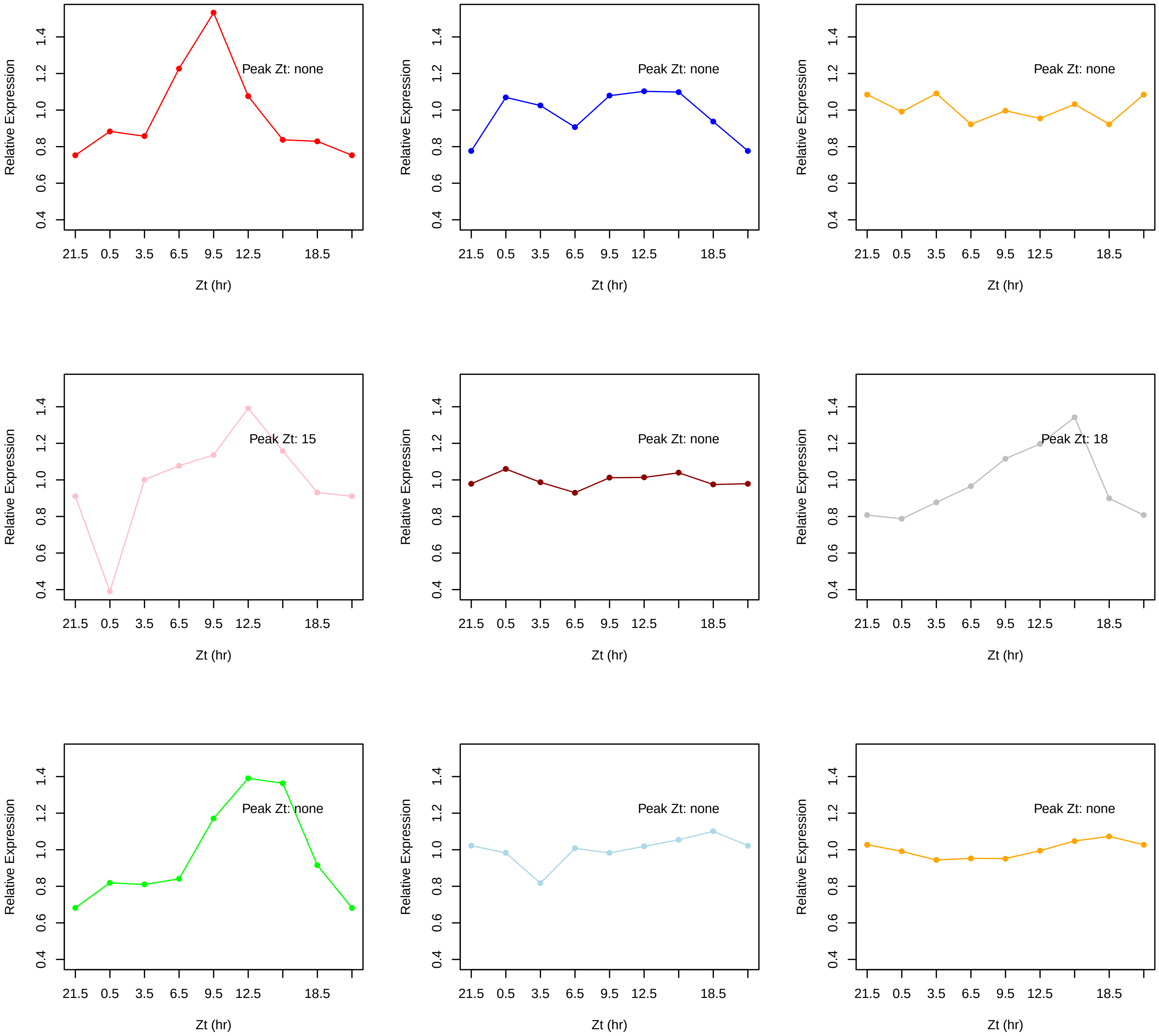

Singleton: ENSXMAG00000017782 ncoa1

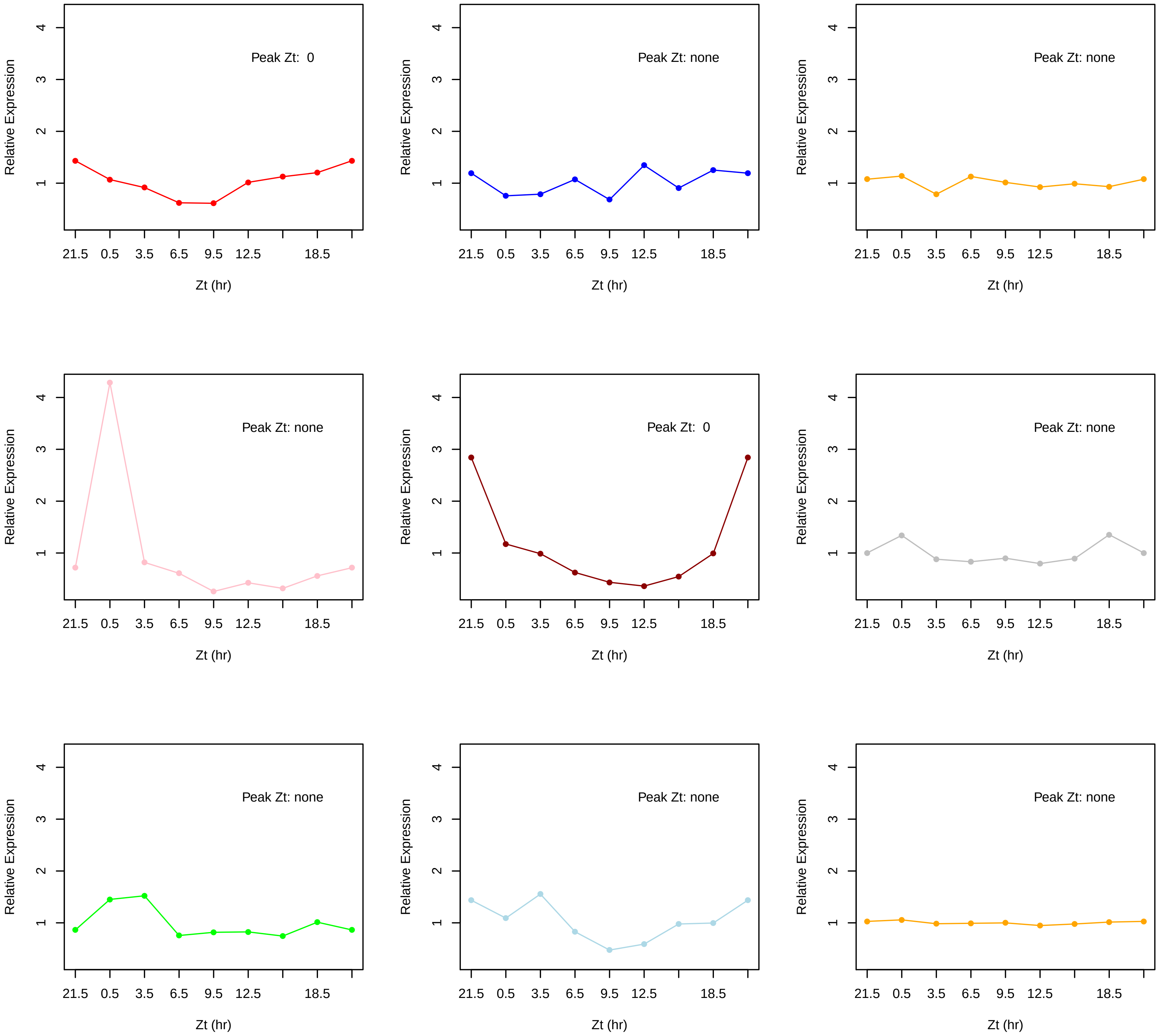

Singeleton: ENSXMAG00000006563 b3galnt2

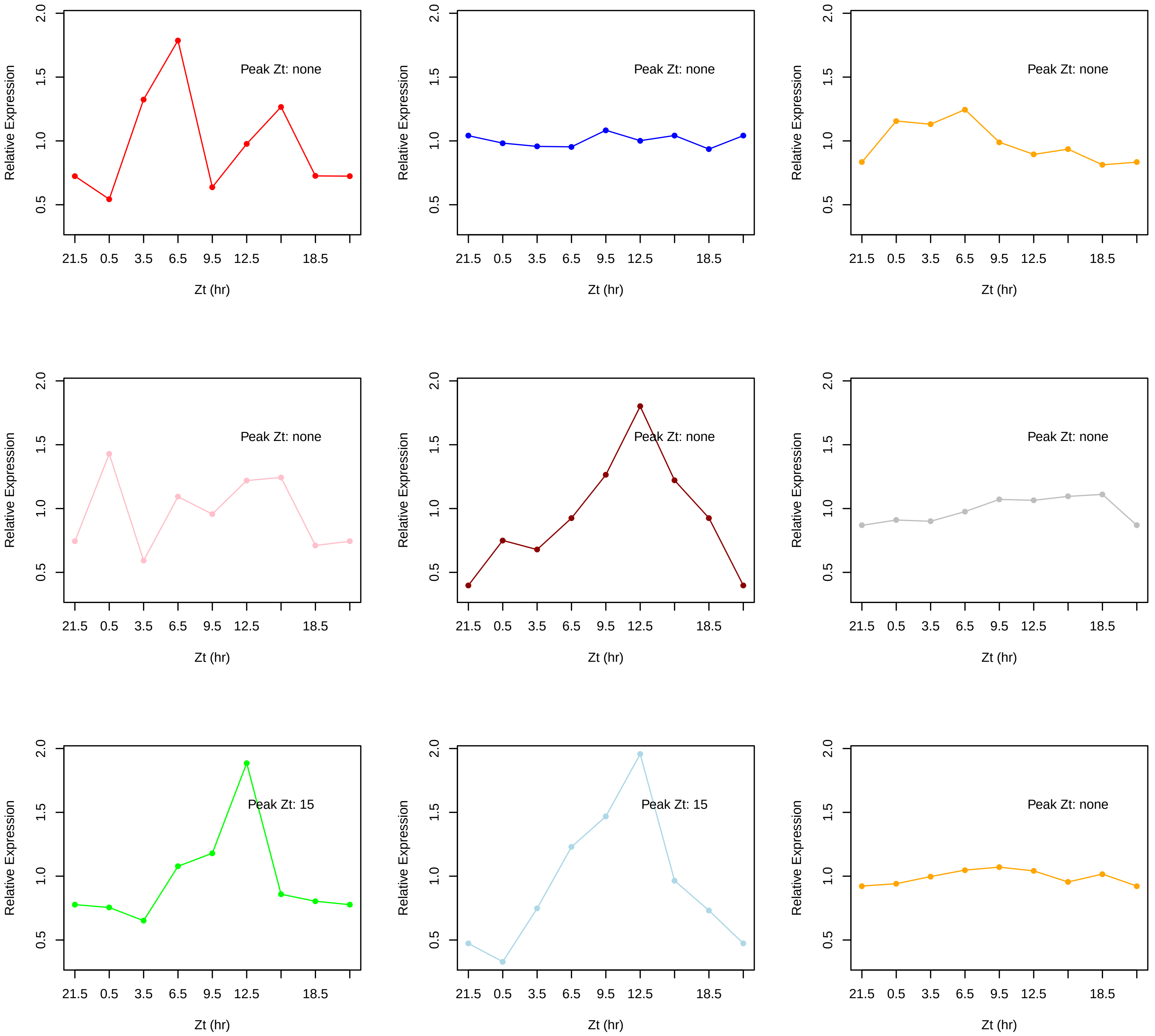

Singeleton: ENSXMAG00000006538 tomm20a

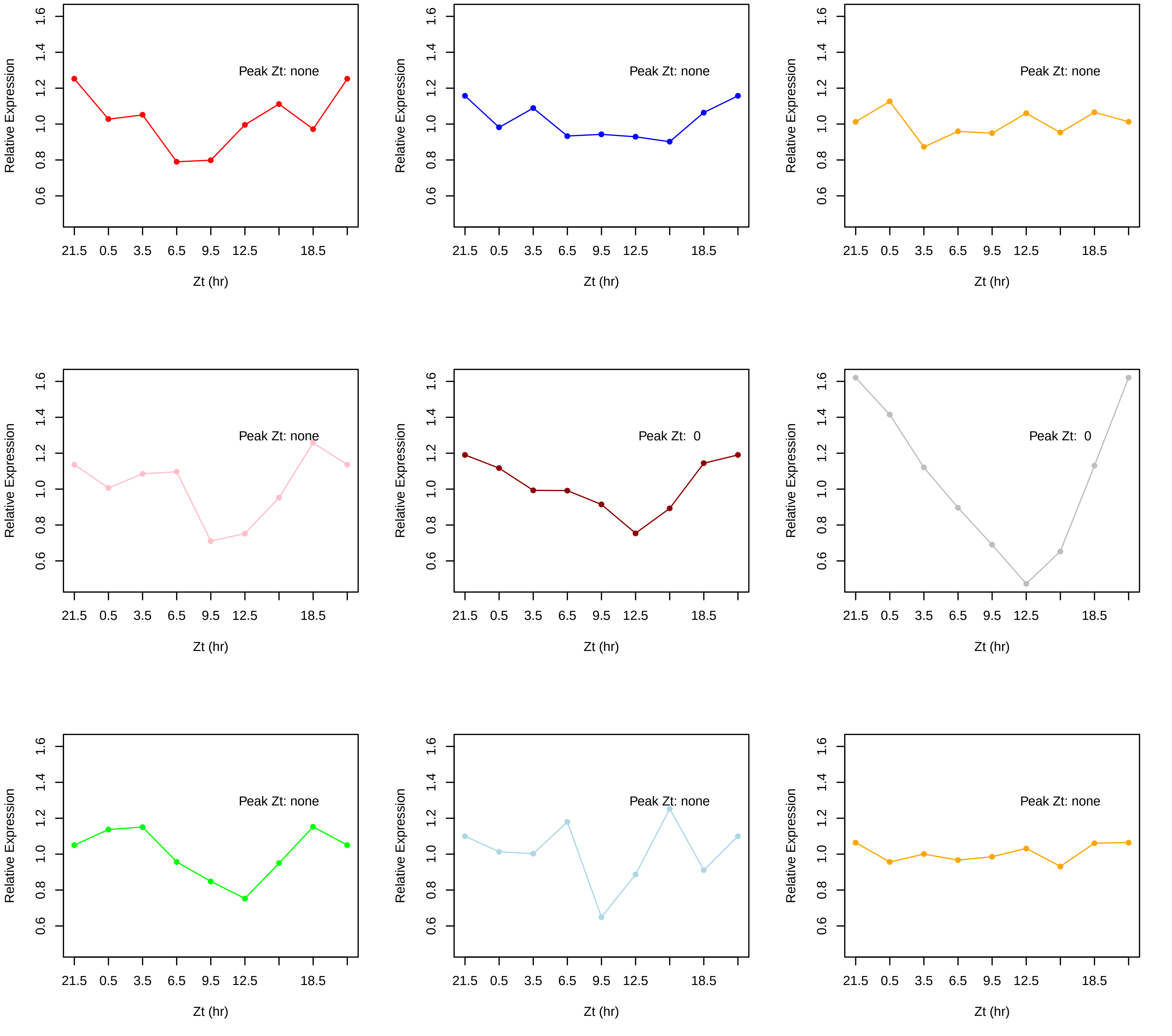

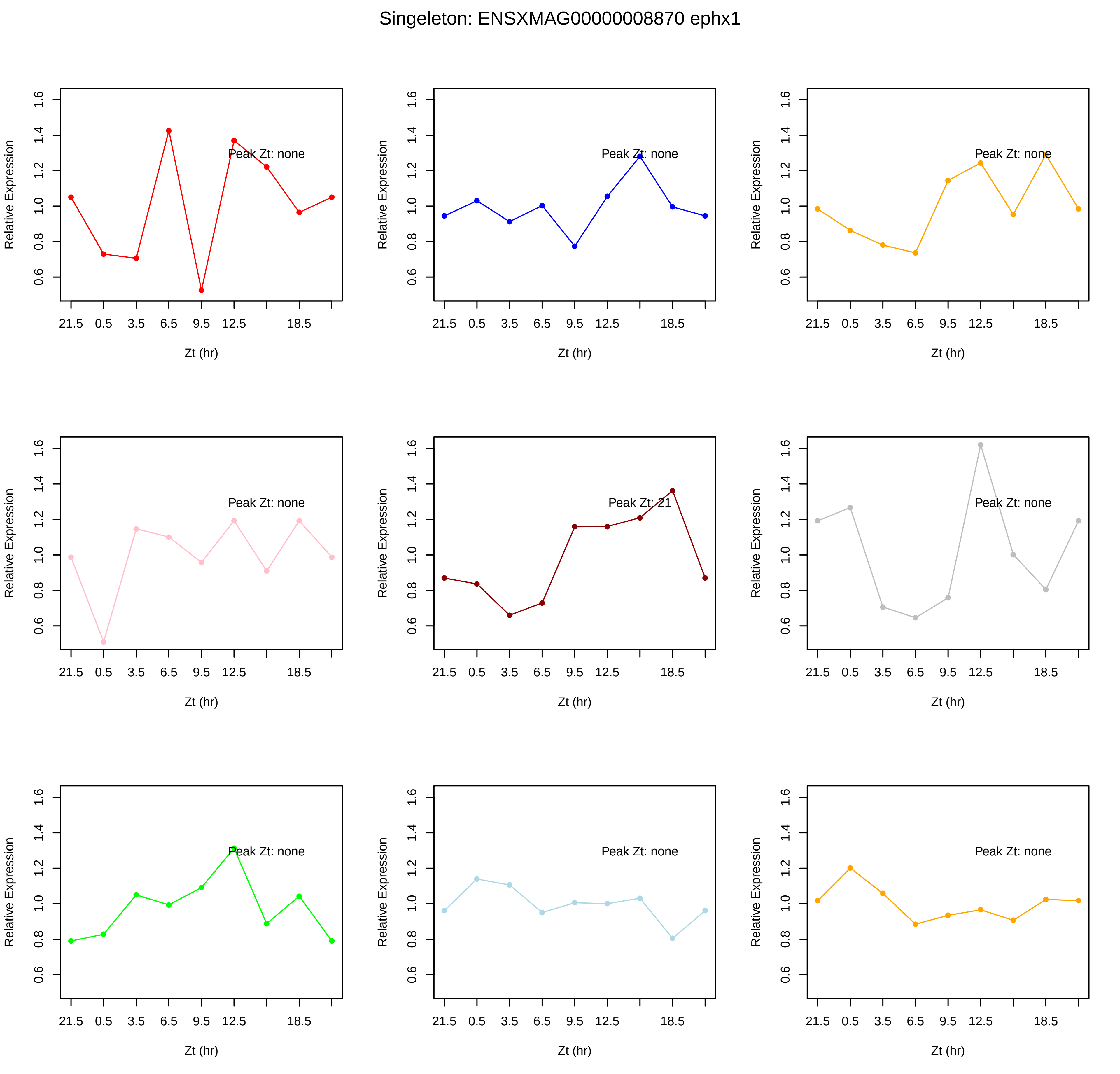
Singleton: ENSXMAG00000008870 ephx1
Relative Expression
Zt (hr)
21.5 0.5 3.5 6.5 9.5 12.5 18.5
0.6 0.8 1.0 1.2 1.4 1.6
Peak Zt: none
Peak Zt: 21

Singeleton: ENSXMAG00000028206 tomm20a

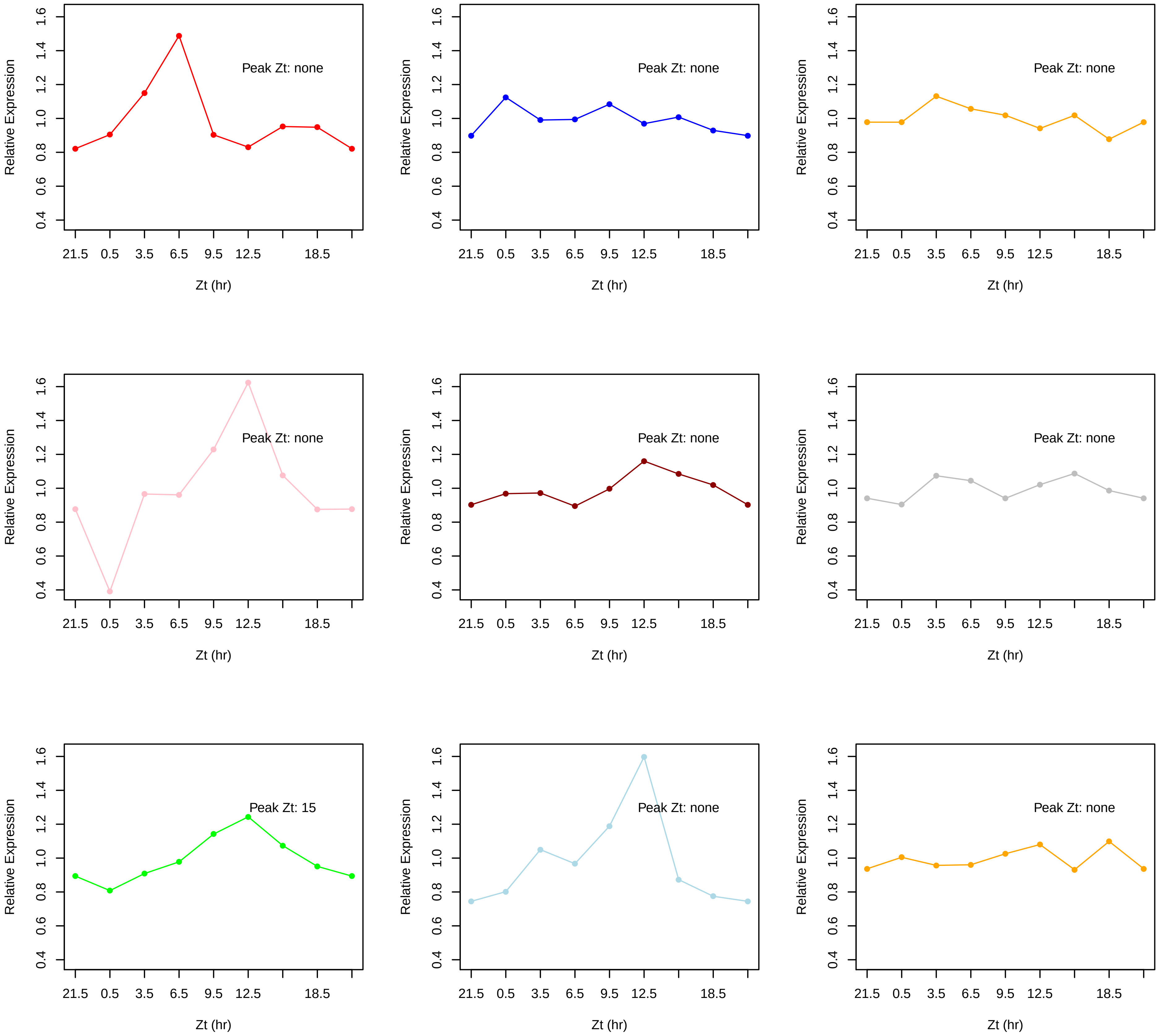

Singleton: ENSXMAG00000023651 cnih3

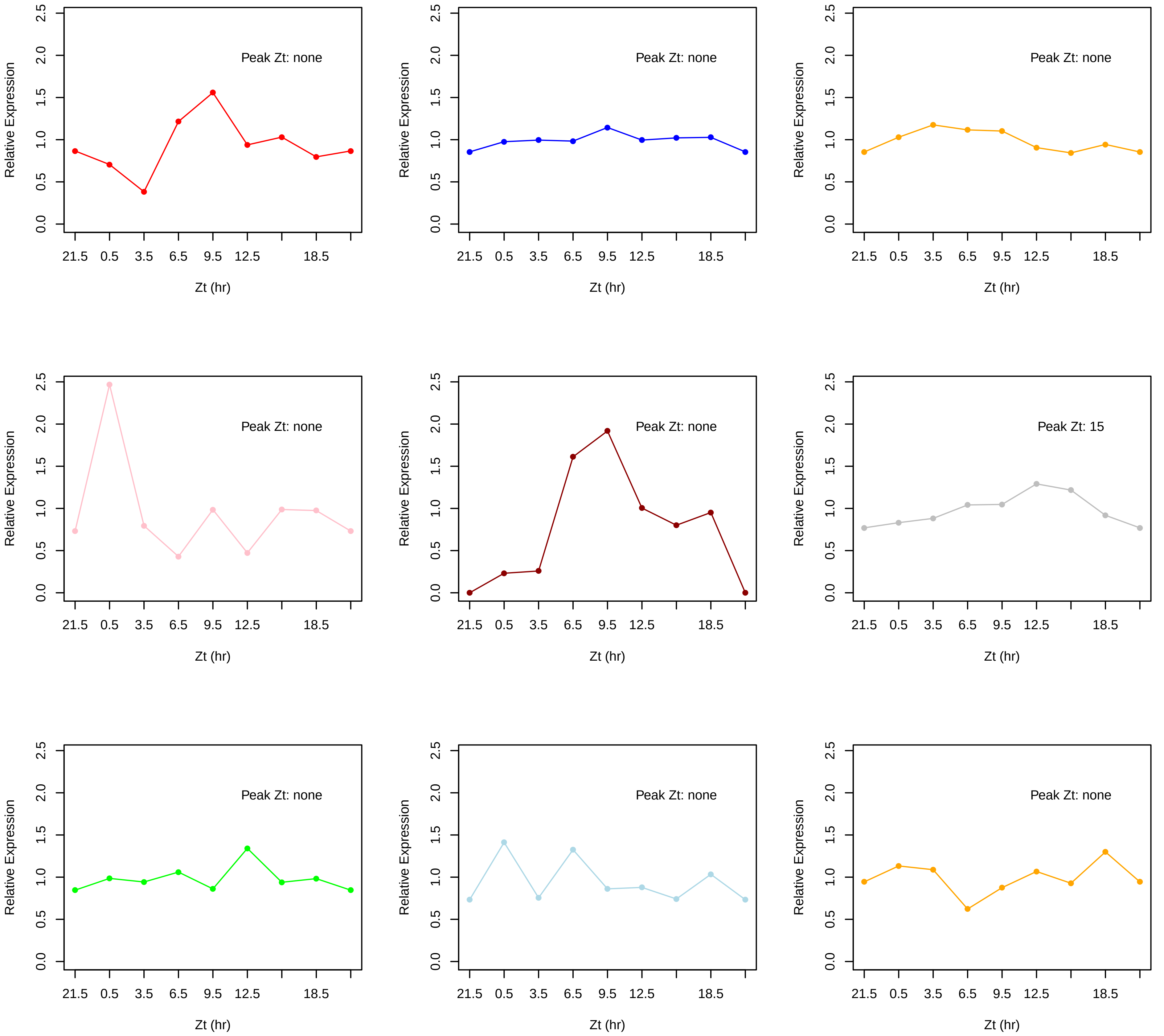

Singleton: ENSXMAG00000004364 ADCY3

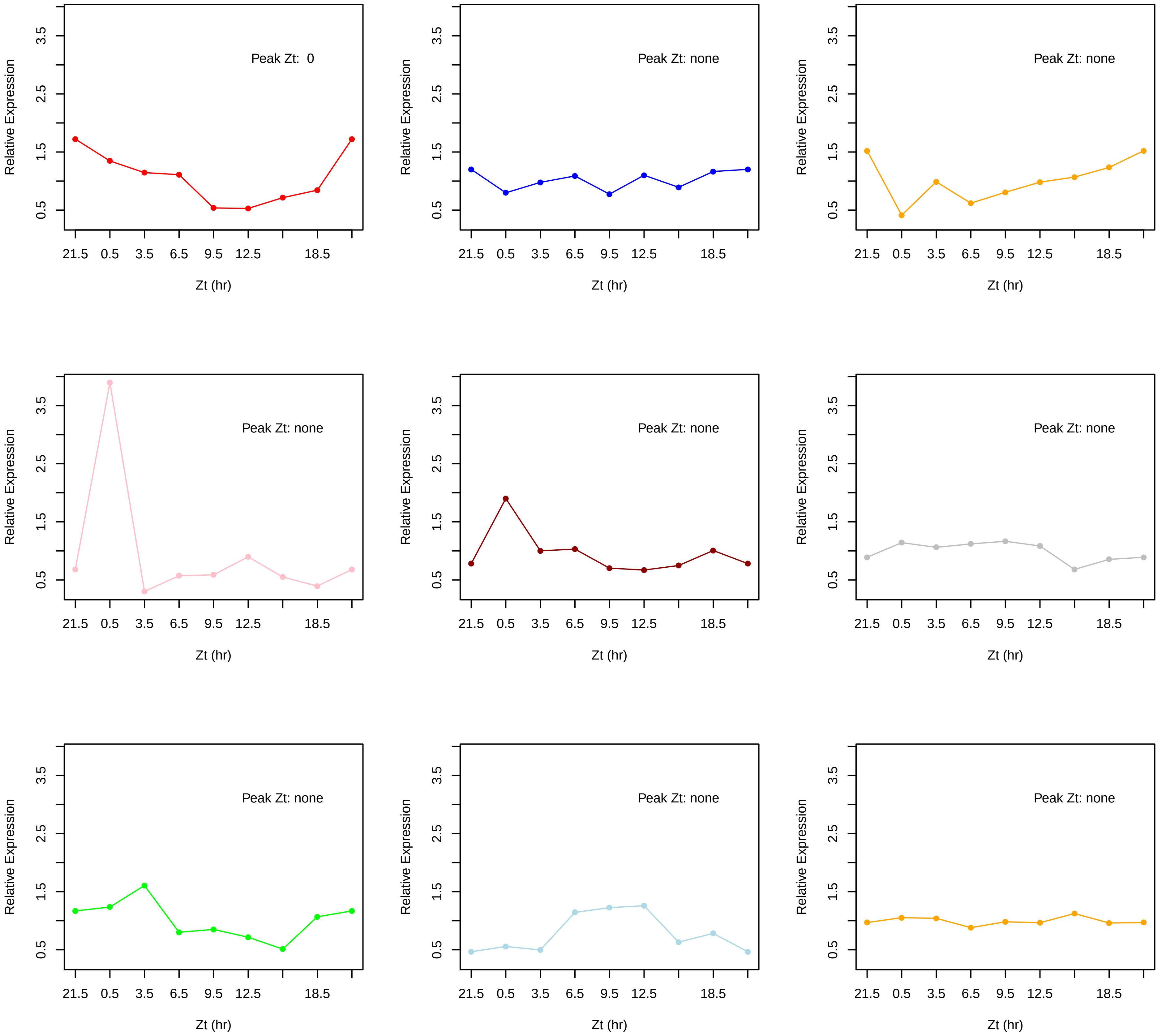

Singeleton: ENSXMAG00000022833 wdr26b

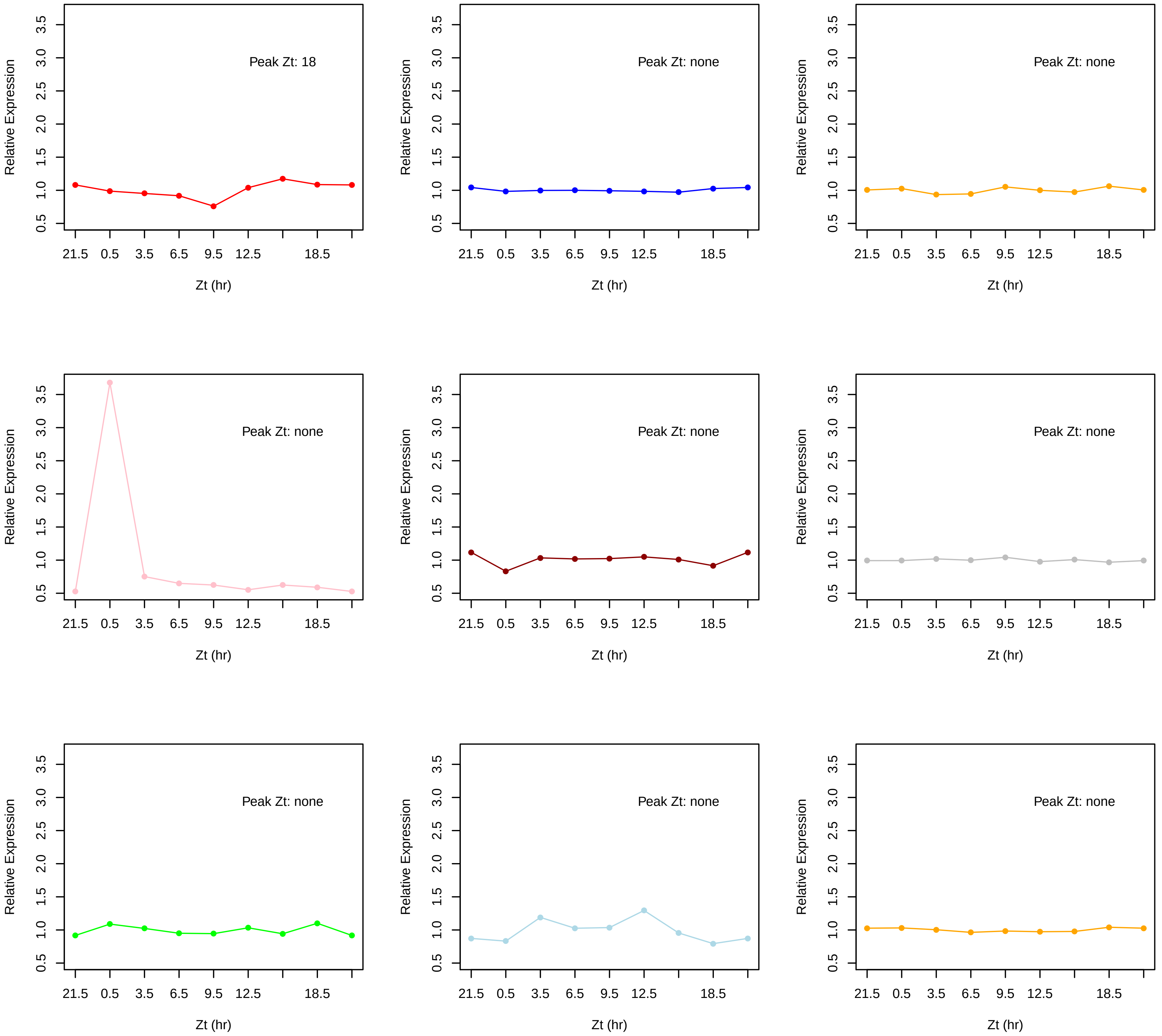

Singeleton: ENSXMAG00000006446 VIT

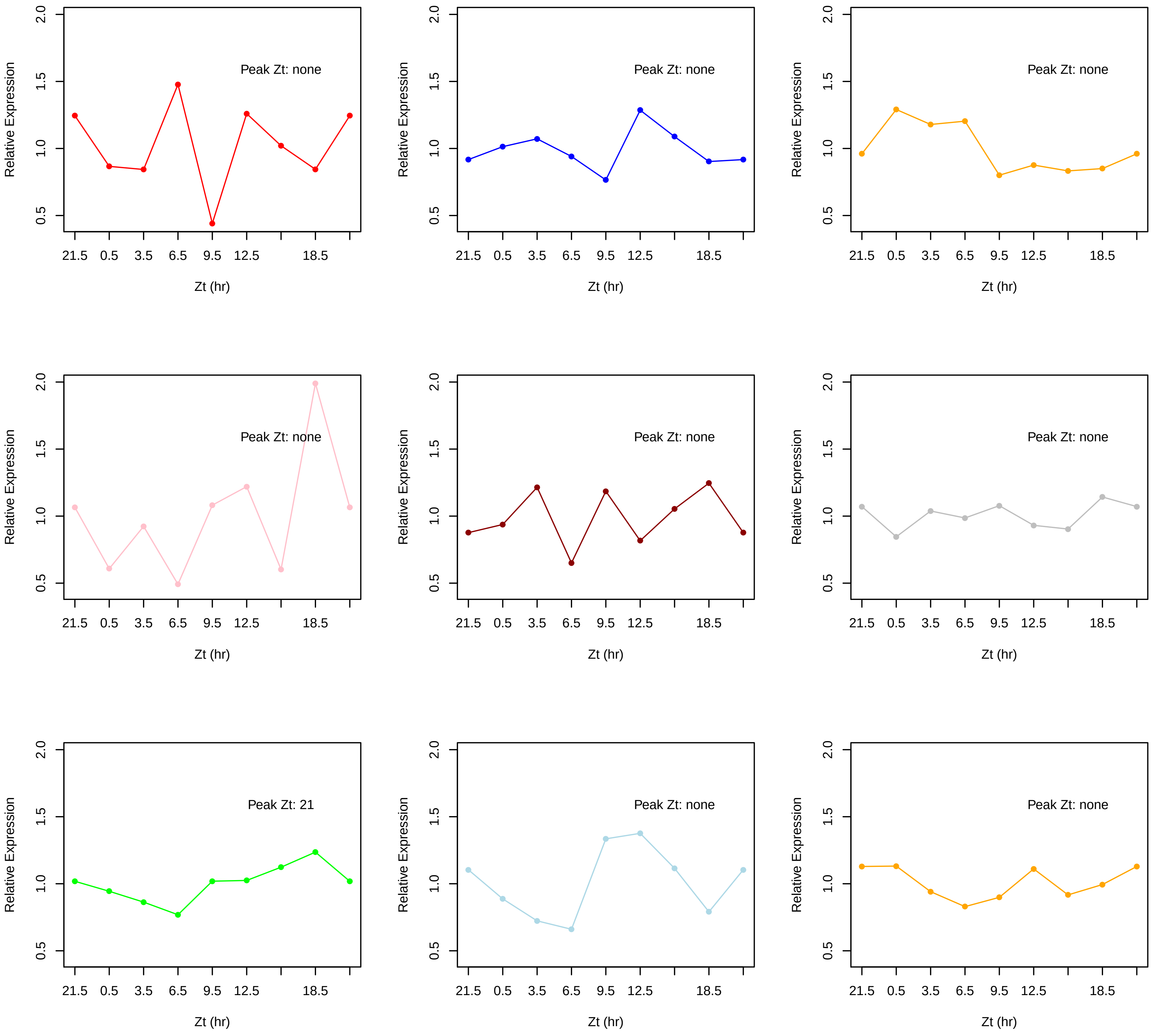

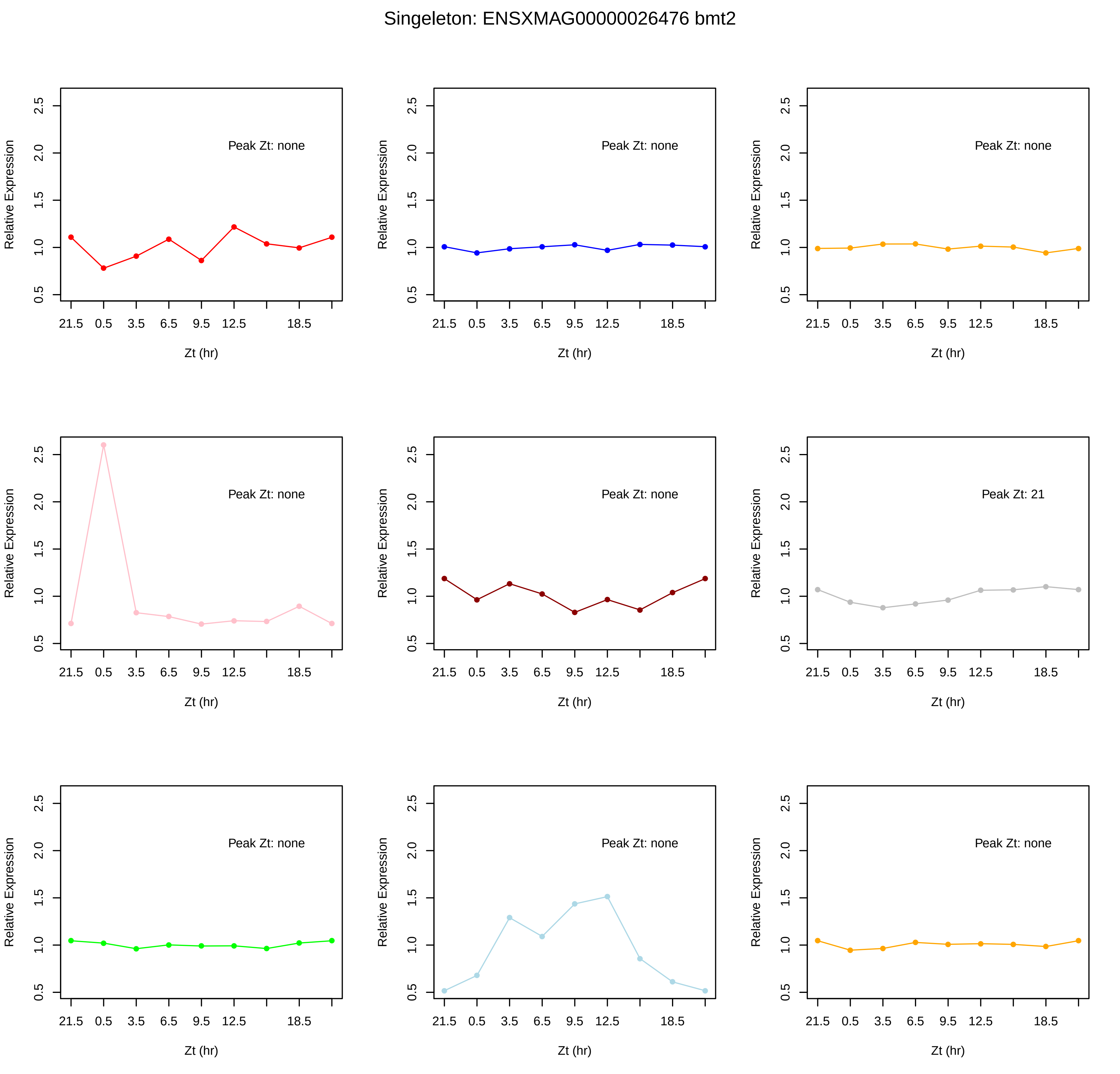
Singeleton: ENSXMAG00000026476 bmt2
Peak Zt: none
Peak Zt: none
Peak Zt: none
Peak Zt: none
Peak Zt: none
Peak Zt: 21
Peak Zt: none
Peak Zt: none
Peak Zt: none
Relative Expression
Zt (hr)
21.5 0.5 3.5 6.5 9.5 12.5 18.5
0.5 1.0 1.5 2.0 2.5

Singleton: ENSXMAG00000021637

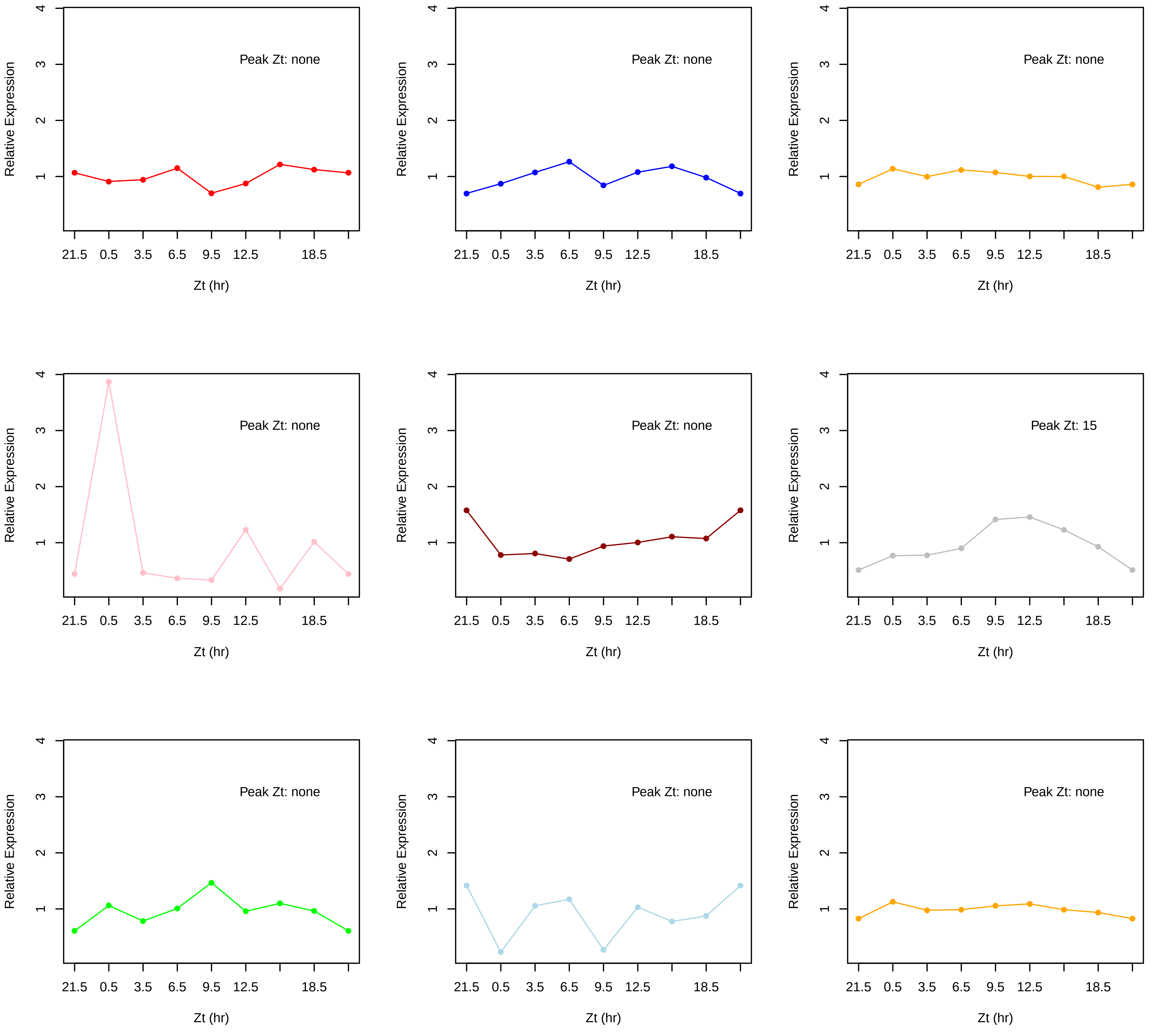

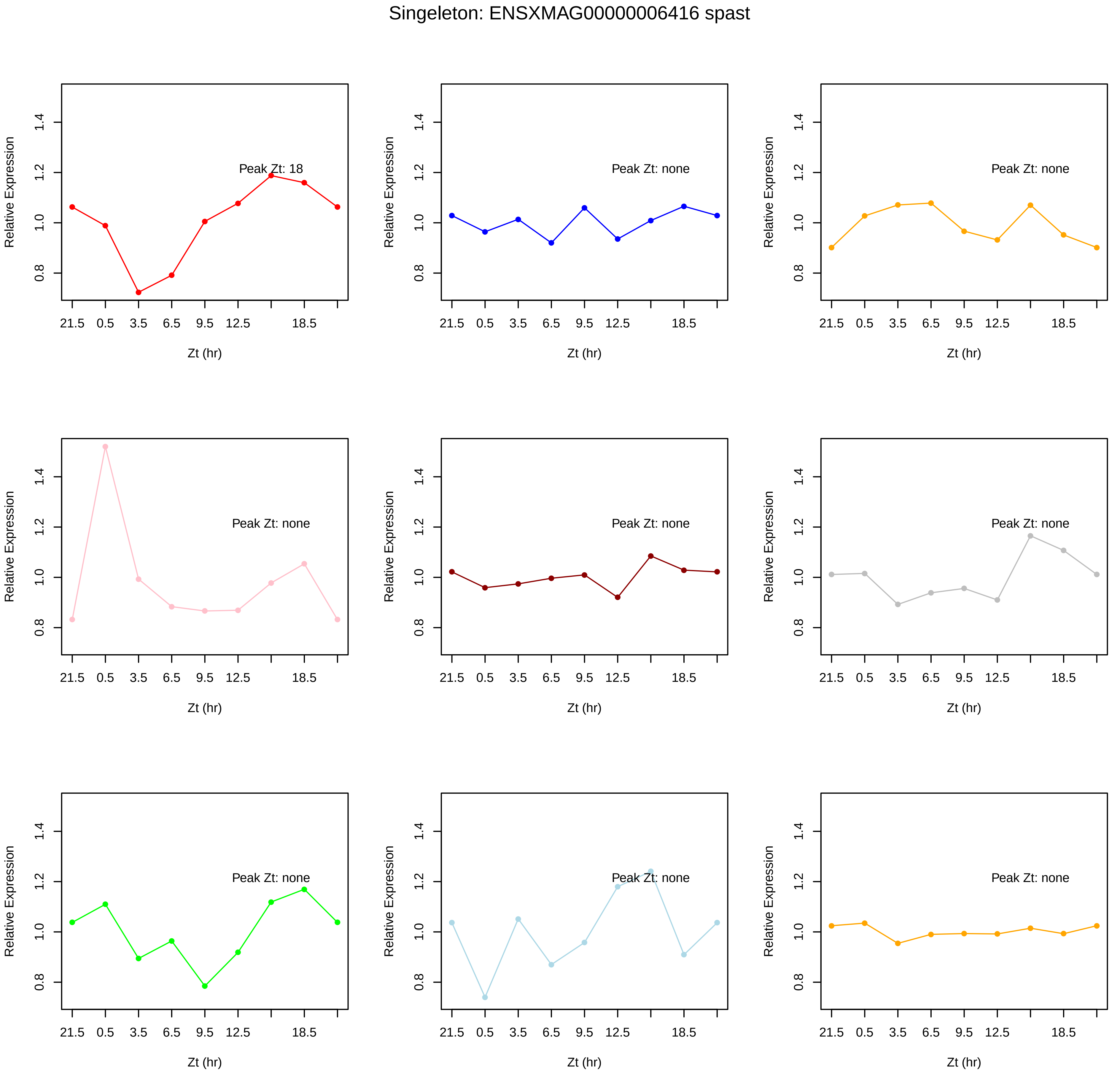
Singeleton: ENSXMAG00000006416 spast
Relative Expression
Zt (hr)
21.5 0.5 3.5 6.5 9.5 12.5 18.5
0.8 1.0 1.2 1.4
Peak Zt: 18
Peak Zt: none
Peak Zt: none
Peak Zt: none
Peak Zt: none
Peak Zt: none
Peak Zt: none
Peak Zt: none
Peak Zt: none

Singeleton: ENSXMAG00000007741 ankef1a

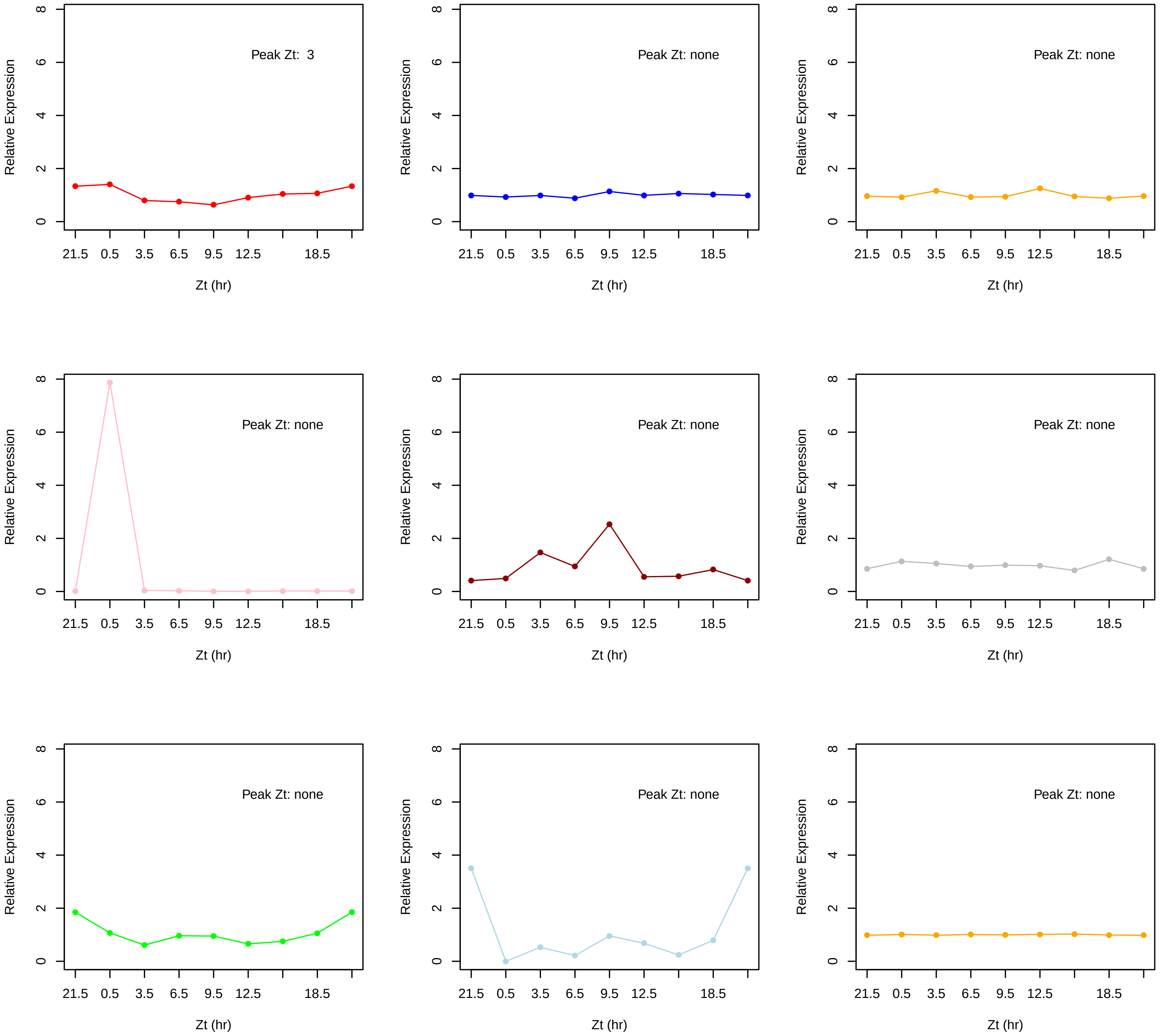

Singeleton: ENSXMAG00000012187 khk

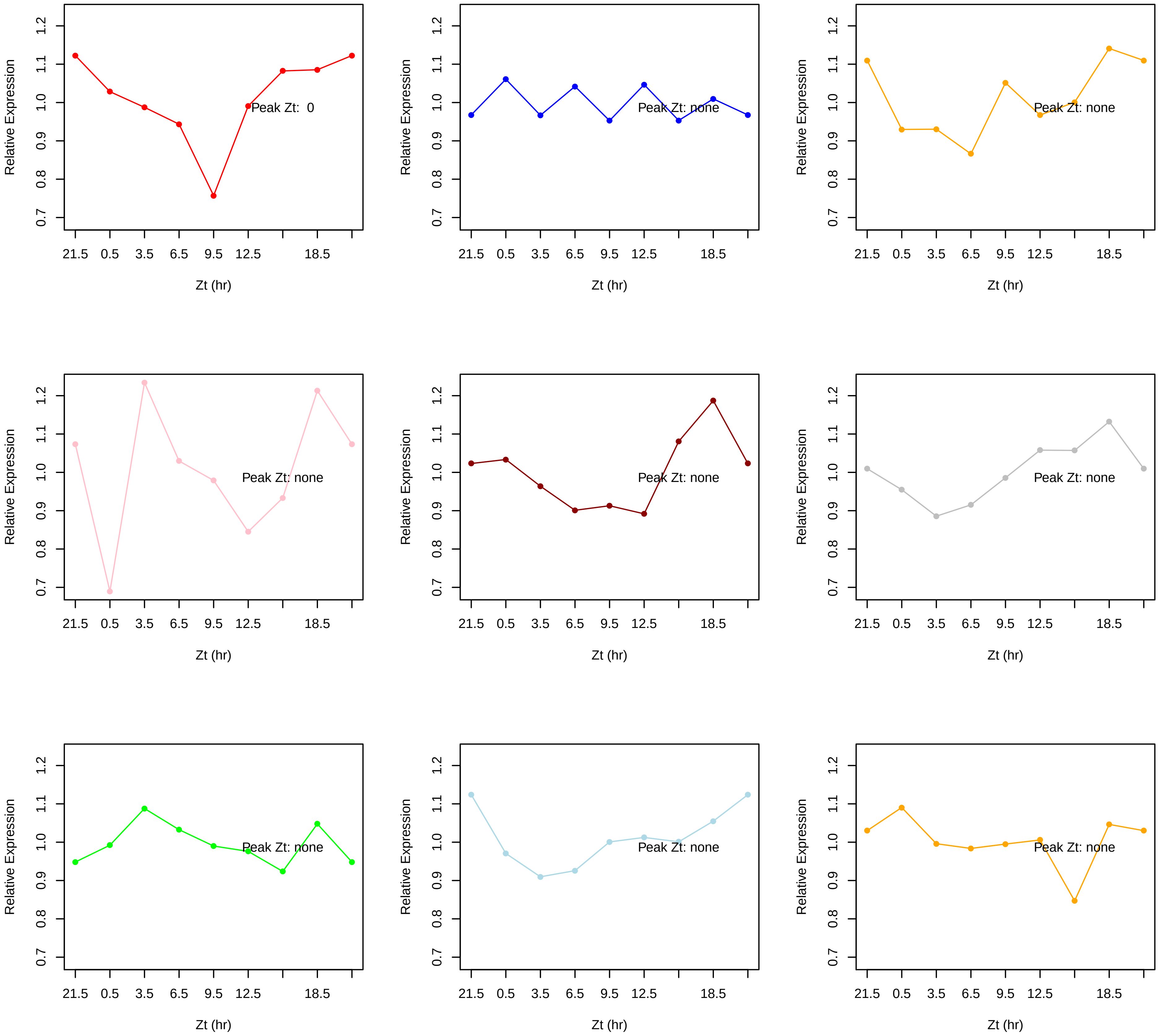

Singeleton: ENSXMAG00000009449 cpa4

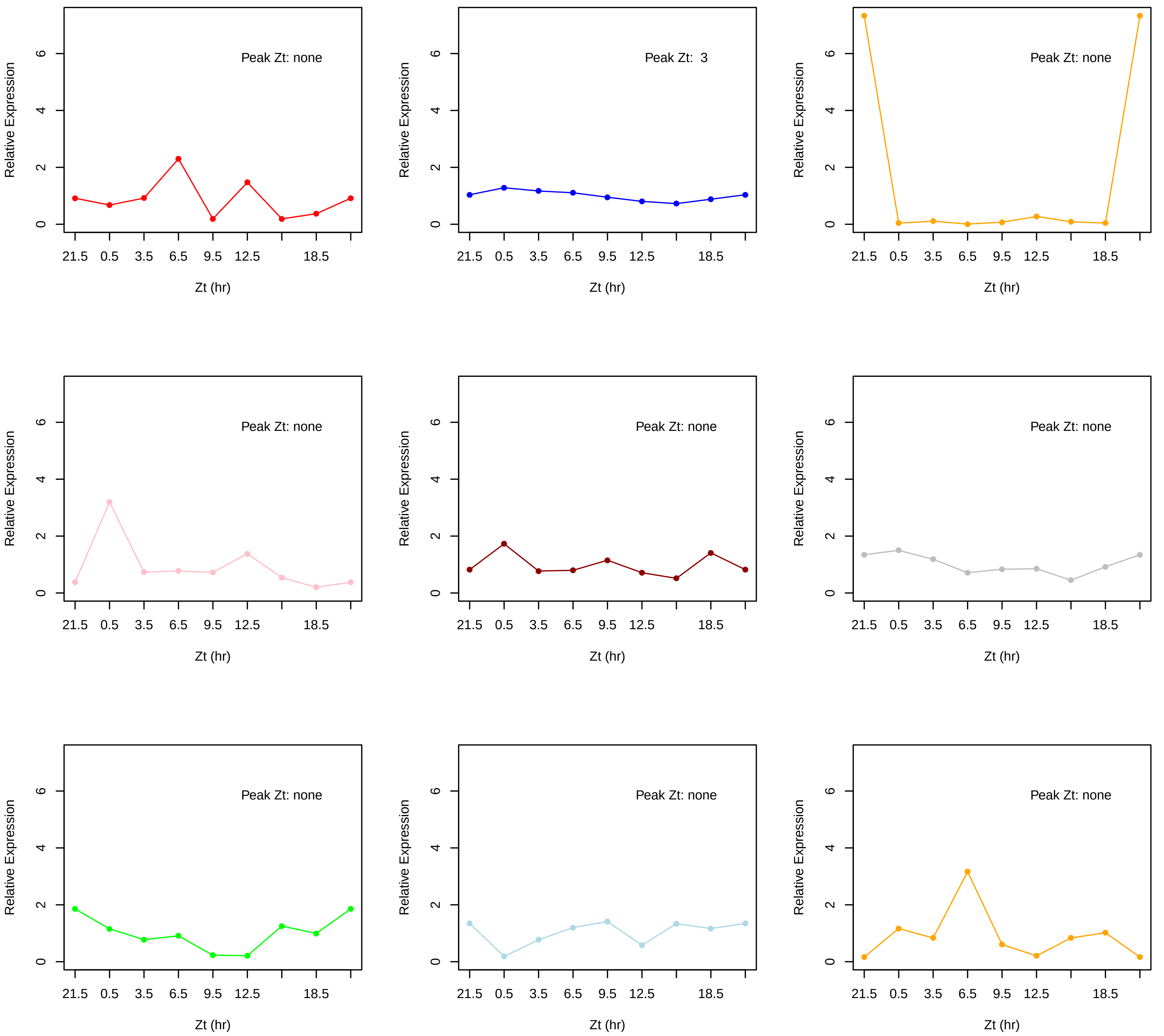

Singeleton: ENSXMAG00000022052 dpy30

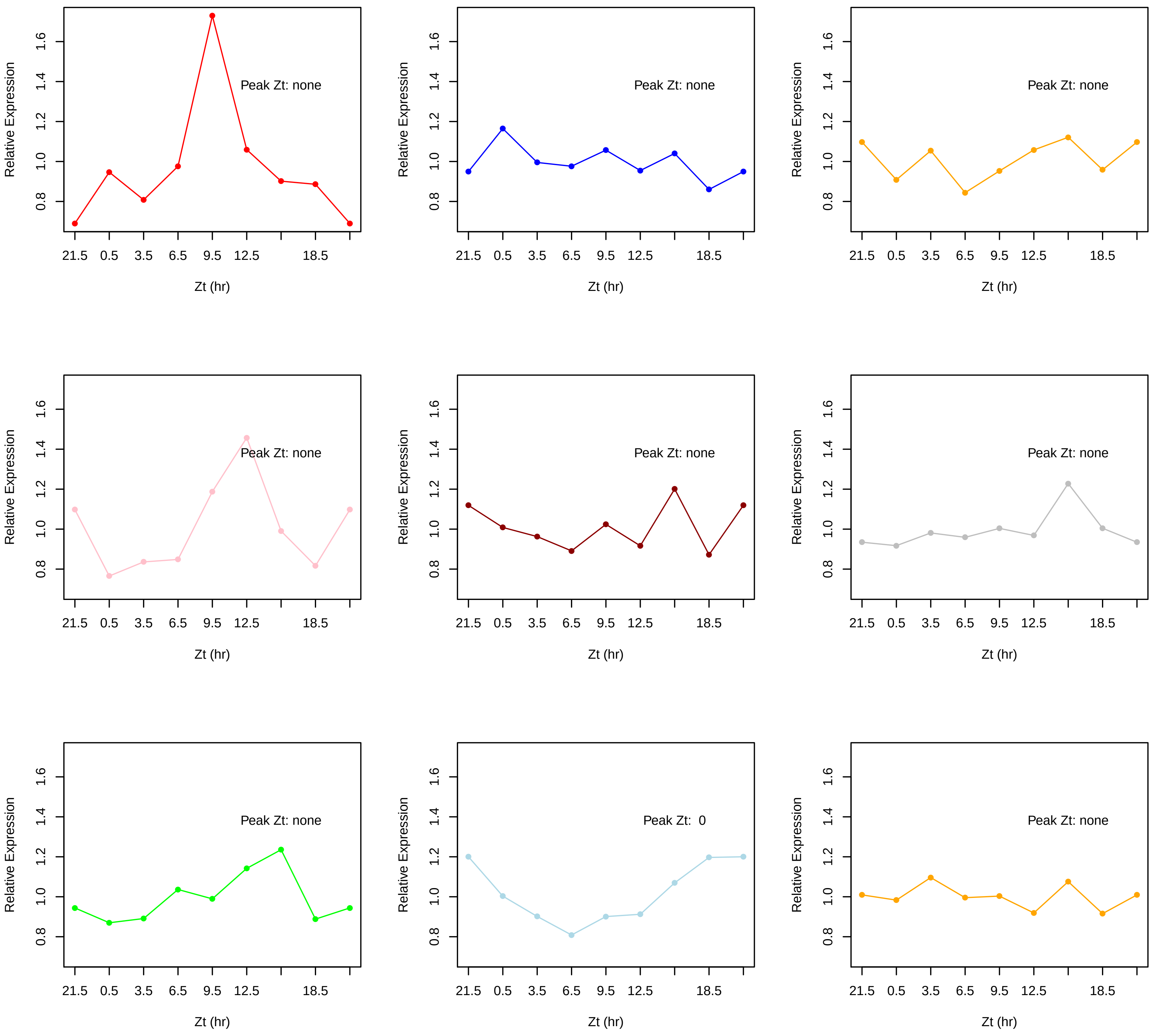

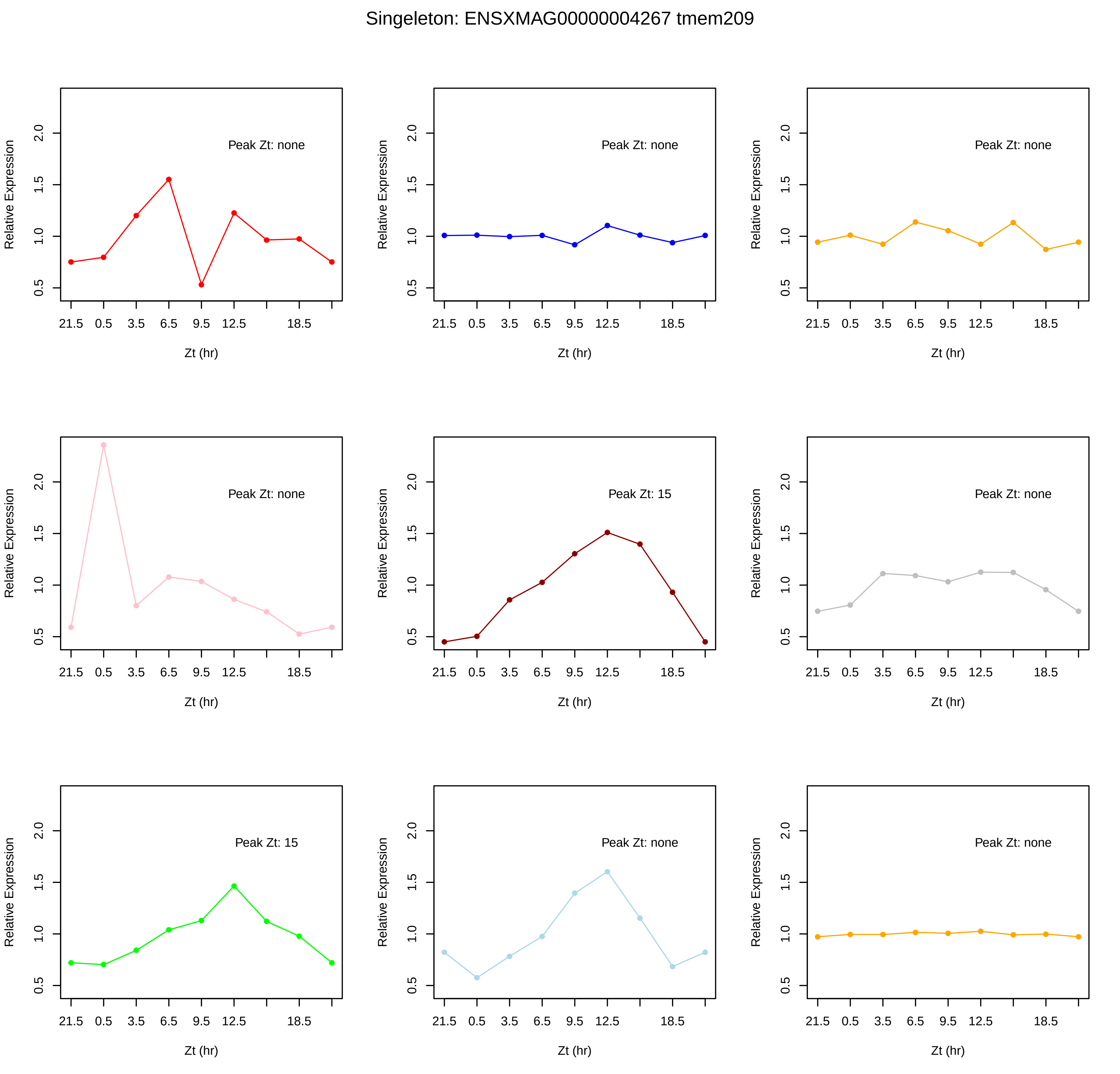

Singeleton: ENSXMAG00000004267 tmem209
Peak Zt: none
Peak Zt: none
Peak Zt: none
Peak Zt: none
Peak Zt: 15
Peak Zt: none
Peak Zt: 15
Peak Zt: none
Peak Zt: none
Relative Expression
Zt (hr)

Singeleton: ENSXMAG00000005436 gpn1

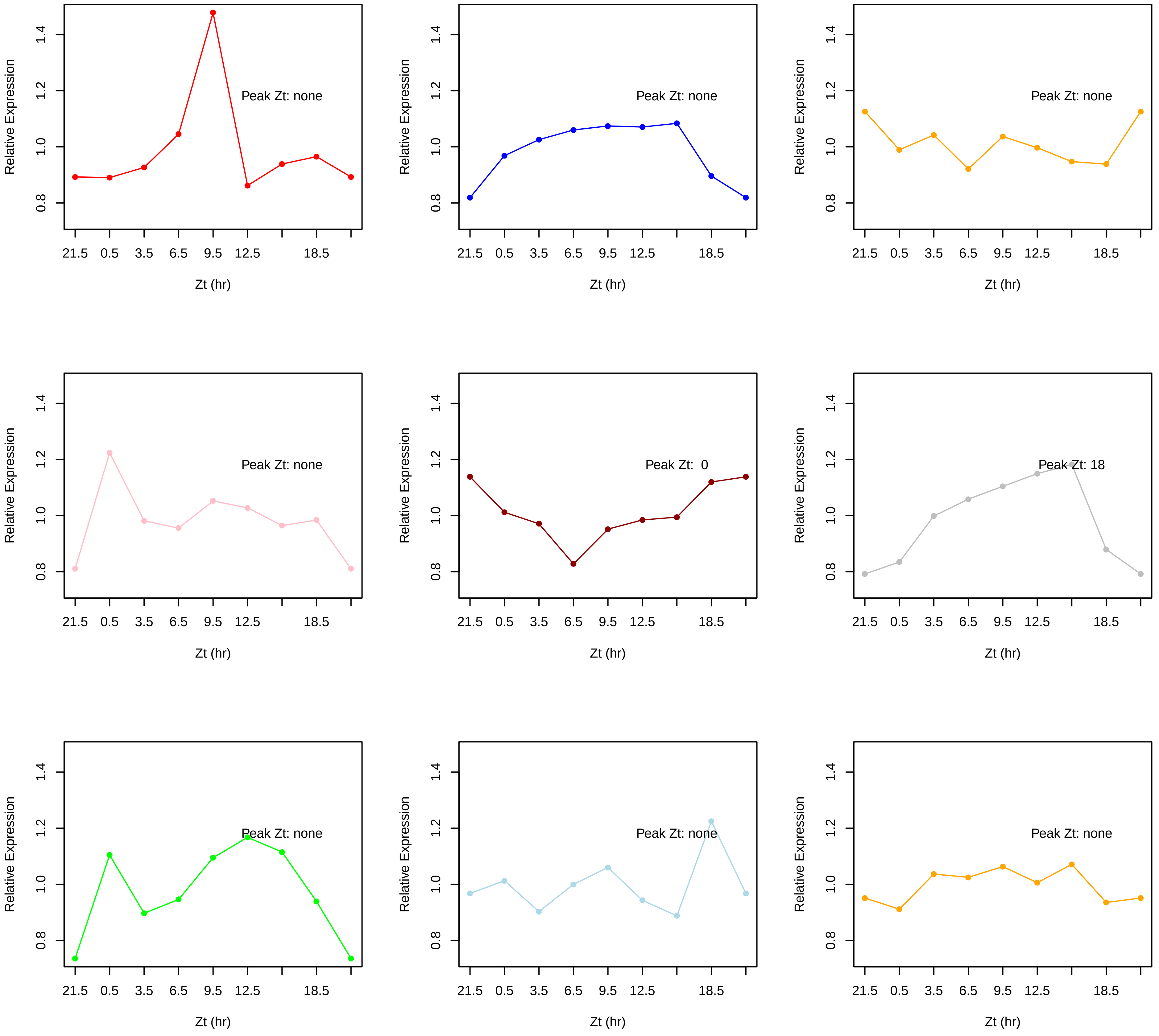

Singeleton: ENSXMAG00000001575 ahcyl2

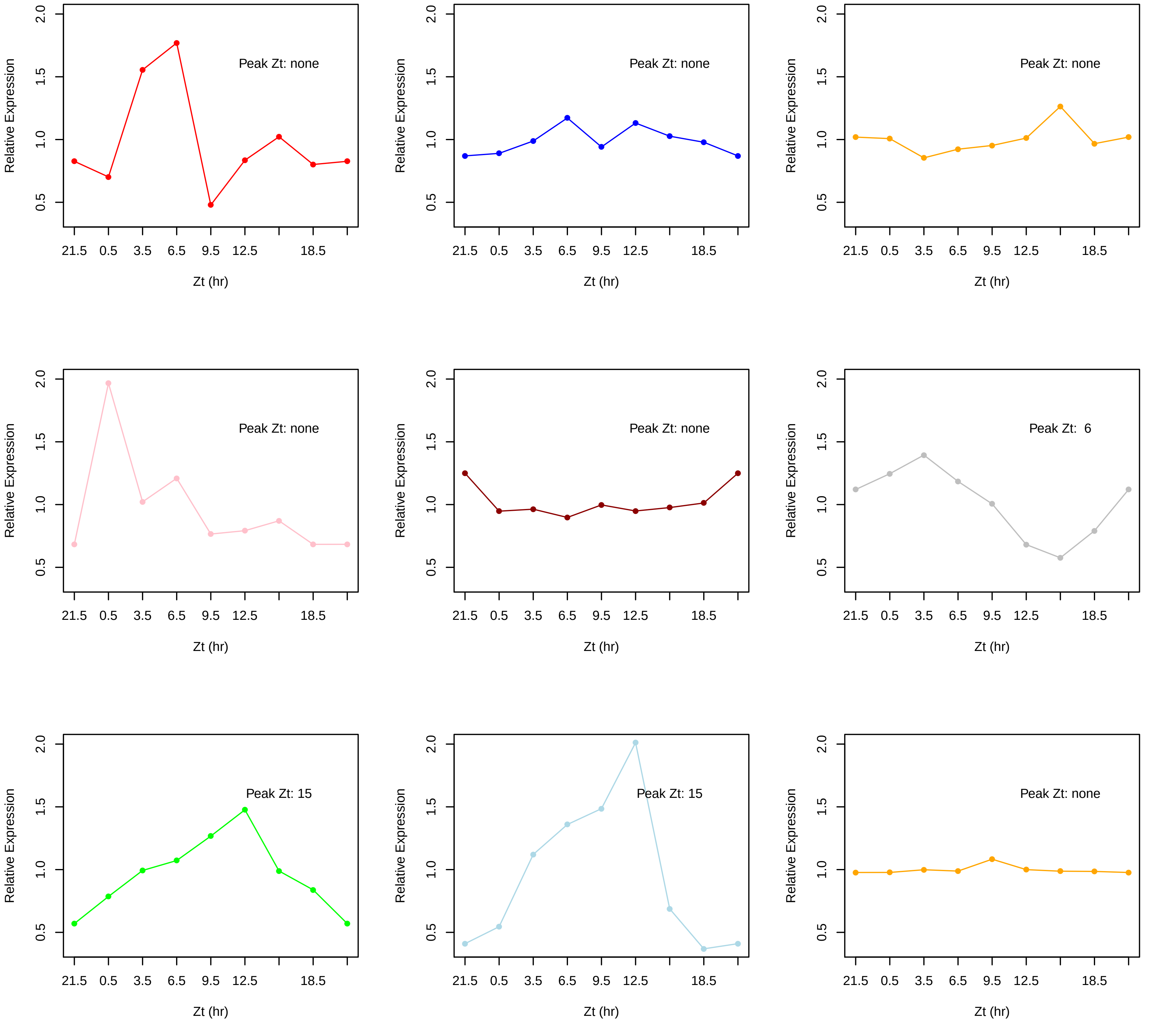

Singeleton: ENSXMAG00000004176 strip2

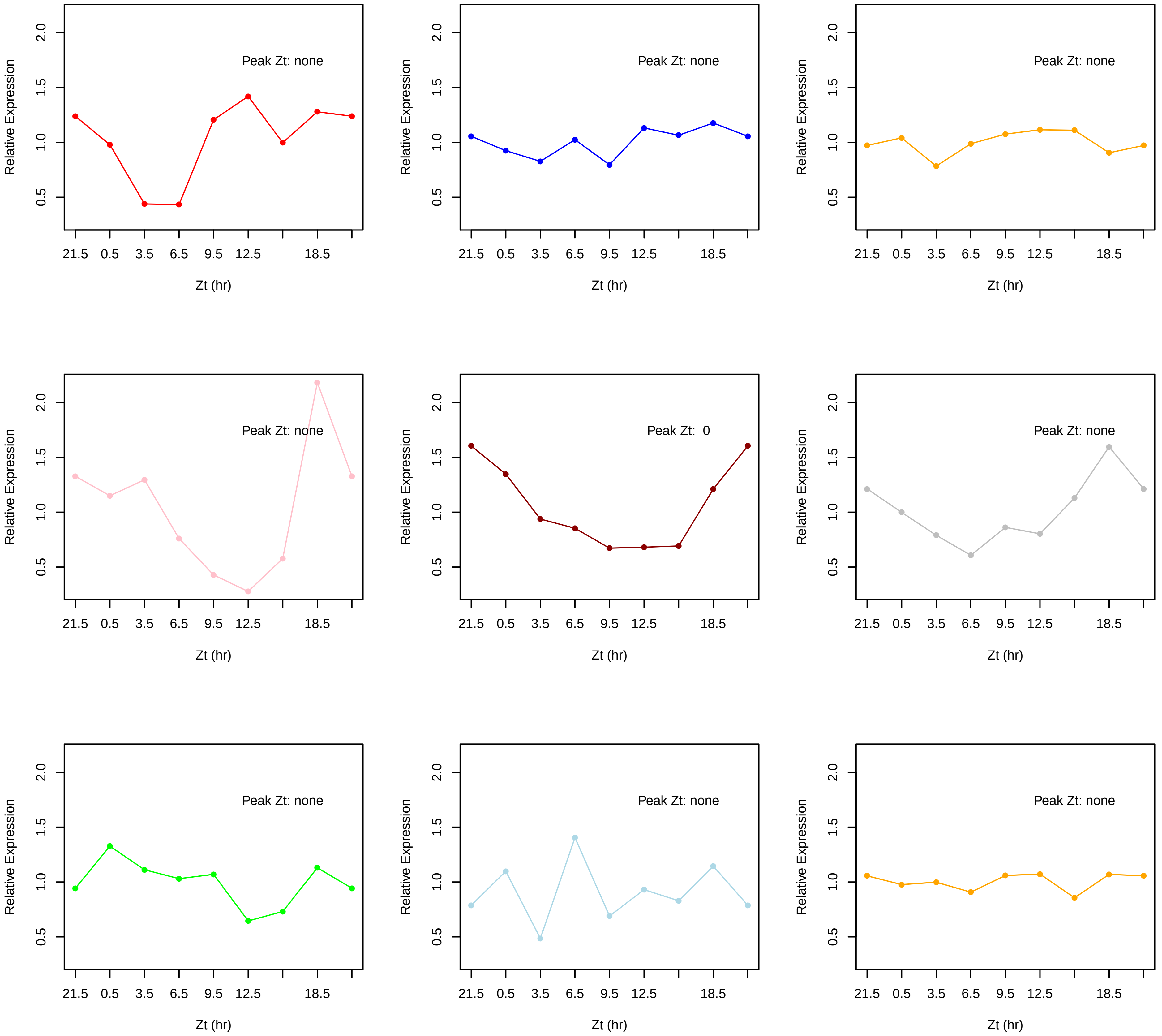

Singeleton: ENSXMAG00000006388 srd5a2b

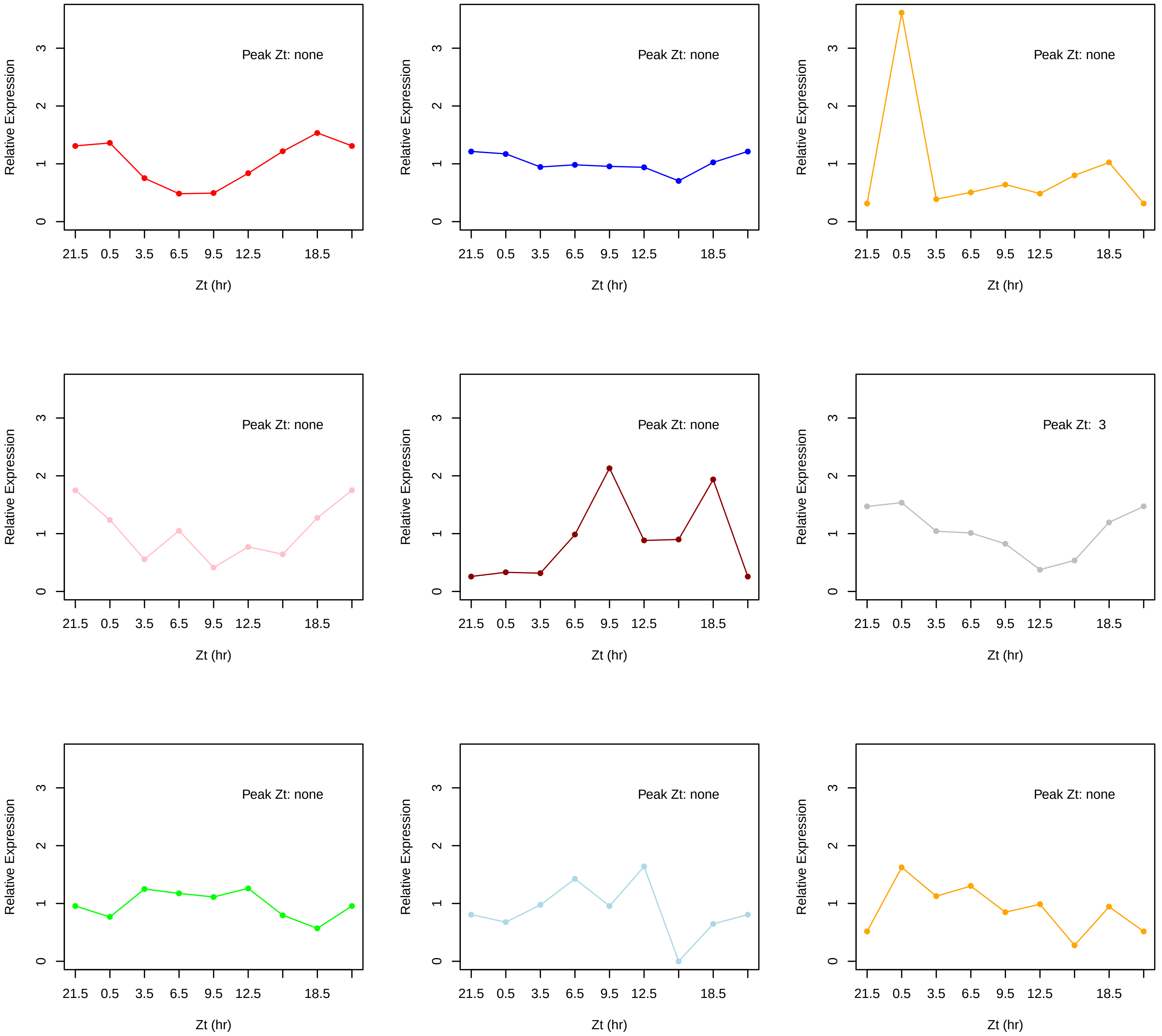

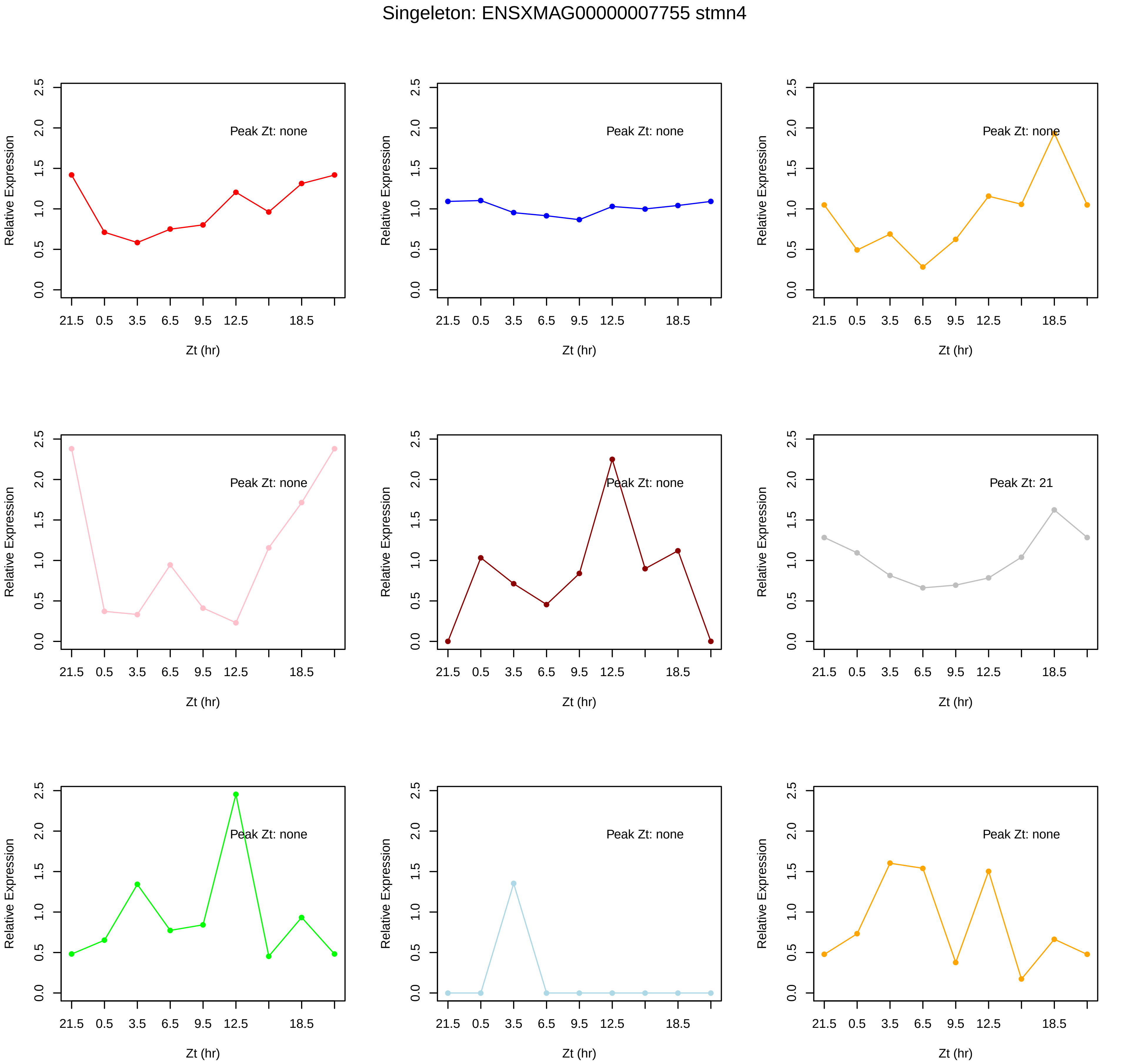
Singleton: ENSXMAG00000007755 stmn4
Peak Zt: none
Peak Zt: none
Peak Zt: none
Peak Zt: none
Peak Zt: none
Peak Zt: 21
Peak Zt: none
Peak Zt: none
Peak Zt: none
Relative Expression
Zt (hr)
21.5
0.5
3.5
6.5
9.5
12.5
18.5
0.0
0.5
1.0
1.5
2.0
2.5

Singeleton: ENSXMAG00000010946 nrf1

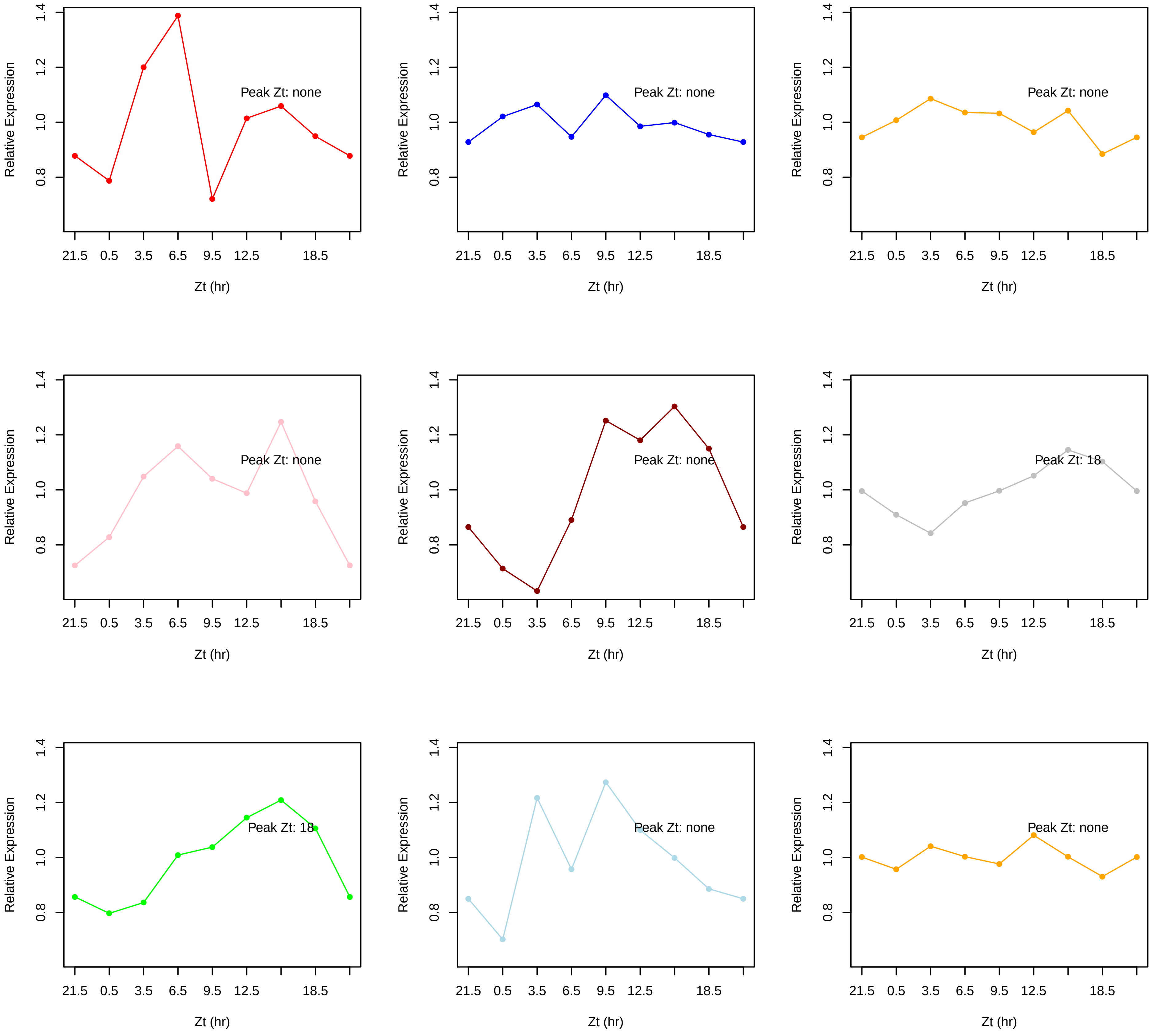

Singeleton: ENSXMAG00000010979 zc3hc1

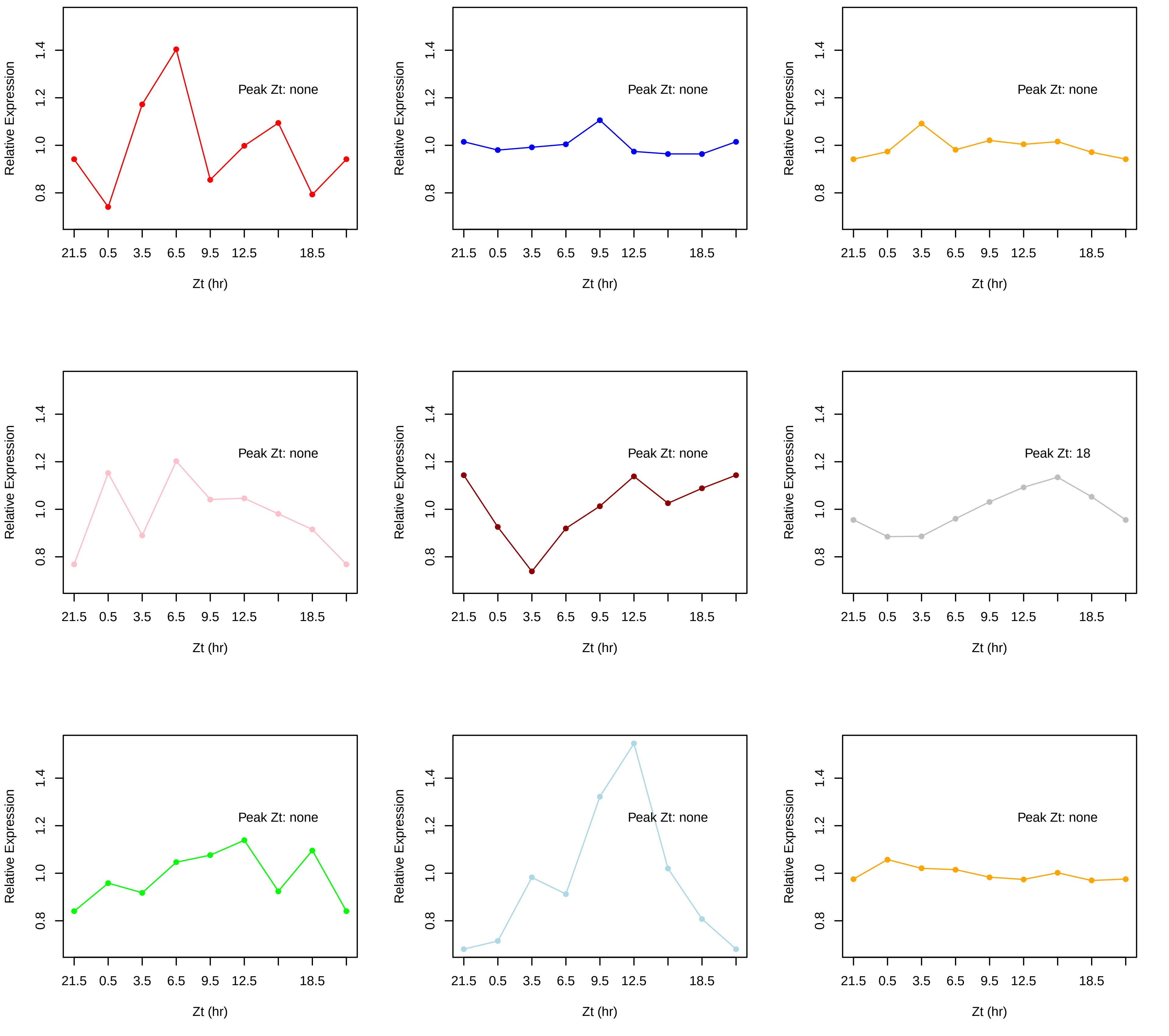

Singleton: ENSXMAG00000029893 sptlc3

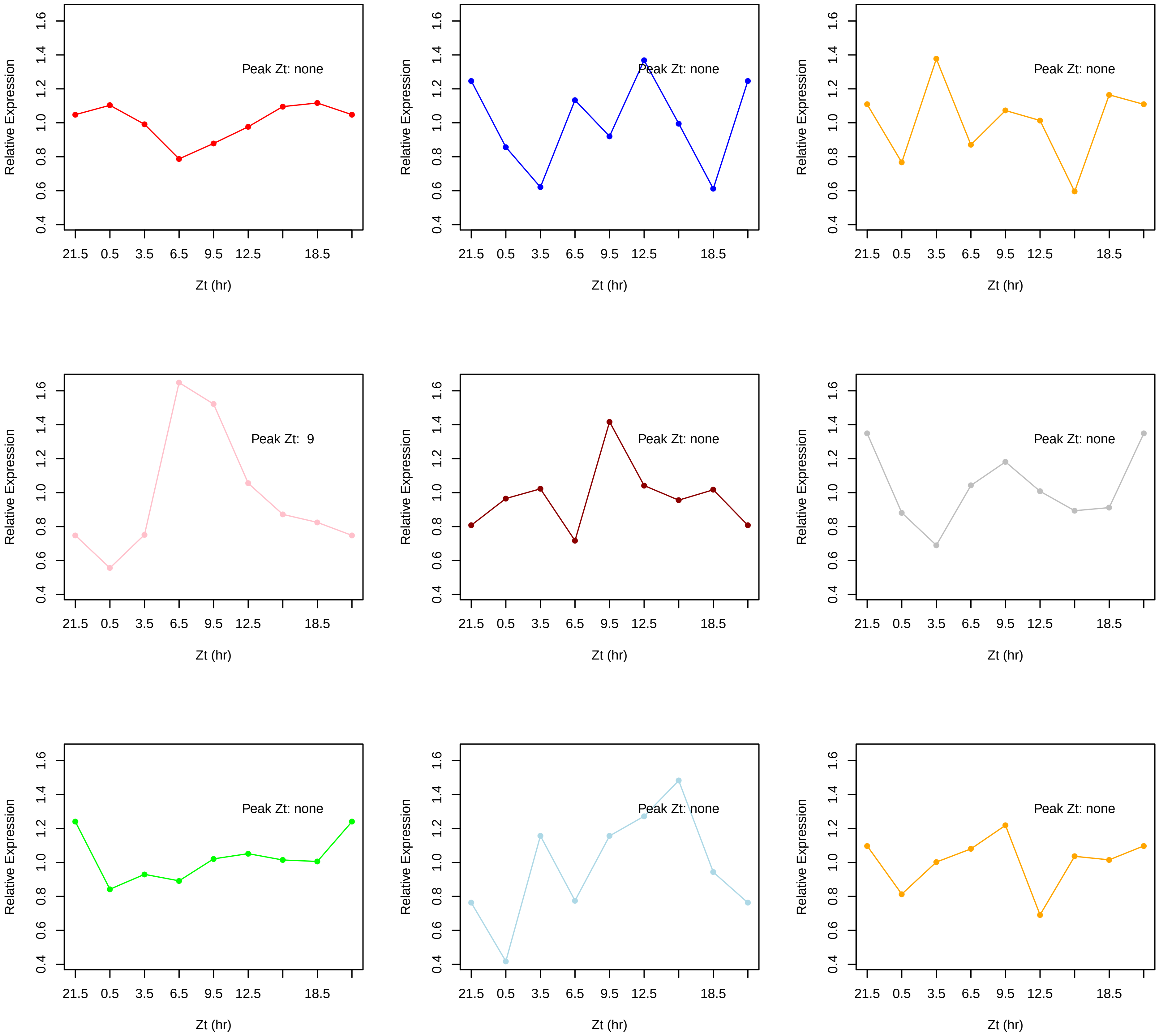

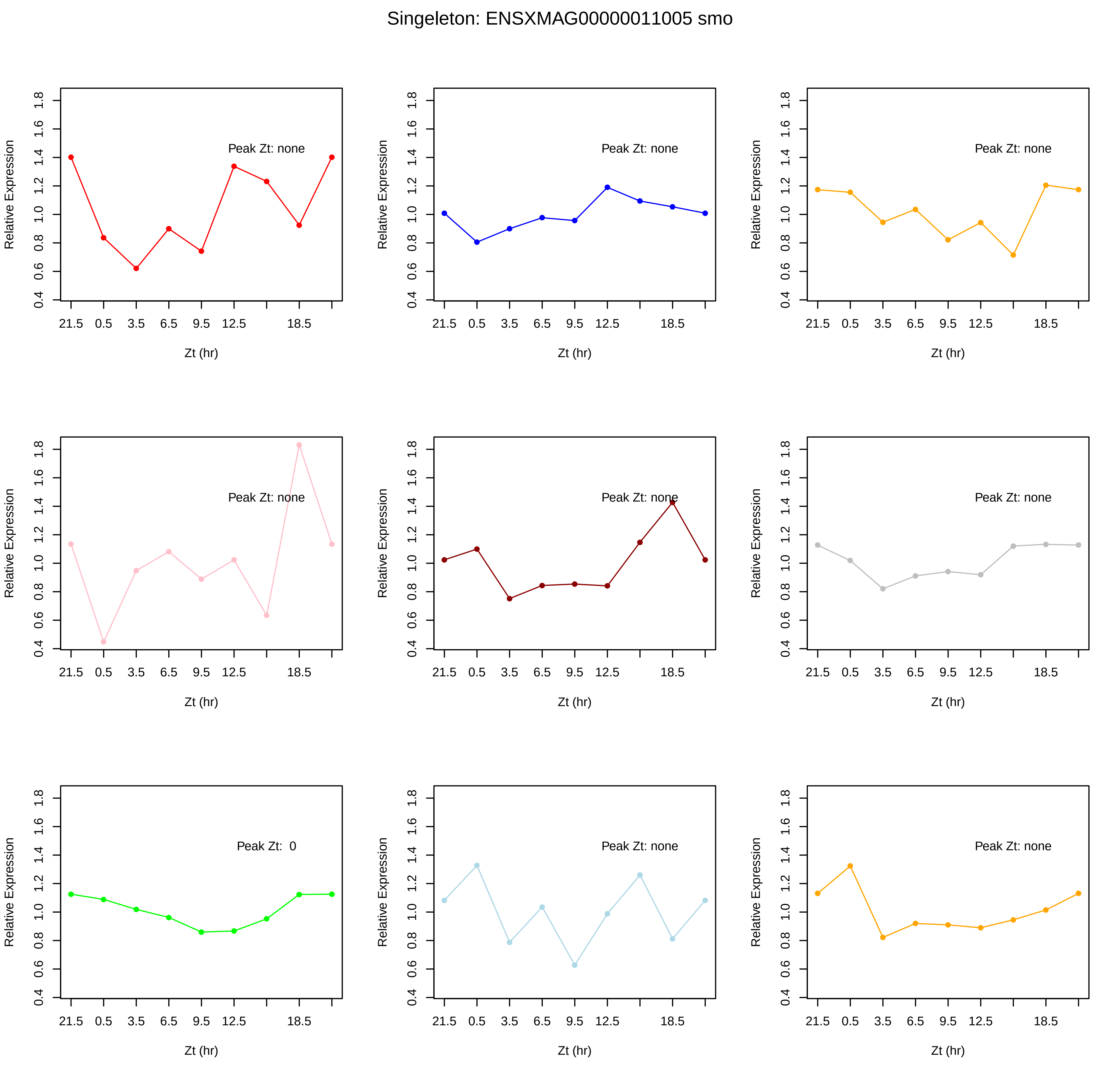

Singeleton: ENSXMAG00000011005 smo
Relative Expression
Zt (hr)
Peak Zt: none
Peak Zt: 0
21.5 0.5 3.5 6.5 9.5 12.5 18.5
0.4 0.6 0.8 1.0 1.2 1.4 1.6 1.8

Singeleton: ENSXMAG00000006423 SLC26A5

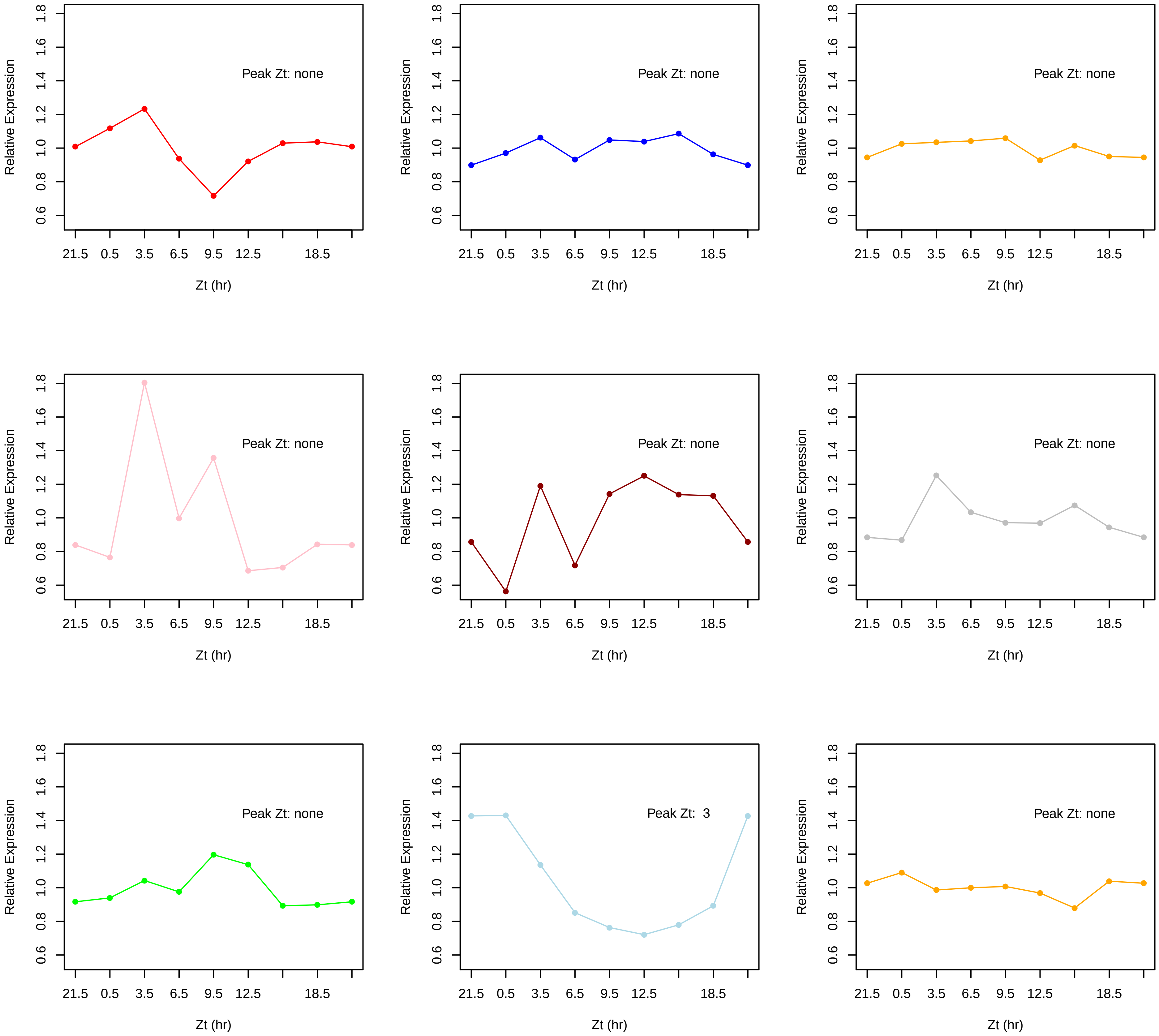

Singleton: ENSXMAG00000006253 tasp1

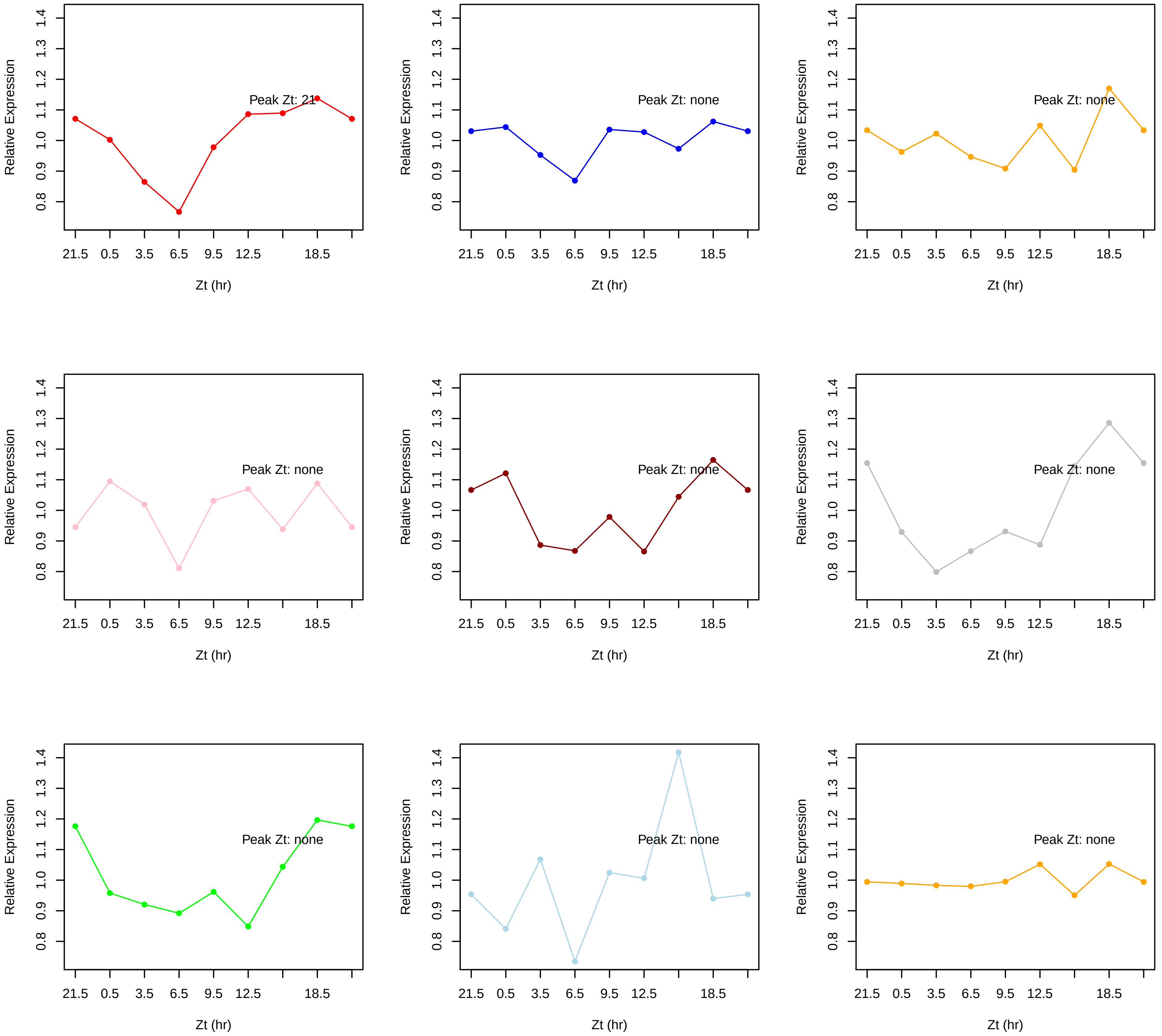

Singeleton: ENSXMAG00000006406 psmc2

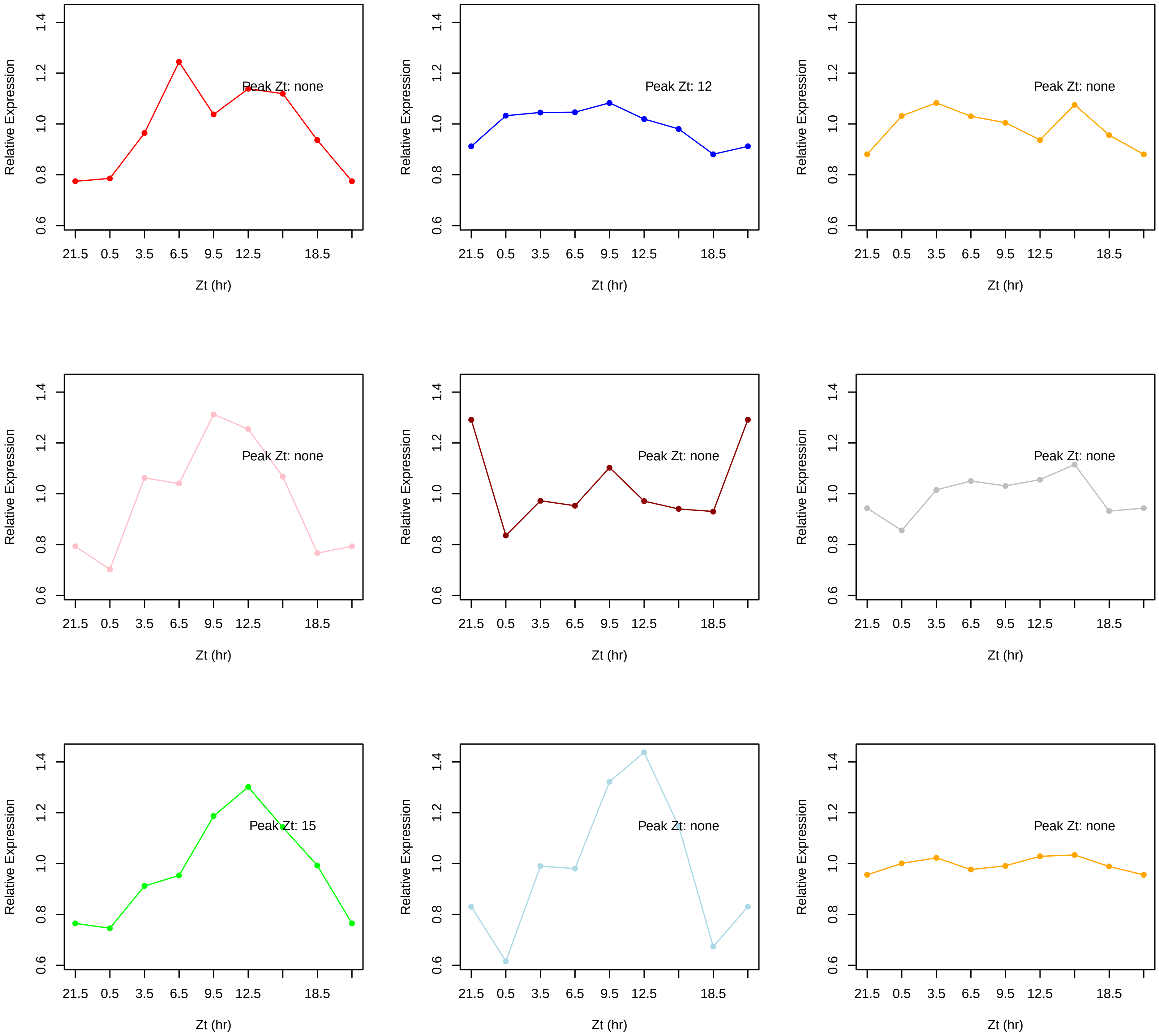

Singeleton: ENSXMAG00000006390 dnajc2

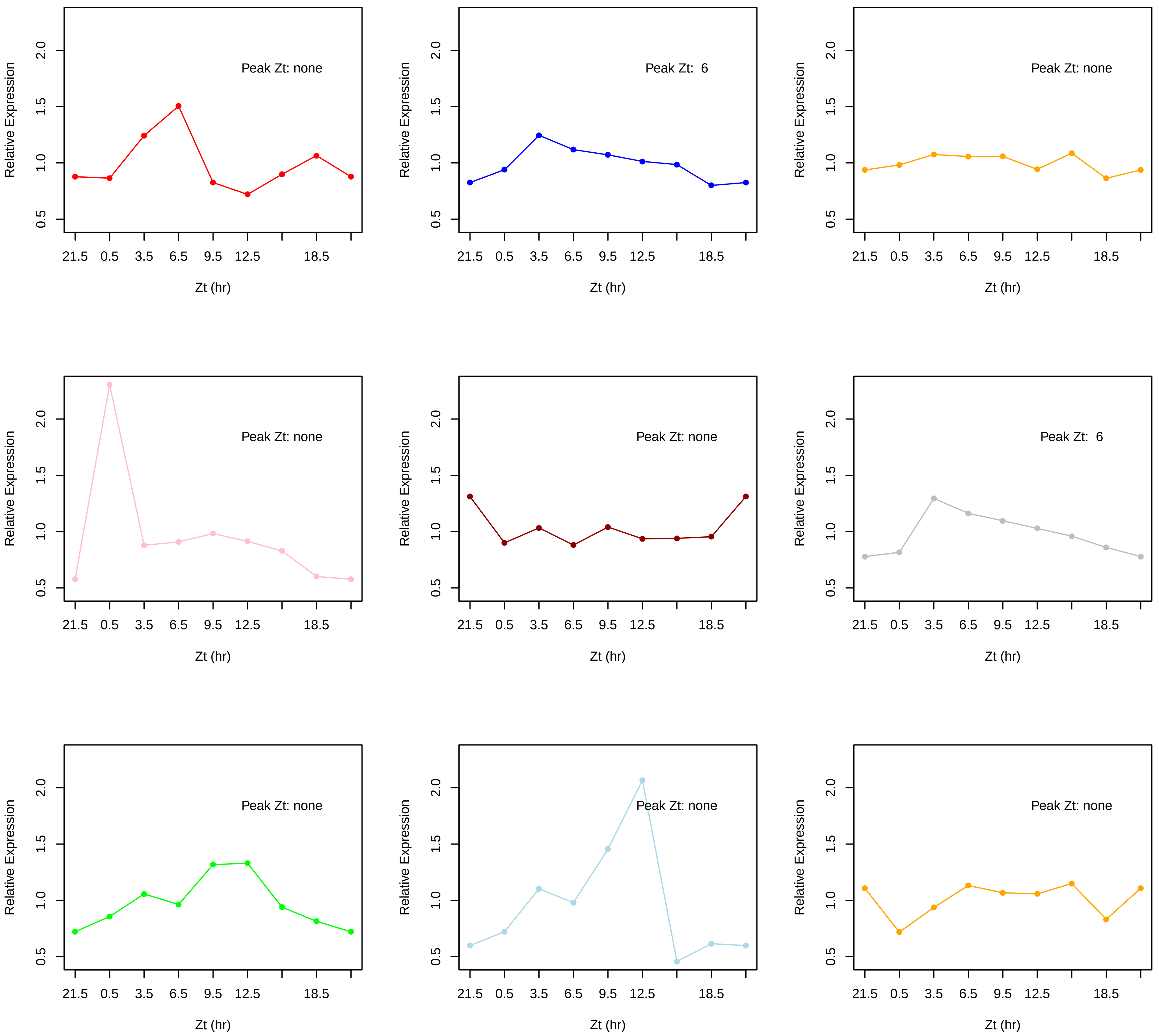

Singeleton: ENSXMAG00000003491 NAPEPLD

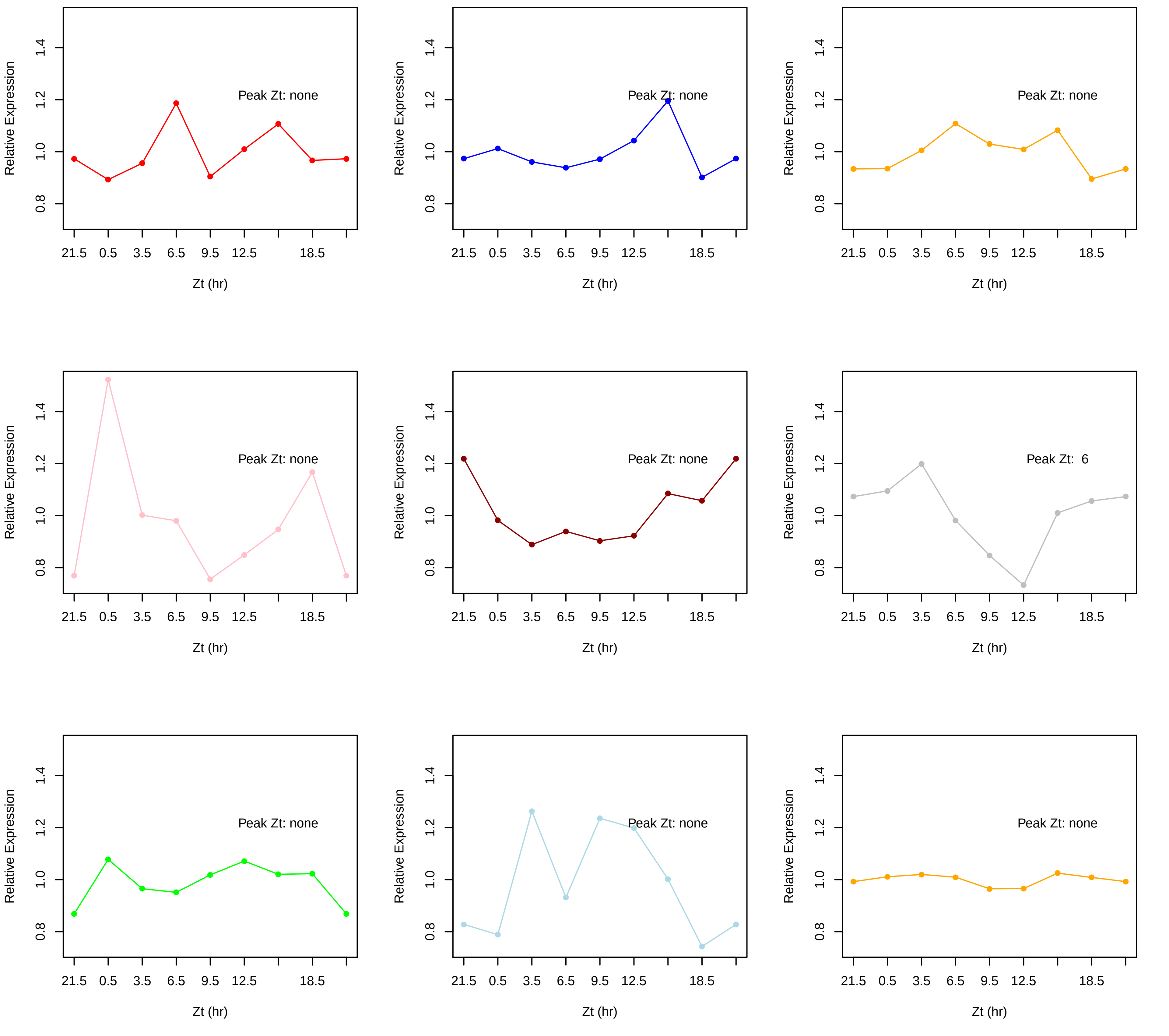

Singeleton: ENSXMAG00000009099 armc10

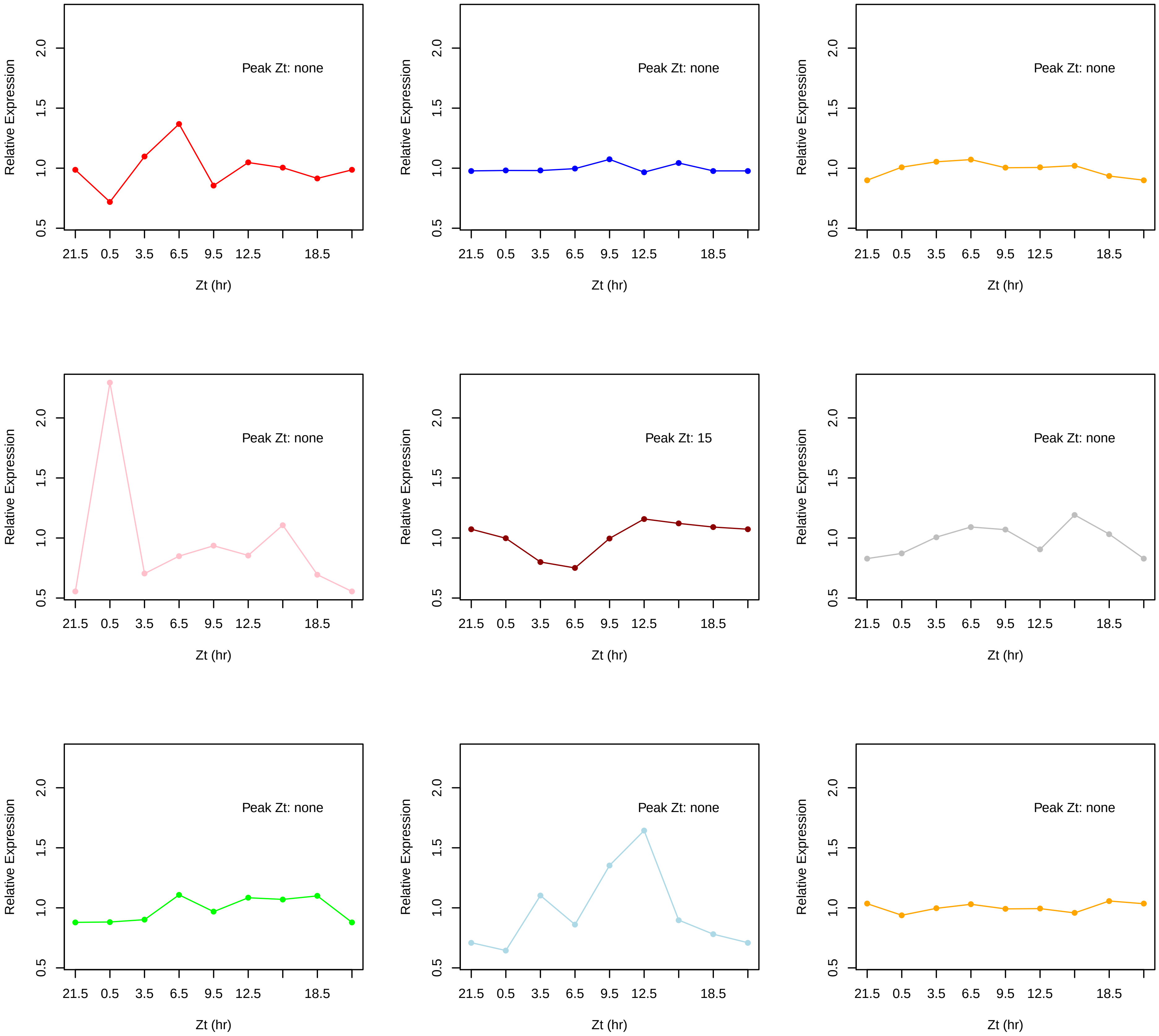

Singleton: ENSXMAG00000009110 fbxl13

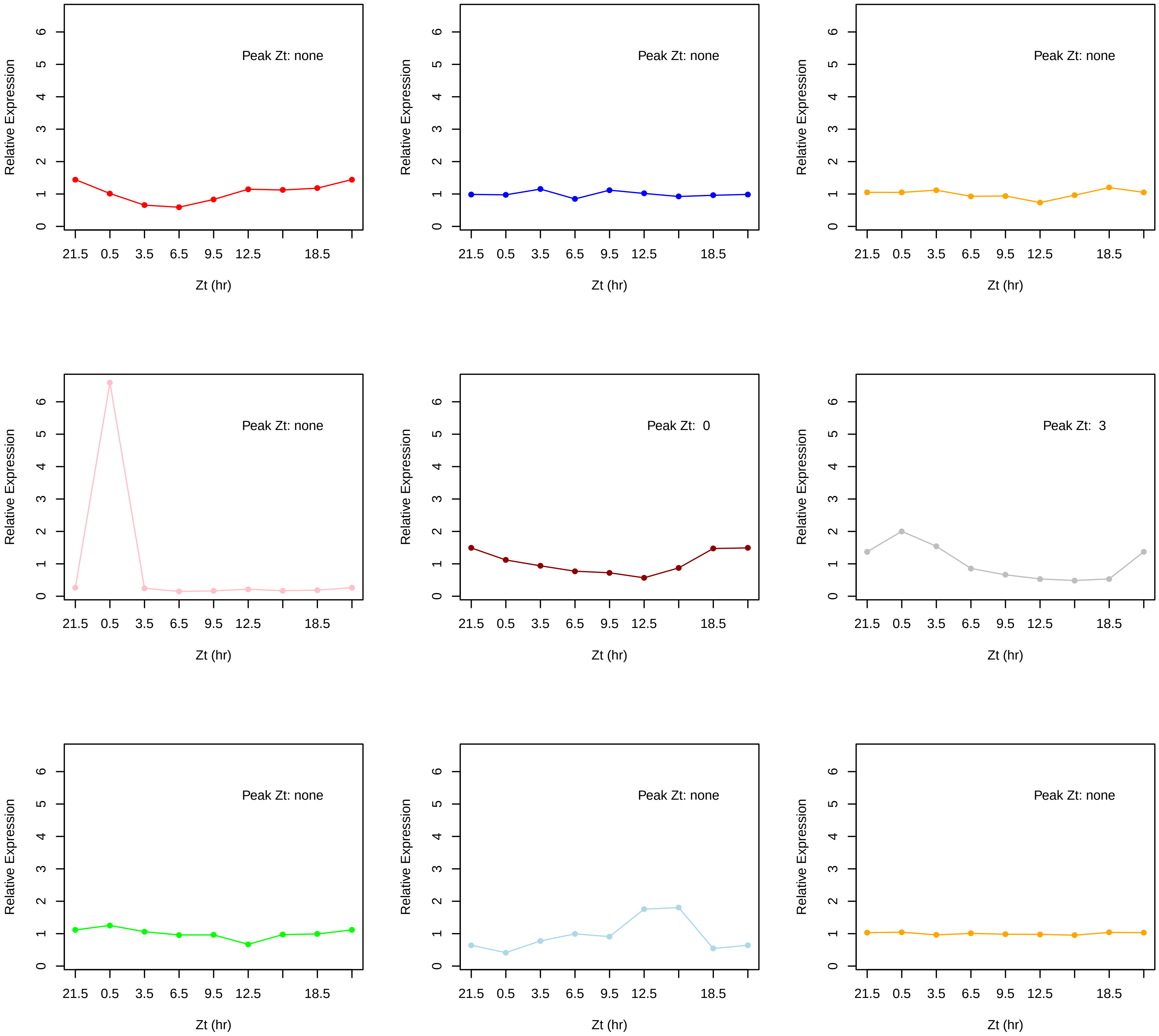

Singeleton: ENSXMAG00000009116 fam185a

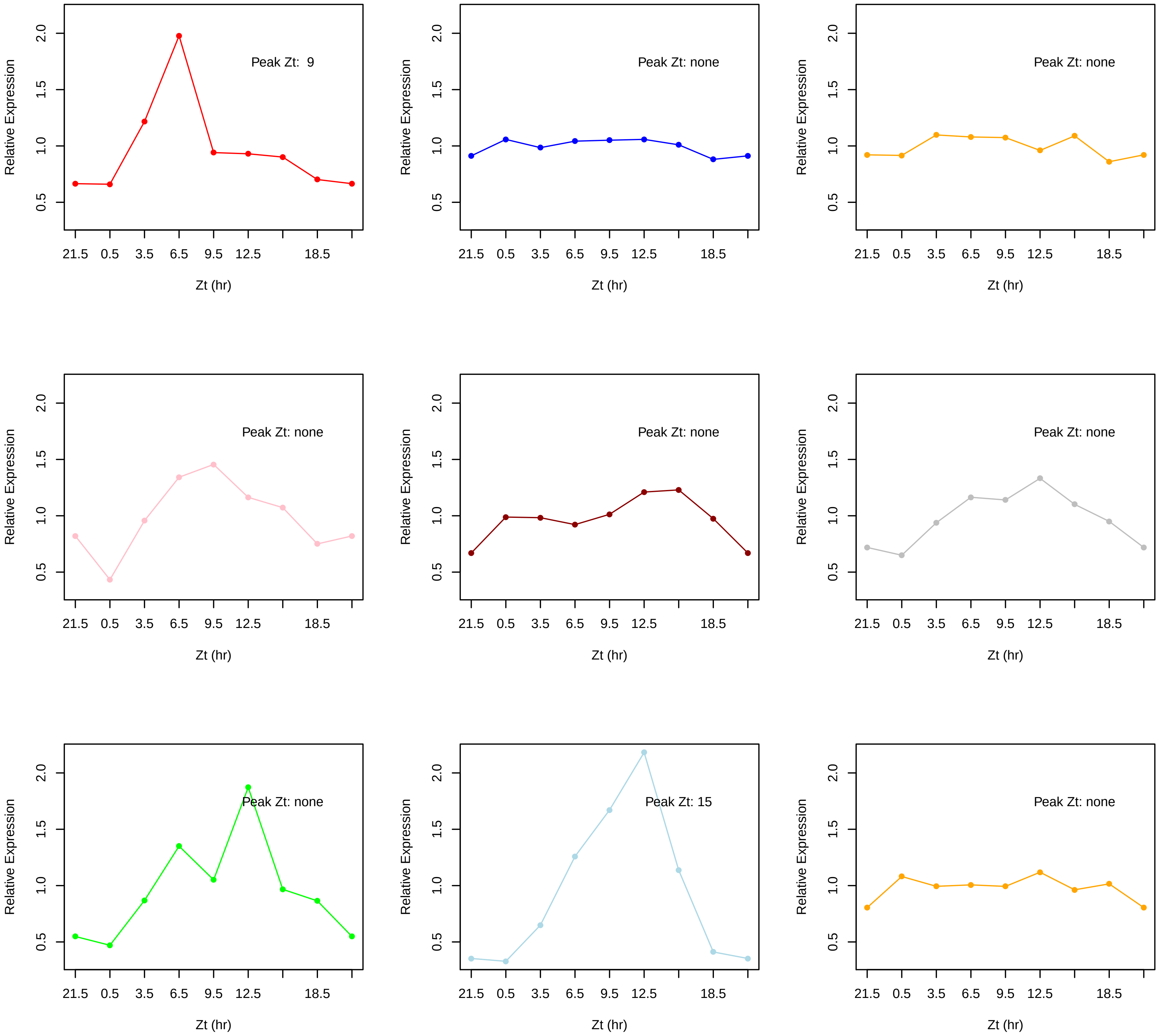

Singeleton: ENSXMAG00000000699 snrpb2

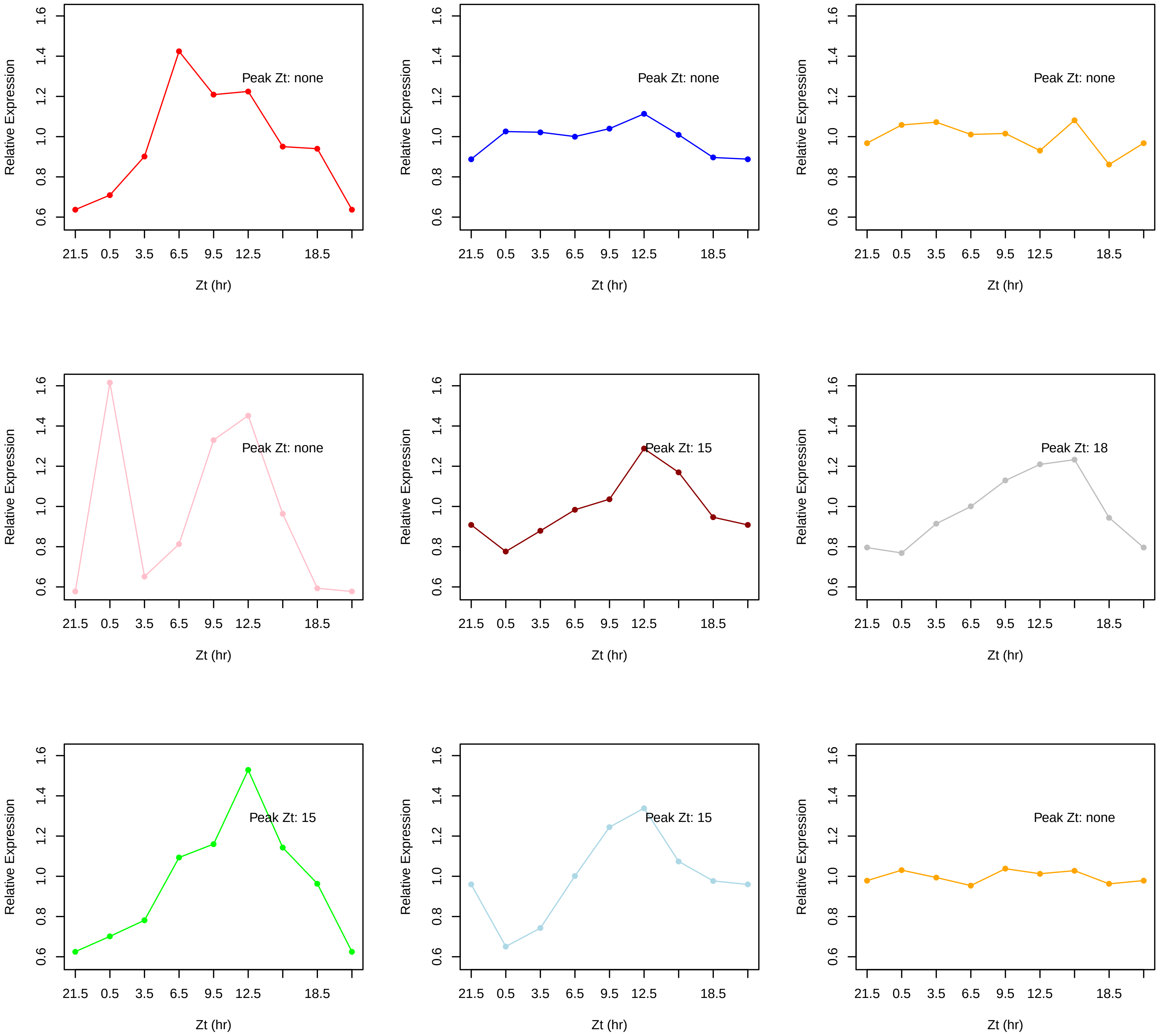

Singeleton: ENSXMAG00000028311 OTOR

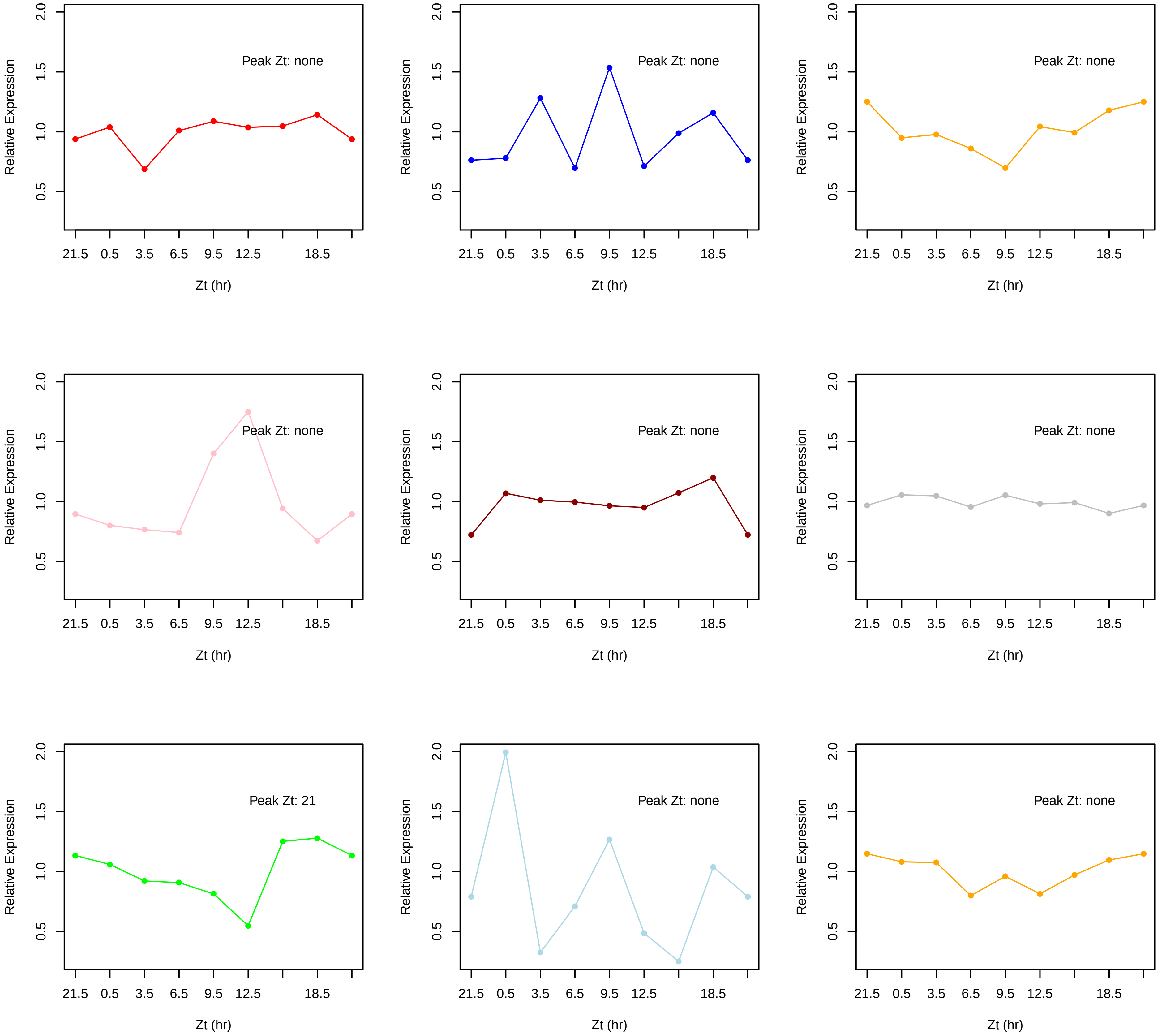

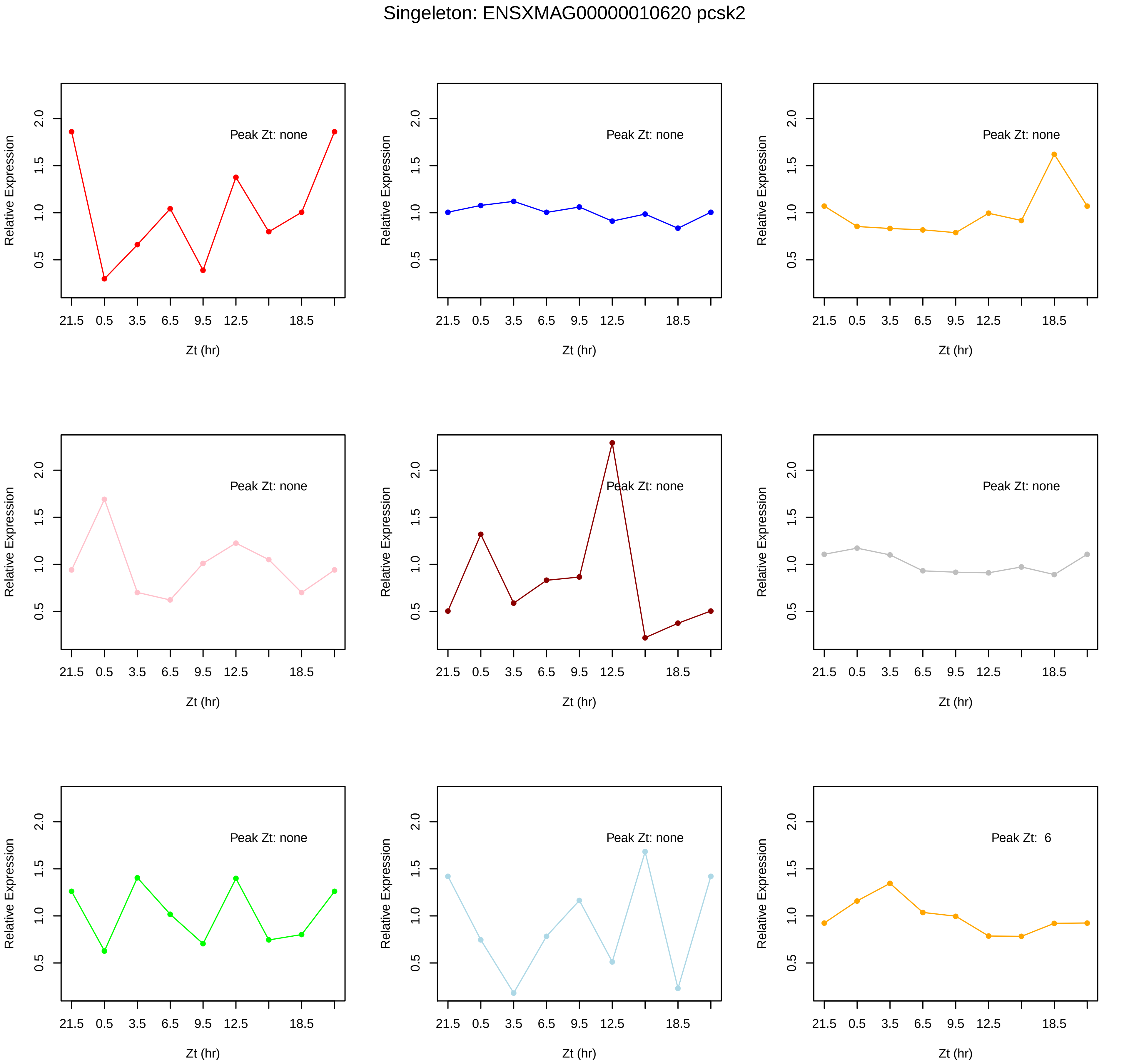
Singleton: ENSXMAG00000010620 pcsk2
Peak Zt: none
Peak Zt: none
Peak Zt: none
Peak Zt: none
Peak Zt: none
Peak Zt: none
Peak Zt: none
Peak Zt: none
Peak Zt: 6
Relative Expression
Zt (hr)
21.5
0.5
3.5
6.5
9.5
12.5
18.5
0.5
1.0
1.5
2.0

Singeleton: ENSXMAG00000028704 BFSP1

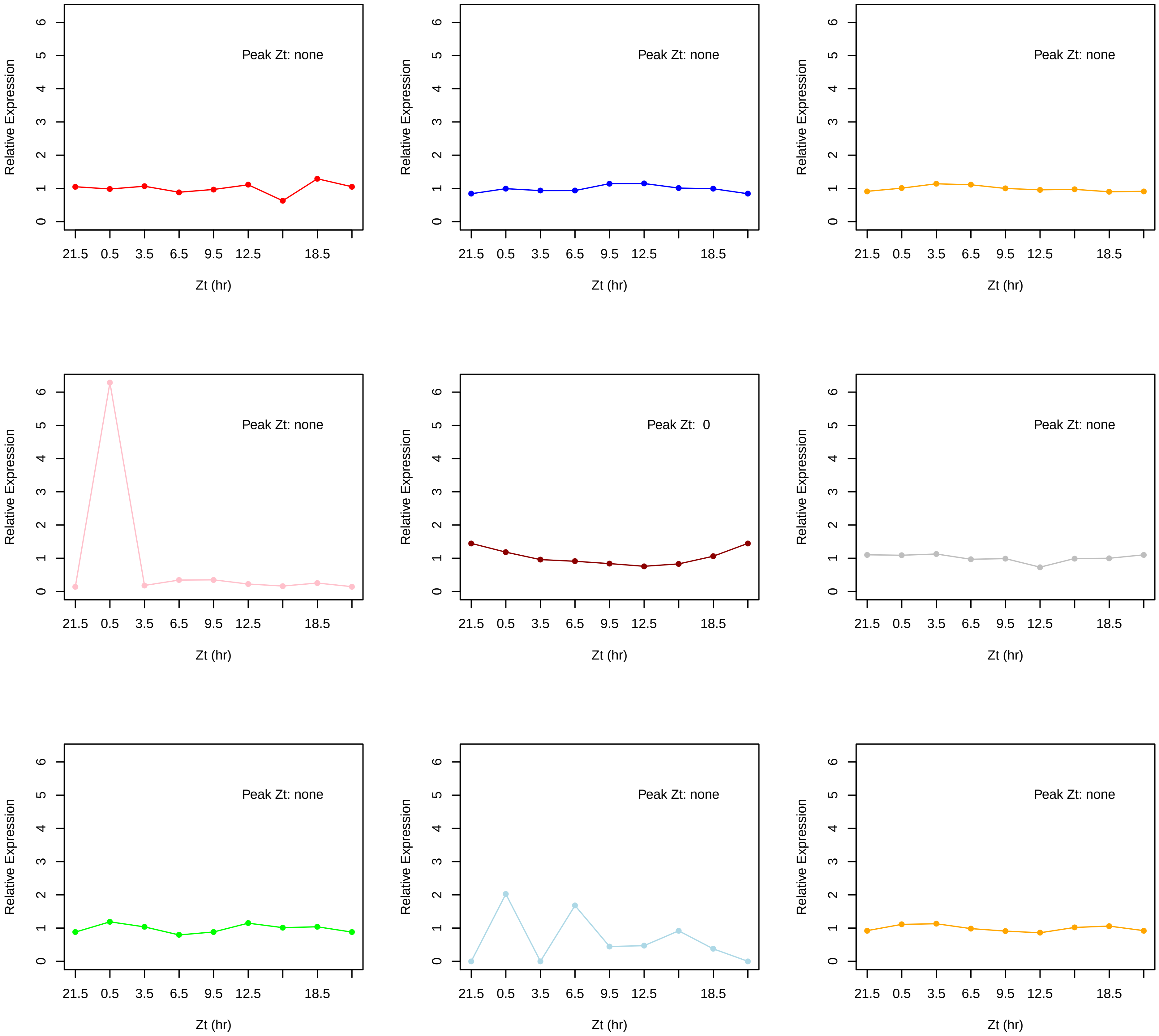

Singeleton: ENSXMAG00000017017 extl3

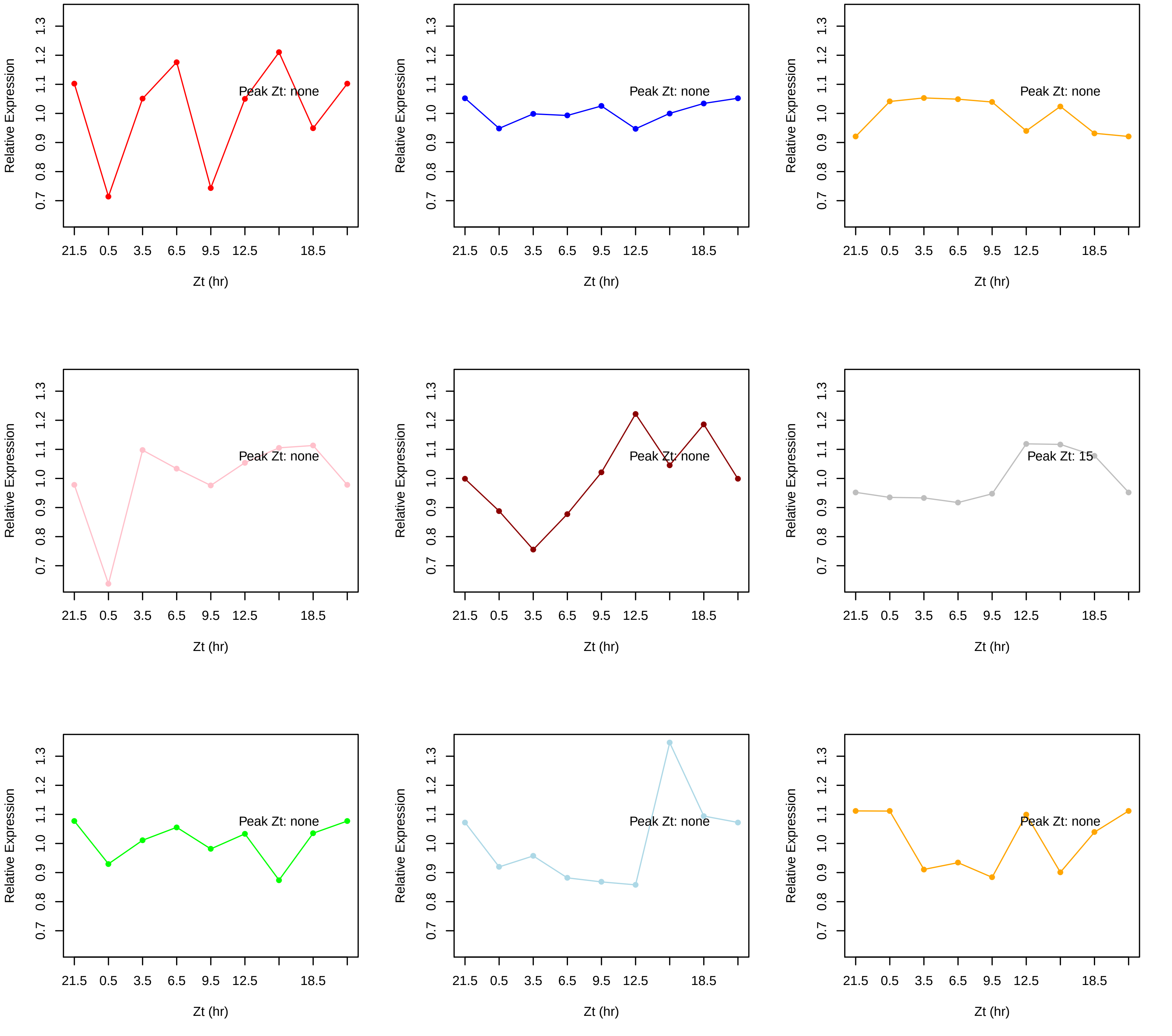

Singeleton: ENSXMAG00000026751 vegfab

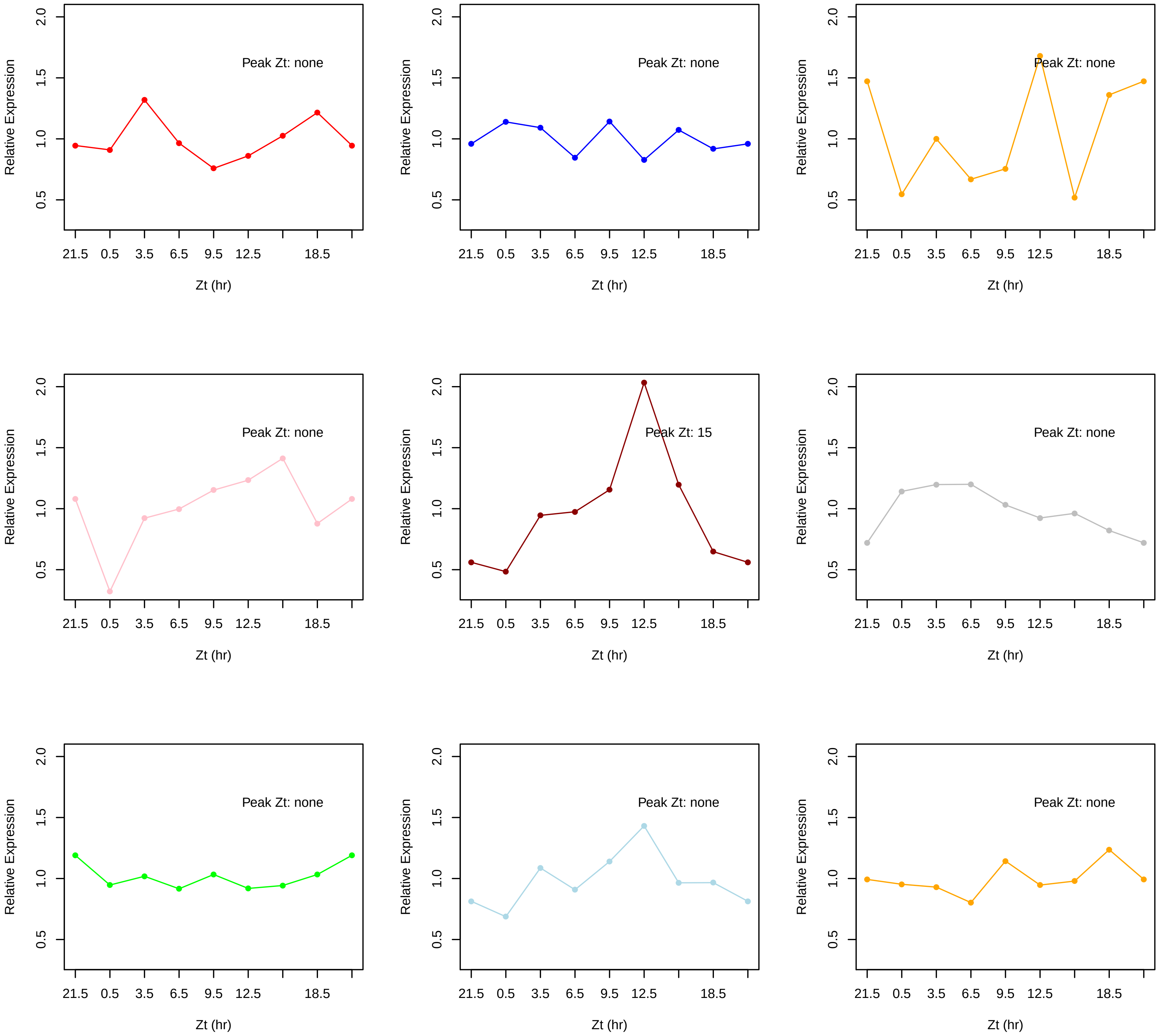

Singeleton: ENSXMAG00000004100 phtf2

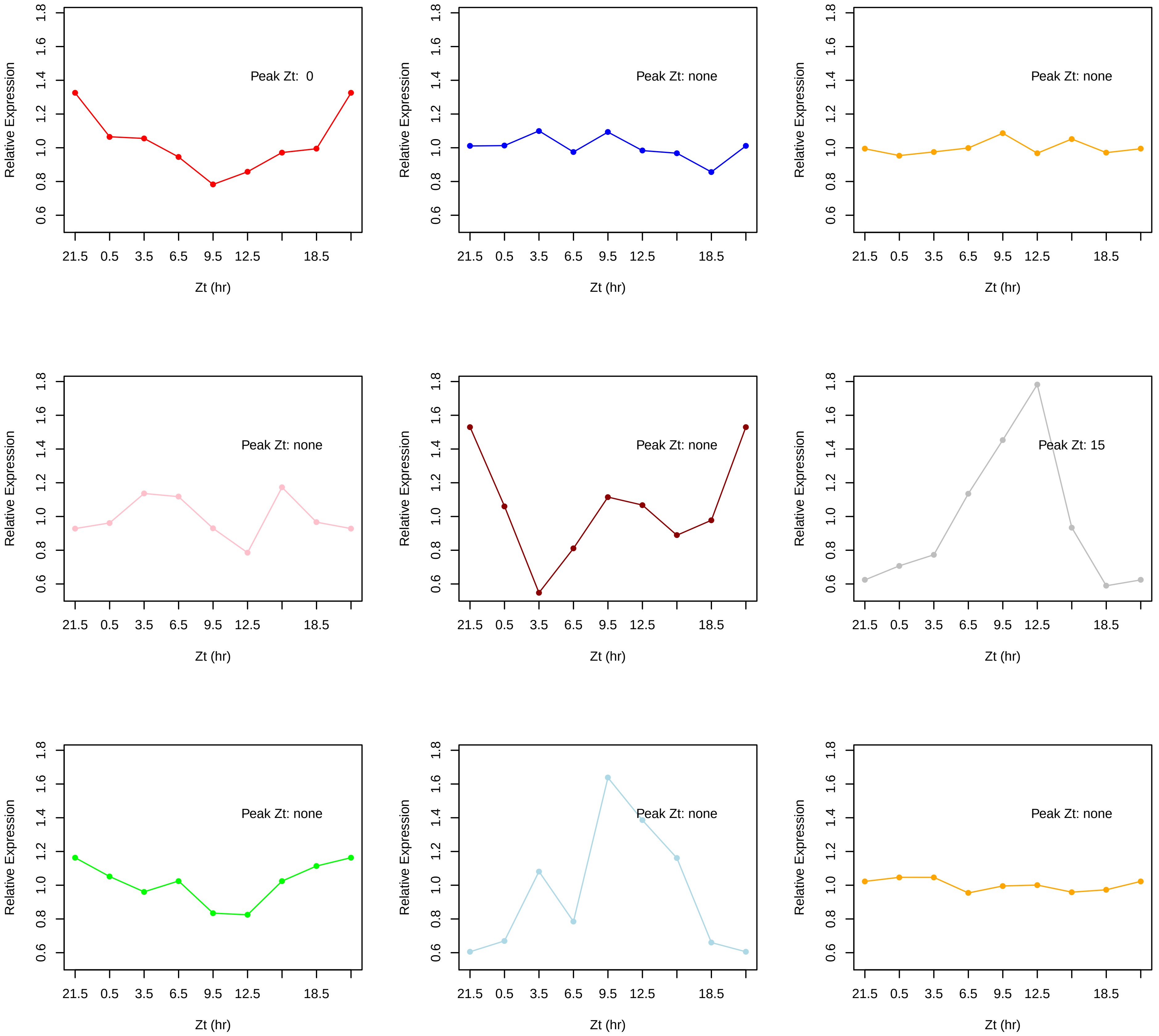

Singeleton: ENSXMAG00000004877 tmem63a

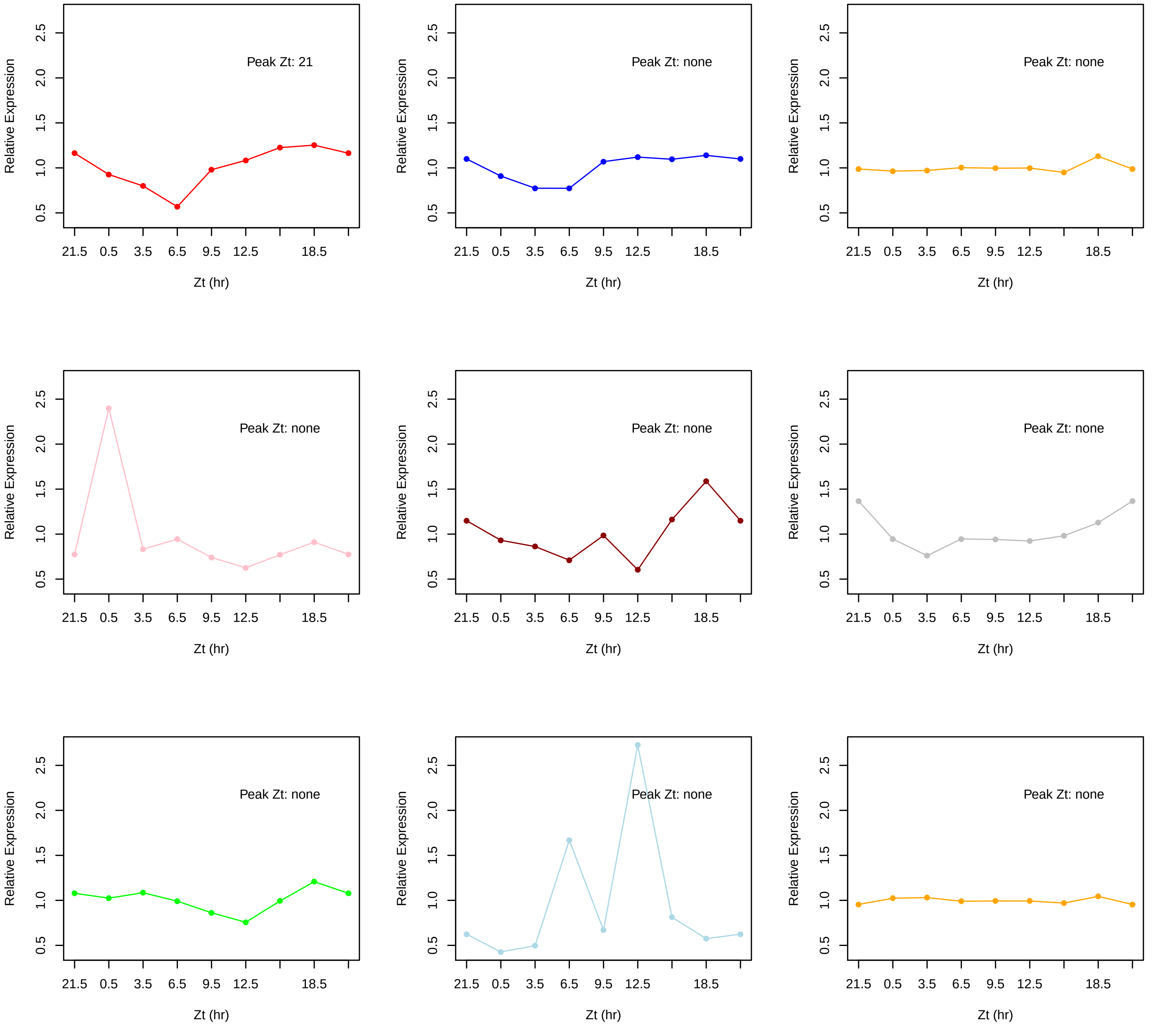

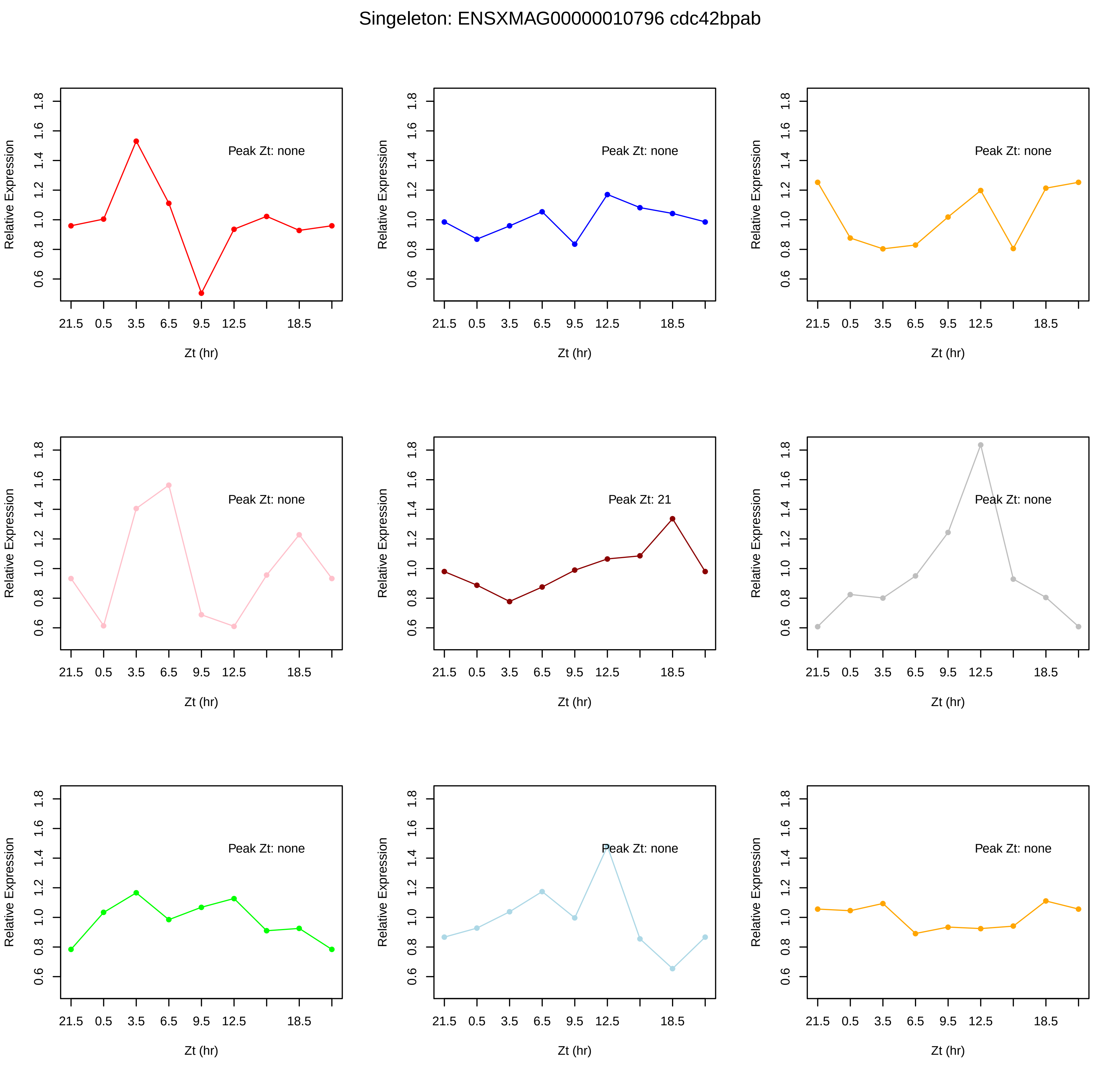
Singeleton: ENSXMAG00000010796 cdc42bpab
Peak Zt: none
Peak Zt: none
Peak Zt: none
Peak Zt: none
Peak Zt: 21
Peak Zt: none
Peak Zt: none
Peak Zt: none
Peak Zt: none
Relative Expression
Zt (hr)
21.5
0.5
3.5
6.5
9.5
12.5
18.5
0.6
0.8
1.0
1.2
1.4
1.6
1.8

Singleton: ENSXMAG00000004792 sema3c

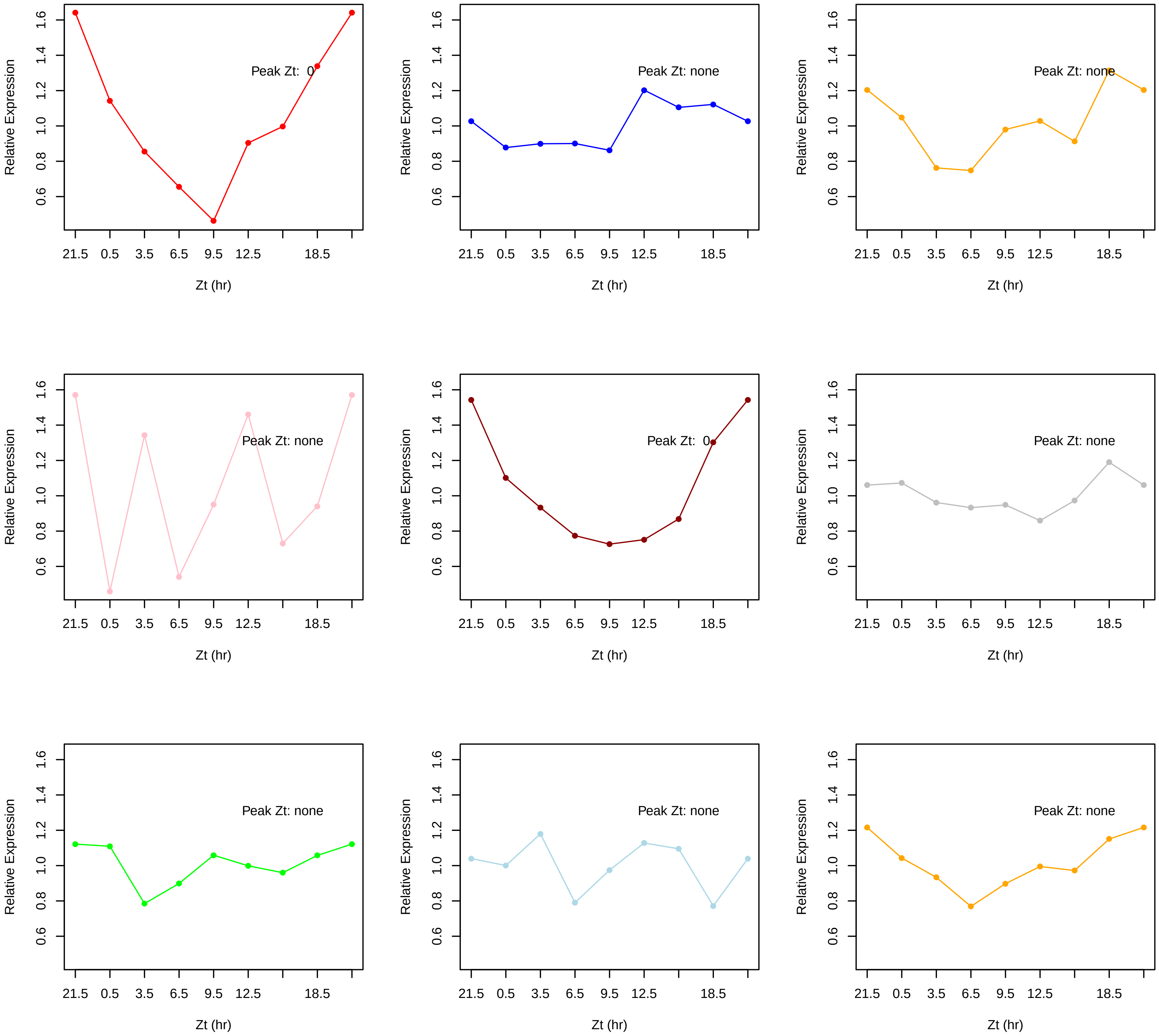

Singeleton: ENSXMAG00000022273 c1d

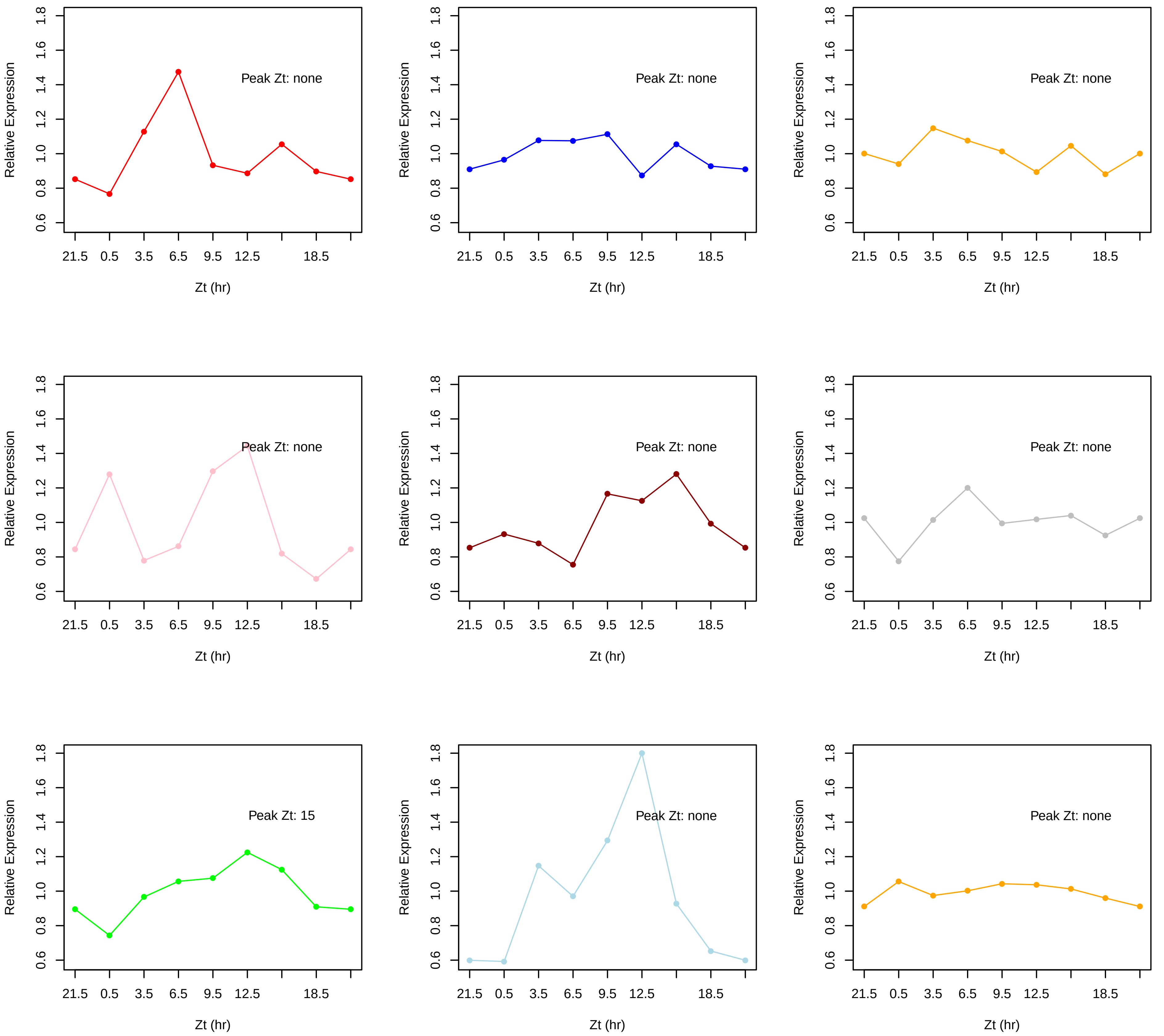

Singleton: ENSXMAG00000027364

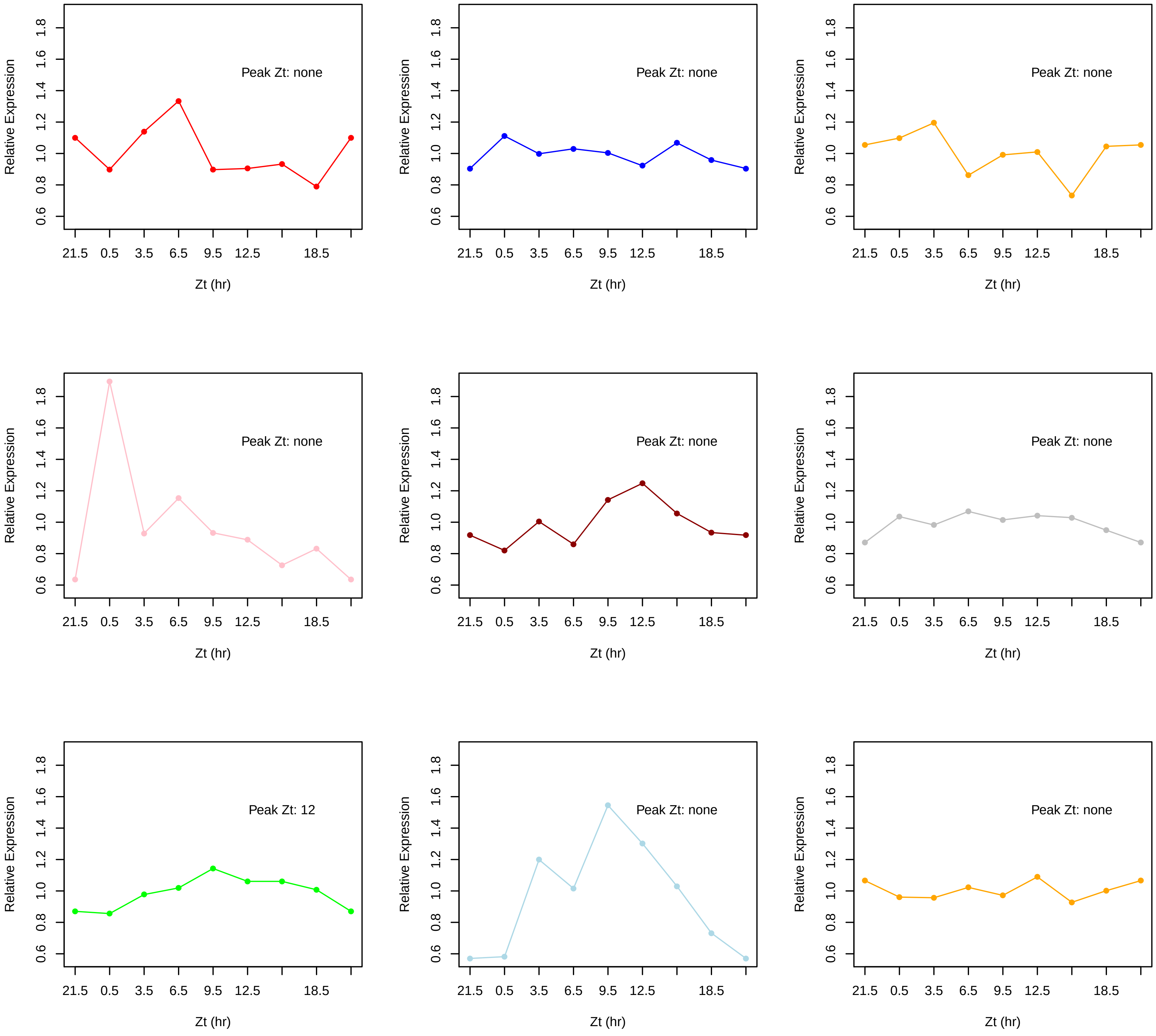

Singeleton: ENSXMAG00000011739 meis1b

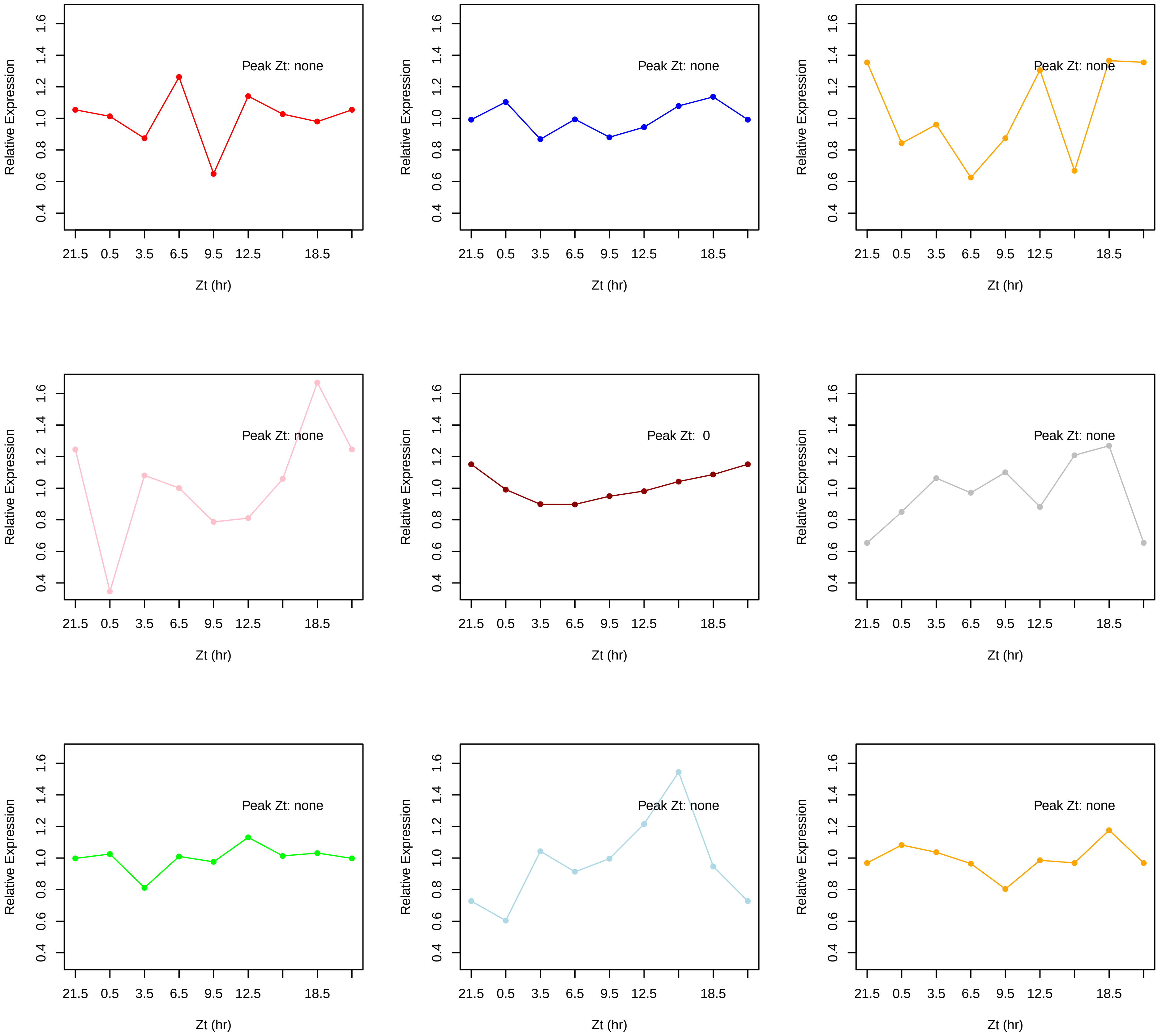

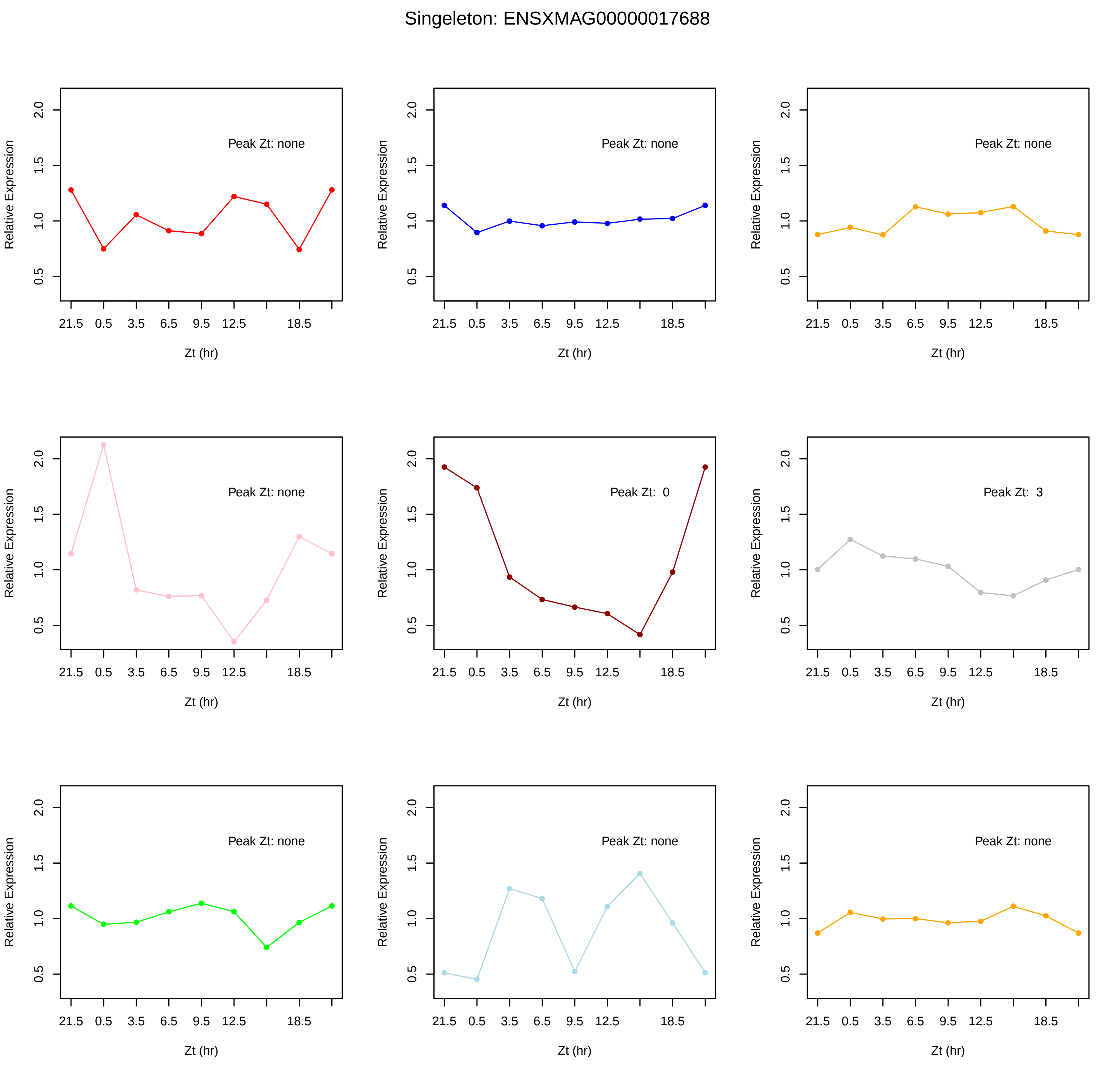
Singeleton: ENSXMAG00000017688
Peak Zt: none
Peak Zt: none
Peak Zt: none
Peak Zt: none
Peak Zt: 0
Peak Zt: 3
Peak Zt: none
Peak Zt: none
Peak Zt: none
Relative Expression
Zt (hr)
21.5
0.5
3.5
6.5
9.5
12.5
18.5
0.5
1.0
1.5
2.0

Singeleton: ENSXMAG00000021554

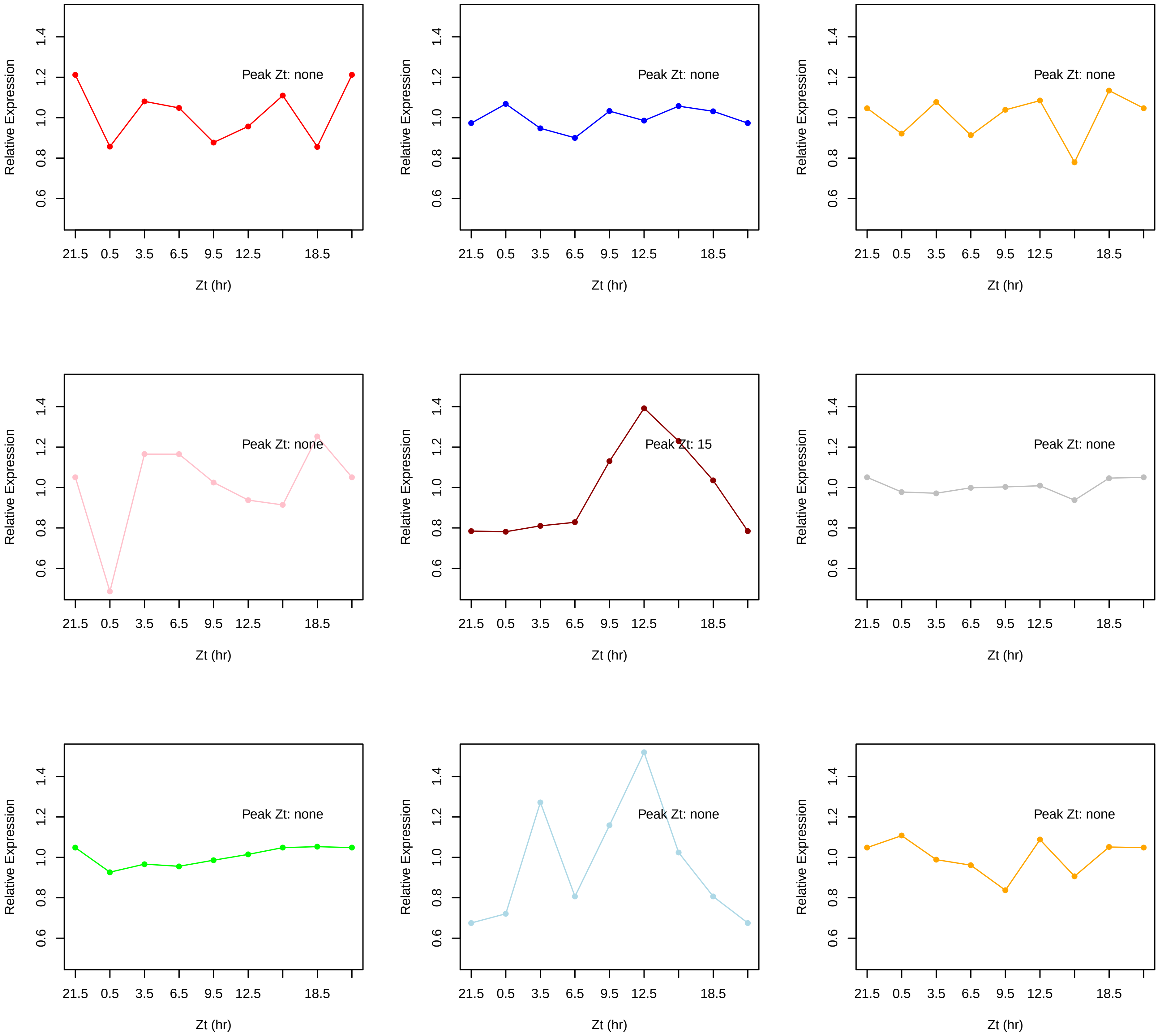

Singeleton: ENSXMAG00000017642 kctd3

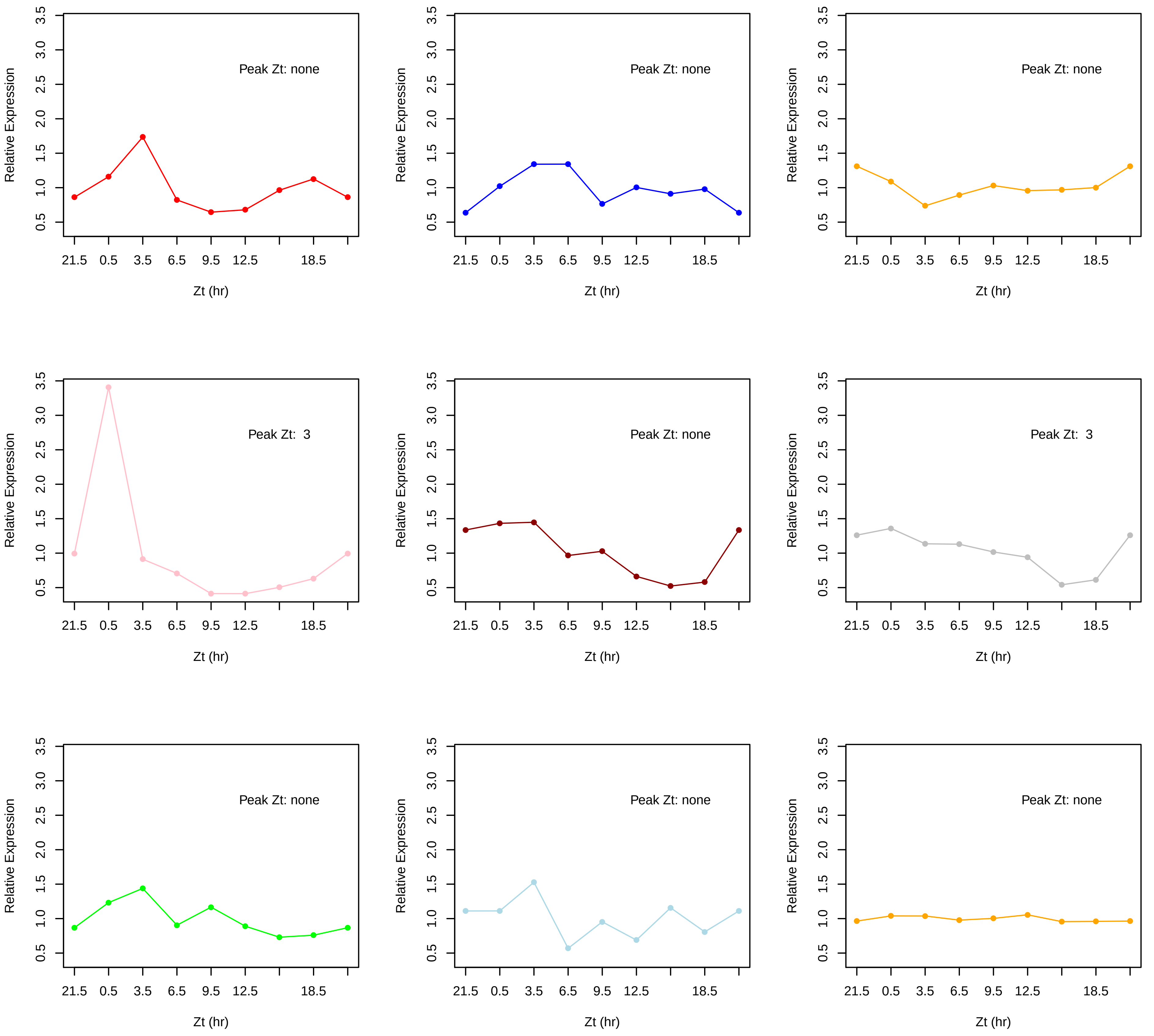

Singeleton: ENSXMAG00000004577 pim3

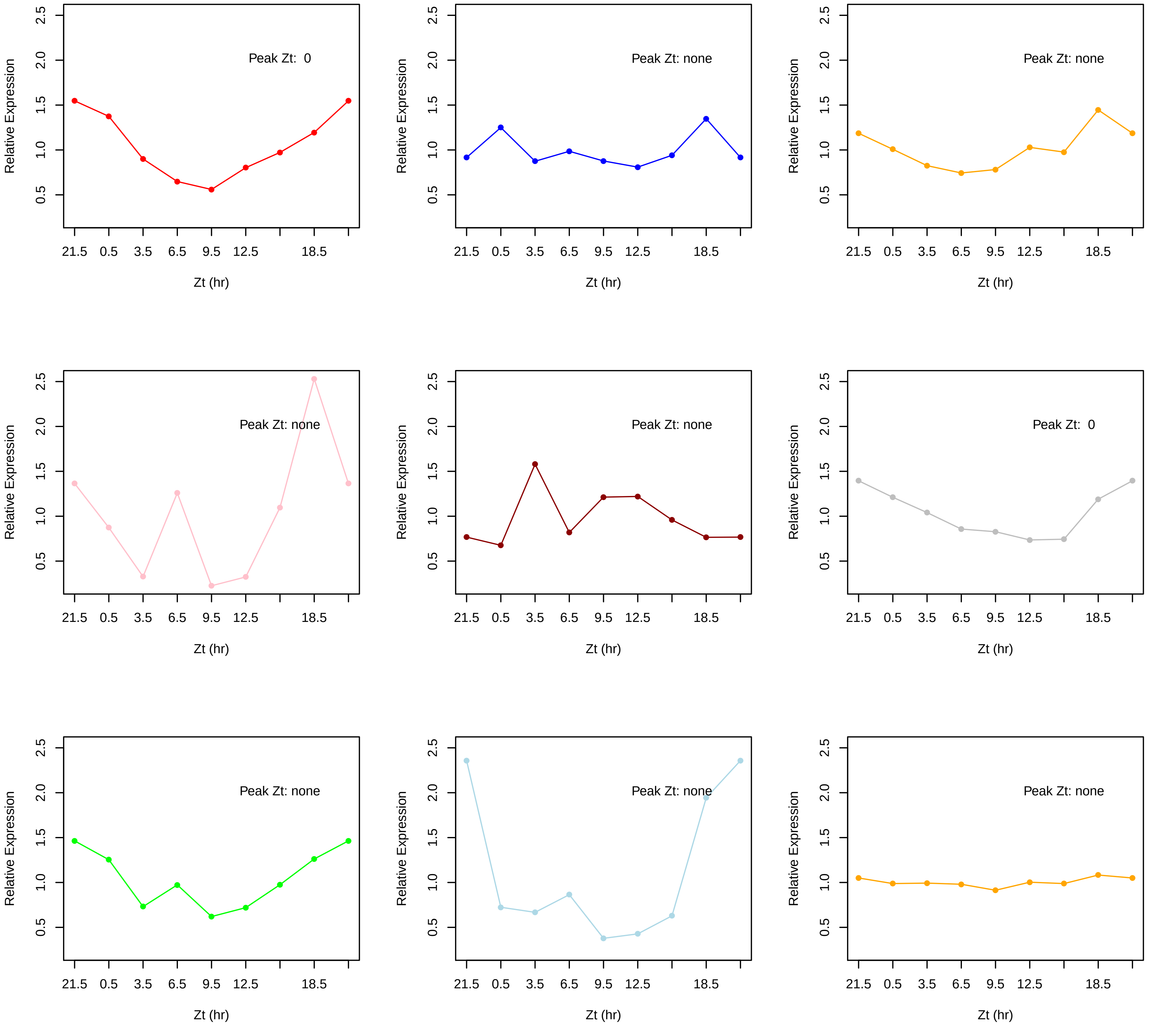

Singleton: ENSXMAG00000004593 creld2

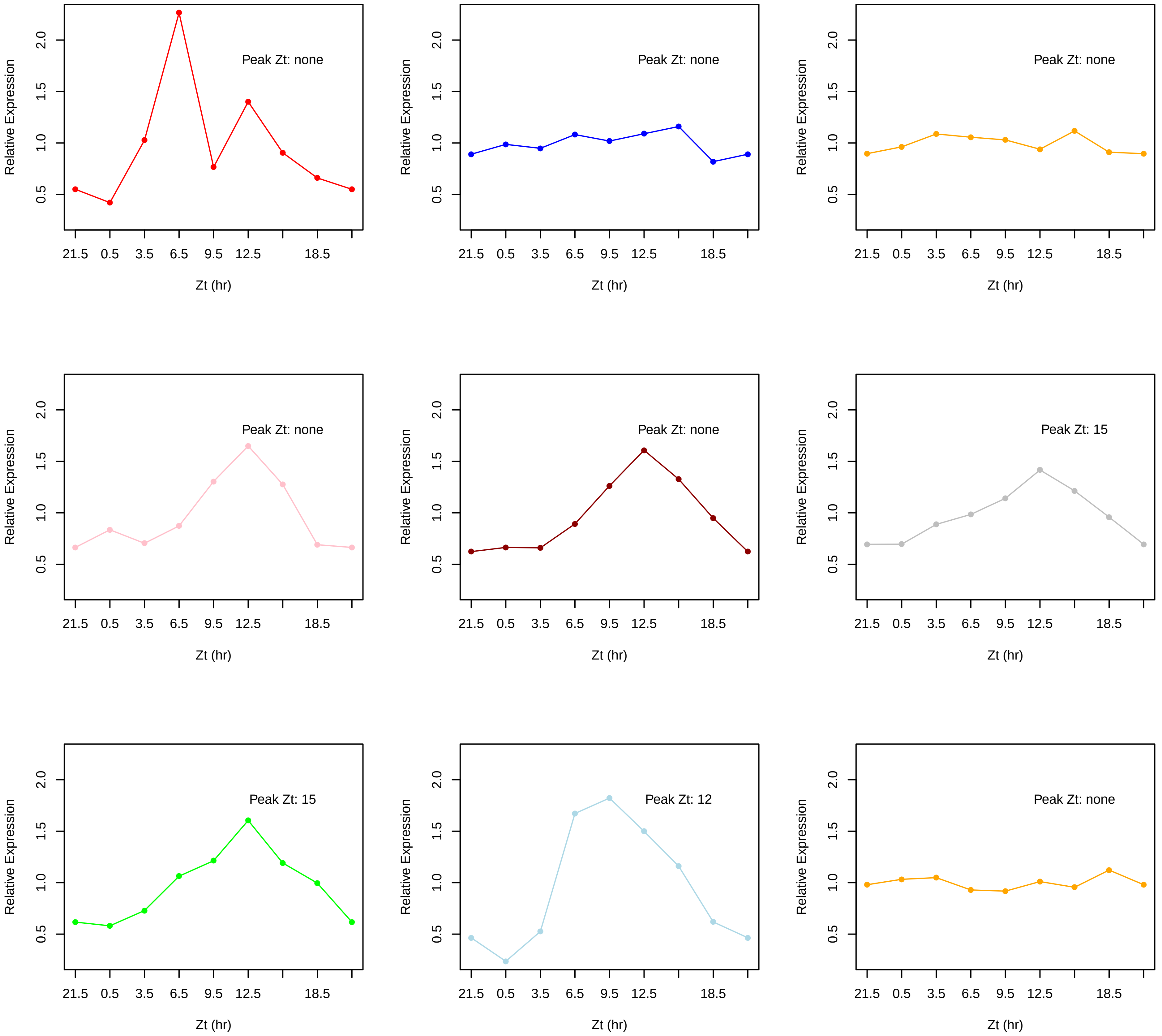

Singleton: ENSXMAG00000017603 ush2a

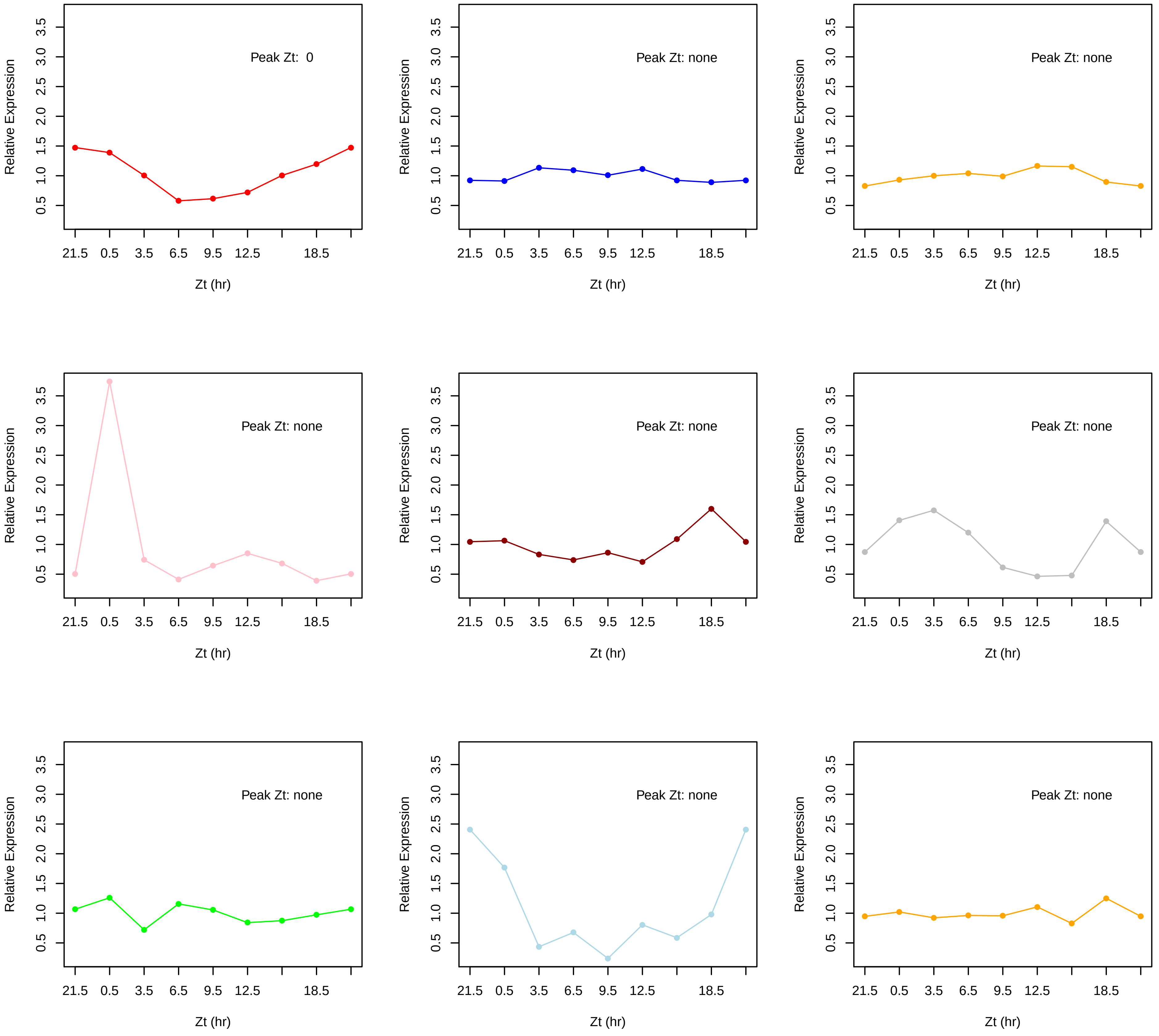

Singeleton: ENSXMAG00000004610 alg12

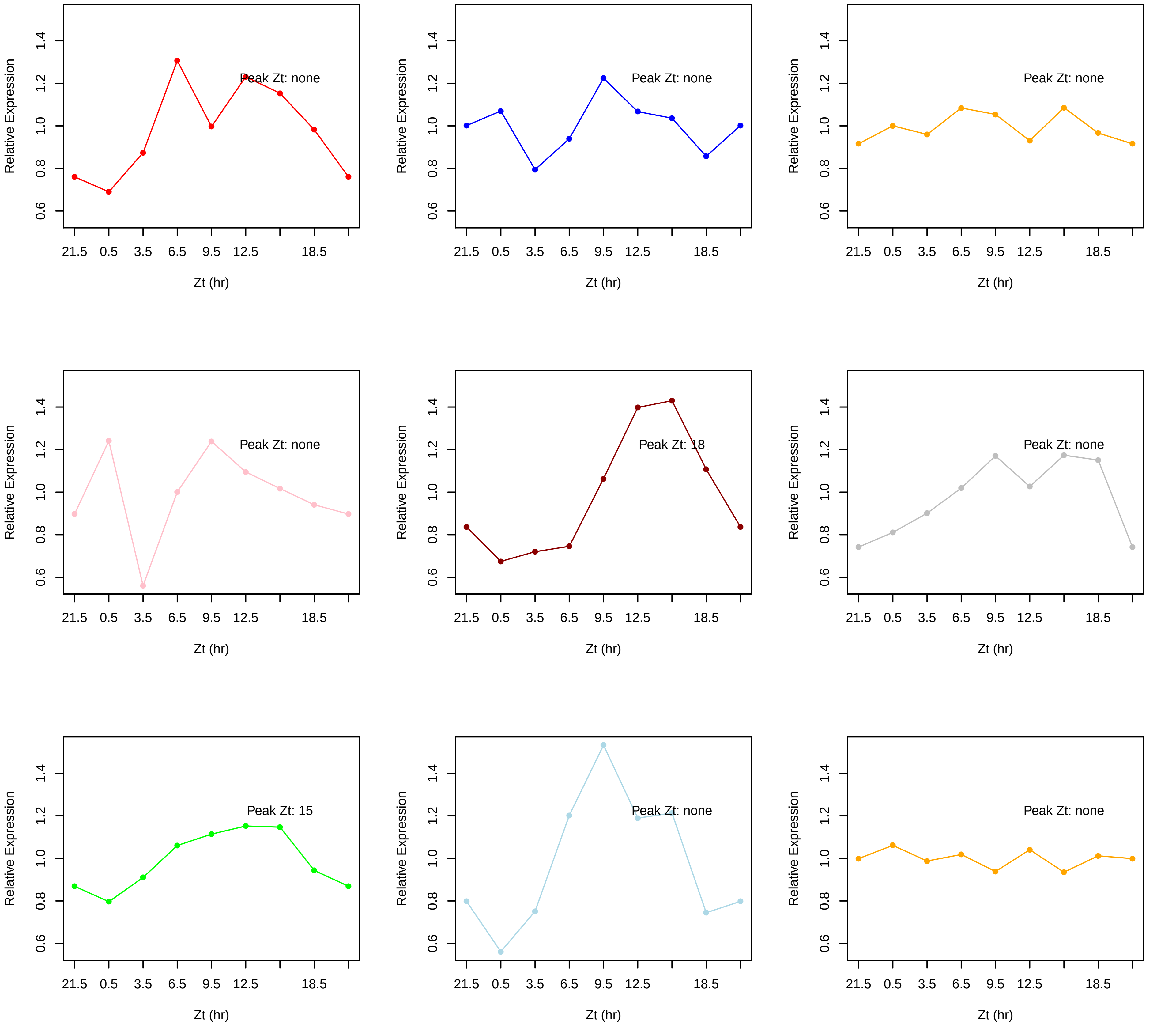

Singeleton: ENSXMAG00000027229

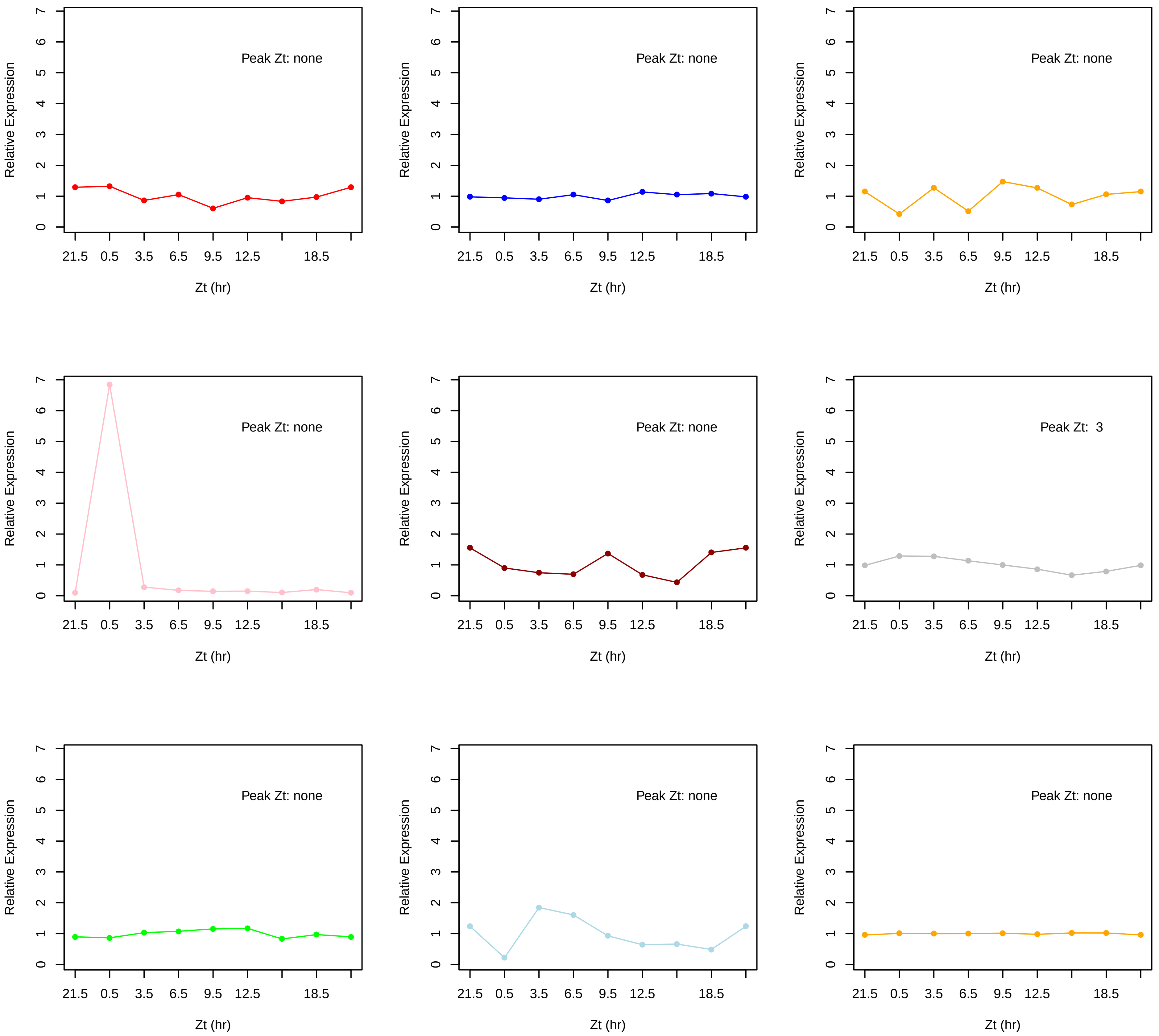

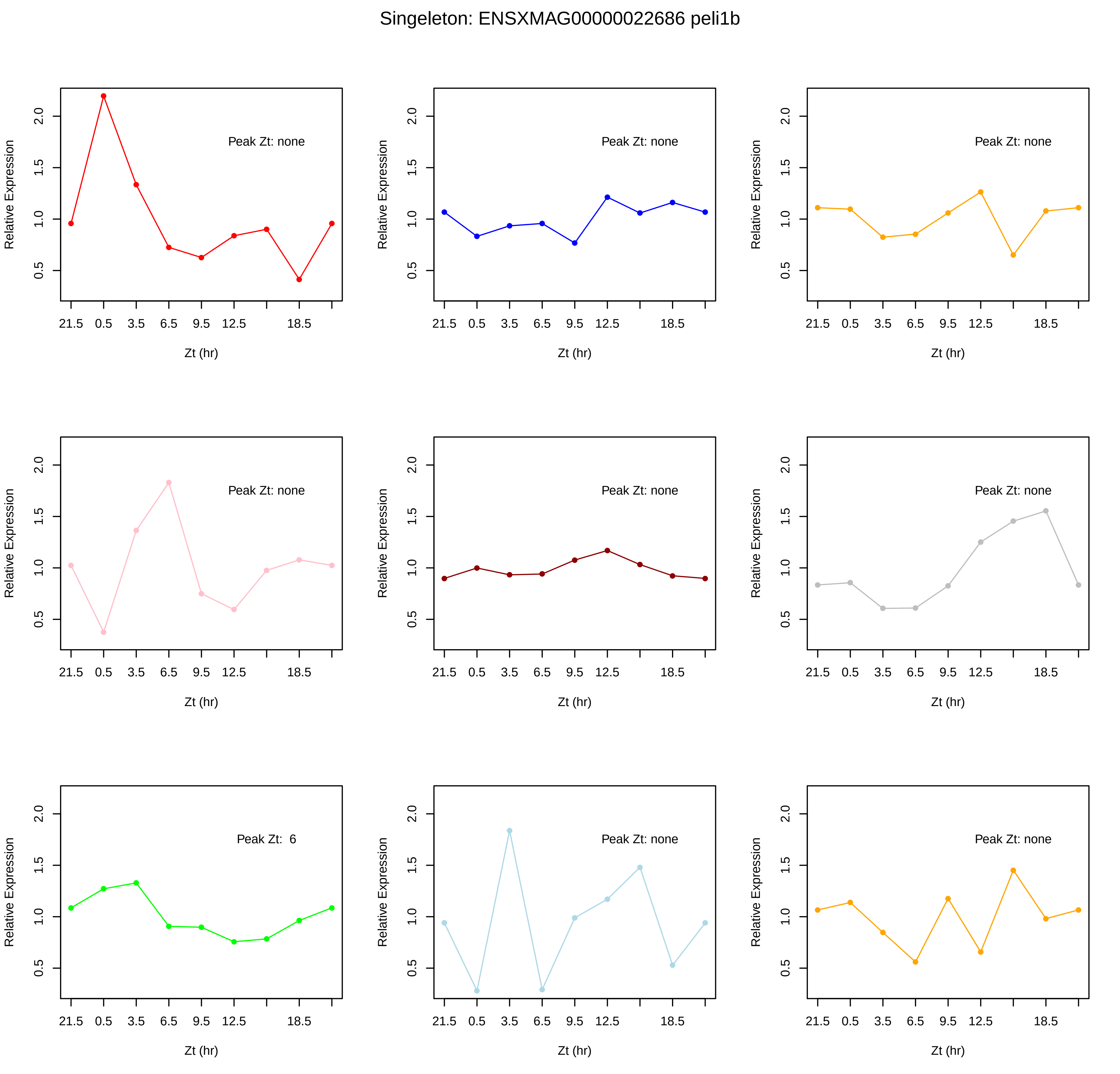
Singeleton: ENSXMAG00000022686 peli1b
Peak Zt: none
Peak Zt: none
Peak Zt: none
Peak Zt: none
Peak Zt: none
Peak Zt: none
Peak Zt: 6
Peak Zt: none
Peak Zt: none
Relative Expression
Zt (hr)
21.5
0.5
3.5
6.5
9.5
12.5
18.5
0.5
1.0
1.5
2.0

Singeleton: ENSXMAG00000017600 esrrga

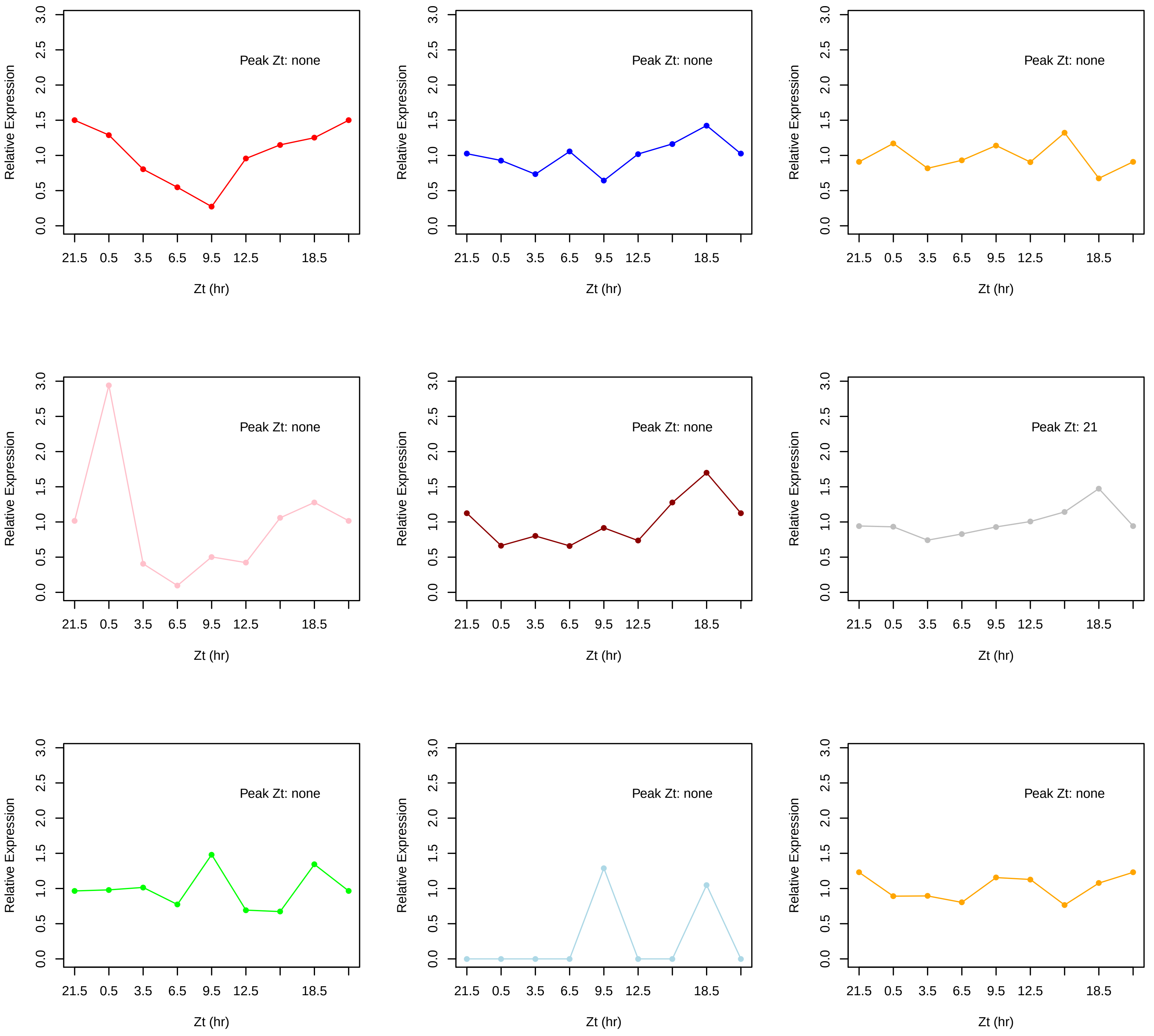

Singeleton: ENSXMAG00000004679 si:ch73−21k16.4

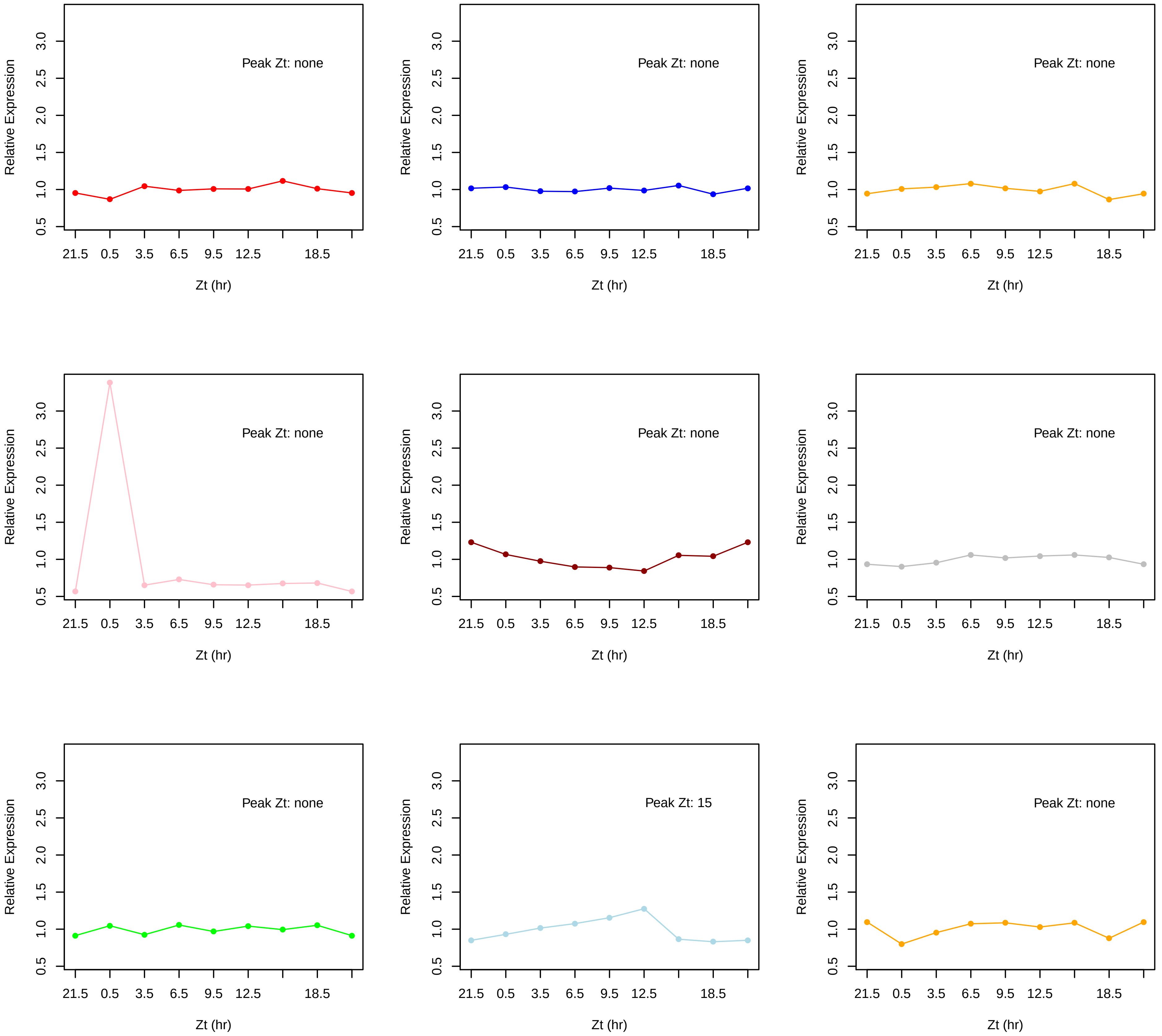

Singeleton: ENSXMAG00000006721 celsr1a

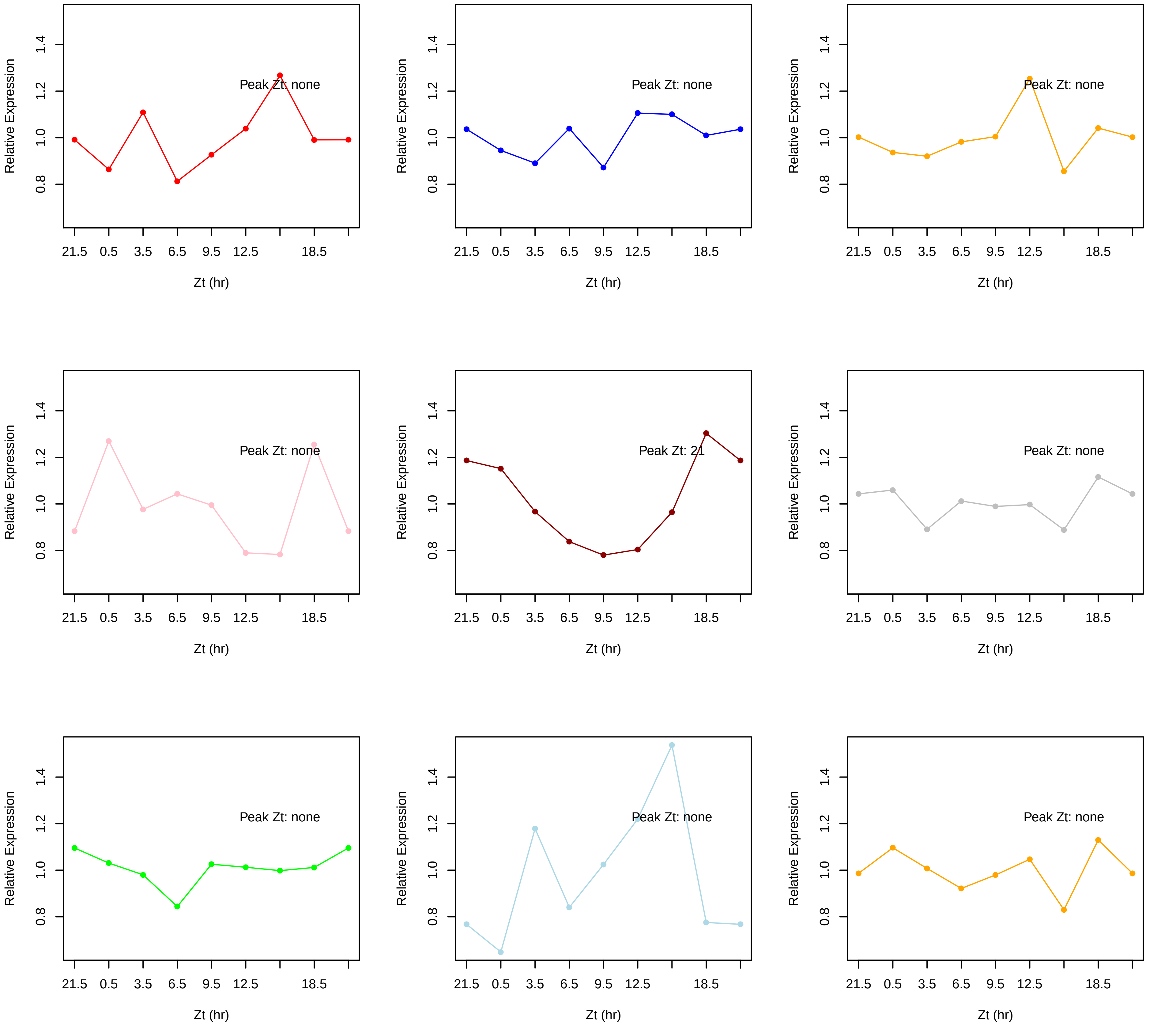

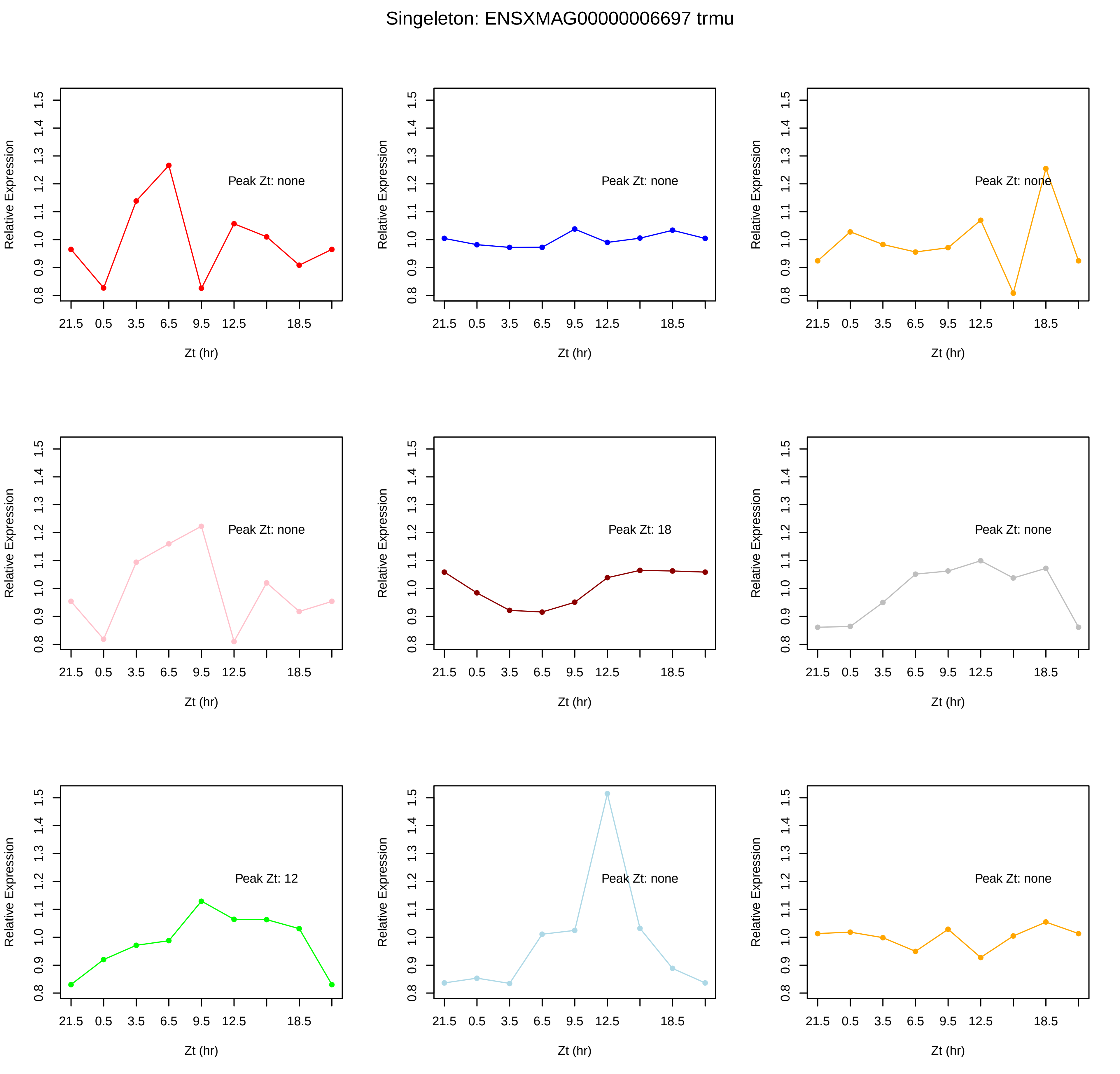
Singeleton: ENSXMAG00000006697 trmu
Peak Zt: none
Peak Zt: none
Peak Zt: none
Peak Zt: none
Peak Zt: 18
Peak Zt: none
Peak Zt: 12
Peak Zt: none
Peak Zt: none
Relative Expression
Zt (hr)

Singleton: ENSXMAG00000011708 gtse1

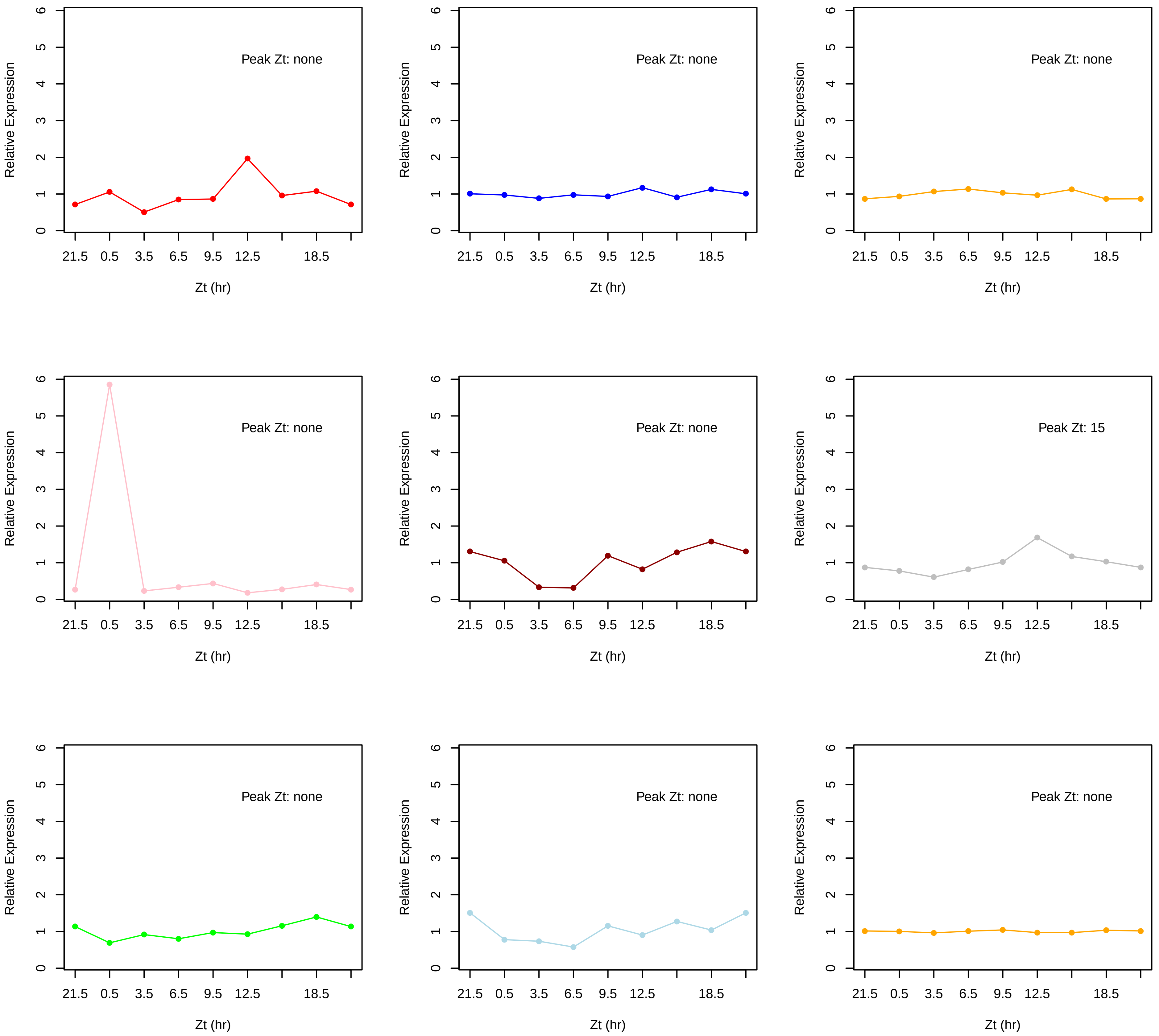

Singeleton: ENSXMAG00000005604 otx1

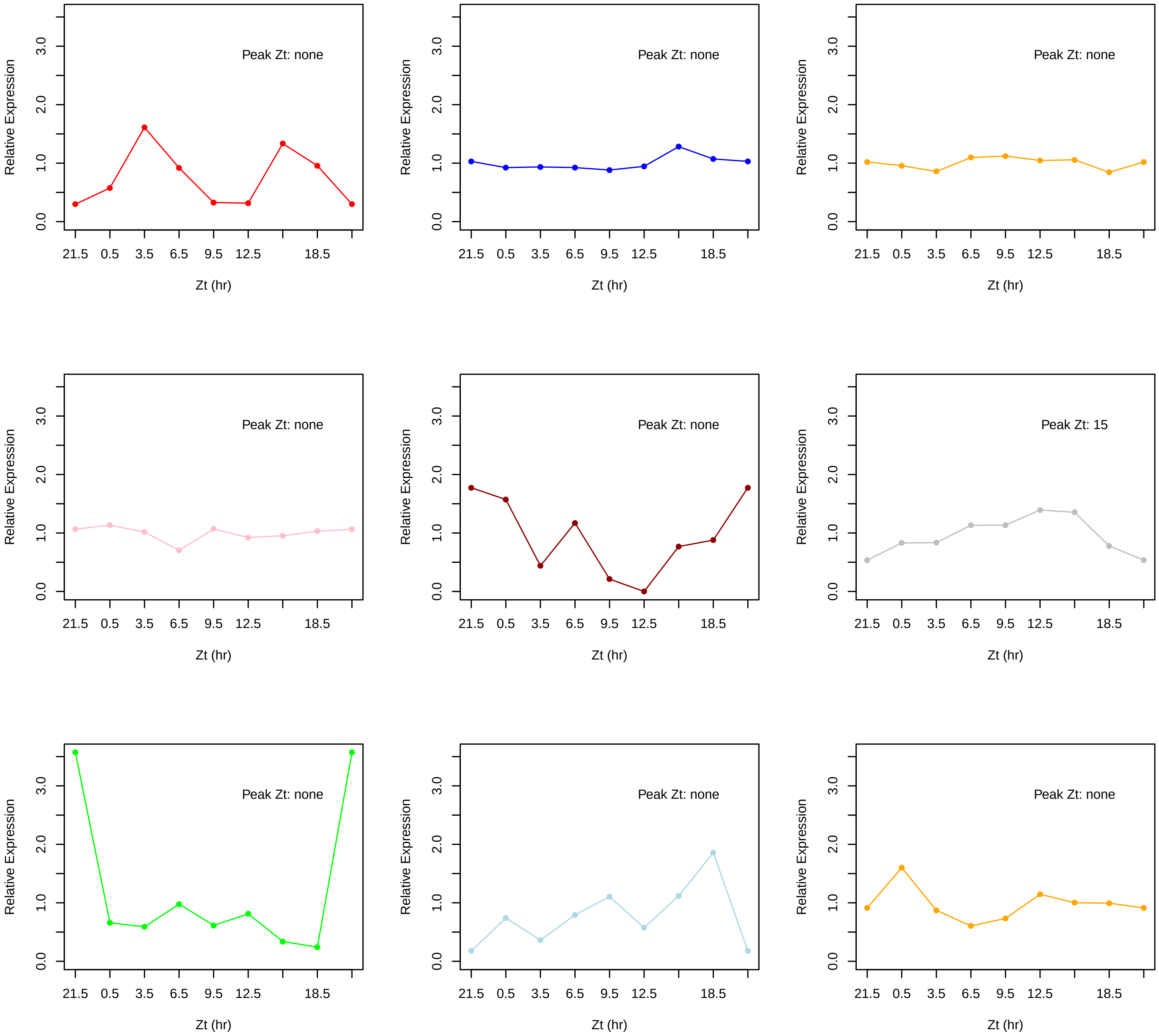

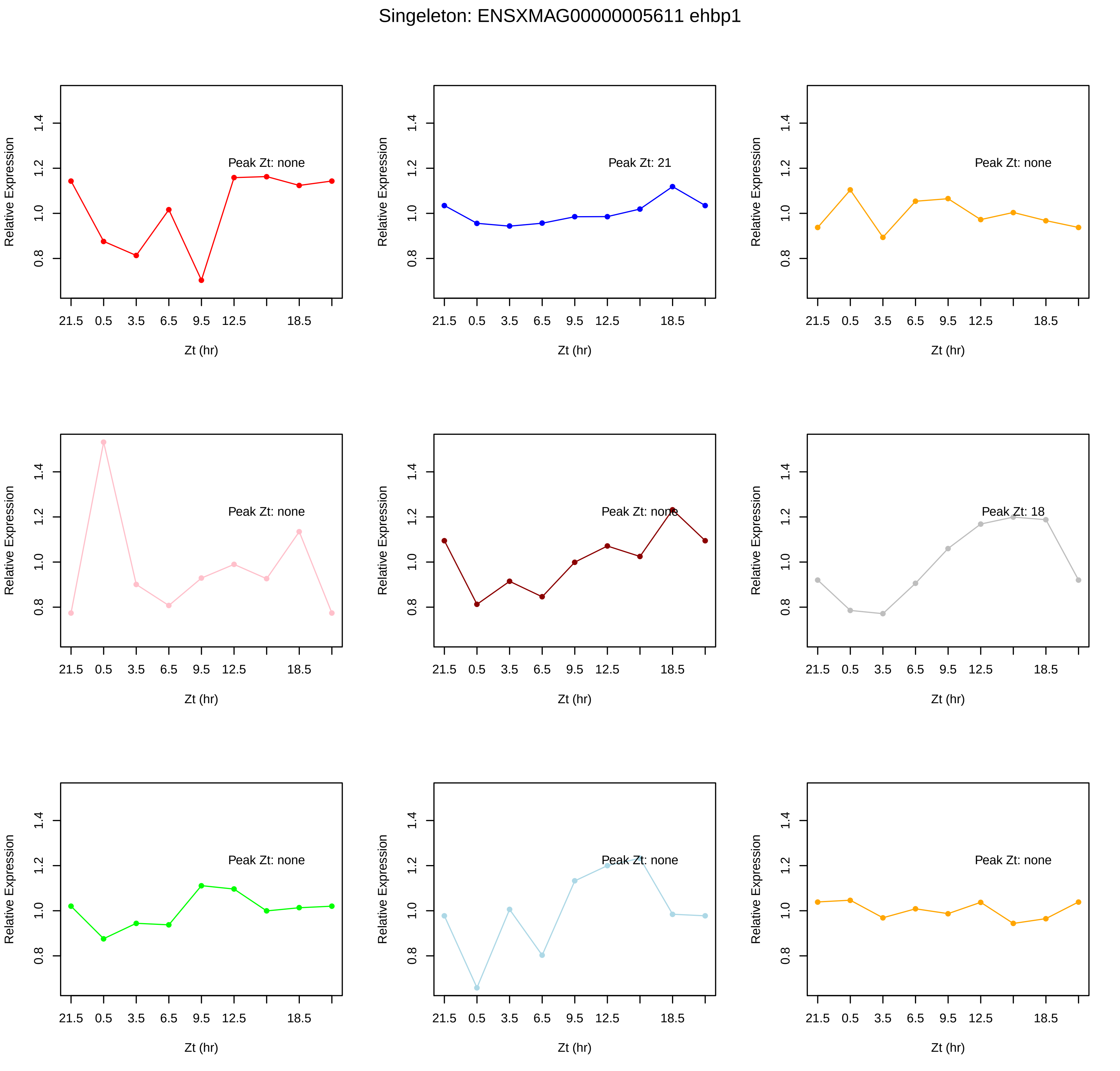
Singleton: ENSXMAG00000005611 ehbp1
Peak Zt: none
Peak Zt: 21
Peak Zt: none
Peak Zt: none
Peak Zt: none
Peak Zt: 18
Peak Zt: none
Peak Zt: none
Peak Zt: none
Relative Expression
Zt (hr)
21.5
0.5
3.5
6.5
9.5
12.5
18.5
0.8
1.0
1.2
1.4

Singeleton: ENSXMAG00000001872 polr3d

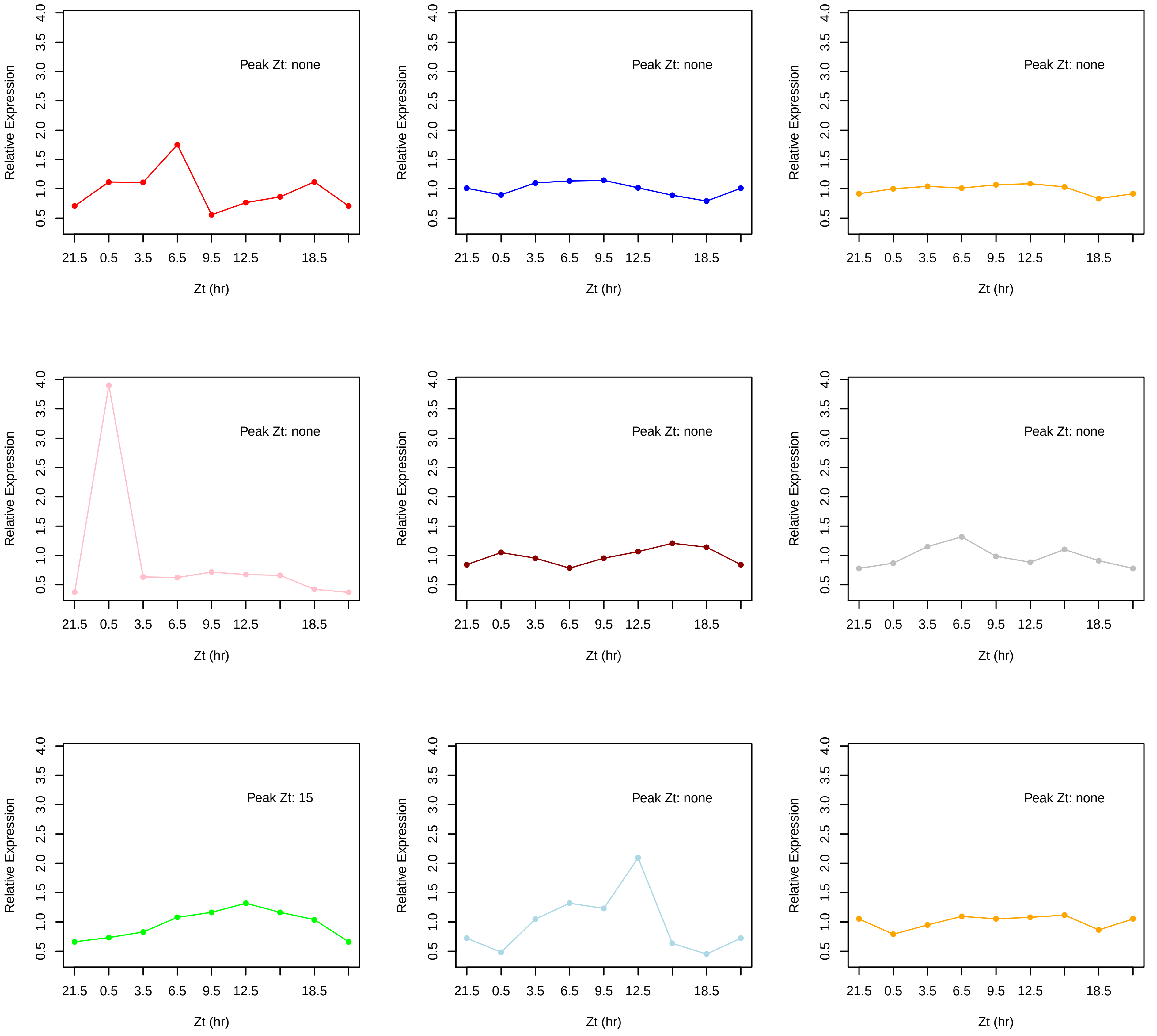

Singeleton: ENSXMAG00000001878 zgc:77112

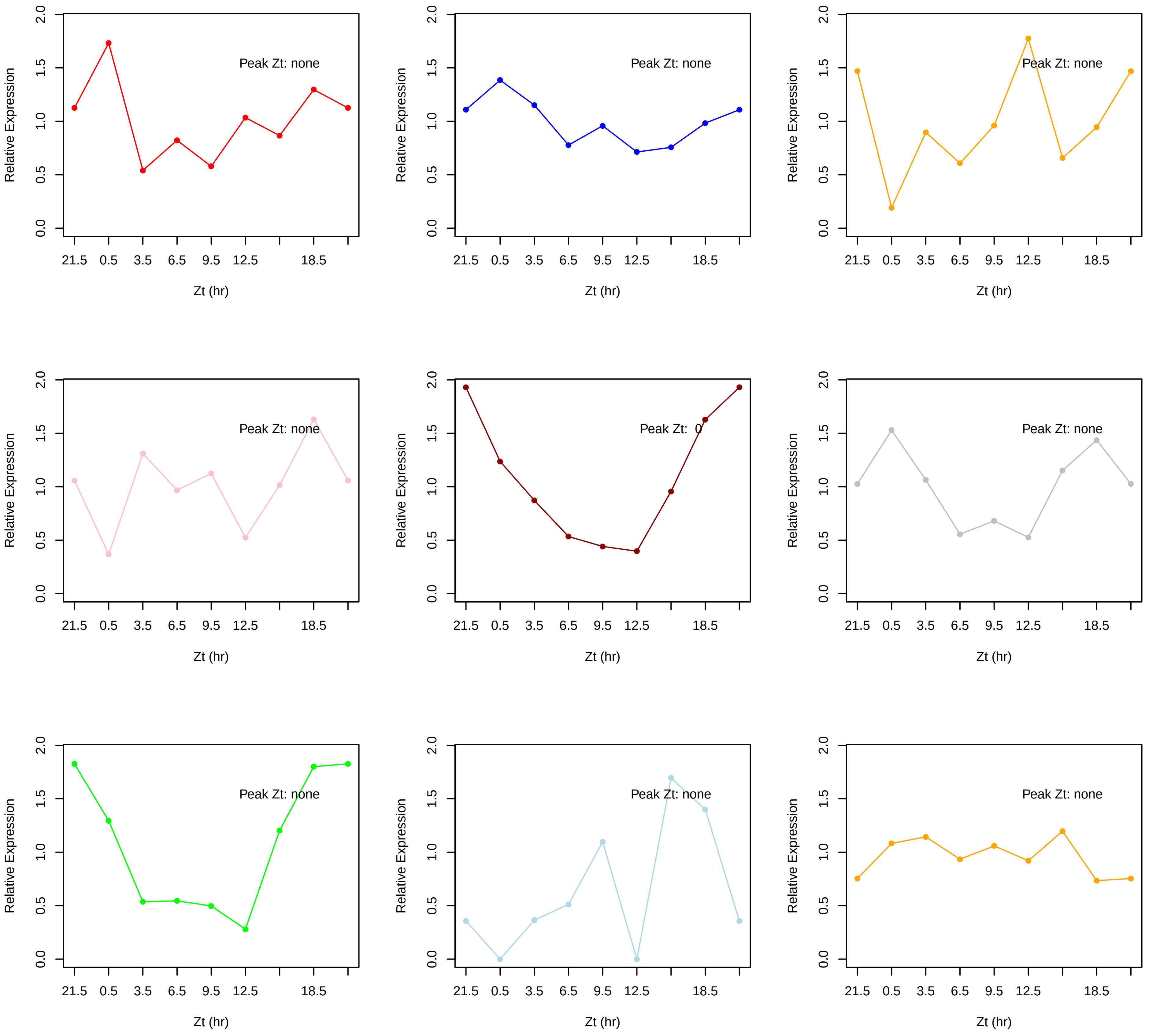

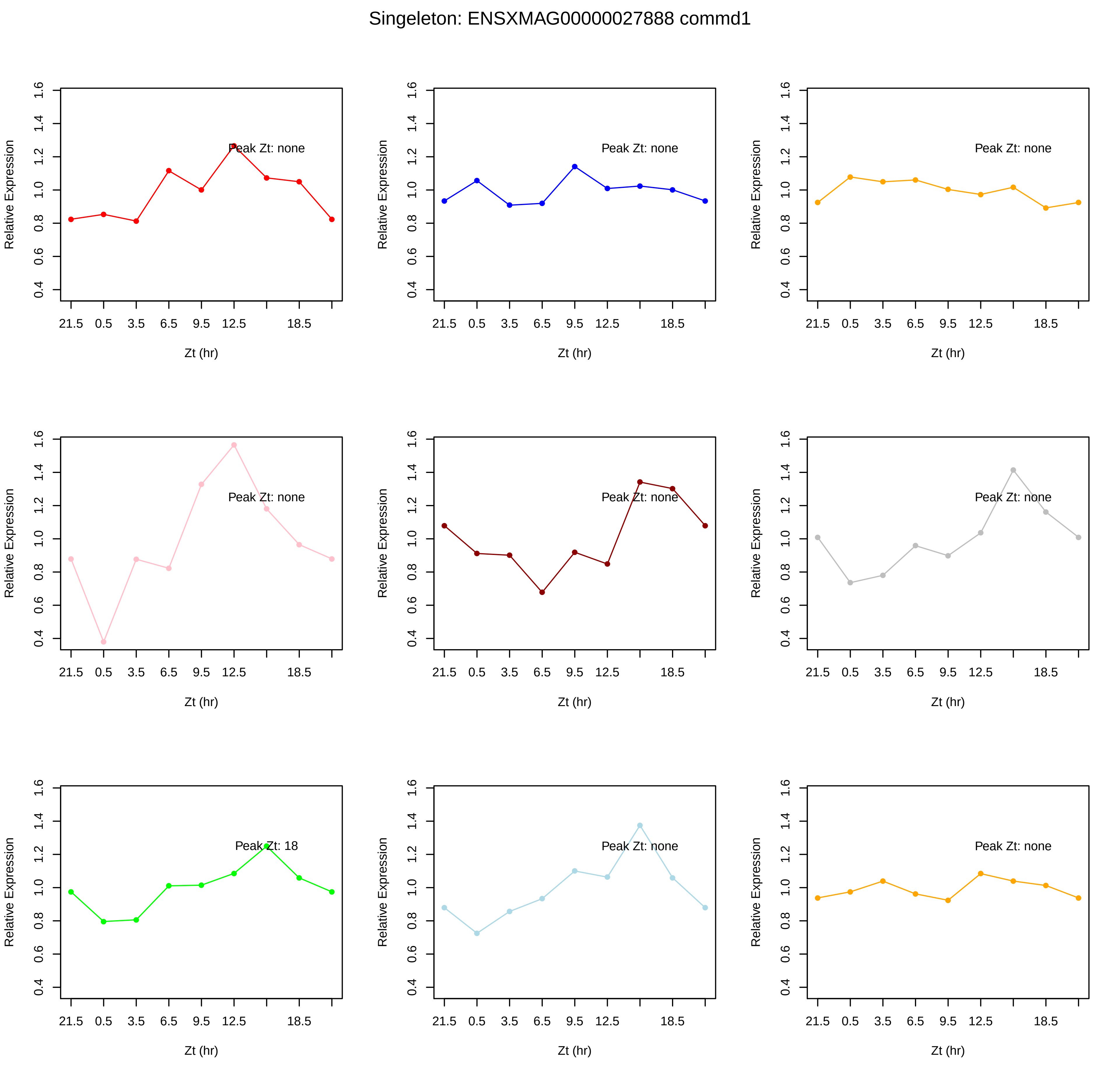

Singeleton: ENSXMAG00000027888 commd1
Peak Zt: none
Peak Zt: none
Peak Zt: none
Peak Zt: none
Peak Zt: none
Peak Zt: none
Peak Zt: 18
Peak Zt: none
Peak Zt: none
Relative Expression
Zt (hr)
21.5
0.5
3.5
6.5
9.5
12.5
18.5

Singeleton: ENSXMAG00000002046 es1

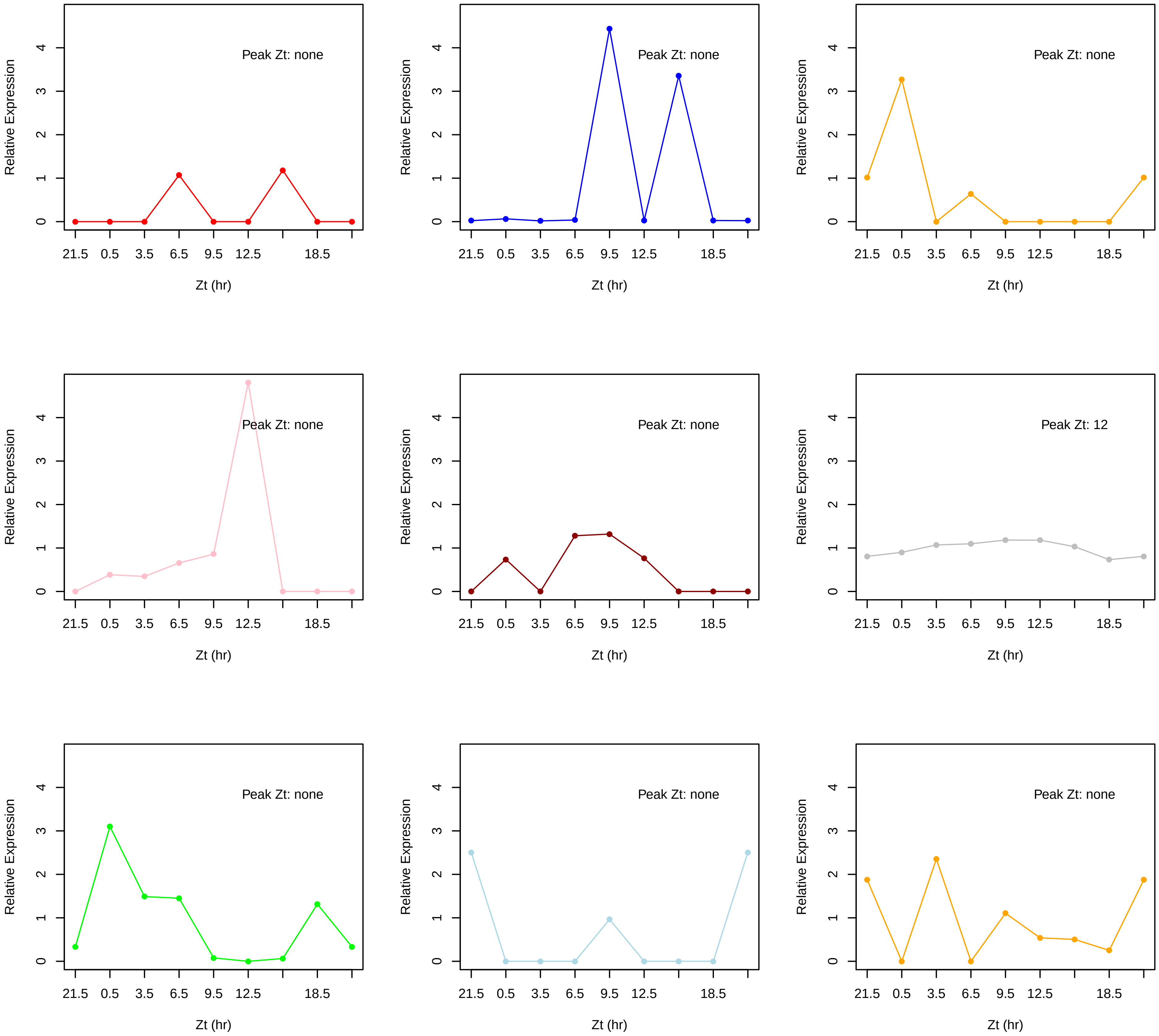

Singeleton: ENSXMAG00000005787 fam161a

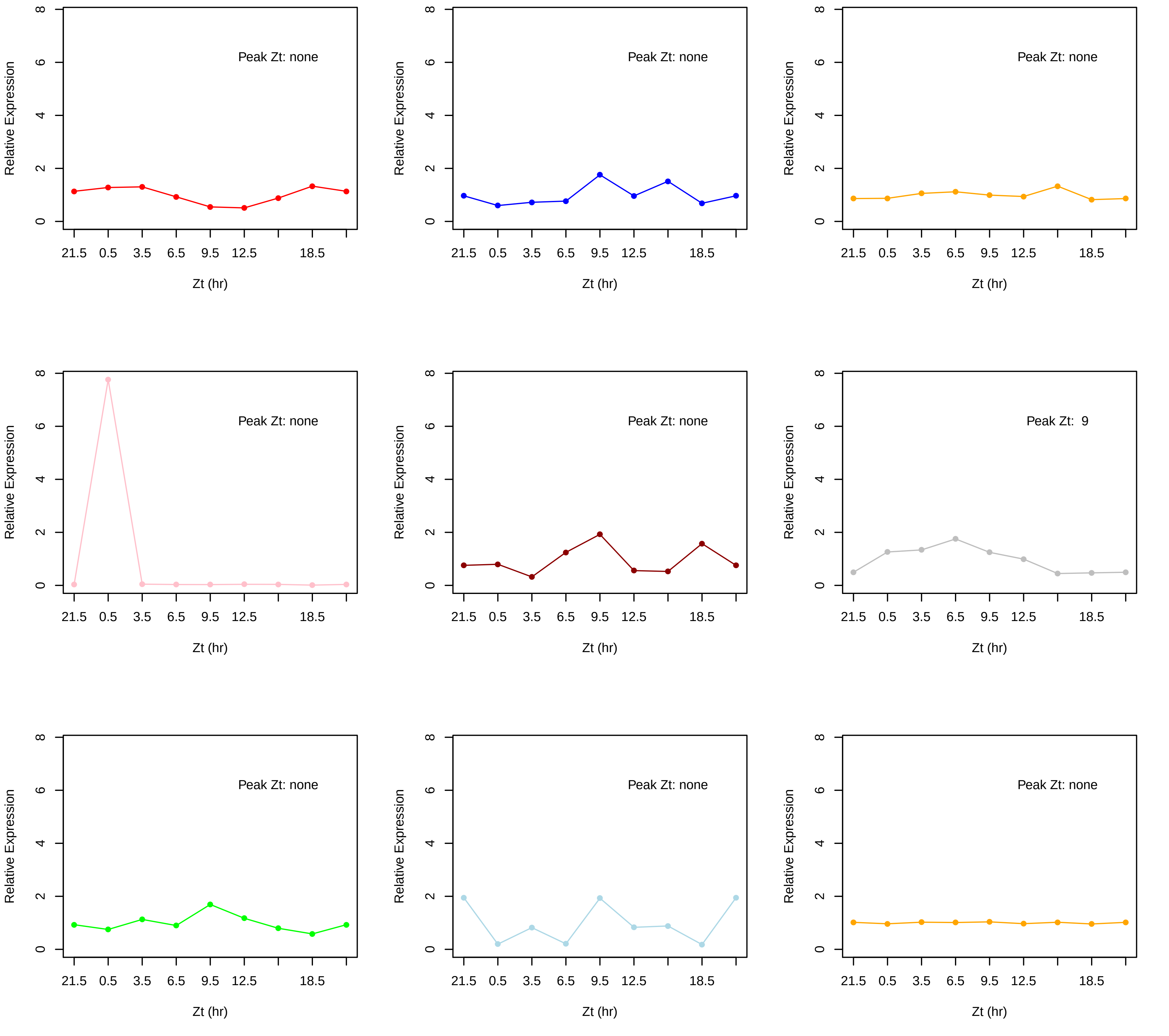

Singeleton: ENSXMAG00000005819 USP34

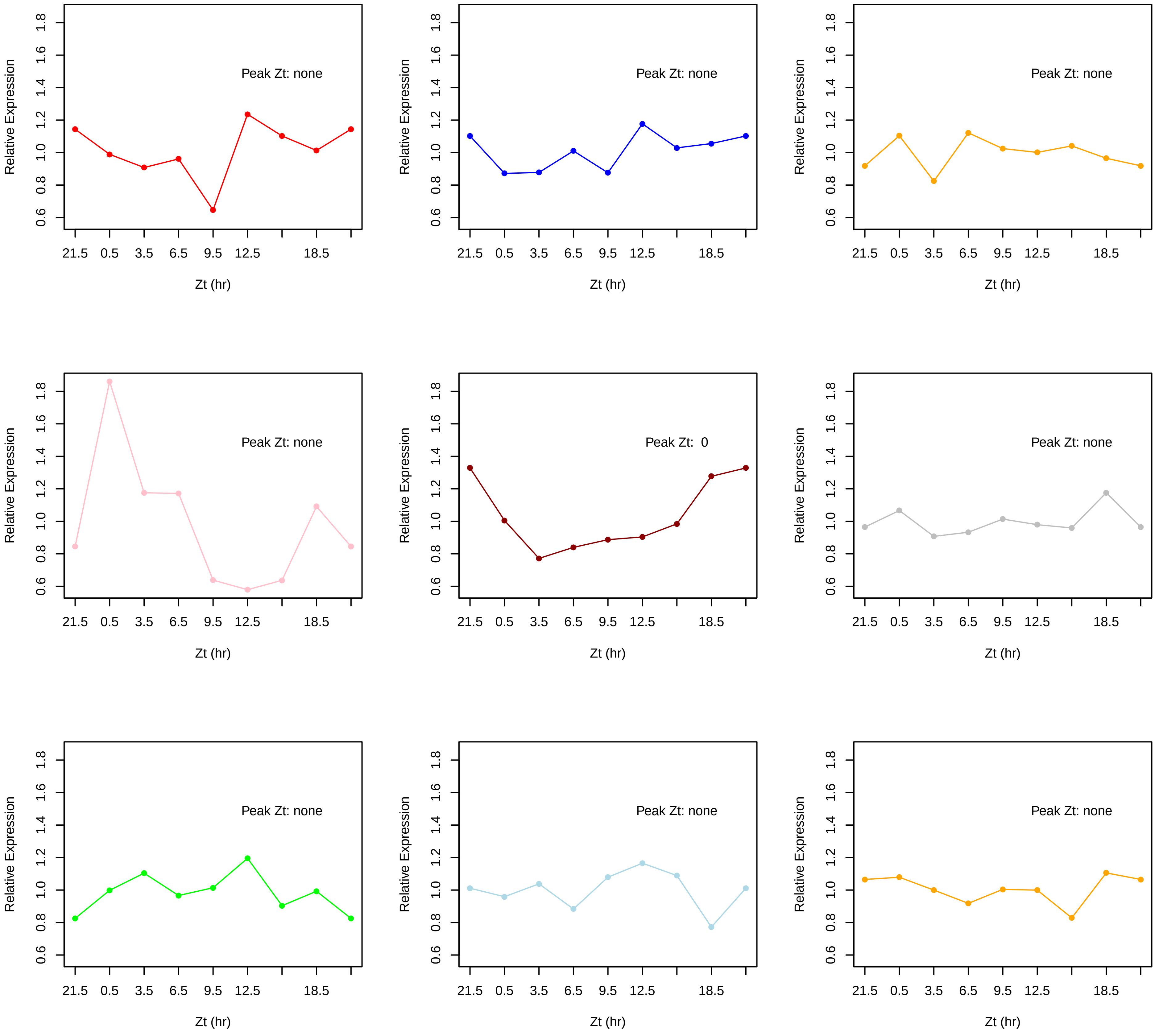

Singeleton: ENSXMAG00000002775 lrrk2

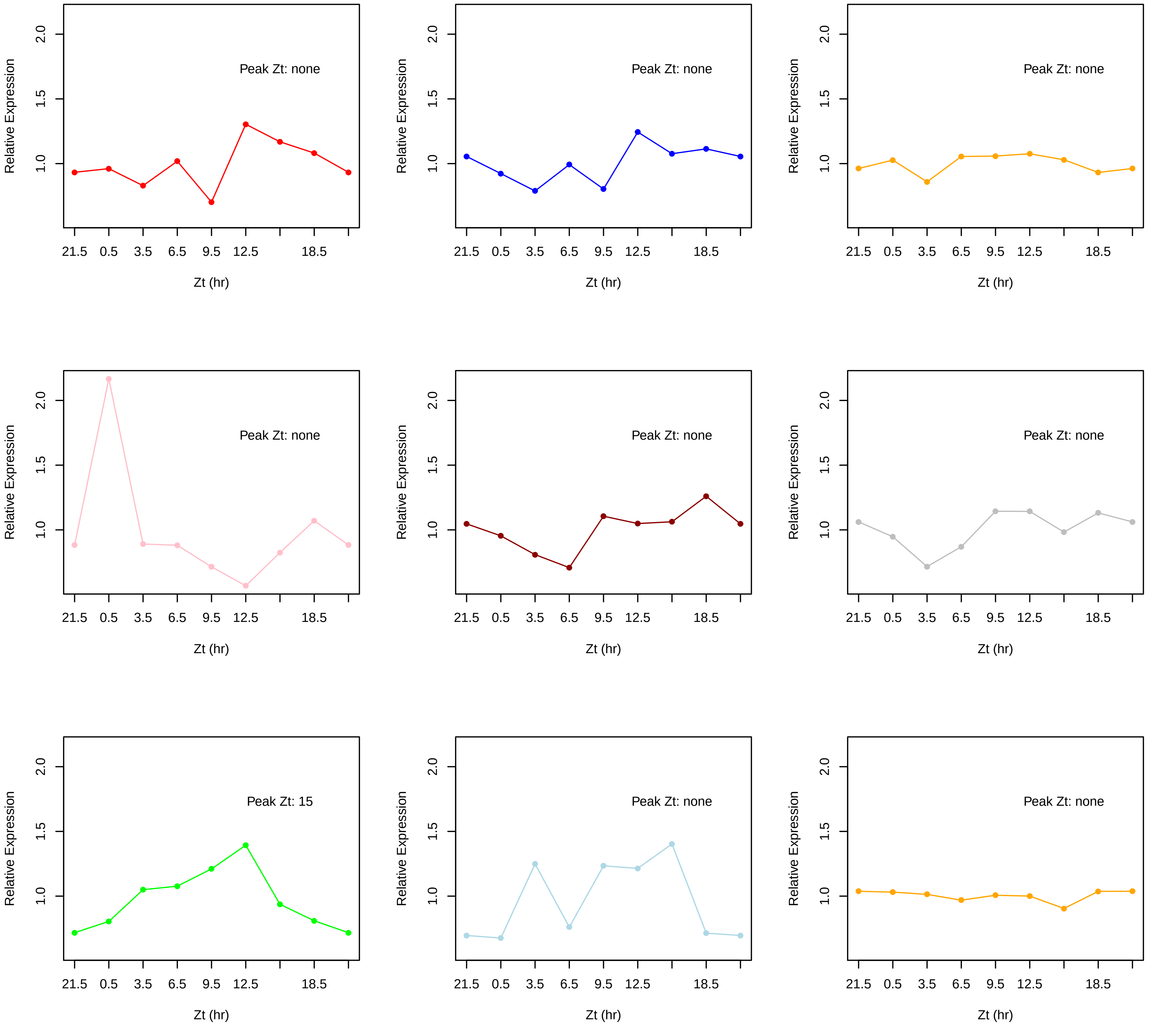

Singeleton: ENSXMAG00000001828 ppp2r2ab

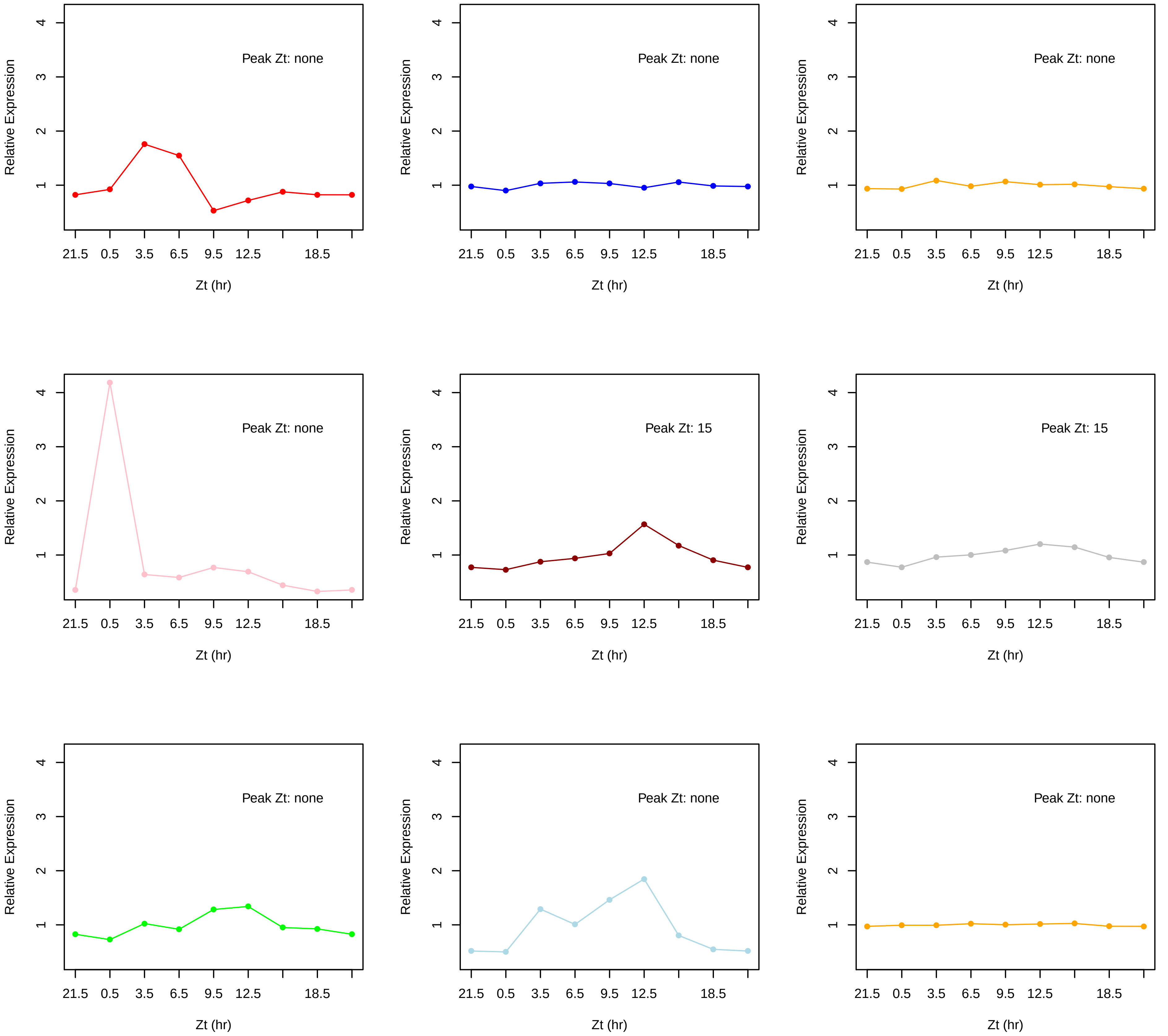

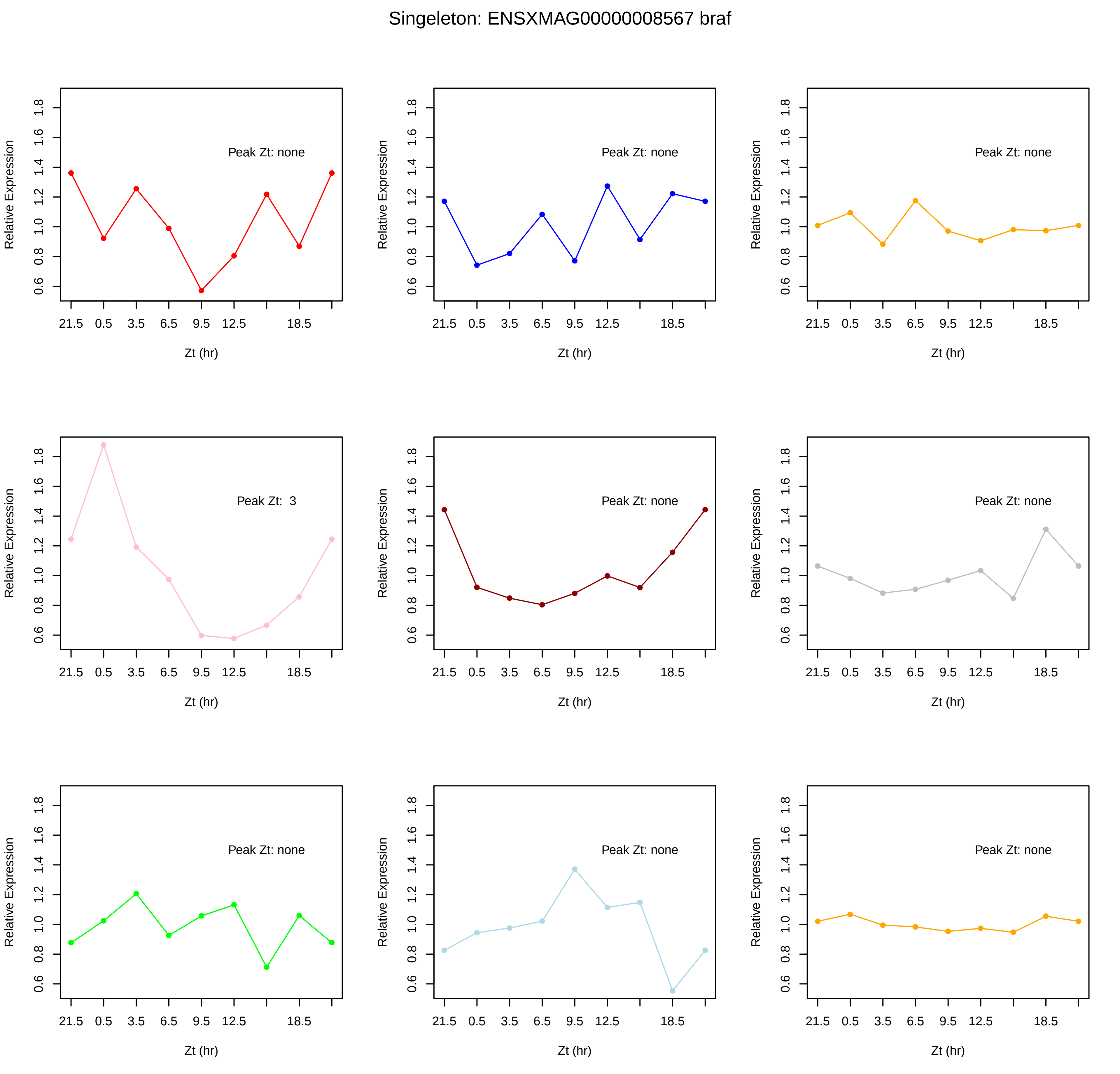

Singeleton: ENSXMAG00000008567 braf
Peak Zt: none
Peak Zt: none
Peak Zt: none
Peak Zt: 3
Peak Zt: none
Peak Zt: none
Peak Zt: none
Peak Zt: none
Peak Zt: none
Relative Expression
Zt (hr)

Singeleton: ENSXMAG00000016984 cnot4a

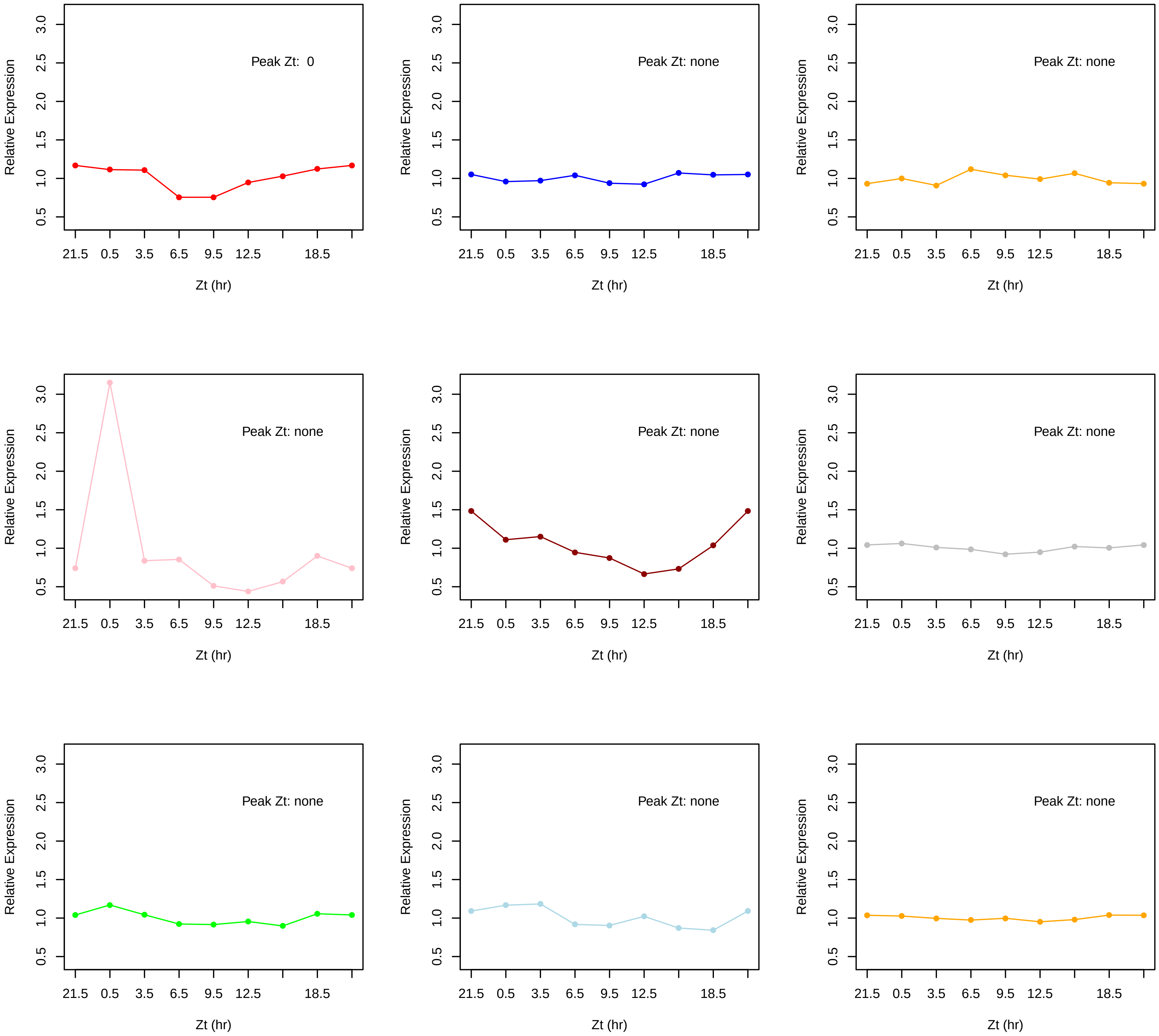

Singleton: ENSXMAG00000005086 mkrn1

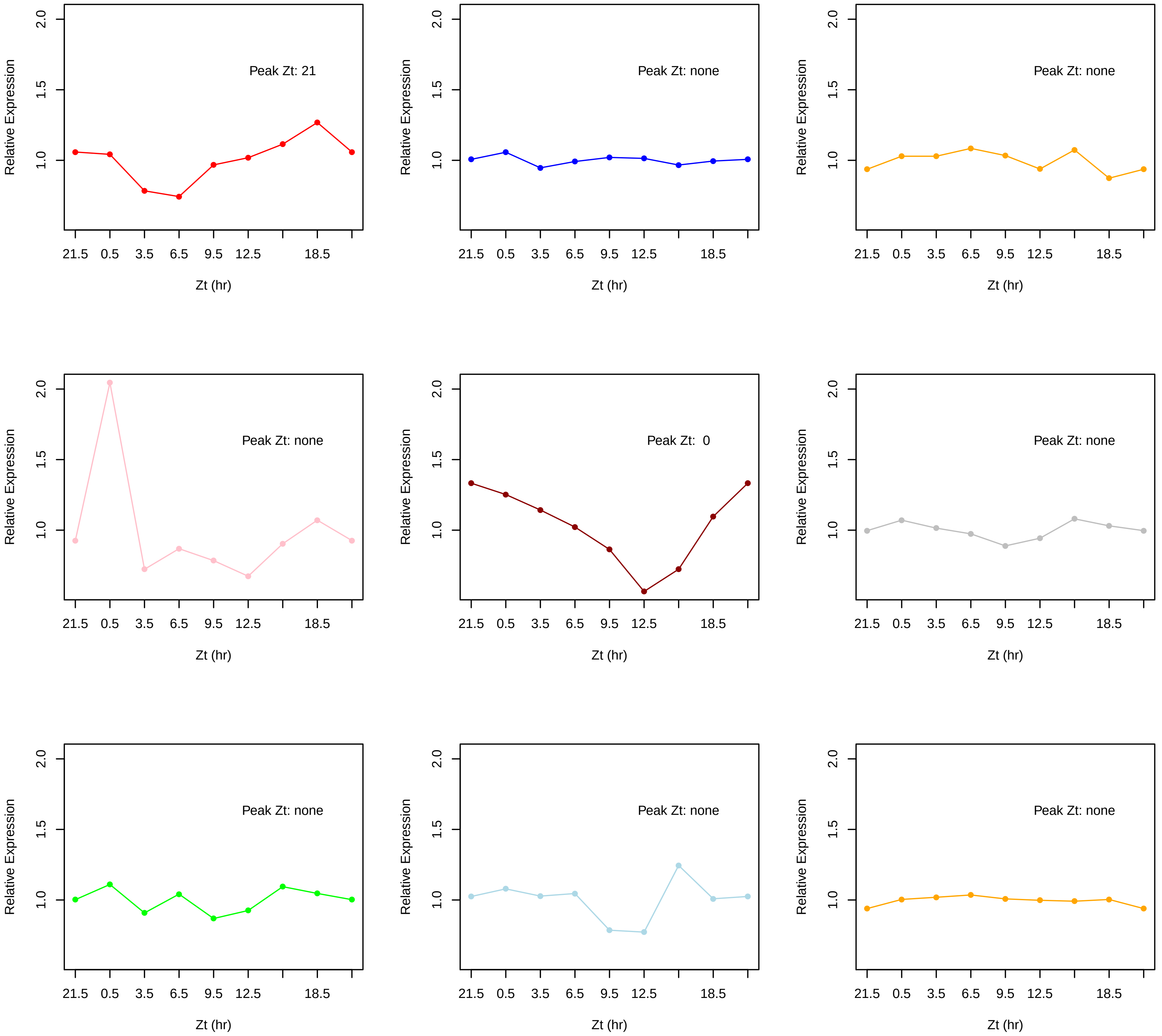

Singeleton: ENSXMAG00000005072 arhgef39

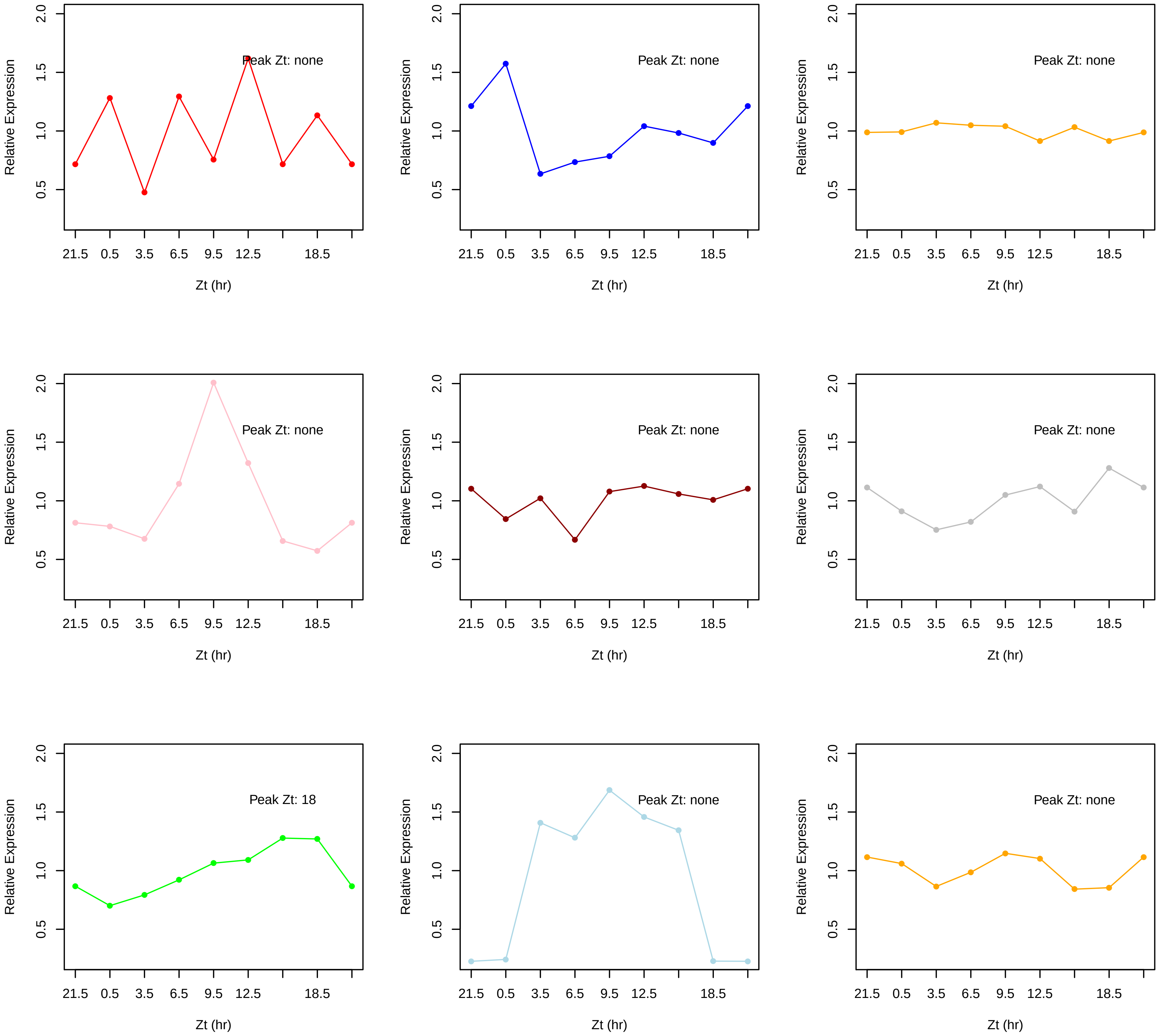

Singeleton: ENSXMAG00000007201 vrk2

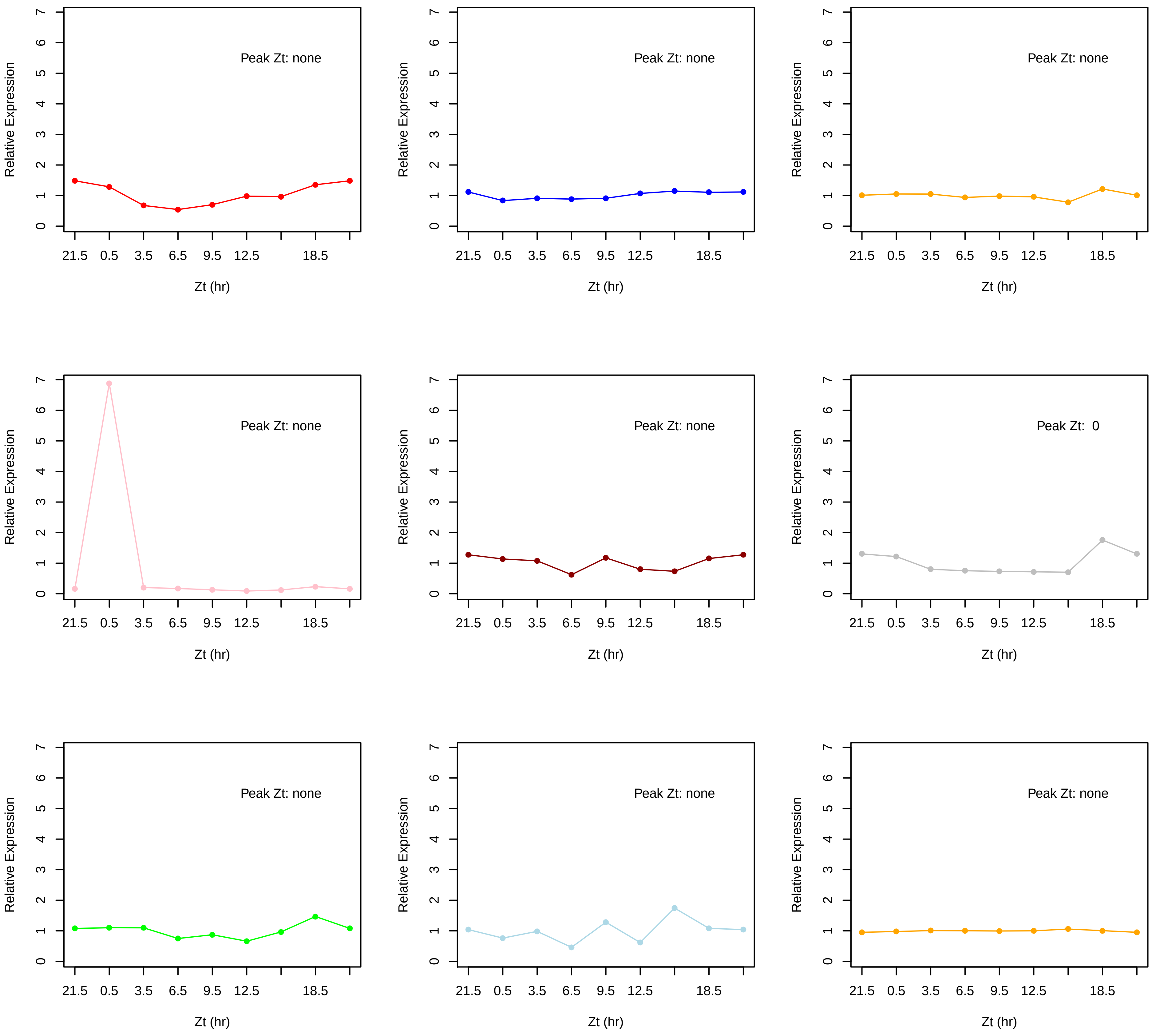

Singeleton: ENSXMAG00000002972 adgb

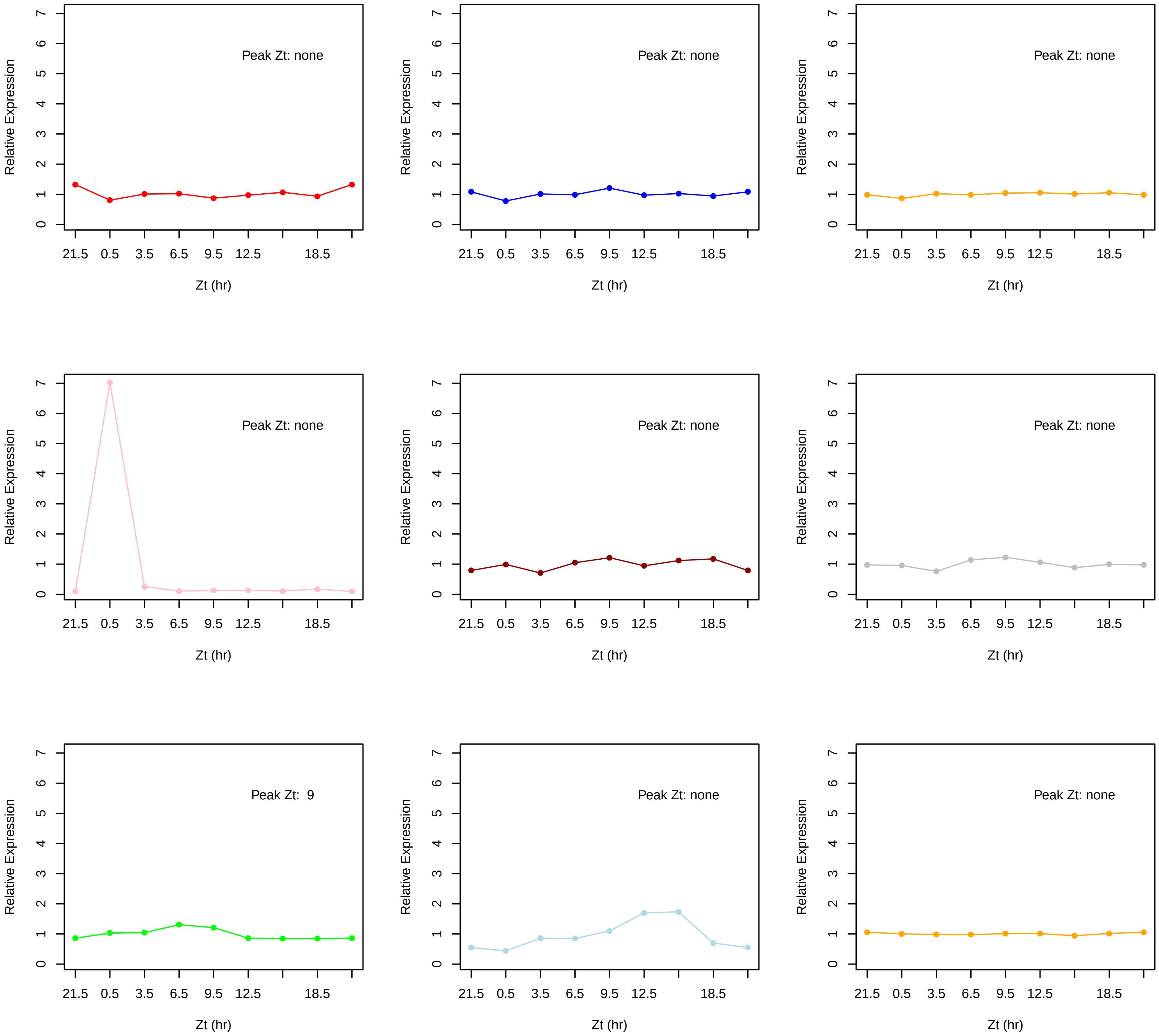

Singeleton: ENSXMAG00000005055 dera

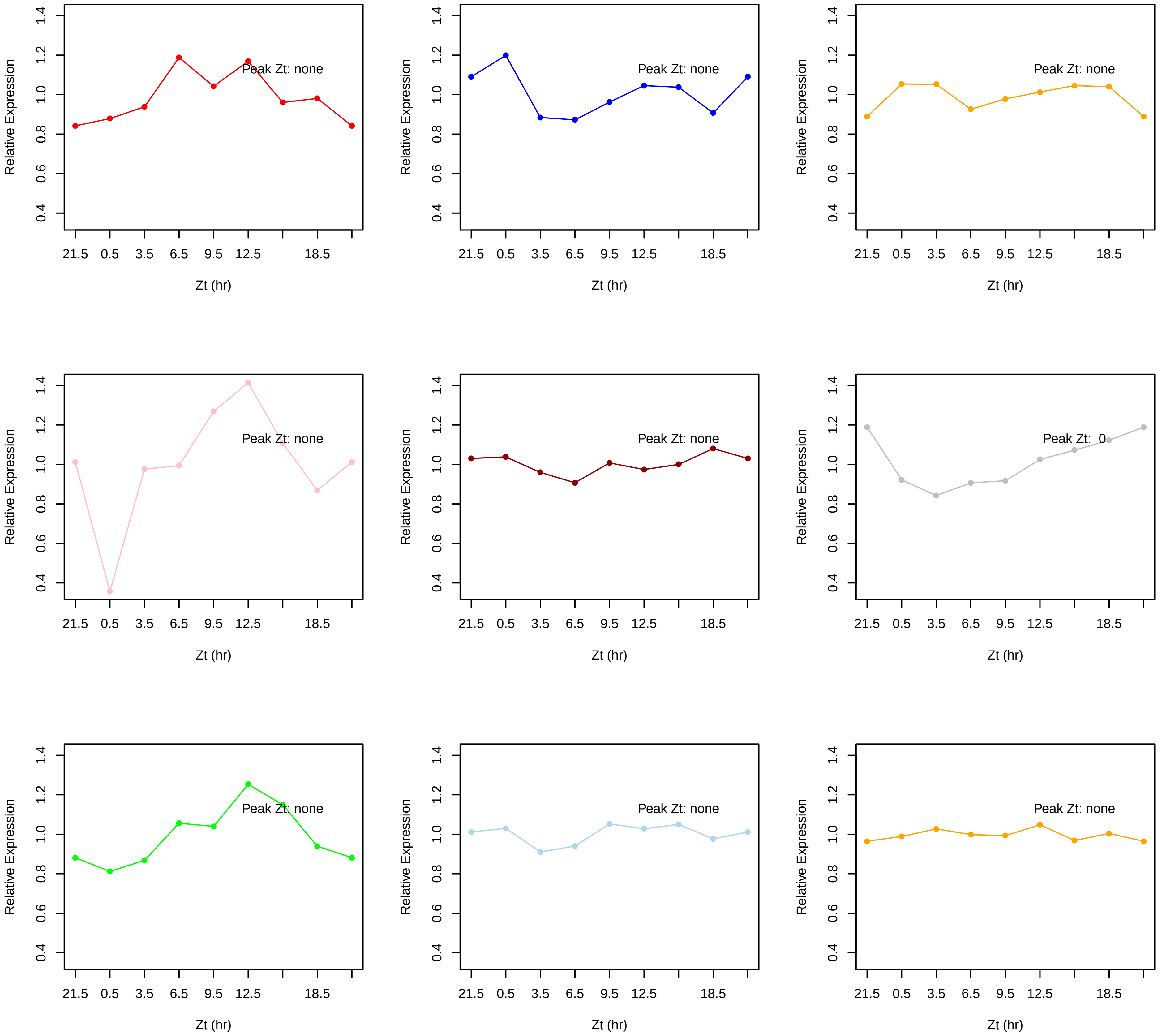

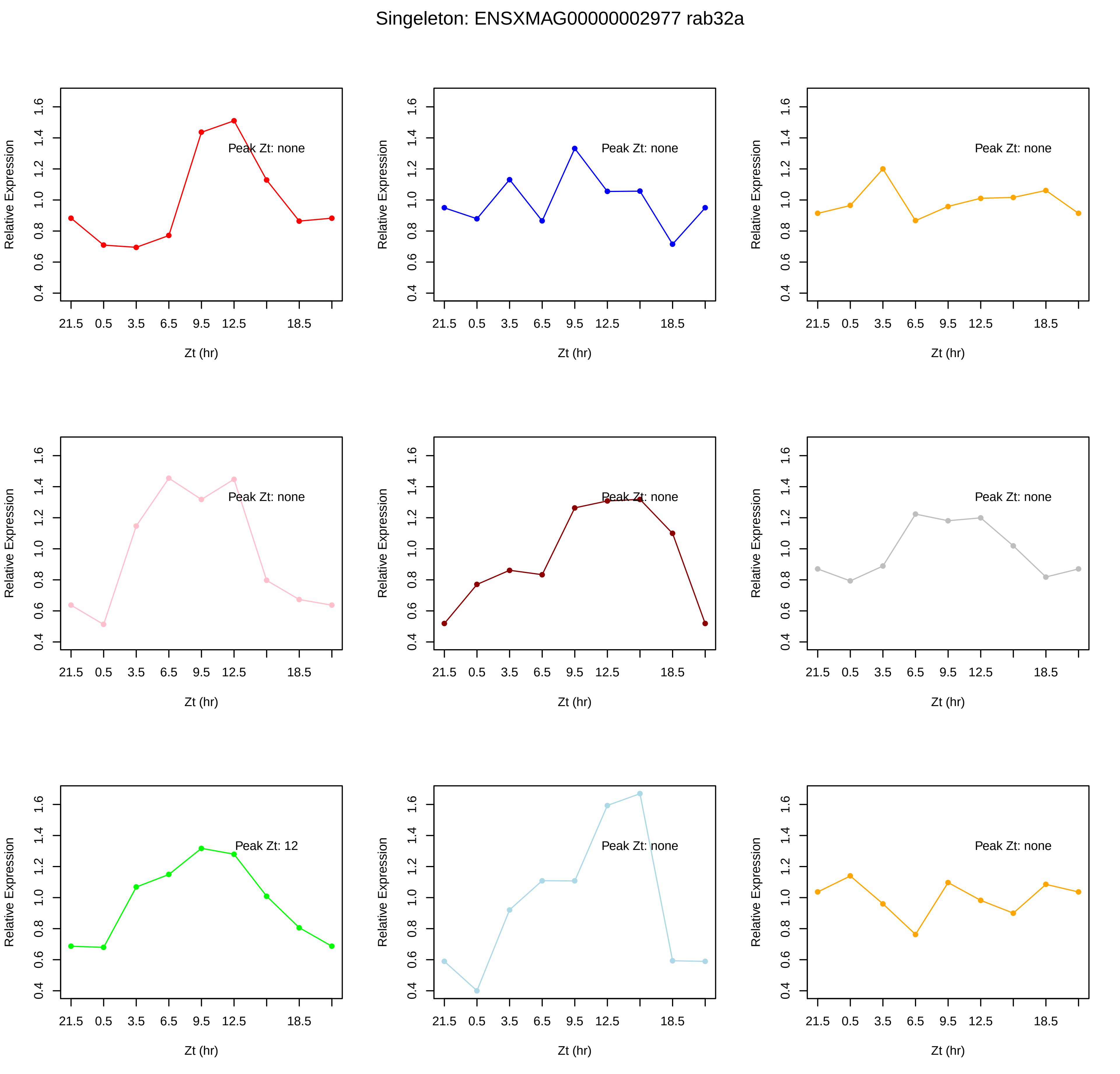

Singeleton: ENSXMAG00000002977 rab32a
Peak Zt: none
Peak Zt: none
Peak Zt: none
Peak Zt: none
Peak Zt: none
Peak Zt: none
Peak Zt: 12
Peak Zt: none
Peak Zt: none
Relative Expression
Zt (hr)
21.5
0.5
3.5
6.5
9.5
12.5
18.5
0.4
0.6
0.8
1.0
1.2
1.4
1.6

Singleton: ENSXMAG00000005030 lemd3

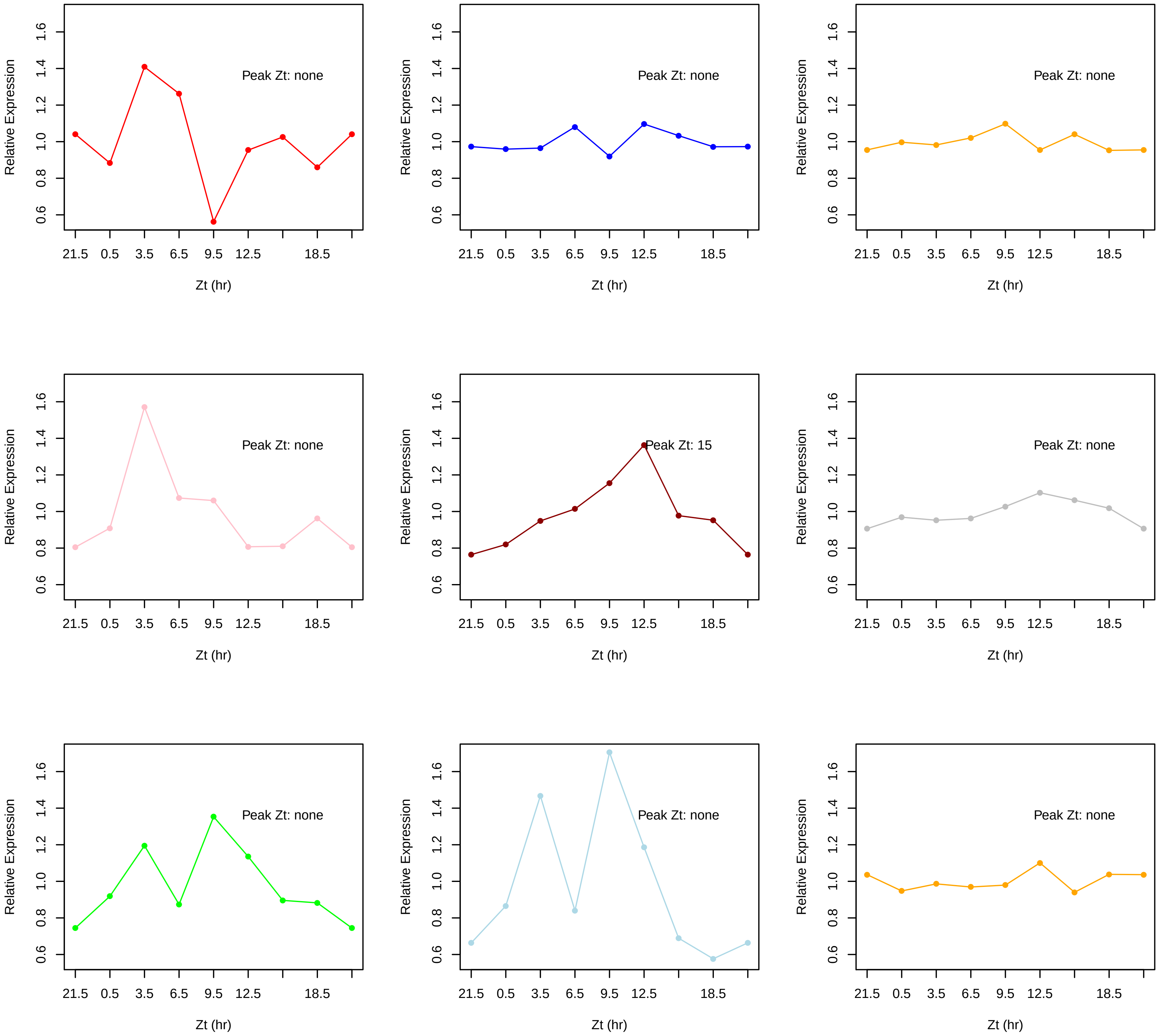

Singeleton: ENSXMAG00000010278 dtd1

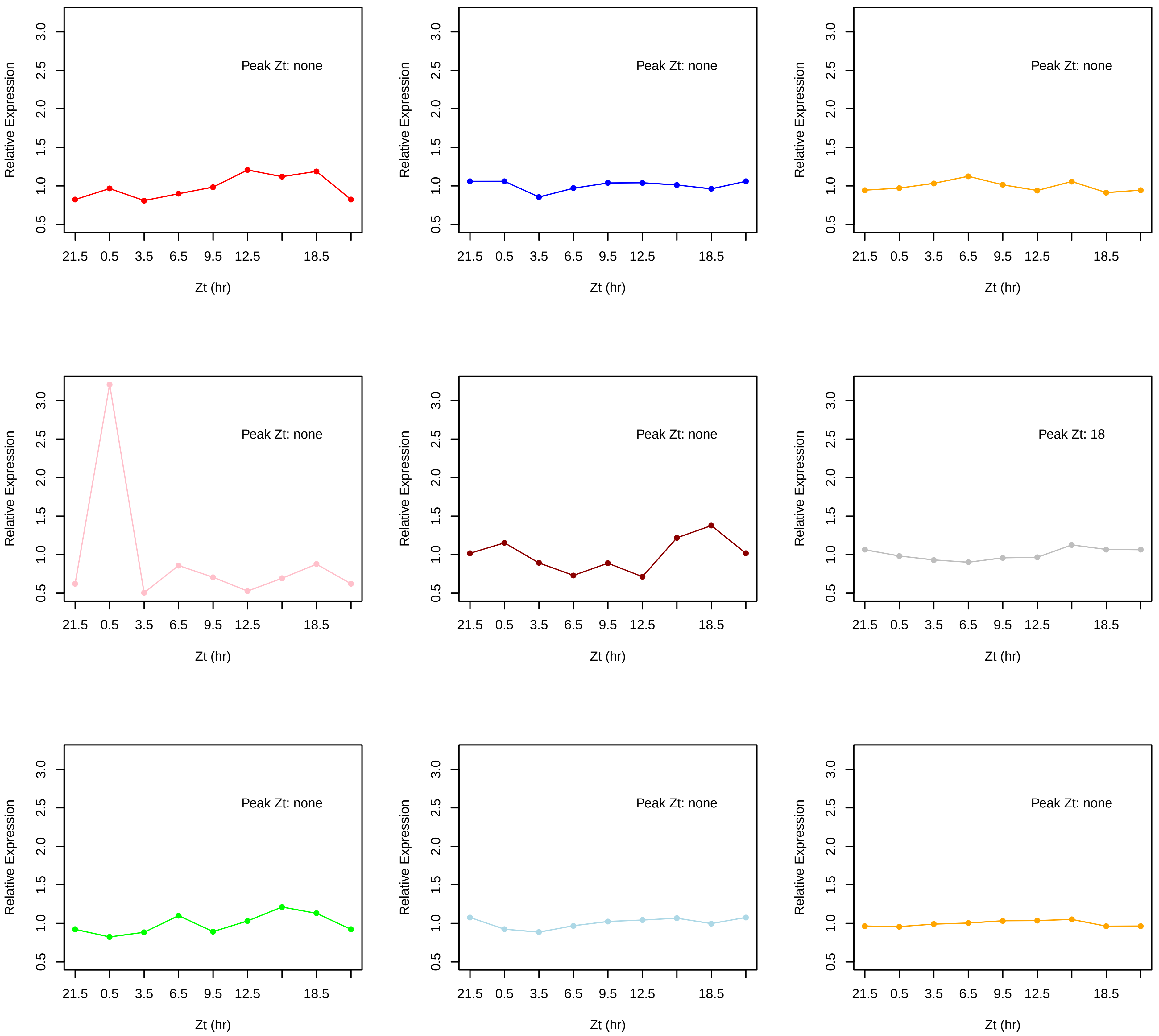

Singeleton: ENSXMAG00000015378 fbxo30a

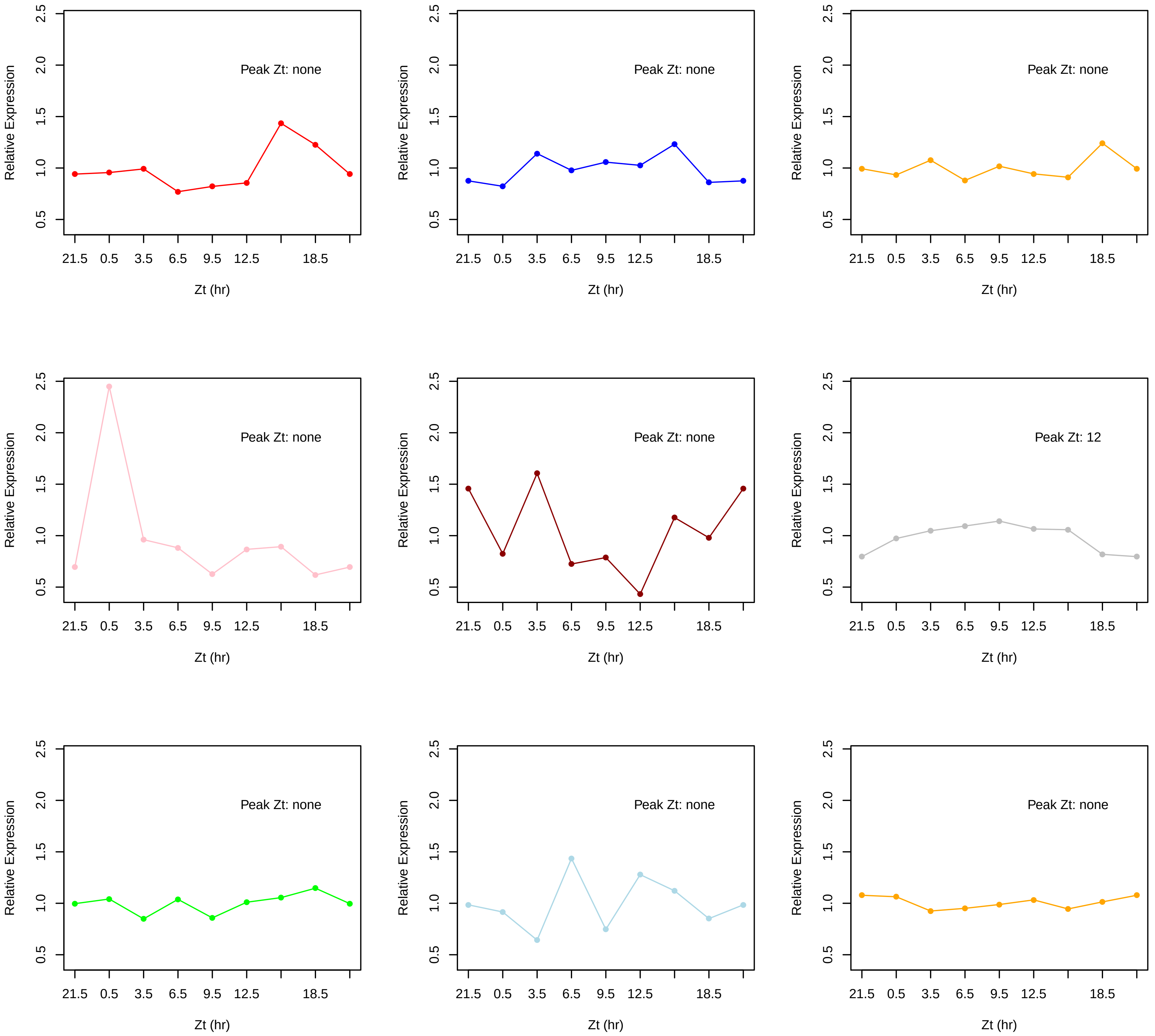

Singleton: ENSXMAG00000017162

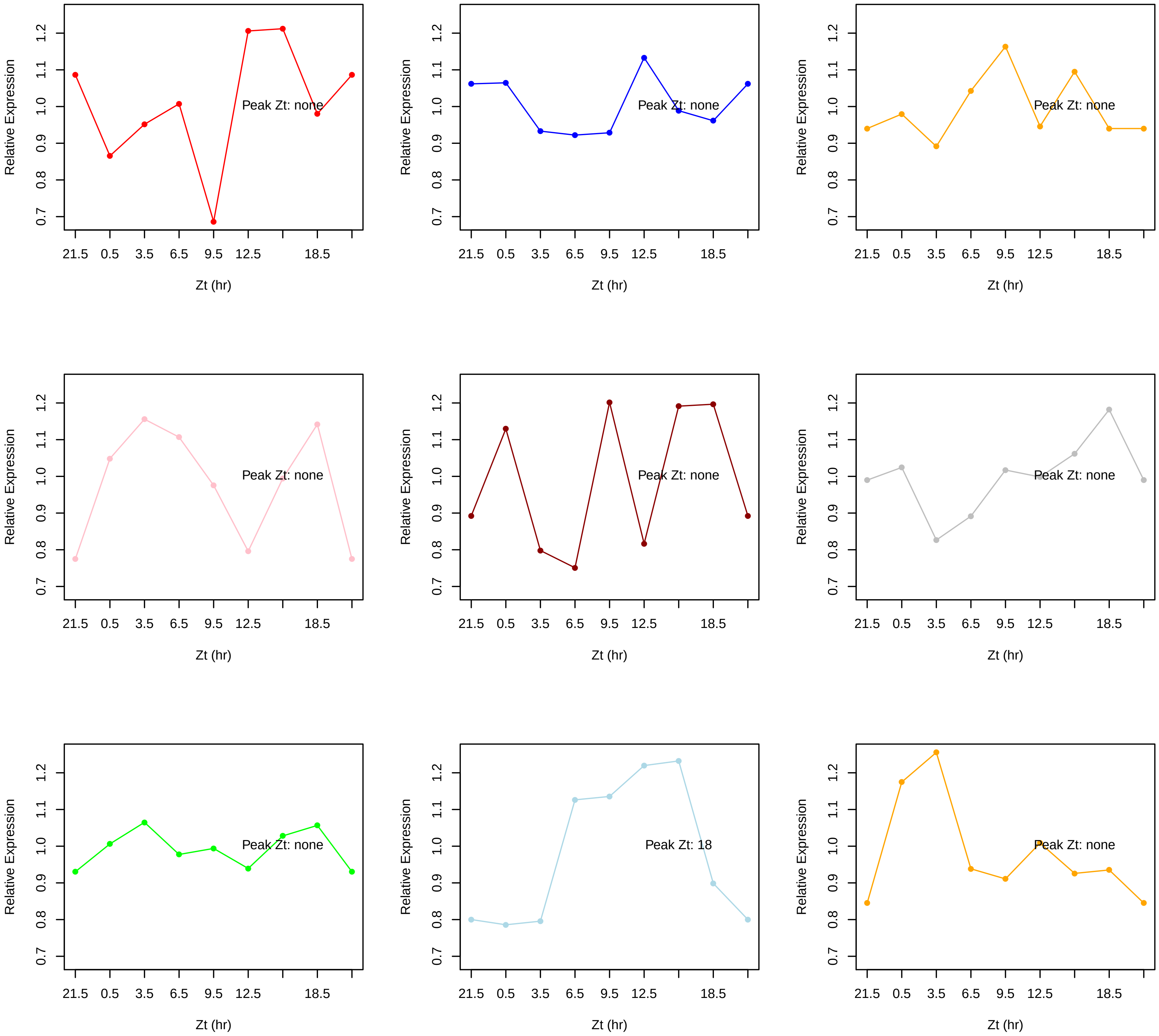

Singeleton: ENSXMAG00000017243 slc35e3

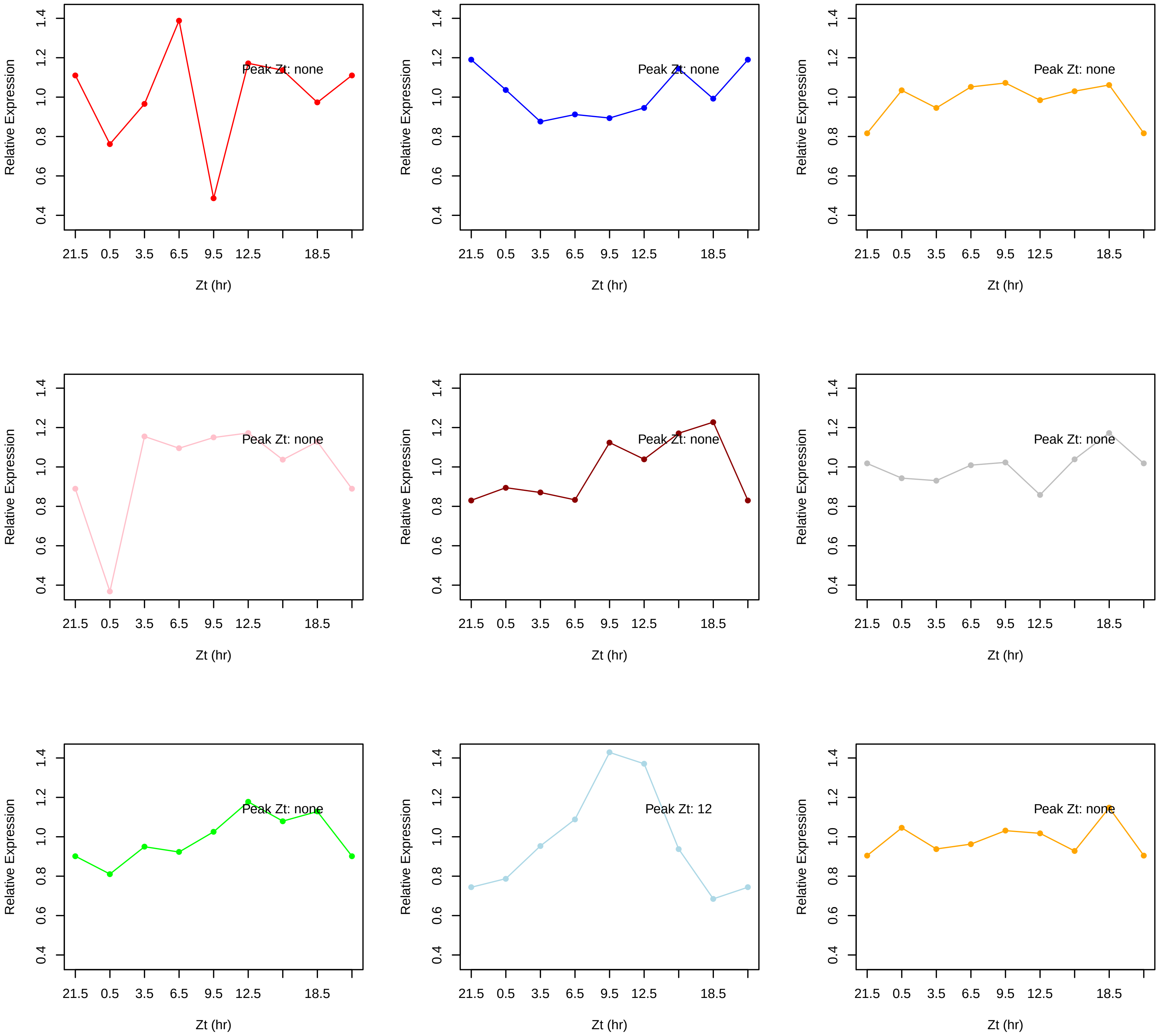

Singeleton: ENSXMAG00000003322 fndc1

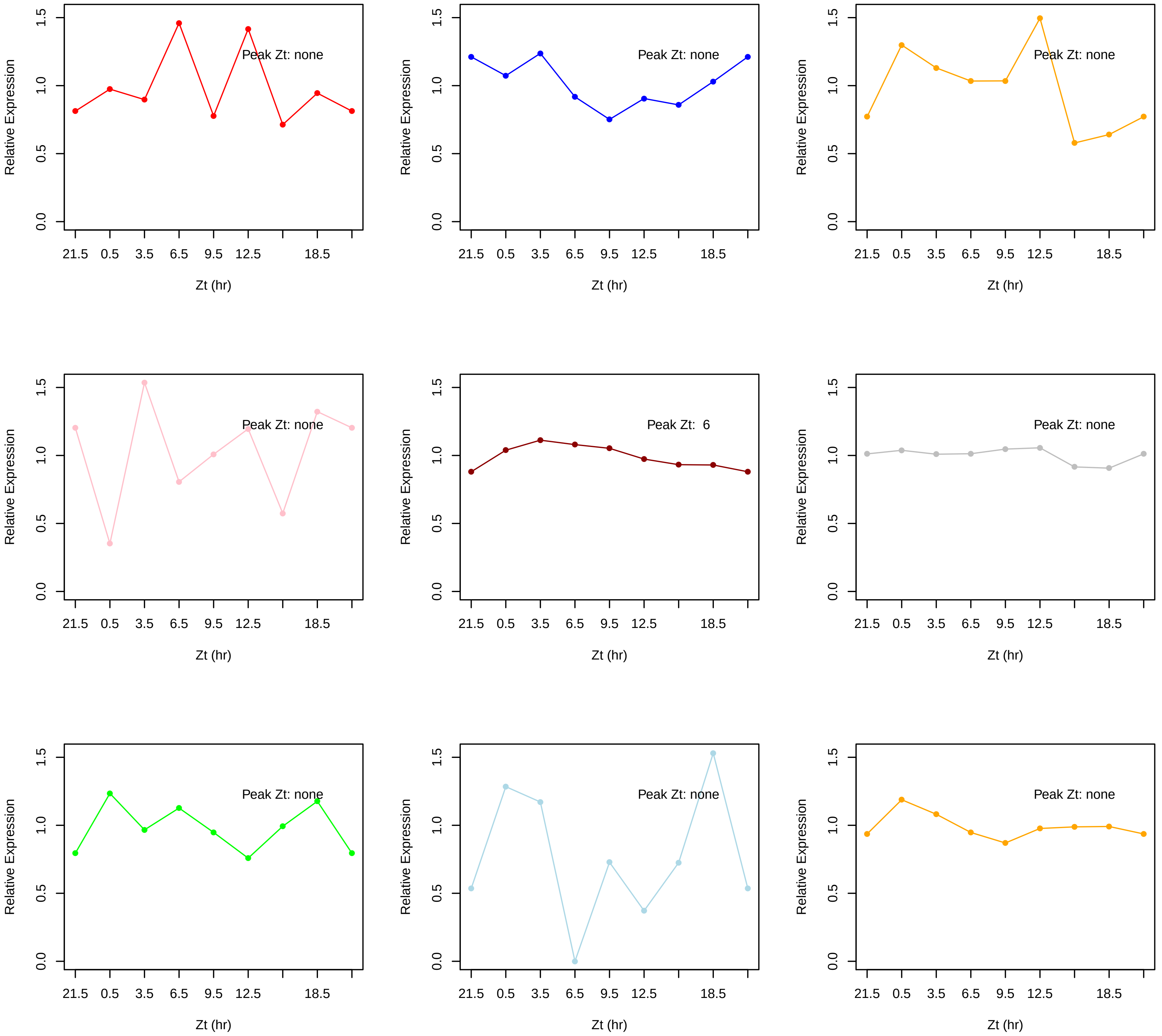

Singeleton: ENSXMAG00000003325 sod2

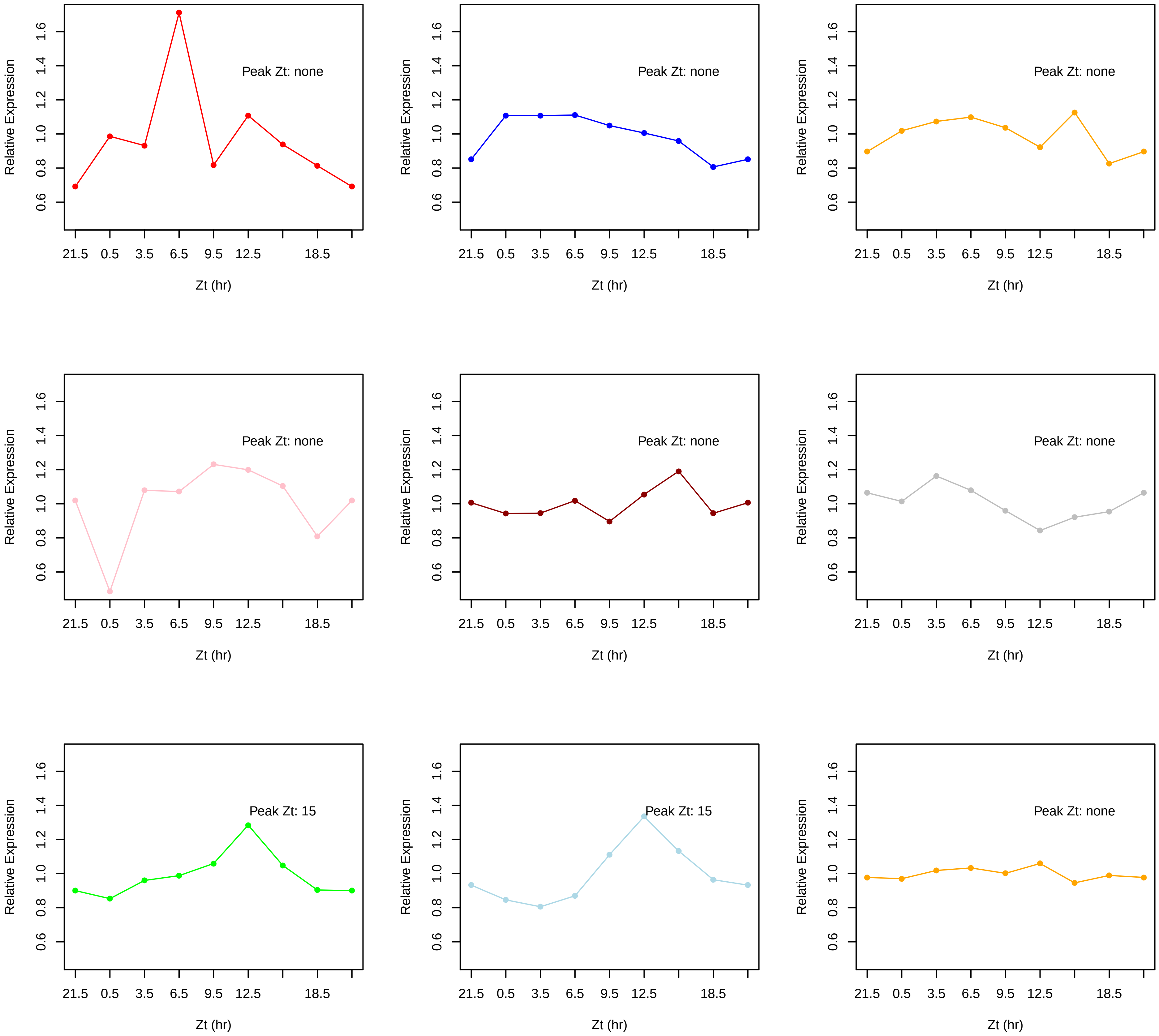

Singleton: ENSXMAG00000020852 asrgl1

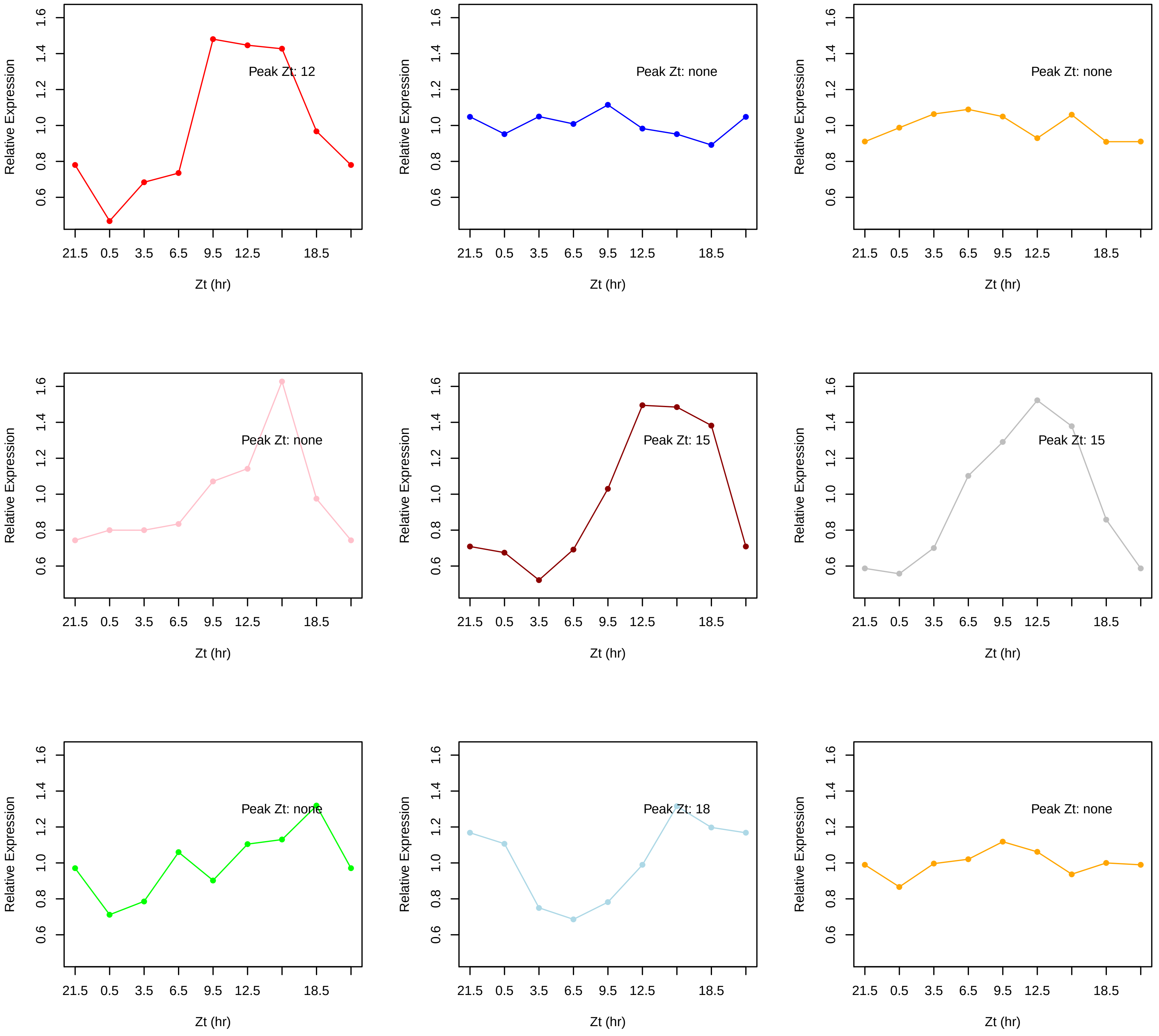

Singeleton: ENSXMAG00000003474 yeats4

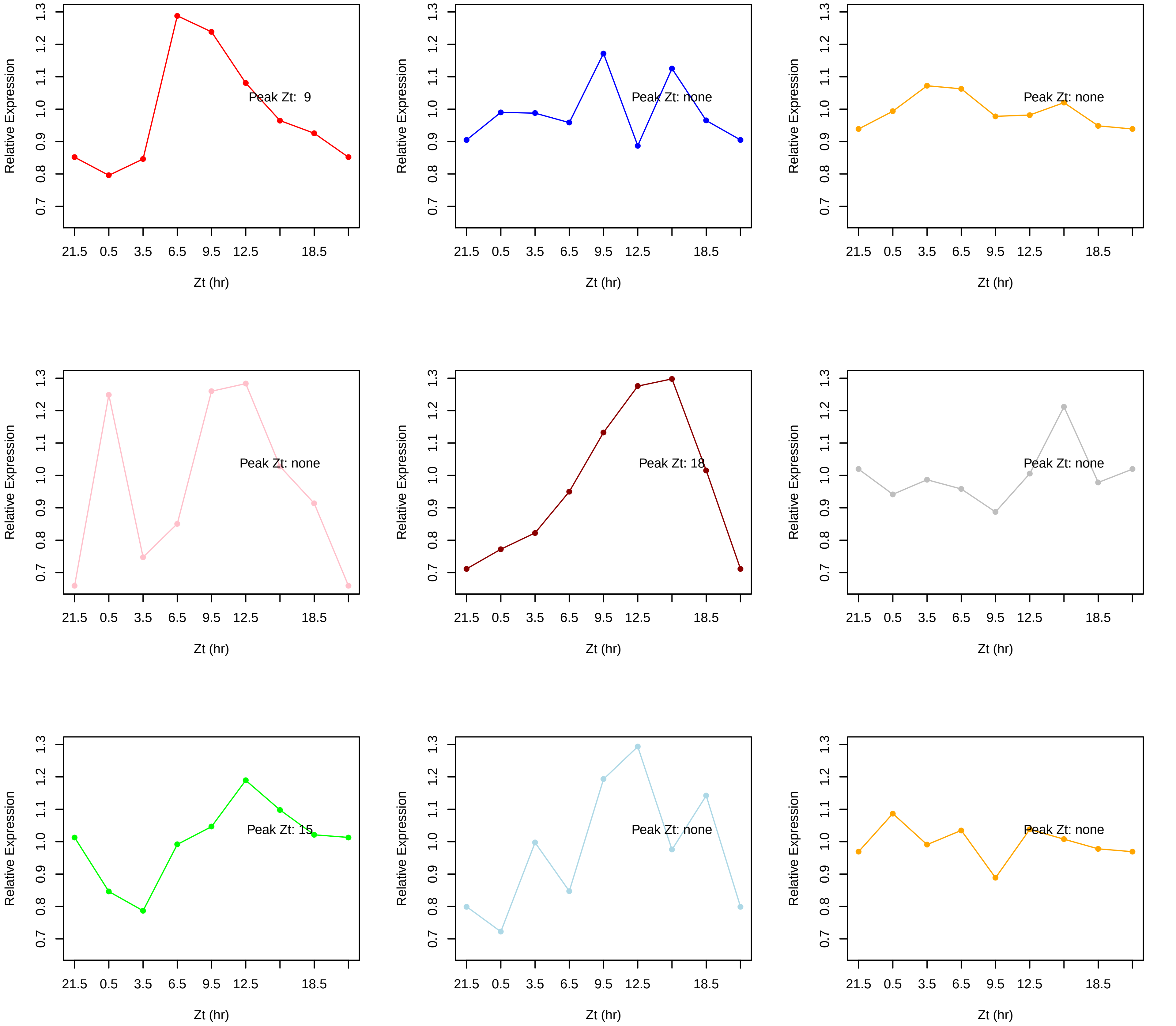

Singeleton: ENSXMAG00000026801 ppp1r14c

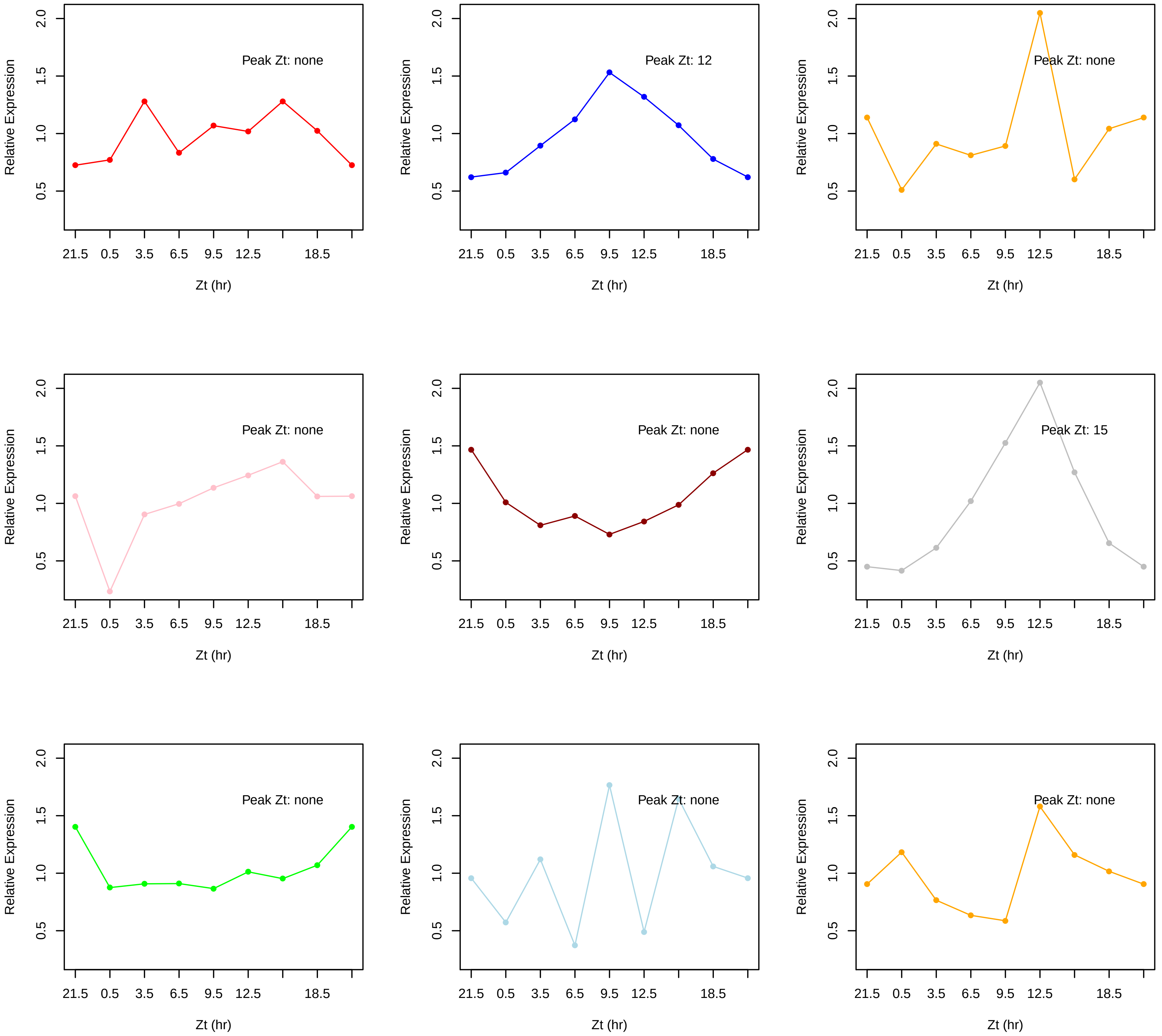

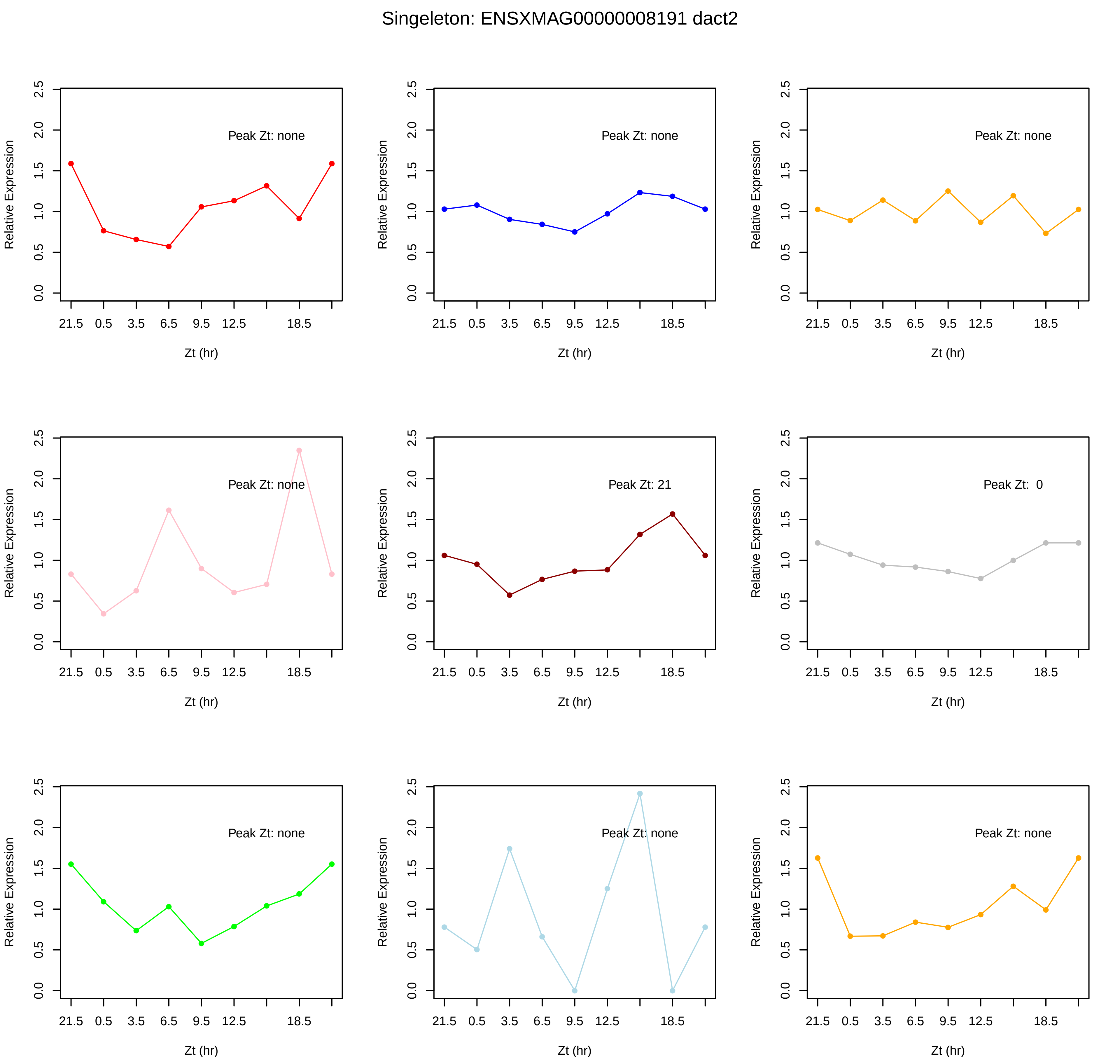

Singeleton: ENSXMAG00000008191 dact2
Peak Zt: none
Peak Zt: none
Peak Zt: none
Peak Zt: none
Peak Zt: 21
Peak Zt: 0
Peak Zt: none
Peak Zt: none
Peak Zt: none
Relative Expression
Zt (hr)
21.5 0.5 3.5 6.5 9.5 12.5 18.5
0.0 0.5 1.0 1.5 2.0 2.5

Singleton: ENSXMAG00000025600

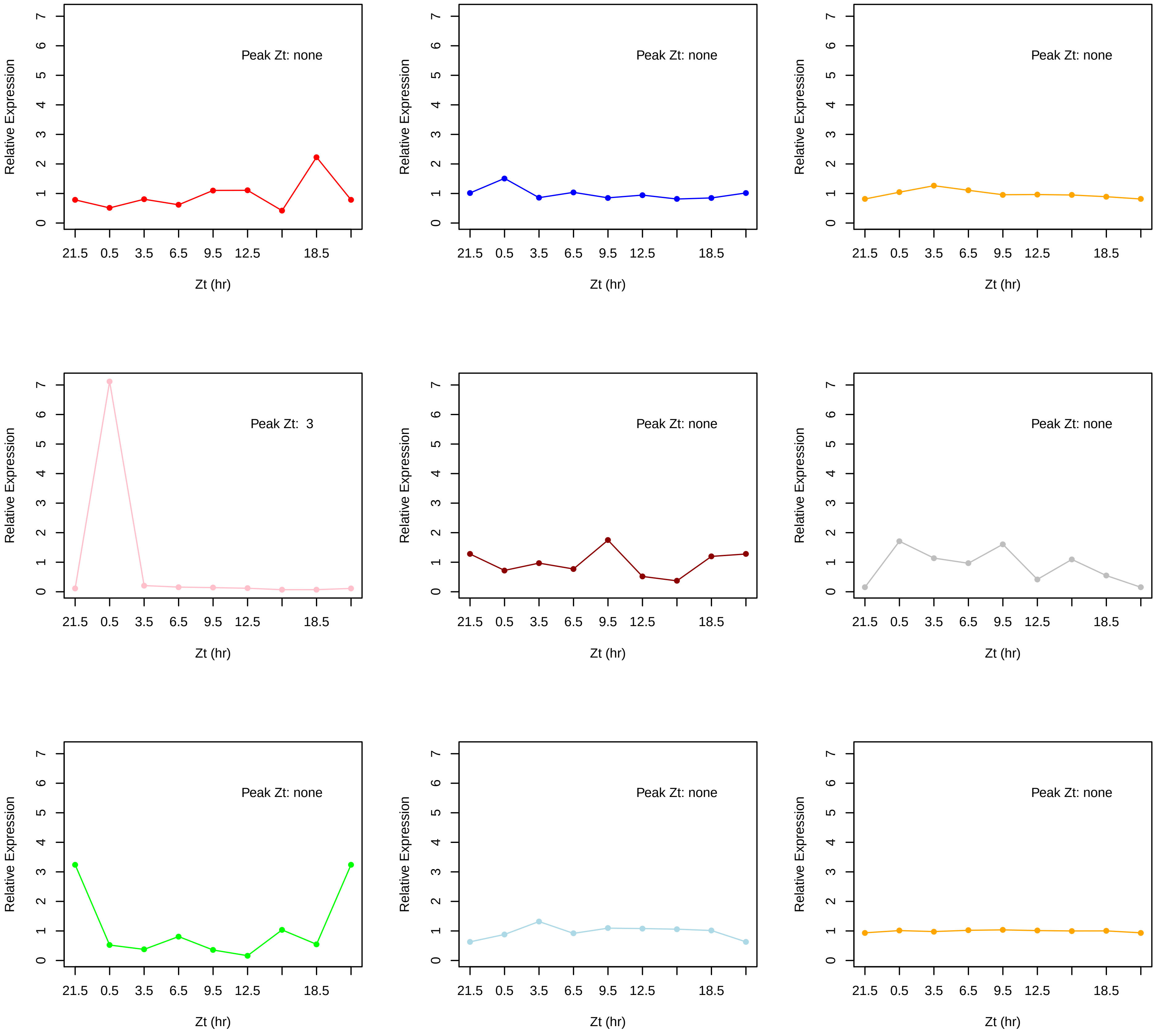

Singeleton: ENSXMAG00000004423

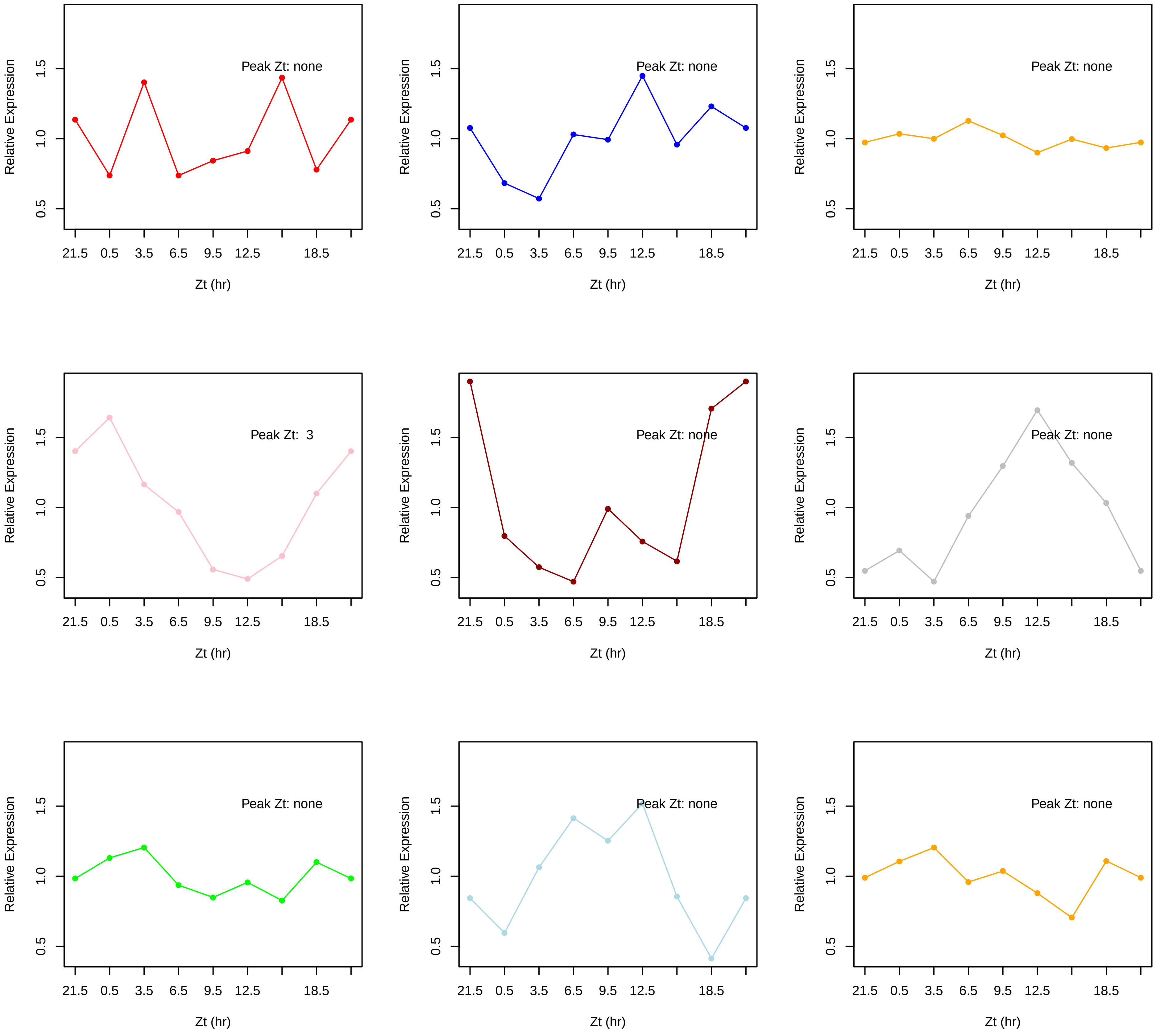

Singeleton: ENSXMAG00000009600 si:dkey−85a20.4

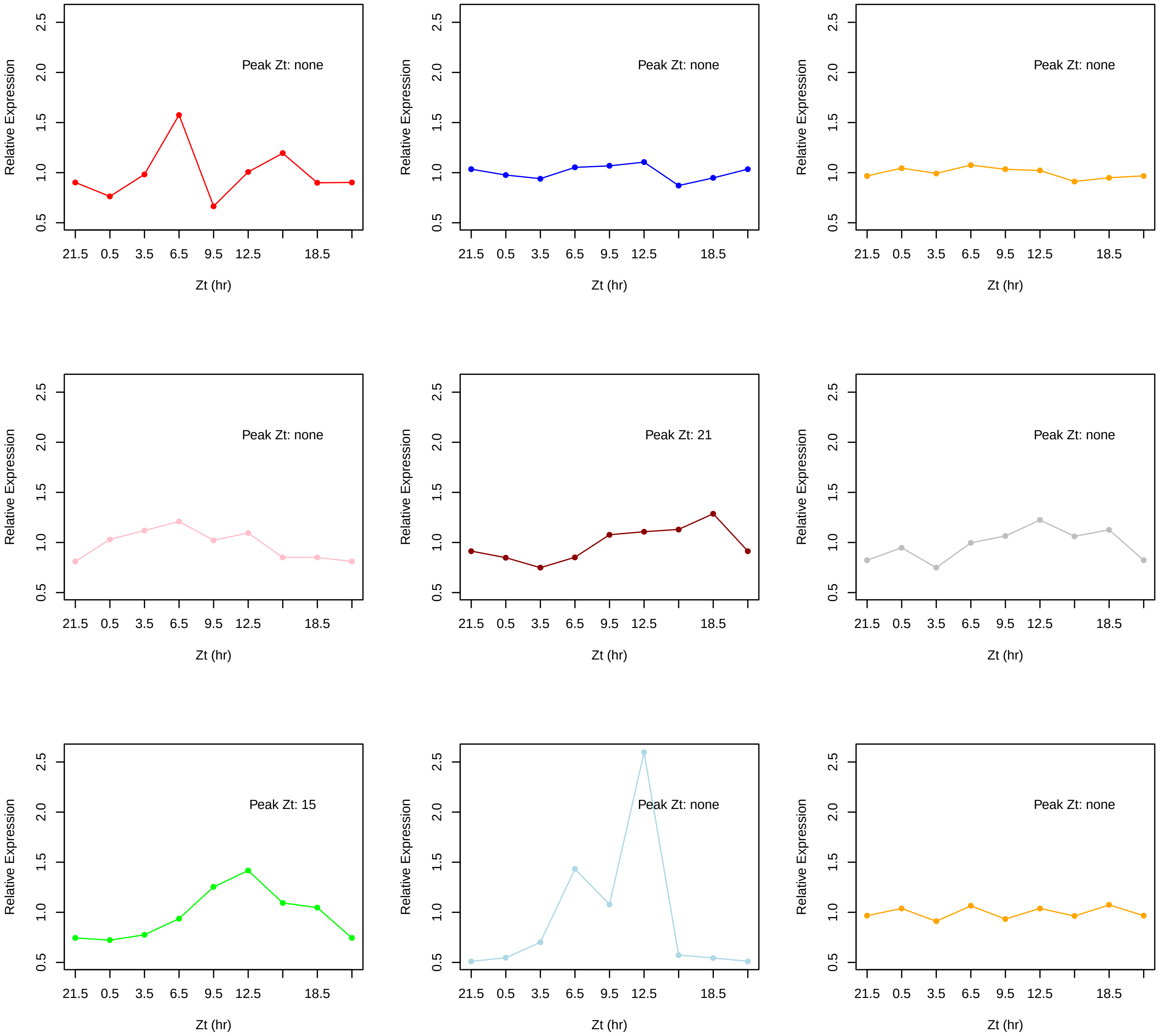

Singeleton: ENSXMAG00000029185 KCNMB4

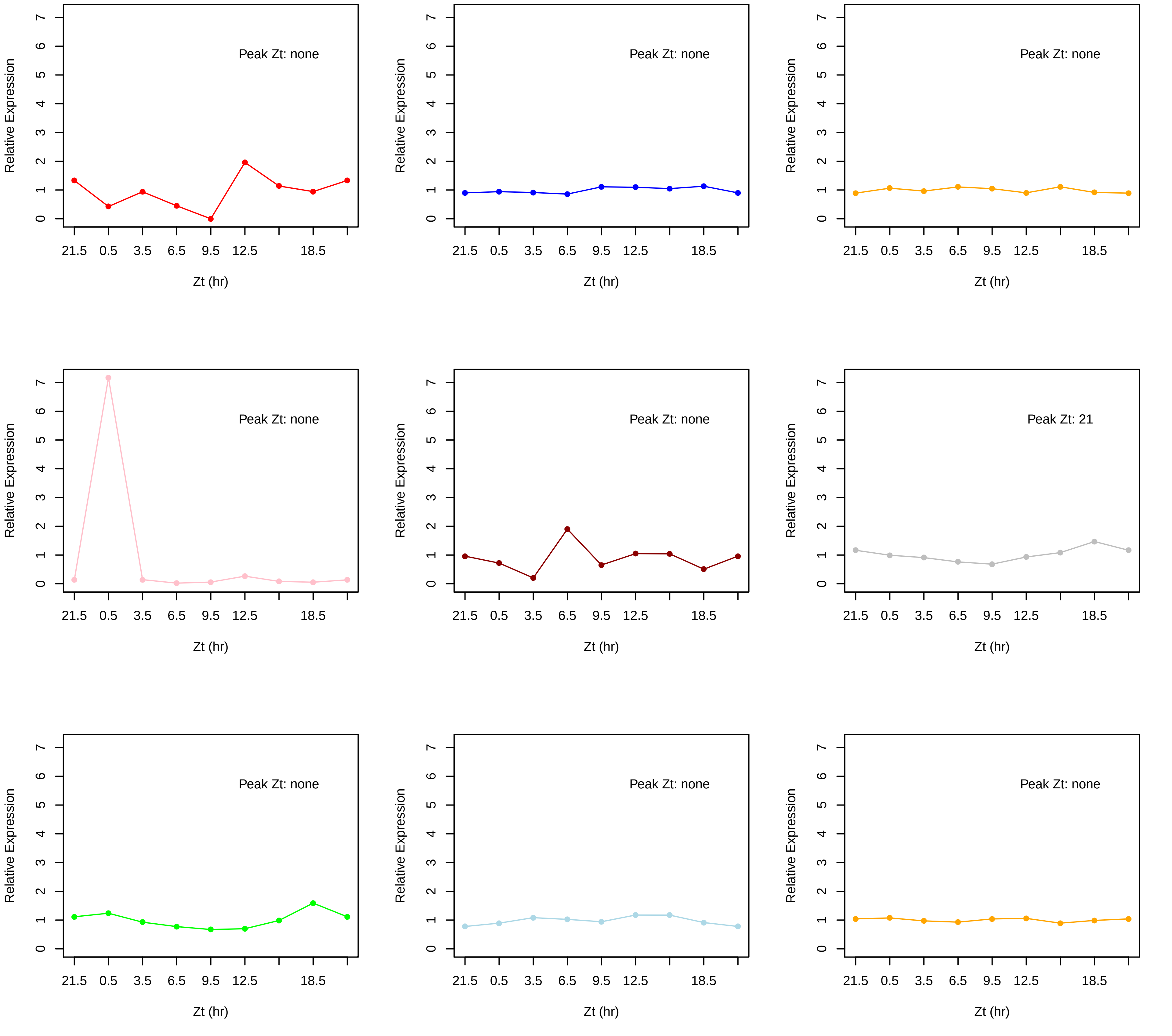

Singleton: ENSXMAG00000004339 SGK1

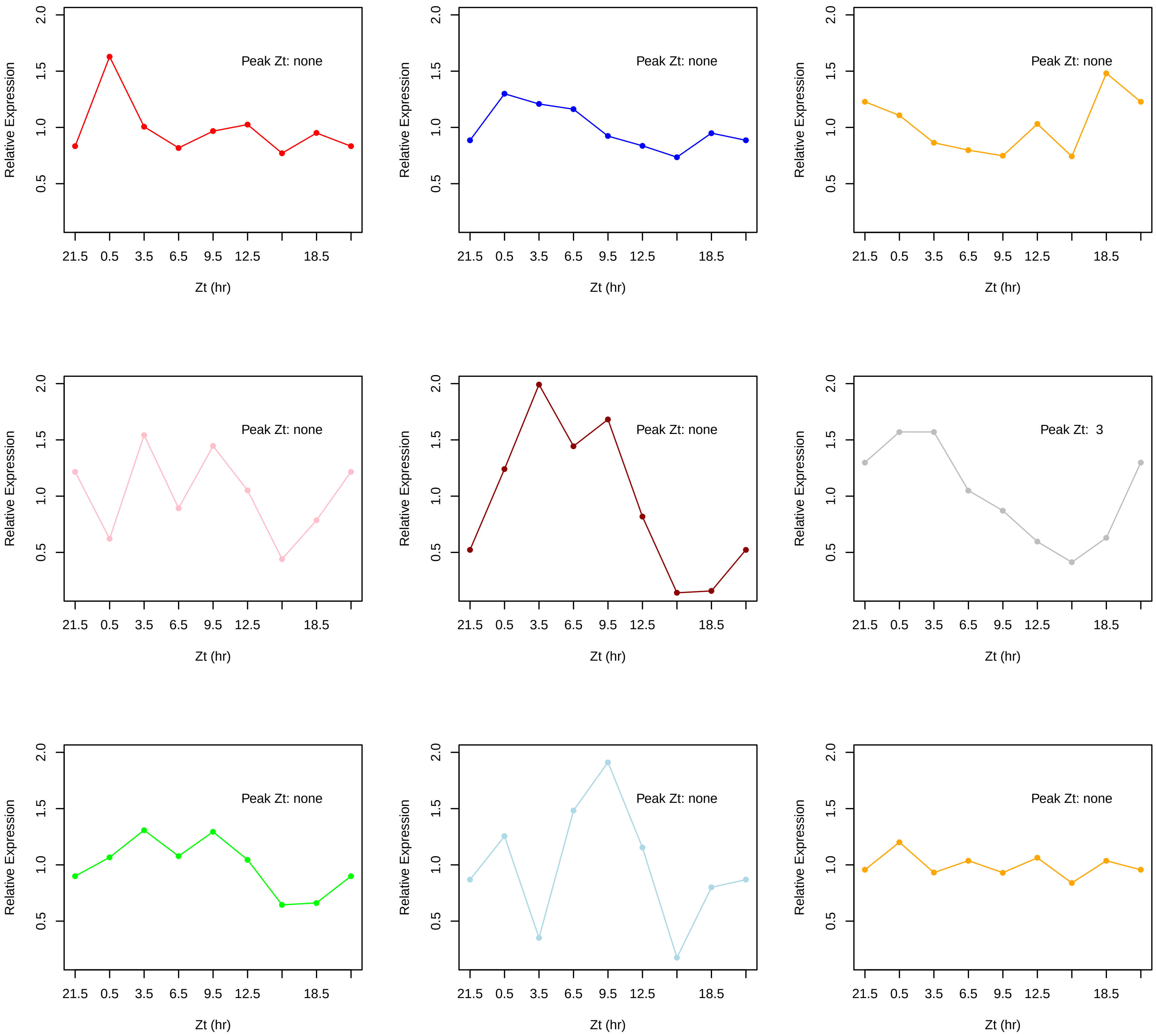

Singeleton: ENSXMAG00000004337 slc2a12

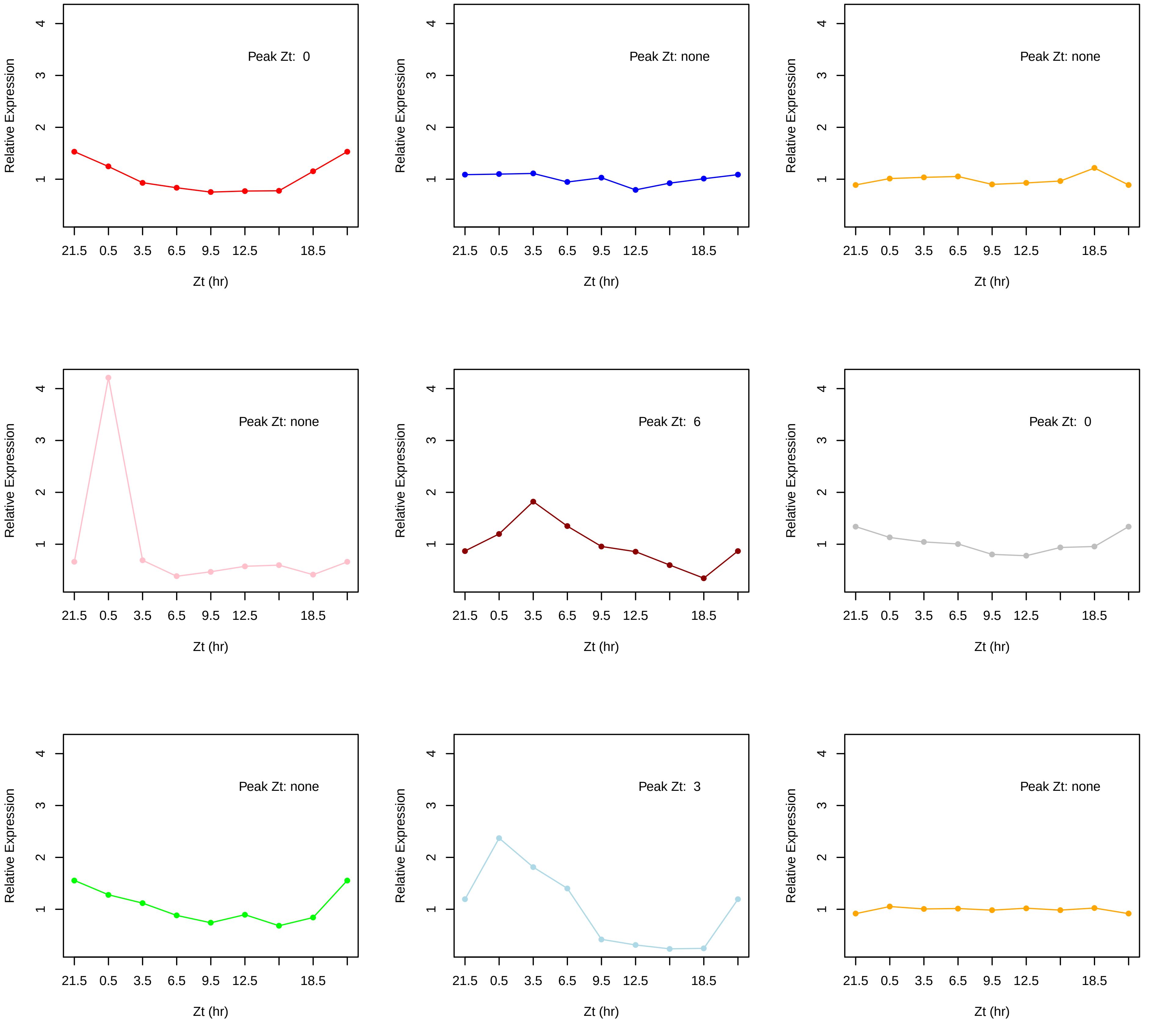

Singeleton: ENSXMAG00000006933

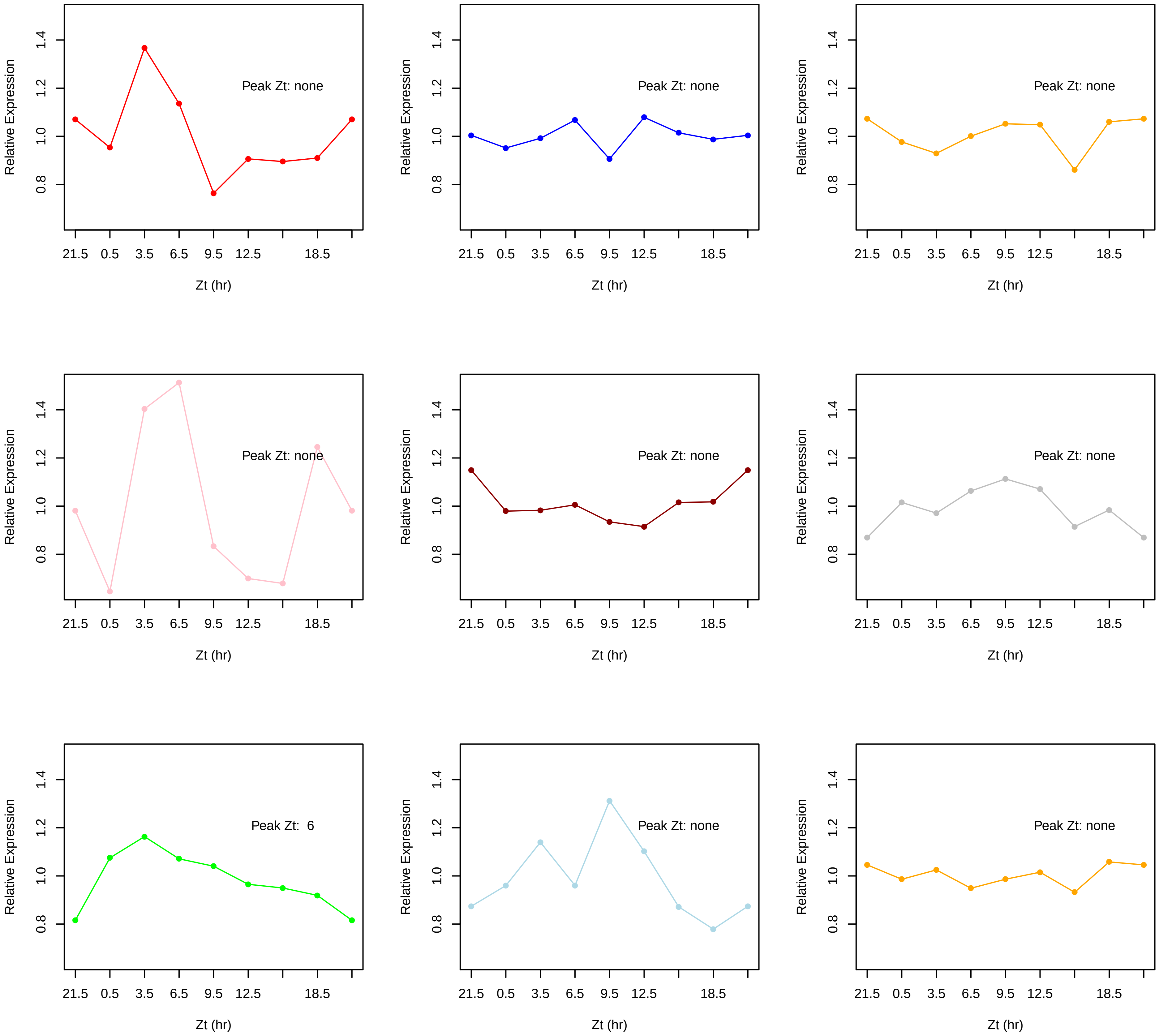

Singeleton: ENSXMAG00000003209 thbs2a

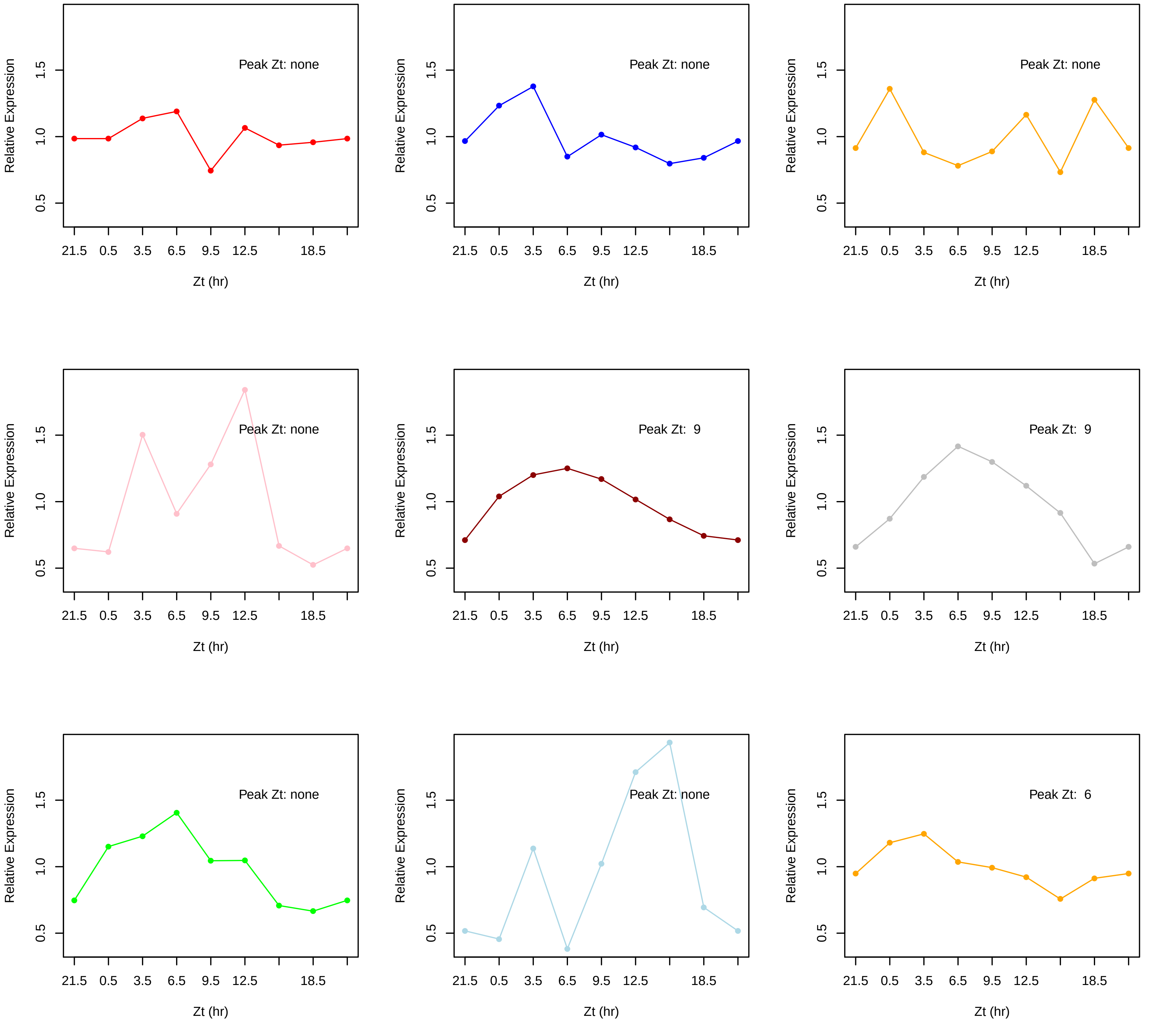

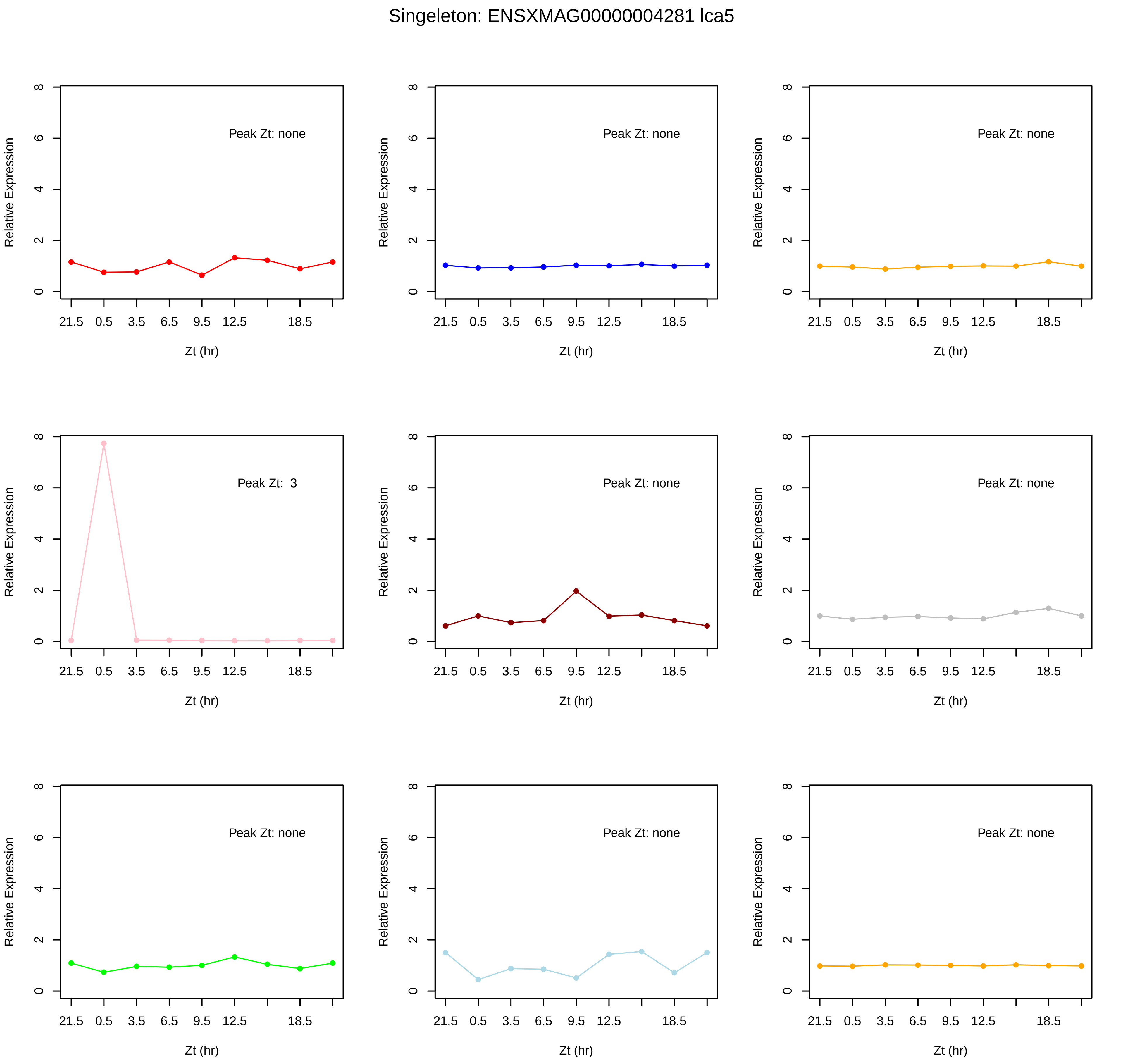

Singeleton: ENSXMAG00000004281 lca5
Peak Zt: none
Peak Zt: none
Peak Zt: none
Peak Zt: 3
Peak Zt: none
Peak Zt: none
Peak Zt: none
Peak Zt: none
Peak Zt: none
Relative Expression
Zt (hr)
21.5
0.5
3.5
6.5
9.5
12.5
18.5
0
2
4
6
8

Singeleton: ENSXMAG00000004875 phip

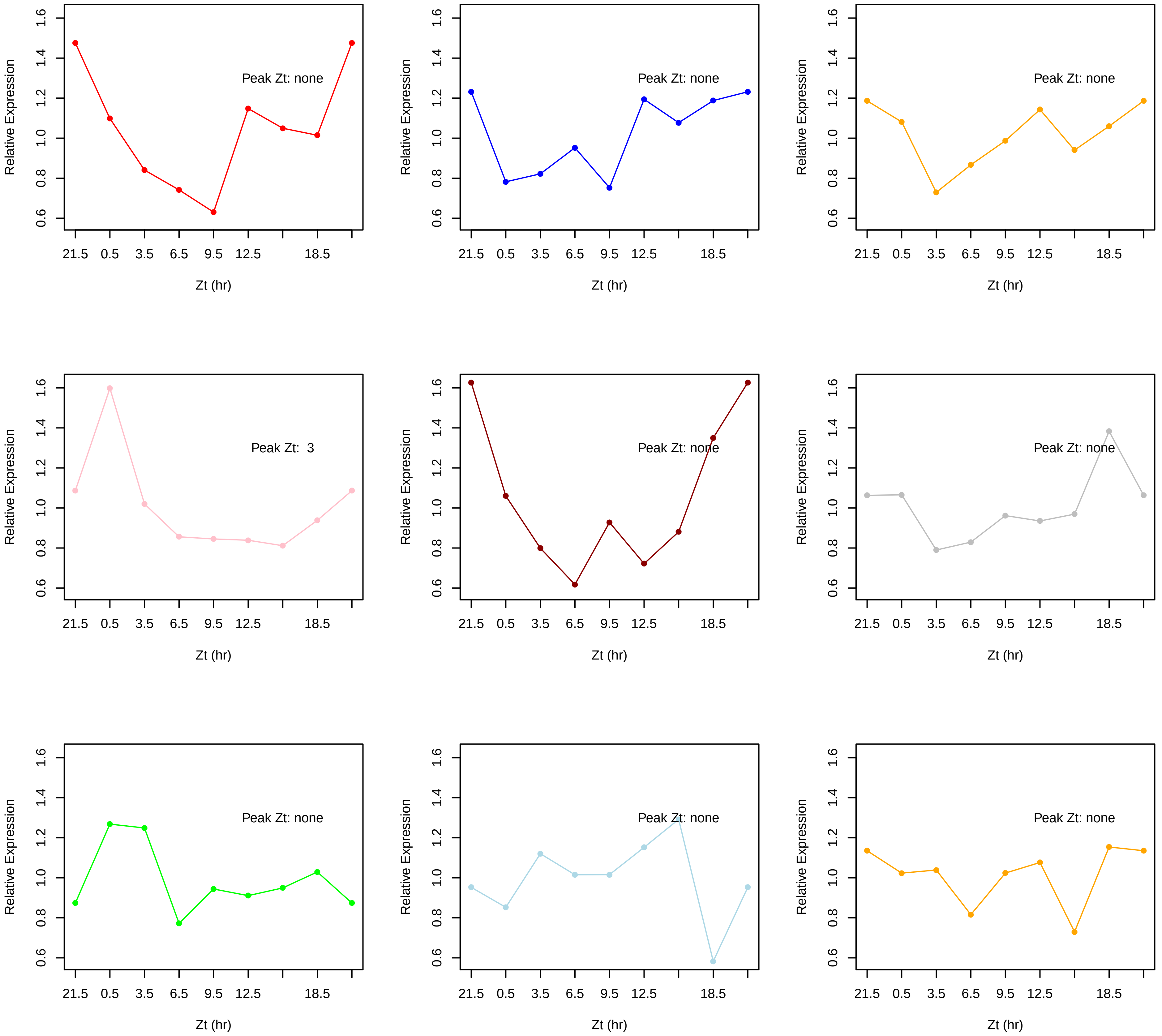

Singeleton: ENSXMAG00000017191 TMEM19

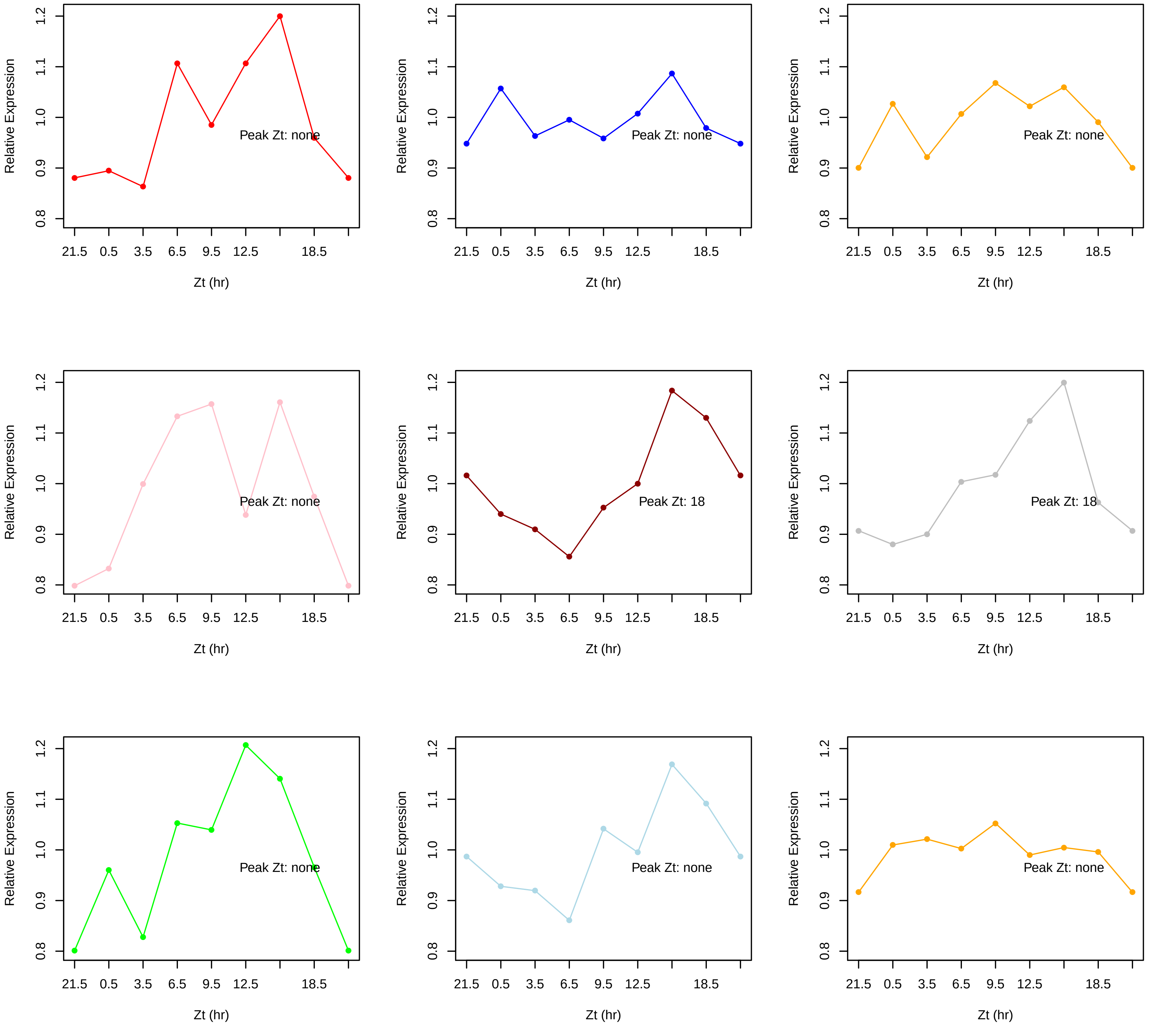

Singeleton: ENSXMAG00000029463 irak1bp1

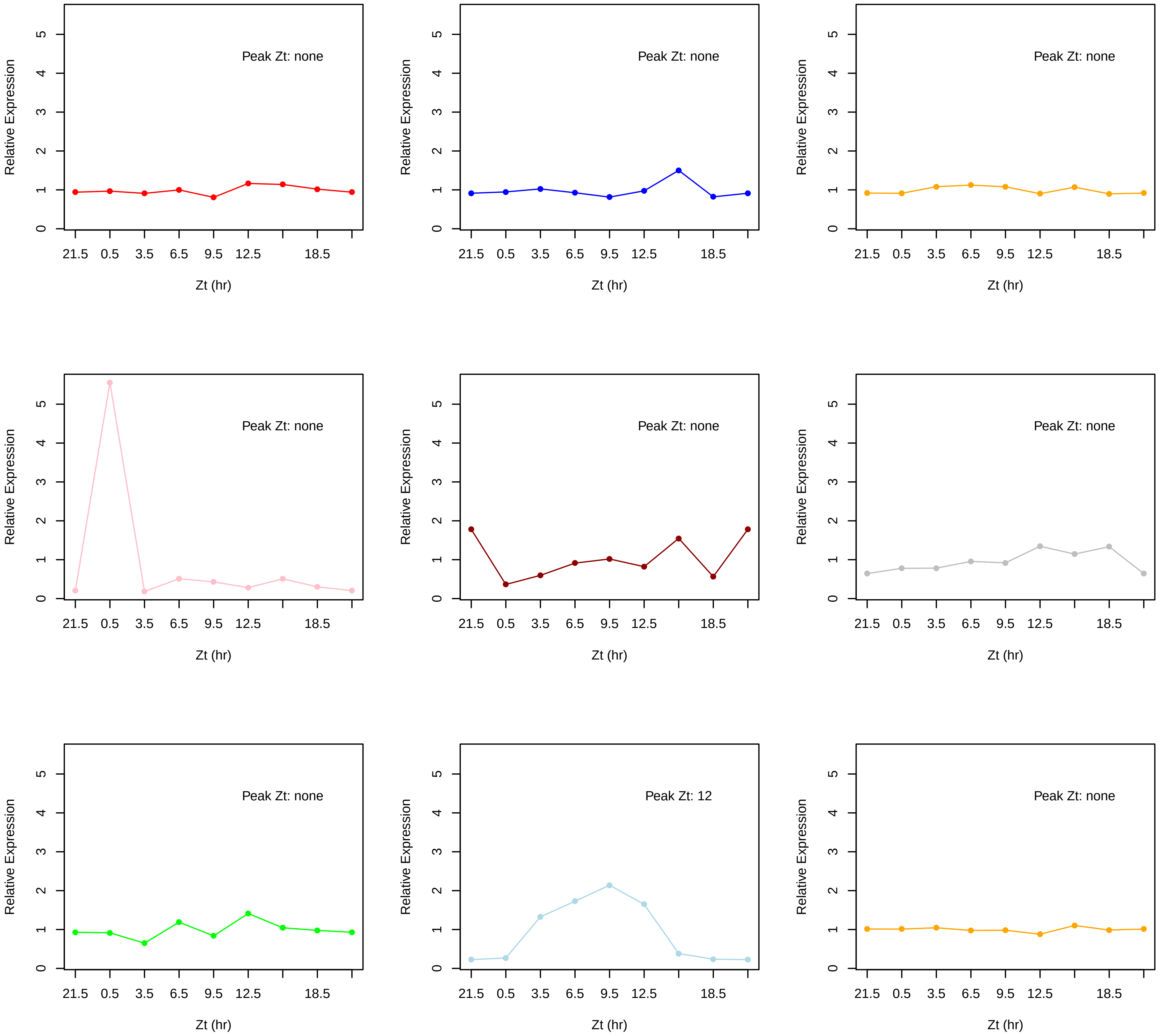

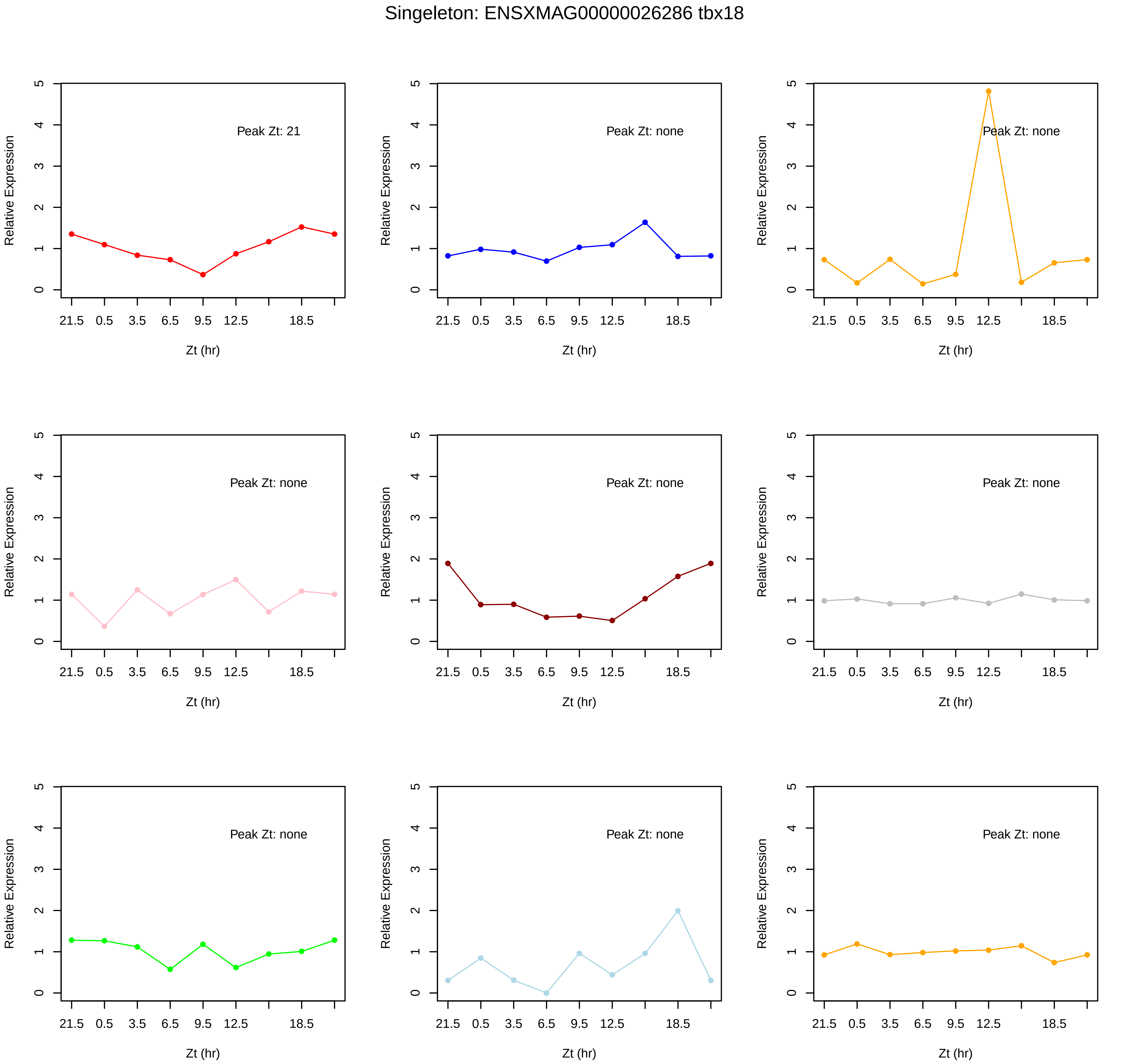
Singleton: ENSXMAG00000026286 tbx18
Peak Zt: 21
Peak Zt: none
Relative Expression
Zt (hr)
21.5
0.5
3.5
6.5
9.5
12.5
18.5
0
1
2
3
4
5

Singleton: ENSXMAG00000003205 dynlt1

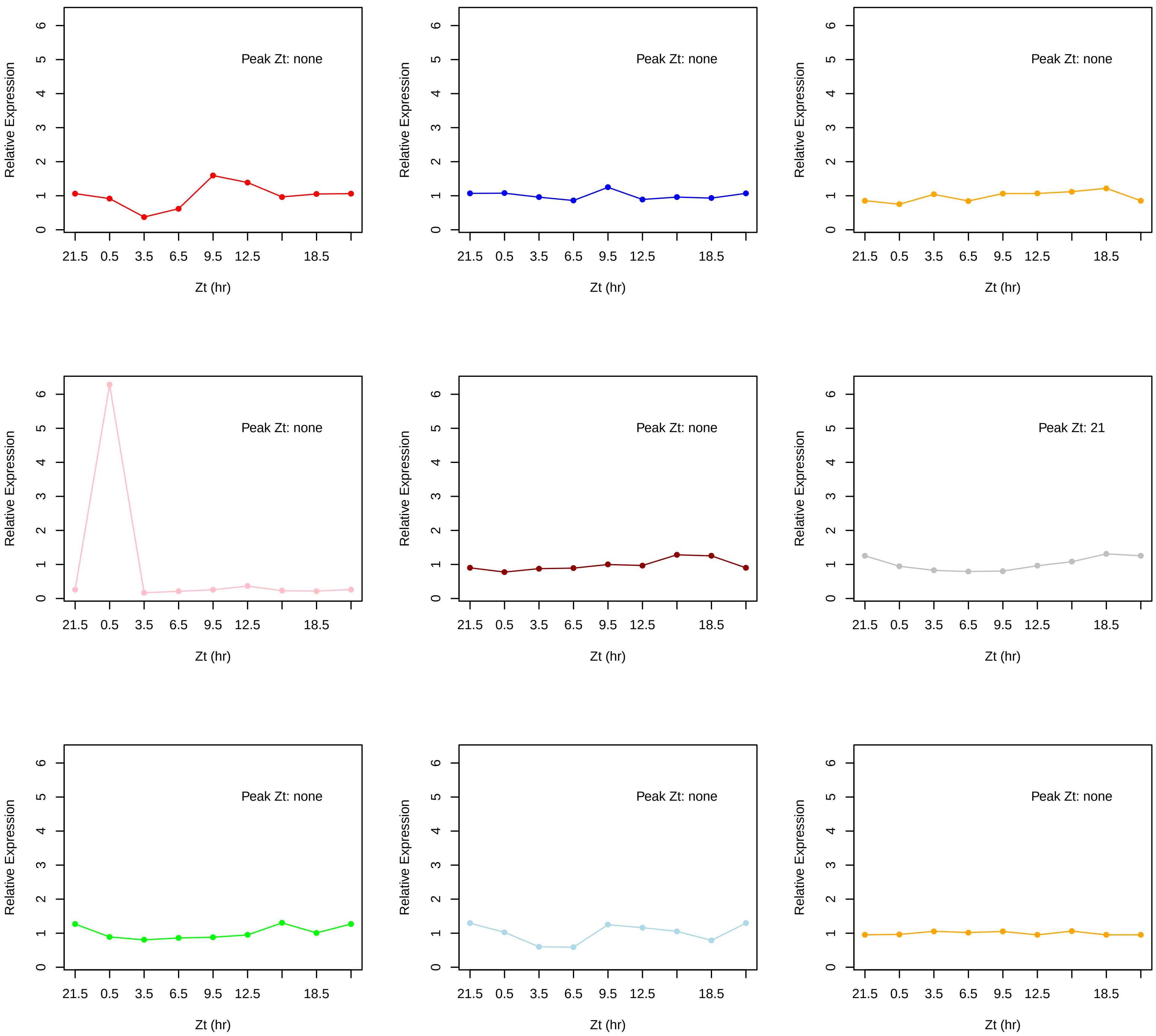

Singeleton: ENSXMAG00000023624 krr1

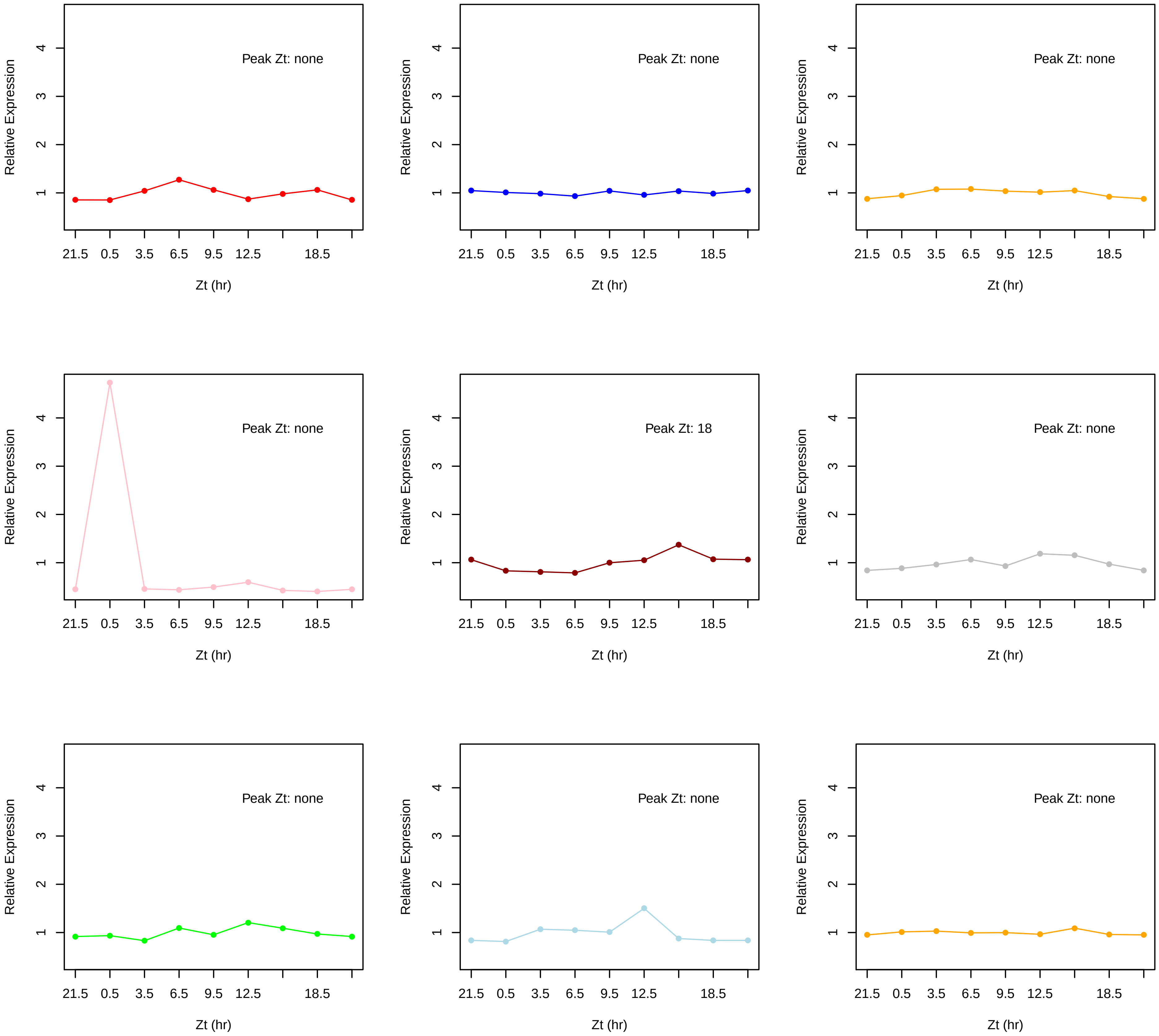

Singleton: ENSXMAG00000010822 erlec1

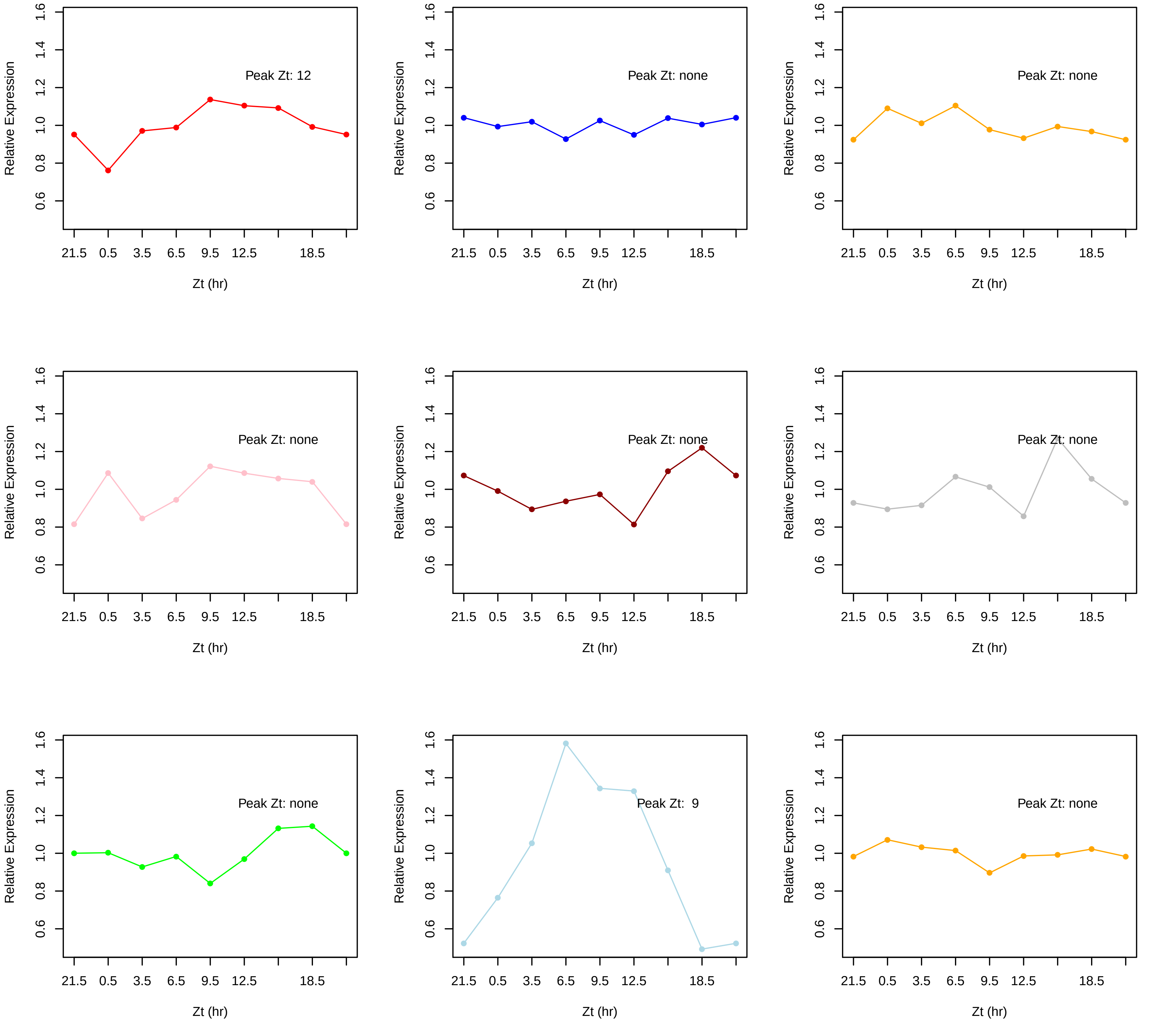

Singeleton: ENSXMAG00000025006 nap1l1

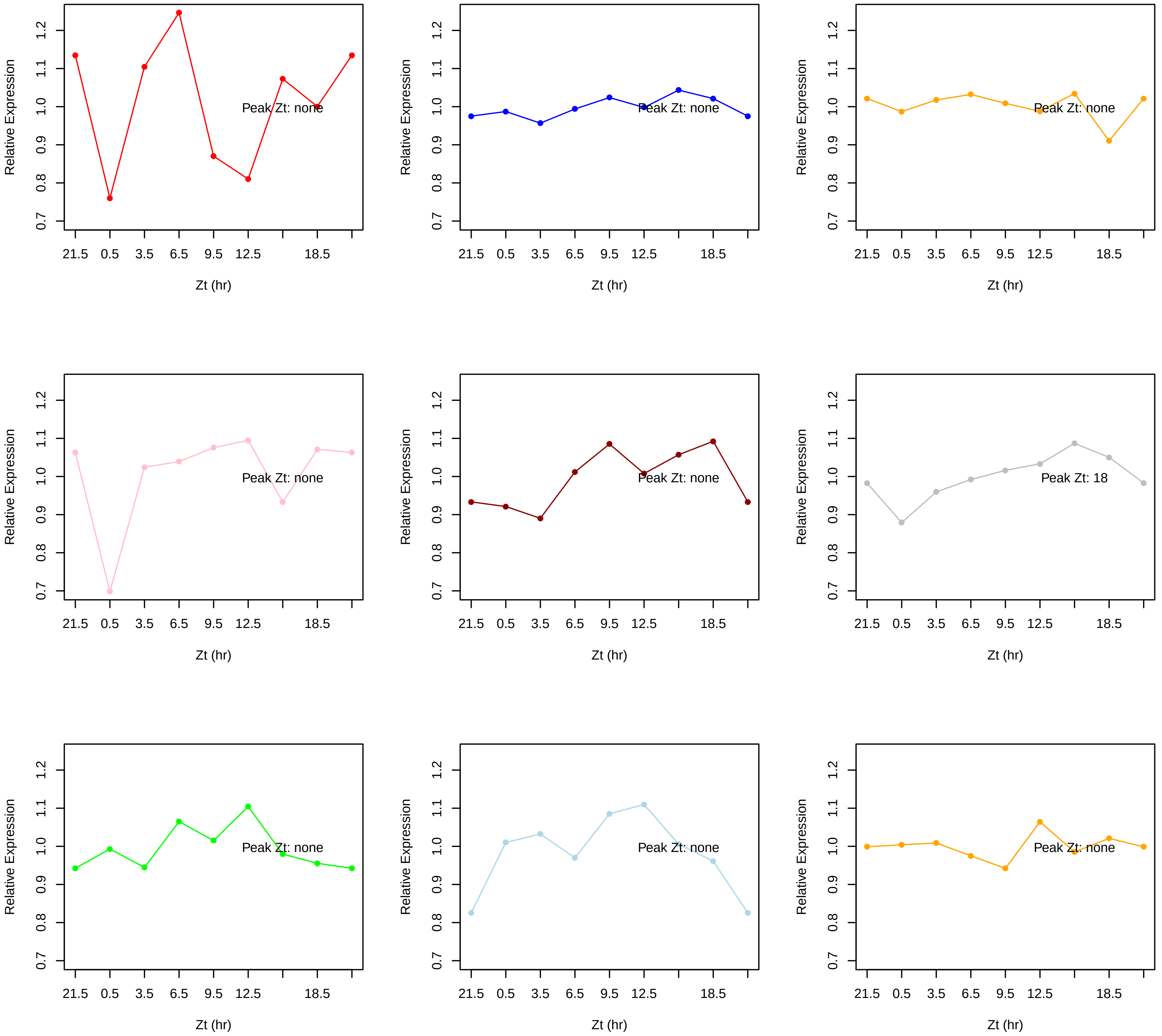

Singeleton: ENSXMAG00000016161 chac2

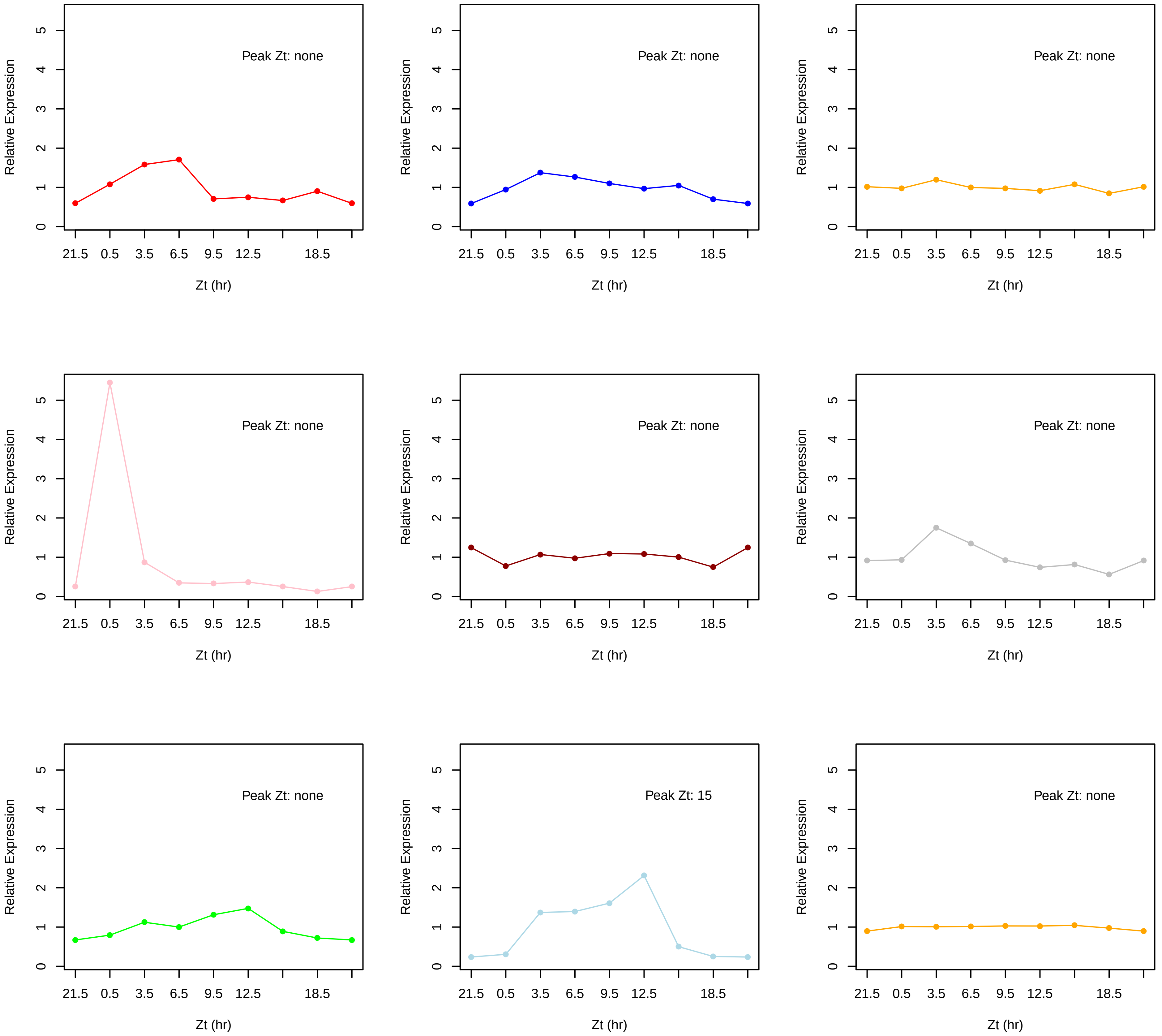

Singeleton: ENSXMAG00000006300 OSBPL8

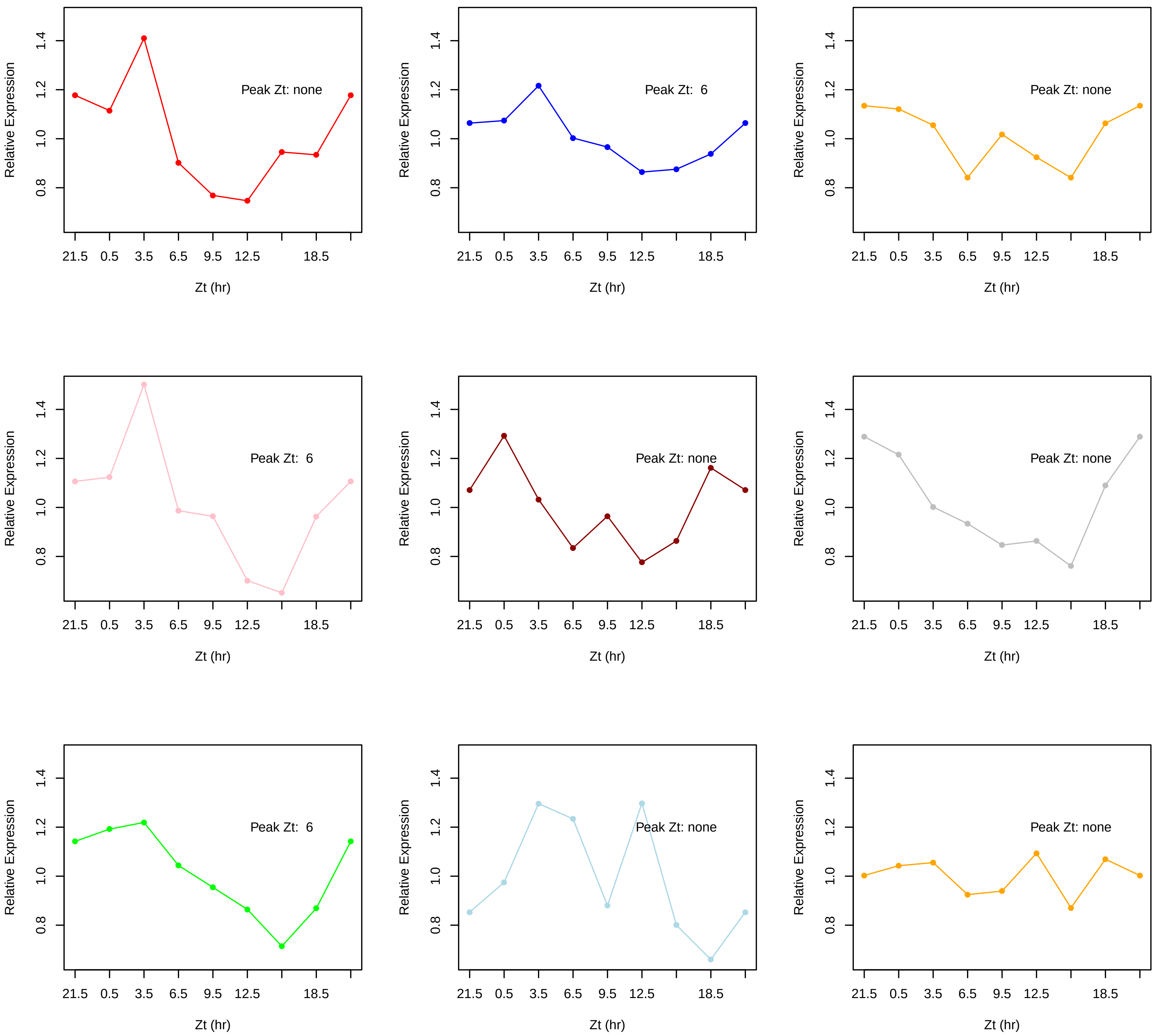

Singeleton: ENSXMAG00000006257 csrp2

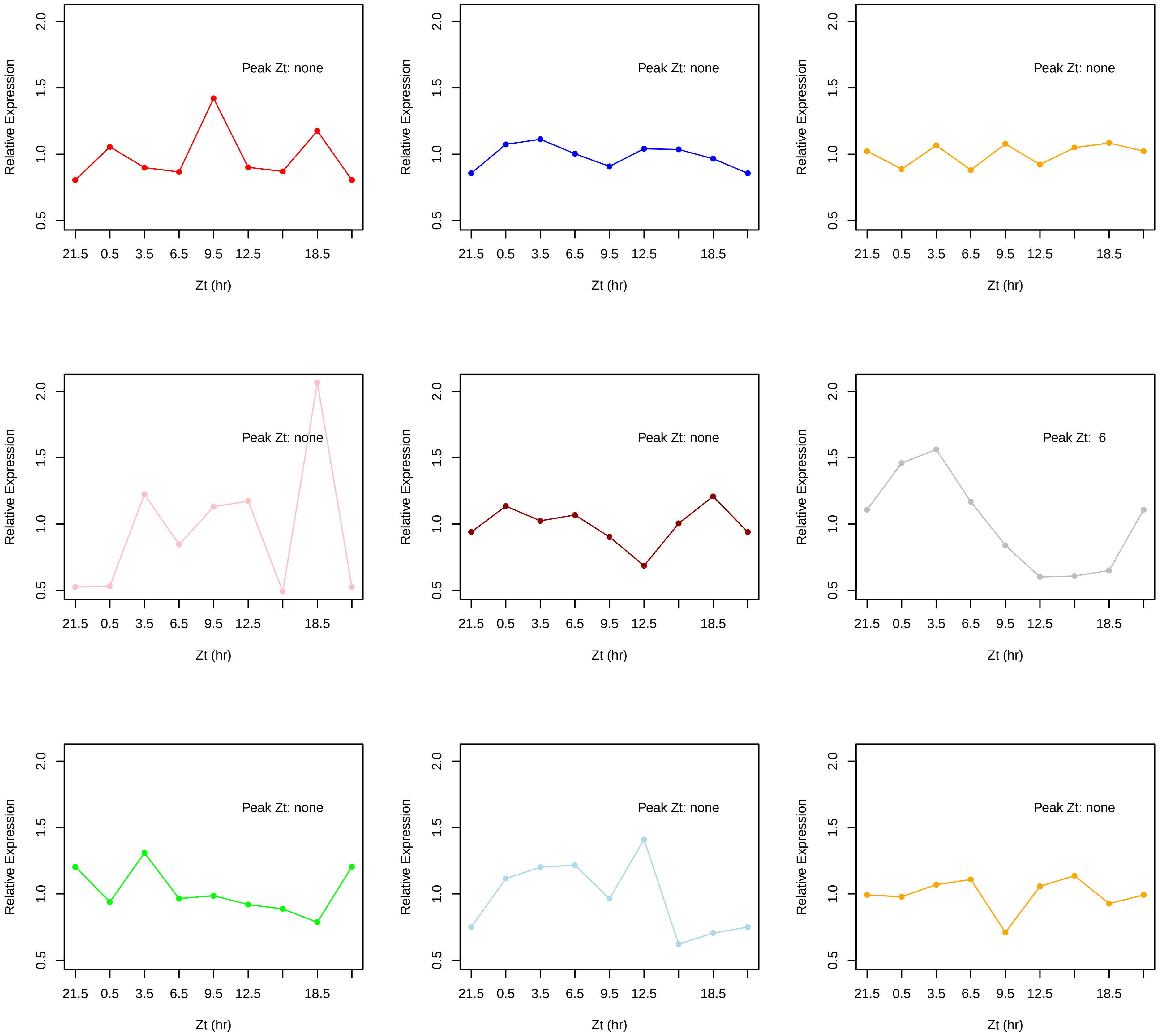

Singeleton: ENSXMAG00000010942 ppp1r21

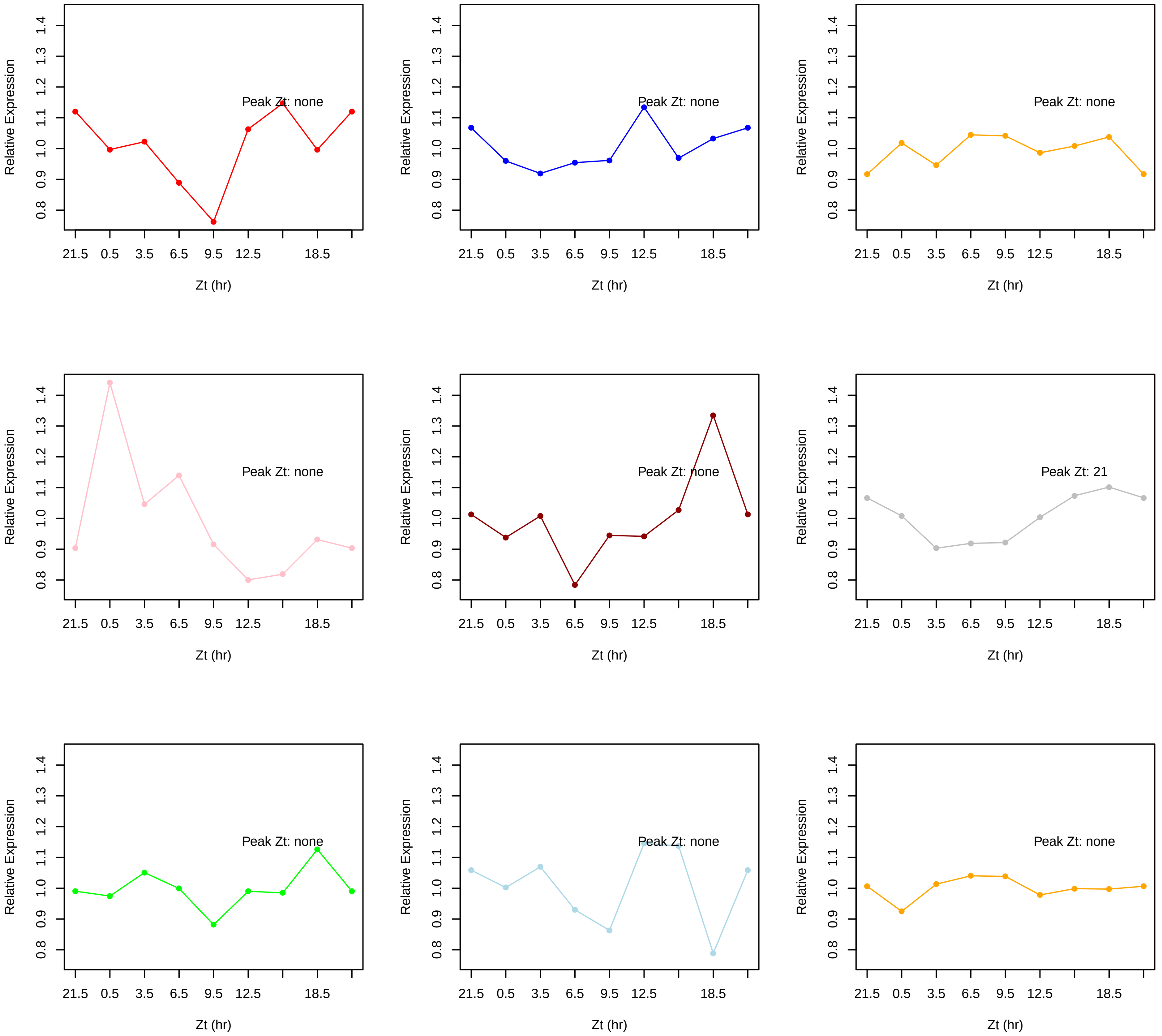

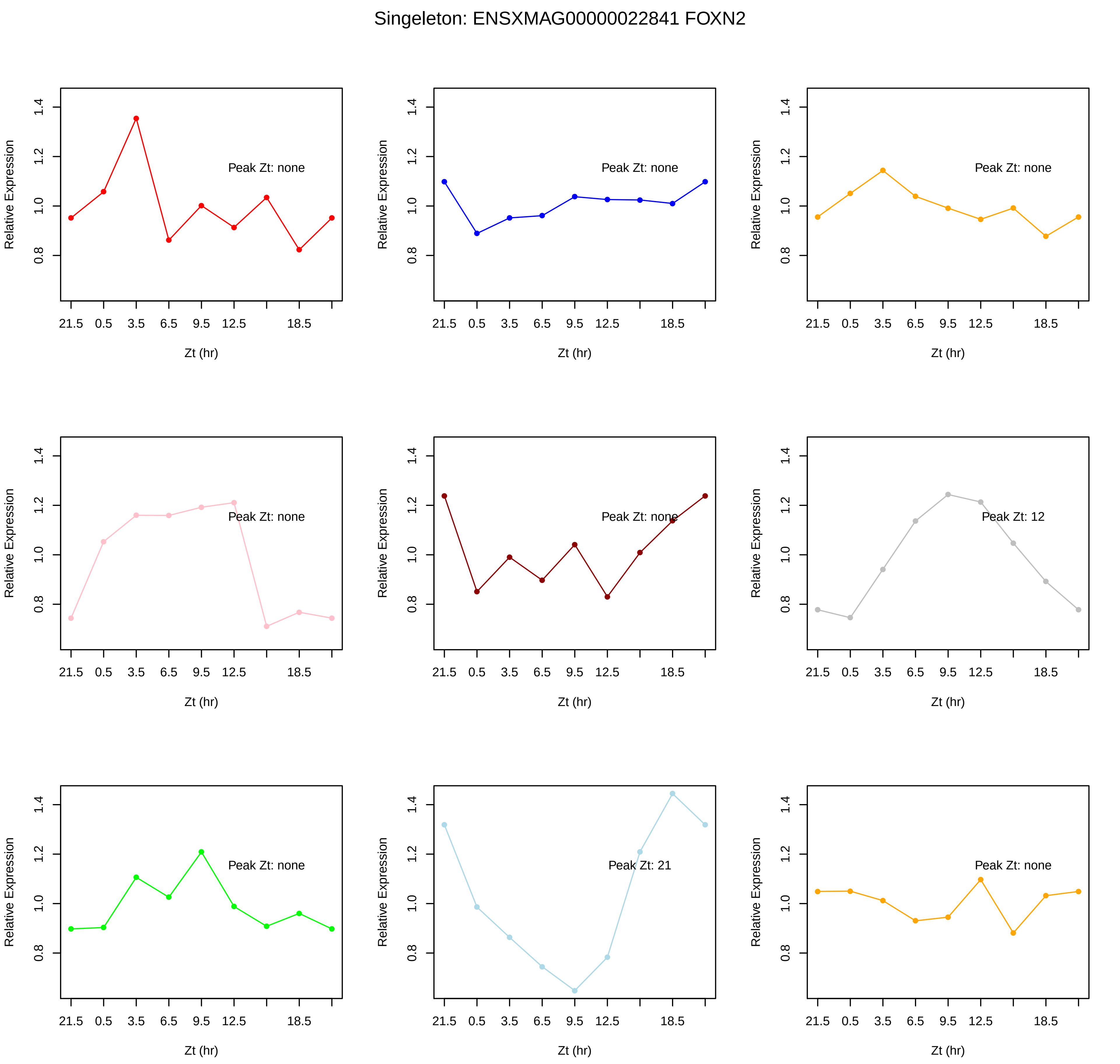
Singeleton: ENSXMAG00000022841 FOXN2
Peak Zt: none
Peak Zt: none
Peak Zt: none
Peak Zt: none
Peak Zt: none
Peak Zt: 12
Peak Zt: none
Peak Zt: 21
Peak Zt: none
Relative Expression
Zt (hr)
21.5
0.5
3.5
6.5
9.5
12.5
18.5
0.8
1.0
1.2
1.4

Singeleton: ENSXMAG00000015383 rmnd1

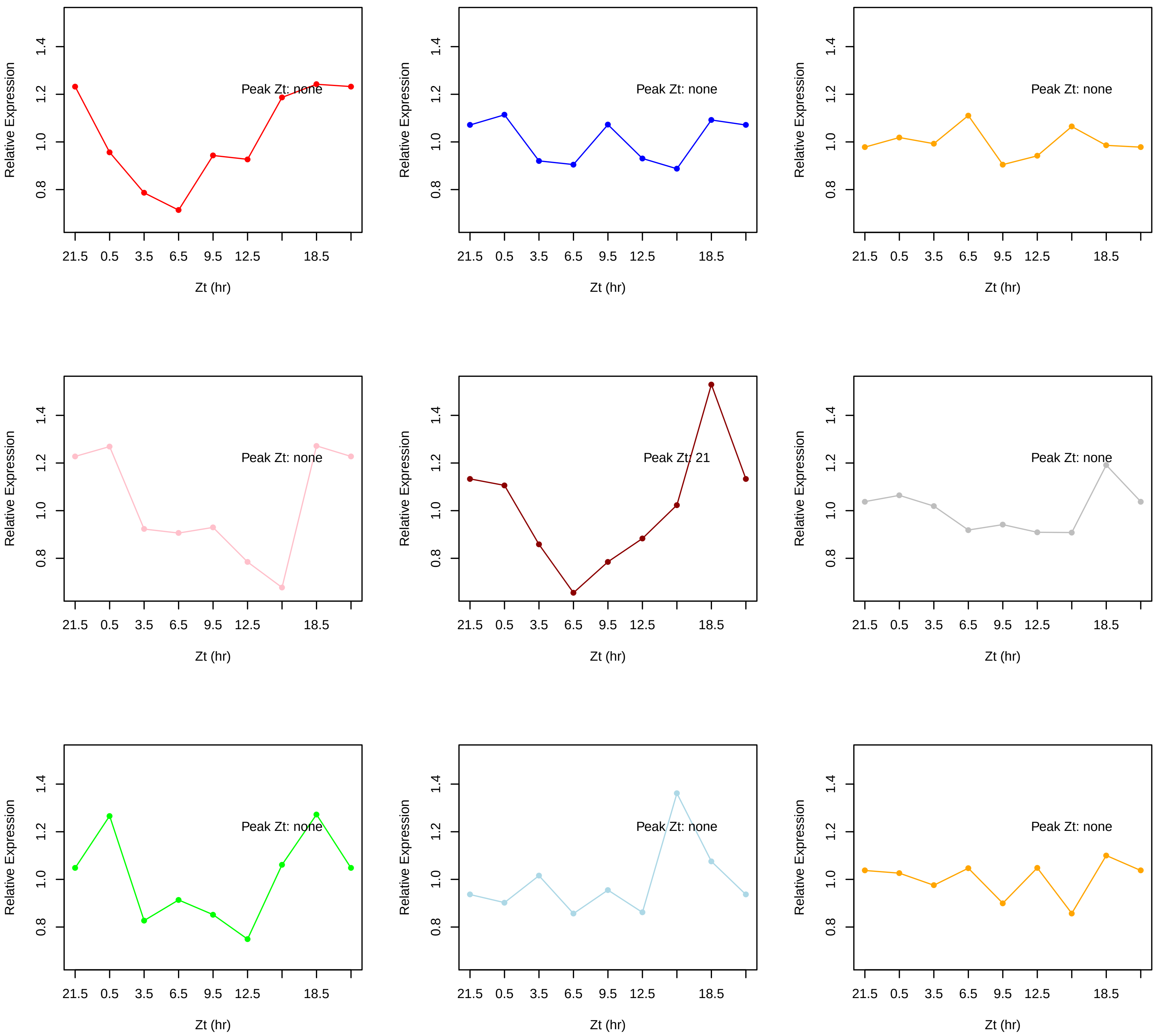

Singeleton: ENSXMAG00000025504 zbtb2b

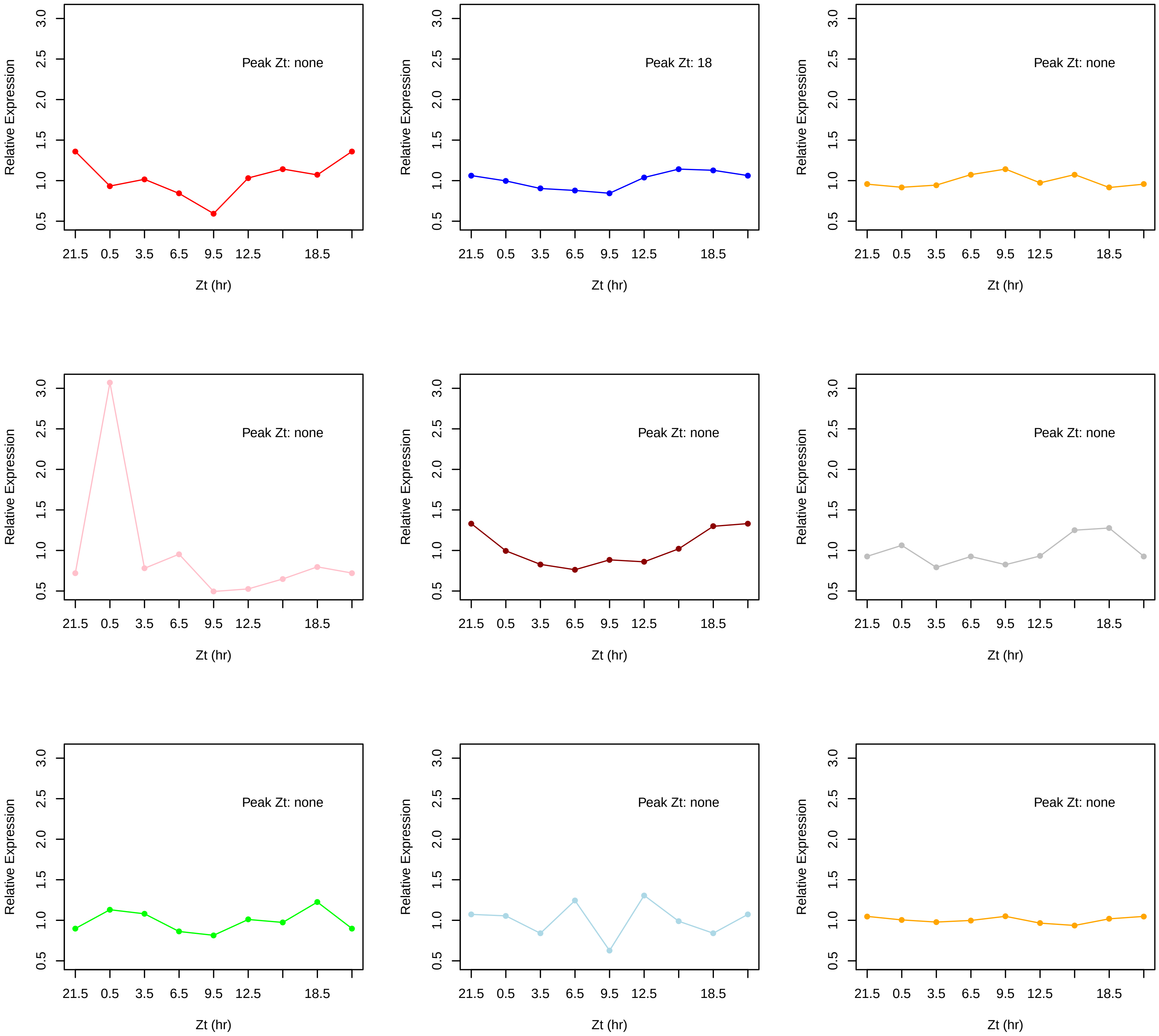

Singeleton: ENSXMAG00000024248 akap12b

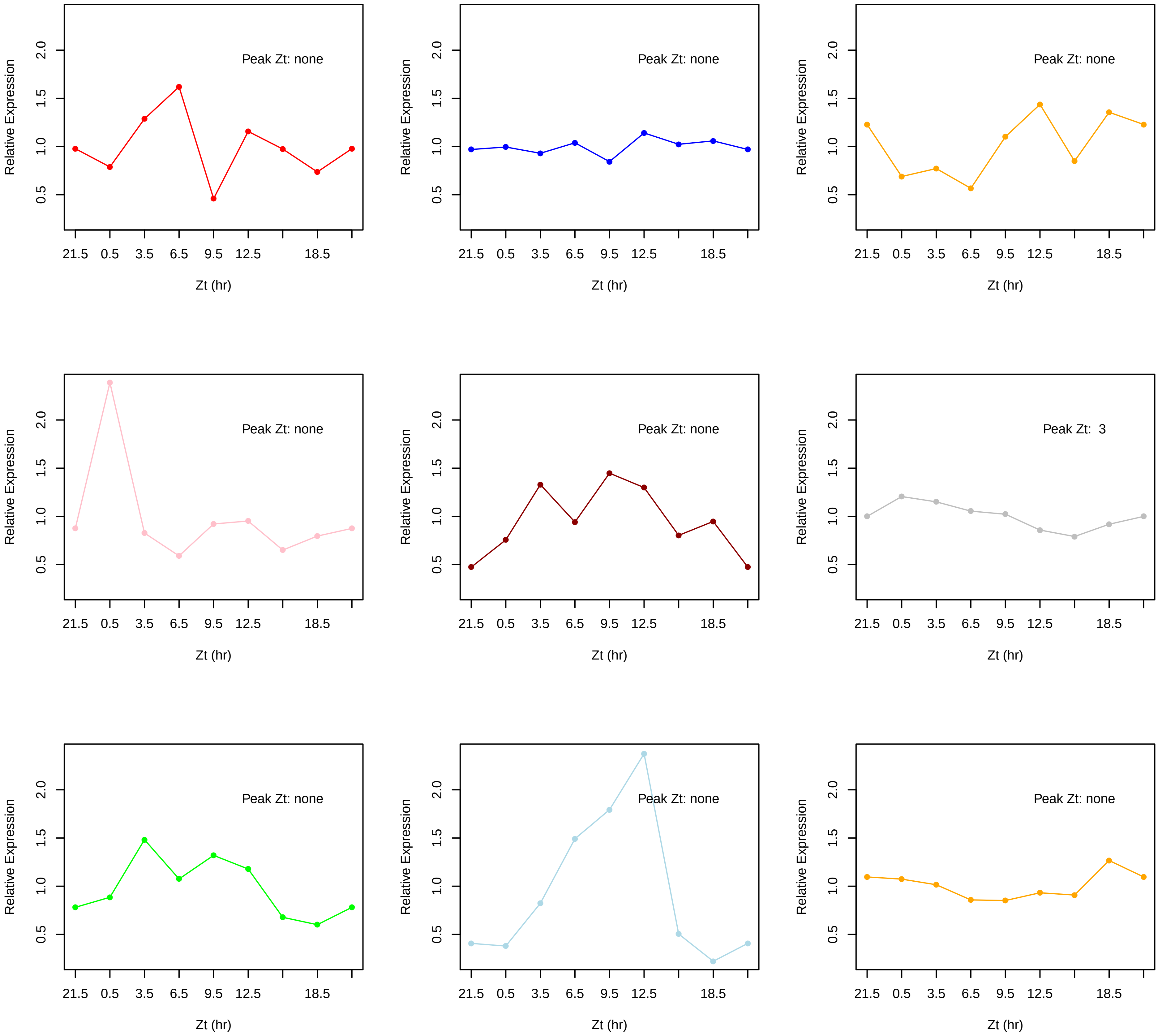

Singeleton: ENSXMAG00000027669 mthfd1l

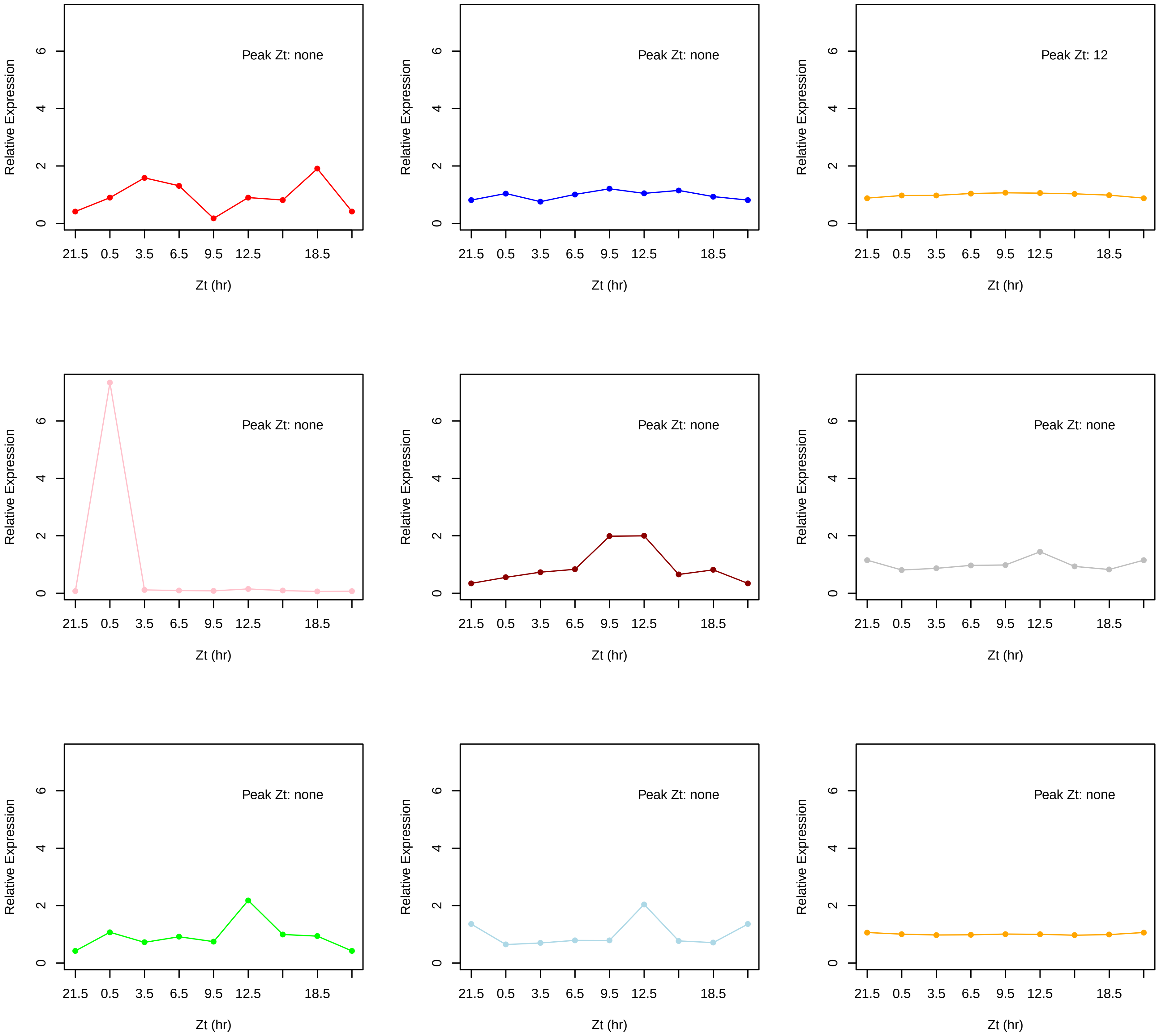

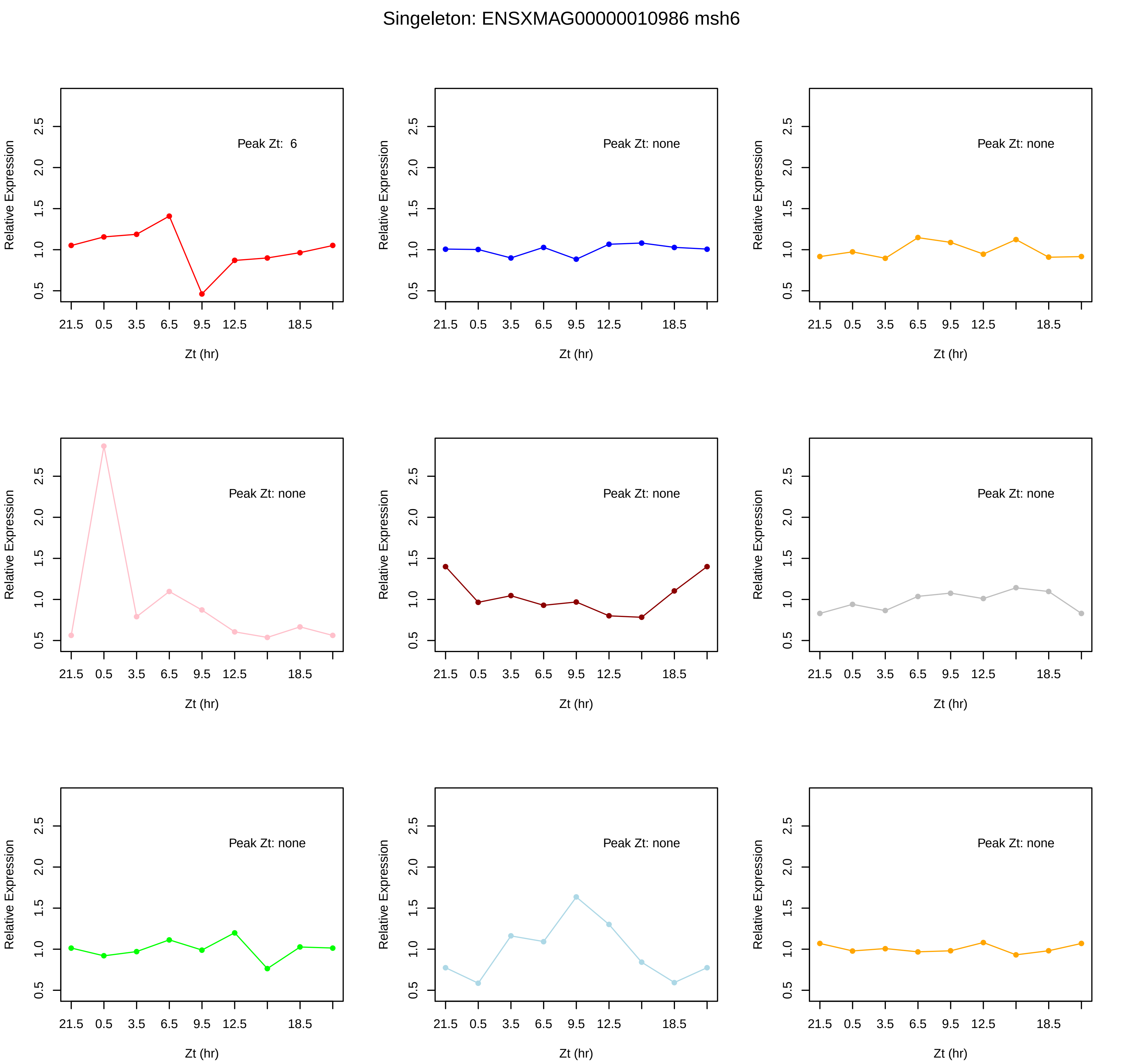
Singeleton: ENSXMAG00000010986 msh6
Peak Zt: 6
Peak Zt: none
Peak Zt: none
Peak Zt: none
Peak Zt: none
Peak Zt: none
Peak Zt: none
Peak Zt: none
Peak Zt: none
Relative Expression
Zt (hr)
21.5
0.5
3.5
6.5
9.5
12.5
18.5
0.5
1.0
1.5
2.0
2.5

Singeleton: ENSXMAG00000026246 lin7a

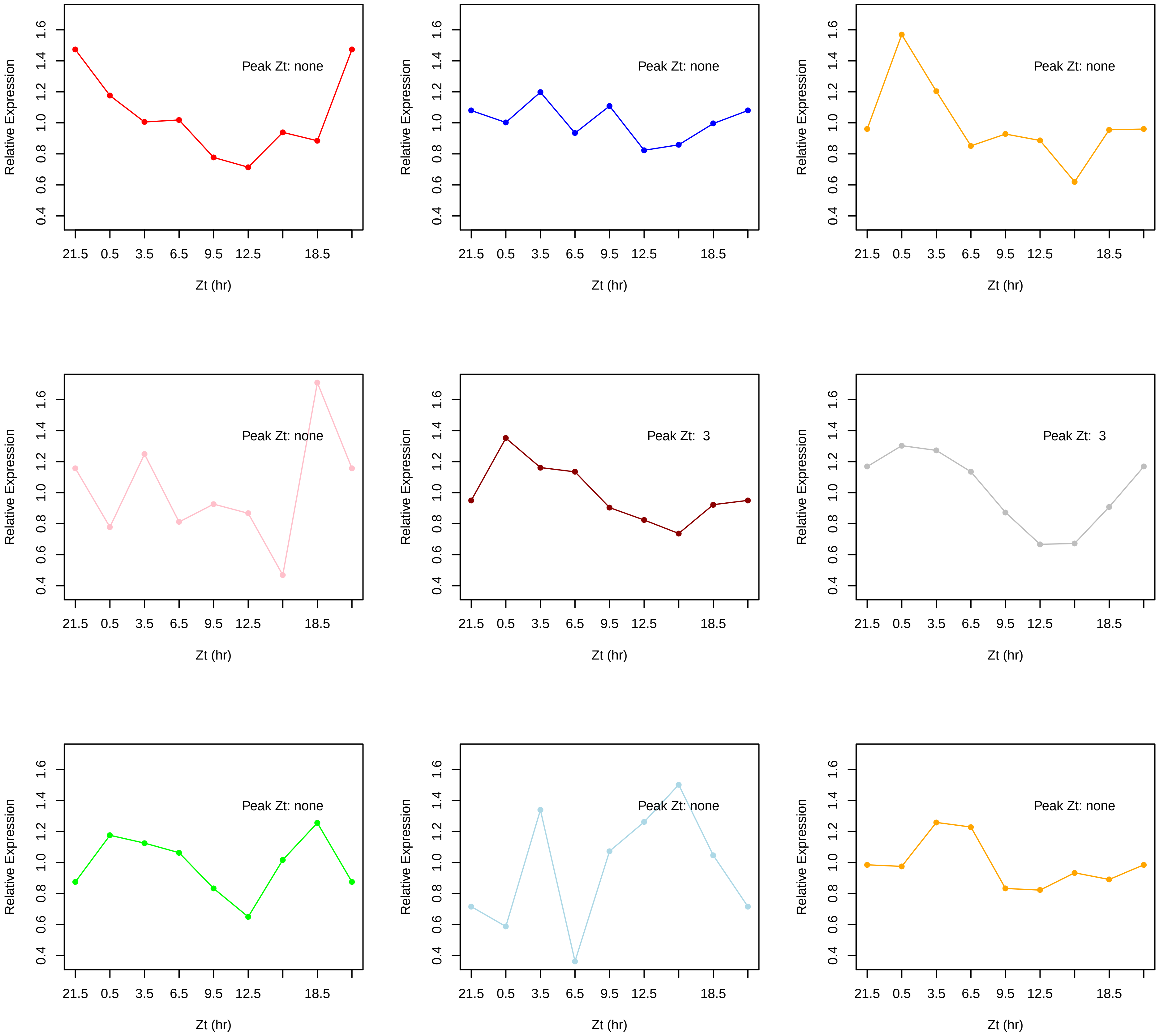

Singleton: ENSXMAG00000007455 epcam

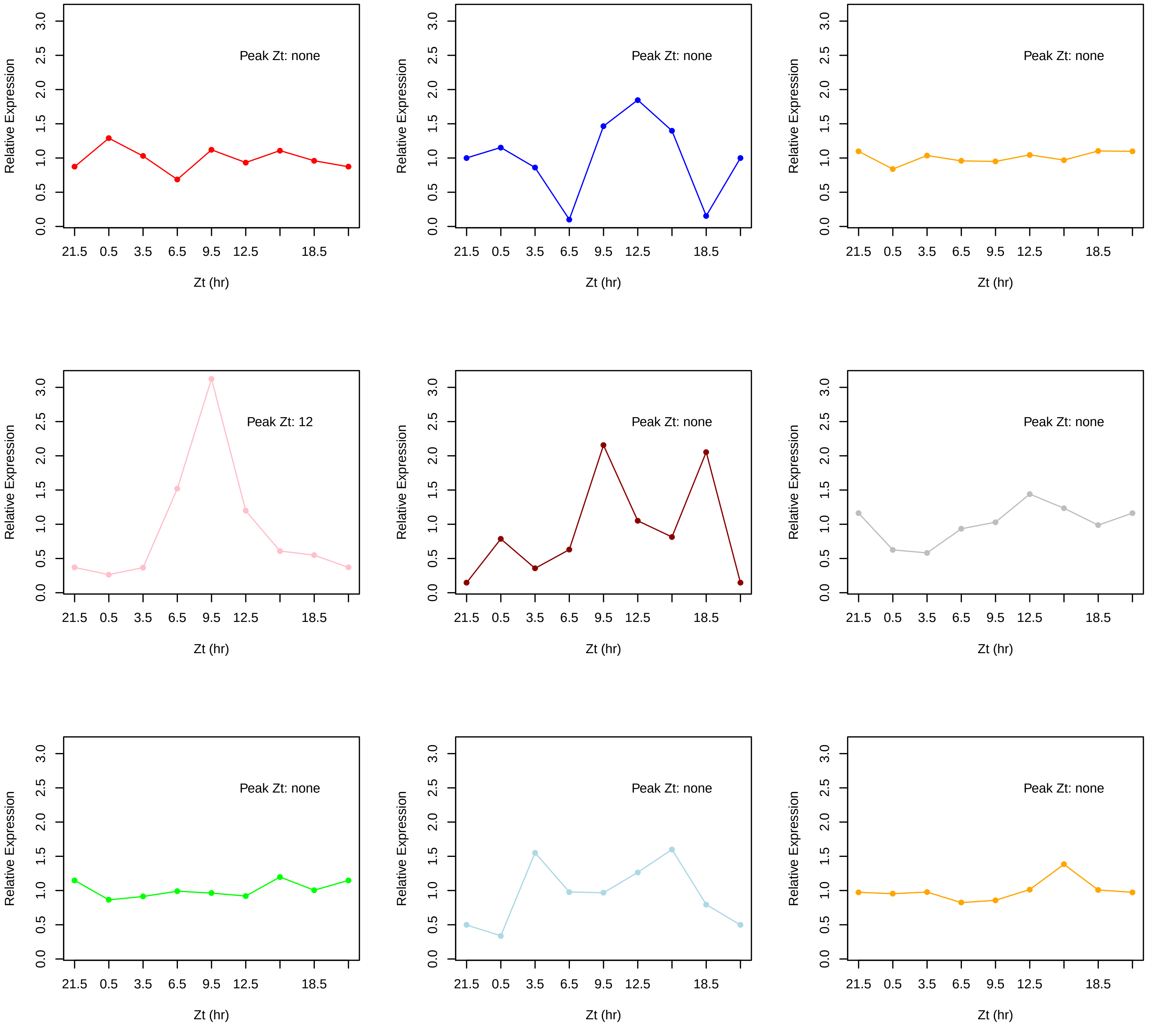

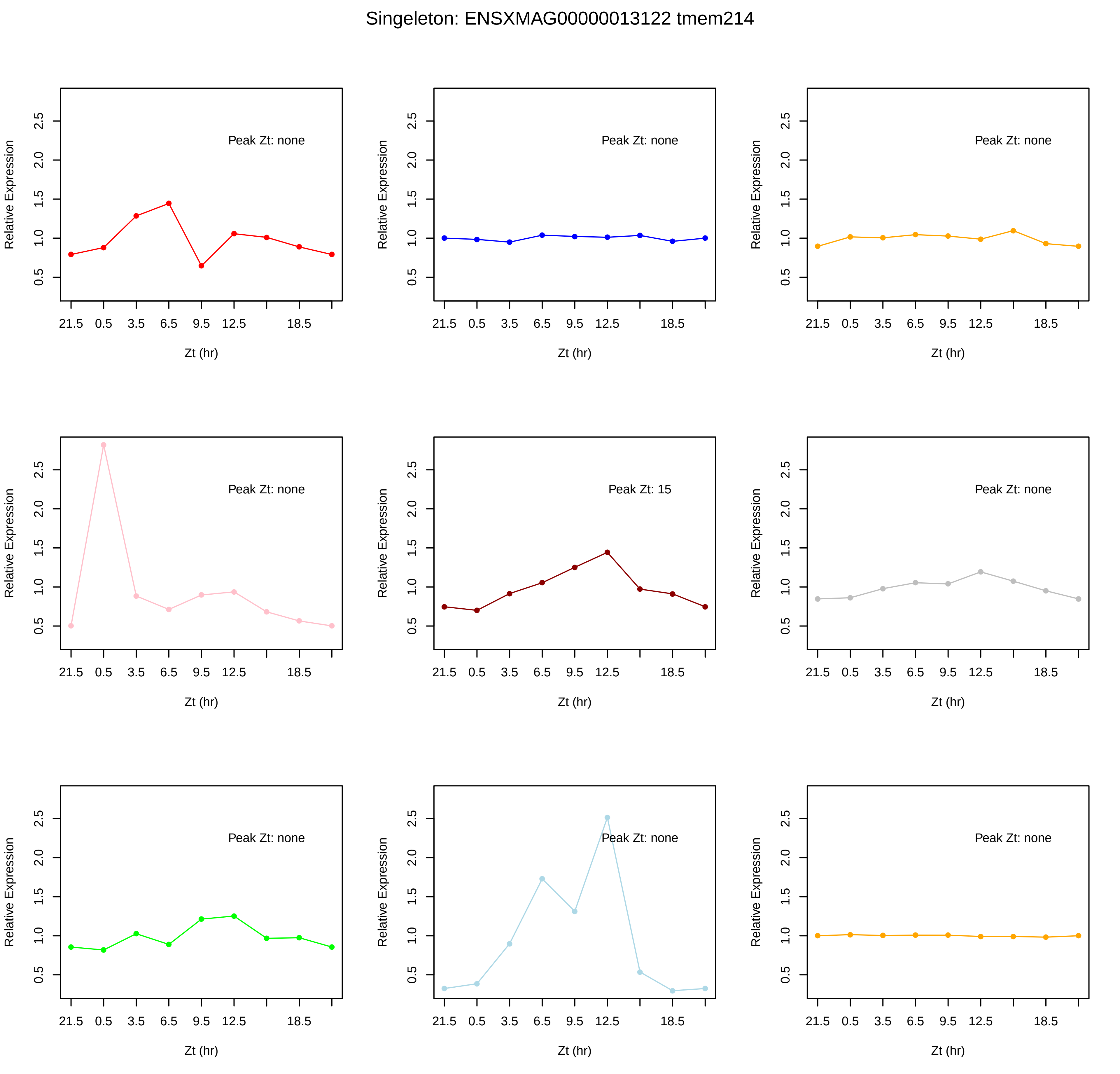
Singeleton: ENSXMAG00000013122 tmem214
Peak Zt: none
Peak Zt: none
Peak Zt: none
Peak Zt: none
Peak Zt: 15
Peak Zt: none
Peak Zt: none
Peak Zt: none
Peak Zt: none
Relative Expression
Zt (hr)
21.5
0.5
3.5
6.5
9.5
12.5
18.5
0.5
1.0
1.5
2.0
2.5

Singeleton: ENSXMAG00000015588 mapre3a

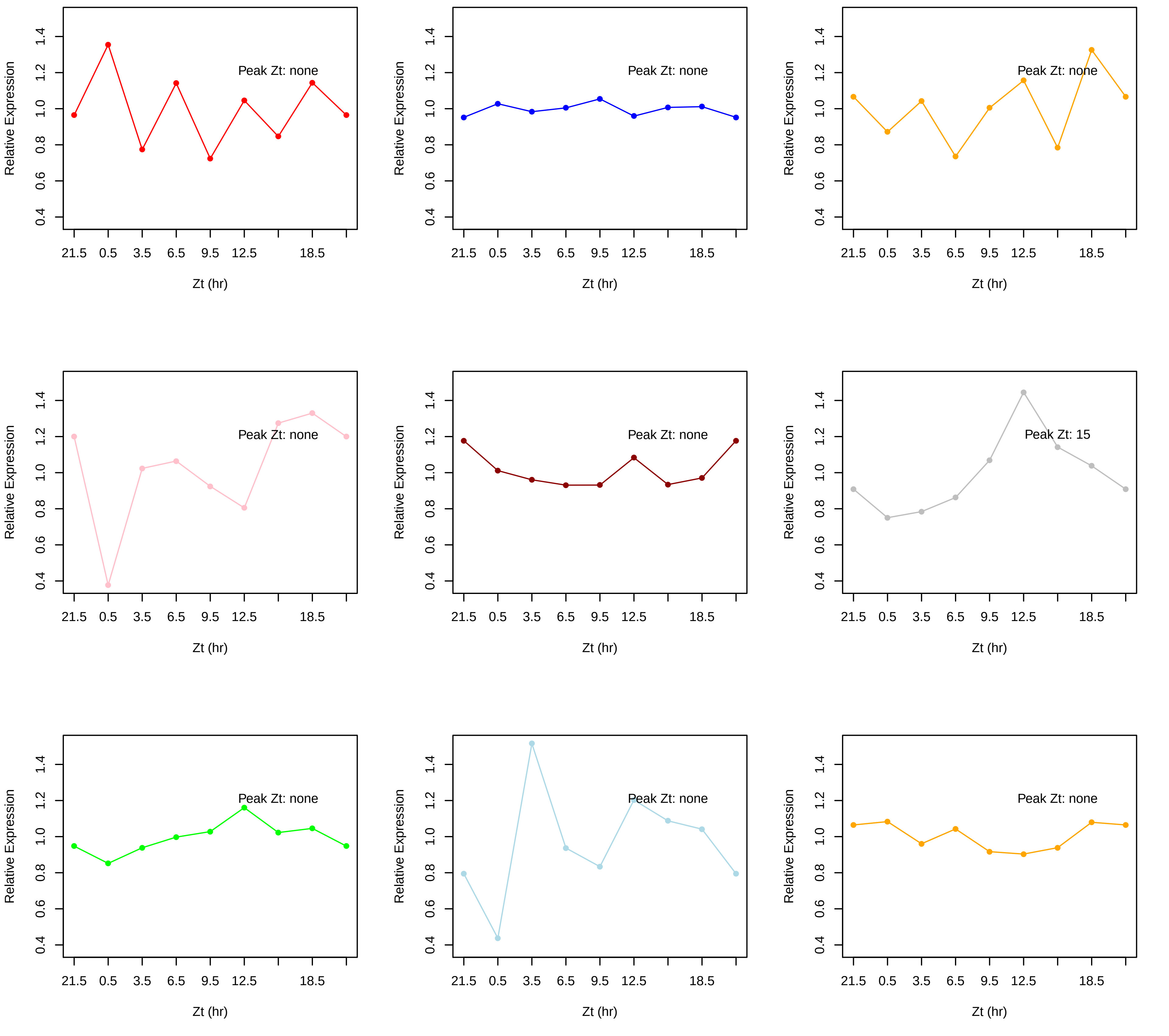

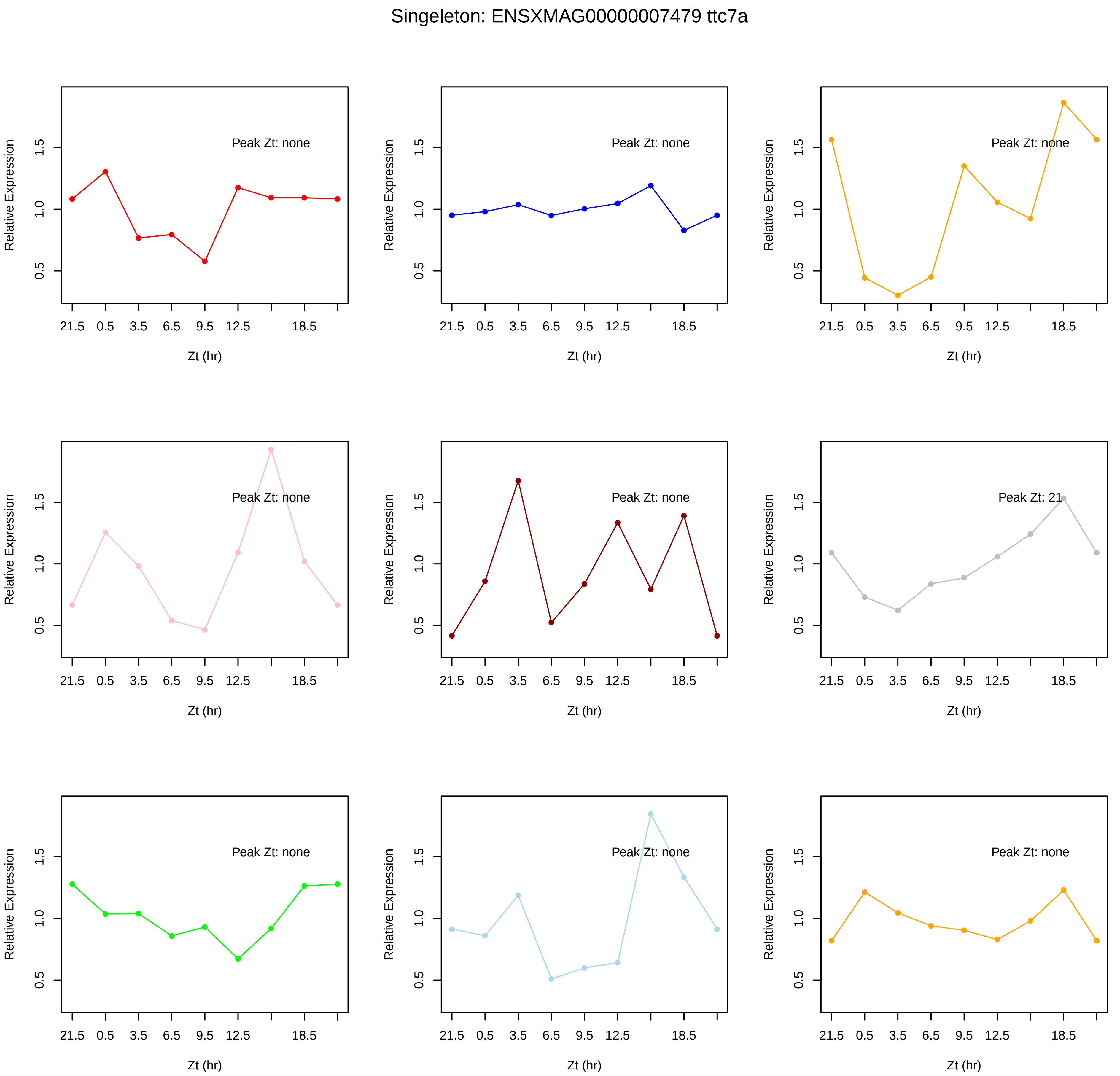

Singeleton: ENSXMAG00000007479 ttc7a
Peak Zt: none
Peak Zt: 21
Relative Expression
Zt (hr)
21.5
0.5
3.5
6.5
9.5
12.5
18.5
0.5
1.0
1.5

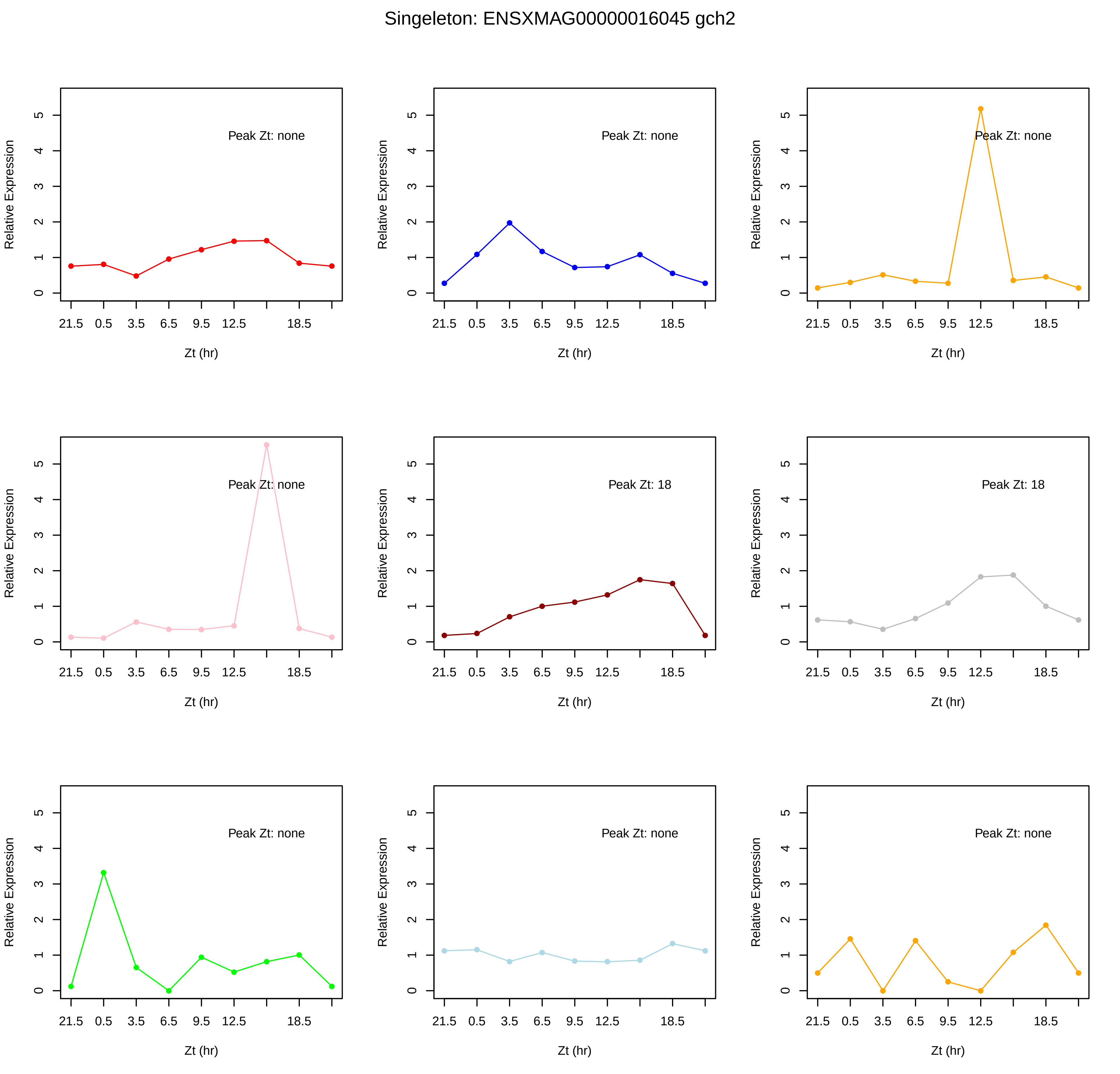
Singeleton: ENSXMAG00000016045 gch2
Peak Zt: none
Peak Zt: none
Peak Zt: none
Peak Zt: none
Peak Zt: 18
Peak Zt: 18
Peak Zt: none
Peak Zt: none
Peak Zt: none
Relative Expression
Zt (hr)
21.5 0.5 3.5 6.5 9.5 12.5 18.5

Singeleton: ENSXMAG00000011194 mettl25

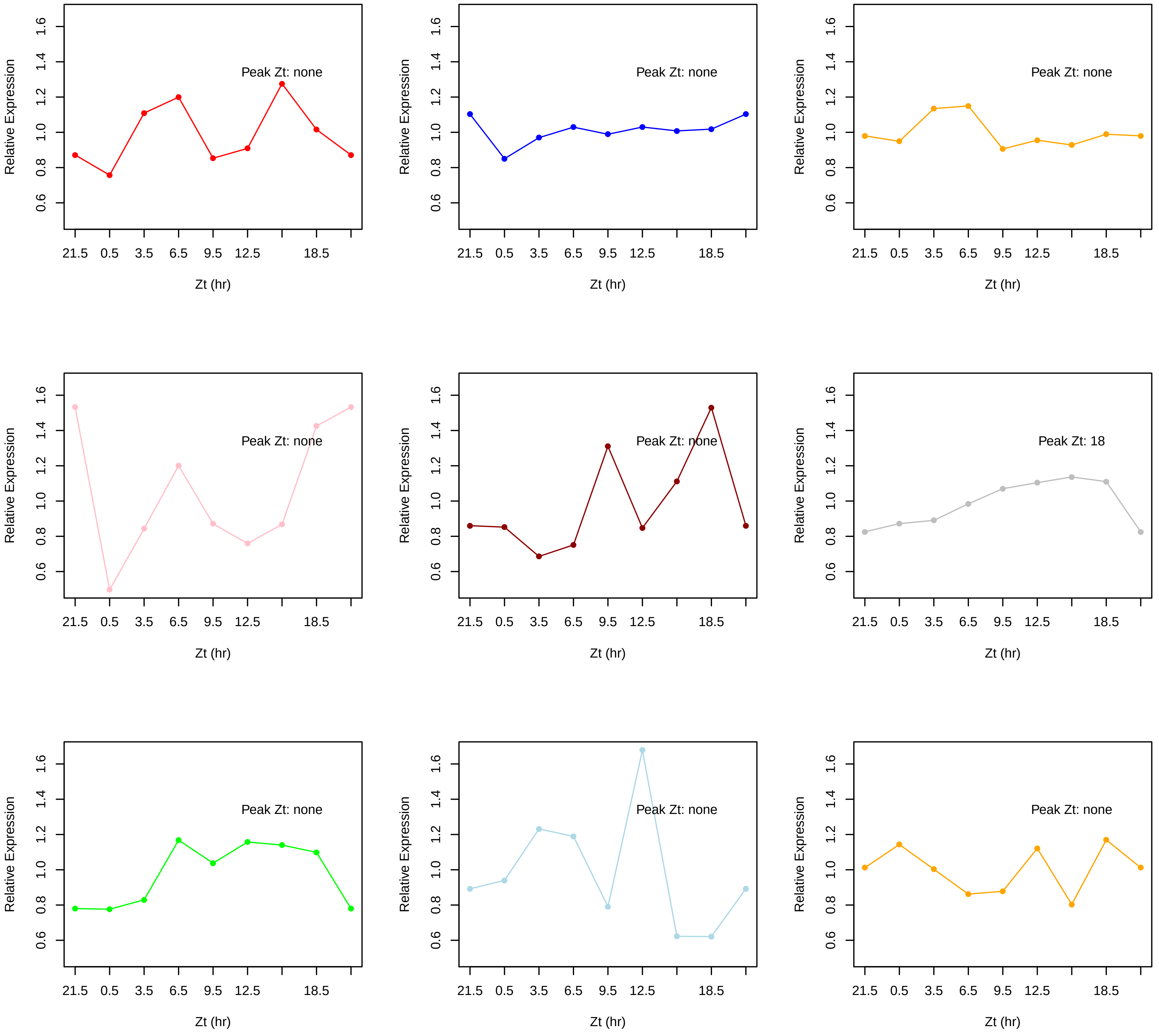

Singeleton: ENSXMAG00000016036 pigf

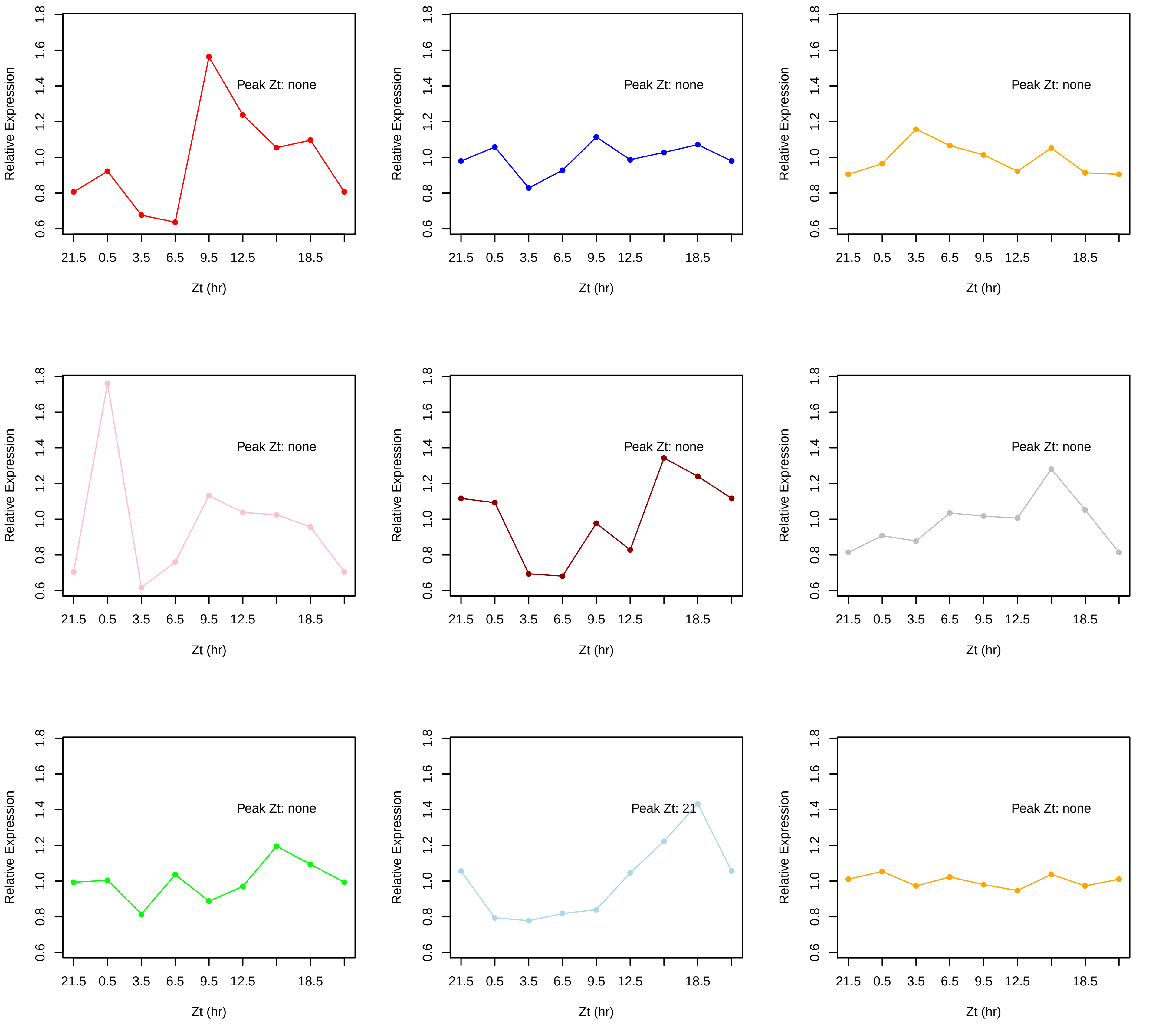

Singeleton: ENSXMAG00000006169 hadhb

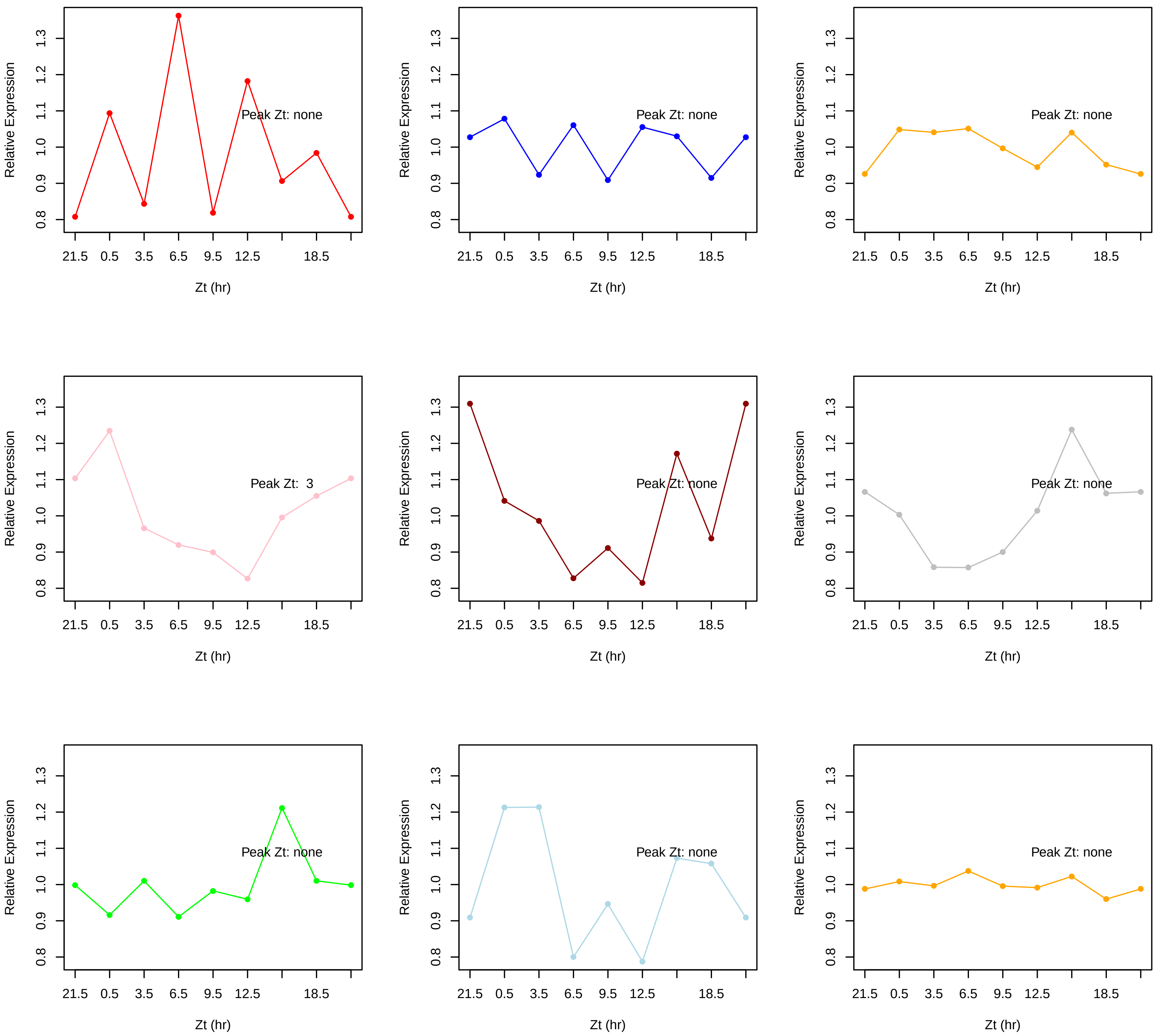

Singeleton: ENSXMAG00000007515 rhoq

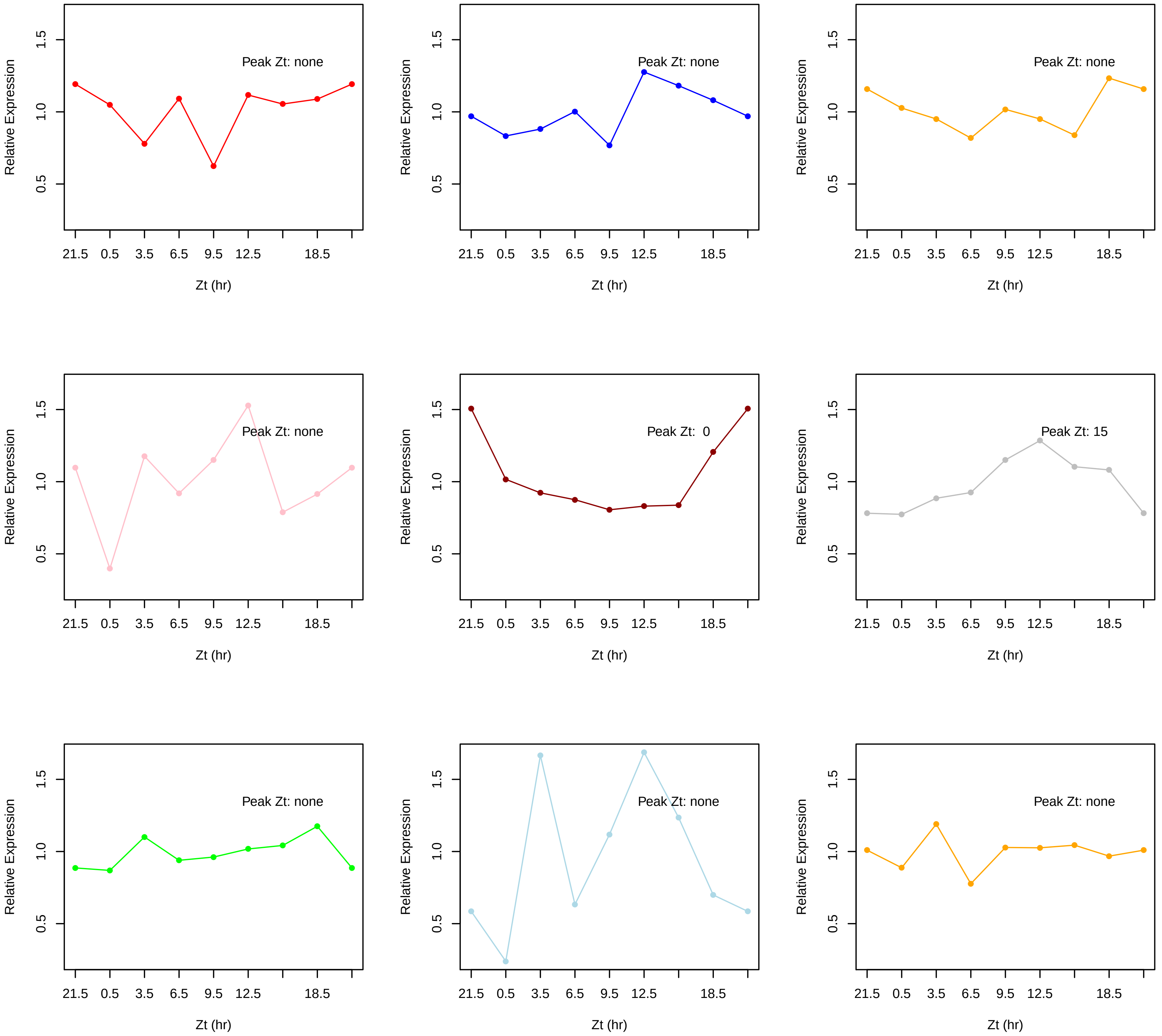

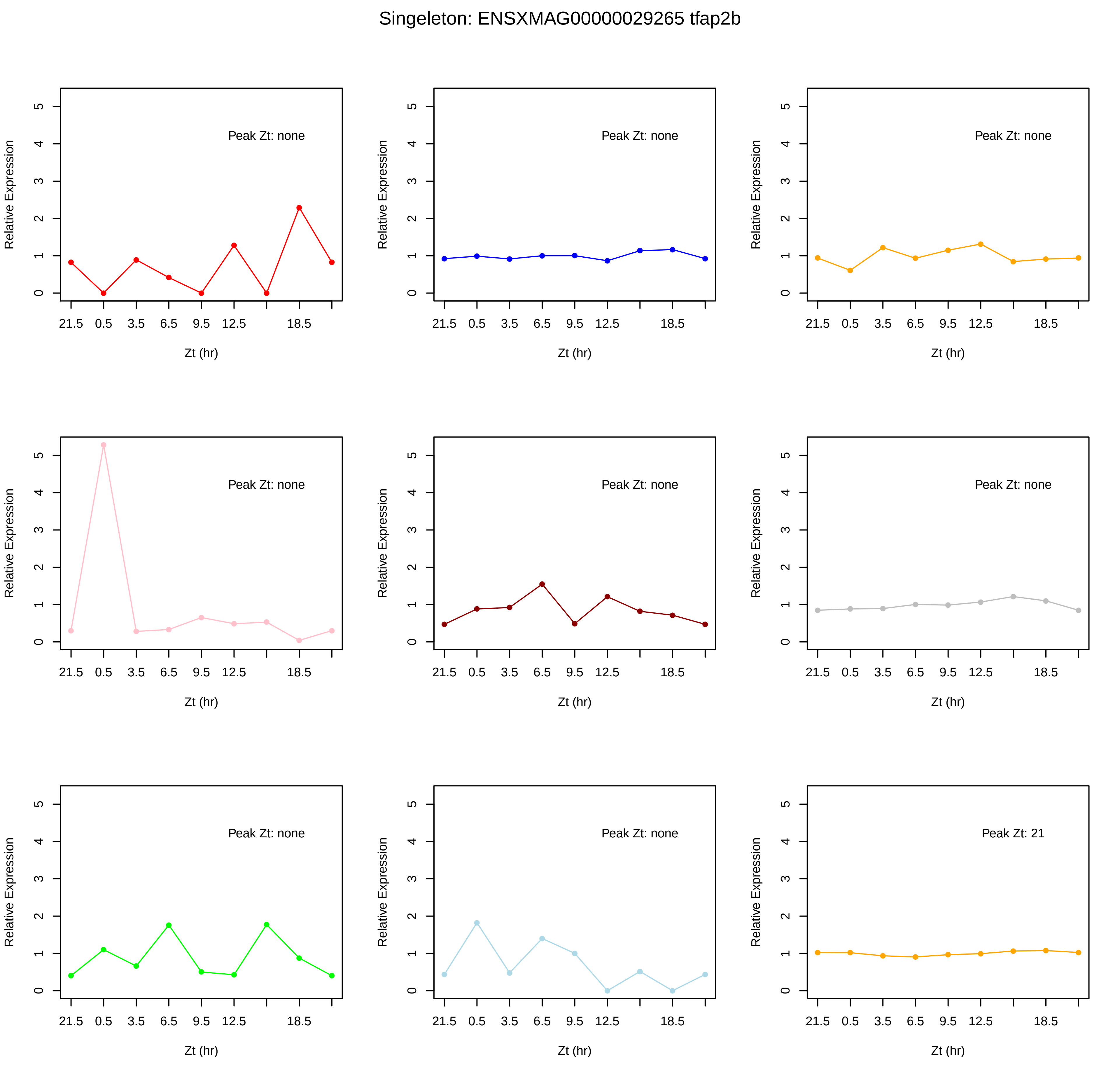

Singleton: ENSXMAG00000029265 tfap2b
Peak Zt: none
Peak Zt: none
Peak Zt: none
Peak Zt: none
Peak Zt: none
Peak Zt: none
Peak Zt: none
Peak Zt: none
Peak Zt: 21
Relative Expression
Zt (hr)
21.5 0.5 3.5 6.5 9.5 12.5 18.5

Singeleton: ENSXMAG00000013519 cep290

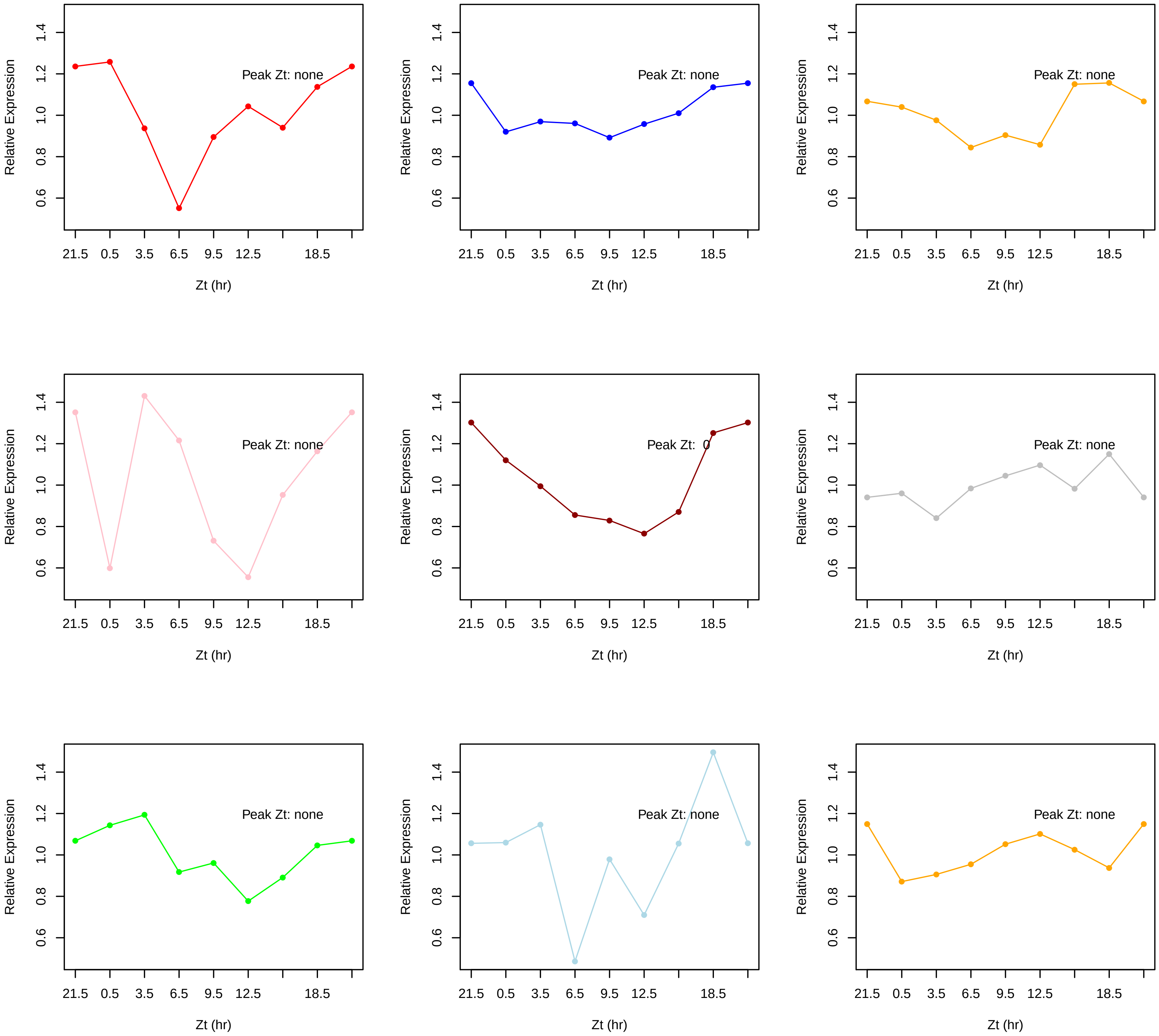

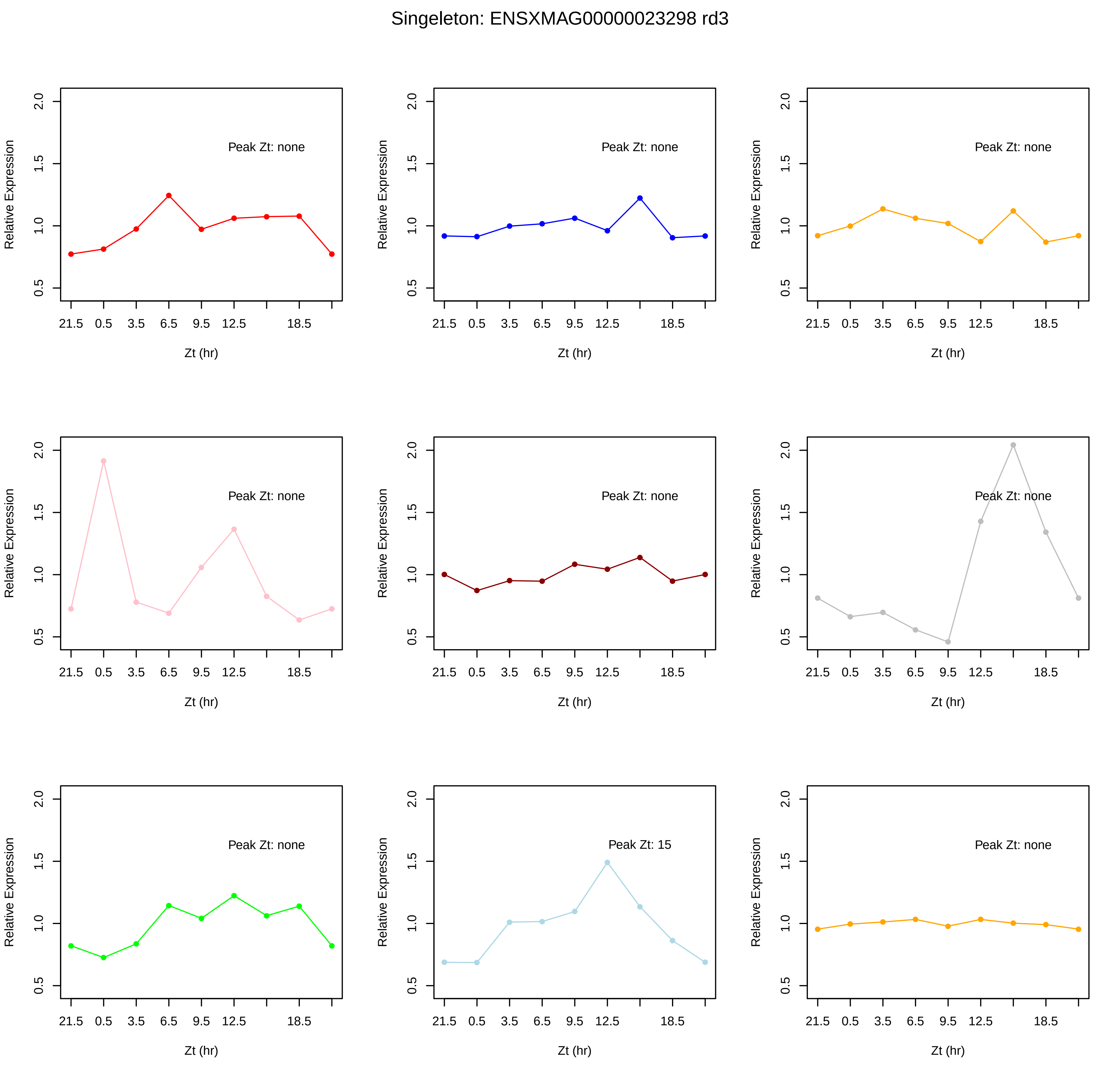
Singeleton: ENSXMAG00000023298 rd3
Peak Zt: none
Peak Zt: none
Peak Zt: none
Peak Zt: none
Peak Zt: none
Peak Zt: none
Peak Zt: none
Peak Zt: 15
Peak Zt: none
Relative Expression
Zt (hr)
21.5
0.5
3.5
6.5
9.5
12.5
18.5
0.5
1.0
1.5
2.0

Singeleton: ENSXMAG00000017560 nek2

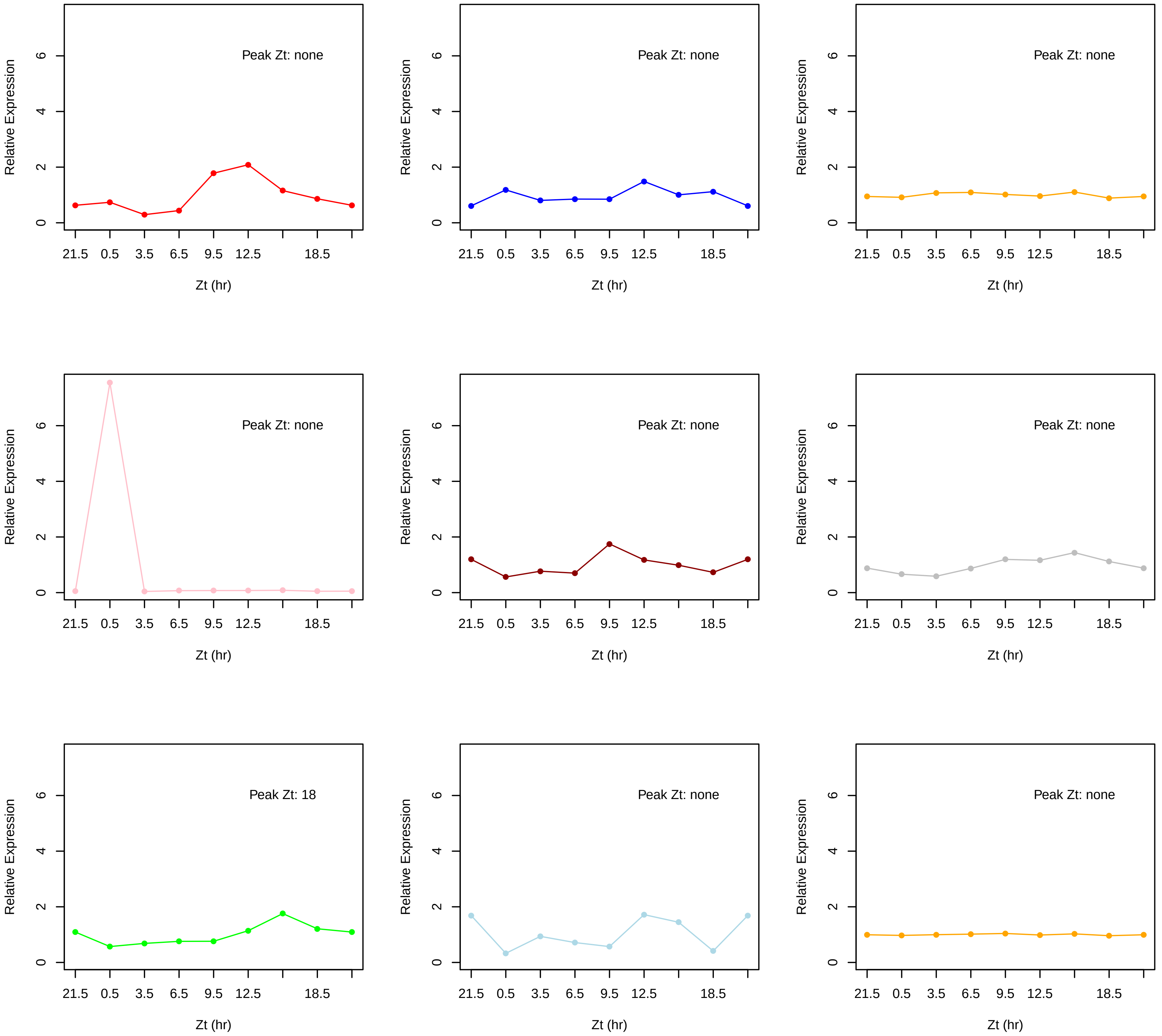

Singeleton: ENSXMAG00000017549 lpgat1

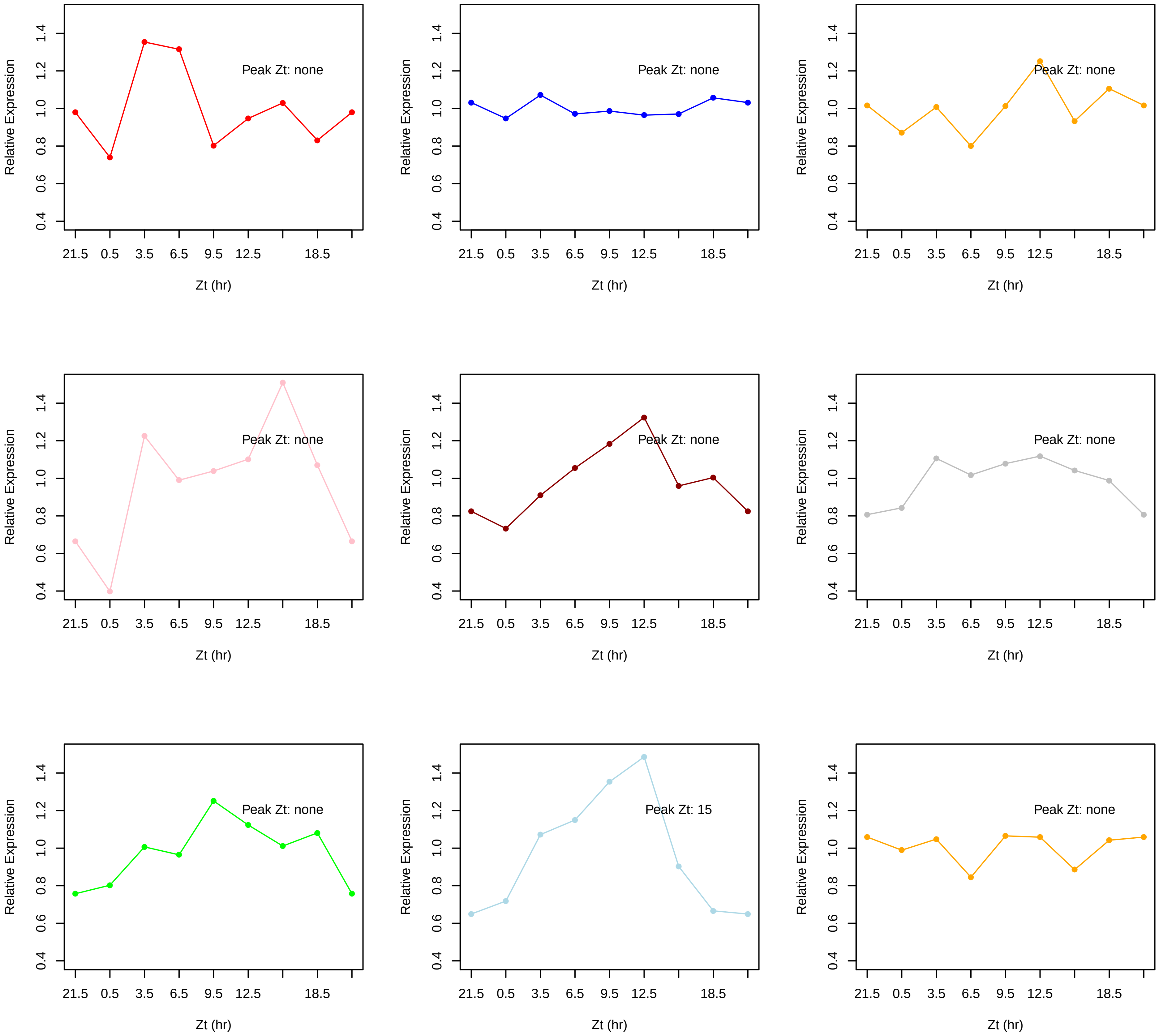

Singeleton: ENSXMAG00000017503 ints7

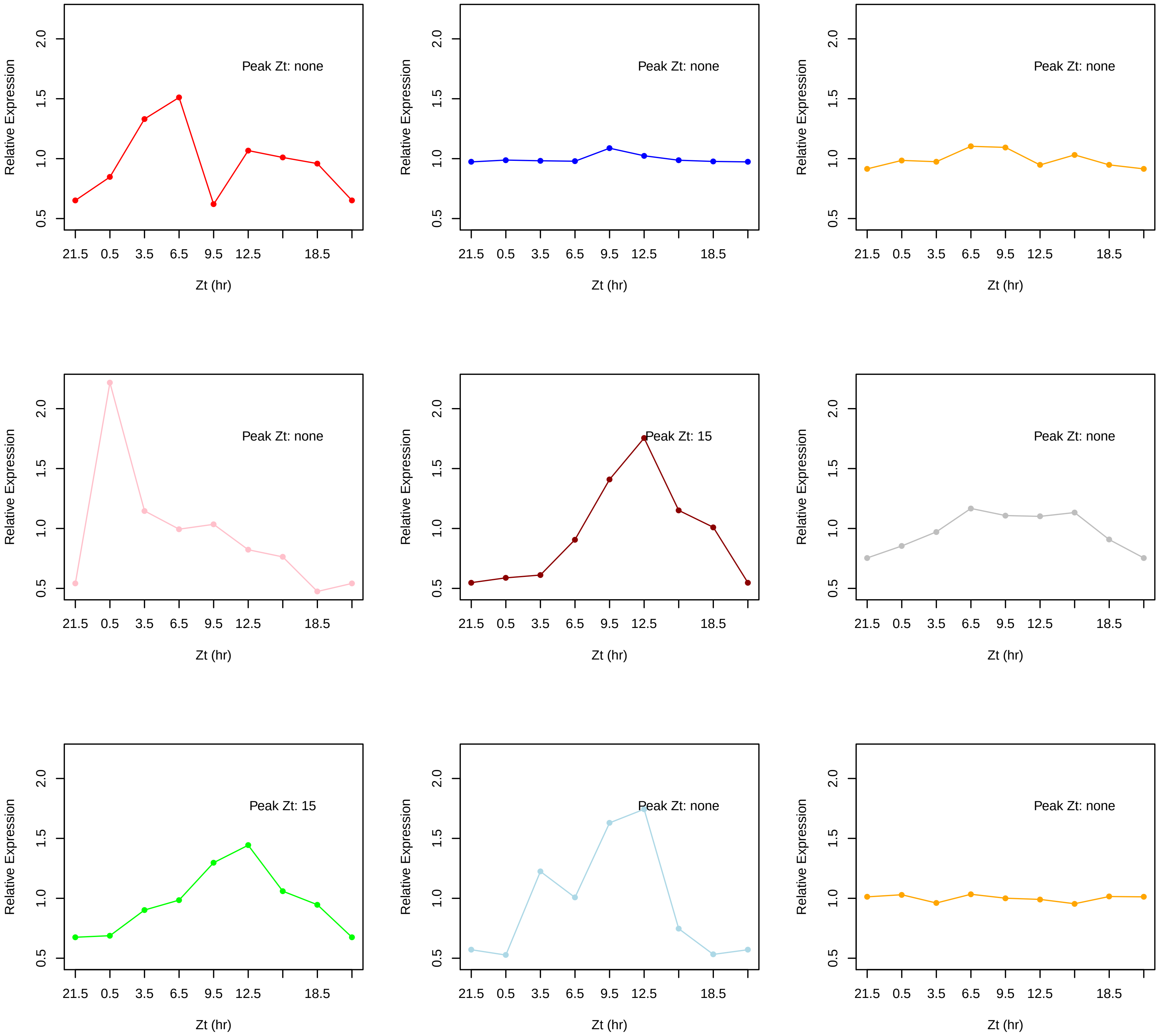

Singeleton: ENSXMAG00000016861 SRBD1

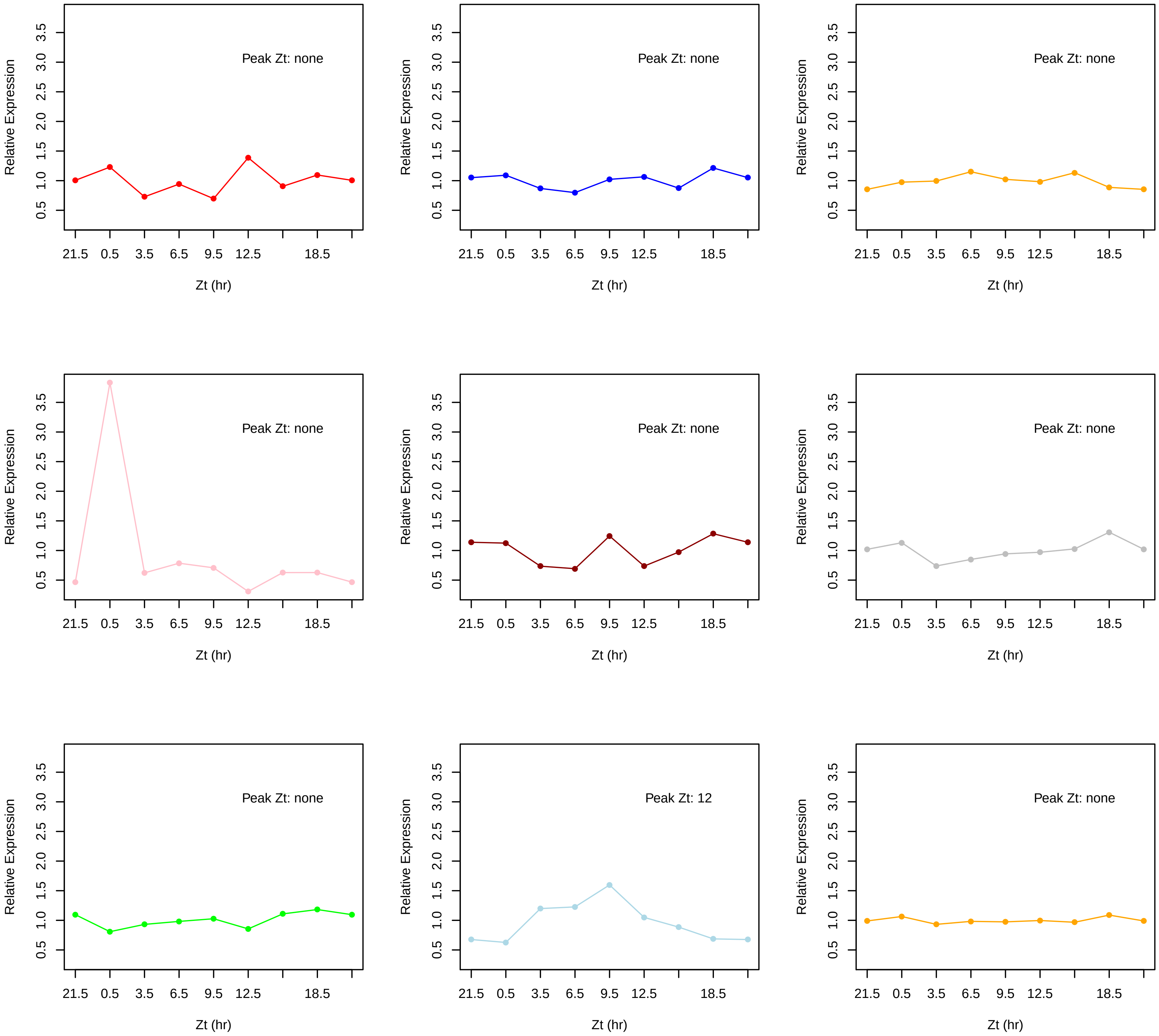

Singeleton: ENSXMAG00000009436 tmem206

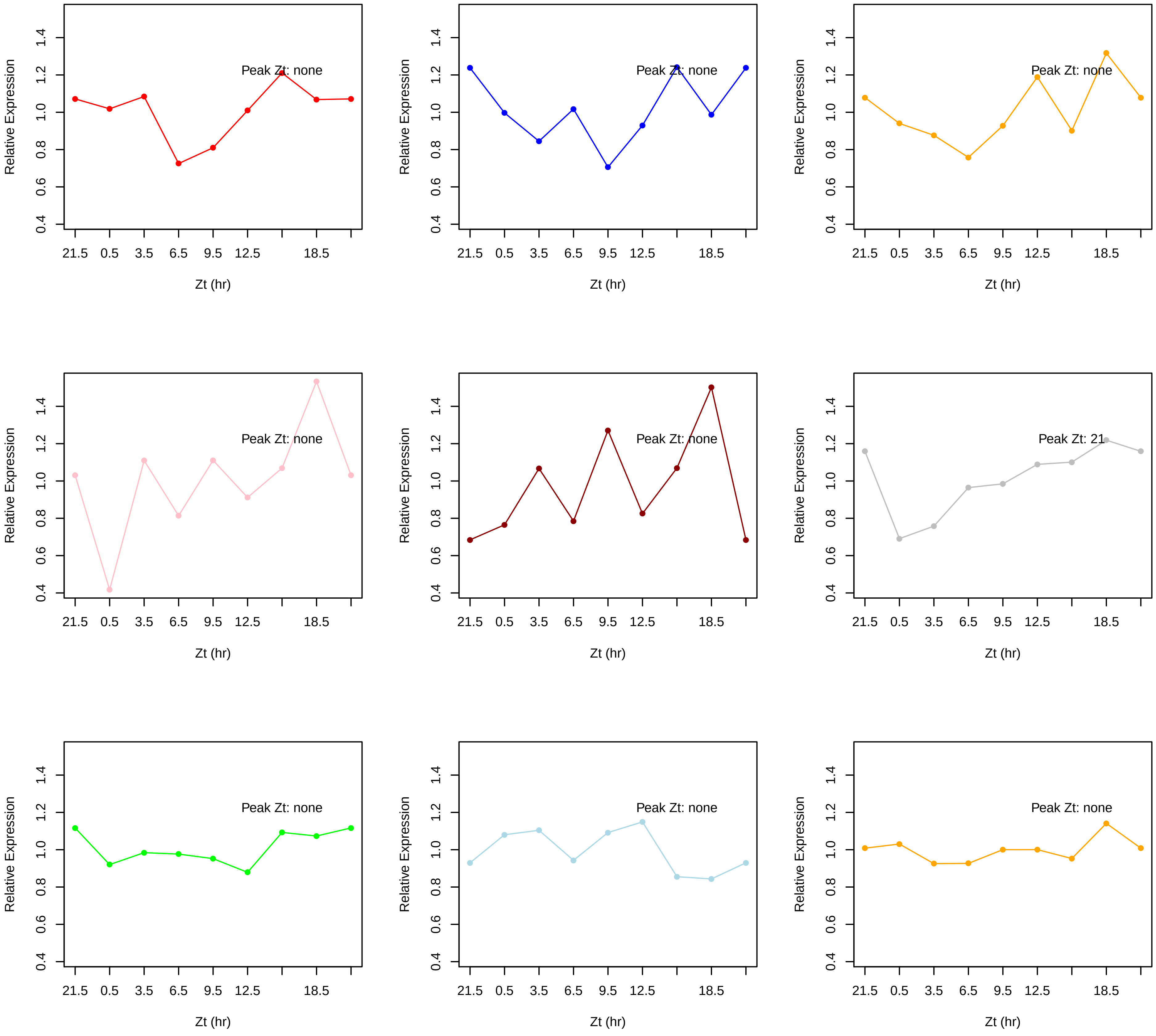

Singeleton: ENSXMAG00000017458 tagapb

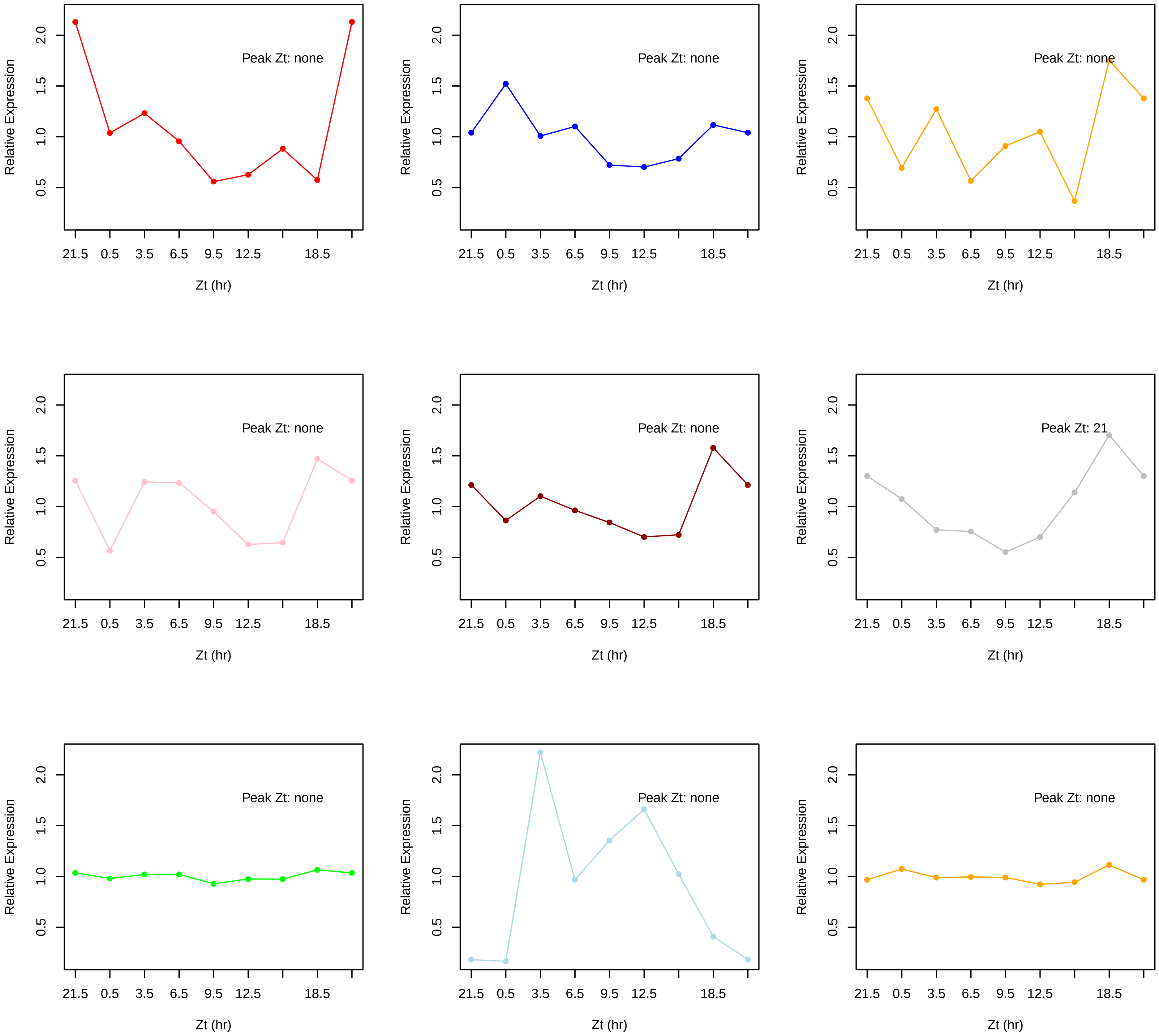

Singeleton: ENSXMAG00000025648 six2a

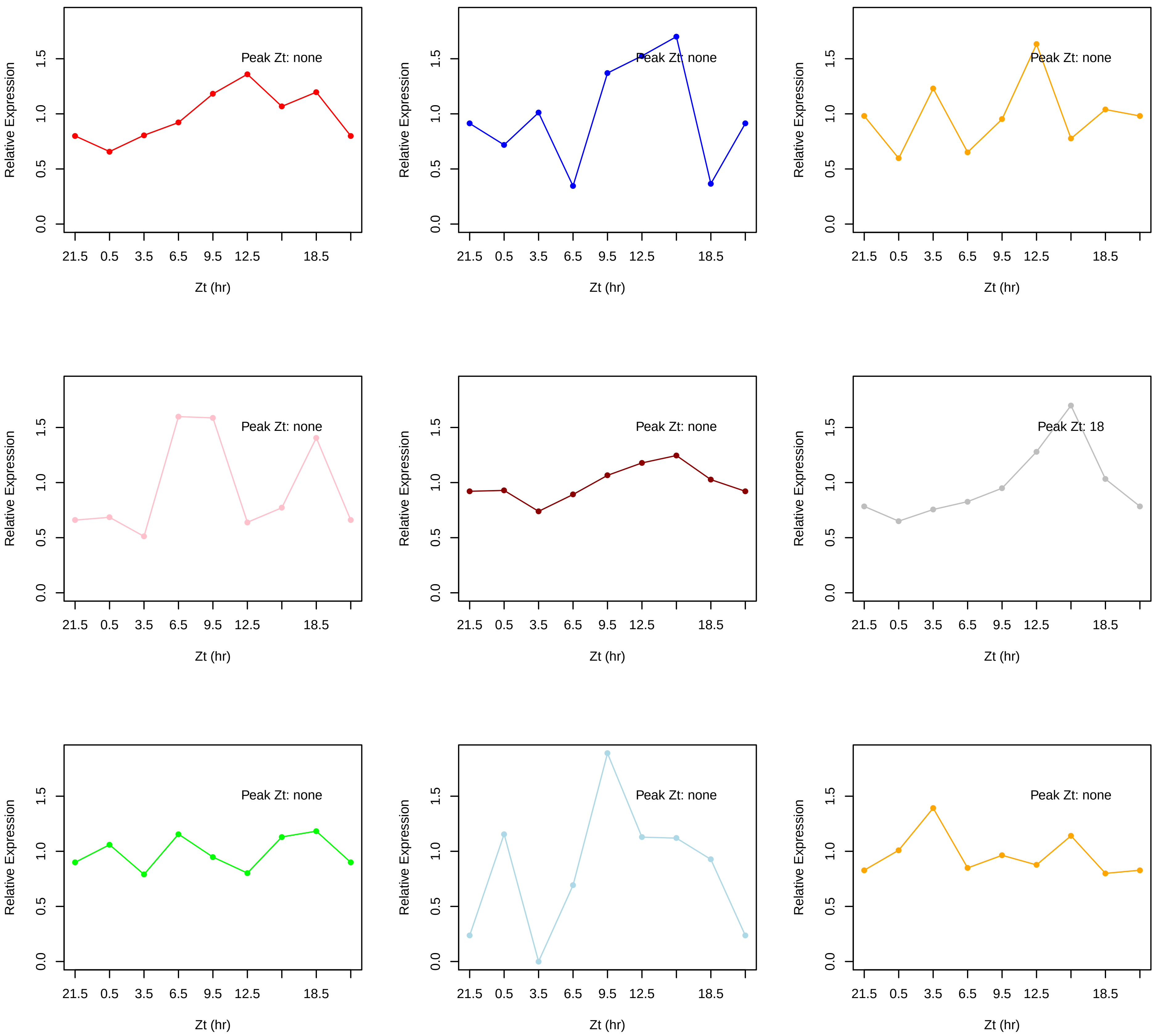

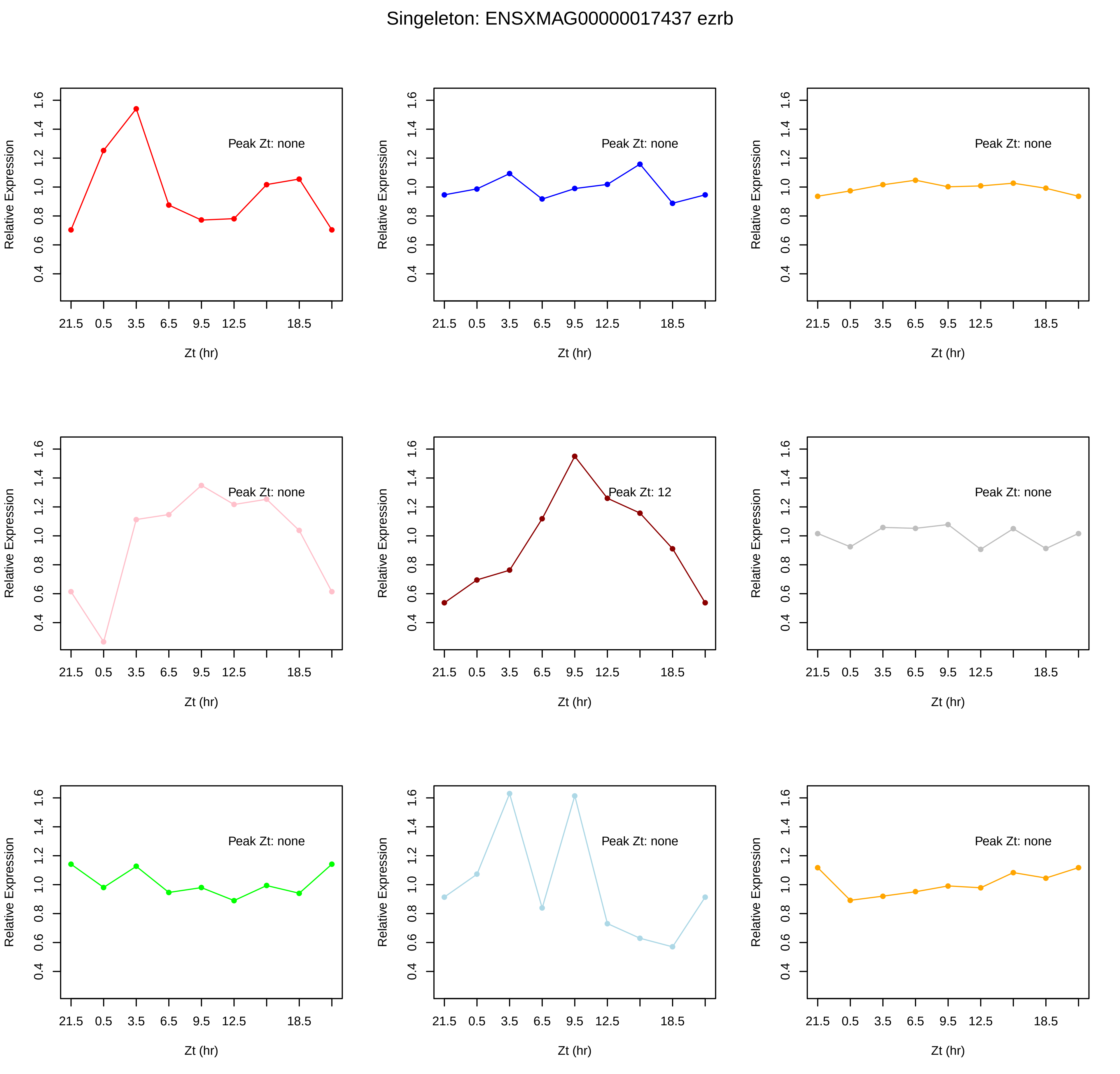
Singeleton: ENSXMAG00000017437 ezrb
Relative Expression
Zt (hr)
Peak Zt: none
Peak Zt: none
Peak Zt: none
Peak Zt: none
Peak Zt: 12
Peak Zt: none
Peak Zt: none
Peak Zt: none
Peak Zt: none
21.5 0.5 3.5 6.5 9.5 12.5 18.5
0.4 0.6 0.8 1.0 1.2 1.4 1.6

Singleton: ENSXMAG00000024628 CAMKMT

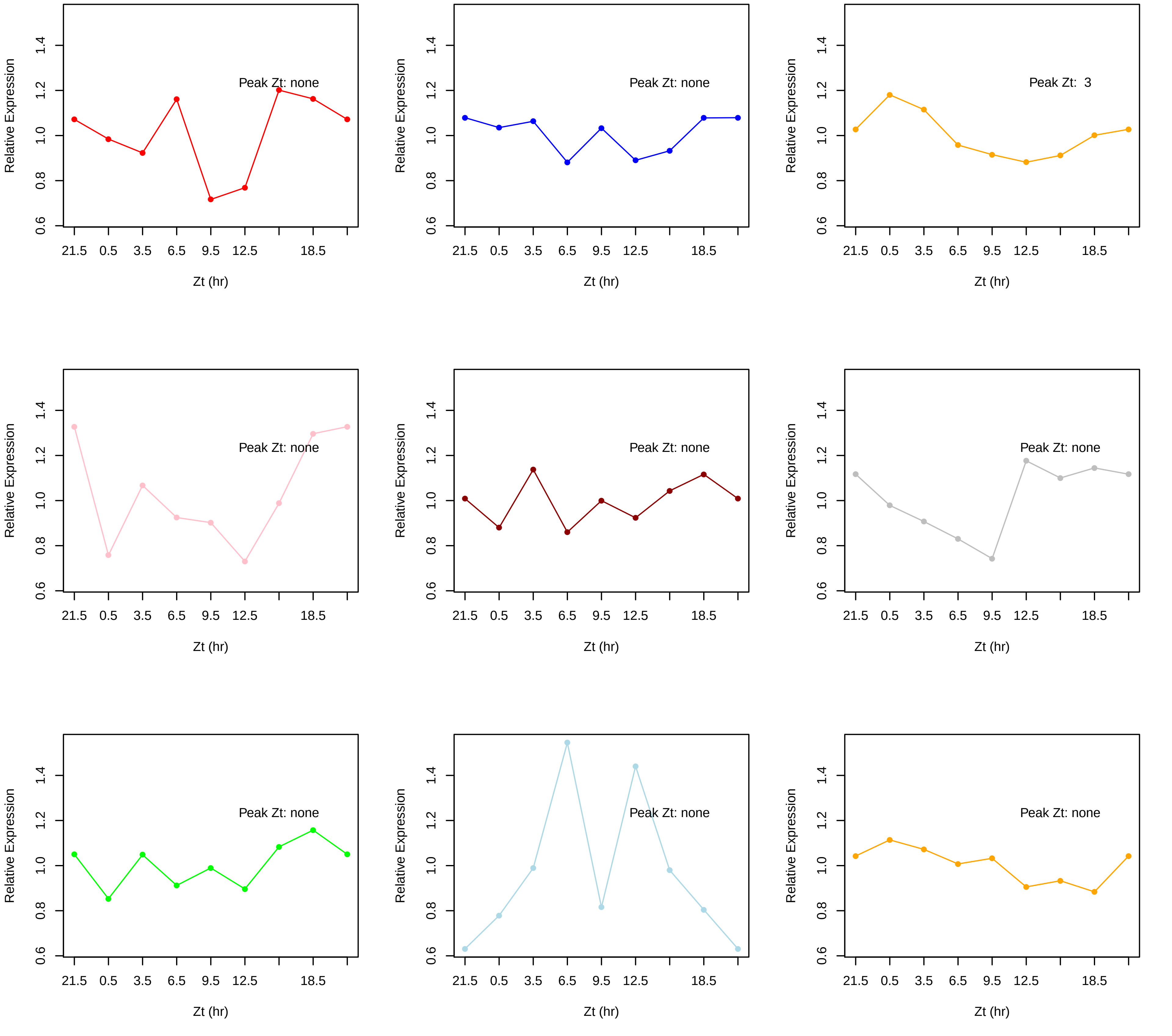

Singleton: ENSXMAG00000004999 dusp6

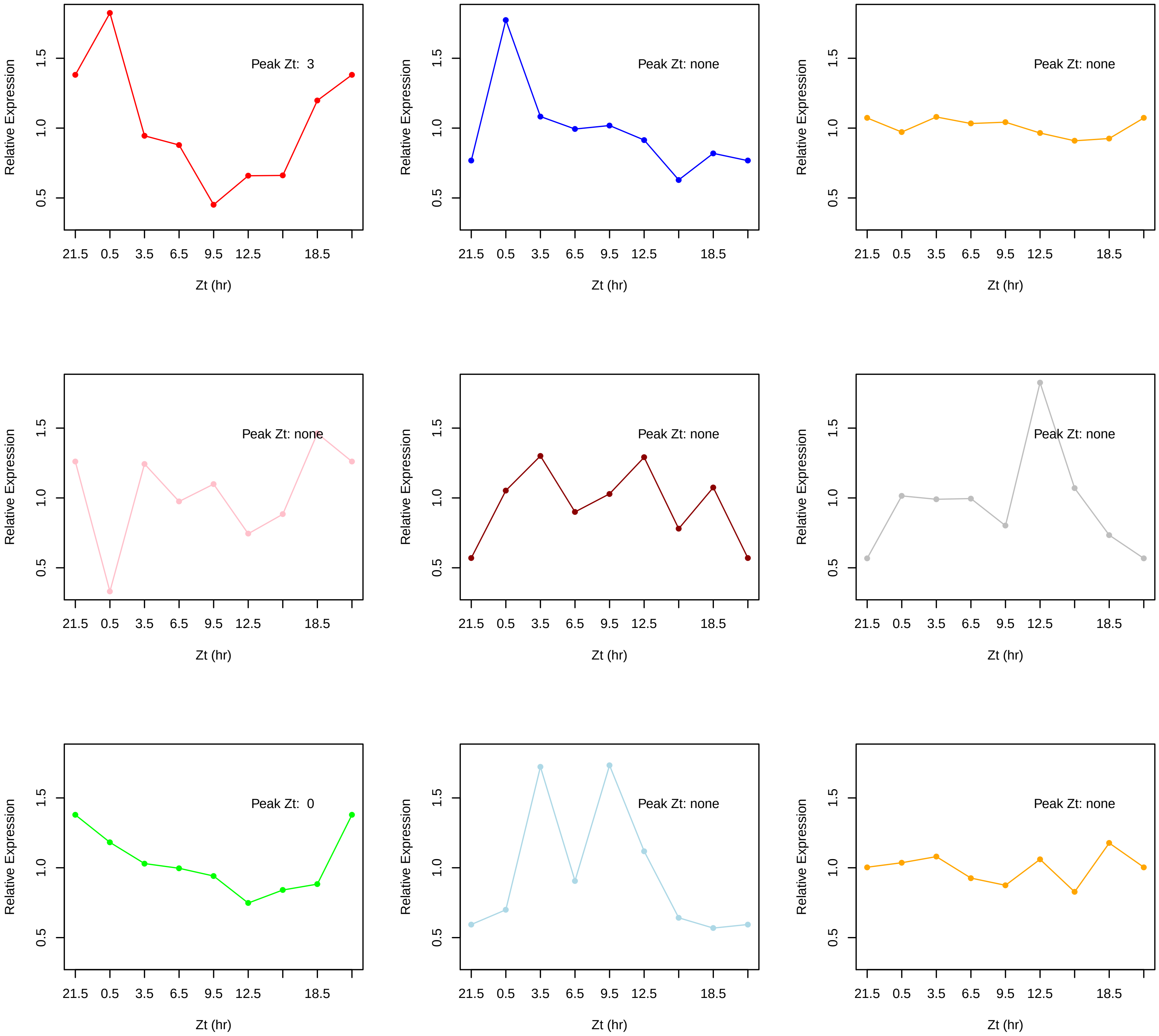

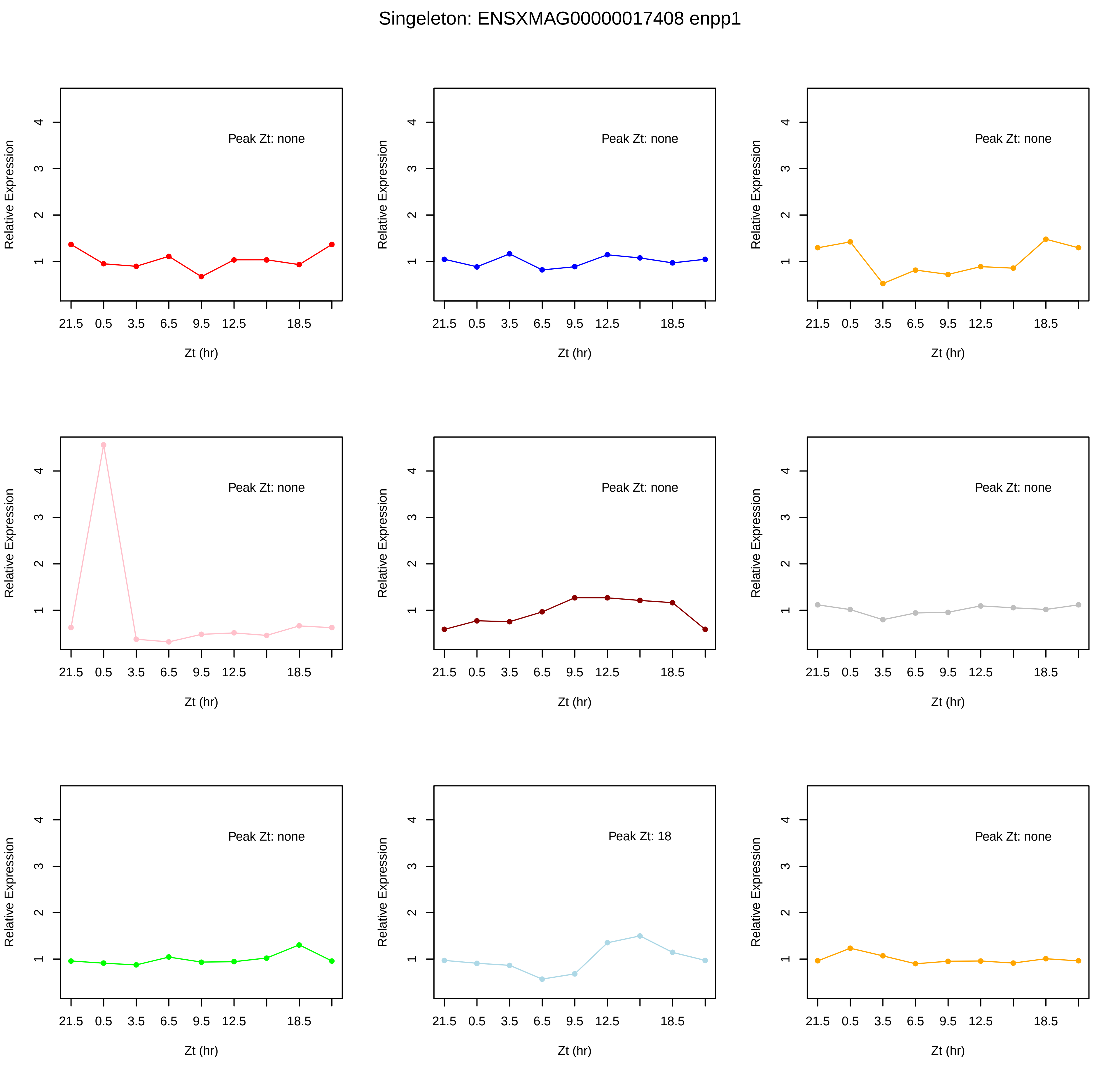
Singleton: ENSXMAG00000017408 enpp1
Peak Zt: none
Peak Zt: none
Peak Zt: none
Peak Zt: none
Peak Zt: none
Peak Zt: none
Peak Zt: none
Peak Zt: 18
Peak Zt: none
Relative Expression
Zt (hr)
21.5
0.5
3.5
6.5
9.5
12.5
18.5

Singeleton: ENSXMAG00000005331

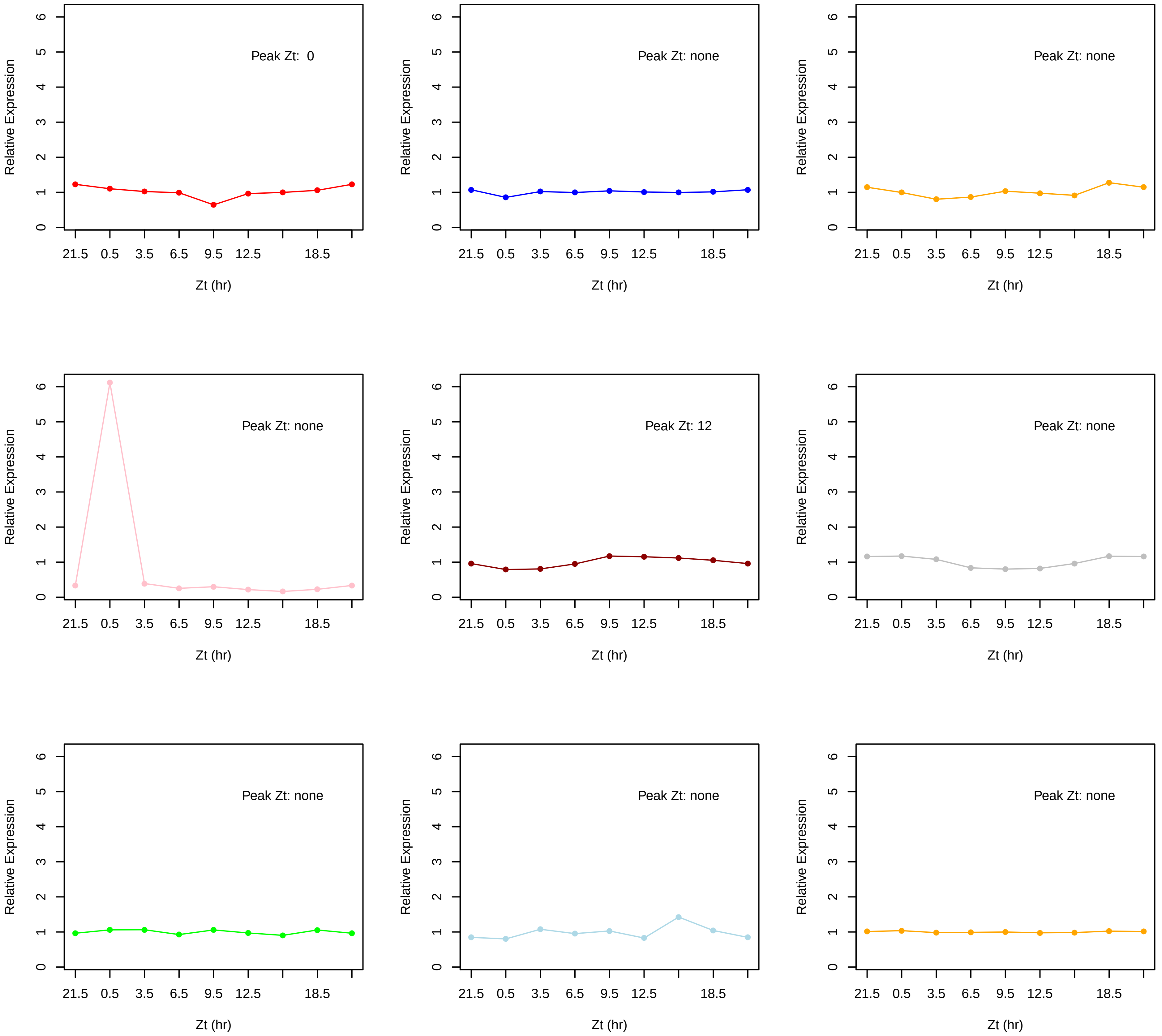

Singeleton: ENSXMAG00000018588 epyc

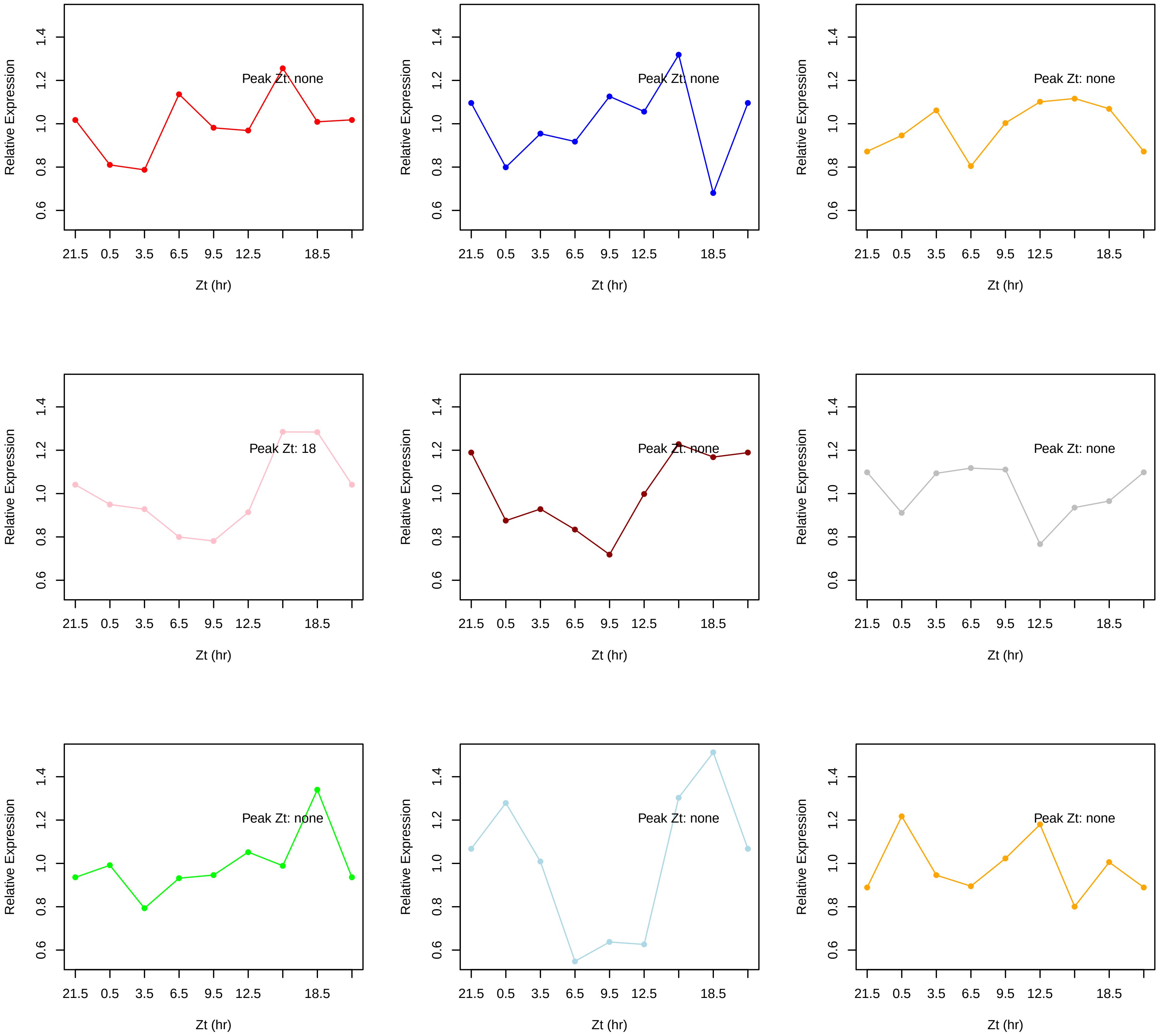

Singeleton: ENSXMAG00000026974 slc35f6

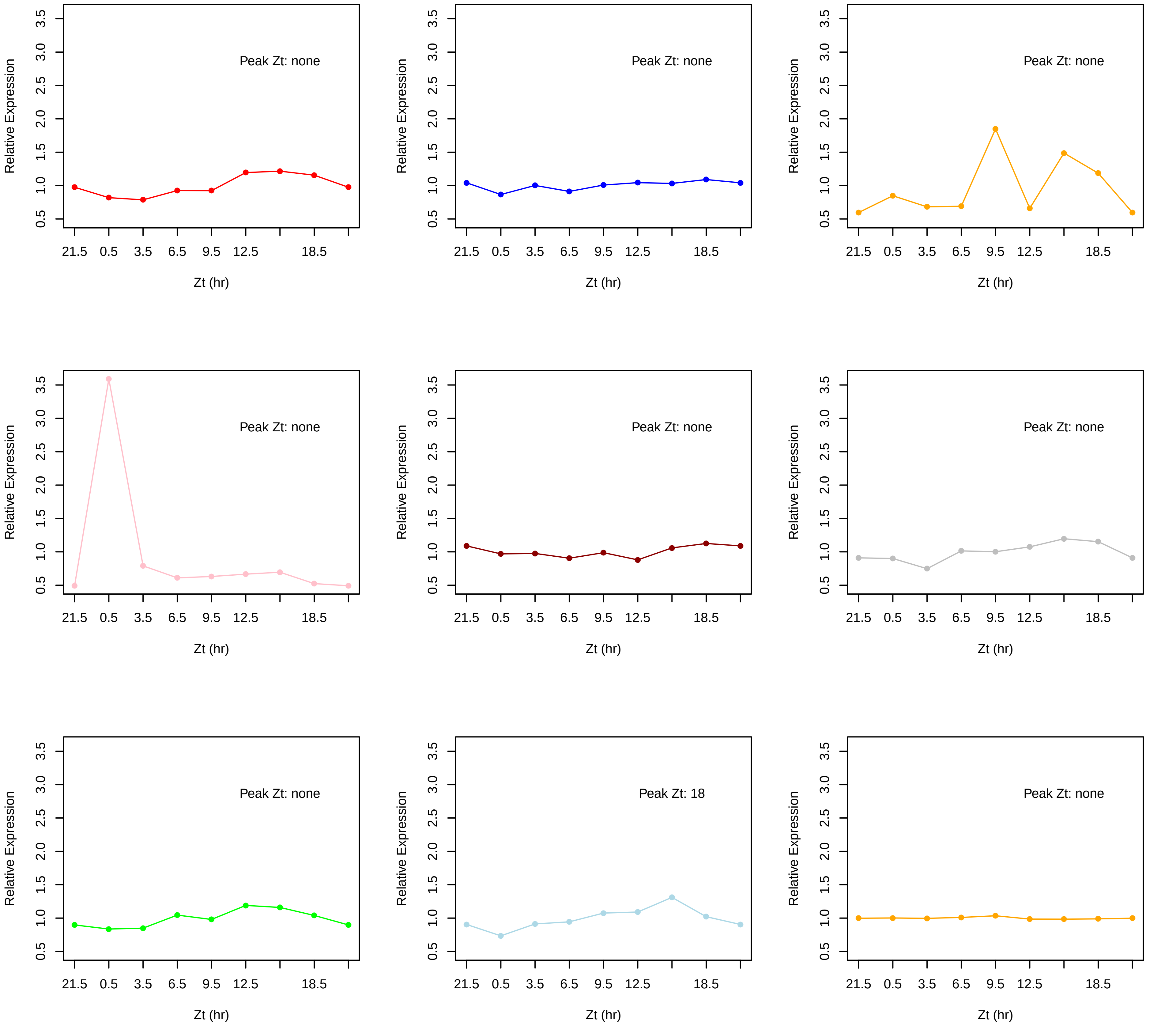

Singeleton: ENSXMAG00000015990 ppm1bb

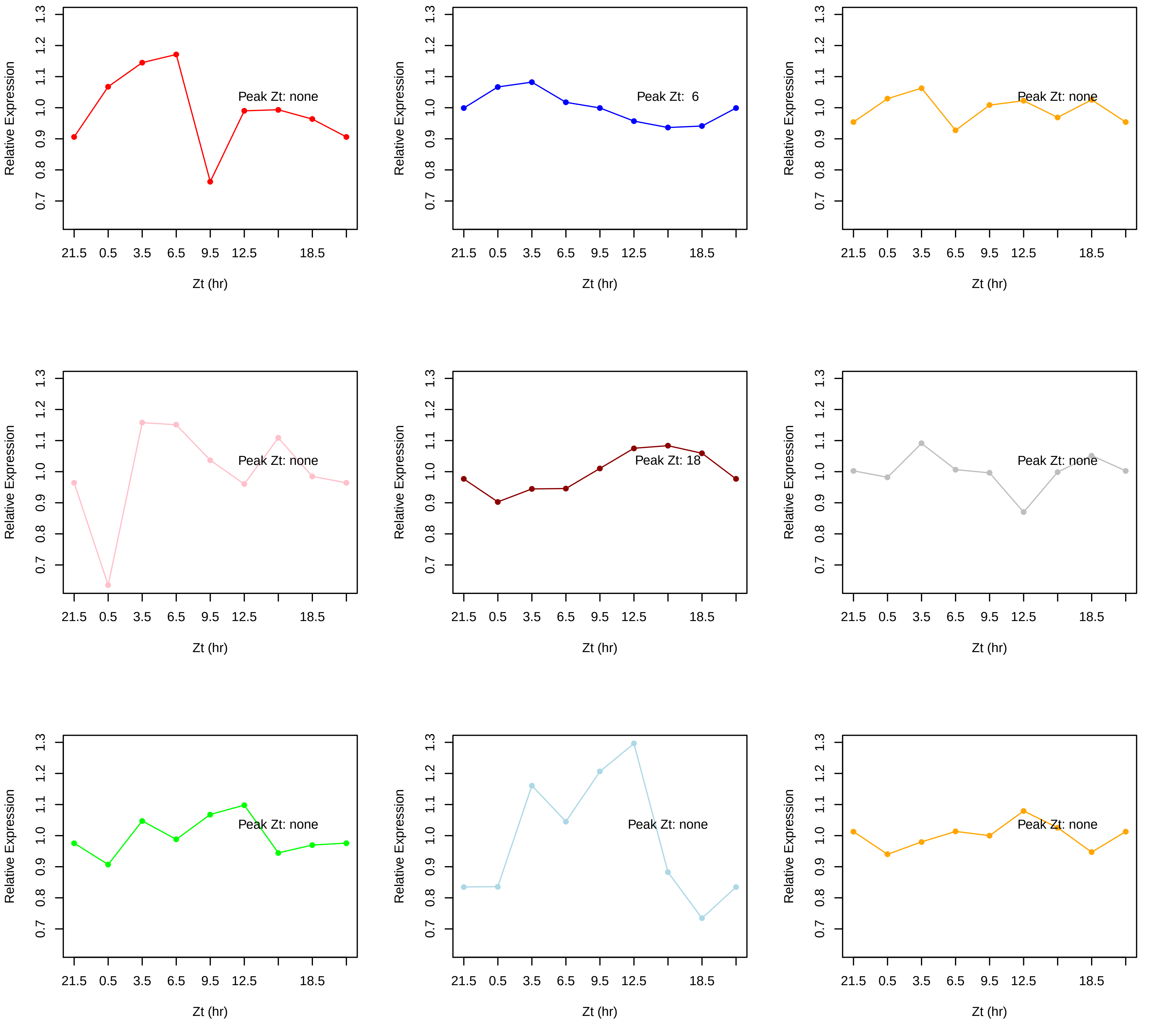

Singeleton: ENSXMAG00000023151 dusp10

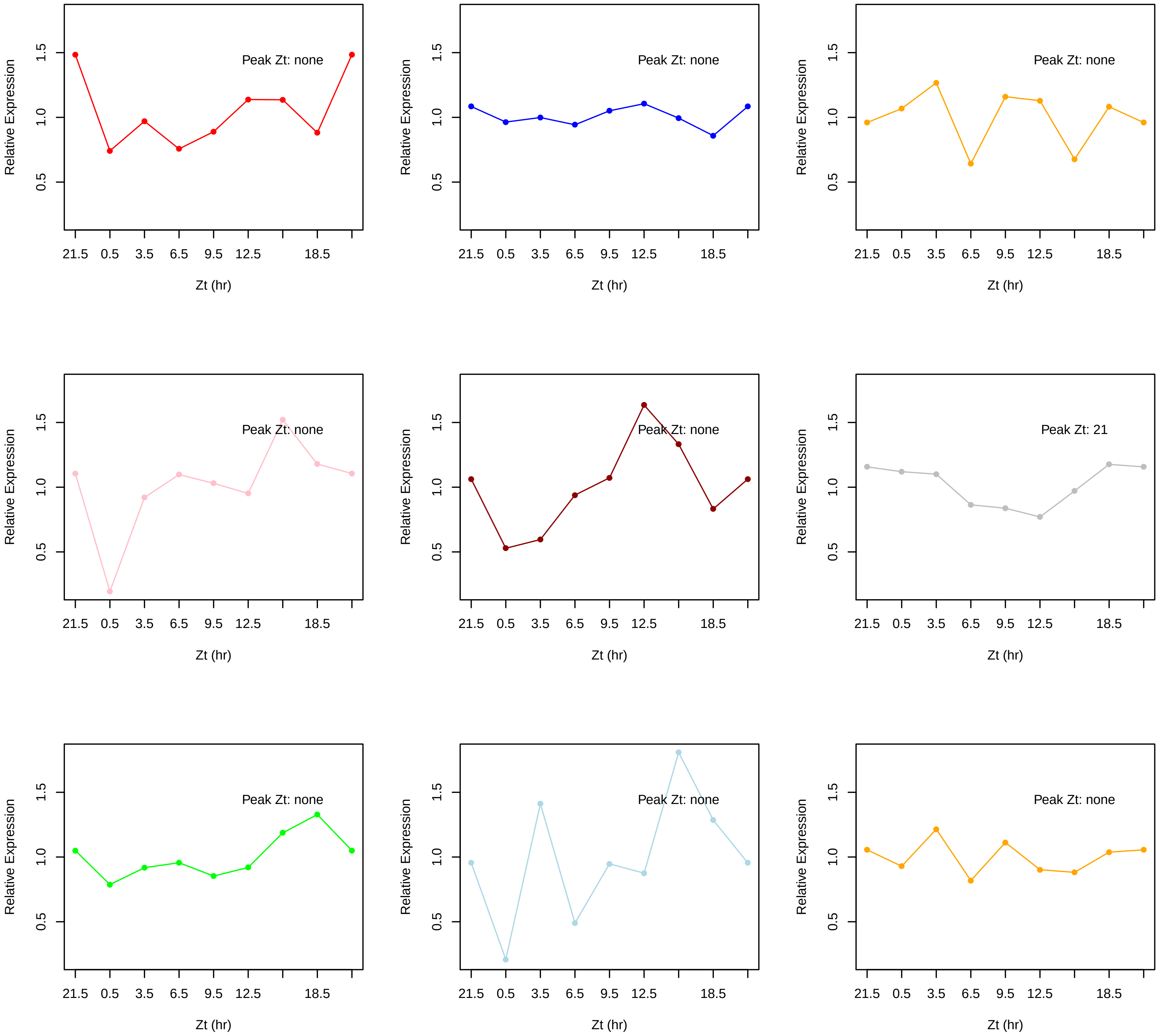

Singleton: ENSXMAG00000024291 nfkbie

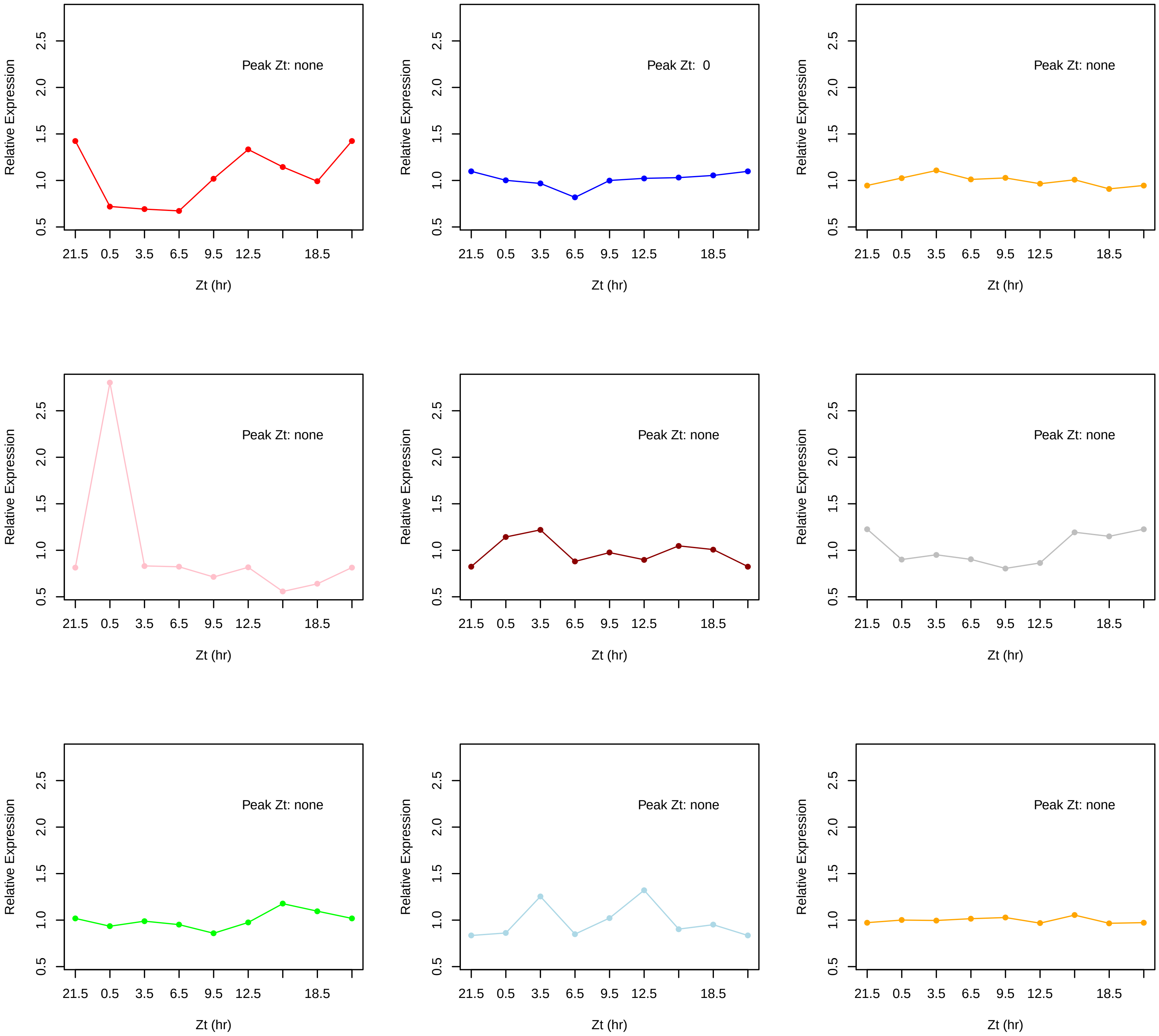

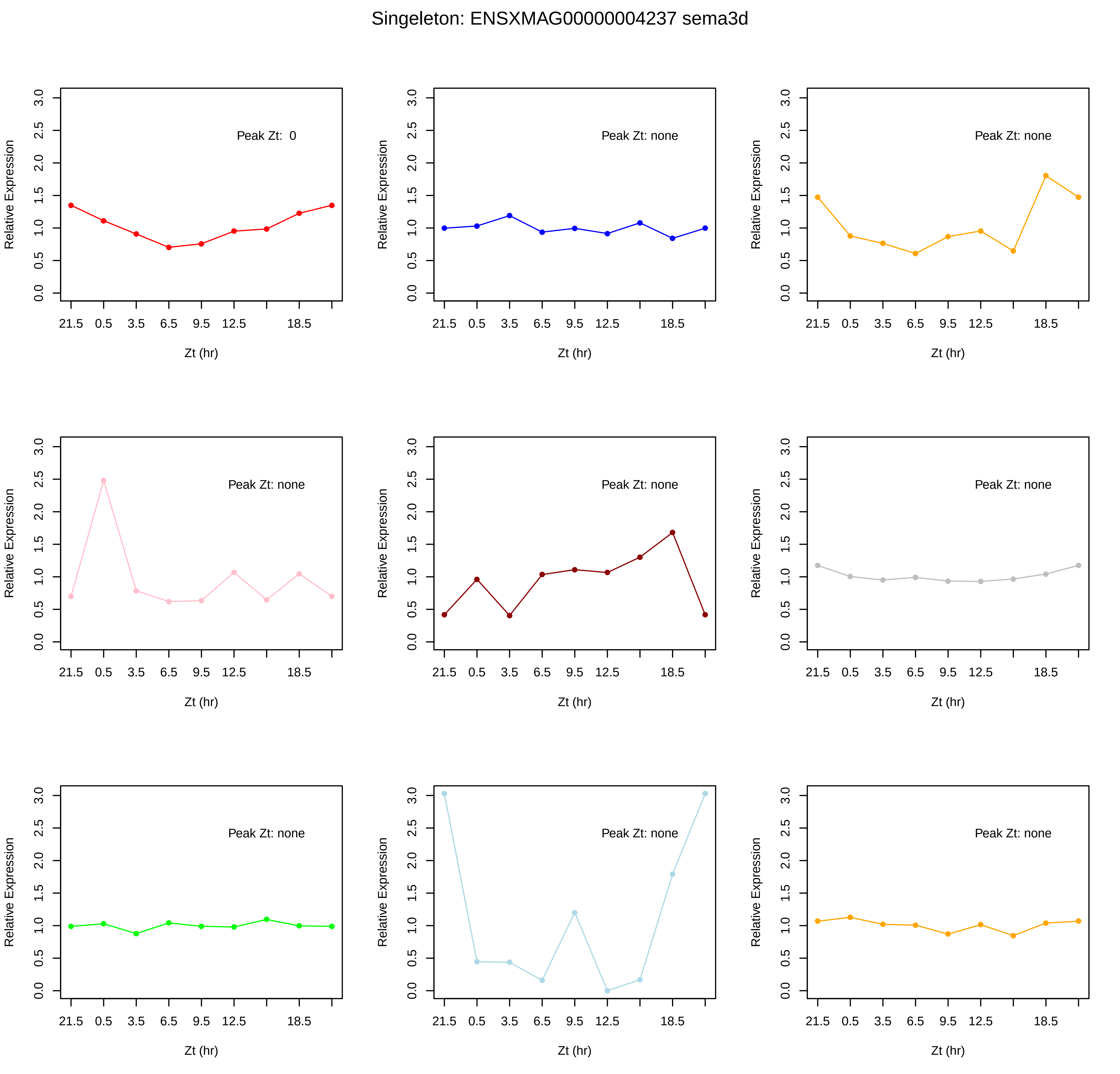
Singeleton: ENSXMAG00000004237 sema3d
Peak Zt: 0
Peak Zt: none
Peak Zt: none
Peak Zt: none
Peak Zt: none
Peak Zt: none
Peak Zt: none
Peak Zt: none
Peak Zt: none
Relative Expression
Zt (hr)
21.5
0.5
3.5
6.5
9.5
12.5
18.5
0.0
0.5
1.0
1.5
2.0
2.5
3.0

Singeleton: ENSXMAG00000001789 disp1

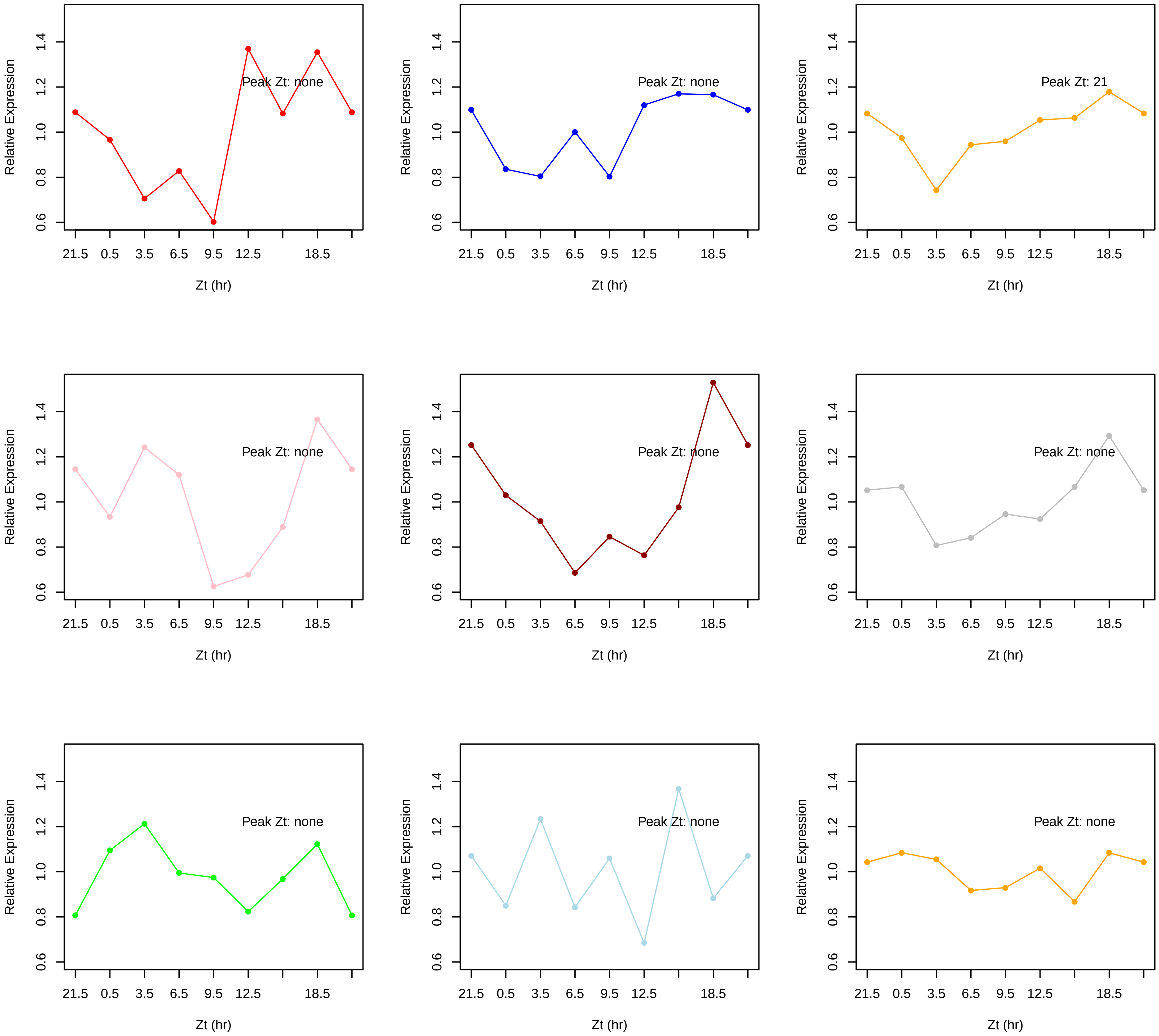

Singeleton: ENSXMAG00000006133 slc38a4

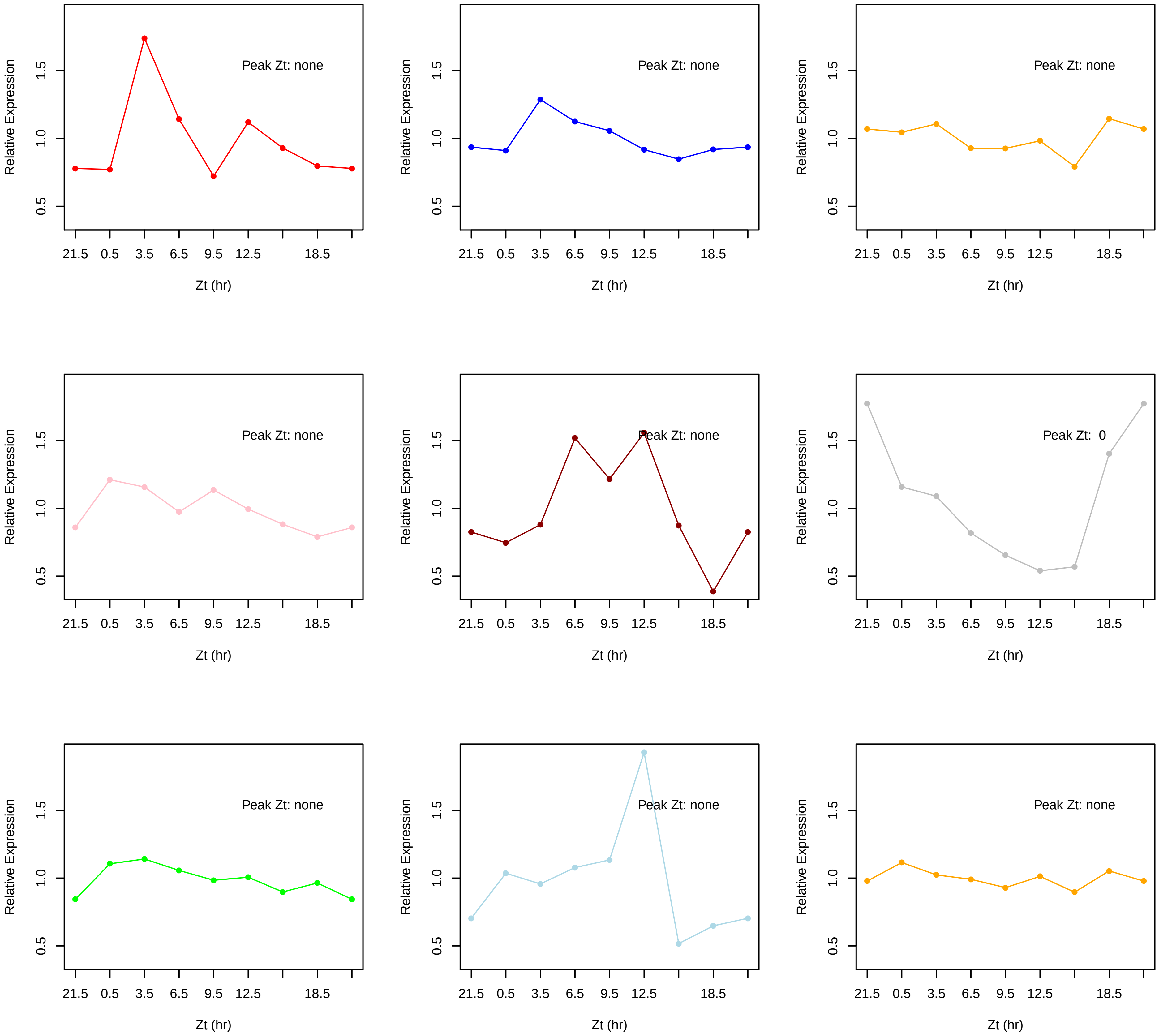

Singeleton: ENSXMAG00000017367 aida

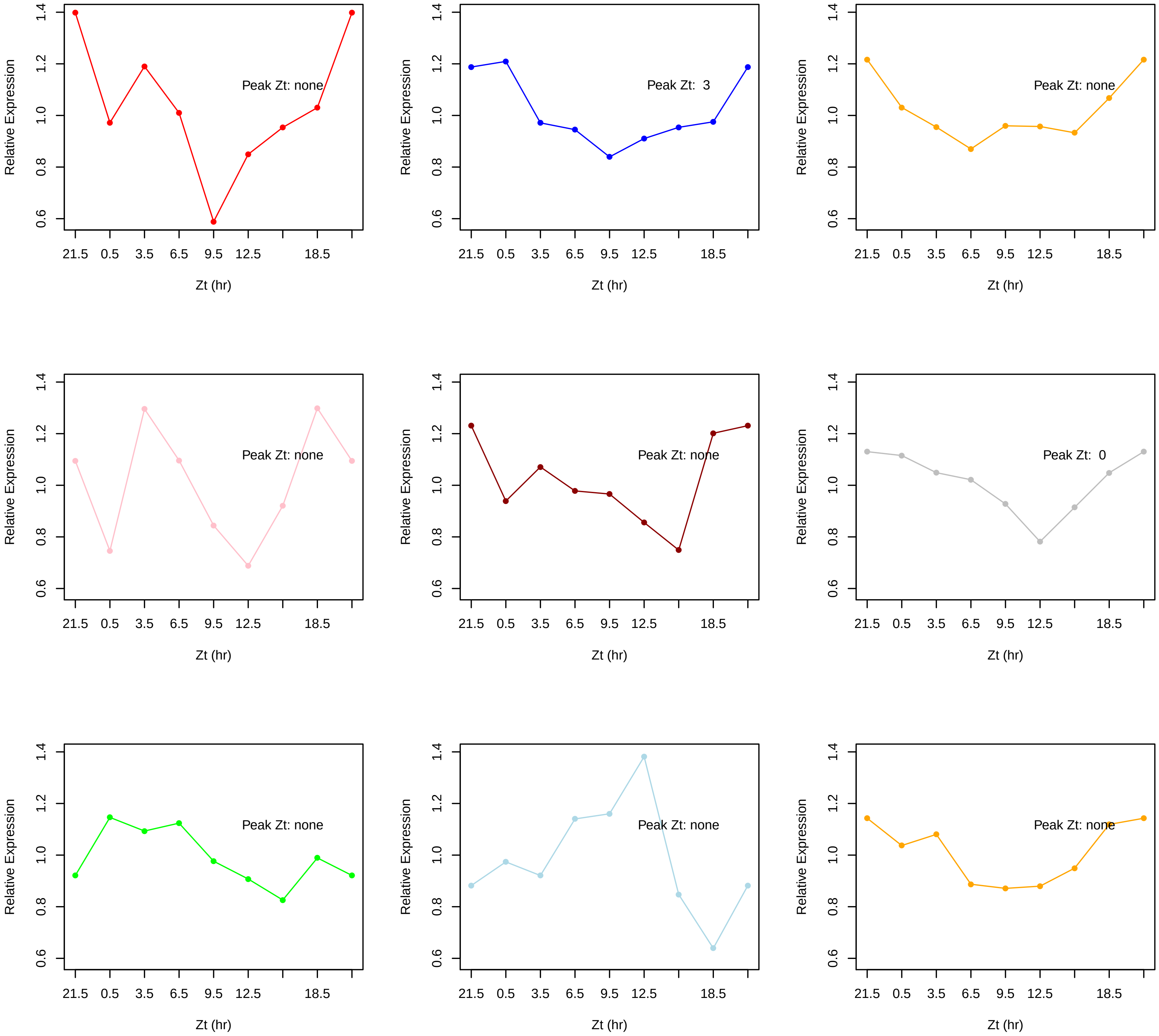

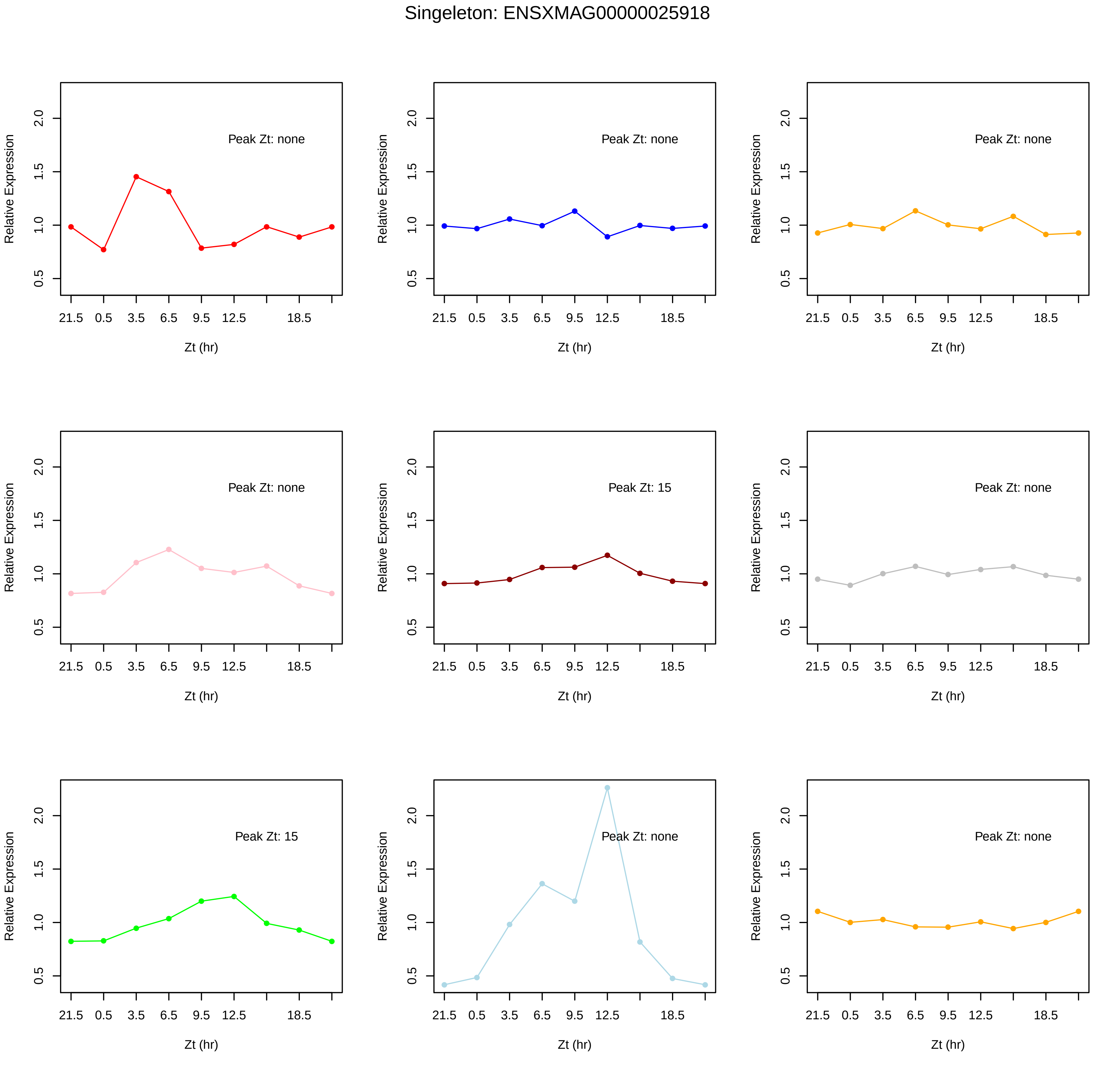
Singeleton: ENSXMAG00000025918
Peak Zt: none
Peak Zt: none
Peak Zt: none
Peak Zt: none
Peak Zt: 15
Peak Zt: none
Peak Zt: 15
Peak Zt: none
Peak Zt: none
Relative Expression
Zt (hr)
21.5 0.5 3.5 6.5 9.5 12.5 18.5
0.5 1.0 1.5 2.0

Singleton: ENSXMAG00000004892 taf1a

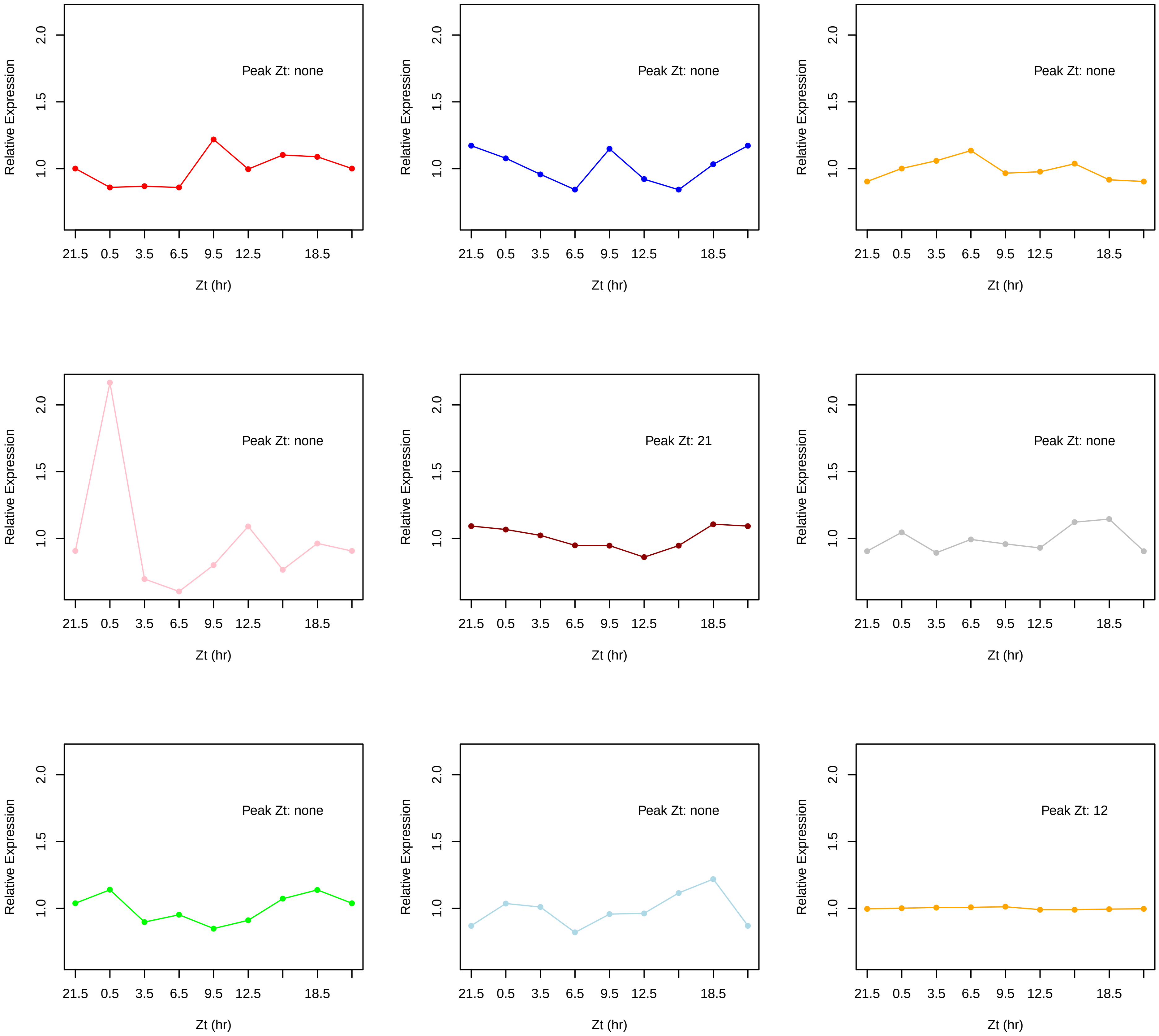

Singleton: ENSXMAG00000015225

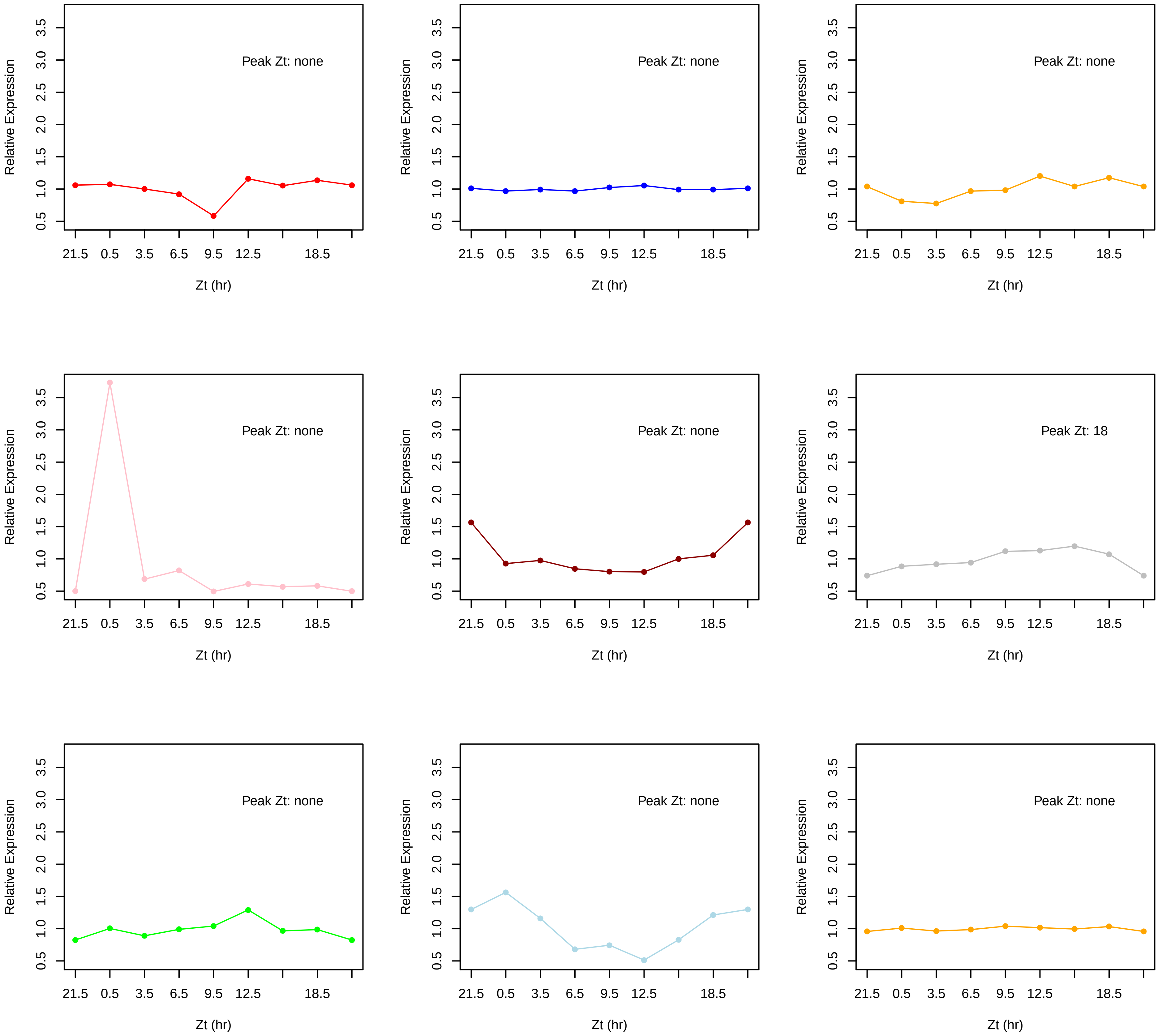

Singeleton: ENSXMAG00000004021 ano6

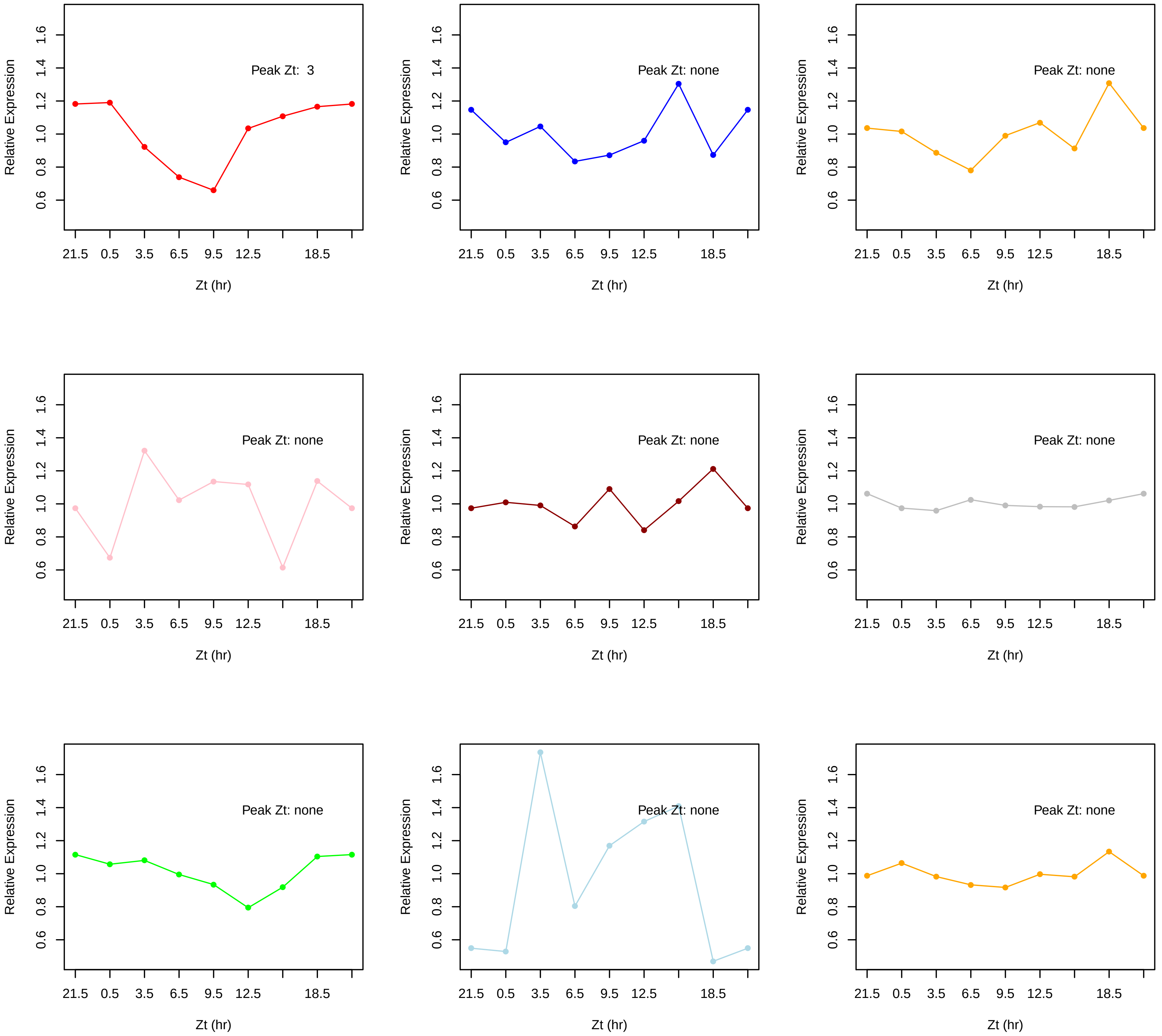

Singeleton: ENSXMAG00000008469 pak7

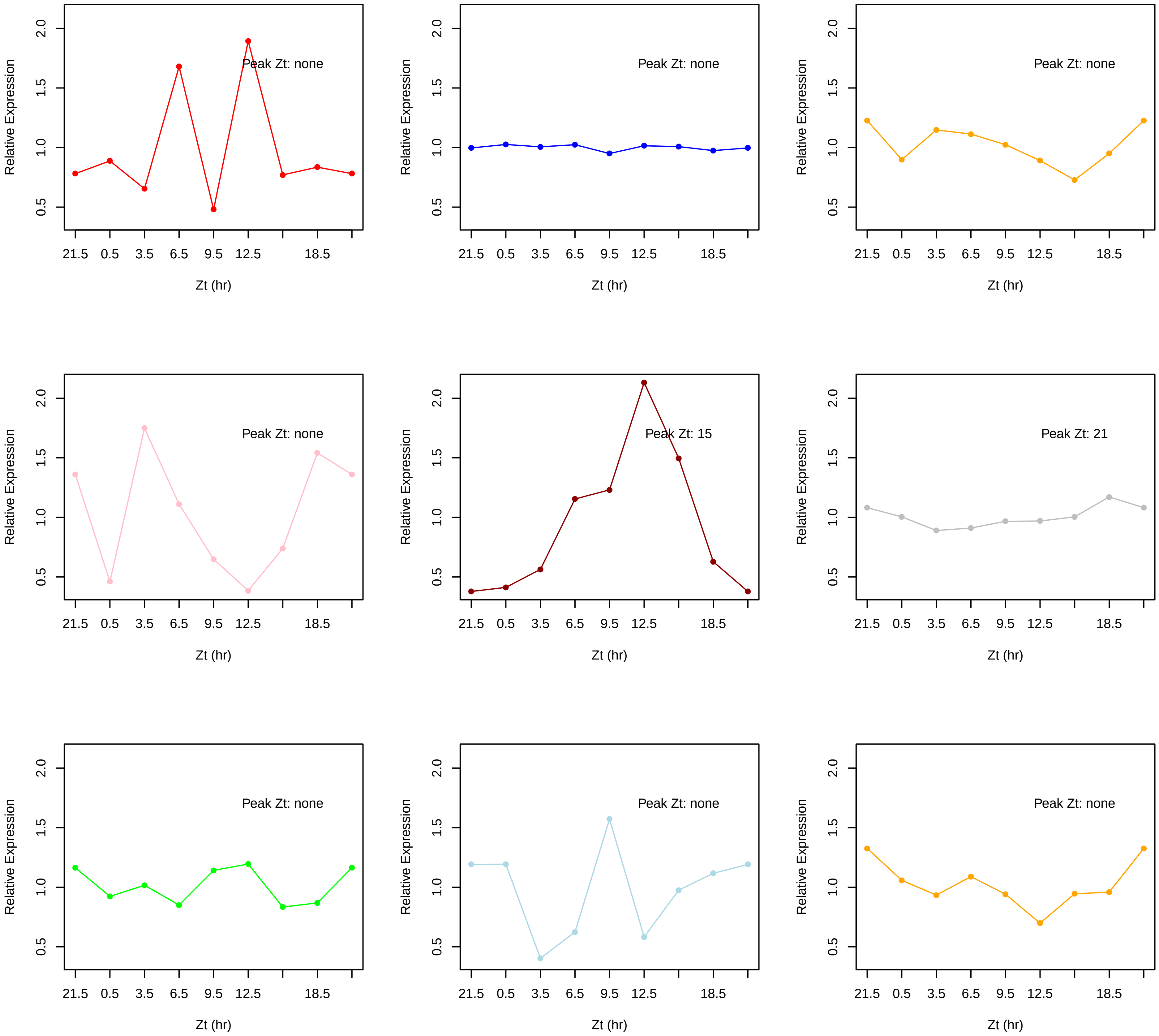

Singeleton: ENSXMAG00000007422 THADA

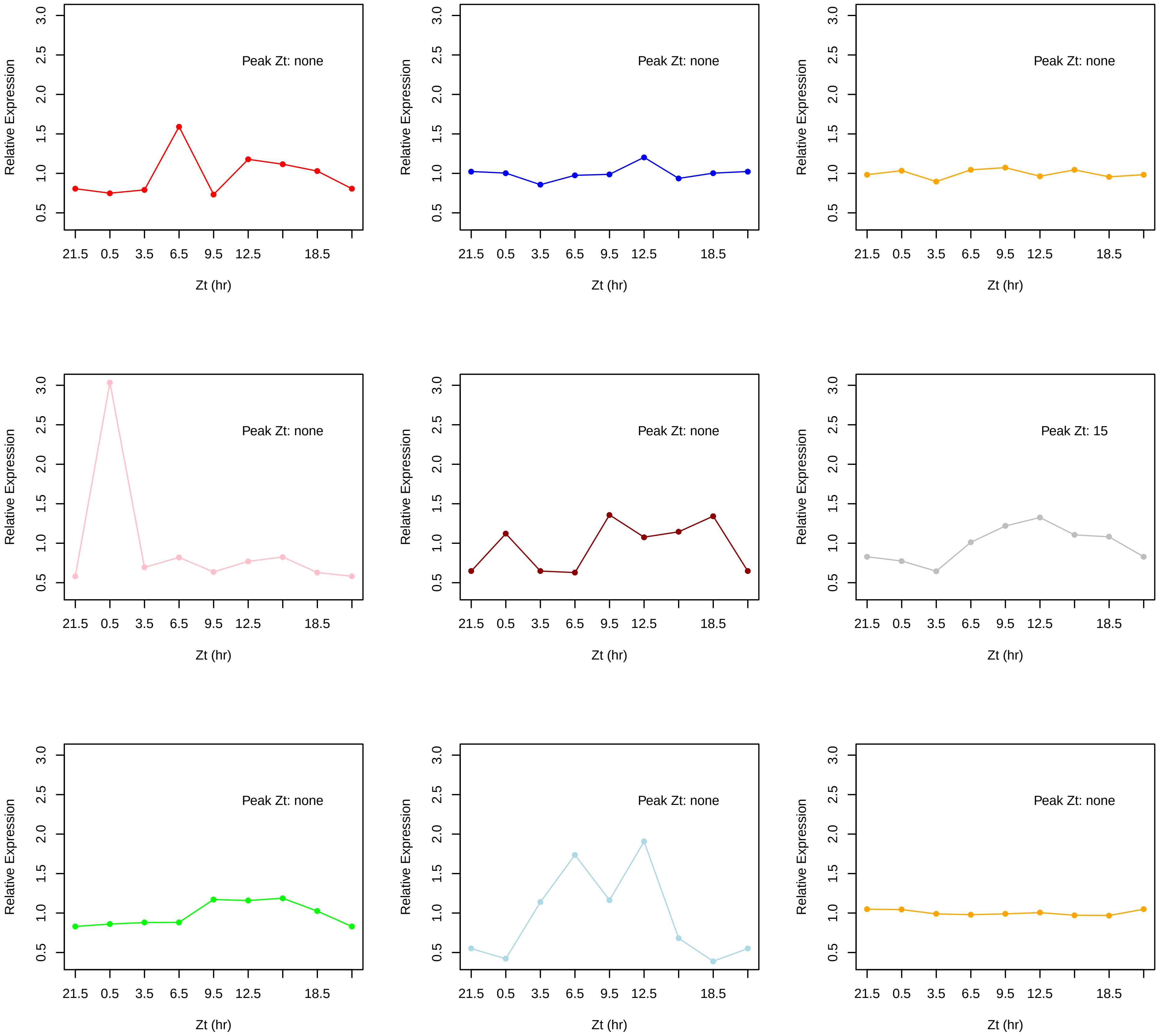

Singeleton: ENSXMAG00000004689

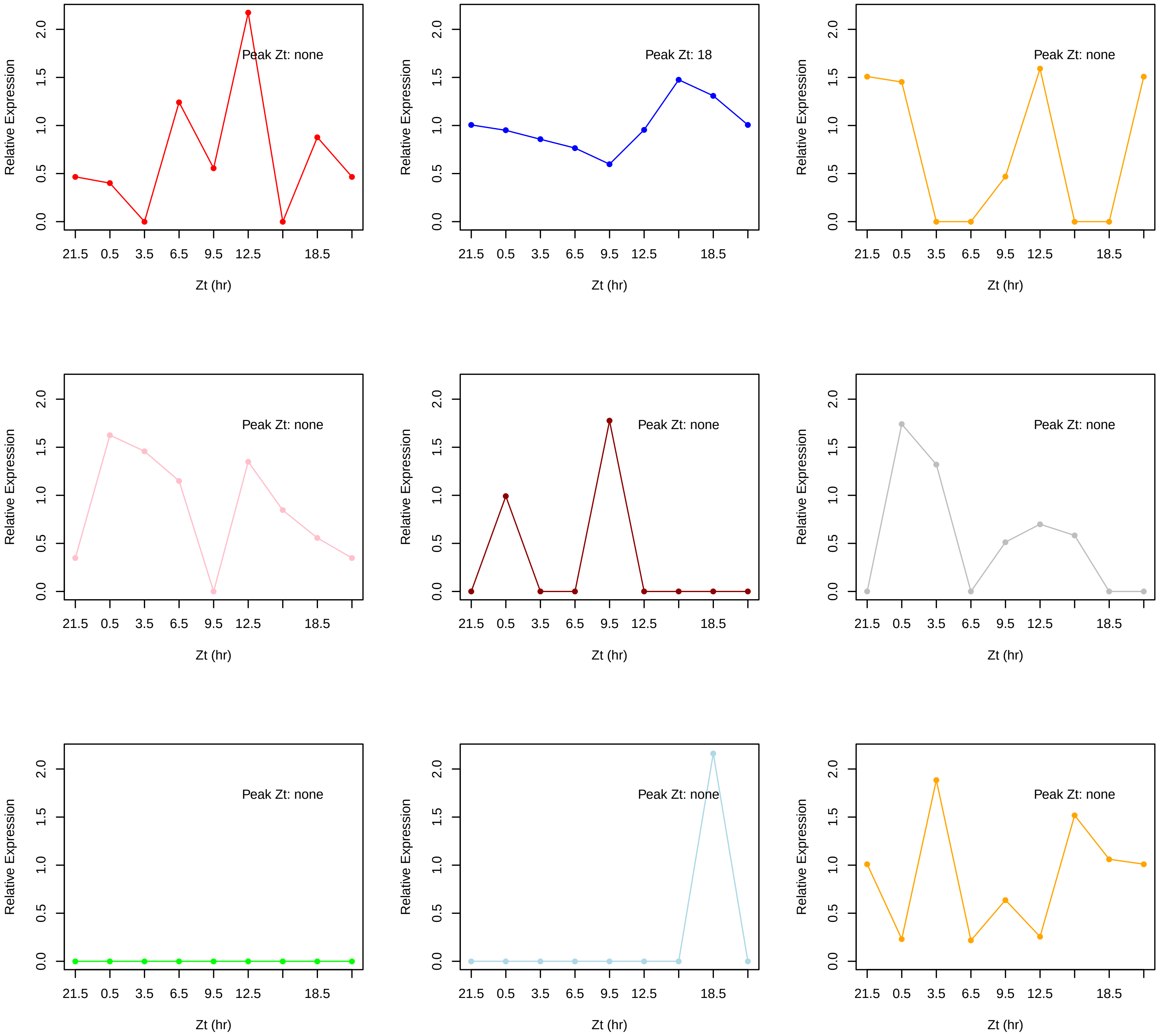

Singeleton: ENSXMAG00000008481 PLCB4

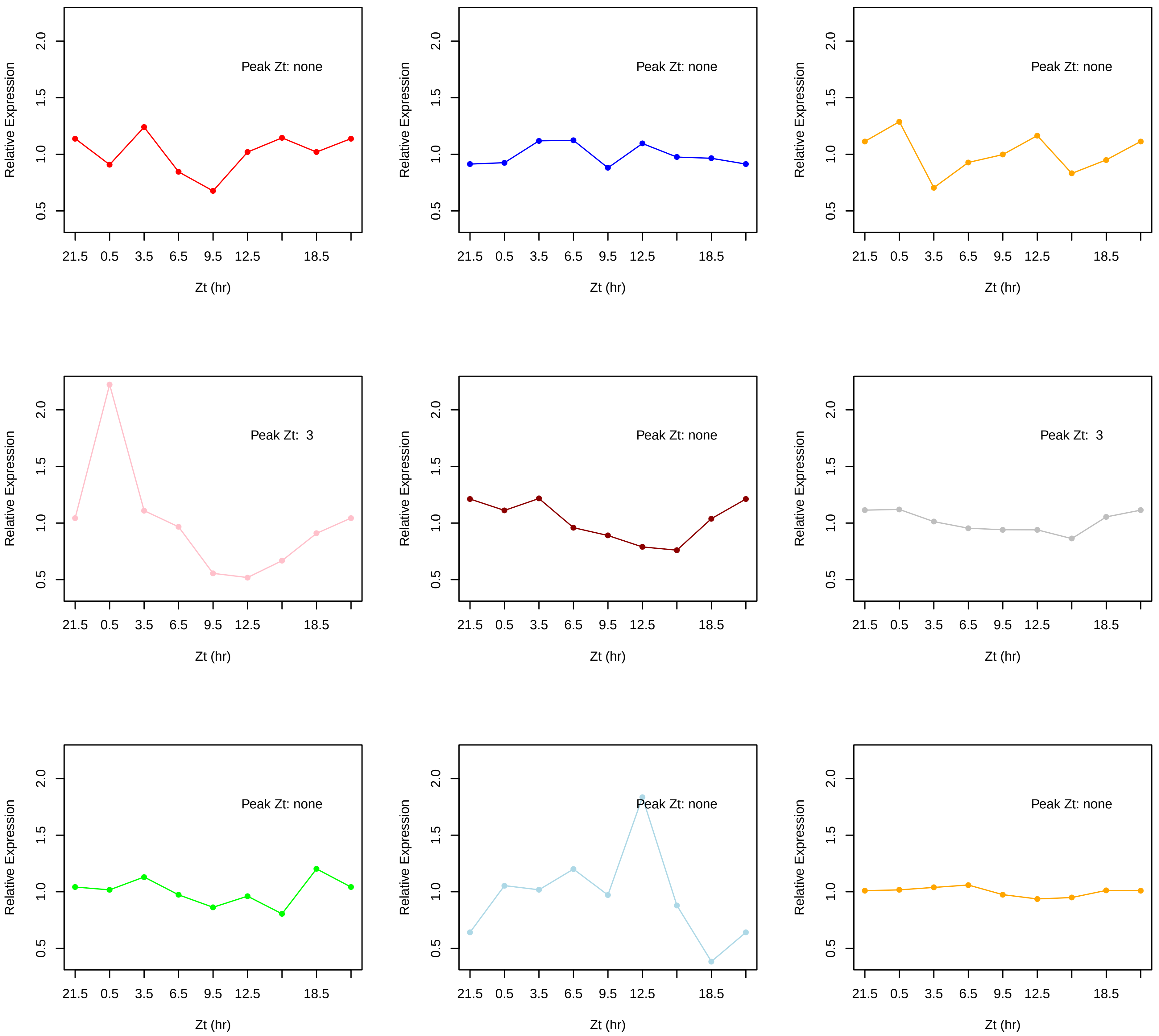

Singeleton: ENSXMAG00000001297 gdf3

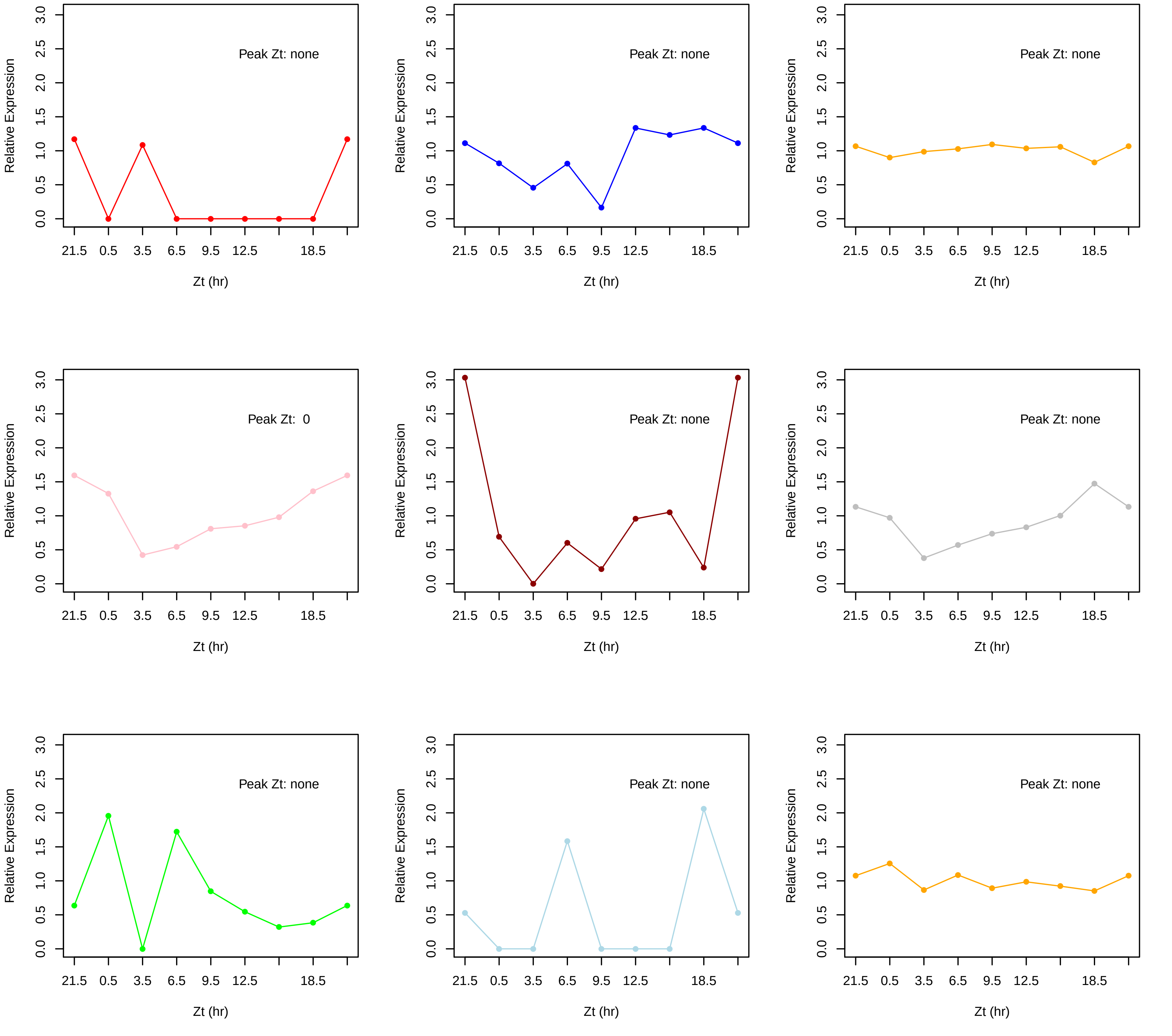

Singeleton: ENSXMAG00000027513

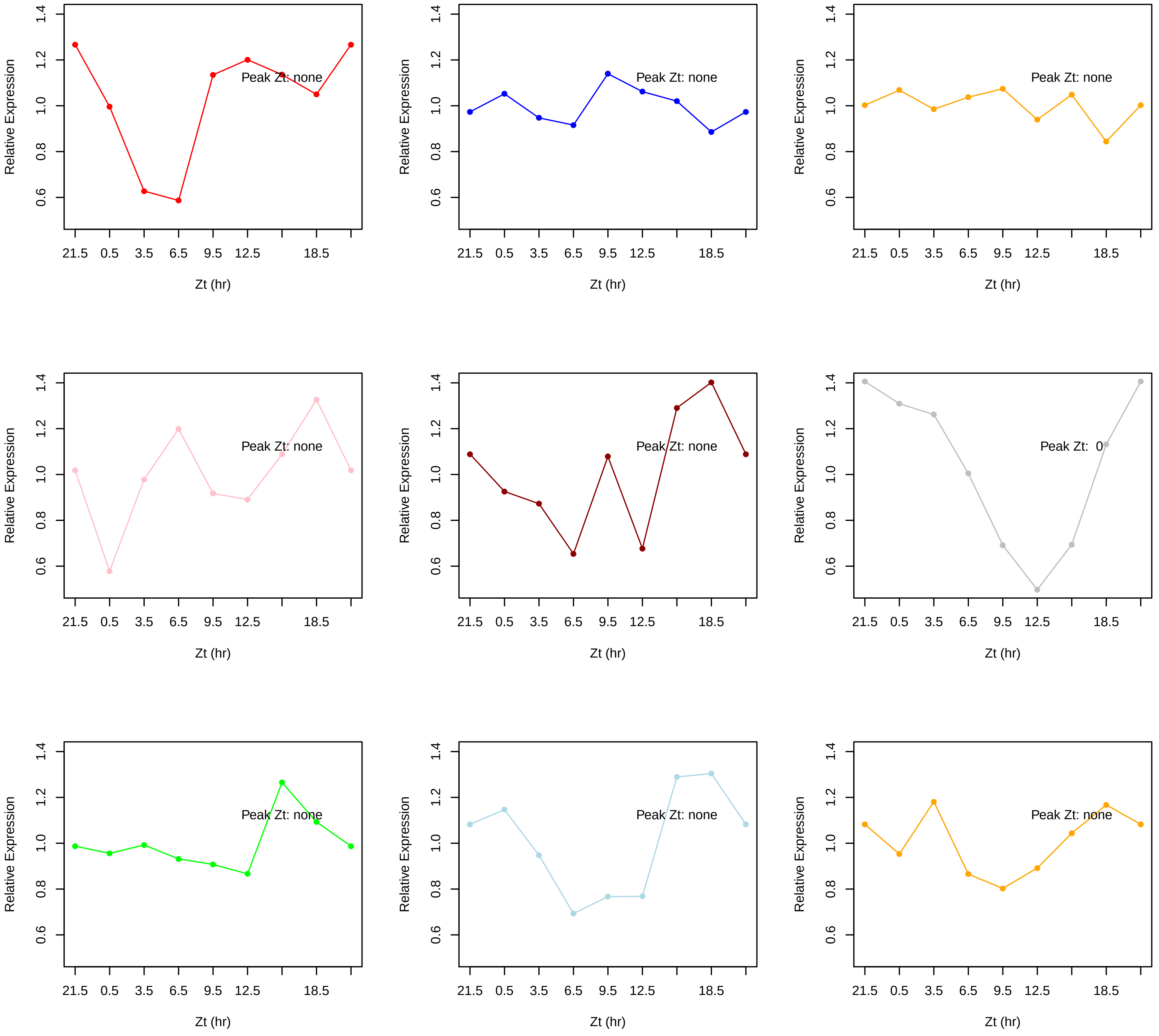

Singleton: ENSXMAG00000024497 HAAO

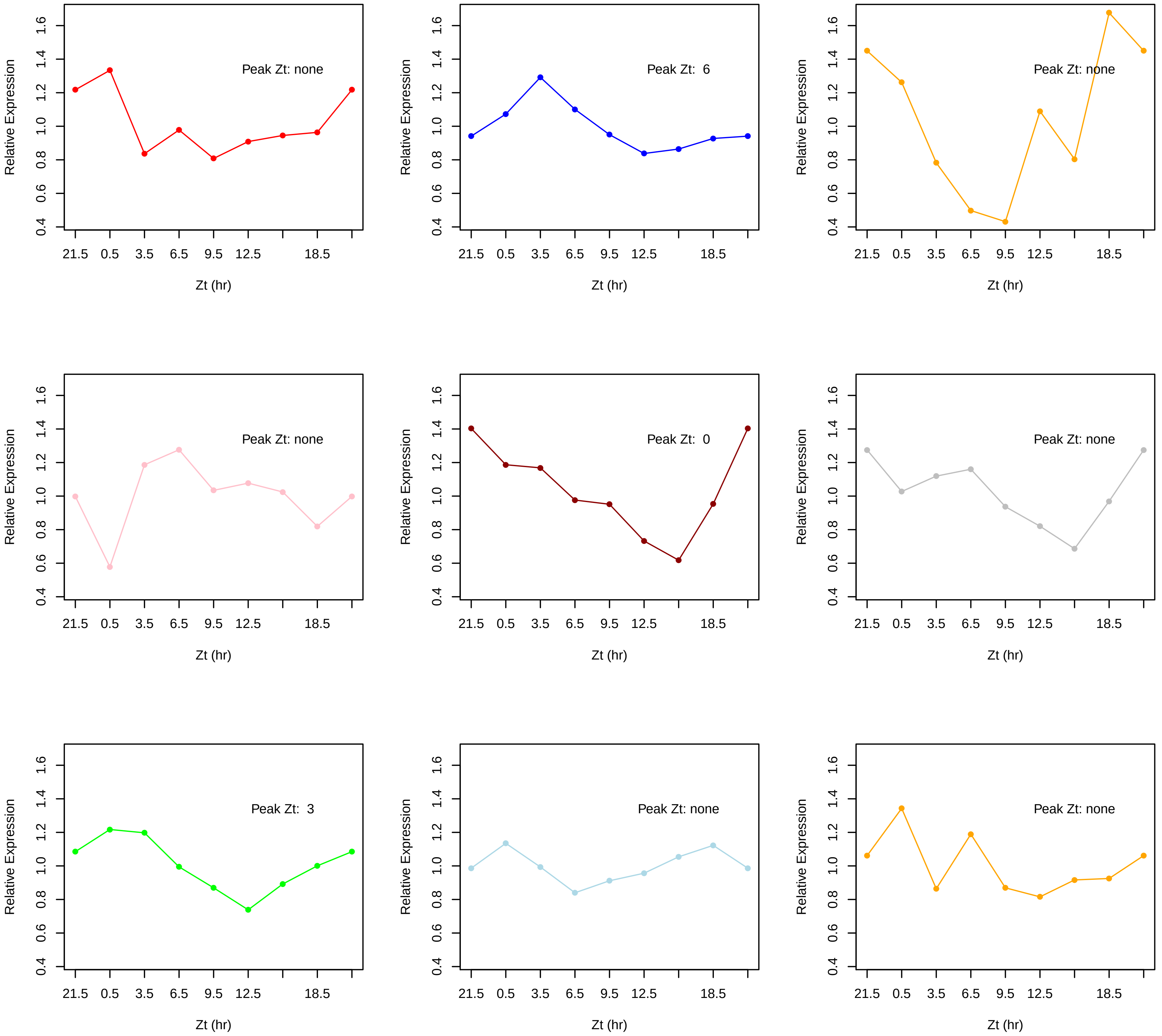

Singeleton: ENSXMAG00000024193 crls1

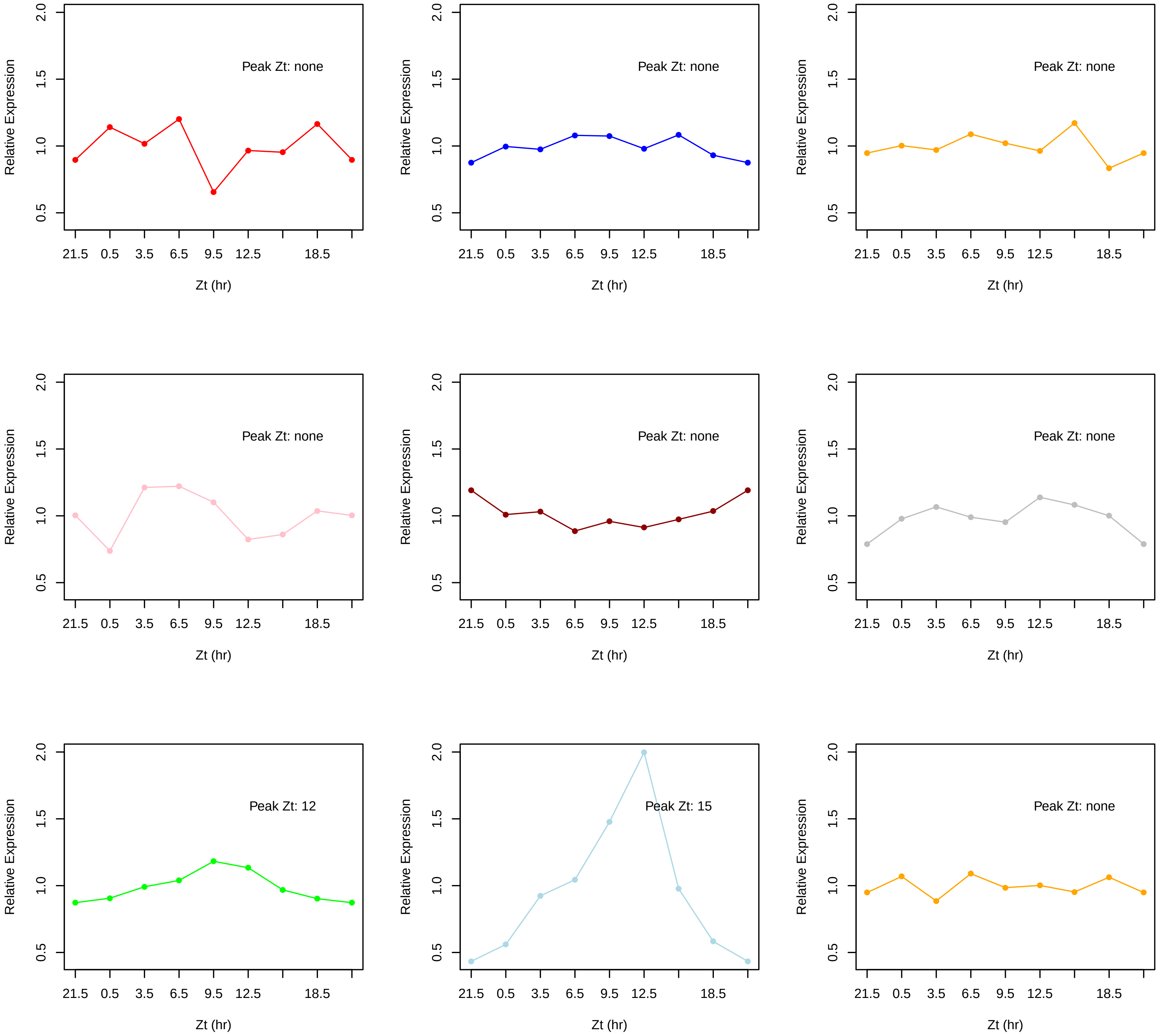

Singleton: ENSXMAG00000008592 trmt6

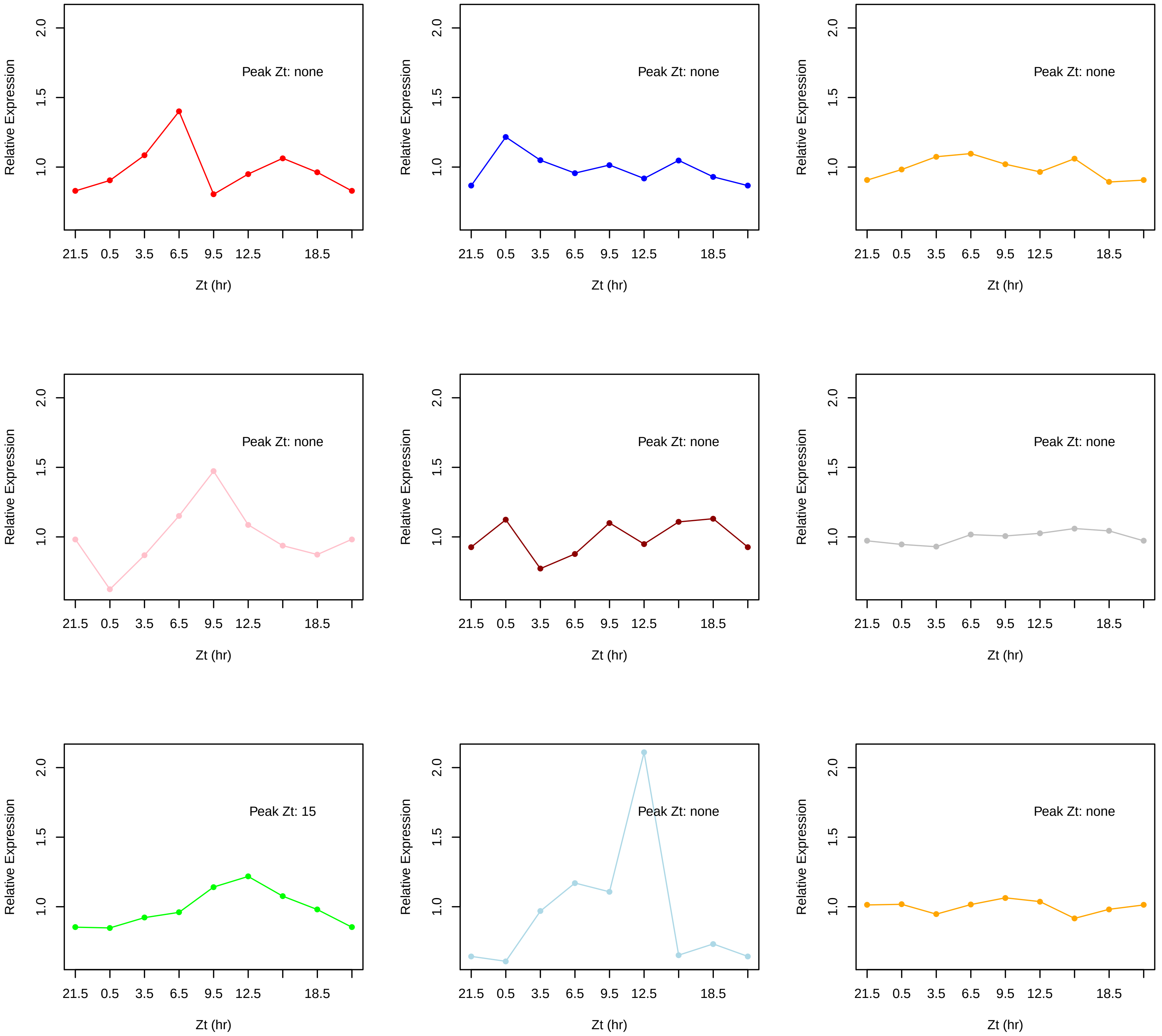

Singeleton: ENSXMAG00000015994 mta3

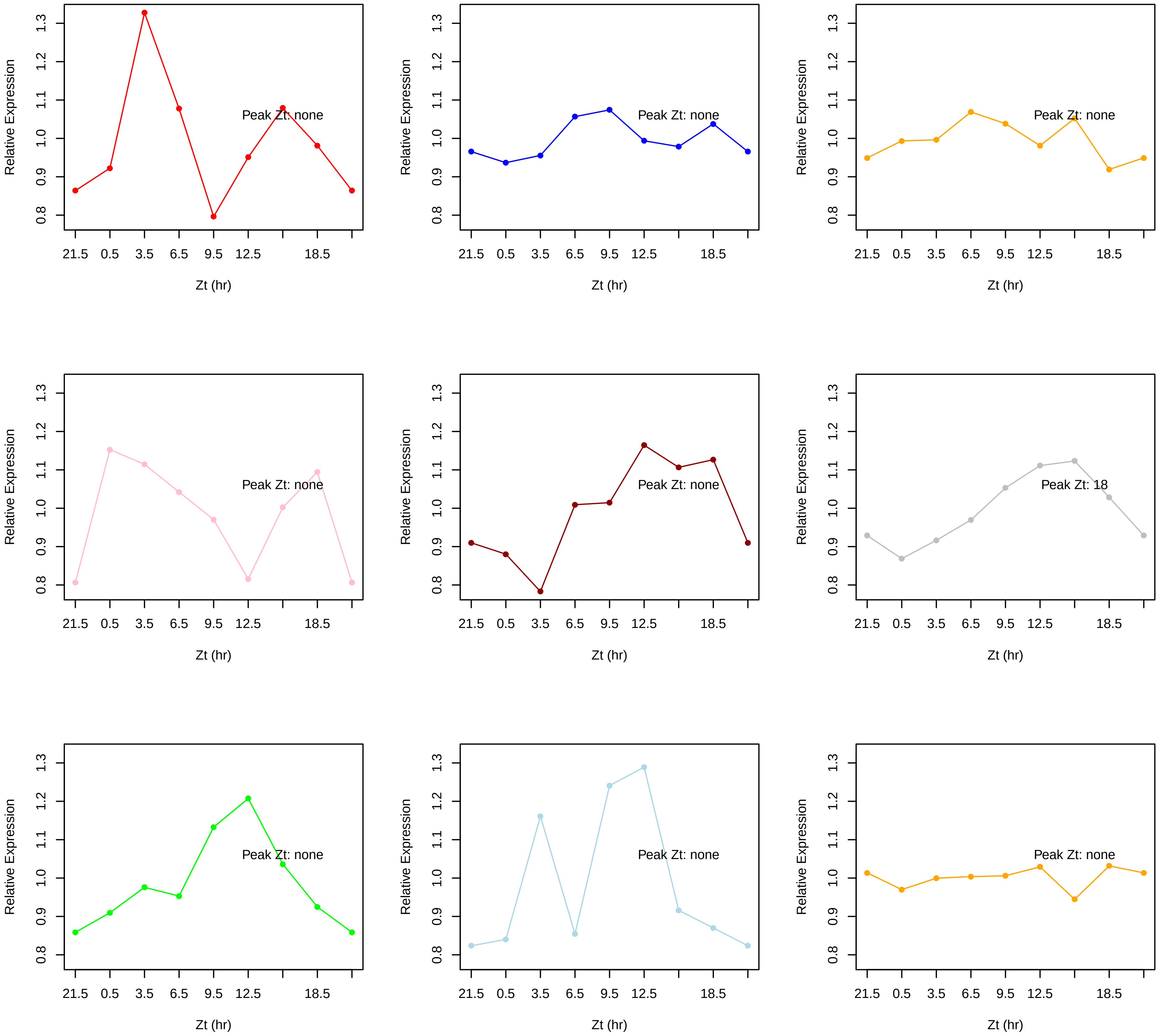

Singeleton: ENSXMAG00000008610 gpcpd1

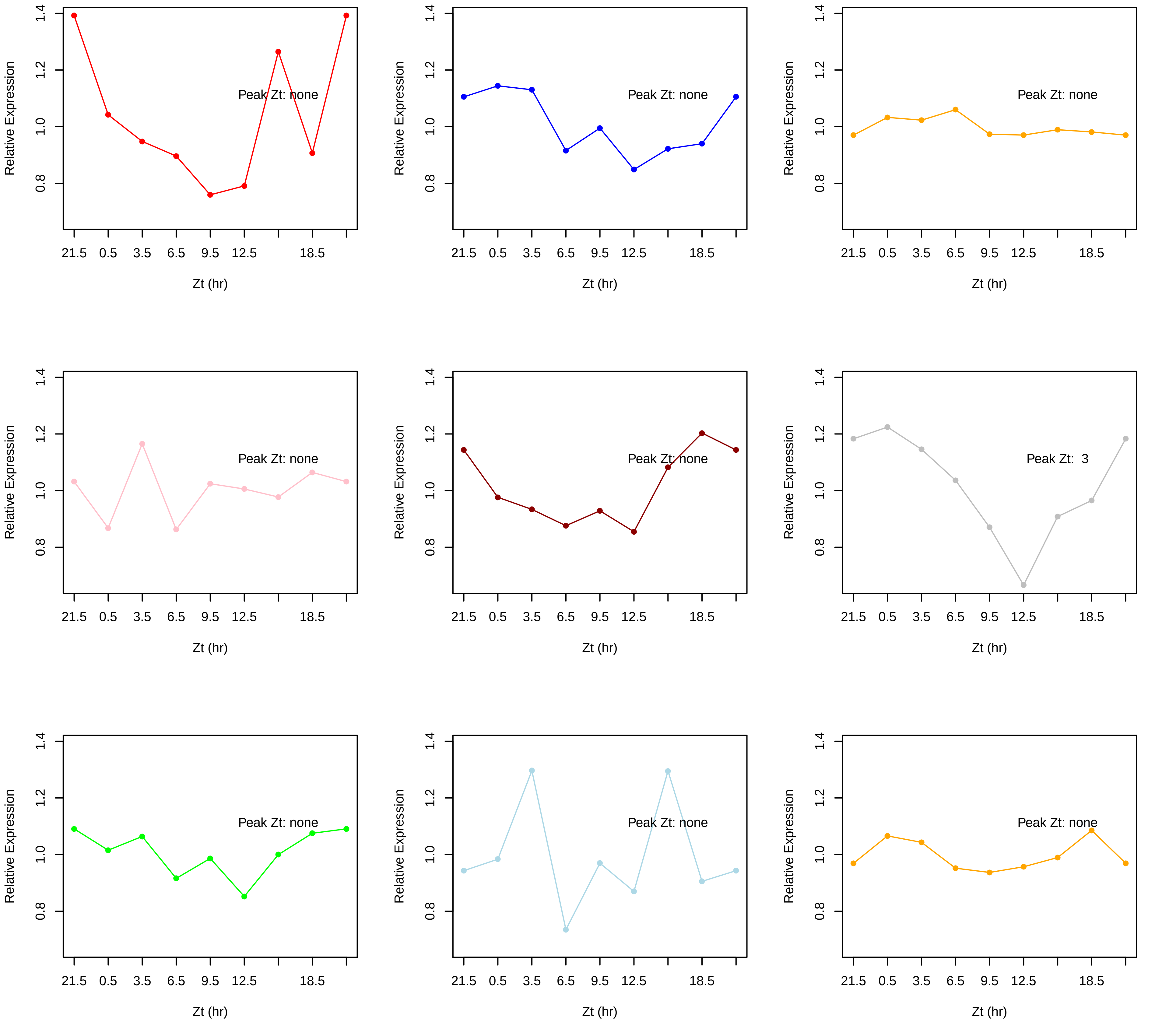

Singleton: ENSXMAG00000021060 TIMP3

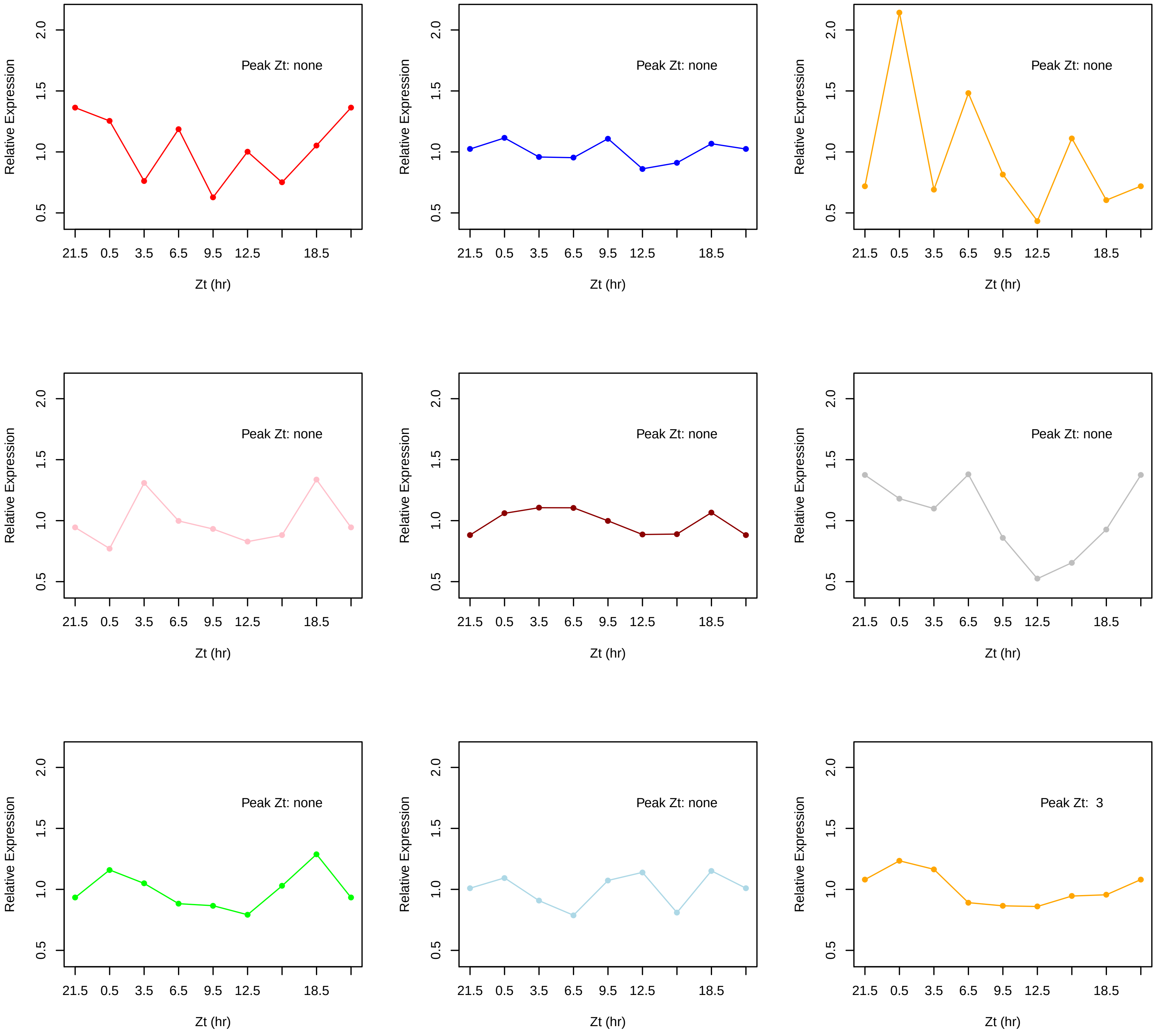

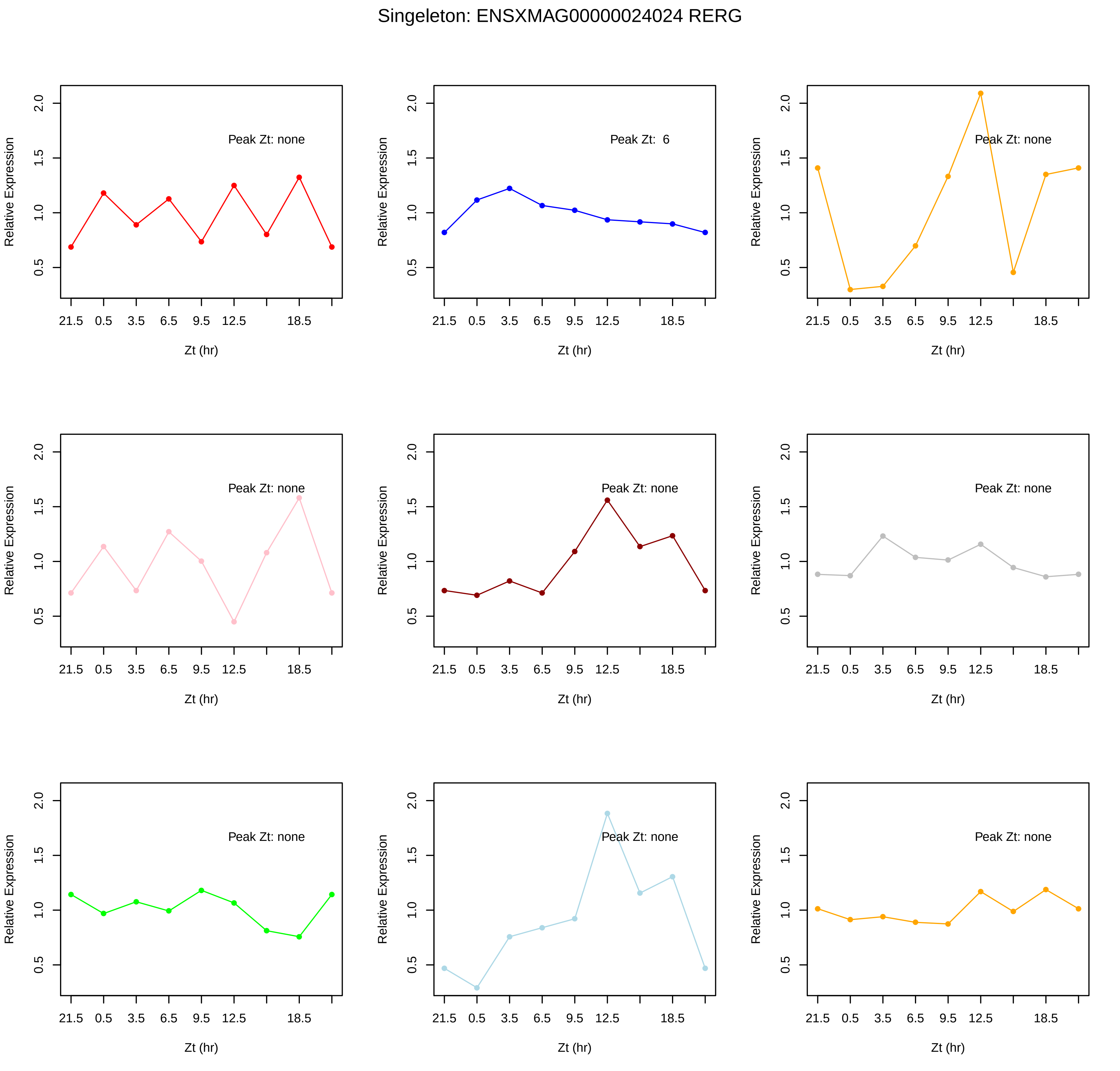

Singeleton: ENSXMAG00000024024 RERG
Peak Zt: none
Peak Zt: 6
Peak Zt: none
Peak Zt: none
Peak Zt: none
Peak Zt: none
Peak Zt: none
Peak Zt: none
Peak Zt: none
Relative Expression
Zt (hr)
21.5
0.5
3.5
6.5
9.5
12.5
18.5

Singeleton: ENSXMAG00000009306

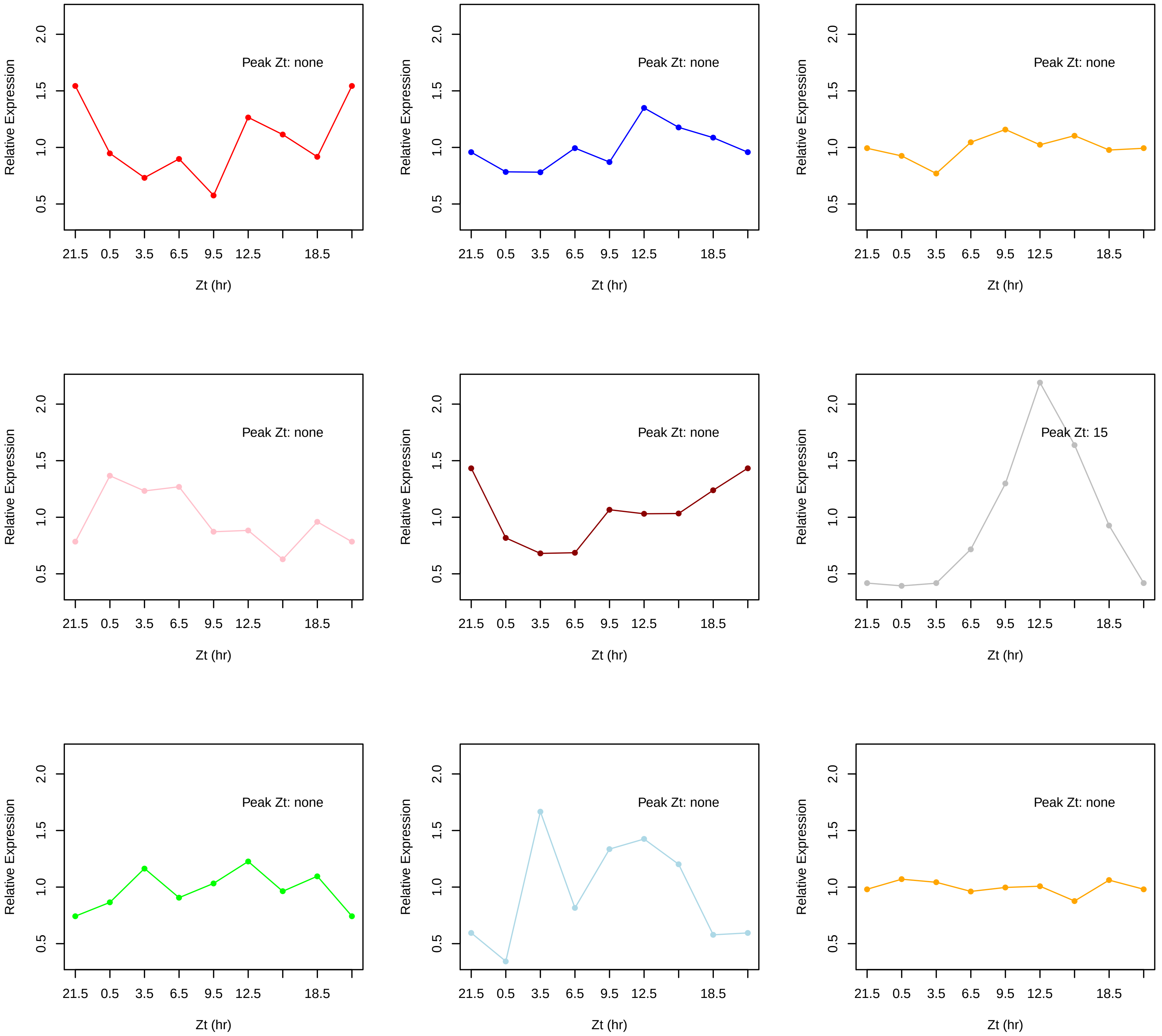

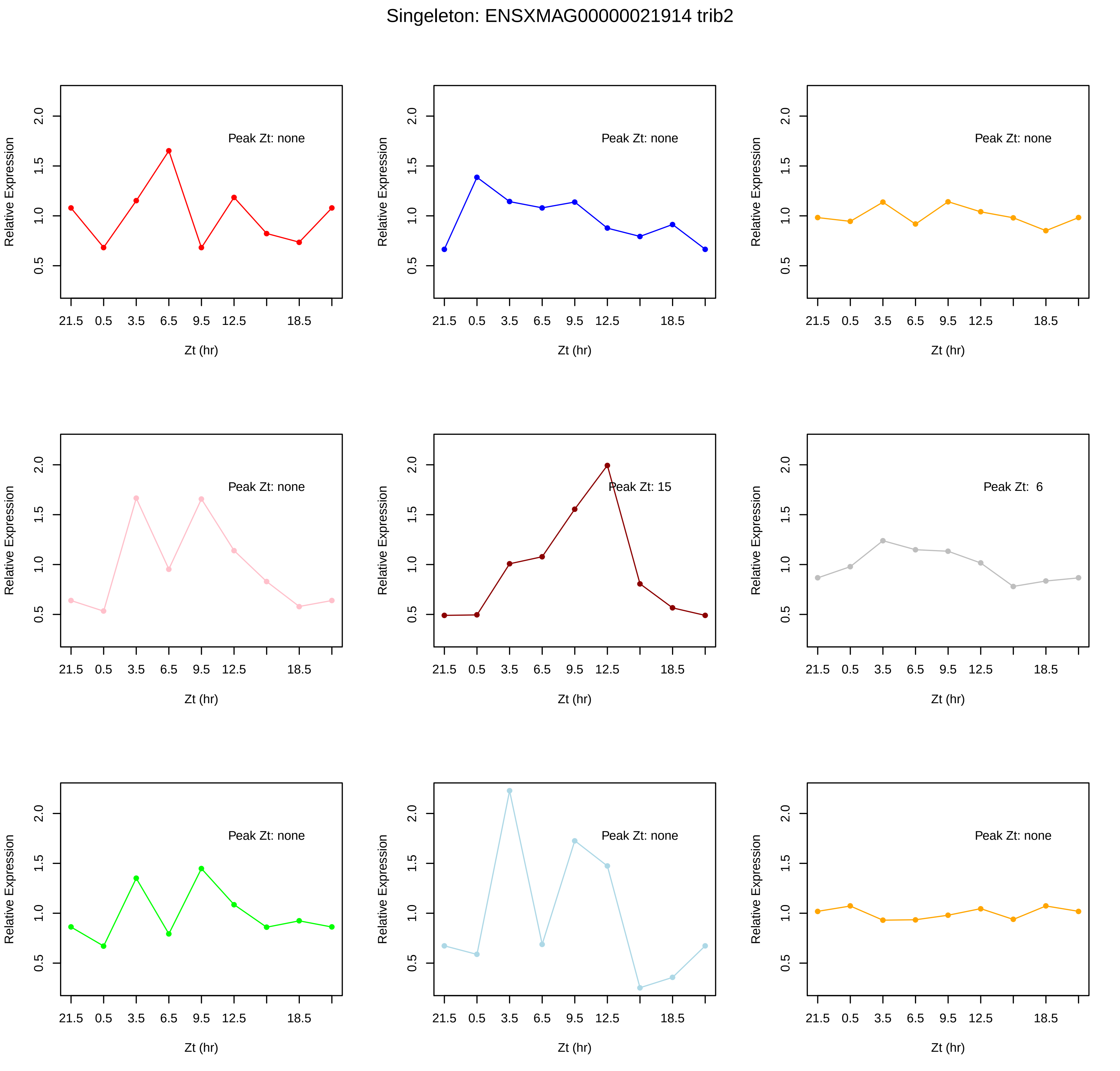
Singeleton: ENSXMAG00000021914 trib2
Peak Zt: none
Peak Zt: none
Peak Zt: none
Peak Zt: none
Peak Zt: 15
Peak Zt: 6
Peak Zt: none
Peak Zt: none
Peak Zt: none
Relative Expression
Zt (hr)
21.5
0.5
3.5
6.5
9.5
12.5
18.5
0.5
1.0
1.5
2.0

Singeleton: ENSXMAG00000009050

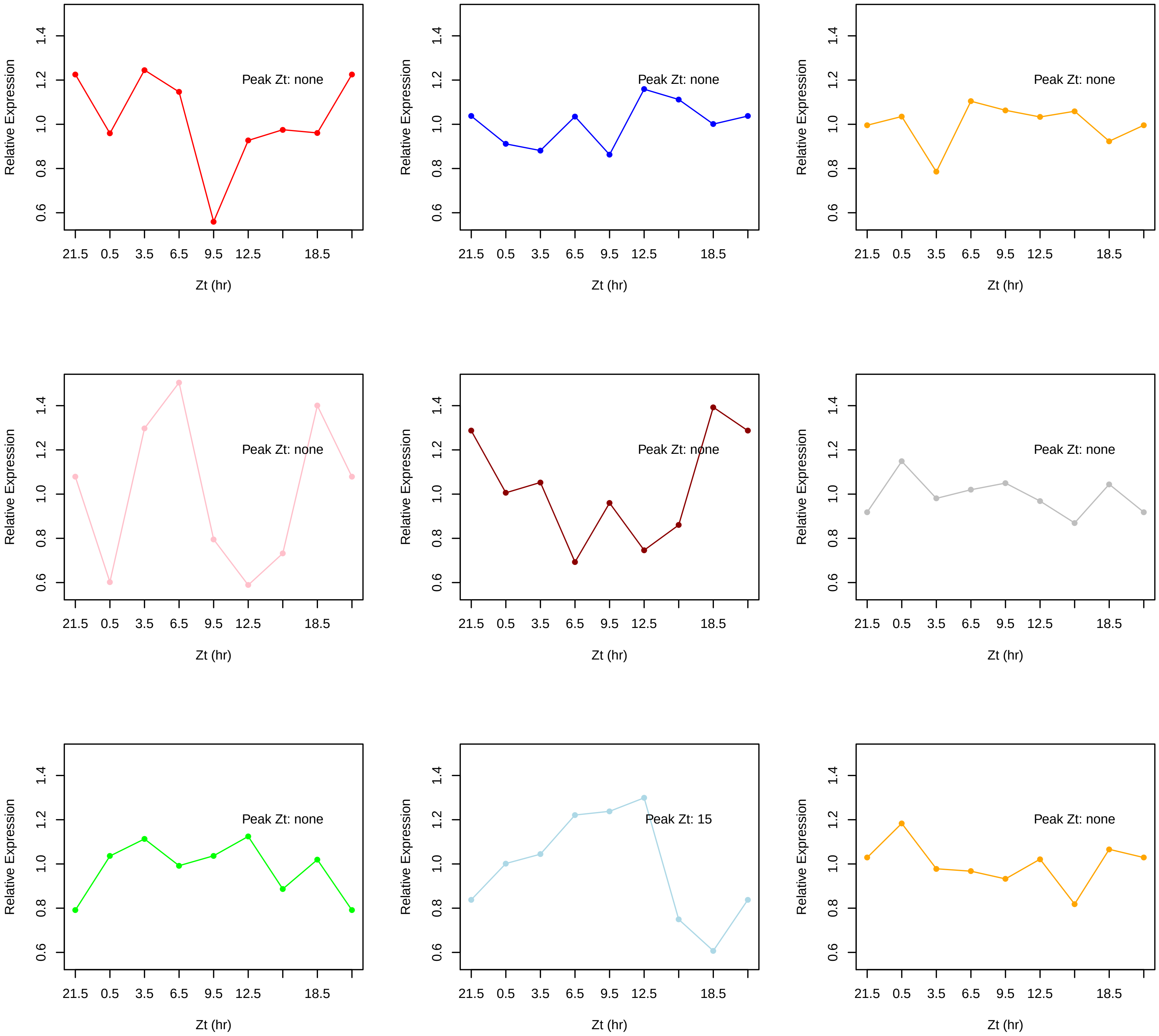

Singeleton: ENSXMAG00000004555 ddx1

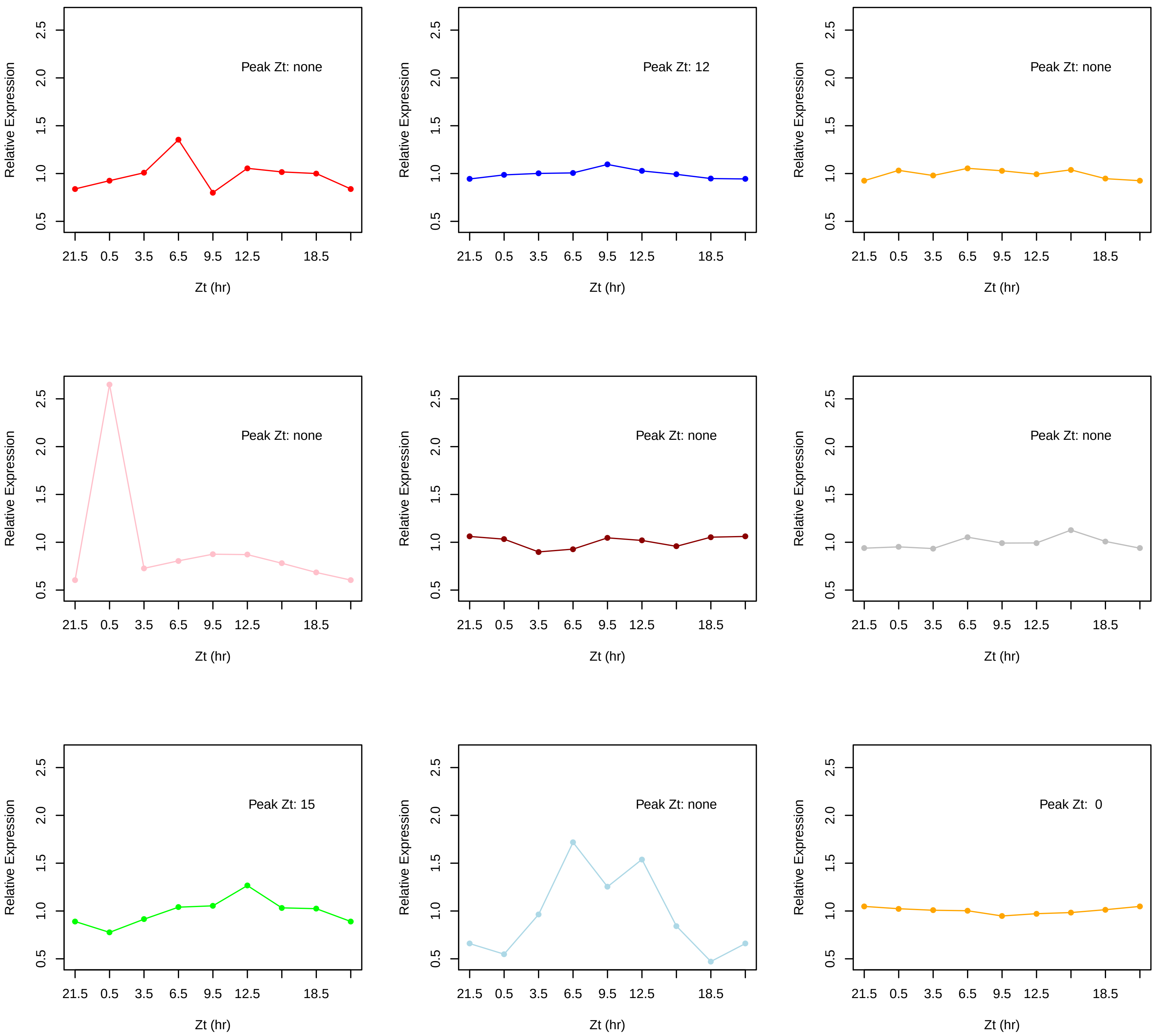

Singeleton: ENSXMAG00000026375

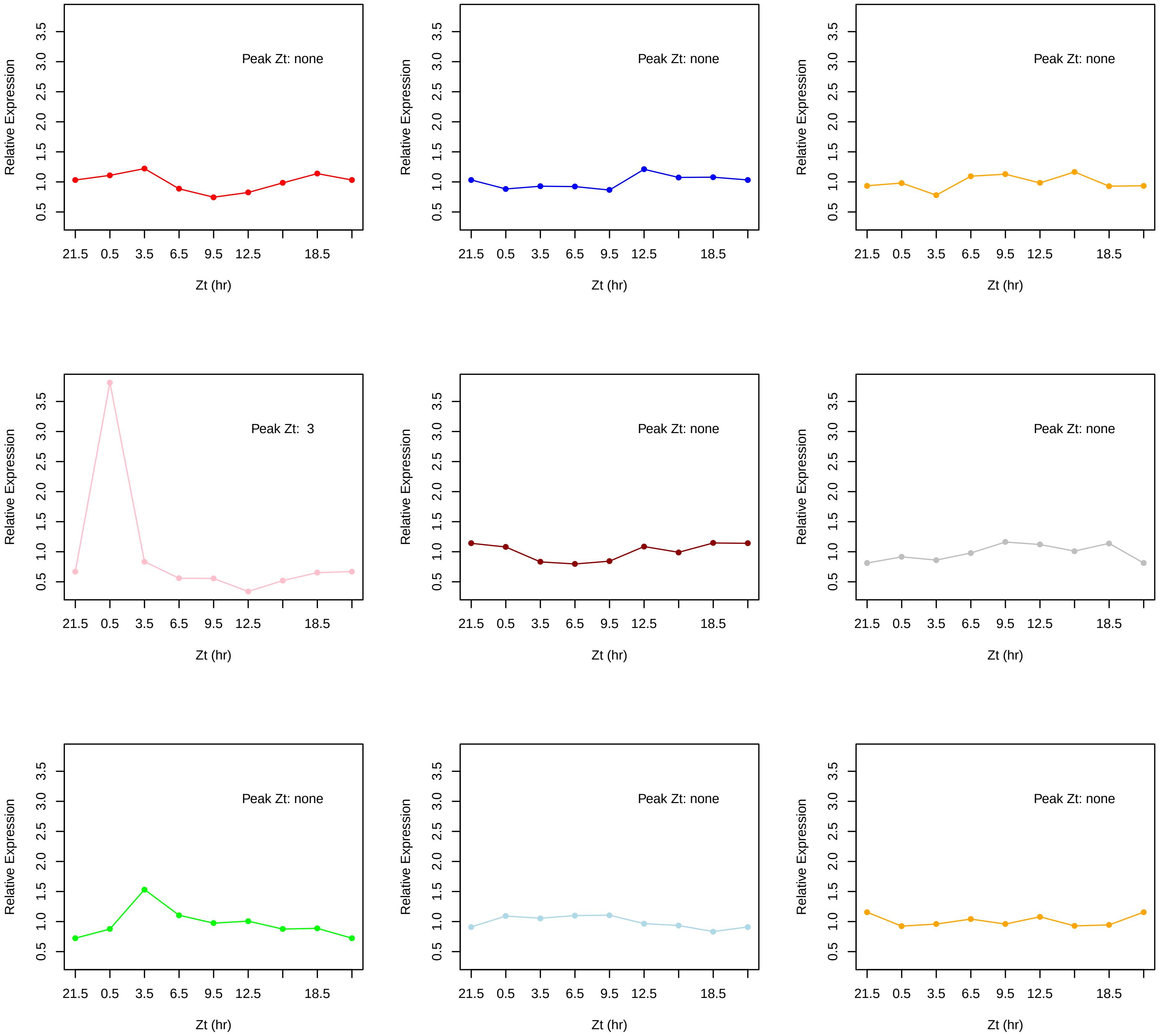

Singeleton: ENSXMAG00000003386 mycn

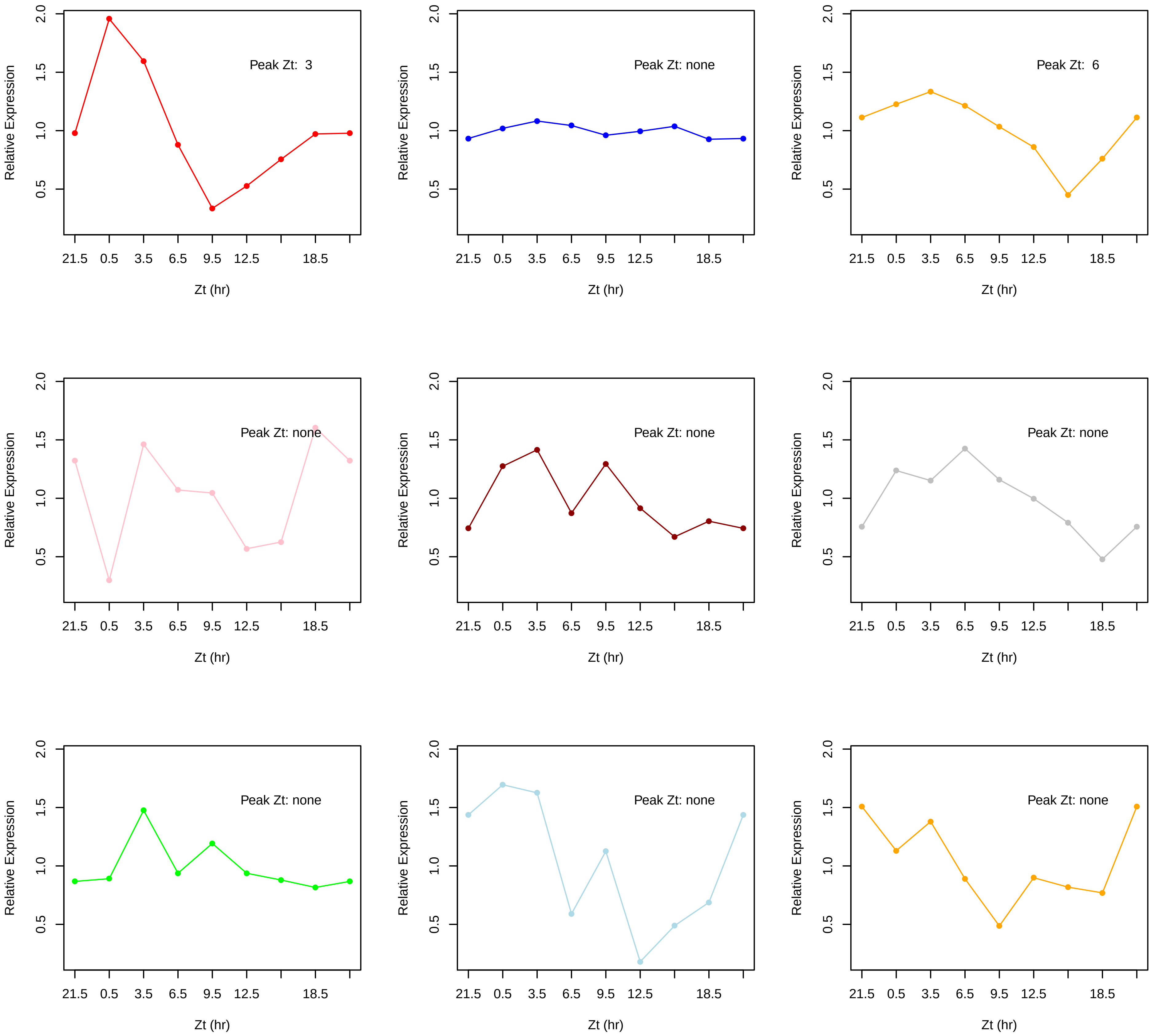

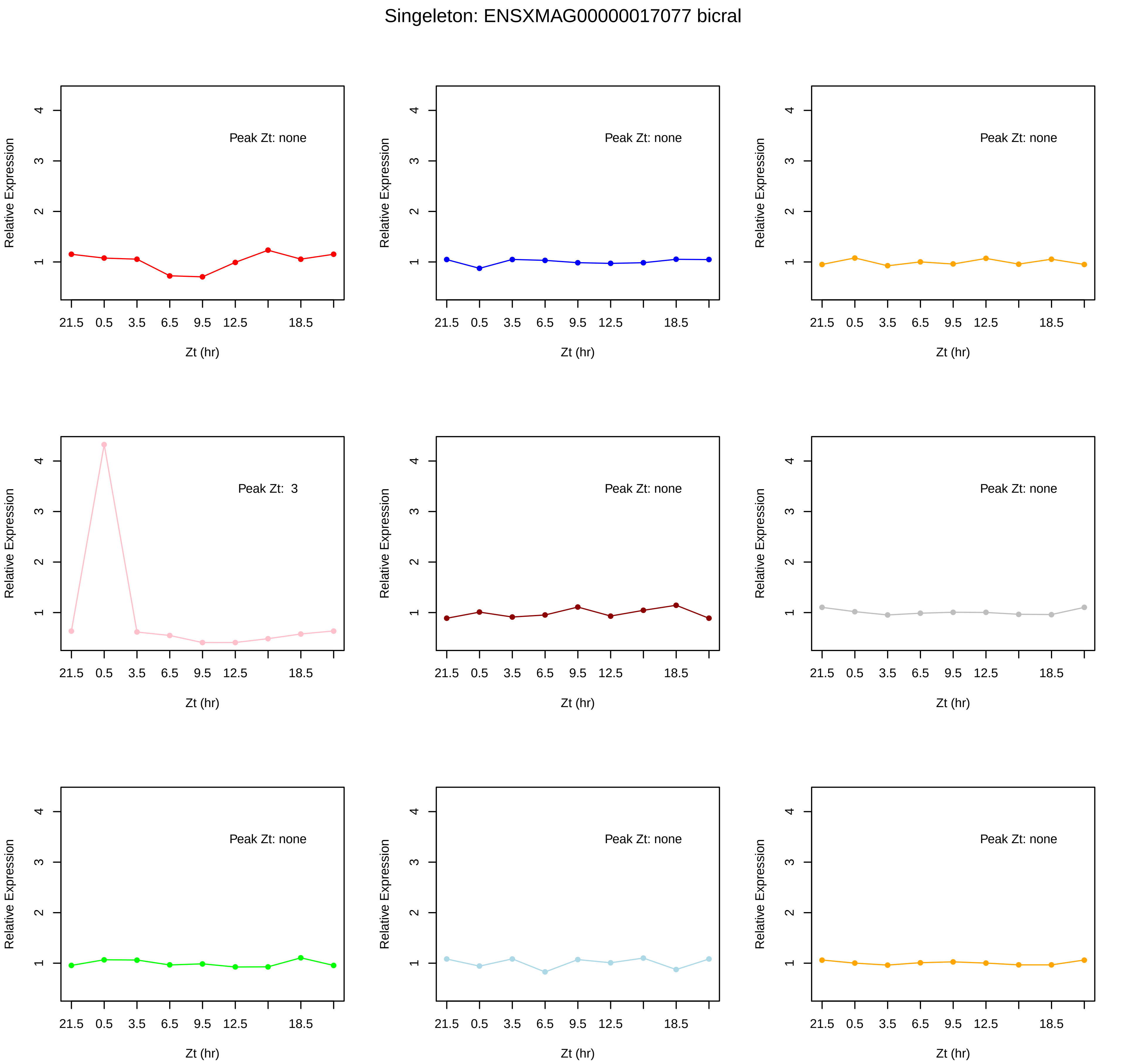
Singeleton: ENSXMAG00000017077 bicral
Peak Zt: none
Peak Zt: none
Peak Zt: none
Peak Zt: 3
Peak Zt: none
Peak Zt: none
Peak Zt: none
Peak Zt: none
Peak Zt: none
Relative Expression
Zt (hr)
21.5
0.5
3.5
6.5
9.5
12.5
18.5

Singeleton: ENSXMAG00000010759 fam49a

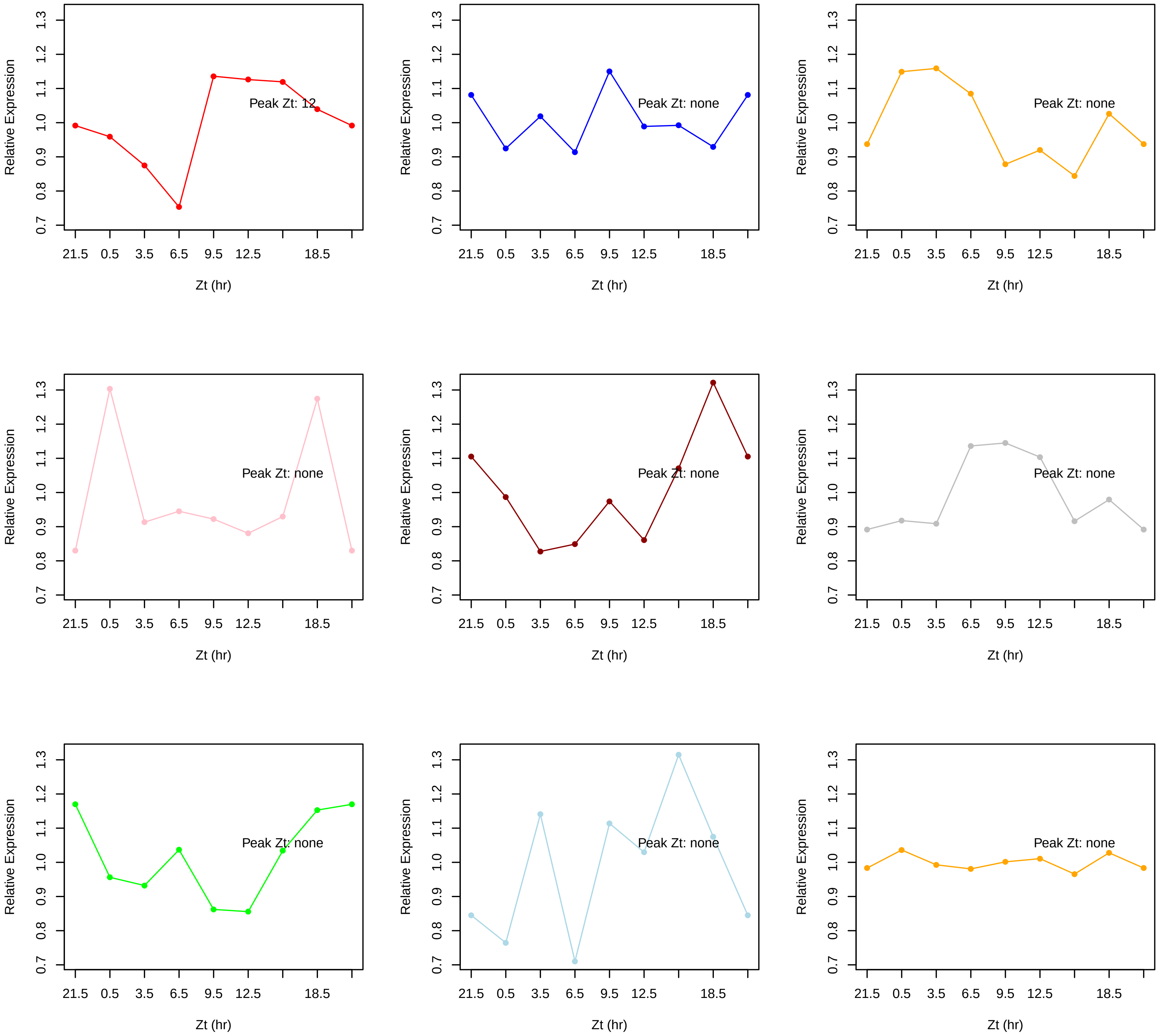

Singeleton: ENSXMAG00000010747 sf3b6

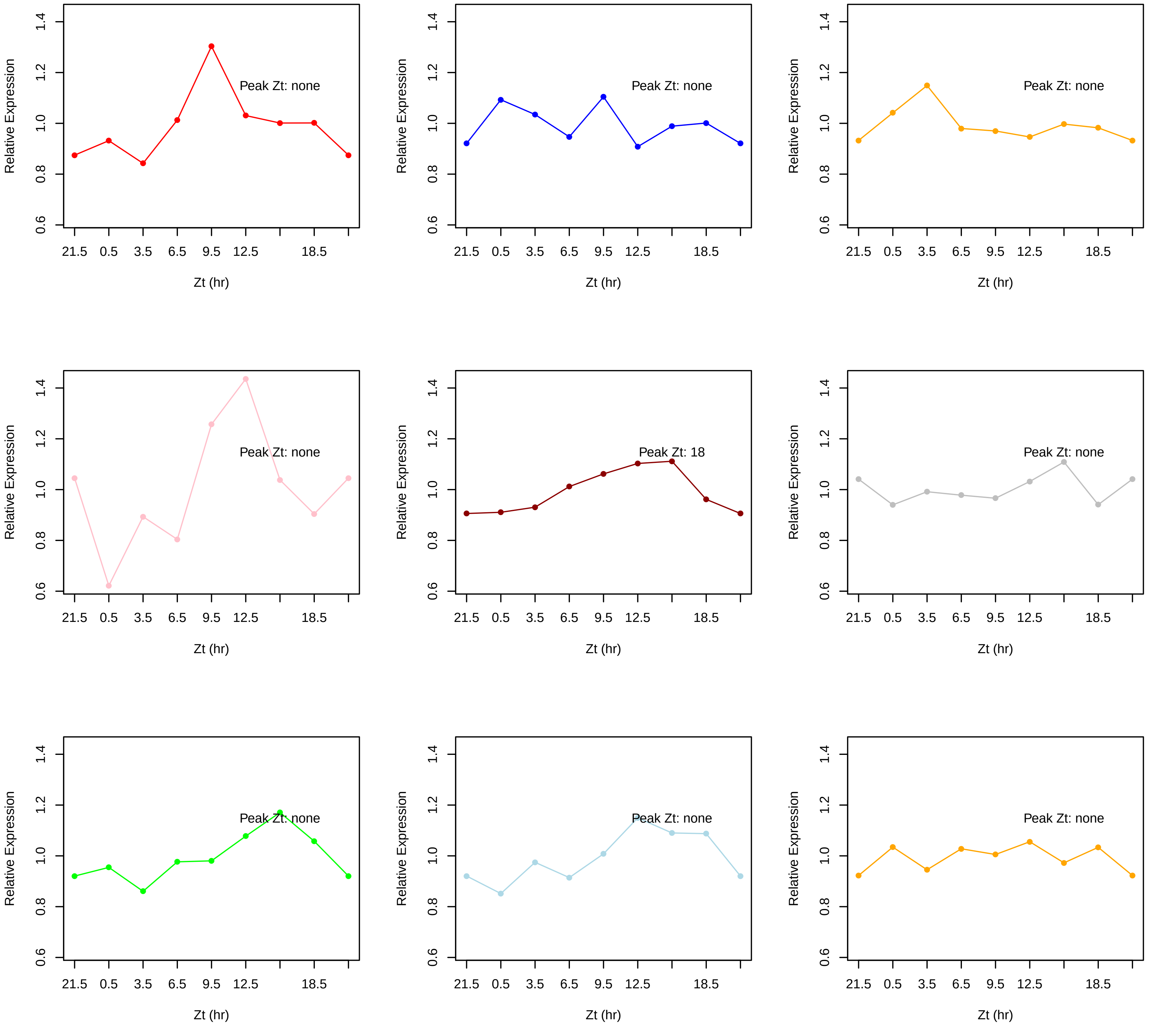

Singeleton: ENSXMAG00000010731 pla2g7

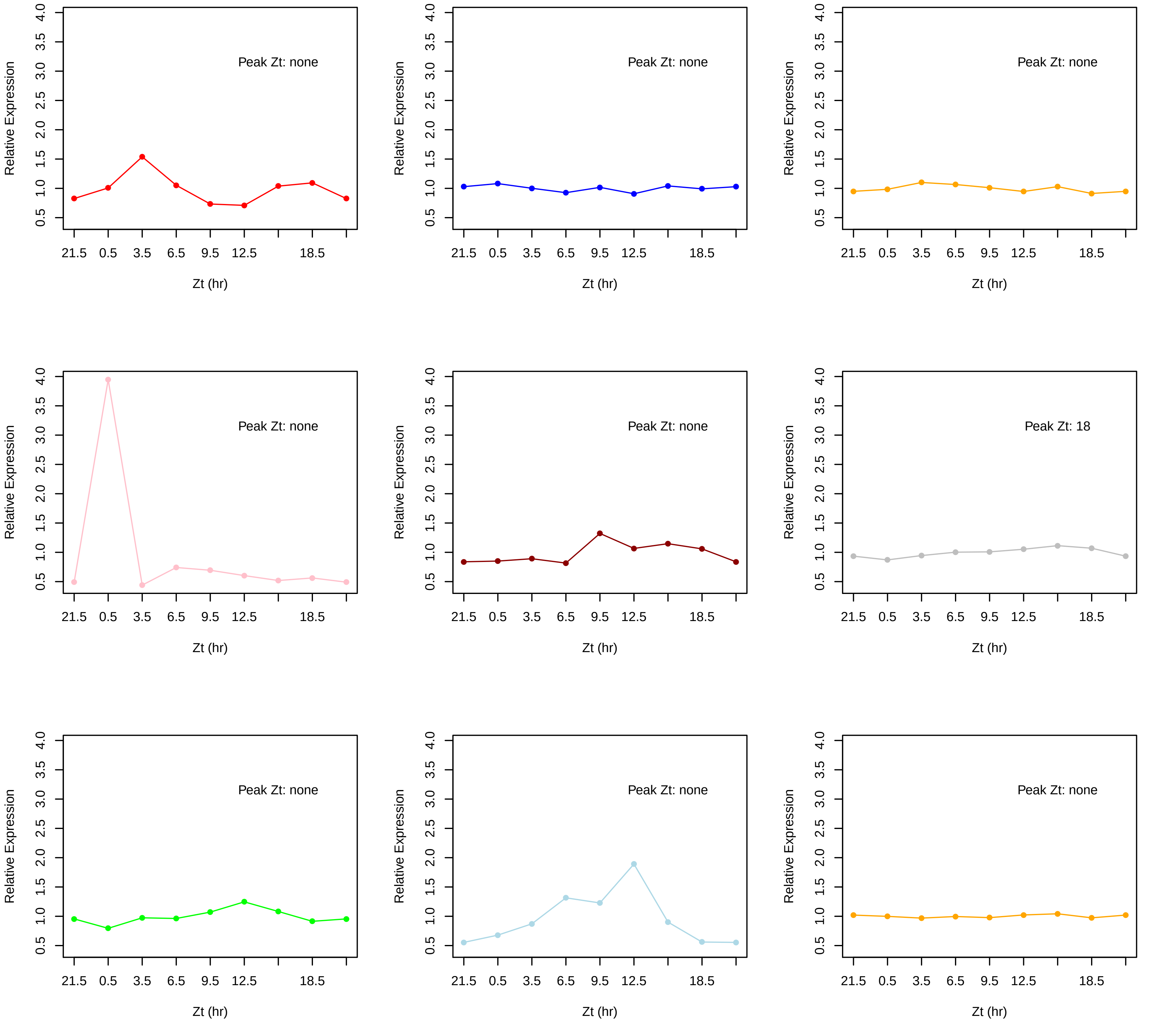

Singeleton: ENSXMAG00000026975 borcs5

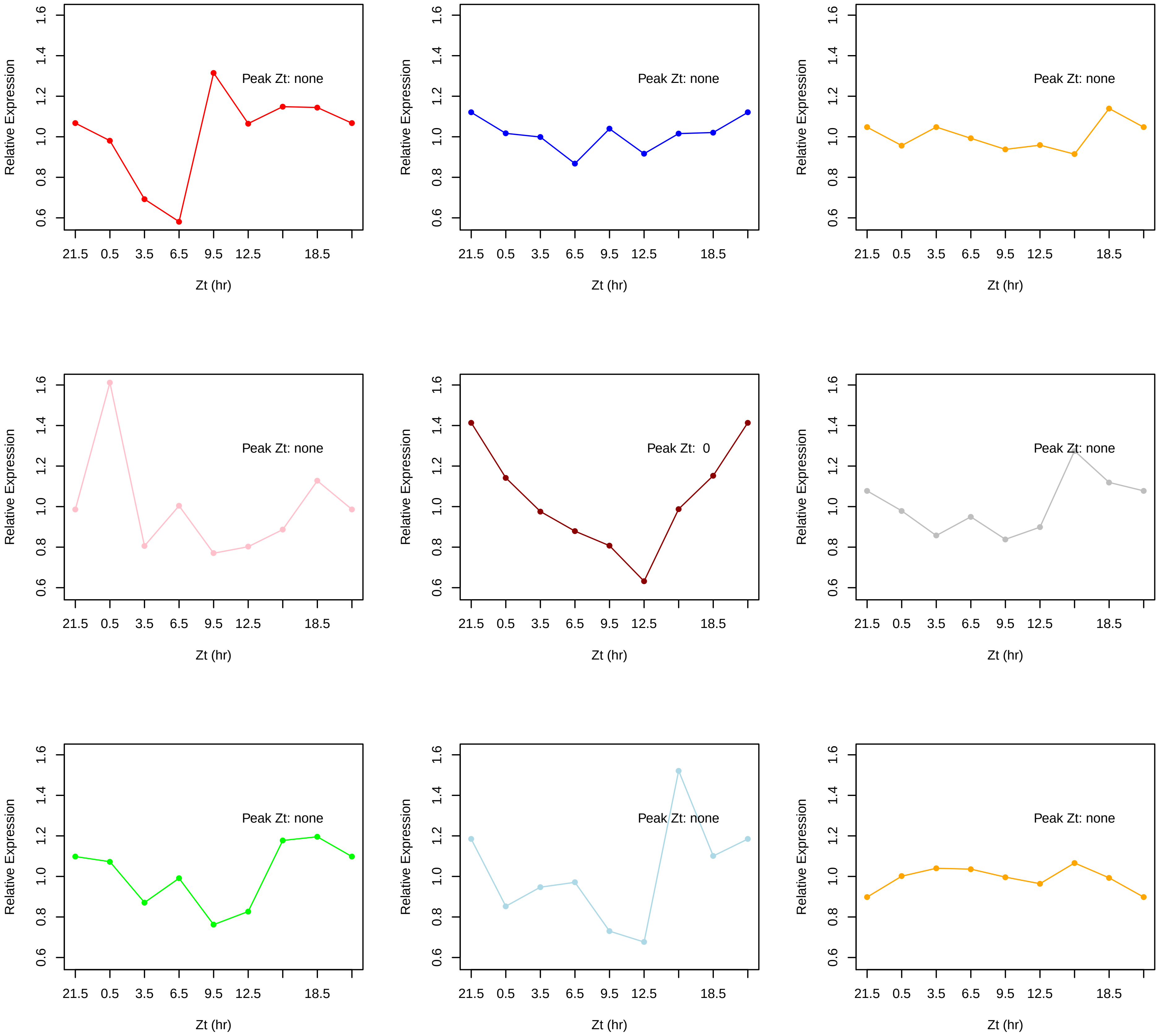

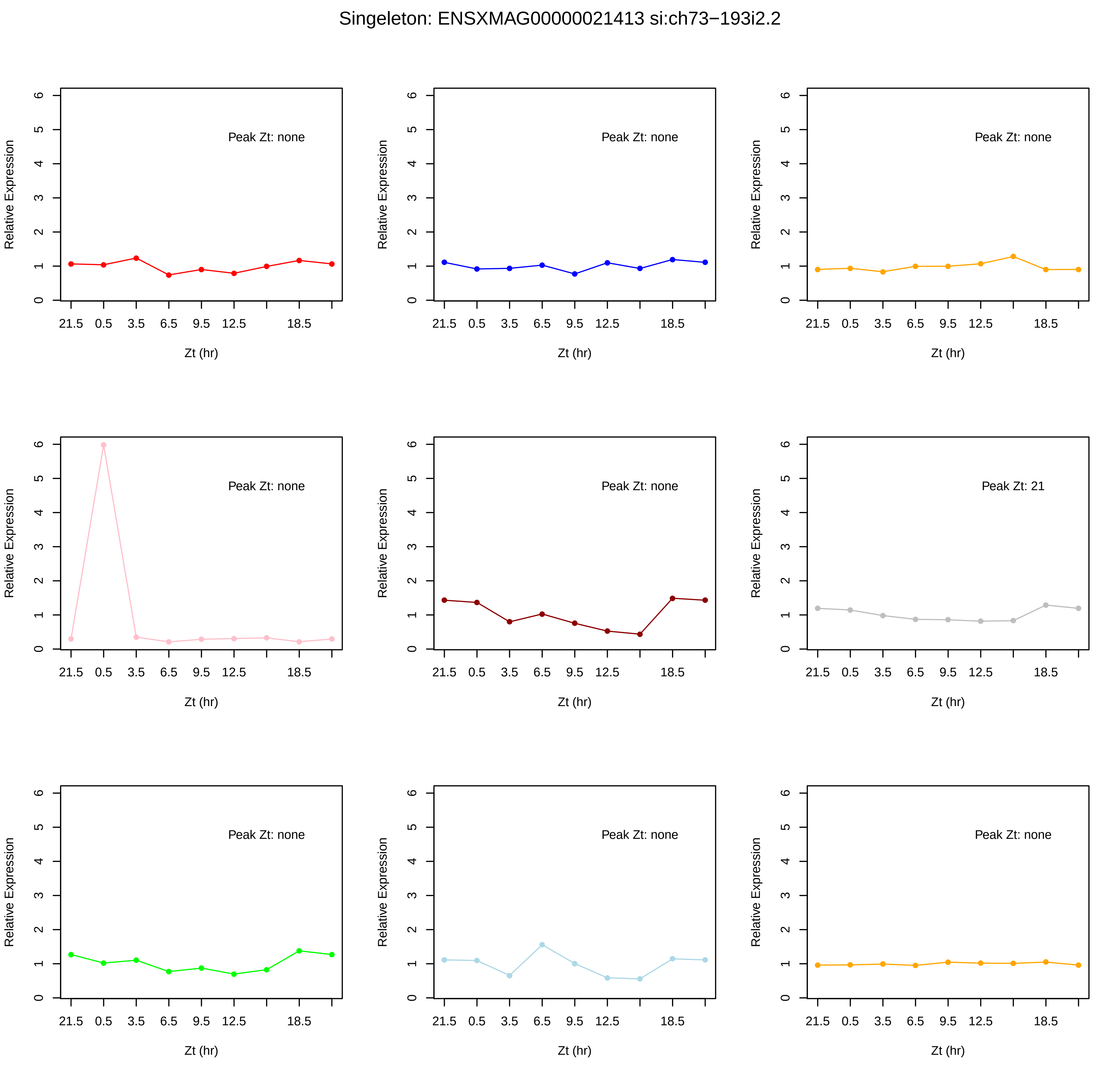

Singeleton: ENSXMAG00000021413 si:ch73−193i2.2
Peak Zt: none
Peak Zt: none
Peak Zt: none
Peak Zt: none
Peak Zt: none
Peak Zt: 21
Peak Zt: none
Peak Zt: none
Peak Zt: none
Relative Expression
Zt (hr)
21.5 0.5 3.5 6.5 9.5 12.5 18.5
0 1 2 3 4 5 6

Singeleton: ENSXMAG00000023022

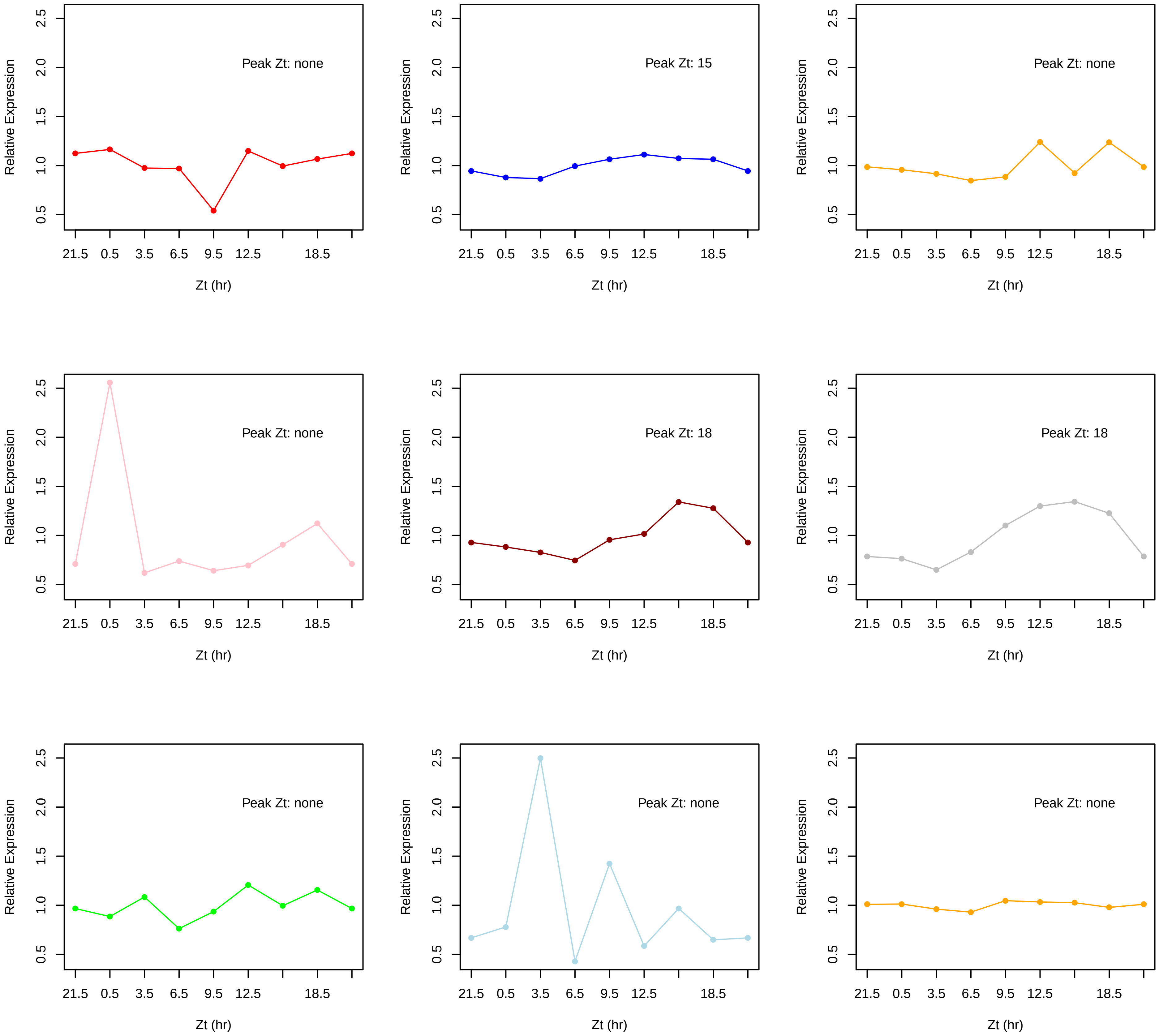

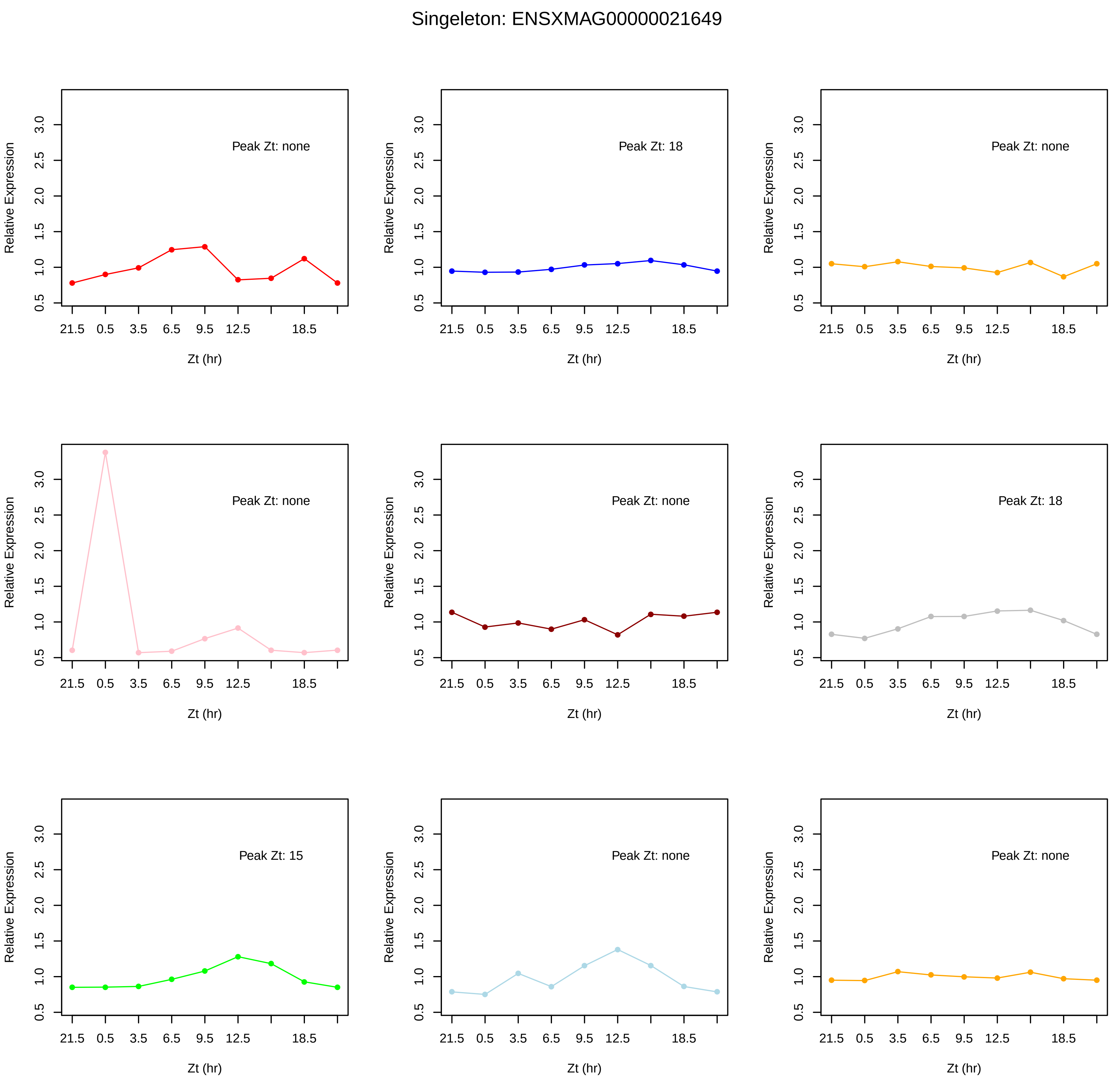

Singleton: ENSXMAG00000021649
Peak Zt: none
Peak Zt: 18
Peak Zt: none
Peak Zt: none
Peak Zt: none
Peak Zt: 18
Peak Zt: 15
Peak Zt: none
Peak Zt: none
Relative Expression
Zt (hr)
21.5
0.5
3.5
6.5
9.5
12.5
18.5
0.5
1.0
1.5
2.0
2.5
3.0

Singleton: ENSXMAG00000008926 ptchd4

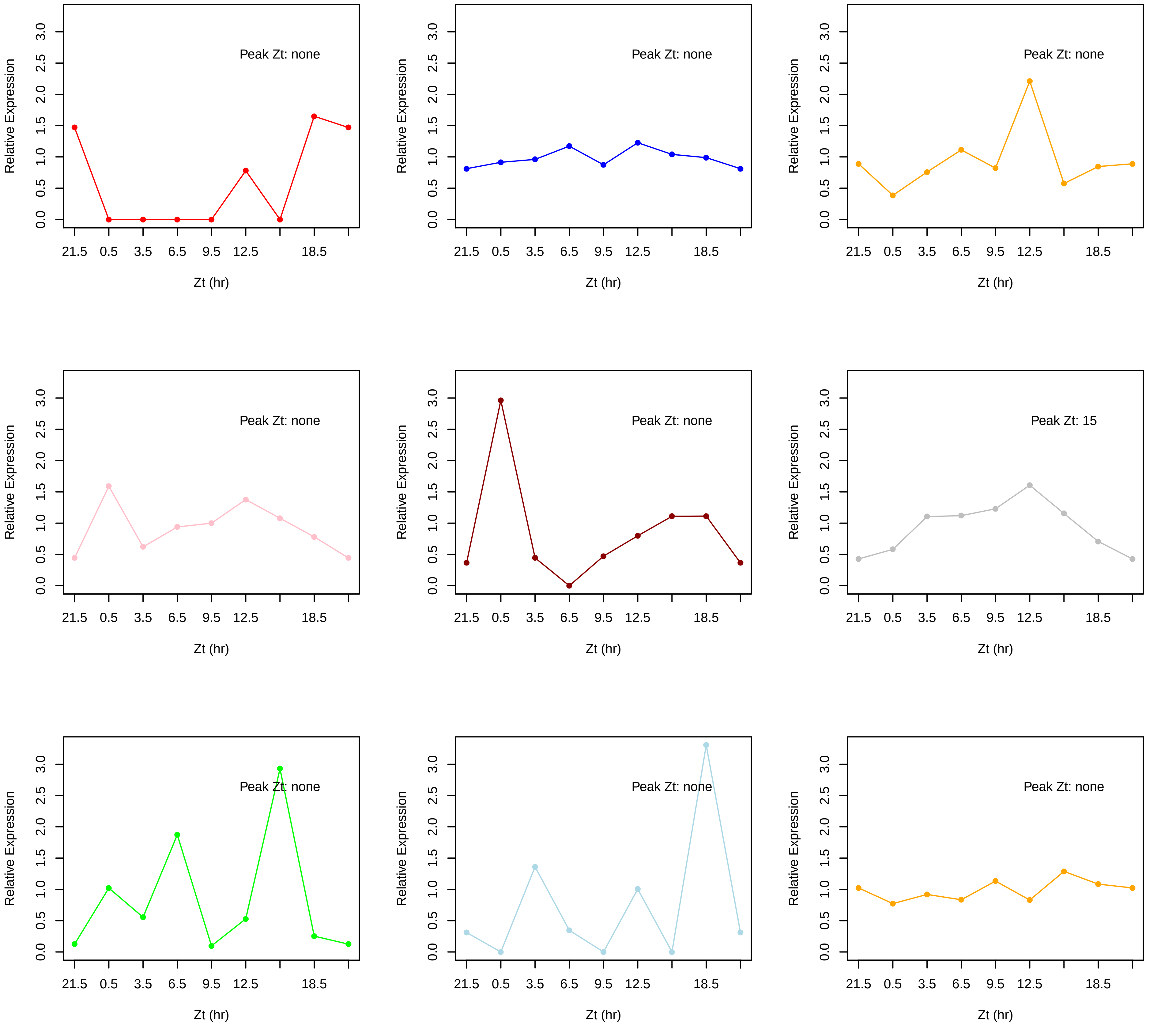

Singeleton: ENSXMAG00000008677 ECHDC1

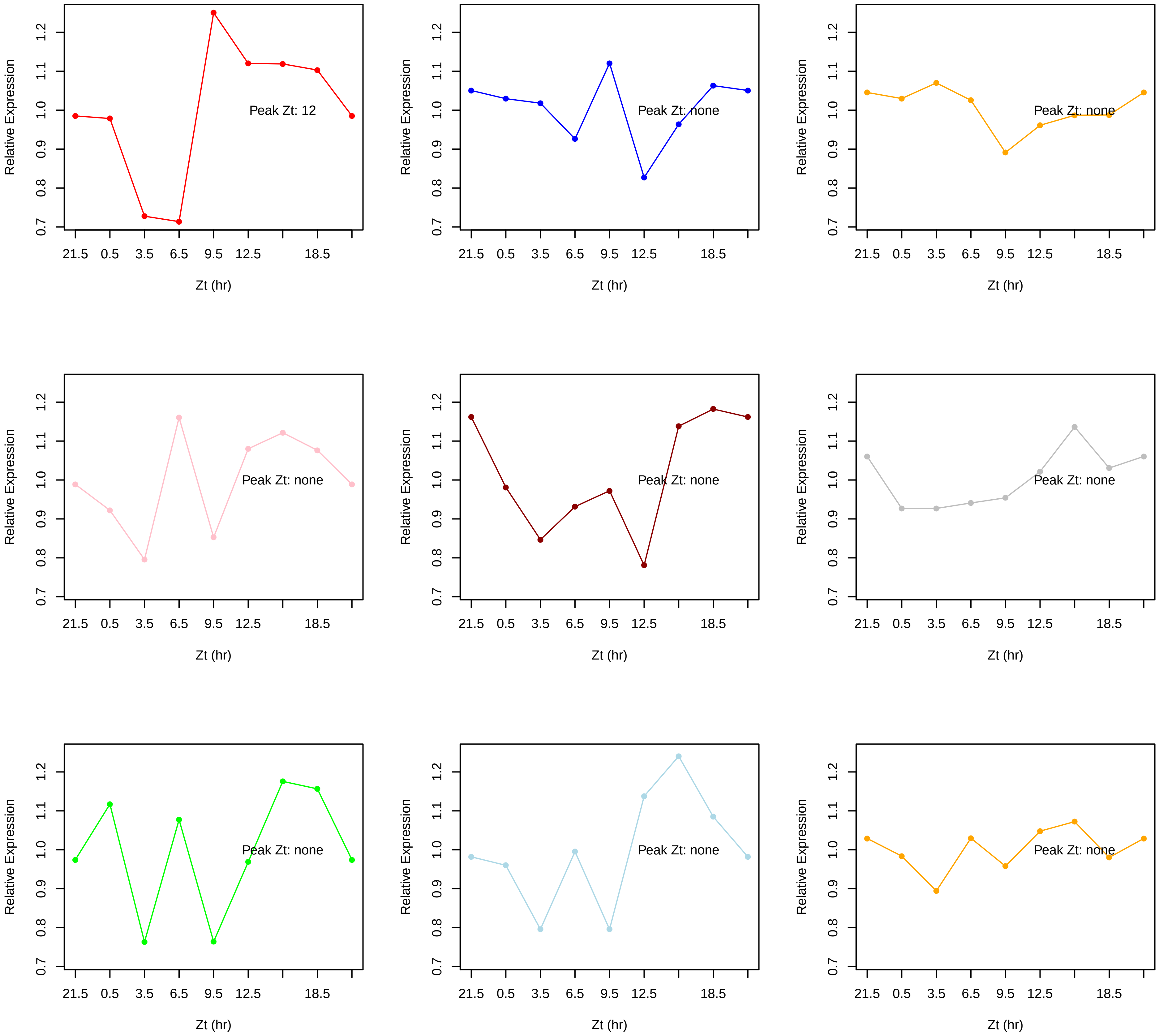

Singeleton: ENSXMAG00000002827 xpot

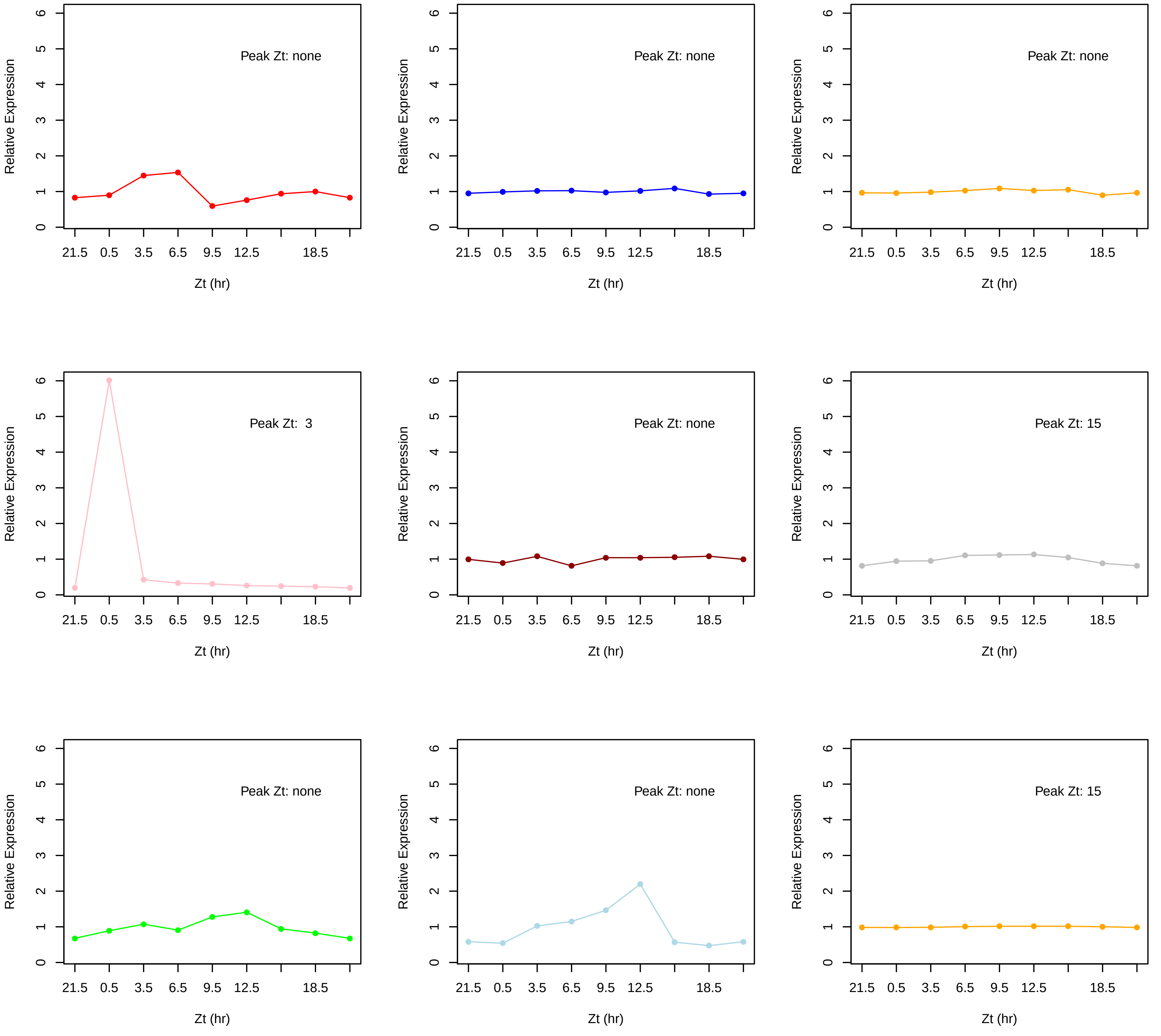

Singeleton: ENSXMAG00000002855 rtcb

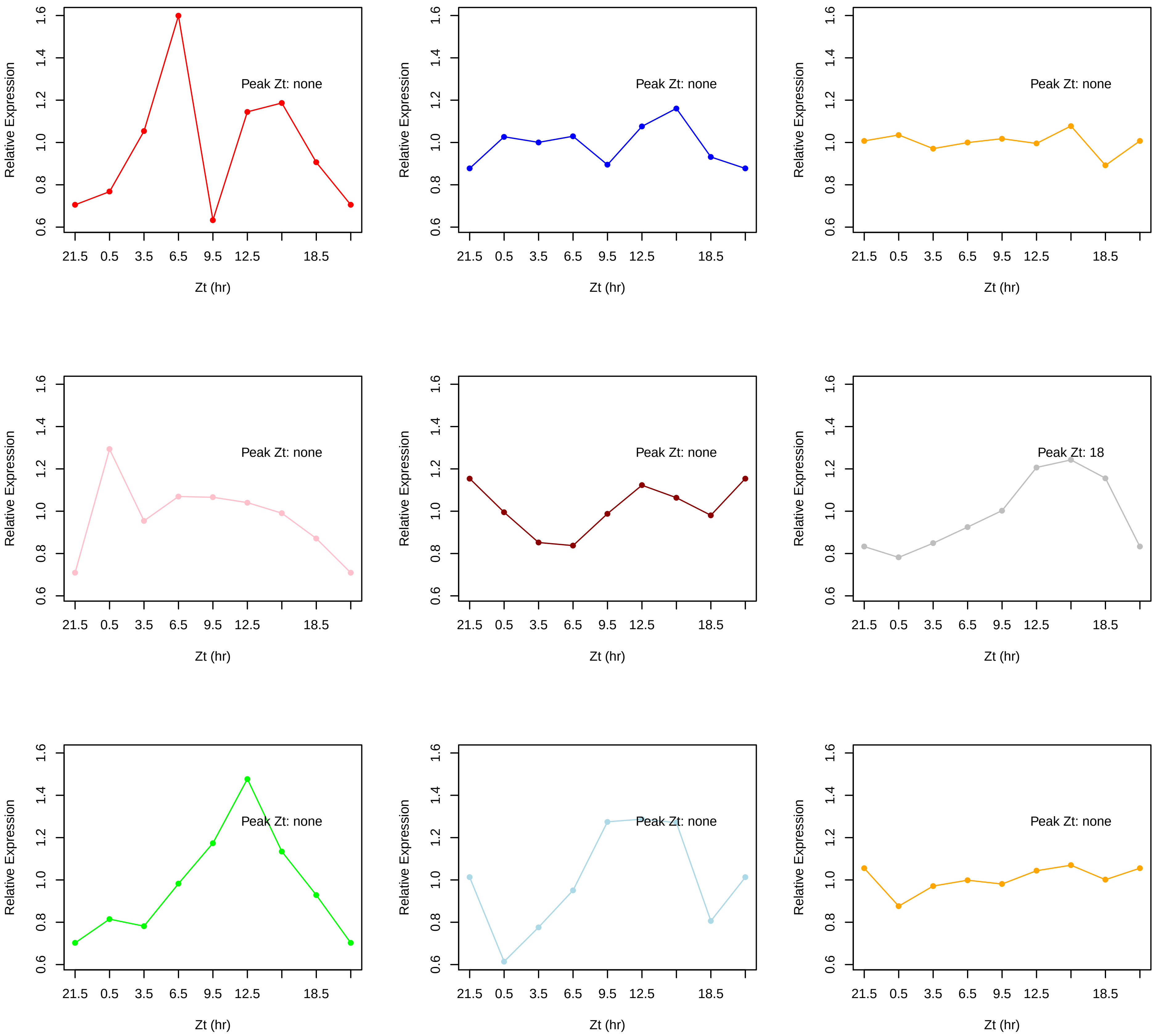

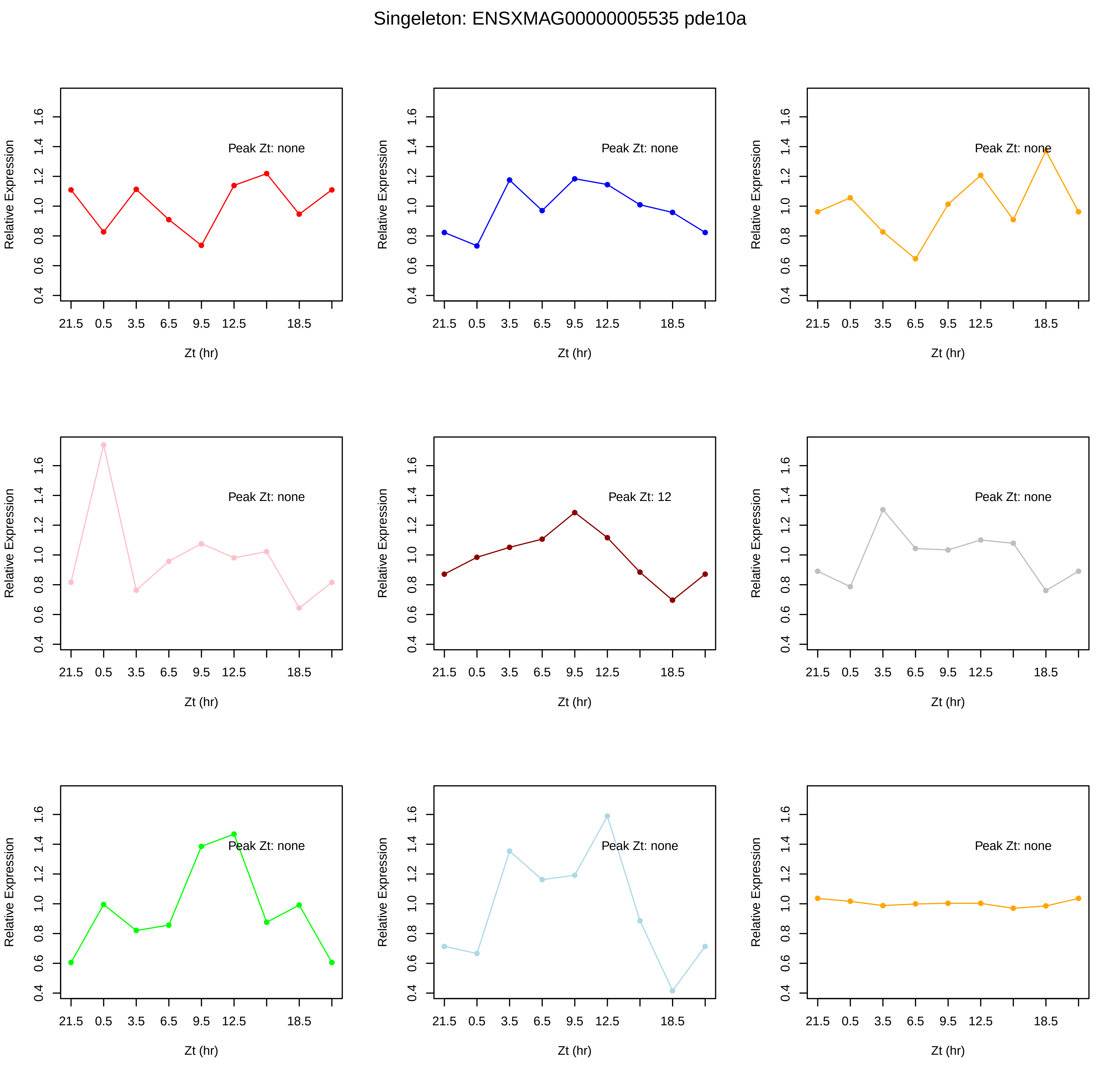
Singeleton: ENSXMAG00000005535 pde10a
Peak Zt: none
Peak Zt: none
Peak Zt: none
Peak Zt: none
Peak Zt: 12
Peak Zt: none
Peak Zt: none
Peak Zt: none
Peak Zt: none
Relative Expression
Zt (hr)
21.5 0.5 3.5 6.5 9.5 12.5 18.5
0.4 0.6 0.8 1.0 1.2 1.4 1.6

Singeleton: ENSXMAG00000013351 prdm1a

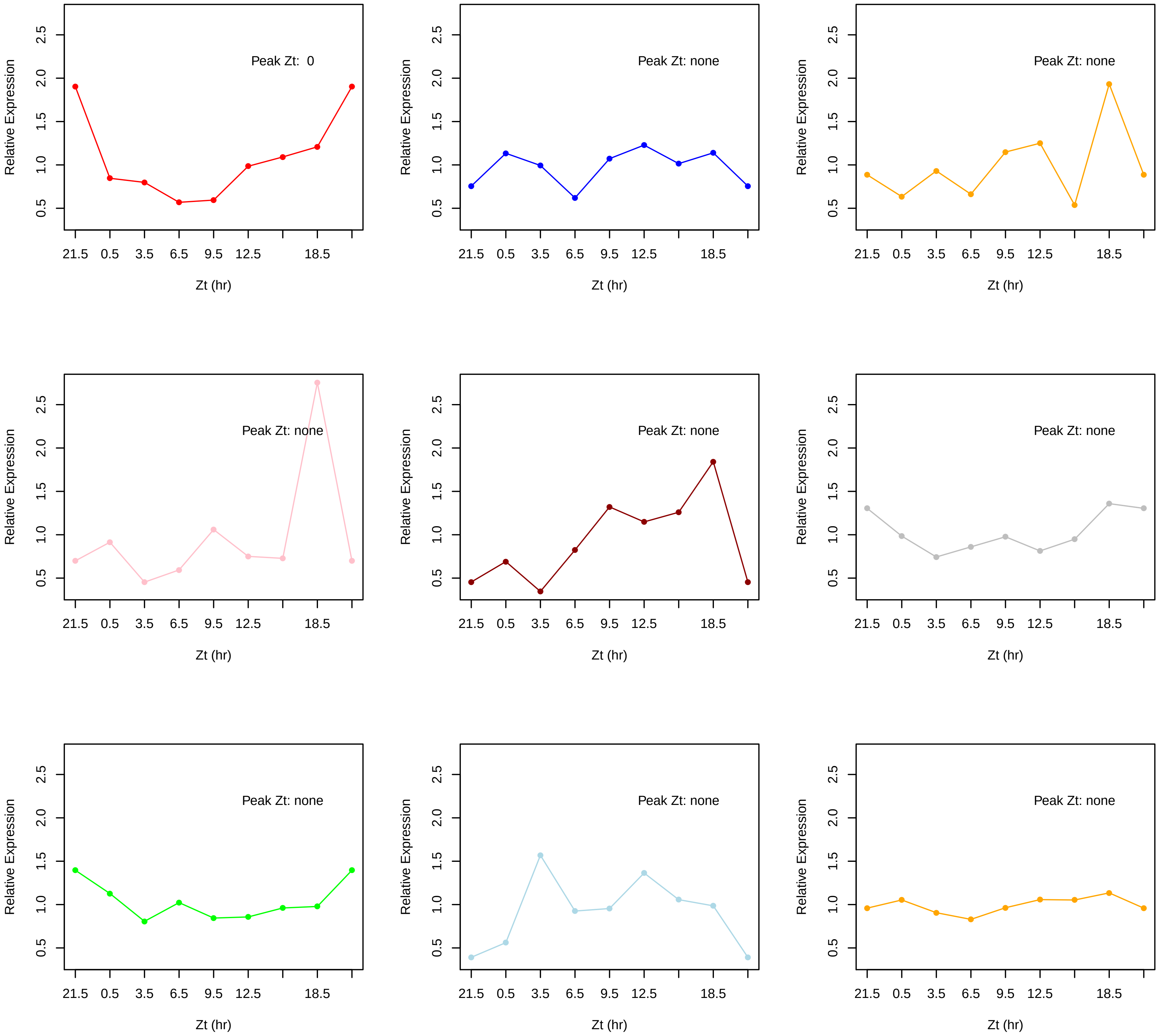

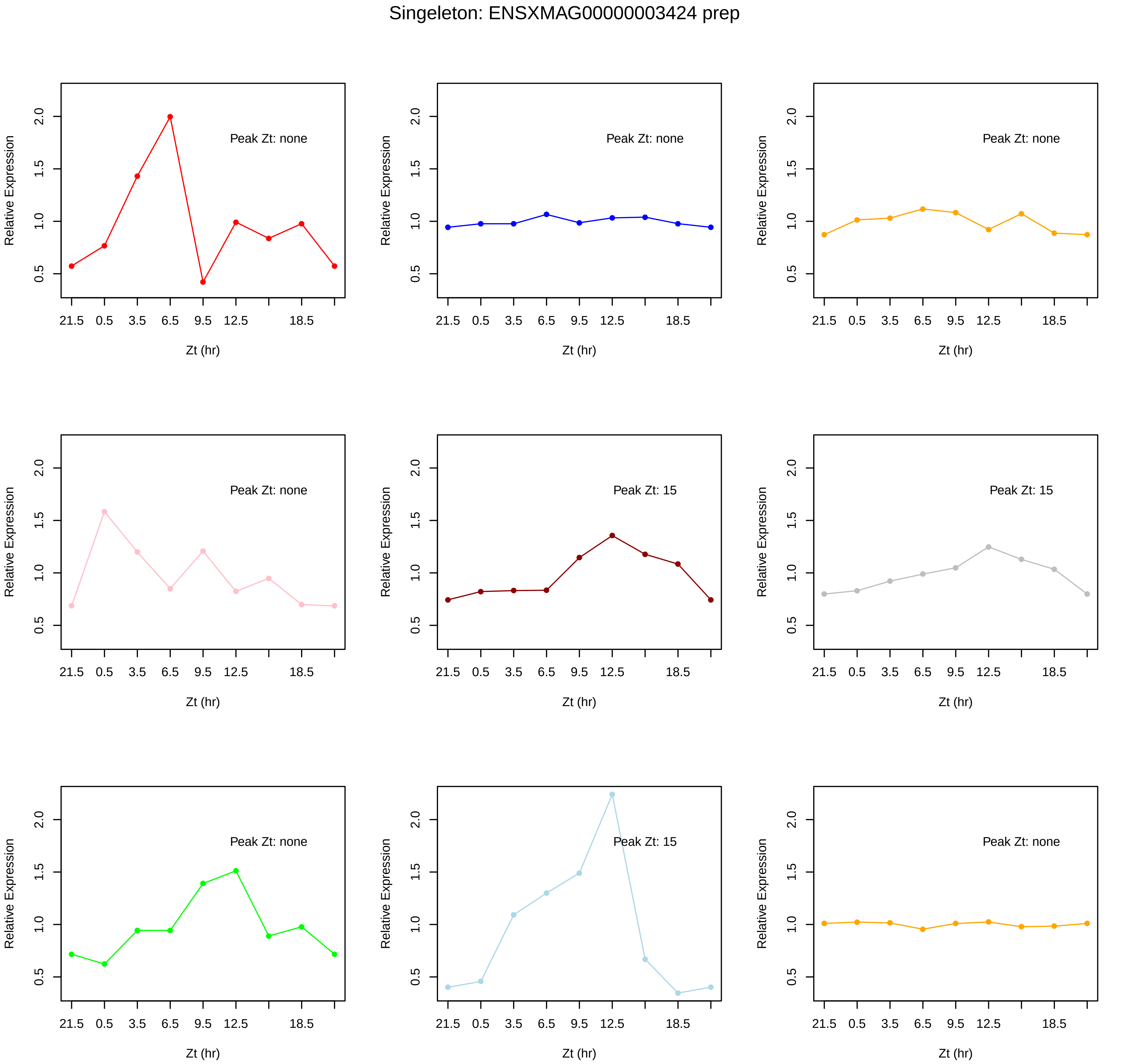
Singeleton: ENSXMAG00000003424 prep
Peak Zt: none
Peak Zt: none
Peak Zt: none
Peak Zt: none
Peak Zt: 15
Peak Zt: 15
Peak Zt: none
Peak Zt: 15
Peak Zt: none
Relative Expression
Zt (hr)

Singeleton: ENSXMAG00000010312 nup50

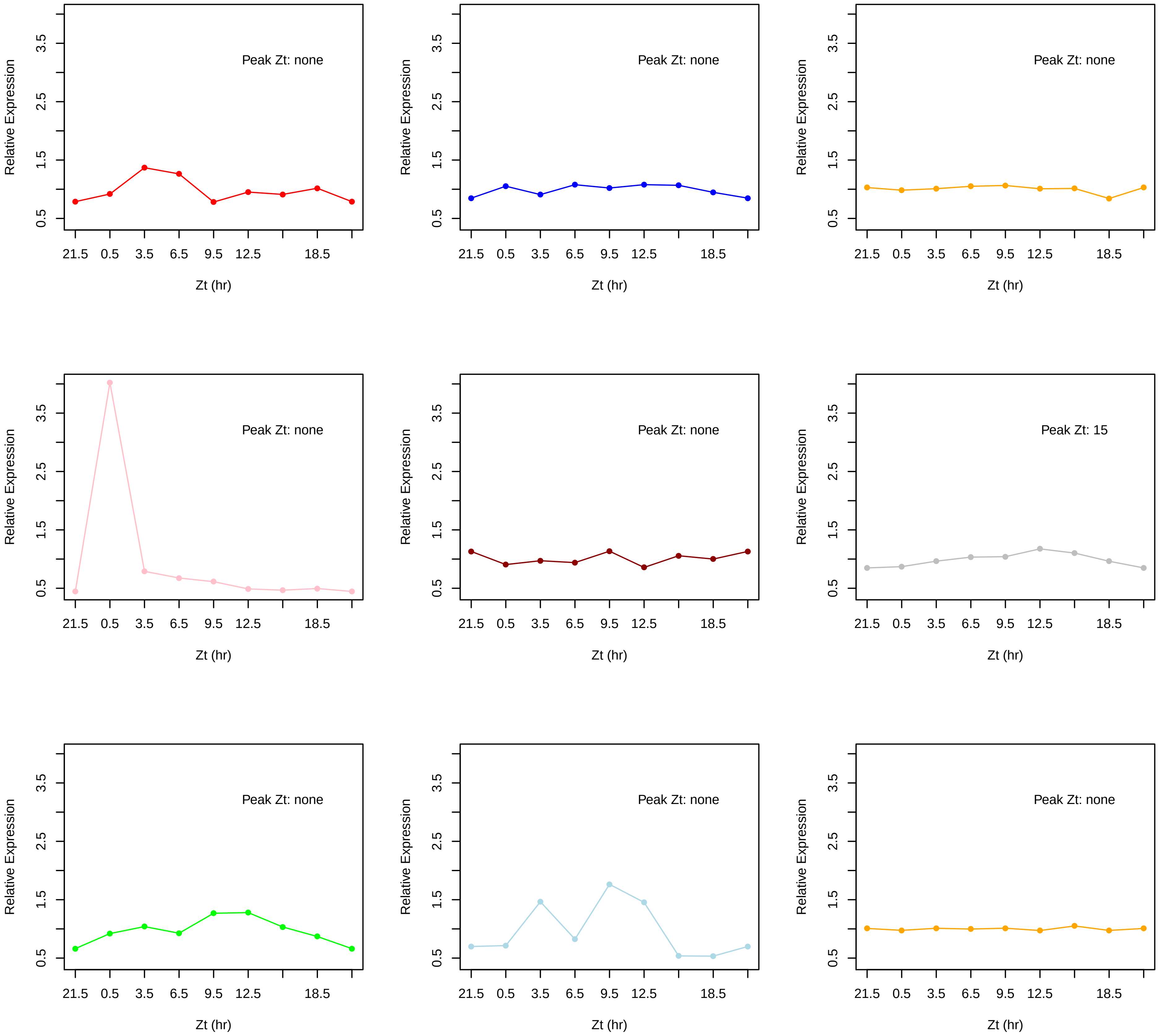

Singleton: ENSXMAG00000017328 hace1

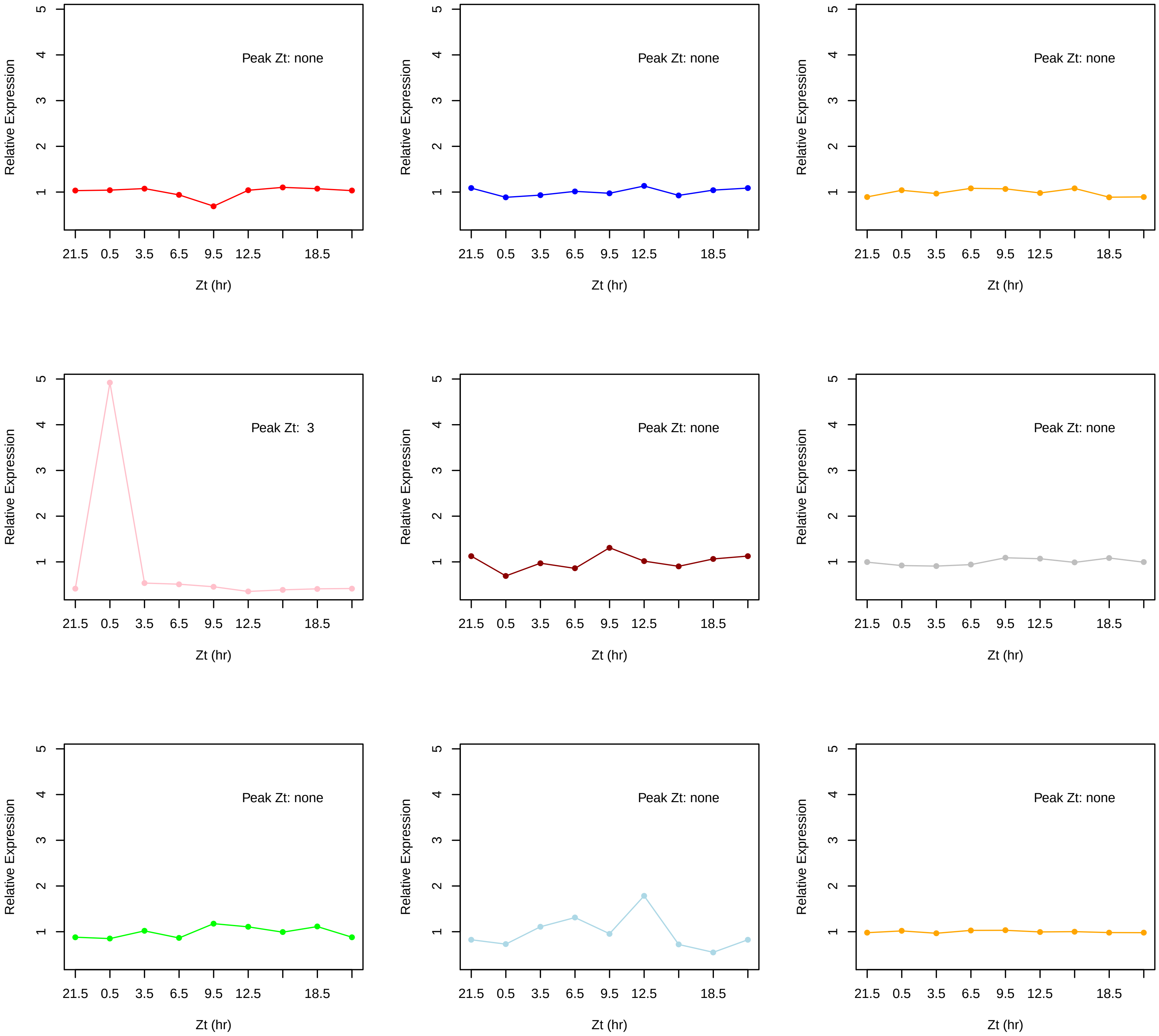

Singleton: ENSXMAG00000010196 atp23

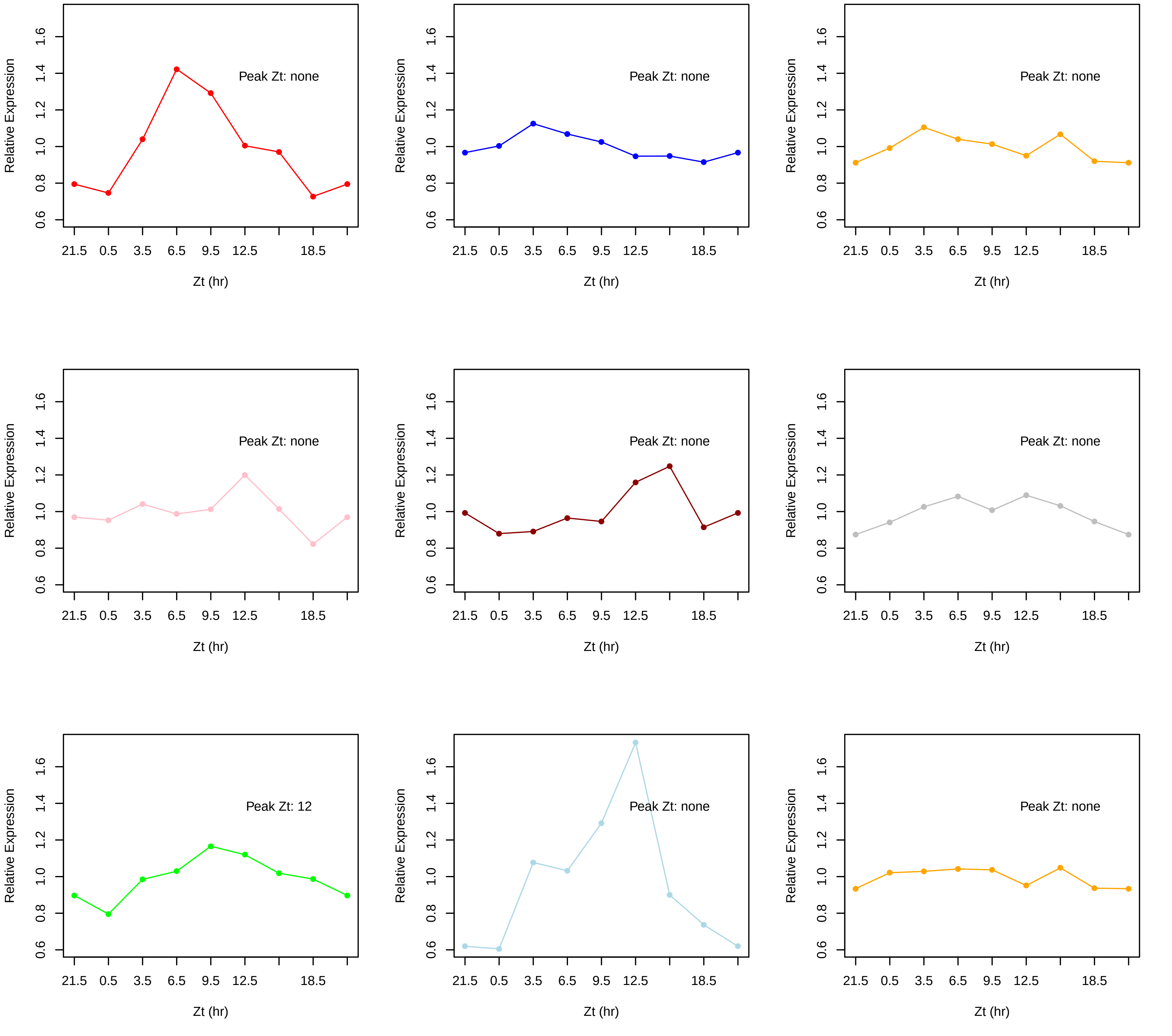

Singeleton: ENSXMAG00000010178 lrig3

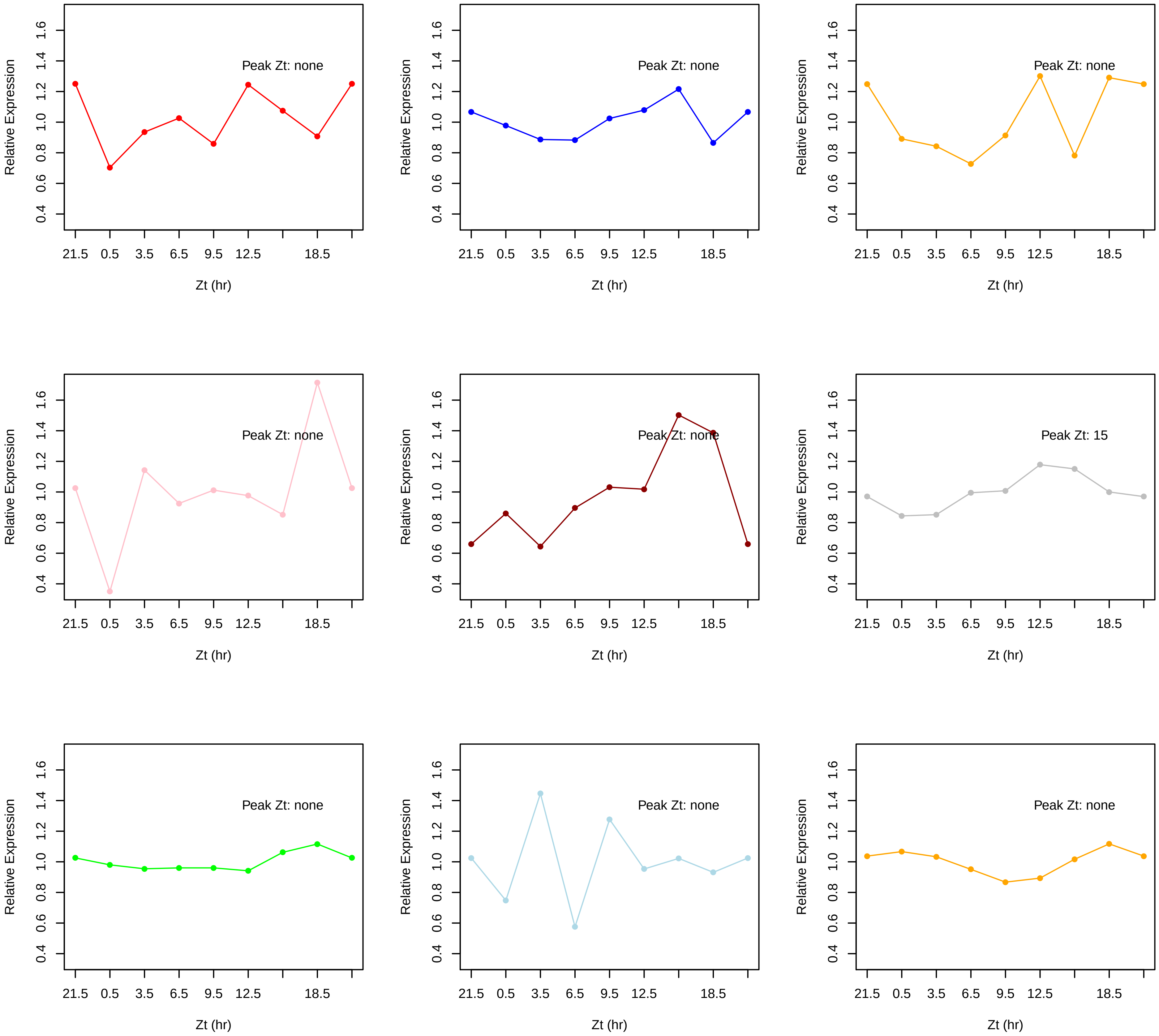

Singleton: ENSXMAG00000010168 SLC16A7

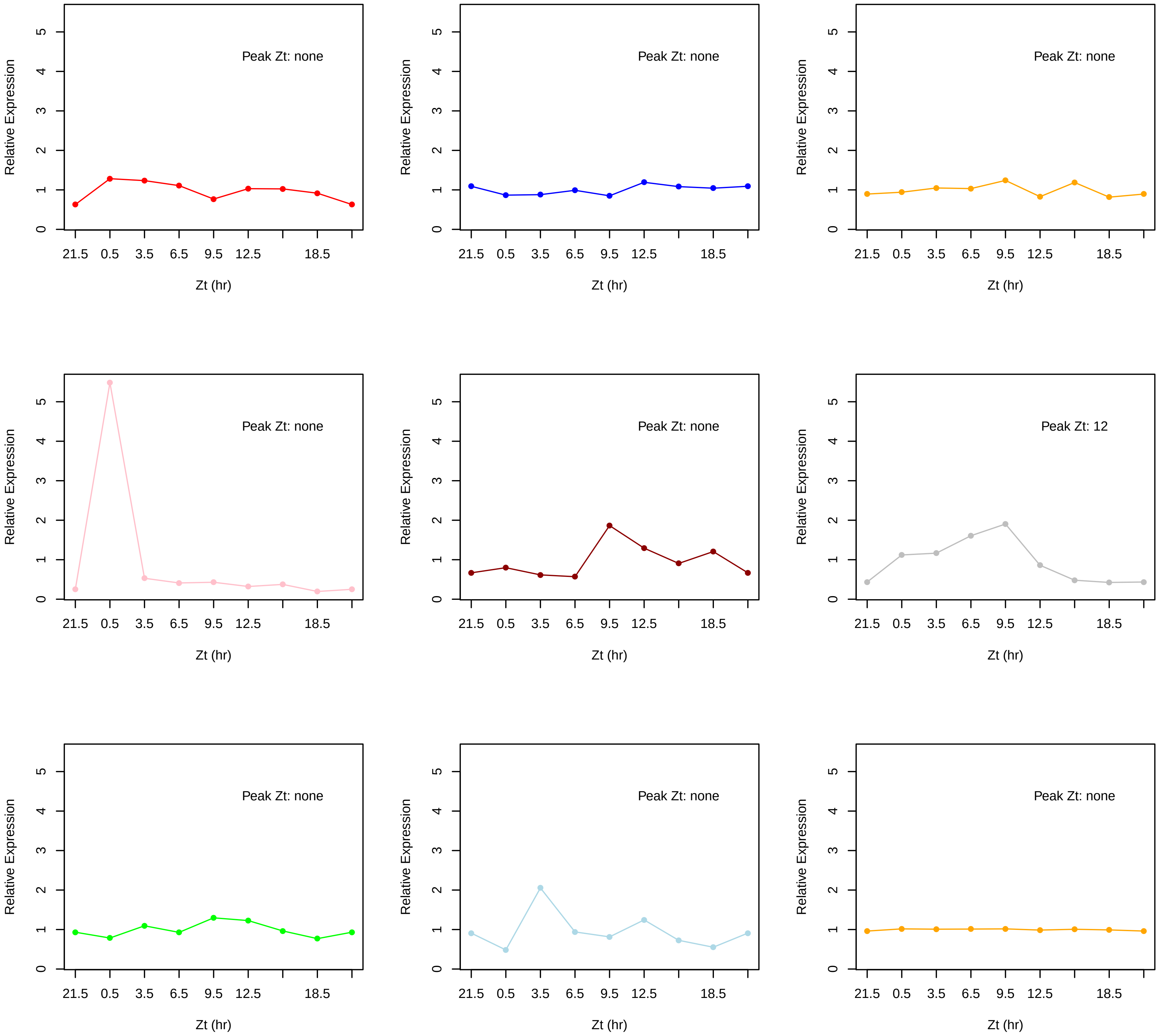

Singleton: ENSXMAG00000020822 zc3h6

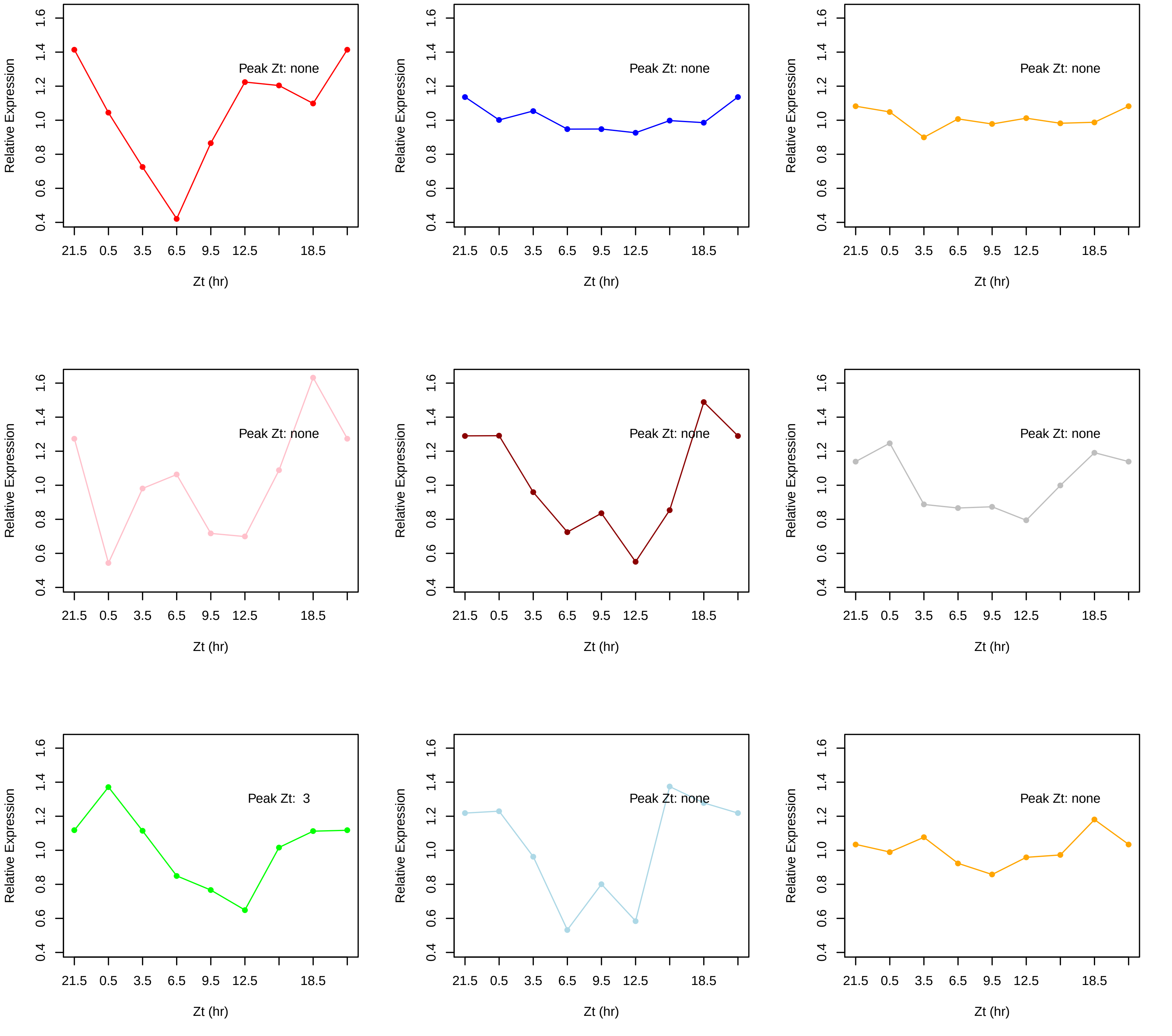

Singeleton: ENSXMAG00000029793 ptn

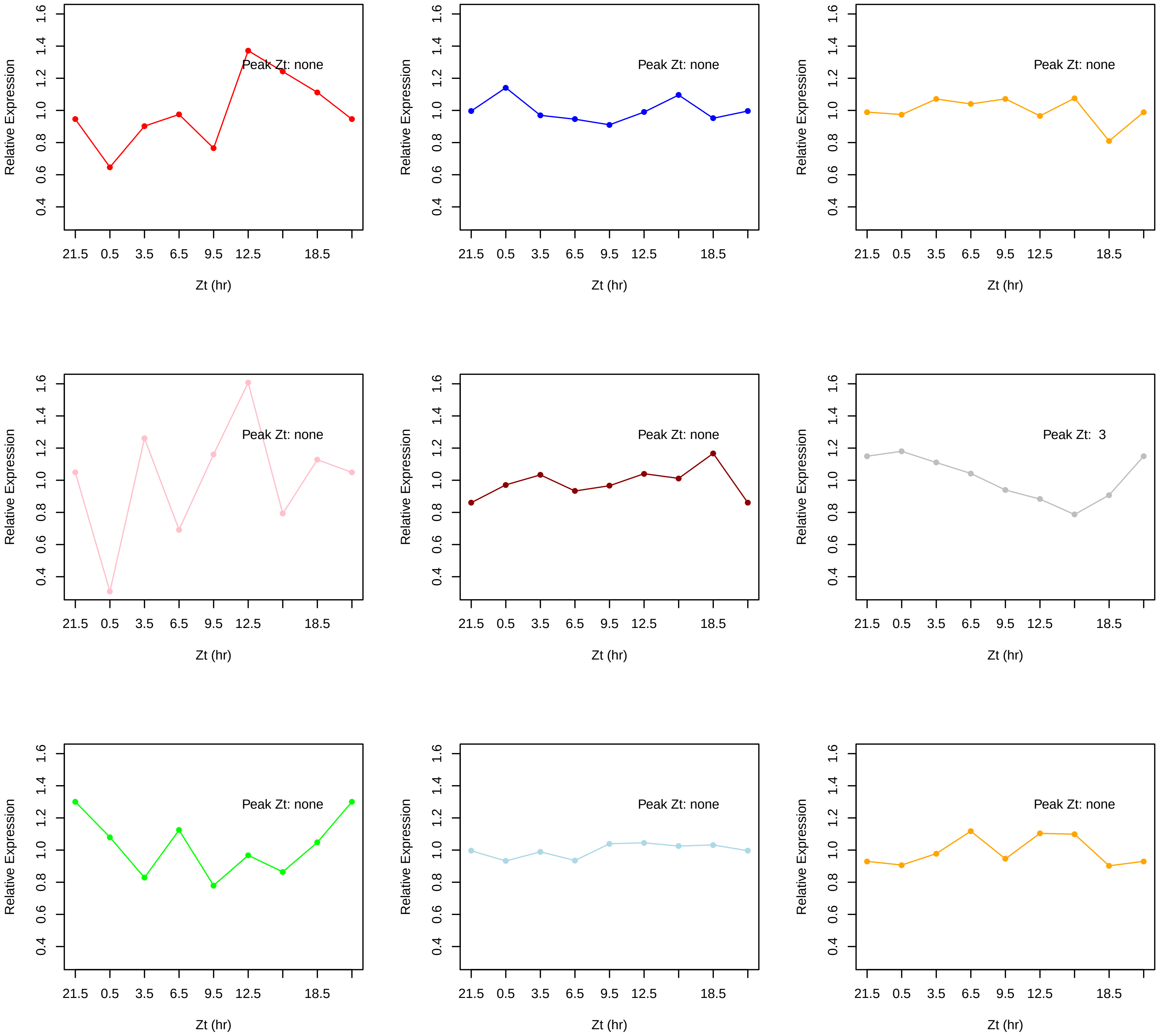

Singleton: ENSXMAG00000000071 ascc3

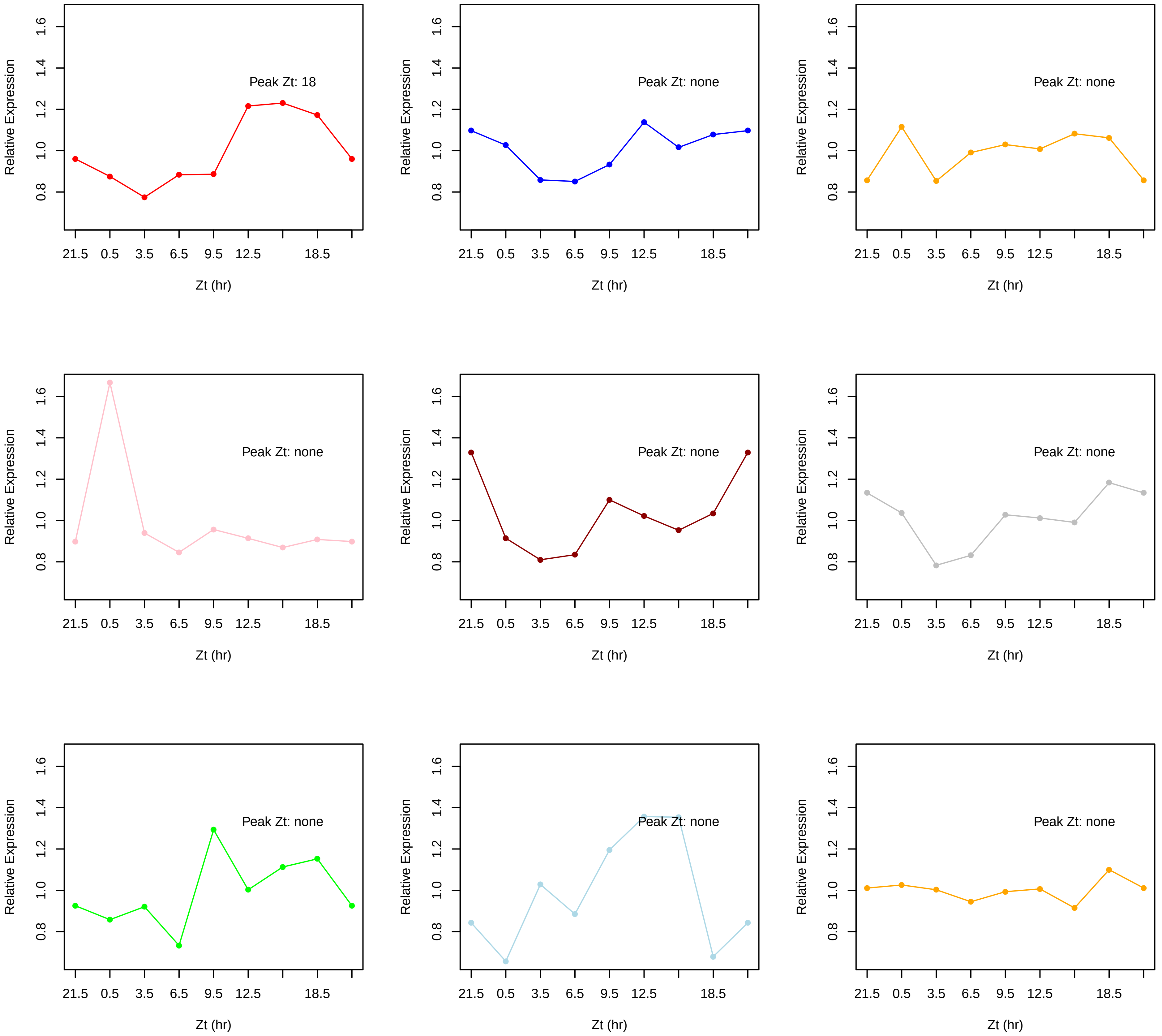

Singeleton: ENSXMAG00000008085 ttl

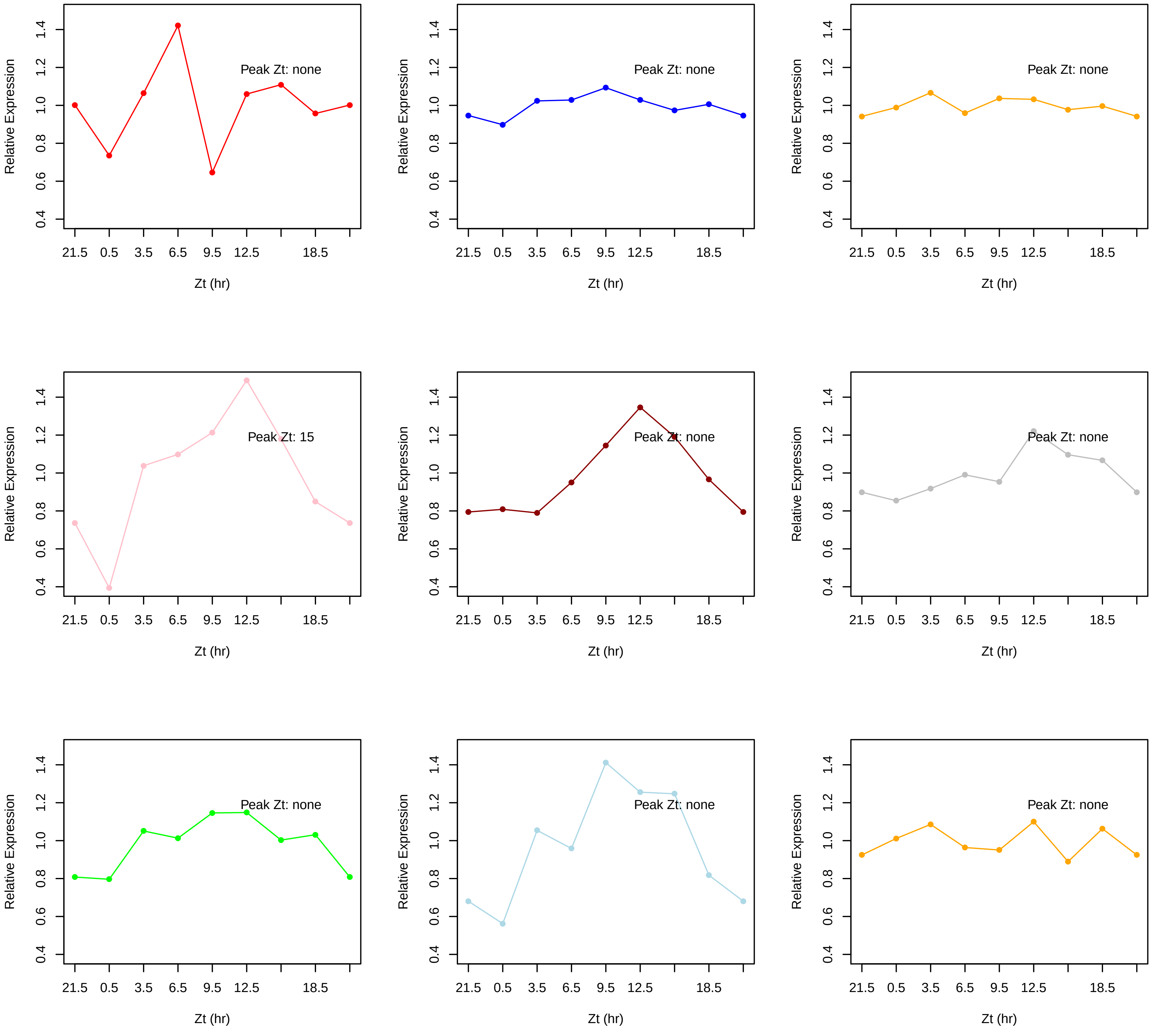

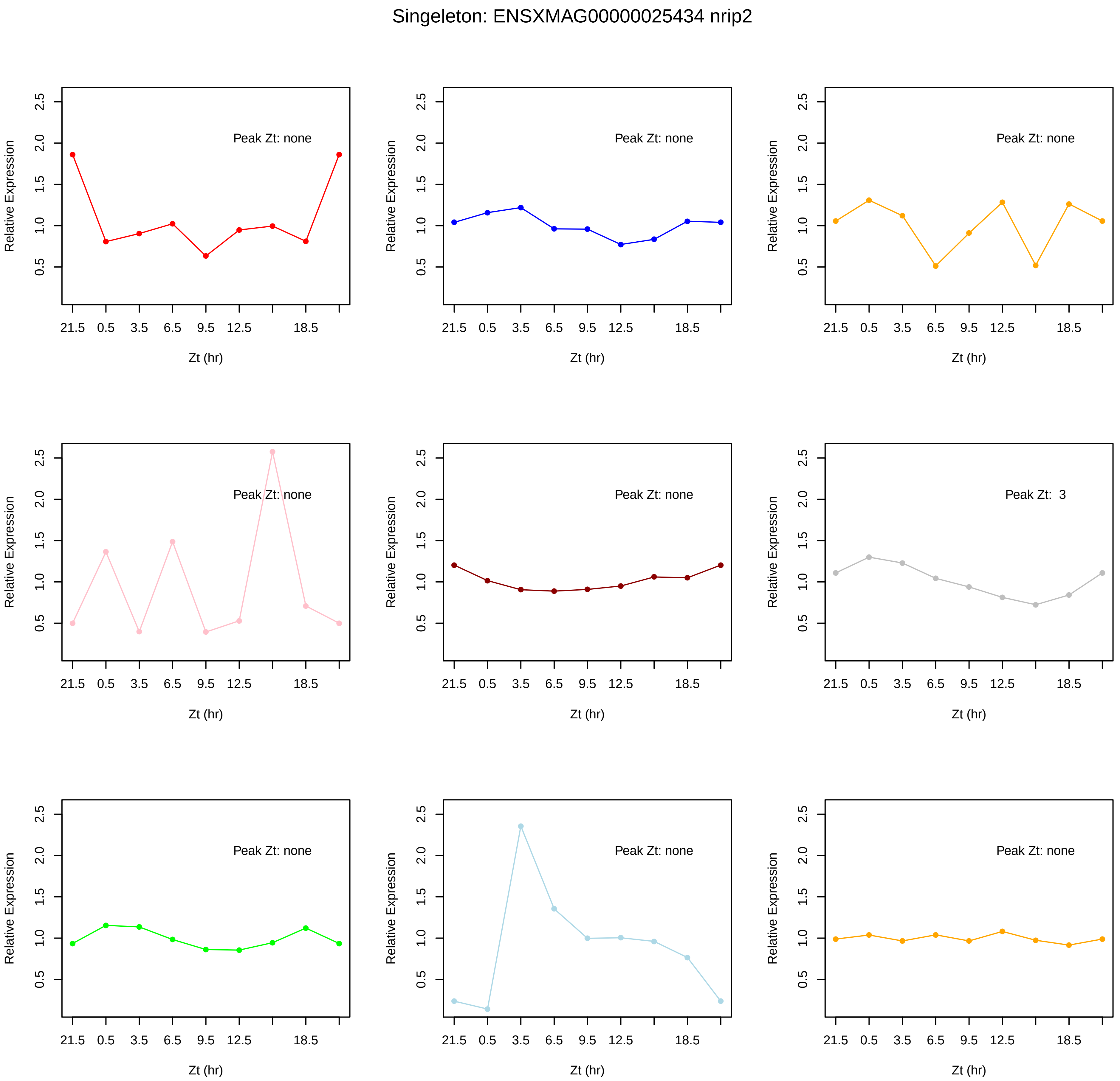
Singeleton: ENSXMAG00000025434 nrip2
Peak Zt: none
Peak Zt: none
Peak Zt: none
Peak Zt: none
Peak Zt: none
Peak Zt: 3
Peak Zt: none
Peak Zt: none
Peak Zt: none
Relative Expression
Zt (hr)
21.5
0.5
3.5
6.5
9.5
12.5
18.5
0.5
1.0
1.5
2.0
2.5

Singeleton: ENSXMAG00000017228 itfg2

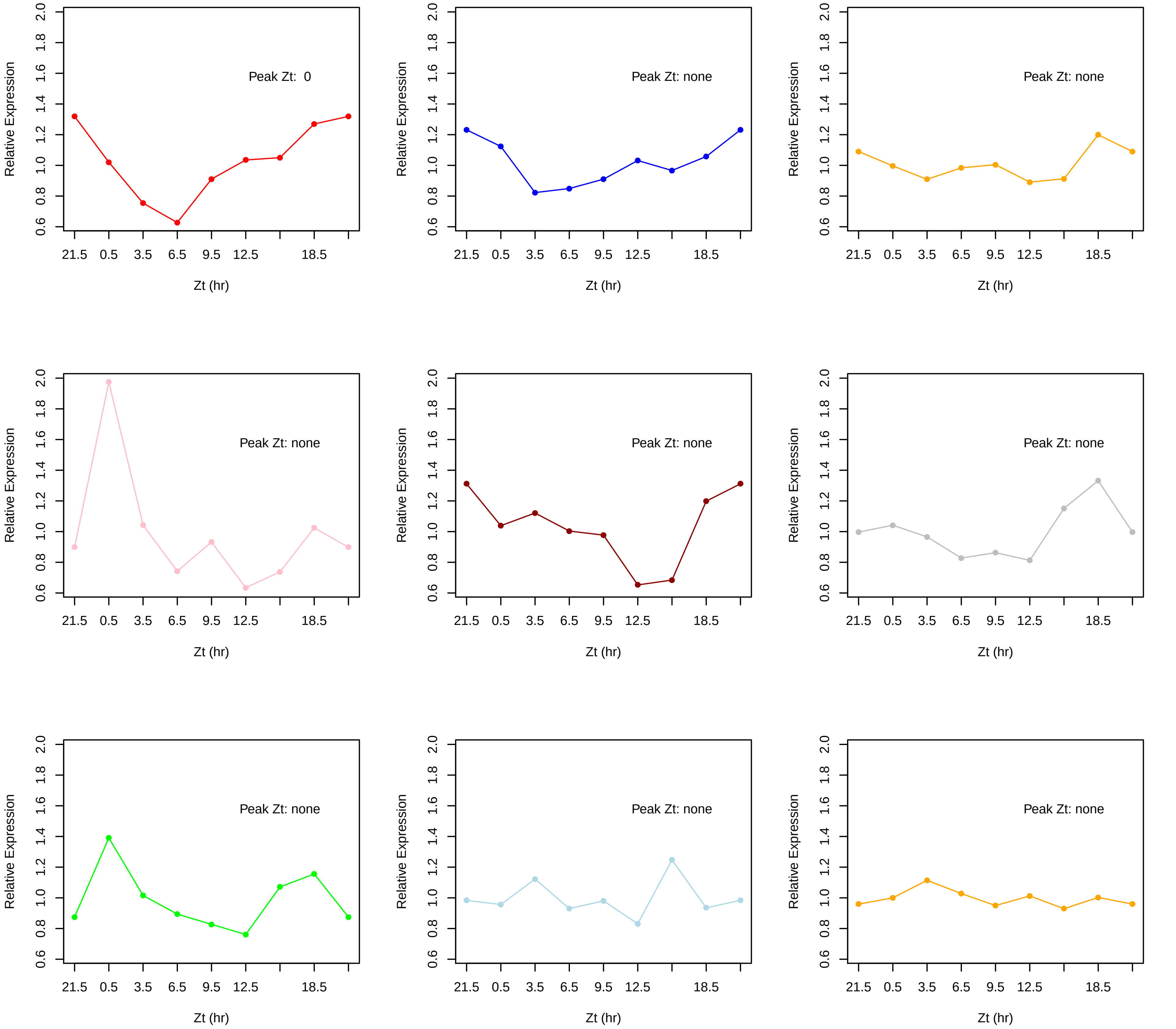

Singeleton: ENSXMAG00000007712 rmdn2

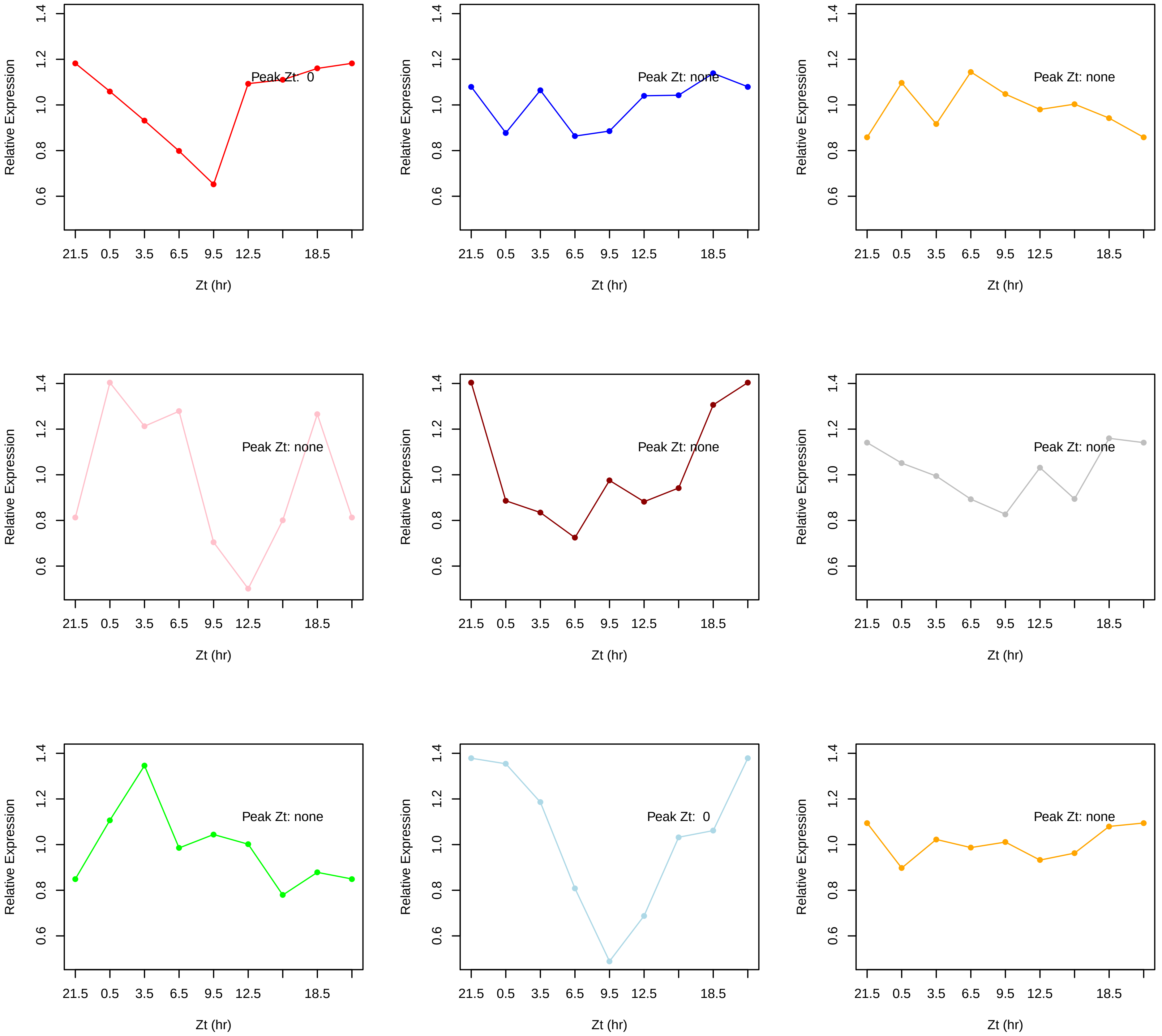

Singleton: ENSXMAG00000021149 cdc42ep3

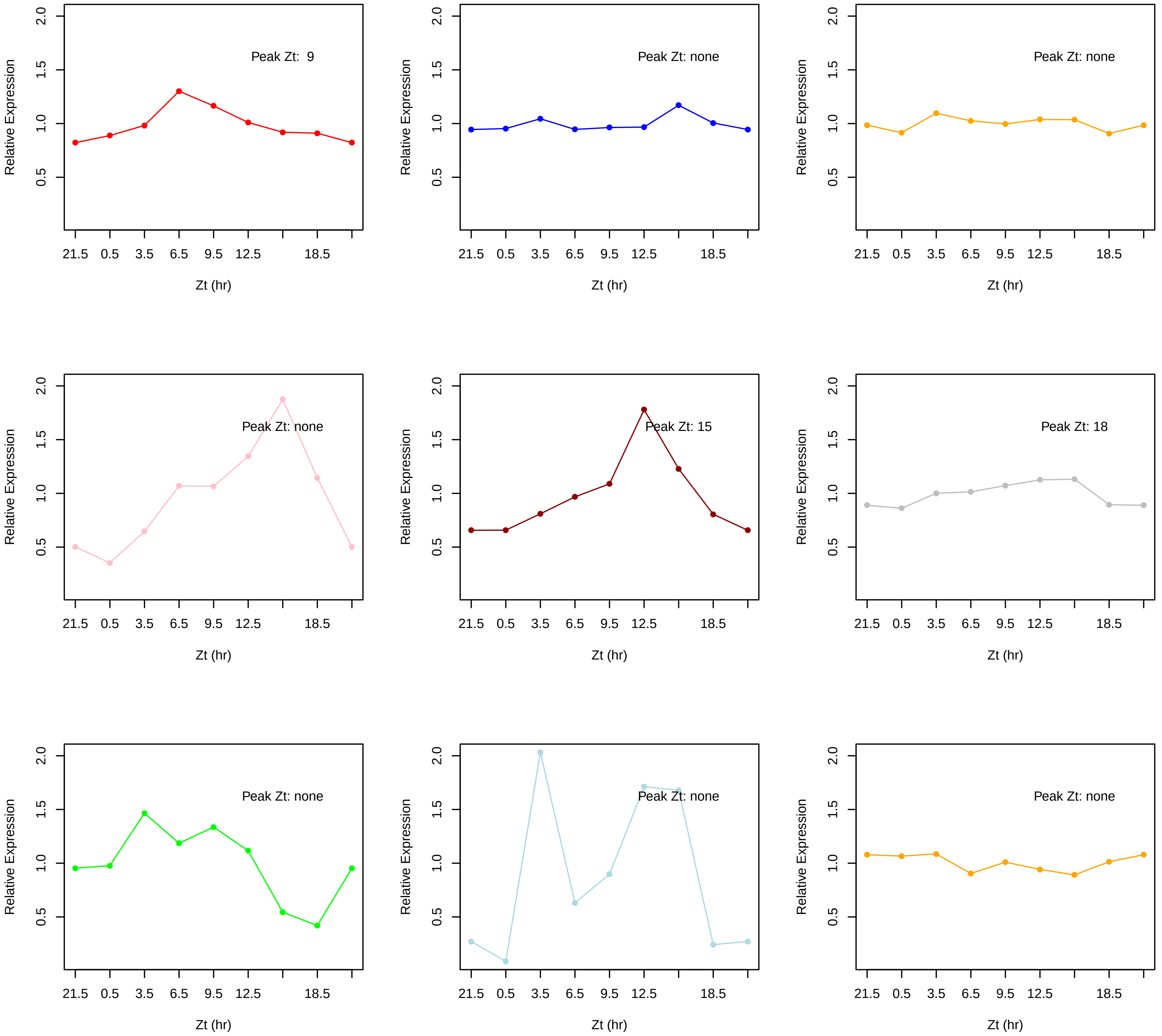

Singeleton: ENSXMAG00000007707 opn3

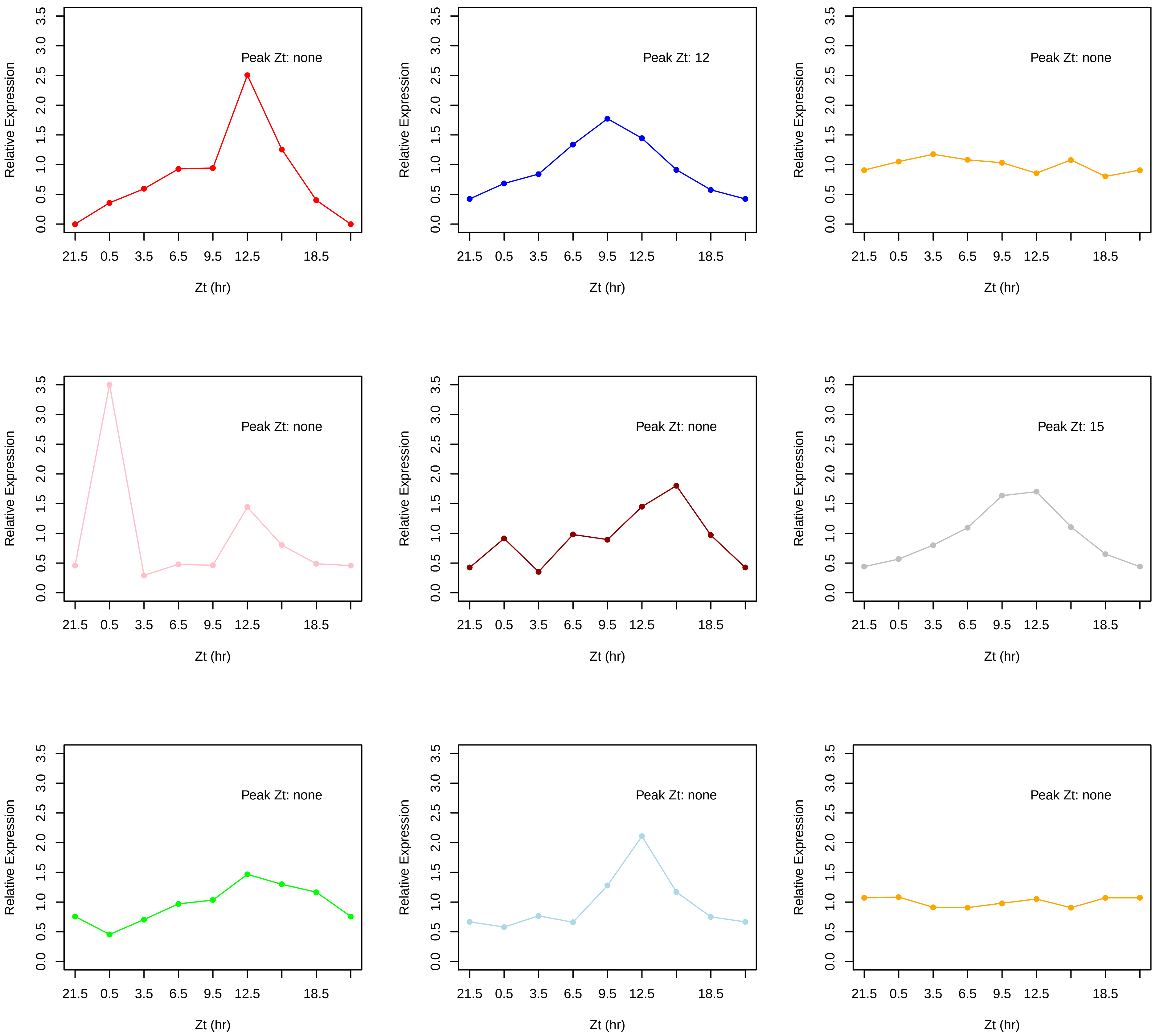

Singeleton: ENSXMAG00000005401

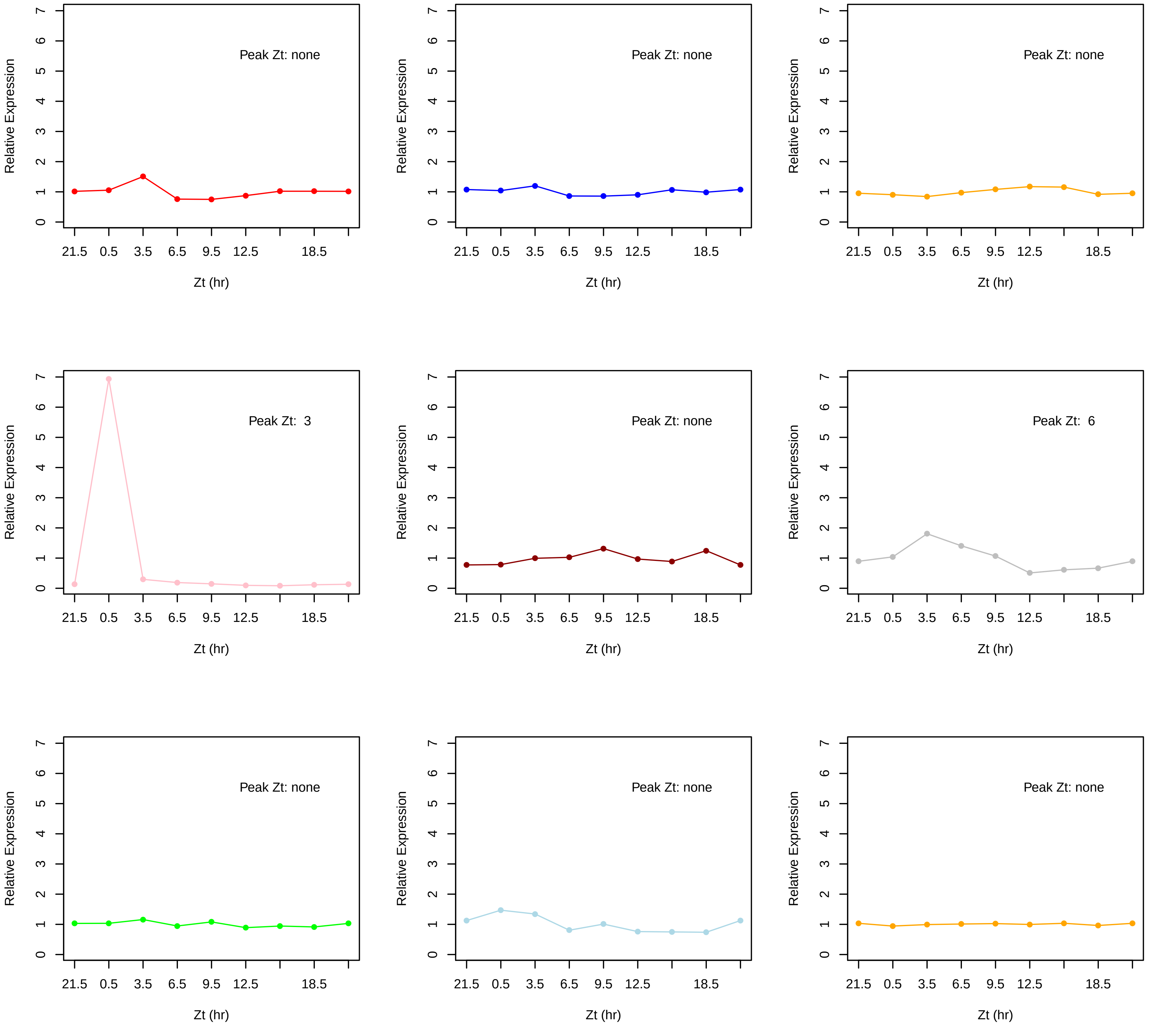

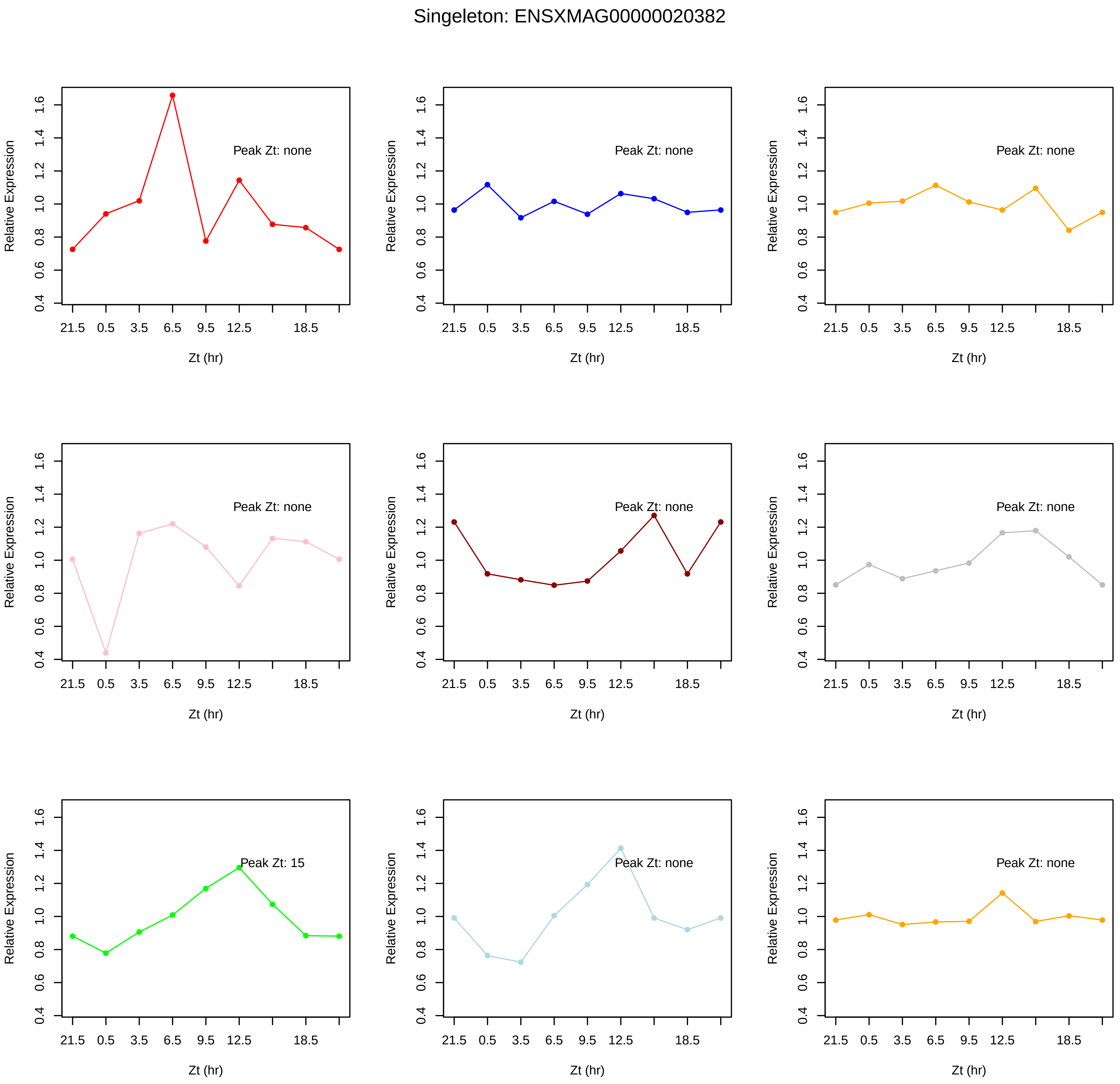
Singeleton: ENSXMAG00000020382
Peak Zt: none
Peak Zt: none
Peak Zt: none
Peak Zt: none
Peak Zt: none
Peak Zt: none
Peak Zt: 15
Peak Zt: none
Peak Zt: none
Relative Expression
Zt (hr)
21.5
0.5
3.5
6.5
9.5
12.5
18.5
0.4
0.6
0.8
1.0
1.2
1.4
1.6

Singeleton: ENSXMAG00000003800 rgs7a

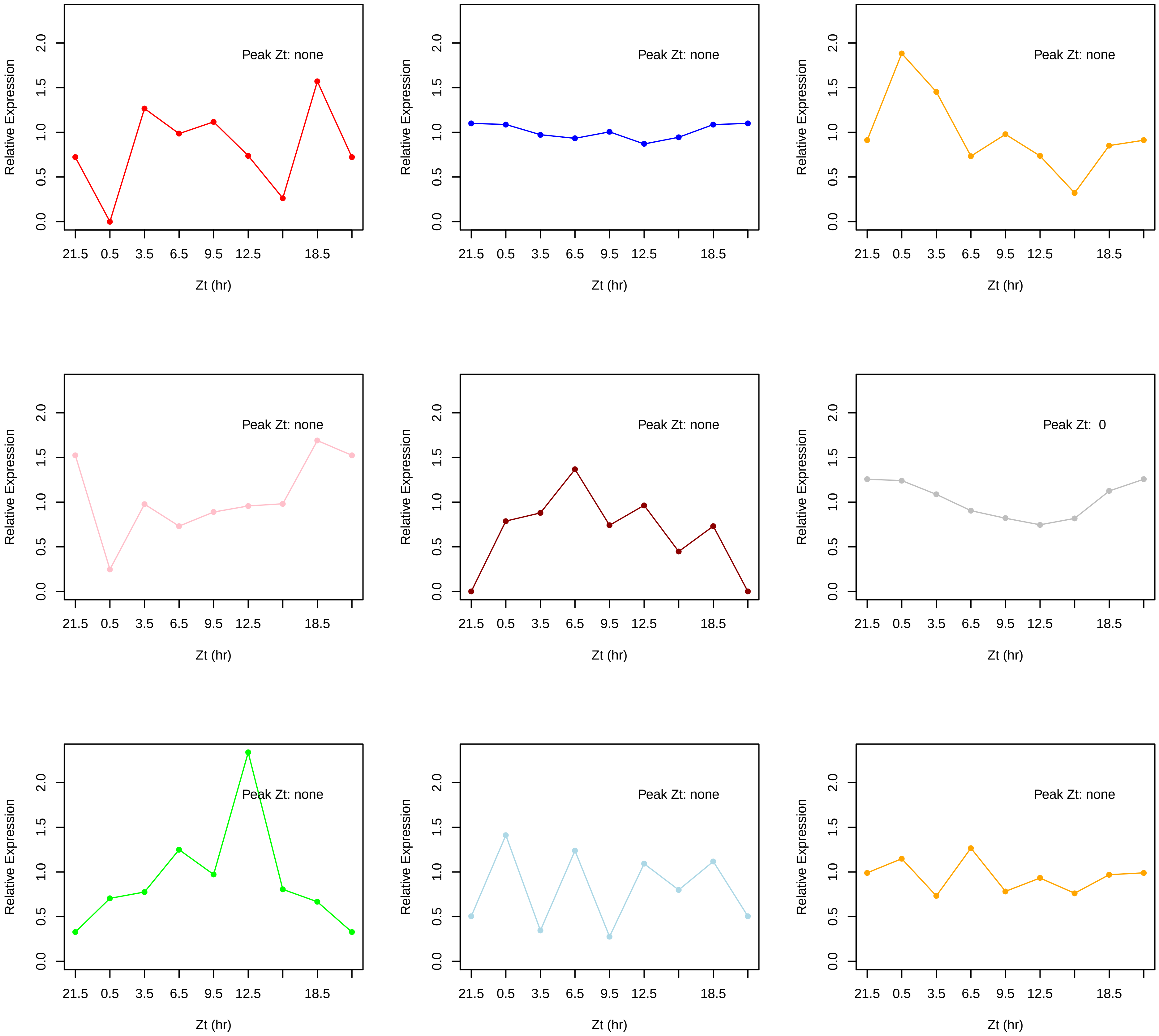

Singleton: ENSXMAG00000005435 wee2

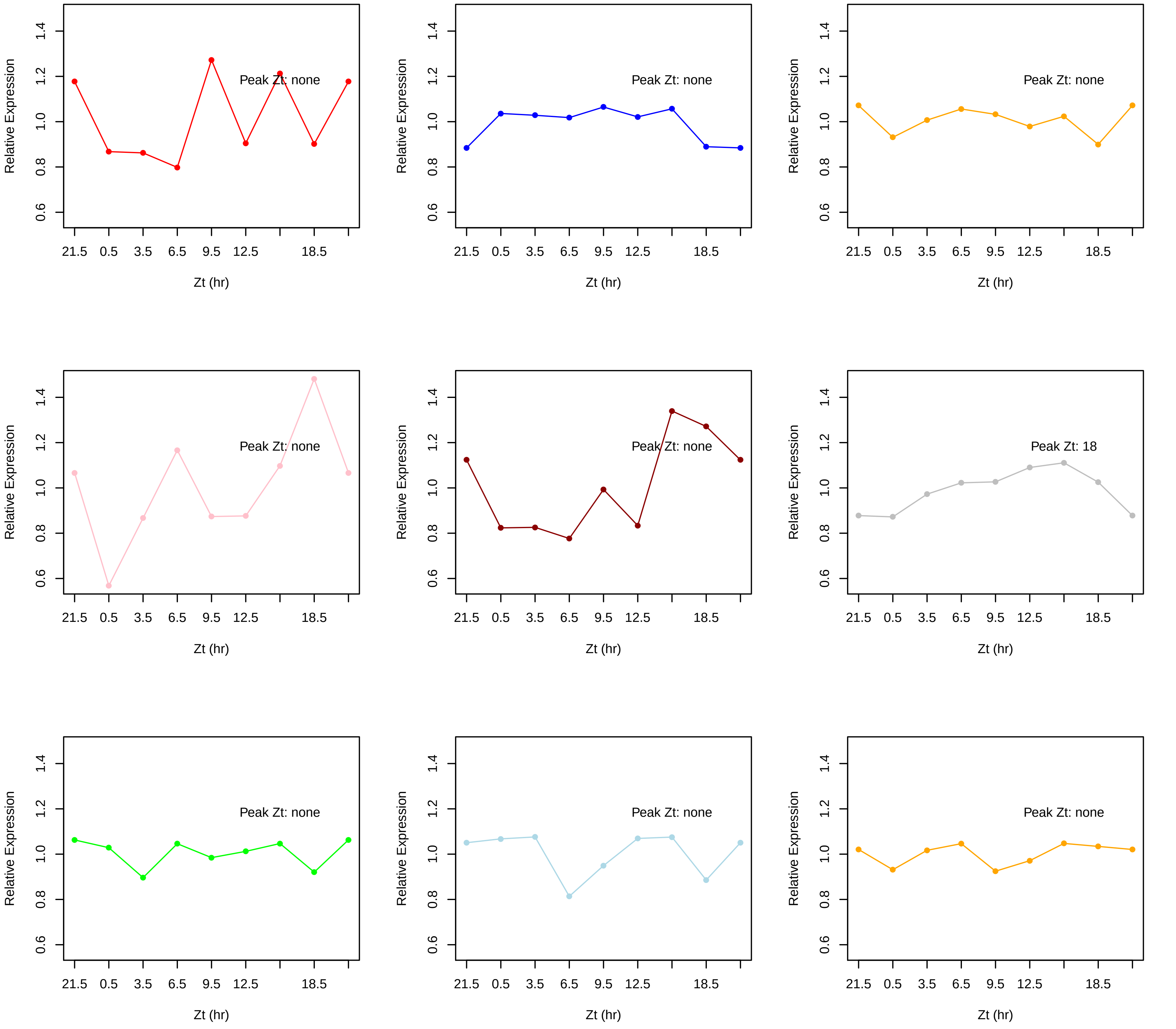

Singeleton: ENSXMAG00000003816

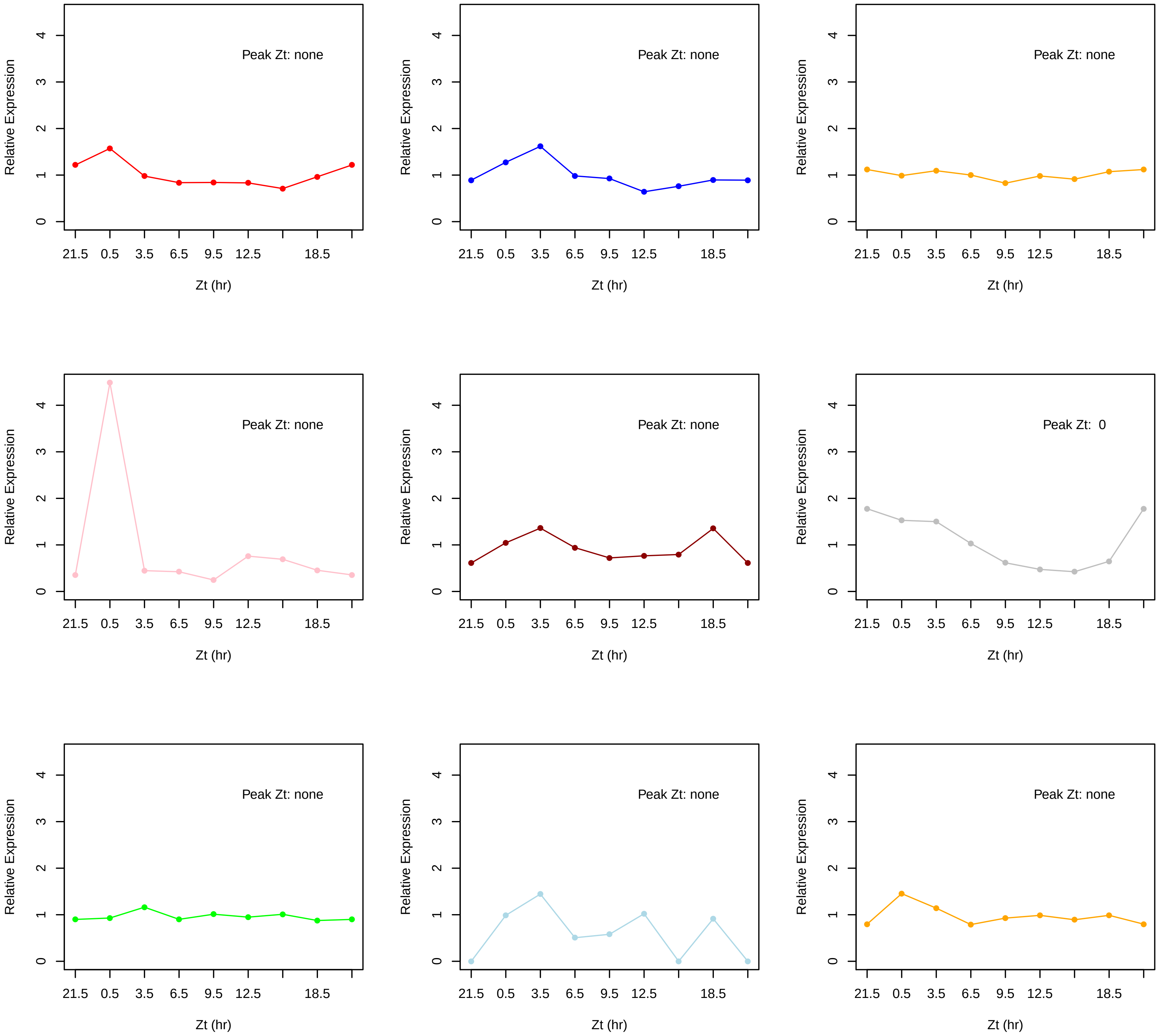

Singleton: ENSXMAG00000014335 RYR2

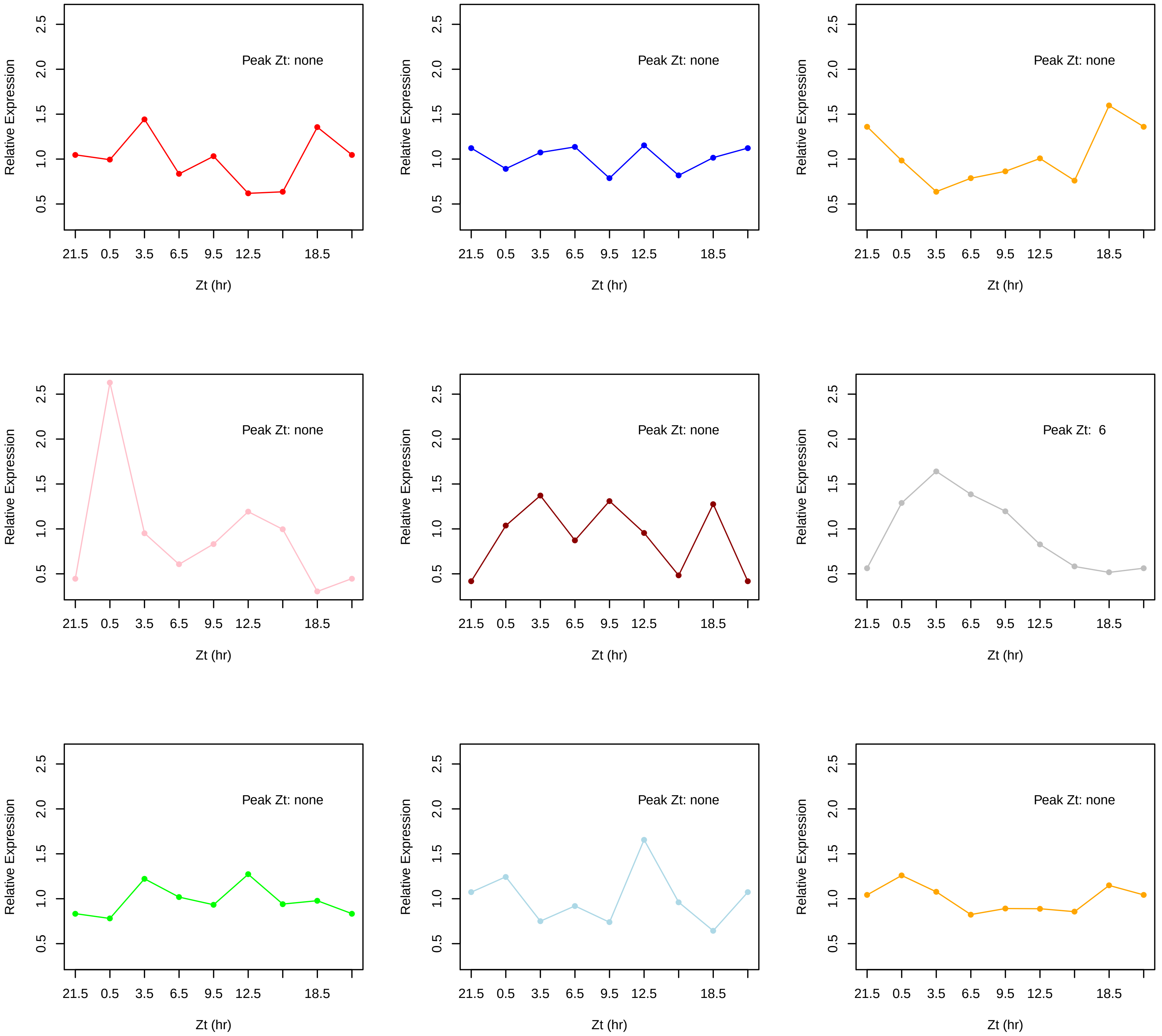

Singeleton: ENSXMAG00000004462 sec63

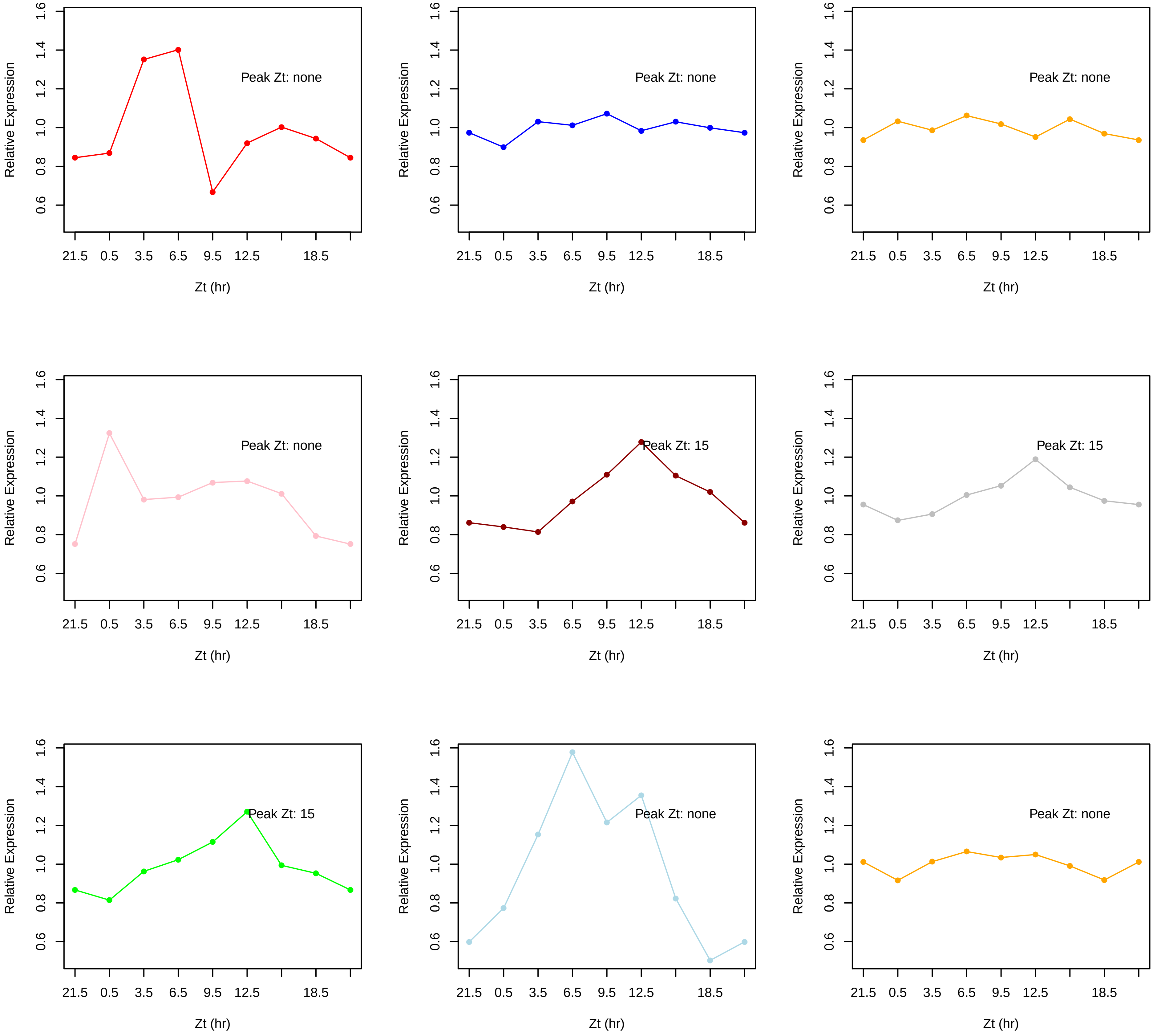

Singleton: ENSXMAG00000023765

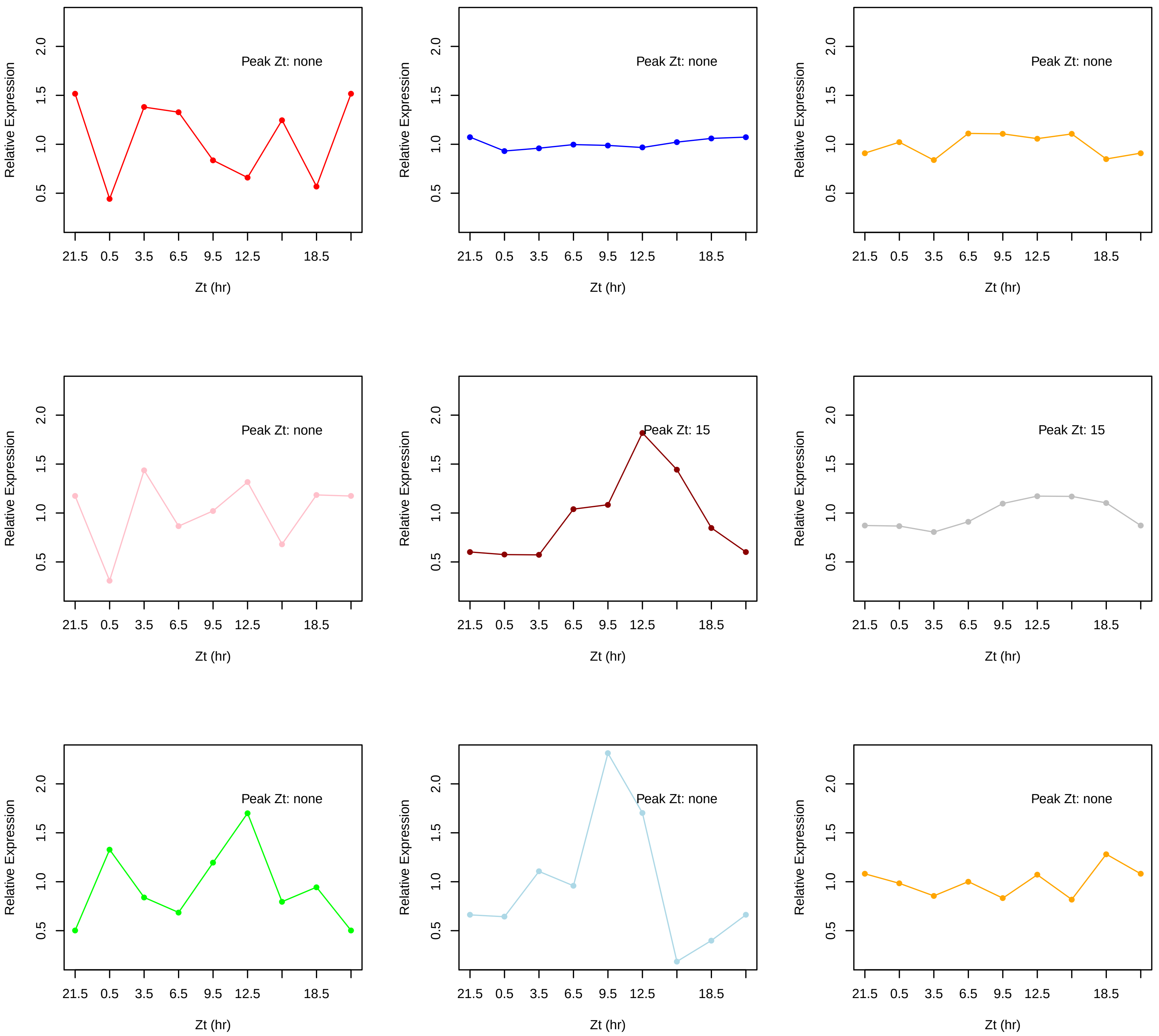

Singleton: ENSXMAG00000028518 sesn1

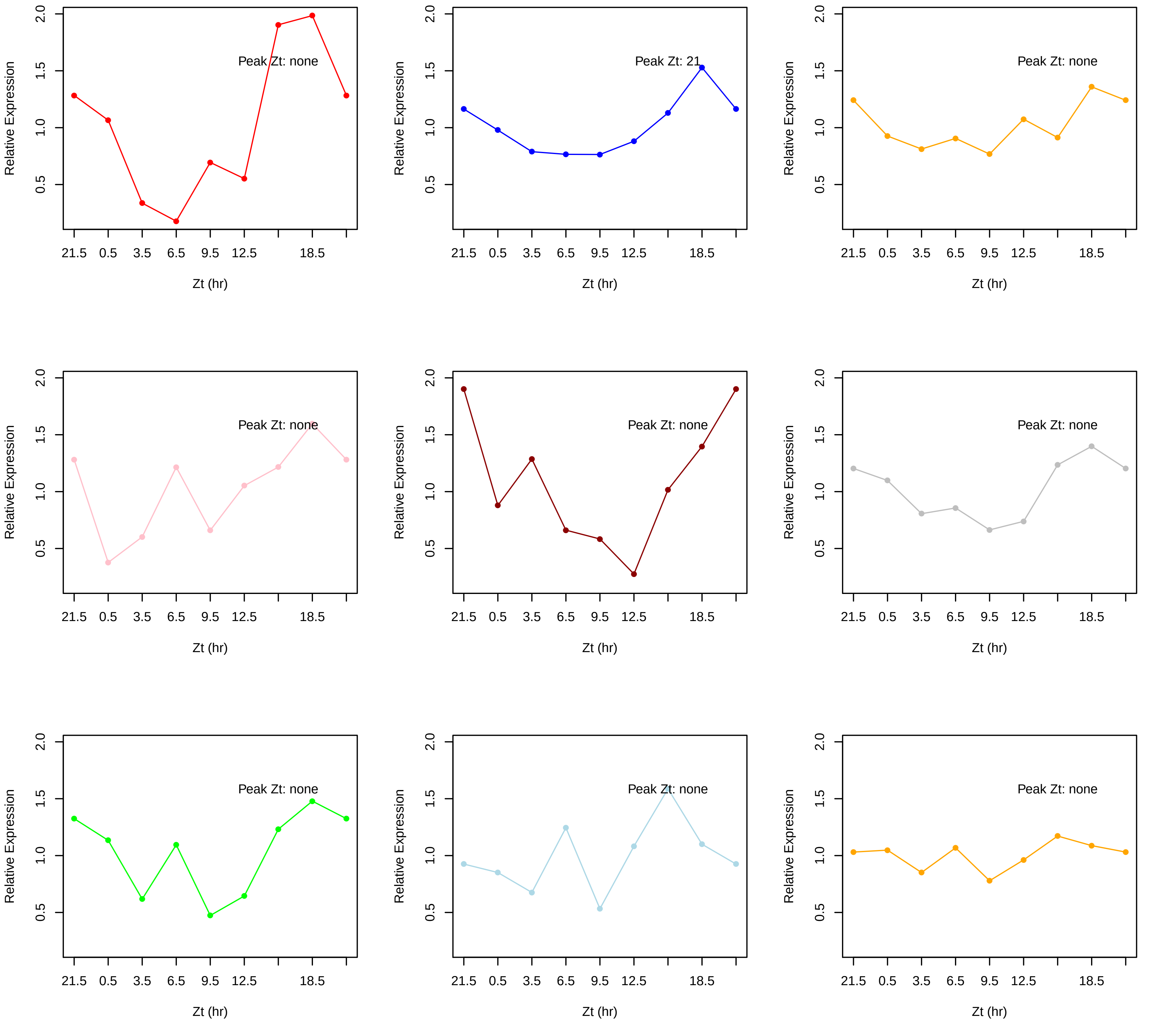

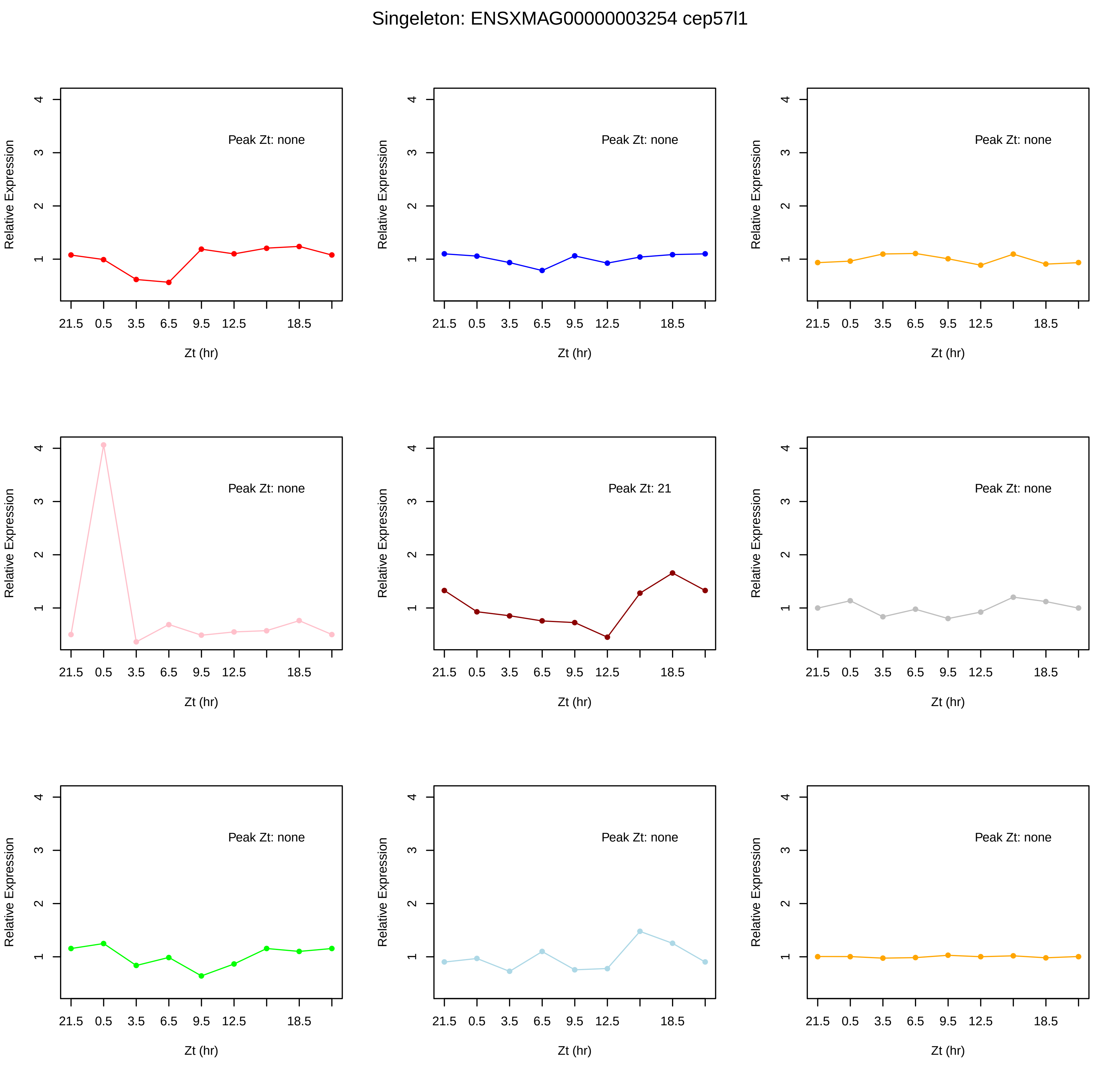

Singeleton: ENSXMAG00000003254 cep57l1
Peak Zt: none
Peak Zt: none
Peak Zt: none
Peak Zt: none
Peak Zt: 21
Peak Zt: none
Peak Zt: none
Peak Zt: none
Peak Zt: none
Relative Expression
Zt (hr)
21.5 0.5 3.5 6.5 9.5 12.5 18.5

Singeleton: ENSXMAG00000014278 mtr

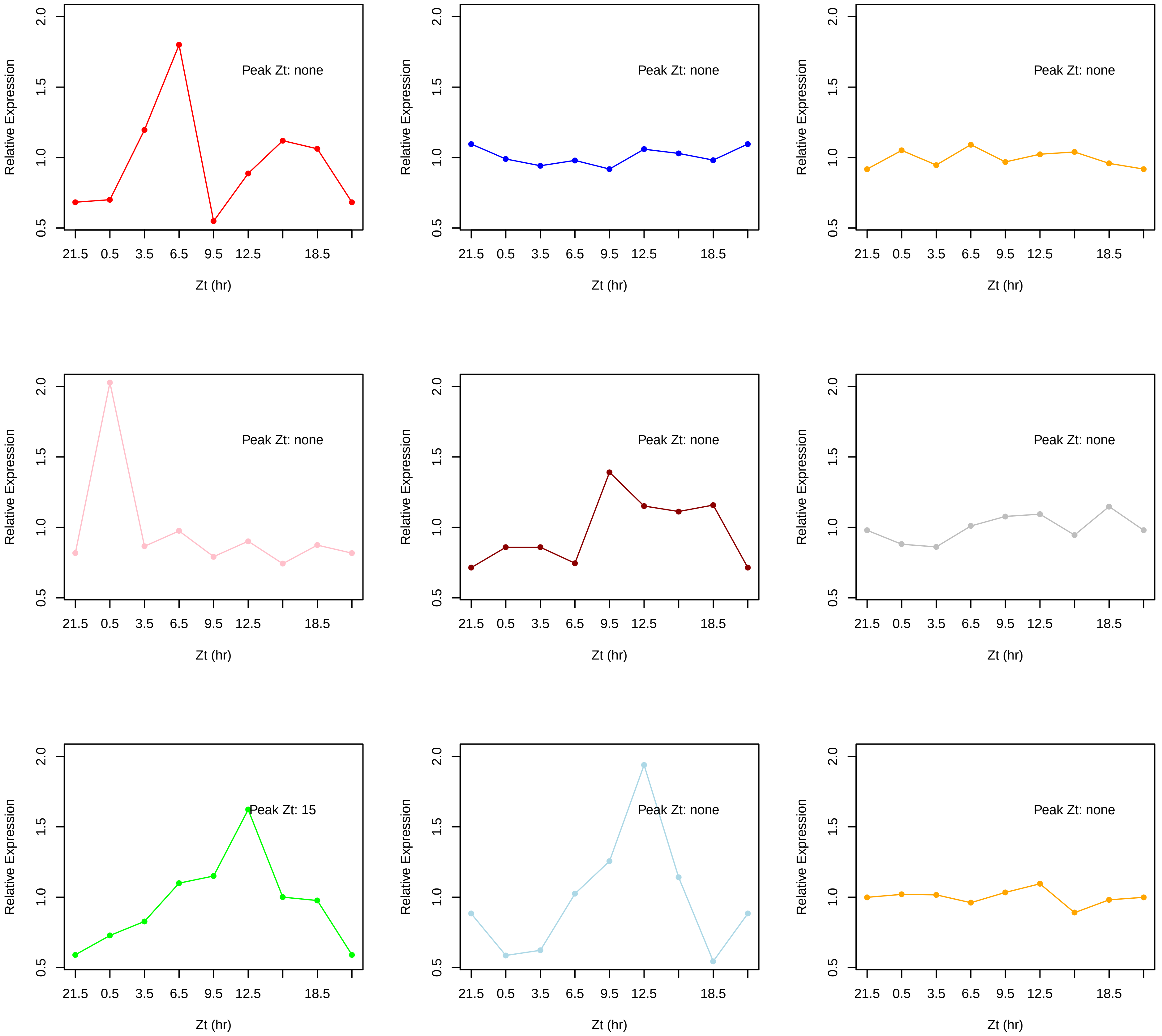

Singleton: ENSXMAG00000003497 klhl29

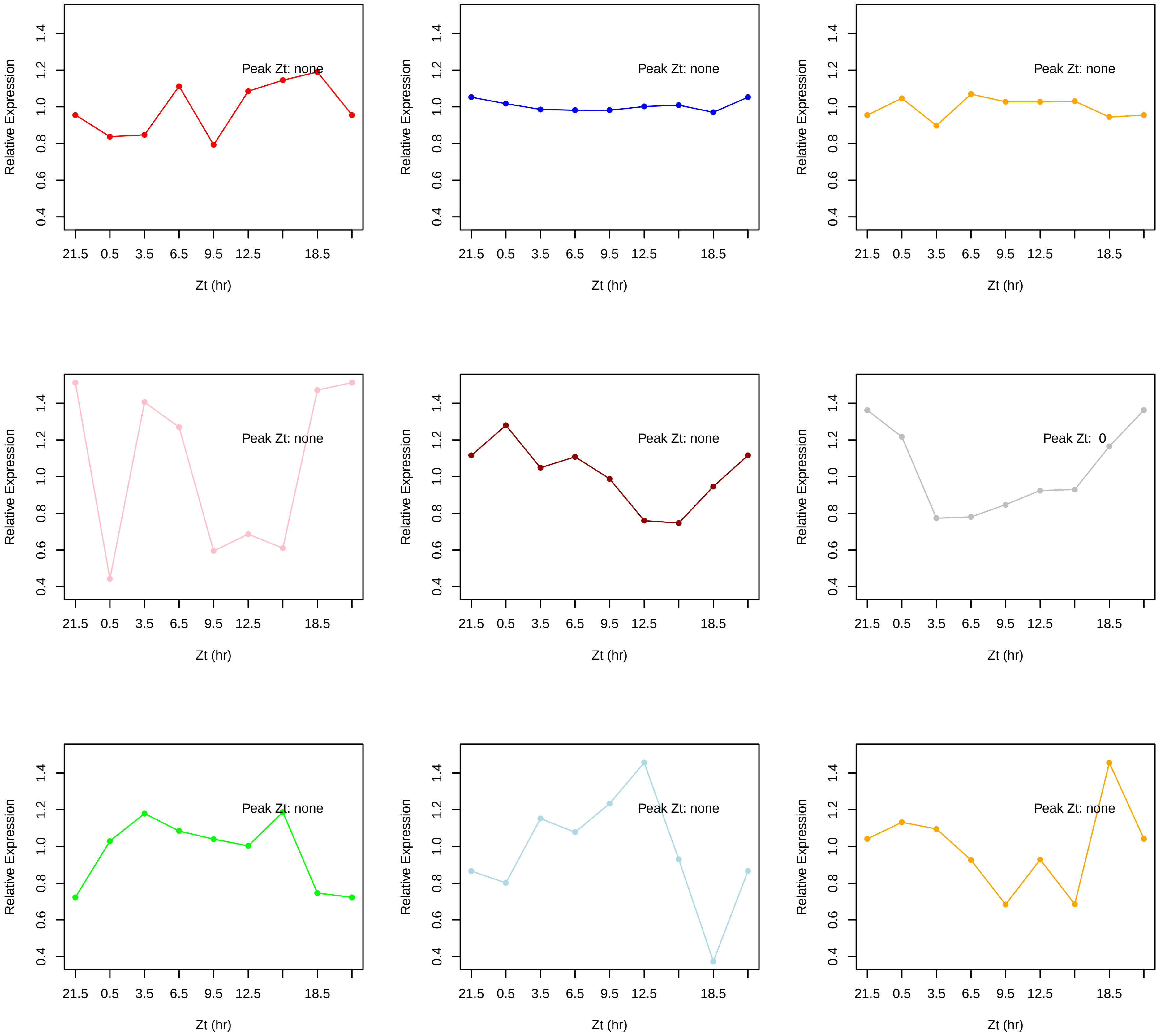

Singeleton: ENSXMAG00000003479 atad2b

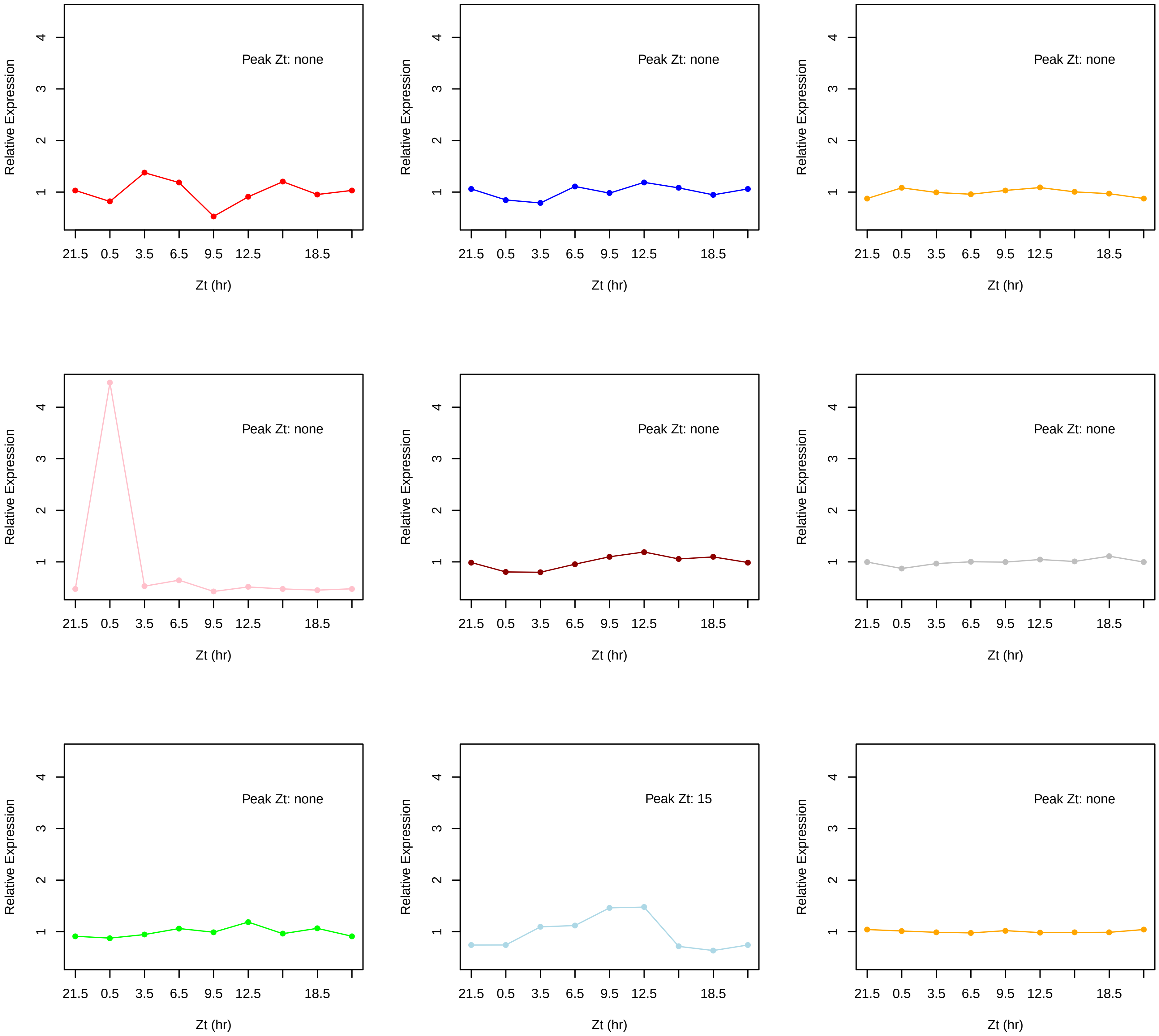

Singleton: ENSXMAG00000003439 mfsd2b

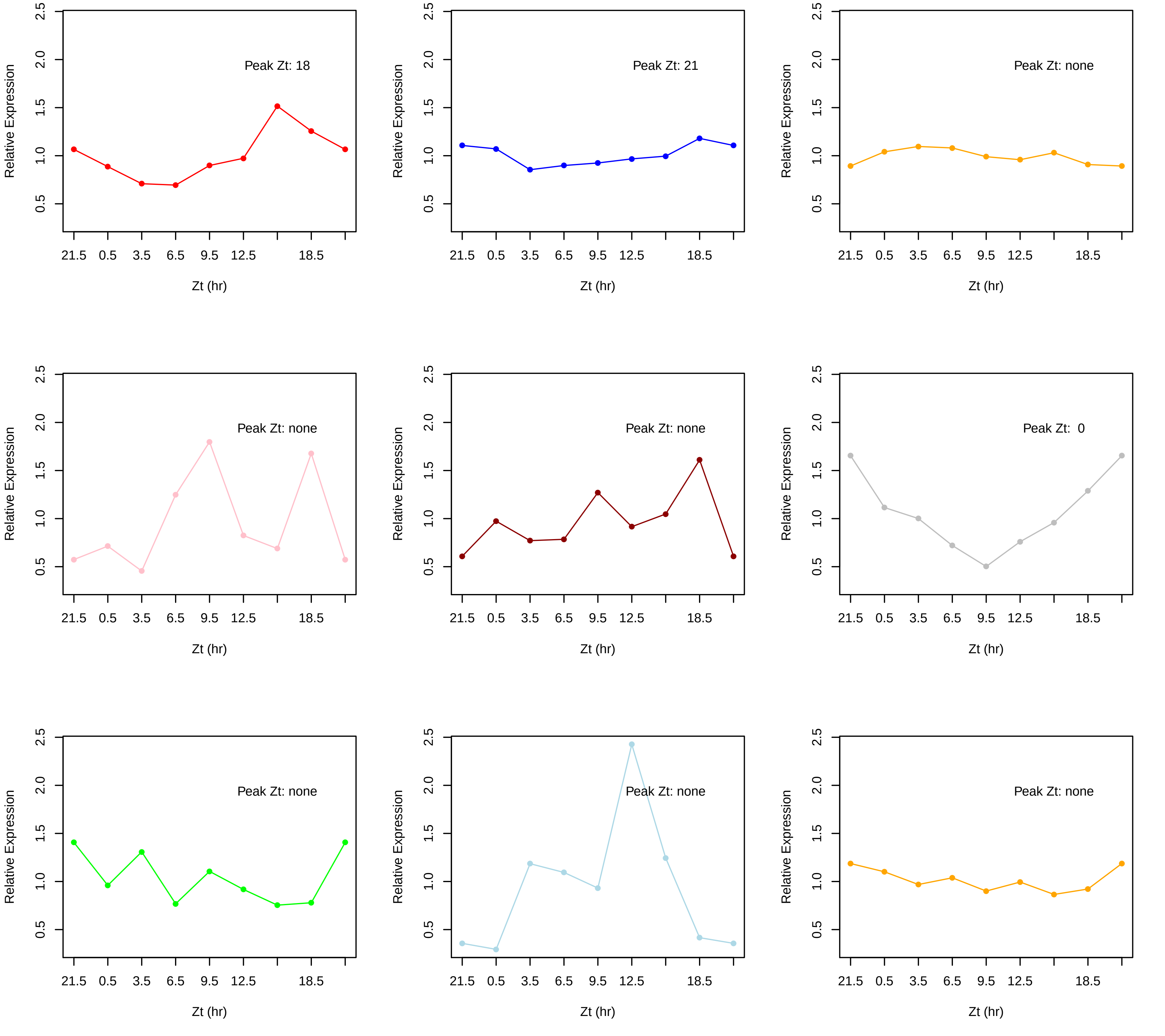

Singeleton: ENSXMAG00000003436 wdcp

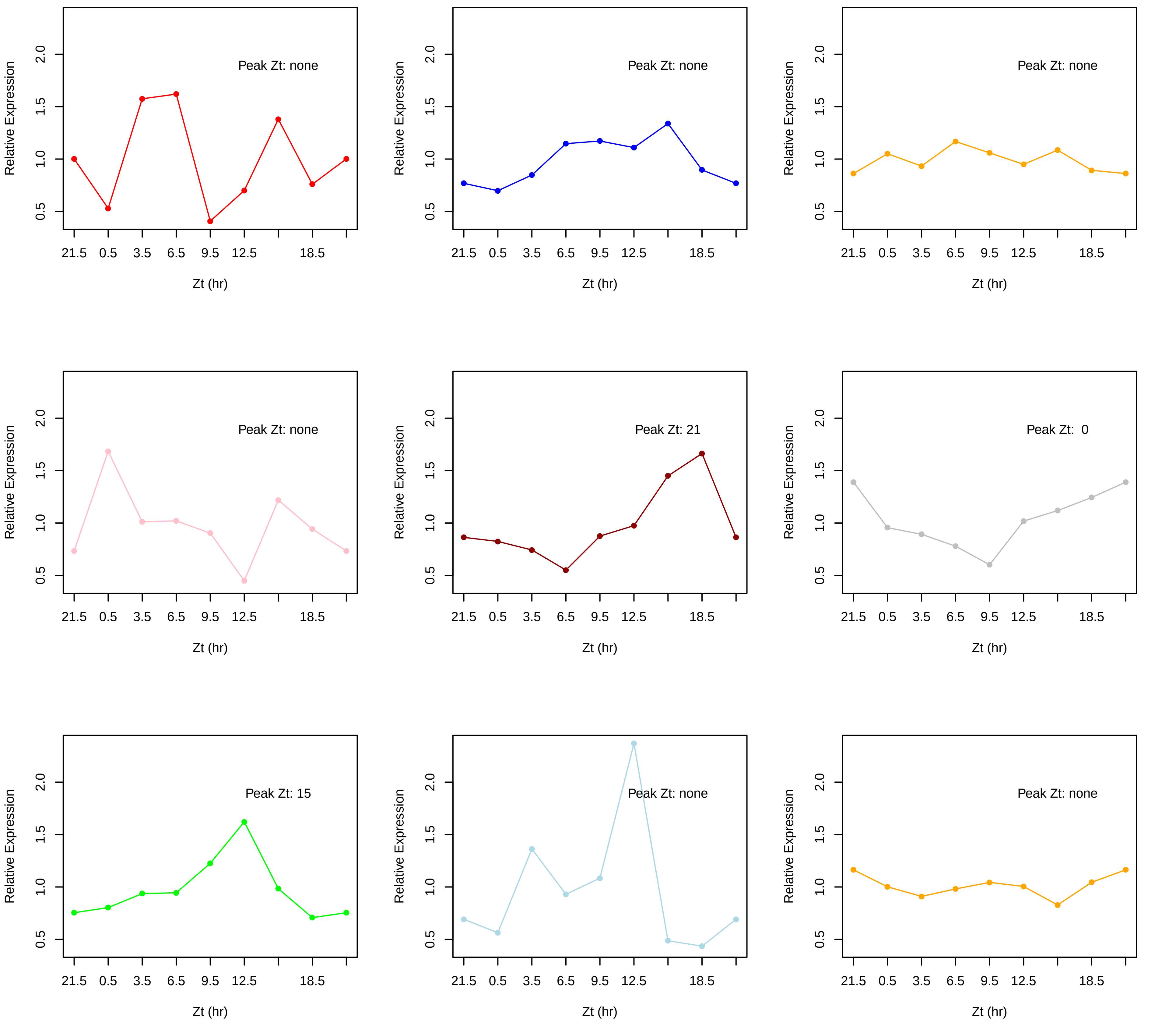

Singeleton: ENSXMAG00000027946 fkbp1b

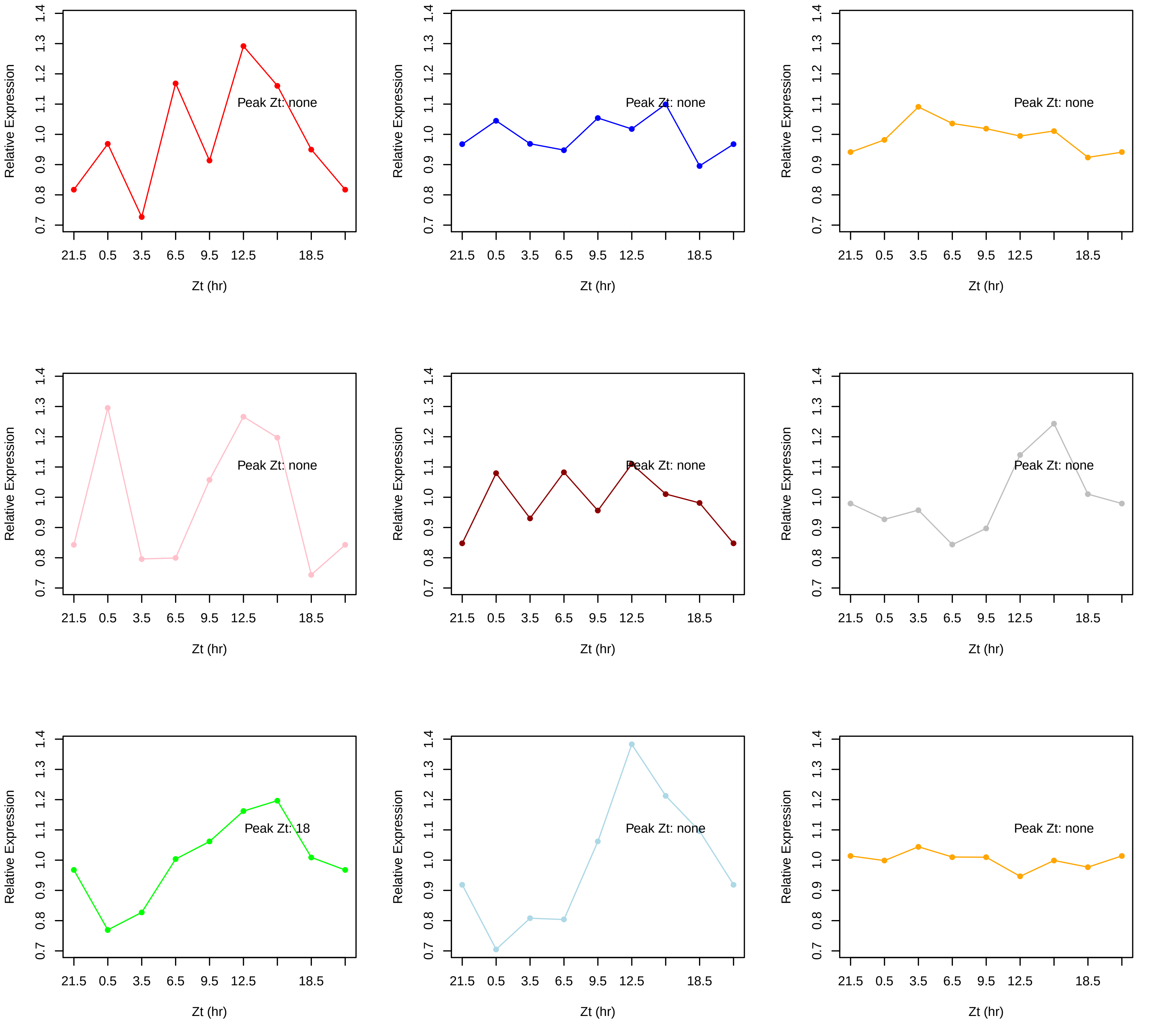

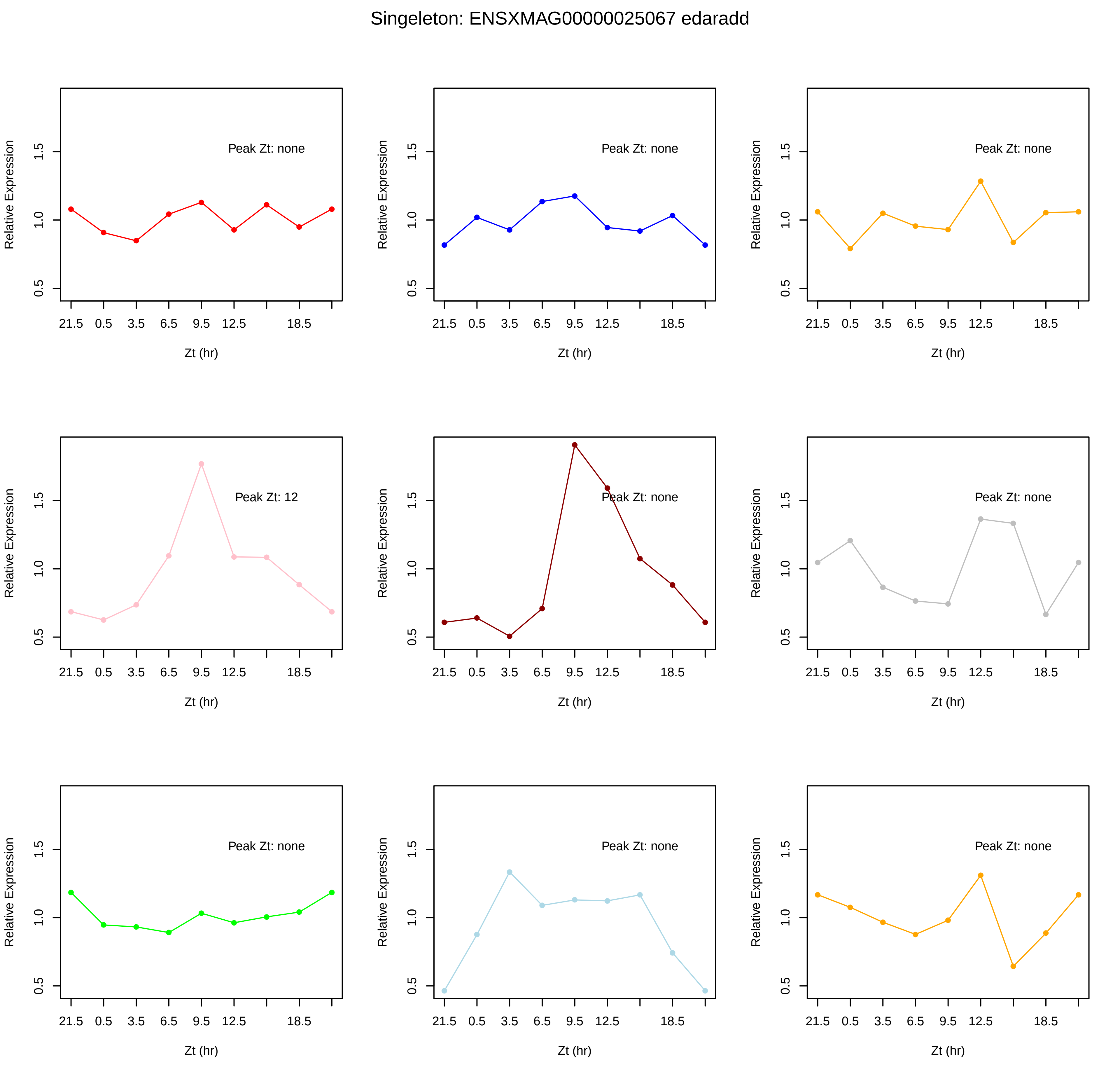
Singeleton: ENSXMAG00000025067 edaradd
Peak Zt: none
Peak Zt: none
Peak Zt: none
Peak Zt: 12
Peak Zt: none
Peak Zt: none
Peak Zt: none
Peak Zt: none
Peak Zt: none
Relative Expression
Zt (hr)
21.5
0.5
3.5
6.5
9.5
12.5
18.5
0.5
1.0
1.5

Singeleton: ENSXMAG00000010356 hnrnpua

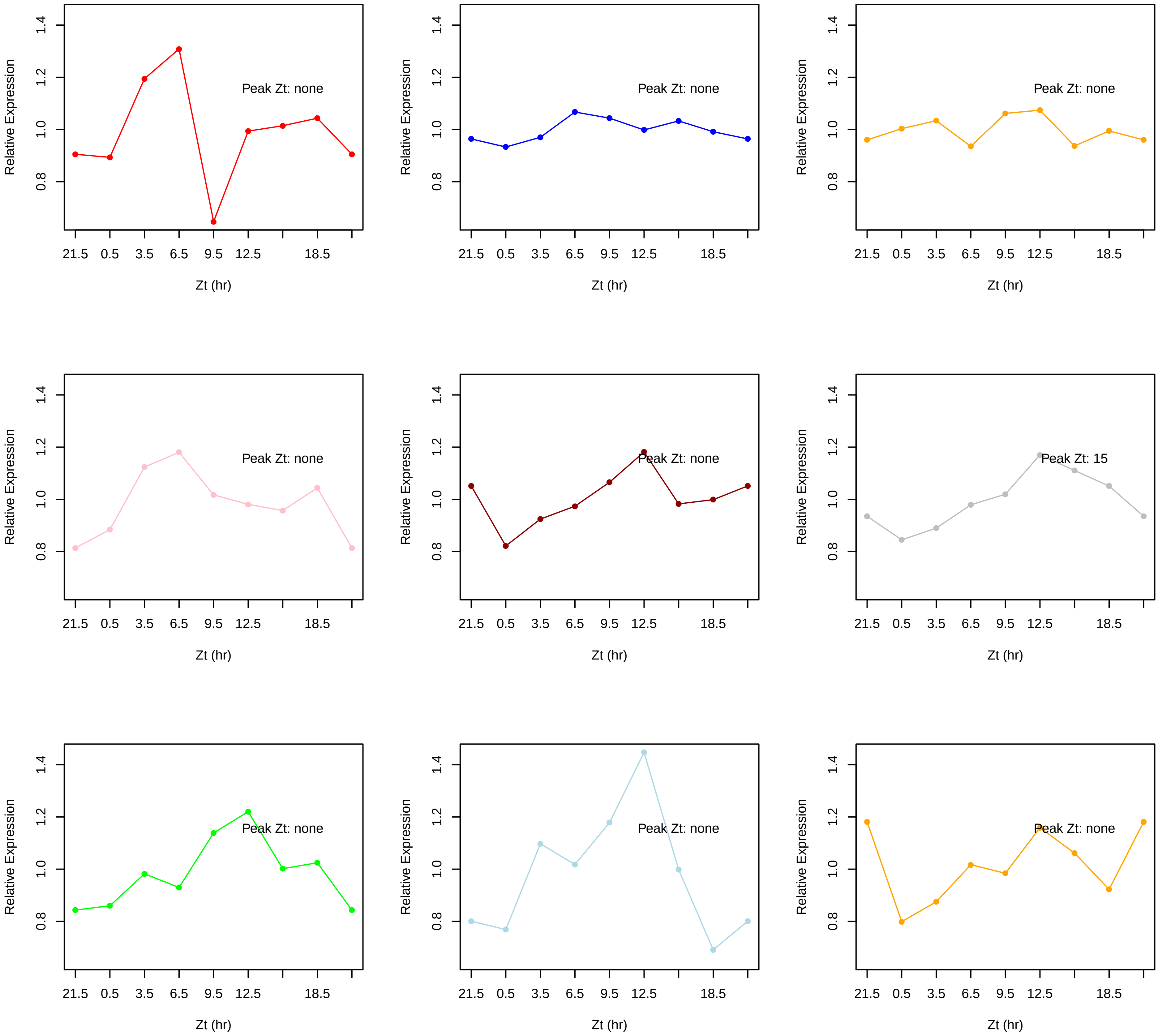

Singeleton: ENSXMAG00000015149 adpgk2

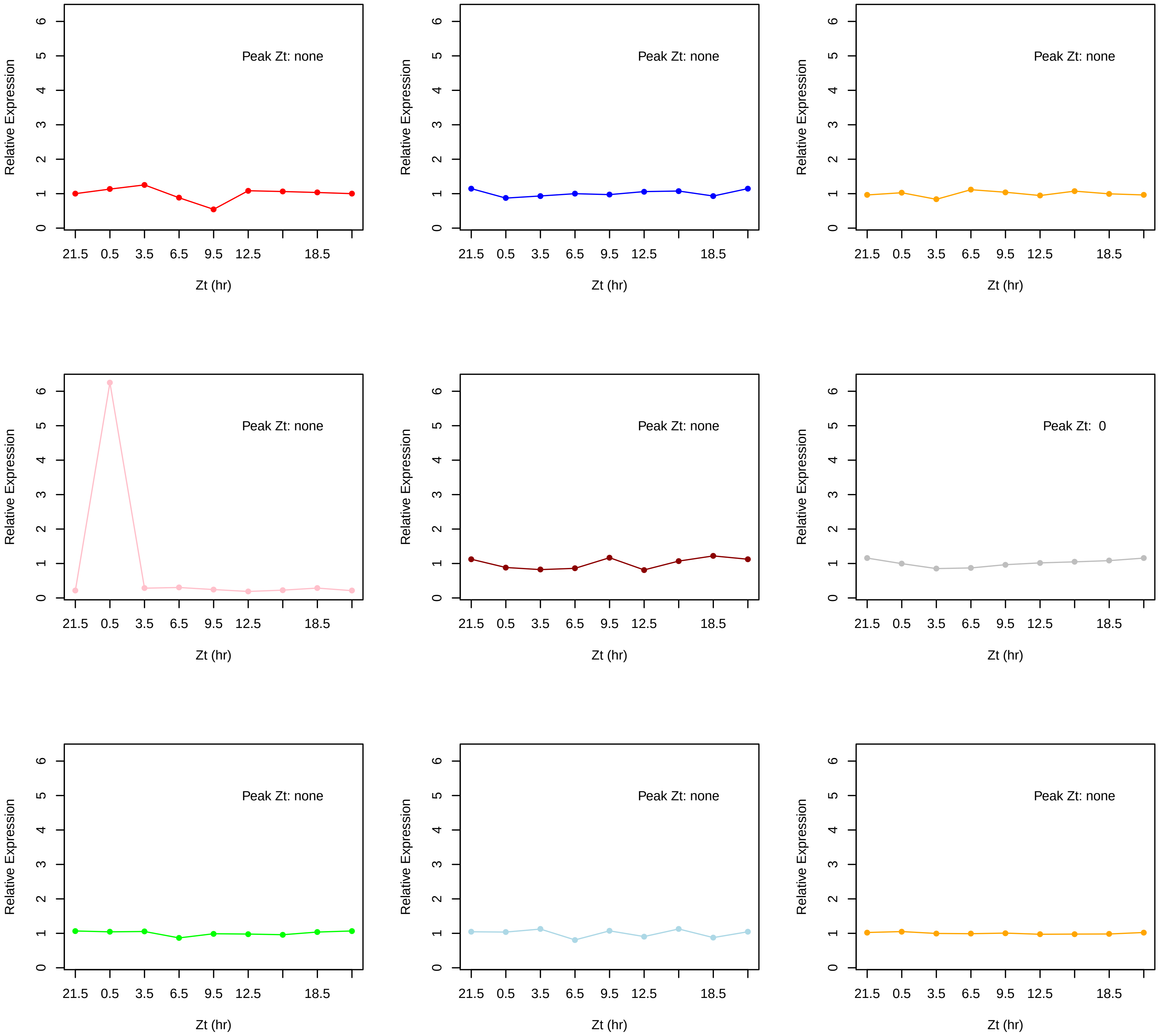

Singleton: ENSXMAG00000018498 nol10

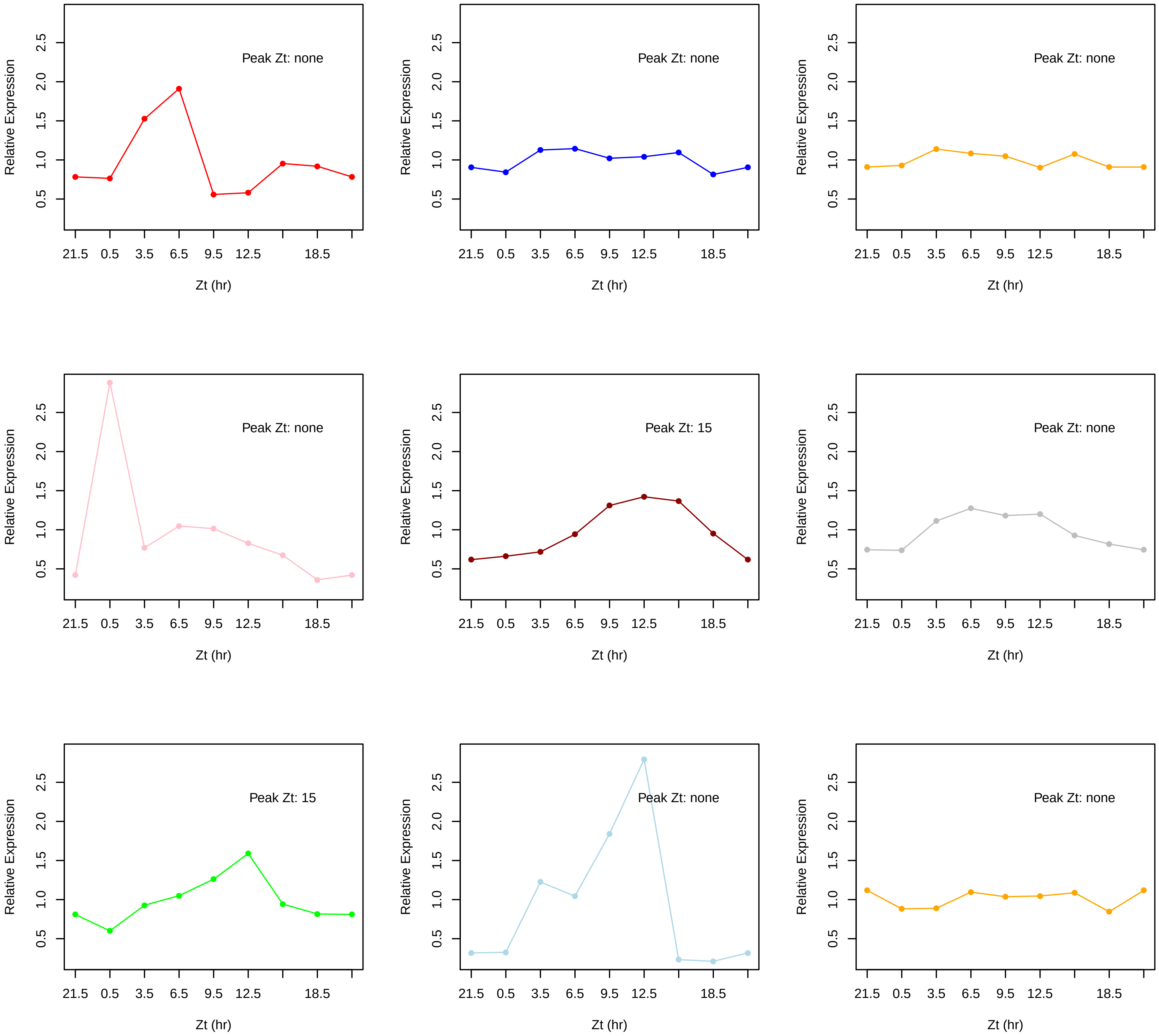

Singeleton: ENSXMAG00000000103 dyrk4

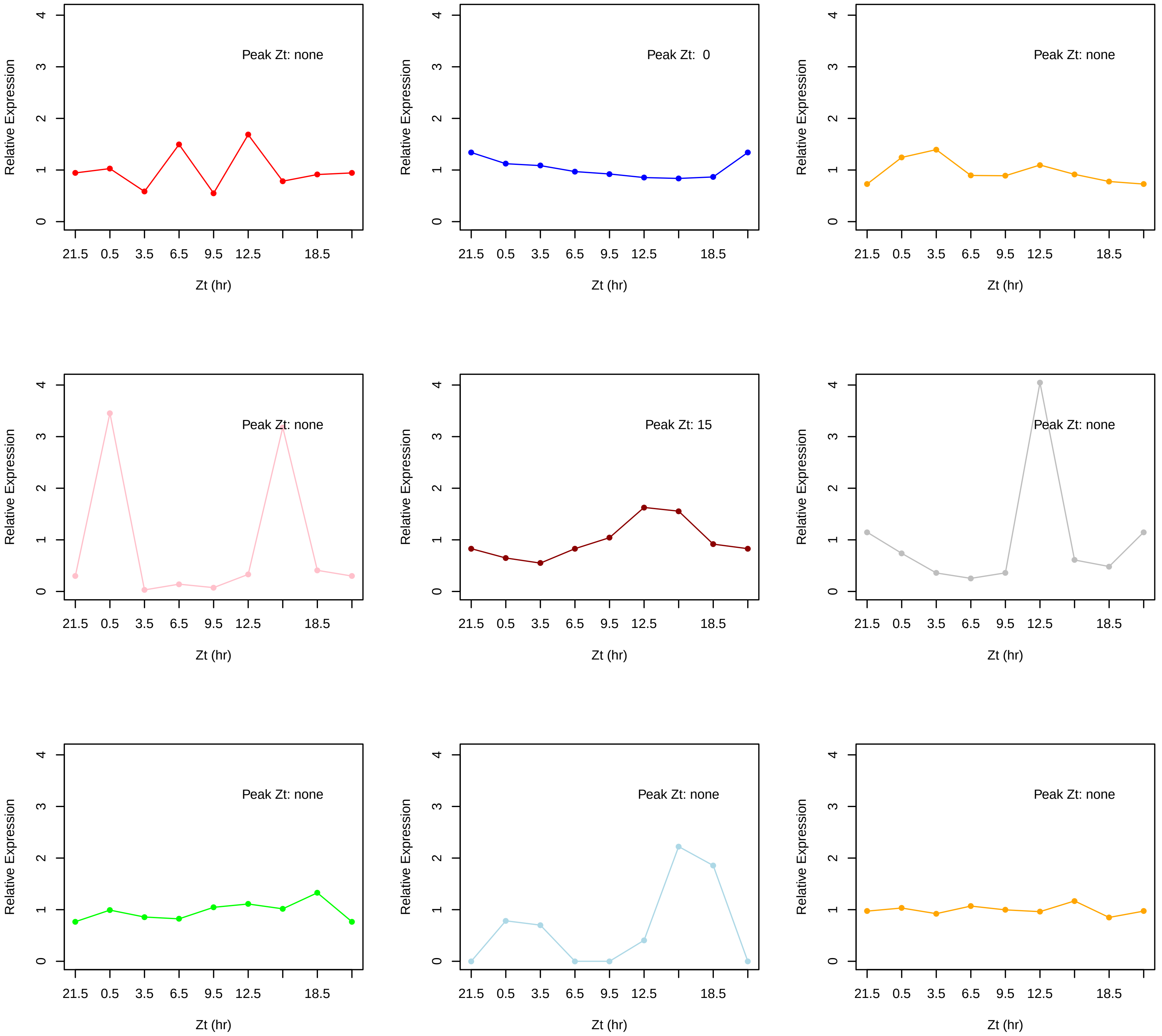

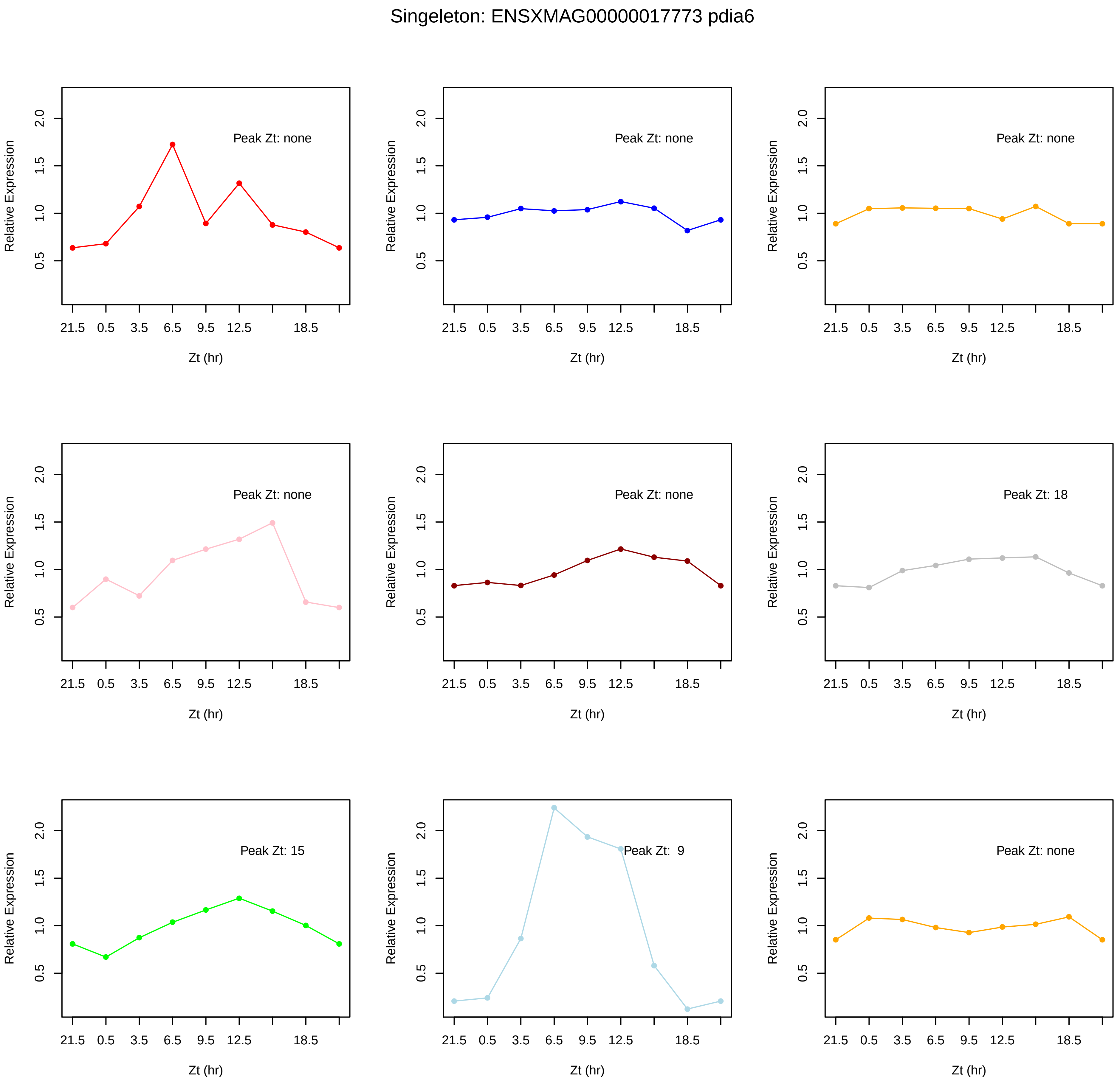
Singeleton: ENSXMAG00000017773 pdia6
Relative Expression
Zt (hr)
21.5 0.5 3.5 6.5 9.5 12.5 18.5
0.5 1.0 1.5 2.0
Peak Zt: none
Peak Zt: none
Peak Zt: none
Peak Zt: none
Peak Zt: none
Peak Zt: 18
Peak Zt: 15
Peak Zt: 9
Peak Zt: none

Singeleton: ENSXMAG00000022964 zgc:154077

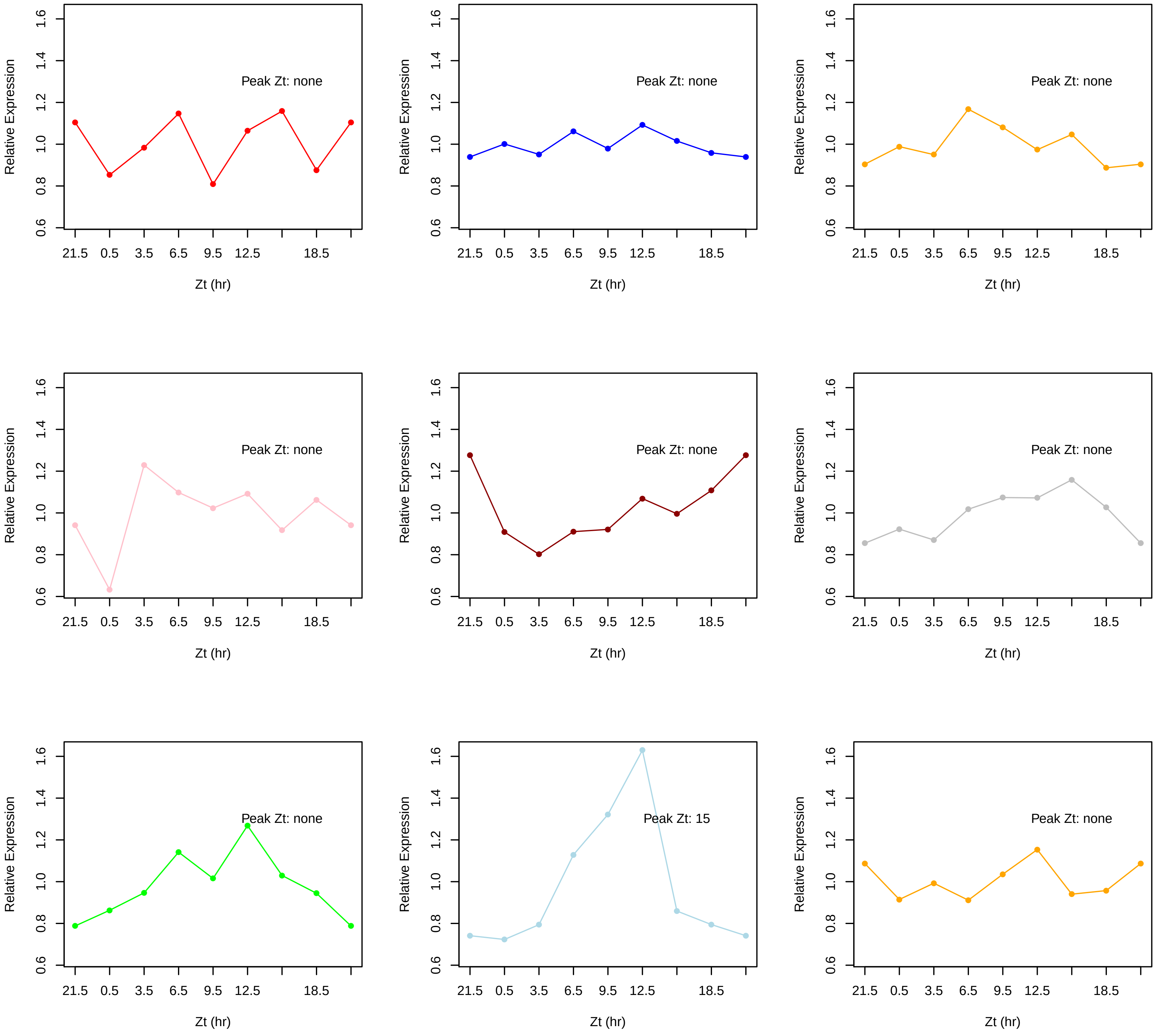

Singeleton: ENSXMAG00000028226 zgc:77158

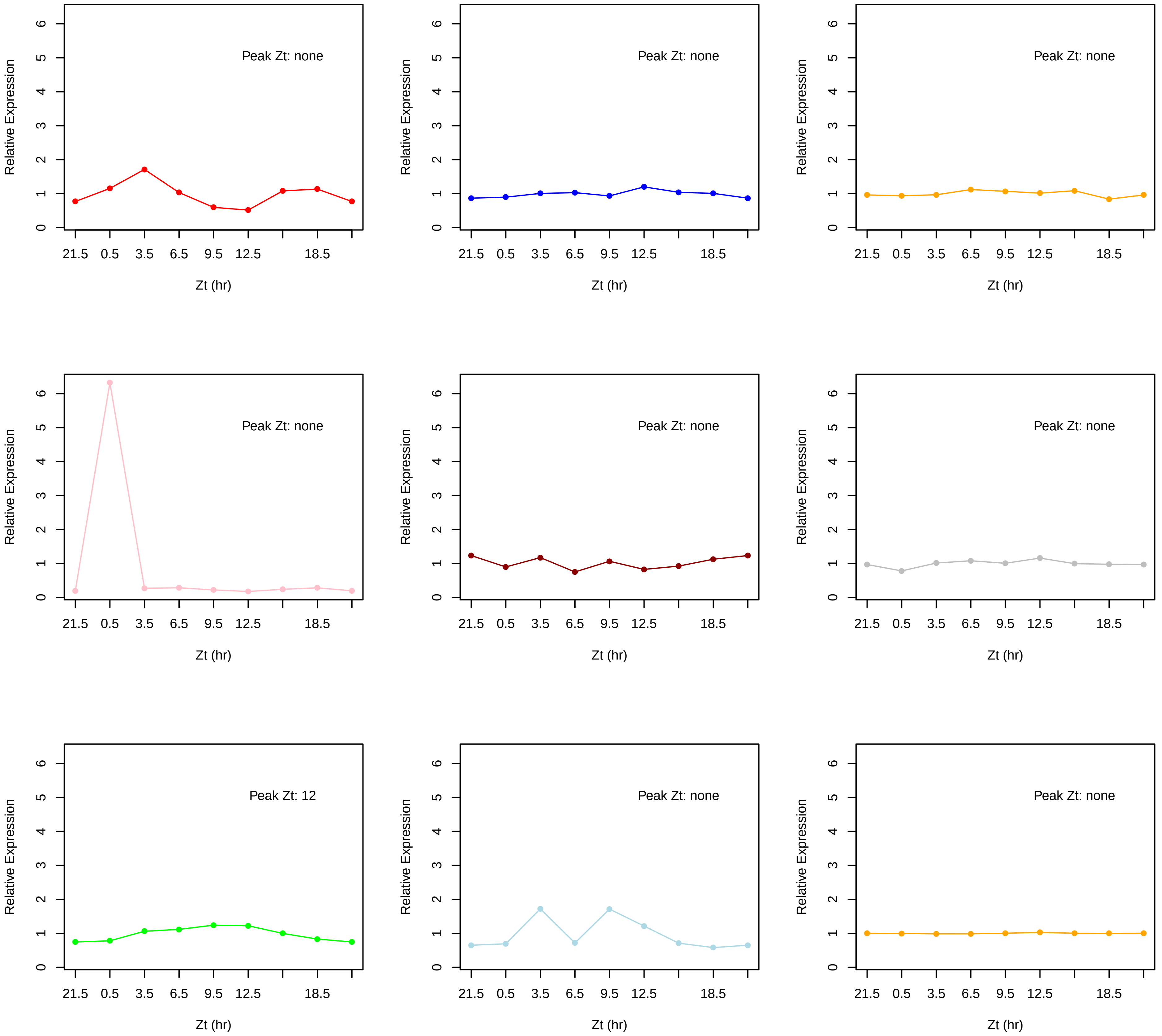

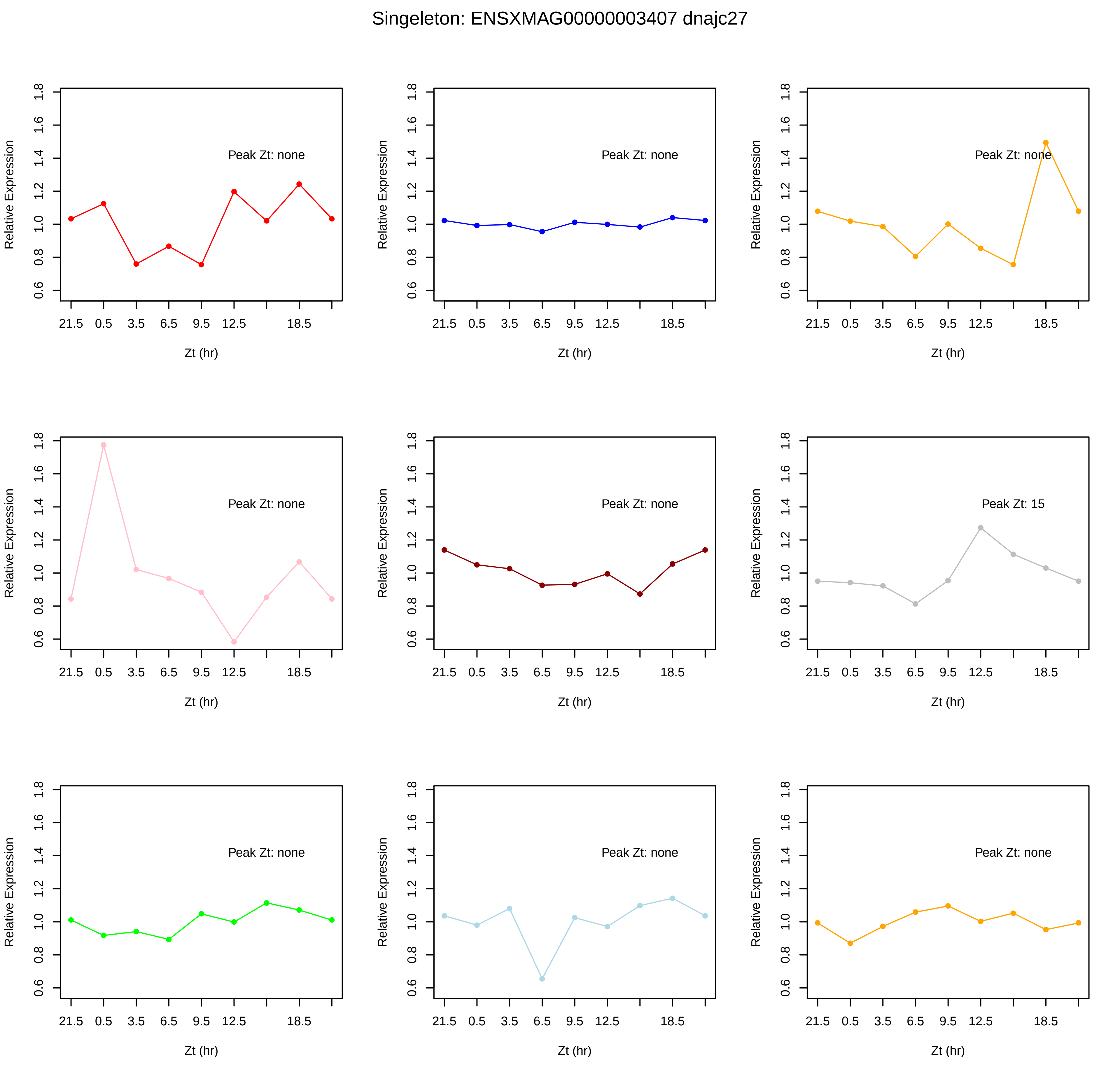
Singeleton: ENSXMAG00000003407 dnajc27
Peak Zt: none
Peak Zt: none
Peak Zt: none
Peak Zt: none
Peak Zt: none
Peak Zt: 15
Peak Zt: none
Peak Zt: none
Peak Zt: none
Relative Expression
Zt (hr)
21.5 0.5 3.5 6.5 9.5 12.5 18.5
0.6 0.8 1.0 1.2 1.4 1.6 1.8

Singeleton: ENSXMAG00000010575 ttc27

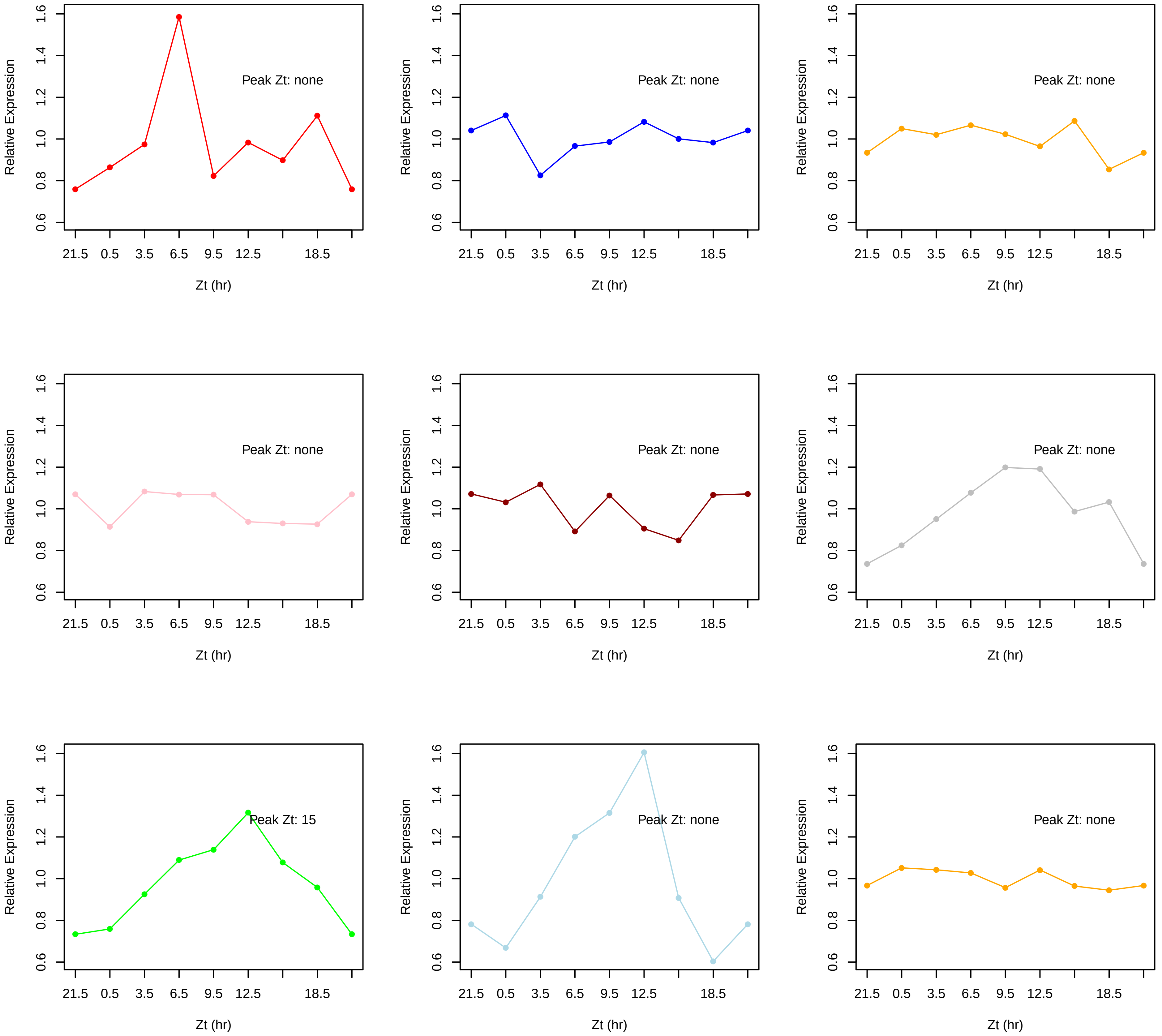

Singeleton: ENSXMAG00000007292 TEAD4

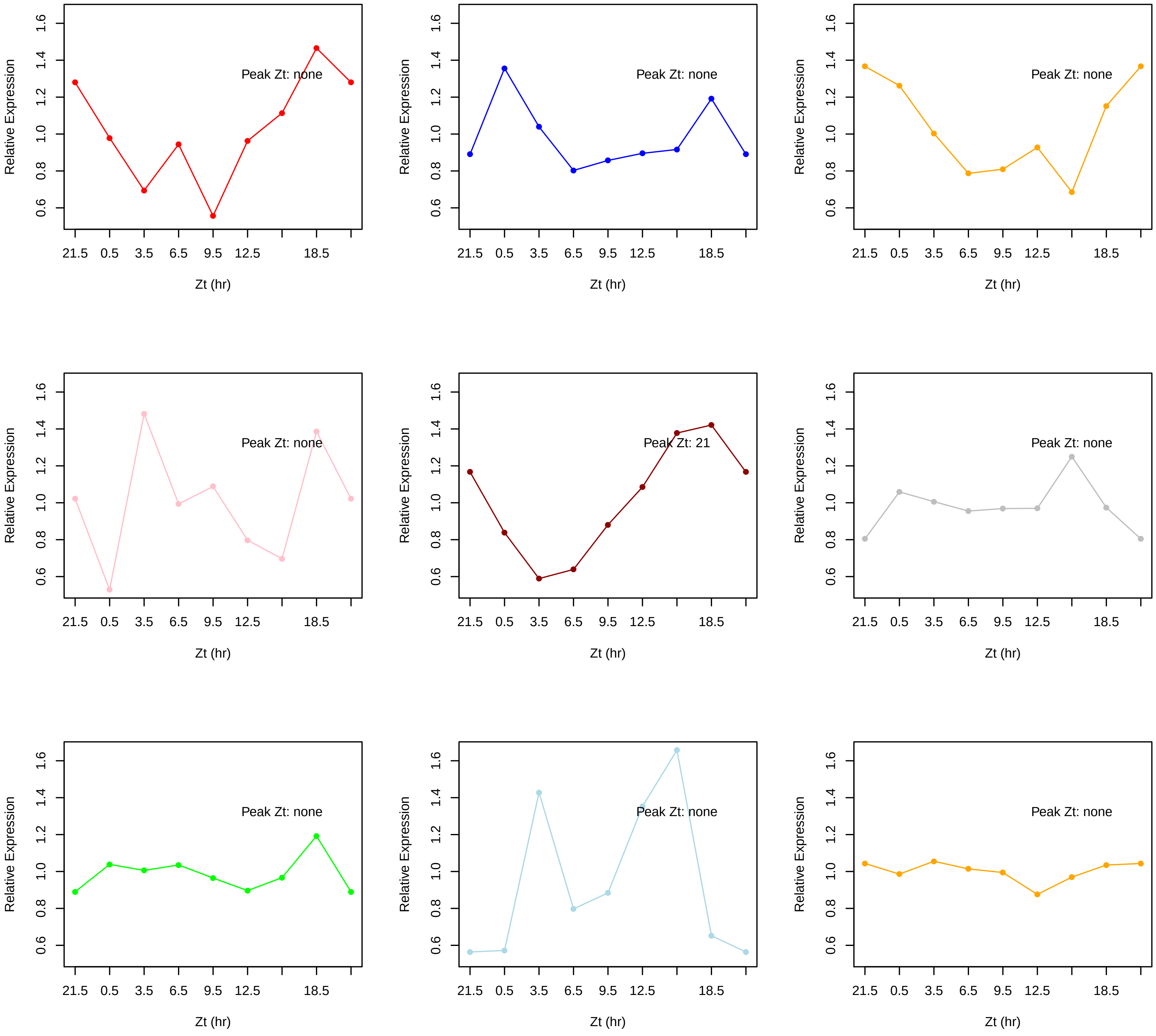

Singeleton: ENSXMAG00000004422 rasgrp3

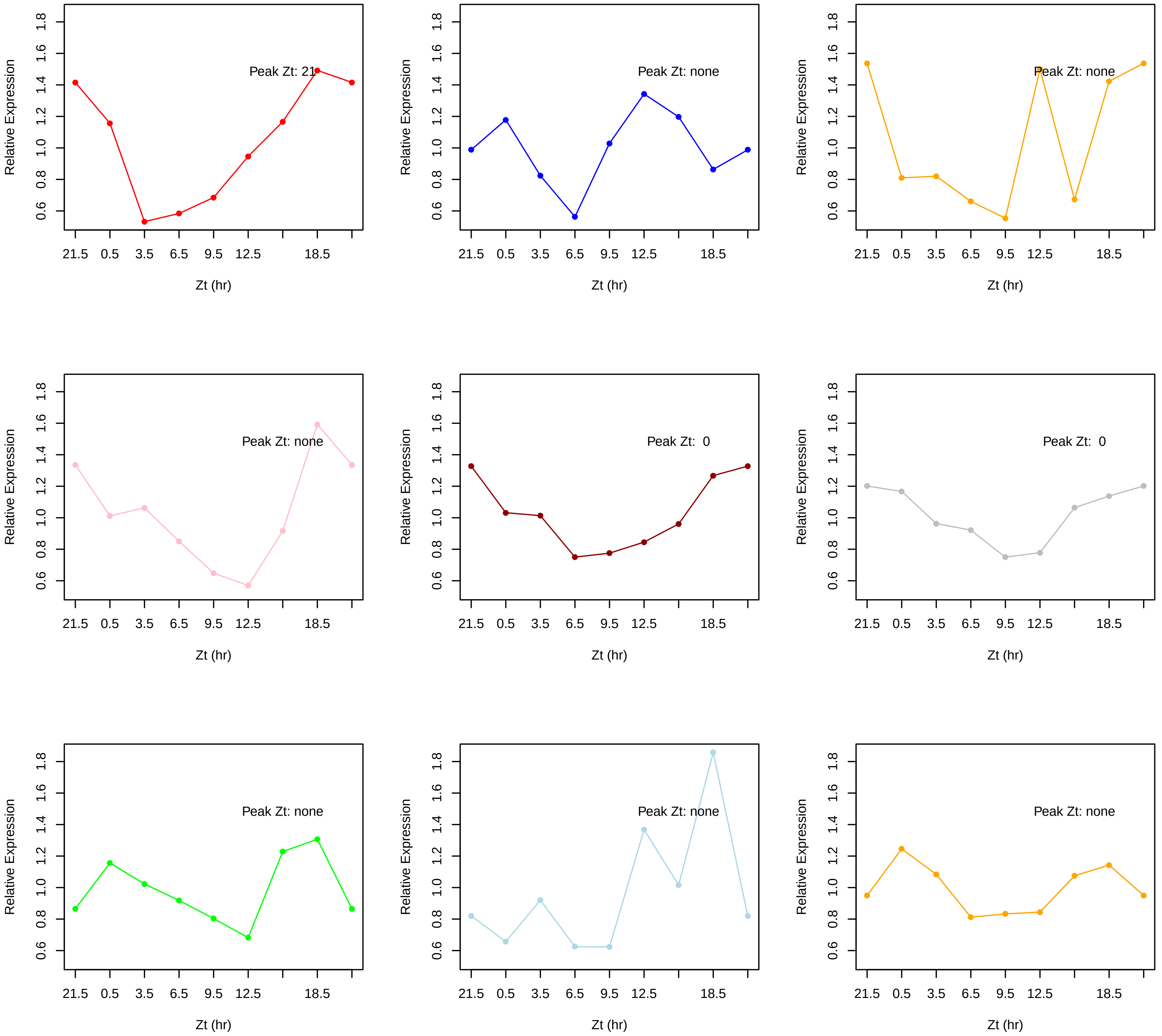

Singeleton: ENSXMAG00000003715 rps6kc1

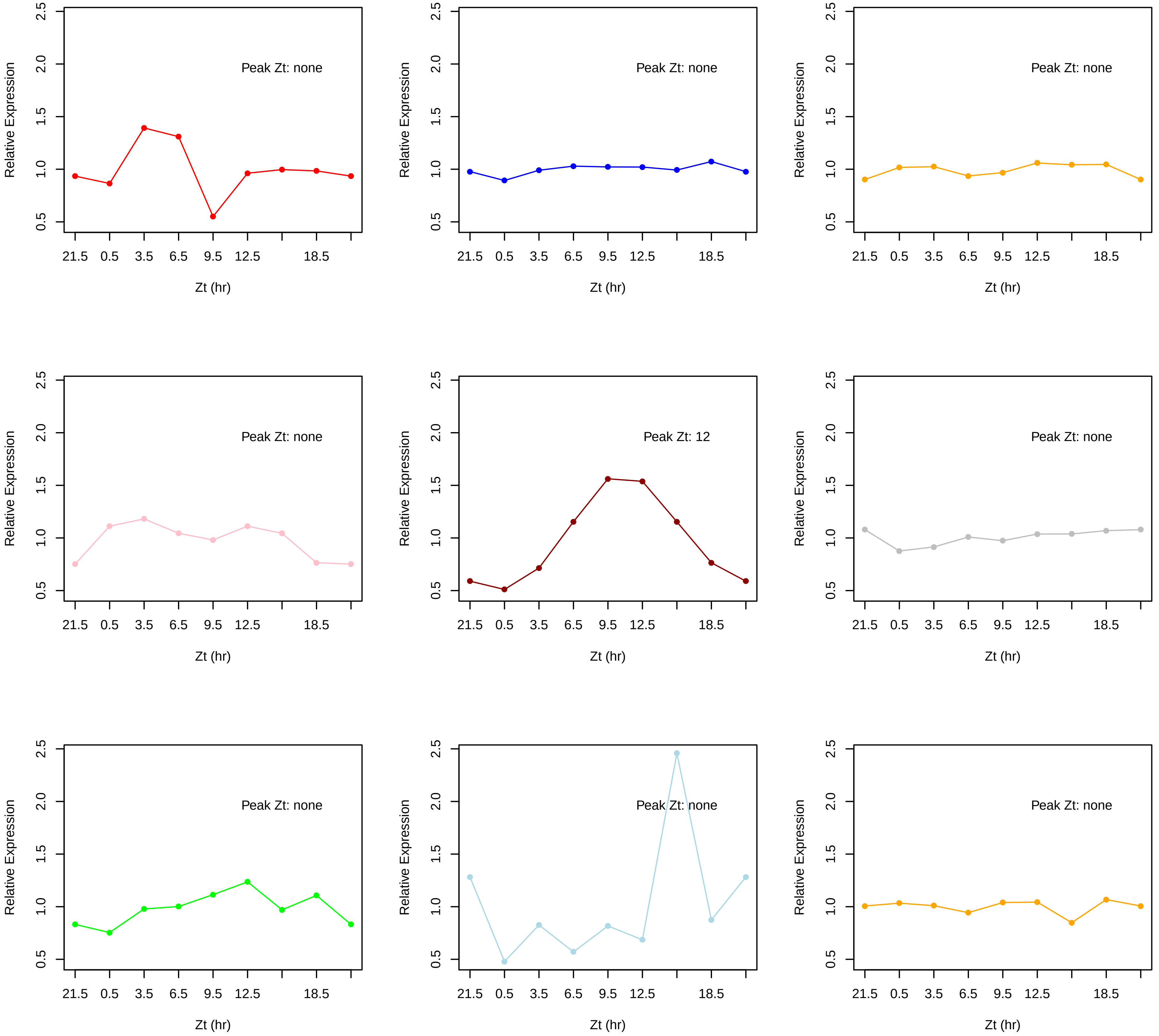

Singeleton: ENSXMAG00000009648 cdkn1bb

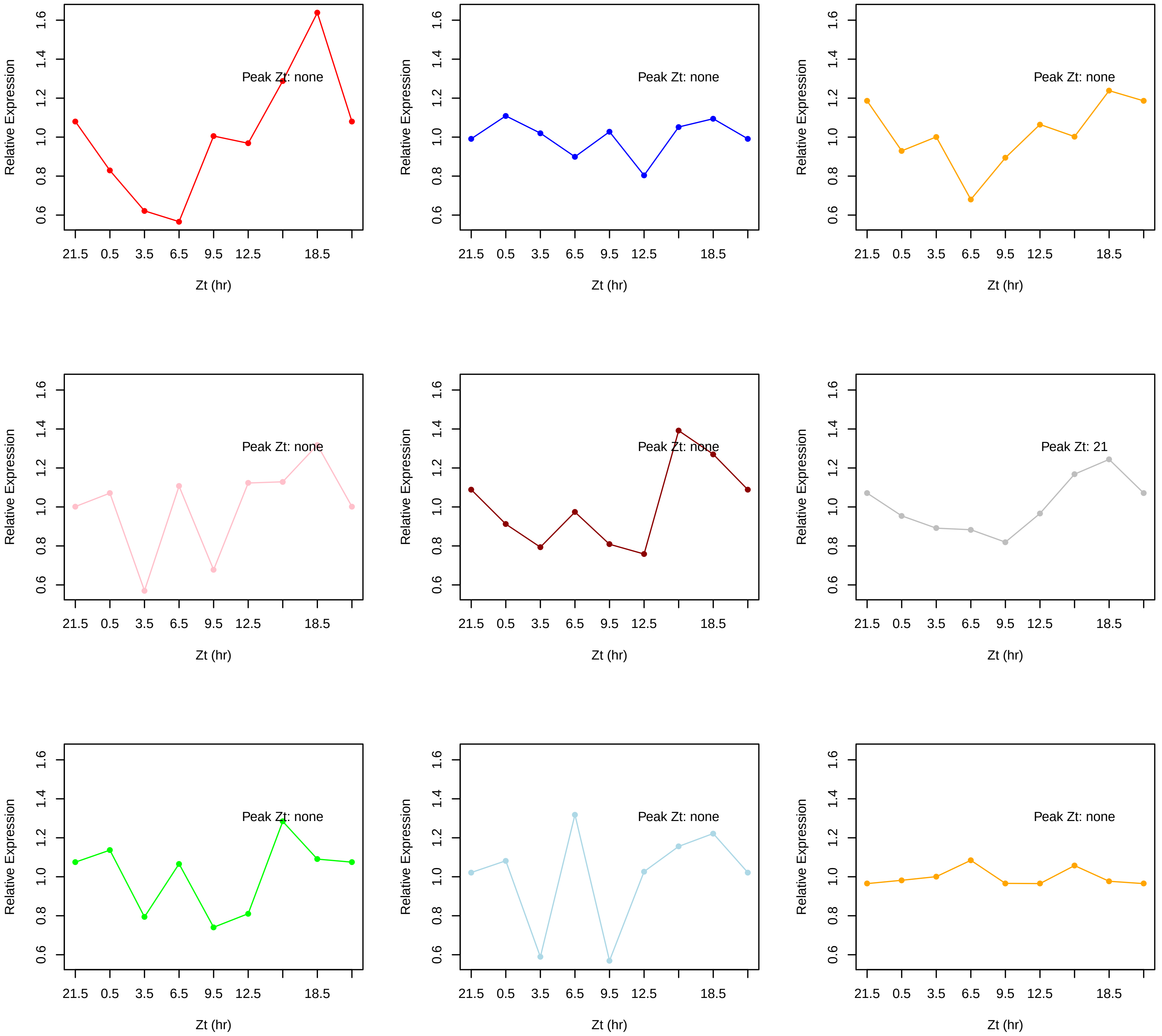

Singeleton: ENSXMAG00000028526 smyd2a

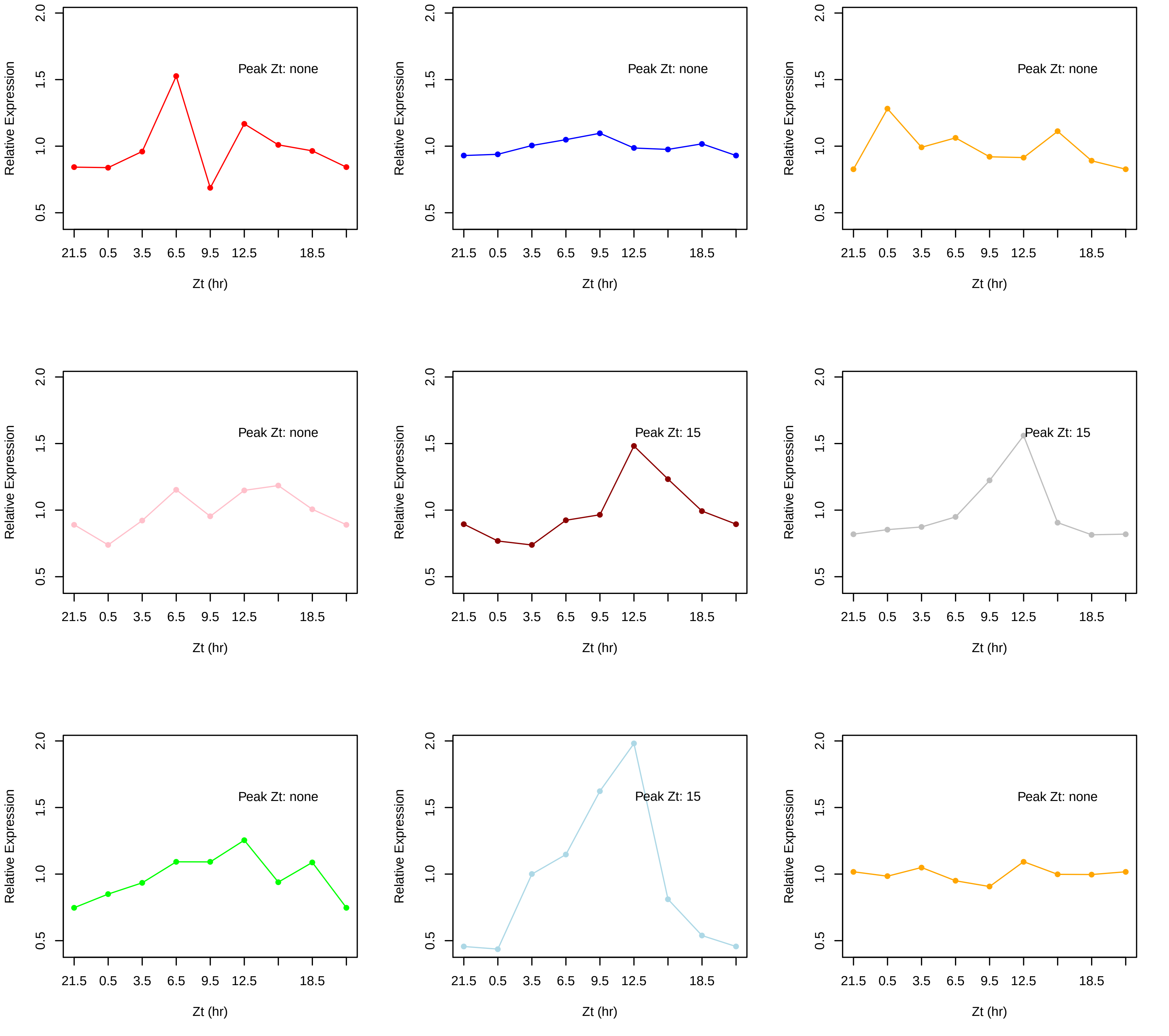

Singeleton: ENSXMAG00000003682 PTPN14

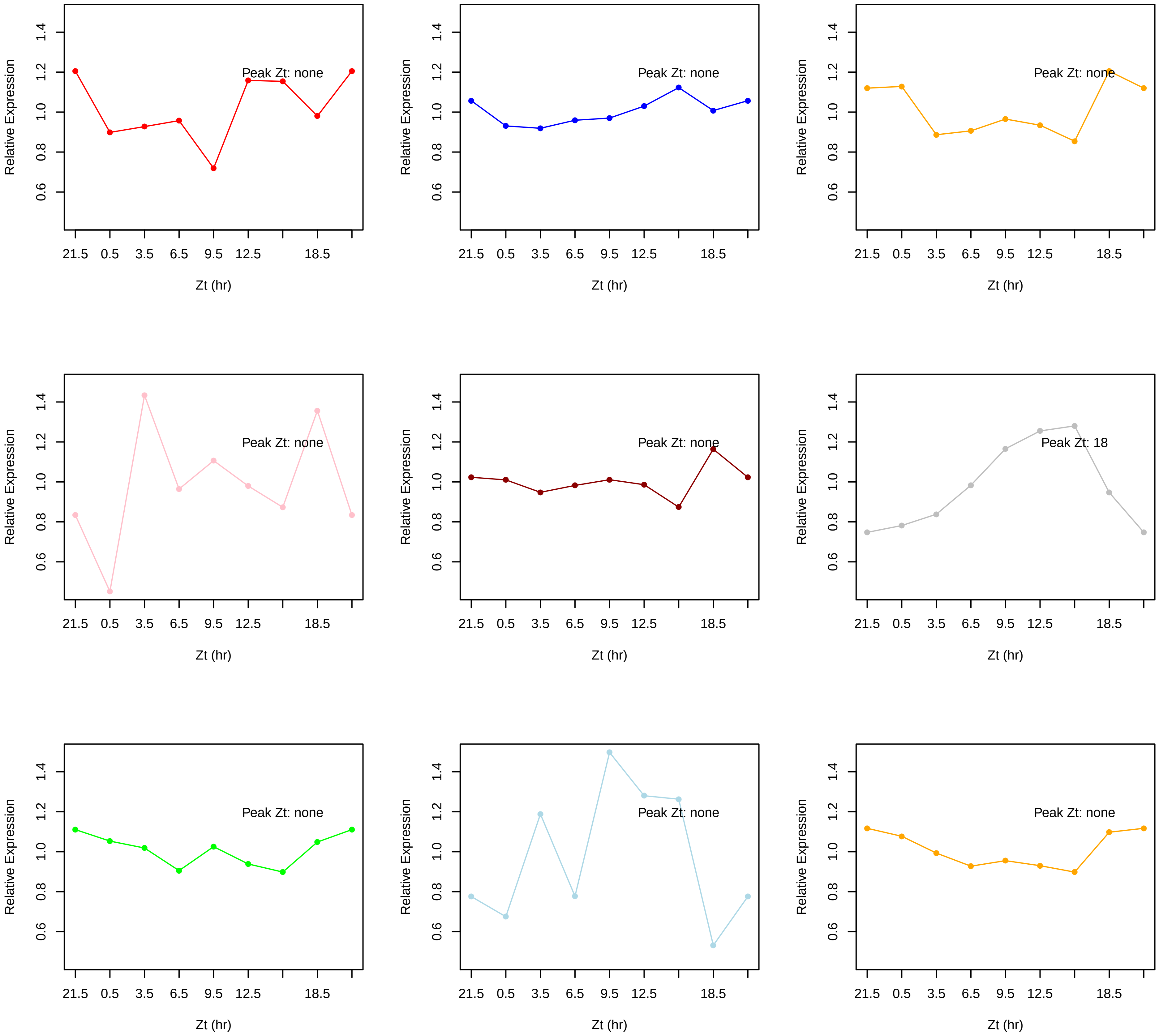

Singeleton: ENSXMAG00000015125 cenpf

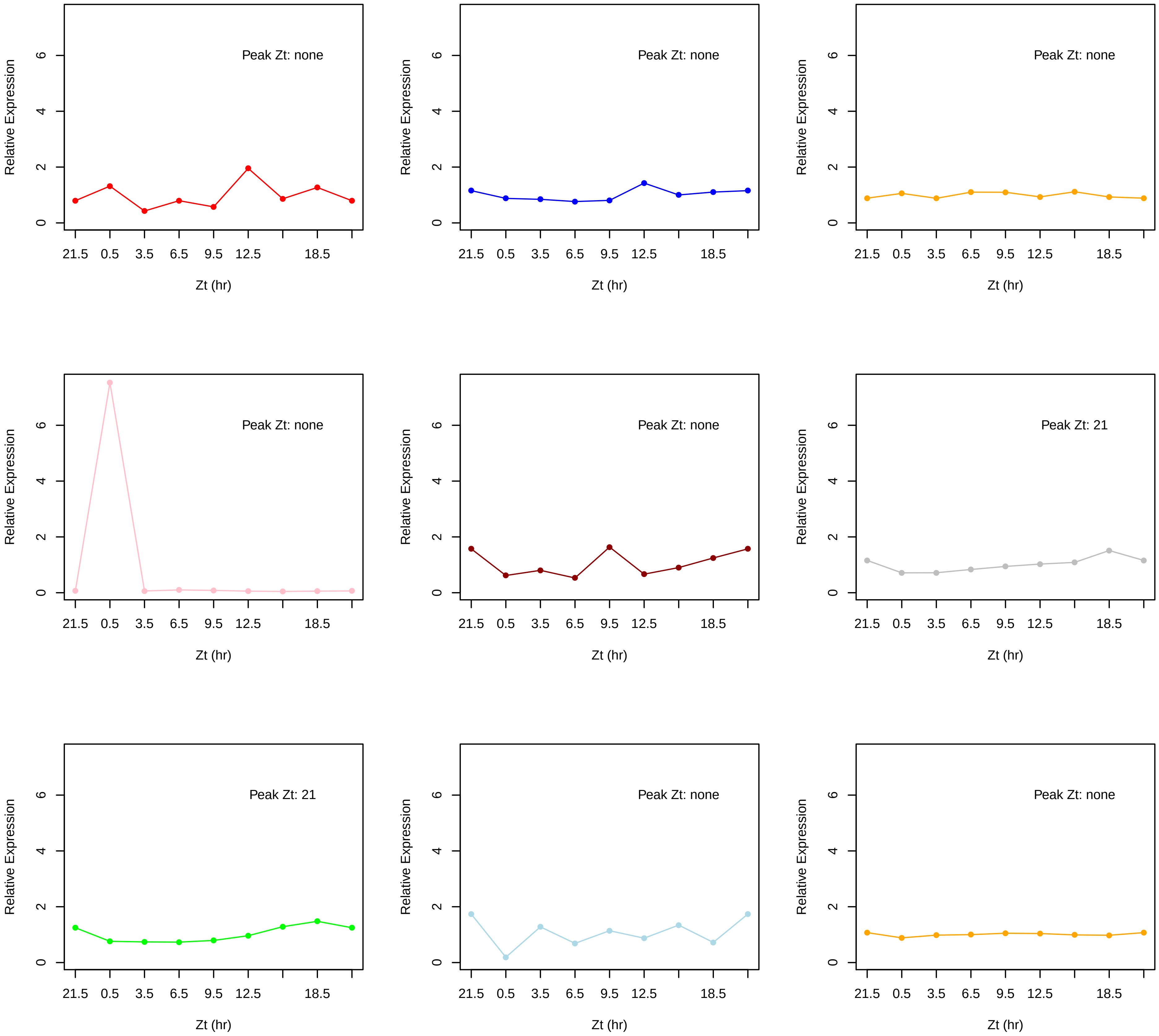

Singleton: ENSXMAG00000029834 degs1

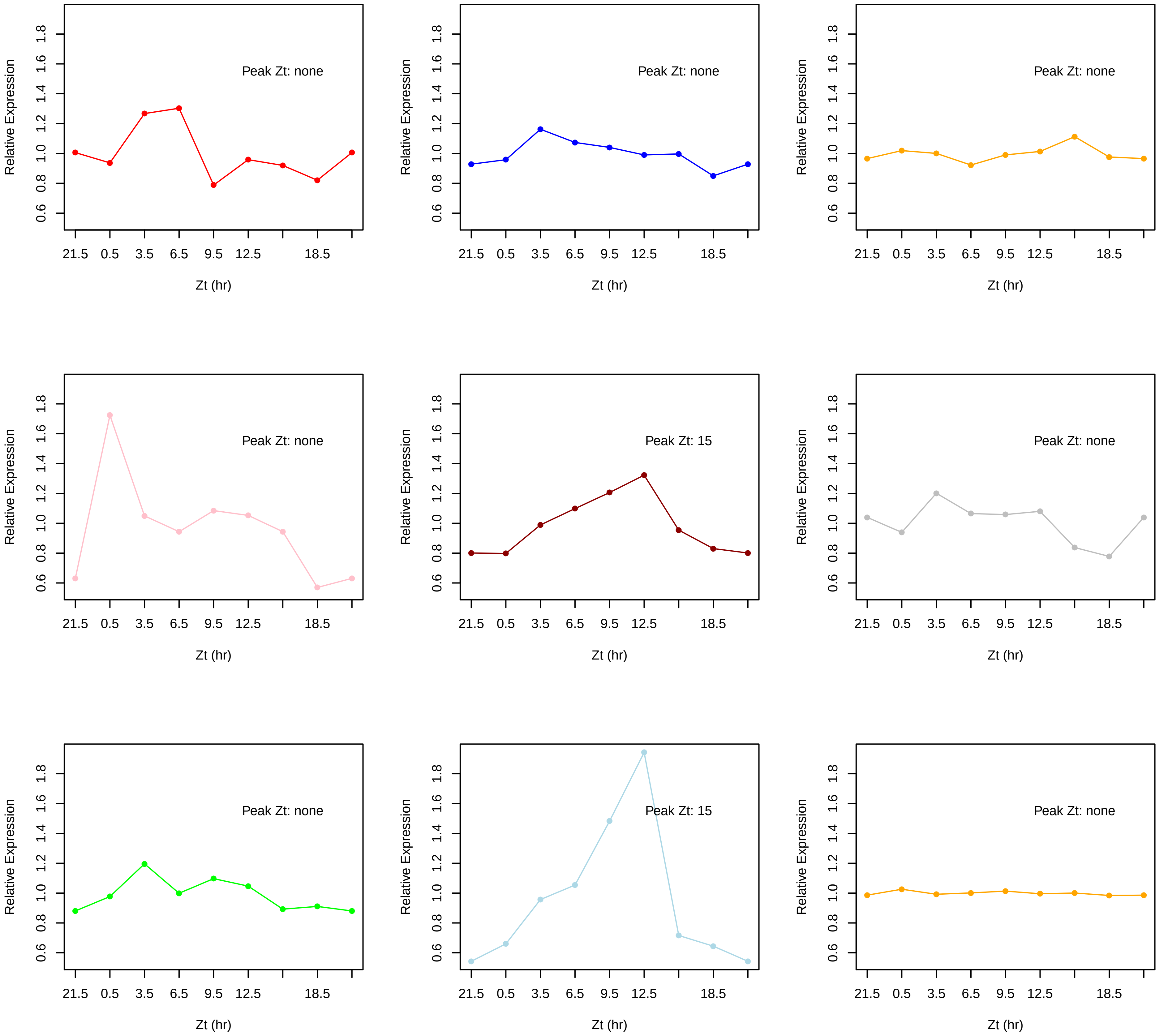

Singleton: ENSXMAG00000015076 polr1c

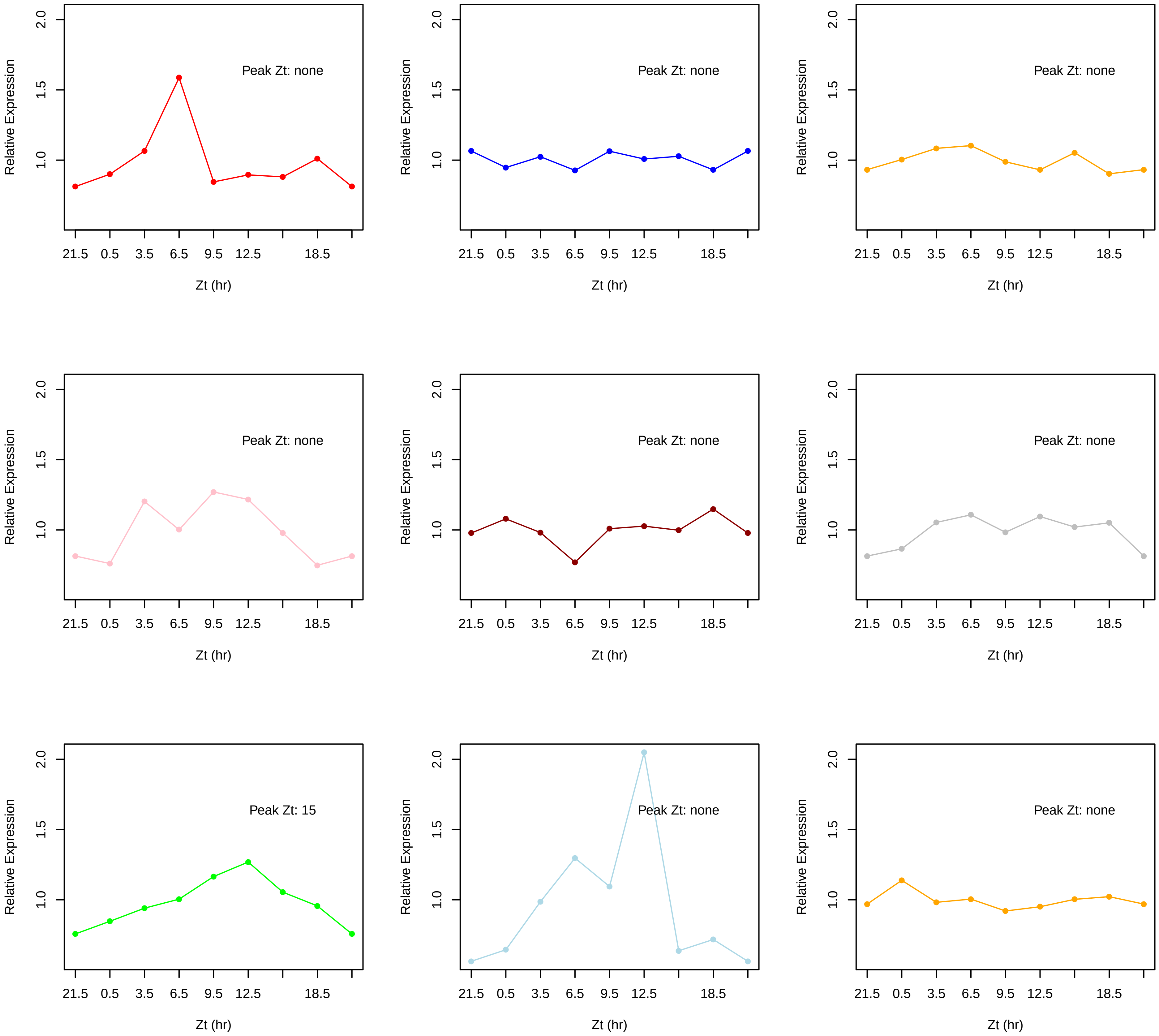

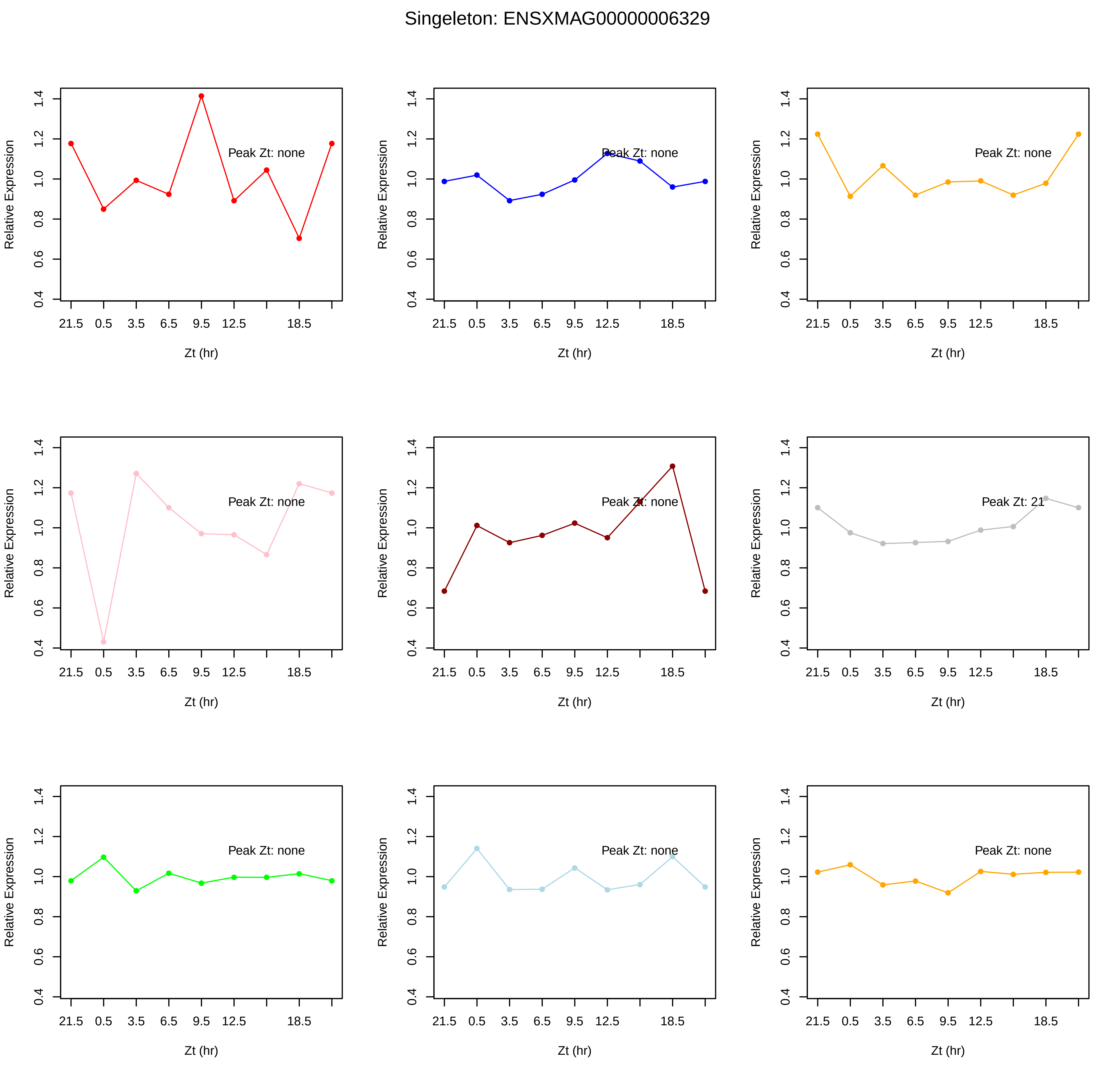

Singleton: ENSXMAG00000006329
Peak Zt: none
Peak Zt: none
Peak Zt: none
Peak Zt: none
Peak Zt: none
Peak Zt: 21
Peak Zt: none
Peak Zt: none
Peak Zt: none
Relative Expression
Zt (hr)
21.5
0.5
3.5
6.5
9.5
12.5
18.5
0.4
0.6
0.8
1.0
1.2
1.4

Singeleton: ENSXMAG00000022186 yipf3

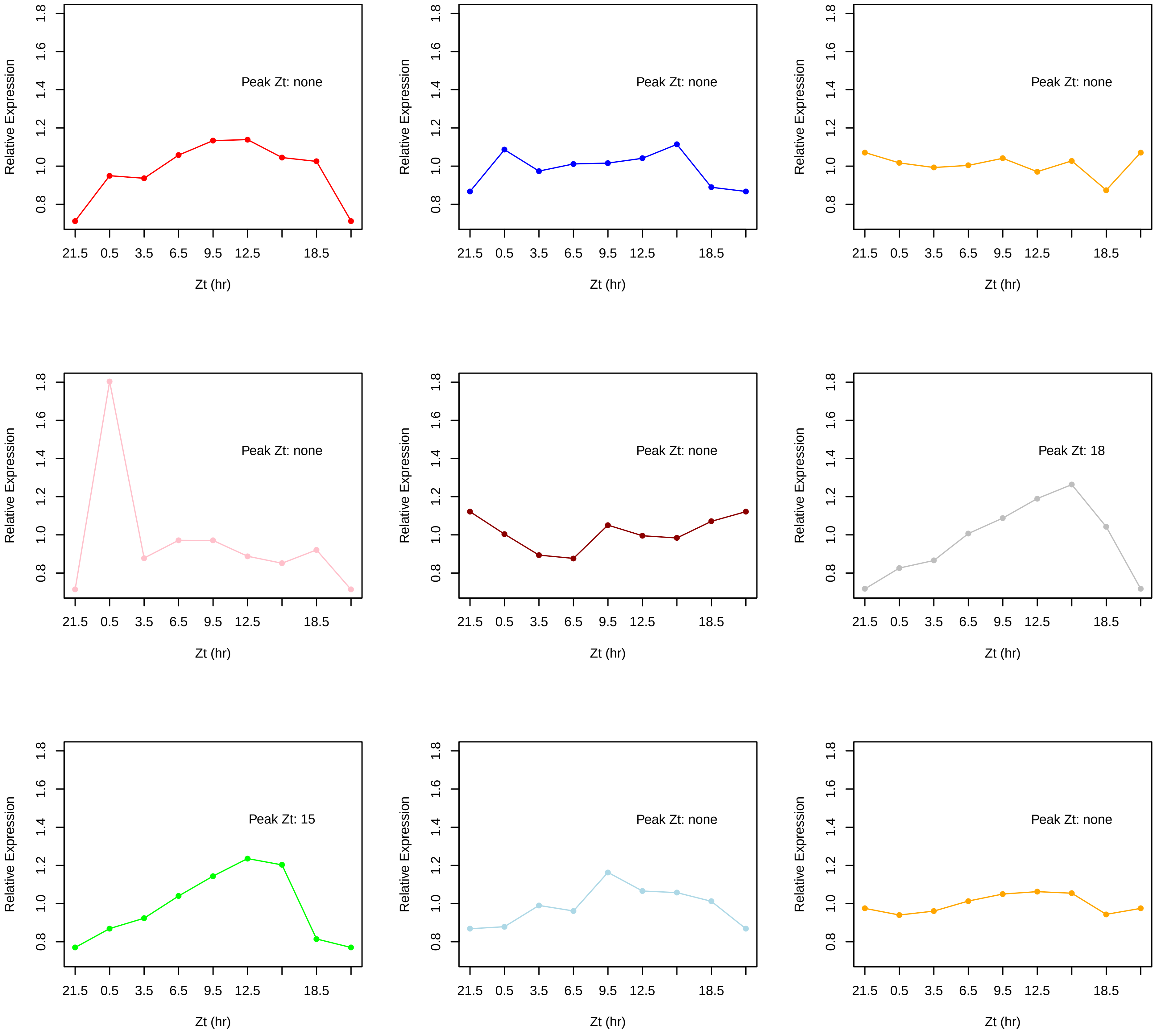

Singeleton: ENSXMAG00000024559 dmtf1

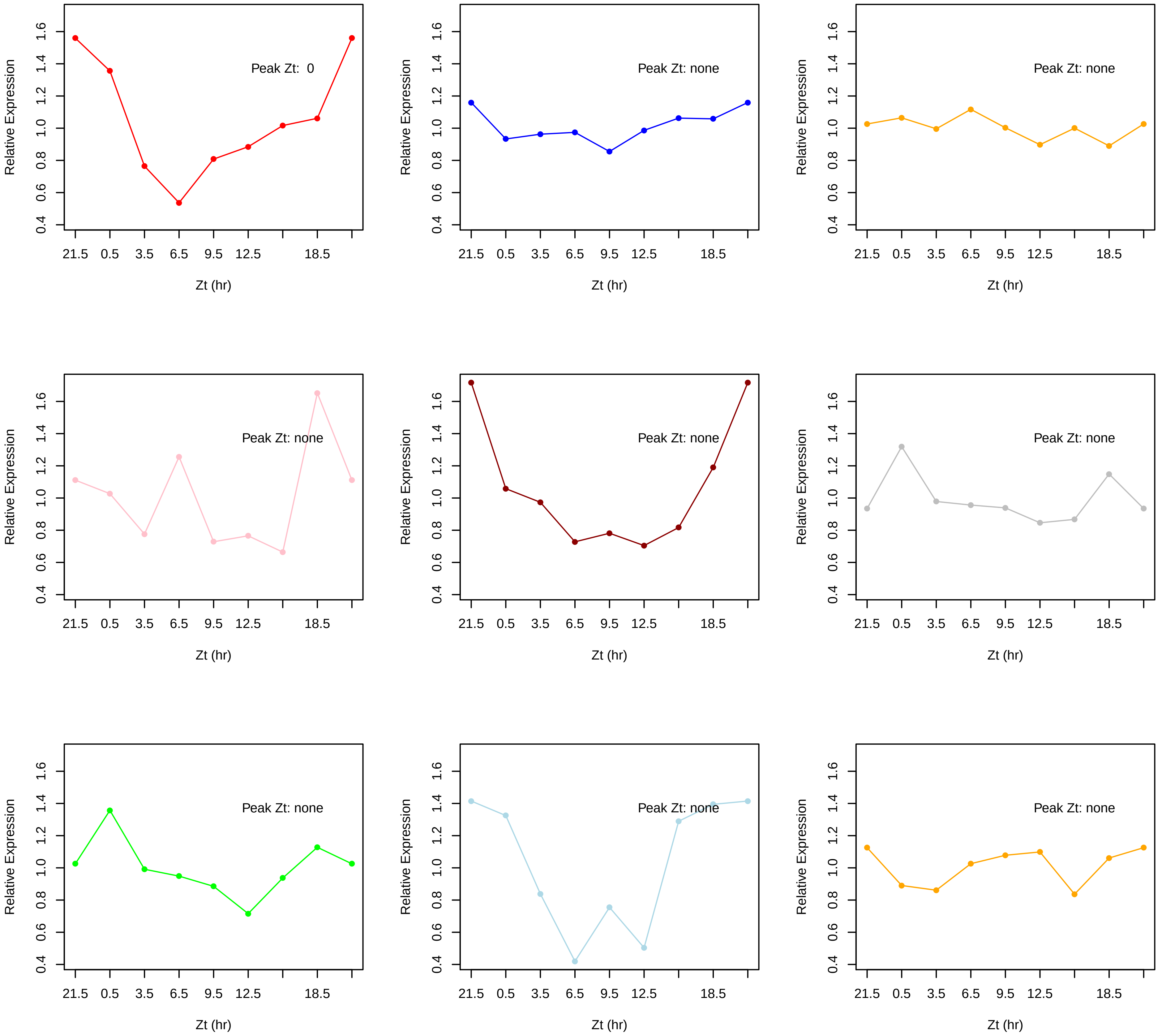

Singeleton: ENSXMAG00000016136 tjap1

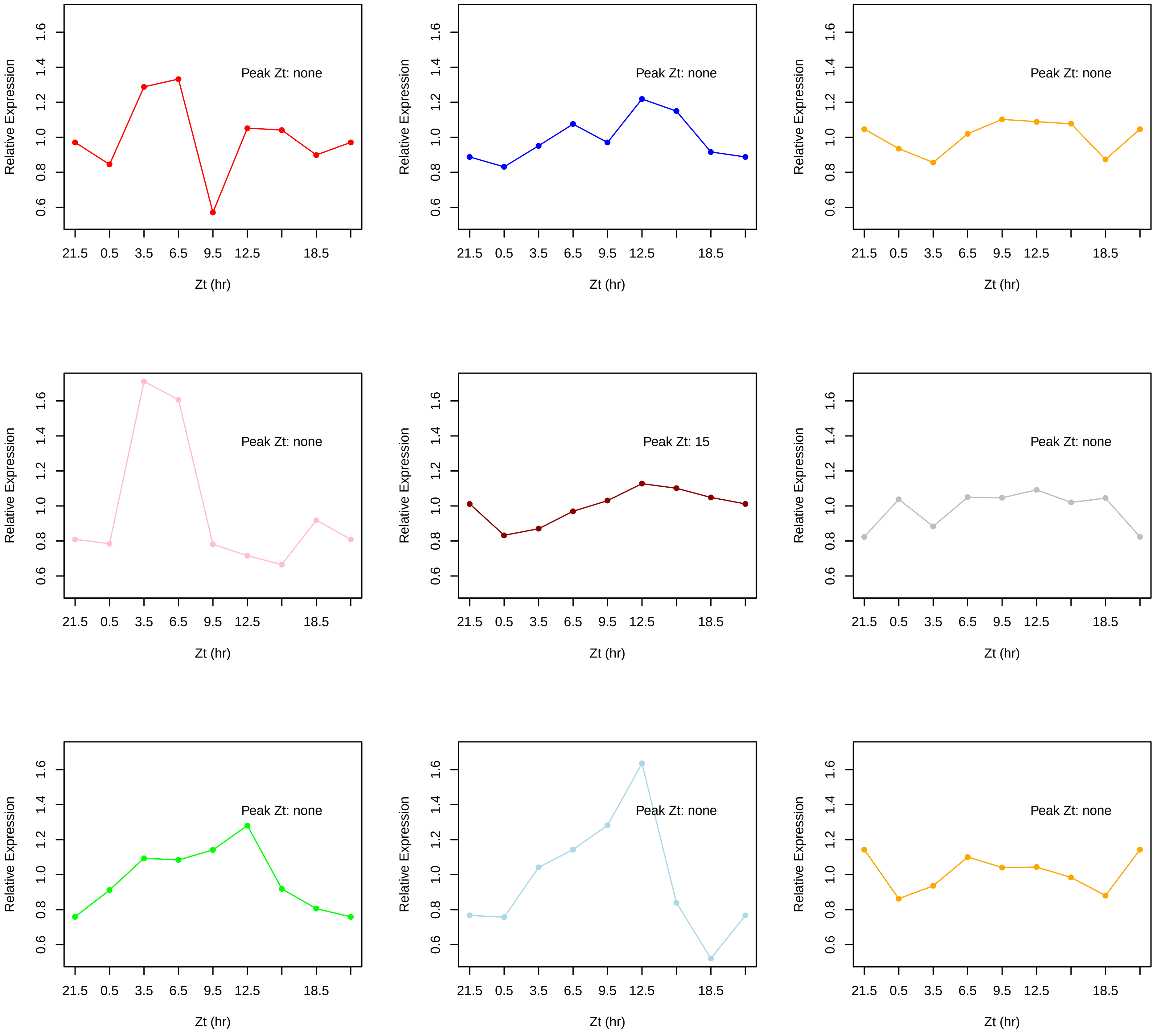

Singeleton: ENSXMAG00000009446 abcc10

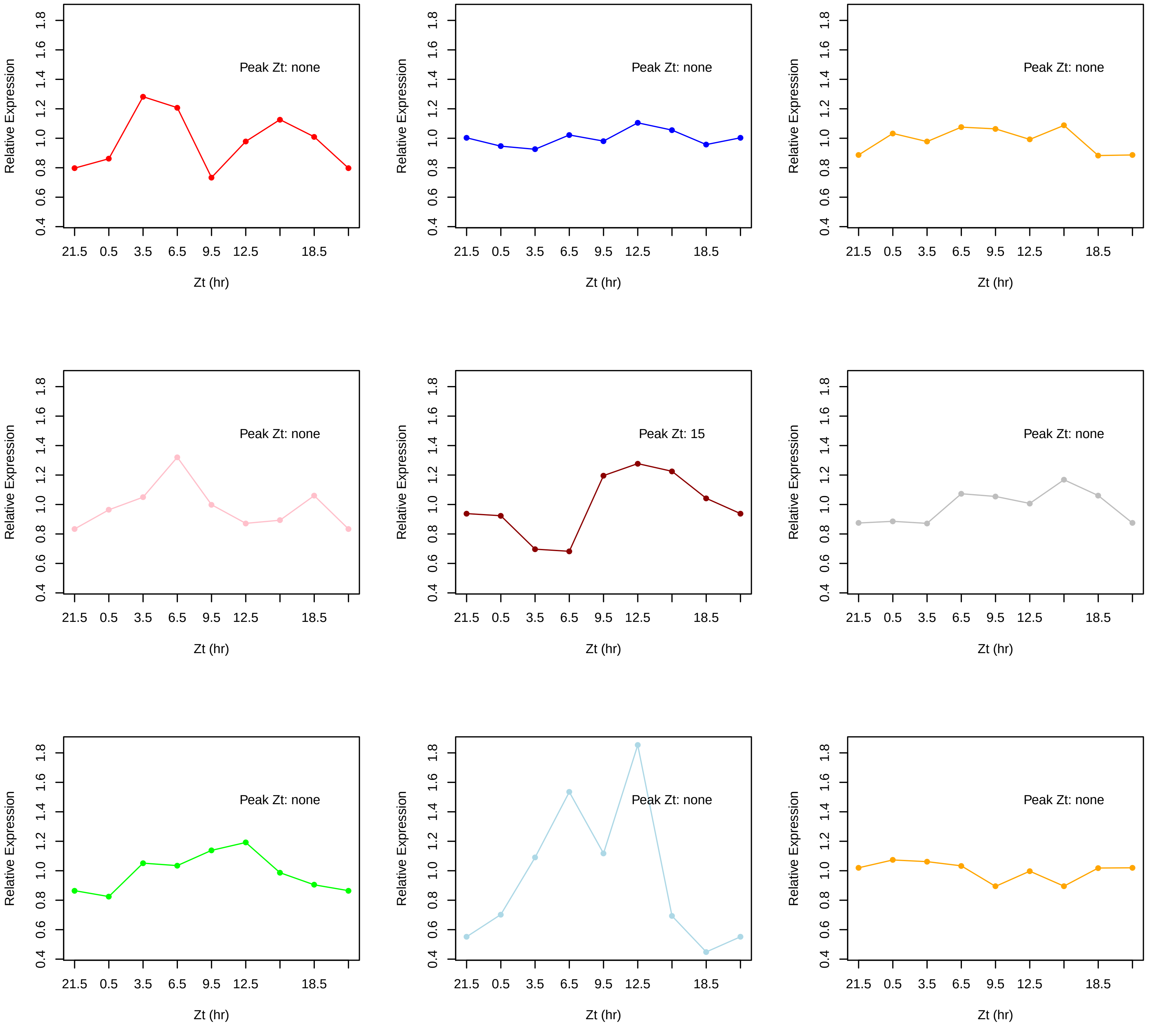

Singeleton: ENSXMAG00000004460

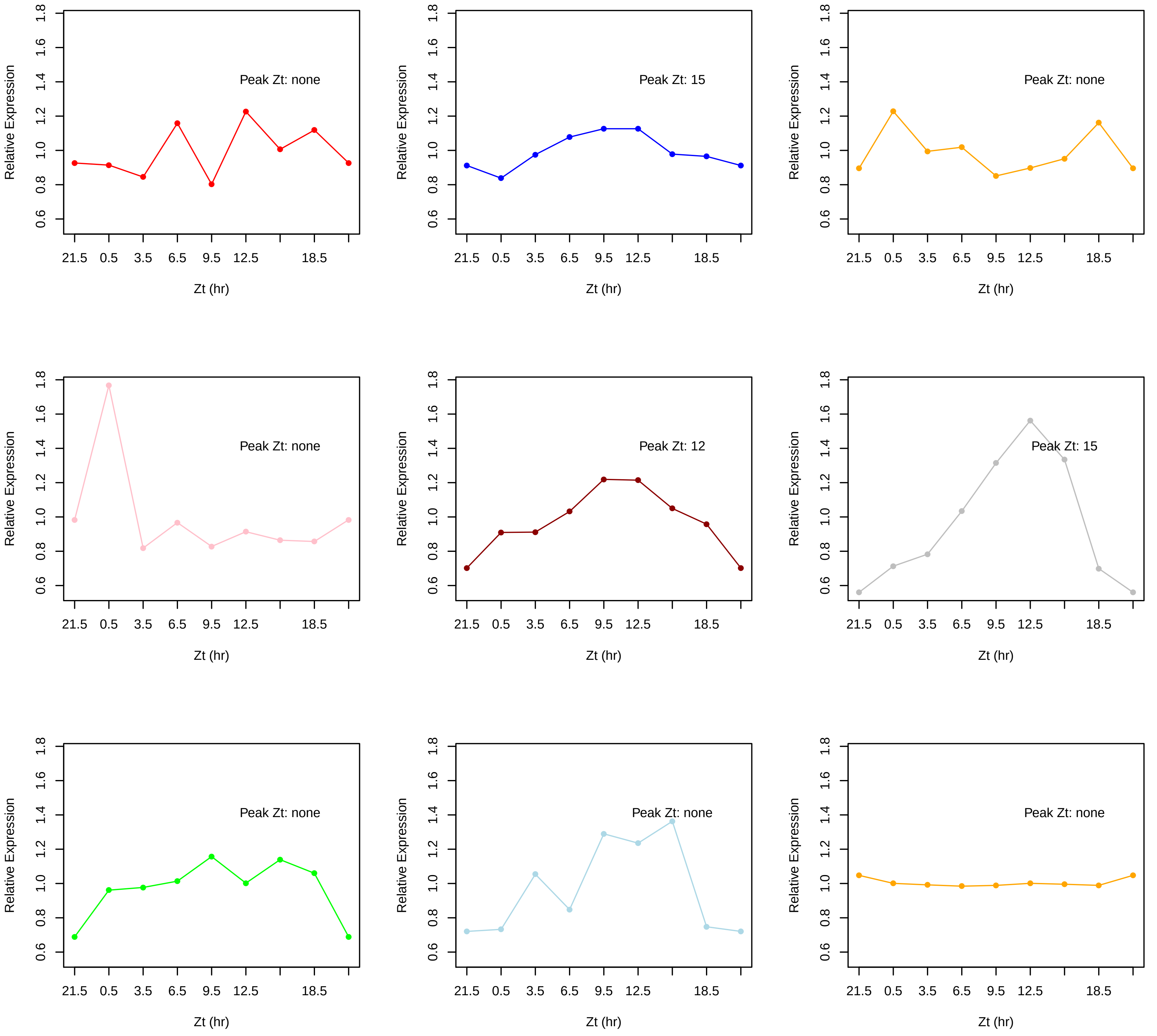

Singleton: ENSXMAG00000004713 prpf18

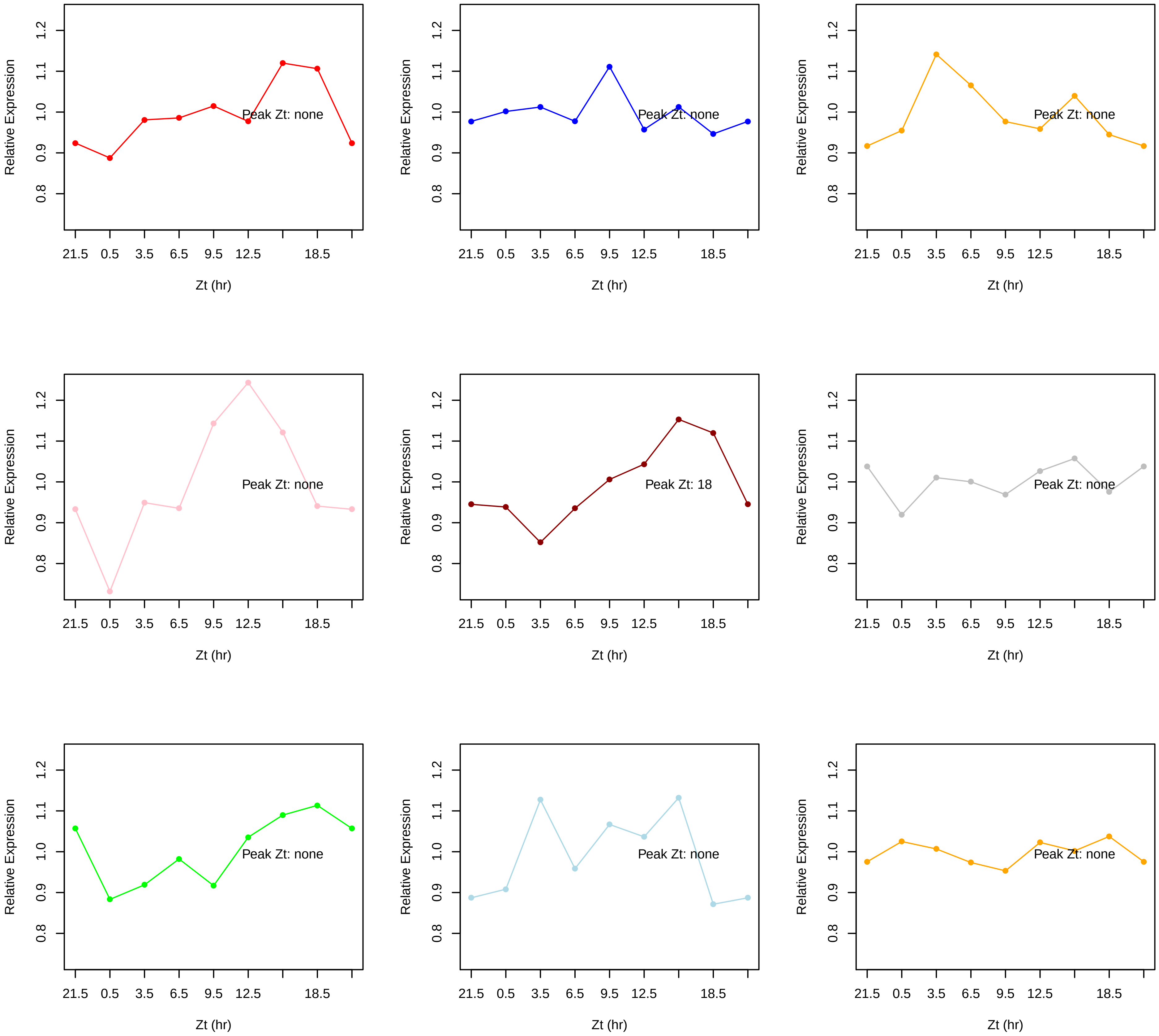

Singleton: ENSXMAG00000025860 dnph1

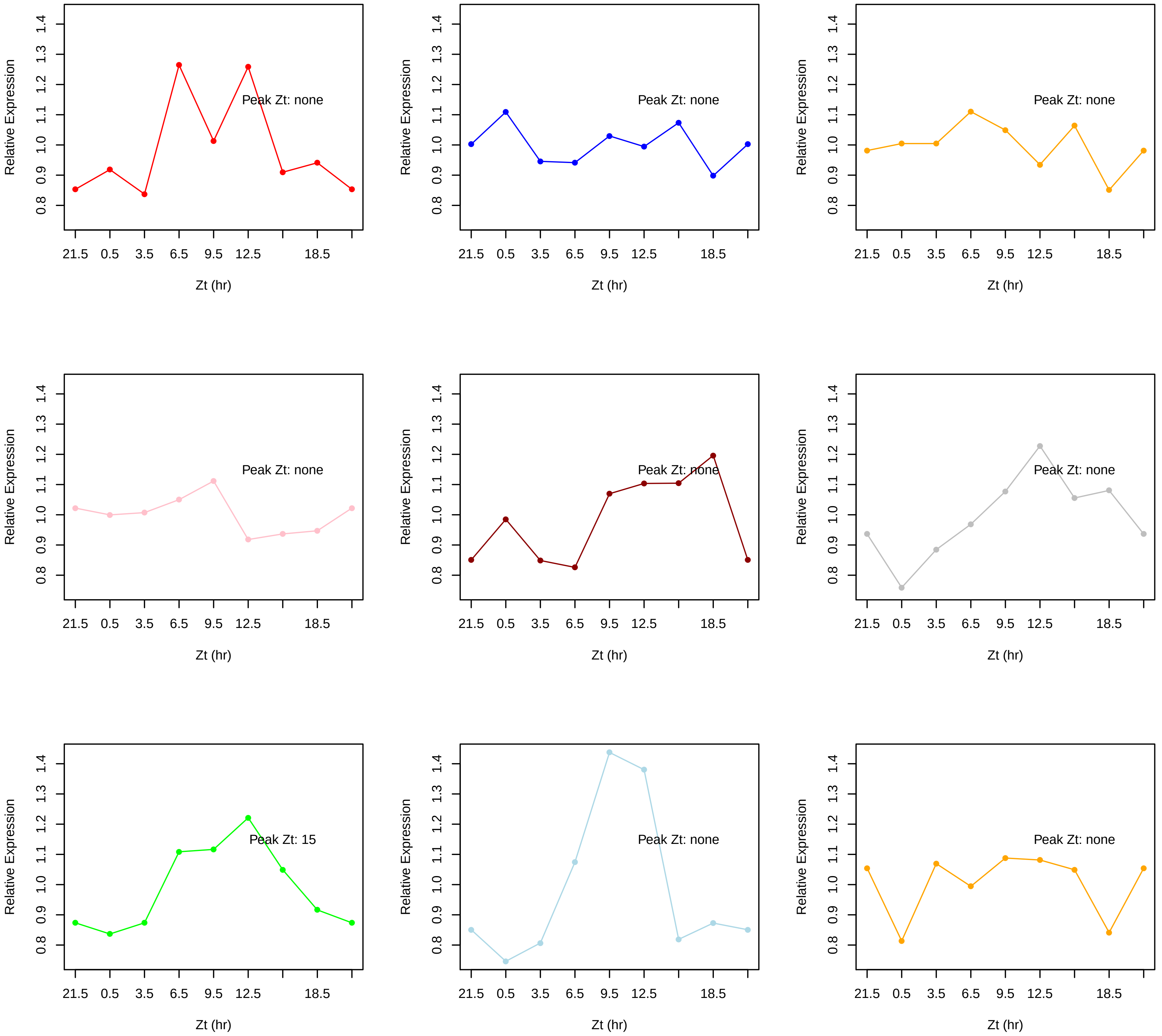

Singeleton: ENSXMAG00000004219 si:ch211−278j3.3

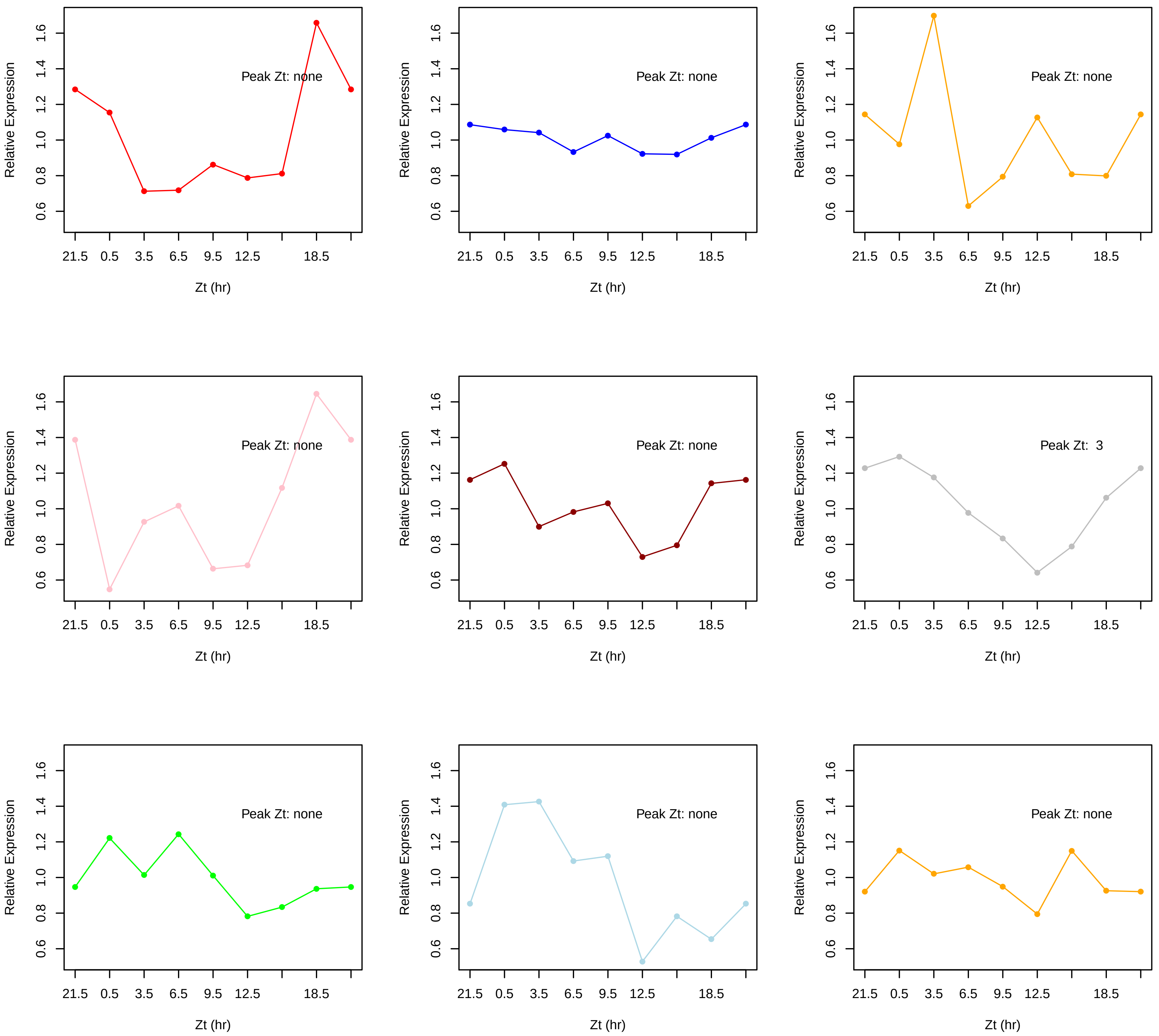

Singeleton: ENSXMAG00000004677 sephs1

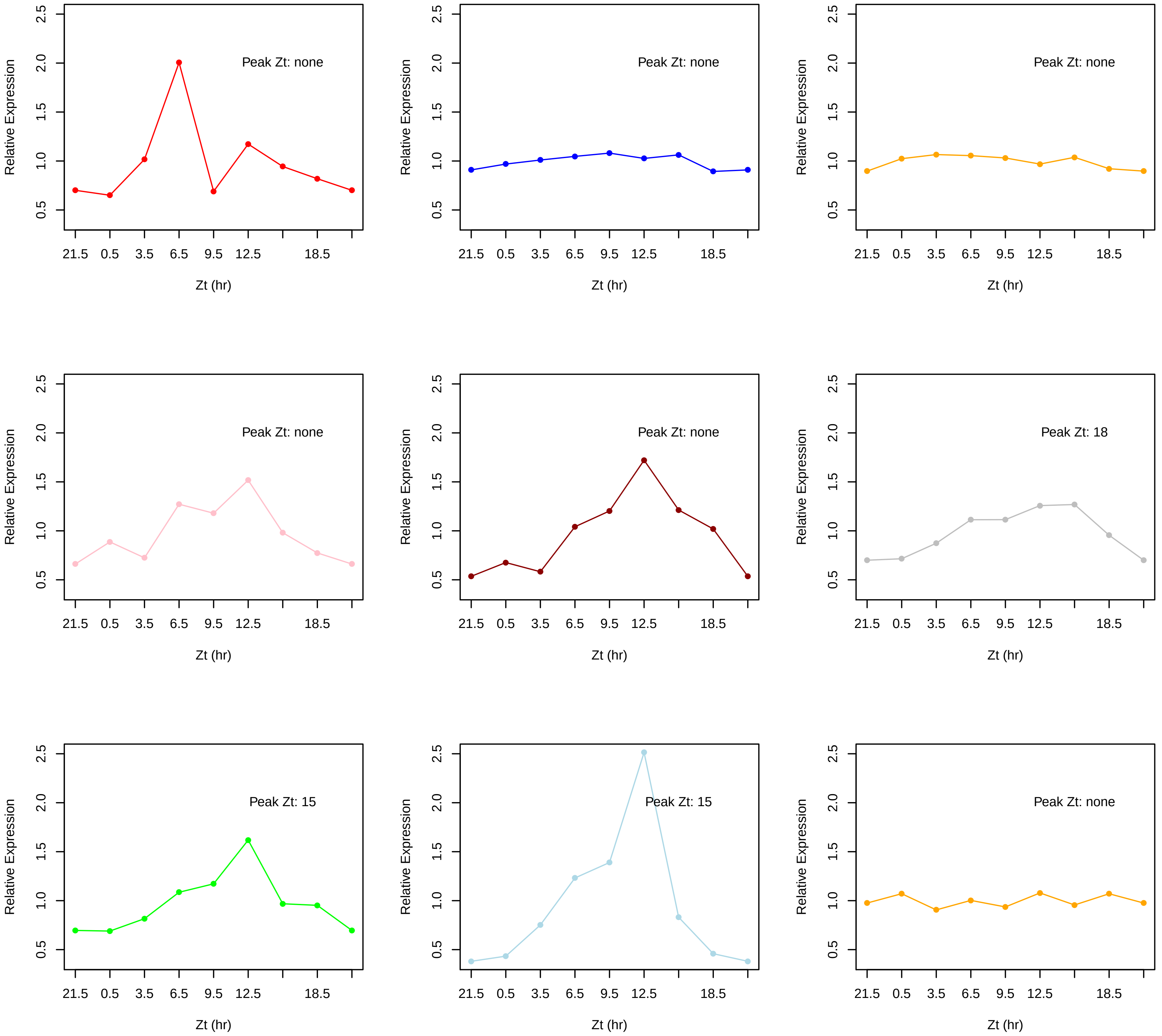

Singeleton: ENSXMAG00000004656 cpt1b

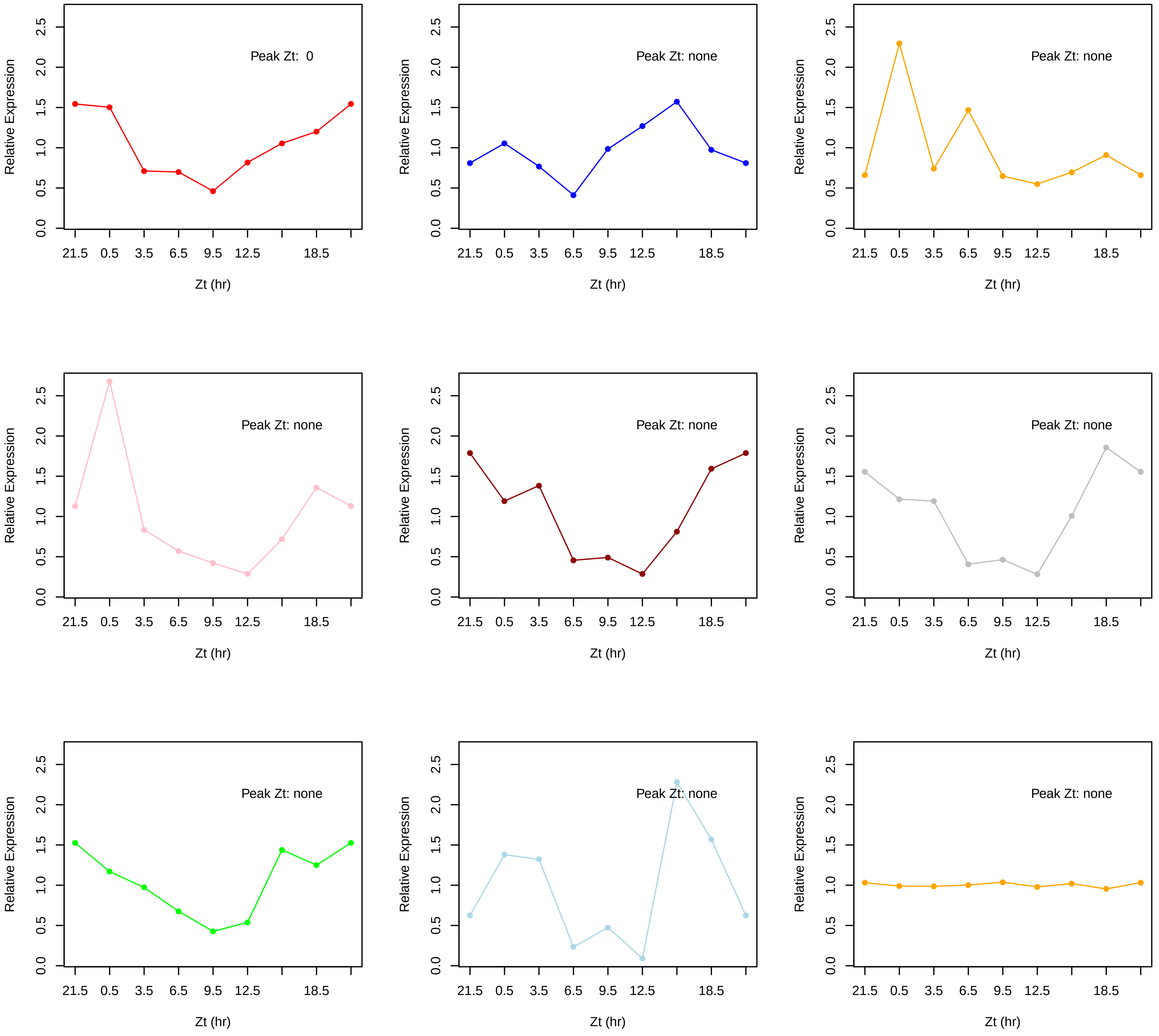

Singeleton: ENSXMAG00000007815

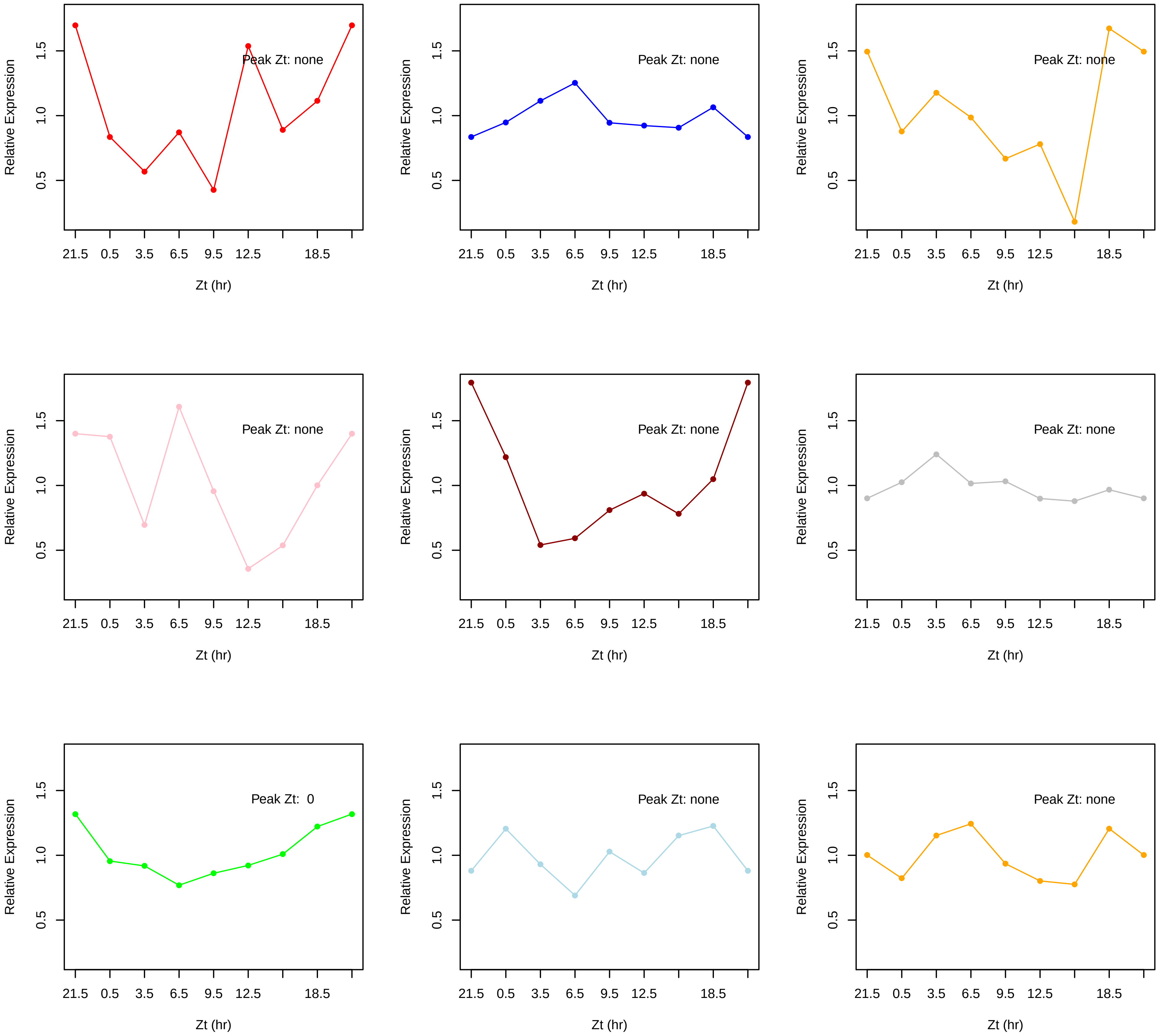

Singeleton: ENSXMAG00000027487

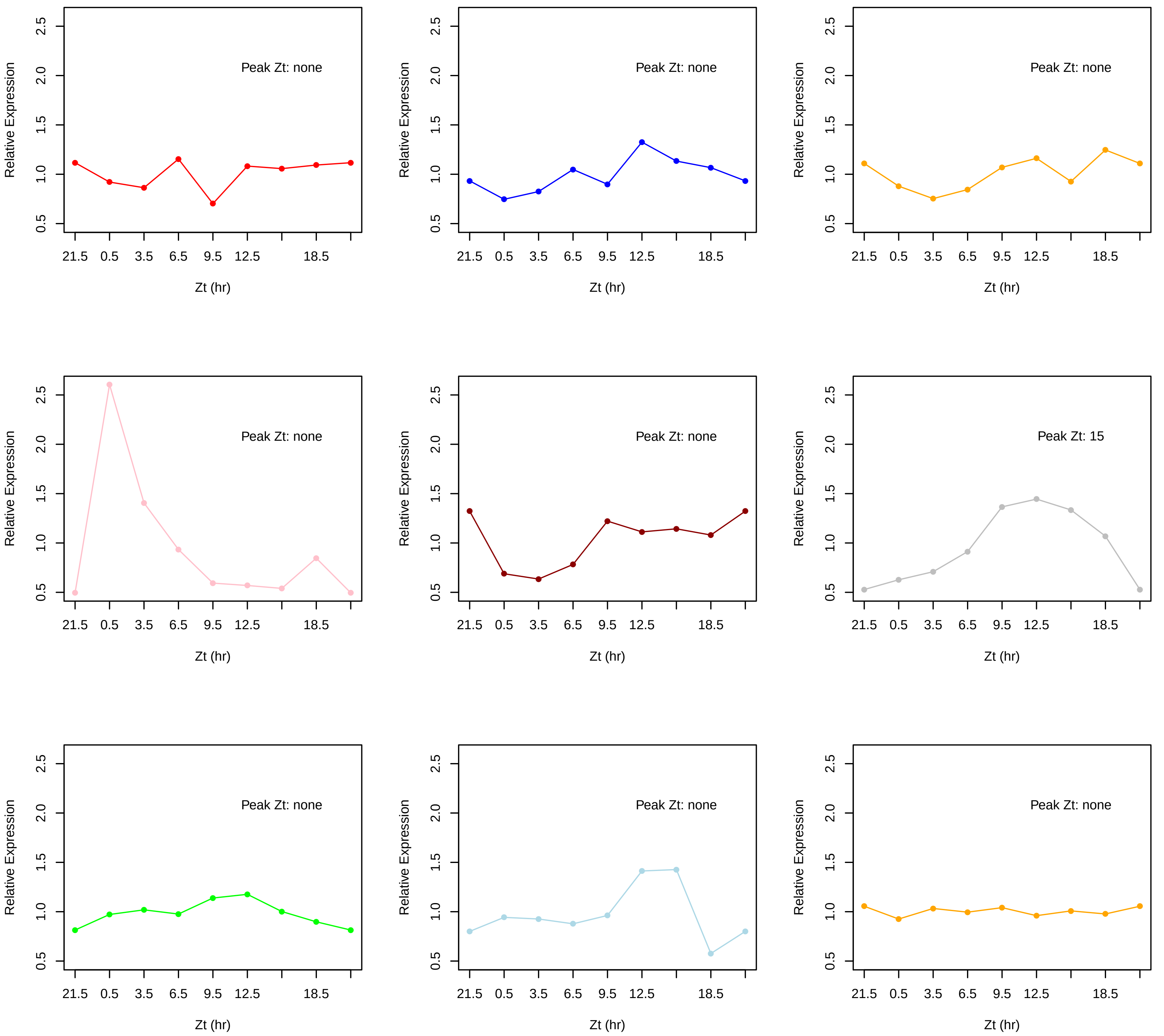

Singeleton: ENSXMAG00000022940

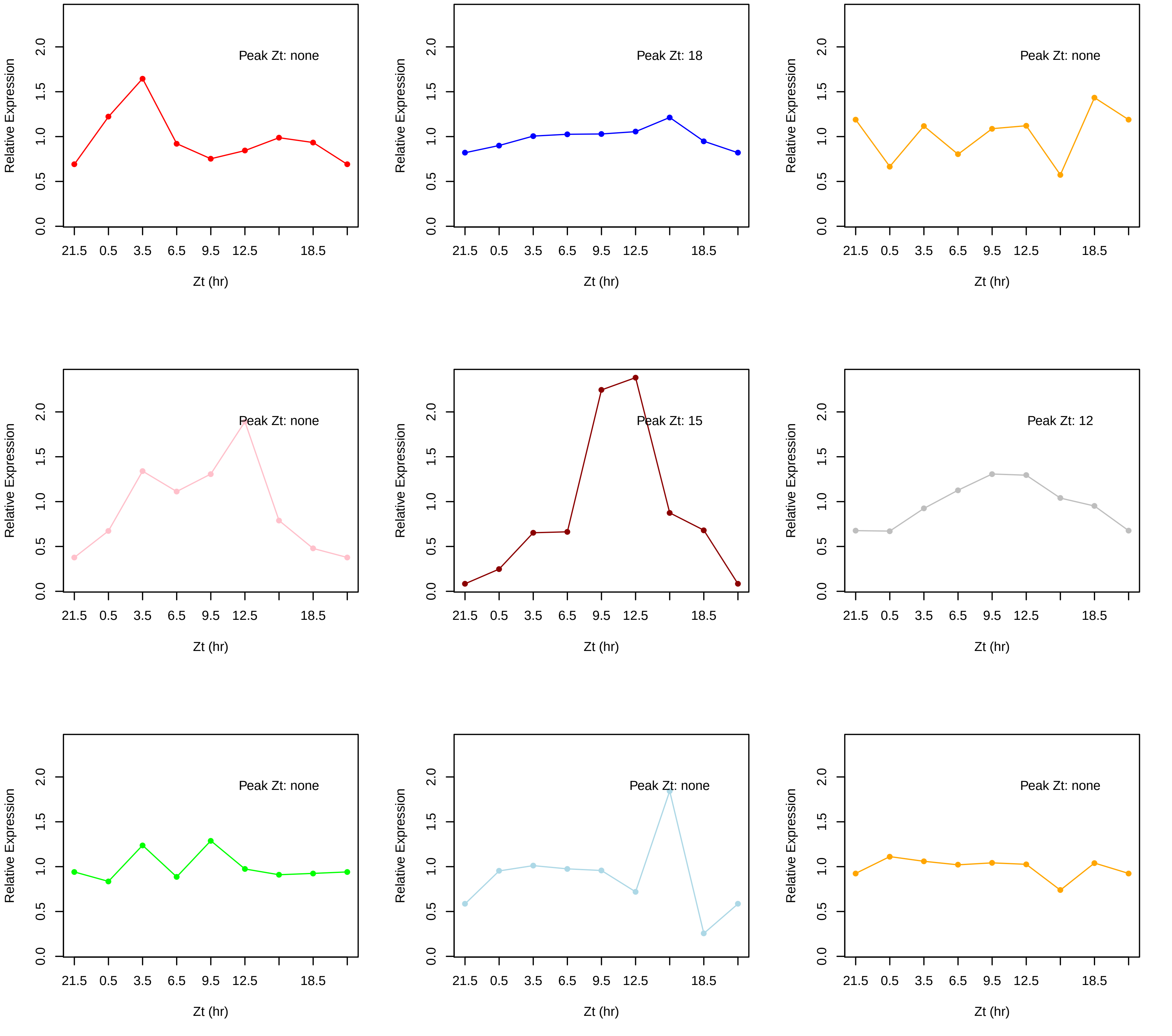

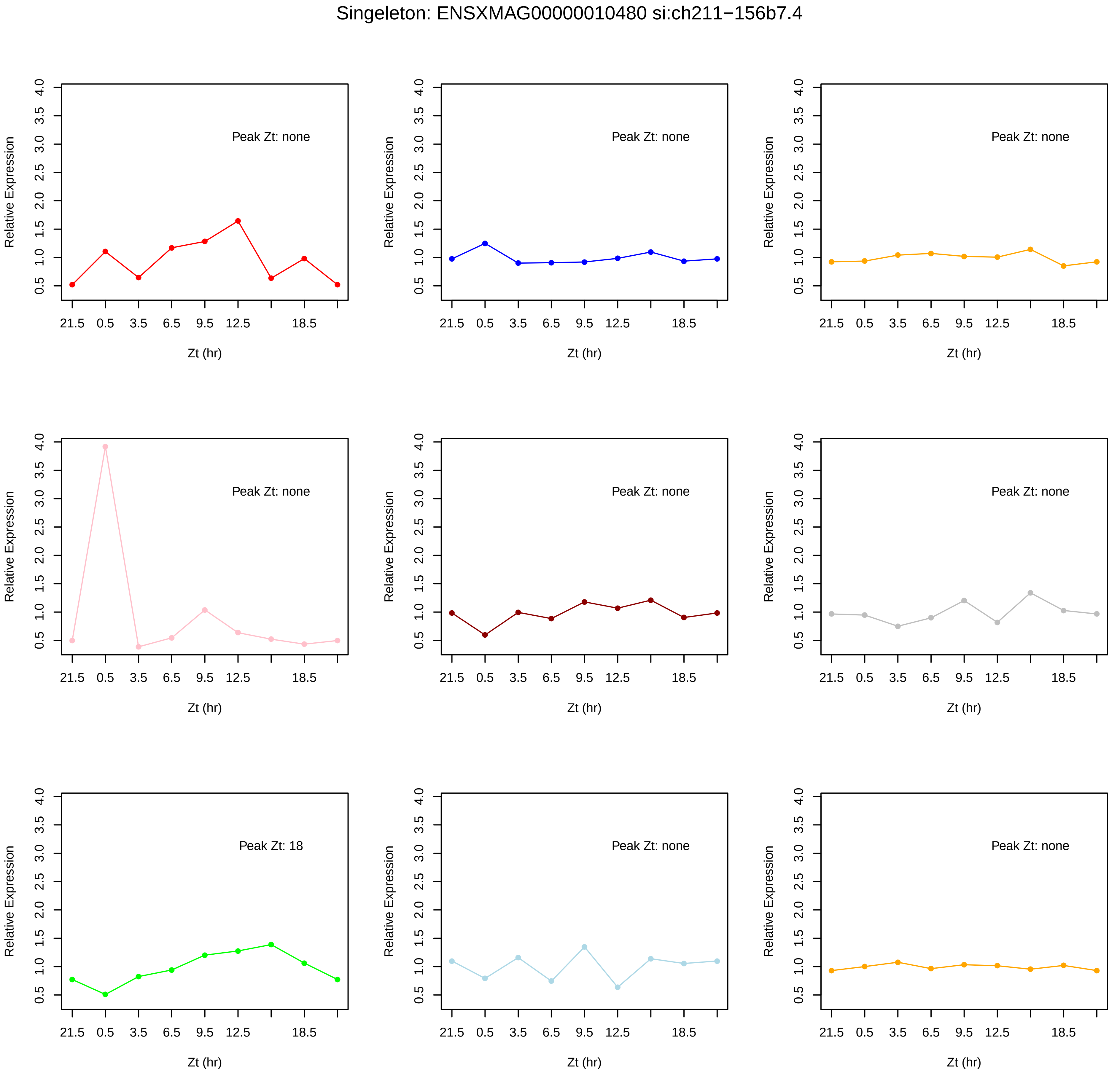
Singleton: ENSXMAG00000010480 si:ch211−156b7.4
Peak Zt: none
Peak Zt: none
Peak Zt: none
Peak Zt: none
Peak Zt: none
Peak Zt: none
Peak Zt: 18
Peak Zt: none
Peak Zt: none
Relative Expression
Zt (hr)
21.5 0.5 3.5 6.5 9.5 12.5 18.5
0.5 1.0 1.5 2.0 2.5 3.0 3.5 4.0

Singeleton: ENSXMAG00000007812

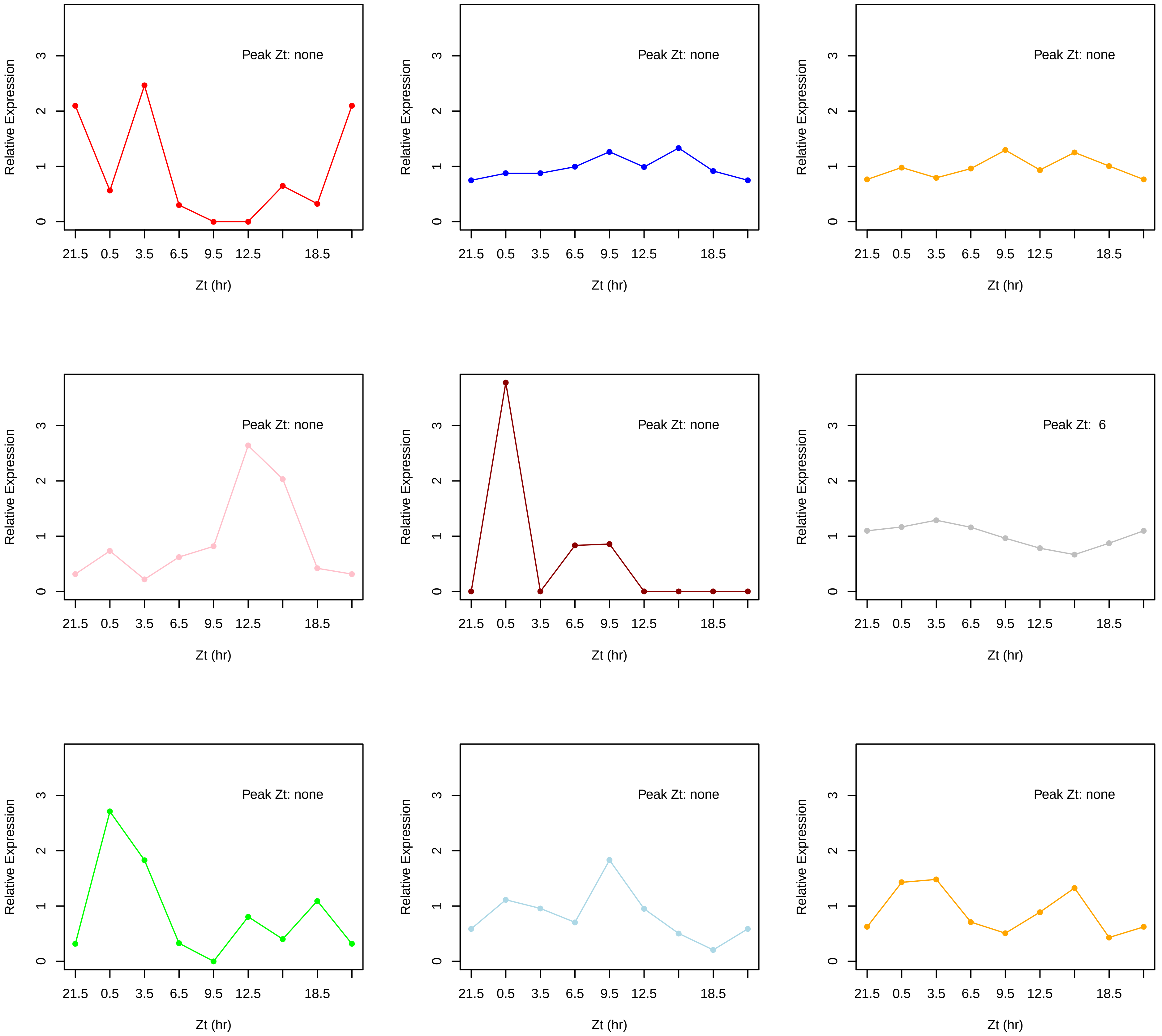

Singeleton: ENSXMAG00000004618 arsa

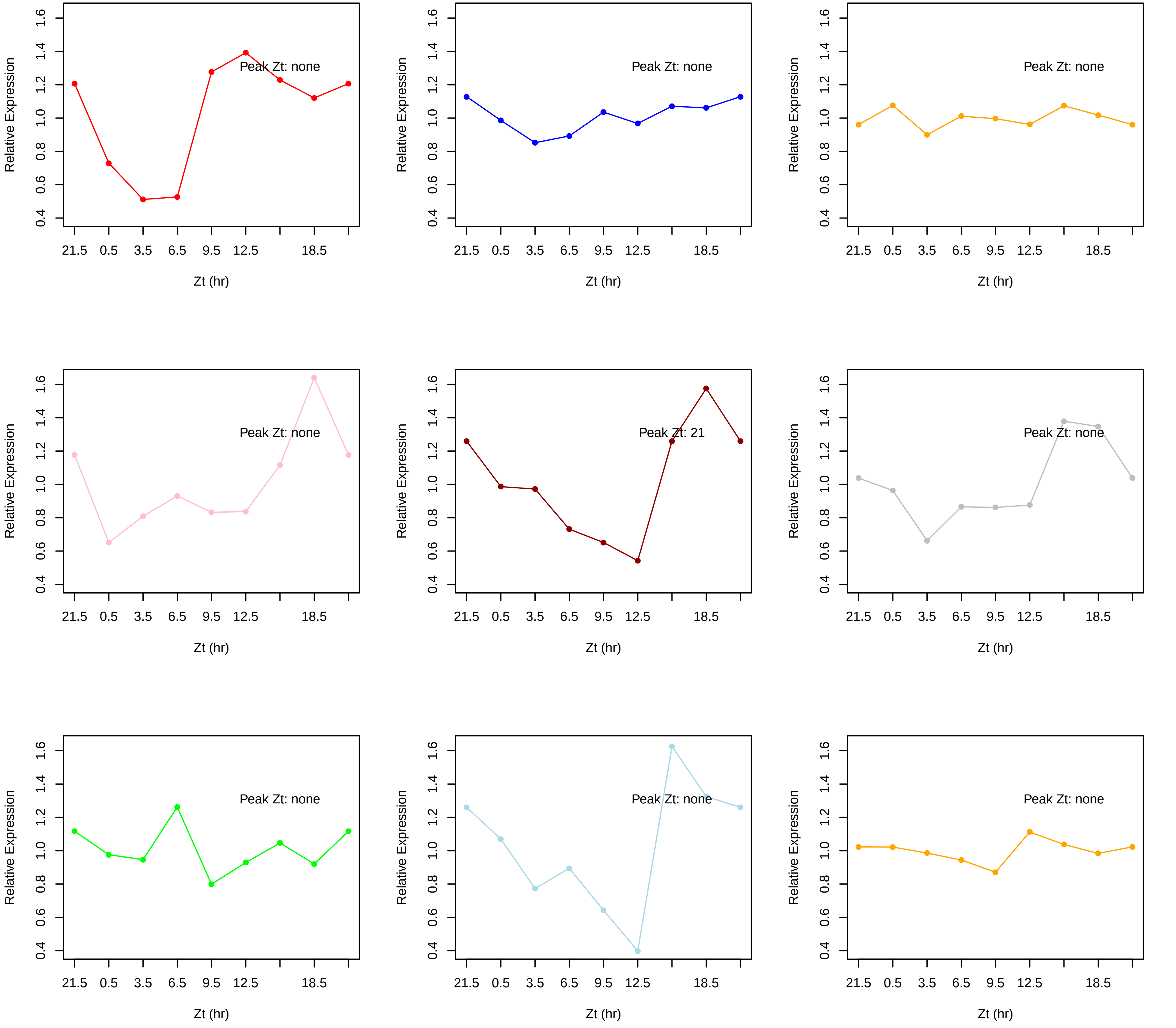

Singeleton: ENSXMAG00000027197 pinx1

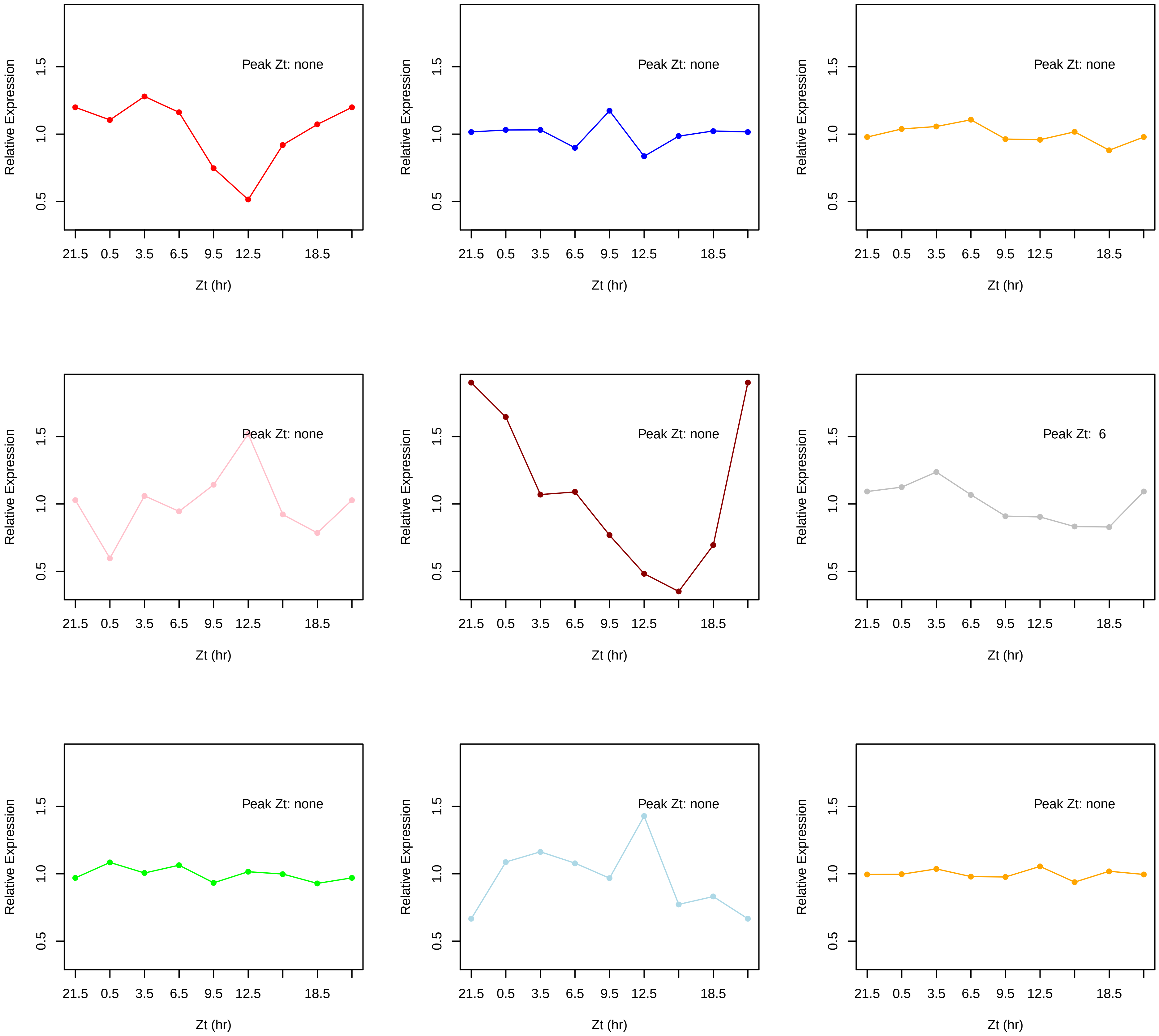

Singeleton: ENSXMAG00000004234 tdh

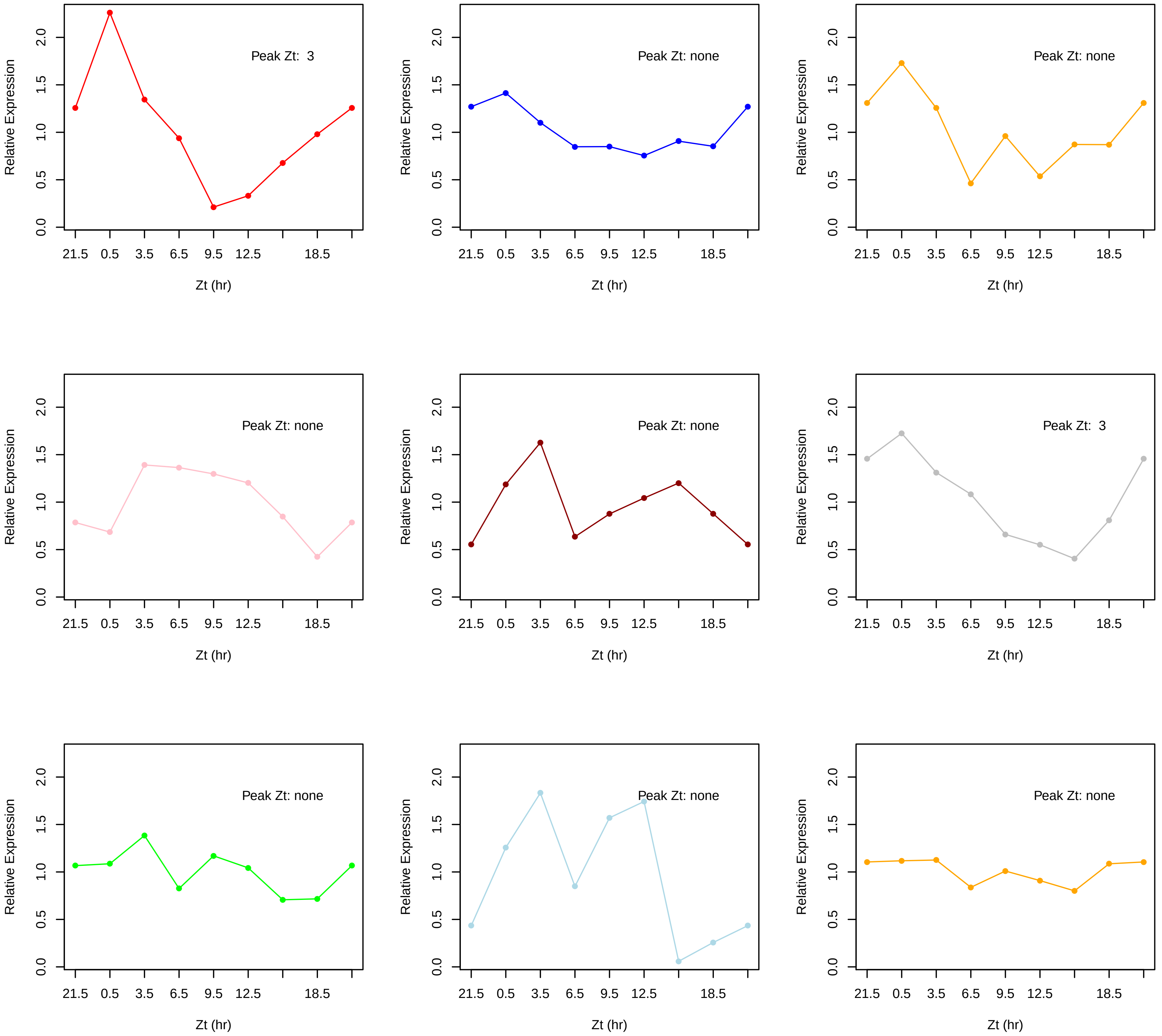

Singeleton: ENSXMAG00000016077 abhd12

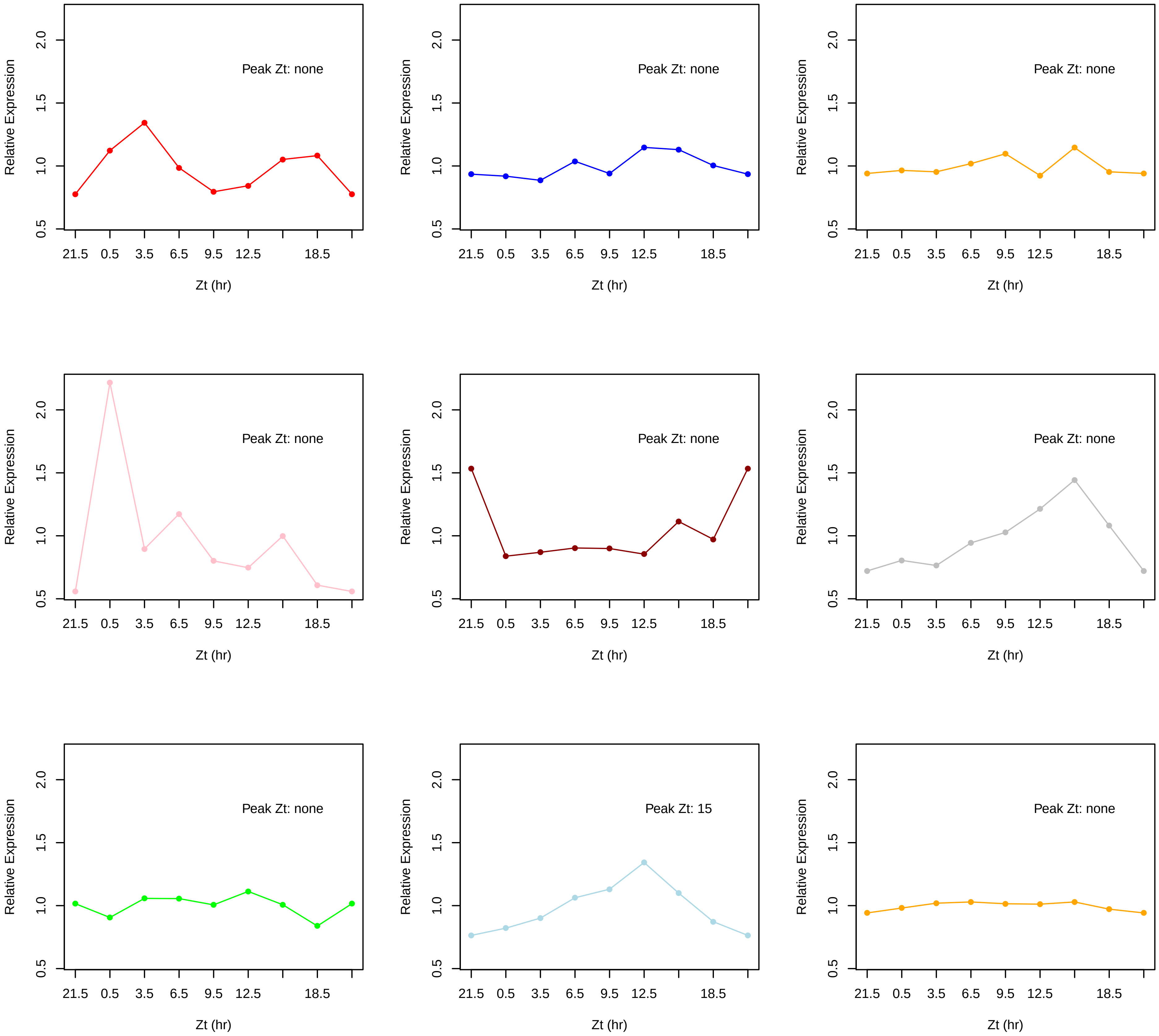

Singeleton: ENSXMAG00000004240 mtmr9

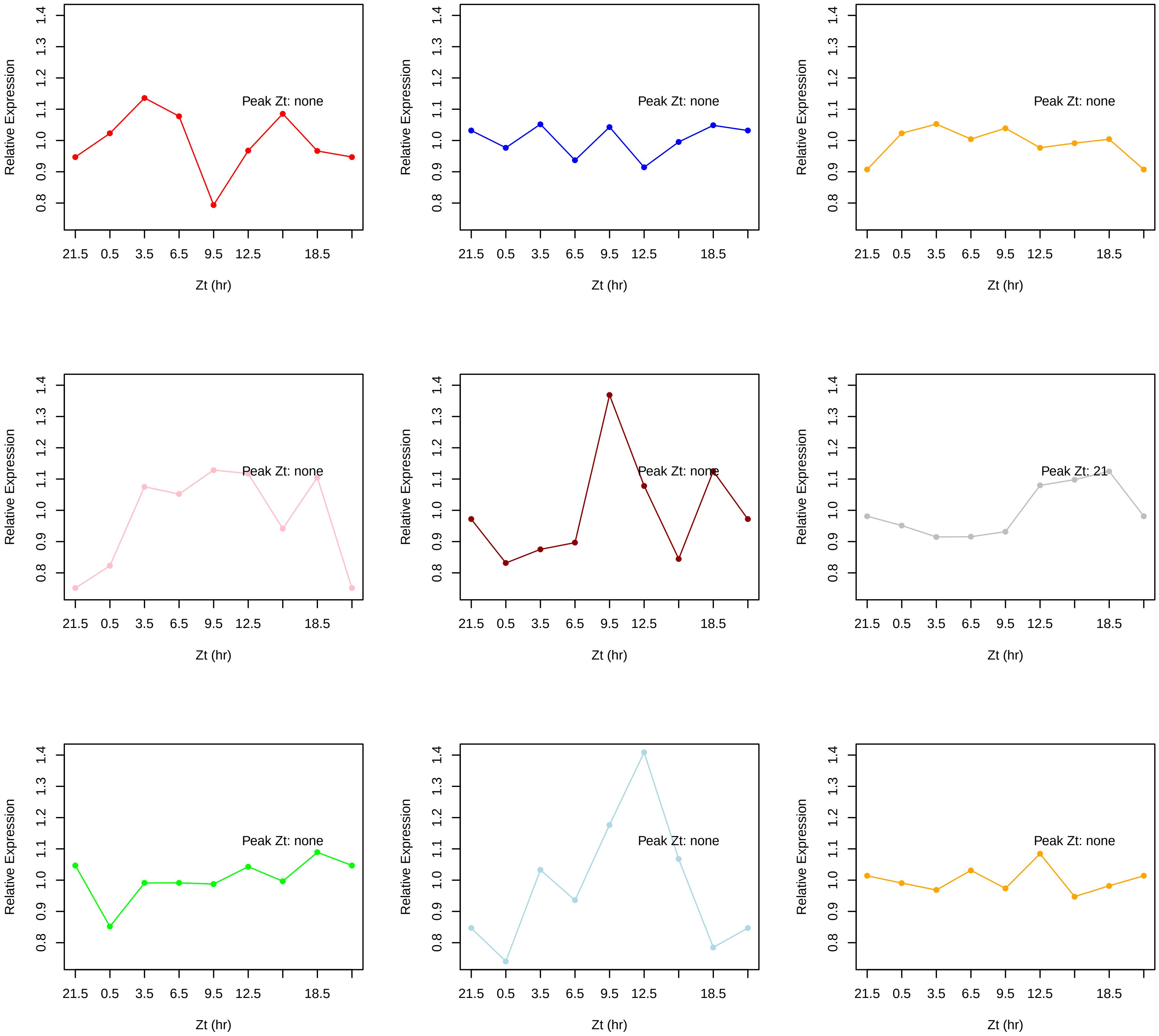

Singeleton: ENSXMAG00000010835 ccdc25

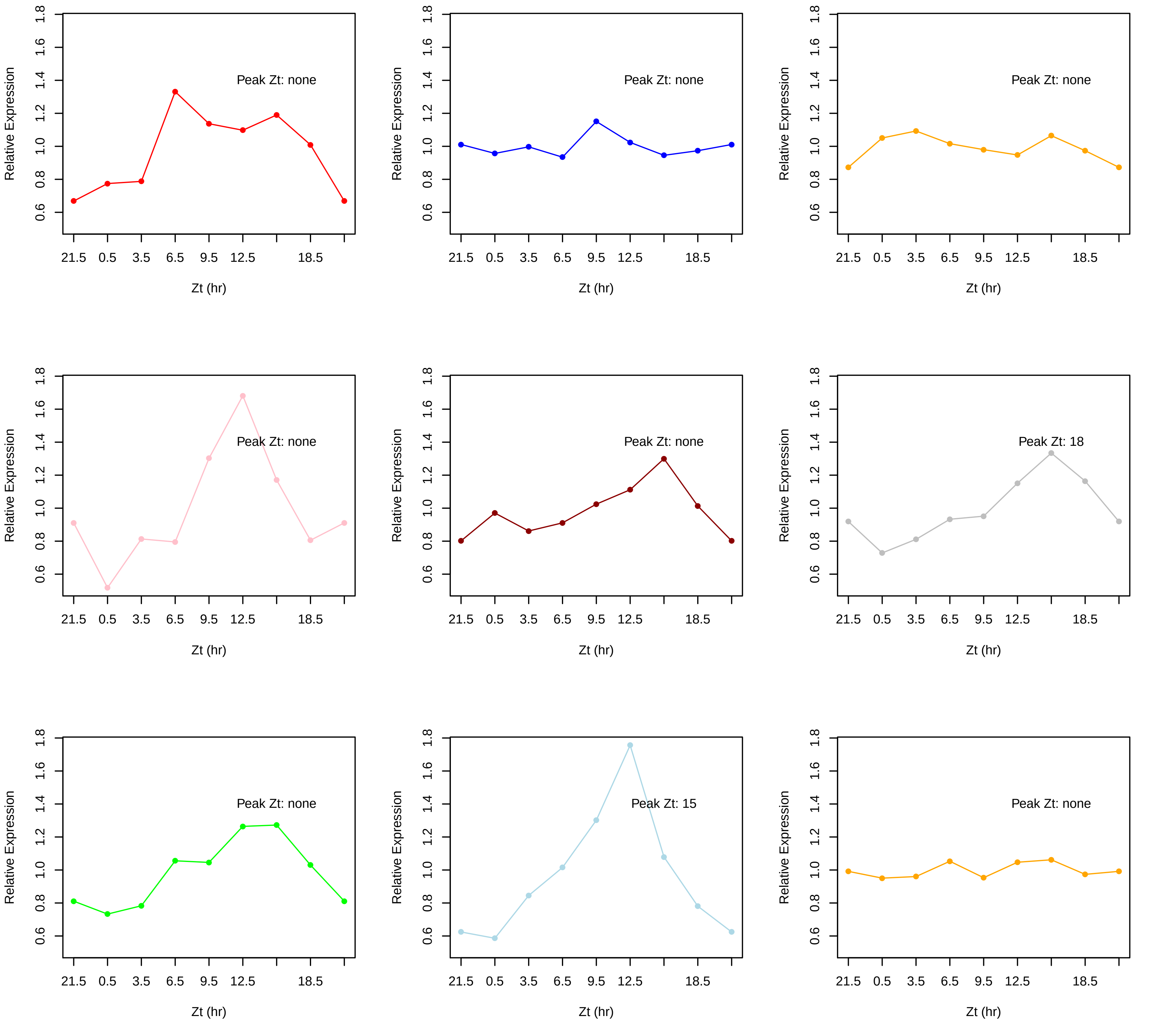

Singleton: ENSXMAG00000027828 lamtor4

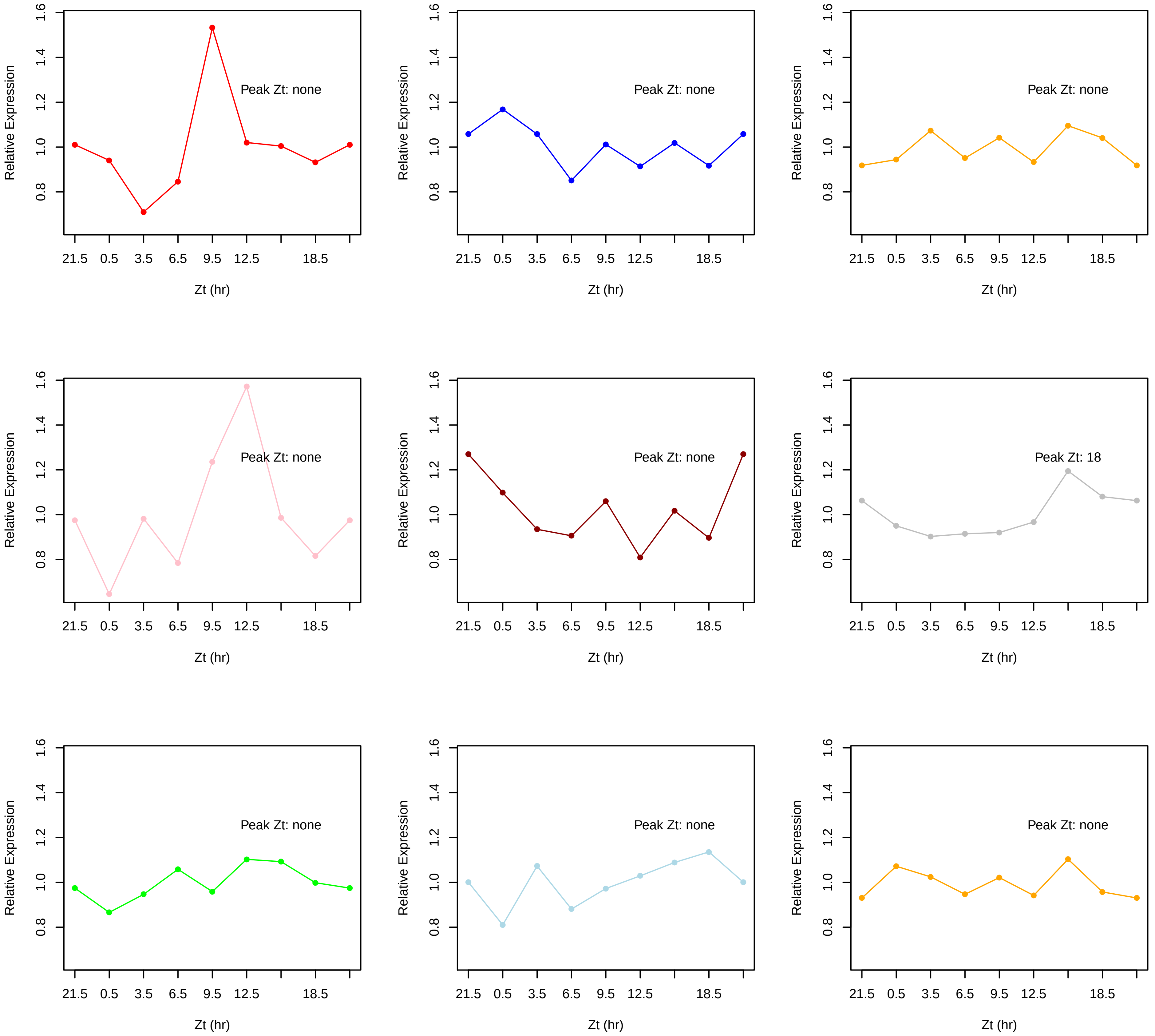

Singeleton: ENSXMAG00000004556 si:dkey−30c15.2

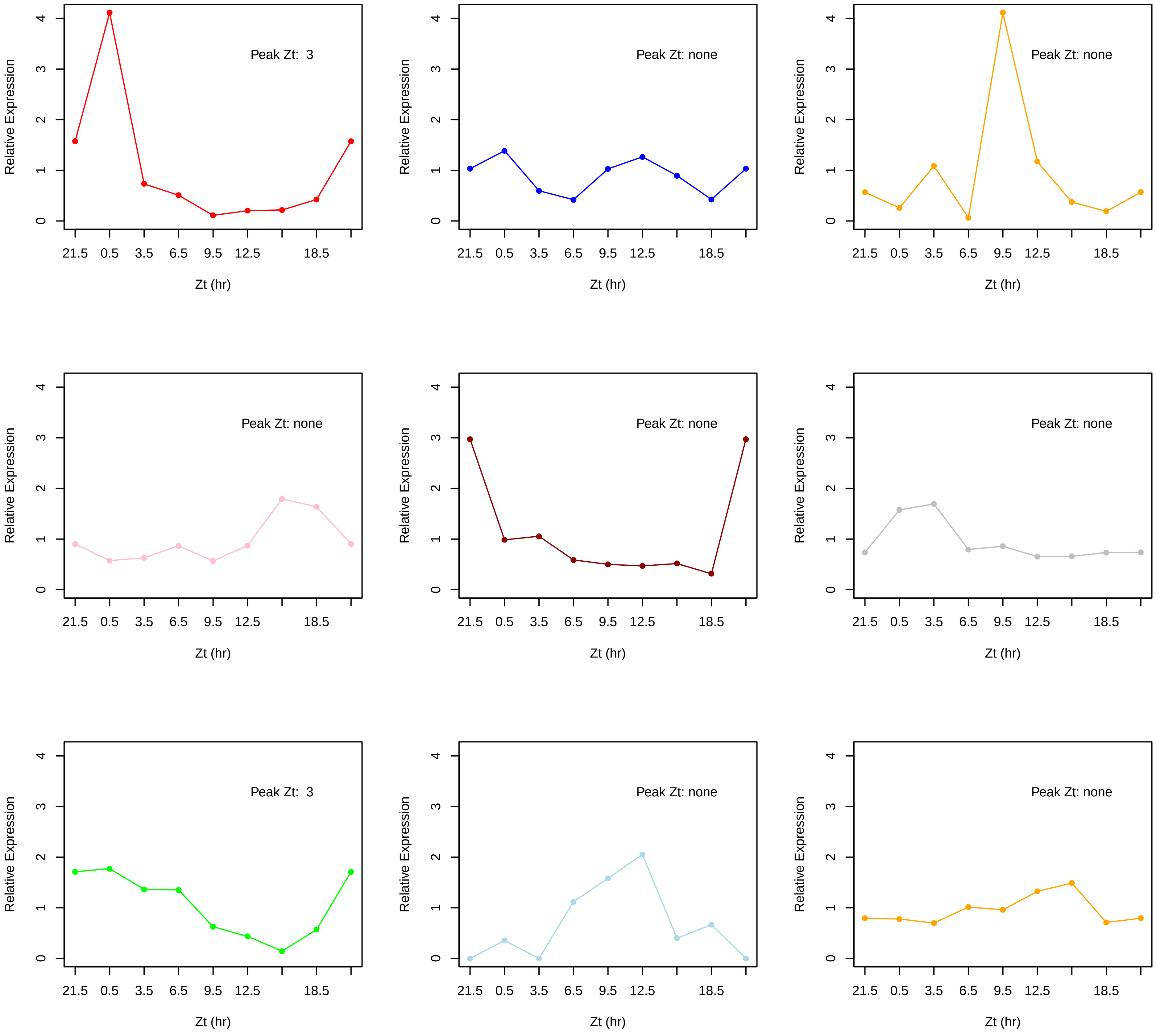

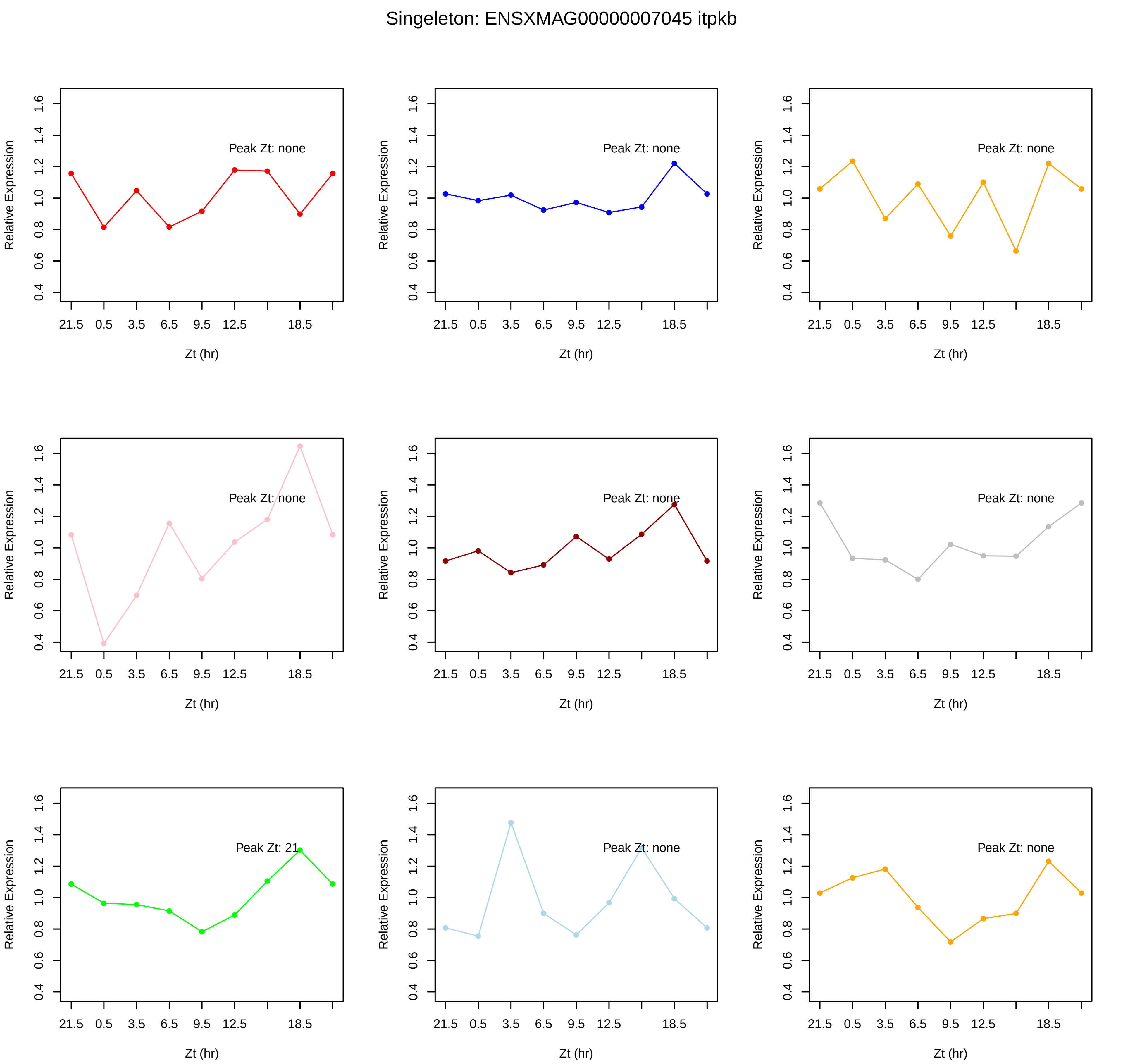
Singeleton: ENSXMAG00000007045 itpkb
Peak Zt: none
Peak Zt: none
Peak Zt: none
Peak Zt: none
Peak Zt: none
Peak Zt: none
Peak Zt: 21
Peak Zt: none
Peak Zt: none
Relative Expression
Zt (hr)
21.5
0.5
3.5
6.5
9.5
12.5
18.5
0.4
0.6
0.8
1.0
1.2
1.4
1.6

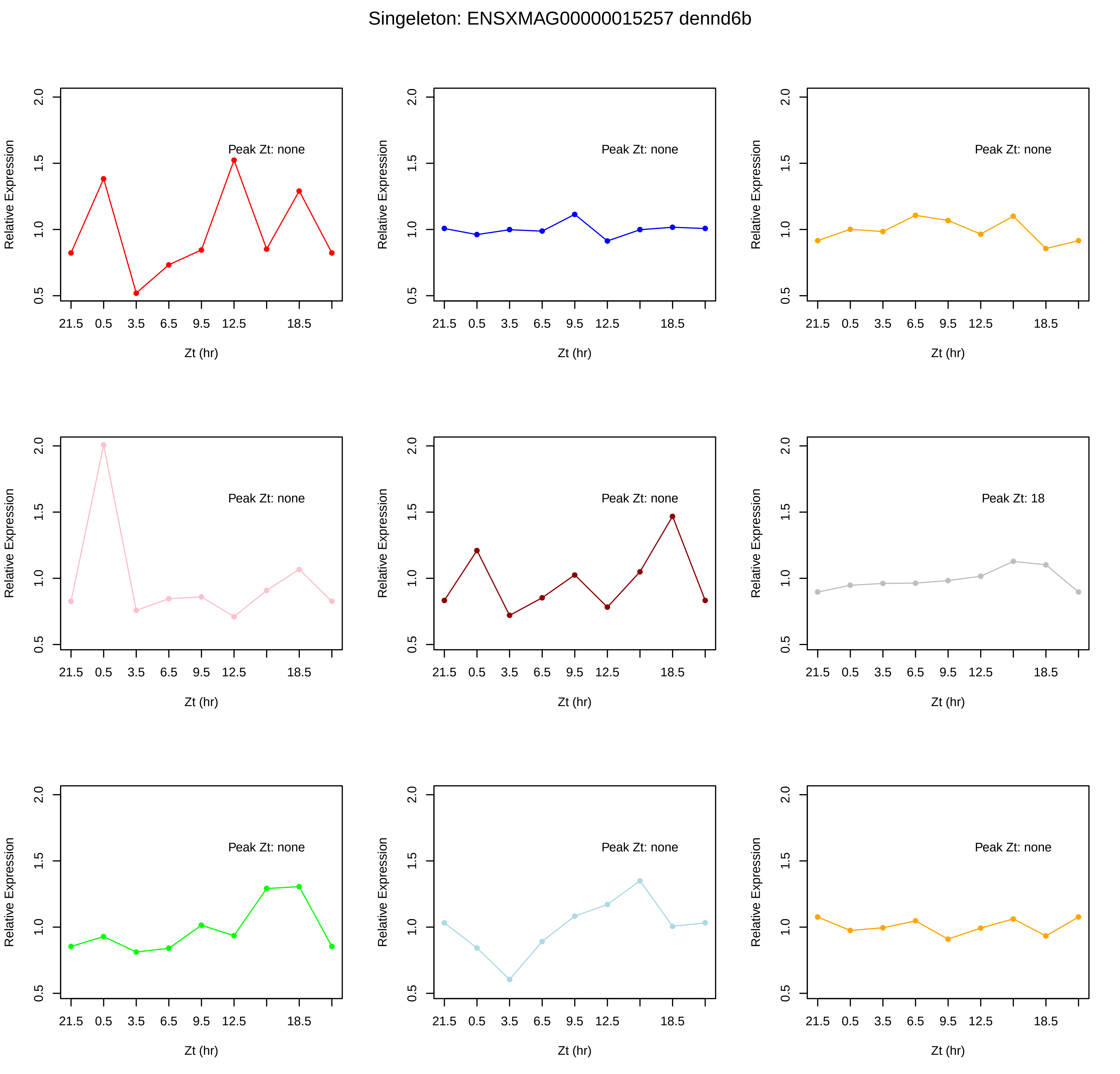
Singeleton: ENSXMAG00000015257 dennd6b
Peak Zt: none
Peak Zt: none
Peak Zt: none
Peak Zt: none
Peak Zt: none
Peak Zt: 18
Peak Zt: none
Peak Zt: none
Peak Zt: none
Relative Expression
Zt (hr)
21.5
0.5
3.5
6.5
9.5
12.5
18.5
0.5
1.0
1.5
2.0

Singleton: ENSXMAG00000016104 entpd6

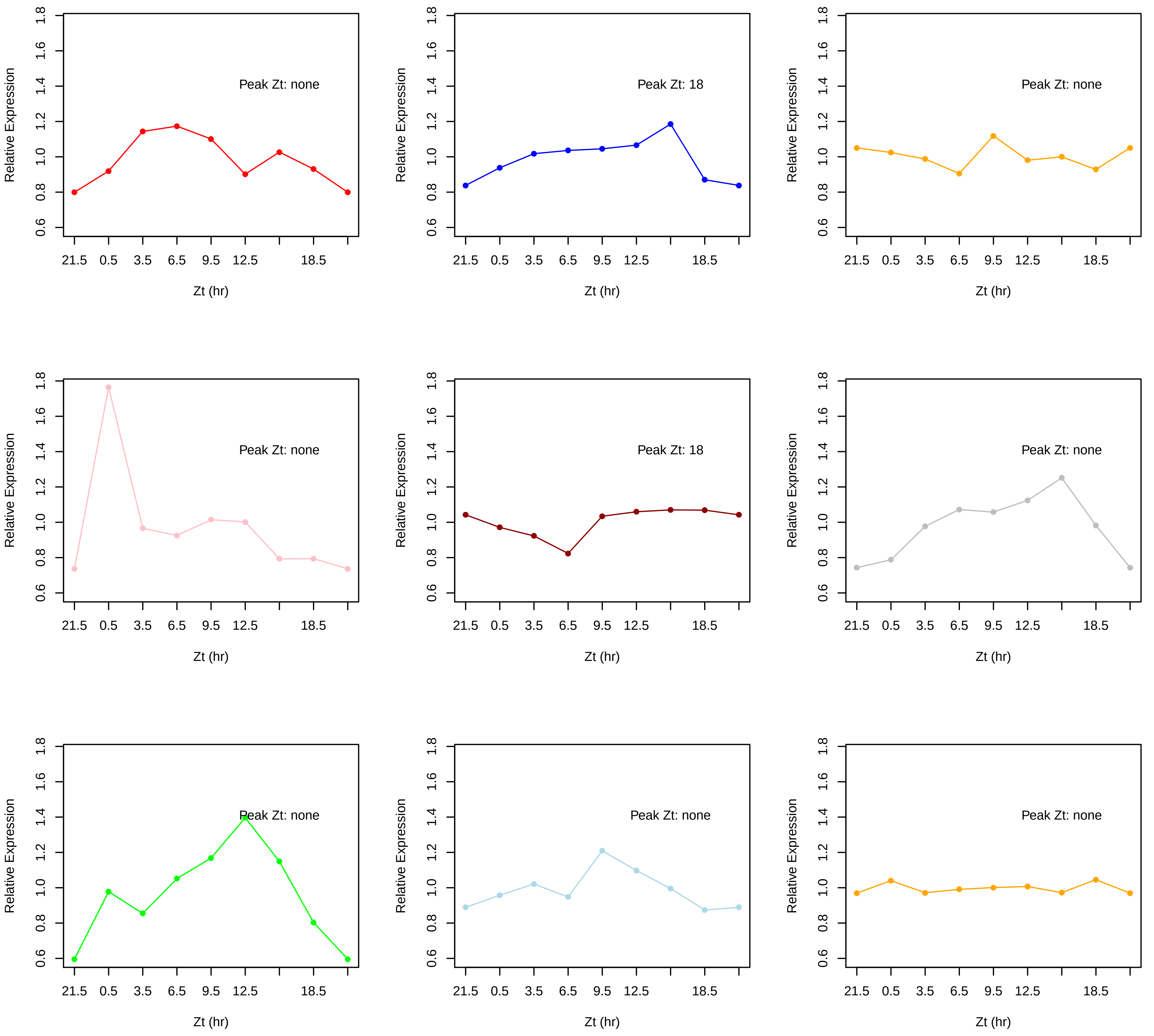

Singeleton: ENSXMAG00000025261 ehd3

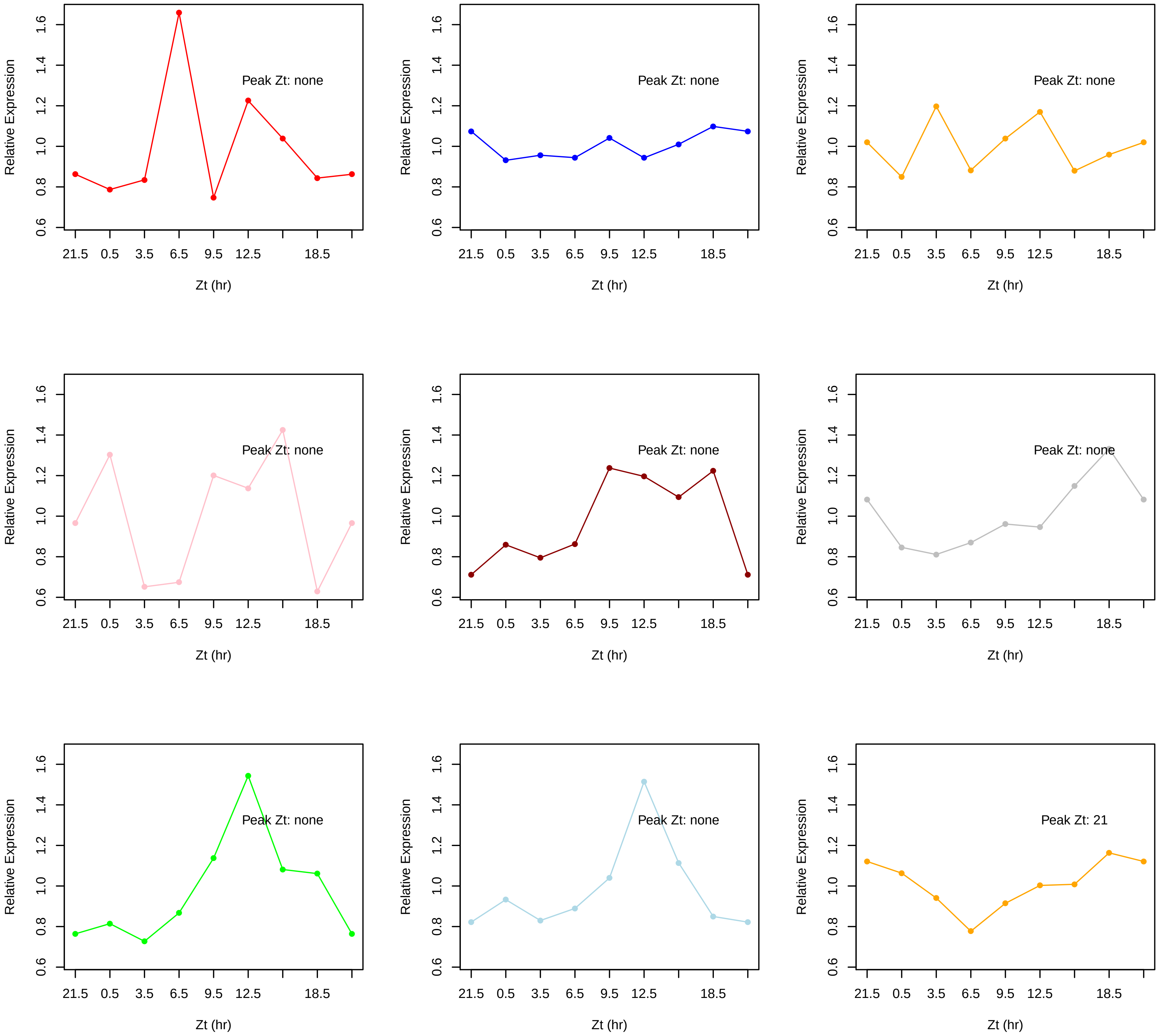

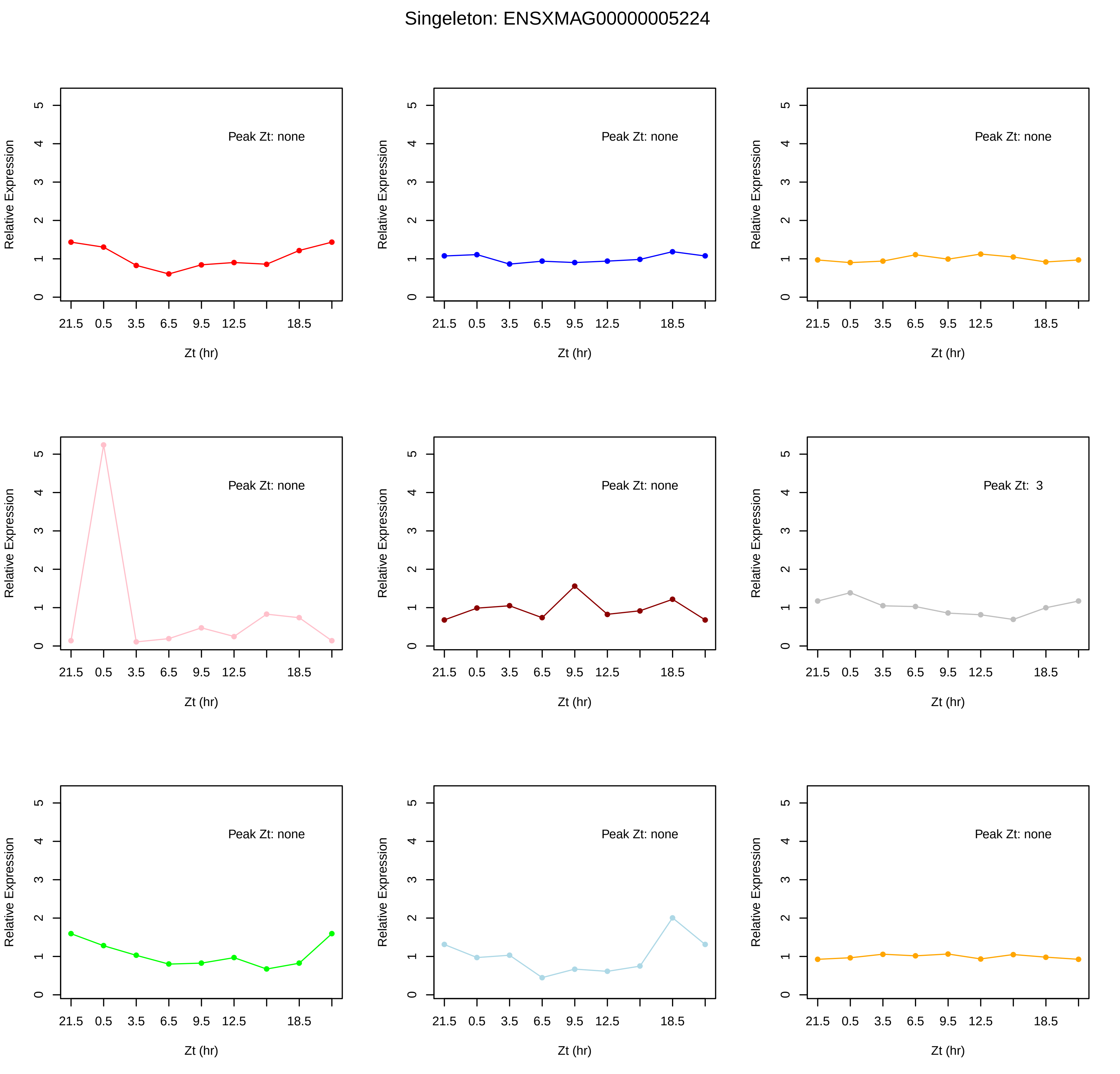

Singeleton: ENSXMAG00000005224
Peak Zt: none
Peak Zt: none
Peak Zt: none
Peak Zt: none
Peak Zt: none
Peak Zt: 3
Peak Zt: none
Peak Zt: none
Peak Zt: none
Relative Expression
Zt (hr)
21.5 0.5 3.5 6.5 9.5 12.5 18.5
0 1 2 3 4 5

Singeleton: ENSXMAG00000005218 angel2

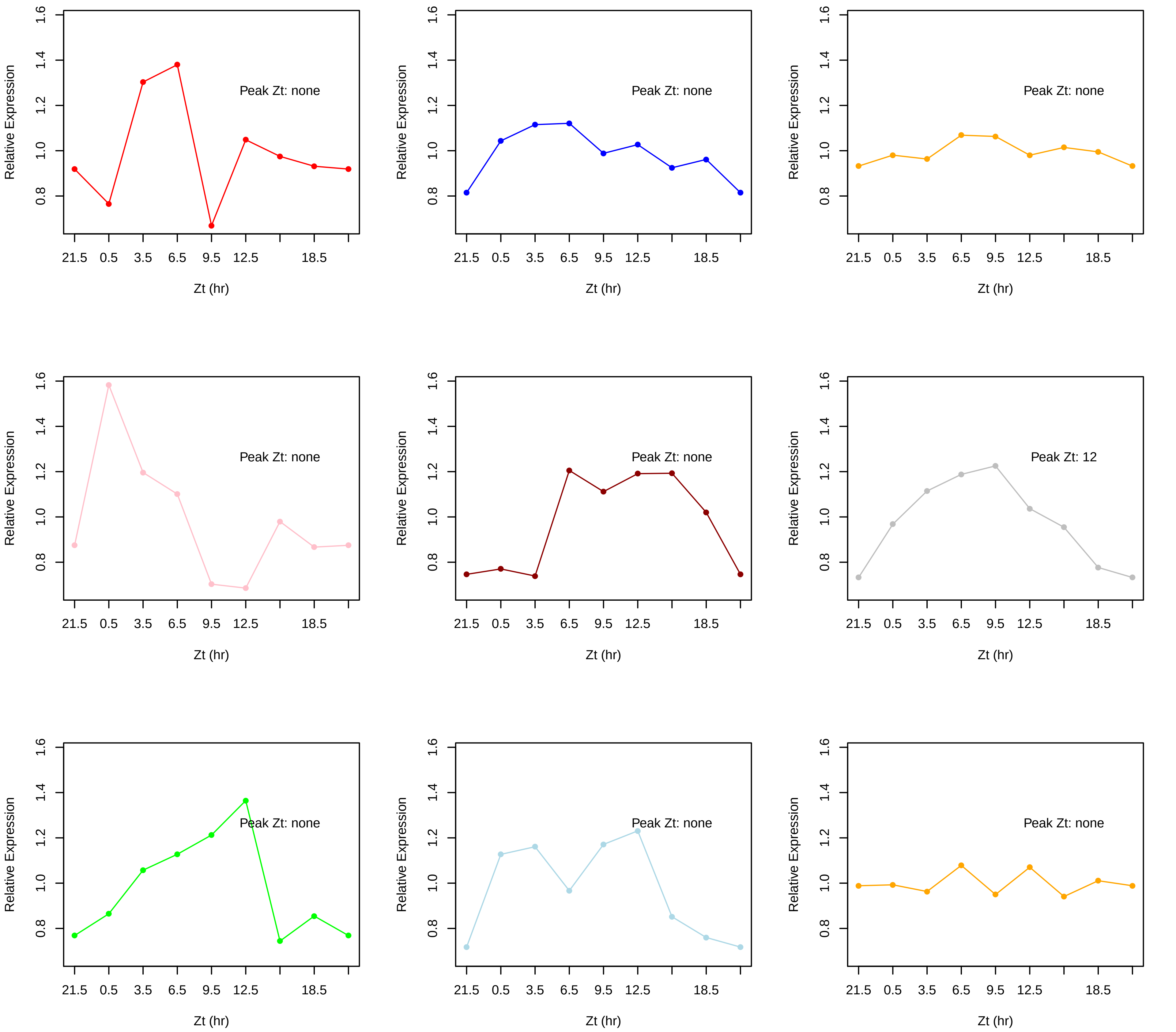

Singeleton: ENSXMAG00000009152

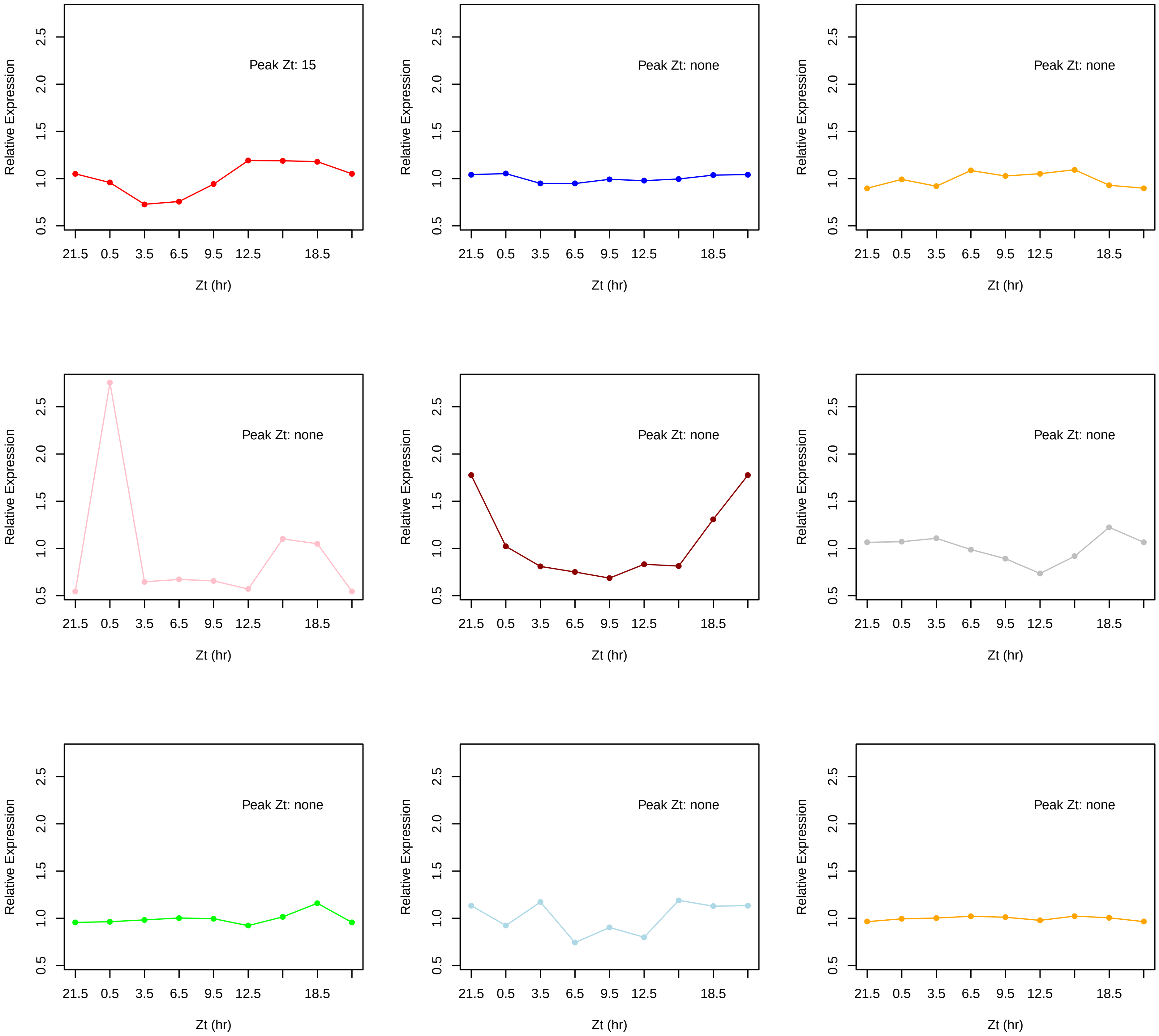

Singeleton: ENSXMAG00000004076 si:dkey−266m15.5

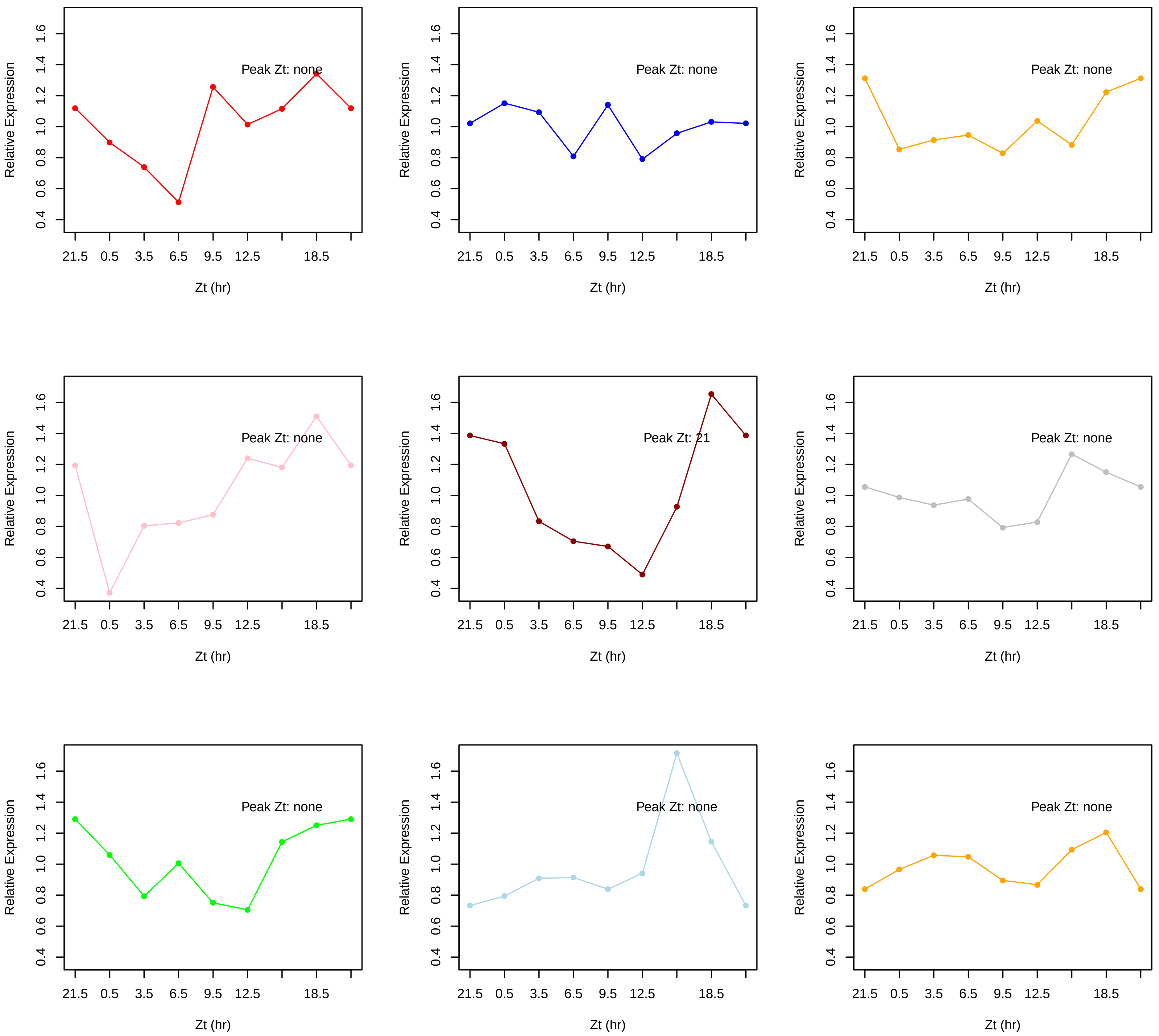

Singeleton: ENSXMAG00000028111 rnf144aa

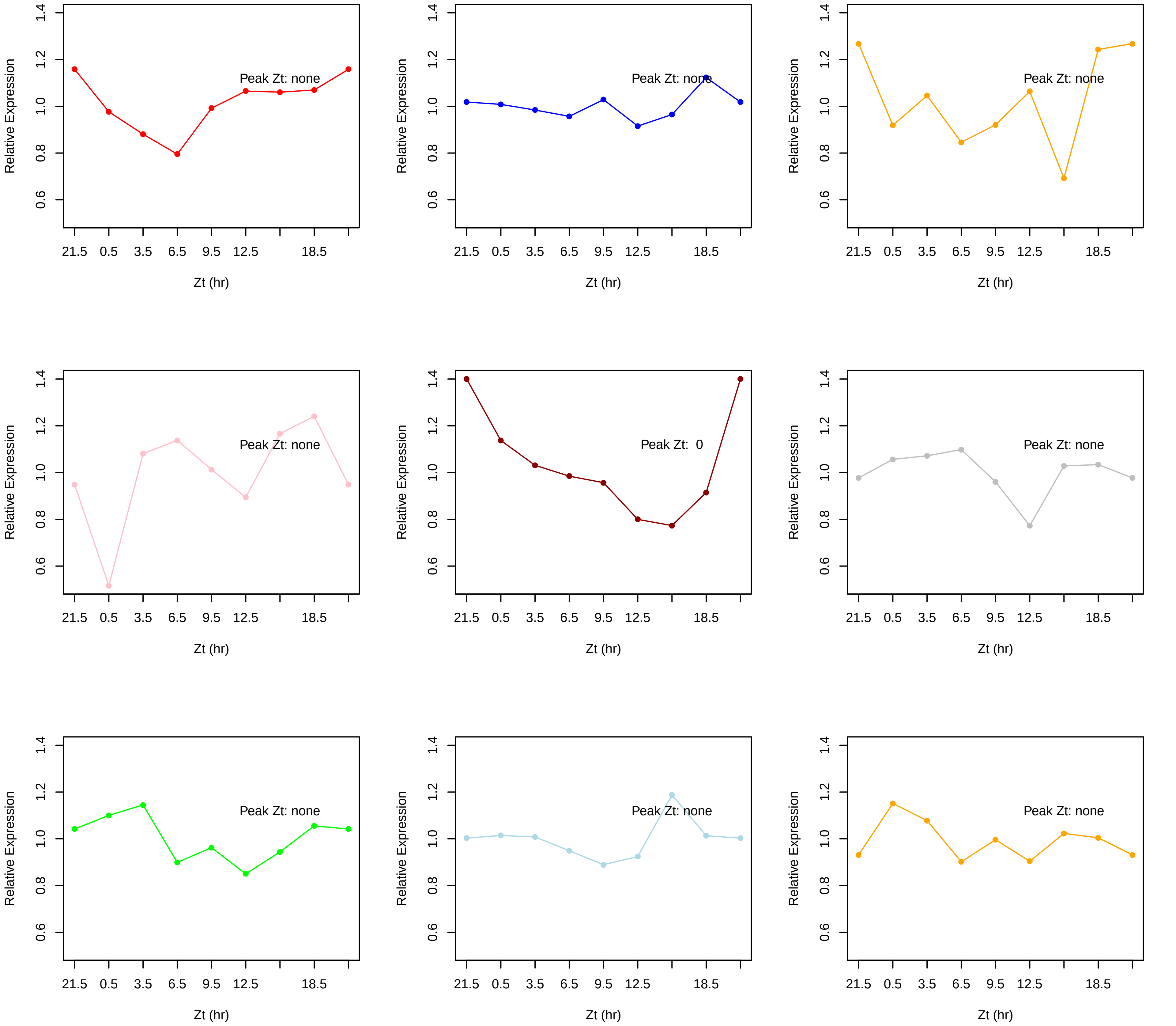

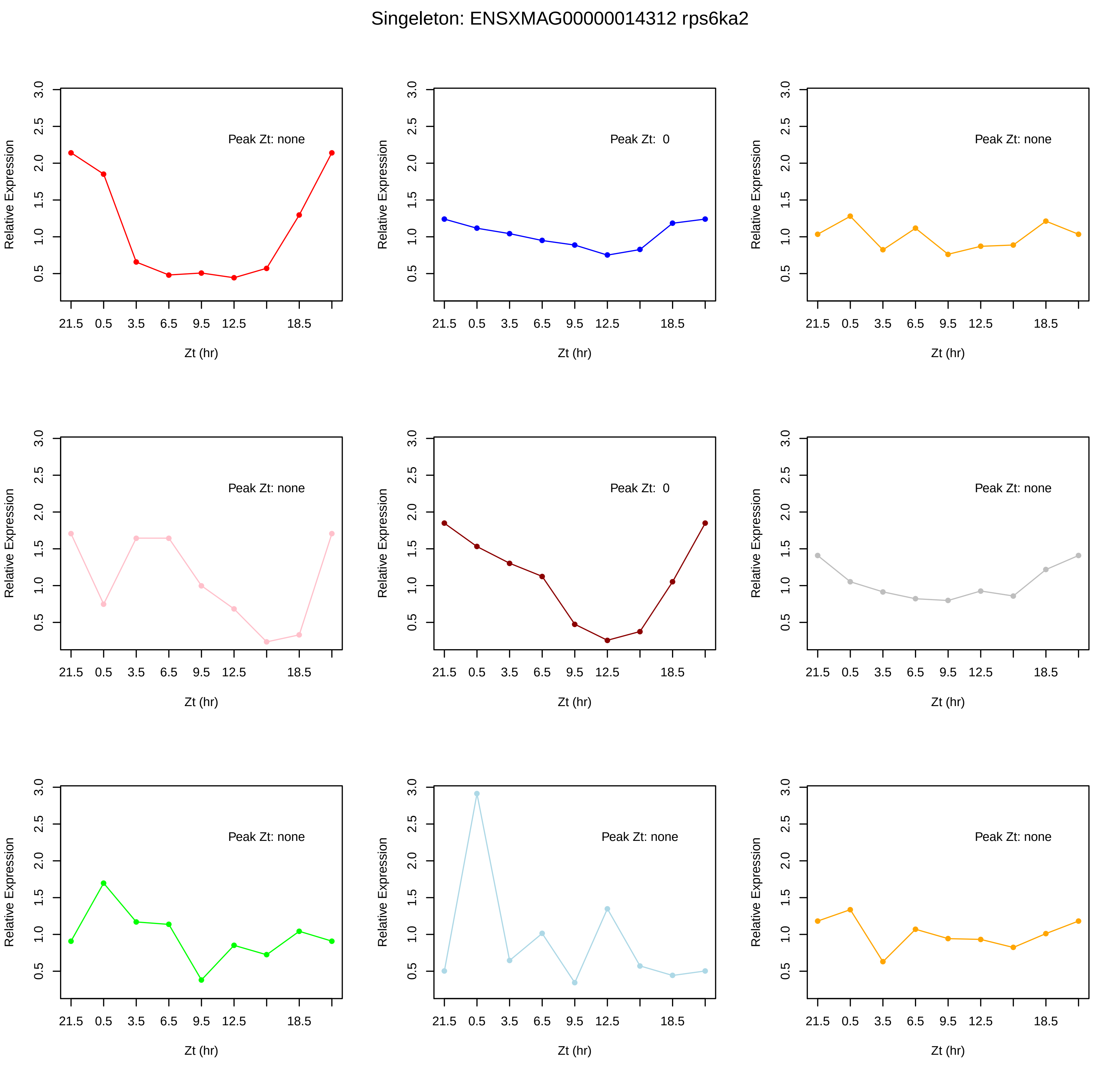

Singeleton: ENSXMAG00000014312 rps6ka2
Peak Zt: none
Peak Zt: 0
Peak Zt: none
Peak Zt: none
Peak Zt: 0
Peak Zt: none
Peak Zt: none
Peak Zt: none
Peak Zt: none
Relative Expression
Zt (hr)
21.5
0.5
3.5
6.5
9.5
12.5
18.5

Singeleton: ENSXMAG00000004061 miox

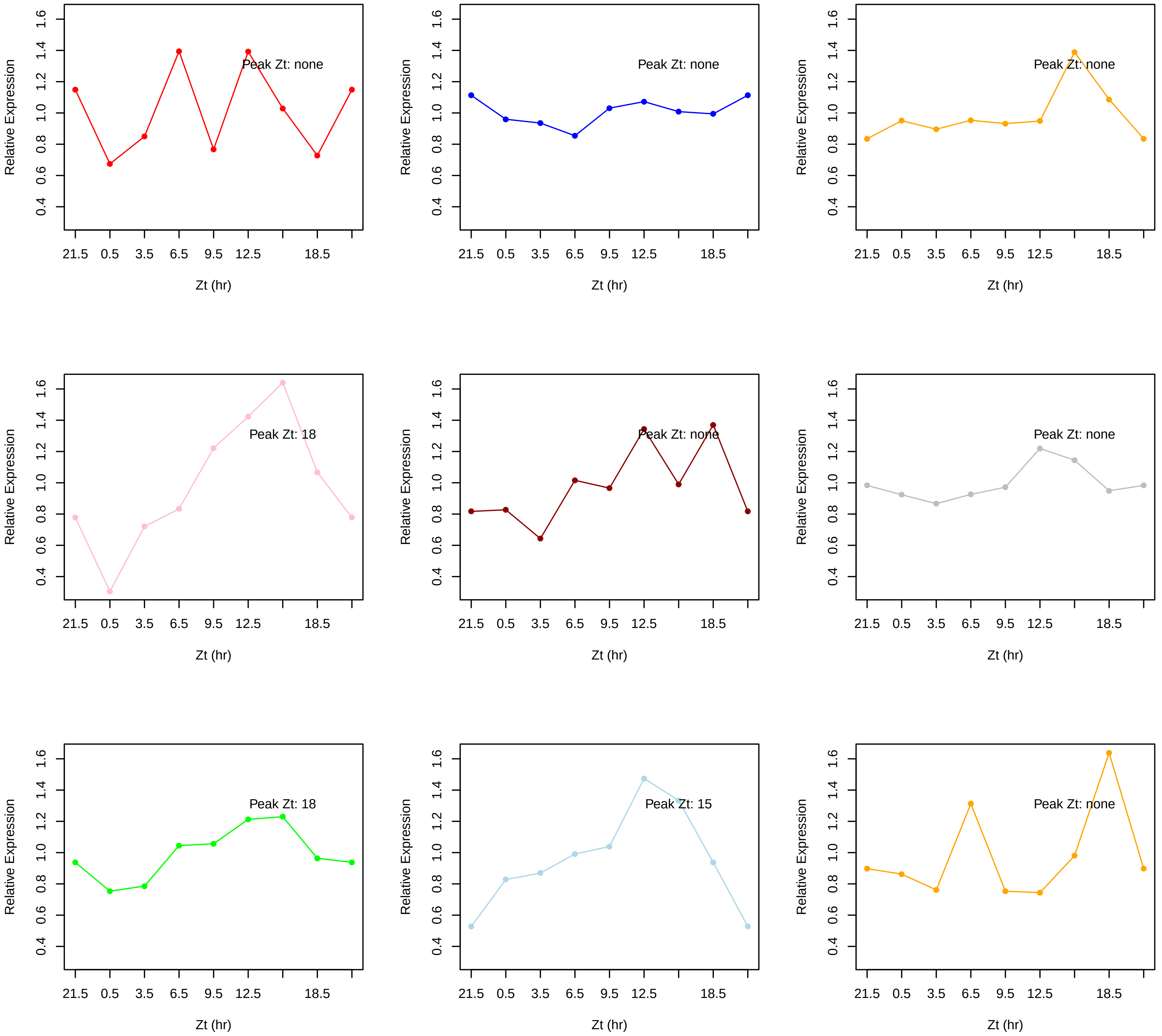

Singeleton: ENSXMAG00000004047 lmf2a

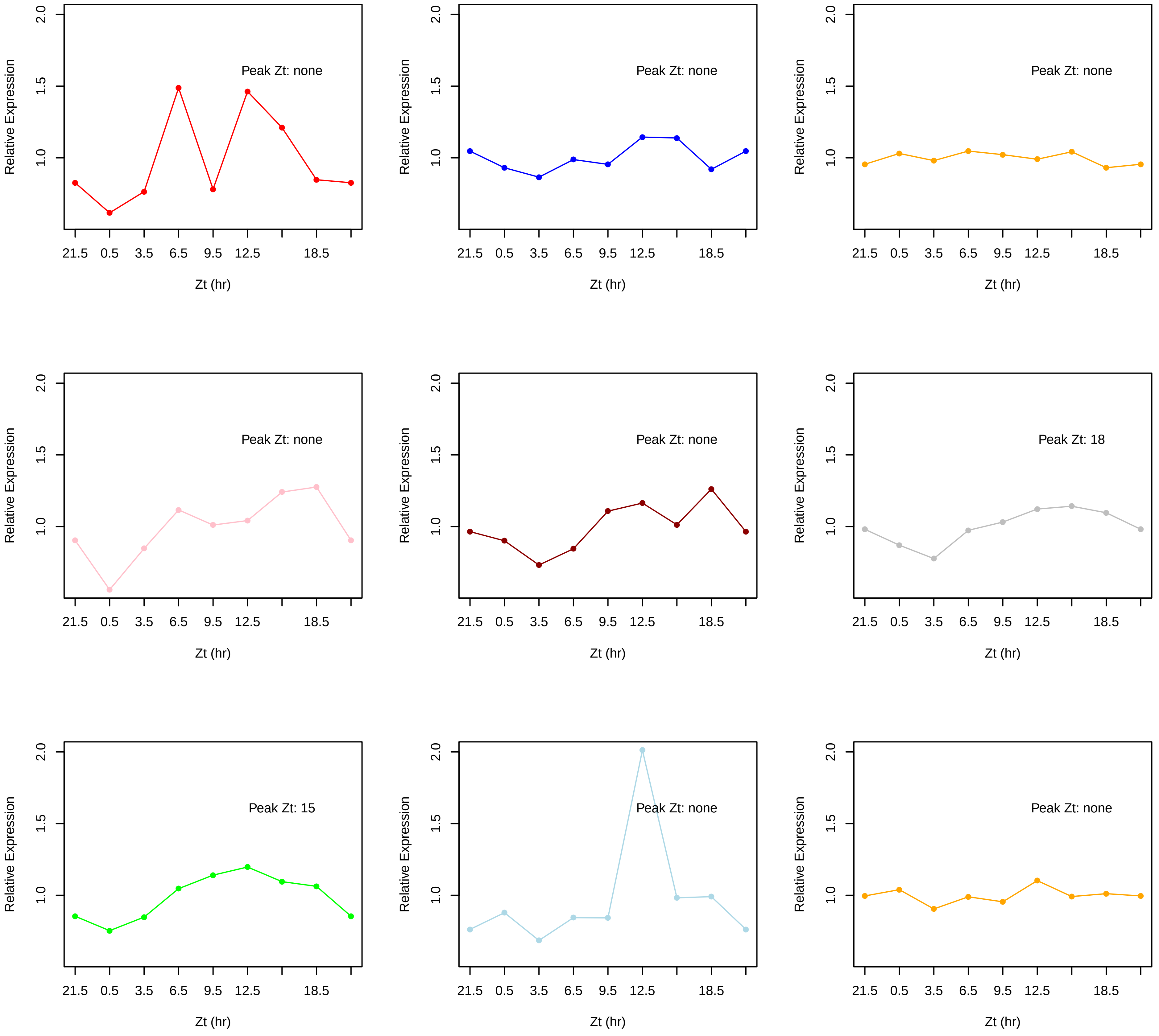

Singeleton: ENSXMAG00000002959 ywhaqb

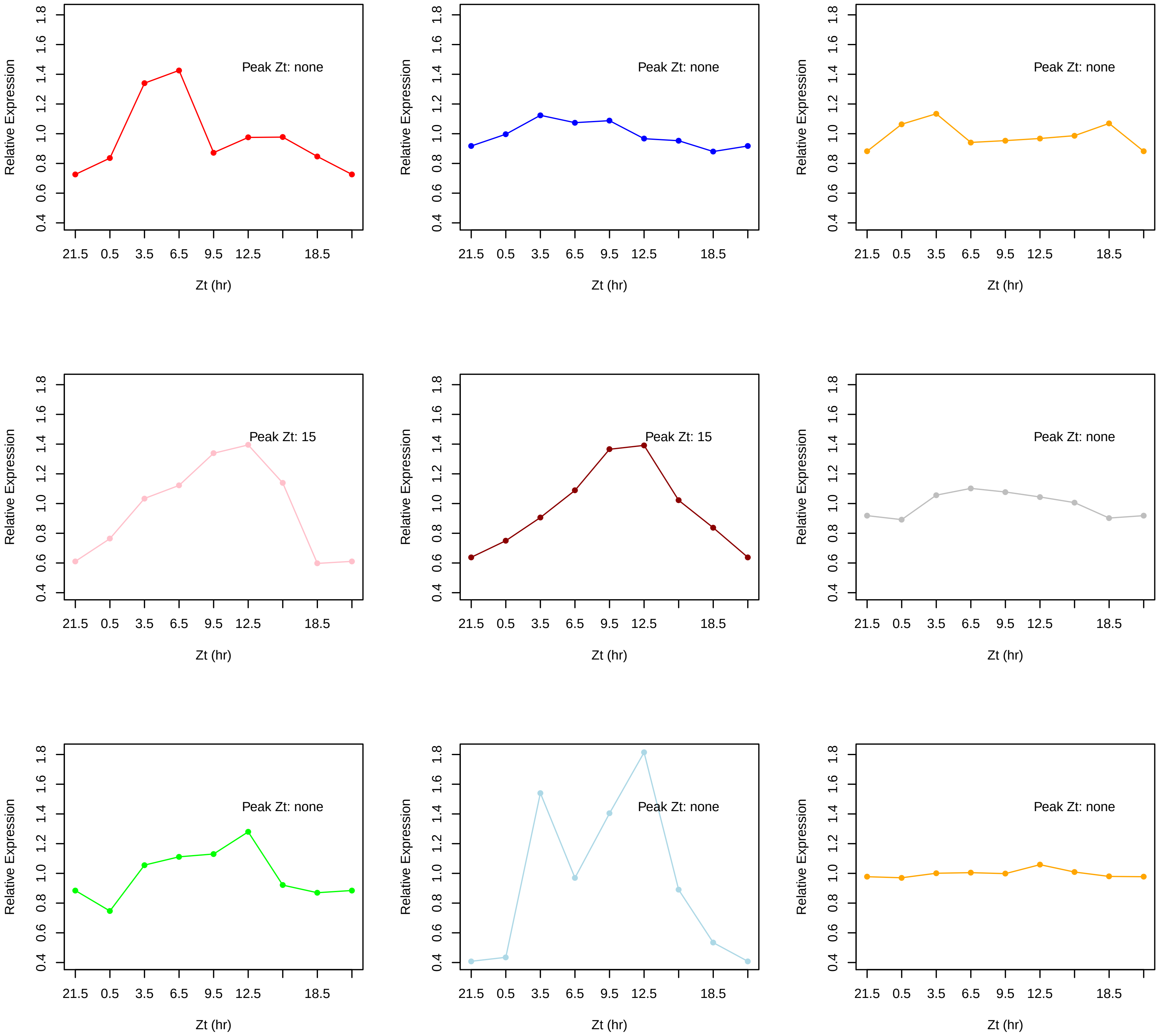

Singleton: ENSXMAG00000009858 taf1b

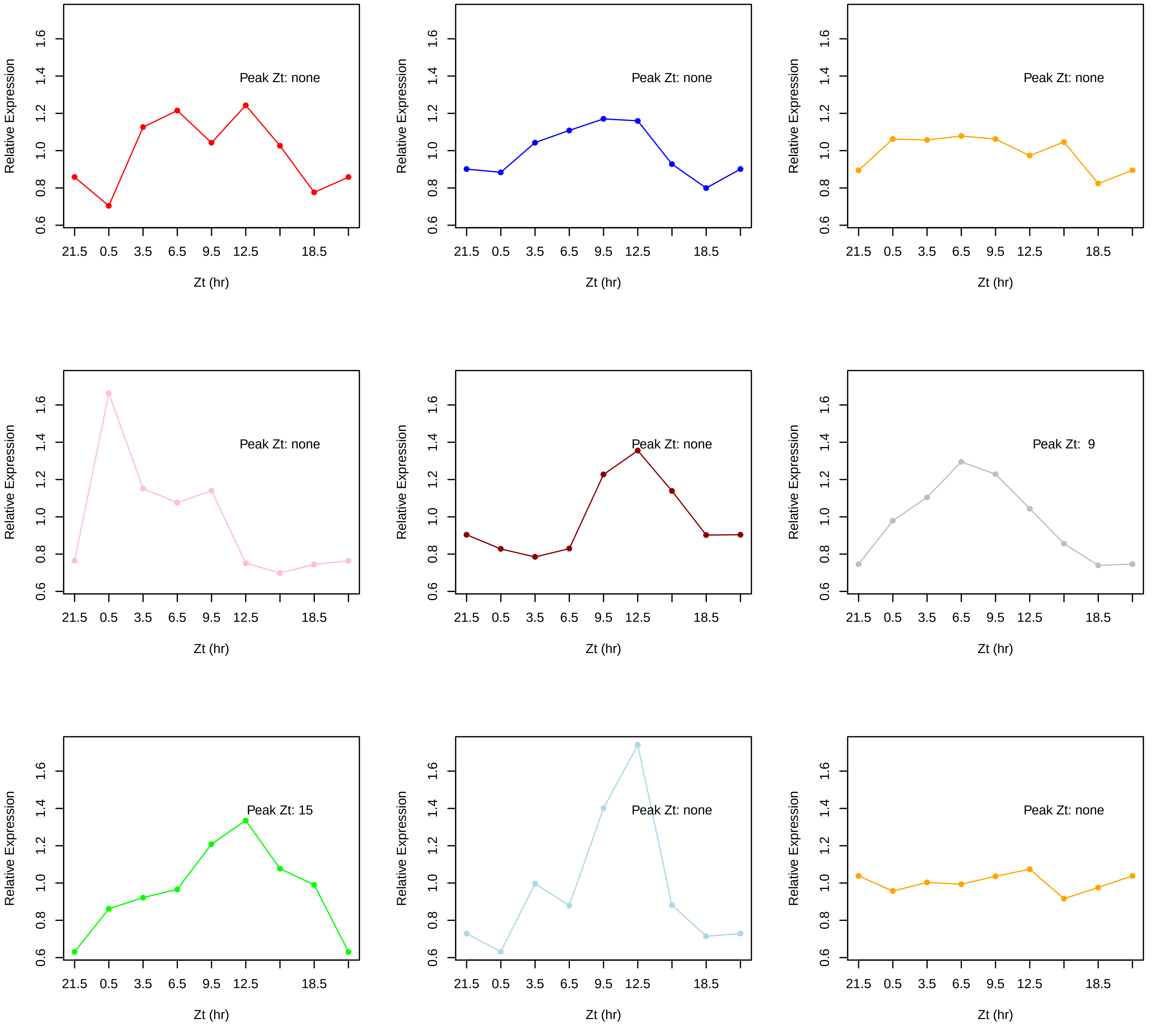

Singeleton: ENSXMAG00000013267 grhl1

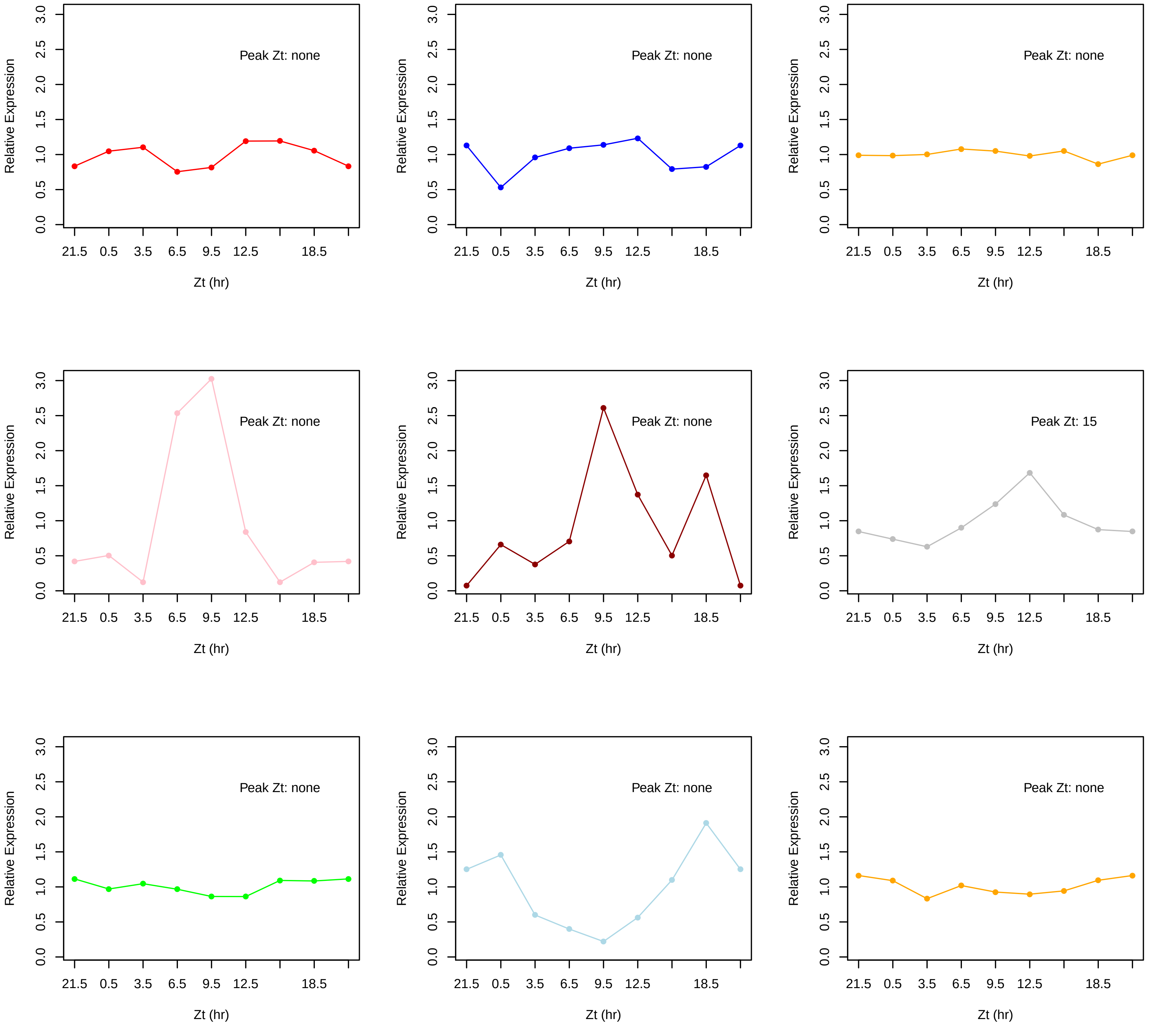

Singeleton: ENSXMAG00000005640 ttc38

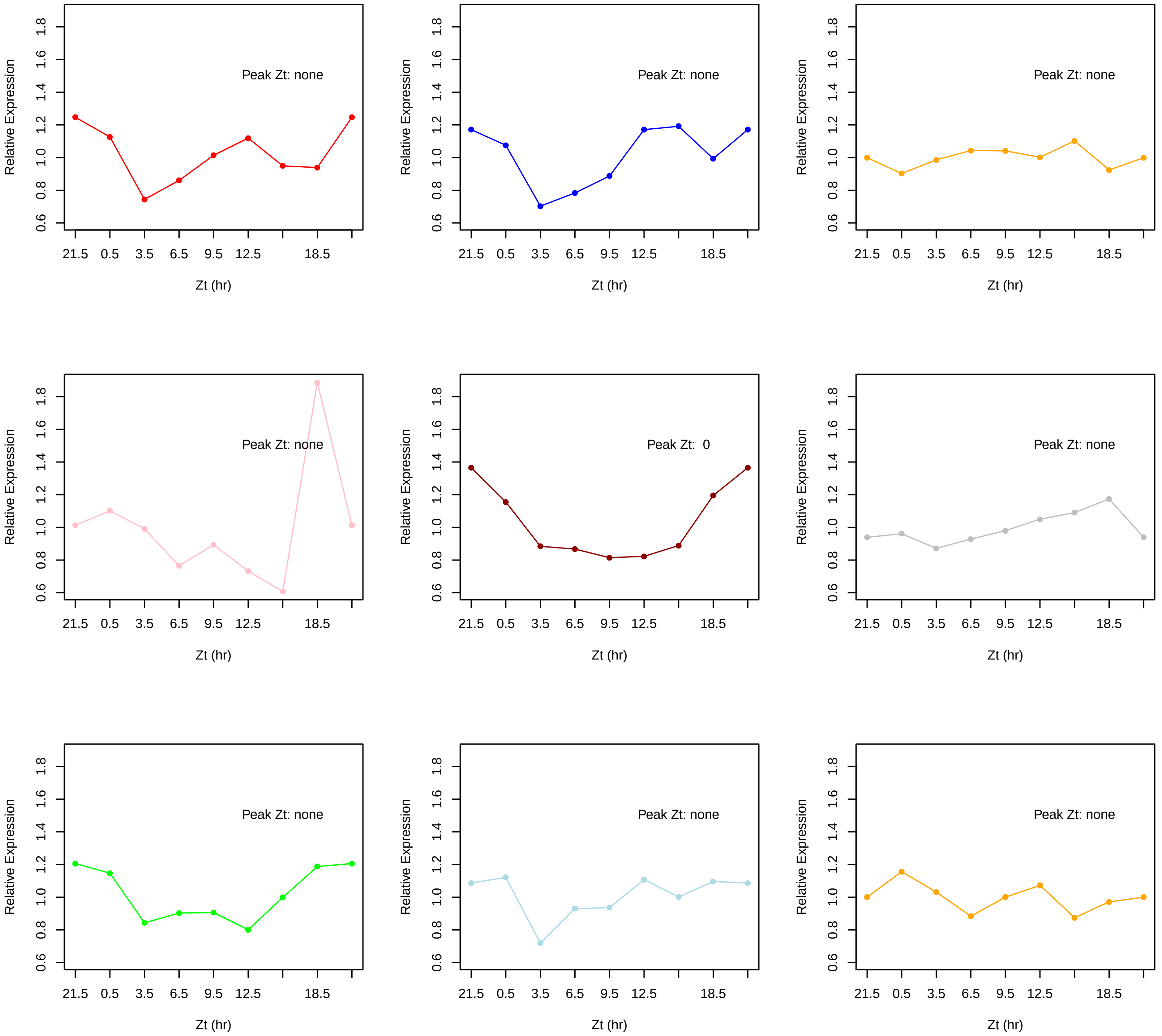

Singeleton: ENSXMAG00000011220 tspan11

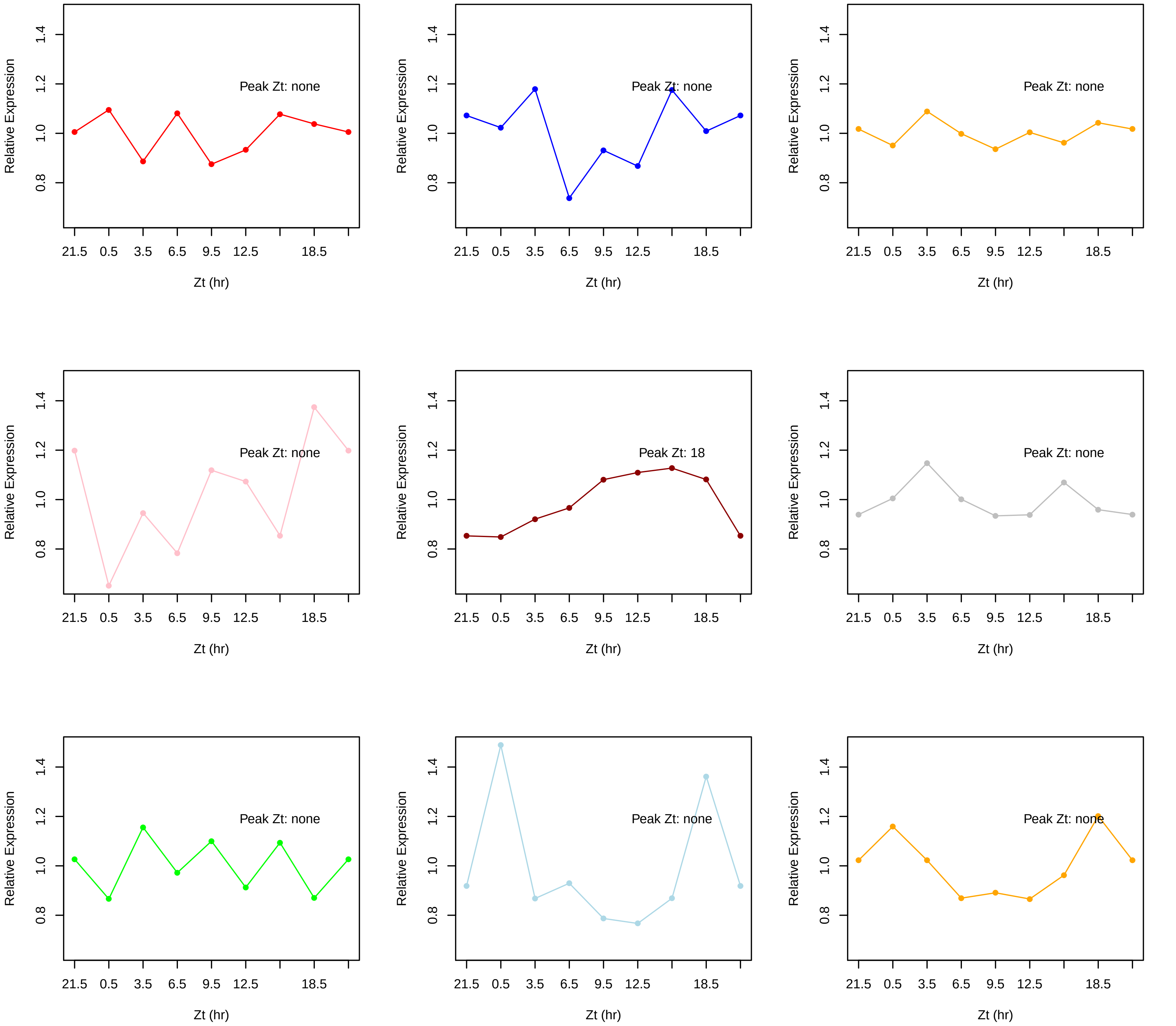

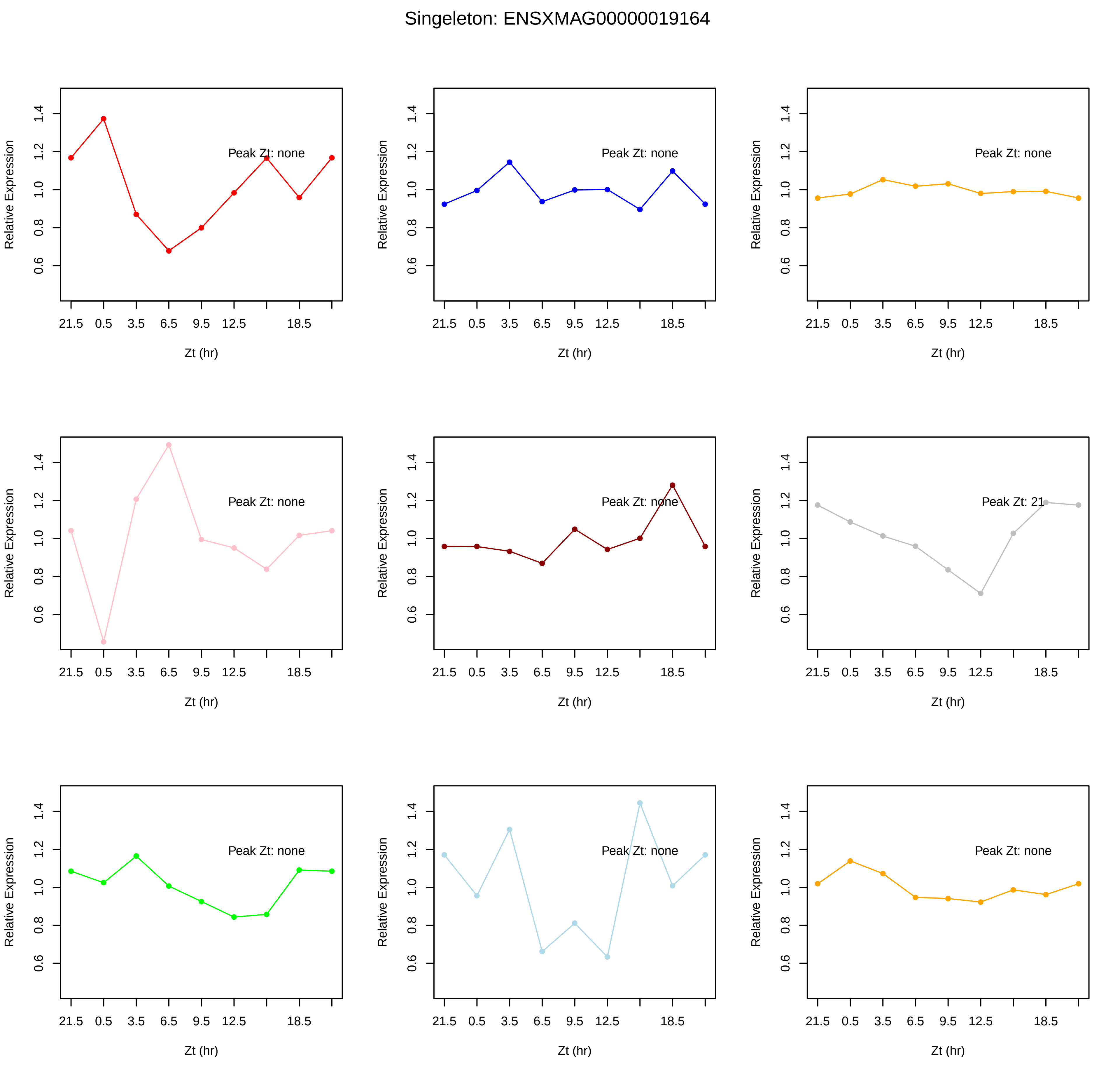

Singeleton: ENSXMAG00000019164
Peak Zt: none
Peak Zt: 21
Relative Expression
Zt (hr)
21.5
0.5
3.5
6.5
9.5
12.5
18.5
0.6
0.8
1.0
1.2
1.4

Singeleton: ENSXMAG00000010331 mcph1

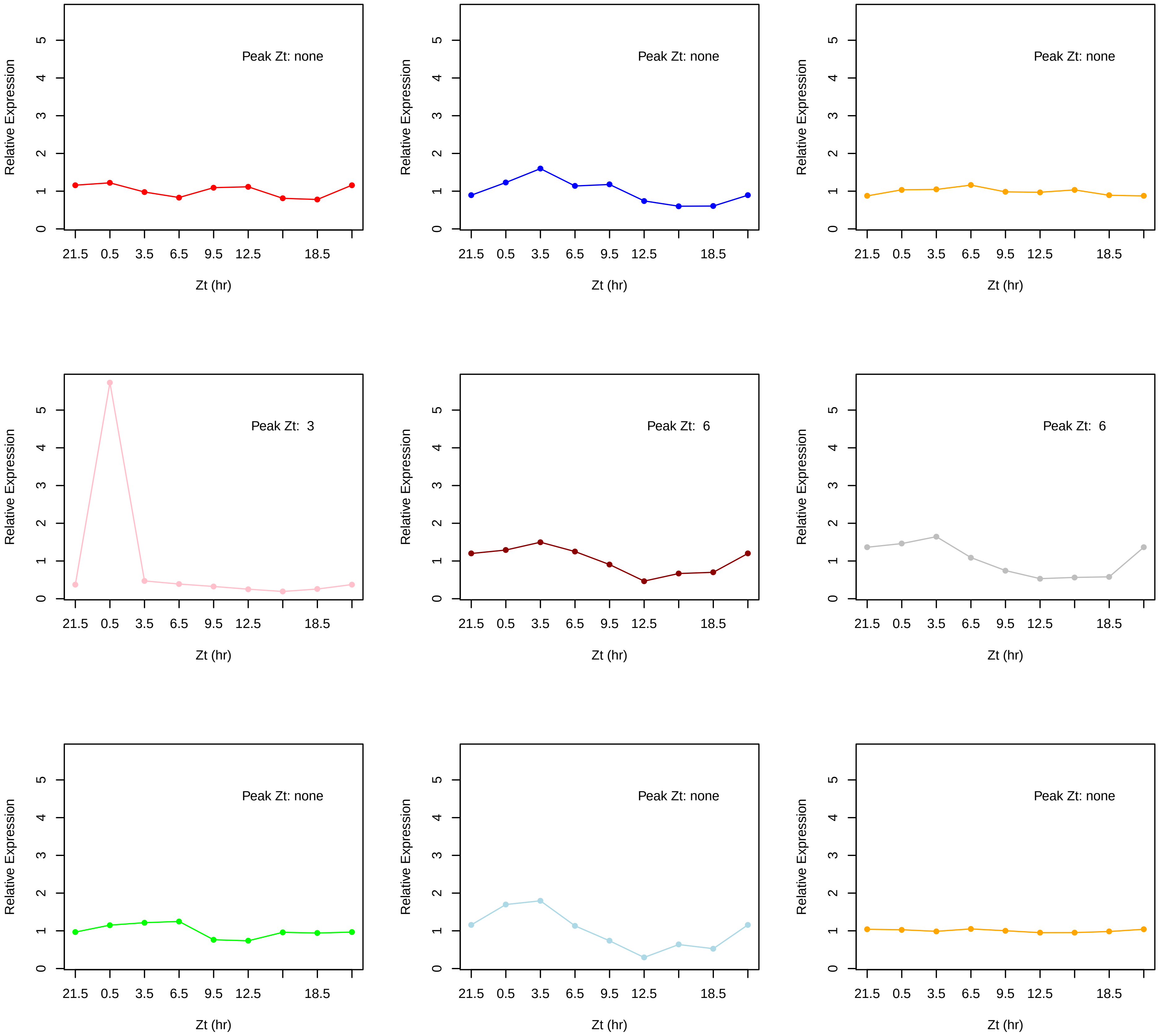

Singeleton: ENSXMAG00000010360 angpt2a

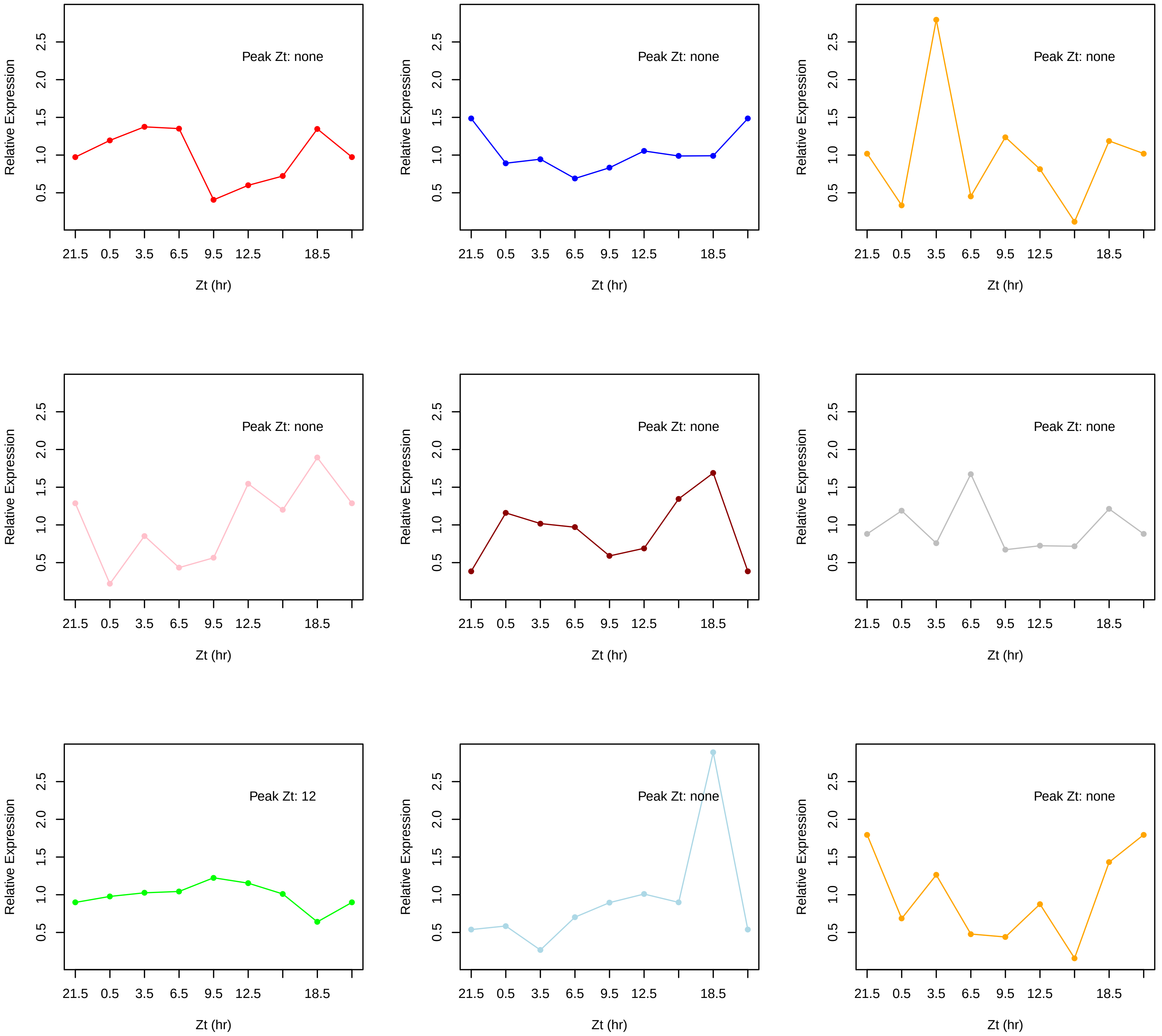

Singeleton: ENSXMAG00000007499 cebpz

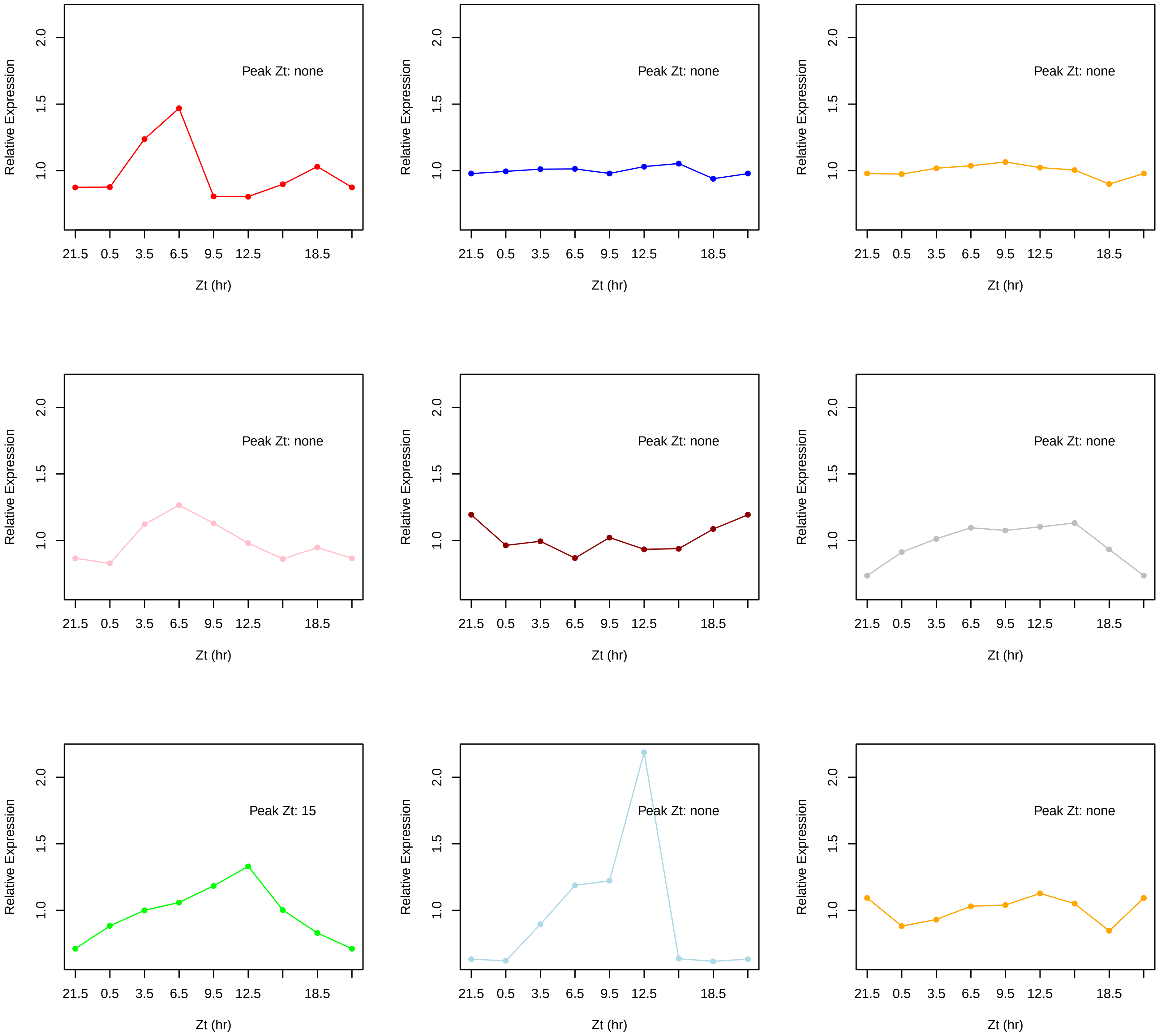

Singeleton: ENSXMAG00000000242 xrn2

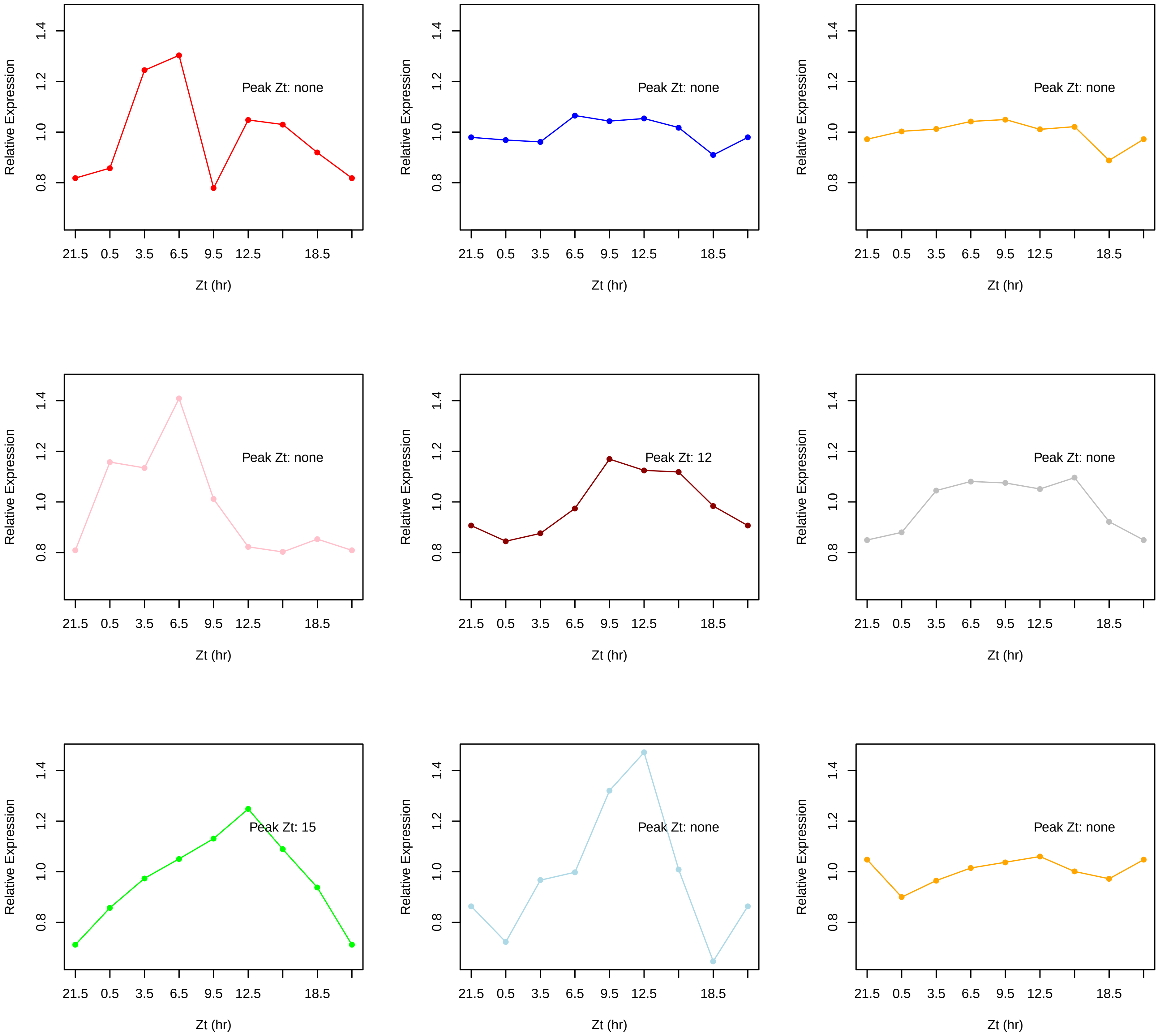

Singeleton: ENSXMAG00000003963 fkbp4

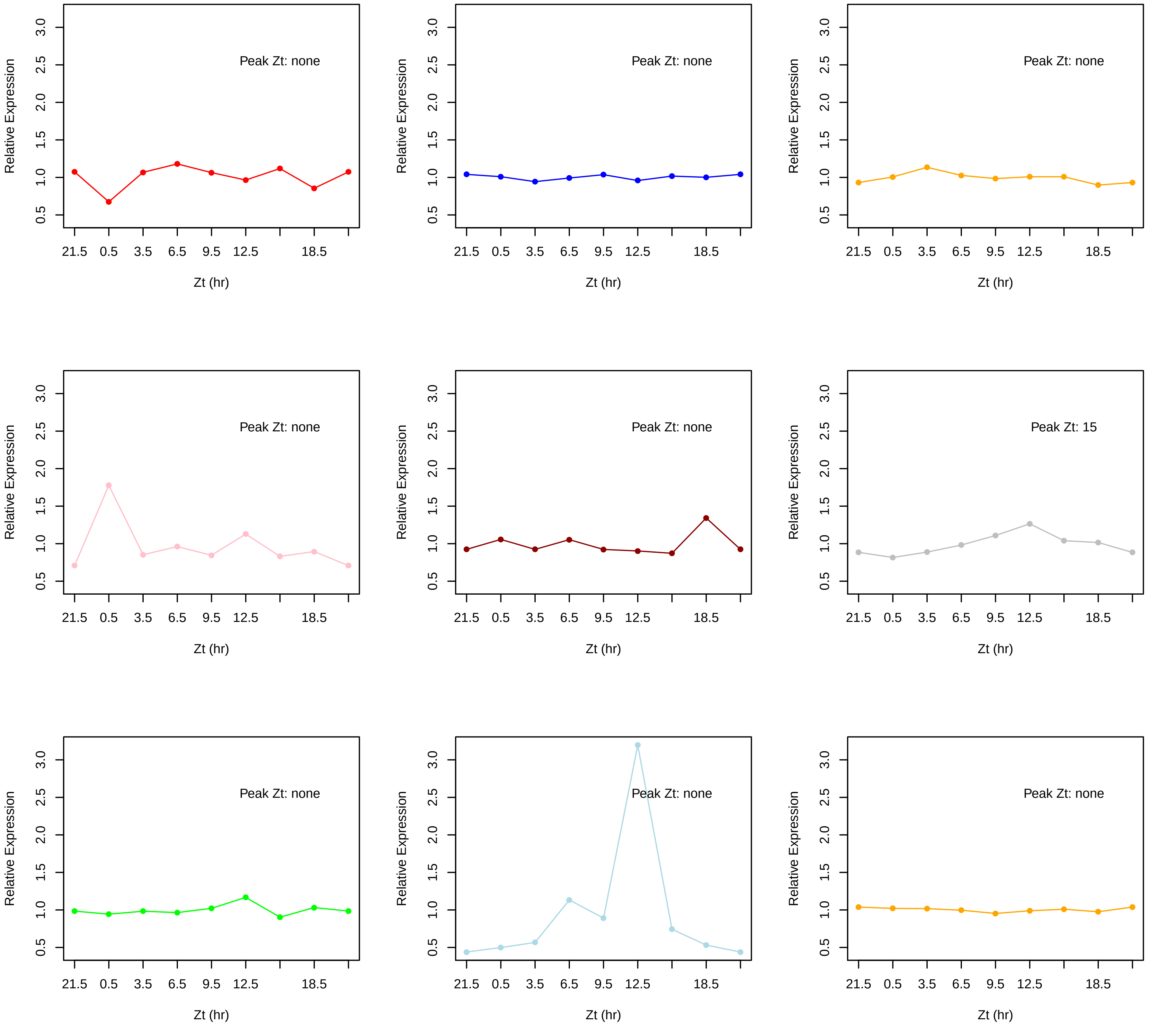

Singeleton: ENSXMAG00000019103 kiz

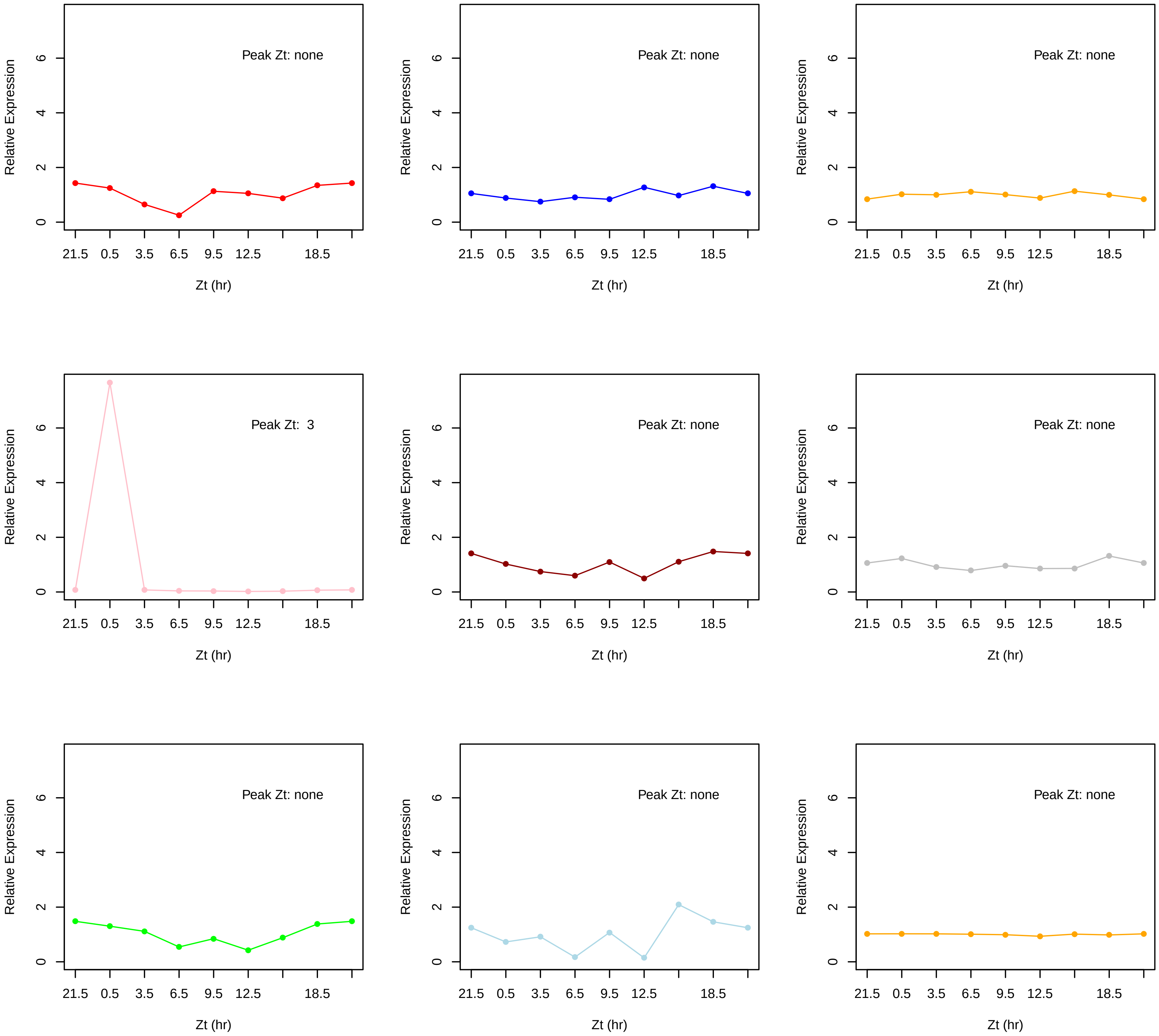

Singleton: ENSXMAG00000010261 elovl5

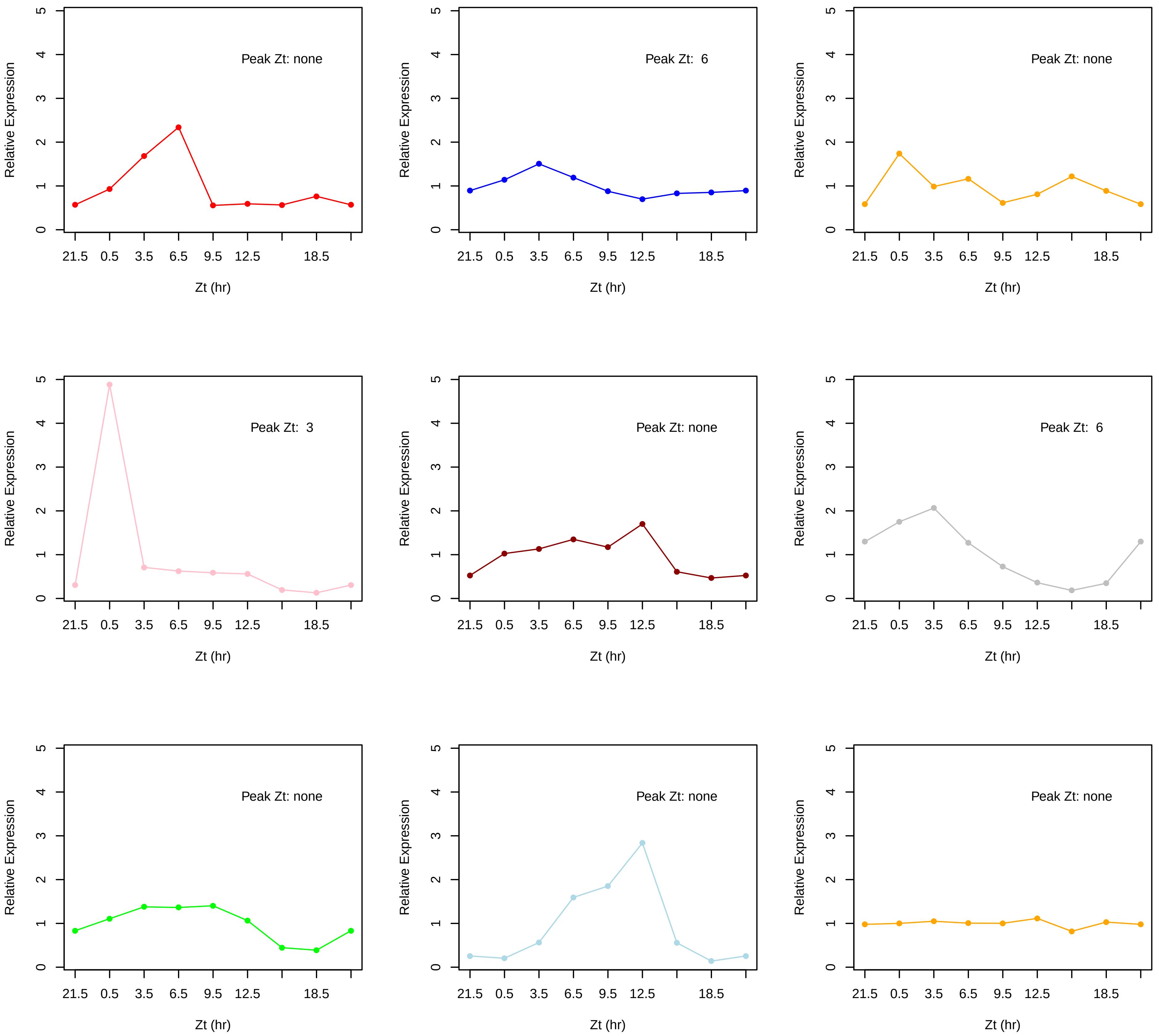

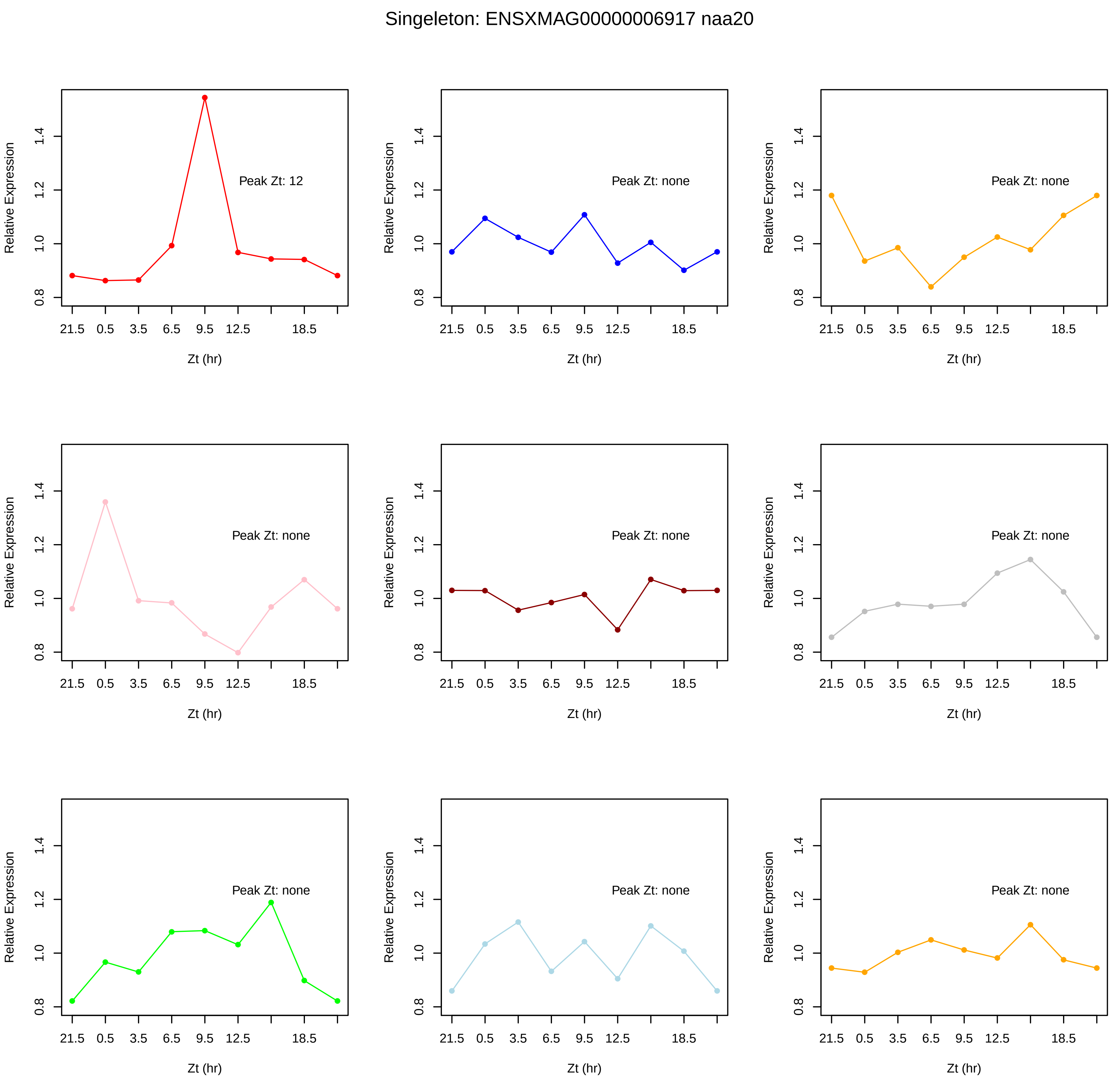
Singleton: ENSXMAG00000006917 naa20
Peak Zt: 12
Peak Zt: none
Relative Expression
Zt (hr)
21.5
0.5
3.5
6.5
9.5
12.5
18.5
0.8
1.0
1.2
1.4

Singeleton: ENSXMAG00000010214 lrrc1

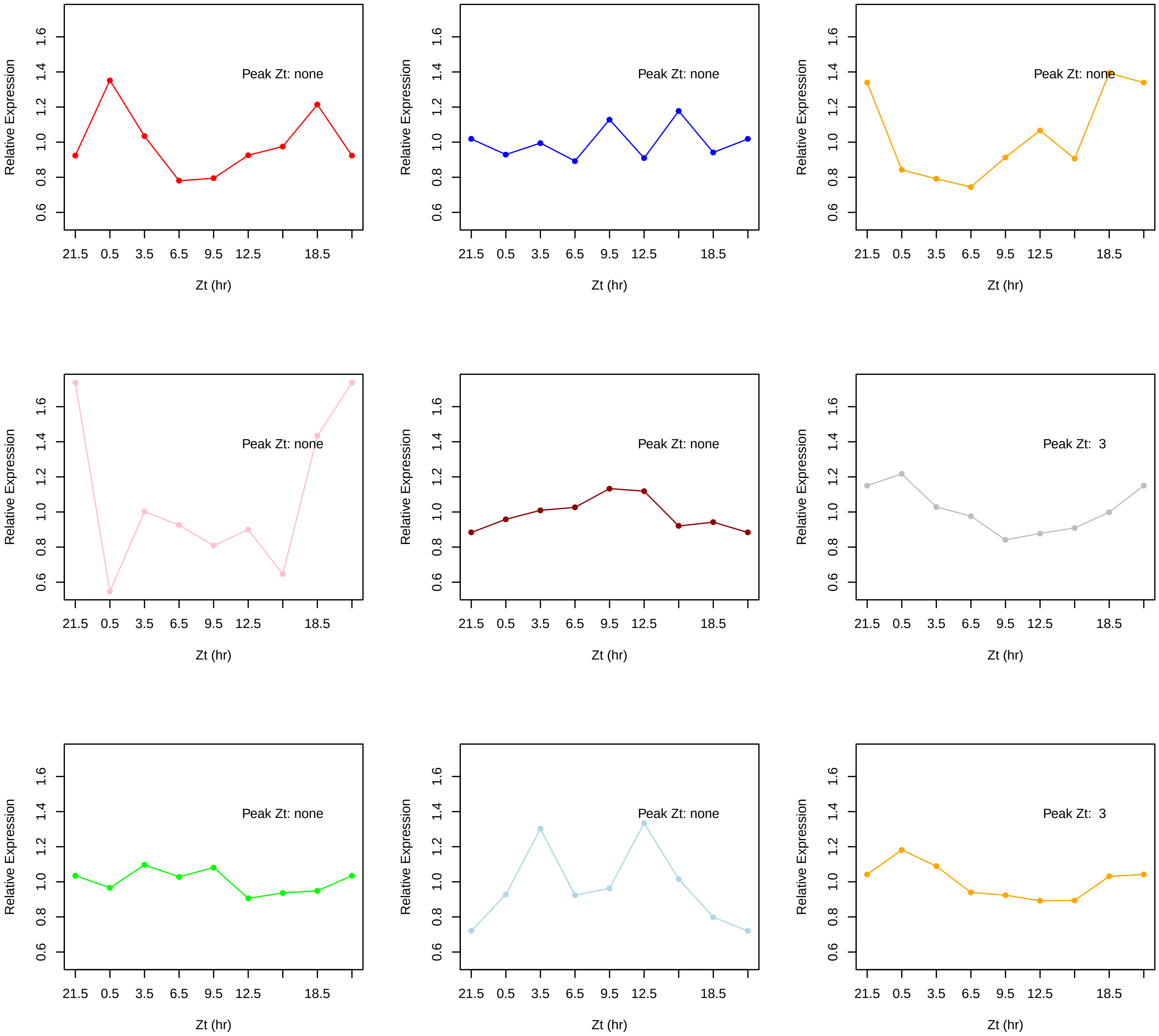

Singeleton: ENSXMAG00000004499 COX7A2

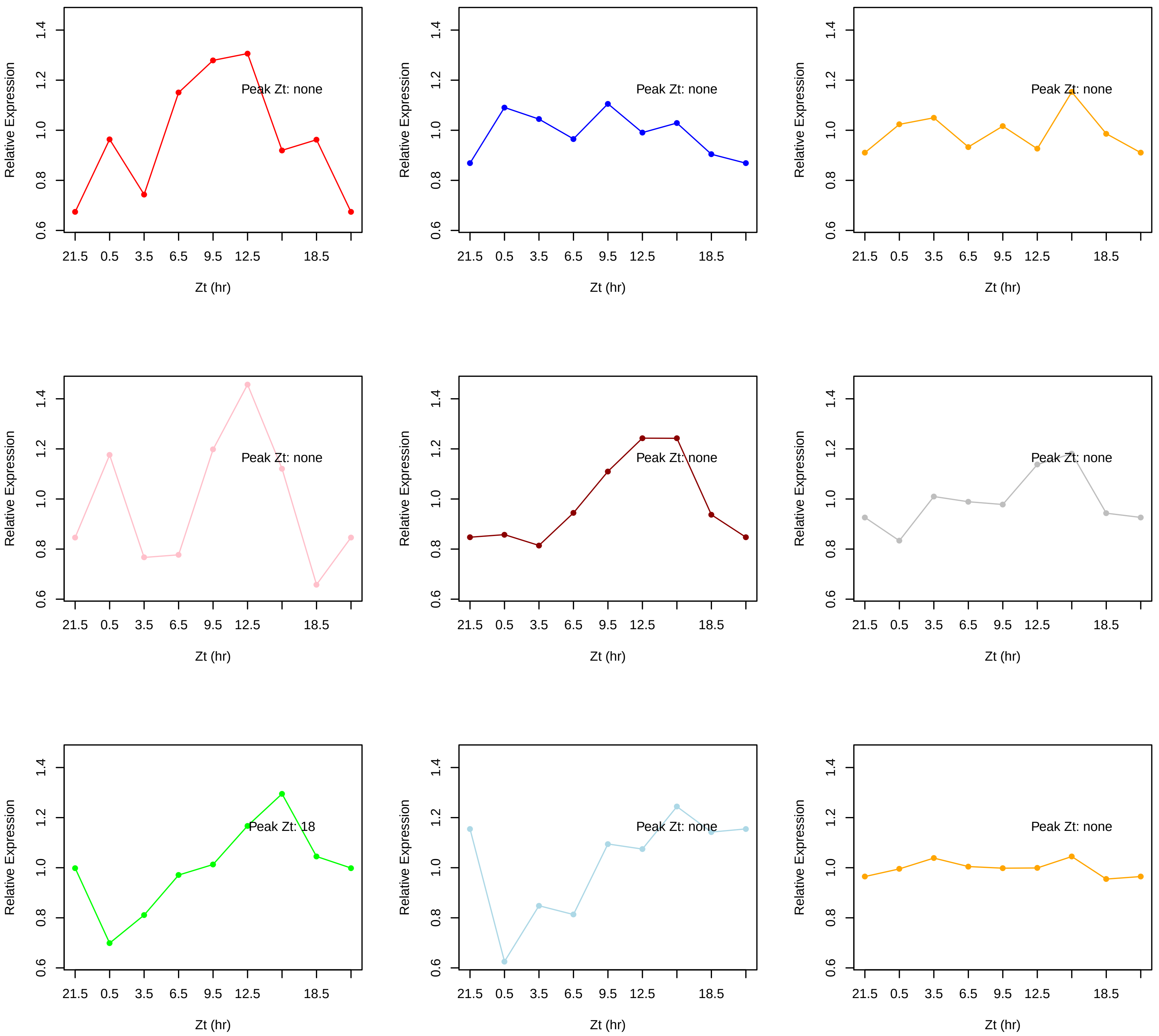

Singeleton: ENSXMAG00000004575

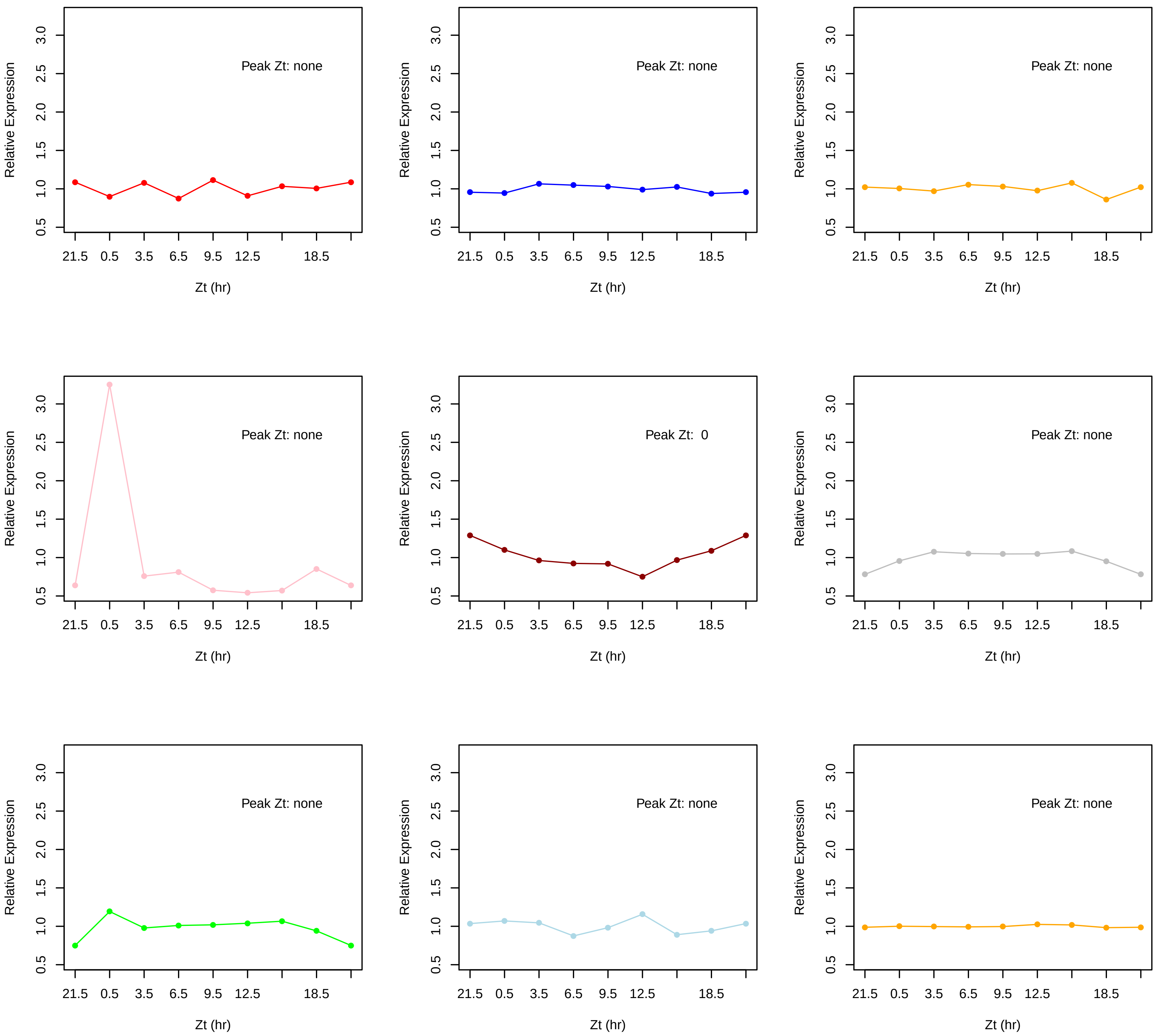

Singleton: ENSXMAG00000025549 bmp5

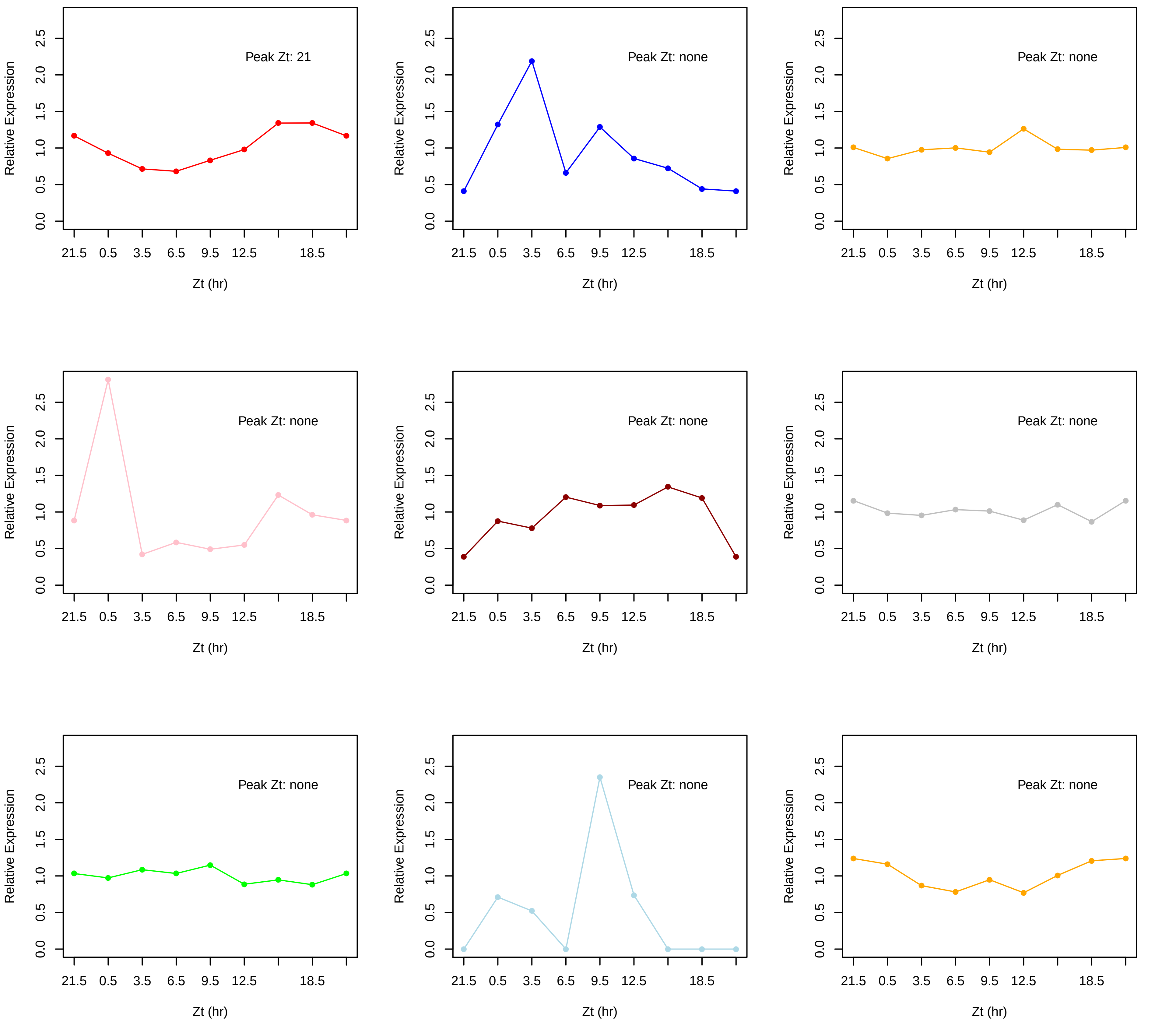

Singeleton: ENSXMAG00000007584

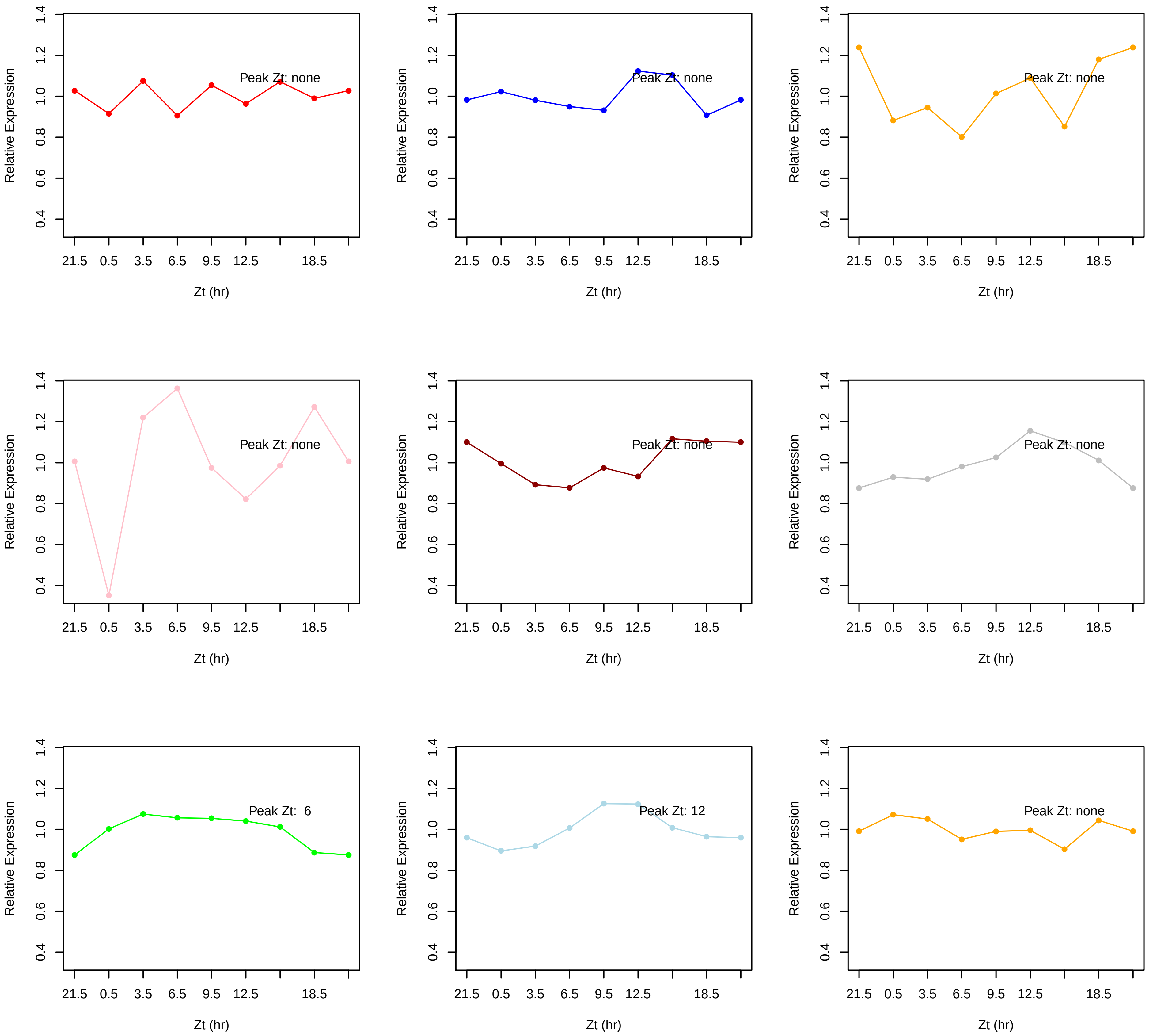

Singleton: ENSXMAG00000027263 zmp:0000000760

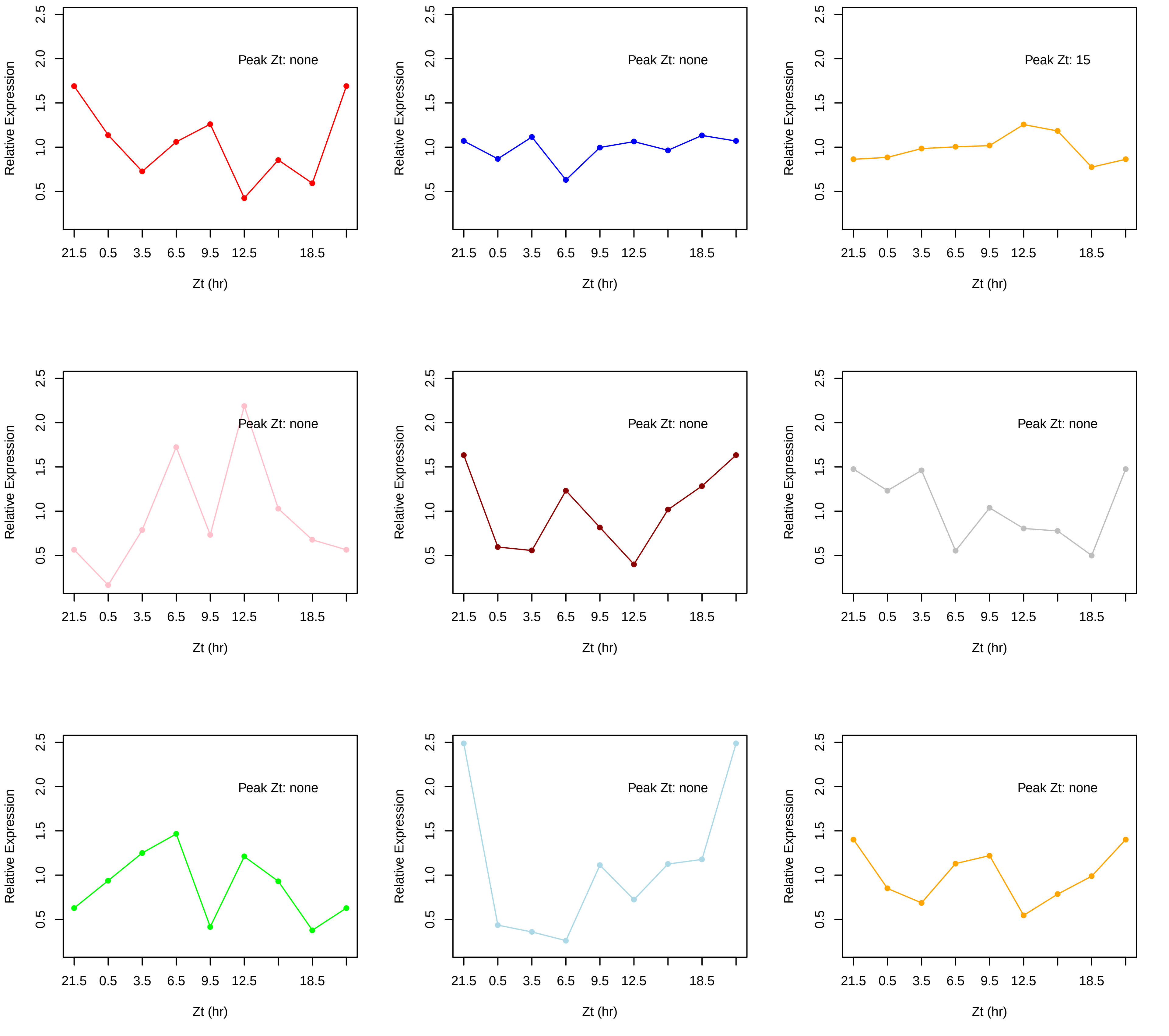

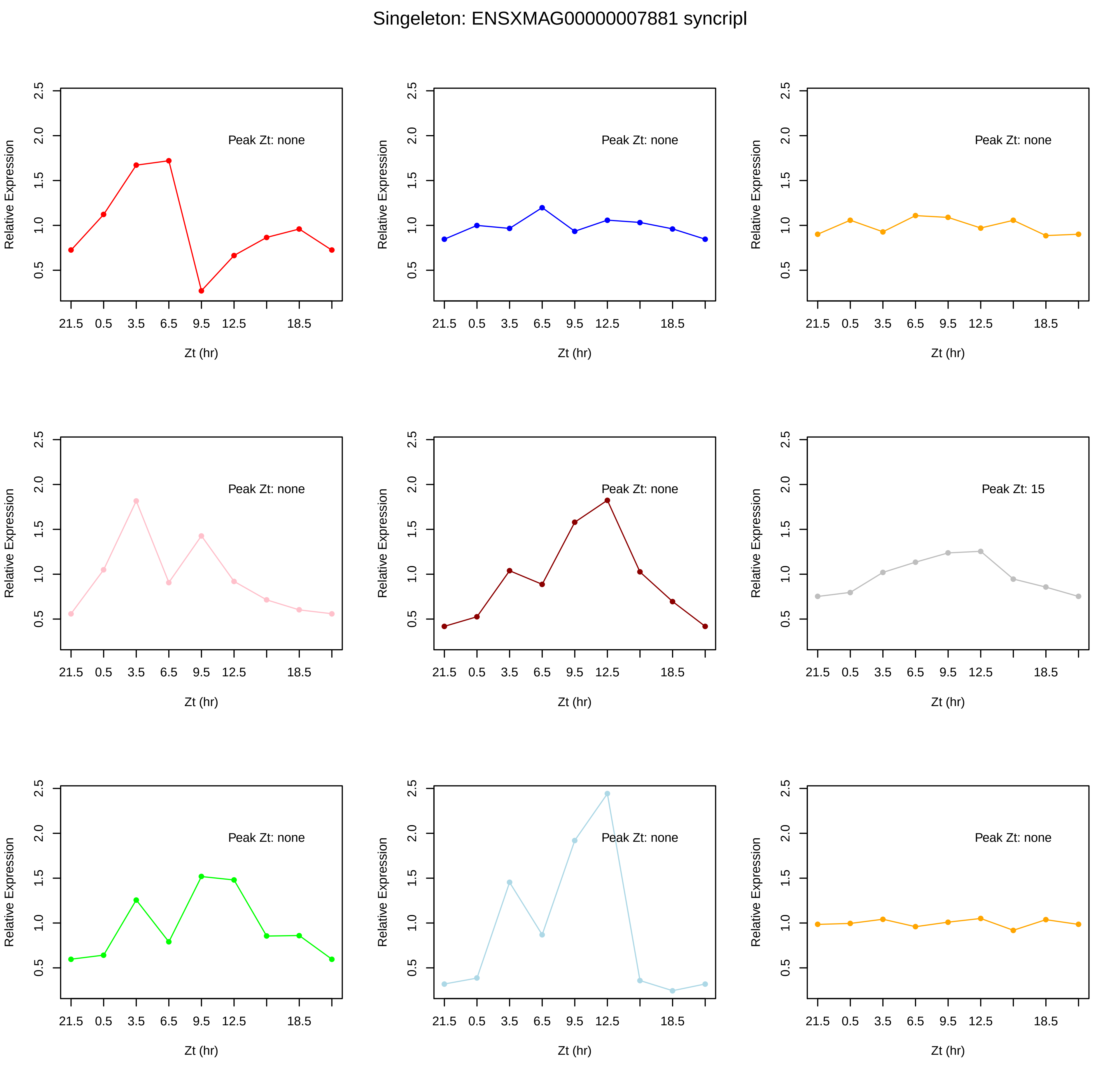

Singeleton: ENSXMAG00000007881 syncripl
Peak Zt: none
Peak Zt: none
Peak Zt: none
Peak Zt: none
Peak Zt: none
Peak Zt: 15
Peak Zt: none
Peak Zt: none
Peak Zt: none
Relative Expression
Zt (hr)
21.5
0.5
3.5
6.5
9.5
12.5
18.5

Singeleton: ENSXMAG00000005352 smim8

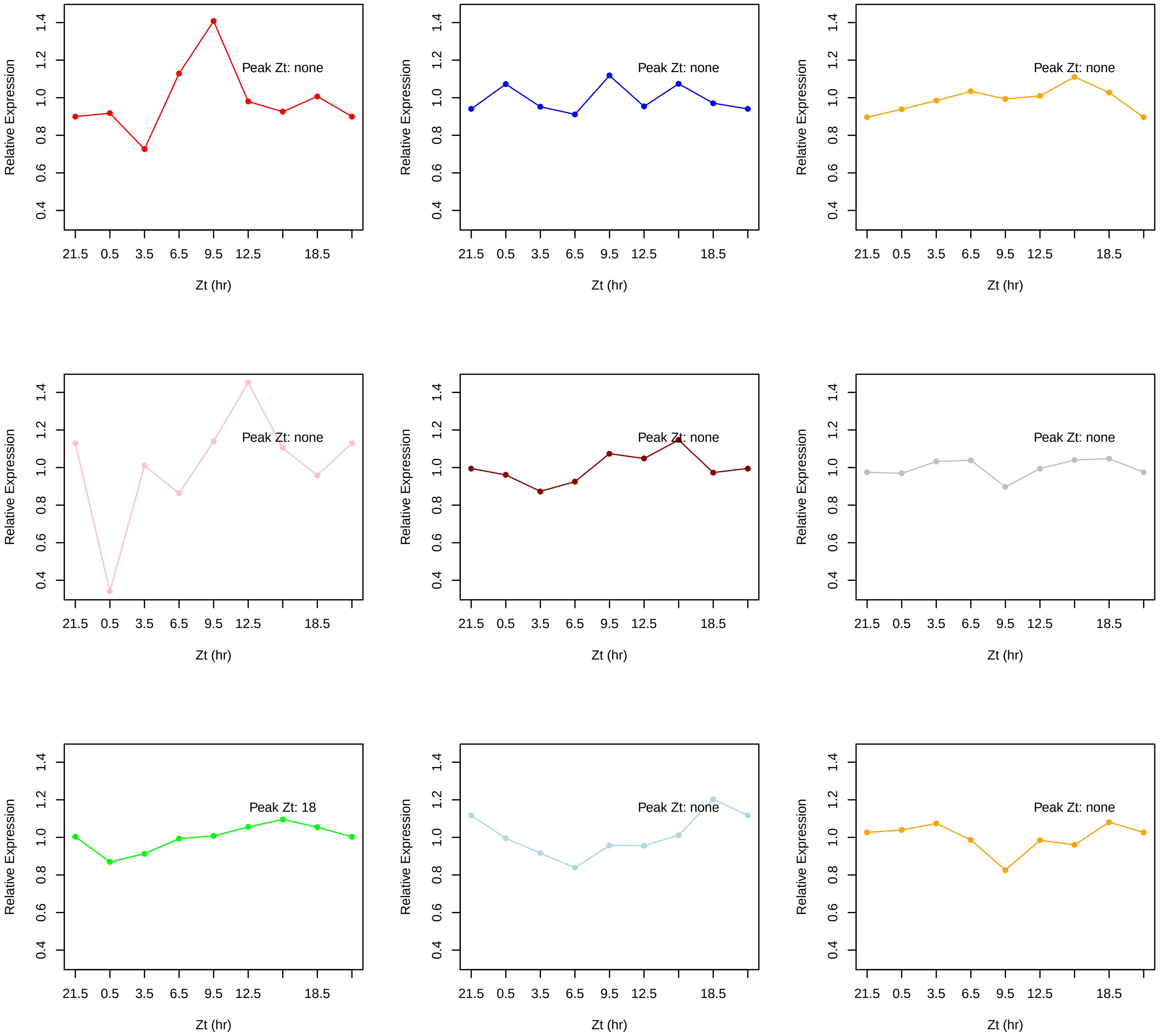

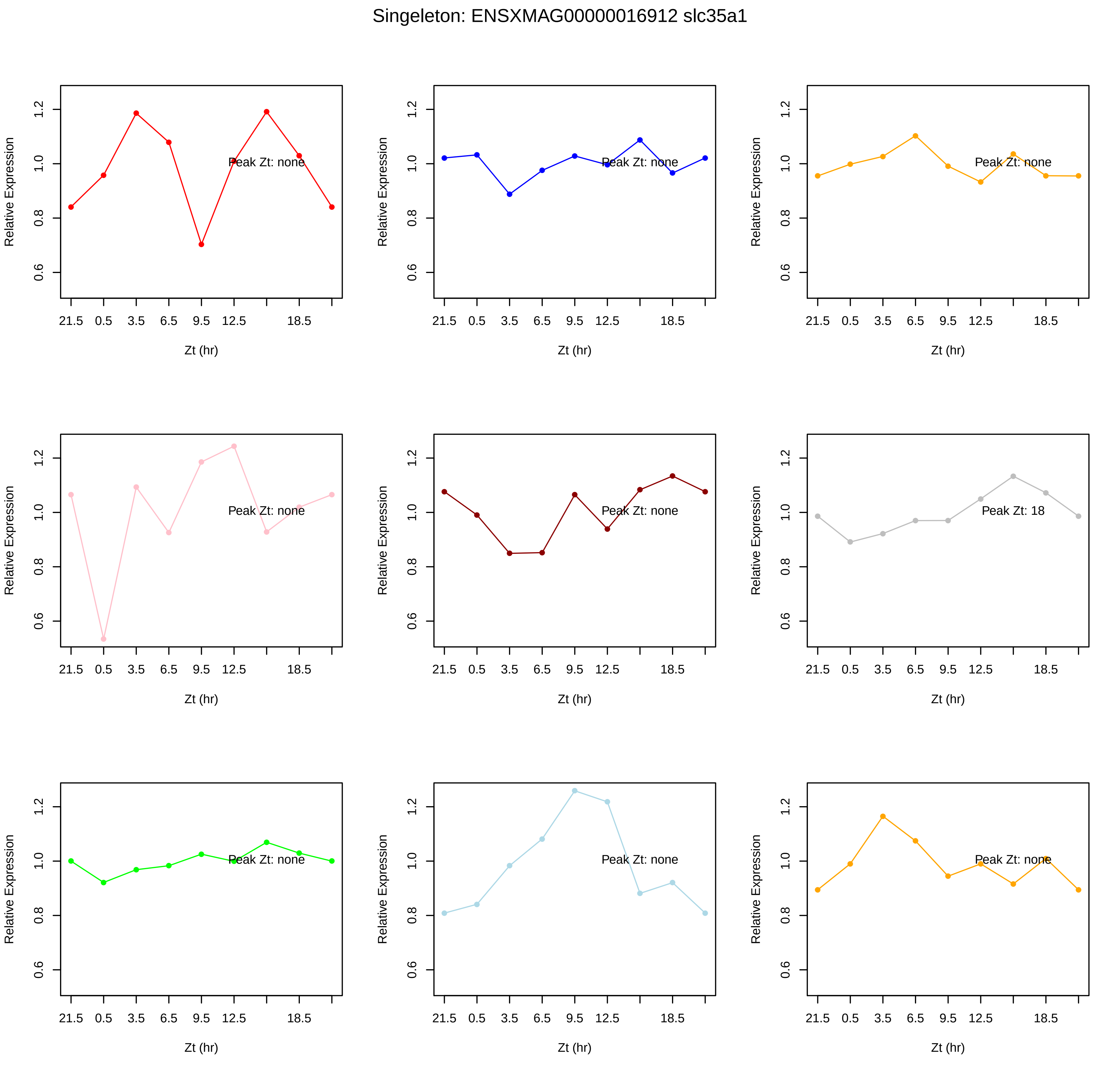
Singeleton: ENSXMAG00000016912 slc35a1
Relative Expression
Zt (hr)
21.5 0.5 3.5 6.5 9.5 12.5 18.5
0.6 0.8 1.0 1.2
Peak Zt: none
Peak Zt: none
Peak Zt: none
Peak Zt: none
Peak Zt: none
Peak Zt: 18
Peak Zt: none
Peak Zt: none
Peak Zt: none

Singeleton: ENSXMAG00000016921 rars2

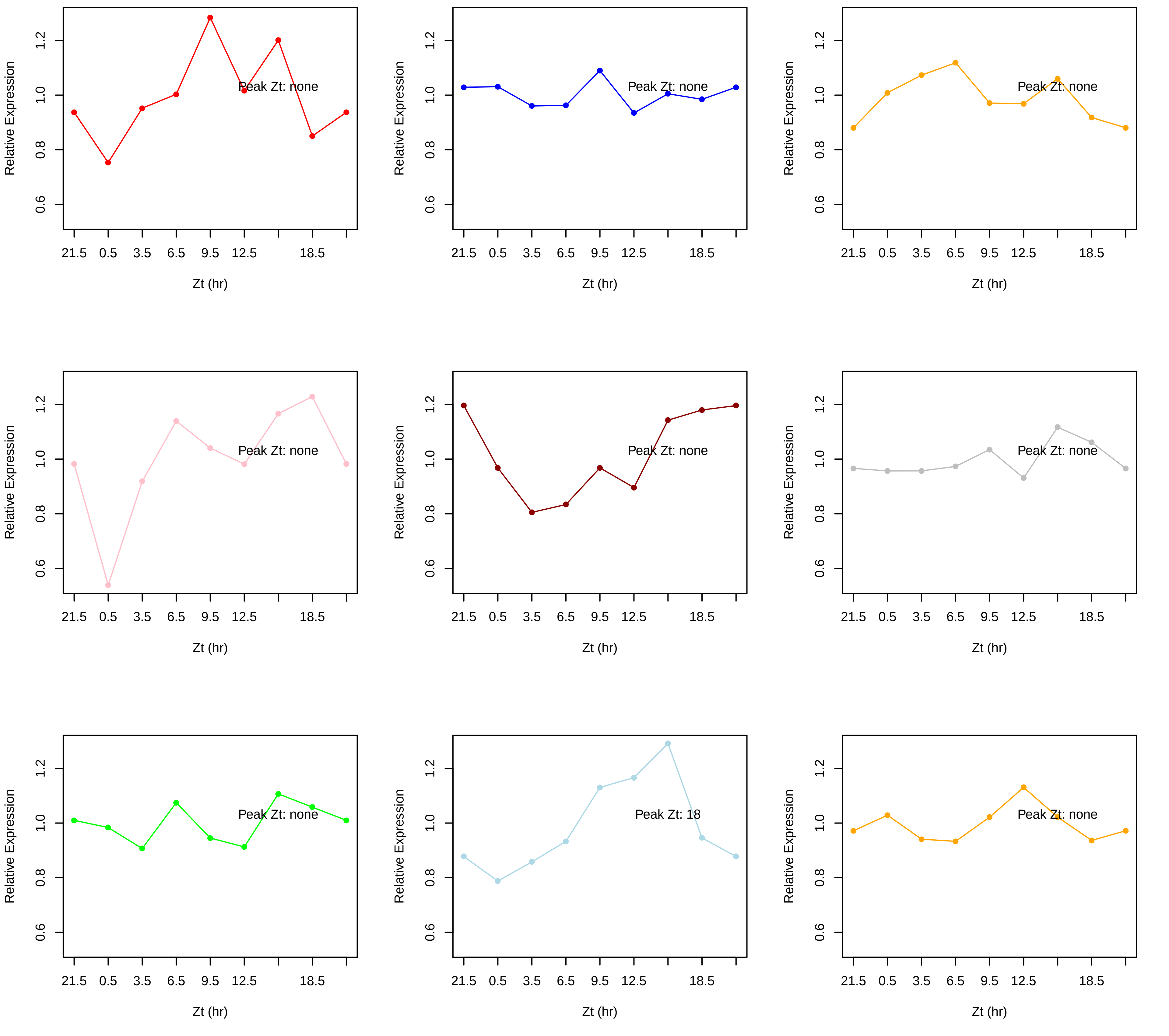

Singeleton: ENSXMAG00000016967 rngtt

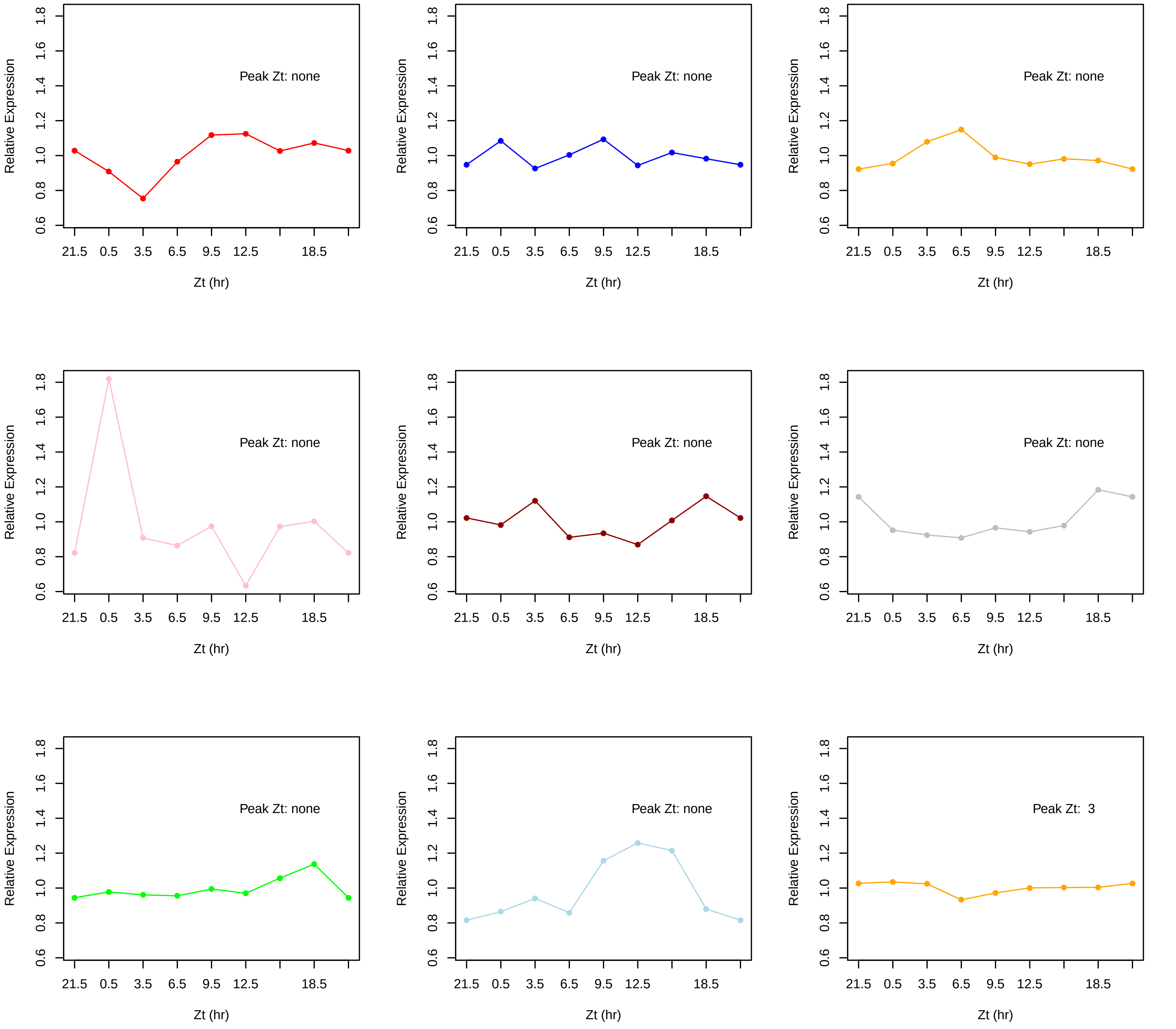

Singeleton: ENSXMAG00000026707 PM20D2

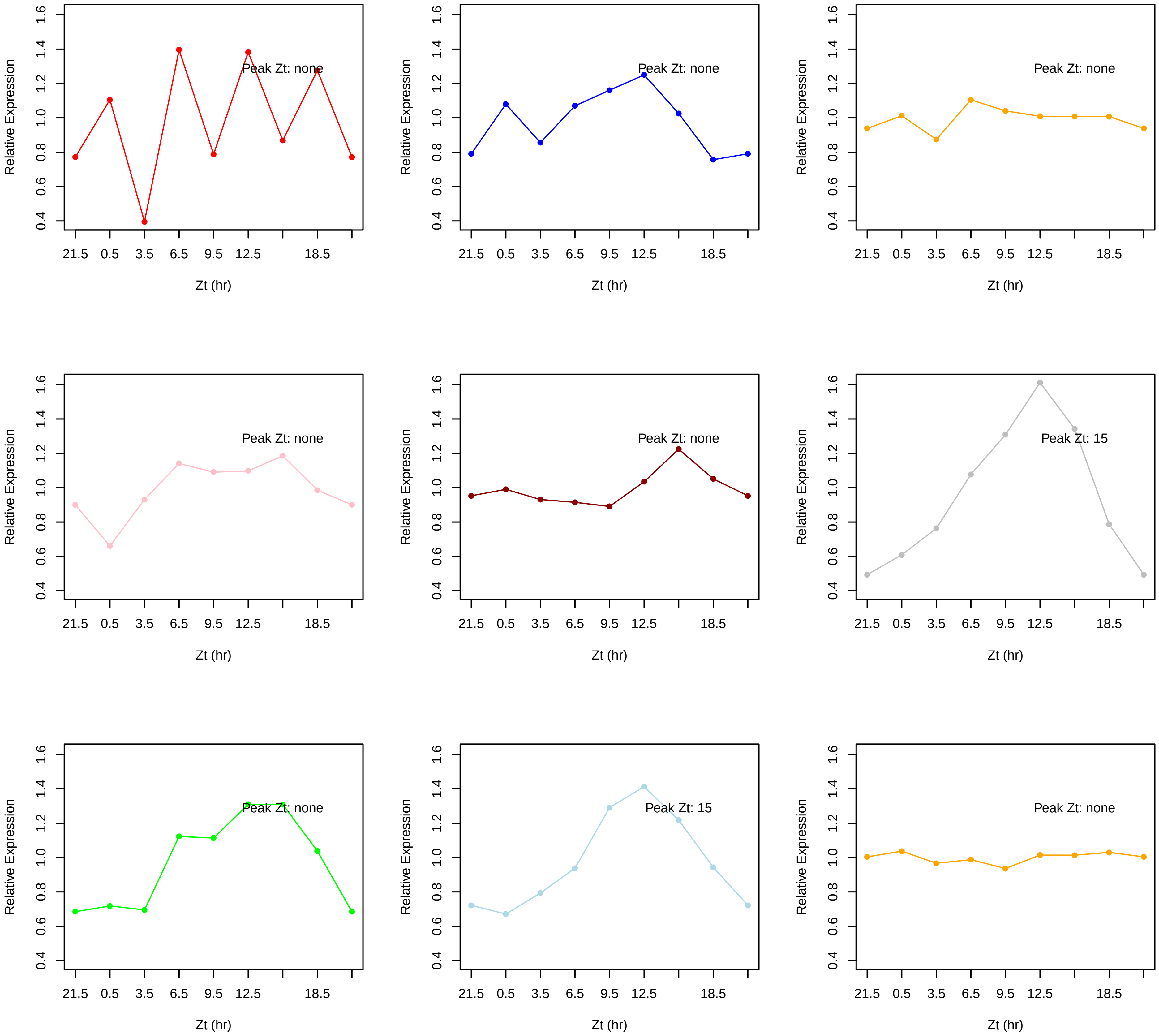

Singeleton: ENSXMAG00000003487

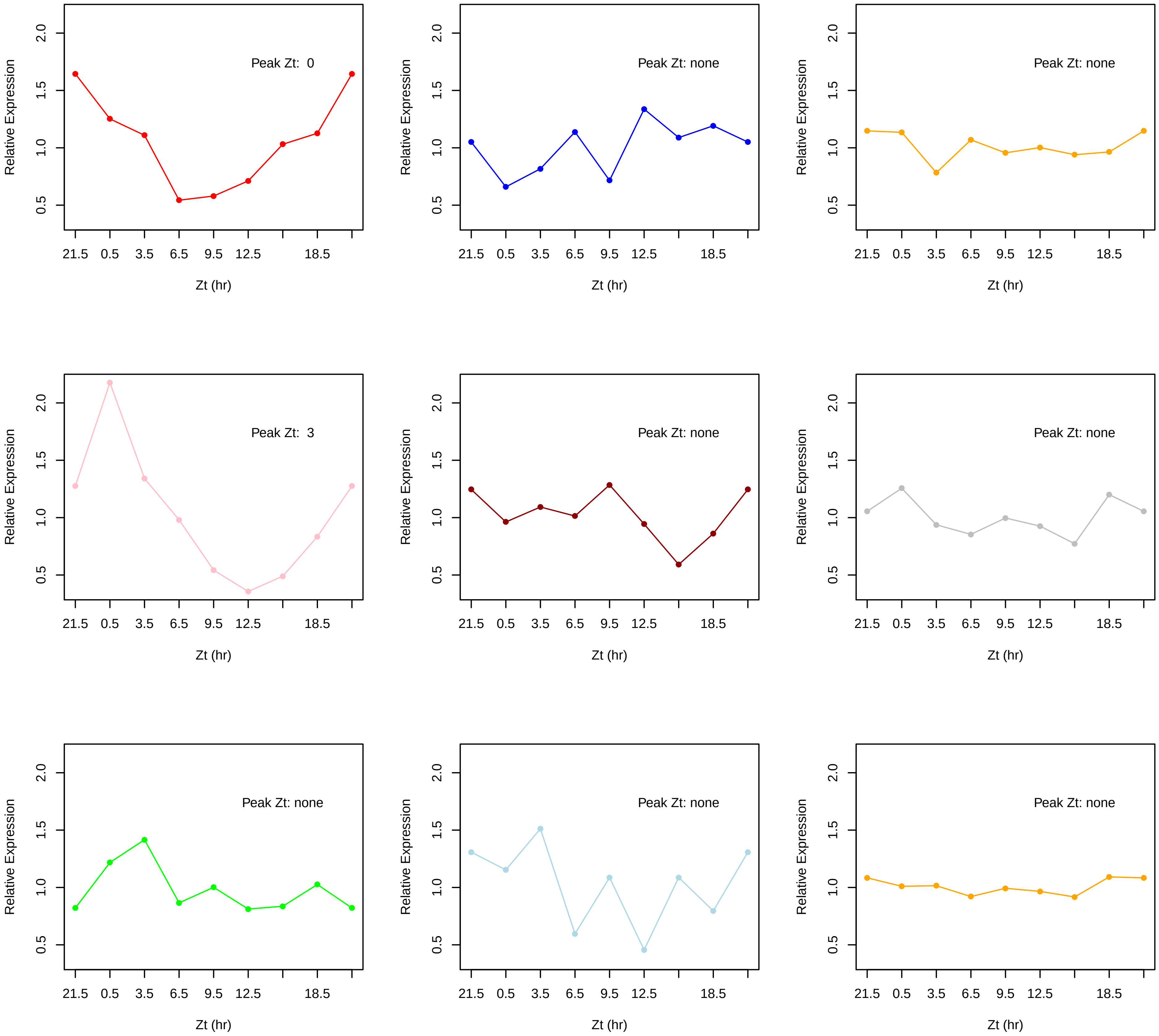

Singeleton: ENSXMAG00000000600

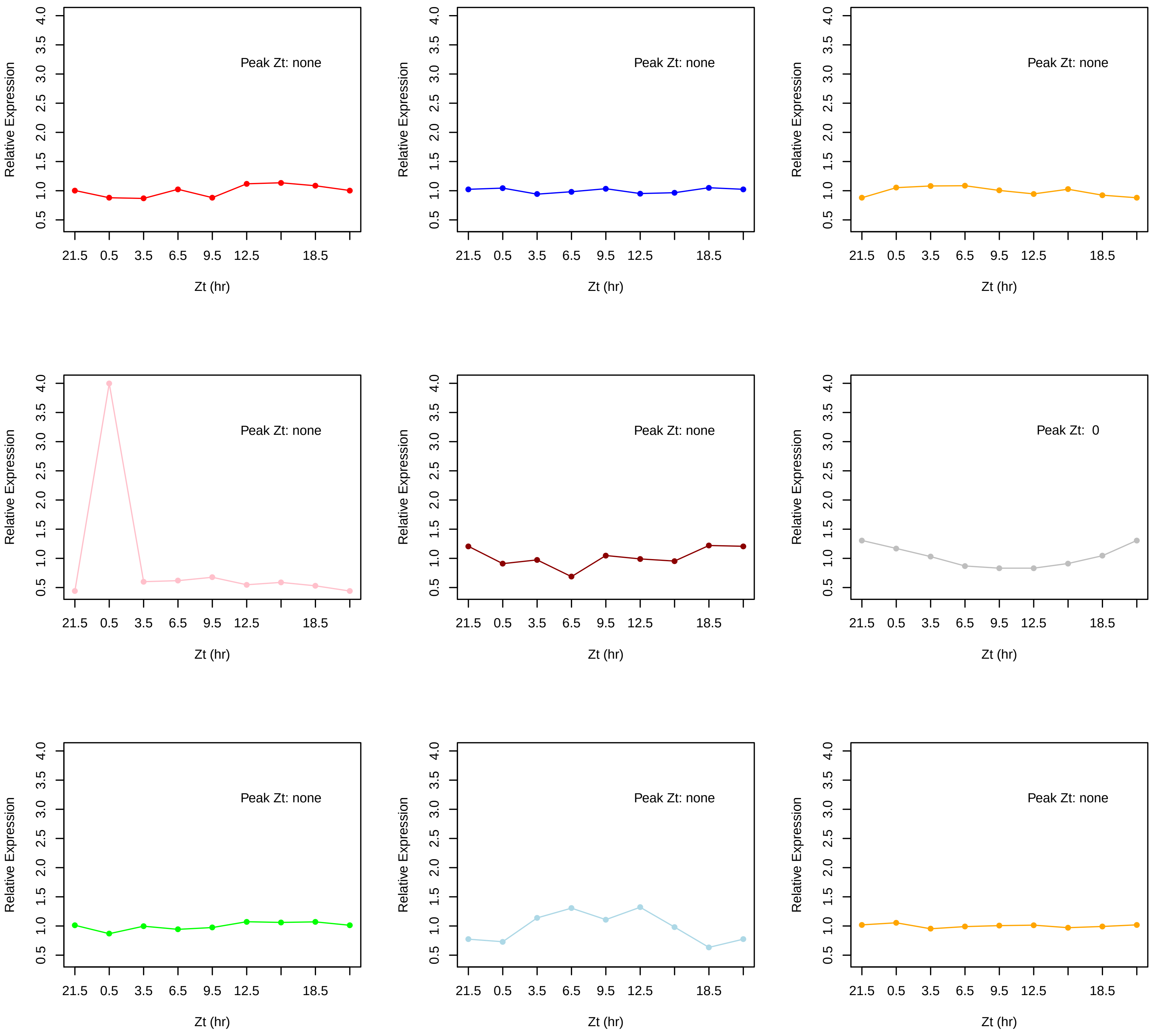

Singeleton: ENSXMAG00000013764 ankrd6a

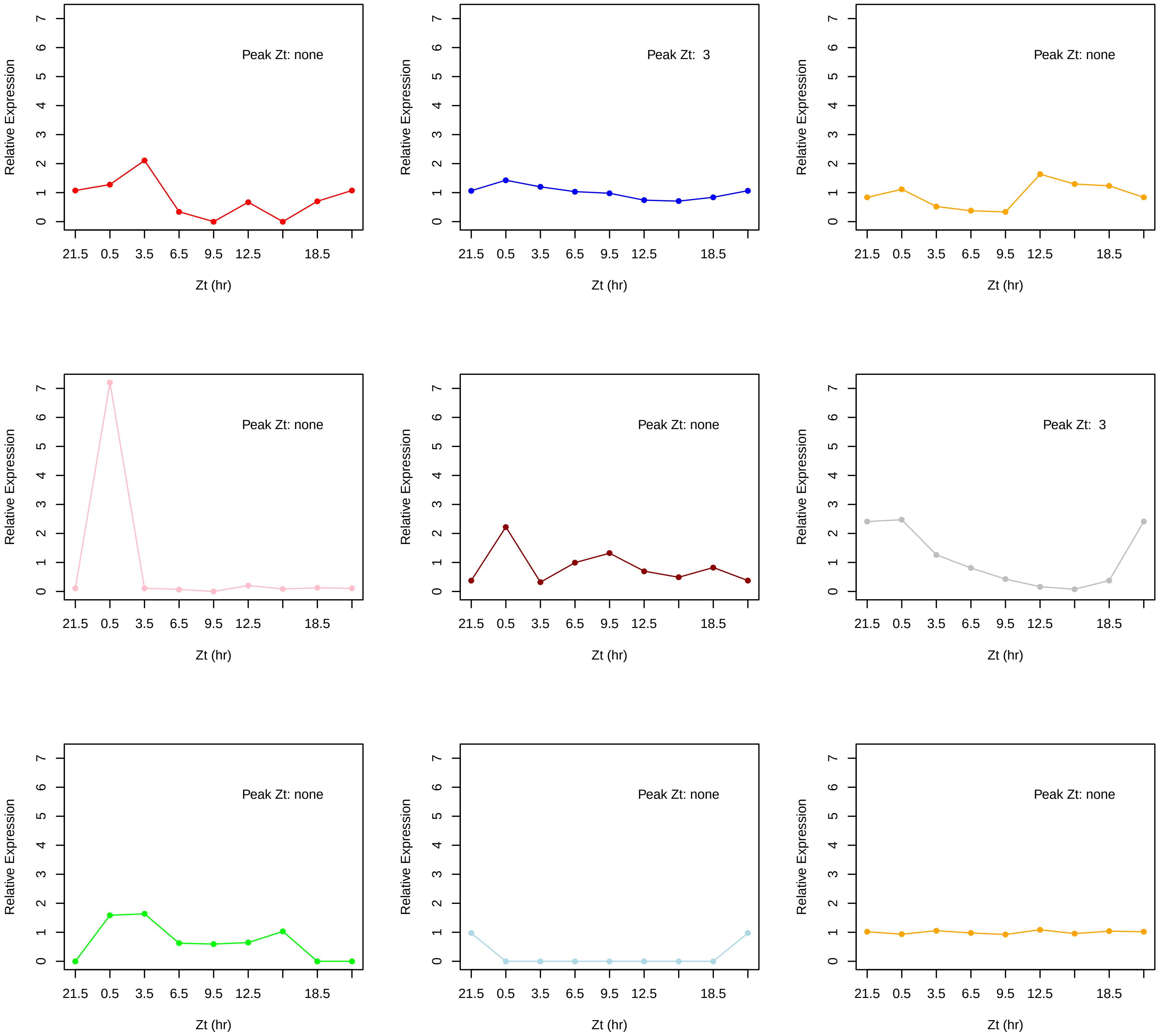

Singeleton: ENSXMAG00000009709 manea

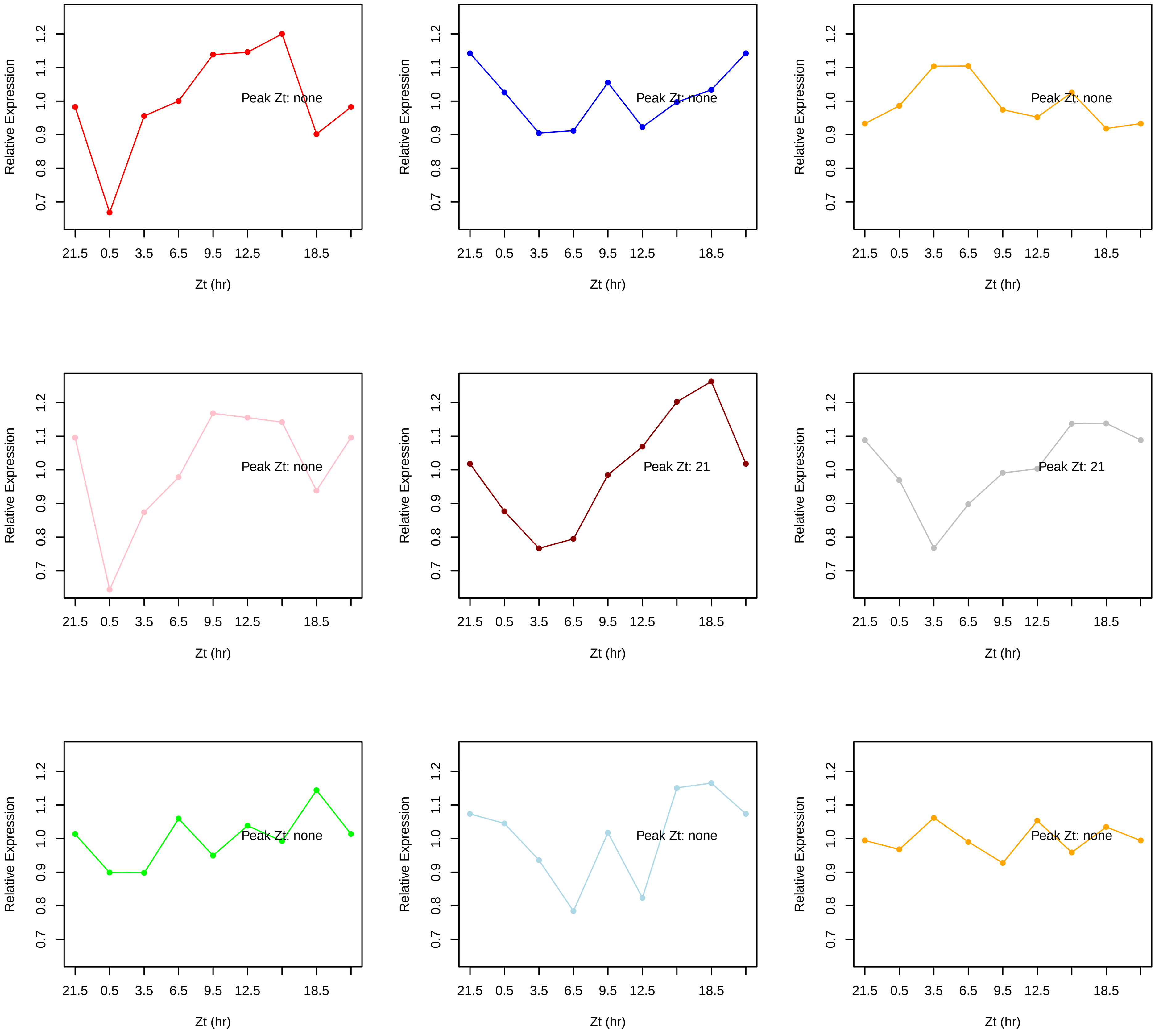

Singeleton: ENSXMAG00000025685 ufl1

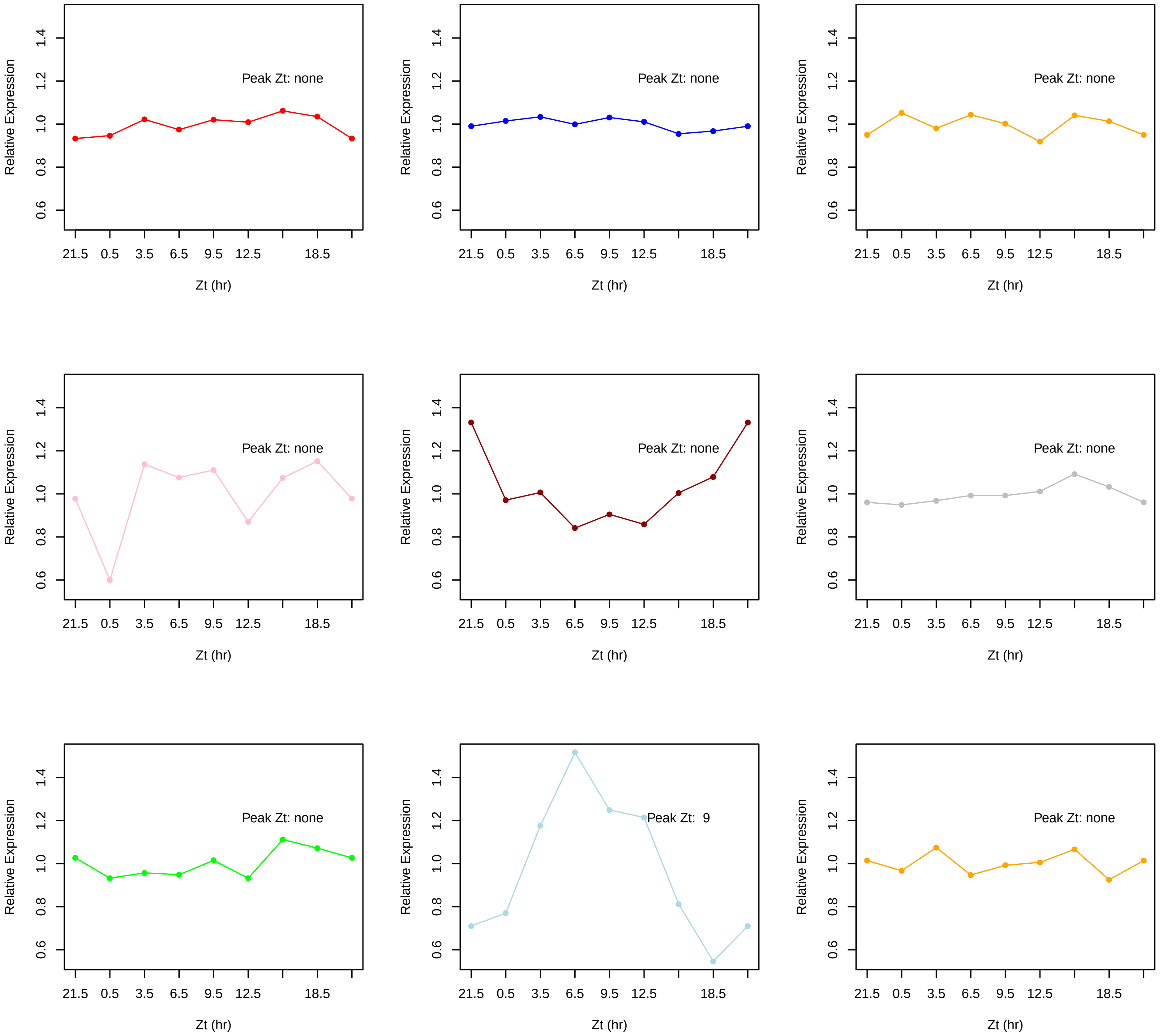

Singeleton: ENSXMAG00000009005 dse

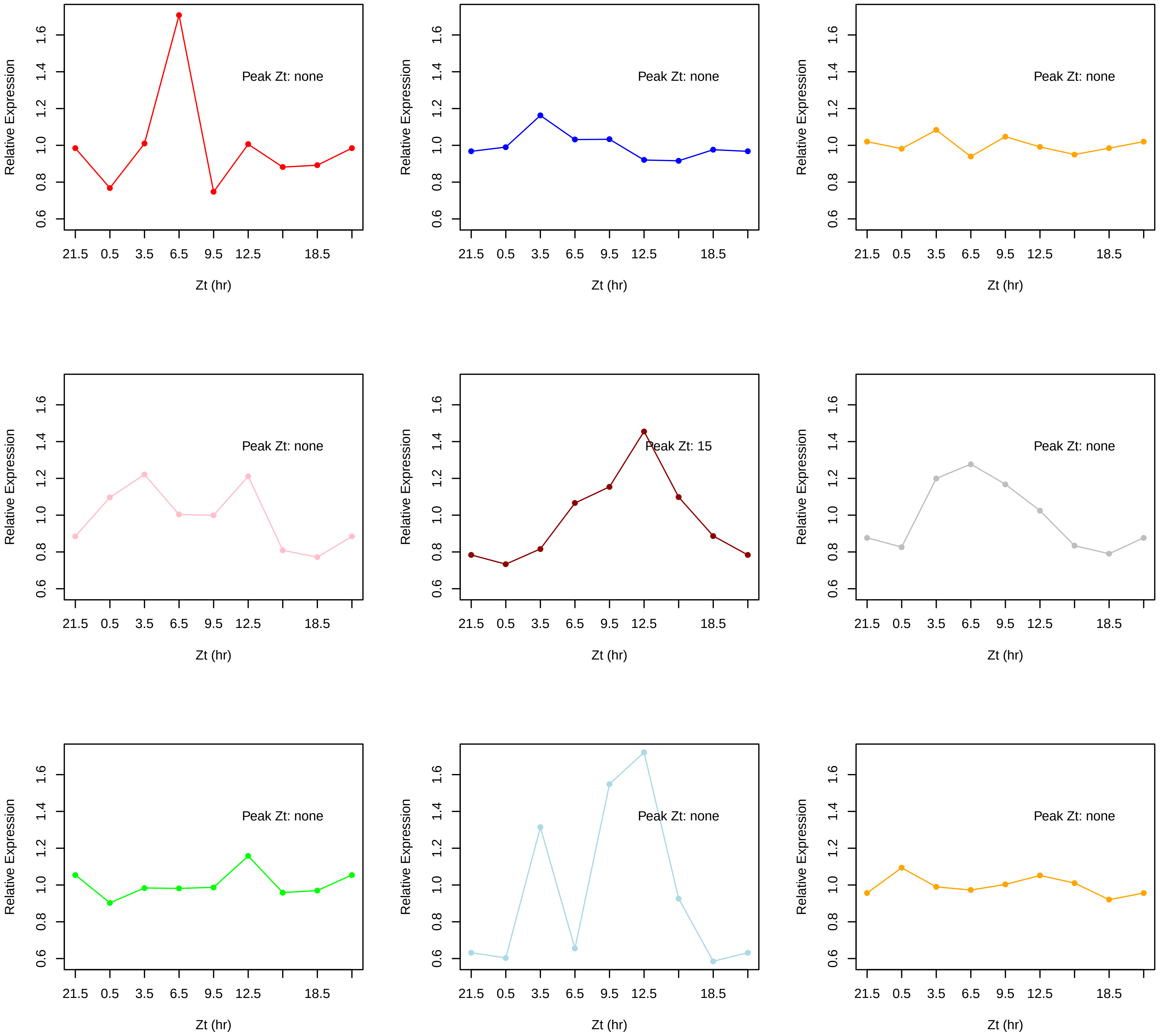

Singeleton: ENSXMAG00000005199 HDAC2

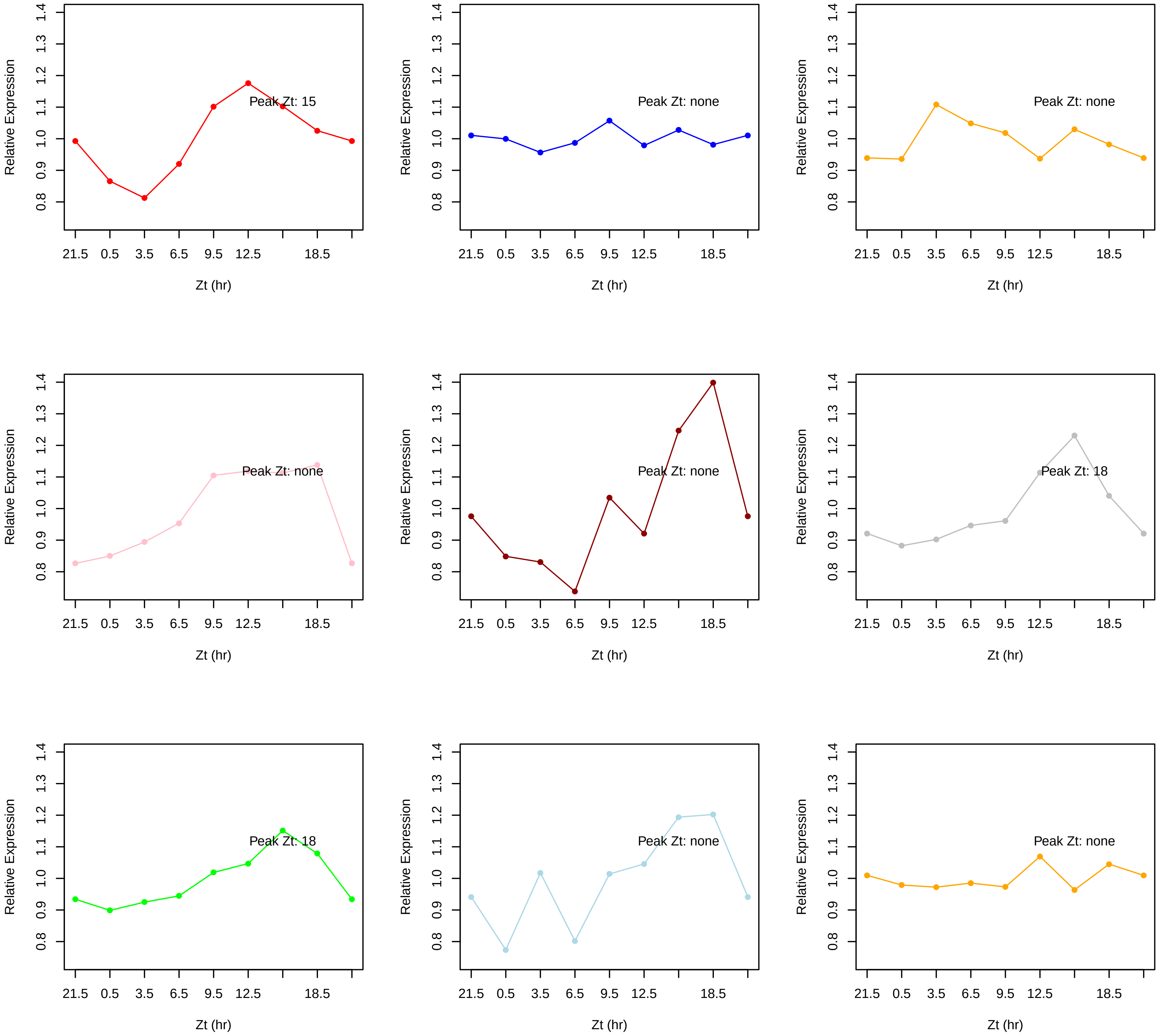

Singleton: ENSXMAG00000005197 MARCKS

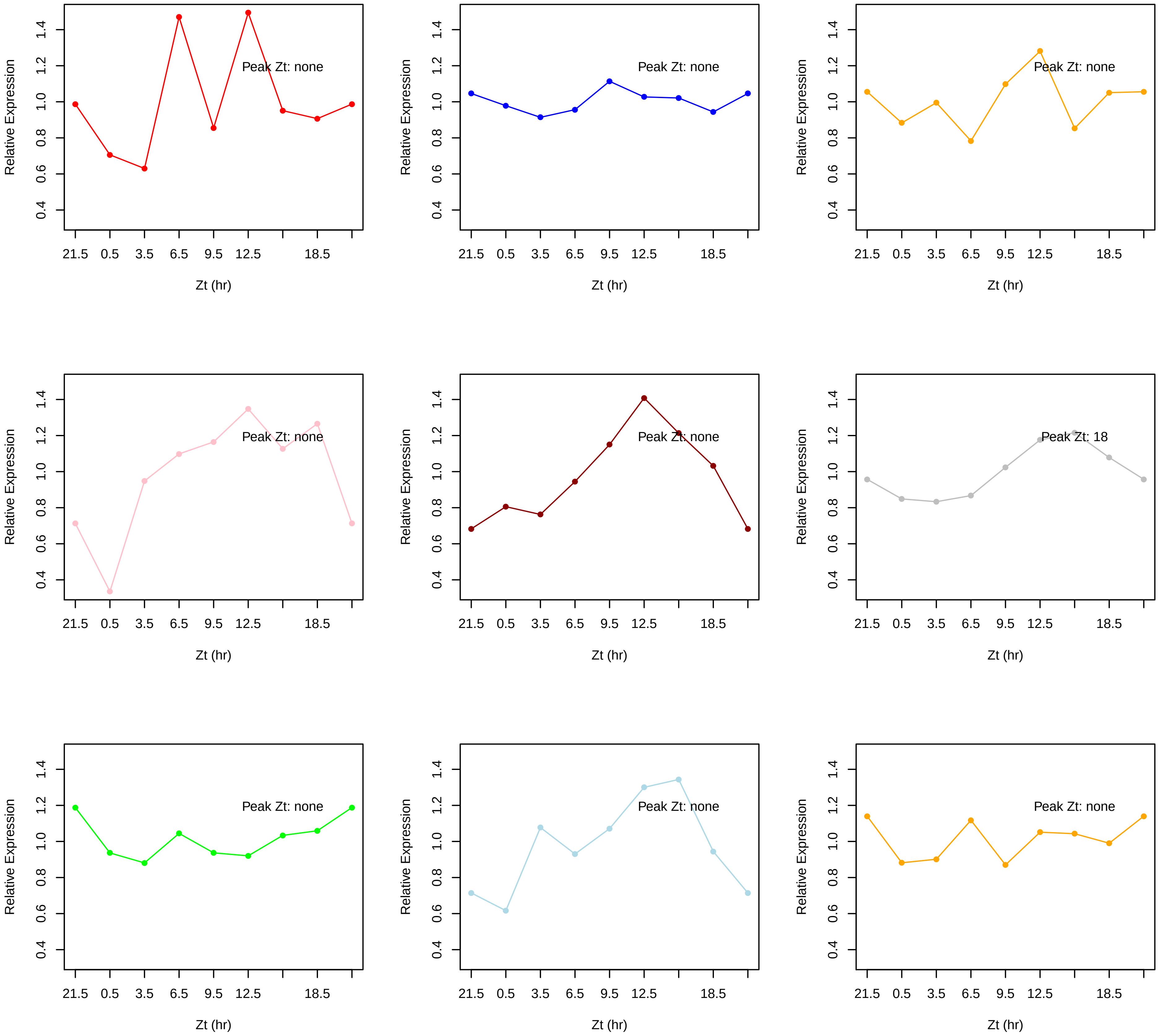

Singleton: ENSXMAG00000005194 LAMA4

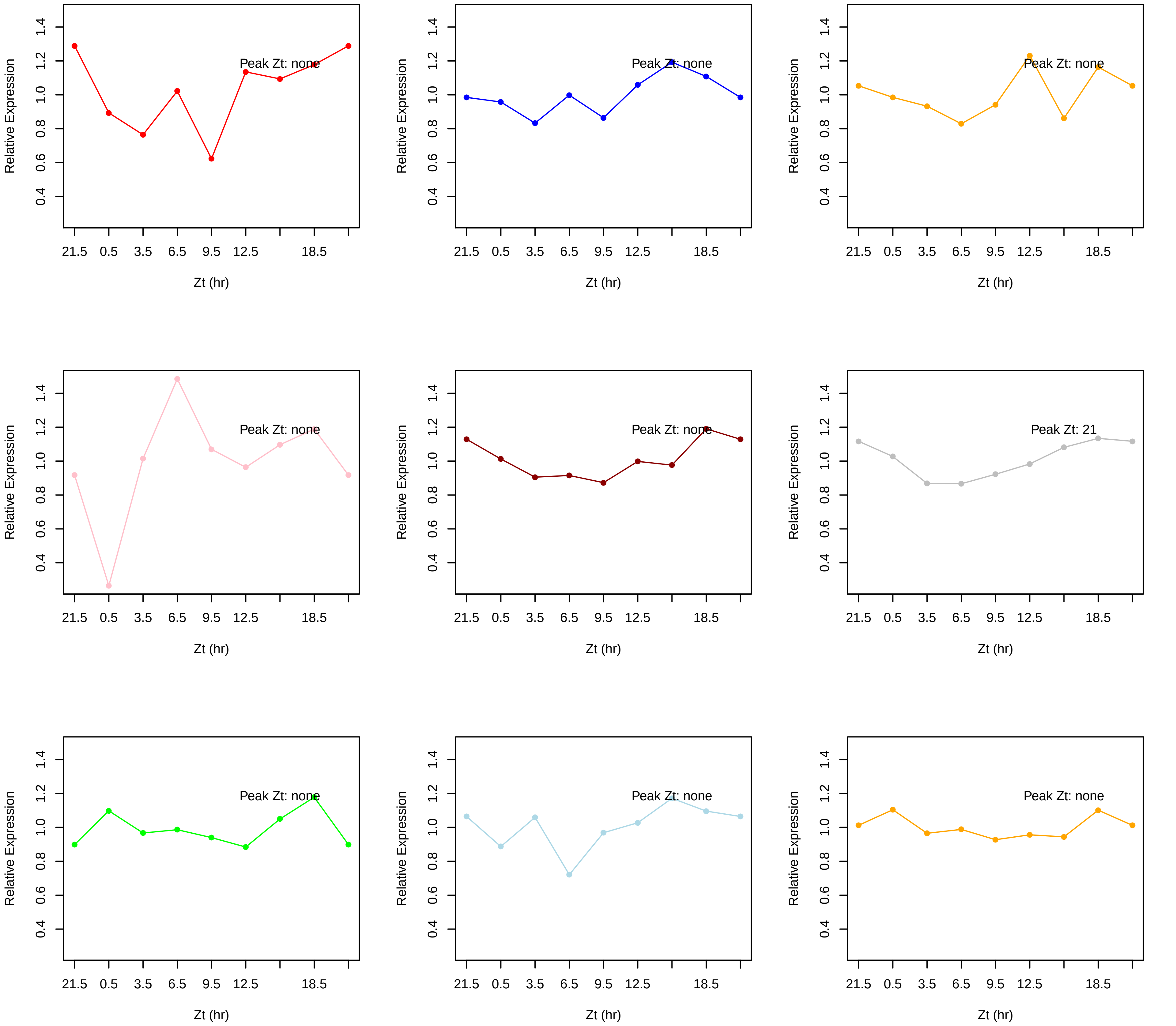

Singeleton: ENSXMAG00000019131 rxylt1

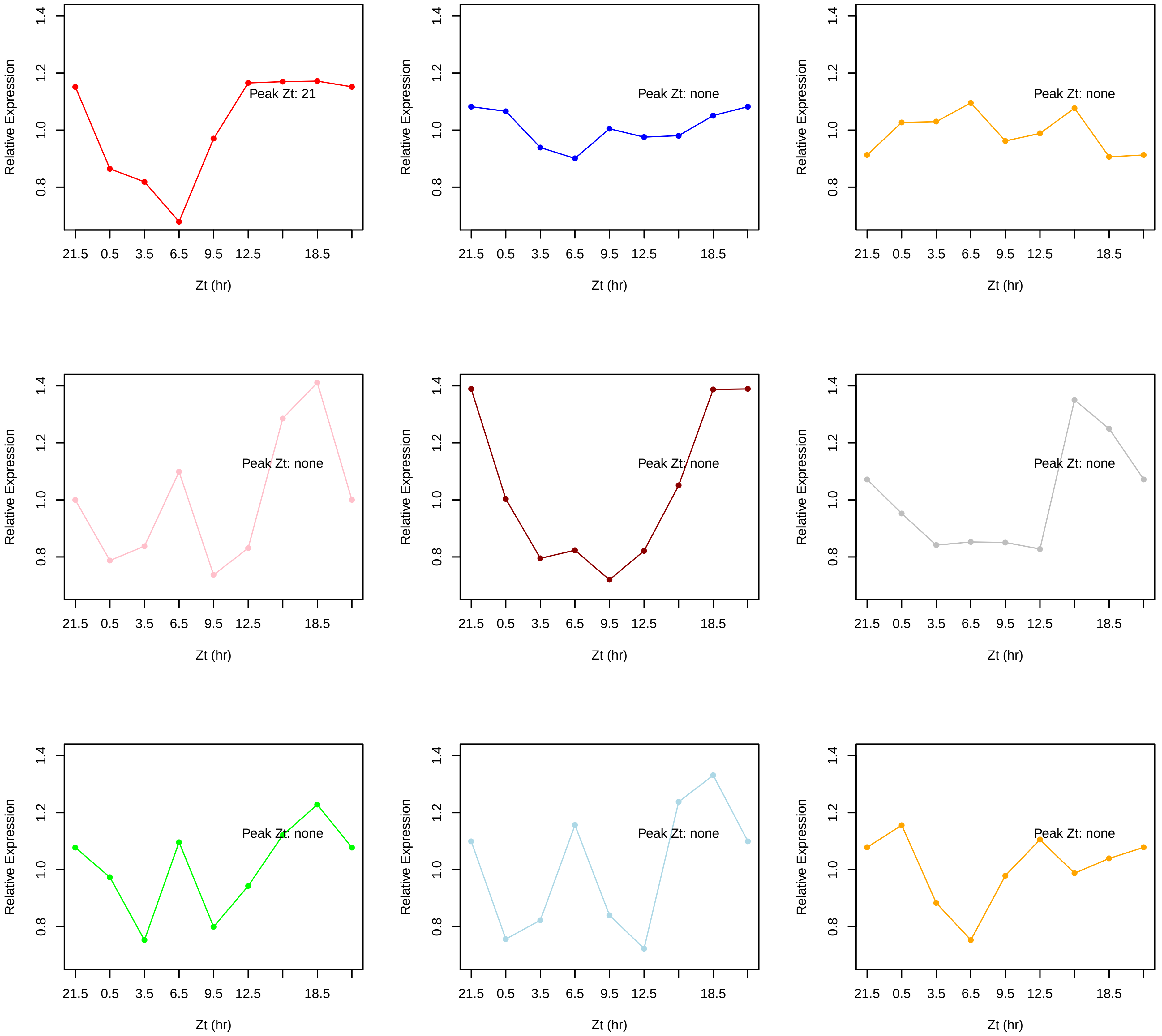

Singeleton: ENSXMAG00000022599 rev3l

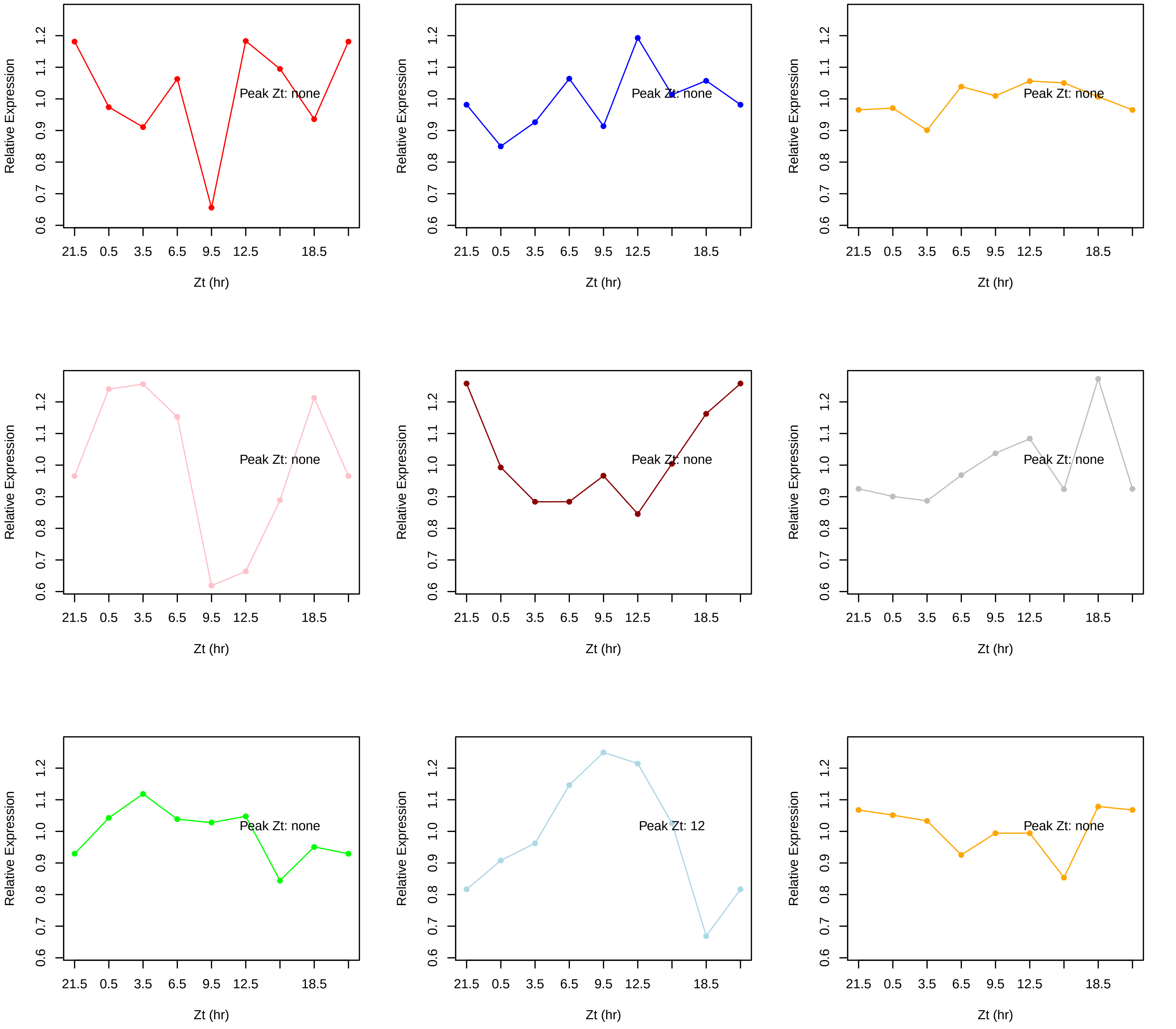

Singeleton: ENSXMAG00000004421 USP15

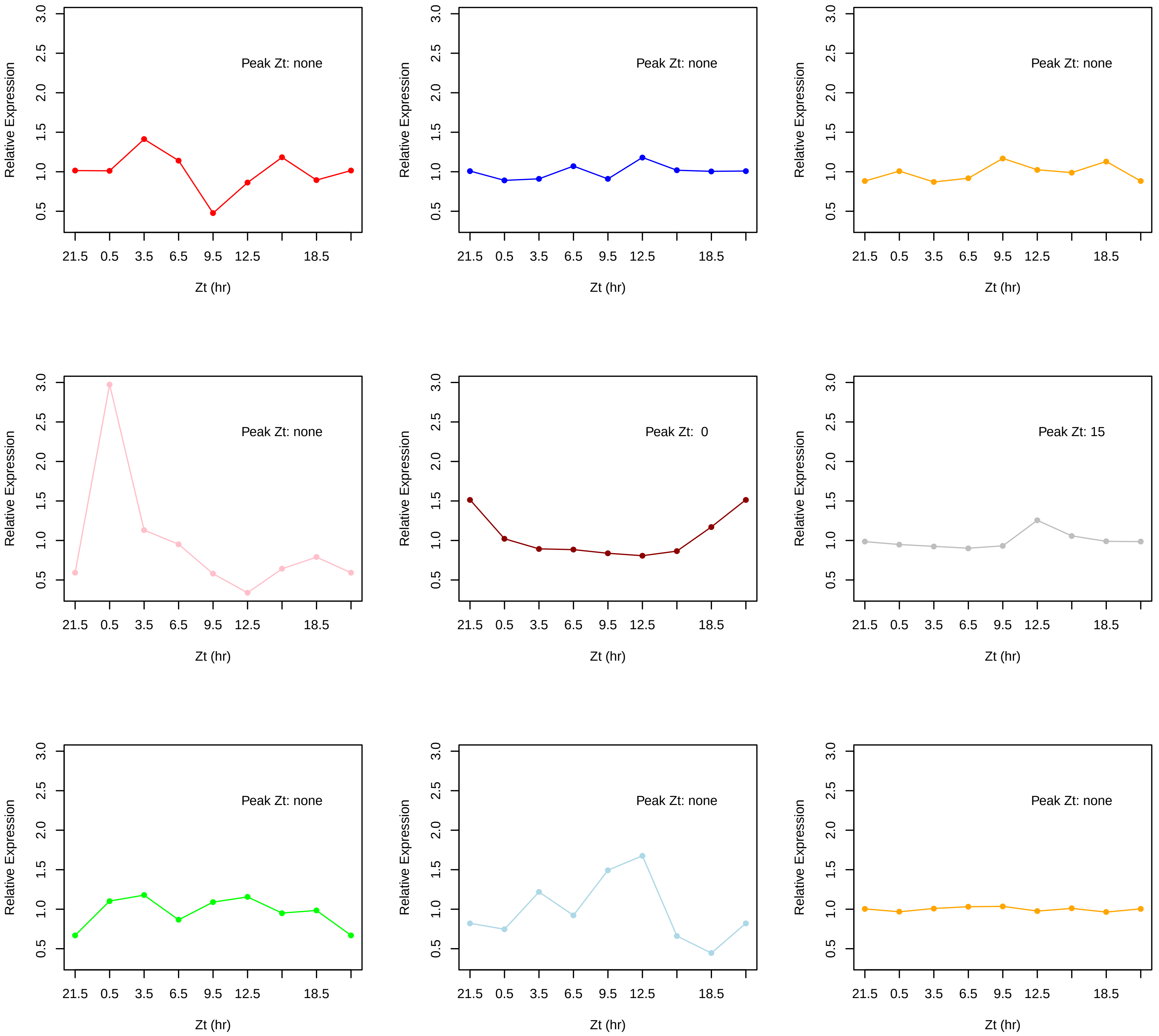

Singeleton: ENSXMAG00000021796 slc16a10

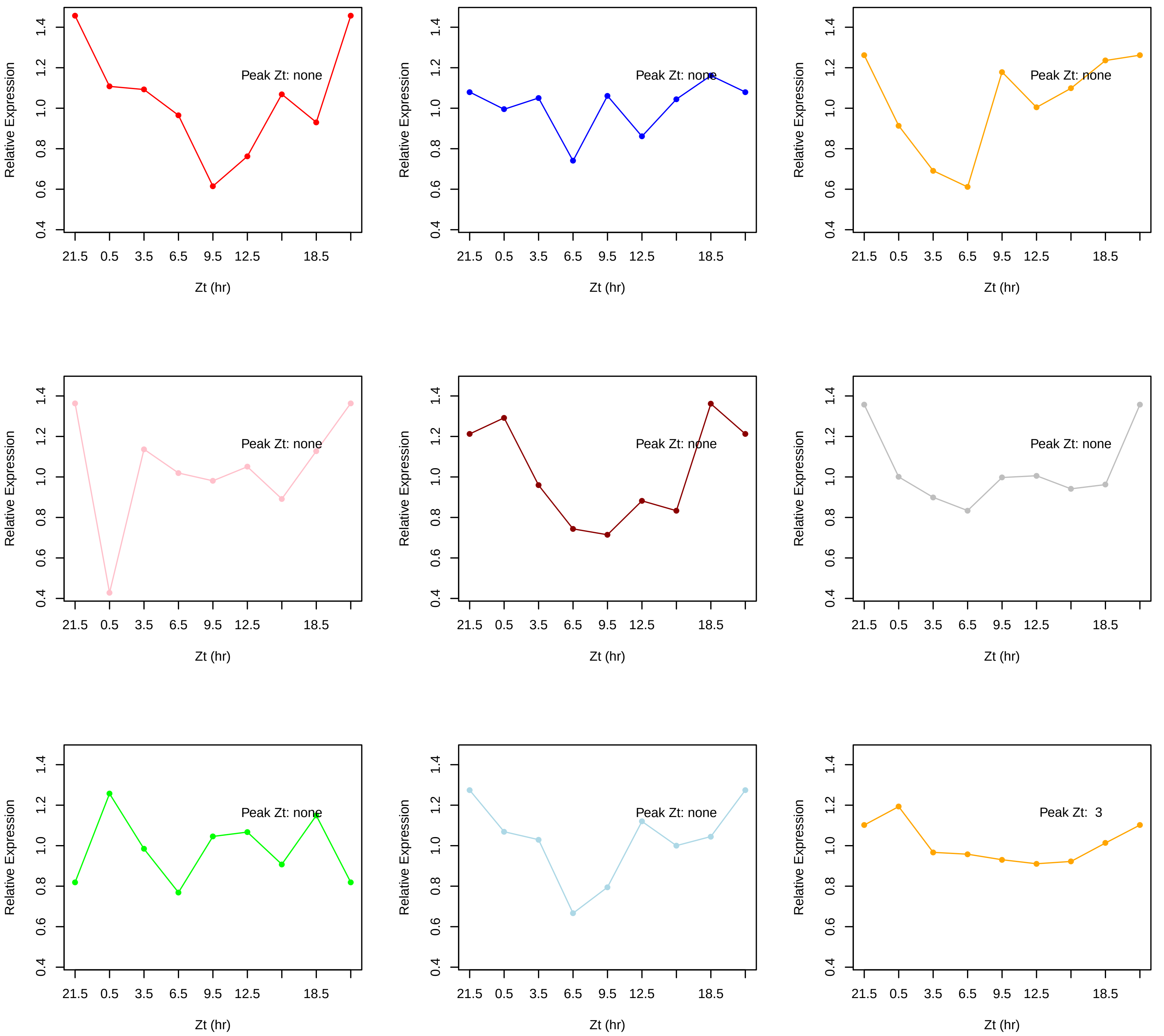

Singleton: ENSXMAG00000005067 amd1

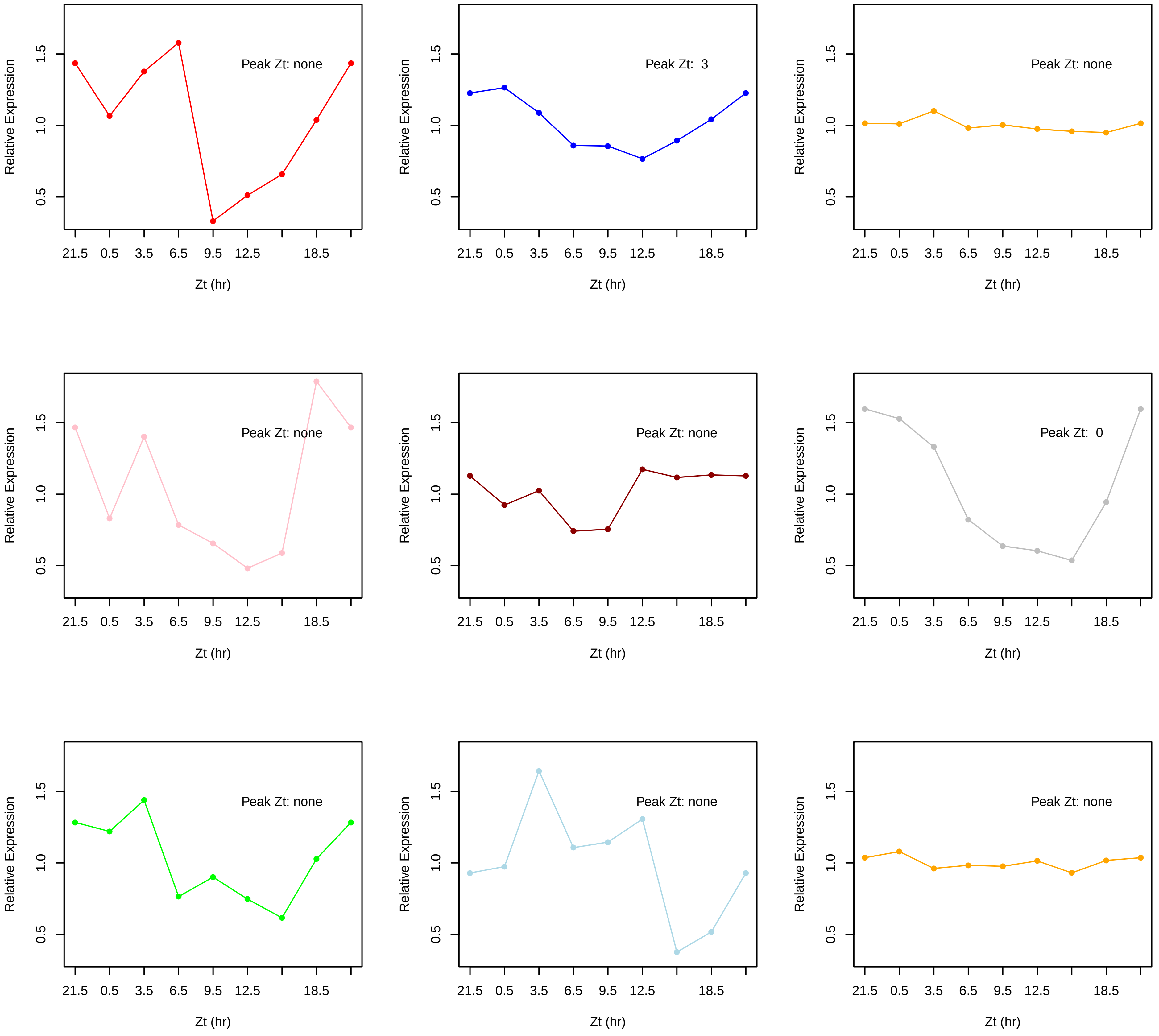

Singeleton: ENSXMAG00000005023

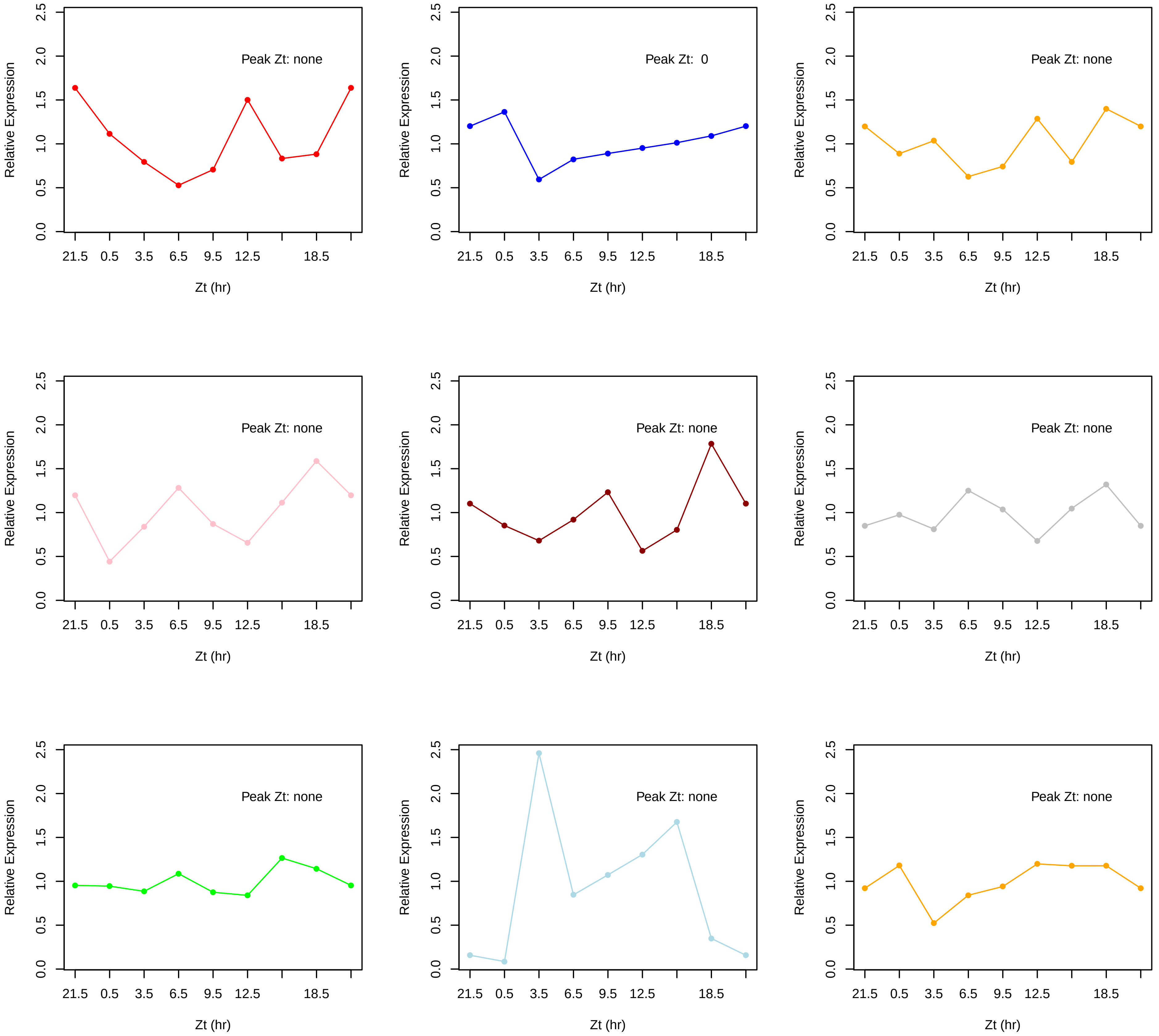

Singeleton: ENSXMAG00000004521 KDM5A

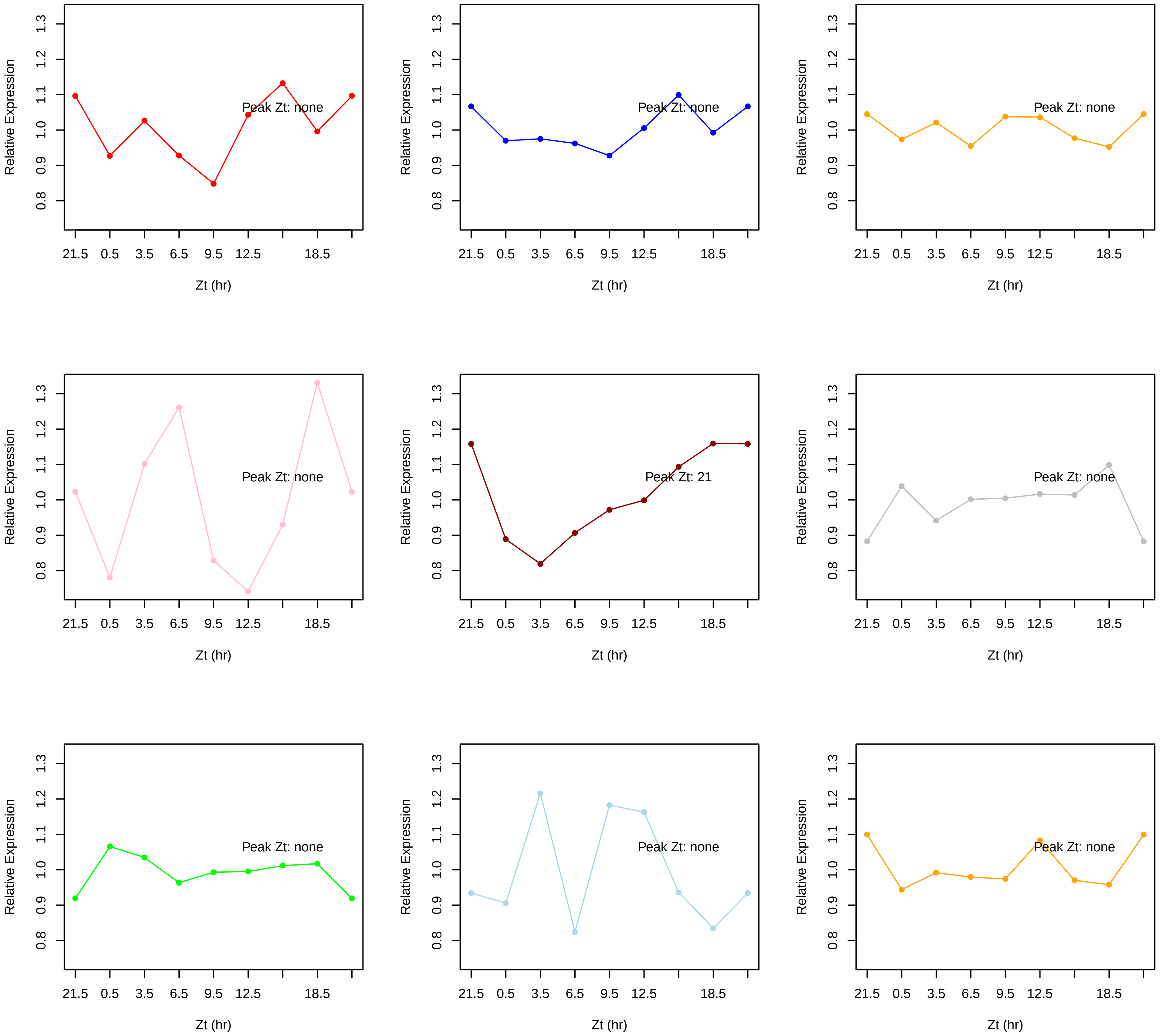

Singeleton: ENSXMAG00000004998 ppil4

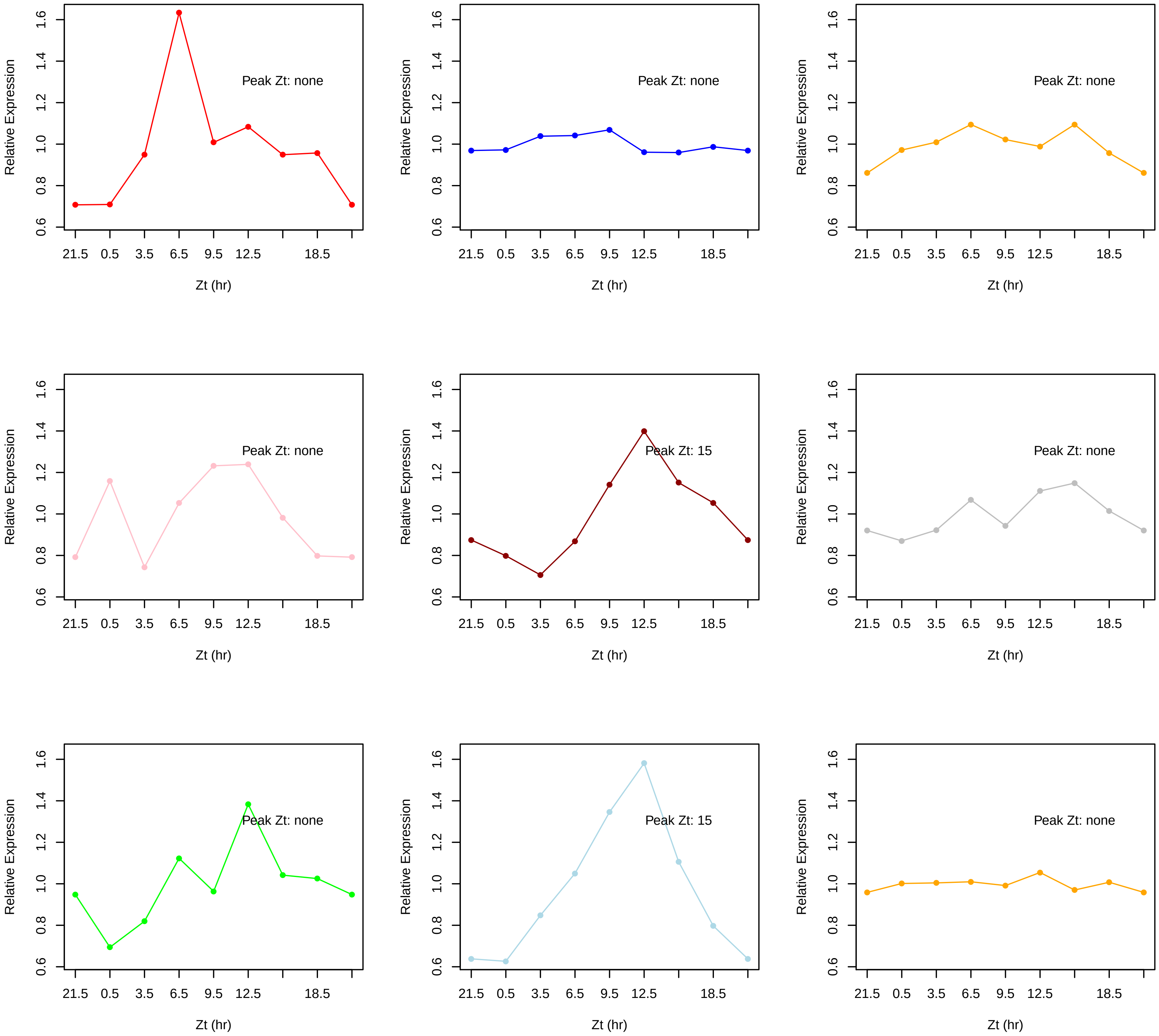

Singeleton: ENSXMAG00000006278 lats1

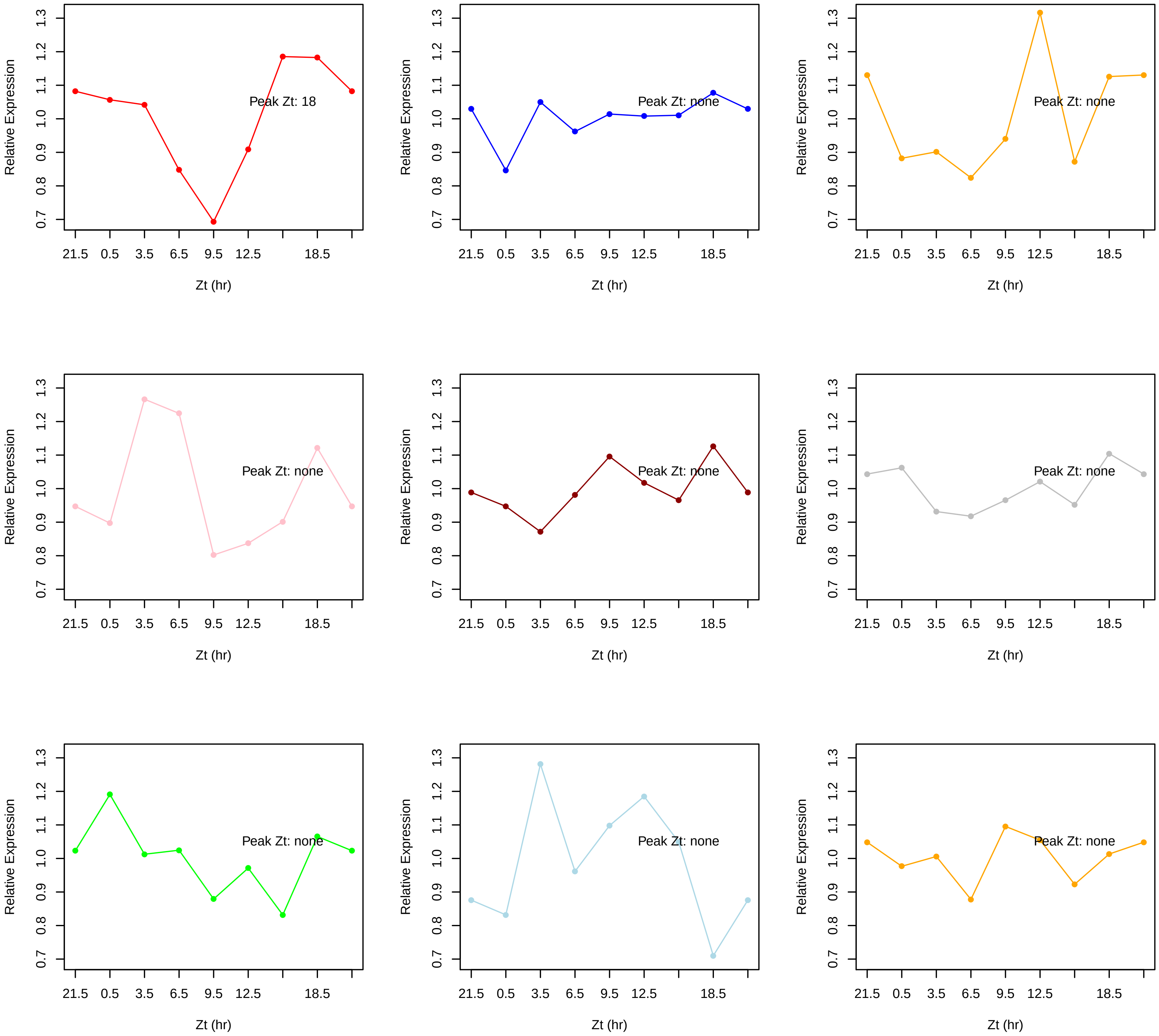

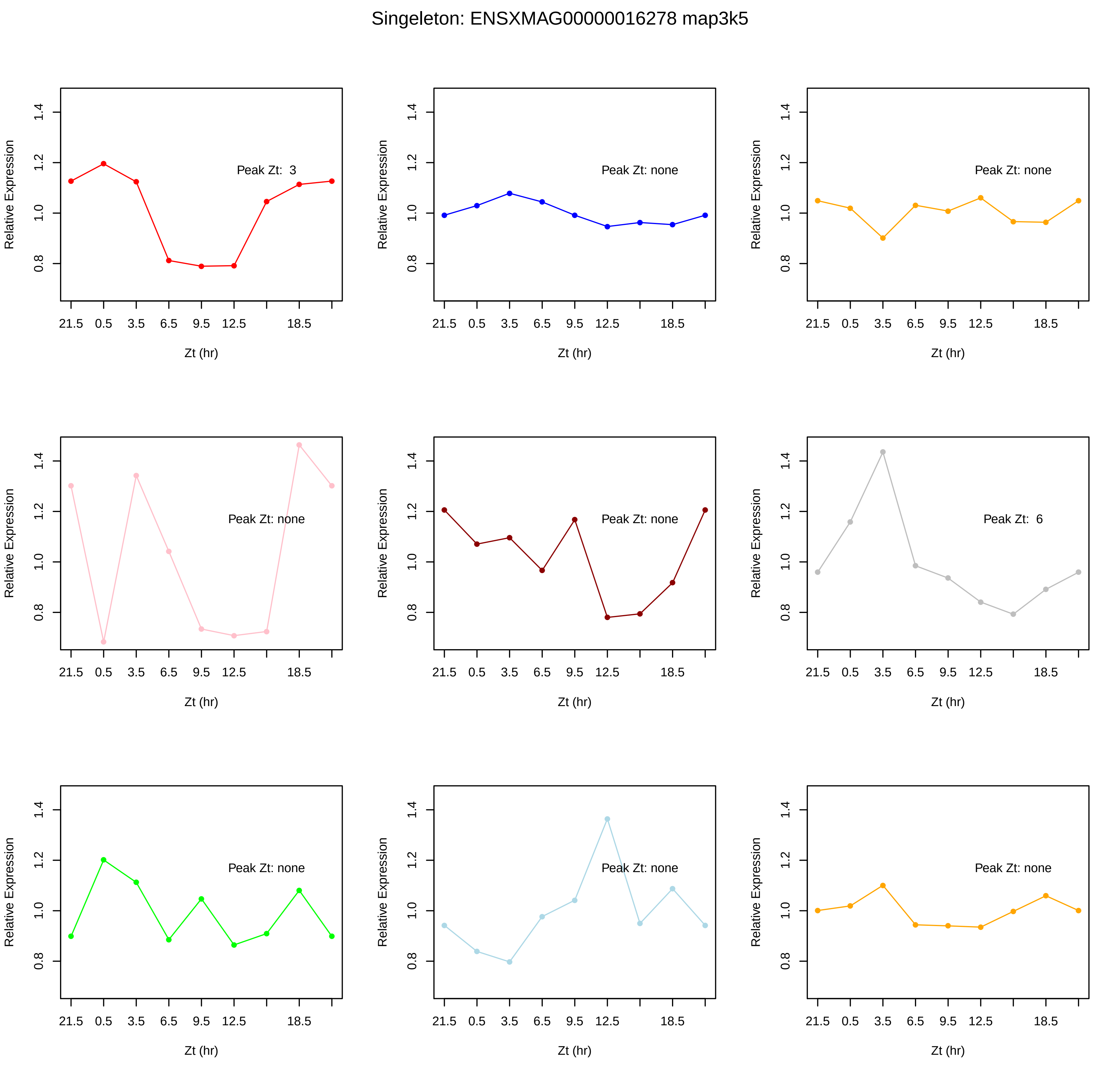
Singeleton: ENSXMAG00000016278 map3k5
Relative Expression
Zt (hr)
21.5
0.5
3.5
6.5
9.5
12.5
18.5
0.8
1.0
1.2
1.4
Peak Zt: 3
Peak Zt: none
Peak Zt: 6

Singeleton: ENSXMAG00000006892 ctgfa

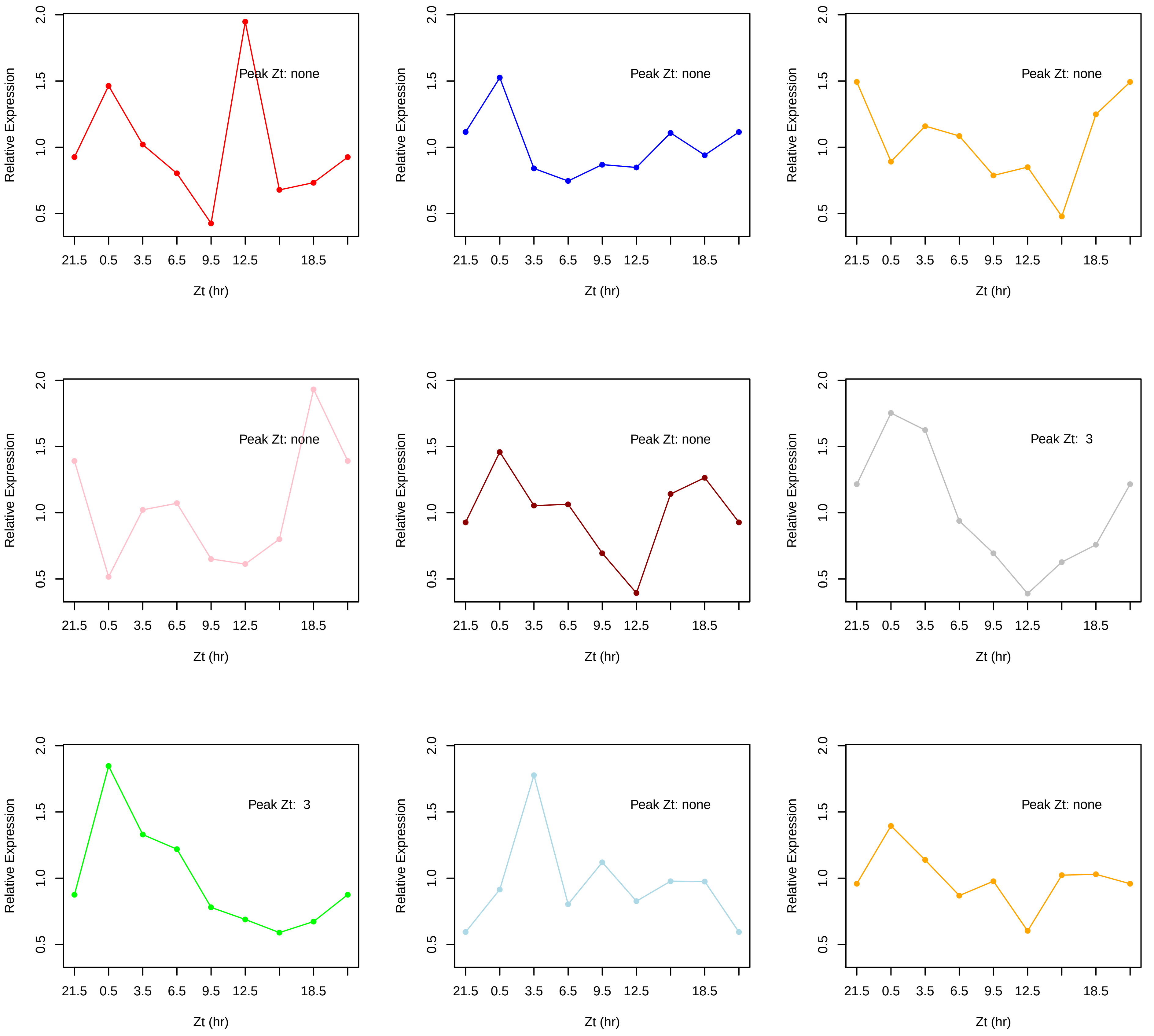

Singleton: ENSXMAG00000010309

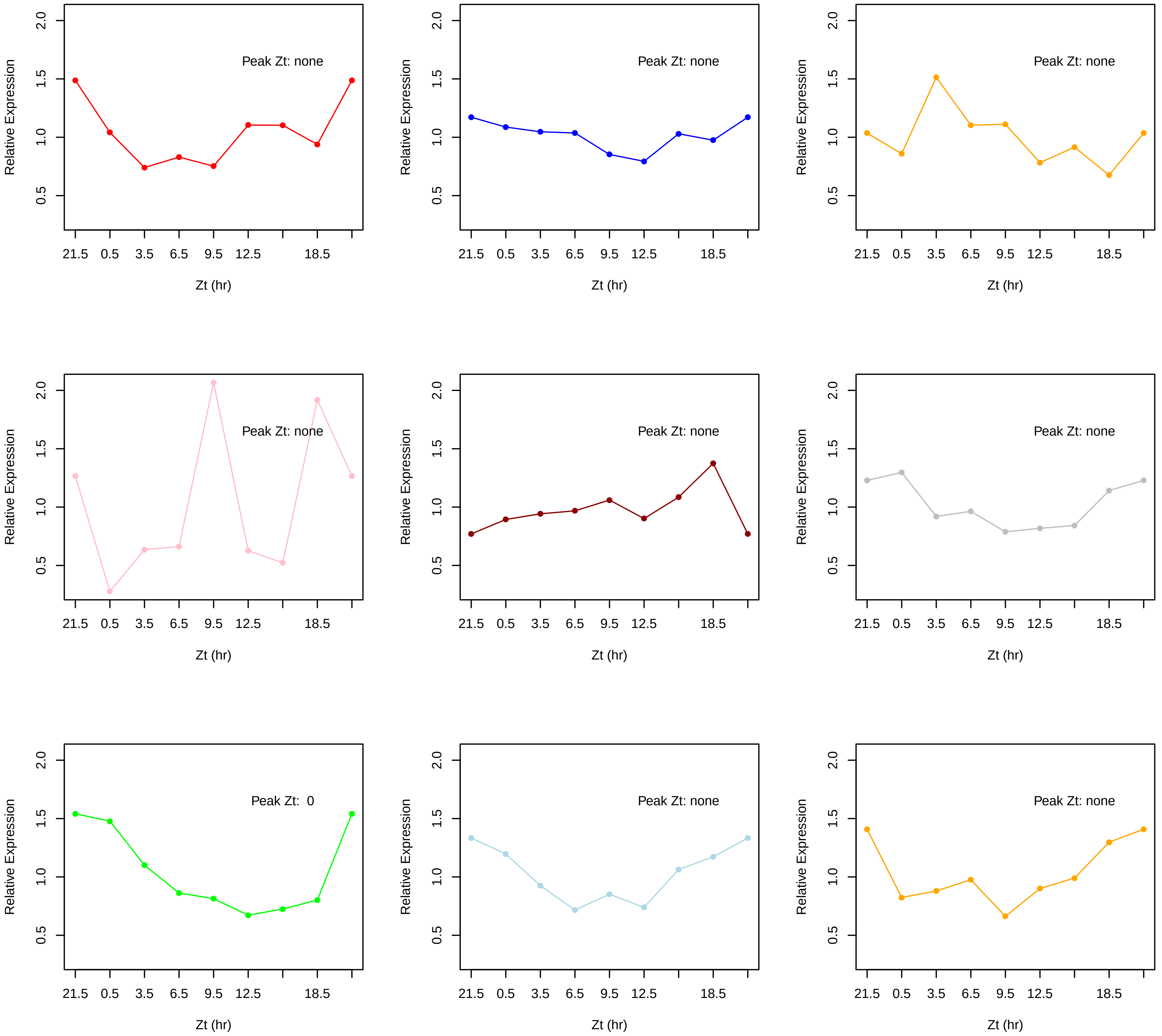

Singeleton: ENSXMAG00000010304 wnt7bb

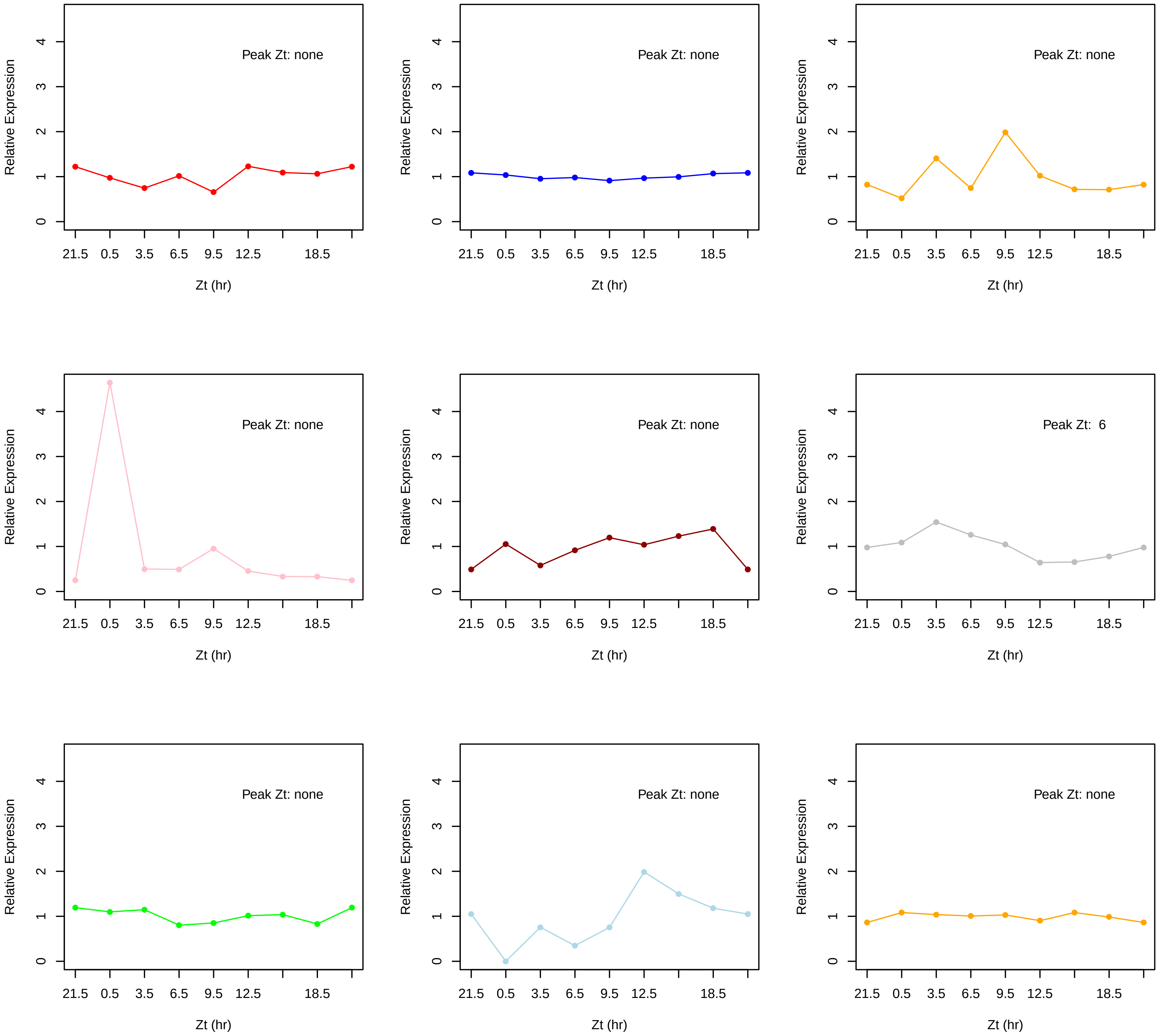

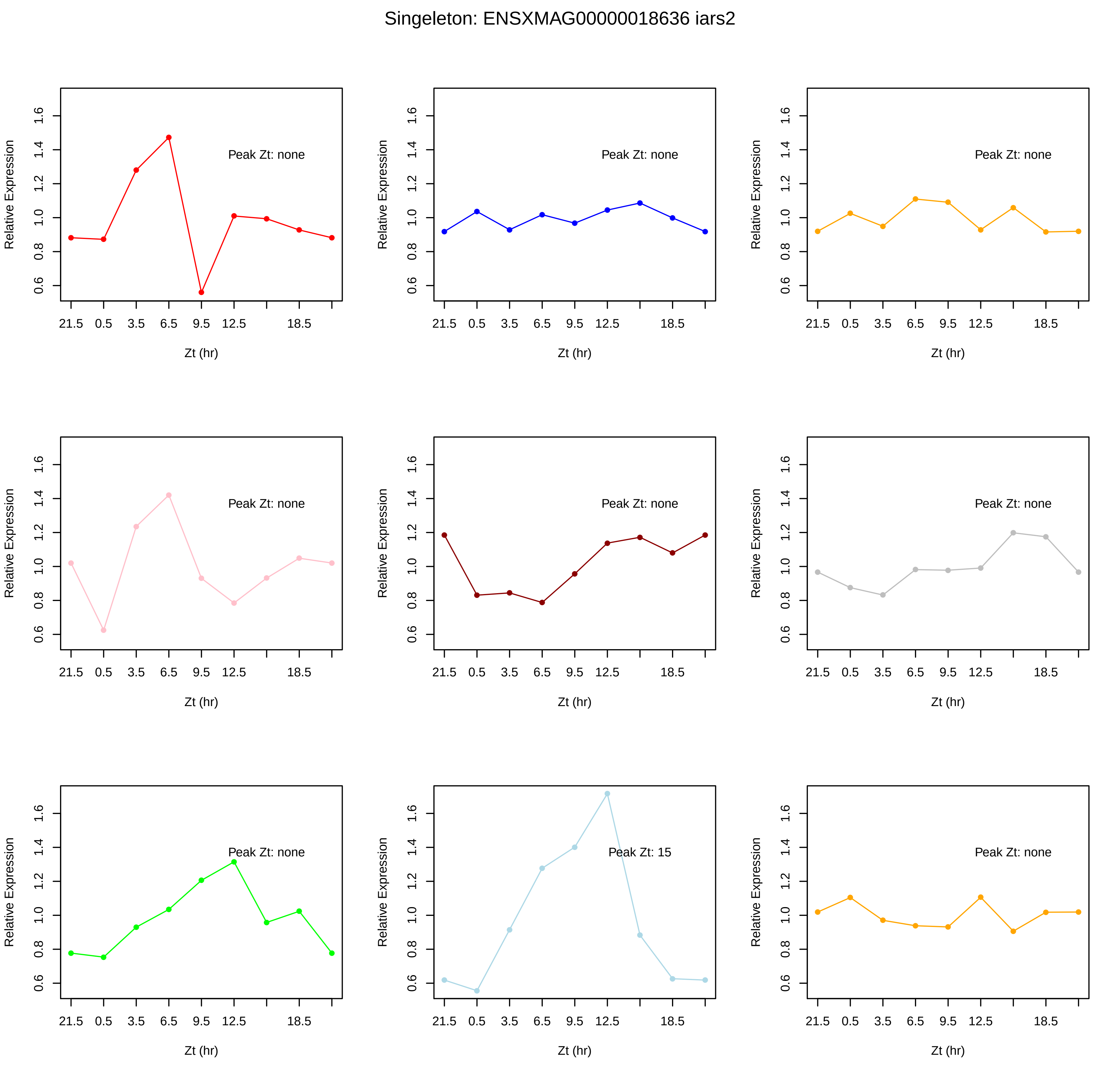
Singeleton: ENSXMAG00000018636 iars2
Relative Expression
Zt (hr)
21.5
0.5
3.5
6.5
9.5
12.5
18.5
0.6
0.8
1.0
1.2
1.4
1.6
Peak Zt: none
Peak Zt: 15

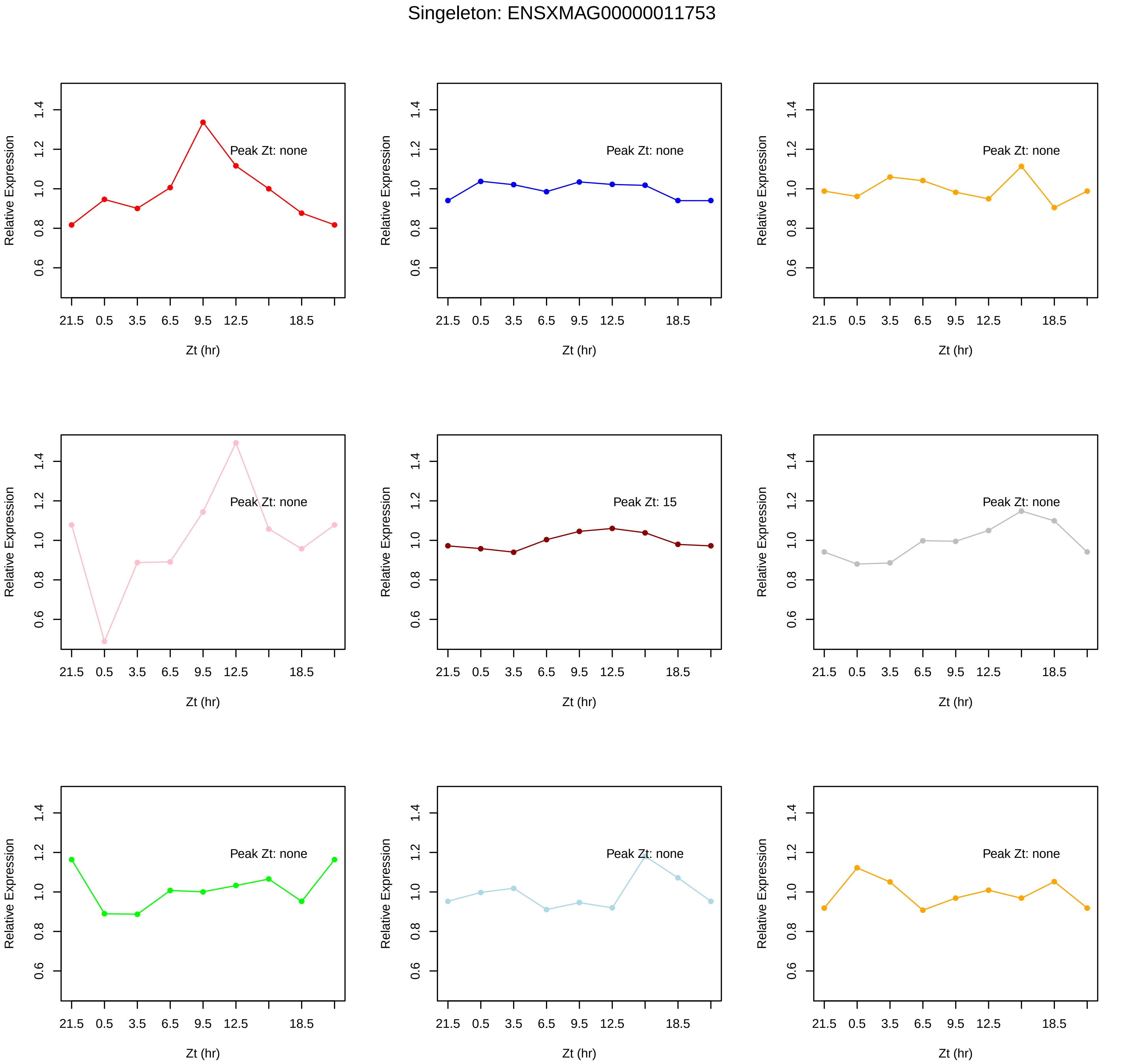
Singleton: ENSXMAG00000011753
Peak Zt: none
Peak Zt: none
Peak Zt: none
Peak Zt: none
Peak Zt: 15
Peak Zt: none
Peak Zt: none
Peak Zt: none
Peak Zt: none
Relative Expression
Zt (hr)
21.5
0.5
3.5
6.5
9.5
12.5
18.5
0.6
0.8
1.0
1.2
1.4

Singleton: ENSXMAG00000005267 btbd9

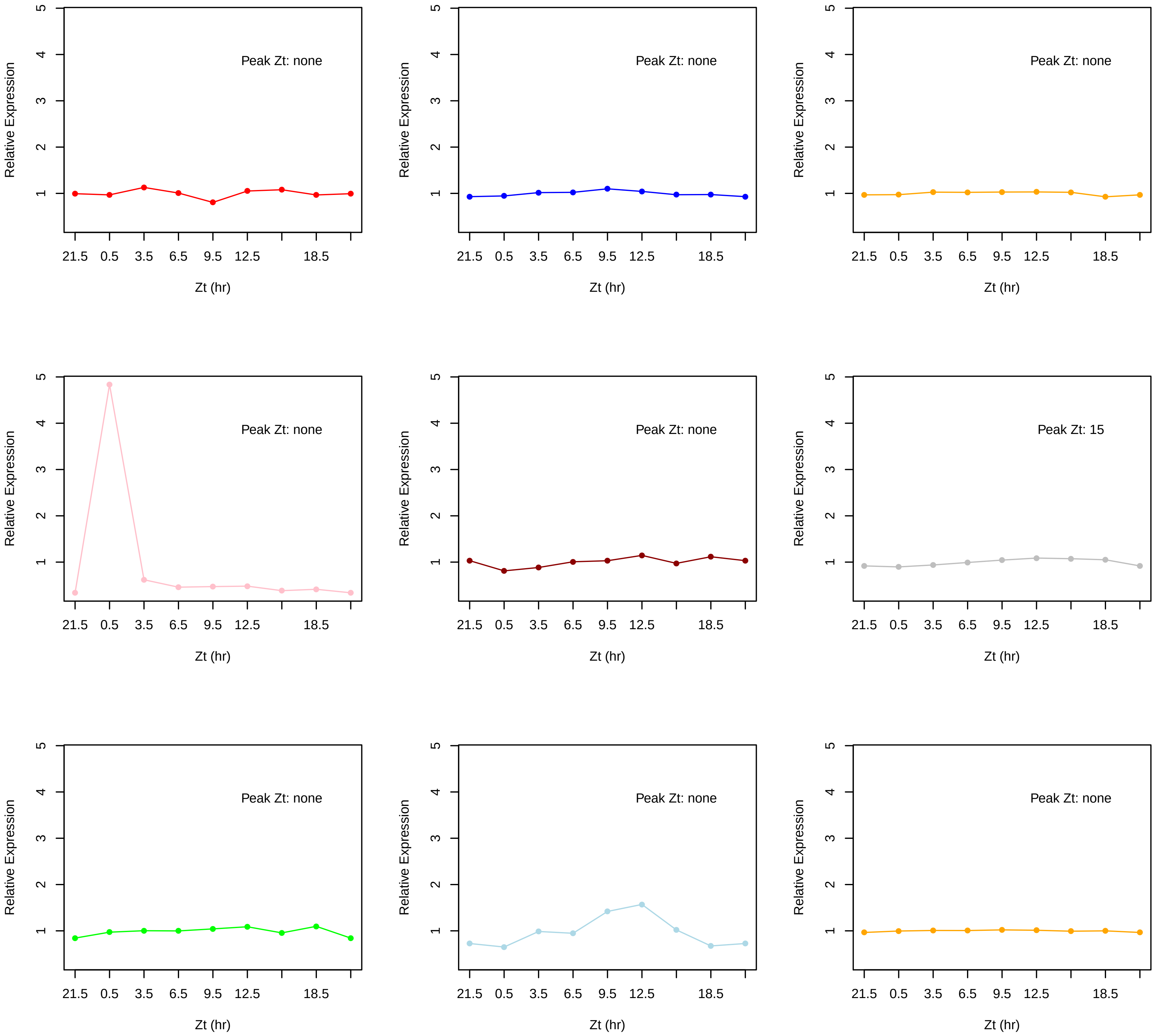

Singleton: ENSXMAG00000007486 cmtr1

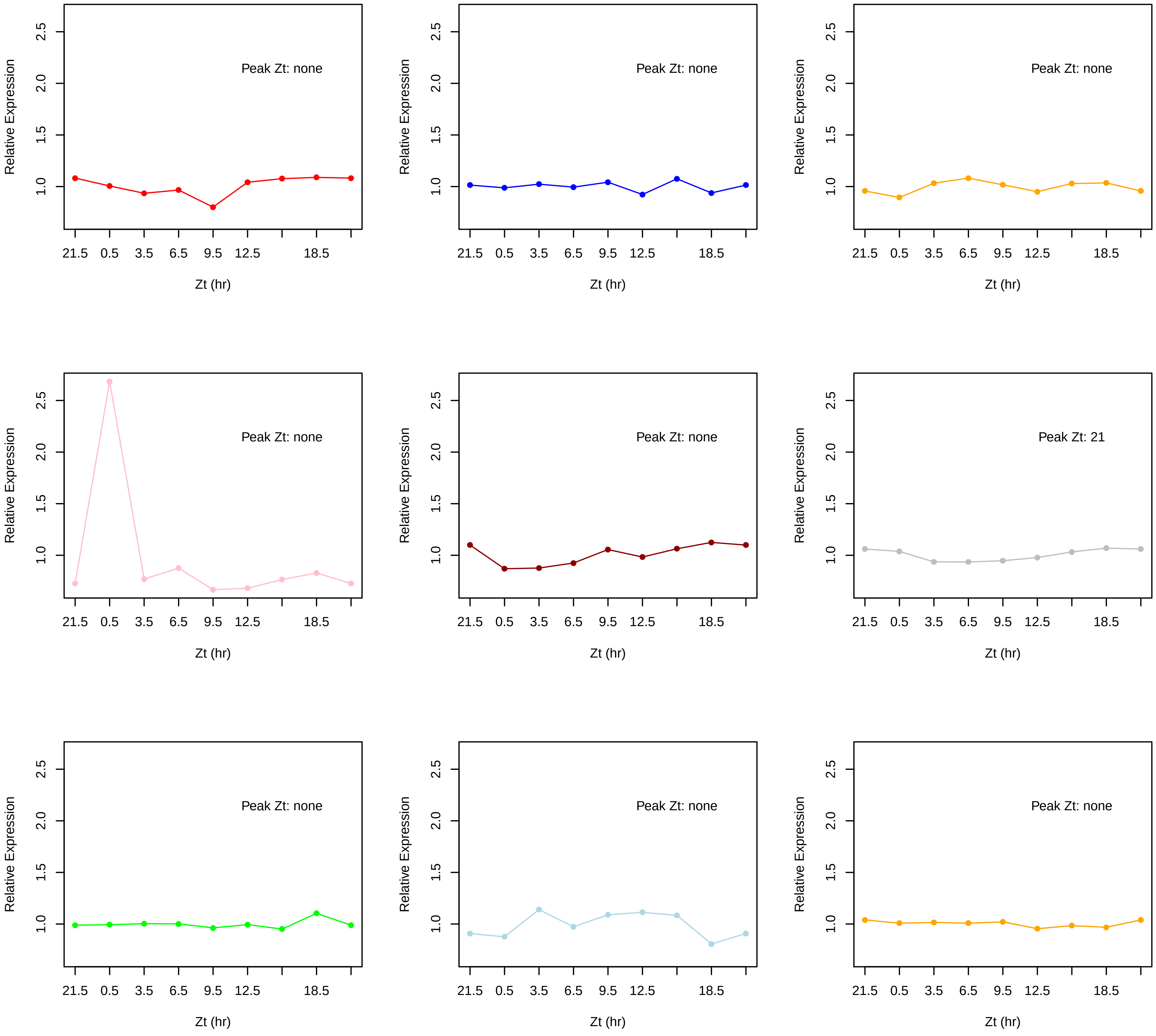

Singeleton: ENSXMAG00000013321 lmbrd1

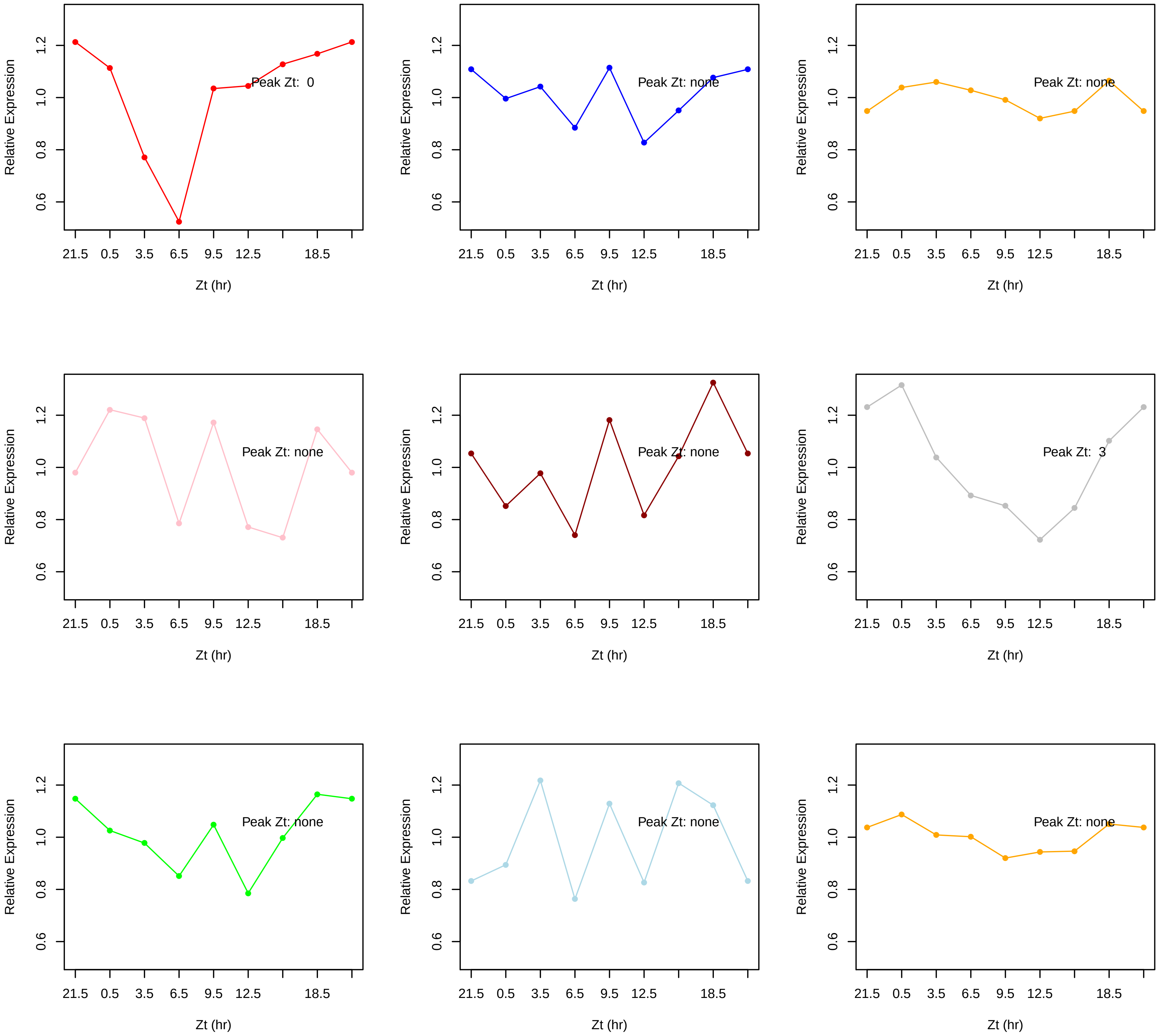

Singeleton: ENSXMAG00000007075 ttk

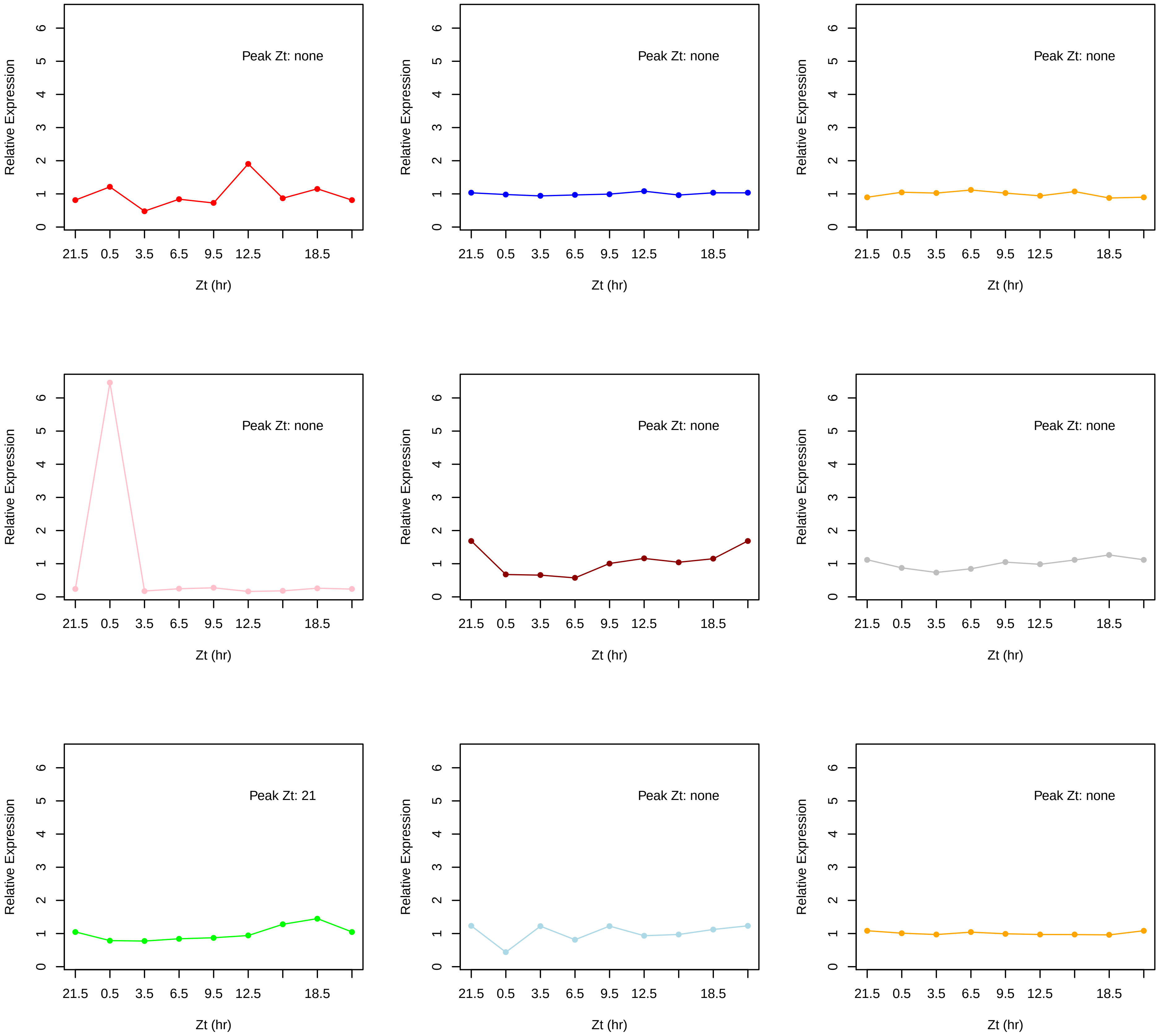

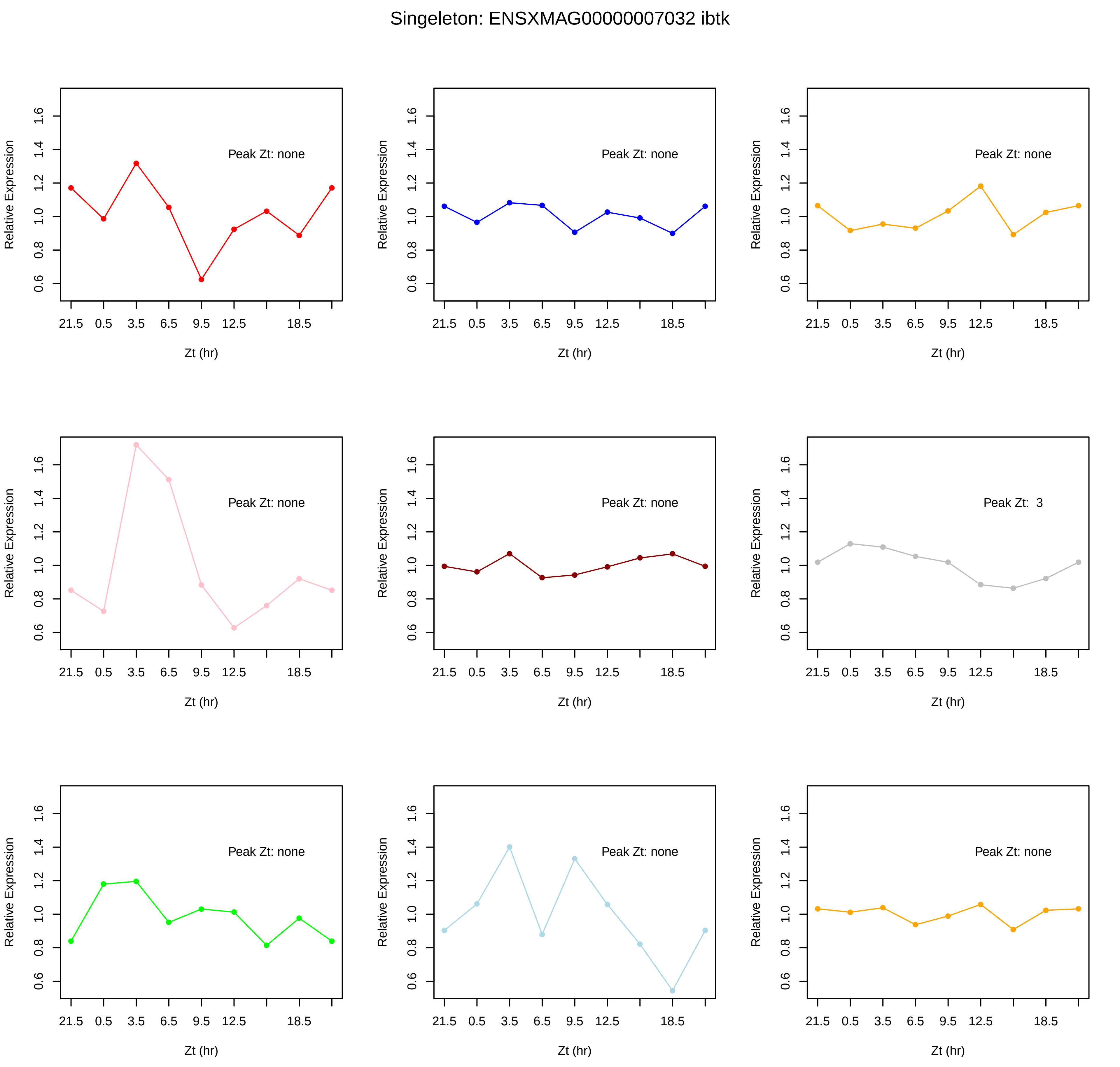
Singeleton: ENSXMAG00000007032 ibtk
Peak Zt: none
Peak Zt: none
Peak Zt: none
Peak Zt: none
Peak Zt: none
Peak Zt: 3
Peak Zt: none
Peak Zt: none
Peak Zt: none
Relative Expression
Zt (hr)
21.5 0.5 3.5 6.5 9.5 12.5 18.5
0.6 0.8 1.0 1.2 1.4 1.6

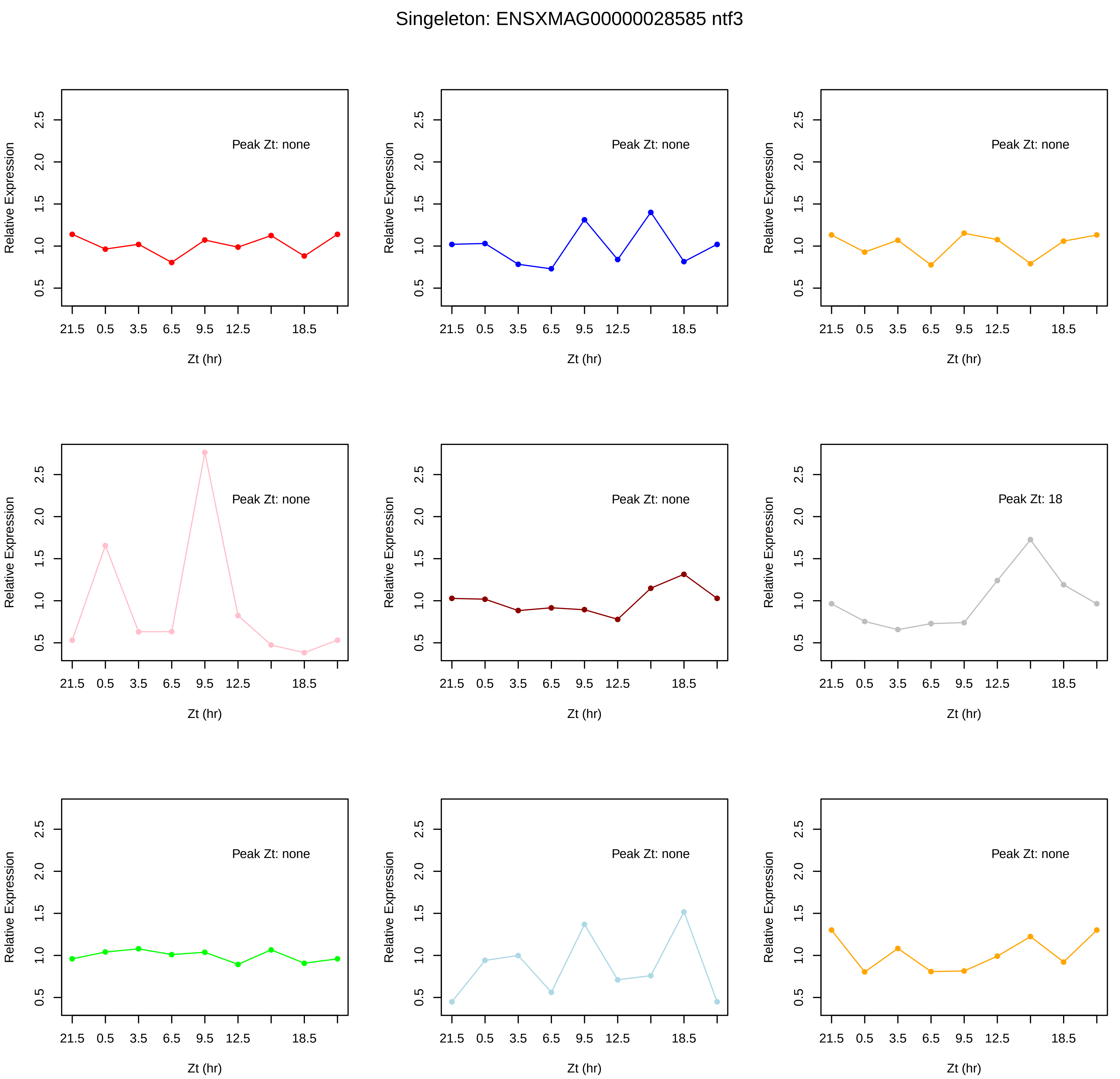
Singeleton: ENSXMAG00000028585 ntf3
Peak Zt: none
Peak Zt: none
Peak Zt: none
Peak Zt: none
Peak Zt: none
Peak Zt: 18
Peak Zt: none
Peak Zt: none
Peak Zt: none
Relative Expression
Zt (hr)
21.5
0.5
3.5
6.5
9.5
12.5
18.5
0.5
1.0
1.5
2.0
2.5

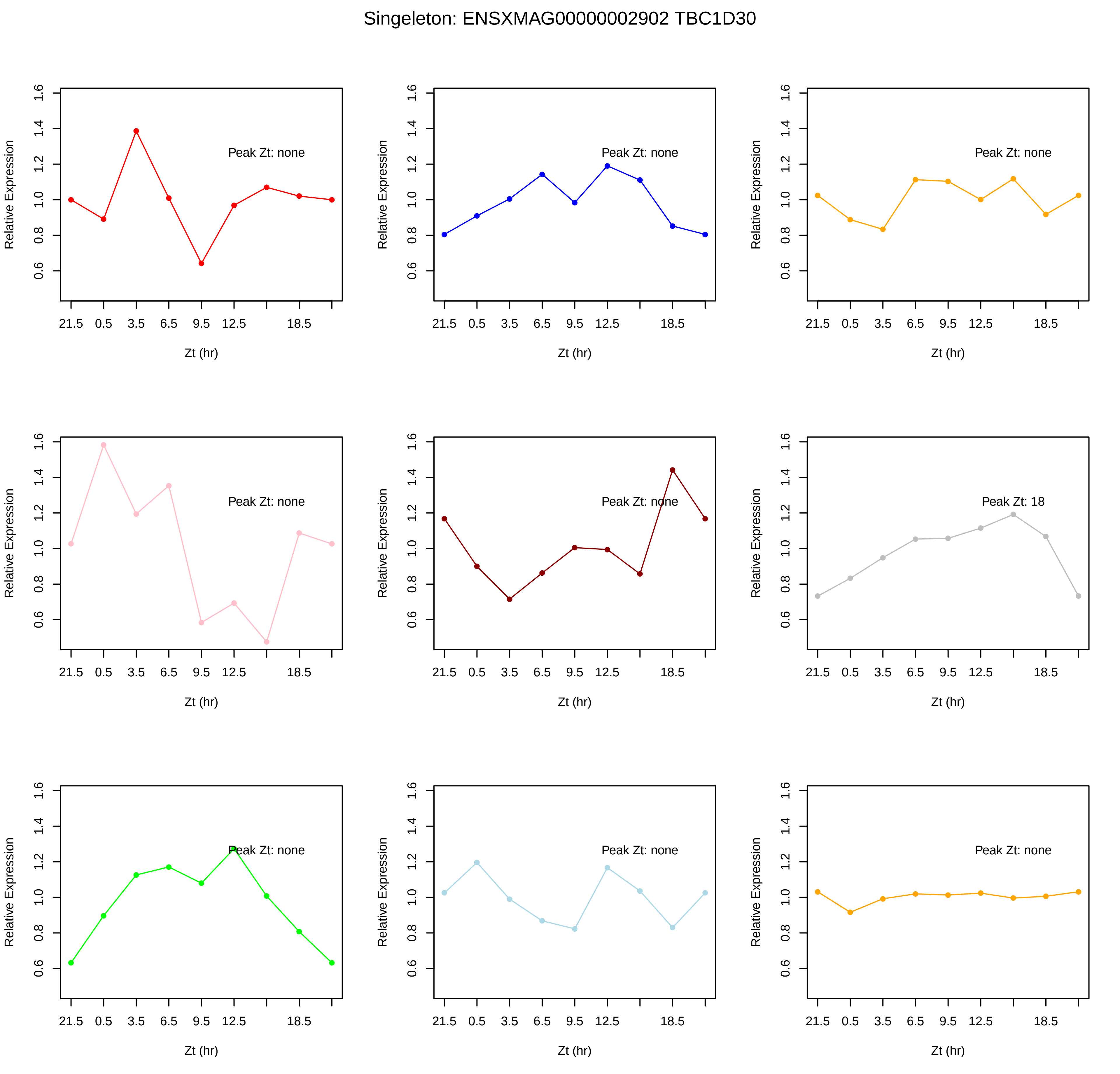

Singleton: ENSXMAG00000002902 TBC1D30
Peak Zt: none
Peak Zt: none
Peak Zt: none
Peak Zt: none
Peak Zt: none
Peak Zt: 18
Peak Zt: none
Peak Zt: none
Peak Zt: none
Relative Expression
Zt (hr)
21.5
0.5
3.5
6.5
9.5
12.5
18.5

Singeleton: ENSXMAG00000007024 ube3d

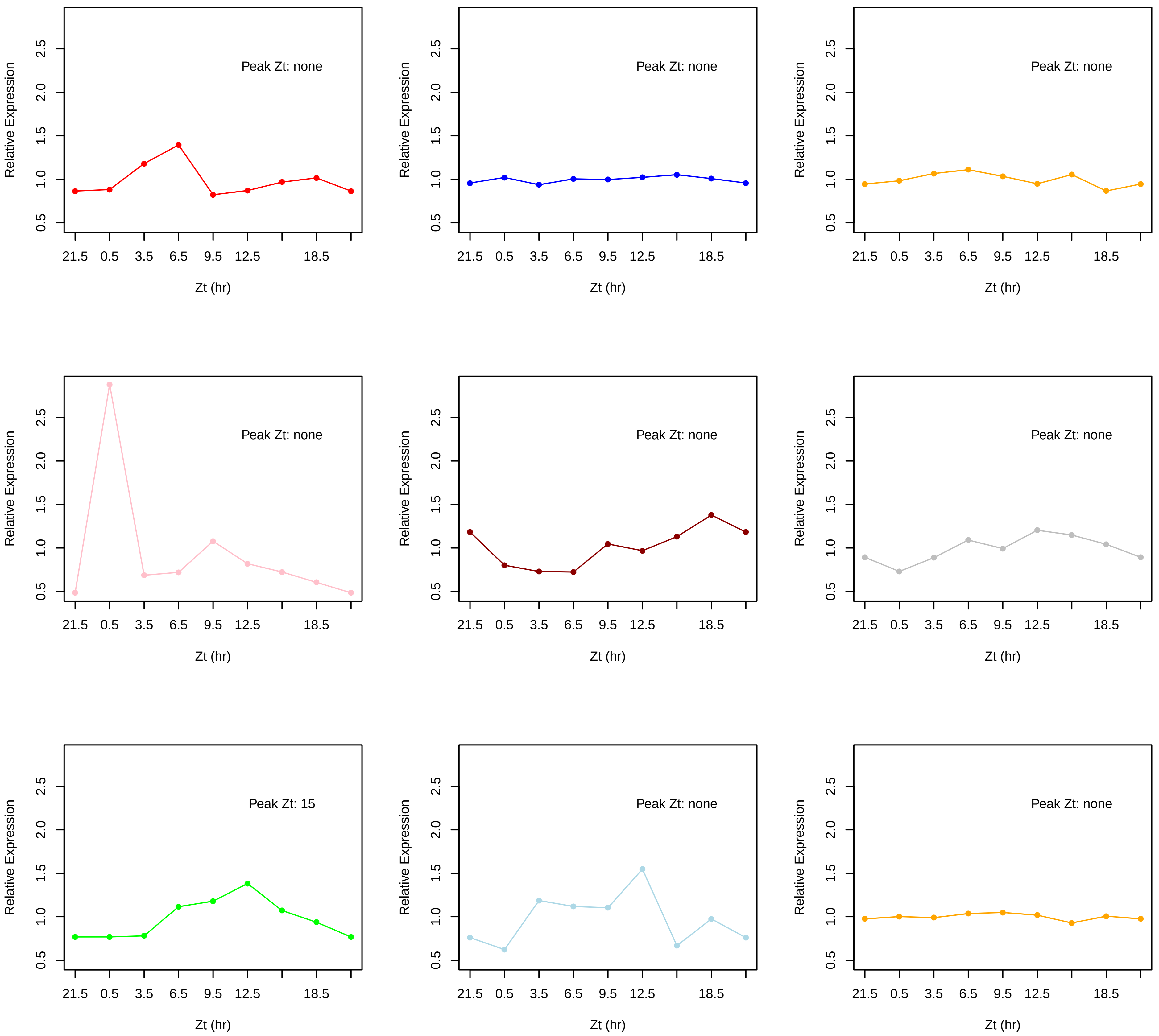

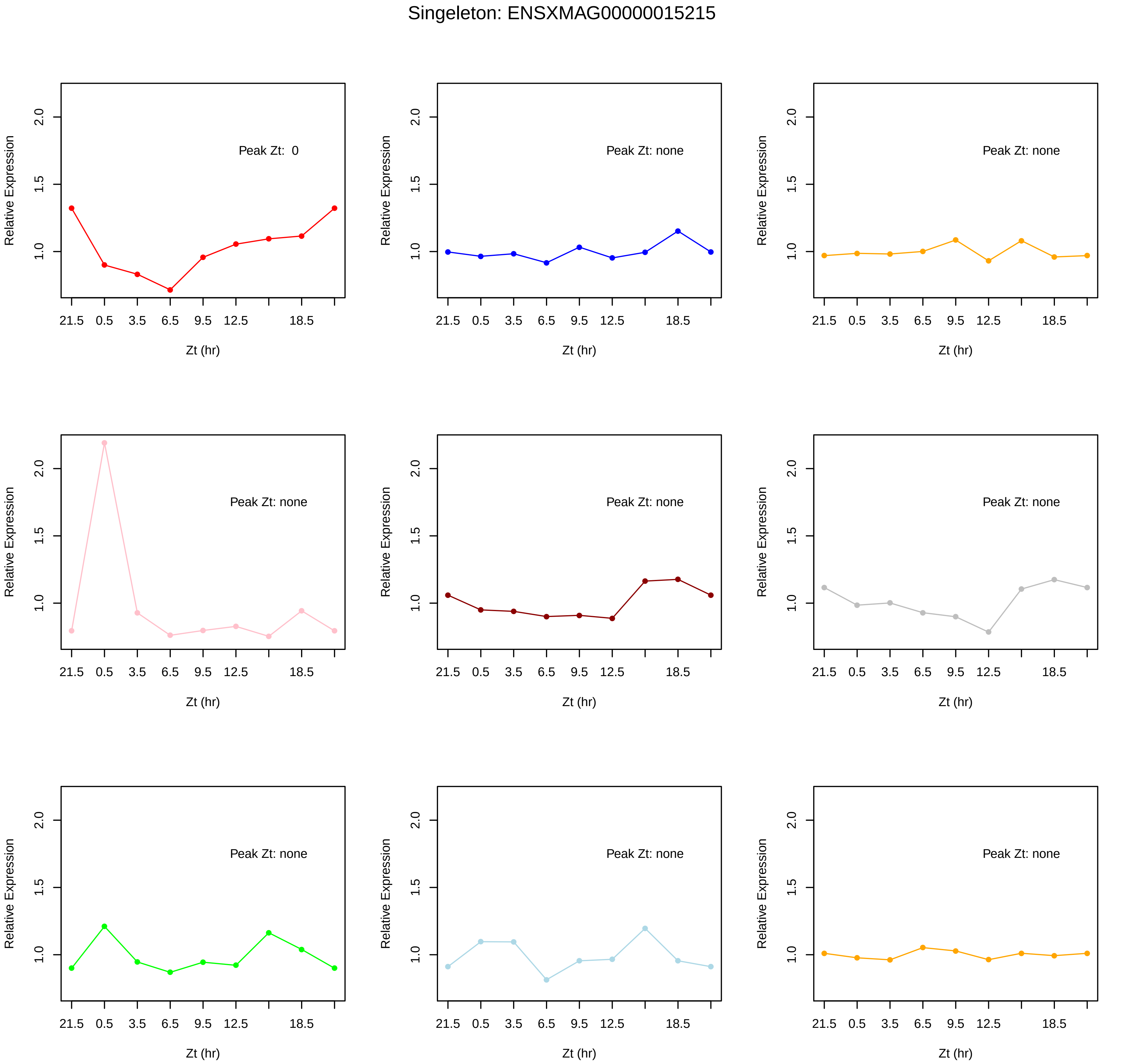

Singeleton: ENSXMAG00000015215
Peak Zt: 0
Peak Zt: none
Peak Zt: none
Peak Zt: none
Peak Zt: none
Peak Zt: none
Peak Zt: none
Peak Zt: none
Peak Zt: none
Relative Expression
Zt (hr)
21.5
0.5
3.5
6.5
9.5
12.5
18.5
1.0
1.5
2.0

Singeleton: ENSXMAG00000007222 pgm3

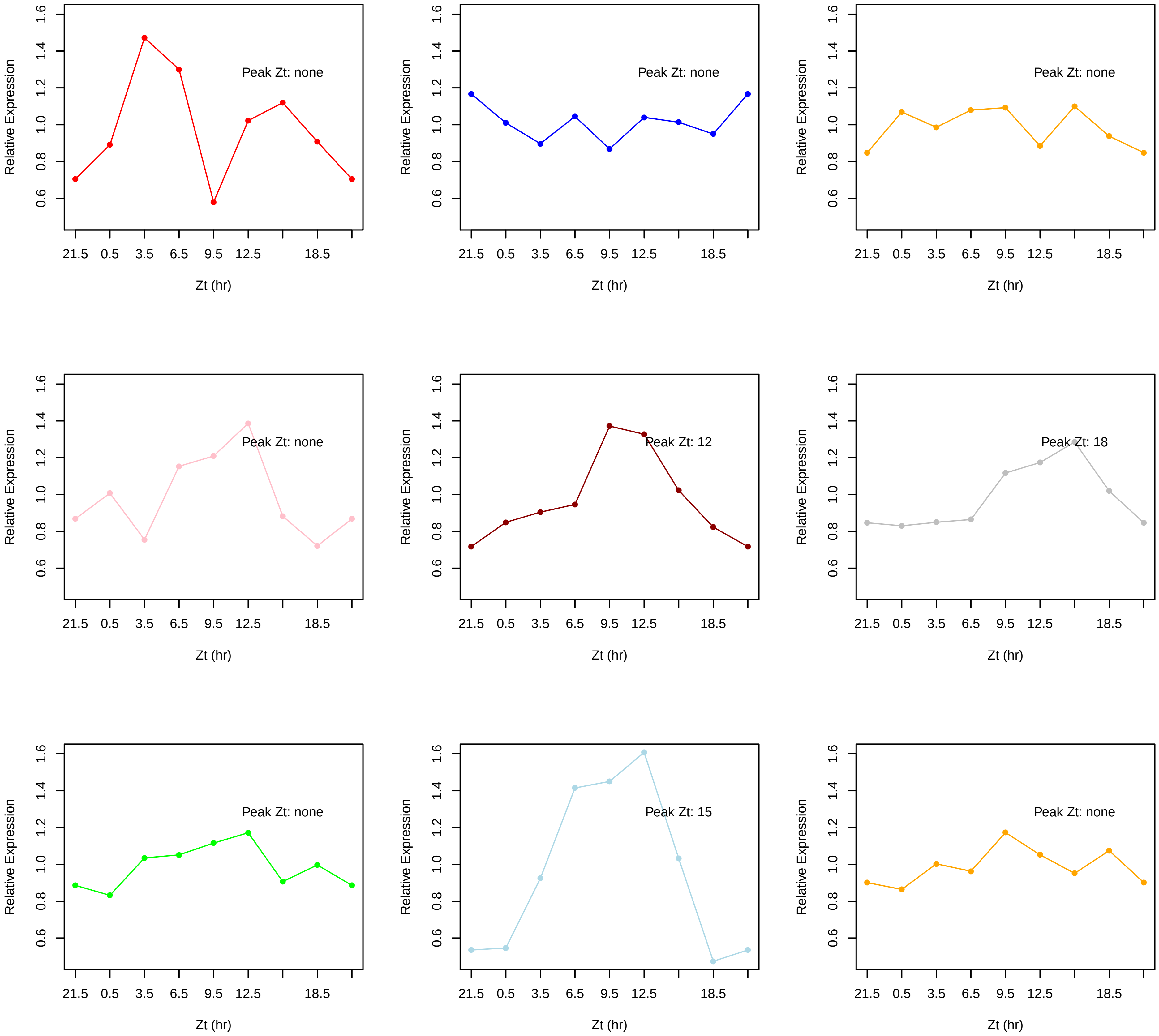

Singeleton: ENSXMAG00000006865 me1

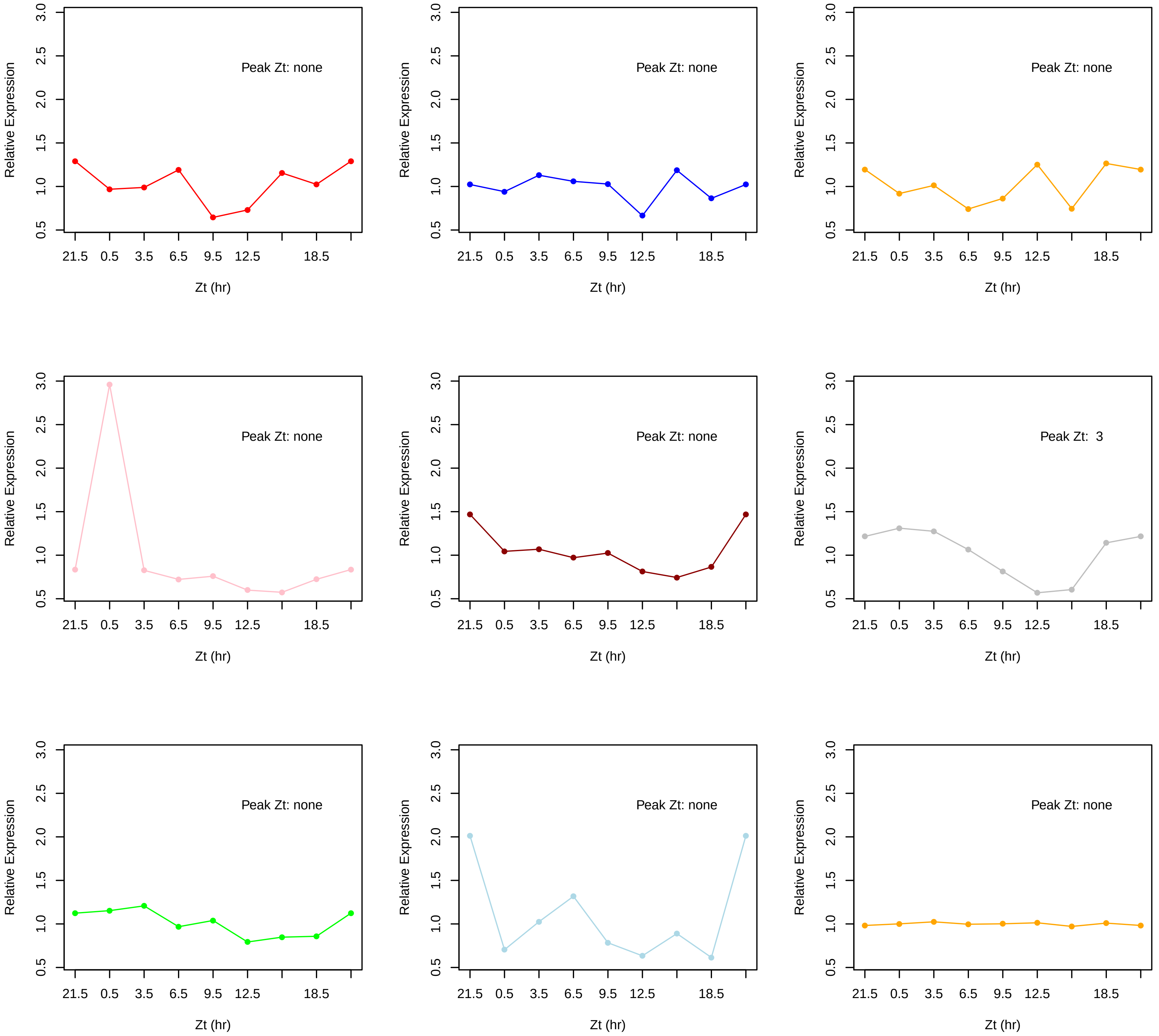

Singleton: ENSXMAG00000026207 rassf3

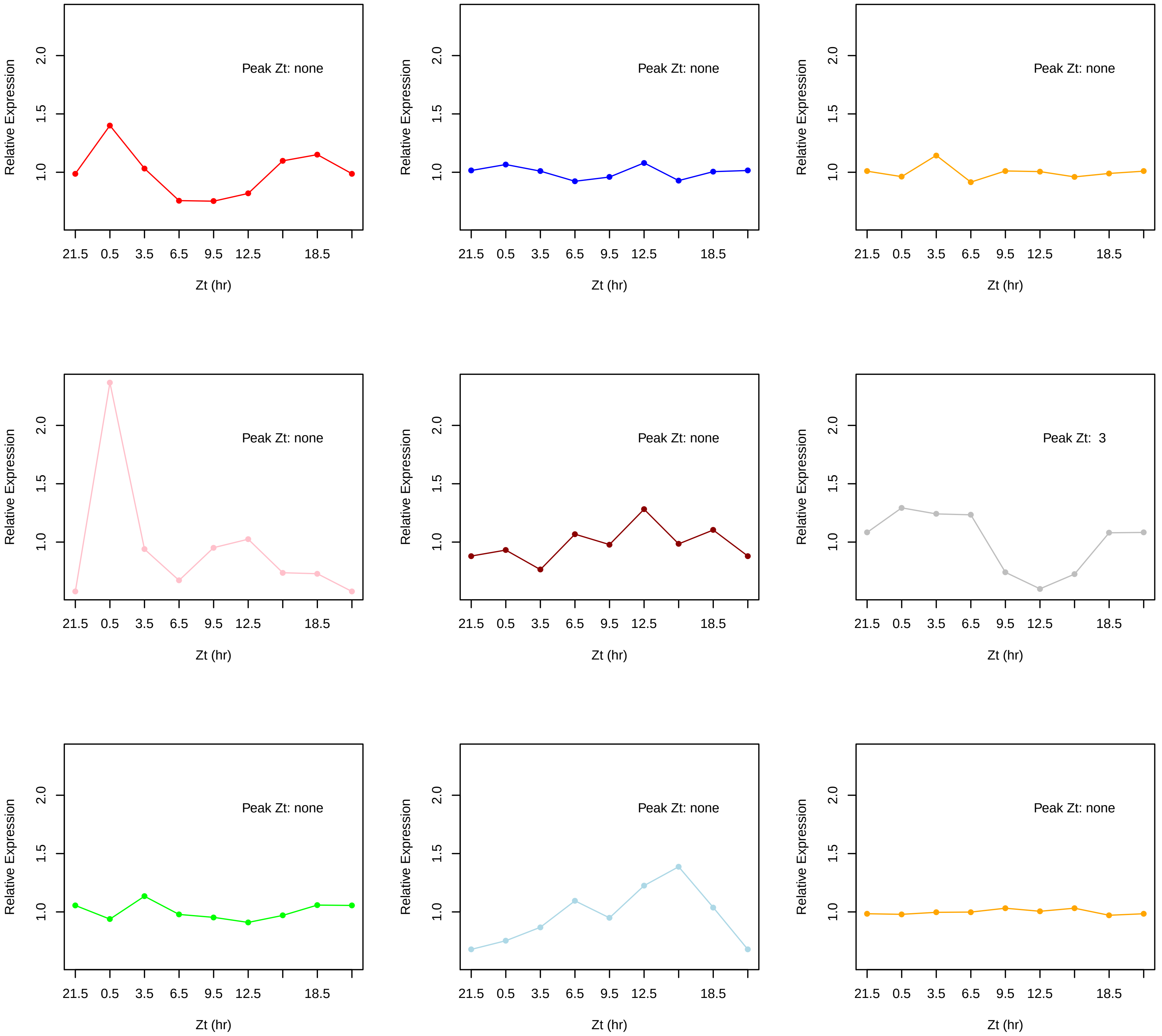

Singleton: ENSXMAG00000019263 hdhd5

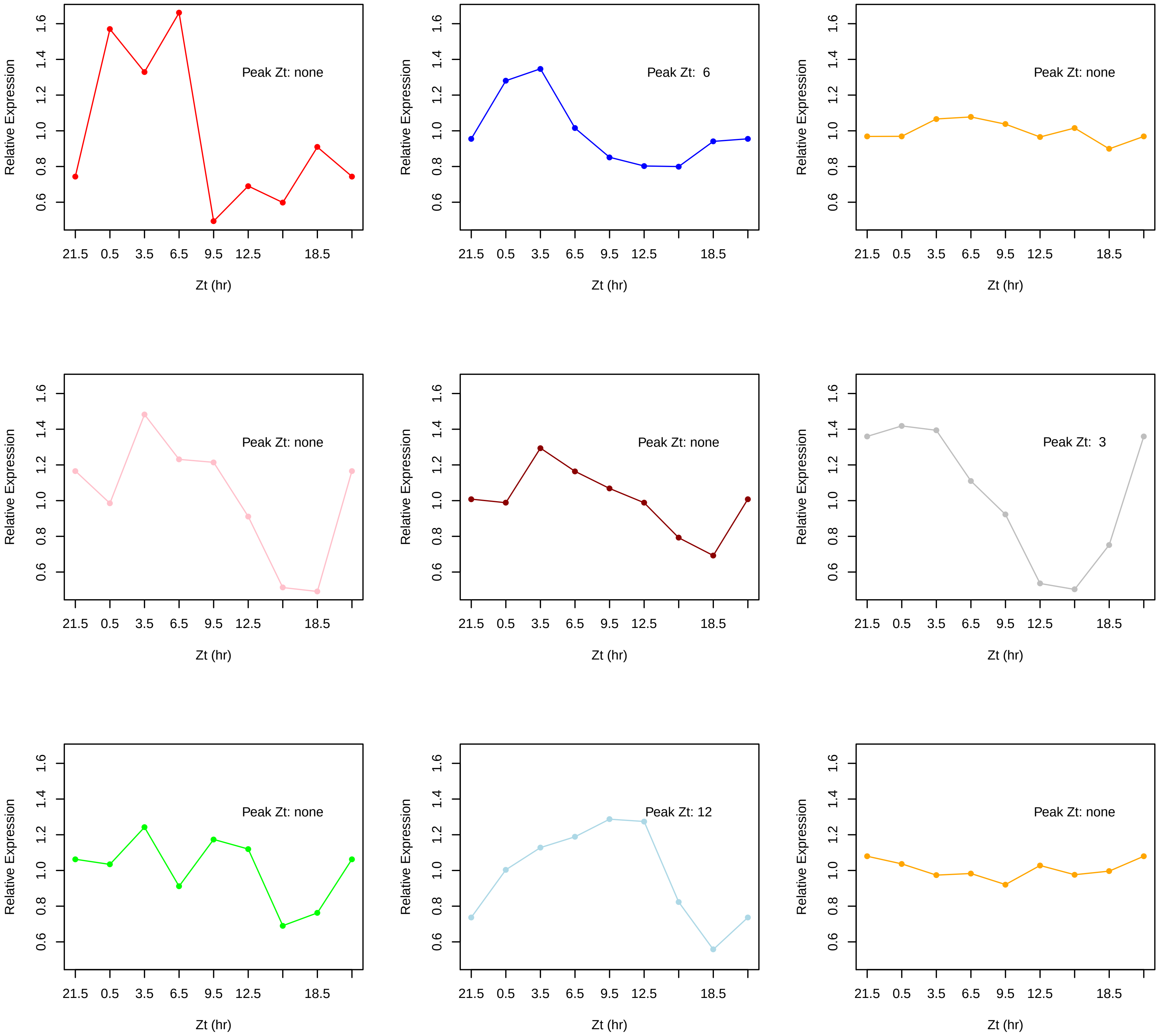

Singeleton: ENSXMAG00000004156 usp45

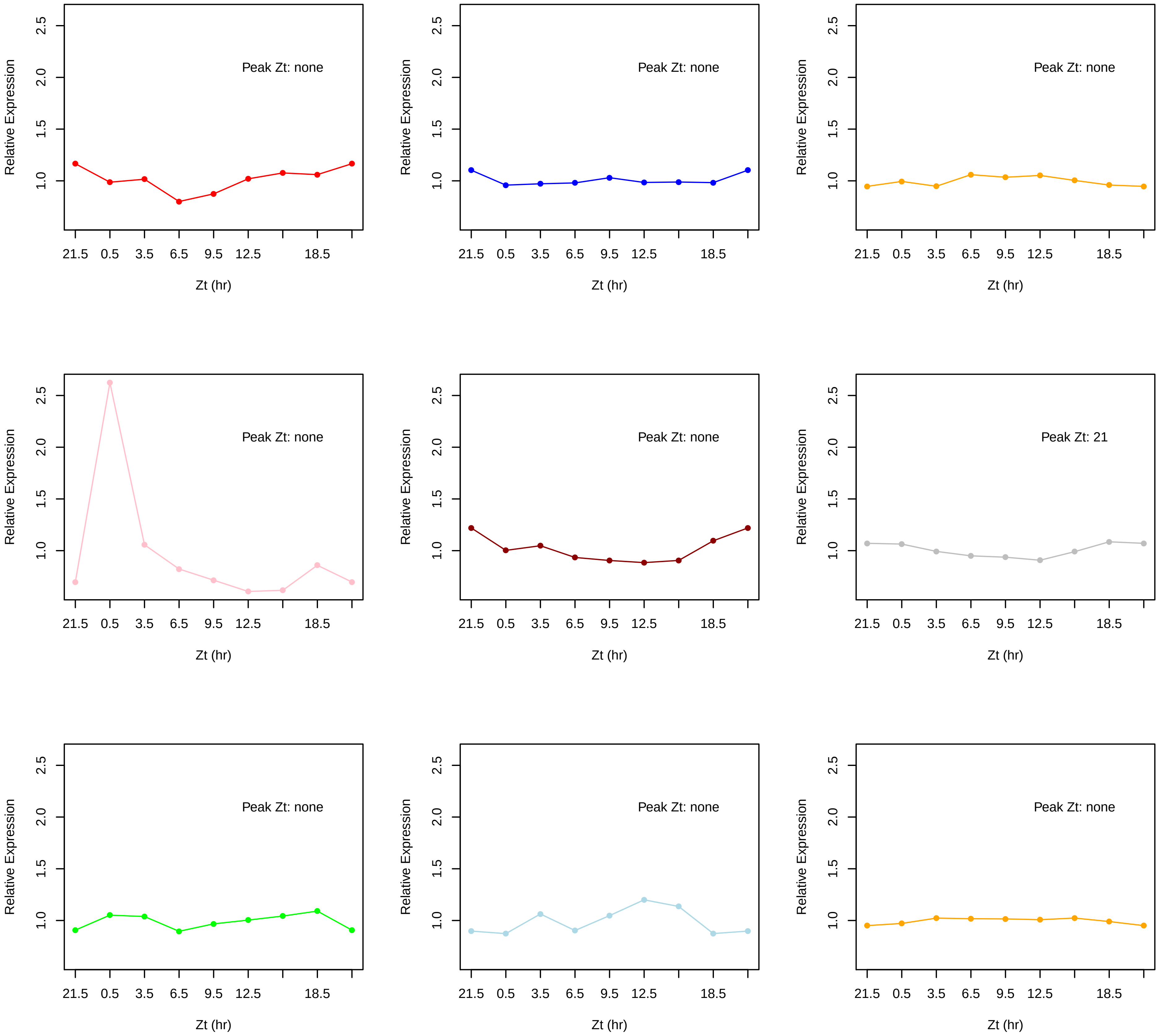

Singeleton: ENSXMAG00000004098 fbxl4

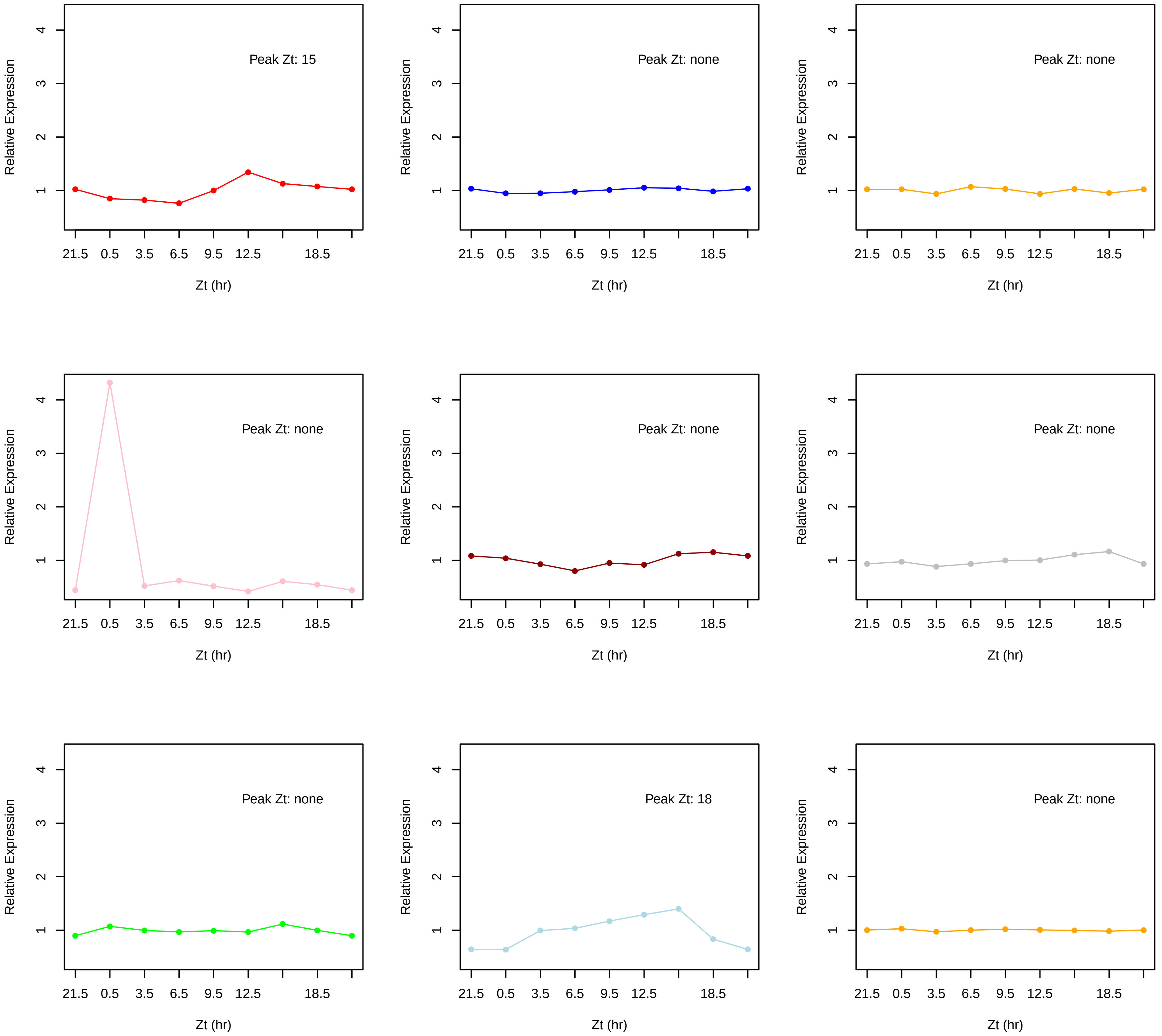

Singeleton: ENSXMAG00000008857 coa6

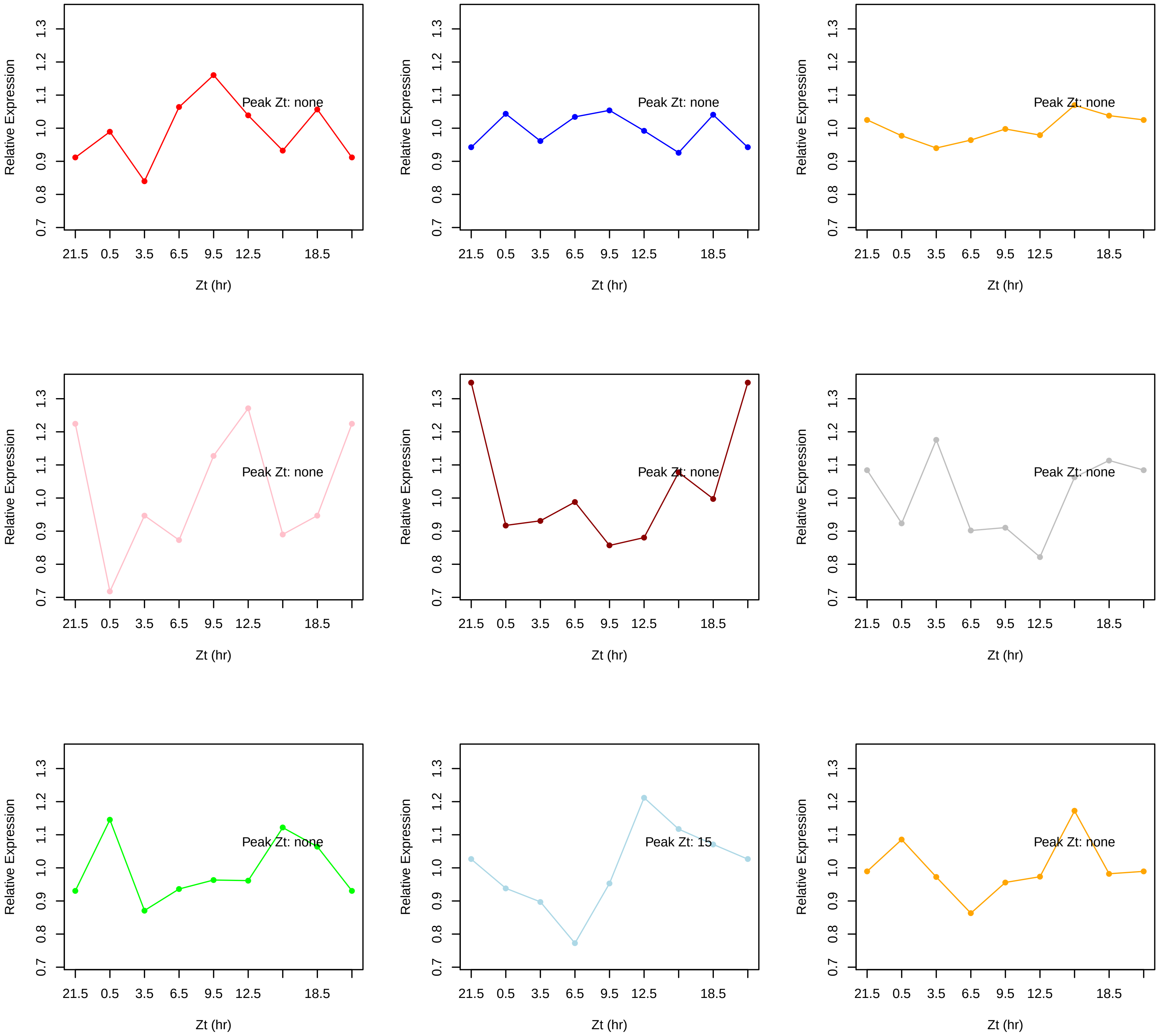

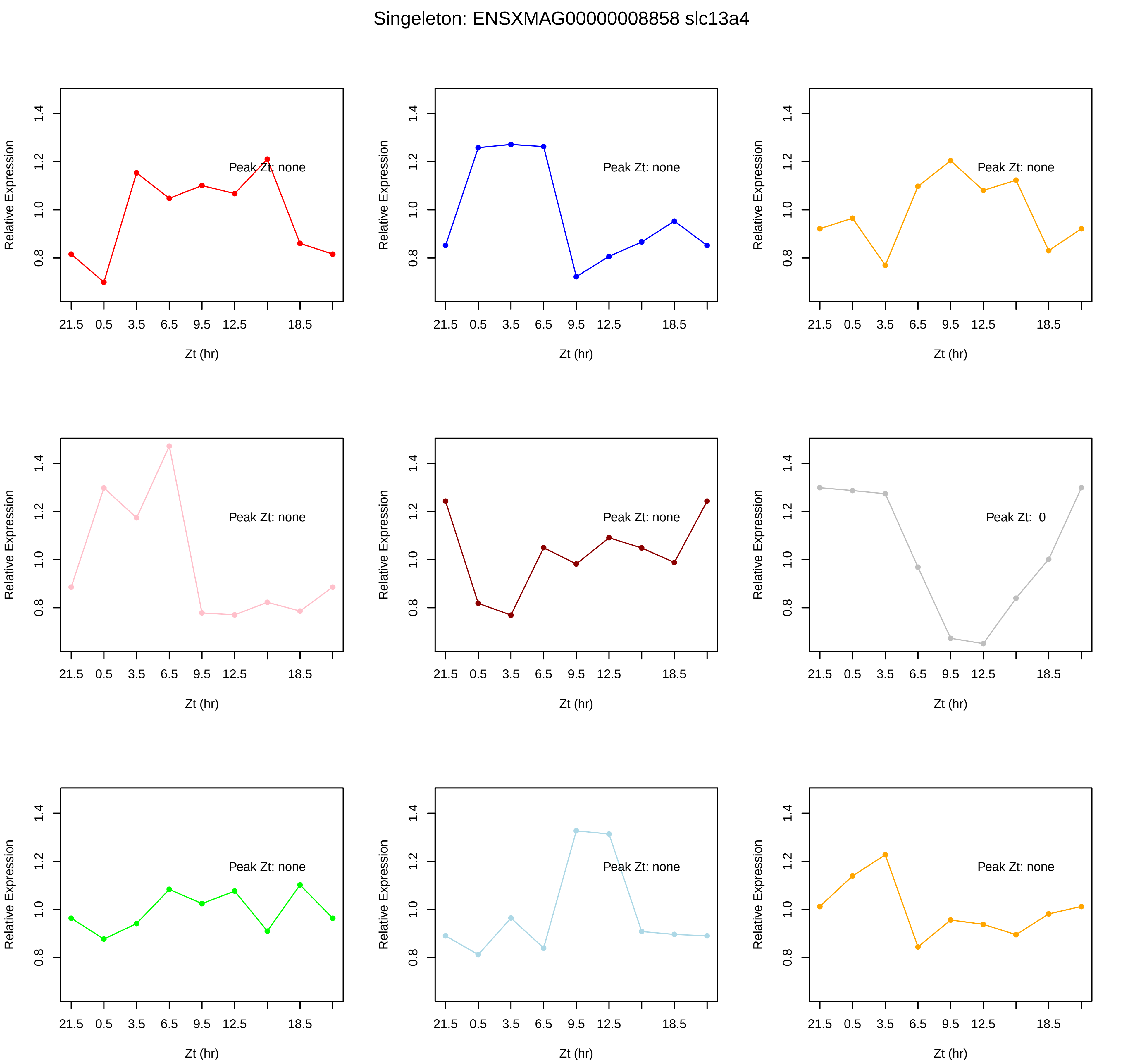
Singeleton: ENSXMAG00000008858 slc13a4
Relative Expression
Zt (hr)
21.5
0.5
3.5
6.5
9.5
12.5
18.5
0.8
1.0
1.2
1.4
Peak Zt: none
Peak Zt: none
Peak Zt: none
Peak Zt: none
Peak Zt: none
Peak Zt: 0
Peak Zt: none
Peak Zt: none
Peak Zt: none

Singleton: ENSXMAG00000024949 ssbp1

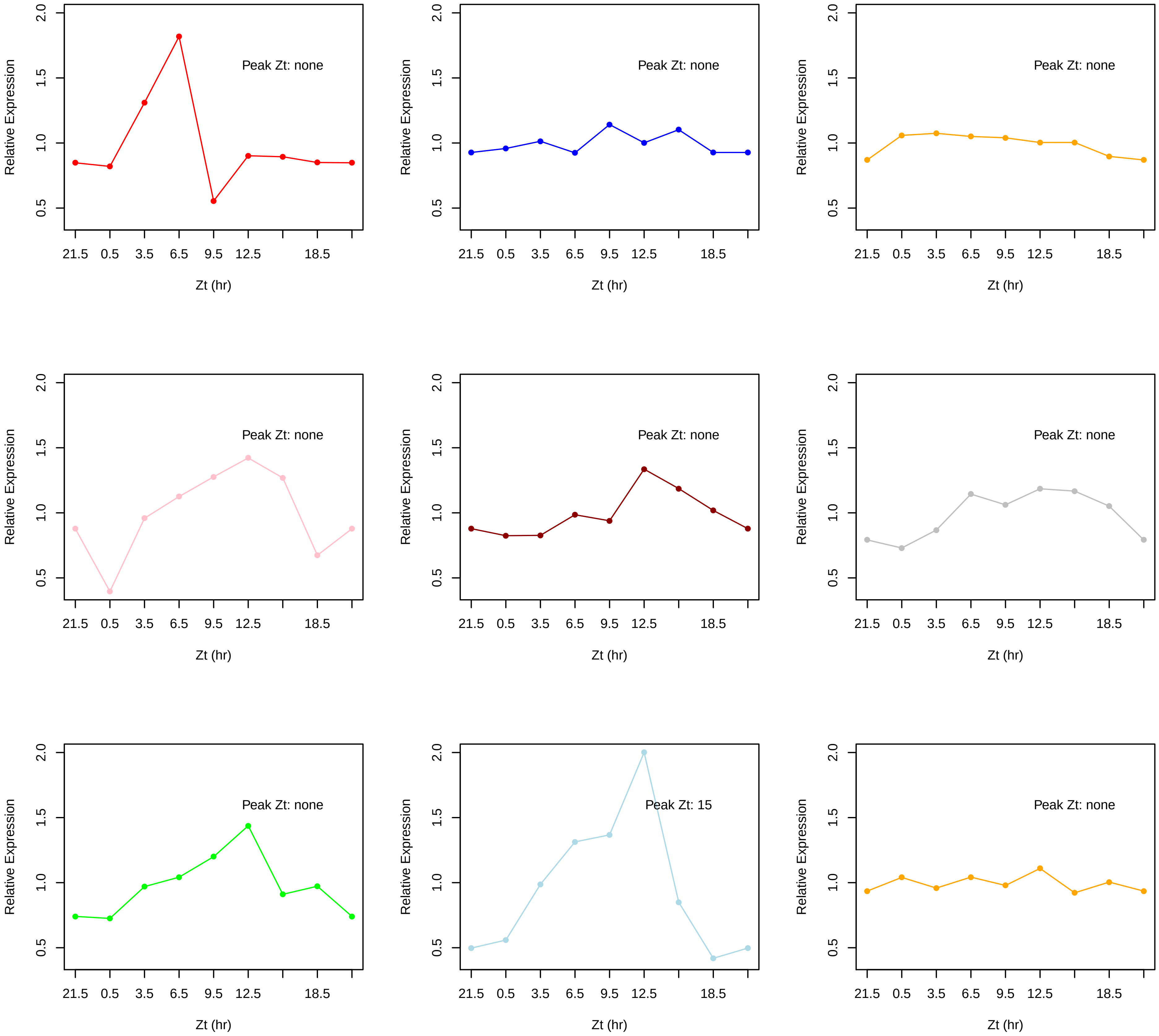

Singleton: ENSXMAG00000004046 klhl32

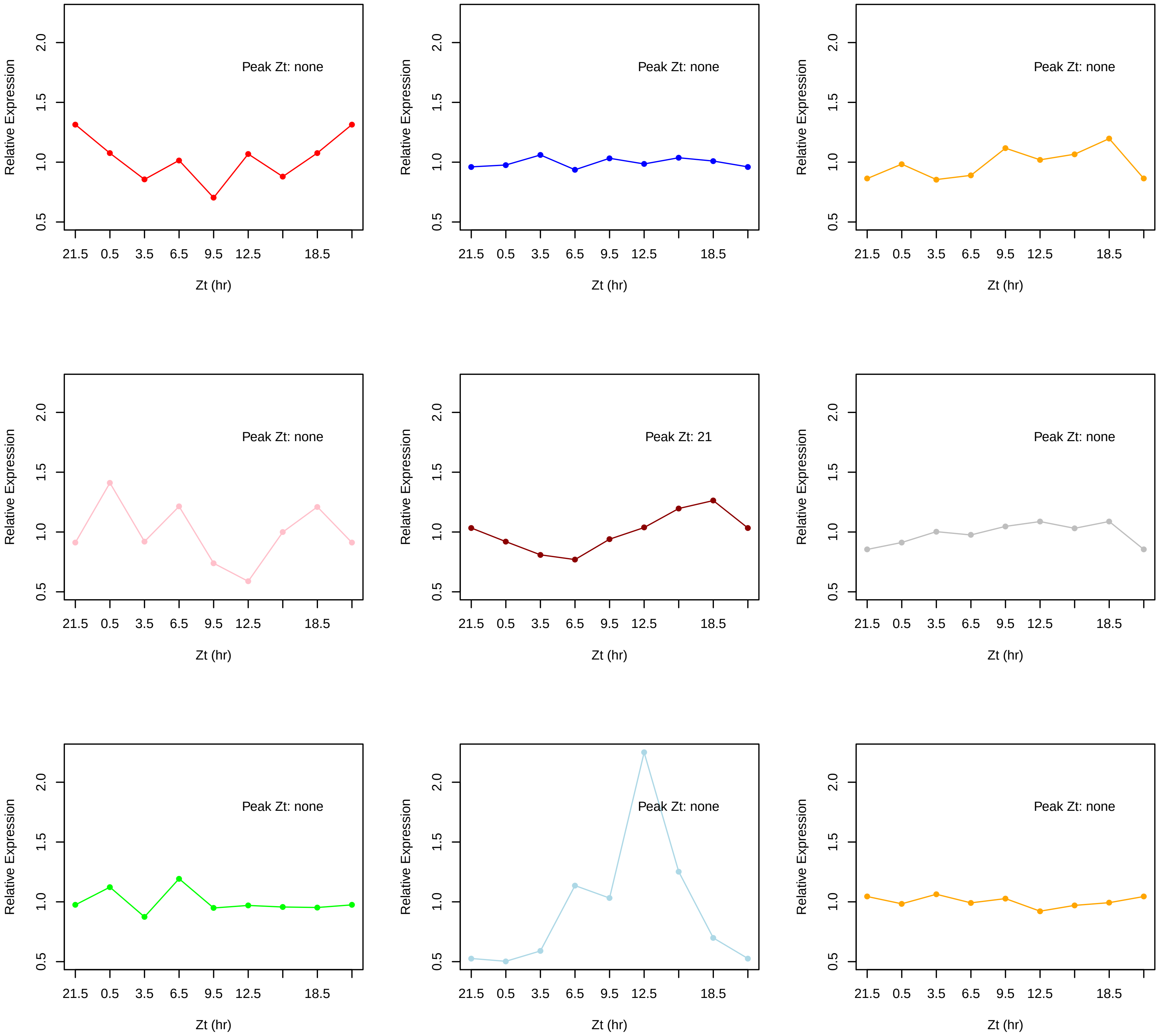

Singleton: ENSXMAG00000007288 ogfrl1

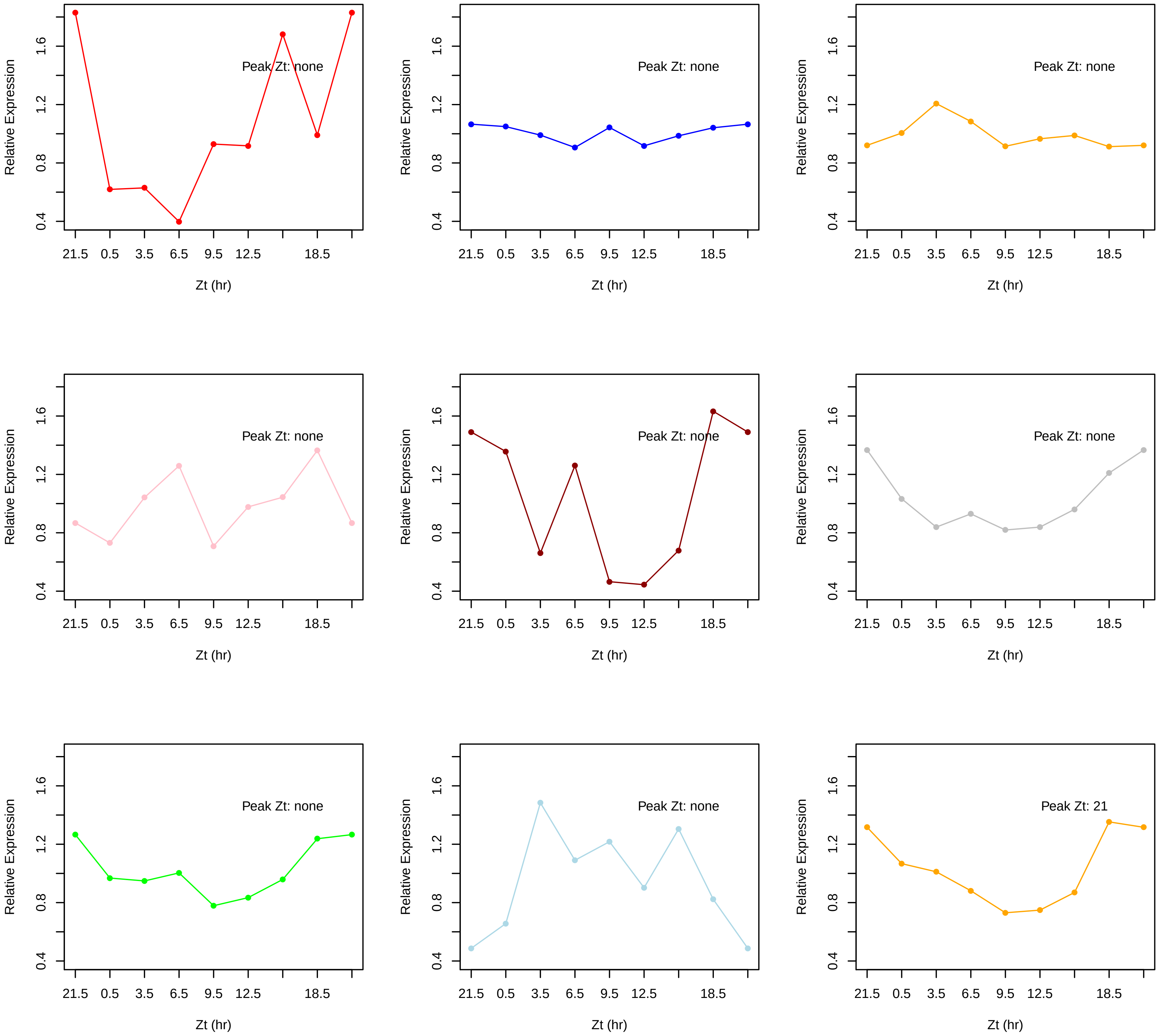

Singleton: ENSXMAG00000005605 vgll2a

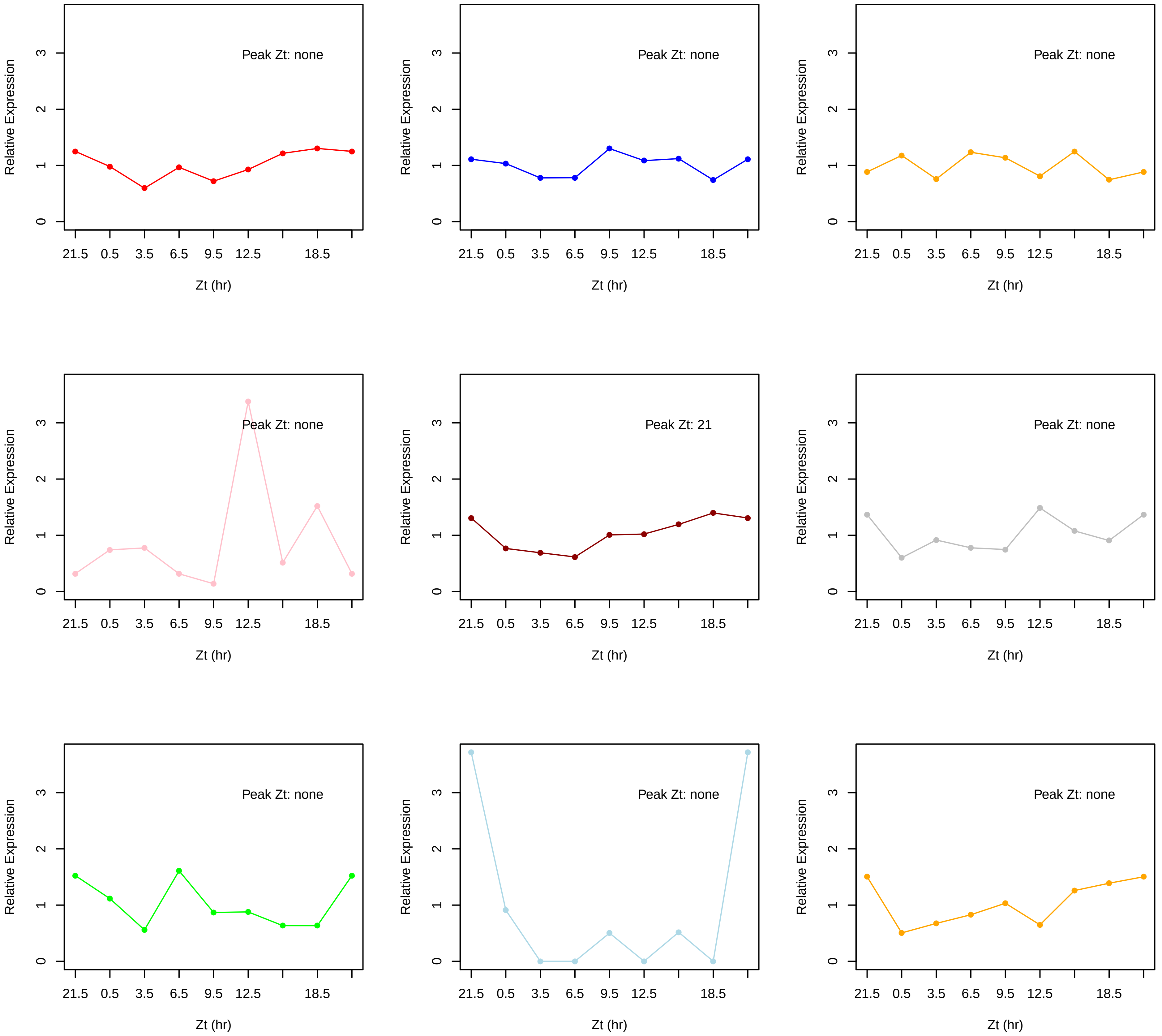

Singeleton: ENSXMAG00000005293 nus1

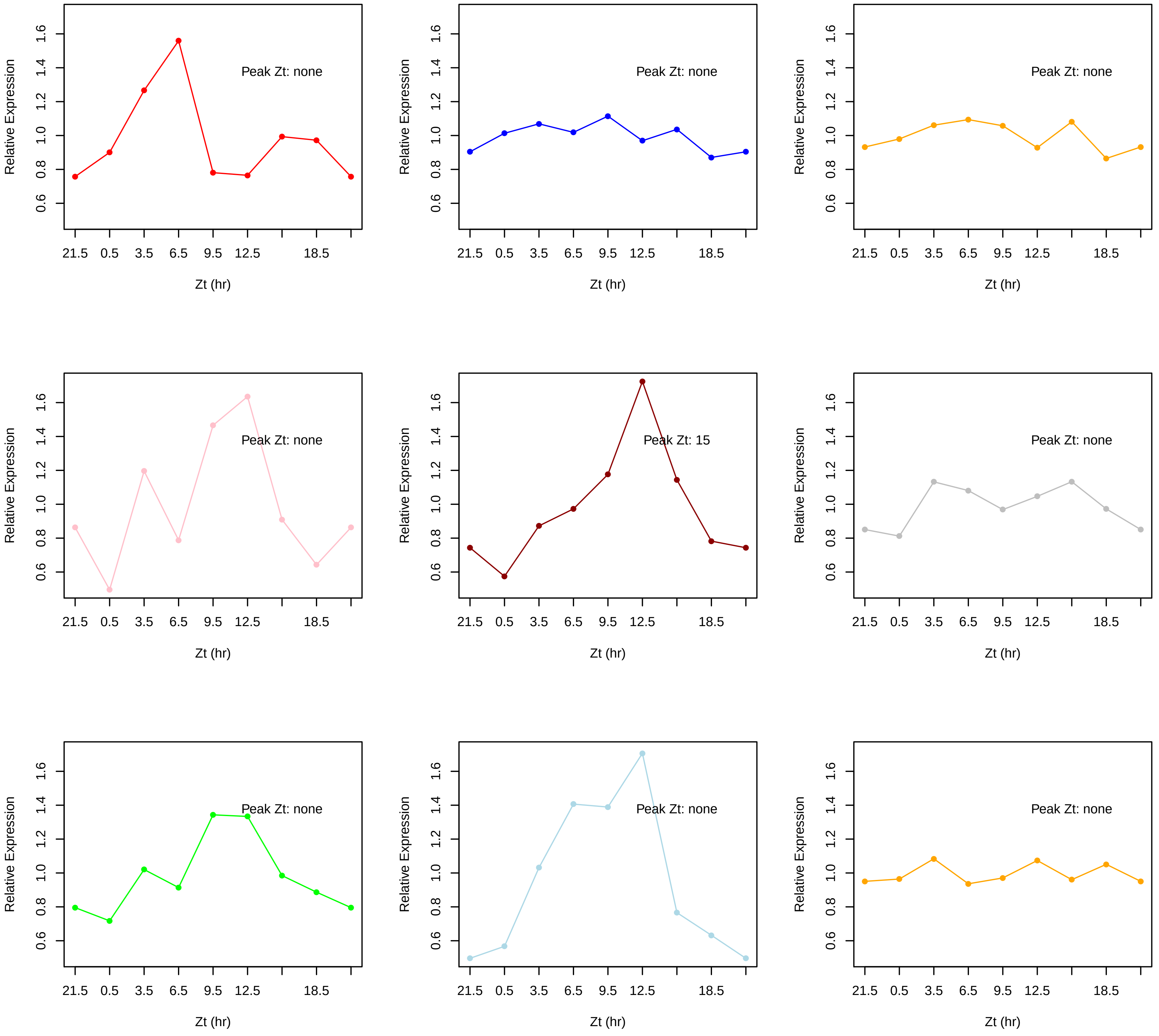

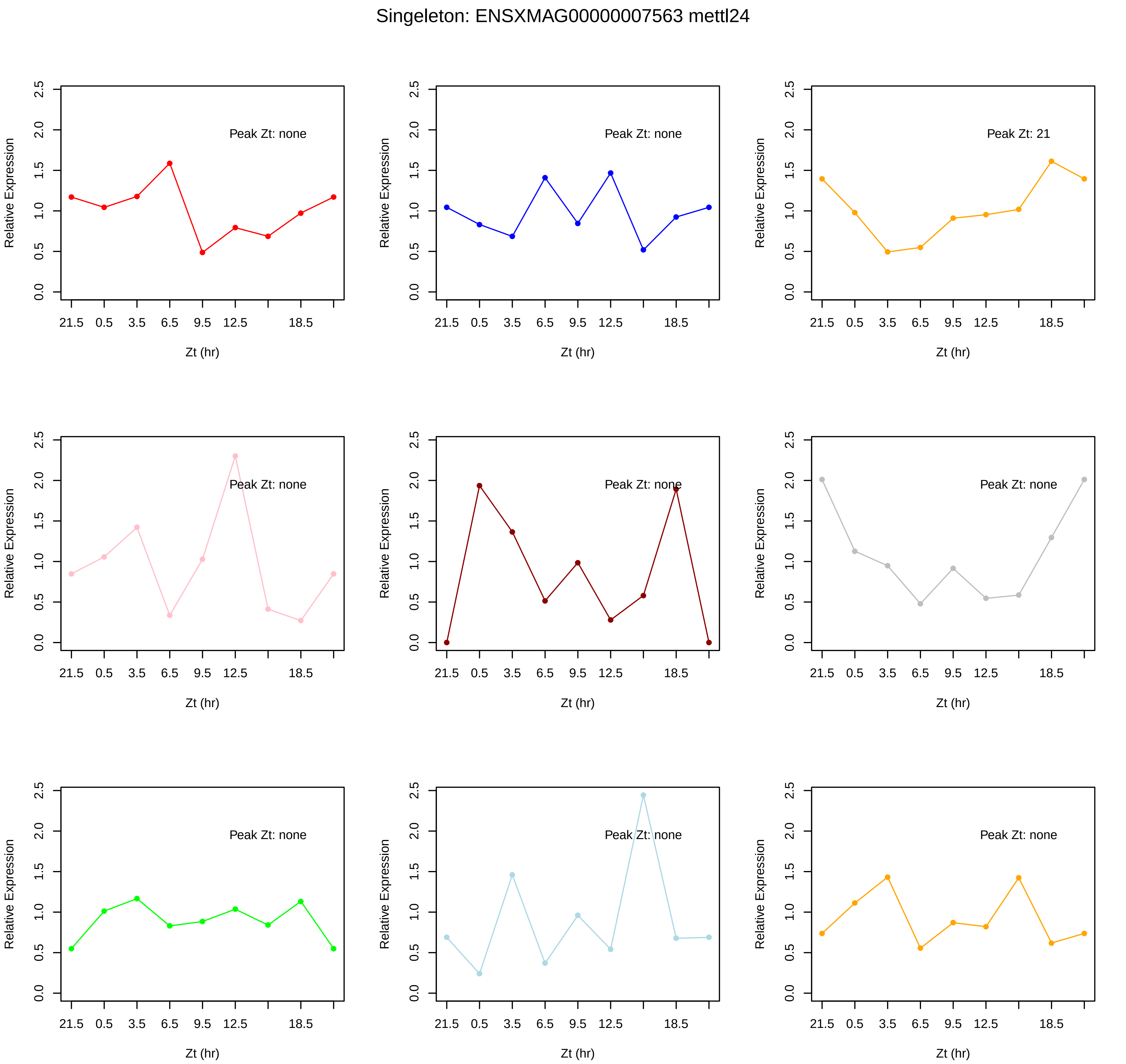
Singeleton: ENSXMAG00000007563 mettl24
Peak Zt: none
Peak Zt: none
Peak Zt: 21
Peak Zt: none
Peak Zt: none
Peak Zt: none
Peak Zt: none
Peak Zt: none
Peak Zt: none
Relative Expression
Zt (hr)
21.5
0.5
3.5
6.5
9.5
12.5
18.5
0.0
0.5
1.0
1.5
2.0
2.5

Singleton: ENSXMAG00000029677 wasf1

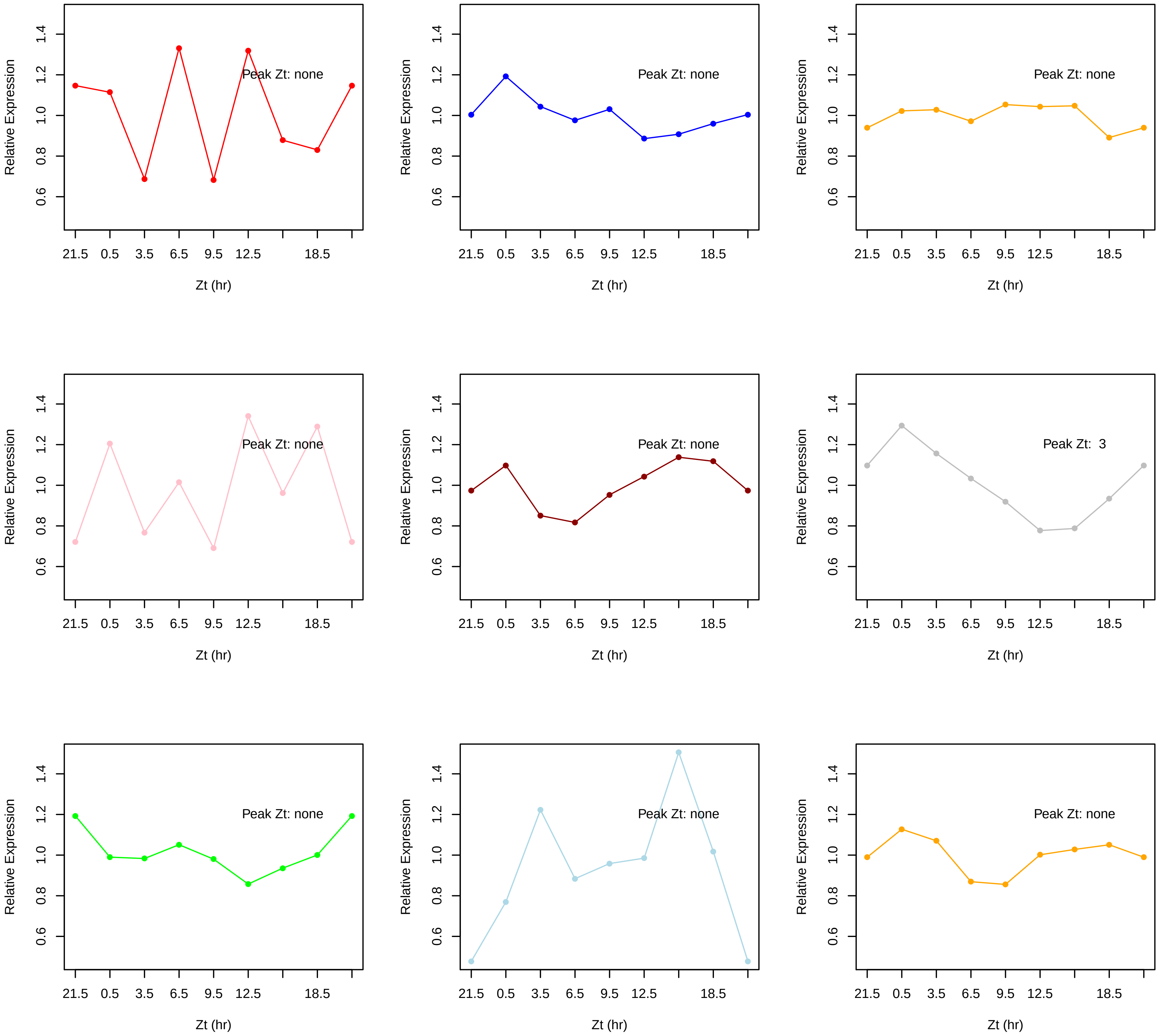

Singeleton: ENSXMAG00000007529 FIG4

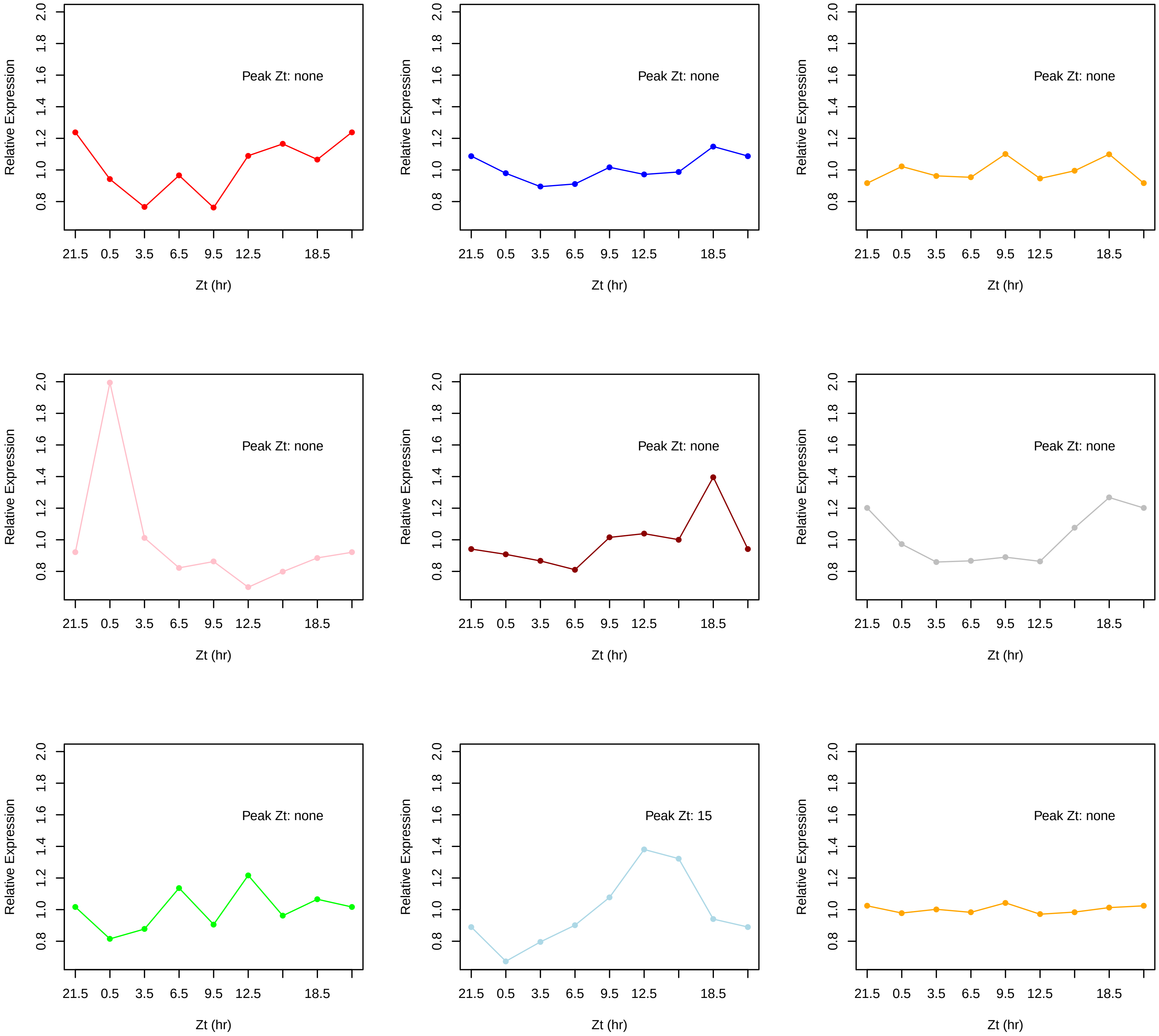

Singeleton: ENSXMAG00000018409 supt7l

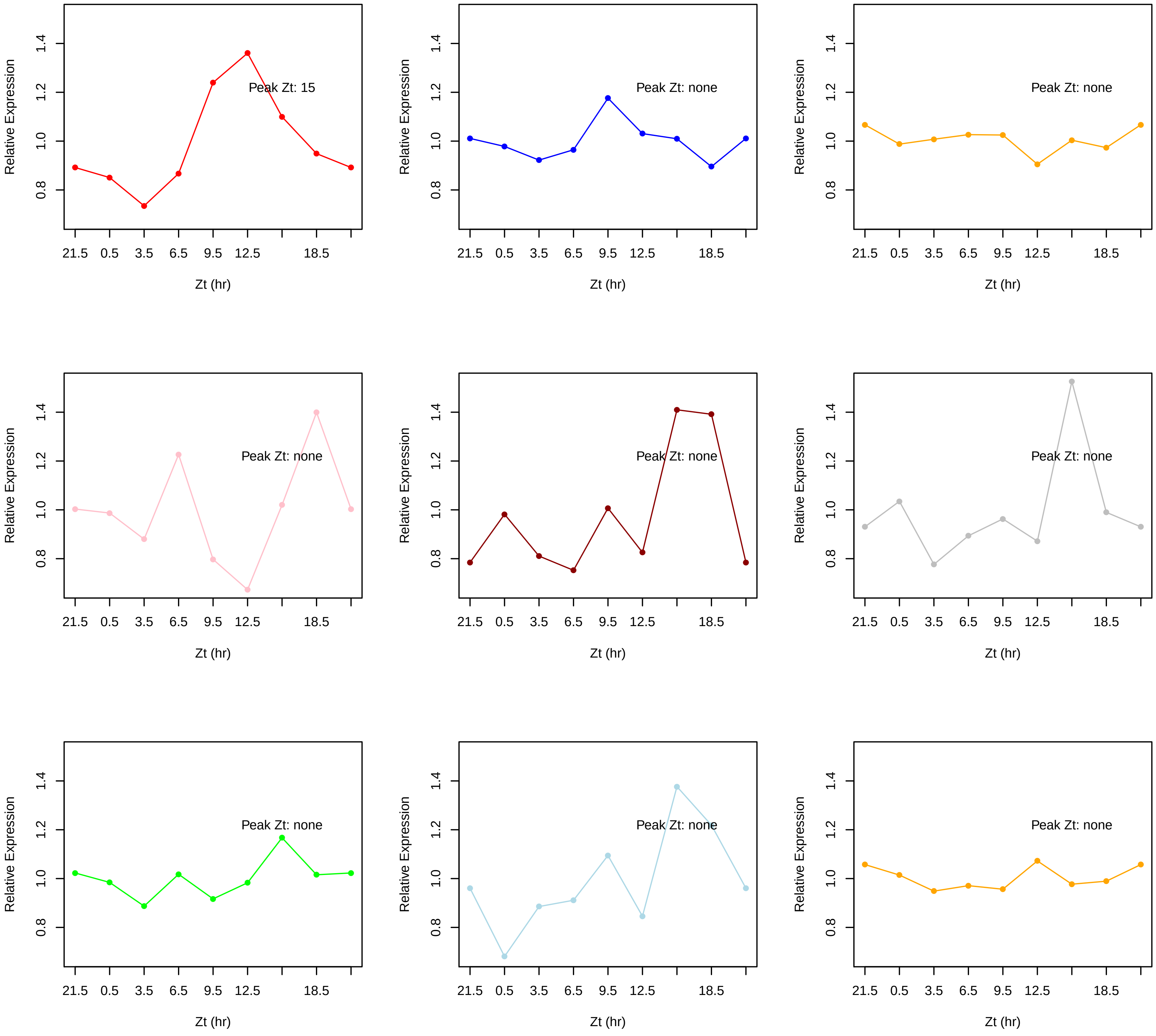

Singeleton: ENSXMAG00000002604 tspan4b

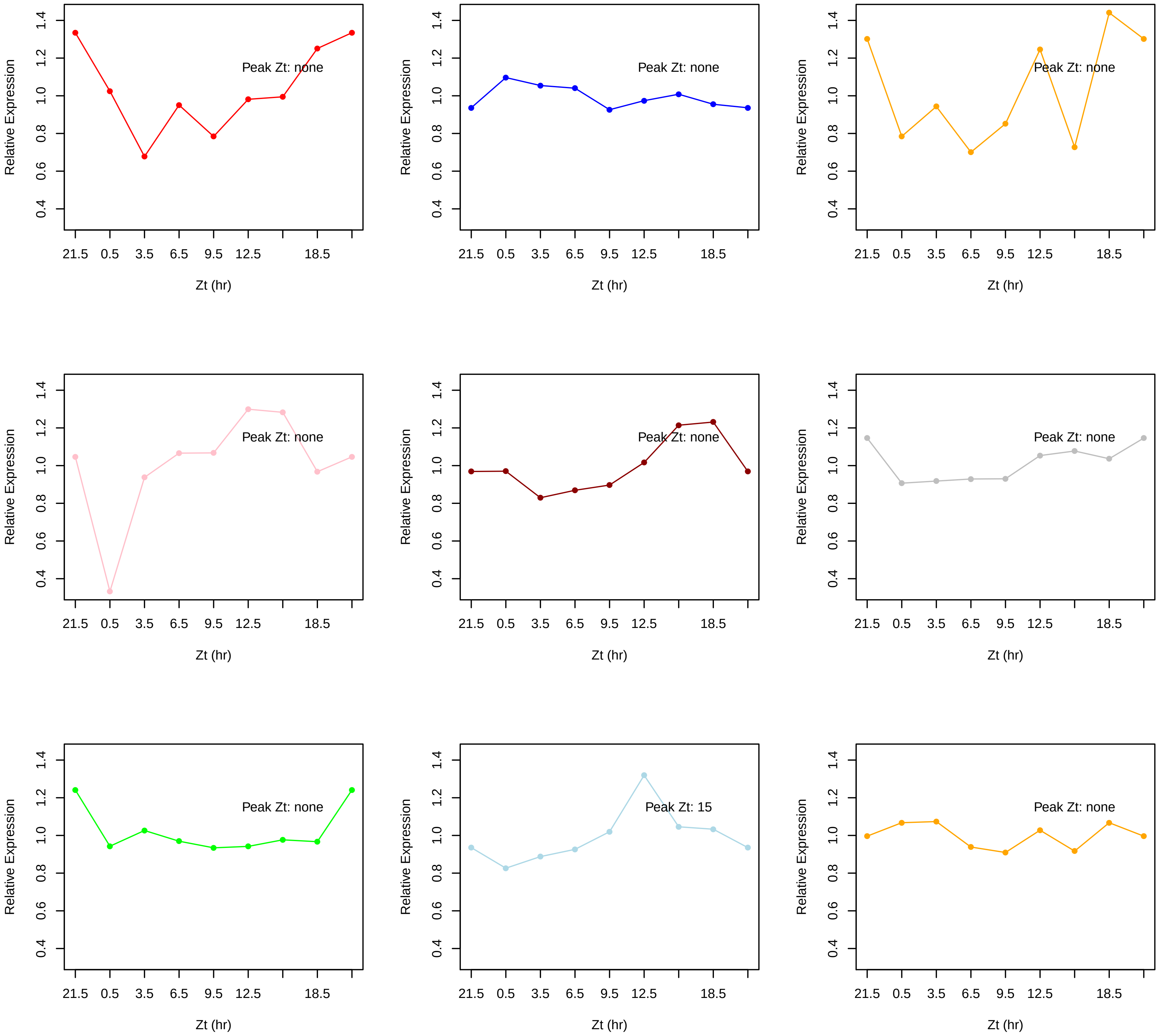

Singeleton: ENSXMAG00000018428 rbks

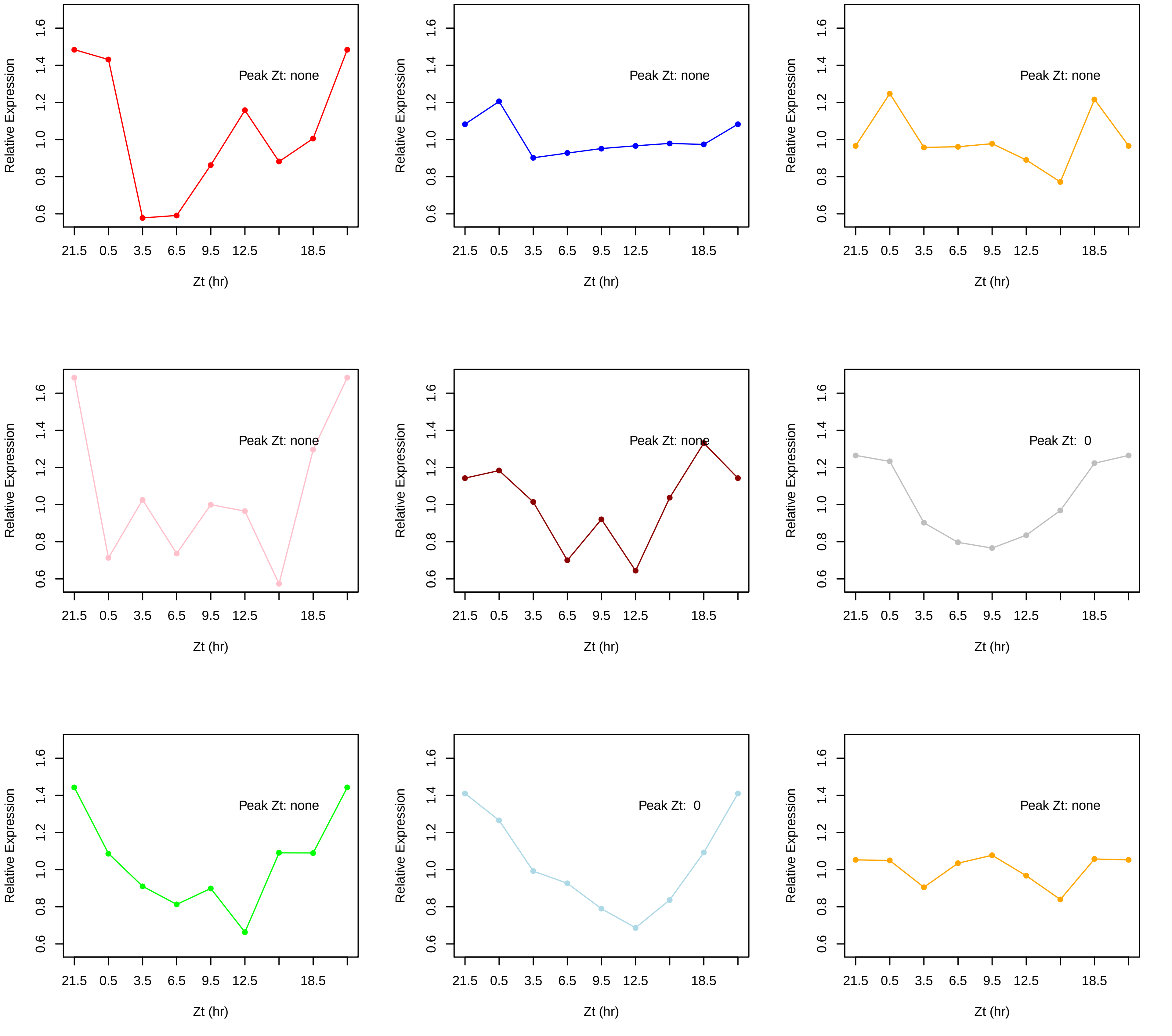

Singleton: ENSXMAG00000023180 si:ch73−248e21.1

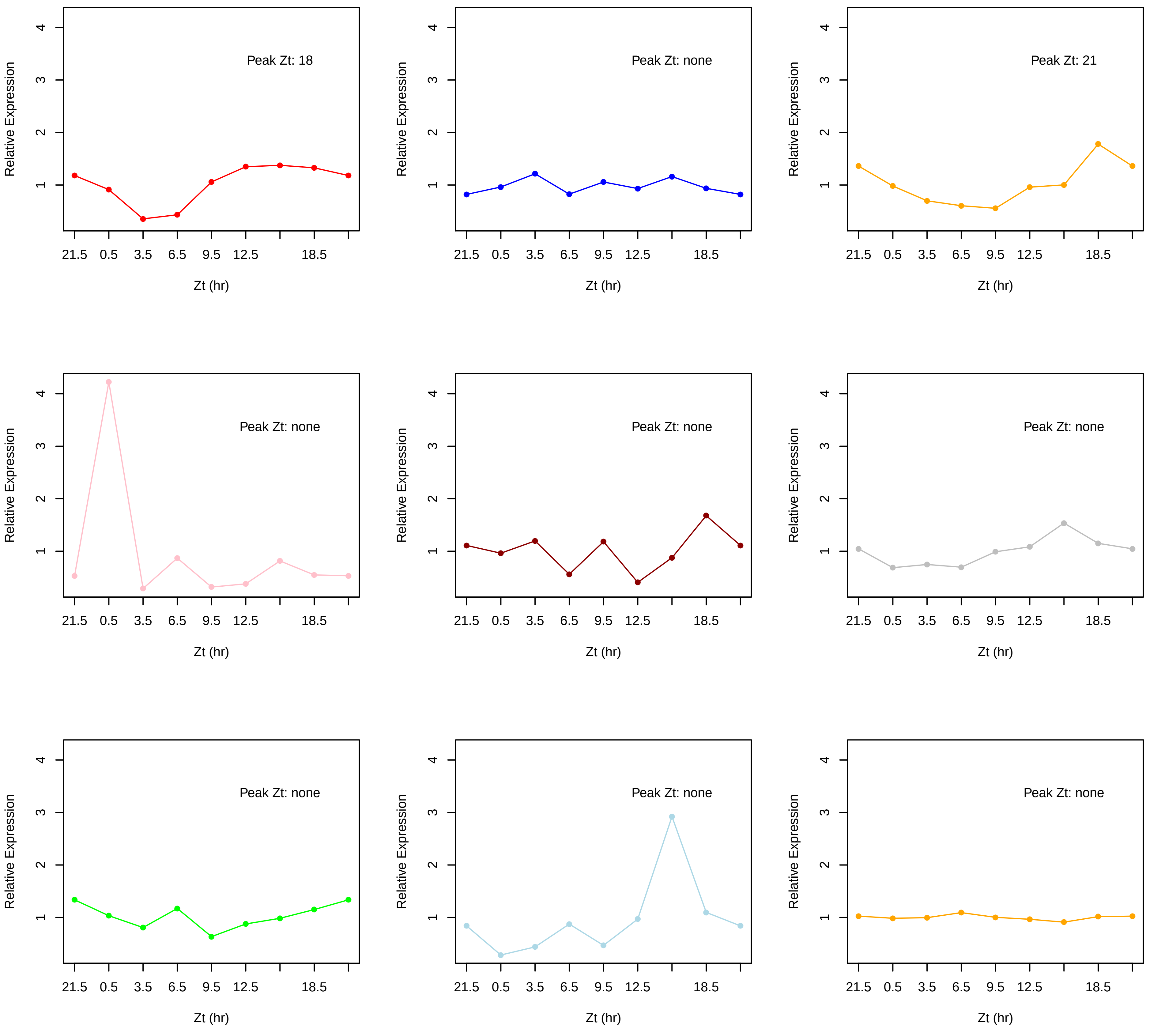

Singeleton: ENSXMAG00000018440 babam2

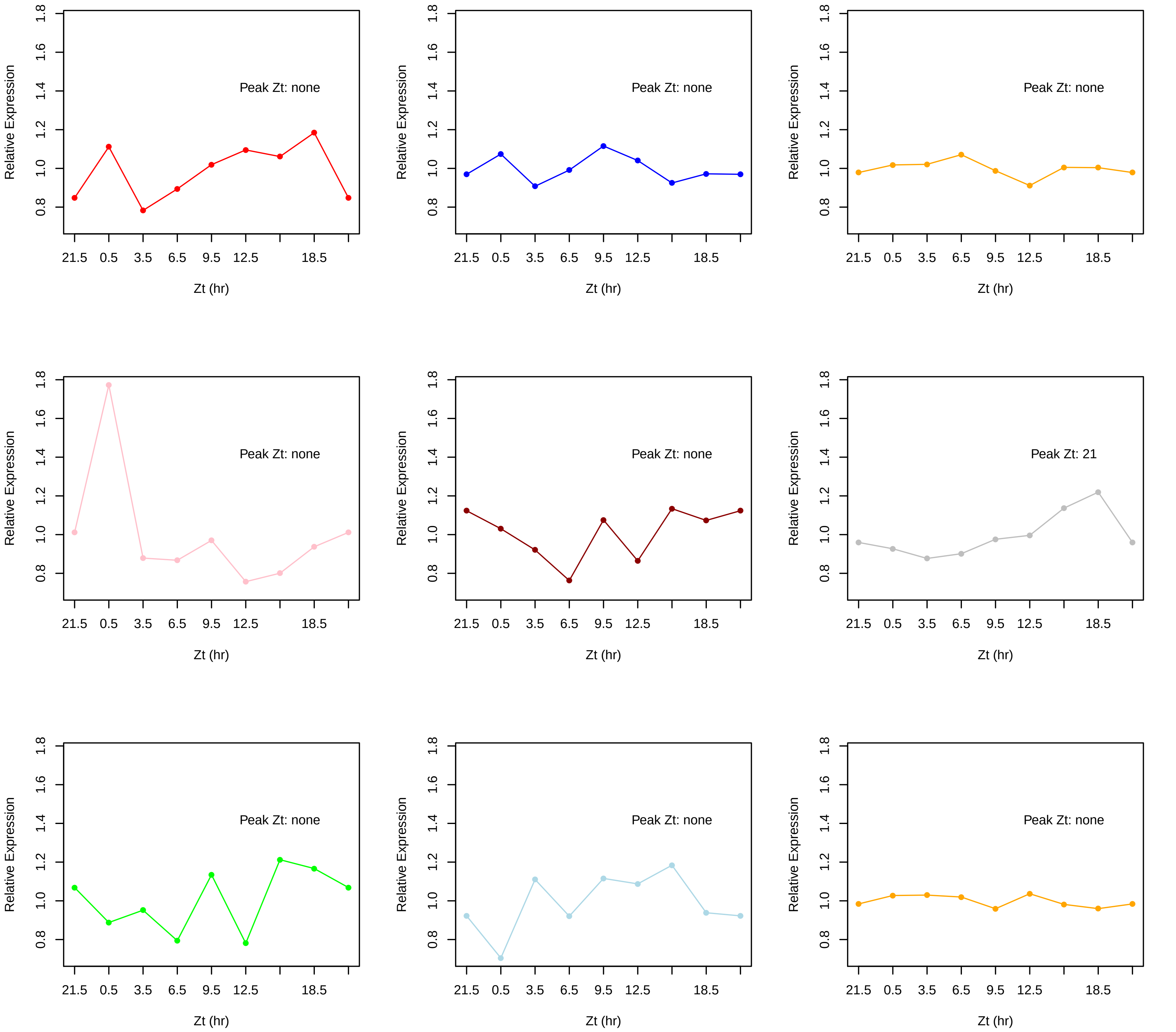

Singeleton: ENSXMAG00000026947 fosl2

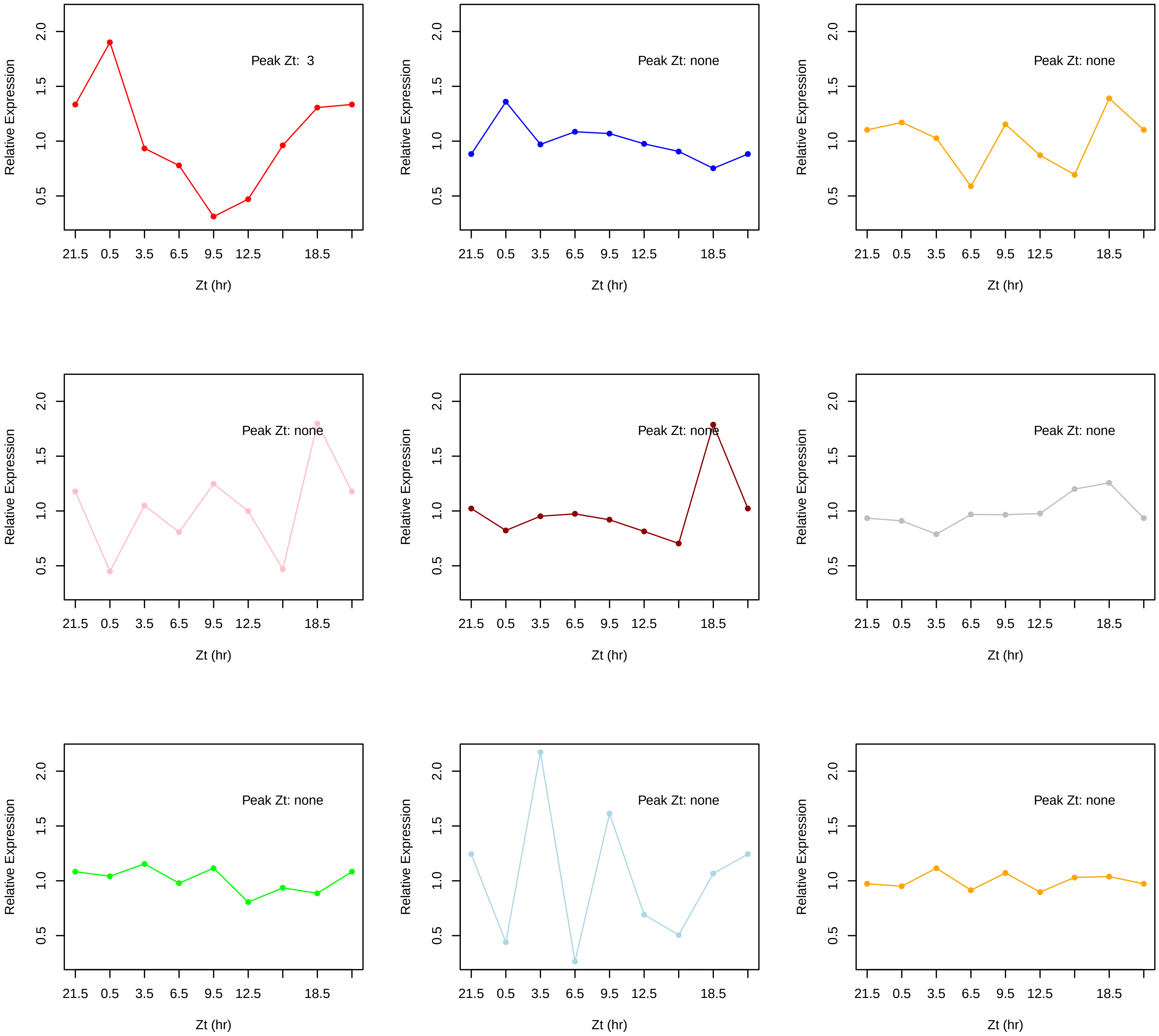

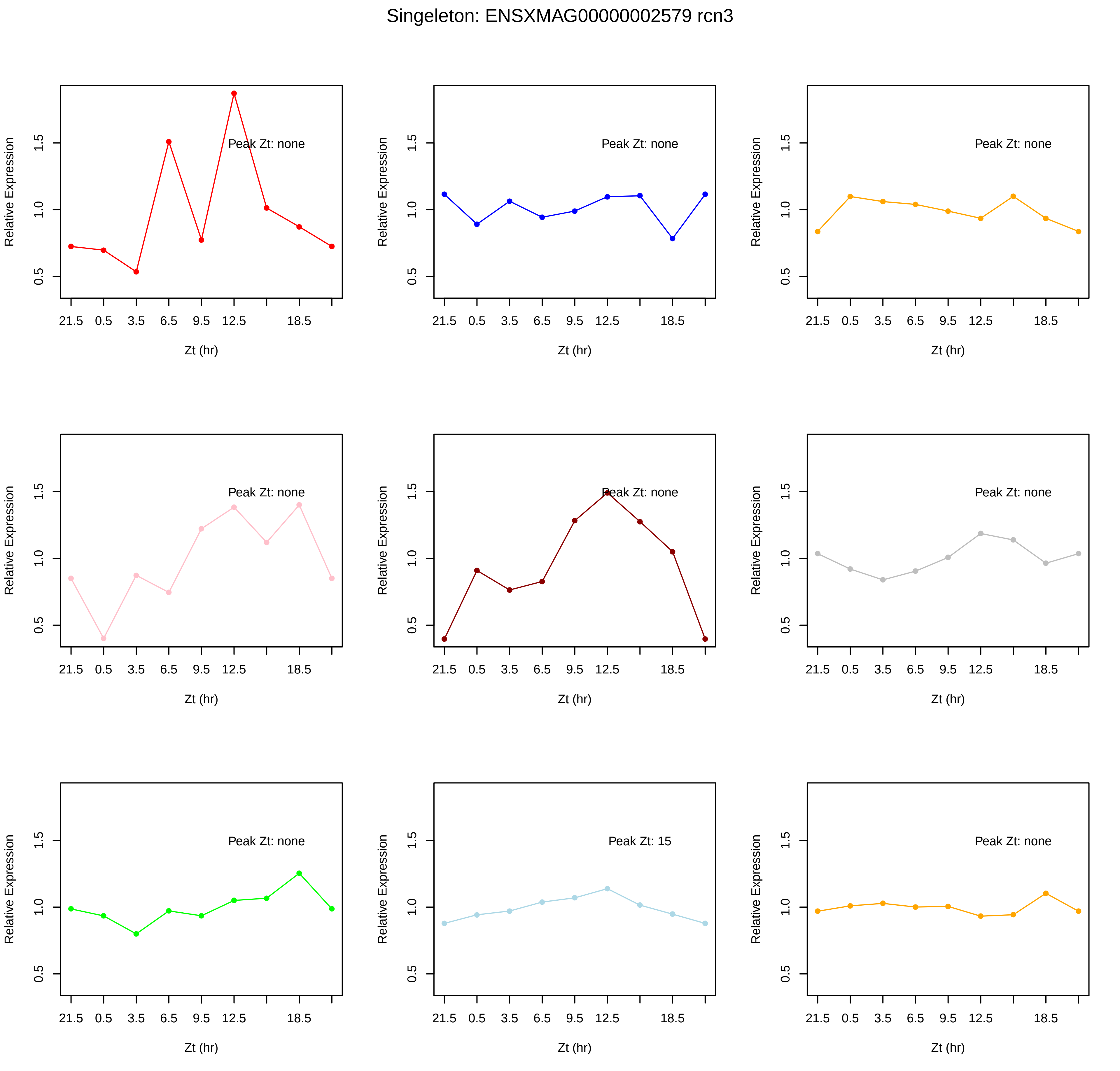

Singeleton: ENSXMAG00000002579 rcn3
Peak Zt: none
Peak Zt: none
Peak Zt: none
Peak Zt: none
Peak Zt: none
Peak Zt: none
Peak Zt: none
Peak Zt: 15
Peak Zt: none
Relative Expression
Zt (hr)
21.5
0.5
3.5
6.5
9.5
12.5
18.5
0.5
1.0
1.5

Singeleton: ENSXMAG00000002594 nosip

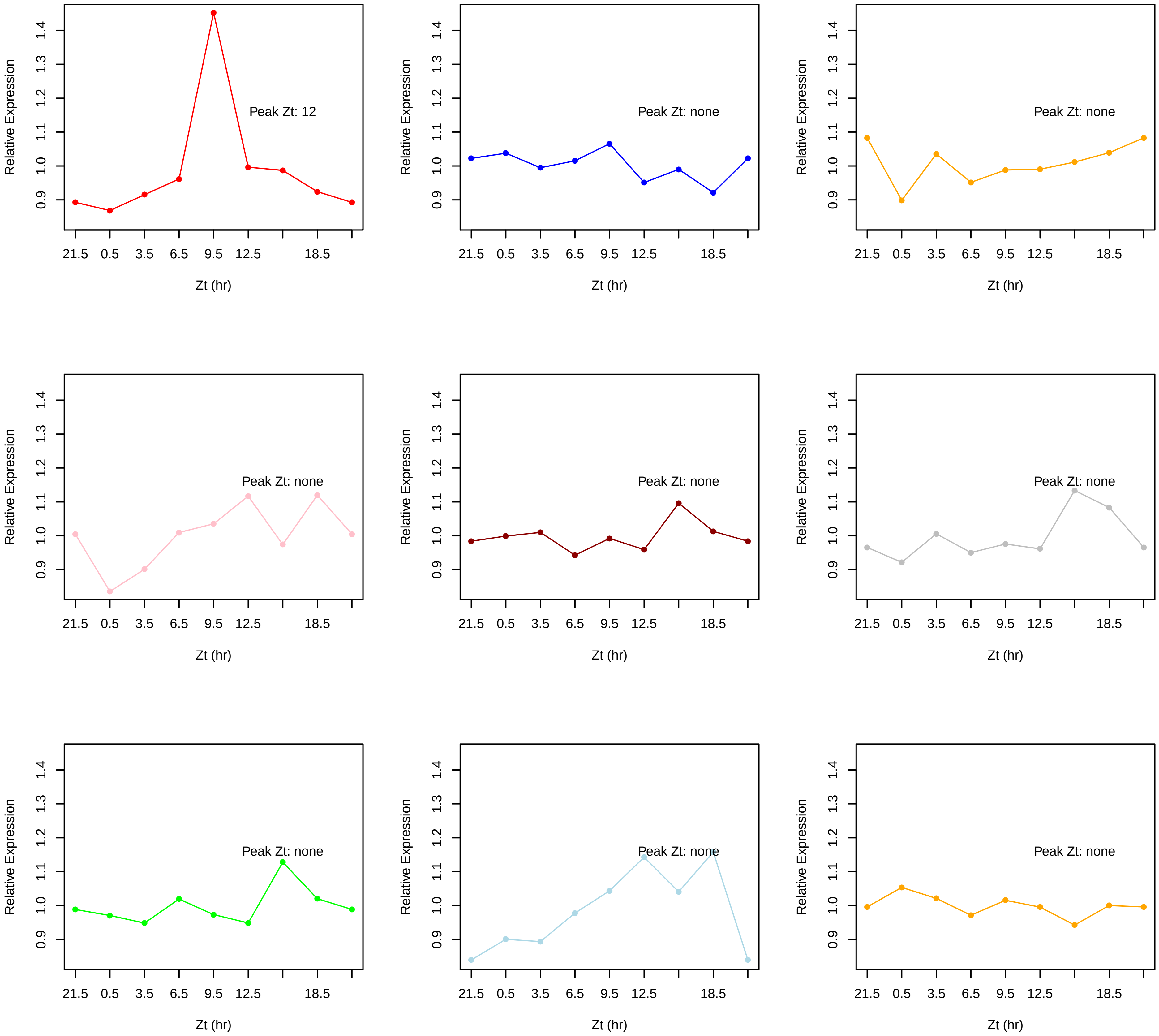

Singeleton: ENSXMAG00000018547 daam2

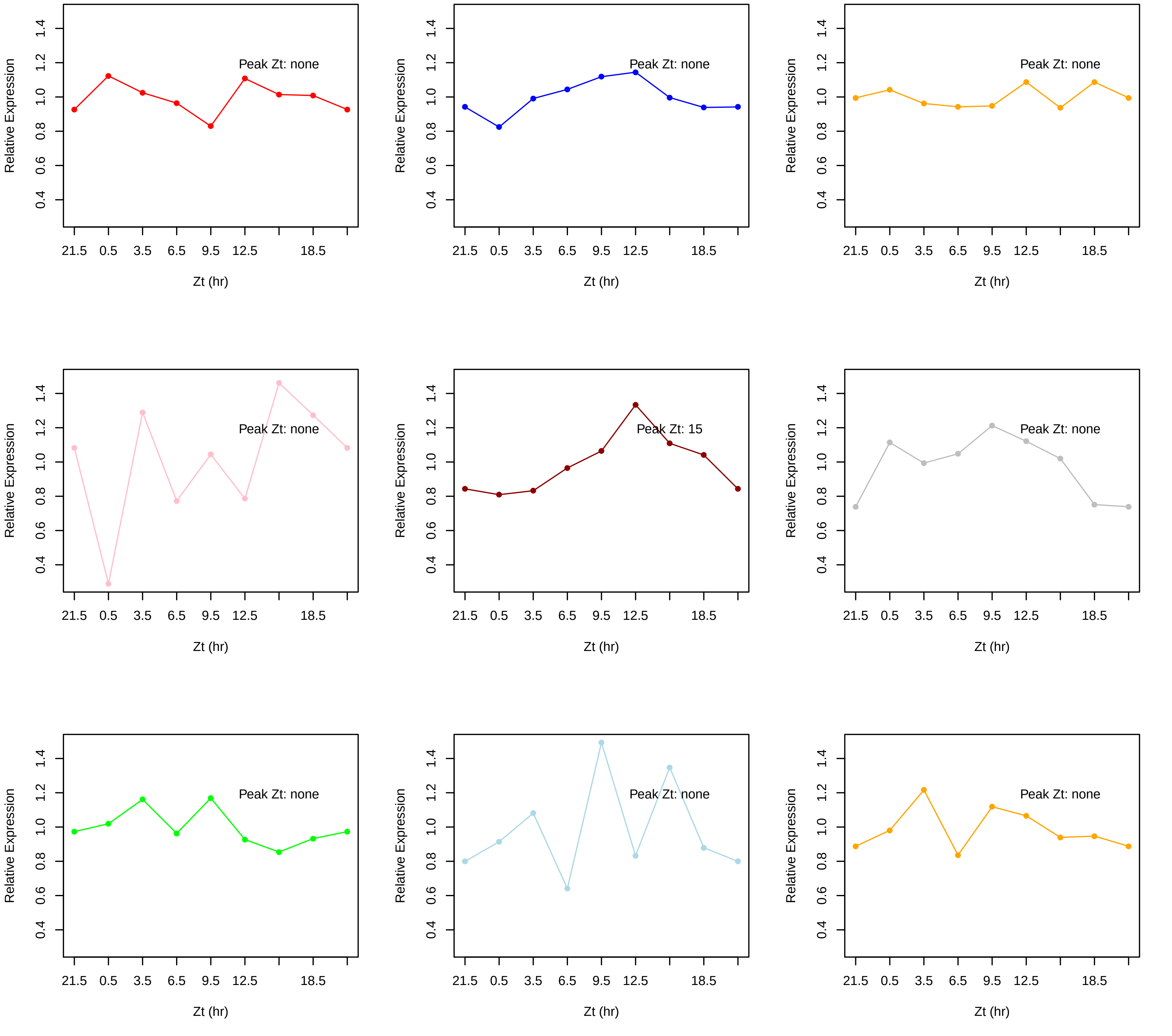

Singeleton: ENSXMAG00000000189 gnmt

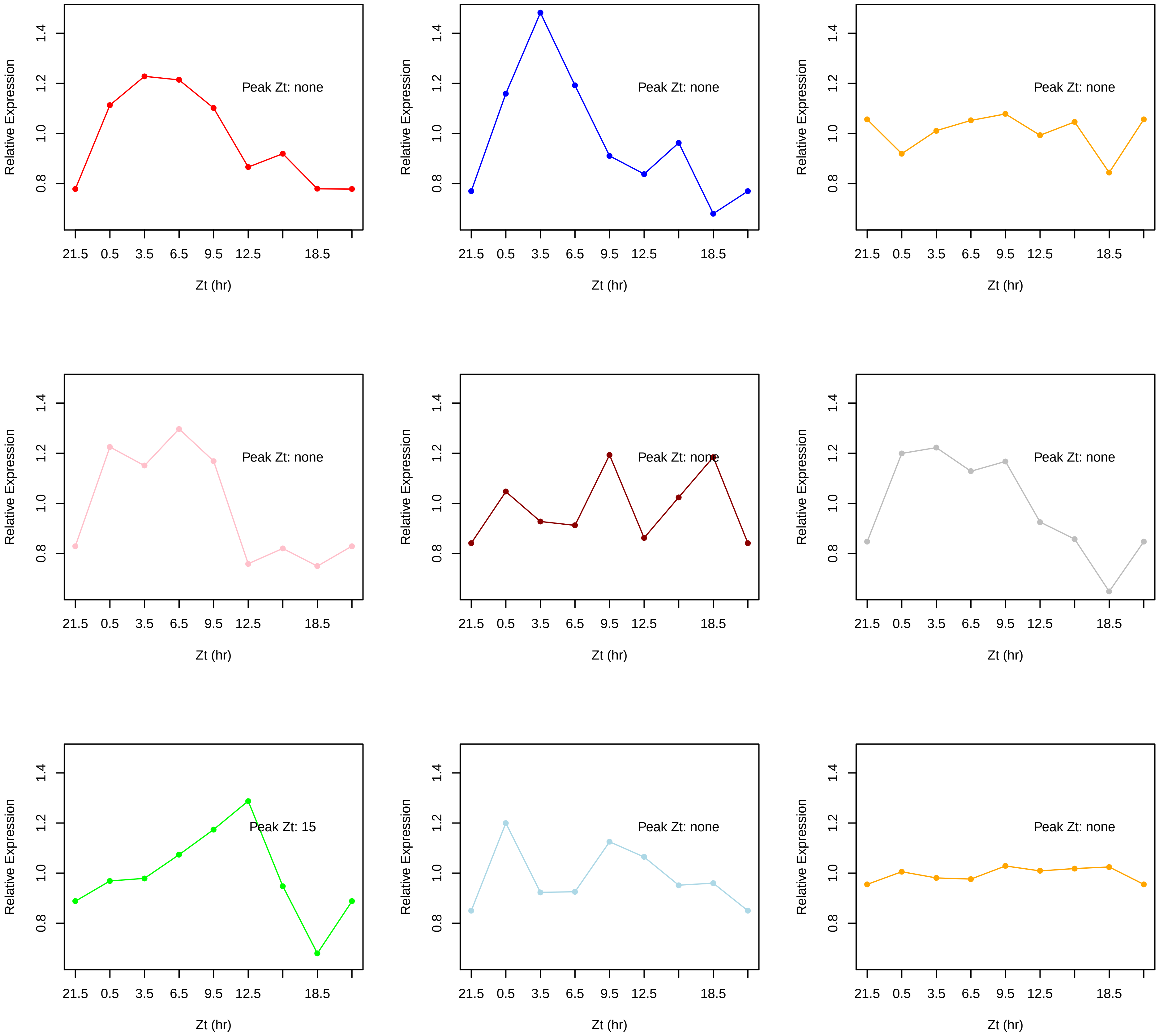

Singeleton: ENSXMAG00000025868 syt14a

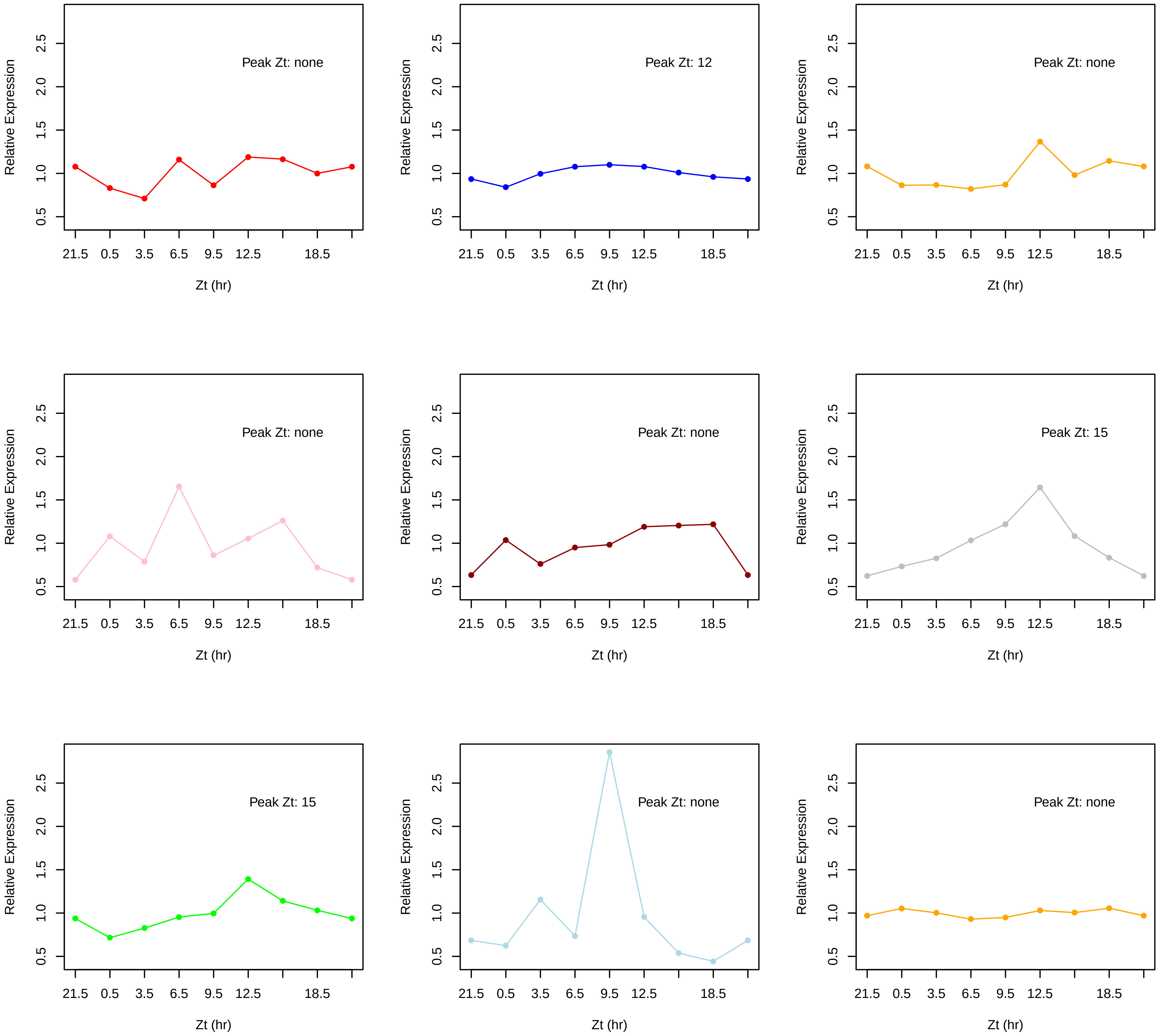

Singeleton: ENSXMAG00000016994 nt5c1bb

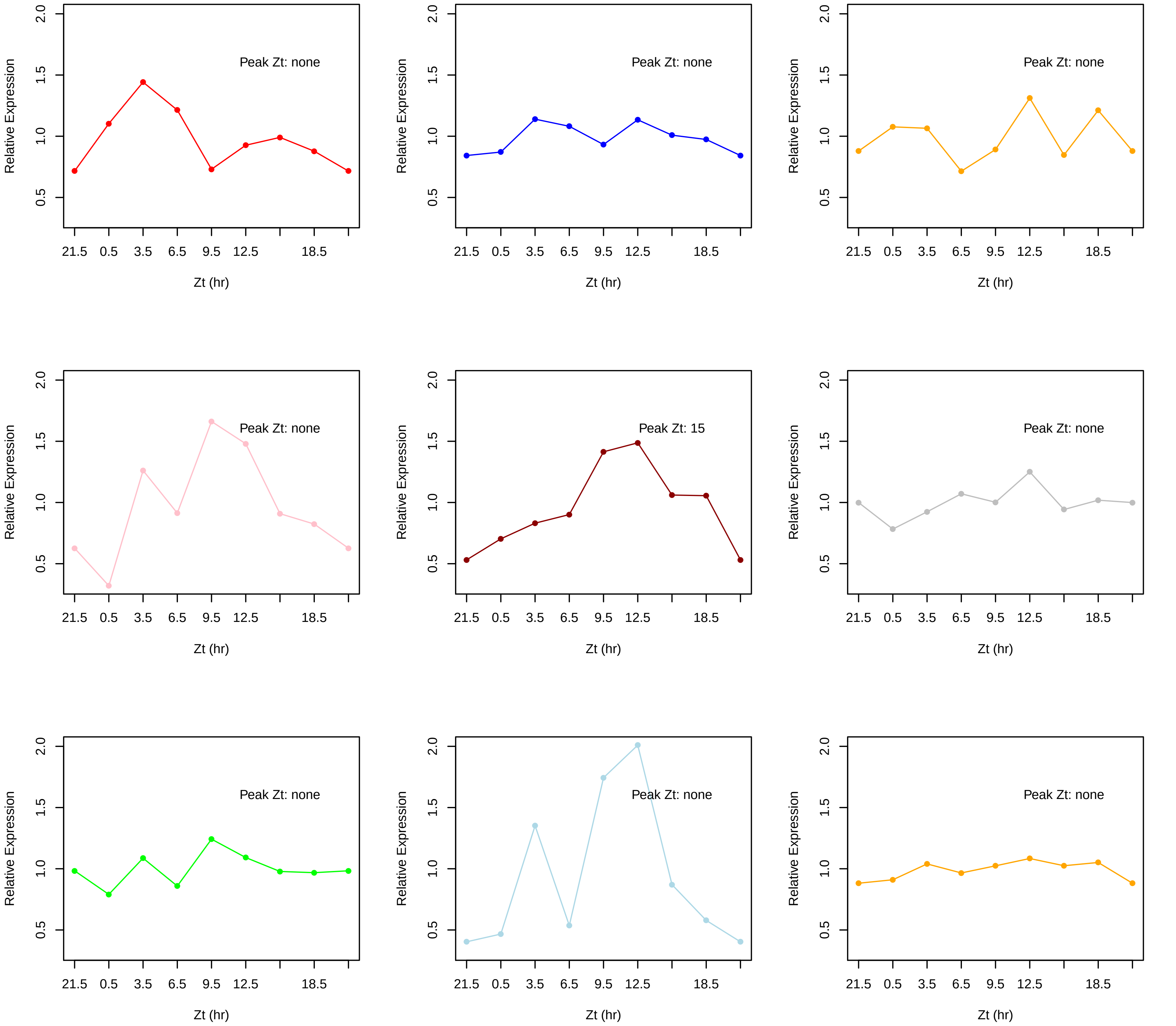

Singeleton: ENSXMAG00000028047 laptm4a

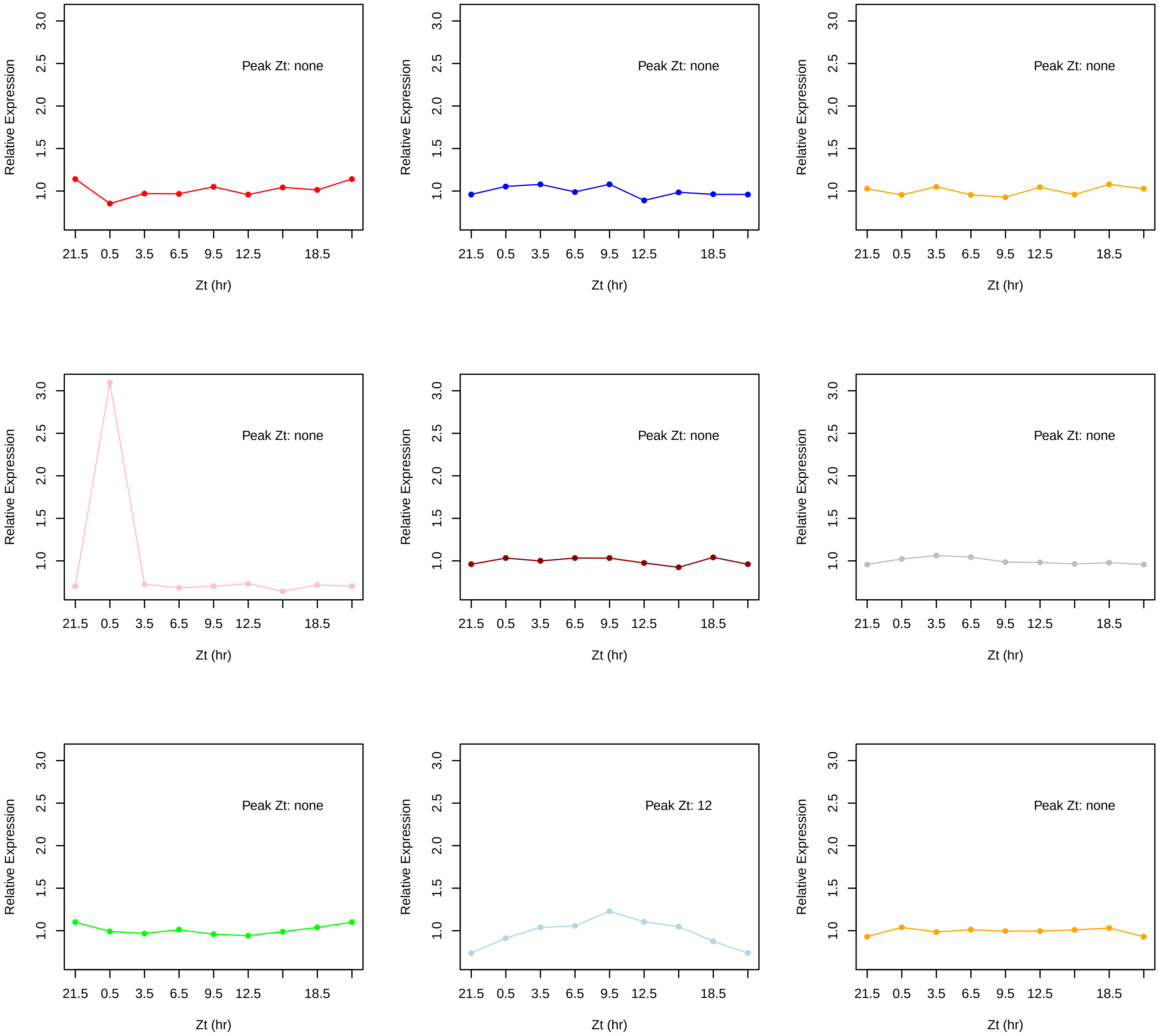

Singeleton: ENSXMAG00000017682 pum2

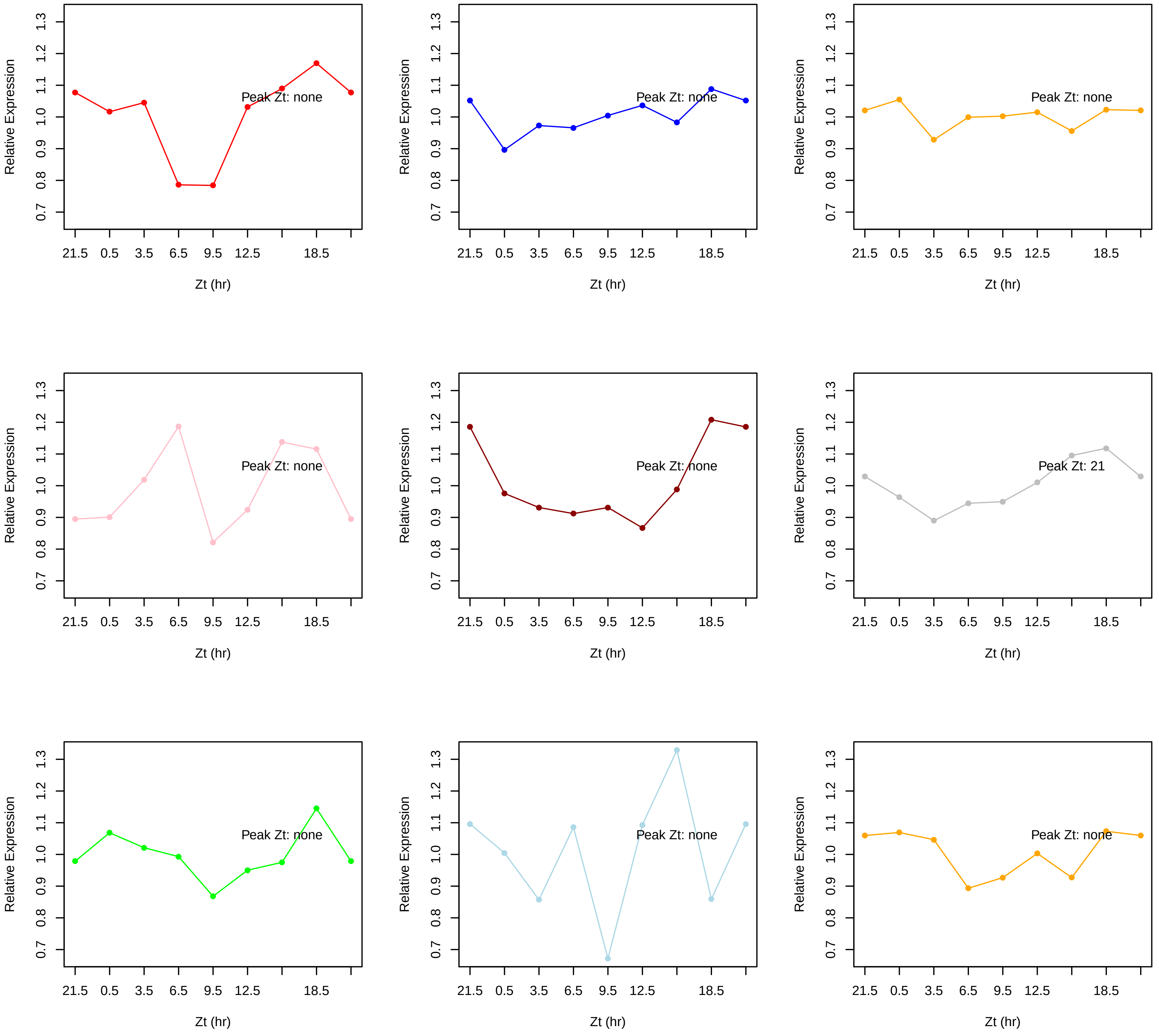

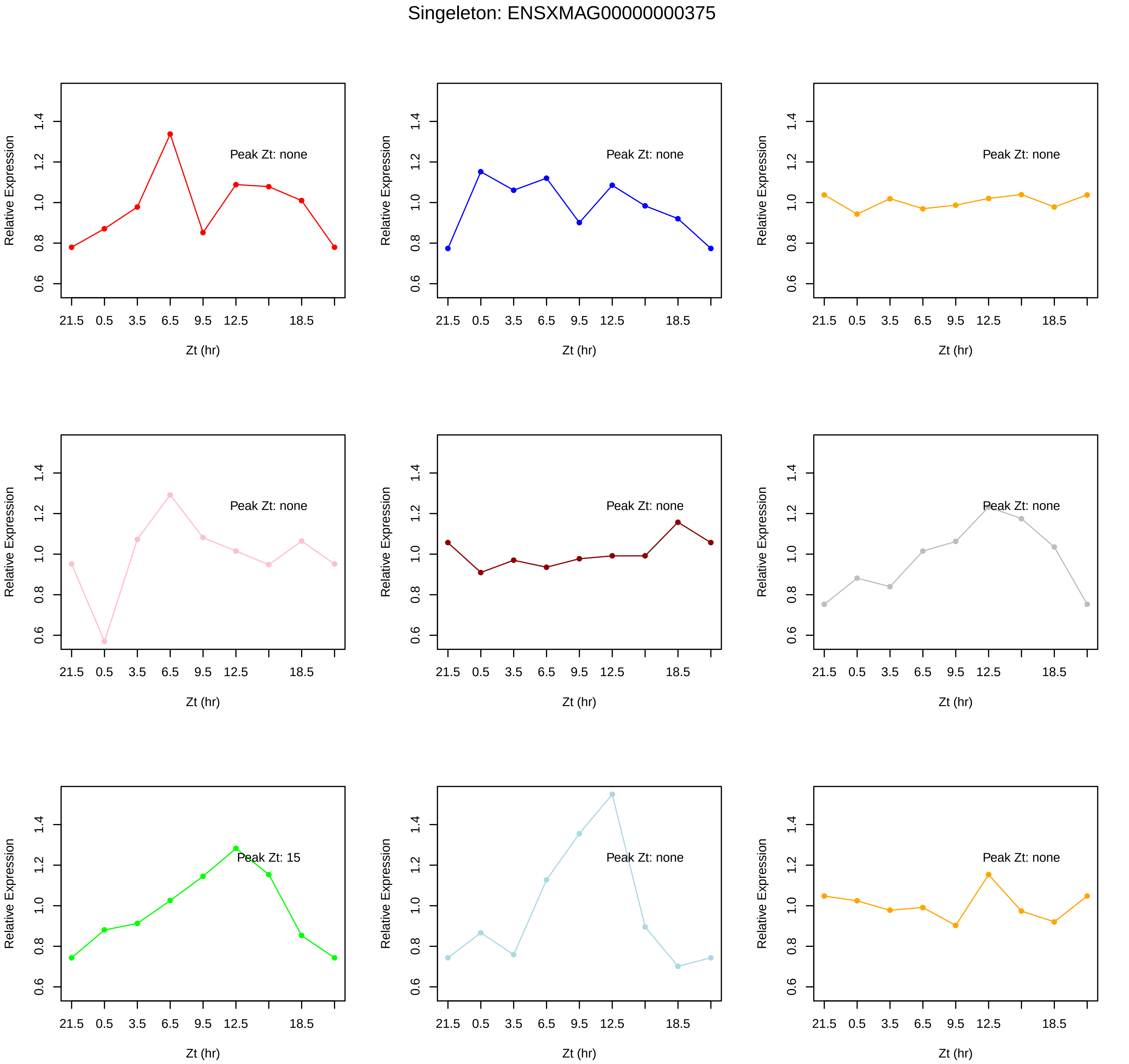
Singeleton: ENSXMAG00000000375
Peak Zt: none
Peak Zt: none
Peak Zt: none
Peak Zt: none
Peak Zt: none
Peak Zt: none
Peak Zt: 15
Peak Zt: none
Peak Zt: none
Relative Expression
Zt (hr)

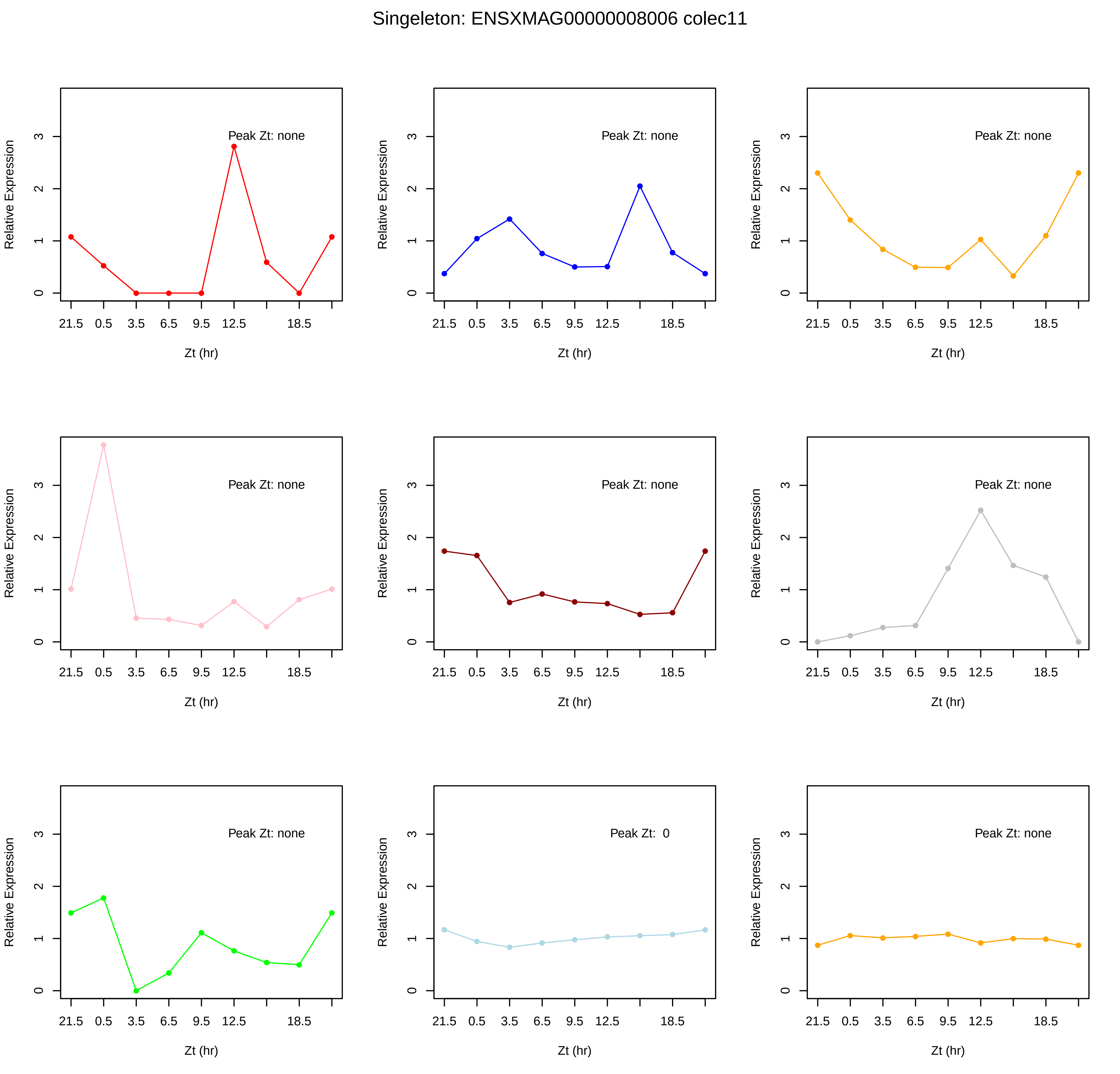

Singeleton: ENSXMAG00000008006 colec11
Peak Zt: none
Peak Zt: none
Peak Zt: none
Peak Zt: none
Peak Zt: none
Peak Zt: none
Peak Zt: none
Peak Zt: 0
Peak Zt: none
Relative Expression
Zt (hr)
21.5
0.5
3.5
6.5
9.5
12.5
18.5
0
1
2
3

Singeleton: ENSXMAG00000002346 rps7

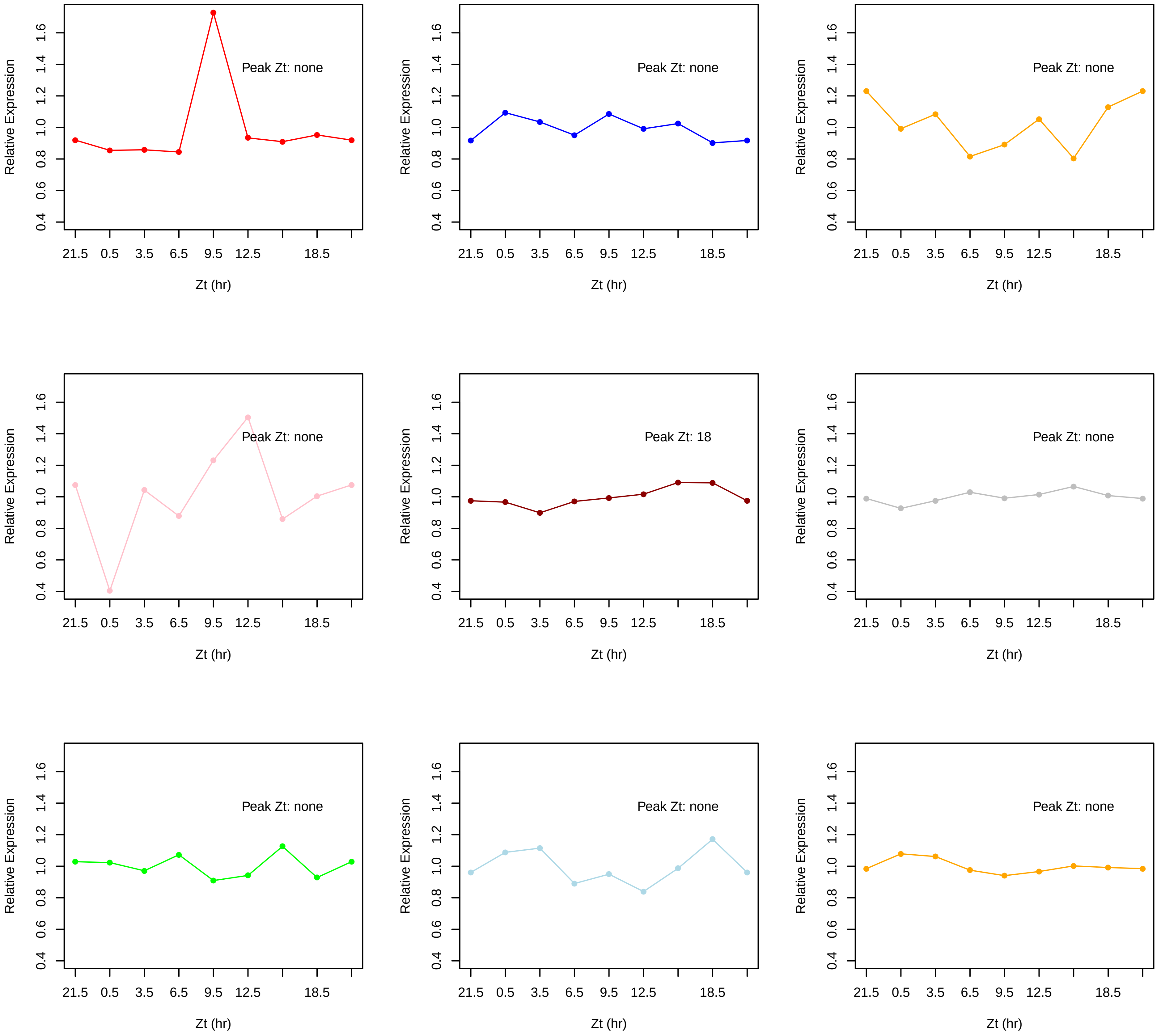

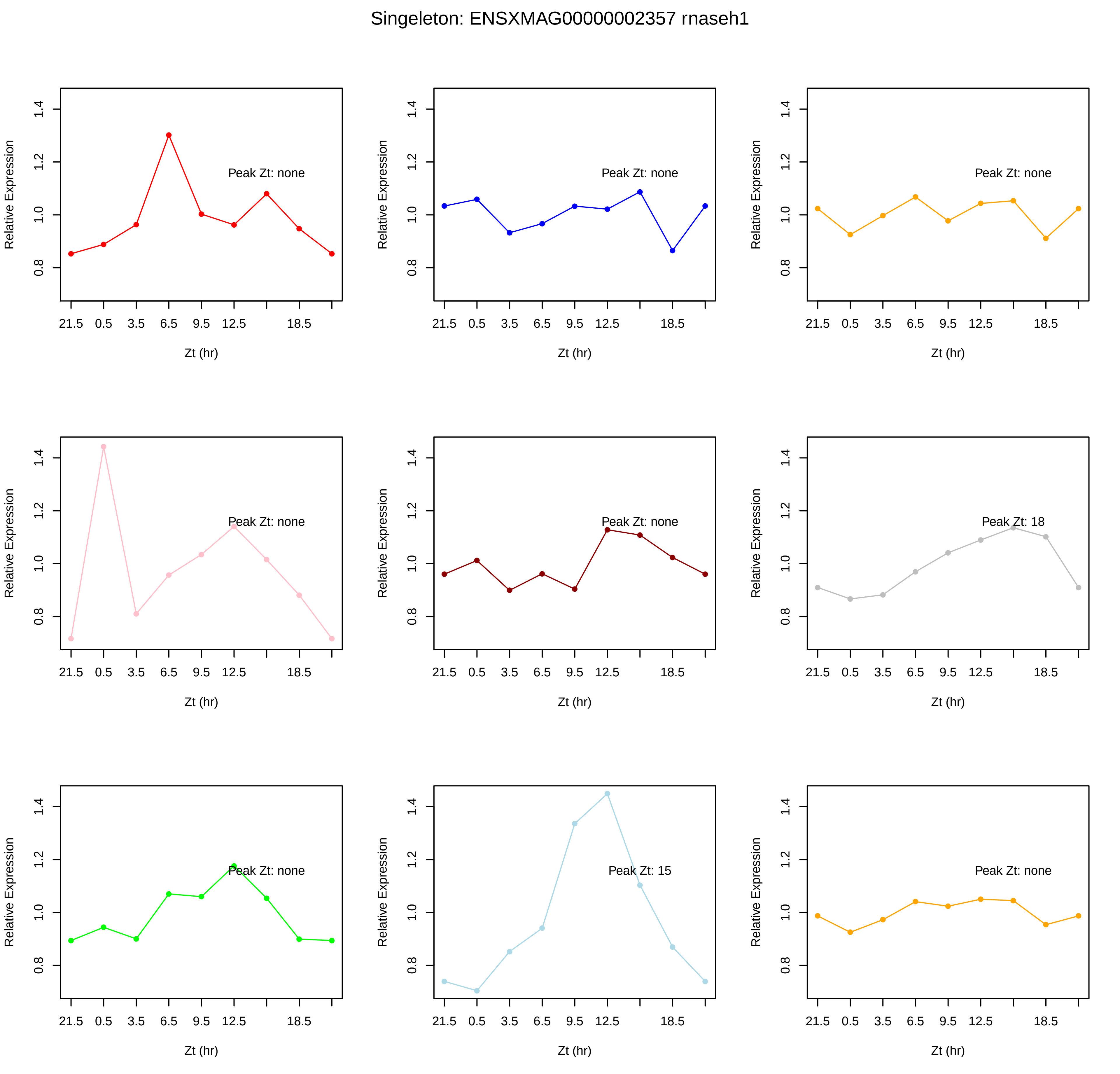
Singeleton: ENSXMAG00000002357 rnaseh1
Peak Zt: none
Peak Zt: none
Peak Zt: none
Peak Zt: none
Peak Zt: none
Peak Zt: 18
Peak Zt: none
Peak Zt: 15
Peak Zt: none
Relative Expression
Zt (hr)
21.5
0.5
3.5
6.5
9.5
12.5
18.5
0.8
1.0
1.2
1.4

Singeleton: ENSXMAG00000028792 ADI1

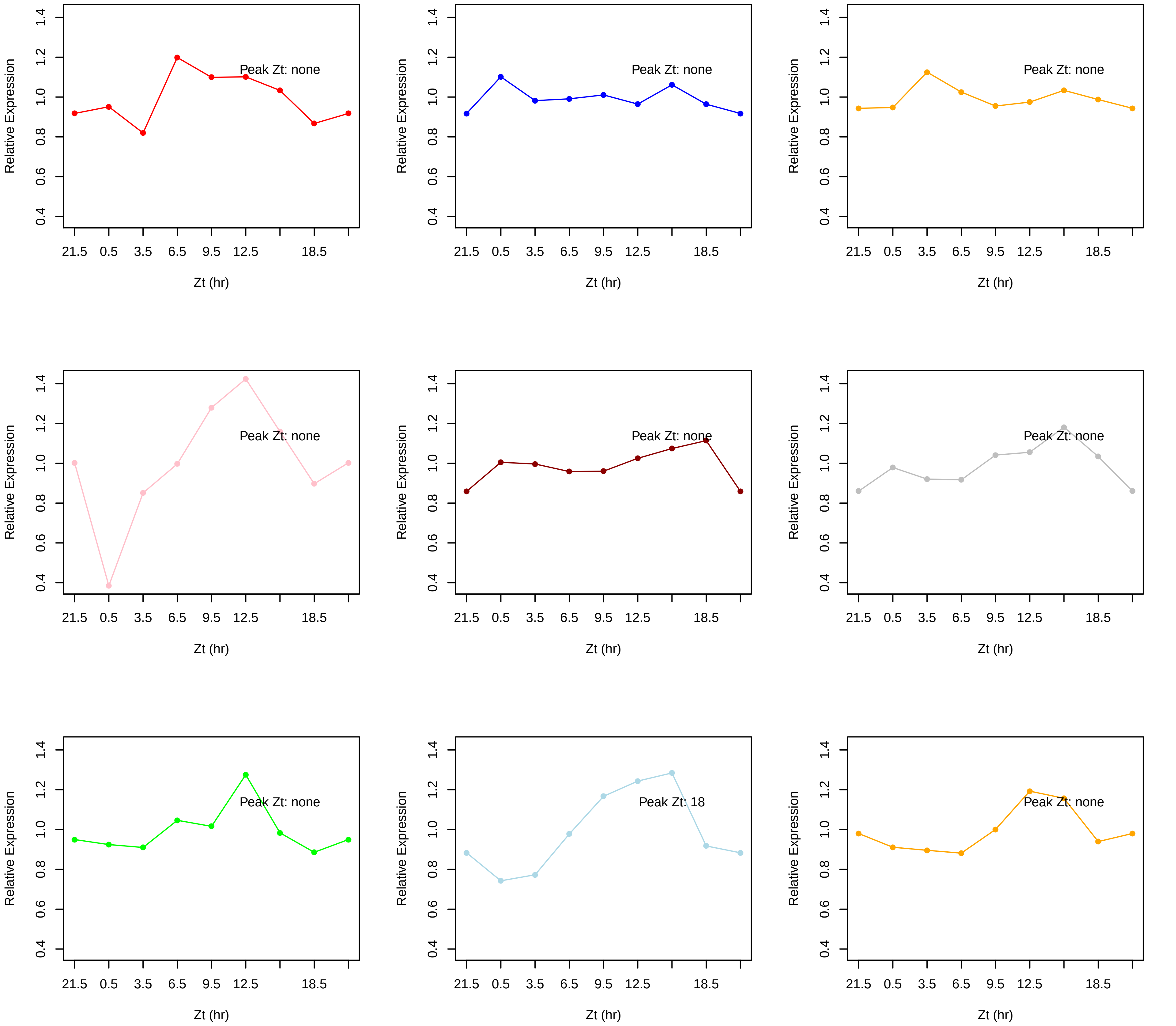

Singeleton: ENSXMAG00000018716 trappc12

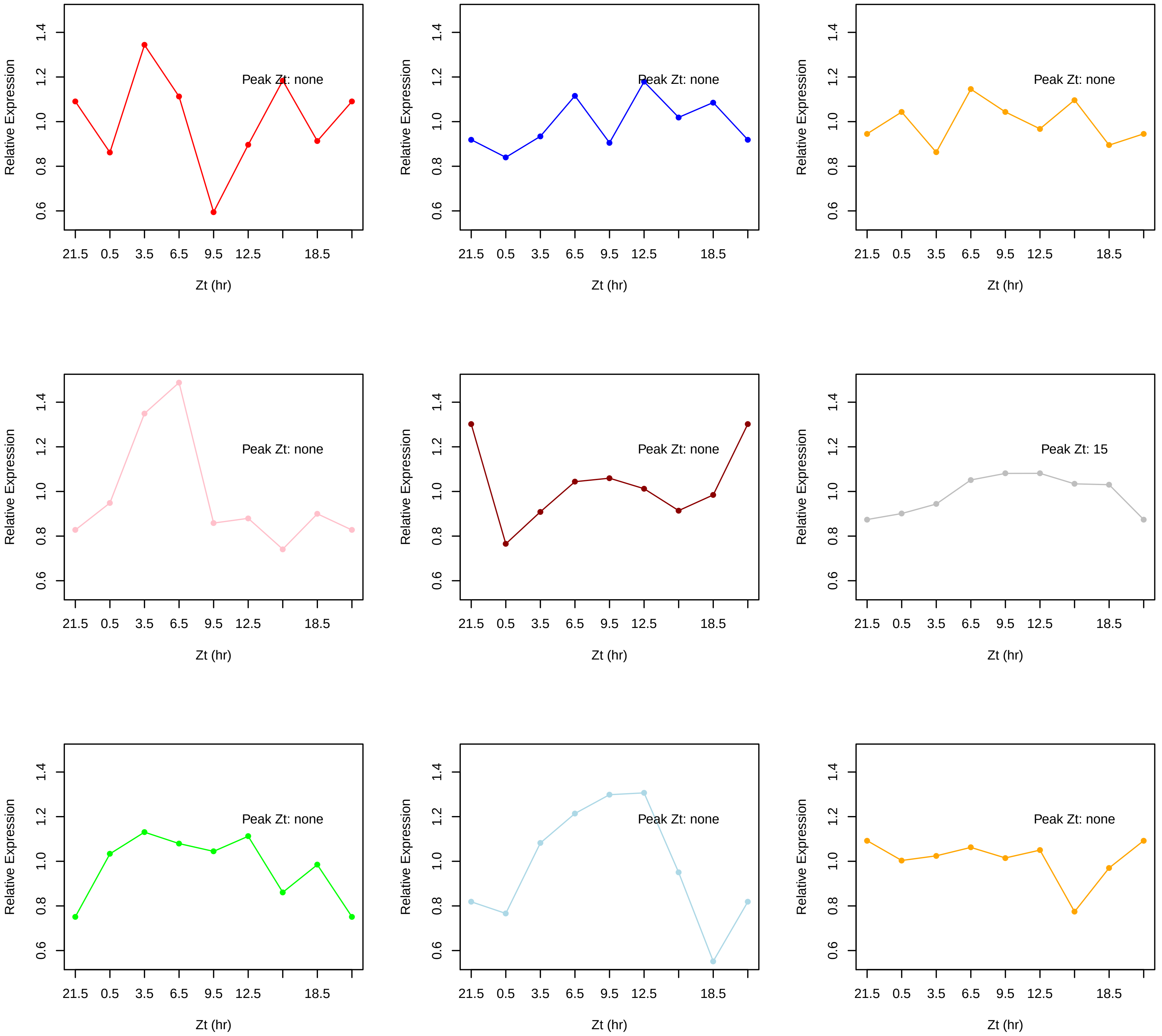

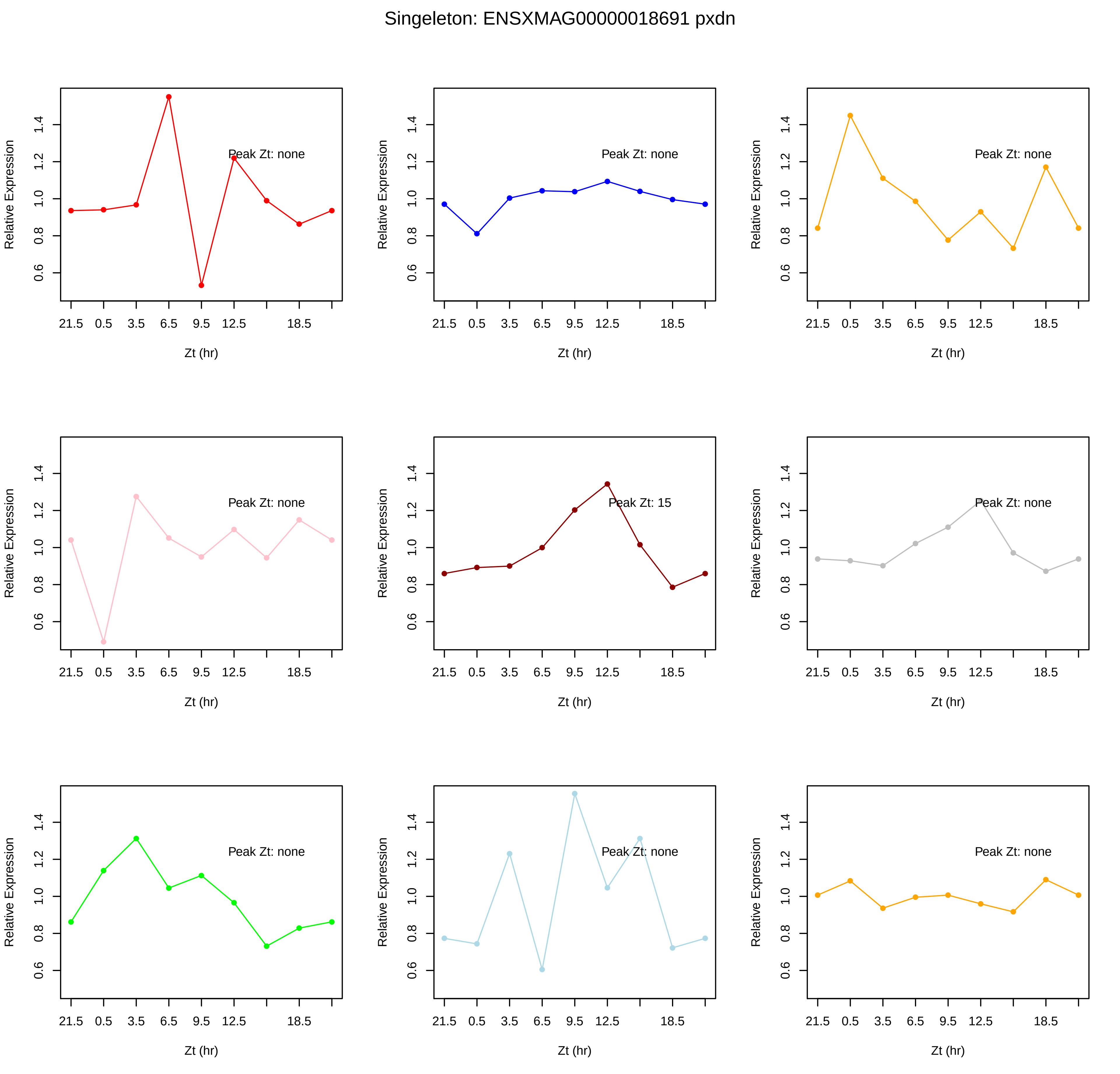
Singeleton: ENSXMAG00000018691 pxdn
Relative Expression
Zt (hr)
21.5 0.5 3.5 6.5 9.5 12.5 18.5
0.6 0.8 1.0 1.2 1.4
Peak Zt: none
Peak Zt: none
Peak Zt: none
Peak Zt: none
Peak Zt: 15
Peak Zt: none
Peak Zt: none
Peak Zt: none
Peak Zt: none

Singeleton: ENSXMAG00000009667 fgfr1op

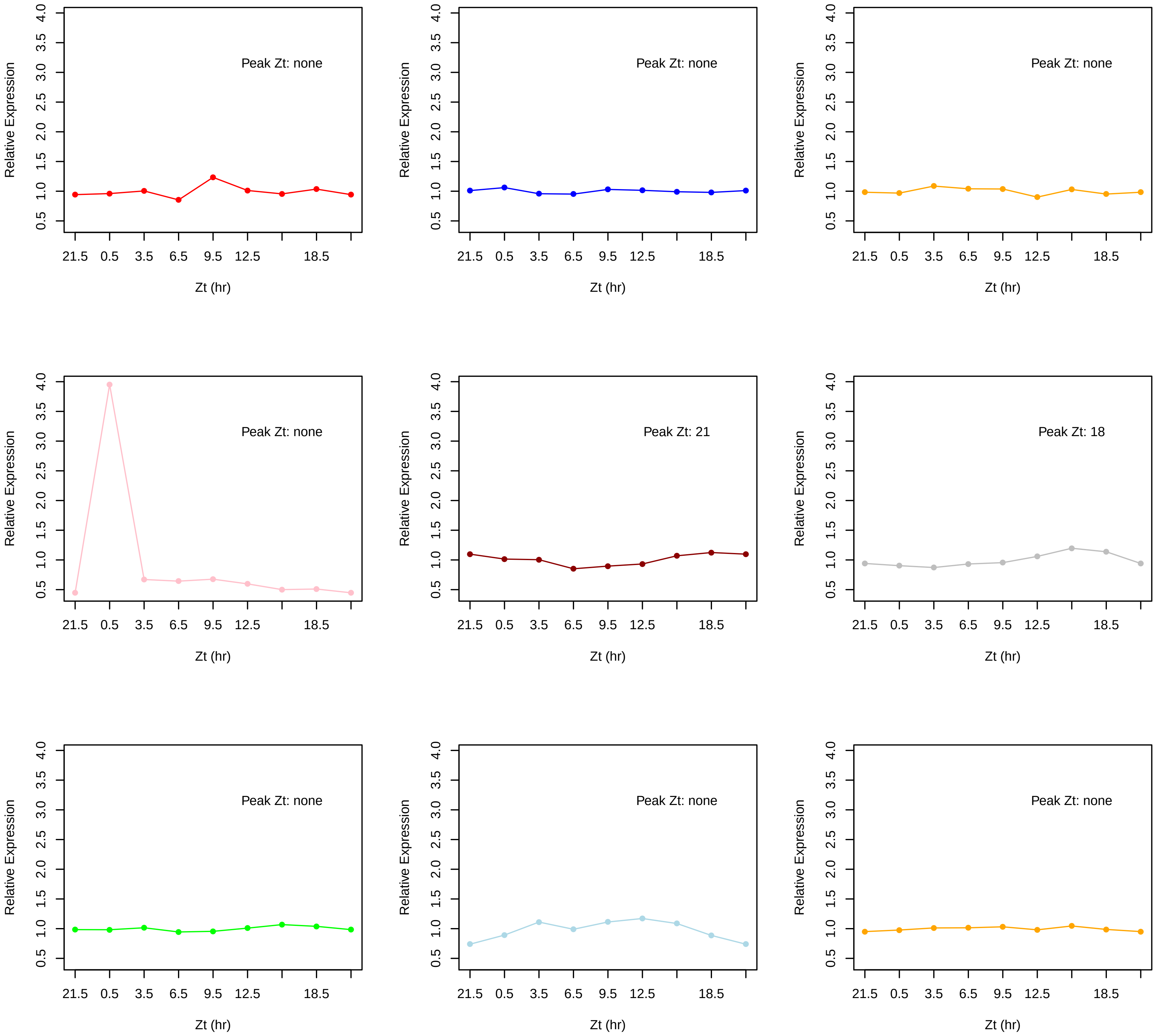

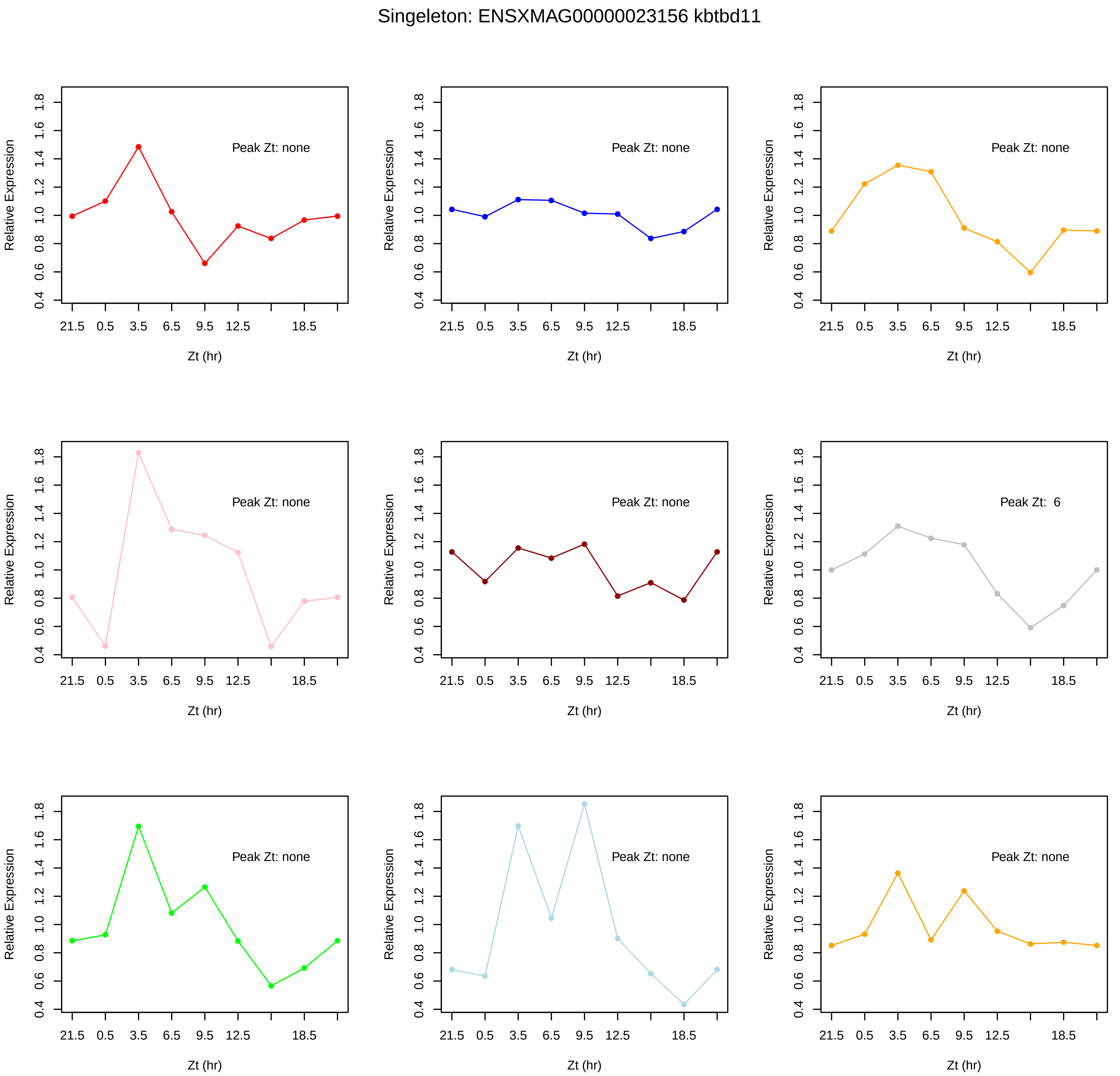

Singeleton: ENSXMAG00000023156 kbtbd11
Peak Zt: none
Peak Zt: none
Peak Zt: none
Peak Zt: none
Peak Zt: none
Peak Zt: 6
Peak Zt: none
Peak Zt: none
Peak Zt: none
Relative Expression
Zt (hr)
21.5
0.5
3.5
6.5
9.5
12.5
18.5
0.4
0.6
0.8
1.0
1.2
1.4
1.6
1.8

Singeleton: ENSXMAG00000000276

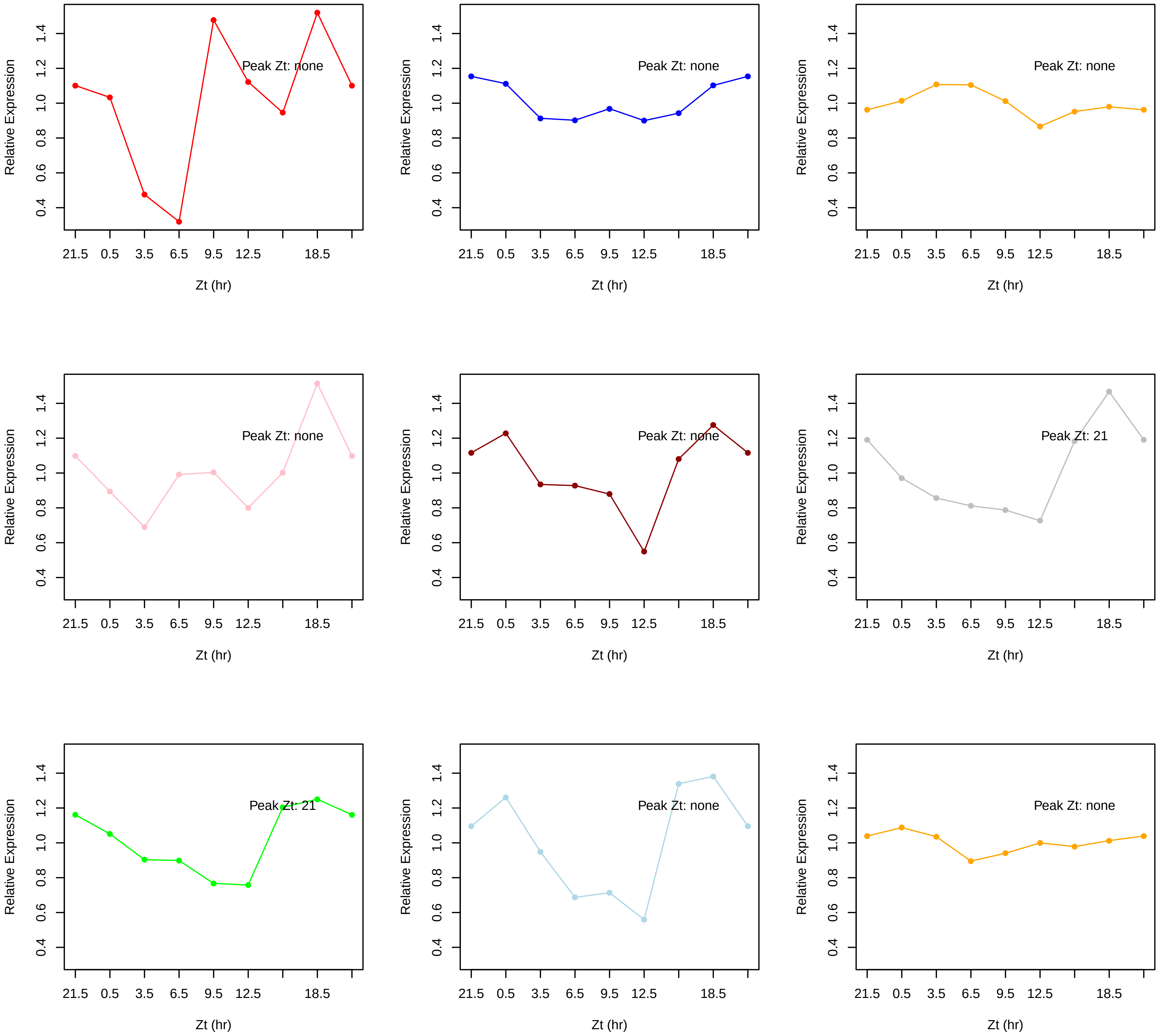

Singleton: ENSXMAG00000007731 fbxo25

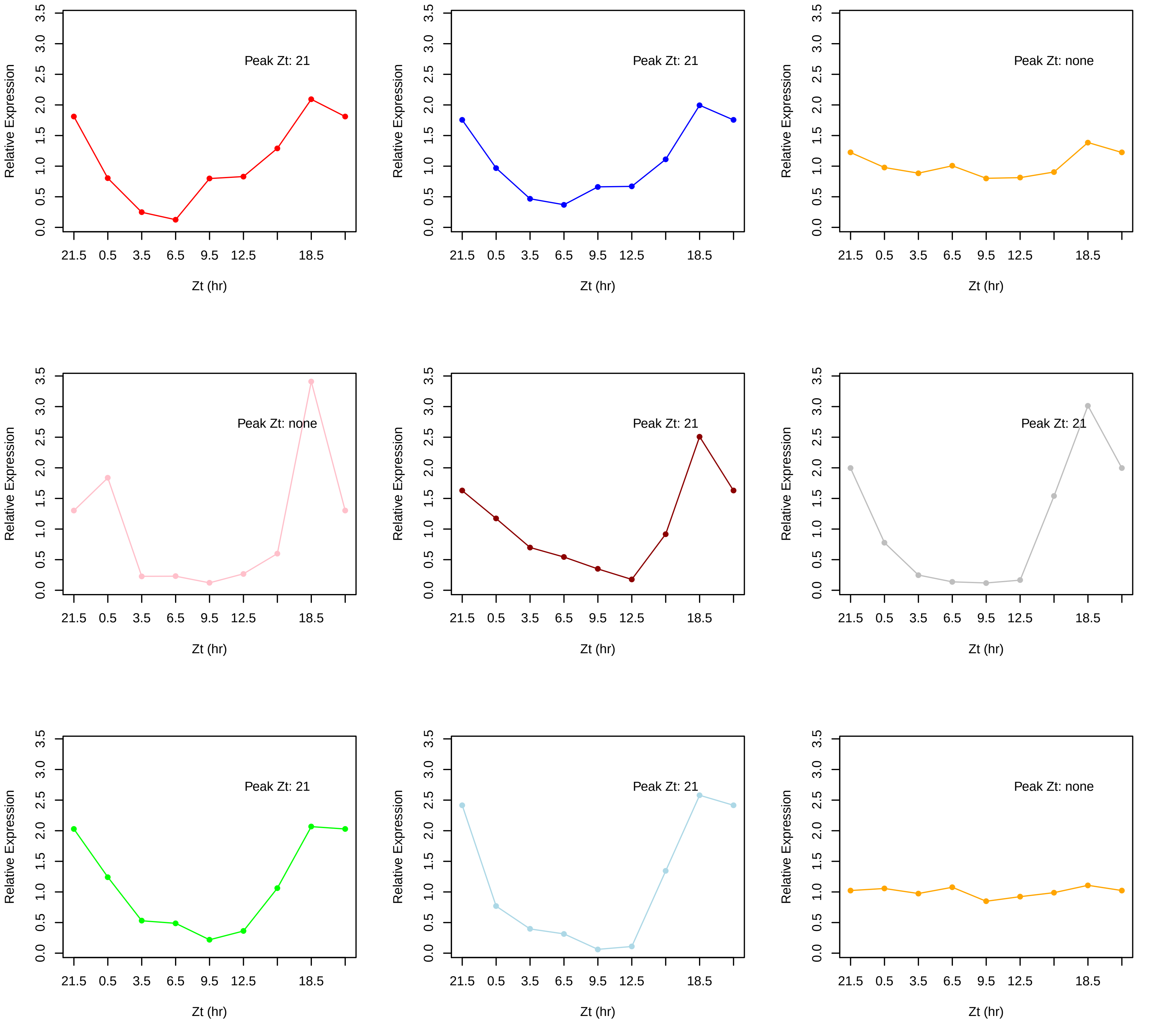

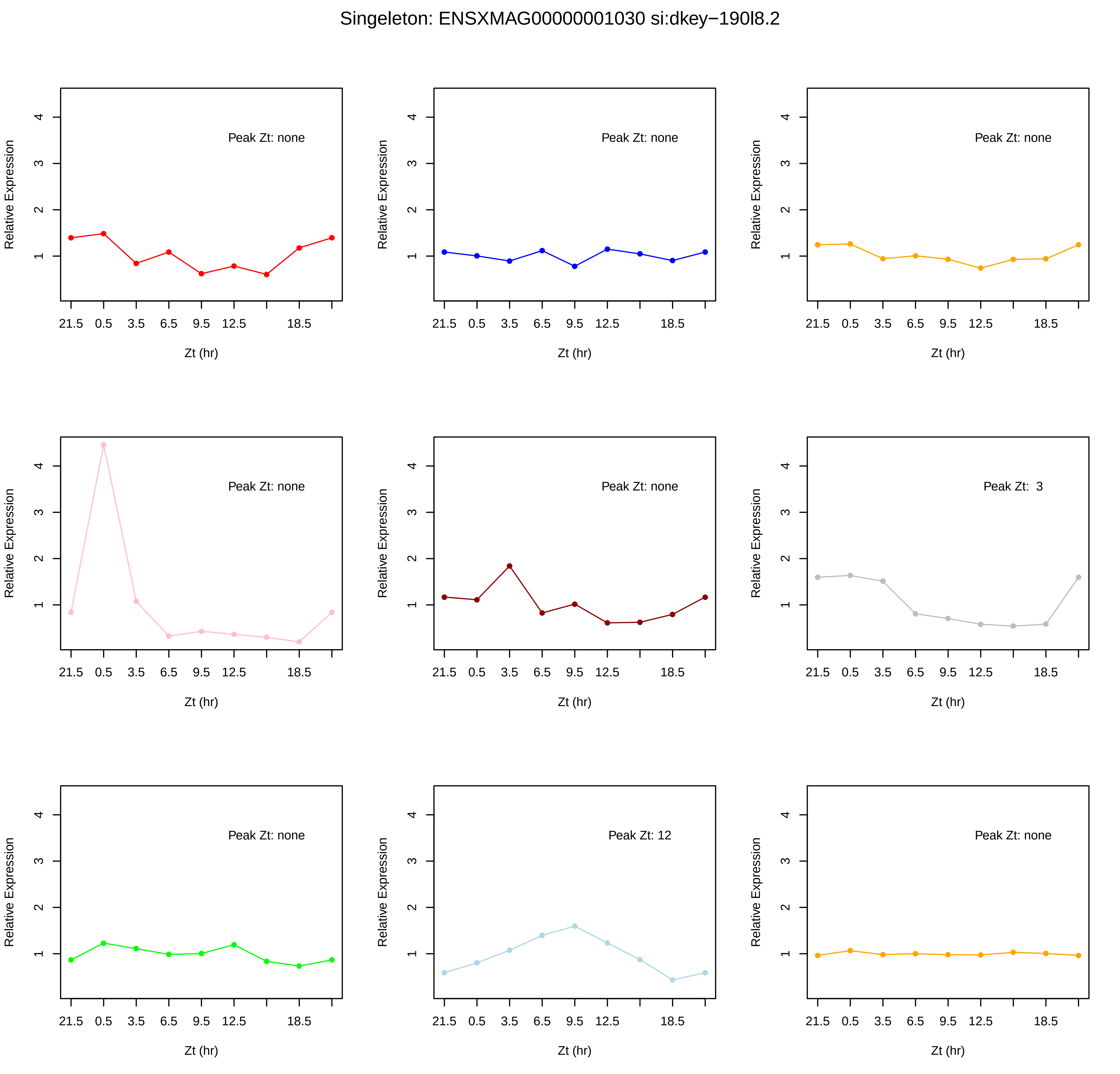

Singeleton: ENSXMAG00000001030 si:dkey−190l8.2
Peak Zt: none
Peak Zt: none
Peak Zt: none
Peak Zt: none
Peak Zt: none
Peak Zt: 3
Peak Zt: none
Peak Zt: 12
Peak Zt: none
Relative Expression
Zt (hr)
21.5
0.5
3.5
6.5
9.5
12.5
18.5

Singeleton: ENSXMAG00000001042 sh3yl1

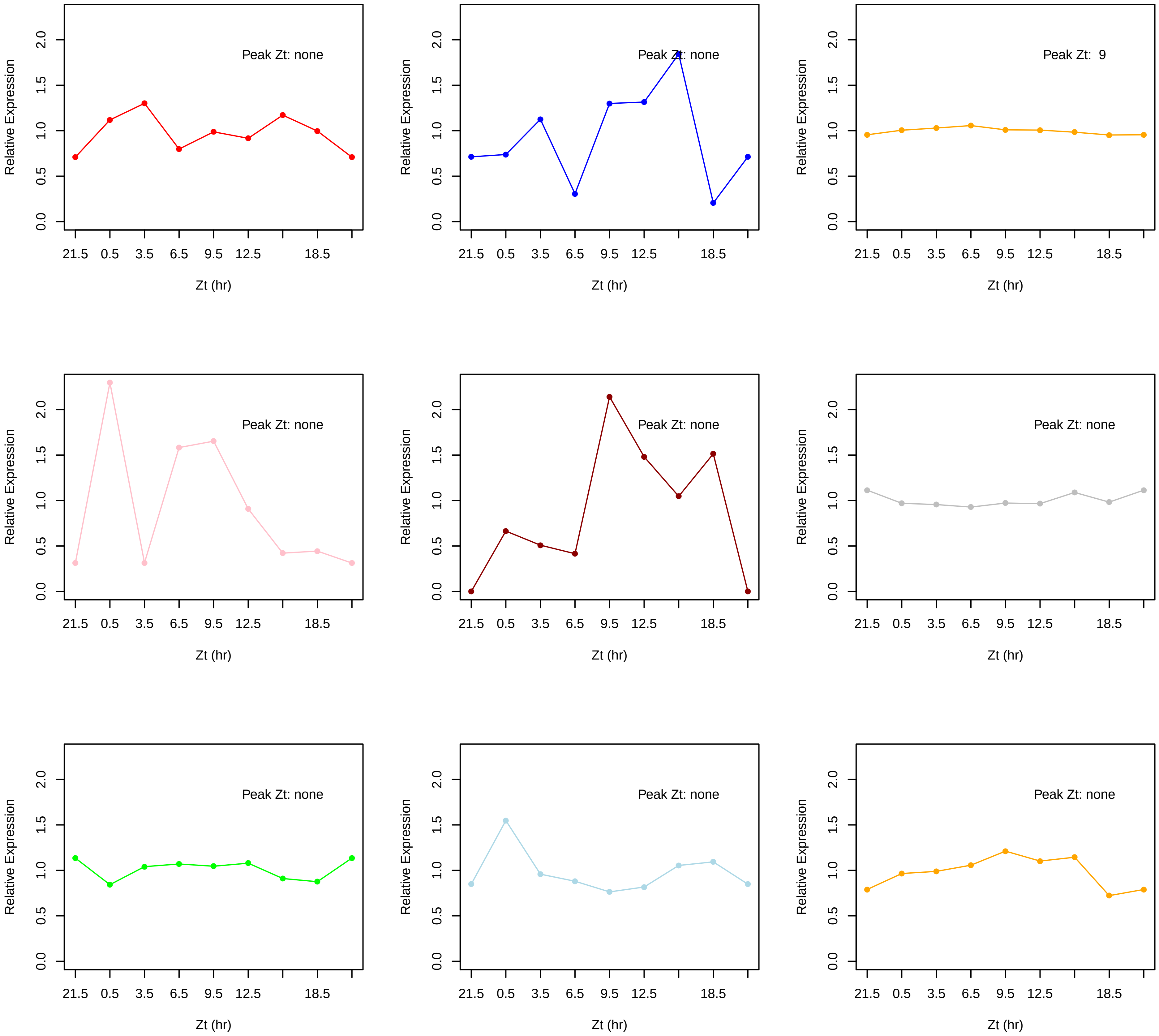

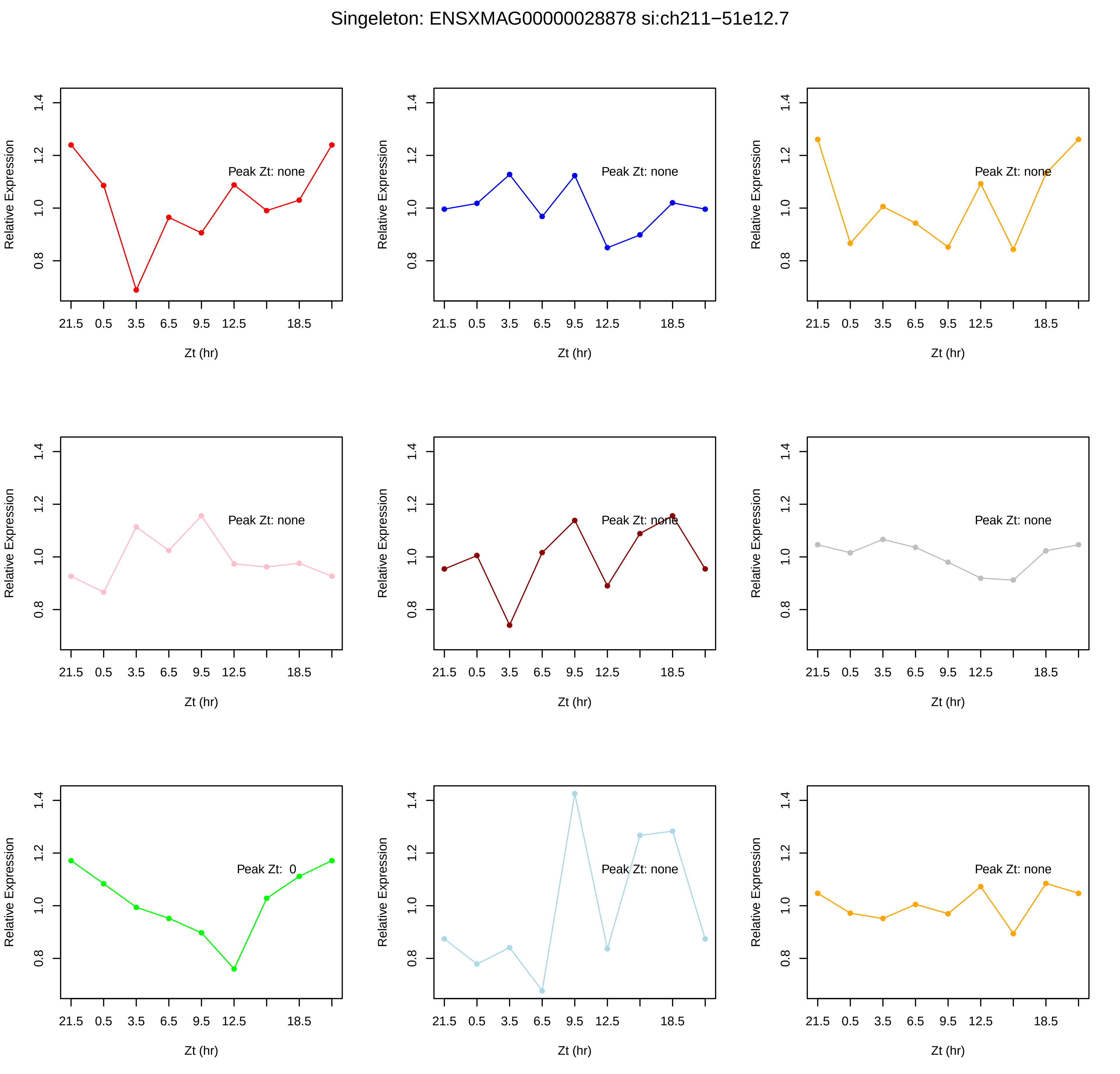

Singleton: ENSXMAG00000028878 si:ch211−51e12.7
Peak Zt: none
Peak Zt: none
Peak Zt: none
Peak Zt: none
Peak Zt: none
Peak Zt: none
Peak Zt: 0
Peak Zt: none
Peak Zt: none
Relative Expression
Zt (hr)

Singleton: ENSXMAG00000003237 mrps10

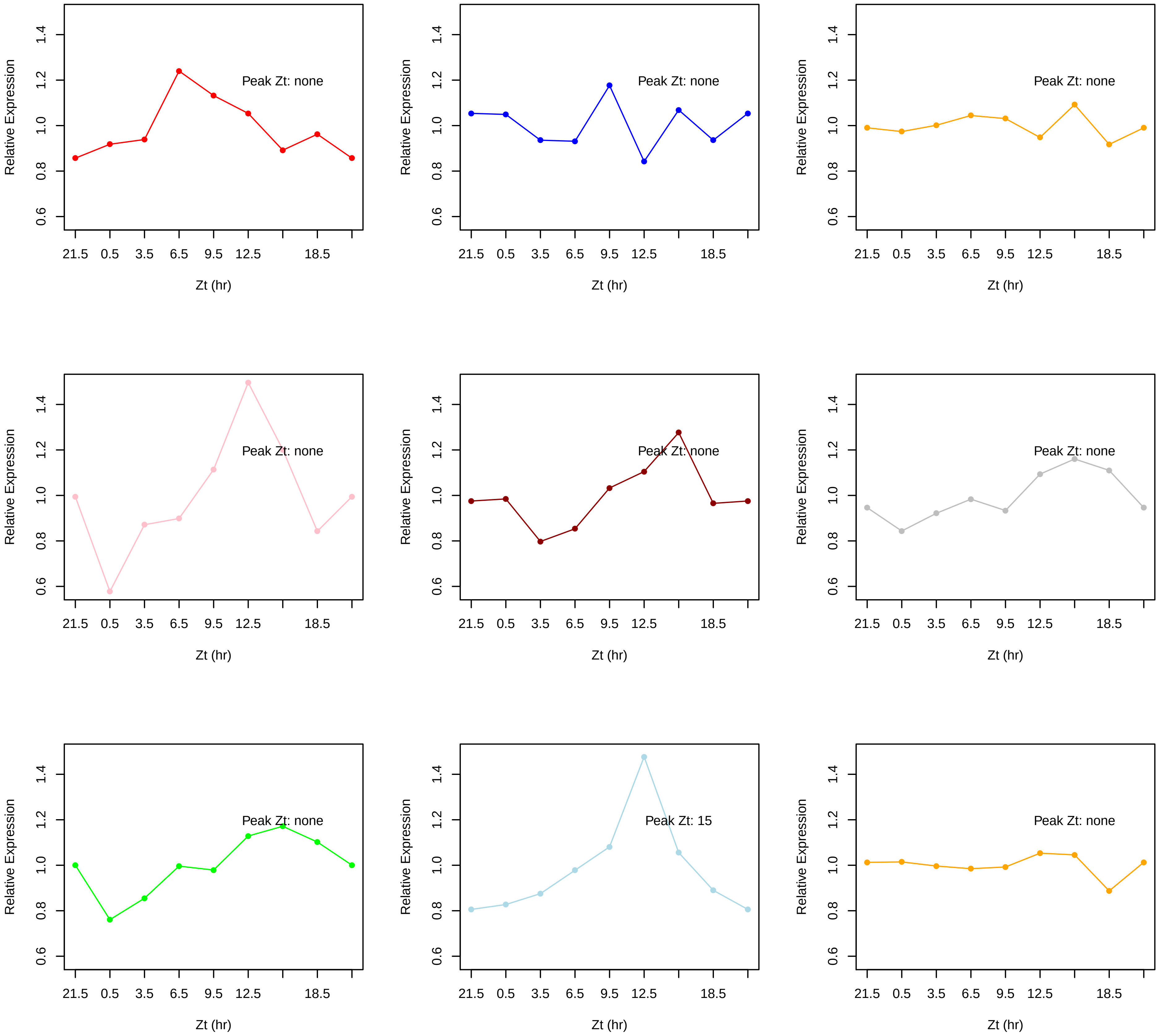

Singeleton: ENSXMAG00000004108 chrna2b

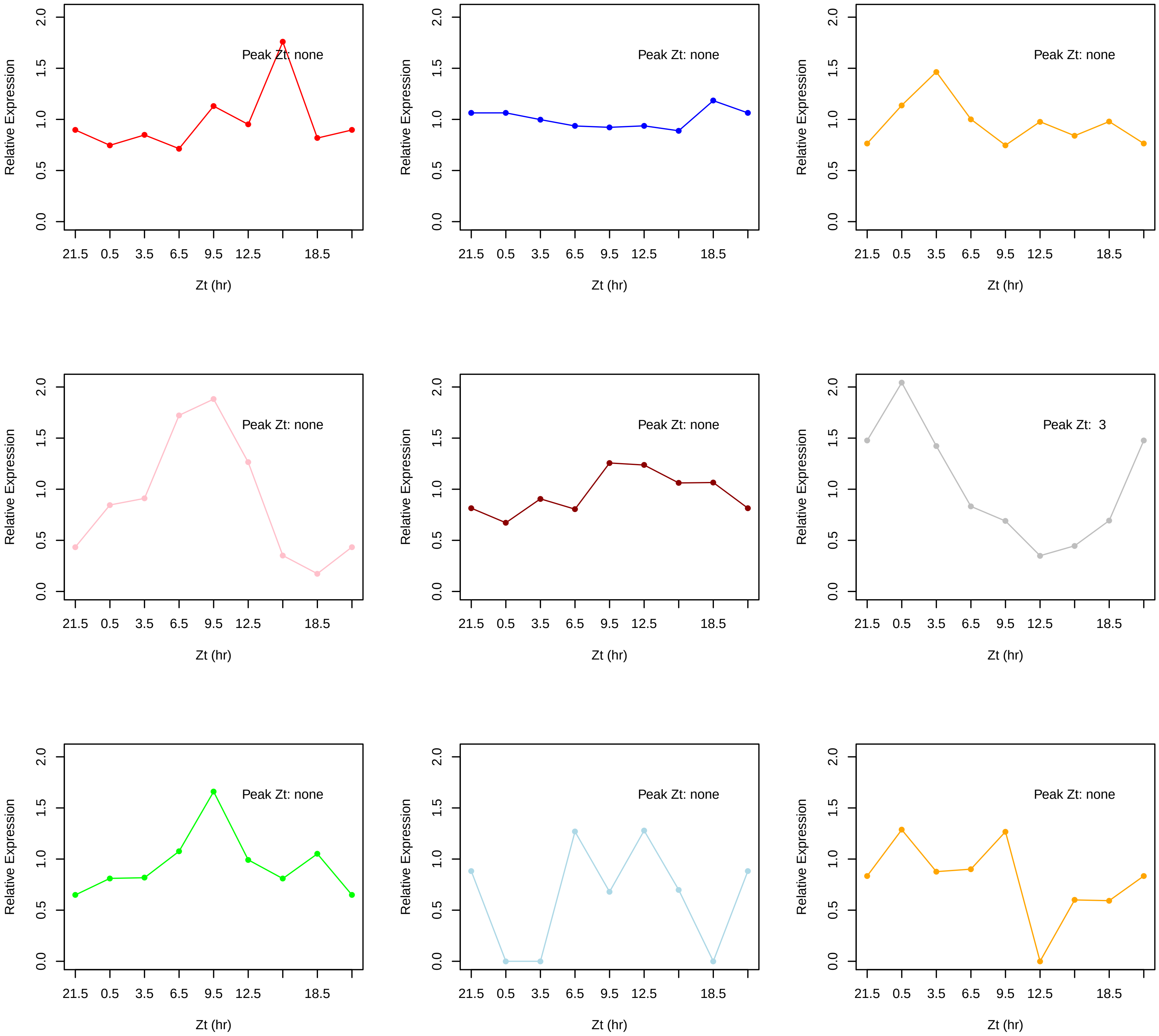

Singeleton: ENSXMAG00000005255 cep85l

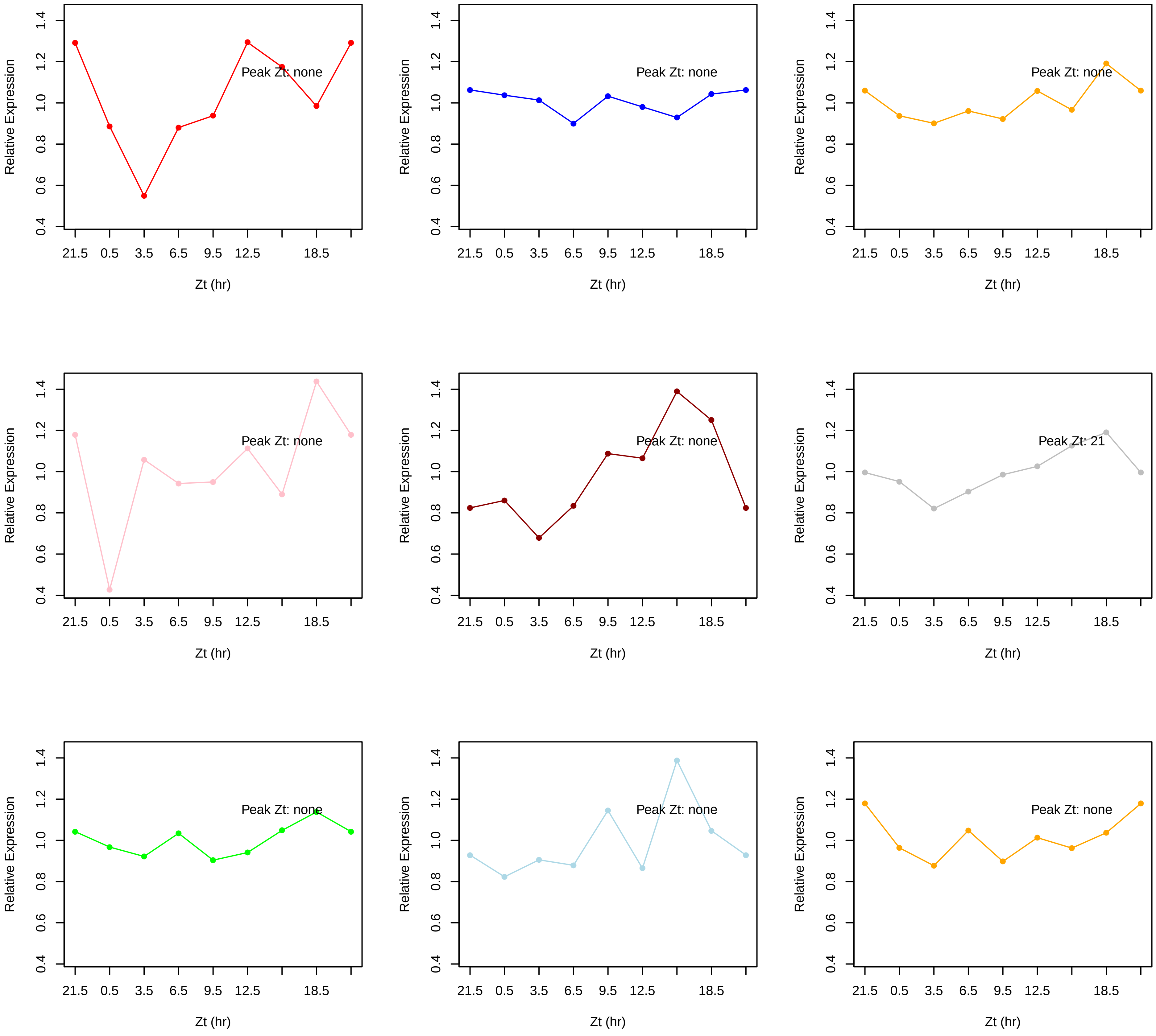

Singeleton: ENSXMAG00000005270

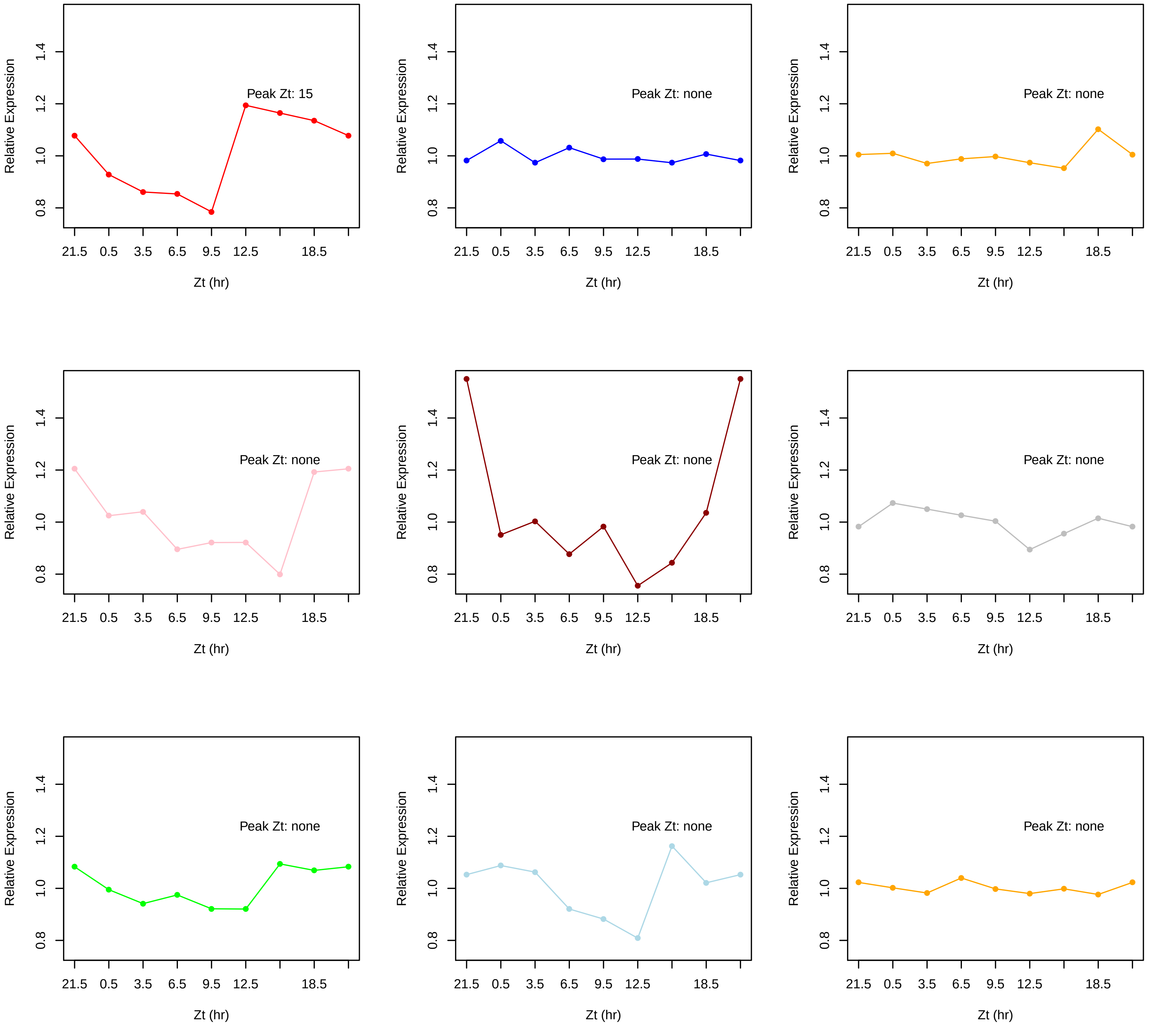

Singeleton: ENSXMAG00000025931 tpd52l1

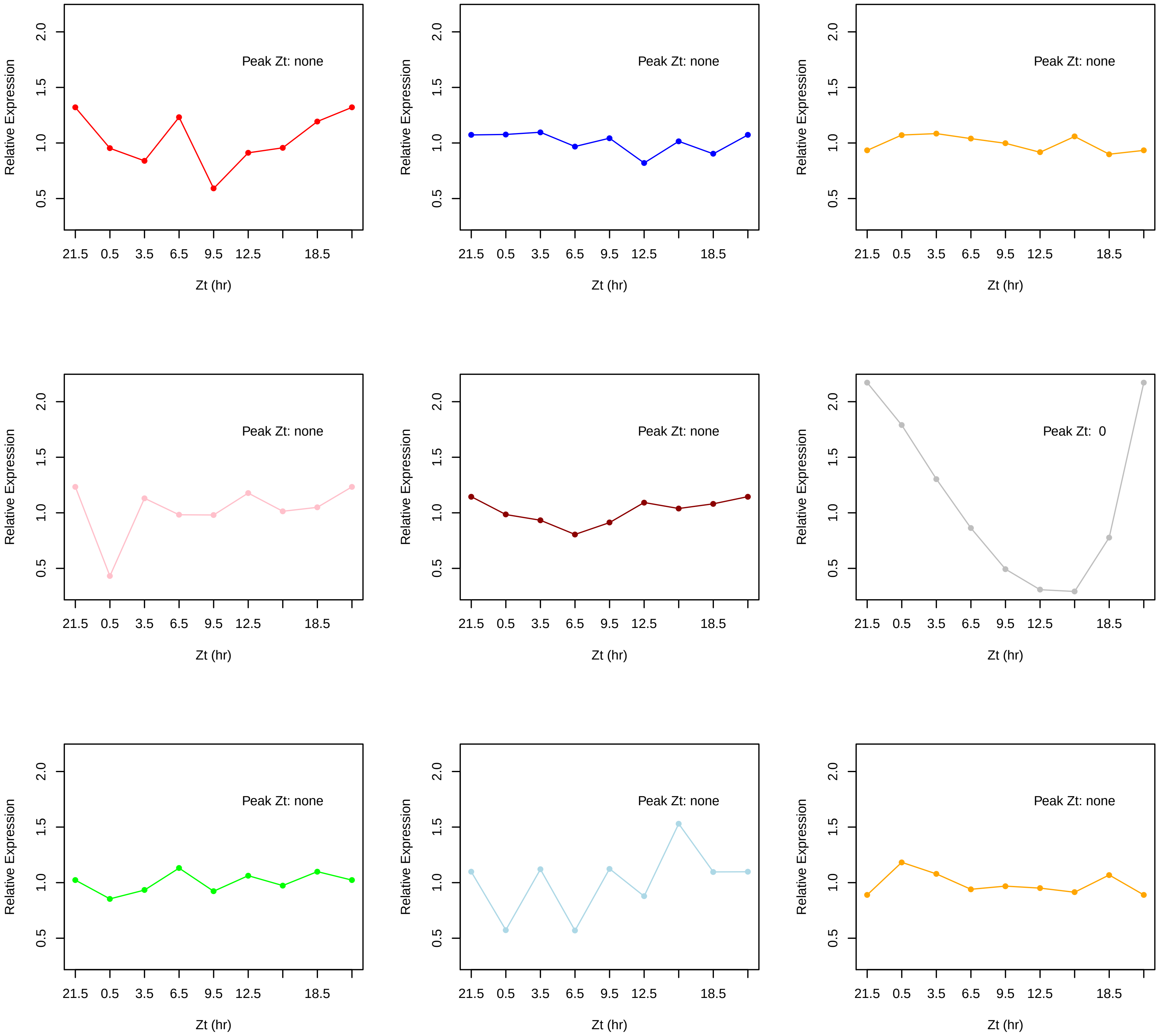

Singeleton: ENSXMAG00000017572 ptprk

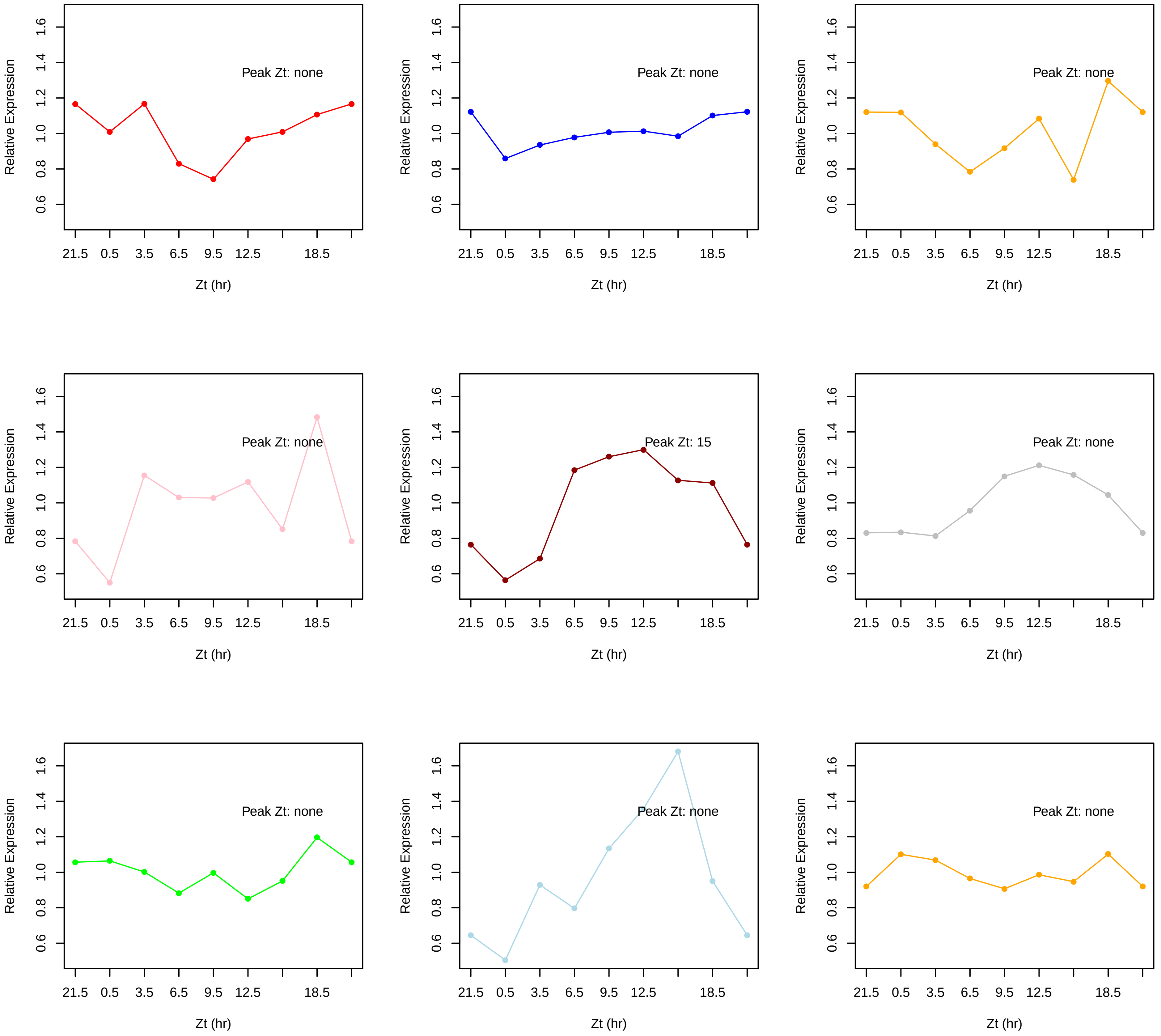

Singeleton: ENSXMAG00000028133 alkbh3

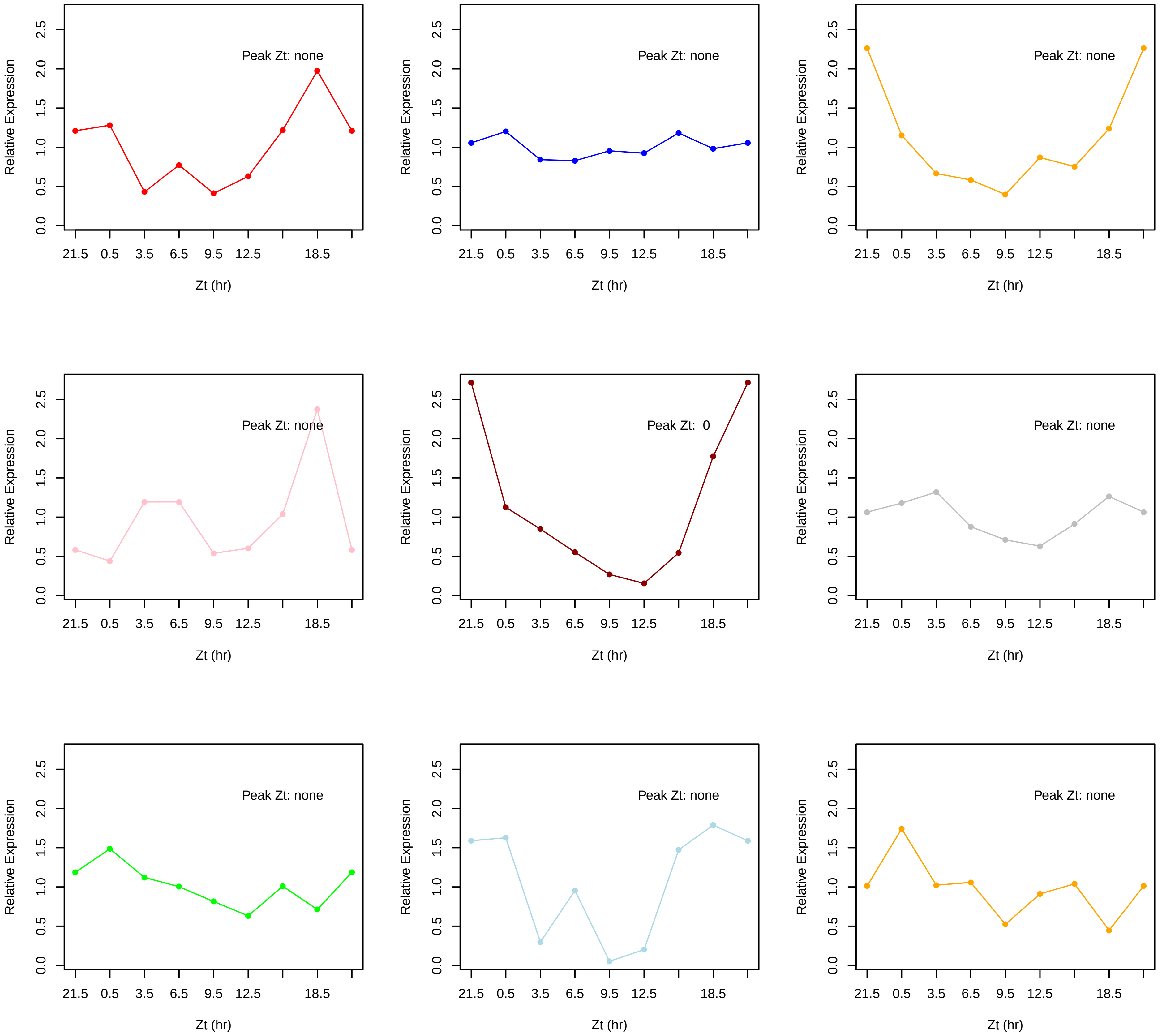

Singeleton: ENSXMAG00000020250 fjx1

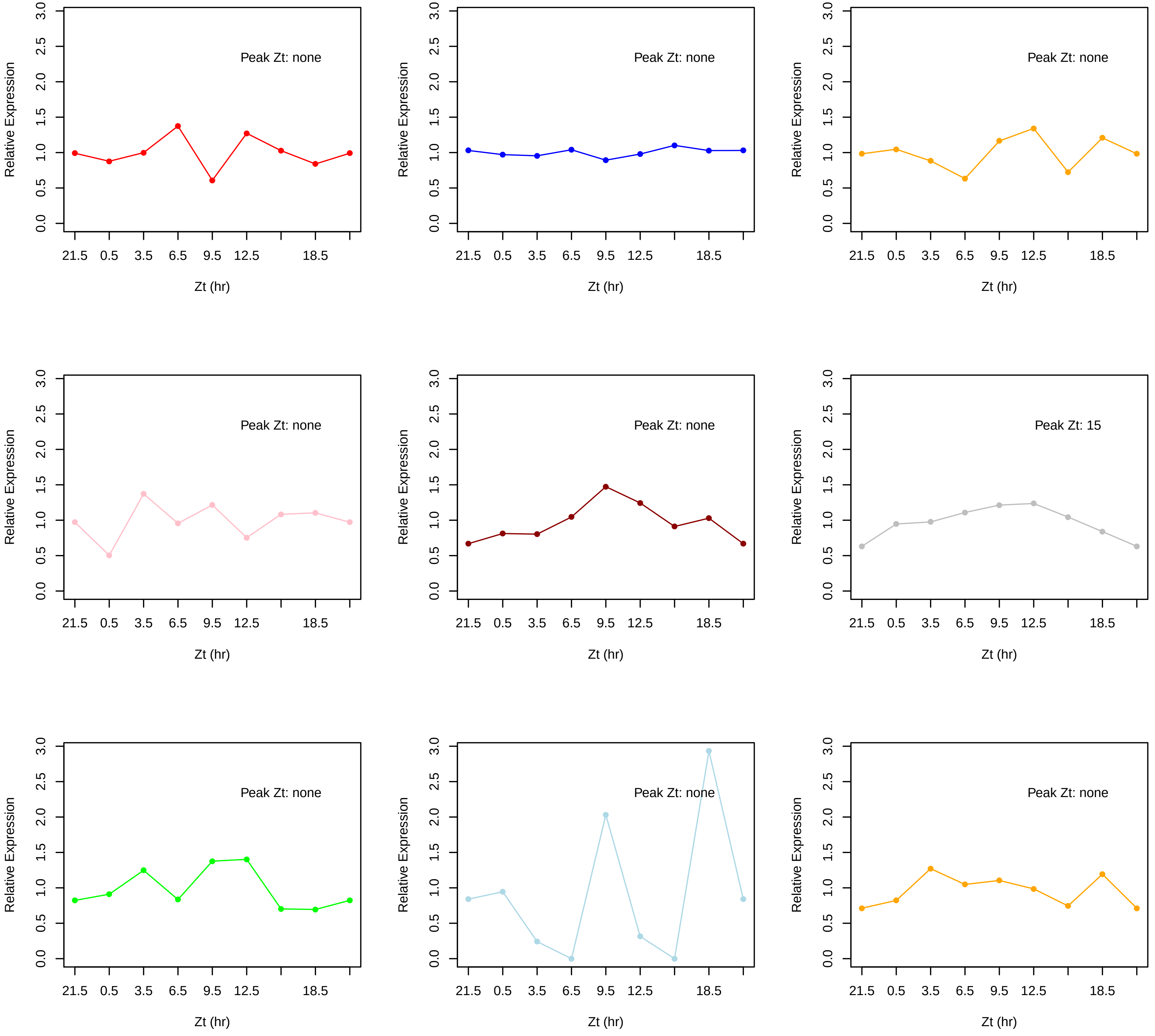

Singleton: ENSXMAG00000015418 RASSF10

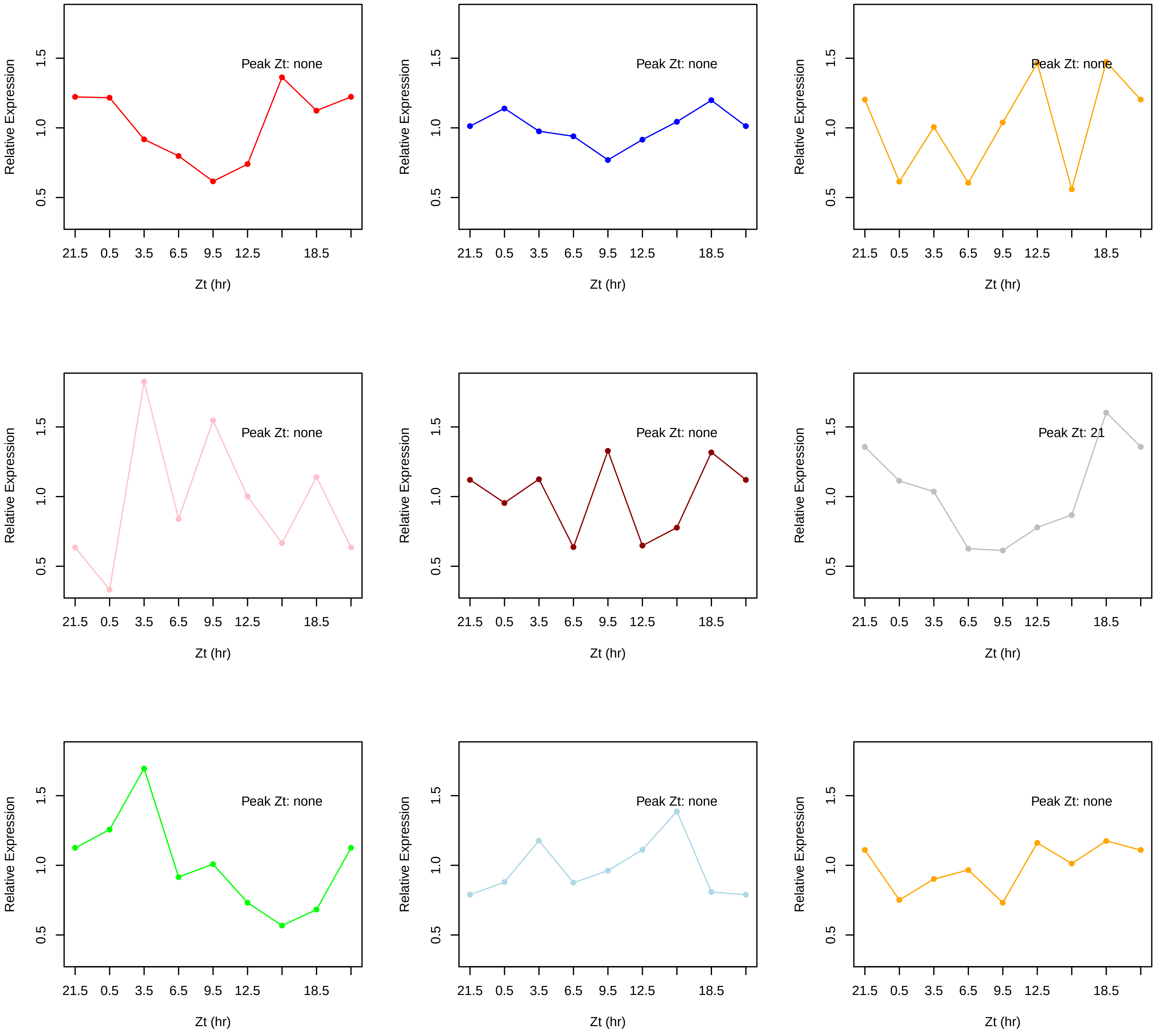

Singeleton: ENSXMAG00000020315 CHST1

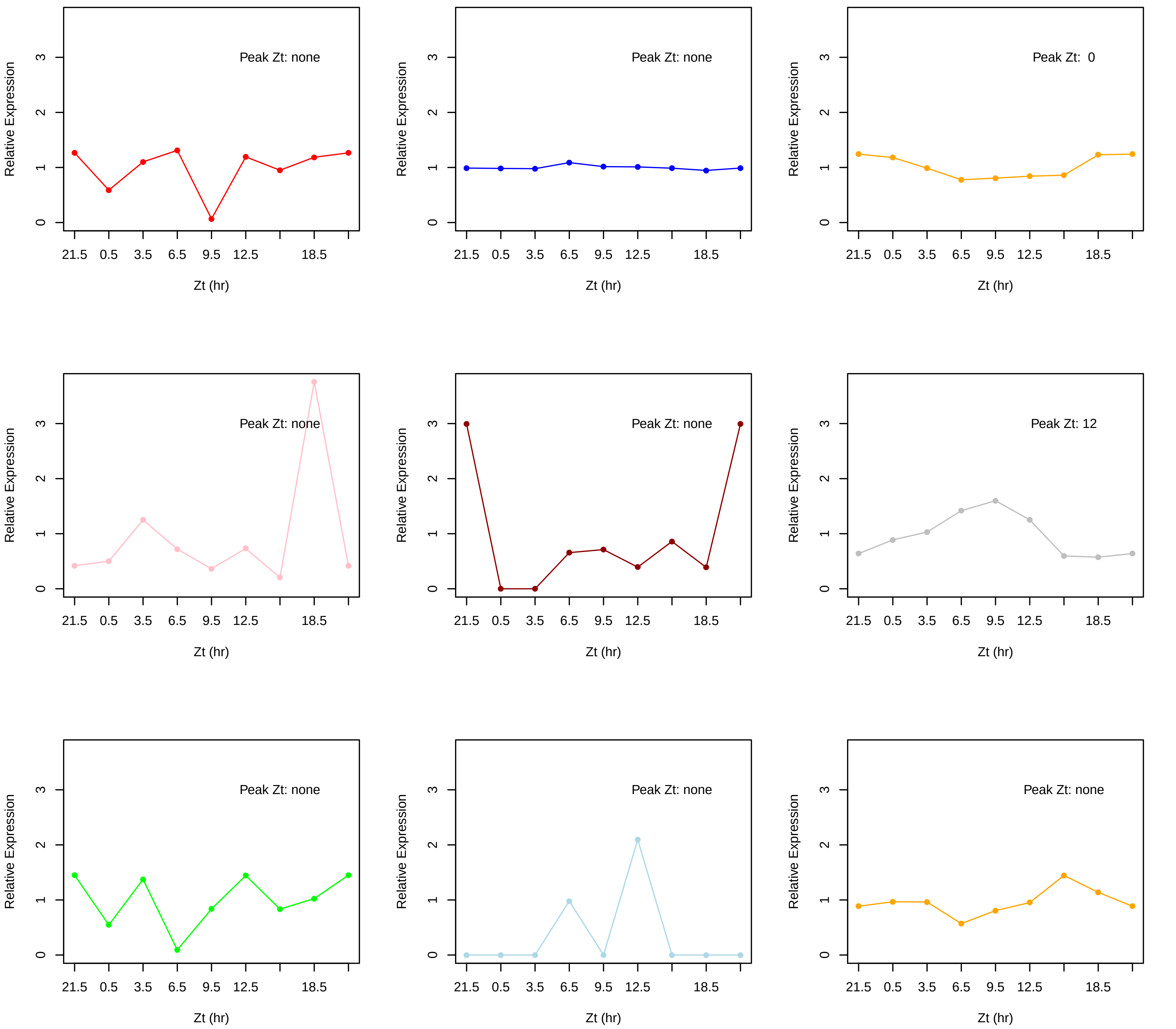

Singeleton: ENSXMAG00000022652 RNF208

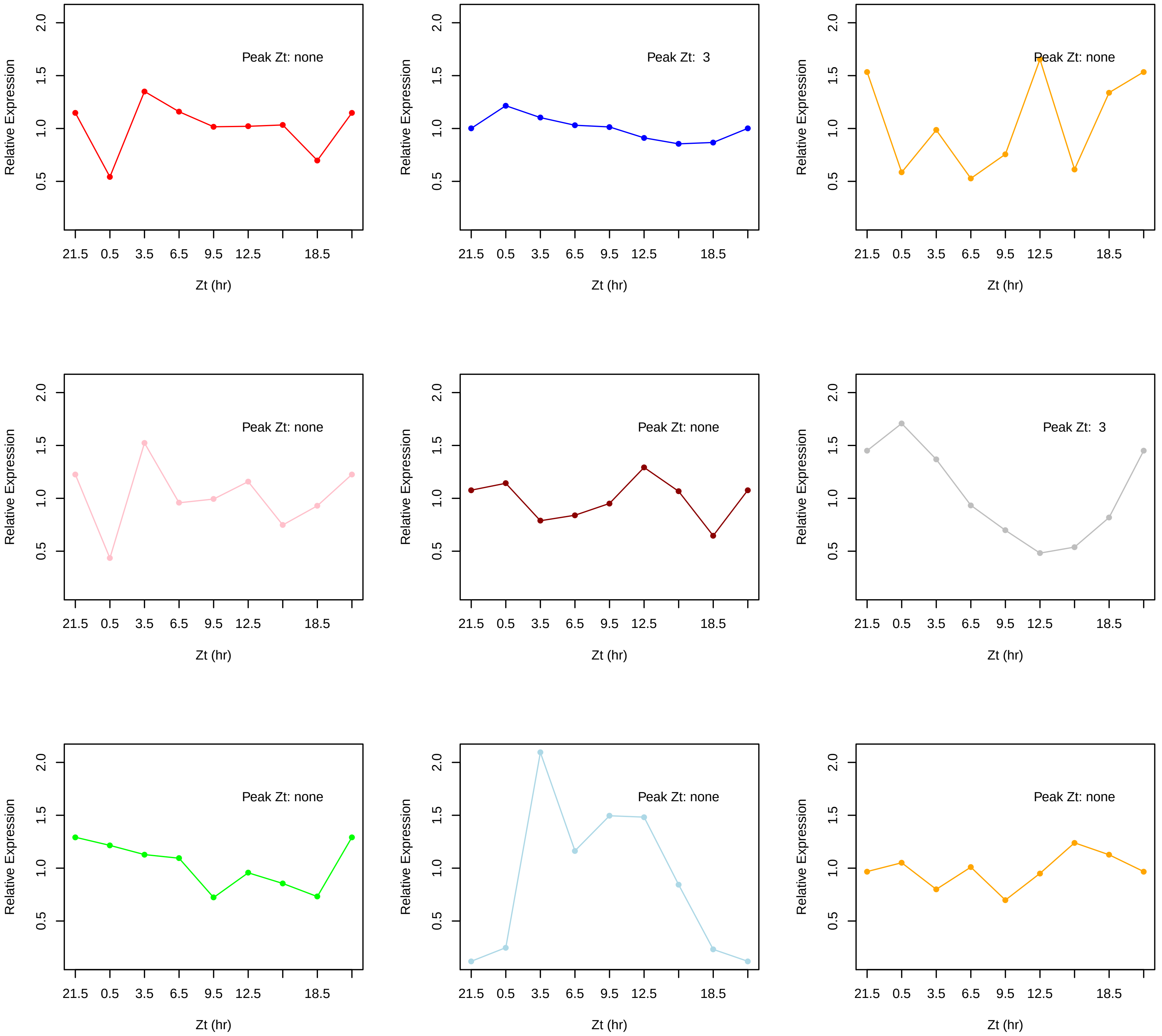

Singeleton: ENSXMAG00000019701 tmem203

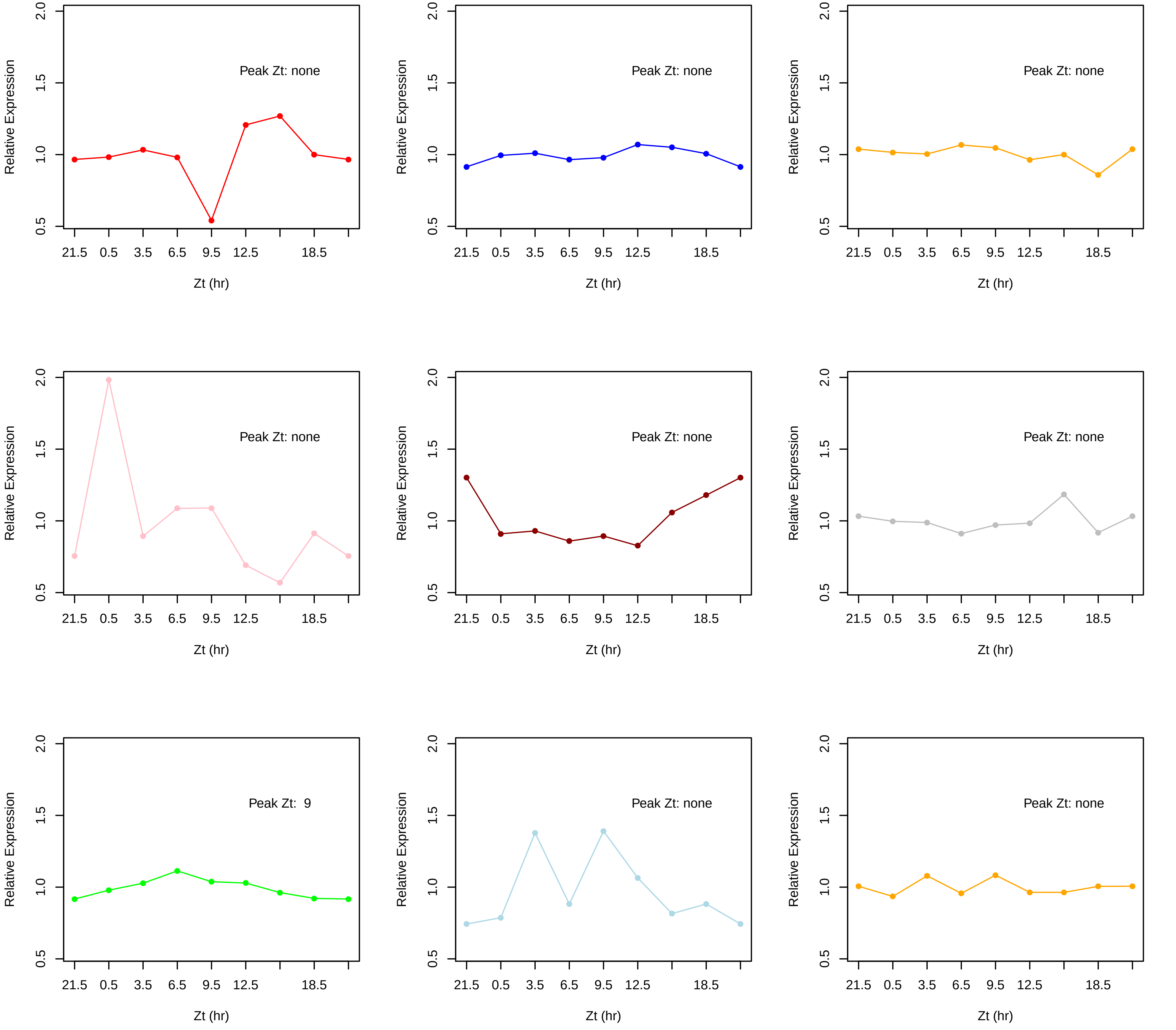

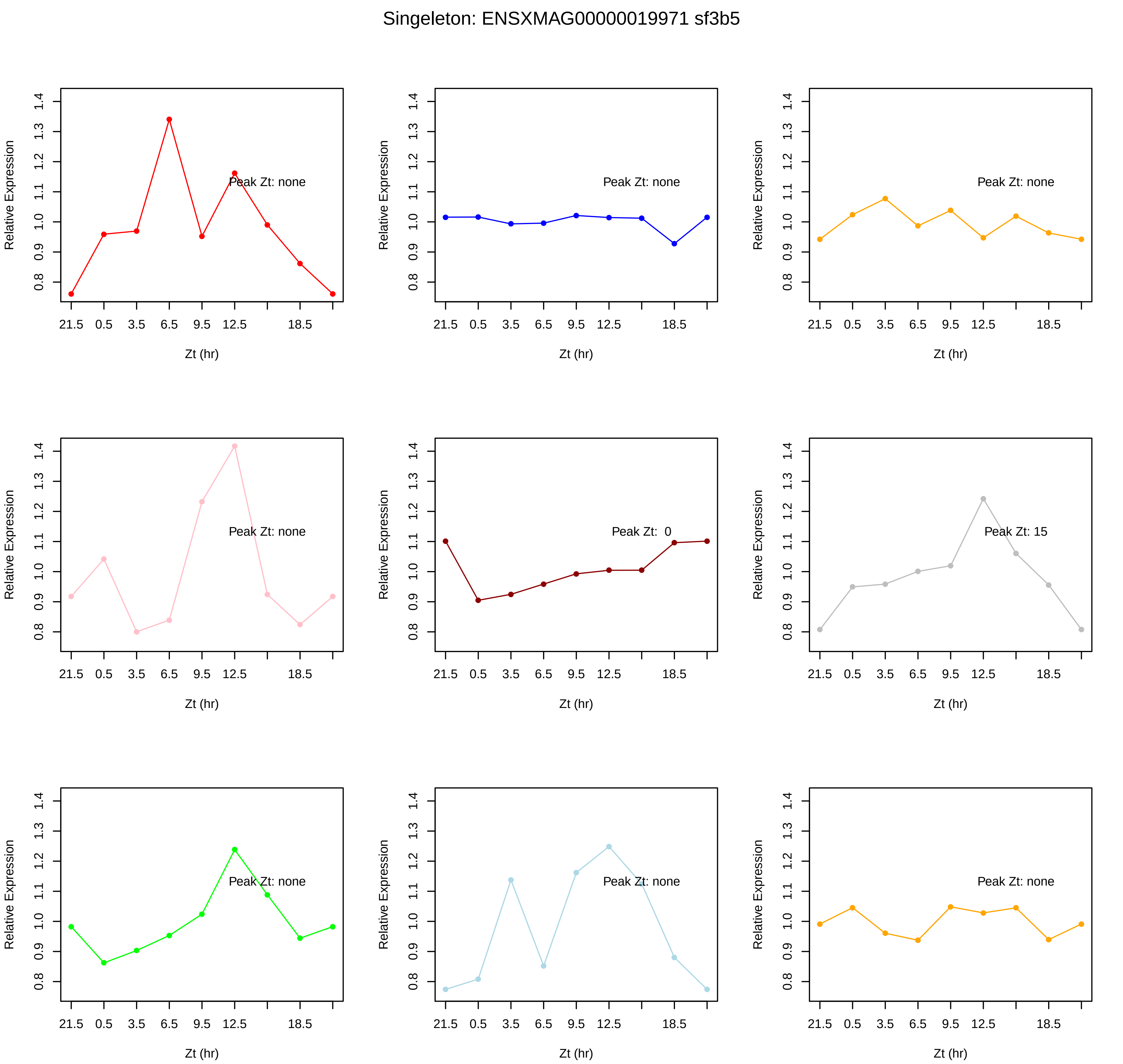
Singeleton: ENSXMAG00000019971 sf3b5
Peak Zt: none
Peak Zt: none
Peak Zt: none
Peak Zt: none
Peak Zt: 0
Peak Zt: 15
Peak Zt: none
Peak Zt: none
Peak Zt: none
Relative Expression
Zt (hr)
21.5
0.5
3.5
6.5
9.5
12.5
18.5

Singeleton: ENSXMAG00000020296 zgc:77929

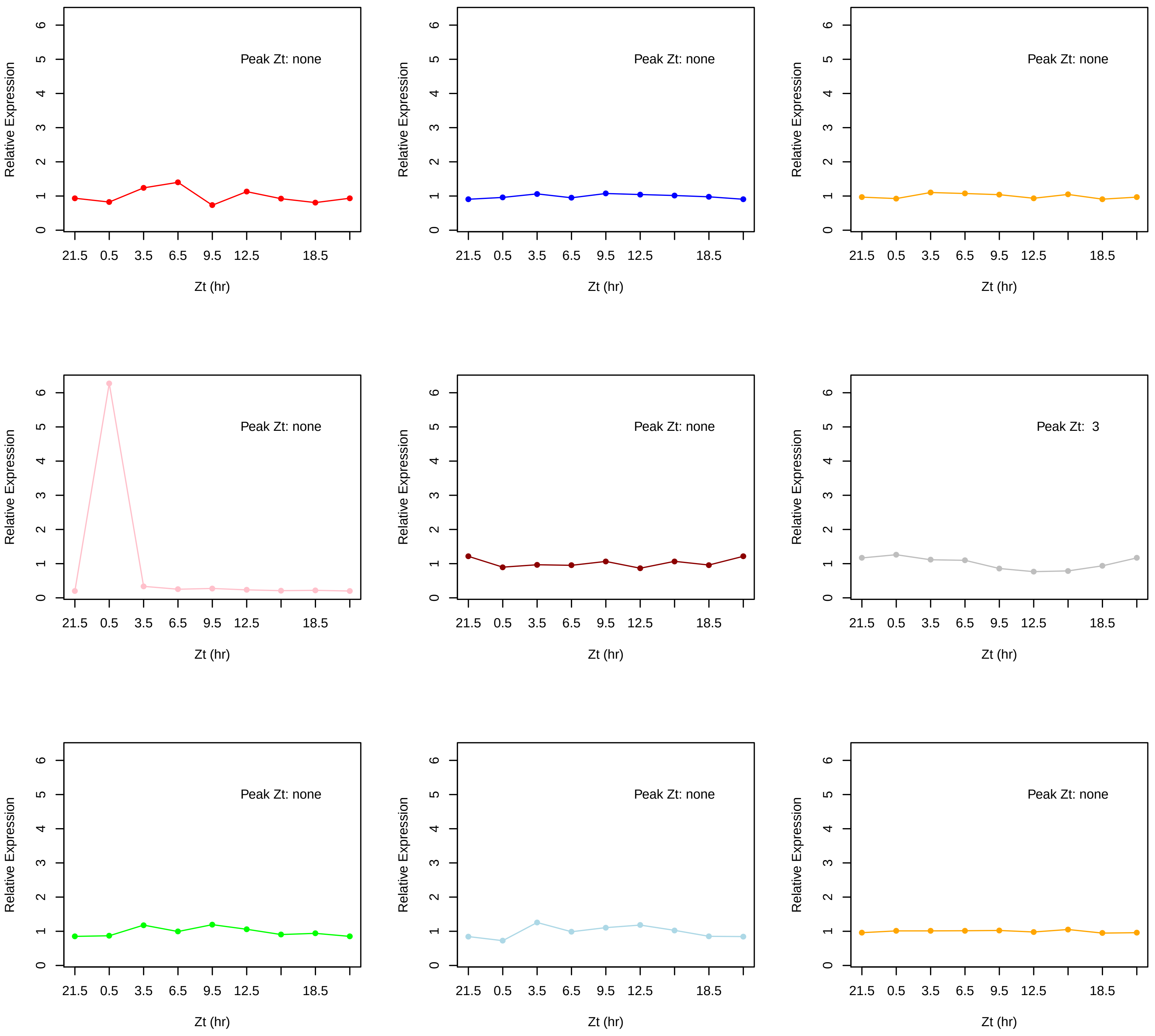

Singleton: ENSXMAG00000025973

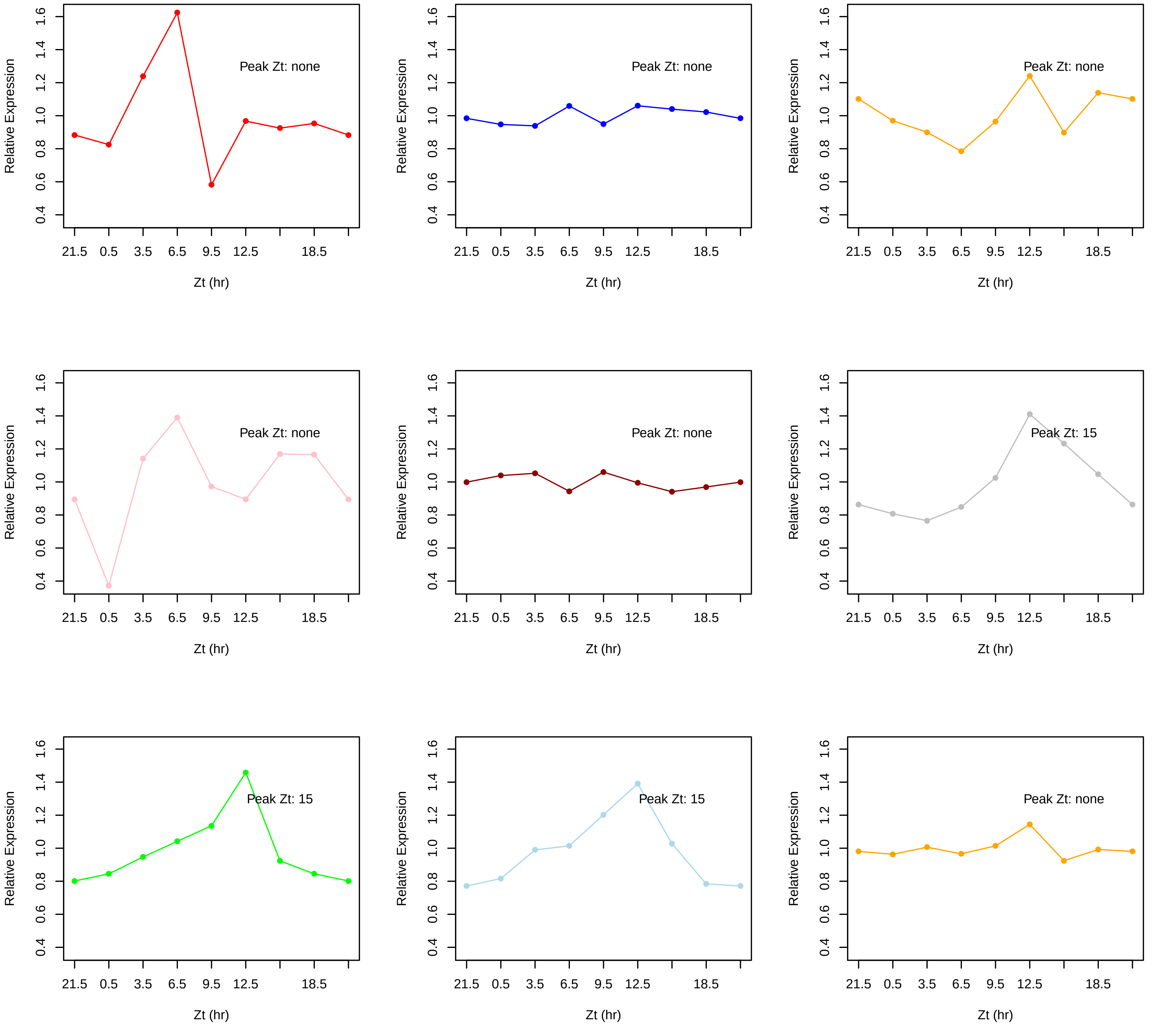

Singeleton: ENSXMAG00000019821 flrt3

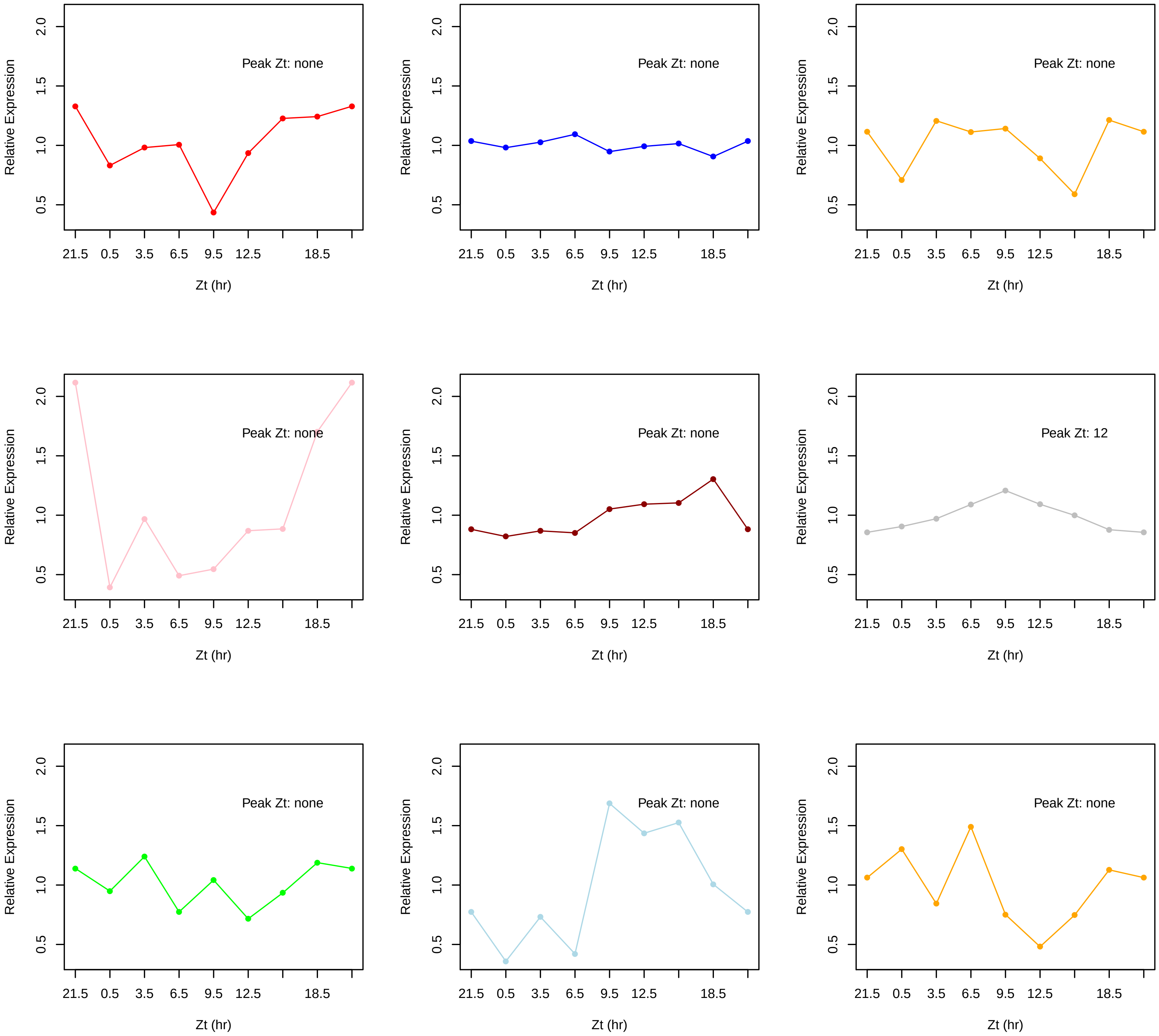

Singeleton: ENSXMAG00000019825 sertad2b

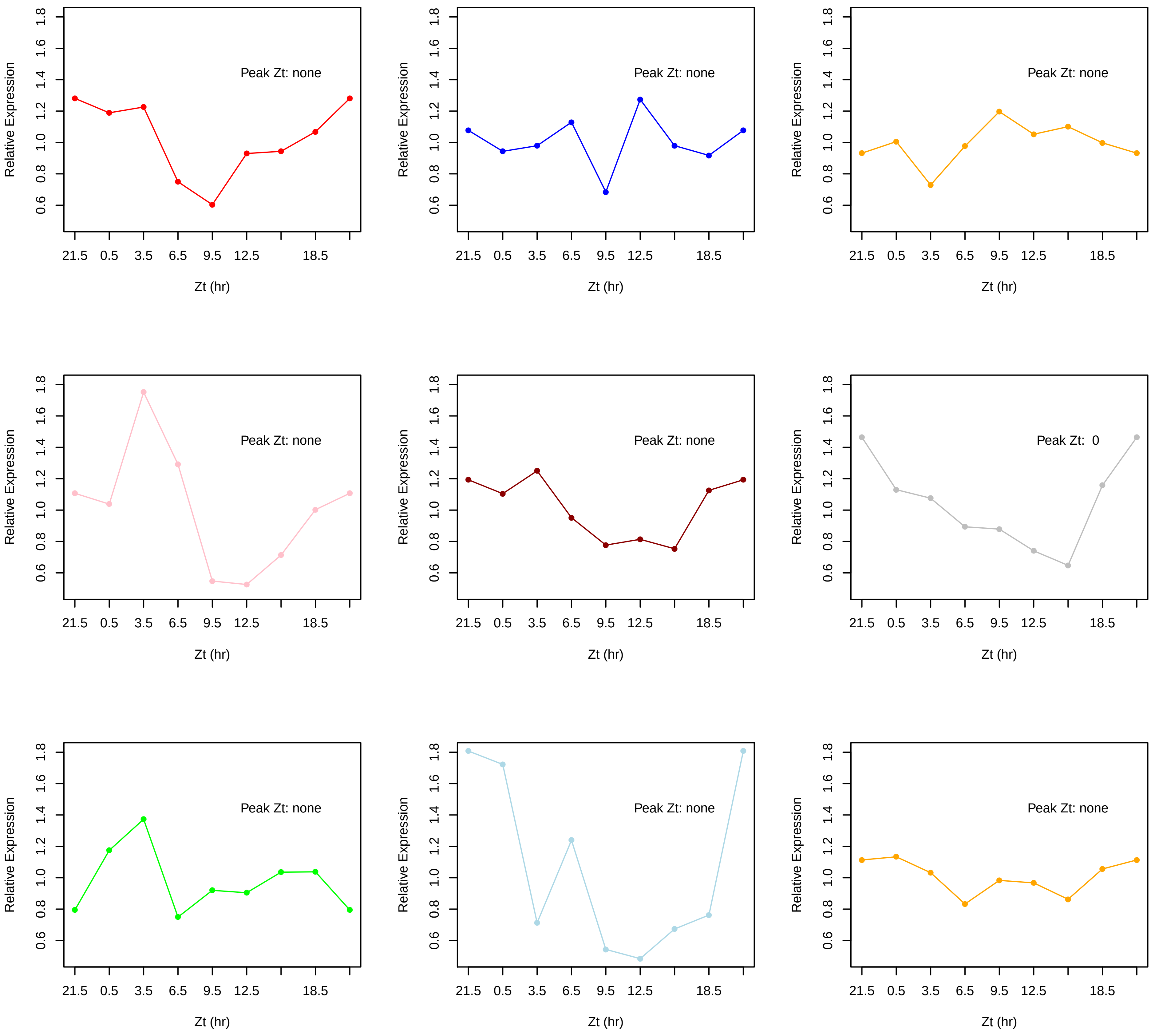

Singeleton: ENSXMAG00000019917 prr18

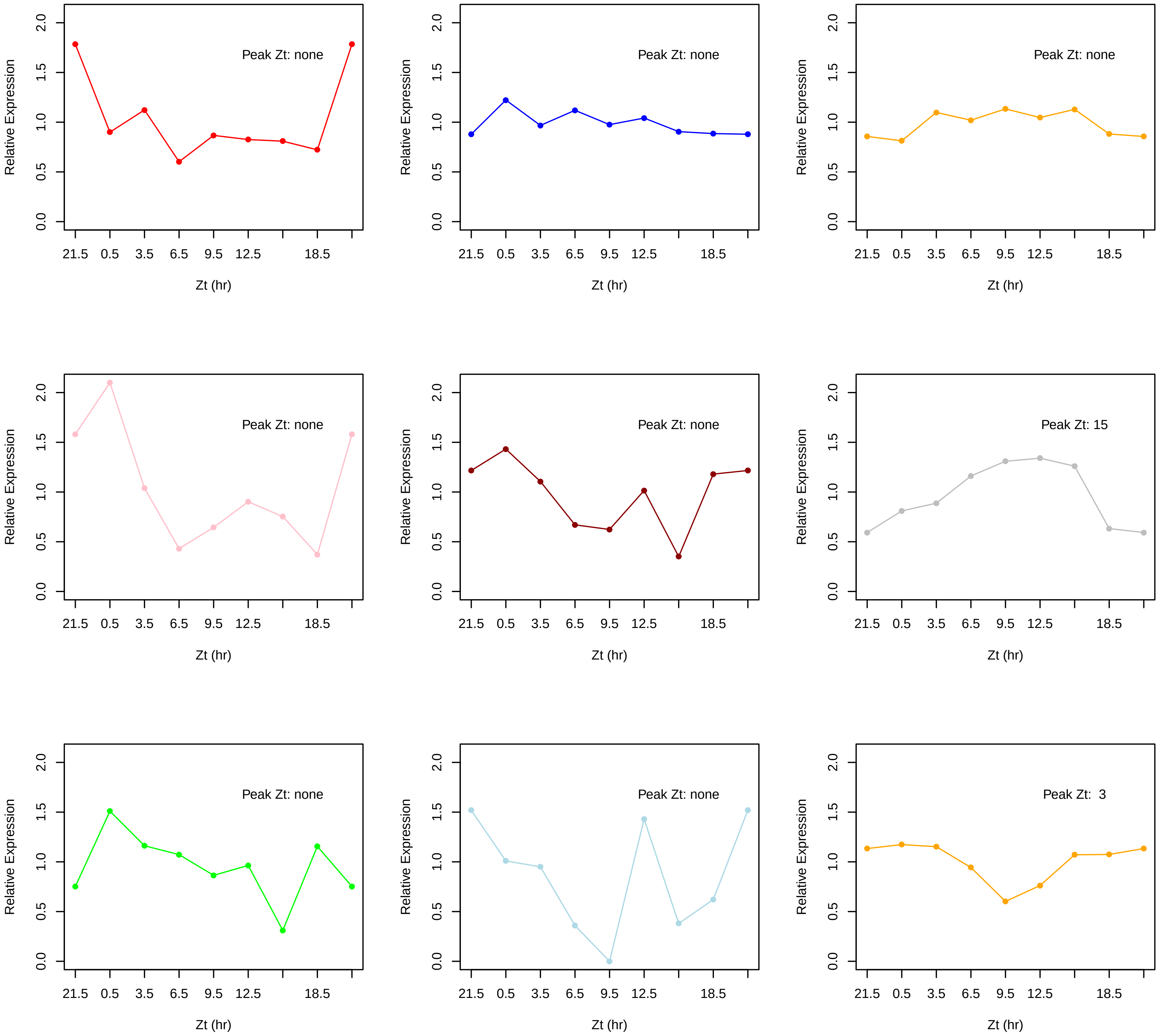

Singeleton: ENSXMAG00000020156 slc35g2b

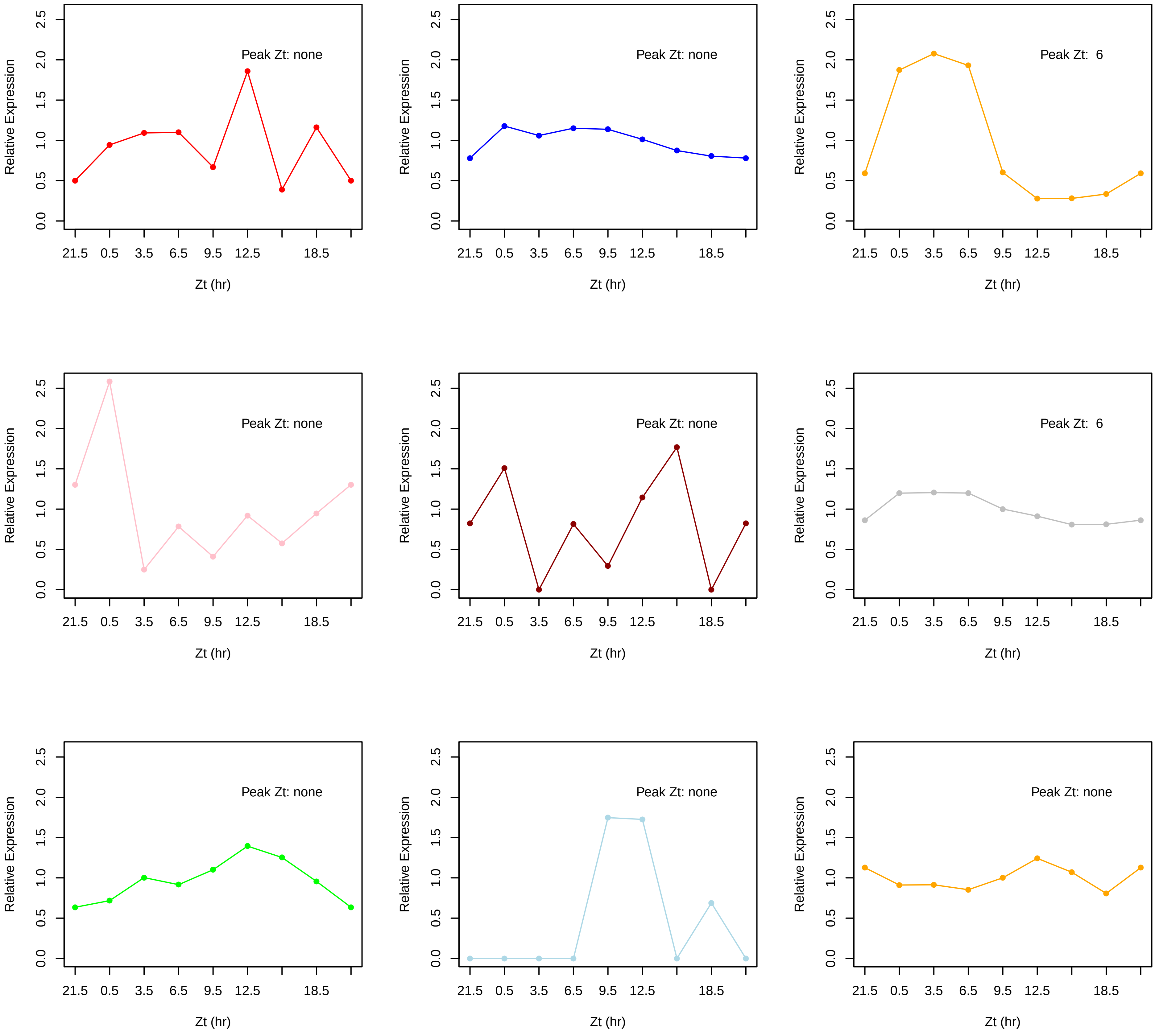

Singeleton: ENSXMAG00000020056 chst2b

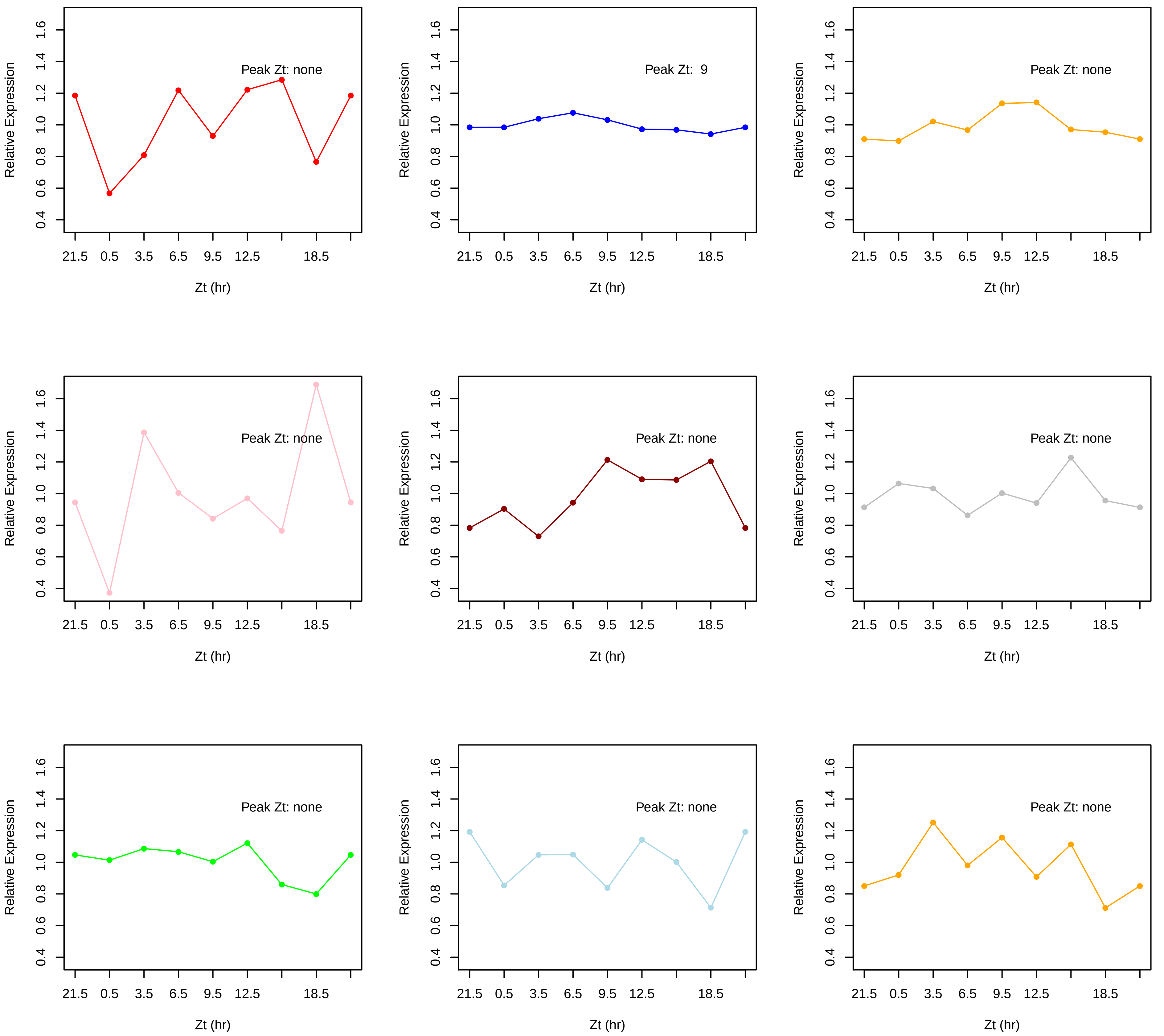

Singleton: ENSXMAG00000022901 sft2d3

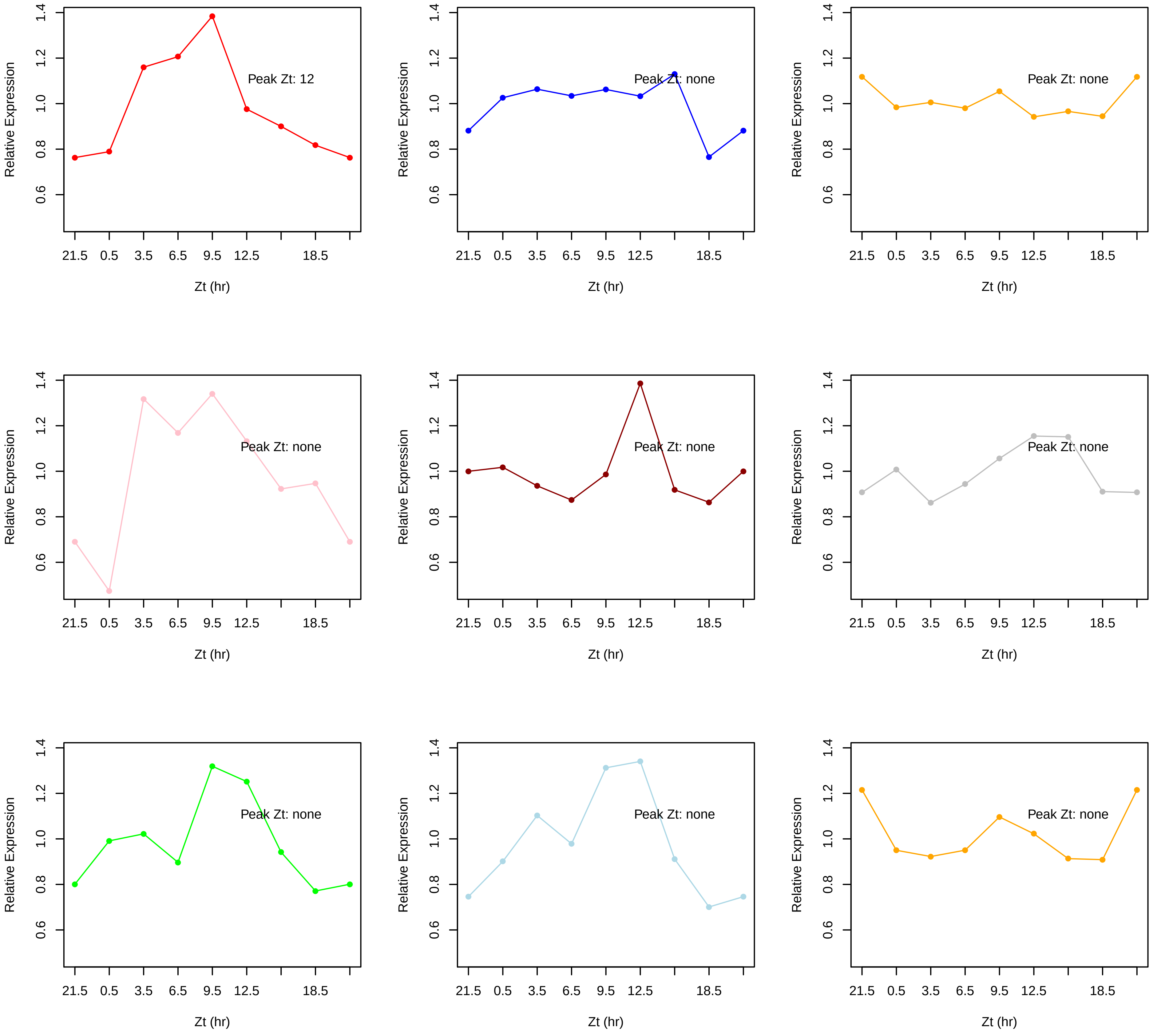

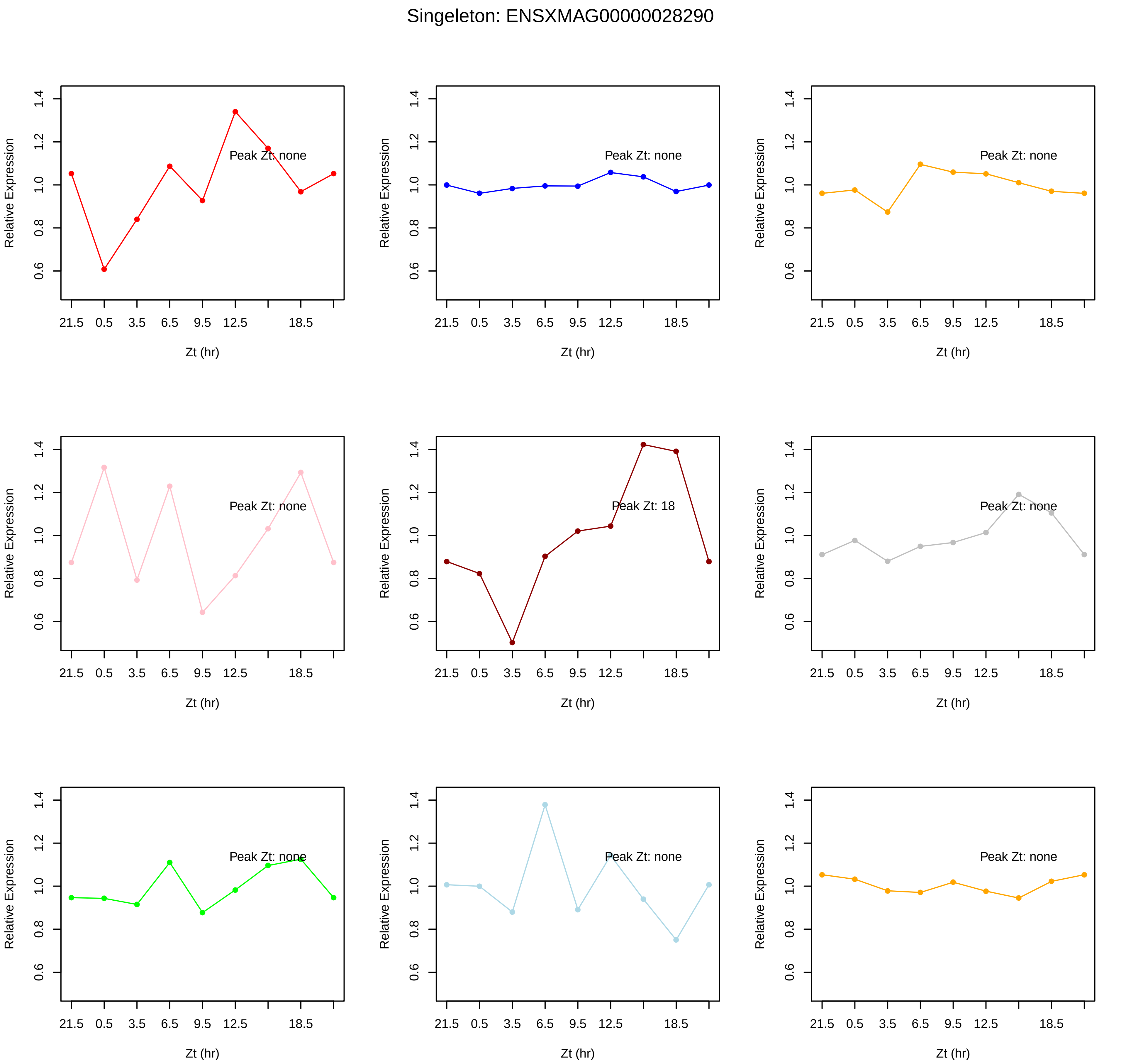

Singeleton: ENSXMAG00000028290
Peak Zt: none
Peak Zt: none
Peak Zt: none
Peak Zt: none
Peak Zt: 18
Peak Zt: none
Peak Zt: none
Peak Zt: none
Peak Zt: none
Relative Expression
Zt (hr)
21.5
0.5
3.5
6.5
9.5
12.5
18.5
0.6
0.8
1.0
1.2
1.4

Singeleton: ENSXMAG00000024329

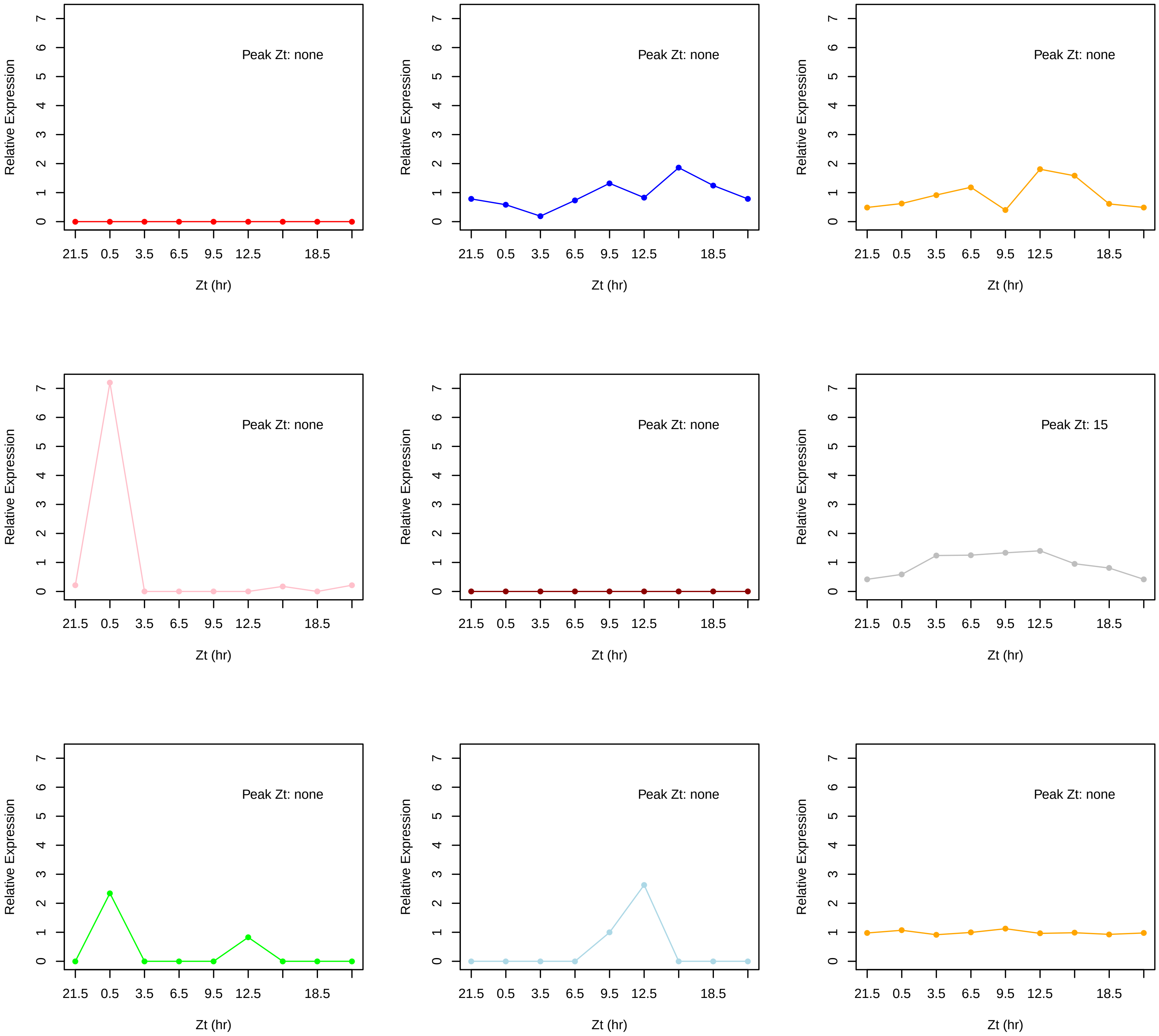

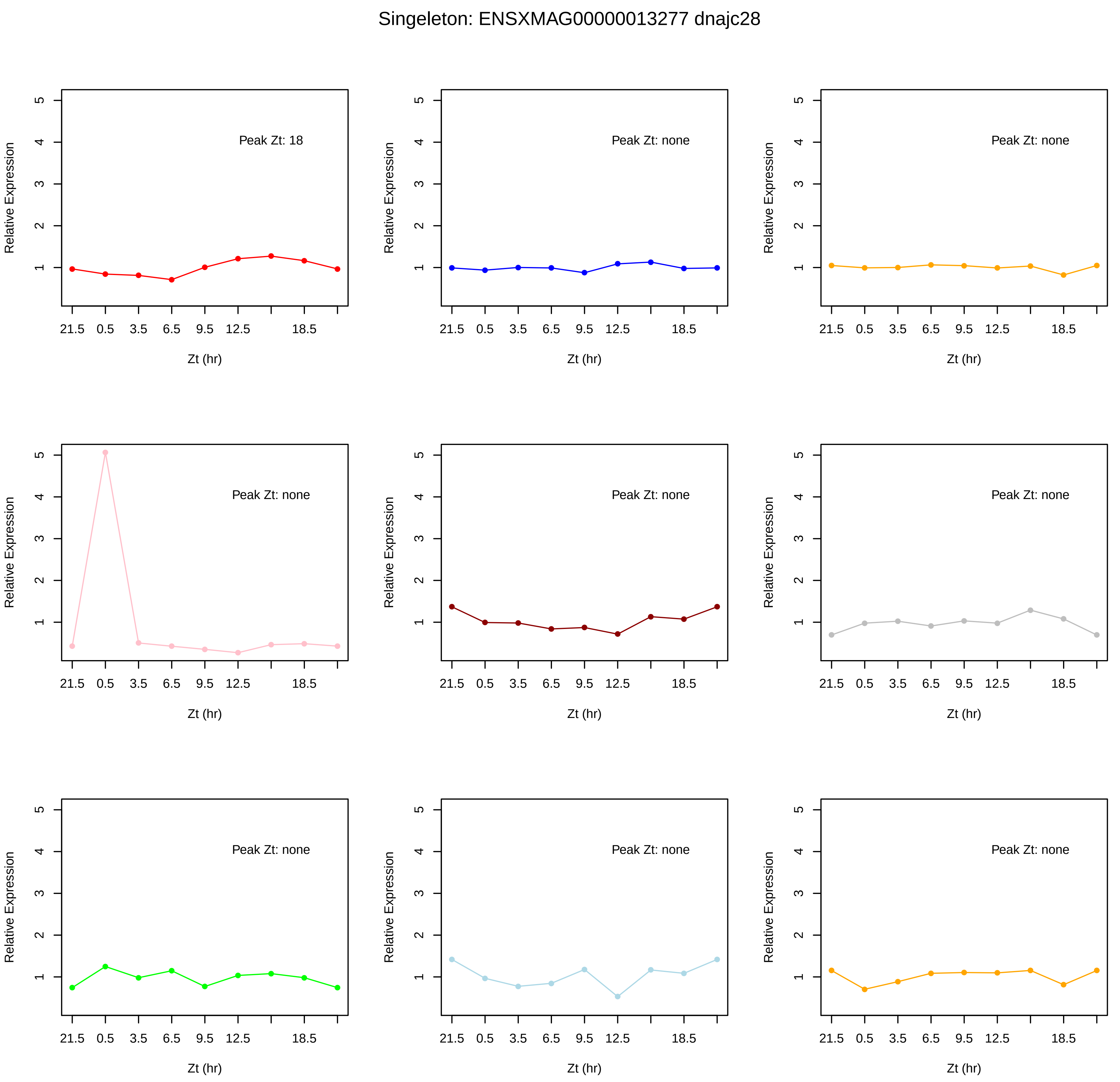
Singeleton: ENSXMAG00000013277 dnajc28
Peak Zt: 18
Peak Zt: none
Peak Zt: none
Peak Zt: none
Peak Zt: none
Peak Zt: none
Peak Zt: none
Peak Zt: none
Peak Zt: none
Relative Expression
Zt (hr)
21.5 0.5 3.5 6.5 9.5 12.5 18.5

Singeleton: ENSXMAG00000014894

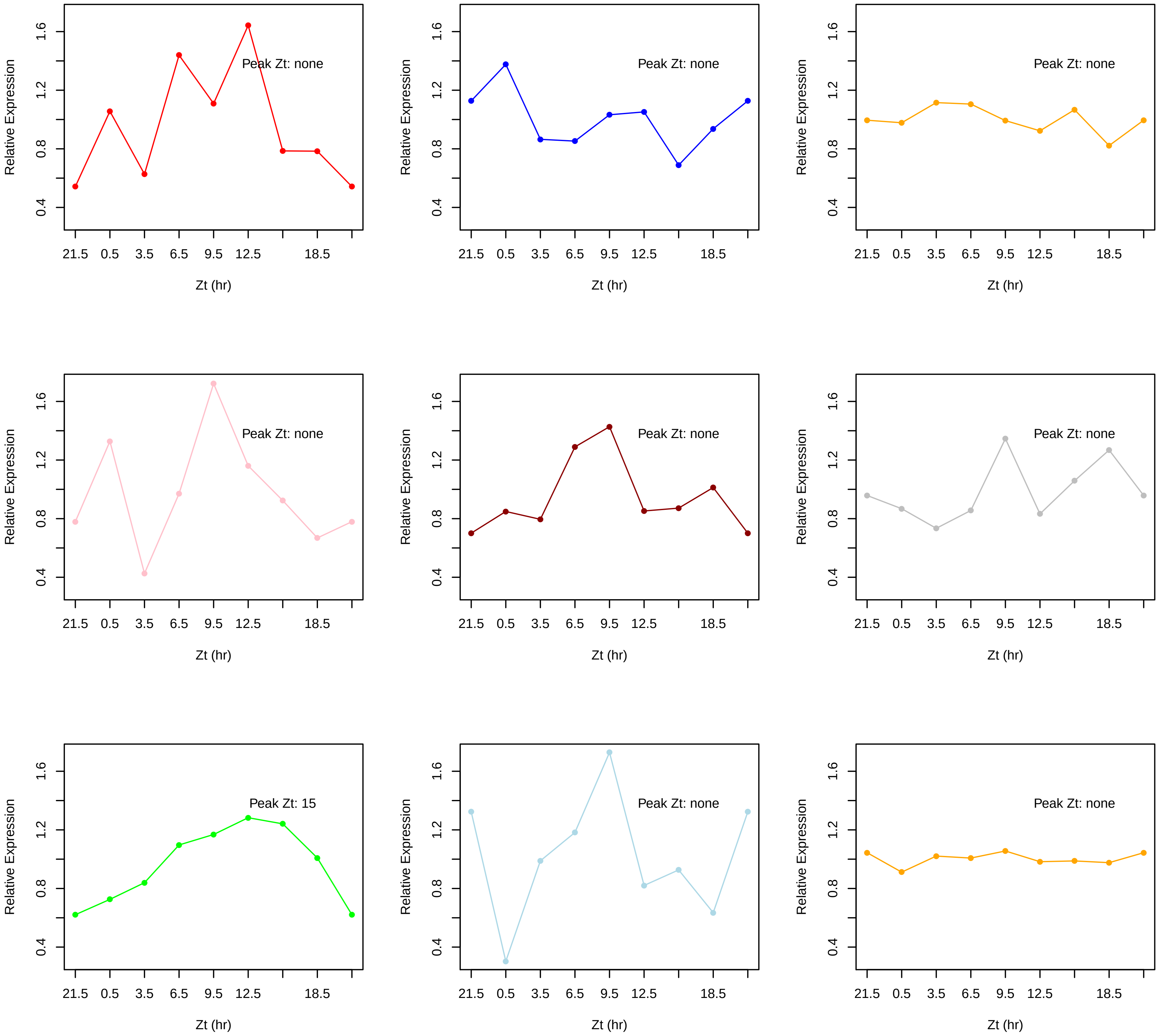

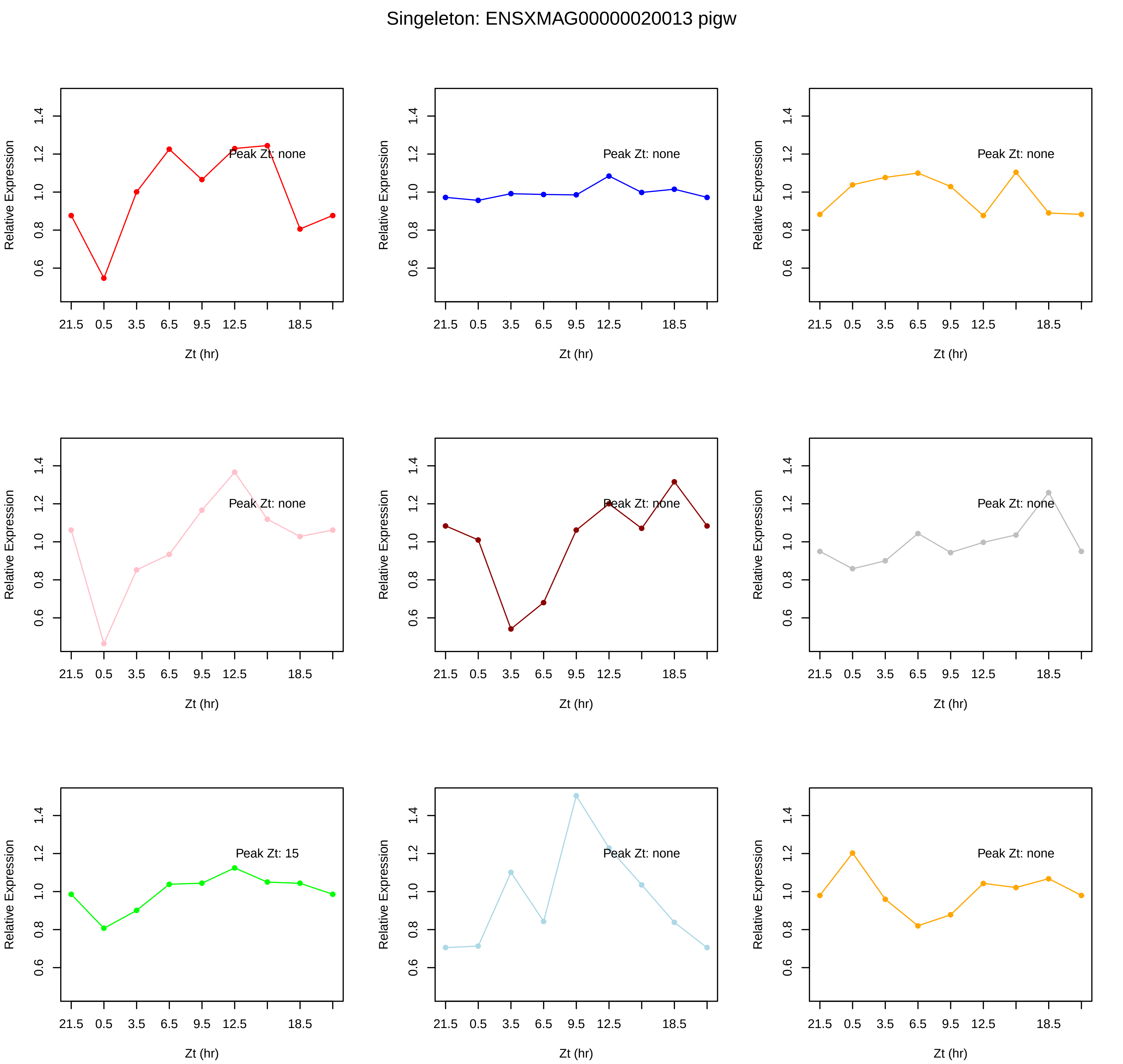
Singeleton: ENSXMAG00000020013 pigw
Relative Expression
Zt (hr)
21.5 0.5 3.5 6.5 9.5 12.5 18.5
0.6 0.8 1.0 1.2 1.4
Peak Zt: none
Peak Zt: none
Peak Zt: none
Peak Zt: none
Peak Zt: none
Peak Zt: none
Peak Zt: 15
Peak Zt: none
Peak Zt: none

Singleton: ENSXMAG00000020012 emc6

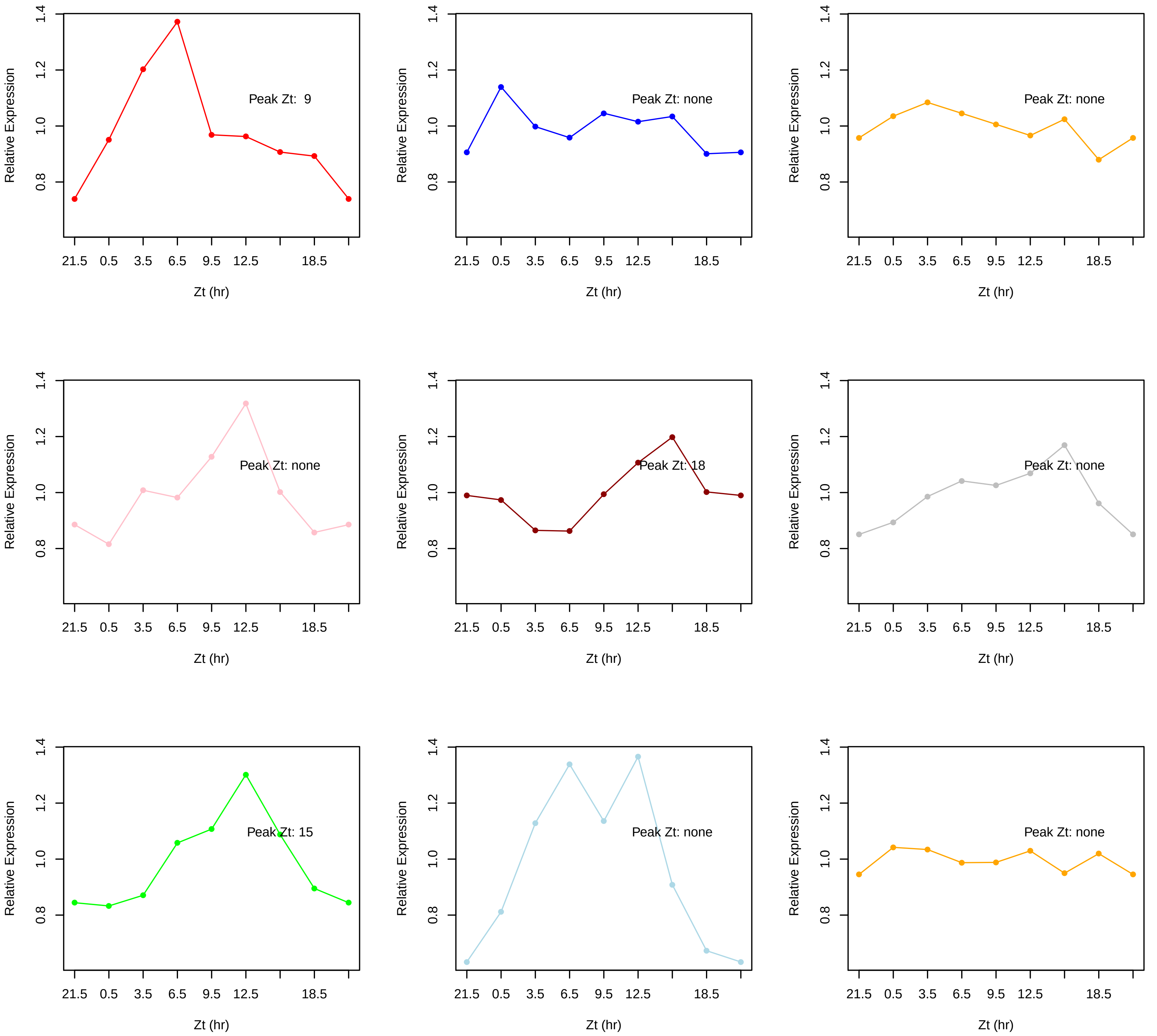

Singleton: ENSXMAG00000020094 ptrh2

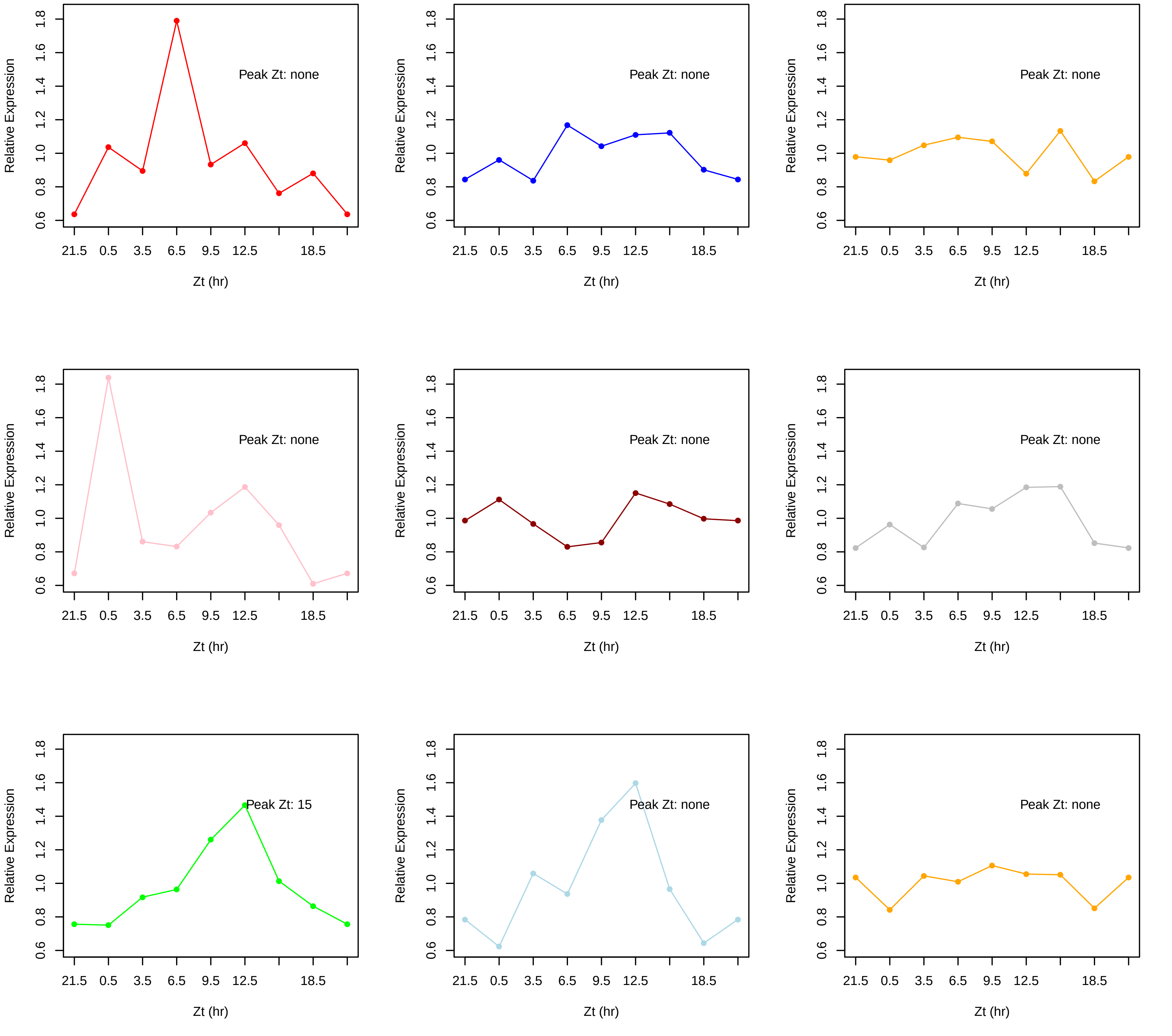

Singeleton: ENSXMAG00000019675 dusp14

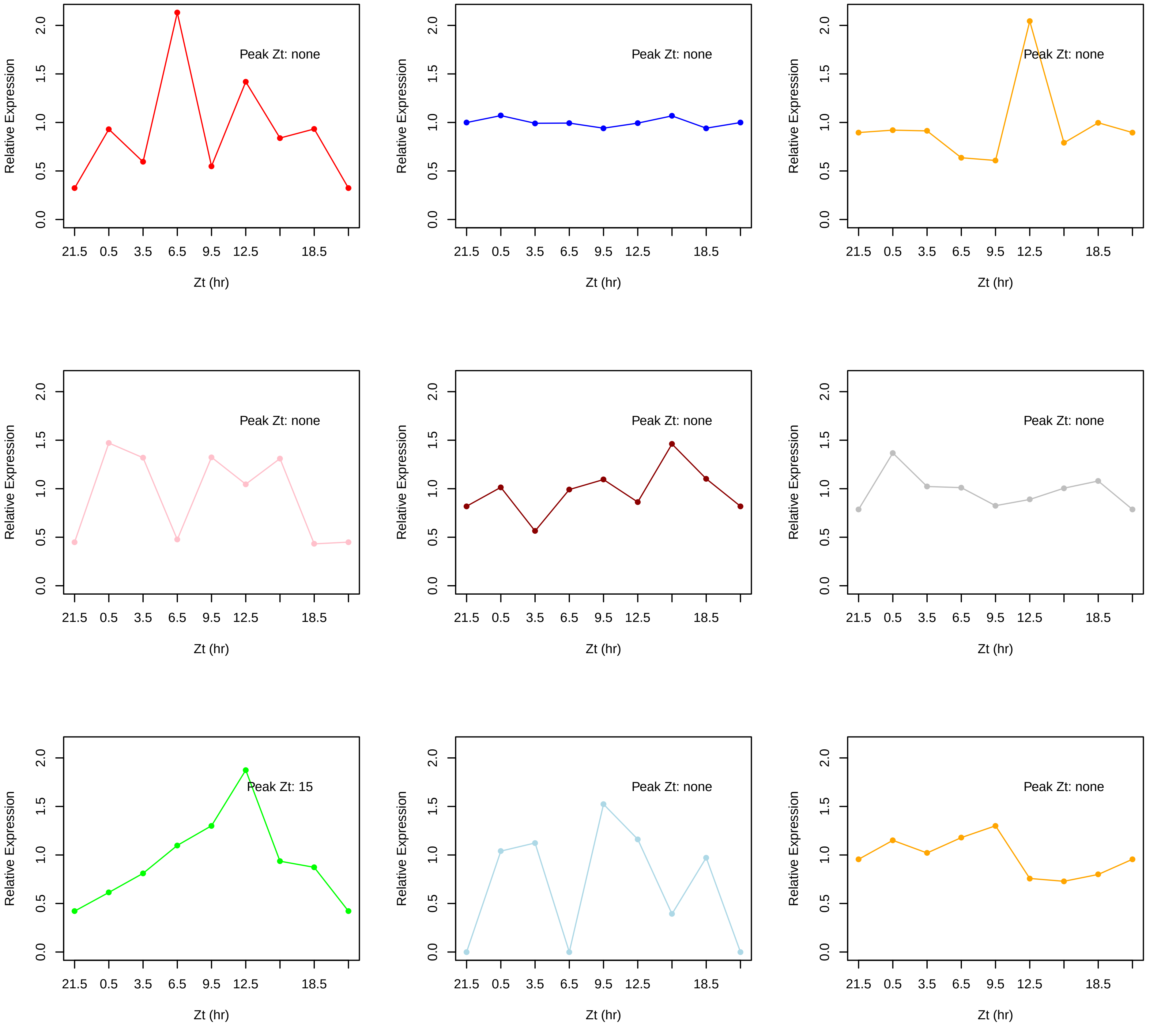

Singeleton: ENSXMAG00000011919 agtr2

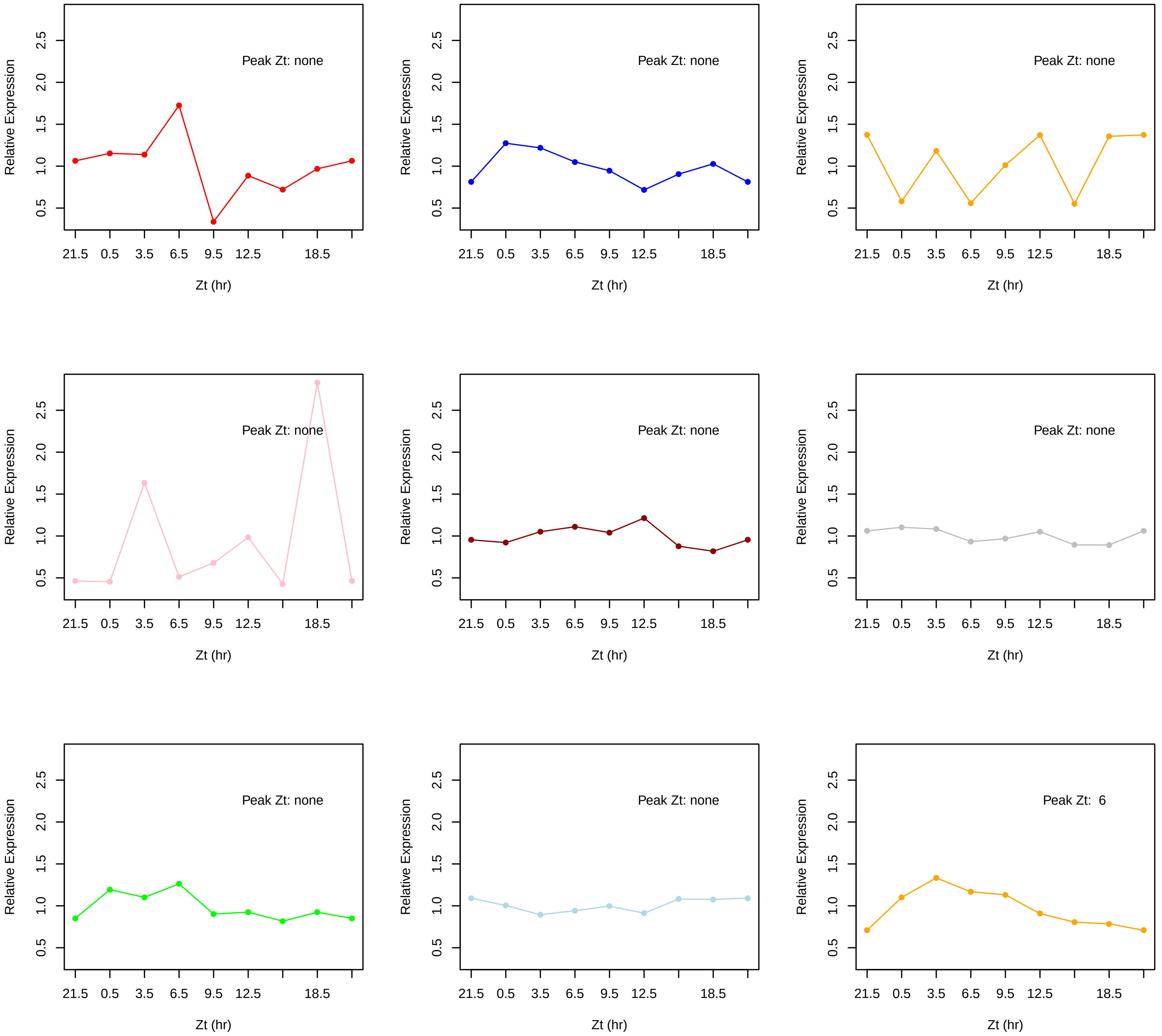

Singeleton: ENSXMAG00000020370 tmem229b

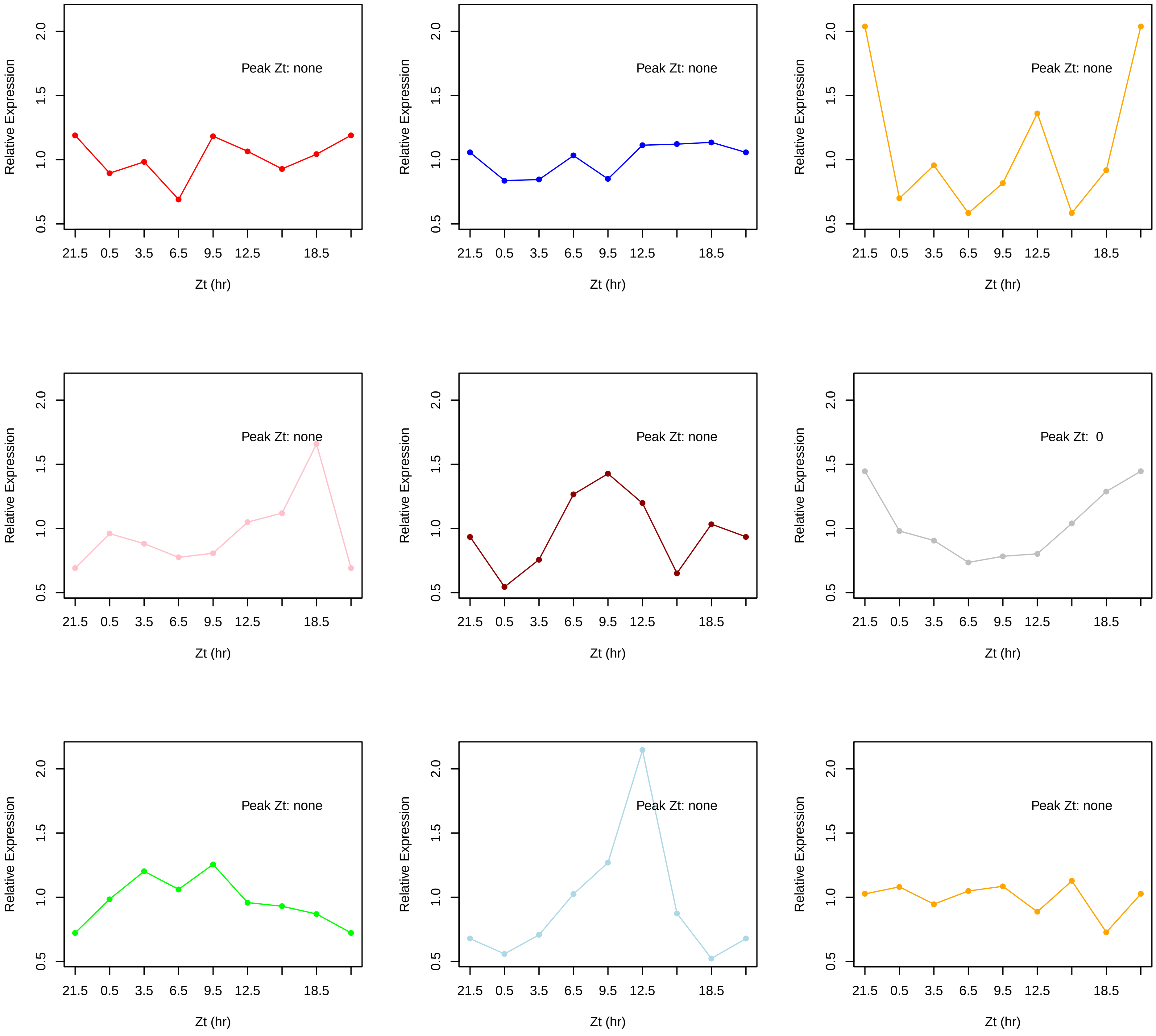

Singleton: ENSXMAG00000025214 cited1

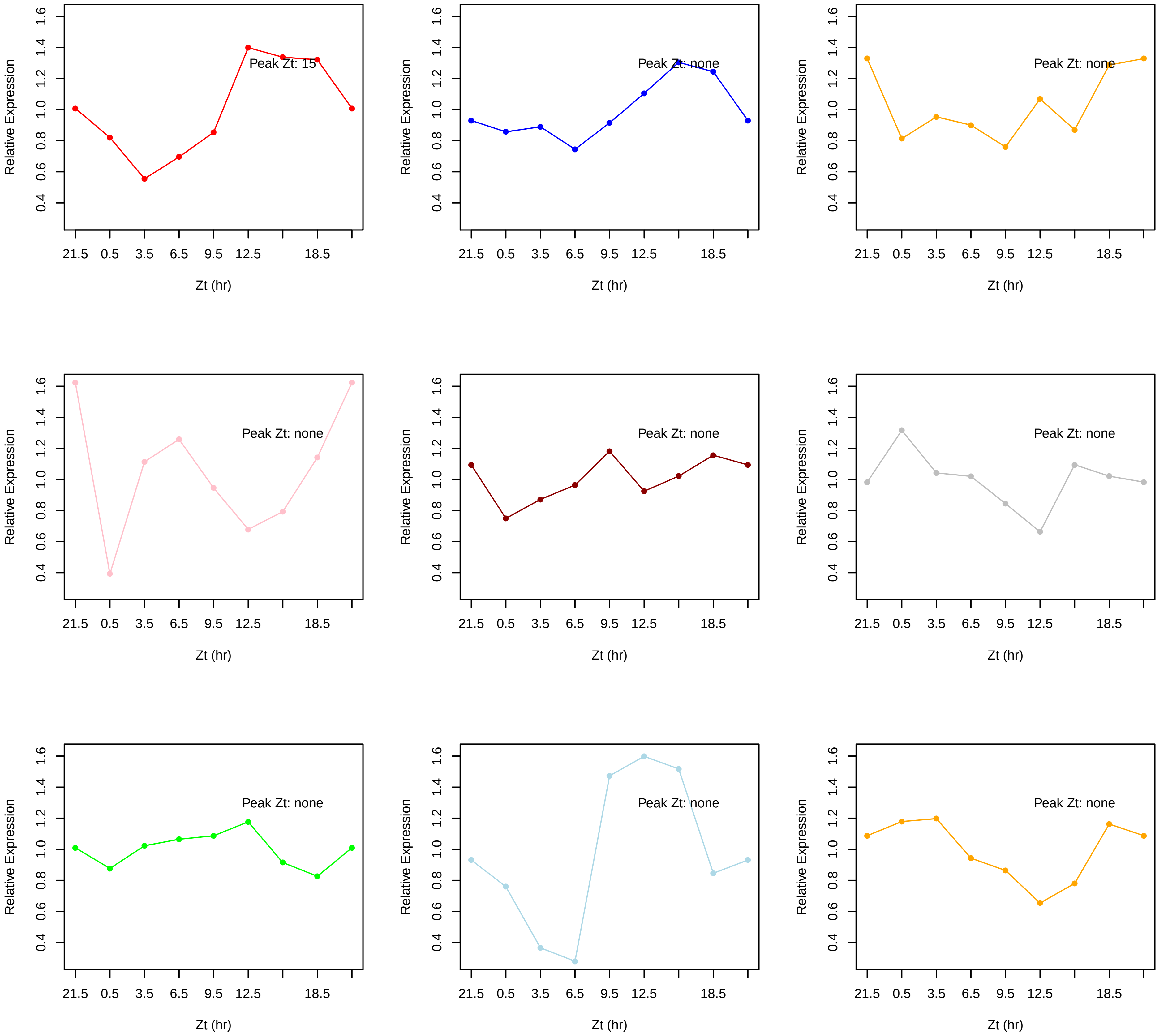

Singeleton: ENSXMAG00000019515 sox3

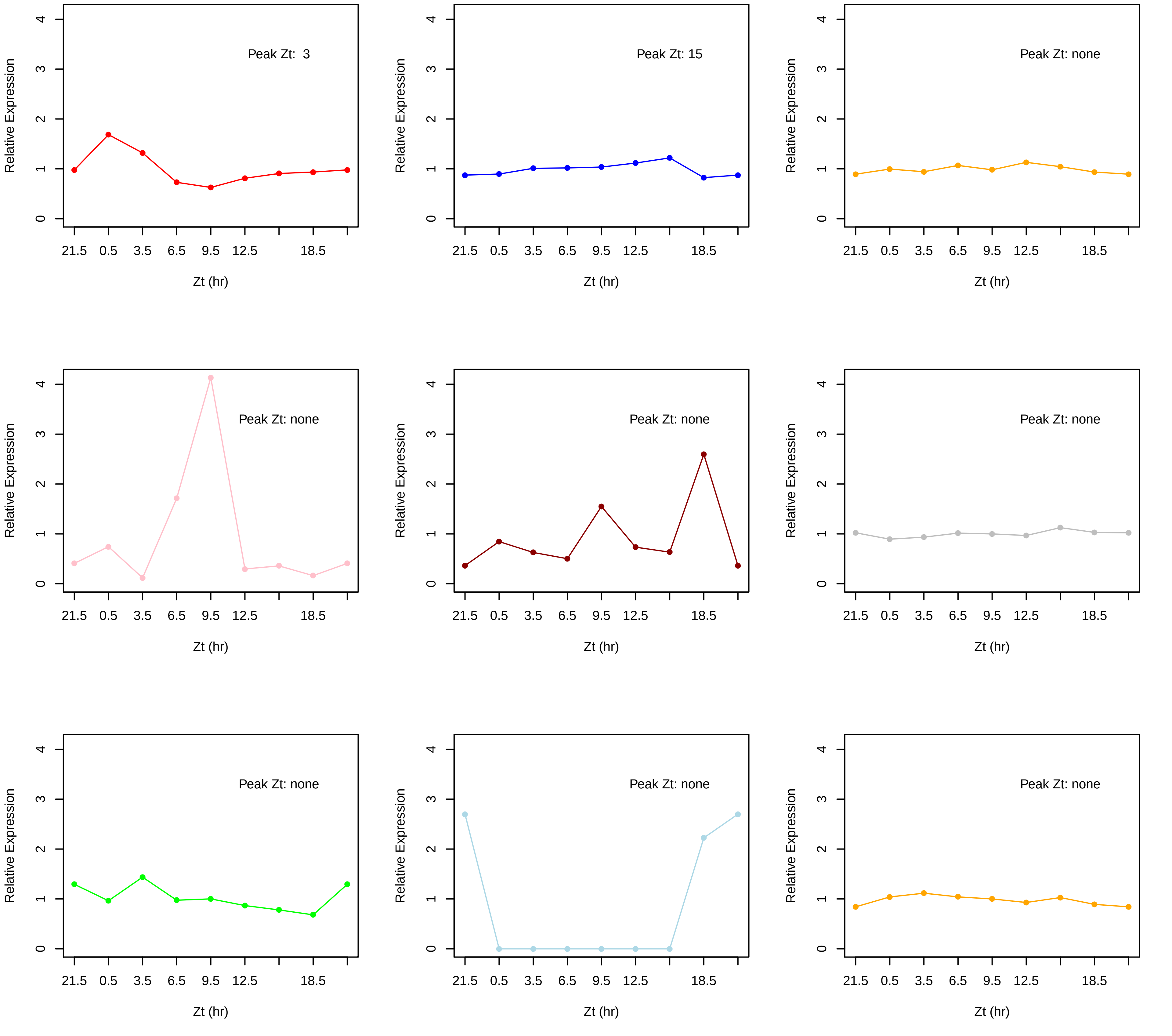

Singleton: ENSXMAG00000013810 slitrk2

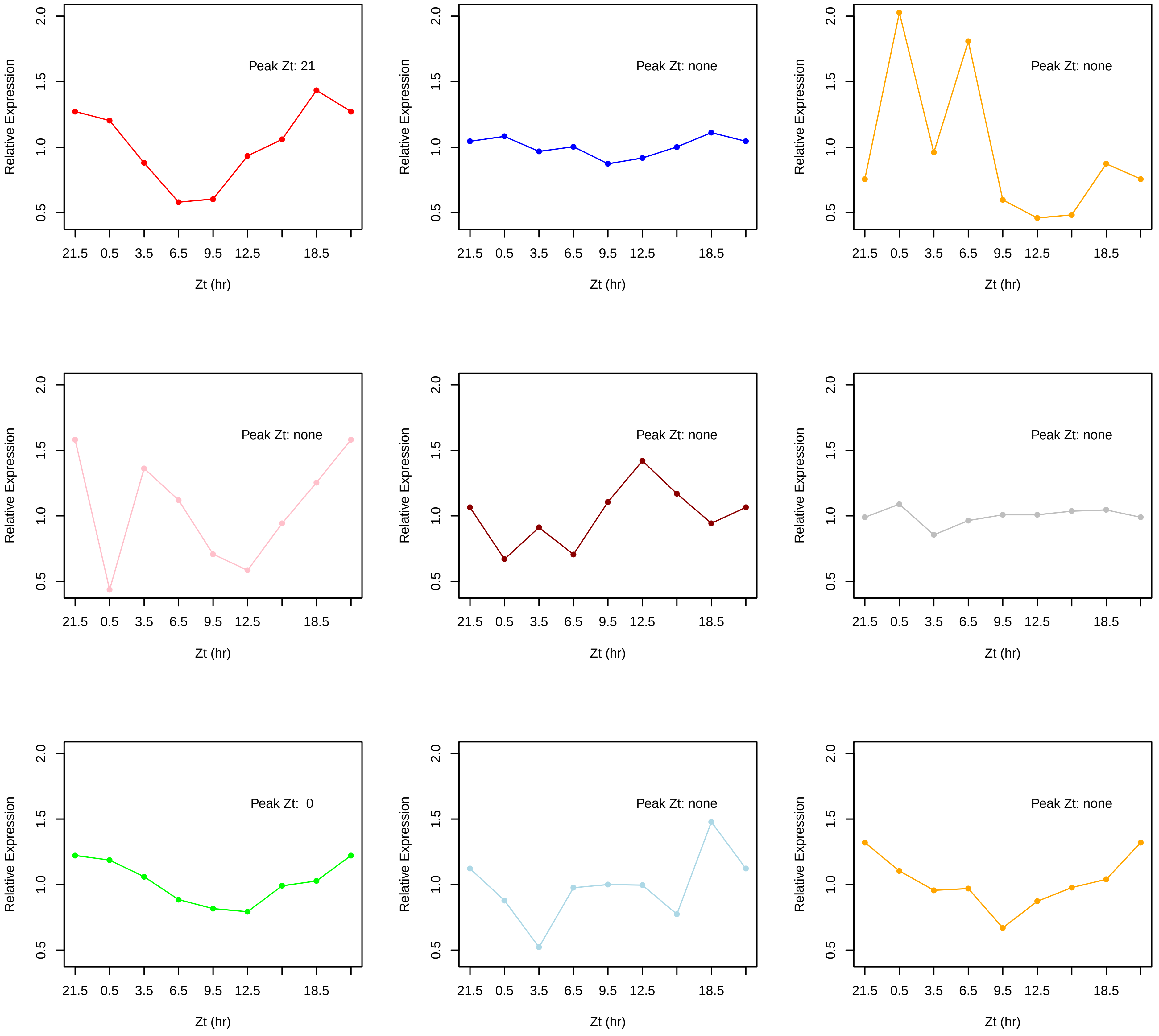

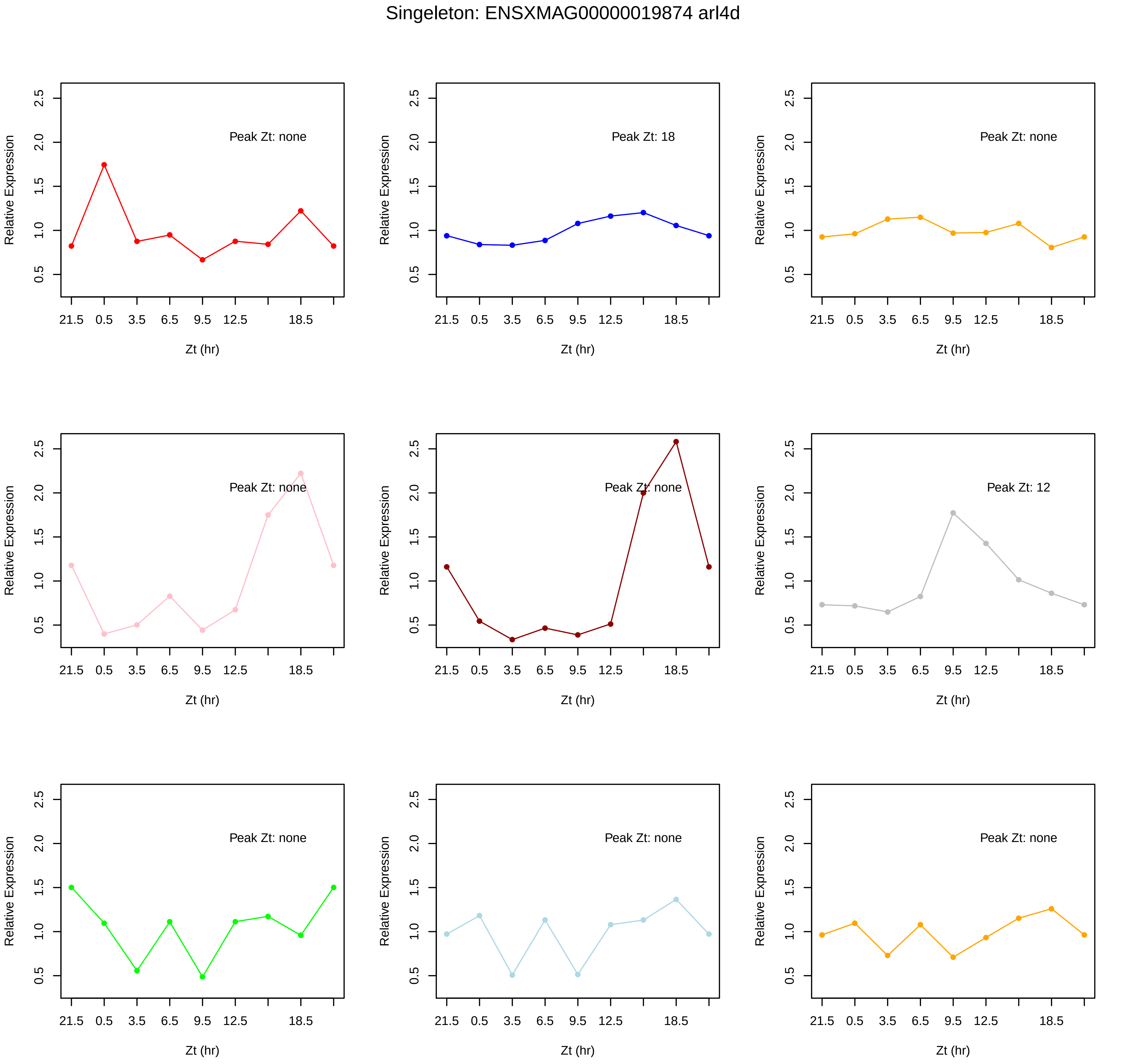
Singeleton: ENSXMAG00000019874 arl4d
Peak Zt: none
Peak Zt: 18
Peak Zt: none
Peak Zt: none
Peak Zt: none
Peak Zt: 12
Peak Zt: none
Peak Zt: none
Peak Zt: none
Relative Expression
Zt (hr)

Singeleton: ENSXMAG00000020021 ora5

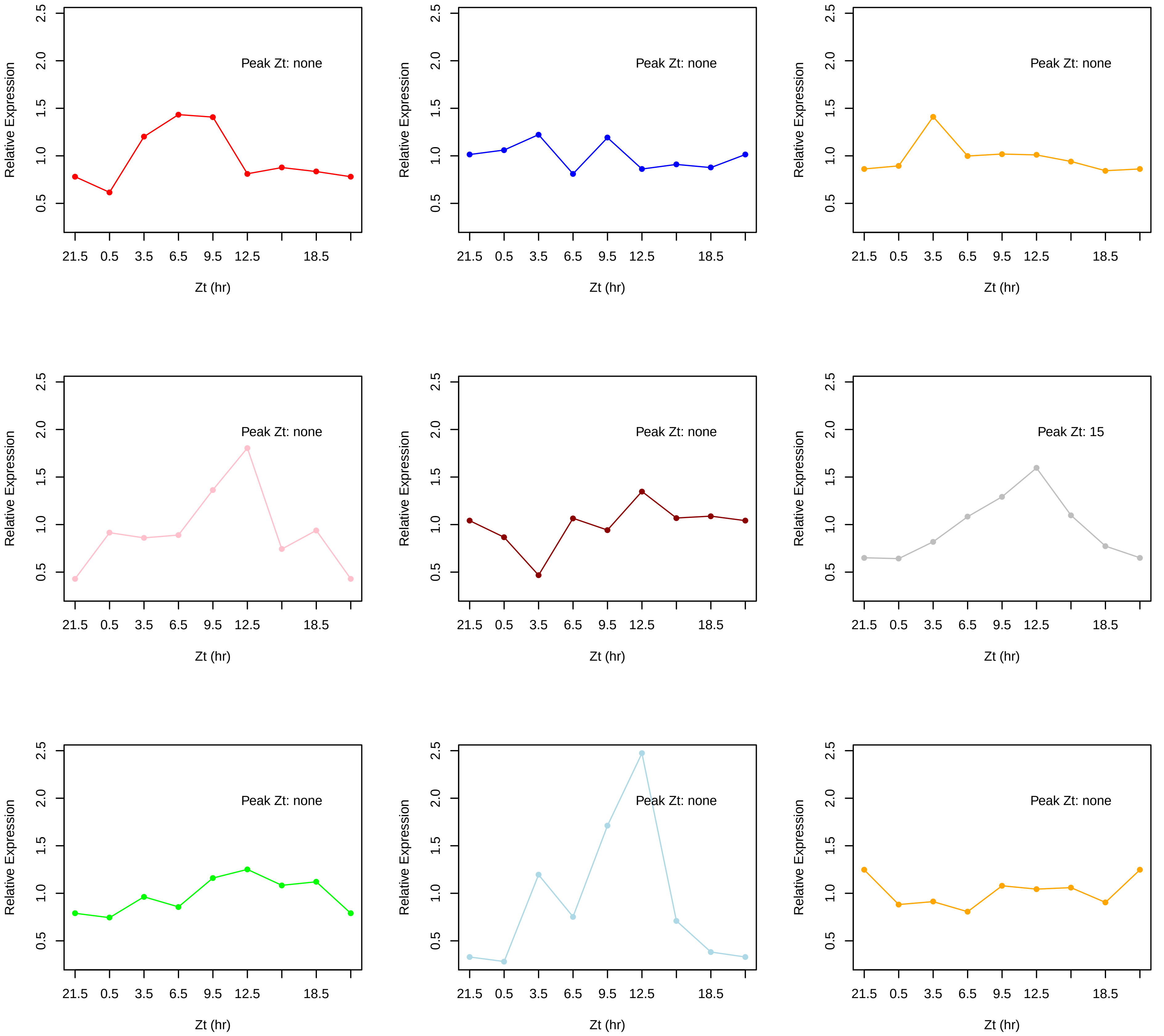

Singeleton: ENSXMAG00000019746 pop7

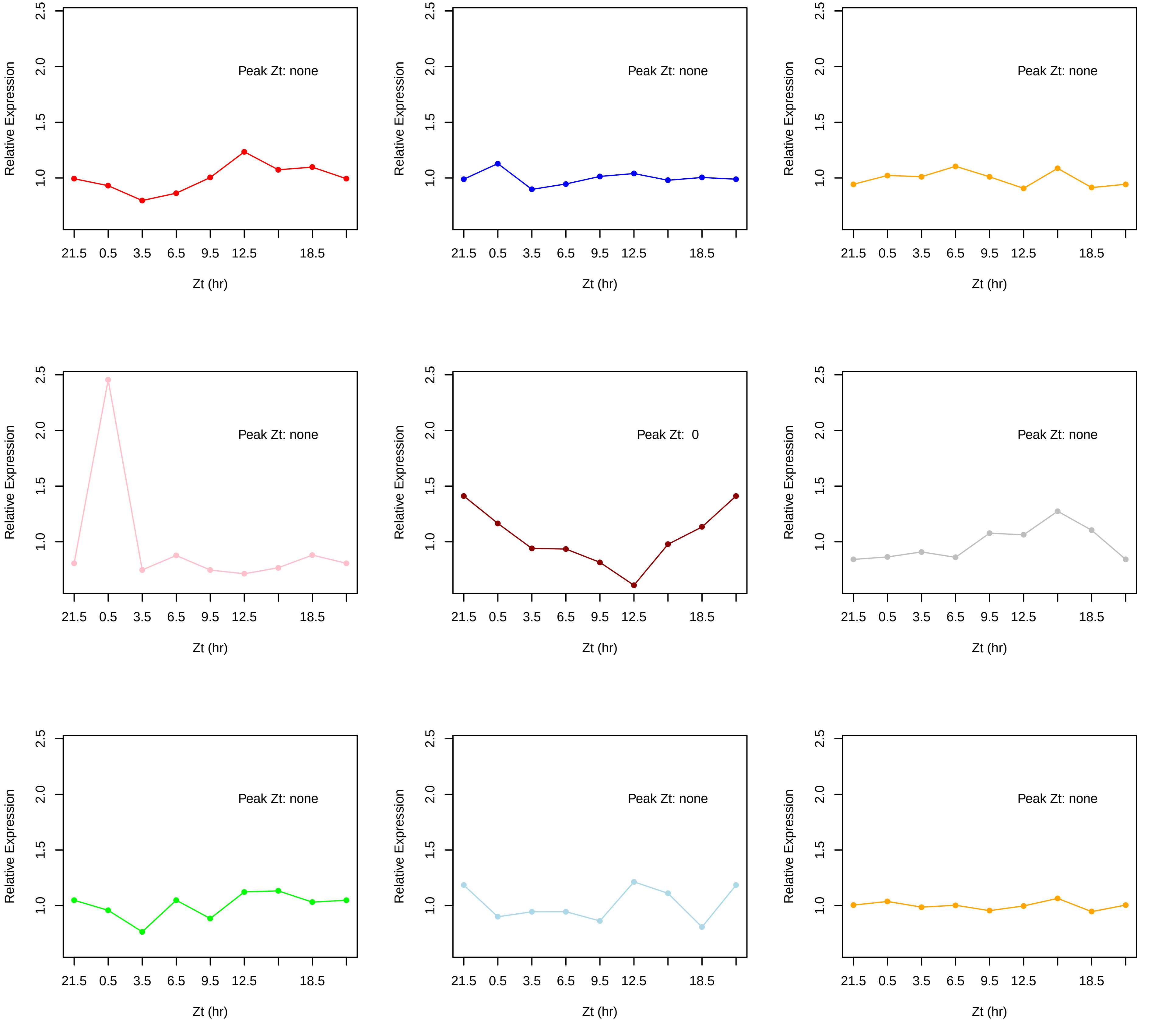

Singeleton: ENSXMAG00000019564 zgc:154093

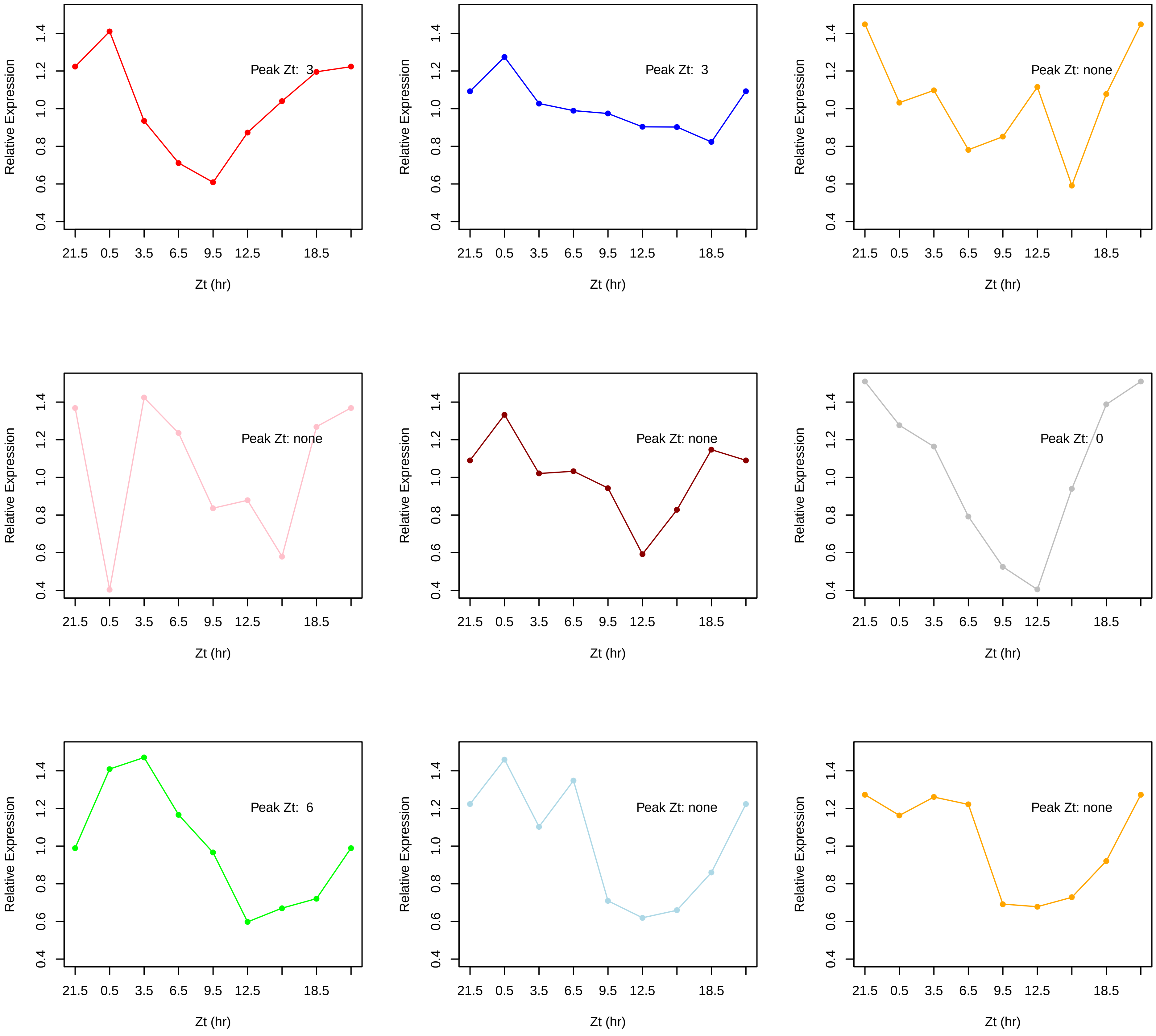

Singleton: ENSXMAG00000021787

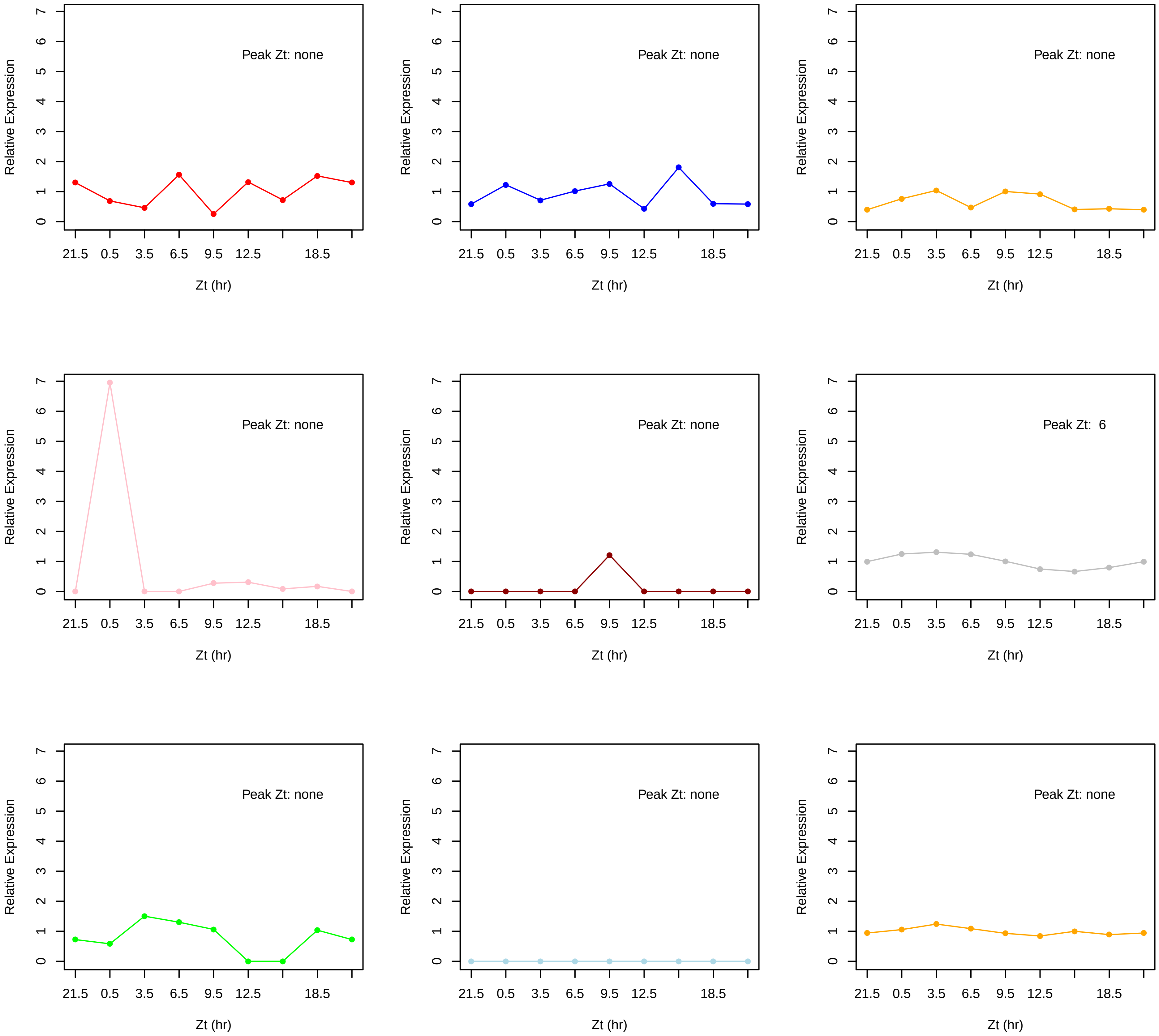

Singeleton: ENSXMAG00000019691 cebpg

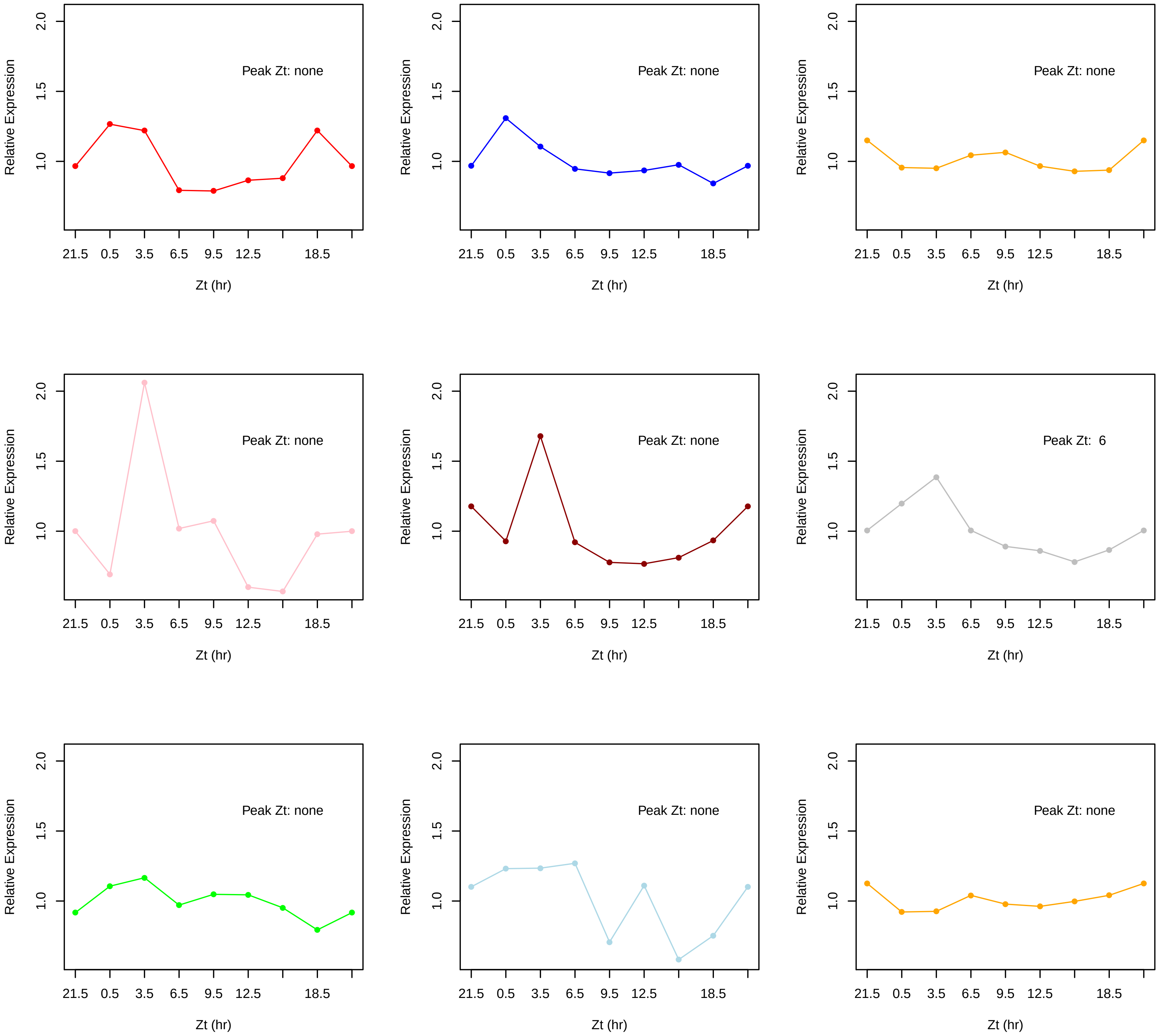

Singeleton: ENSXMAG00000020295 slc10a3

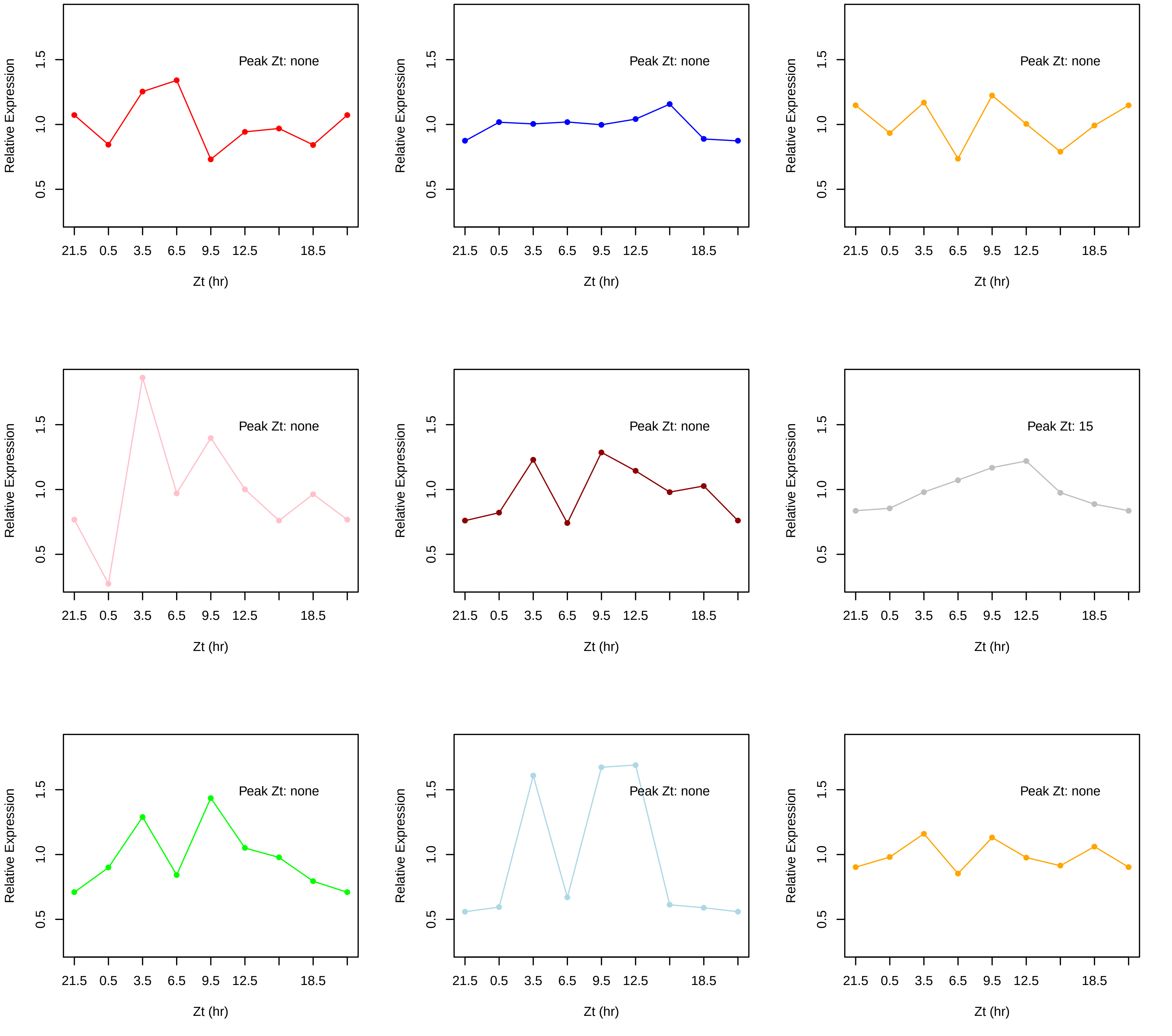

Singeleton: ENSXMAG00000019795 thap11

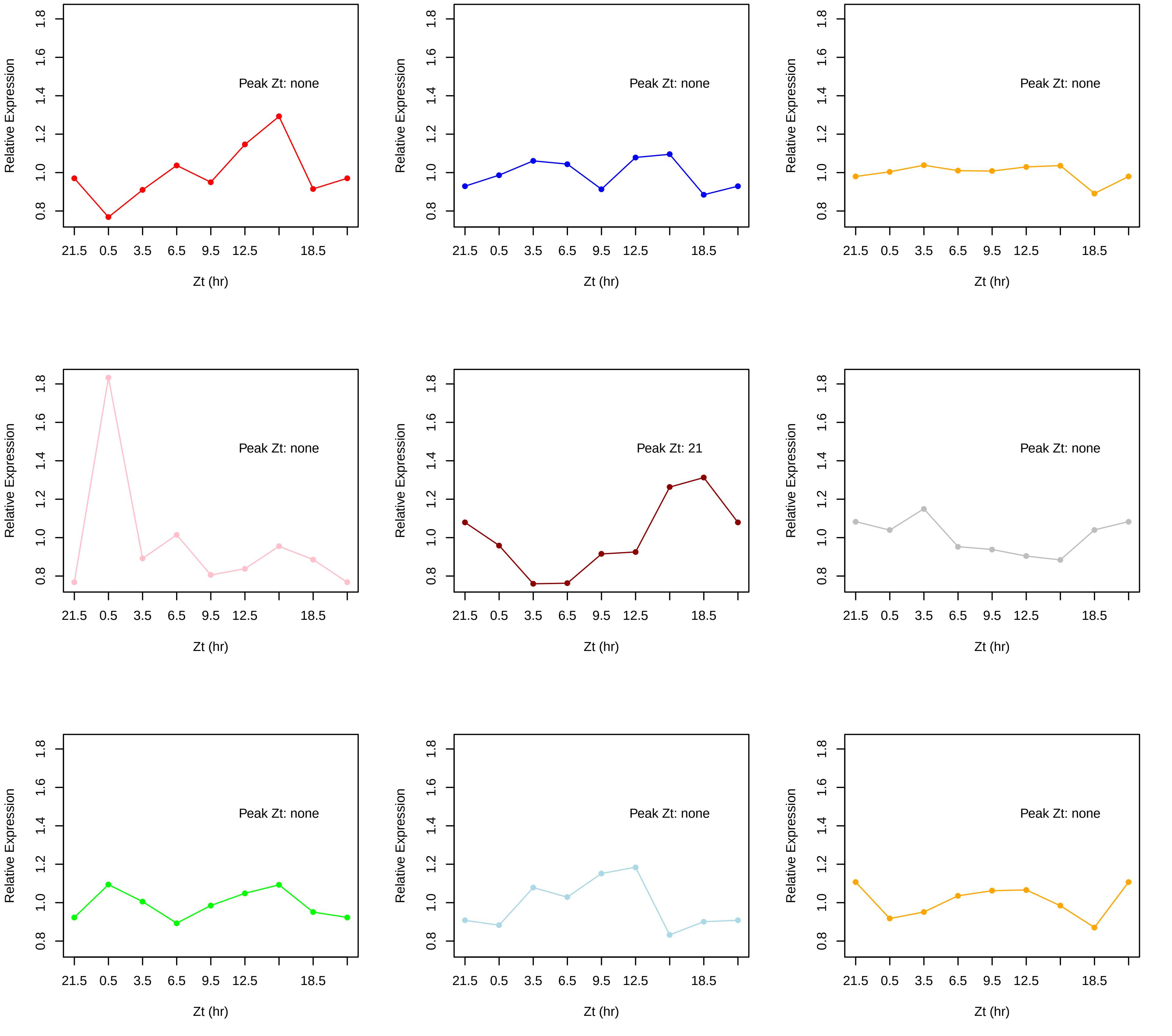

Singleton: ENSXMAG00000019603 ddx28

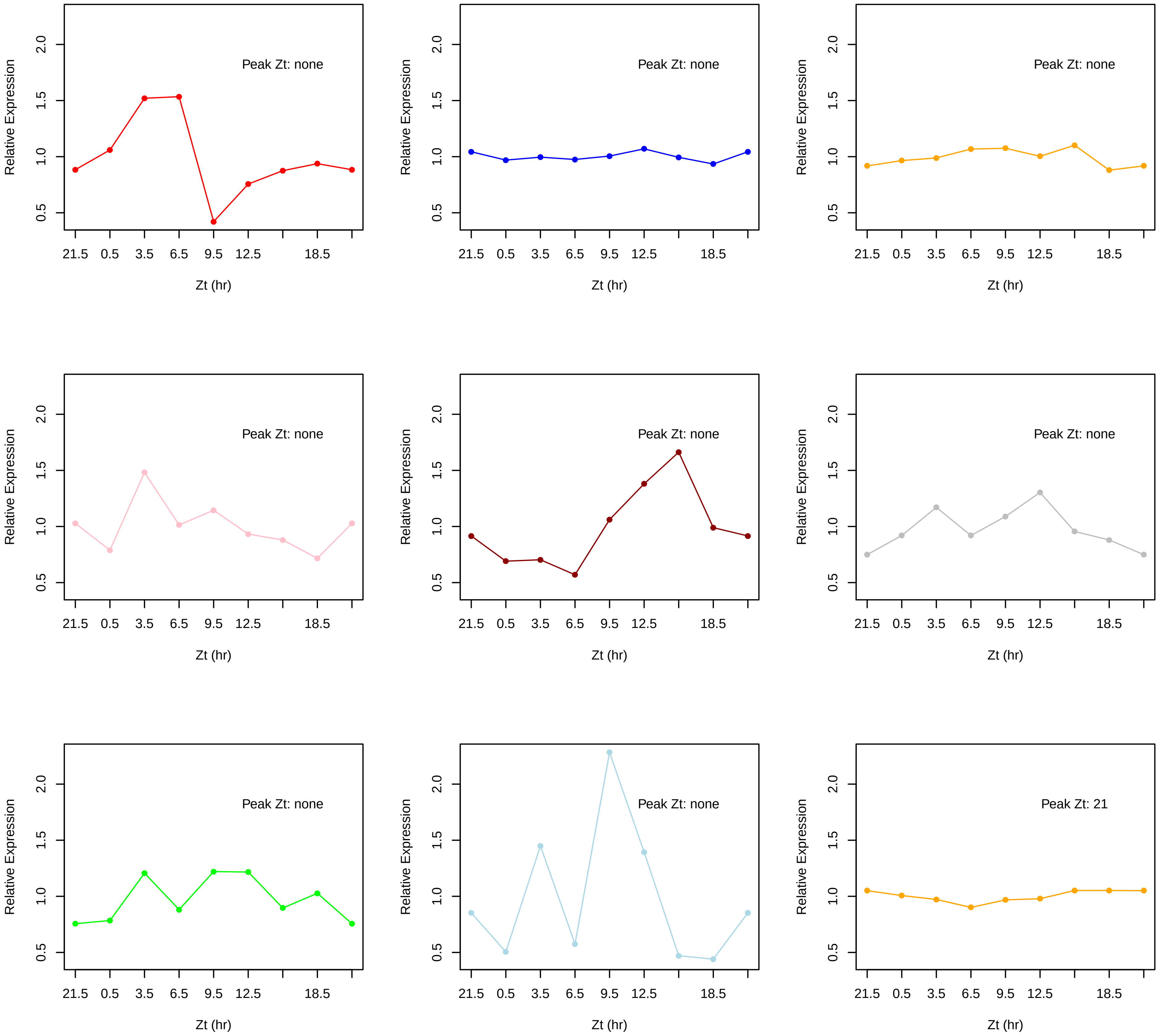

Singeleton: ENSXMAG00000025753 ppp1r14bb

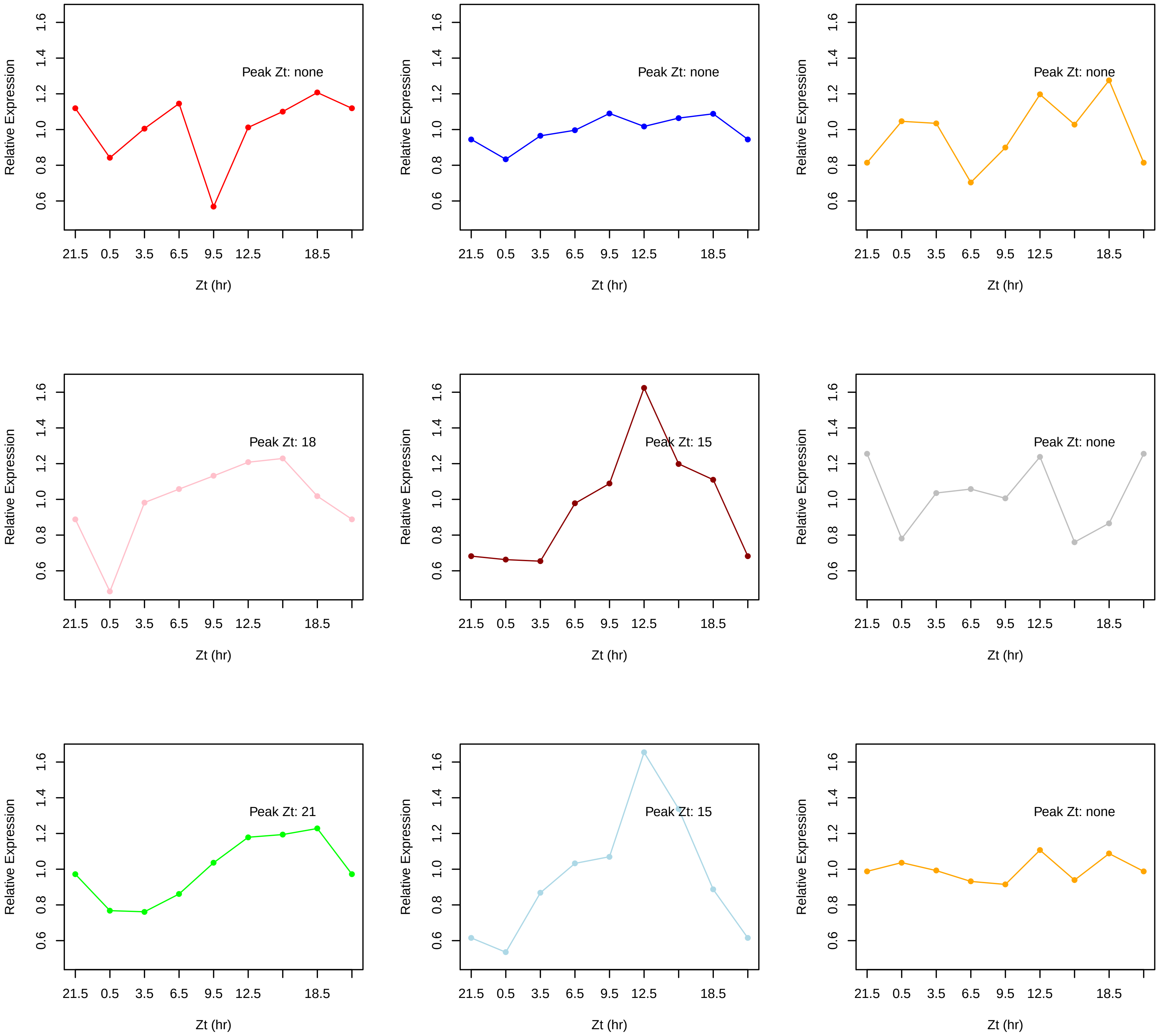

Singeleton: ENSXMAG00000005888

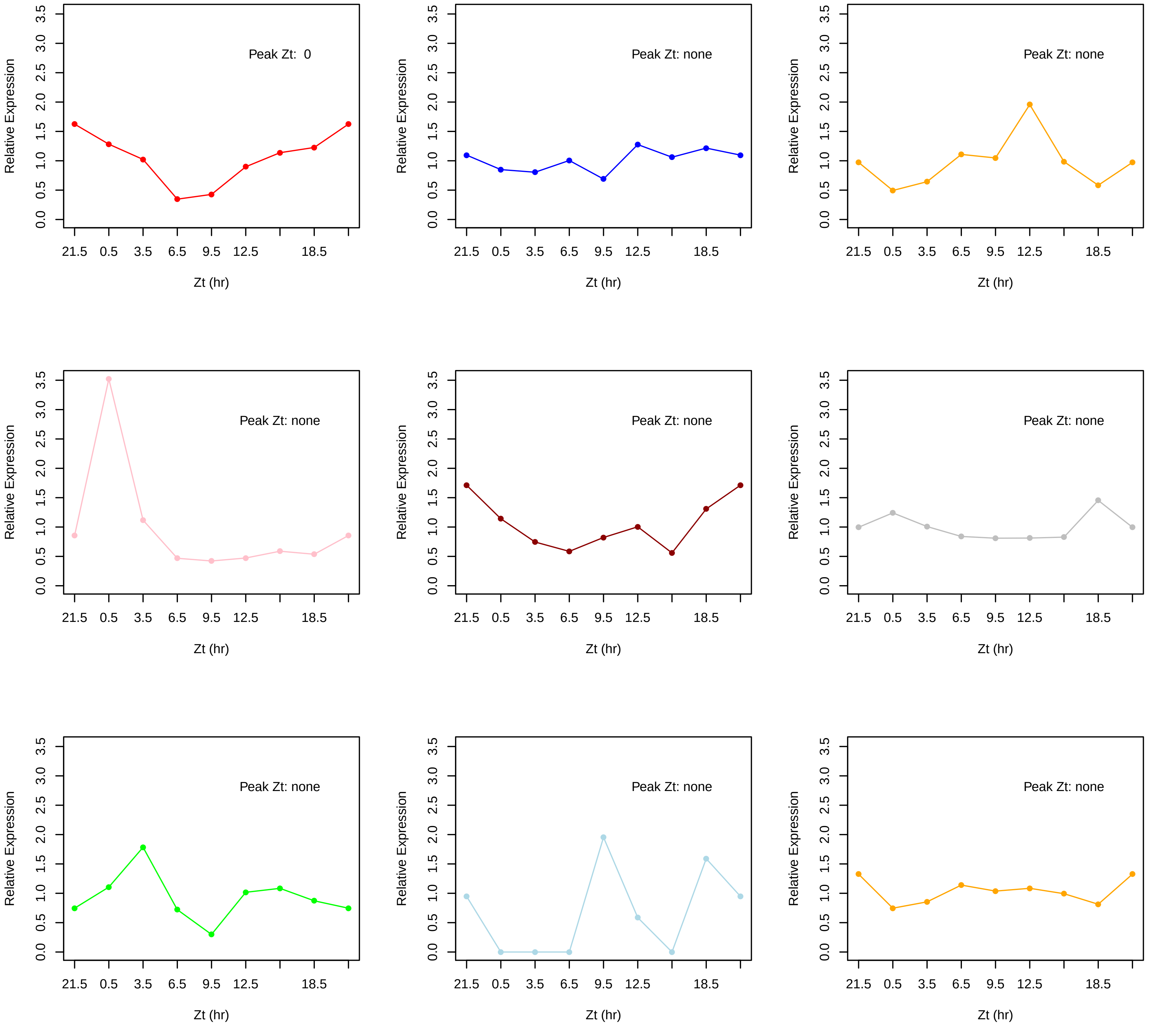

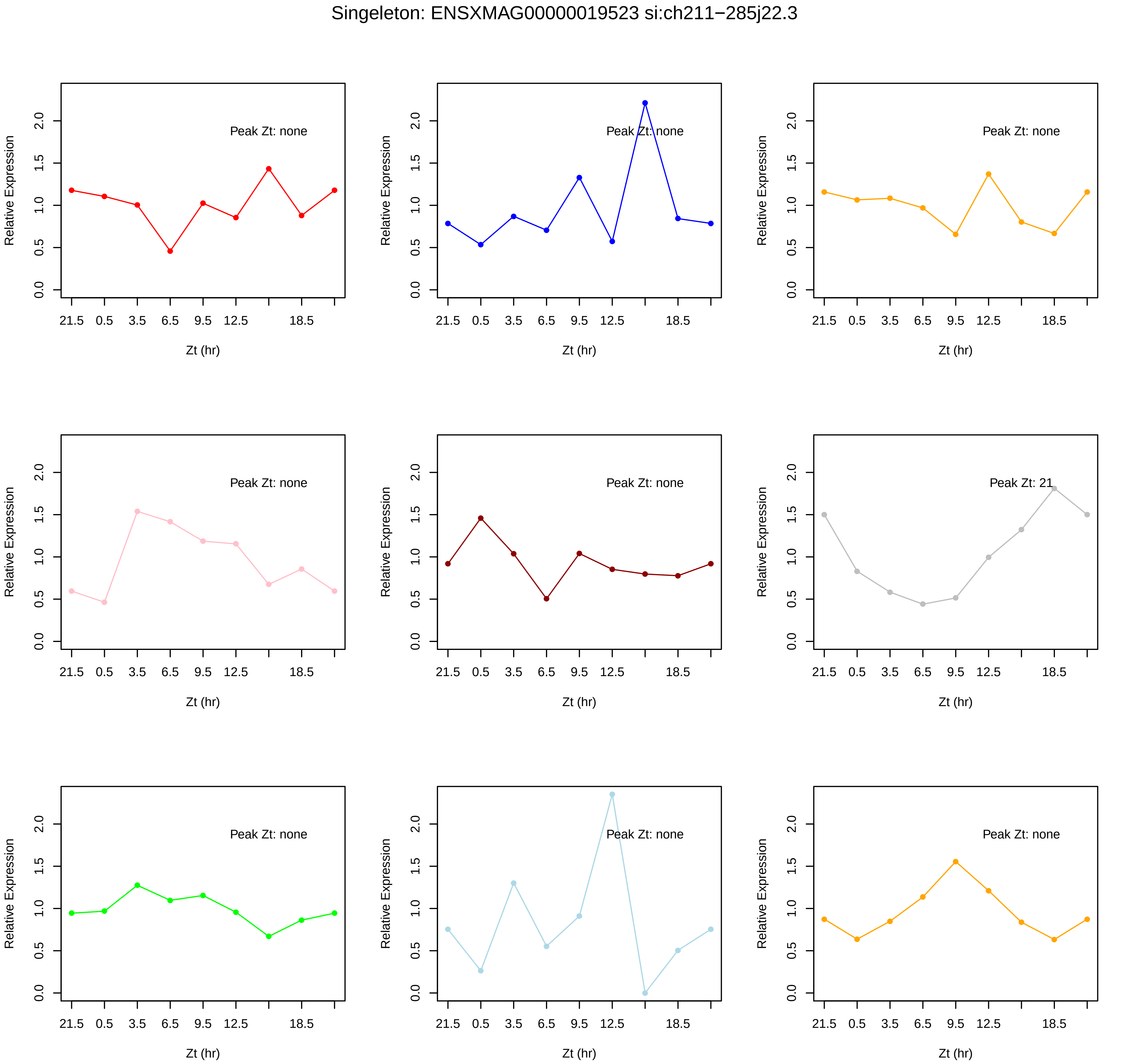

Singeleton: ENSXMAG00000019523 si:ch211−285j22.3
Peak Zt: none
Peak Zt: none
Peak Zt: none
Peak Zt: none
Peak Zt: none
Peak Zt: 21
Peak Zt: none
Peak Zt: none
Peak Zt: none
Relative Expression
Zt (hr)
21.5
0.5
3.5
6.5
9.5
12.5
18.5

Singeleton: ENSXMAG00000021418

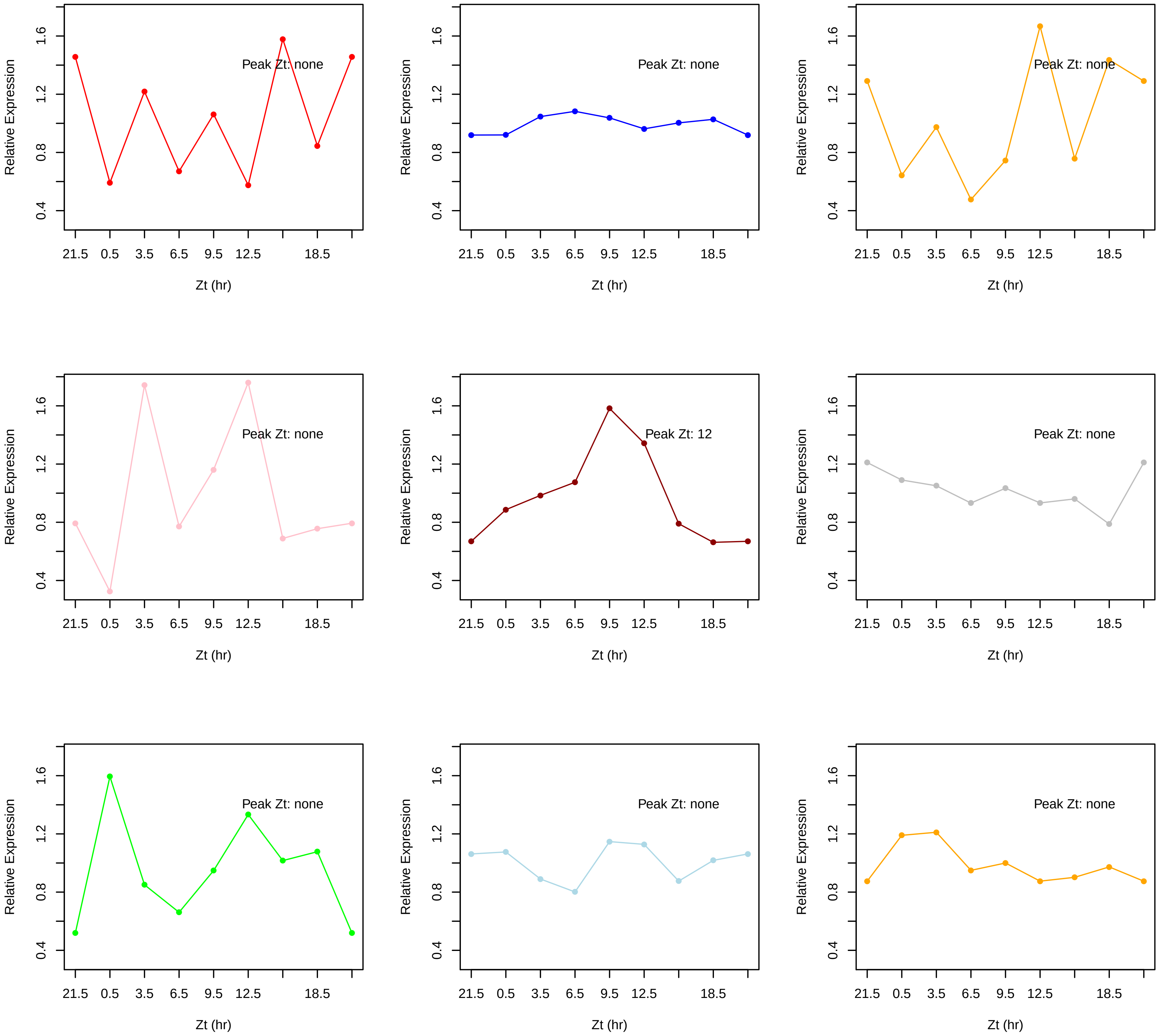

Singeleton: ENSXMAG00000018635 enc3

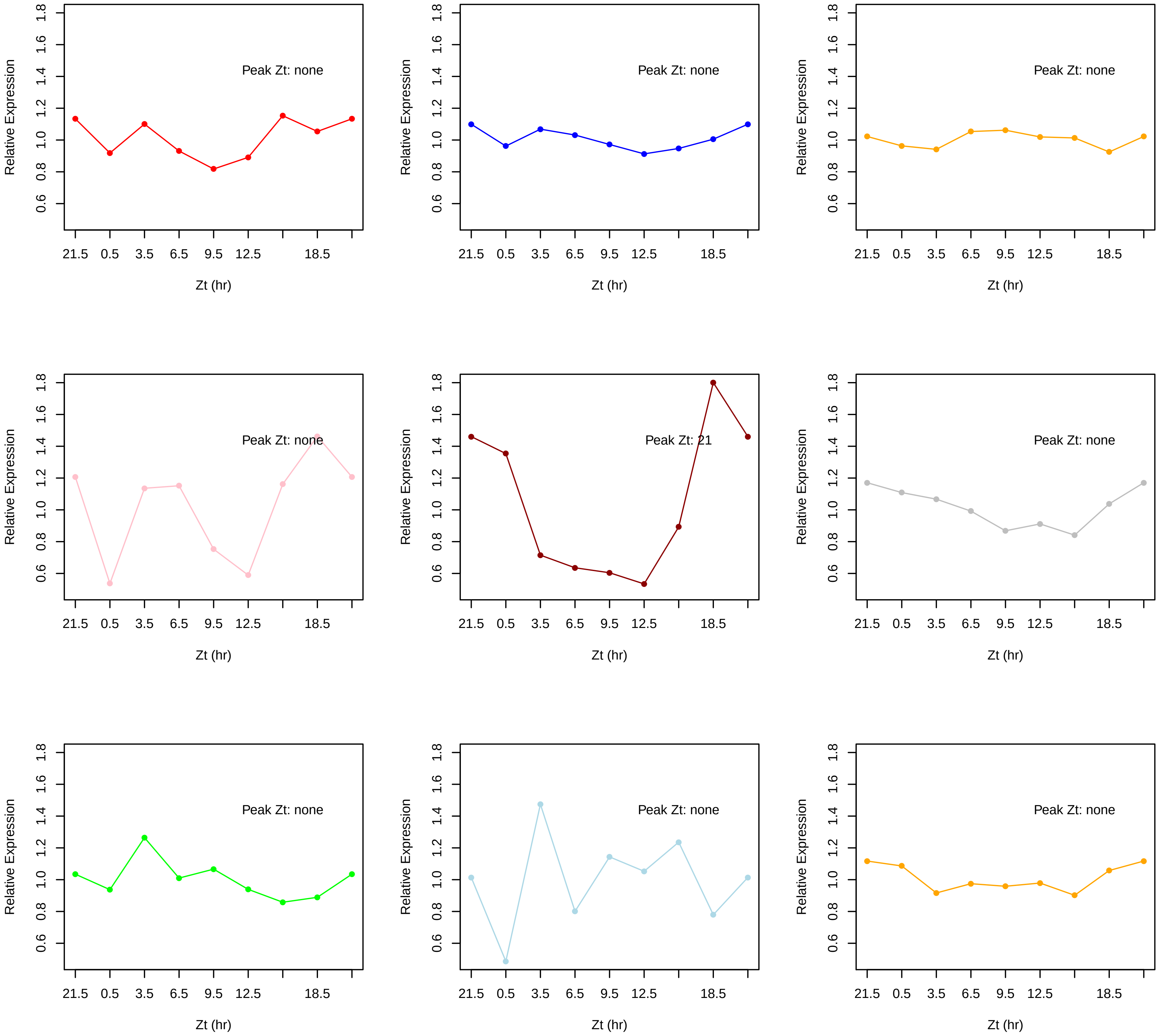

Singeleton: ENSXMAG00000006087 dad1

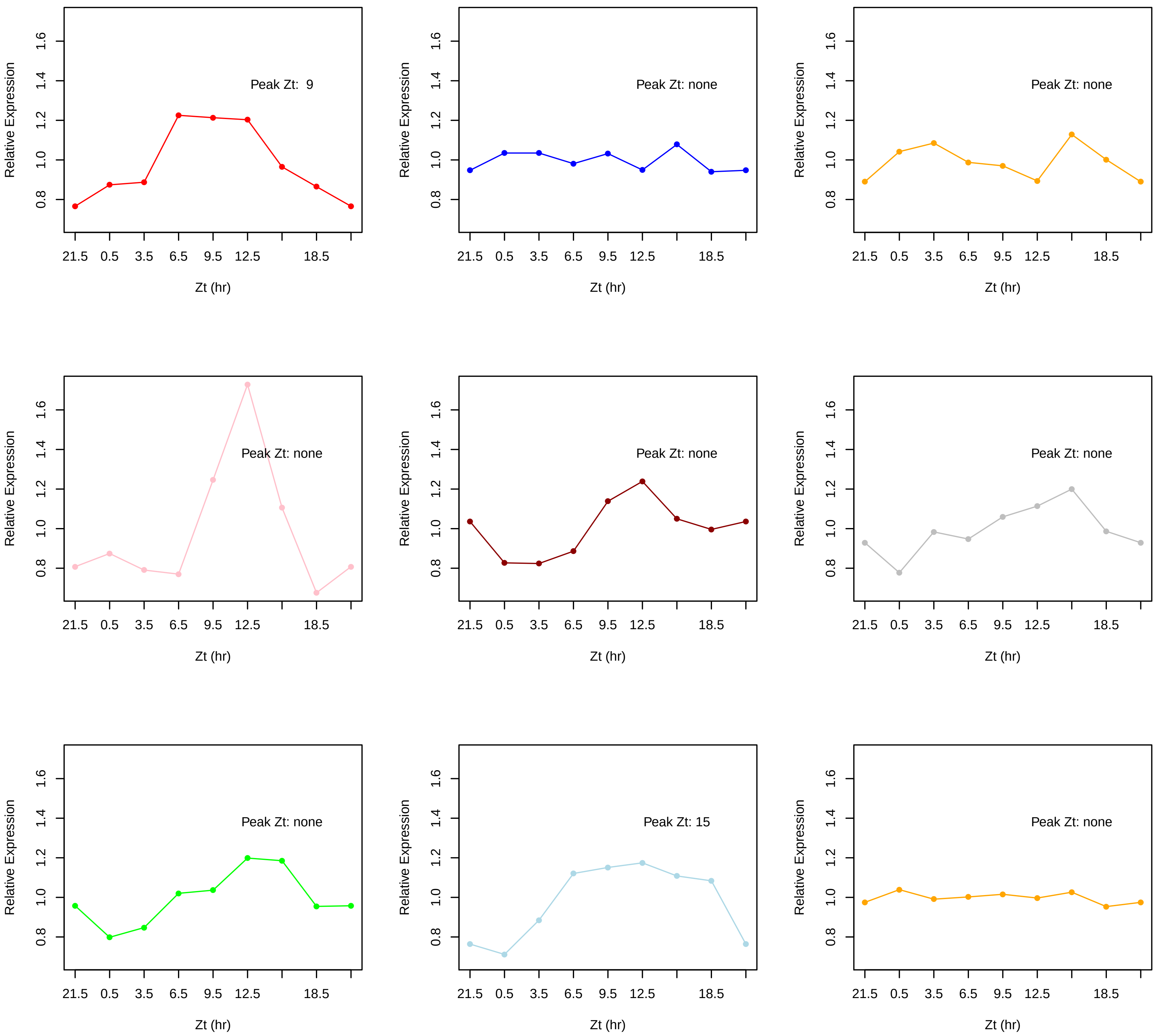

Singleton: ENSXMAG00000020287 dpm3

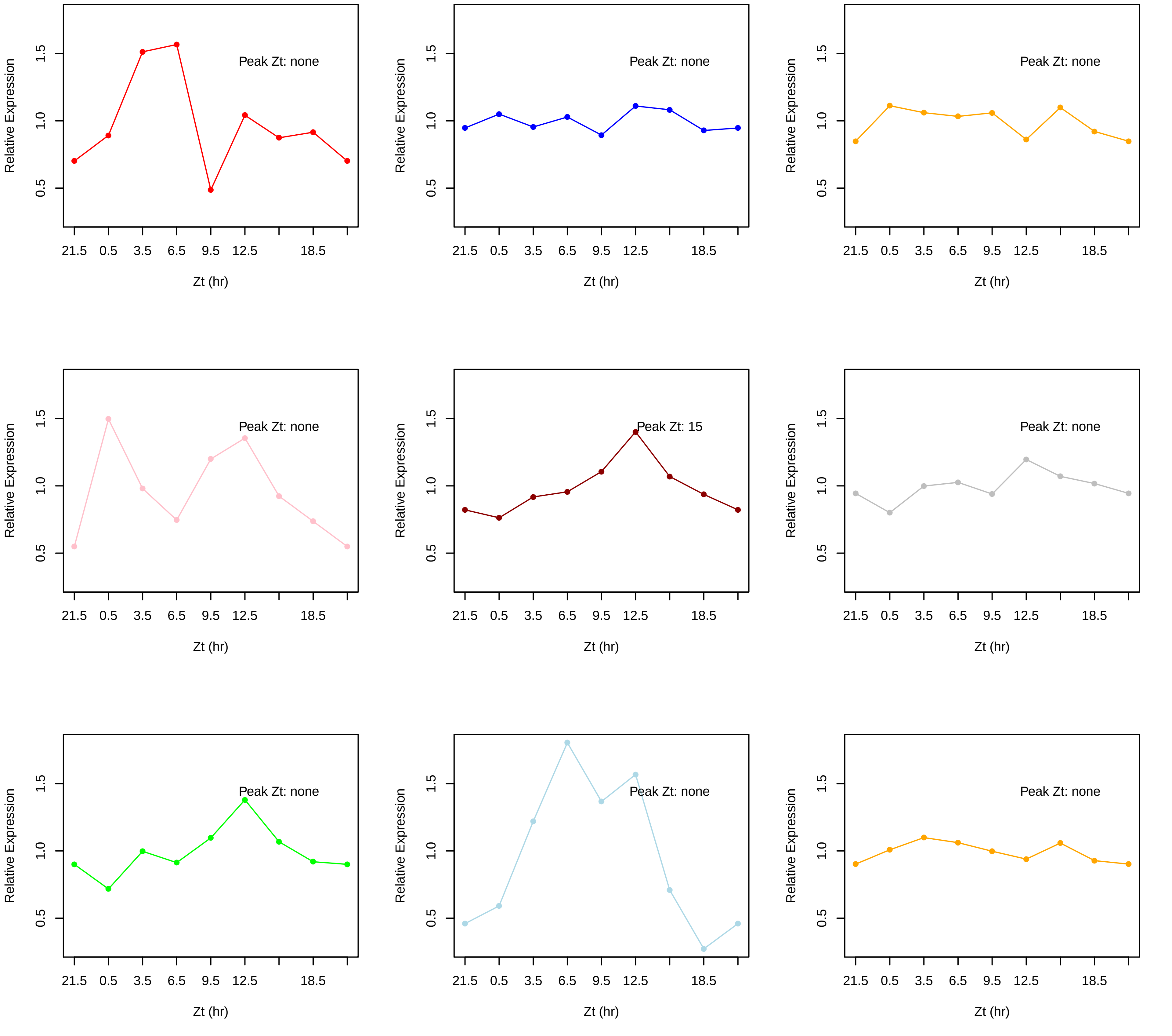

Singleton: ENSXMAG00000023595 mllt11

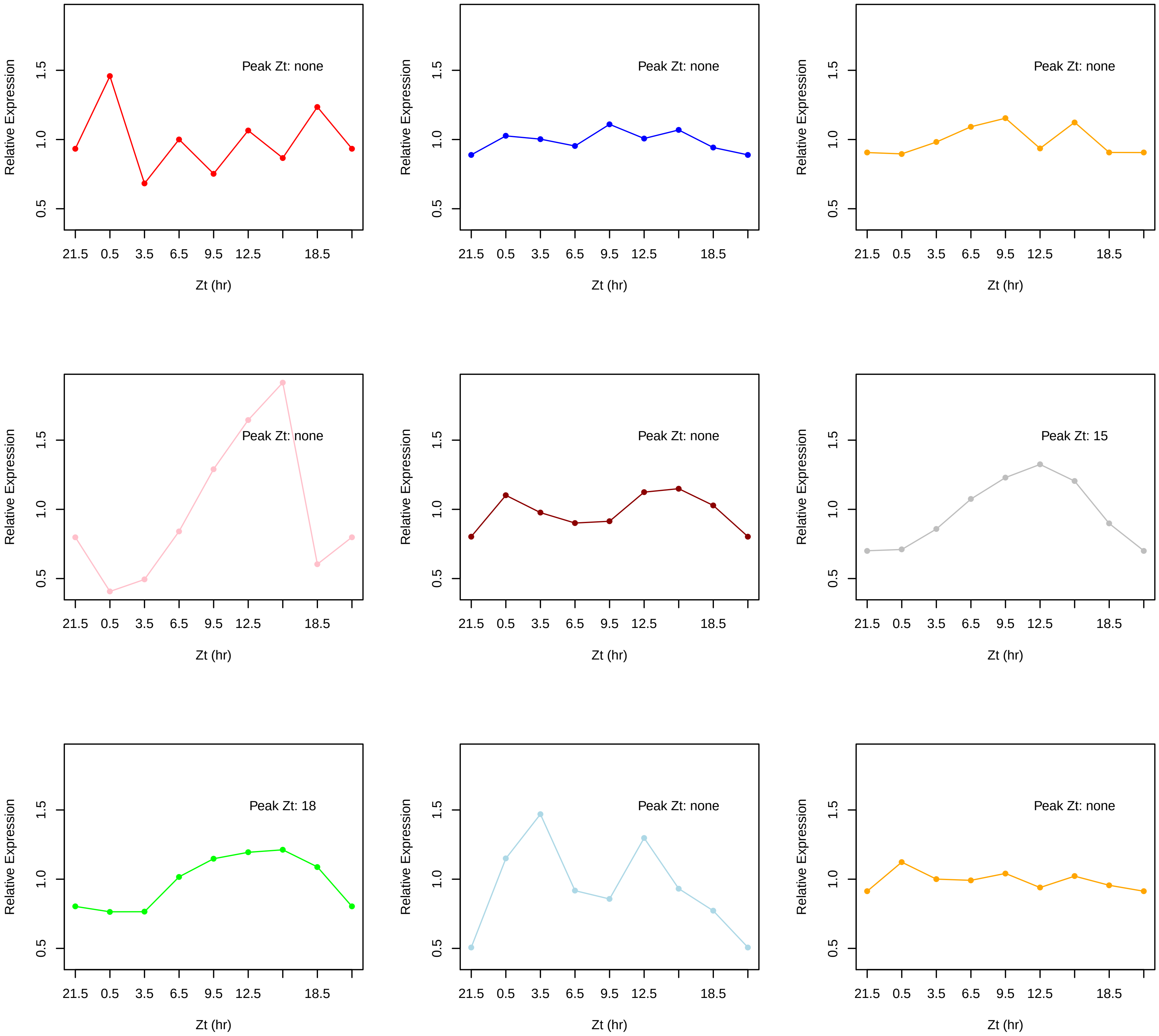

Singeleton: ENSXMAG00000019538 bola1

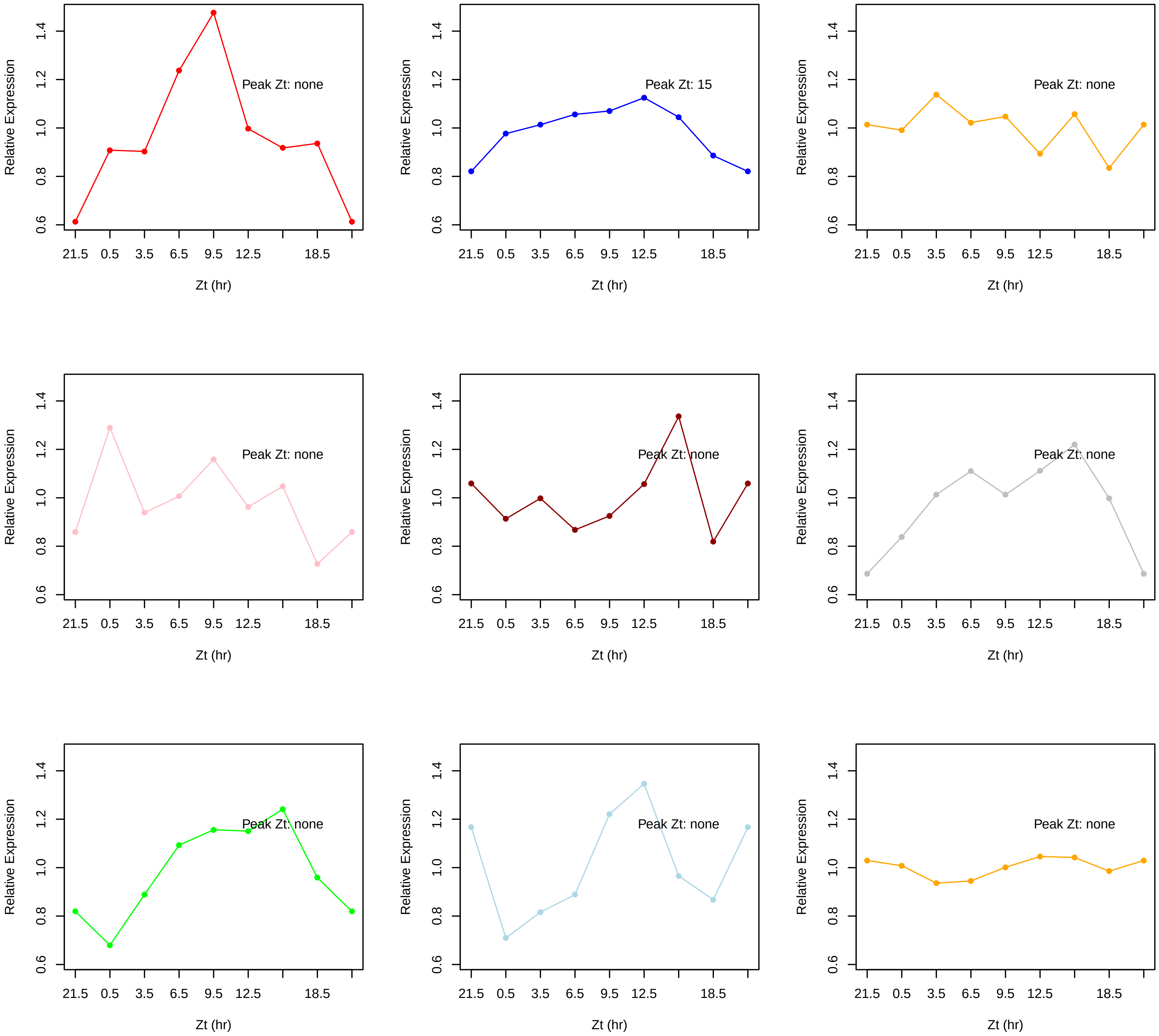

Singleton: ENSXMAG00000019477 mgat2

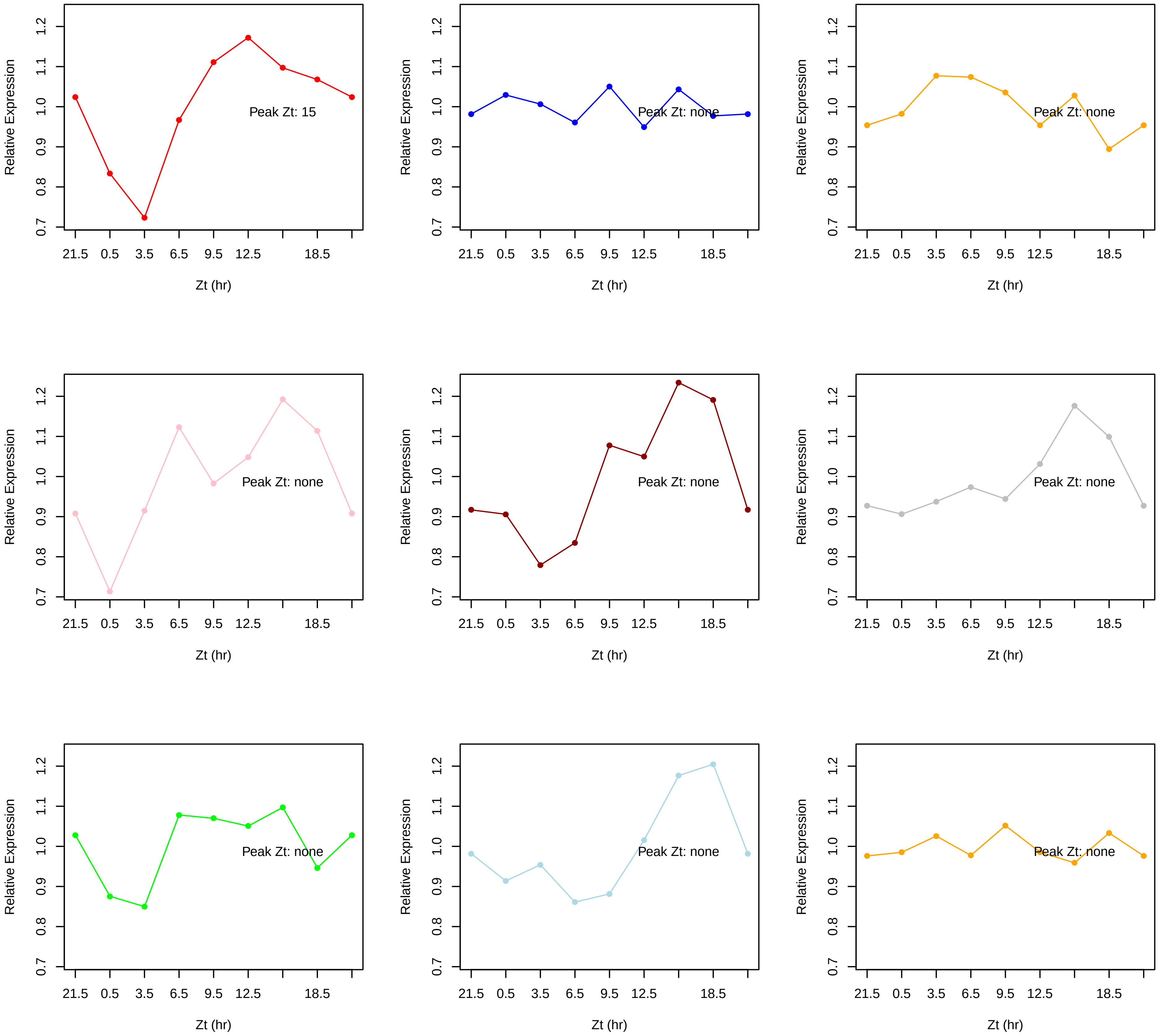

Singleton: ENSXMAG00000029698

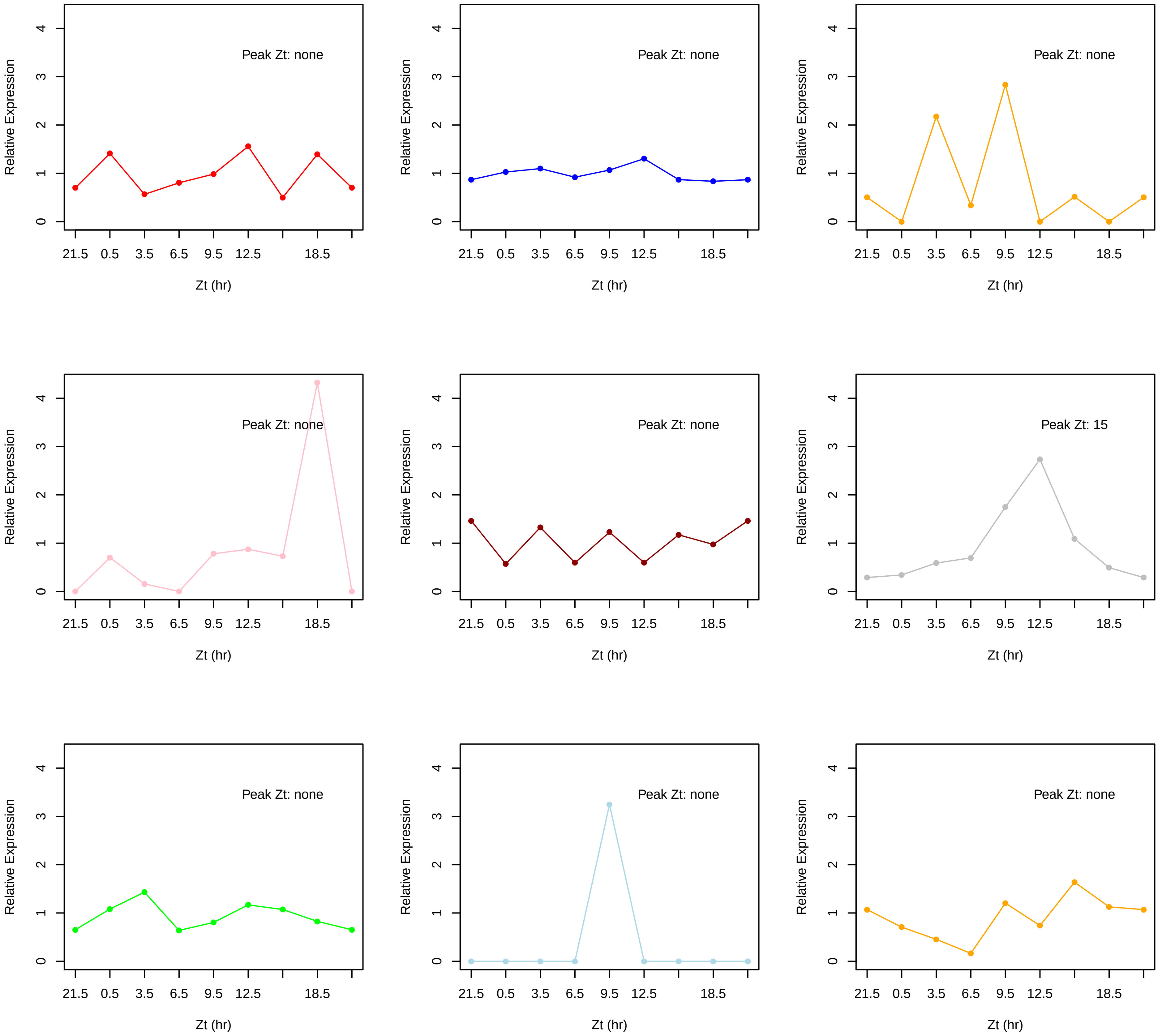

Singeleton: ENSXMAG00000020208 cyp1c1

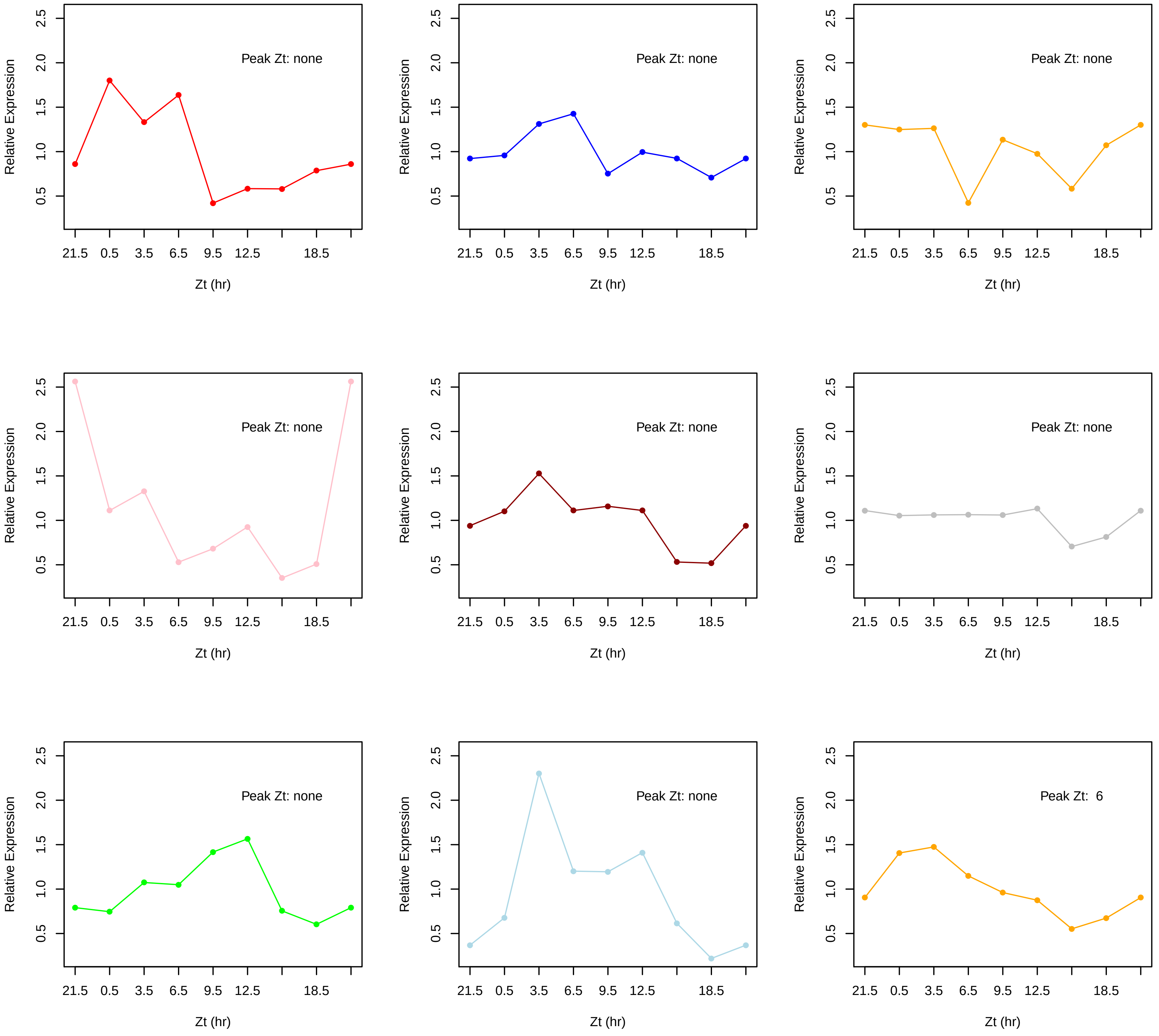

Singleton: ENSXMAG00000020178 zbtb1

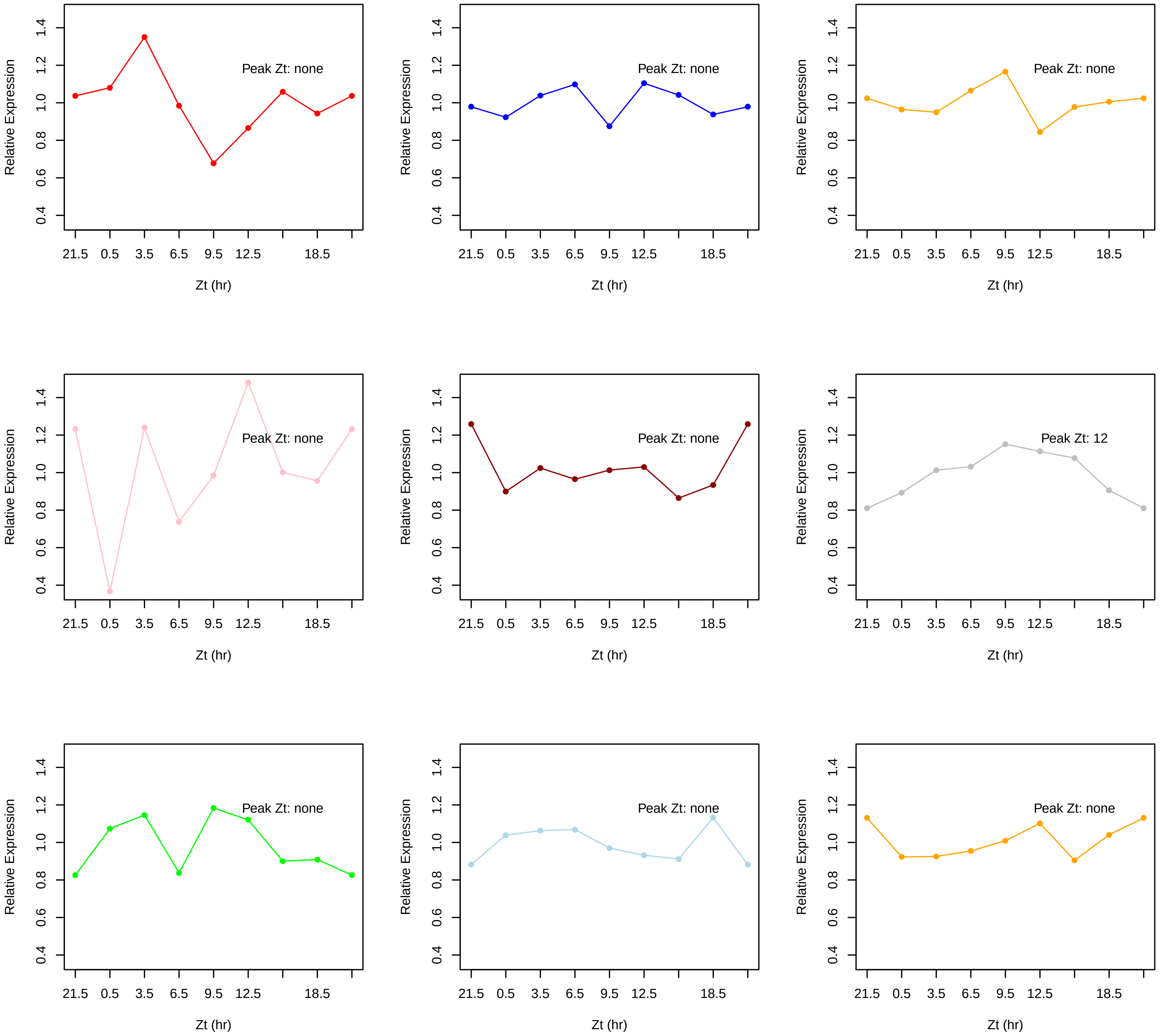

Singleton: ENSXMAG00000019446 cdca4

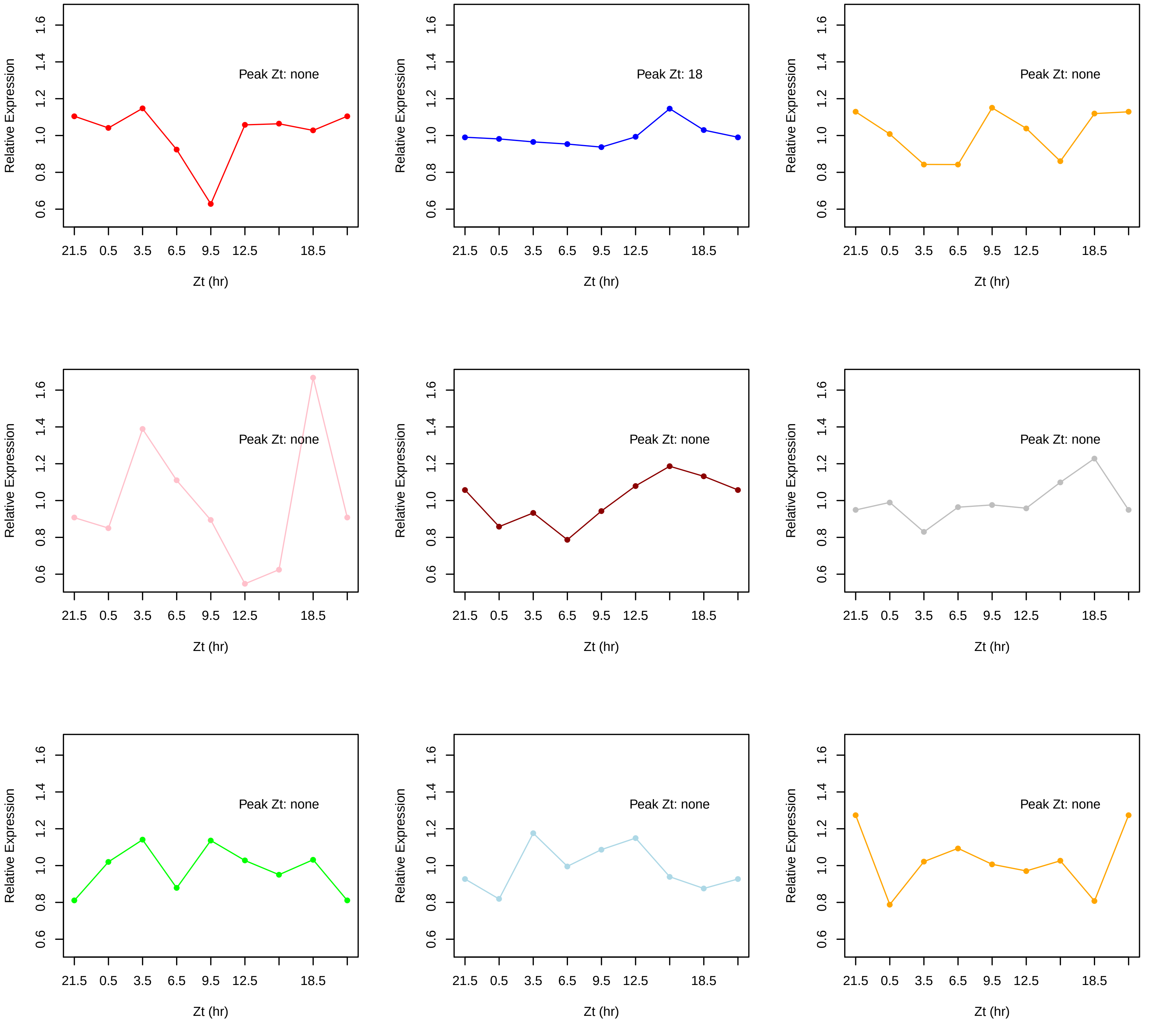

Singeleton: ENSXMAG00000026298 GPR68

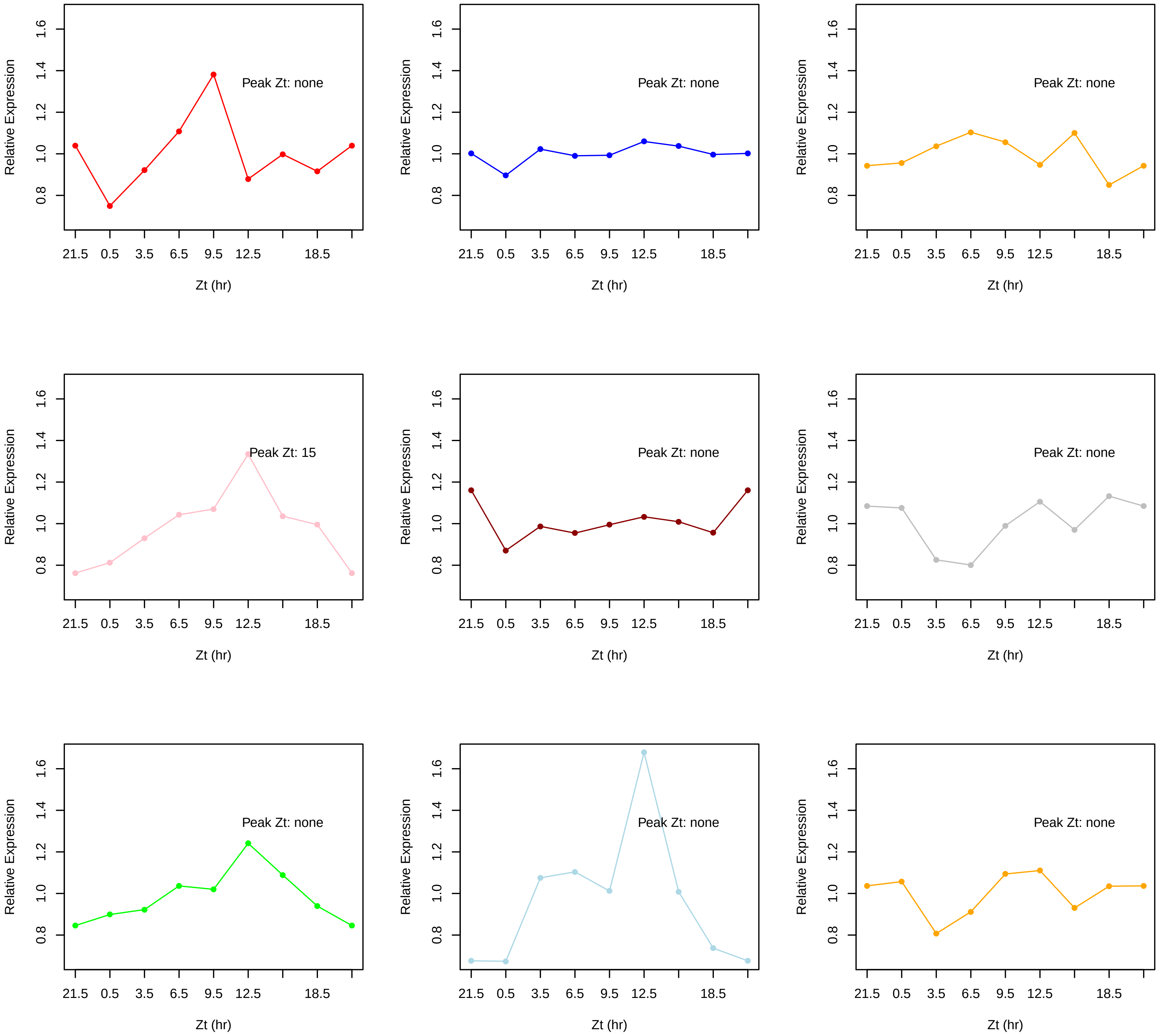

Singleton: ENSXMAG00000024240 si:ch211−137a8.4

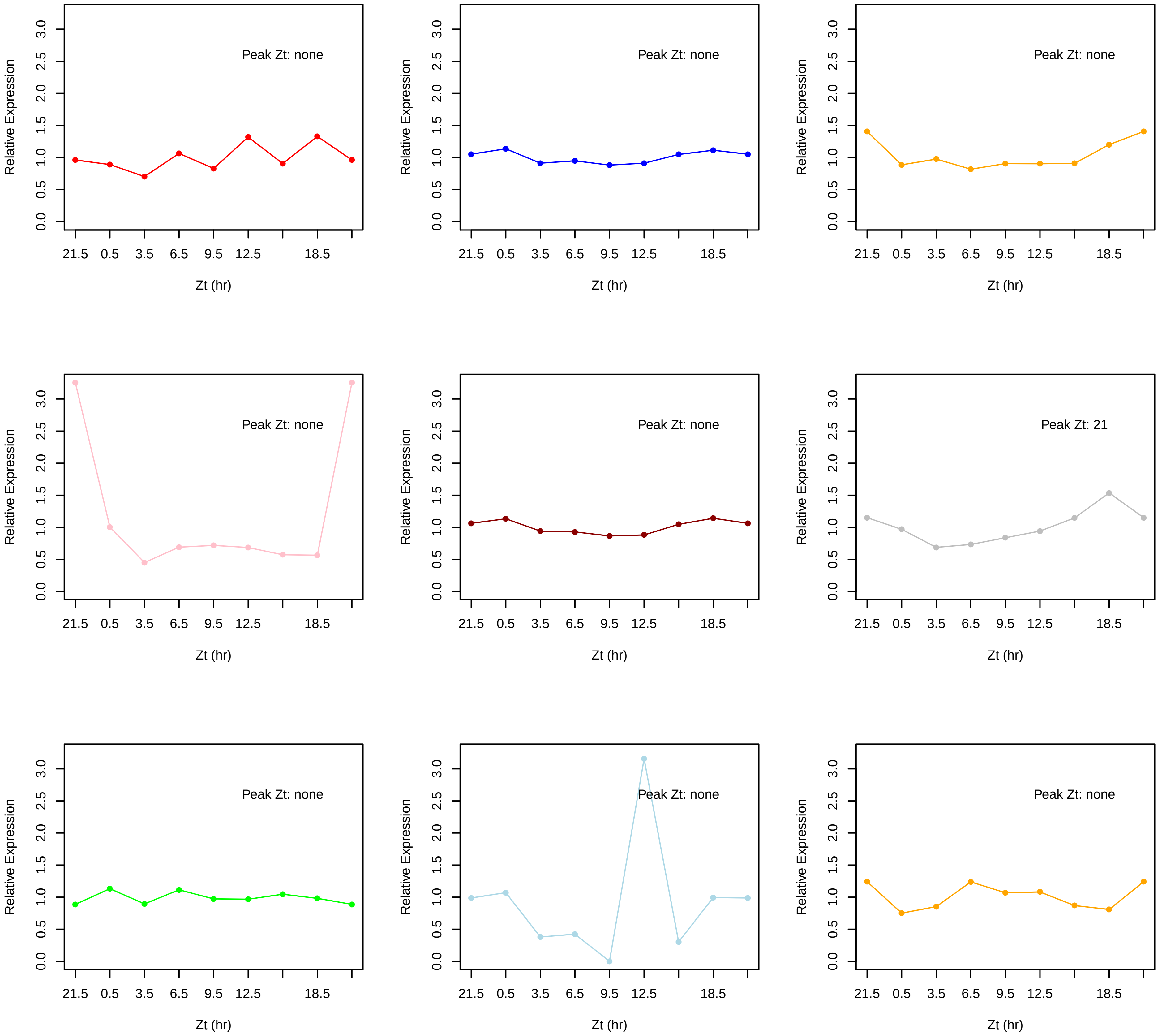

Singeleton: ENSXMAG00000004081

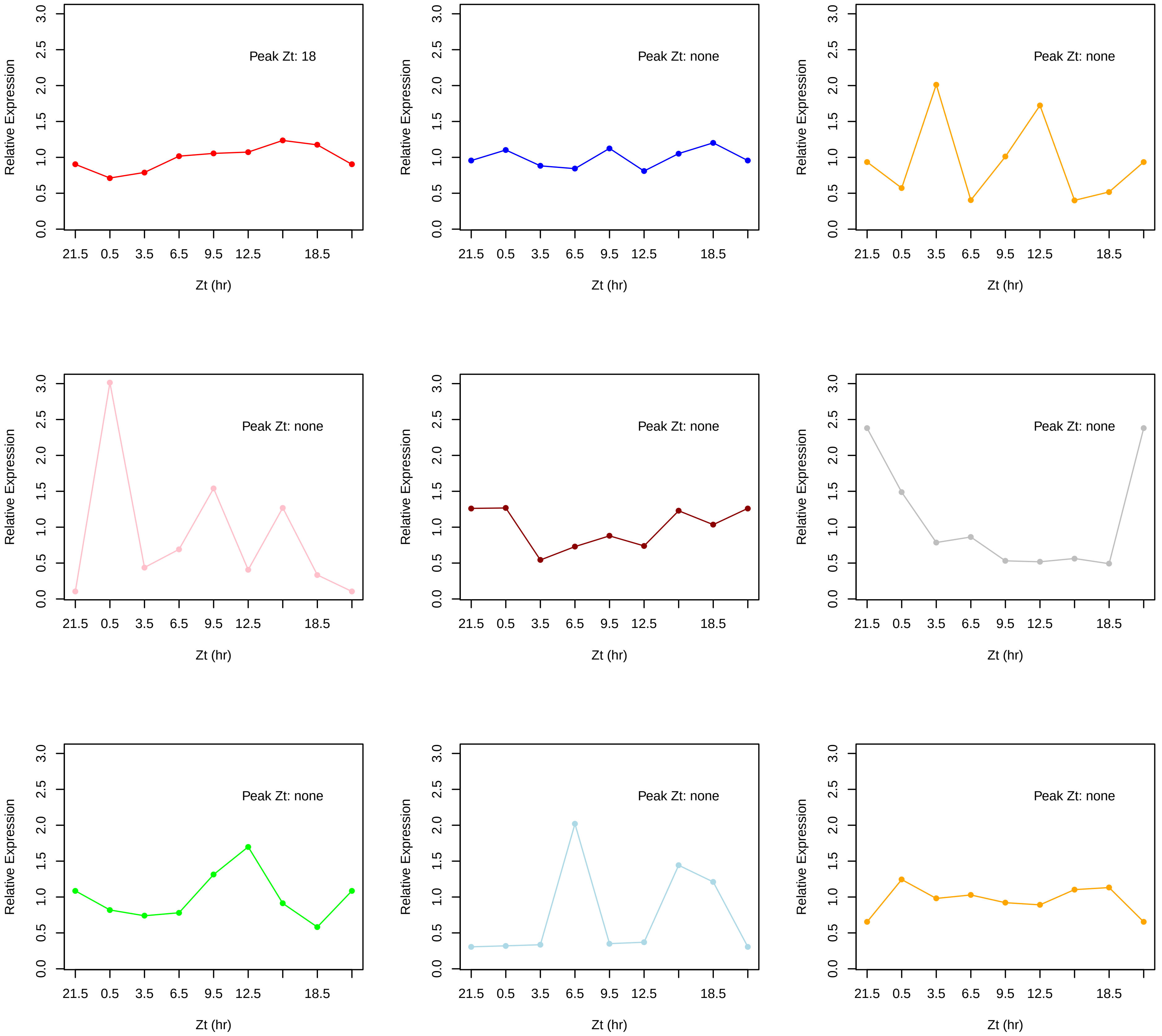

Singeleton: ENSXMAG00000004087 gpr184

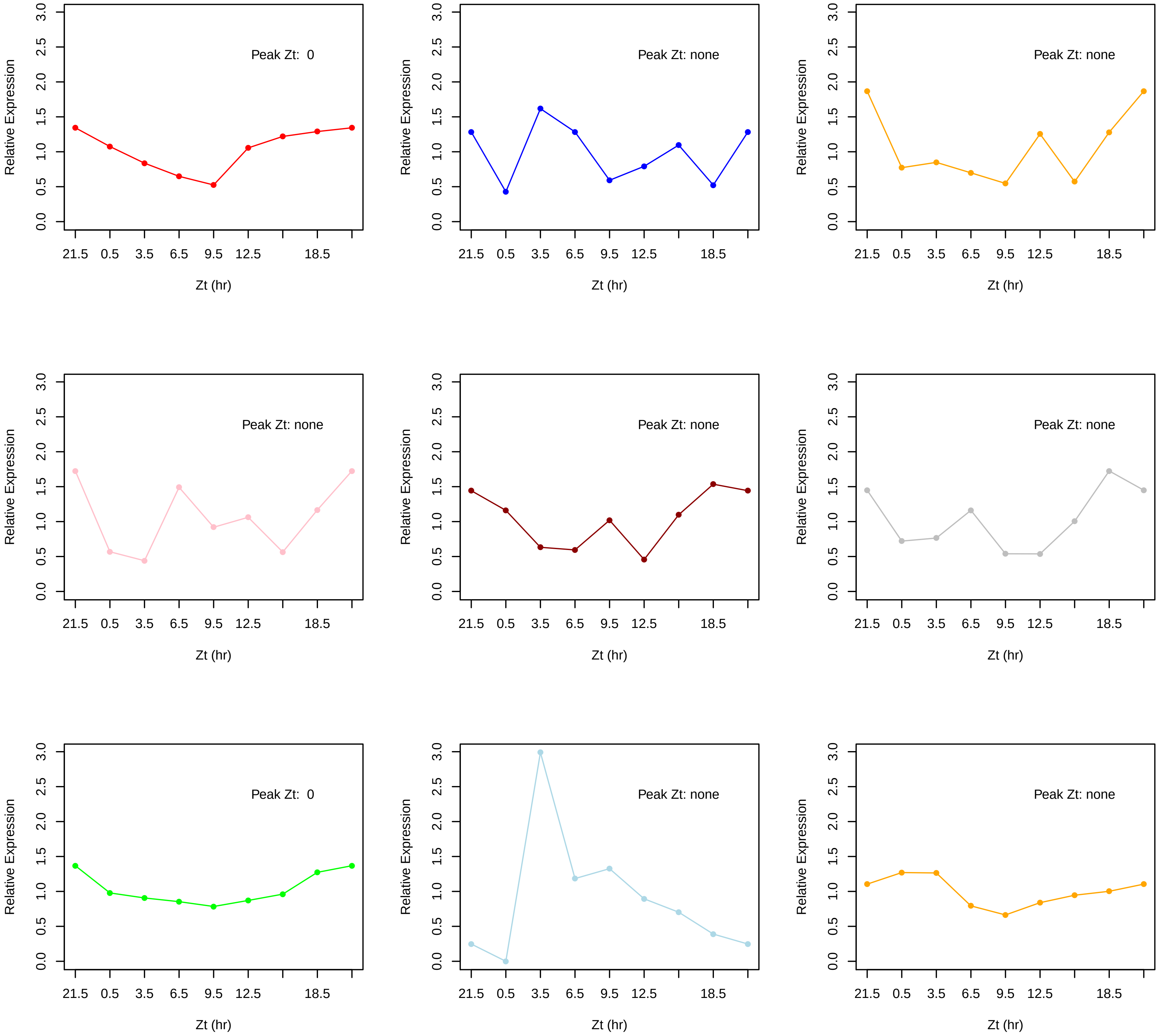

Singeleton: ENSXMAG00000019558 lrrc8da

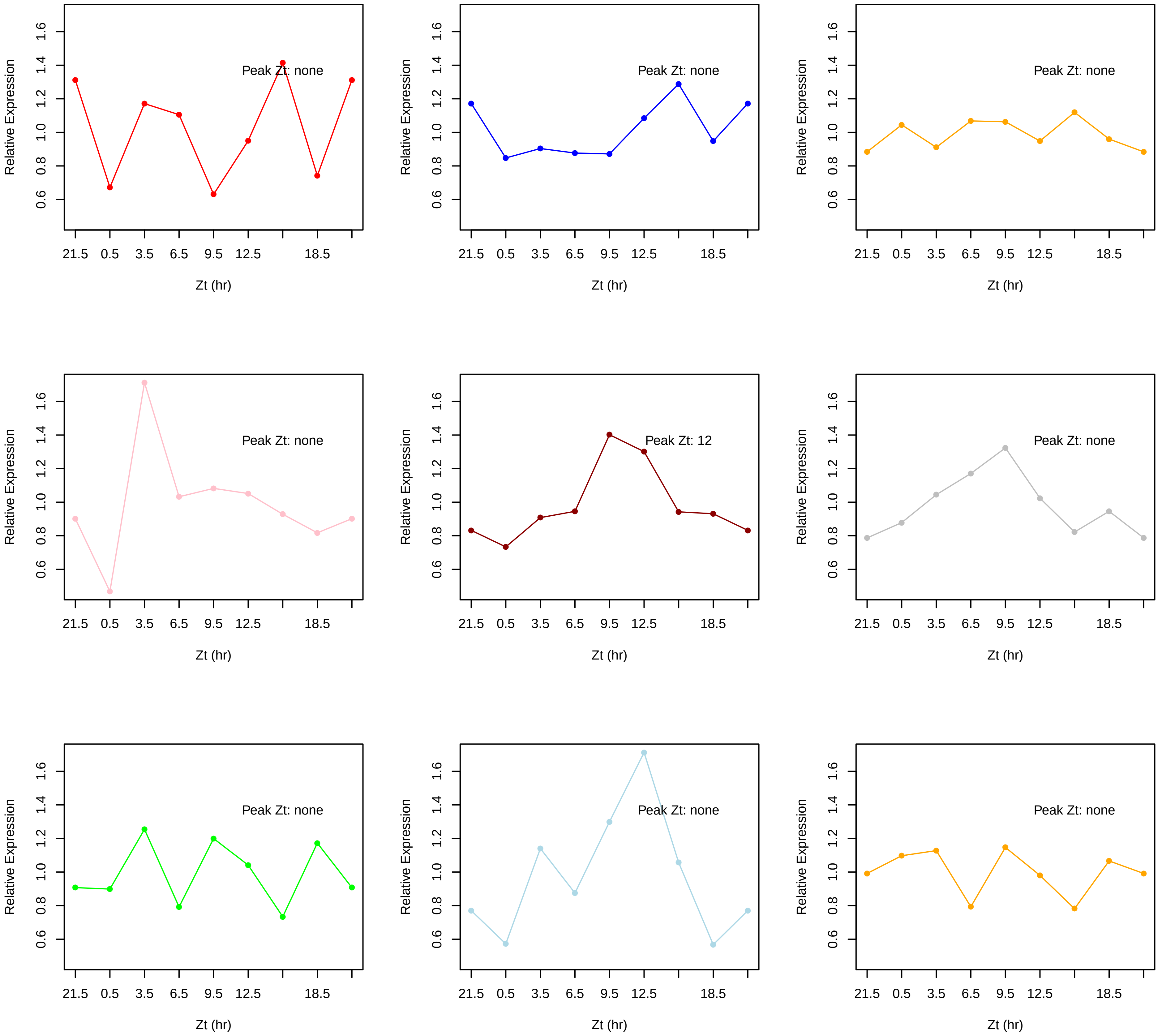

Singleton: ENSXMAG00000029818 foxd3

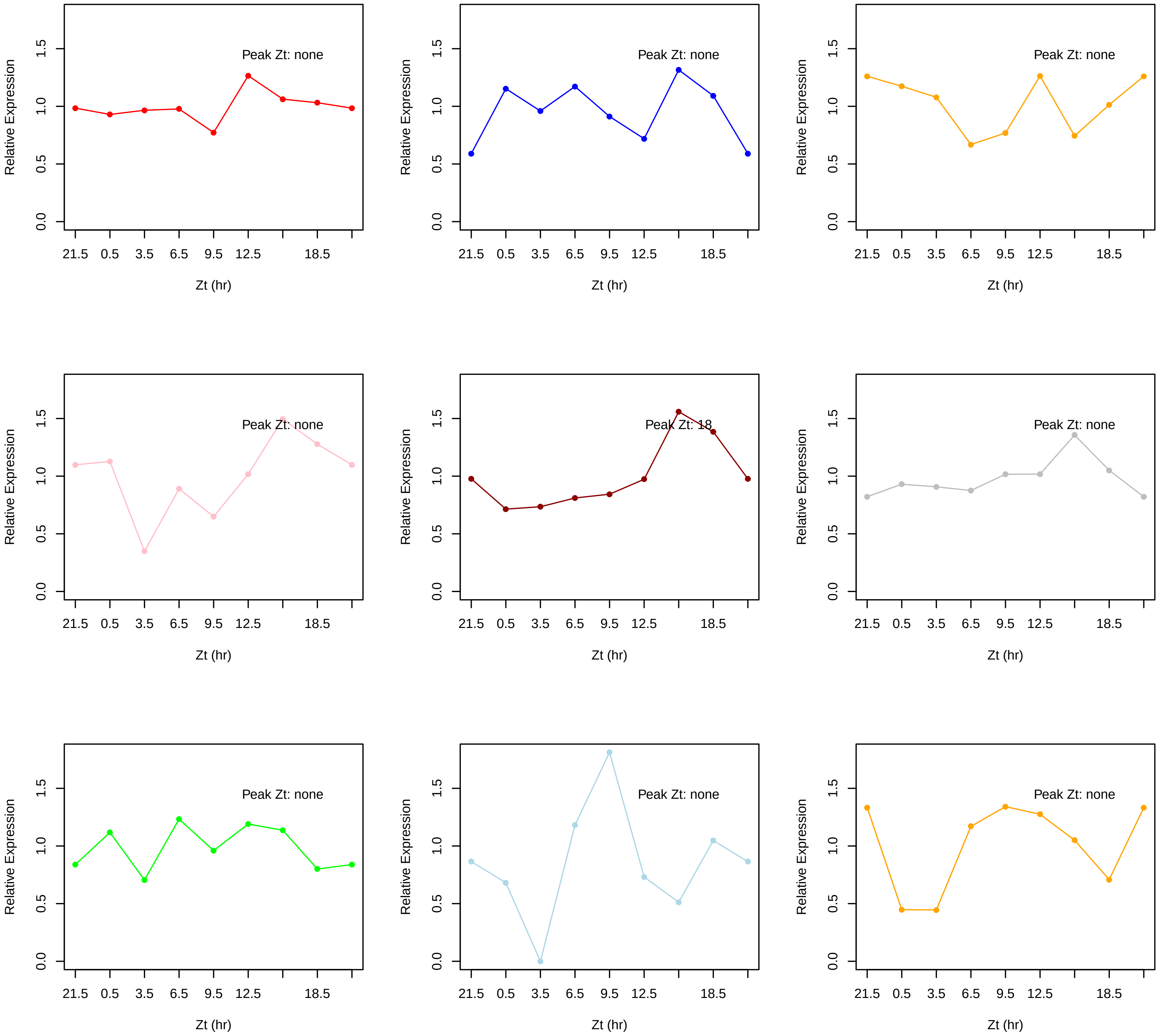

Singeleton: ENSXMAG00000027316

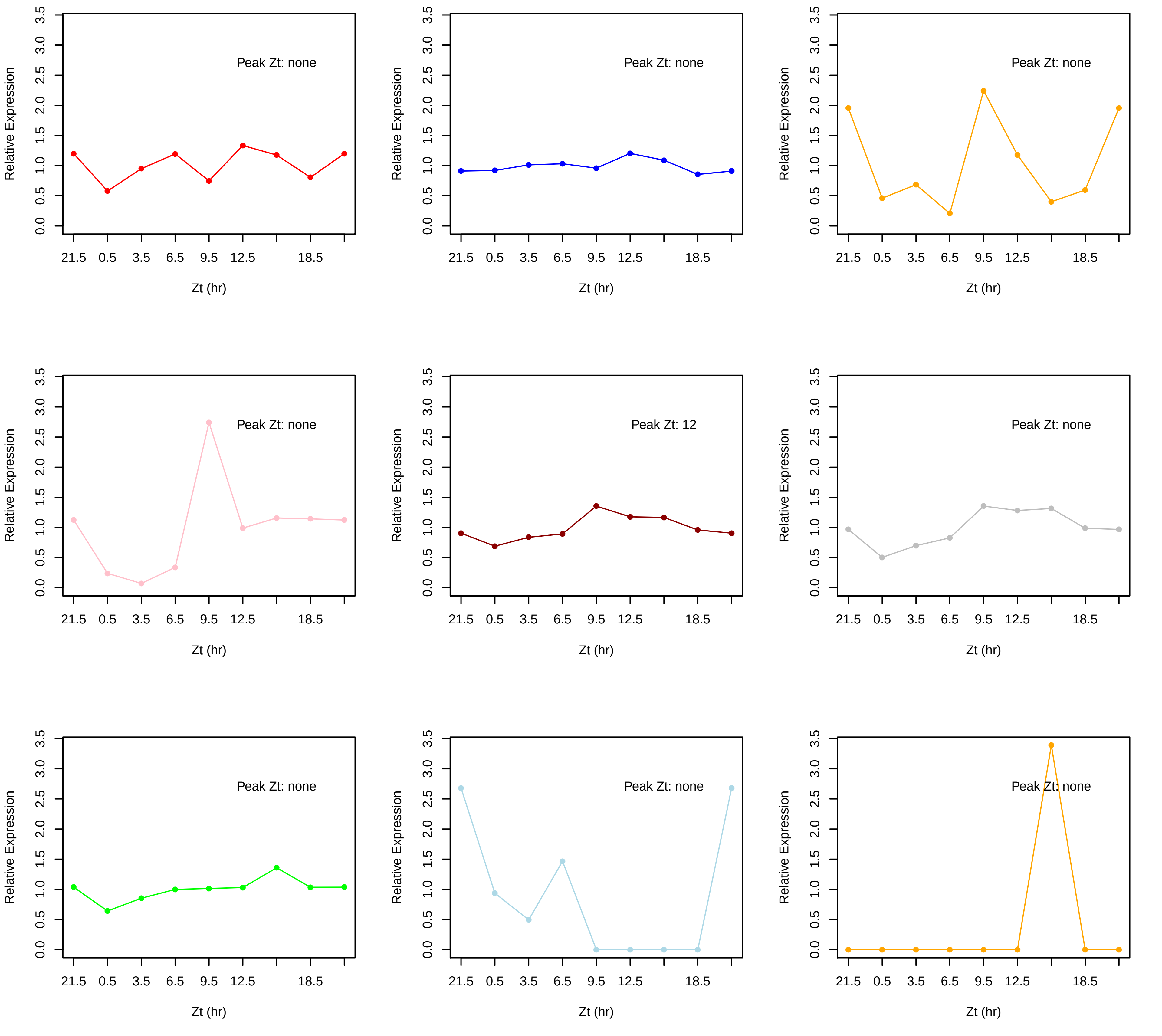

Singeleton: ENSXMAG00000001025 lipt1

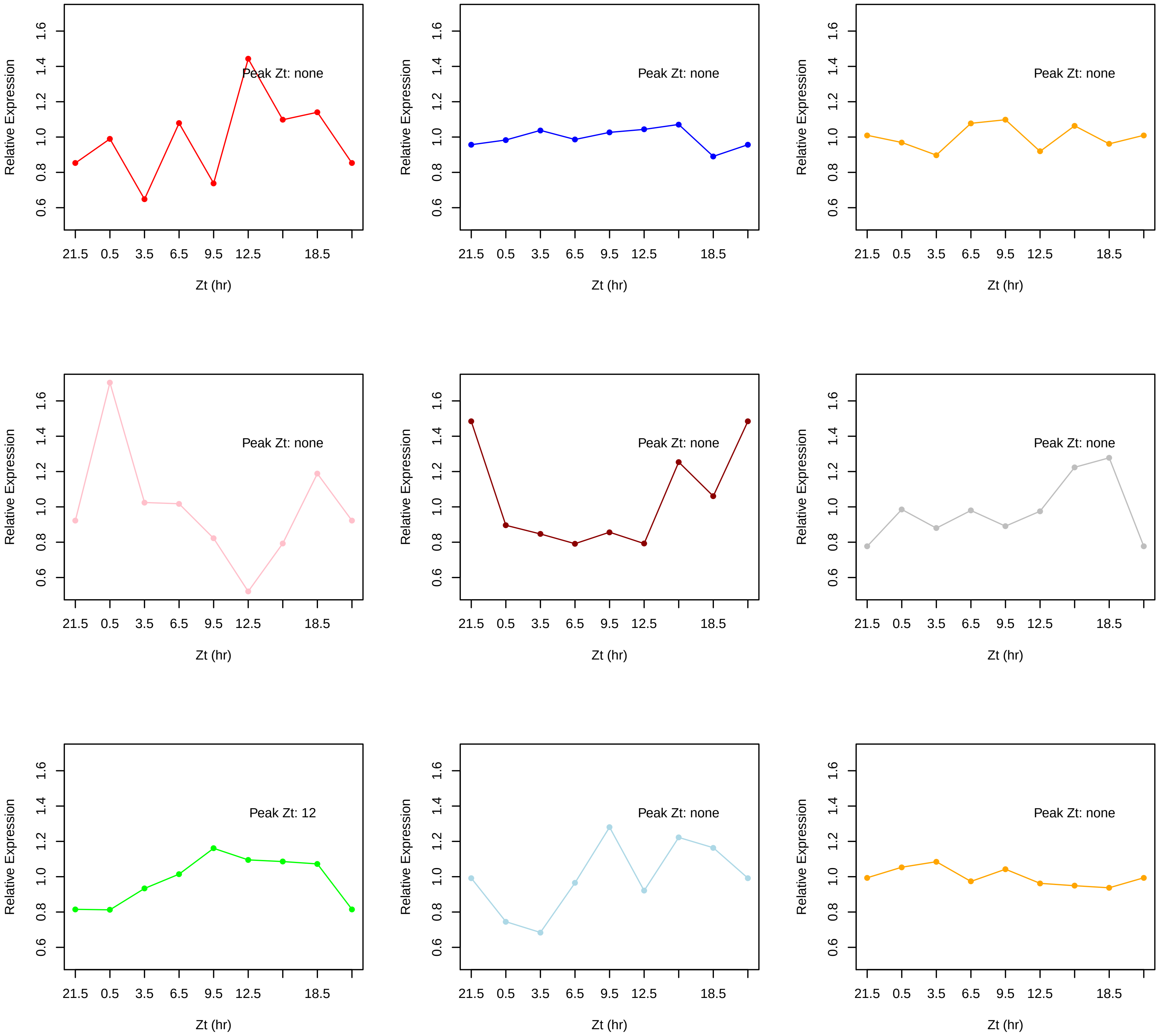

Singeleton: ENSXMAG00000019500

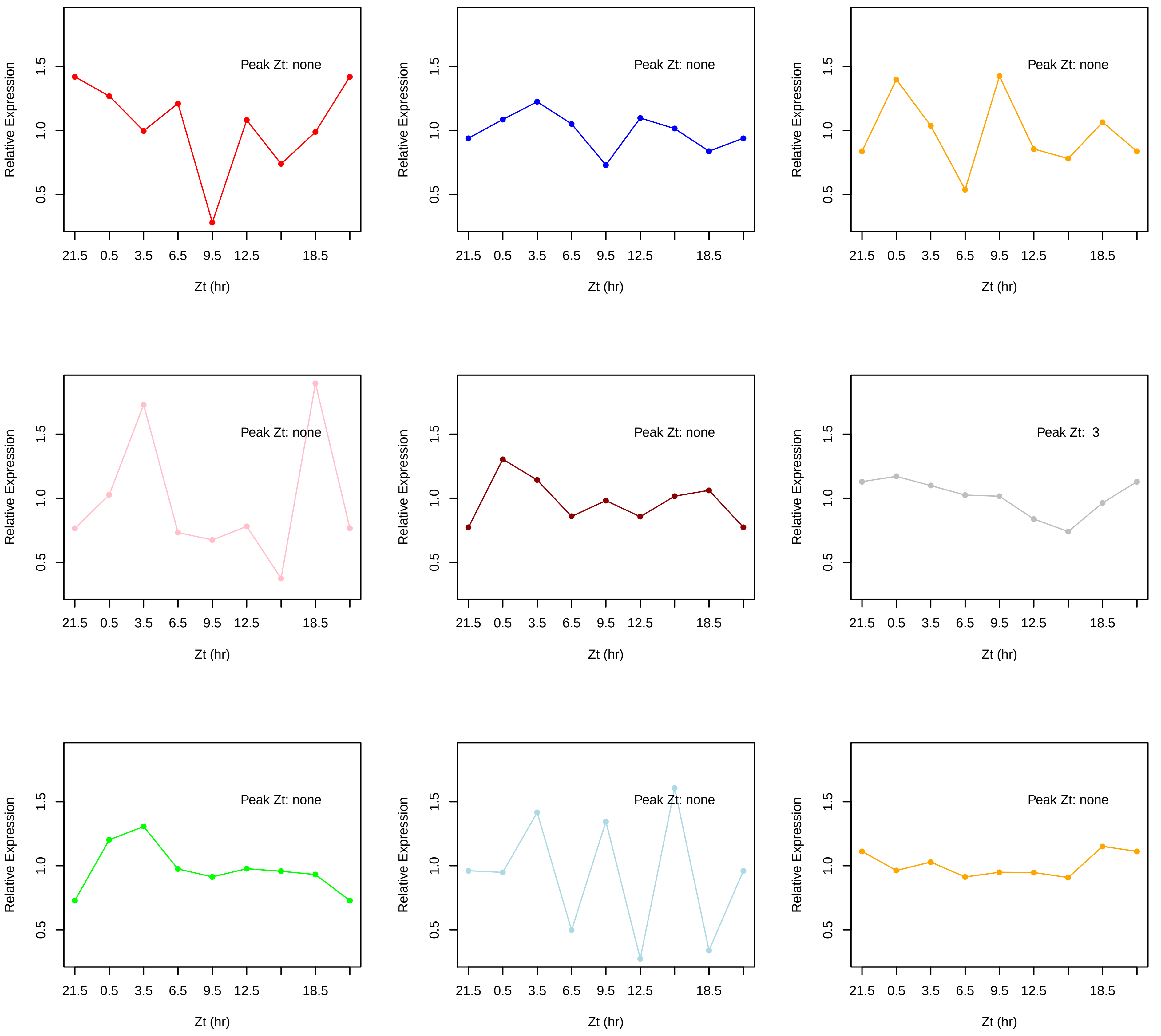

Singleton: ENSXMAG00000025172 slitrk6

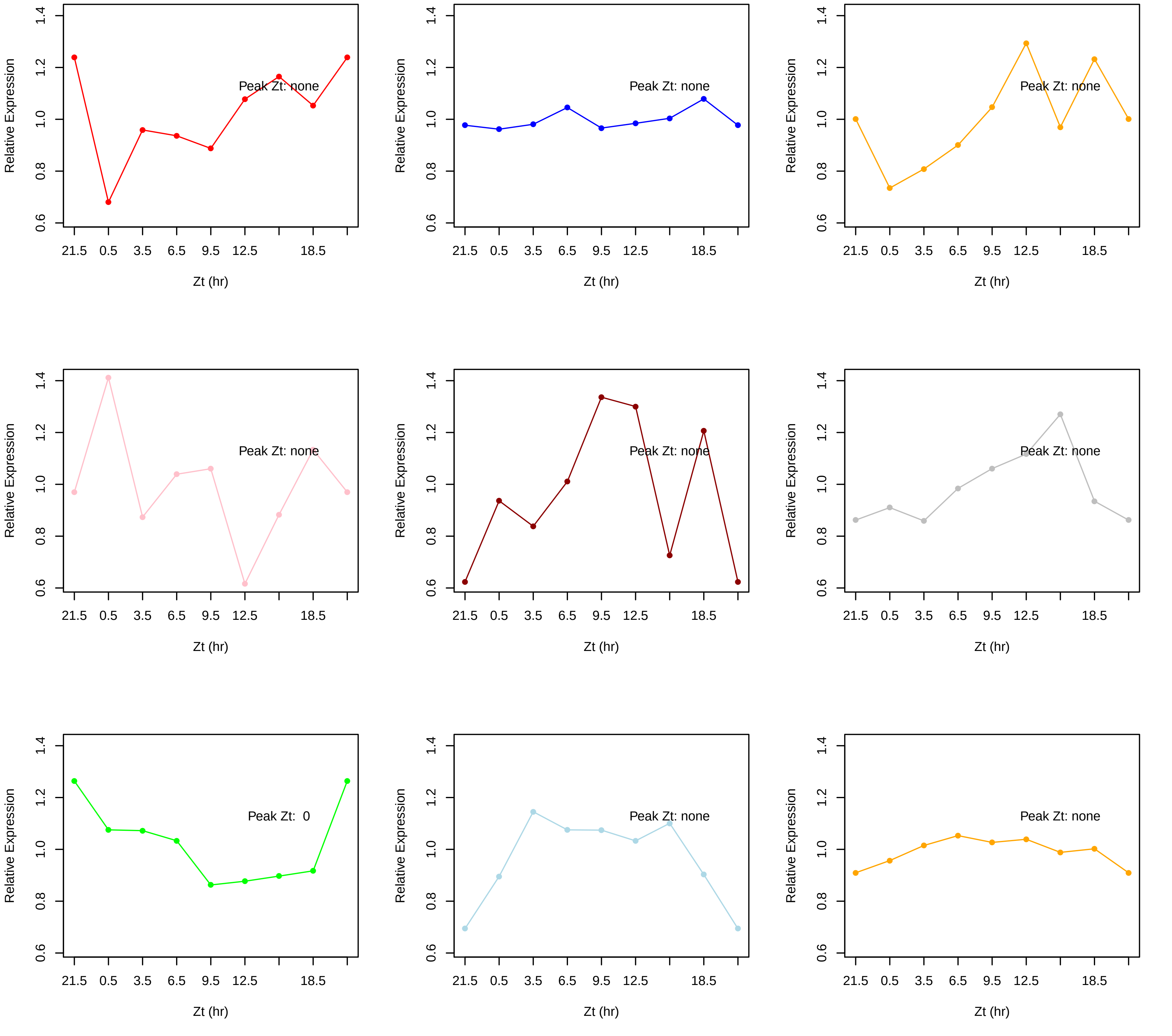

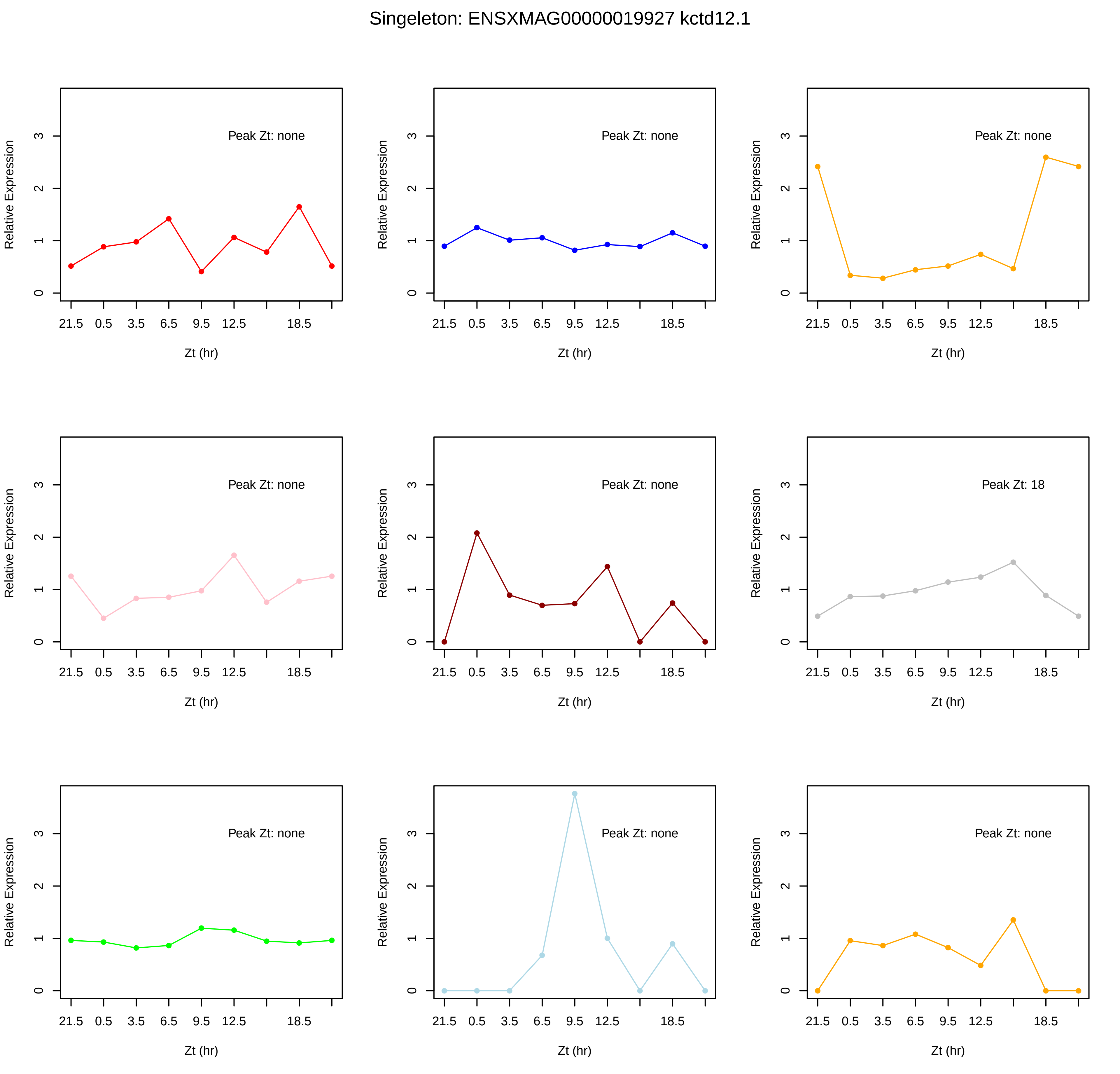
Singeleton: ENSXMAG00000019927 kctd12.1
Peak Zt: none
Peak Zt: none
Peak Zt: none
Peak Zt: none
Peak Zt: none
Peak Zt: 18
Peak Zt: none
Peak Zt: none
Peak Zt: none
Relative Expression
Zt (hr)
21.5 0.5 3.5 6.5 9.5 12.5 18.5

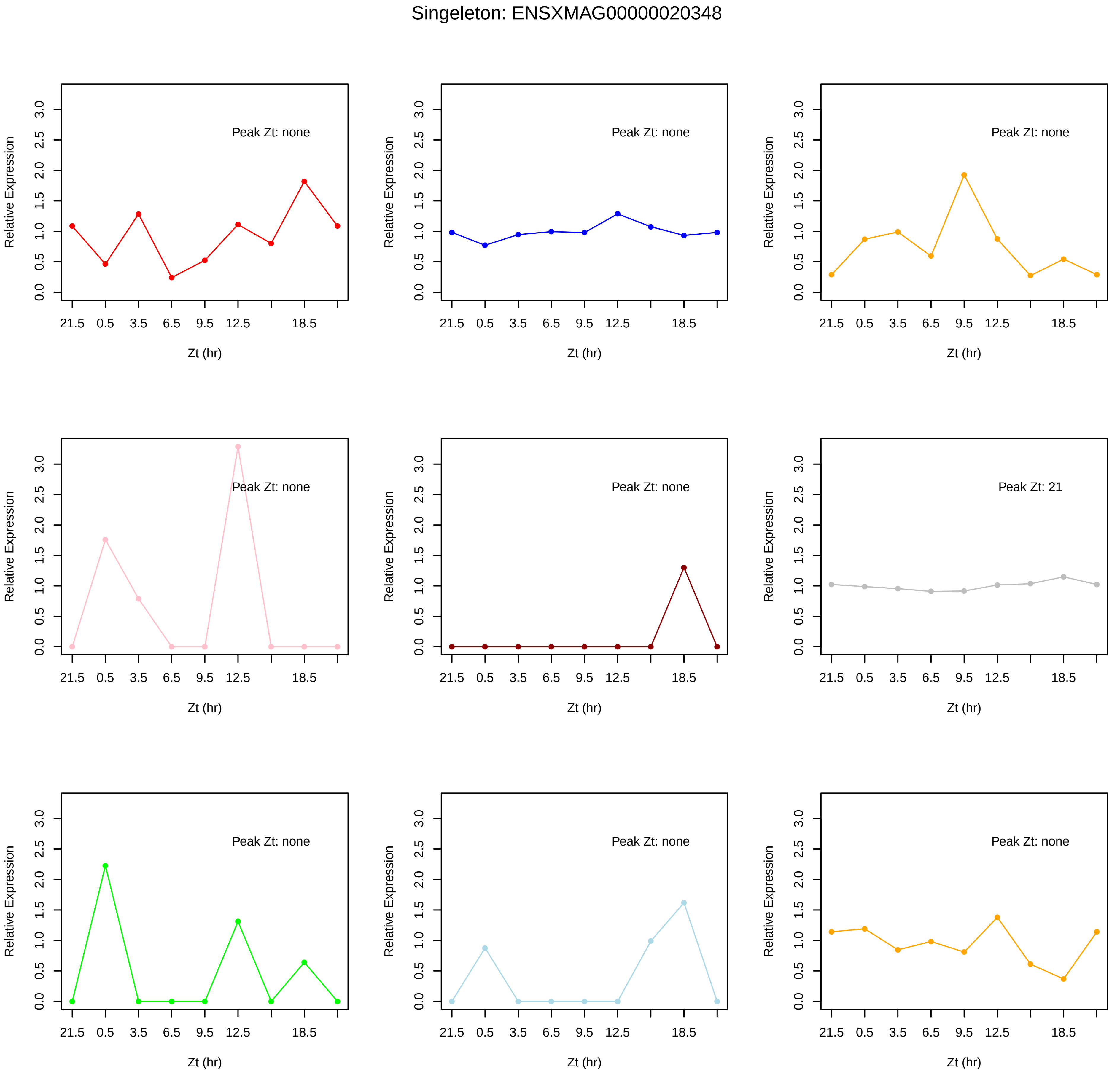
Singeleton: ENSXMAG00000020348
Peak Zt: none
Peak Zt: none
Peak Zt: none
Peak Zt: none
Peak Zt: none
Peak Zt: 21
Peak Zt: none
Peak Zt: none
Peak Zt: none
Relative Expression
Zt (hr)
21.5 0.5 3.5 6.5 9.5 12.5 18.5
0.0 0.5 1.0 1.5 2.0 2.5 3.0

Singeleton: ENSXMAG00000019936 chst7

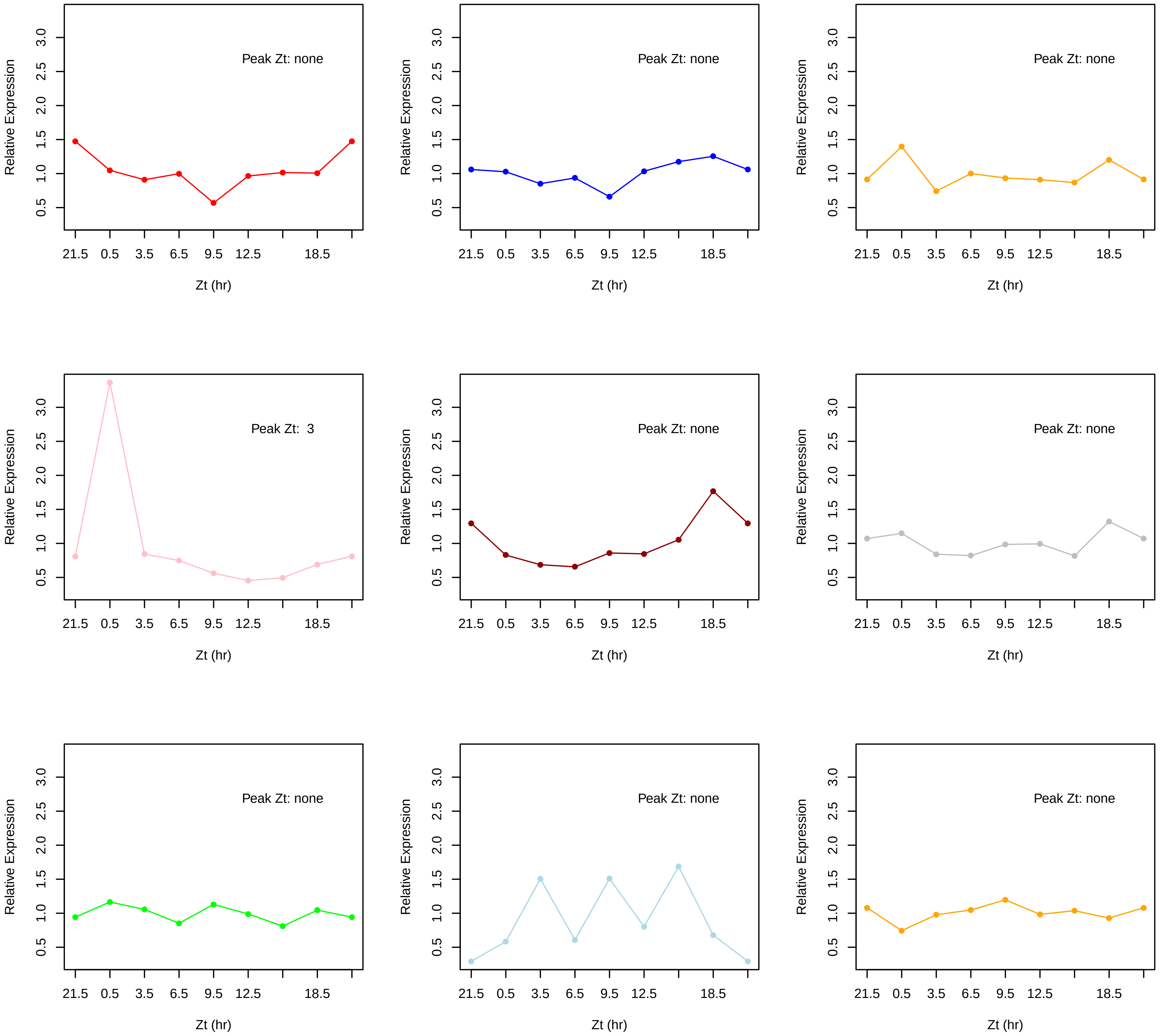

Singeleton: ENSXMAG00000019937 abhd13

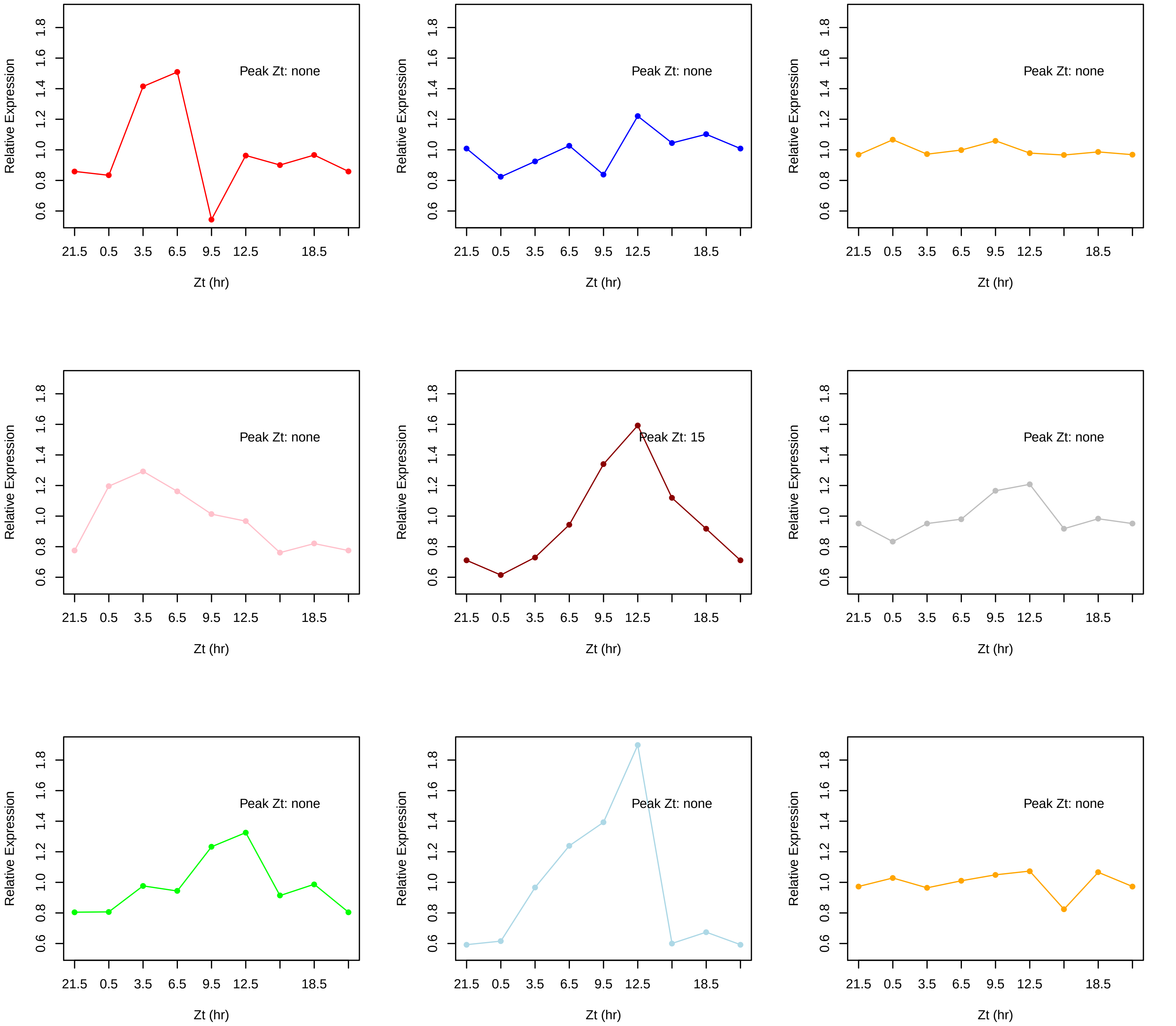

Singeleton: ENSXMAG00000028981

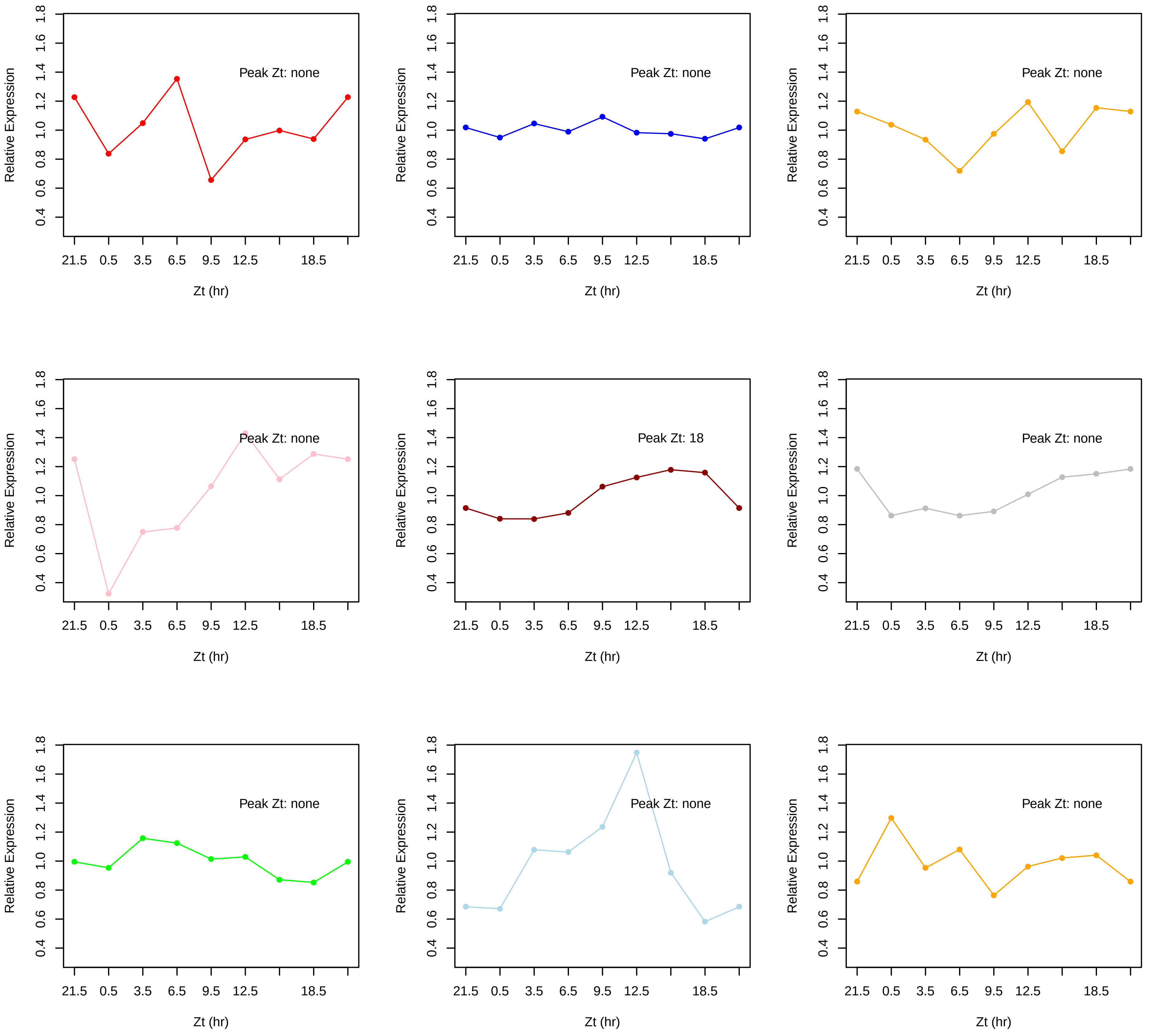

Singeleton: ENSXMAG00000023917 ZBTB14

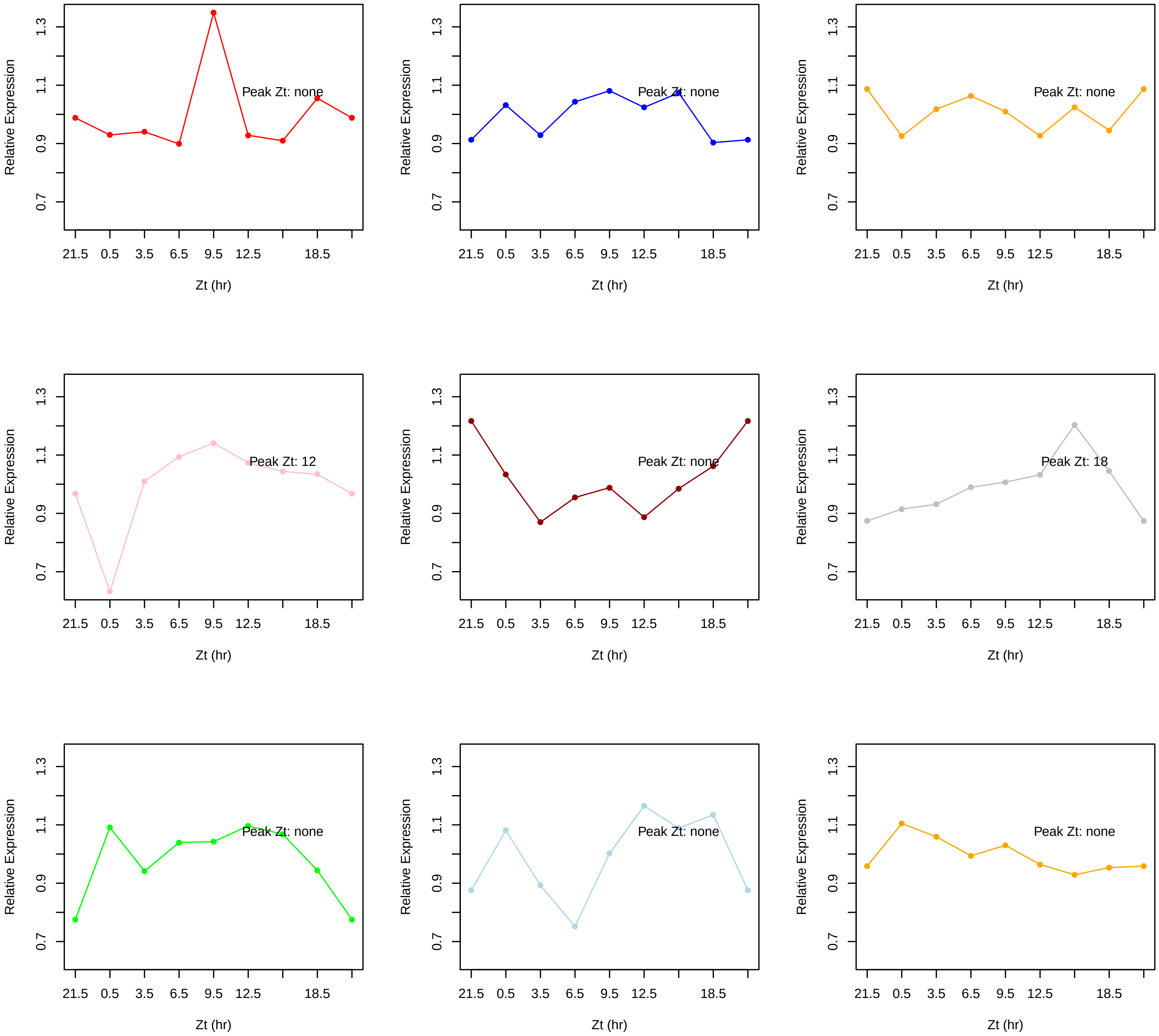

Singeleton: ENSXMAG00000001961 tmem177

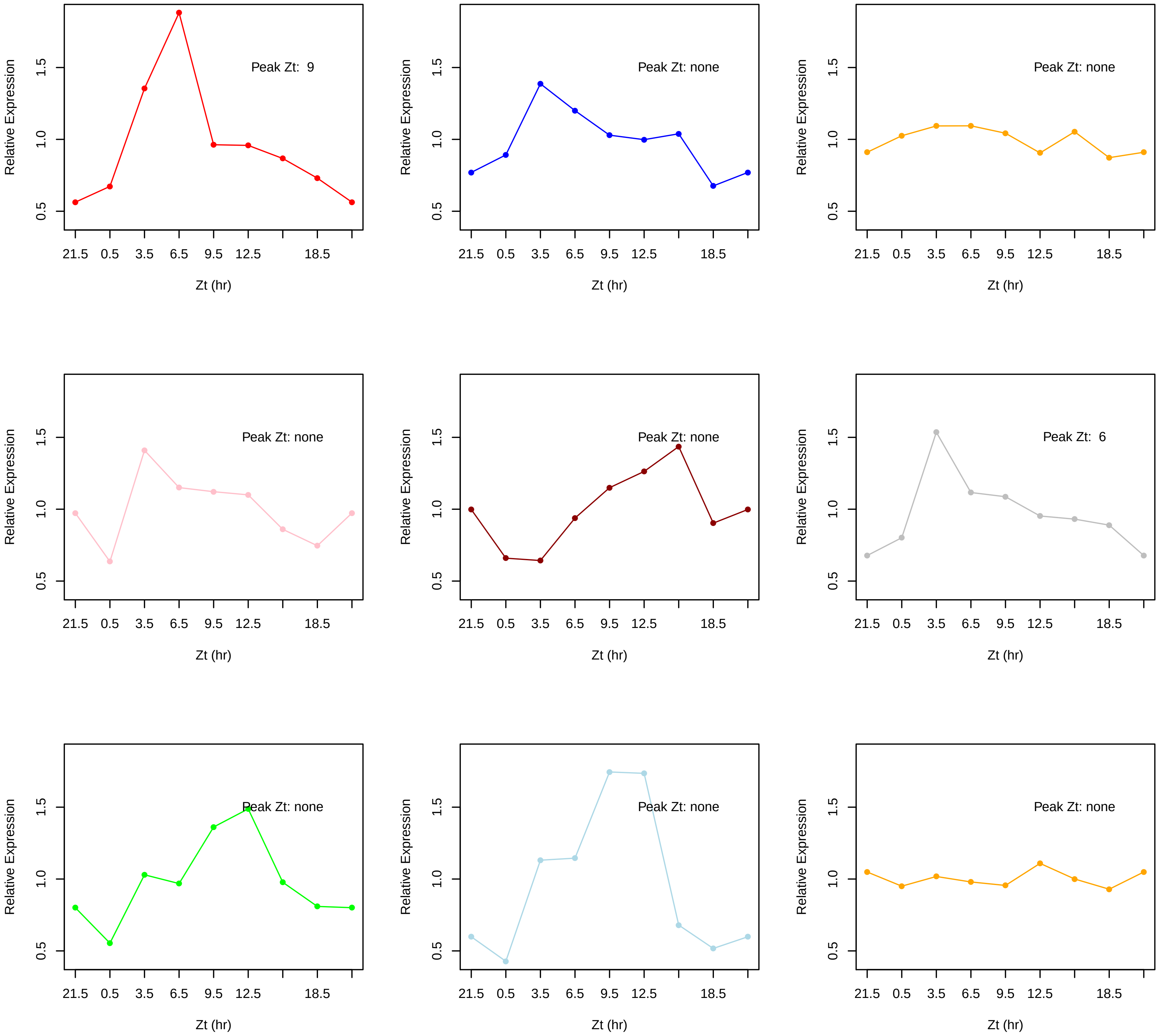

Singeleton: ENSXMAG00000020059 cdk5r2b

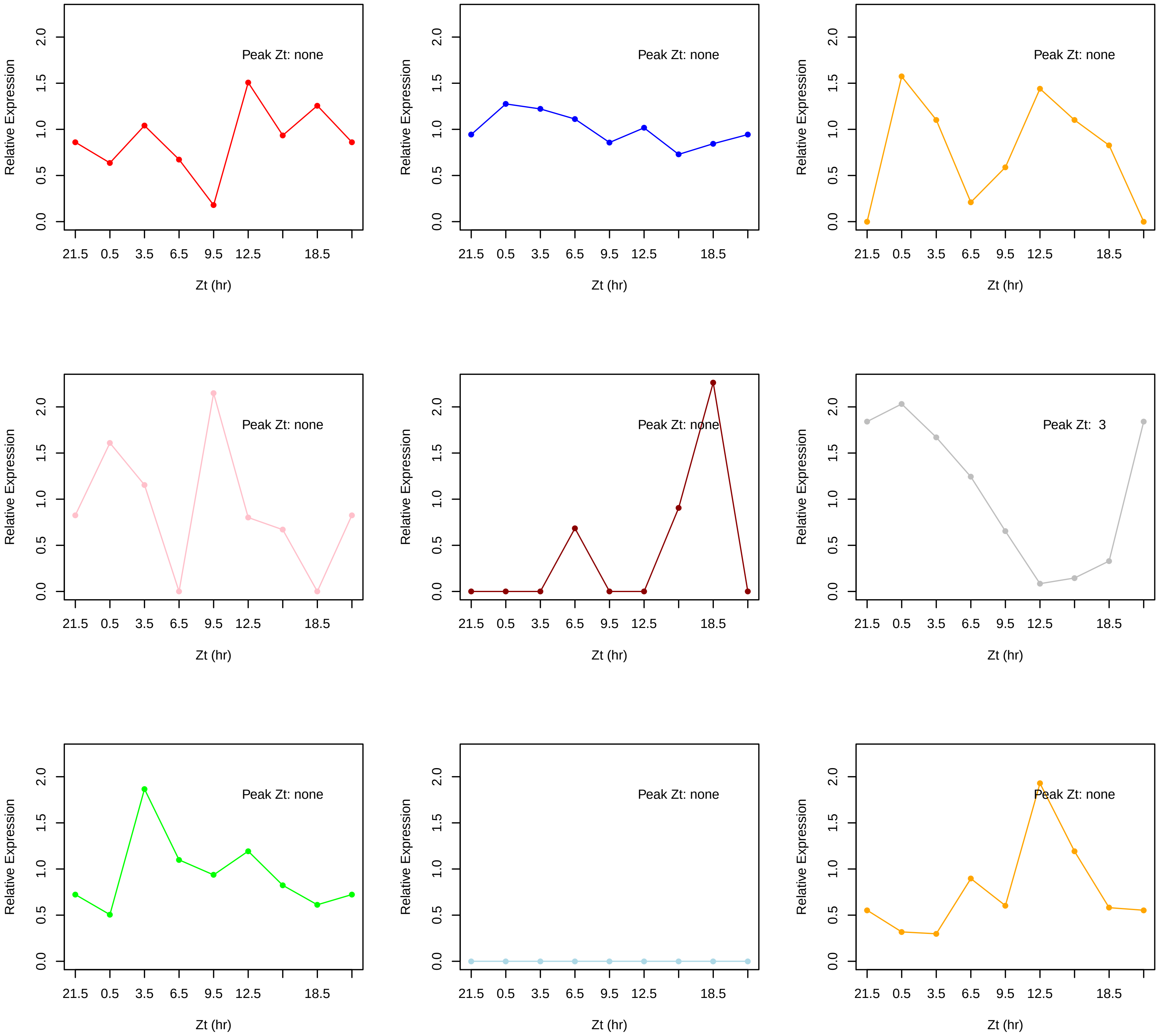

Singleton: ENSXMAG00000012162 phospho2

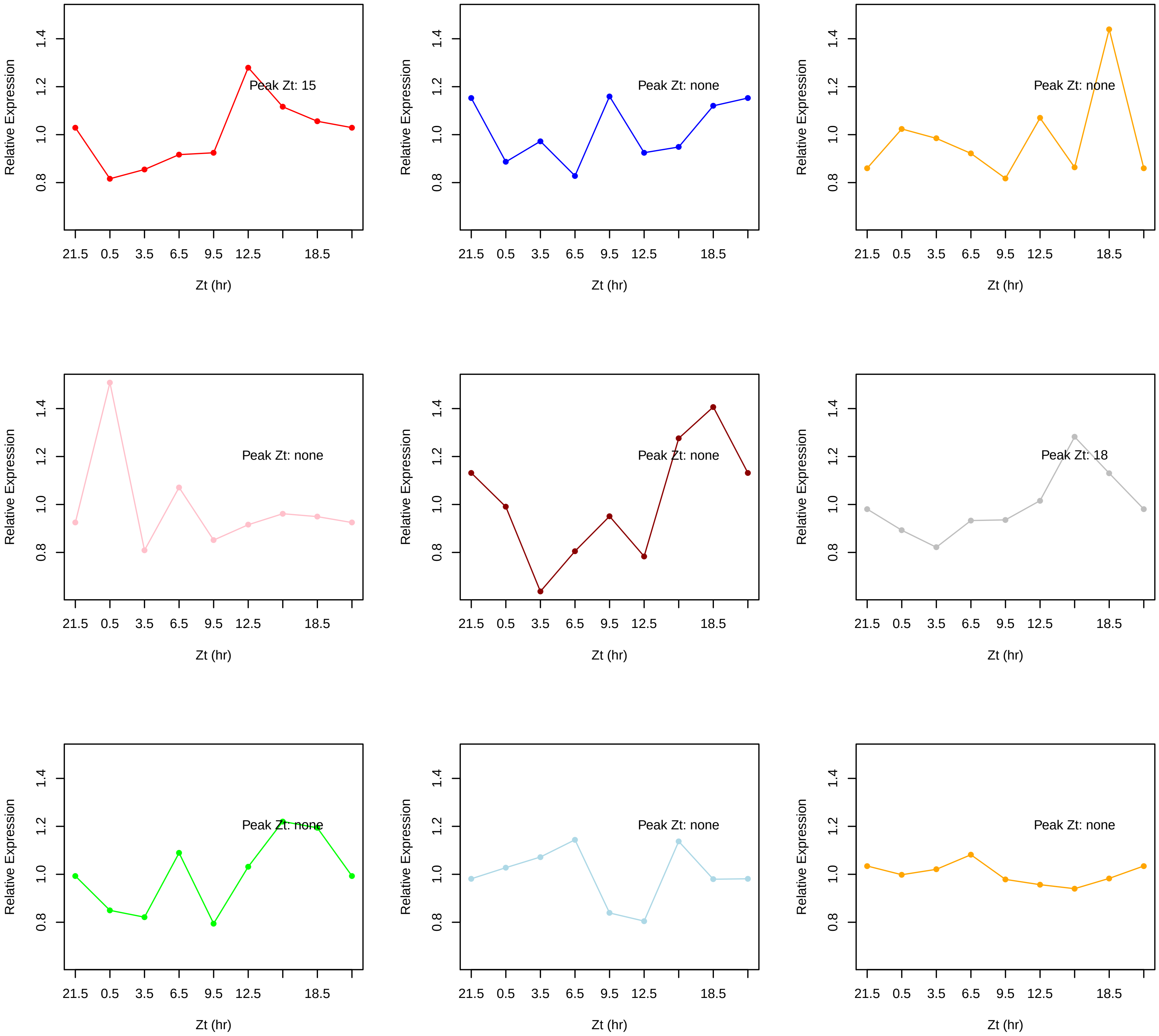

Singeleton: ENSXMAG00000026653

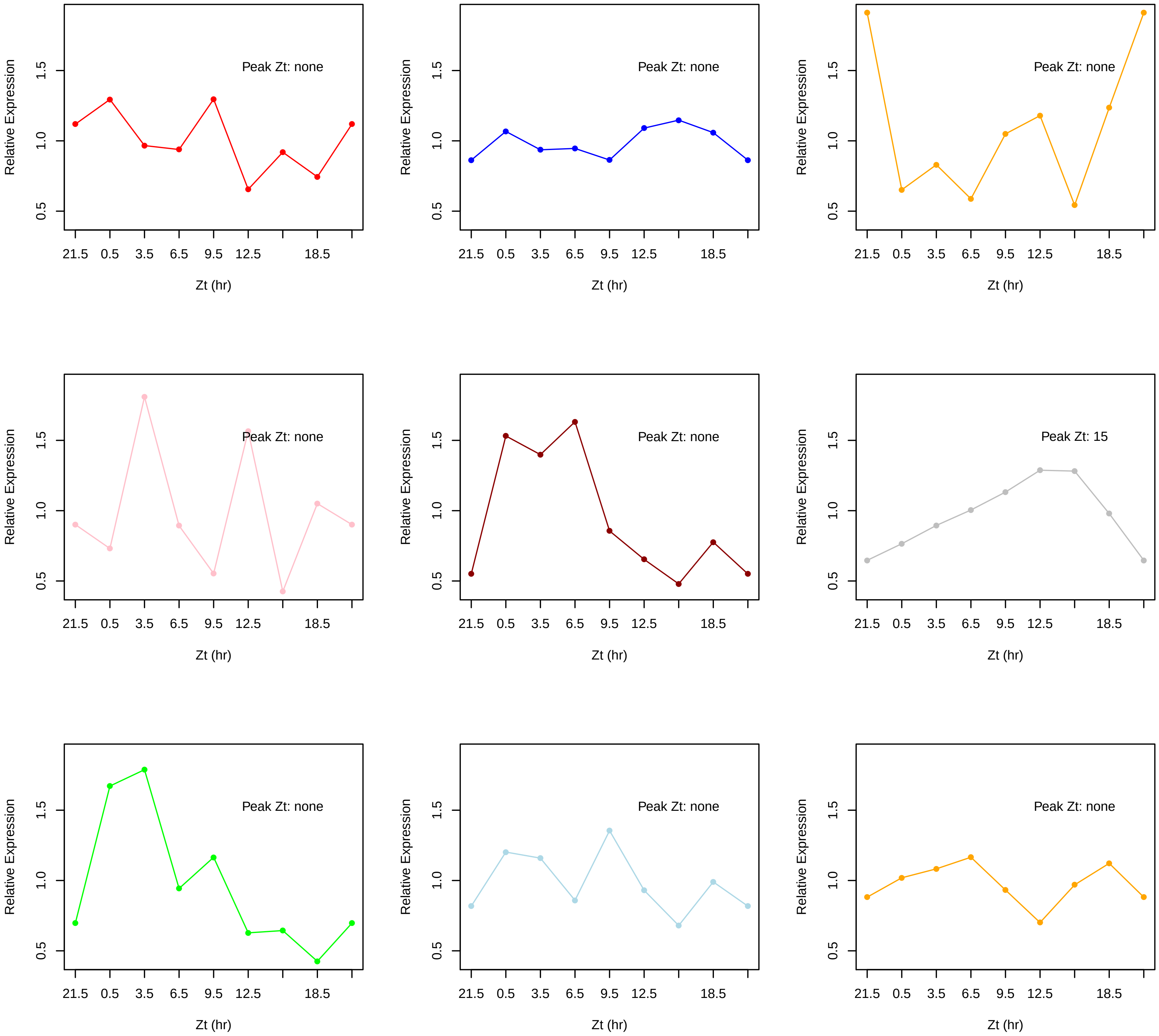

Singleton: ENSXMAG00000017101 fzd9b

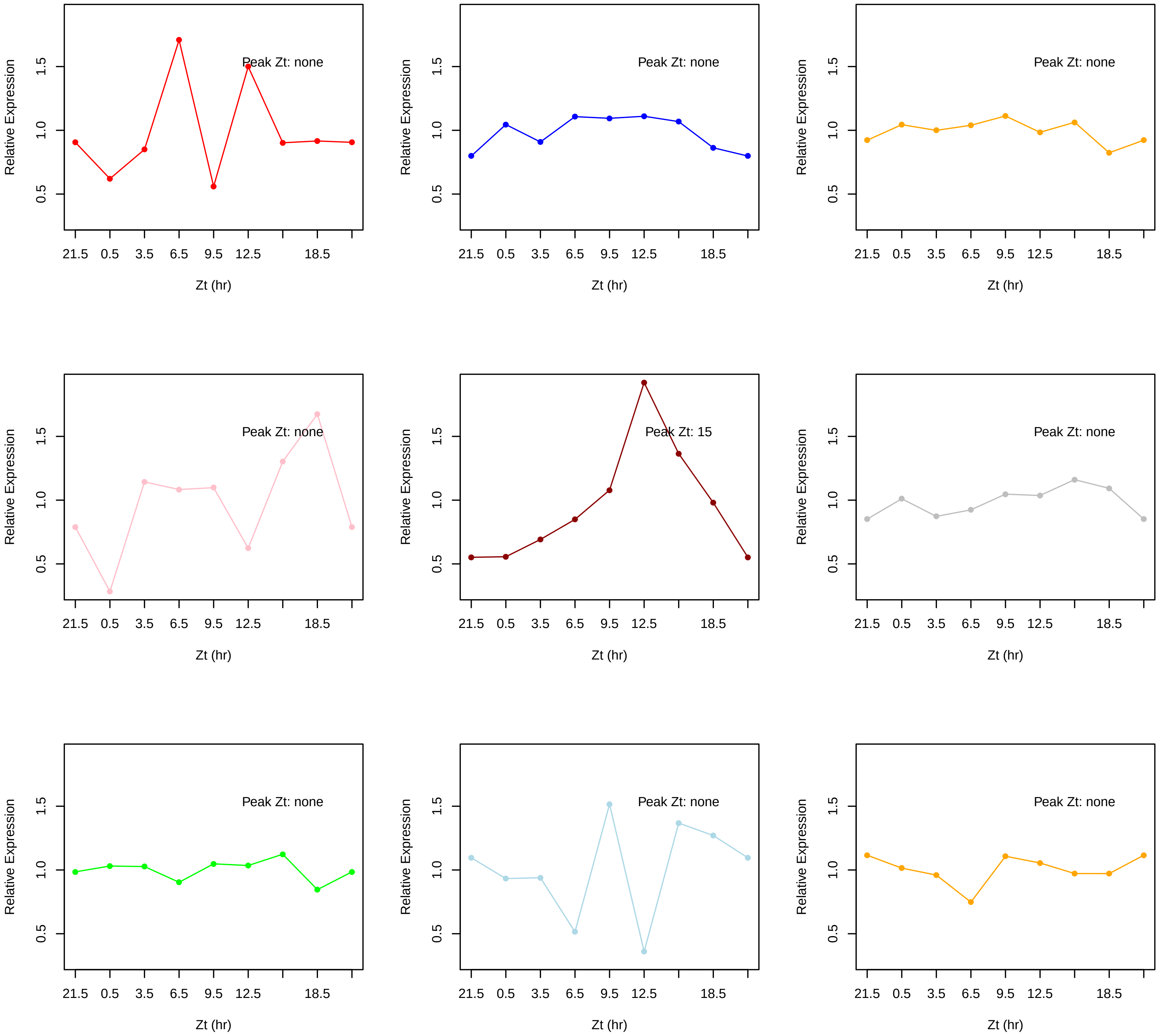

Singeleton: ENSXMAG00000005269 cx43

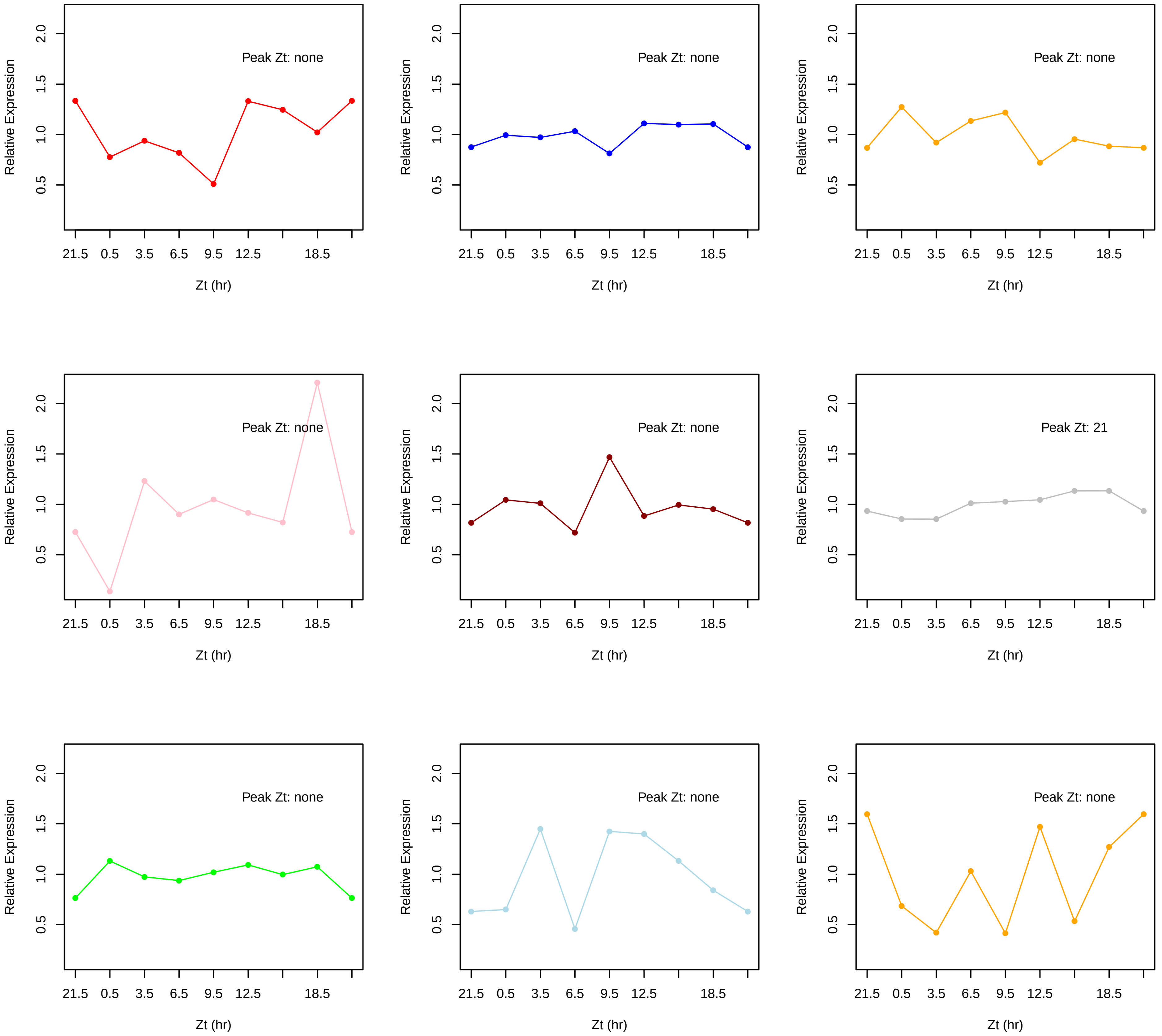

Singleton: ENSXMAG00000027173 si:ch211−219a15.3

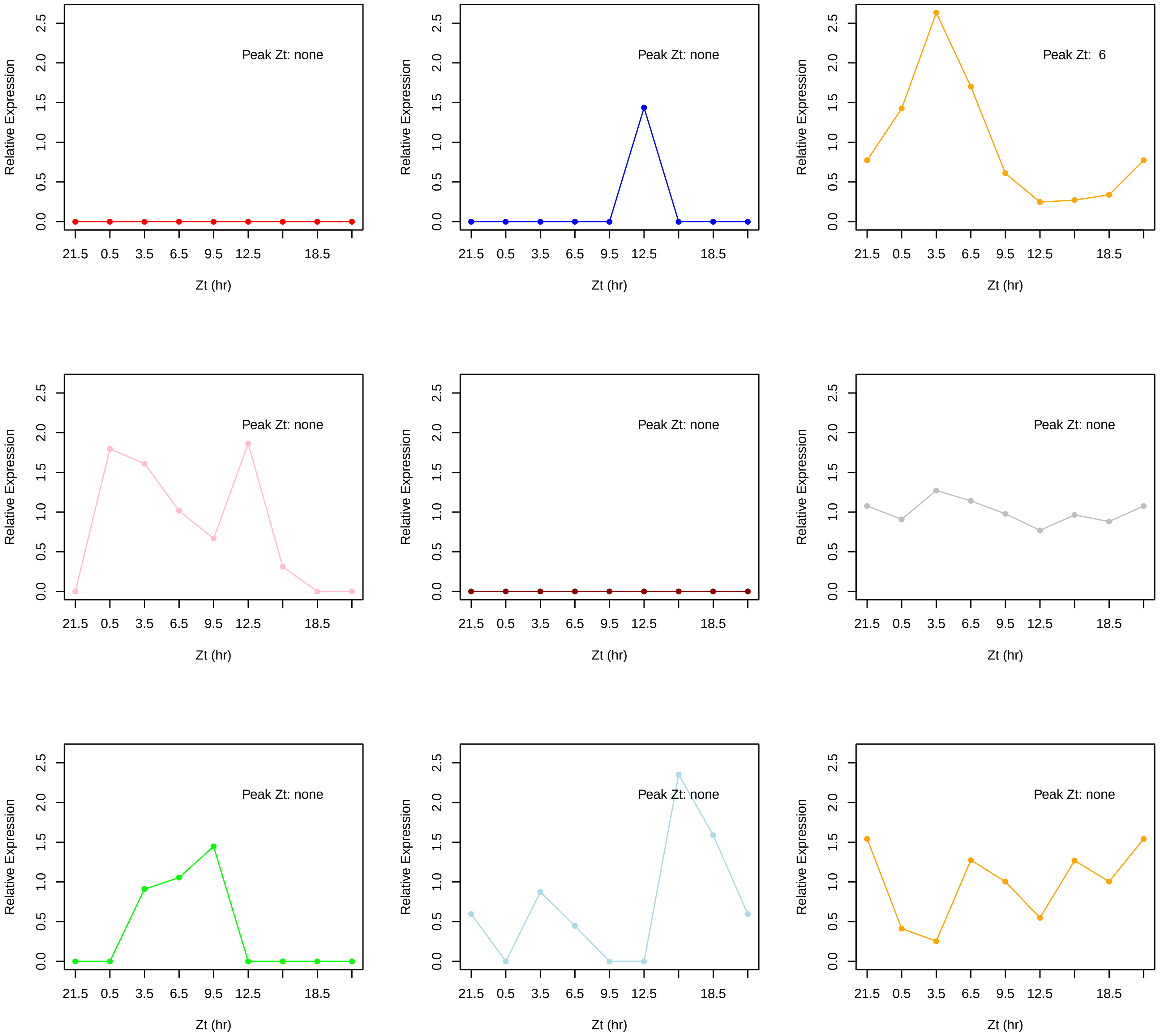

Singeleton: ENSXMAG00000026259 TMEM229A

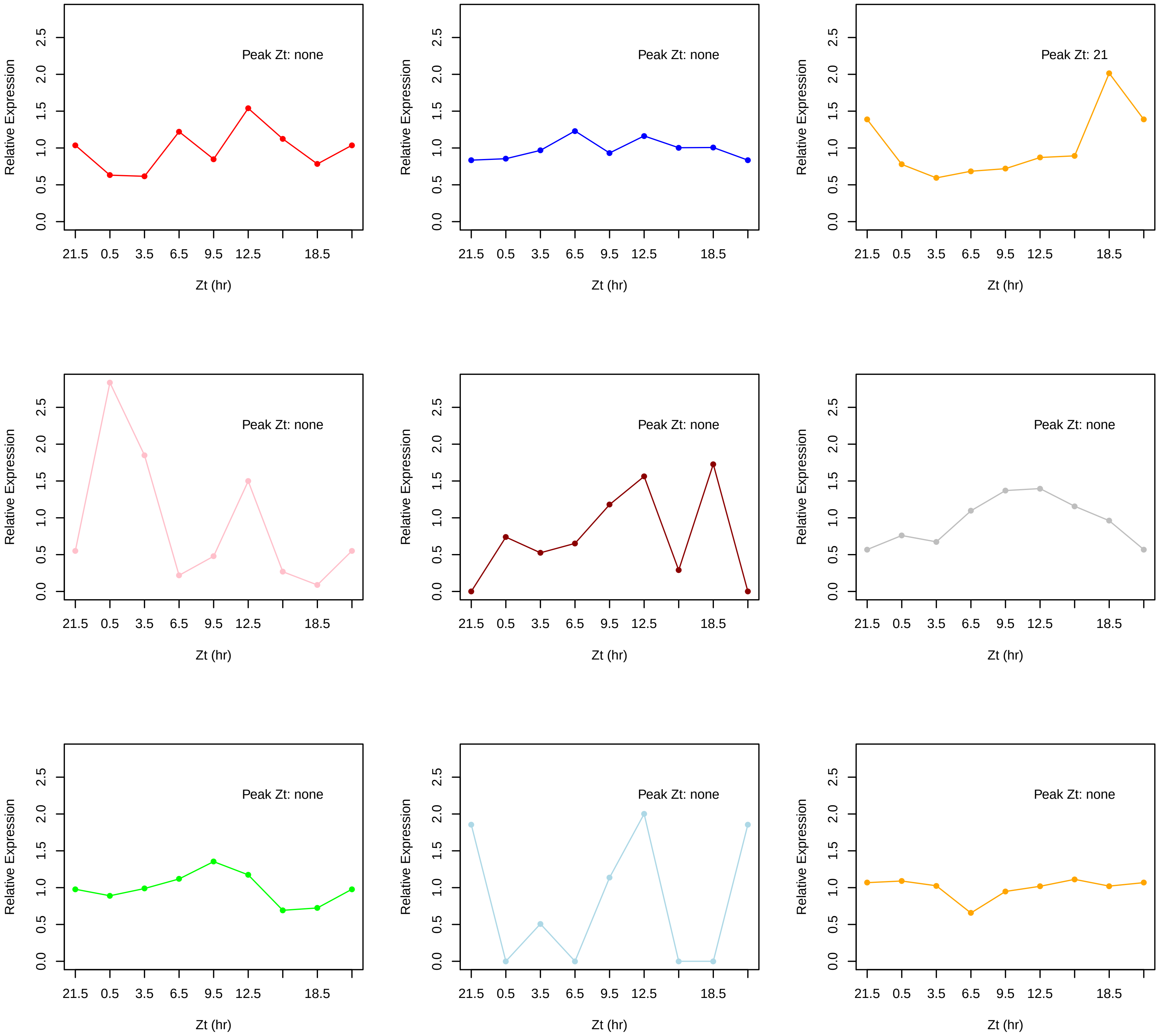

Singleton: ENSXMAG00000020020 tmem121b

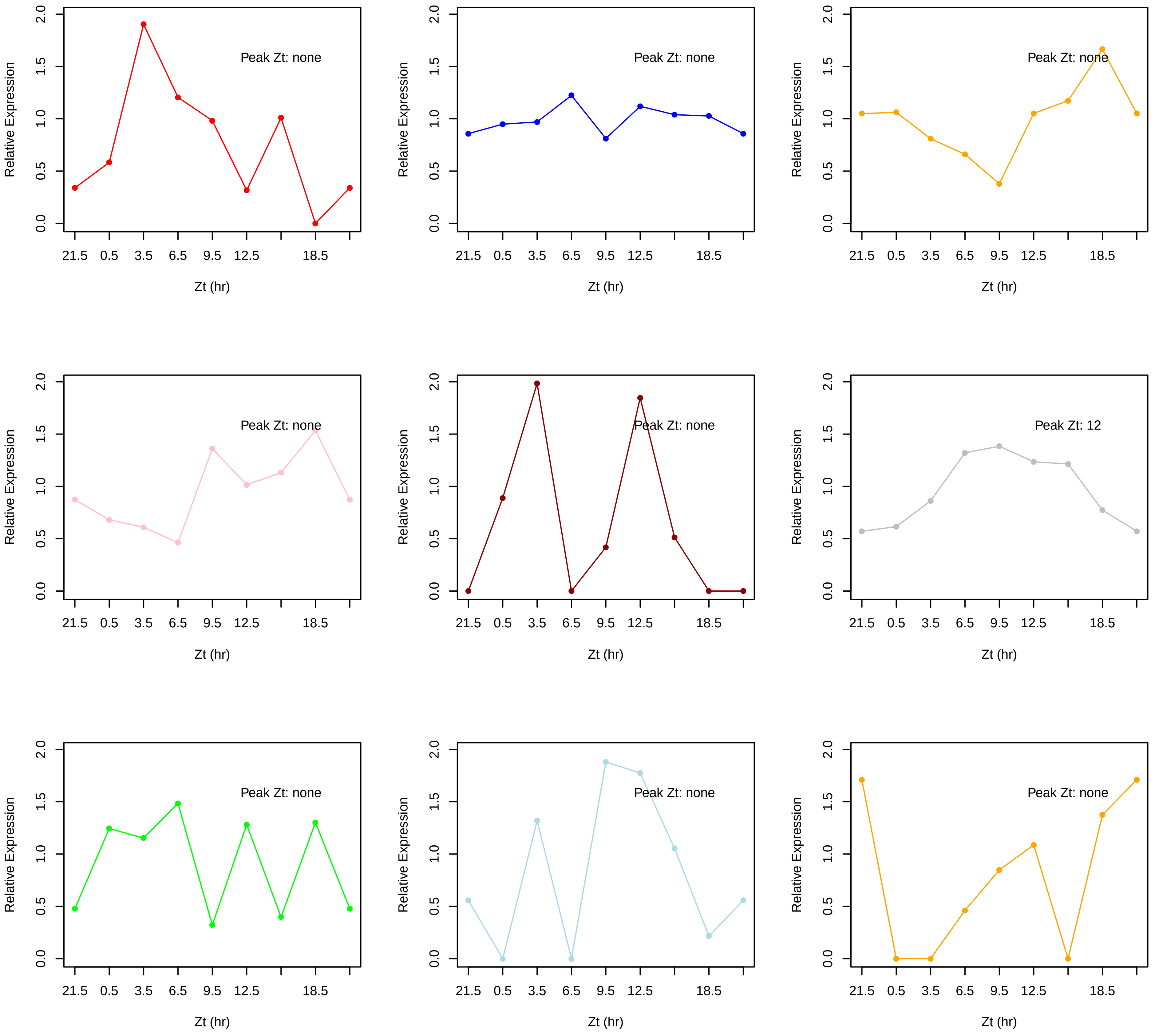

Singeleton: ENSXMAG00000016729

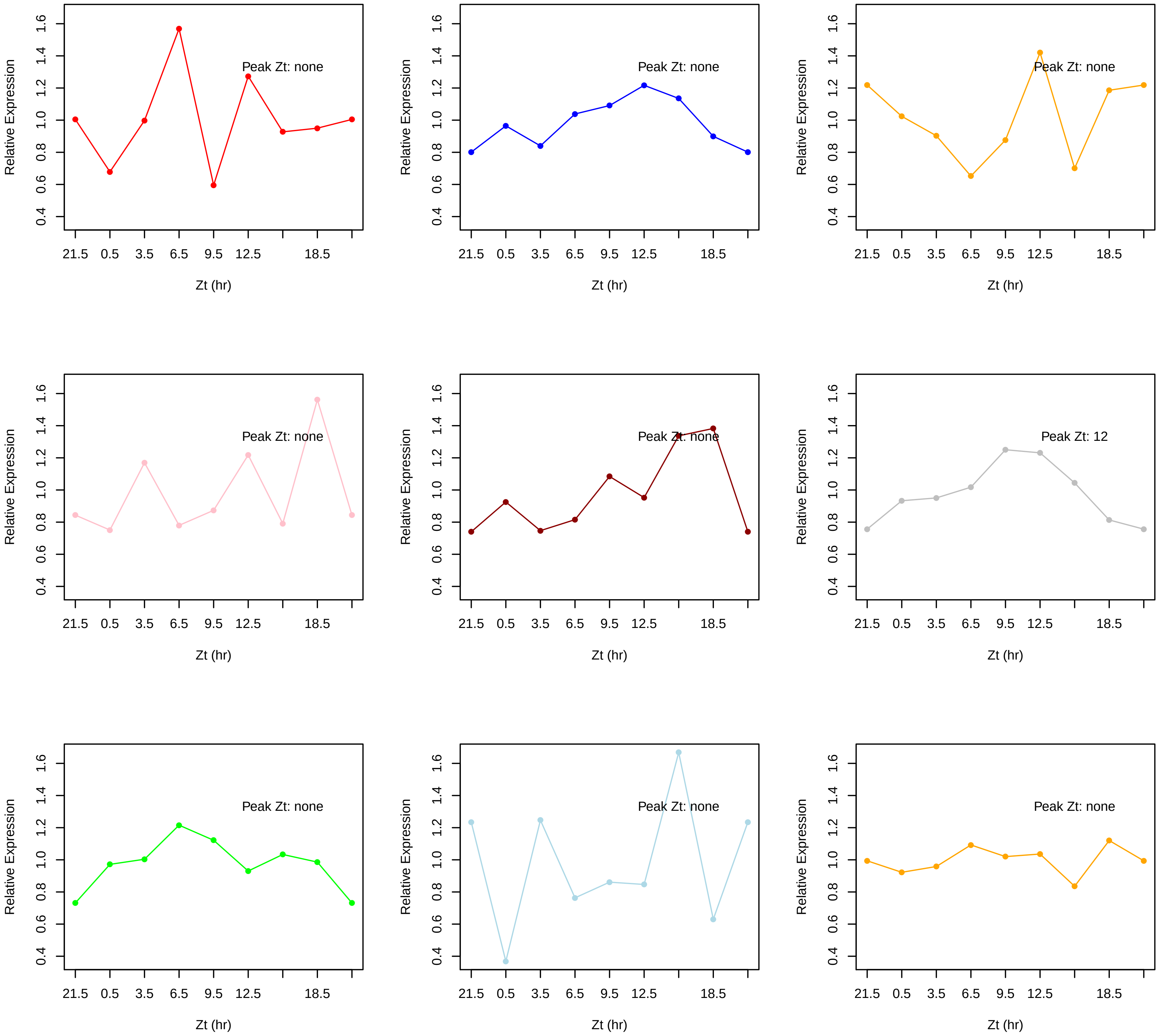

Singeleton: ENSXMAG00000003906 june

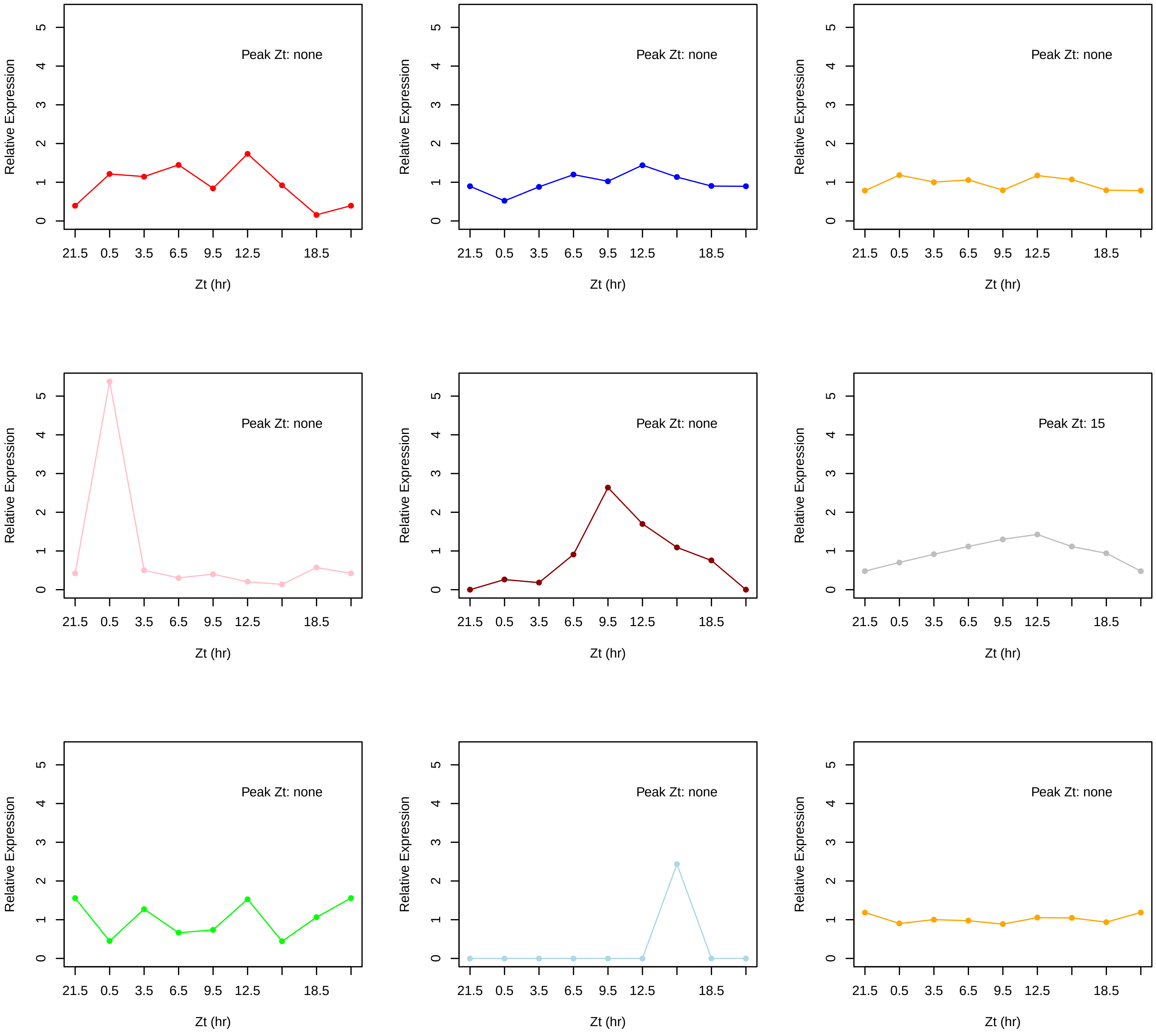

Singeleton: ENSXMAG00000026614 P2RY6

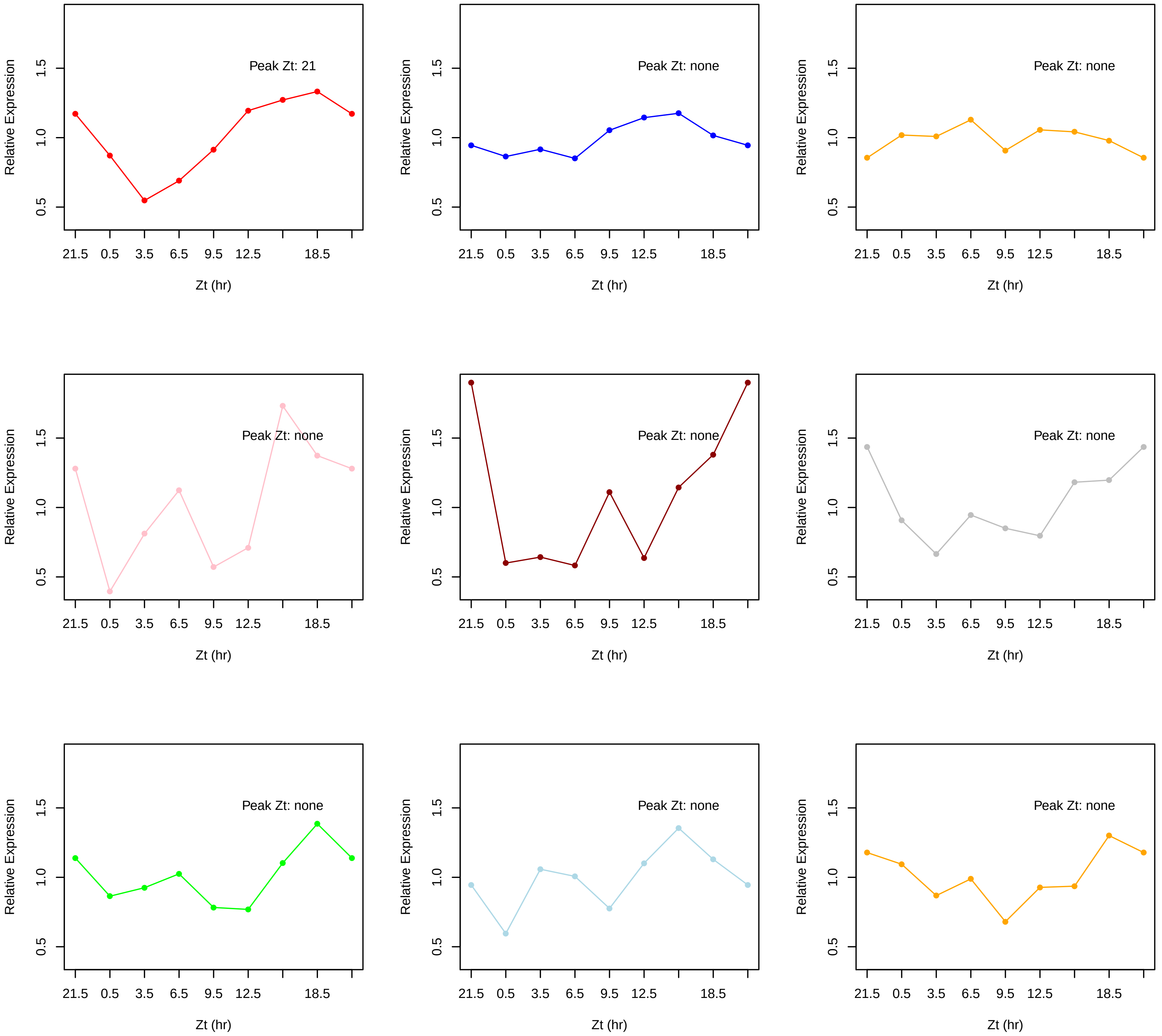

Singeleton: ENSXMAG00000001787 si:ch73−91k6.2

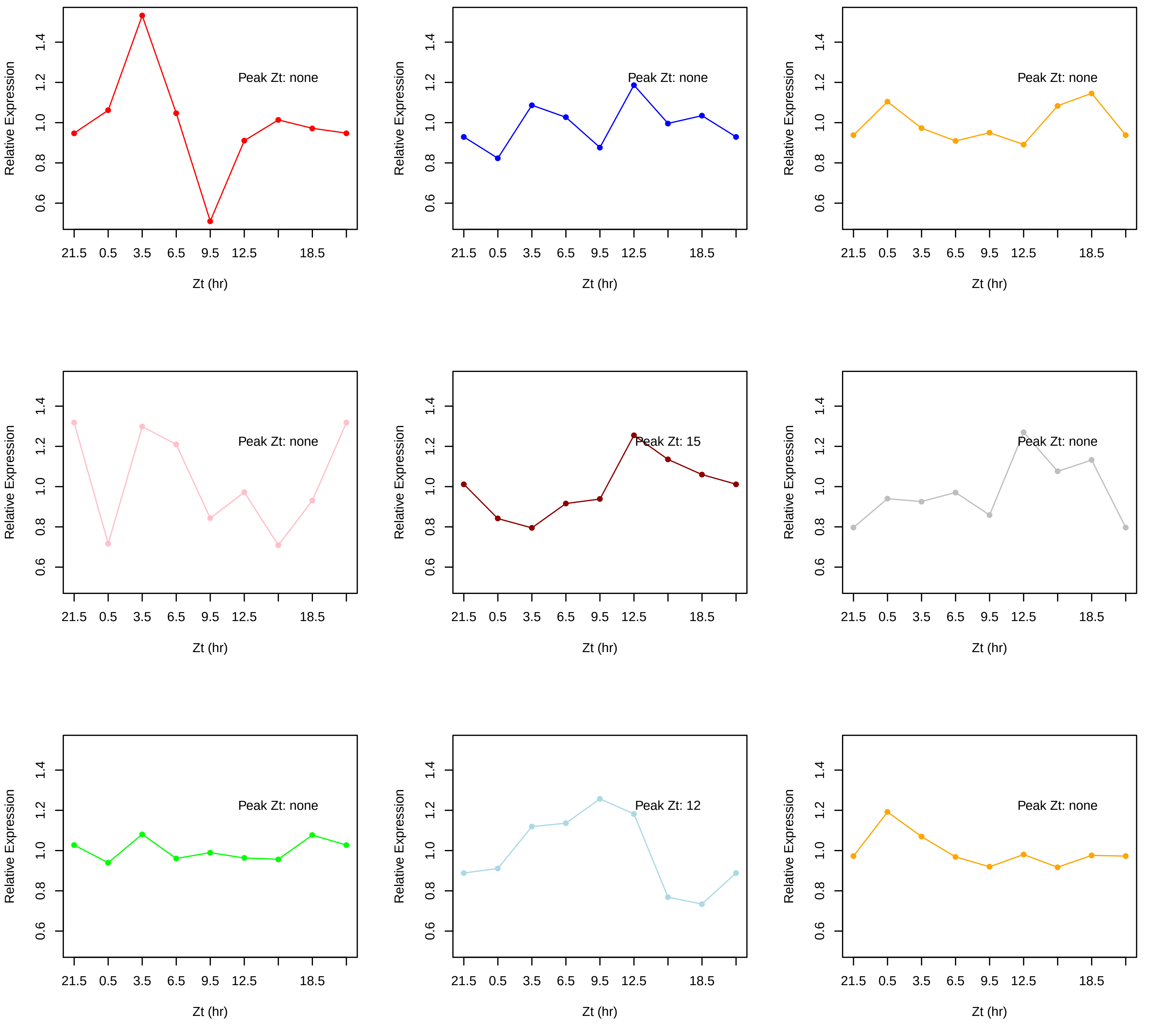

Singeleton: ENSXMAG00000020057 thbd

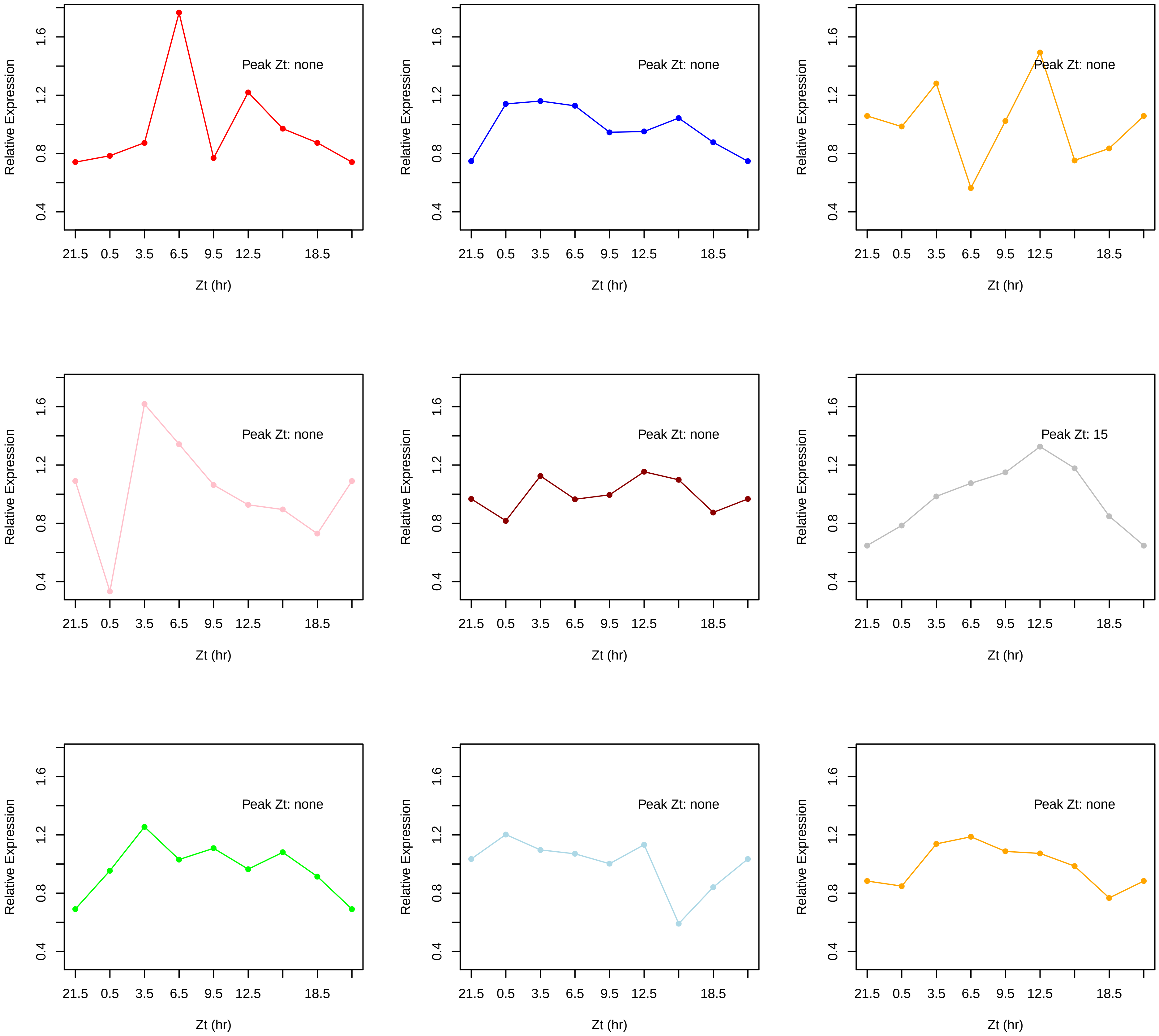

Singeleton: ENSXMAG00000020065

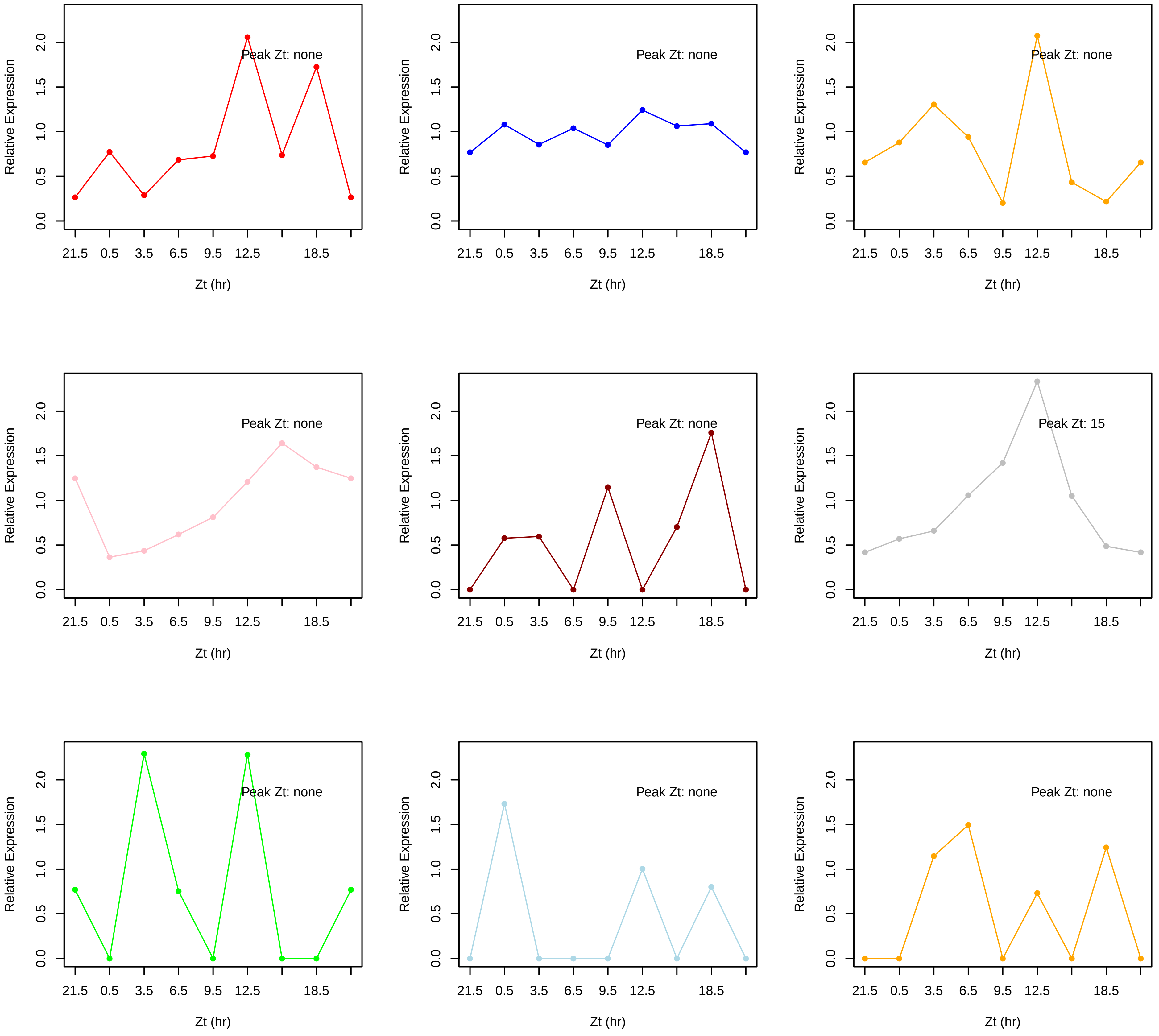

Singeleton: ENSXMAG00000024326 mab21l1

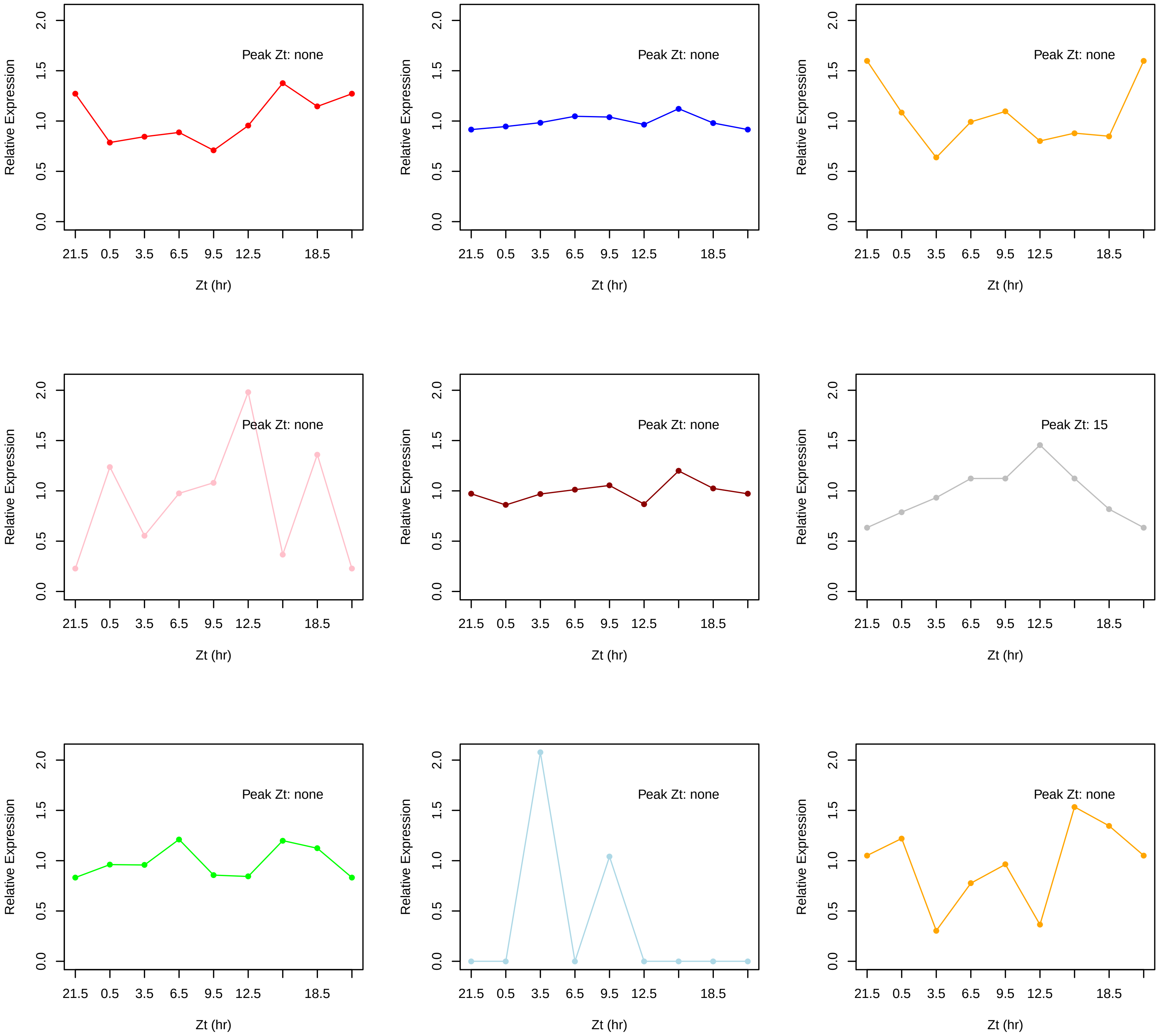

Singeleton: ENSXMAG00000021011

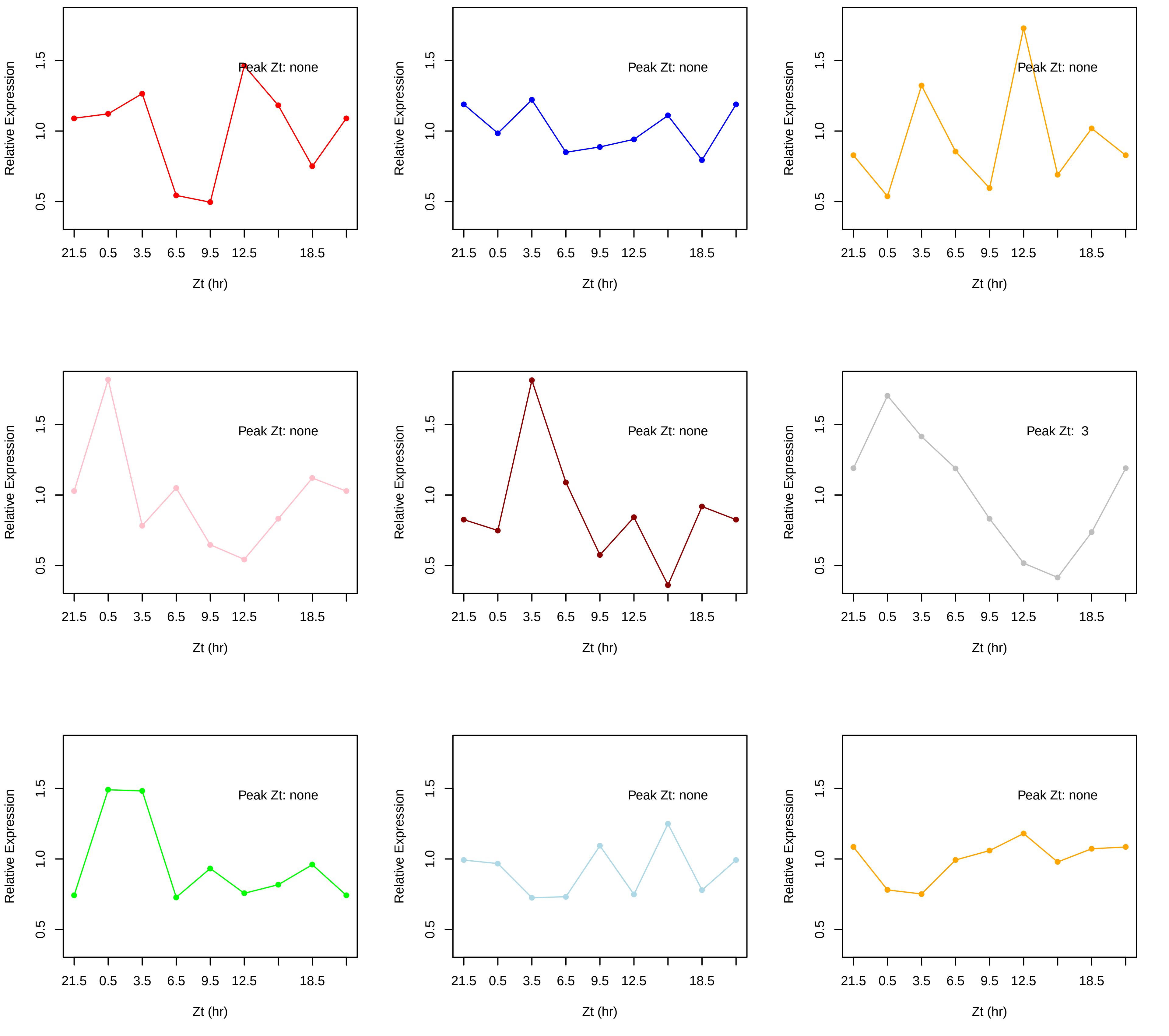

Singeleton: ENSXMAG00000029540 inka2

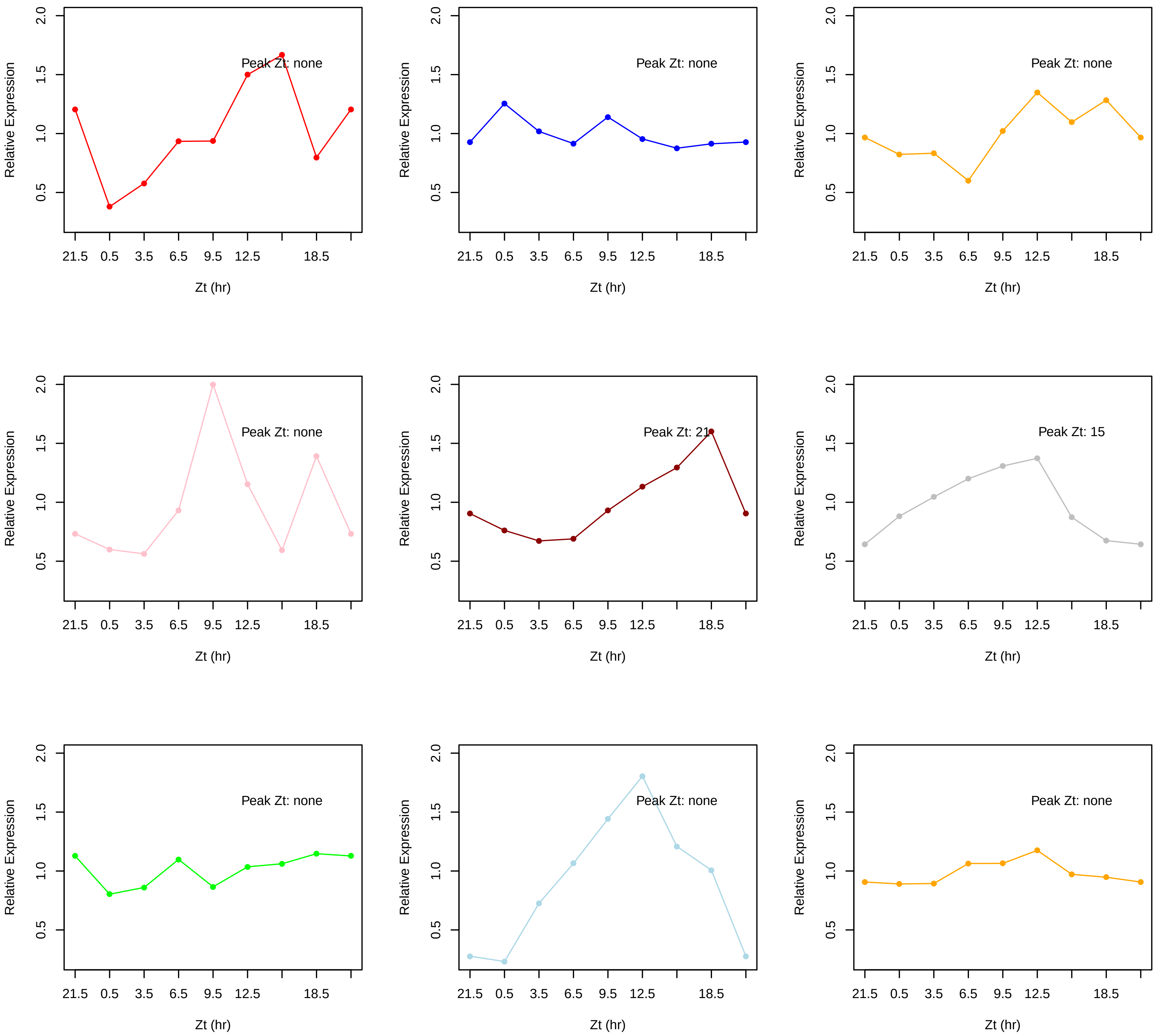

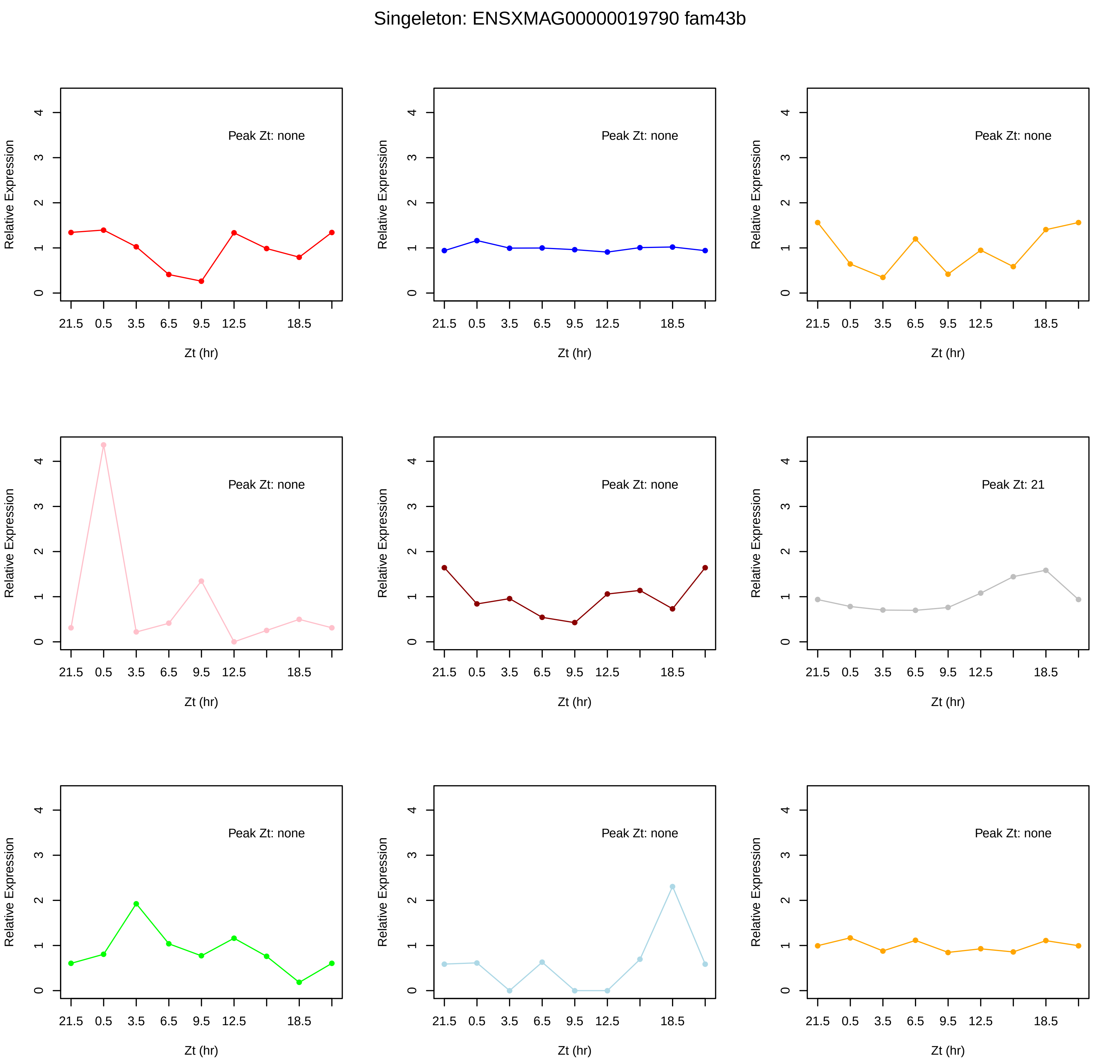
Singeleton: ENSXMAG00000019790 fam43b
Peak Zt: none
Peak Zt: none
Peak Zt: none
Peak Zt: none
Peak Zt: none
Peak Zt: 21
Peak Zt: none
Peak Zt: none
Peak Zt: none
Relative Expression
Zt (hr)
21.5
0.5
3.5
6.5
9.5
12.5
18.5

Singeleton: ENSXMAG00000019660 kcnj12a

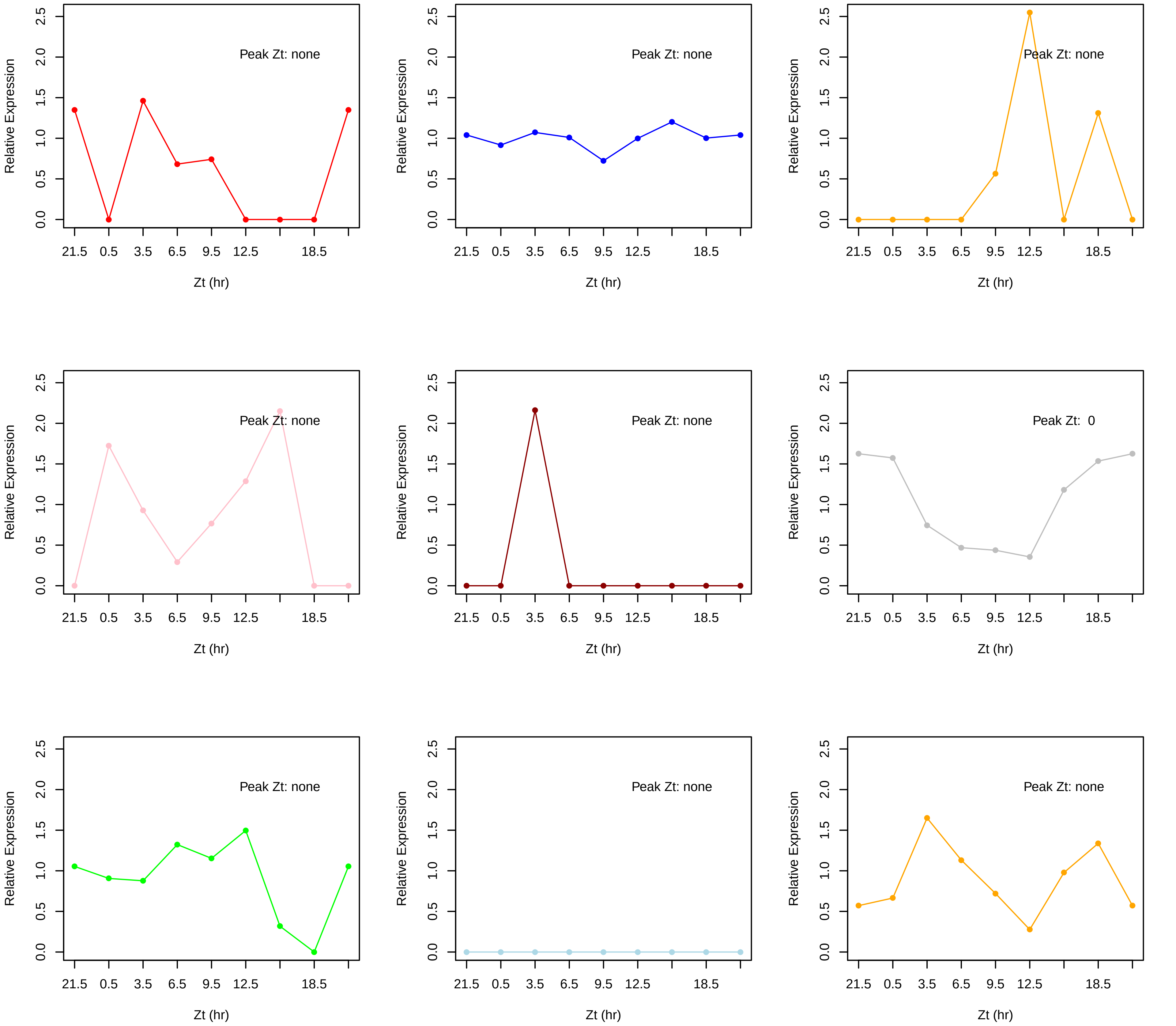

Singeleton: ENSXMAG00000026379 chst12a

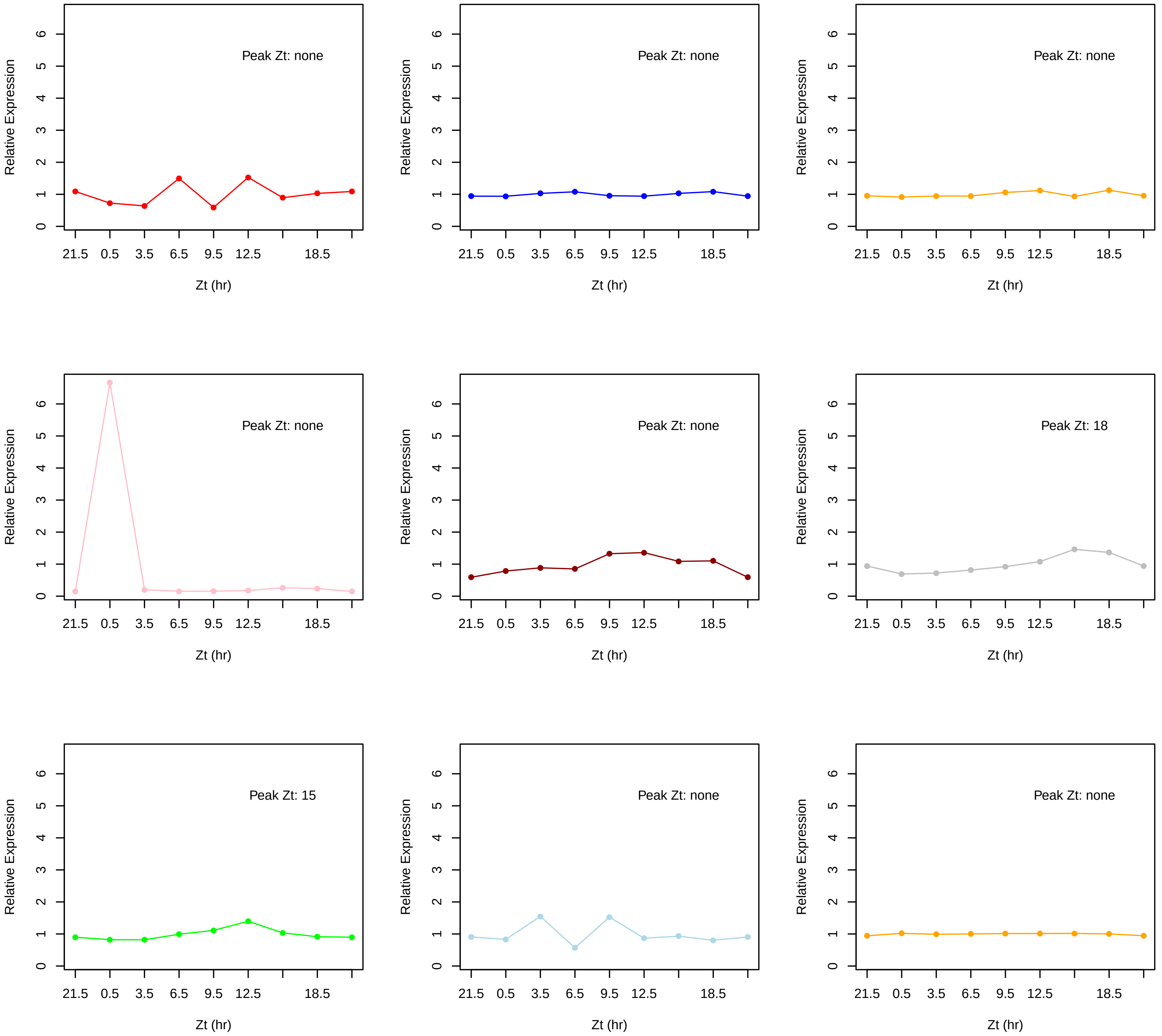

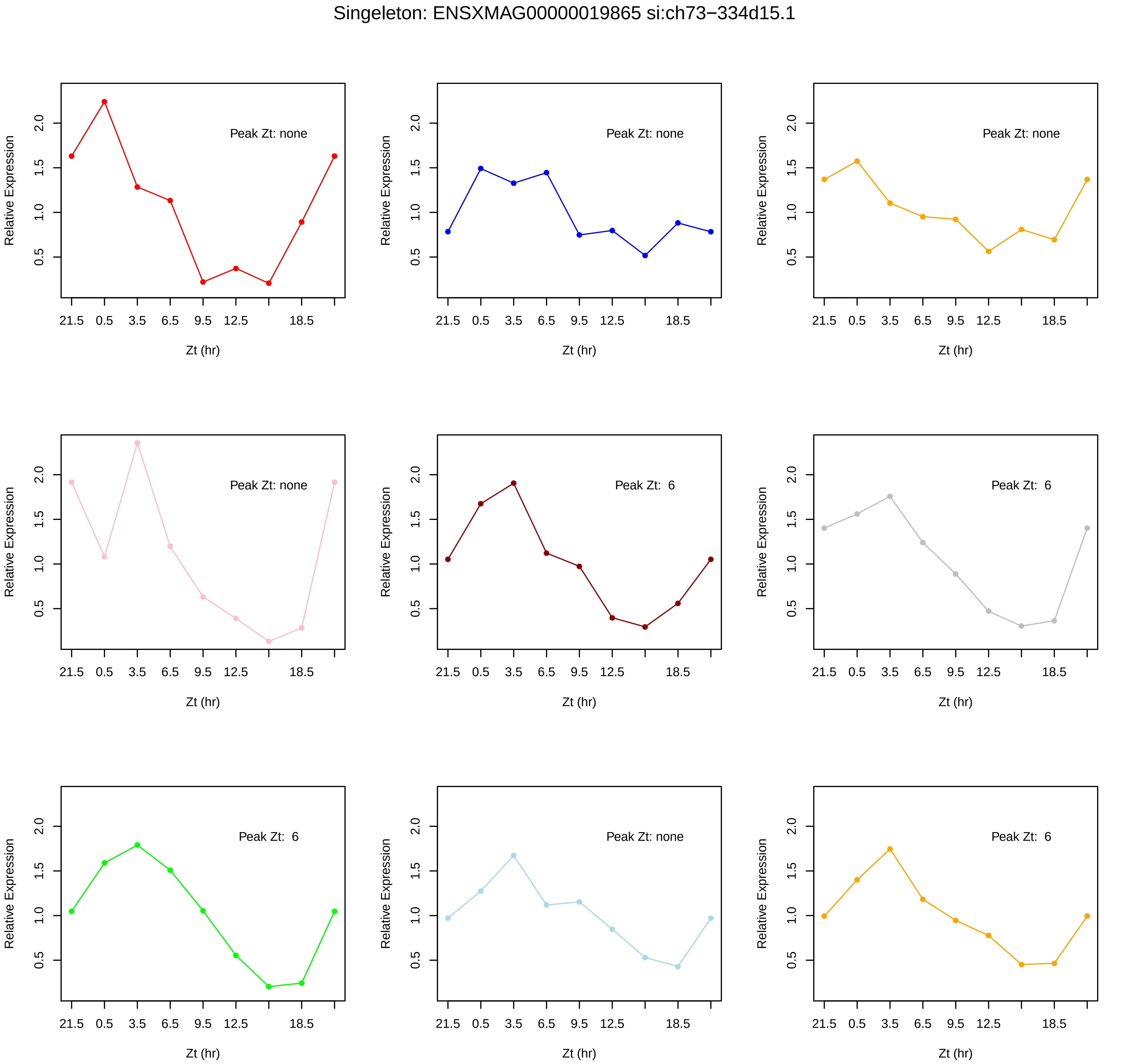
Singleton: ENSXMAG00000019865 si:ch73−334d15.1
Peak Zt: none
Peak Zt: none
Peak Zt: none
Peak Zt: none
Peak Zt: 6
Peak Zt: 6
Peak Zt: 6
Peak Zt: none
Peak Zt: 6
Relative Expression
Zt (hr)
21.5
0.5
3.5
6.5
9.5
12.5
18.5

Singleton: ENSXMAG00000019533 nog2

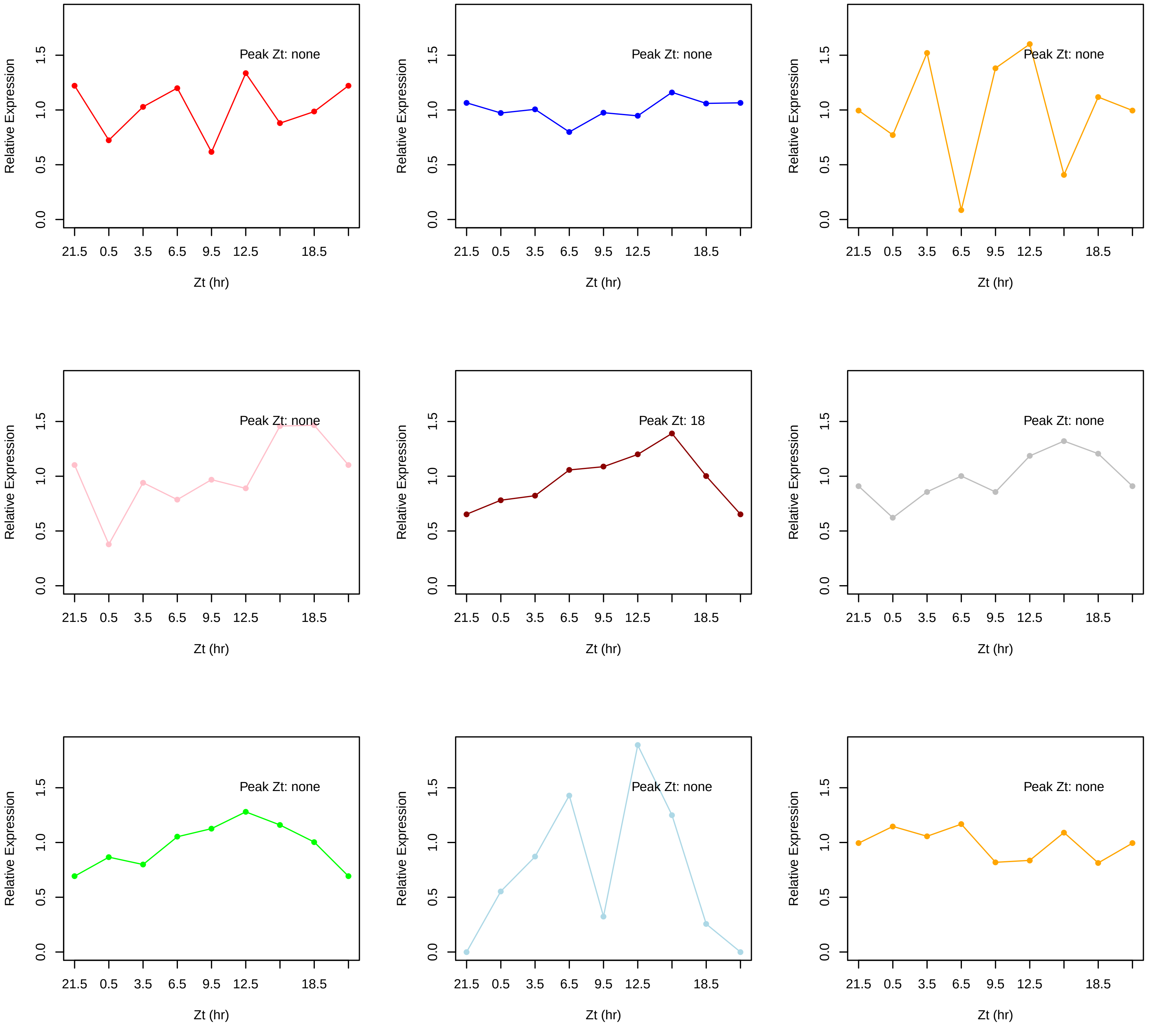

Singeleton: ENSXMAG00000019823 tmem264

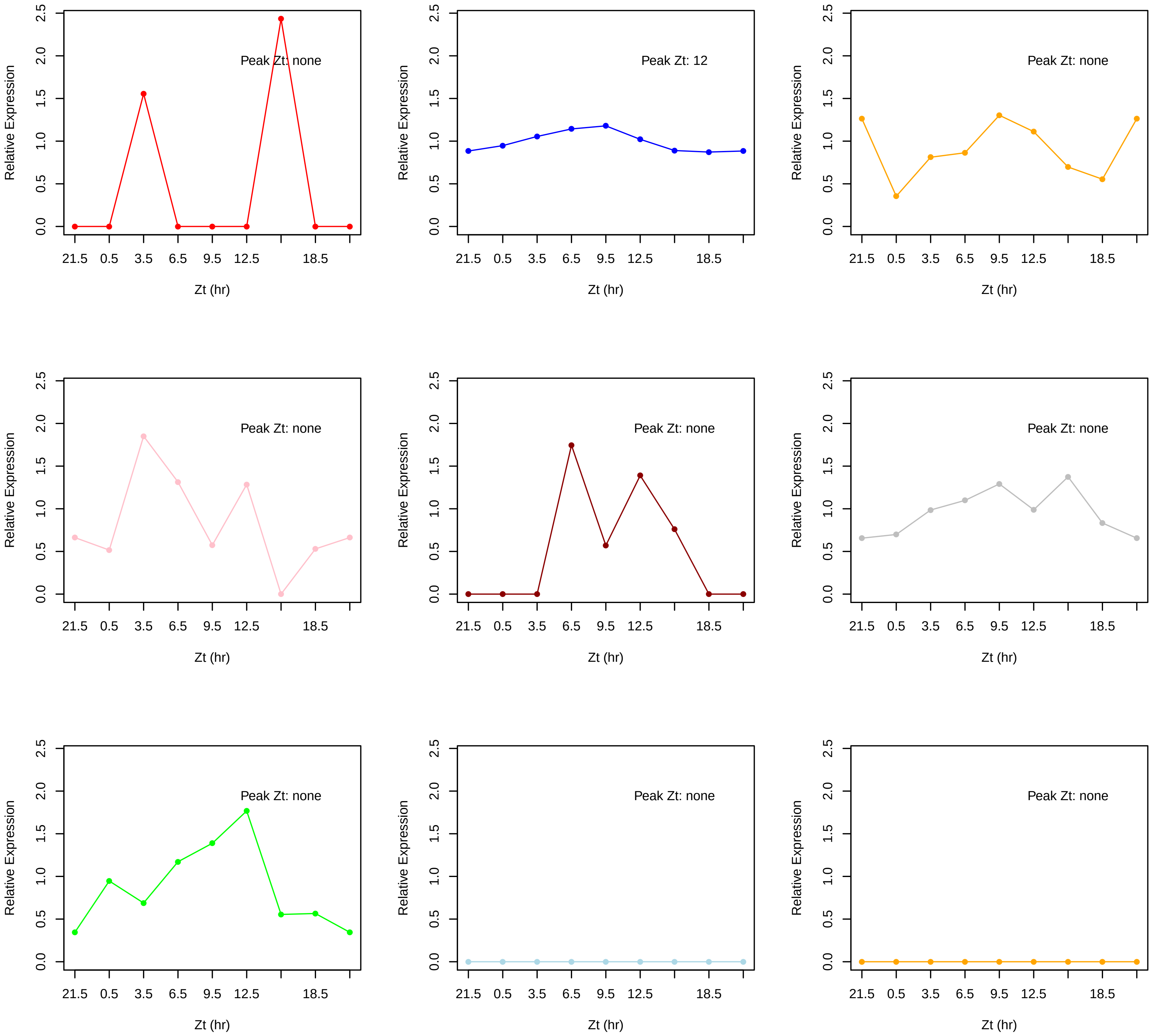

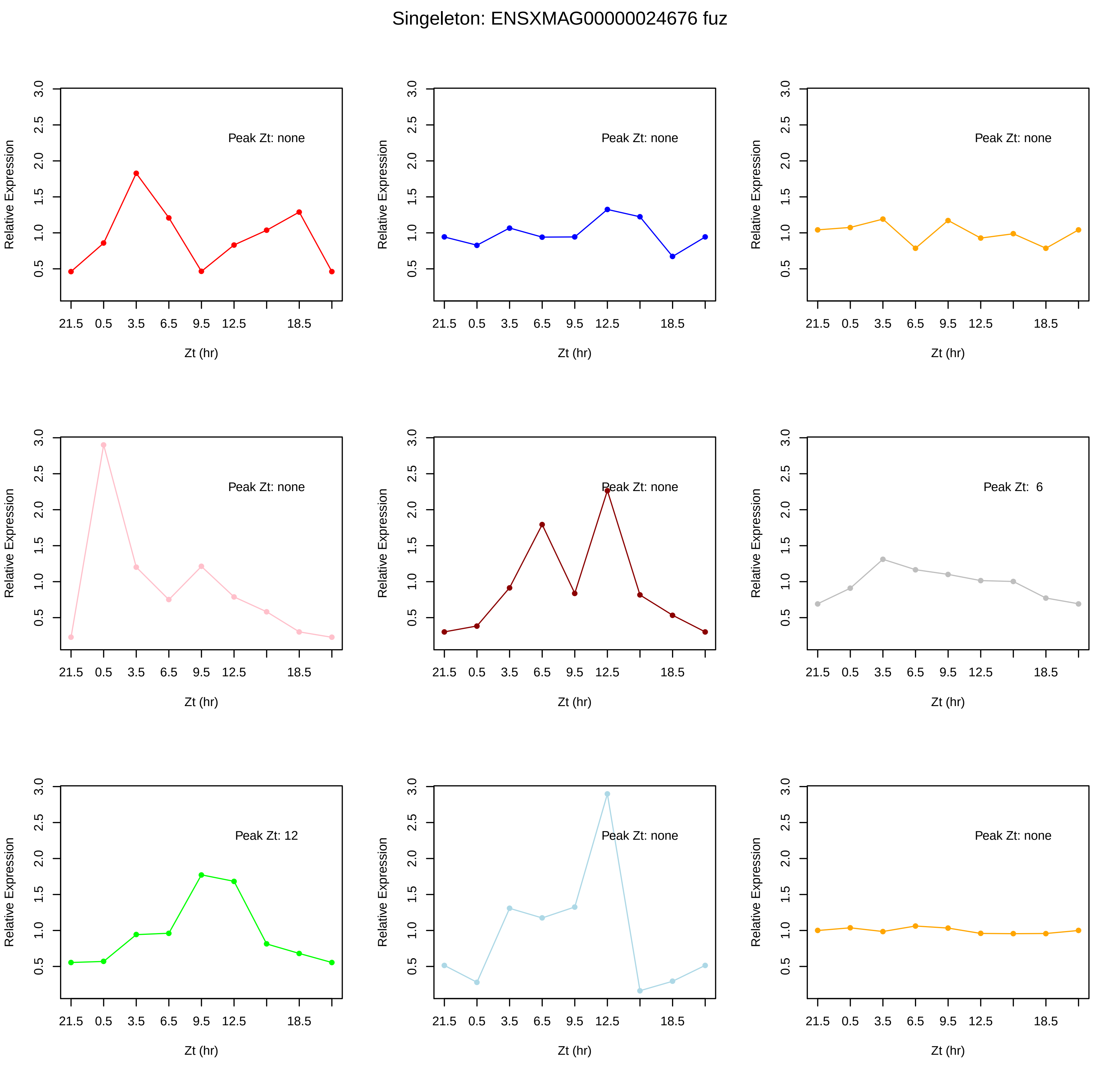
Singeleton: ENSXMAG00000024676 fuz
Peak Zt: none
Peak Zt: none
Peak Zt: none
Peak Zt: none
Peak Zt: none
Peak Zt: 6
Peak Zt: 12
Peak Zt: none
Peak Zt: none
Relative Expression
Zt (hr)
21.5
0.5
3.5
6.5
9.5
12.5
18.5

Singeleton: ENSXMAG00000005815 cx47.1

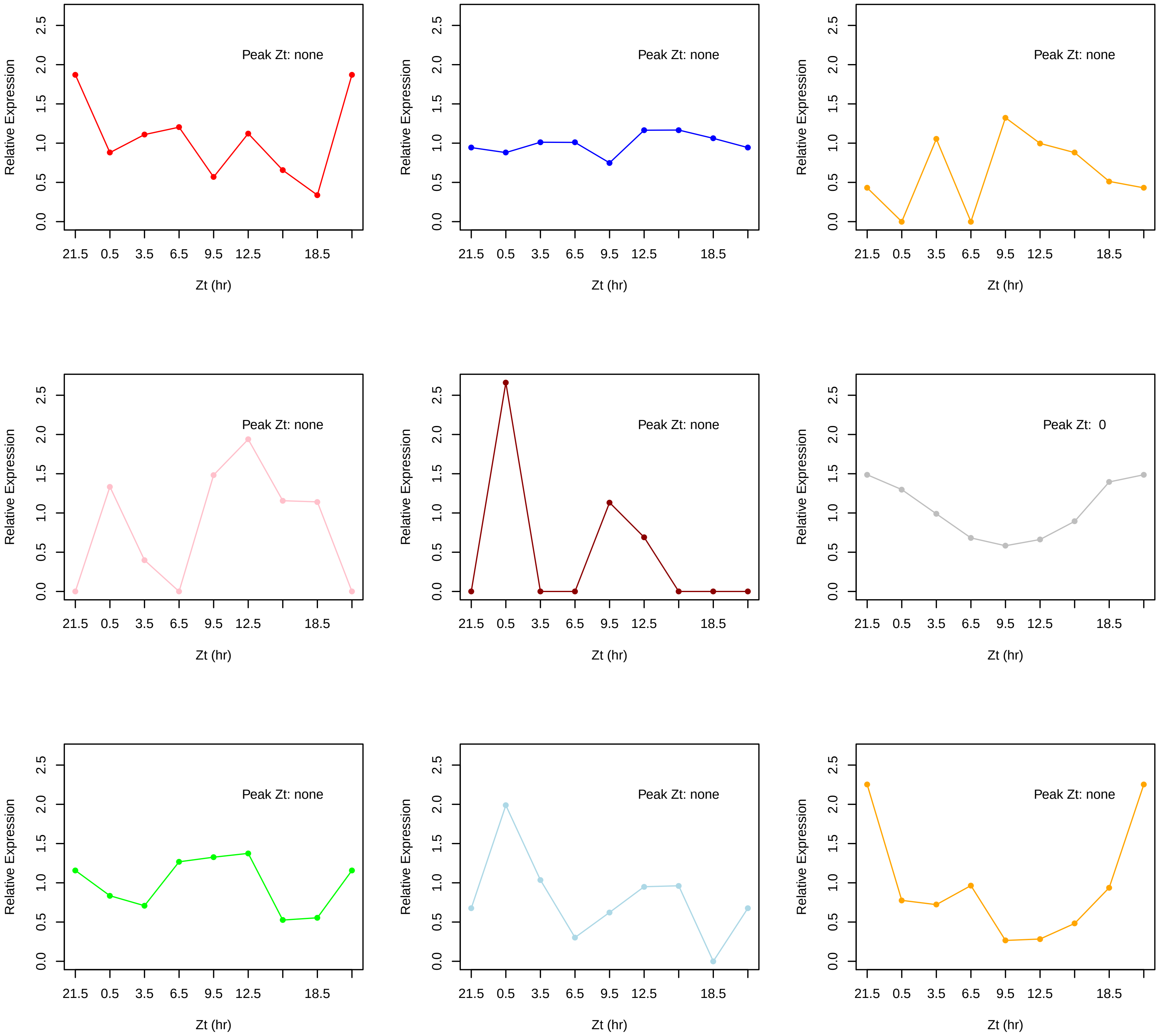

Singeleton: ENSXMAG00000019994 opn4.1

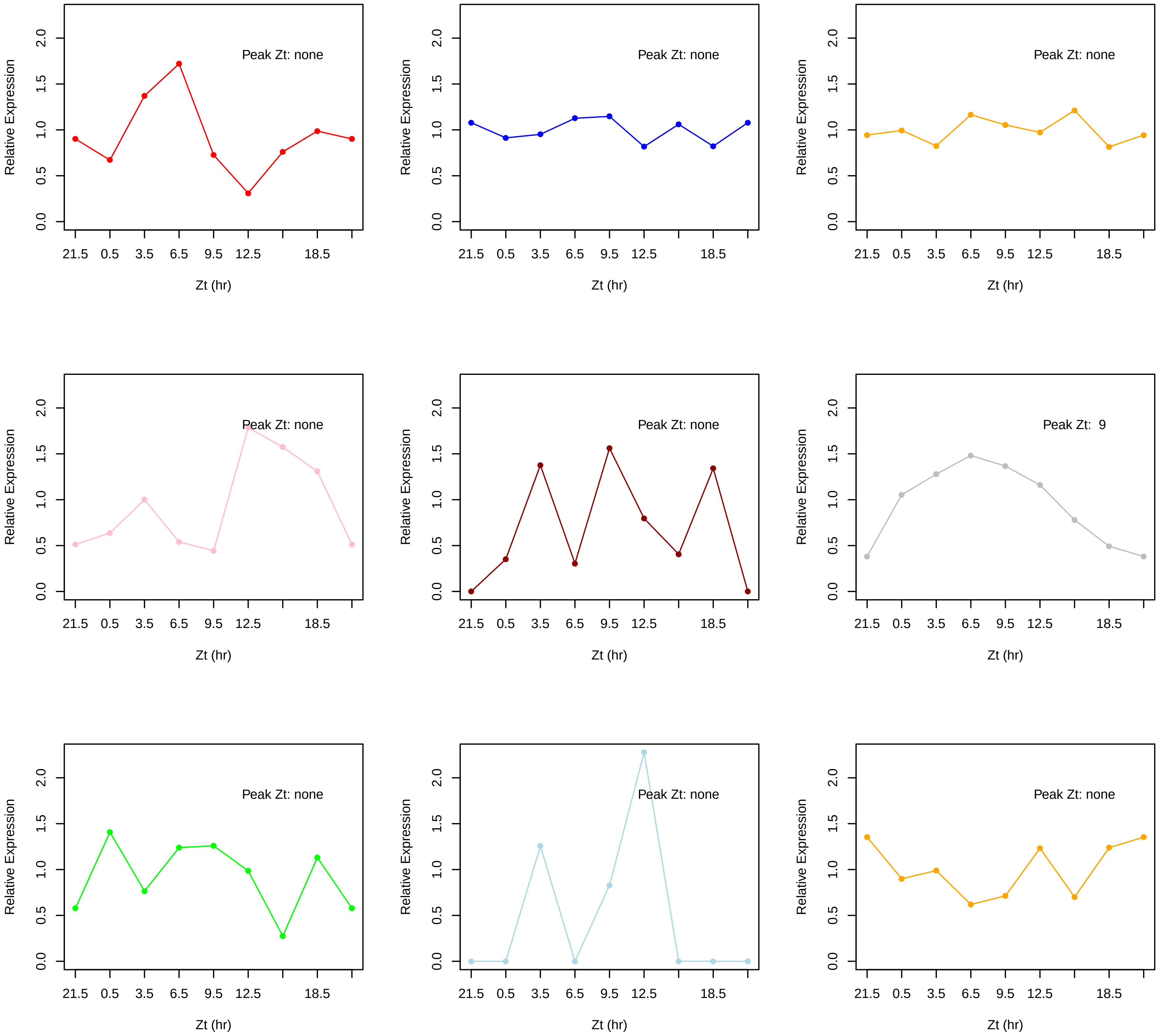

Singeleton: ENSXMAG00000019885 snrnp35

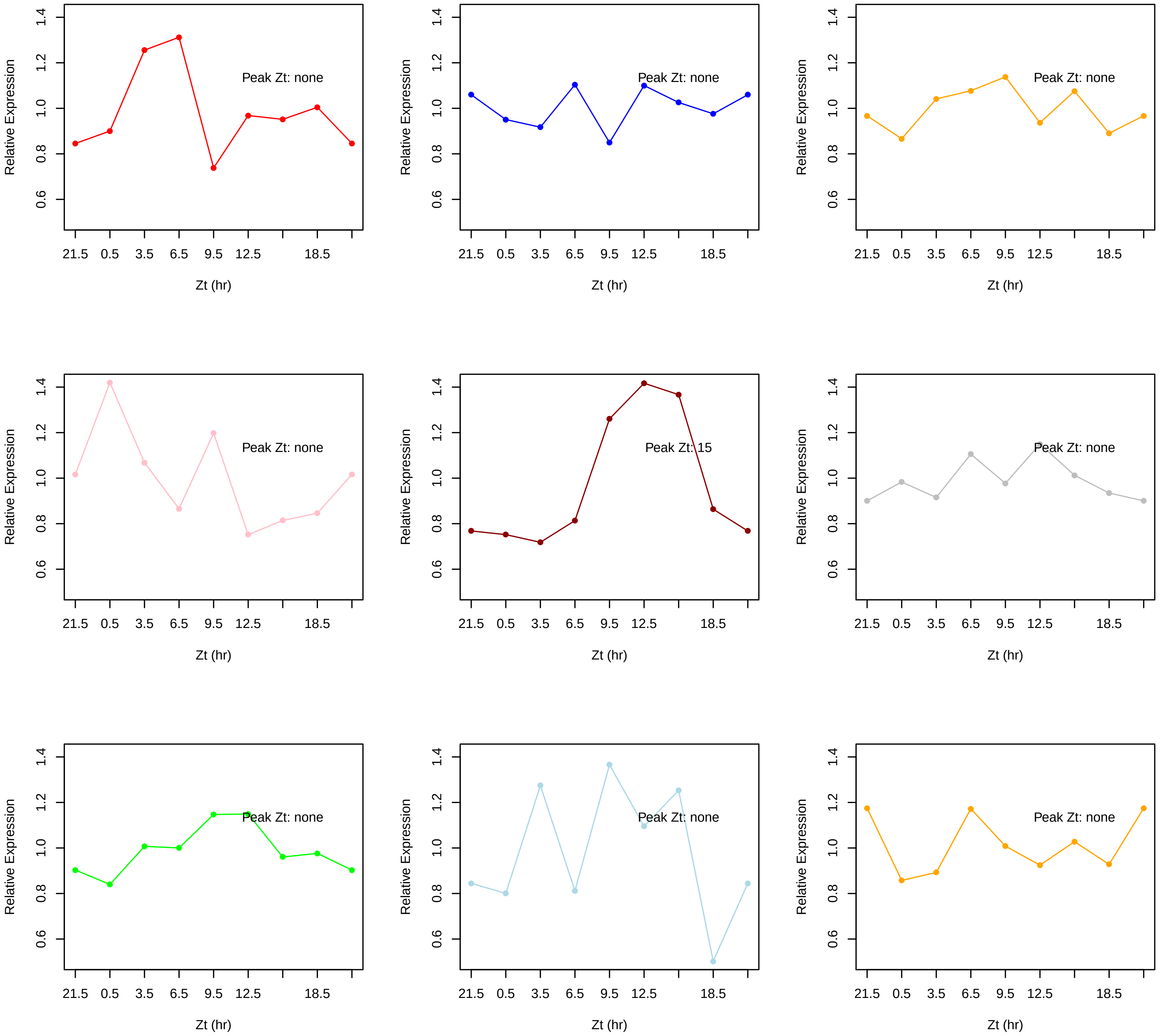

Singeleton: ENSXMAG00000020058

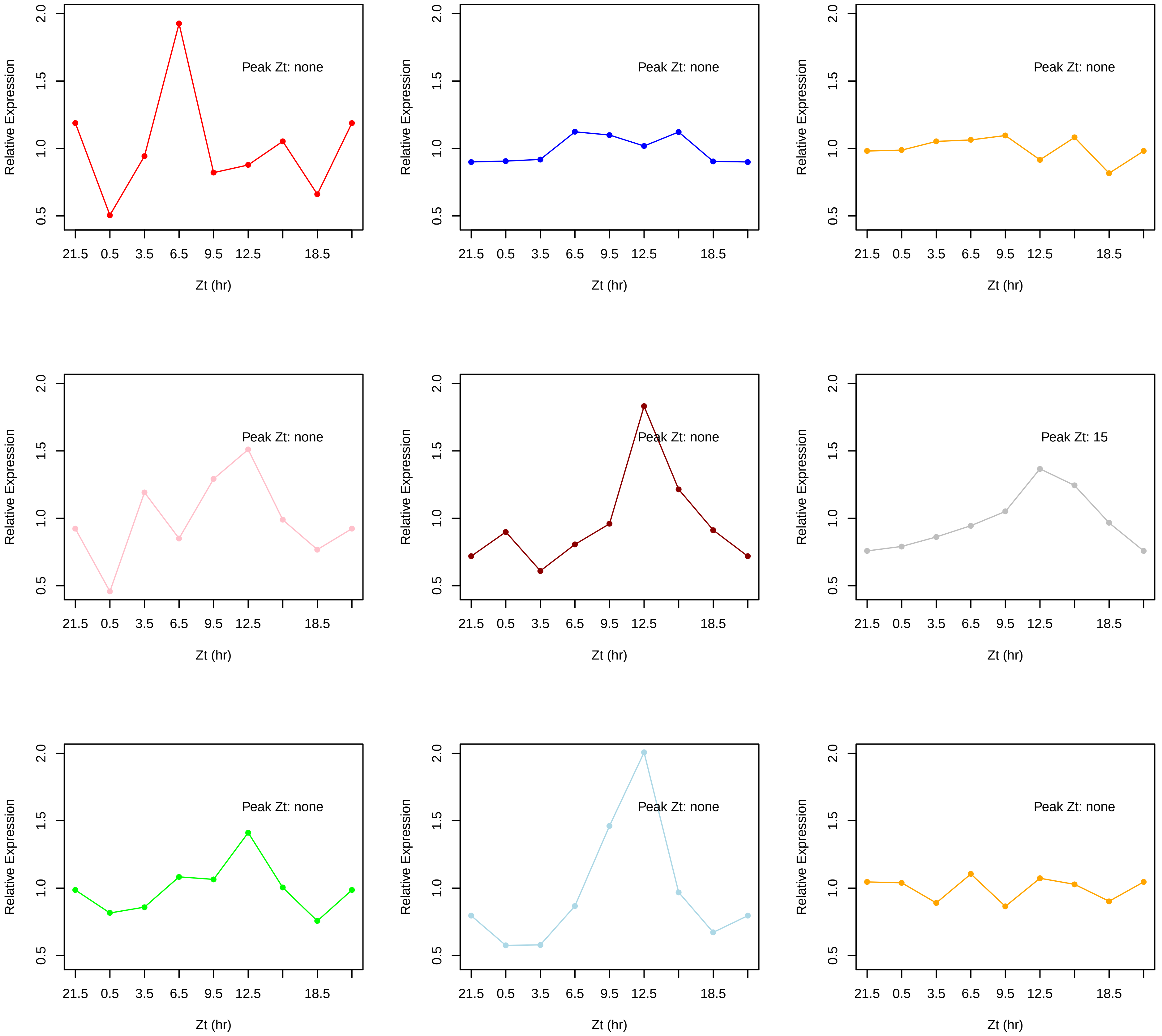

Singleton: ENSXMAG00000015247 aplnra

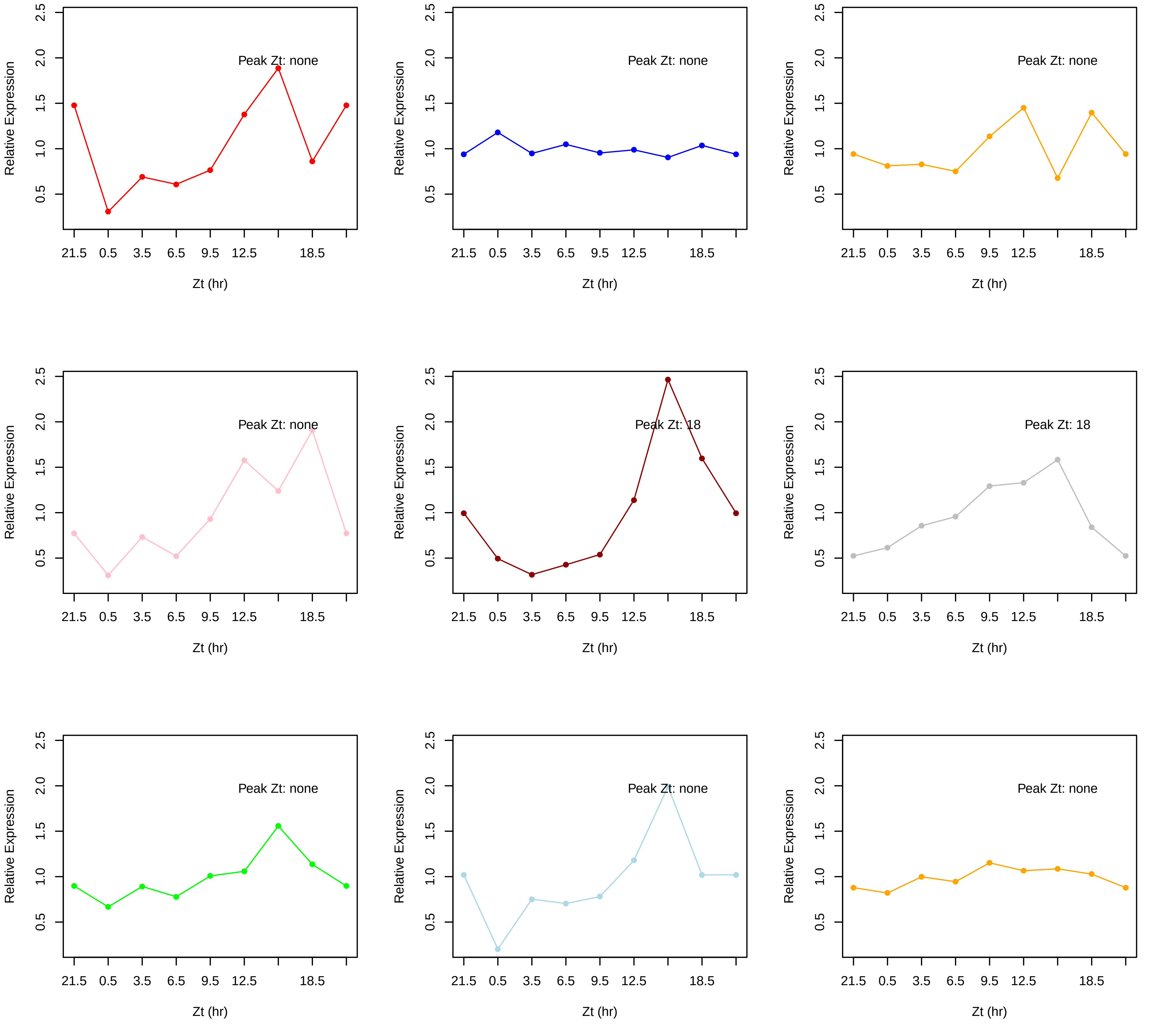

Singeleton: ENSXMAG00000019594 rnf152

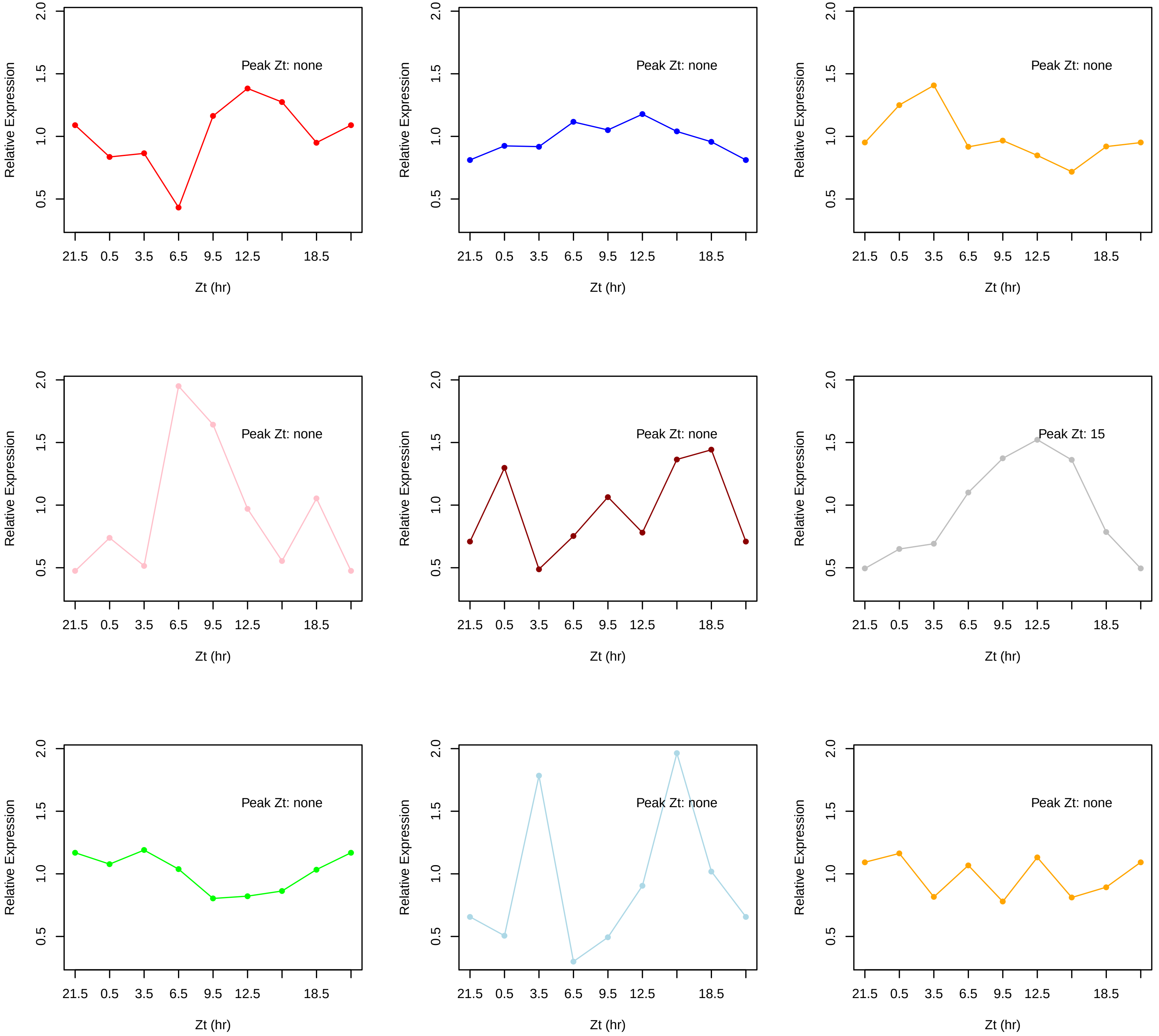

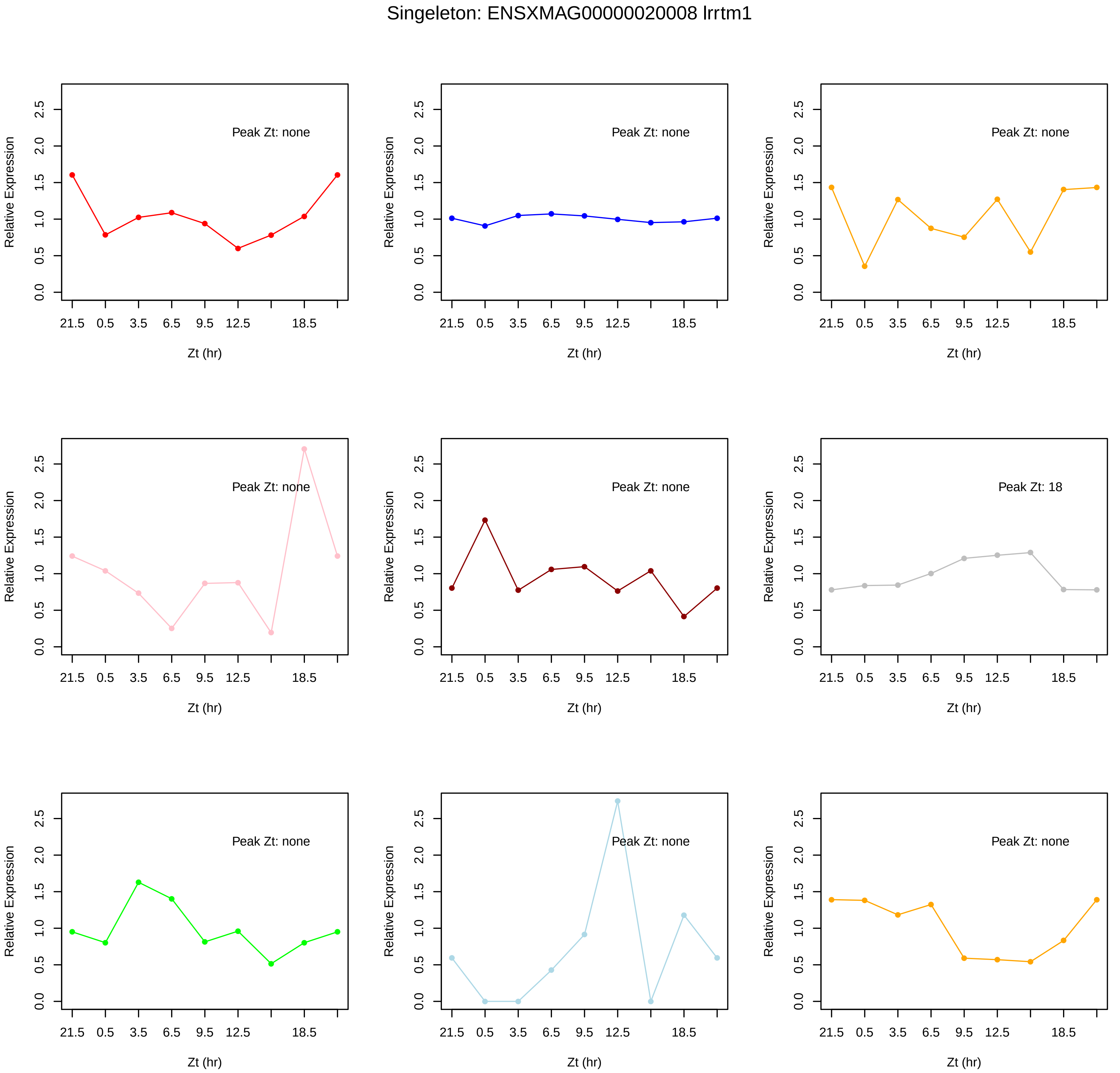
Singleton: ENSXMAG00000020008 lrrtm1
Peak Zt: none
Peak Zt: none
Peak Zt: none
Peak Zt: none
Peak Zt: none
Peak Zt: 18
Peak Zt: none
Peak Zt: none
Peak Zt: none
Relative Expression
Zt (hr)
21.5
0.5
3.5
6.5
9.5
12.5
18.5
0.0
0.5
1.0
1.5
2.0
2.5

Singeleton: ENSXMAG00000019535 gpr182

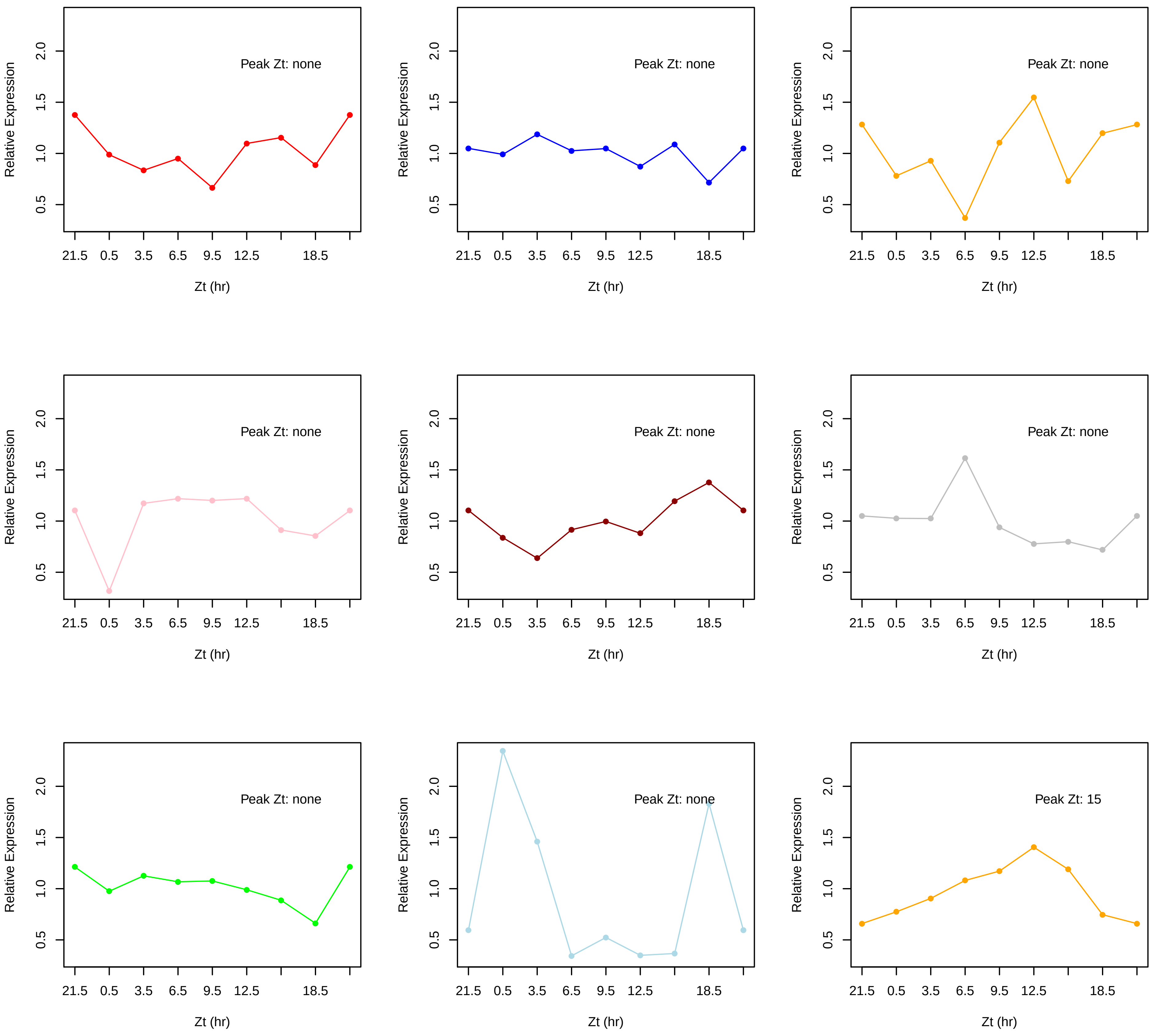

Singleton: ENSXMAG00000023558 SUOX

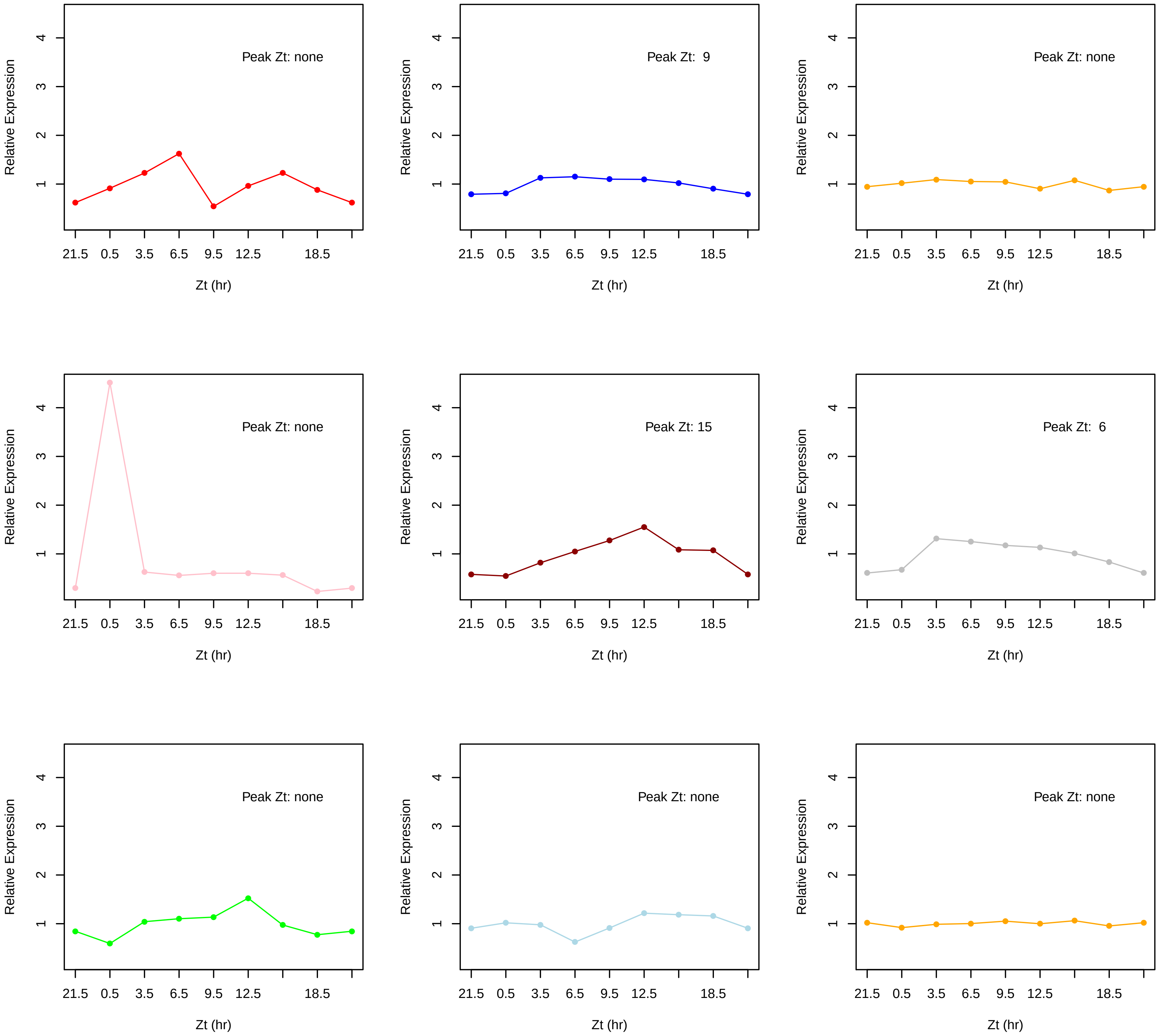

Singleton: ENSXMAG00000019666 si:ch211−210c8.7

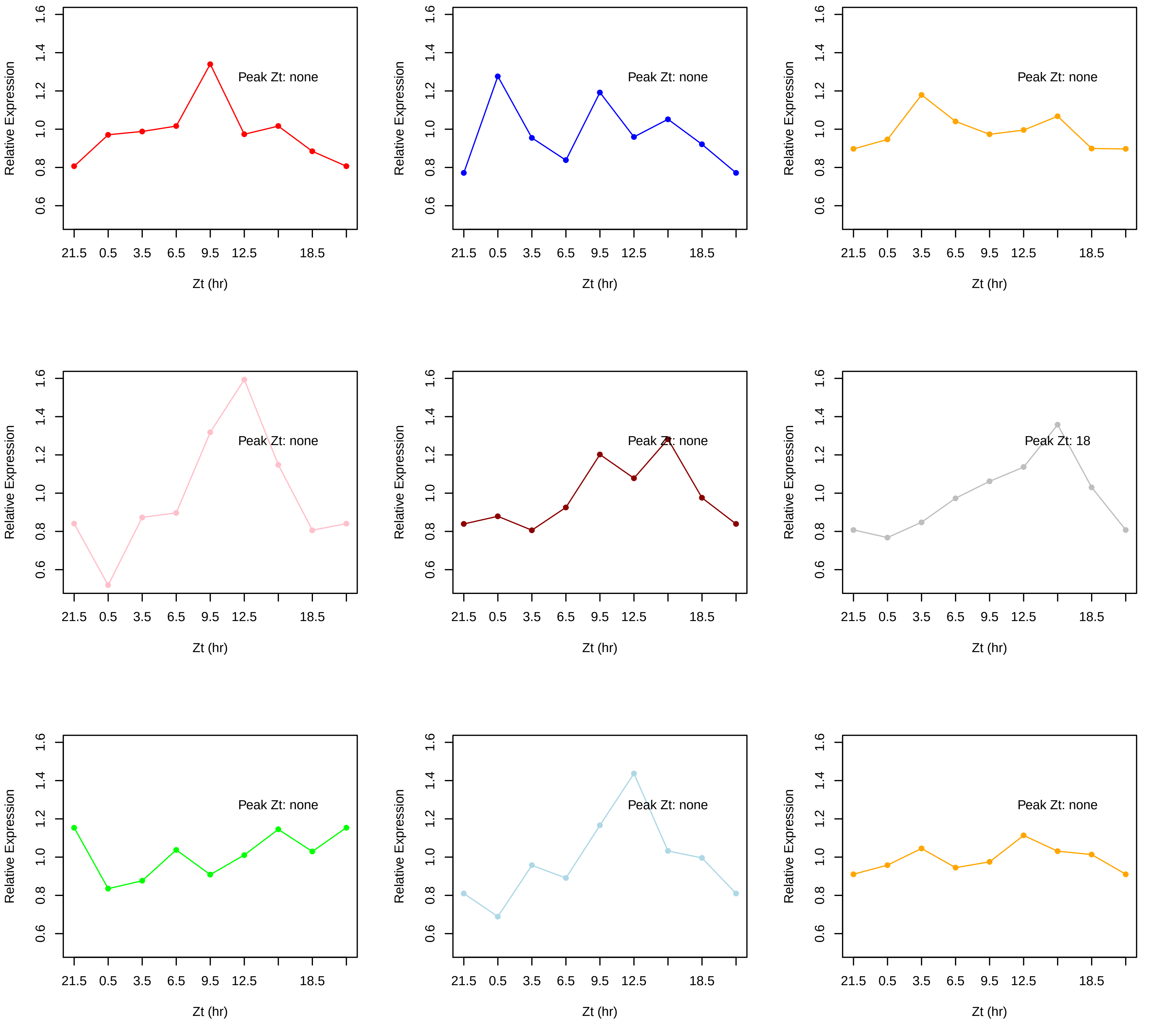

Singleton: ENSXMAG00000025050 mfsd5

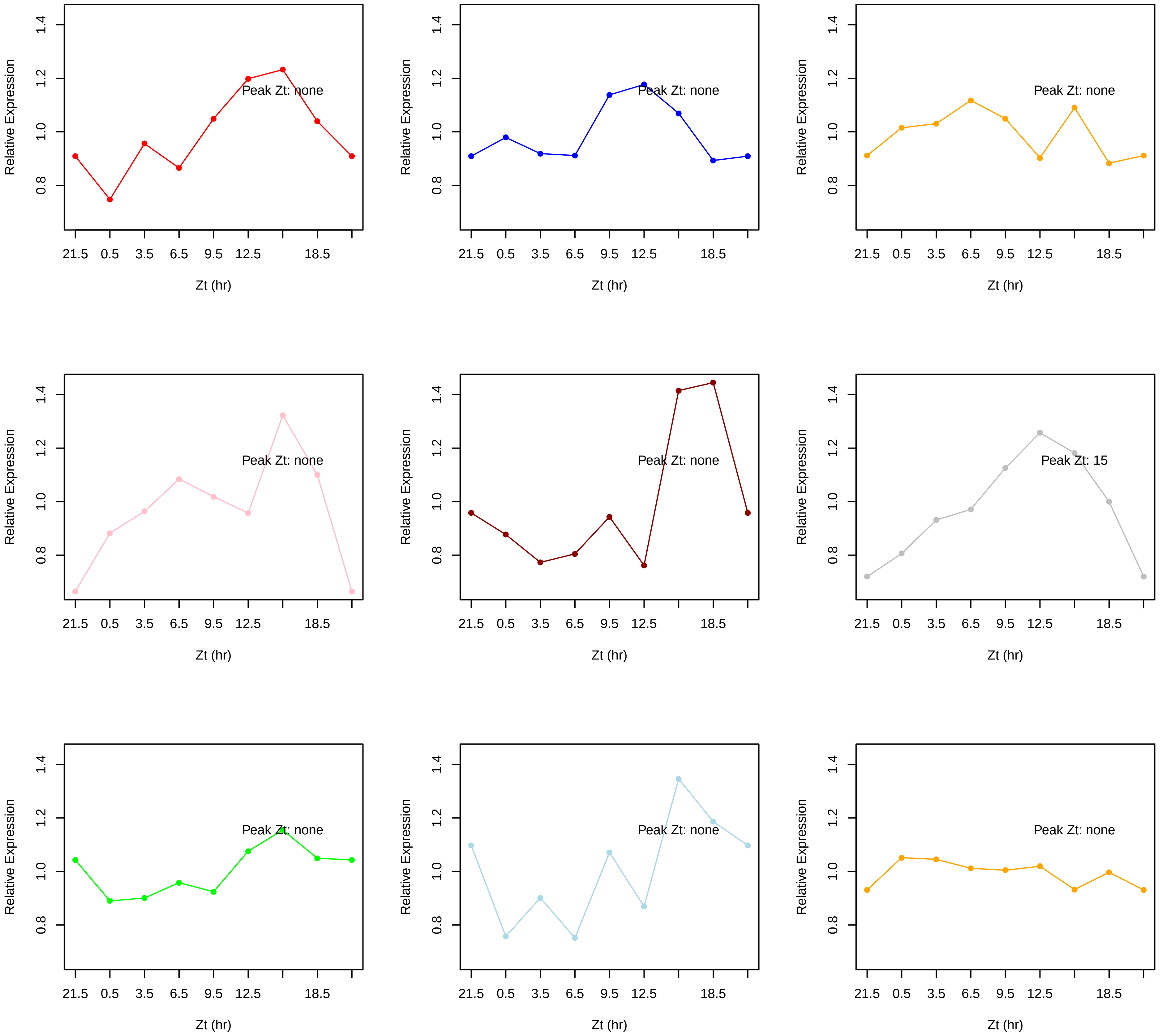

Singeleton: ENSXMAG00000020034 si:dkeyp−66d1.7

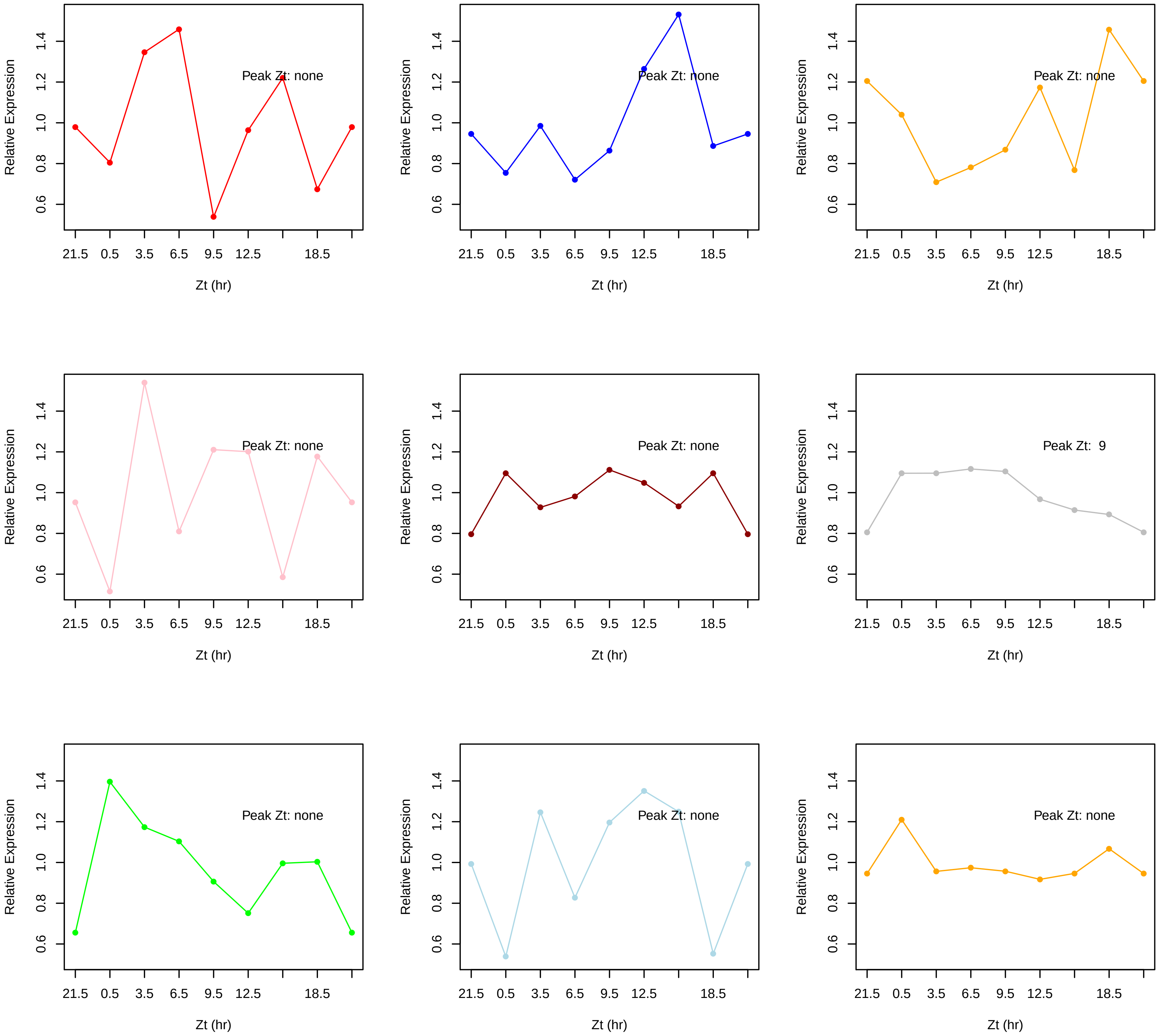

Singleton: ENSXMAG00000025311 METTL18

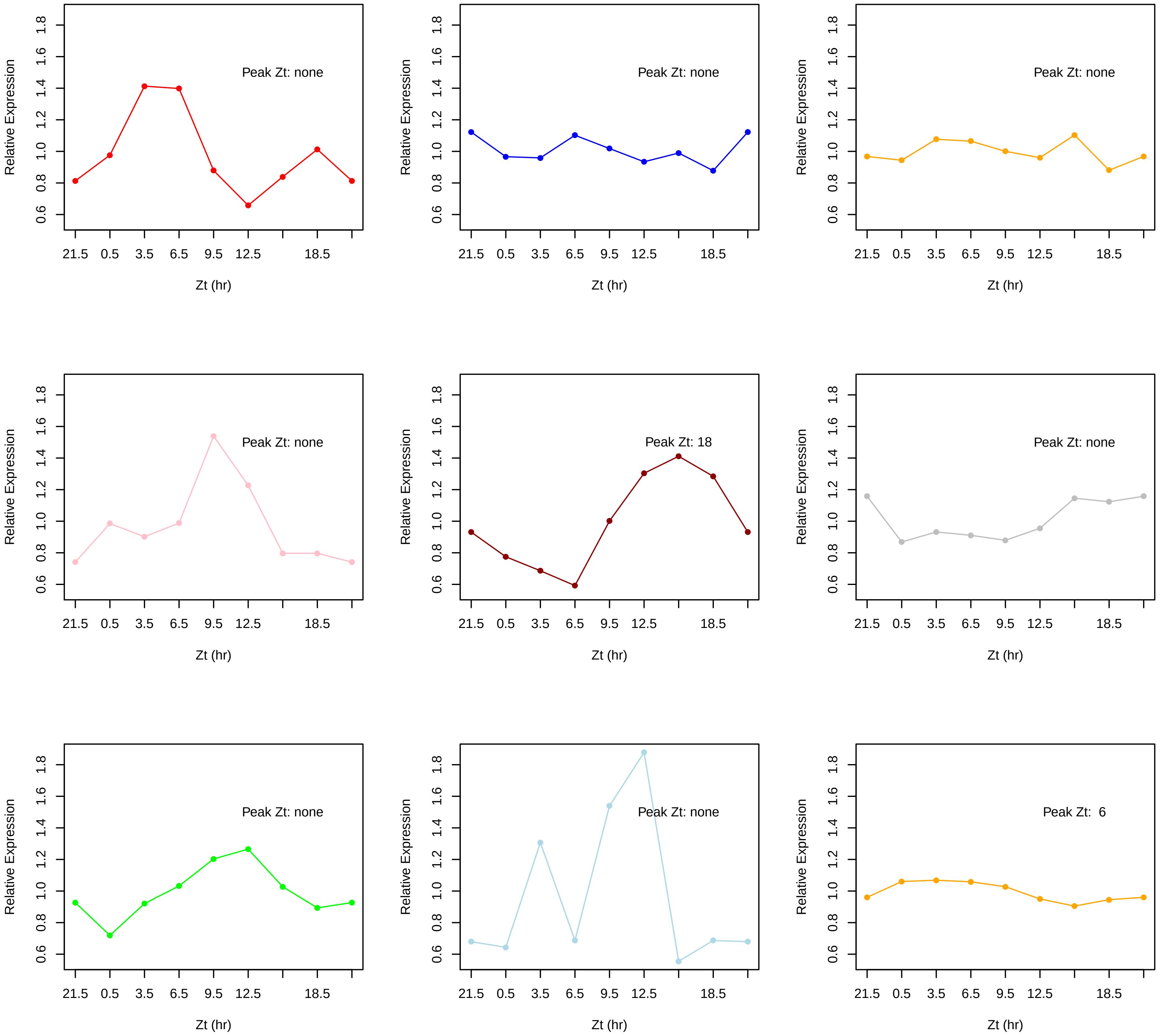

Singeleton: ENSXMAG00000019635 rho

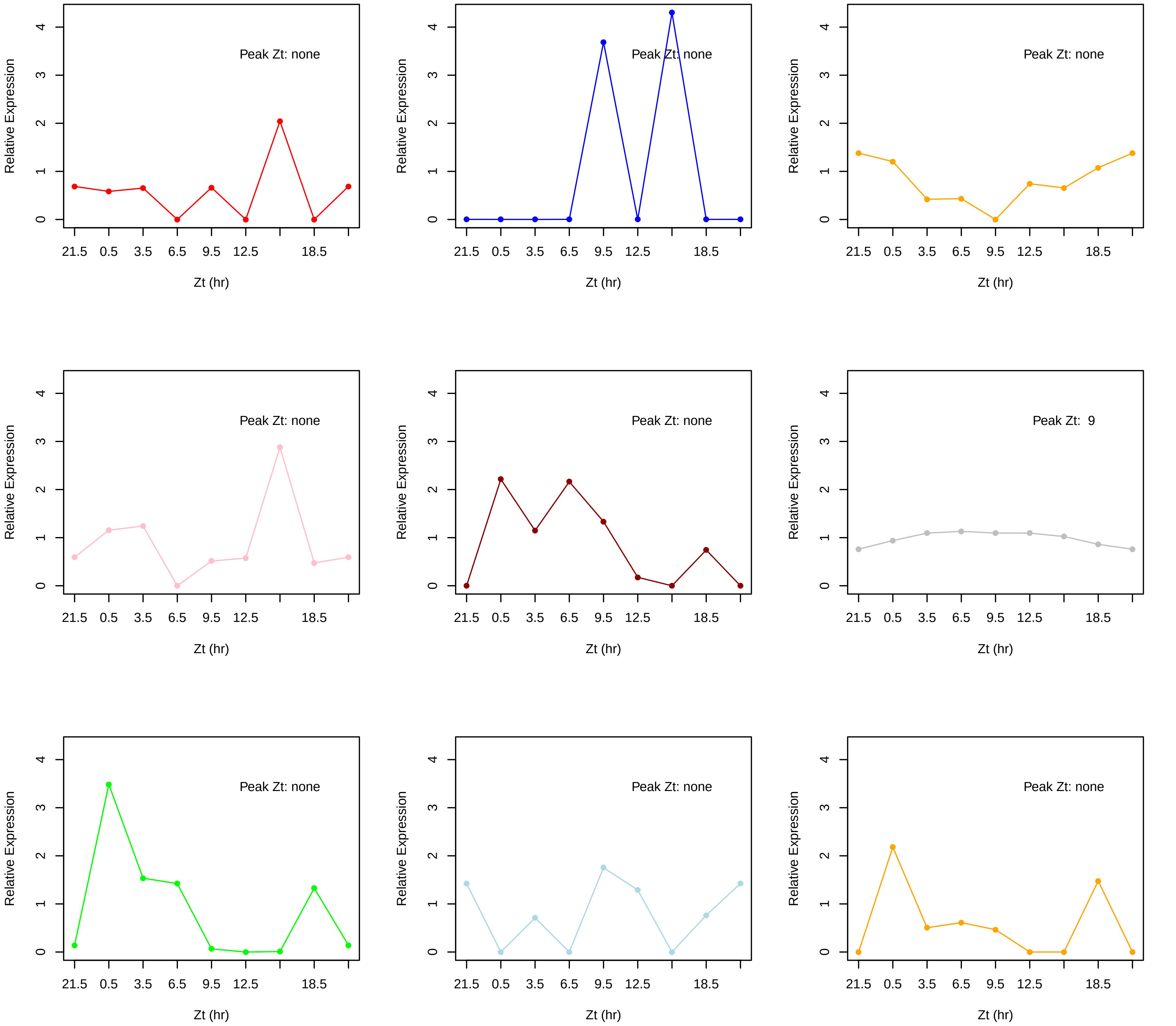

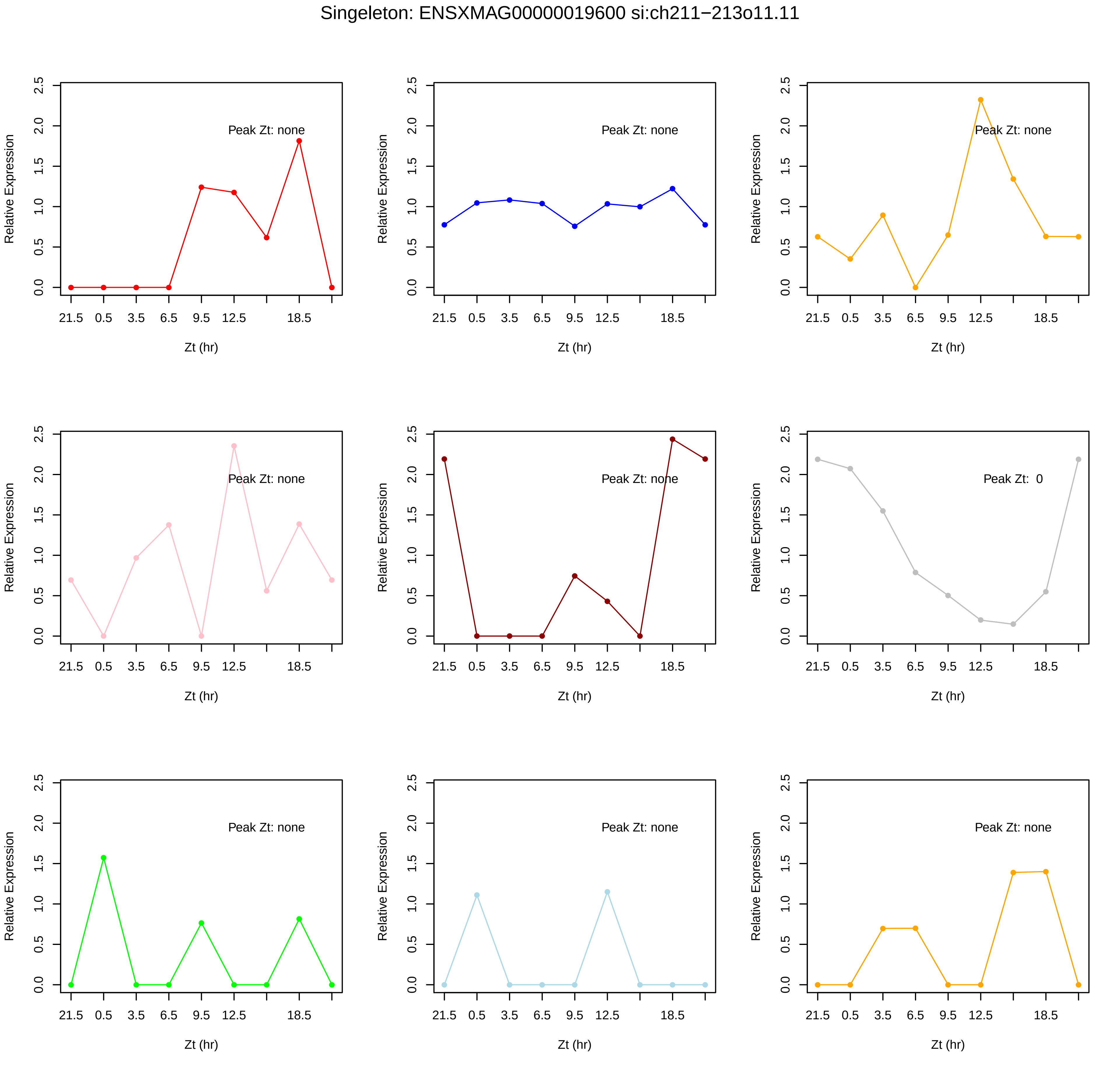

Singeleton: ENSXMAG00000019600 si:ch211−213o11.11
Relative Expression
Zt (hr)
Peak Zt: none
Peak Zt: 0
21.5
0.5
3.5
6.5
9.5
12.5
18.5
0.0
0.5
1.0
1.5
2.0
2.5

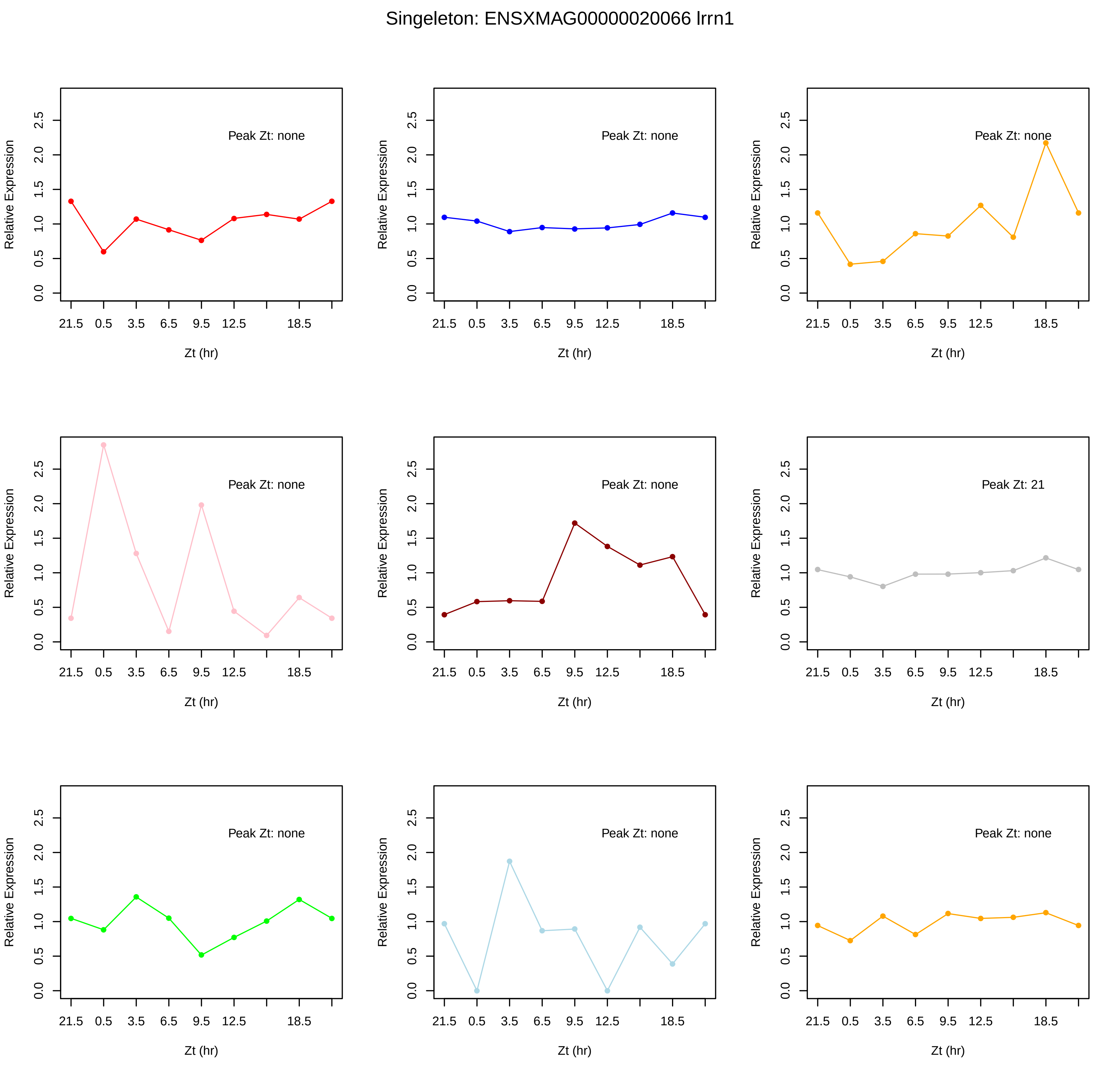
Singeleton: ENSXMAG00000020066 lrrn1
Peak Zt: none
Peak Zt: none
Peak Zt: none
Peak Zt: none
Peak Zt: none
Peak Zt: 21
Peak Zt: none
Peak Zt: none
Peak Zt: none
Relative Expression
Zt (hr)
21.5
0.5
3.5
6.5
9.5
12.5
18.5
0.0
0.5
1.0
1.5
2.0
2.5

Singleton: ENSXMAG00000010901 amigo3

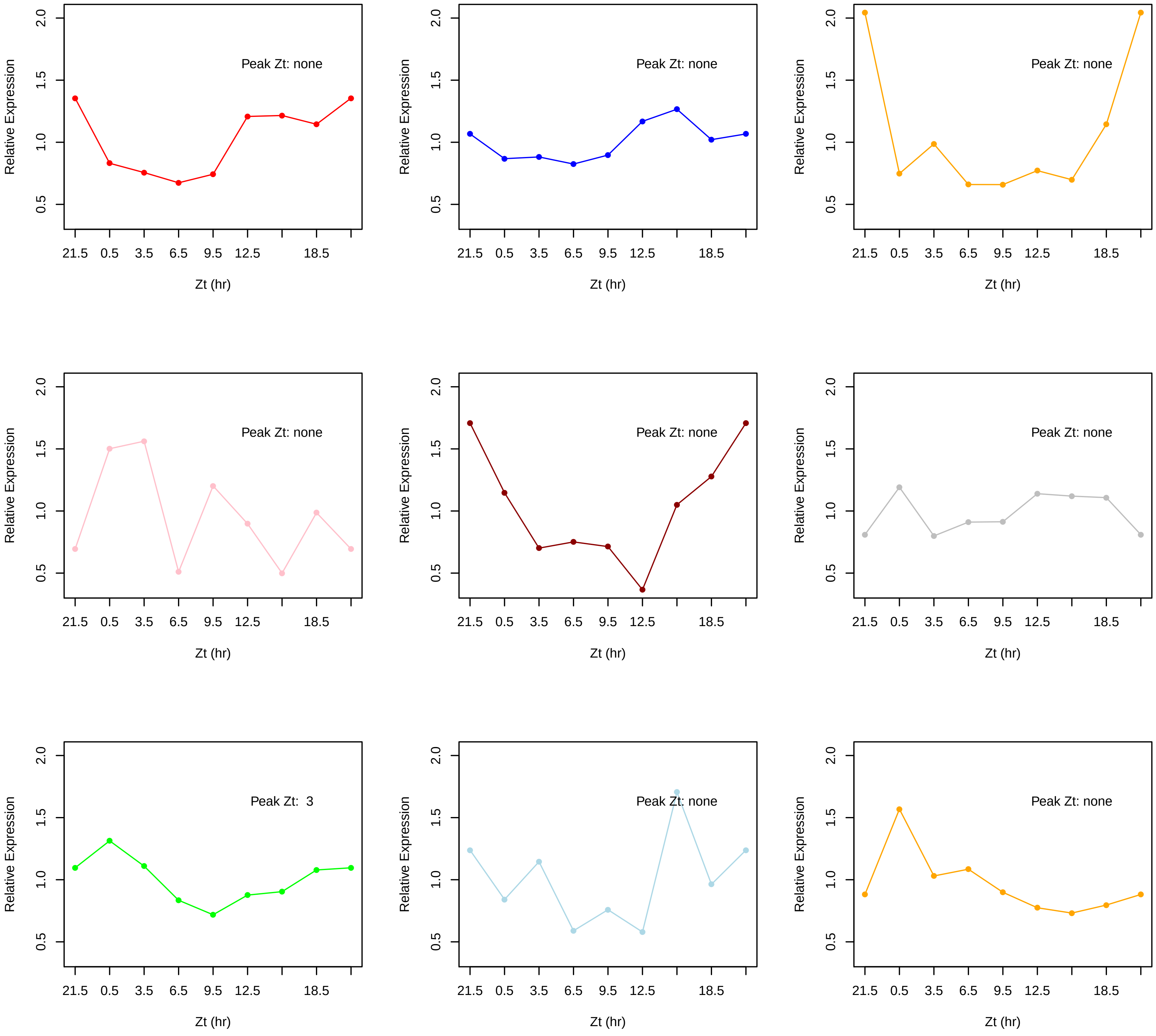

Singeleton: ENSXMAG00000019863 paqr7b

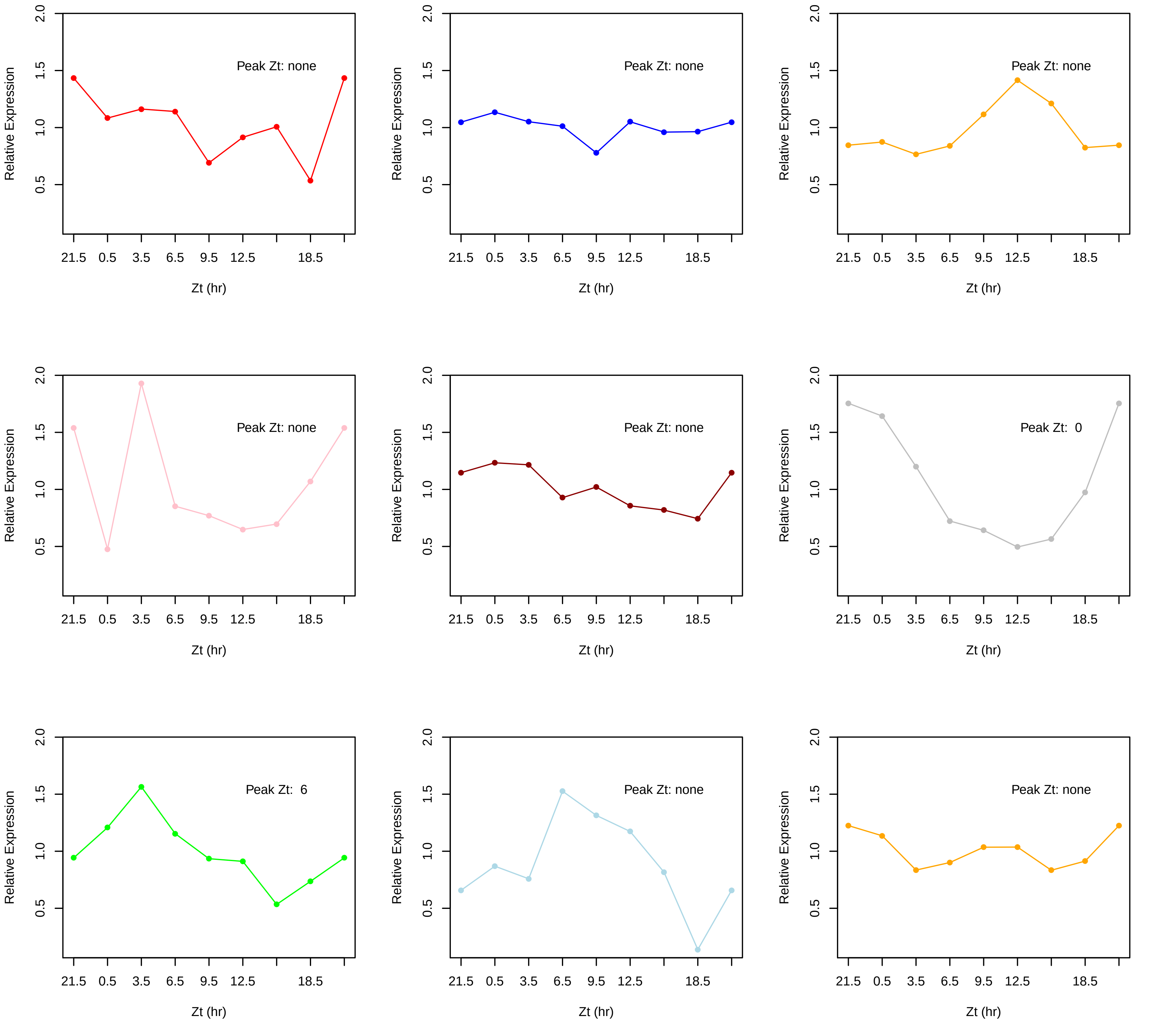

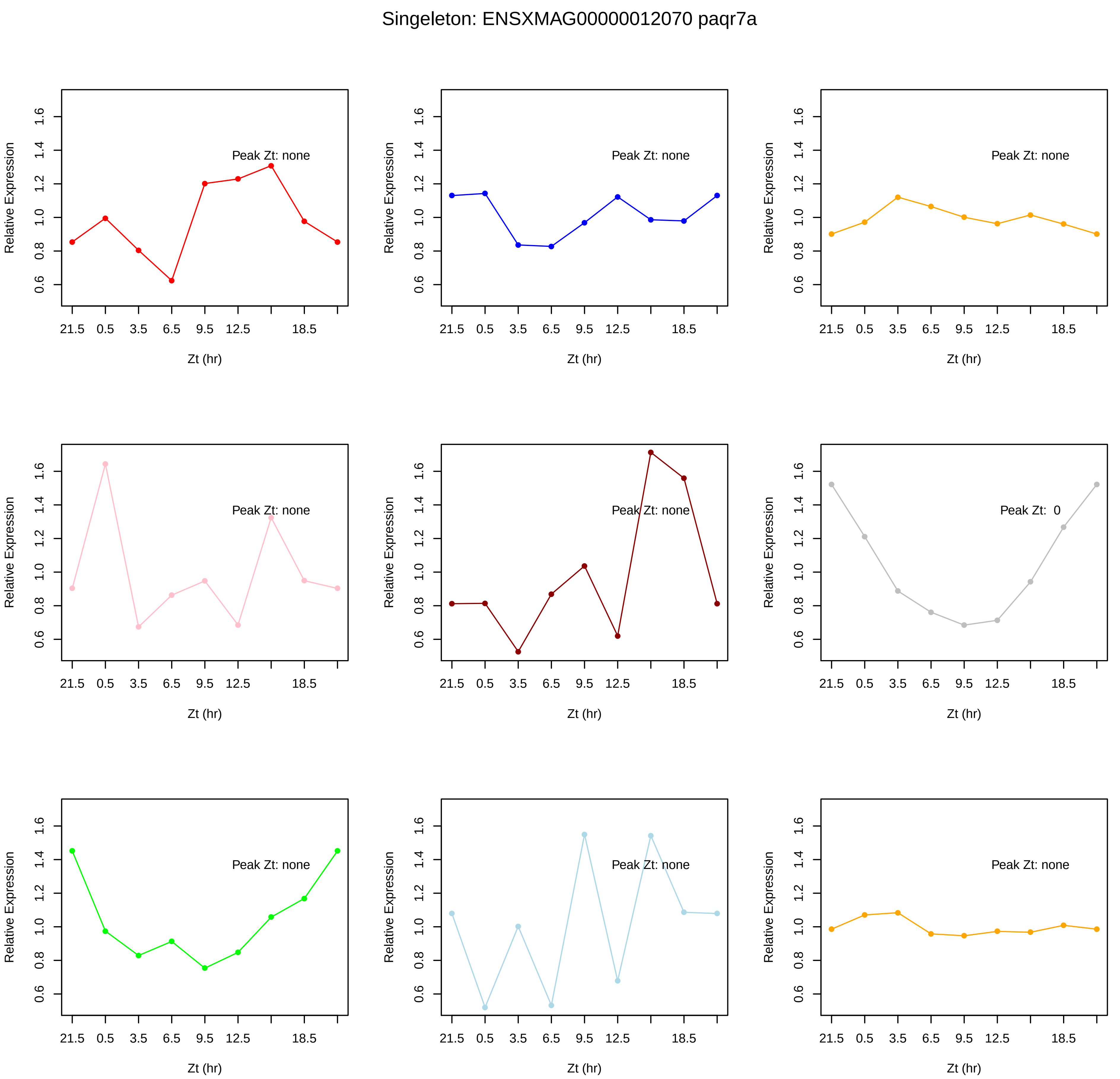
Singeleton: ENSXMAG00000012070 paqr7a
Relative Expression
Zt (hr)
Peak Zt: none
Peak Zt: 0
21.5
0.5
3.5
6.5
9.5
12.5
18.5
0.6
0.8
1.0
1.2
1.4
1.6

Singeleton: ENSXMAG00000013637 si:dkey−221l4.11

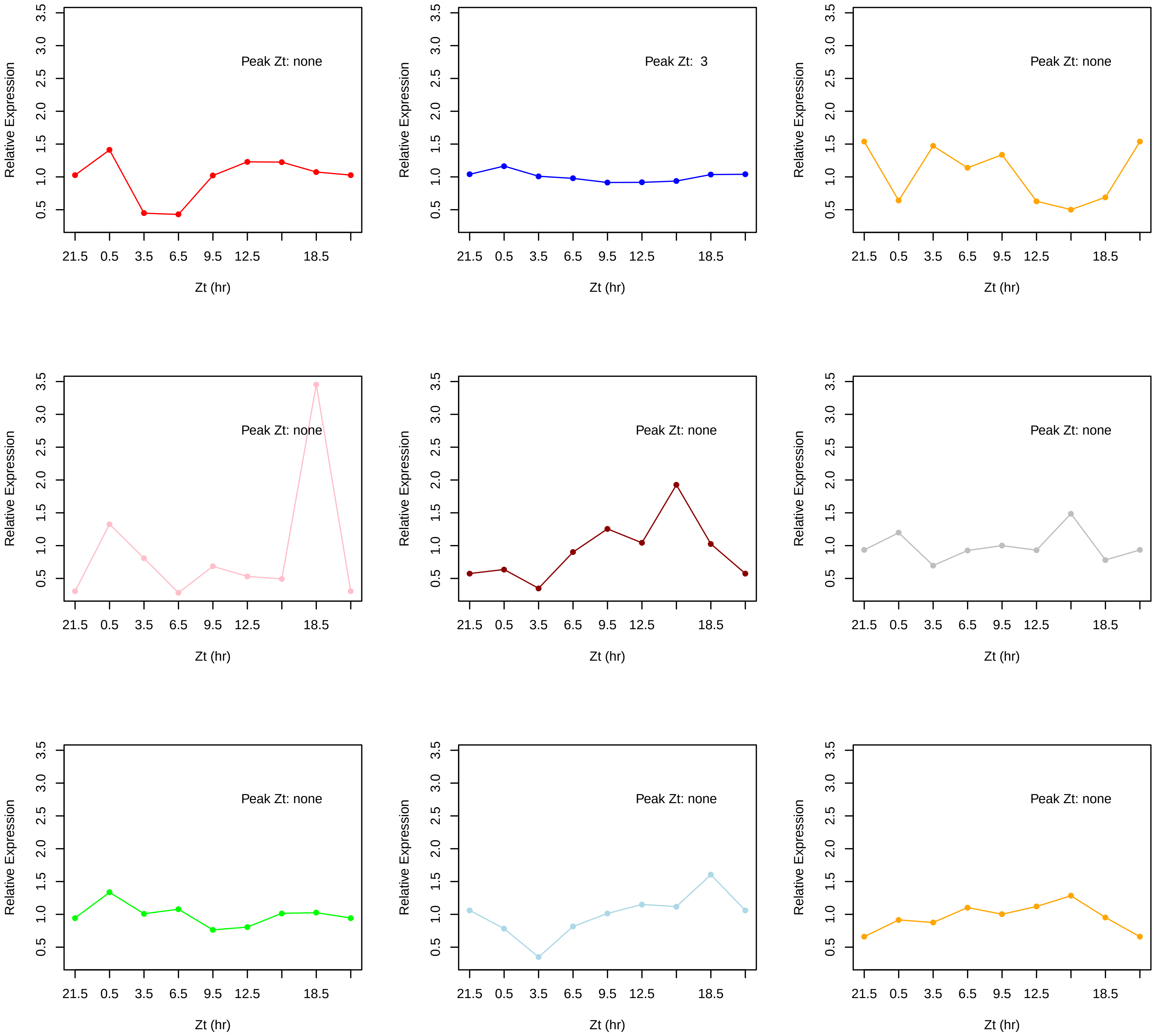

Singeleton: ENSXMAG00000019531 pnrc2

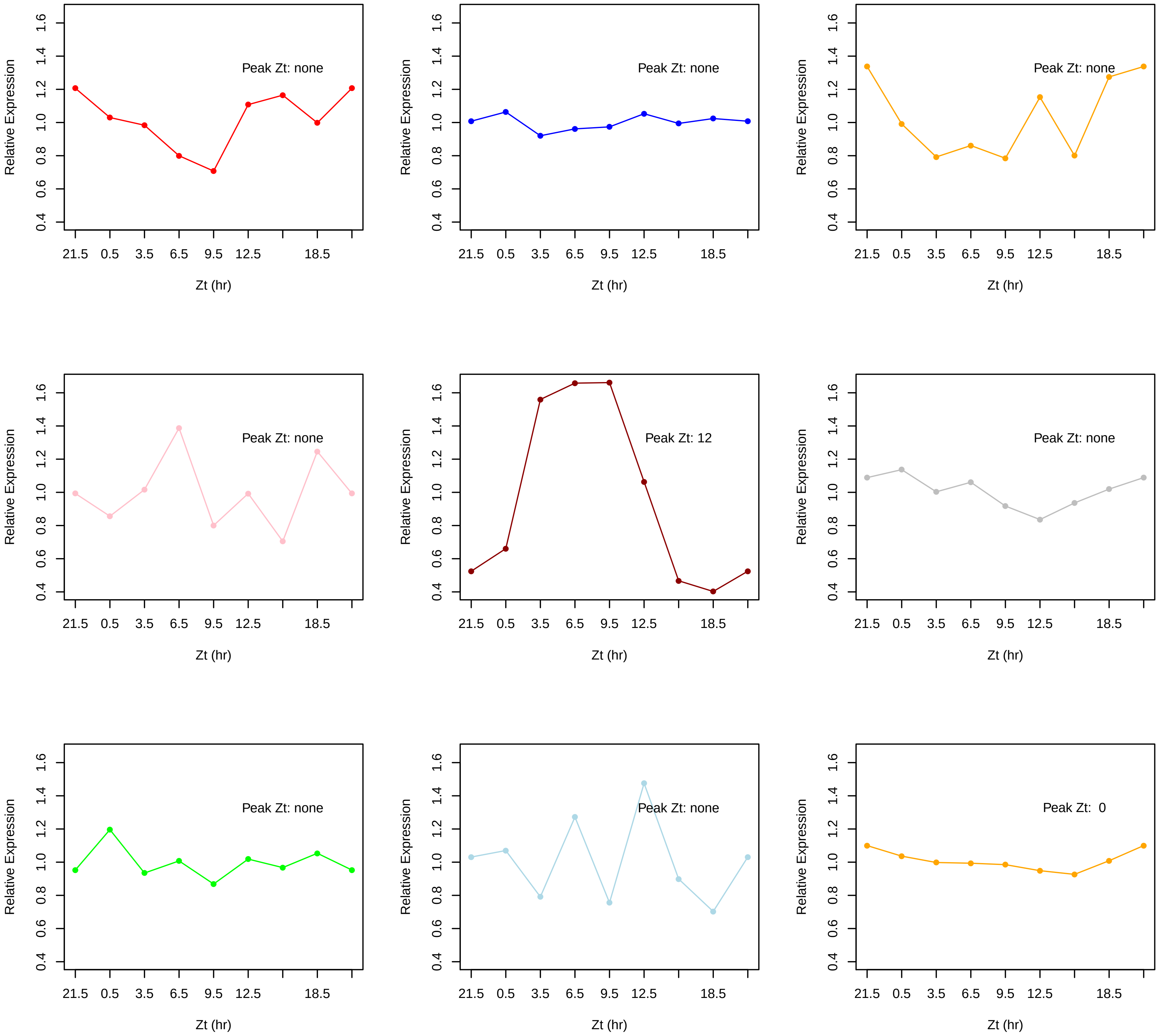

Singeleton: ENSXMAG00000019487 SLC35A4

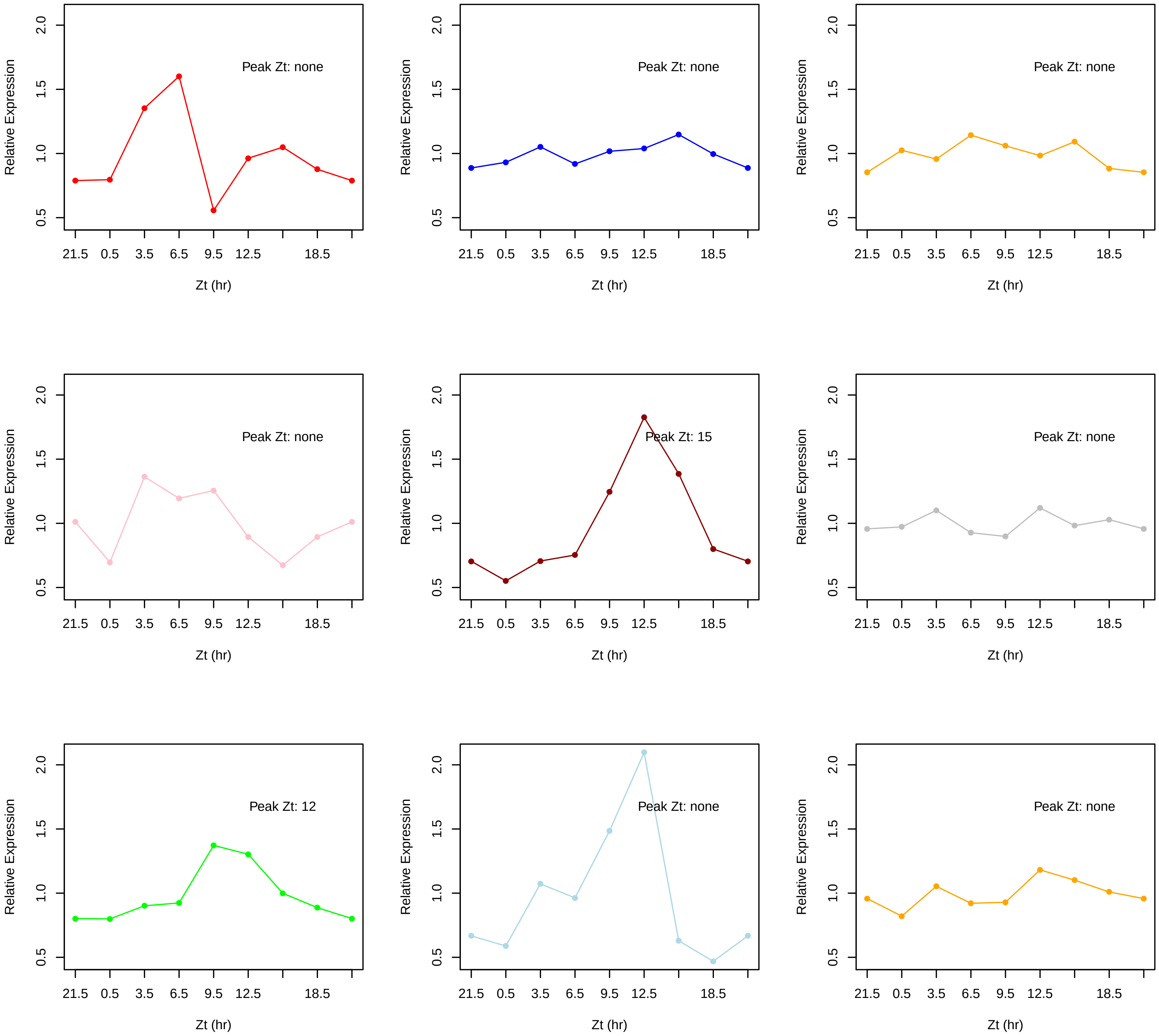

Singeleton: ENSXMAG00000021577 rpp25l

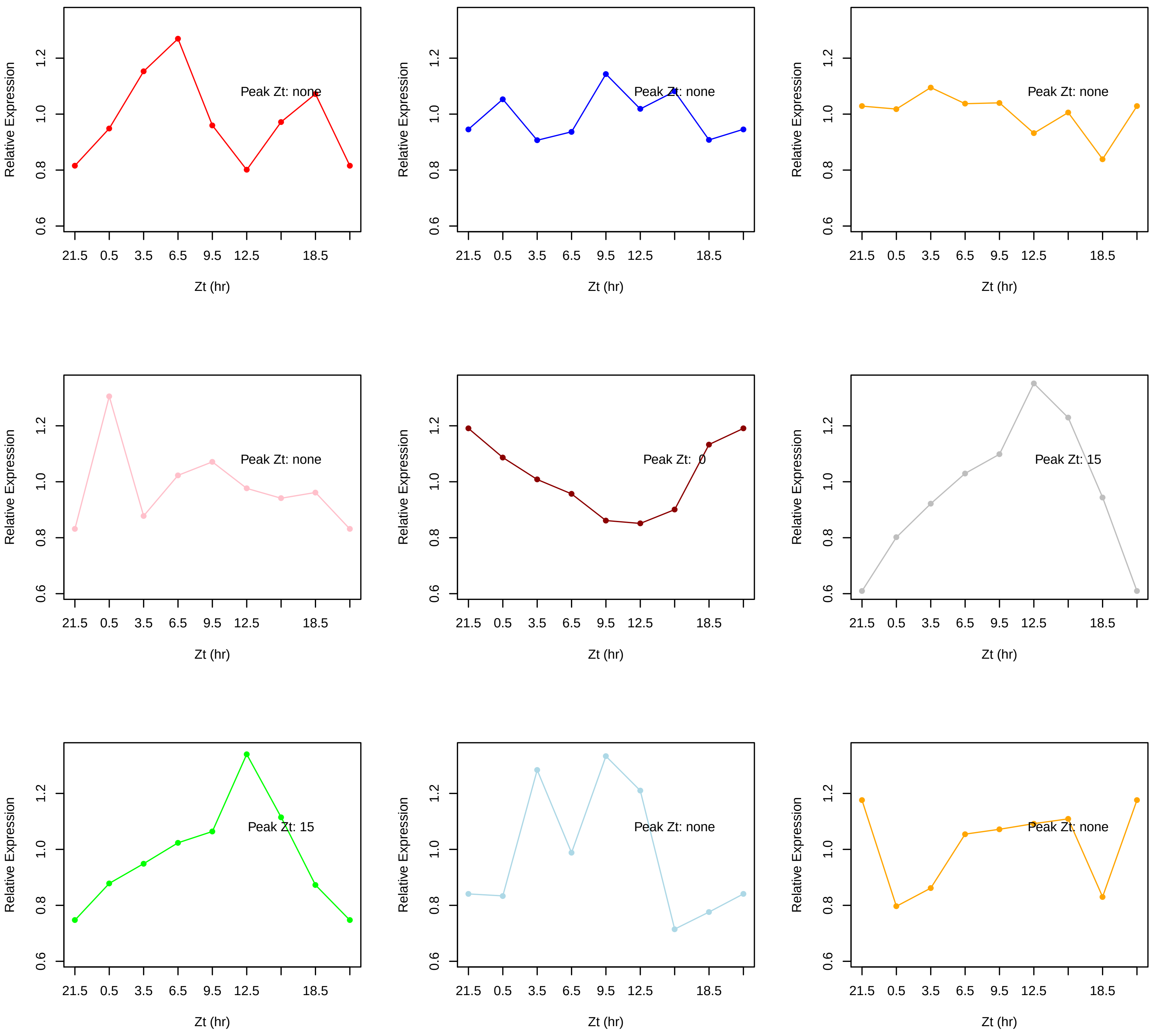

Singeleton: ENSXMAG00000025221 gbp

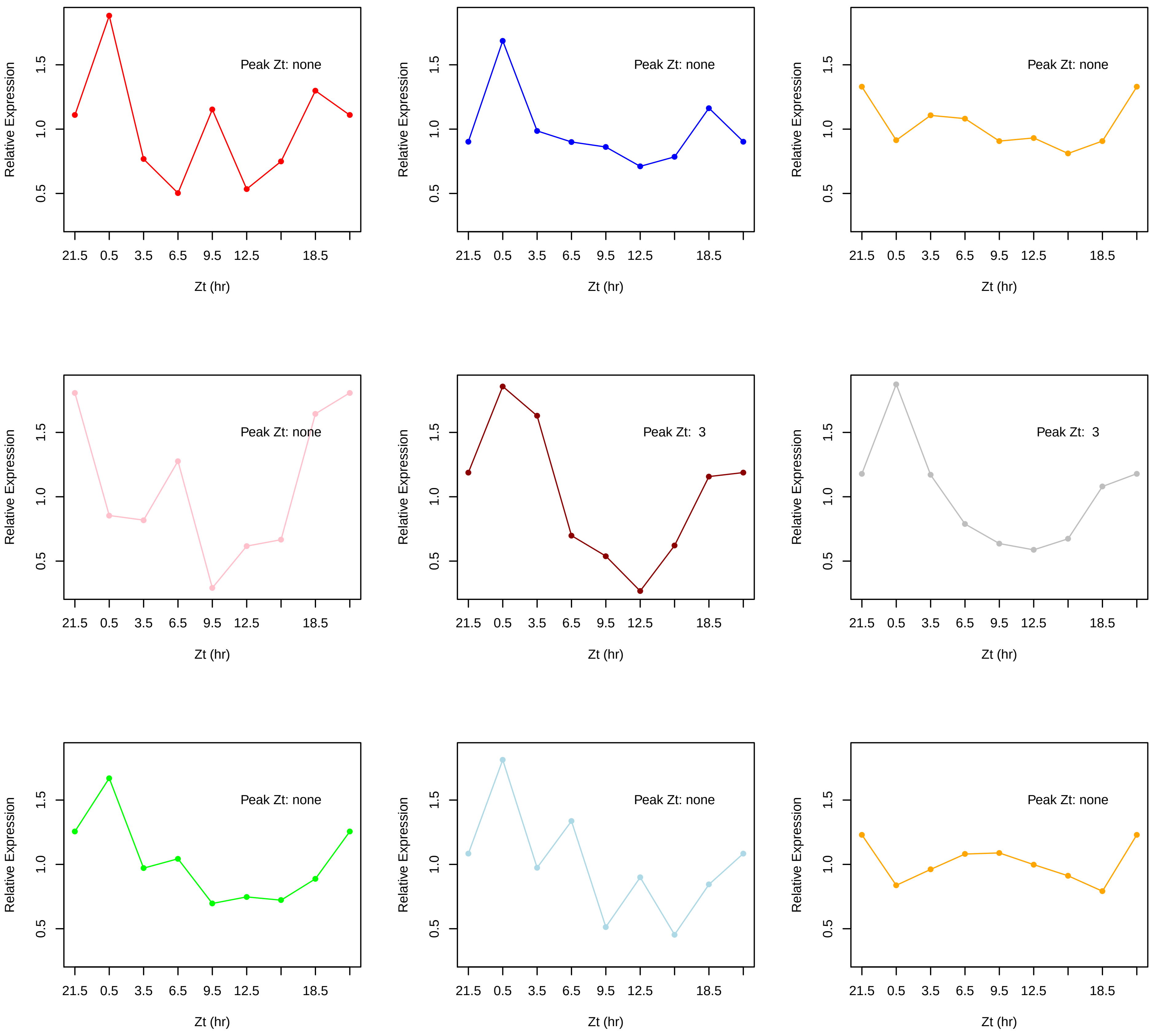

Singeleton: ENSXMAG00000020143

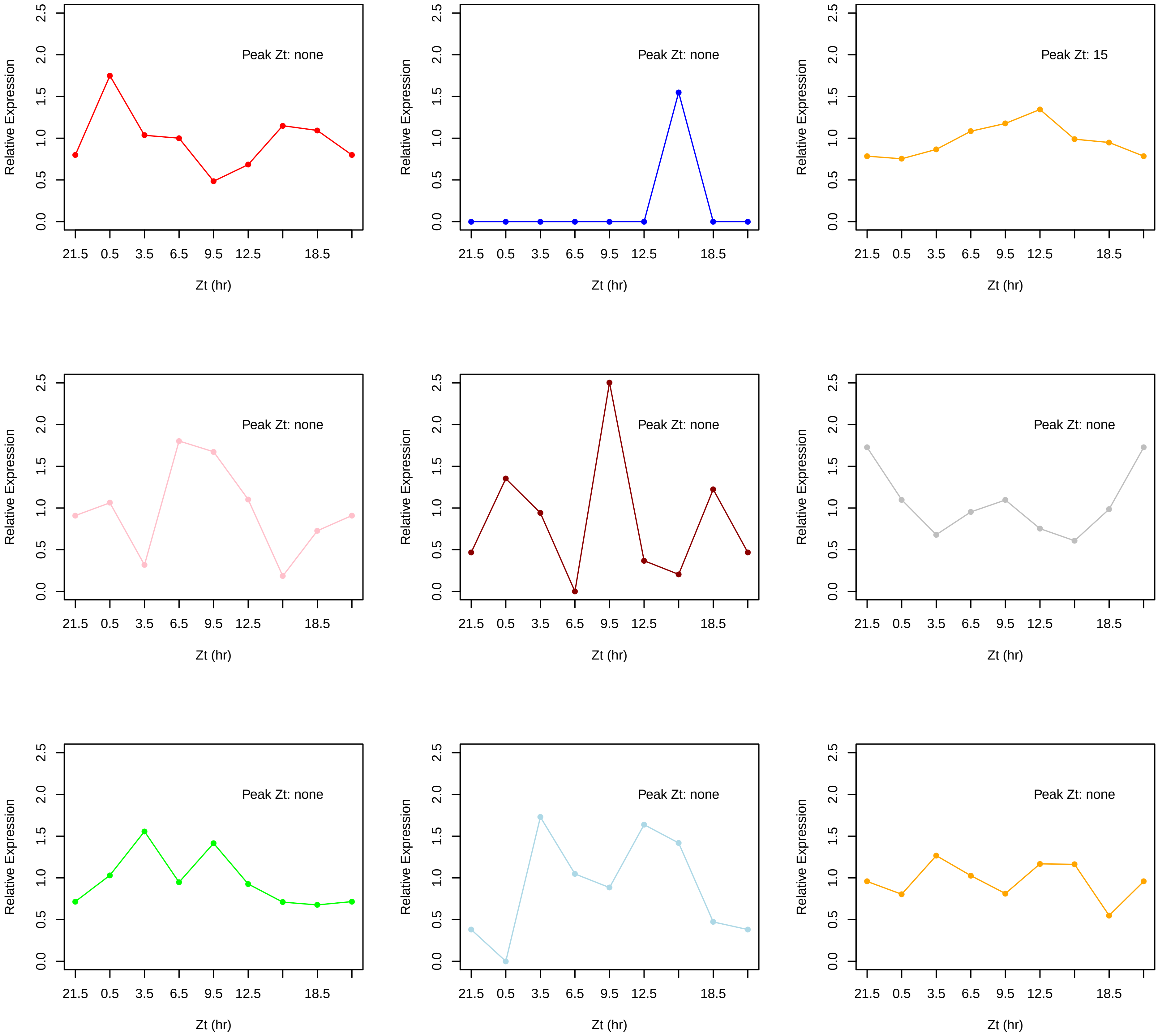

Singeleton: ENSXMAG00000019788 NHLRC1

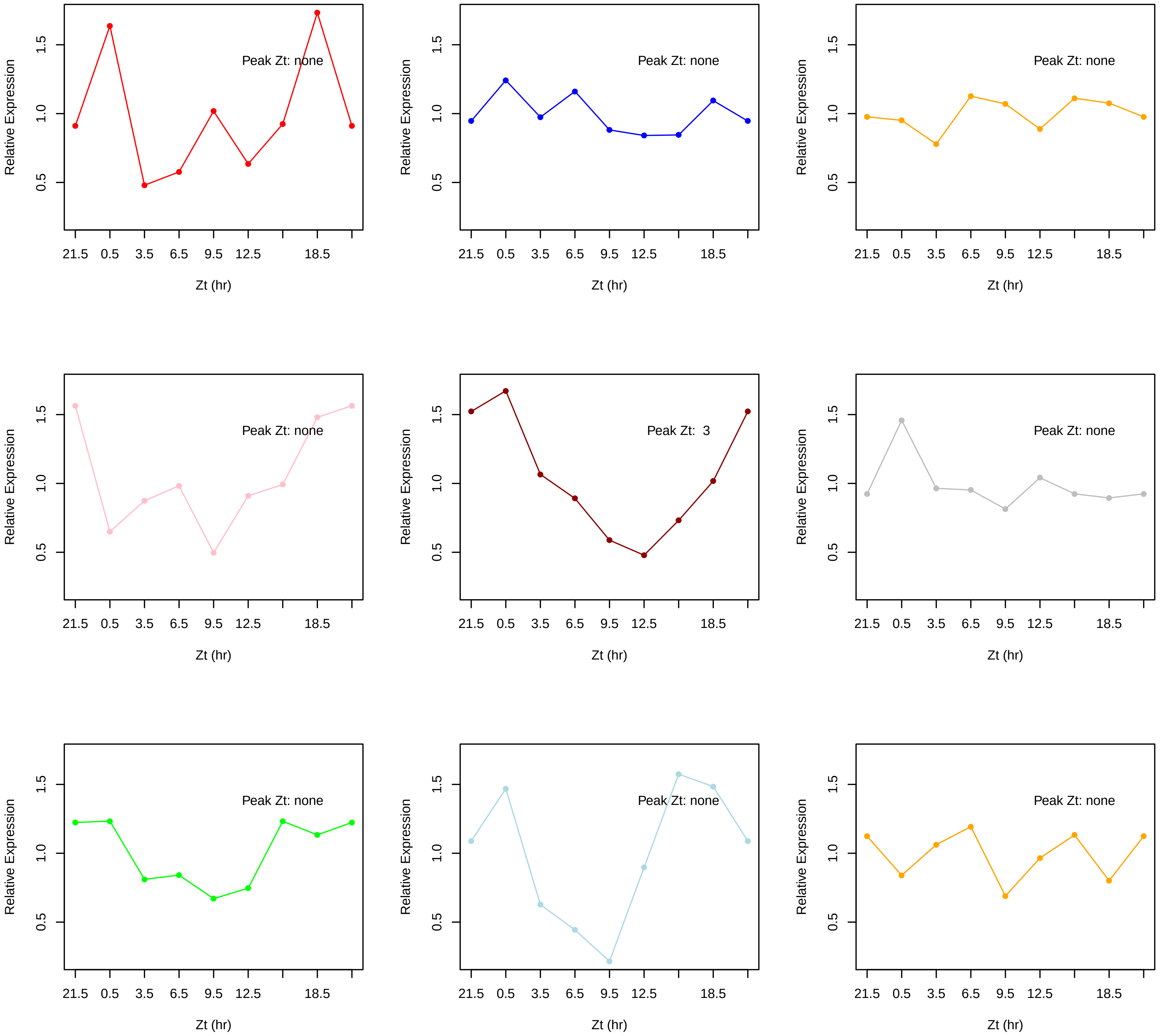

Singeleton: ENSXMAG00000020015 KCNS2

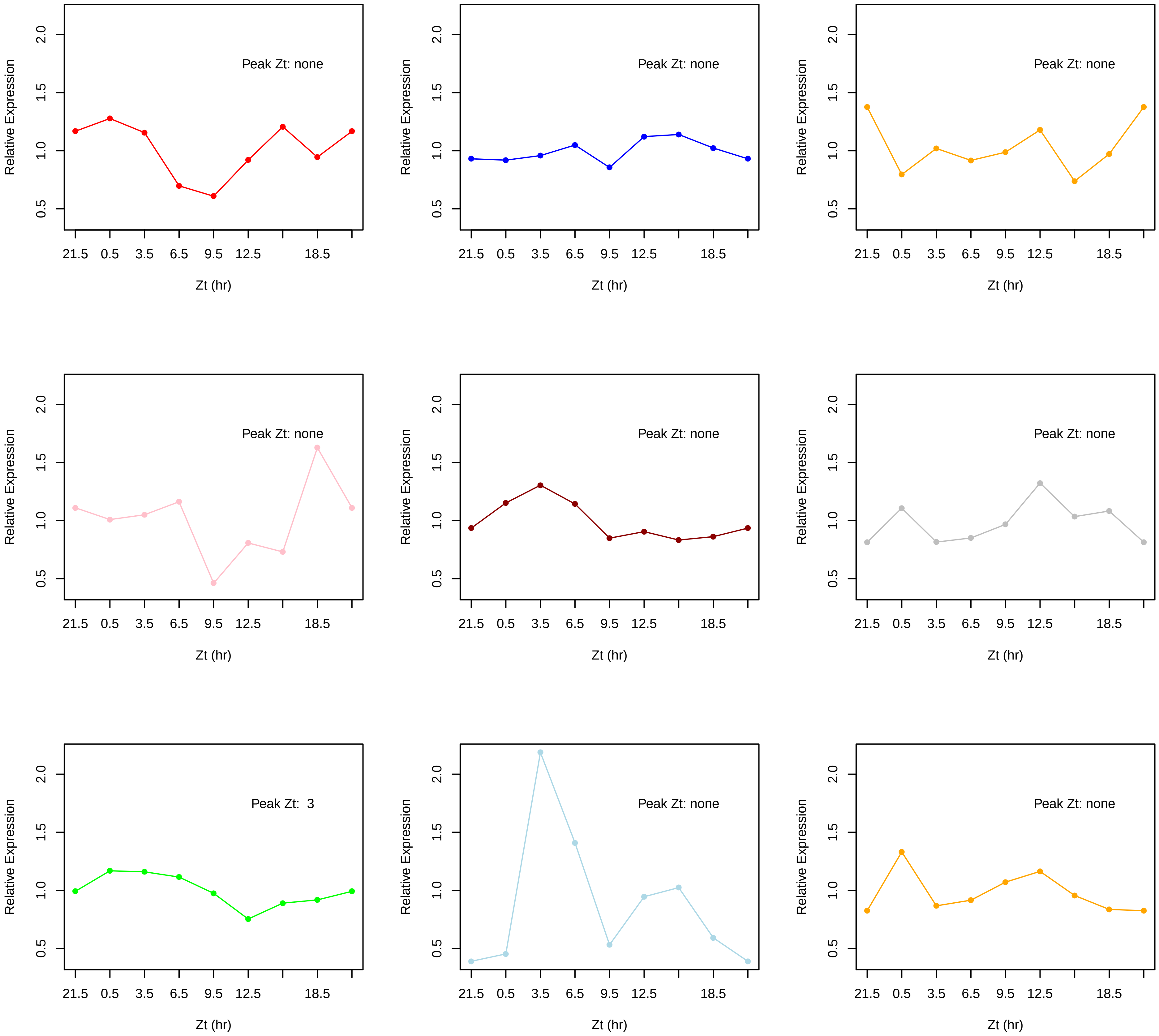

Singeleton: ENSXMAG00000013155 pdp1

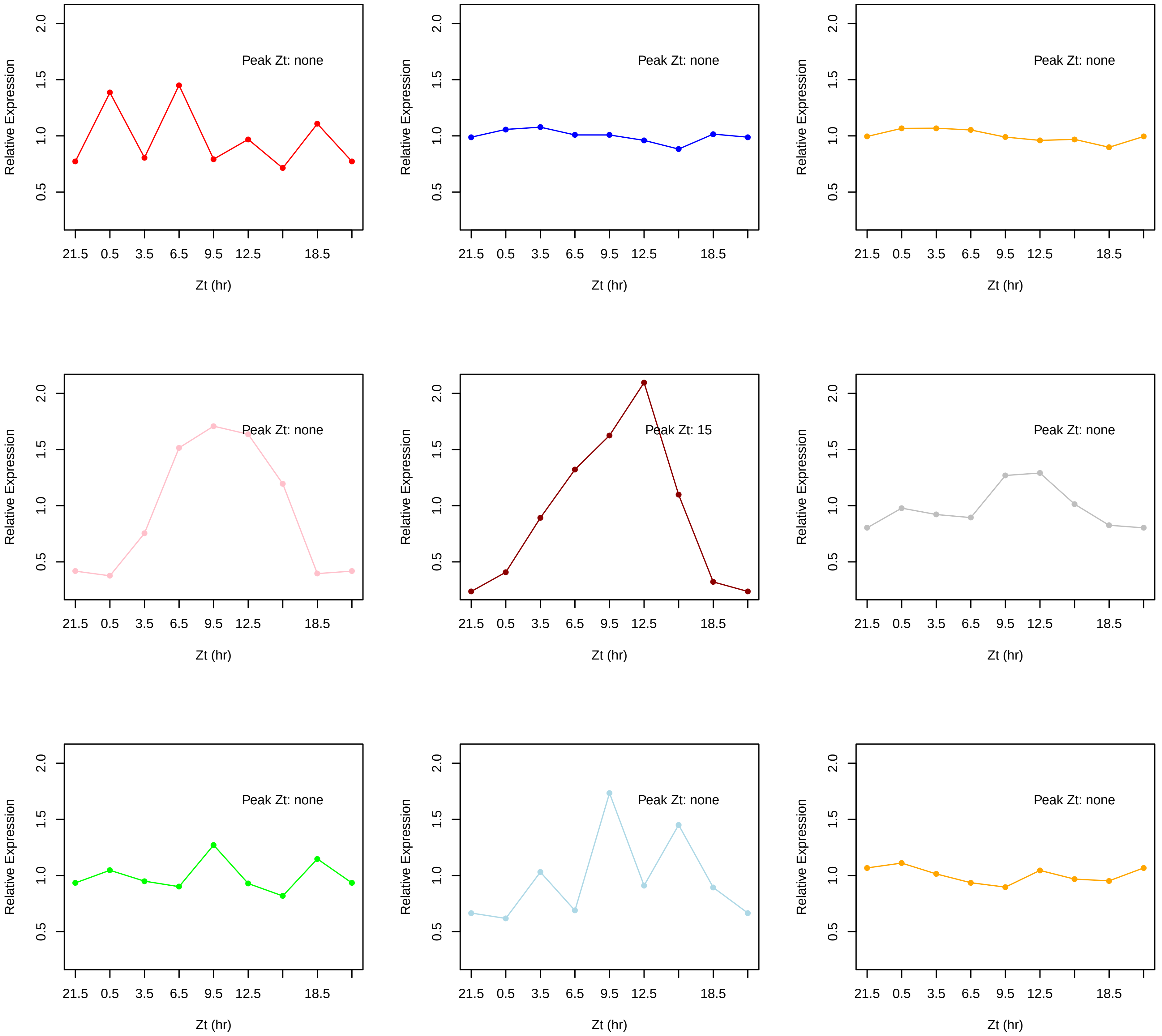

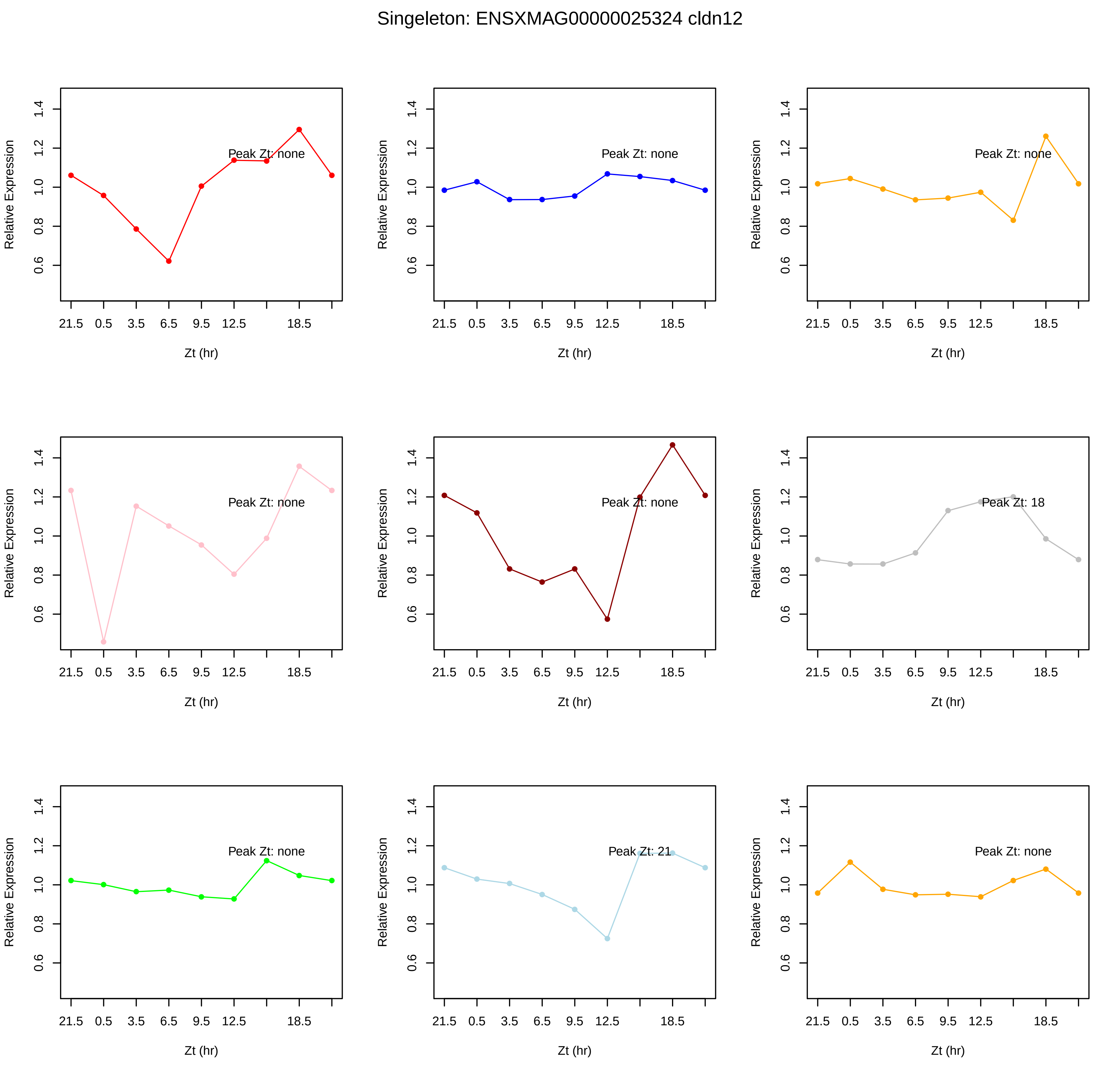
Singeleton: ENSXMAG00000025324 cldn12
Peak Zt: none
Peak Zt: none
Peak Zt: none
Peak Zt: none
Peak Zt: none
Peak Zt: 18
Peak Zt: none
Peak Zt: 21
Peak Zt: none
Relative Expression
Zt (hr)
21.5
0.5
3.5
6.5
9.5
12.5
18.5
0.6
0.8
1.0
1.2
1.4

Singleton: ENSXMAG00000019995 arl4aa

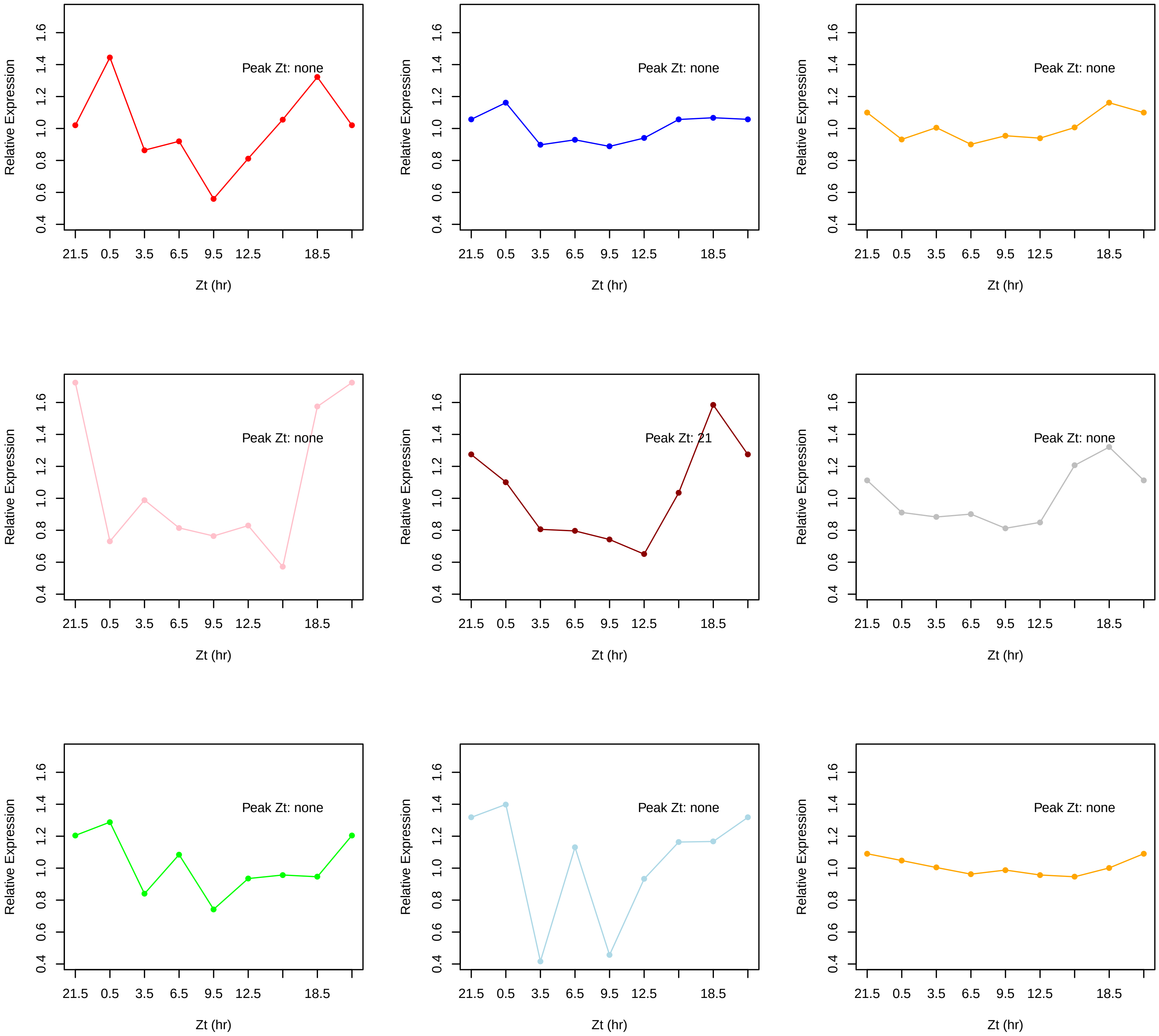

Singleton: ENSXMAG00000019665 cebpb

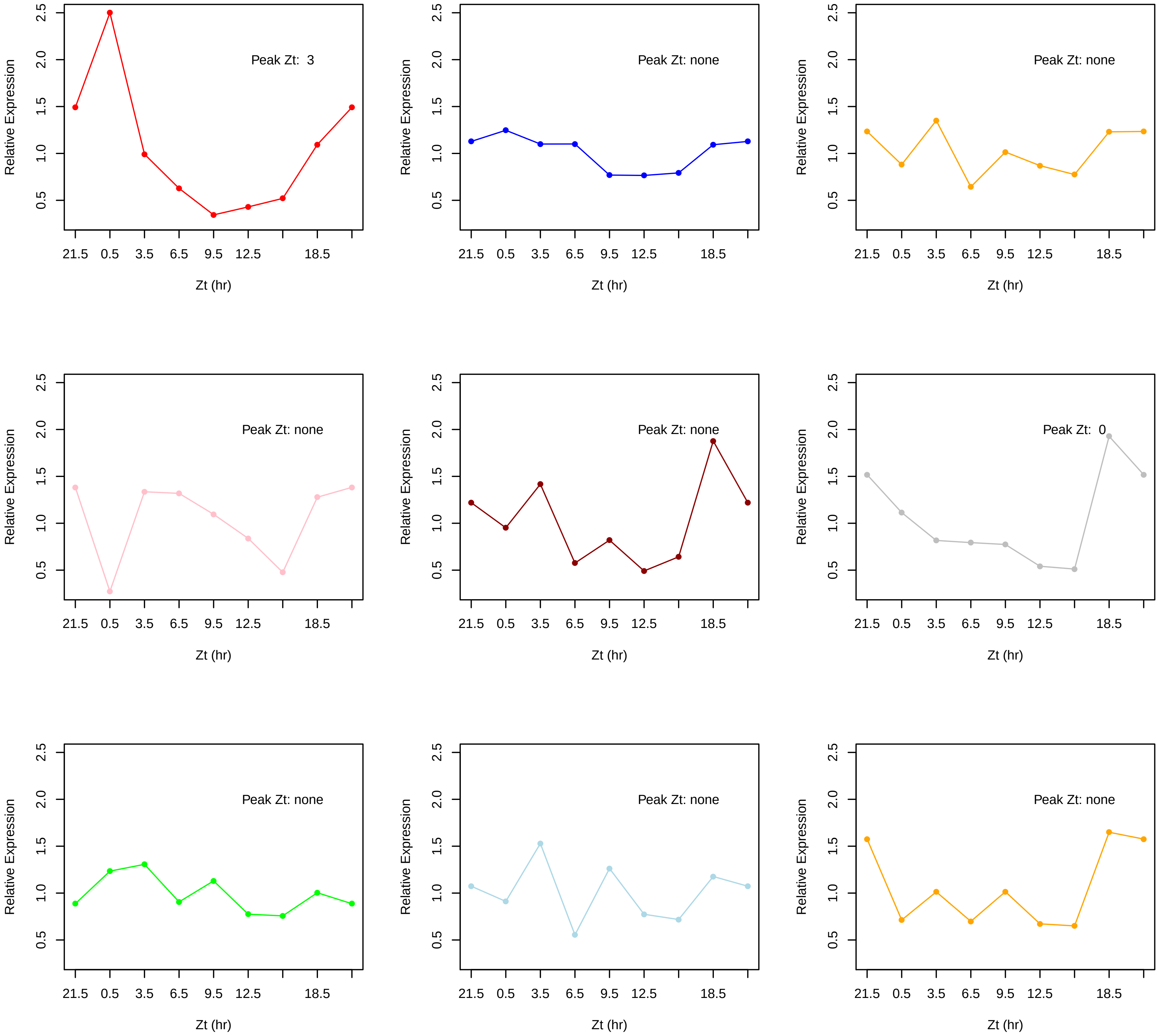

Singleton: ENSXMAG00000021148

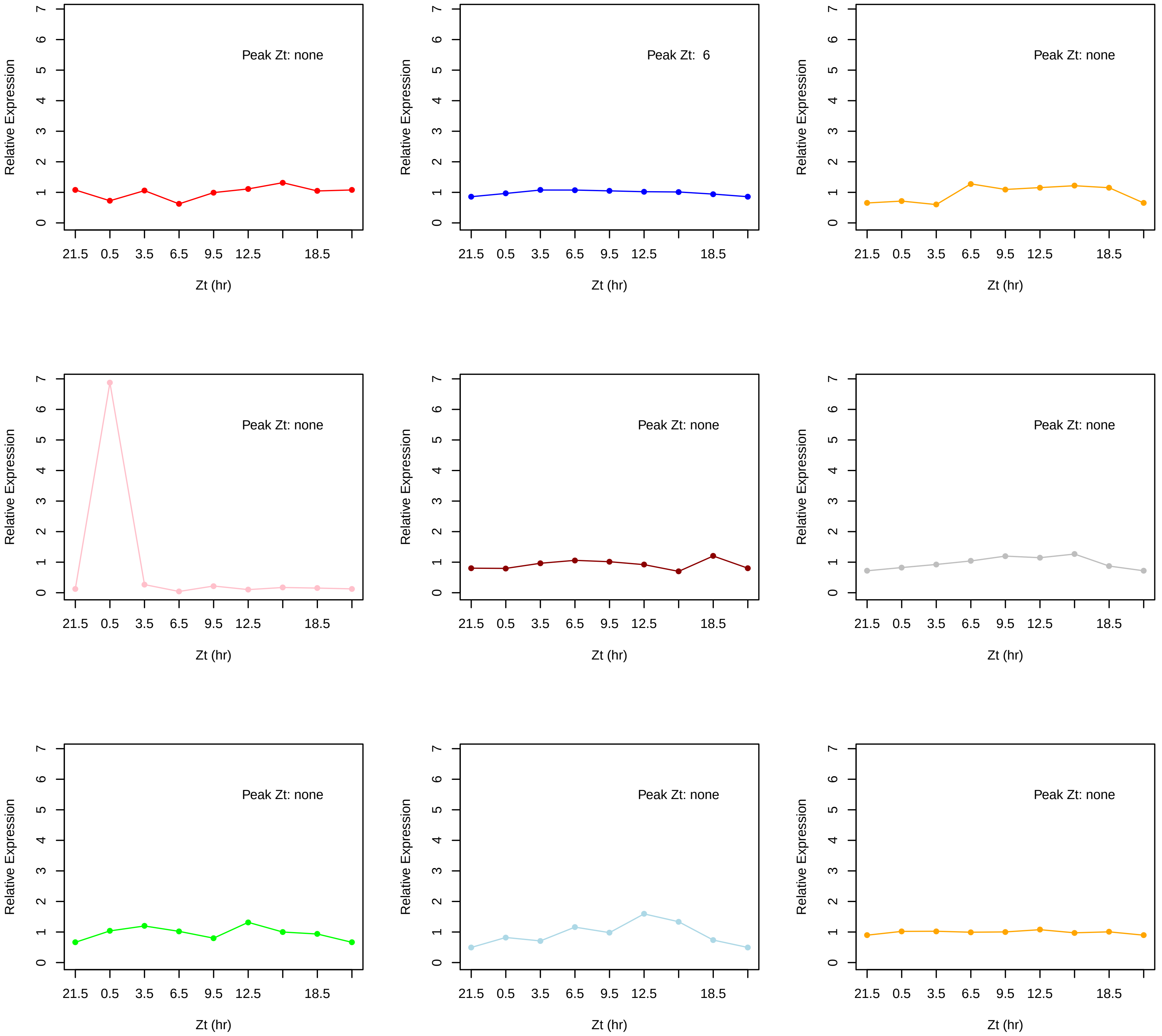

Singeleton: ENSXMAG00000024192 RF00443

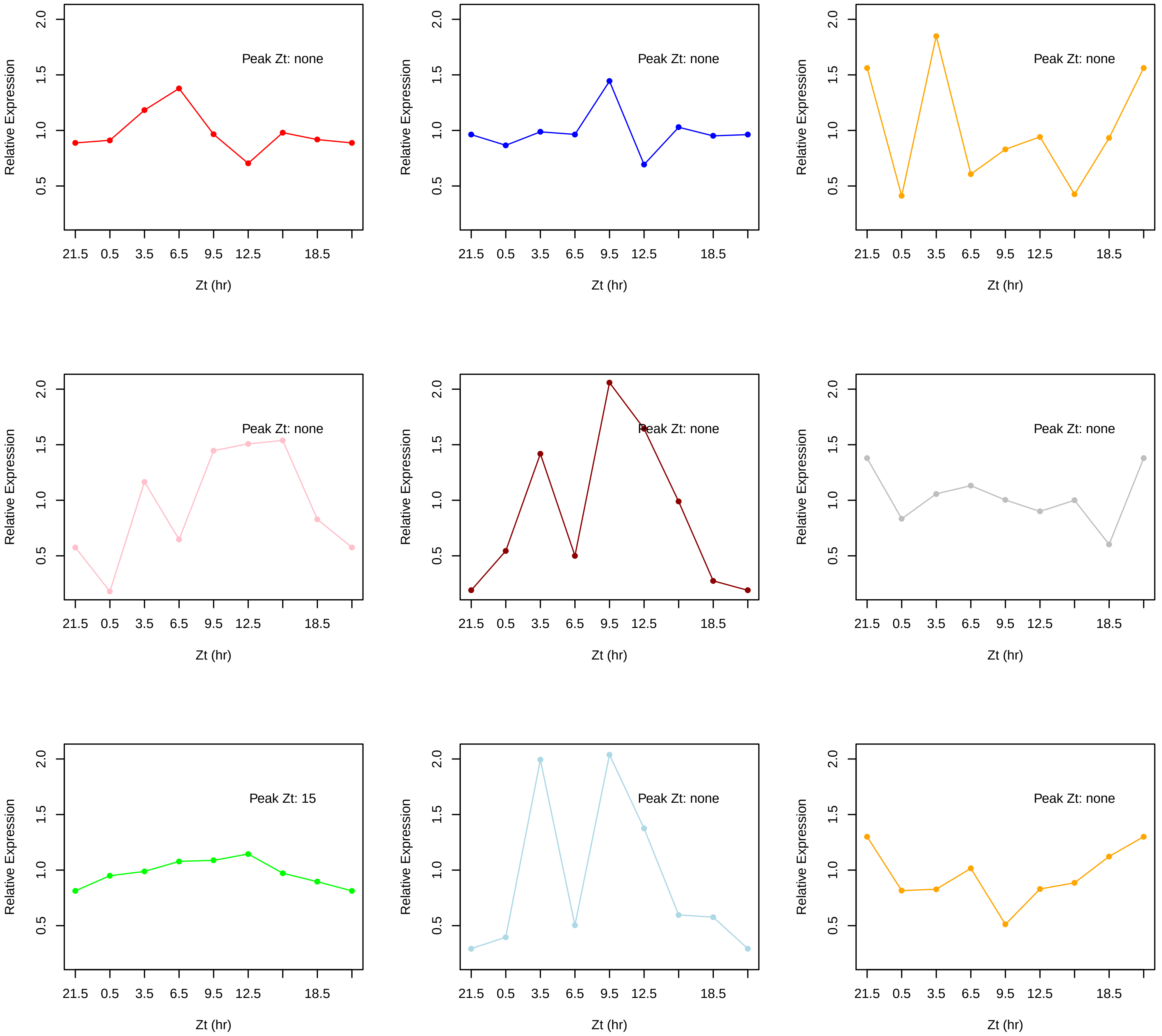

Singeleton: ENSXMAG00000020519 MIR133B

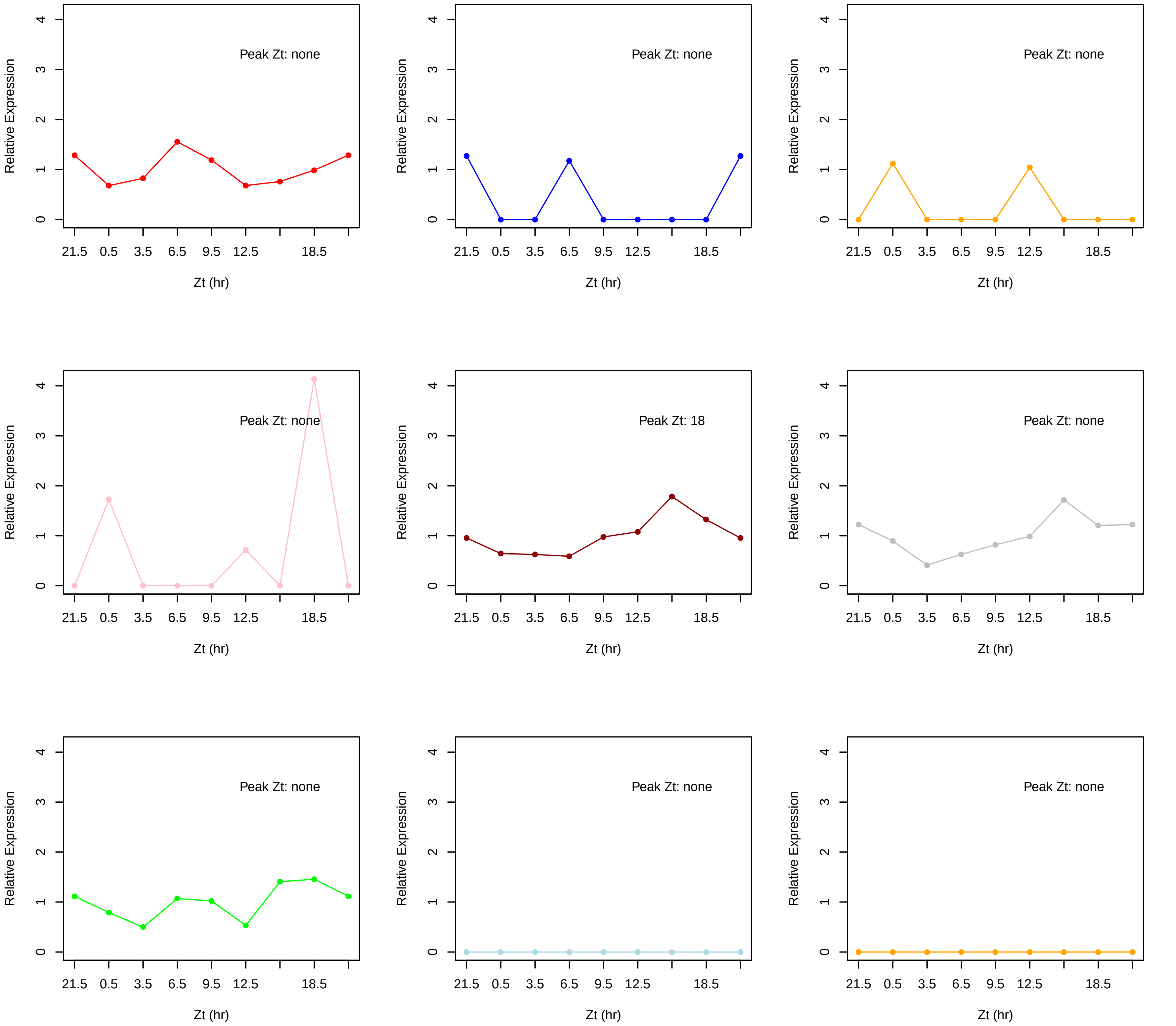

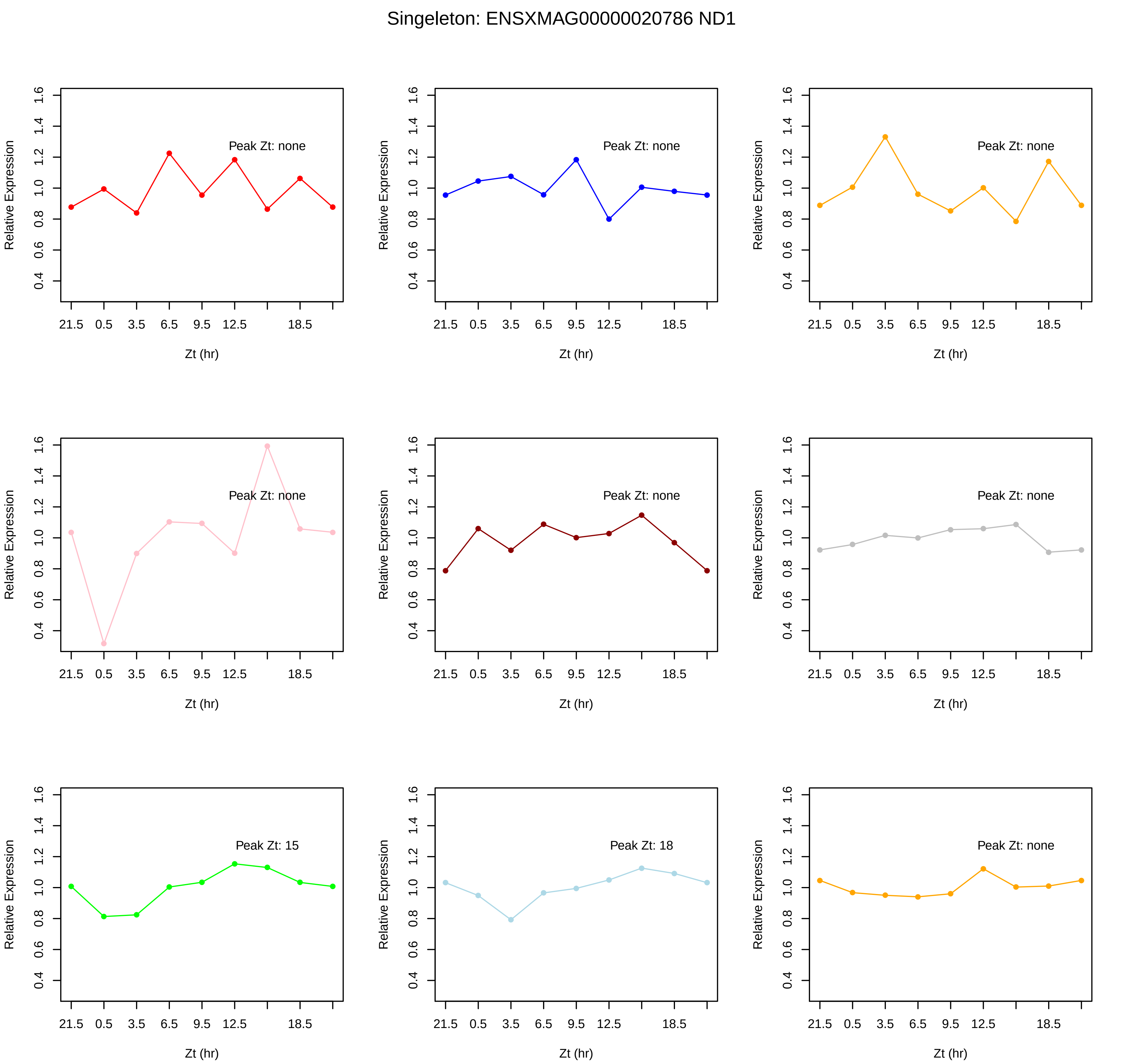
Singeleton: ENSXMAG00000020786 ND1
Relative Expression
Zt (hr)
21.5
0.5
3.5
6.5
9.5
12.5
18.5
0.4
0.6
0.8
1.0
1.2
1.4
1.6
Peak Zt: none
Peak Zt: 15
Peak Zt: 18
